# Loss of the chromatin modifier Kdm2aa causes Braf^V600E^-independent spontaneous melanoma in zebrafish

**DOI:** 10.1371/journal.pgen.1006959

**Published:** 2017-08-14

**Authors:** Catherine M. Scahill, Zsofia Digby, Ian M. Sealy, Sonia Wojciechowska, Richard J. White, John E. Collins, Derek L. Stemple, Till Bartke, Marie E. Mathers, E. Elizabeth Patton, Elisabeth M. Busch-Nentwich

**Affiliations:** 1 Wellcome Trust Sanger Institute, Wellcome Genome Campus, Hinxton, United Kingdom; 2 MRC Institute of Genetics and Molecular Medicine, MRC Human Genetics Unit & The University of Edinburgh Cancer Research UK Centre, University of Edinburgh, Edinburgh, United Kingdom; 3 MRC London Institute of Medical Sciences (LMS), London, United Kingdom; 4 Institute of Clinical Sciences (ICS), Faculty of Medicine, Imperial College London, London, United Kingdom; 5 Institute of Functional Epigenetics, Helmholtz Zentrum München, Neuherberg, Germany; 6 Department of Pathology, Western General Hospital, Edinburgh, United Kingdom; 7 Department of Medicine, University of Cambridge, Cambridge, United Kingdom; Weill Cornell Medical College, UNITED STATES

## Abstract

KDM2A is a histone demethylase associated with transcriptional silencing, however very little is known about its *in vivo* role in development and disease. Here we demonstrate that loss of the orthologue *kdm2aa* in zebrafish causes widespread transcriptional disruption and leads to spontaneous melanomas at a high frequency. Fish homozygous for two independent premature stop codon alleles show reduced growth and survival, a strong male sex bias, and homozygous females exhibit a progressive oogenesis defect. *kdm2aa* mutant fish also develop melanomas from early adulthood onwards which are independent from mutations in *braf* and other common oncogenes and tumour suppressors as revealed by deep whole exome sequencing. In addition to effects on translation and DNA replication gene expression, high-replicate RNA-seq in morphologically normal individuals demonstrates a stable regulatory response of epigenetic modifiers and the specific de-repression of a group of zinc finger genes residing in constitutive heterochromatin. Together our data reveal a complex role for Kdm2aa in regulating normal mRNA levels and carcinogenesis. These findings establish *kdm2aa* mutants as the first single gene knockout model of melanoma biology.

## Author summary

Epigenetic modifications of DNA and histones, the major components of chromatin, play a central role in transcriptional regulation. KDM2A is a histone demethylase that integrates DNA and histone modification signatures and is involved in transcriptional silencing through heterochromatin maintenance. Here we show that adult zebrafish homozygous for the orthologue *kdm2aa* develop melanomas, a malignant form of skin cancer, independently from oncogenes known to drive melanoma formation. We observe that transcript abundance is widely affected in *kdm2aa* mutants and find that gene expression of several DNA- and histone- modifying enzymes is stably altered. We furthermore demonstrate a specific de-repression of a group of genes encoding zinc finger-containing proteins that has the potential to be involved in transcriptional regulation. We suggest that these molecular disruptions underlie the melanoma formation, as well as the other observed phenotypes such as reduced growth and survival, a male sex bias and an oogenesis defect. This work demonstrates *in vivo* a role for Kdm2aa as a tumour suppressor and establishes, to our knowledge, *kdm2aa*-deficient fish as the first single gene knockout vertebrate model of melanoma.

## Introduction

The World Health Organisation (WHO) reports that 132,000 melanoma skin cancers occur each year across the globe, with increasing incidence rates. Melanomas are cancers of the melanocytes, which are neural crest-derived pigment-producing cells in vertebrates. Accumulation of mutations, often due to UV damage, leads to the transformation of melanocytes to become a melanoma (reviewed in [1]).

Zebrafish models of melanoma provide a tractable resource to study melanoma biology, however current models require lineage-specific overexpression of an activated oncogene such as *BRAF*^*V600E*^, often in a *tp53* or *mitfa* mutant background, to induce melanoma [2–6]. These models have enabled the identification of additional genes implicated in melanoma development by assessing a candidate gene’s ability to accelerate or delay onset of tumour formation. For example, both the histone H3 lysine 9 methyltransferase *SETDB1* [7] and the transcription factor *SOX10* [8] accelerate melanoma onset when coexpressed with *BRAF*^*V600E*^ in a *tp53* mutant line, whereas overexpression of *HEXIM1* in this system suppresses tumour formation [9].

Setdb1 belongs to the class of chromatin-modifying enzymes that enable the same DNA sequence in every cell to produce distinct transcriptional outputs in different tissues. Chromatin-modifying enzymes function through the chemical modification of DNA or histone proteins to promote transcriptional activation or repression, either through direct alteration of overall chromatin structure, or by altering the ability of effector molecules to bind [10]. Whereas the primary modification found on DNA is cytosine methylation, histones can have a wide variety of post-translational modifications on various amino acid residues [11]. Due to their profound involvement in transcriptional regulation it is not surprising that mutations in chromatin modifiers have been implicated in cancers and developmental defects [12–14]. The general importance of chromatin-modifying enzymes also limits the *in vivo* study of their function in mammalian models since mice homozygous mutant for a number of different chromatin modifiers are embryonic lethal [15–19].

In order to gain insight into the *in vivo* function of chromatin regulators we have studied a zebrafish knockout model of the lysine de-methylase KDM2A. KDM2A specifically removes mono- and di-methyl marks on H3K36 [20]. KDM2A has been implicated in the regulation of CpG island promoters [21] and in the silencing of heterochromatin and rDNA repeats [22, 23]. KDM2A is recruited to H3K9me3-modified chromatin in cooperation with HP1 [24] and this interaction is blocked by DNA methylation [21, 25]. KDM2A knockout mice are embryonic lethal at E10.5–12.5 and exhibit severe growth defects [16] pointing to a role for KDM2A during development. Furthermore, cell culture studies suggest a role for KDM2A in cancer development, but there is conflicting evidence as to whether it acts to promote or inhibit tumourigenesis [26–31].

Here we highlight the complexity of the function of *KDM2A* by demonstrating that the zebrafish orthologue *kdm2aa* is required at multiple stages throughout the life of the zebrafish. Zygotic homozygous zebrafish carrying mutations in one of the KDM2A orthologues, *kdm2aa*, escape early embryonic defects and thus enable the interrogation of both embryonic and adult phenotypes. We show that *kdm2aa*-deficient fish have reduced growth and survival, a strong male sex bias and that females exhibit a progressive oogenesis defect. Furthermore, *kdm2aa*-deficient fish develop *braf*-independent, spontaneous melanoma, providing, to our knowledge, the first single gene knockout model of melanoma. Transcriptome analysis of individual *kdm2aa* mutant embryos reveals widespread effects on transcript abundance as well as stable regulatory responses of epigenetic modifiers of both histones and DNA, and a specific upregulation of a group of previously uncharacterised zinc finger (ZnF) genes located in constitutive heterochromatin. Our results provide insights into the *in vivo* function of KDM2A throughout the complete life span of a vertebrate model organism and establish *kdm2aa*-deficient zebrafish as a new model to study the aetiology of triple wild-type melanoma.

## Results

### Kdm2aa but not Kdm2ab loss of function zebrafish show growth deficiency and reduced viability

**Fig 1:**
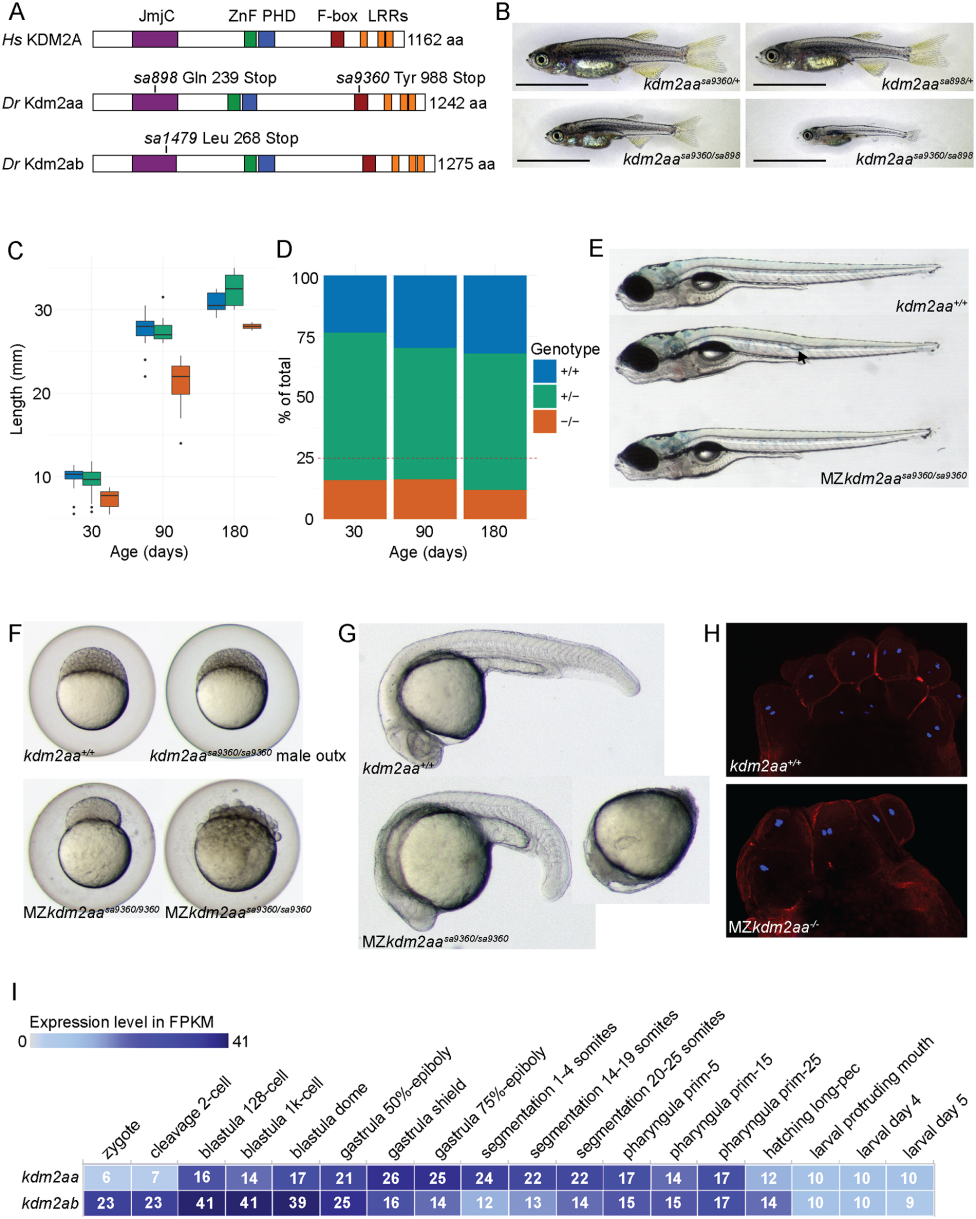
Loss of Kdm2aa function leads to growth and oogenesis defects. (A) The zebrafish paralogues Kdm2aa and Kdm2ab share the protein domain structure with their human orthologue KDM2A. The mutations *kdm2aa*^*sa898*^ and *kdm2aa*^*sa9360*^ produce premature stop codons in the JmjC and F-box domain, respectively. The allele *kdm2ab*^*sa1479*^ produces a premature stop codon in the JmjC domain. (B) Juvenile fish compound heterozygous for *kdm2aa*^*sa898*^ and *kdm2aa*^*sa9360*^ are shorter and thinner at 30 d.p.f. than their heterozygous siblings. Scale bar: 5 mm. (C) Box plots showing length in mm of each of the three sibling groups for a representative family at 30, 90 and 180 d.p.f. Fish homozygous for *kdm2aa*^*sa898*^ are smaller than their siblings. (D) Fish homozygous for premature stop codons in *kdm2aa* represent less than the expected 25% of the group. (E) A small proportion of maternal zygotic *kdm2aa*^*sa9360*^ embryos survive to 5 d.p.f. with mild (arrow in second panel) or no malformations (bottom panel). (F) Embryos derived from *kdm2aa*^*sa9360/sa9360*^ intercrosses show severe defects and lethality during the first 24 h.p.f. whereas *kdm2aa*^*sa9360/sa9360*^ male outcrosses are normal. (G) Two examples of embryos derived from *kdm2aa*^*sa9360/sa9360*^ female outcrosses displaying severe defects at 24 h.p.f. (H) TRITC-Phalloidin and DAPI staining on MZ*kdm2aa*^-/-^ embryos derived from intercrosses of *kdm2aa*^*sa898/sa9360*^ fish at the 8–16 cell stage showed that despite cell asymmetry and asynchronous division, nuclei seemed overall normal. (I) Expression data from Expression Atlas http://www.ebi.ac.uk/gxa/experiments/E-ERAD-475 showing normalised counts (FPKMs) from RNA-seq data for *kdm2aa* and *kdm2ab*. There is maternal contribution of both genes, but *kdm2ab* polyA transcript abundance peaks at early blastula stages, whereas *kdm2aa* polyA transcript abundance peaks later at the gastrula shield stage.

We assessed the *in vivo* function of KDM2A using zebrafish mutants generated by the Zebrafish Mutation Project [32]. KDM2A has two paralogues in zebrafish, *kdm2aa* (ENSDARG00000059653) on chromosome 1 (chr1) and *kdm2ab* (ENSDARG00000078133) on chr14 (Fig 1A). Embryonic expression of *kdm2ab* peaks during blastula stages, whereas *kdm2aa* expression is highest later in embryogenesis, during gastrula and early segmentation stages (Fig 1I). We raised two premature stop codon alleles affecting *kdm2aa* and one premature stop codon allele affecting *kdm2ab* (Fig 1A). *kdm2aa*^*sa898*^ and *kdm2ab*^*sa1479*^ are assumed to produce non-functional protein. *kdm2aa*^*sa9360*^ may produce a partially functional protein lacking the F-box and LRRs.

Fish homozygous for *kdm2ab*^*sa1479*^ showed no phenotype by 5 days post fertilisation (d.p.f.), grew to adulthood in the expected Mendelian ratio and had healthy offspring. We therefore concluded that *kdm2ab* loss of function (LOF) does not produce an overt embryonic or adult phenotype. Equally, both *kdm2aa*^*sa898*^ and *kdm2aa*^*sa9360*^ homozygous embryos did not display morphological defects at 5 d.p.f. (S1A and S1B Fig). We also generated double mutants between *kdm2aa*^*sa898*^ and *kdm2ab*^*sa1479*^ to test whether there was compensation between the paralogues, but embryos homozygous mutant for both genes also showed no phenotypic difference to their siblings at 5 d.p.f. (S1 Table).

However, by 30 d.p.f. juvenile fish homozygous for either *kdm2aa* allele were thinner and shorter compared to their siblings (Fig 1B and 1C, S2 Table and S1 File). The size difference persisted into adulthood at 180 d.p.f. (Fig 1C). We confirmed that this phenotype was due to the loss of *kdm2aa* function by raising two clutches containing compound heterozygous *kdm2aa*^*sa898/sa9360*^ fish (Fig 1B and S1 File). In addition, survival of homozygotes was reduced at 30 d.p.f. from the expected 25% to below 20% and fell further by 90 d.p.f. (Fig 1D, S2 Table and S1 File). Furthermore, incrosses for either *kdm2aa* allele produced at most two or three females out of a maximum of 20 homozygotes.

### *kdm2aa*^*-/-*^ females have a progressive oogenesis defect

We next assessed whether homozygous mutant *kdm2aa* adults were fertile. Initial intercrosses of *kdm2aa*^*sa9360/sa9360*^ or compound heterozygous *kdm2aa*^*sa898/sa9360*^ adults produced phenotypically diverse clutches in which some embryos successfully inflated their swimbladders and either developed phenotypically normally (Fig 1E bottom panel), or with only mild defects (Fig 1E middle panel). Later crosses of the same females produced clutches in which over half of the eggs either failed to fertilise or did not divide beyond four cells (S1C Fig). The remaining eggs showed severe cleavage defects with asymmetric division, detaching cells, and slower division rate (Fig 1F). By 24 hours post fertilisation (h.p.f.) about a third of the maternal-zygotic mutant (MZ) *kdm2aa*^*-/-*^ embryos had died and those that survived displayed degrees of generalised developmental defects (Fig 1G). This indicated that subsequent intercrosses from the same females displayed a progressive worsening of egg quality, with fewer eggs being fertilised and fewer embryos surviving beyond 24 h.p.f. Double labelling with DAPI and TRITC-conjugated phalloidin of 8–32 cell wild-type and MZ*kdm2aa*^*-/-*^ embryos confirmed asymmetric cells and unsynchronised division (Fig 1H).

To confirm that this phenotype was caused by *kdm2aa* LOF in the female, we outcrossed male and female *kdm2aa*^*sa9360/sa9360*^ fish to wild-type fish of the same genetic background. Offspring from three *kdm2aa*^*sa9360/sa9360*^ males were normal (Fig 1F and S1C Fig). By contrast, the majority of embryos from initial outcrosses of two *kdm2aa*^*sa9360/sa9360*^ females died before 5 d.p.f., however some (12/64) embryos survived to 5 d.p.f. with 6 out of 12 showing no obvious phenotype (S1D Fig top panel) and the remaining 6 displaying only localised malformations (S1D Fig). Subsequent homozygous female outcrosses produced clutches with low fertilisation rates and embryos with severe defects very similar to MZ*kdm2aa*^*-/-*^ embryos (S1C and S1E Fig).

This demonstrates that embryos from oocytes devoid of functional *kdm2aa* mRNA or protein can develop normally and that the maternally deposited mRNA (Fig 1I) [33] does not explain the lack of phenotype in zygotic homozygous mutants. Instead the increase in unfertilized eggs and severity of the phenotype in the remaining embryos point to a role for Kdm2aa in maintaining the production of healthy oocytes.

### Kdm2aa-deficient fish develop melanoma

**Fig 2:**
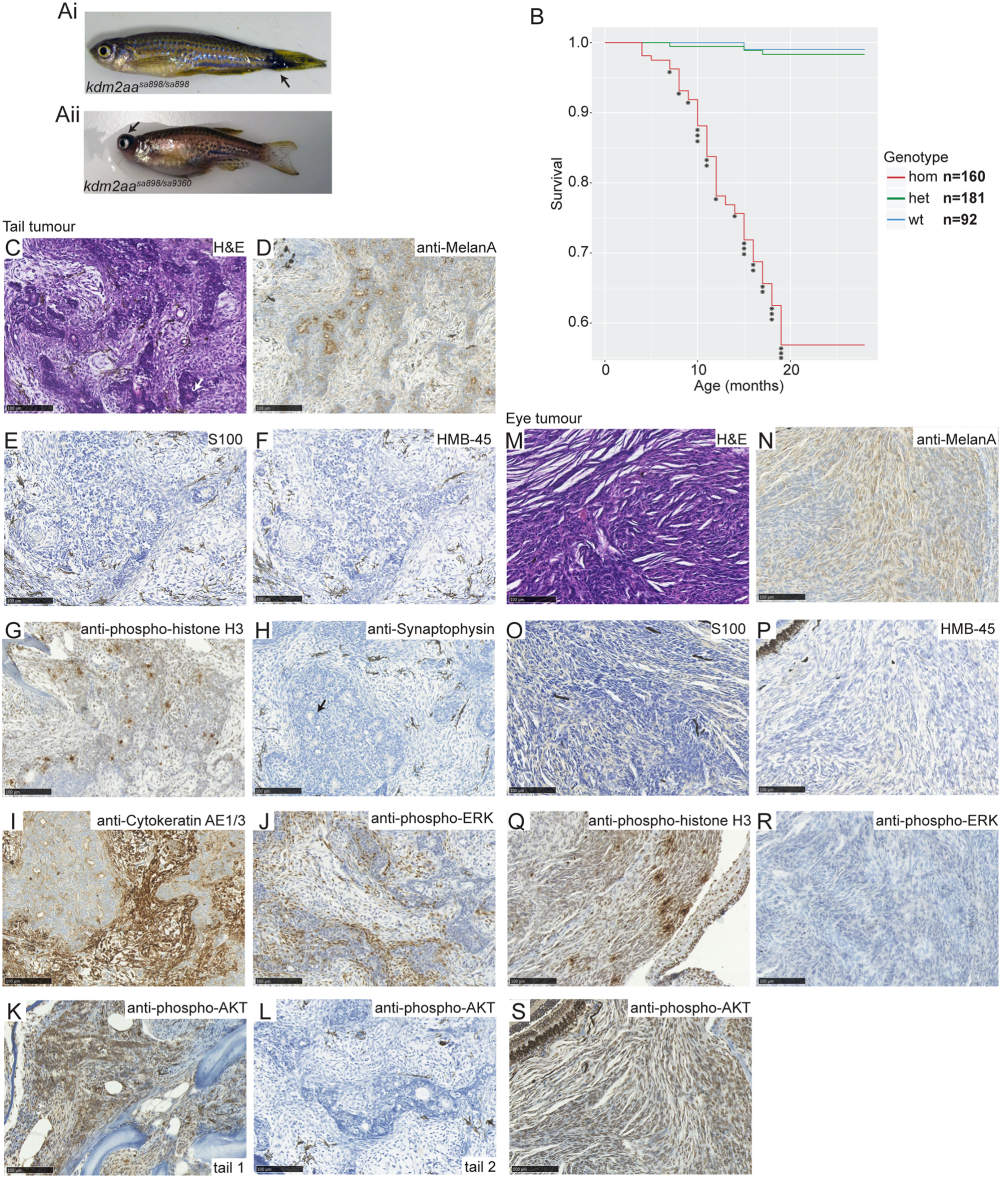
Kdm2aa-deficient fish develop melanoma. Aberrant melanocytic pigmentation on the tail (Ai, arrow) and eye tumours (Aii, arrow) are observed in Kdm2aa-deficient fish. (B) Survival graph for *kdm2aa*^*sa898*^ showing incidence of suspected cancer. Each (*) indicates a single culled fish due to suspected cancer. No wild-type or heterozygous siblings developed any suspected cancers. (C) H and E stained section of a tail tumour showing a biphasic appearance with pseudoglandular features (arrow shows an example) alternating with areas of spindle cell growth. (D) Section of tail tumour staining positive for the melanoma marker Melan-A (brown). (E) Section of tail tumour negative for the melanoma marker S100. (F) Section of tail tumour staining negative for the melanoma marker HMB-45. (G) Section of tail tumour staining positive for mitotic marker phospho-histone H3 (brown). (H) Section of tail tumour showing negative staining for the neuroendocrine marker Synaptophysin. Arrow points to example of pseudoglandular structures. (I) Section of tail tumour showing positive staining for the epithelial marker Cytokeratin (brown) in both the pseudoglandular and spindle cell elements. (J) Section of tail tumour with positive phospho-ERK staining (brown). (K) Section of tail tumour with positive phospho-AKT staining (brown) (L) Section of a second tail tumour which did not stain positive for phospho-AKT. (M) H and E stained section from an eye tumour revealing spindle cell morphology. (N) Section of eye tumour staining positive for Melan-A (brown). (O) Eye tumour section showing negative staining for the S100. (P) Section of eye tumour negative for HMB-45. (Q) Section of eye tumour staining positive for phospho-histone H3 (brown). (R) Phospho-ERK staining of eye tumour sections was negative. (S) Section of eye tumour showing positive staining for phospho-AKT (brown). Scale bar in C-S is 100 μm.

From the age of 7 months, we observed that *kdm2aa*-deficient fish (homozygotes for either allele and also compound heterozygous fish) began to develop suspected cancers. We observed aberrant melanocytic pigmentation at the base of the tail extending into the tail fin (Fig 2Ai), masses behind one eye causing it to protrude (Fig 2Aii) and masses on the body (S2B Fig). 23/92 (25%) of *kdm2aa*^*sa898/sa898*^ (Fig 2B) and 20/204 (10%) of *kdm2aa*^*sa9360/sa9360*^ (S2A Fig) fish developed these suspected cancers within the first 28 months, whereas none of the heterozygous or wild-type siblings did. Of the 43 fish with potentially cancerous phenotypes, 10 fish had excessive melanocytic pigmentation on their tail, 12 fish had a tumour behind one eye causing it to protrude, 18 fish had a mass on their body, and 3 fish were found to have both excessive melanocytic pigmentation on their tail and a mass on their body.

To confirm that these growths were cancerous, tissue sections from 10 affected fish and 2 control siblings were haematoxylin and eosin (H&E) stained and analysed by two independent clinical histopathologists. Seven of the fish had excessive melanocytic pigmentation on their tails, and all of these fish were diagnosed with spindle cell malignant melanoma on the tail, invading the surrounding skeletal muscle and bone to varying degrees (Fig 2C). Furthermore pigmented melanophages were present in half of the tumours and these cells have previously been reported in zebrafish melanomas [34]. Two of the fish analysed had eye tumours, which confirmed as either spindle cell, or epithelioid and spindle cell melanoma and pigmented melanophages were present in one of the two tumours. A single fish was analysed with a suspected abdominal tumour and this was found to have a nodular lesion around the ultimobranchial body, vena cava and pancreas, composed of epithelioid and spindle cells (S2C Fig). No pigmented melanophages were present. Additionally in internal sections from one of the fish with excessive melanocytic pigmentation on the tail an abnormal spindle-cell proliferation within the proximal intestinal epithelium and the pancreas was found. Given the pigmentation, spindle cell morphology and malignant proliferation these two abdominal tumours are consistent with melanoma, but further analysis would be required for a firm diagnosis.

**Fig 3:**
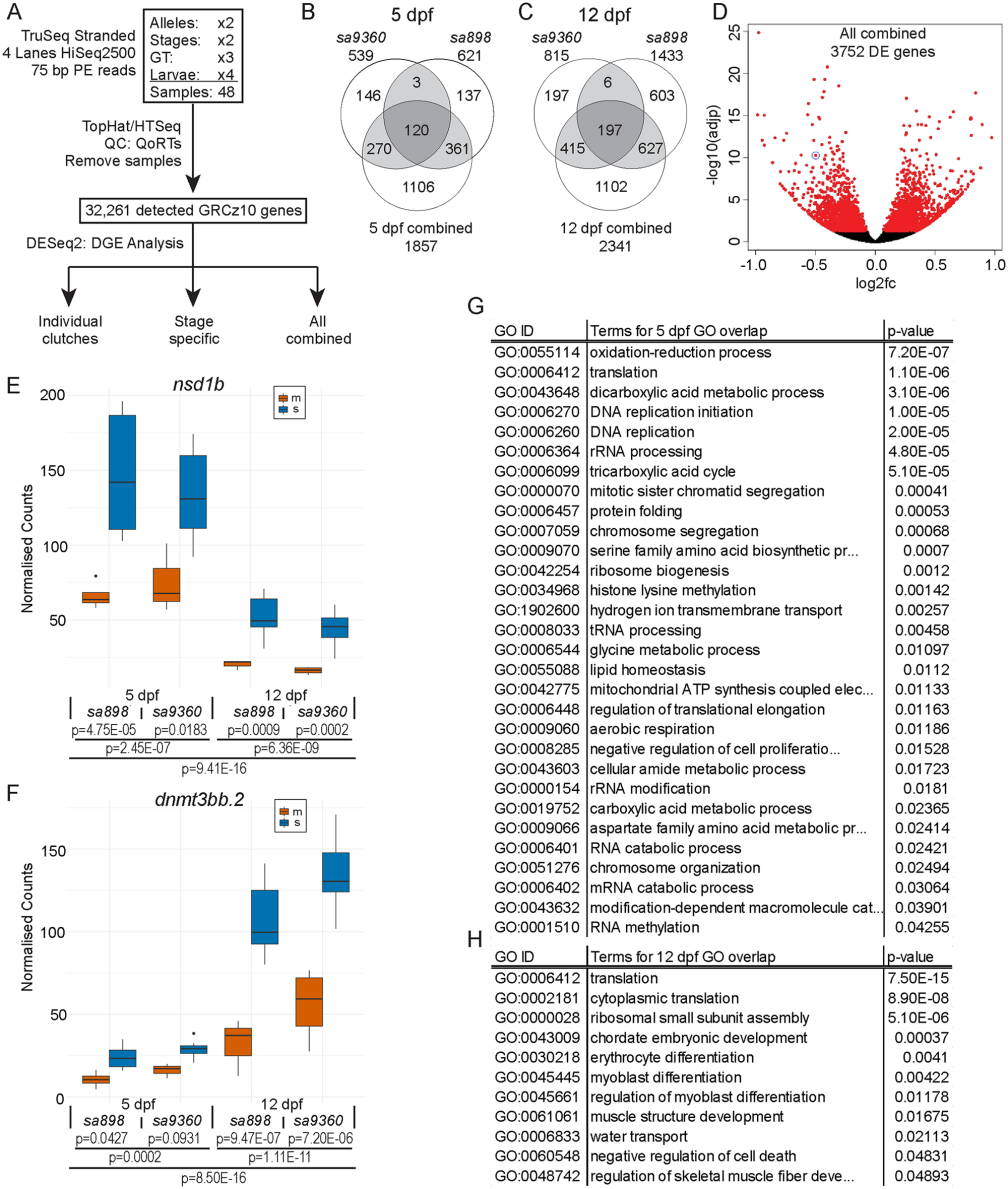
RNA-seq profiling of individual *kdm2aa*-deficient embryos and their siblings. (A) Summary of experimental design. TruSeq stranded mRNA sequencing libraries were prepared from four individual embryos from each genotype at both 5 d.p.f. and 12 d.p.f., totalling 48 libraries. Paired end sequencing with a read length of 75 bp was performed on four lanes of Illumina HiSeq 2500 machines. Sequence was aligned to the GRCz10 reference genome with TopHat and read counts were obtained with HTseq-count. Quality control was performed using QoRTs and outliers removed. 32,261 genes were detected. DESeq2 was used to determine DE genes in each individual clutch, DE genes at each stage by combining the two clutches for each stage whilst controlling for clutch in the DESeq2 model, and also in a combined analysis combining all 4 clutches whilst controlling for stage and clutch. (B) Venn diagram showing the overlap in DE genes between the two 5 d.p.f. experiments and the combined 5 d.p.f. analysis. The increase in power due to the increased sample size enabled an additional 1106 DE genes to be detected. (C) Venn diagram displaying the overlap in DE genes between the two 12 d.p.f. experiments and the combined 12 d.p.f. analysis. An additional 1102 DE genes were detected in the combined analysis. (D) Volcano plot of all detected genes in the combined RNA-seq analysis with the 3752 DE genes shown in red and *kdm2aa* circled. (E) Box plot of normalised counts for the chromatin modifier *nsd1b*, with adjusted p-values for individual experiments, stage-specific and combined analysis as indicated by horizontal bars. Data for heterozygous and wild-type siblings are combined. In the figure legend ‘s’ denotes siblings and ‘m’ homozygous mutants. (F) Box plot of normalised counts for *dnmt3bb*.*2*, a *de novo* DNA methyl transferase, with adjusted p-values for individual experiments, stage-specific and combined analysis as indicated by horizontal bars. Data for heterozygous and wild-type siblings are combined. In the figure legend ‘s’ denotes siblings and ‘m’ homozygous mutants. (G) Table of enriched Gene Ontology (GO) terms of the biological process (BP) GO domain which overlap in both 5 d.p.f. experiments and also the 5 d.p.f. combined analysis. P-values shown are for the combined analysis. (H) Table of enriched GO terms that overlap in both individual 12 d.p.f. experiments and the 12 d.p.f. combined analysis, with p-values for the combined analysis shown.

Three additional affected fish, two with excessive melanocytic pigmentation on the tail and one with an eye tumour, were analysed further using immunohistochemistry. H&E staining of both tail tumours revealed a biphasic appearance, with pseudoglandular or rosette-like structures alternating with areas of spindle cell growth (Fig 2C) and both the pseudoglandular and spindle cell elements stained positive for the melanoma marker Melan-A (Fig 2D) but negative for two alternative melanoma markers S100 and HMB-45 (Fig 2E and 2F). These tumours were also diagnosed as melanoma showing divergent differentiation. Both tumours stained positive for phospho-histone H3 (Fig 2G) indicating that they were mitotically active. To further characterise the pseudoglandular differentiation the tail tumours were stained for the neuroendocrine marker Synaptophysin (Fig 2H) which was negative and for the epithelial marker Cytokeratin (Fig 2I) which was positive. The eye tumour shared many characteristics with the tail tumours and was diagnosed as invasive melanoma; H&E staining revealed spindle cell morphology (Fig 2M), Melan-A and phospho-histone H3 were positive (Fig 2N and 2Q) and S100 and HMB-45 were negative (Fig 2O and 2P). Interestingly, both tail tumours stained positively for phospho-ERK (Fig 2J) indicating activation of the MAPK signalling pathway, whereas the eye tumour was phospho-ERK negative (Fig 2R). One tail tumour and the eye tumour stained positive for phospho-AKT (Fig 3K and 3S) indicating that PI3K signalling was activated, whereas the second tail tumour was phospho-AKT negative (Fig 2L).

### Melanoma development in *kdm2aa*-deficient zebrafish is independent of coding mutations in common oncogenes and tumour suppressors

To assess the mutational landscape in *kdm2aa*-deficient fish melanomas, we performed whole exome sequencing on four dissected tumours, adjacent non-tumour control tissue and sibling tissue, and called the single nucleotide variants (SNVs) and small insertions/deletions (indels) present. Across the 11 samples we obtained exome coverage of 50x (S3 Table). Laboratory zebrafish are not inbred and consequently there is a high level of natural variation [35]. We therefore used exome data from 3,811 individual fish generated in the Zebrafish Mutation Project [32] to define a common variant catalogue of 61,276,211 SNVs and filtered the SNVs found in sibling, control and tumour tissues using this variant set. This revealed on average 951 SNVs between sibling fish and control tissues (Table 1). Tumour tissues harboured on average 517 SNVs compared to non-tumour tissue from the same fish demonstrating that the tumours had accumulated mutations and increased their SNV burden by the equivalent of 50% of the normal sibling variation. By contrast the tumours had not increased their indel frequency with each tumour only showing one additional indel when compared to control tissue (Table 1).

**Table 1:** Mutations present in tumour samples.

Comparison	# SNVs	# indels
sibling_tissue_1.vs.control_tissue_1	944	7221
control_tissue_1.vs.tumour_tissue_1	485	1
sibling_tissue_2.vs.control_tissue_2a	995	6959
sibling_tissue_2.vs.control_tissue_2b	1001	9747
control_tissue_2a.vs.tumour_tissue_2a	592	1
control_tissue_2b.vs.tumour_tissue_2b	526	1
sibling_tissue_3.vs.control_tissue_3	866	7829
control_tissue_3.vs.tumour_tissue_3	465	1

Whole exome sequencing was performed on dissected tumours, adjacent non-tumour control tissue and siblings. The number of SNVs and indels detected in sibling vs control tissue and control vs tumour tissue are shown.

Next, we sought to identify potential protein-disrupting mutations. Filtering the mutations for those which are predicted to disrupt the protein (for details see Methods) revealed between 9 and 21 disruptive mutations per tumour compared to control tissue, and there was no overlap between these acquired disruptive mutations in the different tumours (S3 Table). Whole genome sequencing would be needed in order to assess whether mutations were present in non-coding regions.

Although we did not detect a common mutational signature in the tumours analysed, the exome data showed that none of the oncogenes or tumour suppressors most commonly mutated in human melanomas had accumulated exonic mutations (S3 Table). We looked specifically at the 13 significantly mutated genes in human cutaneous melanomas identified by The Cancer Genome Atlas, as well as *HRAS* and *KRAS* which were used to categorise the samples and also *GNAQ*, *GNA11*, *KIT*, *CTNNB1* and *EZH2* which were found to carry mutations at low frequencies in the triple wild-type set of melanomas [36]. We used the Ensembl Compara database [37] to detect orthologues for these genes in our whole exome sequencing data, but none were found to carry potentially disruptive exonic mutations (S3 Table). Analysis of four tumours is not enough to prove conclusively that *kdm2aa*-deficient melanomas never harbour mutations in common oncogenes. However if mutations were present at the same frequency as in human melanomas there is, for example, a 94.7% probability that we would have detected a *braf* mutation (present in 52% of human melanomas [36]) and a 73.1% probability that we would have found *nras* mutations which are present in 28% of human melanomas [36]. Since *BRAF* and *NRAS* hotspot mutations are almost mutually exclusive [36] there is close to a 97.6% probability that we would have found either a *BRAF* or an *NRAS* hotspot mutation in at least one of the tumours. Therefore the absence of potentially disruptive mutations in any of the 20 genes assessed across the four melanomas supports a role for Kdm2aa in melanoma development independent of common oncogenes and tumour suppressors.

### mRNA expression profiling in individual embryos reveals widespread effects on transcript abundances in *kdm2aa* LOF fish

Given the role of Kdm2aa in chromatin regulation [20, 21, 26] we investigated whether loss of Kdm2aa resulted in an altered transcriptional profile which might explain the observed phenotypes. We performed a comparative transcriptome analysis (polyA RNA-seq) on four individual homozygous mutant, heterozygous and homozygous wild-type siblings each from both alleles at 5 d.p.f. and 12 d.p.f. giving us four mRNA expression profiles (Fig 3A). We chose these time points and individual embryos from two different alleles to capture changes at the mRNA level in morphologically normal individuals rather than secondary transcriptional deviations due to size differences, developmental delay and genetic background. Using DESeq2 [38] we determined differential transcript abundance as significant at an adjusted p-value <0.05. This revealed that *kdm2aa* transcripts were present at lower levels in fish homozygous for either allele and at both stages (log_2_ fold change between -0.34 and -0.73), indicating nonsense-mediated decay [39] had occurred. We tested for haploinsufficiency in heterozygous animals by running differential analysis between heterozygous and wild-type embryos in both alleles at 5 d.p.f. and 12 d.p.f. This yielded 0 and 1 differentially expressed (DE) genes for *kdm2aa*^*sa9360/+*^ at 5 d.p.f. and 12 d.p.f., respectively. By contrast, *kdm2aa*^*sa898/+*^ heterozygosity led to 29 (5 d.p.f.) and 80 (12 d.p.f.) DE genes, suggesting a mild haploinsufficiency effect of that allele on mRNA levels (S4 Table). When comparing homozygous mutants with siblings (Fig 3B–3D and S4 Table) between 539 and 1433 genes out of 32,261 detected genes were significantly differentially abundant in the four clutches. The four DE gene lists only had 19 genes in common (S3A and S3B Fig) with an additional 76 DE genes being significant in at least three of the four clutches (S3A Fig). The discrepancy between the clutches could either be due to clutch-specific and/or stochastic effects on transcript abundance or the fact that four biological replicates per condition do not provide sufficient power to detect differential gene expression above individual embryo variability. To test this we combined the samples for each stage and ran the differential analysis of homozygous mutants against siblings while controlling for clutch in the DESeq2 model (Fig 3A and Methods). The combined stage-specific analyses showed large overlap with their respective individual experiments (77% and 72% at 5 d.p.f., 57% and 75% at 12 d.p.f.), confirming that the majority of discrepancy was due to detection power rather than clutch difference (Fig 3B and 3C). The increase in power due to the larger sample size also enabled us to detect over 1100 additional DE genes for each stage (Fig 3B and 3C). Combining all four experiments in the analysis while controlling for stage and clutch identified 3,752 DE genes (Fig 3D). These results are consistent with previous findings that the number of biological replicates is the main factor in the ability to identify differentially expressed genes [40].

### Genes involved in translation, DNA replication and chromatin regulation respond to loss of *kdm2aa*

Gene ontology (GO) analysis of DE genes using topGO [41] revealed enrichment of a large number of terms relating to translation, DNA replication, energy metabolism, and chromosome organisation in the biological process (BP) domain in the four separate and the combined 5 d.p.f. RNA-seq analyses (S5 Table). The translation enrichment was driven mostly by upregulation of genes encoding ribosomal proteins (19/72 contributing genes in the 5 d.p.f. analysis), translation elongation or initiation factors (14/72) and mitochondrial ribosomal proteins (9/72), which is consistent with KDM2A’s described function in repressing ribosomal RNA genes [23]. This upregulation of ribosomal genes and energy generation processes together with differential expression of DNA replication genes suggests cellular stress.

We also found stage-specific differences. While different terms relating to translation, chromosome organisation and metabolism appeared in all individual analyses, the GO enrichment at 12 d.p.f. also included a large number of terms related to development of different tissues. This is very likely to reflect the emerging growth retardation observed morphologically from 30 d.p.f. onwards. To visualise this stage difference we filtered the lists for terms that are present in the stage analysis as well as their individual experiments (Fig 3G and 3H and S3C and S3D Fig). This showed a dominance of translation, DNA replication and chromosome segregation at 5 d.p.f., whereas the list at 12 d.p.f. contains mostly translation- and development-related terms.

In accordance with the role of Kdm2aa in chromatin regulation the theme of DNA replication and chromatin remodelling represents the core gene expression profile even in the comparatively small set of 95 DE genes that overlapped between at least three individual clutches across both stages and alleles (S3E Fig). Included in this core set are chromatin modifiers such as *nsd1b*, a methyltransferase for the KDM2A target H3 lysine 36, and the *de novo* DNA methyltransferase *dnmt3bb*.*2* which is recruited to DNA by H3K36me3 [42], both of which were downregulated (Fig 3E and 3F). By contrast, the gene encoding the Snf2-related CREBBP activator protein Srcap, the catalytic subunit of a protein complex that incorporates the histone variant H2A.Z at promoters and eu- and heterochromatin boundaries was upregulated (S3F Fig) (S2 File for all count plots). This gene expression signature suggests a compensation for loss of H3K36-demethylase activity and a wider concerted response to chromatin disruption.

### Kdm2aa represses specific genes in heterochromatic regions

**Fig 4:**
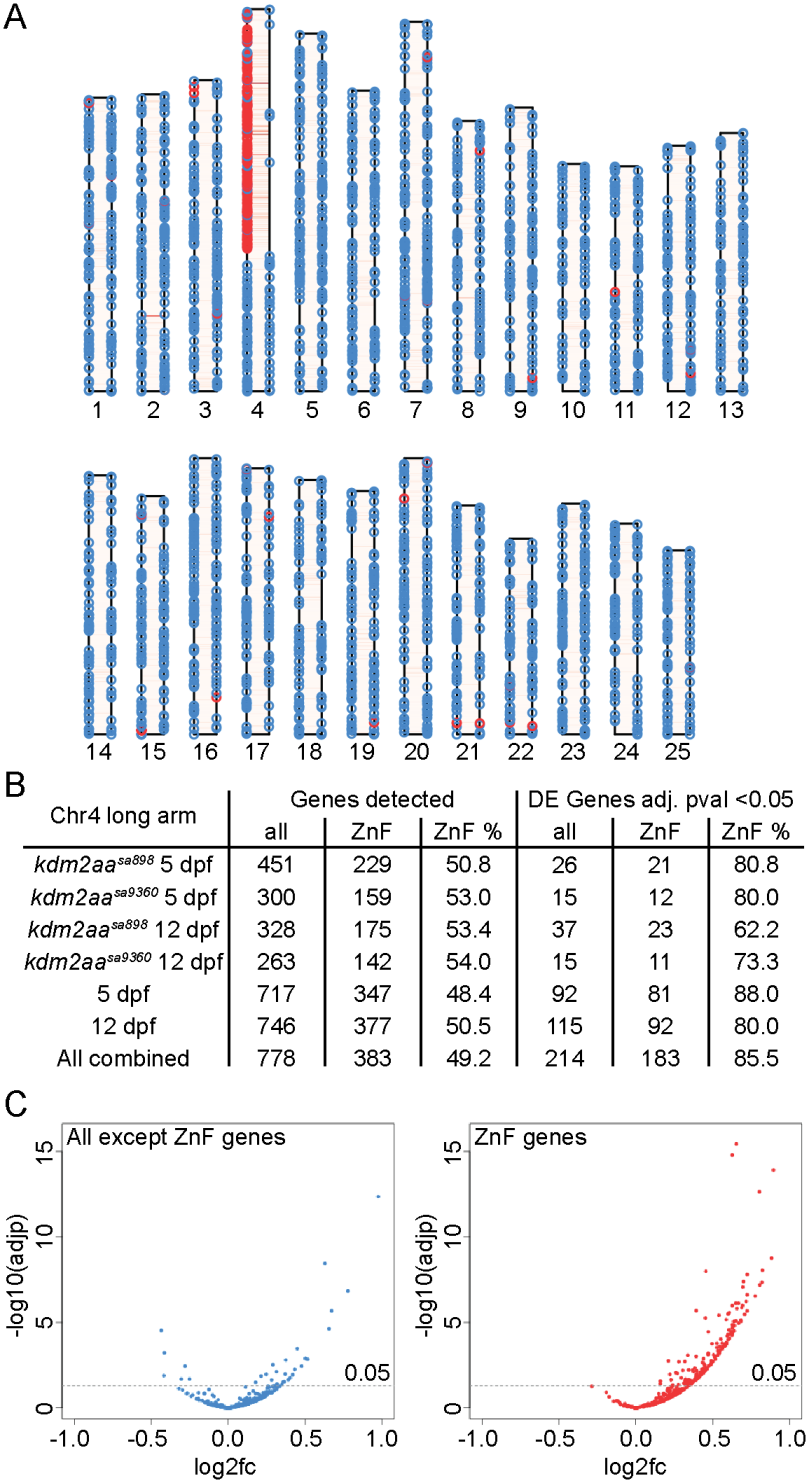
Specific de-repression of a group of ZnF genes on the long arm of chromosome 4. (A) Ideogram of all DE genes in the combined analysis. Upregulated genes are plotted on the left, down-regulated genes on the right of each chromosome. Genes containing ZnF domains are indicated in red. Red shading of chromosomes shows type I transposons. (B) Table of genes detected and differentially expressed on the long arm of chr4 showing enrichment for ZnF domain-containing genes in the differentially expressed sets. (C) Volcano plots of genes on the long arm of chr4 demonstrate that genes without a ZnF domain are both up- and downregulated, whereas ZnF genes are exclusively upregulated.

When plotting up- and downregulated genes separately onto their chromosomes, we noticed enrichment of upregulated genes on the long arm of chr4 (Fig 4A). This region is repeat rich (Fig 4A), and contains extensive constitutive heterochromatin [43, 44]. We therefore speculated that *kdm2aa* LOF causes generalised de-repression of genes located within this heterochromatin stretch. However, of the 208 genes that were upregulated on the long arm of chr4 in the combined analysis, 183 genes were annotated as containing a zinc finger (ZnF) domain. ZnF domain-containing genes represented 49.2% of detected genes on the long arm of chr4, but rose to 85.5% in the DE gene set and thus demonstrated specific enrichment (Fig 4B). Furthermore, none of the 183 DE ZnF genes on the long arm were downregulated whereas this was the case for 6 of the other 31 DE genes (Fig 4C). Kdm2aa therefore seems to have a function in repressing heterochromatic ZnF genes on the long arm of chr4 in a gene-specific manner. We have shown previously that these genes are normally expressed in a sharp peak at zygotic genome activation [33], pointing to a role for these genes in regulating zygotic transcription.

## Discussion

In this study we have used two non-complementing point mutations to identify a complex set of phenotypes caused by *kdm2aa* LOF, which affect different stages of development and adulthood: oogenesis is impaired, juveniles display reduced survival and grow to smaller adults with a strong male sex bias. We also demonstrate that Kdm2aa is not required for early embryonic development as a proportion of embryos from early clutches devoid of maternal wild-type transcript or protein develop normally. Furthermore, while oogenesis is abnormal, Kdm2aa is not required for meiosis *per se*, since embryos from homozygous male outcrosses are phenotypically wild type. Importantly, a significant proportion of mutants develop cancerous growths. All of the tumours analysed were diagnosed as melanomas, however they are atypical given their unusual histologic and immunologic characteristics and the absence of a mutational signature common to human melanomas. Cell culture studies have pointed to a role for KDM2A and other histone demethylases [45] in the development of human cancers, but it is unclear whether KDM2A acts to promote or suppress carcinogenesis [26–31]. Here we demonstrate that *in vivo kdm2aa* acts as a tumour suppressor. This is consistent with previous studies identifying chromatin modifiers as key players in cancer development [14, 46–49] and makes the *kdm2aa* mutant the first single gene knockout animal model of melanoma.

It has been shown previously that fish homozygous mutant for genes known to be involved in DNA damage repair, such as *brca2*, develop as all males [50]. The female-to-male sex reversal is caused by oocyte death, presumably due to an inability to repair the damage caused by recombination during meiosis [50, 51]. The strong male sex bias that we observe in homozygous mutant Kdm2aa adults raises the possibility that the DNA damage response might also be impaired in Kdm2aa-deficient fish.

A defect in DNA damage repair would also fit with the incidence of melanoma, since patients with Xeroderma Pigmentosum (XP) have a vastly increased risk of skin cancer [52]. XP is caused by mutations in genes involved in the nucleotide excision repair pathway which functions to repair bulky DNA helix distorting lesions such as those produced as a result of UV irradiation or endogenous reactive oxygen species [53, 54]. The effects of *kdm2aa* loss of function on the DNA damage repair pathway thus warrants further investigation.

Our RNA-seq analysis was carried out at 5 d.p.f. and 12 d.p.f. time points where the mutants do not display any discernible morphological phenotype. Nevertheless, we discovered significant effects on mRNA levels, indicating that we were able to identify the transcriptional profile underlying the later observed morphological phenotypes. We were able to confirm the core DNA replication and chromatin remodelling gene signature by examining the DE genes common to either all 4 or at least 3 of the 4 experiments. Out of the 19 DE genes significant in all four sets six genes are known to be involved in chromatin structure and function (*rbbp5*, *smg9*, *chd3*, *rad23aa*, *kdm2aa* and *nsd1b*). This reproducible gene signature suggests that *kdm2aa* LOF generally affects chromatin structure and function which is a main factor in transcriptional control. The de-repression of ZnF genes in heterochromatin on chromosome 4, which are normally expressed in a sharp peak at zygotic genome activation [33] and therefore likely to be involved in regulation of transcription at that stage, could also contribute to impaired control of gene expression. Consistent with our observations, disruption of transcriptional control is emerging as a key feature of cancer development and is proposed to favour malignancy [49, 55–58].

Disruption to chromatin has been shown to play a role in melanoma development. For example reduced acetylation and H3K4me2/3 marks at specific regions have been observed in a tumourigenic melanocyte cell model system [59]. Furthermore altered expression of chromatin modifiers has been associated with melanoma development. The histone demethylase KDM5B is highly expressed in many cancers [60] including melanoma cell lines and patient tumours and causes a slowing of the cell cycle which promotes resistance to chemotherapeutic drugs [61]. In a zebrafish melanoma model, overexpression of the histone methylase *SETDB1* accelerates the onset of melanoma development [7]. Our Kdm2aa-deficient zebrafish model identifies *kdm2aa*^*sa898*^ and *kdm2aa*^*sa9360*^ as driver mutations in melanoma and therefore fits with current models demonstrating an important involvement of chromatin modifiers in melanoma. In further support of this, a recent study analysing whole genome sequences from cutaneous, acral and mucosal melanomas identified a number of chromatin modifiers as candidate driver genes harbouring protein-disrupting aberrations [62]. KDM2A is not among the commonly mutated chromatin modifiers in melanoma, but code-disrupting mutations have been identified in melanomas and other cancers [63, 64]. We also cannot exclude the possibility that *kdm2aa*-deficient fish additionally develop other types of cancers which were not assessed in this study.

Immunohistochemistry of Kdm2aa-deficient tumours with antibodies routinely used for clinical melanoma diagnoses revealed that they stained positive for Melan-A, but negative for two other melanoma markers S100 and HMB-45. Whilst this is unusual, a number of human melanomas do not stain positively for all three markers [65–67]. Additionally H&E staining revealed pseudoglandular or rosette-like features alternating with areas of spindle cell growth, and both tail tumours stained focally positive for the epithelial marker Cytokeratin, suggesting divergent epithelial differentiation within a melanoma. Divergent differentiation towards a range of cell types is a well-recognised although rare phenomenon in human melanoma [68] but the significance of this finding in several of our tumours is uncertain. At this time, with the diagnosis of two independent pathologists, these tumours are best classified as melanoma with divergent differentiation, although the atypical nature of the tumours, and the lack of similarity with human and other zebrafish melanomas suggest that additional evidence is needed to confirm the cell of origin.

All three tumours assessed were mitotically active, shown by phospho-histone H3 antibody staining. The rate of mitoses within a tumour has been identified as the second most powerful predictor of patient survival; a mitotic rate of 1 or more per square millimetre is associated with reduced survival [69, 70]. Furthermore MAPK signalling is activated in over 90% of human melanomas [71] and our immunohistochemical analysis showed that despite an absence of exonic mutations in *braf* or *nras*, both tail tumours but not the eye tumour had activated MAPK signalling. The eye tumour and one tail tumour however showed activated PI3K signalling. This suggests that there is not a uniform pathway to melanoma development in *kdm2aa*-deficient fish, but instead activation of either of the two major pathways known to be involved in human melanomas [62] leads to melanoma development in these fish.

This mutant provides an alternative genetic system to study melanoma development to previous zebrafish and mouse models which require overexpression of an activated oncogene or use xenografts [2, 4, 5] (reviewed in [72]). Our RNA-seq data show that key genes in melanocyte development, including *mitfa* and *sox10*, are expressed at normal levels. This is in contrast to fish that overexpress activated BRAF in a *tp53*-deficient background which already show altered expression of neural crest genes by 80 h.p.f. [57]. We also do not find a significant overlap between our core set of 95 genes DE in at least 3 of the 4 clutches and the gene signatures of either MITF high expressing or AXL high expressing human melanoma cells determined by single cell RNA-seq [73]. Taken together this suggests that the emergence of melanoma at later stages is not due to a direct effect on genes involved in melanocyte development. The melanoma predisposition due to a single gene knockout is comparable to deleterious germline variants in a number of genes such as *CDKN2A* and *POT1* that have been shown to underlie familial melanoma cases in human patients [74, 75].

Due to the disparity between common human melanomas and Kdm2aa-deficient tumours this melanoma model is different from classic *BRAF* mutation model systems. It does not mimic all hallmarks of common melanomas, but it provides a unique opportunity to interrogate the relationship between chromatin regulation and cancer development. Indeed, transcriptional fluctuations rather than acquired mutations have recently been identified to underlie drug resistance in melanoma cells [76] and chromatin regulators have been demonstrated to function not only in melanoma development but also specifically in the emergence of resistance to BRAF inhibitors ([77] and reviewed in [48]).

Taken together, our work interrogates for the first time *in vivo* and across the vertebrate life span the role of Kdm2aa in development and disease. We uncover a function for Kdm2aa in oogenesis as opposed to embryogenesis and identify its role as a tumour suppressor. This loss of function model will be invaluable to further dissect the interplay of chromatin structure and transcription, and its impact on cancer.

## Materials and methods

### Husbandry

Zebrafish were maintained in accordance with UK Home Office regulations, UK Animals (Scientific Procedures) Act 1986, under project licence 70/7606, which was reviewed by the Wellcome Trust Sanger Institute Ethical Review Committee. Embryos were obtained either through natural matings or *in vitro* fertilisation and maintained in an incubator at 28.5°C up to 5 days post fertilisation (d.p.f.). The mutant alleles *kdm2aa*^*sa898*^, *kdm2aa*^*sa9360*^ and *kdm2ab*^*sa1479*^ were obtained from the Zebrafish Mutation Project [32].

### Length measurements

Standard length (SL) and height at the anterior margin of the anal fin (HAA) of anaesthetised offspring from heterozygous intercrosses were measured at 30, 90 and 180 d.p.f. Measurements were taken as previously described [78]. Tissue samples were taken from each measured fish for genotyping either by sacrificing whole individuals at 30 d.p.f. or by caudal fin biopsies at 90 and 180 d.p.f. To test whether there is a difference in SL or HAA as a function of genotype, we performed ANOVA on each clutch to check for significant differences between the three genotype groups of homozygous mutant, heterozygous and homozygous wild-type fish. Post-hoc testing (Tukey HSD) was used to assess which groups differed significantly.

### Genotyping

DNA from embryos or fin biopsies was extracted and DNA samples were genotyped for *kdm2aa*^*sa898*^, *kdm2aa*^*sa9360*^ or *kdm2ab*^*sa1479*^ using KASP genotyping as previously described [79].

### Histology

Fish samples were either collected in formalin and sent to Advance Histopathology Laboratory Ltd, 75 Harley Street, London, UK, for H&E staining and analysis, or fixed, processed and stained as described in [80]. Briefly, fish tissue was fixed in 4% PFA at 4°C for 3 days, decalcified in 0.5M EDTA (pH 8) at 4°C for 5 days and transferred to 70% ethanol. It was then processed in 95% ethanol, absolute alcohol, xylene and paraffin wax, embedded in wax blocks, cut into 5 μm thick sections and placed onto glass slides.

Hematoxylin and eosin staining and immunohistochemistry were performed as described in [80]. The slides were de-waxed by xylene and ethanol washes, stained, dehydrated and mounted with DPX. Antigen retrieval for IHC was performed in 0.01 M citrate buffer (1.8 mM citric acid, 8.2 mM sodium citrate, distilled water—pH 6) in a microwave pressure cooker. The samples were stained with the primary antibody (monoclonal mouse anti-human Melan-A clone A103, DAKO, Cat. No. M7196 concentration 1:75, anti-phospho-Histone 3, Cell Signalling Technology, rabbit, 1:200, anti-phospho-p44/42 MAPK (Erk1/2), Cell Signalling Technology, rabbit, 1:400 and anti-phospho-Akt, Cell Signalling Technology, rabbit, 1:50) overnight at 4°C and secondary antibody (HRP rabbit/mouse, DAKO) for 30 min at room temperature. DAKO Real EnVision Detection System (Peroxidase/DAB+, Rabbit/Mouse, Cat. No. K5007) was used to visualise the IHC staining. S100, HMB-45, Cytokeratin AE1/3 and Synaptophysin antibody stainings were performed under standard laboratory conditions at the Immunohistochemistry Laboratory in the Department of Pathology, Royal Infirmary of Edinburgh.

The stained slides were imaged using Pathology Nanozoomer SlideScanner and the images were processed using NDP.2 software.

### Whole mount staining of zebrafish embryos

For DAPI and TRITC-Phalloidin staining, embryos at the 8–32 cell stage were fixed in 4%PFA/PBS overnight at 4°C, washed in PBST (0.1% Tween-20 in PBS) and dechorionated. After 4 x 30 minute washes in 2% Triton/PBS they were incubated with 4',6-diamidino-2-phenylindole (DAPI) (1:300) in PBST and TRITC-Phalloidin (1:200) in PBST in the dark at 4°C overnight. Embryos were washed 3-4x in PBST, mounted in Vectashield Antifade Mounting Medium and imaged using a Leica SP5 confocal microscope.

### Illumina library preparation for RNA-seq

Using Sera Mag beads, total nucleic acid was isolated from 96 larvae from heterozygous sibling intercrosses for both *kdm2aa* alleles at 5 d.p.f. and 12 d.p.f. resulting in four experiments. KASP genotyping was performed on all samples to identify 4 individual homozygous mutant, heterozygous and wild-type sibling samples for each of the four experiments. From these 48 samples 300 ng total RNA were used to prepare sequencing libraries with Ambion ERCC spike-in mix 1 (Cat. No. 4456740) according to the manufacturer’s instructions using the Illumina TruSeq Stranded mRNA Sample Prep Kit Set A and B (RS-122-2101 and RS-122-2102). Paired end sequencing with a read length of 75 bp was performed on four lanes of Illumina HiSeq 2500 machines.

### Read mapping and differential expression analysis

Quality control of sequenced samples was performed using QoRTs [81] and 7 libraries showing characteristics of RNA degradation were excluded from further analysis. Sequence was aligned to the GRCz10 reference genome with TopHat 2.0.13, using a known transcripts file from Ensembl v87 (ftp://ftp.ensembl.org/pub/release-87/gtf/danio_rerio/Danio_rerio.Zv9.87.gtf.gz) and the "fr-firststrand" library type option. Read counts were obtained with htseq-count and used as input for differential expression analysis with DESeq2. For the analyses of individual clutch experiments, the DESeq2 model was “~ condition” where the condition is either “hom” or “het_wt”. For the stage-specific analyses, the model was “~ group + condition” with the same conditions as previously and where the group is either “sa898” or “sa9360”, corresponding to the different alleles. For the combined analysis, the model was also “~ group + condition” with the same conditions as previously and where the groups are “sa898_day5”, “sa898_day12”, “sa9360_day5” or “sa9360_day12”, corresponding to the different alleles and stages. Enrichment analysis for Gene Ontology terms from Ensembl v87 annotation was performed with topGO [41] using the Kolmogorov-Smirnov test and the "elim" algorithm with a nodeSize of 10. RNA-seq data were submitted to ENA under Study Accession Number: ERP007082 and to ArrayExpress under Accession Number: E-ERAD-326.

### Whole exome sequencing

Biopsies were taken from tumours and adjacent non-tumour control tissue of homozygous mutants and from corresponding tissues of wild-type or heterozygous siblings. Dissected tissues were placed in 400 μl of 100 μg/ml proteinase K overnight at 55°C, followed by 30 min at 80°C to heat inactivate the proteinase K. DNA was precipitated by adding 400 μl of isopropanol and centrifuging for 40 min at 4100 rpm at room temperature. DNA pellets were washed twice with 400 μl of 70% ethanol followed by centrifugation at 4100 rpm for 25 min and 10 min, and resuspended in ddH_2_0. The isolated DNA was whole exome enriched using Agilent SureSelect and used to generate standard Illumina sequencing libraries, which were paired end sequenced with a read length of 75 bp using two lanes of Illumina HiSeq 2500 machines. SNVs were called using MuTect [82] and indels were called using Strelka [83]. Known SNPs, obtained from the Zebrafish Mutation Project [32], were removed from the MuTect output. Potential protein-disrupting SNVs were identified using the Ensembl Variant Effect Predictor (VEP) [84] and filtering the output for stop_gained, missense_variant, transcript_ablation, splice_acceptor_variant, splice_donor_variant and frameshift_variant consequences. Whole exome sequencing data were submitted to ENA under Study Accession Number: ERP016095.

## Supporting information

S1 File *kdm2aa* homozygous mutant or compound heterozygous fish have reduced size and survival compared to siblings. (A) Box plots of length versus genotype for 30 d.p.f. fish. (B) Box plots of height versus genotype for 30 d.p.f. fish. (C) Box plots of length versus genotype for fish aged 30, 90, 150 and 180 d.p.f. (D) Frequency of genotypes at 30 d.p.f. across 4 different clutches. Homozygous mutant *kdm2aa* fish are consistently present below 25% (red line). (E) Frequency of genotypes at 90 d.p.f. showing that survival of homozygous mutant fish has dropped even further below 25% (red line). (F and G) Box plots of length (F) or height (G) versus genotype for two compound heterozygous incrosses showing that compound heterozygous fish have reduced length and height compared to their siblings. (H) Table of frequency of homozygous fish and siblings at 30, 90 and 180 d.p.f. along with p-values from a binomial test indicating that homozygous mutant fish are present significantly below 25%.
(PDF)

S2 File Normalised count plots for significantly differentially expressed genes. Genes identified to be significantly differentially expressed (p<0.05) in the combined analysis are shown, with the normalised counts displayed across all four experiments.
(PDF)

S1 Fig Zygotic and maternal-zygotic *kdm2aa* mutants at 5 d.p.f. (related to Fig 1). (A, B) Homozygous mutants for either *kdm2aa*^*sa898*^ or *kdm2aa*^*sa9360*^ are morphologically normal at 5 d.p.f. (C) Survival rates up to 5 d.p.f. of embryos resulting from intercrosses and outcrosses of homozygous *kdm2aa*^*sa9360*^ mutants. (D) A small number of embryos from initial *kdm2aa*^*sa9360/sa9360*^ female outcrosses survive to 5 d.p.f. albeit with malformations (arrow) or missing tissue (arrowhead). (E) Embryos from *kdm2aa*^*sa9360/sa9360*^ female outcrosses display the same phenotypes as MZ*kdm2aa*^*-/-*^ mutants.
(TIF)

S2 Fig Kdm2aa-deficient fish develop melanoma. (related to Fig 2). (A) Survival graph for *kdm2aa*^*sa9360*^ showing incidence of suspected cancer. Each (*) indicates a single culled fish due to suspected cancer. No wild-type or heterozygous siblings developed any suspected cancers. (B) *kdm2aa*^*sa898*^ homozygous fish with a mass on its body (arrow). (C) H and E stained section through the abdominal mass of a *kdm2aa*^*s8980/sa898*^ fish showing epithelioid and spindle cells with a nested pattern involving skeletal muscle.
(TIF)

S3 Fig RNA-seq profiling of individual *kdm2aa*-deficient embryos and their siblings. (related to Fig 3). (A) Venn diagram showing the overlap of DE genes in each of the four individual RNA-seq experiments. Regions shaded in grey are DE genes common to at least 3 out of 4 experiments. (B) Bar graph of the log2 fold change in mRNA levels of the 19 DE genes common to all 4 experiments. (C) Venn diagram showing the overlap in enriched GO terms in the BP domain between the two 5 d.p.f. experiments and the 5 d.p.f. combined analysis. Shaded in grey are the 30 terms common to all three analyses which are shown in Fig 3G. (D) Venn diagram showing the overlap in enriched GO terms in the BP domain between the two 12 d.p.f. experiments and the 12 d.p.f. combined analysis. Shaded in grey are the 11 GO terms common to all 3 analyses, which are shown in Fig 3H. (E) Table of enriched GO terms in the BP domain from GO analysis of the 95 DE genes common to at least 3 out of 4 individual experiments. The 30 terms with the lowest p-values are shown. See S5 Table for full list. (F) Box plot of normalised counts for *srcap*, with adjusted p-values for individual experiments, stage-specific and combined analysis as indicated by horizontal bars. Data for heterozygous and wild-type siblings are combined. In the figure legend ‘s’ denotes siblings and ‘m’ homozygous mutants.
(TIF)

S1 Table *kdm2aa*^*sa898/sa898*^; *kdm2ab*^*sa1479/sa1479*^ double mutants are not phenotypically different to their siblings at 5 d.p.f. Genotyping 5 d.p.f. embryos from an intercross of *kdm2aa*^*sa898/+*^; *kdm2ab*^*sa1479/+*^ double heterozygous parents revealed that *kdm2aa*^*sa898/sa898*^; *kdm2ab*^*sa1479/sa1479*^ double homozygous mutant offspring were not phenotypically different to their siblings.
(XLSX)

S2 Table Kdm2aa-deficient fish have reduced survival. The number of fish at 30, 90, 150 and 180 d.p.f. is given for each genotype from intercrosses of either *kdm2aa*^*sa898/+*^ or *kdm2aa*^*sa9360/+*^ heterozygous parents. Kdm2aa-deficient fish are consistently present below the expected 25%.
(XLSX)

S3 Table Kdm2aa-deficient melanomas do not harbour mutations in genes commonly mutated in human melanoma. Average exome coverage is given for each sample. A list of zebrafish genes and their human orthologues containing SNVs or indels that are predicted to disrupt the protein in sibling vs control tissue comparisons is shown. Zebrafish orthologues and exome sequencing coverage are given for the 13 commonly mutated genes in human melanoma identified by The Cancer Genome Atlas [36] in addition to *KRAS*, *HRAS*, *GNAQ*, *GNA11*, *KIT*, *CTNNB1* and *EZH2*. None of these were found to harbour predicted disruptive mutations.
(XLSX)

S4 Table Differentially abundant genes determined from RNA-seq data. Lists of differentially abundant genes (adjusted p-value <0.05) from RNA-seq data are provided for individual experiments as well as 5 d.p.f. combined, 12 d.p.f. combined and all experiments combined. The first tab shows which outliers were removed from the analysis and includes a table indicating which stage and allele each zmp_ph number corresponds to.
(XLSX)

S5 Table Gene Ontology enrichment from RNA-seq data. Lists of significantly enriched (elimKS<0.05) Gene Ontology (GO) terms in the biological process (BP), cellular component (CC) and molecular function (MF) domains are given for day 5 combined, day 12 combined, all combined and each individual experiment. Enriched GO terms are also provided for the 95 genes which are differentially expressed in at least 3 out of the 4 individual experiments.
(XLSX)
