# Supplementary material for: Loss of the chromatin modifier Kdm2aa causes BrafV600E-independent spontaneous melanoma in zebrafish
Source: PLoS Genet. 2017 Aug 14;13(8):e1006959. doi: 10.1371/journal.pgen.1006959 (PMC5570503; doi:10.1371/journal.pgen.1006959)
Supplement: S1 File — (A) Box plots of length versus genotype for 30 d.p.f. fish. (B) Box plots of height versus genotype for 30 d.p.f. fish. (C) Box plots of length versus genotype for fish aged 30, 90, 150 and 180 d.p.f. (D) Frequency of genotypes at 30 d.p.f. across 4 different clutches. Homozygous mutant kdm2aa fish are consistently present below 25% (red line). (E) Frequency of genotypes at 90 d.p.f. showing that survival of homozygous mutant fish has dropped even further below 25% (red line). (F and G) Box plots of length (F) or height (G) versus genotype for two compound heterozygous incrosses showing that compound heterozygous fish have reduced length and height compared to their siblings. (H) Table of frequency of homozygous fish and siblings at 30, 90 and 180 d.p.f. along with p-values from a binomial test indicating that homozygous mutant fish are present significantly below 25%. (PDF) [file pgen.1006959.s001.pdf]

A

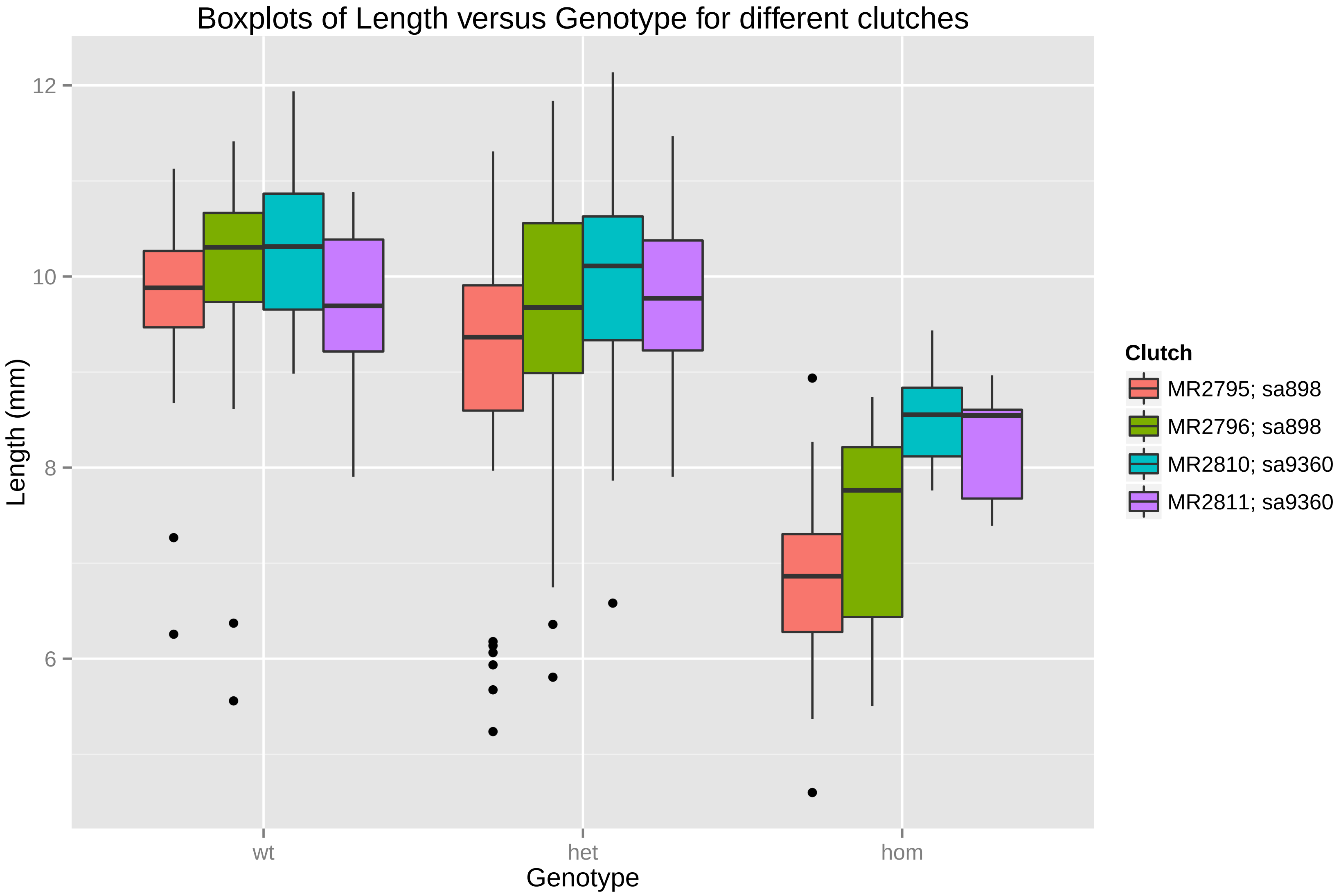

Boxplots of Length versus Genotype for different clutches
Length (mm)
12
10
8
6
wt
het
hom
Genotype
Clutch
MR2795; sa898
MR2796; sa898
MR2810; sa9360
MR2811; sa9360

B

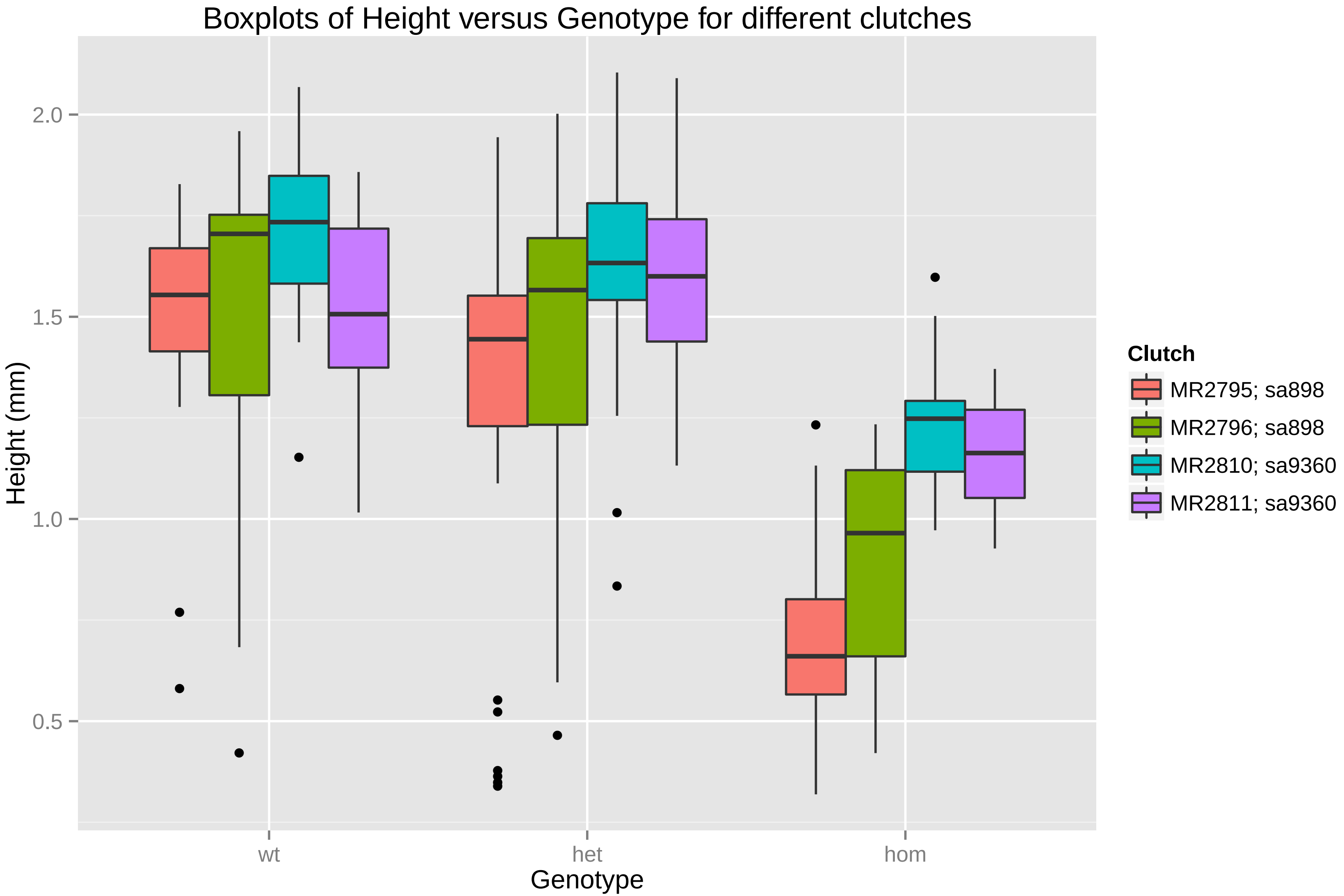
Boxplots of Height versus Genotype for different clutches
Height (mm)
2.0
1.5
1.0
0.5
wt
het
hom
Genotype
Clutch
MR2795; sa898
MR2796; sa898
MR2810; sa9360
MR2811; sa9360

C

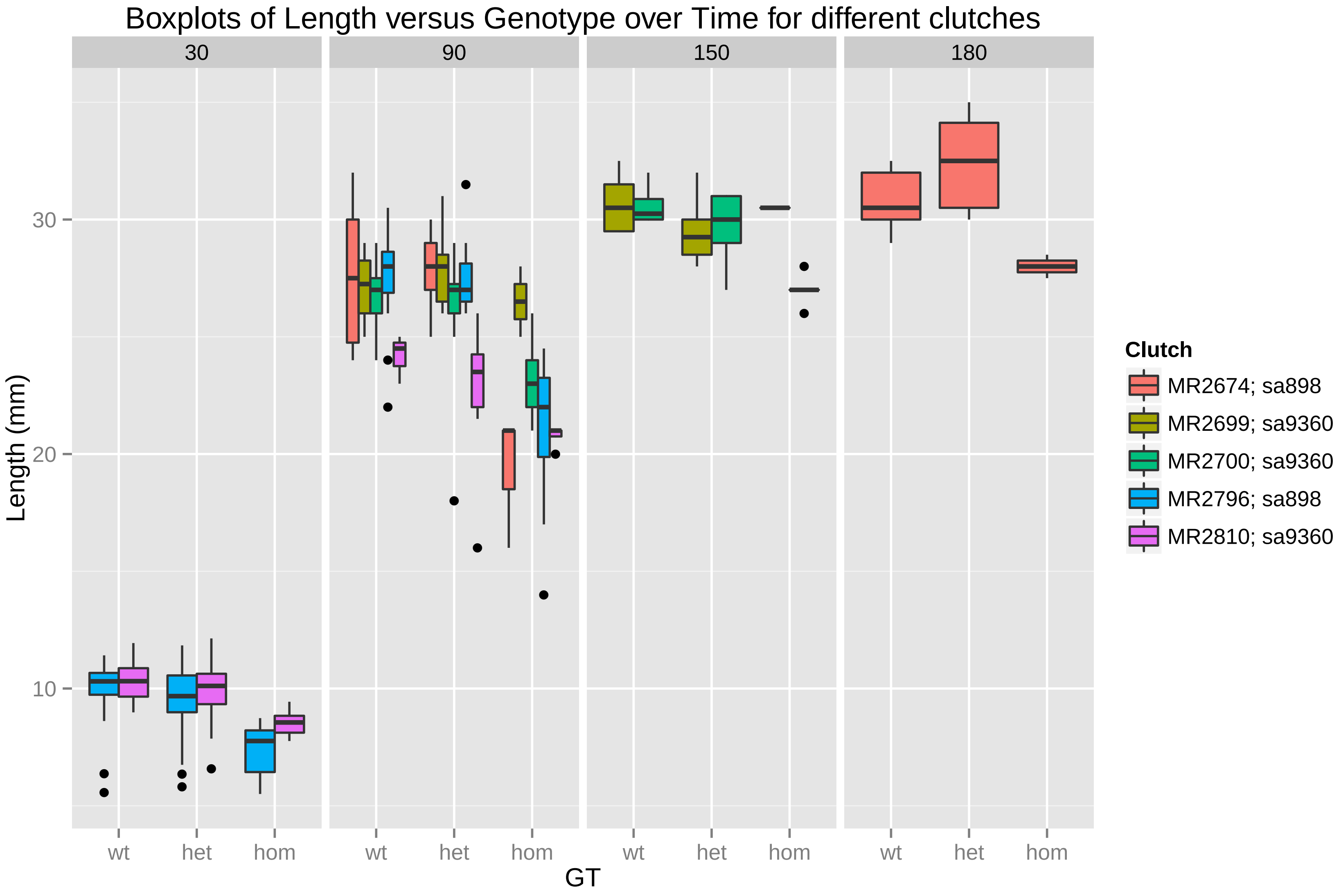

Boxplots of Length versus Genotype over Time for different clutches
30
90
150
180
Length (mm)
10
20
30
wt
het
hom
GT
Clutch
MR2674; sa898
MR2699; sa9360
MR2700; sa9360
MR2796; sa898
MR2810; sa9360

D

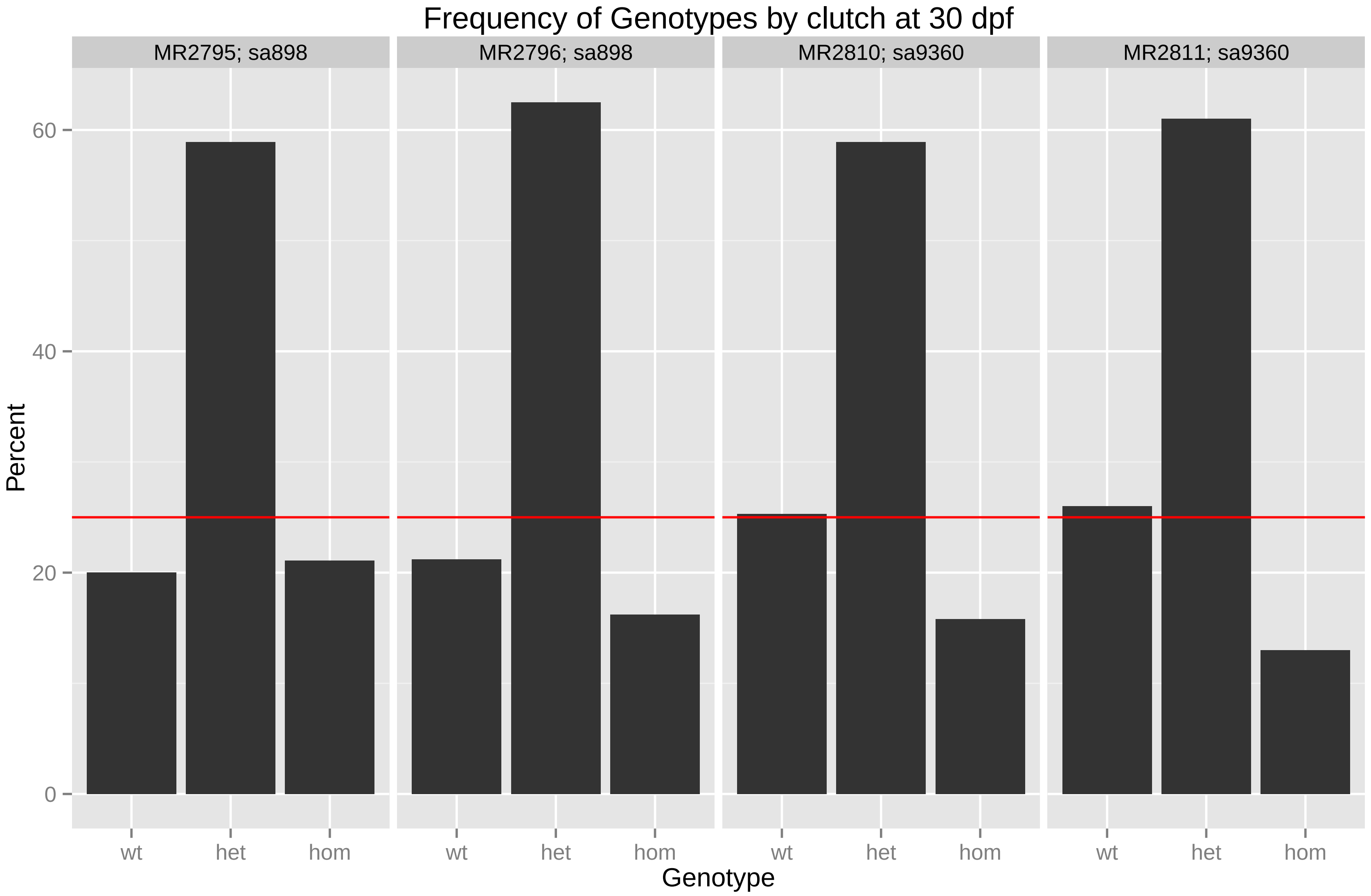
Frequency of Genotypes by clutch at 30 dpf
MR2795; sa898
MR2796; sa898
MR2810; sa9360
MR2811; sa9360
Percent
0
20
40
60
wt
het
hom
Genotype

E

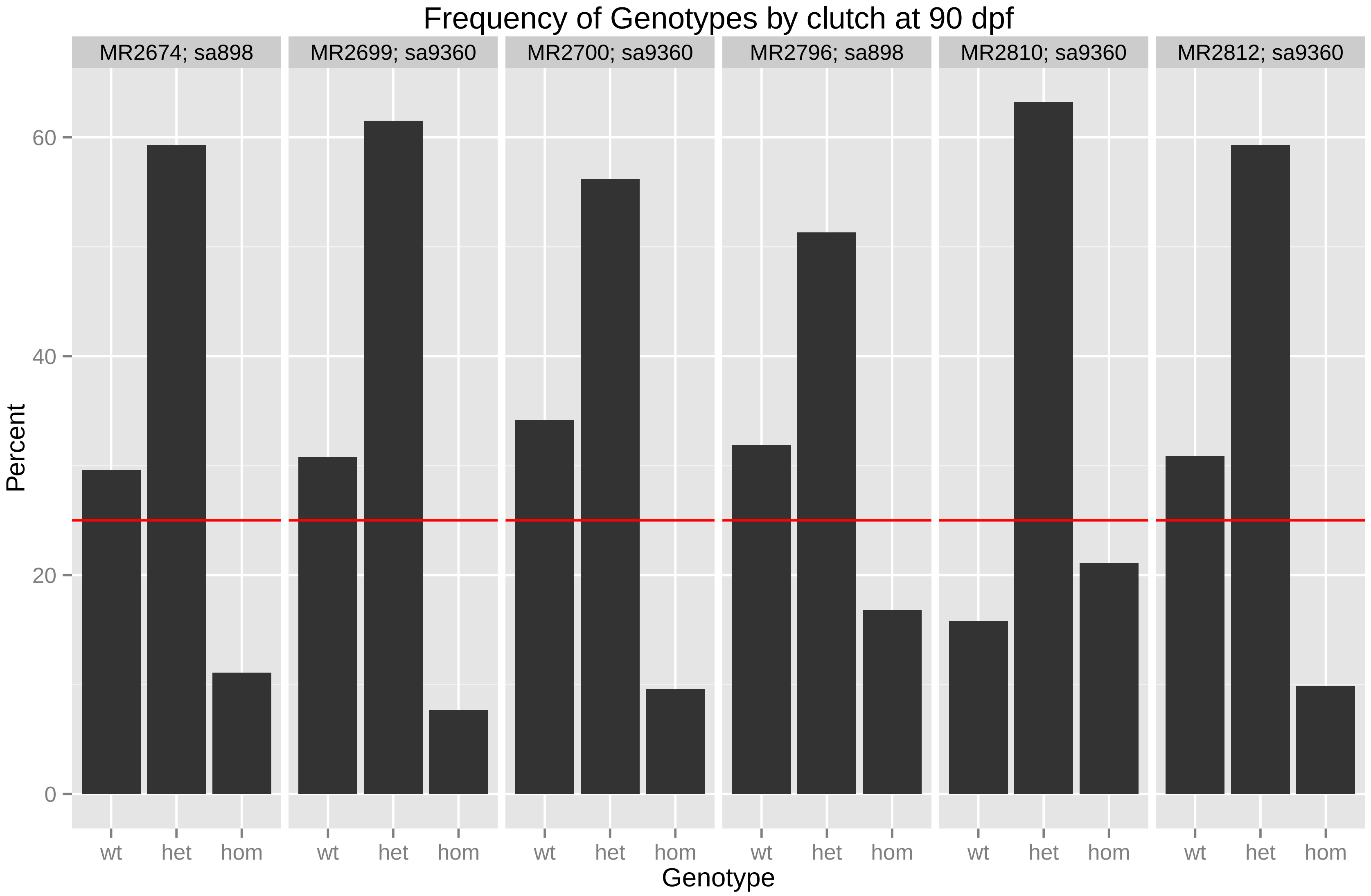
Frequency of Genotypes by clutch at 90 dpf
MR2674; sa898
MR2699; sa9360
MR2700; sa9360
MR2796; sa898
MR2810; sa9360
MR2812; sa9360
Percent
0
20
40
60
wt
het
hom
Genotype

F

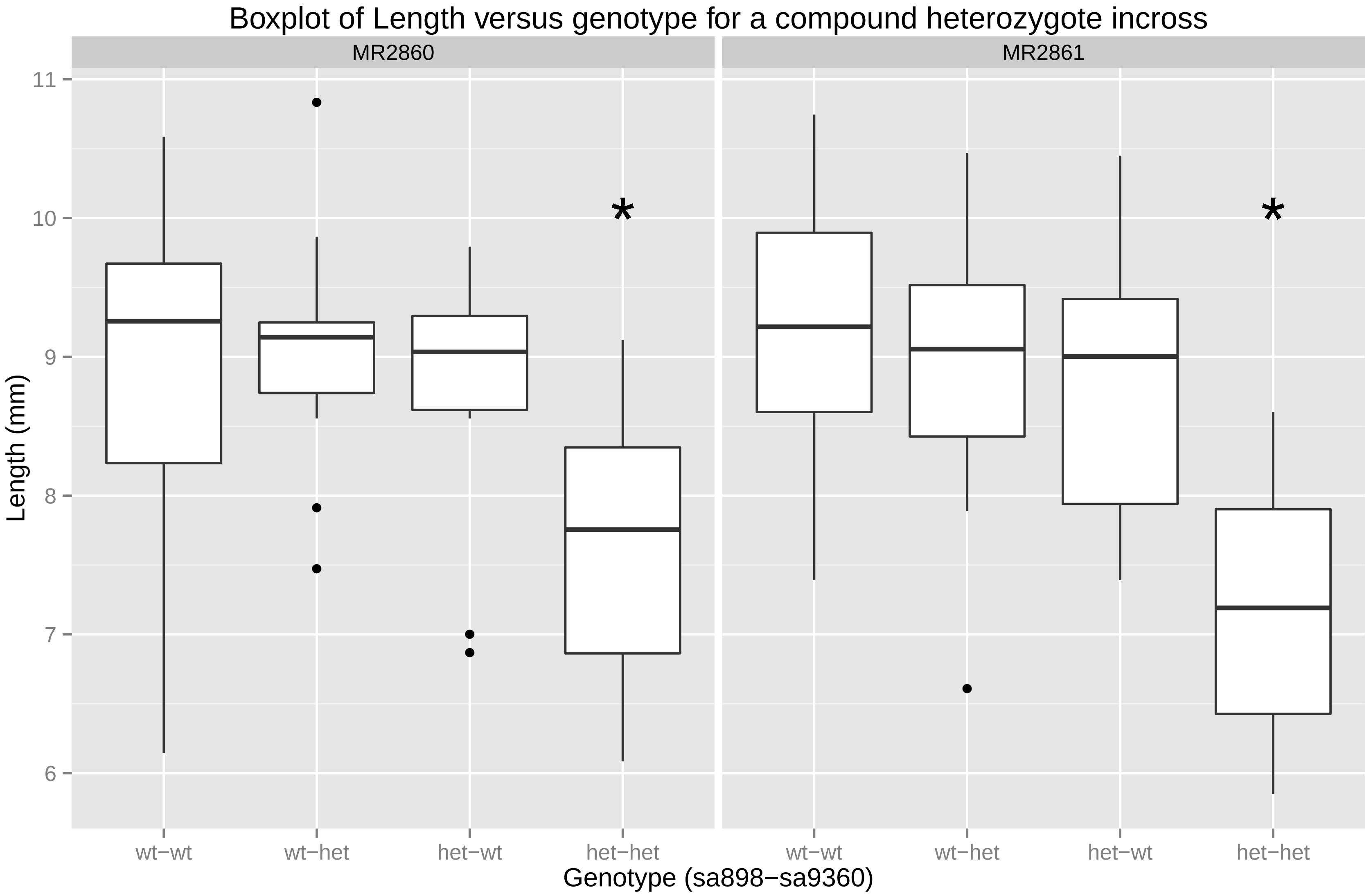
Boxplot of Length versus genotype for a compound heterozygote incross
MR2860
MR2861
Length (mm)
11
10
9
8
7
6
*
*
wt−wt
wt−het
het−wt
het−het
wt−wt
wt−het
het−wt
het−het
Genotype (sa898−sa9360)

G

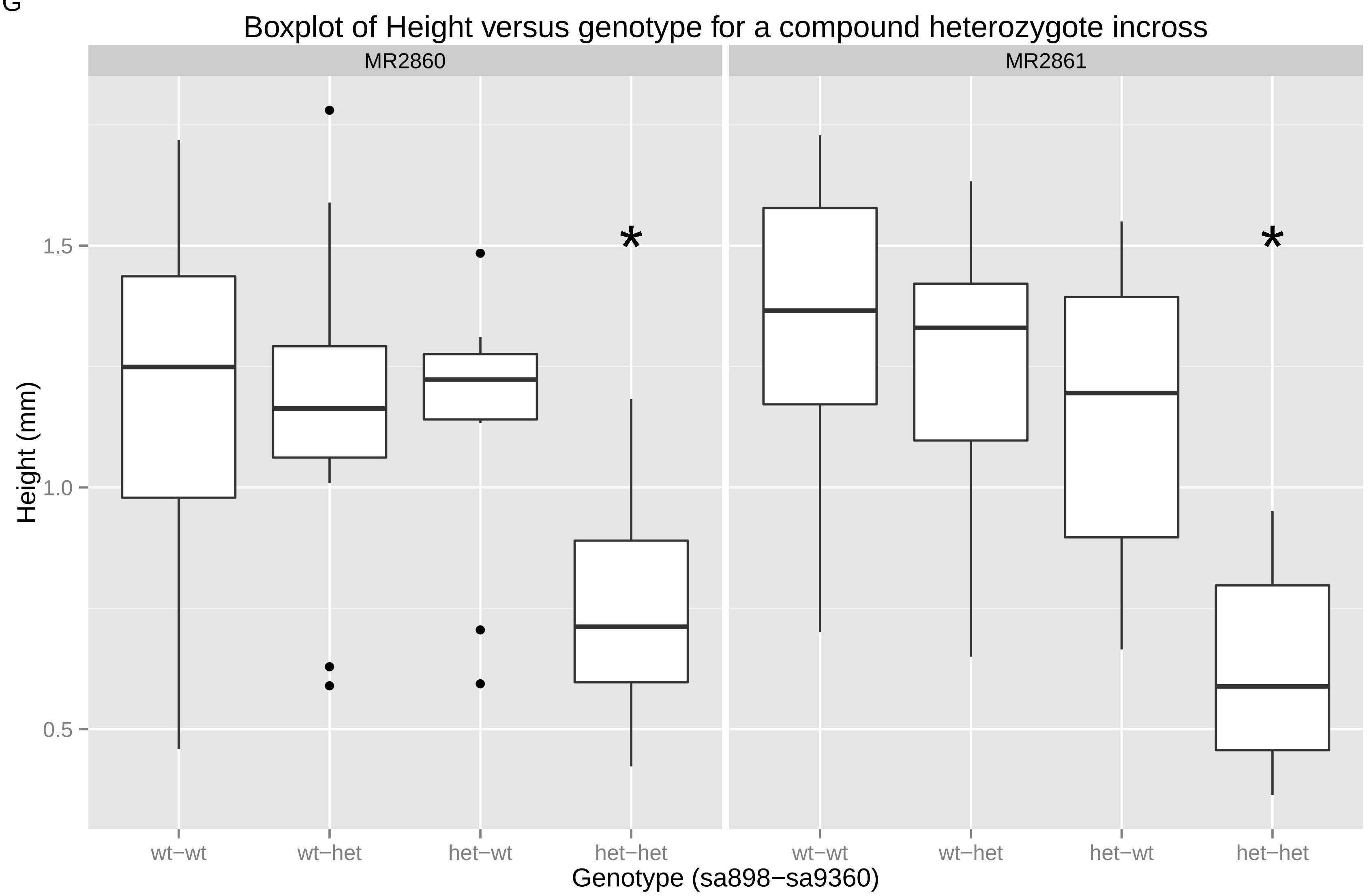
Boxplot of Height versus genotype for a compound heterozygote incross
MR2860
MR2861
Height (mm)
1.5
1.0
0.5
*
*
wt−wt
wt−het
het−wt
het−het
wt−wt
wt−het
het−wt
het−het
Genotype (sa898−sa9360)

H

Age (dpf)	Count (sibs)	Count (homs)	p−value
30	289	58	0.000244
90	313	45	0.000000
180	22	3	0.167542
