# Supplementary figures and images for: Loss of the chromatin modifier Kdm2aa causes BrafV600E-independent spontaneous melanoma in zebrafish
Source: PLoS Genet. 2017 Aug 14;13(8):e1006959. doi: 10.1371/journal.pgen.1006959 (PMC5570503; doi:10.1371/journal.pgen.1006959)

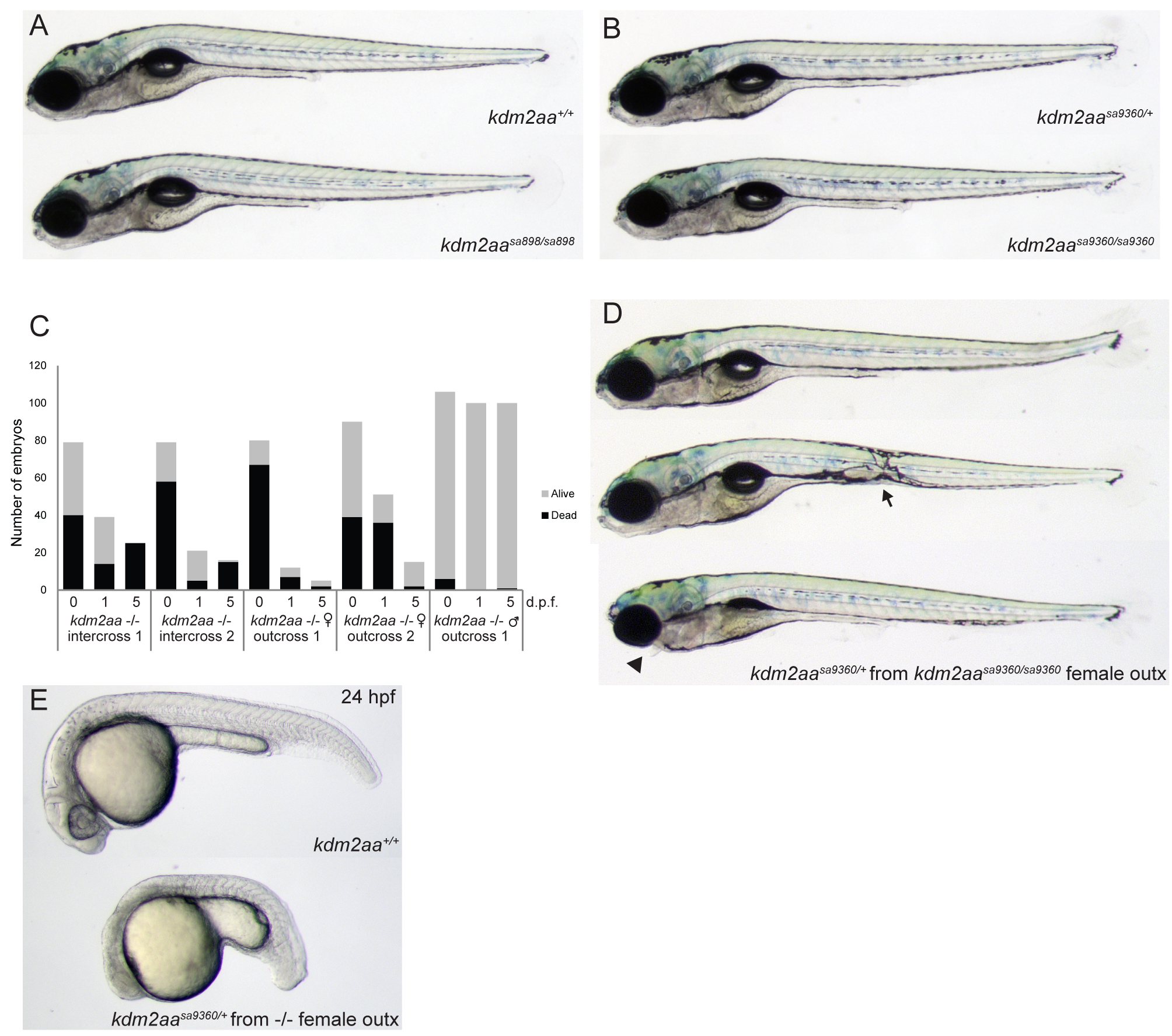

Supplement: S1 Fig — (A, B) Homozygous mutants for either kdm2aasa898 or kdm2aasa9360 are morphologically normal at 5 d.p.f. (C) Survival rates up to 5 d.p.f. of embryos resulting from intercrosses and outcrosses of homozygous kdm2aasa9360 mutants. (D) A small number of embryos from initial kdm2aasa9360/sa9360 female outcrosses survive to 5 d.p.f. albeit with malformations (arrow) or missing tissue (arrowhead). (E) Embryos from kdm2aasa9360/sa9360 female outcrosses display the same phenotypes as MZkdm2aa-/- mutants. (TIF) [file pgen.1006959.s003.tif]

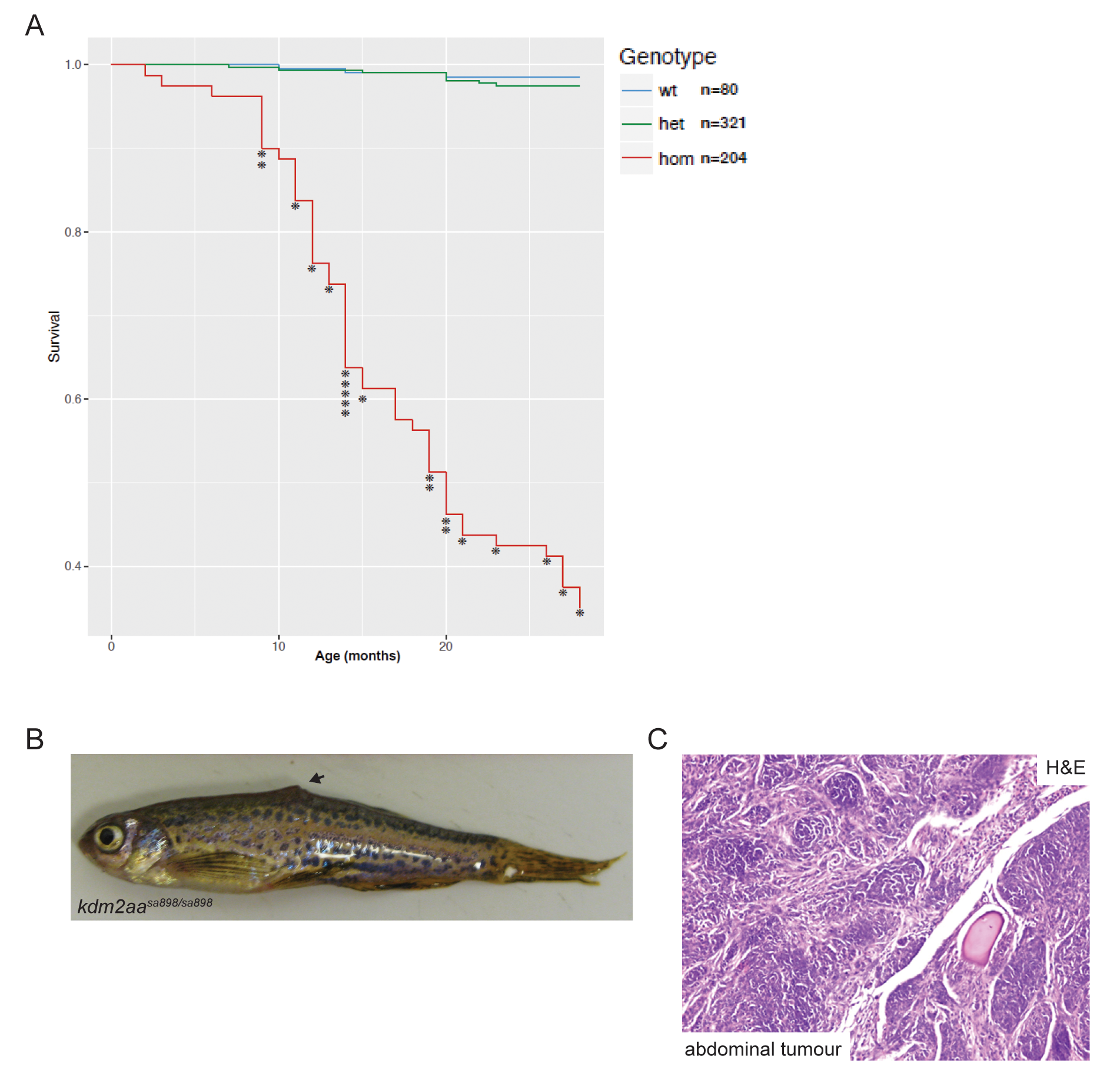

Supplement: S2 Fig — (A) Survival graph for kdm2aasa9360 showing incidence of suspected cancer. Each (*) indicates a single culled fish due to suspected cancer. No wild-type or heterozygous siblings developed any suspected cancers. (B) kdm2aasa898 homozygous fish with a mass on its body (arrow). (C) H and E stained section through the abdominal mass of a kdm2aas8980/sa898 fish showing epithelioid and spindle cells with a nested pattern involving skeletal muscle. (TIF) [file pgen.1006959.s004.tif]

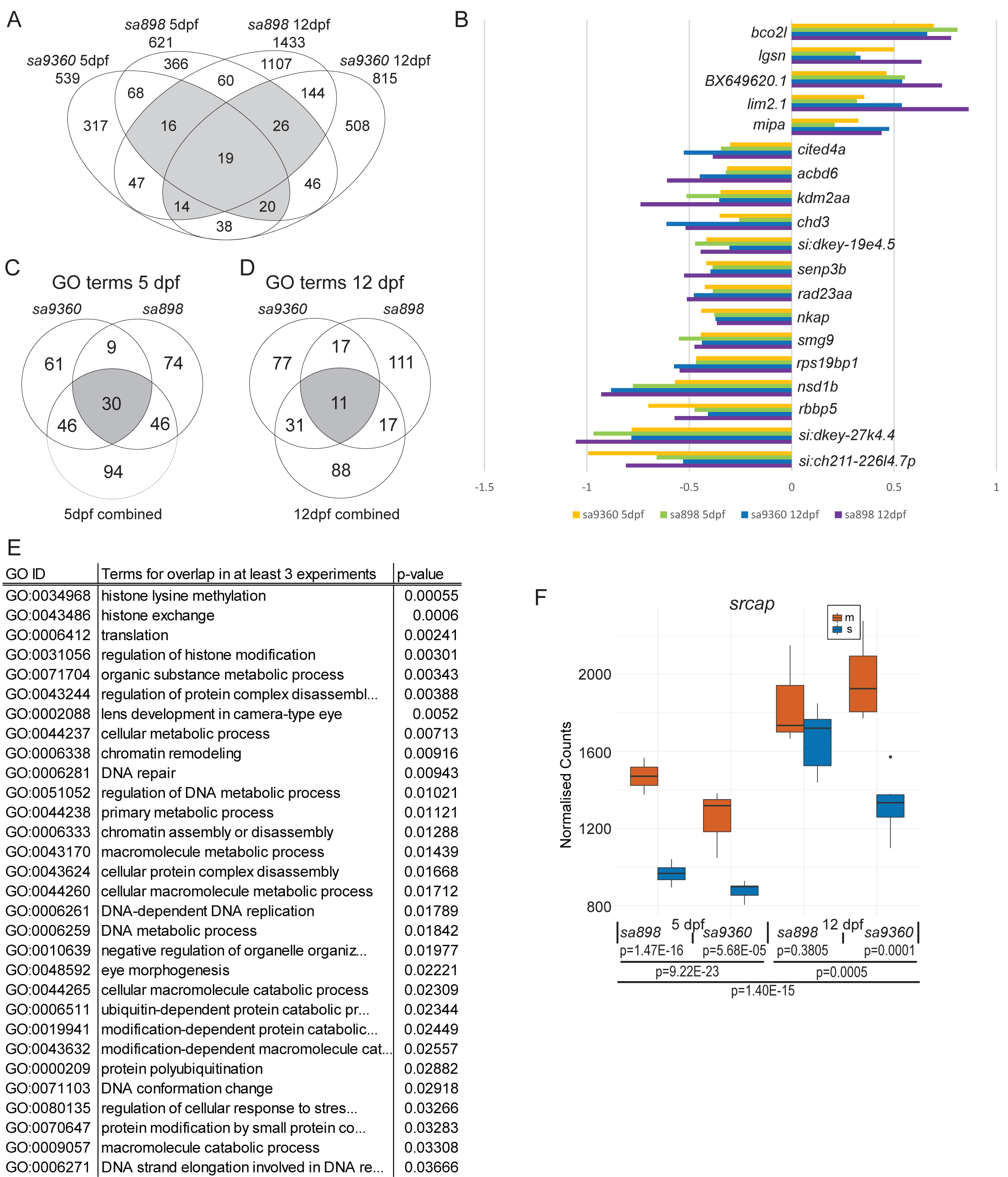

Supplement: S3 Fig — (A) Venn diagram showing the overlap of DE genes in each of the four individual RNA-seq experiments. Regions shaded in grey are DE genes common to at least 3 out of 4 experiments. (B) Bar graph of the log2 fold change in mRNA levels of the 19 DE genes common to all 4 experiments. (C) Venn diagram showing the overlap in enriched GO terms in the BP domain between the two 5 d.p.f. experiments and the 5 d.p.f. combined analysis. Shaded in grey are the 30 terms common to all three analyses which are shown in Fig 3G. (D) Venn diagram showing the overlap in enriched GO terms in the BP domain between the two 12 d.p.f. experiments and the 12 d.p.f. combined analysis. Shaded in grey are the 11 GO terms common to all 3 analyses, which are shown in Fig 3H. (E) Table of enriched GO terms in the BP domain from GO analysis of the 95 DE genes common to at least 3 out of 4 individual experiments. The 30 terms with the lowest p-values are shown. See S5 Table for full list. (F) Box plot of normalised counts for srcap, with adjusted p-values for individual experiments, stage-specific and combined analysis as indicated by horizontal bars. Data for heterozygous and wild-type siblings are combined. In the figure legend ‘s’ denotes siblings and ‘m’ homozygous mutants. (TIF) [file pgen.1006959.s005.tif]
