# Supplementary material for: Loss of the chromatin modifier Kdm2aa causes BrafV600E-independent spontaneous melanoma in zebrafish
Source: PLoS Genet. 2017 Aug 14;13(8):e1006959. doi: 10.1371/journal.pgen.1006959 (PMC5570503; doi:10.1371/journal.pgen.1006959)
Supplement: S2 File — Genes identified to be significantly differentially expressed (p<0.05) in the combined analysis are shown, with the normalised counts displayed across all four experiments. (PDF) [file pgen.1006959.s002.pdf]

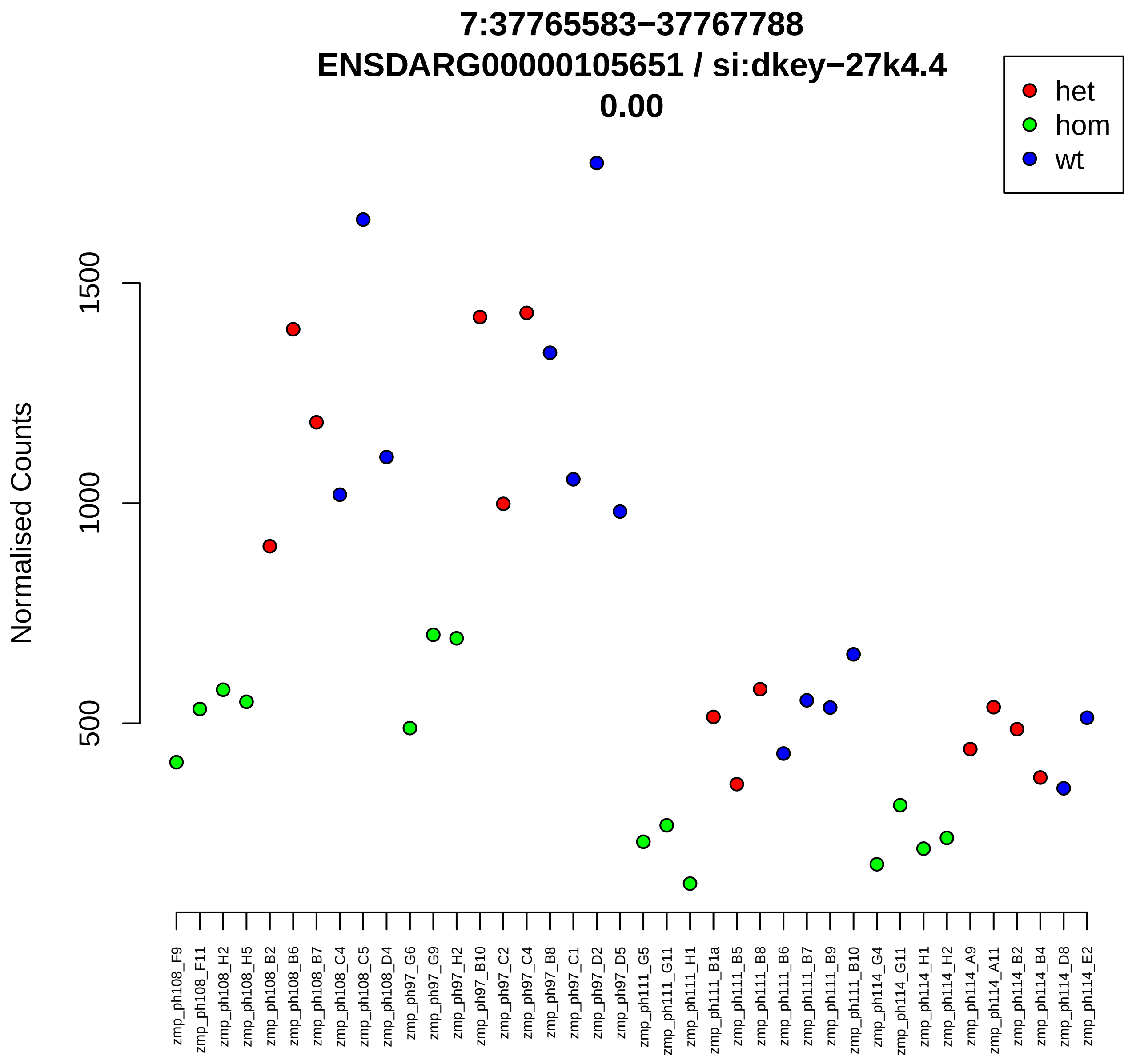
7:37765583−37767788
ENSDARG00000105651 / si:dkey−27k4.4
0.00
het
hom
wt
Normalised Counts
500
1000
1500
zmp_ph108_F9
zmp_ph108_F11
zmp_ph108_H2
zmp_ph108_H5
zmp_ph108_B2
zmp_ph108_B6
zmp_ph108_B7
zmp_ph108_C4
zmp_ph108_C5
zmp_ph108_D4
zmp_ph97_G6
zmp_ph97_G9
zmp_ph97_H2
zmp_ph97_B10
zmp_ph97_C2
zmp_ph97_C4
zmp_ph97_B8
zmp_ph97_C1
zmp_ph97_D2
zmp_ph97_D5
zmp_ph111_G5
zmp_ph111_G11
zmp_ph111_H1
zmp_ph111_B1a
zmp_ph111_B5
zmp_ph111_B8
zmp_ph111_B6
zmp_ph111_B7
zmp_ph111_B9
zmp_ph111_B10
zmp_ph114_G4
zmp_ph114_G11
zmp_ph114_H1
zmp_ph114_H2
zmp_ph114_A9
zmp_ph114_A11
zmp_ph114_B2
zmp_ph114_B4
zmp_ph114_D8
zmp_ph114_E2

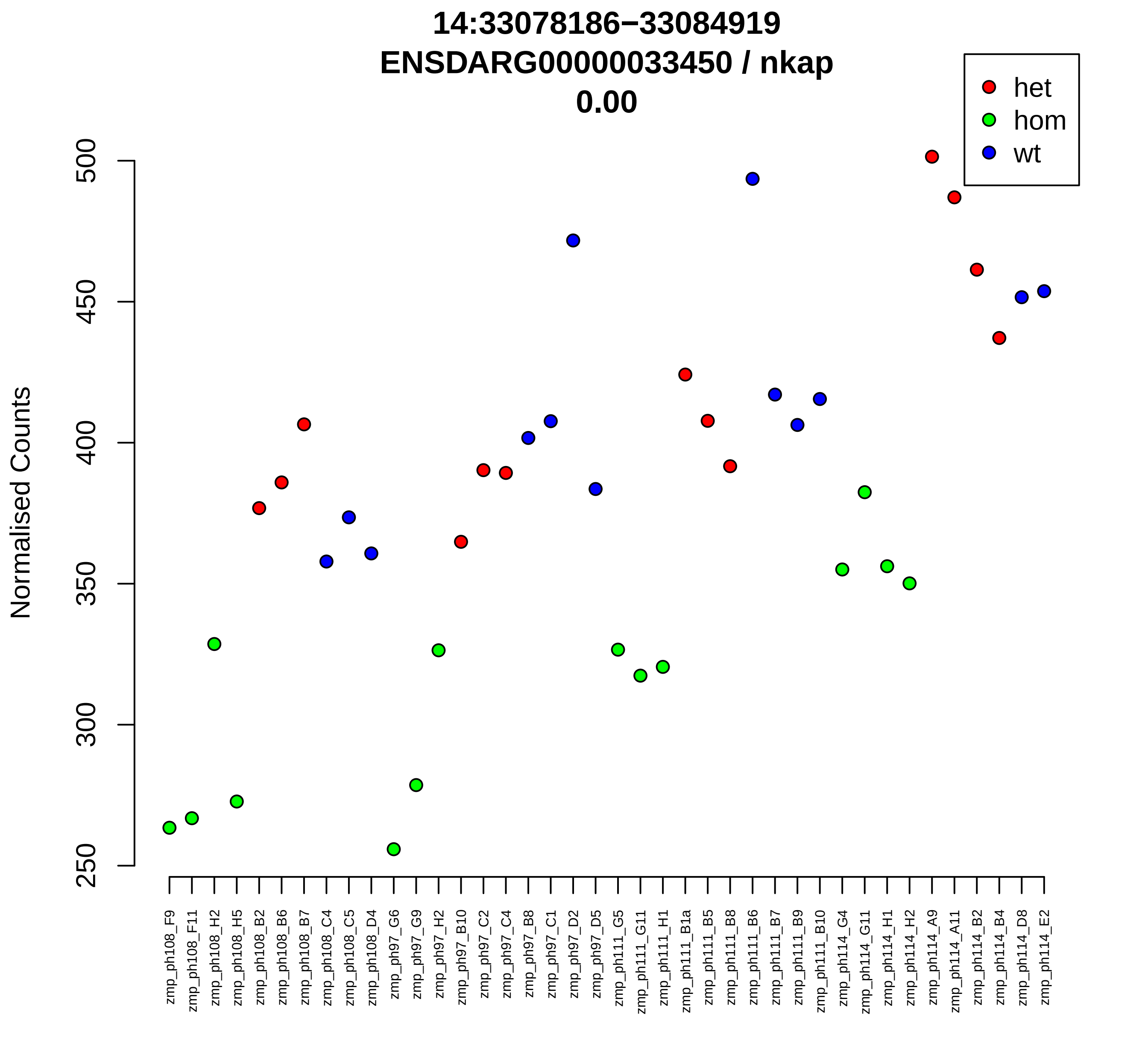
14:33078186−33084919
ENSDARG00000033450 / nkap
0.00
het
hom
wt
Normalised Counts
250
300
350
400
450
500
zmp_ph108_F9
zmp_ph108_F11
zmp_ph108_H2
zmp_ph108_H5
zmp_ph108_B2
zmp_ph108_B6
zmp_ph108_B7
zmp_ph108_C4
zmp_ph108_C5
zmp_ph108_D4
zmp_ph97_G6
zmp_ph97_G9
zmp_ph97_H2
zmp_ph97_B10
zmp_ph97_C2
zmp_ph97_C4
zmp_ph97_B8
zmp_ph97_C1
zmp_ph97_D2
zmp_ph97_D5
zmp_ph111_G5
zmp_ph111_G11
zmp_ph111_H1
zmp_ph111_B1a
zmp_ph111_B5
zmp_ph111_B8
zmp_ph111_B6
zmp_ph111_B7
zmp_ph111_B9
zmp_ph111_B10
zmp_ph114_G4
zmp_ph114_G11
zmp_ph114_H1
zmp_ph114_H2
zmp_ph114_A9
zmp_ph114_A11
zmp_ph114_B2
zmp_ph114_B4
zmp_ph114_D8
zmp_ph114_E2

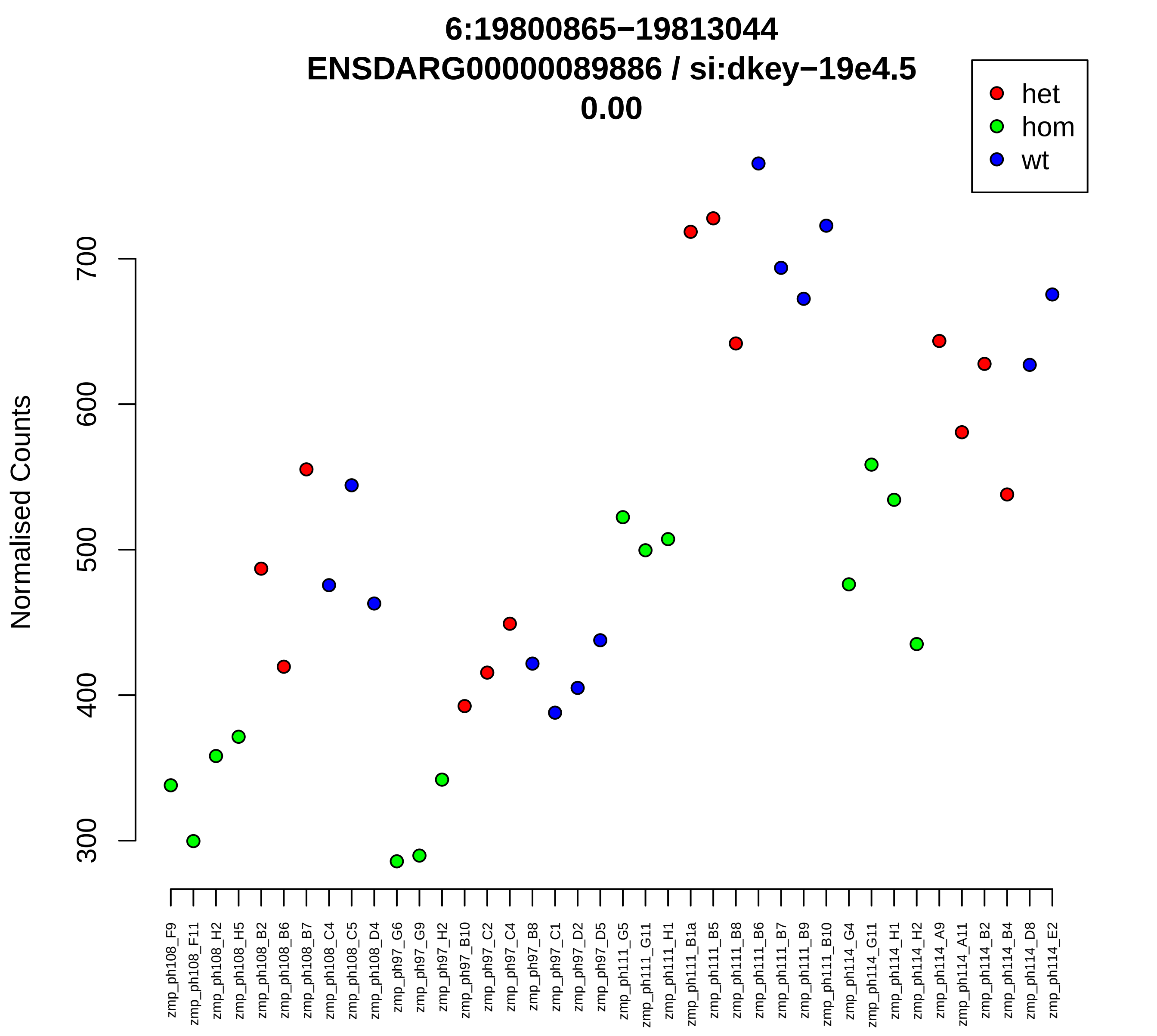
6:19800865−19813044
ENSDARG00000089886 / si:dkey−19e4.5
0.00
het
hom
wt
Normalised Counts
300
400
500
600
700
zmp_ph108_F9
zmp_ph108_F11
zmp_ph108_H2
zmp_ph108_H5
zmp_ph108_B2
zmp_ph108_B6
zmp_ph108_B7
zmp_ph108_C4
zmp_ph108_C5
zmp_ph108_D4
zmp_ph97_G6
zmp_ph97_G9
zmp_ph97_H2
zmp_ph97_B10
zmp_ph97_C2
zmp_ph97_C4
zmp_ph97_B8
zmp_ph97_C1
zmp_ph97_D2
zmp_ph97_D5
zmp_ph111_G5
zmp_ph111_G11
zmp_ph111_H1
zmp_ph111_B1a
zmp_ph111_B5
zmp_ph111_B8
zmp_ph111_B6
zmp_ph111_B7
zmp_ph111_B9
zmp_ph111_B10
zmp_ph114_G4
zmp_ph114_G11
zmp_ph114_H1
zmp_ph114_H2
zmp_ph114_A9
zmp_ph114_A11
zmp_ph114_B2
zmp_ph114_B4
zmp_ph114_D8
zmp_ph114_E2

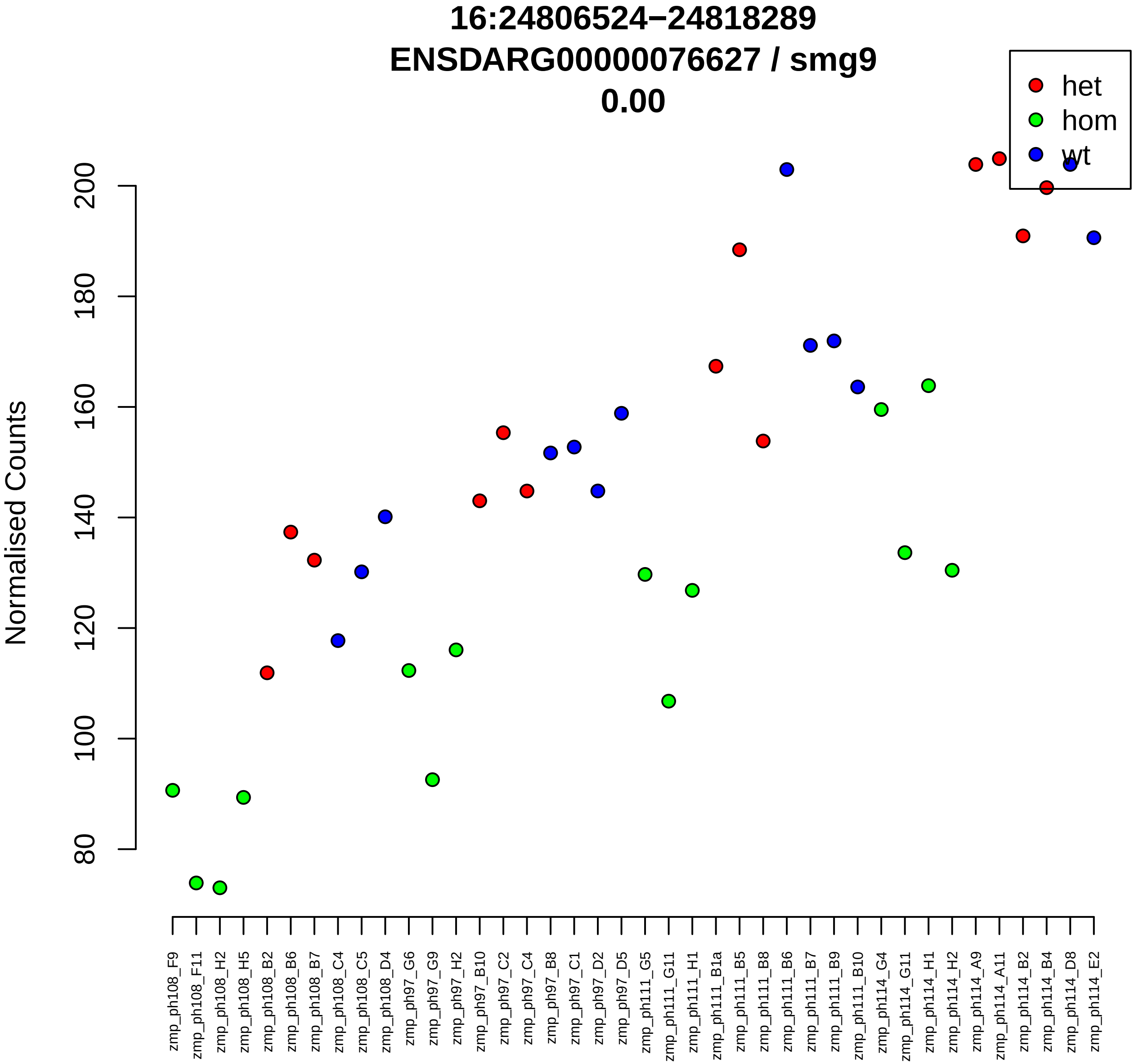

16:24806524−24818289
ENSDARG00000076627 / smg9
0.00
het
hom
wt
Normalised Counts
80
100
120
140
160
180
200
zmp_ph108_F9
zmp_ph108_F11
zmp_ph108_H2
zmp_ph108_H5
zmp_ph108_B2
zmp_ph108_B6
zmp_ph108_B7
zmp_ph108_C4
zmp_ph108_C5
zmp_ph108_D4
zmp_ph97_G6
zmp_ph97_G9
zmp_ph97_H2
zmp_ph97_B10
zmp_ph97_C2
zmp_ph97_C4
zmp_ph97_B8
zmp_ph97_C1
zmp_ph97_D2
zmp_ph97_D5
zmp_ph111_G5
zmp_ph111_G11
zmp_ph111_H1
zmp_ph111_B1a
zmp_ph111_B5
zmp_ph111_B8
zmp_ph111_B6
zmp_ph111_B7
zmp_ph111_B9
zmp_ph111_B10
zmp_ph114_G4
zmp_ph114_G11
zmp_ph114_H1
zmp_ph114_H2
zmp_ph114_A9
zmp_ph114_A11
zmp_ph114_B2
zmp_ph114_B4
zmp_ph114_D8
zmp_ph114_E2

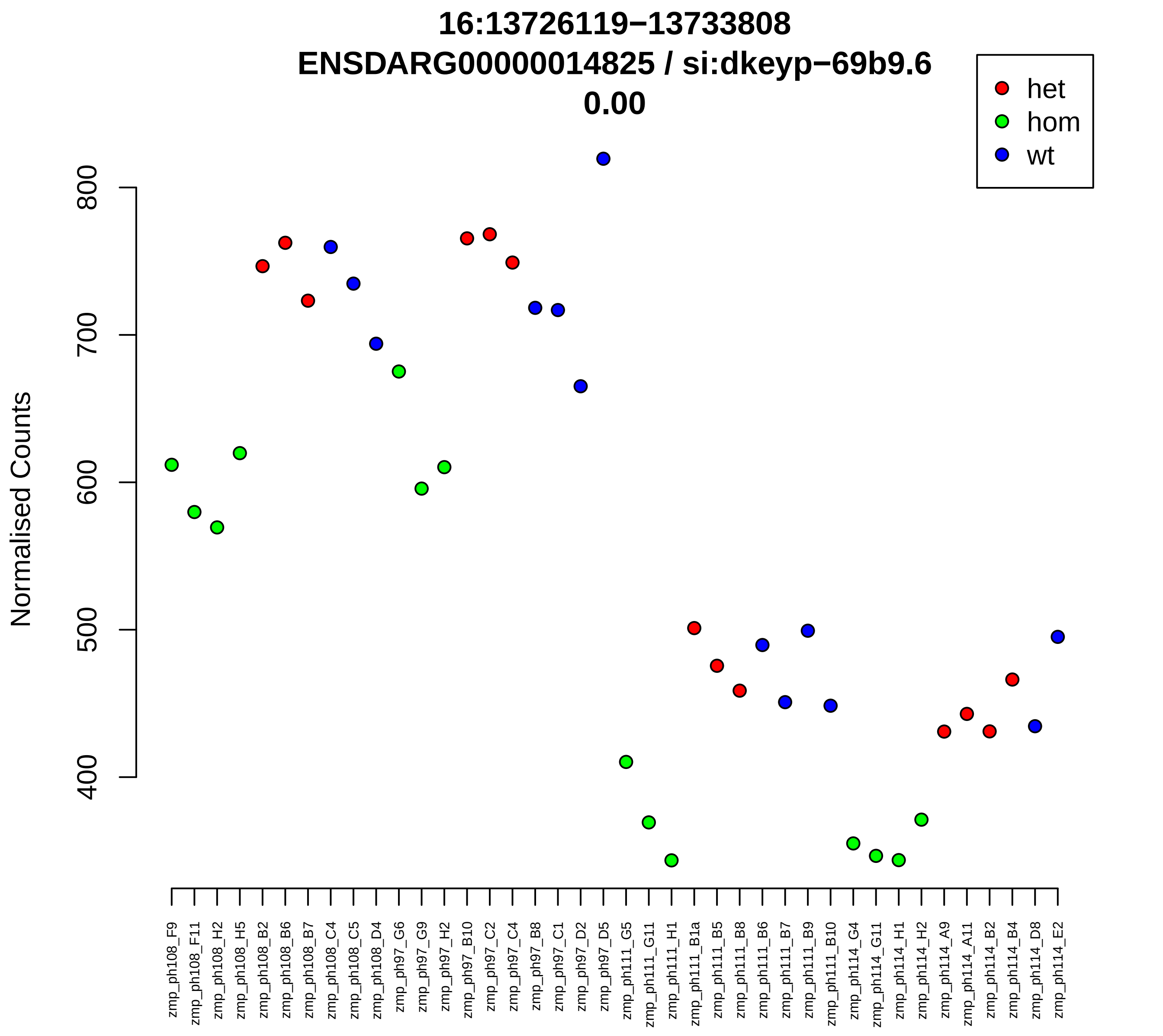

16:13726119−13733808
ENSDARG00000014825 / si:dkeyp−69b9.6
0.00
het
hom
wt
Normalised Counts
400
500
600
700
800
zmp_ph108_F9
zmp_ph108_F11
zmp_ph108_H2
zmp_ph108_H5
zmp_ph108_B2
zmp_ph108_B6
zmp_ph108_B7
zmp_ph108_C4
zmp_ph108_C5
zmp_ph108_D4
zmp_ph97_G6
zmp_ph97_G9
zmp_ph97_H2
zmp_ph97_B10
zmp_ph97_C2
zmp_ph97_C4
zmp_ph97_B8
zmp_ph97_C1
zmp_ph97_D2
zmp_ph97_D5
zmp_ph111_G5
zmp_ph111_G11
zmp_ph111_H1
zmp_ph111_B1a
zmp_ph111_B5
zmp_ph111_B8
zmp_ph111_B6
zmp_ph111_B7
zmp_ph111_B9
zmp_ph111_B10
zmp_ph114_G4
zmp_ph114_G11
zmp_ph114_H1
zmp_ph114_H2
zmp_ph114_A9
zmp_ph114_A11
zmp_ph114_B2
zmp_ph114_B4
zmp_ph114_D8
zmp_ph114_E2

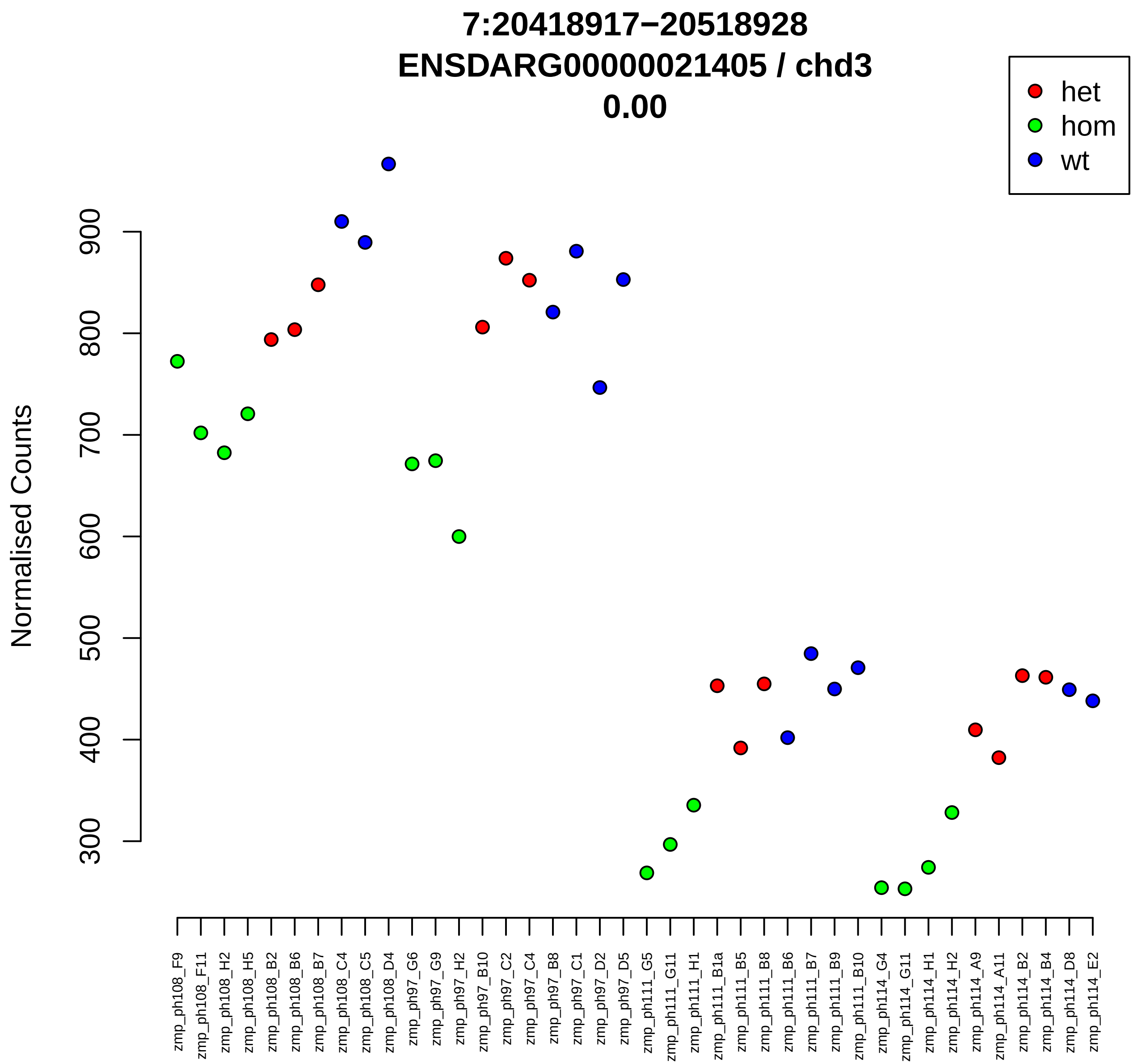

7:20418917−20518928
ENSDARG00000021405 / chd3
0.00
het
hom
wt
Normalised Counts
300
400
500
600
700
800
900
zmp_ph108_F9
zmp_ph108_F11
zmp_ph108_H2
zmp_ph108_H5
zmp_ph108_B2
zmp_ph108_B6
zmp_ph108_B7
zmp_ph108_C4
zmp_ph108_C5
zmp_ph108_D4
zmp_ph97_G6
zmp_ph97_G9
zmp_ph97_H2
zmp_ph97_B10
zmp_ph97_C2
zmp_ph97_C4
zmp_ph97_B8
zmp_ph97_C1
zmp_ph97_D2
zmp_ph97_D5
zmp_ph111_G5
zmp_ph111_G11
zmp_ph111_H1
zmp_ph111_B1a
zmp_ph111_B5
zmp_ph111_B8
zmp_ph111_B6
zmp_ph111_B7
zmp_ph111_B9
zmp_ph111_B10
zmp_ph114_G4
zmp_ph114_G11
zmp_ph114_H1
zmp_ph114_H2
zmp_ph114_A9
zmp_ph114_A11
zmp_ph114_B2
zmp_ph114_B4
zmp_ph114_D8
zmp_ph114_E2

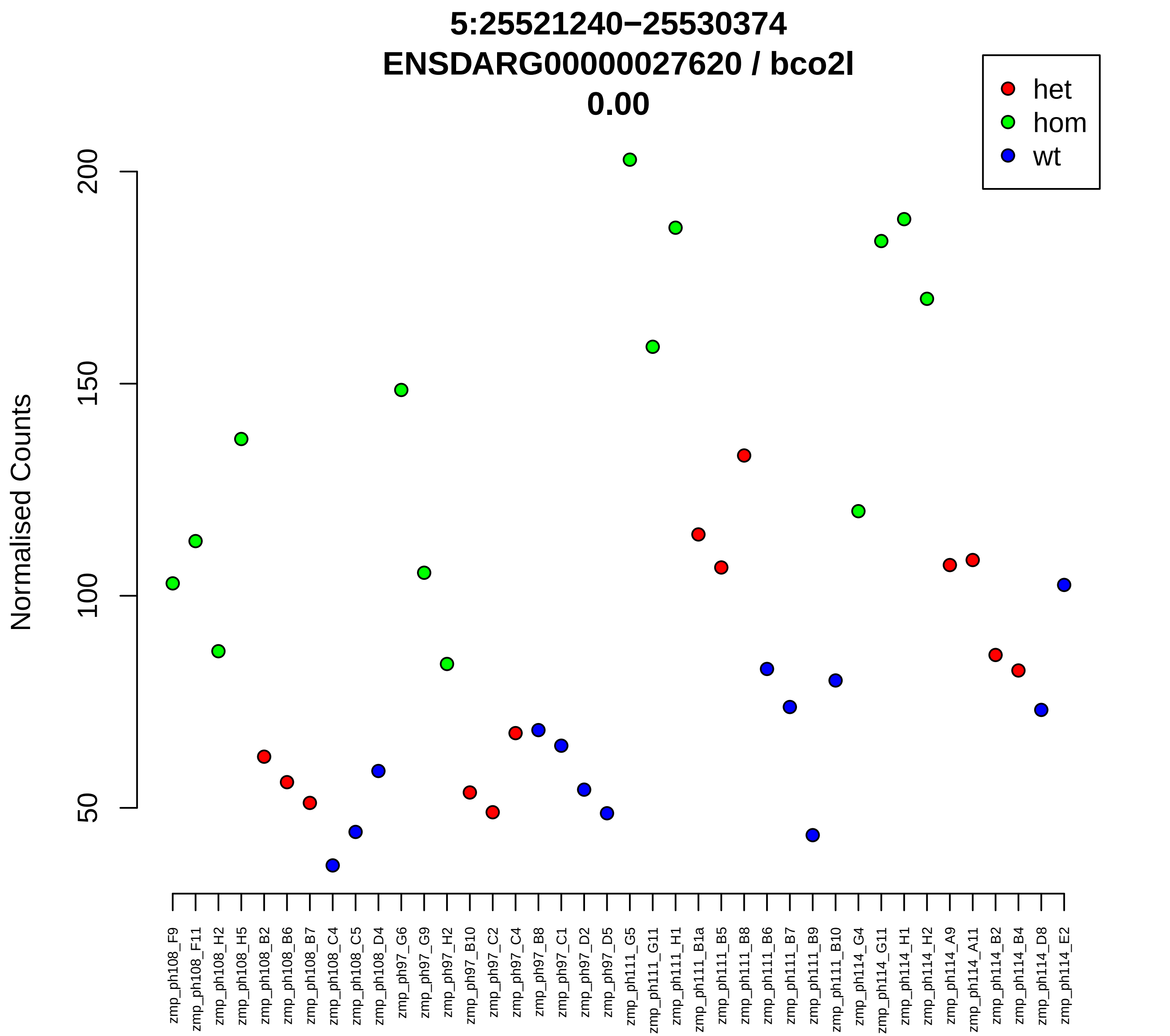

5:25521240−25530374
ENSDARG00000027620 / bco2l
0.00
het
hom
wt
Normalised Counts
200
150
100
50
zmp_ph108_F9
zmp_ph108_F11
zmp_ph108_H2
zmp_ph108_H5
zmp_ph108_B2
zmp_ph108_B6
zmp_ph108_B7
zmp_ph108_C4
zmp_ph108_C5
zmp_ph108_D4
zmp_ph97_G6
zmp_ph97_G9
zmp_ph97_H2
zmp_ph97_B10
zmp_ph97_C2
zmp_ph97_C4
zmp_ph97_B8
zmp_ph97_C1
zmp_ph97_D2
zmp_ph97_D5
zmp_ph111_G5
zmp_ph111_G11
zmp_ph111_H1
zmp_ph111_B1a
zmp_ph111_B5
zmp_ph111_B8
zmp_ph111_B6
zmp_ph111_B7
zmp_ph111_B9
zmp_ph111_B10
zmp_ph114_G4
zmp_ph114_G11
zmp_ph114_H1
zmp_ph114_H2
zmp_ph114_A9
zmp_ph114_A11
zmp_ph114_B2
zmp_ph114_B4
zmp_ph114_D8
zmp_ph114_E2

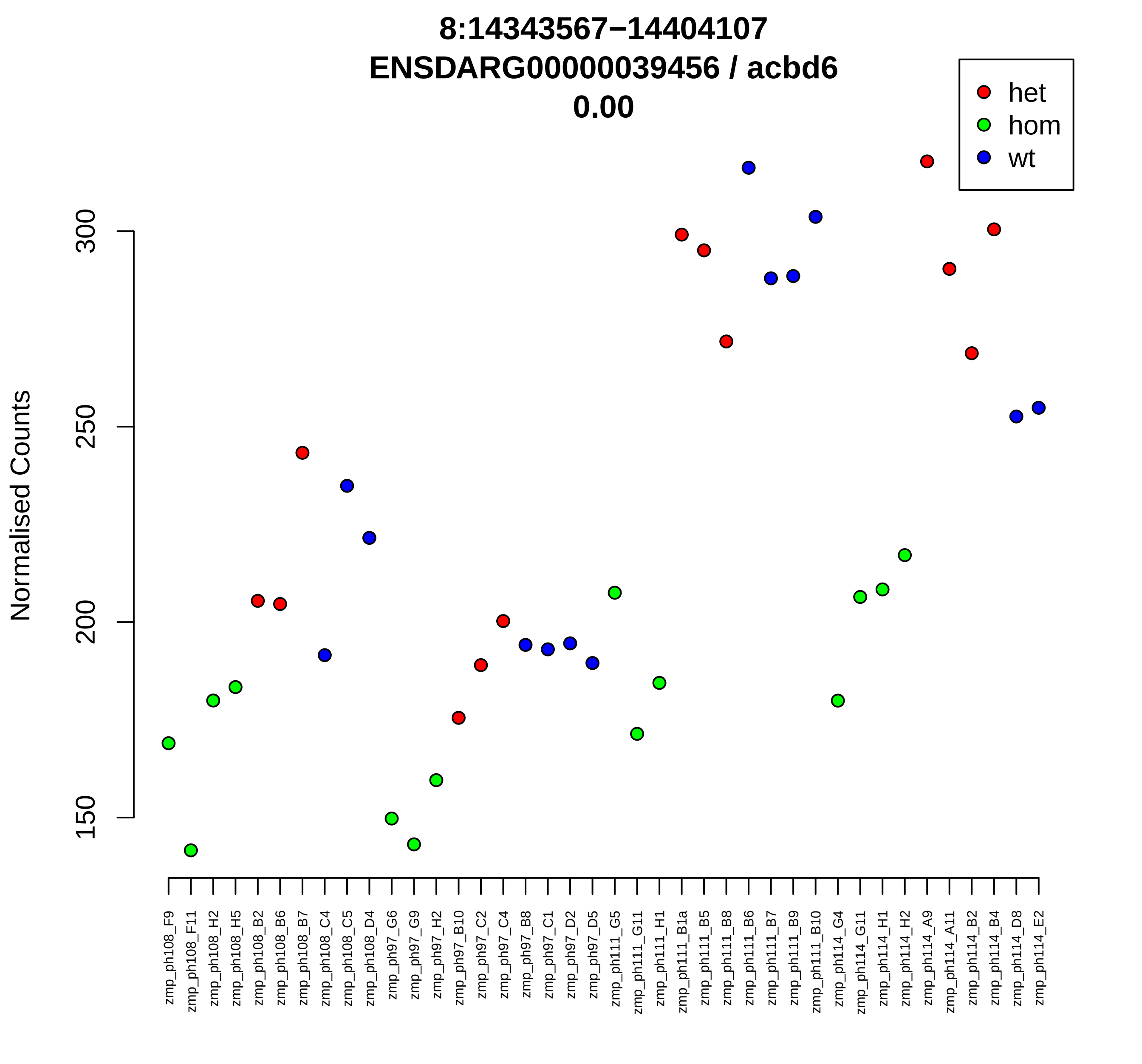

8:14343567−14404107
ENSDARG00000039456 / acbd6
0.00
het
hom
wt
Normalised Counts
150
200
250
300
zmp_ph108_F9
zmp_ph108_F11
zmp_ph108_H2
zmp_ph108_H5
zmp_ph108_B2
zmp_ph108_B6
zmp_ph108_B7
zmp_ph108_C4
zmp_ph108_C5
zmp_ph108_D4
zmp_ph97_G6
zmp_ph97_G9
zmp_ph97_H2
zmp_ph97_B10
zmp_ph97_C2
zmp_ph97_C4
zmp_ph97_B8
zmp_ph97_C1
zmp_ph97_D2
zmp_ph97_D5
zmp_ph111_G5
zmp_ph111_G11
zmp_ph111_H1
zmp_ph111_B1a
zmp_ph111_B5
zmp_ph111_B8
zmp_ph111_B6
zmp_ph111_B7
zmp_ph111_B9
zmp_ph111_B10
zmp_ph114_G4
zmp_ph114_G11
zmp_ph114_H1
zmp_ph114_H2
zmp_ph114_A9
zmp_ph114_A11
zmp_ph114_B2
zmp_ph114_B4
zmp_ph114_D8
zmp_ph114_E2

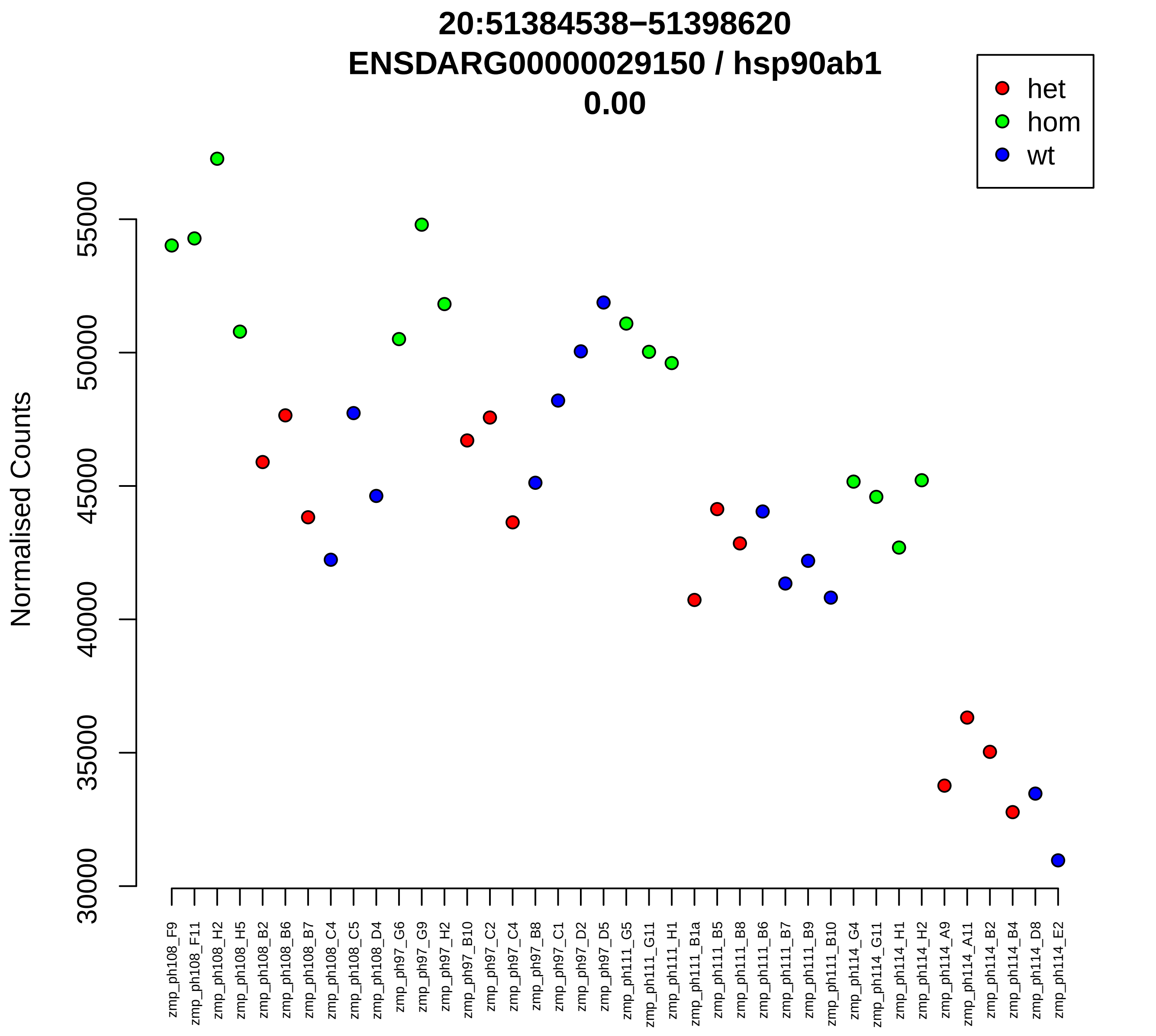
20:51384538−51398620
ENSDARG00000029150 / hsp90ab1
0.00
het
hom
wt
Normalised Counts
30000
35000
40000
45000
50000
55000
zmp_ph108_F9
zmp_ph108_F11
zmp_ph108_H2
zmp_ph108_H5
zmp_ph108_B2
zmp_ph108_B6
zmp_ph108_B7
zmp_ph108_C4
zmp_ph108_C5
zmp_ph108_D4
zmp_ph97_G6
zmp_ph97_G9
zmp_ph97_H2
zmp_ph97_B10
zmp_ph97_C2
zmp_ph97_C4
zmp_ph97_B8
zmp_ph97_C1
zmp_ph97_D2
zmp_ph97_D5
zmp_ph111_G5
zmp_ph111_G11
zmp_ph111_H1
zmp_ph111_B1a
zmp_ph111_B5
zmp_ph111_B8
zmp_ph111_B6
zmp_ph111_B7
zmp_ph111_B9
zmp_ph111_B10
zmp_ph114_G4
zmp_ph114_G11
zmp_ph114_H1
zmp_ph114_H2
zmp_ph114_A9
zmp_ph114_A11
zmp_ph114_B2
zmp_ph114_B4
zmp_ph114_D8
zmp_ph114_E2

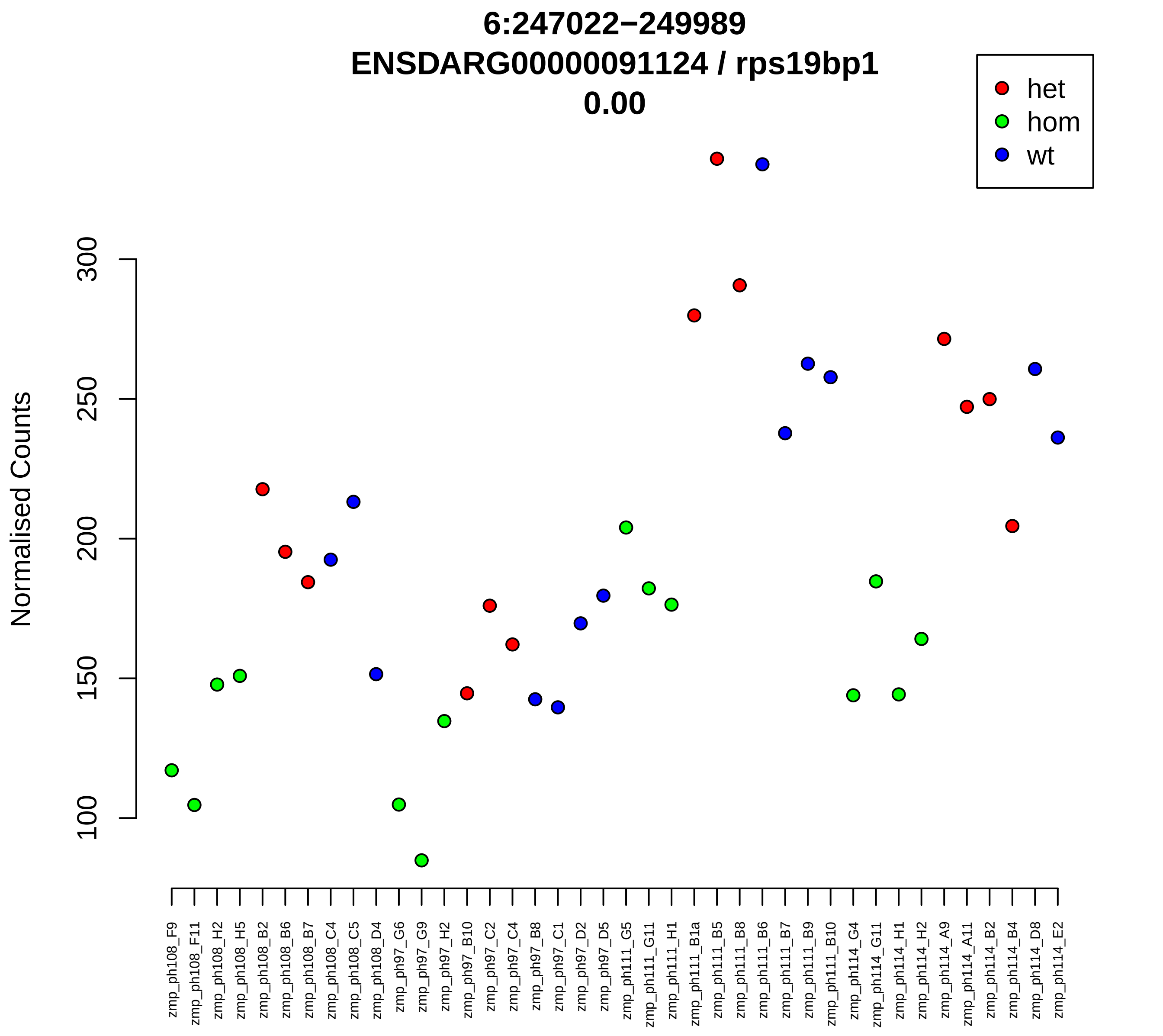

6:247022−249989
ENSDARG00000091124 / rps19bp1
0.00
het
hom
wt
Normalised Counts
100
150
200
250
300
zmp_ph108_F9
zmp_ph108_F11
zmp_ph108_H2
zmp_ph108_H5
zmp_ph108_B2
zmp_ph108_B6
zmp_ph108_B7
zmp_ph108_C4
zmp_ph108_C5
zmp_ph108_D4
zmp_ph97_G6
zmp_ph97_G9
zmp_ph97_H2
zmp_ph97_B10
zmp_ph97_C2
zmp_ph97_C4
zmp_ph97_B8
zmp_ph97_C1
zmp_ph97_D2
zmp_ph97_D5
zmp_ph111_G5
zmp_ph111_G11
zmp_ph111_H1
zmp_ph111_B1a
zmp_ph111_B5
zmp_ph111_B8
zmp_ph111_B6
zmp_ph111_B7
zmp_ph111_B9
zmp_ph111_B10
zmp_ph114_G4
zmp_ph114_G11
zmp_ph114_H1
zmp_ph114_H2
zmp_ph114_A9
zmp_ph114_A11
zmp_ph114_B2
zmp_ph114_B4
zmp_ph114_D8
zmp_ph114_E2

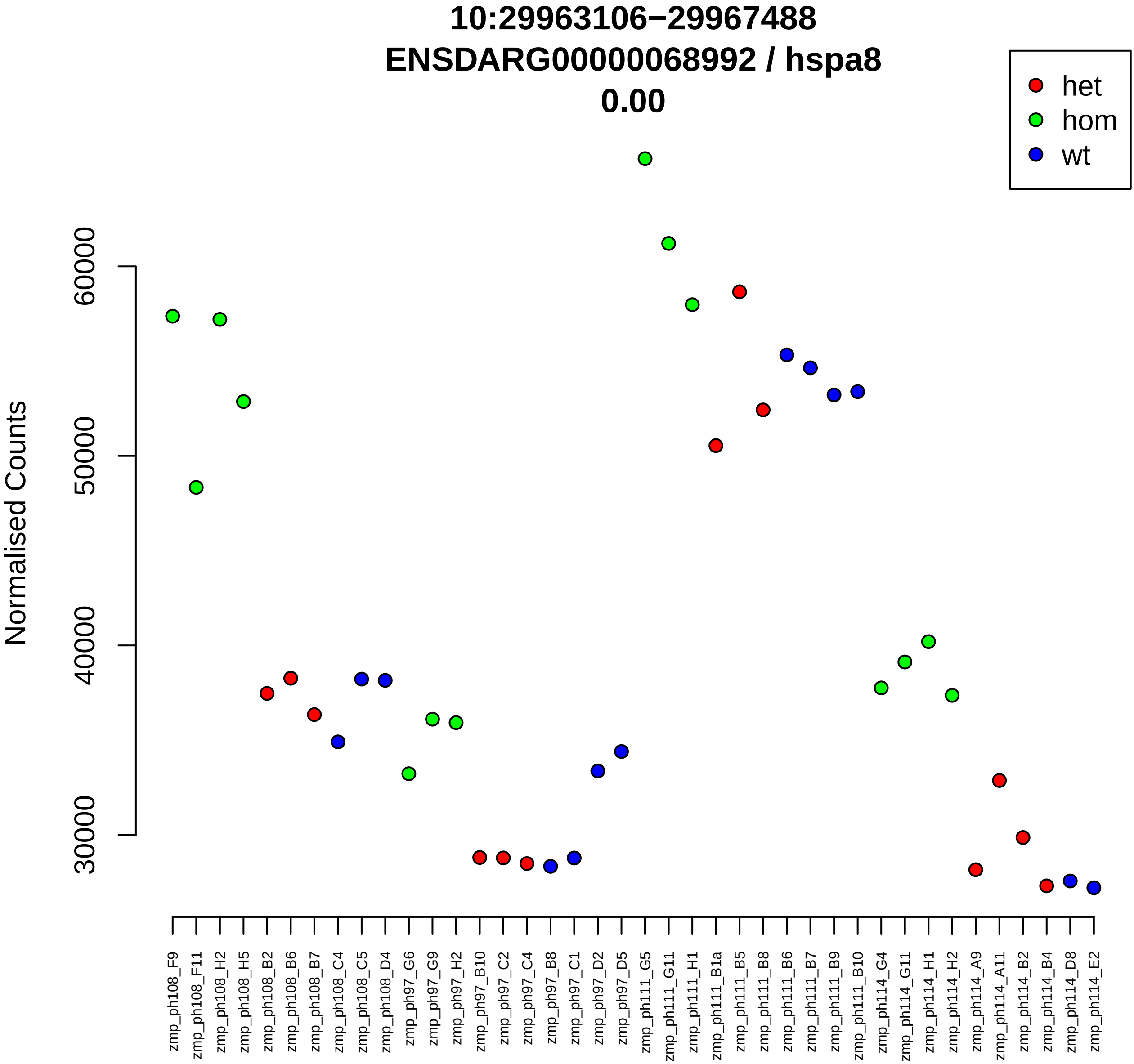
10:29963106−29967488
ENSDARG00000068992 / hspa8
0.00
het
hom
wt
Normalised Counts
30000
40000
50000
60000
zmp_ph108_F9
zmp_ph108_F11
zmp_ph108_H2
zmp_ph108_H5
zmp_ph108_B2
zmp_ph108_B6
zmp_ph108_B7
zmp_ph108_C4
zmp_ph108_C5
zmp_ph108_D4
zmp_ph97_G6
zmp_ph97_G9
zmp_ph97_H2
zmp_ph97_B10
zmp_ph97_C2
zmp_ph97_C4
zmp_ph97_B8
zmp_ph97_C1
zmp_ph97_D2
zmp_ph97_D5
zmp_ph111_G5
zmp_ph111_G11
zmp_ph111_H1
zmp_ph111_B1a
zmp_ph111_B5
zmp_ph111_B8
zmp_ph111_B6
zmp_ph111_B7
zmp_ph111_B9
zmp_ph111_B10
zmp_ph114_G4
zmp_ph114_G11
zmp_ph114_H1
zmp_ph114_H2
zmp_ph114_A9
zmp_ph114_A11
zmp_ph114_B2
zmp_ph114_B4
zmp_ph114_D8
zmp_ph114_E2

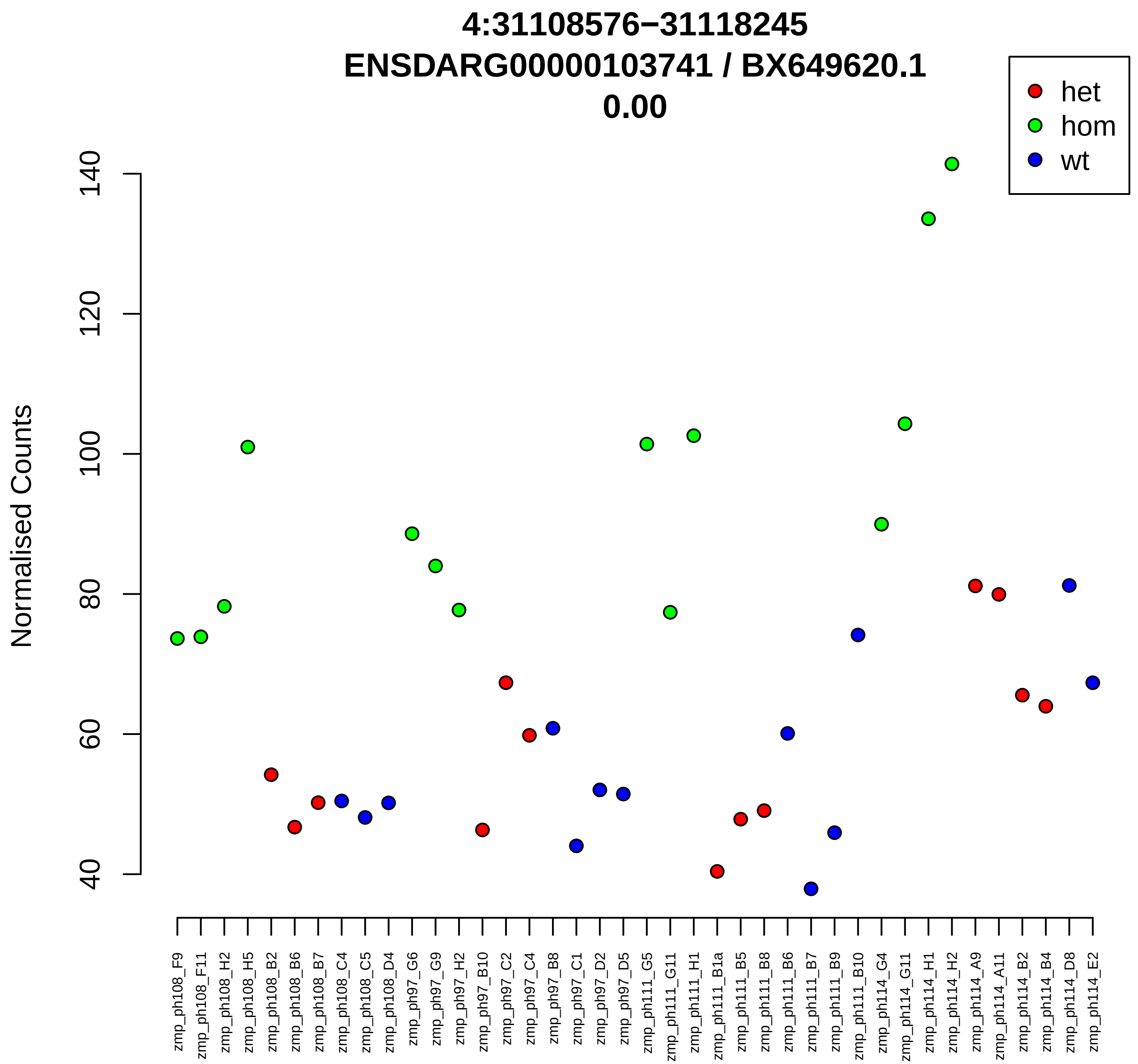
4:31108576−31118245
ENSDARG00000103741 / BX649620.1
0.00
het
hom
wt
Normalised Counts
40
60
80
100
120
140
zmp_ph108_F9
zmp_ph108_F11
zmp_ph108_H2
zmp_ph108_H5
zmp_ph108_B2
zmp_ph108_B6
zmp_ph108_B7
zmp_ph108_C4
zmp_ph108_C5
zmp_ph108_D4
zmp_ph97_G6
zmp_ph97_G9
zmp_ph97_H2
zmp_ph97_B10
zmp_ph97_C2
zmp_ph97_C4
zmp_ph97_B8
zmp_ph97_C1
zmp_ph97_D2
zmp_ph97_D5
zmp_ph111_G5
zmp_ph111_G11
zmp_ph111_H1
zmp_ph111_B1a
zmp_ph111_B5
zmp_ph111_B8
zmp_ph111_B6
zmp_ph111_B7
zmp_ph111_B9
zmp_ph111_B10
zmp_ph114_G4
zmp_ph114_G11
zmp_ph114_H1
zmp_ph114_H2
zmp_ph114_A9
zmp_ph114_A11
zmp_ph114_B2
zmp_ph114_B4
zmp_ph114_D8
zmp_ph114_E2

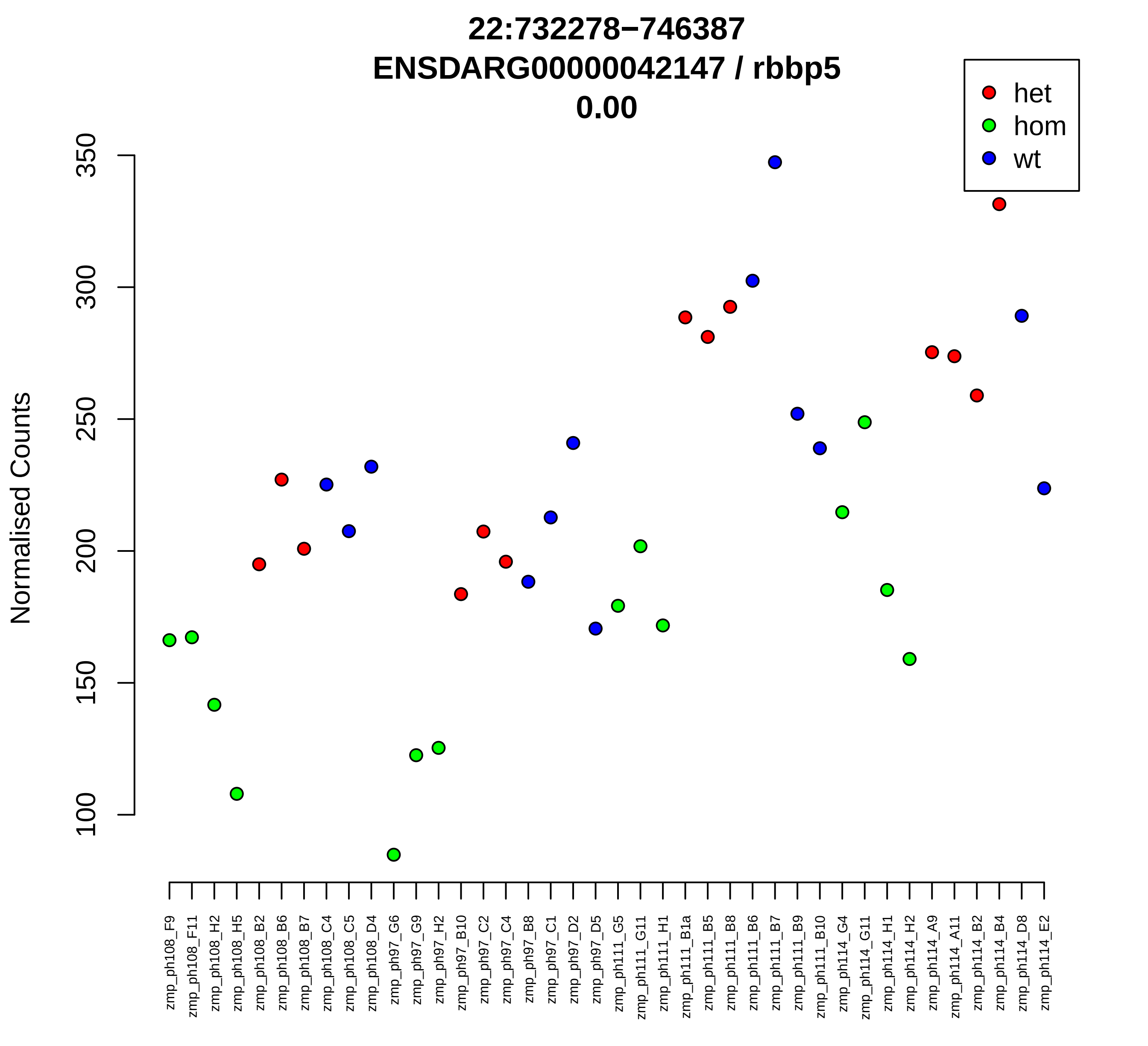
22:732278−746387
ENSDARG00000042147 / rbbp5
0.00
het
hom
wt
Normalised Counts
100
150
200
250
300
350
zmp_ph108_F9
zmp_ph108_F11
zmp_ph108_H2
zmp_ph108_H5
zmp_ph108_B2
zmp_ph108_B6
zmp_ph108_B7
zmp_ph108_C4
zmp_ph108_C5
zmp_ph108_D4
zmp_ph97_G6
zmp_ph97_G9
zmp_ph97_H2
zmp_ph97_B10
zmp_ph97_C2
zmp_ph97_C4
zmp_ph97_B8
zmp_ph97_C1
zmp_ph97_D2
zmp_ph97_D5
zmp_ph111_G5
zmp_ph111_G11
zmp_ph111_H1
zmp_ph111_B1a
zmp_ph111_B5
zmp_ph111_B8
zmp_ph111_B6
zmp_ph111_B7
zmp_ph111_B9
zmp_ph111_B10
zmp_ph114_G4
zmp_ph114_G11
zmp_ph114_H1
zmp_ph114_H2
zmp_ph114_A9
zmp_ph114_A11
zmp_ph114_B2
zmp_ph114_B4
zmp_ph114_D8
zmp_ph114_E2

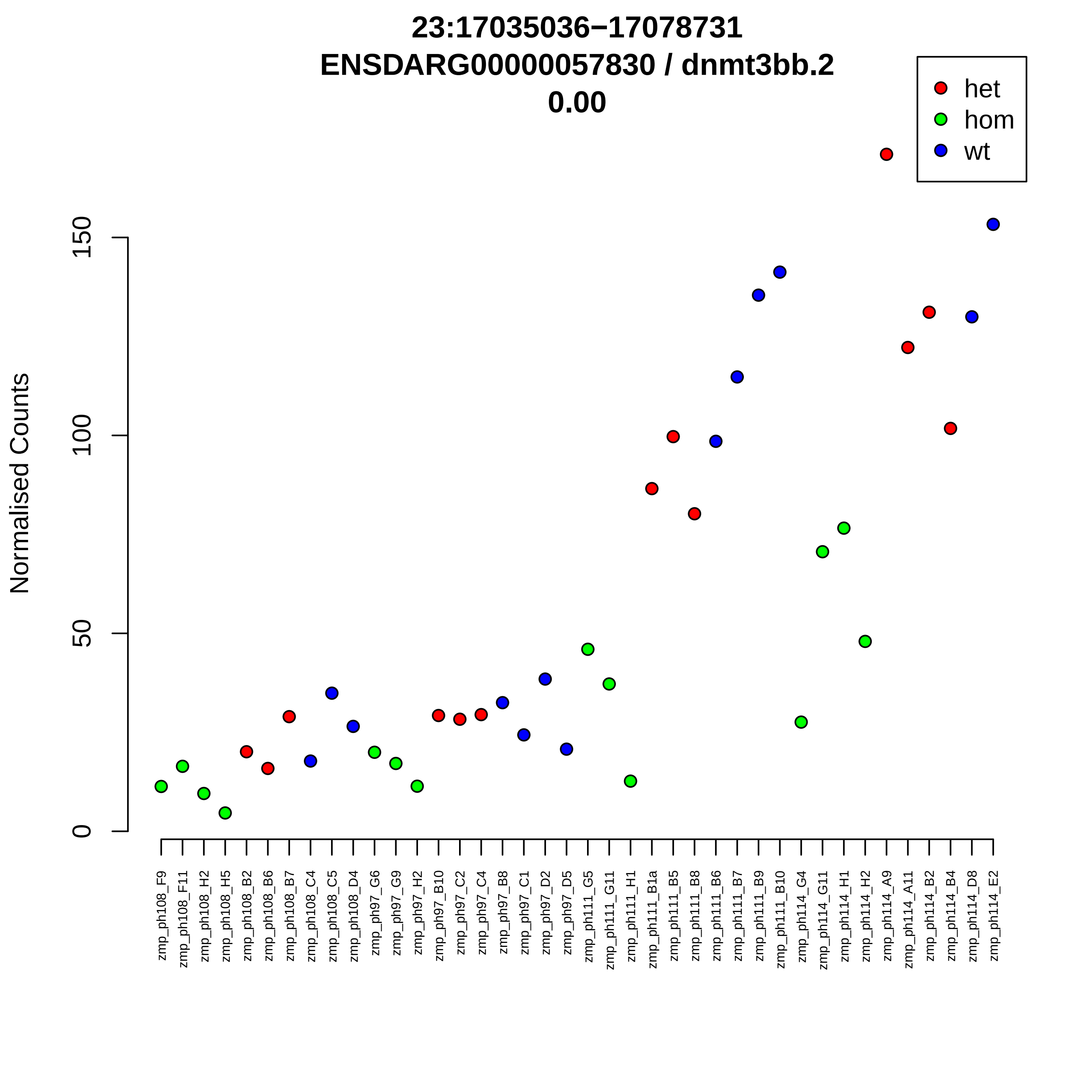

23:17035036−17078731
ENSDARG00000057830 / dnmt3bb.2
0.00
het
hom
wt
Normalised Counts
0
50
100
150
zmp_ph108_F9
zmp_ph108_F11
zmp_ph108_H2
zmp_ph108_H5
zmp_ph108_B2
zmp_ph108_B6
zmp_ph108_B7
zmp_ph108_C4
zmp_ph108_C5
zmp_ph108_D4
zmp_ph97_G6
zmp_ph97_G9
zmp_ph97_H2
zmp_ph97_B10
zmp_ph97_C2
zmp_ph97_C4
zmp_ph97_B8
zmp_ph97_C1
zmp_ph97_D2
zmp_ph97_D5
zmp_ph111_G5
zmp_ph111_G11
zmp_ph111_H1
zmp_ph111_B1a
zmp_ph111_B5
zmp_ph111_B8
zmp_ph111_B6
zmp_ph111_B7
zmp_ph111_B9
zmp_ph111_B10
zmp_ph114_G4
zmp_ph114_G11
zmp_ph114_H1
zmp_ph114_H2
zmp_ph114_A9
zmp_ph114_A11
zmp_ph114_B2
zmp_ph114_B4
zmp_ph114_D8
zmp_ph114_E2

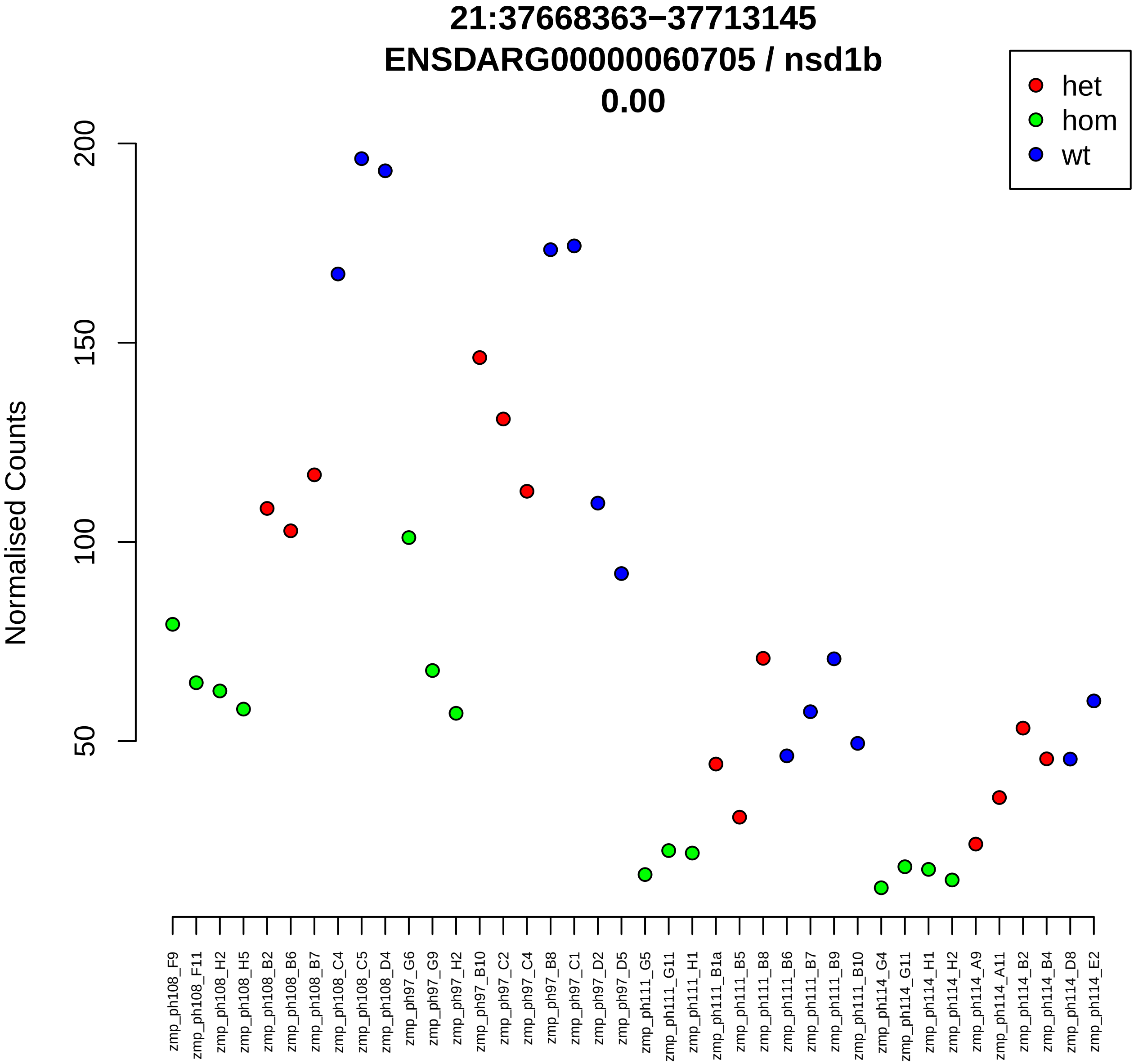

21:37668363−37713145
ENSDARG00000060705 / nsd1b
0.00
Normalised Counts
200
150
100
50
het
hom
wt
zmp_ph108_F9
zmp_ph108_F11
zmp_ph108_H2
zmp_ph108_H5
zmp_ph108_B2
zmp_ph108_B6
zmp_ph108_B7
zmp_ph108_C4
zmp_ph108_C5
zmp_ph108_D4
zmp_ph97_G6
zmp_ph97_G9
zmp_ph97_H2
zmp_ph97_B10
zmp_ph97_C2
zmp_ph97_C4
zmp_ph97_B8
zmp_ph97_C1
zmp_ph97_D2
zmp_ph97_D5
zmp_ph111_G5
zmp_ph111_G11
zmp_ph111_H1
zmp_ph111_B1a
zmp_ph111_B5
zmp_ph111_B8
zmp_ph111_B6
zmp_ph111_B7
zmp_ph111_B9
zmp_ph111_B10
zmp_ph114_G4
zmp_ph114_G11
zmp_ph114_H1
zmp_ph114_H2
zmp_ph114_A9
zmp_ph114_A11
zmp_ph114_B2
zmp_ph114_B4
zmp_ph114_D8
zmp_ph114_E2

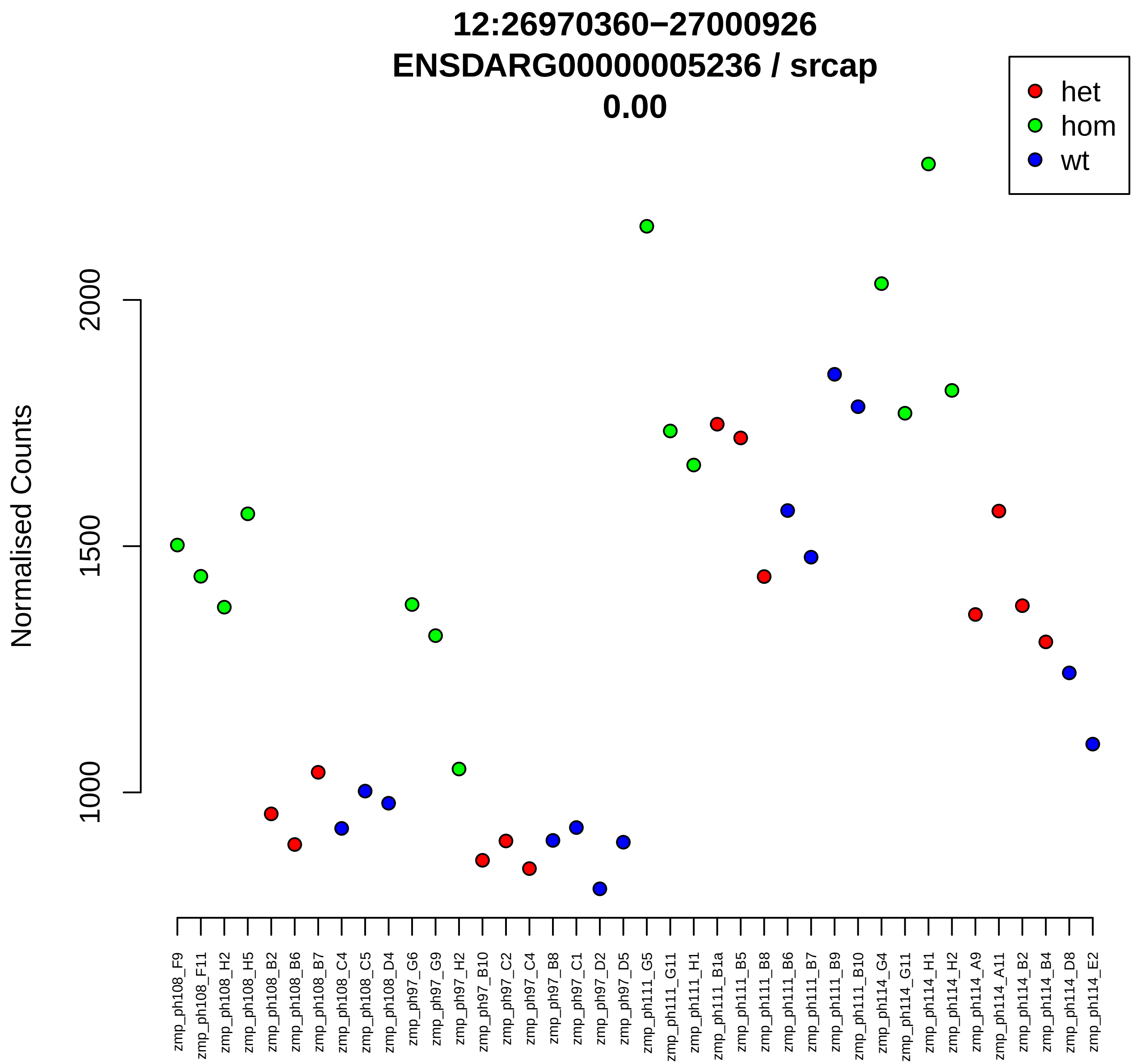
12:26970360−27000926
ENSDARG00000005236 / srcap
0.00
het
hom
wt
Normalised Counts
1000
1500
2000
zmp_ph108_F9
zmp_ph108_F11
zmp_ph108_H2
zmp_ph108_H5
zmp_ph108_B2
zmp_ph108_B6
zmp_ph108_B7
zmp_ph108_C4
zmp_ph108_C5
zmp_ph108_D4
zmp_ph97_G6
zmp_ph97_G9
zmp_ph97_H2
zmp_ph97_B10
zmp_ph97_C2
zmp_ph97_C4
zmp_ph97_B8
zmp_ph97_C1
zmp_ph97_D2
zmp_ph97_D5
zmp_ph111_G5
zmp_ph111_G11
zmp_ph111_H1
zmp_ph111_B1a
zmp_ph111_B5
zmp_ph111_B8
zmp_ph111_B6
zmp_ph111_B7
zmp_ph111_B9
zmp_ph111_B10
zmp_ph114_G4
zmp_ph114_G11
zmp_ph114_H1
zmp_ph114_H2
zmp_ph114_A9
zmp_ph114_A11
zmp_ph114_B2
zmp_ph114_B4
zmp_ph114_D8
zmp_ph114_E2

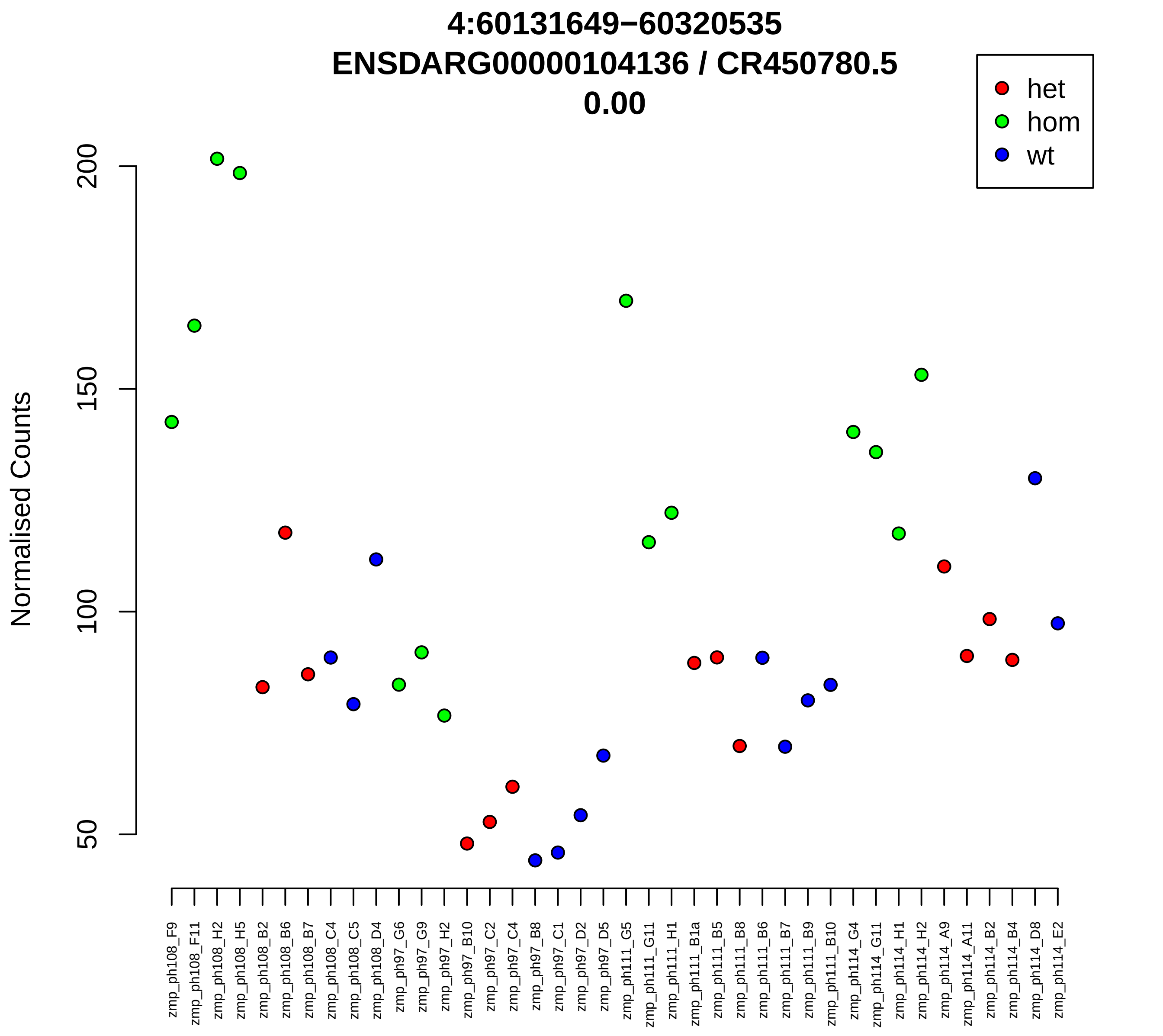

4:60131649−60320535
ENSDARG00000104136 / CR450780.5
0.00
het
hom
wt
Normalised Counts
50
100
150
200
zmp_ph108_F9
zmp_ph108_F11
zmp_ph108_H2
zmp_ph108_H5
zmp_ph108_B2
zmp_ph108_B6
zmp_ph108_B7
zmp_ph108_C4
zmp_ph108_C5
zmp_ph108_D4
zmp_ph97_G6
zmp_ph97_G9
zmp_ph97_H2
zmp_ph97_B10
zmp_ph97_C2
zmp_ph97_C4
zmp_ph97_B8
zmp_ph97_C1
zmp_ph97_D2
zmp_ph97_D5
zmp_ph111_G5
zmp_ph111_G11
zmp_ph111_H1
zmp_ph111_B1a
zmp_ph111_B5
zmp_ph111_B8
zmp_ph111_B6
zmp_ph111_B7
zmp_ph111_B9
zmp_ph111_B10
zmp_ph114_G4
zmp_ph114_G11
zmp_ph114_H1
zmp_ph114_H2
zmp_ph114_A9
zmp_ph114_A11
zmp_ph114_B2
zmp_ph114_B4
zmp_ph114_D8
zmp_ph114_E2

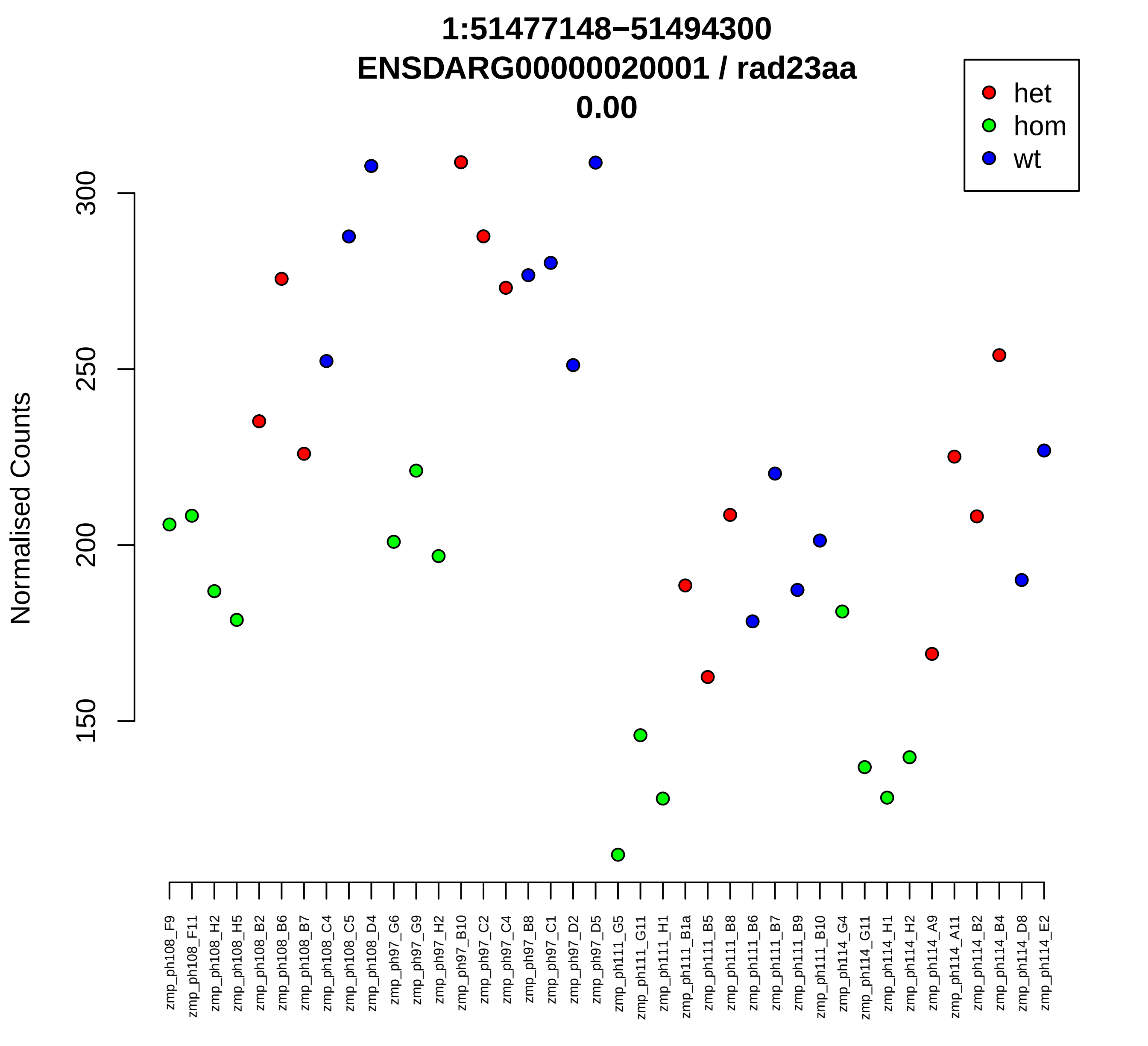

1:51477148−51494300
ENSDARG00000020001 / rad23aa
0.00
het
hom
wt
Normalised Counts
300
250
200
150
zmp_ph108_F9
zmp_ph108_F11
zmp_ph108_H2
zmp_ph108_H5
zmp_ph108_B2
zmp_ph108_B6
zmp_ph108_B7
zmp_ph108_C4
zmp_ph108_C5
zmp_ph108_D4
zmp_ph97_G6
zmp_ph97_G9
zmp_ph97_H2
zmp_ph97_B10
zmp_ph97_C2
zmp_ph97_C4
zmp_ph97_B8
zmp_ph97_C1
zmp_ph97_D2
zmp_ph97_D5
zmp_ph111_G5
zmp_ph111_G11
zmp_ph111_H1
zmp_ph111_B1a
zmp_ph111_B5
zmp_ph111_B8
zmp_ph111_B6
zmp_ph111_B7
zmp_ph111_B9
zmp_ph111_B10
zmp_ph114_G4
zmp_ph114_G11
zmp_ph114_H1
zmp_ph114_H2
zmp_ph114_A9
zmp_ph114_A11
zmp_ph114_B2
zmp_ph114_B4
zmp_ph114_D8
zmp_ph114_E2

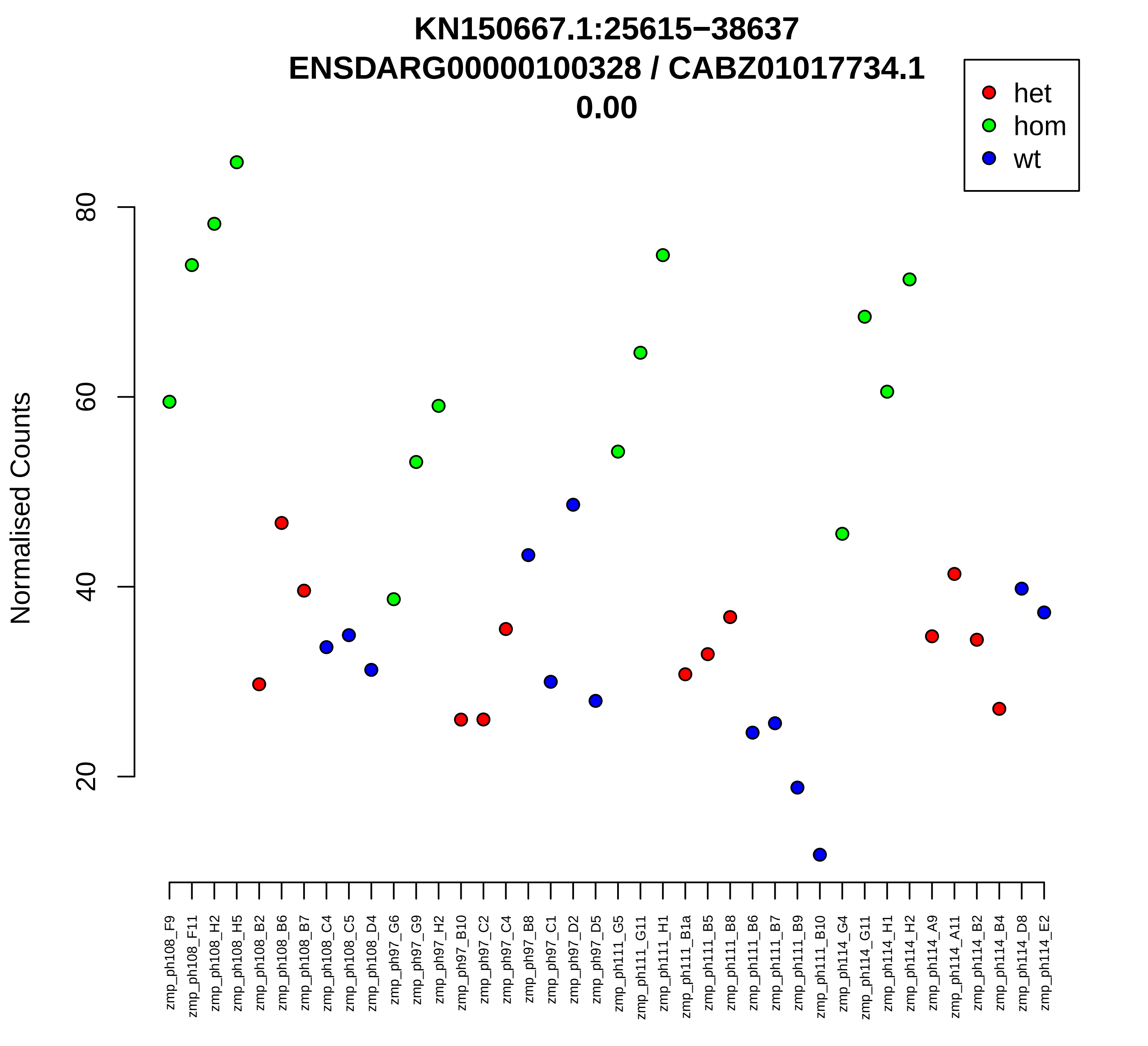
KN150667.1:25615−38637
ENSDARG00000100328 / CABZ01017734.1
0.00
het
hom
wt
Normalised Counts
20
40
60
80
zmp_ph108_F9
zmp_ph108_F11
zmp_ph108_H2
zmp_ph108_H5
zmp_ph108_B2
zmp_ph108_B6
zmp_ph108_B7
zmp_ph108_C4
zmp_ph108_C5
zmp_ph108_D4
zmp_ph97_G6
zmp_ph97_G9
zmp_ph97_H2
zmp_ph97_B10
zmp_ph97_C2
zmp_ph97_C4
zmp_ph97_B8
zmp_ph97_C1
zmp_ph97_D2
zmp_ph97_D5
zmp_ph111_G5
zmp_ph111_G11
zmp_ph111_H1
zmp_ph111_B1a
zmp_ph111_B5
zmp_ph111_B8
zmp_ph111_B6
zmp_ph111_B7
zmp_ph111_B9
zmp_ph111_B10
zmp_ph114_G4
zmp_ph114_G11
zmp_ph114_H1
zmp_ph114_H2
zmp_ph114_A9
zmp_ph114_A11
zmp_ph114_B2
zmp_ph114_B4
zmp_ph114_D8
zmp_ph114_E2

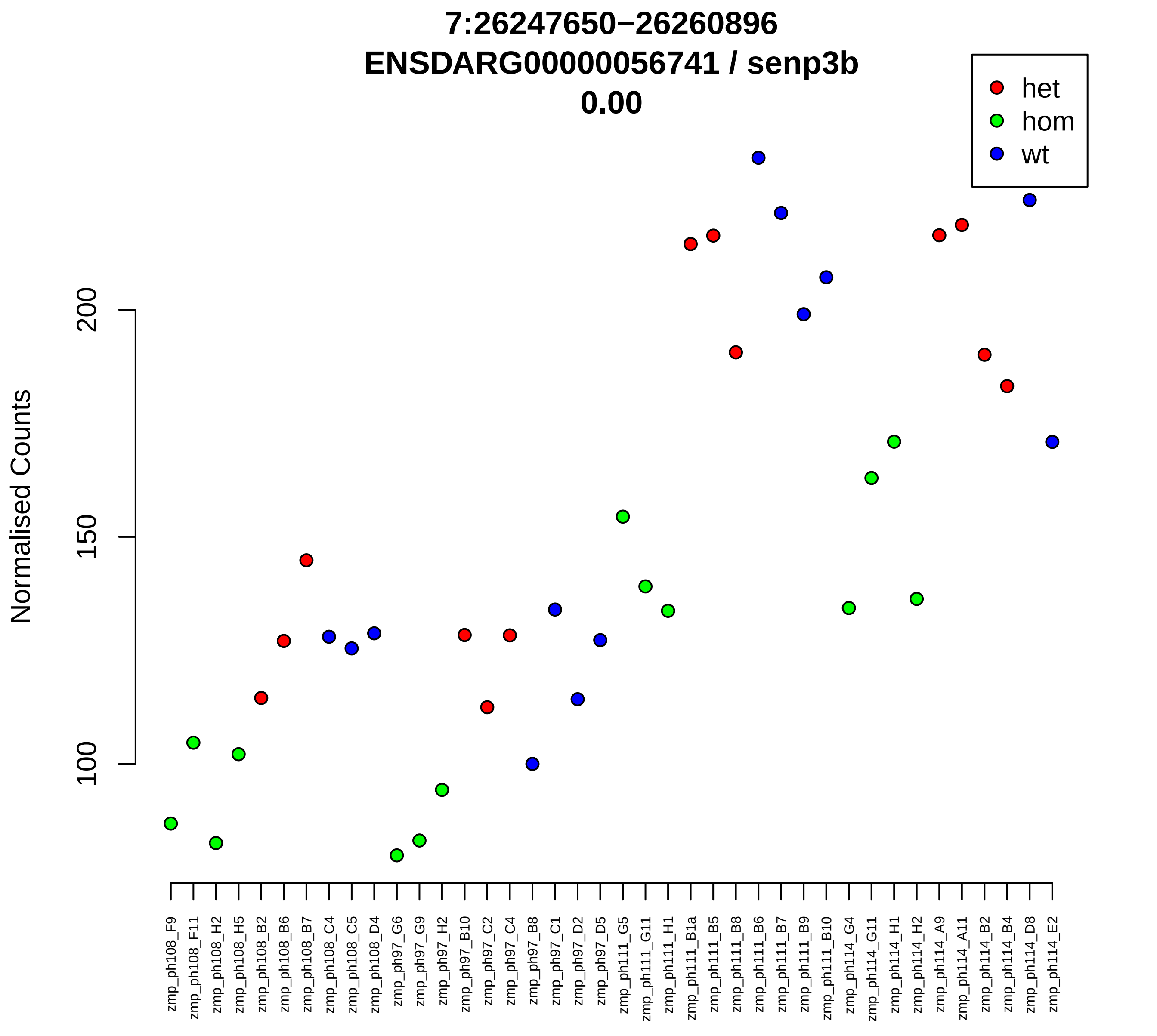

7:26247650−26260896
ENSDARG00000056741 / senp3b
0.00
het
hom
wt
Normalised Counts
100
150
200
zmp_ph108_F9
zmp_ph108_F11
zmp_ph108_H2
zmp_ph108_H5
zmp_ph108_B2
zmp_ph108_B6
zmp_ph108_B7
zmp_ph108_C4
zmp_ph108_C5
zmp_ph108_D4
zmp_ph97_G6
zmp_ph97_G9
zmp_ph97_H2
zmp_ph97_B10
zmp_ph97_C2
zmp_ph97_C4
zmp_ph97_B8
zmp_ph97_C1
zmp_ph97_D2
zmp_ph97_D5
zmp_ph111_G5
zmp_ph111_G11
zmp_ph111_H1
zmp_ph111_B1a
zmp_ph111_B5
zmp_ph111_B8
zmp_ph111_B6
zmp_ph111_B7
zmp_ph111_B9
zmp_ph111_B10
zmp_ph114_G4
zmp_ph114_G11
zmp_ph114_H1
zmp_ph114_H2
zmp_ph114_A9
zmp_ph114_A11
zmp_ph114_B2
zmp_ph114_B4
zmp_ph114_D8
zmp_ph114_E2

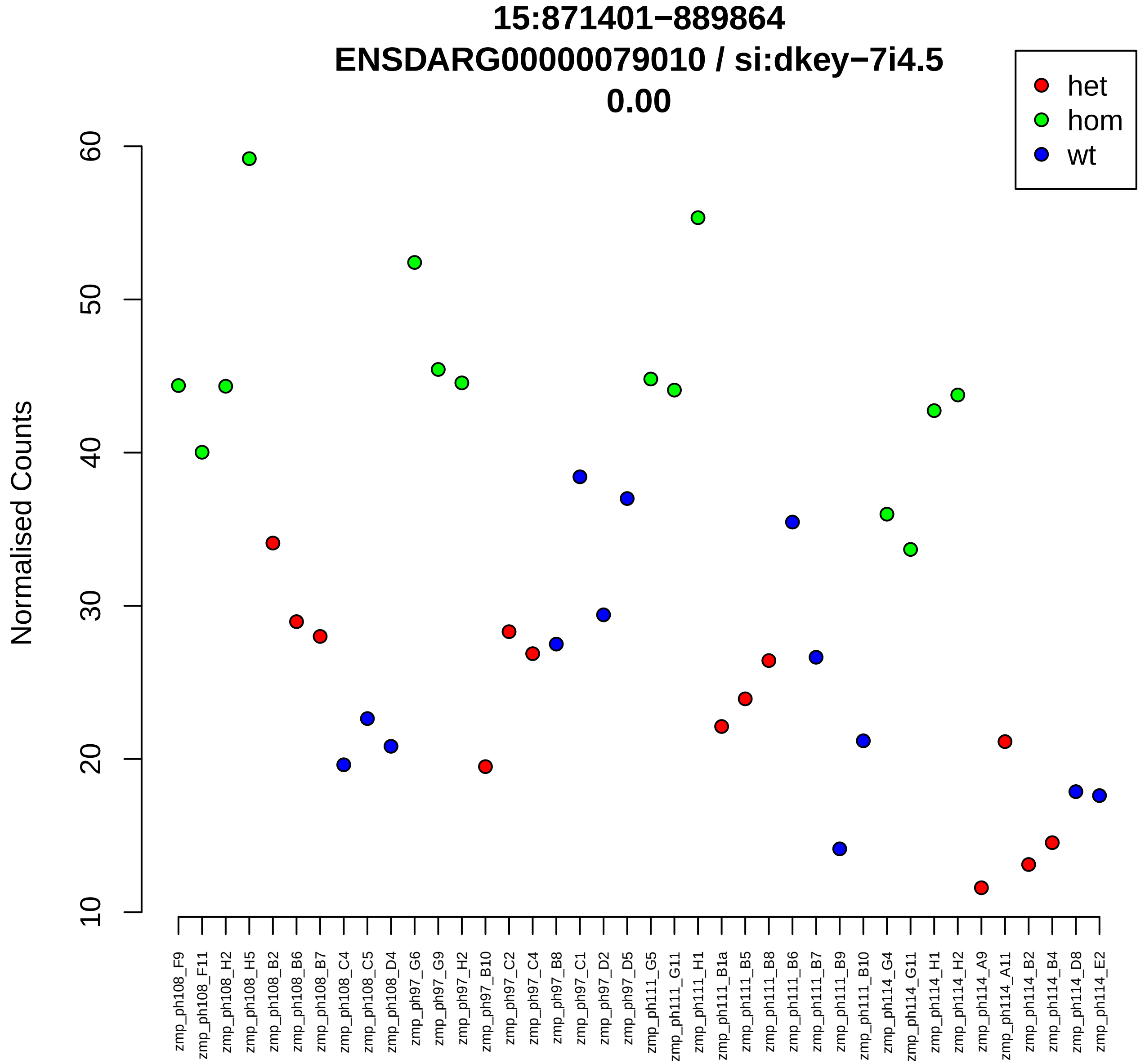

15:871401−889864
ENSDARG00000079010 / si:dkey−7i4.5
0.00
het
hom
wt
Normalised Counts
10
20
30
40
50
60
zmp_ph108_F9
zmp_ph108_F11
zmp_ph108_H2
zmp_ph108_H5
zmp_ph108_B2
zmp_ph108_B6
zmp_ph108_B7
zmp_ph108_C4
zmp_ph108_C5
zmp_ph108_D4
zmp_ph97_G6
zmp_ph97_G9
zmp_ph97_H2
zmp_ph97_B10
zmp_ph97_C2
zmp_ph97_C4
zmp_ph97_B8
zmp_ph97_C1
zmp_ph97_D2
zmp_ph97_D5
zmp_ph111_G5
zmp_ph111_G11
zmp_ph111_H1
zmp_ph111_B1a
zmp_ph111_B5
zmp_ph111_B8
zmp_ph111_B6
zmp_ph111_B7
zmp_ph111_B9
zmp_ph111_B10
zmp_ph114_G4
zmp_ph114_G11
zmp_ph114_H1
zmp_ph114_H2
zmp_ph114_A9
zmp_ph114_A11
zmp_ph114_B2
zmp_ph114_B4
zmp_ph114_D8
zmp_ph114_E2

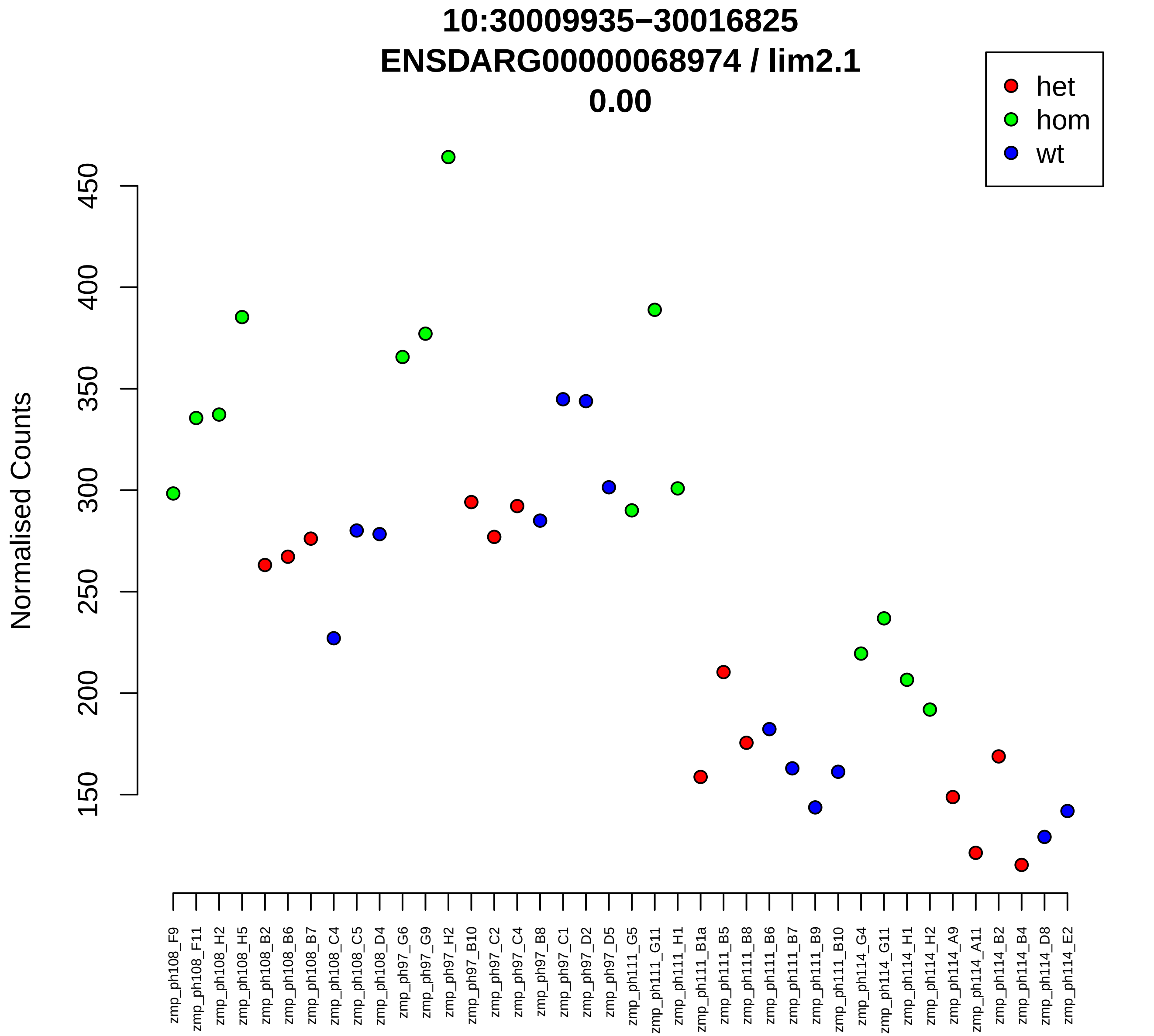

10:30009935−30016825
ENSDARG00000068974 / lim2.1
0.00
het
hom
wt
Normalised Counts
450
400
350
300
250
200
150
zmp_ph108_F9
zmp_ph108_F11
zmp_ph108_H2
zmp_ph108_H5
zmp_ph108_B2
zmp_ph108_B6
zmp_ph108_B7
zmp_ph108_C4
zmp_ph108_C5
zmp_ph108_D4
zmp_ph97_G6
zmp_ph97_G9
zmp_ph97_H2
zmp_ph97_B10
zmp_ph97_C2
zmp_ph97_C4
zmp_ph97_B8
zmp_ph97_C1
zmp_ph97_D2
zmp_ph97_D5
zmp_ph111_G5
zmp_ph111_G11
zmp_ph111_H1
zmp_ph111_B1a
zmp_ph111_B5
zmp_ph111_B8
zmp_ph111_B6
zmp_ph111_B7
zmp_ph111_B9
zmp_ph111_B10
zmp_ph114_G4
zmp_ph114_G11
zmp_ph114_H1
zmp_ph114_H2
zmp_ph114_A9
zmp_ph114_A11
zmp_ph114_B2
zmp_ph114_B4
zmp_ph114_D8
zmp_ph114_E2

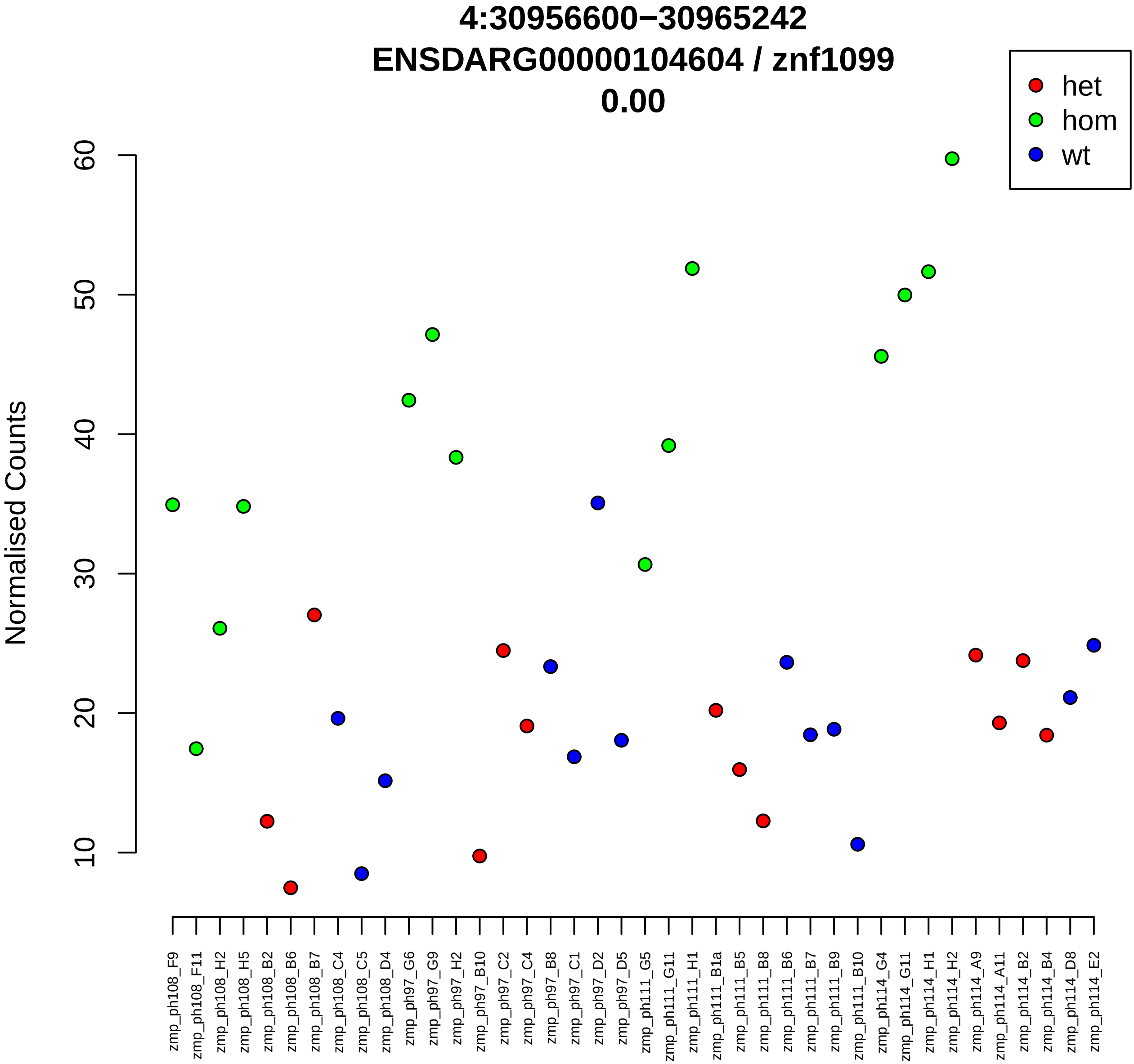
4:30956600−30965242
ENSDARG00000104604 / znf1099
0.00
het
hom
wt
Normalised Counts
10
20
30
40
50
60
zmp_ph108_F9
zmp_ph108_F11
zmp_ph108_H2
zmp_ph108_H5
zmp_ph108_B2
zmp_ph108_B6
zmp_ph108_B7
zmp_ph108_C4
zmp_ph108_C5
zmp_ph108_D4
zmp_ph97_G6
zmp_ph97_G9
zmp_ph97_H2
zmp_ph97_B10
zmp_ph97_C2
zmp_ph97_C4
zmp_ph97_B8
zmp_ph97_C1
zmp_ph97_D2
zmp_ph97_D5
zmp_ph111_G5
zmp_ph111_G11
zmp_ph111_H1
zmp_ph111_B1a
zmp_ph111_B5
zmp_ph111_B8
zmp_ph111_B6
zmp_ph111_B7
zmp_ph111_B9
zmp_ph111_B10
zmp_ph114_G4
zmp_ph114_G11
zmp_ph114_H1
zmp_ph114_H2
zmp_ph114_A9
zmp_ph114_A11
zmp_ph114_B2
zmp_ph114_B4
zmp_ph114_D8
zmp_ph114_E2

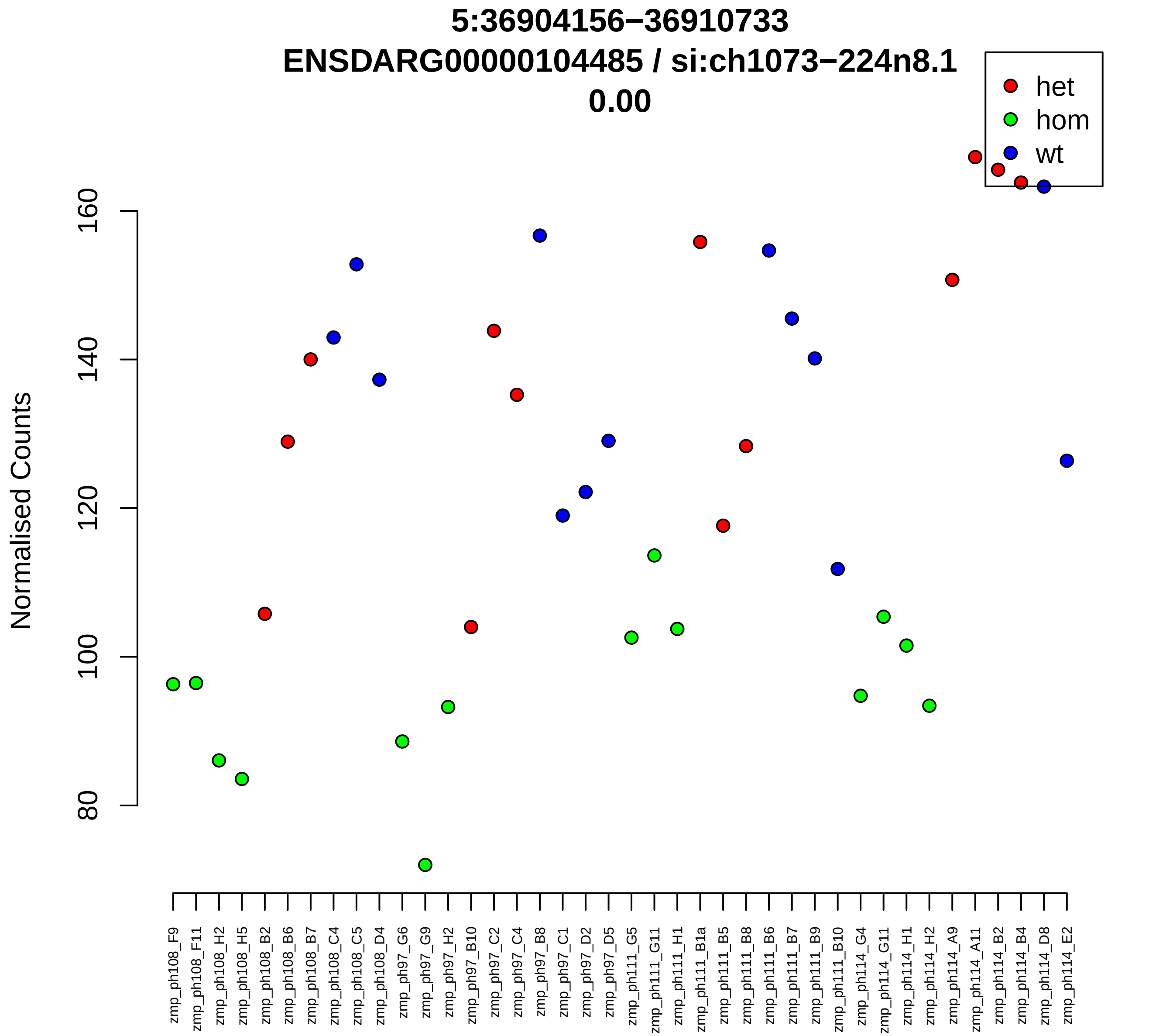

5:36904156−36910733
ENSDARG00000104485 / si:ch1073−224n8.1
0.00
het
hom
wt
Normalised Counts
80
100
120
140
160
zmp_ph108_F9
zmp_ph108_F11
zmp_ph108_H2
zmp_ph108_H5
zmp_ph108_B2
zmp_ph108_B6
zmp_ph108_B7
zmp_ph108_C4
zmp_ph108_C5
zmp_ph108_D4
zmp_ph97_G6
zmp_ph97_G9
zmp_ph97_H2
zmp_ph97_B10
zmp_ph97_C2
zmp_ph97_C4
zmp_ph97_B8
zmp_ph97_C1
zmp_ph97_D2
zmp_ph97_D5
zmp_ph111_G5
zmp_ph111_G11
zmp_ph111_H1
zmp_ph111_B1a
zmp_ph111_B5
zmp_ph111_B8
zmp_ph111_B6
zmp_ph111_B7
zmp_ph111_B9
zmp_ph111_B10
zmp_ph114_G4
zmp_ph114_G11
zmp_ph114_H1
zmp_ph114_H2
zmp_ph114_A9
zmp_ph114_A11
zmp_ph114_B2
zmp_ph114_B4
zmp_ph114_D8
zmp_ph114_E2

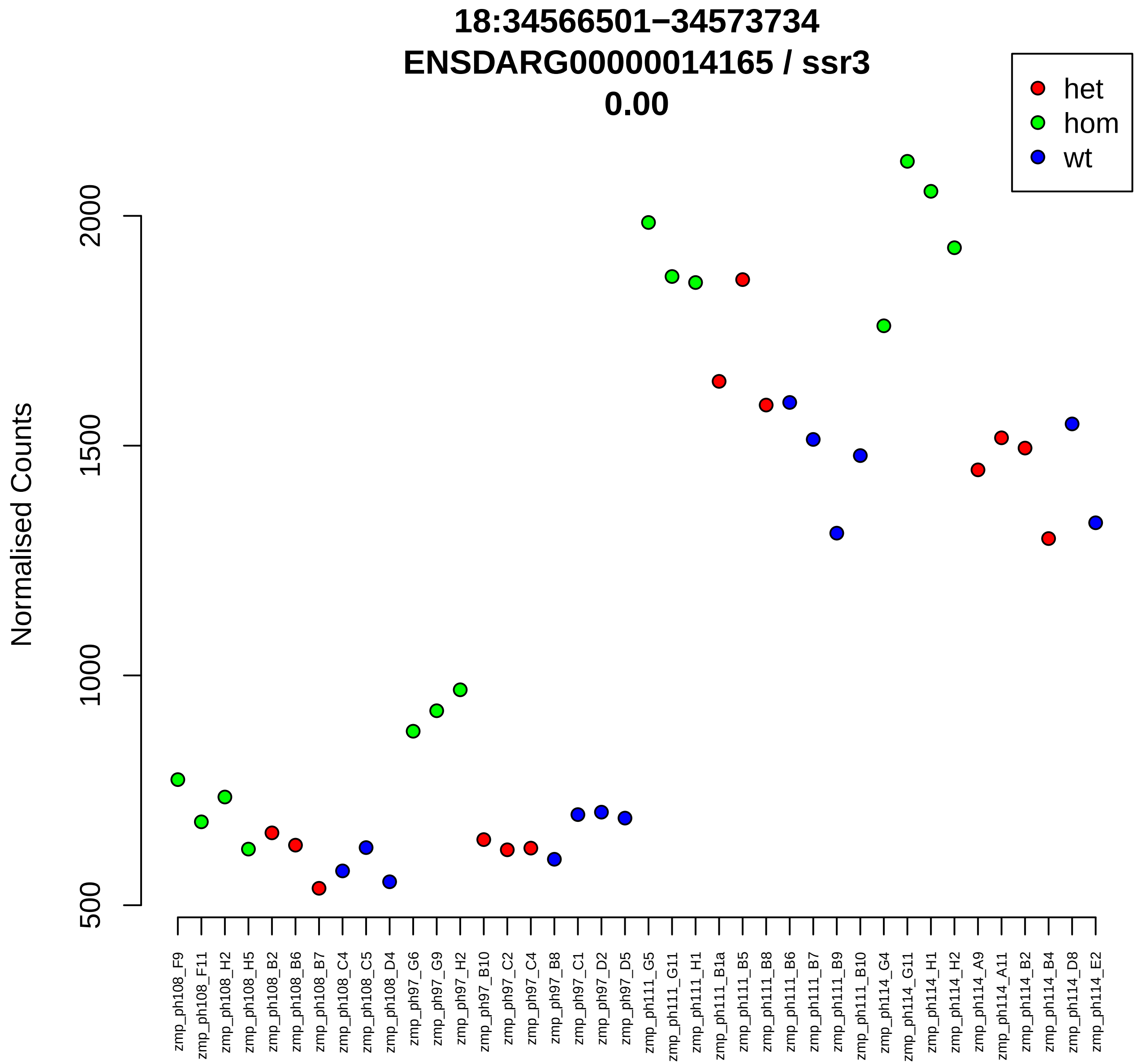

18:34566501−34573734
ENSDARG00000014165 / ssr3
0.00
het
hom
wt
Normalised Counts
500
1000
1500
2000
zmp_ph108_F9
zmp_ph108_F11
zmp_ph108_H2
zmp_ph108_H5
zmp_ph108_B2
zmp_ph108_B6
zmp_ph108_B7
zmp_ph108_C4
zmp_ph108_C5
zmp_ph108_D4
zmp_ph97_G6
zmp_ph97_G9
zmp_ph97_H2
zmp_ph97_B10
zmp_ph97_C2
zmp_ph97_C4
zmp_ph97_B8
zmp_ph97_C1
zmp_ph97_D2
zmp_ph97_D5
zmp_ph111_G5
zmp_ph111_G11
zmp_ph111_H1
zmp_ph111_B1a
zmp_ph111_B5
zmp_ph111_B8
zmp_ph111_B6
zmp_ph111_B7
zmp_ph111_B9
zmp_ph111_B10
zmp_ph114_G4
zmp_ph114_G11
zmp_ph114_H1
zmp_ph114_H2
zmp_ph114_A9
zmp_ph114_A11
zmp_ph114_B2
zmp_ph114_B4
zmp_ph114_D8
zmp_ph114_E2

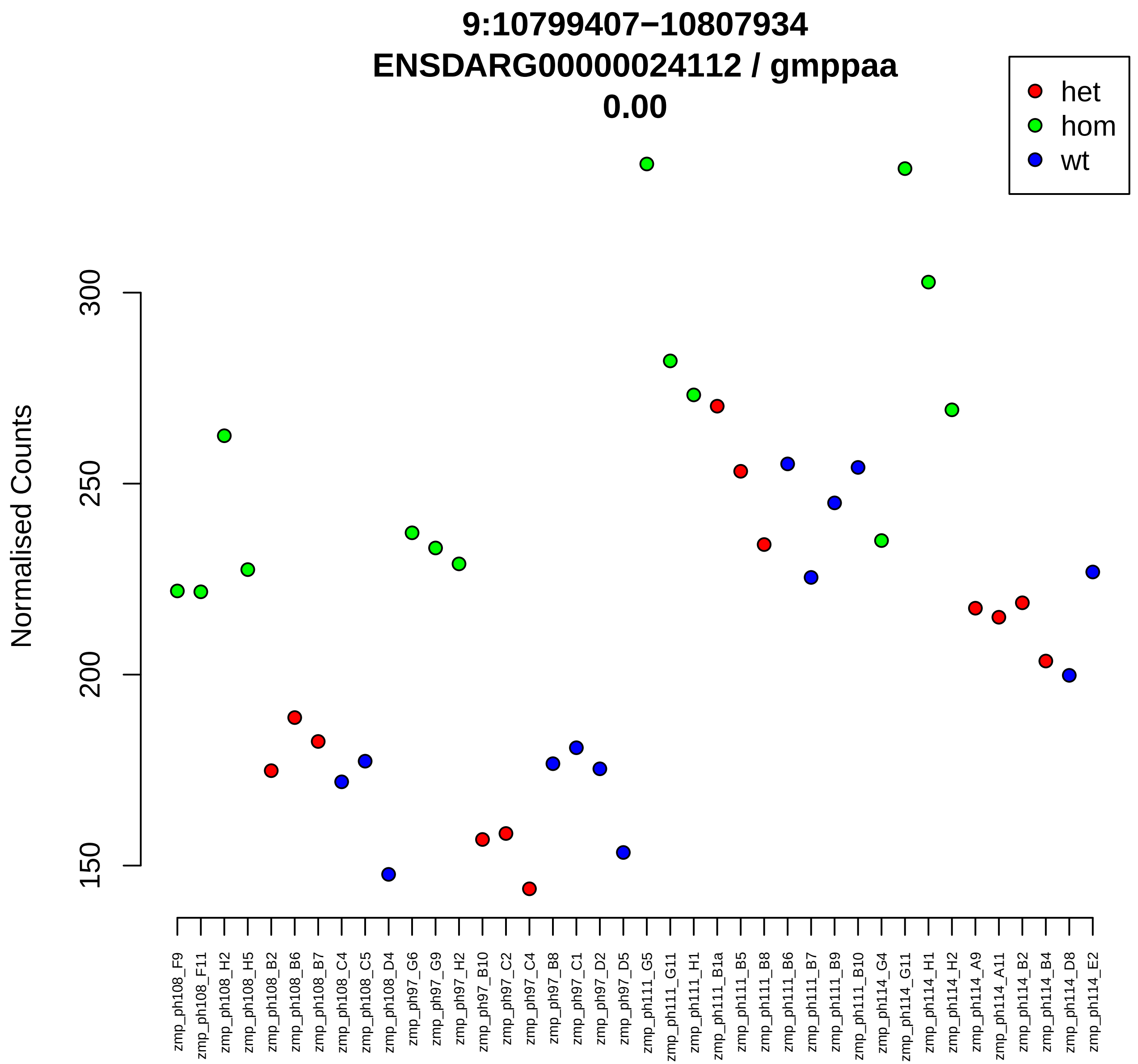

9:10799407−10807934
ENSDARG00000024112 / gmppaa
0.00
het
hom
wt
Normalised Counts
150
200
250
300
zmp_ph108_F9
zmp_ph108_F11
zmp_ph108_H2
zmp_ph108_H5
zmp_ph108_B2
zmp_ph108_B6
zmp_ph108_B7
zmp_ph108_C4
zmp_ph108_C5
zmp_ph108_D4
zmp_ph97_G6
zmp_ph97_G9
zmp_ph97_H2
zmp_ph97_B10
zmp_ph97_C2
zmp_ph97_C4
zmp_ph97_B8
zmp_ph97_C1
zmp_ph97_D2
zmp_ph97_D5
zmp_ph111_G5
zmp_ph111_G11
zmp_ph111_H1
zmp_ph111_B1a
zmp_ph111_B5
zmp_ph111_B8
zmp_ph111_B6
zmp_ph111_B7
zmp_ph111_B9
zmp_ph111_B10
zmp_ph114_G4
zmp_ph114_G11
zmp_ph114_H1
zmp_ph114_H2
zmp_ph114_A9
zmp_ph114_A11
zmp_ph114_B2
zmp_ph114_B4
zmp_ph114_D8
zmp_ph114_E2

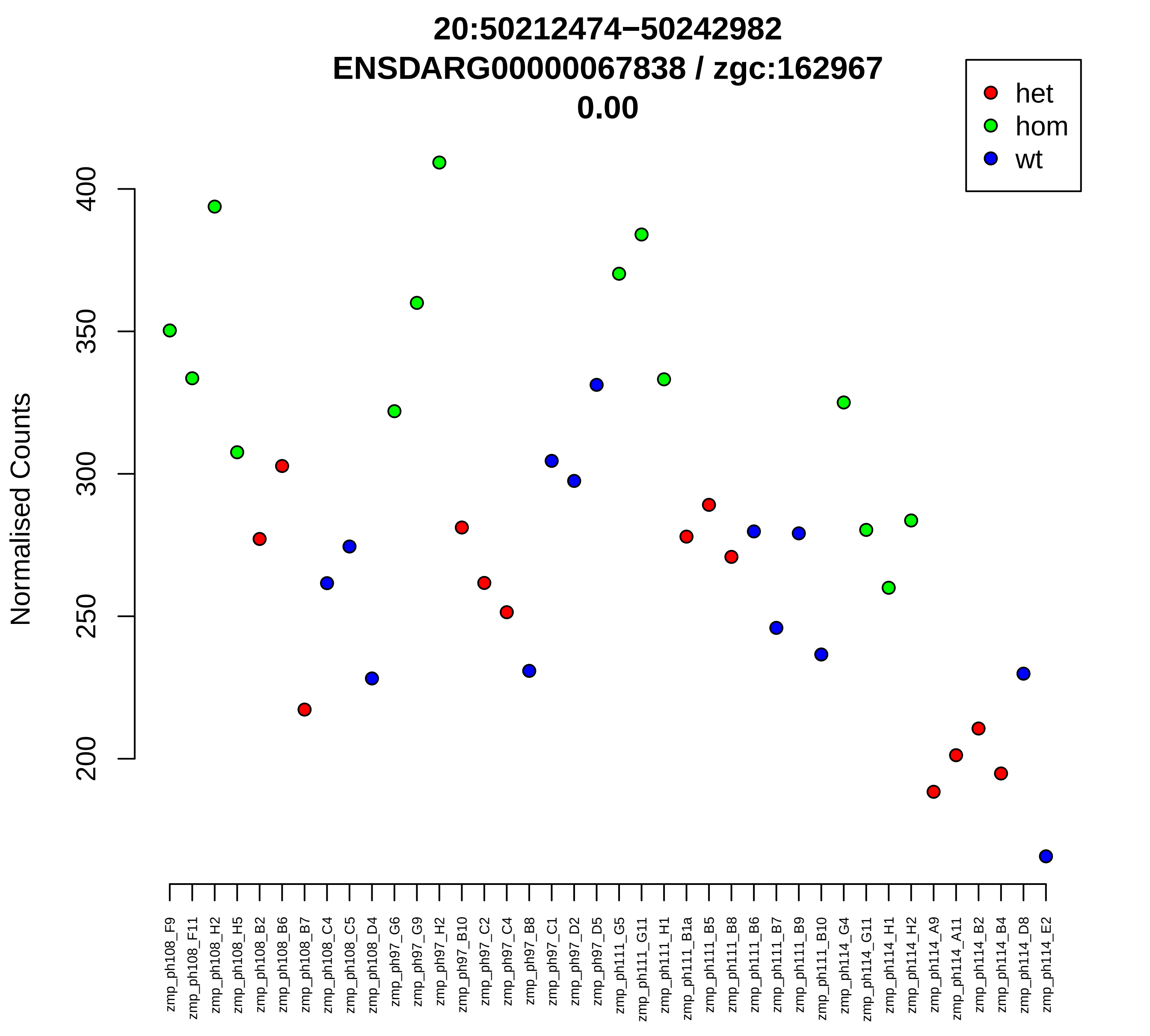

20:50212474−50242982
ENSDARG00000067838 / zgc:162967
0.00
het
hom
wt
Normalised Counts
400
350
300
250
200
zmp_ph108_F9
zmp_ph108_F11
zmp_ph108_H2
zmp_ph108_H5
zmp_ph108_B2
zmp_ph108_B6
zmp_ph108_B7
zmp_ph108_C4
zmp_ph108_C5
zmp_ph108_D4
zmp_ph97_G6
zmp_ph97_G9
zmp_ph97_H2
zmp_ph97_B10
zmp_ph97_C2
zmp_ph97_C4
zmp_ph97_B8
zmp_ph97_C1
zmp_ph97_D2
zmp_ph97_D5
zmp_ph111_G5
zmp_ph111_G11
zmp_ph111_H1
zmp_ph111_B1a
zmp_ph111_B5
zmp_ph111_B8
zmp_ph111_B6
zmp_ph111_B7
zmp_ph111_B9
zmp_ph111_B10
zmp_ph114_G4
zmp_ph114_G11
zmp_ph114_H1
zmp_ph114_H2
zmp_ph114_A9
zmp_ph114_A11
zmp_ph114_B2
zmp_ph114_B4
zmp_ph114_D8
zmp_ph114_E2

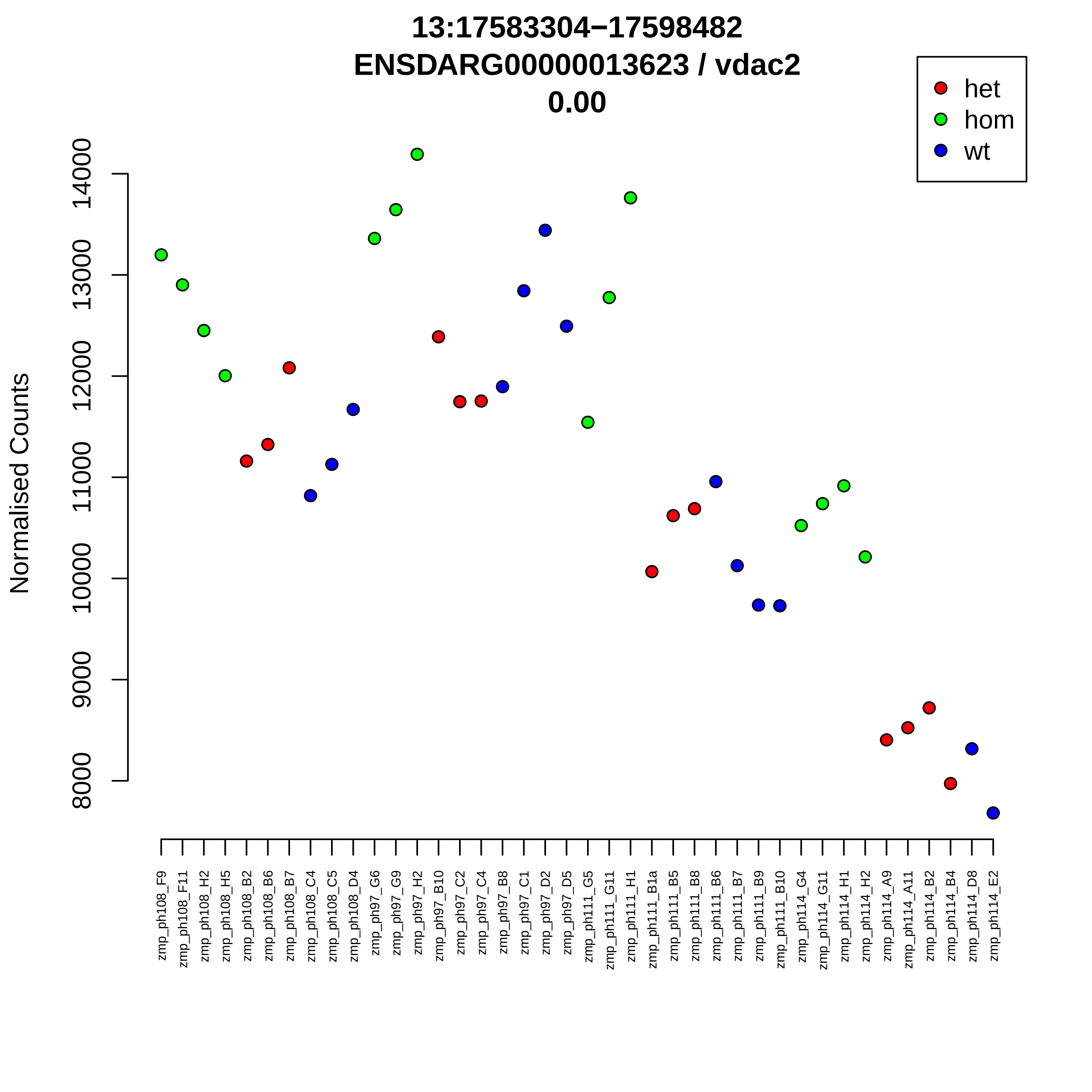

13:17583304−17598482
ENSDARG00000013623 / vdac2
0.00
het
hom
wt
Normalised Counts
8000
9000
10000
11000
12000
13000
14000
zmp_ph108_F9
zmp_ph108_F11
zmp_ph108_H2
zmp_ph108_H5
zmp_ph108_B2
zmp_ph108_B6
zmp_ph108_B7
zmp_ph108_C4
zmp_ph108_C5
zmp_ph108_D4
zmp_ph97_G6
zmp_ph97_G9
zmp_ph97_H2
zmp_ph97_B10
zmp_ph97_C2
zmp_ph97_C4
zmp_ph97_B8
zmp_ph97_C1
zmp_ph97_D2
zmp_ph97_D5
zmp_ph111_G5
zmp_ph111_G11
zmp_ph111_H1
zmp_ph111_B1a
zmp_ph111_B5
zmp_ph111_B8
zmp_ph111_B6
zmp_ph111_B7
zmp_ph111_B9
zmp_ph111_B10
zmp_ph114_G4
zmp_ph114_G11
zmp_ph114_H1
zmp_ph114_H2
zmp_ph114_A9
zmp_ph114_A11
zmp_ph114_B2
zmp_ph114_B4
zmp_ph114_D8
zmp_ph114_E2

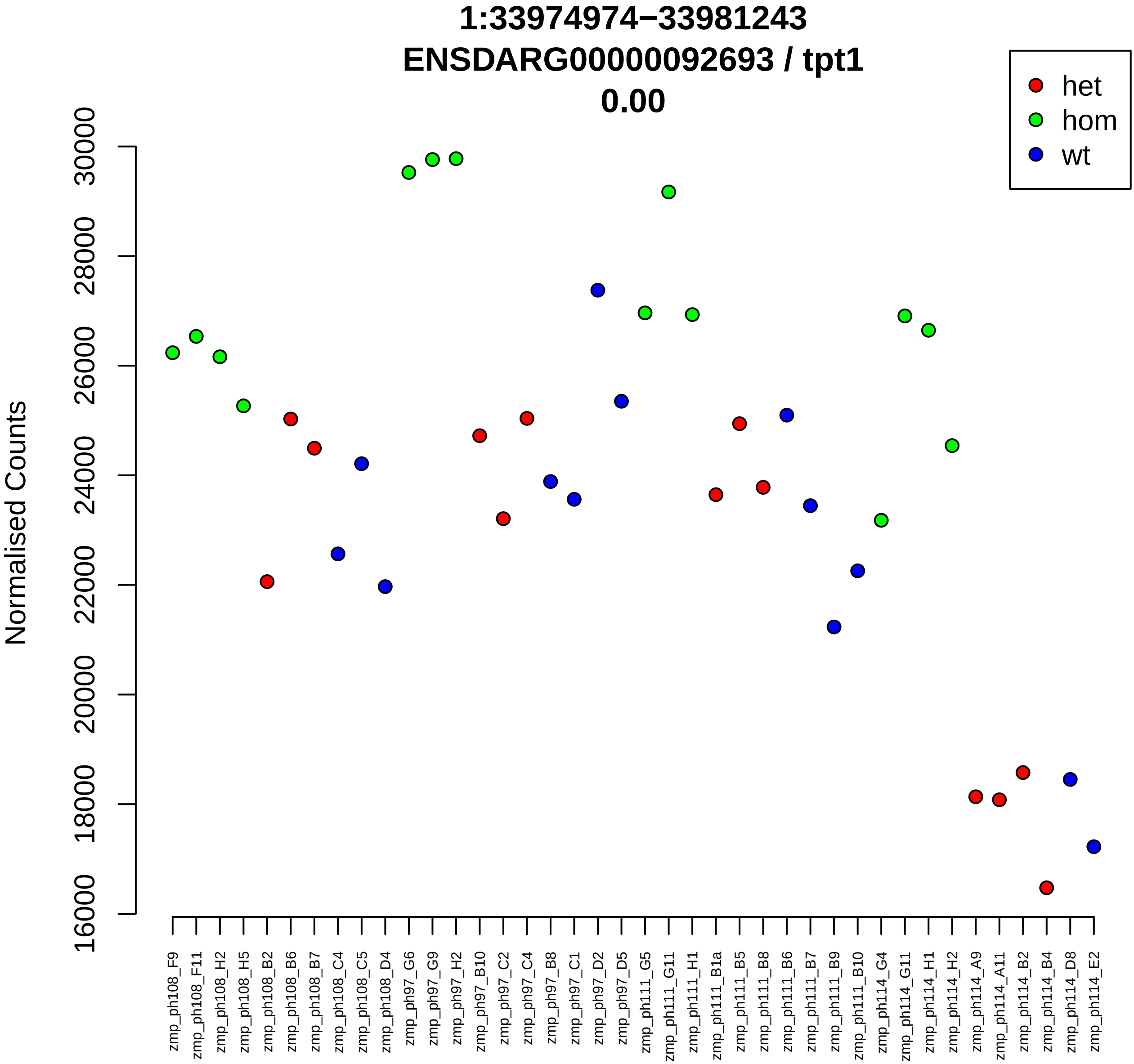

1:33974974−33981243
ENSDARG00000092693 / tpt1
0.00
het
hom
wt
Normalised Counts
16000
18000
20000
22000
24000
26000
28000
30000
zmp_ph108_F9
zmp_ph108_F11
zmp_ph108_H2
zmp_ph108_H5
zmp_ph108_B2
zmp_ph108_B6
zmp_ph108_B7
zmp_ph108_C4
zmp_ph108_C5
zmp_ph108_D4
zmp_ph97_G6
zmp_ph97_G9
zmp_ph97_H2
zmp_ph97_B10
zmp_ph97_C2
zmp_ph97_C4
zmp_ph97_B8
zmp_ph97_C1
zmp_ph97_D2
zmp_ph97_D5
zmp_ph111_G5
zmp_ph111_G11
zmp_ph111_H1
zmp_ph111_B1a
zmp_ph111_B5
zmp_ph111_B8
zmp_ph111_B6
zmp_ph111_B7
zmp_ph111_B9
zmp_ph111_B10
zmp_ph114_G4
zmp_ph114_G11
zmp_ph114_H1
zmp_ph114_H2
zmp_ph114_A9
zmp_ph114_A11
zmp_ph114_B2
zmp_ph114_B4
zmp_ph114_D8
zmp_ph114_E2

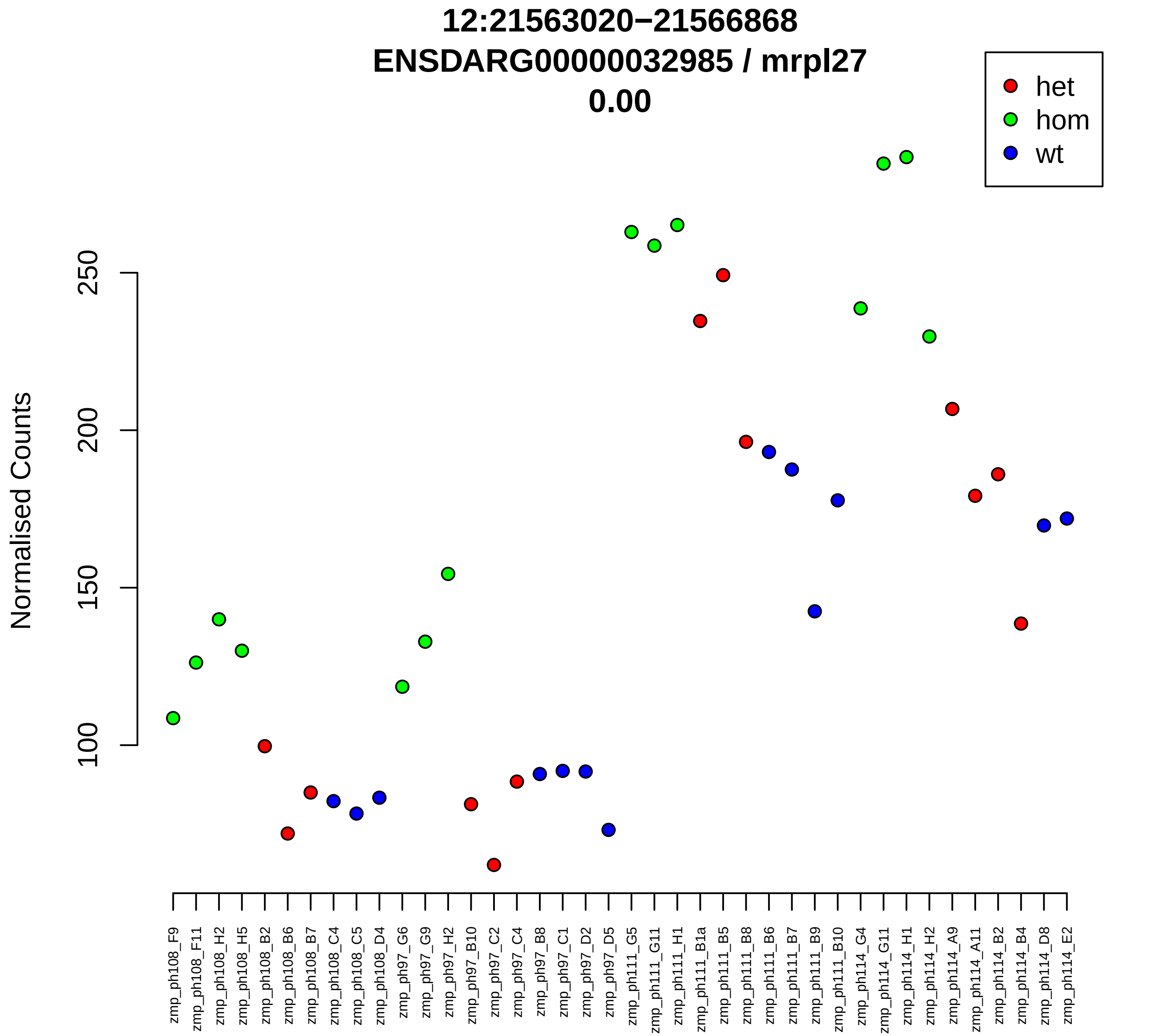
12:21563020−21566868
ENSDARG00000032985 / mrpl27
0.00
het
hom
wt
Normalised Counts
100
150
200
250
zmp_ph108_F9
zmp_ph108_F11
zmp_ph108_H2
zmp_ph108_H5
zmp_ph108_B2
zmp_ph108_B6
zmp_ph108_B7
zmp_ph108_C4
zmp_ph108_C5
zmp_ph108_D4
zmp_ph97_G6
zmp_ph97_G9
zmp_ph97_H2
zmp_ph97_B10
zmp_ph97_C2
zmp_ph97_C4
zmp_ph97_B8
zmp_ph97_C1
zmp_ph97_D2
zmp_ph97_D5
zmp_ph111_G5
zmp_ph111_G11
zmp_ph111_H1
zmp_ph111_B1a
zmp_ph111_B5
zmp_ph111_B8
zmp_ph111_B6
zmp_ph111_B7
zmp_ph111_B9
zmp_ph111_B10
zmp_ph114_G4
zmp_ph114_G11
zmp_ph114_H1
zmp_ph114_H2
zmp_ph114_A9
zmp_ph114_A11
zmp_ph114_B2
zmp_ph114_B4
zmp_ph114_D8
zmp_ph114_E2

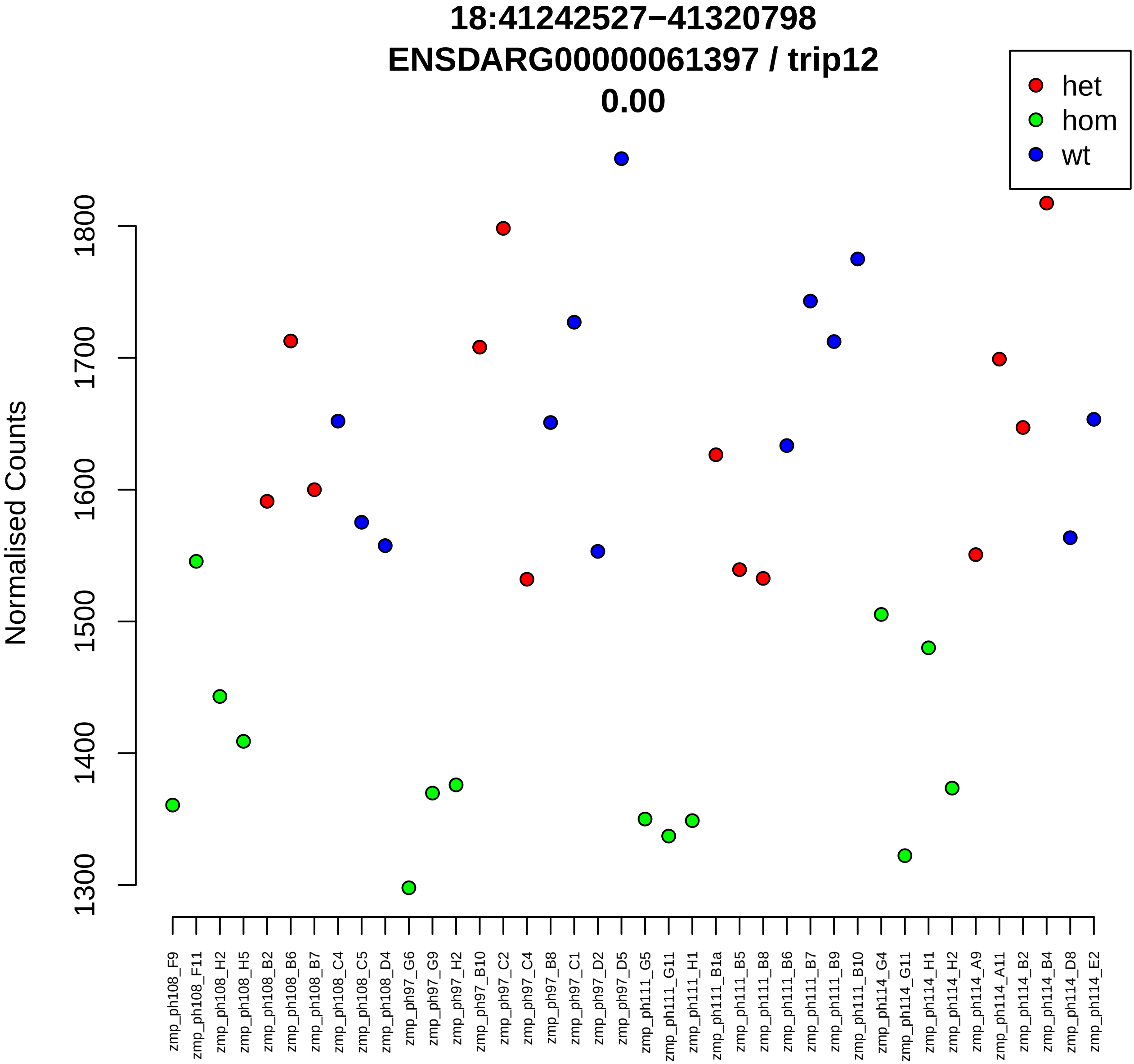

18:41242527−41320798
ENSDARG00000061397 / trip12
0.00
het
hom
wt
Normalised Counts
1300
1400
1500
1600
1700
1800
zmp_ph108_F9
zmp_ph108_F11
zmp_ph108_H2
zmp_ph108_H5
zmp_ph108_B2
zmp_ph108_B6
zmp_ph108_B7
zmp_ph108_C4
zmp_ph108_C5
zmp_ph108_D4
zmp_ph97_G6
zmp_ph97_G9
zmp_ph97_H2
zmp_ph97_B10
zmp_ph97_C2
zmp_ph97_C4
zmp_ph97_B8
zmp_ph97_C1
zmp_ph97_D2
zmp_ph97_D5
zmp_ph111_G5
zmp_ph111_G11
zmp_ph111_H1
zmp_ph111_B1a
zmp_ph111_B5
zmp_ph111_B8
zmp_ph111_B6
zmp_ph111_B7
zmp_ph111_B9
zmp_ph111_B10
zmp_ph114_G4
zmp_ph114_G11
zmp_ph114_H1
zmp_ph114_H2
zmp_ph114_A9
zmp_ph114_A11
zmp_ph114_B2
zmp_ph114_B4
zmp_ph114_D8
zmp_ph114_E2

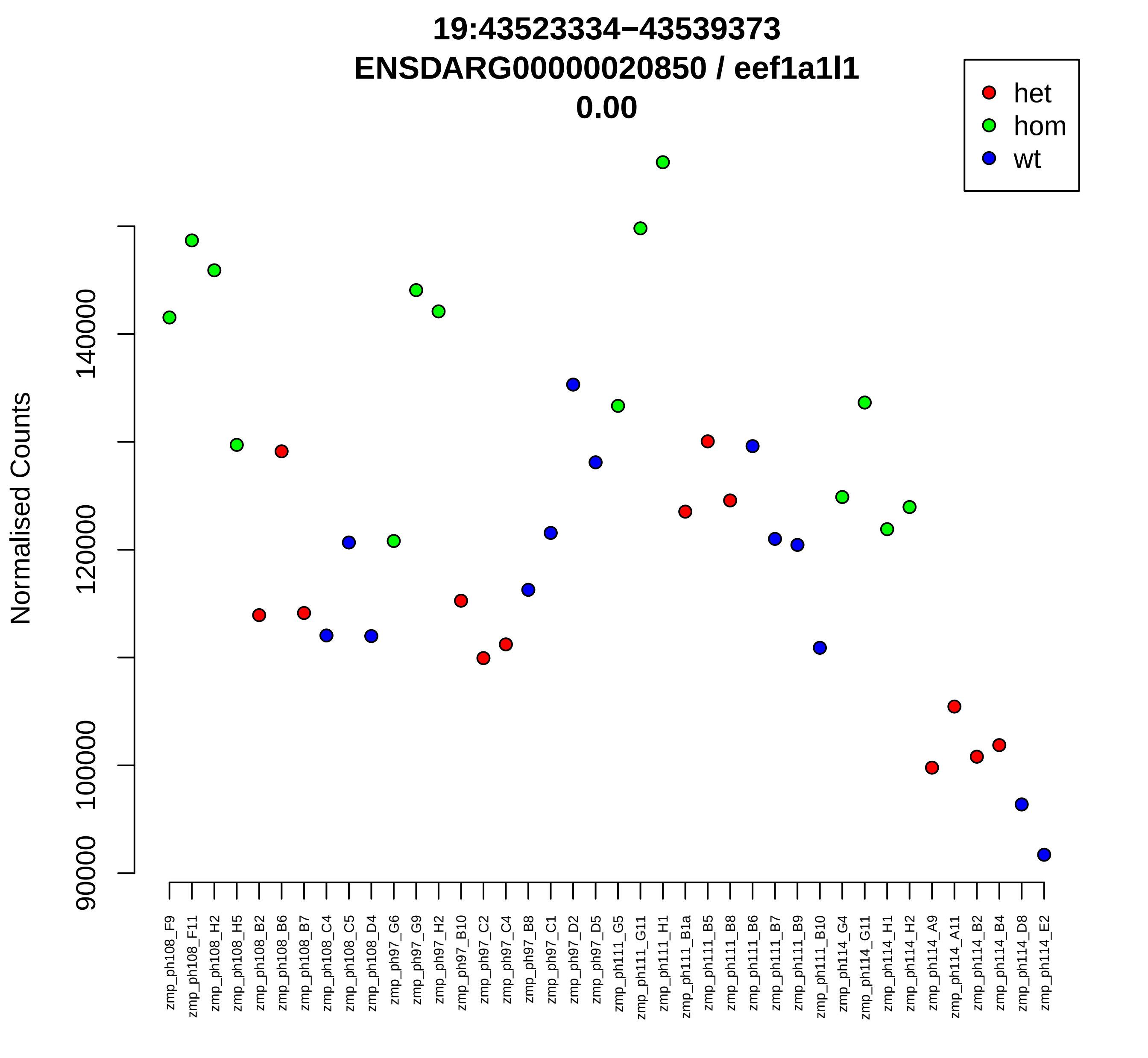

19:43523334−43539373
ENSDARG00000020850 / eef1a1l1
0.00
het
hom
wt
Normalised Counts
90000
100000
120000
140000
zmp_ph108_F9
zmp_ph108_F11
zmp_ph108_H2
zmp_ph108_H5
zmp_ph108_B2
zmp_ph108_B6
zmp_ph108_B7
zmp_ph108_C4
zmp_ph108_C5
zmp_ph108_D4
zmp_ph97_G6
zmp_ph97_G9
zmp_ph97_H2
zmp_ph97_B10
zmp_ph97_C2
zmp_ph97_C4
zmp_ph97_B8
zmp_ph97_C1
zmp_ph97_D2
zmp_ph97_D5
zmp_ph111_G5
zmp_ph111_G11
zmp_ph111_H1
zmp_ph111_B1a
zmp_ph111_B5
zmp_ph111_B8
zmp_ph111_B6
zmp_ph111_B7
zmp_ph111_B9
zmp_ph111_B10
zmp_ph114_G4
zmp_ph114_G11
zmp_ph114_H1
zmp_ph114_H2
zmp_ph114_A9
zmp_ph114_A11
zmp_ph114_B2
zmp_ph114_B4
zmp_ph114_D8
zmp_ph114_E2

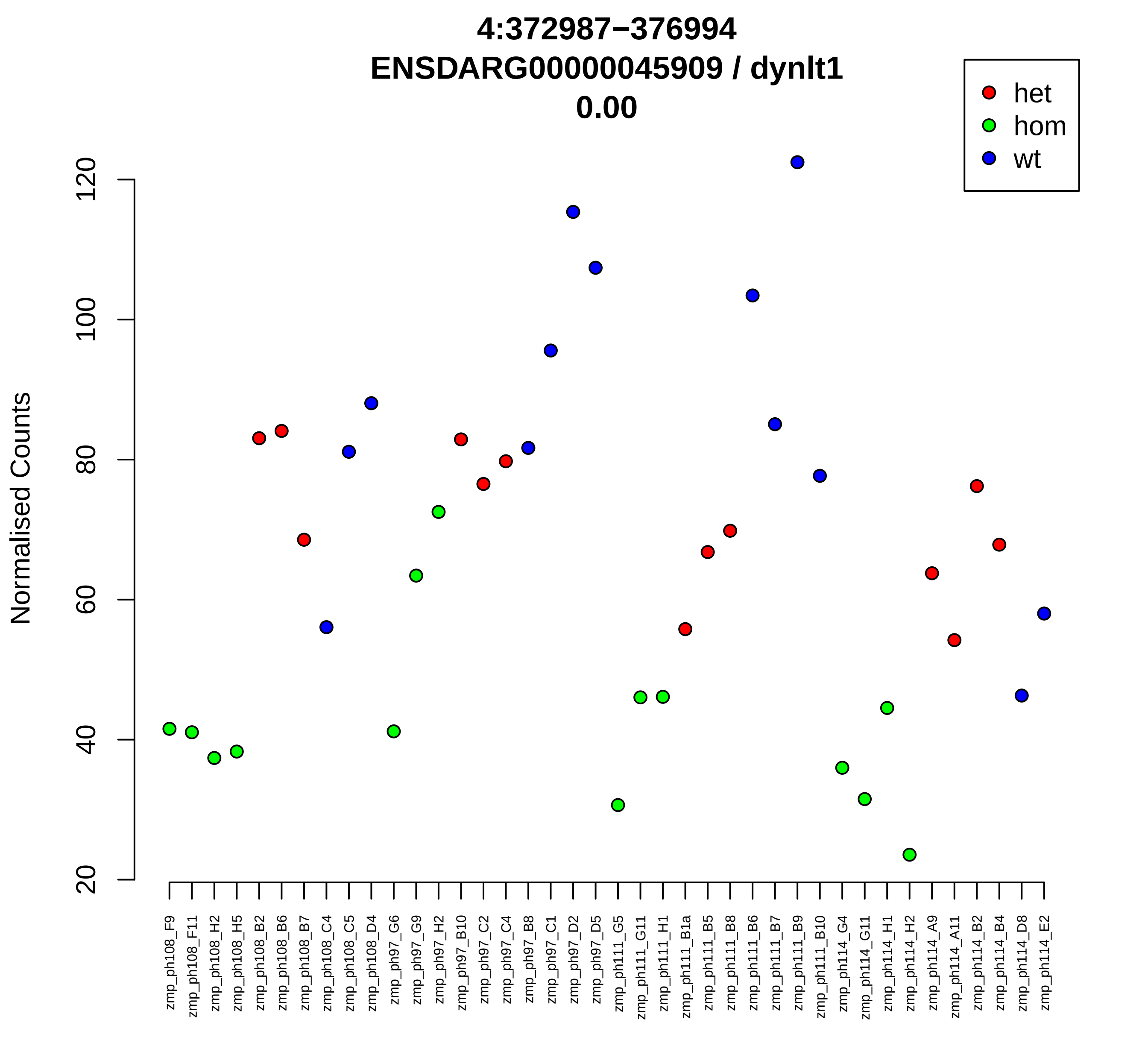
4:372987−376994
ENSDARG00000045909 / dynlt1
0.00
het
hom
wt
Normalised Counts
20
40
60
80
100
120
zmp_ph108_F9
zmp_ph108_F11
zmp_ph108_H2
zmp_ph108_H5
zmp_ph108_B2
zmp_ph108_B6
zmp_ph108_B7
zmp_ph108_C4
zmp_ph108_C5
zmp_ph108_D4
zmp_ph97_G6
zmp_ph97_G9
zmp_ph97_H2
zmp_ph97_B10
zmp_ph97_C2
zmp_ph97_C4
zmp_ph97_B8
zmp_ph97_C1
zmp_ph97_D2
zmp_ph97_D5
zmp_ph111_G5
zmp_ph111_G11
zmp_ph111_H1
zmp_ph111_B1a
zmp_ph111_B5
zmp_ph111_B8
zmp_ph111_B6
zmp_ph111_B7
zmp_ph111_B9
zmp_ph111_B10
zmp_ph114_G4
zmp_ph114_G11
zmp_ph114_H1
zmp_ph114_H2
zmp_ph114_A9
zmp_ph114_A11
zmp_ph114_B2
zmp_ph114_B4
zmp_ph114_D8
zmp_ph114_E2

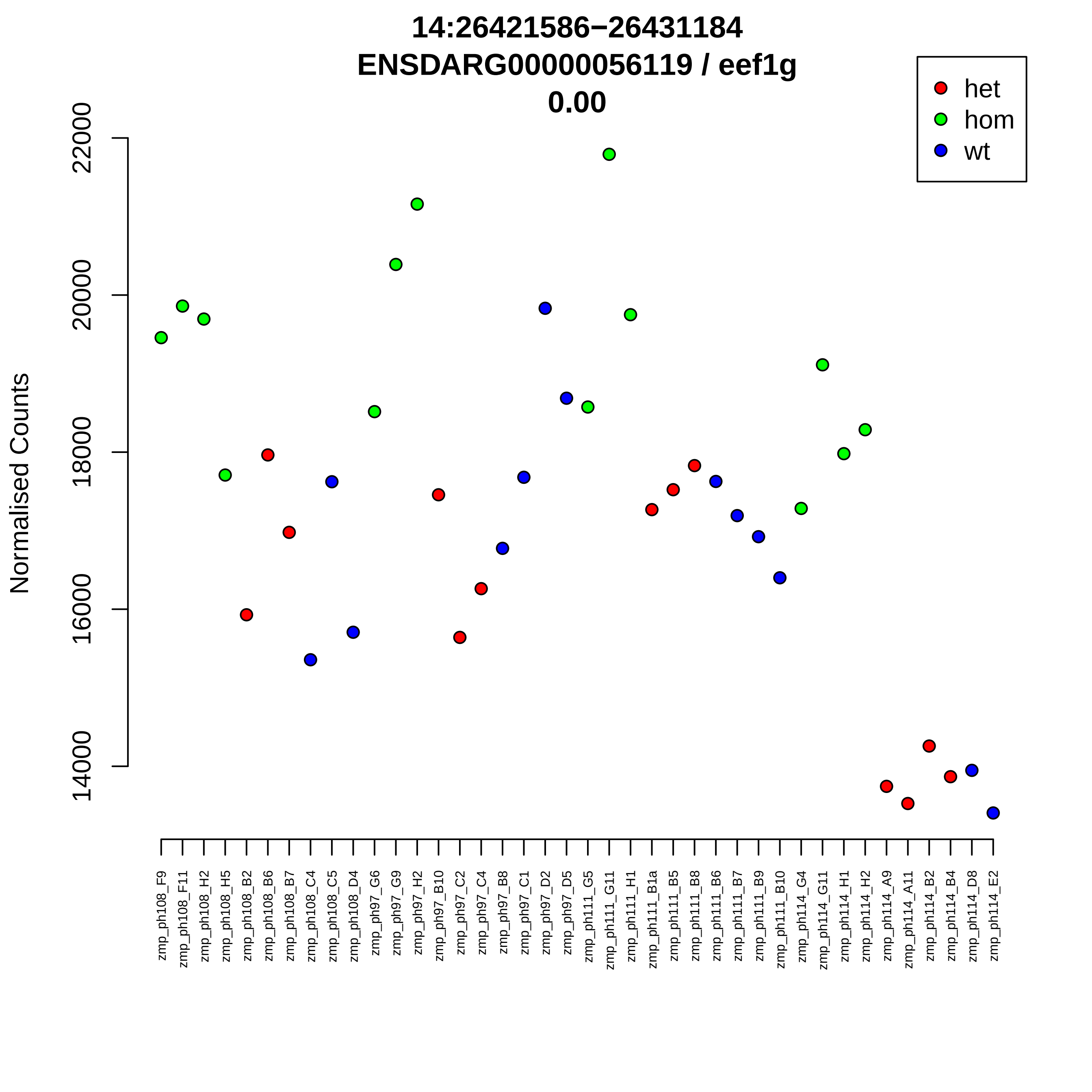
14:26421586−26431184
ENSDARG00000056119 / eef1g
0.00
het
hom
wt
Normalised Counts
22000
20000
18000
16000
14000
zmp_ph108_F9
zmp_ph108_F11
zmp_ph108_H2
zmp_ph108_H5
zmp_ph108_B2
zmp_ph108_B6
zmp_ph108_B7
zmp_ph108_C4
zmp_ph108_C5
zmp_ph108_D4
zmp_ph97_G6
zmp_ph97_G9
zmp_ph97_H2
zmp_ph97_B10
zmp_ph97_C2
zmp_ph97_C4
zmp_ph97_B8
zmp_ph97_C1
zmp_ph97_D2
zmp_ph97_D5
zmp_ph111_G5
zmp_ph111_G11
zmp_ph111_H1
zmp_ph111_B1a
zmp_ph111_B5
zmp_ph111_B8
zmp_ph111_B6
zmp_ph111_B7
zmp_ph111_B9
zmp_ph111_B10
zmp_ph114_G4
zmp_ph114_G11
zmp_ph114_H1
zmp_ph114_H2
zmp_ph114_A9
zmp_ph114_A11
zmp_ph114_B2
zmp_ph114_B4
zmp_ph114_D8
zmp_ph114_E2

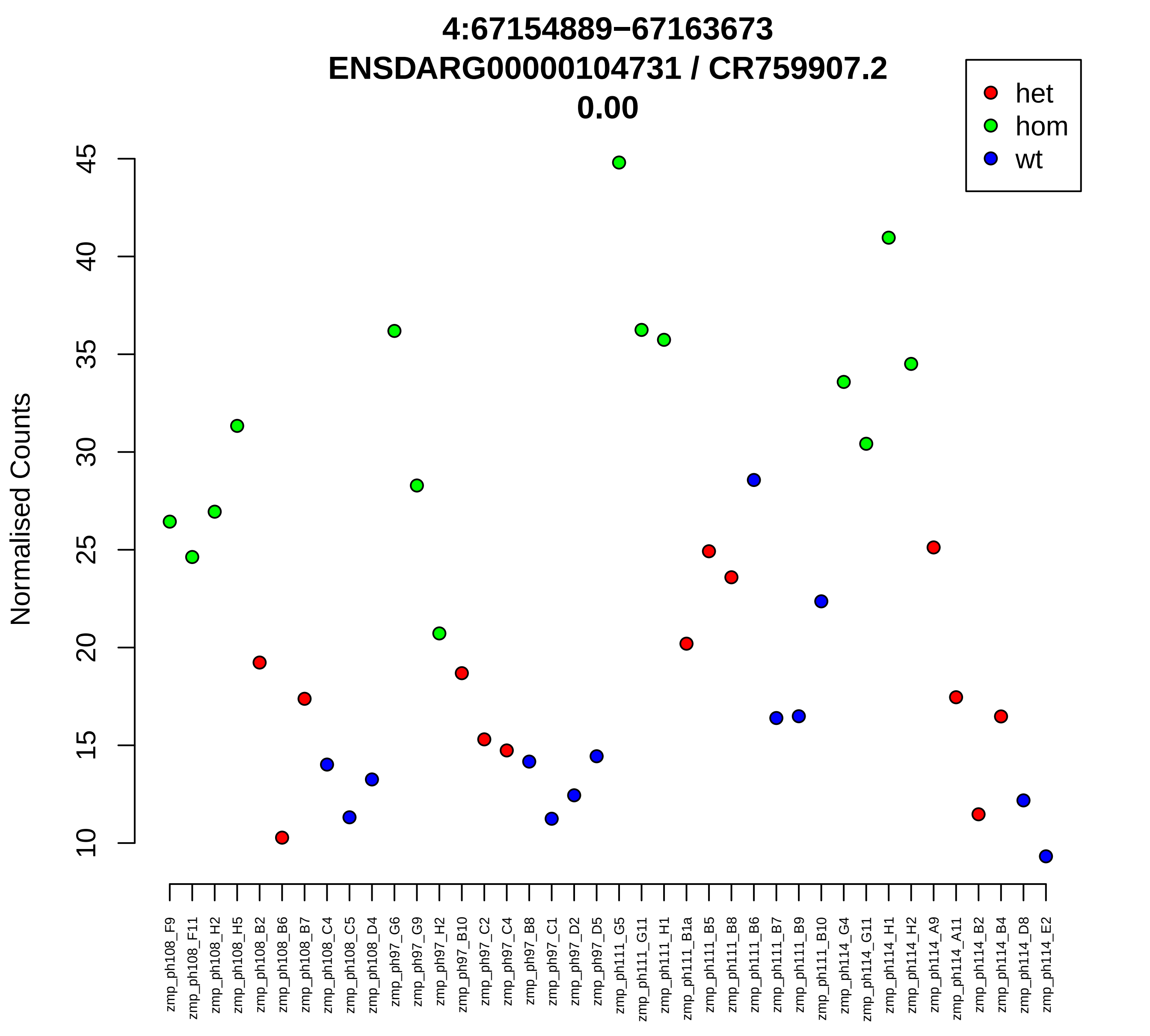
4:67154889−67163673
ENSDARG00000104731 / CR759907.2
0.00
het
hom
wt
Normalised Counts
10
15
20
25
30
35
40
45
zmp_ph108_F9
zmp_ph108_F11
zmp_ph108_H2
zmp_ph108_H5
zmp_ph108_B2
zmp_ph108_B6
zmp_ph108_B7
zmp_ph108_C4
zmp_ph108_C5
zmp_ph108_D4
zmp_ph97_G6
zmp_ph97_G9
zmp_ph97_H2
zmp_ph97_B10
zmp_ph97_C2
zmp_ph97_C4
zmp_ph97_B8
zmp_ph97_C1
zmp_ph97_D2
zmp_ph97_D5
zmp_ph111_G5
zmp_ph111_G11
zmp_ph111_H1
zmp_ph111_B1a
zmp_ph111_B5
zmp_ph111_B8
zmp_ph111_B6
zmp_ph111_B7
zmp_ph111_B9
zmp_ph111_B10
zmp_ph114_G4
zmp_ph114_G11
zmp_ph114_H1
zmp_ph114_H2
zmp_ph114_A9
zmp_ph114_A11
zmp_ph114_B2
zmp_ph114_B4
zmp_ph114_D8
zmp_ph114_E2

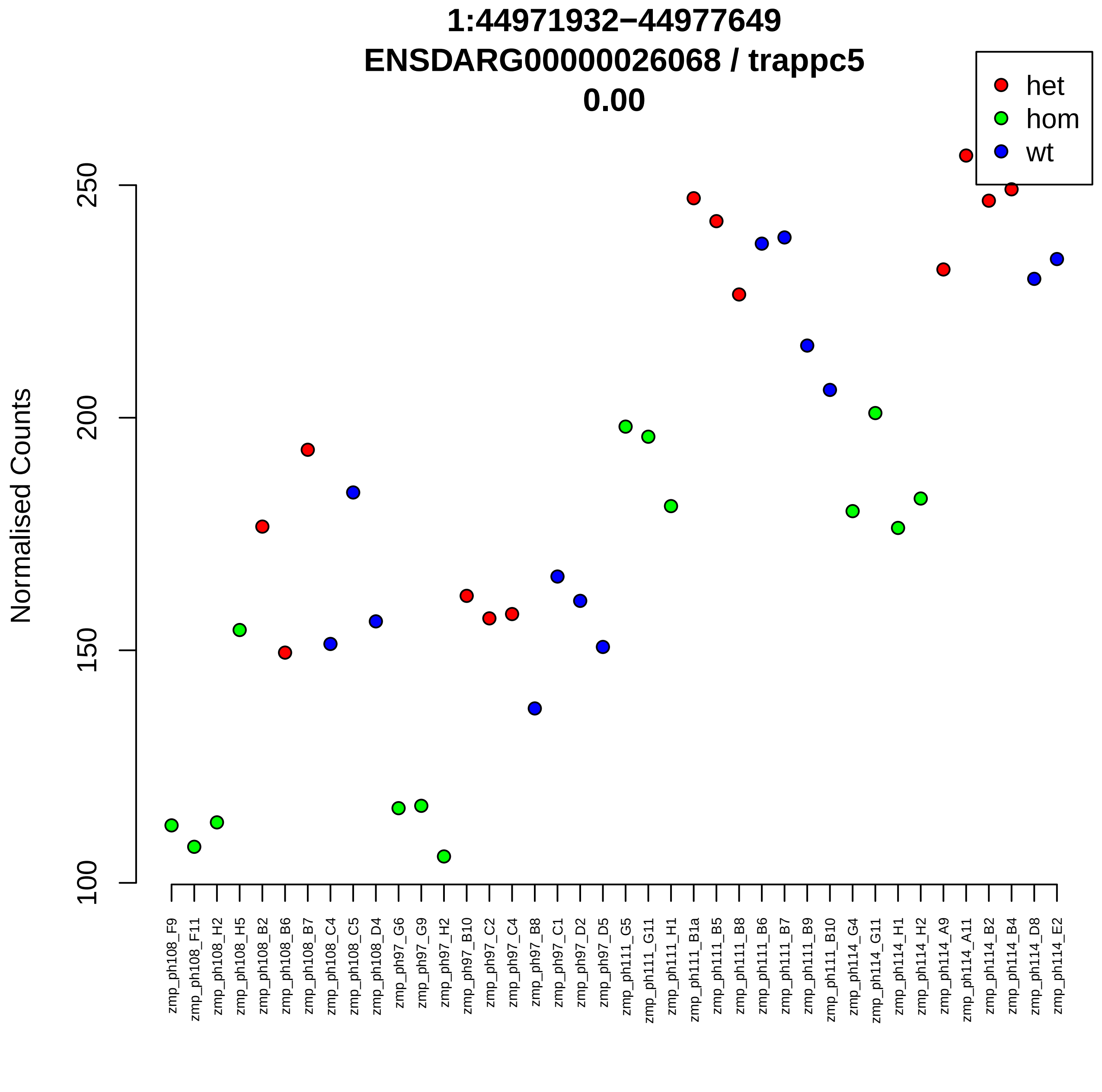

1:44971932−44977649
ENSDARG00000026068 / trappc5
0.00
het
hom
wt
Normalised Counts
100
150
200
250
zmp_ph108_F9
zmp_ph108_F11
zmp_ph108_H2
zmp_ph108_H5
zmp_ph108_B2
zmp_ph108_B6
zmp_ph108_B7
zmp_ph108_C4
zmp_ph108_C5
zmp_ph108_D4
zmp_ph97_G6
zmp_ph97_G9
zmp_ph97_H2
zmp_ph97_B10
zmp_ph97_C2
zmp_ph97_C4
zmp_ph97_B8
zmp_ph97_C1
zmp_ph97_D2
zmp_ph97_D5
zmp_ph111_G5
zmp_ph111_G11
zmp_ph111_H1
zmp_ph111_B1a
zmp_ph111_B5
zmp_ph111_B8
zmp_ph111_B6
zmp_ph111_B7
zmp_ph111_B9
zmp_ph111_B10
zmp_ph114_G4
zmp_ph114_G11
zmp_ph114_H1
zmp_ph114_H2
zmp_ph114_A9
zmp_ph114_A11
zmp_ph114_B2
zmp_ph114_B4
zmp_ph114_D8
zmp_ph114_E2

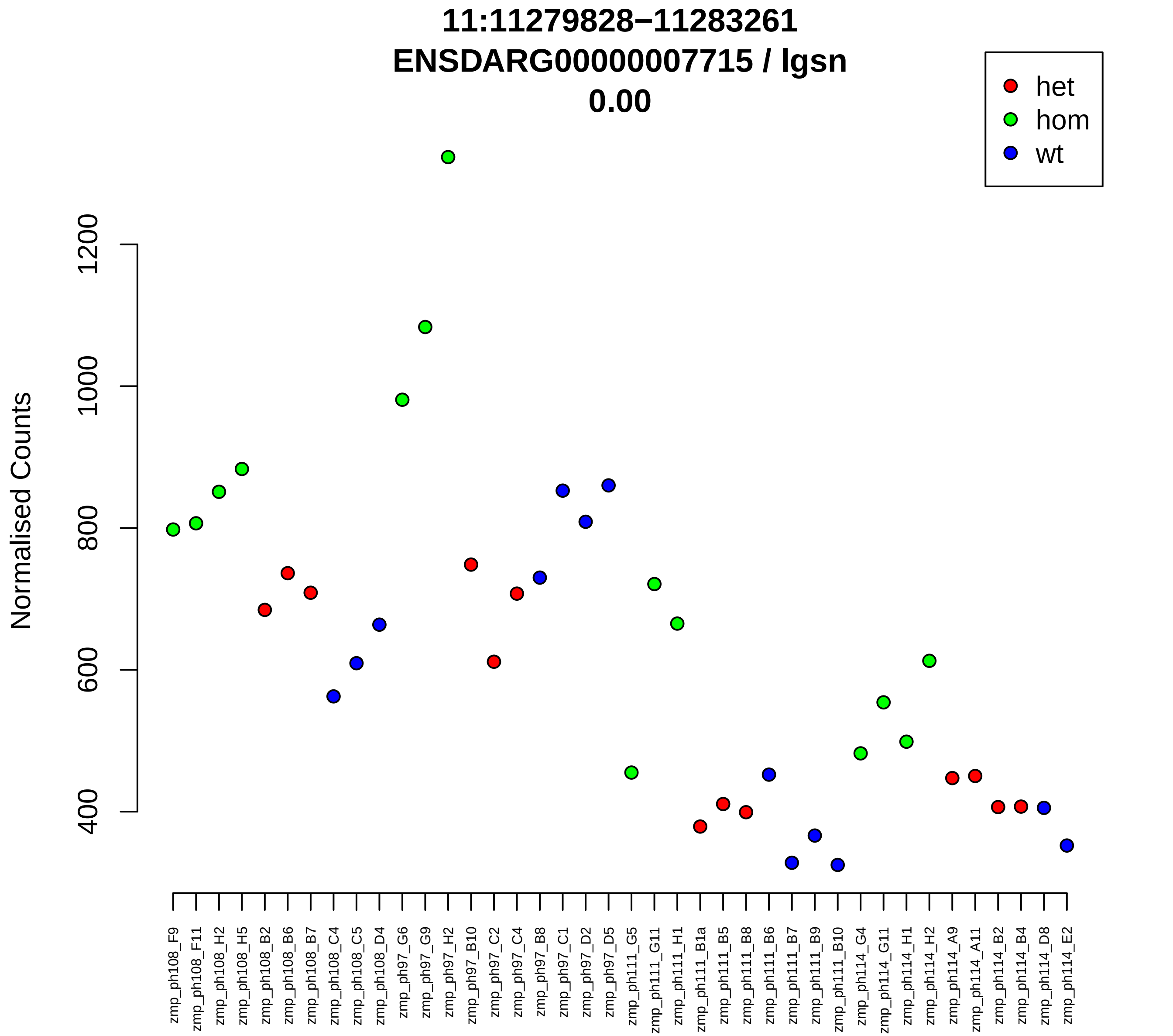
11:11279828−11283261
ENSDARG00000007715 / lgsn
0.00
het
hom
wt
Normalised Counts
1200
1000
800
600
400
zmp_ph108_F9
zmp_ph108_F11
zmp_ph108_H2
zmp_ph108_H5
zmp_ph108_B2
zmp_ph108_B6
zmp_ph108_B7
zmp_ph108_C4
zmp_ph108_C5
zmp_ph108_D4
zmp_ph97_G6
zmp_ph97_G9
zmp_ph97_H2
zmp_ph97_B10
zmp_ph97_C2
zmp_ph97_C4
zmp_ph97_B8
zmp_ph97_C1
zmp_ph97_D2
zmp_ph97_D5
zmp_ph111_G5
zmp_ph111_G11
zmp_ph111_H1
zmp_ph111_B1a
zmp_ph111_B5
zmp_ph111_B8
zmp_ph111_B6
zmp_ph111_B7
zmp_ph111_B9
zmp_ph111_B10
zmp_ph114_G4
zmp_ph114_G11
zmp_ph114_H1
zmp_ph114_H2
zmp_ph114_A9
zmp_ph114_A11
zmp_ph114_B2
zmp_ph114_B4
zmp_ph114_D8
zmp_ph114_E2

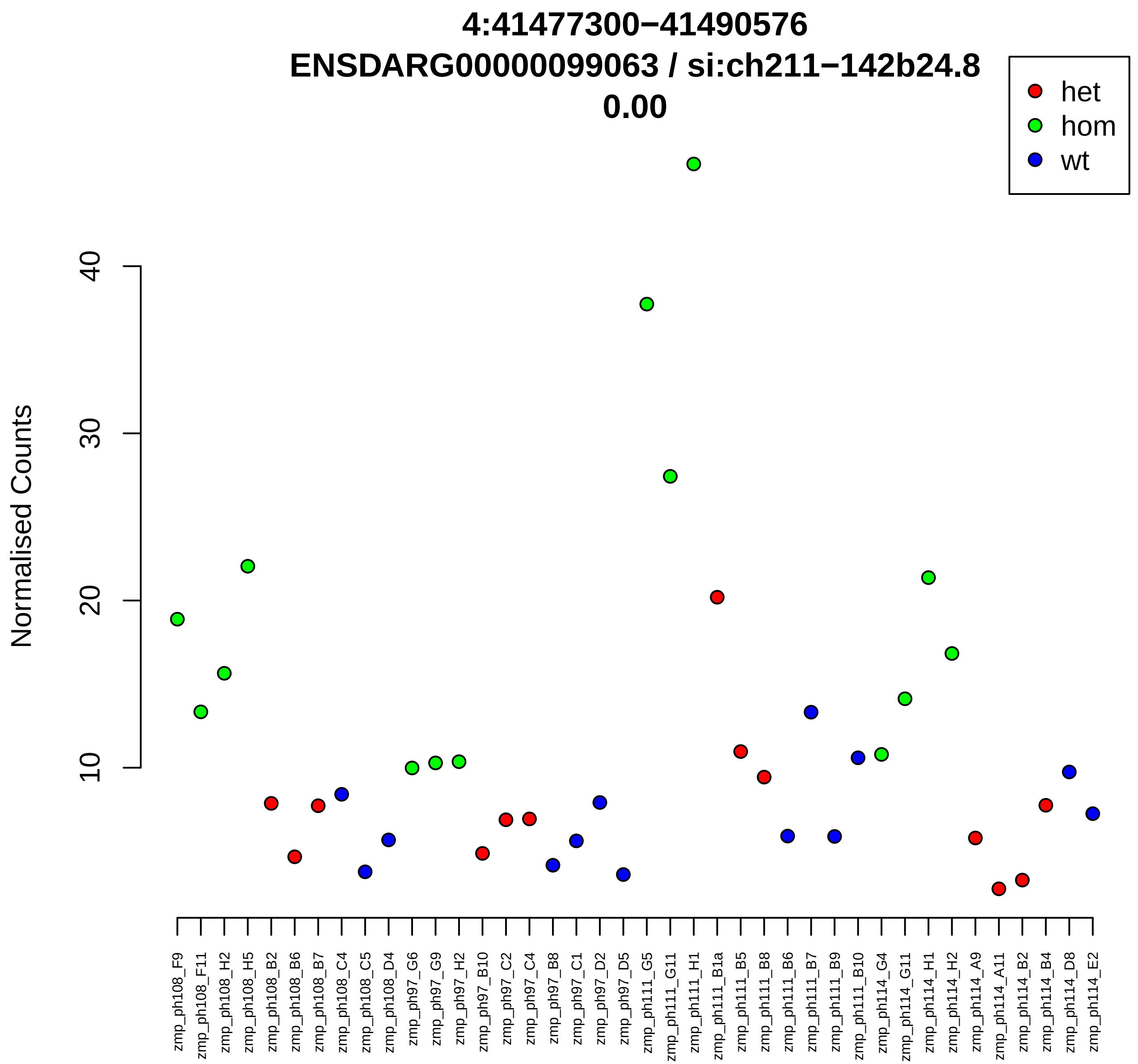

4:41477300−41490576
ENSDARG00000099063 / si:ch211−142b24.8
0.00
het
hom
wt
Normalised Counts
10
20
30
40
zmp_ph108_F9
zmp_ph108_F11
zmp_ph108_H2
zmp_ph108_H5
zmp_ph108_B2
zmp_ph108_B6
zmp_ph108_B7
zmp_ph108_C4
zmp_ph108_C5
zmp_ph108_D4
zmp_ph97_G6
zmp_ph97_G9
zmp_ph97_H2
zmp_ph97_B10
zmp_ph97_C2
zmp_ph97_C4
zmp_ph97_B8
zmp_ph97_C1
zmp_ph97_D2
zmp_ph97_D5
zmp_ph111_G5
zmp_ph111_G11
zmp_ph111_H1
zmp_ph111_B1a
zmp_ph111_B5
zmp_ph111_B8
zmp_ph111_B6
zmp_ph111_B7
zmp_ph111_B9
zmp_ph111_B10
zmp_ph114_G4
zmp_ph114_G11
zmp_ph114_H1
zmp_ph114_H2
zmp_ph114_A9
zmp_ph114_A11
zmp_ph114_B2
zmp_ph114_B4
zmp_ph114_D8
zmp_ph114_E2

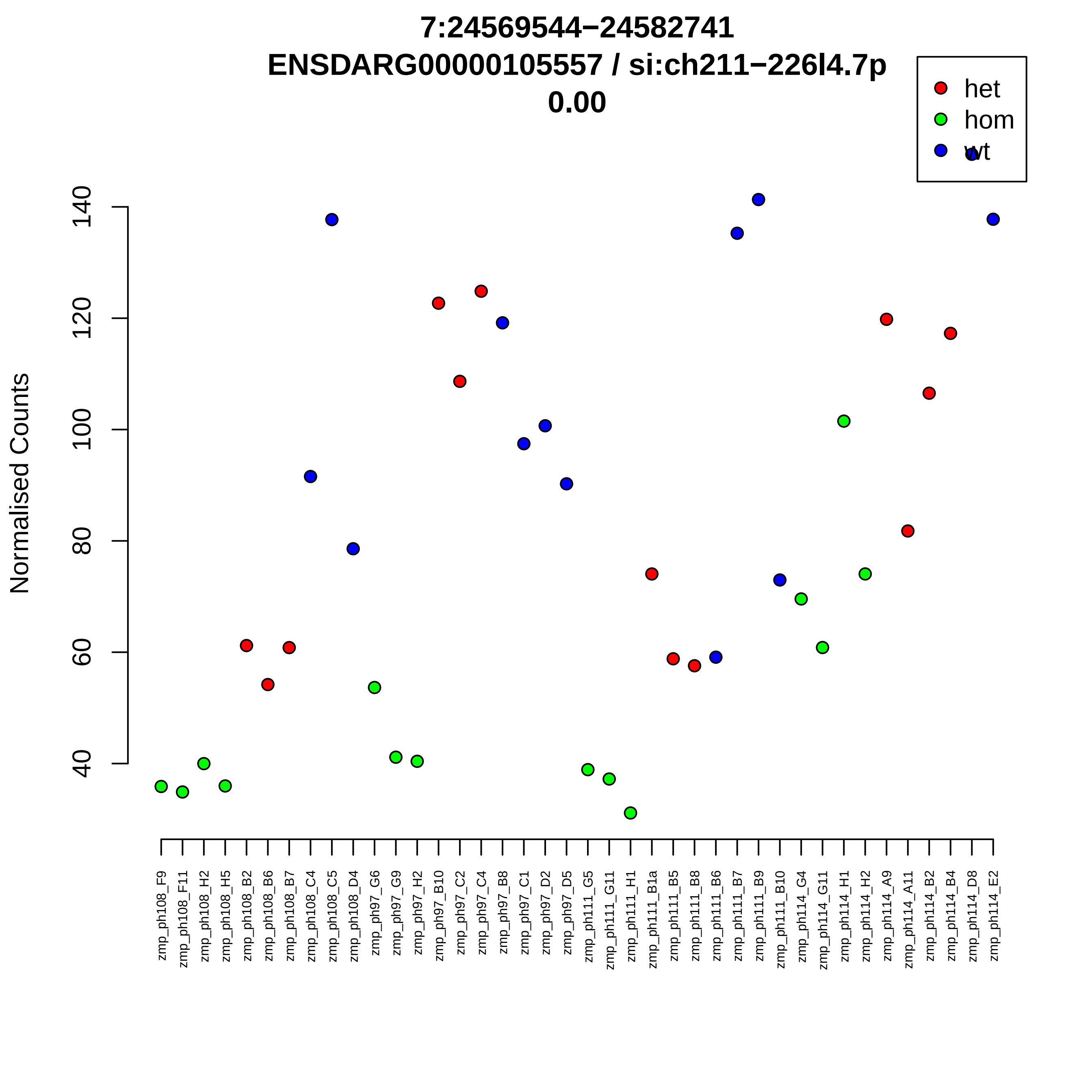
7:24569544−24582741
ENSDARG00000105557 / si:ch211−226l4.7p
0.00
het
hom
wt
Normalised Counts
40
60
80
100
120
140
zmp_ph108_F9
zmp_ph108_F11
zmp_ph108_H2
zmp_ph108_H5
zmp_ph108_B2
zmp_ph108_B6
zmp_ph108_B7
zmp_ph108_C4
zmp_ph108_C5
zmp_ph108_D4
zmp_ph97_G6
zmp_ph97_G9
zmp_ph97_H2
zmp_ph97_B10
zmp_ph97_C2
zmp_ph97_C4
zmp_ph97_B8
zmp_ph97_C1
zmp_ph97_D2
zmp_ph97_D5
zmp_ph111_G5
zmp_ph111_G11
zmp_ph111_H1
zmp_ph111_B1a
zmp_ph111_B5
zmp_ph111_B8
zmp_ph111_B6
zmp_ph111_B7
zmp_ph111_B9
zmp_ph111_B10
zmp_ph114_G4
zmp_ph114_G11
zmp_ph114_H1
zmp_ph114_H2
zmp_ph114_A9
zmp_ph114_A11
zmp_ph114_B2
zmp_ph114_B4
zmp_ph114_D8
zmp_ph114_E2

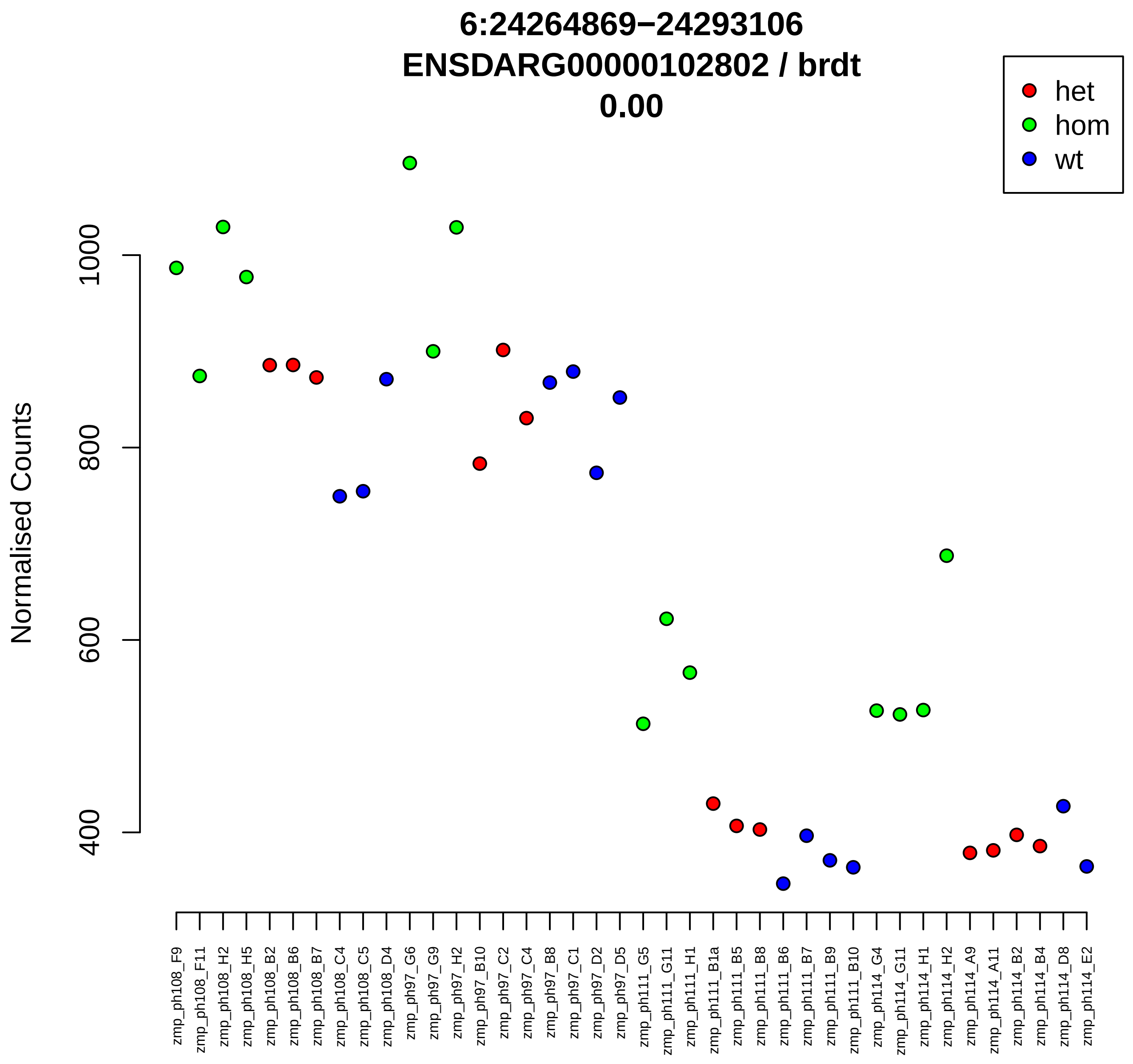
6:24264869−24293106
ENSDARG00000102802 / brdt
0.00
het
hom
wt
Normalised Counts
400
600
800
1000
zmp_ph108_F9
zmp_ph108_F11
zmp_ph108_H2
zmp_ph108_H5
zmp_ph108_B2
zmp_ph108_B6
zmp_ph108_B7
zmp_ph108_C4
zmp_ph108_C5
zmp_ph108_D4
zmp_ph97_G6
zmp_ph97_G9
zmp_ph97_H2
zmp_ph97_B10
zmp_ph97_C2
zmp_ph97_C4
zmp_ph97_B8
zmp_ph97_C1
zmp_ph97_D2
zmp_ph97_D5
zmp_ph111_G5
zmp_ph111_G11
zmp_ph111_H1
zmp_ph111_B1a
zmp_ph111_B5
zmp_ph111_B8
zmp_ph111_B6
zmp_ph111_B7
zmp_ph111_B9
zmp_ph111_B10
zmp_ph114_G4
zmp_ph114_G11
zmp_ph114_H1
zmp_ph114_H2
zmp_ph114_A9
zmp_ph114_A11
zmp_ph114_B2
zmp_ph114_B4
zmp_ph114_D8
zmp_ph114_E2

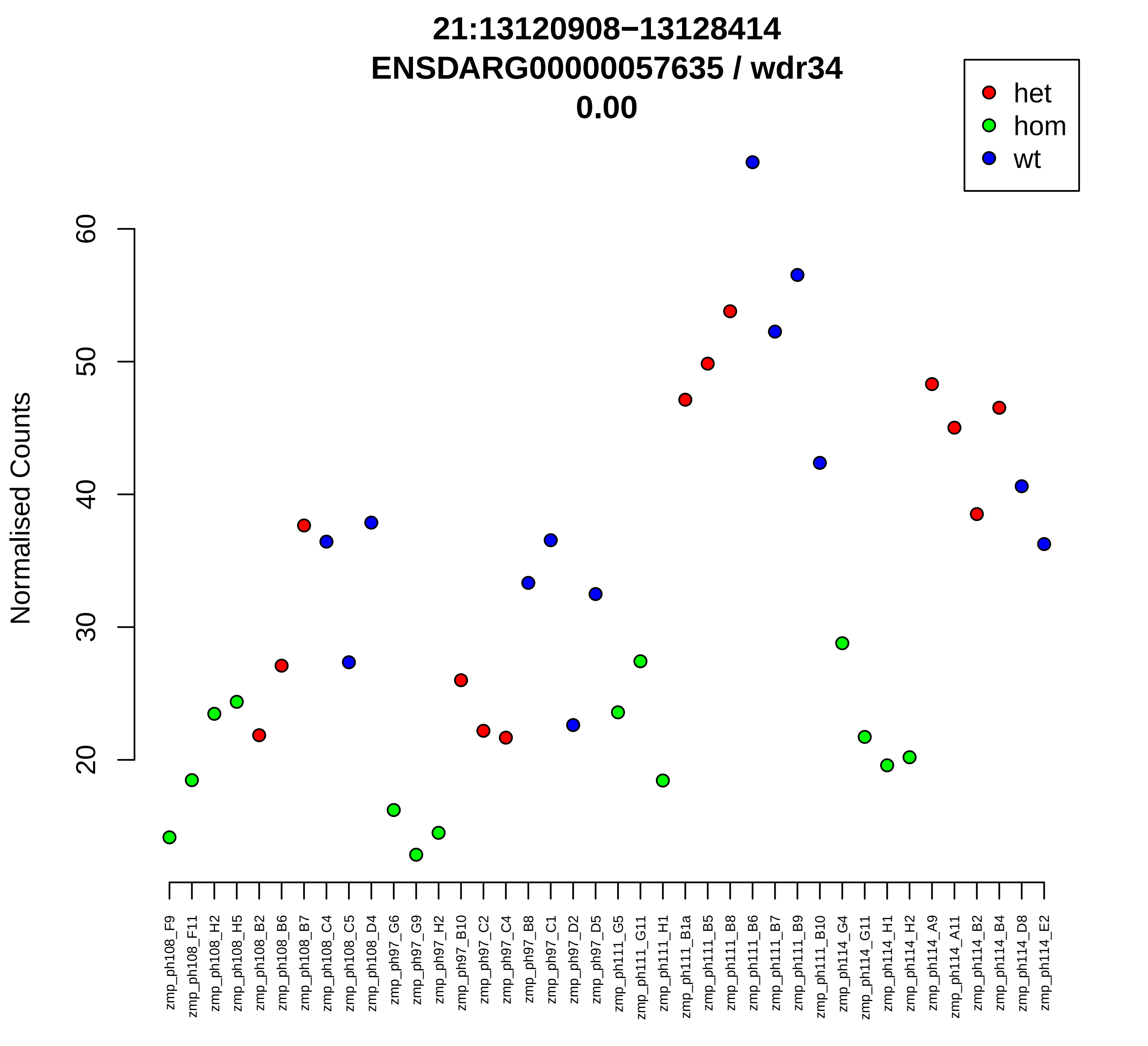

21:13120908−13128414
ENSDARG00000057635 / wdr34
0.00
het
hom
wt
Normalised Counts
20
30
40
50
60
zmp_ph108_F9
zmp_ph108_F11
zmp_ph108_H2
zmp_ph108_H5
zmp_ph108_B2
zmp_ph108_B6
zmp_ph108_B7
zmp_ph108_C4
zmp_ph108_C5
zmp_ph108_D4
zmp_ph97_G6
zmp_ph97_G9
zmp_ph97_H2
zmp_ph97_B10
zmp_ph97_C2
zmp_ph97_C4
zmp_ph97_B8
zmp_ph97_C1
zmp_ph97_D2
zmp_ph97_D5
zmp_ph111_G5
zmp_ph111_G11
zmp_ph111_H1
zmp_ph111_B1a
zmp_ph111_B5
zmp_ph111_B8
zmp_ph111_B6
zmp_ph111_B7
zmp_ph111_B9
zmp_ph111_B10
zmp_ph114_G4
zmp_ph114_G11
zmp_ph114_H1
zmp_ph114_H2
zmp_ph114_A9
zmp_ph114_A11
zmp_ph114_B2
zmp_ph114_B4
zmp_ph114_D8
zmp_ph114_E2

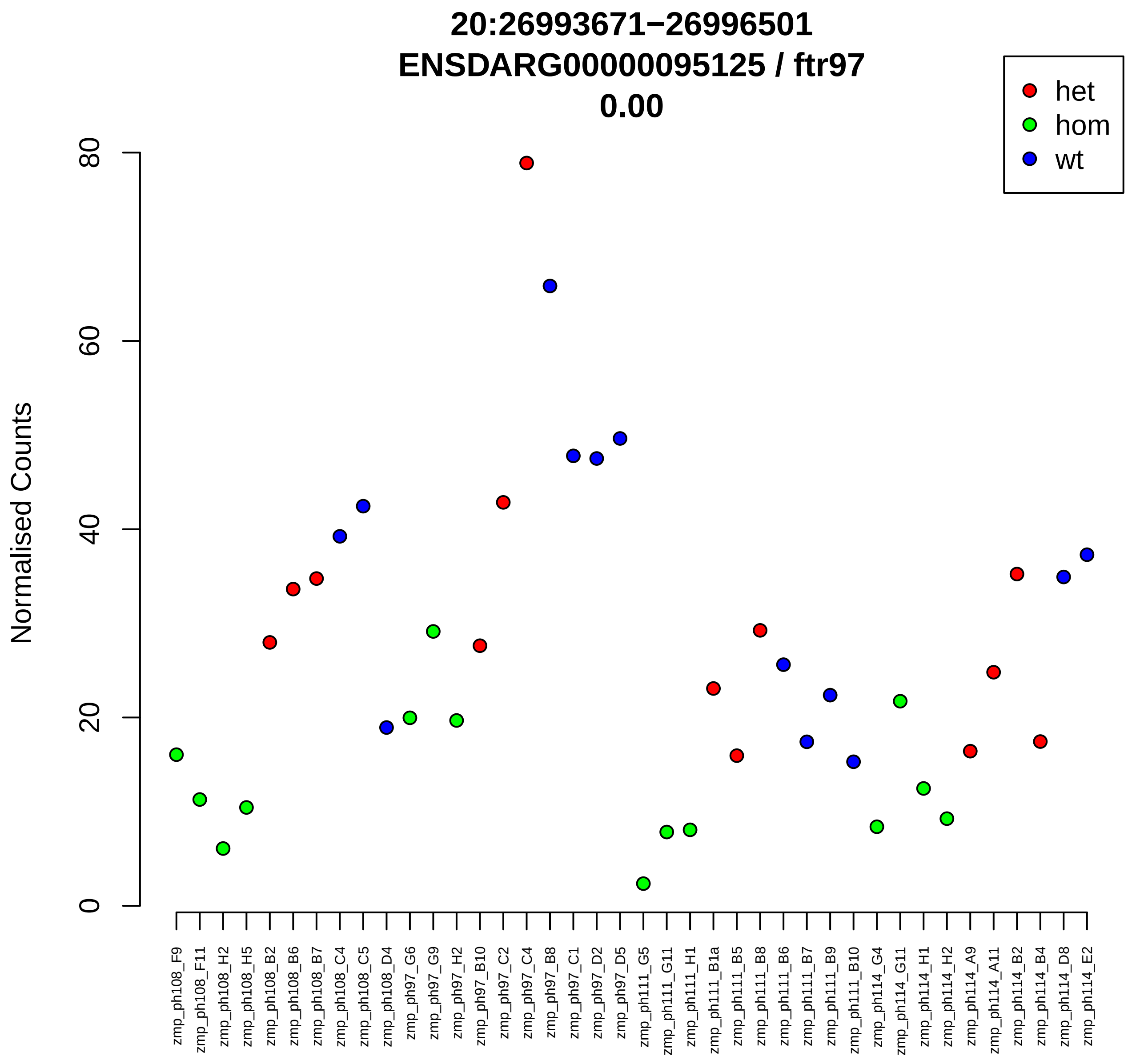

20:26993671−26996501
ENSDARG00000095125 / ftr97
0.00
het
hom
wt
Normalised Counts
0
20
40
60
80
zmp_ph108_F9
zmp_ph108_F11
zmp_ph108_H2
zmp_ph108_H5
zmp_ph108_B2
zmp_ph108_B6
zmp_ph108_B7
zmp_ph108_C4
zmp_ph108_C5
zmp_ph108_D4
zmp_ph97_G6
zmp_ph97_G9
zmp_ph97_H2
zmp_ph97_B10
zmp_ph97_C2
zmp_ph97_C4
zmp_ph97_B8
zmp_ph97_C1
zmp_ph97_D2
zmp_ph97_D5
zmp_ph111_G5
zmp_ph111_G11
zmp_ph111_H1
zmp_ph111_B1a
zmp_ph111_B5
zmp_ph111_B8
zmp_ph111_B6
zmp_ph111_B7
zmp_ph111_B9
zmp_ph111_B10
zmp_ph114_G4
zmp_ph114_G11
zmp_ph114_H1
zmp_ph114_H2
zmp_ph114_A9
zmp_ph114_A11
zmp_ph114_B2
zmp_ph114_B4
zmp_ph114_D8
zmp_ph114_E2

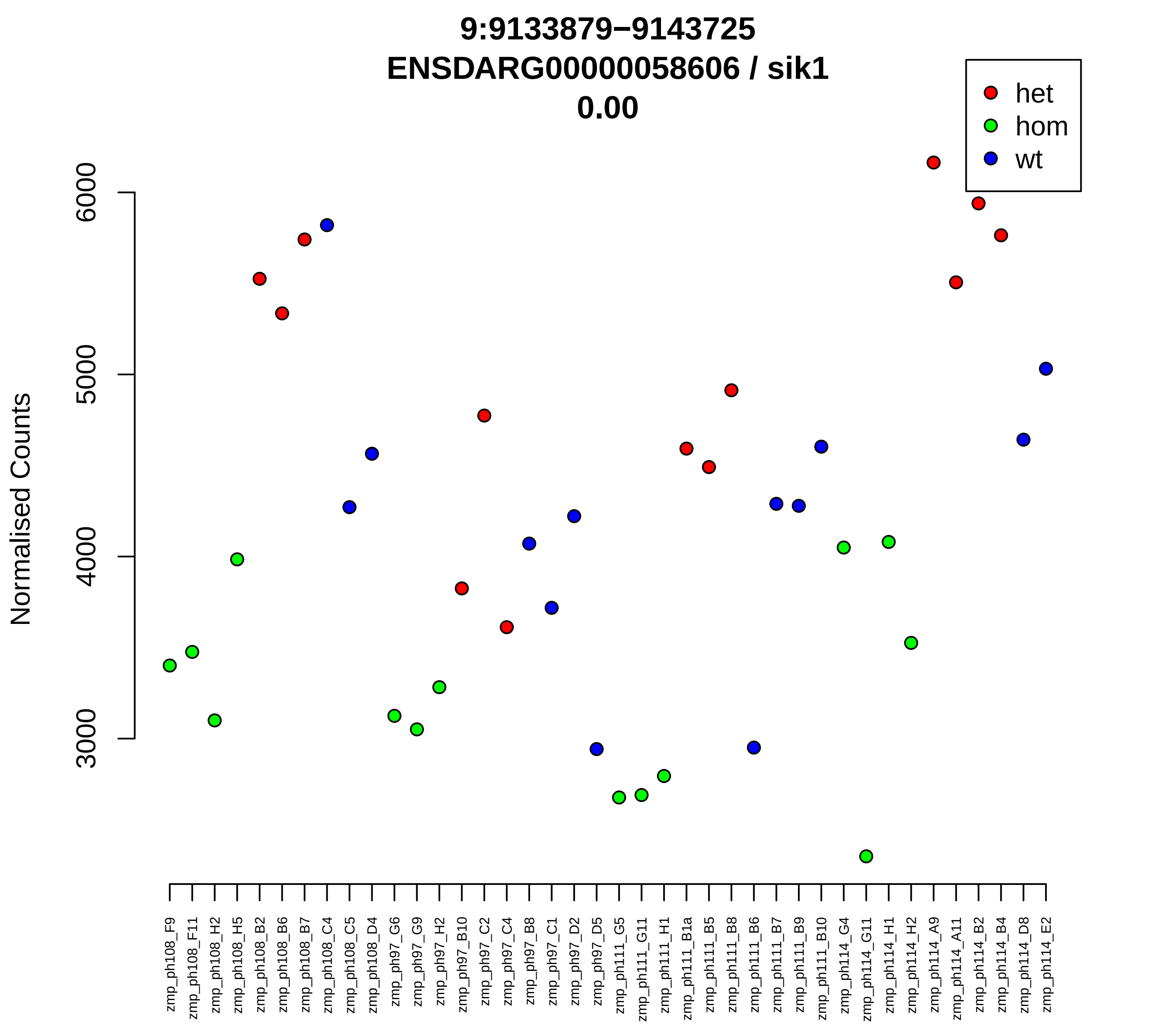

9:9133879−9143725
ENSDARG00000058606 / sik1
0.00
het
hom
wt
Normalised Counts
3000
4000
5000
6000
zmp_ph108_F9
zmp_ph108_F11
zmp_ph108_H2
zmp_ph108_H5
zmp_ph108_B2
zmp_ph108_B6
zmp_ph108_B7
zmp_ph108_C4
zmp_ph108_C5
zmp_ph108_D4
zmp_ph97_G6
zmp_ph97_G9
zmp_ph97_H2
zmp_ph97_B10
zmp_ph97_C2
zmp_ph97_C4
zmp_ph97_B8
zmp_ph97_C1
zmp_ph97_D2
zmp_ph97_D5
zmp_ph111_G5
zmp_ph111_G11
zmp_ph111_H1
zmp_ph111_B1a
zmp_ph111_B5
zmp_ph111_B8
zmp_ph111_B6
zmp_ph111_B7
zmp_ph111_B9
zmp_ph111_B10
zmp_ph114_G4
zmp_ph114_G11
zmp_ph114_H1
zmp_ph114_H2
zmp_ph114_A9
zmp_ph114_A11
zmp_ph114_B2
zmp_ph114_B4
zmp_ph114_D8
zmp_ph114_E2

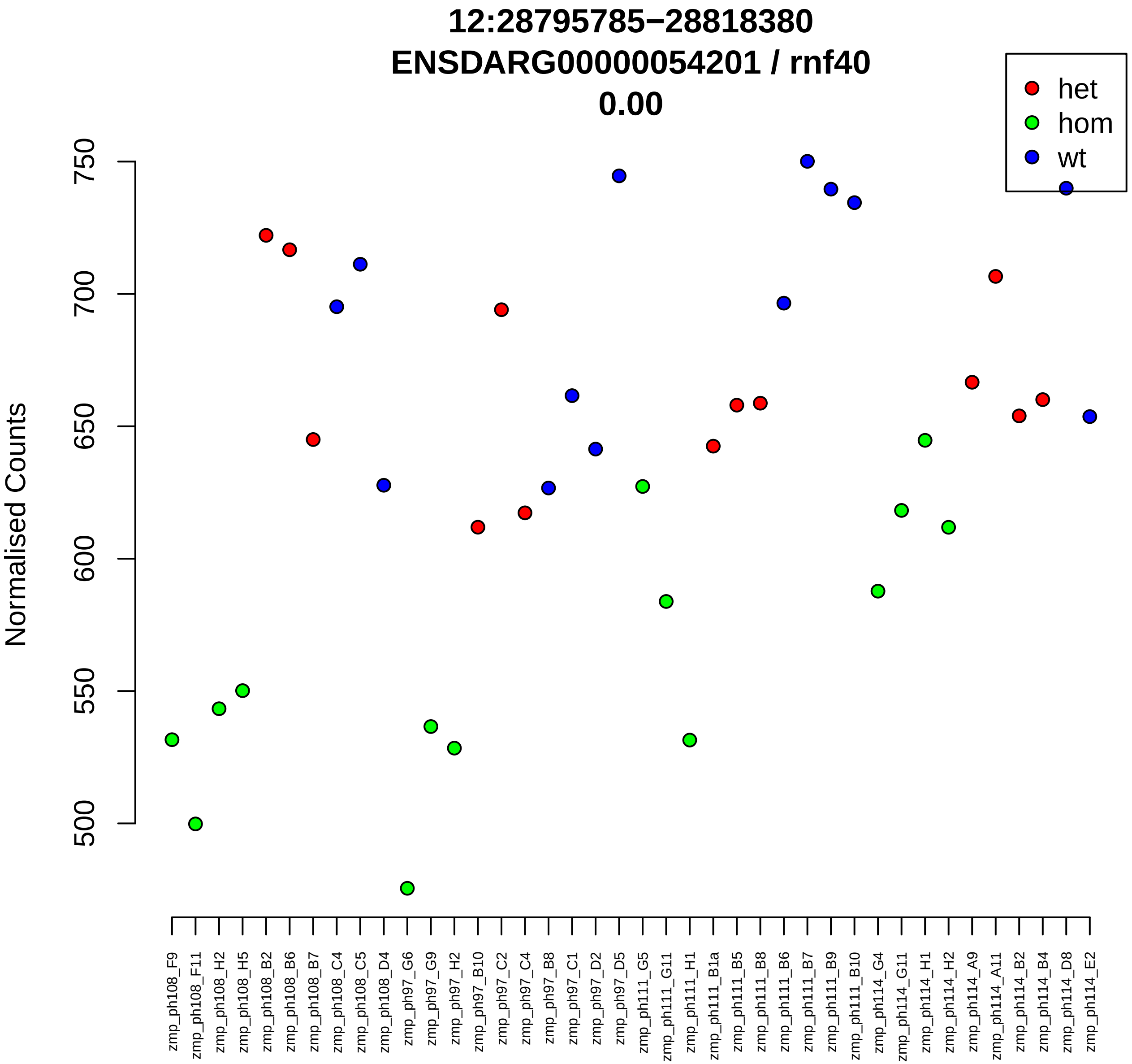

12:28795785−28818380
ENSDARG00000054201 / rnf40
0.00
het
hom
wt
Normalised Counts
500
550
600
650
700
750
zmp_ph108_F9
zmp_ph108_F11
zmp_ph108_H2
zmp_ph108_H5
zmp_ph108_B2
zmp_ph108_B6
zmp_ph108_B7
zmp_ph108_C4
zmp_ph108_C5
zmp_ph108_D4
zmp_ph97_G6
zmp_ph97_G9
zmp_ph97_H2
zmp_ph97_B10
zmp_ph97_C2
zmp_ph97_C4
zmp_ph97_B8
zmp_ph97_C1
zmp_ph97_D2
zmp_ph97_D5
zmp_ph111_G5
zmp_ph111_G11
zmp_ph111_H1
zmp_ph111_B1a
zmp_ph111_B5
zmp_ph111_B8
zmp_ph111_B6
zmp_ph111_B7
zmp_ph111_B9
zmp_ph111_B10
zmp_ph114_G4
zmp_ph114_G11
zmp_ph114_H1
zmp_ph114_H2
zmp_ph114_A9
zmp_ph114_A11
zmp_ph114_B2
zmp_ph114_B4
zmp_ph114_D8
zmp_ph114_E2

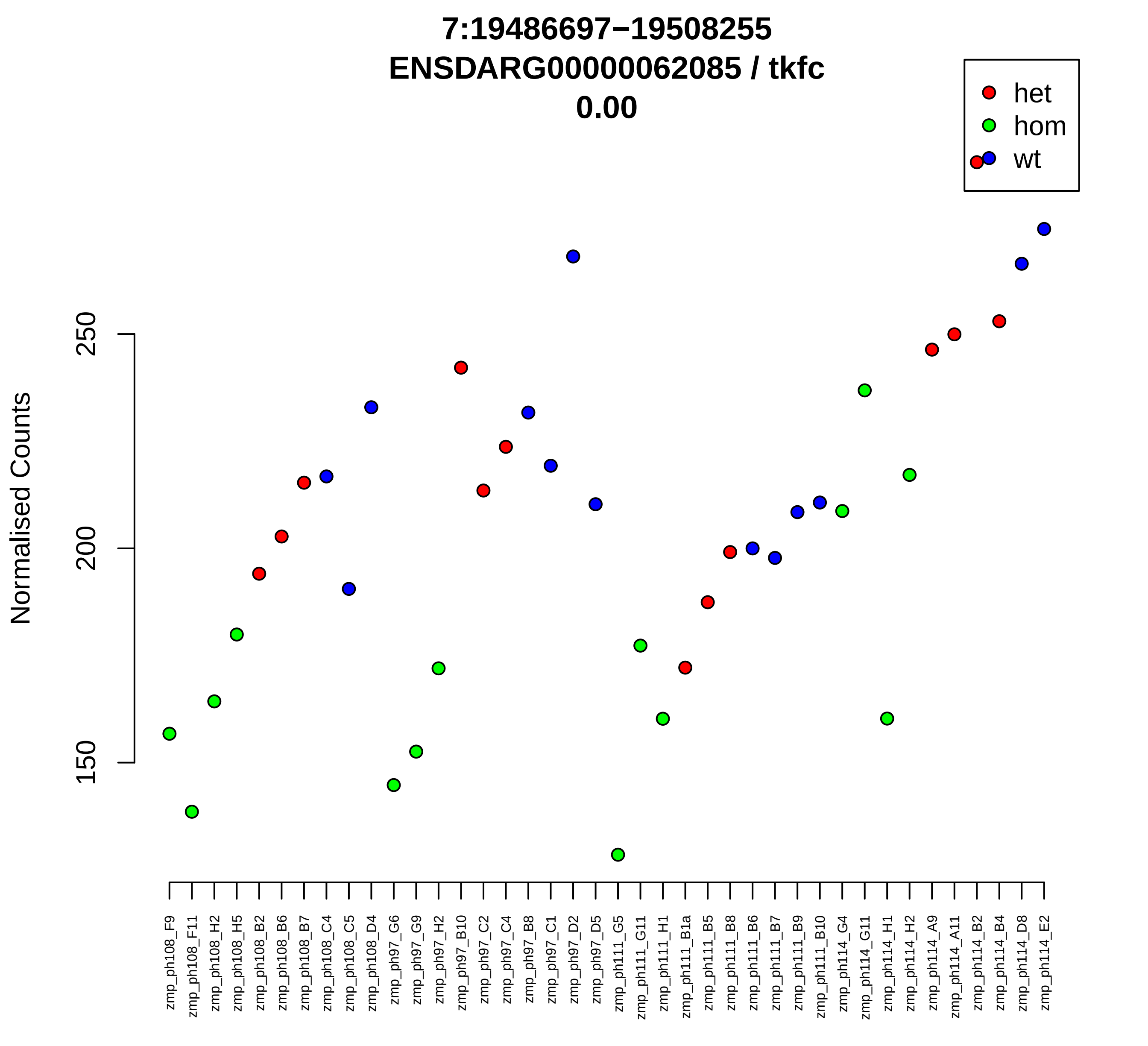

7:19486697−19508255
ENSDARG00000062085 / tkfc
0.00
het
hom
wt
Normalised Counts
150
200
250
zmp_ph108_F9
zmp_ph108_F11
zmp_ph108_H2
zmp_ph108_H5
zmp_ph108_B2
zmp_ph108_B6
zmp_ph108_B7
zmp_ph108_C4
zmp_ph108_C5
zmp_ph108_D4
zmp_ph97_G6
zmp_ph97_G9
zmp_ph97_H2
zmp_ph97_B10
zmp_ph97_C2
zmp_ph97_C4
zmp_ph97_B8
zmp_ph97_C1
zmp_ph97_D2
zmp_ph97_D5
zmp_ph111_G5
zmp_ph111_G11
zmp_ph111_H1
zmp_ph111_B1a
zmp_ph111_B5
zmp_ph111_B8
zmp_ph111_B6
zmp_ph111_B7
zmp_ph111_B9
zmp_ph111_B10
zmp_ph114_G4
zmp_ph114_G11
zmp_ph114_H1
zmp_ph114_H2
zmp_ph114_A9
zmp_ph114_A11
zmp_ph114_B2
zmp_ph114_B4
zmp_ph114_D8
zmp_ph114_E2

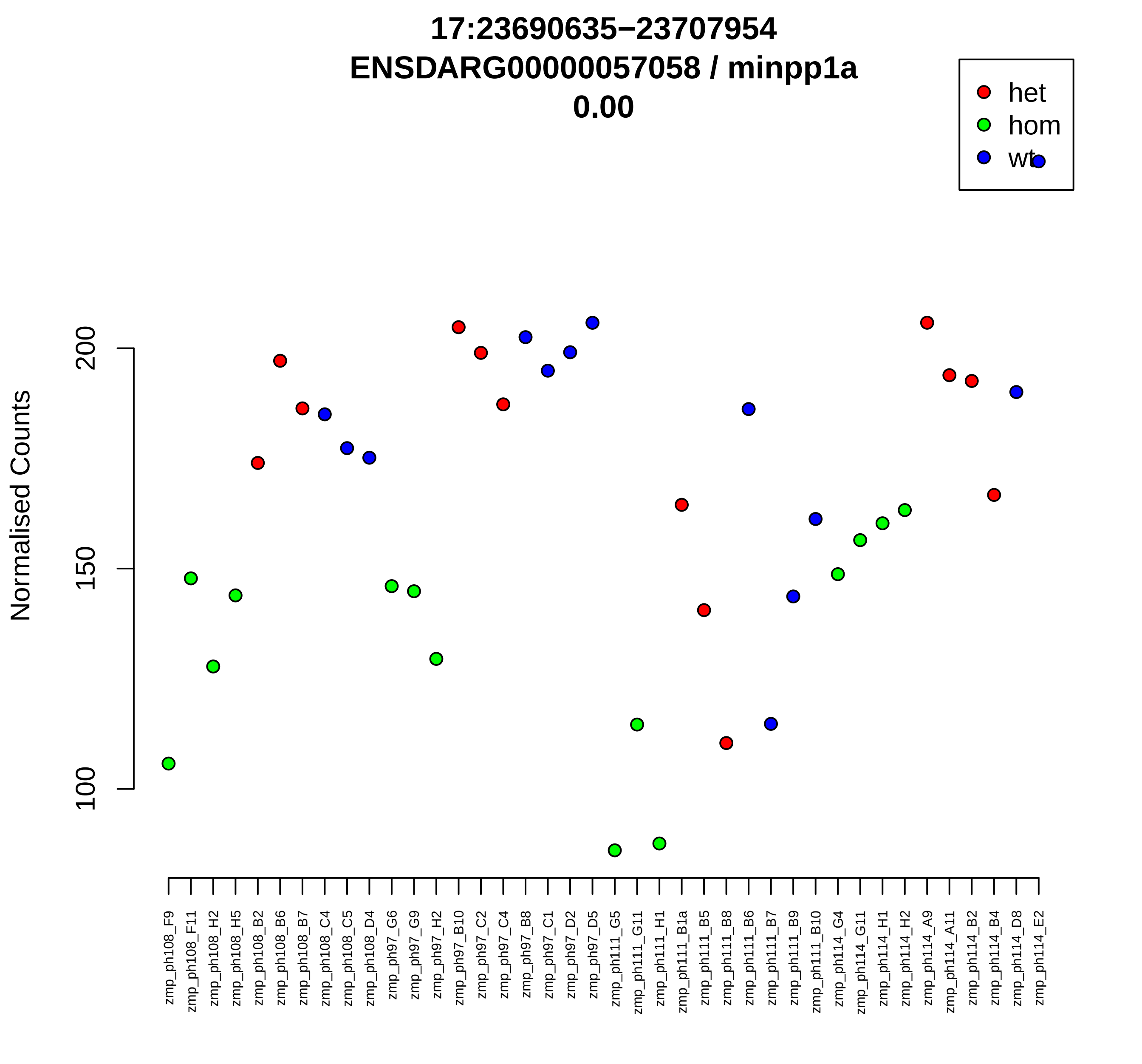

17:23690635−23707954
ENSDARG00000057058 / minpp1a
0.00
het
hom
wt
Normalised Counts
100
150
200
zmp_ph108_F9
zmp_ph108_F11
zmp_ph108_H2
zmp_ph108_H5
zmp_ph108_B2
zmp_ph108_B6
zmp_ph108_B7
zmp_ph108_C4
zmp_ph108_C5
zmp_ph108_D4
zmp_ph97_G6
zmp_ph97_G9
zmp_ph97_H2
zmp_ph97_B10
zmp_ph97_C2
zmp_ph97_C4
zmp_ph97_B8
zmp_ph97_C1
zmp_ph97_D2
zmp_ph97_D5
zmp_ph111_G5
zmp_ph111_G11
zmp_ph111_H1
zmp_ph111_B1a
zmp_ph111_B5
zmp_ph111_B8
zmp_ph111_B6
zmp_ph111_B7
zmp_ph111_B9
zmp_ph111_B10
zmp_ph114_G4
zmp_ph114_G11
zmp_ph114_H1
zmp_ph114_H2
zmp_ph114_A9
zmp_ph114_A11
zmp_ph114_B2
zmp_ph114_B4
zmp_ph114_D8
zmp_ph114_E2

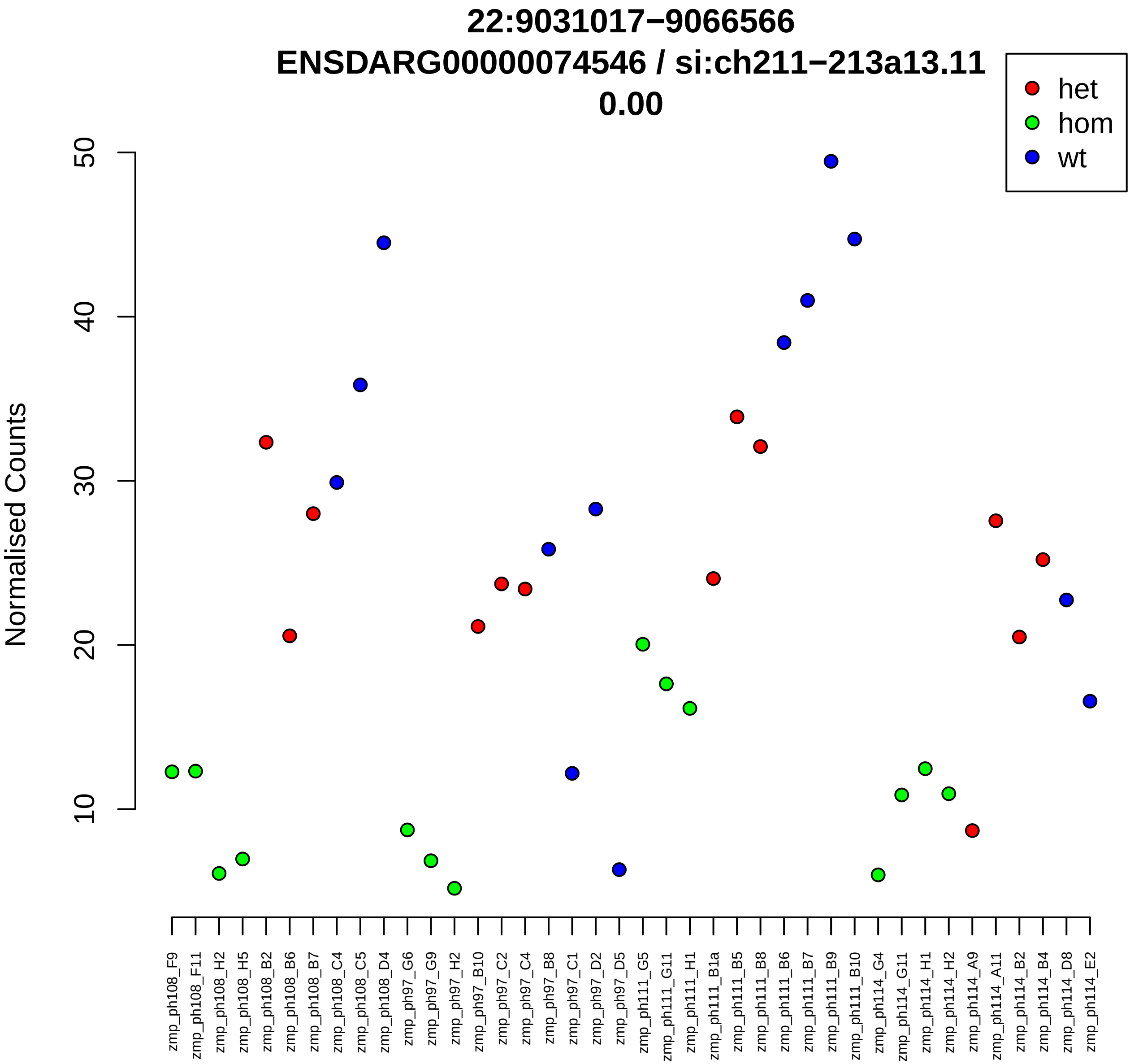
22:9031017−9066566
ENSDARG00000074546 / si:ch211−213a13.11
0.00
het
hom
wt
Normalised Counts
10
20
30
40
50
zmp_ph108_F9
zmp_ph108_F11
zmp_ph108_H2
zmp_ph108_H5
zmp_ph108_B2
zmp_ph108_B6
zmp_ph108_B7
zmp_ph108_C4
zmp_ph108_C5
zmp_ph108_D4
zmp_ph97_G6
zmp_ph97_G9
zmp_ph97_H2
zmp_ph97_B10
zmp_ph97_C2
zmp_ph97_C4
zmp_ph97_B8
zmp_ph97_C1
zmp_ph97_D2
zmp_ph97_D5
zmp_ph111_G5
zmp_ph111_G11
zmp_ph111_H1
zmp_ph111_B1a
zmp_ph111_B5
zmp_ph111_B8
zmp_ph111_B6
zmp_ph111_B7
zmp_ph111_B9
zmp_ph111_B10
zmp_ph114_G4
zmp_ph114_G11
zmp_ph114_H1
zmp_ph114_H2
zmp_ph114_A9
zmp_ph114_A11
zmp_ph114_B2
zmp_ph114_B4
zmp_ph114_D8
zmp_ph114_E2

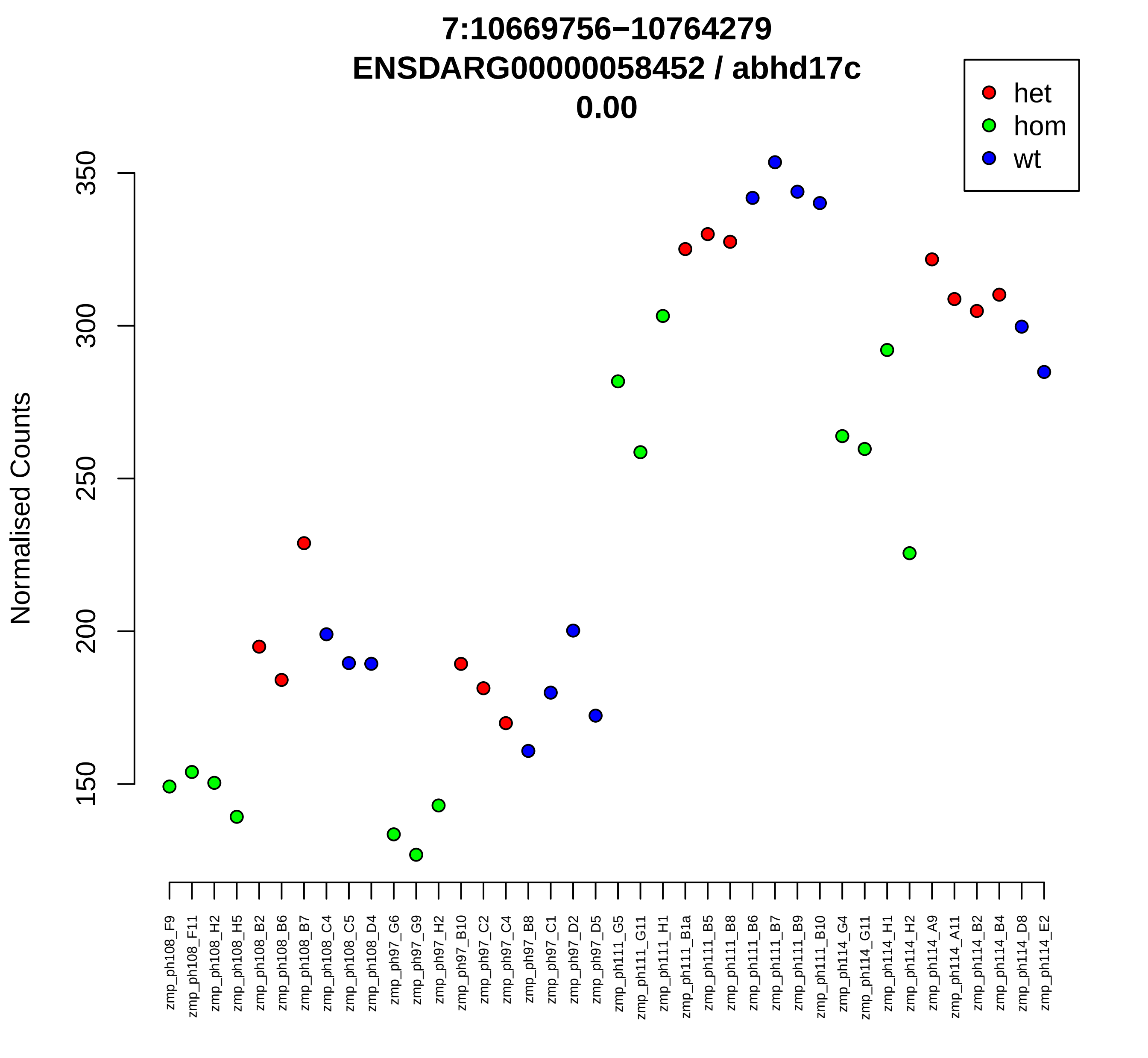

7:10669756−10764279
ENSDARG00000058452 / abhd17c
0.00
het
hom
wt
Normalised Counts
150
200
250
300
350
zmp_ph108_F9
zmp_ph108_F11
zmp_ph108_H2
zmp_ph108_H5
zmp_ph108_B2
zmp_ph108_B6
zmp_ph108_B7
zmp_ph108_C4
zmp_ph108_C5
zmp_ph108_D4
zmp_ph97_G6
zmp_ph97_G9
zmp_ph97_H2
zmp_ph97_B10
zmp_ph97_C2
zmp_ph97_C4
zmp_ph97_B8
zmp_ph97_C1
zmp_ph97_D2
zmp_ph97_D5
zmp_ph111_G5
zmp_ph111_G11
zmp_ph111_H1
zmp_ph111_B1a
zmp_ph111_B5
zmp_ph111_B8
zmp_ph111_B6
zmp_ph111_B7
zmp_ph111_B9
zmp_ph111_B10
zmp_ph114_G4
zmp_ph114_G11
zmp_ph114_H1
zmp_ph114_H2
zmp_ph114_A9
zmp_ph114_A11
zmp_ph114_B2
zmp_ph114_B4
zmp_ph114_D8
zmp_ph114_E2

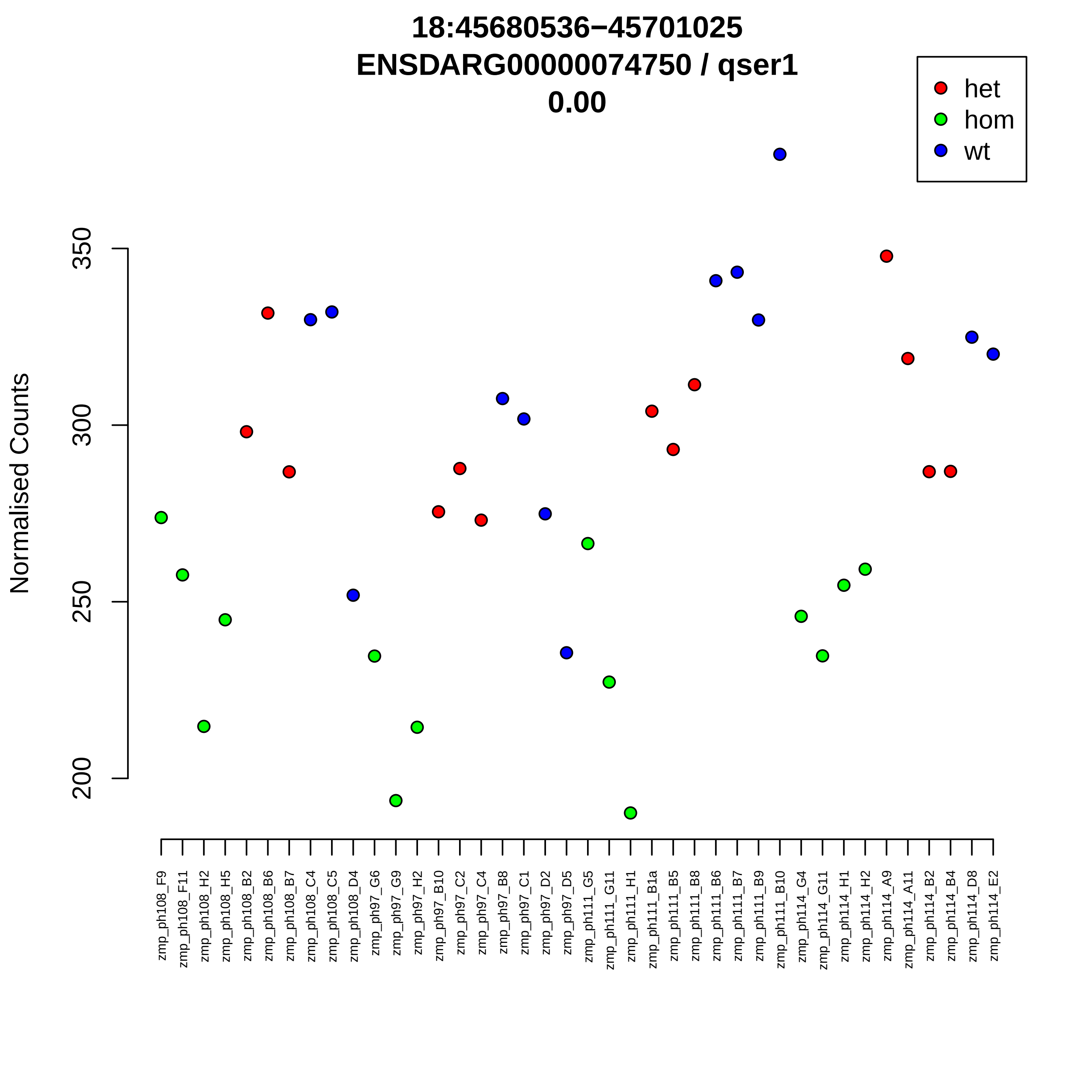

18:45680536−45701025
ENSDARG00000074750 / qser1
0.00
het
hom
wt
Normalised Counts
350
300
250
200
zmp_ph108_F9
zmp_ph108_F11
zmp_ph108_H2
zmp_ph108_H5
zmp_ph108_B2
zmp_ph108_B6
zmp_ph108_B7
zmp_ph108_C4
zmp_ph108_C5
zmp_ph108_D4
zmp_ph97_G6
zmp_ph97_G9
zmp_ph97_H2
zmp_ph97_B10
zmp_ph97_C2
zmp_ph97_C4
zmp_ph97_B8
zmp_ph97_C1
zmp_ph97_D2
zmp_ph97_D5
zmp_ph111_G5
zmp_ph111_G11
zmp_ph111_H1
zmp_ph111_B1a
zmp_ph111_B5
zmp_ph111_B8
zmp_ph111_B6
zmp_ph111_B7
zmp_ph111_B9
zmp_ph111_B10
zmp_ph114_G4
zmp_ph114_G11
zmp_ph114_H1
zmp_ph114_H2
zmp_ph114_A9
zmp_ph114_A11
zmp_ph114_B2
zmp_ph114_B4
zmp_ph114_D8
zmp_ph114_E2

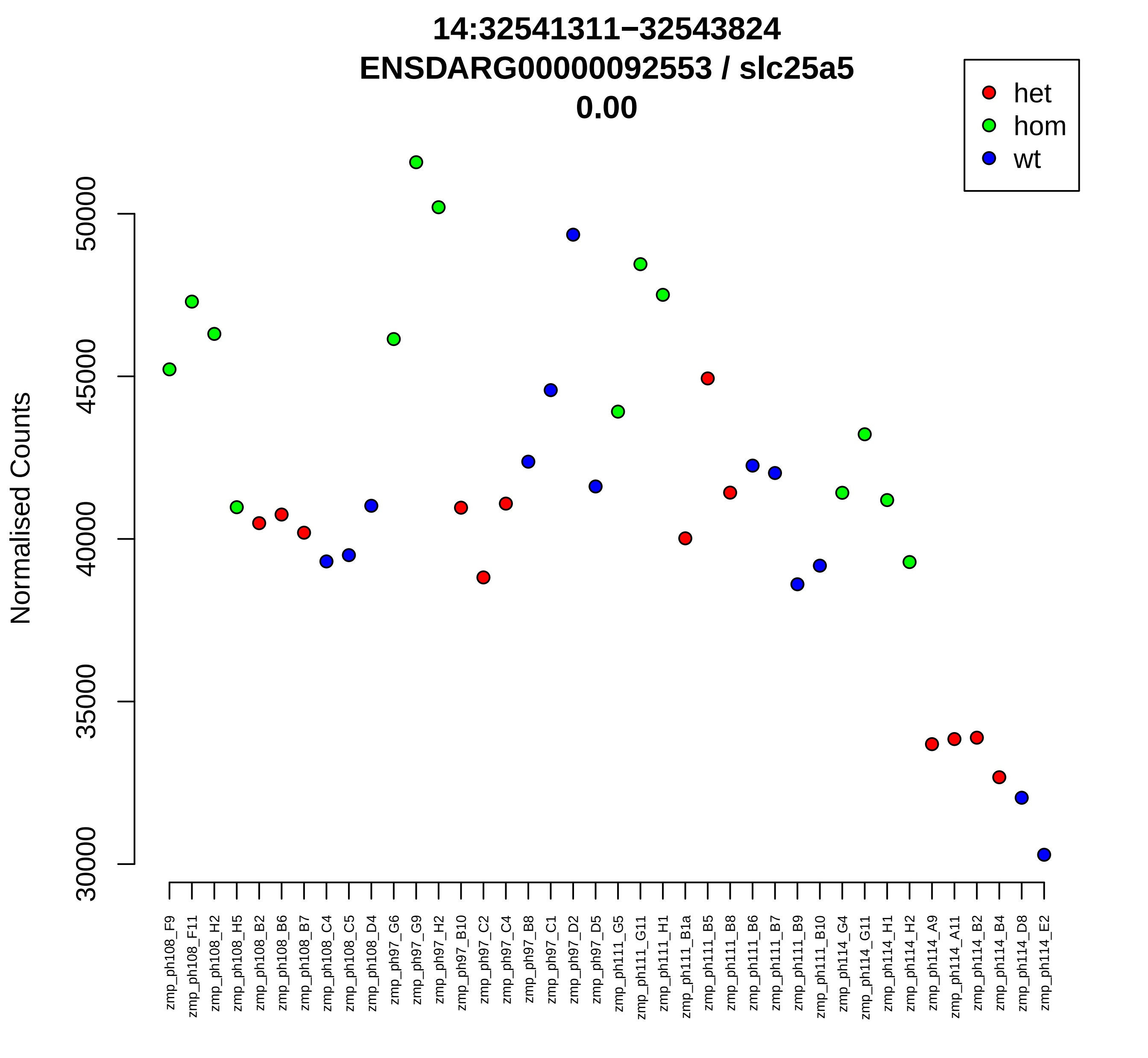

14:32541311−32543824
ENSDARG00000092553 / slc25a5
0.00
het
hom
wt
Normalised Counts
30000
35000
40000
45000
50000
zmp_ph108_F9
zmp_ph108_F11
zmp_ph108_H2
zmp_ph108_H5
zmp_ph108_B2
zmp_ph108_B6
zmp_ph108_B7
zmp_ph108_C4
zmp_ph108_C5
zmp_ph108_D4
zmp_ph97_G6
zmp_ph97_G9
zmp_ph97_H2
zmp_ph97_B10
zmp_ph97_C2
zmp_ph97_C4
zmp_ph97_B8
zmp_ph97_C1
zmp_ph97_D2
zmp_ph97_D5
zmp_ph111_G5
zmp_ph111_G11
zmp_ph111_H1
zmp_ph111_B1a
zmp_ph111_B5
zmp_ph111_B8
zmp_ph111_B6
zmp_ph111_B7
zmp_ph111_B9
zmp_ph111_B10
zmp_ph114_G4
zmp_ph114_G11
zmp_ph114_H1
zmp_ph114_H2
zmp_ph114_A9
zmp_ph114_A11
zmp_ph114_B2
zmp_ph114_B4
zmp_ph114_D8
zmp_ph114_E2

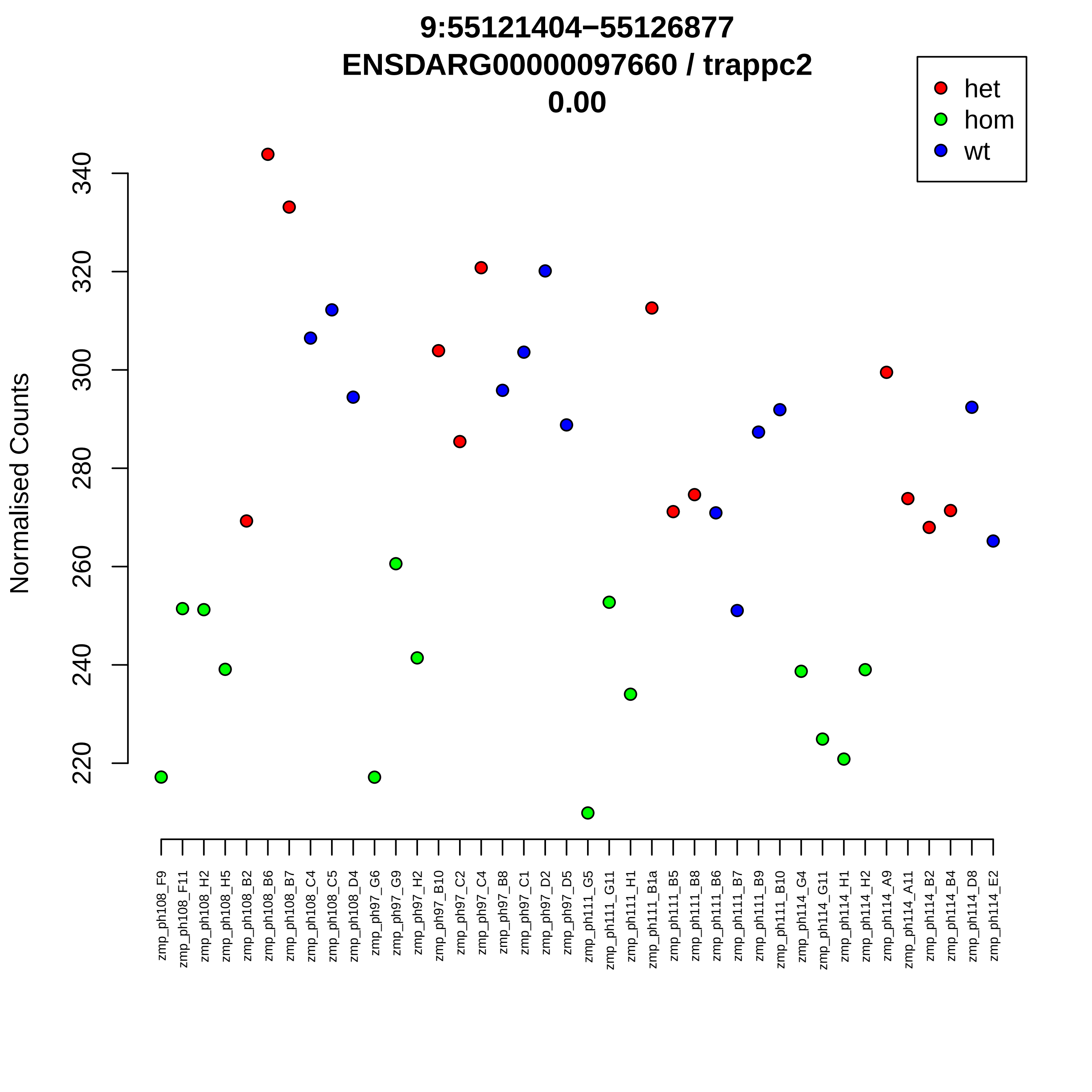

9:55121404−55126877
ENSDARG00000097660 / trappc2
0.00
het
hom
wt
Normalised Counts
220
240
260
280
300
320
340
zmp_ph108_F9
zmp_ph108_F11
zmp_ph108_H2
zmp_ph108_H5
zmp_ph108_B2
zmp_ph108_B6
zmp_ph108_B7
zmp_ph108_C4
zmp_ph108_C5
zmp_ph108_D4
zmp_ph97_G6
zmp_ph97_G9
zmp_ph97_H2
zmp_ph97_B10
zmp_ph97_C2
zmp_ph97_C4
zmp_ph97_B8
zmp_ph97_C1
zmp_ph97_D2
zmp_ph97_D5
zmp_ph111_G5
zmp_ph111_G11
zmp_ph111_H1
zmp_ph111_B1a
zmp_ph111_B5
zmp_ph111_B8
zmp_ph111_B6
zmp_ph111_B7
zmp_ph111_B9
zmp_ph111_B10
zmp_ph114_G4
zmp_ph114_G11
zmp_ph114_H1
zmp_ph114_H2
zmp_ph114_A9
zmp_ph114_A11
zmp_ph114_B2
zmp_ph114_B4
zmp_ph114_D8
zmp_ph114_E2

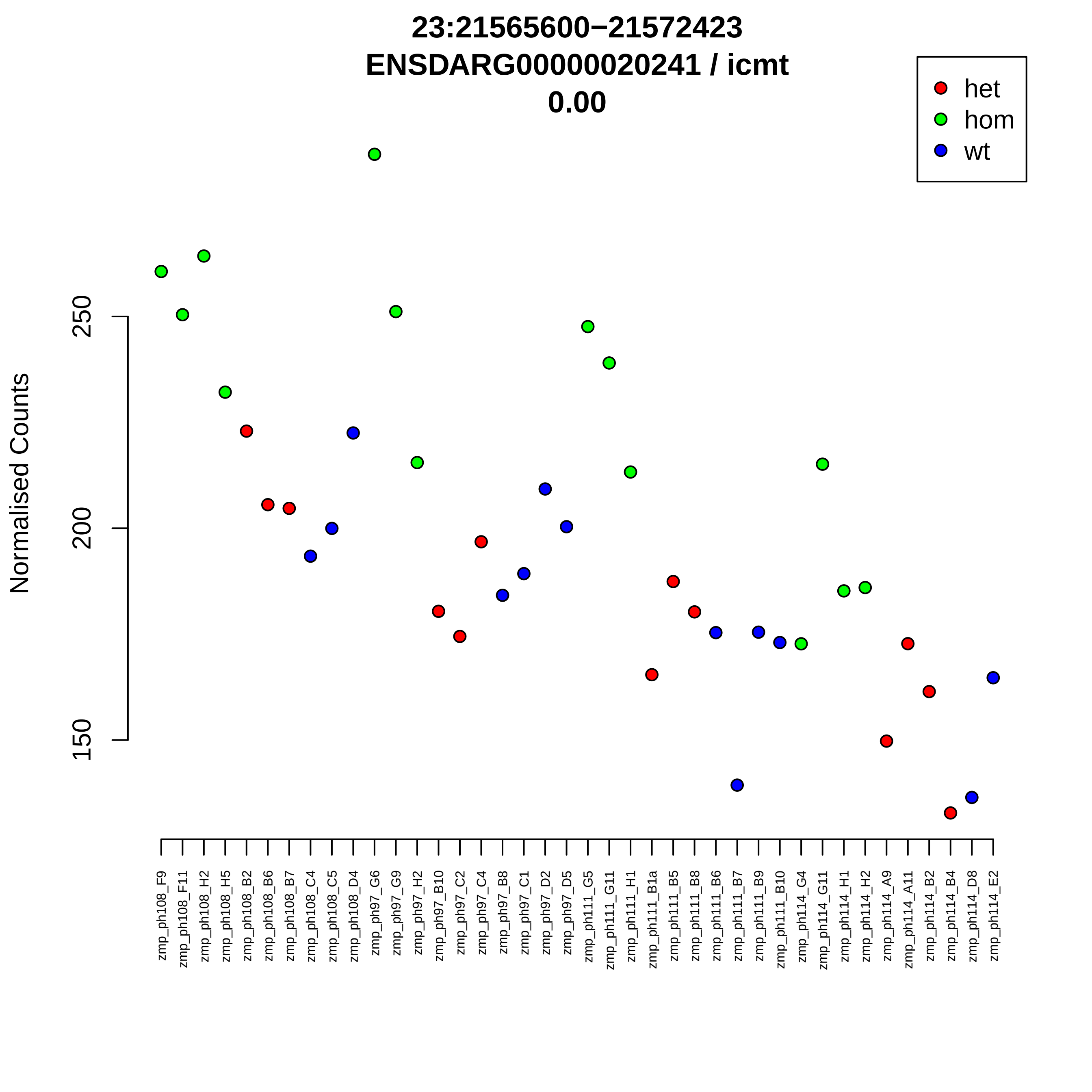

23:21565600−21572423
ENSDARG00000020241 / icmt
0.00
het
hom
wt
Normalised Counts
150
200
250
zmp_ph108_F9
zmp_ph108_F11
zmp_ph108_H2
zmp_ph108_H5
zmp_ph108_B2
zmp_ph108_B6
zmp_ph108_B7
zmp_ph108_C4
zmp_ph108_C5
zmp_ph108_D4
zmp_ph97_G6
zmp_ph97_G9
zmp_ph97_H2
zmp_ph97_B10
zmp_ph97_C2
zmp_ph97_C4
zmp_ph97_B8
zmp_ph97_C1
zmp_ph97_D2
zmp_ph97_D5
zmp_ph111_G5
zmp_ph111_G11
zmp_ph111_H1
zmp_ph111_B1a
zmp_ph111_B5
zmp_ph111_B8
zmp_ph111_B6
zmp_ph111_B7
zmp_ph111_B9
zmp_ph111_B10
zmp_ph114_G4
zmp_ph114_G11
zmp_ph114_H1
zmp_ph114_H2
zmp_ph114_A9
zmp_ph114_A11
zmp_ph114_B2
zmp_ph114_B4
zmp_ph114_D8
zmp_ph114_E2

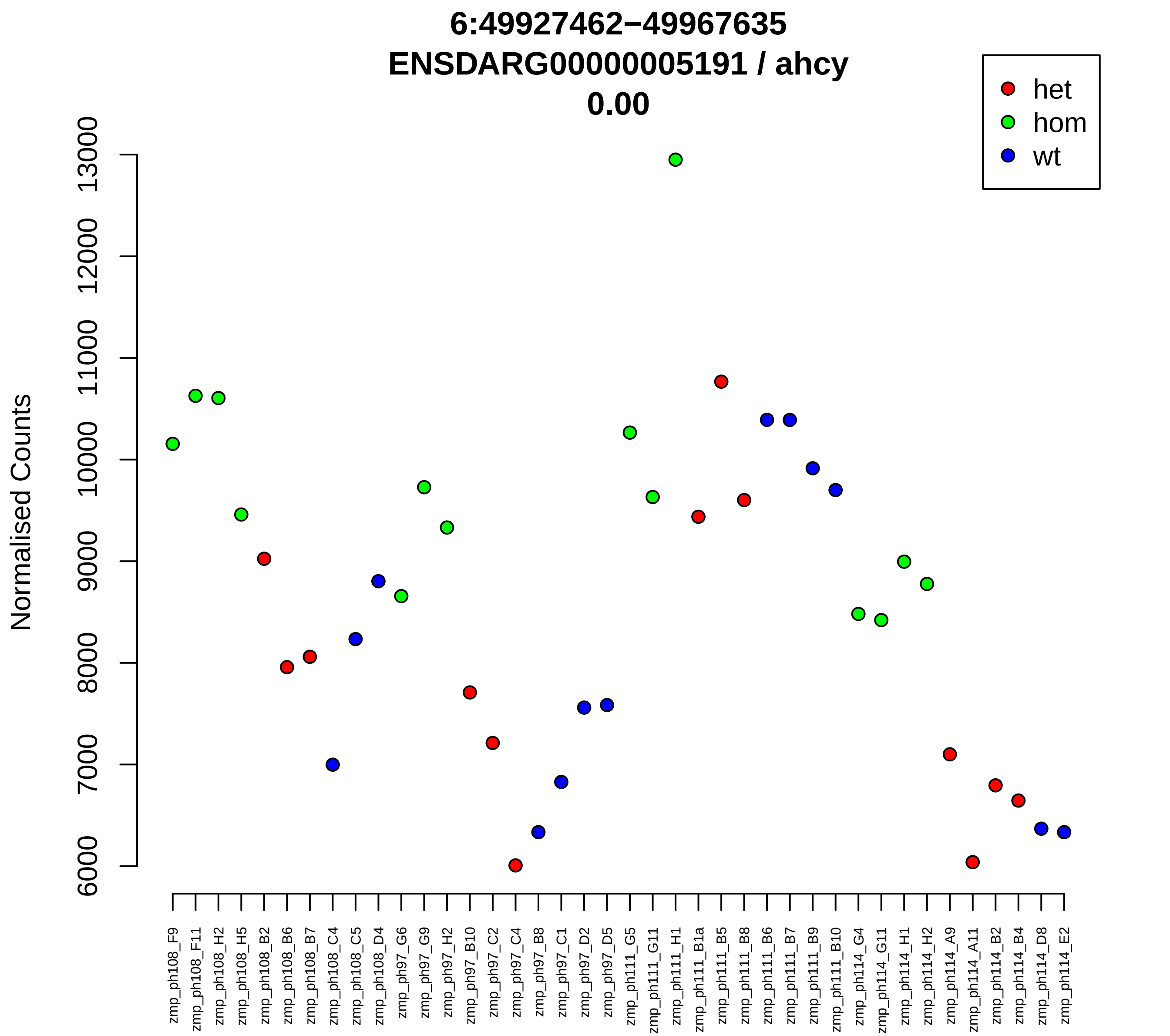

6:49927462−49967635
ENSDARG00000005191 / ahcy
0.00
het
hom
wt
Normalised Counts
6000
7000
8000
9000
10000
11000
12000
13000
zmp_ph108_F9
zmp_ph108_F11
zmp_ph108_H2
zmp_ph108_H5
zmp_ph108_B2
zmp_ph108_B6
zmp_ph108_B7
zmp_ph108_C4
zmp_ph108_C5
zmp_ph108_D4
zmp_ph97_G6
zmp_ph97_G9
zmp_ph97_H2
zmp_ph97_B10
zmp_ph97_C2
zmp_ph97_C4
zmp_ph97_B8
zmp_ph97_C1
zmp_ph97_D2
zmp_ph97_D5
zmp_ph111_G5
zmp_ph111_G11
zmp_ph111_H1
zmp_ph111_B1a
zmp_ph111_B5
zmp_ph111_B8
zmp_ph111_B6
zmp_ph111_B7
zmp_ph111_B9
zmp_ph111_B10
zmp_ph114_G4
zmp_ph114_G11
zmp_ph114_H1
zmp_ph114_H2
zmp_ph114_A9
zmp_ph114_A11
zmp_ph114_B2
zmp_ph114_B4
zmp_ph114_D8
zmp_ph114_E2

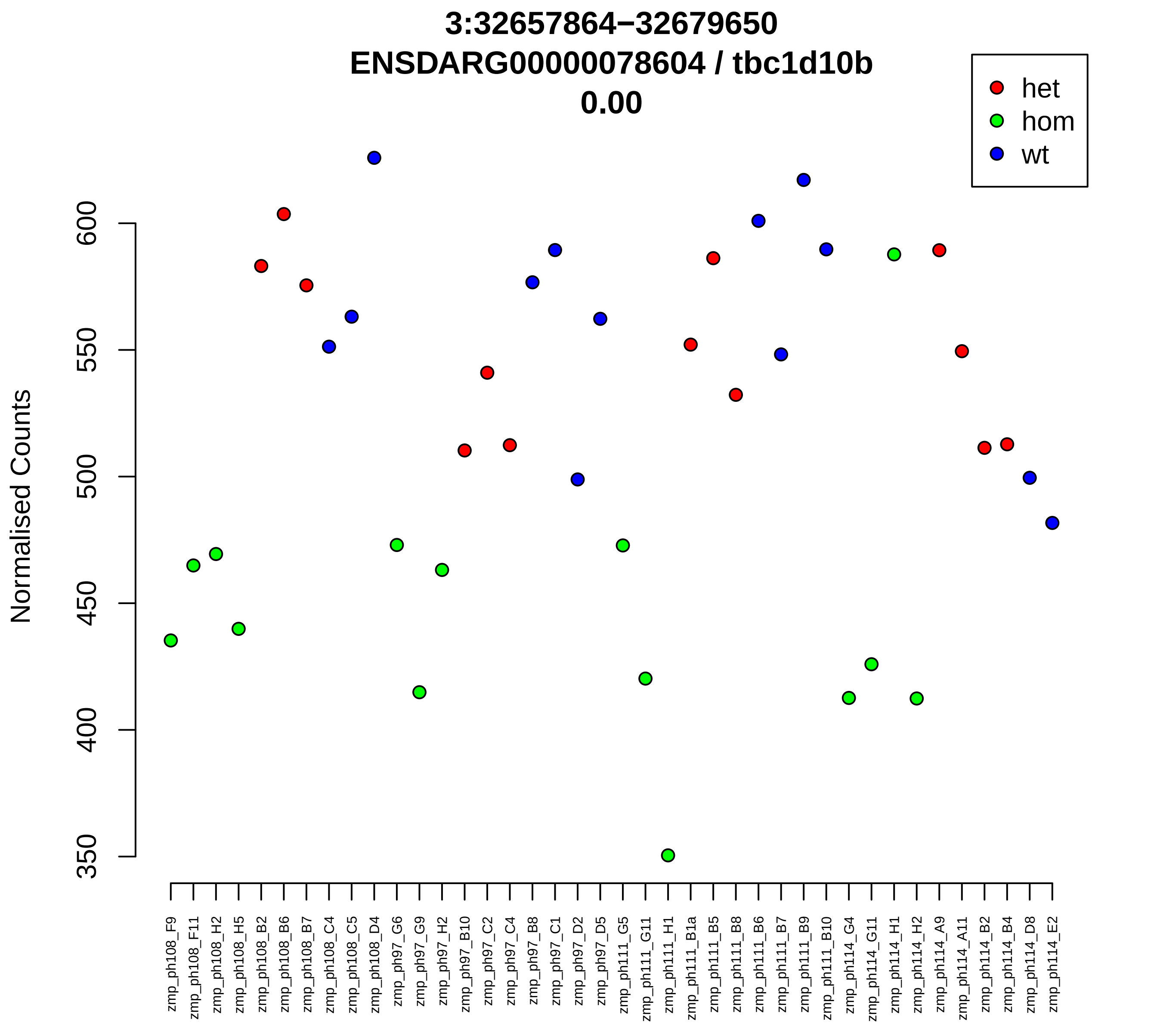
3:32657864−32679650
ENSDARG00000078604 / tbc1d10b
0.00
het
hom
wt
Normalised Counts
350
400
450
500
550
600
zmp_ph108_F9
zmp_ph108_F11
zmp_ph108_H2
zmp_ph108_H5
zmp_ph108_B2
zmp_ph108_B6
zmp_ph108_B7
zmp_ph108_C4
zmp_ph108_C5
zmp_ph108_D4
zmp_ph97_G6
zmp_ph97_G9
zmp_ph97_H2
zmp_ph97_B10
zmp_ph97_C2
zmp_ph97_C4
zmp_ph97_B8
zmp_ph97_C1
zmp_ph97_D2
zmp_ph97_D5
zmp_ph111_G5
zmp_ph111_G11
zmp_ph111_H1
zmp_ph111_B1a
zmp_ph111_B5
zmp_ph111_B8
zmp_ph111_B6
zmp_ph111_B7
zmp_ph111_B9
zmp_ph111_B10
zmp_ph114_G4
zmp_ph114_G11
zmp_ph114_H1
zmp_ph114_H2
zmp_ph114_A9
zmp_ph114_A11
zmp_ph114_B2
zmp_ph114_B4
zmp_ph114_D8
zmp_ph114_E2

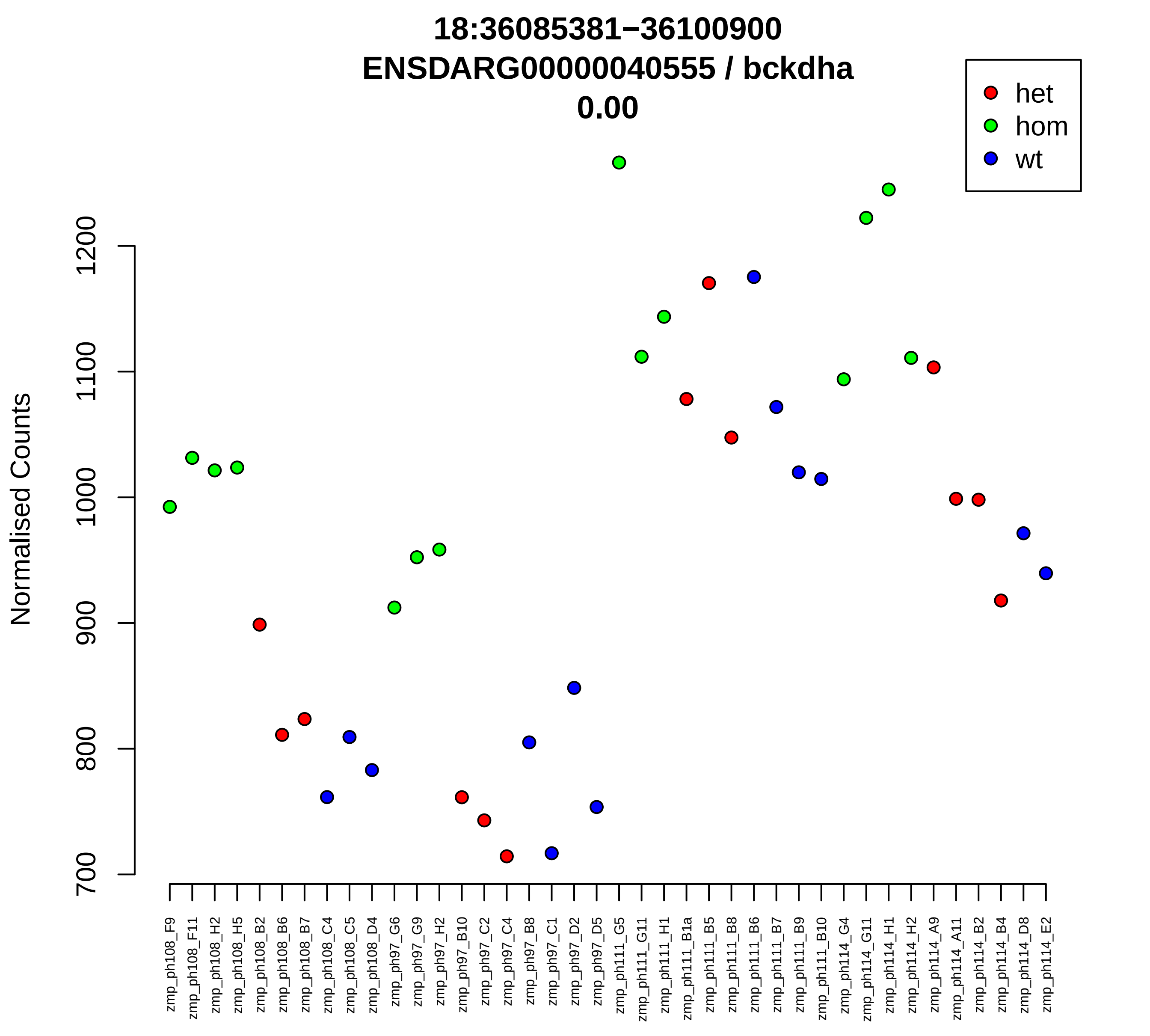

18:36085381−36100900
ENSDARG00000040555 / bckdha
0.00
het
hom
wt
Normalised Counts
700
800
900
1000
1100
1200
zmp_ph108_F9
zmp_ph108_F11
zmp_ph108_H2
zmp_ph108_H5
zmp_ph108_B2
zmp_ph108_B6
zmp_ph108_B7
zmp_ph108_C4
zmp_ph108_C5
zmp_ph108_D4
zmp_ph97_G6
zmp_ph97_G9
zmp_ph97_H2
zmp_ph97_B10
zmp_ph97_C2
zmp_ph97_C4
zmp_ph97_B8
zmp_ph97_C1
zmp_ph97_D2
zmp_ph97_D5
zmp_ph111_G5
zmp_ph111_G11
zmp_ph111_H1
zmp_ph111_B1a
zmp_ph111_B5
zmp_ph111_B8
zmp_ph111_B6
zmp_ph111_B7
zmp_ph111_B9
zmp_ph111_B10
zmp_ph114_G4
zmp_ph114_G11
zmp_ph114_H1
zmp_ph114_H2
zmp_ph114_A9
zmp_ph114_A11
zmp_ph114_B2
zmp_ph114_B4
zmp_ph114_D8
zmp_ph114_E2

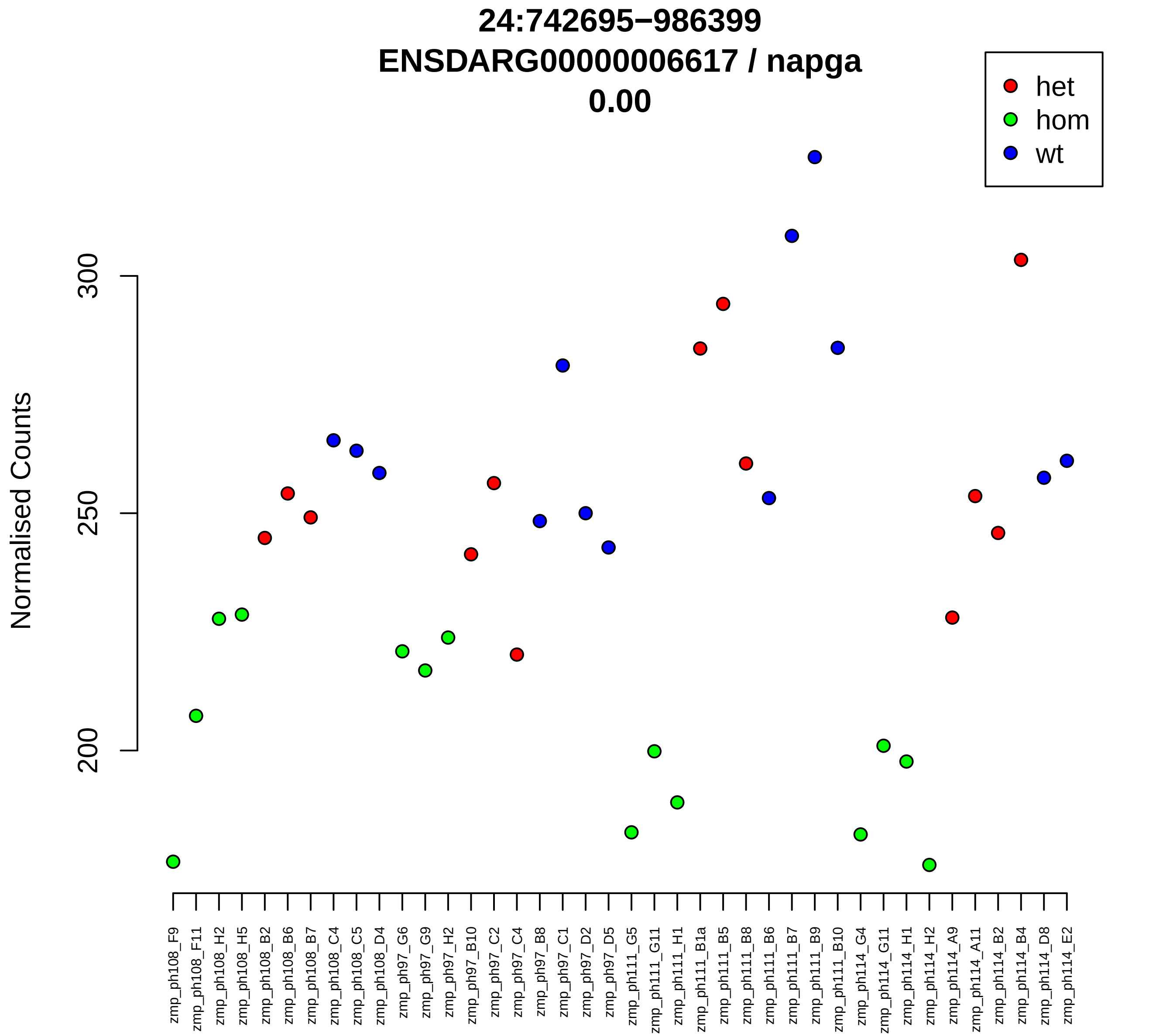
24:742695−986399
ENSDARG00000006617 / napga
0.00
het
hom
wt
Normalised Counts
200
250
300
zmp_ph108_F9
zmp_ph108_F11
zmp_ph108_H2
zmp_ph108_H5
zmp_ph108_B2
zmp_ph108_B6
zmp_ph108_B7
zmp_ph108_C4
zmp_ph108_C5
zmp_ph108_D4
zmp_ph97_G6
zmp_ph97_G9
zmp_ph97_H2
zmp_ph97_B10
zmp_ph97_C2
zmp_ph97_C4
zmp_ph97_B8
zmp_ph97_C1
zmp_ph97_D2
zmp_ph97_D5
zmp_ph111_G5
zmp_ph111_G11
zmp_ph111_H1
zmp_ph111_B1a
zmp_ph111_B5
zmp_ph111_B8
zmp_ph111_B6
zmp_ph111_B7
zmp_ph111_B9
zmp_ph111_B10
zmp_ph114_G4
zmp_ph114_G11
zmp_ph114_H1
zmp_ph114_H2
zmp_ph114_A9
zmp_ph114_A11
zmp_ph114_B2
zmp_ph114_B4
zmp_ph114_D8
zmp_ph114_E2

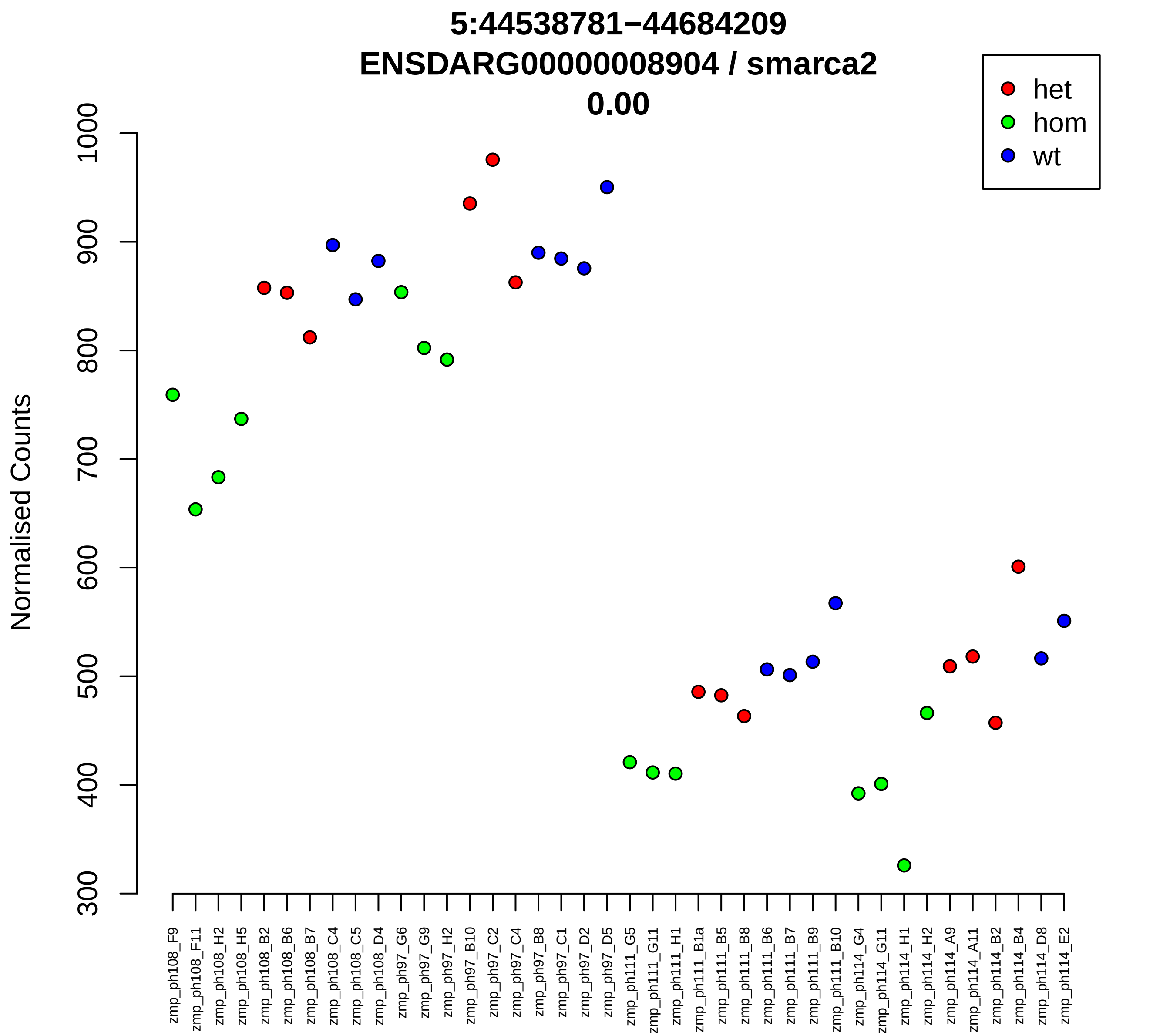

5:44538781−44684209
ENSDARG00000008904 / smarca2
0.00
het
hom
wt
Normalised Counts
300
400
500
600
700
800
900
1000
zmp_ph108_F9
zmp_ph108_F11
zmp_ph108_H2
zmp_ph108_H5
zmp_ph108_B2
zmp_ph108_B6
zmp_ph108_B7
zmp_ph108_C4
zmp_ph108_C5
zmp_ph108_D4
zmp_ph97_G6
zmp_ph97_G9
zmp_ph97_H2
zmp_ph97_B10
zmp_ph97_C2
zmp_ph97_C4
zmp_ph97_B8
zmp_ph97_C1
zmp_ph97_D2
zmp_ph97_D5
zmp_ph111_G5
zmp_ph111_G11
zmp_ph111_H1
zmp_ph111_B1a
zmp_ph111_B5
zmp_ph111_B8
zmp_ph111_B6
zmp_ph111_B7
zmp_ph111_B9
zmp_ph111_B10
zmp_ph114_G4
zmp_ph114_G11
zmp_ph114_H1
zmp_ph114_H2
zmp_ph114_A9
zmp_ph114_A11
zmp_ph114_B2
zmp_ph114_B4
zmp_ph114_D8
zmp_ph114_E2

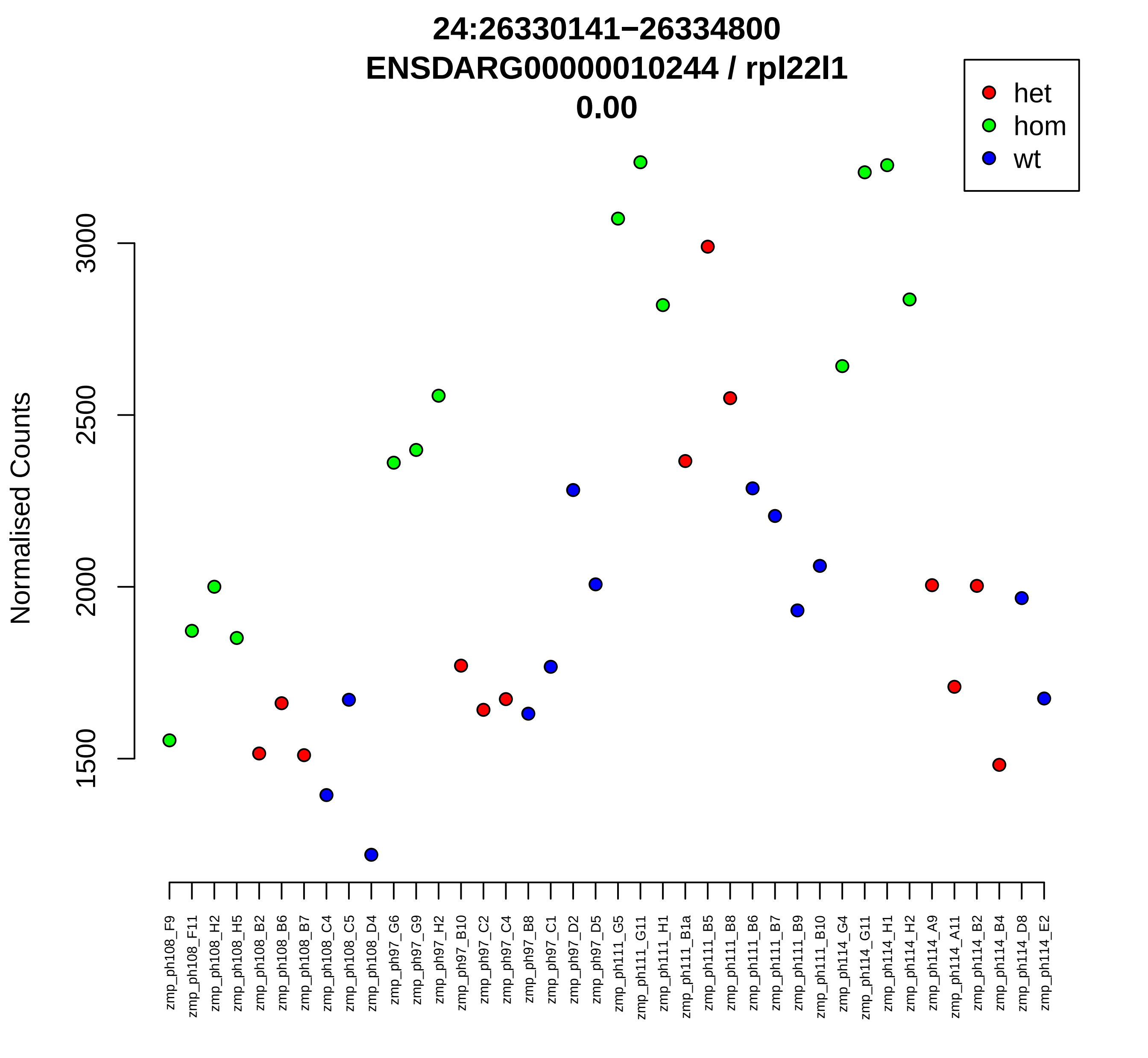
24:26330141−26334800
ENSDARG00000010244 / rpl22l1
0.00
het
hom
wt
Normalised Counts
1500
2000
2500
3000
zmp_ph108_F9
zmp_ph108_F11
zmp_ph108_H2
zmp_ph108_H5
zmp_ph108_B2
zmp_ph108_B6
zmp_ph108_B7
zmp_ph108_C4
zmp_ph108_C5
zmp_ph108_D4
zmp_ph97_G6
zmp_ph97_G9
zmp_ph97_H2
zmp_ph97_B10
zmp_ph97_C2
zmp_ph97_C4
zmp_ph97_B8
zmp_ph97_C1
zmp_ph97_D2
zmp_ph97_D5
zmp_ph111_G5
zmp_ph111_G11
zmp_ph111_H1
zmp_ph111_B1a
zmp_ph111_B5
zmp_ph111_B8
zmp_ph111_B6
zmp_ph111_B7
zmp_ph111_B9
zmp_ph111_B10
zmp_ph114_G4
zmp_ph114_G11
zmp_ph114_H1
zmp_ph114_H2
zmp_ph114_A9
zmp_ph114_A11
zmp_ph114_B2
zmp_ph114_B4
zmp_ph114_D8
zmp_ph114_E2

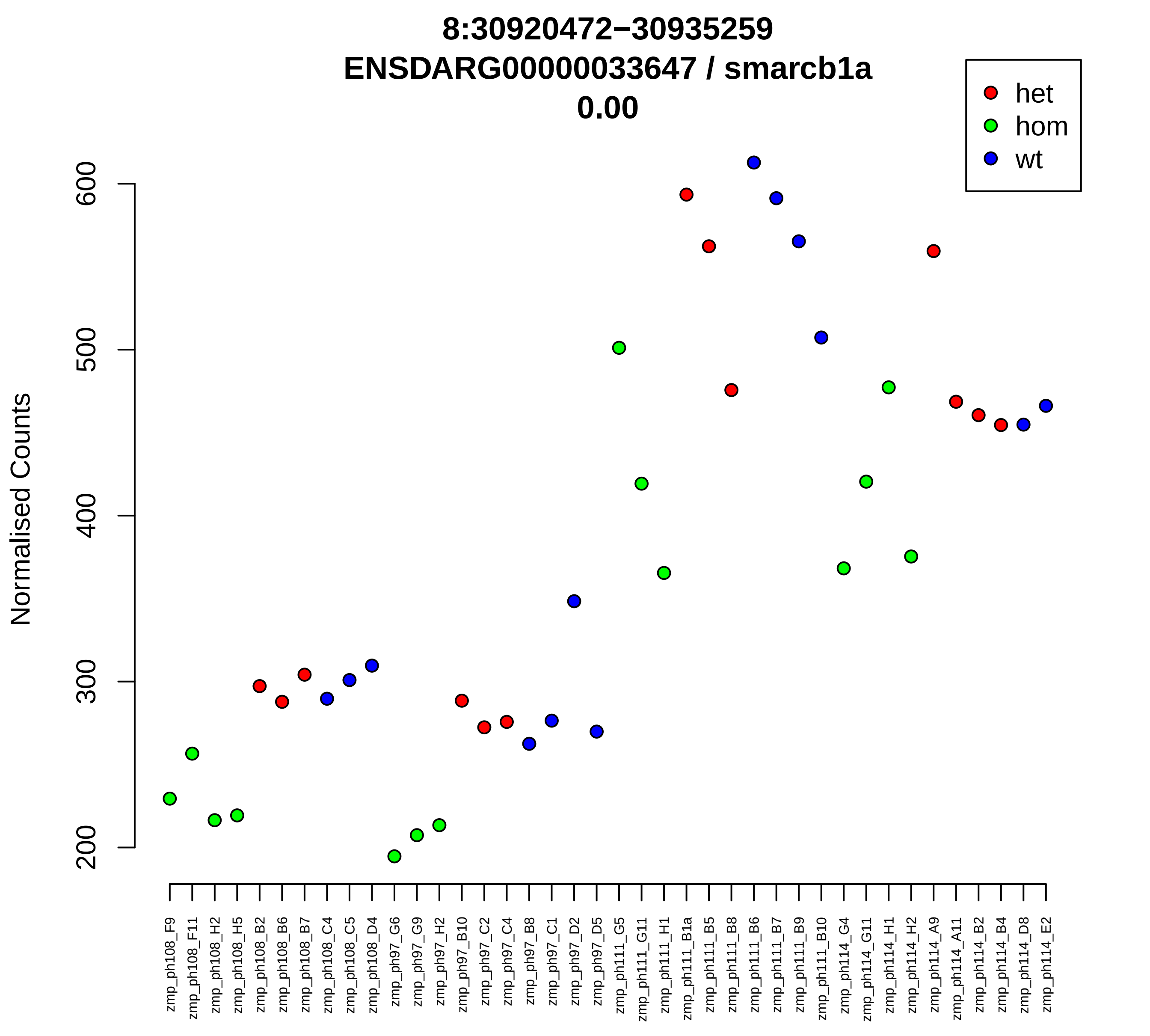

8:30920472−30935259
ENSDARG00000033647 / smarcb1a
0.00
het
hom
wt
Normalised Counts
200
300
400
500
600
zmp_ph108_F9
zmp_ph108_F11
zmp_ph108_H2
zmp_ph108_H5
zmp_ph108_B2
zmp_ph108_B6
zmp_ph108_B7
zmp_ph108_C4
zmp_ph108_C5
zmp_ph108_D4
zmp_ph97_G6
zmp_ph97_G9
zmp_ph97_H2
zmp_ph97_B10
zmp_ph97_C2
zmp_ph97_C4
zmp_ph97_B8
zmp_ph97_C1
zmp_ph97_D2
zmp_ph97_D5
zmp_ph111_G5
zmp_ph111_G11
zmp_ph111_H1
zmp_ph111_B1a
zmp_ph111_B5
zmp_ph111_B8
zmp_ph111_B6
zmp_ph111_B7
zmp_ph111_B9
zmp_ph111_B10
zmp_ph114_G4
zmp_ph114_G11
zmp_ph114_H1
zmp_ph114_H2
zmp_ph114_A9
zmp_ph114_A11
zmp_ph114_B2
zmp_ph114_B4
zmp_ph114_D8
zmp_ph114_E2

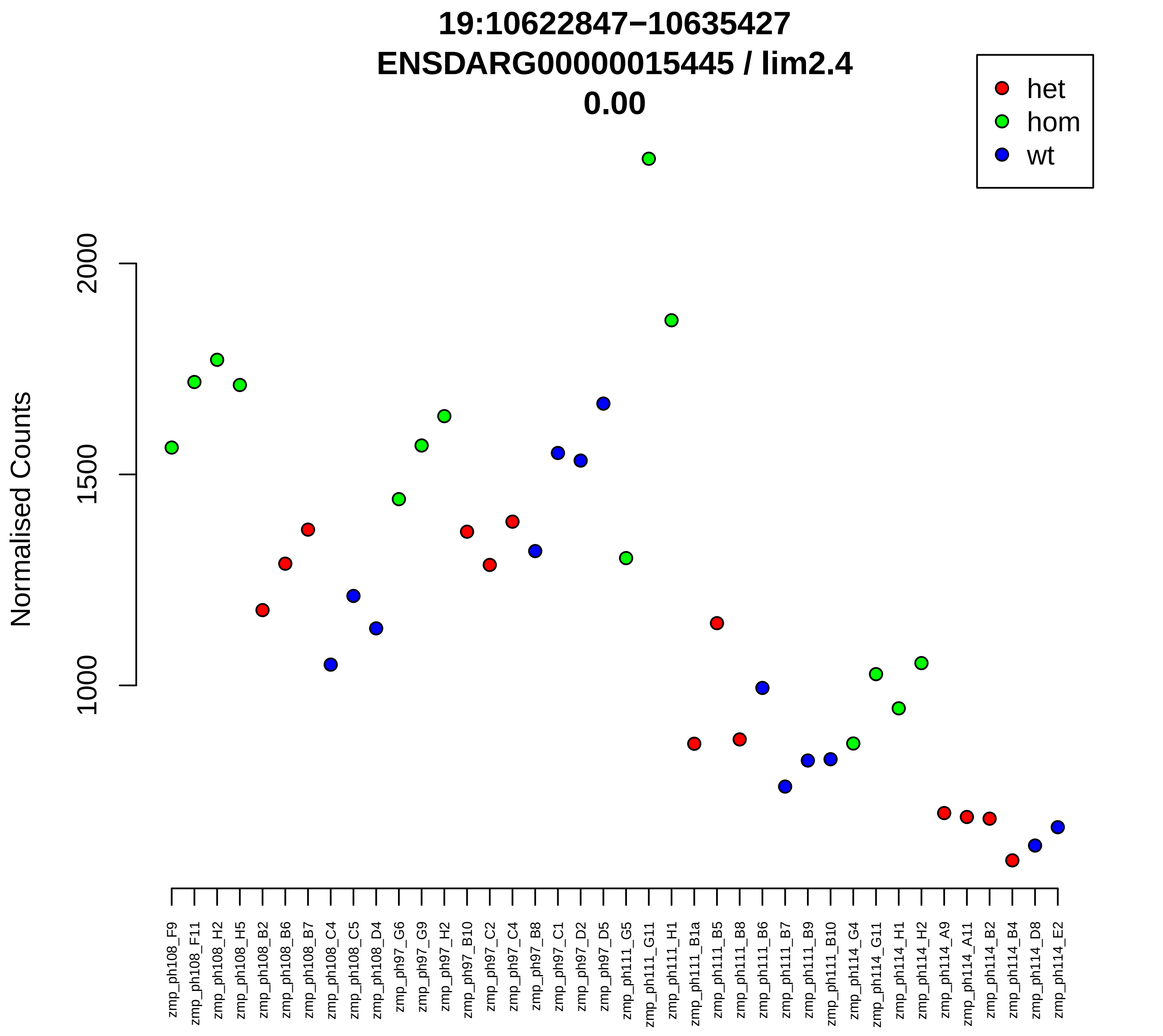

19:10622847−10635427
ENSDARG00000015445 / lim2.4
0.00
het
hom
wt
Normalised Counts
2000
1500
1000
zmp_ph108_F9
zmp_ph108_F11
zmp_ph108_H2
zmp_ph108_H5
zmp_ph108_B2
zmp_ph108_B6
zmp_ph108_B7
zmp_ph108_C4
zmp_ph108_C5
zmp_ph108_D4
zmp_ph97_G6
zmp_ph97_G9
zmp_ph97_H2
zmp_ph97_B10
zmp_ph97_C2
zmp_ph97_C4
zmp_ph97_B8
zmp_ph97_C1
zmp_ph97_D2
zmp_ph97_D5
zmp_ph111_G5
zmp_ph111_G11
zmp_ph111_H1
zmp_ph111_B1a
zmp_ph111_B5
zmp_ph111_B8
zmp_ph111_B6
zmp_ph111_B7
zmp_ph111_B9
zmp_ph111_B10
zmp_ph114_G4
zmp_ph114_G11
zmp_ph114_H1
zmp_ph114_H2
zmp_ph114_A9
zmp_ph114_A11
zmp_ph114_B2
zmp_ph114_B4
zmp_ph114_D8
zmp_ph114_E2

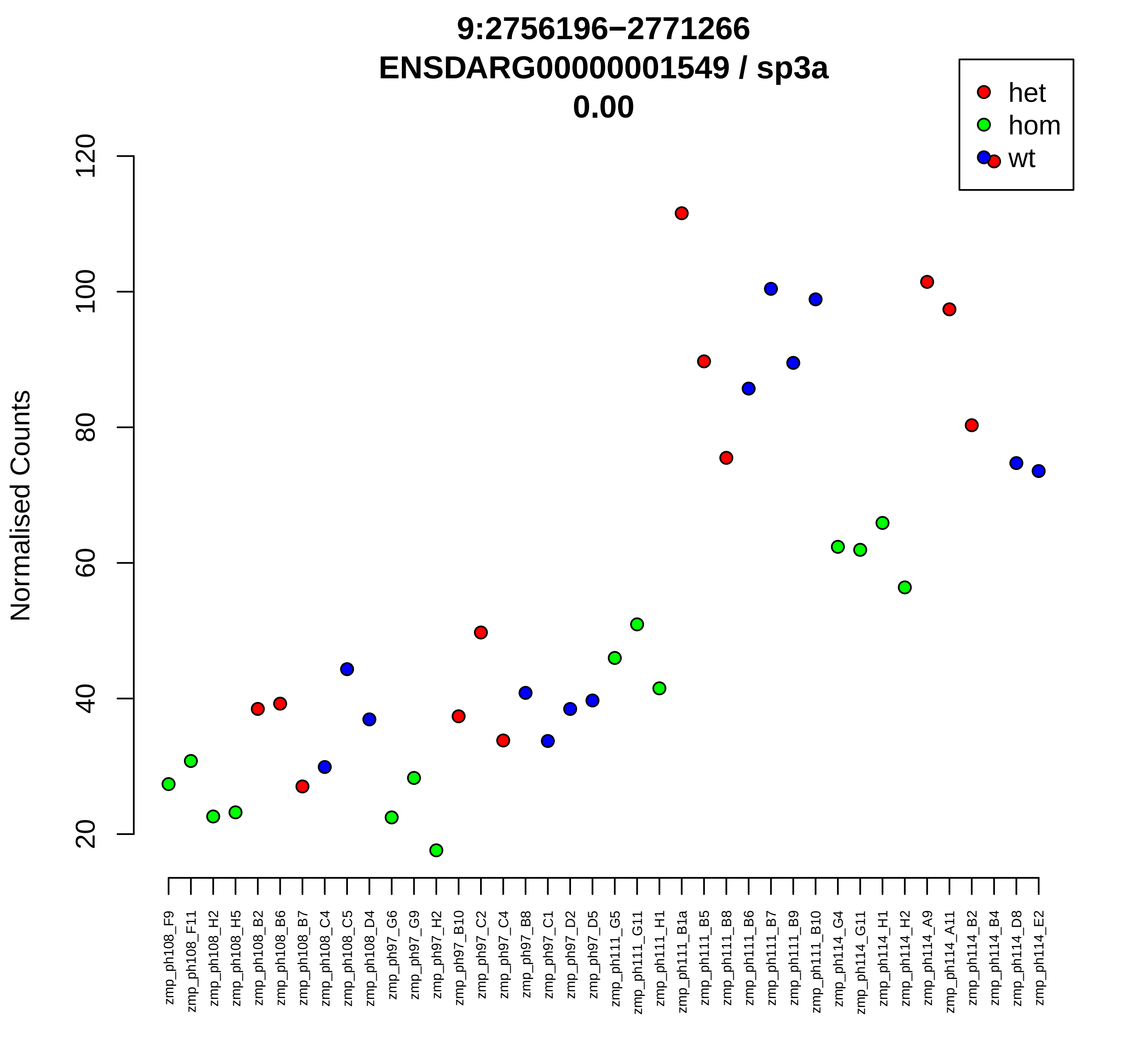

9:2756196−2771266
ENSDARG00000001549 / sp3a
0.00
het
hom
wt
Normalised Counts
20
40
60
80
100
120
zmp_ph108_F9
zmp_ph108_F11
zmp_ph108_H2
zmp_ph108_H5
zmp_ph108_B2
zmp_ph108_B6
zmp_ph108_B7
zmp_ph108_C4
zmp_ph108_C5
zmp_ph108_D4
zmp_ph97_G6
zmp_ph97_G9
zmp_ph97_H2
zmp_ph97_B10
zmp_ph97_C2
zmp_ph97_C4
zmp_ph97_B8
zmp_ph97_C1
zmp_ph97_D2
zmp_ph97_D5
zmp_ph111_G5
zmp_ph111_G11
zmp_ph111_H1
zmp_ph111_B1a
zmp_ph111_B5
zmp_ph111_B8
zmp_ph111_B6
zmp_ph111_B7
zmp_ph111_B9
zmp_ph111_B10
zmp_ph114_G4
zmp_ph114_G11
zmp_ph114_H1
zmp_ph114_H2
zmp_ph114_A9
zmp_ph114_A11
zmp_ph114_B2
zmp_ph114_B4
zmp_ph114_D8
zmp_ph114_E2

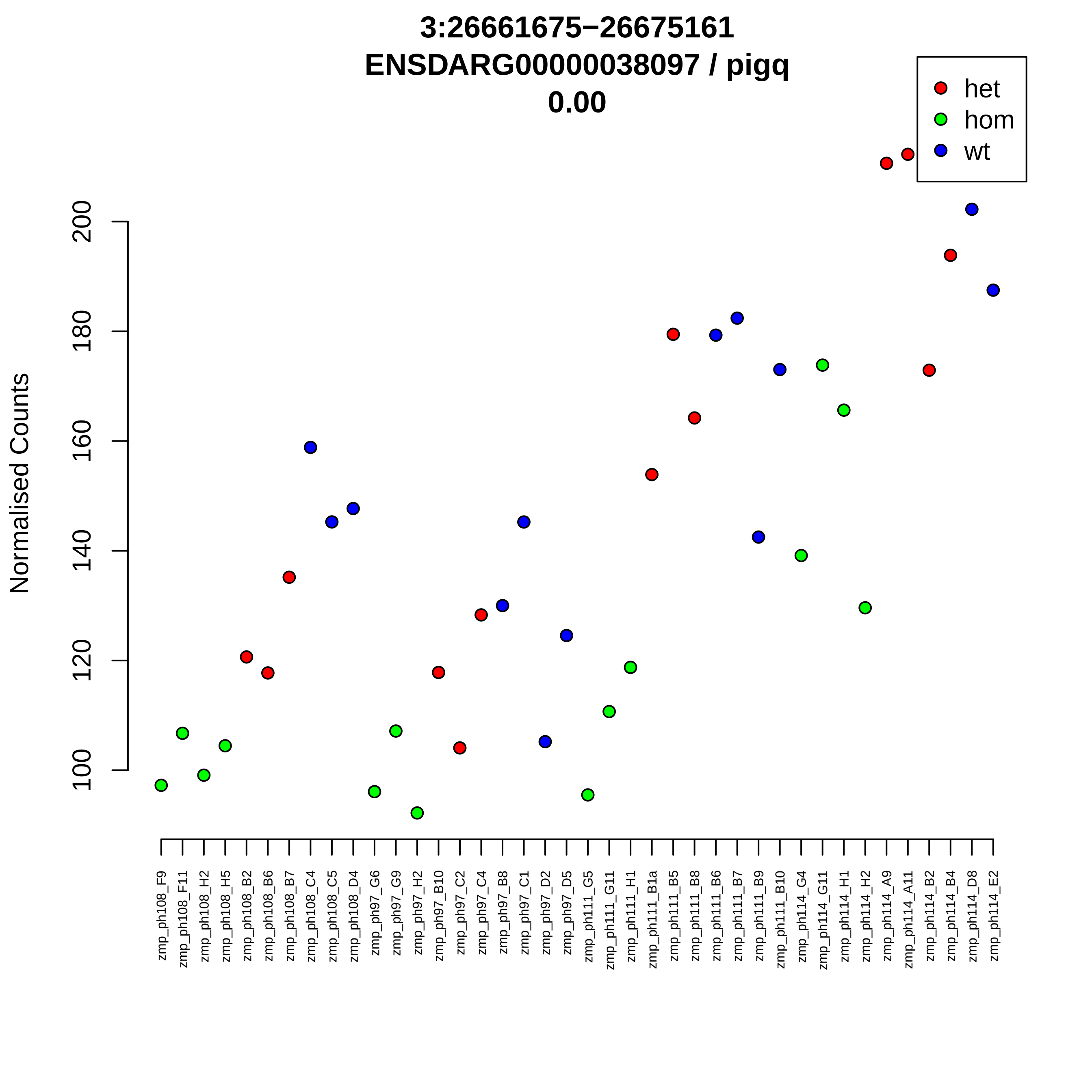

3:26661675−26675161
ENSDARG00000038097 / pigq
0.00
het
hom
wt
Normalised Counts
100
120
140
160
180
200
zmp_ph108_F9
zmp_ph108_F11
zmp_ph108_H2
zmp_ph108_H5
zmp_ph108_B2
zmp_ph108_B6
zmp_ph108_B7
zmp_ph108_C4
zmp_ph108_C5
zmp_ph108_D4
zmp_ph97_G6
zmp_ph97_G9
zmp_ph97_H2
zmp_ph97_B10
zmp_ph97_C2
zmp_ph97_C4
zmp_ph97_B8
zmp_ph97_C1
zmp_ph97_D2
zmp_ph97_D5
zmp_ph111_G5
zmp_ph111_G11
zmp_ph111_H1
zmp_ph111_B1a
zmp_ph111_B5
zmp_ph111_B8
zmp_ph111_B6
zmp_ph111_B7
zmp_ph111_B9
zmp_ph111_B10
zmp_ph114_G4
zmp_ph114_G11
zmp_ph114_H1
zmp_ph114_H2
zmp_ph114_A9
zmp_ph114_A11
zmp_ph114_B2
zmp_ph114_B4
zmp_ph114_D8
zmp_ph114_E2

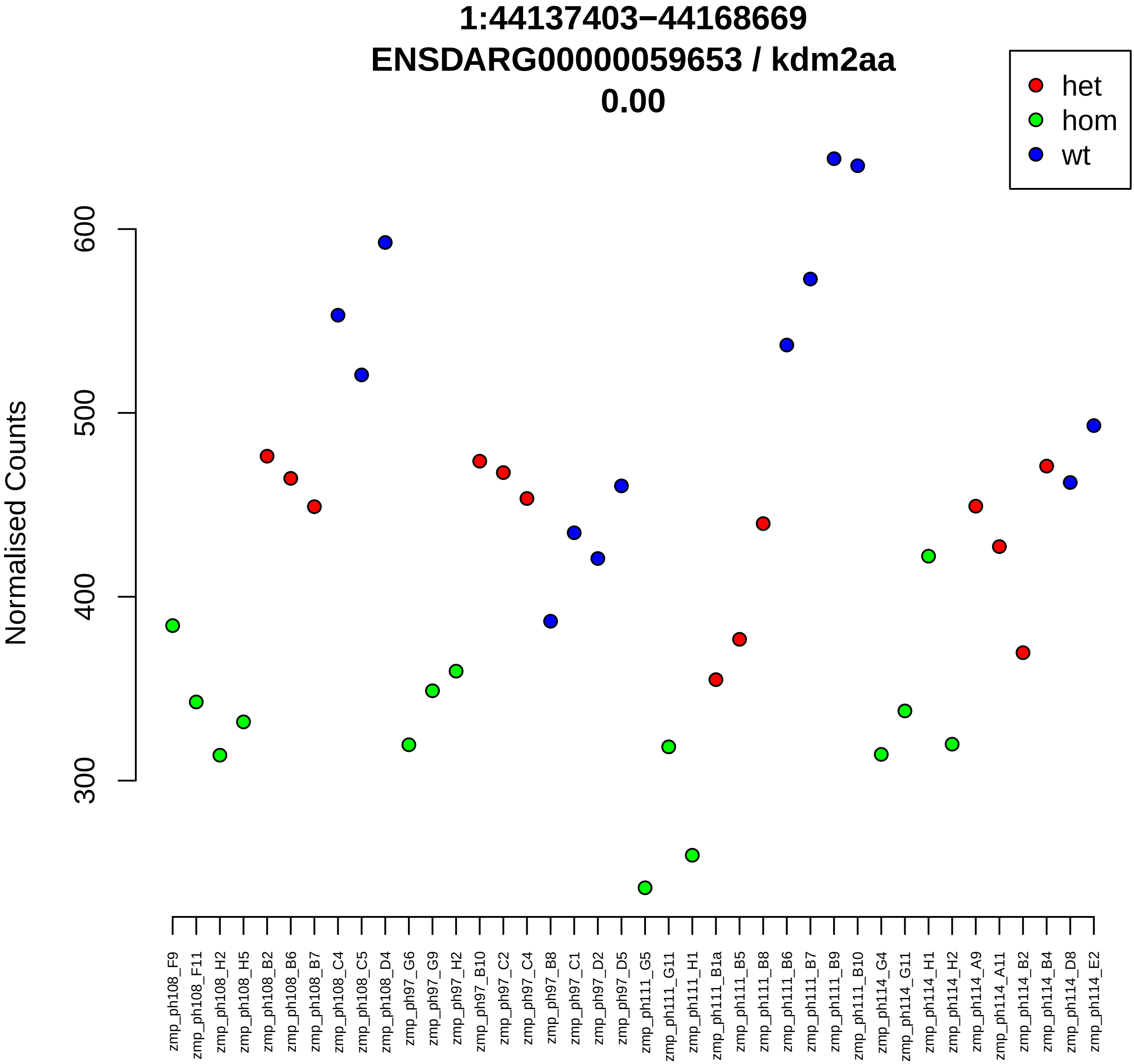
1:44137403−44168669
ENSDARG00000059653 / kdm2aa
0.00
het
hom
wt
Normalised Counts
300
400
500
600
zmp_ph108_F9
zmp_ph108_F11
zmp_ph108_H2
zmp_ph108_H5
zmp_ph108_B2
zmp_ph108_B6
zmp_ph108_B7
zmp_ph108_C4
zmp_ph108_C5
zmp_ph108_D4
zmp_ph97_G6
zmp_ph97_G9
zmp_ph97_H2
zmp_ph97_B10
zmp_ph97_C2
zmp_ph97_C4
zmp_ph97_B8
zmp_ph97_C1
zmp_ph97_D2
zmp_ph97_D5
zmp_ph111_G5
zmp_ph111_G11
zmp_ph111_H1
zmp_ph111_B1a
zmp_ph111_B5
zmp_ph111_B8
zmp_ph111_B6
zmp_ph111_B7
zmp_ph111_B9
zmp_ph111_B10
zmp_ph114_G4
zmp_ph114_G11
zmp_ph114_H1
zmp_ph114_H2
zmp_ph114_A9
zmp_ph114_A11
zmp_ph114_B2
zmp_ph114_B4
zmp_ph114_D8
zmp_ph114_E2

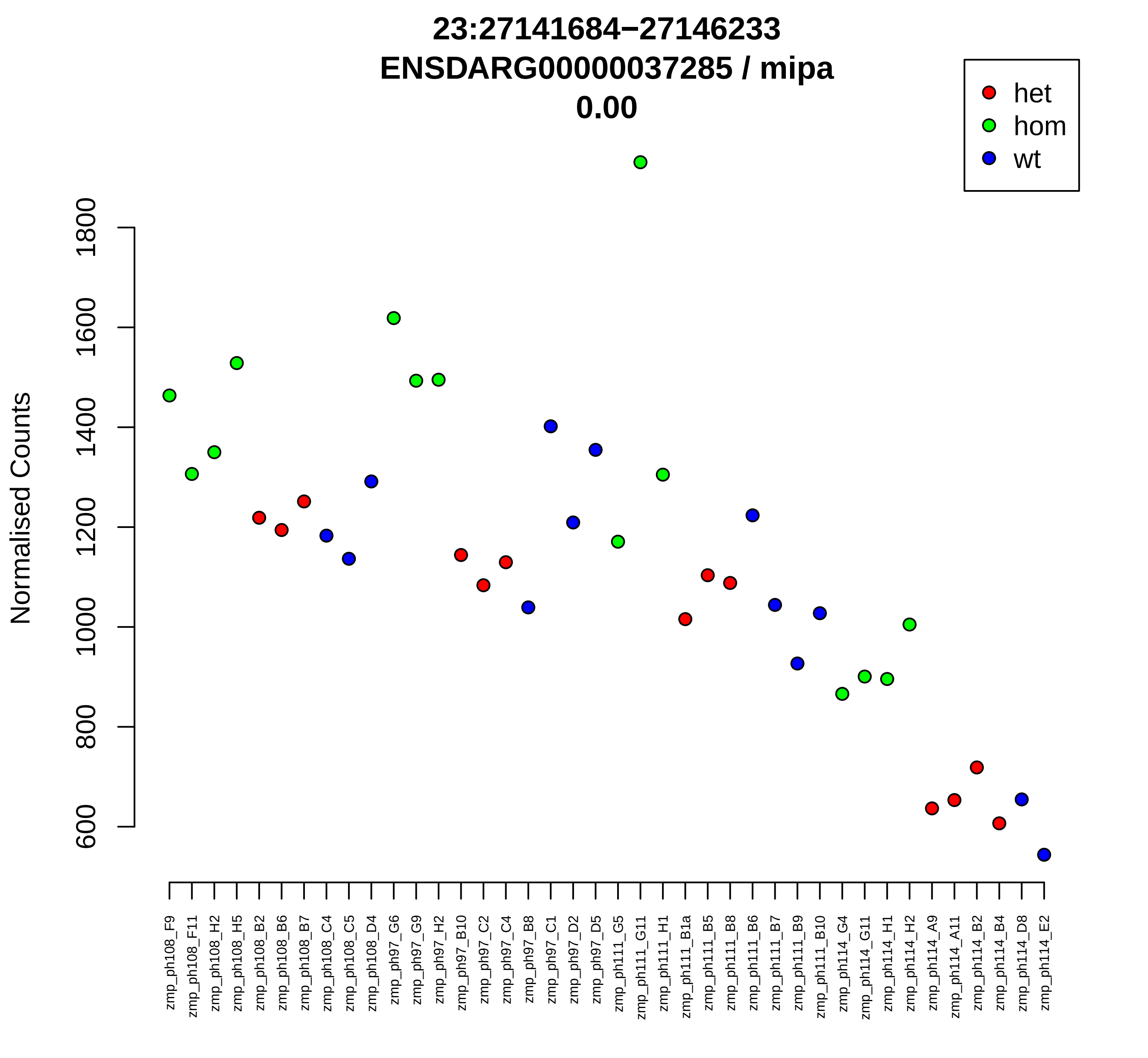
23:27141684−27146233
ENSDARG00000037285 / mipa
0.00
het
hom
wt
Normalised Counts
600
800
1000
1200
1400
1600
1800
zmp_ph108_F9
zmp_ph108_F11
zmp_ph108_H2
zmp_ph108_H5
zmp_ph108_B2
zmp_ph108_B6
zmp_ph108_B7
zmp_ph108_C4
zmp_ph108_C5
zmp_ph108_D4
zmp_ph97_G6
zmp_ph97_G9
zmp_ph97_H2
zmp_ph97_B10
zmp_ph97_C2
zmp_ph97_C4
zmp_ph97_B8
zmp_ph97_C1
zmp_ph97_D2
zmp_ph97_D5
zmp_ph111_G5
zmp_ph111_G11
zmp_ph111_H1
zmp_ph111_B1a
zmp_ph111_B5
zmp_ph111_B8
zmp_ph111_B6
zmp_ph111_B7
zmp_ph111_B9
zmp_ph111_B10
zmp_ph114_G4
zmp_ph114_G11
zmp_ph114_H1
zmp_ph114_H2
zmp_ph114_A9
zmp_ph114_A11
zmp_ph114_B2
zmp_ph114_B4
zmp_ph114_D8
zmp_ph114_E2

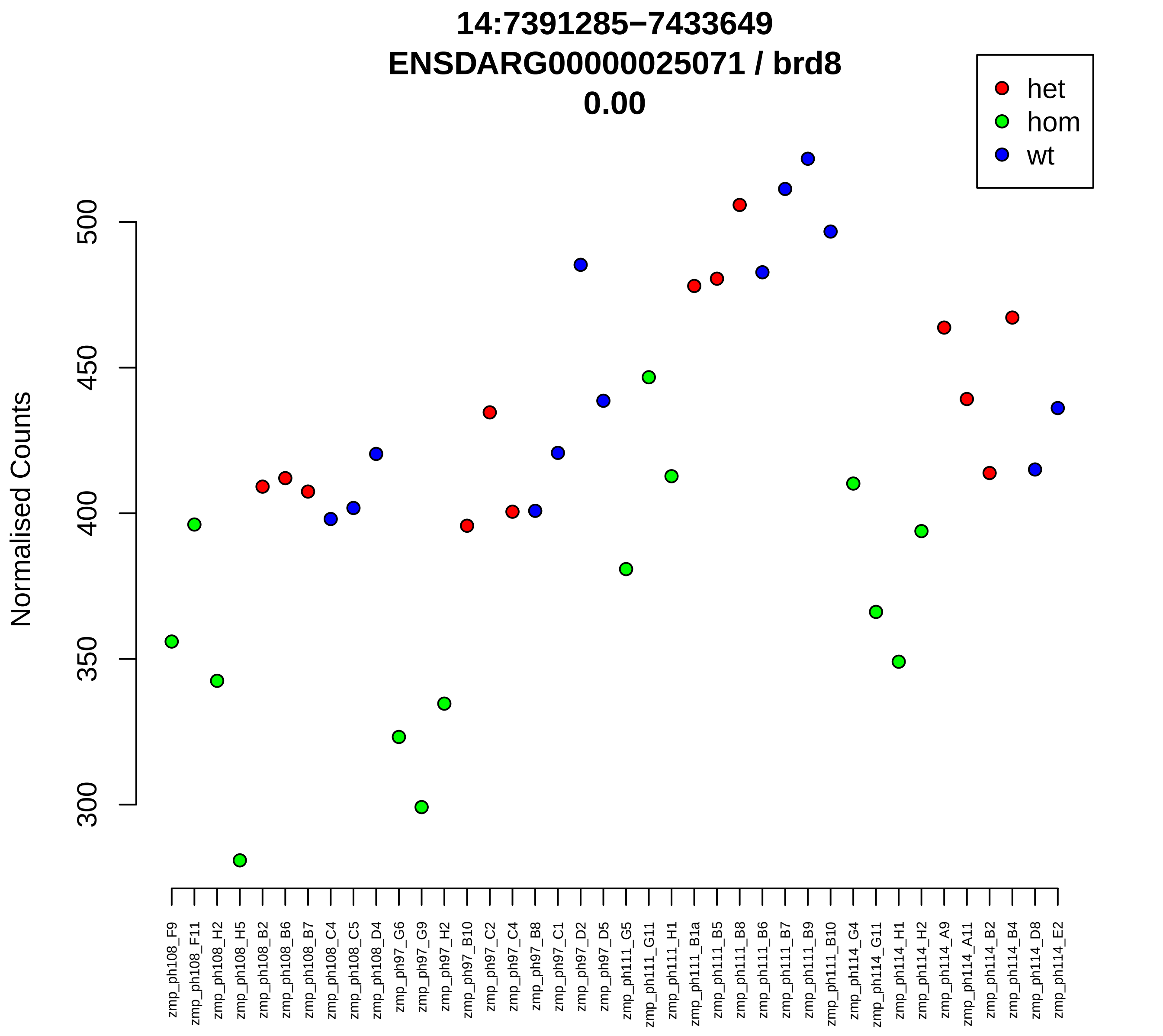
14:7391285−7433649
ENSDARG00000025071 / brd8
0.00
het
hom
wt
Normalised Counts
300
350
400
450
500
zmp_ph108_F9
zmp_ph108_F11
zmp_ph108_H2
zmp_ph108_H5
zmp_ph108_B2
zmp_ph108_B6
zmp_ph108_B7
zmp_ph108_C4
zmp_ph108_C5
zmp_ph108_D4
zmp_ph97_G6
zmp_ph97_G9
zmp_ph97_H2
zmp_ph97_B10
zmp_ph97_C2
zmp_ph97_C4
zmp_ph97_B8
zmp_ph97_C1
zmp_ph97_D2
zmp_ph97_D5
zmp_ph111_G5
zmp_ph111_G11
zmp_ph111_H1
zmp_ph111_B1a
zmp_ph111_B5
zmp_ph111_B8
zmp_ph111_B6
zmp_ph111_B7
zmp_ph111_B9
zmp_ph111_B10
zmp_ph114_G4
zmp_ph114_G11
zmp_ph114_H1
zmp_ph114_H2
zmp_ph114_A9
zmp_ph114_A11
zmp_ph114_B2
zmp_ph114_B4
zmp_ph114_D8
zmp_ph114_E2

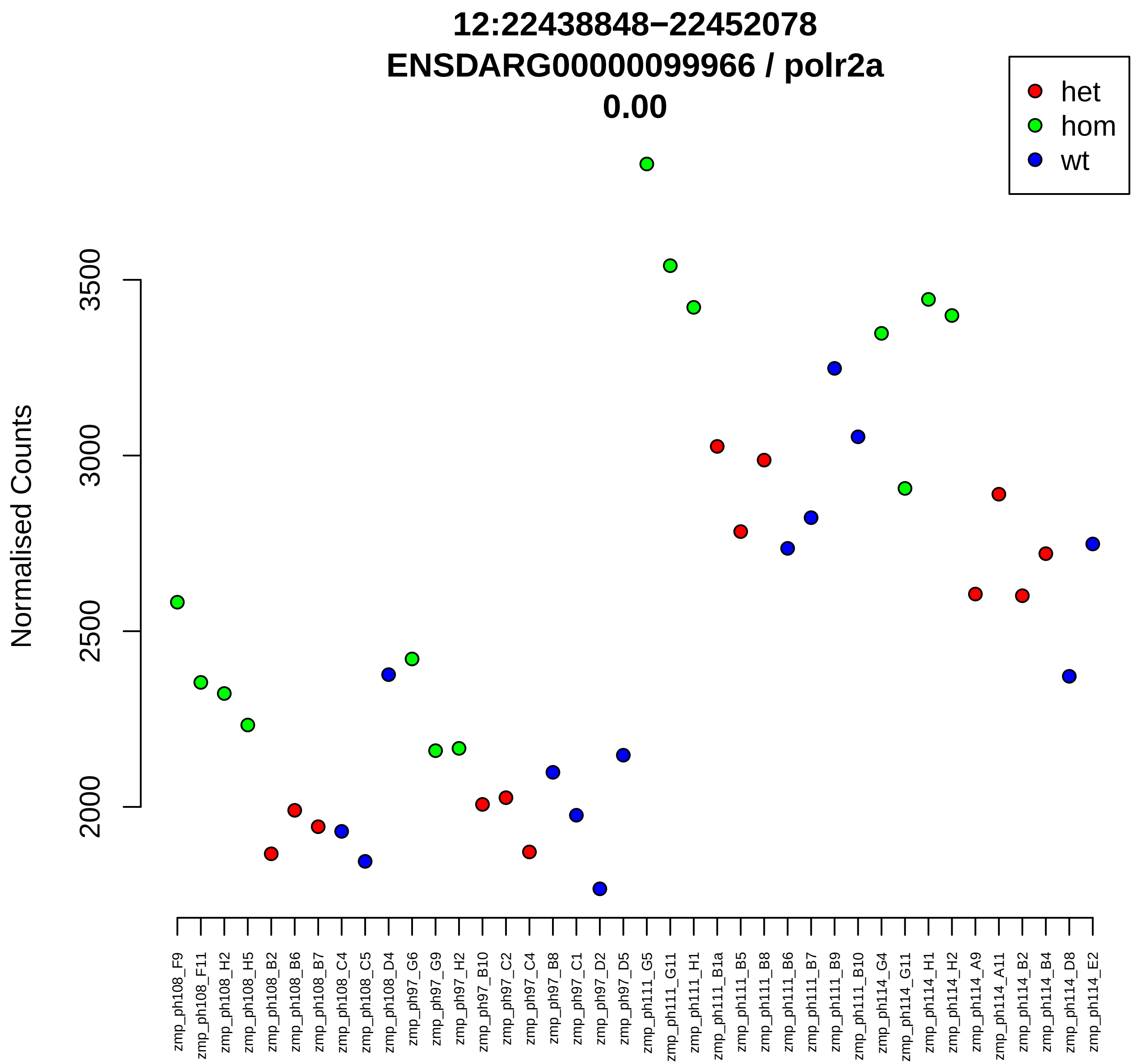
12:22438848−22452078
ENSDARG00000099966 / polr2a
0.00
het
hom
wt
Normalised Counts
2000
2500
3000
3500
zmp_ph108_F9
zmp_ph108_F11
zmp_ph108_H2
zmp_ph108_H5
zmp_ph108_B2
zmp_ph108_B6
zmp_ph108_B7
zmp_ph108_C4
zmp_ph108_C5
zmp_ph108_D4
zmp_ph97_G6
zmp_ph97_G9
zmp_ph97_H2
zmp_ph97_B10
zmp_ph97_C2
zmp_ph97_C4
zmp_ph97_B8
zmp_ph97_C1
zmp_ph97_D2
zmp_ph97_D5
zmp_ph111_G5
zmp_ph111_G11
zmp_ph111_H1
zmp_ph111_B1a
zmp_ph111_B5
zmp_ph111_B8
zmp_ph111_B6
zmp_ph111_B7
zmp_ph111_B9
zmp_ph111_B10
zmp_ph114_G4
zmp_ph114_G11
zmp_ph114_H1
zmp_ph114_H2
zmp_ph114_A9
zmp_ph114_A11
zmp_ph114_B2
zmp_ph114_B4
zmp_ph114_D8
zmp_ph114_E2

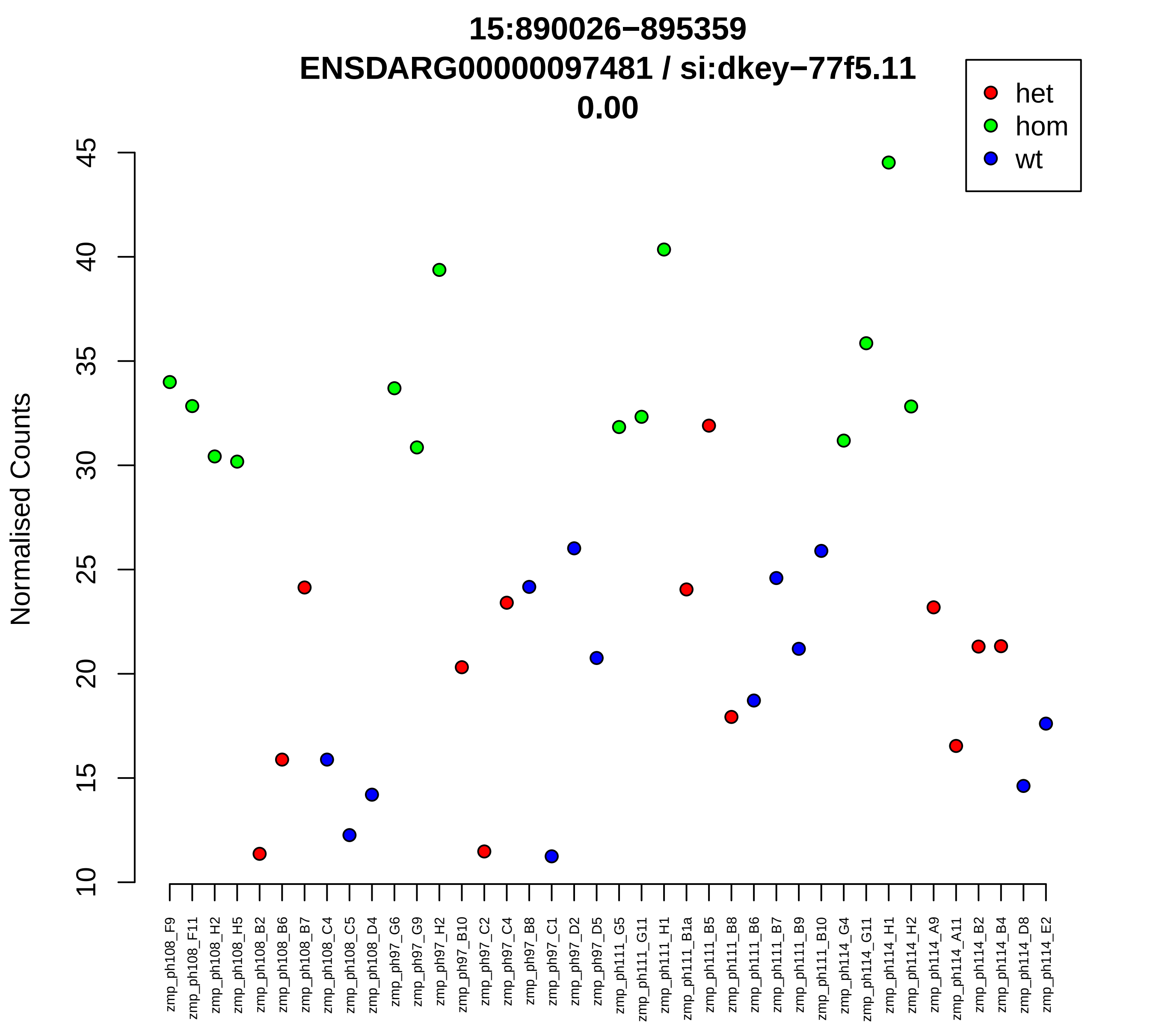
15:890026−895359
ENSDARG00000097481 / si:dkey−77f5.11
0.00
het
hom
wt
Normalised Counts
10
15
20
25
30
35
40
45
zmp_ph108_F9
zmp_ph108_F11
zmp_ph108_H2
zmp_ph108_H5
zmp_ph108_B2
zmp_ph108_B6
zmp_ph108_B7
zmp_ph108_C4
zmp_ph108_C5
zmp_ph108_D4
zmp_ph97_G6
zmp_ph97_G9
zmp_ph97_H2
zmp_ph97_B10
zmp_ph97_C2
zmp_ph97_C4
zmp_ph97_B8
zmp_ph97_C1
zmp_ph97_D2
zmp_ph97_D5
zmp_ph111_G5
zmp_ph111_G11
zmp_ph111_H1
zmp_ph111_B1a
zmp_ph111_B5
zmp_ph111_B8
zmp_ph111_B6
zmp_ph111_B7
zmp_ph111_B9
zmp_ph111_B10
zmp_ph114_G4
zmp_ph114_G11
zmp_ph114_H1
zmp_ph114_H2
zmp_ph114_A9
zmp_ph114_A11
zmp_ph114_B2
zmp_ph114_B4
zmp_ph114_D8
zmp_ph114_E2

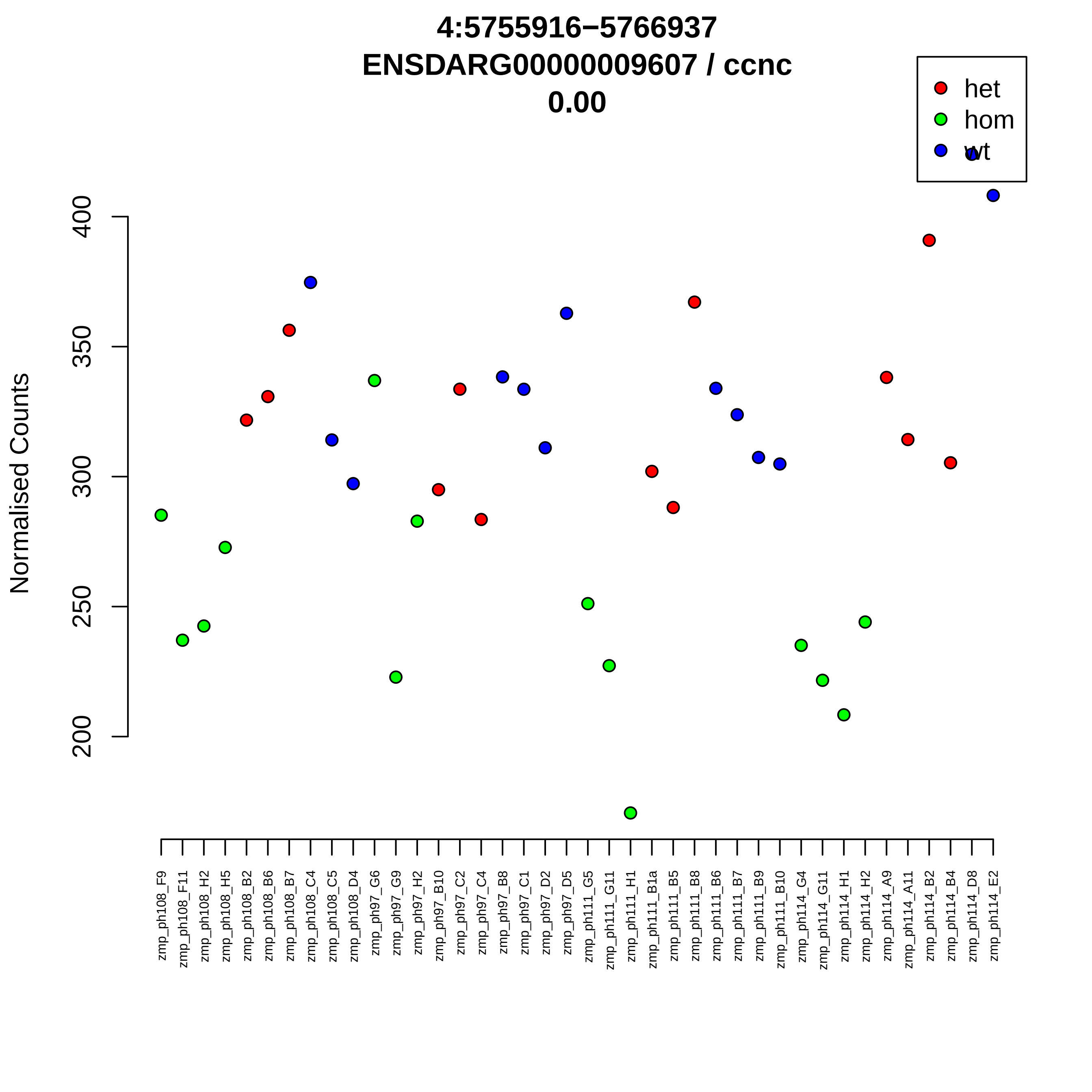
4:5755916−5766937
ENSDARG00000009607 / ccnc
0.00
het
hom
wt
Normalised Counts
200
250
300
350
400
zmp_ph108_F9
zmp_ph108_F11
zmp_ph108_H2
zmp_ph108_H5
zmp_ph108_B2
zmp_ph108_B6
zmp_ph108_B7
zmp_ph108_C4
zmp_ph108_C5
zmp_ph108_D4
zmp_ph97_G6
zmp_ph97_G9
zmp_ph97_H2
zmp_ph97_B10
zmp_ph97_C2
zmp_ph97_C4
zmp_ph97_B8
zmp_ph97_C1
zmp_ph97_D2
zmp_ph97_D5
zmp_ph111_G5
zmp_ph111_G11
zmp_ph111_H1
zmp_ph111_B1a
zmp_ph111_B5
zmp_ph111_B8
zmp_ph111_B6
zmp_ph111_B7
zmp_ph111_B9
zmp_ph111_B10
zmp_ph114_G4
zmp_ph114_G11
zmp_ph114_H1
zmp_ph114_H2
zmp_ph114_A9
zmp_ph114_A11
zmp_ph114_B2
zmp_ph114_B4
zmp_ph114_D8
zmp_ph114_E2

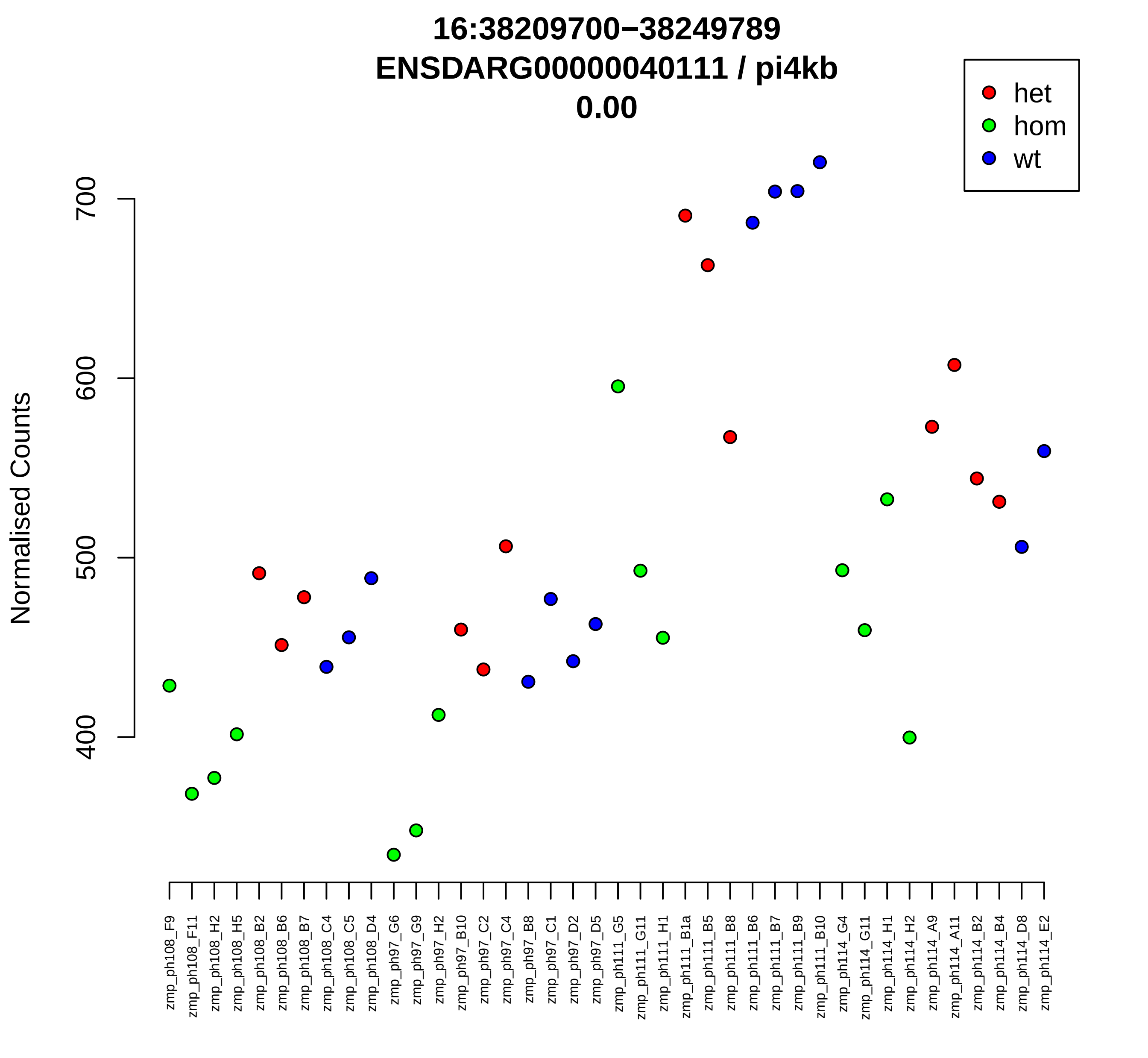
16:38209700−38249789
ENSDARG00000040111 / pi4kb
0.00
het
hom
wt
Normalised Counts
400
500
600
700
zmp_ph108_F9
zmp_ph108_F11
zmp_ph108_H2
zmp_ph108_H5
zmp_ph108_B2
zmp_ph108_B6
zmp_ph108_B7
zmp_ph108_C4
zmp_ph108_C5
zmp_ph108_D4
zmp_ph97_G6
zmp_ph97_G9
zmp_ph97_H2
zmp_ph97_B10
zmp_ph97_C2
zmp_ph97_C4
zmp_ph97_B8
zmp_ph97_C1
zmp_ph97_D2
zmp_ph97_D5
zmp_ph111_G5
zmp_ph111_G11
zmp_ph111_H1
zmp_ph111_B1a
zmp_ph111_B5
zmp_ph111_B8
zmp_ph111_B6
zmp_ph111_B7
zmp_ph111_B9
zmp_ph111_B10
zmp_ph114_G4
zmp_ph114_G11
zmp_ph114_H1
zmp_ph114_H2
zmp_ph114_A9
zmp_ph114_A11
zmp_ph114_B2
zmp_ph114_B4
zmp_ph114_D8
zmp_ph114_E2

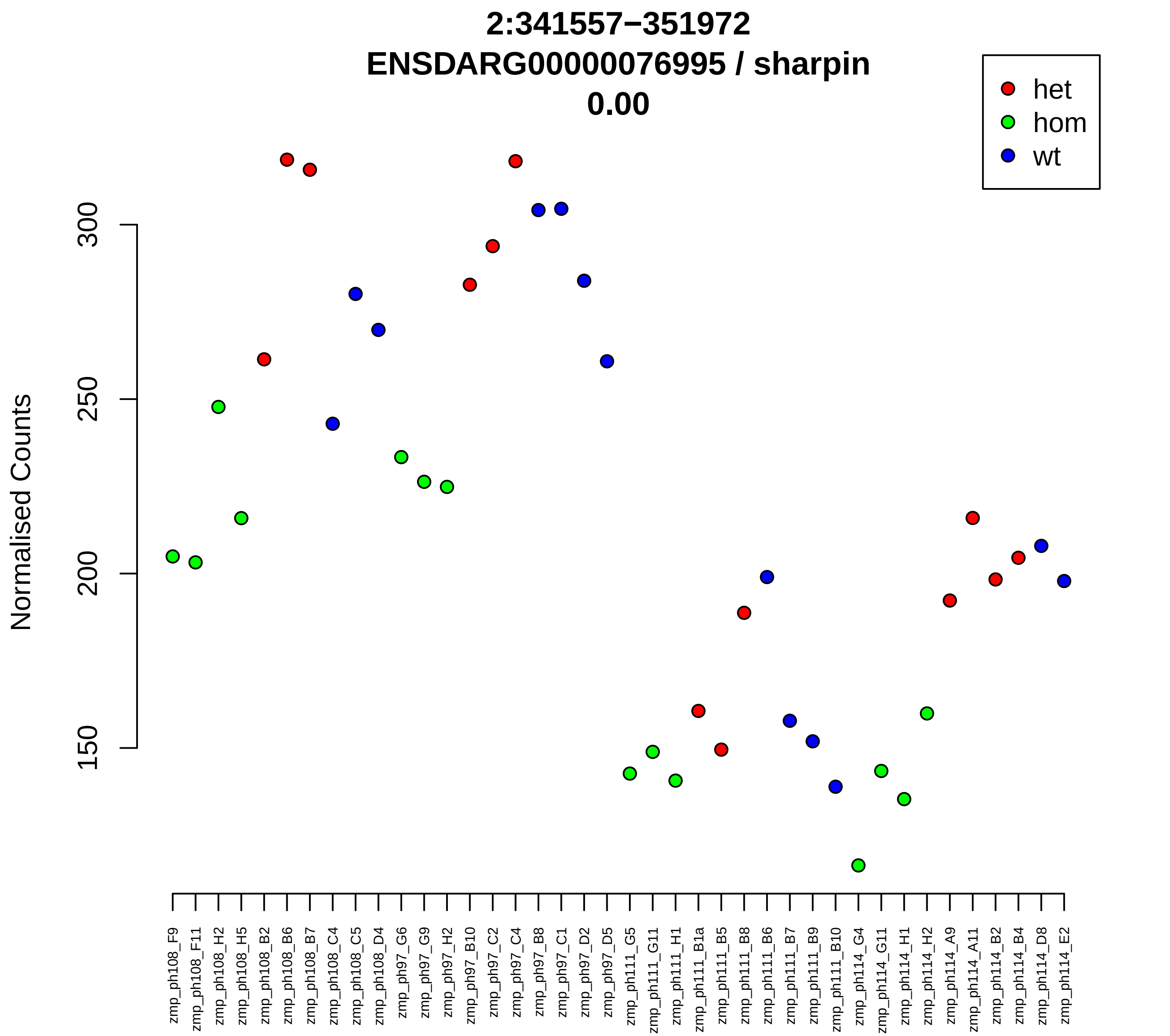

2:341557−351972
ENSDARG00000076995 / sharpin
0.00
het
hom
wt
Normalised Counts
150
200
250
300
zmp_ph108_F9
zmp_ph108_F11
zmp_ph108_H2
zmp_ph108_H5
zmp_ph108_B2
zmp_ph108_B6
zmp_ph108_B7
zmp_ph108_C4
zmp_ph108_C5
zmp_ph108_D4
zmp_ph97_G6
zmp_ph97_G9
zmp_ph97_H2
zmp_ph97_B10
zmp_ph97_C2
zmp_ph97_C4
zmp_ph97_B8
zmp_ph97_C1
zmp_ph97_D2
zmp_ph97_D5
zmp_ph111_G5
zmp_ph111_G11
zmp_ph111_H1
zmp_ph111_B1a
zmp_ph111_B5
zmp_ph111_B8
zmp_ph111_B6
zmp_ph111_B7
zmp_ph111_B9
zmp_ph111_B10
zmp_ph114_G4
zmp_ph114_G11
zmp_ph114_H1
zmp_ph114_H2
zmp_ph114_A9
zmp_ph114_A11
zmp_ph114_B2
zmp_ph114_B4
zmp_ph114_D8
zmp_ph114_E2

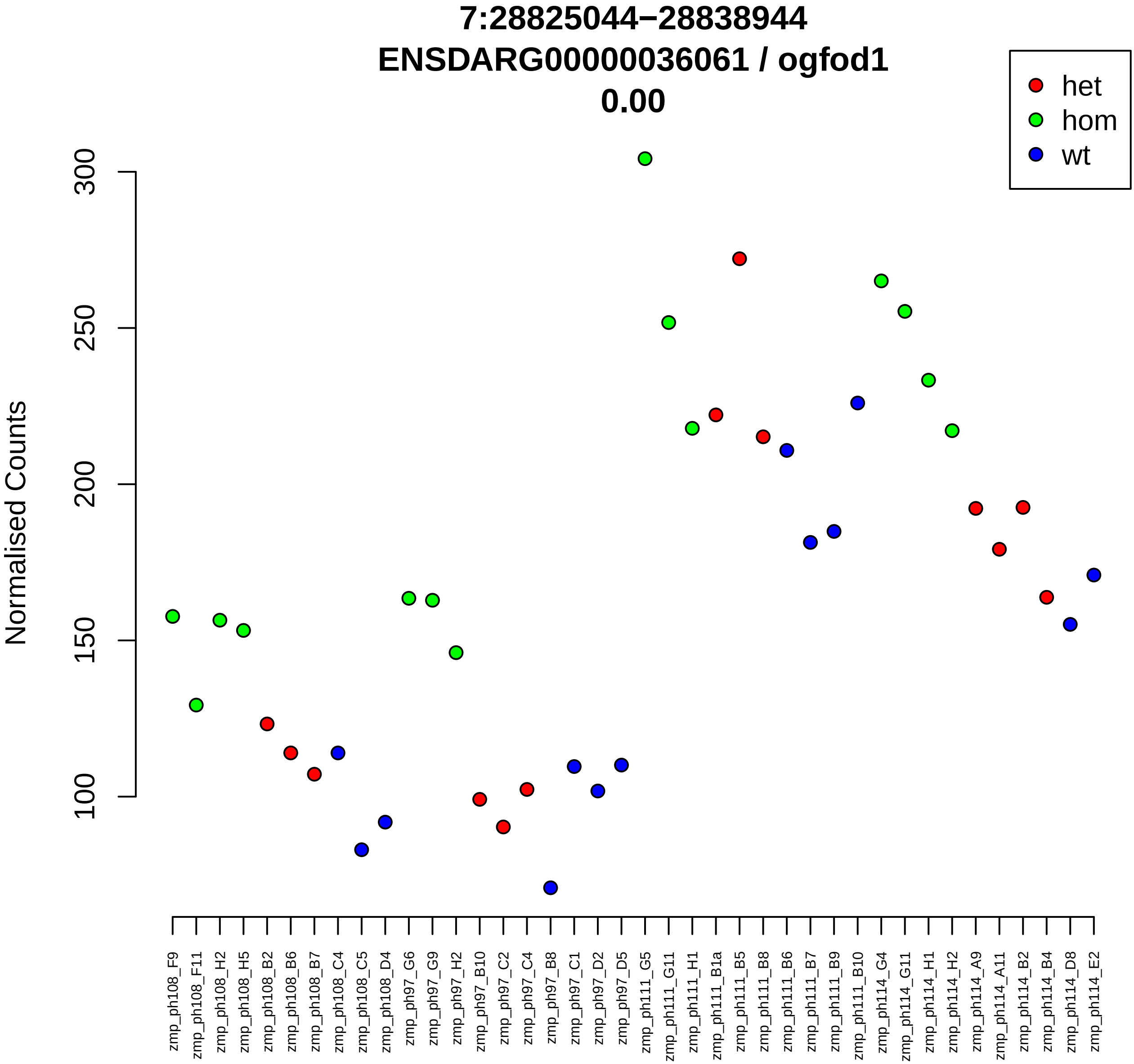

7:28825044−28838944
ENSDARG00000036061 / ogfod1
0.00
het
hom
wt
Normalised Counts
100
150
200
250
300
zmp_ph108_F9
zmp_ph108_F11
zmp_ph108_H2
zmp_ph108_H5
zmp_ph108_B2
zmp_ph108_B6
zmp_ph108_B7
zmp_ph108_C4
zmp_ph108_C5
zmp_ph108_D4
zmp_ph97_G6
zmp_ph97_G9
zmp_ph97_H2
zmp_ph97_B10
zmp_ph97_C2
zmp_ph97_C4
zmp_ph97_B8
zmp_ph97_C1
zmp_ph97_D2
zmp_ph97_D5
zmp_ph111_G5
zmp_ph111_G11
zmp_ph111_H1
zmp_ph111_B1a
zmp_ph111_B5
zmp_ph111_B8
zmp_ph111_B6
zmp_ph111_B7
zmp_ph111_B9
zmp_ph111_B10
zmp_ph114_G4
zmp_ph114_G11
zmp_ph114_H1
zmp_ph114_H2
zmp_ph114_A9
zmp_ph114_A11
zmp_ph114_B2
zmp_ph114_B4
zmp_ph114_D8
zmp_ph114_E2

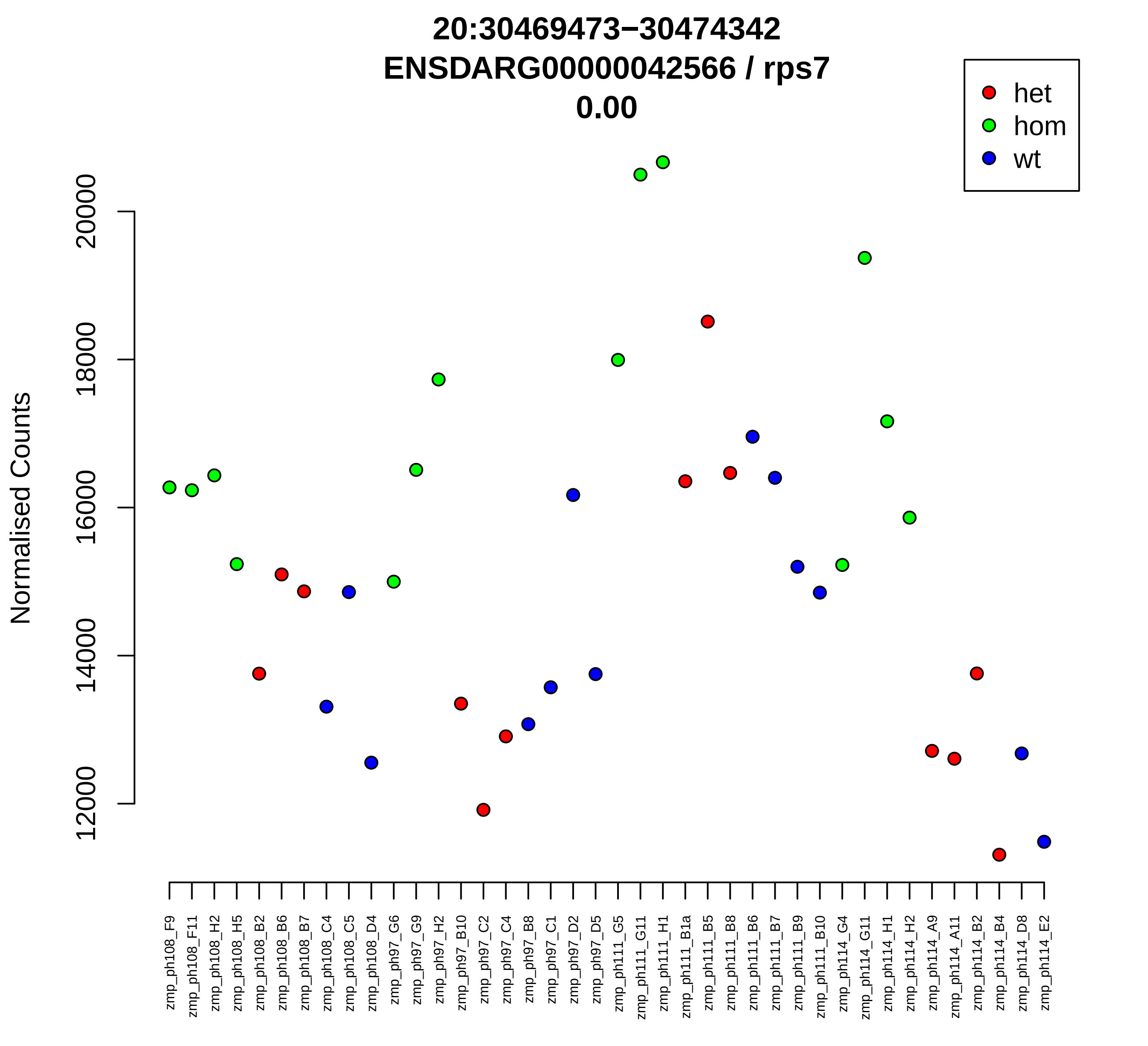
20:30469473−30474342
ENSDARG00000042566 / rps7
0.00
het
hom
wt
Normalised Counts
12000
14000
16000
18000
20000
zmp_ph108_F9
zmp_ph108_F11
zmp_ph108_H2
zmp_ph108_H5
zmp_ph108_B2
zmp_ph108_B6
zmp_ph108_B7
zmp_ph108_C4
zmp_ph108_C5
zmp_ph108_D4
zmp_ph97_G6
zmp_ph97_G9
zmp_ph97_H2
zmp_ph97_B10
zmp_ph97_C2
zmp_ph97_C4
zmp_ph97_B8
zmp_ph97_C1
zmp_ph97_D2
zmp_ph97_D5
zmp_ph111_G5
zmp_ph111_G11
zmp_ph111_H1
zmp_ph111_B1a
zmp_ph111_B5
zmp_ph111_B8
zmp_ph111_B6
zmp_ph111_B7
zmp_ph111_B9
zmp_ph111_B10
zmp_ph114_G4
zmp_ph114_G11
zmp_ph114_H1
zmp_ph114_H2
zmp_ph114_A9
zmp_ph114_A11
zmp_ph114_B2
zmp_ph114_B4
zmp_ph114_D8
zmp_ph114_E2

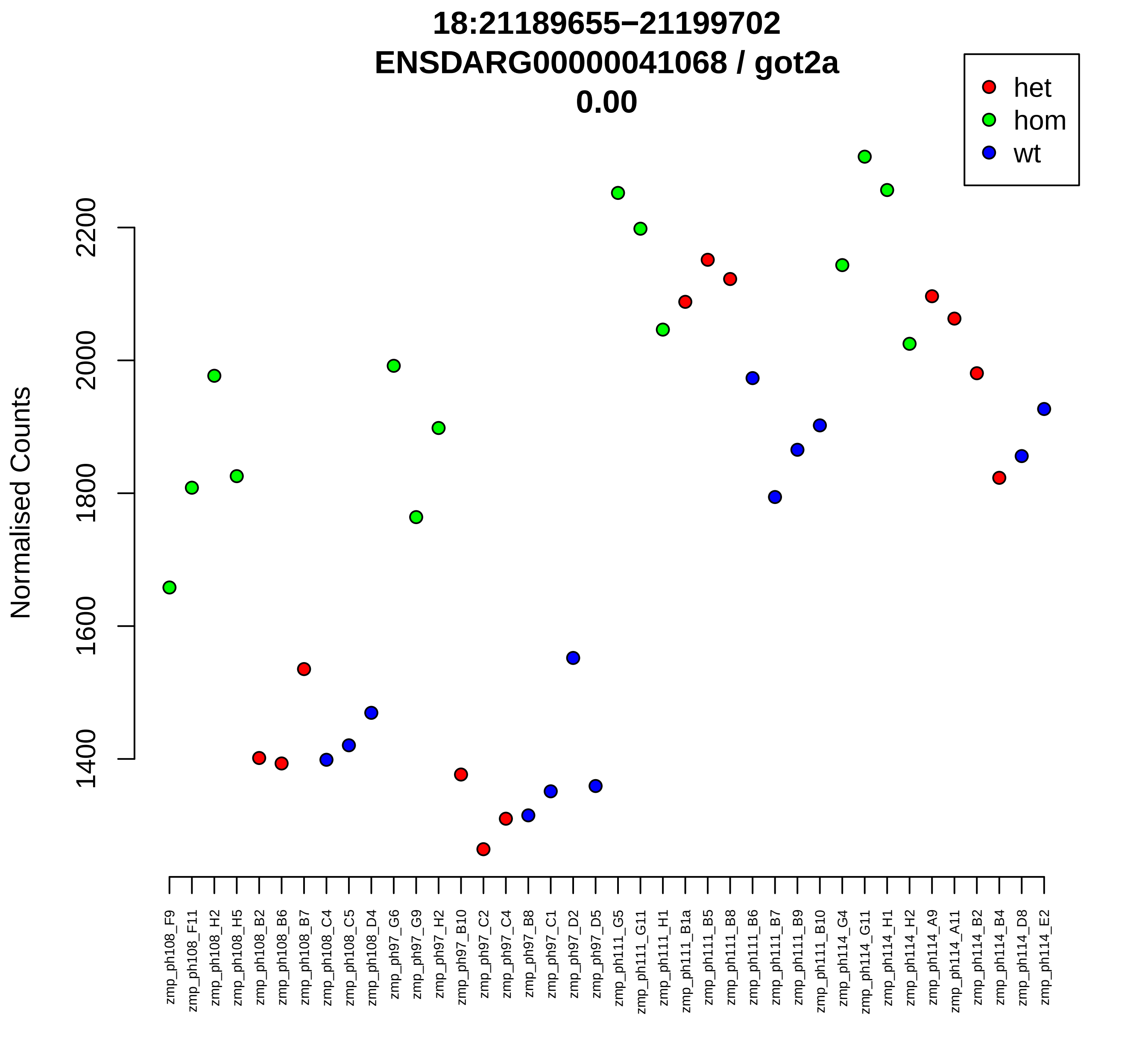

18:21189655−21199702
ENSDARG00000041068 / got2a
0.00
het
hom
wt
Normalised Counts
1400
1600
1800
2000
2200
zmp_ph108_F9
zmp_ph108_F11
zmp_ph108_H2
zmp_ph108_H5
zmp_ph108_B2
zmp_ph108_B6
zmp_ph108_B7
zmp_ph108_C4
zmp_ph108_C5
zmp_ph108_D4
zmp_ph97_G6
zmp_ph97_G9
zmp_ph97_H2
zmp_ph97_B10
zmp_ph97_C2
zmp_ph97_C4
zmp_ph97_B8
zmp_ph97_C1
zmp_ph97_D2
zmp_ph97_D5
zmp_ph111_G5
zmp_ph111_G11
zmp_ph111_H1
zmp_ph111_B1a
zmp_ph111_B5
zmp_ph111_B8
zmp_ph111_B6
zmp_ph111_B7
zmp_ph111_B9
zmp_ph111_B10
zmp_ph114_G4
zmp_ph114_G11
zmp_ph114_H1
zmp_ph114_H2
zmp_ph114_A9
zmp_ph114_A11
zmp_ph114_B2
zmp_ph114_B4
zmp_ph114_D8
zmp_ph114_E2

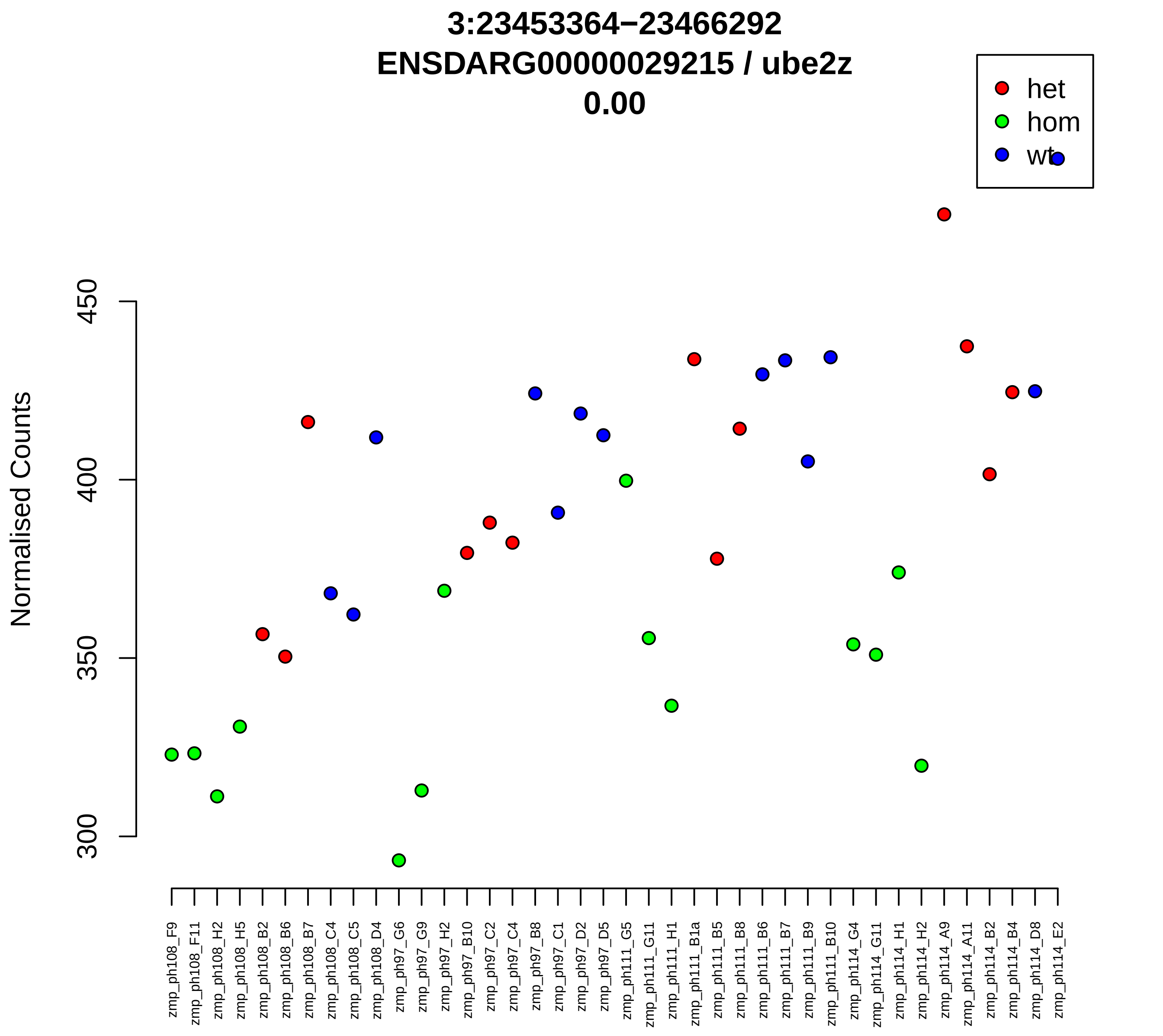

3:23453364−23466292
ENSDARG00000029215 / ube2z
0.00
het
hom
wt
Normalised Counts
300
350
400
450
zmp_ph108_F9
zmp_ph108_F11
zmp_ph108_H2
zmp_ph108_H5
zmp_ph108_B2
zmp_ph108_B6
zmp_ph108_B7
zmp_ph108_C4
zmp_ph108_C5
zmp_ph108_D4
zmp_ph97_G6
zmp_ph97_G9
zmp_ph97_H2
zmp_ph97_B10
zmp_ph97_C2
zmp_ph97_C4
zmp_ph97_B8
zmp_ph97_C1
zmp_ph97_D2
zmp_ph97_D5
zmp_ph111_G5
zmp_ph111_G11
zmp_ph111_H1
zmp_ph111_B1a
zmp_ph111_B5
zmp_ph111_B8
zmp_ph111_B6
zmp_ph111_B7
zmp_ph111_B9
zmp_ph111_B10
zmp_ph114_G4
zmp_ph114_G11
zmp_ph114_H1
zmp_ph114_H2
zmp_ph114_A9
zmp_ph114_A11
zmp_ph114_B2
zmp_ph114_B4
zmp_ph114_D8
zmp_ph114_E2

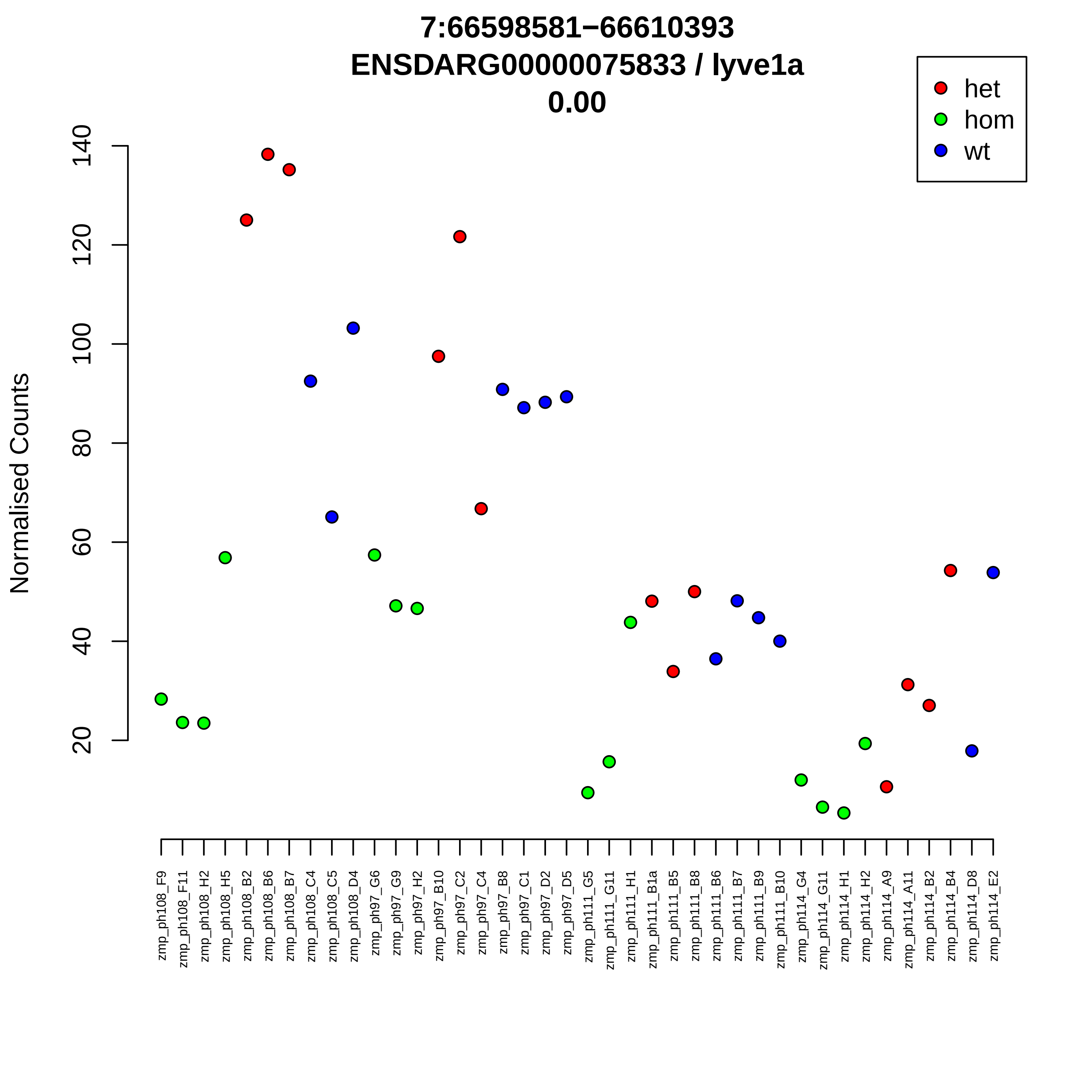

7:66598581−66610393
ENSDARG00000075833 / lyve1a
0.00
het
hom
wt
Normalised Counts
20
40
60
80
100
120
140
zmp_ph108_F9
zmp_ph108_F11
zmp_ph108_H2
zmp_ph108_H5
zmp_ph108_B2
zmp_ph108_B6
zmp_ph108_B7
zmp_ph108_C4
zmp_ph108_C5
zmp_ph108_D4
zmp_ph97_G6
zmp_ph97_G9
zmp_ph97_H2
zmp_ph97_B10
zmp_ph97_C2
zmp_ph97_C4
zmp_ph97_B8
zmp_ph97_C1
zmp_ph97_D2
zmp_ph97_D5
zmp_ph111_G5
zmp_ph111_G11
zmp_ph111_H1
zmp_ph111_B1a
zmp_ph111_B5
zmp_ph111_B8
zmp_ph111_B6
zmp_ph111_B7
zmp_ph111_B9
zmp_ph111_B10
zmp_ph114_G4
zmp_ph114_G11
zmp_ph114_H1
zmp_ph114_H2
zmp_ph114_A9
zmp_ph114_A11
zmp_ph114_B2
zmp_ph114_B4
zmp_ph114_D8
zmp_ph114_E2

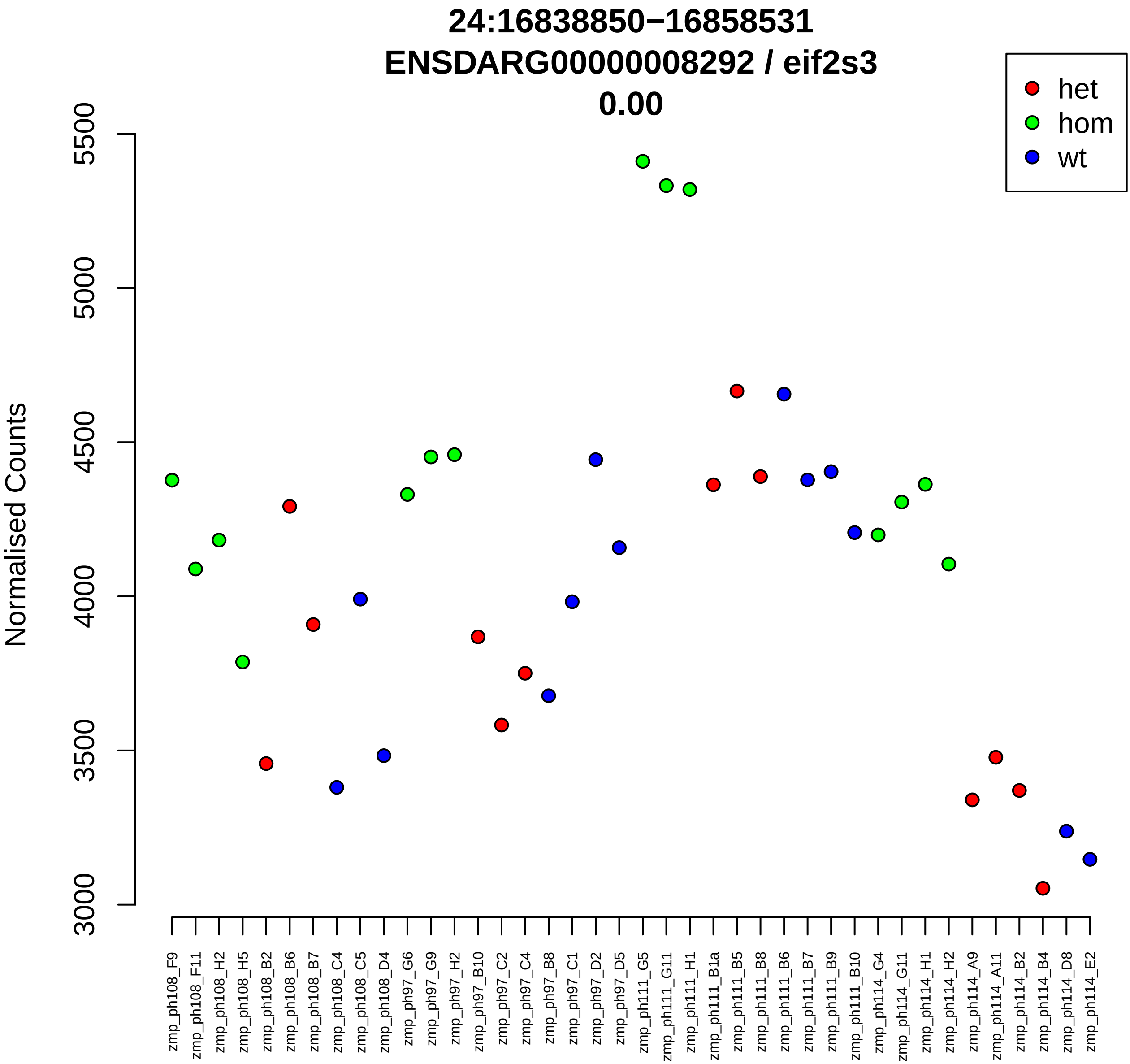

24:16838850−16858531
ENSDARG00000008292 / eif2s3
0.00
het
hom
wt
Normalised Counts
3000
3500
4000
4500
5000
5500
zmp_ph108_F9
zmp_ph108_F11
zmp_ph108_H2
zmp_ph108_H5
zmp_ph108_B2
zmp_ph108_B6
zmp_ph108_B7
zmp_ph108_C4
zmp_ph108_C5
zmp_ph108_D4
zmp_ph97_G6
zmp_ph97_G9
zmp_ph97_H2
zmp_ph97_B10
zmp_ph97_C2
zmp_ph97_C4
zmp_ph97_B8
zmp_ph97_C1
zmp_ph97_D2
zmp_ph97_D5
zmp_ph111_G5
zmp_ph111_G11
zmp_ph111_H1
zmp_ph111_B1a
zmp_ph111_B5
zmp_ph111_B8
zmp_ph111_B6
zmp_ph111_B7
zmp_ph111_B9
zmp_ph111_B10
zmp_ph114_G4
zmp_ph114_G11
zmp_ph114_H1
zmp_ph114_H2
zmp_ph114_A9
zmp_ph114_A11
zmp_ph114_B2
zmp_ph114_B4
zmp_ph114_D8
zmp_ph114_E2

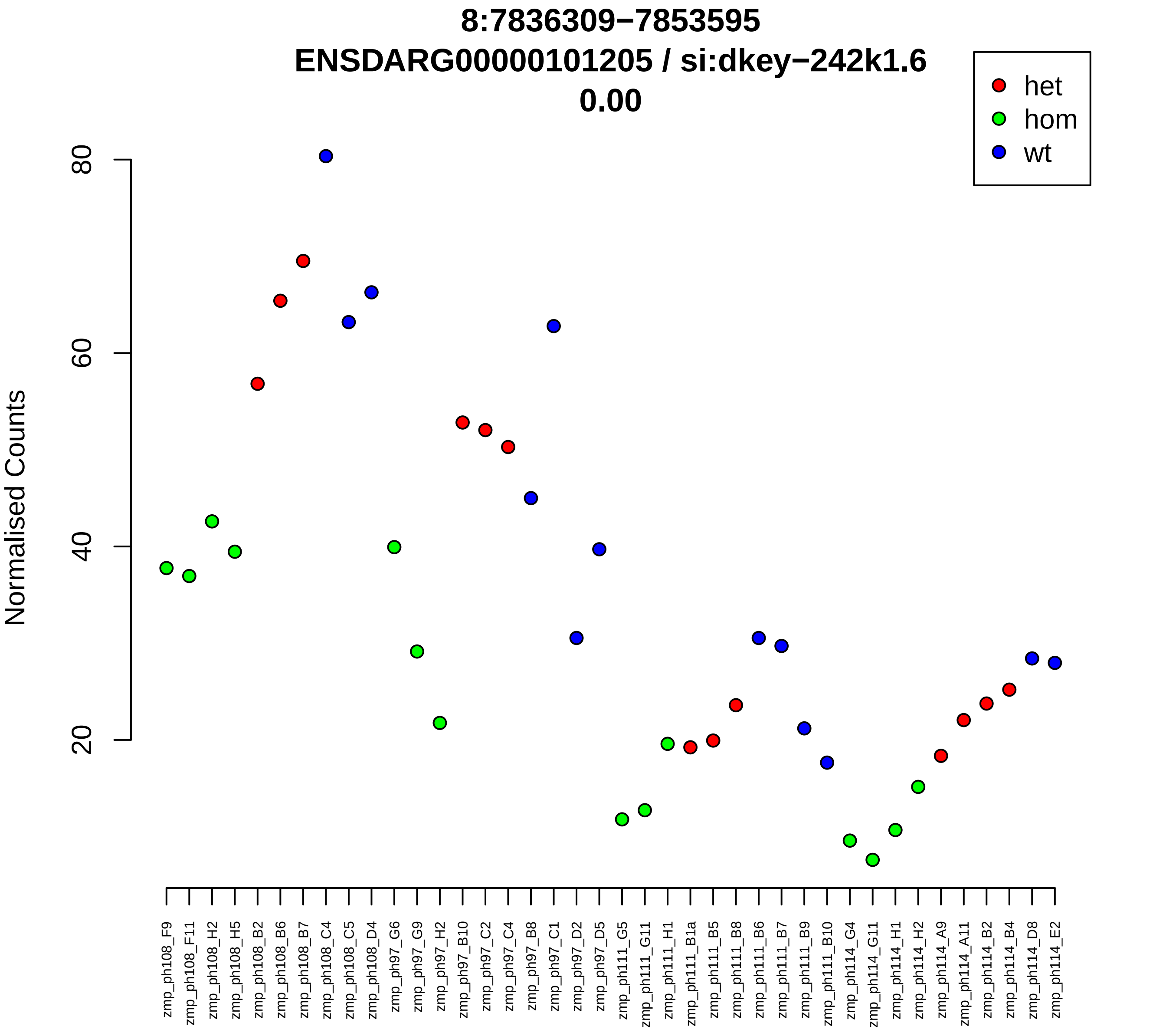
8:7836309−7853595
ENSDARG00000101205 / si:dkey−242k1.6
0.00
het
hom
wt
Normalised Counts
80
60
40
20
zmp_ph108_F9
zmp_ph108_F11
zmp_ph108_H2
zmp_ph108_H5
zmp_ph108_B2
zmp_ph108_B6
zmp_ph108_B7
zmp_ph108_C4
zmp_ph108_C5
zmp_ph108_D4
zmp_ph97_G6
zmp_ph97_G9
zmp_ph97_H2
zmp_ph97_B10
zmp_ph97_C2
zmp_ph97_C4
zmp_ph97_B8
zmp_ph97_C1
zmp_ph97_D2
zmp_ph97_D5
zmp_ph111_G5
zmp_ph111_G11
zmp_ph111_H1
zmp_ph111_B1a
zmp_ph111_B5
zmp_ph111_B8
zmp_ph111_B6
zmp_ph111_B7
zmp_ph111_B9
zmp_ph111_B10
zmp_ph114_G4
zmp_ph114_G11
zmp_ph114_H1
zmp_ph114_H2
zmp_ph114_A9
zmp_ph114_A11
zmp_ph114_B2
zmp_ph114_B4
zmp_ph114_D8
zmp_ph114_E2

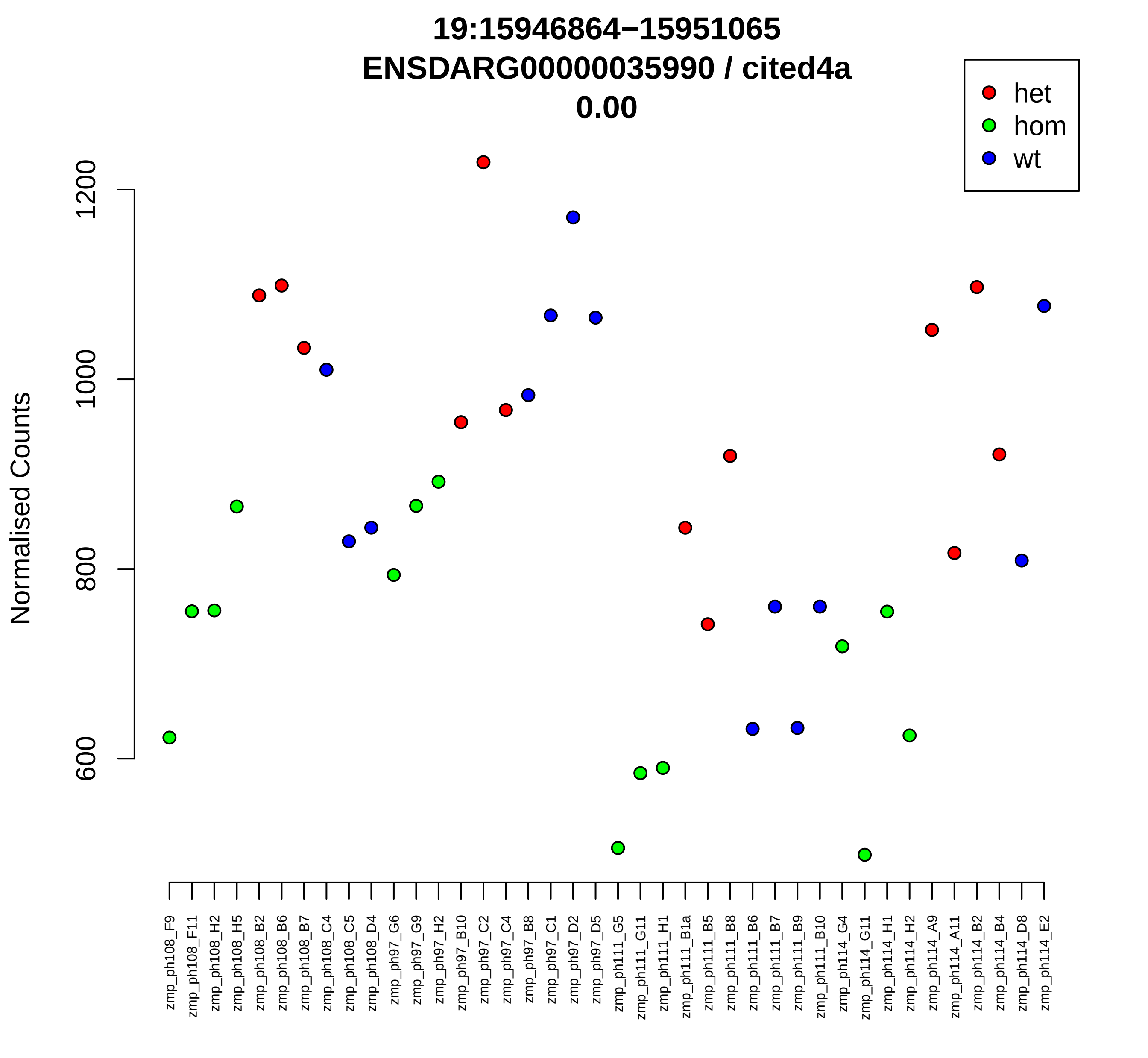

19:15946864−15951065
ENSDARG00000035990 / cited4a
0.00
het
hom
wt
Normalised Counts
600
800
1000
1200
zmp_ph108_F9
zmp_ph108_F11
zmp_ph108_H2
zmp_ph108_H5
zmp_ph108_B2
zmp_ph108_B6
zmp_ph108_B7
zmp_ph108_C4
zmp_ph108_C5
zmp_ph108_D4
zmp_ph97_G6
zmp_ph97_G9
zmp_ph97_H2
zmp_ph97_B10
zmp_ph97_C2
zmp_ph97_C4
zmp_ph97_B8
zmp_ph97_C1
zmp_ph97_D2
zmp_ph97_D5
zmp_ph111_G5
zmp_ph111_G11
zmp_ph111_H1
zmp_ph111_B1a
zmp_ph111_B5
zmp_ph111_B8
zmp_ph111_B6
zmp_ph111_B7
zmp_ph111_B9
zmp_ph111_B10
zmp_ph114_G4
zmp_ph114_G11
zmp_ph114_H1
zmp_ph114_H2
zmp_ph114_A9
zmp_ph114_A11
zmp_ph114_B2
zmp_ph114_B4
zmp_ph114_D8
zmp_ph114_E2

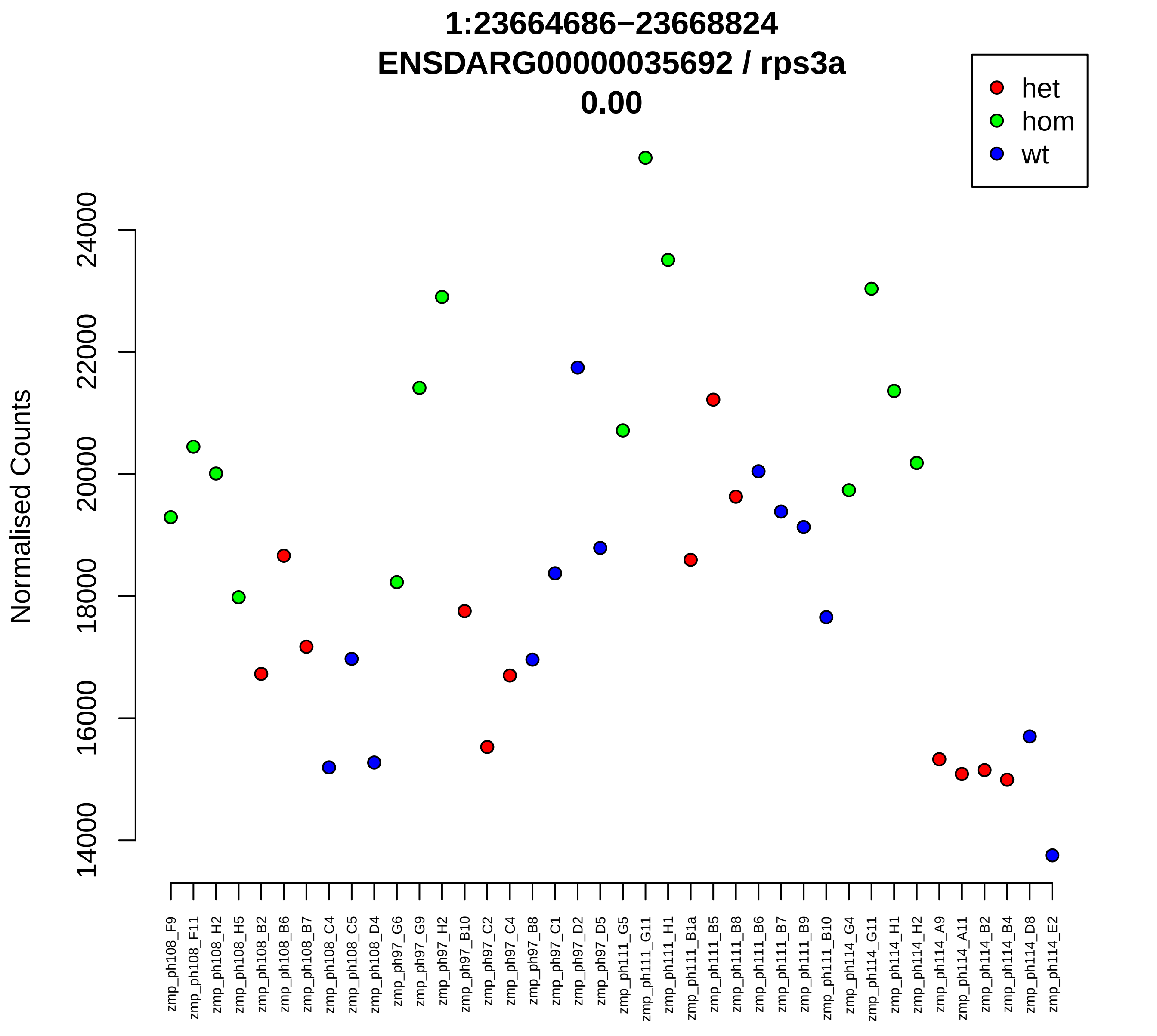
1:23664686−23668824
ENSDARG00000035692 / rps3a
0.00
het
hom
wt
Normalised Counts
14000
16000
18000
20000
22000
24000
zmp_ph108_F9
zmp_ph108_F11
zmp_ph108_H2
zmp_ph108_H5
zmp_ph108_B2
zmp_ph108_B6
zmp_ph108_B7
zmp_ph108_C4
zmp_ph108_C5
zmp_ph108_D4
zmp_ph97_G6
zmp_ph97_G9
zmp_ph97_H2
zmp_ph97_B10
zmp_ph97_C2
zmp_ph97_C4
zmp_ph97_B8
zmp_ph97_C1
zmp_ph97_D2
zmp_ph97_D5
zmp_ph111_G5
zmp_ph111_G11
zmp_ph111_H1
zmp_ph111_B1a
zmp_ph111_B5
zmp_ph111_B8
zmp_ph111_B6
zmp_ph111_B7
zmp_ph111_B9
zmp_ph111_B10
zmp_ph114_G4
zmp_ph114_G11
zmp_ph114_H1
zmp_ph114_H2
zmp_ph114_A9
zmp_ph114_A11
zmp_ph114_B2
zmp_ph114_B4
zmp_ph114_D8
zmp_ph114_E2

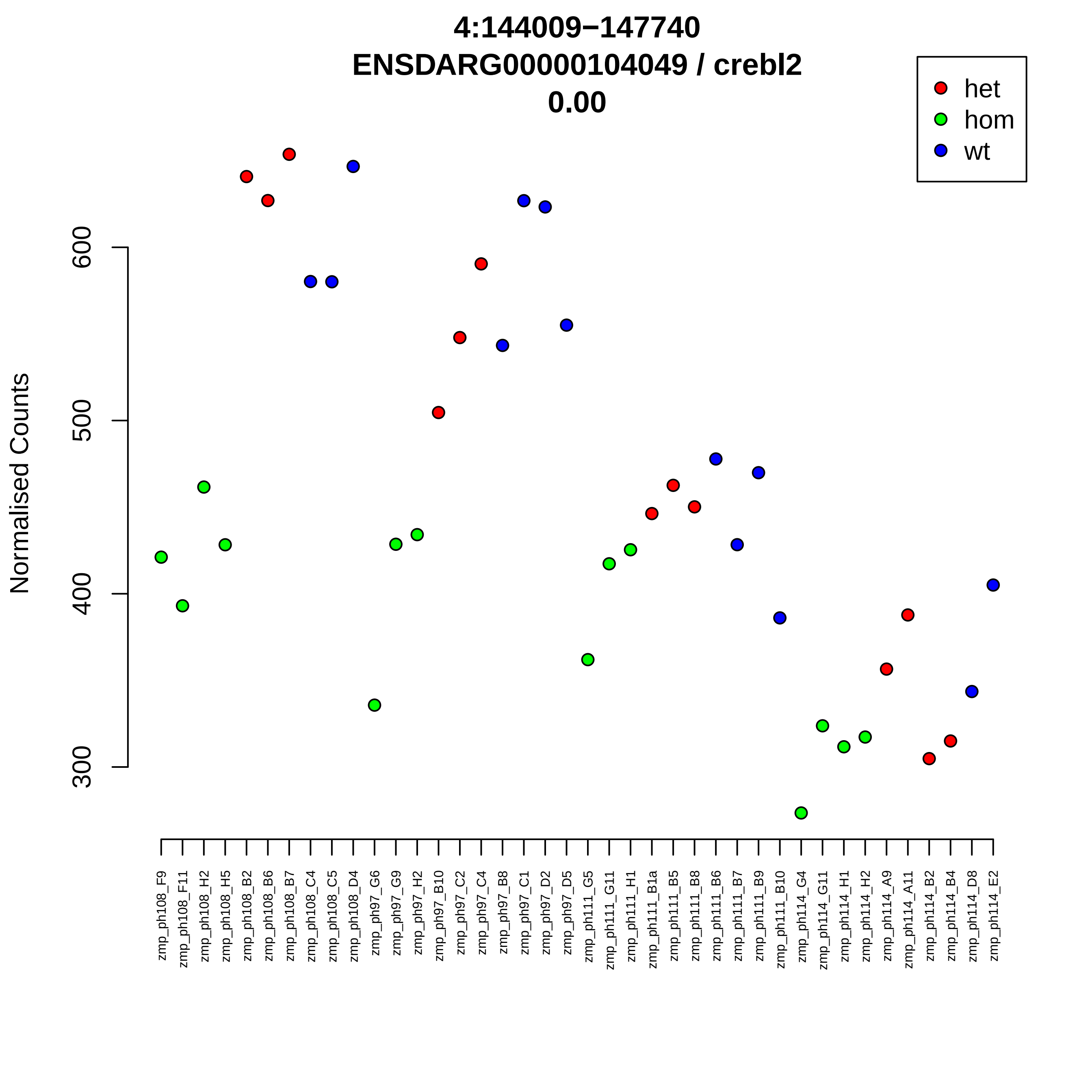
4:144009−147740
ENSDARG00000104049 / crebl2
0.00
het
hom
wt
Normalised Counts
300
400
500
600
zmp_ph108_F9
zmp_ph108_F11
zmp_ph108_H2
zmp_ph108_H5
zmp_ph108_B2
zmp_ph108_B6
zmp_ph108_B7
zmp_ph108_C4
zmp_ph108_C5
zmp_ph108_D4
zmp_ph97_G6
zmp_ph97_G9
zmp_ph97_H2
zmp_ph97_B10
zmp_ph97_C2
zmp_ph97_C4
zmp_ph97_B8
zmp_ph97_C1
zmp_ph97_D2
zmp_ph97_D5
zmp_ph111_G5
zmp_ph111_G11
zmp_ph111_H1
zmp_ph111_B1a
zmp_ph111_B5
zmp_ph111_B8
zmp_ph111_B6
zmp_ph111_B7
zmp_ph111_B9
zmp_ph111_B10
zmp_ph114_G4
zmp_ph114_G11
zmp_ph114_H1
zmp_ph114_H2
zmp_ph114_A9
zmp_ph114_A11
zmp_ph114_B2
zmp_ph114_B4
zmp_ph114_D8
zmp_ph114_E2

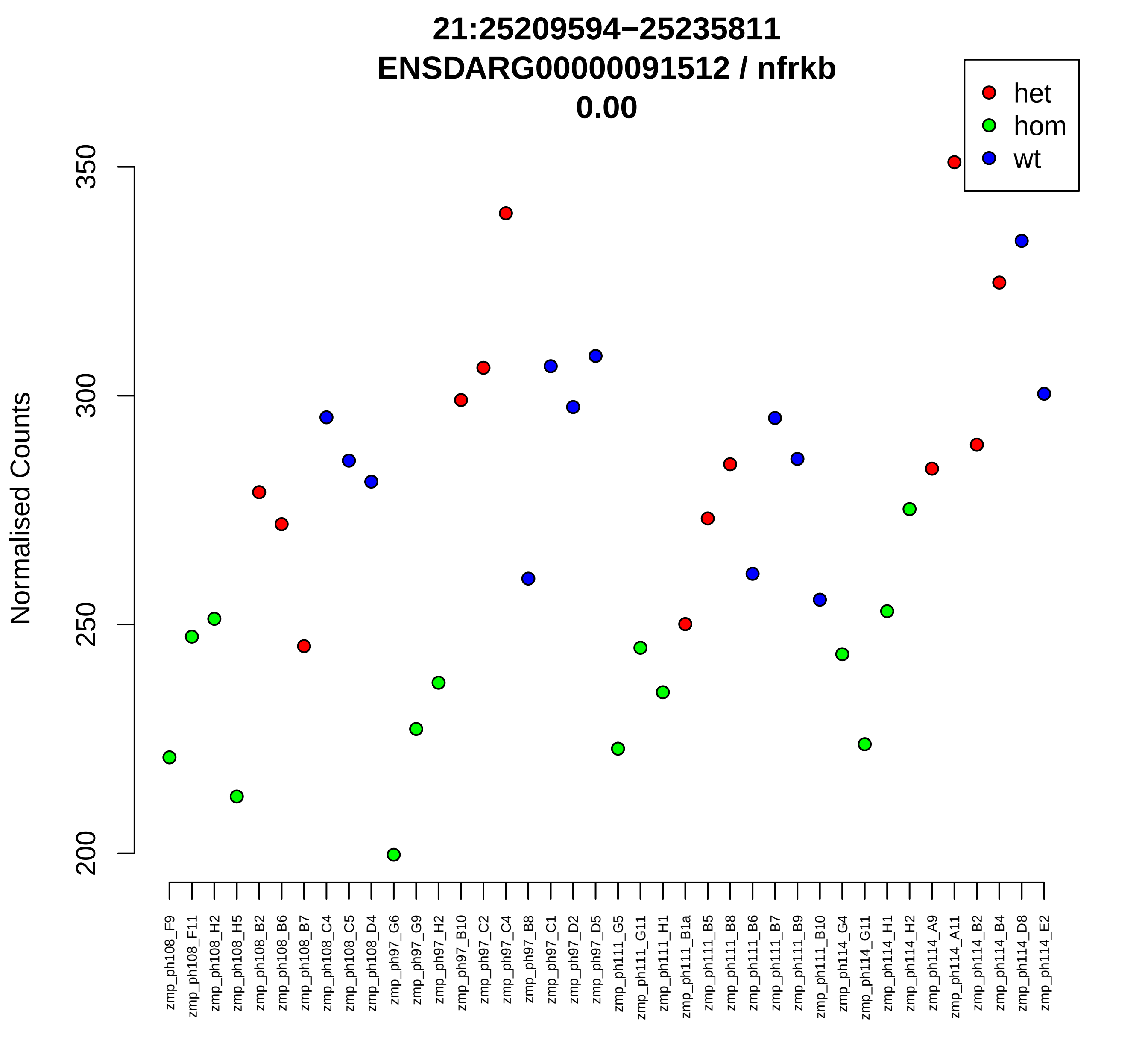
21:25209594−25235811
ENSDARG00000091512 / nfrkb
0.00
het
hom
wt
Normalised Counts
200
250
300
350
zmp_ph108_F9
zmp_ph108_F11
zmp_ph108_H2
zmp_ph108_H5
zmp_ph108_B2
zmp_ph108_B6
zmp_ph108_B7
zmp_ph108_C4
zmp_ph108_C5
zmp_ph108_D4
zmp_ph97_G6
zmp_ph97_G9
zmp_ph97_H2
zmp_ph97_B10
zmp_ph97_C2
zmp_ph97_C4
zmp_ph97_B8
zmp_ph97_C1
zmp_ph97_D2
zmp_ph97_D5
zmp_ph111_G5
zmp_ph111_G11
zmp_ph111_H1
zmp_ph111_B1a
zmp_ph111_B5
zmp_ph111_B8
zmp_ph111_B6
zmp_ph111_B7
zmp_ph111_B9
zmp_ph111_B10
zmp_ph114_G4
zmp_ph114_G11
zmp_ph114_H1
zmp_ph114_H2
zmp_ph114_A9
zmp_ph114_A11
zmp_ph114_B2
zmp_ph114_B4
zmp_ph114_D8
zmp_ph114_E2

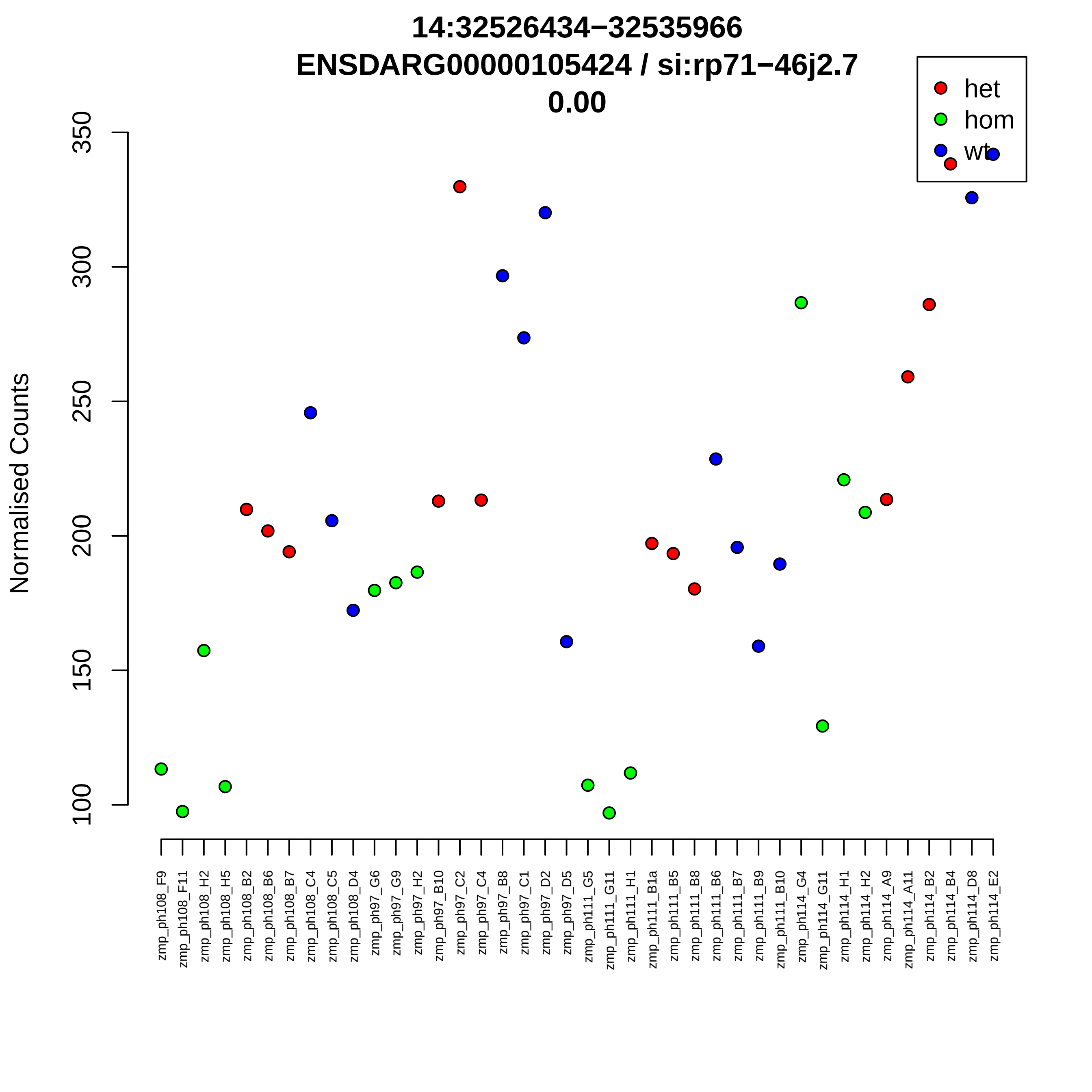

14:32526434−32535966
ENSDARG00000105424 / si:rp71−46j2.7
0.00
het
hom
wt
Normalised Counts
100
150
200
250
300
350
zmp_ph108_F9
zmp_ph108_F11
zmp_ph108_H2
zmp_ph108_H5
zmp_ph108_B2
zmp_ph108_B6
zmp_ph108_B7
zmp_ph108_C4
zmp_ph108_C5
zmp_ph108_D4
zmp_ph97_G6
zmp_ph97_G9
zmp_ph97_H2
zmp_ph97_B10
zmp_ph97_C2
zmp_ph97_C4
zmp_ph97_B8
zmp_ph97_C1
zmp_ph97_D2
zmp_ph97_D5
zmp_ph111_G5
zmp_ph111_G11
zmp_ph111_H1
zmp_ph111_B1a
zmp_ph111_B5
zmp_ph111_B8
zmp_ph111_B6
zmp_ph111_B7
zmp_ph111_B9
zmp_ph111_B10
zmp_ph114_G4
zmp_ph114_G11
zmp_ph114_H1
zmp_ph114_H2
zmp_ph114_A9
zmp_ph114_A11
zmp_ph114_B2
zmp_ph114_B4
zmp_ph114_D8
zmp_ph114_E2

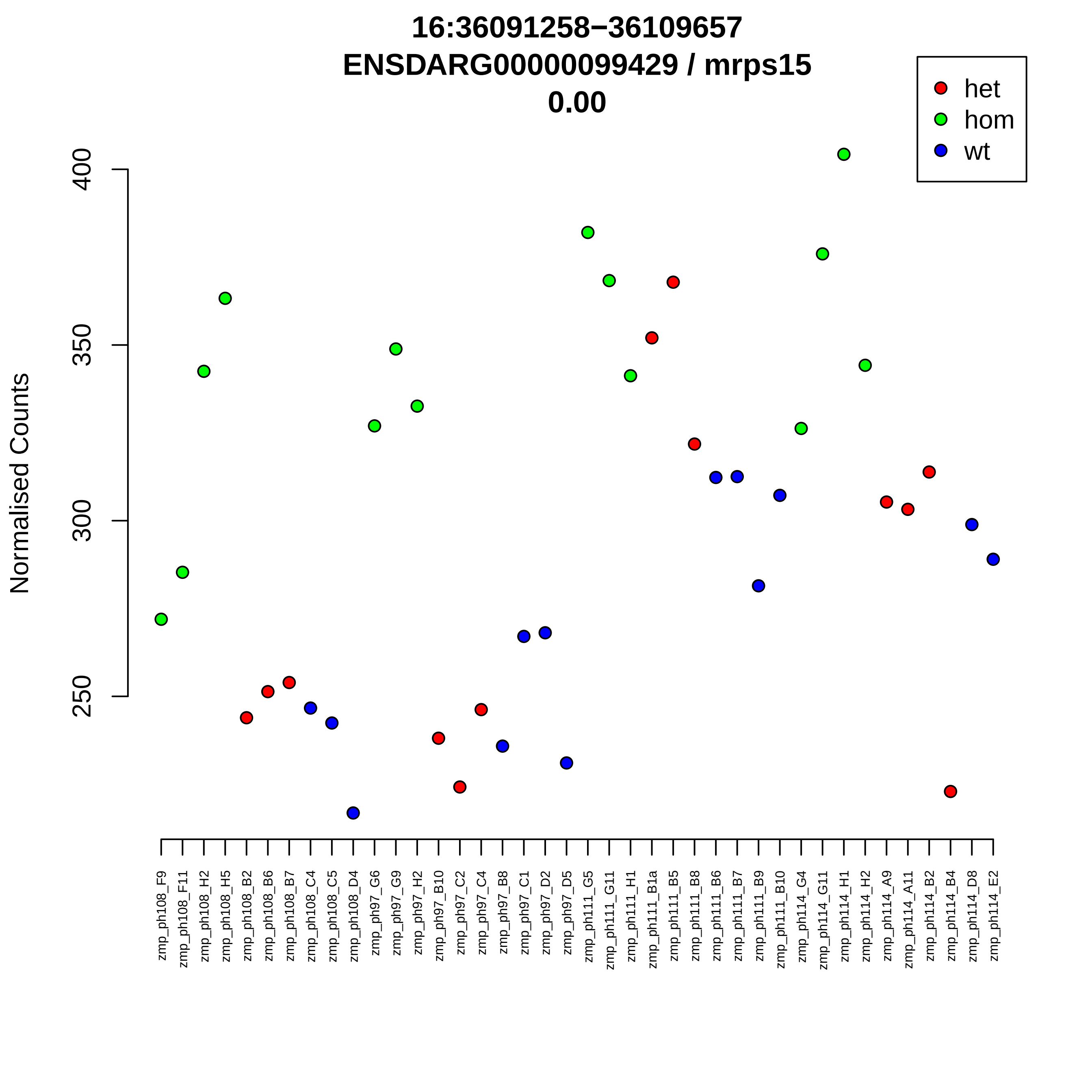

16:36091258−36109657
ENSDARG00000099429 / mrps15
0.00
het
hom
wt
Normalised Counts
400
350
300
250
zmp_ph108_F9
zmp_ph108_F11
zmp_ph108_H2
zmp_ph108_H5
zmp_ph108_B2
zmp_ph108_B6
zmp_ph108_B7
zmp_ph108_C4
zmp_ph108_C5
zmp_ph108_D4
zmp_ph97_G6
zmp_ph97_G9
zmp_ph97_H2
zmp_ph97_B10
zmp_ph97_C2
zmp_ph97_C4
zmp_ph97_B8
zmp_ph97_C1
zmp_ph97_D2
zmp_ph97_D5
zmp_ph111_G5
zmp_ph111_G11
zmp_ph111_H1
zmp_ph111_B1a
zmp_ph111_B5
zmp_ph111_B8
zmp_ph111_B6
zmp_ph111_B7
zmp_ph111_B9
zmp_ph111_B10
zmp_ph114_G4
zmp_ph114_G11
zmp_ph114_H1
zmp_ph114_H2
zmp_ph114_A9
zmp_ph114_A11
zmp_ph114_B2
zmp_ph114_B4
zmp_ph114_D8
zmp_ph114_E2

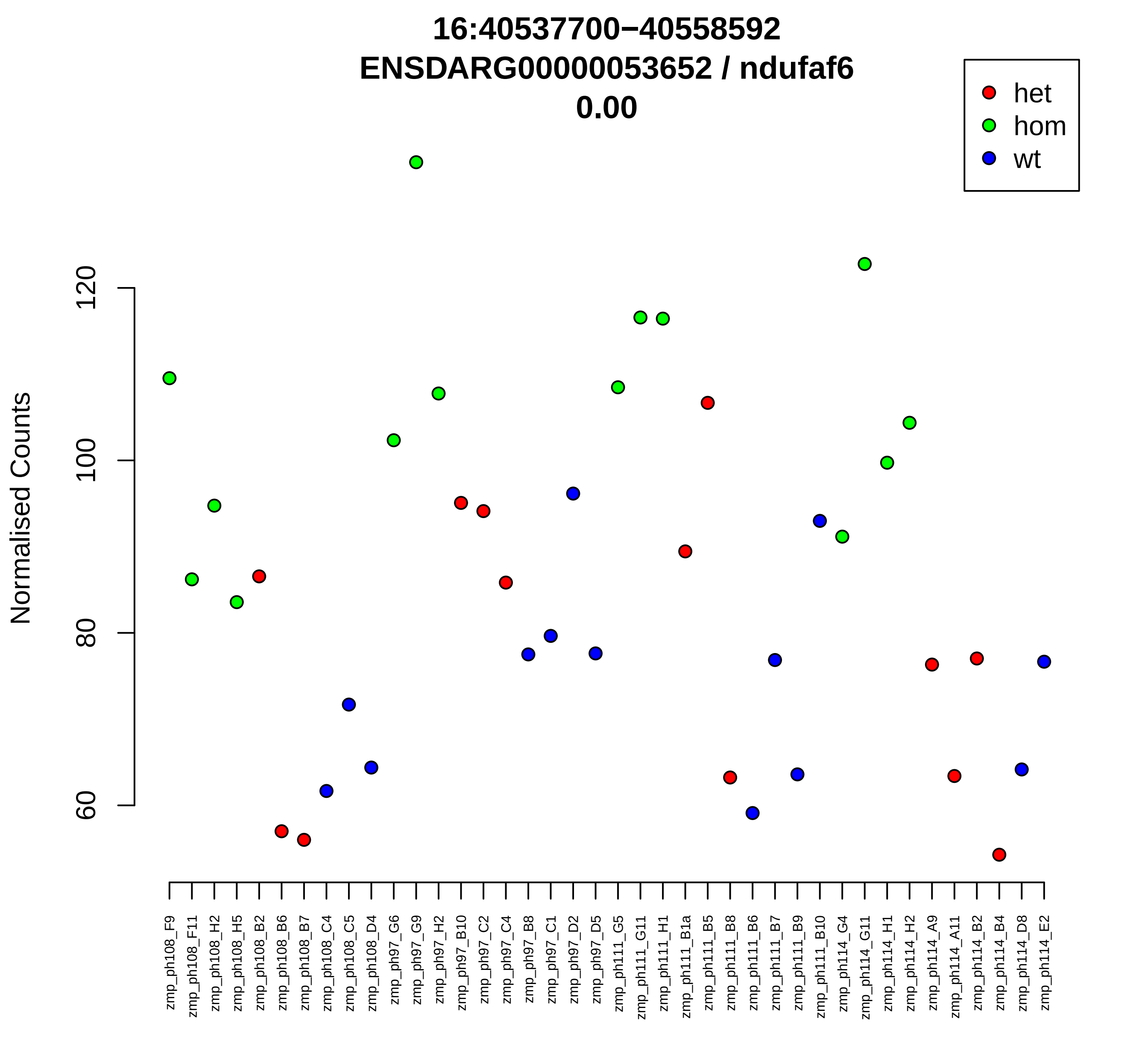
16:40537700−40558592
ENSDARG00000053652 / ndufaf6
0.00
het
hom
wt
Normalised Counts
60
80
100
120
zmp_ph108_F9
zmp_ph108_F11
zmp_ph108_H2
zmp_ph108_H5
zmp_ph108_B2
zmp_ph108_B6
zmp_ph108_B7
zmp_ph108_C4
zmp_ph108_C5
zmp_ph108_D4
zmp_ph97_G6
zmp_ph97_G9
zmp_ph97_H2
zmp_ph97_B10
zmp_ph97_C2
zmp_ph97_C4
zmp_ph97_B8
zmp_ph97_C1
zmp_ph97_D2
zmp_ph97_D5
zmp_ph111_G5
zmp_ph111_G11
zmp_ph111_H1
zmp_ph111_B1a
zmp_ph111_B5
zmp_ph111_B8
zmp_ph111_B6
zmp_ph111_B7
zmp_ph111_B9
zmp_ph111_B10
zmp_ph114_G4
zmp_ph114_G11
zmp_ph114_H1
zmp_ph114_H2
zmp_ph114_A9
zmp_ph114_A11
zmp_ph114_B2
zmp_ph114_B4
zmp_ph114_D8
zmp_ph114_E2

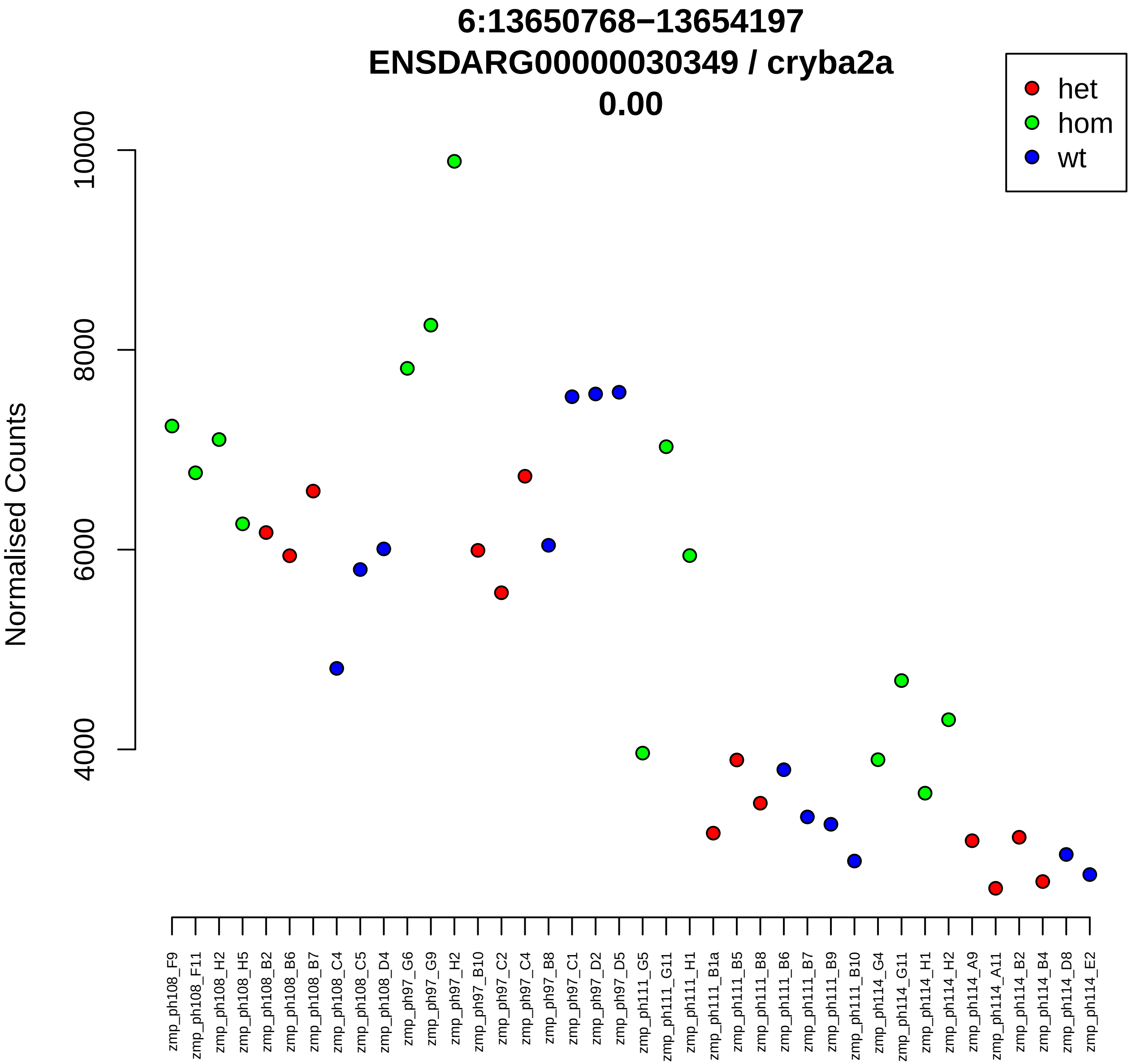

6:13650768−13654197
ENSDARG00000030349 / cryba2a
0.00
Normalised Counts
10000
8000
6000
4000
het
hom
wt
zmp_ph108_F9
zmp_ph108_F11
zmp_ph108_H2
zmp_ph108_H5
zmp_ph108_B2
zmp_ph108_B6
zmp_ph108_B7
zmp_ph108_C4
zmp_ph108_C5
zmp_ph108_D4
zmp_ph97_G6
zmp_ph97_G9
zmp_ph97_H2
zmp_ph97_B10
zmp_ph97_C2
zmp_ph97_C4
zmp_ph97_B8
zmp_ph97_C1
zmp_ph97_D2
zmp_ph97_D5
zmp_ph111_G5
zmp_ph111_G11
zmp_ph111_H1
zmp_ph111_B1a
zmp_ph111_B5
zmp_ph111_B8
zmp_ph111_B6
zmp_ph111_B7
zmp_ph111_B9
zmp_ph111_B10
zmp_ph114_G4
zmp_ph114_G11
zmp_ph114_H1
zmp_ph114_H2
zmp_ph114_A9
zmp_ph114_A11
zmp_ph114_B2
zmp_ph114_B4
zmp_ph114_D8
zmp_ph114_E2

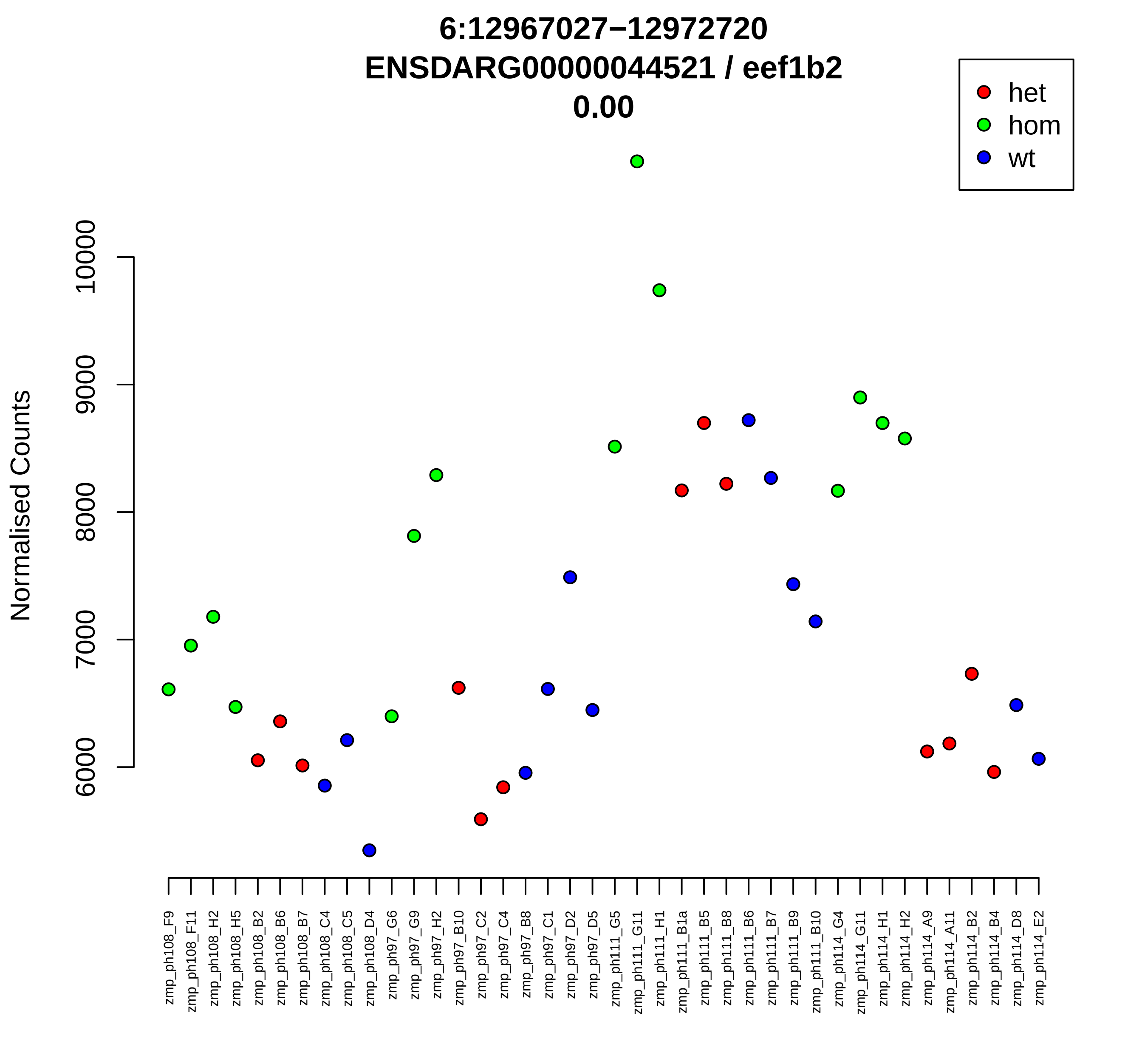

6:12967027−12972720
ENSDARG00000044521 / eef1b2
0.00
het
hom
wt
Normalised Counts
6000
7000
8000
9000
10000
zmp_ph108_F9
zmp_ph108_F11
zmp_ph108_H2
zmp_ph108_H5
zmp_ph108_B2
zmp_ph108_B6
zmp_ph108_B7
zmp_ph108_C4
zmp_ph108_C5
zmp_ph108_D4
zmp_ph97_G6
zmp_ph97_G9
zmp_ph97_H2
zmp_ph97_B10
zmp_ph97_C2
zmp_ph97_C4
zmp_ph97_B8
zmp_ph97_C1
zmp_ph97_D2
zmp_ph97_D5
zmp_ph111_G5
zmp_ph111_G11
zmp_ph111_H1
zmp_ph111_B1a
zmp_ph111_B5
zmp_ph111_B8
zmp_ph111_B6
zmp_ph111_B7
zmp_ph111_B9
zmp_ph111_B10
zmp_ph114_G4
zmp_ph114_G11
zmp_ph114_H1
zmp_ph114_H2
zmp_ph114_A9
zmp_ph114_A11
zmp_ph114_B2
zmp_ph114_B4
zmp_ph114_D8
zmp_ph114_E2

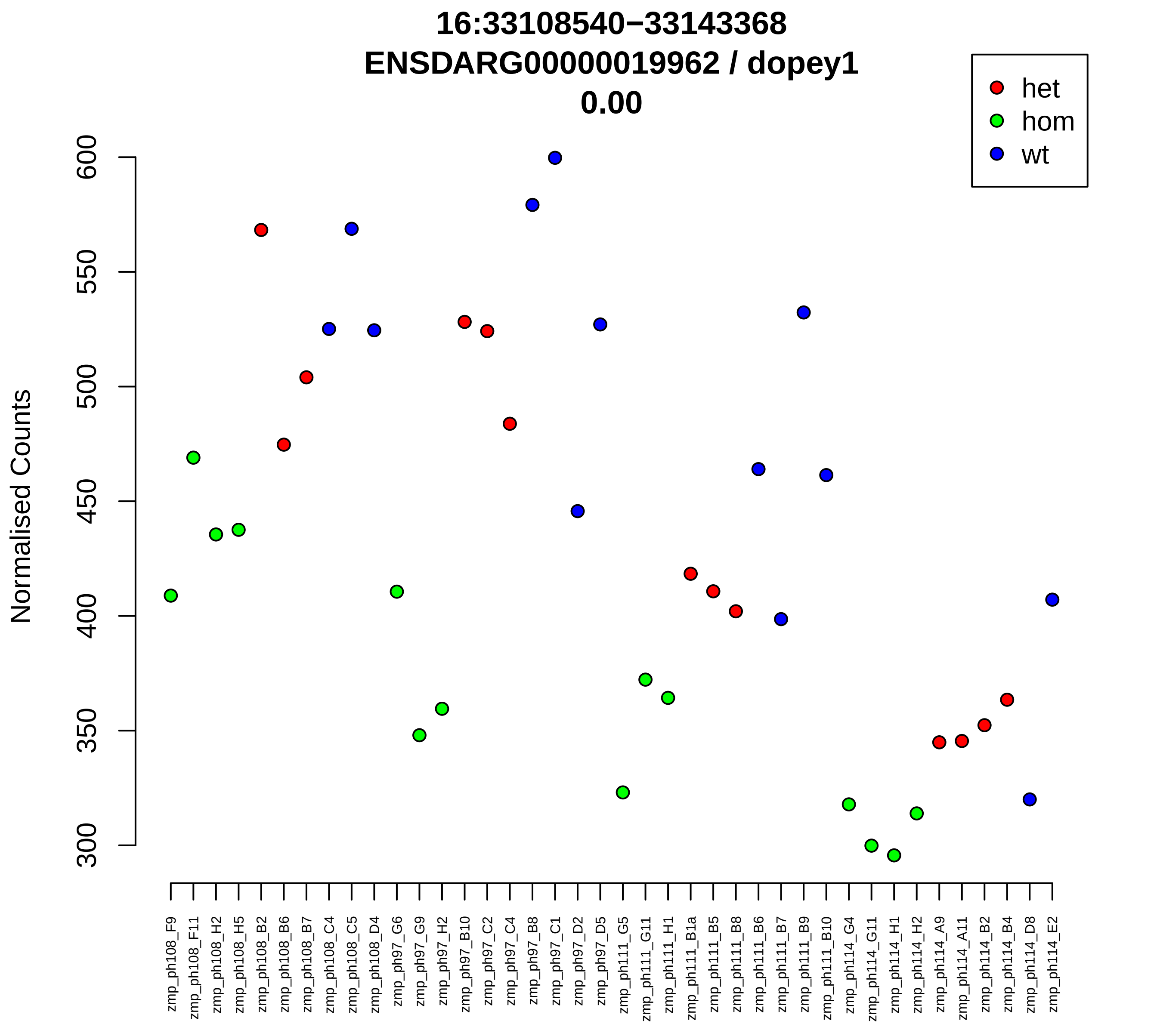

16:33108540−33143368
ENSDARG00000019962 / dopey1
0.00
het
hom
wt
Normalised Counts
300
350
400
450
500
550
600
zmp_ph108_F9
zmp_ph108_F11
zmp_ph108_H2
zmp_ph108_H5
zmp_ph108_B2
zmp_ph108_B6
zmp_ph108_B7
zmp_ph108_C4
zmp_ph108_C5
zmp_ph108_D4
zmp_ph97_G6
zmp_ph97_G9
zmp_ph97_H2
zmp_ph97_B10
zmp_ph97_C2
zmp_ph97_C4
zmp_ph97_B8
zmp_ph97_C1
zmp_ph97_D2
zmp_ph97_D5
zmp_ph111_G5
zmp_ph111_G11
zmp_ph111_H1
zmp_ph111_B1a
zmp_ph111_B5
zmp_ph111_B8
zmp_ph111_B6
zmp_ph111_B7
zmp_ph111_B9
zmp_ph111_B10
zmp_ph114_G4
zmp_ph114_G11
zmp_ph114_H1
zmp_ph114_H2
zmp_ph114_A9
zmp_ph114_A11
zmp_ph114_B2
zmp_ph114_B4
zmp_ph114_D8
zmp_ph114_E2

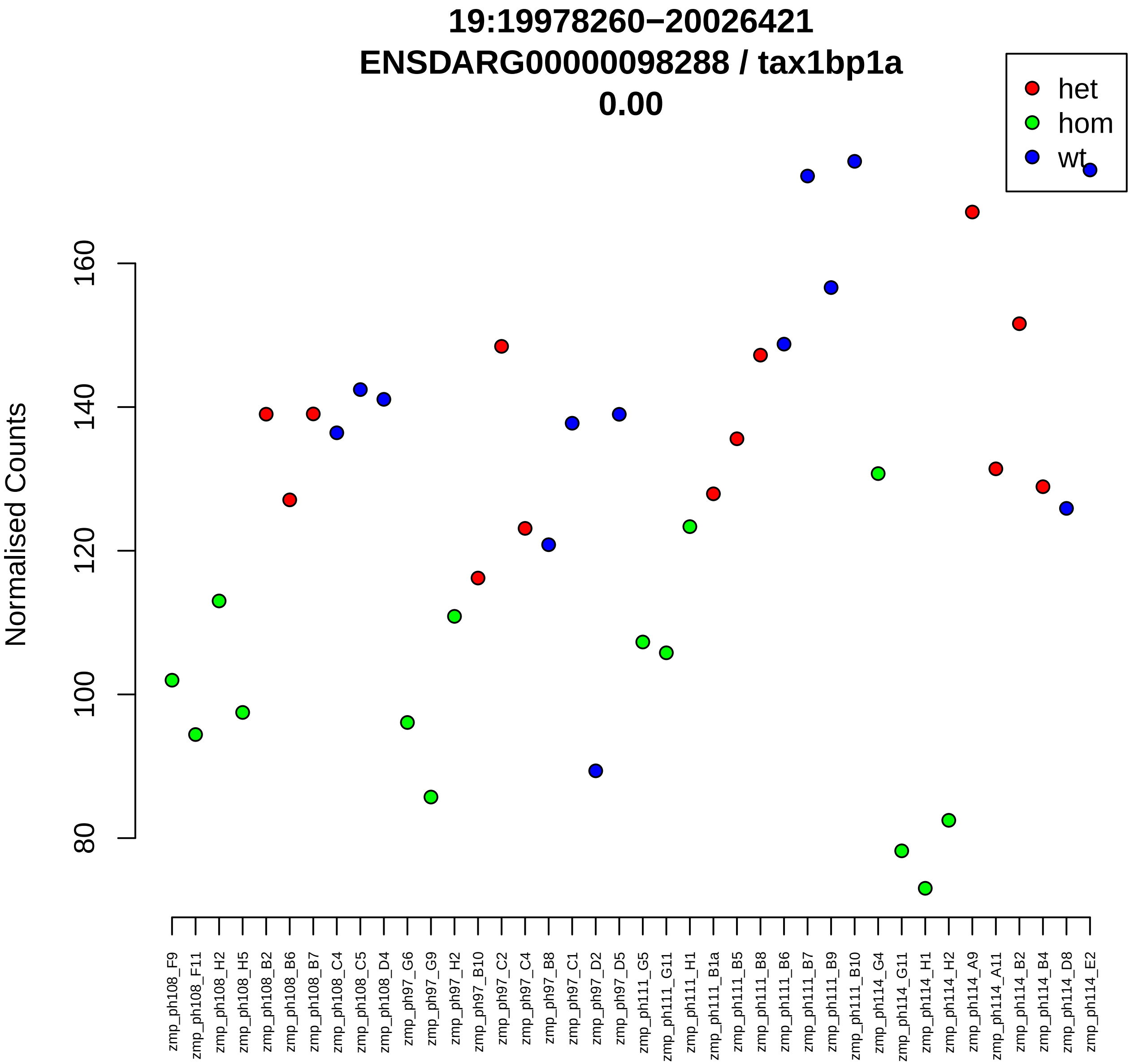

19:19978260−20026421
ENSDARG00000098288 / tax1bp1a
0.00
het
hom
wt
Normalised Counts
80
100
120
140
160
zmp_ph108_F9
zmp_ph108_F11
zmp_ph108_H2
zmp_ph108_H5
zmp_ph108_B2
zmp_ph108_B6
zmp_ph108_B7
zmp_ph108_C4
zmp_ph108_C5
zmp_ph108_D4
zmp_ph97_G6
zmp_ph97_G9
zmp_ph97_H2
zmp_ph97_B10
zmp_ph97_C2
zmp_ph97_C4
zmp_ph97_B8
zmp_ph97_C1
zmp_ph97_D2
zmp_ph97_D5
zmp_ph111_G5
zmp_ph111_G11
zmp_ph111_H1
zmp_ph111_B1a
zmp_ph111_B5
zmp_ph111_B8
zmp_ph111_B6
zmp_ph111_B7
zmp_ph111_B9
zmp_ph111_B10
zmp_ph114_G4
zmp_ph114_G11
zmp_ph114_H1
zmp_ph114_H2
zmp_ph114_A9
zmp_ph114_A11
zmp_ph114_B2
zmp_ph114_B4
zmp_ph114_D8
zmp_ph114_E2

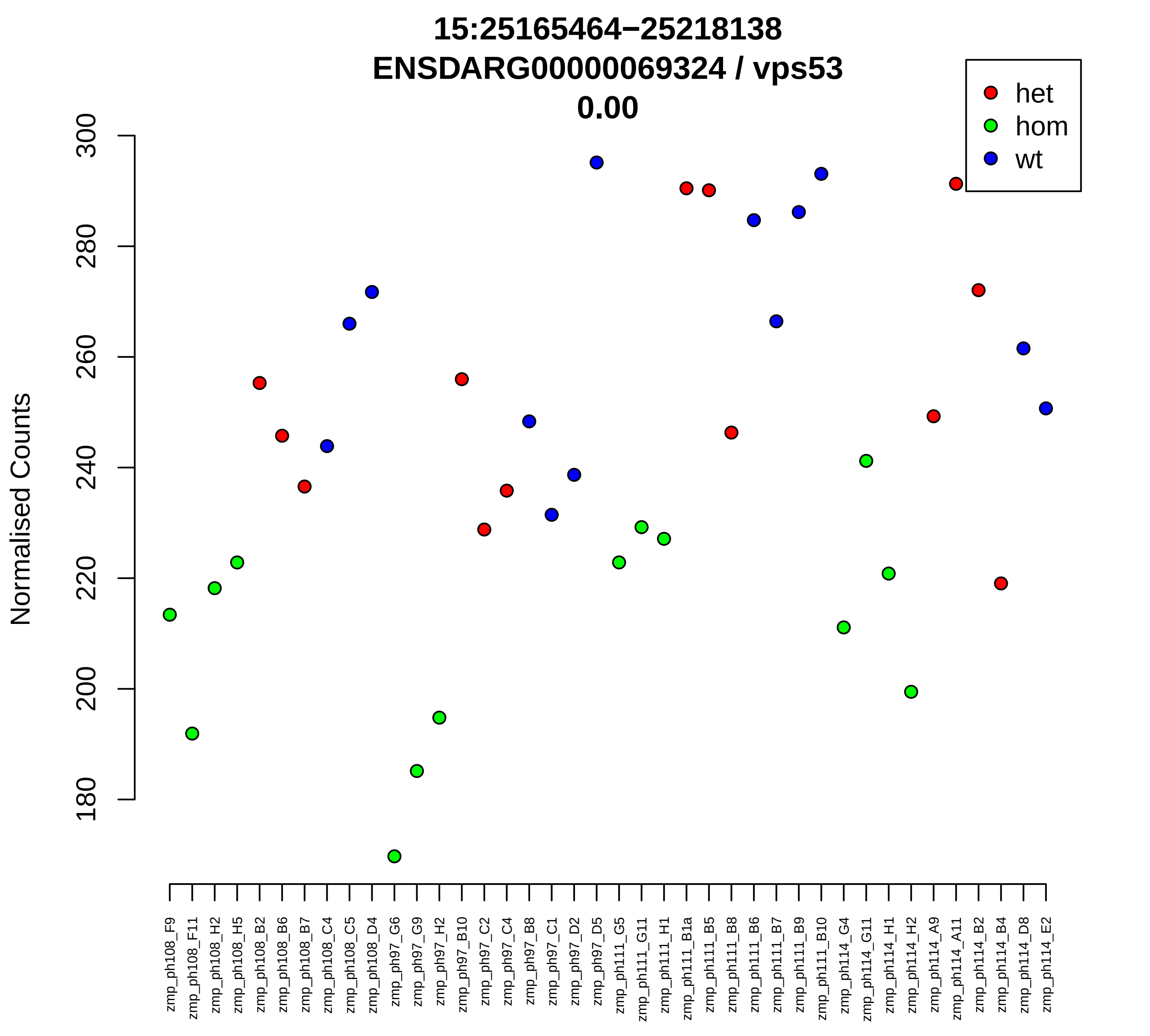
15:25165464−25218138
ENSDARG00000069324 / vps53
0.00
het
hom
wt
Normalised Counts
180
200
220
240
260
280
300
zmp_ph108_F9
zmp_ph108_F11
zmp_ph108_H2
zmp_ph108_H5
zmp_ph108_B2
zmp_ph108_B6
zmp_ph108_B7
zmp_ph108_C4
zmp_ph108_C5
zmp_ph108_D4
zmp_ph97_G6
zmp_ph97_G9
zmp_ph97_H2
zmp_ph97_B10
zmp_ph97_C2
zmp_ph97_C4
zmp_ph97_B8
zmp_ph97_C1
zmp_ph97_D2
zmp_ph97_D5
zmp_ph111_G5
zmp_ph111_G11
zmp_ph111_H1
zmp_ph111_B1a
zmp_ph111_B5
zmp_ph111_B8
zmp_ph111_B6
zmp_ph111_B7
zmp_ph111_B9
zmp_ph111_B10
zmp_ph114_G4
zmp_ph114_G11
zmp_ph114_H1
zmp_ph114_H2
zmp_ph114_A9
zmp_ph114_A11
zmp_ph114_B2
zmp_ph114_B4
zmp_ph114_D8
zmp_ph114_E2

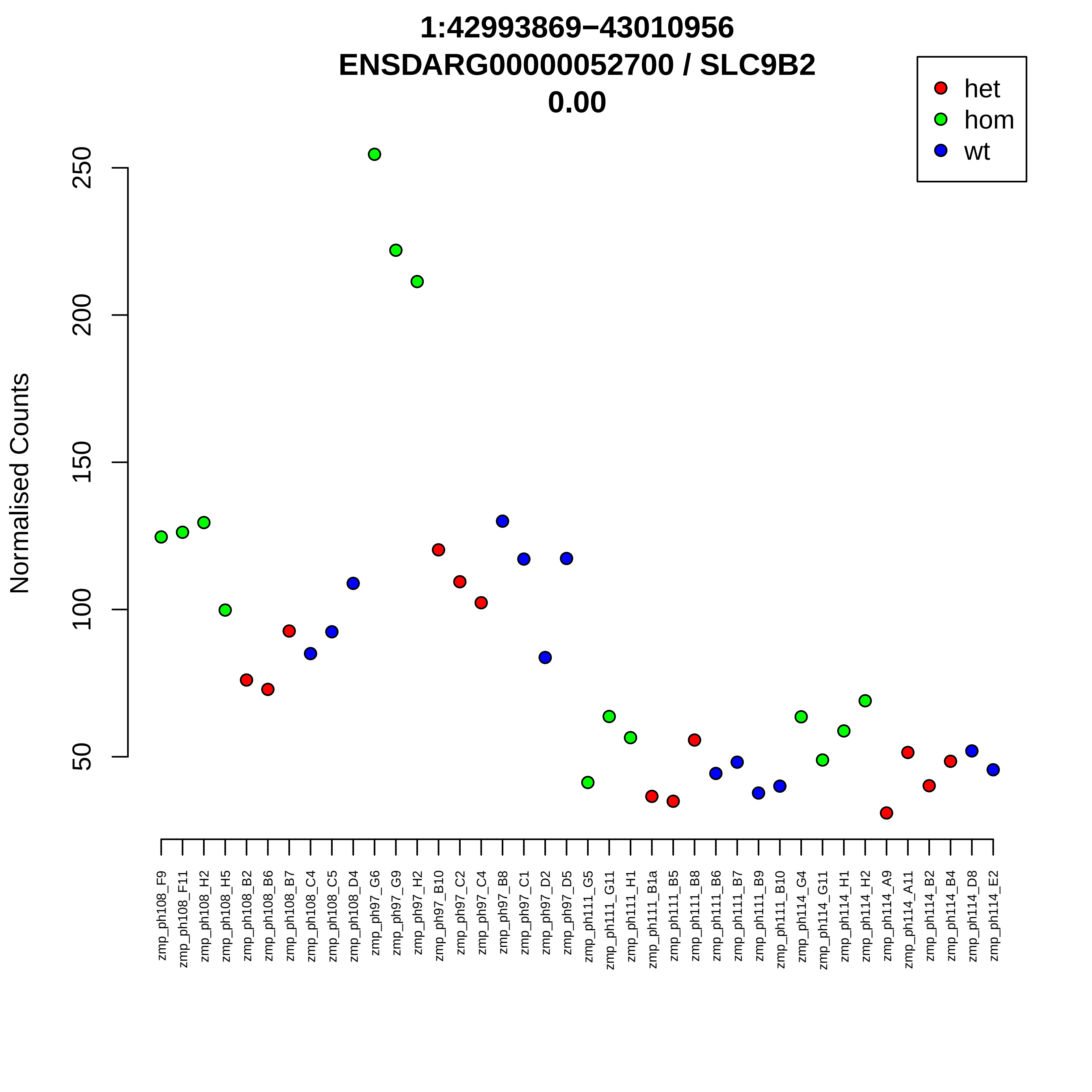

1:42993869−43010956
ENSDARG00000052700 / SLC9B2
0.00
het
hom
wt
Normalised Counts
50
100
150
200
250
zmp_ph108_F9
zmp_ph108_F11
zmp_ph108_H2
zmp_ph108_H5
zmp_ph108_B2
zmp_ph108_B6
zmp_ph108_B7
zmp_ph108_C4
zmp_ph108_C5
zmp_ph108_D4
zmp_ph97_G6
zmp_ph97_G9
zmp_ph97_H2
zmp_ph97_B10
zmp_ph97_C2
zmp_ph97_C4
zmp_ph97_B8
zmp_ph97_C1
zmp_ph97_D2
zmp_ph97_D5
zmp_ph111_G5
zmp_ph111_G11
zmp_ph111_H1
zmp_ph111_B1a
zmp_ph111_B5
zmp_ph111_B8
zmp_ph111_B6
zmp_ph111_B7
zmp_ph111_B9
zmp_ph111_B10
zmp_ph114_G4
zmp_ph114_G11
zmp_ph114_H1
zmp_ph114_H2
zmp_ph114_A9
zmp_ph114_A11
zmp_ph114_B2
zmp_ph114_B4
zmp_ph114_D8
zmp_ph114_E2

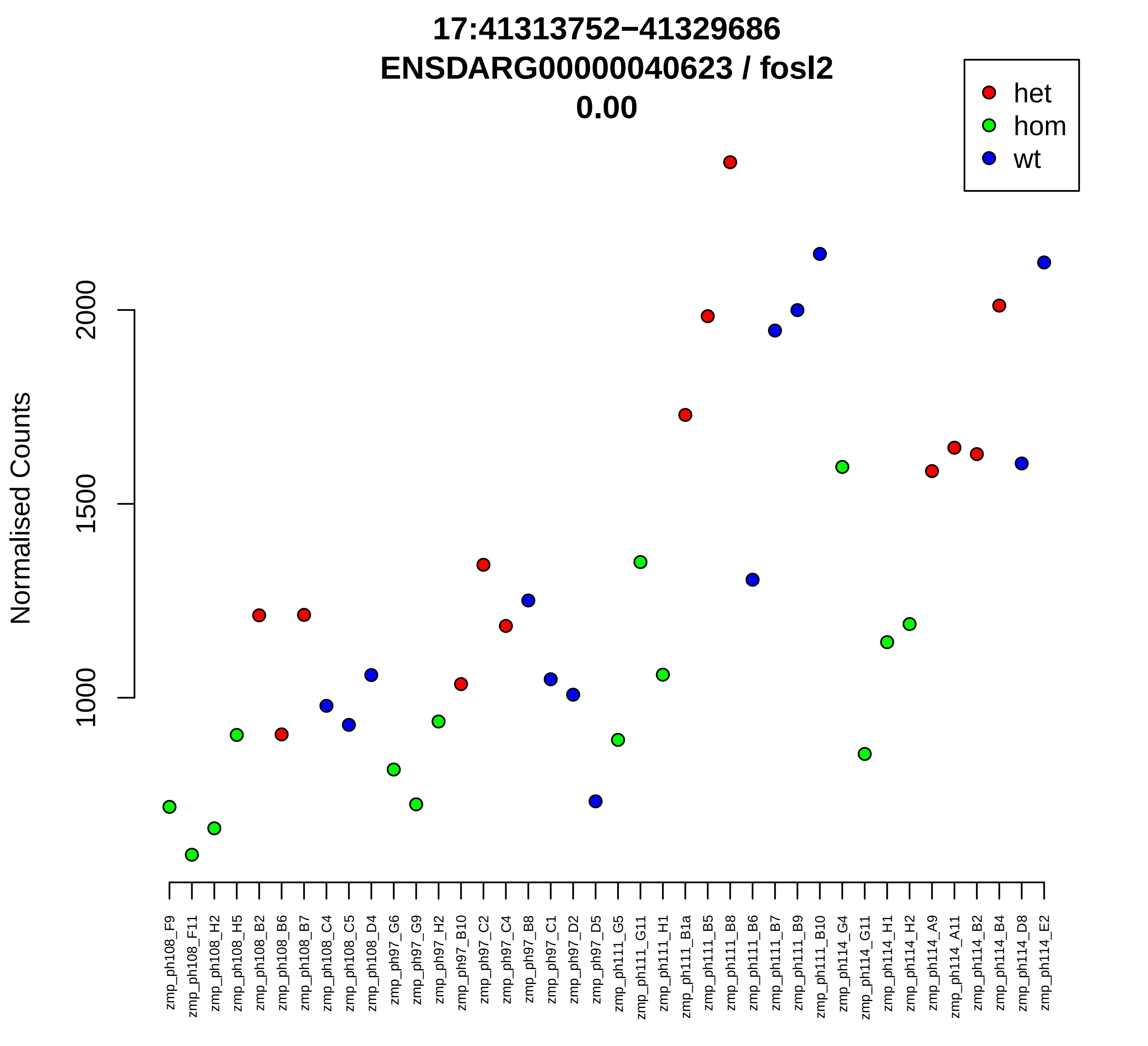

17:41313752−41329686
ENSDARG00000040623 / fosl2
0.00
het
hom
wt
Normalised Counts
1000
1500
2000
zmp_ph108_F9
zmp_ph108_F11
zmp_ph108_H2
zmp_ph108_H5
zmp_ph108_B2
zmp_ph108_B6
zmp_ph108_B7
zmp_ph108_C4
zmp_ph108_C5
zmp_ph108_D4
zmp_ph97_G6
zmp_ph97_G9
zmp_ph97_H2
zmp_ph97_B10
zmp_ph97_C2
zmp_ph97_C4
zmp_ph97_B8
zmp_ph97_C1
zmp_ph97_D2
zmp_ph97_D5
zmp_ph111_G5
zmp_ph111_G11
zmp_ph111_H1
zmp_ph111_B1a
zmp_ph111_B5
zmp_ph111_B8
zmp_ph111_B6
zmp_ph111_B7
zmp_ph111_B9
zmp_ph111_B10
zmp_ph114_G4
zmp_ph114_G11
zmp_ph114_H1
zmp_ph114_H2
zmp_ph114_A9
zmp_ph114_A11
zmp_ph114_B2
zmp_ph114_B4
zmp_ph114_D8
zmp_ph114_E2

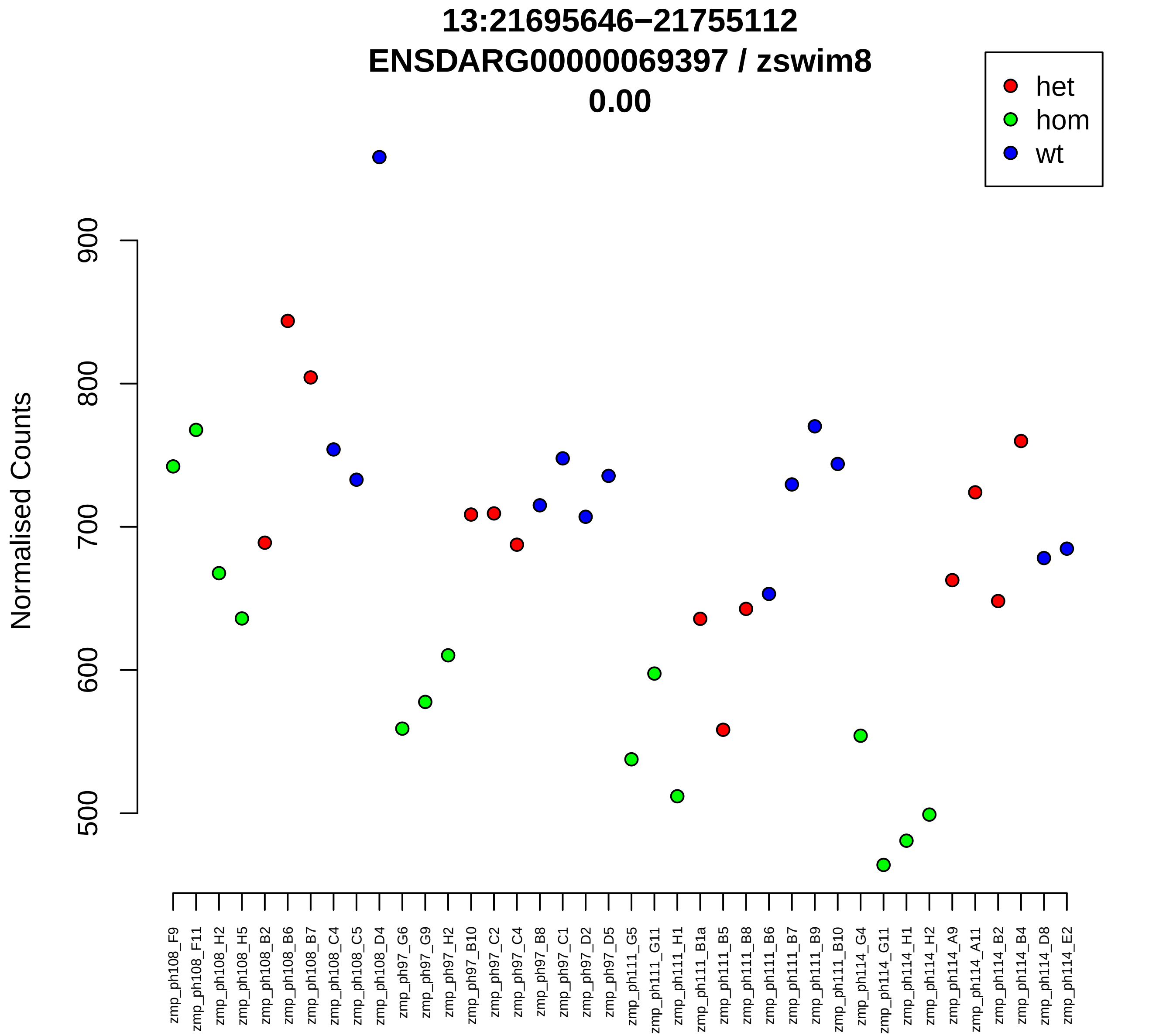
13:21695646−21755112
ENSDARG00000069397 / zswim8
0.00
het
hom
wt
Normalised Counts
500
600
700
800
900
zmp_ph108_F9
zmp_ph108_F11
zmp_ph108_H2
zmp_ph108_H5
zmp_ph108_B2
zmp_ph108_B6
zmp_ph108_B7
zmp_ph108_C4
zmp_ph108_C5
zmp_ph108_D4
zmp_ph97_G6
zmp_ph97_G9
zmp_ph97_H2
zmp_ph97_B10
zmp_ph97_C2
zmp_ph97_C4
zmp_ph97_B8
zmp_ph97_C1
zmp_ph97_D2
zmp_ph97_D5
zmp_ph111_G5
zmp_ph111_G11
zmp_ph111_H1
zmp_ph111_B1a
zmp_ph111_B5
zmp_ph111_B8
zmp_ph111_B6
zmp_ph111_B7
zmp_ph111_B9
zmp_ph111_B10
zmp_ph114_G4
zmp_ph114_G11
zmp_ph114_H1
zmp_ph114_H2
zmp_ph114_A9
zmp_ph114_A11
zmp_ph114_B2
zmp_ph114_B4
zmp_ph114_D8
zmp_ph114_E2

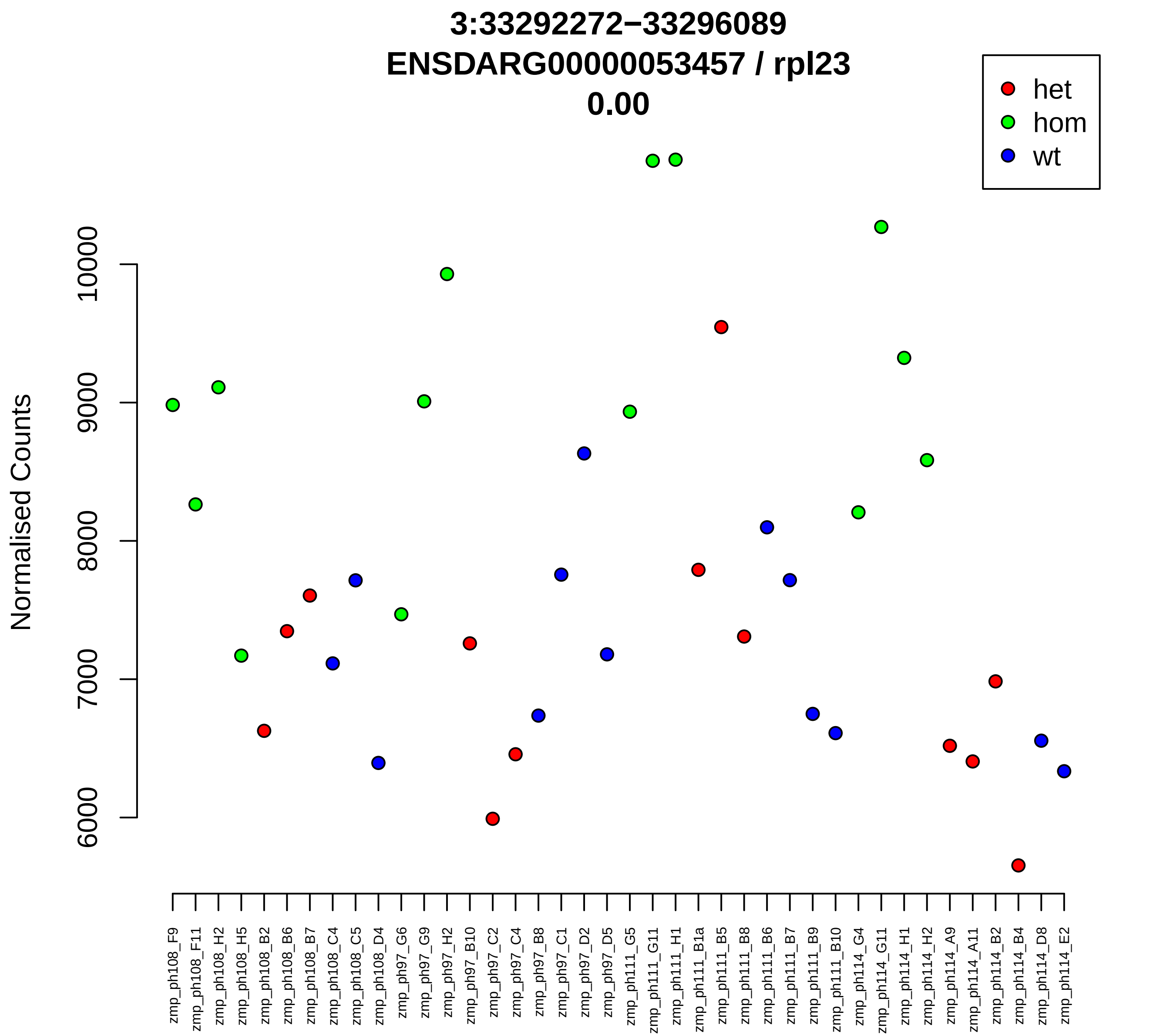

3:33292272−33296089
ENSDARG00000053457 / rpl23
0.00
het
hom
wt
Normalised Counts
6000
7000
8000
9000
10000
zmp_ph108_F9
zmp_ph108_F11
zmp_ph108_H2
zmp_ph108_H5
zmp_ph108_B2
zmp_ph108_B6
zmp_ph108_B7
zmp_ph108_C4
zmp_ph108_C5
zmp_ph108_D4
zmp_ph97_G6
zmp_ph97_G9
zmp_ph97_H2
zmp_ph97_B10
zmp_ph97_C2
zmp_ph97_C4
zmp_ph97_B8
zmp_ph97_C1
zmp_ph97_D2
zmp_ph97_D5
zmp_ph111_G5
zmp_ph111_G11
zmp_ph111_H1
zmp_ph111_B1a
zmp_ph111_B5
zmp_ph111_B8
zmp_ph111_B6
zmp_ph111_B7
zmp_ph111_B9
zmp_ph111_B10
zmp_ph114_G4
zmp_ph114_G11
zmp_ph114_H1
zmp_ph114_H2
zmp_ph114_A9
zmp_ph114_A11
zmp_ph114_B2
zmp_ph114_B4
zmp_ph114_D8
zmp_ph114_E2

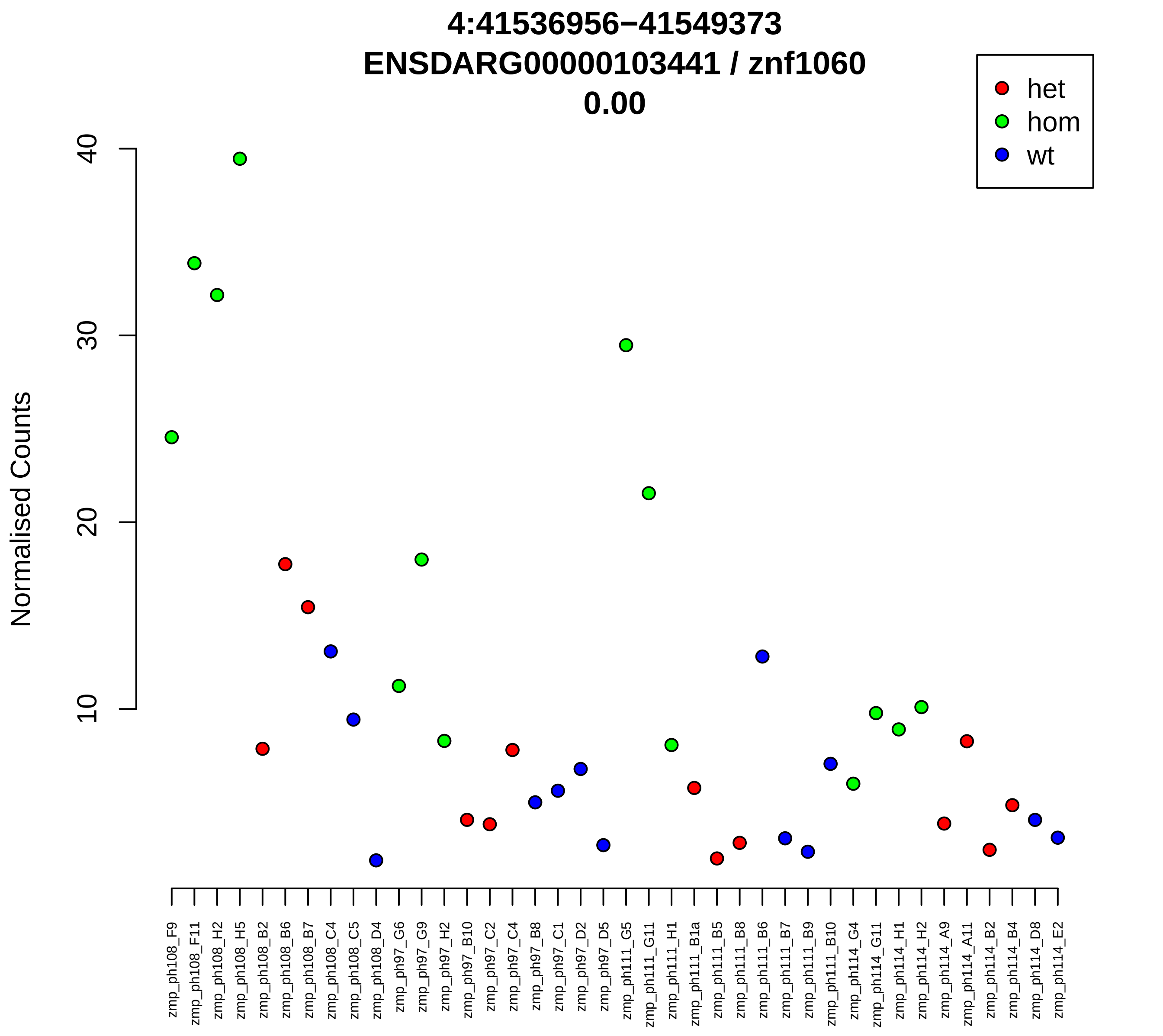
4:41536956−41549373
ENSDARG00000103441 / znf1060
0.00
het
hom
wt
Normalised Counts
10
20
30
40
zmp_ph108_F9
zmp_ph108_F11
zmp_ph108_H2
zmp_ph108_H5
zmp_ph108_B2
zmp_ph108_B6
zmp_ph108_B7
zmp_ph108_C4
zmp_ph108_C5
zmp_ph108_D4
zmp_ph97_G6
zmp_ph97_G9
zmp_ph97_H2
zmp_ph97_B10
zmp_ph97_C2
zmp_ph97_C4
zmp_ph97_B8
zmp_ph97_C1
zmp_ph97_D2
zmp_ph97_D5
zmp_ph111_G5
zmp_ph111_G11
zmp_ph111_H1
zmp_ph111_B1a
zmp_ph111_B5
zmp_ph111_B8
zmp_ph111_B6
zmp_ph111_B7
zmp_ph111_B9
zmp_ph111_B10
zmp_ph114_G4
zmp_ph114_G11
zmp_ph114_H1
zmp_ph114_H2
zmp_ph114_A9
zmp_ph114_A11
zmp_ph114_B2
zmp_ph114_B4
zmp_ph114_D8
zmp_ph114_E2

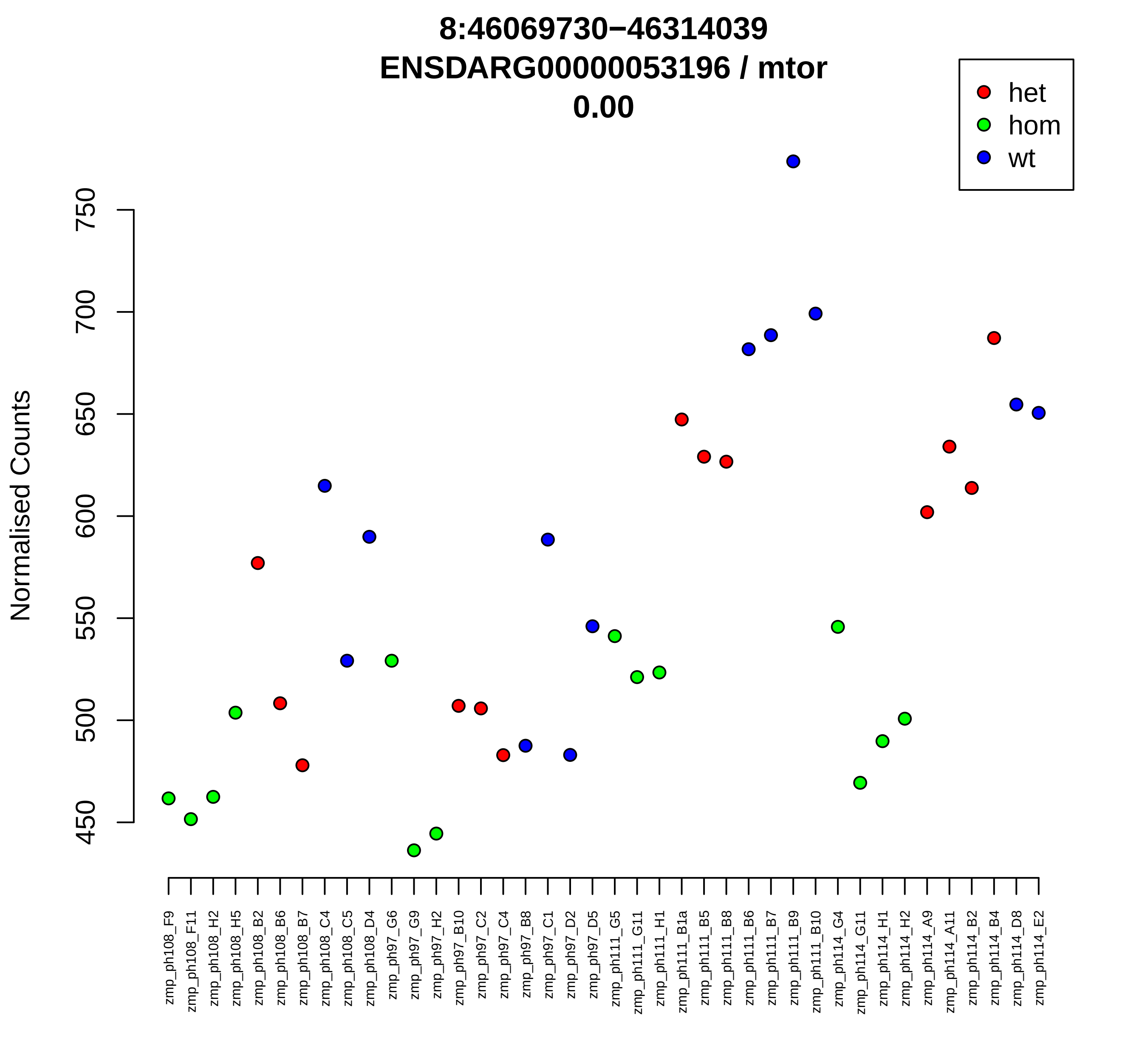

8:46069730−46314039
ENSDARG00000053196 / mtor
0.00
het
hom
wt
Normalised Counts
450
500
550
600
650
700
750
zmp_ph108_F9
zmp_ph108_F11
zmp_ph108_H2
zmp_ph108_H5
zmp_ph108_B2
zmp_ph108_B6
zmp_ph108_B7
zmp_ph108_C4
zmp_ph108_C5
zmp_ph108_D4
zmp_ph97_G6
zmp_ph97_G9
zmp_ph97_H2
zmp_ph97_B10
zmp_ph97_C2
zmp_ph97_C4
zmp_ph97_B8
zmp_ph97_C1
zmp_ph97_D2
zmp_ph97_D5
zmp_ph111_G5
zmp_ph111_G11
zmp_ph111_H1
zmp_ph111_B1a
zmp_ph111_B5
zmp_ph111_B8
zmp_ph111_B6
zmp_ph111_B7
zmp_ph111_B9
zmp_ph111_B10
zmp_ph114_G4
zmp_ph114_G11
zmp_ph114_H1
zmp_ph114_H2
zmp_ph114_A9
zmp_ph114_A11
zmp_ph114_B2
zmp_ph114_B4
zmp_ph114_D8
zmp_ph114_E2

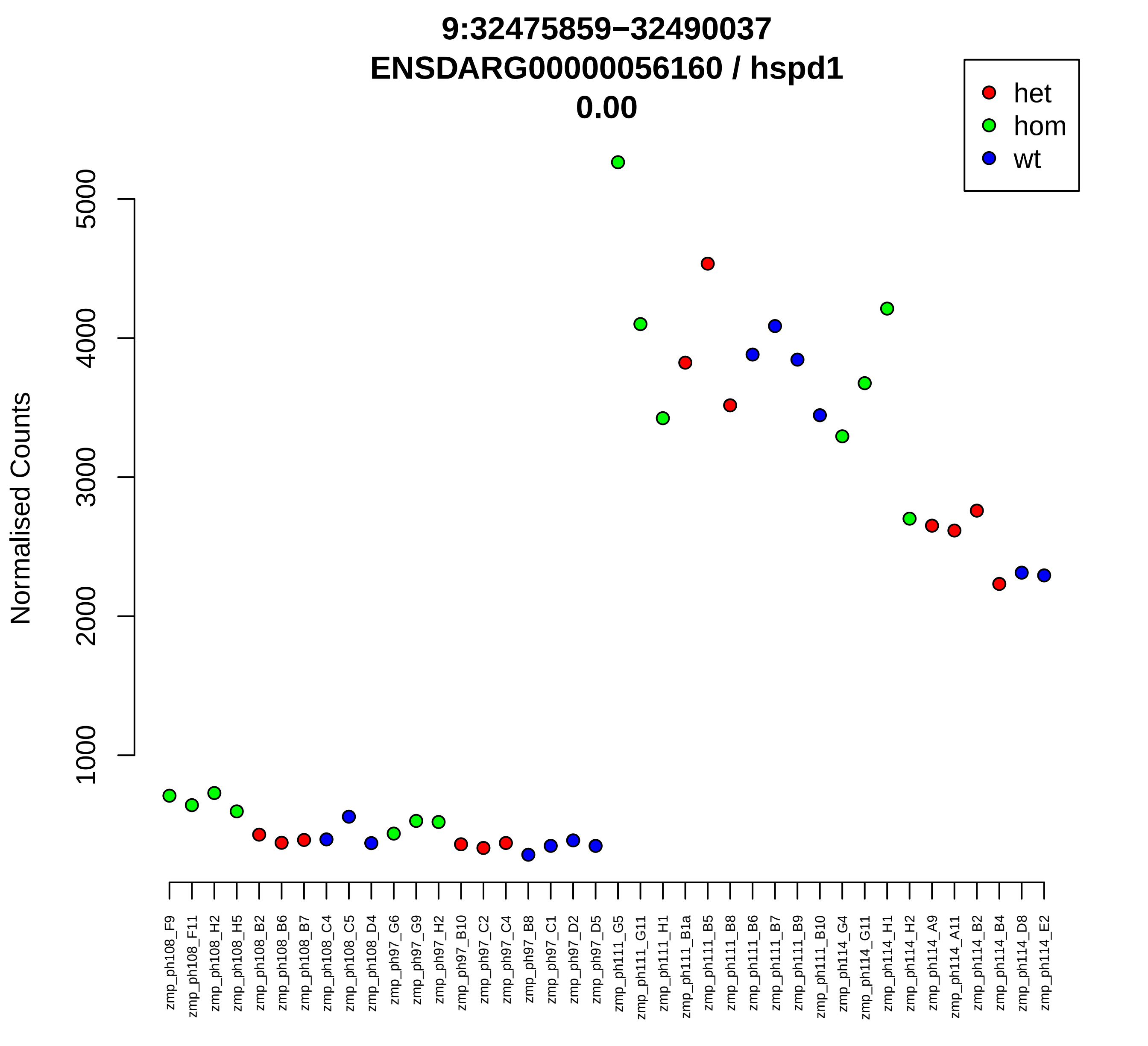

9:32475859−32490037
ENSDARG00000056160 / hspd1
0.00
het
hom
wt
Normalised Counts
1000
2000
3000
4000
5000
zmp_ph108_F9
zmp_ph108_F11
zmp_ph108_H2
zmp_ph108_H5
zmp_ph108_B2
zmp_ph108_B6
zmp_ph108_B7
zmp_ph108_C4
zmp_ph108_C5
zmp_ph108_D4
zmp_ph97_G6
zmp_ph97_G9
zmp_ph97_H2
zmp_ph97_B10
zmp_ph97_C2
zmp_ph97_C4
zmp_ph97_B8
zmp_ph97_C1
zmp_ph97_D2
zmp_ph97_D5
zmp_ph111_G5
zmp_ph111_G11
zmp_ph111_H1
zmp_ph111_B1a
zmp_ph111_B5
zmp_ph111_B8
zmp_ph111_B6
zmp_ph111_B7
zmp_ph111_B9
zmp_ph111_B10
zmp_ph114_G4
zmp_ph114_G11
zmp_ph114_H1
zmp_ph114_H2
zmp_ph114_A9
zmp_ph114_A11
zmp_ph114_B2
zmp_ph114_B4
zmp_ph114_D8
zmp_ph114_E2

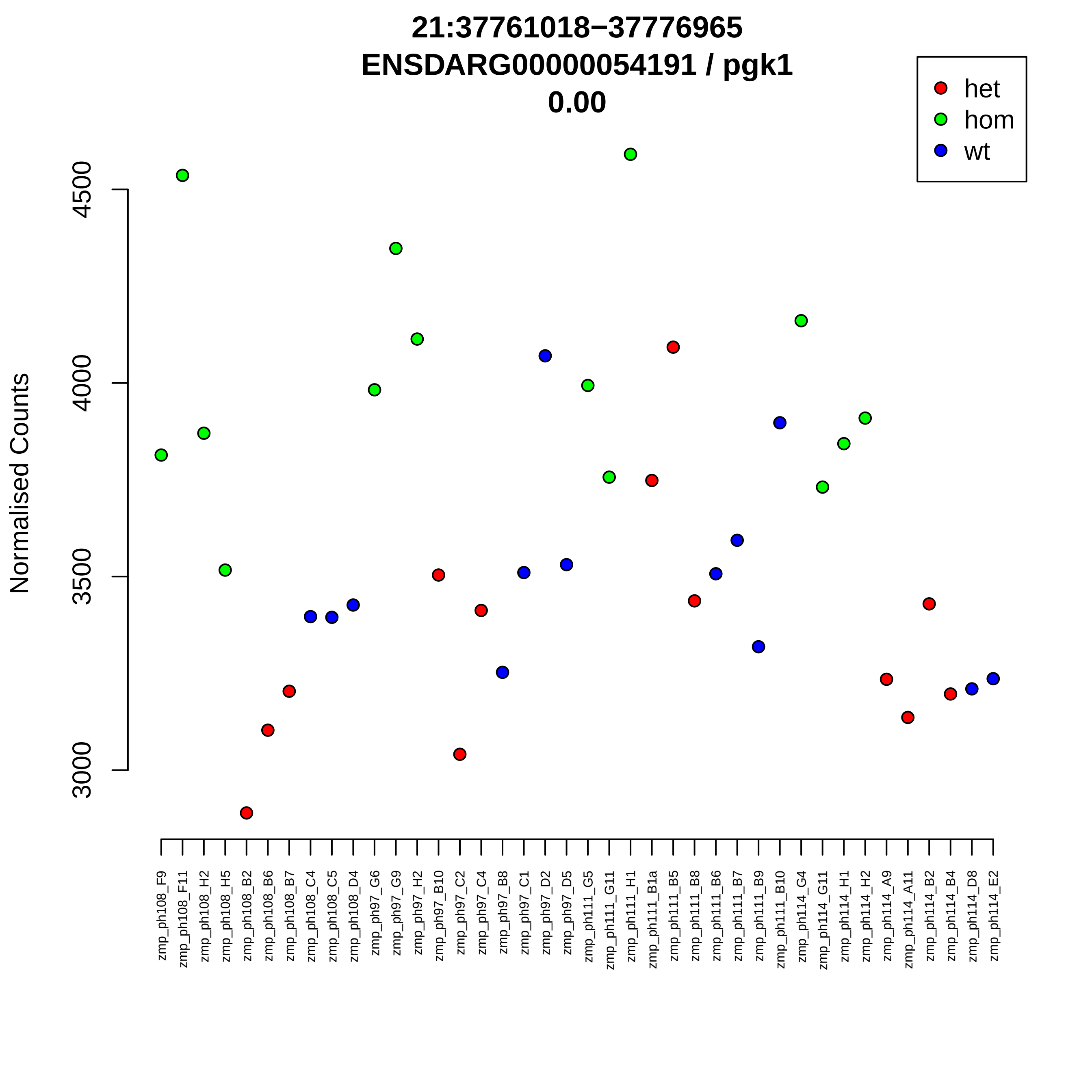

21:37761018−37776965
ENSDARG00000054191 / pgk1
0.00
het
hom
wt
Normalised Counts
3000
3500
4000
4500
zmp_ph108_F9
zmp_ph108_F11
zmp_ph108_H2
zmp_ph108_H5
zmp_ph108_B2
zmp_ph108_B6
zmp_ph108_B7
zmp_ph108_C4
zmp_ph108_C5
zmp_ph108_D4
zmp_ph97_G6
zmp_ph97_G9
zmp_ph97_H2
zmp_ph97_B10
zmp_ph97_C2
zmp_ph97_C4
zmp_ph97_B8
zmp_ph97_C1
zmp_ph97_D2
zmp_ph97_D5
zmp_ph111_G5
zmp_ph111_G11
zmp_ph111_H1
zmp_ph111_B1a
zmp_ph111_B5
zmp_ph111_B8
zmp_ph111_B6
zmp_ph111_B7
zmp_ph111_B9
zmp_ph111_B10
zmp_ph114_G4
zmp_ph114_G11
zmp_ph114_H1
zmp_ph114_H2
zmp_ph114_A9
zmp_ph114_A11
zmp_ph114_B2
zmp_ph114_B4
zmp_ph114_D8
zmp_ph114_E2

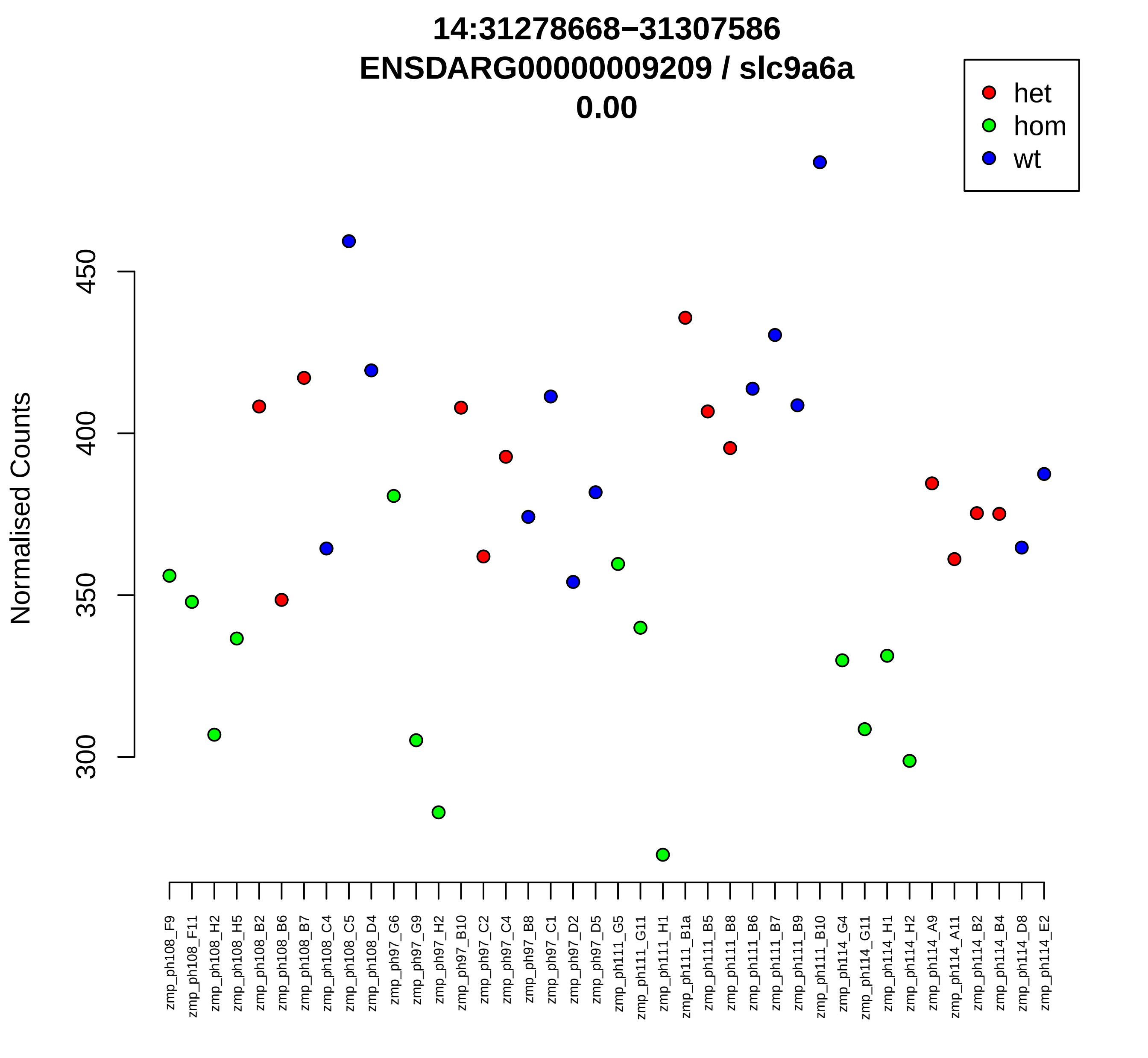

14:31278668−31307586
ENSDARG00000009209 / slc9a6a
0.00
het
hom
wt
Normalised Counts
300
350
400
450
zmp_ph108_F9
zmp_ph108_F11
zmp_ph108_H2
zmp_ph108_H5
zmp_ph108_B2
zmp_ph108_B6
zmp_ph108_B7
zmp_ph108_C4
zmp_ph108_C5
zmp_ph108_D4
zmp_ph97_G6
zmp_ph97_G9
zmp_ph97_H2
zmp_ph97_B10
zmp_ph97_C2
zmp_ph97_C4
zmp_ph97_B8
zmp_ph97_C1
zmp_ph97_D2
zmp_ph97_D5
zmp_ph111_G5
zmp_ph111_G11
zmp_ph111_H1
zmp_ph111_B1a
zmp_ph111_B5
zmp_ph111_B8
zmp_ph111_B6
zmp_ph111_B7
zmp_ph111_B9
zmp_ph111_B10
zmp_ph114_G4
zmp_ph114_G11
zmp_ph114_H1
zmp_ph114_H2
zmp_ph114_A9
zmp_ph114_A11
zmp_ph114_B2
zmp_ph114_B4
zmp_ph114_D8
zmp_ph114_E2

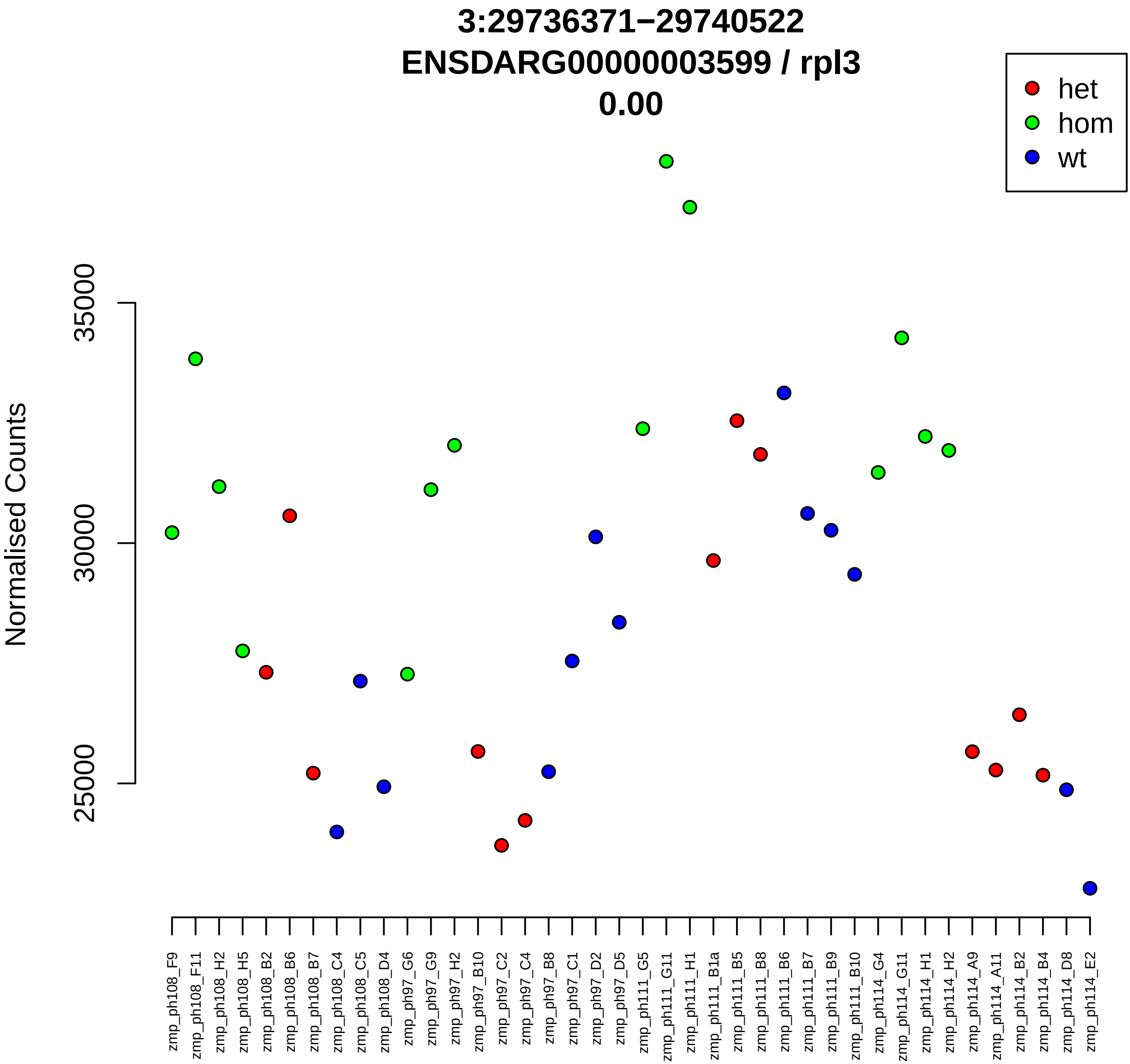

3:29736371−29740522
ENSDARG00000003599 / rpl3
0.00
het
hom
wt
Normalised Counts
25000
30000
35000
zmp_ph108_F9
zmp_ph108_F11
zmp_ph108_H2
zmp_ph108_H5
zmp_ph108_B2
zmp_ph108_B6
zmp_ph108_B7
zmp_ph108_C4
zmp_ph108_C5
zmp_ph108_D4
zmp_ph97_G6
zmp_ph97_G9
zmp_ph97_H2
zmp_ph97_B10
zmp_ph97_C2
zmp_ph97_C4
zmp_ph97_B8
zmp_ph97_C1
zmp_ph97_D2
zmp_ph97_D5
zmp_ph111_G5
zmp_ph111_G11
zmp_ph111_H1
zmp_ph111_B1a
zmp_ph111_B5
zmp_ph111_B8
zmp_ph111_B6
zmp_ph111_B7
zmp_ph111_B9
zmp_ph111_B10
zmp_ph114_G4
zmp_ph114_G11
zmp_ph114_H1
zmp_ph114_H2
zmp_ph114_A9
zmp_ph114_A11
zmp_ph114_B2
zmp_ph114_B4
zmp_ph114_D8
zmp_ph114_E2

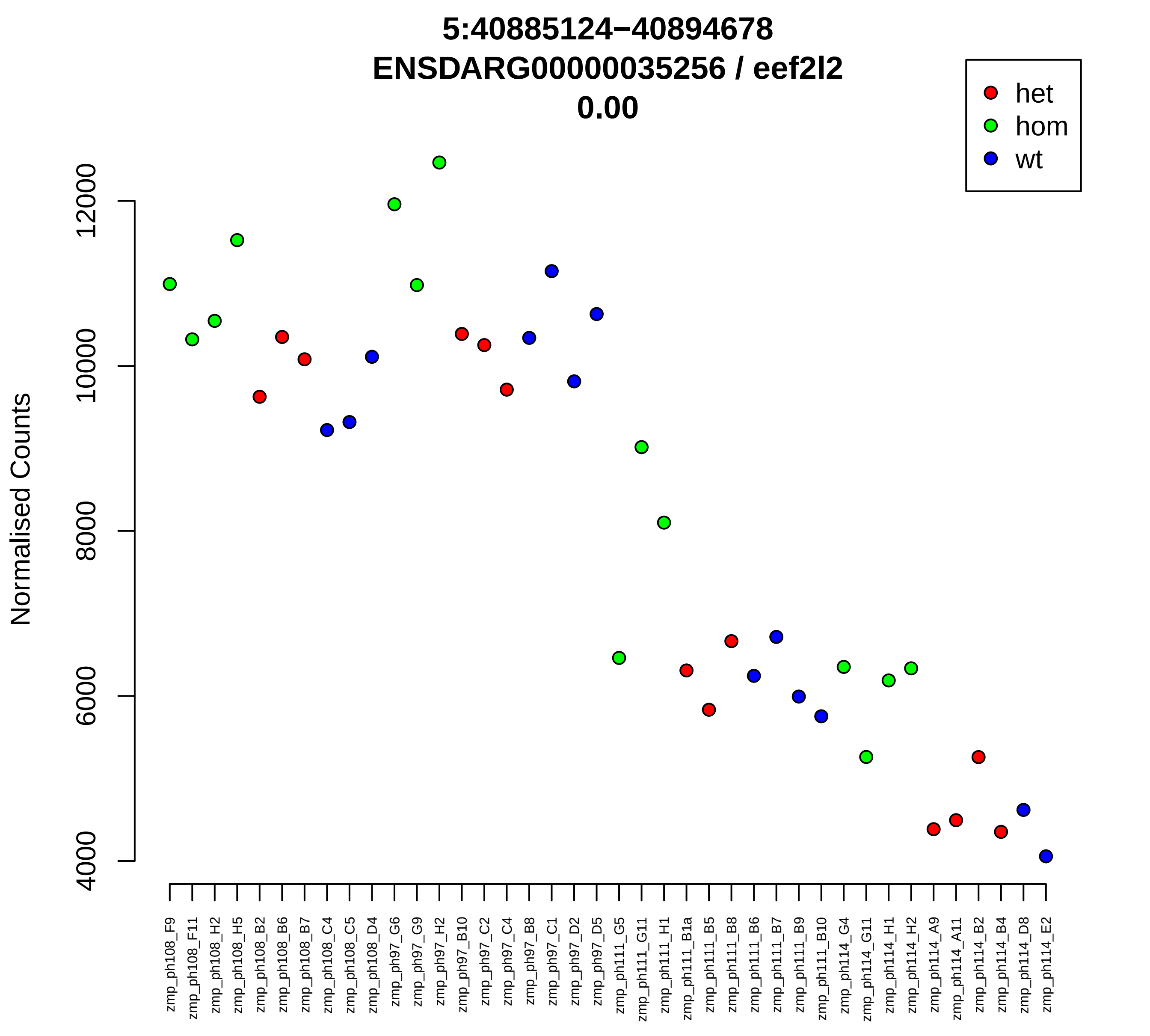
5:40885124−40894678
ENSDARG00000035256 / eef2l2
0.00
het
hom
wt
Normalised Counts
4000
6000
8000
10000
12000
zmp_ph108_F9
zmp_ph108_F11
zmp_ph108_H2
zmp_ph108_H5
zmp_ph108_B2
zmp_ph108_B6
zmp_ph108_B7
zmp_ph108_C4
zmp_ph108_C5
zmp_ph108_D4
zmp_ph97_G6
zmp_ph97_G9
zmp_ph97_H2
zmp_ph97_B10
zmp_ph97_C2
zmp_ph97_C4
zmp_ph97_B8
zmp_ph97_C1
zmp_ph97_D2
zmp_ph97_D5
zmp_ph111_G5
zmp_ph111_G11
zmp_ph111_H1
zmp_ph111_B1a
zmp_ph111_B5
zmp_ph111_B8
zmp_ph111_B6
zmp_ph111_B7
zmp_ph111_B9
zmp_ph111_B10
zmp_ph114_G4
zmp_ph114_G11
zmp_ph114_H1
zmp_ph114_H2
zmp_ph114_A9
zmp_ph114_A11
zmp_ph114_B2
zmp_ph114_B4
zmp_ph114_D8
zmp_ph114_E2

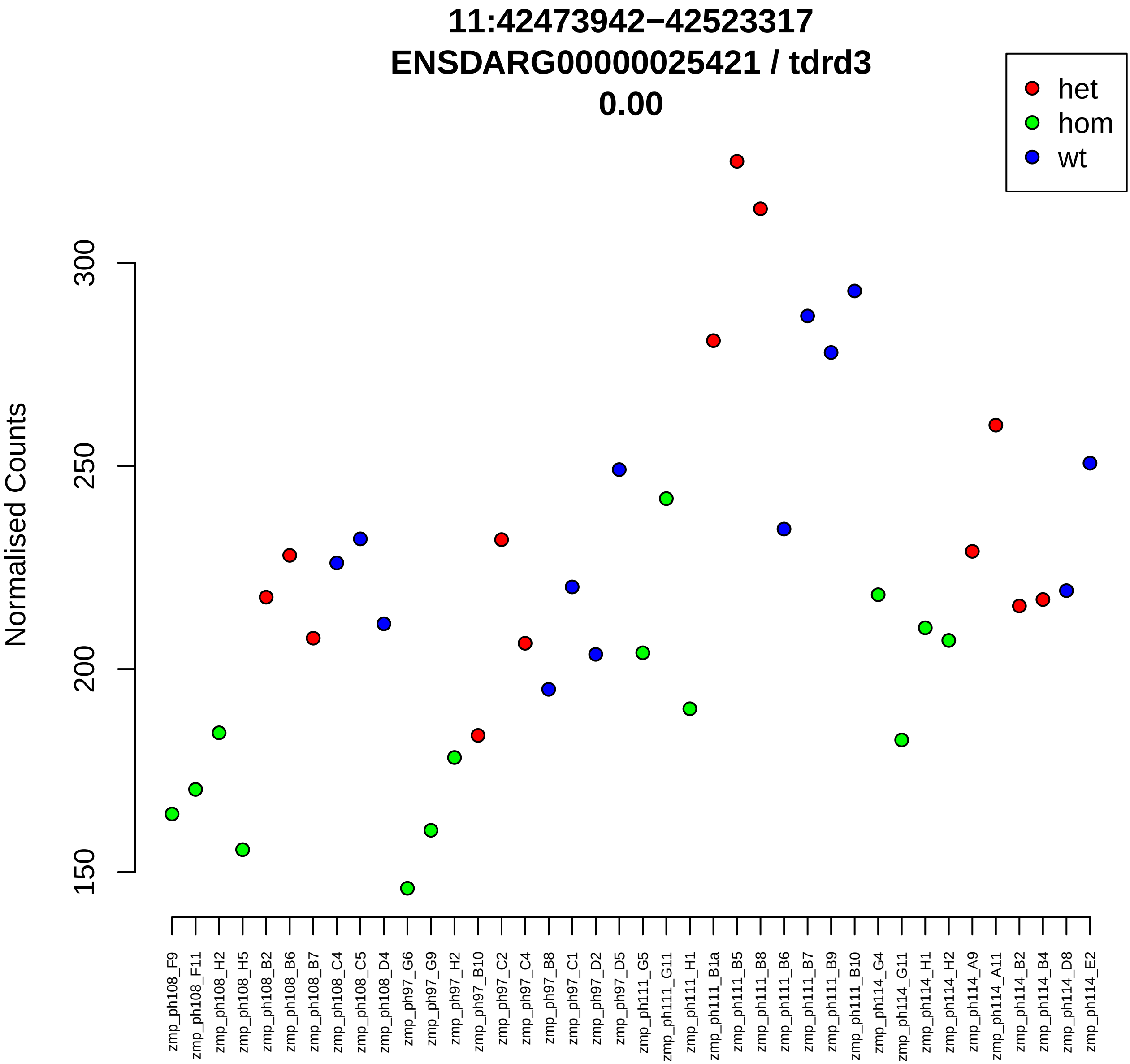
11:42473942−42523317
ENSDARG00000025421 / tdrd3
0.00
het
hom
wt
Normalised Counts
150
200
250
300
zmp_ph108_F9
zmp_ph108_F11
zmp_ph108_H2
zmp_ph108_H5
zmp_ph108_B2
zmp_ph108_B6
zmp_ph108_B7
zmp_ph108_C4
zmp_ph108_C5
zmp_ph108_D4
zmp_ph97_G6
zmp_ph97_G9
zmp_ph97_H2
zmp_ph97_B10
zmp_ph97_C2
zmp_ph97_C4
zmp_ph97_B8
zmp_ph97_C1
zmp_ph97_D2
zmp_ph97_D5
zmp_ph111_G5
zmp_ph111_G11
zmp_ph111_H1
zmp_ph111_B1a
zmp_ph111_B5
zmp_ph111_B8
zmp_ph111_B6
zmp_ph111_B7
zmp_ph111_B9
zmp_ph111_B10
zmp_ph114_G4
zmp_ph114_G11
zmp_ph114_H1
zmp_ph114_H2
zmp_ph114_A9
zmp_ph114_A11
zmp_ph114_B2
zmp_ph114_B4
zmp_ph114_D8
zmp_ph114_E2

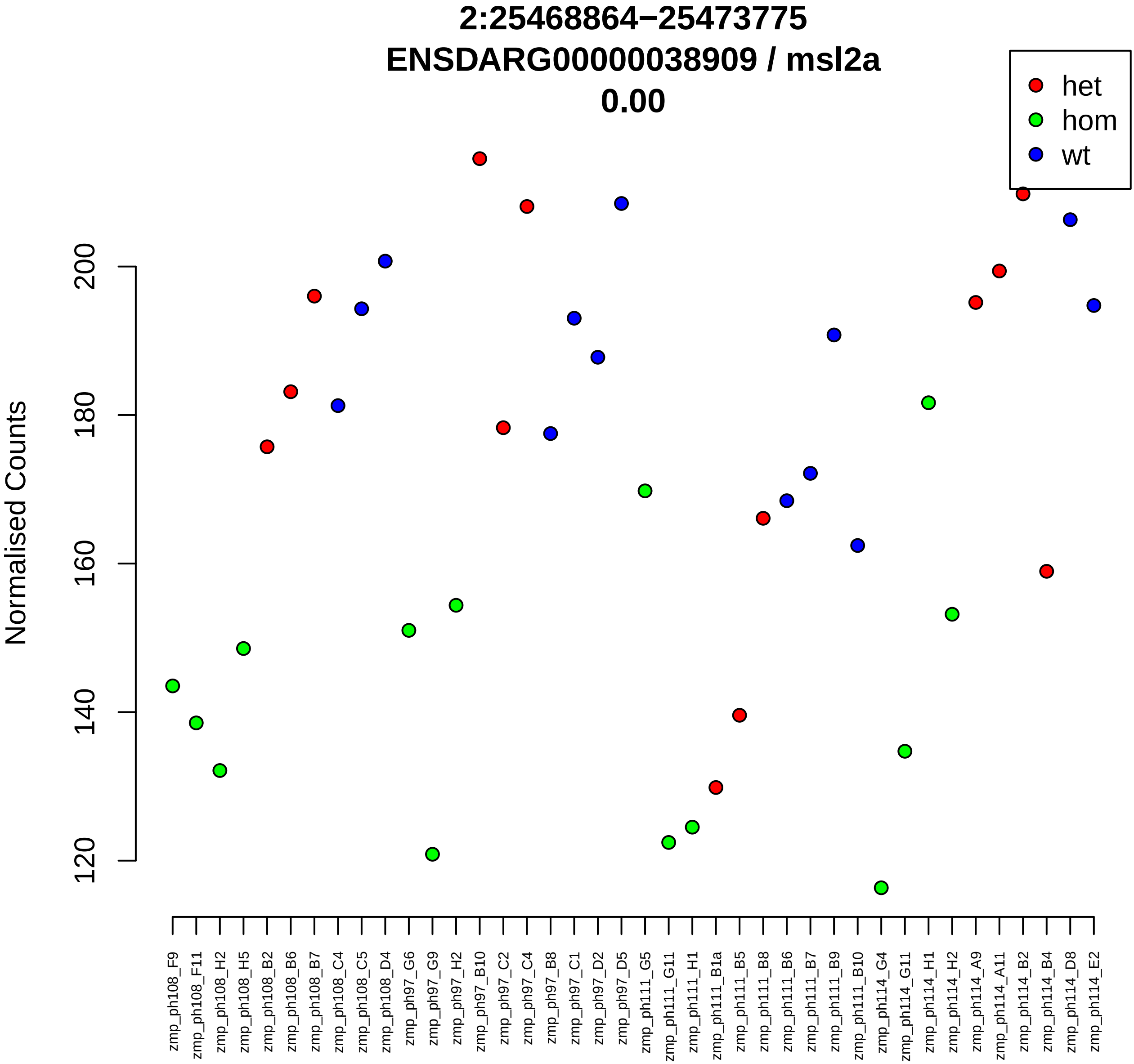
2:25468864−25473775
ENSDARG00000038909 / msl2a
0.00
het
hom
wt
Normalised Counts
120
140
160
180
200
zmp_ph108_F9
zmp_ph108_F11
zmp_ph108_H2
zmp_ph108_H5
zmp_ph108_B2
zmp_ph108_B6
zmp_ph108_B7
zmp_ph108_C4
zmp_ph108_C5
zmp_ph108_D4
zmp_ph97_G6
zmp_ph97_G9
zmp_ph97_H2
zmp_ph97_B10
zmp_ph97_C2
zmp_ph97_C4
zmp_ph97_B8
zmp_ph97_C1
zmp_ph97_D2
zmp_ph97_D5
zmp_ph111_G5
zmp_ph111_G11
zmp_ph111_H1
zmp_ph111_B1a
zmp_ph111_B5
zmp_ph111_B8
zmp_ph111_B6
zmp_ph111_B7
zmp_ph111_B9
zmp_ph111_B10
zmp_ph114_G4
zmp_ph114_G11
zmp_ph114_H1
zmp_ph114_H2
zmp_ph114_A9
zmp_ph114_A11
zmp_ph114_B2
zmp_ph114_B4
zmp_ph114_D8
zmp_ph114_E2

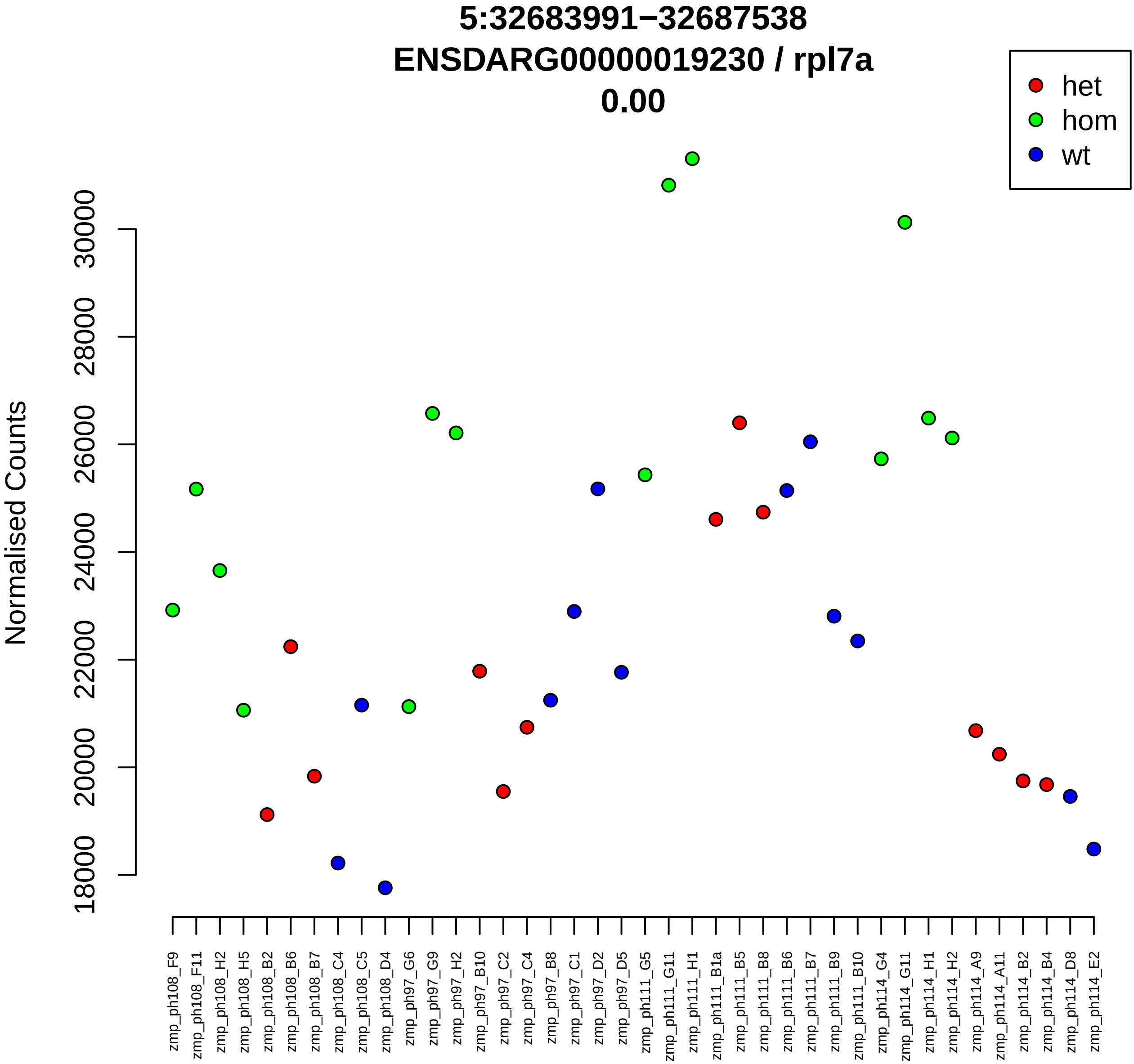
5:32683991−32687538
ENSDARG00000019230 / rpl7a
0.00
het
hom
wt
Normalised Counts
18000
20000
22000
24000
26000
28000
30000
zmp_ph108_F9
zmp_ph108_F11
zmp_ph108_H2
zmp_ph108_H5
zmp_ph108_B2
zmp_ph108_B6
zmp_ph108_B7
zmp_ph108_C4
zmp_ph108_C5
zmp_ph108_D4
zmp_ph97_G6
zmp_ph97_G9
zmp_ph97_H2
zmp_ph97_B10
zmp_ph97_C2
zmp_ph97_C4
zmp_ph97_B8
zmp_ph97_C1
zmp_ph97_D2
zmp_ph97_D5
zmp_ph111_G5
zmp_ph111_G11
zmp_ph111_H1
zmp_ph111_B1a
zmp_ph111_B5
zmp_ph111_B8
zmp_ph111_B6
zmp_ph111_B7
zmp_ph111_B9
zmp_ph111_B10
zmp_ph114_G4
zmp_ph114_G11
zmp_ph114_H1
zmp_ph114_H2
zmp_ph114_A9
zmp_ph114_A11
zmp_ph114_B2
zmp_ph114_B4
zmp_ph114_D8
zmp_ph114_E2

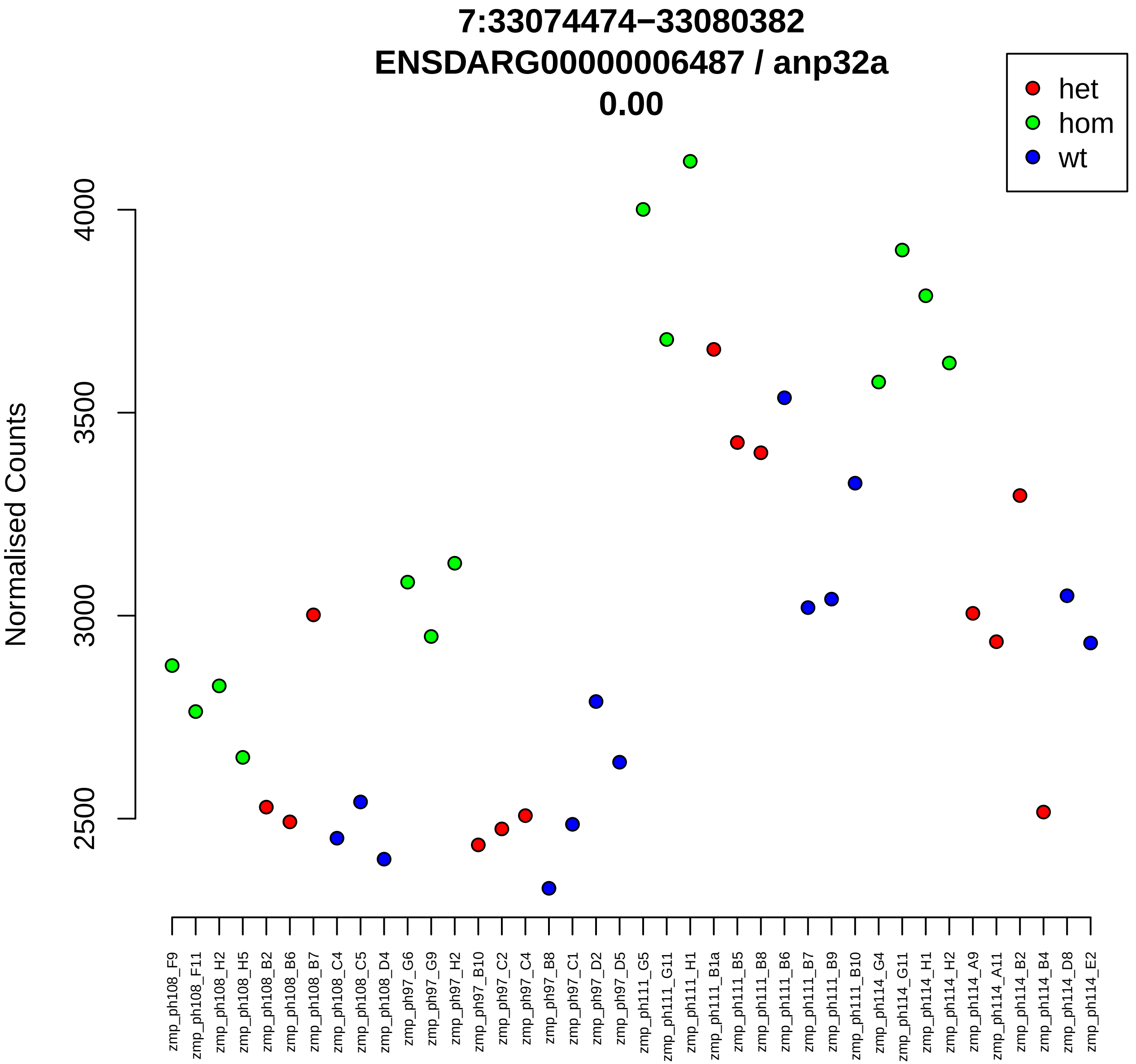
7:33074474−33080382
ENSDARG00000006487 / anp32a
0.00
het
hom
wt
Normalised Counts
4000
3500
3000
2500
zmp_ph108_F9
zmp_ph108_F11
zmp_ph108_H2
zmp_ph108_H5
zmp_ph108_B2
zmp_ph108_B6
zmp_ph108_B7
zmp_ph108_C4
zmp_ph108_C5
zmp_ph108_D4
zmp_ph97_G6
zmp_ph97_G9
zmp_ph97_H2
zmp_ph97_B10
zmp_ph97_C2
zmp_ph97_C4
zmp_ph97_B8
zmp_ph97_C1
zmp_ph97_D2
zmp_ph97_D5
zmp_ph111_G5
zmp_ph111_G11
zmp_ph111_H1
zmp_ph111_B1a
zmp_ph111_B5
zmp_ph111_B8
zmp_ph111_B6
zmp_ph111_B7
zmp_ph111_B9
zmp_ph111_B10
zmp_ph114_G4
zmp_ph114_G11
zmp_ph114_H1
zmp_ph114_H2
zmp_ph114_A9
zmp_ph114_A11
zmp_ph114_B2
zmp_ph114_B4
zmp_ph114_D8
zmp_ph114_E2

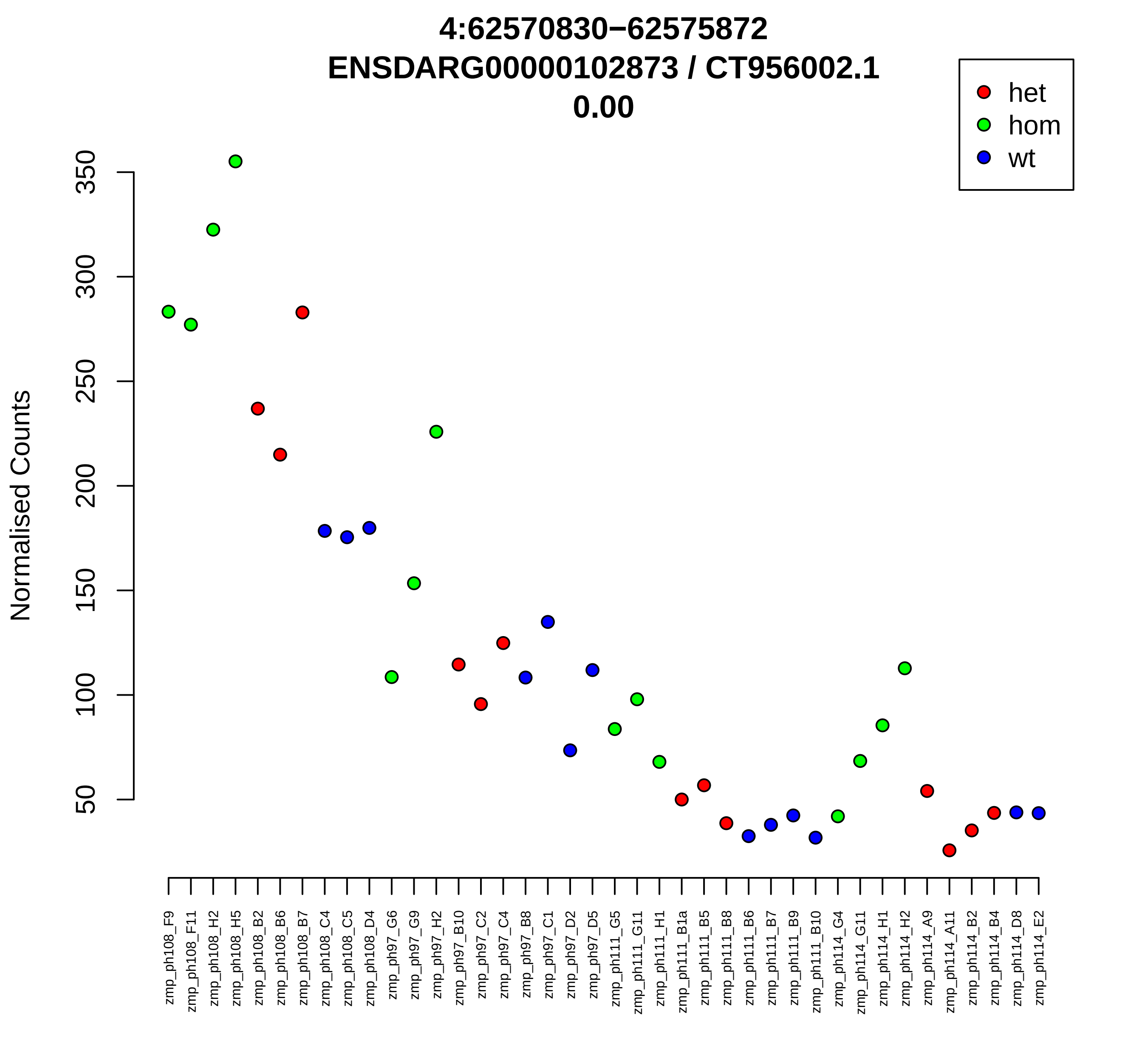
4:62570830−62575872
ENSDARG00000102873 / CT956002.1
0.00
het
hom
wt
Normalised Counts
50
100
150
200
250
300
350
zmp_ph108_F9
zmp_ph108_F11
zmp_ph108_H2
zmp_ph108_H5
zmp_ph108_B2
zmp_ph108_B6
zmp_ph108_B7
zmp_ph108_C4
zmp_ph108_C5
zmp_ph108_D4
zmp_ph97_G6
zmp_ph97_G9
zmp_ph97_H2
zmp_ph97_B10
zmp_ph97_C2
zmp_ph97_C4
zmp_ph97_B8
zmp_ph97_C1
zmp_ph97_D2
zmp_ph97_D5
zmp_ph111_G5
zmp_ph111_G11
zmp_ph111_H1
zmp_ph111_B1a
zmp_ph111_B5
zmp_ph111_B8
zmp_ph111_B6
zmp_ph111_B7
zmp_ph111_B9
zmp_ph111_B10
zmp_ph114_G4
zmp_ph114_G11
zmp_ph114_H1
zmp_ph114_H2
zmp_ph114_A9
zmp_ph114_A11
zmp_ph114_B2
zmp_ph114_B4
zmp_ph114_D8
zmp_ph114_E2

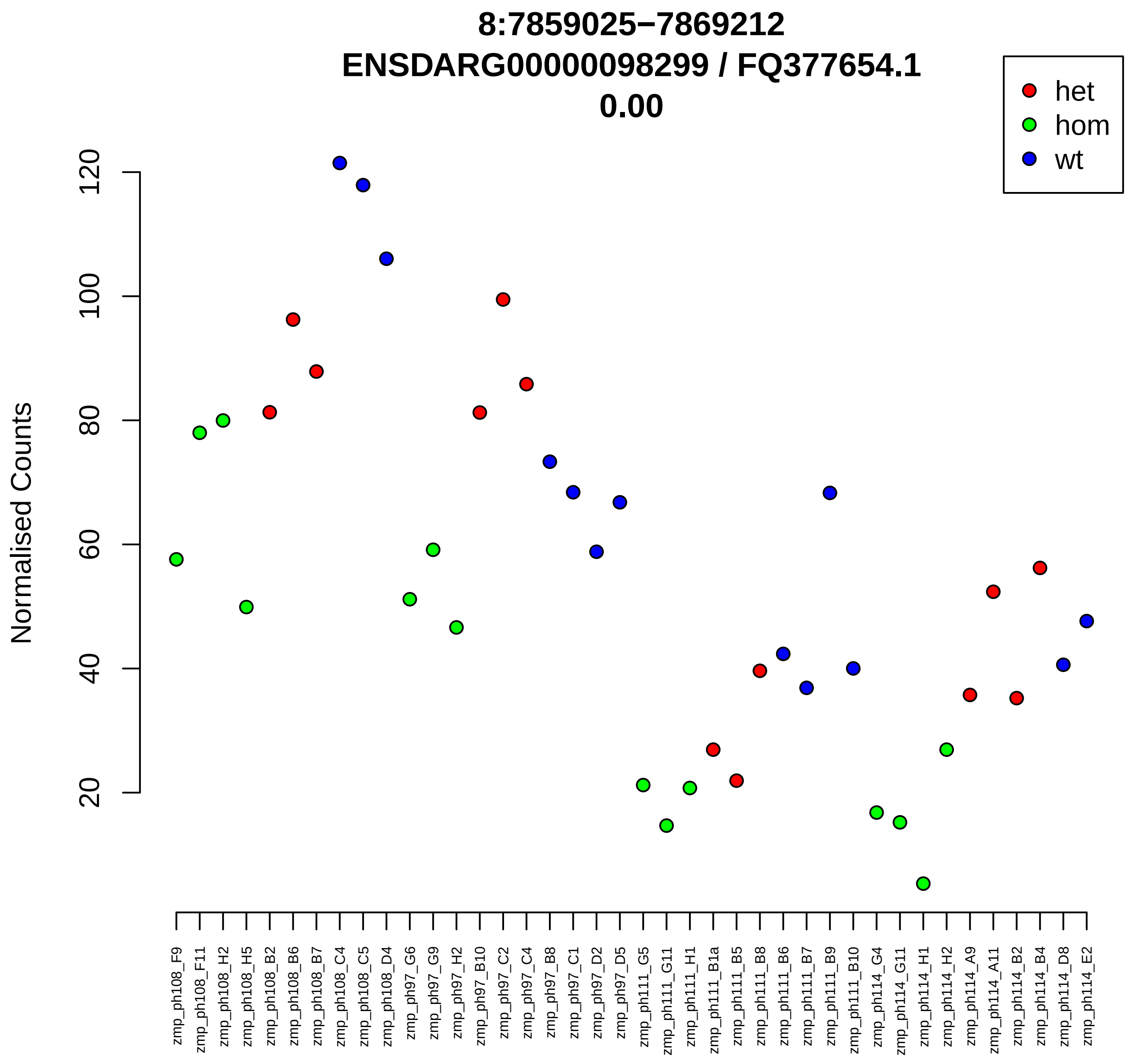

8:7859025−7869212
ENSDARG00000098299 / FQ377654.1
0.00
het
hom
wt
Normalised Counts
20
40
60
80
100
120
zmp_ph108_F9
zmp_ph108_F11
zmp_ph108_H2
zmp_ph108_H5
zmp_ph108_B2
zmp_ph108_B6
zmp_ph108_B7
zmp_ph108_C4
zmp_ph108_C5
zmp_ph108_D4
zmp_ph97_G6
zmp_ph97_G9
zmp_ph97_H2
zmp_ph97_B10
zmp_ph97_C2
zmp_ph97_C4
zmp_ph97_B8
zmp_ph97_C1
zmp_ph97_D2
zmp_ph97_D5
zmp_ph111_G5
zmp_ph111_G11
zmp_ph111_H1
zmp_ph111_B1a
zmp_ph111_B5
zmp_ph111_B8
zmp_ph111_B6
zmp_ph111_B7
zmp_ph111_B9
zmp_ph111_B10
zmp_ph114_G4
zmp_ph114_G11
zmp_ph114_H1
zmp_ph114_H2
zmp_ph114_A9
zmp_ph114_A11
zmp_ph114_B2
zmp_ph114_B4
zmp_ph114_D8
zmp_ph114_E2

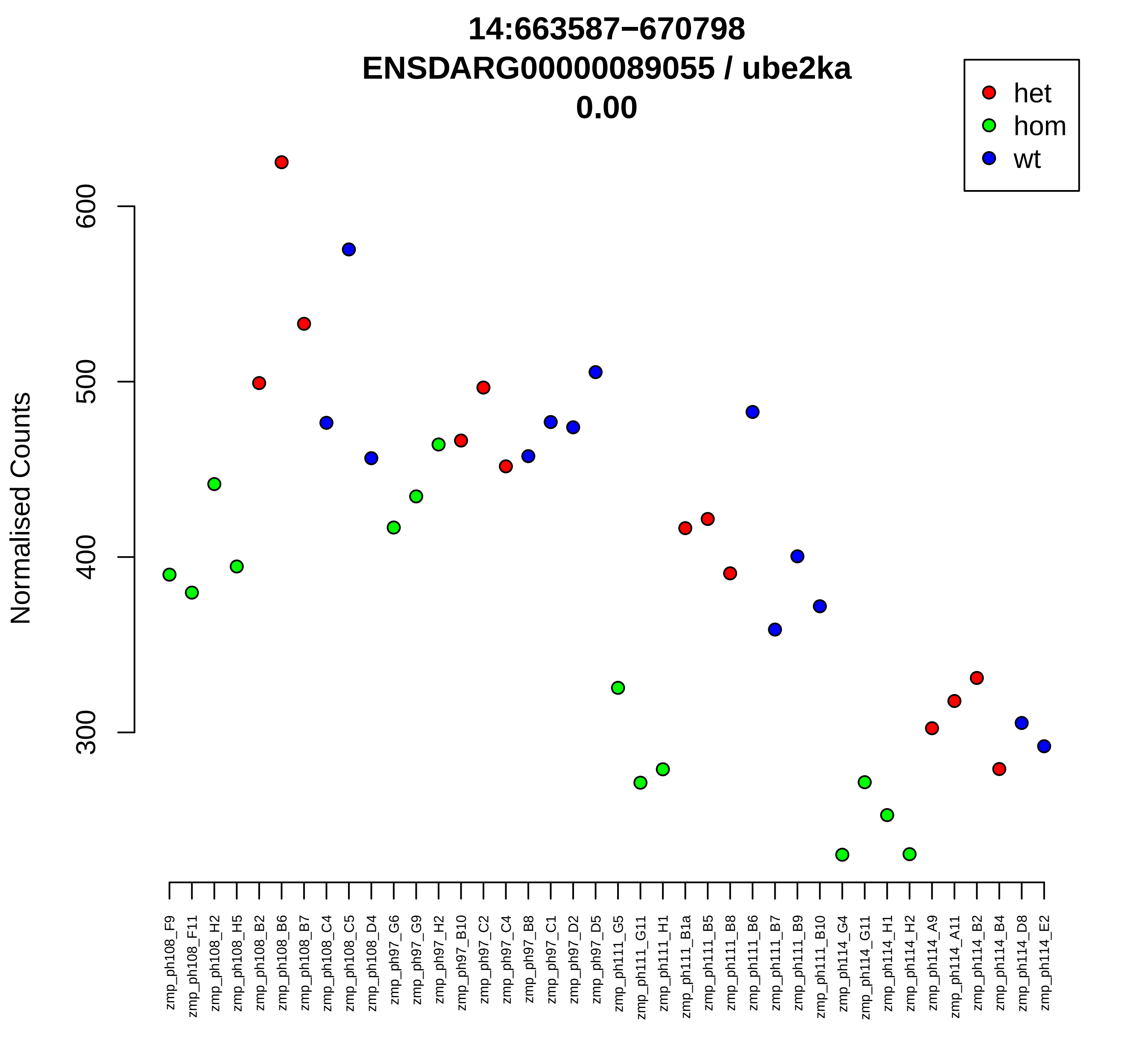
14:663587−670798
ENSDARG00000089055 / ube2ka
0.00
het
hom
wt
Normalised Counts
300
400
500
600
zmp_ph108_F9
zmp_ph108_F11
zmp_ph108_H2
zmp_ph108_H5
zmp_ph108_B2
zmp_ph108_B6
zmp_ph108_B7
zmp_ph108_C4
zmp_ph108_C5
zmp_ph108_D4
zmp_ph97_G6
zmp_ph97_G9
zmp_ph97_H2
zmp_ph97_B10
zmp_ph97_C2
zmp_ph97_C4
zmp_ph97_B8
zmp_ph97_C1
zmp_ph97_D2
zmp_ph97_D5
zmp_ph111_G5
zmp_ph111_G11
zmp_ph111_H1
zmp_ph111_B1a
zmp_ph111_B5
zmp_ph111_B8
zmp_ph111_B6
zmp_ph111_B7
zmp_ph111_B9
zmp_ph111_B10
zmp_ph114_G4
zmp_ph114_G11
zmp_ph114_H1
zmp_ph114_H2
zmp_ph114_A9
zmp_ph114_A11
zmp_ph114_B2
zmp_ph114_B4
zmp_ph114_D8
zmp_ph114_E2

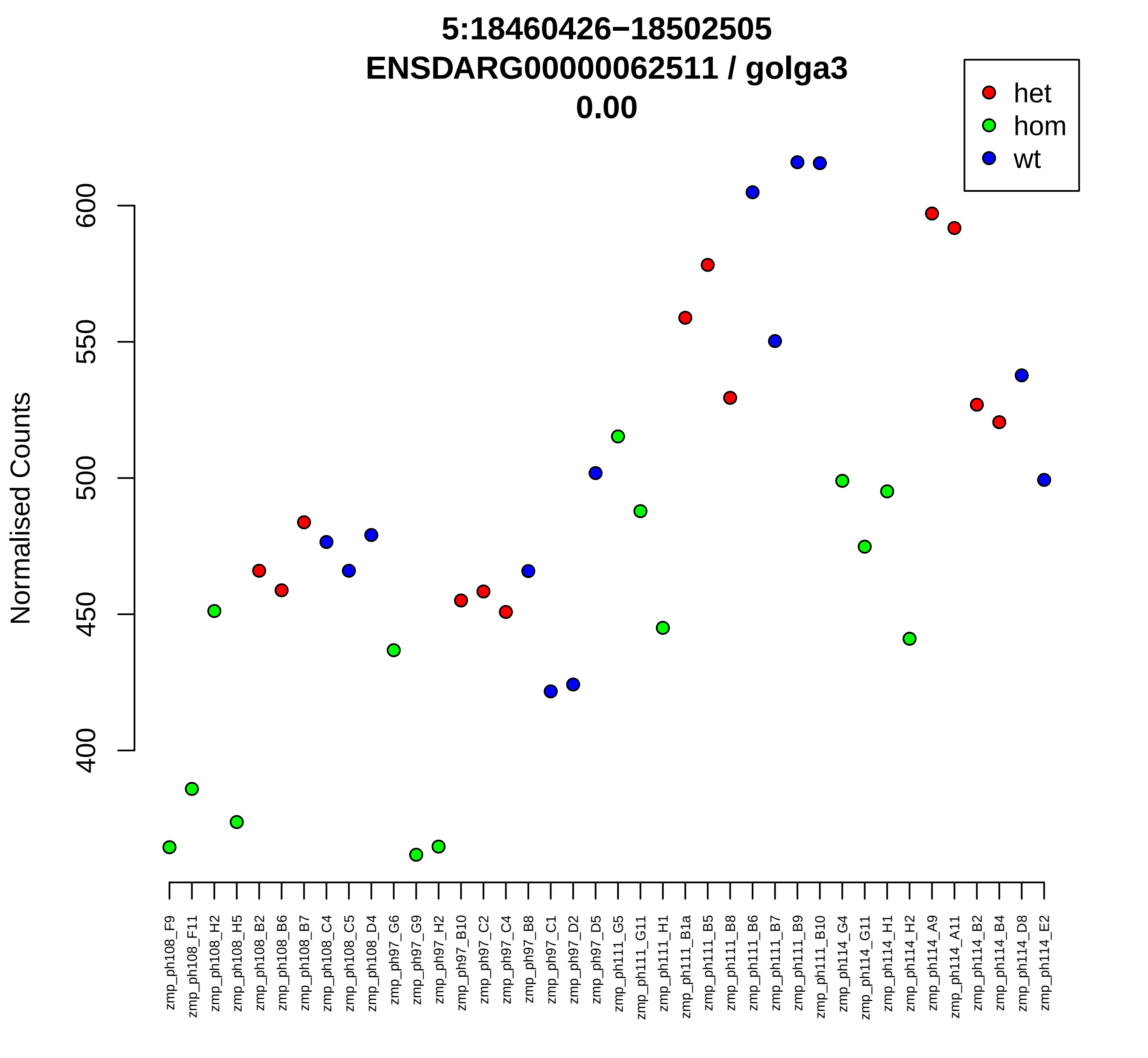
5:18460426−18502505
ENSDARG00000062511 / golga3
0.00
het
hom
wt
Normalised Counts
400
450
500
550
600
zmp_ph108_F9
zmp_ph108_F11
zmp_ph108_H2
zmp_ph108_H5
zmp_ph108_B2
zmp_ph108_B6
zmp_ph108_B7
zmp_ph108_C4
zmp_ph108_C5
zmp_ph108_D4
zmp_ph97_G6
zmp_ph97_G9
zmp_ph97_H2
zmp_ph97_B10
zmp_ph97_C2
zmp_ph97_C4
zmp_ph97_B8
zmp_ph97_C1
zmp_ph97_D2
zmp_ph97_D5
zmp_ph111_G5
zmp_ph111_G11
zmp_ph111_H1
zmp_ph111_B1a
zmp_ph111_B5
zmp_ph111_B8
zmp_ph111_B6
zmp_ph111_B7
zmp_ph111_B9
zmp_ph111_B10
zmp_ph114_G4
zmp_ph114_G11
zmp_ph114_H1
zmp_ph114_H2
zmp_ph114_A9
zmp_ph114_A11
zmp_ph114_B2
zmp_ph114_B4
zmp_ph114_D8
zmp_ph114_E2

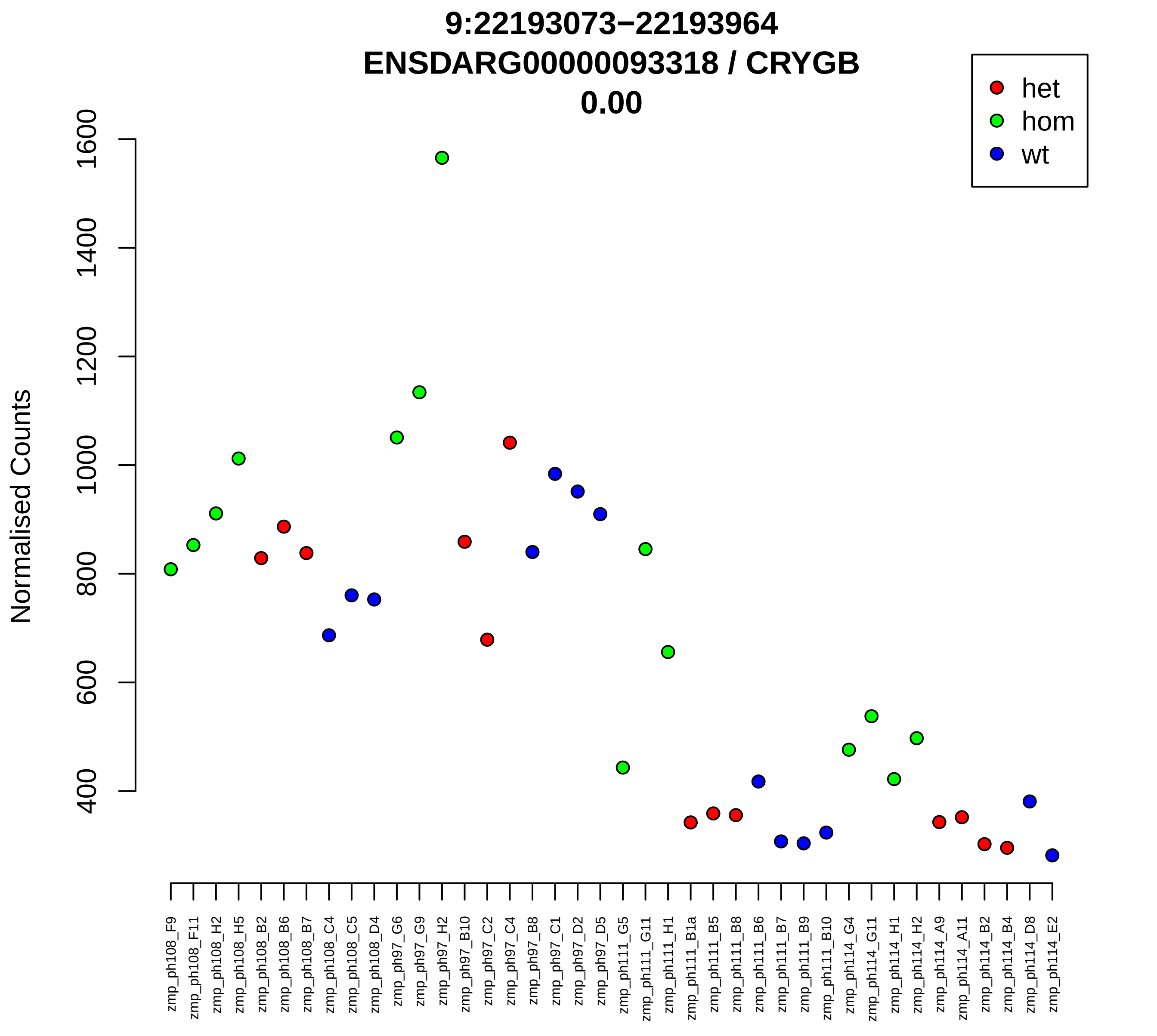
9:22193073−22193964
ENSDARG00000093318 / CRYGB
0.00
het
hom
wt
Normalised Counts
400
600
800
1000
1200
1400
1600
zmp_ph108_F9
zmp_ph108_F11
zmp_ph108_H2
zmp_ph108_H5
zmp_ph108_B2
zmp_ph108_B6
zmp_ph108_B7
zmp_ph108_C4
zmp_ph108_C5
zmp_ph108_D4
zmp_ph97_G6
zmp_ph97_G9
zmp_ph97_H2
zmp_ph97_B10
zmp_ph97_C2
zmp_ph97_C4
zmp_ph97_B8
zmp_ph97_C1
zmp_ph97_D2
zmp_ph97_D5
zmp_ph111_G5
zmp_ph111_G11
zmp_ph111_H1
zmp_ph111_B1a
zmp_ph111_B5
zmp_ph111_B8
zmp_ph111_B6
zmp_ph111_B7
zmp_ph111_B9
zmp_ph111_B10
zmp_ph114_G4
zmp_ph114_G11
zmp_ph114_H1
zmp_ph114_H2
zmp_ph114_A9
zmp_ph114_A11
zmp_ph114_B2
zmp_ph114_B4
zmp_ph114_D8
zmp_ph114_E2

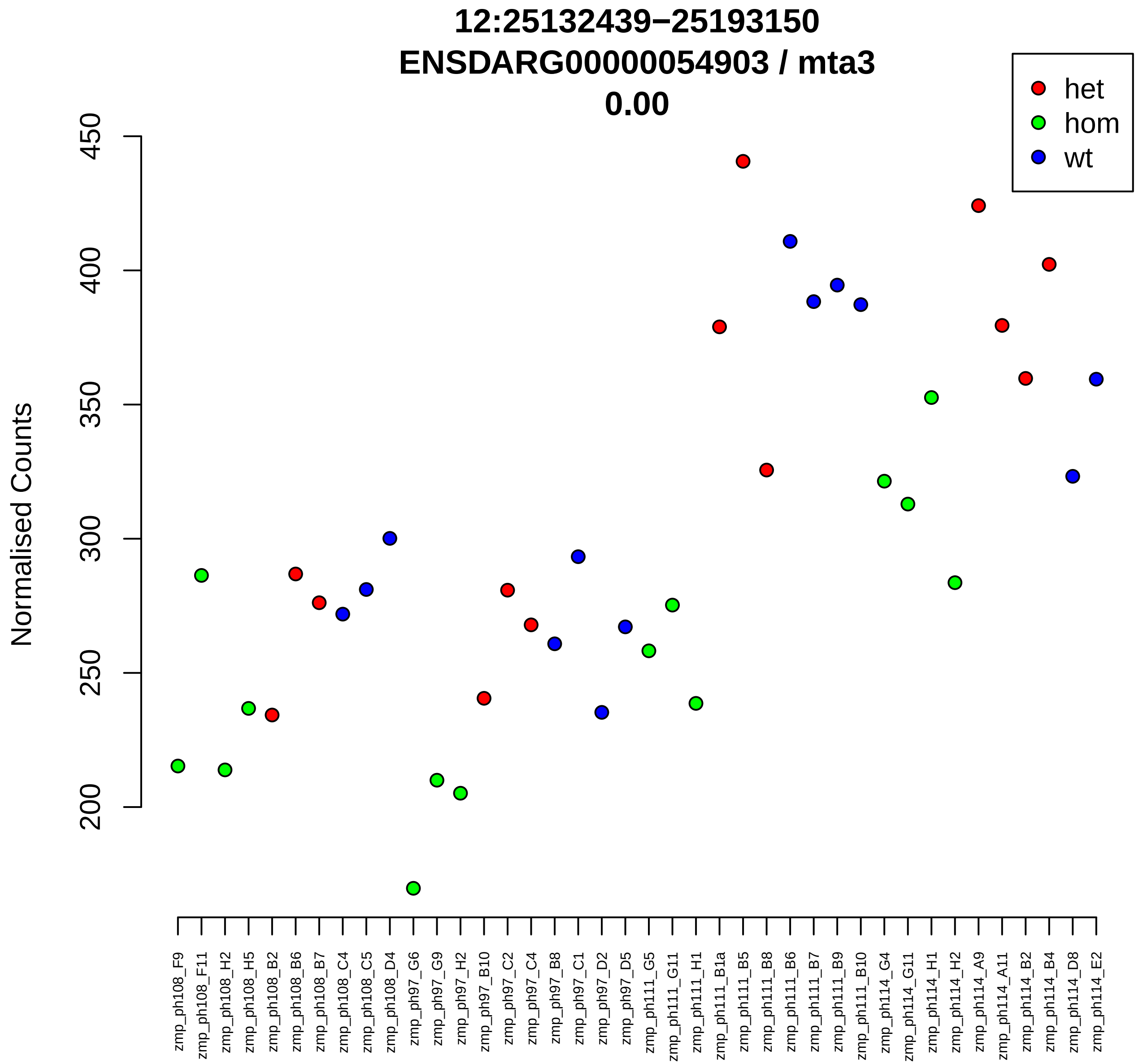
12:25132439−25193150
ENSDARG00000054903 / mta3
0.00
het
hom
wt
Normalised Counts
450
400
350
300
250
200
zmp_ph108_F9
zmp_ph108_F11
zmp_ph108_H2
zmp_ph108_H5
zmp_ph108_B2
zmp_ph108_B6
zmp_ph108_B7
zmp_ph108_C4
zmp_ph108_C5
zmp_ph108_D4
zmp_ph97_G6
zmp_ph97_G9
zmp_ph97_H2
zmp_ph97_B10
zmp_ph97_C2
zmp_ph97_C4
zmp_ph97_B8
zmp_ph97_C1
zmp_ph97_D2
zmp_ph97_D5
zmp_ph111_G5
zmp_ph111_G11
zmp_ph111_H1
zmp_ph111_B1a
zmp_ph111_B5
zmp_ph111_B8
zmp_ph111_B6
zmp_ph111_B7
zmp_ph111_B9
zmp_ph111_B10
zmp_ph114_G4
zmp_ph114_G11
zmp_ph114_H1
zmp_ph114_H2
zmp_ph114_A9
zmp_ph114_A11
zmp_ph114_B2
zmp_ph114_B4
zmp_ph114_D8
zmp_ph114_E2

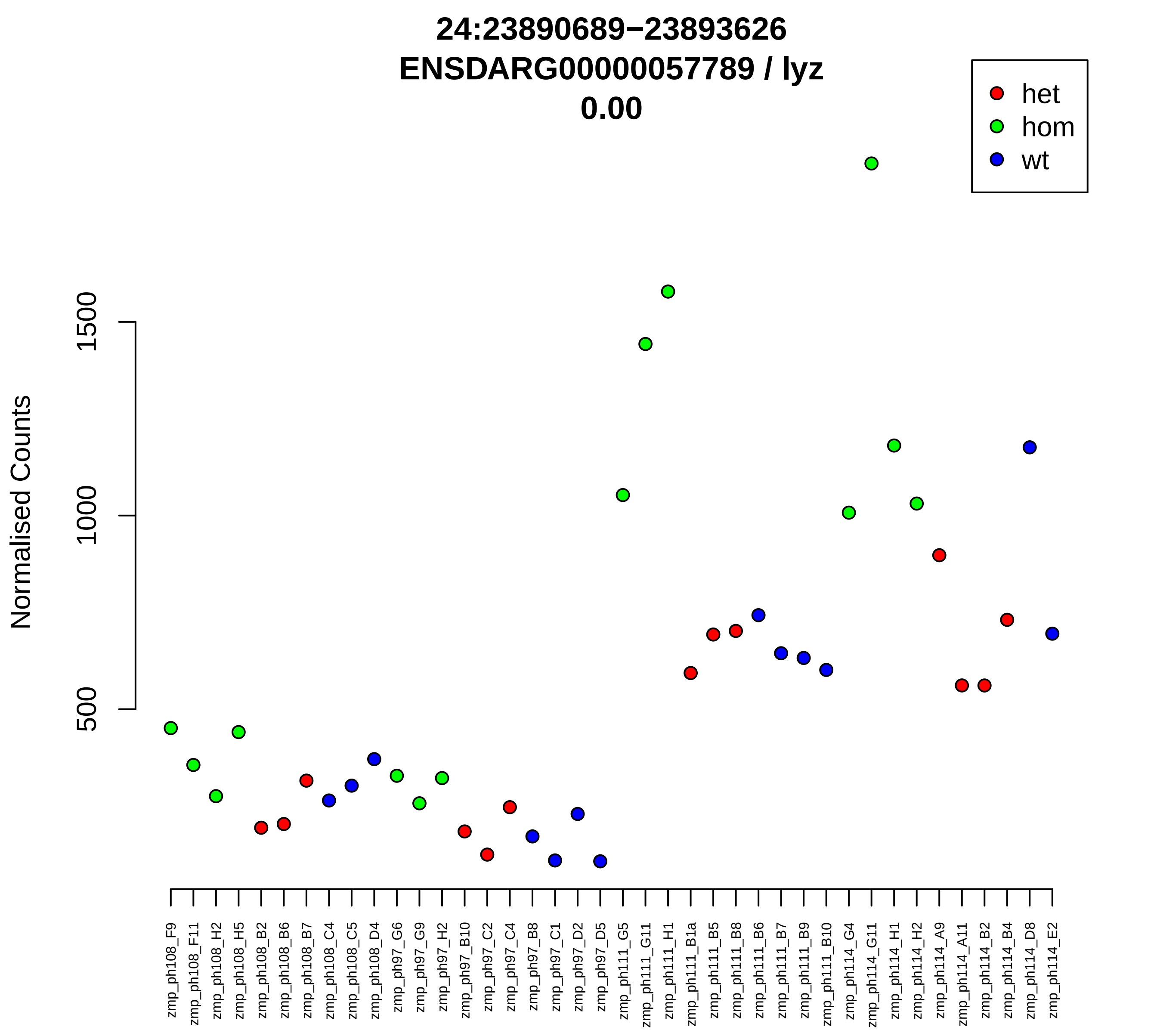

24:23890689−23893626
ENSDARG00000057789 / lyz
0.00
het
hom
wt
Normalised Counts
500
1000
1500
zmp_ph108_F9
zmp_ph108_F11
zmp_ph108_H2
zmp_ph108_H5
zmp_ph108_B2
zmp_ph108_B6
zmp_ph108_B7
zmp_ph108_C4
zmp_ph108_C5
zmp_ph108_D4
zmp_ph97_G6
zmp_ph97_G9
zmp_ph97_H2
zmp_ph97_B10
zmp_ph97_C2
zmp_ph97_C4
zmp_ph97_B8
zmp_ph97_C1
zmp_ph97_D2
zmp_ph97_D5
zmp_ph111_G5
zmp_ph111_G11
zmp_ph111_H1
zmp_ph111_B1a
zmp_ph111_B5
zmp_ph111_B8
zmp_ph111_B6
zmp_ph111_B7
zmp_ph111_B9
zmp_ph111_B10
zmp_ph114_G4
zmp_ph114_G11
zmp_ph114_H1
zmp_ph114_H2
zmp_ph114_A9
zmp_ph114_A11
zmp_ph114_B2
zmp_ph114_B4
zmp_ph114_D8
zmp_ph114_E2

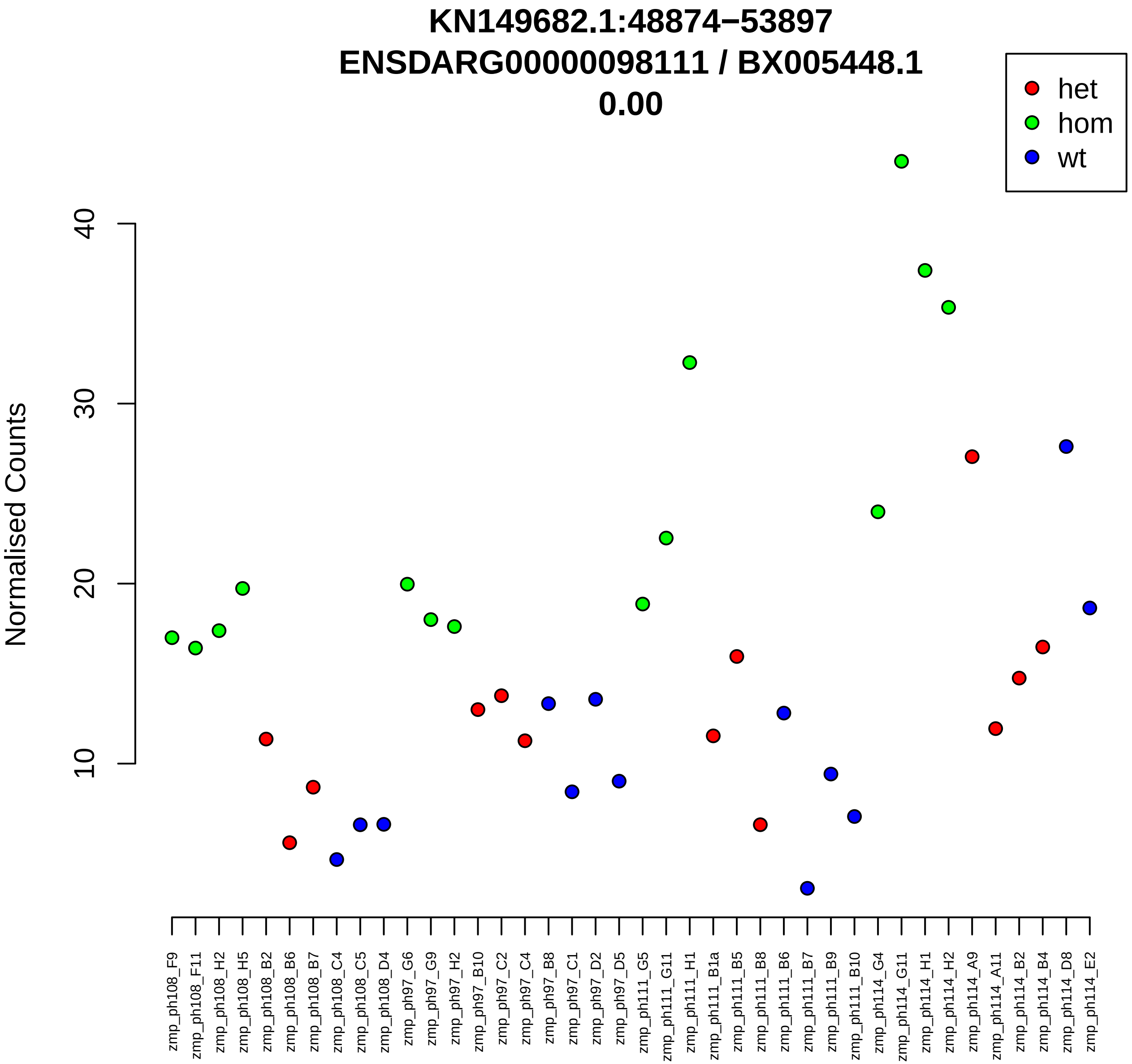

KN149682.1:48874−53897
ENSDARG00000098111 / BX005448.1
0.00
het
hom
wt
Normalised Counts
10
20
30
40
zmp_ph108_F9
zmp_ph108_F11
zmp_ph108_H2
zmp_ph108_H5
zmp_ph108_B2
zmp_ph108_B6
zmp_ph108_B7
zmp_ph108_C4
zmp_ph108_C5
zmp_ph108_D4
zmp_ph97_G6
zmp_ph97_G9
zmp_ph97_H2
zmp_ph97_B10
zmp_ph97_C2
zmp_ph97_C4
zmp_ph97_B8
zmp_ph97_C1
zmp_ph97_D2
zmp_ph97_D5
zmp_ph111_G5
zmp_ph111_G11
zmp_ph111_H1
zmp_ph111_B1a
zmp_ph111_B5
zmp_ph111_B8
zmp_ph111_B6
zmp_ph111_B7
zmp_ph111_B9
zmp_ph111_B10
zmp_ph114_G4
zmp_ph114_G11
zmp_ph114_H1
zmp_ph114_H2
zmp_ph114_A9
zmp_ph114_A11
zmp_ph114_B2
zmp_ph114_B4
zmp_ph114_D8
zmp_ph114_E2

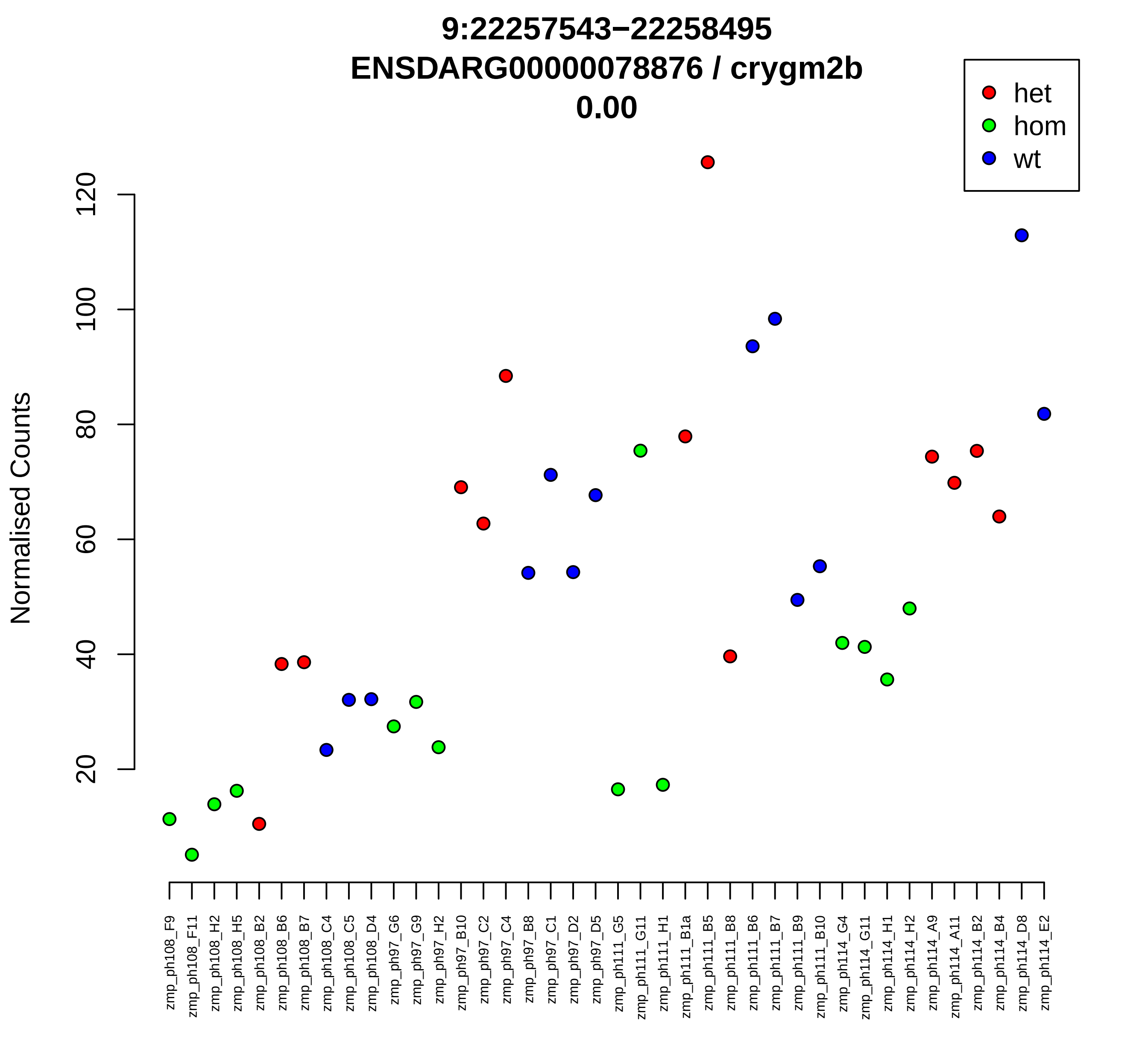
9:22257543−22258495
ENSDARG00000078876 / crygm2b
0.00
het
hom
wt
Normalised Counts
20
40
60
80
100
120
zmp_ph108_F9
zmp_ph108_F11
zmp_ph108_H2
zmp_ph108_H5
zmp_ph108_B2
zmp_ph108_B6
zmp_ph108_B7
zmp_ph108_C4
zmp_ph108_C5
zmp_ph108_D4
zmp_ph97_G6
zmp_ph97_G9
zmp_ph97_H2
zmp_ph97_B10
zmp_ph97_C2
zmp_ph97_C4
zmp_ph97_B8
zmp_ph97_C1
zmp_ph97_D2
zmp_ph97_D5
zmp_ph111_G5
zmp_ph111_G11
zmp_ph111_H1
zmp_ph111_B1a
zmp_ph111_B5
zmp_ph111_B8
zmp_ph111_B6
zmp_ph111_B7
zmp_ph111_B9
zmp_ph111_B10
zmp_ph114_G4
zmp_ph114_G11
zmp_ph114_H1
zmp_ph114_H2
zmp_ph114_A9
zmp_ph114_A11
zmp_ph114_B2
zmp_ph114_B4
zmp_ph114_D8
zmp_ph114_E2

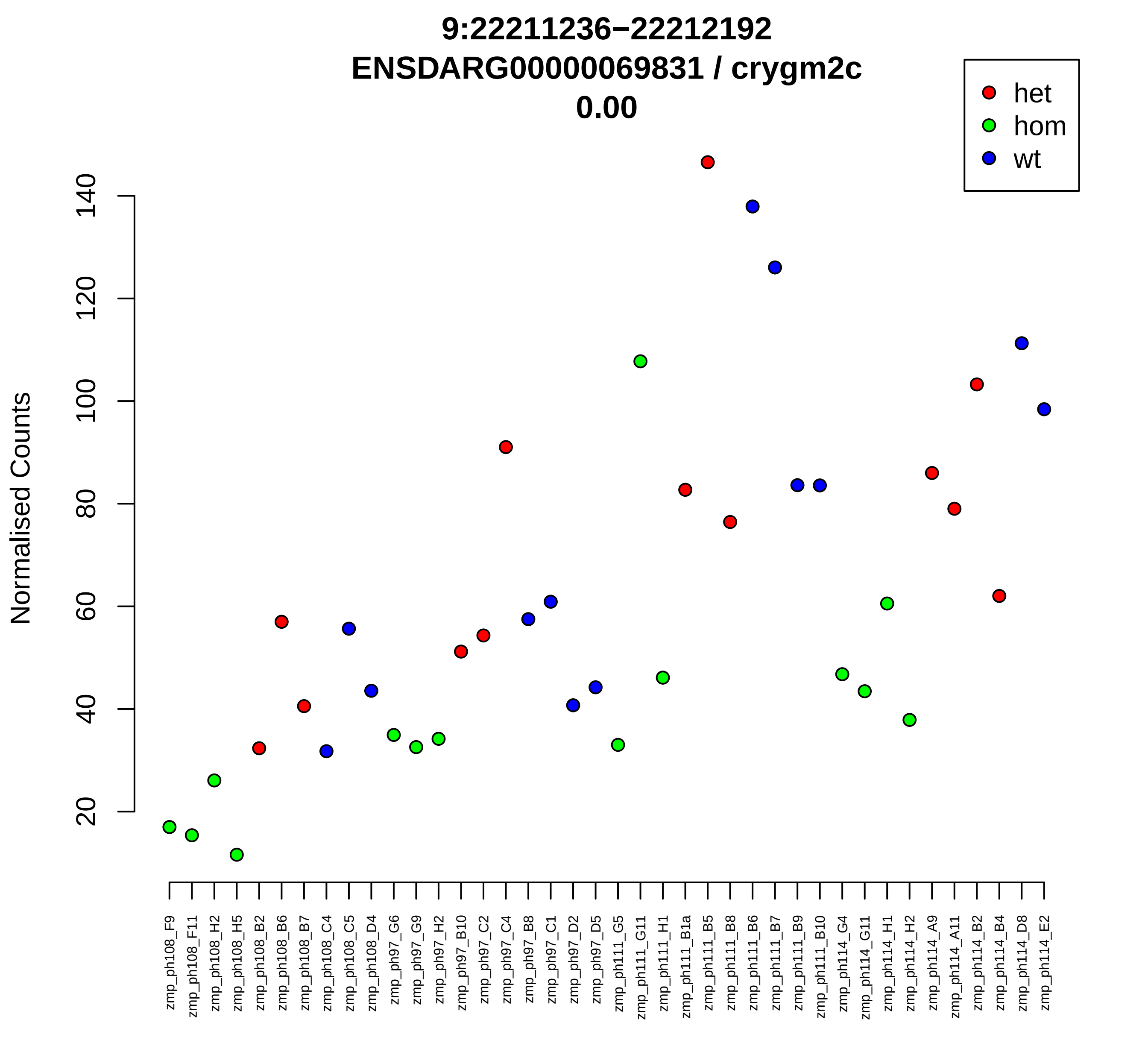
9:22211236−22212192
ENSDARG00000069831 / crygm2c
0.00
het
hom
wt
Normalised Counts
20
40
60
80
100
120
140
zmp_ph108_F9
zmp_ph108_F11
zmp_ph108_H2
zmp_ph108_H5
zmp_ph108_B2
zmp_ph108_B6
zmp_ph108_B7
zmp_ph108_C4
zmp_ph108_C5
zmp_ph108_D4
zmp_ph97_G6
zmp_ph97_G9
zmp_ph97_H2
zmp_ph97_B10
zmp_ph97_C2
zmp_ph97_C4
zmp_ph97_B8
zmp_ph97_C1
zmp_ph97_D2
zmp_ph97_D5
zmp_ph111_G5
zmp_ph111_G11
zmp_ph111_H1
zmp_ph111_B1a
zmp_ph111_B5
zmp_ph111_B8
zmp_ph111_B6
zmp_ph111_B7
zmp_ph111_B9
zmp_ph111_B10
zmp_ph114_G4
zmp_ph114_G11
zmp_ph114_H1
zmp_ph114_H2
zmp_ph114_A9
zmp_ph114_A11
zmp_ph114_B2
zmp_ph114_B4
zmp_ph114_D8
zmp_ph114_E2

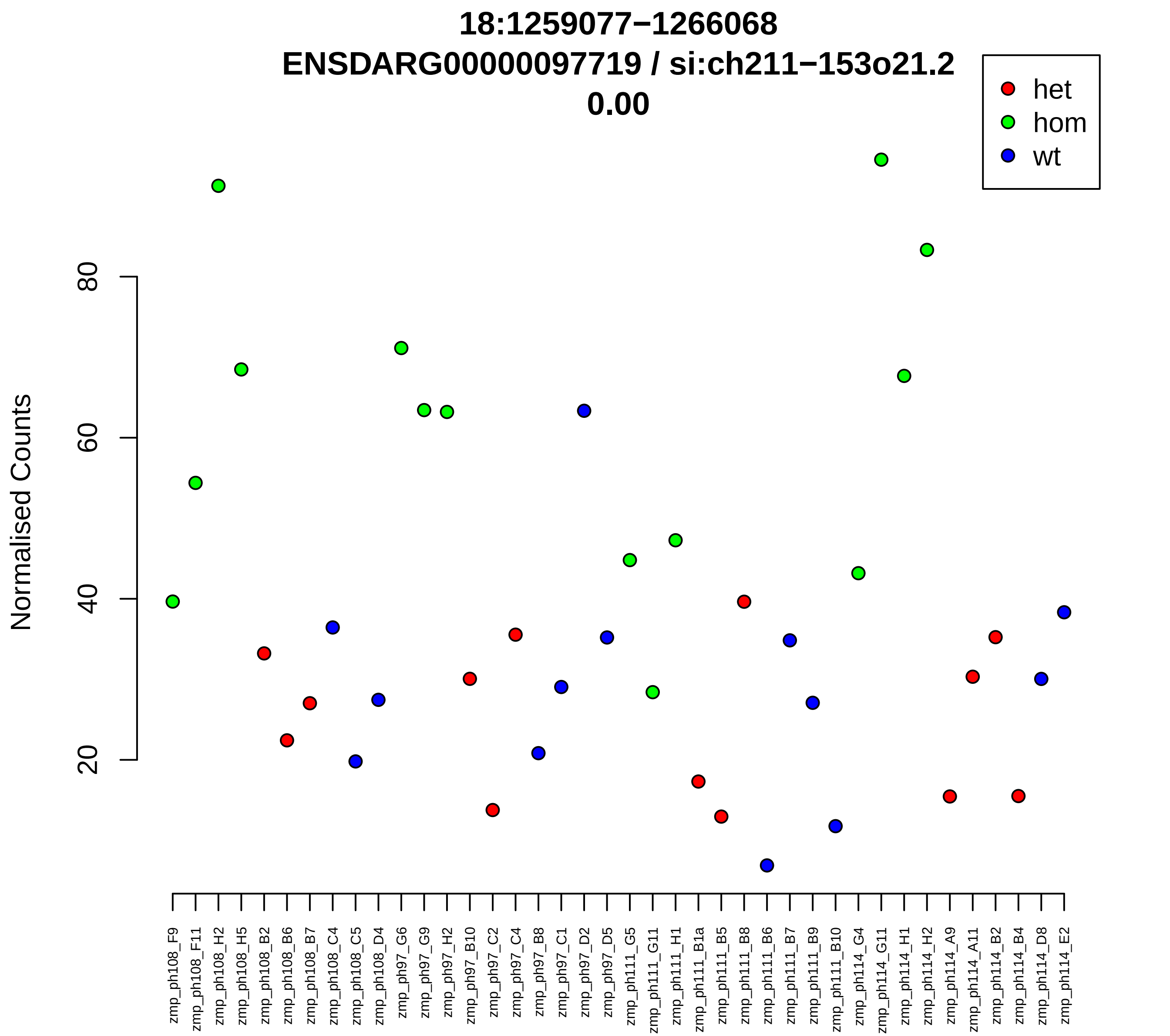

18:1259077−1266068
ENSDARG00000097719 / si:ch211−153o21.2
0.00
het
hom
wt
Normalised Counts
20
40
60
80
zmp_ph108_F9
zmp_ph108_F11
zmp_ph108_H2
zmp_ph108_H5
zmp_ph108_B2
zmp_ph108_B6
zmp_ph108_B7
zmp_ph108_C4
zmp_ph108_C5
zmp_ph108_D4
zmp_ph97_G6
zmp_ph97_G9
zmp_ph97_H2
zmp_ph97_B10
zmp_ph97_C2
zmp_ph97_C4
zmp_ph97_B8
zmp_ph97_C1
zmp_ph97_D2
zmp_ph97_D5
zmp_ph111_G5
zmp_ph111_G11
zmp_ph111_H1
zmp_ph111_B1a
zmp_ph111_B5
zmp_ph111_B8
zmp_ph111_B6
zmp_ph111_B7
zmp_ph111_B9
zmp_ph111_B10
zmp_ph114_G4
zmp_ph114_G11
zmp_ph114_H1
zmp_ph114_H2
zmp_ph114_A9
zmp_ph114_A11
zmp_ph114_B2
zmp_ph114_B4
zmp_ph114_D8
zmp_ph114_E2

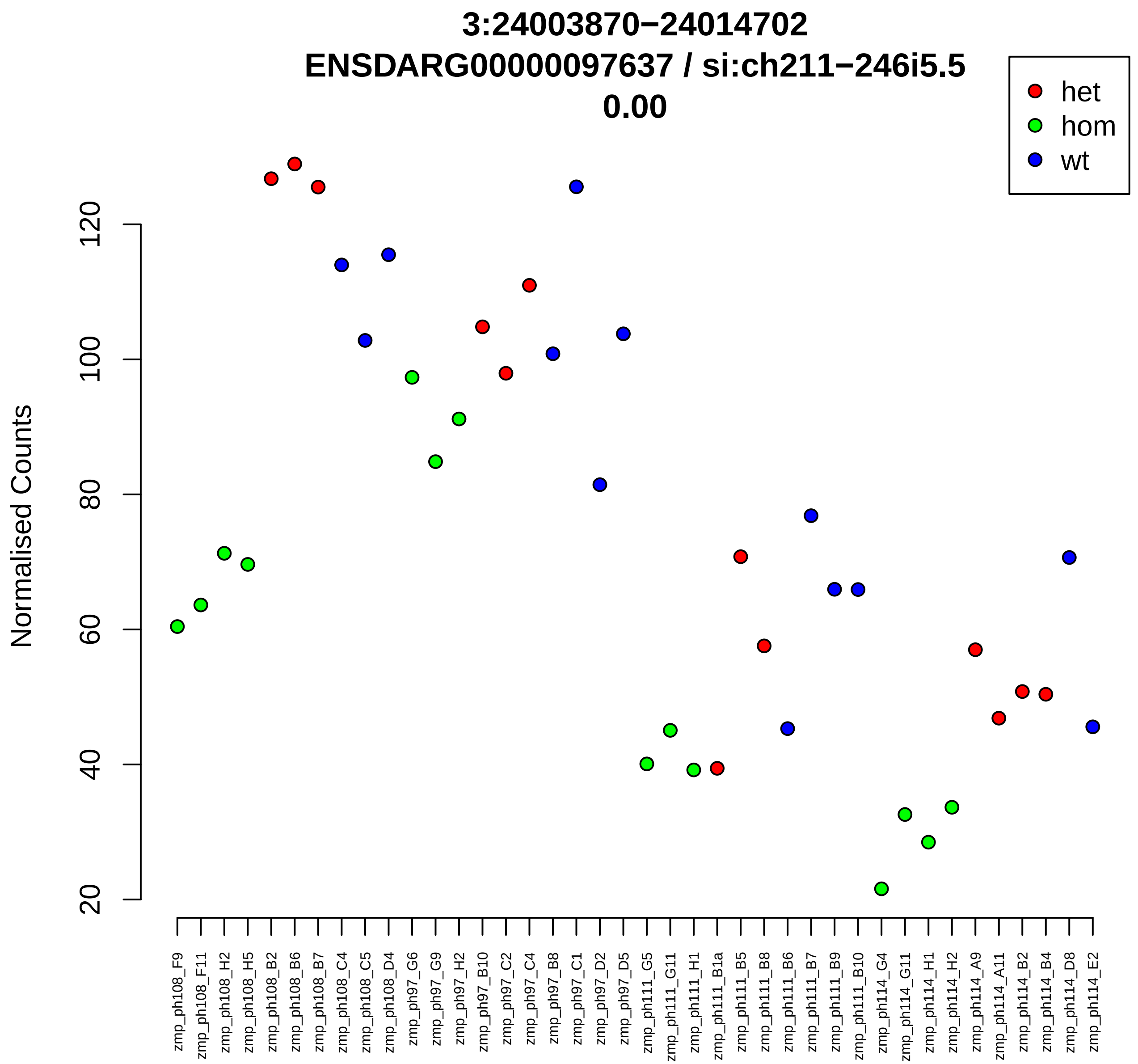
3:24003870−24014702
ENSDARG00000097637 / si:ch211−246i5.5
0.00
het
hom
wt
Normalised Counts
20
40
60
80
100
120
zmp_ph108_F9
zmp_ph108_F11
zmp_ph108_H2
zmp_ph108_H5
zmp_ph108_B2
zmp_ph108_B6
zmp_ph108_B7
zmp_ph108_C4
zmp_ph108_C5
zmp_ph108_D4
zmp_ph97_G6
zmp_ph97_G9
zmp_ph97_H2
zmp_ph97_B10
zmp_ph97_C2
zmp_ph97_C4
zmp_ph97_B8
zmp_ph97_C1
zmp_ph97_D2
zmp_ph97_D5
zmp_ph111_G5
zmp_ph111_G11
zmp_ph111_H1
zmp_ph111_B1a
zmp_ph111_B5
zmp_ph111_B8
zmp_ph111_B6
zmp_ph111_B7
zmp_ph111_B9
zmp_ph111_B10
zmp_ph114_G4
zmp_ph114_G11
zmp_ph114_H1
zmp_ph114_H2
zmp_ph114_A9
zmp_ph114_A11
zmp_ph114_B2
zmp_ph114_B4
zmp_ph114_D8
zmp_ph114_E2

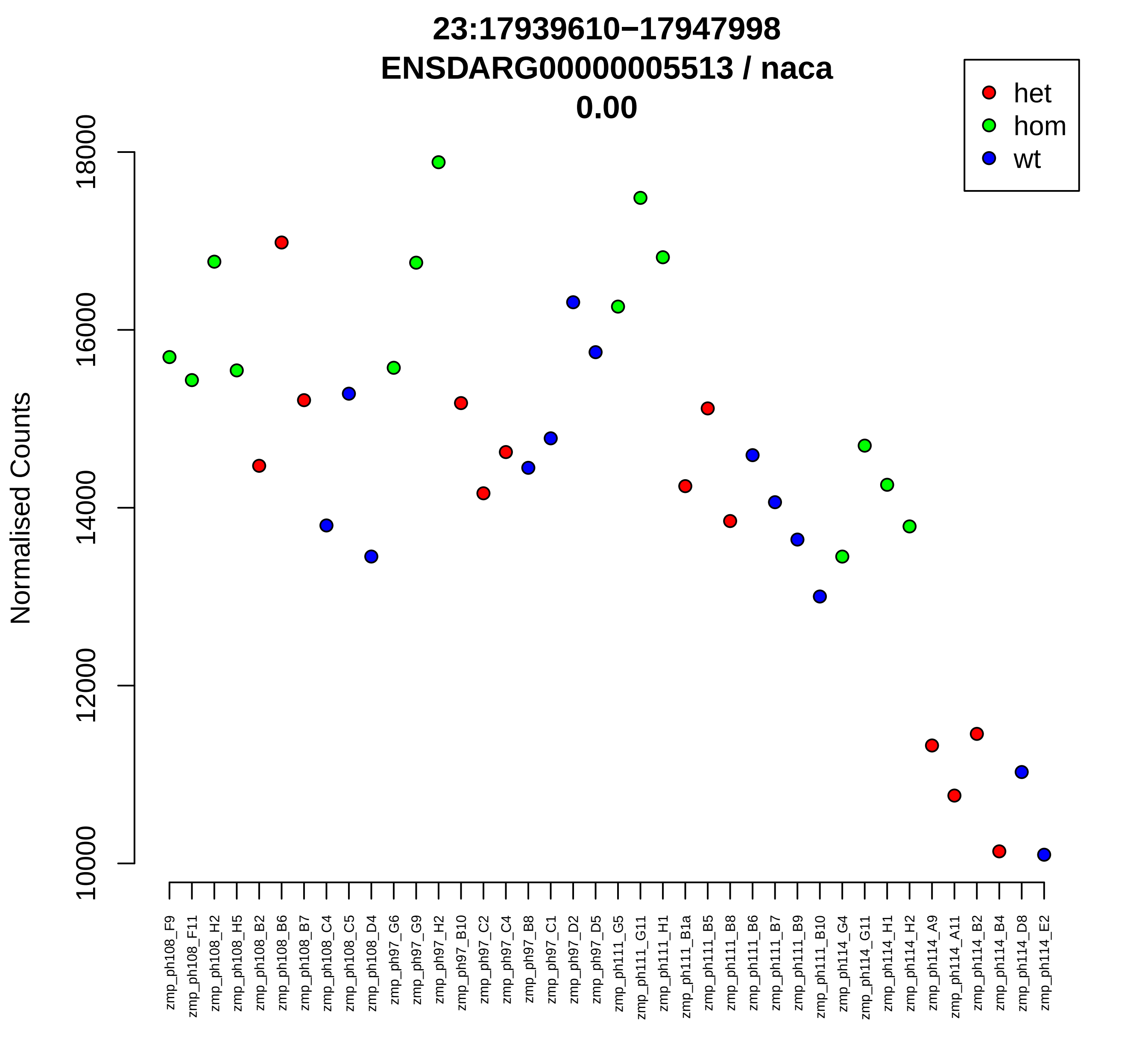

23:17939610−17947998
ENSDARG00000005513 / naca
0.00
het
hom
wt
Normalised Counts
10000
12000
14000
16000
18000
zmp_ph108_F9
zmp_ph108_F11
zmp_ph108_H2
zmp_ph108_H5
zmp_ph108_B2
zmp_ph108_B6
zmp_ph108_B7
zmp_ph108_C4
zmp_ph108_C5
zmp_ph108_D4
zmp_ph97_G6
zmp_ph97_G9
zmp_ph97_H2
zmp_ph97_B10
zmp_ph97_C2
zmp_ph97_C4
zmp_ph97_B8
zmp_ph97_C1
zmp_ph97_D2
zmp_ph97_D5
zmp_ph111_G5
zmp_ph111_G11
zmp_ph111_H1
zmp_ph111_B1a
zmp_ph111_B5
zmp_ph111_B8
zmp_ph111_B6
zmp_ph111_B7
zmp_ph111_B9
zmp_ph111_B10
zmp_ph114_G4
zmp_ph114_G11
zmp_ph114_H1
zmp_ph114_H2
zmp_ph114_A9
zmp_ph114_A11
zmp_ph114_B2
zmp_ph114_B4
zmp_ph114_D8
zmp_ph114_E2

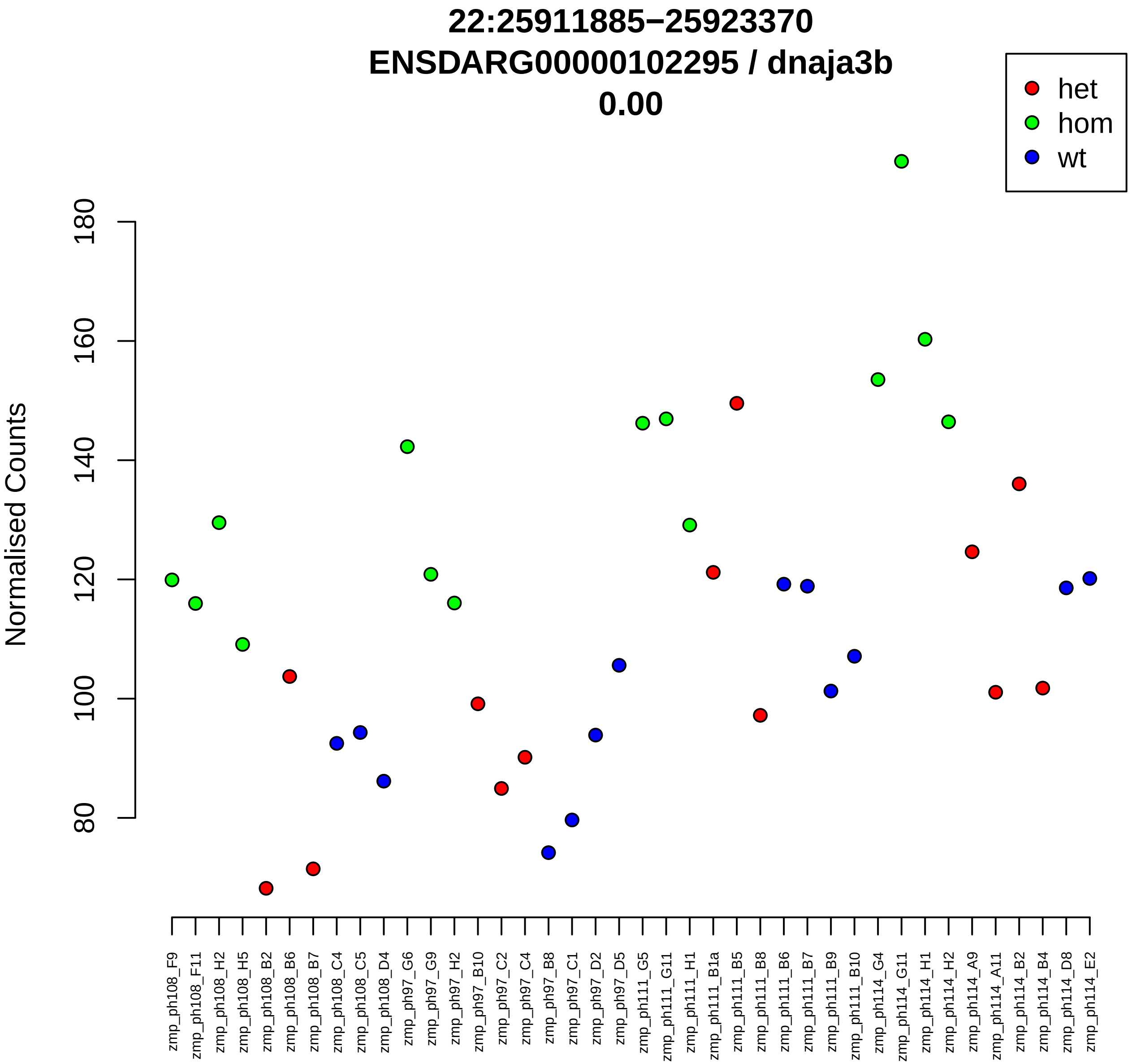

22:25911885−25923370
ENSDARG00000102295 / dnaja3b
0.00
het
hom
wt
Normalised Counts
80
100
120
140
160
180
zmp_ph108_F9
zmp_ph108_F11
zmp_ph108_H2
zmp_ph108_H5
zmp_ph108_B2
zmp_ph108_B6
zmp_ph108_B7
zmp_ph108_C4
zmp_ph108_C5
zmp_ph108_D4
zmp_ph97_G6
zmp_ph97_G9
zmp_ph97_H2
zmp_ph97_B10
zmp_ph97_C2
zmp_ph97_C4
zmp_ph97_B8
zmp_ph97_C1
zmp_ph97_D2
zmp_ph97_D5
zmp_ph111_G5
zmp_ph111_G11
zmp_ph111_H1
zmp_ph111_B1a
zmp_ph111_B5
zmp_ph111_B8
zmp_ph111_B6
zmp_ph111_B7
zmp_ph111_B9
zmp_ph111_B10
zmp_ph114_G4
zmp_ph114_G11
zmp_ph114_H1
zmp_ph114_H2
zmp_ph114_A9
zmp_ph114_A11
zmp_ph114_B2
zmp_ph114_B4
zmp_ph114_D8
zmp_ph114_E2

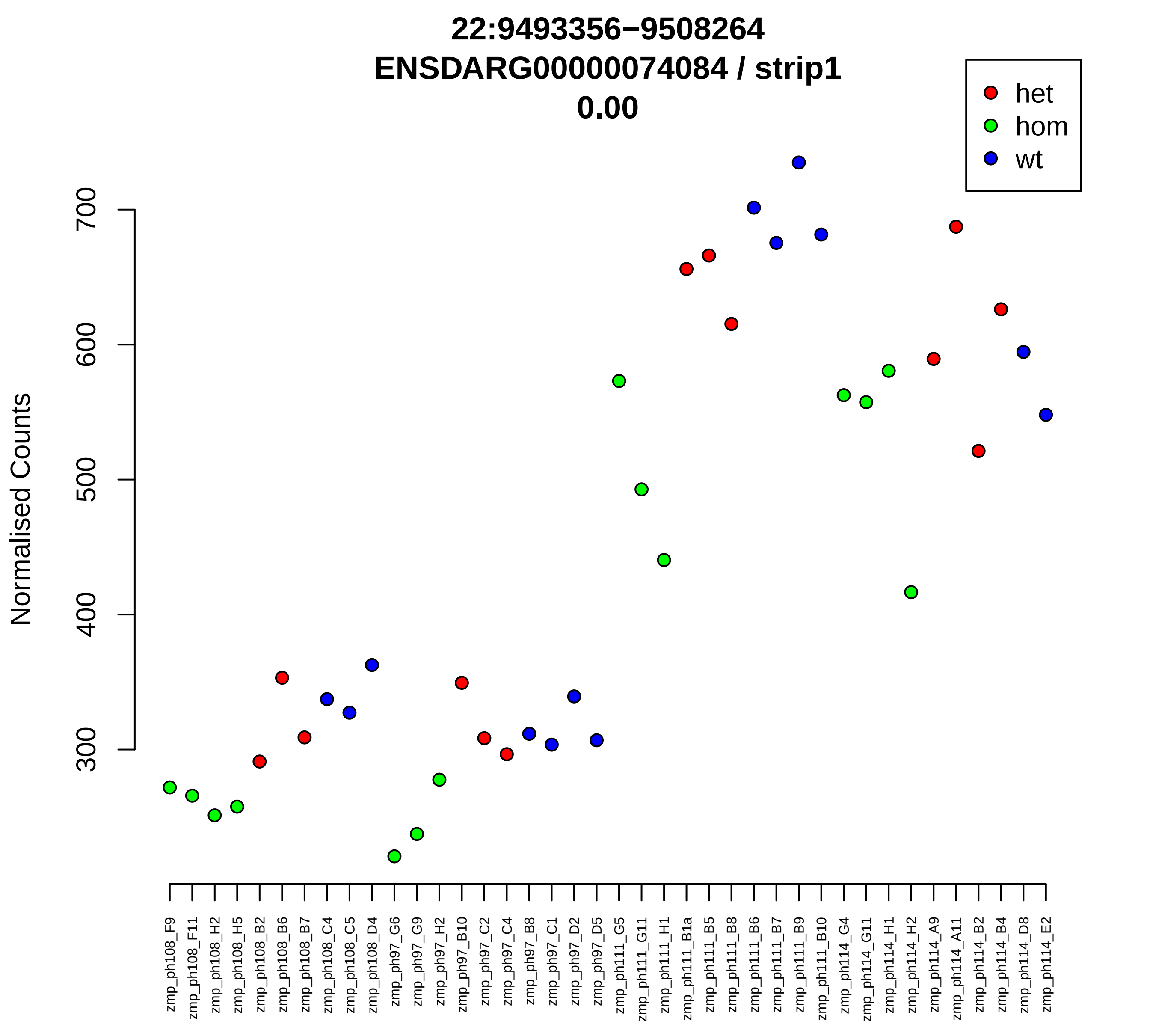
22:9493356−9508264
ENSDARG00000074084 / strip1
0.00
het
hom
wt
Normalised Counts
300
400
500
600
700
zmp_ph108_F9
zmp_ph108_F11
zmp_ph108_H2
zmp_ph108_H5
zmp_ph108_B2
zmp_ph108_B6
zmp_ph108_B7
zmp_ph108_C4
zmp_ph108_C5
zmp_ph108_D4
zmp_ph97_G6
zmp_ph97_G9
zmp_ph97_H2
zmp_ph97_B10
zmp_ph97_C2
zmp_ph97_C4
zmp_ph97_B8
zmp_ph97_C1
zmp_ph97_D2
zmp_ph97_D5
zmp_ph111_G5
zmp_ph111_G11
zmp_ph111_H1
zmp_ph111_B1a
zmp_ph111_B5
zmp_ph111_B8
zmp_ph111_B6
zmp_ph111_B7
zmp_ph111_B9
zmp_ph111_B10
zmp_ph114_G4
zmp_ph114_G11
zmp_ph114_H1
zmp_ph114_H2
zmp_ph114_A9
zmp_ph114_A11
zmp_ph114_B2
zmp_ph114_B4
zmp_ph114_D8
zmp_ph114_E2

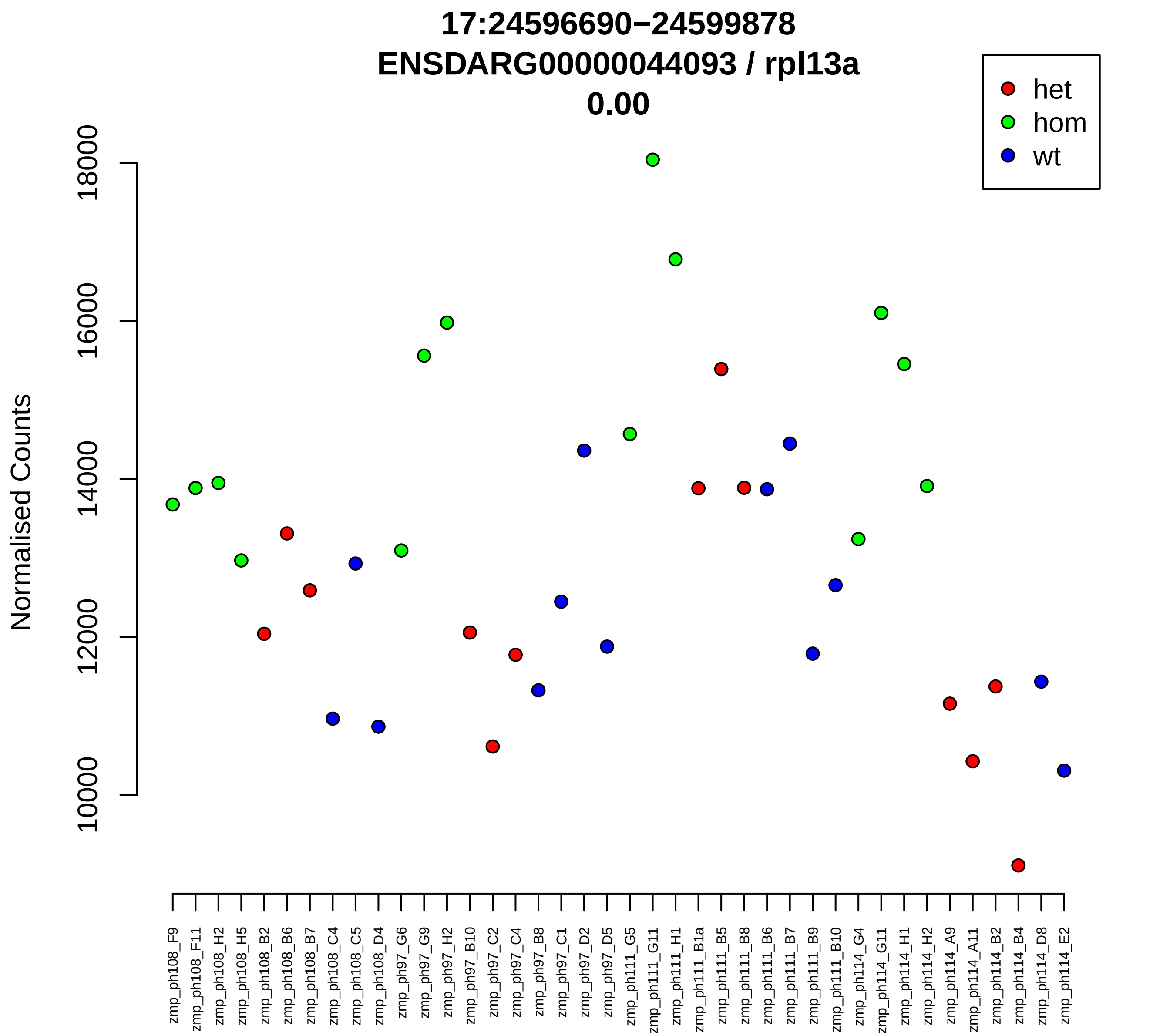

17:24596690−24599878
ENSDARG00000044093 / rpl13a
0.00
het
hom
wt
Normalised Counts
10000
12000
14000
16000
18000
zmp_ph108_F9
zmp_ph108_F11
zmp_ph108_H2
zmp_ph108_H5
zmp_ph108_B2
zmp_ph108_B6
zmp_ph108_B7
zmp_ph108_C4
zmp_ph108_C5
zmp_ph108_D4
zmp_ph97_G6
zmp_ph97_G9
zmp_ph97_H2
zmp_ph97_B10
zmp_ph97_C2
zmp_ph97_C4
zmp_ph97_B8
zmp_ph97_C1
zmp_ph97_D2
zmp_ph97_D5
zmp_ph111_G5
zmp_ph111_G11
zmp_ph111_H1
zmp_ph111_B1a
zmp_ph111_B5
zmp_ph111_B8
zmp_ph111_B6
zmp_ph111_B7
zmp_ph111_B9
zmp_ph111_B10
zmp_ph114_G4
zmp_ph114_G11
zmp_ph114_H1
zmp_ph114_H2
zmp_ph114_A9
zmp_ph114_A11
zmp_ph114_B2
zmp_ph114_B4
zmp_ph114_D8
zmp_ph114_E2

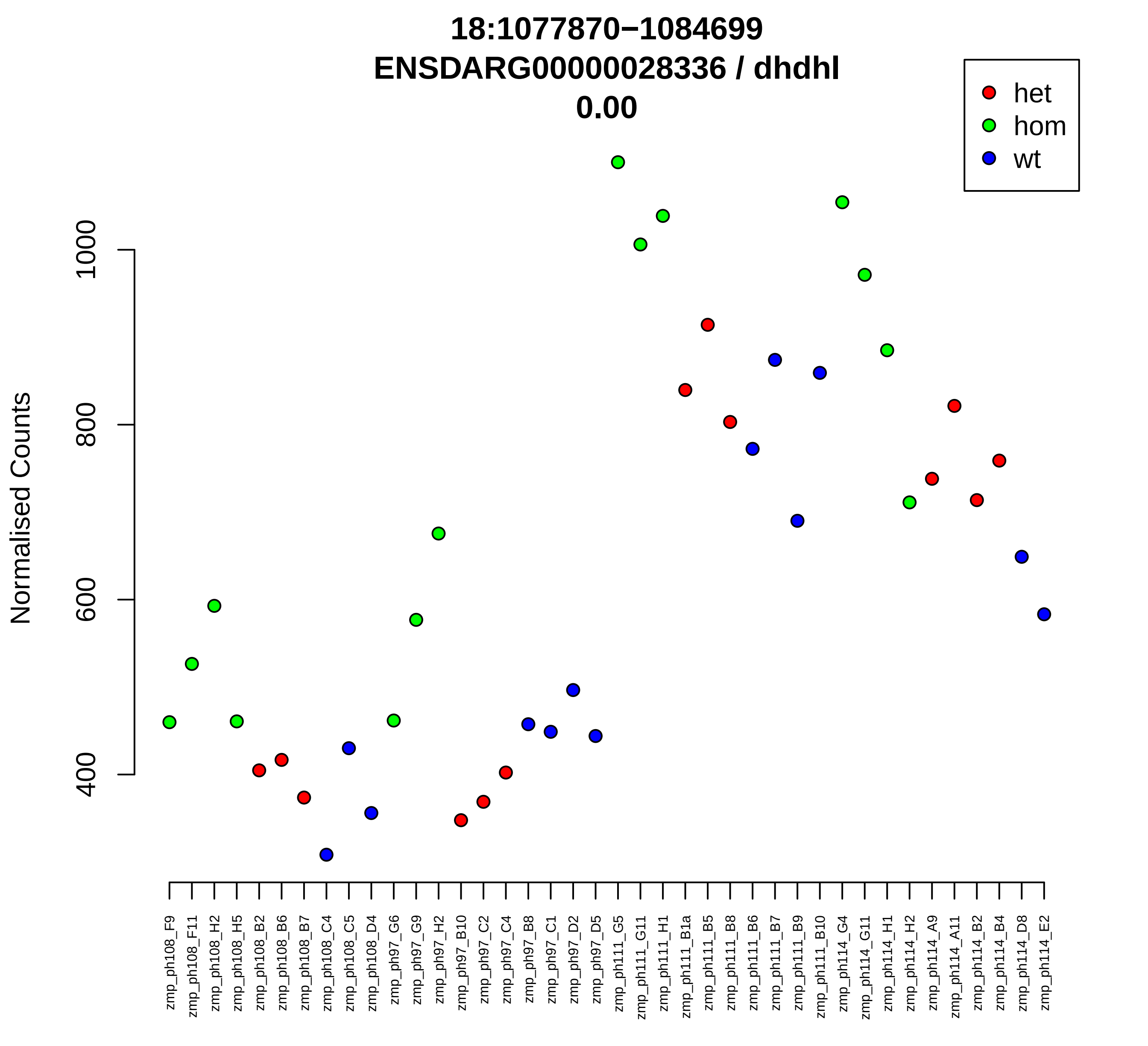

18:1077870−1084699
ENSDARG00000028336 / dhdhl
0.00
het
hom
wt
Normalised Counts
400
600
800
1000
zmp_ph108_F9
zmp_ph108_F11
zmp_ph108_H2
zmp_ph108_H5
zmp_ph108_B2
zmp_ph108_B6
zmp_ph108_B7
zmp_ph108_C4
zmp_ph108_C5
zmp_ph108_D4
zmp_ph97_G6
zmp_ph97_G9
zmp_ph97_H2
zmp_ph97_B10
zmp_ph97_C2
zmp_ph97_C4
zmp_ph97_B8
zmp_ph97_C1
zmp_ph97_D2
zmp_ph97_D5
zmp_ph111_G5
zmp_ph111_G11
zmp_ph111_H1
zmp_ph111_B1a
zmp_ph111_B5
zmp_ph111_B8
zmp_ph111_B6
zmp_ph111_B7
zmp_ph111_B9
zmp_ph111_B10
zmp_ph114_G4
zmp_ph114_G11
zmp_ph114_H1
zmp_ph114_H2
zmp_ph114_A9
zmp_ph114_A11
zmp_ph114_B2
zmp_ph114_B4
zmp_ph114_D8
zmp_ph114_E2

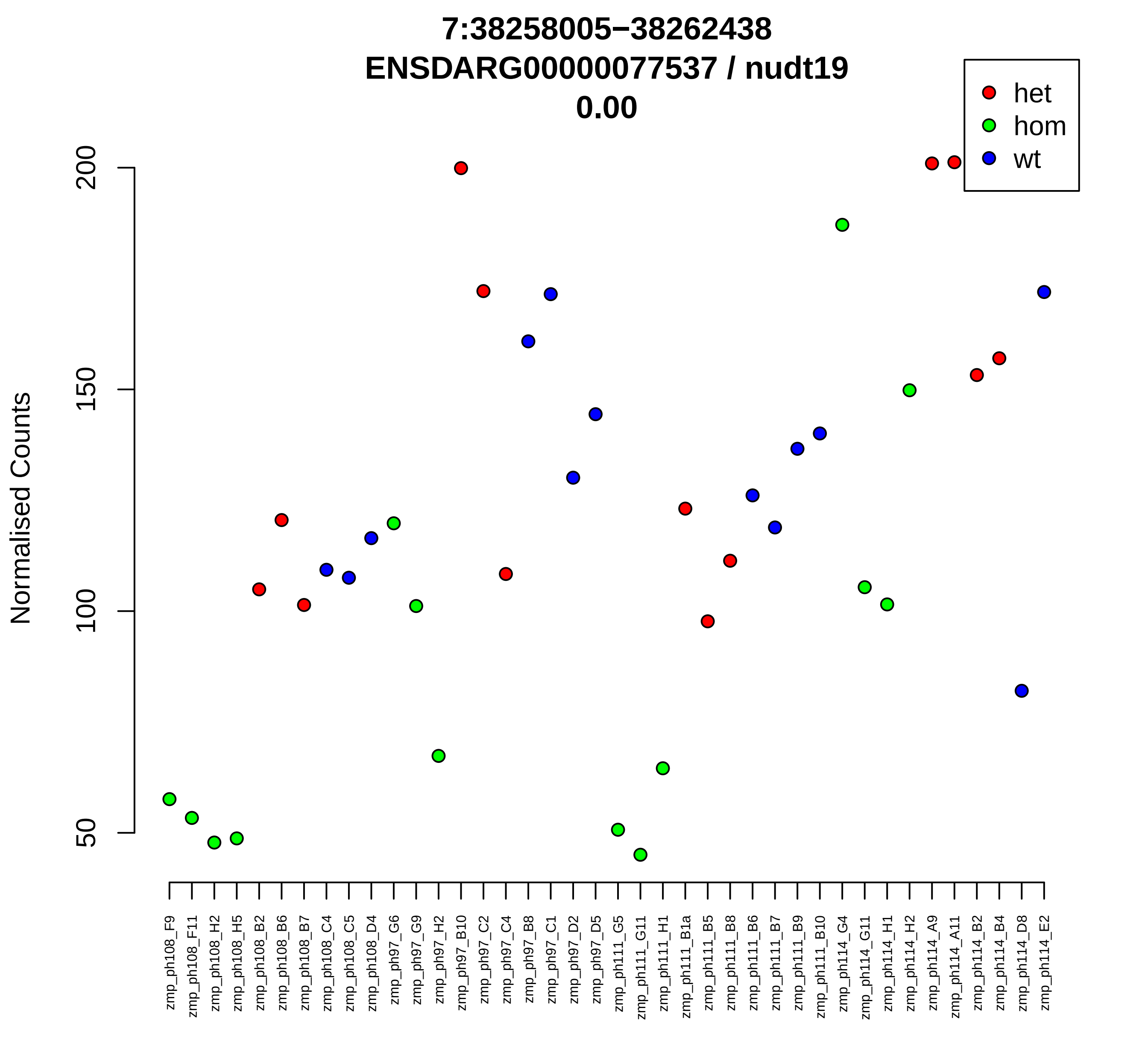

7:38258005−38262438
ENSDARG00000077537 / nudt19
0.00
het
hom
wt
Normalised Counts
200
150
100
50
zmp_ph108_F9
zmp_ph108_F11
zmp_ph108_H2
zmp_ph108_H5
zmp_ph108_B2
zmp_ph108_B6
zmp_ph108_B7
zmp_ph108_C4
zmp_ph108_C5
zmp_ph108_D4
zmp_ph97_G6
zmp_ph97_G9
zmp_ph97_H2
zmp_ph97_B10
zmp_ph97_C2
zmp_ph97_C4
zmp_ph97_B8
zmp_ph97_C1
zmp_ph97_D2
zmp_ph97_D5
zmp_ph111_G5
zmp_ph111_G11
zmp_ph111_H1
zmp_ph111_B1a
zmp_ph111_B5
zmp_ph111_B8
zmp_ph111_B6
zmp_ph111_B7
zmp_ph111_B9
zmp_ph111_B10
zmp_ph114_G4
zmp_ph114_G11
zmp_ph114_H1
zmp_ph114_H2
zmp_ph114_A9
zmp_ph114_A11
zmp_ph114_B2
zmp_ph114_B4
zmp_ph114_D8
zmp_ph114_E2

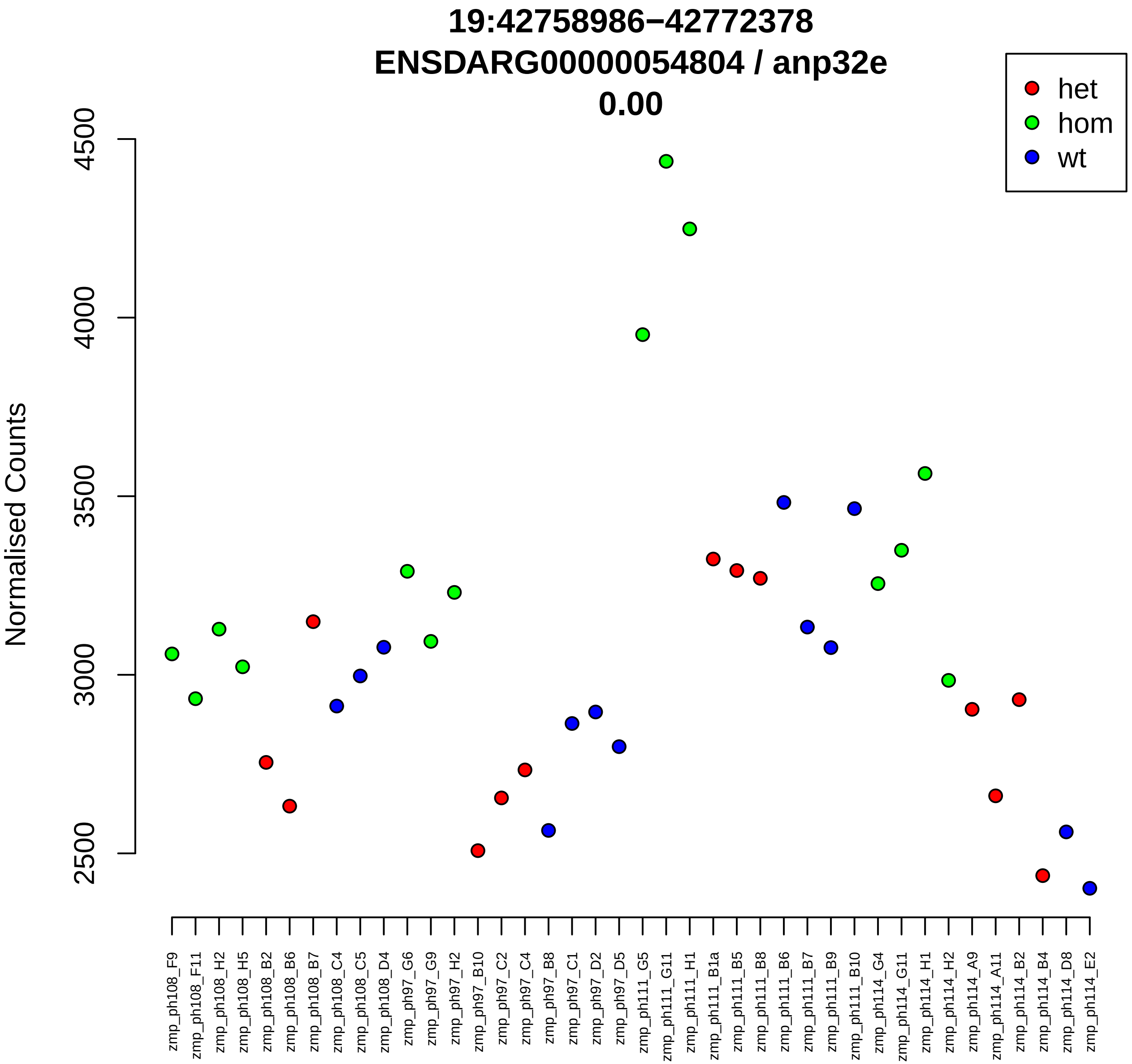

19:42758986−42772378
ENSDARG00000054804 / anp32e
0.00
het
hom
wt
Normalised Counts
4500
4000
3500
3000
2500
zmp_ph108_F9
zmp_ph108_F11
zmp_ph108_H2
zmp_ph108_H5
zmp_ph108_B2
zmp_ph108_B6
zmp_ph108_B7
zmp_ph108_C4
zmp_ph108_C5
zmp_ph108_D4
zmp_ph97_G6
zmp_ph97_G9
zmp_ph97_H2
zmp_ph97_B10
zmp_ph97_C2
zmp_ph97_C4
zmp_ph97_B8
zmp_ph97_C1
zmp_ph97_D2
zmp_ph97_D5
zmp_ph111_G5
zmp_ph111_G11
zmp_ph111_H1
zmp_ph111_B1a
zmp_ph111_B5
zmp_ph111_B8
zmp_ph111_B6
zmp_ph111_B7
zmp_ph111_B9
zmp_ph111_B10
zmp_ph114_G4
zmp_ph114_G11
zmp_ph114_H1
zmp_ph114_H2
zmp_ph114_A9
zmp_ph114_A11
zmp_ph114_B2
zmp_ph114_B4
zmp_ph114_D8
zmp_ph114_E2

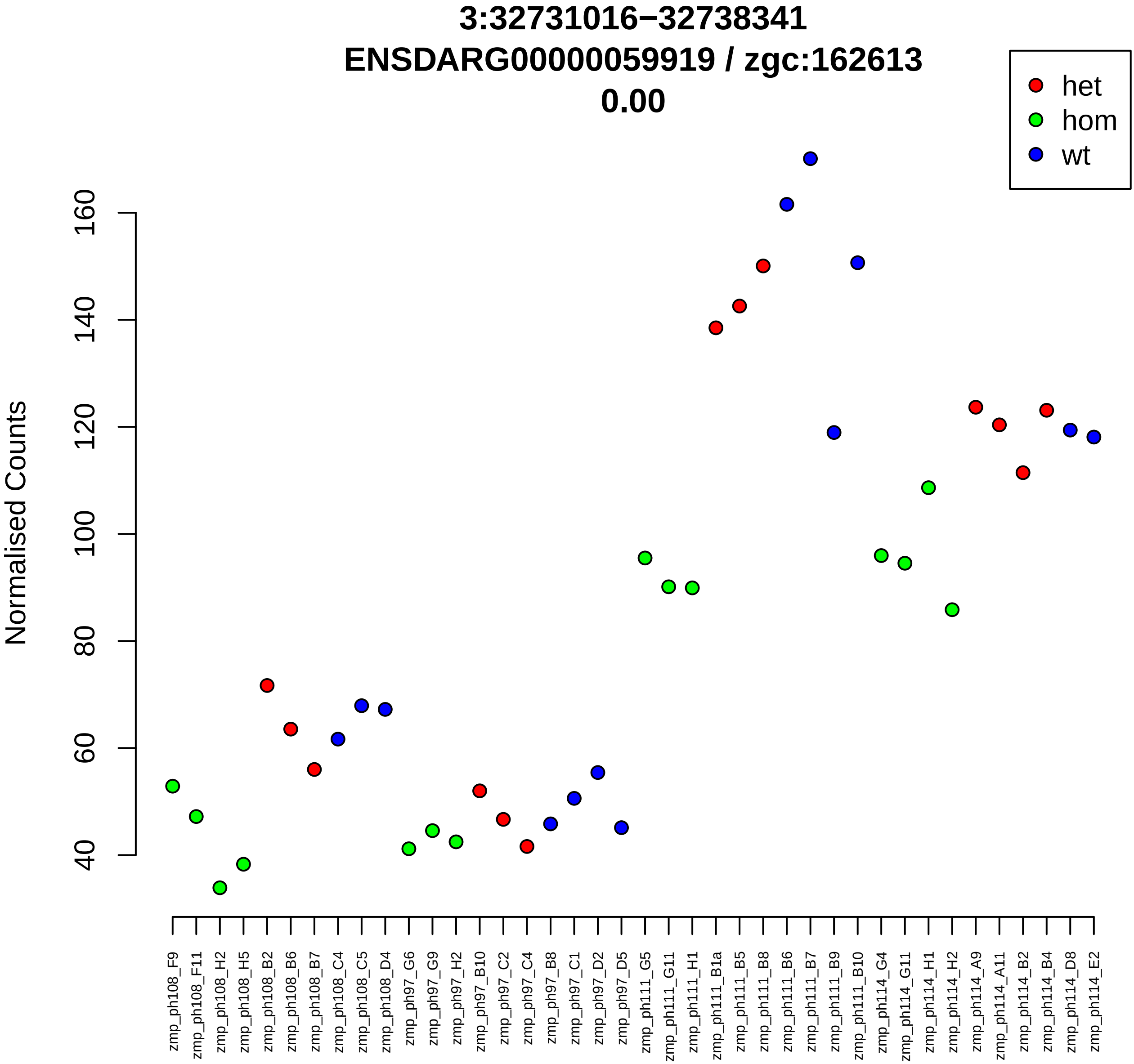
3:32731016−32738341
ENSDARG00000059919 / zgc:162613
0.00
het
hom
wt
Normalised Counts
40
60
80
100
120
140
160
zmp_ph108_F9
zmp_ph108_F11
zmp_ph108_H2
zmp_ph108_H5
zmp_ph108_B2
zmp_ph108_B6
zmp_ph108_B7
zmp_ph108_C4
zmp_ph108_C5
zmp_ph108_D4
zmp_ph97_G6
zmp_ph97_G9
zmp_ph97_H2
zmp_ph97_B10
zmp_ph97_C2
zmp_ph97_C4
zmp_ph97_B8
zmp_ph97_C1
zmp_ph97_D2
zmp_ph97_D5
zmp_ph111_G5
zmp_ph111_G11
zmp_ph111_H1
zmp_ph111_B1a
zmp_ph111_B5
zmp_ph111_B8
zmp_ph111_B6
zmp_ph111_B7
zmp_ph111_B9
zmp_ph111_B10
zmp_ph114_G4
zmp_ph114_G11
zmp_ph114_H1
zmp_ph114_H2
zmp_ph114_A9
zmp_ph114_A11
zmp_ph114_B2
zmp_ph114_B4
zmp_ph114_D8
zmp_ph114_E2

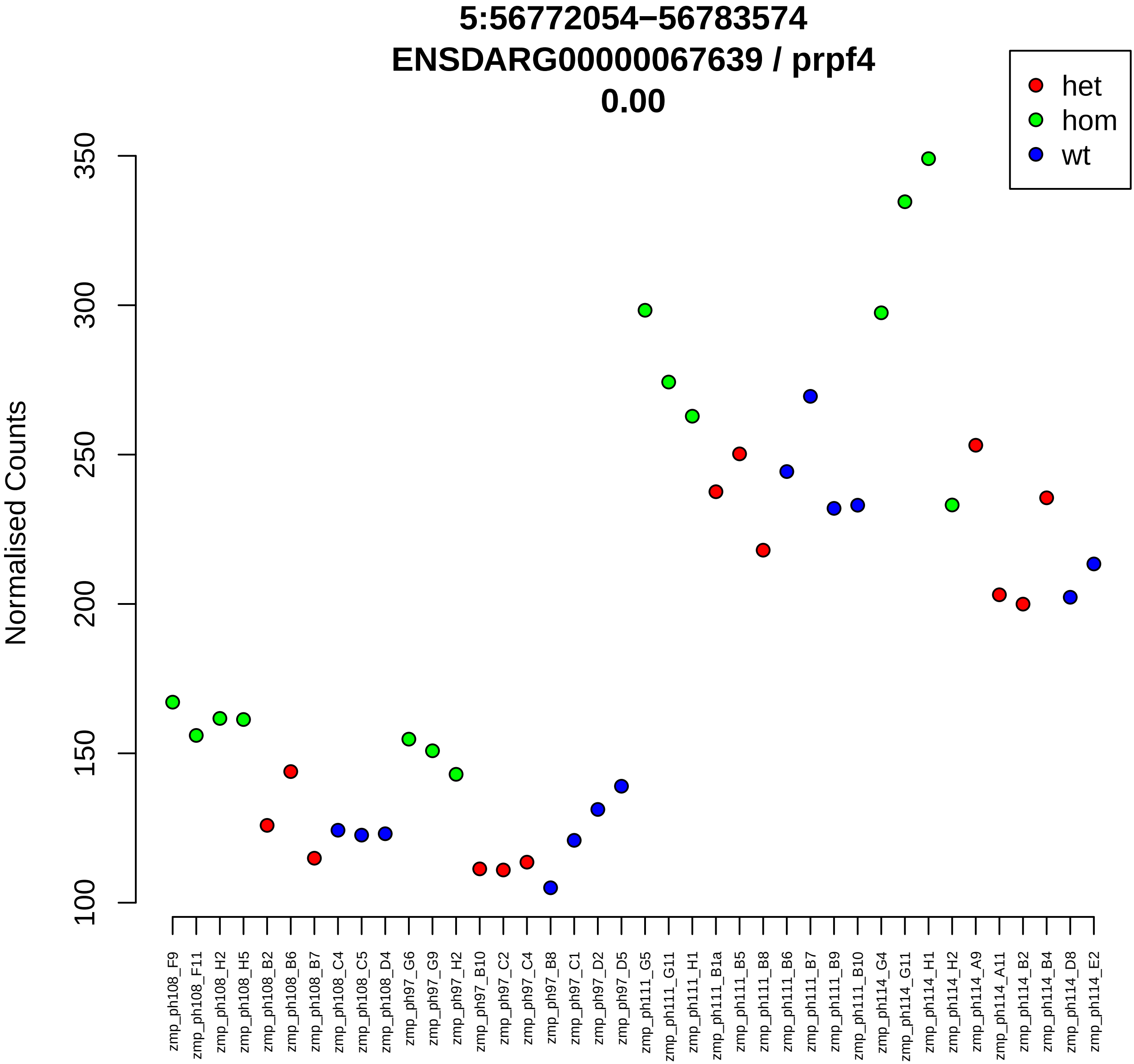
5:56772054−56783574
ENSDARG00000067639 / prpf4
0.00
het
hom
wt
Normalised Counts
100
150
200
250
300
350
zmp_ph108_F9
zmp_ph108_F11
zmp_ph108_H2
zmp_ph108_H5
zmp_ph108_B2
zmp_ph108_B6
zmp_ph108_B7
zmp_ph108_C4
zmp_ph108_C5
zmp_ph108_D4
zmp_ph97_G6
zmp_ph97_G9
zmp_ph97_H2
zmp_ph97_B10
zmp_ph97_C2
zmp_ph97_C4
zmp_ph97_B8
zmp_ph97_C1
zmp_ph97_D2
zmp_ph97_D5
zmp_ph111_G5
zmp_ph111_G11
zmp_ph111_H1
zmp_ph111_B1a
zmp_ph111_B5
zmp_ph111_B8
zmp_ph111_B6
zmp_ph111_B7
zmp_ph111_B9
zmp_ph111_B10
zmp_ph114_G4
zmp_ph114_G11
zmp_ph114_H1
zmp_ph114_H2
zmp_ph114_A9
zmp_ph114_A11
zmp_ph114_B2
zmp_ph114_B4
zmp_ph114_D8
zmp_ph114_E2

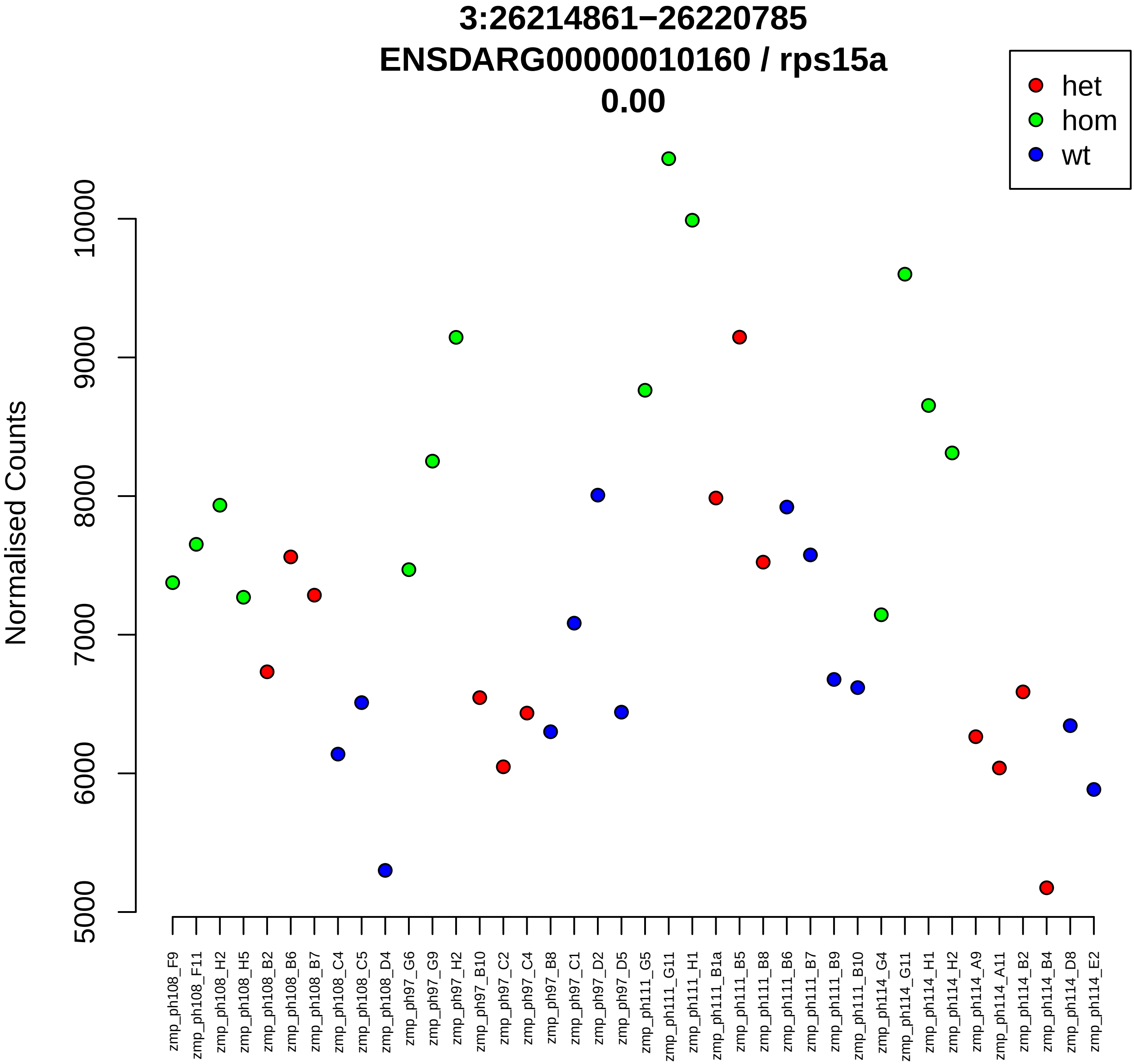

3:26214861−26220785
ENSDARG00000010160 / rps15a
0.00
het
hom
wt
Normalised Counts
5000
6000
7000
8000
9000
10000
zmp_ph108_F9
zmp_ph108_F11
zmp_ph108_H2
zmp_ph108_H5
zmp_ph108_B2
zmp_ph108_B6
zmp_ph108_B7
zmp_ph108_C4
zmp_ph108_C5
zmp_ph108_D4
zmp_ph97_G6
zmp_ph97_G9
zmp_ph97_H2
zmp_ph97_B10
zmp_ph97_C2
zmp_ph97_C4
zmp_ph97_B8
zmp_ph97_C1
zmp_ph97_D2
zmp_ph97_D5
zmp_ph111_G5
zmp_ph111_G11
zmp_ph111_H1
zmp_ph111_B1a
zmp_ph111_B5
zmp_ph111_B8
zmp_ph111_B6
zmp_ph111_B7
zmp_ph111_B9
zmp_ph111_B10
zmp_ph114_G4
zmp_ph114_G11
zmp_ph114_H1
zmp_ph114_H2
zmp_ph114_A9
zmp_ph114_A11
zmp_ph114_B2
zmp_ph114_B4
zmp_ph114_D8
zmp_ph114_E2

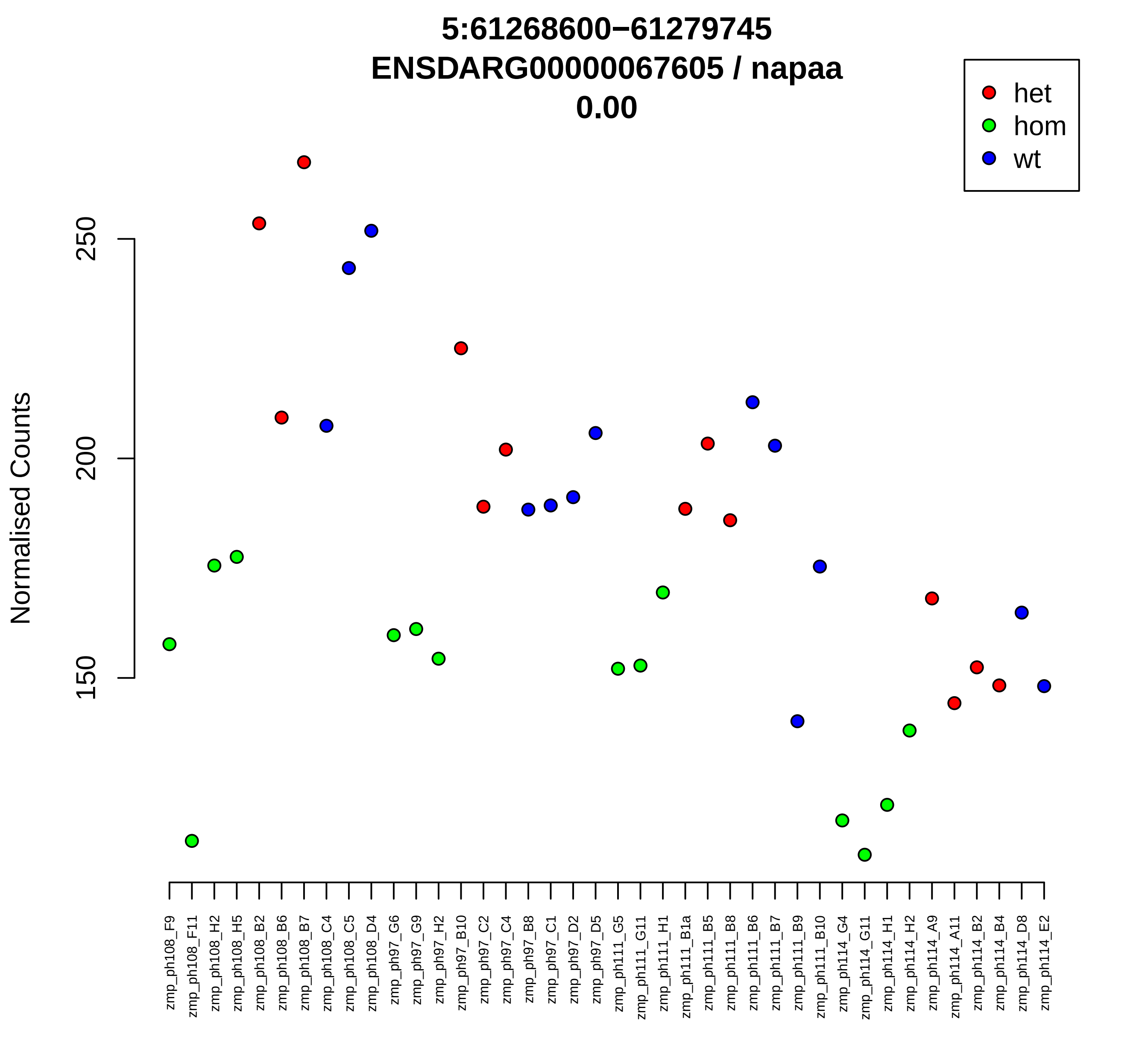
5:61268600−61279745
ENSDARG00000067605 / napaa
0.00
het
hom
wt
Normalised Counts
150
200
250
zmp_ph108_F9
zmp_ph108_F11
zmp_ph108_H2
zmp_ph108_H5
zmp_ph108_B2
zmp_ph108_B6
zmp_ph108_B7
zmp_ph108_C4
zmp_ph108_C5
zmp_ph108_D4
zmp_ph97_G6
zmp_ph97_G9
zmp_ph97_H2
zmp_ph97_B10
zmp_ph97_C2
zmp_ph97_C4
zmp_ph97_B8
zmp_ph97_C1
zmp_ph97_D2
zmp_ph97_D5
zmp_ph111_G5
zmp_ph111_G11
zmp_ph111_H1
zmp_ph111_B1a
zmp_ph111_B5
zmp_ph111_B8
zmp_ph111_B6
zmp_ph111_B7
zmp_ph111_B9
zmp_ph111_B10
zmp_ph114_G4
zmp_ph114_G11
zmp_ph114_H1
zmp_ph114_H2
zmp_ph114_A9
zmp_ph114_A11
zmp_ph114_B2
zmp_ph114_B4
zmp_ph114_D8
zmp_ph114_E2

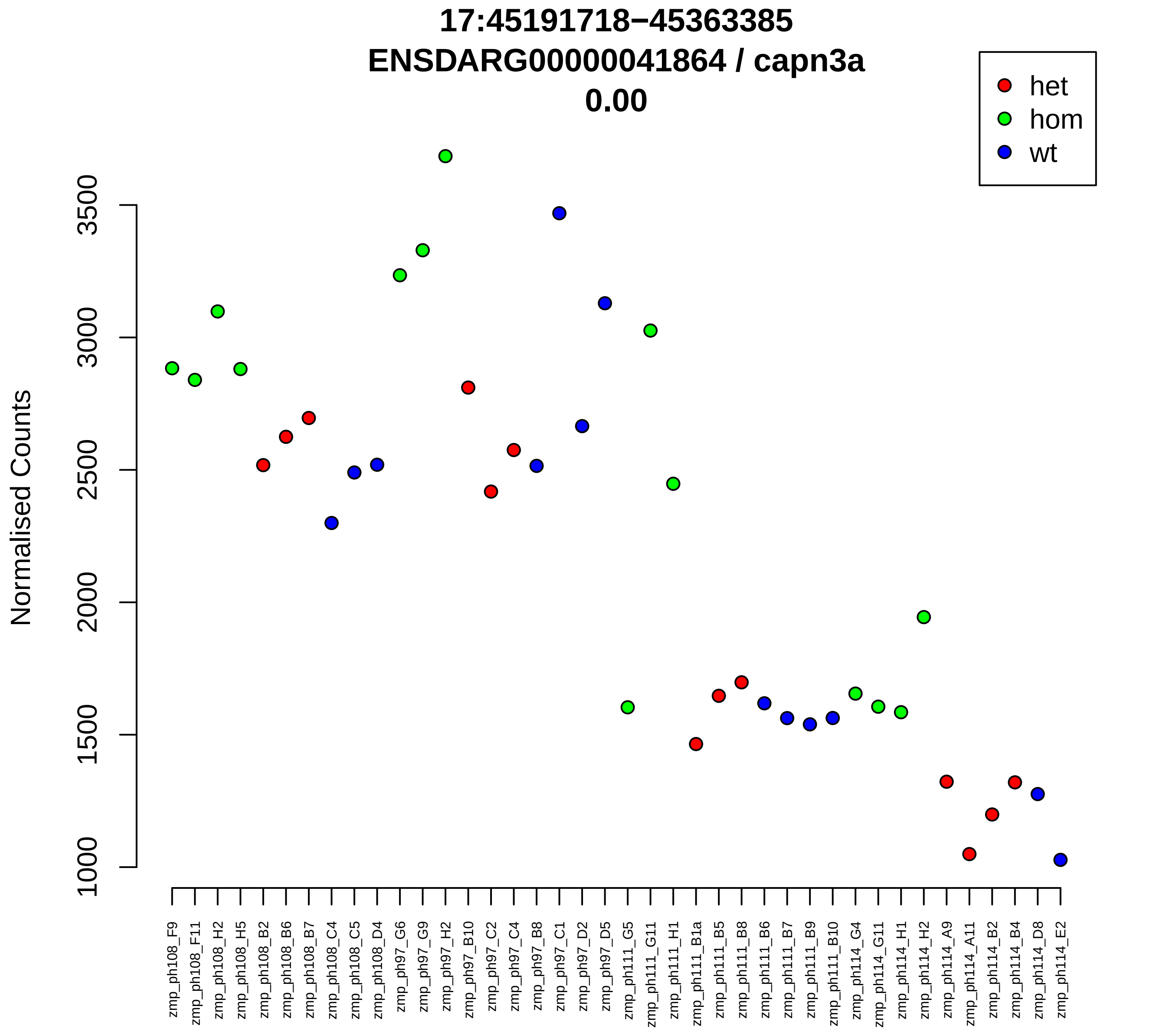

17:45191718−45363385
ENSDARG00000041864 / capn3a
0.00
het
hom
wt
Normalised Counts
1000
1500
2000
2500
3000
3500
zmp_ph108_F9
zmp_ph108_F11
zmp_ph108_H2
zmp_ph108_H5
zmp_ph108_B2
zmp_ph108_B6
zmp_ph108_B7
zmp_ph108_C4
zmp_ph108_C5
zmp_ph108_D4
zmp_ph97_G6
zmp_ph97_G9
zmp_ph97_H2
zmp_ph97_B10
zmp_ph97_C2
zmp_ph97_C4
zmp_ph97_B8
zmp_ph97_C1
zmp_ph97_D2
zmp_ph97_D5
zmp_ph111_G5
zmp_ph111_G11
zmp_ph111_H1
zmp_ph111_B1a
zmp_ph111_B5
zmp_ph111_B8
zmp_ph111_B6
zmp_ph111_B7
zmp_ph111_B9
zmp_ph111_B10
zmp_ph114_G4
zmp_ph114_G11
zmp_ph114_H1
zmp_ph114_H2
zmp_ph114_A9
zmp_ph114_A11
zmp_ph114_B2
zmp_ph114_B4
zmp_ph114_D8
zmp_ph114_E2

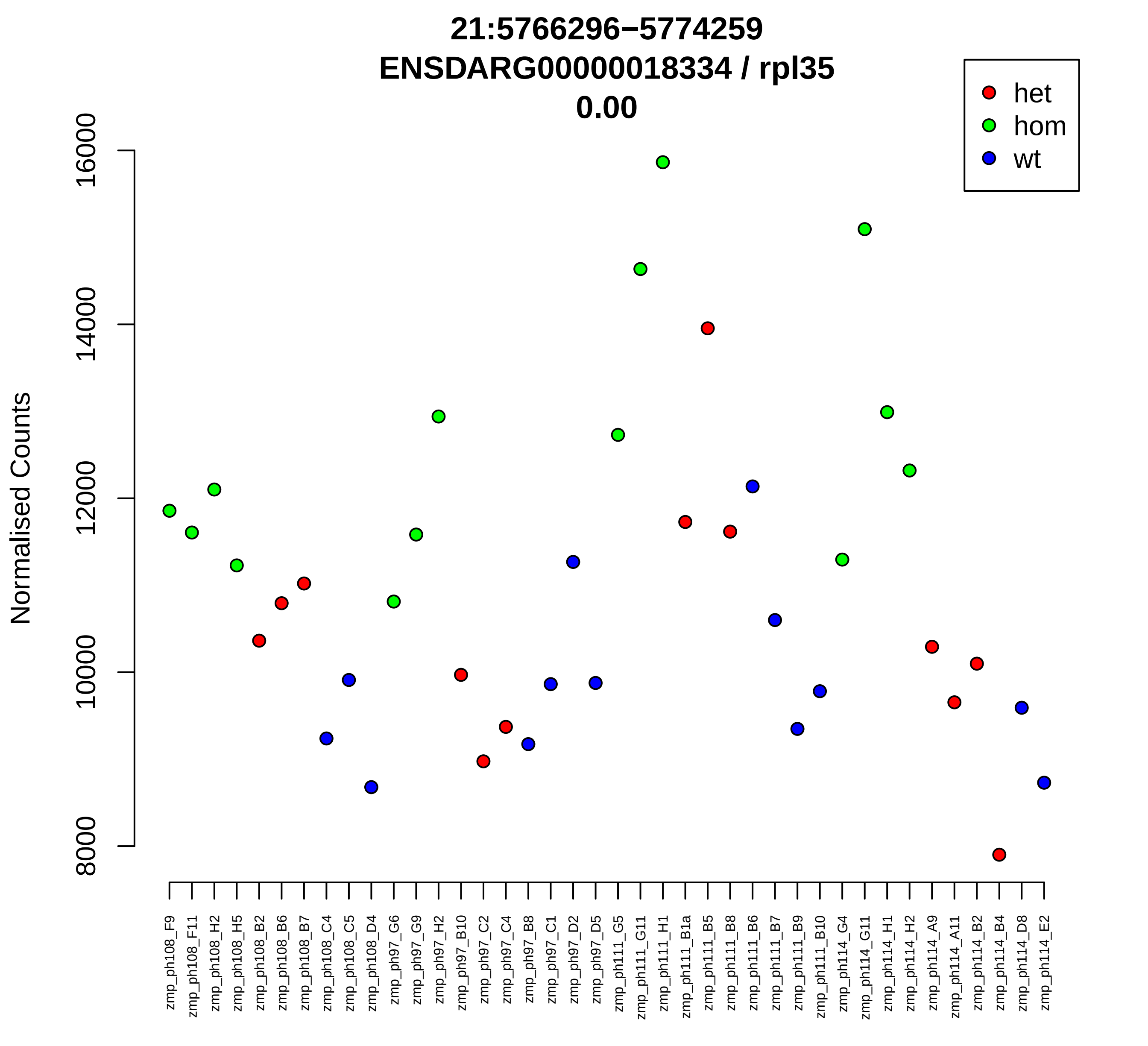

21:5766296−5774259
ENSDARG00000018334 / rpl35
0.00
het
hom
wt
Normalised Counts
8000
10000
12000
14000
16000
zmp_ph108_F9
zmp_ph108_F11
zmp_ph108_H2
zmp_ph108_H5
zmp_ph108_B2
zmp_ph108_B6
zmp_ph108_B7
zmp_ph108_C4
zmp_ph108_C5
zmp_ph108_D4
zmp_ph97_G6
zmp_ph97_G9
zmp_ph97_H2
zmp_ph97_B10
zmp_ph97_C2
zmp_ph97_C4
zmp_ph97_B8
zmp_ph97_C1
zmp_ph97_D2
zmp_ph97_D5
zmp_ph111_G5
zmp_ph111_G11
zmp_ph111_H1
zmp_ph111_B1a
zmp_ph111_B5
zmp_ph111_B8
zmp_ph111_B6
zmp_ph111_B7
zmp_ph111_B9
zmp_ph111_B10
zmp_ph114_G4
zmp_ph114_G11
zmp_ph114_H1
zmp_ph114_H2
zmp_ph114_A9
zmp_ph114_A11
zmp_ph114_B2
zmp_ph114_B4
zmp_ph114_D8
zmp_ph114_E2

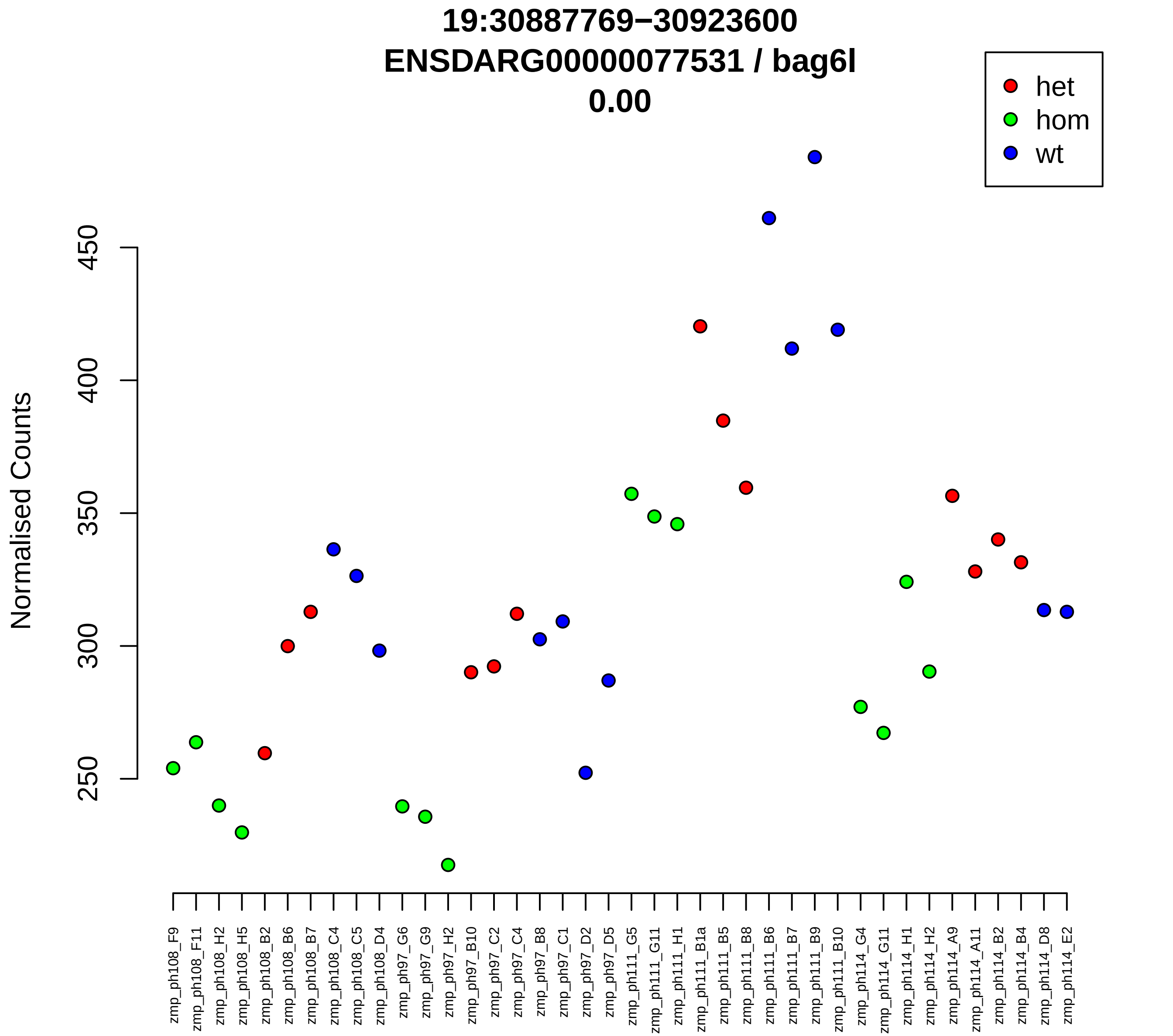
19:30887769−30923600
ENSDARG00000077531 / bag6l
0.00
het
hom
wt
Normalised Counts
250
300
350
400
450
zmp_ph108_F9
zmp_ph108_F11
zmp_ph108_H2
zmp_ph108_H5
zmp_ph108_B2
zmp_ph108_B6
zmp_ph108_B7
zmp_ph108_C4
zmp_ph108_C5
zmp_ph108_D4
zmp_ph97_G6
zmp_ph97_G9
zmp_ph97_H2
zmp_ph97_B10
zmp_ph97_C2
zmp_ph97_C4
zmp_ph97_B8
zmp_ph97_C1
zmp_ph97_D2
zmp_ph97_D5
zmp_ph111_G5
zmp_ph111_G11
zmp_ph111_H1
zmp_ph111_B1a
zmp_ph111_B5
zmp_ph111_B8
zmp_ph111_B6
zmp_ph111_B7
zmp_ph111_B9
zmp_ph111_B10
zmp_ph114_G4
zmp_ph114_G11
zmp_ph114_H1
zmp_ph114_H2
zmp_ph114_A9
zmp_ph114_A11
zmp_ph114_B2
zmp_ph114_B4
zmp_ph114_D8
zmp_ph114_E2

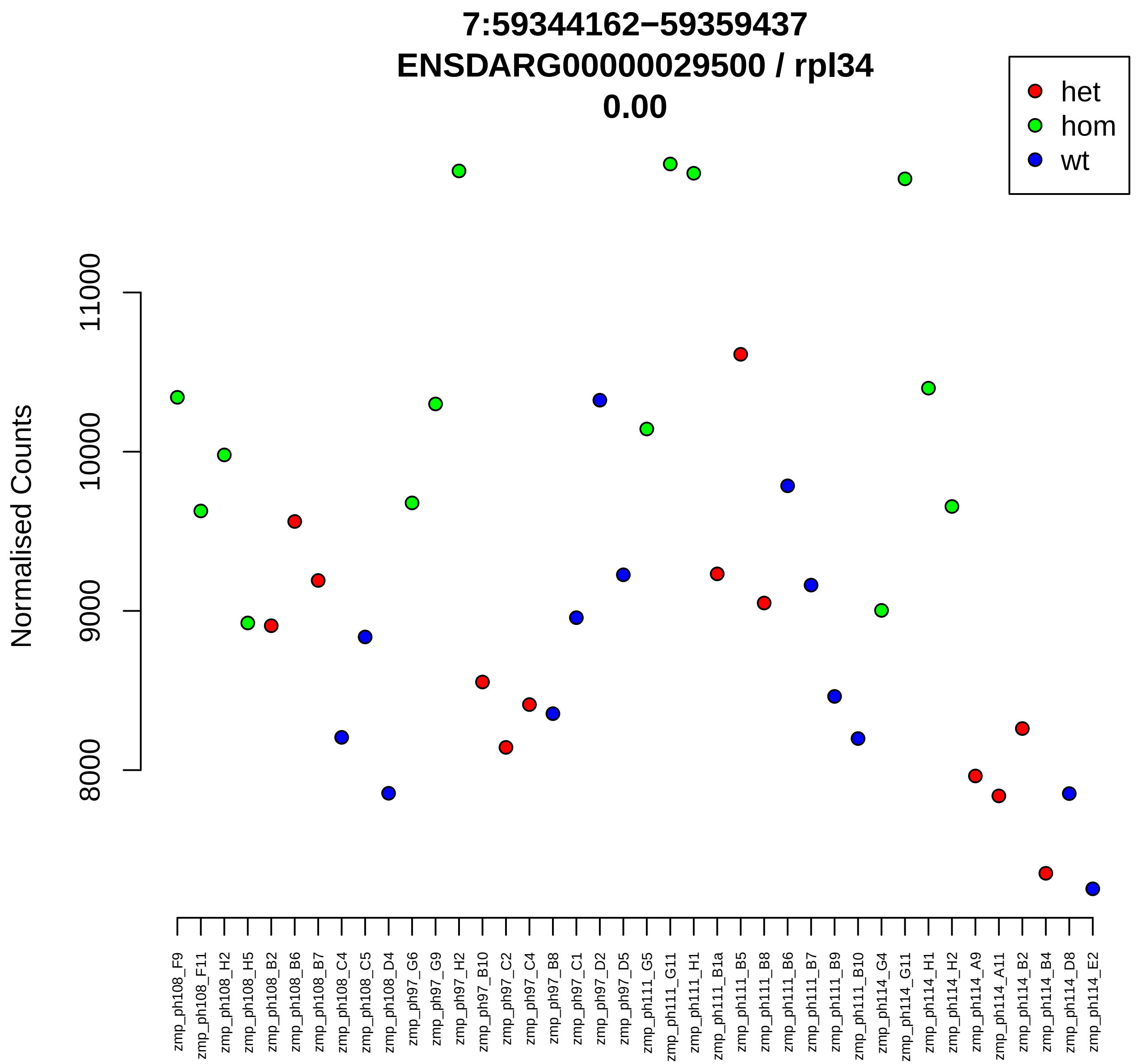

7:59344162−59359437
ENSDARG00000029500 / rpl34
0.00
het
hom
wt
Normalised Counts
8000
9000
10000
11000
zmp_ph108_F9
zmp_ph108_F11
zmp_ph108_H2
zmp_ph108_H5
zmp_ph108_B2
zmp_ph108_B6
zmp_ph108_B7
zmp_ph108_C4
zmp_ph108_C5
zmp_ph108_D4
zmp_ph97_G6
zmp_ph97_G9
zmp_ph97_H2
zmp_ph97_B10
zmp_ph97_C2
zmp_ph97_C4
zmp_ph97_B8
zmp_ph97_C1
zmp_ph97_D2
zmp_ph97_D5
zmp_ph111_G5
zmp_ph111_G11
zmp_ph111_H1
zmp_ph111_B1a
zmp_ph111_B5
zmp_ph111_B8
zmp_ph111_B6
zmp_ph111_B7
zmp_ph111_B9
zmp_ph111_B10
zmp_ph114_G4
zmp_ph114_G11
zmp_ph114_H1
zmp_ph114_H2
zmp_ph114_A9
zmp_ph114_A11
zmp_ph114_B2
zmp_ph114_B4
zmp_ph114_D8
zmp_ph114_E2

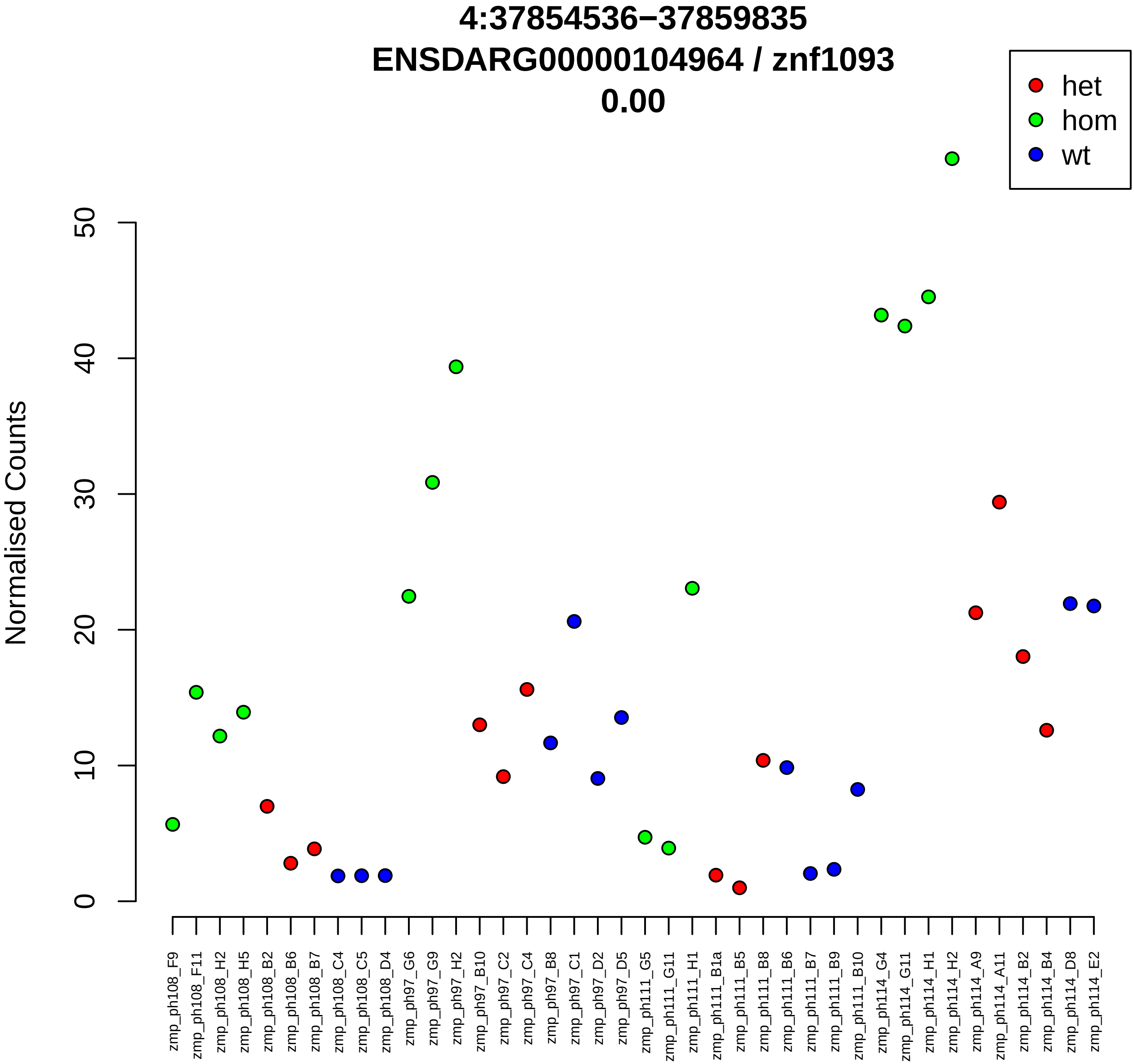

4:37854536−37859835
ENSDARG00000104964 / znf1093
0.00
het
hom
wt
Normalised Counts
0
10
20
30
40
50
zmp_ph108_F9
zmp_ph108_F11
zmp_ph108_H2
zmp_ph108_H5
zmp_ph108_B2
zmp_ph108_B6
zmp_ph108_B7
zmp_ph108_C4
zmp_ph108_C5
zmp_ph108_D4
zmp_ph97_G6
zmp_ph97_G9
zmp_ph97_H2
zmp_ph97_B10
zmp_ph97_C2
zmp_ph97_C4
zmp_ph97_B8
zmp_ph97_C1
zmp_ph97_D2
zmp_ph97_D5
zmp_ph111_G5
zmp_ph111_G11
zmp_ph111_H1
zmp_ph111_B1a
zmp_ph111_B5
zmp_ph111_B8
zmp_ph111_B6
zmp_ph111_B7
zmp_ph111_B9
zmp_ph111_B10
zmp_ph114_G4
zmp_ph114_G11
zmp_ph114_H1
zmp_ph114_H2
zmp_ph114_A9
zmp_ph114_A11
zmp_ph114_B2
zmp_ph114_B4
zmp_ph114_D8
zmp_ph114_E2

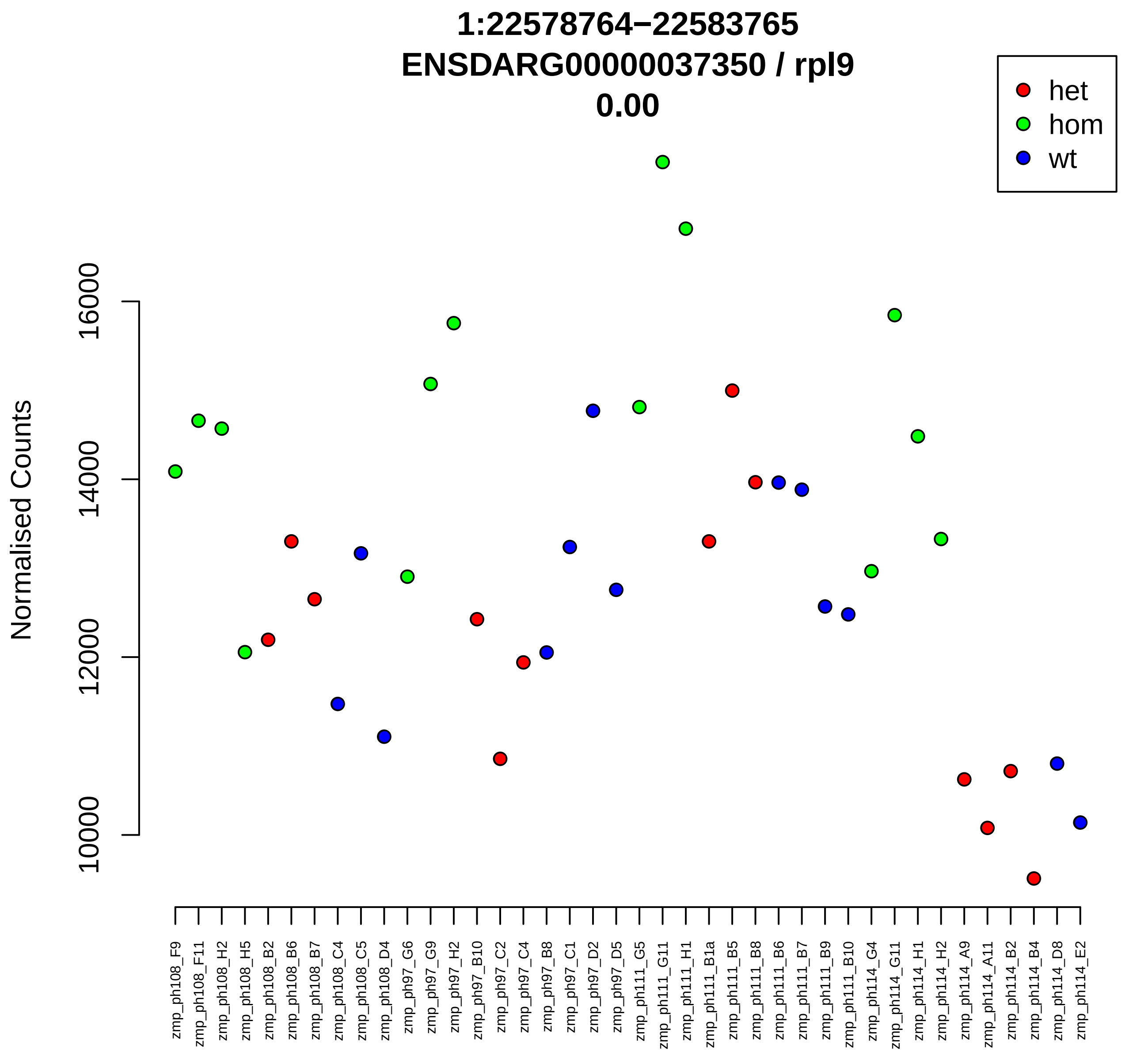
1:22578764−22583765
ENSDARG00000037350 / rpl9
0.00
het
hom
wt
Normalised Counts
10000
12000
14000
16000
zmp_ph108_F9
zmp_ph108_F11
zmp_ph108_H2
zmp_ph108_H5
zmp_ph108_B2
zmp_ph108_B6
zmp_ph108_B7
zmp_ph108_C4
zmp_ph108_C5
zmp_ph108_D4
zmp_ph97_G6
zmp_ph97_G9
zmp_ph97_H2
zmp_ph97_B10
zmp_ph97_C2
zmp_ph97_C4
zmp_ph97_B8
zmp_ph97_C1
zmp_ph97_D2
zmp_ph97_D5
zmp_ph111_G5
zmp_ph111_G11
zmp_ph111_H1
zmp_ph111_B1a
zmp_ph111_B5
zmp_ph111_B8
zmp_ph111_B6
zmp_ph111_B7
zmp_ph111_B9
zmp_ph111_B10
zmp_ph114_G4
zmp_ph114_G11
zmp_ph114_H1
zmp_ph114_H2
zmp_ph114_A9
zmp_ph114_A11
zmp_ph114_B2
zmp_ph114_B4
zmp_ph114_D8
zmp_ph114_E2

15:19717075−19723846
ENSDARG00000037402 / lim2.3
0.00

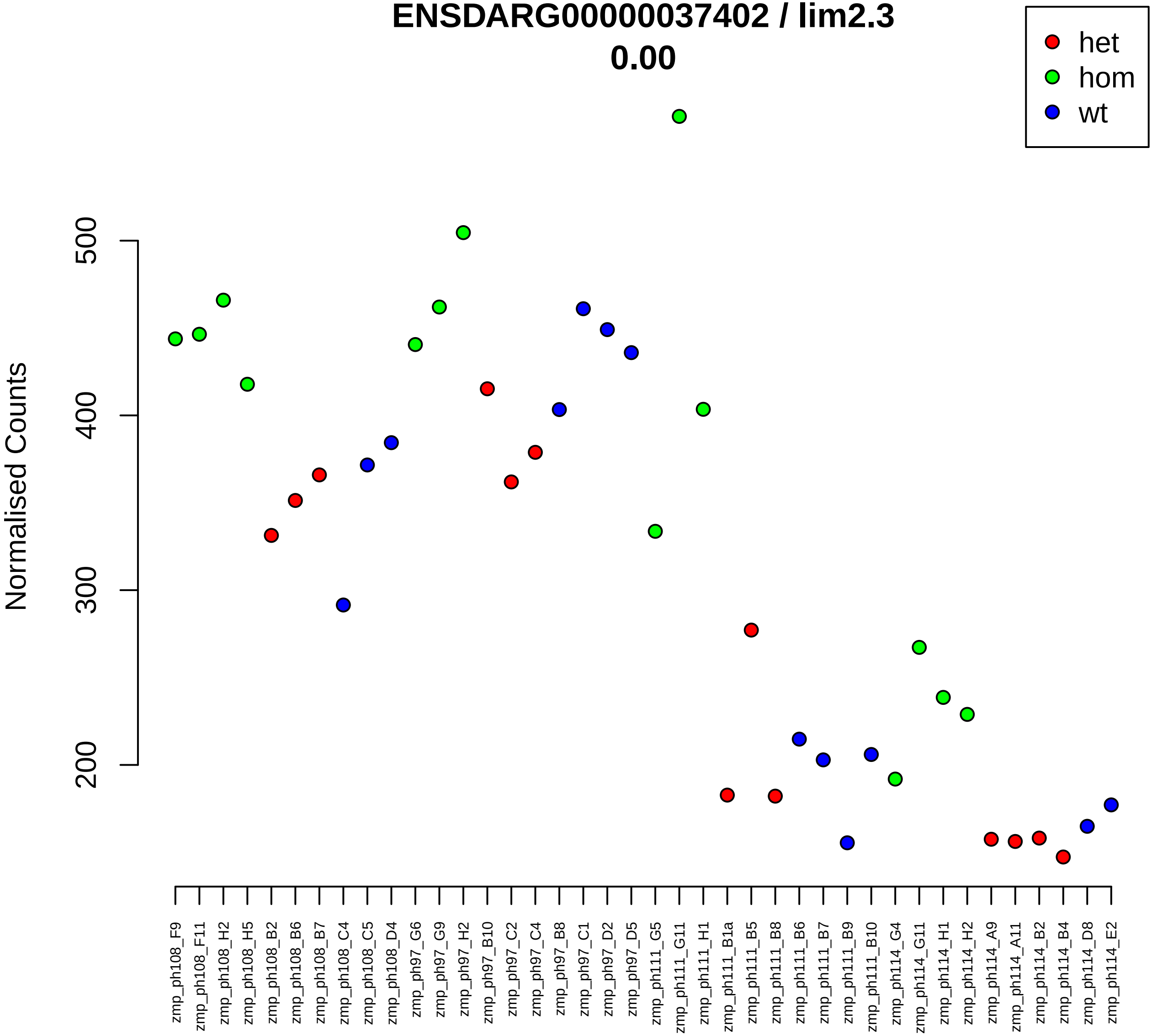

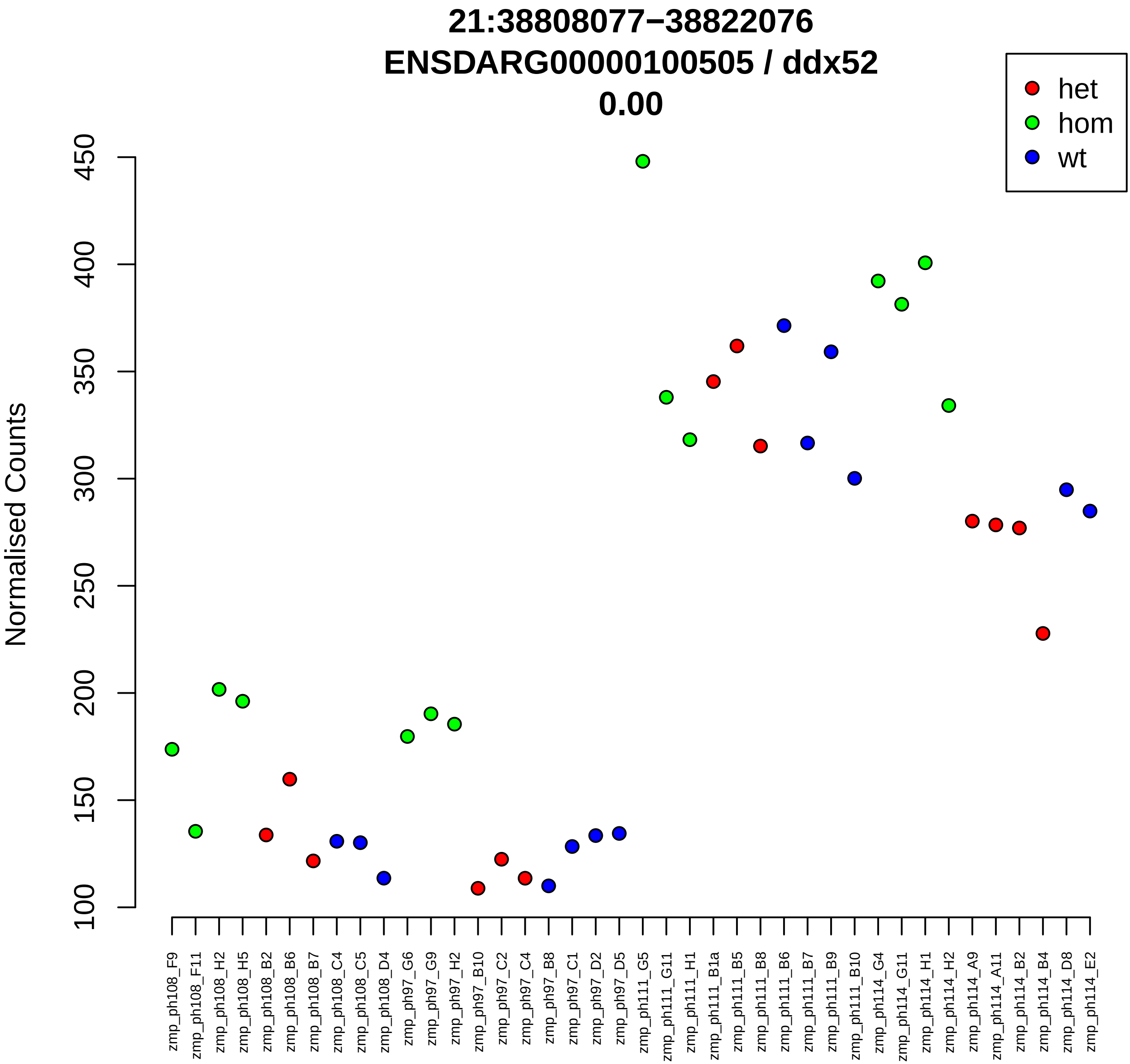

21:38808077−38822076
ENSDARG00000100505 / ddx52
0.00
het
hom
wt
Normalised Counts
100
150
200
250
300
350
400
450
zmp_ph108_F9
zmp_ph108_F11
zmp_ph108_H2
zmp_ph108_H5
zmp_ph108_B2
zmp_ph108_B6
zmp_ph108_B7
zmp_ph108_C4
zmp_ph108_C5
zmp_ph108_D4
zmp_ph97_G6
zmp_ph97_G9
zmp_ph97_H2
zmp_ph97_B10
zmp_ph97_C2
zmp_ph97_C4
zmp_ph97_B8
zmp_ph97_C1
zmp_ph97_D2
zmp_ph97_D5
zmp_ph111_G5
zmp_ph111_G11
zmp_ph111_H1
zmp_ph111_B1a
zmp_ph111_B5
zmp_ph111_B8
zmp_ph111_B6
zmp_ph111_B7
zmp_ph111_B9
zmp_ph111_B10
zmp_ph114_G4
zmp_ph114_G11
zmp_ph114_H1
zmp_ph114_H2
zmp_ph114_A9
zmp_ph114_A11
zmp_ph114_B2
zmp_ph114_B4
zmp_ph114_D8
zmp_ph114_E2

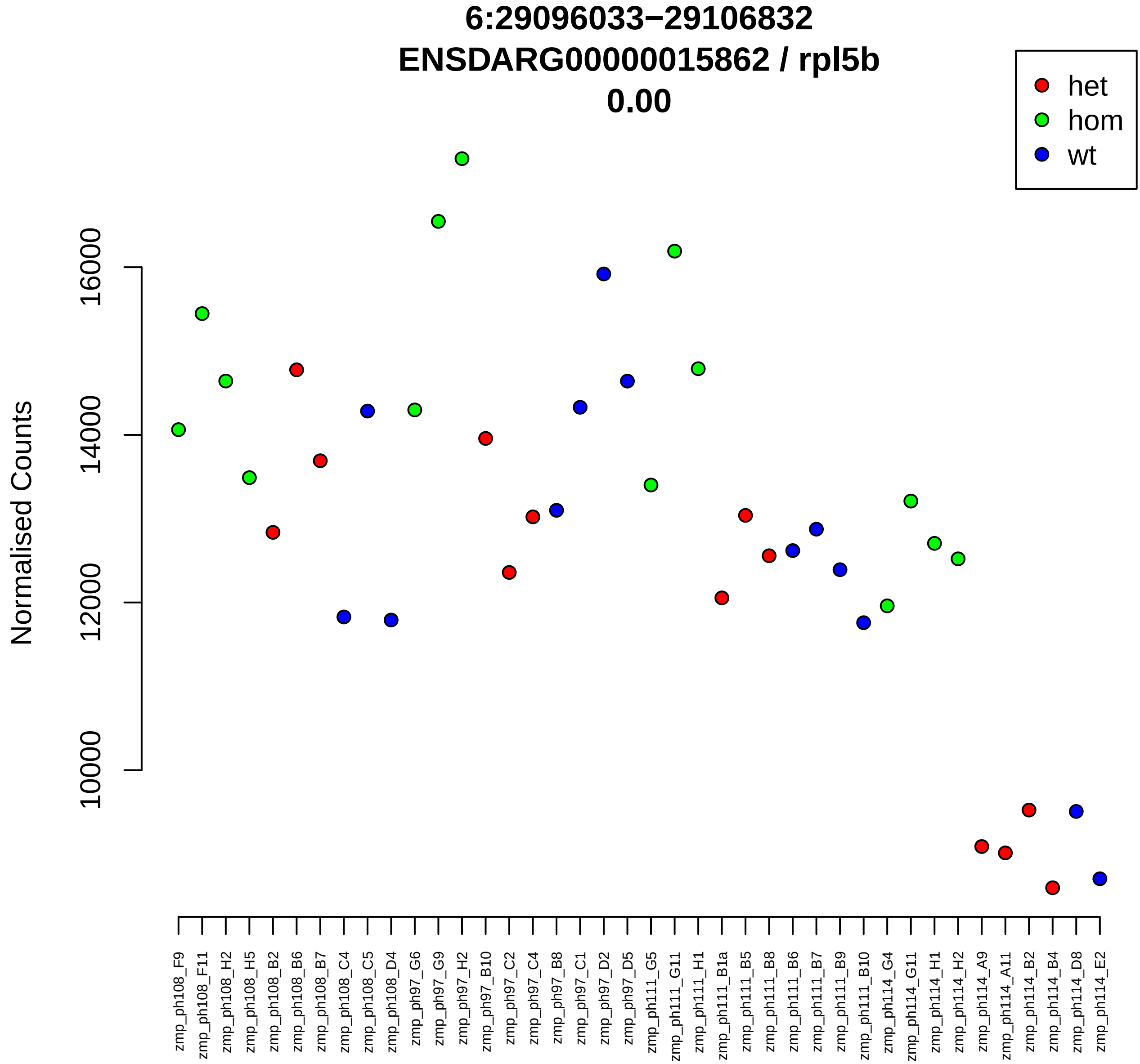

6:29096033−29106832
ENSDARG00000015862 / rpl5b
0.00
het
hom
wt
Normalised Counts
10000
12000
14000
16000
zmp_ph108_F9
zmp_ph108_F11
zmp_ph108_H2
zmp_ph108_H5
zmp_ph108_B2
zmp_ph108_B6
zmp_ph108_B7
zmp_ph108_C4
zmp_ph108_C5
zmp_ph108_D4
zmp_ph97_G6
zmp_ph97_G9
zmp_ph97_H2
zmp_ph97_B10
zmp_ph97_C2
zmp_ph97_C4
zmp_ph97_B8
zmp_ph97_C1
zmp_ph97_D2
zmp_ph97_D5
zmp_ph111_G5
zmp_ph111_G11
zmp_ph111_H1
zmp_ph111_B1a
zmp_ph111_B5
zmp_ph111_B8
zmp_ph111_B6
zmp_ph111_B7
zmp_ph111_B9
zmp_ph111_B10
zmp_ph114_G4
zmp_ph114_G11
zmp_ph114_H1
zmp_ph114_H2
zmp_ph114_A9
zmp_ph114_A11
zmp_ph114_B2
zmp_ph114_B4
zmp_ph114_D8
zmp_ph114_E2

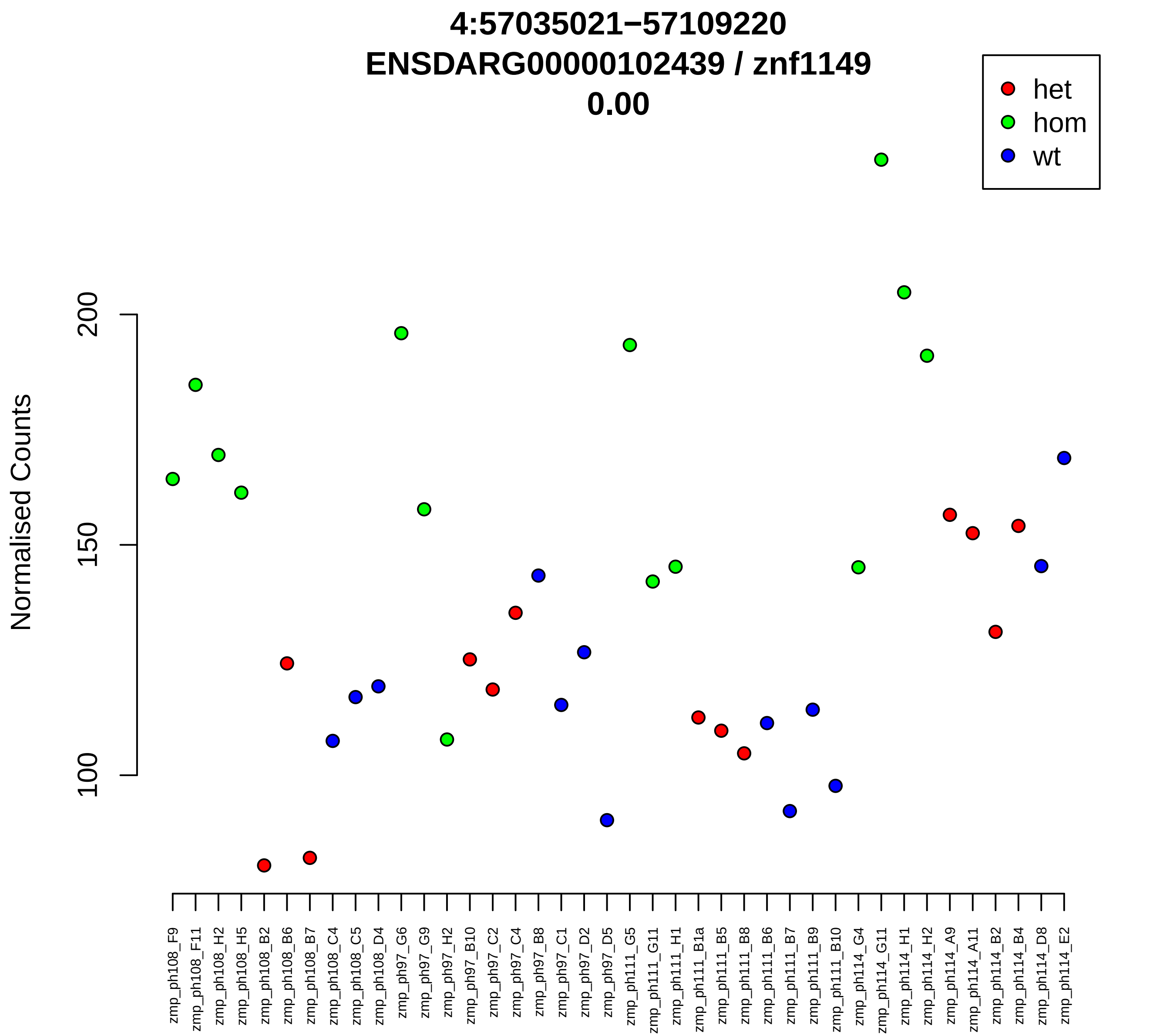
4:57035021−57109220
ENSDARG00000102439 / znf1149
0.00
het
hom
wt
Normalised Counts
100
150
200
zmp_ph108_F9
zmp_ph108_F11
zmp_ph108_H2
zmp_ph108_H5
zmp_ph108_B2
zmp_ph108_B6
zmp_ph108_B7
zmp_ph108_C4
zmp_ph108_C5
zmp_ph108_D4
zmp_ph97_G6
zmp_ph97_G9
zmp_ph97_H2
zmp_ph97_B10
zmp_ph97_C2
zmp_ph97_C4
zmp_ph97_B8
zmp_ph97_C1
zmp_ph97_D2
zmp_ph97_D5
zmp_ph111_G5
zmp_ph111_G11
zmp_ph111_H1
zmp_ph111_B1a
zmp_ph111_B5
zmp_ph111_B8
zmp_ph111_B6
zmp_ph111_B7
zmp_ph111_B9
zmp_ph111_B10
zmp_ph114_G4
zmp_ph114_G11
zmp_ph114_H1
zmp_ph114_H2
zmp_ph114_A9
zmp_ph114_A11
zmp_ph114_B2
zmp_ph114_B4
zmp_ph114_D8
zmp_ph114_E2

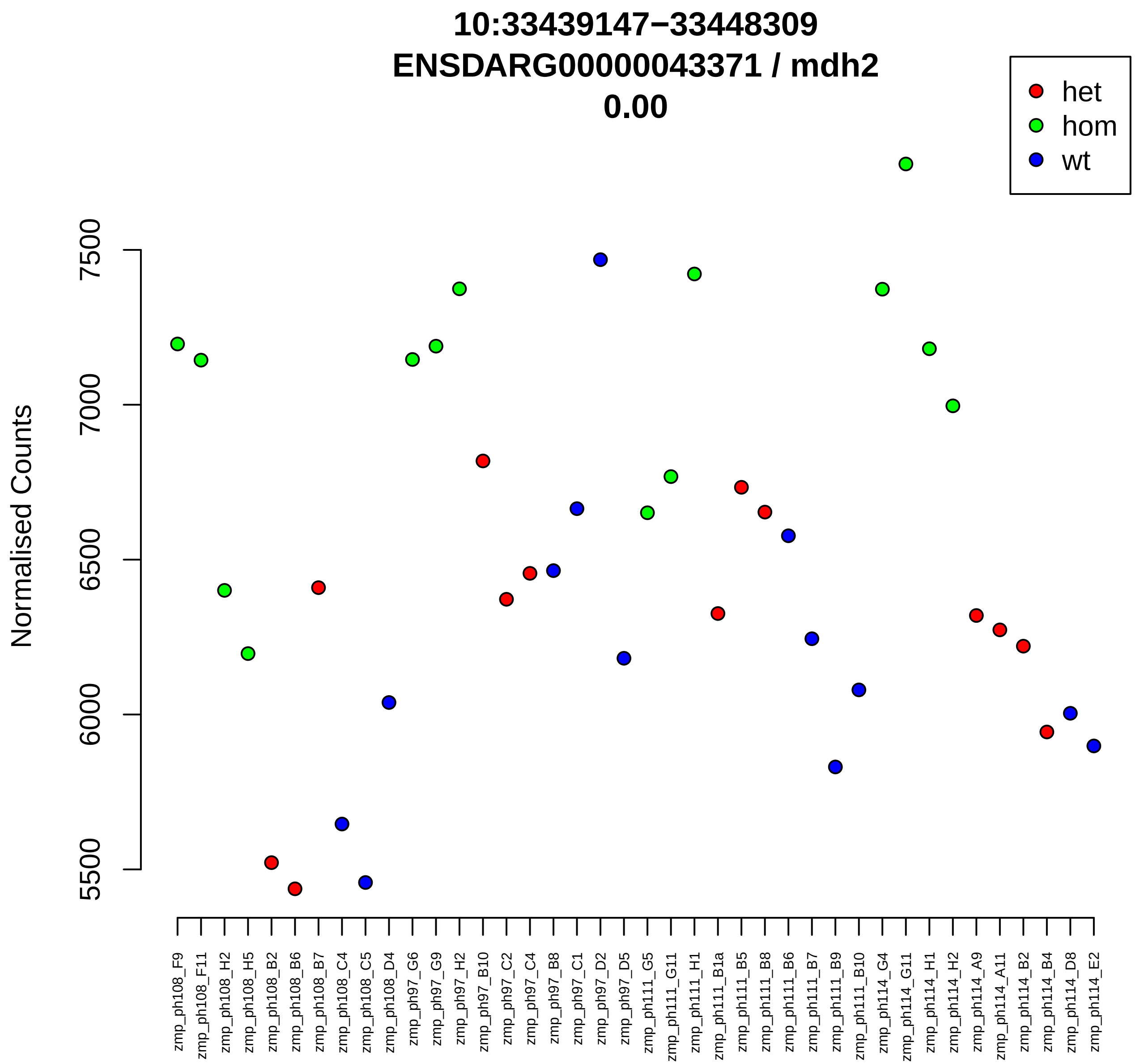

10:33439147−33448309
ENSDARG00000043371 / mdh2
0.00
het
hom
wt
Normalised Counts
5500
6000
6500
7000
7500
zmp_ph108_F9
zmp_ph108_F11
zmp_ph108_H2
zmp_ph108_H5
zmp_ph108_B2
zmp_ph108_B6
zmp_ph108_B7
zmp_ph108_C4
zmp_ph108_C5
zmp_ph108_D4
zmp_ph97_G6
zmp_ph97_G9
zmp_ph97_H2
zmp_ph97_B10
zmp_ph97_C2
zmp_ph97_C4
zmp_ph97_B8
zmp_ph97_C1
zmp_ph97_D2
zmp_ph97_D5
zmp_ph111_G5
zmp_ph111_G11
zmp_ph111_H1
zmp_ph111_B1a
zmp_ph111_B5
zmp_ph111_B8
zmp_ph111_B6
zmp_ph111_B7
zmp_ph111_B9
zmp_ph111_B10
zmp_ph114_G4
zmp_ph114_G11
zmp_ph114_H1
zmp_ph114_H2
zmp_ph114_A9
zmp_ph114_A11
zmp_ph114_B2
zmp_ph114_B4
zmp_ph114_D8
zmp_ph114_E2

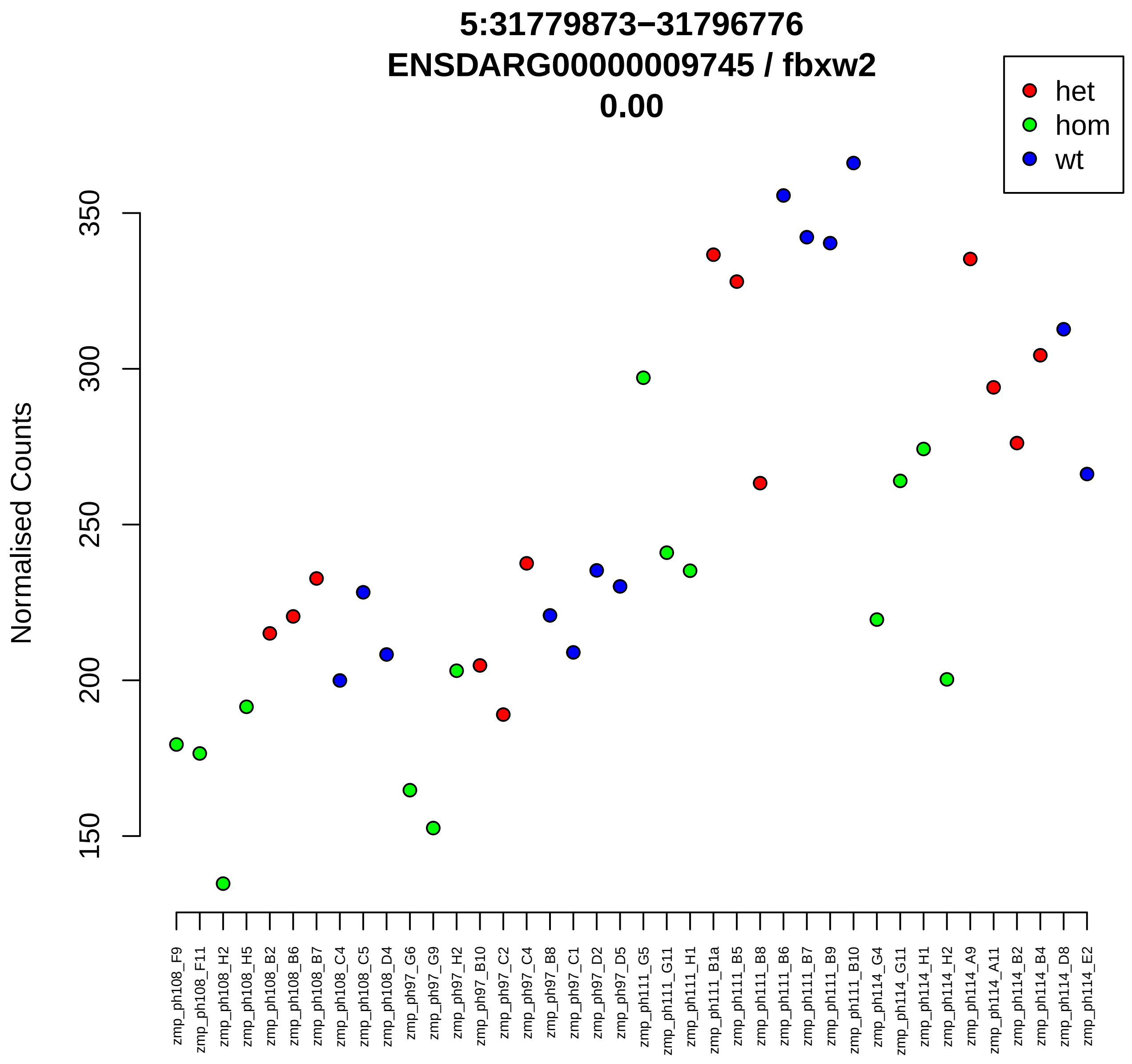
5:31779873−31796776
ENSDARG00000009745 / fbxw2
0.00
het
hom
wt
Normalised Counts
150
200
250
300
350
zmp_ph108_F9
zmp_ph108_F11
zmp_ph108_H2
zmp_ph108_H5
zmp_ph108_B2
zmp_ph108_B6
zmp_ph108_B7
zmp_ph108_C4
zmp_ph108_C5
zmp_ph108_D4
zmp_ph97_G6
zmp_ph97_G9
zmp_ph97_H2
zmp_ph97_B10
zmp_ph97_C2
zmp_ph97_C4
zmp_ph97_B8
zmp_ph97_C1
zmp_ph97_D2
zmp_ph97_D5
zmp_ph111_G5
zmp_ph111_G11
zmp_ph111_H1
zmp_ph111_B1a
zmp_ph111_B5
zmp_ph111_B8
zmp_ph111_B6
zmp_ph111_B7
zmp_ph111_B9
zmp_ph111_B10
zmp_ph114_G4
zmp_ph114_G11
zmp_ph114_H1
zmp_ph114_H2
zmp_ph114_A9
zmp_ph114_A11
zmp_ph114_B2
zmp_ph114_B4
zmp_ph114_D8
zmp_ph114_E2

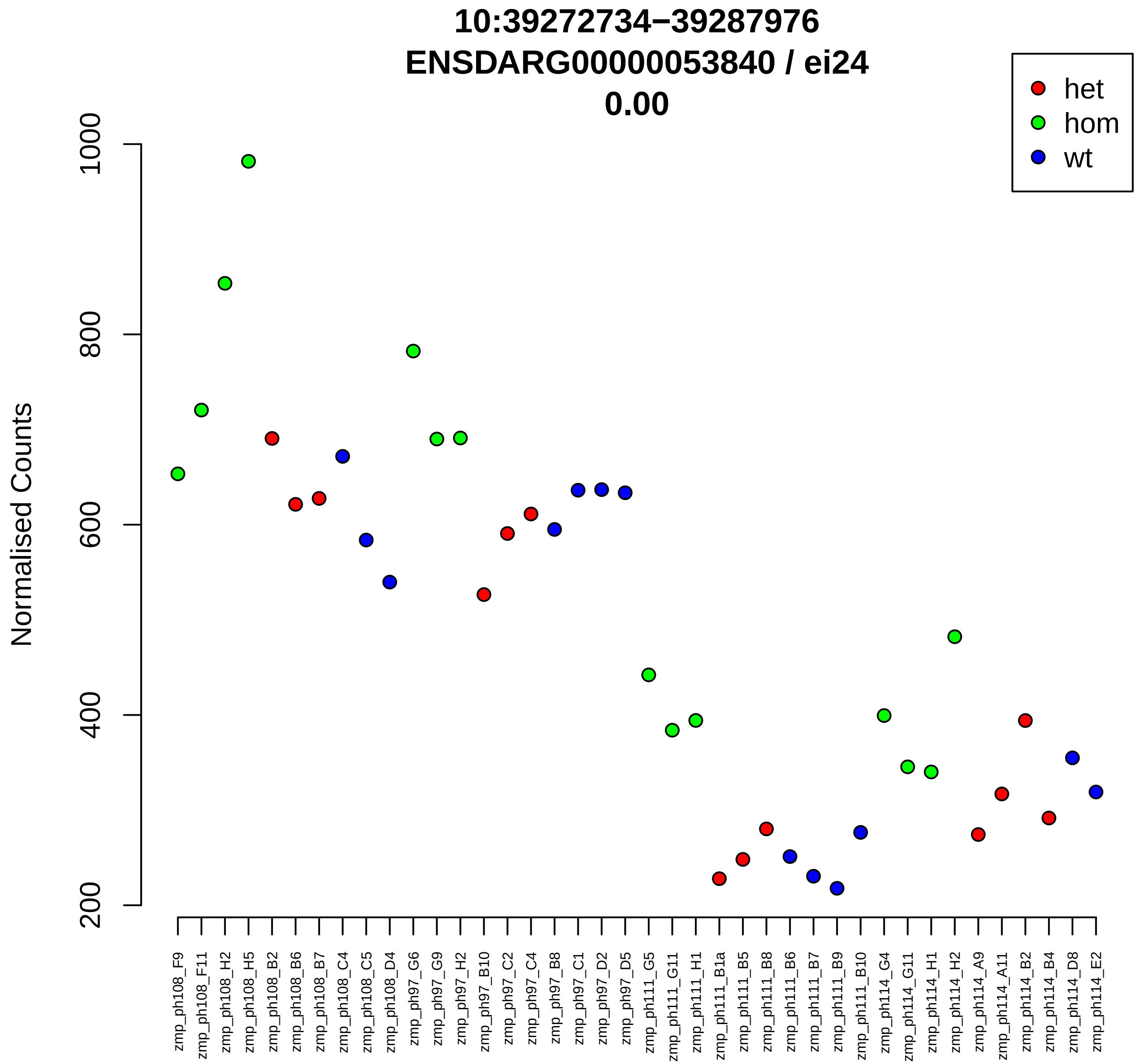
10:39272734−39287976
ENSDARG00000053840 / ei24
0.00
het
hom
wt
Normalised Counts
1000
800
600
400
200
zmp_ph108_F9
zmp_ph108_F11
zmp_ph108_H2
zmp_ph108_H5
zmp_ph108_B2
zmp_ph108_B6
zmp_ph108_B7
zmp_ph108_C4
zmp_ph108_C5
zmp_ph108_D4
zmp_ph97_G6
zmp_ph97_G9
zmp_ph97_H2
zmp_ph97_B10
zmp_ph97_C2
zmp_ph97_C4
zmp_ph97_B8
zmp_ph97_C1
zmp_ph97_D2
zmp_ph97_D5
zmp_ph111_G5
zmp_ph111_G11
zmp_ph111_H1
zmp_ph111_B1a
zmp_ph111_B5
zmp_ph111_B8
zmp_ph111_B6
zmp_ph111_B7
zmp_ph111_B9
zmp_ph111_B10
zmp_ph114_G4
zmp_ph114_G11
zmp_ph114_H1
zmp_ph114_H2
zmp_ph114_A9
zmp_ph114_A11
zmp_ph114_B2
zmp_ph114_B4
zmp_ph114_D8
zmp_ph114_E2

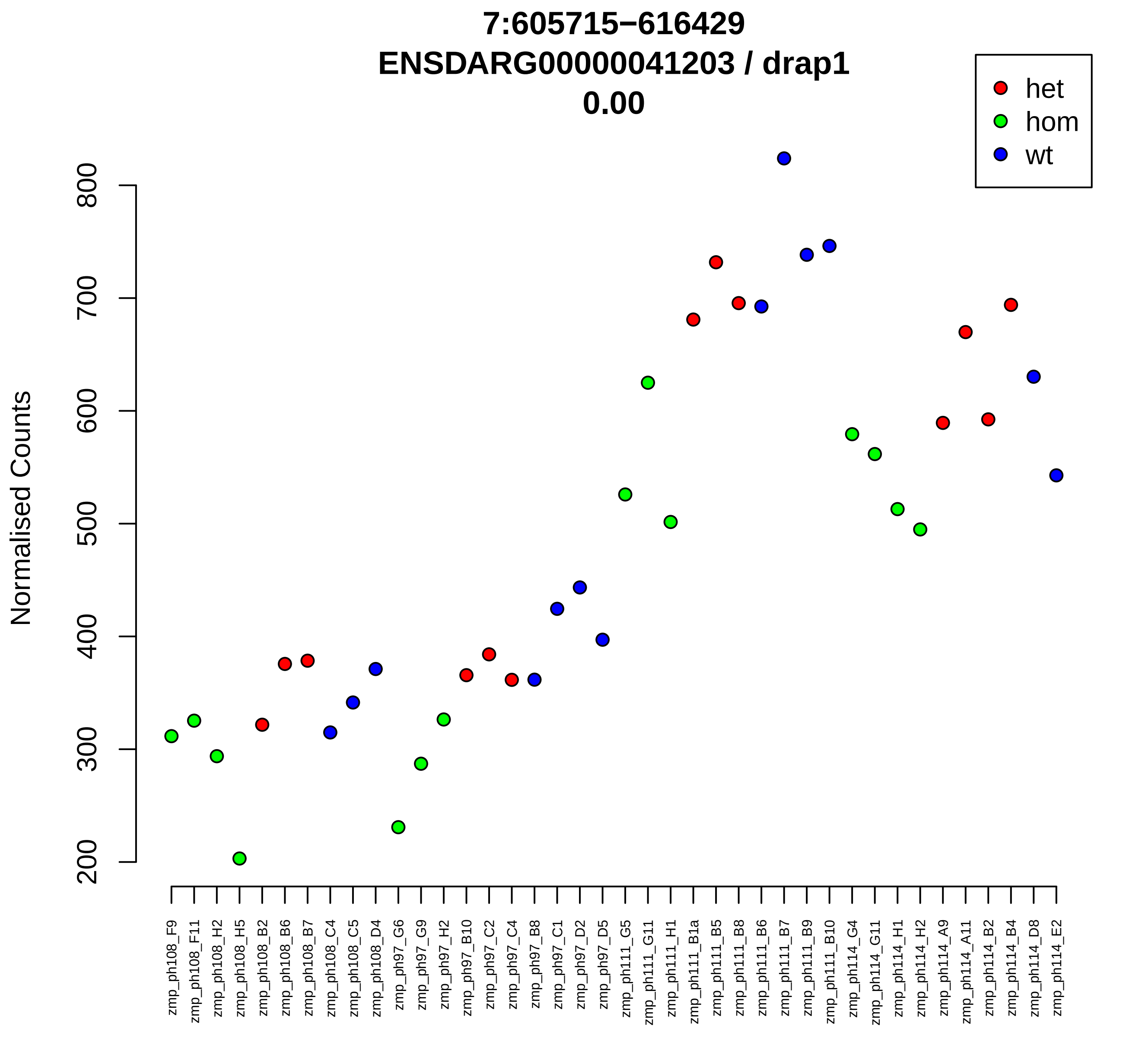
7:605715−616429
ENSDARG00000041203 / drap1
0.00
het
hom
wt
Normalised Counts
200
300
400
500
600
700
800
zmp_ph108_F9
zmp_ph108_F11
zmp_ph108_H2
zmp_ph108_H5
zmp_ph108_B2
zmp_ph108_B6
zmp_ph108_B7
zmp_ph108_C4
zmp_ph108_C5
zmp_ph108_D4
zmp_ph97_G6
zmp_ph97_G9
zmp_ph97_H2
zmp_ph97_B10
zmp_ph97_C2
zmp_ph97_C4
zmp_ph97_B8
zmp_ph97_C1
zmp_ph97_D2
zmp_ph97_D5
zmp_ph111_G5
zmp_ph111_G11
zmp_ph111_H1
zmp_ph111_B1a
zmp_ph111_B5
zmp_ph111_B8
zmp_ph111_B6
zmp_ph111_B7
zmp_ph111_B9
zmp_ph111_B10
zmp_ph114_G4
zmp_ph114_G11
zmp_ph114_H1
zmp_ph114_H2
zmp_ph114_A9
zmp_ph114_A11
zmp_ph114_B2
zmp_ph114_B4
zmp_ph114_D8
zmp_ph114_E2

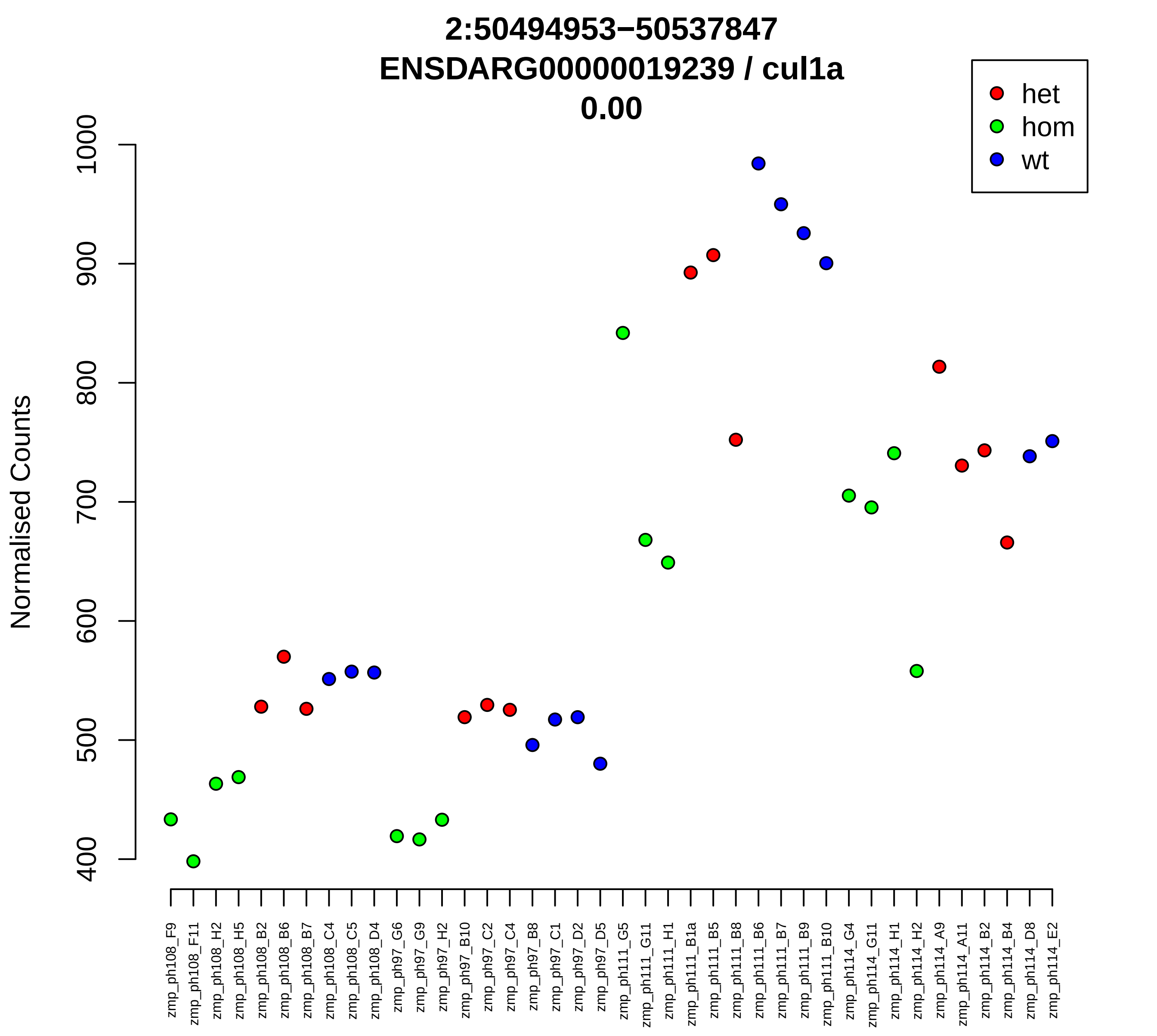

2:50494953−50537847
ENSDARG00000019239 / cul1a
0.00
het
hom
wt
Normalised Counts
1000
900
800
700
600
500
400
zmp_ph108_F9
zmp_ph108_F11
zmp_ph108_H2
zmp_ph108_H5
zmp_ph108_B2
zmp_ph108_B6
zmp_ph108_B7
zmp_ph108_C4
zmp_ph108_C5
zmp_ph108_D4
zmp_ph97_G6
zmp_ph97_G9
zmp_ph97_H2
zmp_ph97_B10
zmp_ph97_C2
zmp_ph97_C4
zmp_ph97_B8
zmp_ph97_C1
zmp_ph97_D2
zmp_ph97_D5
zmp_ph111_G5
zmp_ph111_G11
zmp_ph111_H1
zmp_ph111_B1a
zmp_ph111_B5
zmp_ph111_B8
zmp_ph111_B6
zmp_ph111_B7
zmp_ph111_B9
zmp_ph111_B10
zmp_ph114_G4
zmp_ph114_G11
zmp_ph114_H1
zmp_ph114_H2
zmp_ph114_A9
zmp_ph114_A11
zmp_ph114_B2
zmp_ph114_B4
zmp_ph114_D8
zmp_ph114_E2

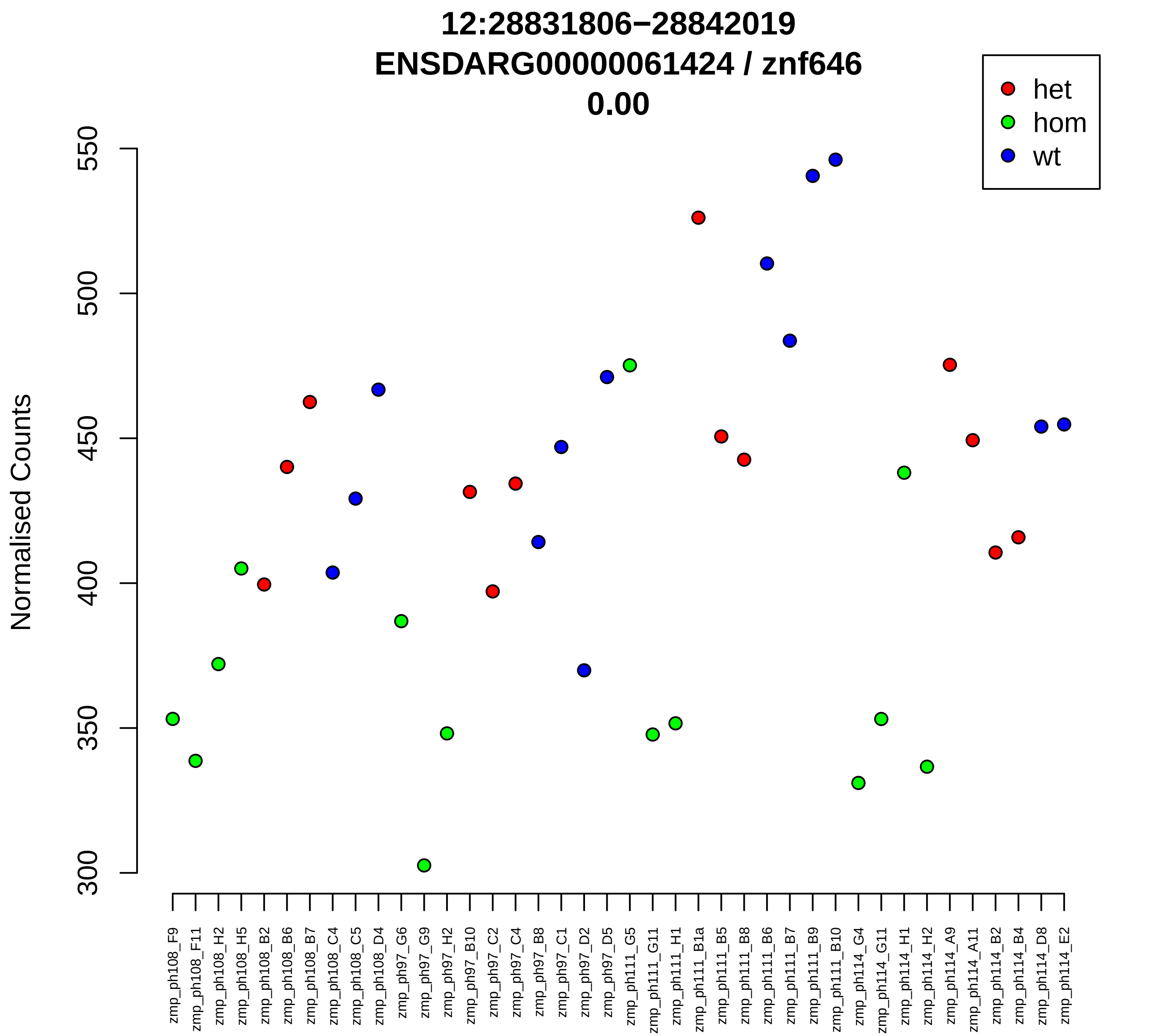
12:28831806−28842019
ENSDARG00000061424 / znf646
0.00
het
hom
wt
Normalised Counts
300
350
400
450
500
550
zmp_ph108_F9
zmp_ph108_F11
zmp_ph108_H2
zmp_ph108_H5
zmp_ph108_B2
zmp_ph108_B6
zmp_ph108_B7
zmp_ph108_C4
zmp_ph108_C5
zmp_ph108_D4
zmp_ph97_G6
zmp_ph97_G9
zmp_ph97_H2
zmp_ph97_B10
zmp_ph97_C2
zmp_ph97_C4
zmp_ph97_B8
zmp_ph97_C1
zmp_ph97_D2
zmp_ph97_D5
zmp_ph111_G5
zmp_ph111_G11
zmp_ph111_H1
zmp_ph111_B1a
zmp_ph111_B5
zmp_ph111_B8
zmp_ph111_B6
zmp_ph111_B7
zmp_ph111_B9
zmp_ph111_B10
zmp_ph114_G4
zmp_ph114_G11
zmp_ph114_H1
zmp_ph114_H2
zmp_ph114_A9
zmp_ph114_A11
zmp_ph114_B2
zmp_ph114_B4
zmp_ph114_D8
zmp_ph114_E2

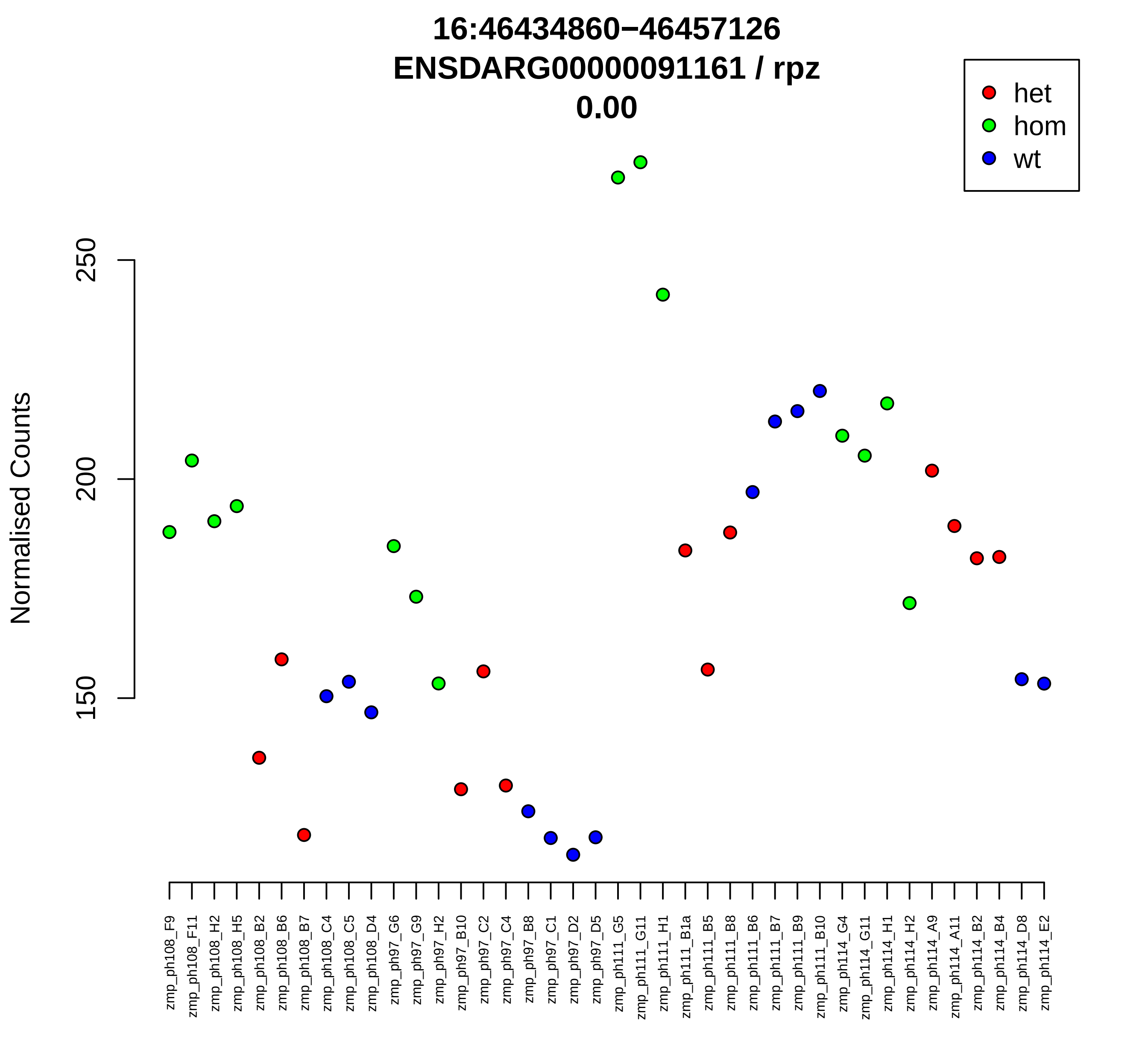

16:46434860−46457126
ENSDARG00000091161 / rpz
0.00
het
hom
wt
Normalised Counts
150
200
250
zmp_ph108_F9
zmp_ph108_F11
zmp_ph108_H2
zmp_ph108_H5
zmp_ph108_B2
zmp_ph108_B6
zmp_ph108_B7
zmp_ph108_C4
zmp_ph108_C5
zmp_ph108_D4
zmp_ph97_G6
zmp_ph97_G9
zmp_ph97_H2
zmp_ph97_B10
zmp_ph97_C2
zmp_ph97_C4
zmp_ph97_B8
zmp_ph97_C1
zmp_ph97_D2
zmp_ph97_D5
zmp_ph111_G5
zmp_ph111_G11
zmp_ph111_H1
zmp_ph111_B1a
zmp_ph111_B5
zmp_ph111_B8
zmp_ph111_B6
zmp_ph111_B7
zmp_ph111_B9
zmp_ph111_B10
zmp_ph114_G4
zmp_ph114_G11
zmp_ph114_H1
zmp_ph114_H2
zmp_ph114_A9
zmp_ph114_A11
zmp_ph114_B2
zmp_ph114_B4
zmp_ph114_D8
zmp_ph114_E2

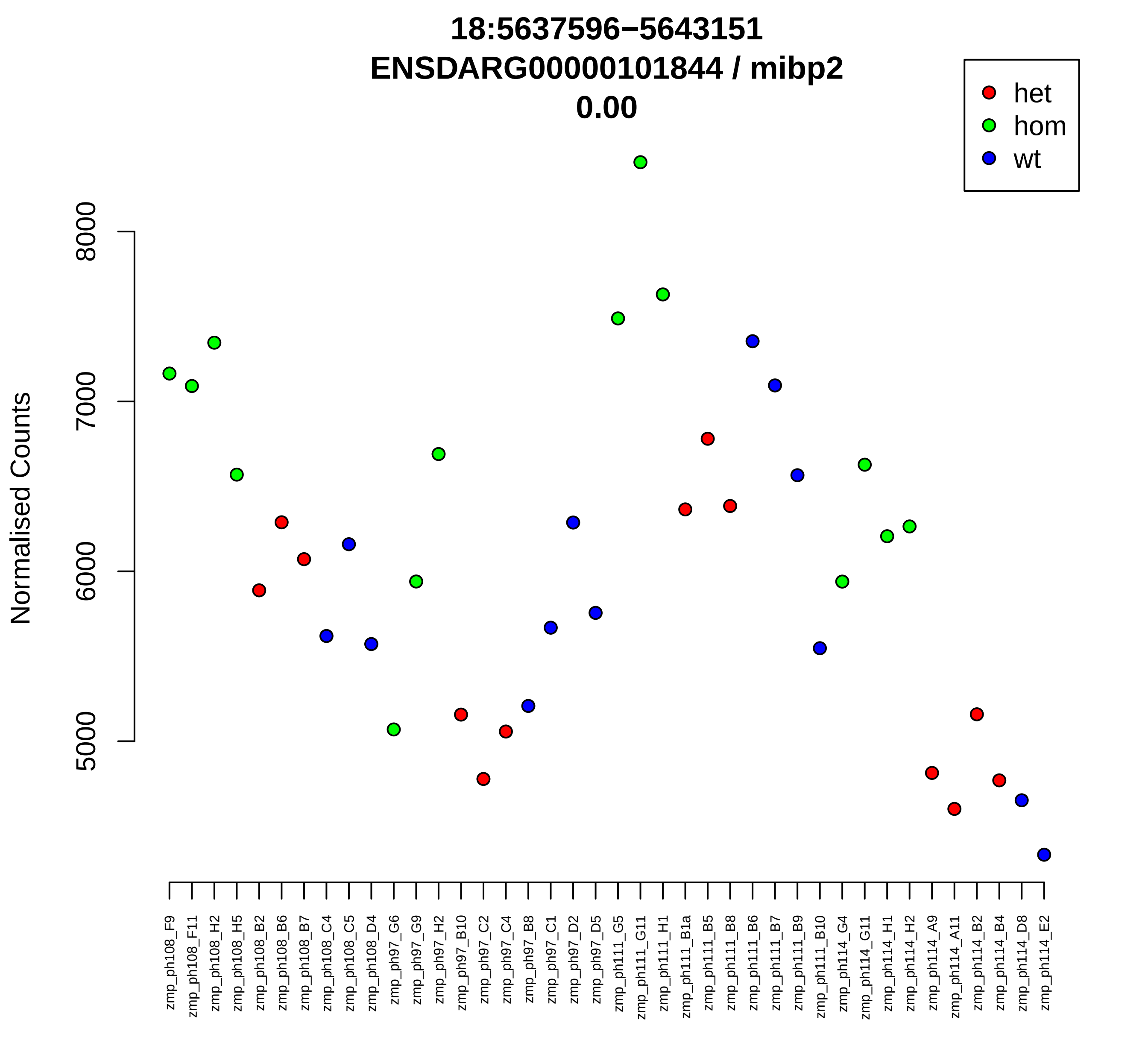
18:5637596−5643151
ENSDARG00000101844 / mibp2
0.00
het
hom
wt
Normalised Counts
5000
6000
7000
8000
zmp_ph108_F9
zmp_ph108_F11
zmp_ph108_H2
zmp_ph108_H5
zmp_ph108_B2
zmp_ph108_B6
zmp_ph108_B7
zmp_ph108_C4
zmp_ph108_C5
zmp_ph108_D4
zmp_ph97_G6
zmp_ph97_G9
zmp_ph97_H2
zmp_ph97_B10
zmp_ph97_C2
zmp_ph97_C4
zmp_ph97_B8
zmp_ph97_C1
zmp_ph97_D2
zmp_ph97_D5
zmp_ph111_G5
zmp_ph111_G11
zmp_ph111_H1
zmp_ph111_B1a
zmp_ph111_B5
zmp_ph111_B8
zmp_ph111_B6
zmp_ph111_B7
zmp_ph111_B9
zmp_ph111_B10
zmp_ph114_G4
zmp_ph114_G11
zmp_ph114_H1
zmp_ph114_H2
zmp_ph114_A9
zmp_ph114_A11
zmp_ph114_B2
zmp_ph114_B4
zmp_ph114_D8
zmp_ph114_E2

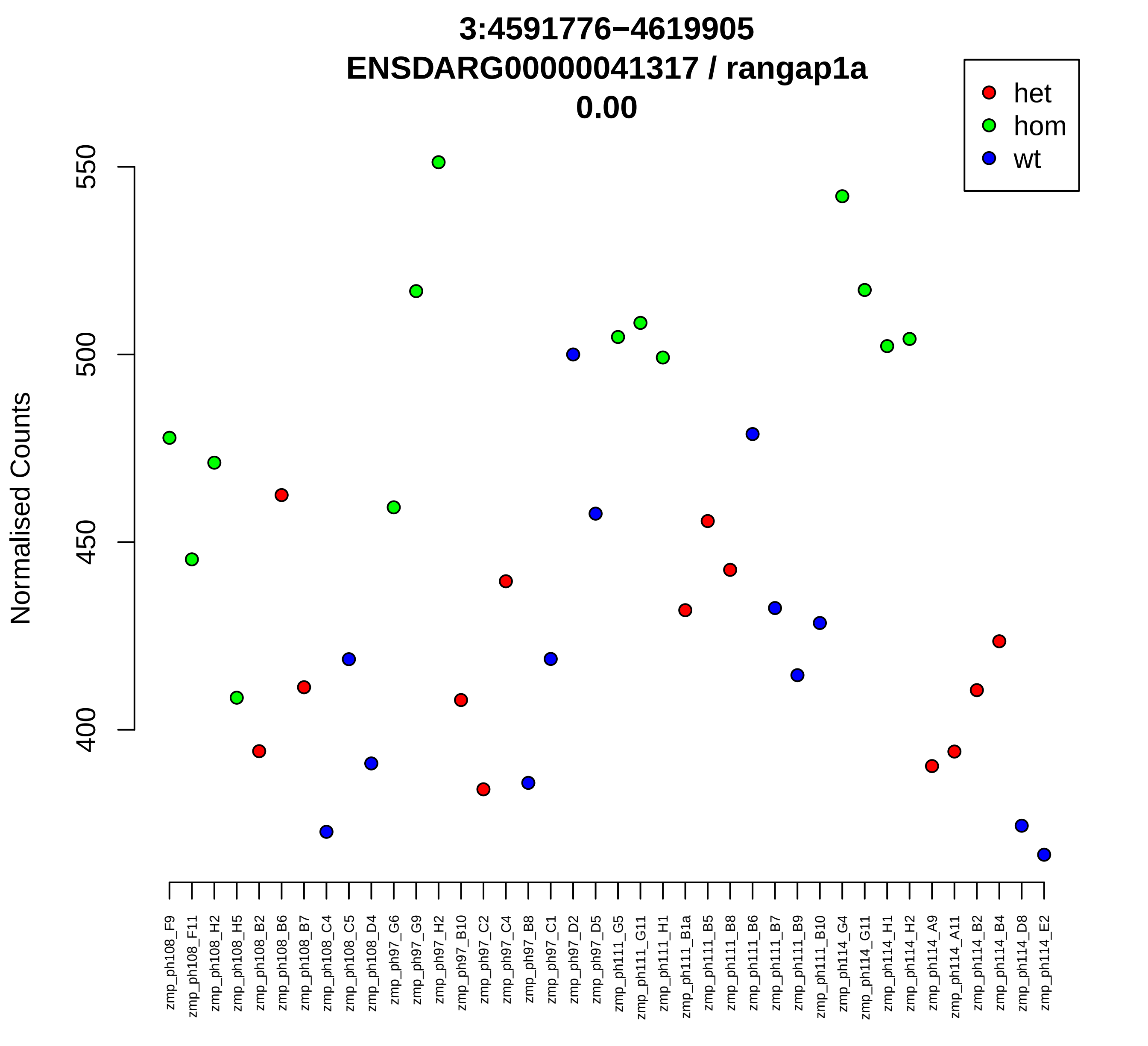
3:4591776−4619905
ENSDARG00000041317 / rangap1a
0.00
het
hom
wt
Normalised Counts
400
450
500
550
zmp_ph108_F9
zmp_ph108_F11
zmp_ph108_H2
zmp_ph108_H5
zmp_ph108_B2
zmp_ph108_B6
zmp_ph108_B7
zmp_ph108_C4
zmp_ph108_C5
zmp_ph108_D4
zmp_ph97_G6
zmp_ph97_G9
zmp_ph97_H2
zmp_ph97_B10
zmp_ph97_C2
zmp_ph97_C4
zmp_ph97_B8
zmp_ph97_C1
zmp_ph97_D2
zmp_ph97_D5
zmp_ph111_G5
zmp_ph111_G11
zmp_ph111_H1
zmp_ph111_B1a
zmp_ph111_B5
zmp_ph111_B8
zmp_ph111_B6
zmp_ph111_B7
zmp_ph111_B9
zmp_ph111_B10
zmp_ph114_G4
zmp_ph114_G11
zmp_ph114_H1
zmp_ph114_H2
zmp_ph114_A9
zmp_ph114_A11
zmp_ph114_B2
zmp_ph114_B4
zmp_ph114_D8
zmp_ph114_E2

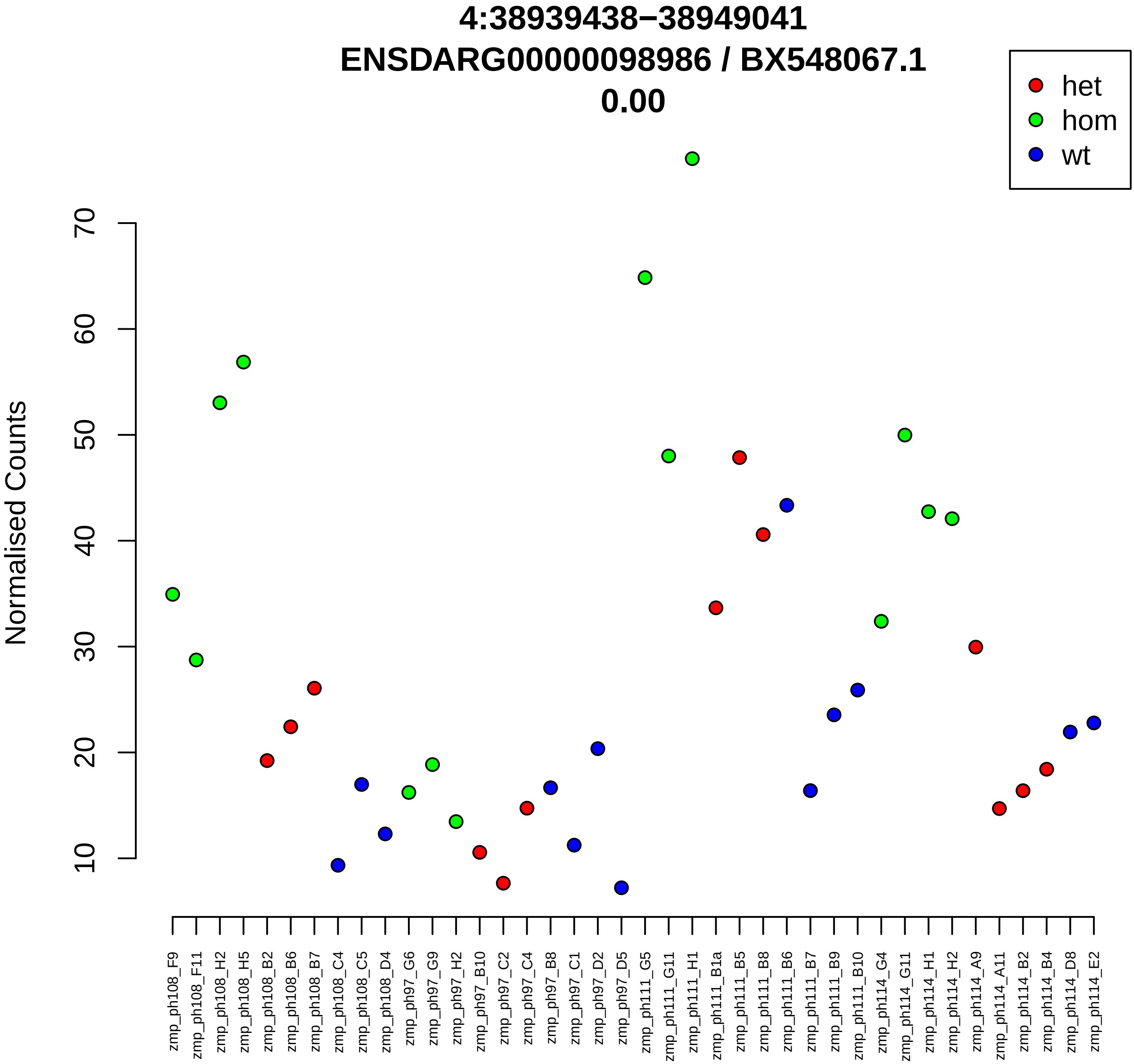
4:38939438−38949041
ENSDARG00000098986 / BX548067.1
0.00
het
hom
wt
Normalised Counts
10
20
30
40
50
60
70
zmp_ph108_F9
zmp_ph108_F11
zmp_ph108_H2
zmp_ph108_H5
zmp_ph108_B2
zmp_ph108_B6
zmp_ph108_B7
zmp_ph108_C4
zmp_ph108_C5
zmp_ph108_D4
zmp_ph97_G6
zmp_ph97_G9
zmp_ph97_H2
zmp_ph97_B10
zmp_ph97_C2
zmp_ph97_C4
zmp_ph97_B8
zmp_ph97_C1
zmp_ph97_D2
zmp_ph97_D5
zmp_ph111_G5
zmp_ph111_G11
zmp_ph111_H1
zmp_ph111_B1a
zmp_ph111_B5
zmp_ph111_B8
zmp_ph111_B6
zmp_ph111_B7
zmp_ph111_B9
zmp_ph111_B10
zmp_ph114_G4
zmp_ph114_G11
zmp_ph114_H1
zmp_ph114_H2
zmp_ph114_A9
zmp_ph114_A11
zmp_ph114_B2
zmp_ph114_B4
zmp_ph114_D8
zmp_ph114_E2

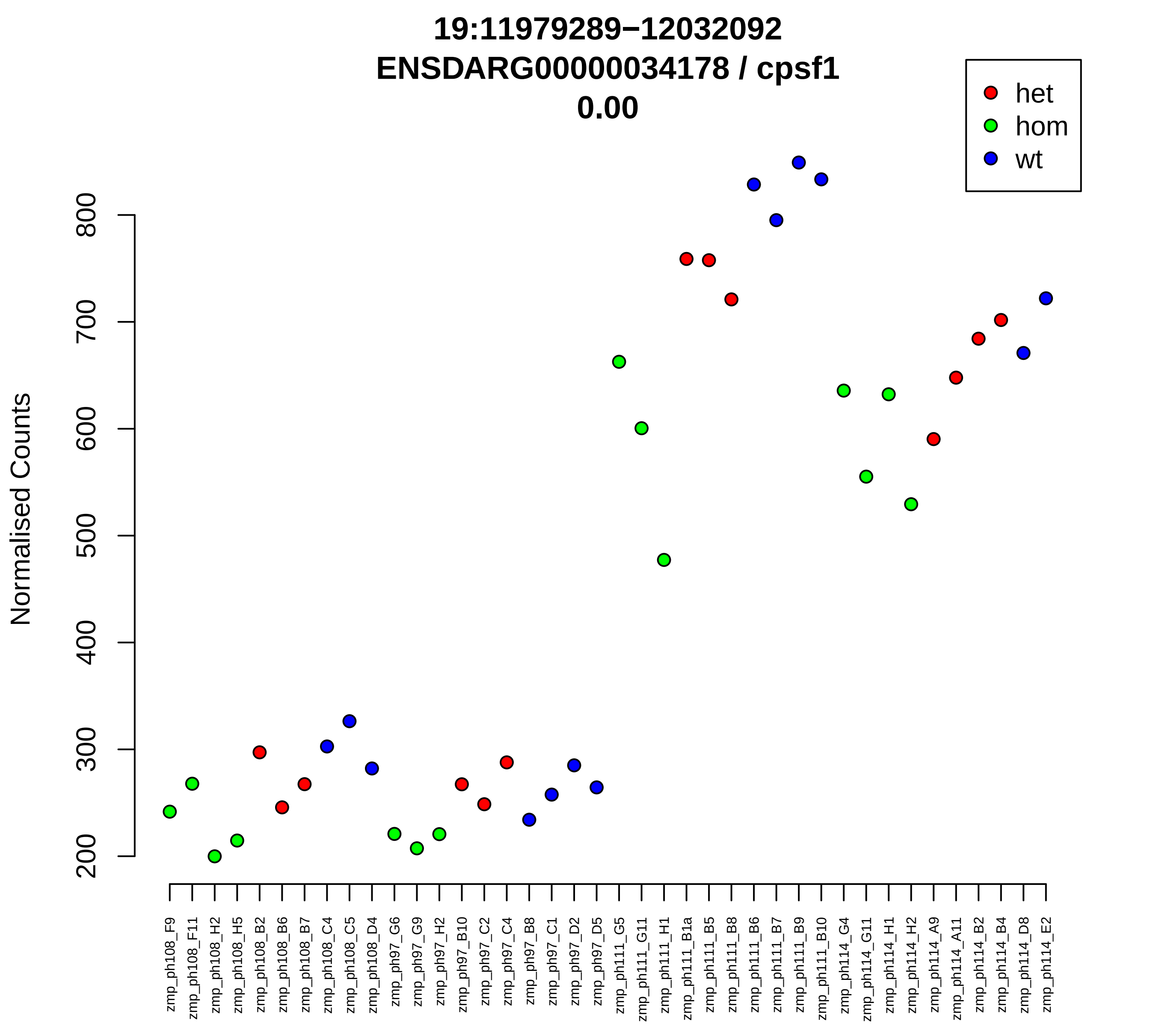

19:11979289−12032092
ENSDARG00000034178 / cpsf1
0.00
het
hom
wt
Normalised Counts
200
300
400
500
600
700
800
zmp_ph108_F9
zmp_ph108_F11
zmp_ph108_H2
zmp_ph108_H5
zmp_ph108_B2
zmp_ph108_B6
zmp_ph108_B7
zmp_ph108_C4
zmp_ph108_C5
zmp_ph108_D4
zmp_ph97_G6
zmp_ph97_G9
zmp_ph97_H2
zmp_ph97_B10
zmp_ph97_C2
zmp_ph97_C4
zmp_ph97_B8
zmp_ph97_C1
zmp_ph97_D2
zmp_ph97_D5
zmp_ph111_G5
zmp_ph111_G11
zmp_ph111_H1
zmp_ph111_B1a
zmp_ph111_B5
zmp_ph111_B8
zmp_ph111_B6
zmp_ph111_B7
zmp_ph111_B9
zmp_ph111_B10
zmp_ph114_G4
zmp_ph114_G11
zmp_ph114_H1
zmp_ph114_H2
zmp_ph114_A9
zmp_ph114_A11
zmp_ph114_B2
zmp_ph114_B4
zmp_ph114_D8
zmp_ph114_E2

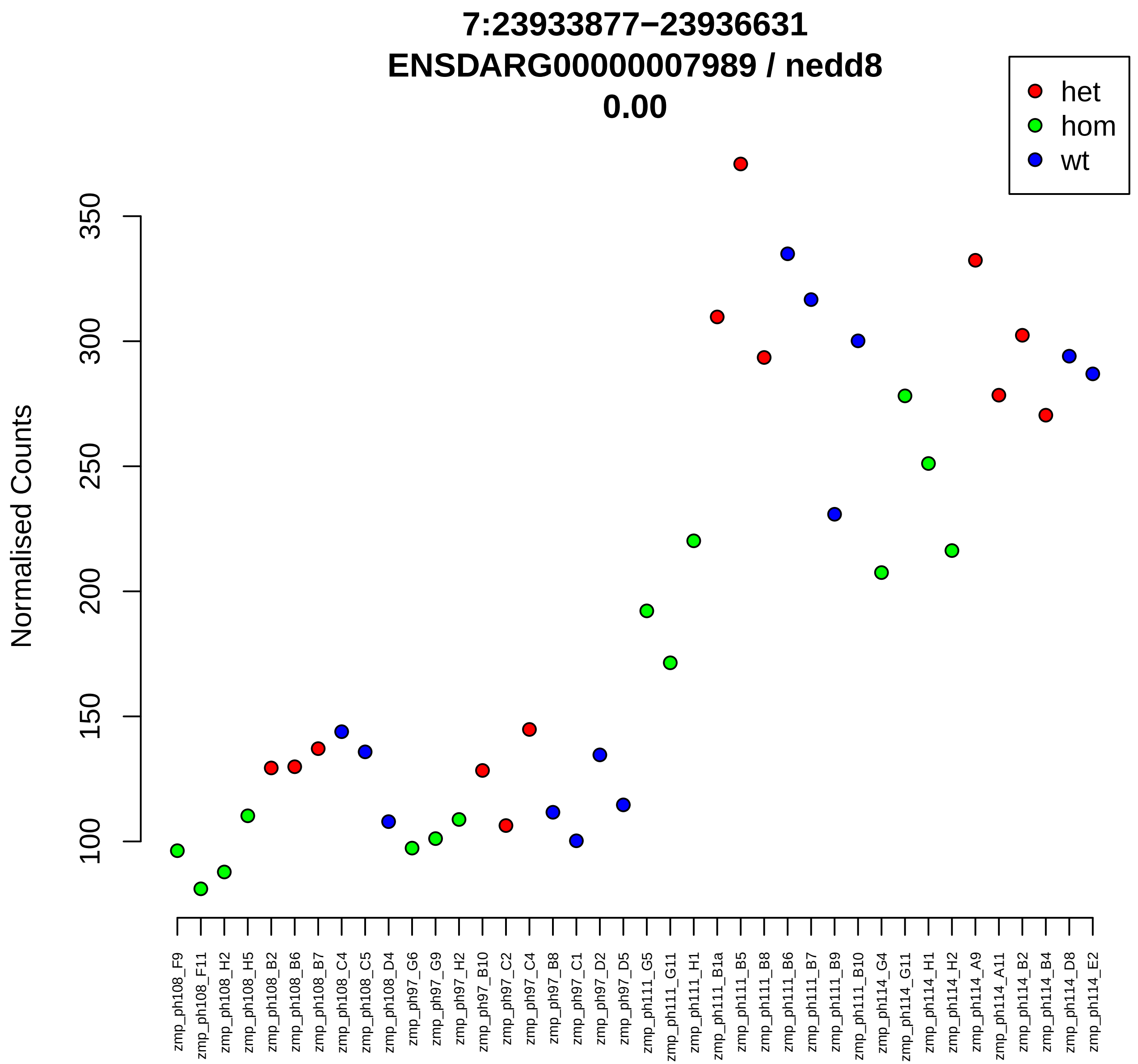

7:23933877−23936631
ENSDARG00000007989 / nedd8
0.00
het
hom
wt
Normalised Counts
100
150
200
250
300
350
zmp_ph108_F9
zmp_ph108_F11
zmp_ph108_H2
zmp_ph108_H5
zmp_ph108_B2
zmp_ph108_B6
zmp_ph108_B7
zmp_ph108_C4
zmp_ph108_C5
zmp_ph108_D4
zmp_ph97_G6
zmp_ph97_G9
zmp_ph97_H2
zmp_ph97_B10
zmp_ph97_C2
zmp_ph97_C4
zmp_ph97_B8
zmp_ph97_C1
zmp_ph97_D2
zmp_ph97_D5
zmp_ph111_G5
zmp_ph111_G11
zmp_ph111_H1
zmp_ph111_B1a
zmp_ph111_B5
zmp_ph111_B8
zmp_ph111_B6
zmp_ph111_B7
zmp_ph111_B9
zmp_ph111_B10
zmp_ph114_G4
zmp_ph114_G11
zmp_ph114_H1
zmp_ph114_H2
zmp_ph114_A9
zmp_ph114_A11
zmp_ph114_B2
zmp_ph114_B4
zmp_ph114_D8
zmp_ph114_E2

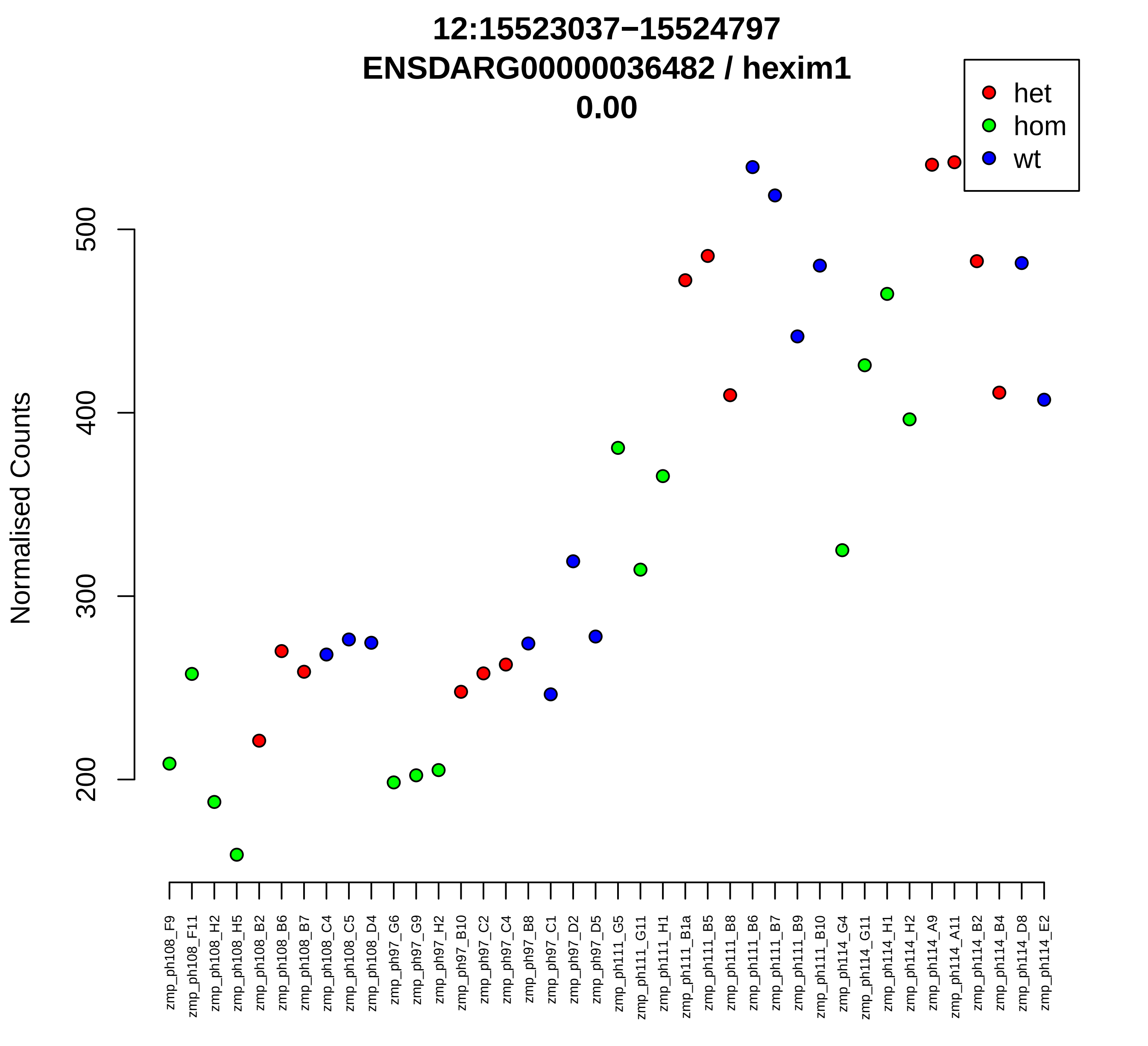
12:15523037−15524797
ENSDARG00000036482 / hexim1
0.00
het
hom
wt
Normalised Counts
200
300
400
500
zmp_ph108_F9
zmp_ph108_F11
zmp_ph108_H2
zmp_ph108_H5
zmp_ph108_B2
zmp_ph108_B6
zmp_ph108_B7
zmp_ph108_C4
zmp_ph108_C5
zmp_ph108_D4
zmp_ph97_G6
zmp_ph97_G9
zmp_ph97_H2
zmp_ph97_B10
zmp_ph97_C2
zmp_ph97_C4
zmp_ph97_B8
zmp_ph97_C1
zmp_ph97_D2
zmp_ph97_D5
zmp_ph111_G5
zmp_ph111_G11
zmp_ph111_H1
zmp_ph111_B1a
zmp_ph111_B5
zmp_ph111_B8
zmp_ph111_B6
zmp_ph111_B7
zmp_ph111_B9
zmp_ph111_B10
zmp_ph114_G4
zmp_ph114_G11
zmp_ph114_H1
zmp_ph114_H2
zmp_ph114_A9
zmp_ph114_A11
zmp_ph114_B2
zmp_ph114_B4
zmp_ph114_D8
zmp_ph114_E2

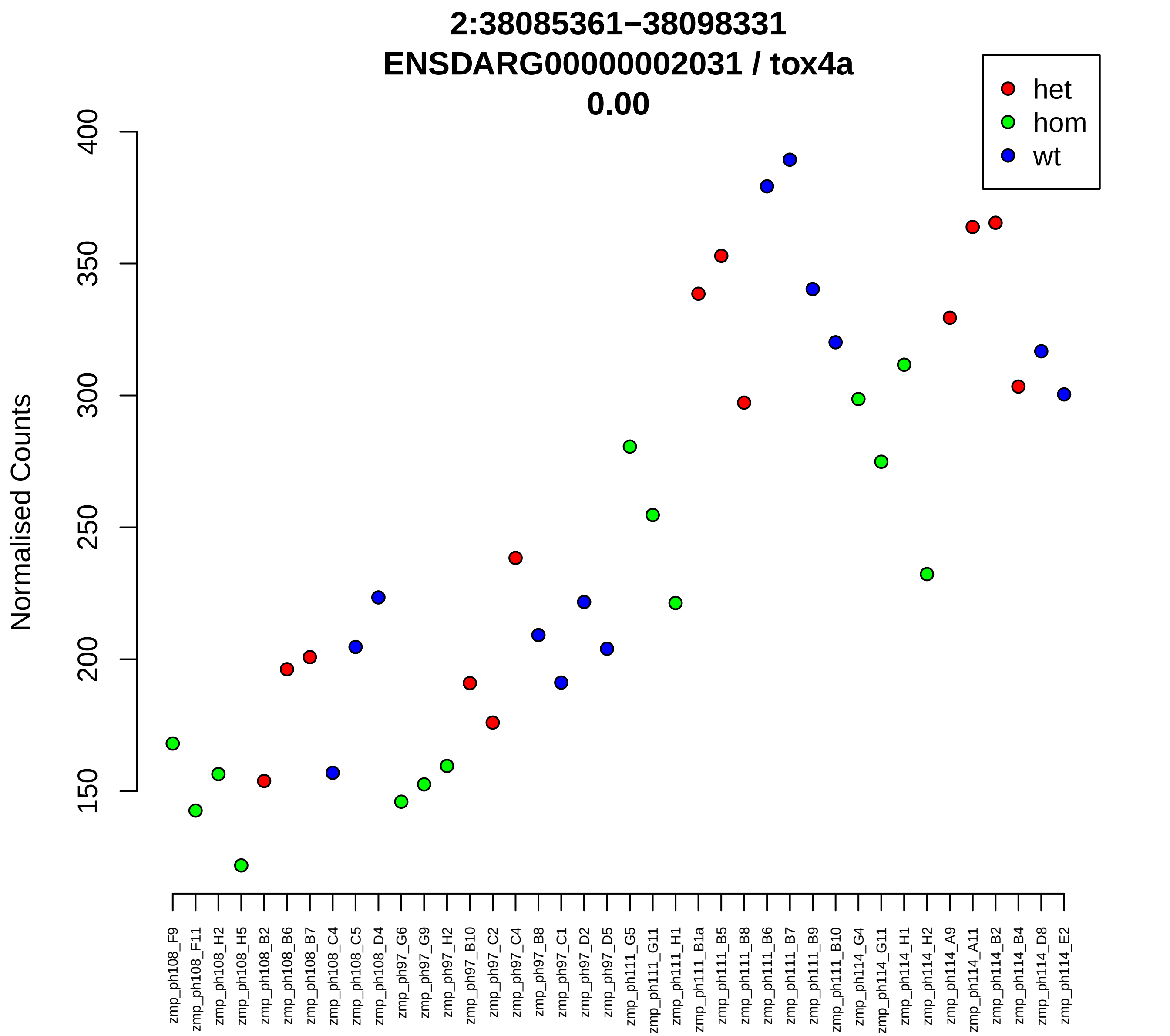
2:38085361−38098331
ENSDARG00000002031 / tox4a
0.00
het
hom
wt
Normalised Counts
150
200
250
300
350
400
zmp_ph108_F9
zmp_ph108_F11
zmp_ph108_H2
zmp_ph108_H5
zmp_ph108_B2
zmp_ph108_B6
zmp_ph108_B7
zmp_ph108_C4
zmp_ph108_C5
zmp_ph108_D4
zmp_ph97_G6
zmp_ph97_G9
zmp_ph97_H2
zmp_ph97_B10
zmp_ph97_C2
zmp_ph97_C4
zmp_ph97_B8
zmp_ph97_C1
zmp_ph97_D2
zmp_ph97_D5
zmp_ph111_G5
zmp_ph111_G11
zmp_ph111_H1
zmp_ph111_B1a
zmp_ph111_B5
zmp_ph111_B8
zmp_ph111_B6
zmp_ph111_B7
zmp_ph111_B9
zmp_ph111_B10
zmp_ph114_G4
zmp_ph114_G11
zmp_ph114_H1
zmp_ph114_H2
zmp_ph114_A9
zmp_ph114_A11
zmp_ph114_B2
zmp_ph114_B4
zmp_ph114_D8
zmp_ph114_E2

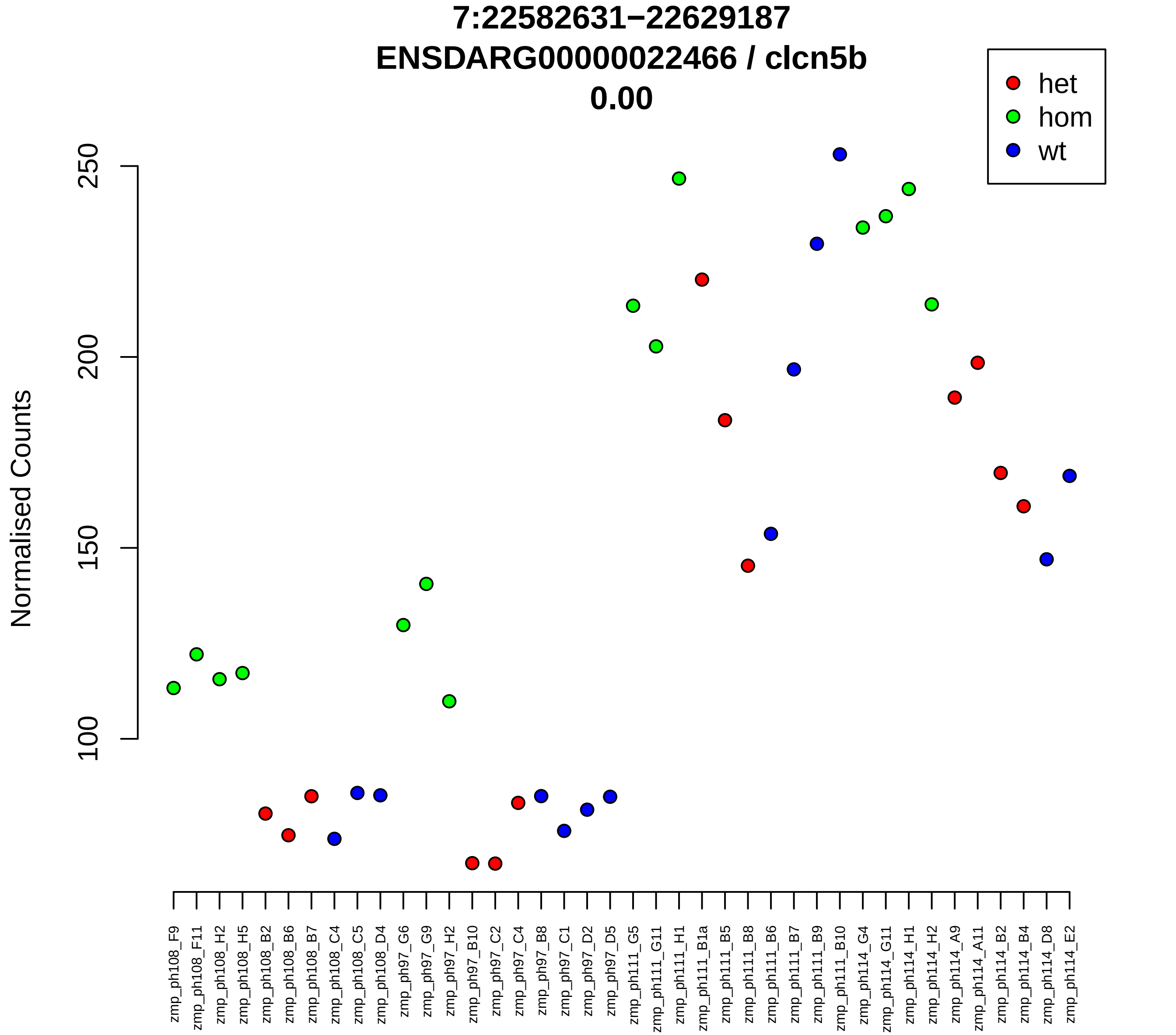
7:22582631−22629187
ENSDARG00000022466 / clcn5b
0.00
het
hom
wt
Normalised Counts
100
150
200
250
zmp_ph108_F9
zmp_ph108_F11
zmp_ph108_H2
zmp_ph108_H5
zmp_ph108_B2
zmp_ph108_B6
zmp_ph108_B7
zmp_ph108_C4
zmp_ph108_C5
zmp_ph108_D4
zmp_ph97_G6
zmp_ph97_G9
zmp_ph97_H2
zmp_ph97_B10
zmp_ph97_C2
zmp_ph97_C4
zmp_ph97_B8
zmp_ph97_C1
zmp_ph97_D2
zmp_ph97_D5
zmp_ph111_G5
zmp_ph111_G11
zmp_ph111_H1
zmp_ph111_B1a
zmp_ph111_B5
zmp_ph111_B8
zmp_ph111_B6
zmp_ph111_B7
zmp_ph111_B9
zmp_ph111_B10
zmp_ph114_G4
zmp_ph114_G11
zmp_ph114_H1
zmp_ph114_H2
zmp_ph114_A9
zmp_ph114_A11
zmp_ph114_B2
zmp_ph114_B4
zmp_ph114_D8
zmp_ph114_E2

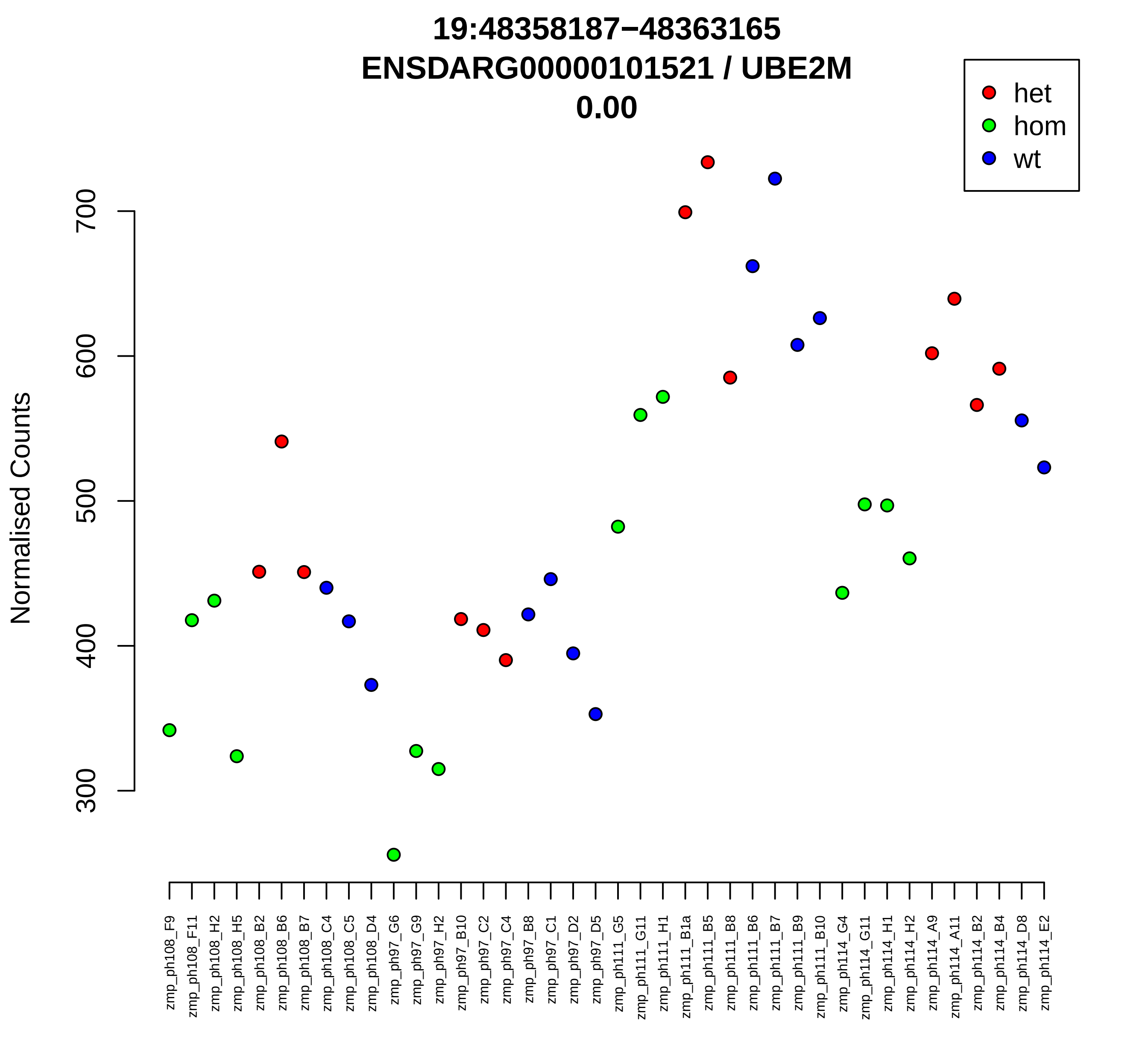
19:48358187−48363165
ENSDARG00000101521 / UBE2M
0.00
het
hom
wt
Normalised Counts
300
400
500
600
700
zmp_ph108_F9
zmp_ph108_F11
zmp_ph108_H2
zmp_ph108_H5
zmp_ph108_B2
zmp_ph108_B6
zmp_ph108_B7
zmp_ph108_C4
zmp_ph108_C5
zmp_ph108_D4
zmp_ph97_G6
zmp_ph97_G9
zmp_ph97_H2
zmp_ph97_B10
zmp_ph97_C2
zmp_ph97_C4
zmp_ph97_B8
zmp_ph97_C1
zmp_ph97_D2
zmp_ph97_D5
zmp_ph111_G5
zmp_ph111_G11
zmp_ph111_H1
zmp_ph111_B1a
zmp_ph111_B5
zmp_ph111_B8
zmp_ph111_B6
zmp_ph111_B7
zmp_ph111_B9
zmp_ph111_B10
zmp_ph114_G4
zmp_ph114_G11
zmp_ph114_H1
zmp_ph114_H2
zmp_ph114_A9
zmp_ph114_A11
zmp_ph114_B2
zmp_ph114_B4
zmp_ph114_D8
zmp_ph114_E2

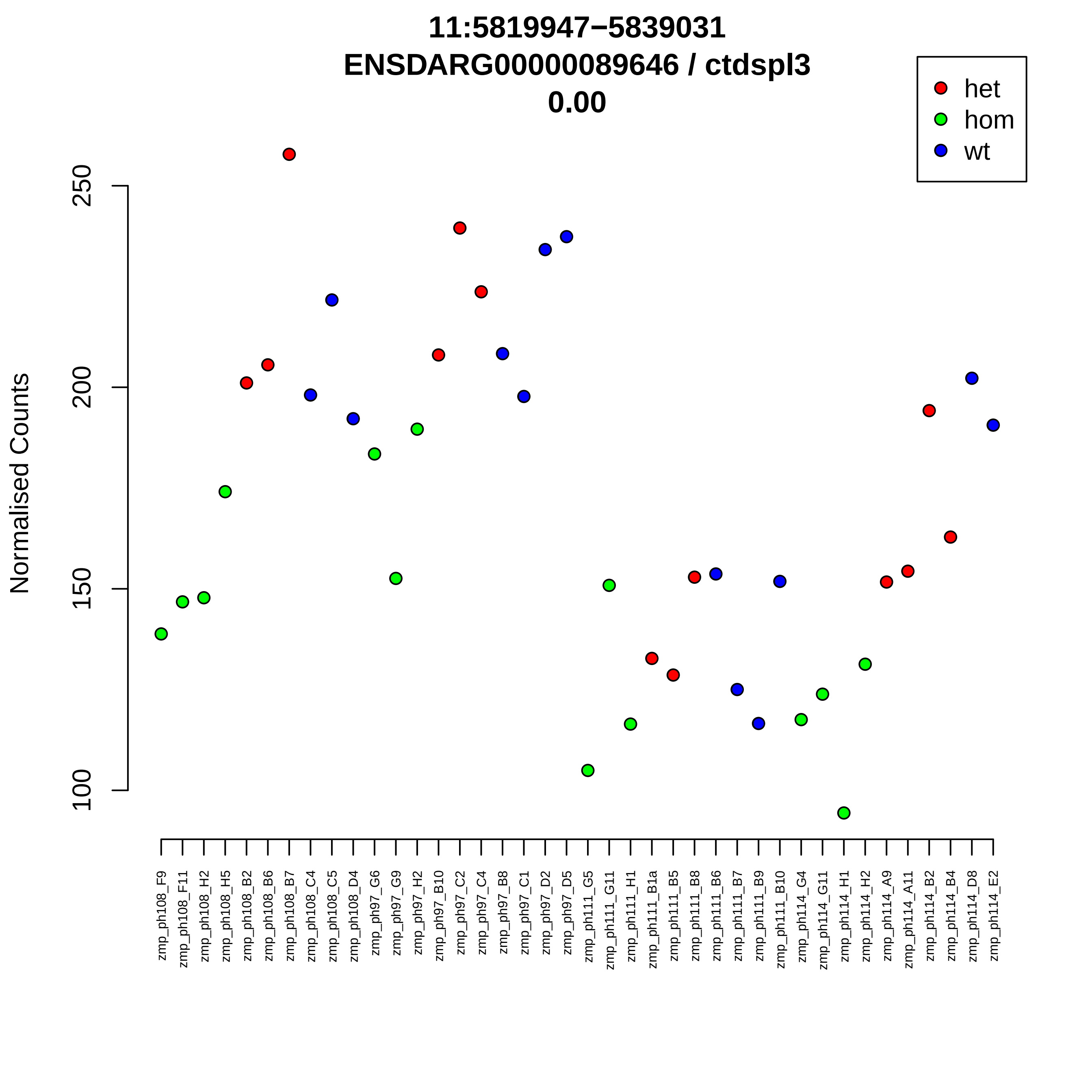
11:5819947−5839031
ENSDARG00000089646 / ctdspl3
0.00
het
hom
wt
Normalised Counts
100
150
200
250
zmp_ph108_F9
zmp_ph108_F11
zmp_ph108_H2
zmp_ph108_H5
zmp_ph108_B2
zmp_ph108_B6
zmp_ph108_B7
zmp_ph108_C4
zmp_ph108_C5
zmp_ph108_D4
zmp_ph97_G6
zmp_ph97_G9
zmp_ph97_H2
zmp_ph97_B10
zmp_ph97_C2
zmp_ph97_C4
zmp_ph97_B8
zmp_ph97_C1
zmp_ph97_D2
zmp_ph97_D5
zmp_ph111_G5
zmp_ph111_G11
zmp_ph111_H1
zmp_ph111_B1a
zmp_ph111_B5
zmp_ph111_B8
zmp_ph111_B6
zmp_ph111_B7
zmp_ph111_B9
zmp_ph111_B10
zmp_ph114_G4
zmp_ph114_G11
zmp_ph114_H1
zmp_ph114_H2
zmp_ph114_A9
zmp_ph114_A11
zmp_ph114_B2
zmp_ph114_B4
zmp_ph114_D8
zmp_ph114_E2

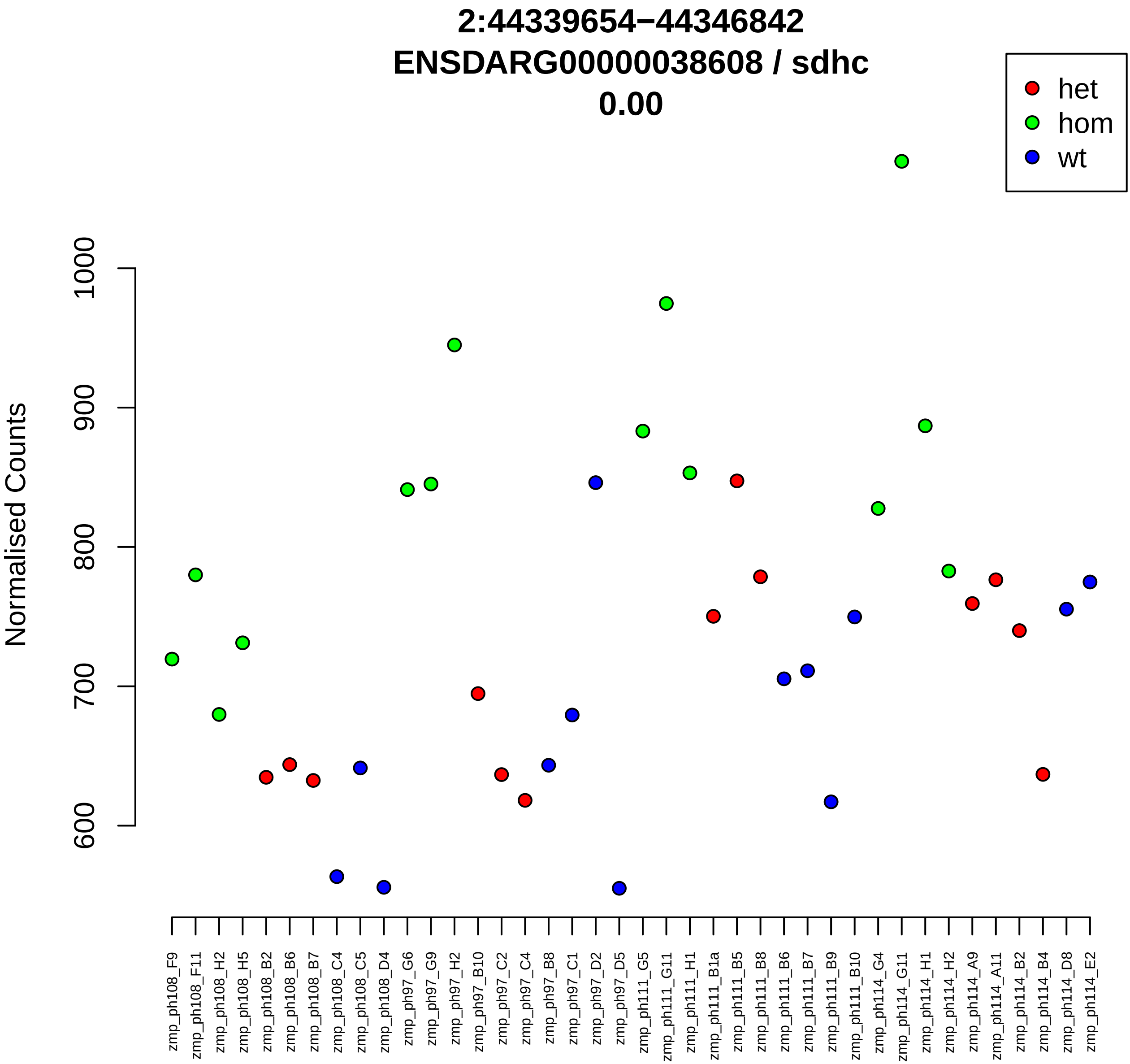

2:44339654−44346842
ENSDARG00000038608 / sdhc
0.00
het
hom
wt
Normalised Counts
600
700
800
900
1000
zmp_ph108_F9
zmp_ph108_F11
zmp_ph108_H2
zmp_ph108_H5
zmp_ph108_B2
zmp_ph108_B6
zmp_ph108_B7
zmp_ph108_C4
zmp_ph108_C5
zmp_ph108_D4
zmp_ph97_G6
zmp_ph97_G9
zmp_ph97_H2
zmp_ph97_B10
zmp_ph97_C2
zmp_ph97_C4
zmp_ph97_B8
zmp_ph97_C1
zmp_ph97_D2
zmp_ph97_D5
zmp_ph111_G5
zmp_ph111_G11
zmp_ph111_H1
zmp_ph111_B1a
zmp_ph111_B5
zmp_ph111_B8
zmp_ph111_B6
zmp_ph111_B7
zmp_ph111_B9
zmp_ph111_B10
zmp_ph114_G4
zmp_ph114_G11
zmp_ph114_H1
zmp_ph114_H2
zmp_ph114_A9
zmp_ph114_A11
zmp_ph114_B2
zmp_ph114_B4
zmp_ph114_D8
zmp_ph114_E2

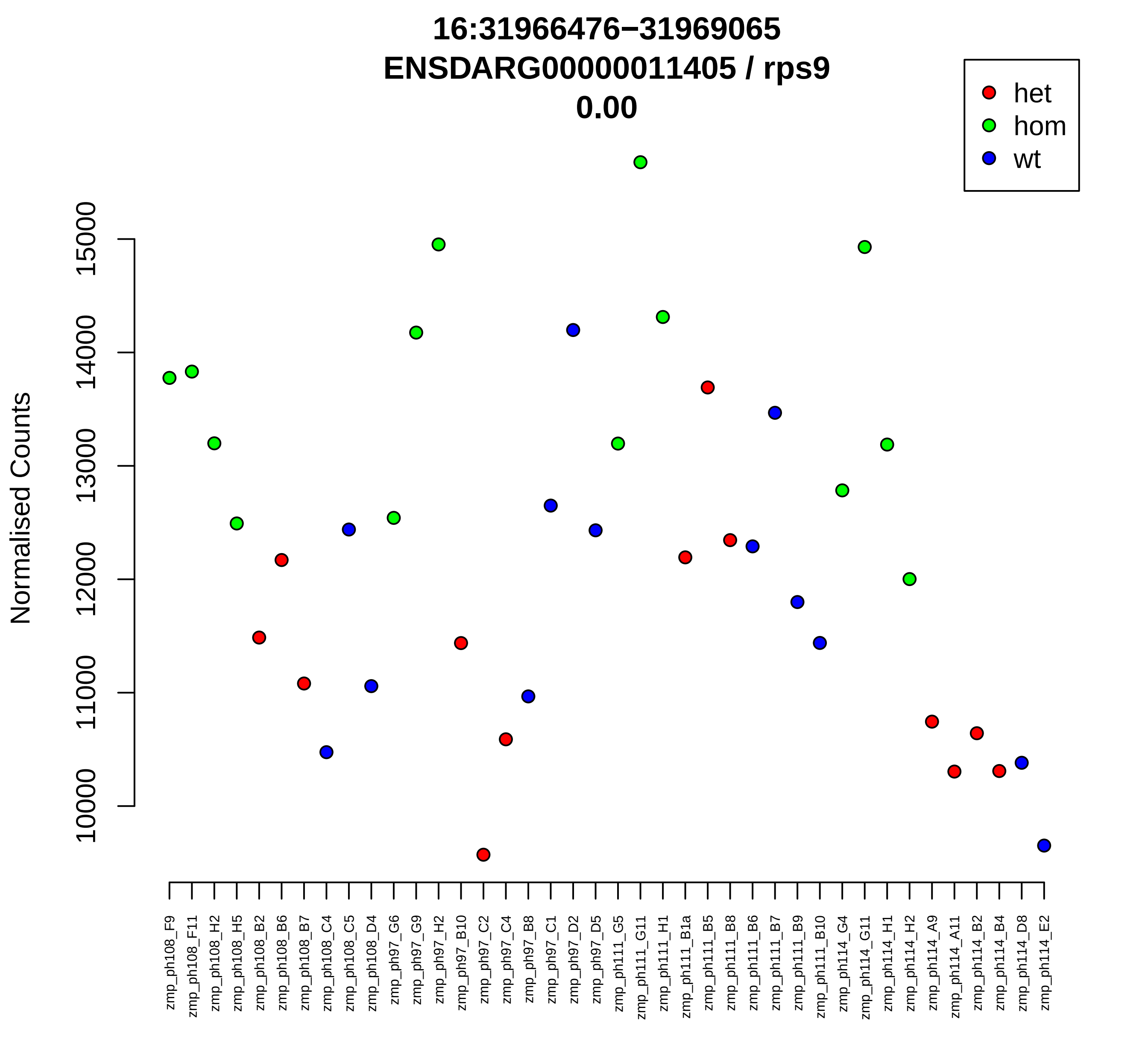
16:31966476−31969065
ENSDARG00000011405 / rps9
0.00
het
hom
wt
Normalised Counts
10000
11000
12000
13000
14000
15000
zmp_ph108_F9
zmp_ph108_F11
zmp_ph108_H2
zmp_ph108_H5
zmp_ph108_B2
zmp_ph108_B6
zmp_ph108_B7
zmp_ph108_C4
zmp_ph108_C5
zmp_ph108_D4
zmp_ph97_G6
zmp_ph97_G9
zmp_ph97_H2
zmp_ph97_B10
zmp_ph97_C2
zmp_ph97_C4
zmp_ph97_B8
zmp_ph97_C1
zmp_ph97_D2
zmp_ph97_D5
zmp_ph111_G5
zmp_ph111_G11
zmp_ph111_H1
zmp_ph111_B1a
zmp_ph111_B5
zmp_ph111_B8
zmp_ph111_B6
zmp_ph111_B7
zmp_ph111_B9
zmp_ph111_B10
zmp_ph114_G4
zmp_ph114_G11
zmp_ph114_H1
zmp_ph114_H2
zmp_ph114_A9
zmp_ph114_A11
zmp_ph114_B2
zmp_ph114_B4
zmp_ph114_D8
zmp_ph114_E2

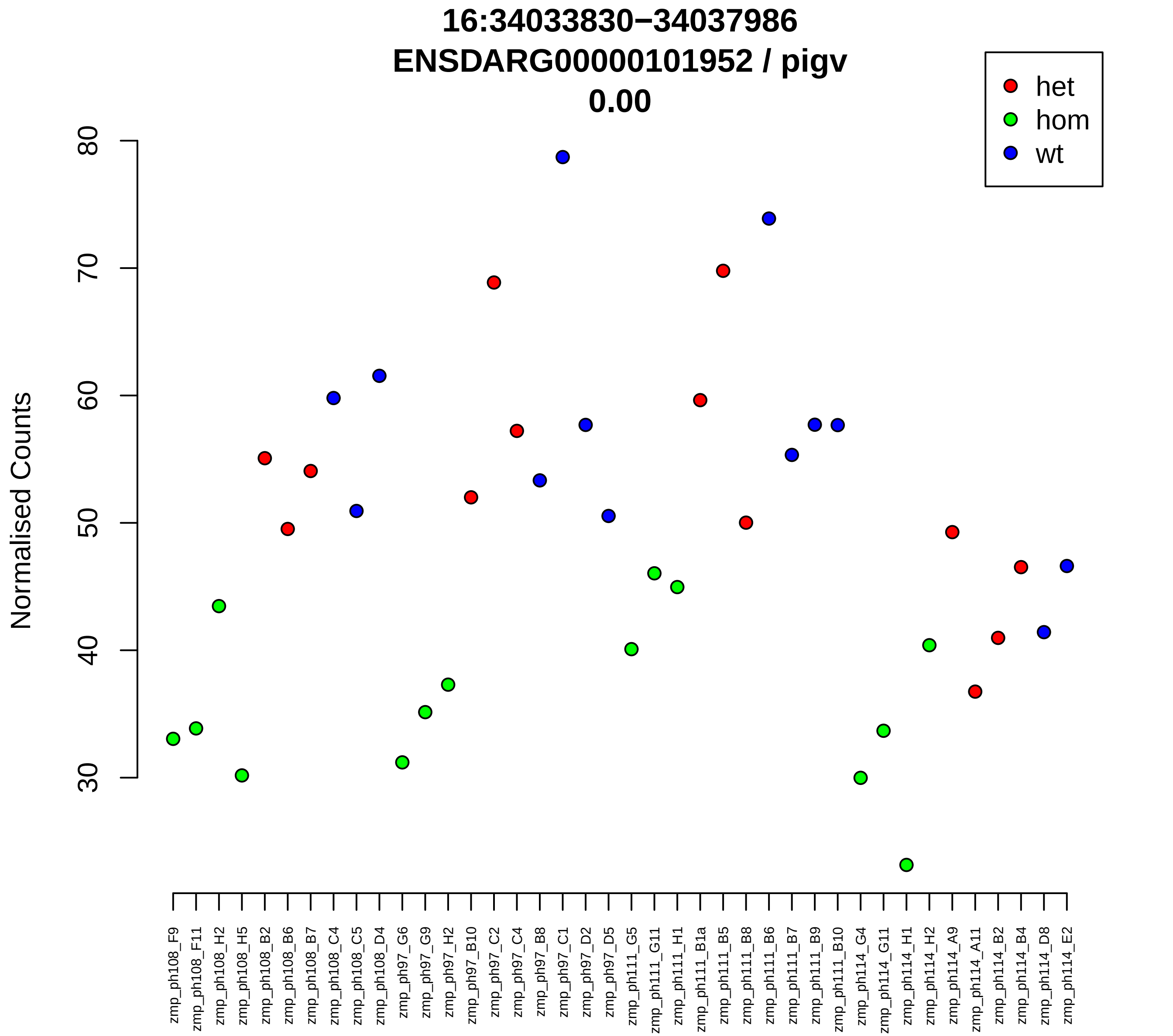
16:34033830−34037986
ENSDARG00000101952 / pigv
0.00
het
hom
wt
Normalised Counts
30
40
50
60
70
80
zmp_ph108_F9
zmp_ph108_F11
zmp_ph108_H2
zmp_ph108_H5
zmp_ph108_B2
zmp_ph108_B6
zmp_ph108_B7
zmp_ph108_C4
zmp_ph108_C5
zmp_ph108_D4
zmp_ph97_G6
zmp_ph97_G9
zmp_ph97_H2
zmp_ph97_B10
zmp_ph97_C2
zmp_ph97_C4
zmp_ph97_B8
zmp_ph97_C1
zmp_ph97_D2
zmp_ph97_D5
zmp_ph111_G5
zmp_ph111_G11
zmp_ph111_H1
zmp_ph111_B1a
zmp_ph111_B5
zmp_ph111_B8
zmp_ph111_B6
zmp_ph111_B7
zmp_ph111_B9
zmp_ph111_B10
zmp_ph114_G4
zmp_ph114_G11
zmp_ph114_H1
zmp_ph114_H2
zmp_ph114_A9
zmp_ph114_A11
zmp_ph114_B2
zmp_ph114_B4
zmp_ph114_D8
zmp_ph114_E2

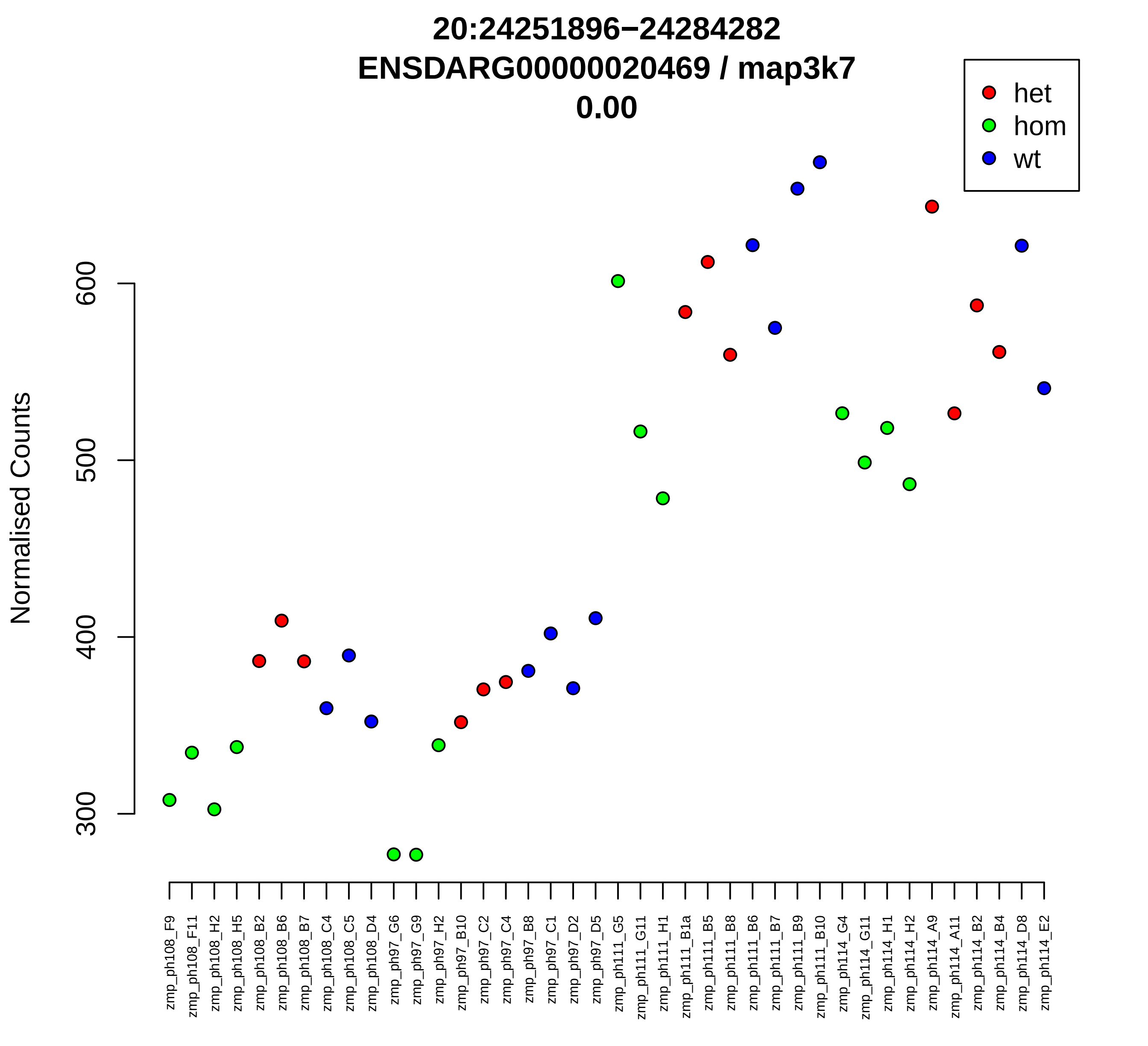
20:24251896−24284282
ENSDARG00000020469 / map3k7
0.00
het
hom
wt
Normalised Counts
300
400
500
600
zmp_ph108_F9
zmp_ph108_F11
zmp_ph108_H2
zmp_ph108_H5
zmp_ph108_B2
zmp_ph108_B6
zmp_ph108_B7
zmp_ph108_C4
zmp_ph108_C5
zmp_ph108_D4
zmp_ph97_G6
zmp_ph97_G9
zmp_ph97_H2
zmp_ph97_B10
zmp_ph97_C2
zmp_ph97_C4
zmp_ph97_B8
zmp_ph97_C1
zmp_ph97_D2
zmp_ph97_D5
zmp_ph111_G5
zmp_ph111_G11
zmp_ph111_H1
zmp_ph111_B1a
zmp_ph111_B5
zmp_ph111_B8
zmp_ph111_B6
zmp_ph111_B7
zmp_ph111_B9
zmp_ph111_B10
zmp_ph114_G4
zmp_ph114_G11
zmp_ph114_H1
zmp_ph114_H2
zmp_ph114_A9
zmp_ph114_A11
zmp_ph114_B2
zmp_ph114_B4
zmp_ph114_D8
zmp_ph114_E2

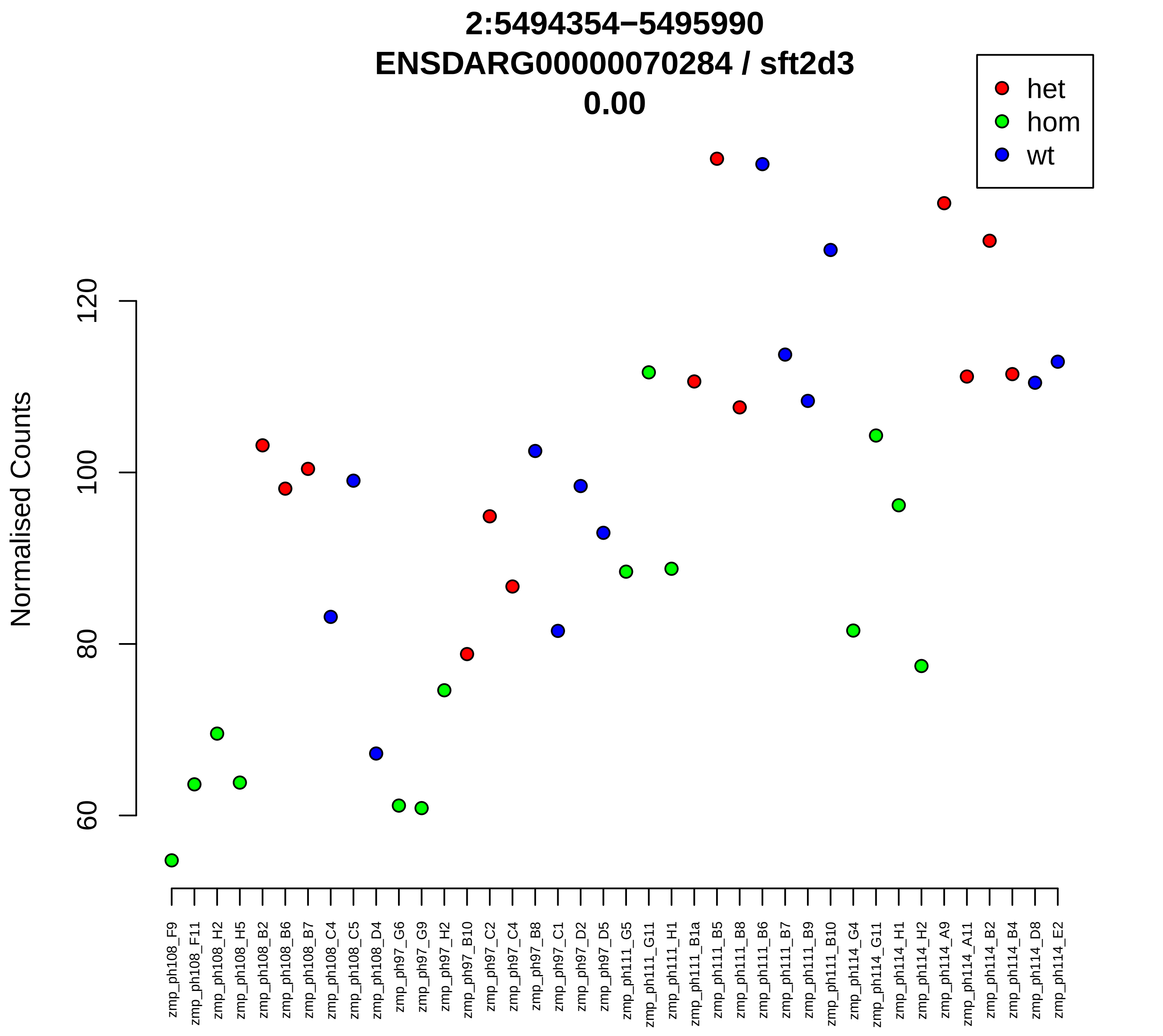

2:5494354−5495990
ENSDARG00000070284 / sft2d3
0.00
het
hom
wt
Normalised Counts
60
80
100
120
zmp_ph108_F9
zmp_ph108_F11
zmp_ph108_H2
zmp_ph108_H5
zmp_ph108_B2
zmp_ph108_B6
zmp_ph108_B7
zmp_ph108_C4
zmp_ph108_C5
zmp_ph108_D4
zmp_ph97_G6
zmp_ph97_G9
zmp_ph97_H2
zmp_ph97_B10
zmp_ph97_C2
zmp_ph97_C4
zmp_ph97_B8
zmp_ph97_C1
zmp_ph97_D2
zmp_ph97_D5
zmp_ph111_G5
zmp_ph111_G11
zmp_ph111_H1
zmp_ph111_B1a
zmp_ph111_B5
zmp_ph111_B8
zmp_ph111_B6
zmp_ph111_B7
zmp_ph111_B9
zmp_ph111_B10
zmp_ph114_G4
zmp_ph114_G11
zmp_ph114_H1
zmp_ph114_H2
zmp_ph114_A9
zmp_ph114_A11
zmp_ph114_B2
zmp_ph114_B4
zmp_ph114_D8
zmp_ph114_E2

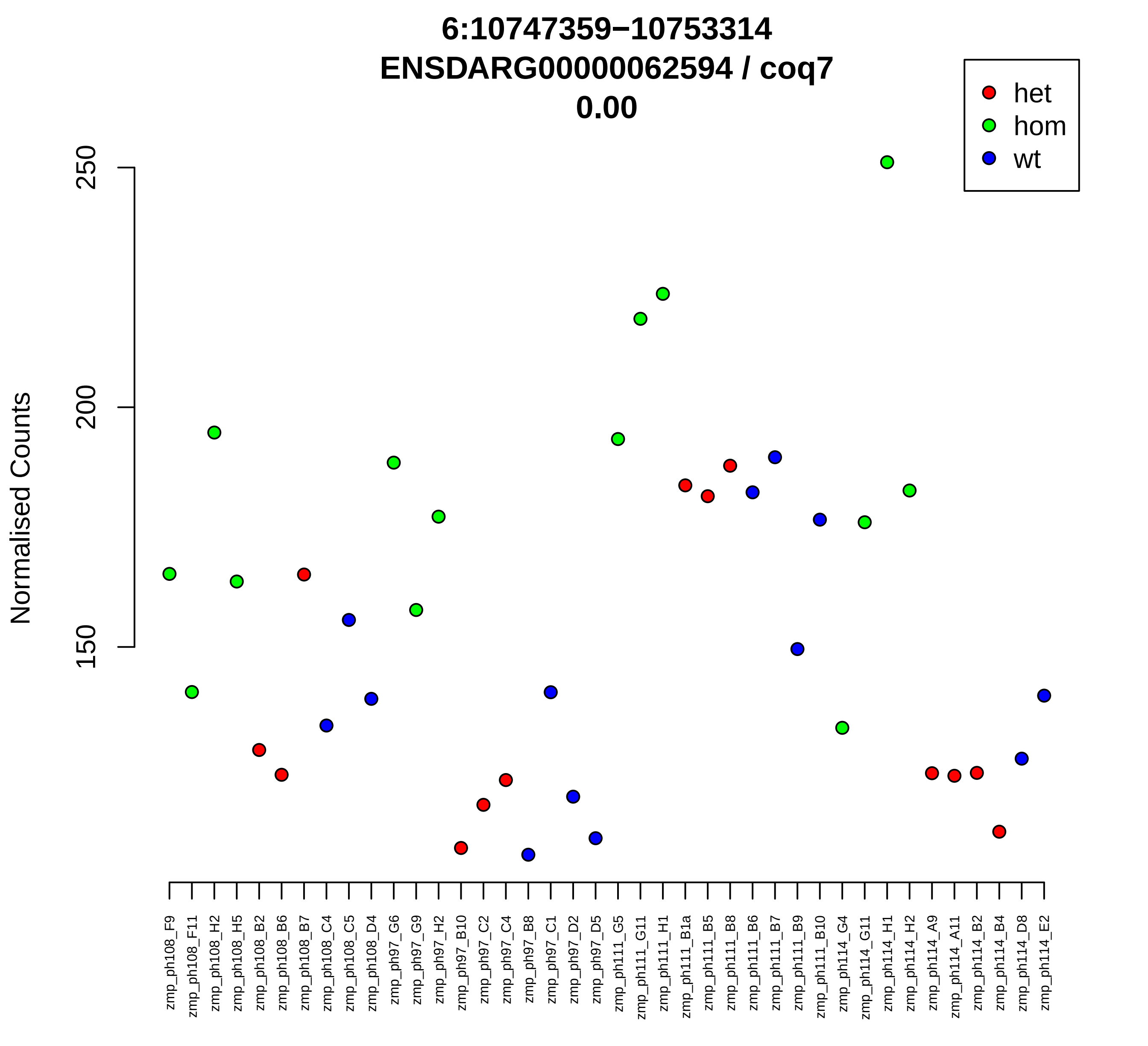
6:10747359−10753314
ENSDARG00000062594 / coq7
0.00
het
hom
wt
Normalised Counts
250
200
150
zmp_ph108_F9
zmp_ph108_F11
zmp_ph108_H2
zmp_ph108_H5
zmp_ph108_B2
zmp_ph108_B6
zmp_ph108_B7
zmp_ph108_C4
zmp_ph108_C5
zmp_ph108_D4
zmp_ph97_G6
zmp_ph97_G9
zmp_ph97_H2
zmp_ph97_B10
zmp_ph97_C2
zmp_ph97_C4
zmp_ph97_B8
zmp_ph97_C1
zmp_ph97_D2
zmp_ph97_D5
zmp_ph111_G5
zmp_ph111_G11
zmp_ph111_H1
zmp_ph111_B1a
zmp_ph111_B5
zmp_ph111_B8
zmp_ph111_B6
zmp_ph111_B7
zmp_ph111_B9
zmp_ph111_B10
zmp_ph114_G4
zmp_ph114_G11
zmp_ph114_H1
zmp_ph114_H2
zmp_ph114_A9
zmp_ph114_A11
zmp_ph114_B2
zmp_ph114_B4
zmp_ph114_D8
zmp_ph114_E2

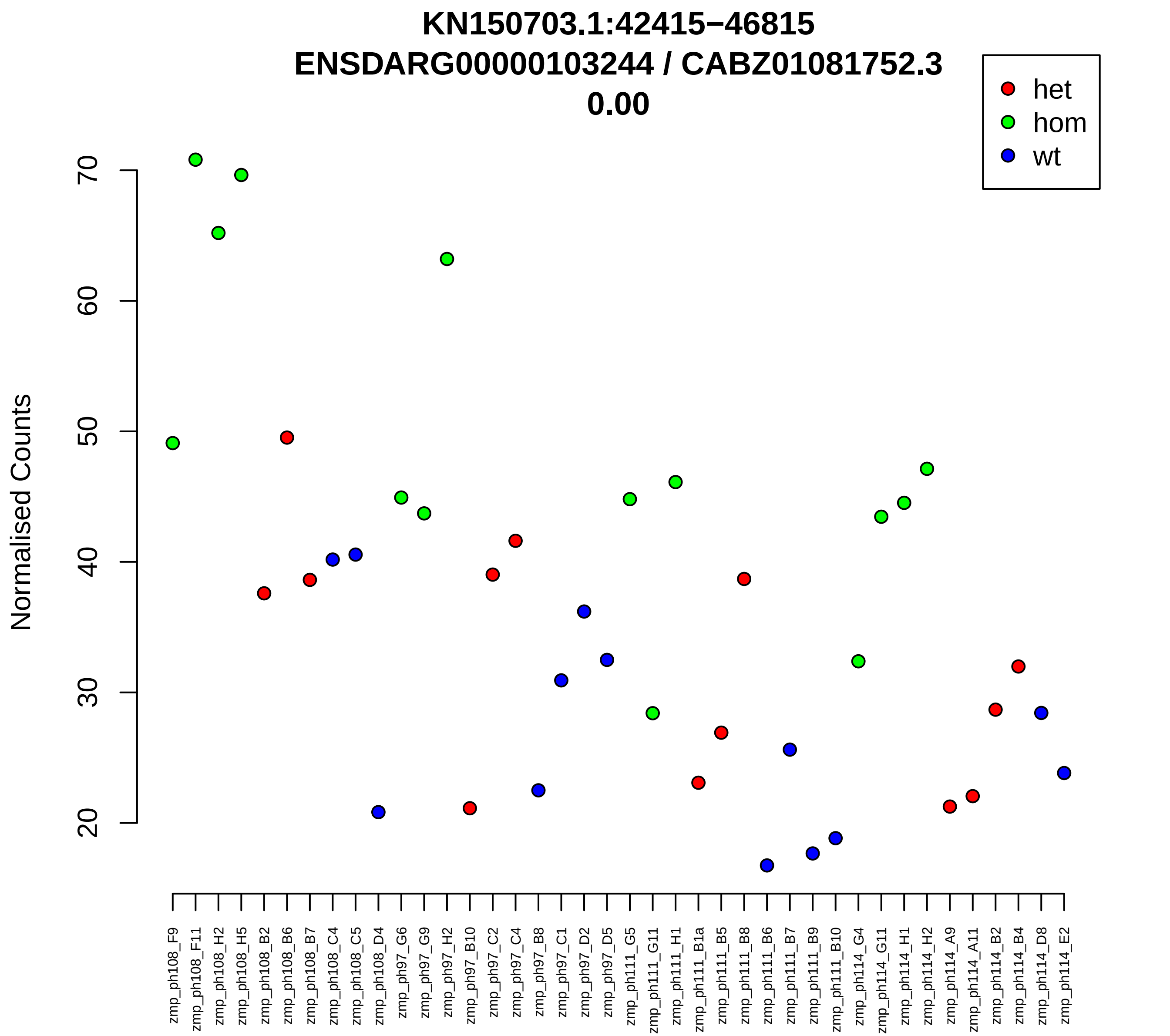
KN150703.1:42415−46815
ENSDARG00000103244 / CABZ01081752.3
0.00
het
hom
wt
Normalised Counts
20
30
40
50
60
70
zmp_ph108_F9
zmp_ph108_F11
zmp_ph108_H2
zmp_ph108_H5
zmp_ph108_B2
zmp_ph108_B6
zmp_ph108_B7
zmp_ph108_C4
zmp_ph108_C5
zmp_ph108_D4
zmp_ph97_G6
zmp_ph97_G9
zmp_ph97_H2
zmp_ph97_B10
zmp_ph97_C2
zmp_ph97_C4
zmp_ph97_B8
zmp_ph97_C1
zmp_ph97_D2
zmp_ph97_D5
zmp_ph111_G5
zmp_ph111_G11
zmp_ph111_H1
zmp_ph111_B1a
zmp_ph111_B5
zmp_ph111_B8
zmp_ph111_B6
zmp_ph111_B7
zmp_ph111_B9
zmp_ph111_B10
zmp_ph114_G4
zmp_ph114_G11
zmp_ph114_H1
zmp_ph114_H2
zmp_ph114_A9
zmp_ph114_A11
zmp_ph114_B2
zmp_ph114_B4
zmp_ph114_D8
zmp_ph114_E2

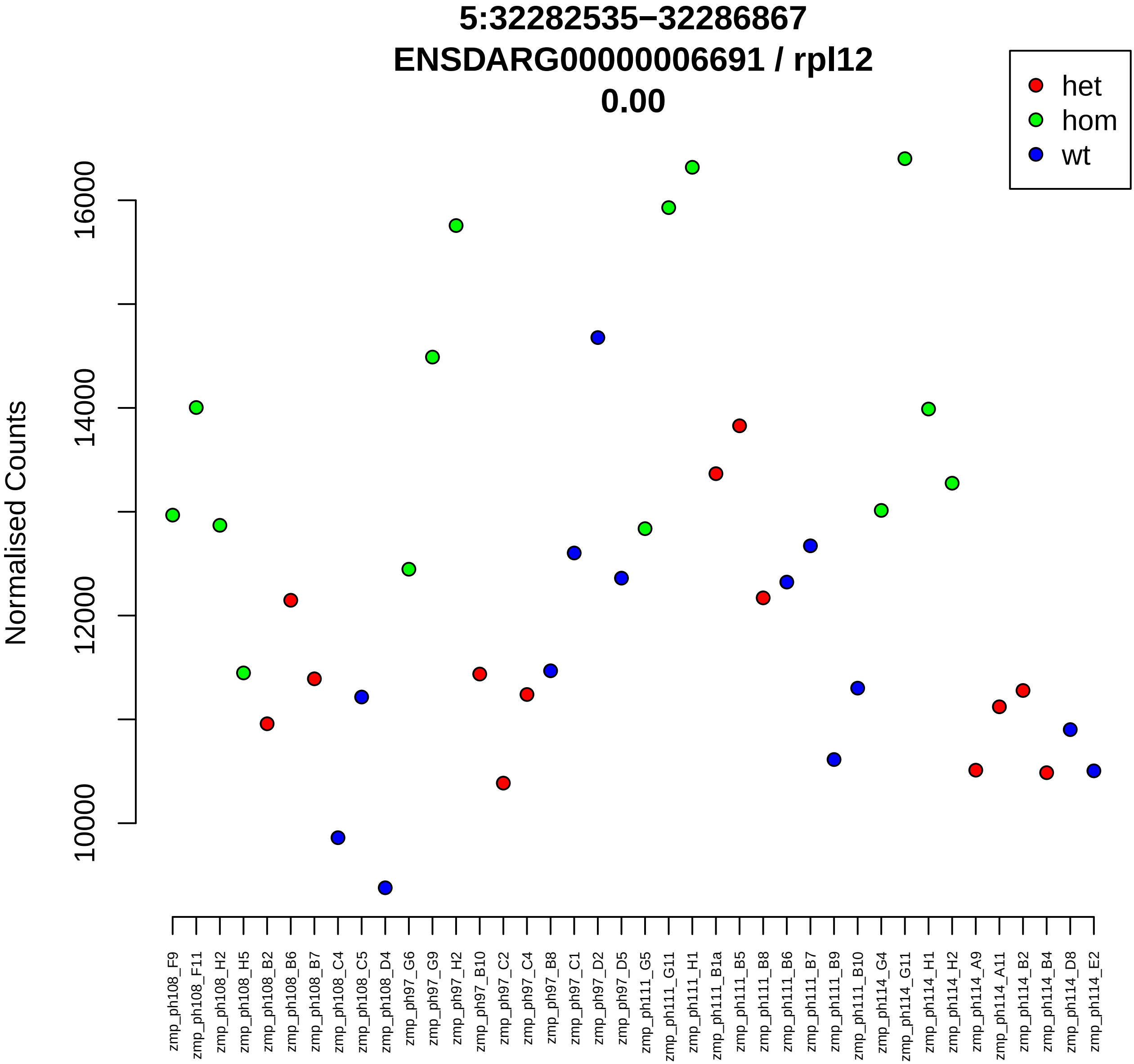

5:32282535−32286867
ENSDARG00000006691 / rpl12
0.00
het
hom
wt
Normalised Counts
10000
12000
14000
16000
zmp_ph108_F9
zmp_ph108_F11
zmp_ph108_H2
zmp_ph108_H5
zmp_ph108_B2
zmp_ph108_B6
zmp_ph108_B7
zmp_ph108_C4
zmp_ph108_C5
zmp_ph108_D4
zmp_ph97_G6
zmp_ph97_G9
zmp_ph97_H2
zmp_ph97_B10
zmp_ph97_C2
zmp_ph97_C4
zmp_ph97_B8
zmp_ph97_C1
zmp_ph97_D2
zmp_ph97_D5
zmp_ph111_G5
zmp_ph111_G11
zmp_ph111_H1
zmp_ph111_B1a
zmp_ph111_B5
zmp_ph111_B8
zmp_ph111_B6
zmp_ph111_B7
zmp_ph111_B9
zmp_ph111_B10
zmp_ph114_G4
zmp_ph114_G11
zmp_ph114_H1
zmp_ph114_H2
zmp_ph114_A9
zmp_ph114_A11
zmp_ph114_B2
zmp_ph114_B4
zmp_ph114_D8
zmp_ph114_E2

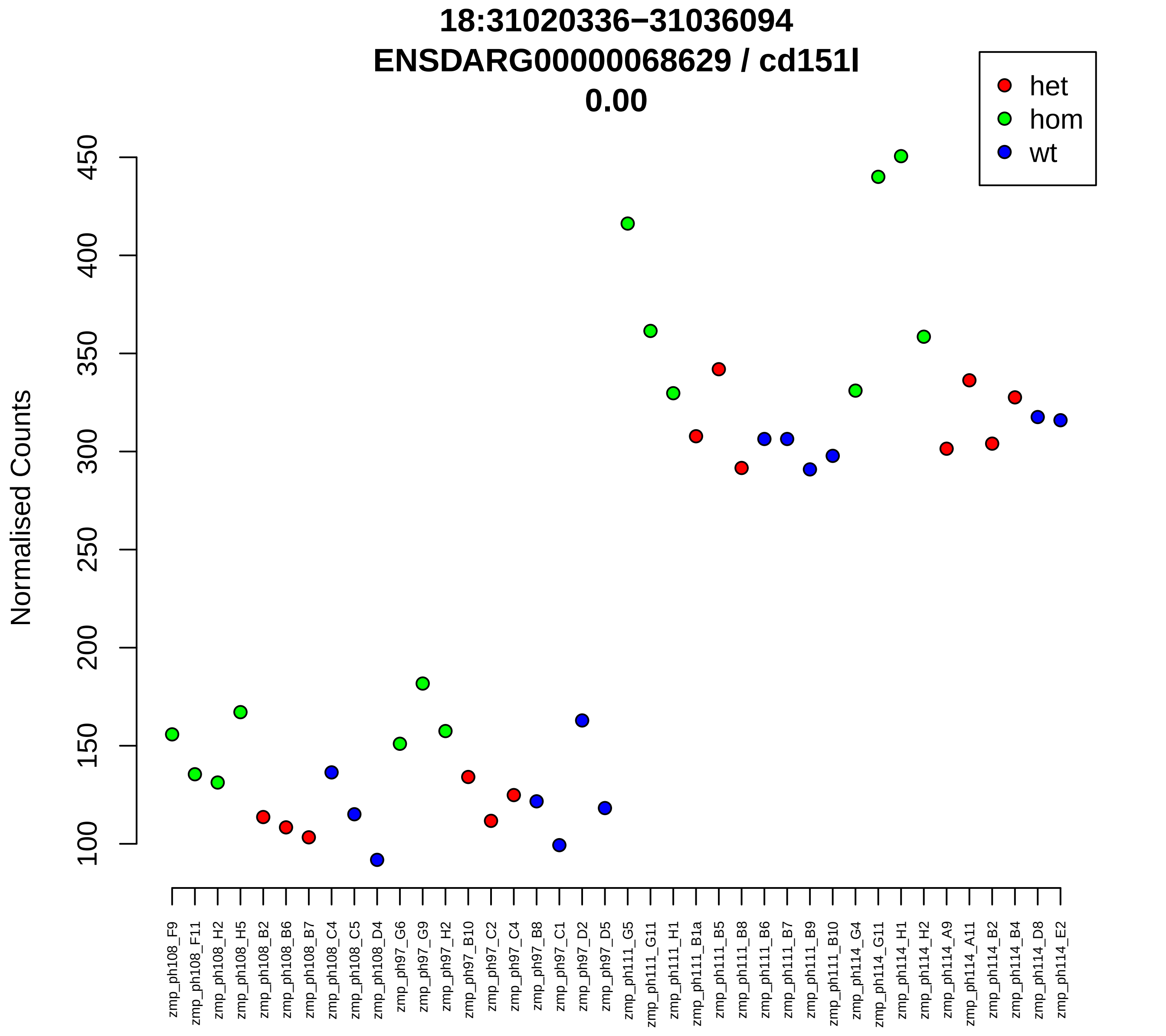
18:31020336−31036094
ENSDARG00000068629 / cd151l
0.00
het
hom
wt
Normalised Counts
100
150
200
250
300
350
400
450
zmp_ph108_F9
zmp_ph108_F11
zmp_ph108_H2
zmp_ph108_H5
zmp_ph108_B2
zmp_ph108_B6
zmp_ph108_B7
zmp_ph108_C4
zmp_ph108_C5
zmp_ph108_D4
zmp_ph97_G6
zmp_ph97_G9
zmp_ph97_H2
zmp_ph97_B10
zmp_ph97_C2
zmp_ph97_C4
zmp_ph97_B8
zmp_ph97_C1
zmp_ph97_D2
zmp_ph97_D5
zmp_ph111_G5
zmp_ph111_G11
zmp_ph111_H1
zmp_ph111_B1a
zmp_ph111_B5
zmp_ph111_B8
zmp_ph111_B6
zmp_ph111_B7
zmp_ph111_B9
zmp_ph111_B10
zmp_ph114_G4
zmp_ph114_G11
zmp_ph114_H1
zmp_ph114_H2
zmp_ph114_A9
zmp_ph114_A11
zmp_ph114_B2
zmp_ph114_B4
zmp_ph114_D8
zmp_ph114_E2

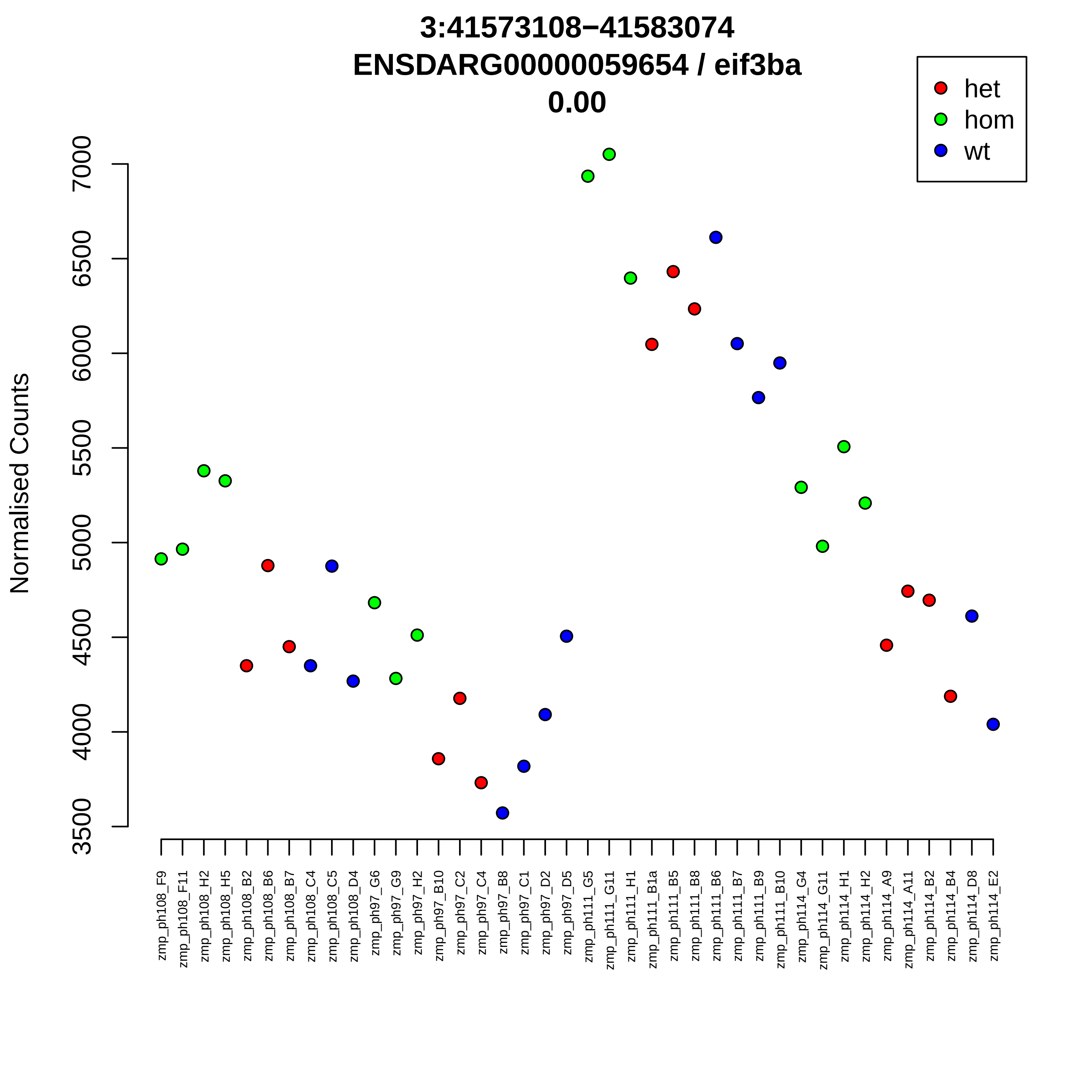

3:41573108−41583074
ENSDARG00000059654 / eif3ba
0.00
het
hom
wt
Normalised Counts
3500
4000
4500
5000
5500
6000
6500
7000
zmp_ph108_F9
zmp_ph108_F11
zmp_ph108_H2
zmp_ph108_H5
zmp_ph108_B2
zmp_ph108_B6
zmp_ph108_B7
zmp_ph108_C4
zmp_ph108_C5
zmp_ph108_D4
zmp_ph97_G6
zmp_ph97_G9
zmp_ph97_H2
zmp_ph97_B10
zmp_ph97_C2
zmp_ph97_C4
zmp_ph97_B8
zmp_ph97_C1
zmp_ph97_D2
zmp_ph97_D5
zmp_ph111_G5
zmp_ph111_G11
zmp_ph111_H1
zmp_ph111_B1a
zmp_ph111_B5
zmp_ph111_B8
zmp_ph111_B6
zmp_ph111_B7
zmp_ph111_B9
zmp_ph111_B10
zmp_ph114_G4
zmp_ph114_G11
zmp_ph114_H1
zmp_ph114_H2
zmp_ph114_A9
zmp_ph114_A11
zmp_ph114_B2
zmp_ph114_B4
zmp_ph114_D8
zmp_ph114_E2

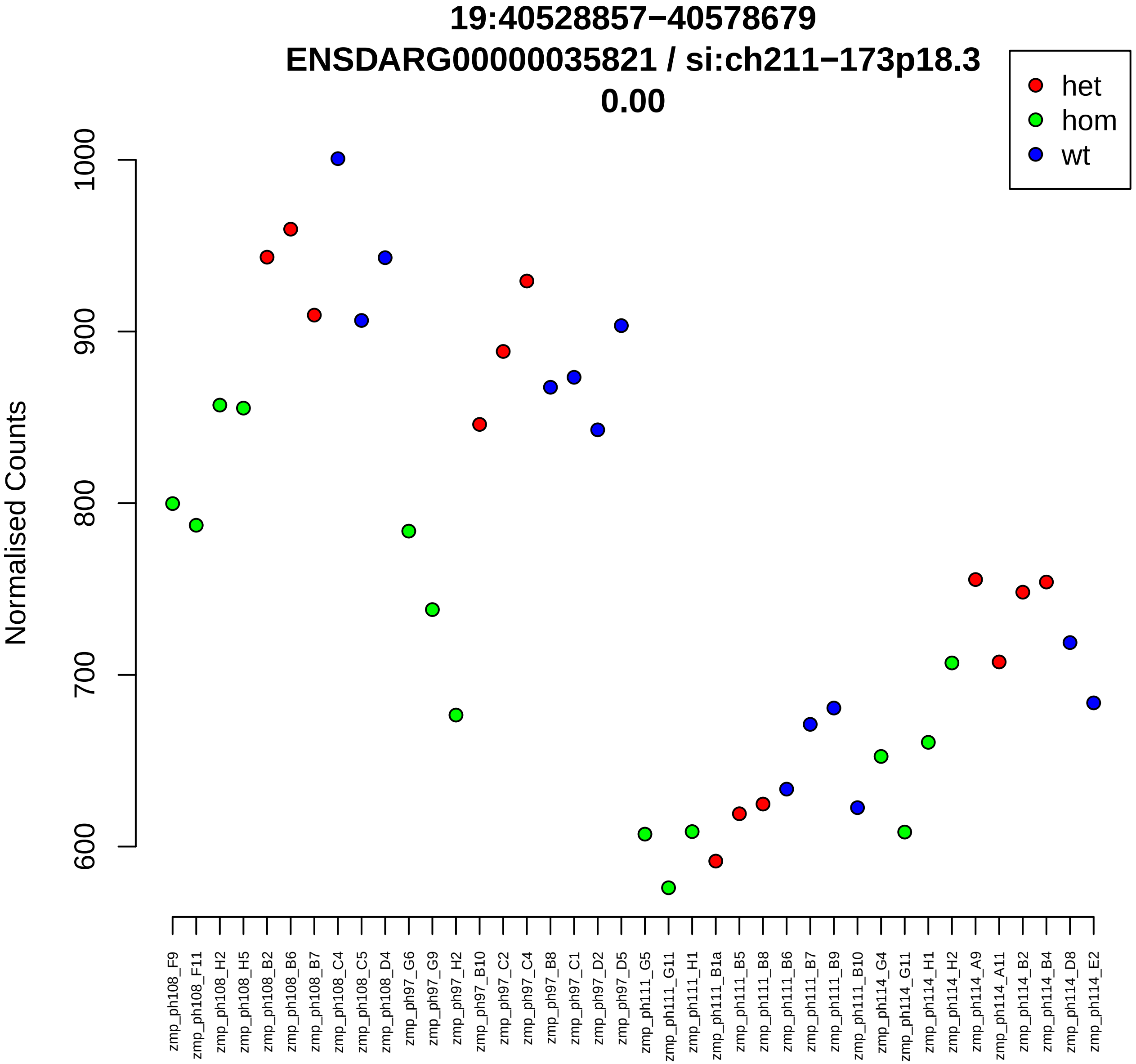

19:40528857−40578679
ENSDARG00000035821 / si:ch211−173p18.3
0.00
het
hom
wt
Normalised Counts
600
700
800
900
1000
zmp_ph108_F9
zmp_ph108_F11
zmp_ph108_H2
zmp_ph108_H5
zmp_ph108_B2
zmp_ph108_B6
zmp_ph108_B7
zmp_ph108_C4
zmp_ph108_C5
zmp_ph108_D4
zmp_ph97_G6
zmp_ph97_G9
zmp_ph97_H2
zmp_ph97_B10
zmp_ph97_C2
zmp_ph97_C4
zmp_ph97_B8
zmp_ph97_C1
zmp_ph97_D2
zmp_ph97_D5
zmp_ph111_G5
zmp_ph111_G11
zmp_ph111_H1
zmp_ph111_B1a
zmp_ph111_B5
zmp_ph111_B8
zmp_ph111_B6
zmp_ph111_B7
zmp_ph111_B9
zmp_ph111_B10
zmp_ph114_G4
zmp_ph114_G11
zmp_ph114_H1
zmp_ph114_H2
zmp_ph114_A9
zmp_ph114_A11
zmp_ph114_B2
zmp_ph114_B4
zmp_ph114_D8
zmp_ph114_E2

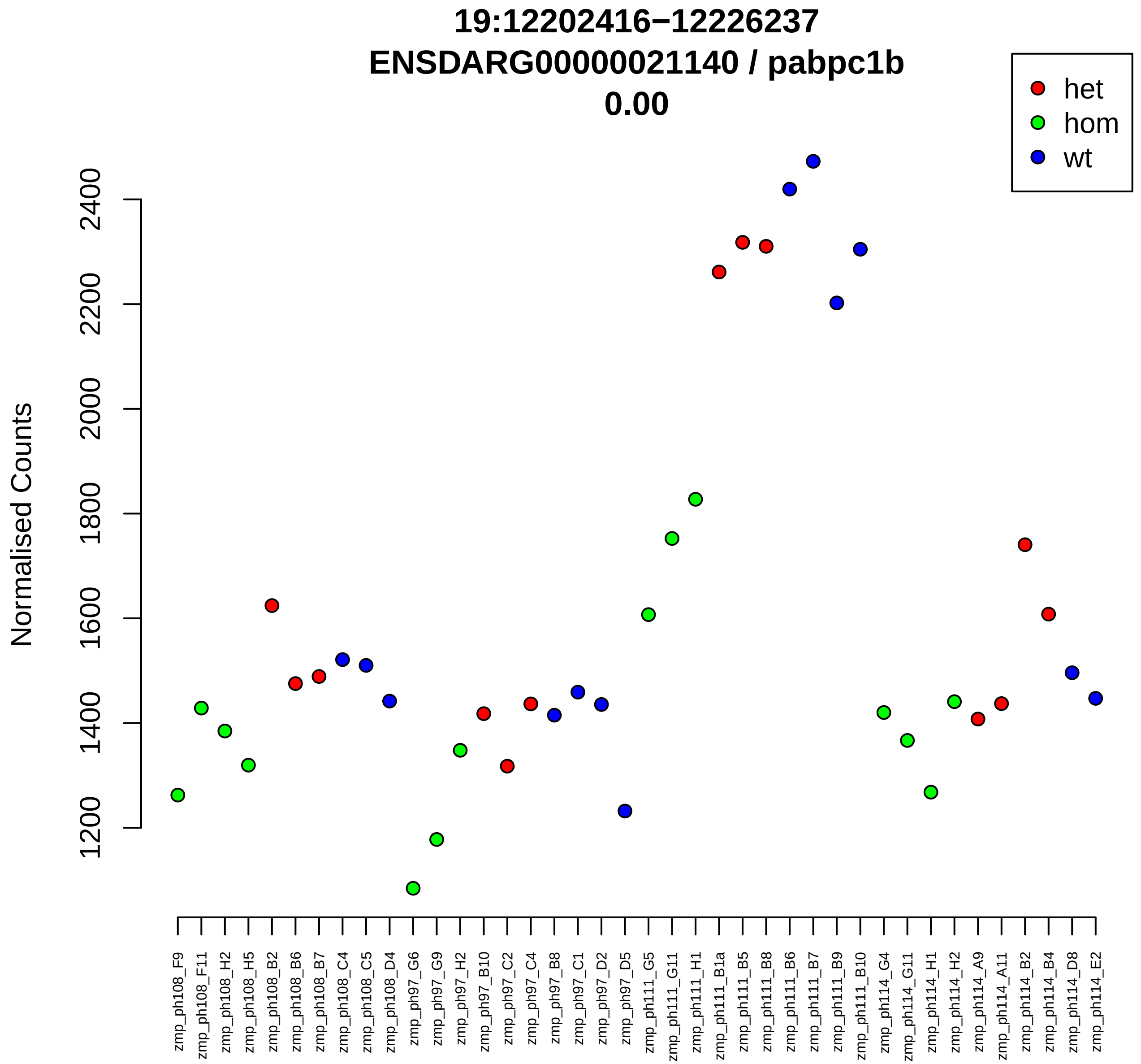

19:12202416−12226237
ENSDARG00000021140 / pabpc1b
0.00
het
hom
wt
Normalised Counts
1200
1400
1600
1800
2000
2200
2400
zmp_ph108_F9
zmp_ph108_F11
zmp_ph108_H2
zmp_ph108_H5
zmp_ph108_B2
zmp_ph108_B6
zmp_ph108_B7
zmp_ph108_C4
zmp_ph108_C5
zmp_ph108_D4
zmp_ph97_G6
zmp_ph97_G9
zmp_ph97_H2
zmp_ph97_B10
zmp_ph97_C2
zmp_ph97_C4
zmp_ph97_B8
zmp_ph97_C1
zmp_ph97_D2
zmp_ph97_D5
zmp_ph111_G5
zmp_ph111_G11
zmp_ph111_H1
zmp_ph111_B1a
zmp_ph111_B5
zmp_ph111_B8
zmp_ph111_B6
zmp_ph111_B7
zmp_ph111_B9
zmp_ph111_B10
zmp_ph114_G4
zmp_ph114_G11
zmp_ph114_H1
zmp_ph114_H2
zmp_ph114_A9
zmp_ph114_A11
zmp_ph114_B2
zmp_ph114_B4
zmp_ph114_D8
zmp_ph114_E2

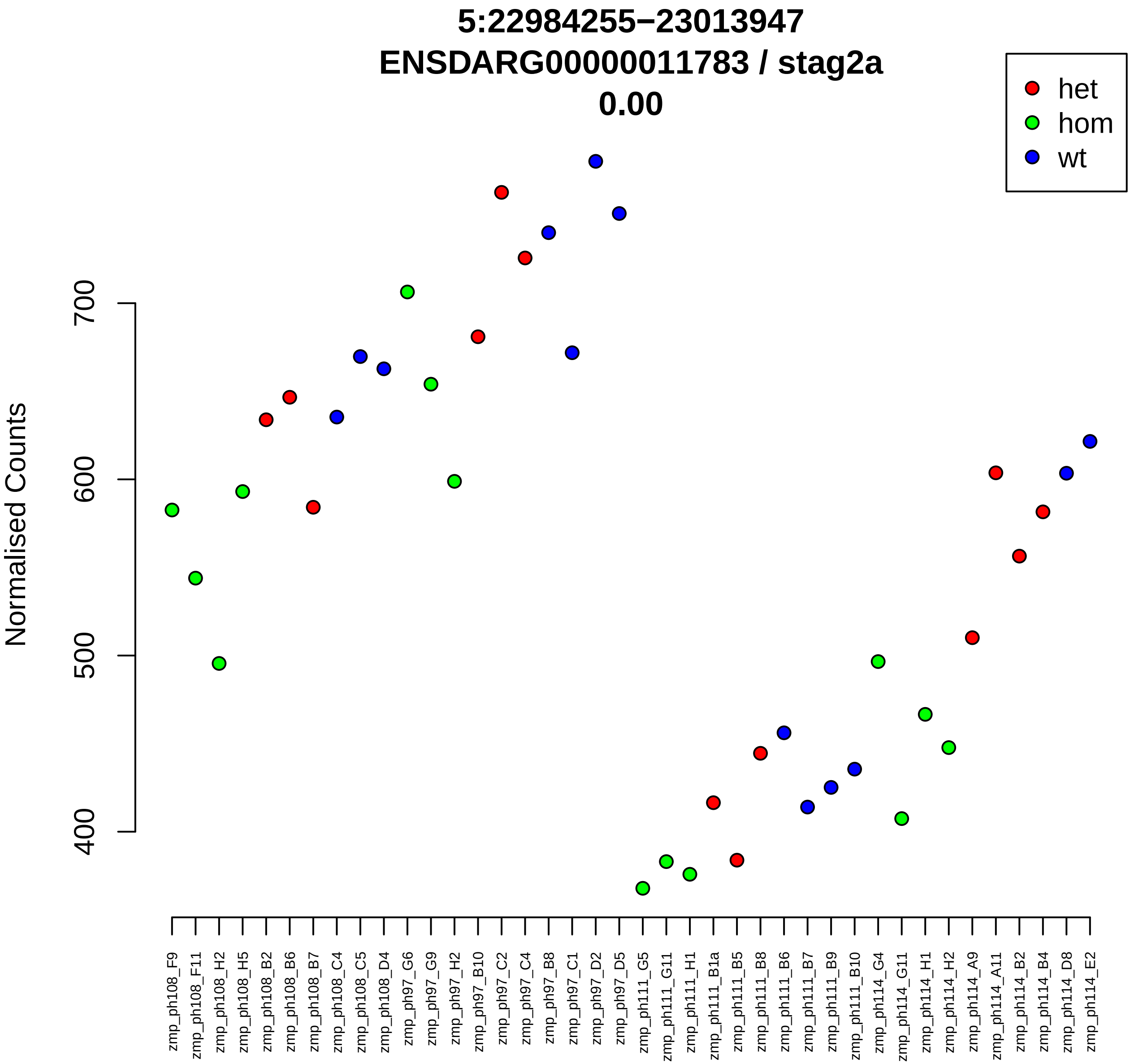

5:22984255−23013947
ENSDARG00000011783 / stag2a
0.00
het
hom
wt
Normalised Counts
400
500
600
700
zmp_ph108_F9
zmp_ph108_F11
zmp_ph108_H2
zmp_ph108_H5
zmp_ph108_B2
zmp_ph108_B6
zmp_ph108_B7
zmp_ph108_C4
zmp_ph108_C5
zmp_ph108_D4
zmp_ph97_G6
zmp_ph97_G9
zmp_ph97_H2
zmp_ph97_B10
zmp_ph97_C2
zmp_ph97_C4
zmp_ph97_B8
zmp_ph97_C1
zmp_ph97_D2
zmp_ph97_D5
zmp_ph111_G5
zmp_ph111_G11
zmp_ph111_H1
zmp_ph111_B1a
zmp_ph111_B5
zmp_ph111_B8
zmp_ph111_B6
zmp_ph111_B7
zmp_ph111_B9
zmp_ph111_B10
zmp_ph114_G4
zmp_ph114_G11
zmp_ph114_H1
zmp_ph114_H2
zmp_ph114_A9
zmp_ph114_A11
zmp_ph114_B2
zmp_ph114_B4
zmp_ph114_D8
zmp_ph114_E2

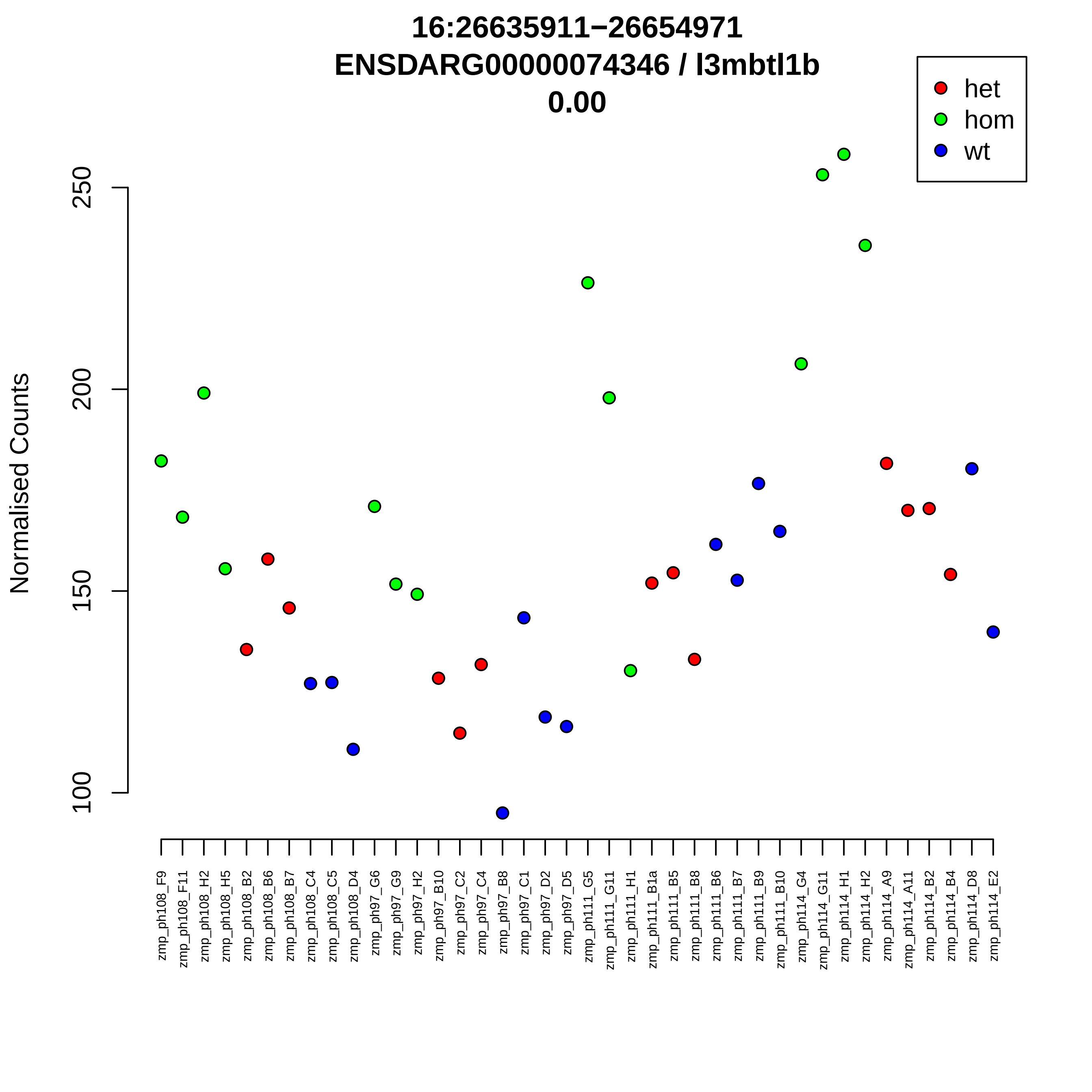
16:26635911−26654971
ENSDARG00000074346 / l3mbtl1b
0.00
het
hom
wt
Normalised Counts
100
150
200
250
zmp_ph108_F9
zmp_ph108_F11
zmp_ph108_H2
zmp_ph108_H5
zmp_ph108_B2
zmp_ph108_B6
zmp_ph108_B7
zmp_ph108_C4
zmp_ph108_C5
zmp_ph108_D4
zmp_ph97_G6
zmp_ph97_G9
zmp_ph97_H2
zmp_ph97_B10
zmp_ph97_C2
zmp_ph97_C4
zmp_ph97_B8
zmp_ph97_C1
zmp_ph97_D2
zmp_ph97_D5
zmp_ph111_G5
zmp_ph111_G11
zmp_ph111_H1
zmp_ph111_B1a
zmp_ph111_B5
zmp_ph111_B8
zmp_ph111_B6
zmp_ph111_B7
zmp_ph111_B9
zmp_ph111_B10
zmp_ph114_G4
zmp_ph114_G11
zmp_ph114_H1
zmp_ph114_H2
zmp_ph114_A9
zmp_ph114_A11
zmp_ph114_B2
zmp_ph114_B4
zmp_ph114_D8
zmp_ph114_E2

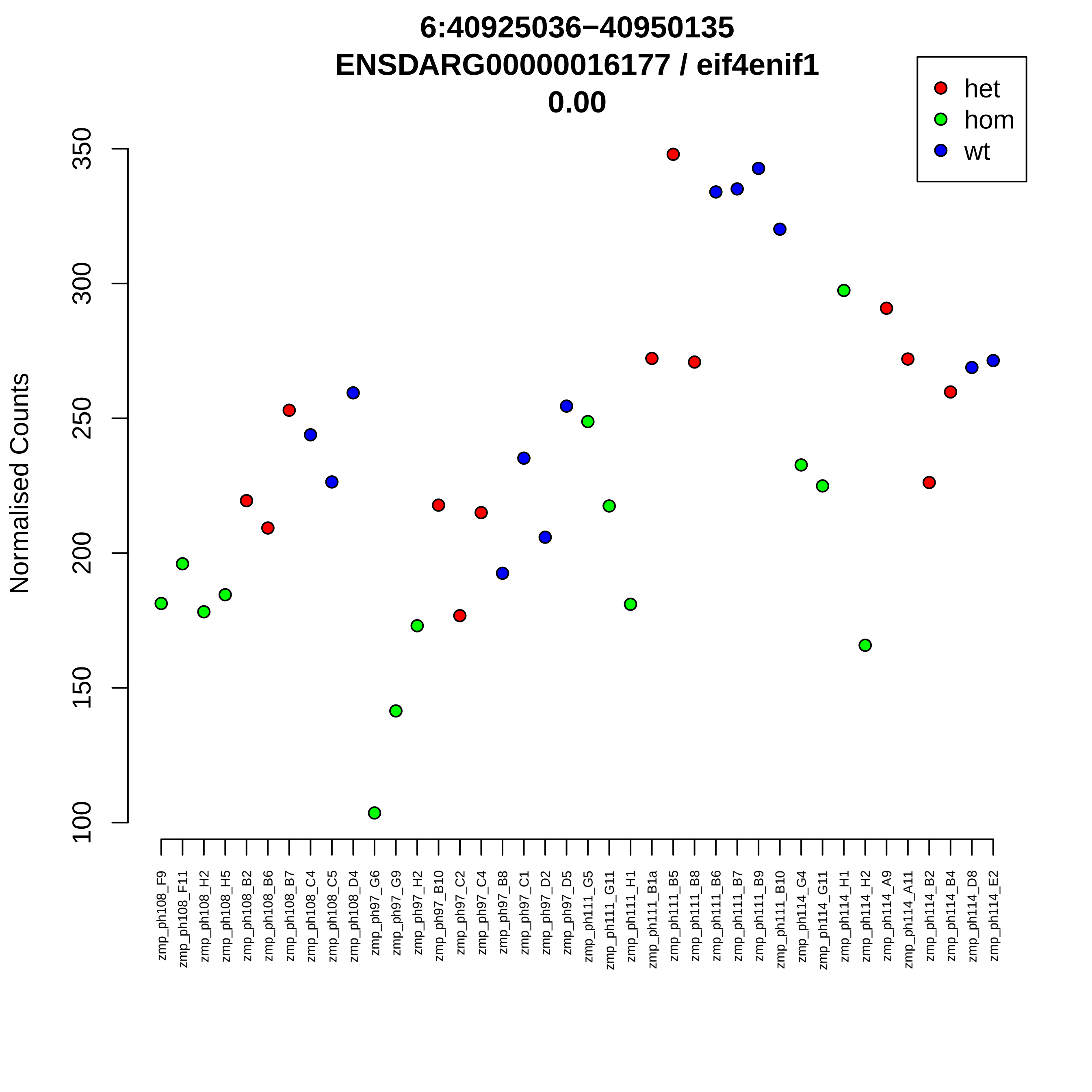

6:40925036−40950135
ENSDARG00000016177 / eif4enif1
0.00
het
hom
wt
Normalised Counts
100
150
200
250
300
350
zmp_ph108_F9
zmp_ph108_F11
zmp_ph108_H2
zmp_ph108_H5
zmp_ph108_B2
zmp_ph108_B6
zmp_ph108_B7
zmp_ph108_C4
zmp_ph108_C5
zmp_ph108_D4
zmp_ph97_G6
zmp_ph97_G9
zmp_ph97_H2
zmp_ph97_B10
zmp_ph97_C2
zmp_ph97_C4
zmp_ph97_B8
zmp_ph97_C1
zmp_ph97_D2
zmp_ph97_D5
zmp_ph111_G5
zmp_ph111_G11
zmp_ph111_H1
zmp_ph111_B1a
zmp_ph111_B5
zmp_ph111_B8
zmp_ph111_B6
zmp_ph111_B7
zmp_ph111_B9
zmp_ph111_B10
zmp_ph114_G4
zmp_ph114_G11
zmp_ph114_H1
zmp_ph114_H2
zmp_ph114_A9
zmp_ph114_A11
zmp_ph114_B2
zmp_ph114_B4
zmp_ph114_D8
zmp_ph114_E2

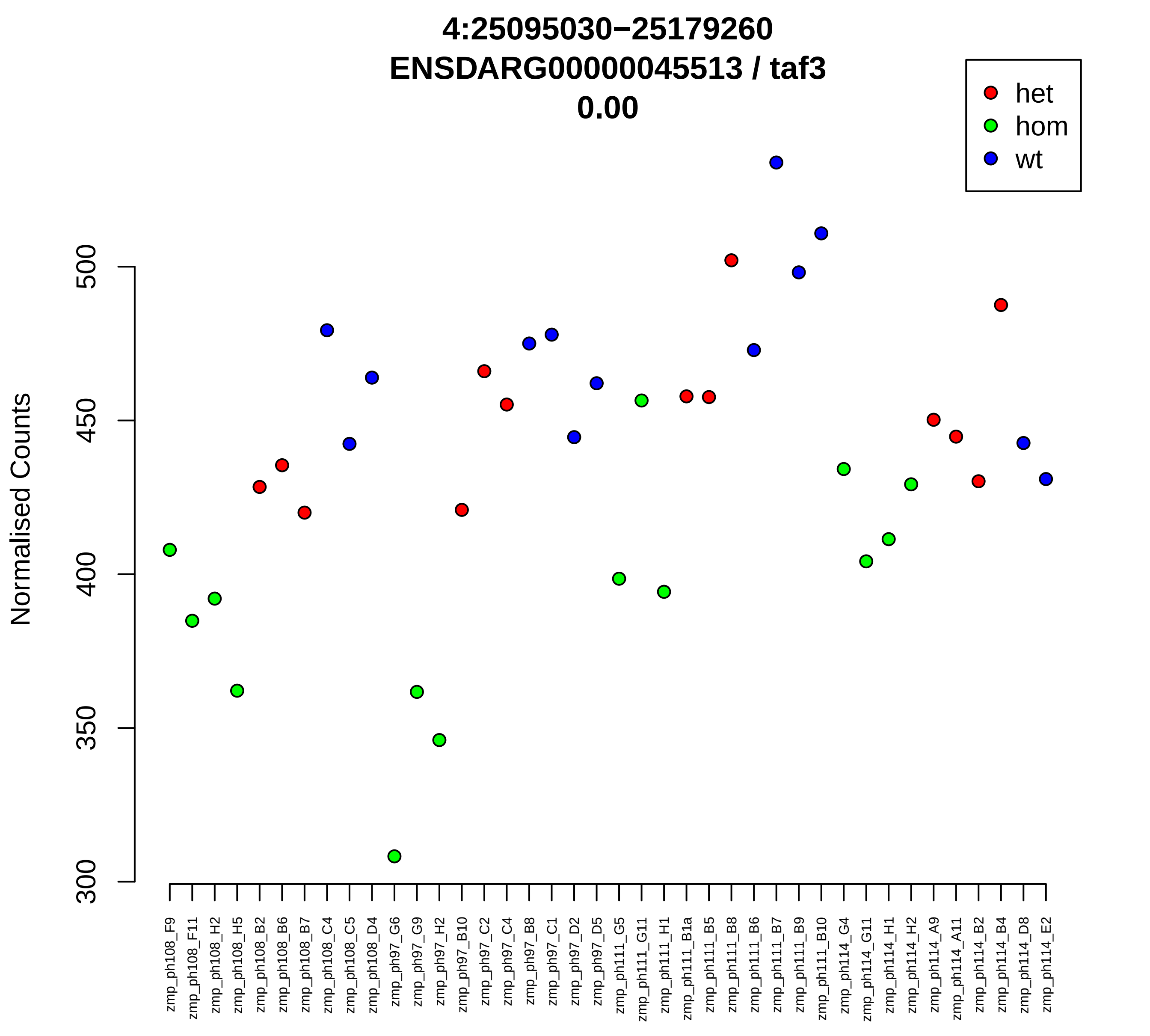

4:25095030−25179260
ENSDARG00000045513 / taf3
0.00
het
hom
wt
Normalised Counts
300
350
400
450
500
zmp_ph108_F9
zmp_ph108_F11
zmp_ph108_H2
zmp_ph108_H5
zmp_ph108_B2
zmp_ph108_B6
zmp_ph108_B7
zmp_ph108_C4
zmp_ph108_C5
zmp_ph108_D4
zmp_ph97_G6
zmp_ph97_G9
zmp_ph97_H2
zmp_ph97_B10
zmp_ph97_C2
zmp_ph97_C4
zmp_ph97_B8
zmp_ph97_C1
zmp_ph97_D2
zmp_ph97_D5
zmp_ph111_G5
zmp_ph111_G11
zmp_ph111_H1
zmp_ph111_B1a
zmp_ph111_B5
zmp_ph111_B8
zmp_ph111_B6
zmp_ph111_B7
zmp_ph111_B9
zmp_ph111_B10
zmp_ph114_G4
zmp_ph114_G11
zmp_ph114_H1
zmp_ph114_H2
zmp_ph114_A9
zmp_ph114_A11
zmp_ph114_B2
zmp_ph114_B4
zmp_ph114_D8
zmp_ph114_E2

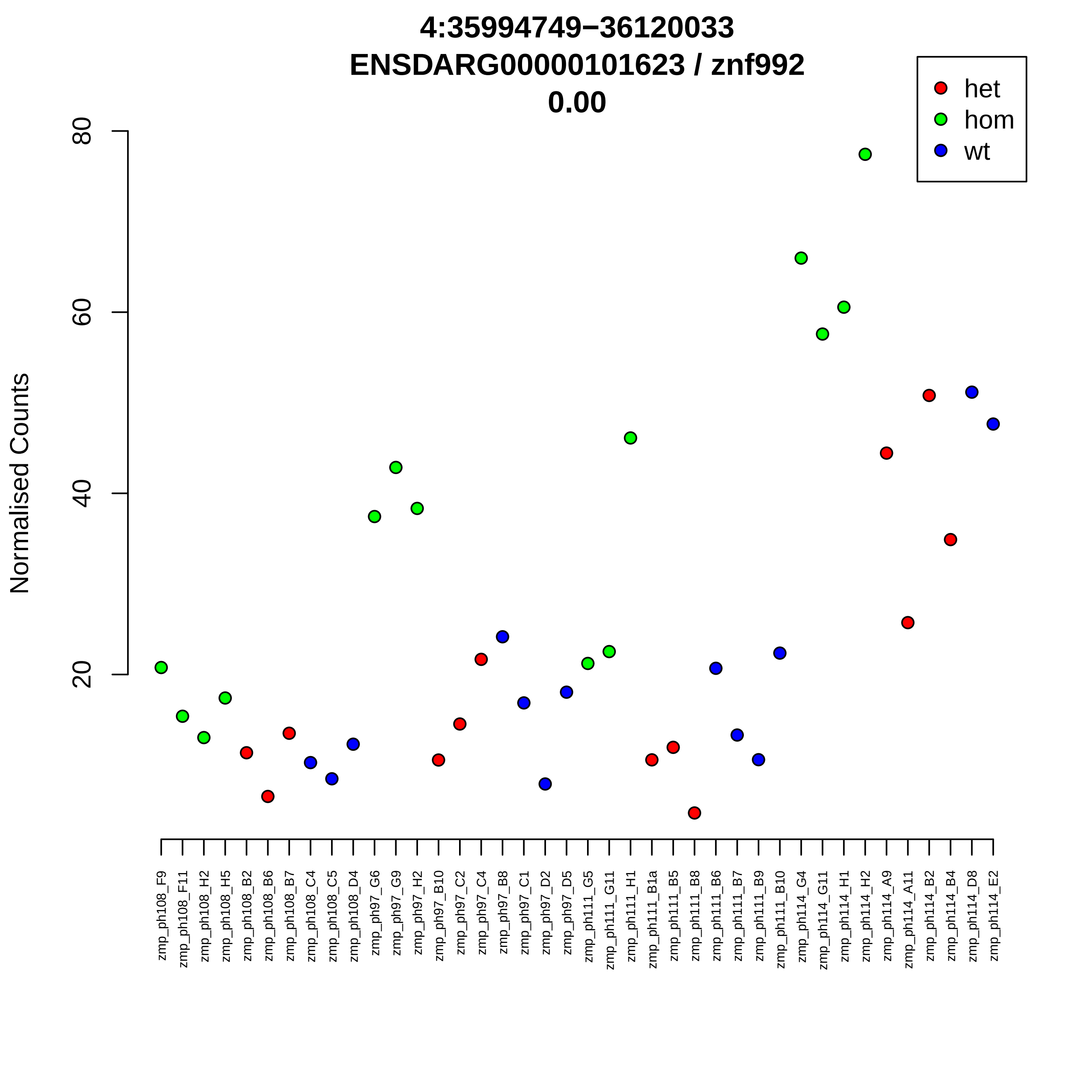

4:35994749−36120033
ENSDARG00000101623 / znf992
0.00
Normalised Counts
20
40
60
80
het
hom
wt
zmp_ph108_F9
zmp_ph108_F11
zmp_ph108_H2
zmp_ph108_H5
zmp_ph108_B2
zmp_ph108_B6
zmp_ph108_B7
zmp_ph108_C4
zmp_ph108_C5
zmp_ph108_D4
zmp_ph97_G6
zmp_ph97_G9
zmp_ph97_H2
zmp_ph97_B10
zmp_ph97_C2
zmp_ph97_C4
zmp_ph97_B8
zmp_ph97_C1
zmp_ph97_D2
zmp_ph97_D5
zmp_ph111_G5
zmp_ph111_G11
zmp_ph111_H1
zmp_ph111_B1a
zmp_ph111_B5
zmp_ph111_B8
zmp_ph111_B6
zmp_ph111_B7
zmp_ph111_B9
zmp_ph111_B10
zmp_ph114_G4
zmp_ph114_G11
zmp_ph114_H1
zmp_ph114_H2
zmp_ph114_A9
zmp_ph114_A11
zmp_ph114_B2
zmp_ph114_B4
zmp_ph114_D8
zmp_ph114_E2

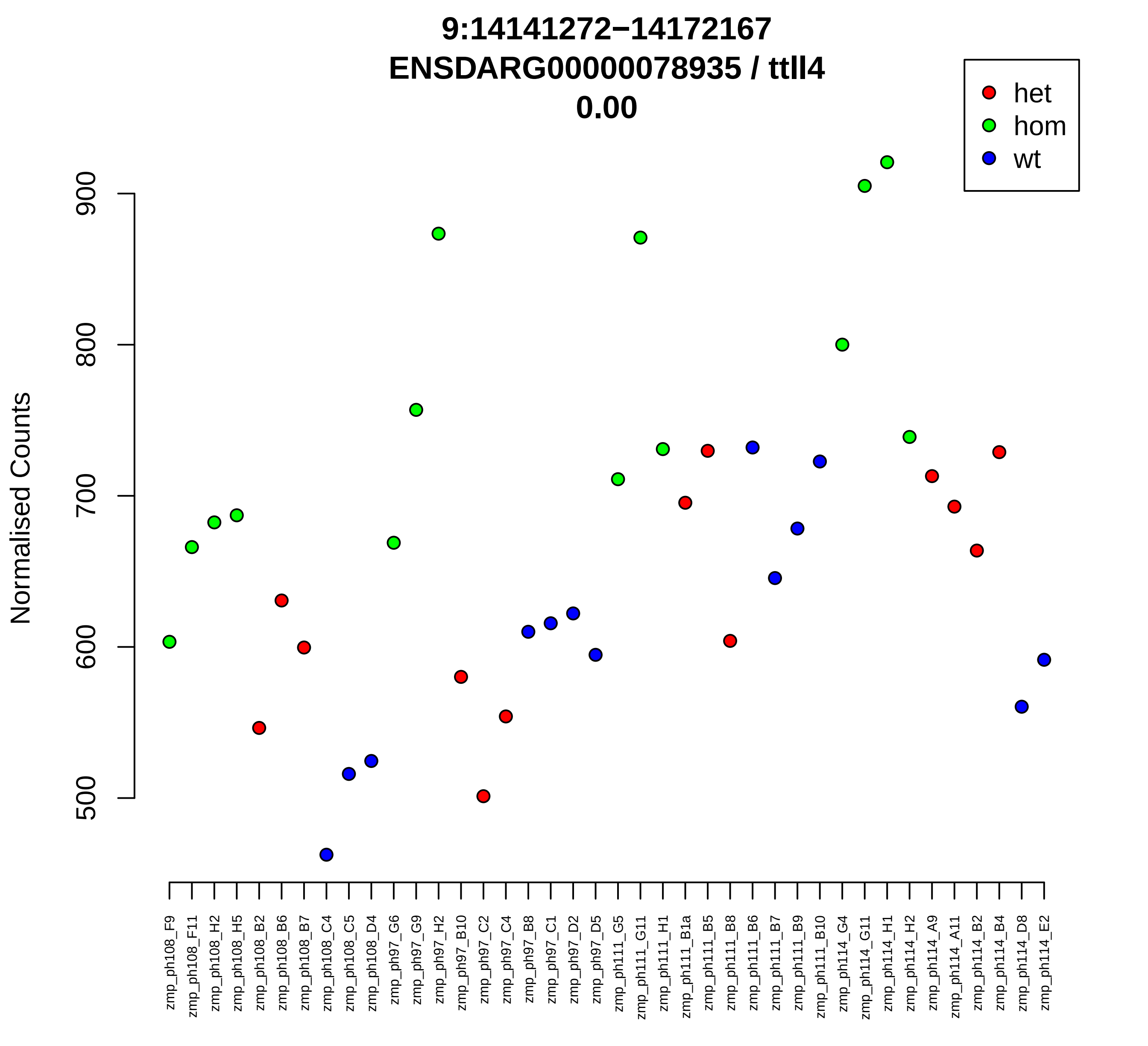
9:14141272−14172167
ENSDARG00000078935 / ttll4
0.00
het
hom
wt
Normalised Counts
500
600
700
800
900
zmp_ph108_F9
zmp_ph108_F11
zmp_ph108_H2
zmp_ph108_H5
zmp_ph108_B2
zmp_ph108_B6
zmp_ph108_B7
zmp_ph108_C4
zmp_ph108_C5
zmp_ph108_D4
zmp_ph97_G6
zmp_ph97_G9
zmp_ph97_H2
zmp_ph97_B10
zmp_ph97_C2
zmp_ph97_C4
zmp_ph97_B8
zmp_ph97_C1
zmp_ph97_D2
zmp_ph97_D5
zmp_ph111_G5
zmp_ph111_G11
zmp_ph111_H1
zmp_ph111_B1a
zmp_ph111_B5
zmp_ph111_B8
zmp_ph111_B6
zmp_ph111_B7
zmp_ph111_B9
zmp_ph111_B10
zmp_ph114_G4
zmp_ph114_G11
zmp_ph114_H1
zmp_ph114_H2
zmp_ph114_A9
zmp_ph114_A11
zmp_ph114_B2
zmp_ph114_B4
zmp_ph114_D8
zmp_ph114_E2

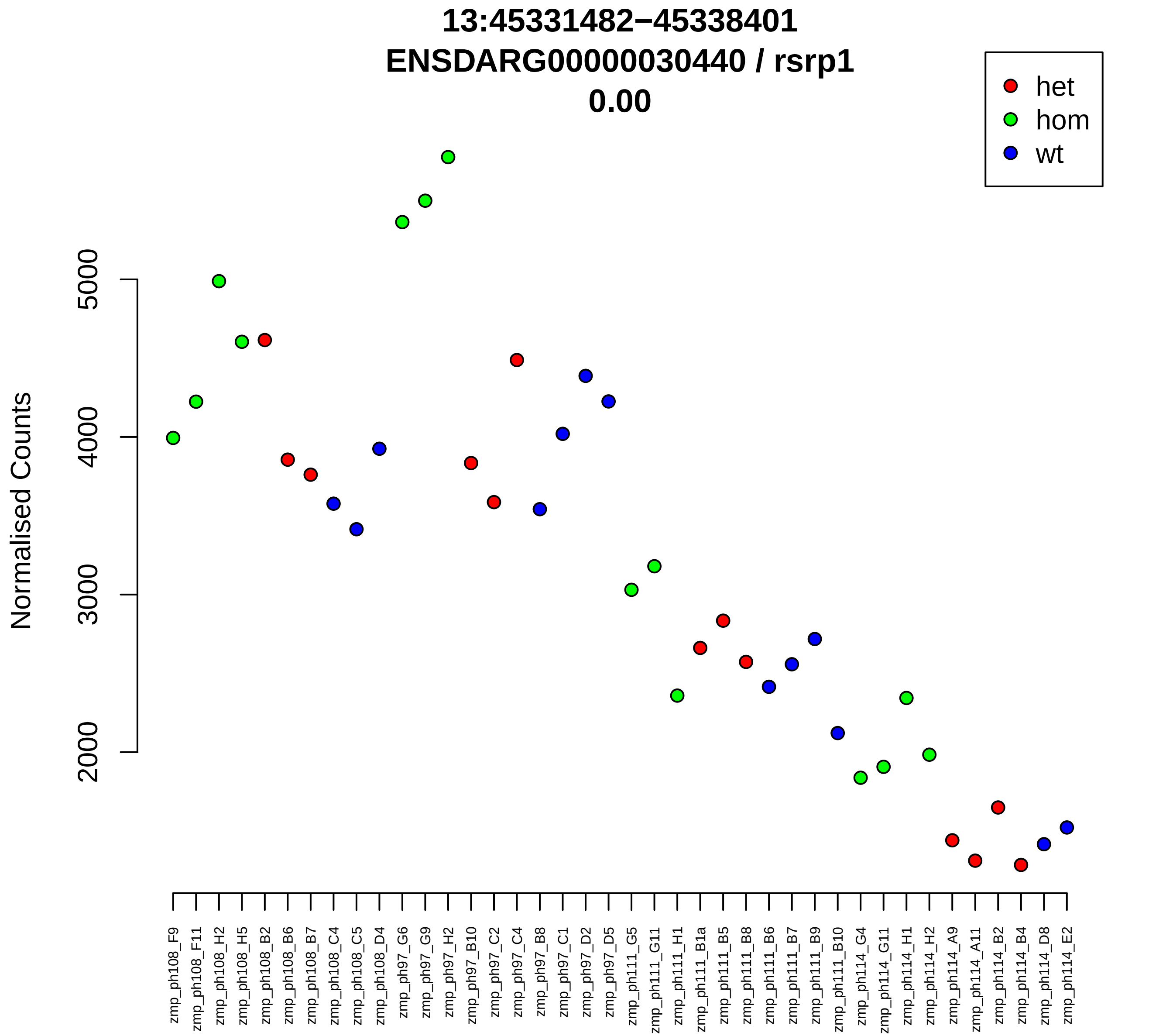

13:45331482−45338401
ENSDARG00000030440 / rsrp1
0.00
het
hom
wt
Normalised Counts
2000
3000
4000
5000
zmp_ph108_F9
zmp_ph108_F11
zmp_ph108_H2
zmp_ph108_H5
zmp_ph108_B2
zmp_ph108_B6
zmp_ph108_B7
zmp_ph108_C4
zmp_ph108_C5
zmp_ph108_D4
zmp_ph97_G6
zmp_ph97_G9
zmp_ph97_H2
zmp_ph97_B10
zmp_ph97_C2
zmp_ph97_C4
zmp_ph97_B8
zmp_ph97_C1
zmp_ph97_D2
zmp_ph97_D5
zmp_ph111_G5
zmp_ph111_G11
zmp_ph111_H1
zmp_ph111_B1a
zmp_ph111_B5
zmp_ph111_B8
zmp_ph111_B6
zmp_ph111_B7
zmp_ph111_B9
zmp_ph111_B10
zmp_ph114_G4
zmp_ph114_G11
zmp_ph114_H1
zmp_ph114_H2
zmp_ph114_A9
zmp_ph114_A11
zmp_ph114_B2
zmp_ph114_B4
zmp_ph114_D8
zmp_ph114_E2

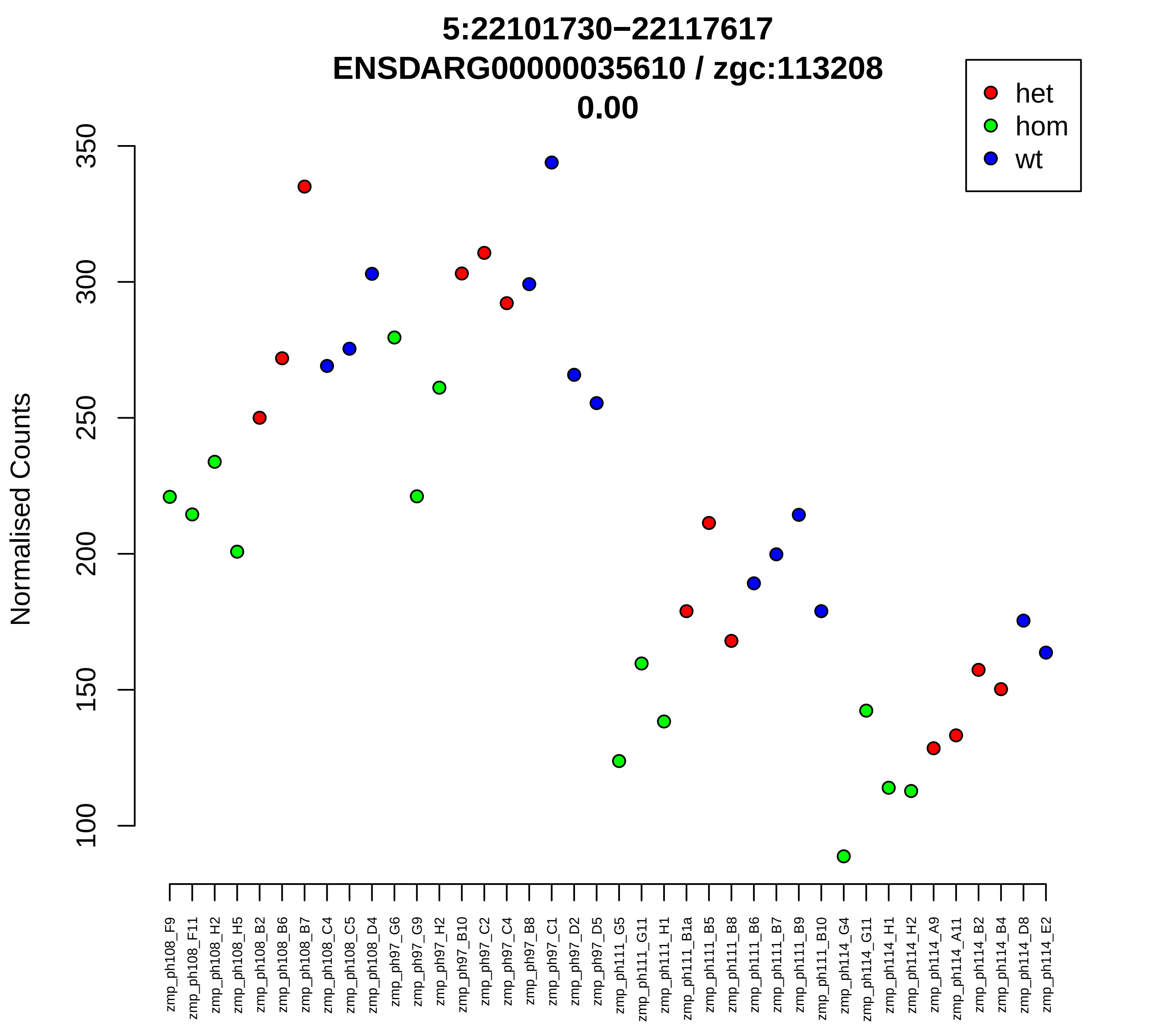

5:22101730−22117617
ENSDARG00000035610 / zgc:113208
0.00
het
hom
wt
Normalised Counts
100
150
200
250
300
350
zmp_ph108_F9
zmp_ph108_F11
zmp_ph108_H2
zmp_ph108_H5
zmp_ph108_B2
zmp_ph108_B6
zmp_ph108_B7
zmp_ph108_C4
zmp_ph108_C5
zmp_ph108_D4
zmp_ph97_G6
zmp_ph97_G9
zmp_ph97_H2
zmp_ph97_B10
zmp_ph97_C2
zmp_ph97_C4
zmp_ph97_B8
zmp_ph97_C1
zmp_ph97_D2
zmp_ph97_D5
zmp_ph111_G5
zmp_ph111_G11
zmp_ph111_H1
zmp_ph111_B1a
zmp_ph111_B5
zmp_ph111_B8
zmp_ph111_B6
zmp_ph111_B7
zmp_ph111_B9
zmp_ph111_B10
zmp_ph114_G4
zmp_ph114_G11
zmp_ph114_H1
zmp_ph114_H2
zmp_ph114_A9
zmp_ph114_A11
zmp_ph114_B2
zmp_ph114_B4
zmp_ph114_D8
zmp_ph114_E2

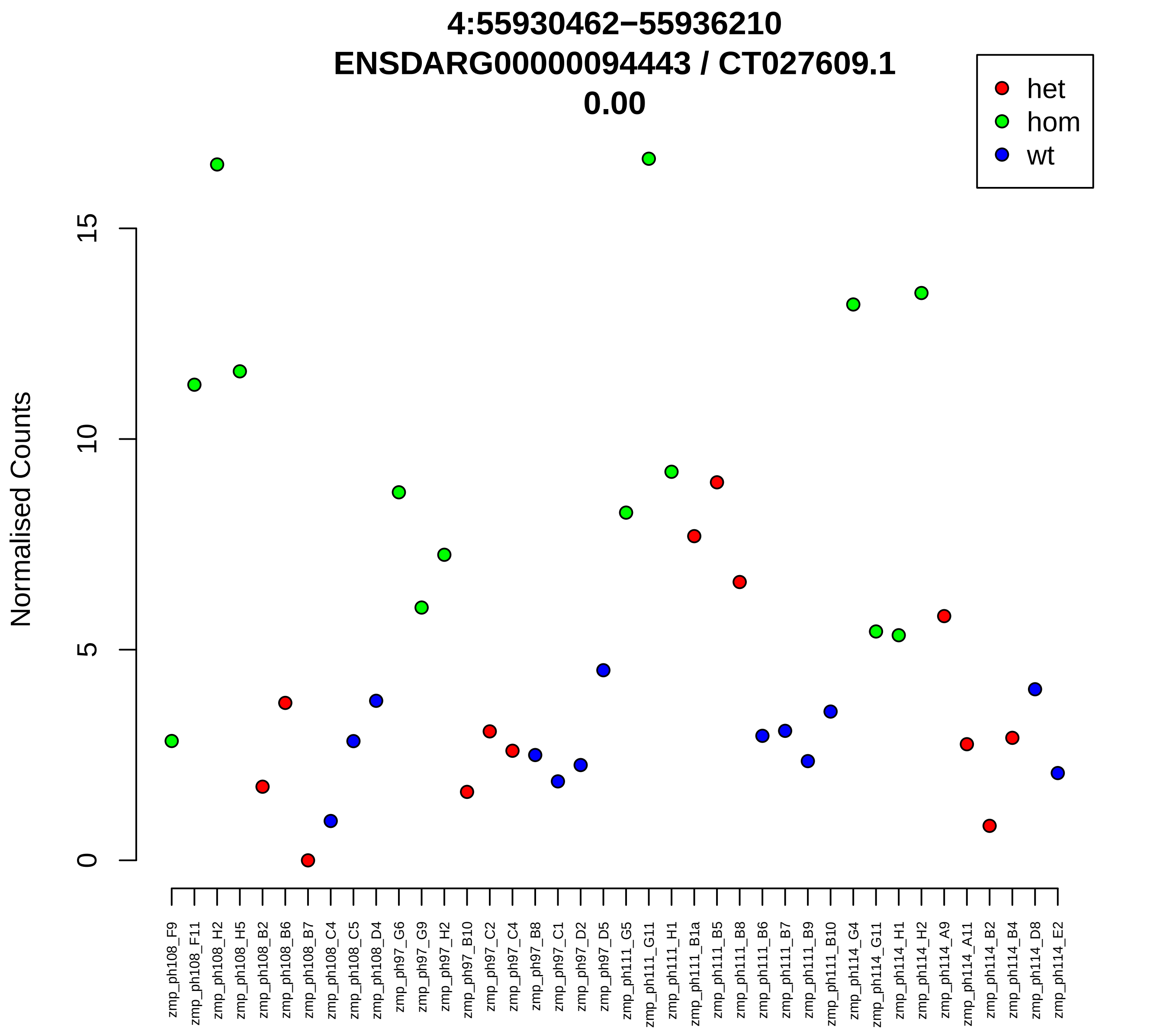

4:55930462−55936210
ENSDARG00000094443 / CT027609.1
0.00
het
hom
wt
Normalised Counts
0
5
10
15
zmp_ph108_F9
zmp_ph108_F11
zmp_ph108_H2
zmp_ph108_H5
zmp_ph108_B2
zmp_ph108_B6
zmp_ph108_B7
zmp_ph108_C4
zmp_ph108_C5
zmp_ph108_D4
zmp_ph97_G6
zmp_ph97_G9
zmp_ph97_H2
zmp_ph97_B10
zmp_ph97_C2
zmp_ph97_C4
zmp_ph97_B8
zmp_ph97_C1
zmp_ph97_D2
zmp_ph97_D5
zmp_ph111_G5
zmp_ph111_G11
zmp_ph111_H1
zmp_ph111_B1a
zmp_ph111_B5
zmp_ph111_B8
zmp_ph111_B6
zmp_ph111_B7
zmp_ph111_B9
zmp_ph111_B10
zmp_ph114_G4
zmp_ph114_G11
zmp_ph114_H1
zmp_ph114_H2
zmp_ph114_A9
zmp_ph114_A11
zmp_ph114_B2
zmp_ph114_B4
zmp_ph114_D8
zmp_ph114_E2

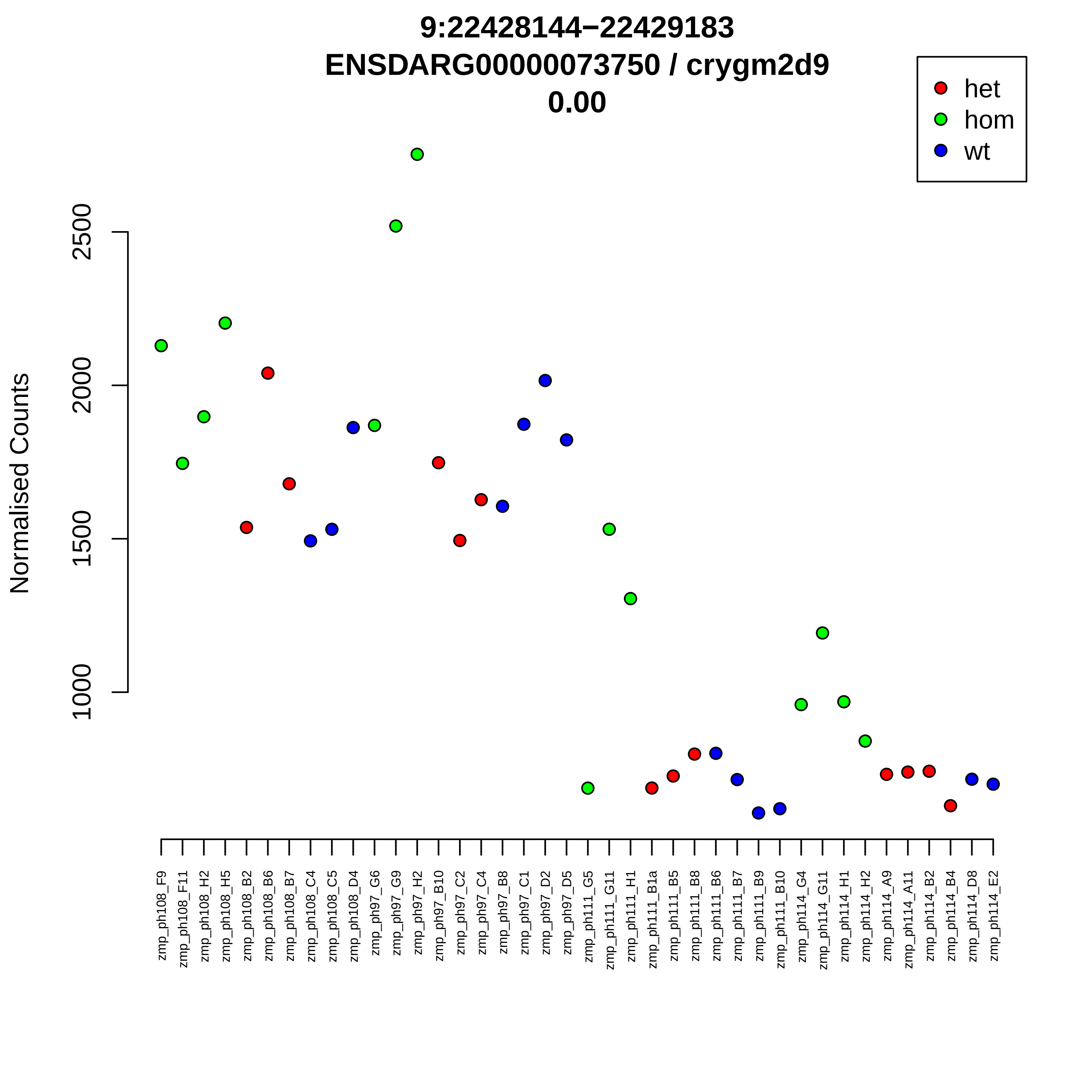
9:22428144−22429183
ENSDARG00000073750 / crygm2d9
0.00
het
hom
wt
Normalised Counts
1000
1500
2000
2500
zmp_ph108_F9
zmp_ph108_F11
zmp_ph108_H2
zmp_ph108_H5
zmp_ph108_B2
zmp_ph108_B6
zmp_ph108_B7
zmp_ph108_C4
zmp_ph108_C5
zmp_ph108_D4
zmp_ph97_G6
zmp_ph97_G9
zmp_ph97_H2
zmp_ph97_B10
zmp_ph97_C2
zmp_ph97_C4
zmp_ph97_B8
zmp_ph97_C1
zmp_ph97_D2
zmp_ph97_D5
zmp_ph111_G5
zmp_ph111_G11
zmp_ph111_H1
zmp_ph111_B1a
zmp_ph111_B5
zmp_ph111_B8
zmp_ph111_B6
zmp_ph111_B7
zmp_ph111_B9
zmp_ph111_B10
zmp_ph114_G4
zmp_ph114_G11
zmp_ph114_H1
zmp_ph114_H2
zmp_ph114_A9
zmp_ph114_A11
zmp_ph114_B2
zmp_ph114_B4
zmp_ph114_D8
zmp_ph114_E2

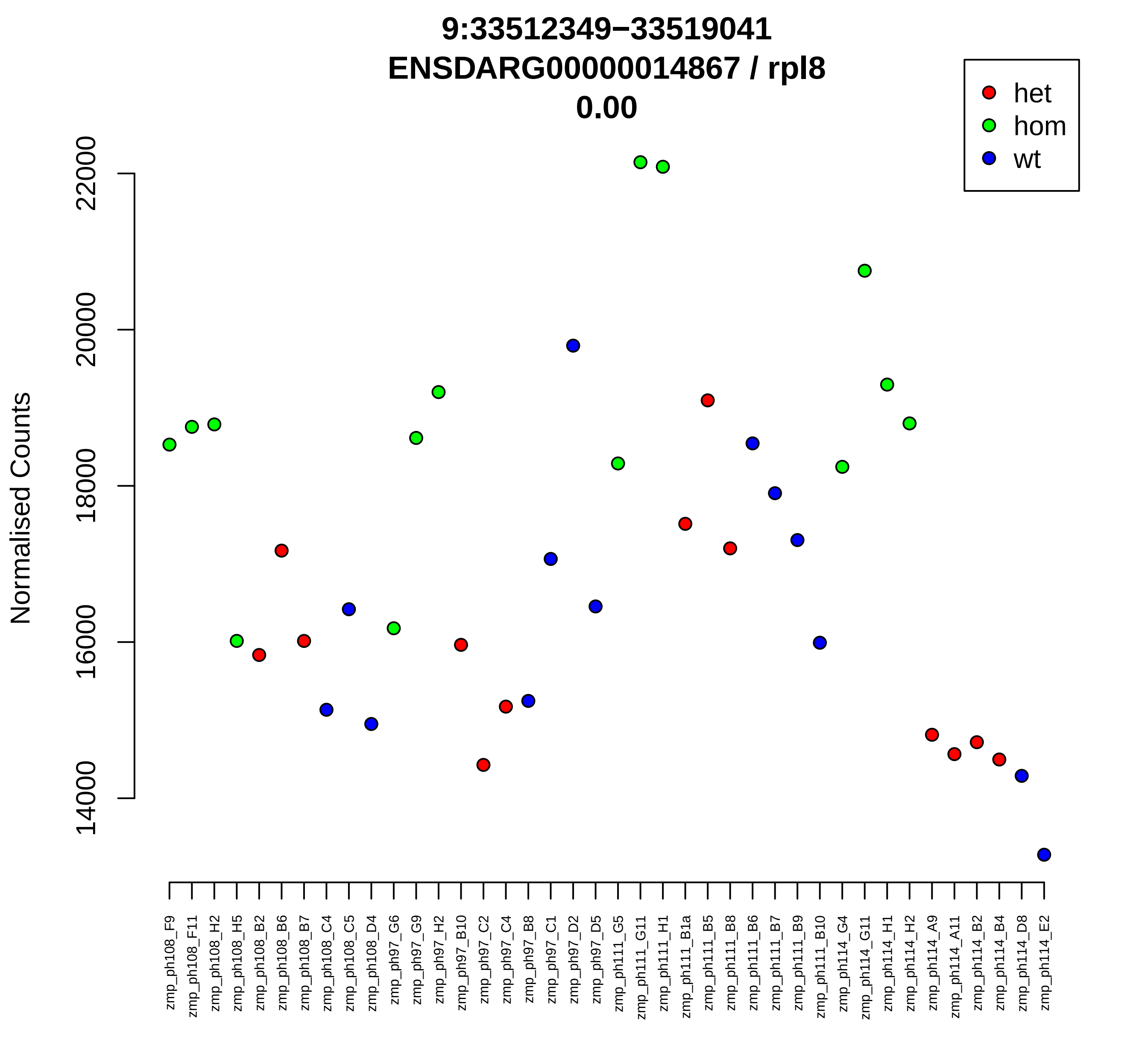

9:33512349−33519041
ENSDARG00000014867 / rpl8
0.00
het
hom
wt
Normalised Counts
14000
16000
18000
20000
22000
zmp_ph108_F9
zmp_ph108_F11
zmp_ph108_H2
zmp_ph108_H5
zmp_ph108_B2
zmp_ph108_B6
zmp_ph108_B7
zmp_ph108_C4
zmp_ph108_C5
zmp_ph108_D4
zmp_ph97_G6
zmp_ph97_G9
zmp_ph97_H2
zmp_ph97_B10
zmp_ph97_C2
zmp_ph97_C4
zmp_ph97_B8
zmp_ph97_C1
zmp_ph97_D2
zmp_ph97_D5
zmp_ph111_G5
zmp_ph111_G11
zmp_ph111_H1
zmp_ph111_B1a
zmp_ph111_B5
zmp_ph111_B8
zmp_ph111_B6
zmp_ph111_B7
zmp_ph111_B9
zmp_ph111_B10
zmp_ph114_G4
zmp_ph114_G11
zmp_ph114_H1
zmp_ph114_H2
zmp_ph114_A9
zmp_ph114_A11
zmp_ph114_B2
zmp_ph114_B4
zmp_ph114_D8
zmp_ph114_E2

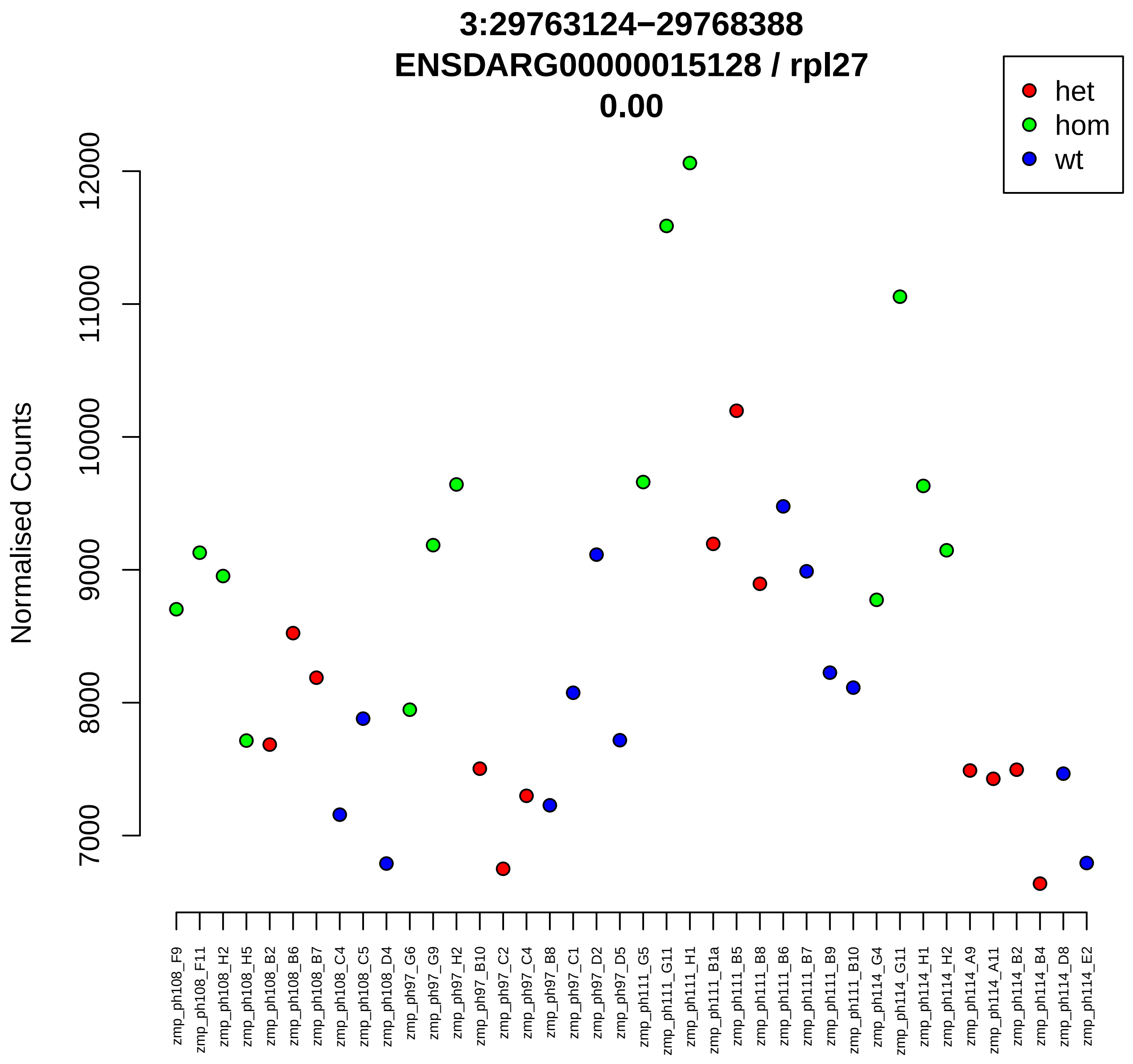

3:29763124−29768388
ENSDARG00000015128 / rpl27
0.00
het
hom
wt
Normalised Counts
7000
8000
9000
10000
11000
12000
zmp_ph108_F9
zmp_ph108_F11
zmp_ph108_H2
zmp_ph108_H5
zmp_ph108_B2
zmp_ph108_B6
zmp_ph108_B7
zmp_ph108_C4
zmp_ph108_C5
zmp_ph108_D4
zmp_ph97_G6
zmp_ph97_G9
zmp_ph97_H2
zmp_ph97_B10
zmp_ph97_C2
zmp_ph97_C4
zmp_ph97_B8
zmp_ph97_C1
zmp_ph97_D2
zmp_ph97_D5
zmp_ph111_G5
zmp_ph111_G11
zmp_ph111_H1
zmp_ph111_B1a
zmp_ph111_B5
zmp_ph111_B8
zmp_ph111_B6
zmp_ph111_B7
zmp_ph111_B9
zmp_ph111_B10
zmp_ph114_G4
zmp_ph114_G11
zmp_ph114_H1
zmp_ph114_H2
zmp_ph114_A9
zmp_ph114_A11
zmp_ph114_B2
zmp_ph114_B4
zmp_ph114_D8
zmp_ph114_E2

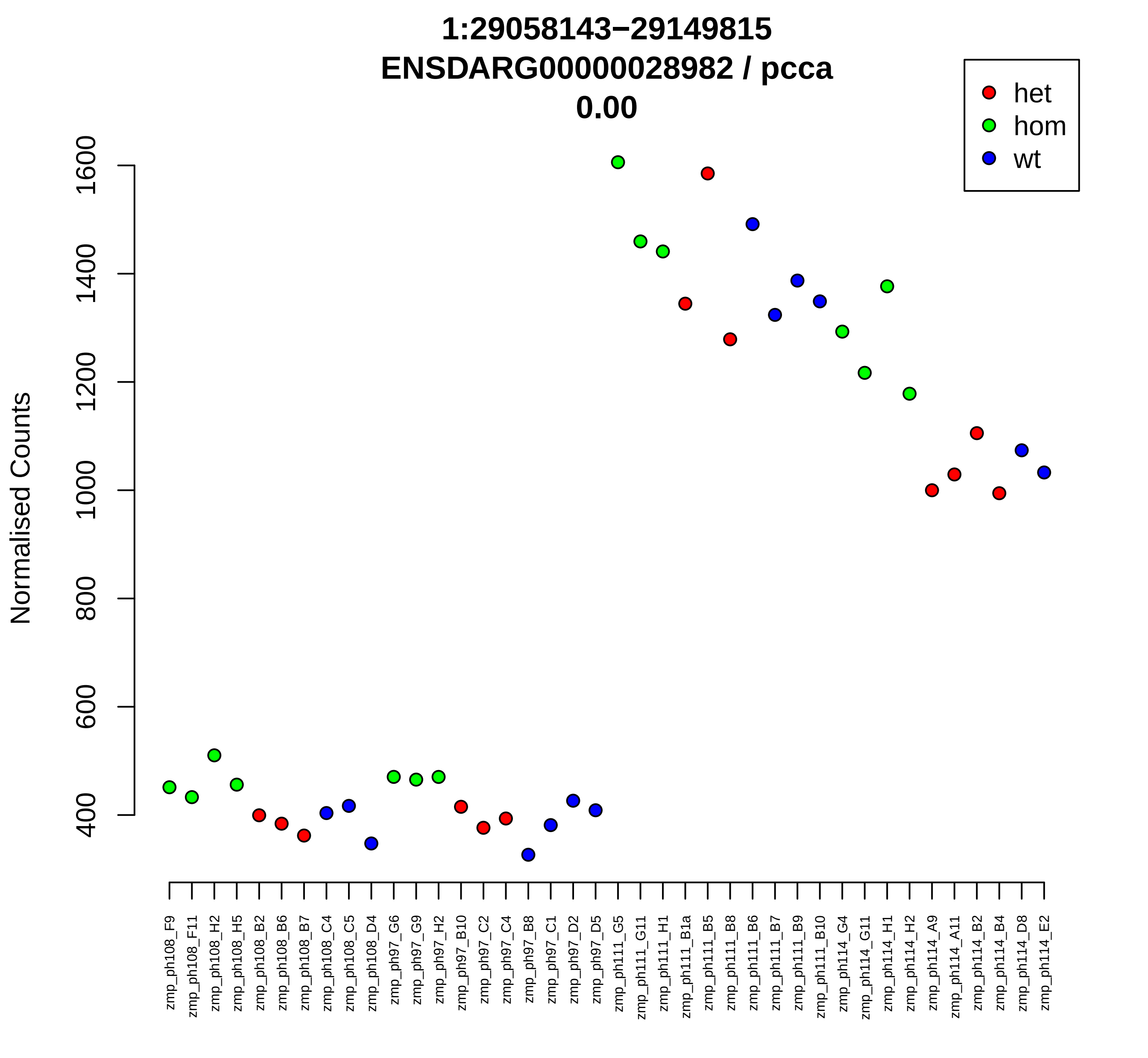
1:29058143−29149815
ENSDARG00000028982 / pcca
0.00
het
hom
wt
Normalised Counts
400
600
800
1000
1200
1400
1600
zmp_ph108_F9
zmp_ph108_F11
zmp_ph108_H2
zmp_ph108_H5
zmp_ph108_B2
zmp_ph108_B6
zmp_ph108_B7
zmp_ph108_C4
zmp_ph108_C5
zmp_ph108_D4
zmp_ph97_G6
zmp_ph97_G9
zmp_ph97_H2
zmp_ph97_B10
zmp_ph97_C2
zmp_ph97_C4
zmp_ph97_B8
zmp_ph97_C1
zmp_ph97_D2
zmp_ph97_D5
zmp_ph111_G5
zmp_ph111_G11
zmp_ph111_H1
zmp_ph111_B1a
zmp_ph111_B5
zmp_ph111_B8
zmp_ph111_B6
zmp_ph111_B7
zmp_ph111_B9
zmp_ph111_B10
zmp_ph114_G4
zmp_ph114_G11
zmp_ph114_H1
zmp_ph114_H2
zmp_ph114_A9
zmp_ph114_A11
zmp_ph114_B2
zmp_ph114_B4
zmp_ph114_D8
zmp_ph114_E2

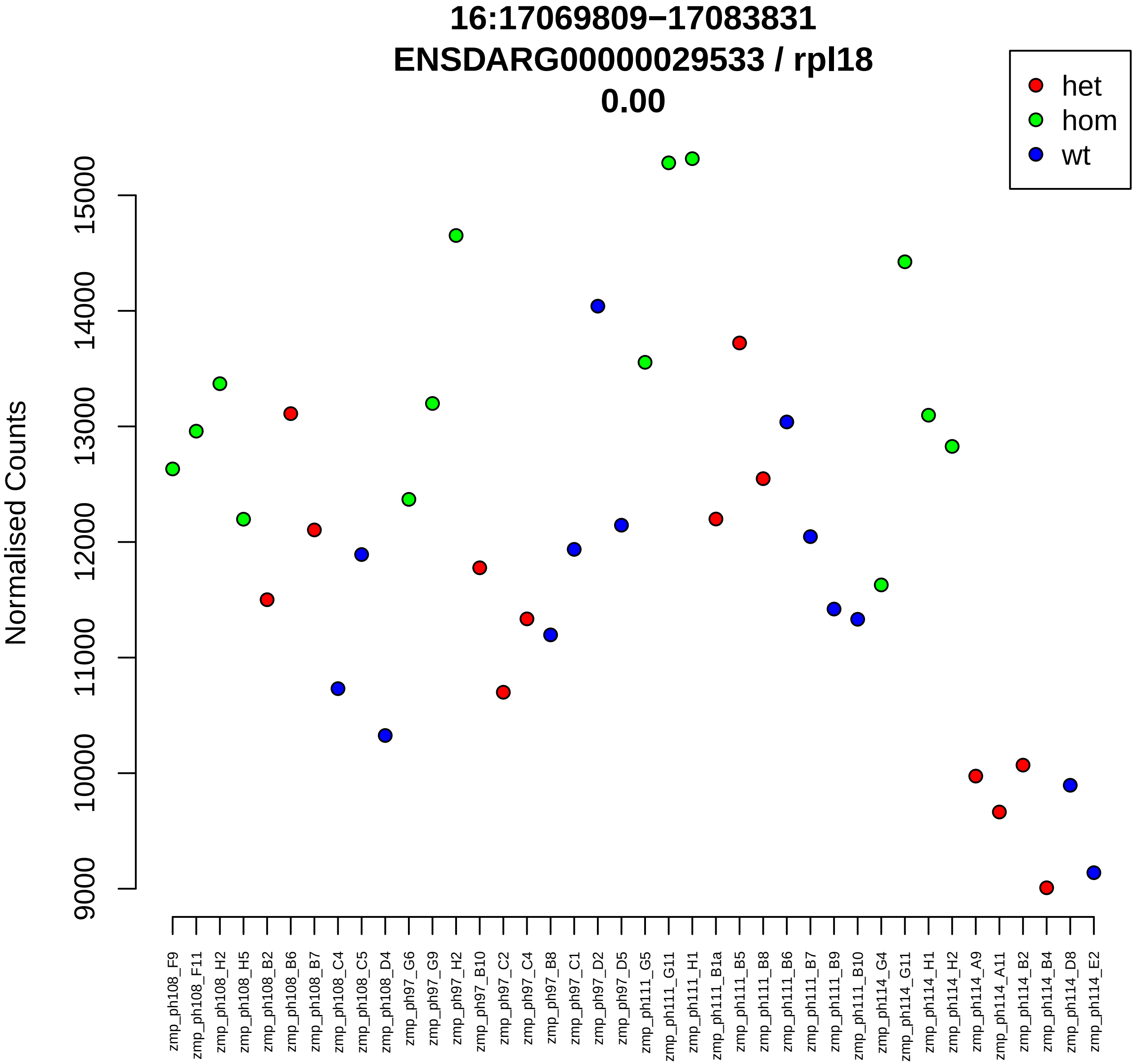

16:17069809−17083831
ENSDARG00000029533 / rpl18
0.00
het
hom
wt
Normalised Counts
9000
10000
11000
12000
13000
14000
15000
zmp_ph108_F9
zmp_ph108_F11
zmp_ph108_H2
zmp_ph108_H5
zmp_ph108_B2
zmp_ph108_B6
zmp_ph108_B7
zmp_ph108_C4
zmp_ph108_C5
zmp_ph108_D4
zmp_ph97_G6
zmp_ph97_G9
zmp_ph97_H2
zmp_ph97_B10
zmp_ph97_C2
zmp_ph97_C4
zmp_ph97_B8
zmp_ph97_C1
zmp_ph97_D2
zmp_ph97_D5
zmp_ph111_G5
zmp_ph111_G11
zmp_ph111_H1
zmp_ph111_B1a
zmp_ph111_B5
zmp_ph111_B8
zmp_ph111_B6
zmp_ph111_B7
zmp_ph111_B9
zmp_ph111_B10
zmp_ph114_G4
zmp_ph114_G11
zmp_ph114_H1
zmp_ph114_H2
zmp_ph114_A9
zmp_ph114_A11
zmp_ph114_B2
zmp_ph114_B4
zmp_ph114_D8
zmp_ph114_E2

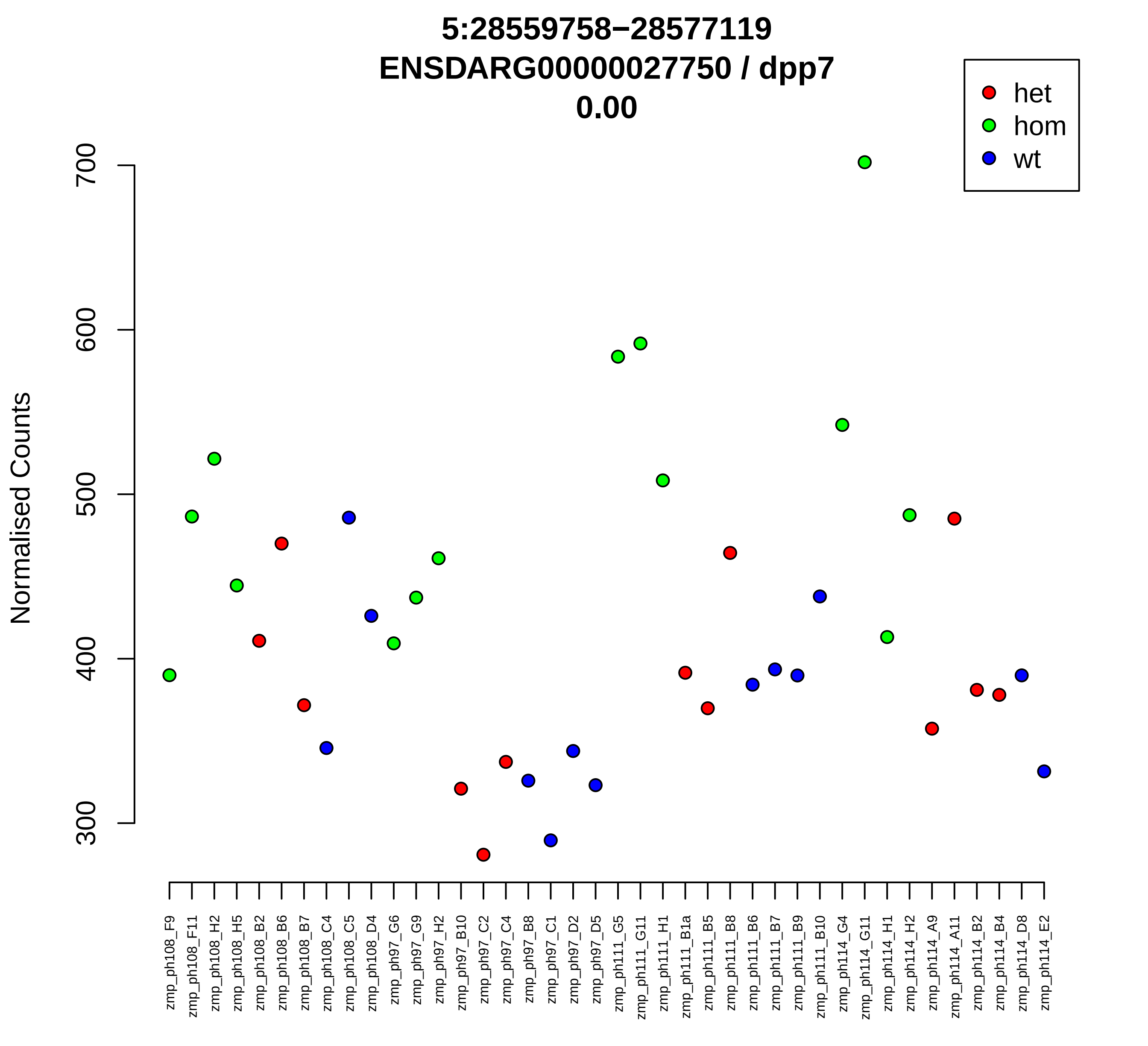
5:28559758−28577119
ENSDARG00000027750 / dpp7
0.00
het
hom
wt
Normalised Counts
700
600
500
400
300
zmp_ph108_F9
zmp_ph108_F11
zmp_ph108_H2
zmp_ph108_H5
zmp_ph108_B2
zmp_ph108_B6
zmp_ph108_B7
zmp_ph108_C4
zmp_ph108_C5
zmp_ph108_D4
zmp_ph97_G6
zmp_ph97_G9
zmp_ph97_H2
zmp_ph97_B10
zmp_ph97_C2
zmp_ph97_C4
zmp_ph97_B8
zmp_ph97_C1
zmp_ph97_D2
zmp_ph97_D5
zmp_ph111_G5
zmp_ph111_G11
zmp_ph111_H1
zmp_ph111_B1a
zmp_ph111_B5
zmp_ph111_B8
zmp_ph111_B6
zmp_ph111_B7
zmp_ph111_B9
zmp_ph111_B10
zmp_ph114_G4
zmp_ph114_G11
zmp_ph114_H1
zmp_ph114_H2
zmp_ph114_A9
zmp_ph114_A11
zmp_ph114_B2
zmp_ph114_B4
zmp_ph114_D8
zmp_ph114_E2

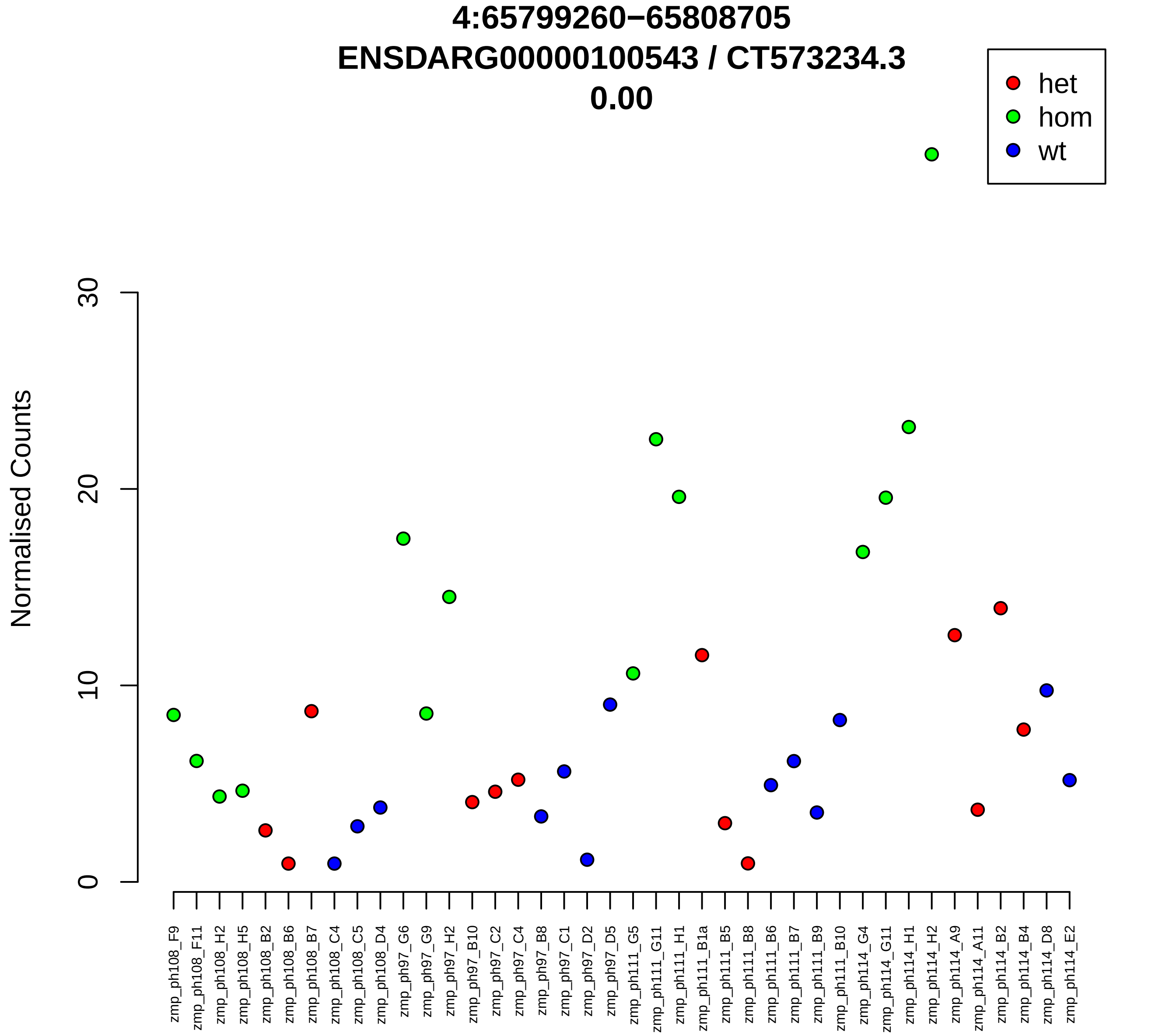

4:65799260−65808705
ENSDARG00000100543 / CT573234.3
0.00
het
hom
wt
Normalised Counts
0
10
20
30
zmp_ph108_F9
zmp_ph108_F11
zmp_ph108_H2
zmp_ph108_H5
zmp_ph108_B2
zmp_ph108_B6
zmp_ph108_B7
zmp_ph108_C4
zmp_ph108_C5
zmp_ph108_D4
zmp_ph97_G6
zmp_ph97_G9
zmp_ph97_H2
zmp_ph97_B10
zmp_ph97_C2
zmp_ph97_C4
zmp_ph97_B8
zmp_ph97_C1
zmp_ph97_D2
zmp_ph97_D5
zmp_ph111_G5
zmp_ph111_G11
zmp_ph111_H1
zmp_ph111_B1a
zmp_ph111_B5
zmp_ph111_B8
zmp_ph111_B6
zmp_ph111_B7
zmp_ph111_B9
zmp_ph111_B10
zmp_ph114_G4
zmp_ph114_G11
zmp_ph114_H1
zmp_ph114_H2
zmp_ph114_A9
zmp_ph114_A11
zmp_ph114_B2
zmp_ph114_B4
zmp_ph114_D8
zmp_ph114_E2

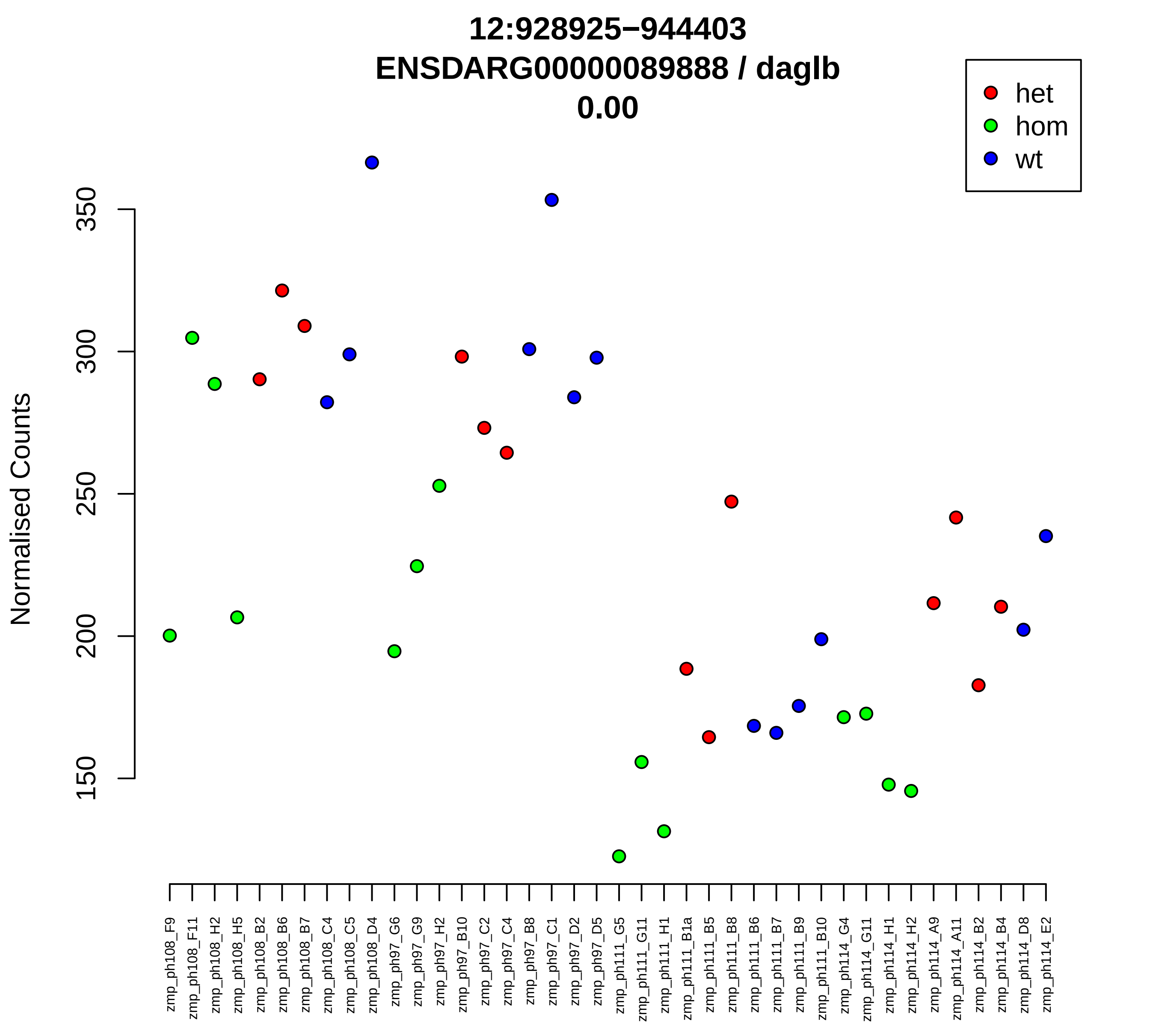
12:928925−944403
ENSDARG00000089888 / daglb
0.00
het
hom
wt
Normalised Counts
150
200
250
300
350
zmp_ph108_F9
zmp_ph108_F11
zmp_ph108_H2
zmp_ph108_H5
zmp_ph108_B2
zmp_ph108_B6
zmp_ph108_B7
zmp_ph108_C4
zmp_ph108_C5
zmp_ph108_D4
zmp_ph97_G6
zmp_ph97_G9
zmp_ph97_H2
zmp_ph97_B10
zmp_ph97_C2
zmp_ph97_C4
zmp_ph97_B8
zmp_ph97_C1
zmp_ph97_D2
zmp_ph97_D5
zmp_ph111_G5
zmp_ph111_G11
zmp_ph111_H1
zmp_ph111_B1a
zmp_ph111_B5
zmp_ph111_B8
zmp_ph111_B6
zmp_ph111_B7
zmp_ph111_B9
zmp_ph111_B10
zmp_ph114_G4
zmp_ph114_G11
zmp_ph114_H1
zmp_ph114_H2
zmp_ph114_A9
zmp_ph114_A11
zmp_ph114_B2
zmp_ph114_B4
zmp_ph114_D8
zmp_ph114_E2

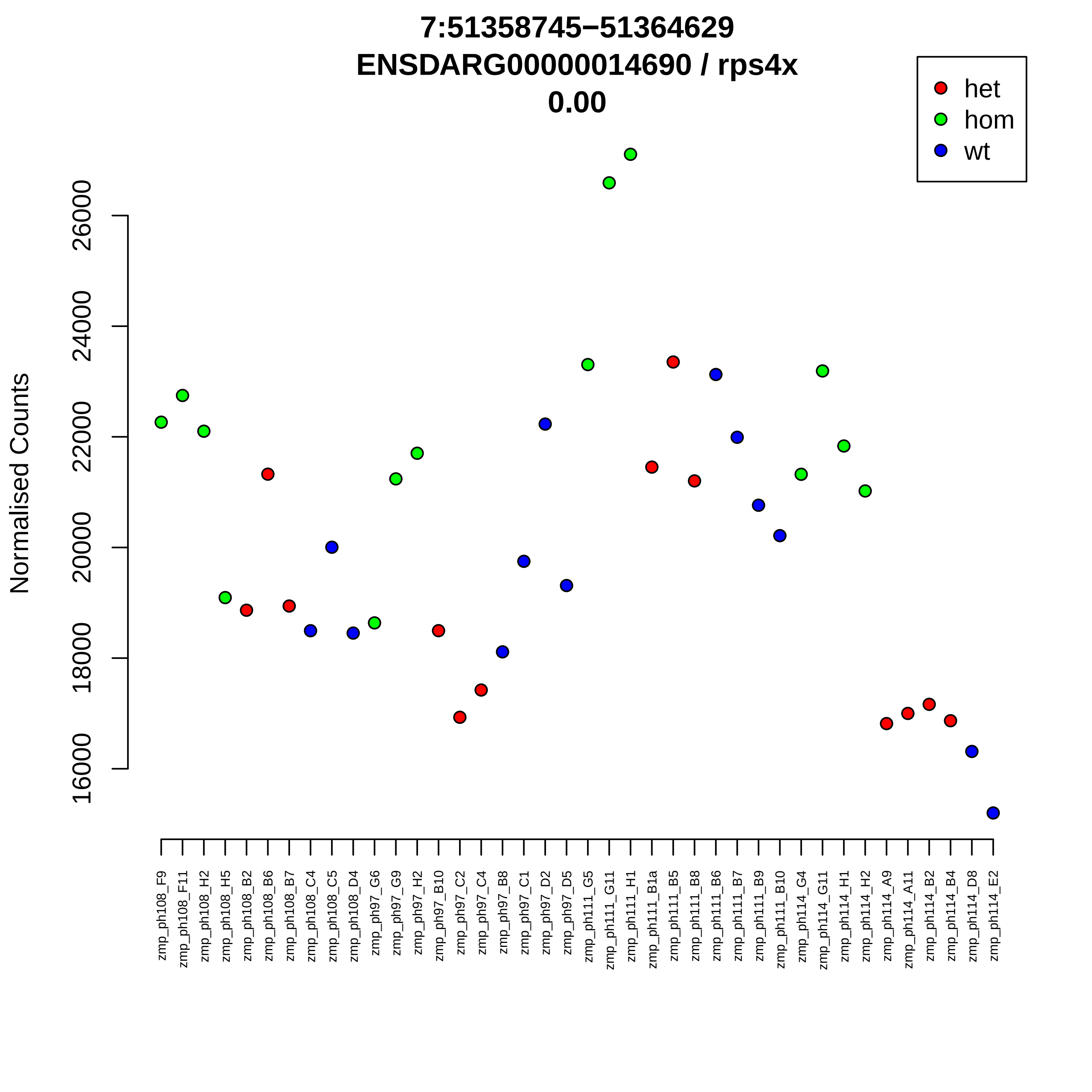
7:51358745−51364629
ENSDARG00000014690 / rps4x
0.00
het
hom
wt
Normalised Counts
16000
18000
20000
22000
24000
26000
zmp_ph108_F9
zmp_ph108_F11
zmp_ph108_H2
zmp_ph108_H5
zmp_ph108_B2
zmp_ph108_B6
zmp_ph108_B7
zmp_ph108_C4
zmp_ph108_C5
zmp_ph108_D4
zmp_ph97_G6
zmp_ph97_G9
zmp_ph97_H2
zmp_ph97_B10
zmp_ph97_C2
zmp_ph97_C4
zmp_ph97_B8
zmp_ph97_C1
zmp_ph97_D2
zmp_ph97_D5
zmp_ph111_G5
zmp_ph111_G11
zmp_ph111_H1
zmp_ph111_B1a
zmp_ph111_B5
zmp_ph111_B8
zmp_ph111_B6
zmp_ph111_B7
zmp_ph111_B9
zmp_ph111_B10
zmp_ph114_G4
zmp_ph114_G11
zmp_ph114_H1
zmp_ph114_H2
zmp_ph114_A9
zmp_ph114_A11
zmp_ph114_B2
zmp_ph114_B4
zmp_ph114_D8
zmp_ph114_E2

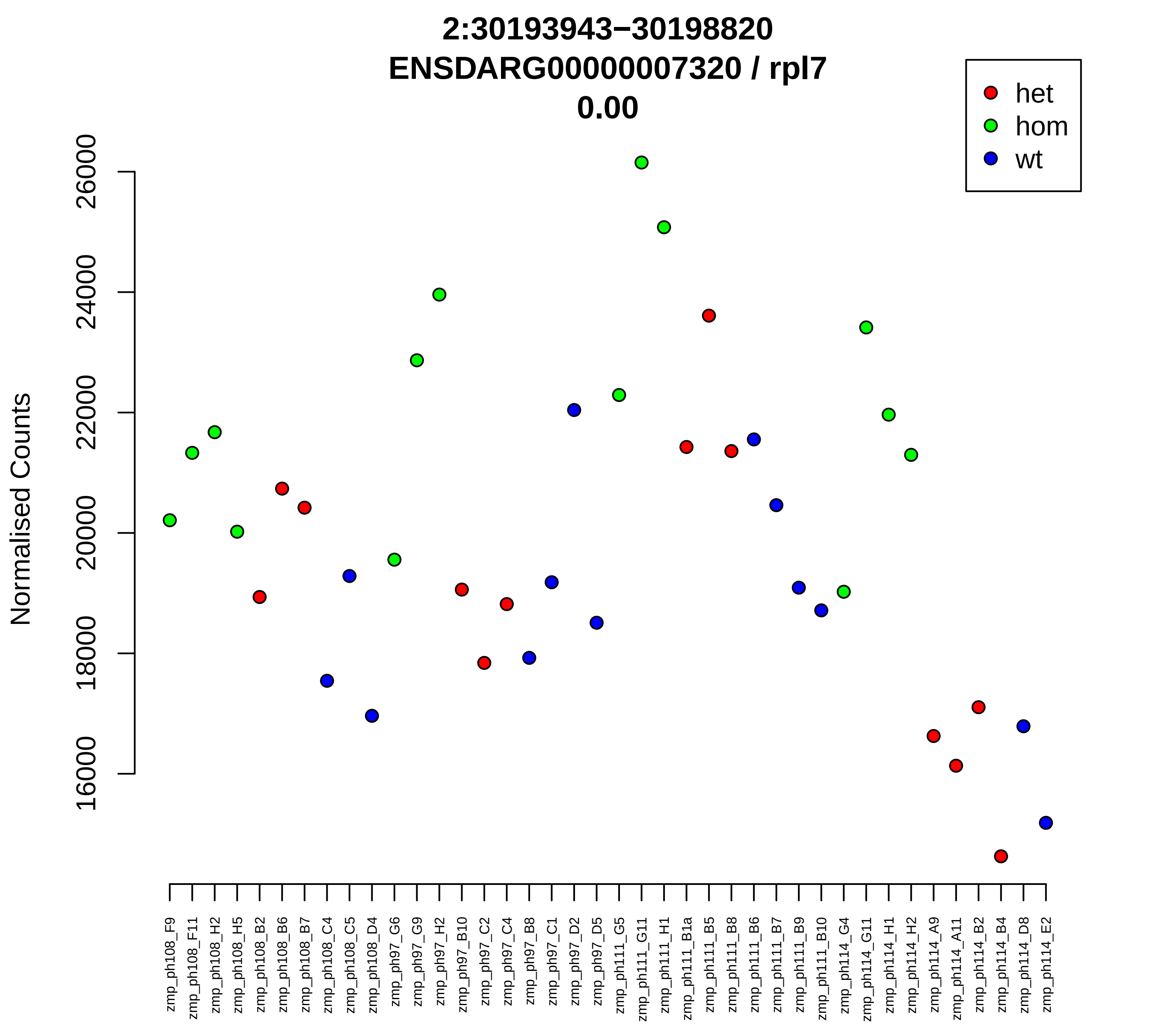

2:30193943−30198820
ENSDARG00000007320 / rpl7
0.00
het
hom
wt
Normalised Counts
16000
18000
20000
22000
24000
26000
zmp_ph108_F9
zmp_ph108_F11
zmp_ph108_H2
zmp_ph108_H5
zmp_ph108_B2
zmp_ph108_B6
zmp_ph108_B7
zmp_ph108_C4
zmp_ph108_C5
zmp_ph108_D4
zmp_ph97_G6
zmp_ph97_G9
zmp_ph97_H2
zmp_ph97_B10
zmp_ph97_C2
zmp_ph97_C4
zmp_ph97_B8
zmp_ph97_C1
zmp_ph97_D2
zmp_ph97_D5
zmp_ph111_G5
zmp_ph111_G11
zmp_ph111_H1
zmp_ph111_B1a
zmp_ph111_B5
zmp_ph111_B8
zmp_ph111_B6
zmp_ph111_B7
zmp_ph111_B9
zmp_ph111_B10
zmp_ph114_G4
zmp_ph114_G11
zmp_ph114_H1
zmp_ph114_H2
zmp_ph114_A9
zmp_ph114_A11
zmp_ph114_B2
zmp_ph114_B4
zmp_ph114_D8
zmp_ph114_E2

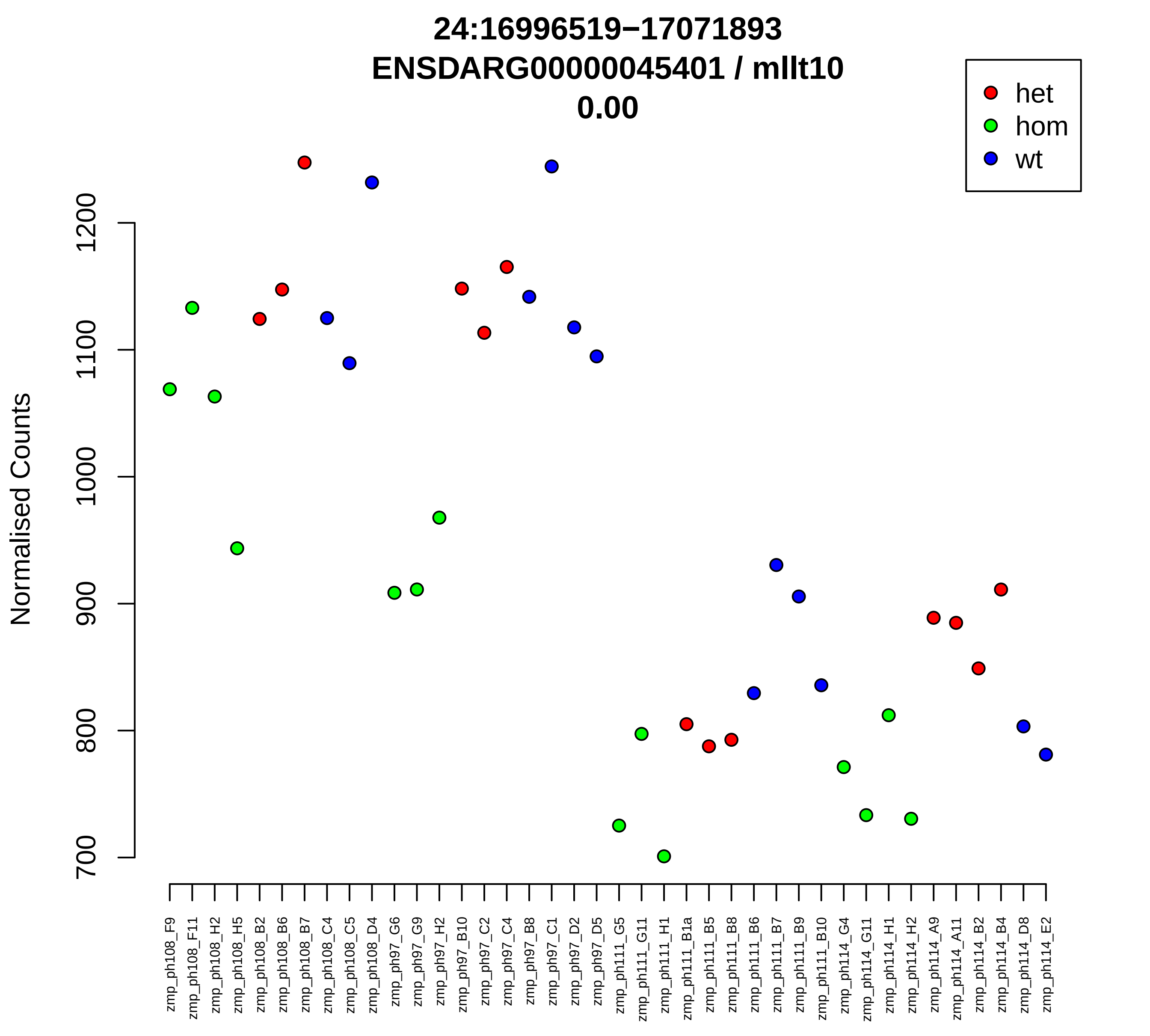
24:16996519−17071893
ENSDARG00000045401 / mllt10
0.00
het
hom
wt
Normalised Counts
700
800
900
1000
1100
1200
zmp_ph108_F9
zmp_ph108_F11
zmp_ph108_H2
zmp_ph108_H5
zmp_ph108_B2
zmp_ph108_B6
zmp_ph108_B7
zmp_ph108_C4
zmp_ph108_C5
zmp_ph108_D4
zmp_ph97_G6
zmp_ph97_G9
zmp_ph97_H2
zmp_ph97_B10
zmp_ph97_C2
zmp_ph97_C4
zmp_ph97_B8
zmp_ph97_C1
zmp_ph97_D2
zmp_ph97_D5
zmp_ph111_G5
zmp_ph111_G11
zmp_ph111_H1
zmp_ph111_B1a
zmp_ph111_B5
zmp_ph111_B8
zmp_ph111_B6
zmp_ph111_B7
zmp_ph111_B9
zmp_ph111_B10
zmp_ph114_G4
zmp_ph114_G11
zmp_ph114_H1
zmp_ph114_H2
zmp_ph114_A9
zmp_ph114_A11
zmp_ph114_B2
zmp_ph114_B4
zmp_ph114_D8
zmp_ph114_E2

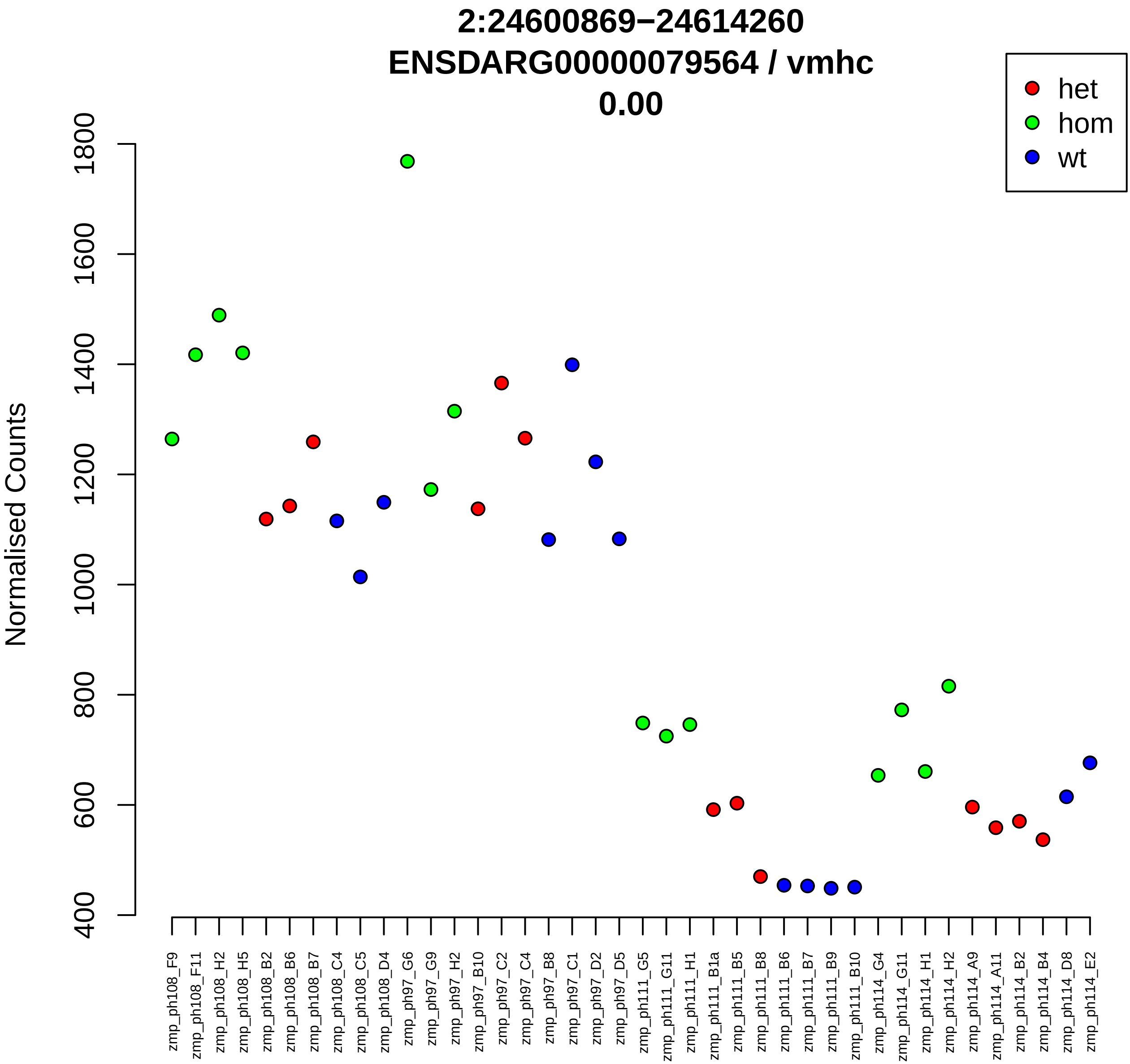
2:24600869−24614260
ENSDARG00000079564 / vmhc
0.00
het
hom
wt
Normalised Counts
400
600
800
1000
1200
1400
1600
1800
zmp_ph108_F9
zmp_ph108_F11
zmp_ph108_H2
zmp_ph108_H5
zmp_ph108_B2
zmp_ph108_B6
zmp_ph108_B7
zmp_ph108_C4
zmp_ph108_C5
zmp_ph108_D4
zmp_ph97_G6
zmp_ph97_G9
zmp_ph97_H2
zmp_ph97_B10
zmp_ph97_C2
zmp_ph97_C4
zmp_ph97_B8
zmp_ph97_C1
zmp_ph97_D2
zmp_ph97_D5
zmp_ph111_G5
zmp_ph111_G11
zmp_ph111_H1
zmp_ph111_B1a
zmp_ph111_B5
zmp_ph111_B8
zmp_ph111_B6
zmp_ph111_B7
zmp_ph111_B9
zmp_ph111_B10
zmp_ph114_G4
zmp_ph114_G11
zmp_ph114_H1
zmp_ph114_H2
zmp_ph114_A9
zmp_ph114_A11
zmp_ph114_B2
zmp_ph114_B4
zmp_ph114_D8
zmp_ph114_E2

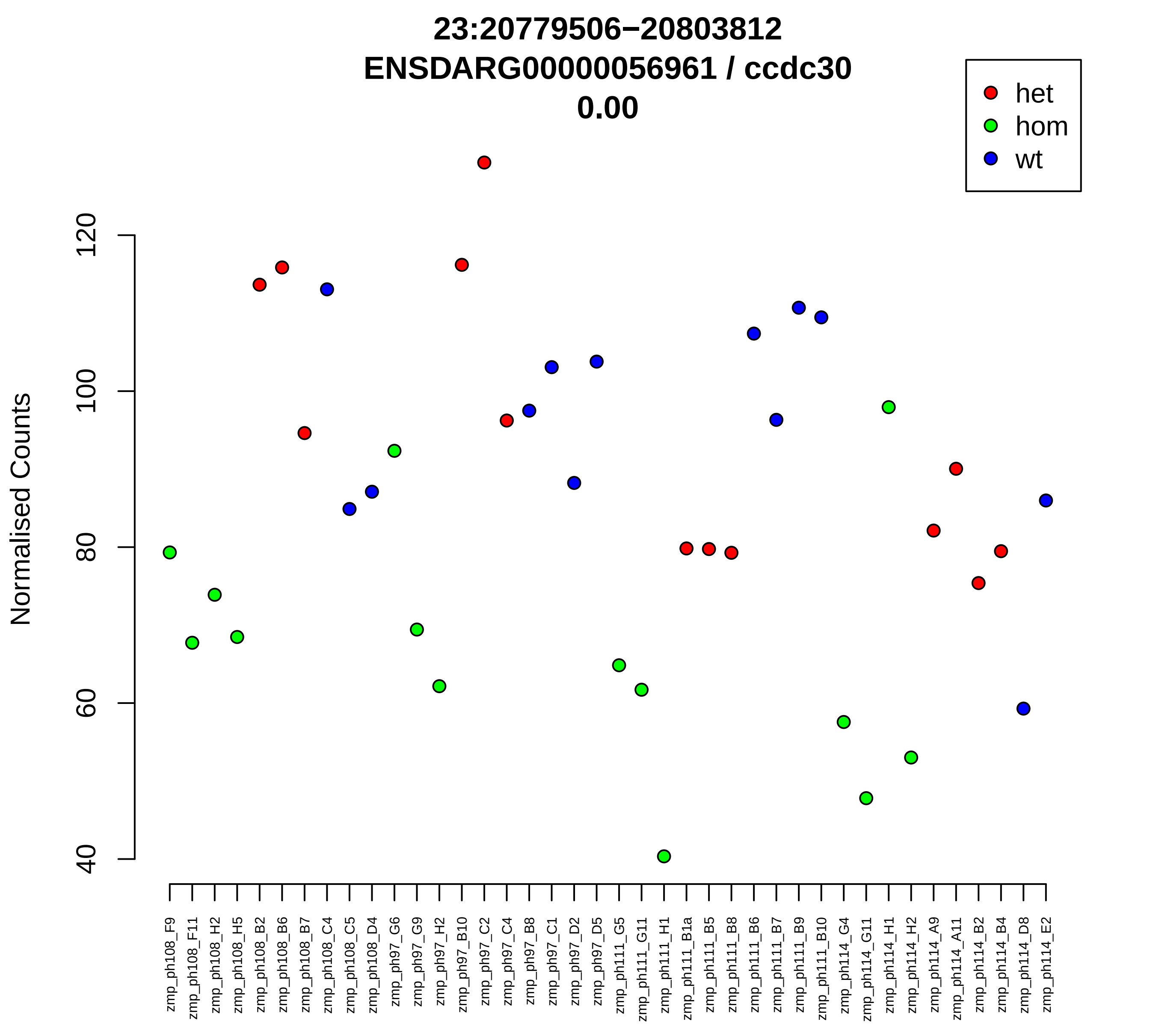

23:20779506−20803812
ENSDARG00000056961 / ccdc30
0.00
het
hom
wt
Normalised Counts
40
60
80
100
120
zmp_ph108_F9
zmp_ph108_F11
zmp_ph108_H2
zmp_ph108_H5
zmp_ph108_B2
zmp_ph108_B6
zmp_ph108_B7
zmp_ph108_C4
zmp_ph108_C5
zmp_ph108_D4
zmp_ph97_G6
zmp_ph97_G9
zmp_ph97_H2
zmp_ph97_B10
zmp_ph97_C2
zmp_ph97_C4
zmp_ph97_B8
zmp_ph97_C1
zmp_ph97_D2
zmp_ph97_D5
zmp_ph111_G5
zmp_ph111_G11
zmp_ph111_H1
zmp_ph111_B1a
zmp_ph111_B5
zmp_ph111_B8
zmp_ph111_B6
zmp_ph111_B7
zmp_ph111_B9
zmp_ph111_B10
zmp_ph114_G4
zmp_ph114_G11
zmp_ph114_H1
zmp_ph114_H2
zmp_ph114_A9
zmp_ph114_A11
zmp_ph114_B2
zmp_ph114_B4
zmp_ph114_D8
zmp_ph114_E2

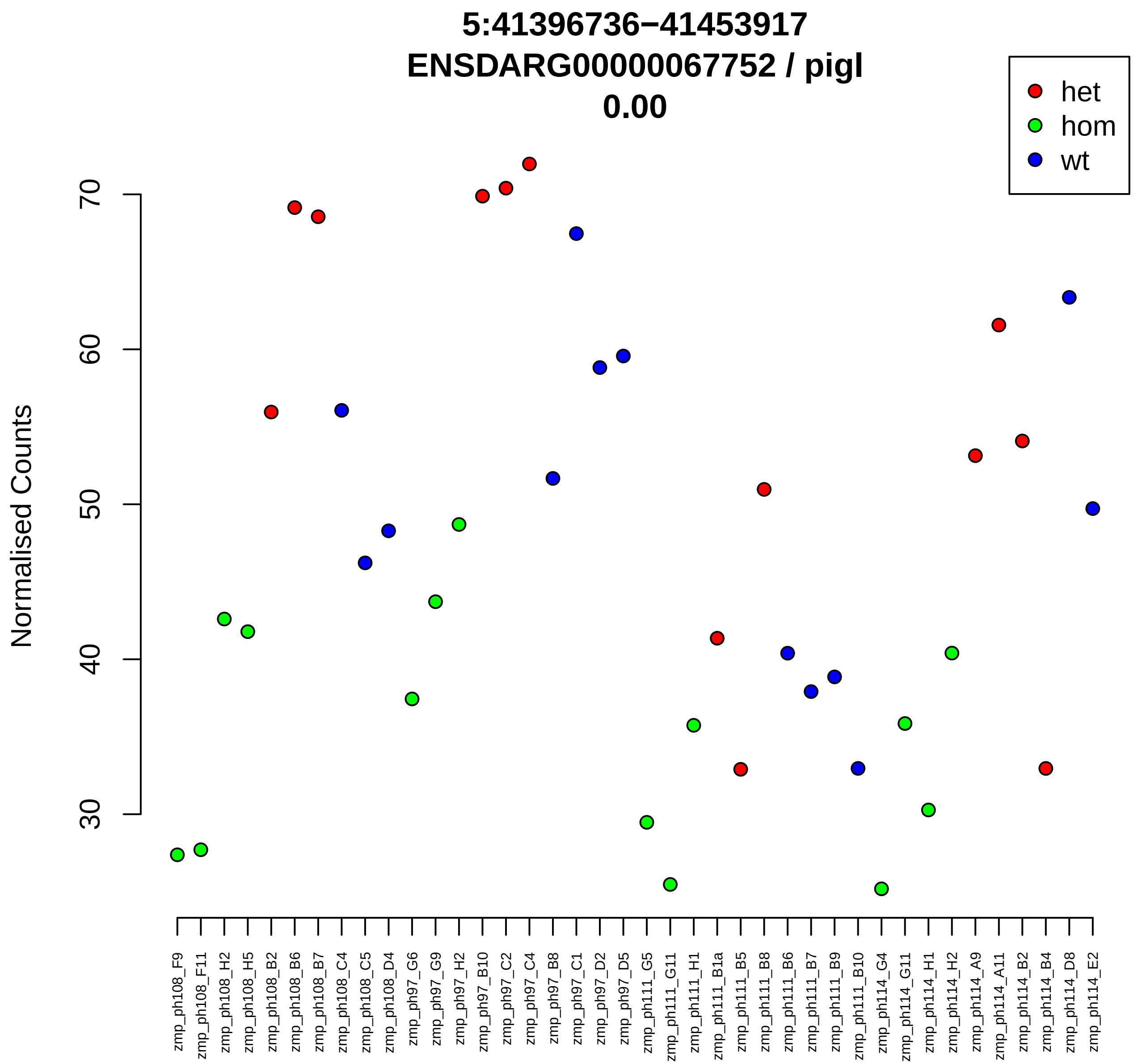

5:41396736−41453917
ENSDARG00000067752 / pigl
0.00
het
hom
wt
Normalised Counts
30
40
50
60
70
zmp_ph108_F9
zmp_ph108_F11
zmp_ph108_H2
zmp_ph108_H5
zmp_ph108_B2
zmp_ph108_B6
zmp_ph108_B7
zmp_ph108_C4
zmp_ph108_C5
zmp_ph108_D4
zmp_ph97_G6
zmp_ph97_G9
zmp_ph97_H2
zmp_ph97_B10
zmp_ph97_C2
zmp_ph97_C4
zmp_ph97_B8
zmp_ph97_C1
zmp_ph97_D2
zmp_ph97_D5
zmp_ph111_G5
zmp_ph111_G11
zmp_ph111_H1
zmp_ph111_B1a
zmp_ph111_B5
zmp_ph111_B8
zmp_ph111_B6
zmp_ph111_B7
zmp_ph111_B9
zmp_ph111_B10
zmp_ph114_G4
zmp_ph114_G11
zmp_ph114_H1
zmp_ph114_H2
zmp_ph114_A9
zmp_ph114_A11
zmp_ph114_B2
zmp_ph114_B4
zmp_ph114_D8
zmp_ph114_E2

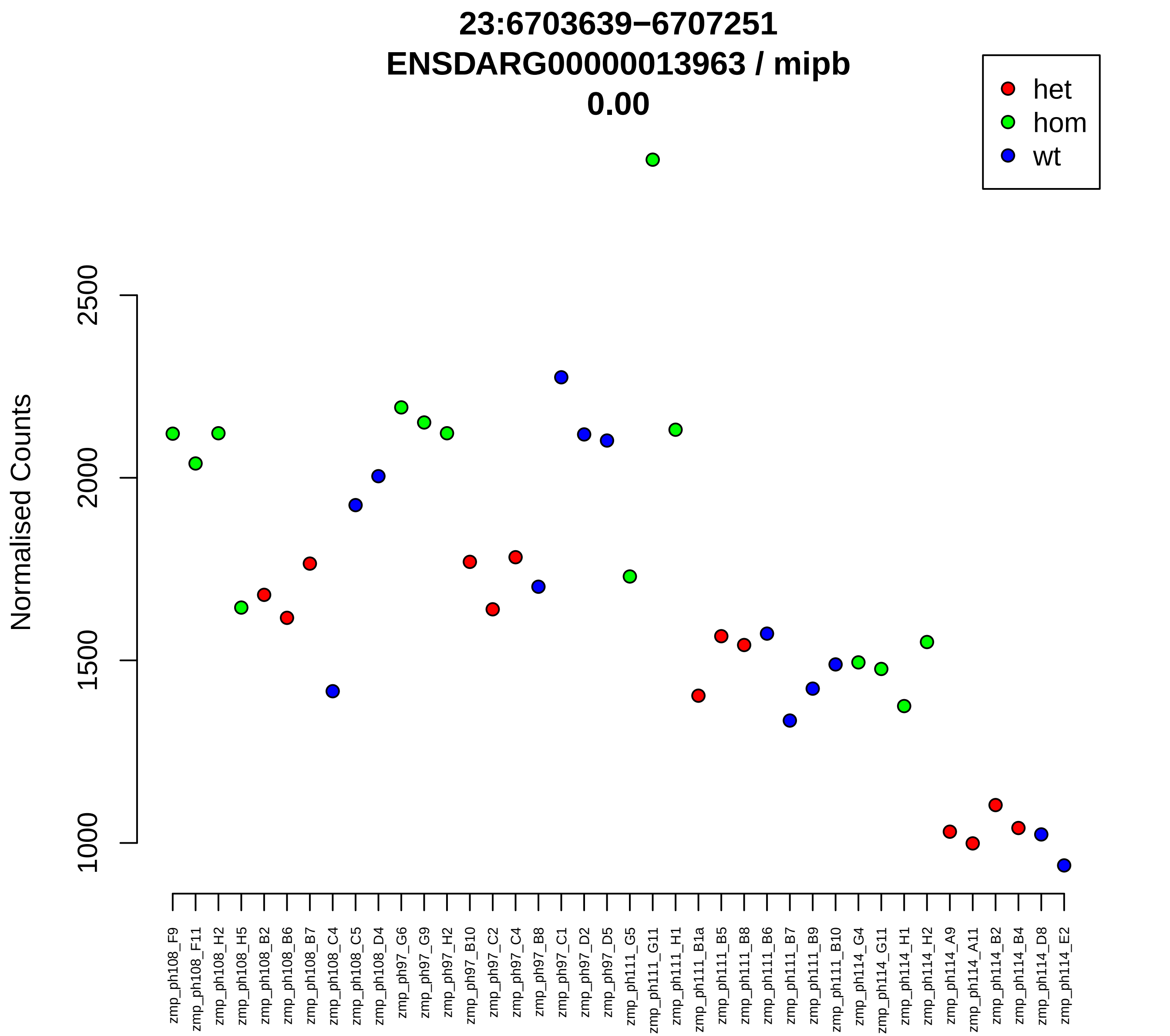
23:6703639−6707251
ENSDARG00000013963 / mipb
0.00
het
hom
wt
Normalised Counts
1000
1500
2000
2500
zmp_ph108_F9
zmp_ph108_F11
zmp_ph108_H2
zmp_ph108_H5
zmp_ph108_B2
zmp_ph108_B6
zmp_ph108_B7
zmp_ph108_C4
zmp_ph108_C5
zmp_ph108_D4
zmp_ph97_G6
zmp_ph97_G9
zmp_ph97_H2
zmp_ph97_B10
zmp_ph97_C2
zmp_ph97_C4
zmp_ph97_B8
zmp_ph97_C1
zmp_ph97_D2
zmp_ph97_D5
zmp_ph111_G5
zmp_ph111_G11
zmp_ph111_H1
zmp_ph111_B1a
zmp_ph111_B5
zmp_ph111_B8
zmp_ph111_B6
zmp_ph111_B7
zmp_ph111_B9
zmp_ph111_B10
zmp_ph114_G4
zmp_ph114_G11
zmp_ph114_H1
zmp_ph114_H2
zmp_ph114_A9
zmp_ph114_A11
zmp_ph114_B2
zmp_ph114_B4
zmp_ph114_D8
zmp_ph114_E2

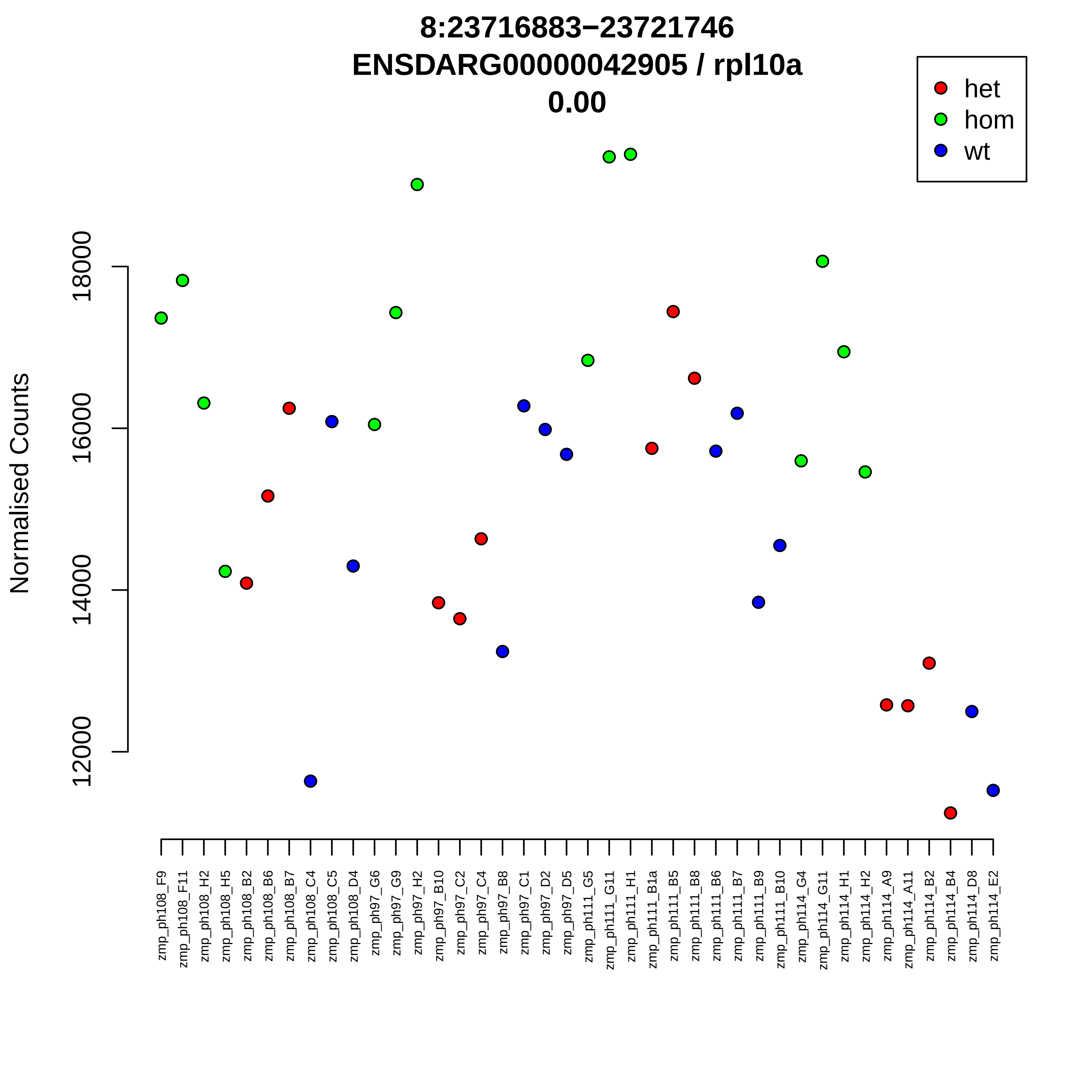

8:23716883−23721746
ENSDARG00000042905 / rpl10a
0.00
het
hom
wt
Normalised Counts
12000
14000
16000
18000
zmp_ph108_F9
zmp_ph108_F11
zmp_ph108_H2
zmp_ph108_H5
zmp_ph108_B2
zmp_ph108_B6
zmp_ph108_B7
zmp_ph108_C4
zmp_ph108_C5
zmp_ph108_D4
zmp_ph97_G6
zmp_ph97_G9
zmp_ph97_H2
zmp_ph97_B10
zmp_ph97_C2
zmp_ph97_C4
zmp_ph97_B8
zmp_ph97_C1
zmp_ph97_D2
zmp_ph97_D5
zmp_ph111_G5
zmp_ph111_G11
zmp_ph111_H1
zmp_ph111_B1a
zmp_ph111_B5
zmp_ph111_B8
zmp_ph111_B6
zmp_ph111_B7
zmp_ph111_B9
zmp_ph111_B10
zmp_ph114_G4
zmp_ph114_G11
zmp_ph114_H1
zmp_ph114_H2
zmp_ph114_A9
zmp_ph114_A11
zmp_ph114_B2
zmp_ph114_B4
zmp_ph114_D8
zmp_ph114_E2

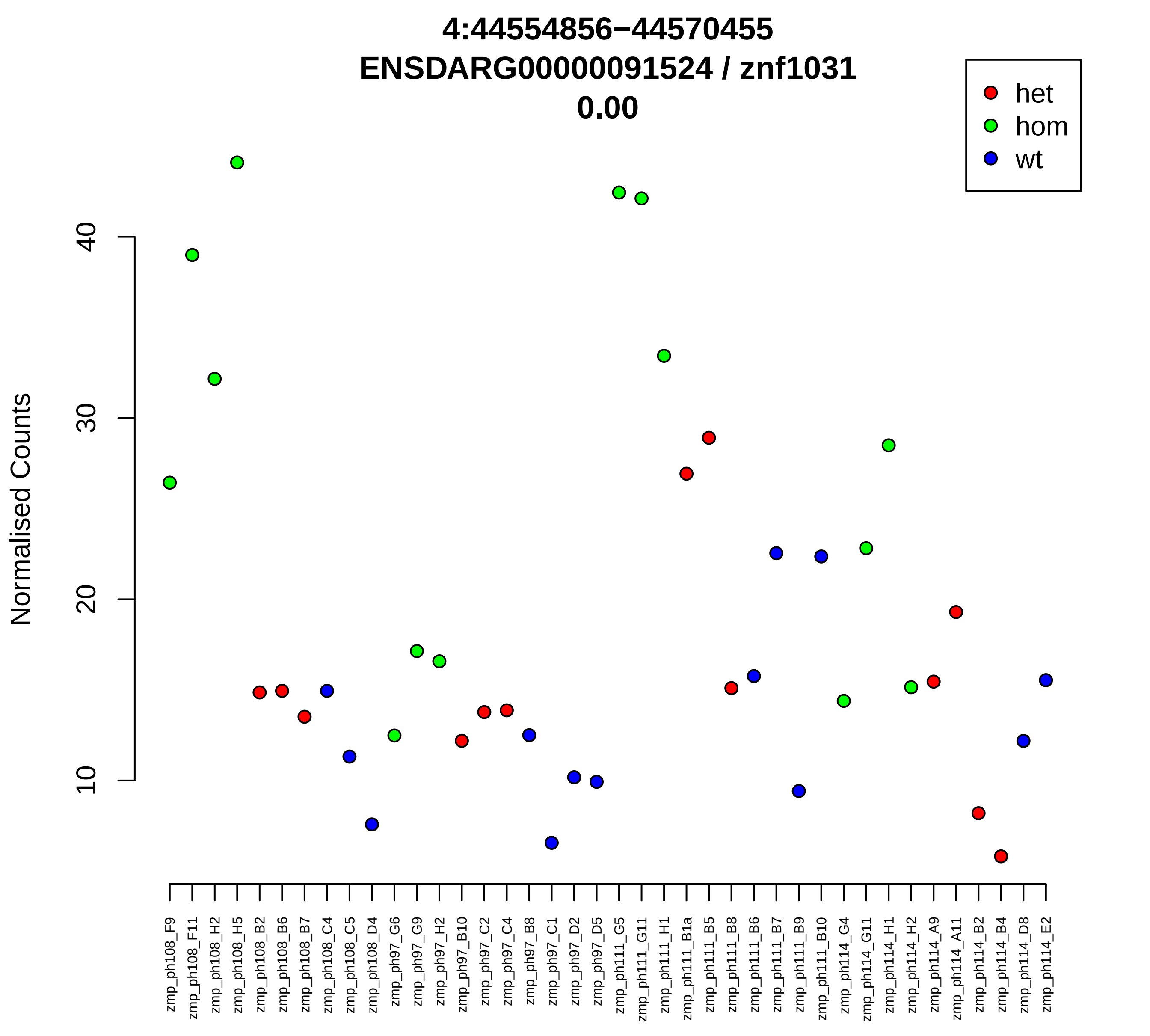

4:44554856−44570455
ENSDARG00000091524 / znf1031
0.00
het
hom
wt
Normalised Counts
10
20
30
40
zmp_ph108_F9
zmp_ph108_F11
zmp_ph108_H2
zmp_ph108_H5
zmp_ph108_B2
zmp_ph108_B6
zmp_ph108_B7
zmp_ph108_C4
zmp_ph108_C5
zmp_ph108_D4
zmp_ph97_G6
zmp_ph97_G9
zmp_ph97_H2
zmp_ph97_B10
zmp_ph97_C2
zmp_ph97_C4
zmp_ph97_B8
zmp_ph97_C1
zmp_ph97_D2
zmp_ph97_D5
zmp_ph111_G5
zmp_ph111_G11
zmp_ph111_H1
zmp_ph111_B1a
zmp_ph111_B5
zmp_ph111_B8
zmp_ph111_B6
zmp_ph111_B7
zmp_ph111_B9
zmp_ph111_B10
zmp_ph114_G4
zmp_ph114_G11
zmp_ph114_H1
zmp_ph114_H2
zmp_ph114_A9
zmp_ph114_A11
zmp_ph114_B2
zmp_ph114_B4
zmp_ph114_D8
zmp_ph114_E2

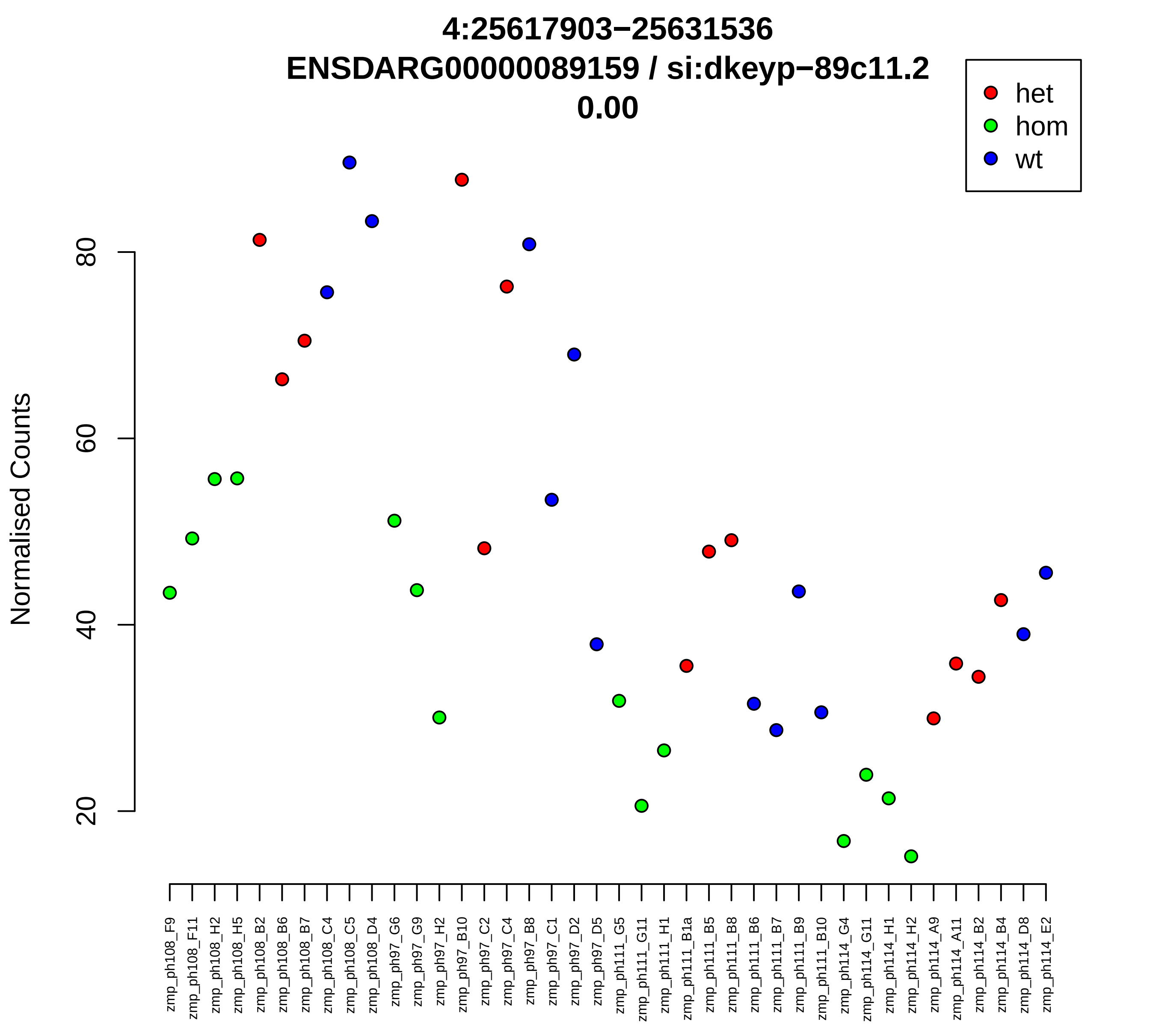
4:25617903−25631536
ENSDARG00000089159 / si:dkeyp−89c11.2
0.00
het
hom
wt
Normalised Counts
20
40
60
80
zmp_ph108_F9
zmp_ph108_F11
zmp_ph108_H2
zmp_ph108_H5
zmp_ph108_B2
zmp_ph108_B6
zmp_ph108_B7
zmp_ph108_C4
zmp_ph108_C5
zmp_ph108_D4
zmp_ph97_G6
zmp_ph97_G9
zmp_ph97_H2
zmp_ph97_B10
zmp_ph97_C2
zmp_ph97_C4
zmp_ph97_B8
zmp_ph97_C1
zmp_ph97_D2
zmp_ph97_D5
zmp_ph111_G5
zmp_ph111_G11
zmp_ph111_H1
zmp_ph111_B1a
zmp_ph111_B5
zmp_ph111_B8
zmp_ph111_B6
zmp_ph111_B7
zmp_ph111_B9
zmp_ph111_B10
zmp_ph114_G4
zmp_ph114_G11
zmp_ph114_H1
zmp_ph114_H2
zmp_ph114_A9
zmp_ph114_A11
zmp_ph114_B2
zmp_ph114_B4
zmp_ph114_D8
zmp_ph114_E2

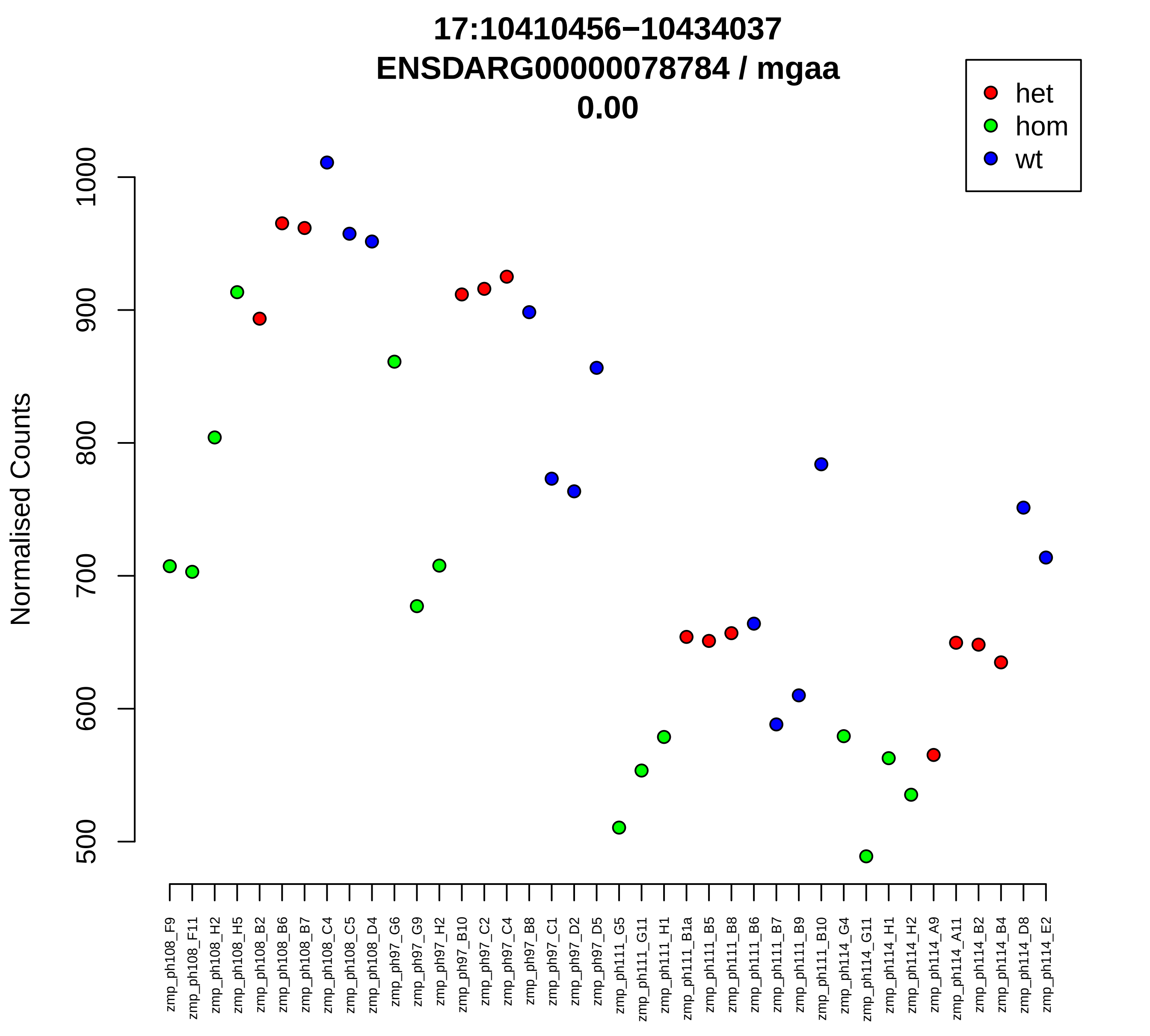
17:10410456−10434037
ENSDARG00000078784 / mgaa
0.00
het
hom
wt
Normalised Counts
500
600
700
800
900
1000
zmp_ph108_F9
zmp_ph108_F11
zmp_ph108_H2
zmp_ph108_H5
zmp_ph108_B2
zmp_ph108_B6
zmp_ph108_B7
zmp_ph108_C4
zmp_ph108_C5
zmp_ph108_D4
zmp_ph97_G6
zmp_ph97_G9
zmp_ph97_H2
zmp_ph97_B10
zmp_ph97_C2
zmp_ph97_C4
zmp_ph97_B8
zmp_ph97_C1
zmp_ph97_D2
zmp_ph97_D5
zmp_ph111_G5
zmp_ph111_G11
zmp_ph111_H1
zmp_ph111_B1a
zmp_ph111_B5
zmp_ph111_B8
zmp_ph111_B6
zmp_ph111_B7
zmp_ph111_B9
zmp_ph111_B10
zmp_ph114_G4
zmp_ph114_G11
zmp_ph114_H1
zmp_ph114_H2
zmp_ph114_A9
zmp_ph114_A11
zmp_ph114_B2
zmp_ph114_B4
zmp_ph114_D8
zmp_ph114_E2

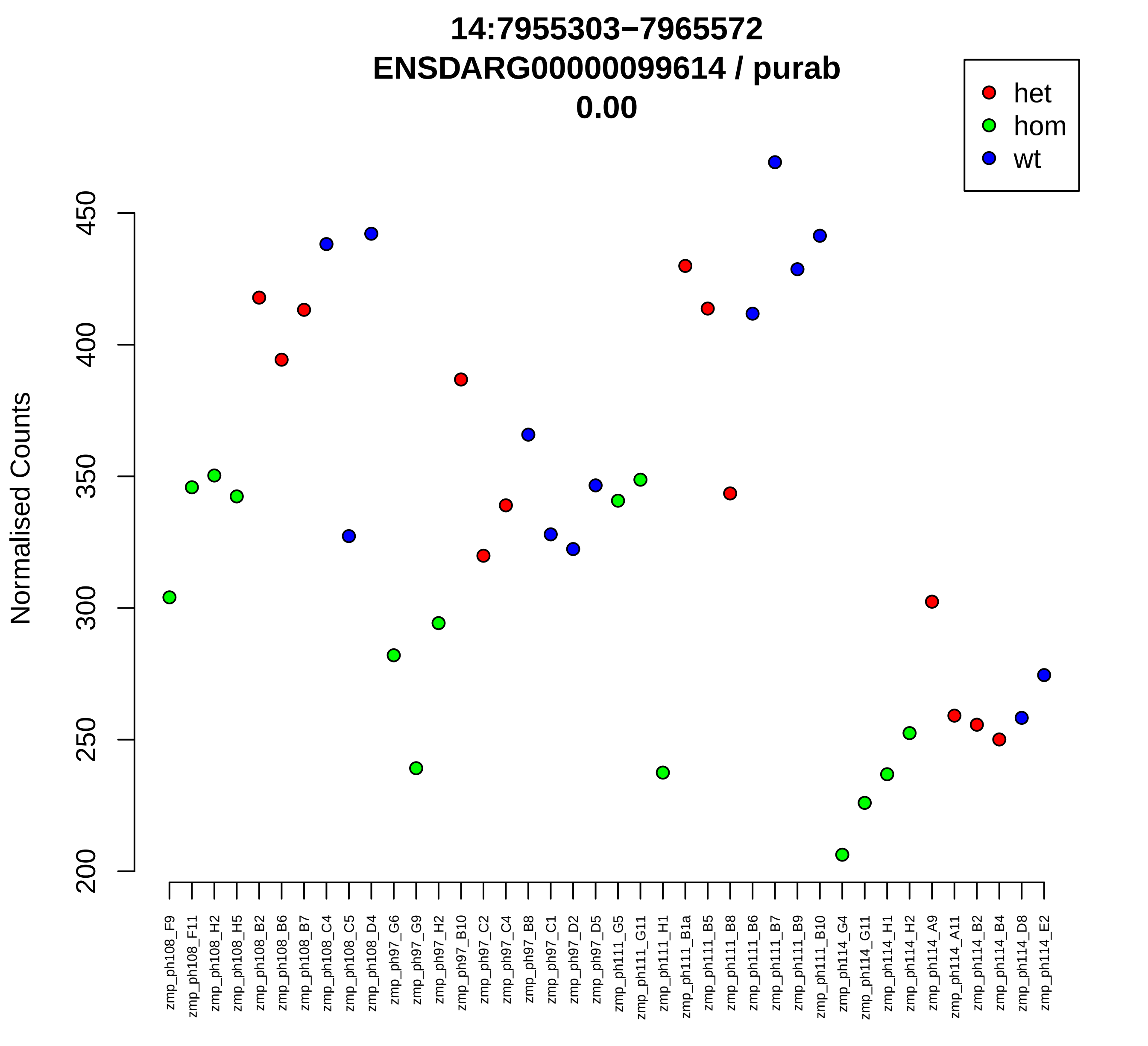
14:7955303−7965572
ENSDARG00000099614 / purab
0.00
het
hom
wt
Normalised Counts
200
250
300
350
400
450
zmp_ph108_F9
zmp_ph108_F11
zmp_ph108_H2
zmp_ph108_H5
zmp_ph108_B2
zmp_ph108_B6
zmp_ph108_B7
zmp_ph108_C4
zmp_ph108_C5
zmp_ph108_D4
zmp_ph97_G6
zmp_ph97_G9
zmp_ph97_H2
zmp_ph97_B10
zmp_ph97_C2
zmp_ph97_C4
zmp_ph97_B8
zmp_ph97_C1
zmp_ph97_D2
zmp_ph97_D5
zmp_ph111_G5
zmp_ph111_G11
zmp_ph111_H1
zmp_ph111_B1a
zmp_ph111_B5
zmp_ph111_B8
zmp_ph111_B6
zmp_ph111_B7
zmp_ph111_B9
zmp_ph111_B10
zmp_ph114_G4
zmp_ph114_G11
zmp_ph114_H1
zmp_ph114_H2
zmp_ph114_A9
zmp_ph114_A11
zmp_ph114_B2
zmp_ph114_B4
zmp_ph114_D8
zmp_ph114_E2

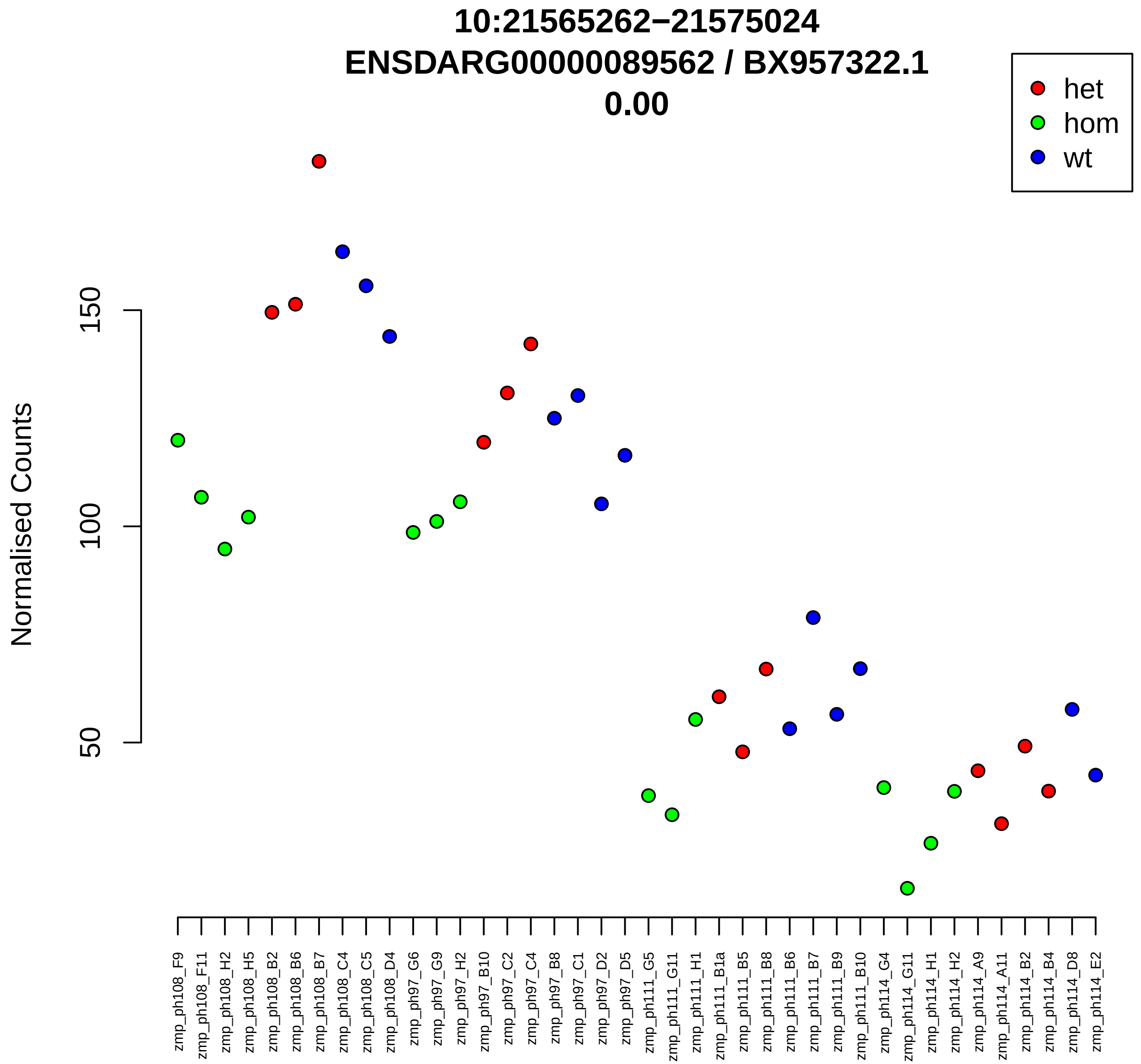

10:21565262−21575024
ENSDARG00000089562 / BX957322.1
0.00
het
hom
wt
Normalised Counts
50
100
150
zmp_ph108_F9
zmp_ph108_F11
zmp_ph108_H2
zmp_ph108_H5
zmp_ph108_B2
zmp_ph108_B6
zmp_ph108_B7
zmp_ph108_C4
zmp_ph108_C5
zmp_ph108_D4
zmp_ph97_G6
zmp_ph97_G9
zmp_ph97_H2
zmp_ph97_B10
zmp_ph97_C2
zmp_ph97_C4
zmp_ph97_B8
zmp_ph97_C1
zmp_ph97_D2
zmp_ph97_D5
zmp_ph111_G5
zmp_ph111_G11
zmp_ph111_H1
zmp_ph111_B1a
zmp_ph111_B5
zmp_ph111_B8
zmp_ph111_B6
zmp_ph111_B7
zmp_ph111_B9
zmp_ph111_B10
zmp_ph114_G4
zmp_ph114_G11
zmp_ph114_H1
zmp_ph114_H2
zmp_ph114_A9
zmp_ph114_A11
zmp_ph114_B2
zmp_ph114_B4
zmp_ph114_D8
zmp_ph114_E2

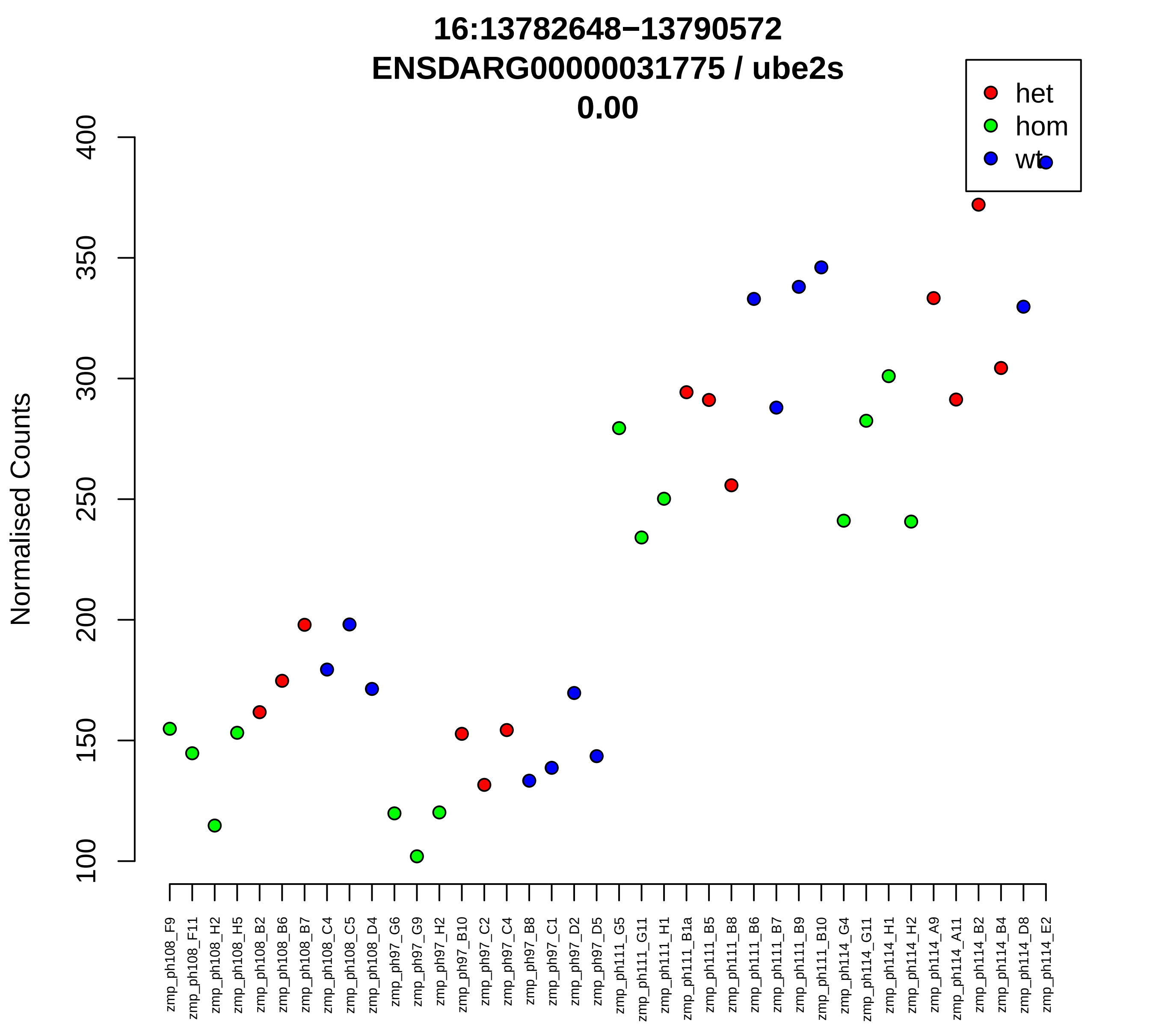
16:13782648−13790572
ENSDARG00000031775 / ube2s
0.00
Normalised Counts
het
hom
wt
100
150
200
250
300
350
400
zmp_ph108_F9
zmp_ph108_F11
zmp_ph108_H2
zmp_ph108_H5
zmp_ph108_B2
zmp_ph108_B6
zmp_ph108_B7
zmp_ph108_C4
zmp_ph108_C5
zmp_ph108_D4
zmp_ph97_G6
zmp_ph97_G9
zmp_ph97_H2
zmp_ph97_B10
zmp_ph97_C2
zmp_ph97_C4
zmp_ph97_B8
zmp_ph97_C1
zmp_ph97_D2
zmp_ph97_D5
zmp_ph111_G5
zmp_ph111_G11
zmp_ph111_H1
zmp_ph111_B1a
zmp_ph111_B5
zmp_ph111_B8
zmp_ph111_B6
zmp_ph111_B7
zmp_ph111_B9
zmp_ph111_B10
zmp_ph114_G4
zmp_ph114_G11
zmp_ph114_H1
zmp_ph114_H2
zmp_ph114_A9
zmp_ph114_A11
zmp_ph114_B2
zmp_ph114_B4
zmp_ph114_D8
zmp_ph114_E2

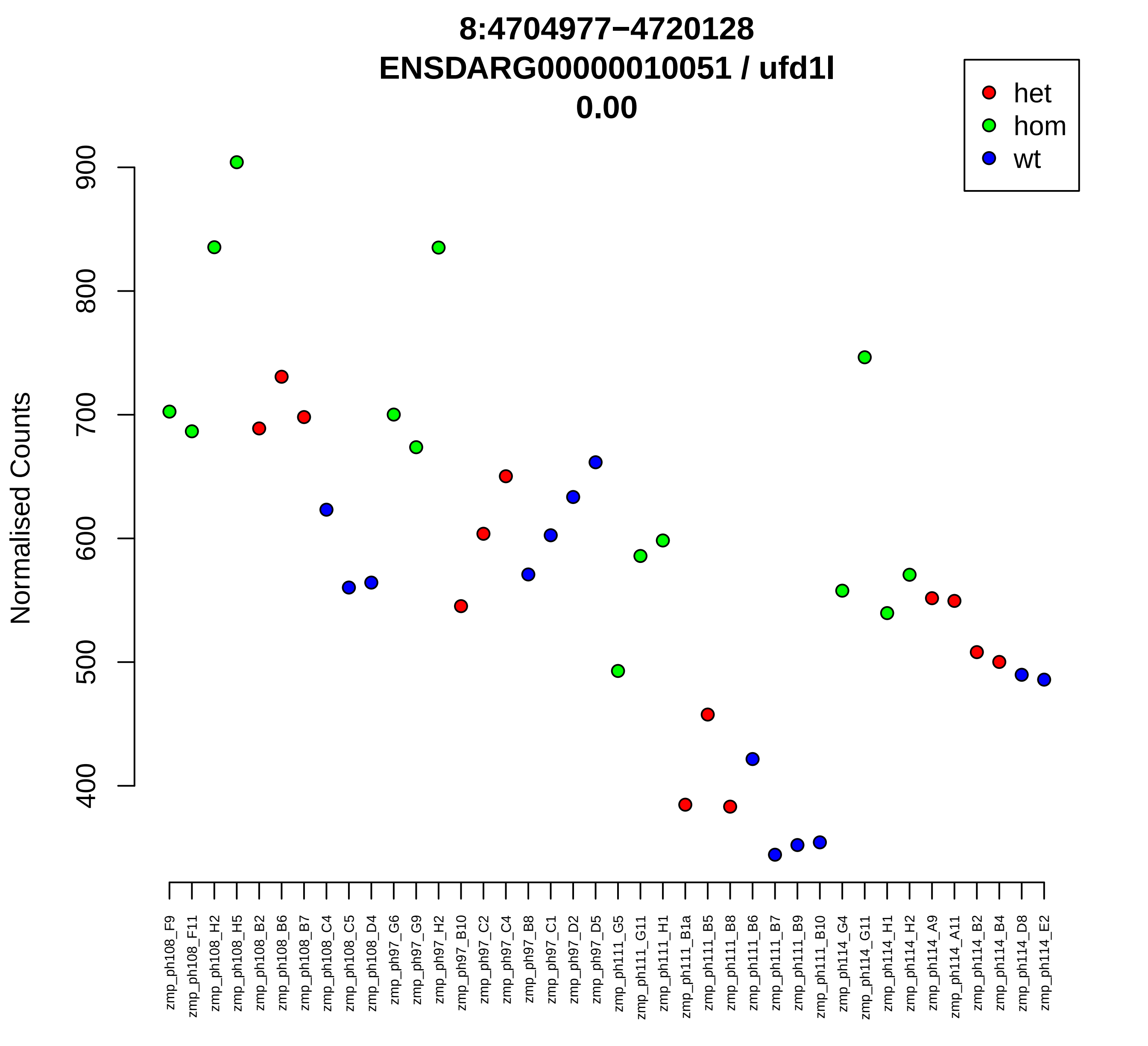
8:4704977−4720128
ENSDARG00000010051 / ufd1l
0.00
het
hom
wt
Normalised Counts
900
800
700
600
500
400
zmp_ph108_F9
zmp_ph108_F11
zmp_ph108_H2
zmp_ph108_H5
zmp_ph108_B2
zmp_ph108_B6
zmp_ph108_B7
zmp_ph108_C4
zmp_ph108_C5
zmp_ph108_D4
zmp_ph97_G6
zmp_ph97_G9
zmp_ph97_H2
zmp_ph97_B10
zmp_ph97_C2
zmp_ph97_C4
zmp_ph97_B8
zmp_ph97_C1
zmp_ph97_D2
zmp_ph97_D5
zmp_ph111_G5
zmp_ph111_G11
zmp_ph111_H1
zmp_ph111_B1a
zmp_ph111_B5
zmp_ph111_B8
zmp_ph111_B6
zmp_ph111_B7
zmp_ph111_B9
zmp_ph111_B10
zmp_ph114_G4
zmp_ph114_G11
zmp_ph114_H1
zmp_ph114_H2
zmp_ph114_A9
zmp_ph114_A11
zmp_ph114_B2
zmp_ph114_B4
zmp_ph114_D8
zmp_ph114_E2

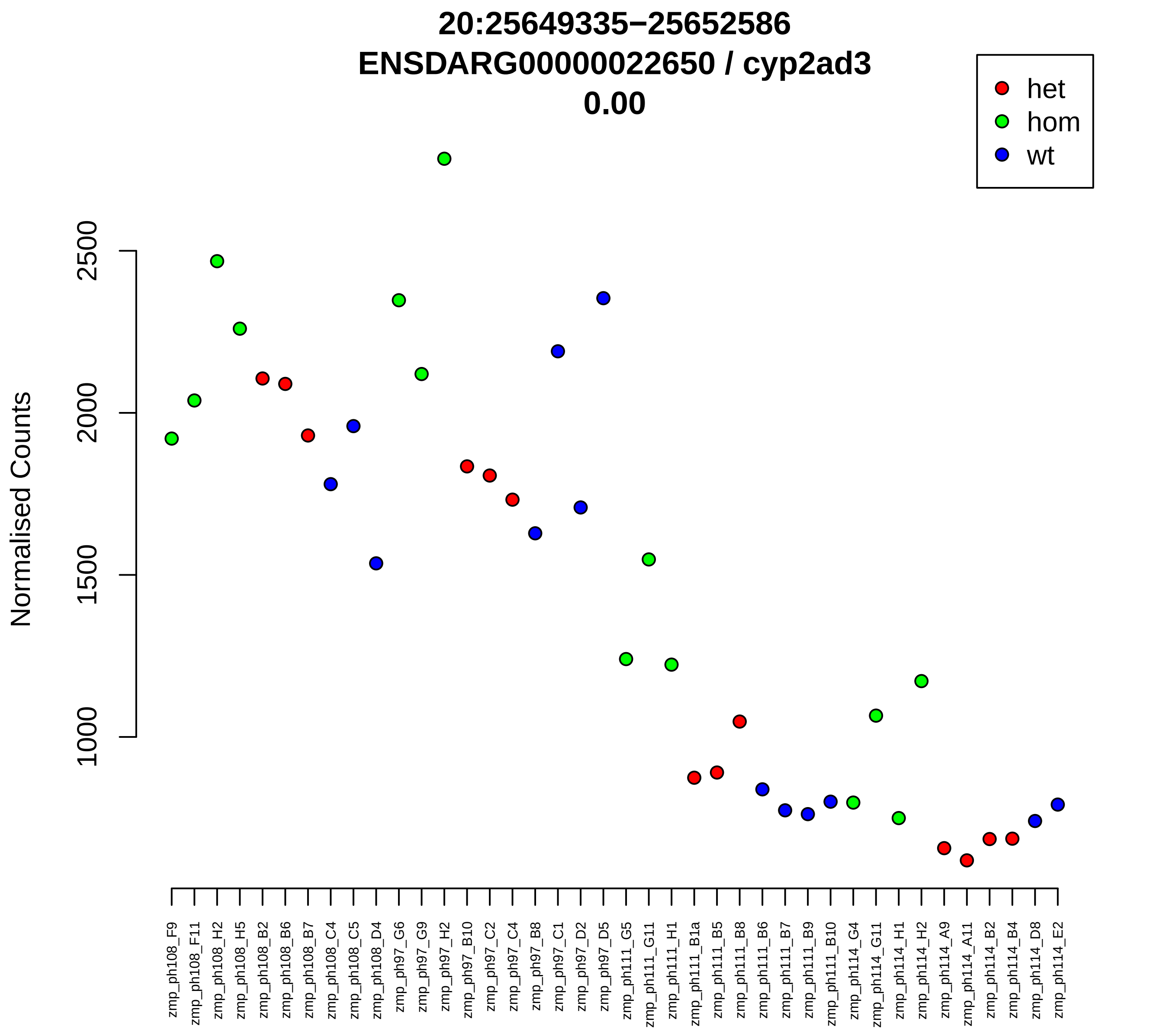
20:25649335−25652586
ENSDARG00000022650 / cyp2ad3
0.00
het
hom
wt
Normalised Counts
1000
1500
2000
2500
zmp_ph108_F9
zmp_ph108_F11
zmp_ph108_H2
zmp_ph108_H5
zmp_ph108_B2
zmp_ph108_B6
zmp_ph108_B7
zmp_ph108_C4
zmp_ph108_C5
zmp_ph108_D4
zmp_ph97_G6
zmp_ph97_G9
zmp_ph97_H2
zmp_ph97_B10
zmp_ph97_C2
zmp_ph97_C4
zmp_ph97_B8
zmp_ph97_C1
zmp_ph97_D2
zmp_ph97_D5
zmp_ph111_G5
zmp_ph111_G11
zmp_ph111_H1
zmp_ph111_B1a
zmp_ph111_B5
zmp_ph111_B8
zmp_ph111_B6
zmp_ph111_B7
zmp_ph111_B9
zmp_ph111_B10
zmp_ph114_G4
zmp_ph114_G11
zmp_ph114_H1
zmp_ph114_H2
zmp_ph114_A9
zmp_ph114_A11
zmp_ph114_B2
zmp_ph114_B4
zmp_ph114_D8
zmp_ph114_E2

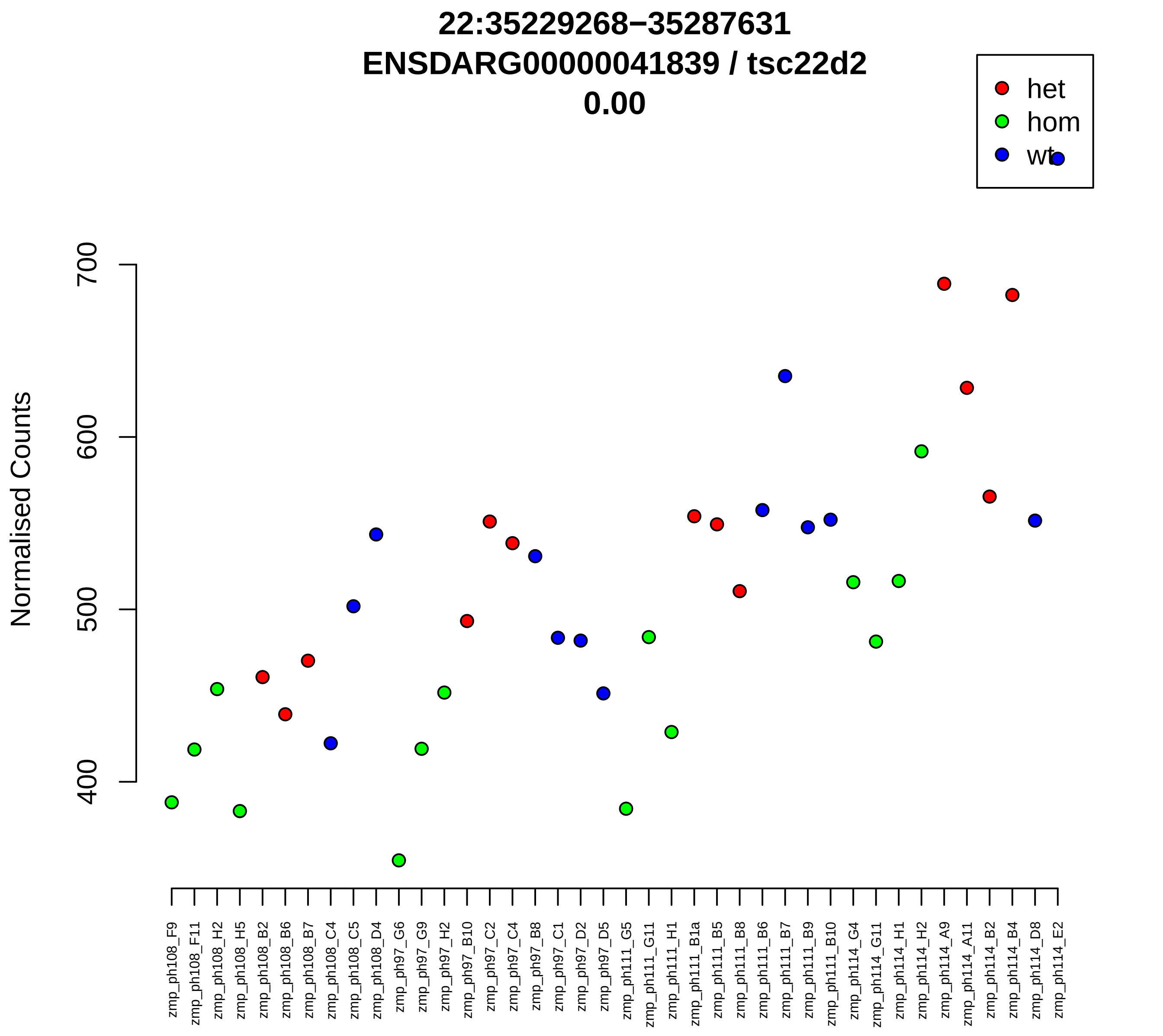

22:35229268−35287631
ENSDARG00000041839 / tsc22d2
0.00
het
hom
wt
Normalised Counts
400
500
600
700
zmp_ph108_F9
zmp_ph108_F11
zmp_ph108_H2
zmp_ph108_H5
zmp_ph108_B2
zmp_ph108_B6
zmp_ph108_B7
zmp_ph108_C4
zmp_ph108_C5
zmp_ph108_D4
zmp_ph97_G6
zmp_ph97_G9
zmp_ph97_H2
zmp_ph97_B10
zmp_ph97_C2
zmp_ph97_C4
zmp_ph97_B8
zmp_ph97_C1
zmp_ph97_D2
zmp_ph97_D5
zmp_ph111_G5
zmp_ph111_G11
zmp_ph111_H1
zmp_ph111_B1a
zmp_ph111_B5
zmp_ph111_B8
zmp_ph111_B6
zmp_ph111_B7
zmp_ph111_B9
zmp_ph111_B10
zmp_ph114_G4
zmp_ph114_G11
zmp_ph114_H1
zmp_ph114_H2
zmp_ph114_A9
zmp_ph114_A11
zmp_ph114_B2
zmp_ph114_B4
zmp_ph114_D8
zmp_ph114_E2

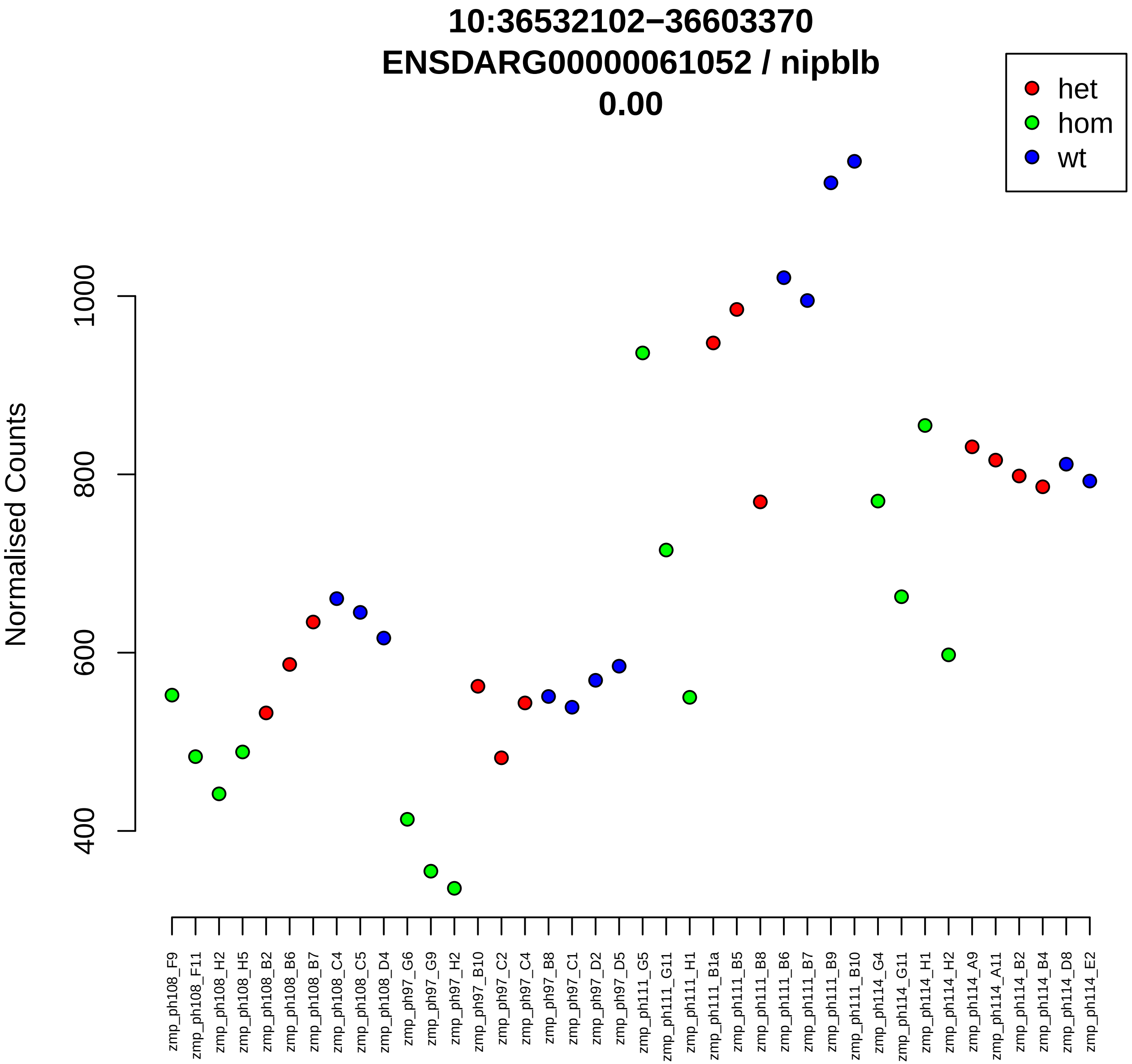
10:36532102−36603370
ENSDARG00000061052 / nipblb
0.00
het
hom
wt
Normalised Counts
400
600
800
1000
zmp_ph108_F9
zmp_ph108_F11
zmp_ph108_H2
zmp_ph108_H5
zmp_ph108_B2
zmp_ph108_B6
zmp_ph108_B7
zmp_ph108_C4
zmp_ph108_C5
zmp_ph108_D4
zmp_ph97_G6
zmp_ph97_G9
zmp_ph97_H2
zmp_ph97_B10
zmp_ph97_C2
zmp_ph97_C4
zmp_ph97_B8
zmp_ph97_C1
zmp_ph97_D2
zmp_ph97_D5
zmp_ph111_G5
zmp_ph111_G11
zmp_ph111_H1
zmp_ph111_B1a
zmp_ph111_B5
zmp_ph111_B8
zmp_ph111_B6
zmp_ph111_B7
zmp_ph111_B9
zmp_ph111_B10
zmp_ph114_G4
zmp_ph114_G11
zmp_ph114_H1
zmp_ph114_H2
zmp_ph114_A9
zmp_ph114_A11
zmp_ph114_B2
zmp_ph114_B4
zmp_ph114_D8
zmp_ph114_E2

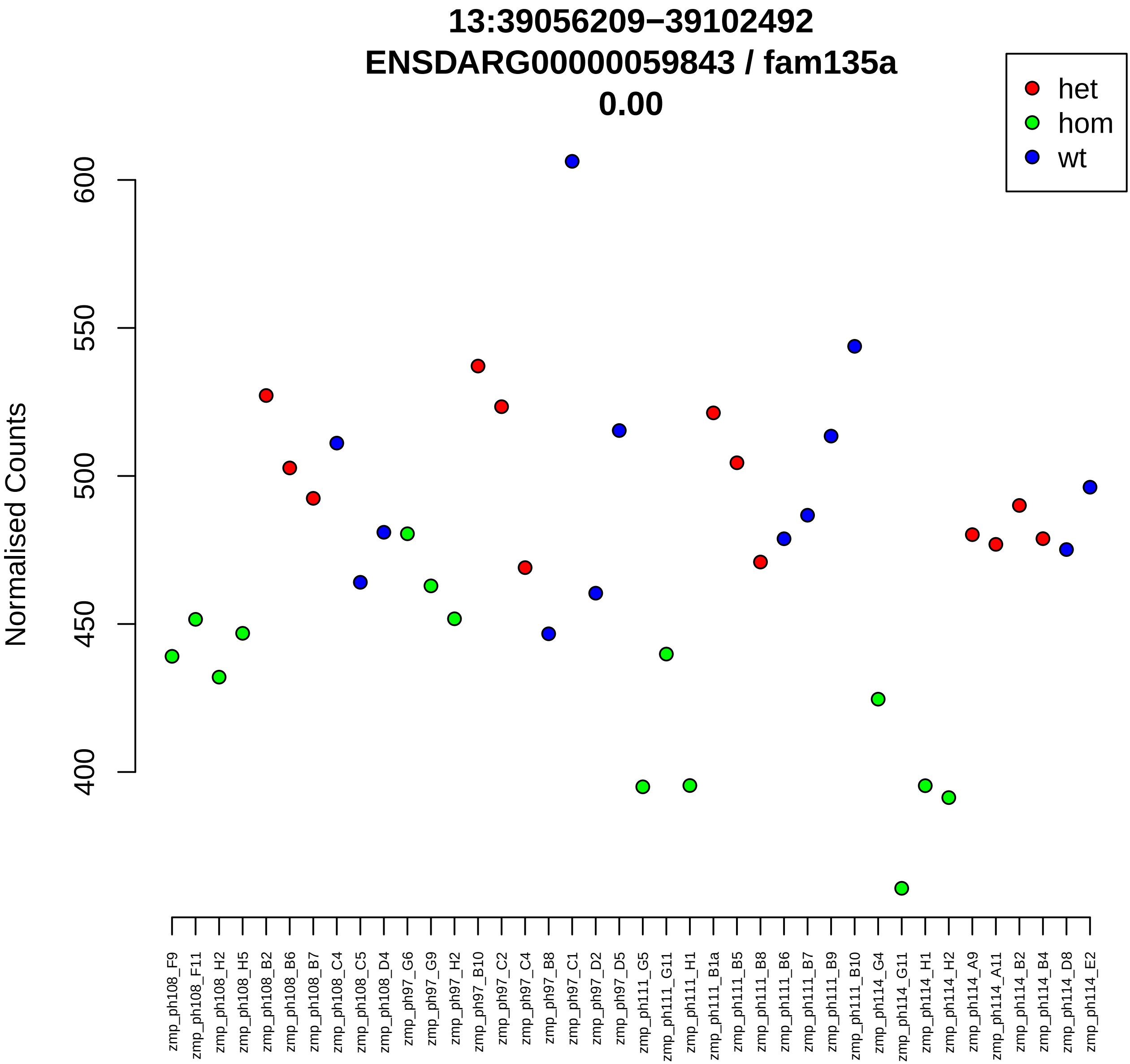

13:39056209−39102492
ENSDARG00000059843 / fam135a
0.00
het
hom
wt
Normalised Counts
400
450
500
550
600
zmp_ph108_F9
zmp_ph108_F11
zmp_ph108_H2
zmp_ph108_H5
zmp_ph108_B2
zmp_ph108_B6
zmp_ph108_B7
zmp_ph108_C4
zmp_ph108_C5
zmp_ph108_D4
zmp_ph97_G6
zmp_ph97_G9
zmp_ph97_H2
zmp_ph97_B10
zmp_ph97_C2
zmp_ph97_C4
zmp_ph97_B8
zmp_ph97_C1
zmp_ph97_D2
zmp_ph97_D5
zmp_ph111_G5
zmp_ph111_G11
zmp_ph111_H1
zmp_ph111_B1a
zmp_ph111_B5
zmp_ph111_B8
zmp_ph111_B6
zmp_ph111_B7
zmp_ph111_B9
zmp_ph111_B10
zmp_ph114_G4
zmp_ph114_G11
zmp_ph114_H1
zmp_ph114_H2
zmp_ph114_A9
zmp_ph114_A11
zmp_ph114_B2
zmp_ph114_B4
zmp_ph114_D8
zmp_ph114_E2

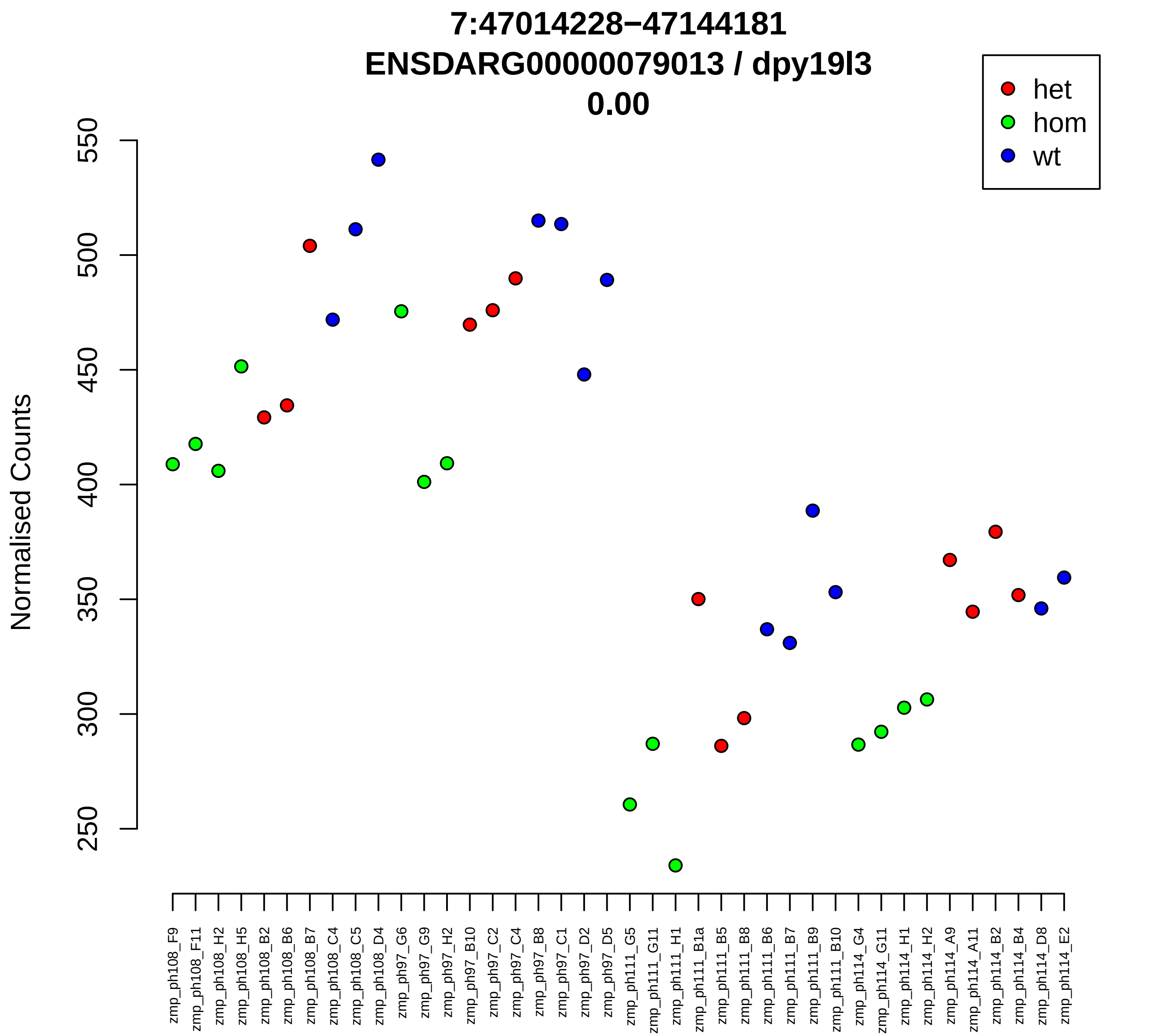
7:47014228−47144181
ENSDARG00000079013 / dpy19l3
0.00
het
hom
wt
Normalised Counts
250
300
350
400
450
500
550
zmp_ph108_F9
zmp_ph108_F11
zmp_ph108_H2
zmp_ph108_H5
zmp_ph108_B2
zmp_ph108_B6
zmp_ph108_B7
zmp_ph108_C4
zmp_ph108_C5
zmp_ph108_D4
zmp_ph97_G6
zmp_ph97_G9
zmp_ph97_H2
zmp_ph97_B10
zmp_ph97_C2
zmp_ph97_C4
zmp_ph97_B8
zmp_ph97_C1
zmp_ph97_D2
zmp_ph97_D5
zmp_ph111_G5
zmp_ph111_G11
zmp_ph111_H1
zmp_ph111_B1a
zmp_ph111_B5
zmp_ph111_B8
zmp_ph111_B6
zmp_ph111_B7
zmp_ph111_B9
zmp_ph111_B10
zmp_ph114_G4
zmp_ph114_G11
zmp_ph114_H1
zmp_ph114_H2
zmp_ph114_A9
zmp_ph114_A11
zmp_ph114_B2
zmp_ph114_B4
zmp_ph114_D8
zmp_ph114_E2

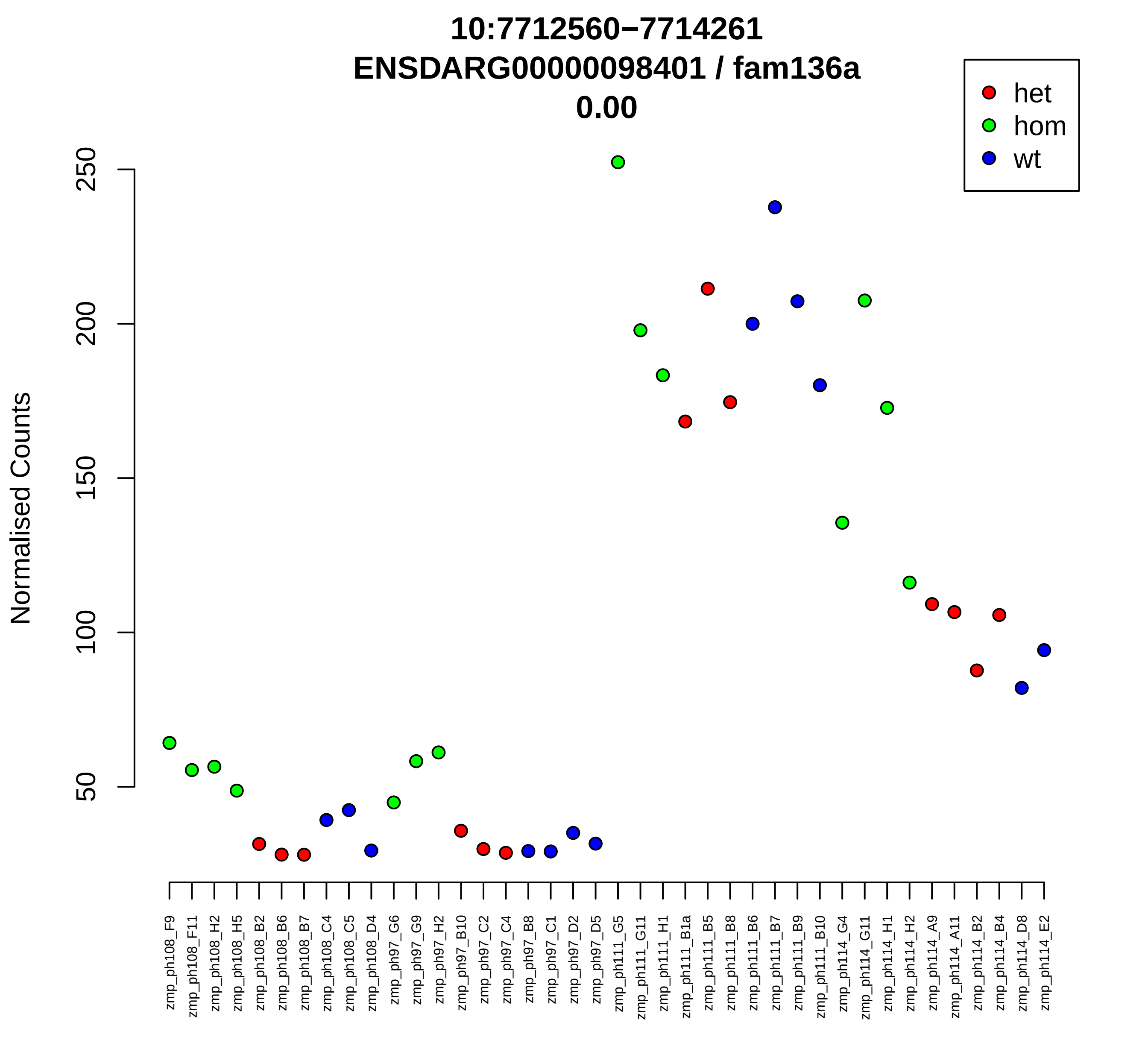

10:7712560−7714261
ENSDARG00000098401 / fam136a
0.00
het
hom
wt
Normalised Counts
50
100
150
200
250
zmp_ph108_F9
zmp_ph108_F11
zmp_ph108_H2
zmp_ph108_H5
zmp_ph108_B2
zmp_ph108_B6
zmp_ph108_B7
zmp_ph108_C4
zmp_ph108_C5
zmp_ph108_D4
zmp_ph97_G6
zmp_ph97_G9
zmp_ph97_H2
zmp_ph97_B10
zmp_ph97_C2
zmp_ph97_C4
zmp_ph97_B8
zmp_ph97_C1
zmp_ph97_D2
zmp_ph97_D5
zmp_ph111_G5
zmp_ph111_G11
zmp_ph111_H1
zmp_ph111_B1a
zmp_ph111_B5
zmp_ph111_B8
zmp_ph111_B6
zmp_ph111_B7
zmp_ph111_B9
zmp_ph111_B10
zmp_ph114_G4
zmp_ph114_G11
zmp_ph114_H1
zmp_ph114_H2
zmp_ph114_A9
zmp_ph114_A11
zmp_ph114_B2
zmp_ph114_B4
zmp_ph114_D8
zmp_ph114_E2

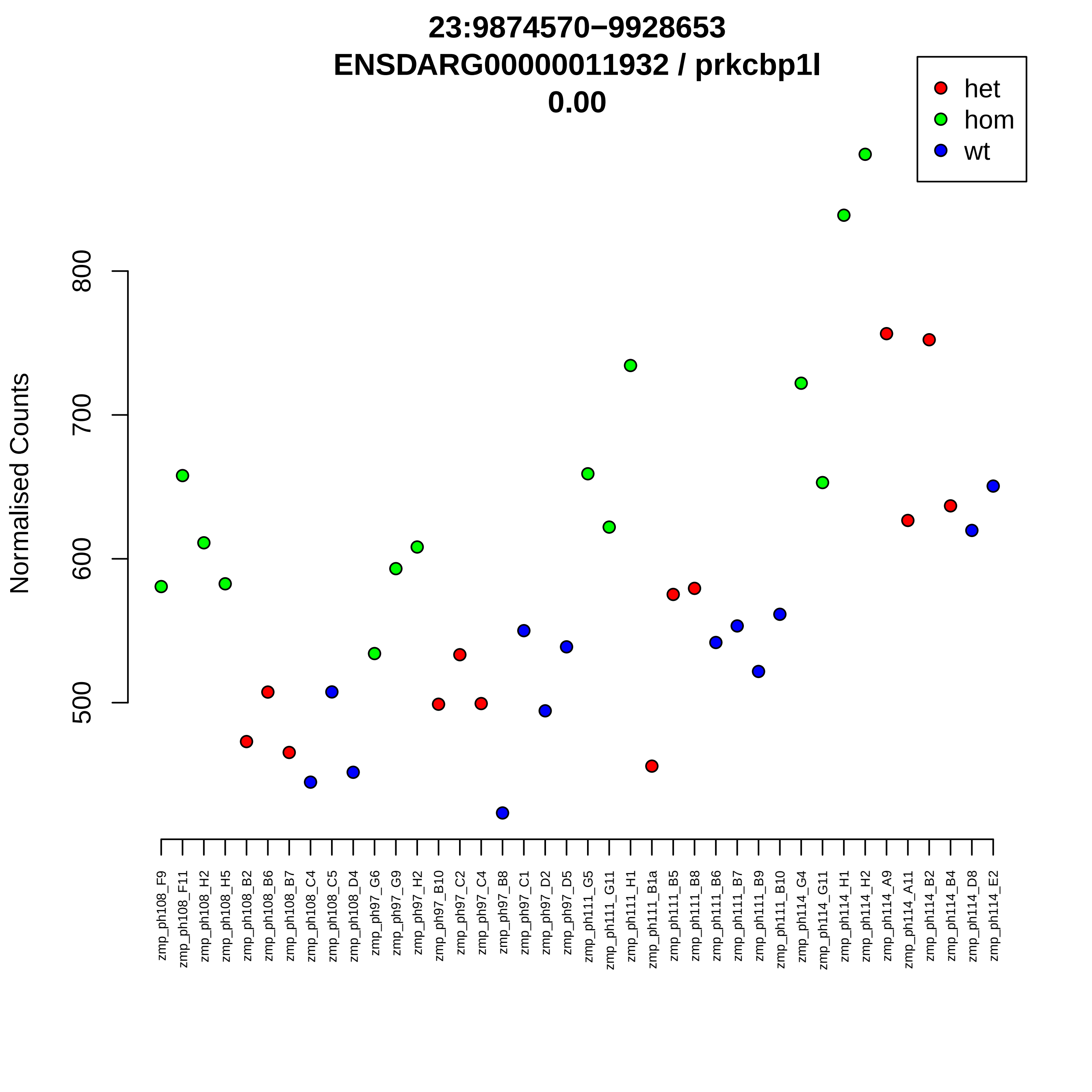
23:9874570−9928653
ENSDARG00000011932 / prkcbp1l
0.00
het
hom
wt
Normalised Counts
500
600
700
800
zmp_ph108_F9
zmp_ph108_F11
zmp_ph108_H2
zmp_ph108_H5
zmp_ph108_B2
zmp_ph108_B6
zmp_ph108_B7
zmp_ph108_C4
zmp_ph108_C5
zmp_ph108_D4
zmp_ph97_G6
zmp_ph97_G9
zmp_ph97_H2
zmp_ph97_B10
zmp_ph97_C2
zmp_ph97_C4
zmp_ph97_B8
zmp_ph97_C1
zmp_ph97_D2
zmp_ph97_D5
zmp_ph111_G5
zmp_ph111_G11
zmp_ph111_H1
zmp_ph111_B1a
zmp_ph111_B5
zmp_ph111_B8
zmp_ph111_B6
zmp_ph111_B7
zmp_ph111_B9
zmp_ph111_B10
zmp_ph114_G4
zmp_ph114_G11
zmp_ph114_H1
zmp_ph114_H2
zmp_ph114_A9
zmp_ph114_A11
zmp_ph114_B2
zmp_ph114_B4
zmp_ph114_D8
zmp_ph114_E2

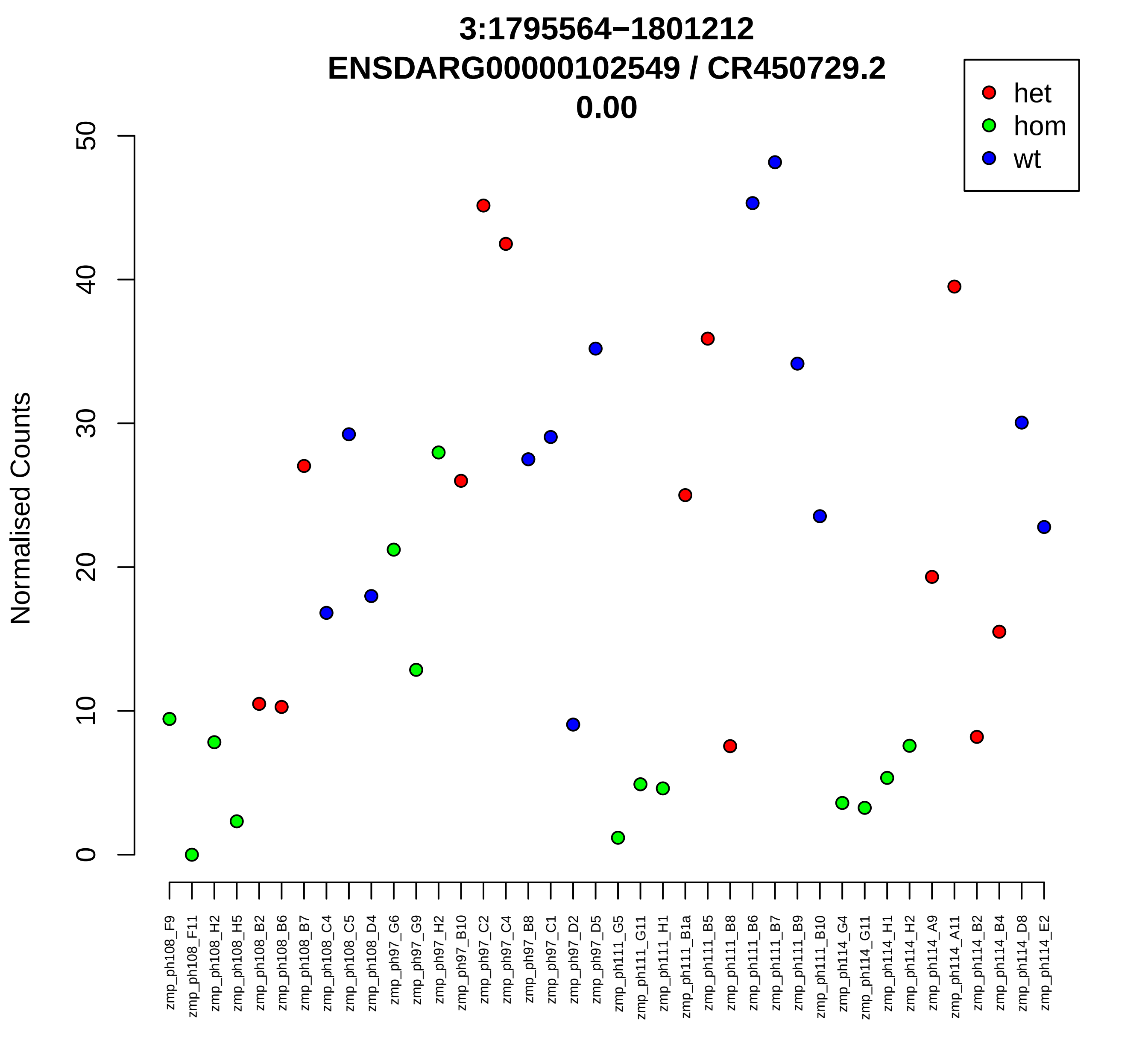

3:1795564−1801212
ENSDARG00000102549 / CR450729.2
0.00
het
hom
wt
Normalised Counts
0
10
20
30
40
50
zmp_ph108_F9
zmp_ph108_F11
zmp_ph108_H2
zmp_ph108_H5
zmp_ph108_B2
zmp_ph108_B6
zmp_ph108_B7
zmp_ph108_C4
zmp_ph108_C5
zmp_ph108_D4
zmp_ph97_G6
zmp_ph97_G9
zmp_ph97_H2
zmp_ph97_B10
zmp_ph97_C2
zmp_ph97_C4
zmp_ph97_B8
zmp_ph97_C1
zmp_ph97_D2
zmp_ph97_D5
zmp_ph111_G5
zmp_ph111_G11
zmp_ph111_H1
zmp_ph111_B1a
zmp_ph111_B5
zmp_ph111_B8
zmp_ph111_B6
zmp_ph111_B7
zmp_ph111_B9
zmp_ph111_B10
zmp_ph114_G4
zmp_ph114_G11
zmp_ph114_H1
zmp_ph114_H2
zmp_ph114_A9
zmp_ph114_A11
zmp_ph114_B2
zmp_ph114_B4
zmp_ph114_D8
zmp_ph114_E2

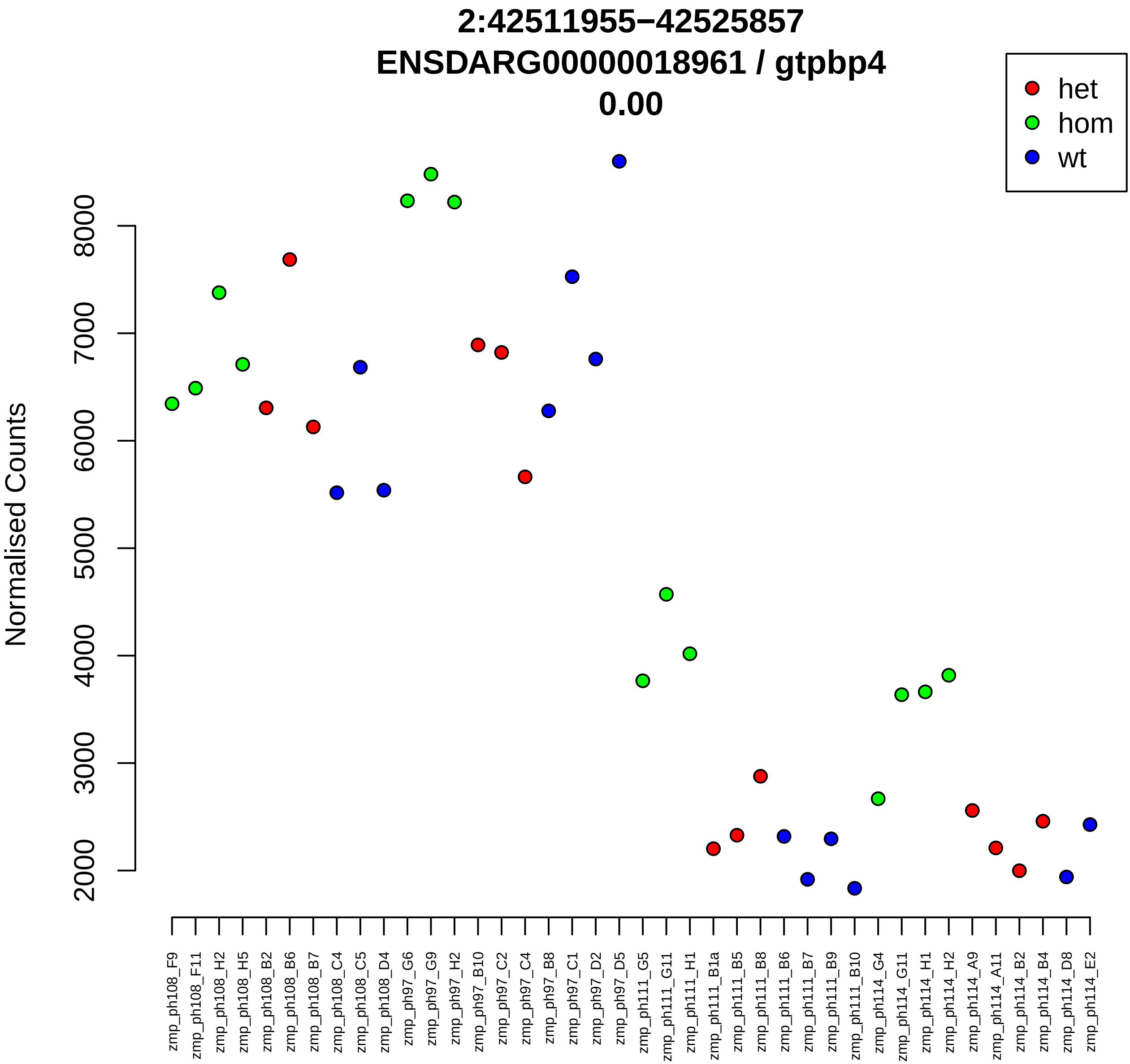

2:42511955−42525857
ENSDARG00000018961 / gtpbp4
0.00
het
hom
wt
Normalised Counts
2000
3000
4000
5000
6000
7000
8000
zmp_ph108_F9
zmp_ph108_F11
zmp_ph108_H2
zmp_ph108_H5
zmp_ph108_B2
zmp_ph108_B6
zmp_ph108_B7
zmp_ph108_C4
zmp_ph108_C5
zmp_ph108_D4
zmp_ph97_G6
zmp_ph97_G9
zmp_ph97_H2
zmp_ph97_B10
zmp_ph97_C2
zmp_ph97_C4
zmp_ph97_B8
zmp_ph97_C1
zmp_ph97_D2
zmp_ph97_D5
zmp_ph111_G5
zmp_ph111_G11
zmp_ph111_H1
zmp_ph111_B1a
zmp_ph111_B5
zmp_ph111_B8
zmp_ph111_B6
zmp_ph111_B7
zmp_ph111_B9
zmp_ph111_B10
zmp_ph114_G4
zmp_ph114_G11
zmp_ph114_H1
zmp_ph114_H2
zmp_ph114_A9
zmp_ph114_A11
zmp_ph114_B2
zmp_ph114_B4
zmp_ph114_D8
zmp_ph114_E2

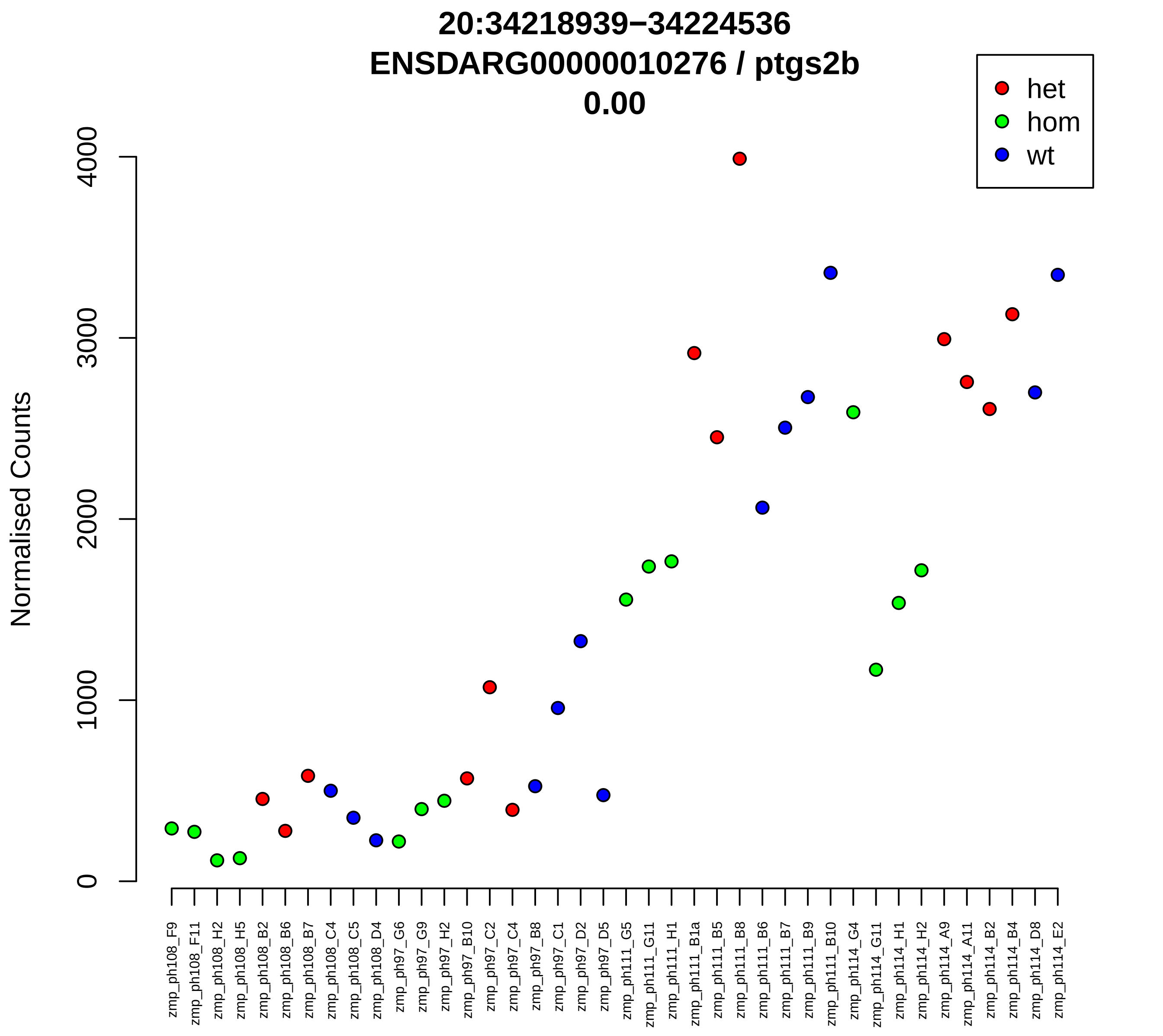

20:34218939−34224536
ENSDARG00000010276 / ptgs2b
0.00
het
hom
wt
Normalised Counts
0
1000
2000
3000
4000
zmp_ph108_F9
zmp_ph108_F11
zmp_ph108_H2
zmp_ph108_H5
zmp_ph108_B2
zmp_ph108_B6
zmp_ph108_B7
zmp_ph108_C4
zmp_ph108_C5
zmp_ph108_D4
zmp_ph97_G6
zmp_ph97_G9
zmp_ph97_H2
zmp_ph97_B10
zmp_ph97_C2
zmp_ph97_C4
zmp_ph97_B8
zmp_ph97_C1
zmp_ph97_D2
zmp_ph97_D5
zmp_ph111_G5
zmp_ph111_G11
zmp_ph111_H1
zmp_ph111_B1a
zmp_ph111_B5
zmp_ph111_B8
zmp_ph111_B6
zmp_ph111_B7
zmp_ph111_B9
zmp_ph111_B10
zmp_ph114_G4
zmp_ph114_G11
zmp_ph114_H1
zmp_ph114_H2
zmp_ph114_A9
zmp_ph114_A11
zmp_ph114_B2
zmp_ph114_B4
zmp_ph114_D8
zmp_ph114_E2

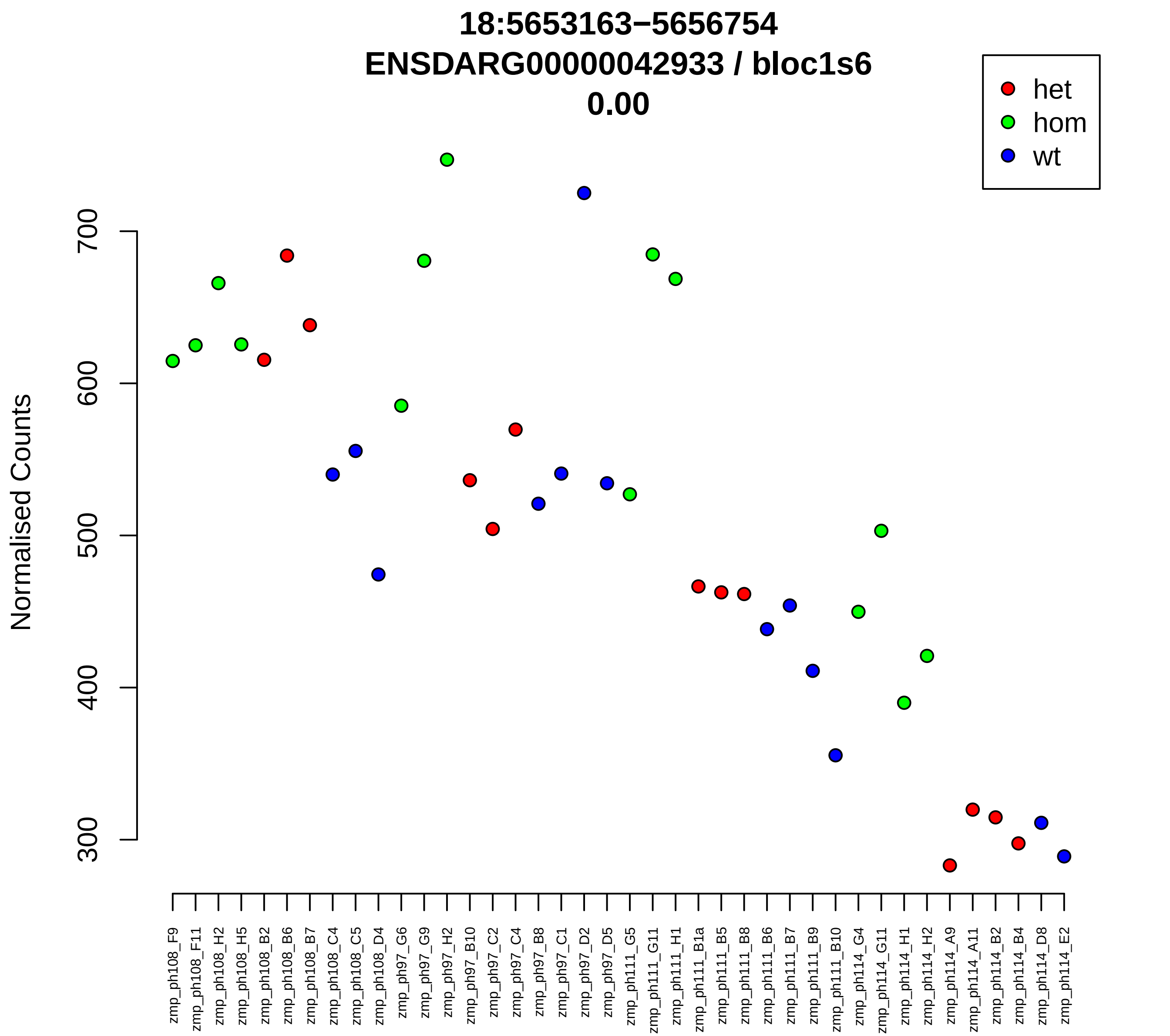

18:5653163−5656754
ENSDARG00000042933 / bloc1s6
0.00
het
hom
wt
Normalised Counts
300
400
500
600
700
zmp_ph108_F9
zmp_ph108_F11
zmp_ph108_H2
zmp_ph108_H5
zmp_ph108_B2
zmp_ph108_B6
zmp_ph108_B7
zmp_ph108_C4
zmp_ph108_C5
zmp_ph108_D4
zmp_ph97_G6
zmp_ph97_G9
zmp_ph97_H2
zmp_ph97_B10
zmp_ph97_C2
zmp_ph97_C4
zmp_ph97_B8
zmp_ph97_C1
zmp_ph97_D2
zmp_ph97_D5
zmp_ph111_G5
zmp_ph111_G11
zmp_ph111_H1
zmp_ph111_B1a
zmp_ph111_B5
zmp_ph111_B8
zmp_ph111_B6
zmp_ph111_B7
zmp_ph111_B9
zmp_ph111_B10
zmp_ph114_G4
zmp_ph114_G11
zmp_ph114_H1
zmp_ph114_H2
zmp_ph114_A9
zmp_ph114_A11
zmp_ph114_B2
zmp_ph114_B4
zmp_ph114_D8
zmp_ph114_E2

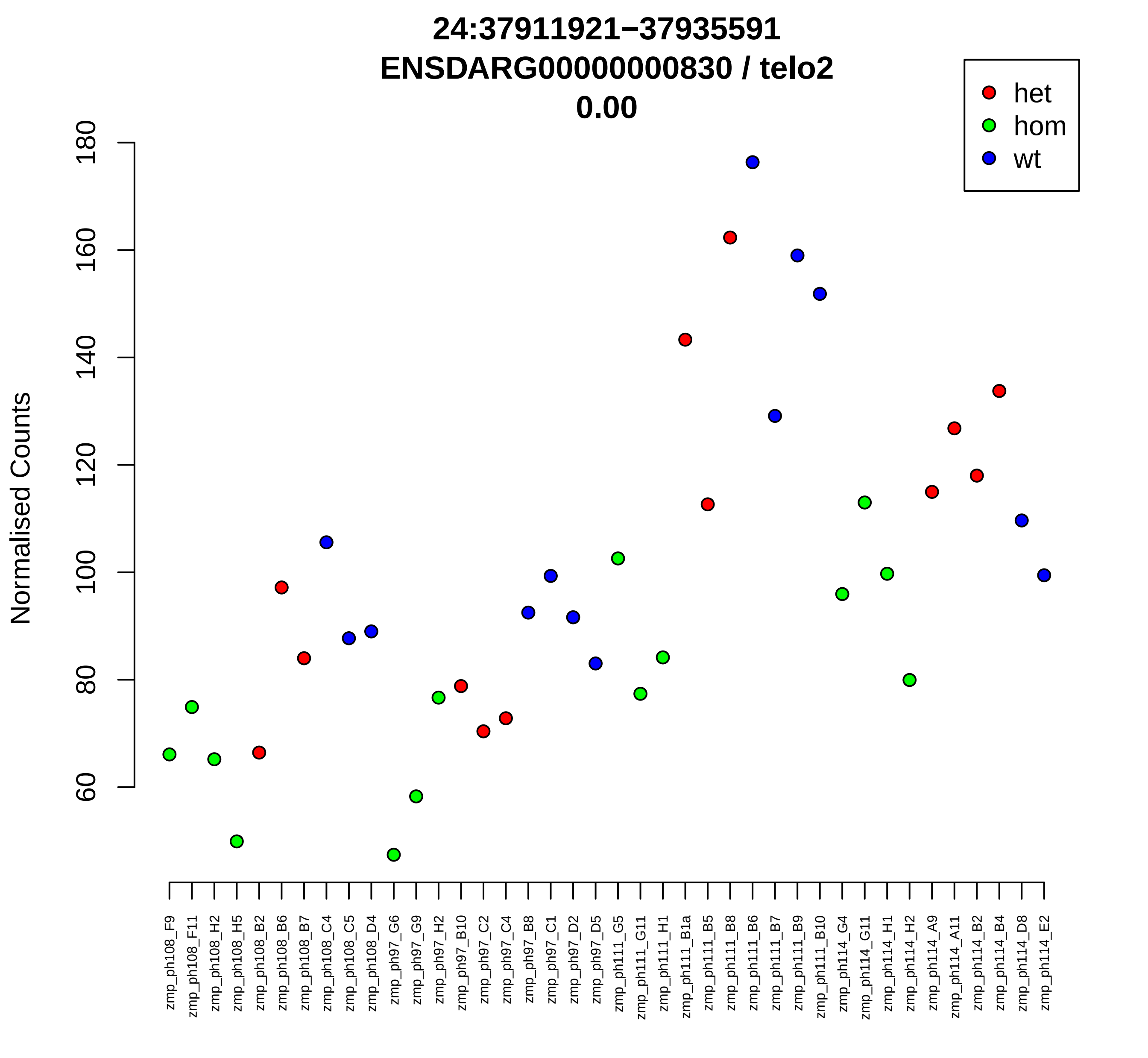

24:37911921−37935591
ENSDARG00000000830 / telo2
0.00
het
hom
wt
Normalised Counts
60
80
100
120
140
160
180
zmp_ph108_F9
zmp_ph108_F11
zmp_ph108_H2
zmp_ph108_H5
zmp_ph108_B2
zmp_ph108_B6
zmp_ph108_B7
zmp_ph108_C4
zmp_ph108_C5
zmp_ph108_D4
zmp_ph97_G6
zmp_ph97_G9
zmp_ph97_H2
zmp_ph97_B10
zmp_ph97_C2
zmp_ph97_C4
zmp_ph97_B8
zmp_ph97_C1
zmp_ph97_D2
zmp_ph97_D5
zmp_ph111_G5
zmp_ph111_G11
zmp_ph111_H1
zmp_ph111_B1a
zmp_ph111_B5
zmp_ph111_B8
zmp_ph111_B6
zmp_ph111_B7
zmp_ph111_B9
zmp_ph111_B10
zmp_ph114_G4
zmp_ph114_G11
zmp_ph114_H1
zmp_ph114_H2
zmp_ph114_A9
zmp_ph114_A11
zmp_ph114_B2
zmp_ph114_B4
zmp_ph114_D8
zmp_ph114_E2

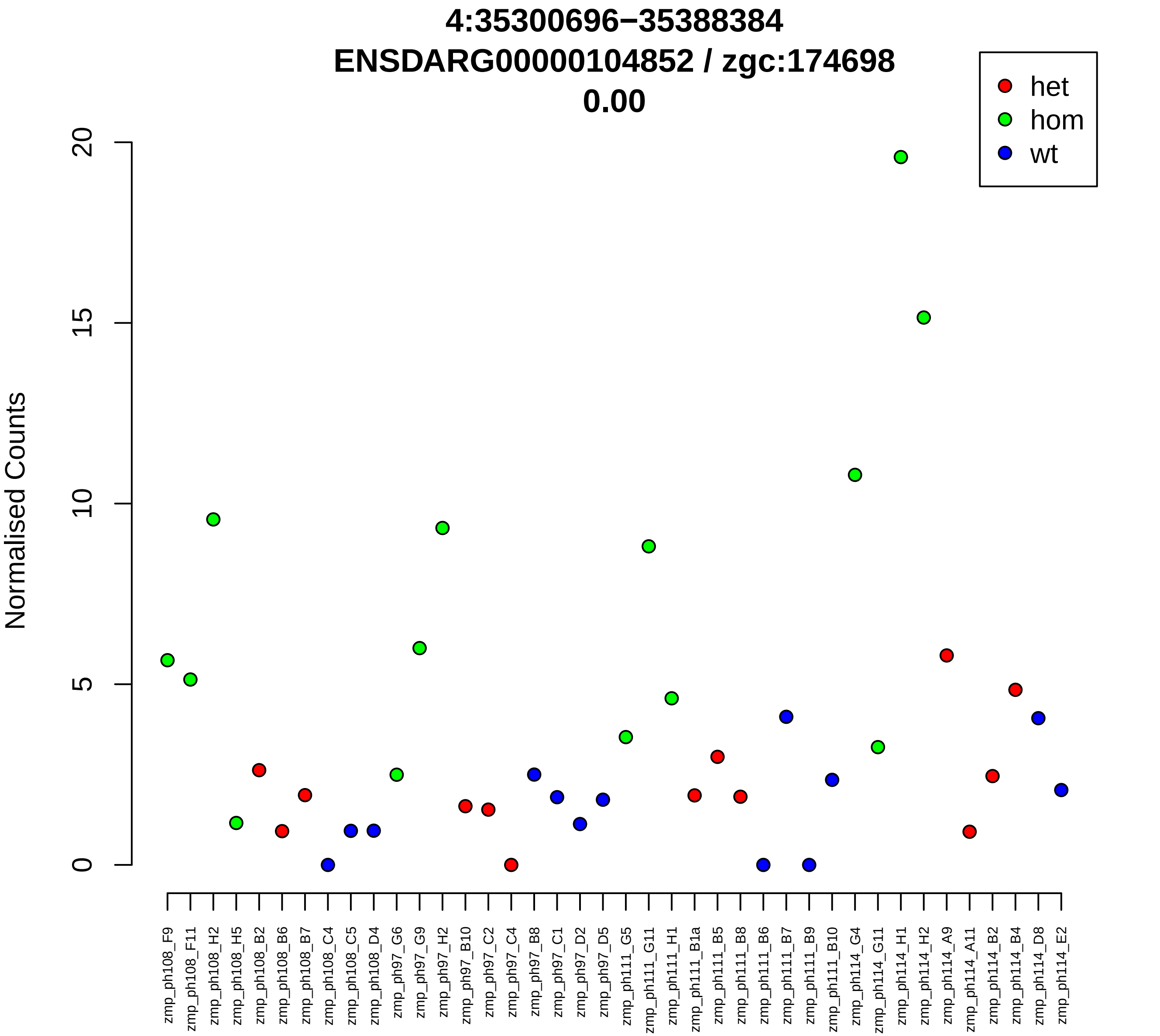

4:35300696−35388384
ENSDARG00000104852 / zgc:174698
0.00
het
hom
wt
Normalised Counts
0
5
10
15
20
zmp_ph108_F9
zmp_ph108_F11
zmp_ph108_H2
zmp_ph108_H5
zmp_ph108_B2
zmp_ph108_B6
zmp_ph108_B7
zmp_ph108_C4
zmp_ph108_C5
zmp_ph108_D4
zmp_ph97_G6
zmp_ph97_G9
zmp_ph97_H2
zmp_ph97_B10
zmp_ph97_C2
zmp_ph97_C4
zmp_ph97_B8
zmp_ph97_C1
zmp_ph97_D2
zmp_ph97_D5
zmp_ph111_G5
zmp_ph111_G11
zmp_ph111_H1
zmp_ph111_B1a
zmp_ph111_B5
zmp_ph111_B8
zmp_ph111_B6
zmp_ph111_B7
zmp_ph111_B9
zmp_ph111_B10
zmp_ph114_G4
zmp_ph114_G11
zmp_ph114_H1
zmp_ph114_H2
zmp_ph114_A9
zmp_ph114_A11
zmp_ph114_B2
zmp_ph114_B4
zmp_ph114_D8
zmp_ph114_E2

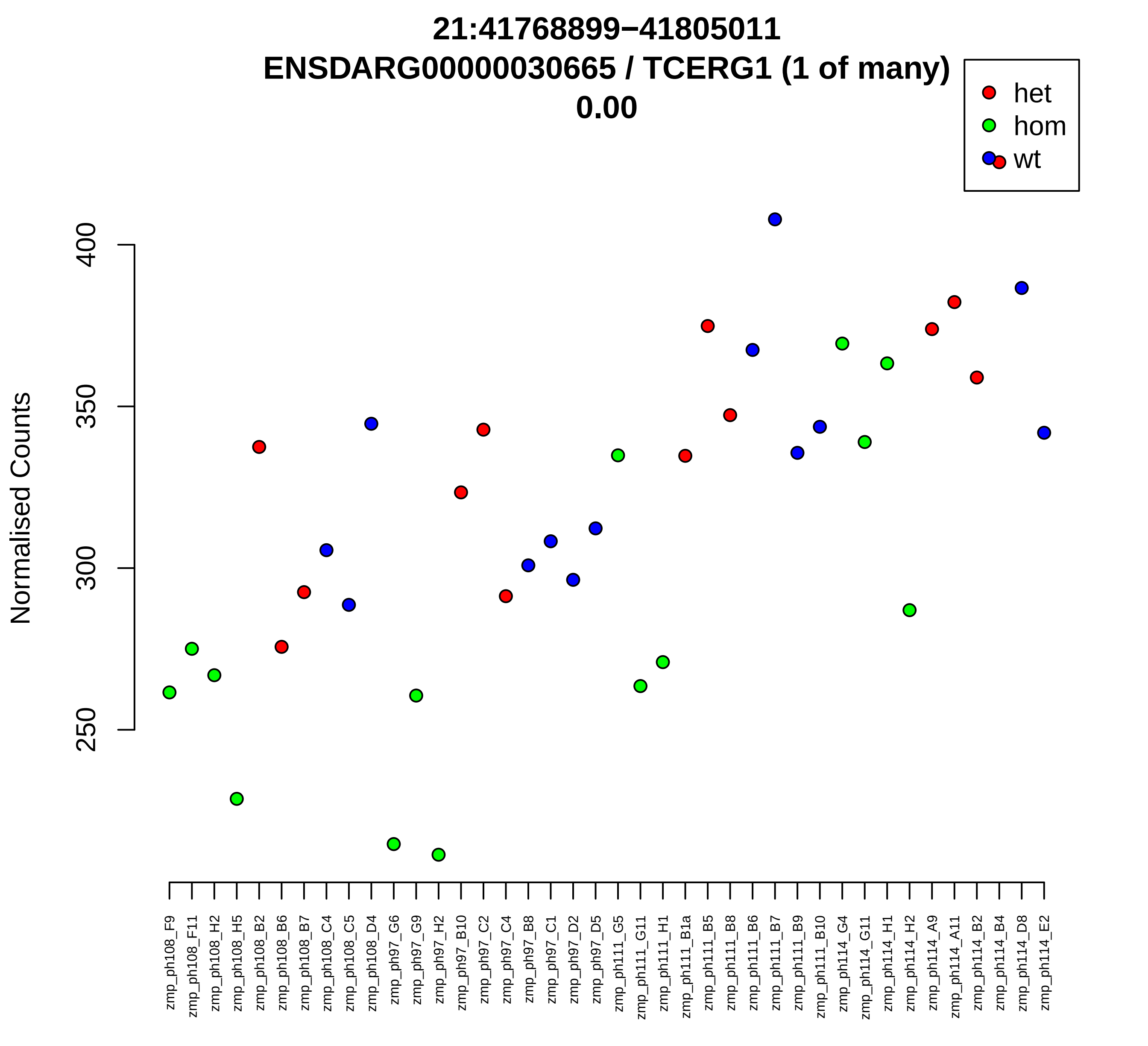
21:41768899−41805011
ENSDARG00000030665 / TCERG1 (1 of many)
0.00
het
hom
wt
Normalised Counts
250
300
350
400
zmp_ph108_F9
zmp_ph108_F11
zmp_ph108_H2
zmp_ph108_H5
zmp_ph108_B2
zmp_ph108_B6
zmp_ph108_B7
zmp_ph108_C4
zmp_ph108_C5
zmp_ph108_D4
zmp_ph97_G6
zmp_ph97_G9
zmp_ph97_H2
zmp_ph97_B10
zmp_ph97_C2
zmp_ph97_C4
zmp_ph97_B8
zmp_ph97_C1
zmp_ph97_D2
zmp_ph97_D5
zmp_ph111_G5
zmp_ph111_G11
zmp_ph111_H1
zmp_ph111_B1a
zmp_ph111_B5
zmp_ph111_B8
zmp_ph111_B6
zmp_ph111_B7
zmp_ph111_B9
zmp_ph111_B10
zmp_ph114_G4
zmp_ph114_G11
zmp_ph114_H1
zmp_ph114_H2
zmp_ph114_A9
zmp_ph114_A11
zmp_ph114_B2
zmp_ph114_B4
zmp_ph114_D8
zmp_ph114_E2

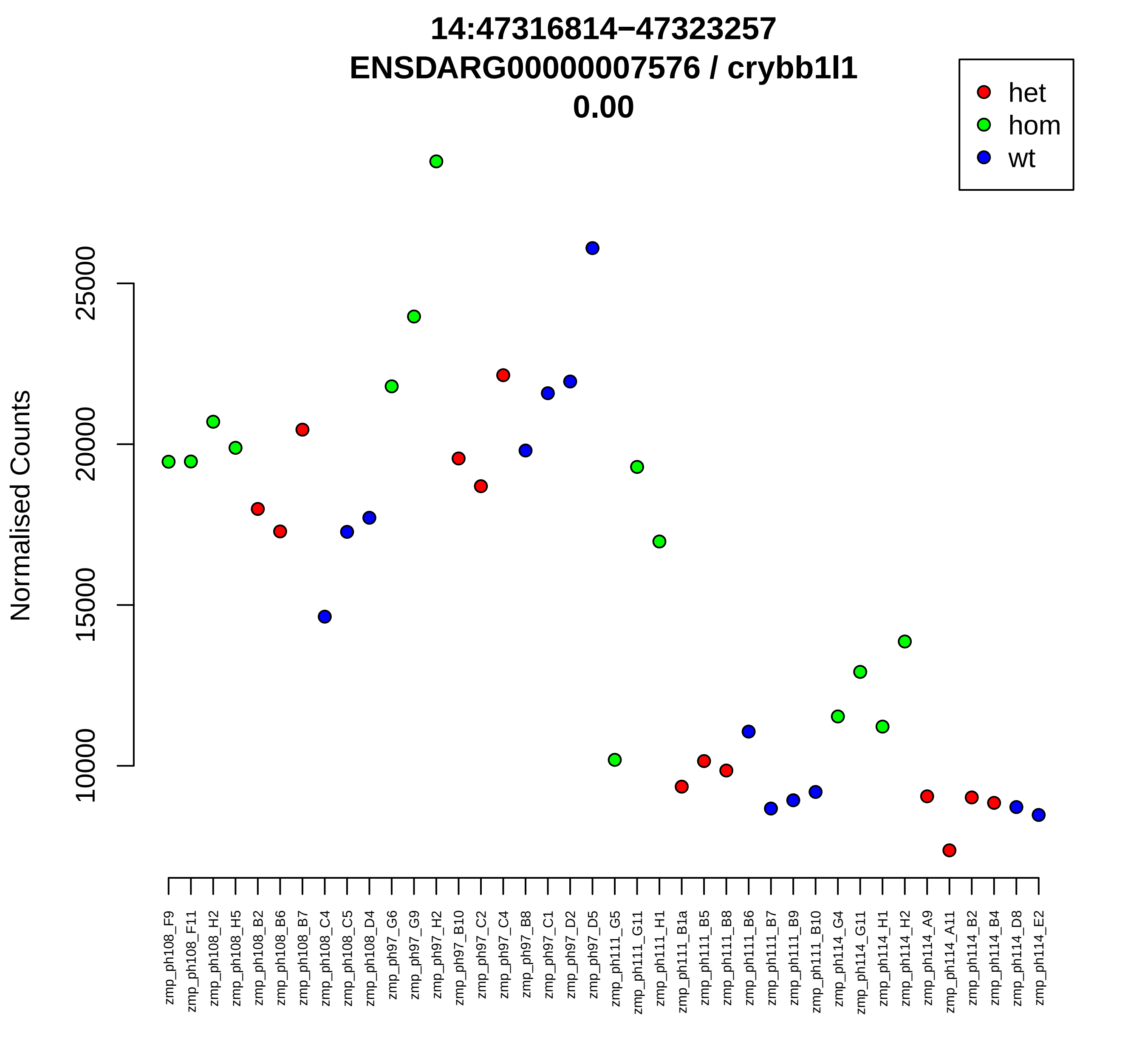

14:47316814−47323257
ENSDARG00000007576 / crybb1l1
0.00
het
hom
wt
Normalised Counts
10000
15000
20000
25000
zmp_ph108_F9
zmp_ph108_F11
zmp_ph108_H2
zmp_ph108_H5
zmp_ph108_B2
zmp_ph108_B6
zmp_ph108_B7
zmp_ph108_C4
zmp_ph108_C5
zmp_ph108_D4
zmp_ph97_G6
zmp_ph97_G9
zmp_ph97_H2
zmp_ph97_B10
zmp_ph97_C2
zmp_ph97_C4
zmp_ph97_B8
zmp_ph97_C1
zmp_ph97_D2
zmp_ph97_D5
zmp_ph111_G5
zmp_ph111_G11
zmp_ph111_H1
zmp_ph111_B1a
zmp_ph111_B5
zmp_ph111_B8
zmp_ph111_B6
zmp_ph111_B7
zmp_ph111_B9
zmp_ph111_B10
zmp_ph114_G4
zmp_ph114_G11
zmp_ph114_H1
zmp_ph114_H2
zmp_ph114_A9
zmp_ph114_A11
zmp_ph114_B2
zmp_ph114_B4
zmp_ph114_D8
zmp_ph114_E2

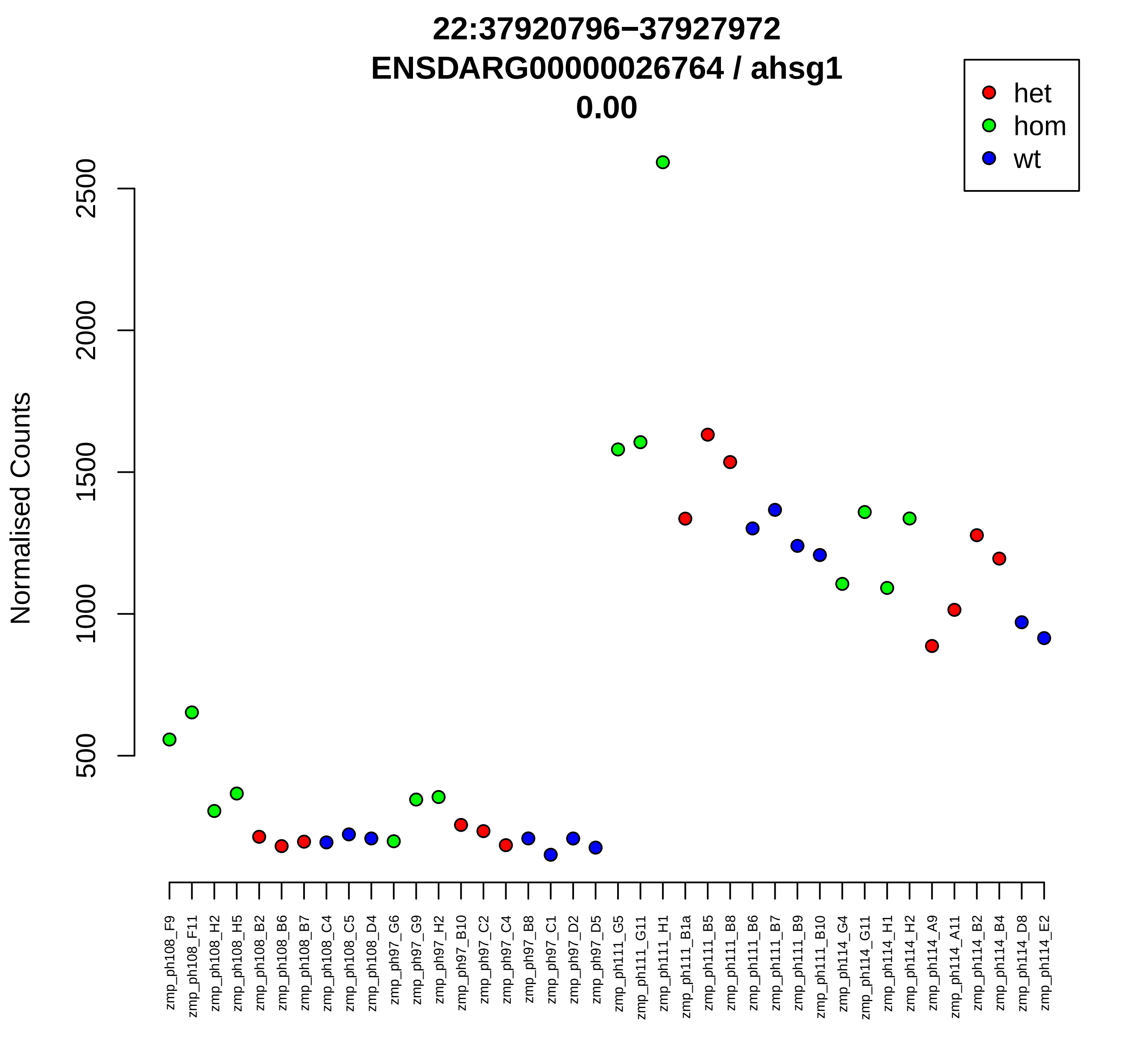

22:37920796−37927972
ENSDARG00000026764 / ahsg1
0.00
het
hom
wt
Normalised Counts
500
1000
1500
2000
2500
zmp_ph108_F9
zmp_ph108_F11
zmp_ph108_H2
zmp_ph108_H5
zmp_ph108_B2
zmp_ph108_B6
zmp_ph108_B7
zmp_ph108_C4
zmp_ph108_C5
zmp_ph108_D4
zmp_ph97_G6
zmp_ph97_G9
zmp_ph97_H2
zmp_ph97_B10
zmp_ph97_C2
zmp_ph97_C4
zmp_ph97_B8
zmp_ph97_C1
zmp_ph97_D2
zmp_ph97_D5
zmp_ph111_G5
zmp_ph111_G11
zmp_ph111_H1
zmp_ph111_B1a
zmp_ph111_B5
zmp_ph111_B8
zmp_ph111_B6
zmp_ph111_B7
zmp_ph111_B9
zmp_ph111_B10
zmp_ph114_G4
zmp_ph114_G11
zmp_ph114_H1
zmp_ph114_H2
zmp_ph114_A9
zmp_ph114_A11
zmp_ph114_B2
zmp_ph114_B4
zmp_ph114_D8
zmp_ph114_E2

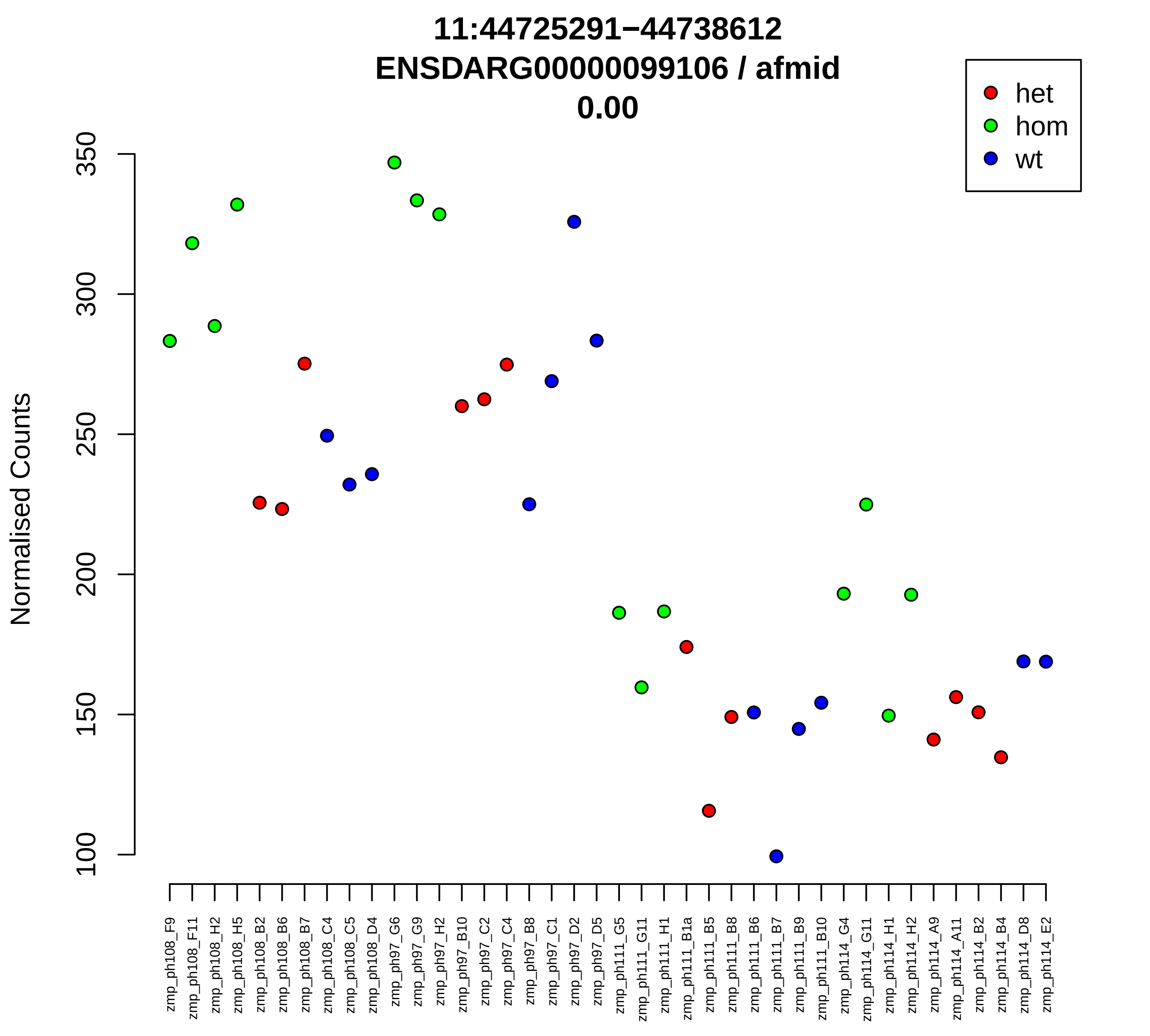

11:44725291−44738612
ENSDARG00000099106 / afmid
0.00
het
hom
wt
Normalised Counts
100
150
200
250
300
350
zmp_ph108_F9
zmp_ph108_F11
zmp_ph108_H2
zmp_ph108_H5
zmp_ph108_B2
zmp_ph108_B6
zmp_ph108_B7
zmp_ph108_C4
zmp_ph108_C5
zmp_ph108_D4
zmp_ph97_G6
zmp_ph97_G9
zmp_ph97_H2
zmp_ph97_B10
zmp_ph97_C2
zmp_ph97_C4
zmp_ph97_B8
zmp_ph97_C1
zmp_ph97_D2
zmp_ph97_D5
zmp_ph111_G5
zmp_ph111_G11
zmp_ph111_H1
zmp_ph111_B1a
zmp_ph111_B5
zmp_ph111_B8
zmp_ph111_B6
zmp_ph111_B7
zmp_ph111_B9
zmp_ph111_B10
zmp_ph114_G4
zmp_ph114_G11
zmp_ph114_H1
zmp_ph114_H2
zmp_ph114_A9
zmp_ph114_A11
zmp_ph114_B2
zmp_ph114_B4
zmp_ph114_D8
zmp_ph114_E2

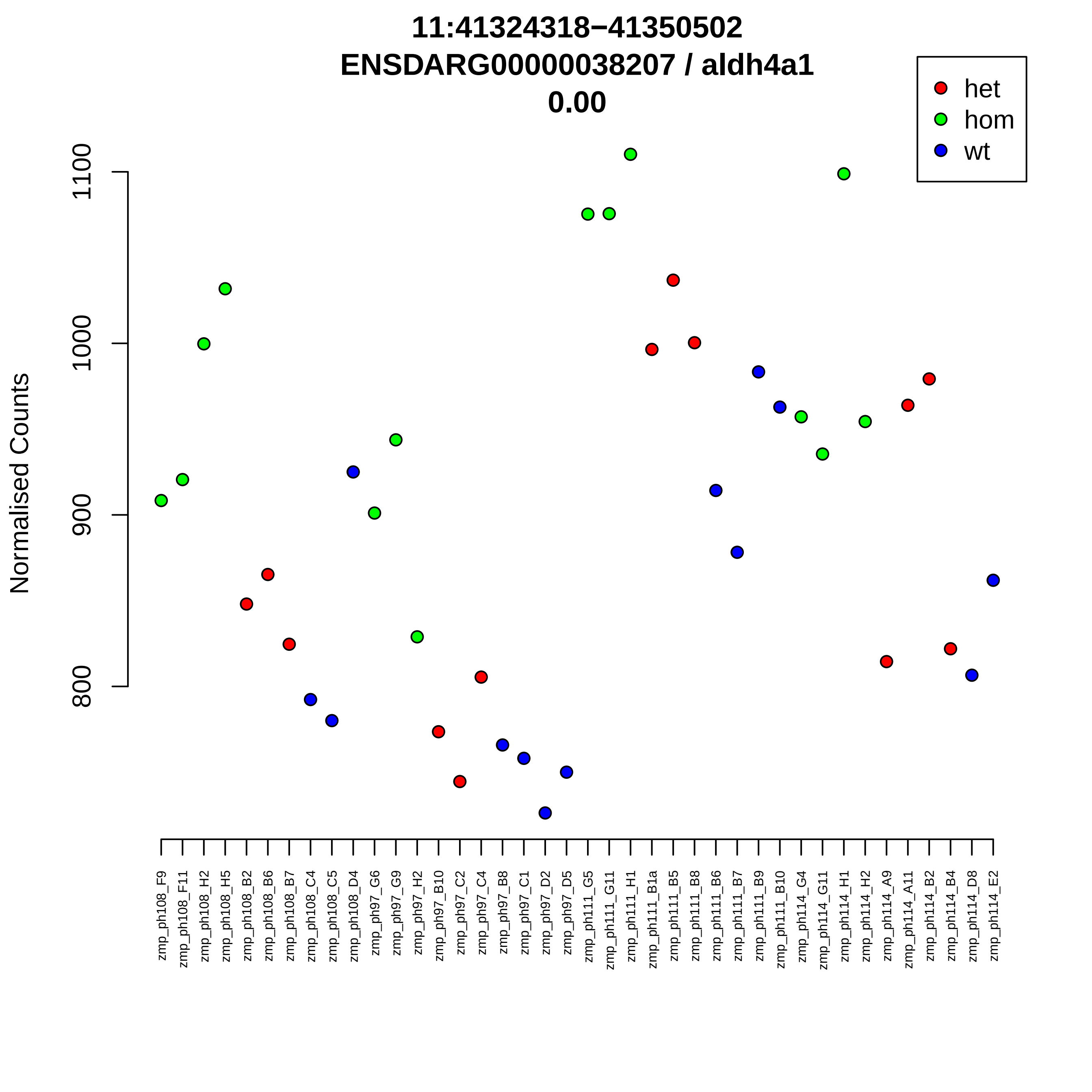

11:41324318−41350502
ENSDARG00000038207 / aldh4a1
0.00
het
hom
wt
Normalised Counts
800
900
1000
1100
zmp_ph108_F9
zmp_ph108_F11
zmp_ph108_H2
zmp_ph108_H5
zmp_ph108_B2
zmp_ph108_B6
zmp_ph108_B7
zmp_ph108_C4
zmp_ph108_C5
zmp_ph108_D4
zmp_ph97_G6
zmp_ph97_G9
zmp_ph97_H2
zmp_ph97_B10
zmp_ph97_C2
zmp_ph97_C4
zmp_ph97_B8
zmp_ph97_C1
zmp_ph97_D2
zmp_ph97_D5
zmp_ph111_G5
zmp_ph111_G11
zmp_ph111_H1
zmp_ph111_B1a
zmp_ph111_B5
zmp_ph111_B8
zmp_ph111_B6
zmp_ph111_B7
zmp_ph111_B9
zmp_ph111_B10
zmp_ph114_G4
zmp_ph114_G11
zmp_ph114_H1
zmp_ph114_H2
zmp_ph114_A9
zmp_ph114_A11
zmp_ph114_B2
zmp_ph114_B4
zmp_ph114_D8
zmp_ph114_E2

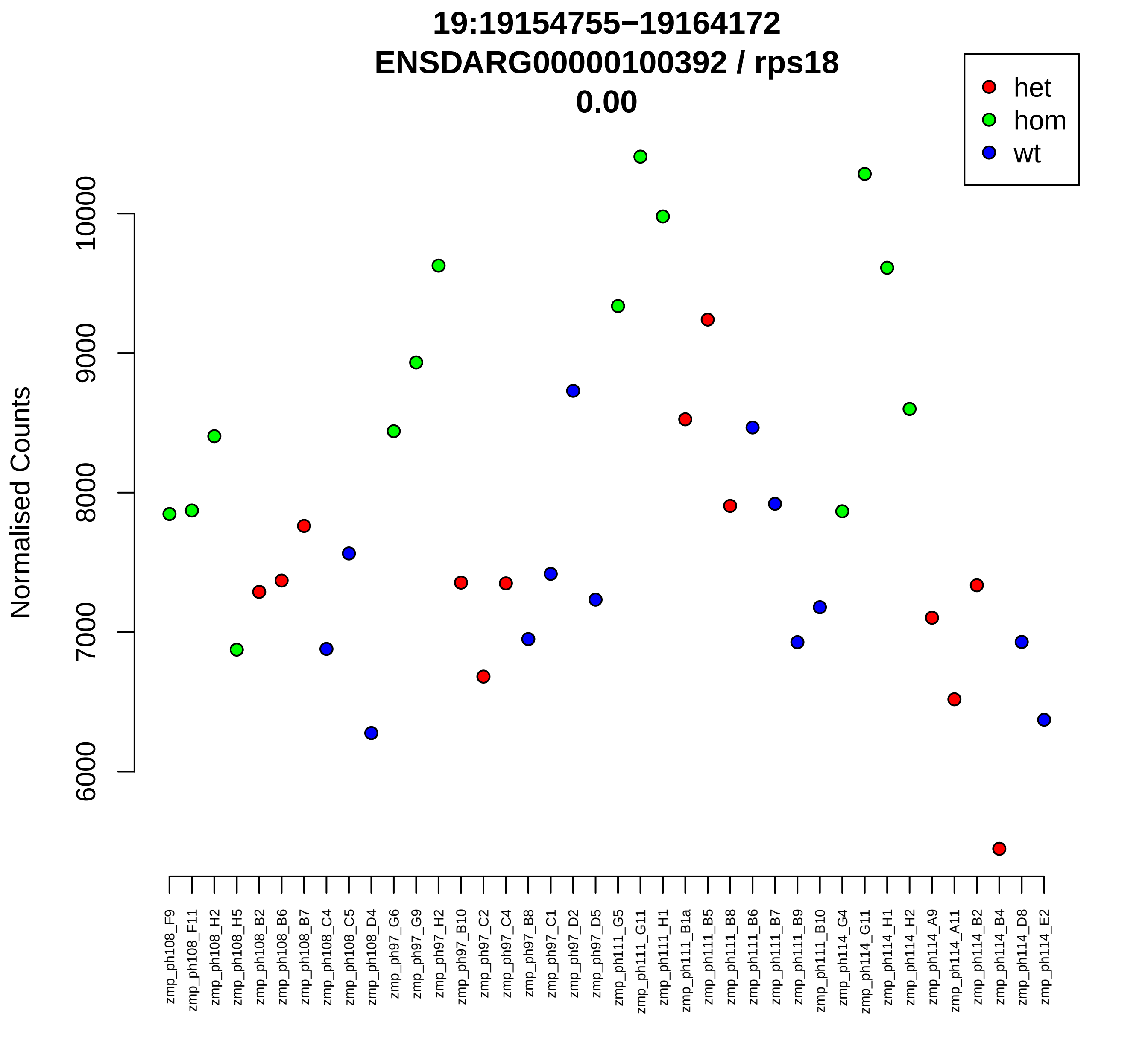

19:19154755−19164172
ENSDARG00000100392 / rps18
0.00
het
hom
wt
Normalised Counts
6000
7000
8000
9000
10000
zmp_ph108_F9
zmp_ph108_F11
zmp_ph108_H2
zmp_ph108_H5
zmp_ph108_B2
zmp_ph108_B6
zmp_ph108_B7
zmp_ph108_C4
zmp_ph108_C5
zmp_ph108_D4
zmp_ph97_G6
zmp_ph97_G9
zmp_ph97_H2
zmp_ph97_B10
zmp_ph97_C2
zmp_ph97_C4
zmp_ph97_B8
zmp_ph97_C1
zmp_ph97_D2
zmp_ph97_D5
zmp_ph111_G5
zmp_ph111_G11
zmp_ph111_H1
zmp_ph111_B1a
zmp_ph111_B5
zmp_ph111_B8
zmp_ph111_B6
zmp_ph111_B7
zmp_ph111_B9
zmp_ph111_B10
zmp_ph114_G4
zmp_ph114_G11
zmp_ph114_H1
zmp_ph114_H2
zmp_ph114_A9
zmp_ph114_A11
zmp_ph114_B2
zmp_ph114_B4
zmp_ph114_D8
zmp_ph114_E2

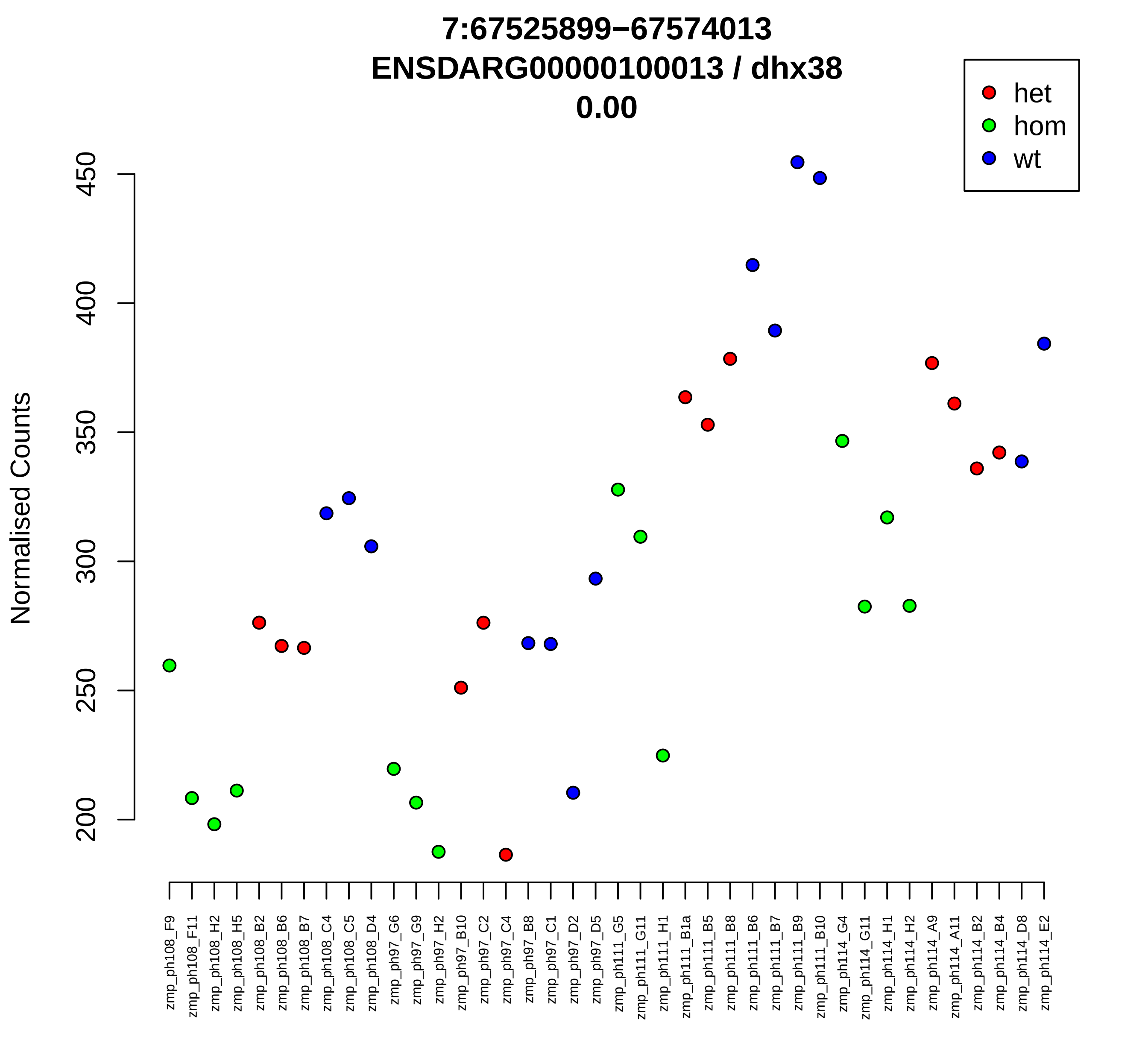

7:67525899−67574013
ENSDARG00000100013 / dhx38
0.00
het
hom
wt
Normalised Counts
200
250
300
350
400
450
zmp_ph108_F9
zmp_ph108_F11
zmp_ph108_H2
zmp_ph108_H5
zmp_ph108_B2
zmp_ph108_B6
zmp_ph108_B7
zmp_ph108_C4
zmp_ph108_C5
zmp_ph108_D4
zmp_ph97_G6
zmp_ph97_G9
zmp_ph97_H2
zmp_ph97_B10
zmp_ph97_C2
zmp_ph97_C4
zmp_ph97_B8
zmp_ph97_C1
zmp_ph97_D2
zmp_ph97_D5
zmp_ph111_G5
zmp_ph111_G11
zmp_ph111_H1
zmp_ph111_B1a
zmp_ph111_B5
zmp_ph111_B8
zmp_ph111_B6
zmp_ph111_B7
zmp_ph111_B9
zmp_ph111_B10
zmp_ph114_G4
zmp_ph114_G11
zmp_ph114_H1
zmp_ph114_H2
zmp_ph114_A9
zmp_ph114_A11
zmp_ph114_B2
zmp_ph114_B4
zmp_ph114_D8
zmp_ph114_E2

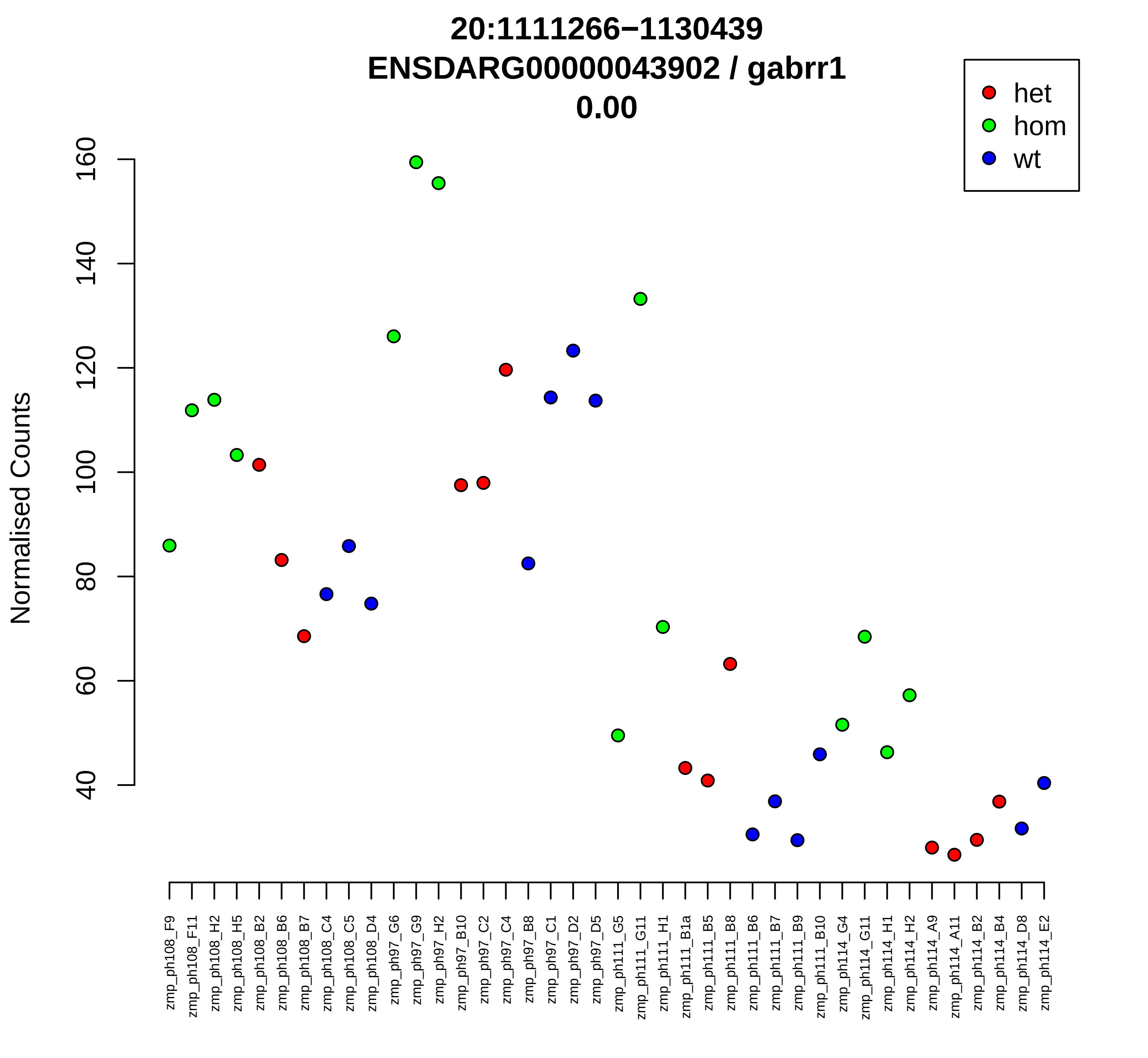
20:1111266−1130439
ENSDARG00000043902 / gabrr1
0.00
het
hom
wt
Normalised Counts
40
60
80
100
120
140
160
zmp_ph108_F9
zmp_ph108_F11
zmp_ph108_H2
zmp_ph108_H5
zmp_ph108_B2
zmp_ph108_B6
zmp_ph108_B7
zmp_ph108_C4
zmp_ph108_C5
zmp_ph108_D4
zmp_ph97_G6
zmp_ph97_G9
zmp_ph97_H2
zmp_ph97_B10
zmp_ph97_C2
zmp_ph97_C4
zmp_ph97_B8
zmp_ph97_C1
zmp_ph97_D2
zmp_ph97_D5
zmp_ph111_G5
zmp_ph111_G11
zmp_ph111_H1
zmp_ph111_B1a
zmp_ph111_B5
zmp_ph111_B8
zmp_ph111_B6
zmp_ph111_B7
zmp_ph111_B9
zmp_ph111_B10
zmp_ph114_G4
zmp_ph114_G11
zmp_ph114_H1
zmp_ph114_H2
zmp_ph114_A9
zmp_ph114_A11
zmp_ph114_B2
zmp_ph114_B4
zmp_ph114_D8
zmp_ph114_E2

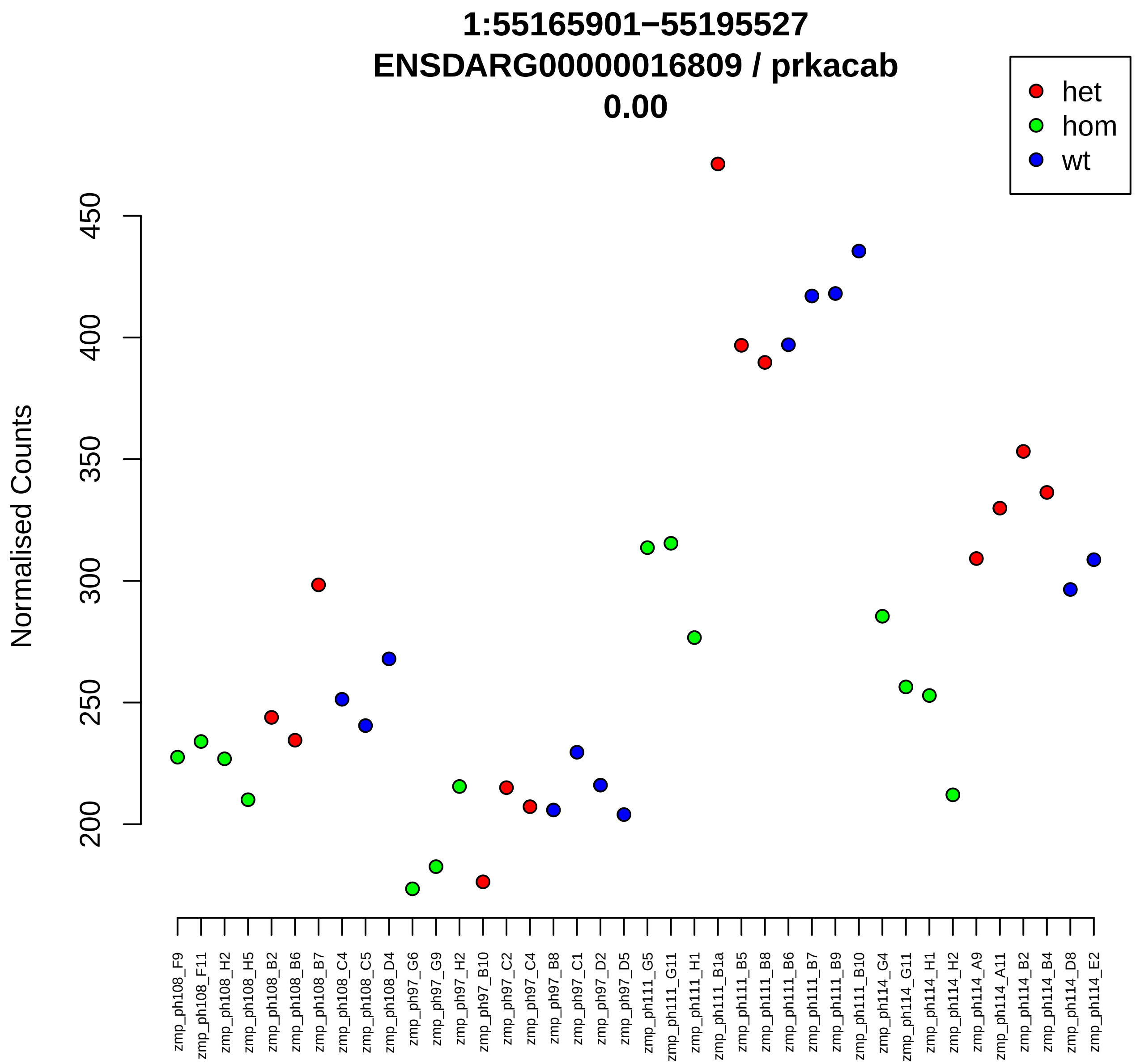
1:55165901−55195527
ENSDARG00000016809 / prkacab
0.00
het
hom
wt
Normalised Counts
200
250
300
350
400
450
zmp_ph108_F9
zmp_ph108_F11
zmp_ph108_H2
zmp_ph108_H5
zmp_ph108_B2
zmp_ph108_B6
zmp_ph108_B7
zmp_ph108_C4
zmp_ph108_C5
zmp_ph108_D4
zmp_ph97_G6
zmp_ph97_G9
zmp_ph97_H2
zmp_ph97_B10
zmp_ph97_C2
zmp_ph97_C4
zmp_ph97_B8
zmp_ph97_C1
zmp_ph97_D2
zmp_ph97_D5
zmp_ph111_G5
zmp_ph111_G11
zmp_ph111_H1
zmp_ph111_B1a
zmp_ph111_B5
zmp_ph111_B8
zmp_ph111_B6
zmp_ph111_B7
zmp_ph111_B9
zmp_ph111_B10
zmp_ph114_G4
zmp_ph114_G11
zmp_ph114_H1
zmp_ph114_H2
zmp_ph114_A9
zmp_ph114_A11
zmp_ph114_B2
zmp_ph114_B4
zmp_ph114_D8
zmp_ph114_E2

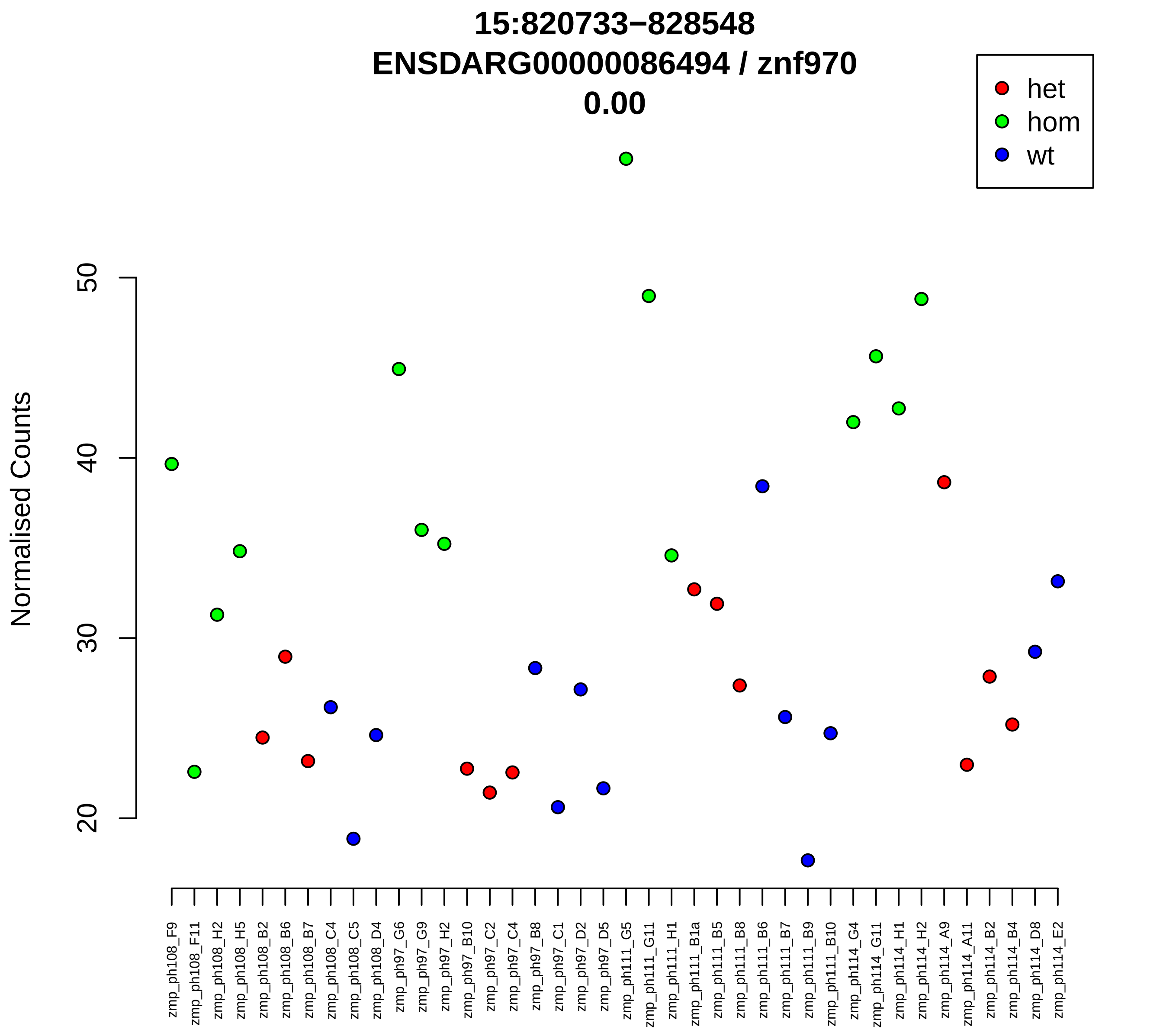
15:820733−828548
ENSDARG00000086494 / znf970
0.00
het
hom
wt
Normalised Counts
20
30
40
50
zmp_ph108_F9
zmp_ph108_F11
zmp_ph108_H2
zmp_ph108_H5
zmp_ph108_B2
zmp_ph108_B6
zmp_ph108_B7
zmp_ph108_C4
zmp_ph108_C5
zmp_ph108_D4
zmp_ph97_G6
zmp_ph97_G9
zmp_ph97_H2
zmp_ph97_B10
zmp_ph97_C2
zmp_ph97_C4
zmp_ph97_B8
zmp_ph97_C1
zmp_ph97_D2
zmp_ph97_D5
zmp_ph111_G5
zmp_ph111_G11
zmp_ph111_H1
zmp_ph111_B1a
zmp_ph111_B5
zmp_ph111_B8
zmp_ph111_B6
zmp_ph111_B7
zmp_ph111_B9
zmp_ph111_B10
zmp_ph114_G4
zmp_ph114_G11
zmp_ph114_H1
zmp_ph114_H2
zmp_ph114_A9
zmp_ph114_A11
zmp_ph114_B2
zmp_ph114_B4
zmp_ph114_D8
zmp_ph114_E2

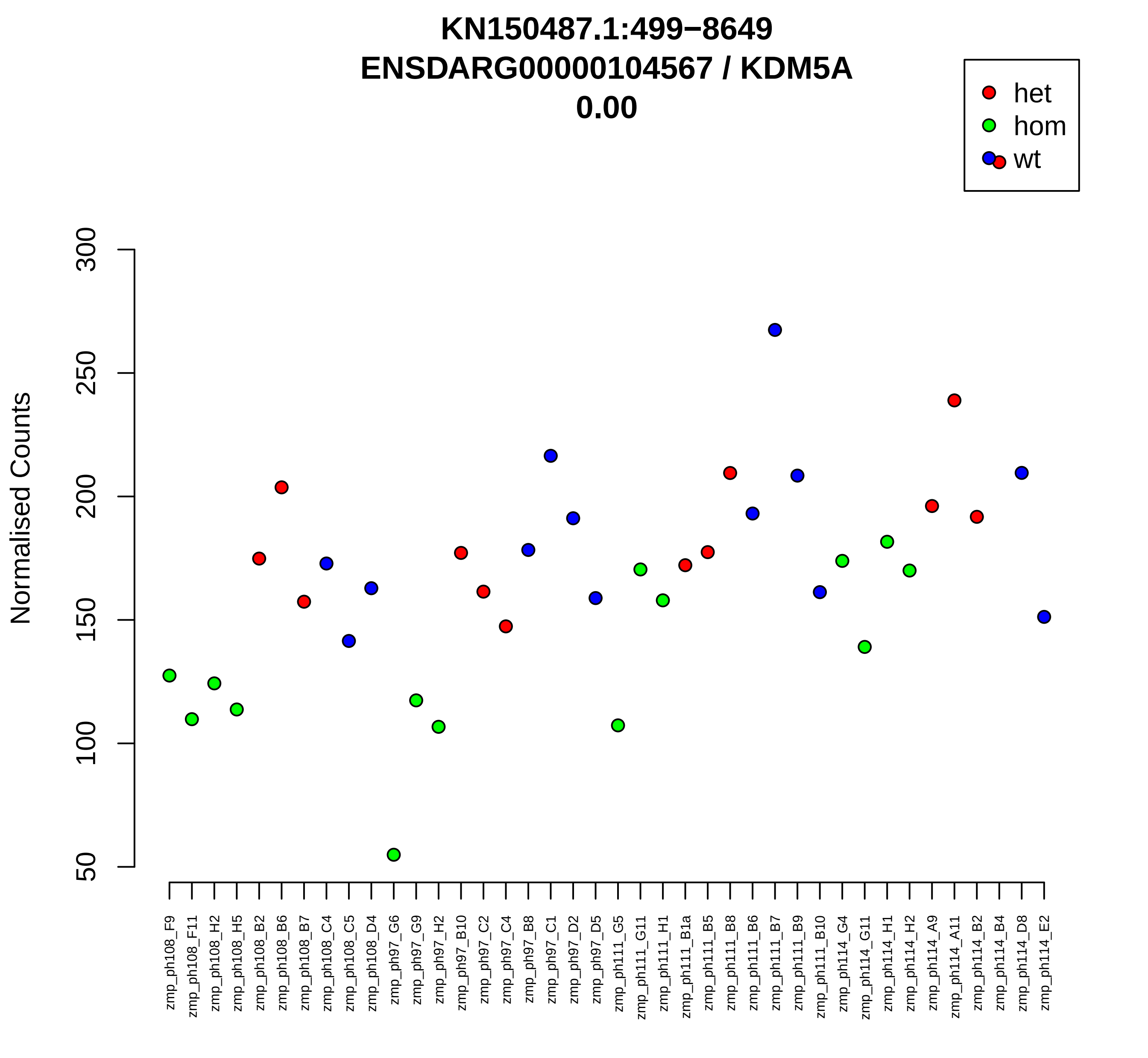
KN150487.1:499−8649
ENSDARG00000104567 / KDM5A
0.00
het
hom
wt
Normalised Counts
50
100
150
200
250
300
zmp_ph108_F9
zmp_ph108_F11
zmp_ph108_H2
zmp_ph108_H5
zmp_ph108_B2
zmp_ph108_B6
zmp_ph108_B7
zmp_ph108_C4
zmp_ph108_C5
zmp_ph108_D4
zmp_ph97_G6
zmp_ph97_G9
zmp_ph97_H2
zmp_ph97_B10
zmp_ph97_C2
zmp_ph97_C4
zmp_ph97_B8
zmp_ph97_C1
zmp_ph97_D2
zmp_ph97_D5
zmp_ph111_G5
zmp_ph111_G11
zmp_ph111_H1
zmp_ph111_B1a
zmp_ph111_B5
zmp_ph111_B8
zmp_ph111_B6
zmp_ph111_B7
zmp_ph111_B9
zmp_ph111_B10
zmp_ph114_G4
zmp_ph114_G11
zmp_ph114_H1
zmp_ph114_H2
zmp_ph114_A9
zmp_ph114_A11
zmp_ph114_B2
zmp_ph114_B4
zmp_ph114_D8
zmp_ph114_E2

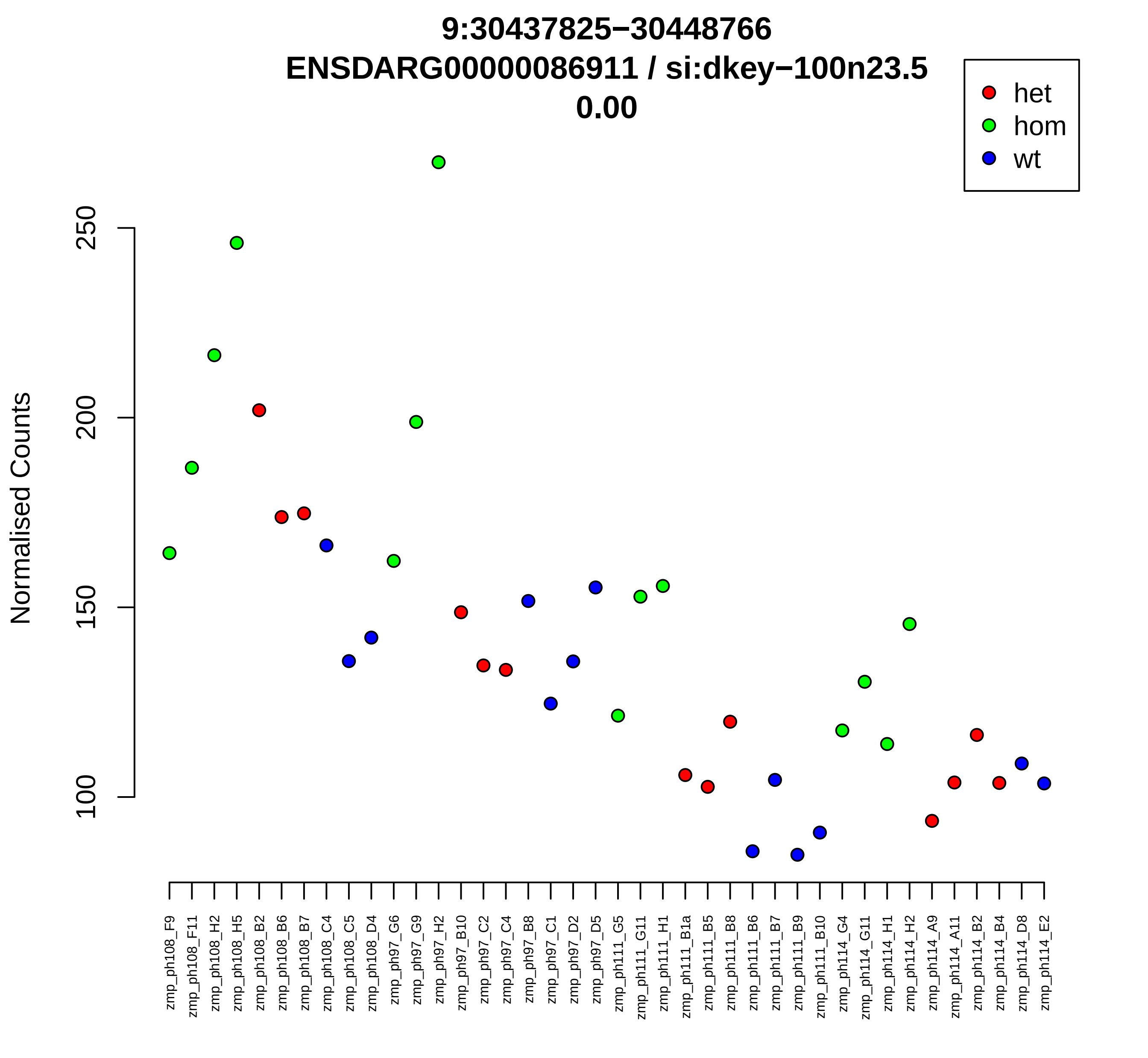
9:30437825−30448766
ENSDARG00000086911 / si:dkey−100n23.5
0.00
het
hom
wt
Normalised Counts
100
150
200
250
zmp_ph108_F9
zmp_ph108_F11
zmp_ph108_H2
zmp_ph108_H5
zmp_ph108_B2
zmp_ph108_B6
zmp_ph108_B7
zmp_ph108_C4
zmp_ph108_C5
zmp_ph108_D4
zmp_ph97_G6
zmp_ph97_G9
zmp_ph97_H2
zmp_ph97_B10
zmp_ph97_C2
zmp_ph97_C4
zmp_ph97_B8
zmp_ph97_C1
zmp_ph97_D2
zmp_ph97_D5
zmp_ph111_G5
zmp_ph111_G11
zmp_ph111_H1
zmp_ph111_B1a
zmp_ph111_B5
zmp_ph111_B8
zmp_ph111_B6
zmp_ph111_B7
zmp_ph111_B9
zmp_ph111_B10
zmp_ph114_G4
zmp_ph114_G11
zmp_ph114_H1
zmp_ph114_H2
zmp_ph114_A9
zmp_ph114_A11
zmp_ph114_B2
zmp_ph114_B4
zmp_ph114_D8
zmp_ph114_E2

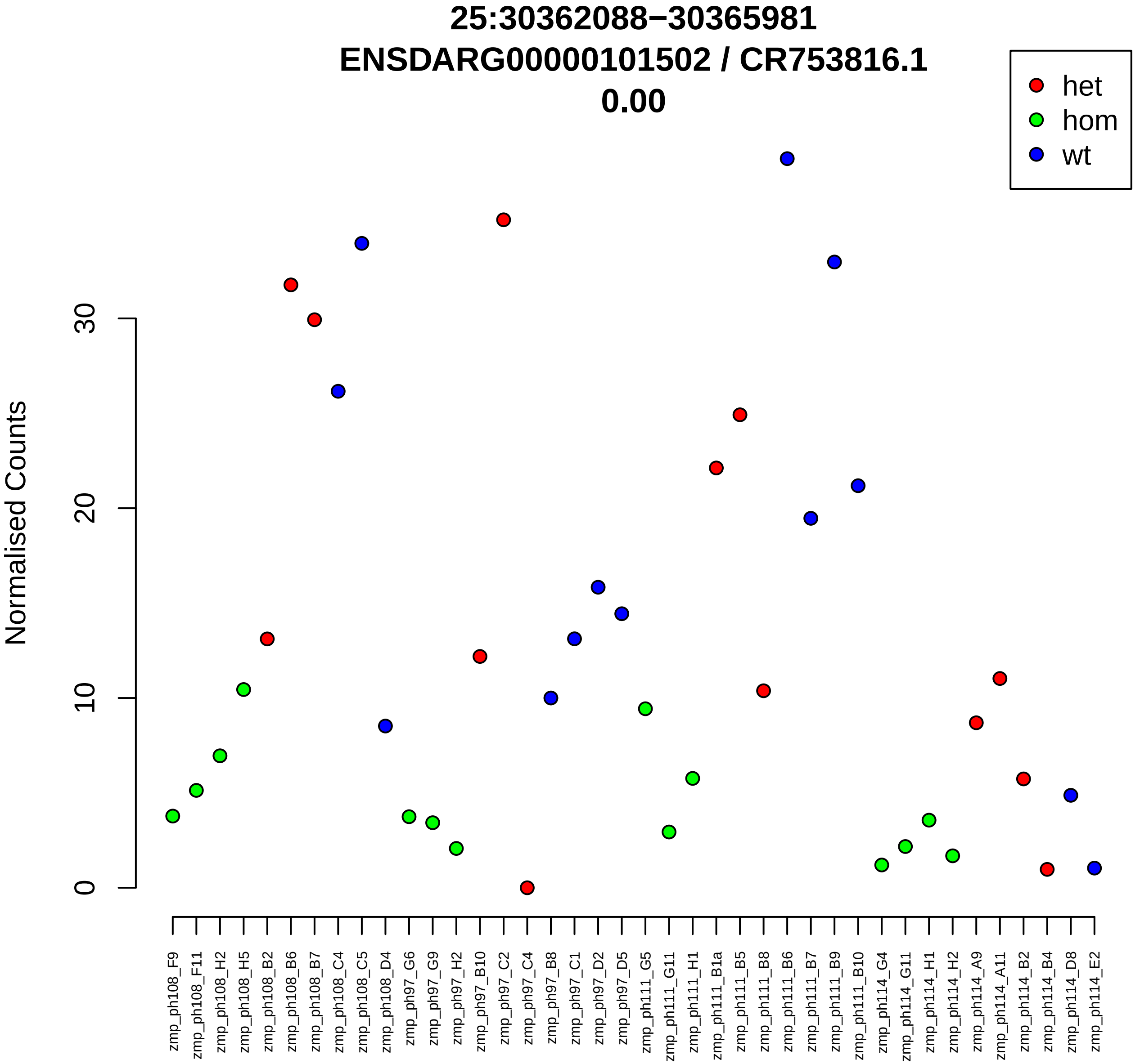

25:30362088−30365981
ENSDARG00000101502 / CR753816.1
0.00
het
hom
wt
Normalised Counts
0
10
20
30
zmp_ph108_F9
zmp_ph108_F11
zmp_ph108_H2
zmp_ph108_H5
zmp_ph108_B2
zmp_ph108_B6
zmp_ph108_B7
zmp_ph108_C4
zmp_ph108_C5
zmp_ph108_D4
zmp_ph97_G6
zmp_ph97_G9
zmp_ph97_H2
zmp_ph97_B10
zmp_ph97_C2
zmp_ph97_C4
zmp_ph97_B8
zmp_ph97_C1
zmp_ph97_D2
zmp_ph97_D5
zmp_ph111_G5
zmp_ph111_G11
zmp_ph111_H1
zmp_ph111_B1a
zmp_ph111_B5
zmp_ph111_B8
zmp_ph111_B6
zmp_ph111_B7
zmp_ph111_B9
zmp_ph111_B10
zmp_ph114_G4
zmp_ph114_G11
zmp_ph114_H1
zmp_ph114_H2
zmp_ph114_A9
zmp_ph114_A11
zmp_ph114_B2
zmp_ph114_B4
zmp_ph114_D8
zmp_ph114_E2

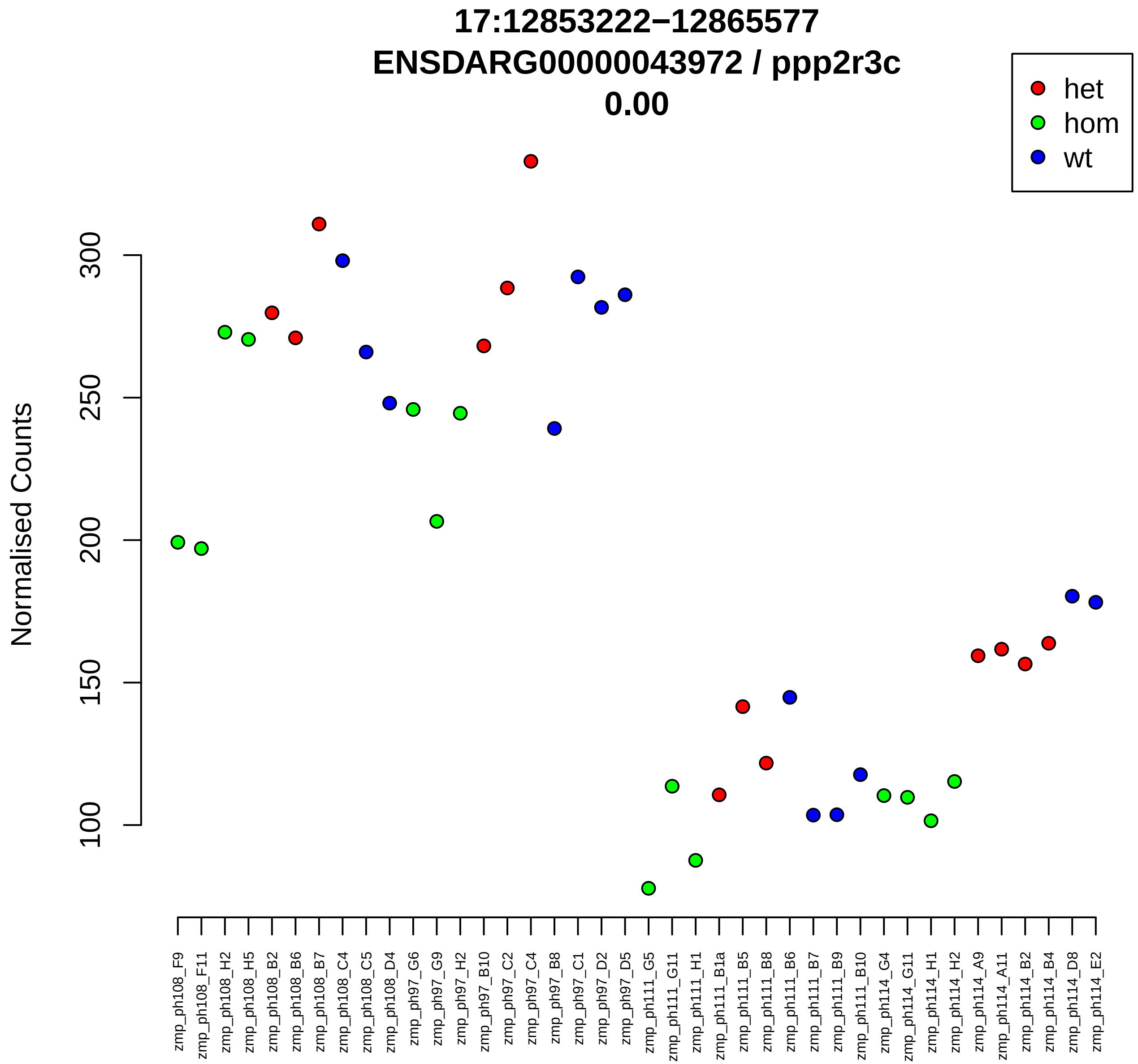
17:12853222−12865577
ENSDARG00000043972 / ppp2r3c
0.00
het
hom
wt
Normalised Counts
100
150
200
250
300
zmp_ph108_F9
zmp_ph108_F11
zmp_ph108_H2
zmp_ph108_H5
zmp_ph108_B2
zmp_ph108_B6
zmp_ph108_B7
zmp_ph108_C4
zmp_ph108_C5
zmp_ph108_D4
zmp_ph97_G6
zmp_ph97_G9
zmp_ph97_H2
zmp_ph97_B10
zmp_ph97_C2
zmp_ph97_C4
zmp_ph97_B8
zmp_ph97_C1
zmp_ph97_D2
zmp_ph97_D5
zmp_ph111_G5
zmp_ph111_G11
zmp_ph111_H1
zmp_ph111_B1a
zmp_ph111_B5
zmp_ph111_B8
zmp_ph111_B6
zmp_ph111_B7
zmp_ph111_B9
zmp_ph111_B10
zmp_ph114_G4
zmp_ph114_G11
zmp_ph114_H1
zmp_ph114_H2
zmp_ph114_A9
zmp_ph114_A11
zmp_ph114_B2
zmp_ph114_B4
zmp_ph114_D8
zmp_ph114_E2

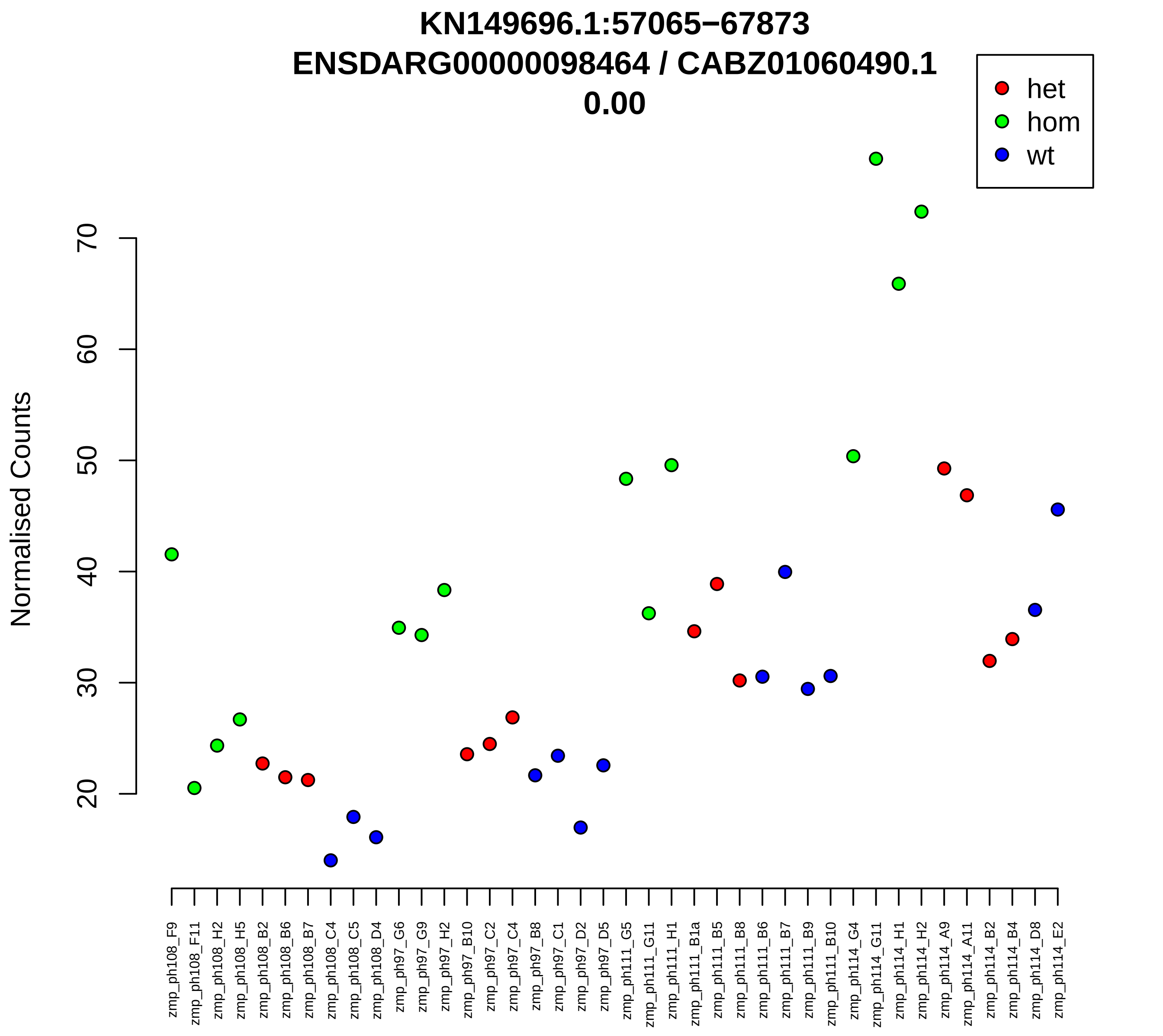

KN149696.1:57065−67873
ENSDARG00000098464 / CABZ01060490.1
0.00
het
hom
wt
Normalised Counts
20
30
40
50
60
70
zmp_ph108_F9
zmp_ph108_F11
zmp_ph108_H2
zmp_ph108_H5
zmp_ph108_B2
zmp_ph108_B6
zmp_ph108_B7
zmp_ph108_C4
zmp_ph108_C5
zmp_ph108_D4
zmp_ph97_G6
zmp_ph97_G9
zmp_ph97_H2
zmp_ph97_B10
zmp_ph97_C2
zmp_ph97_C4
zmp_ph97_B8
zmp_ph97_C1
zmp_ph97_D2
zmp_ph97_D5
zmp_ph111_G5
zmp_ph111_G11
zmp_ph111_H1
zmp_ph111_B1a
zmp_ph111_B5
zmp_ph111_B8
zmp_ph111_B6
zmp_ph111_B7
zmp_ph111_B9
zmp_ph111_B10
zmp_ph114_G4
zmp_ph114_G11
zmp_ph114_H1
zmp_ph114_H2
zmp_ph114_A9
zmp_ph114_A11
zmp_ph114_B2
zmp_ph114_B4
zmp_ph114_D8
zmp_ph114_E2

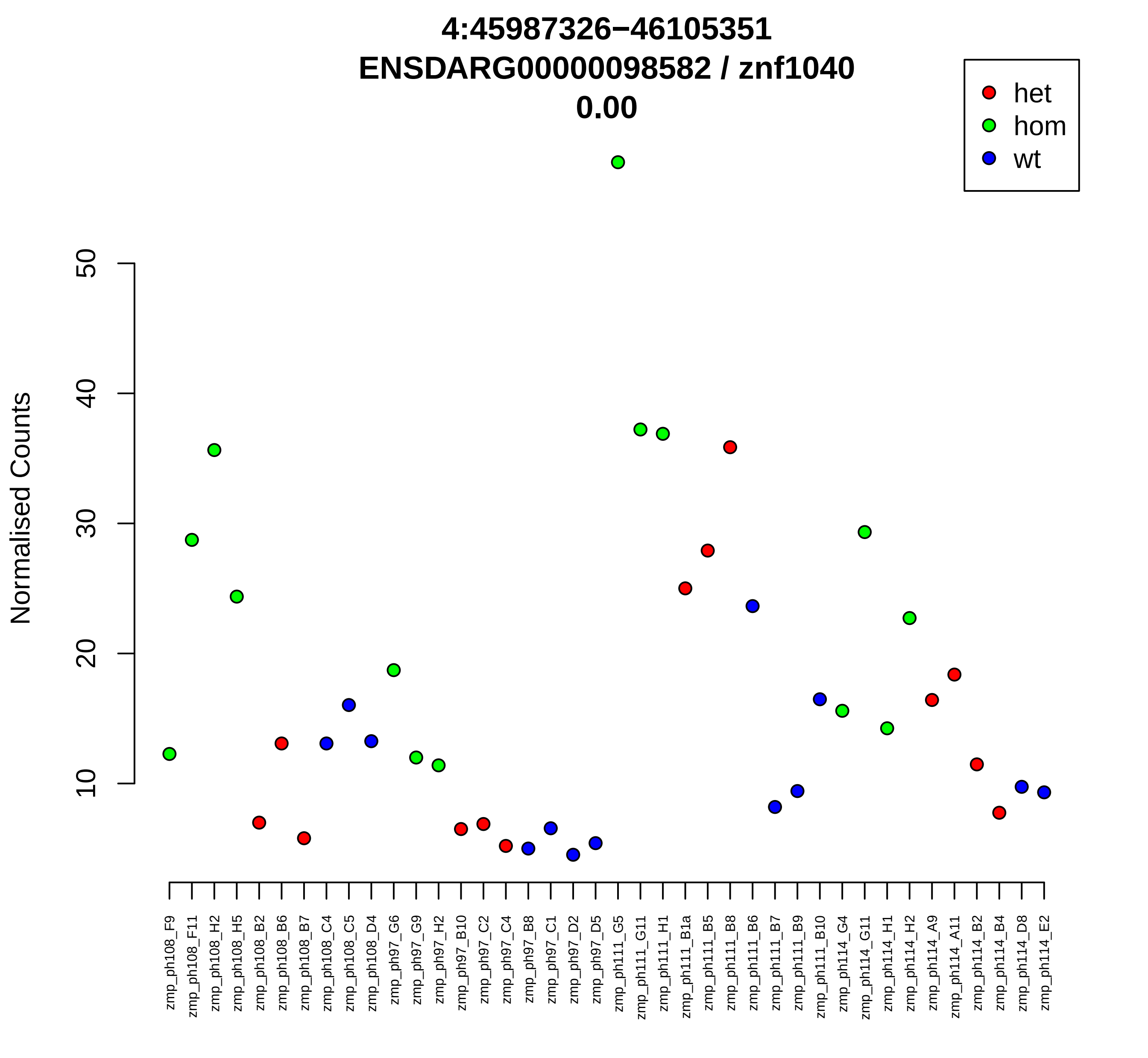

4:45987326−46105351
ENSDARG00000098582 / znf1040
0.00
het
hom
wt
Normalised Counts
10
20
30
40
50
zmp_ph108_F9
zmp_ph108_F11
zmp_ph108_H2
zmp_ph108_H5
zmp_ph108_B2
zmp_ph108_B6
zmp_ph108_B7
zmp_ph108_C4
zmp_ph108_C5
zmp_ph108_D4
zmp_ph97_G6
zmp_ph97_G9
zmp_ph97_H2
zmp_ph97_B10
zmp_ph97_C2
zmp_ph97_C4
zmp_ph97_B8
zmp_ph97_C1
zmp_ph97_D2
zmp_ph97_D5
zmp_ph111_G5
zmp_ph111_G11
zmp_ph111_H1
zmp_ph111_B1a
zmp_ph111_B5
zmp_ph111_B8
zmp_ph111_B6
zmp_ph111_B7
zmp_ph111_B9
zmp_ph111_B10
zmp_ph114_G4
zmp_ph114_G11
zmp_ph114_H1
zmp_ph114_H2
zmp_ph114_A9
zmp_ph114_A11
zmp_ph114_B2
zmp_ph114_B4
zmp_ph114_D8
zmp_ph114_E2

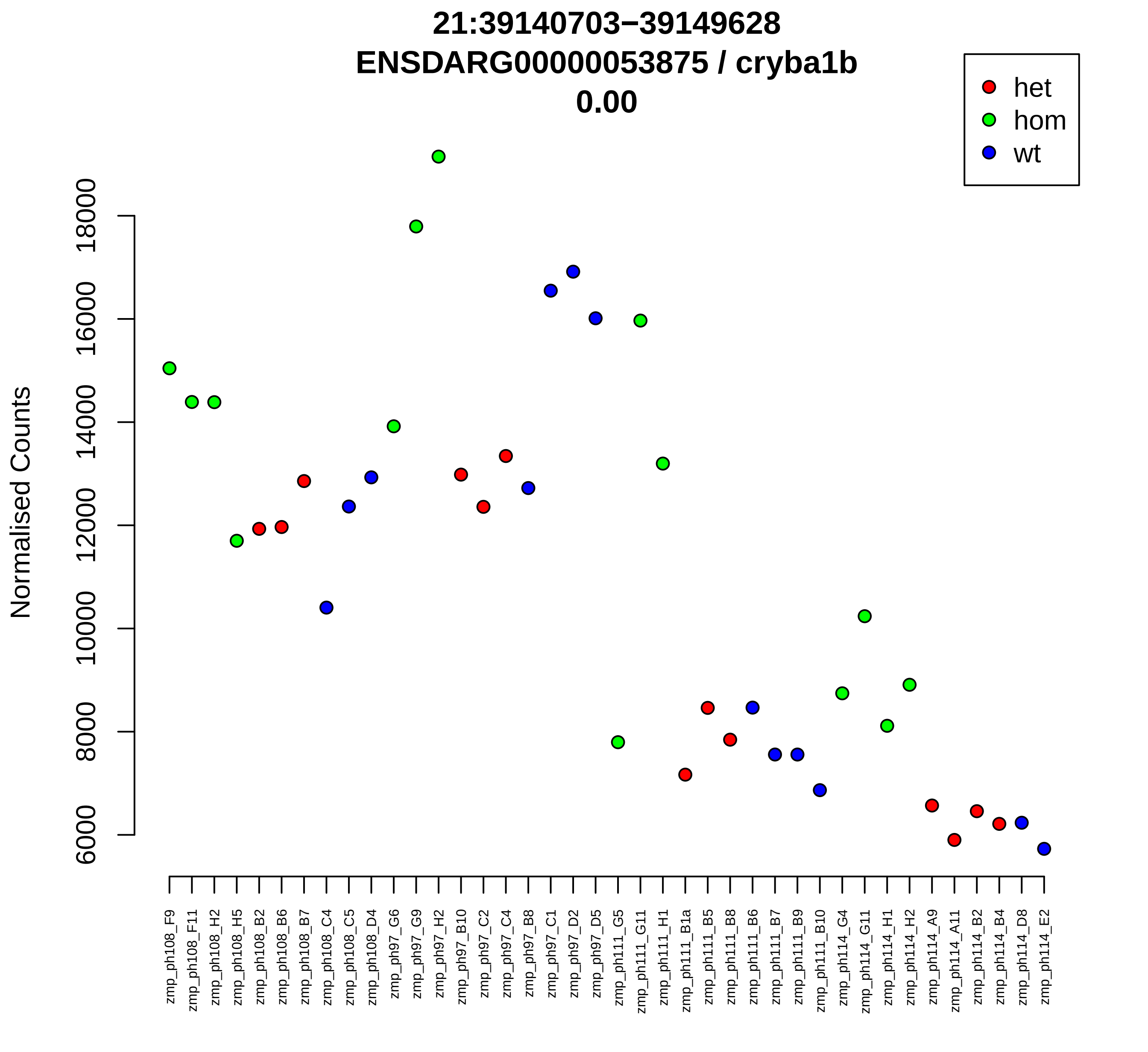
21:39140703−39149628
ENSDARG00000053875 / cryba1b
0.00
het
hom
wt
Normalised Counts
6000
8000
10000
12000
14000
16000
18000
zmp_ph108_F9
zmp_ph108_F11
zmp_ph108_H2
zmp_ph108_H5
zmp_ph108_B2
zmp_ph108_B6
zmp_ph108_B7
zmp_ph108_C4
zmp_ph108_C5
zmp_ph108_D4
zmp_ph97_G6
zmp_ph97_G9
zmp_ph97_H2
zmp_ph97_B10
zmp_ph97_C2
zmp_ph97_C4
zmp_ph97_B8
zmp_ph97_C1
zmp_ph97_D2
zmp_ph97_D5
zmp_ph111_G5
zmp_ph111_G11
zmp_ph111_H1
zmp_ph111_B1a
zmp_ph111_B5
zmp_ph111_B8
zmp_ph111_B6
zmp_ph111_B7
zmp_ph111_B9
zmp_ph111_B10
zmp_ph114_G4
zmp_ph114_G11
zmp_ph114_H1
zmp_ph114_H2
zmp_ph114_A9
zmp_ph114_A11
zmp_ph114_B2
zmp_ph114_B4
zmp_ph114_D8
zmp_ph114_E2

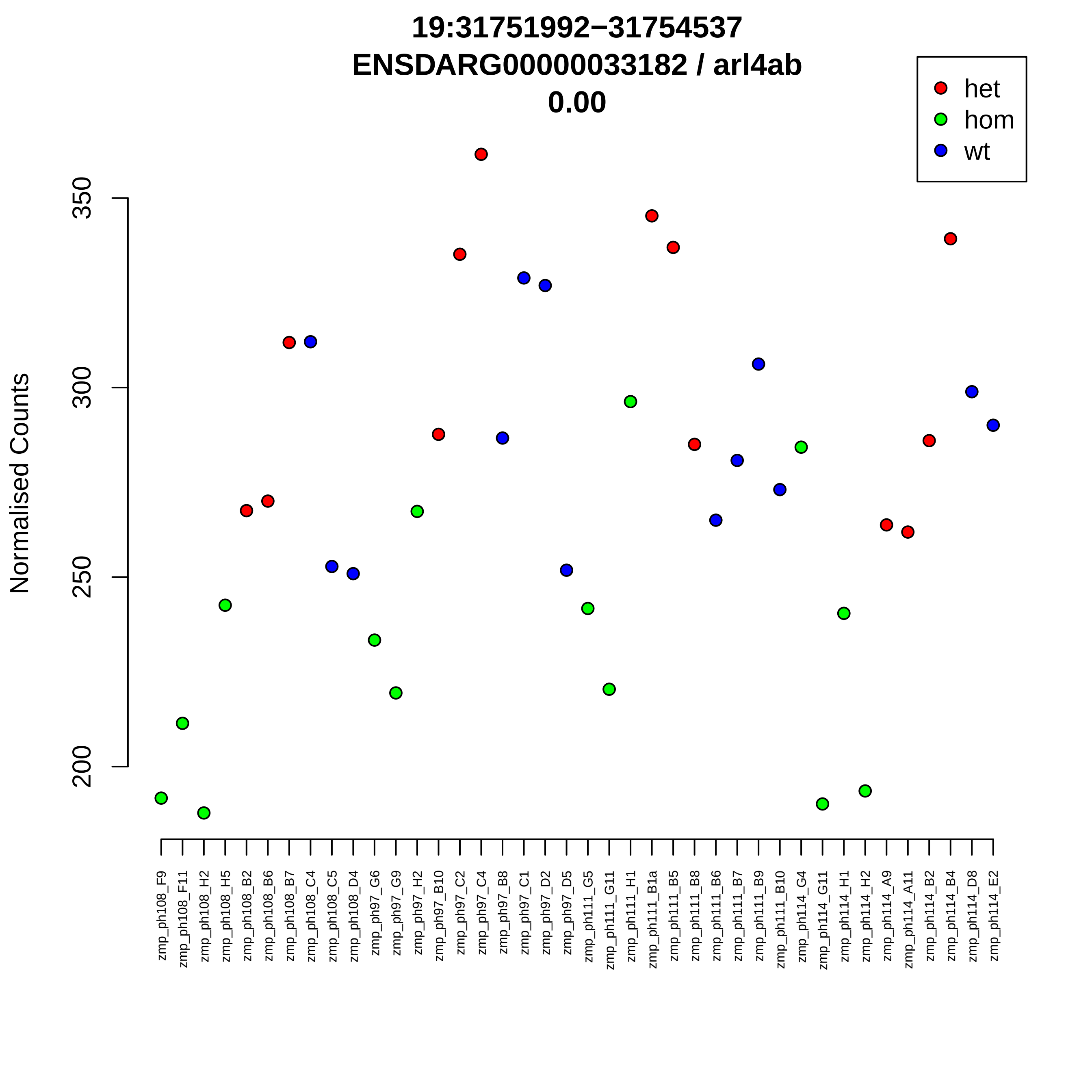
19:31751992−31754537
ENSDARG00000033182 / arl4ab
0.00
het
hom
wt
Normalised Counts
200
250
300
350
zmp_ph108_F9
zmp_ph108_F11
zmp_ph108_H2
zmp_ph108_H5
zmp_ph108_B2
zmp_ph108_B6
zmp_ph108_B7
zmp_ph108_C4
zmp_ph108_C5
zmp_ph108_D4
zmp_ph97_G6
zmp_ph97_G9
zmp_ph97_H2
zmp_ph97_B10
zmp_ph97_C2
zmp_ph97_C4
zmp_ph97_B8
zmp_ph97_C1
zmp_ph97_D2
zmp_ph97_D5
zmp_ph111_G5
zmp_ph111_G11
zmp_ph111_H1
zmp_ph111_B1a
zmp_ph111_B5
zmp_ph111_B8
zmp_ph111_B6
zmp_ph111_B7
zmp_ph111_B9
zmp_ph111_B10
zmp_ph114_G4
zmp_ph114_G11
zmp_ph114_H1
zmp_ph114_H2
zmp_ph114_A9
zmp_ph114_A11
zmp_ph114_B2
zmp_ph114_B4
zmp_ph114_D8
zmp_ph114_E2

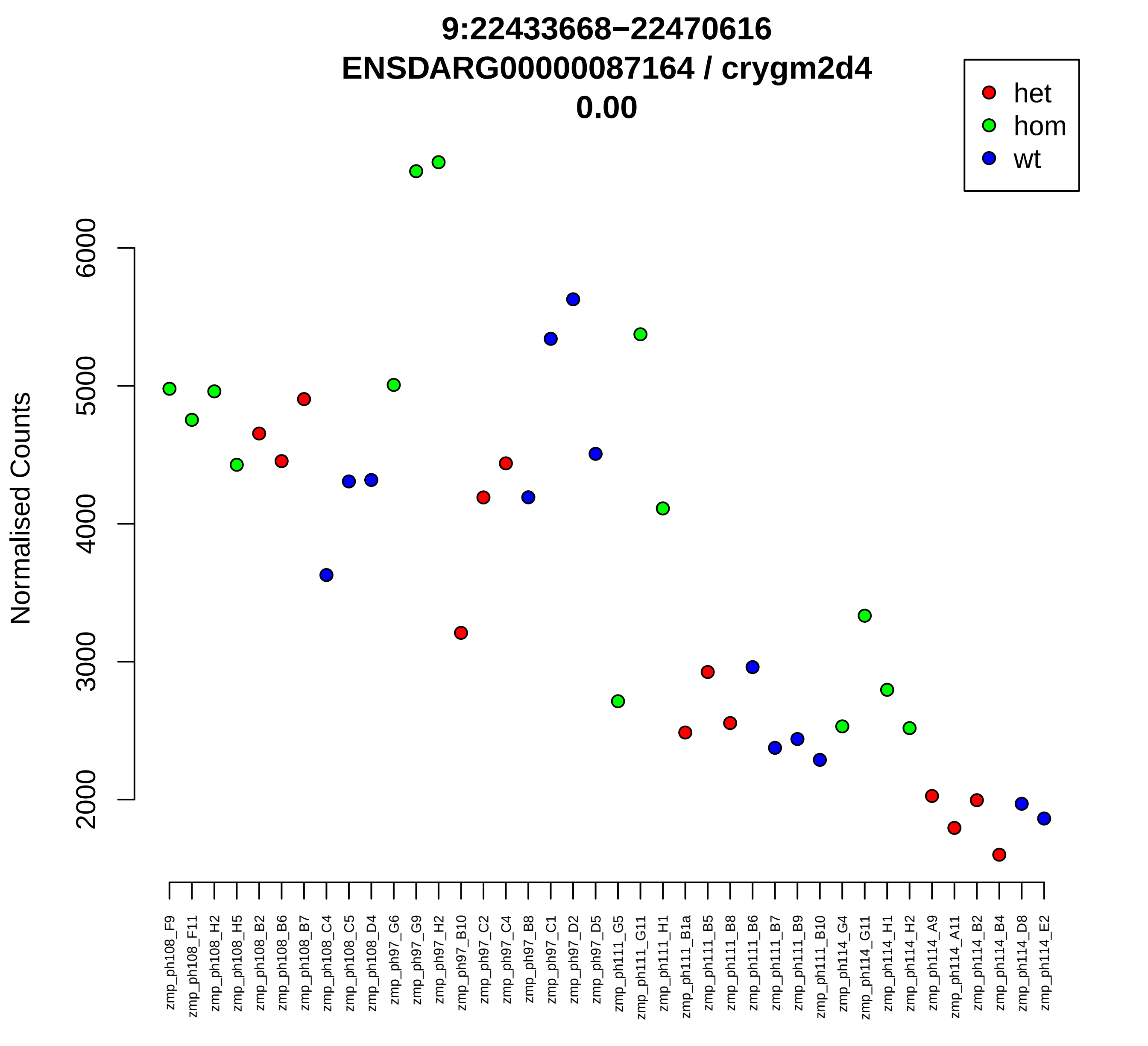
9:22433668−22470616
ENSDARG00000087164 / crygm2d4
0.00
het
hom
wt
Normalised Counts
2000
3000
4000
5000
6000
zmp_ph108_F9
zmp_ph108_F11
zmp_ph108_H2
zmp_ph108_H5
zmp_ph108_B2
zmp_ph108_B6
zmp_ph108_B7
zmp_ph108_C4
zmp_ph108_C5
zmp_ph108_D4
zmp_ph97_G6
zmp_ph97_G9
zmp_ph97_H2
zmp_ph97_B10
zmp_ph97_C2
zmp_ph97_C4
zmp_ph97_B8
zmp_ph97_C1
zmp_ph97_D2
zmp_ph97_D5
zmp_ph111_G5
zmp_ph111_G11
zmp_ph111_H1
zmp_ph111_B1a
zmp_ph111_B5
zmp_ph111_B8
zmp_ph111_B6
zmp_ph111_B7
zmp_ph111_B9
zmp_ph111_B10
zmp_ph114_G4
zmp_ph114_G11
zmp_ph114_H1
zmp_ph114_H2
zmp_ph114_A9
zmp_ph114_A11
zmp_ph114_B2
zmp_ph114_B4
zmp_ph114_D8
zmp_ph114_E2

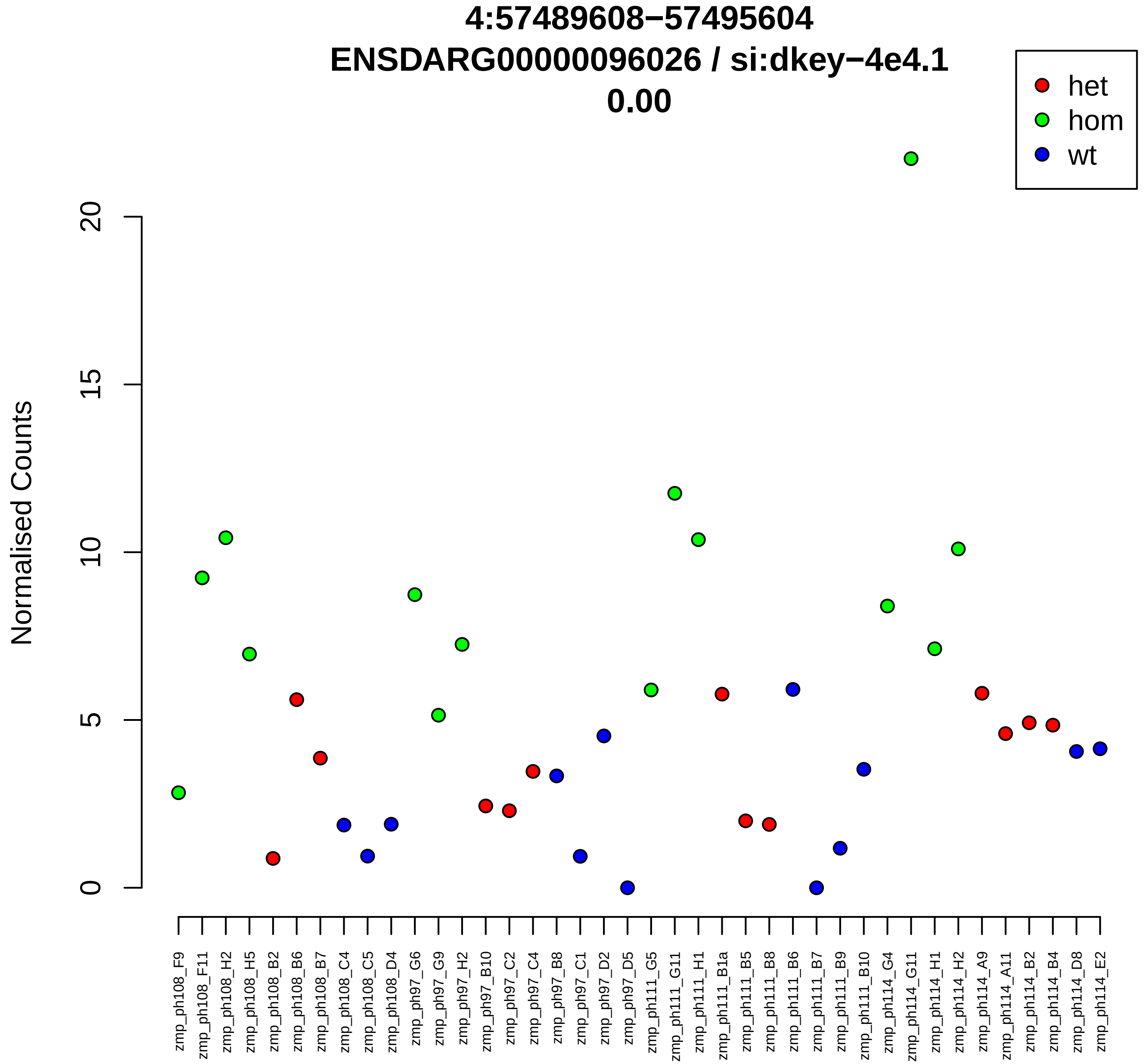
4:57489608−57495604
ENSDARG00000096026 / si:dkey−4e4.1
0.00
het
hom
wt
Normalised Counts
0
5
10
15
20
zmp_ph108_F9
zmp_ph108_F11
zmp_ph108_H2
zmp_ph108_H5
zmp_ph108_B2
zmp_ph108_B6
zmp_ph108_B7
zmp_ph108_C4
zmp_ph108_C5
zmp_ph108_D4
zmp_ph97_G6
zmp_ph97_G9
zmp_ph97_H2
zmp_ph97_B10
zmp_ph97_C2
zmp_ph97_C4
zmp_ph97_B8
zmp_ph97_C1
zmp_ph97_D2
zmp_ph97_D5
zmp_ph111_G5
zmp_ph111_G11
zmp_ph111_H1
zmp_ph111_B1a
zmp_ph111_B5
zmp_ph111_B8
zmp_ph111_B6
zmp_ph111_B7
zmp_ph111_B9
zmp_ph111_B10
zmp_ph114_G4
zmp_ph114_G11
zmp_ph114_H1
zmp_ph114_H2
zmp_ph114_A9
zmp_ph114_A11
zmp_ph114_B2
zmp_ph114_B4
zmp_ph114_D8
zmp_ph114_E2

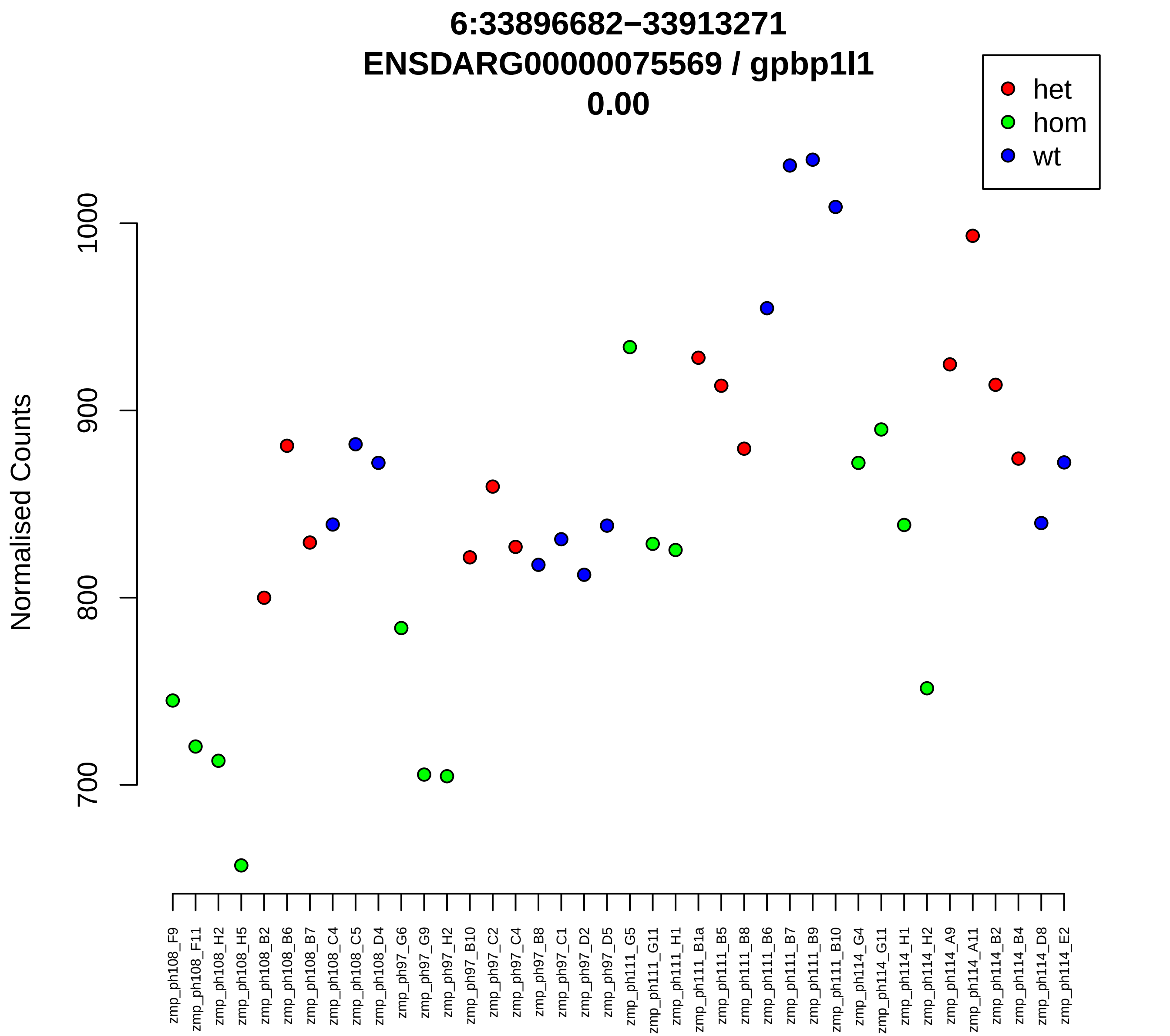

6:33896682−33913271
ENSDARG00000075569 / gpbp1l1
0.00
het
hom
wt
Normalised Counts
700
800
900
1000
zmp_ph108_F9
zmp_ph108_F11
zmp_ph108_H2
zmp_ph108_H5
zmp_ph108_B2
zmp_ph108_B6
zmp_ph108_B7
zmp_ph108_C4
zmp_ph108_C5
zmp_ph108_D4
zmp_ph97_G6
zmp_ph97_G9
zmp_ph97_H2
zmp_ph97_B10
zmp_ph97_C2
zmp_ph97_C4
zmp_ph97_B8
zmp_ph97_C1
zmp_ph97_D2
zmp_ph97_D5
zmp_ph111_G5
zmp_ph111_G11
zmp_ph111_H1
zmp_ph111_B1a
zmp_ph111_B5
zmp_ph111_B8
zmp_ph111_B6
zmp_ph111_B7
zmp_ph111_B9
zmp_ph111_B10
zmp_ph114_G4
zmp_ph114_G11
zmp_ph114_H1
zmp_ph114_H2
zmp_ph114_A9
zmp_ph114_A11
zmp_ph114_B2
zmp_ph114_B4
zmp_ph114_D8
zmp_ph114_E2

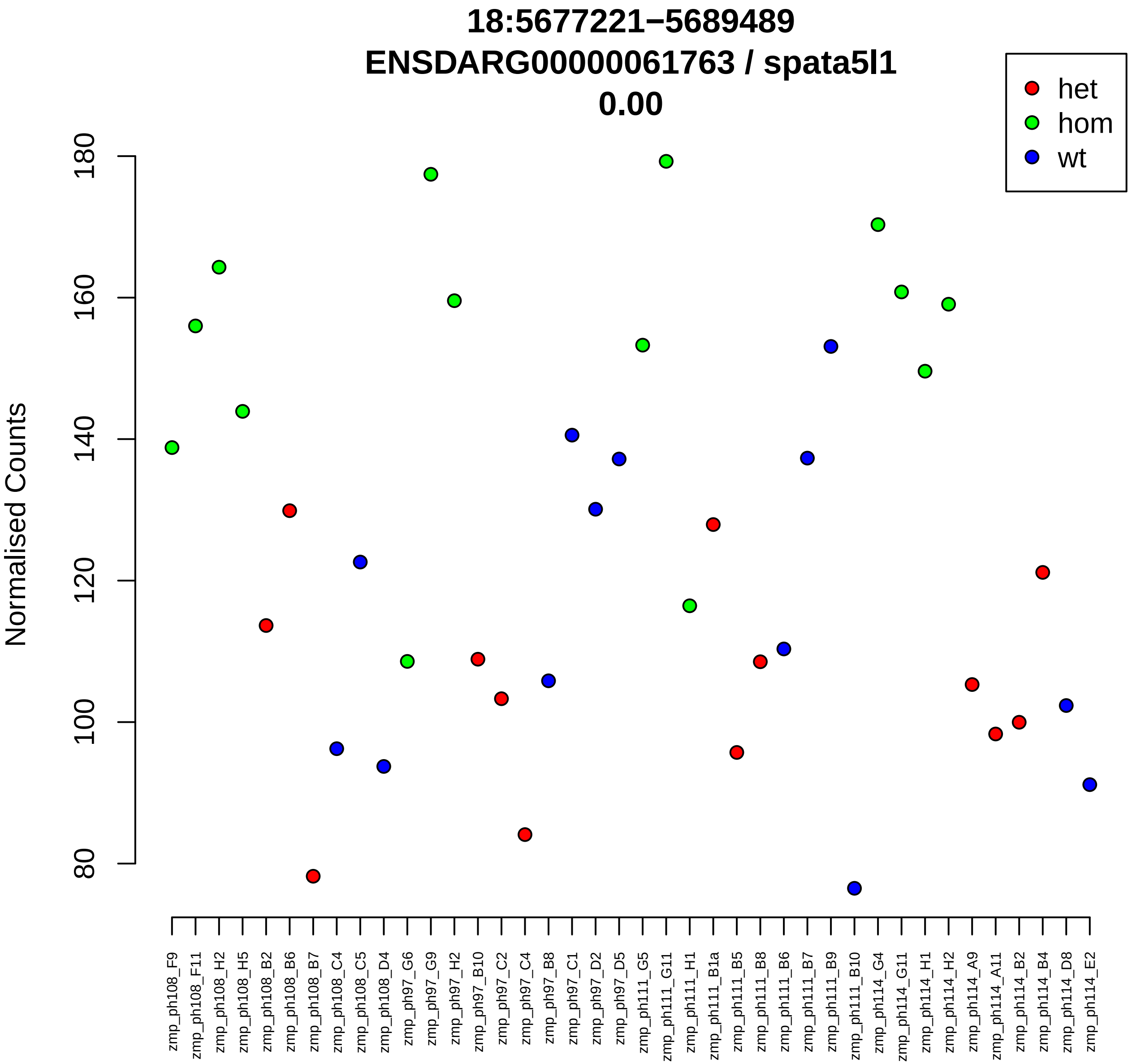

18:5677221−5689489
ENSDARG00000061763 / spata5l1
0.00
het
hom
wt
Normalised Counts
80
100
120
140
160
180
zmp_ph108_F9
zmp_ph108_F11
zmp_ph108_H2
zmp_ph108_H5
zmp_ph108_B2
zmp_ph108_B6
zmp_ph108_B7
zmp_ph108_C4
zmp_ph108_C5
zmp_ph108_D4
zmp_ph97_G6
zmp_ph97_G9
zmp_ph97_H2
zmp_ph97_B10
zmp_ph97_C2
zmp_ph97_C4
zmp_ph97_B8
zmp_ph97_C1
zmp_ph97_D2
zmp_ph97_D5
zmp_ph111_G5
zmp_ph111_G11
zmp_ph111_H1
zmp_ph111_B1a
zmp_ph111_B5
zmp_ph111_B8
zmp_ph111_B6
zmp_ph111_B7
zmp_ph111_B9
zmp_ph111_B10
zmp_ph114_G4
zmp_ph114_G11
zmp_ph114_H1
zmp_ph114_H2
zmp_ph114_A9
zmp_ph114_A11
zmp_ph114_B2
zmp_ph114_B4
zmp_ph114_D8
zmp_ph114_E2

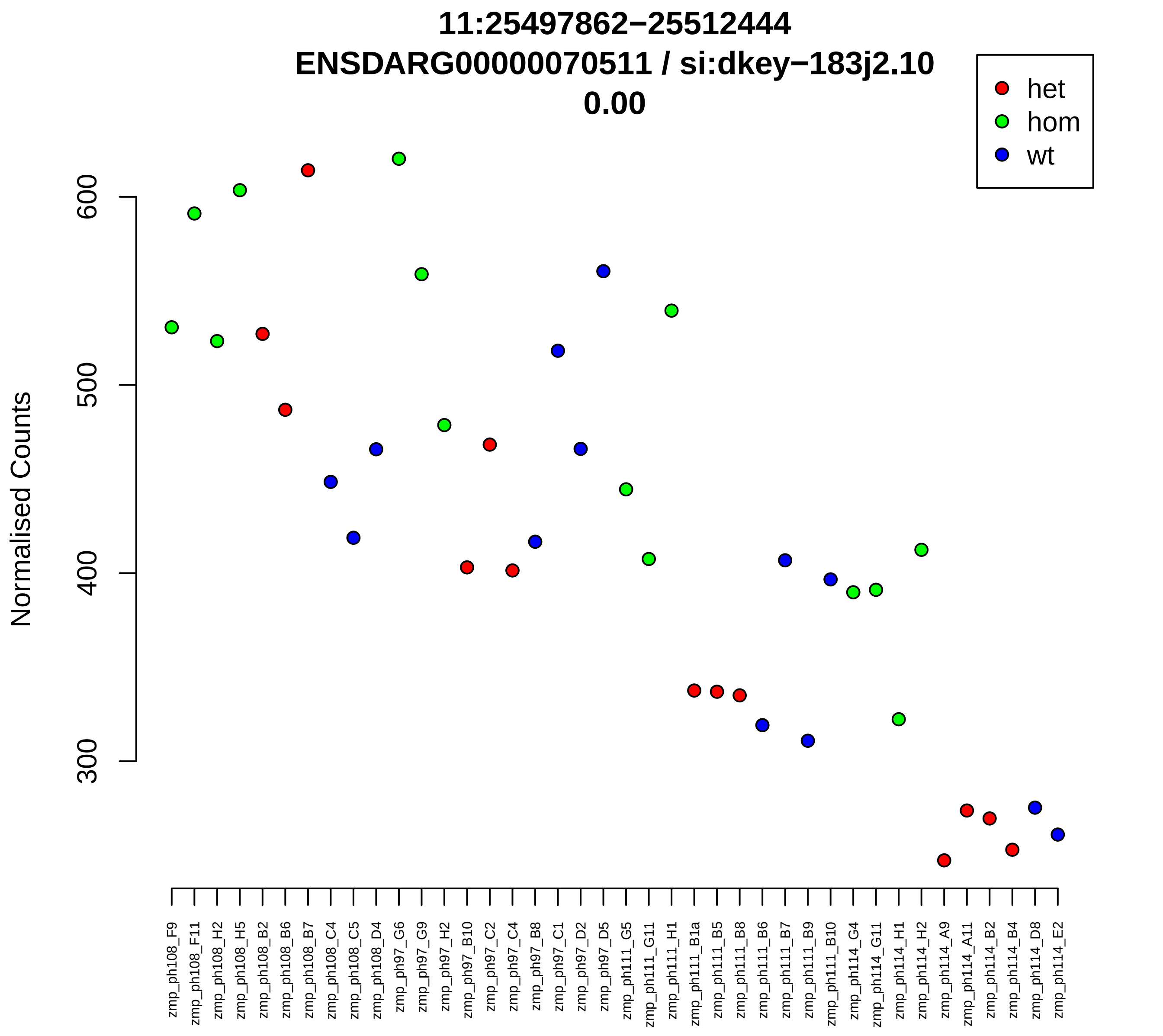
11:25497862−25512444
ENSDARG00000070511 / si:dkey−183j2.10
0.00
het
hom
wt
Normalised Counts
300
400
500
600
zmp_ph108_F9
zmp_ph108_F11
zmp_ph108_H2
zmp_ph108_H5
zmp_ph108_B2
zmp_ph108_B6
zmp_ph108_B7
zmp_ph108_C4
zmp_ph108_C5
zmp_ph108_D4
zmp_ph97_G6
zmp_ph97_G9
zmp_ph97_H2
zmp_ph97_B10
zmp_ph97_C2
zmp_ph97_C4
zmp_ph97_B8
zmp_ph97_C1
zmp_ph97_D2
zmp_ph97_D5
zmp_ph111_G5
zmp_ph111_G11
zmp_ph111_H1
zmp_ph111_B1a
zmp_ph111_B5
zmp_ph111_B8
zmp_ph111_B6
zmp_ph111_B7
zmp_ph111_B9
zmp_ph111_B10
zmp_ph114_G4
zmp_ph114_G11
zmp_ph114_H1
zmp_ph114_H2
zmp_ph114_A9
zmp_ph114_A11
zmp_ph114_B2
zmp_ph114_B4
zmp_ph114_D8
zmp_ph114_E2

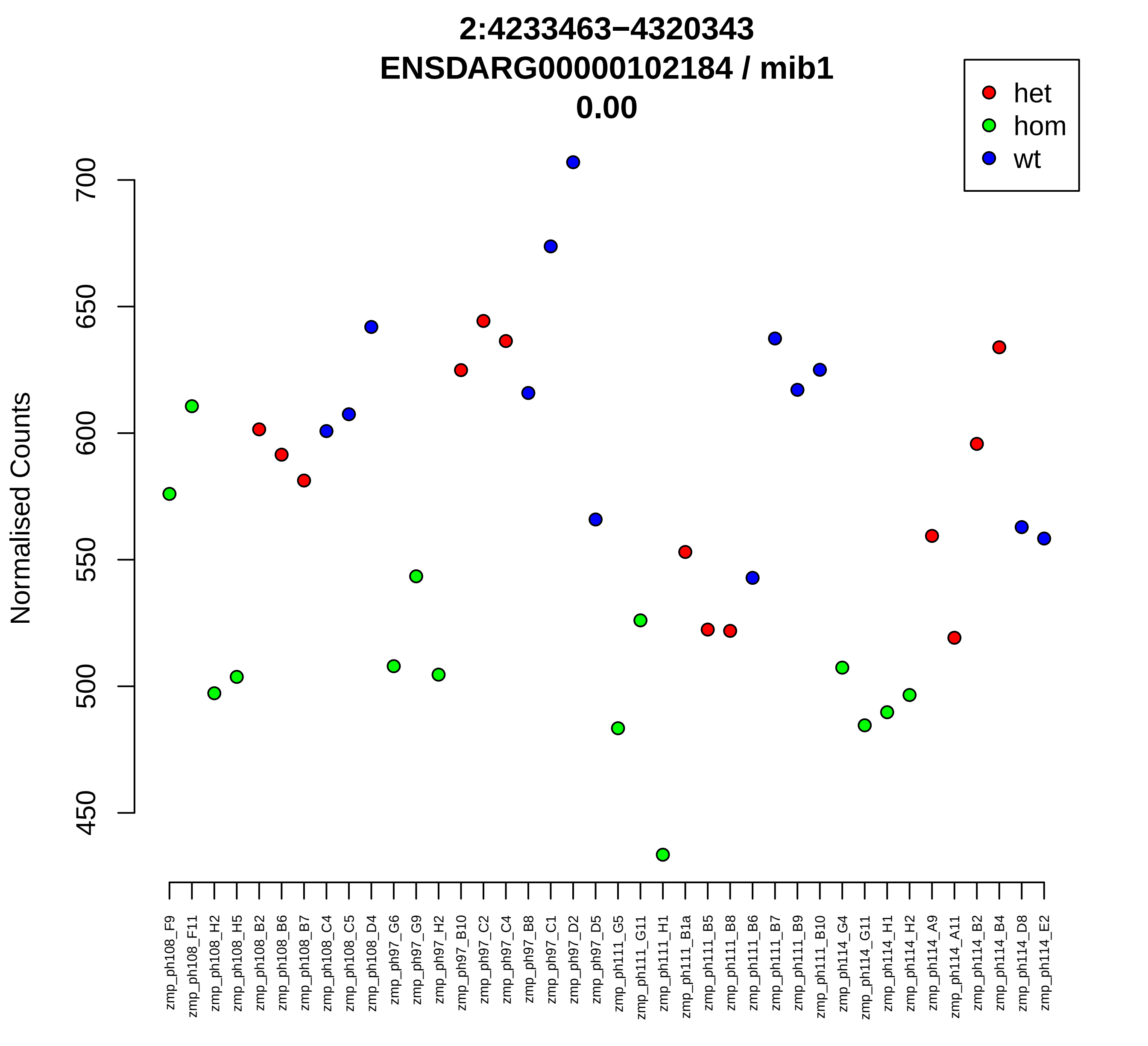
2:4233463−4320343
ENSDARG00000102184 / mib1
0.00
het
hom
wt
Normalised Counts
450
500
550
600
650
700
zmp_ph108_F9
zmp_ph108_F11
zmp_ph108_H2
zmp_ph108_H5
zmp_ph108_B2
zmp_ph108_B6
zmp_ph108_B7
zmp_ph108_C4
zmp_ph108_C5
zmp_ph108_D4
zmp_ph97_G6
zmp_ph97_G9
zmp_ph97_H2
zmp_ph97_B10
zmp_ph97_C2
zmp_ph97_C4
zmp_ph97_B8
zmp_ph97_C1
zmp_ph97_D2
zmp_ph97_D5
zmp_ph111_G5
zmp_ph111_G11
zmp_ph111_H1
zmp_ph111_B1a
zmp_ph111_B5
zmp_ph111_B8
zmp_ph111_B6
zmp_ph111_B7
zmp_ph111_B9
zmp_ph111_B10
zmp_ph114_G4
zmp_ph114_G11
zmp_ph114_H1
zmp_ph114_H2
zmp_ph114_A9
zmp_ph114_A11
zmp_ph114_B2
zmp_ph114_B4
zmp_ph114_D8
zmp_ph114_E2

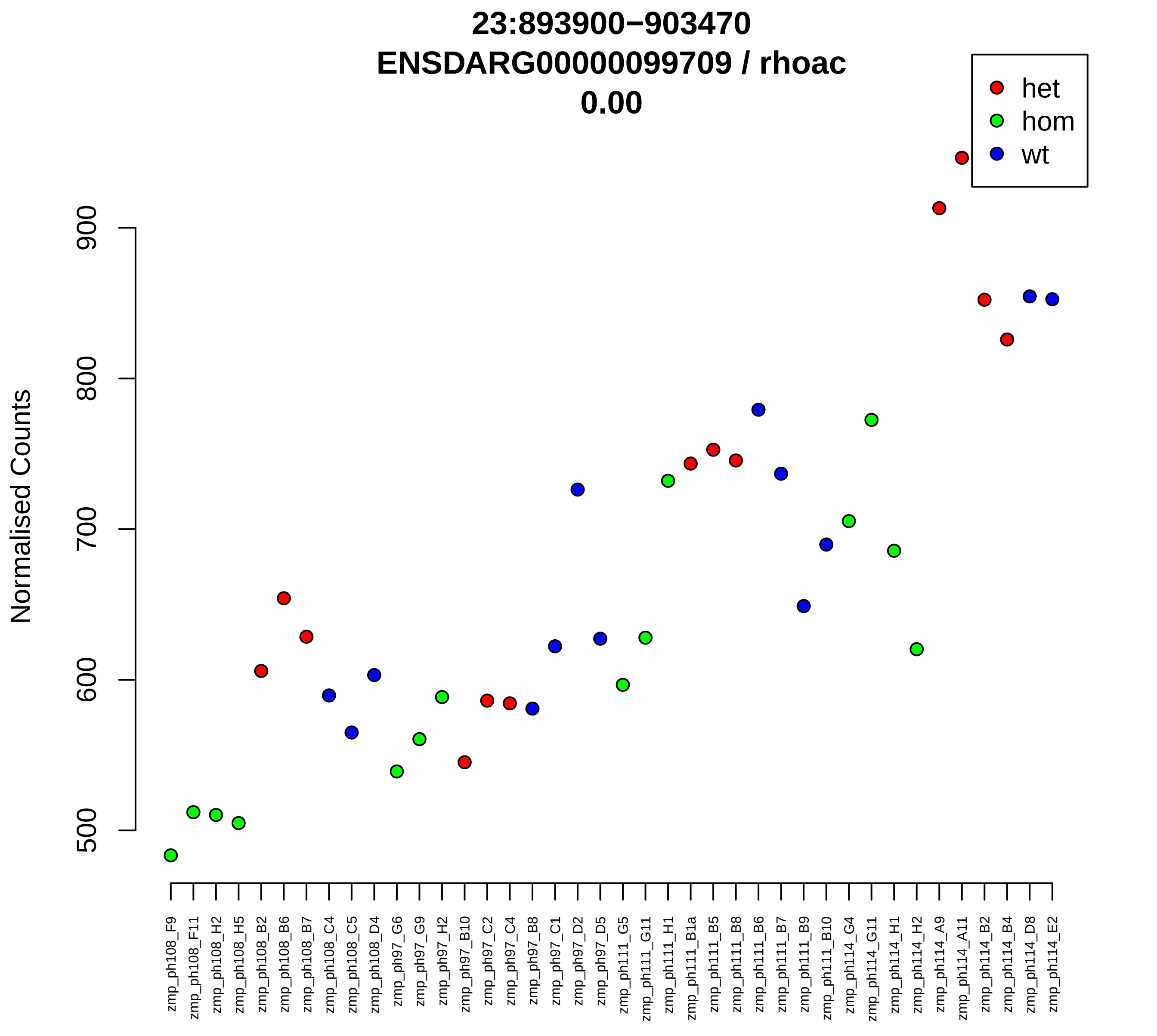

23:893900−903470
ENSDARG00000099709 / rhoac
0.00
het
hom
wt
Normalised Counts
500
600
700
800
900
zmp_ph108_F9
zmp_ph108_F11
zmp_ph108_H2
zmp_ph108_H5
zmp_ph108_B2
zmp_ph108_B6
zmp_ph108_B7
zmp_ph108_C4
zmp_ph108_C5
zmp_ph108_D4
zmp_ph97_G6
zmp_ph97_G9
zmp_ph97_H2
zmp_ph97_B10
zmp_ph97_C2
zmp_ph97_C4
zmp_ph97_B8
zmp_ph97_C1
zmp_ph97_D2
zmp_ph97_D5
zmp_ph111_G5
zmp_ph111_G11
zmp_ph111_H1
zmp_ph111_B1a
zmp_ph111_B5
zmp_ph111_B8
zmp_ph111_B6
zmp_ph111_B7
zmp_ph111_B9
zmp_ph111_B10
zmp_ph114_G4
zmp_ph114_G11
zmp_ph114_H1
zmp_ph114_H2
zmp_ph114_A9
zmp_ph114_A11
zmp_ph114_B2
zmp_ph114_B4
zmp_ph114_D8
zmp_ph114_E2

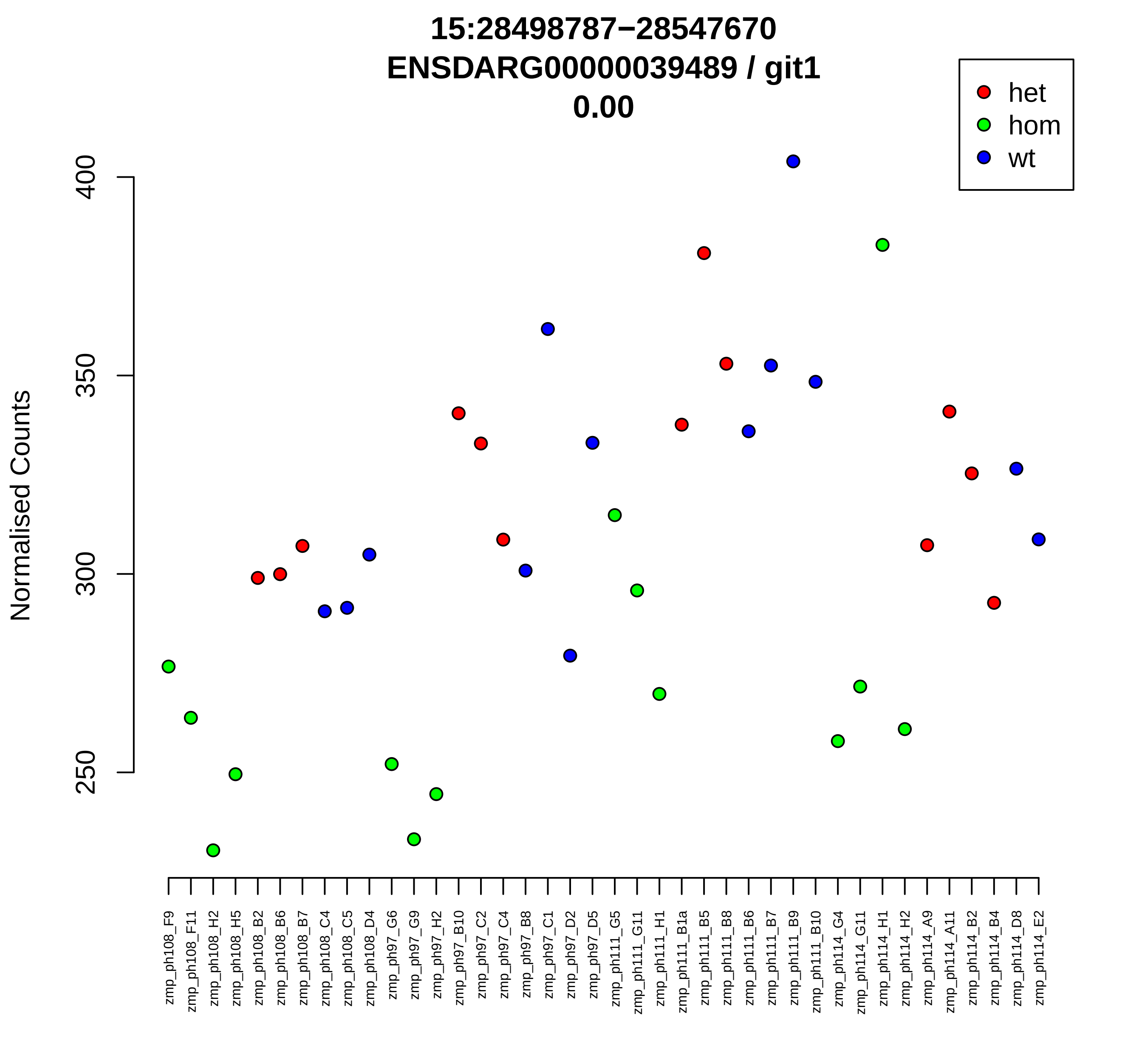

15:28498787−28547670
ENSDARG00000039489 / git1
0.00
het
hom
wt
Normalised Counts
400
350
300
250
zmp_ph108_F9
zmp_ph108_F11
zmp_ph108_H2
zmp_ph108_H5
zmp_ph108_B2
zmp_ph108_B6
zmp_ph108_B7
zmp_ph108_C4
zmp_ph108_C5
zmp_ph108_D4
zmp_ph97_G6
zmp_ph97_G9
zmp_ph97_H2
zmp_ph97_B10
zmp_ph97_C2
zmp_ph97_C4
zmp_ph97_B8
zmp_ph97_C1
zmp_ph97_D2
zmp_ph97_D5
zmp_ph111_G5
zmp_ph111_G11
zmp_ph111_H1
zmp_ph111_B1a
zmp_ph111_B5
zmp_ph111_B8
zmp_ph111_B6
zmp_ph111_B7
zmp_ph111_B9
zmp_ph111_B10
zmp_ph114_G4
zmp_ph114_G11
zmp_ph114_H1
zmp_ph114_H2
zmp_ph114_A9
zmp_ph114_A11
zmp_ph114_B2
zmp_ph114_B4
zmp_ph114_D8
zmp_ph114_E2

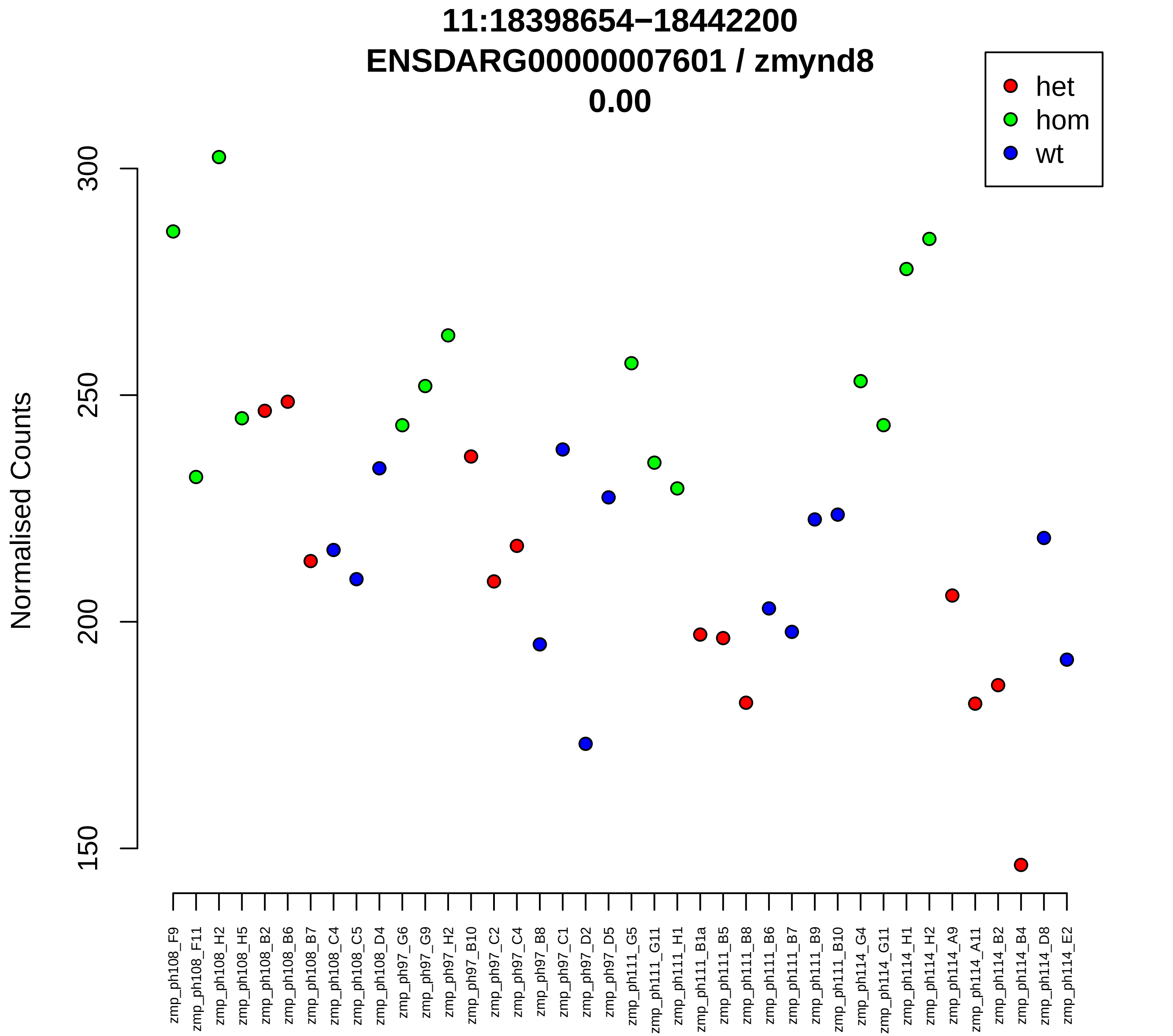
11:18398654−18442200
ENSDARG00000007601 / zmynd8
0.00
het
hom
wt
Normalised Counts
300
250
200
150
zmp_ph108_F9
zmp_ph108_F11
zmp_ph108_H2
zmp_ph108_H5
zmp_ph108_B2
zmp_ph108_B6
zmp_ph108_B7
zmp_ph108_C4
zmp_ph108_C5
zmp_ph108_D4
zmp_ph97_G6
zmp_ph97_G9
zmp_ph97_H2
zmp_ph97_B10
zmp_ph97_C2
zmp_ph97_C4
zmp_ph97_B8
zmp_ph97_C1
zmp_ph97_D2
zmp_ph97_D5
zmp_ph111_G5
zmp_ph111_G11
zmp_ph111_H1
zmp_ph111_B1a
zmp_ph111_B5
zmp_ph111_B8
zmp_ph111_B6
zmp_ph111_B7
zmp_ph111_B9
zmp_ph111_B10
zmp_ph114_G4
zmp_ph114_G11
zmp_ph114_H1
zmp_ph114_H2
zmp_ph114_A9
zmp_ph114_A11
zmp_ph114_B2
zmp_ph114_B4
zmp_ph114_D8
zmp_ph114_E2

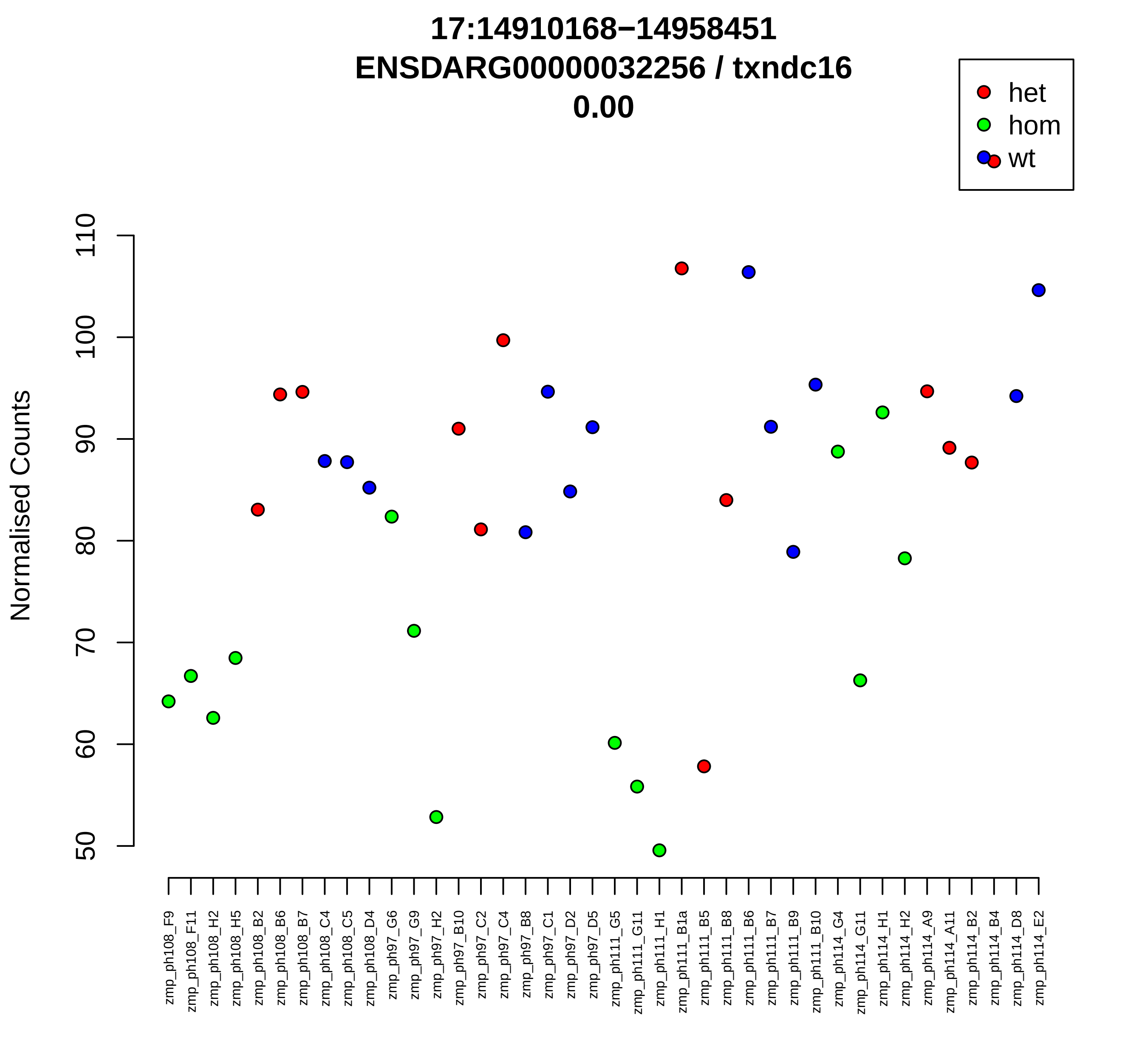
17:14910168−14958451
ENSDARG00000032256 / txndc16
0.00
het
hom
wt
Normalised Counts
50
60
70
80
90
100
110
zmp_ph108_F9
zmp_ph108_F11
zmp_ph108_H2
zmp_ph108_H5
zmp_ph108_B2
zmp_ph108_B6
zmp_ph108_B7
zmp_ph108_C4
zmp_ph108_C5
zmp_ph108_D4
zmp_ph97_G6
zmp_ph97_G9
zmp_ph97_H2
zmp_ph97_B10
zmp_ph97_C2
zmp_ph97_C4
zmp_ph97_B8
zmp_ph97_C1
zmp_ph97_D2
zmp_ph97_D5
zmp_ph111_G5
zmp_ph111_G11
zmp_ph111_H1
zmp_ph111_B1a
zmp_ph111_B5
zmp_ph111_B8
zmp_ph111_B6
zmp_ph111_B7
zmp_ph111_B9
zmp_ph111_B10
zmp_ph114_G4
zmp_ph114_G11
zmp_ph114_H1
zmp_ph114_H2
zmp_ph114_A9
zmp_ph114_A11
zmp_ph114_B2
zmp_ph114_B4
zmp_ph114_D8
zmp_ph114_E2

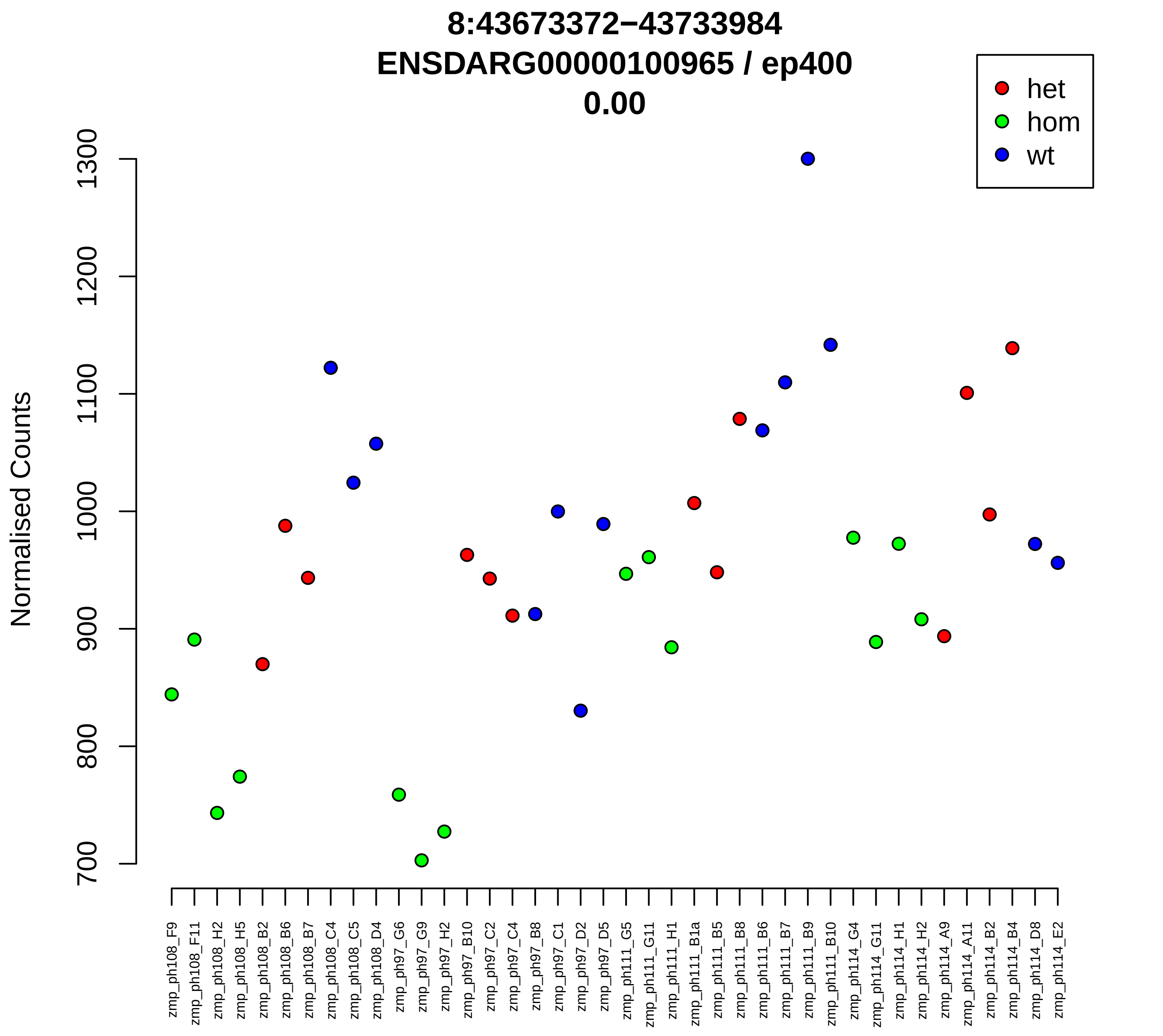

8:43673372−43733984
ENSDARG00000100965 / ep400
0.00
het
hom
wt
Normalised Counts
700
800
900
1000
1100
1200
1300
zmp_ph108_F9
zmp_ph108_F11
zmp_ph108_H2
zmp_ph108_H5
zmp_ph108_B2
zmp_ph108_B6
zmp_ph108_B7
zmp_ph108_C4
zmp_ph108_C5
zmp_ph108_D4
zmp_ph97_G6
zmp_ph97_G9
zmp_ph97_H2
zmp_ph97_B10
zmp_ph97_C2
zmp_ph97_C4
zmp_ph97_B8
zmp_ph97_C1
zmp_ph97_D2
zmp_ph97_D5
zmp_ph111_G5
zmp_ph111_G11
zmp_ph111_H1
zmp_ph111_B1a
zmp_ph111_B5
zmp_ph111_B8
zmp_ph111_B6
zmp_ph111_B7
zmp_ph111_B9
zmp_ph111_B10
zmp_ph114_G4
zmp_ph114_G11
zmp_ph114_H1
zmp_ph114_H2
zmp_ph114_A9
zmp_ph114_A11
zmp_ph114_B2
zmp_ph114_B4
zmp_ph114_D8
zmp_ph114_E2

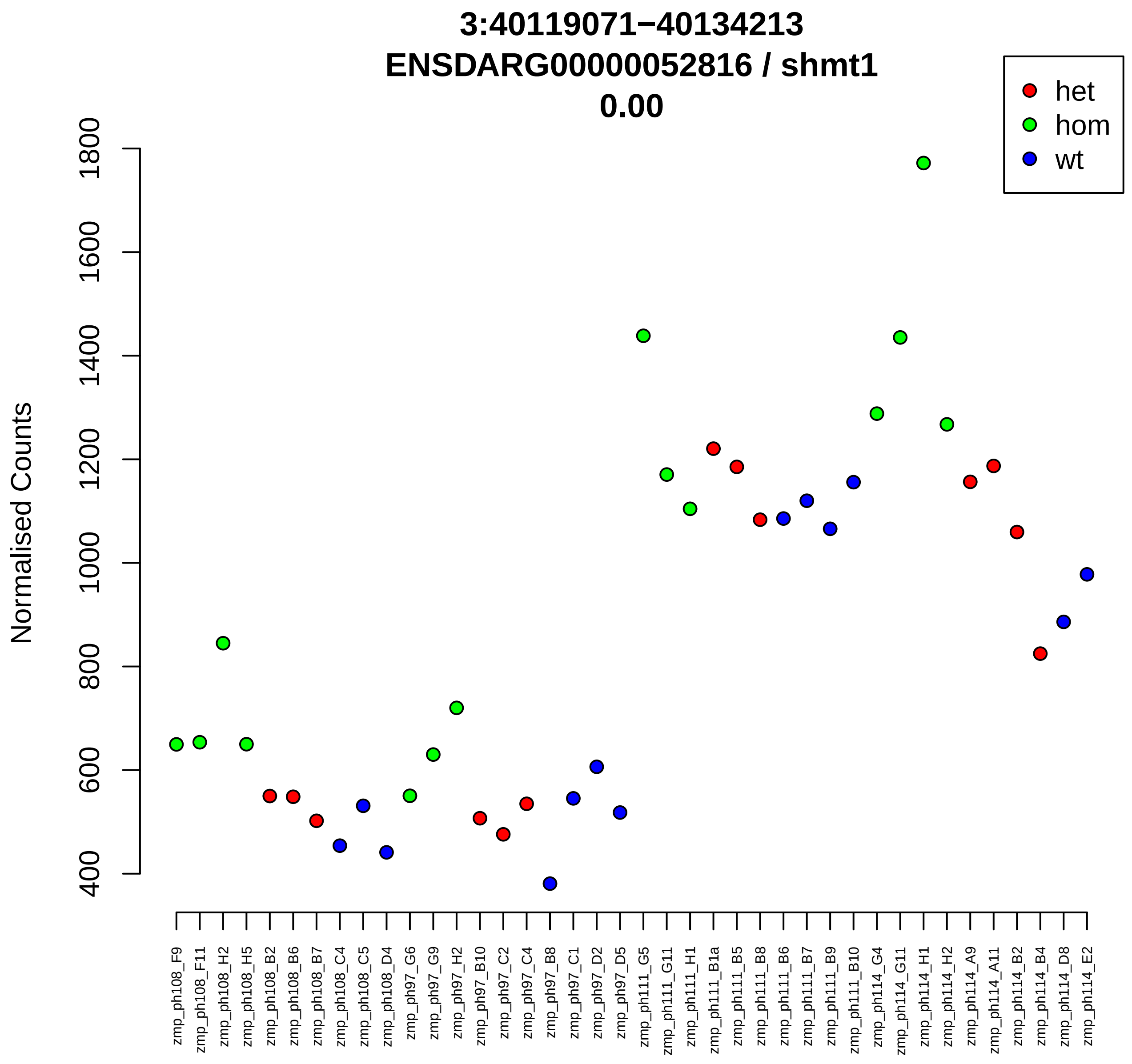
3:40119071−40134213
ENSDARG00000052816 / shmt1
0.00
het
hom
wt
Normalised Counts
400
600
800
1000
1200
1400
1600
1800
zmp_ph108_F9
zmp_ph108_F11
zmp_ph108_H2
zmp_ph108_H5
zmp_ph108_B2
zmp_ph108_B6
zmp_ph108_B7
zmp_ph108_C4
zmp_ph108_C5
zmp_ph108_D4
zmp_ph97_G6
zmp_ph97_G9
zmp_ph97_H2
zmp_ph97_B10
zmp_ph97_C2
zmp_ph97_C4
zmp_ph97_B8
zmp_ph97_C1
zmp_ph97_D2
zmp_ph97_D5
zmp_ph111_G5
zmp_ph111_G11
zmp_ph111_H1
zmp_ph111_B1a
zmp_ph111_B5
zmp_ph111_B8
zmp_ph111_B6
zmp_ph111_B7
zmp_ph111_B9
zmp_ph111_B10
zmp_ph114_G4
zmp_ph114_G11
zmp_ph114_H1
zmp_ph114_H2
zmp_ph114_A9
zmp_ph114_A11
zmp_ph114_B2
zmp_ph114_B4
zmp_ph114_D8
zmp_ph114_E2

10:44195430−44204668
ENSDARG00000024548 / cryba4
0.00

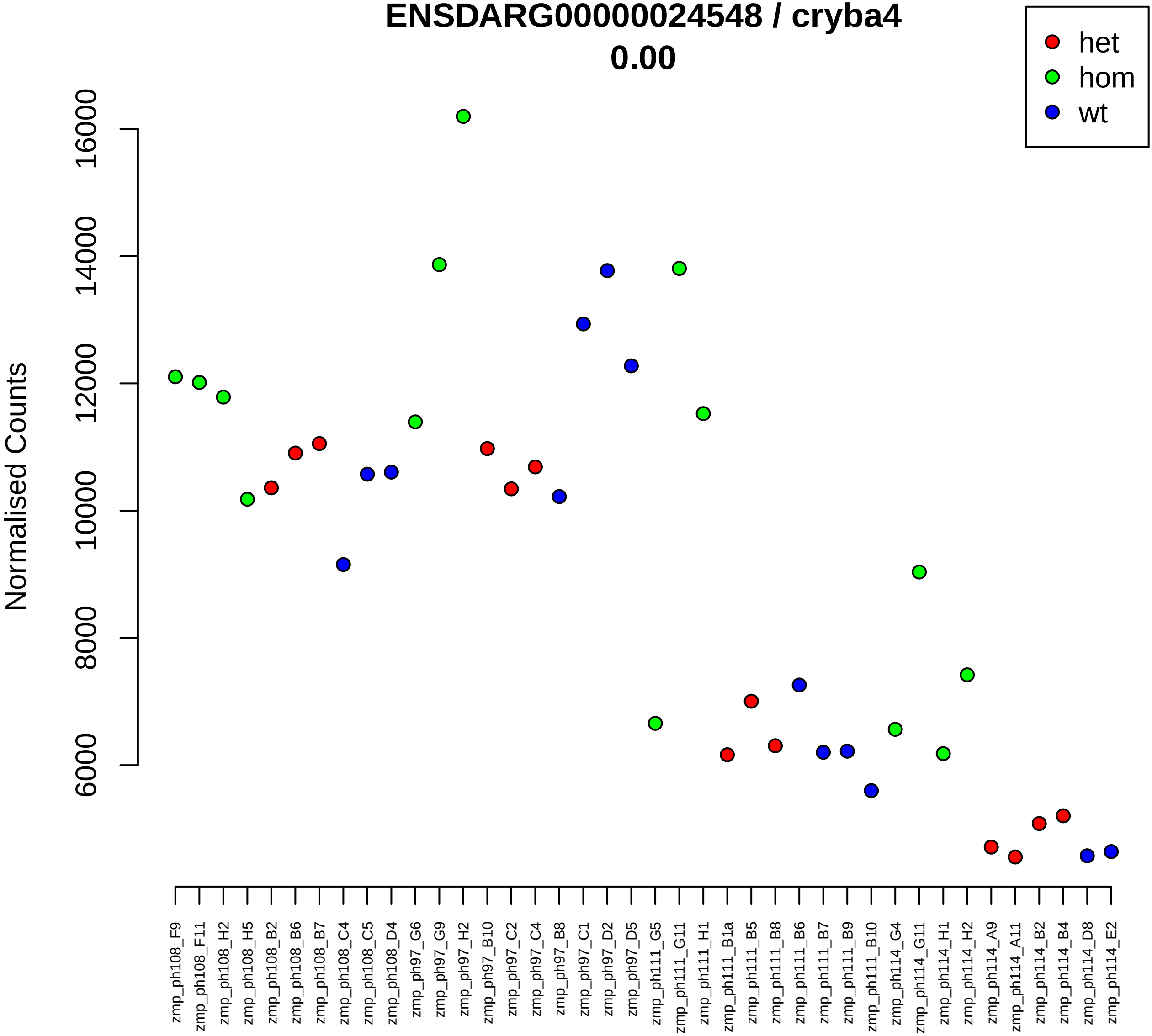

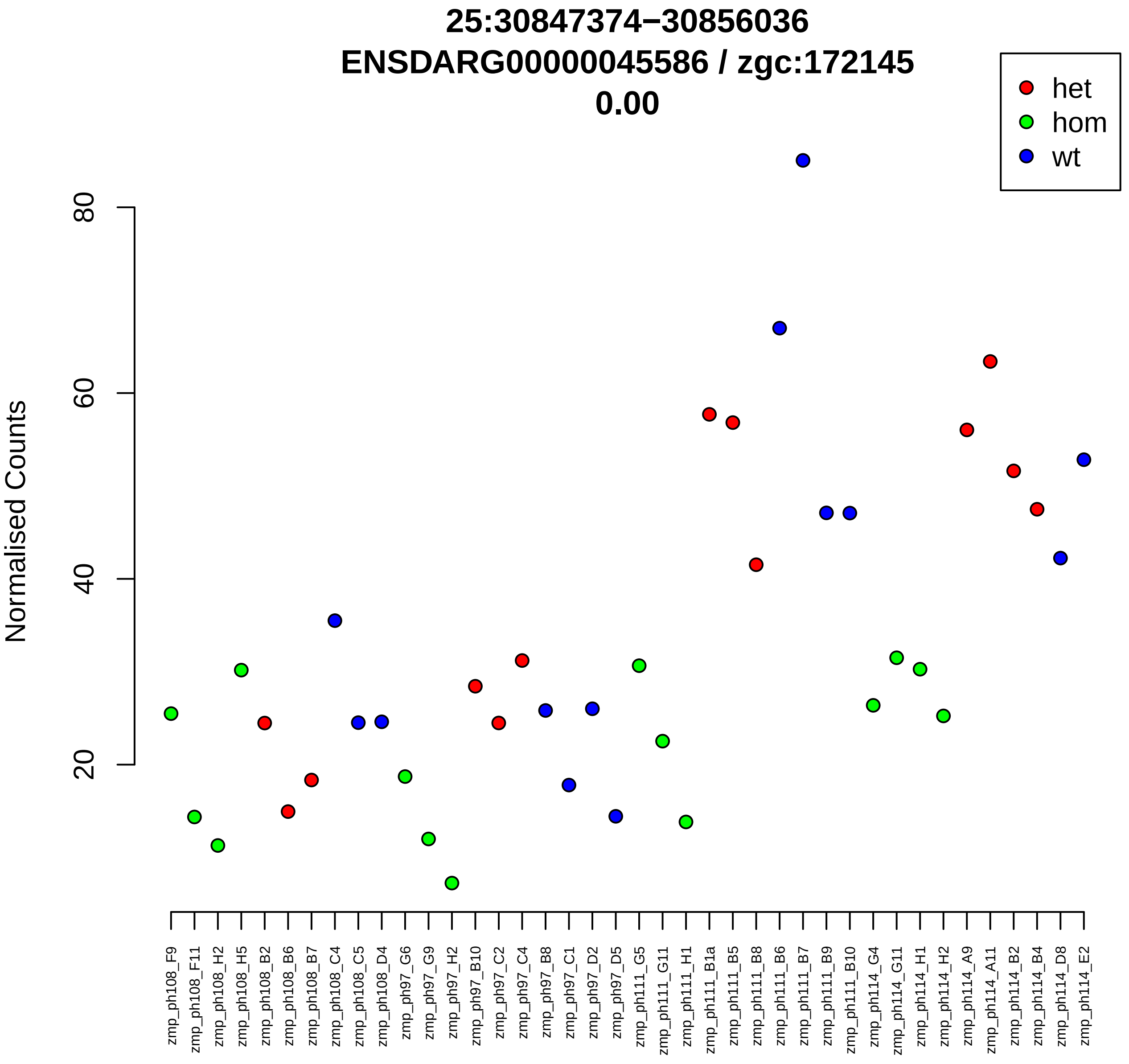
25:30847374−30856036
ENSDARG00000045586 / zgc:172145
0.00
het
hom
wt
Normalised Counts
20
40
60
80
zmp_ph108_F9
zmp_ph108_F11
zmp_ph108_H2
zmp_ph108_H5
zmp_ph108_B2
zmp_ph108_B6
zmp_ph108_B7
zmp_ph108_C4
zmp_ph108_C5
zmp_ph108_D4
zmp_ph97_G6
zmp_ph97_G9
zmp_ph97_H2
zmp_ph97_B10
zmp_ph97_C2
zmp_ph97_C4
zmp_ph97_B8
zmp_ph97_C1
zmp_ph97_D2
zmp_ph97_D5
zmp_ph111_G5
zmp_ph111_G11
zmp_ph111_H1
zmp_ph111_B1a
zmp_ph111_B5
zmp_ph111_B8
zmp_ph111_B6
zmp_ph111_B7
zmp_ph111_B9
zmp_ph111_B10
zmp_ph114_G4
zmp_ph114_G11
zmp_ph114_H1
zmp_ph114_H2
zmp_ph114_A9
zmp_ph114_A11
zmp_ph114_B2
zmp_ph114_B4
zmp_ph114_D8
zmp_ph114_E2

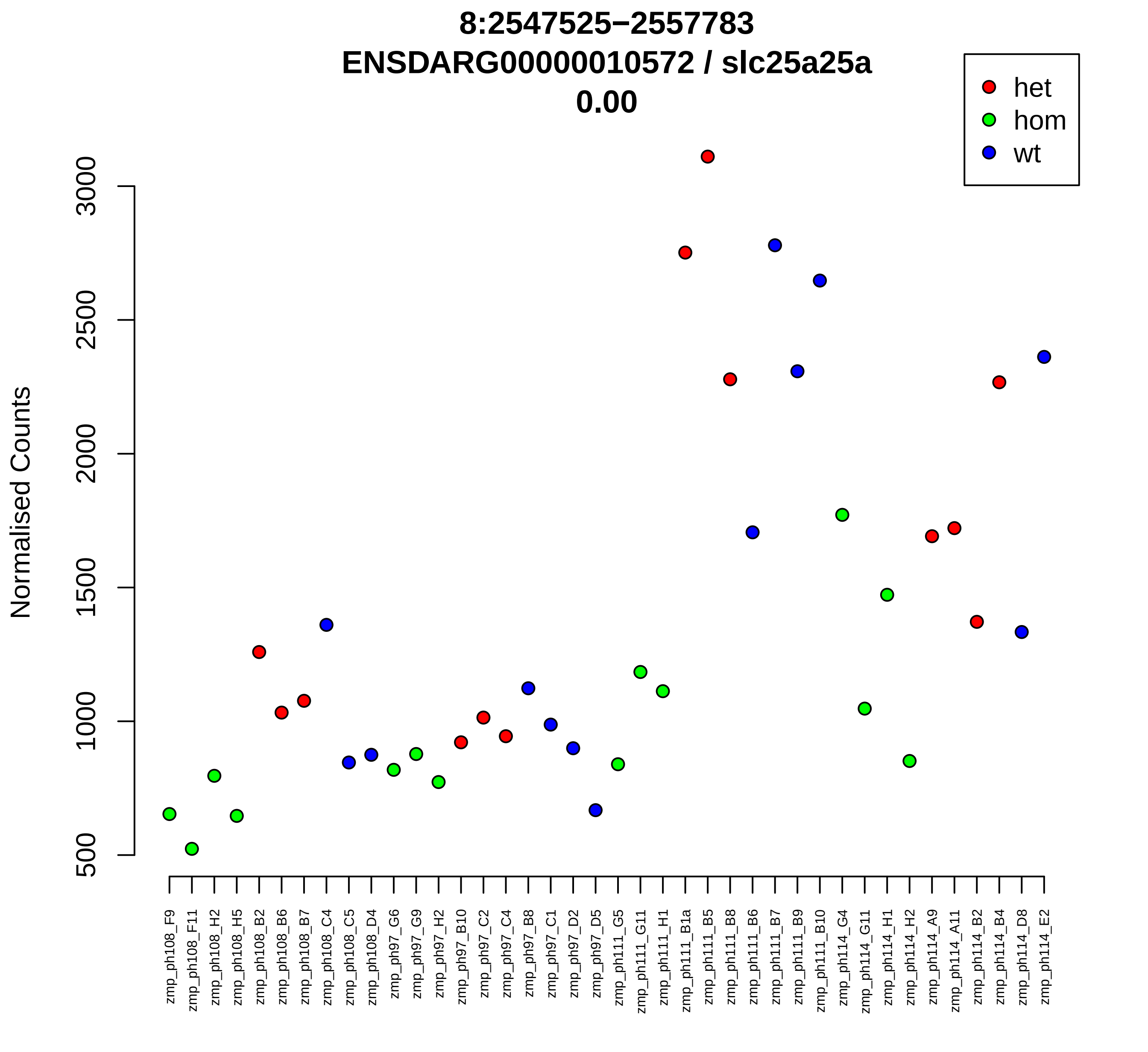

8:2547525−2557783
ENSDARG00000010572 / slc25a25a
0.00
het
hom
wt
Normalised Counts
500
1000
1500
2000
2500
3000
zmp_ph108_F9
zmp_ph108_F11
zmp_ph108_H2
zmp_ph108_H5
zmp_ph108_B2
zmp_ph108_B6
zmp_ph108_B7
zmp_ph108_C4
zmp_ph108_C5
zmp_ph108_D4
zmp_ph97_G6
zmp_ph97_G9
zmp_ph97_H2
zmp_ph97_B10
zmp_ph97_C2
zmp_ph97_C4
zmp_ph97_B8
zmp_ph97_C1
zmp_ph97_D2
zmp_ph97_D5
zmp_ph111_G5
zmp_ph111_G11
zmp_ph111_H1
zmp_ph111_B1a
zmp_ph111_B5
zmp_ph111_B8
zmp_ph111_B6
zmp_ph111_B7
zmp_ph111_B9
zmp_ph111_B10
zmp_ph114_G4
zmp_ph114_G11
zmp_ph114_H1
zmp_ph114_H2
zmp_ph114_A9
zmp_ph114_A11
zmp_ph114_B2
zmp_ph114_B4
zmp_ph114_D8
zmp_ph114_E2

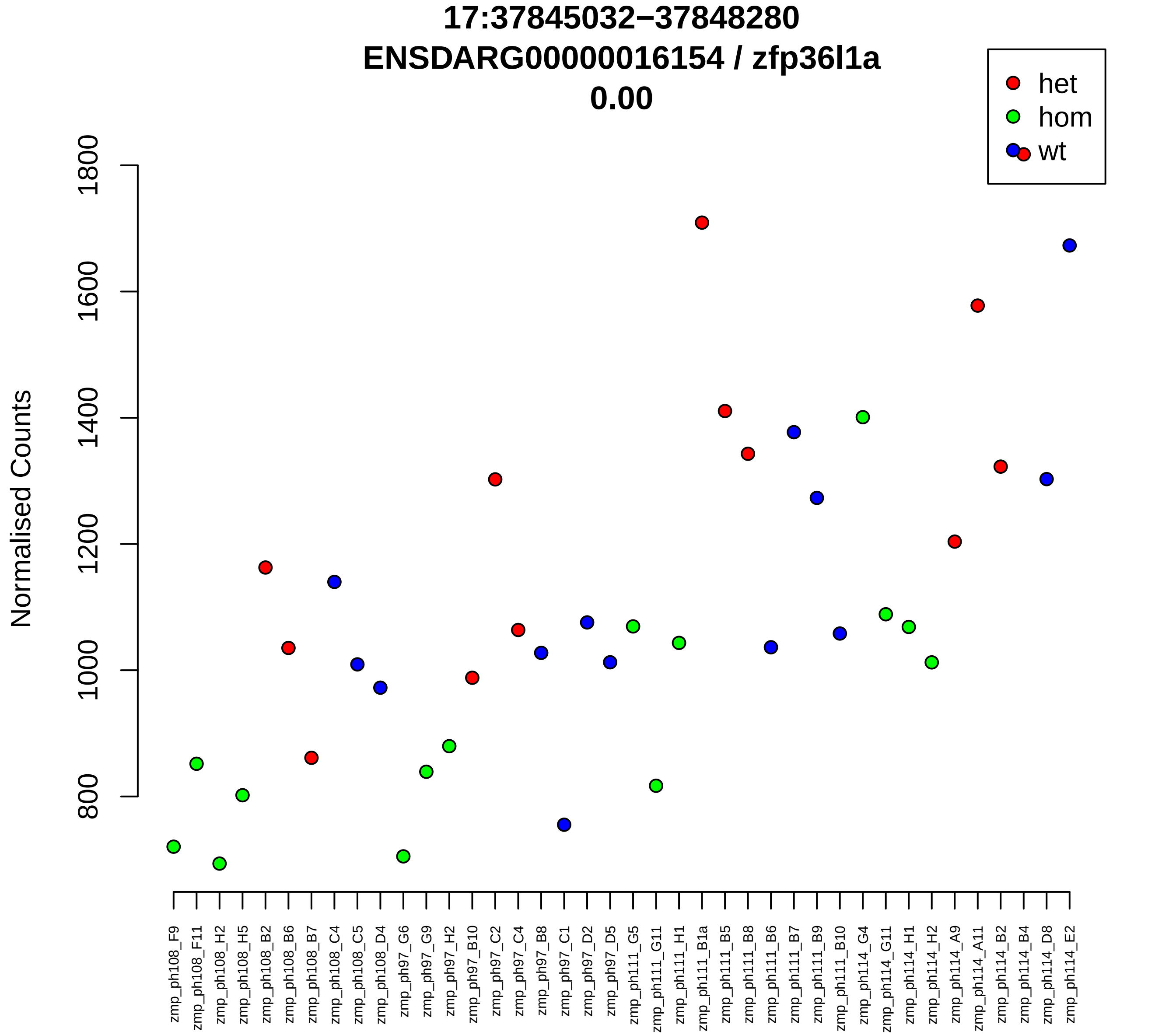
17:37845032−37848280
ENSDARG00000016154 / zfp36l1a
0.00
het
hom
wt
Normalised Counts
800
1000
1200
1400
1600
1800
zmp_ph108_F9
zmp_ph108_F11
zmp_ph108_H2
zmp_ph108_H5
zmp_ph108_B2
zmp_ph108_B6
zmp_ph108_B7
zmp_ph108_C4
zmp_ph108_C5
zmp_ph108_D4
zmp_ph97_G6
zmp_ph97_G9
zmp_ph97_H2
zmp_ph97_B10
zmp_ph97_C2
zmp_ph97_C4
zmp_ph97_B8
zmp_ph97_C1
zmp_ph97_D2
zmp_ph97_D5
zmp_ph111_G5
zmp_ph111_G11
zmp_ph111_H1
zmp_ph111_B1a
zmp_ph111_B5
zmp_ph111_B8
zmp_ph111_B6
zmp_ph111_B7
zmp_ph111_B9
zmp_ph111_B10
zmp_ph114_G4
zmp_ph114_G11
zmp_ph114_H1
zmp_ph114_H2
zmp_ph114_A9
zmp_ph114_A11
zmp_ph114_B2
zmp_ph114_B4
zmp_ph114_D8
zmp_ph114_E2

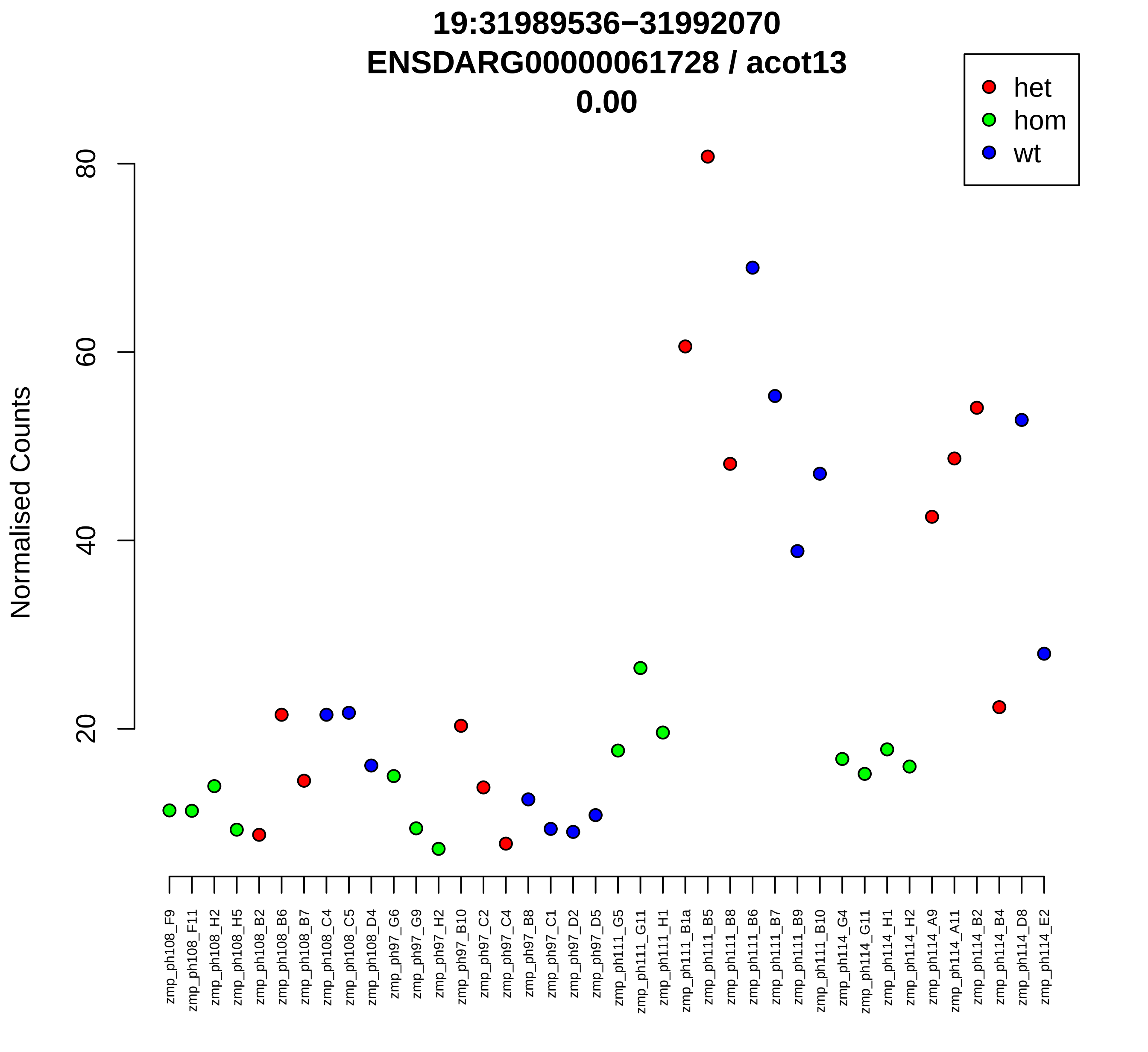
19:31989536−31992070
ENSDARG00000061728 / acot13
0.00
Normalised Counts
80
60
40
20
het
hom
wt
zmp_ph108_F9
zmp_ph108_F11
zmp_ph108_H2
zmp_ph108_H5
zmp_ph108_B2
zmp_ph108_B6
zmp_ph108_B7
zmp_ph108_C4
zmp_ph108_C5
zmp_ph108_D4
zmp_ph97_G6
zmp_ph97_G9
zmp_ph97_H2
zmp_ph97_B10
zmp_ph97_C2
zmp_ph97_C4
zmp_ph97_B8
zmp_ph97_C1
zmp_ph97_D2
zmp_ph97_D5
zmp_ph111_G5
zmp_ph111_G11
zmp_ph111_H1
zmp_ph111_B1a
zmp_ph111_B5
zmp_ph111_B8
zmp_ph111_B6
zmp_ph111_B7
zmp_ph111_B9
zmp_ph111_B10
zmp_ph114_G4
zmp_ph114_G11
zmp_ph114_H1
zmp_ph114_H2
zmp_ph114_A9
zmp_ph114_A11
zmp_ph114_B2
zmp_ph114_B4
zmp_ph114_D8
zmp_ph114_E2

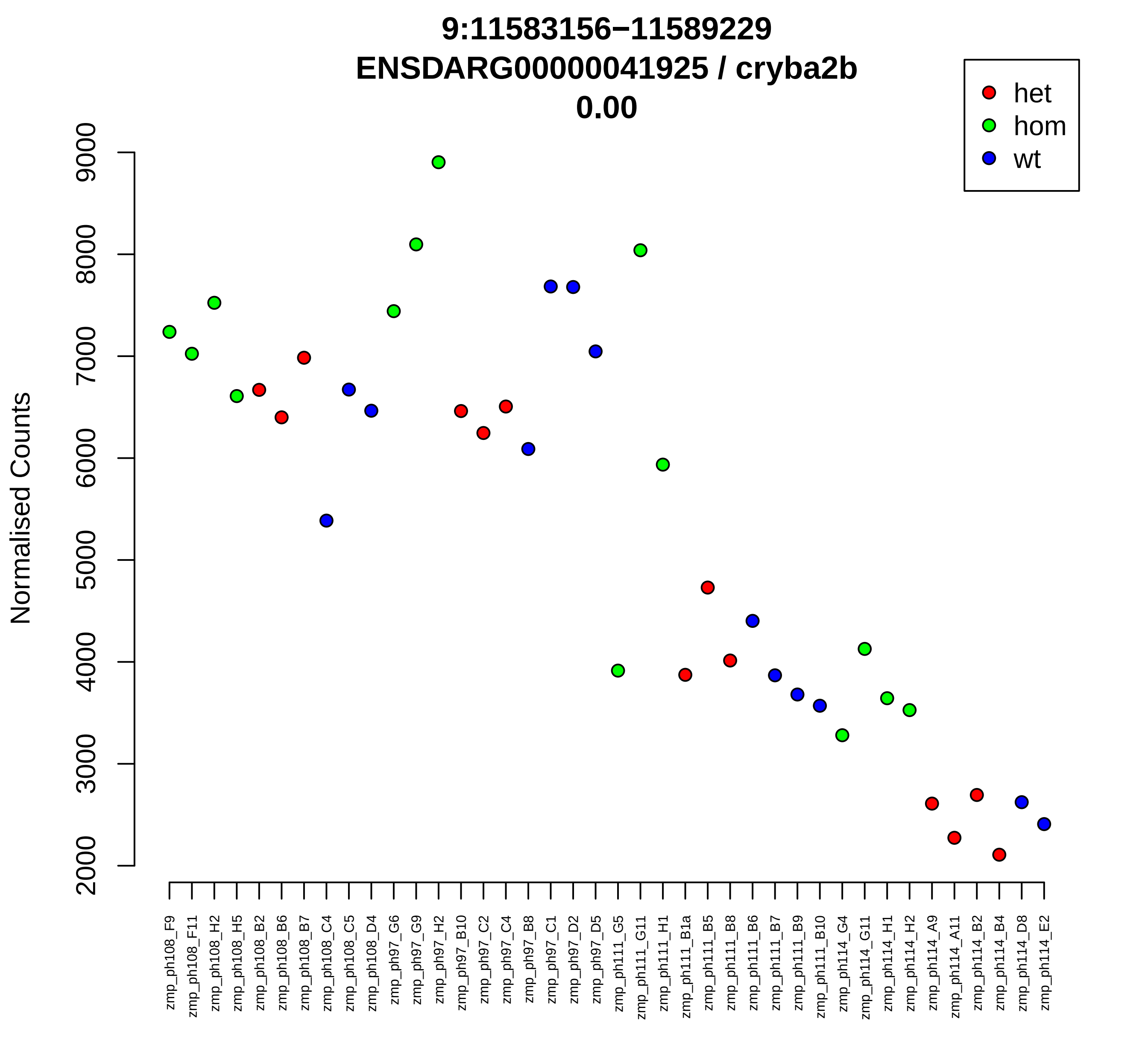

9:11583156−11589229
ENSDARG00000041925 / cryba2b
0.00
het
hom
wt
Normalised Counts
2000
3000
4000
5000
6000
7000
8000
9000
zmp_ph108_F9
zmp_ph108_F11
zmp_ph108_H2
zmp_ph108_H5
zmp_ph108_B2
zmp_ph108_B6
zmp_ph108_B7
zmp_ph108_C4
zmp_ph108_C5
zmp_ph108_D4
zmp_ph97_G6
zmp_ph97_G9
zmp_ph97_H2
zmp_ph97_B10
zmp_ph97_C2
zmp_ph97_C4
zmp_ph97_B8
zmp_ph97_C1
zmp_ph97_D2
zmp_ph97_D5
zmp_ph111_G5
zmp_ph111_G11
zmp_ph111_H1
zmp_ph111_B1a
zmp_ph111_B5
zmp_ph111_B8
zmp_ph111_B6
zmp_ph111_B7
zmp_ph111_B9
zmp_ph111_B10
zmp_ph114_G4
zmp_ph114_G11
zmp_ph114_H1
zmp_ph114_H2
zmp_ph114_A9
zmp_ph114_A11
zmp_ph114_B2
zmp_ph114_B4
zmp_ph114_D8
zmp_ph114_E2

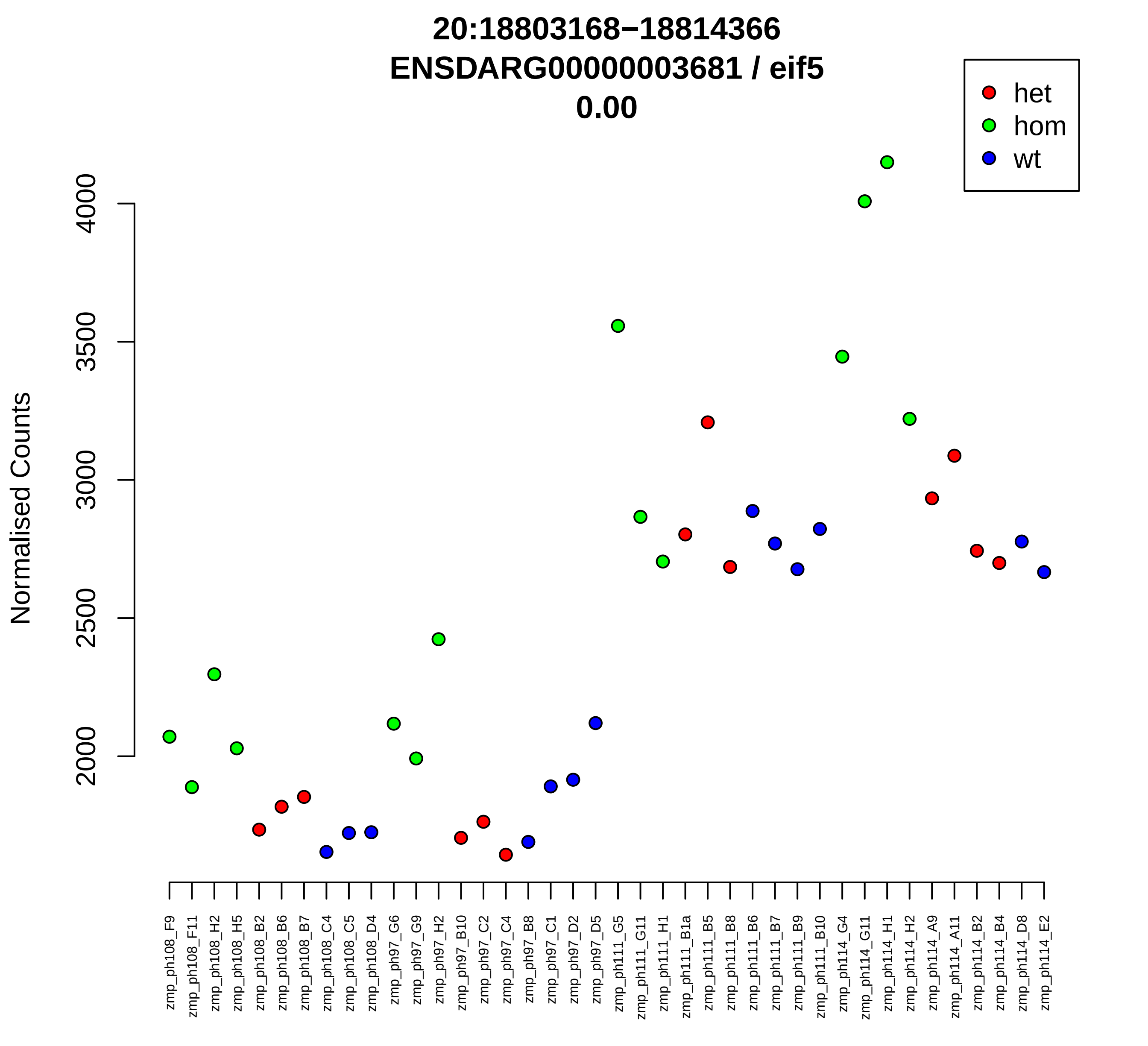

20:18803168−18814366
ENSDARG00000003681 / eif5
0.00
het
hom
wt
Normalised Counts
2000
2500
3000
3500
4000
zmp_ph108_F9
zmp_ph108_F11
zmp_ph108_H2
zmp_ph108_H5
zmp_ph108_B2
zmp_ph108_B6
zmp_ph108_B7
zmp_ph108_C4
zmp_ph108_C5
zmp_ph108_D4
zmp_ph97_G6
zmp_ph97_G9
zmp_ph97_H2
zmp_ph97_B10
zmp_ph97_C2
zmp_ph97_C4
zmp_ph97_B8
zmp_ph97_C1
zmp_ph97_D2
zmp_ph97_D5
zmp_ph111_G5
zmp_ph111_G11
zmp_ph111_H1
zmp_ph111_B1a
zmp_ph111_B5
zmp_ph111_B8
zmp_ph111_B6
zmp_ph111_B7
zmp_ph111_B9
zmp_ph111_B10
zmp_ph114_G4
zmp_ph114_G11
zmp_ph114_H1
zmp_ph114_H2
zmp_ph114_A9
zmp_ph114_A11
zmp_ph114_B2
zmp_ph114_B4
zmp_ph114_D8
zmp_ph114_E2

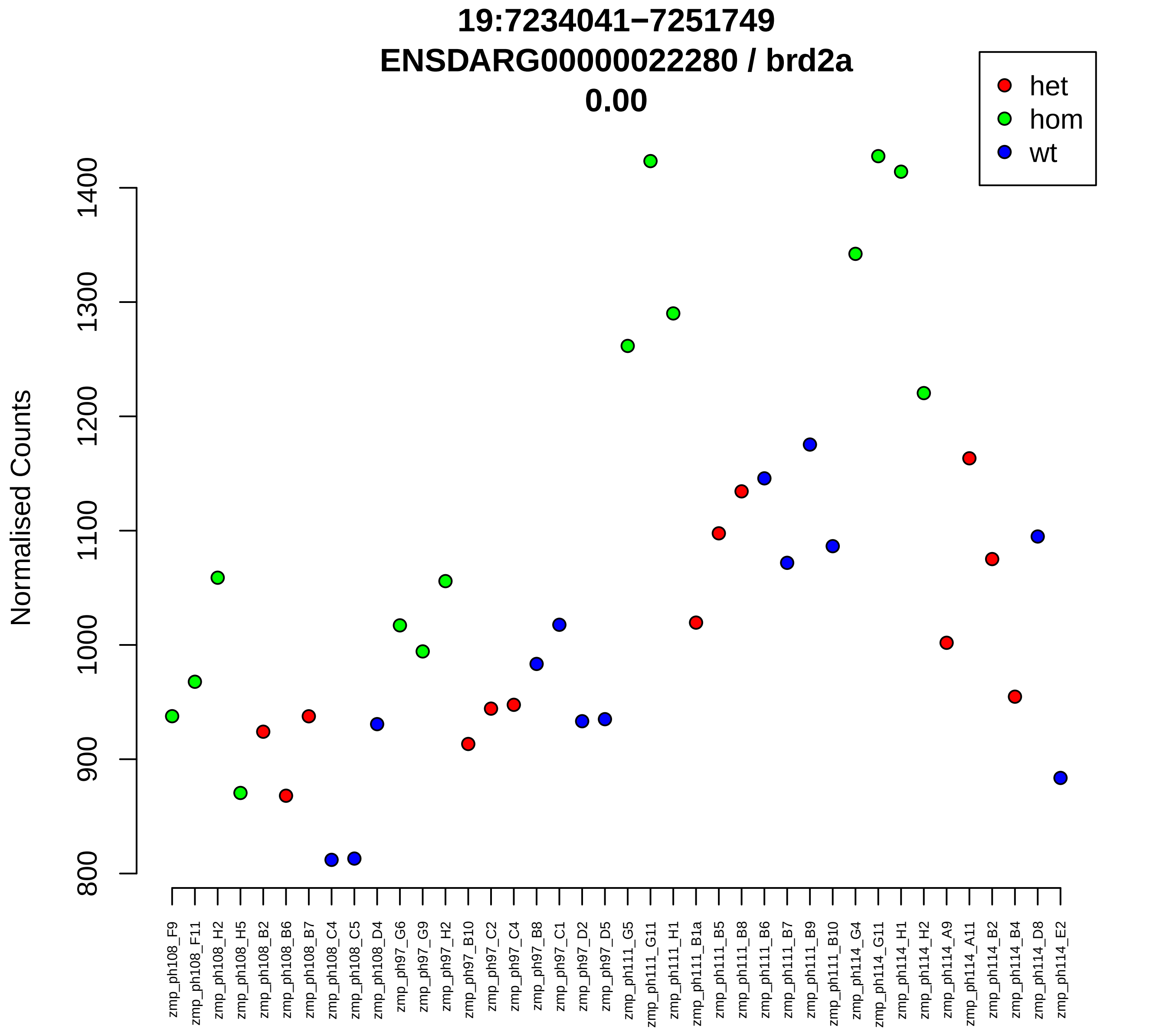
19:7234041−7251749
ENSDARG00000022280 / brd2a
0.00
het
hom
wt
Normalised Counts
800
900
1000
1100
1200
1300
1400
zmp_ph108_F9
zmp_ph108_F11
zmp_ph108_H2
zmp_ph108_H5
zmp_ph108_B2
zmp_ph108_B6
zmp_ph108_B7
zmp_ph108_C4
zmp_ph108_C5
zmp_ph108_D4
zmp_ph97_G6
zmp_ph97_G9
zmp_ph97_H2
zmp_ph97_B10
zmp_ph97_C2
zmp_ph97_C4
zmp_ph97_B8
zmp_ph97_C1
zmp_ph97_D2
zmp_ph97_D5
zmp_ph111_G5
zmp_ph111_G11
zmp_ph111_H1
zmp_ph111_B1a
zmp_ph111_B5
zmp_ph111_B8
zmp_ph111_B6
zmp_ph111_B7
zmp_ph111_B9
zmp_ph111_B10
zmp_ph114_G4
zmp_ph114_G11
zmp_ph114_H1
zmp_ph114_H2
zmp_ph114_A9
zmp_ph114_A11
zmp_ph114_B2
zmp_ph114_B4
zmp_ph114_D8
zmp_ph114_E2

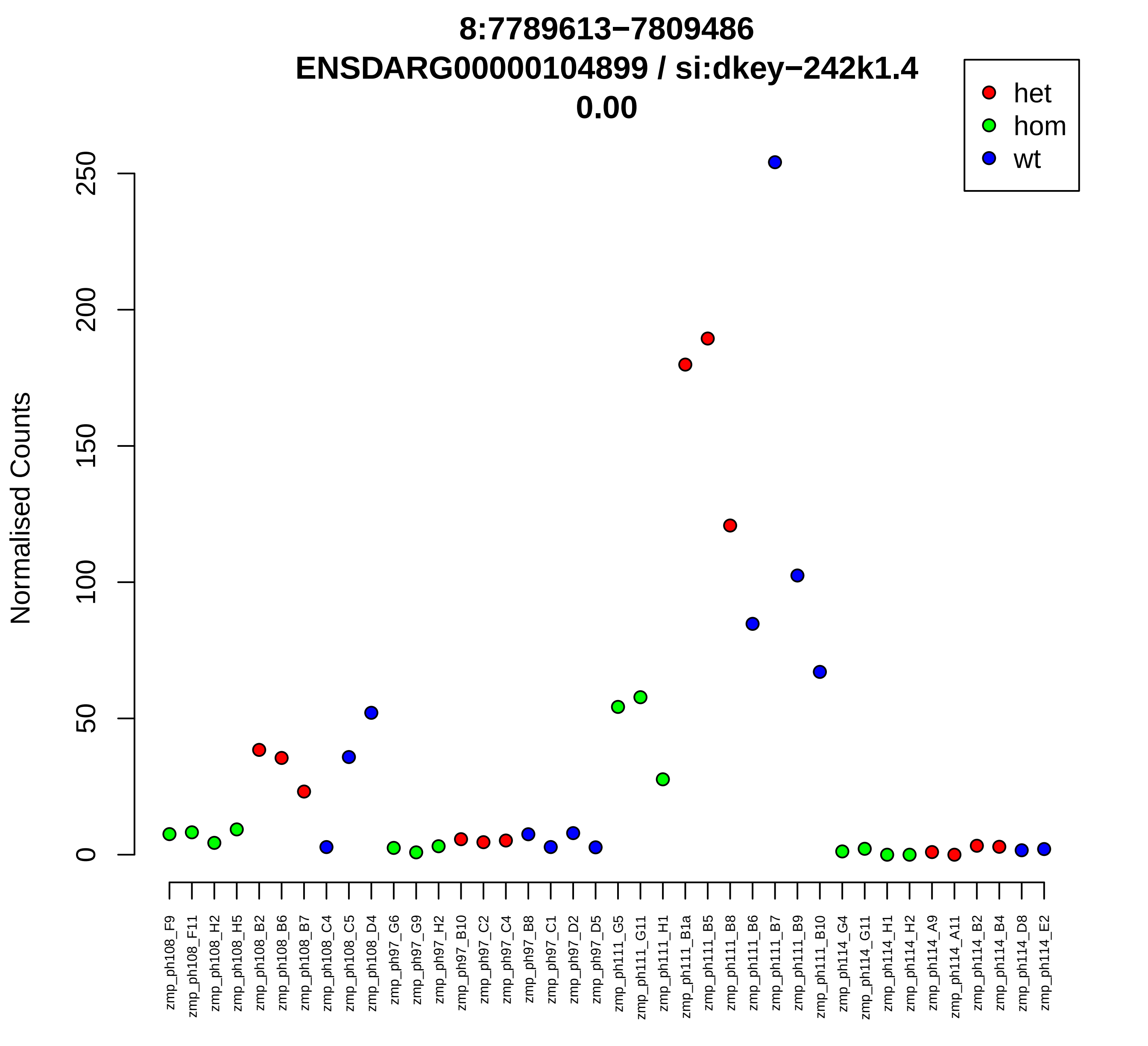

8:7789613−7809486
ENSDARG00000104899 / si:dkey−242k1.4
0.00
het
hom
wt
Normalised Counts
0
50
100
150
200
250
zmp_ph108_F9
zmp_ph108_F11
zmp_ph108_H2
zmp_ph108_H5
zmp_ph108_B2
zmp_ph108_B6
zmp_ph108_B7
zmp_ph108_C4
zmp_ph108_C5
zmp_ph108_D4
zmp_ph97_G6
zmp_ph97_G9
zmp_ph97_H2
zmp_ph97_B10
zmp_ph97_C2
zmp_ph97_C4
zmp_ph97_B8
zmp_ph97_C1
zmp_ph97_D2
zmp_ph97_D5
zmp_ph111_G5
zmp_ph111_G11
zmp_ph111_H1
zmp_ph111_B1a
zmp_ph111_B5
zmp_ph111_B8
zmp_ph111_B6
zmp_ph111_B7
zmp_ph111_B9
zmp_ph111_B10
zmp_ph114_G4
zmp_ph114_G11
zmp_ph114_H1
zmp_ph114_H2
zmp_ph114_A9
zmp_ph114_A11
zmp_ph114_B2
zmp_ph114_B4
zmp_ph114_D8
zmp_ph114_E2

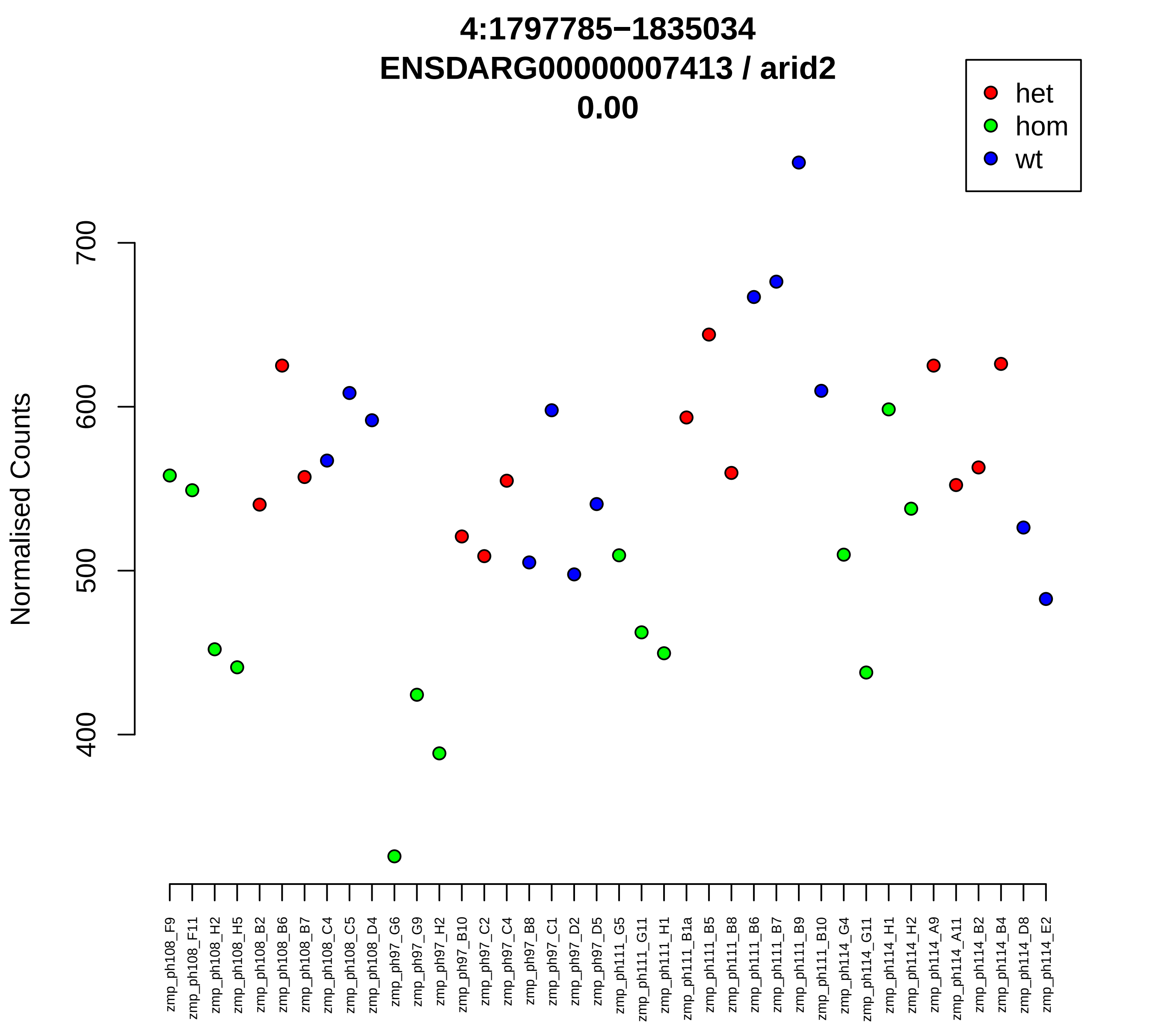
4:1797785−1835034
ENSDARG00000007413 / arid2
0.00
het
hom
wt
Normalised Counts
700
600
500
400
zmp_ph108_F9
zmp_ph108_F11
zmp_ph108_H2
zmp_ph108_H5
zmp_ph108_B2
zmp_ph108_B6
zmp_ph108_B7
zmp_ph108_C4
zmp_ph108_C5
zmp_ph108_D4
zmp_ph97_G6
zmp_ph97_G9
zmp_ph97_H2
zmp_ph97_B10
zmp_ph97_C2
zmp_ph97_C4
zmp_ph97_B8
zmp_ph97_C1
zmp_ph97_D2
zmp_ph97_D5
zmp_ph111_G5
zmp_ph111_G11
zmp_ph111_H1
zmp_ph111_B1a
zmp_ph111_B5
zmp_ph111_B8
zmp_ph111_B6
zmp_ph111_B7
zmp_ph111_B9
zmp_ph111_B10
zmp_ph114_G4
zmp_ph114_G11
zmp_ph114_H1
zmp_ph114_H2
zmp_ph114_A9
zmp_ph114_A11
zmp_ph114_B2
zmp_ph114_B4
zmp_ph114_D8
zmp_ph114_E2

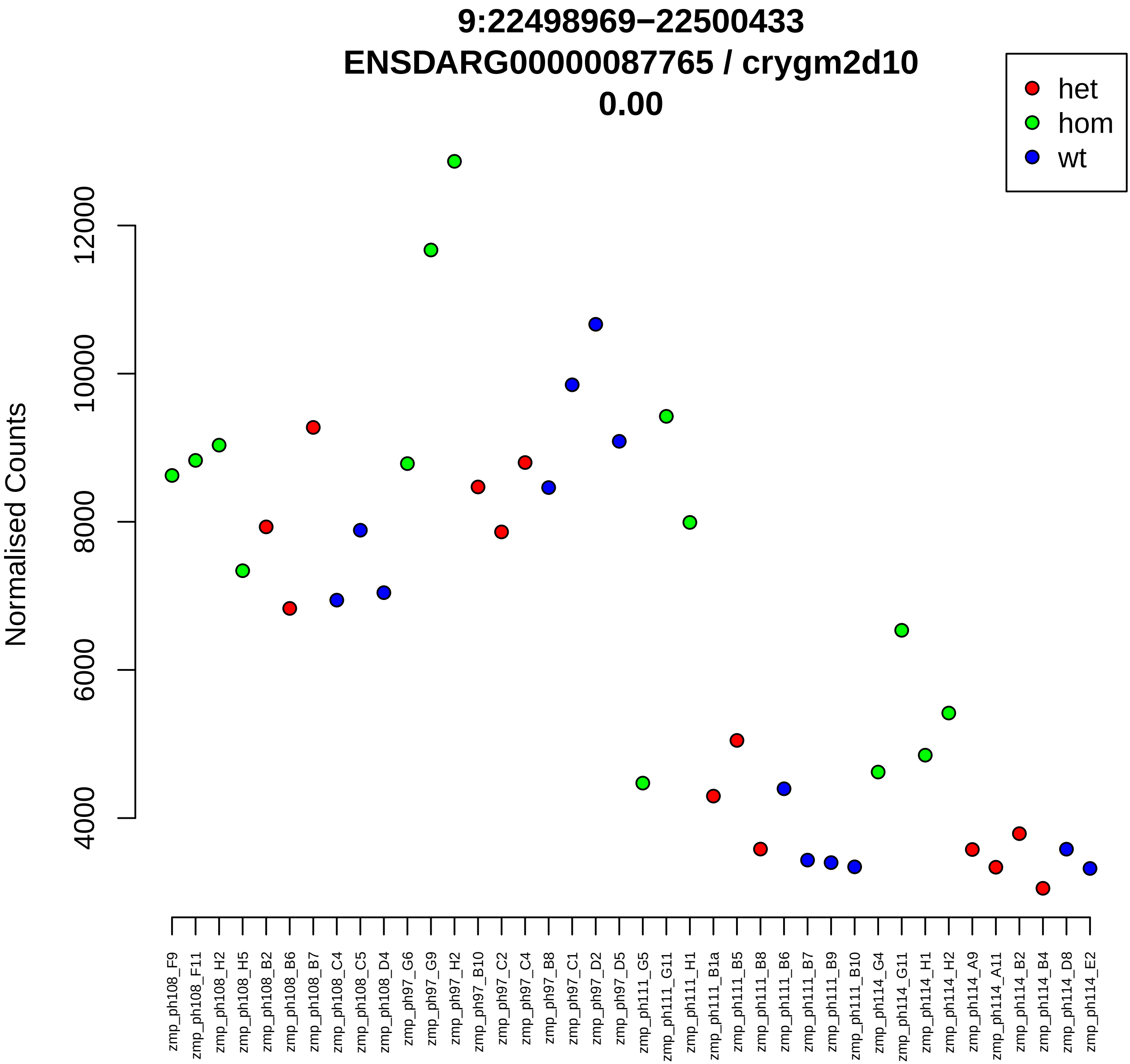

9:22498969−22500433
ENSDARG00000087765 / crygm2d10
0.00
het
hom
wt
Normalised Counts
4000
6000
8000
10000
12000
zmp_ph108_F9
zmp_ph108_F11
zmp_ph108_H2
zmp_ph108_H5
zmp_ph108_B2
zmp_ph108_B6
zmp_ph108_B7
zmp_ph108_C4
zmp_ph108_C5
zmp_ph108_D4
zmp_ph97_G6
zmp_ph97_G9
zmp_ph97_H2
zmp_ph97_B10
zmp_ph97_C2
zmp_ph97_C4
zmp_ph97_B8
zmp_ph97_C1
zmp_ph97_D2
zmp_ph97_D5
zmp_ph111_G5
zmp_ph111_G11
zmp_ph111_H1
zmp_ph111_B1a
zmp_ph111_B5
zmp_ph111_B8
zmp_ph111_B6
zmp_ph111_B7
zmp_ph111_B9
zmp_ph111_B10
zmp_ph114_G4
zmp_ph114_G11
zmp_ph114_H1
zmp_ph114_H2
zmp_ph114_A9
zmp_ph114_A11
zmp_ph114_B2
zmp_ph114_B4
zmp_ph114_D8
zmp_ph114_E2

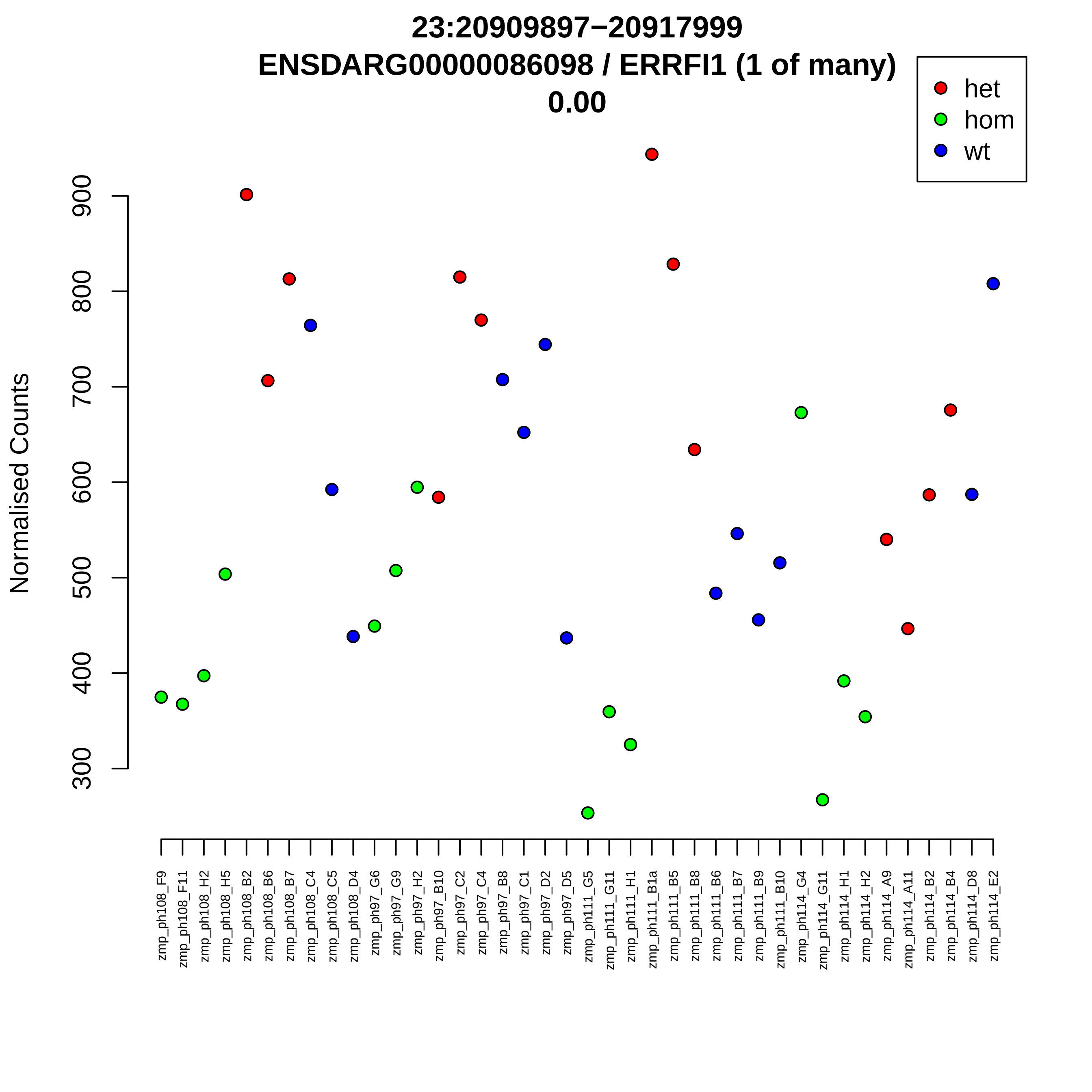

23:20909897−20917999
ENSDARG00000086098 / ERRFI1 (1 of many)
0.00
het
hom
wt
Normalised Counts
300
400
500
600
700
800
900
zmp_ph108_F9
zmp_ph108_F11
zmp_ph108_H2
zmp_ph108_H5
zmp_ph108_B2
zmp_ph108_B6
zmp_ph108_B7
zmp_ph108_C4
zmp_ph108_C5
zmp_ph108_D4
zmp_ph97_G6
zmp_ph97_G9
zmp_ph97_H2
zmp_ph97_B10
zmp_ph97_C2
zmp_ph97_C4
zmp_ph97_B8
zmp_ph97_C1
zmp_ph97_D2
zmp_ph97_D5
zmp_ph111_G5
zmp_ph111_G11
zmp_ph111_H1
zmp_ph111_B1a
zmp_ph111_B5
zmp_ph111_B8
zmp_ph111_B6
zmp_ph111_B7
zmp_ph111_B9
zmp_ph111_B10
zmp_ph114_G4
zmp_ph114_G11
zmp_ph114_H1
zmp_ph114_H2
zmp_ph114_A9
zmp_ph114_A11
zmp_ph114_B2
zmp_ph114_B4
zmp_ph114_D8
zmp_ph114_E2

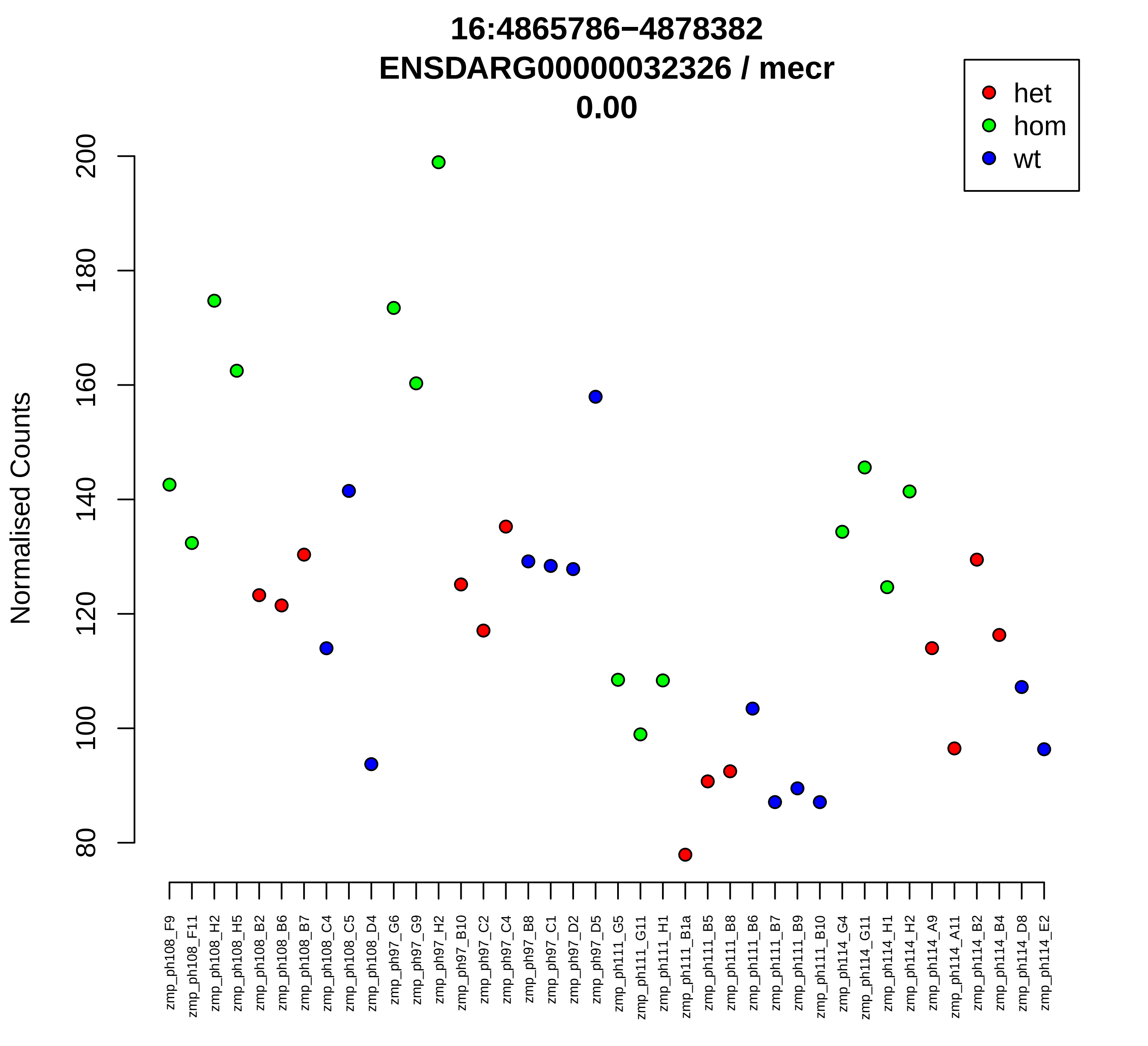

16:4865786−4878382
ENSDARG00000032326 / mecr
0.00
het
hom
wt
Normalised Counts
80
100
120
140
160
180
200
zmp_ph108_F9
zmp_ph108_F11
zmp_ph108_H2
zmp_ph108_H5
zmp_ph108_B2
zmp_ph108_B6
zmp_ph108_B7
zmp_ph108_C4
zmp_ph108_C5
zmp_ph108_D4
zmp_ph97_G6
zmp_ph97_G9
zmp_ph97_H2
zmp_ph97_B10
zmp_ph97_C2
zmp_ph97_C4
zmp_ph97_B8
zmp_ph97_C1
zmp_ph97_D2
zmp_ph97_D5
zmp_ph111_G5
zmp_ph111_G11
zmp_ph111_H1
zmp_ph111_B1a
zmp_ph111_B5
zmp_ph111_B8
zmp_ph111_B6
zmp_ph111_B7
zmp_ph111_B9
zmp_ph111_B10
zmp_ph114_G4
zmp_ph114_G11
zmp_ph114_H1
zmp_ph114_H2
zmp_ph114_A9
zmp_ph114_A11
zmp_ph114_B2
zmp_ph114_B4
zmp_ph114_D8
zmp_ph114_E2

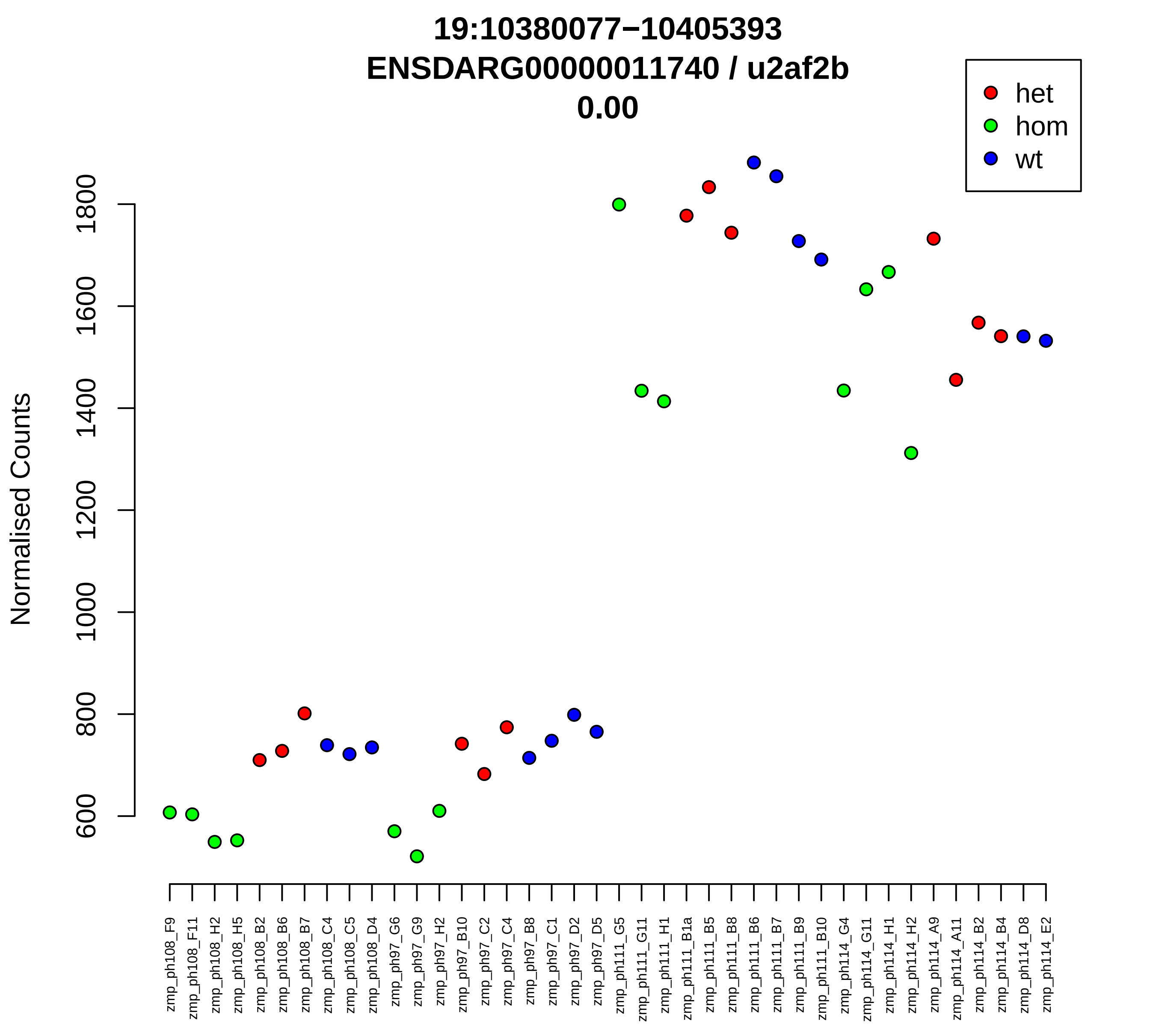

19:10380077−10405393
ENSDARG00000011740 / u2af2b
0.00
het
hom
wt
Normalised Counts
600
800
1000
1200
1400
1600
1800
zmp_ph108_F9
zmp_ph108_F11
zmp_ph108_H2
zmp_ph108_H5
zmp_ph108_B2
zmp_ph108_B6
zmp_ph108_B7
zmp_ph108_C4
zmp_ph108_C5
zmp_ph108_D4
zmp_ph97_G6
zmp_ph97_G9
zmp_ph97_H2
zmp_ph97_B10
zmp_ph97_C2
zmp_ph97_C4
zmp_ph97_B8
zmp_ph97_C1
zmp_ph97_D2
zmp_ph97_D5
zmp_ph111_G5
zmp_ph111_G11
zmp_ph111_H1
zmp_ph111_B1a
zmp_ph111_B5
zmp_ph111_B8
zmp_ph111_B6
zmp_ph111_B7
zmp_ph111_B9
zmp_ph111_B10
zmp_ph114_G4
zmp_ph114_G11
zmp_ph114_H1
zmp_ph114_H2
zmp_ph114_A9
zmp_ph114_A11
zmp_ph114_B2
zmp_ph114_B4
zmp_ph114_D8
zmp_ph114_E2

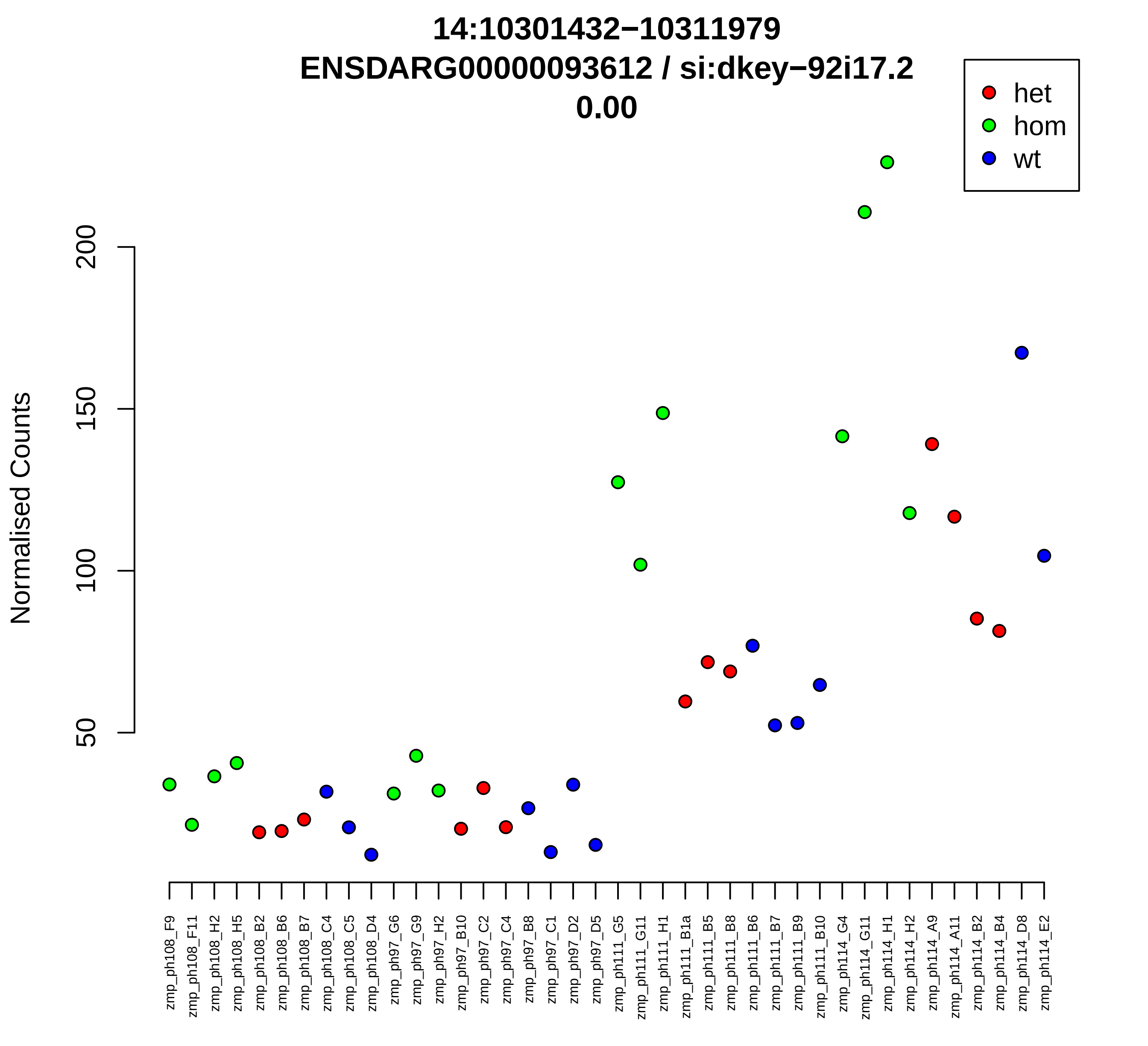
14:10301432−10311979
ENSDARG00000093612 / si:dkey−92i17.2
0.00
het
hom
wt
Normalised Counts
50
100
150
200
zmp_ph108_F9
zmp_ph108_F11
zmp_ph108_H2
zmp_ph108_H5
zmp_ph108_B2
zmp_ph108_B6
zmp_ph108_B7
zmp_ph108_C4
zmp_ph108_C5
zmp_ph108_D4
zmp_ph97_G6
zmp_ph97_G9
zmp_ph97_H2
zmp_ph97_B10
zmp_ph97_C2
zmp_ph97_C4
zmp_ph97_B8
zmp_ph97_C1
zmp_ph97_D2
zmp_ph97_D5
zmp_ph111_G5
zmp_ph111_G11
zmp_ph111_H1
zmp_ph111_B1a
zmp_ph111_B5
zmp_ph111_B8
zmp_ph111_B6
zmp_ph111_B7
zmp_ph111_B9
zmp_ph111_B10
zmp_ph114_G4
zmp_ph114_G11
zmp_ph114_H1
zmp_ph114_H2
zmp_ph114_A9
zmp_ph114_A11
zmp_ph114_B2
zmp_ph114_B4
zmp_ph114_D8
zmp_ph114_E2

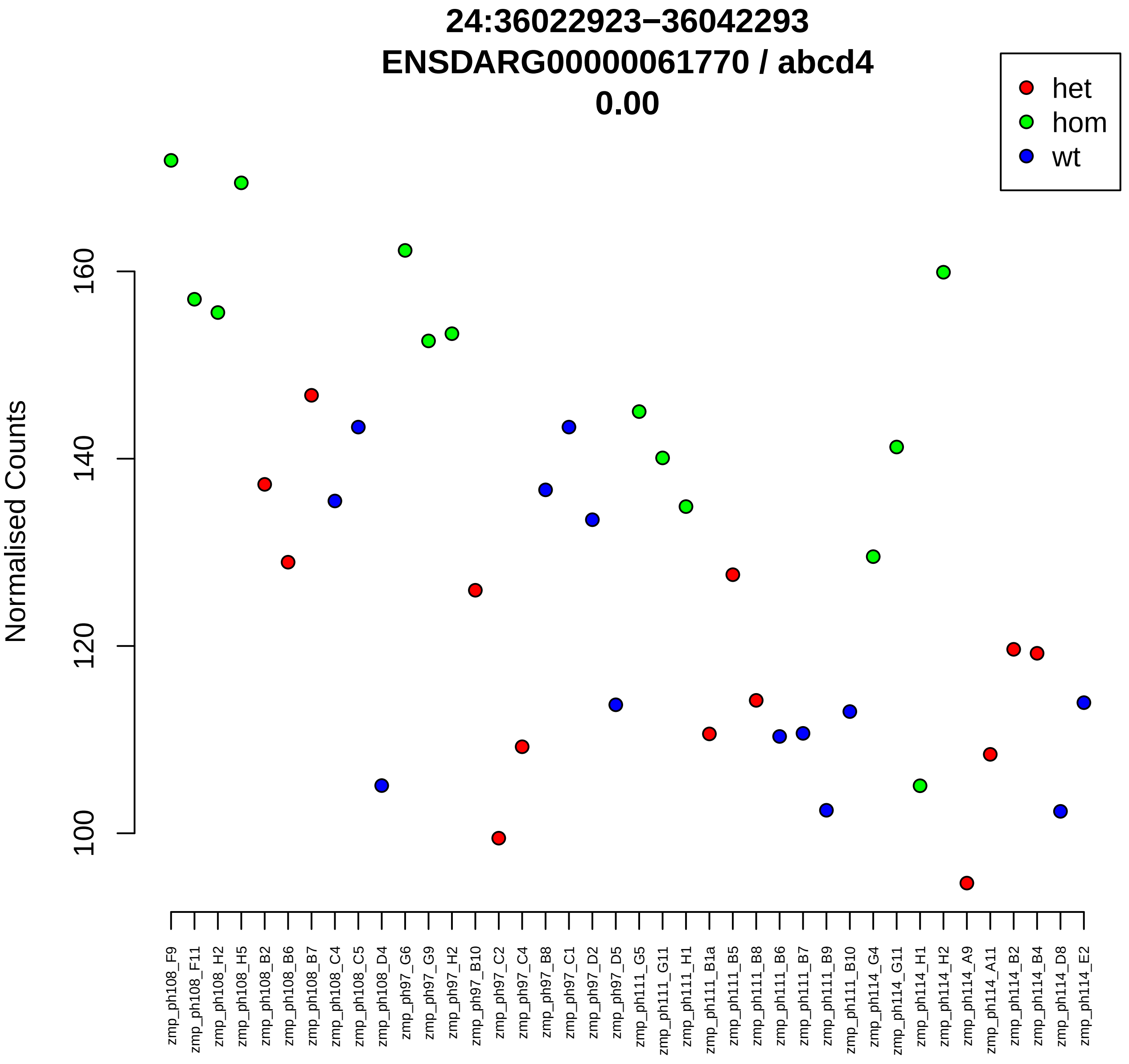
24:36022923−36042293
ENSDARG00000061770 / abcd4
0.00
het
hom
wt
Normalised Counts
100
120
140
160
zmp_ph108_F9
zmp_ph108_F11
zmp_ph108_H2
zmp_ph108_H5
zmp_ph108_B2
zmp_ph108_B6
zmp_ph108_B7
zmp_ph108_C4
zmp_ph108_C5
zmp_ph108_D4
zmp_ph97_G6
zmp_ph97_G9
zmp_ph97_H2
zmp_ph97_B10
zmp_ph97_C2
zmp_ph97_C4
zmp_ph97_B8
zmp_ph97_C1
zmp_ph97_D2
zmp_ph97_D5
zmp_ph111_G5
zmp_ph111_G11
zmp_ph111_H1
zmp_ph111_B1a
zmp_ph111_B5
zmp_ph111_B8
zmp_ph111_B6
zmp_ph111_B7
zmp_ph111_B9
zmp_ph111_B10
zmp_ph114_G4
zmp_ph114_G11
zmp_ph114_H1
zmp_ph114_H2
zmp_ph114_A9
zmp_ph114_A11
zmp_ph114_B2
zmp_ph114_B4
zmp_ph114_D8
zmp_ph114_E2

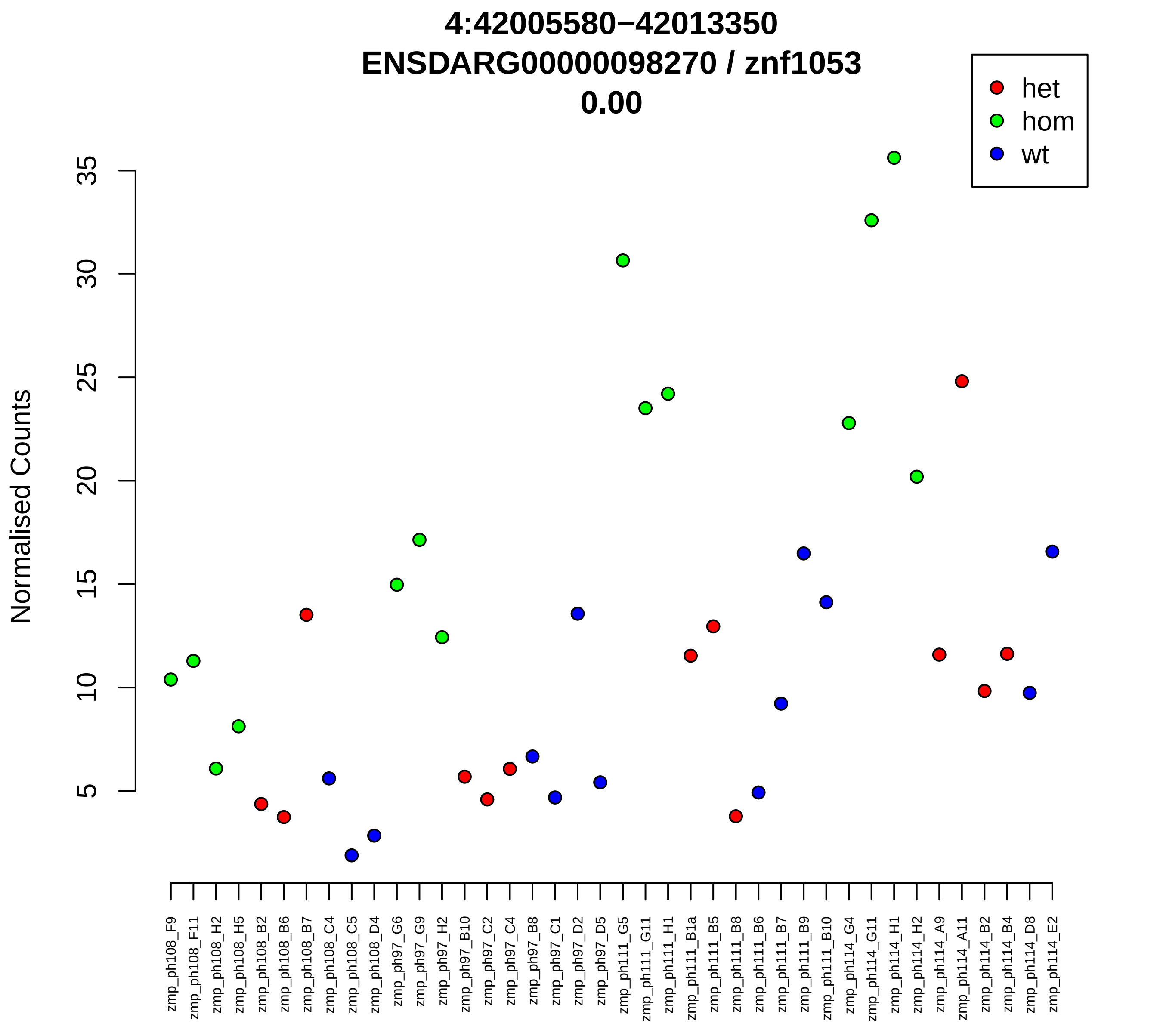

4:42005580−42013350
ENSDARG00000098270 / znf1053
0.00
het
hom
wt
Normalised Counts
5
10
15
20
25
30
35
zmp_ph108_F9
zmp_ph108_F11
zmp_ph108_H2
zmp_ph108_H5
zmp_ph108_B2
zmp_ph108_B6
zmp_ph108_B7
zmp_ph108_C4
zmp_ph108_C5
zmp_ph108_D4
zmp_ph97_G6
zmp_ph97_G9
zmp_ph97_H2
zmp_ph97_B10
zmp_ph97_C2
zmp_ph97_C4
zmp_ph97_B8
zmp_ph97_C1
zmp_ph97_D2
zmp_ph97_D5
zmp_ph111_G5
zmp_ph111_G11
zmp_ph111_H1
zmp_ph111_B1a
zmp_ph111_B5
zmp_ph111_B8
zmp_ph111_B6
zmp_ph111_B7
zmp_ph111_B9
zmp_ph111_B10
zmp_ph114_G4
zmp_ph114_G11
zmp_ph114_H1
zmp_ph114_H2
zmp_ph114_A9
zmp_ph114_A11
zmp_ph114_B2
zmp_ph114_B4
zmp_ph114_D8
zmp_ph114_E2

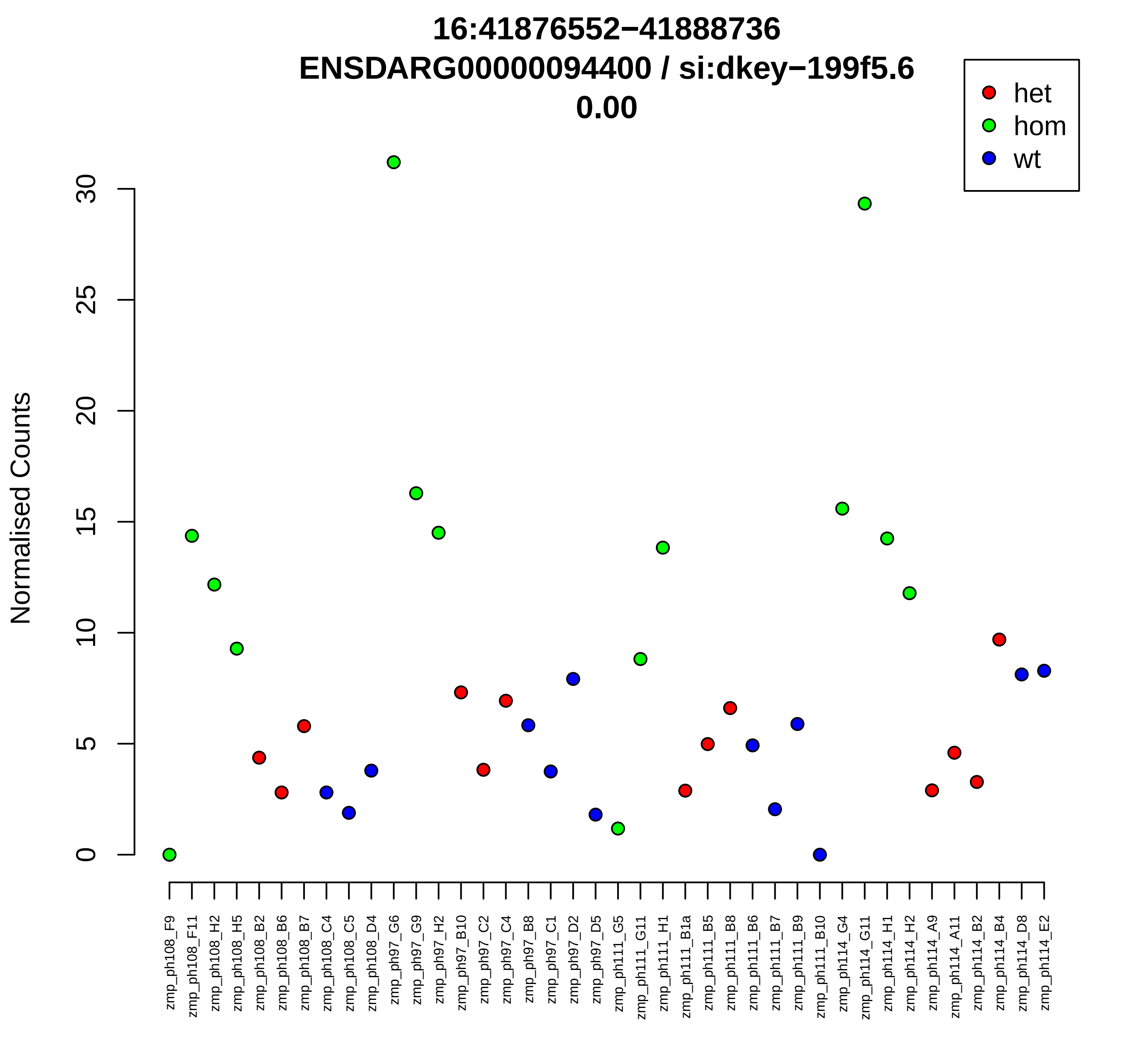

16:41876552−41888736
ENSDARG00000094400 / si:dkey−199f5.6
0.00
het
hom
wt
Normalised Counts
0
5
10
15
20
25
30
zmp_ph108_F9
zmp_ph108_F11
zmp_ph108_H2
zmp_ph108_H5
zmp_ph108_B2
zmp_ph108_B6
zmp_ph108_B7
zmp_ph108_C4
zmp_ph108_C5
zmp_ph108_D4
zmp_ph97_G6
zmp_ph97_G9
zmp_ph97_H2
zmp_ph97_B10
zmp_ph97_C2
zmp_ph97_C4
zmp_ph97_B8
zmp_ph97_C1
zmp_ph97_D2
zmp_ph97_D5
zmp_ph111_G5
zmp_ph111_G11
zmp_ph111_H1
zmp_ph111_B1a
zmp_ph111_B5
zmp_ph111_B8
zmp_ph111_B6
zmp_ph111_B7
zmp_ph111_B9
zmp_ph111_B10
zmp_ph114_G4
zmp_ph114_G11
zmp_ph114_H1
zmp_ph114_H2
zmp_ph114_A9
zmp_ph114_A11
zmp_ph114_B2
zmp_ph114_B4
zmp_ph114_D8
zmp_ph114_E2

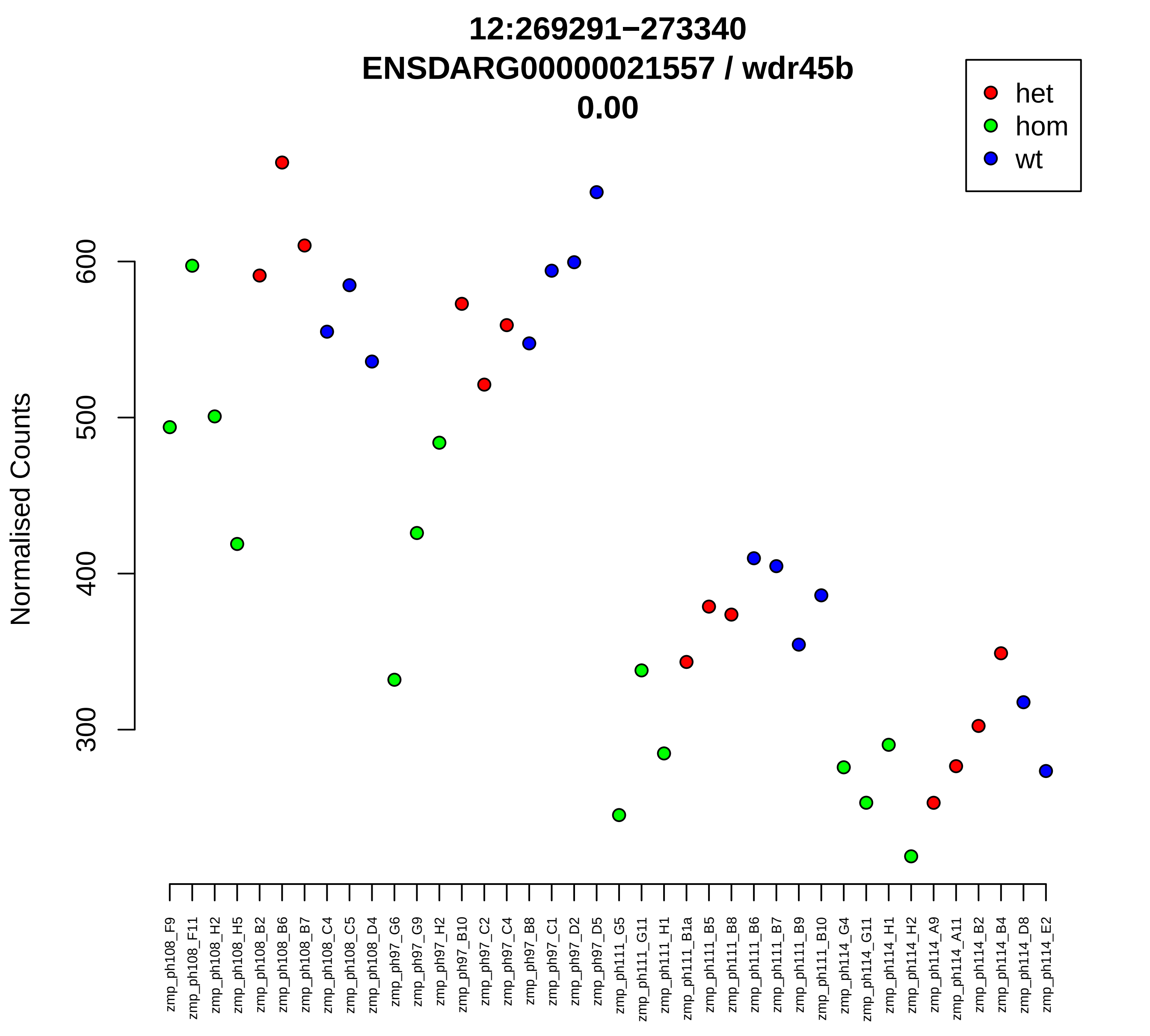

12:269291−273340
ENSDARG00000021557 / wdr45b
0.00
het
hom
wt
Normalised Counts
300
400
500
600
zmp_ph108_F9
zmp_ph108_F11
zmp_ph108_H2
zmp_ph108_H5
zmp_ph108_B2
zmp_ph108_B6
zmp_ph108_B7
zmp_ph108_C4
zmp_ph108_C5
zmp_ph108_D4
zmp_ph97_G6
zmp_ph97_G9
zmp_ph97_H2
zmp_ph97_B10
zmp_ph97_C2
zmp_ph97_C4
zmp_ph97_B8
zmp_ph97_C1
zmp_ph97_D2
zmp_ph97_D5
zmp_ph111_G5
zmp_ph111_G11
zmp_ph111_H1
zmp_ph111_B1a
zmp_ph111_B5
zmp_ph111_B8
zmp_ph111_B6
zmp_ph111_B7
zmp_ph111_B9
zmp_ph111_B10
zmp_ph114_G4
zmp_ph114_G11
zmp_ph114_H1
zmp_ph114_H2
zmp_ph114_A9
zmp_ph114_A11
zmp_ph114_B2
zmp_ph114_B4
zmp_ph114_D8
zmp_ph114_E2

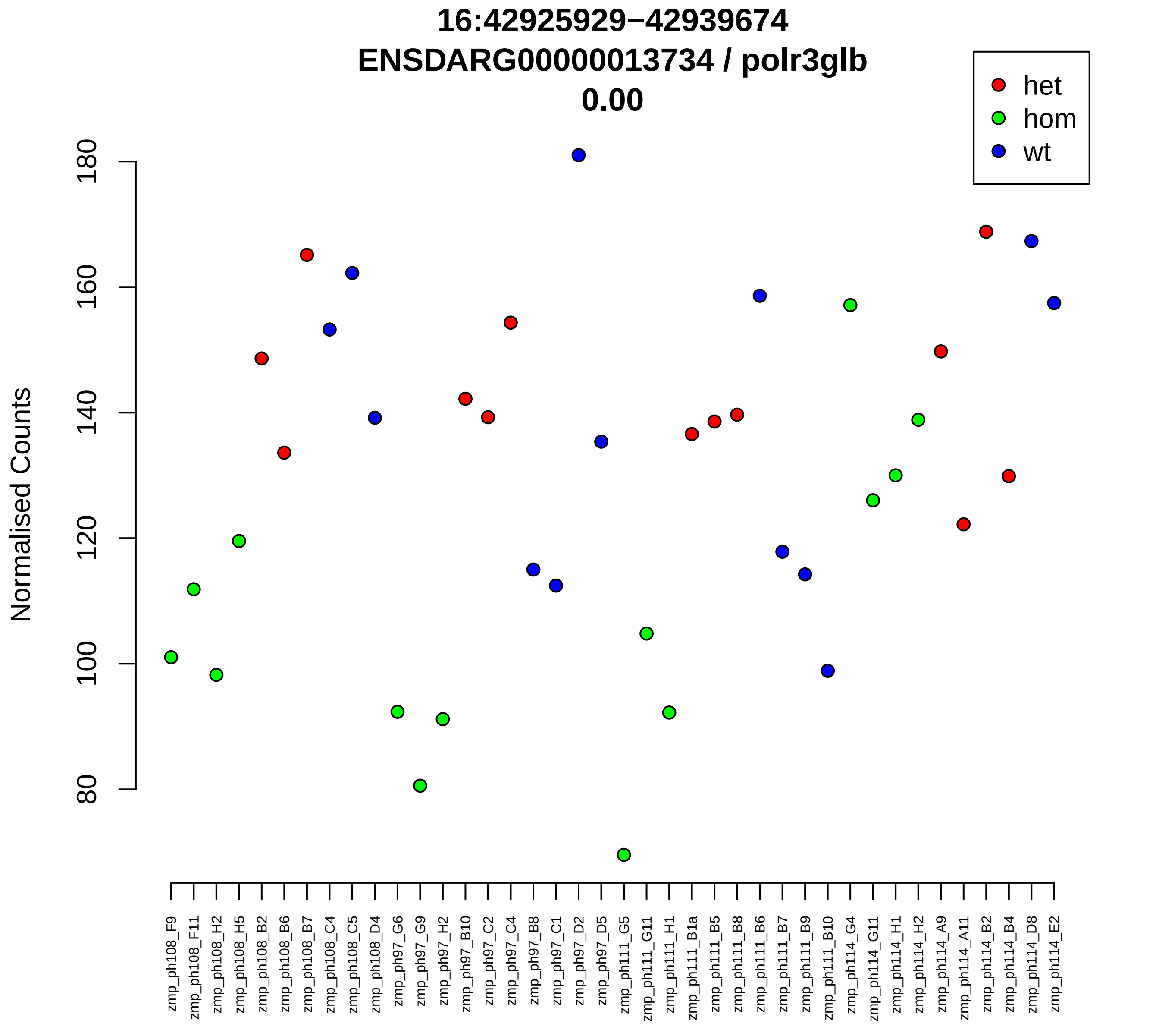

16:42925929−42939674
ENSDARG00000013734 / polr3glb
0.00
het
hom
wt
Normalised Counts
80
100
120
140
160
180
zmp_ph108_F9
zmp_ph108_F11
zmp_ph108_H2
zmp_ph108_H5
zmp_ph108_B2
zmp_ph108_B6
zmp_ph108_B7
zmp_ph108_C4
zmp_ph108_C5
zmp_ph108_D4
zmp_ph97_G6
zmp_ph97_G9
zmp_ph97_H2
zmp_ph97_B10
zmp_ph97_C2
zmp_ph97_C4
zmp_ph97_B8
zmp_ph97_C1
zmp_ph97_D2
zmp_ph97_D5
zmp_ph111_G5
zmp_ph111_G11
zmp_ph111_H1
zmp_ph111_B1a
zmp_ph111_B5
zmp_ph111_B8
zmp_ph111_B6
zmp_ph111_B7
zmp_ph111_B9
zmp_ph111_B10
zmp_ph114_G4
zmp_ph114_G11
zmp_ph114_H1
zmp_ph114_H2
zmp_ph114_A9
zmp_ph114_A11
zmp_ph114_B2
zmp_ph114_B4
zmp_ph114_D8
zmp_ph114_E2

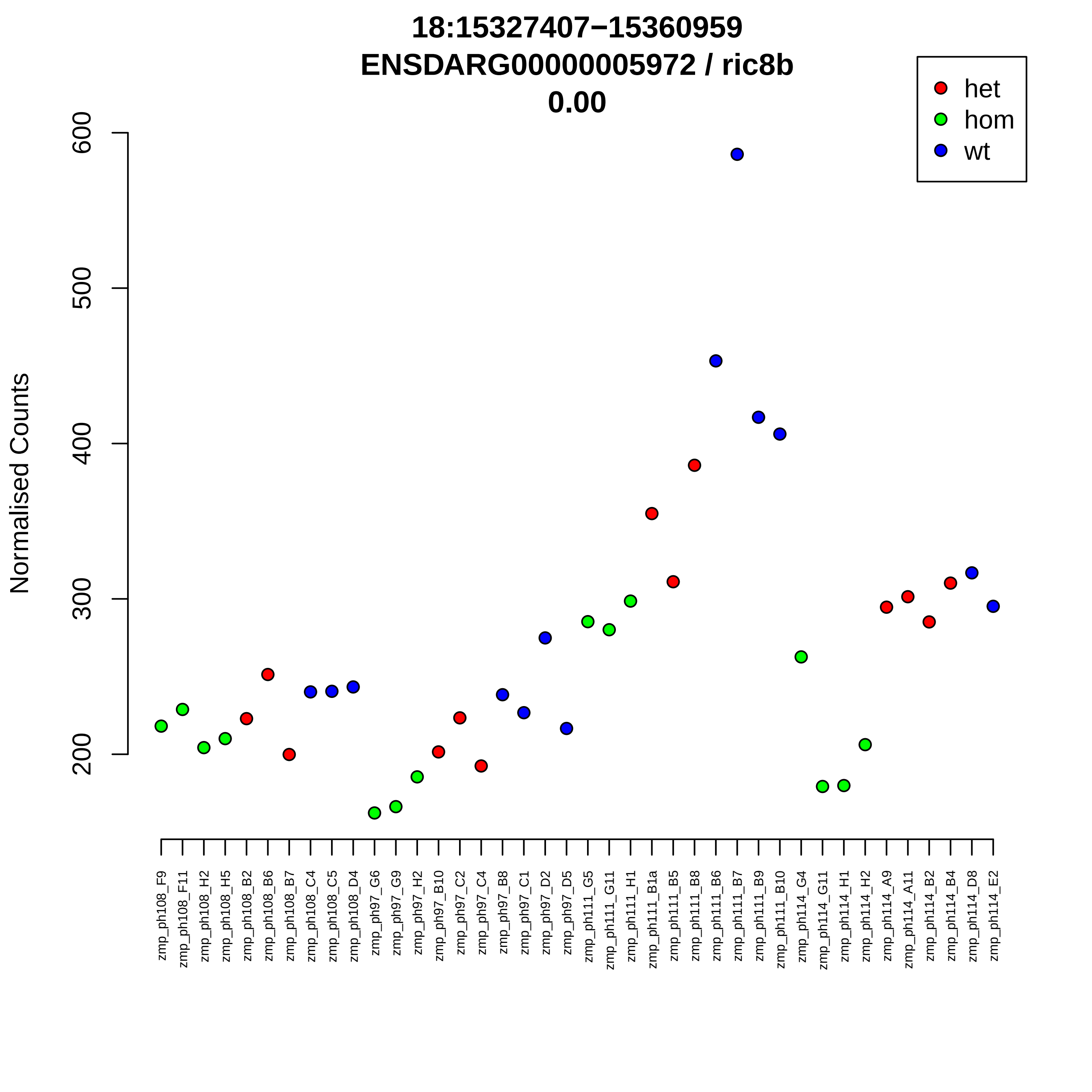

18:15327407−15360959
ENSDARG00000005972 / ric8b
0.00
het
hom
wt
Normalised Counts
200
300
400
500
600
zmp_ph108_F9
zmp_ph108_F11
zmp_ph108_H2
zmp_ph108_H5
zmp_ph108_B2
zmp_ph108_B6
zmp_ph108_B7
zmp_ph108_C4
zmp_ph108_C5
zmp_ph108_D4
zmp_ph97_G6
zmp_ph97_G9
zmp_ph97_H2
zmp_ph97_B10
zmp_ph97_C2
zmp_ph97_C4
zmp_ph97_B8
zmp_ph97_C1
zmp_ph97_D2
zmp_ph97_D5
zmp_ph111_G5
zmp_ph111_G11
zmp_ph111_H1
zmp_ph111_B1a
zmp_ph111_B5
zmp_ph111_B8
zmp_ph111_B6
zmp_ph111_B7
zmp_ph111_B9
zmp_ph111_B10
zmp_ph114_G4
zmp_ph114_G11
zmp_ph114_H1
zmp_ph114_H2
zmp_ph114_A9
zmp_ph114_A11
zmp_ph114_B2
zmp_ph114_B4
zmp_ph114_D8
zmp_ph114_E2

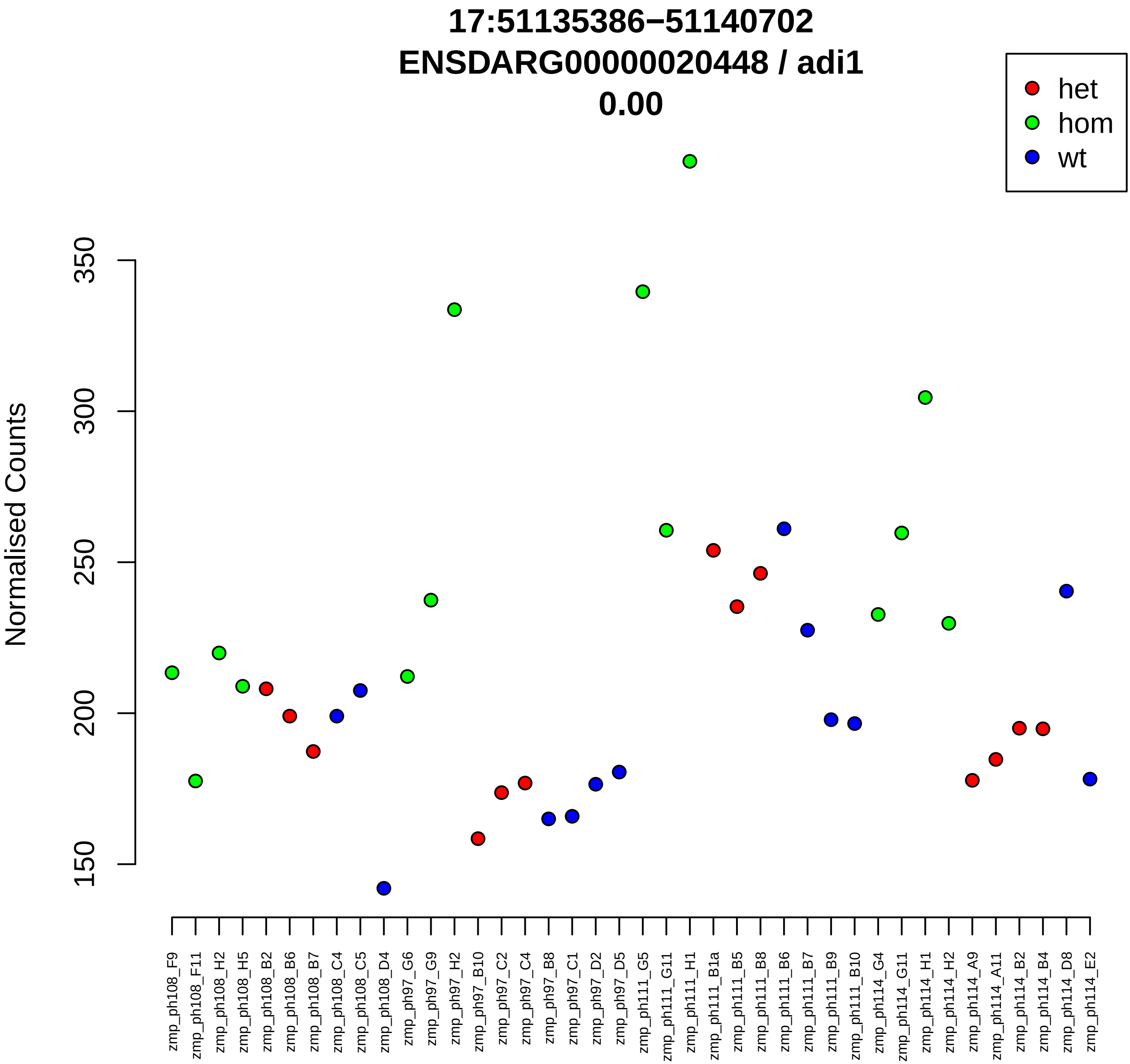
17:51135386−51140702
ENSDARG00000020448 / adi1
0.00
het
hom
wt
Normalised Counts
150
200
250
300
350
zmp_ph108_F9
zmp_ph108_F11
zmp_ph108_H2
zmp_ph108_H5
zmp_ph108_B2
zmp_ph108_B6
zmp_ph108_B7
zmp_ph108_C4
zmp_ph108_C5
zmp_ph108_D4
zmp_ph97_G6
zmp_ph97_G9
zmp_ph97_H2
zmp_ph97_B10
zmp_ph97_C2
zmp_ph97_C4
zmp_ph97_B8
zmp_ph97_C1
zmp_ph97_D2
zmp_ph97_D5
zmp_ph111_G5
zmp_ph111_G11
zmp_ph111_H1
zmp_ph111_B1a
zmp_ph111_B5
zmp_ph111_B8
zmp_ph111_B6
zmp_ph111_B7
zmp_ph111_B9
zmp_ph111_B10
zmp_ph114_G4
zmp_ph114_G11
zmp_ph114_H1
zmp_ph114_H2
zmp_ph114_A9
zmp_ph114_A11
zmp_ph114_B2
zmp_ph114_B4
zmp_ph114_D8
zmp_ph114_E2

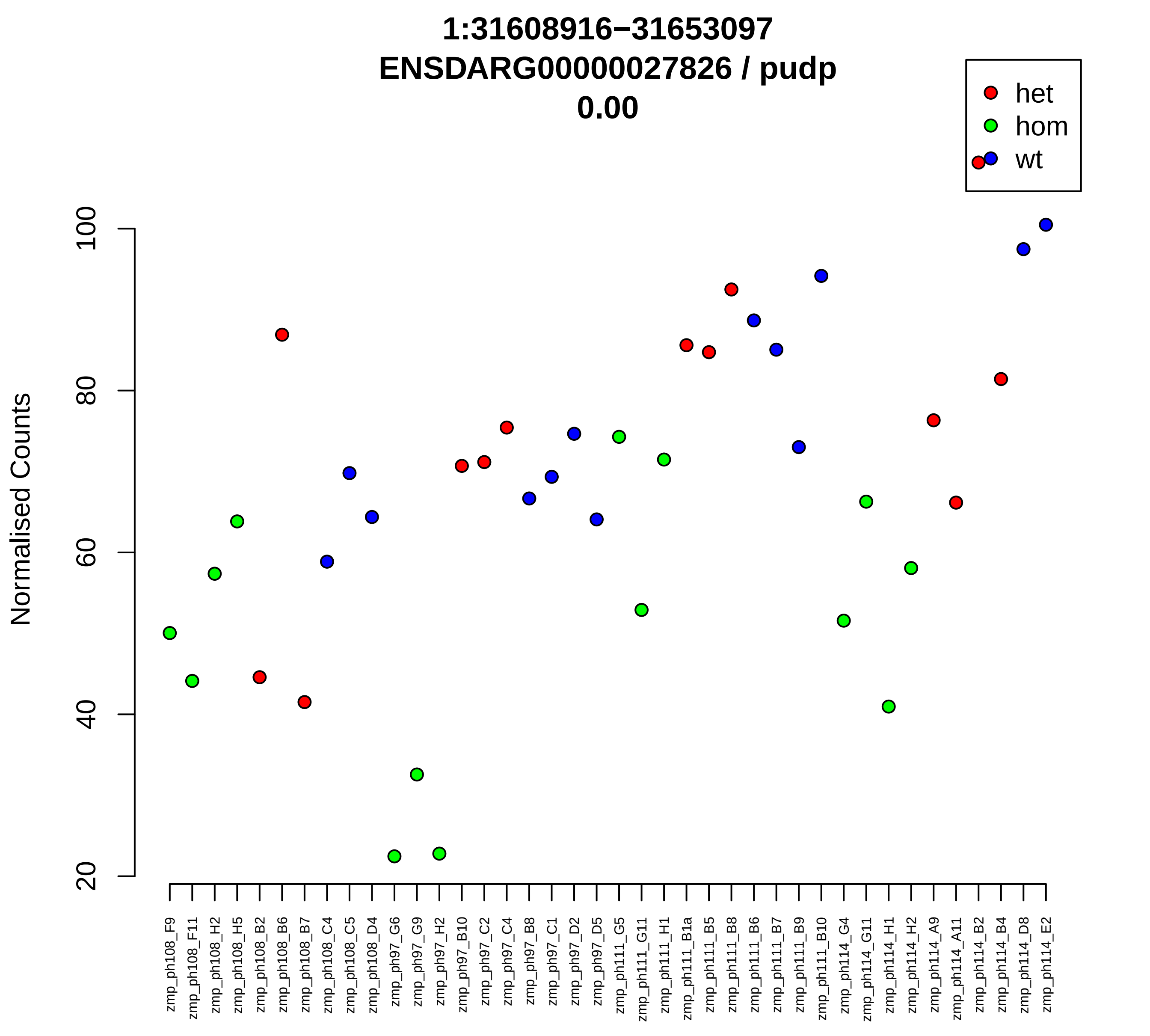
1:31608916−31653097
ENSDARG00000027826 / pudp
0.00
het
hom
wt
Normalised Counts
20
40
60
80
100
zmp_ph108_F9
zmp_ph108_F11
zmp_ph108_H2
zmp_ph108_H5
zmp_ph108_B2
zmp_ph108_B6
zmp_ph108_B7
zmp_ph108_C4
zmp_ph108_C5
zmp_ph108_D4
zmp_ph97_G6
zmp_ph97_G9
zmp_ph97_H2
zmp_ph97_B10
zmp_ph97_C2
zmp_ph97_C4
zmp_ph97_B8
zmp_ph97_C1
zmp_ph97_D2
zmp_ph97_D5
zmp_ph111_G5
zmp_ph111_G11
zmp_ph111_H1
zmp_ph111_B1a
zmp_ph111_B5
zmp_ph111_B8
zmp_ph111_B6
zmp_ph111_B7
zmp_ph111_B9
zmp_ph111_B10
zmp_ph114_G4
zmp_ph114_G11
zmp_ph114_H1
zmp_ph114_H2
zmp_ph114_A9
zmp_ph114_A11
zmp_ph114_B2
zmp_ph114_B4
zmp_ph114_D8
zmp_ph114_E2

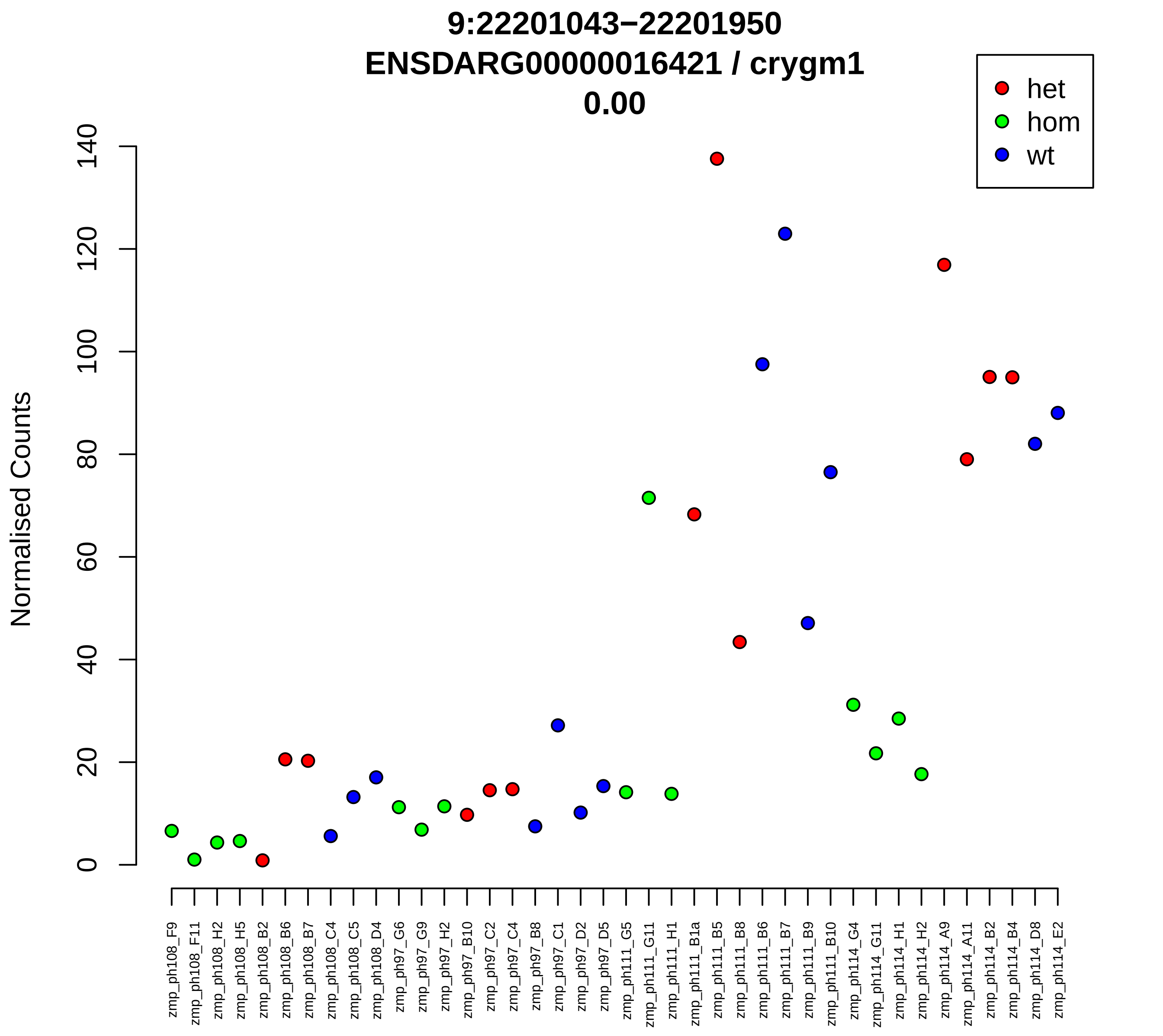
9:22201043−22201950
ENSDARG00000016421 / crygm1
0.00
het
hom
wt
Normalised Counts
0
20
40
60
80
100
120
140
zmp_ph108_F9
zmp_ph108_F11
zmp_ph108_H2
zmp_ph108_H5
zmp_ph108_B2
zmp_ph108_B6
zmp_ph108_B7
zmp_ph108_C4
zmp_ph108_C5
zmp_ph108_D4
zmp_ph97_G6
zmp_ph97_G9
zmp_ph97_H2
zmp_ph97_B10
zmp_ph97_C2
zmp_ph97_C4
zmp_ph97_B8
zmp_ph97_C1
zmp_ph97_D2
zmp_ph97_D5
zmp_ph111_G5
zmp_ph111_G11
zmp_ph111_H1
zmp_ph111_B1a
zmp_ph111_B5
zmp_ph111_B8
zmp_ph111_B6
zmp_ph111_B7
zmp_ph111_B9
zmp_ph111_B10
zmp_ph114_G4
zmp_ph114_G11
zmp_ph114_H1
zmp_ph114_H2
zmp_ph114_A9
zmp_ph114_A11
zmp_ph114_B2
zmp_ph114_B4
zmp_ph114_D8
zmp_ph114_E2

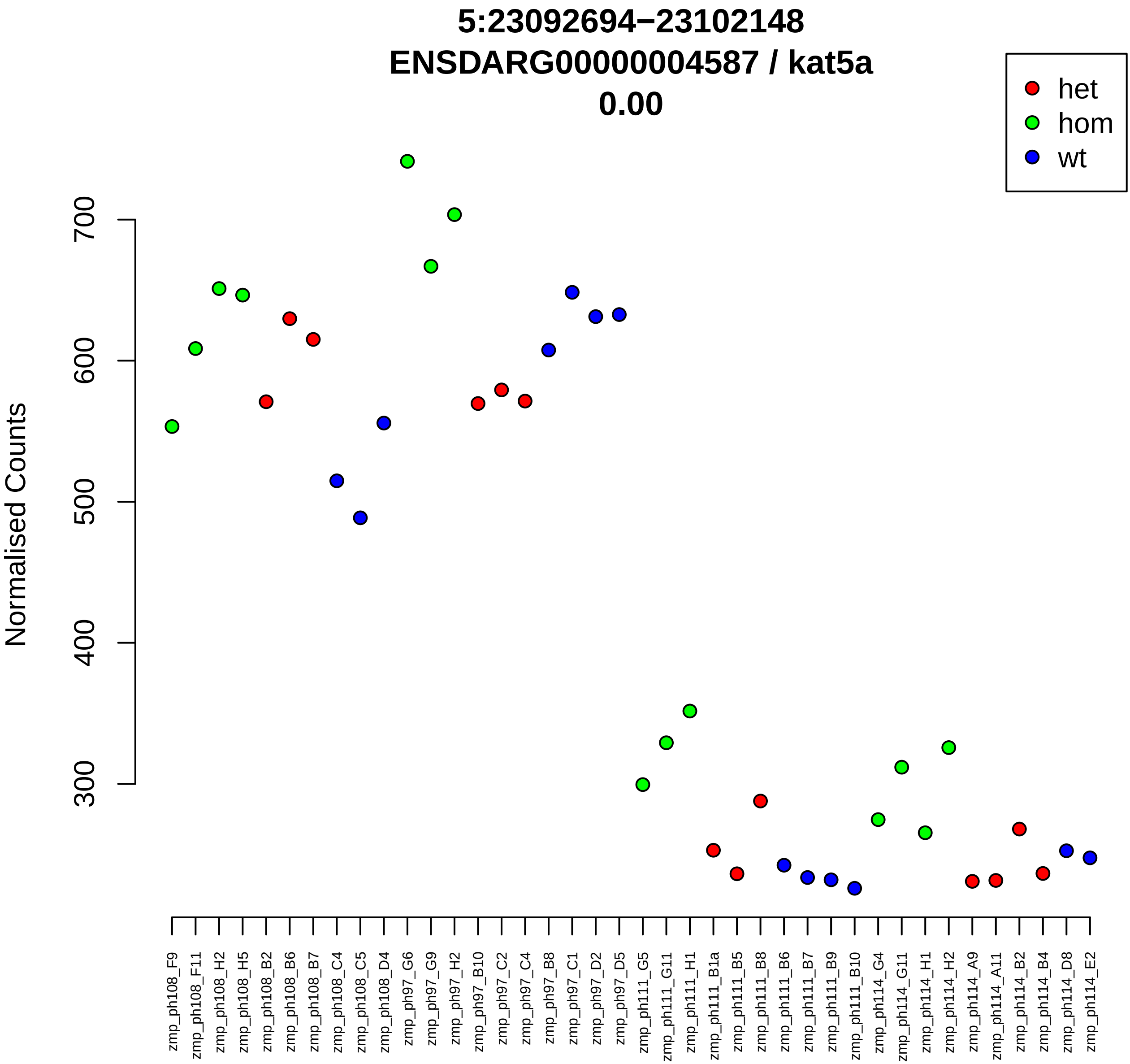
5:23092694−23102148
ENSDARG00000004587 / kat5a
0.00
het
hom
wt
Normalised Counts
300
400
500
600
700
zmp_ph108_F9
zmp_ph108_F11
zmp_ph108_H2
zmp_ph108_H5
zmp_ph108_B2
zmp_ph108_B6
zmp_ph108_B7
zmp_ph108_C4
zmp_ph108_C5
zmp_ph108_D4
zmp_ph97_G6
zmp_ph97_G9
zmp_ph97_H2
zmp_ph97_B10
zmp_ph97_C2
zmp_ph97_C4
zmp_ph97_B8
zmp_ph97_C1
zmp_ph97_D2
zmp_ph97_D5
zmp_ph111_G5
zmp_ph111_G11
zmp_ph111_H1
zmp_ph111_B1a
zmp_ph111_B5
zmp_ph111_B8
zmp_ph111_B6
zmp_ph111_B7
zmp_ph111_B9
zmp_ph111_B10
zmp_ph114_G4
zmp_ph114_G11
zmp_ph114_H1
zmp_ph114_H2
zmp_ph114_A9
zmp_ph114_A11
zmp_ph114_B2
zmp_ph114_B4
zmp_ph114_D8
zmp_ph114_E2

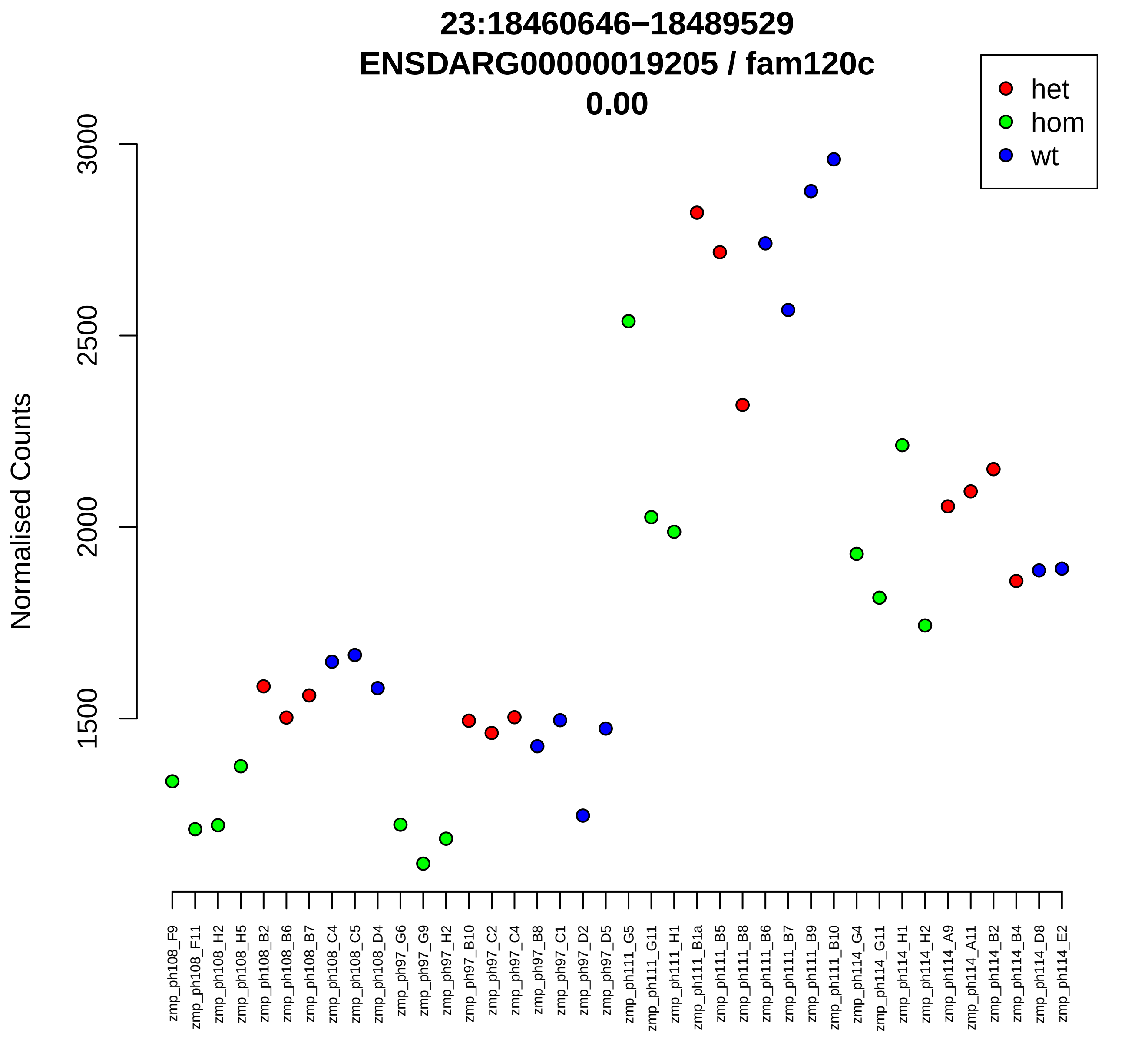
23:18460646−18489529
ENSDARG00000019205 / fam120c
0.00
het
hom
wt
Normalised Counts
1500
2000
2500
3000
zmp_ph108_F9
zmp_ph108_F11
zmp_ph108_H2
zmp_ph108_H5
zmp_ph108_B2
zmp_ph108_B6
zmp_ph108_B7
zmp_ph108_C4
zmp_ph108_C5
zmp_ph108_D4
zmp_ph97_G6
zmp_ph97_G9
zmp_ph97_H2
zmp_ph97_B10
zmp_ph97_C2
zmp_ph97_C4
zmp_ph97_B8
zmp_ph97_C1
zmp_ph97_D2
zmp_ph97_D5
zmp_ph111_G5
zmp_ph111_G11
zmp_ph111_H1
zmp_ph111_B1a
zmp_ph111_B5
zmp_ph111_B8
zmp_ph111_B6
zmp_ph111_B7
zmp_ph111_B9
zmp_ph111_B10
zmp_ph114_G4
zmp_ph114_G11
zmp_ph114_H1
zmp_ph114_H2
zmp_ph114_A9
zmp_ph114_A11
zmp_ph114_B2
zmp_ph114_B4
zmp_ph114_D8
zmp_ph114_E2

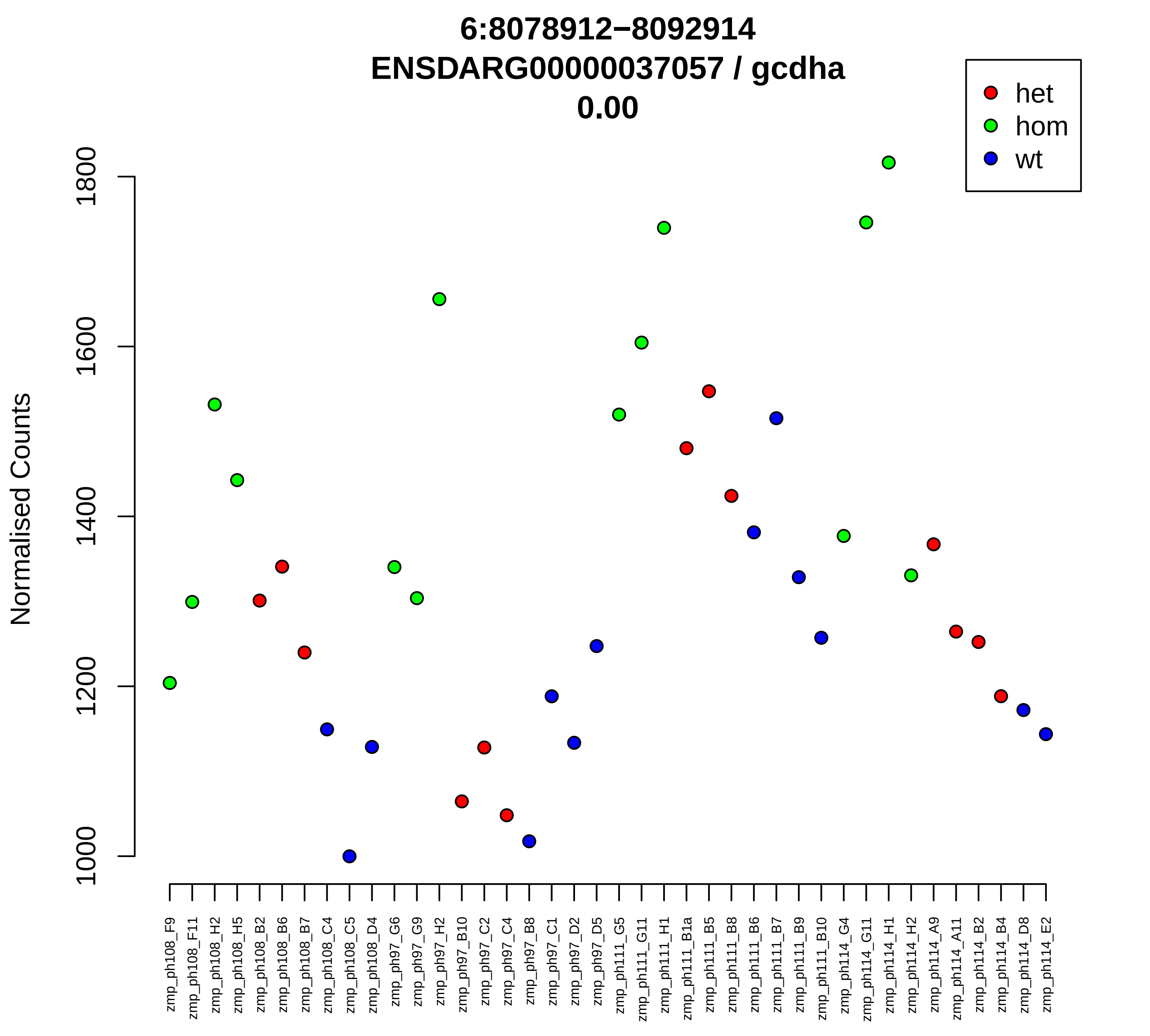

6:8078912−8092914
ENSDARG00000037057 / gcdha
0.00
het
hom
wt
Normalised Counts
1000
1200
1400
1600
1800
zmp_ph108_F9
zmp_ph108_F11
zmp_ph108_H2
zmp_ph108_H5
zmp_ph108_B2
zmp_ph108_B6
zmp_ph108_B7
zmp_ph108_C4
zmp_ph108_C5
zmp_ph108_D4
zmp_ph97_G6
zmp_ph97_G9
zmp_ph97_H2
zmp_ph97_B10
zmp_ph97_C2
zmp_ph97_C4
zmp_ph97_B8
zmp_ph97_C1
zmp_ph97_D2
zmp_ph97_D5
zmp_ph111_G5
zmp_ph111_G11
zmp_ph111_H1
zmp_ph111_B1a
zmp_ph111_B5
zmp_ph111_B8
zmp_ph111_B6
zmp_ph111_B7
zmp_ph111_B9
zmp_ph111_B10
zmp_ph114_G4
zmp_ph114_G11
zmp_ph114_H1
zmp_ph114_H2
zmp_ph114_A9
zmp_ph114_A11
zmp_ph114_B2
zmp_ph114_B4
zmp_ph114_D8
zmp_ph114_E2

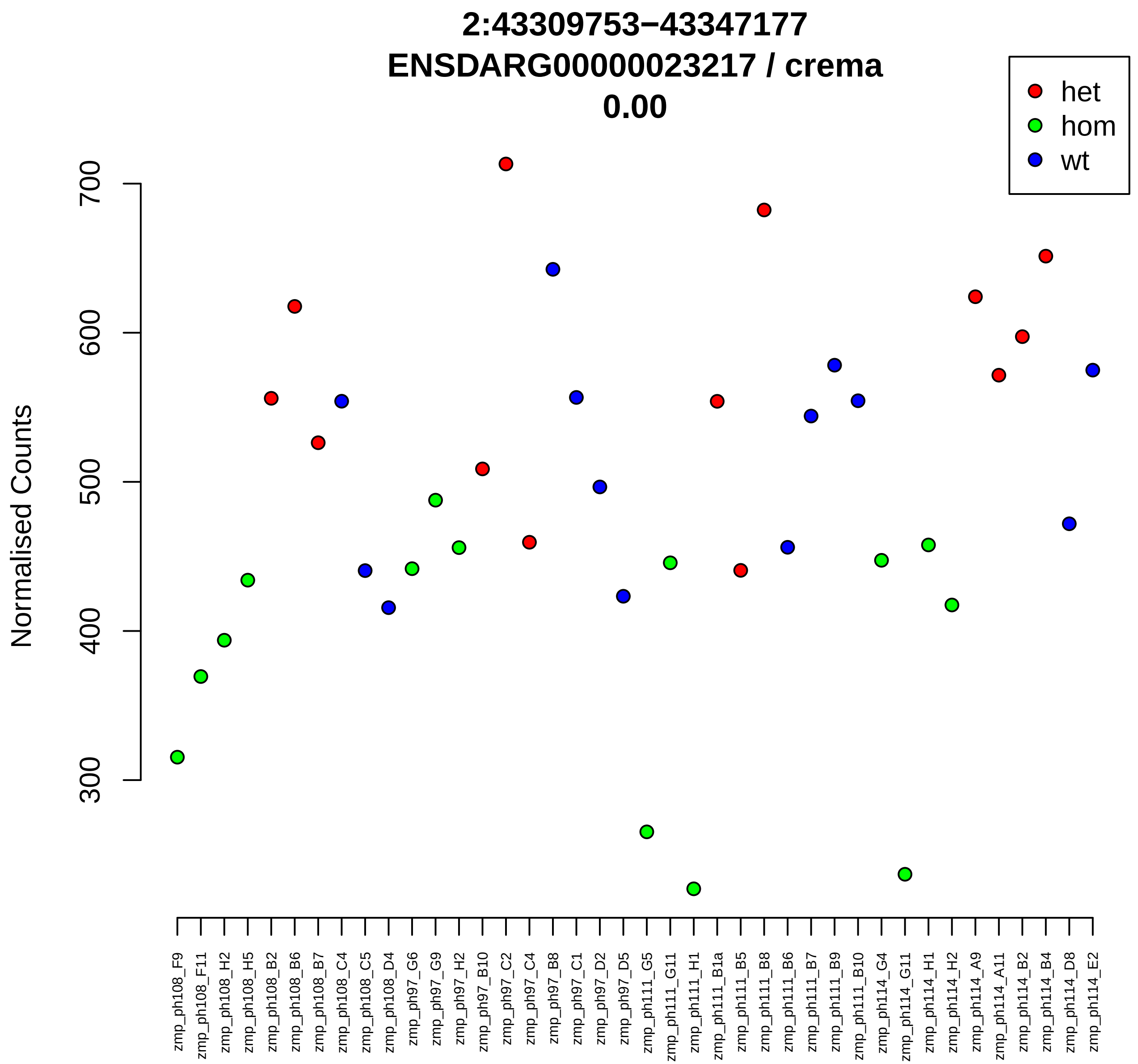
2:43309753−43347177
ENSDARG00000023217 / crema
0.00
het
hom
wt
Normalised Counts
300
400
500
600
700
zmp_ph108_F9
zmp_ph108_F11
zmp_ph108_H2
zmp_ph108_H5
zmp_ph108_B2
zmp_ph108_B6
zmp_ph108_B7
zmp_ph108_C4
zmp_ph108_C5
zmp_ph108_D4
zmp_ph97_G6
zmp_ph97_G9
zmp_ph97_H2
zmp_ph97_B10
zmp_ph97_C2
zmp_ph97_C4
zmp_ph97_B8
zmp_ph97_C1
zmp_ph97_D2
zmp_ph97_D5
zmp_ph111_G5
zmp_ph111_G11
zmp_ph111_H1
zmp_ph111_B1a
zmp_ph111_B5
zmp_ph111_B8
zmp_ph111_B6
zmp_ph111_B7
zmp_ph111_B9
zmp_ph111_B10
zmp_ph114_G4
zmp_ph114_G11
zmp_ph114_H1
zmp_ph114_H2
zmp_ph114_A9
zmp_ph114_A11
zmp_ph114_B2
zmp_ph114_B4
zmp_ph114_D8
zmp_ph114_E2

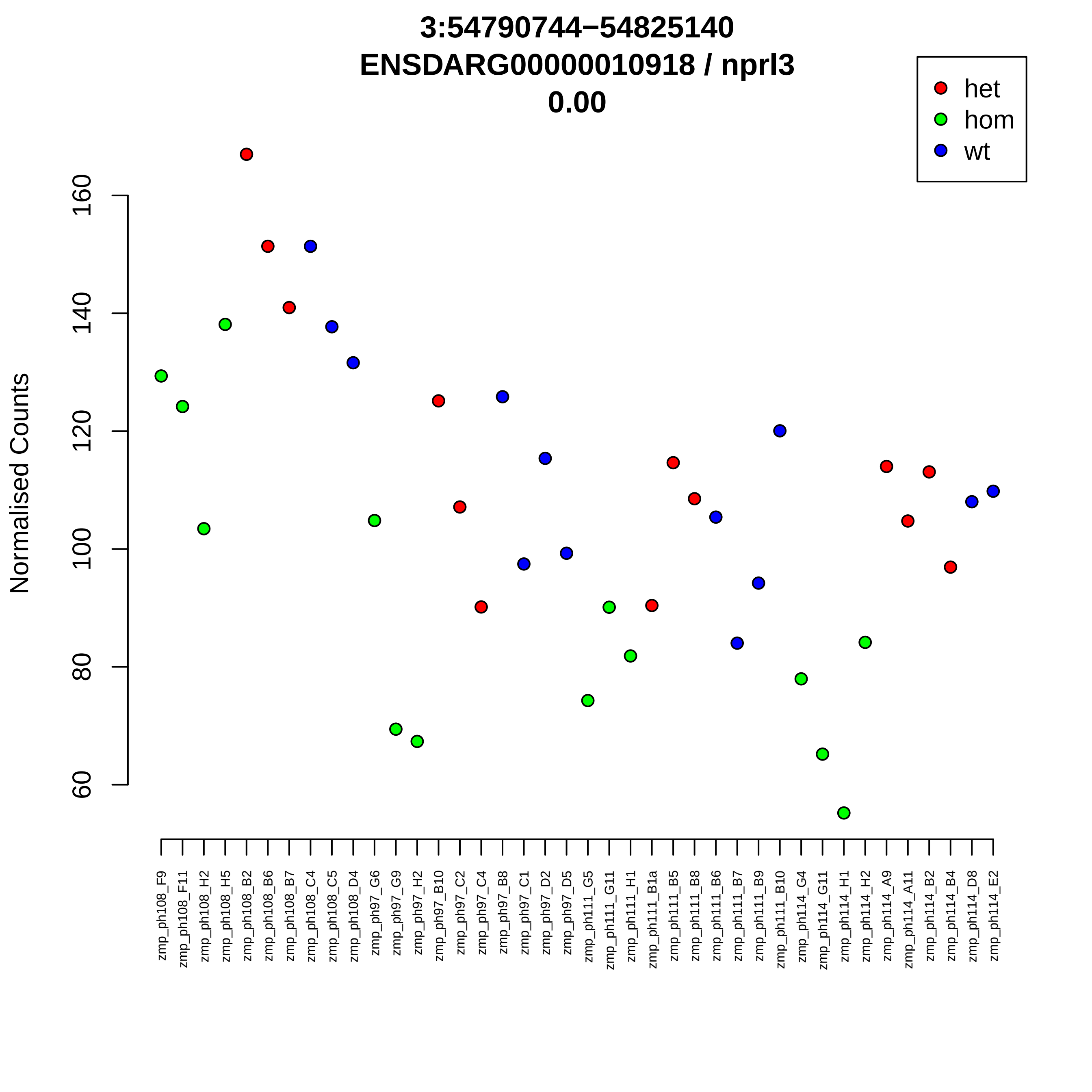
3:54790744−54825140
ENSDARG00000010918 / nprl3
0.00
het
hom
wt
Normalised Counts
60
80
100
120
140
160
zmp_ph108_F9
zmp_ph108_F11
zmp_ph108_H2
zmp_ph108_H5
zmp_ph108_B2
zmp_ph108_B6
zmp_ph108_B7
zmp_ph108_C4
zmp_ph108_C5
zmp_ph108_D4
zmp_ph97_G6
zmp_ph97_G9
zmp_ph97_H2
zmp_ph97_B10
zmp_ph97_C2
zmp_ph97_C4
zmp_ph97_B8
zmp_ph97_C1
zmp_ph97_D2
zmp_ph97_D5
zmp_ph111_G5
zmp_ph111_G11
zmp_ph111_H1
zmp_ph111_B1a
zmp_ph111_B5
zmp_ph111_B8
zmp_ph111_B6
zmp_ph111_B7
zmp_ph111_B9
zmp_ph111_B10
zmp_ph114_G4
zmp_ph114_G11
zmp_ph114_H1
zmp_ph114_H2
zmp_ph114_A9
zmp_ph114_A11
zmp_ph114_B2
zmp_ph114_B4
zmp_ph114_D8
zmp_ph114_E2

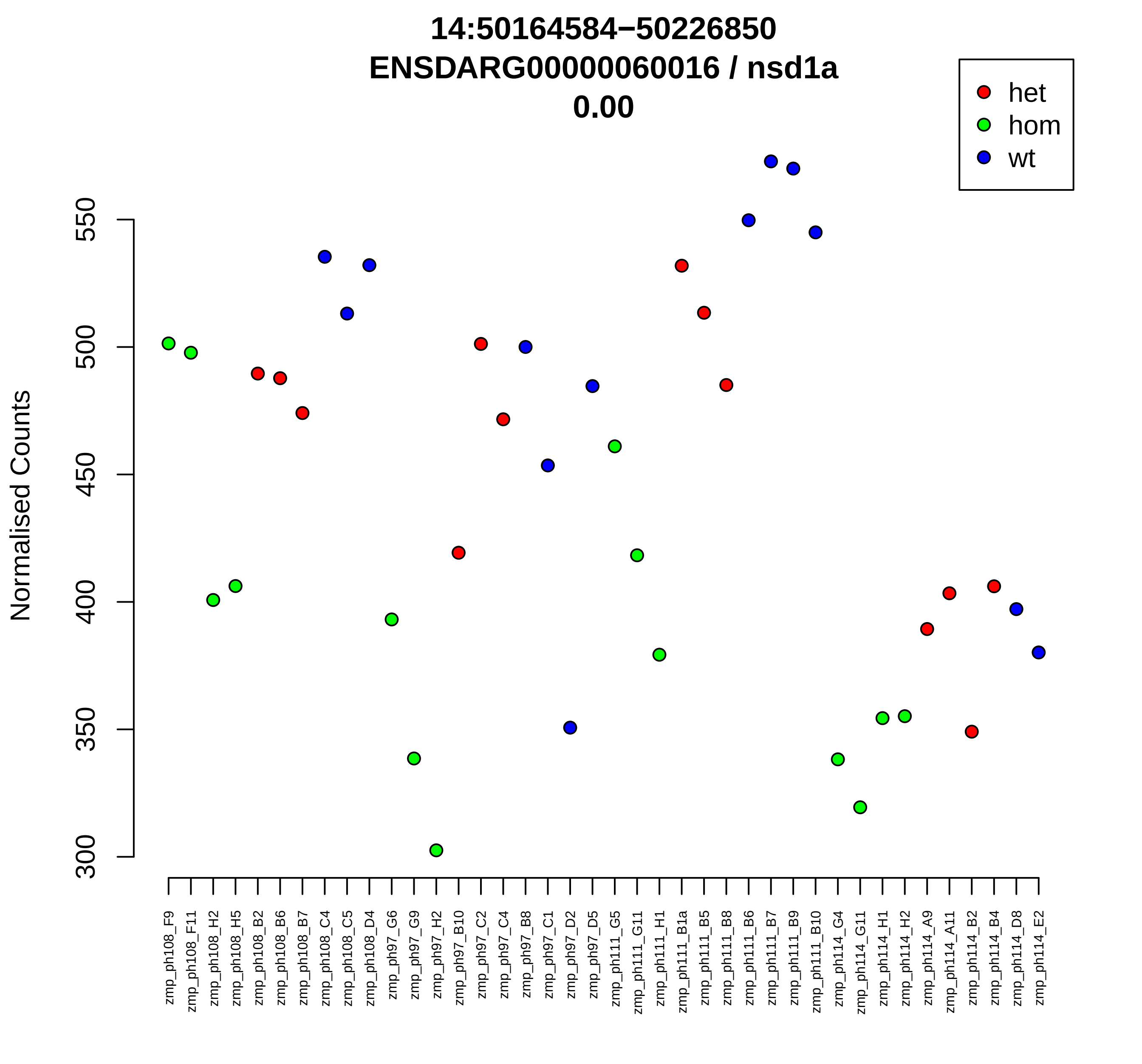

14:50164584−50226850
ENSDARG00000060016 / nsd1a
0.00
het
hom
wt
Normalised Counts
300
350
400
450
500
550
zmp_ph108_F9
zmp_ph108_F11
zmp_ph108_H2
zmp_ph108_H5
zmp_ph108_B2
zmp_ph108_B6
zmp_ph108_B7
zmp_ph108_C4
zmp_ph108_C5
zmp_ph108_D4
zmp_ph97_G6
zmp_ph97_G9
zmp_ph97_H2
zmp_ph97_B10
zmp_ph97_C2
zmp_ph97_C4
zmp_ph97_B8
zmp_ph97_C1
zmp_ph97_D2
zmp_ph97_D5
zmp_ph111_G5
zmp_ph111_G11
zmp_ph111_H1
zmp_ph111_B1a
zmp_ph111_B5
zmp_ph111_B8
zmp_ph111_B6
zmp_ph111_B7
zmp_ph111_B9
zmp_ph111_B10
zmp_ph114_G4
zmp_ph114_G11
zmp_ph114_H1
zmp_ph114_H2
zmp_ph114_A9
zmp_ph114_A11
zmp_ph114_B2
zmp_ph114_B4
zmp_ph114_D8
zmp_ph114_E2

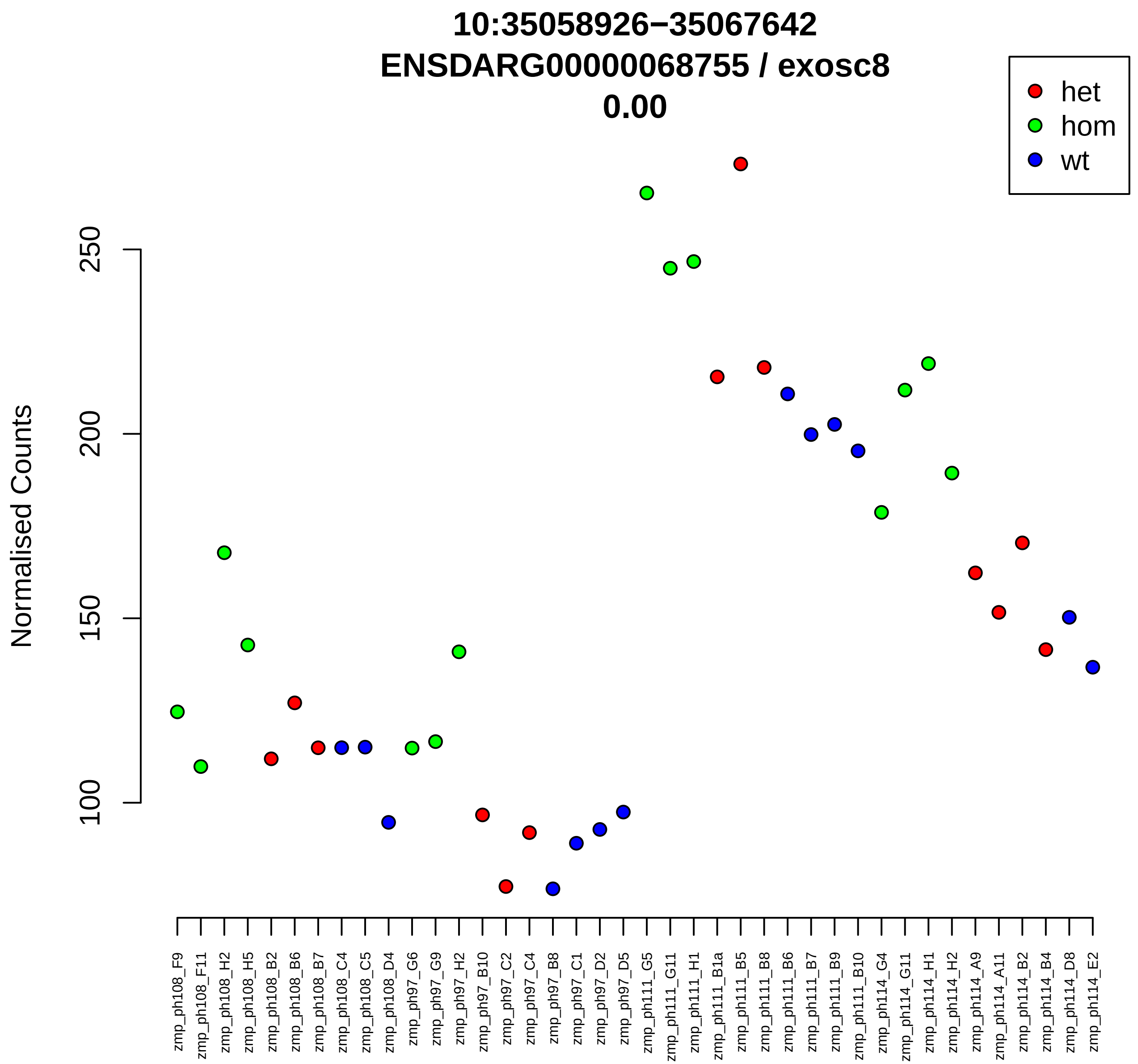
10:35058926−35067642
ENSDARG00000068755 / exosc8
0.00
het
hom
wt
Normalised Counts
100
150
200
250
zmp_ph108_F9
zmp_ph108_F11
zmp_ph108_H2
zmp_ph108_H5
zmp_ph108_B2
zmp_ph108_B6
zmp_ph108_B7
zmp_ph108_C4
zmp_ph108_C5
zmp_ph108_D4
zmp_ph97_G6
zmp_ph97_G9
zmp_ph97_H2
zmp_ph97_B10
zmp_ph97_C2
zmp_ph97_C4
zmp_ph97_B8
zmp_ph97_C1
zmp_ph97_D2
zmp_ph97_D5
zmp_ph111_G5
zmp_ph111_G11
zmp_ph111_H1
zmp_ph111_B1a
zmp_ph111_B5
zmp_ph111_B8
zmp_ph111_B6
zmp_ph111_B7
zmp_ph111_B9
zmp_ph111_B10
zmp_ph114_G4
zmp_ph114_G11
zmp_ph114_H1
zmp_ph114_H2
zmp_ph114_A9
zmp_ph114_A11
zmp_ph114_B2
zmp_ph114_B4
zmp_ph114_D8
zmp_ph114_E2

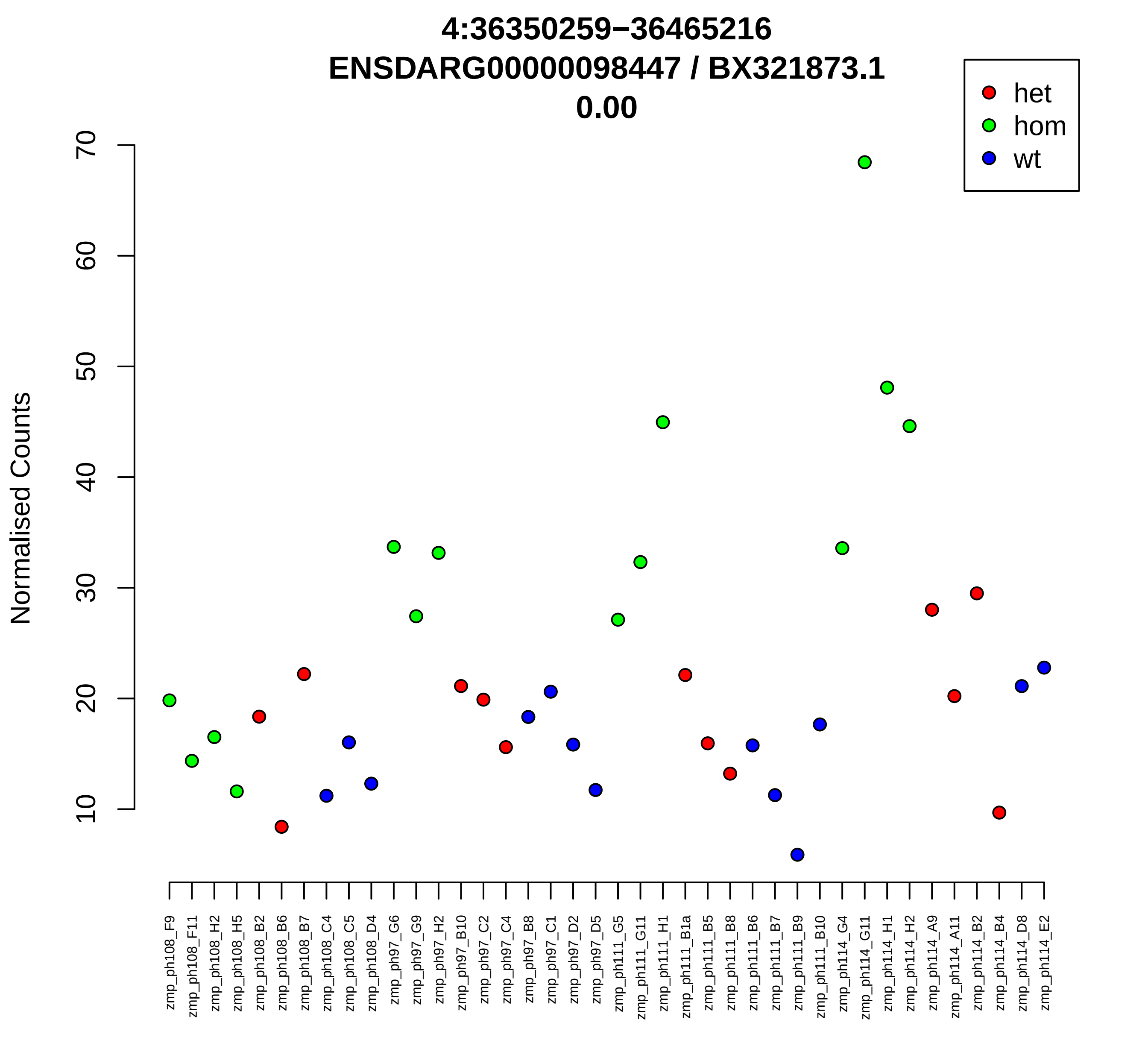
4:36350259−36465216
ENSDARG00000098447 / BX321873.1
0.00
het
hom
wt
Normalised Counts
10
20
30
40
50
60
70
zmp_ph108_F9
zmp_ph108_F11
zmp_ph108_H2
zmp_ph108_H5
zmp_ph108_B2
zmp_ph108_B6
zmp_ph108_B7
zmp_ph108_C4
zmp_ph108_C5
zmp_ph108_D4
zmp_ph97_G6
zmp_ph97_G9
zmp_ph97_H2
zmp_ph97_B10
zmp_ph97_C2
zmp_ph97_C4
zmp_ph97_B8
zmp_ph97_C1
zmp_ph97_D2
zmp_ph97_D5
zmp_ph111_G5
zmp_ph111_G11
zmp_ph111_H1
zmp_ph111_B1a
zmp_ph111_B5
zmp_ph111_B8
zmp_ph111_B6
zmp_ph111_B7
zmp_ph111_B9
zmp_ph111_B10
zmp_ph114_G4
zmp_ph114_G11
zmp_ph114_H1
zmp_ph114_H2
zmp_ph114_A9
zmp_ph114_A11
zmp_ph114_B2
zmp_ph114_B4
zmp_ph114_D8
zmp_ph114_E2

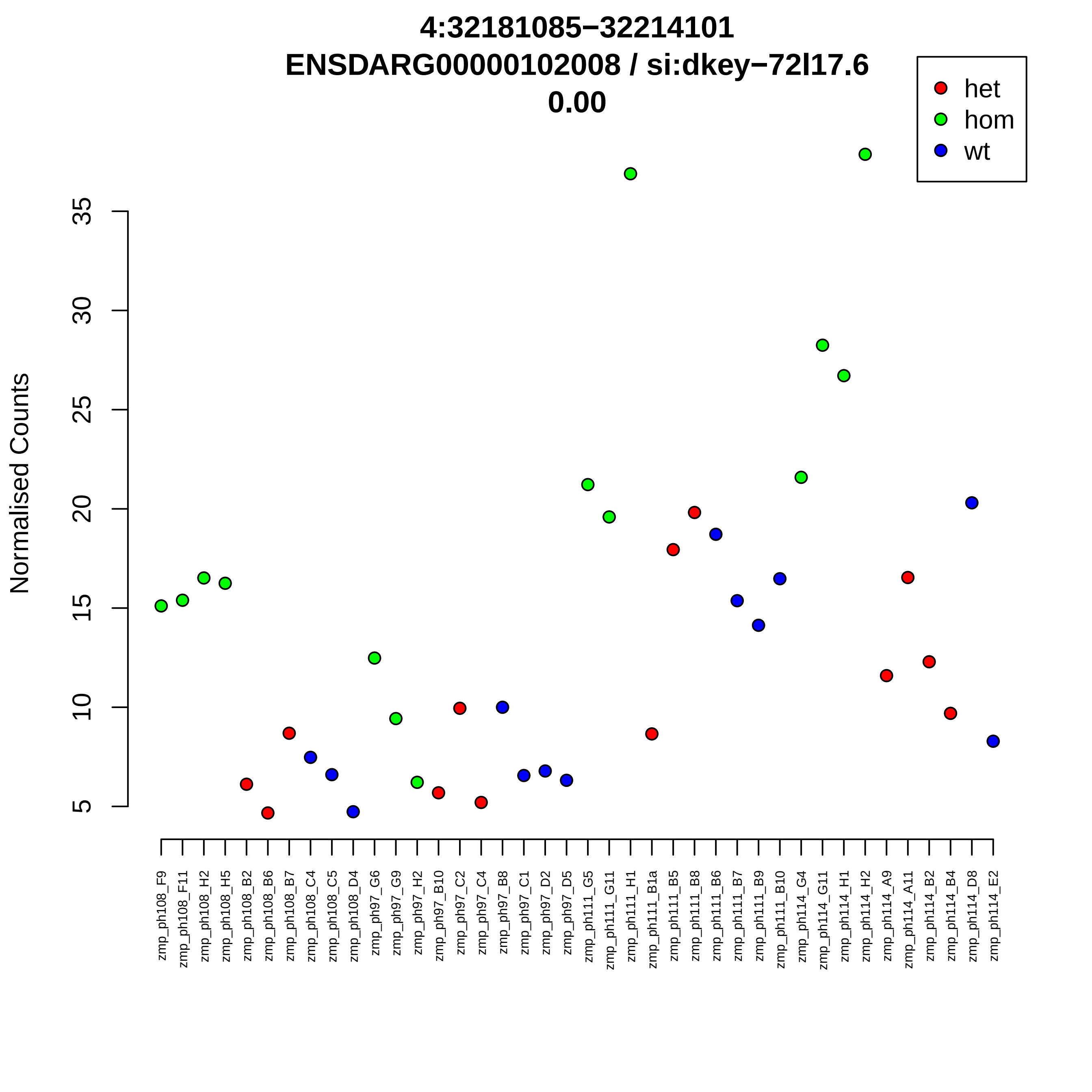

4:32181085−32214101
ENSDARG00000102008 / si:dkey−72l17.6
0.00
het
hom
wt
Normalised Counts
5
10
15
20
25
30
35
zmp_ph108_F9
zmp_ph108_F11
zmp_ph108_H2
zmp_ph108_H5
zmp_ph108_B2
zmp_ph108_B6
zmp_ph108_B7
zmp_ph108_C4
zmp_ph108_C5
zmp_ph108_D4
zmp_ph97_G6
zmp_ph97_G9
zmp_ph97_H2
zmp_ph97_B10
zmp_ph97_C2
zmp_ph97_C4
zmp_ph97_B8
zmp_ph97_C1
zmp_ph97_D2
zmp_ph97_D5
zmp_ph111_G5
zmp_ph111_G11
zmp_ph111_H1
zmp_ph111_B1a
zmp_ph111_B5
zmp_ph111_B8
zmp_ph111_B6
zmp_ph111_B7
zmp_ph111_B9
zmp_ph111_B10
zmp_ph114_G4
zmp_ph114_G11
zmp_ph114_H1
zmp_ph114_H2
zmp_ph114_A9
zmp_ph114_A11
zmp_ph114_B2
zmp_ph114_B4
zmp_ph114_D8
zmp_ph114_E2

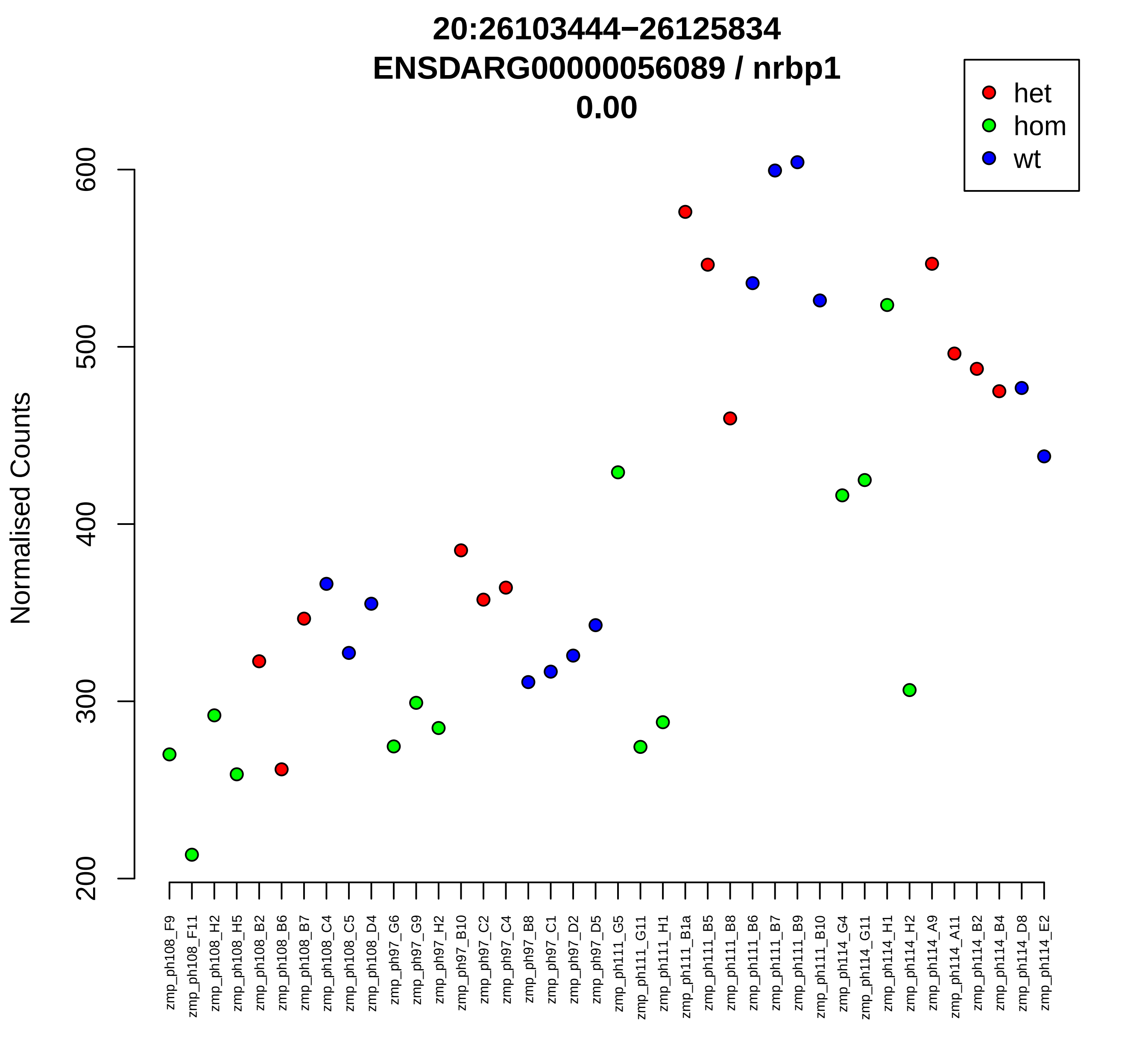

20:26103444−26125834
ENSDARG00000056089 / nrbp1
0.00
het
hom
wt
Normalised Counts
200
300
400
500
600
zmp_ph108_F9
zmp_ph108_F11
zmp_ph108_H2
zmp_ph108_H5
zmp_ph108_B2
zmp_ph108_B6
zmp_ph108_B7
zmp_ph108_C4
zmp_ph108_C5
zmp_ph108_D4
zmp_ph97_G6
zmp_ph97_G9
zmp_ph97_H2
zmp_ph97_B10
zmp_ph97_C2
zmp_ph97_C4
zmp_ph97_B8
zmp_ph97_C1
zmp_ph97_D2
zmp_ph97_D5
zmp_ph111_G5
zmp_ph111_G11
zmp_ph111_H1
zmp_ph111_B1a
zmp_ph111_B5
zmp_ph111_B8
zmp_ph111_B6
zmp_ph111_B7
zmp_ph111_B9
zmp_ph111_B10
zmp_ph114_G4
zmp_ph114_G11
zmp_ph114_H1
zmp_ph114_H2
zmp_ph114_A9
zmp_ph114_A11
zmp_ph114_B2
zmp_ph114_B4
zmp_ph114_D8
zmp_ph114_E2

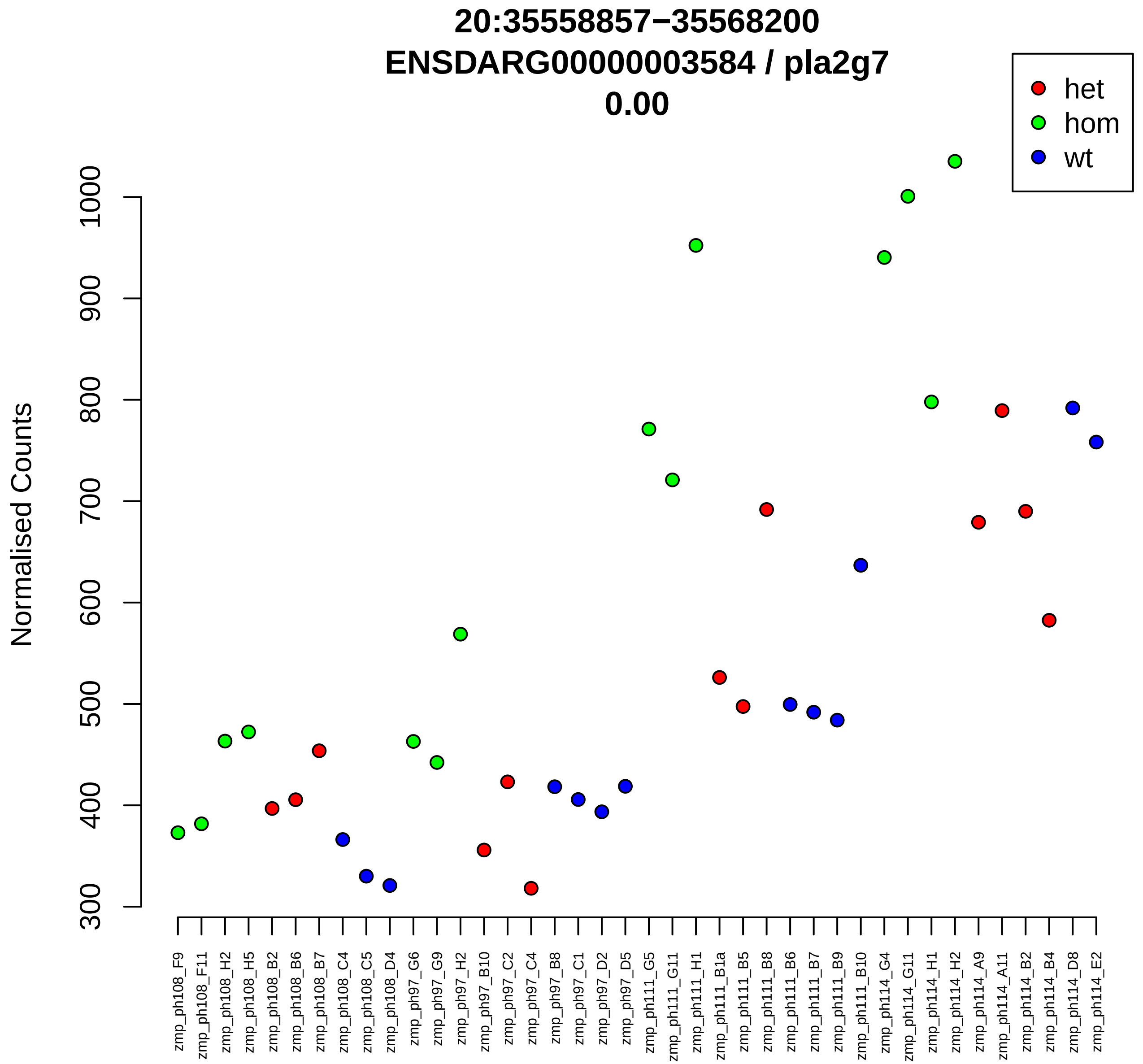
20:35558857−35568200
ENSDARG00000003584 / pla2g7
0.00
het
hom
wt
Normalised Counts
300
400
500
600
700
800
900
1000
zmp_ph108_F9
zmp_ph108_F11
zmp_ph108_H2
zmp_ph108_H5
zmp_ph108_B2
zmp_ph108_B6
zmp_ph108_B7
zmp_ph108_C4
zmp_ph108_C5
zmp_ph108_D4
zmp_ph97_G6
zmp_ph97_G9
zmp_ph97_H2
zmp_ph97_B10
zmp_ph97_C2
zmp_ph97_C4
zmp_ph97_B8
zmp_ph97_C1
zmp_ph97_D2
zmp_ph97_D5
zmp_ph111_G5
zmp_ph111_G11
zmp_ph111_H1
zmp_ph111_B1a
zmp_ph111_B5
zmp_ph111_B8
zmp_ph111_B6
zmp_ph111_B7
zmp_ph111_B9
zmp_ph111_B10
zmp_ph114_G4
zmp_ph114_G11
zmp_ph114_H1
zmp_ph114_H2
zmp_ph114_A9
zmp_ph114_A11
zmp_ph114_B2
zmp_ph114_B4
zmp_ph114_D8
zmp_ph114_E2

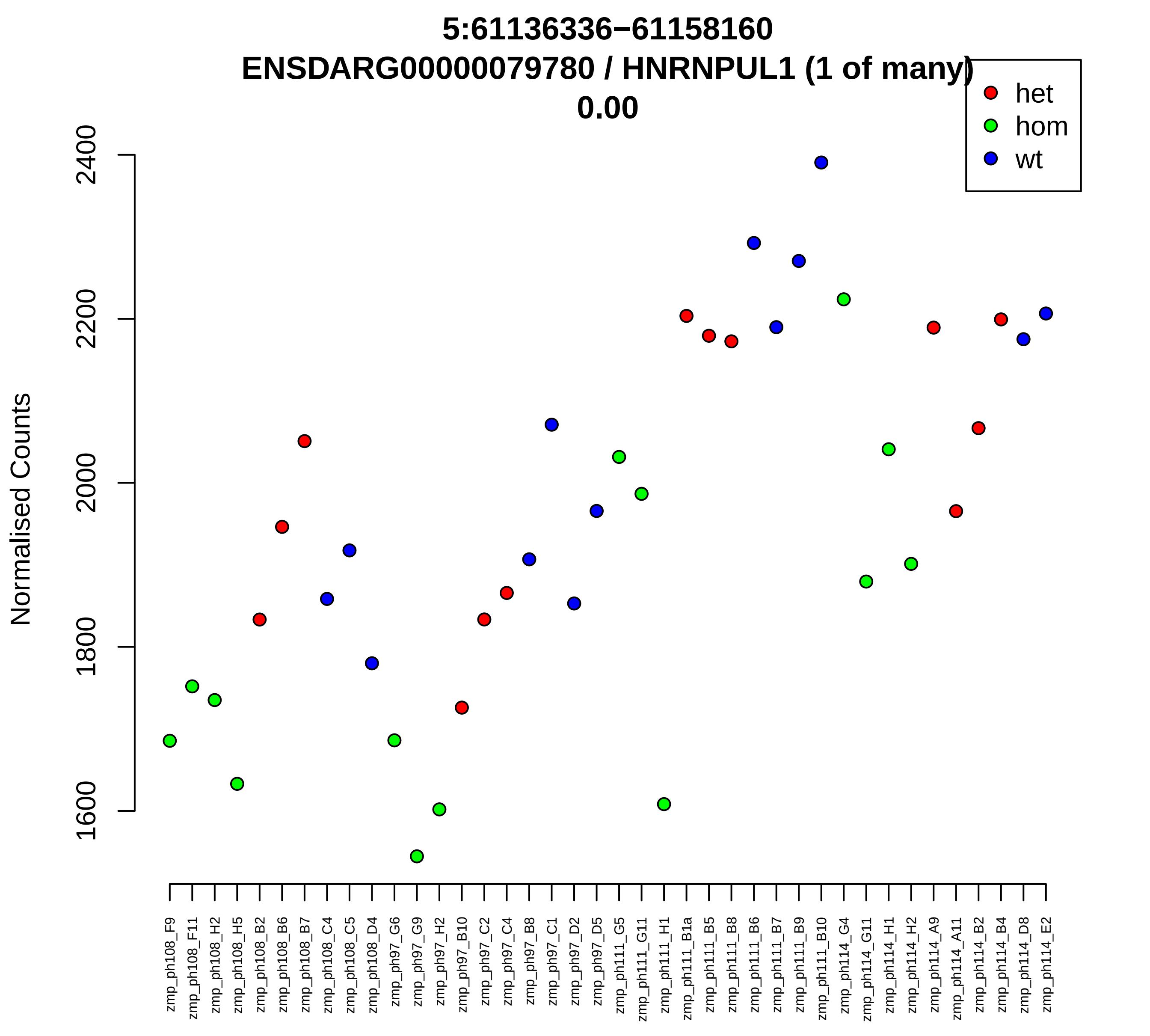
5:61136336−61158160
ENSDARG00000079780 / HNRNPUL1 (1 of many)
0.00
het
hom
wt
Normalised Counts
1600
1800
2000
2200
2400
zmp_ph108_F9
zmp_ph108_F11
zmp_ph108_H2
zmp_ph108_H5
zmp_ph108_B2
zmp_ph108_B6
zmp_ph108_B7
zmp_ph108_C4
zmp_ph108_C5
zmp_ph108_D4
zmp_ph97_G6
zmp_ph97_G9
zmp_ph97_H2
zmp_ph97_B10
zmp_ph97_C2
zmp_ph97_C4
zmp_ph97_B8
zmp_ph97_C1
zmp_ph97_D2
zmp_ph97_D5
zmp_ph111_G5
zmp_ph111_G11
zmp_ph111_H1
zmp_ph111_B1a
zmp_ph111_B5
zmp_ph111_B8
zmp_ph111_B6
zmp_ph111_B7
zmp_ph111_B9
zmp_ph111_B10
zmp_ph114_G4
zmp_ph114_G11
zmp_ph114_H1
zmp_ph114_H2
zmp_ph114_A9
zmp_ph114_A11
zmp_ph114_B2
zmp_ph114_B4
zmp_ph114_D8
zmp_ph114_E2

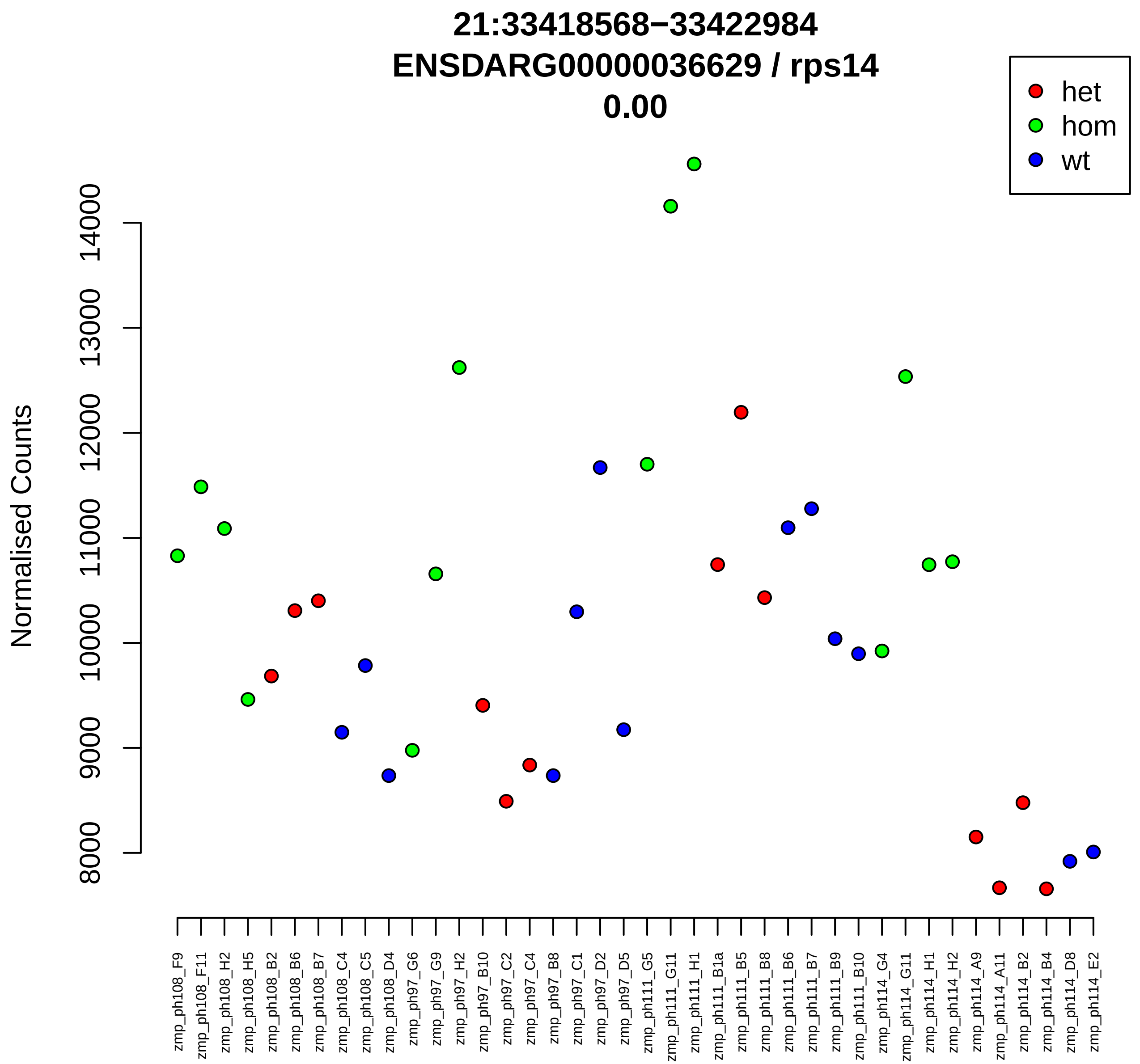

21:33418568−33422984
ENSDARG00000036629 / rps14
0.00
het
hom
wt
Normalised Counts
8000
9000
10000
11000
12000
13000
14000
zmp_ph108_F9
zmp_ph108_F11
zmp_ph108_H2
zmp_ph108_H5
zmp_ph108_B2
zmp_ph108_B6
zmp_ph108_B7
zmp_ph108_C4
zmp_ph108_C5
zmp_ph108_D4
zmp_ph97_G6
zmp_ph97_G9
zmp_ph97_H2
zmp_ph97_B10
zmp_ph97_C2
zmp_ph97_C4
zmp_ph97_B8
zmp_ph97_C1
zmp_ph97_D2
zmp_ph97_D5
zmp_ph111_G5
zmp_ph111_G11
zmp_ph111_H1
zmp_ph111_B1a
zmp_ph111_B5
zmp_ph111_B8
zmp_ph111_B6
zmp_ph111_B7
zmp_ph111_B9
zmp_ph111_B10
zmp_ph114_G4
zmp_ph114_G11
zmp_ph114_H1
zmp_ph114_H2
zmp_ph114_A9
zmp_ph114_A11
zmp_ph114_B2
zmp_ph114_B4
zmp_ph114_D8
zmp_ph114_E2

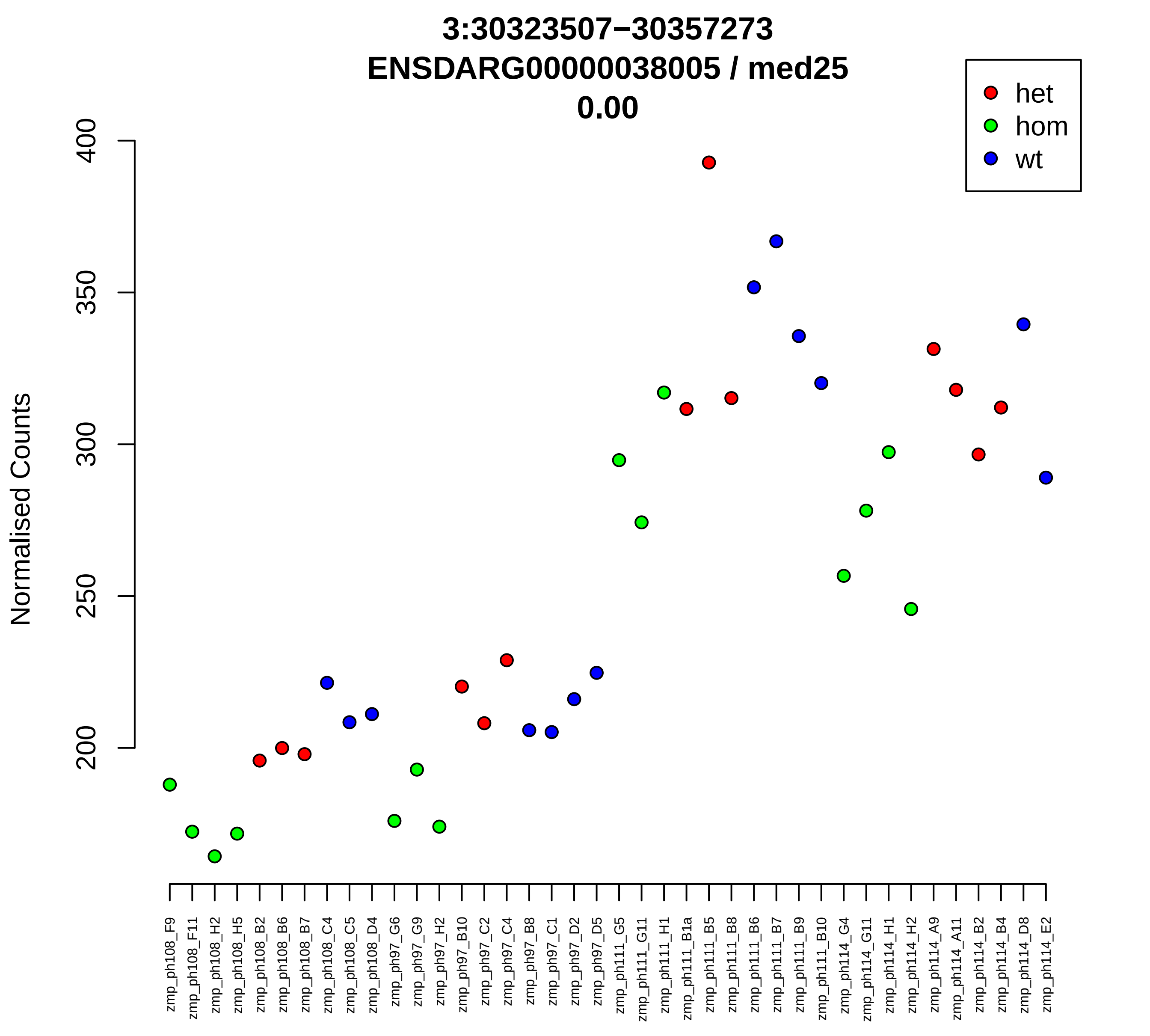

3:30323507−30357273
ENSDARG00000038005 / med25
0.00
het
hom
wt
Normalised Counts
400
350
300
250
200
zmp_ph108_F9
zmp_ph108_F11
zmp_ph108_H2
zmp_ph108_H5
zmp_ph108_B2
zmp_ph108_B6
zmp_ph108_B7
zmp_ph108_C4
zmp_ph108_C5
zmp_ph108_D4
zmp_ph97_G6
zmp_ph97_G9
zmp_ph97_H2
zmp_ph97_B10
zmp_ph97_C2
zmp_ph97_C4
zmp_ph97_B8
zmp_ph97_C1
zmp_ph97_D2
zmp_ph97_D5
zmp_ph111_G5
zmp_ph111_G11
zmp_ph111_H1
zmp_ph111_B1a
zmp_ph111_B5
zmp_ph111_B8
zmp_ph111_B6
zmp_ph111_B7
zmp_ph111_B9
zmp_ph111_B10
zmp_ph114_G4
zmp_ph114_G11
zmp_ph114_H1
zmp_ph114_H2
zmp_ph114_A9
zmp_ph114_A11
zmp_ph114_B2
zmp_ph114_B4
zmp_ph114_D8
zmp_ph114_E2

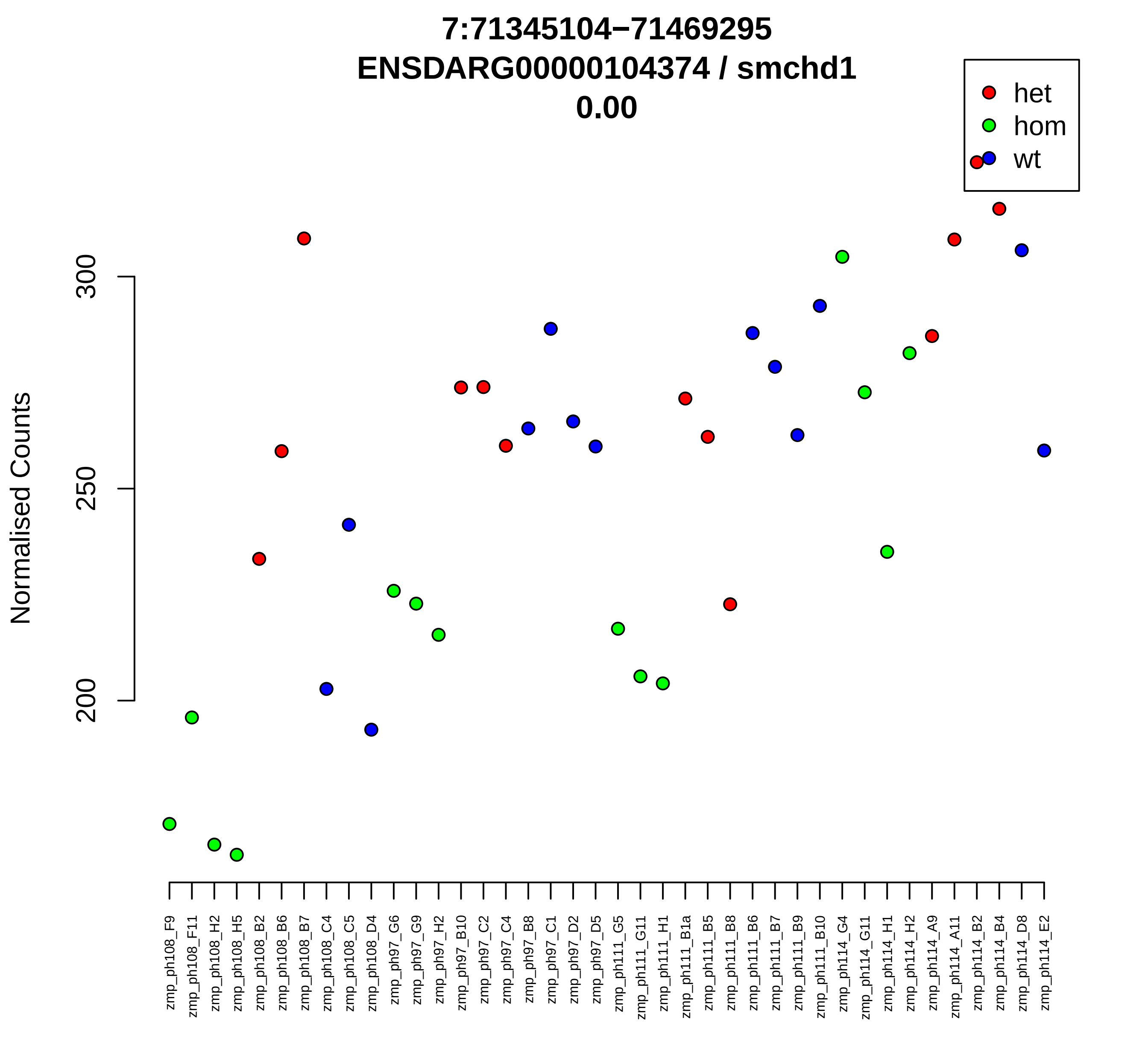

7:71345104−71469295
ENSDARG00000104374 / smchd1
0.00
het
hom
wt
Normalised Counts
200
250
300
zmp_ph108_F9
zmp_ph108_F11
zmp_ph108_H2
zmp_ph108_H5
zmp_ph108_B2
zmp_ph108_B6
zmp_ph108_B7
zmp_ph108_C4
zmp_ph108_C5
zmp_ph108_D4
zmp_ph97_G6
zmp_ph97_G9
zmp_ph97_H2
zmp_ph97_B10
zmp_ph97_C2
zmp_ph97_C4
zmp_ph97_B8
zmp_ph97_C1
zmp_ph97_D2
zmp_ph97_D5
zmp_ph111_G5
zmp_ph111_G11
zmp_ph111_H1
zmp_ph111_B1a
zmp_ph111_B5
zmp_ph111_B8
zmp_ph111_B6
zmp_ph111_B7
zmp_ph111_B9
zmp_ph111_B10
zmp_ph114_G4
zmp_ph114_G11
zmp_ph114_H1
zmp_ph114_H2
zmp_ph114_A9
zmp_ph114_A11
zmp_ph114_B2
zmp_ph114_B4
zmp_ph114_D8
zmp_ph114_E2

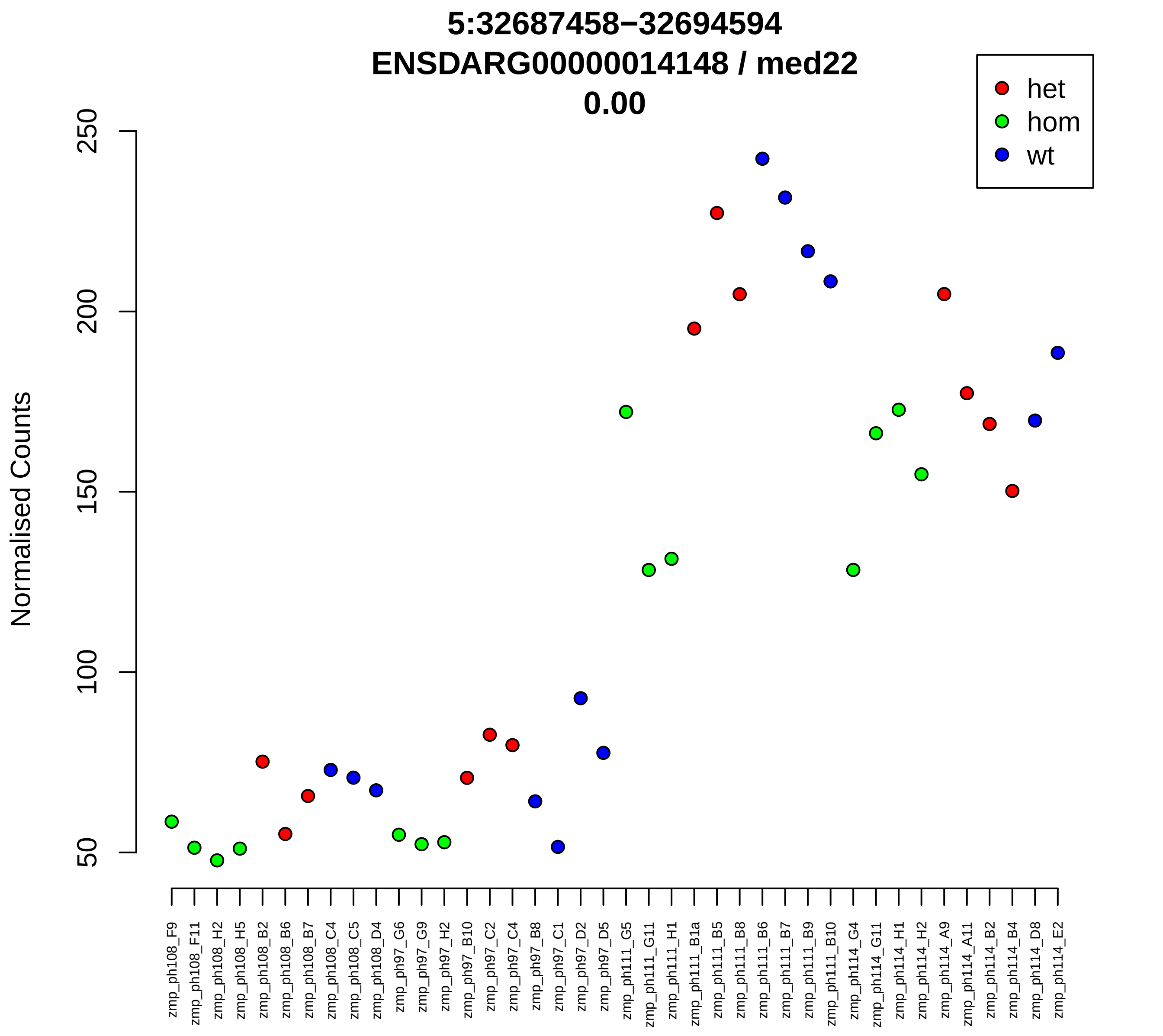

5:32687458−32694594
ENSDARG00000014148 / med22
0.00
het
hom
wt
Normalised Counts
50
100
150
200
250
zmp_ph108_F9
zmp_ph108_F11
zmp_ph108_H2
zmp_ph108_H5
zmp_ph108_B2
zmp_ph108_B6
zmp_ph108_B7
zmp_ph108_C4
zmp_ph108_C5
zmp_ph108_D4
zmp_ph97_G6
zmp_ph97_G9
zmp_ph97_H2
zmp_ph97_B10
zmp_ph97_C2
zmp_ph97_C4
zmp_ph97_B8
zmp_ph97_C1
zmp_ph97_D2
zmp_ph97_D5
zmp_ph111_G5
zmp_ph111_G11
zmp_ph111_H1
zmp_ph111_B1a
zmp_ph111_B5
zmp_ph111_B8
zmp_ph111_B6
zmp_ph111_B7
zmp_ph111_B9
zmp_ph111_B10
zmp_ph114_G4
zmp_ph114_G11
zmp_ph114_H1
zmp_ph114_H2
zmp_ph114_A9
zmp_ph114_A11
zmp_ph114_B2
zmp_ph114_B4
zmp_ph114_D8
zmp_ph114_E2

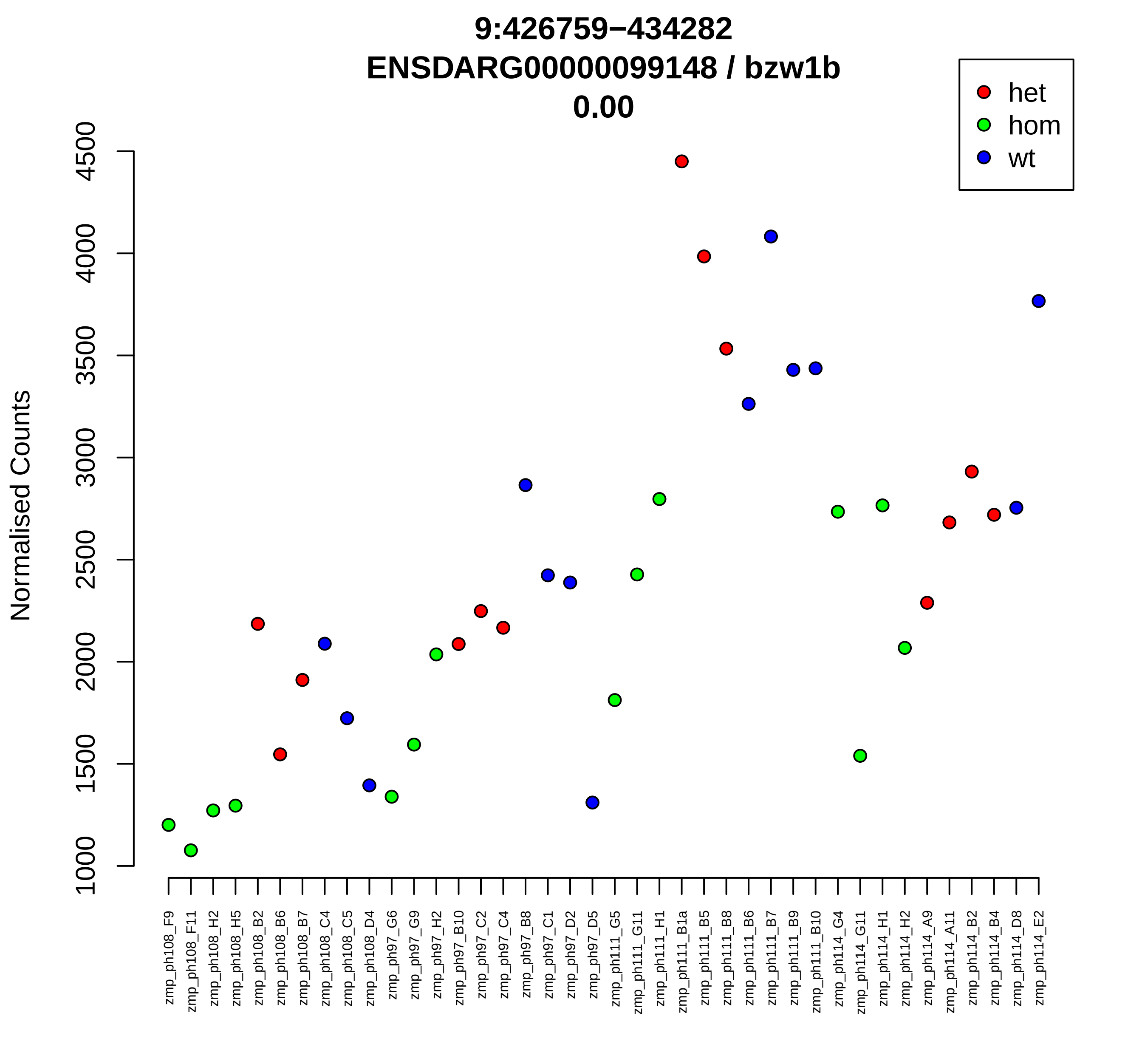

9:426759−434282
ENSDARG00000099148 / bzw1b
0.00
Normalised Counts
het
hom
wt
1000
1500
2000
2500
3000
3500
4000
4500
zmp_ph108_F9
zmp_ph108_F11
zmp_ph108_H2
zmp_ph108_H5
zmp_ph108_B2
zmp_ph108_B6
zmp_ph108_B7
zmp_ph108_C4
zmp_ph108_C5
zmp_ph108_D4
zmp_ph97_G6
zmp_ph97_G9
zmp_ph97_H2
zmp_ph97_B10
zmp_ph97_C2
zmp_ph97_C4
zmp_ph97_B8
zmp_ph97_C1
zmp_ph97_D2
zmp_ph97_D5
zmp_ph111_G5
zmp_ph111_G11
zmp_ph111_H1
zmp_ph111_B1a
zmp_ph111_B5
zmp_ph111_B8
zmp_ph111_B6
zmp_ph111_B7
zmp_ph111_B9
zmp_ph111_B10
zmp_ph114_G4
zmp_ph114_G11
zmp_ph114_H1
zmp_ph114_H2
zmp_ph114_A9
zmp_ph114_A11
zmp_ph114_B2
zmp_ph114_B4
zmp_ph114_D8
zmp_ph114_E2

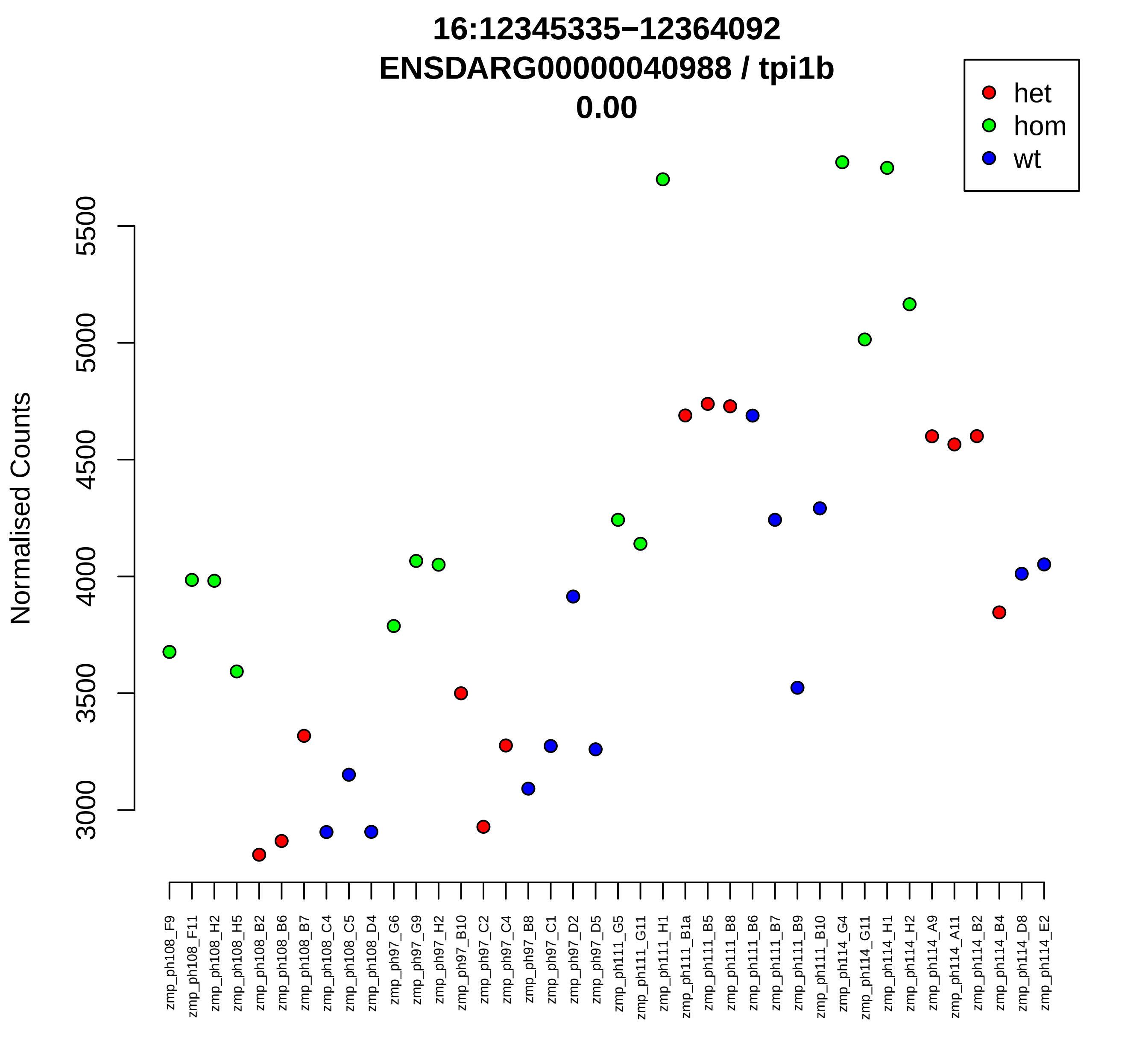

16:12345335−12364092
ENSDARG00000040988 / tpi1b
0.00
het
hom
wt
Normalised Counts
3000
3500
4000
4500
5000
5500
zmp_ph108_F9
zmp_ph108_F11
zmp_ph108_H2
zmp_ph108_H5
zmp_ph108_B2
zmp_ph108_B6
zmp_ph108_B7
zmp_ph108_C4
zmp_ph108_C5
zmp_ph108_D4
zmp_ph97_G6
zmp_ph97_G9
zmp_ph97_H2
zmp_ph97_B10
zmp_ph97_C2
zmp_ph97_C4
zmp_ph97_B8
zmp_ph97_C1
zmp_ph97_D2
zmp_ph97_D5
zmp_ph111_G5
zmp_ph111_G11
zmp_ph111_H1
zmp_ph111_B1a
zmp_ph111_B5
zmp_ph111_B8
zmp_ph111_B6
zmp_ph111_B7
zmp_ph111_B9
zmp_ph111_B10
zmp_ph114_G4
zmp_ph114_G11
zmp_ph114_H1
zmp_ph114_H2
zmp_ph114_A9
zmp_ph114_A11
zmp_ph114_B2
zmp_ph114_B4
zmp_ph114_D8
zmp_ph114_E2

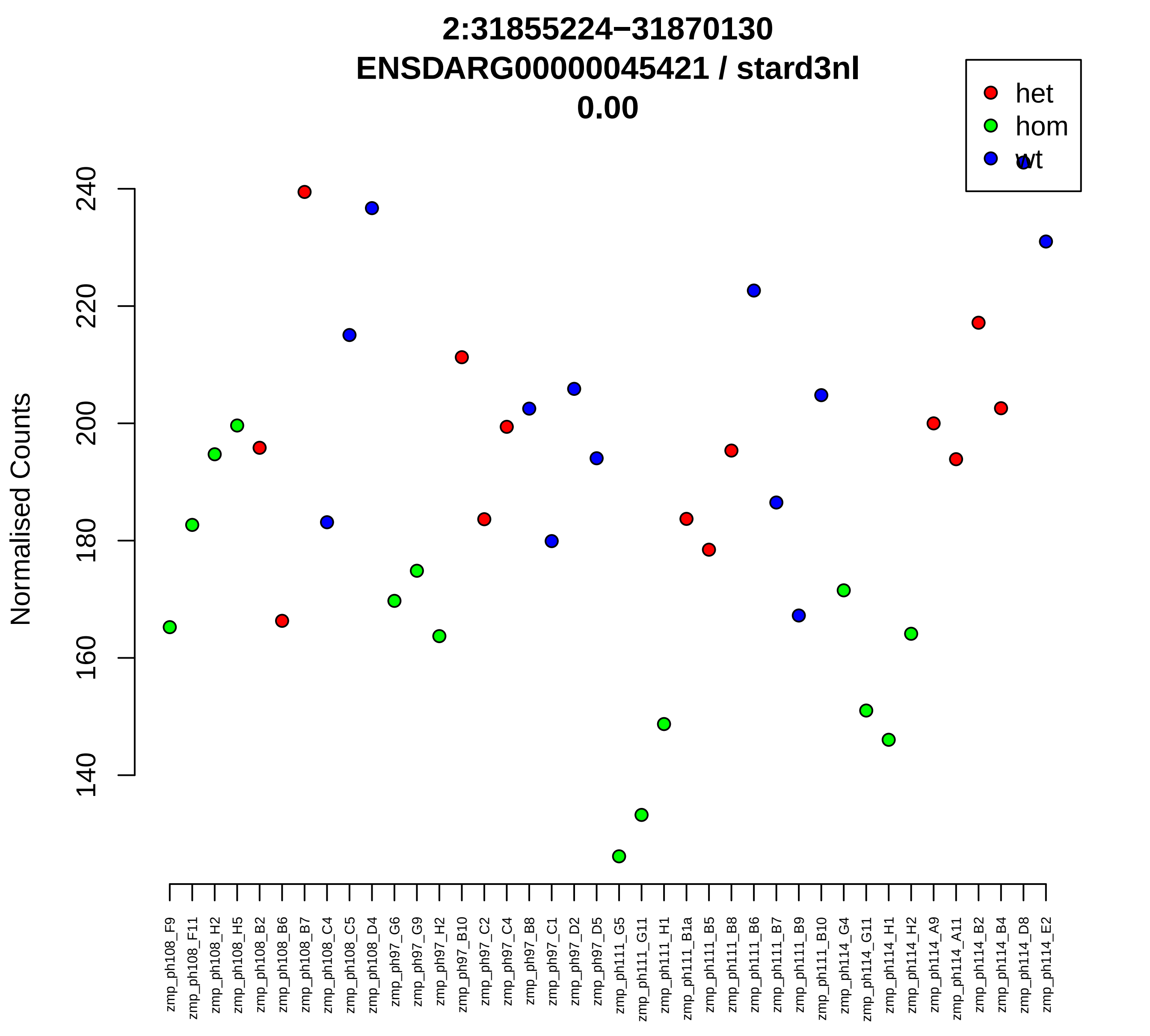
2:31855224−31870130
ENSDARG00000045421 / stard3nl
0.00
het
hom
wt
Normalised Counts
140
160
180
200
220
240
zmp_ph108_F9
zmp_ph108_F11
zmp_ph108_H2
zmp_ph108_H5
zmp_ph108_B2
zmp_ph108_B6
zmp_ph108_B7
zmp_ph108_C4
zmp_ph108_C5
zmp_ph108_D4
zmp_ph97_G6
zmp_ph97_G9
zmp_ph97_H2
zmp_ph97_B10
zmp_ph97_C2
zmp_ph97_C4
zmp_ph97_B8
zmp_ph97_C1
zmp_ph97_D2
zmp_ph97_D5
zmp_ph111_G5
zmp_ph111_G11
zmp_ph111_H1
zmp_ph111_B1a
zmp_ph111_B5
zmp_ph111_B8
zmp_ph111_B6
zmp_ph111_B7
zmp_ph111_B9
zmp_ph111_B10
zmp_ph114_G4
zmp_ph114_G11
zmp_ph114_H1
zmp_ph114_H2
zmp_ph114_A9
zmp_ph114_A11
zmp_ph114_B2
zmp_ph114_B4
zmp_ph114_D8
zmp_ph114_E2

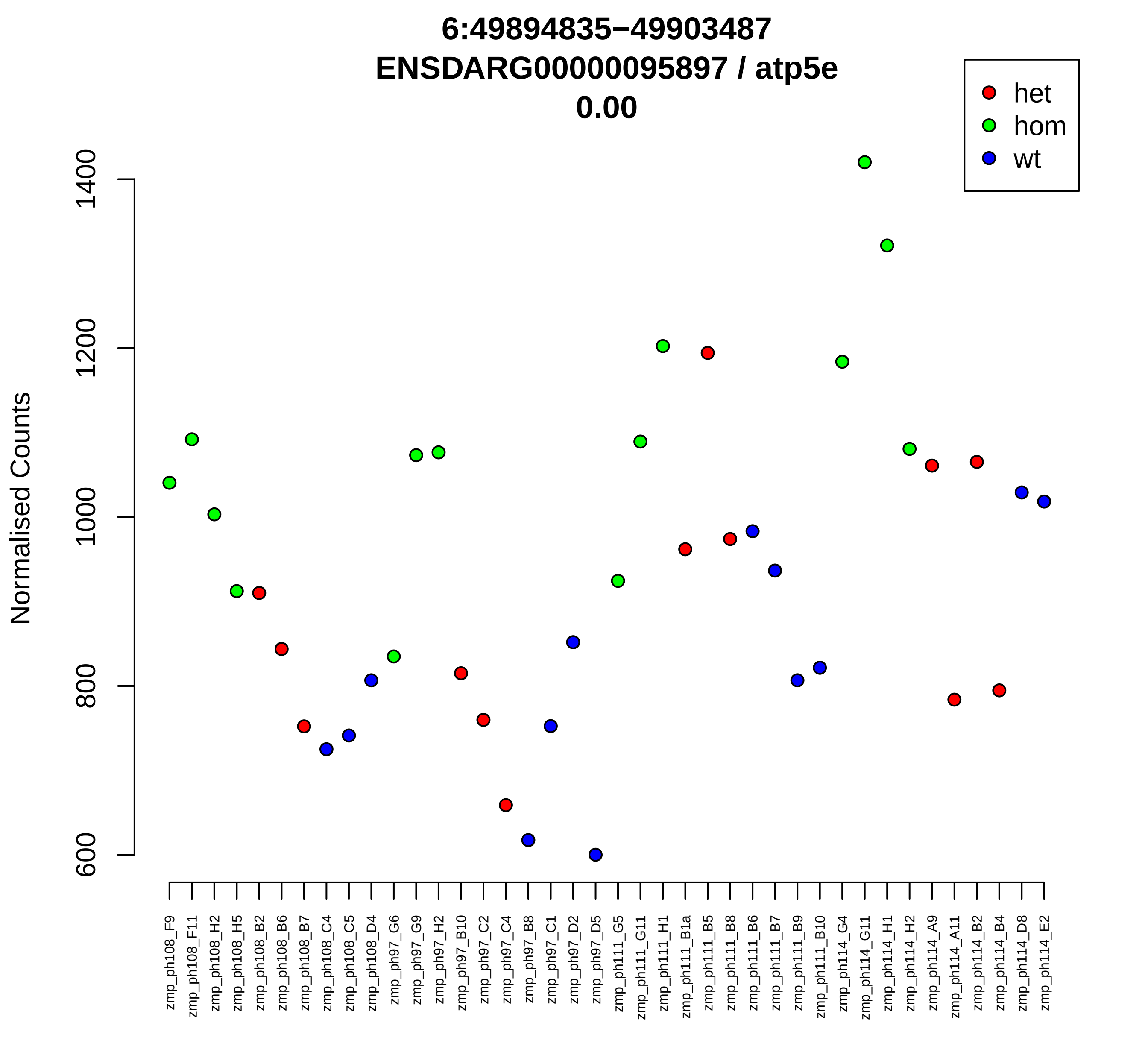

6:49894835−49903487
ENSDARG00000095897 / atp5e
0.00
het
hom
wt
Normalised Counts
1400
1200
1000
800
600
zmp_ph108_F9
zmp_ph108_F11
zmp_ph108_H2
zmp_ph108_H5
zmp_ph108_B2
zmp_ph108_B6
zmp_ph108_B7
zmp_ph108_C4
zmp_ph108_C5
zmp_ph108_D4
zmp_ph97_G6
zmp_ph97_G9
zmp_ph97_H2
zmp_ph97_B10
zmp_ph97_C2
zmp_ph97_C4
zmp_ph97_B8
zmp_ph97_C1
zmp_ph97_D2
zmp_ph97_D5
zmp_ph111_G5
zmp_ph111_G11
zmp_ph111_H1
zmp_ph111_B1a
zmp_ph111_B5
zmp_ph111_B8
zmp_ph111_B6
zmp_ph111_B7
zmp_ph111_B9
zmp_ph111_B10
zmp_ph114_G4
zmp_ph114_G11
zmp_ph114_H1
zmp_ph114_H2
zmp_ph114_A9
zmp_ph114_A11
zmp_ph114_B2
zmp_ph114_B4
zmp_ph114_D8
zmp_ph114_E2

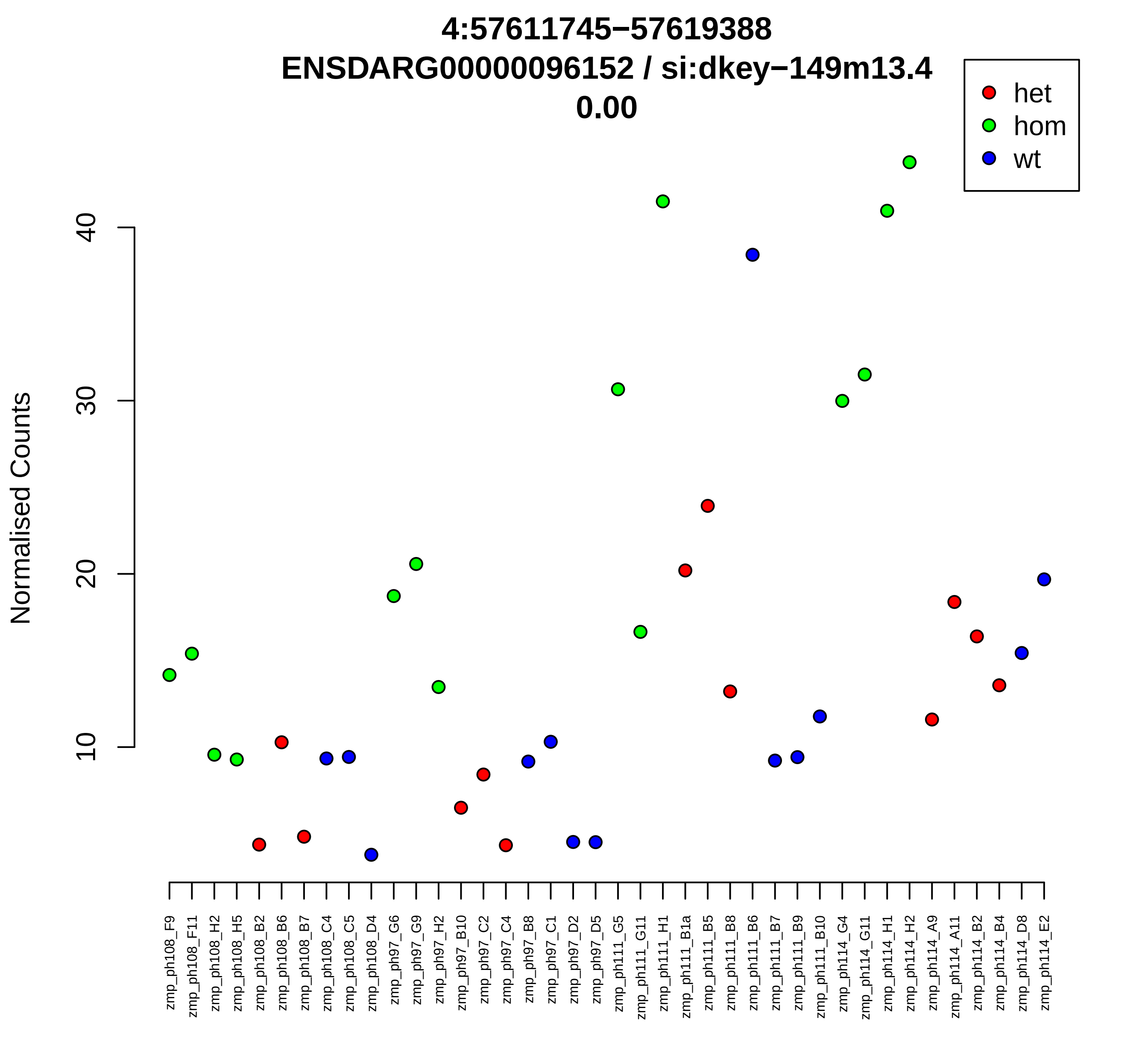
4:57611745−57619388
ENSDARG00000096152 / si:dkey−149m13.4
0.00
het
hom
wt
Normalised Counts
10
20
30
40
zmp_ph108_F9
zmp_ph108_F11
zmp_ph108_H2
zmp_ph108_H5
zmp_ph108_B2
zmp_ph108_B6
zmp_ph108_B7
zmp_ph108_C4
zmp_ph108_C5
zmp_ph108_D4
zmp_ph97_G6
zmp_ph97_G9
zmp_ph97_H2
zmp_ph97_B10
zmp_ph97_C2
zmp_ph97_C4
zmp_ph97_B8
zmp_ph97_C1
zmp_ph97_D2
zmp_ph97_D5
zmp_ph111_G5
zmp_ph111_G11
zmp_ph111_H1
zmp_ph111_B1a
zmp_ph111_B5
zmp_ph111_B8
zmp_ph111_B6
zmp_ph111_B7
zmp_ph111_B9
zmp_ph111_B10
zmp_ph114_G4
zmp_ph114_G11
zmp_ph114_H1
zmp_ph114_H2
zmp_ph114_A9
zmp_ph114_A11
zmp_ph114_B2
zmp_ph114_B4
zmp_ph114_D8
zmp_ph114_E2

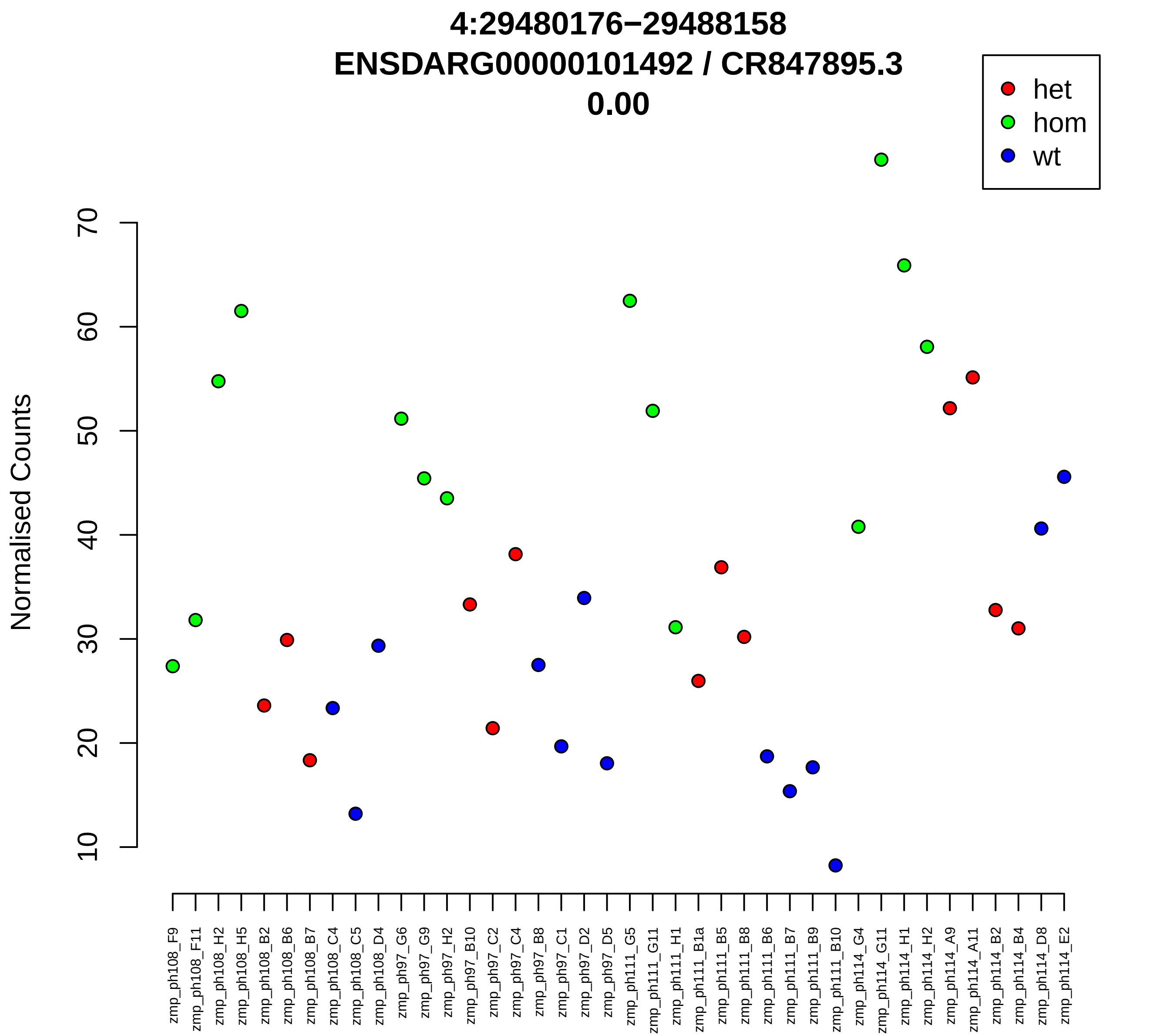
4:29480176−29488158
ENSDARG00000101492 / CR847895.3
0.00
het
hom
wt
Normalised Counts
10
20
30
40
50
60
70
zmp_ph108_F9
zmp_ph108_F11
zmp_ph108_H2
zmp_ph108_H5
zmp_ph108_B2
zmp_ph108_B6
zmp_ph108_B7
zmp_ph108_C4
zmp_ph108_C5
zmp_ph108_D4
zmp_ph97_G6
zmp_ph97_G9
zmp_ph97_H2
zmp_ph97_B10
zmp_ph97_C2
zmp_ph97_C4
zmp_ph97_B8
zmp_ph97_C1
zmp_ph97_D2
zmp_ph97_D5
zmp_ph111_G5
zmp_ph111_G11
zmp_ph111_H1
zmp_ph111_B1a
zmp_ph111_B5
zmp_ph111_B8
zmp_ph111_B6
zmp_ph111_B7
zmp_ph111_B9
zmp_ph111_B10
zmp_ph114_G4
zmp_ph114_G11
zmp_ph114_H1
zmp_ph114_H2
zmp_ph114_A9
zmp_ph114_A11
zmp_ph114_B2
zmp_ph114_B4
zmp_ph114_D8
zmp_ph114_E2

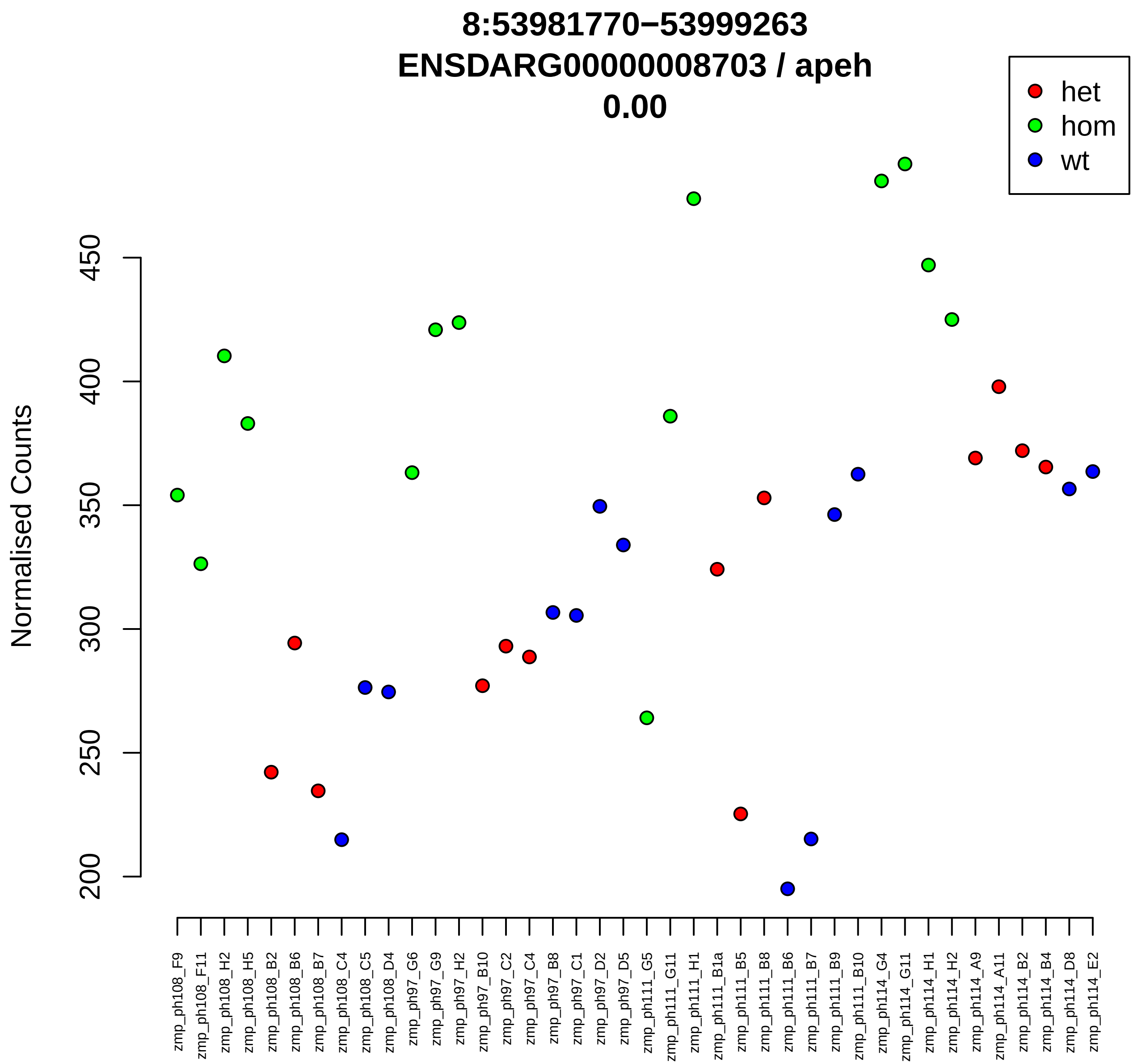
8:53981770−53999263
ENSDARG00000008703 / apeh
0.00
het
hom
wt
Normalised Counts
200
250
300
350
400
450
zmp_ph108_F9
zmp_ph108_F11
zmp_ph108_H2
zmp_ph108_H5
zmp_ph108_B2
zmp_ph108_B6
zmp_ph108_B7
zmp_ph108_C4
zmp_ph108_C5
zmp_ph108_D4
zmp_ph97_G6
zmp_ph97_G9
zmp_ph97_H2
zmp_ph97_B10
zmp_ph97_C2
zmp_ph97_C4
zmp_ph97_B8
zmp_ph97_C1
zmp_ph97_D2
zmp_ph97_D5
zmp_ph111_G5
zmp_ph111_G11
zmp_ph111_H1
zmp_ph111_B1a
zmp_ph111_B5
zmp_ph111_B8
zmp_ph111_B6
zmp_ph111_B7
zmp_ph111_B9
zmp_ph111_B10
zmp_ph114_G4
zmp_ph114_G11
zmp_ph114_H1
zmp_ph114_H2
zmp_ph114_A9
zmp_ph114_A11
zmp_ph114_B2
zmp_ph114_B4
zmp_ph114_D8
zmp_ph114_E2

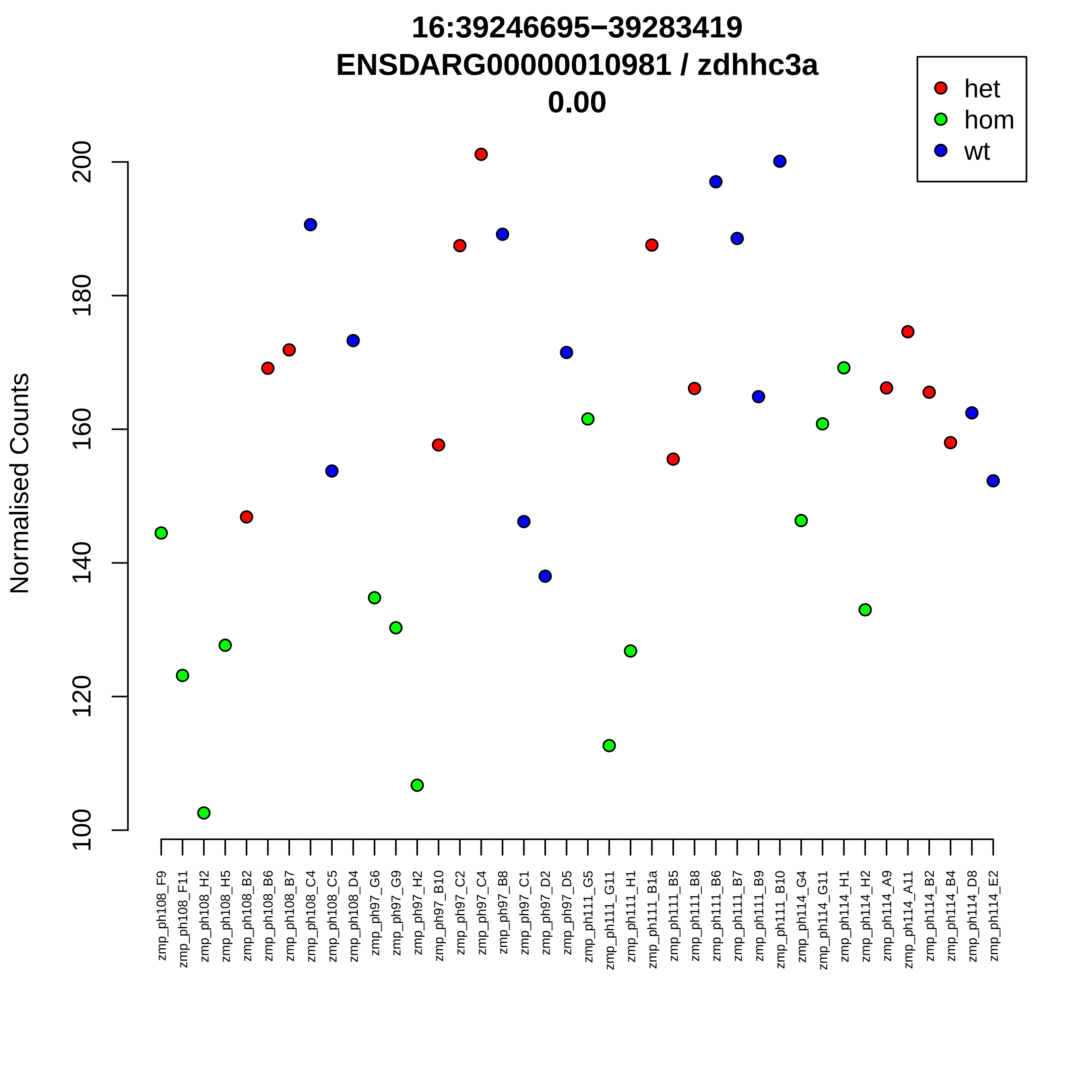
16:39246695−39283419
ENSDARG00000010981 / zdhhc3a
0.00
het
hom
wt
Normalised Counts
100
120
140
160
180
200
zmp_ph108_F9
zmp_ph108_F11
zmp_ph108_H2
zmp_ph108_H5
zmp_ph108_B2
zmp_ph108_B6
zmp_ph108_B7
zmp_ph108_C4
zmp_ph108_C5
zmp_ph108_D4
zmp_ph97_G6
zmp_ph97_G9
zmp_ph97_H2
zmp_ph97_B10
zmp_ph97_C2
zmp_ph97_C4
zmp_ph97_B8
zmp_ph97_C1
zmp_ph97_D2
zmp_ph97_D5
zmp_ph111_G5
zmp_ph111_G11
zmp_ph111_H1
zmp_ph111_B1a
zmp_ph111_B5
zmp_ph111_B8
zmp_ph111_B6
zmp_ph111_B7
zmp_ph111_B9
zmp_ph111_B10
zmp_ph114_G4
zmp_ph114_G11
zmp_ph114_H1
zmp_ph114_H2
zmp_ph114_A9
zmp_ph114_A11
zmp_ph114_B2
zmp_ph114_B4
zmp_ph114_D8
zmp_ph114_E2

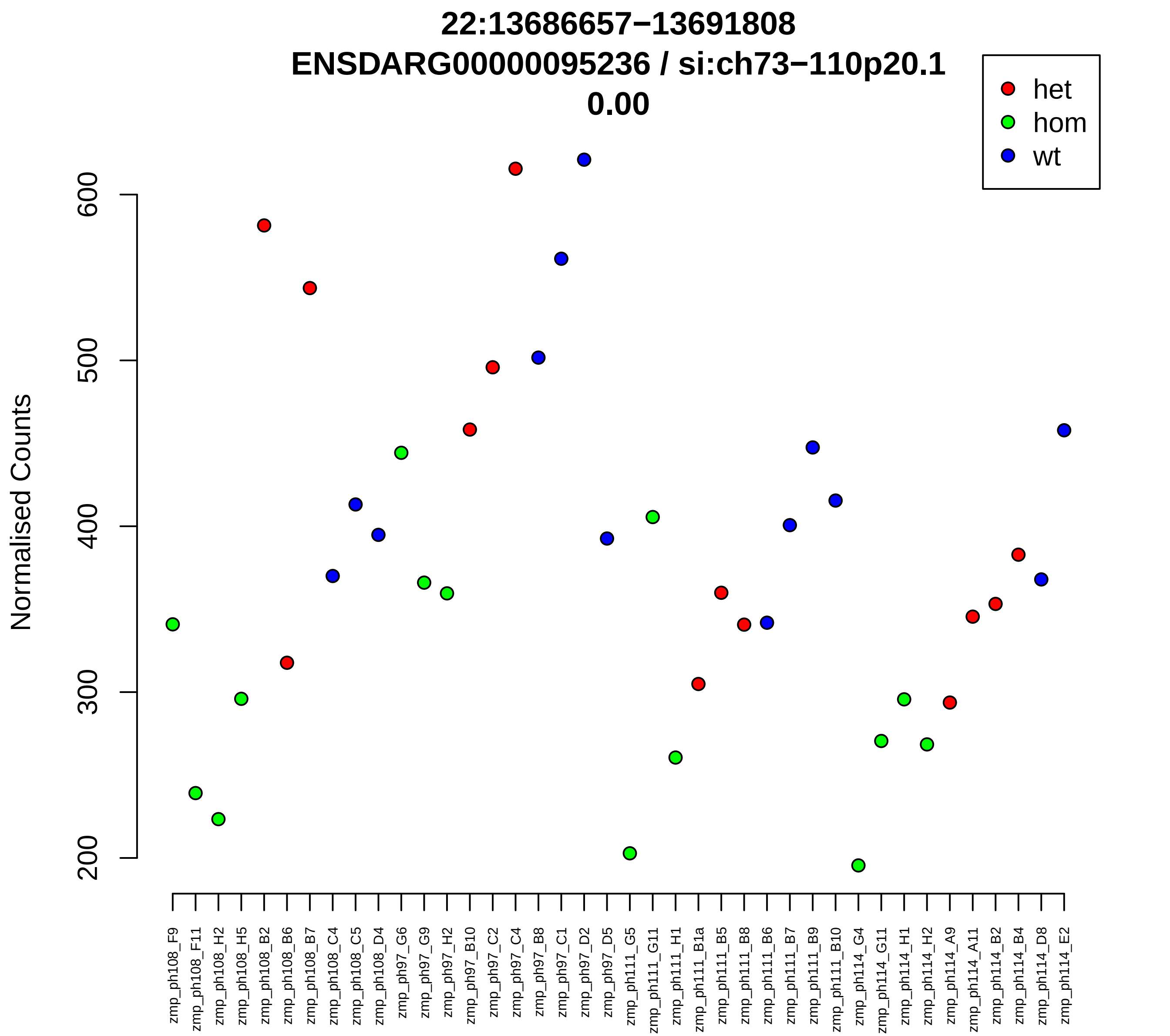
22:13686657−13691808
ENSDARG00000095236 / si:ch73−110p20.1
0.00
het
hom
wt
Normalised Counts
200
300
400
500
600
zmp_ph108_F9
zmp_ph108_F11
zmp_ph108_H2
zmp_ph108_H5
zmp_ph108_B2
zmp_ph108_B6
zmp_ph108_B7
zmp_ph108_C4
zmp_ph108_C5
zmp_ph108_D4
zmp_ph97_G6
zmp_ph97_G9
zmp_ph97_H2
zmp_ph97_B10
zmp_ph97_C2
zmp_ph97_C4
zmp_ph97_B8
zmp_ph97_C1
zmp_ph97_D2
zmp_ph97_D5
zmp_ph111_G5
zmp_ph111_G11
zmp_ph111_H1
zmp_ph111_B1a
zmp_ph111_B5
zmp_ph111_B8
zmp_ph111_B6
zmp_ph111_B7
zmp_ph111_B9
zmp_ph111_B10
zmp_ph114_G4
zmp_ph114_G11
zmp_ph114_H1
zmp_ph114_H2
zmp_ph114_A9
zmp_ph114_A11
zmp_ph114_B2
zmp_ph114_B4
zmp_ph114_D8
zmp_ph114_E2

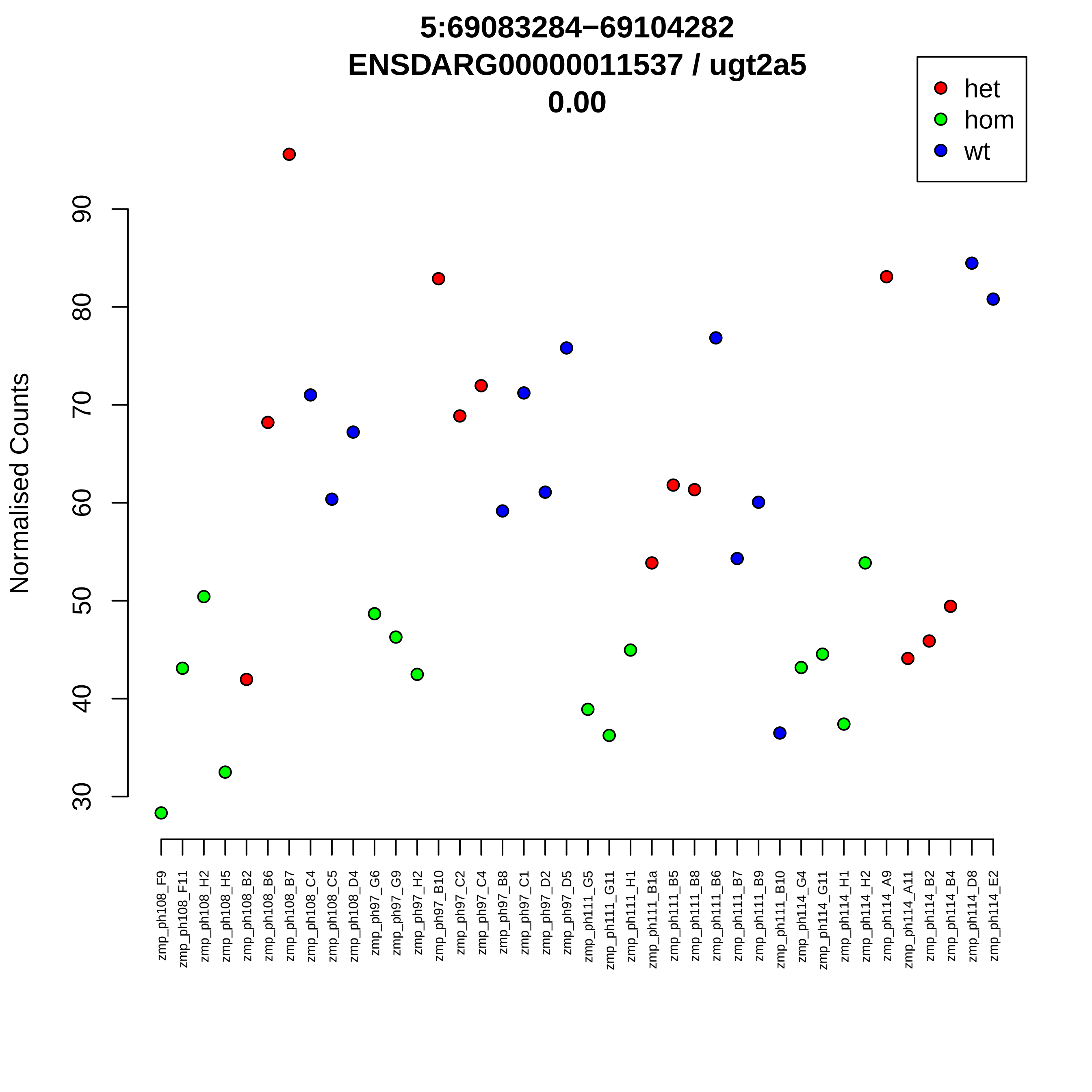

5:69083284−69104282
ENSDARG00000011537 / ugt2a5
0.00
het
hom
wt
Normalised Counts
30
40
50
60
70
80
90
zmp_ph108_F9
zmp_ph108_F11
zmp_ph108_H2
zmp_ph108_H5
zmp_ph108_B2
zmp_ph108_B6
zmp_ph108_B7
zmp_ph108_C4
zmp_ph108_C5
zmp_ph108_D4
zmp_ph97_G6
zmp_ph97_G9
zmp_ph97_H2
zmp_ph97_B10
zmp_ph97_C2
zmp_ph97_C4
zmp_ph97_B8
zmp_ph97_C1
zmp_ph97_D2
zmp_ph97_D5
zmp_ph111_G5
zmp_ph111_G11
zmp_ph111_H1
zmp_ph111_B1a
zmp_ph111_B5
zmp_ph111_B8
zmp_ph111_B6
zmp_ph111_B7
zmp_ph111_B9
zmp_ph111_B10
zmp_ph114_G4
zmp_ph114_G11
zmp_ph114_H1
zmp_ph114_H2
zmp_ph114_A9
zmp_ph114_A11
zmp_ph114_B2
zmp_ph114_B4
zmp_ph114_D8
zmp_ph114_E2

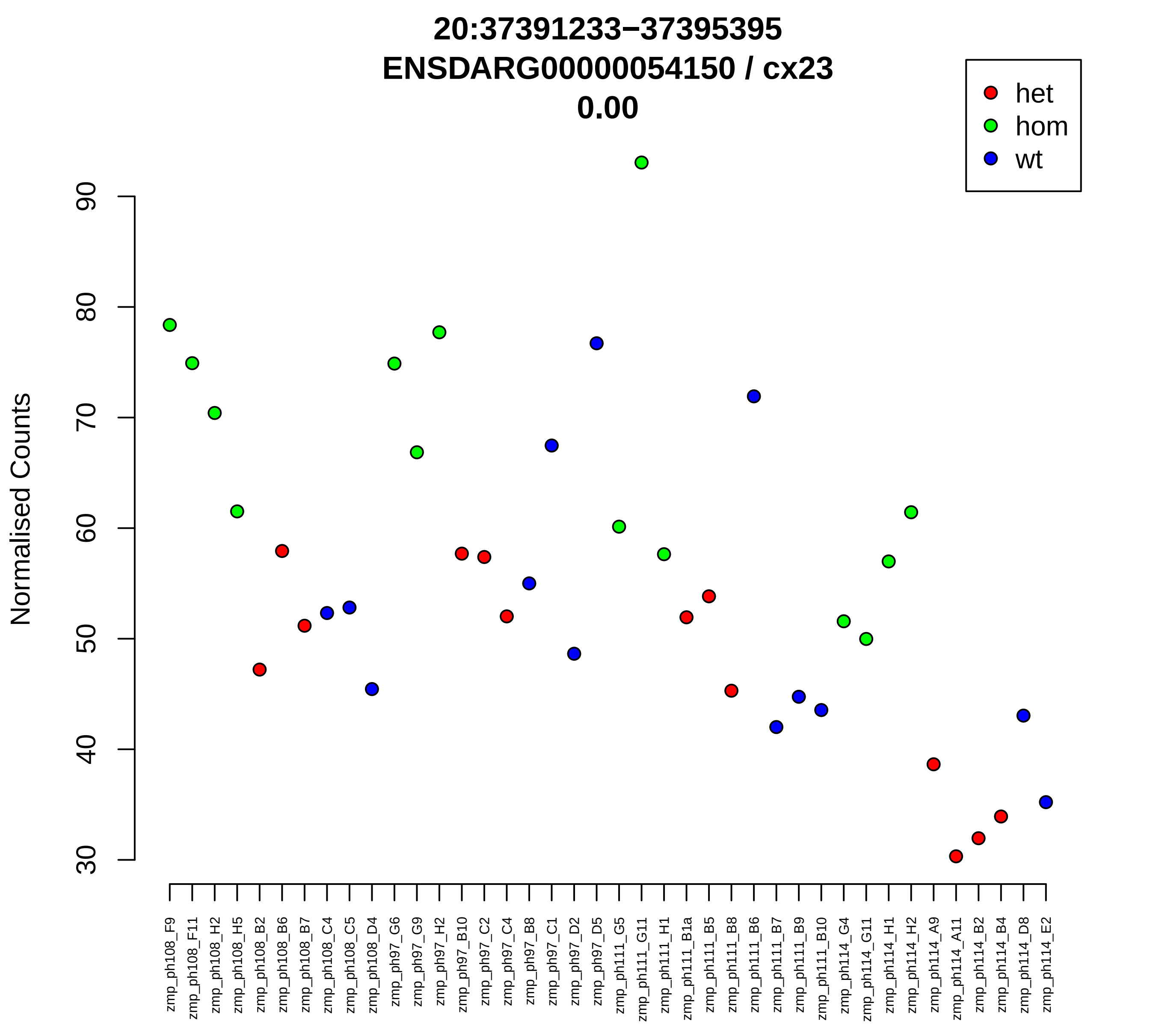
20:37391233−37395395
ENSDARG00000054150 / cx23
0.00
het
hom
wt
Normalised Counts
30
40
50
60
70
80
90
zmp_ph108_F9
zmp_ph108_F11
zmp_ph108_H2
zmp_ph108_H5
zmp_ph108_B2
zmp_ph108_B6
zmp_ph108_B7
zmp_ph108_C4
zmp_ph108_C5
zmp_ph108_D4
zmp_ph97_G6
zmp_ph97_G9
zmp_ph97_H2
zmp_ph97_B10
zmp_ph97_C2
zmp_ph97_C4
zmp_ph97_B8
zmp_ph97_C1
zmp_ph97_D2
zmp_ph97_D5
zmp_ph111_G5
zmp_ph111_G11
zmp_ph111_H1
zmp_ph111_B1a
zmp_ph111_B5
zmp_ph111_B8
zmp_ph111_B6
zmp_ph111_B7
zmp_ph111_B9
zmp_ph111_B10
zmp_ph114_G4
zmp_ph114_G11
zmp_ph114_H1
zmp_ph114_H2
zmp_ph114_A9
zmp_ph114_A11
zmp_ph114_B2
zmp_ph114_B4
zmp_ph114_D8
zmp_ph114_E2

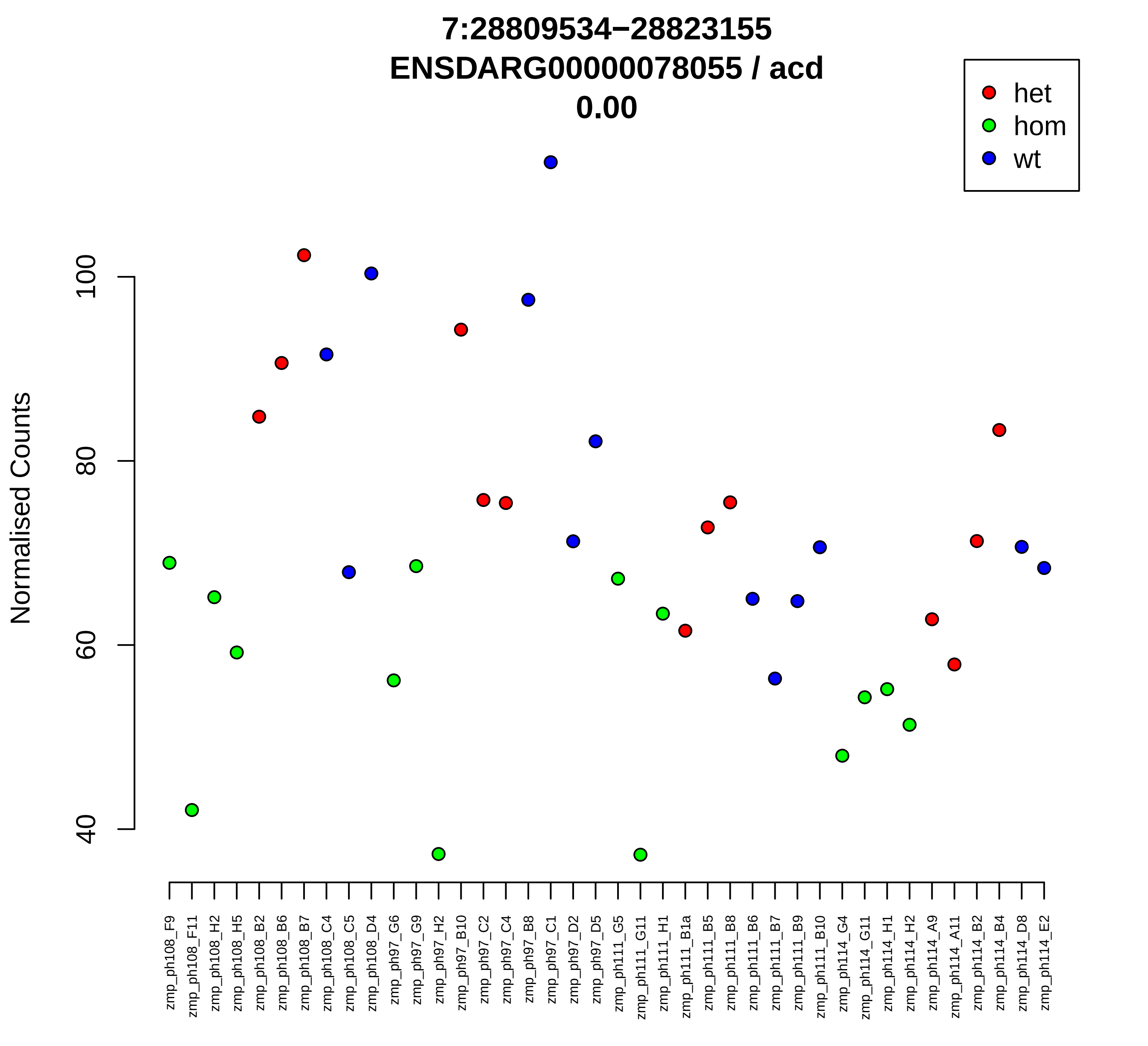
7:28809534−28823155
ENSDARG00000078055 / acd
0.00
het
hom
wt
Normalised Counts
100
80
60
40
zmp_ph108_F9
zmp_ph108_F11
zmp_ph108_H2
zmp_ph108_H5
zmp_ph108_B2
zmp_ph108_B6
zmp_ph108_B7
zmp_ph108_C4
zmp_ph108_C5
zmp_ph108_D4
zmp_ph97_G6
zmp_ph97_G9
zmp_ph97_H2
zmp_ph97_B10
zmp_ph97_C2
zmp_ph97_C4
zmp_ph97_B8
zmp_ph97_C1
zmp_ph97_D2
zmp_ph97_D5
zmp_ph111_G5
zmp_ph111_G11
zmp_ph111_H1
zmp_ph111_B1a
zmp_ph111_B5
zmp_ph111_B8
zmp_ph111_B6
zmp_ph111_B7
zmp_ph111_B9
zmp_ph111_B10
zmp_ph114_G4
zmp_ph114_G11
zmp_ph114_H1
zmp_ph114_H2
zmp_ph114_A9
zmp_ph114_A11
zmp_ph114_B2
zmp_ph114_B4
zmp_ph114_D8
zmp_ph114_E2

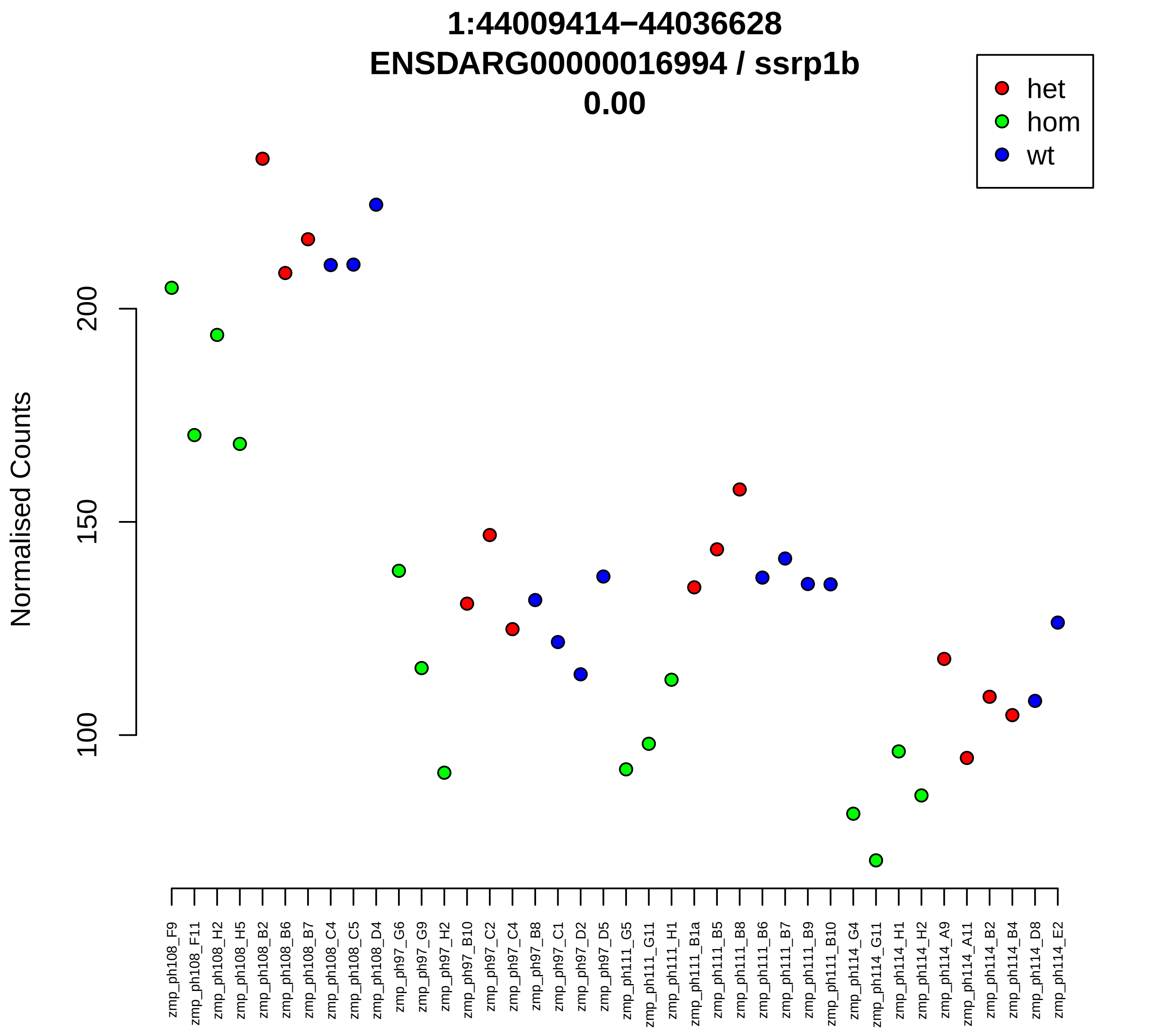
1:44009414−44036628
ENSDARG00000016994 / ssrp1b
0.00
het
hom
wt
Normalised Counts
100
150
200
zmp_ph108_F9
zmp_ph108_F11
zmp_ph108_H2
zmp_ph108_H5
zmp_ph108_B2
zmp_ph108_B6
zmp_ph108_B7
zmp_ph108_C4
zmp_ph108_C5
zmp_ph108_D4
zmp_ph97_G6
zmp_ph97_G9
zmp_ph97_H2
zmp_ph97_B10
zmp_ph97_C2
zmp_ph97_C4
zmp_ph97_B8
zmp_ph97_C1
zmp_ph97_D2
zmp_ph97_D5
zmp_ph111_G5
zmp_ph111_G11
zmp_ph111_H1
zmp_ph111_B1a
zmp_ph111_B5
zmp_ph111_B8
zmp_ph111_B6
zmp_ph111_B7
zmp_ph111_B9
zmp_ph111_B10
zmp_ph114_G4
zmp_ph114_G11
zmp_ph114_H1
zmp_ph114_H2
zmp_ph114_A9
zmp_ph114_A11
zmp_ph114_B2
zmp_ph114_B4
zmp_ph114_D8
zmp_ph114_E2

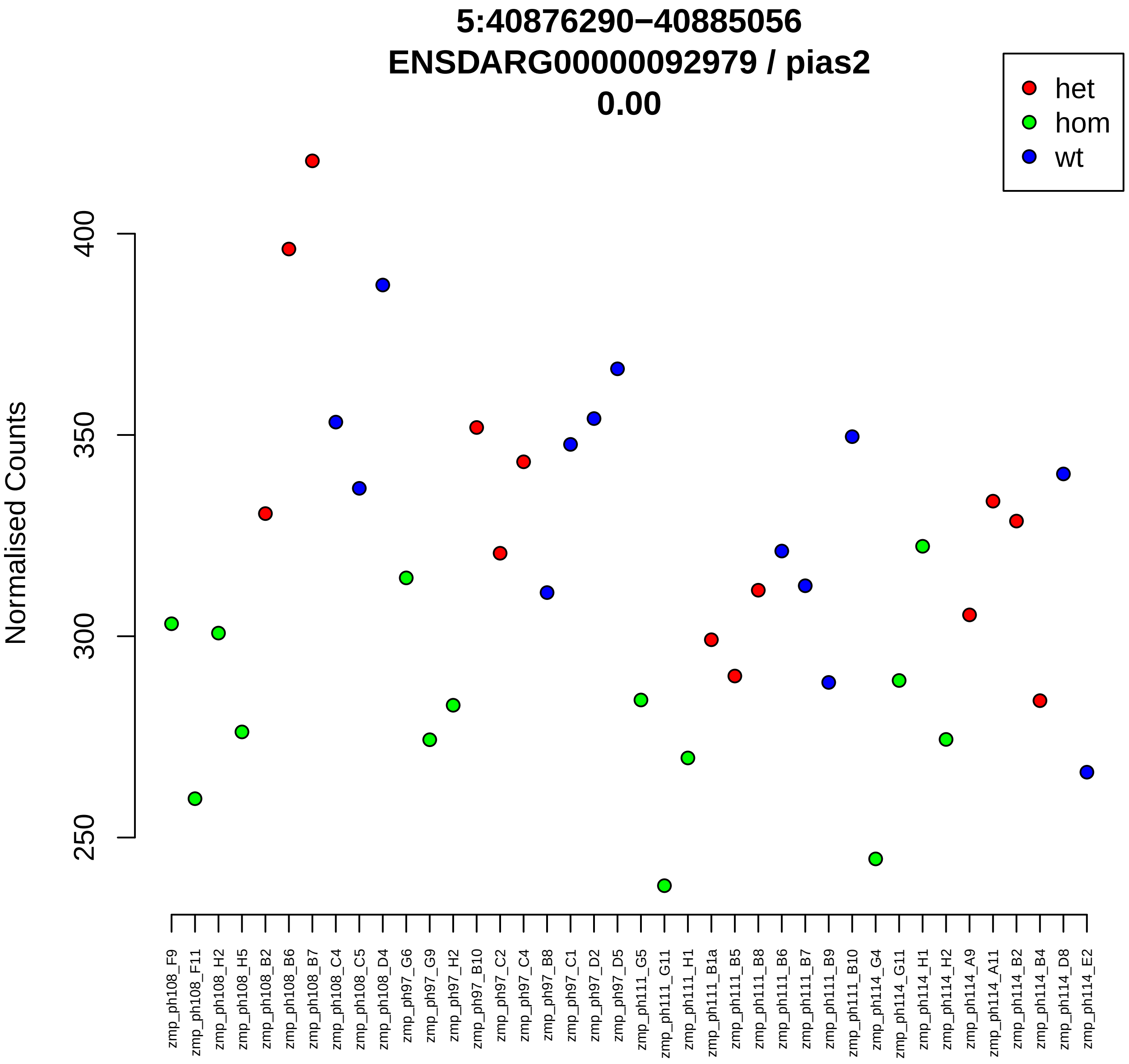

5:40876290−40885056
ENSDARG00000092979 / pias2
0.00
het
hom
wt
Normalised Counts
400
350
300
250
zmp_ph108_F9
zmp_ph108_F11
zmp_ph108_H2
zmp_ph108_H5
zmp_ph108_B2
zmp_ph108_B6
zmp_ph108_B7
zmp_ph108_C4
zmp_ph108_C5
zmp_ph108_D4
zmp_ph97_G6
zmp_ph97_G9
zmp_ph97_H2
zmp_ph97_B10
zmp_ph97_C2
zmp_ph97_C4
zmp_ph97_B8
zmp_ph97_C1
zmp_ph97_D2
zmp_ph97_D5
zmp_ph111_G5
zmp_ph111_G11
zmp_ph111_H1
zmp_ph111_B1a
zmp_ph111_B5
zmp_ph111_B8
zmp_ph111_B6
zmp_ph111_B7
zmp_ph111_B9
zmp_ph111_B10
zmp_ph114_G4
zmp_ph114_G11
zmp_ph114_H1
zmp_ph114_H2
zmp_ph114_A9
zmp_ph114_A11
zmp_ph114_B2
zmp_ph114_B4
zmp_ph114_D8
zmp_ph114_E2

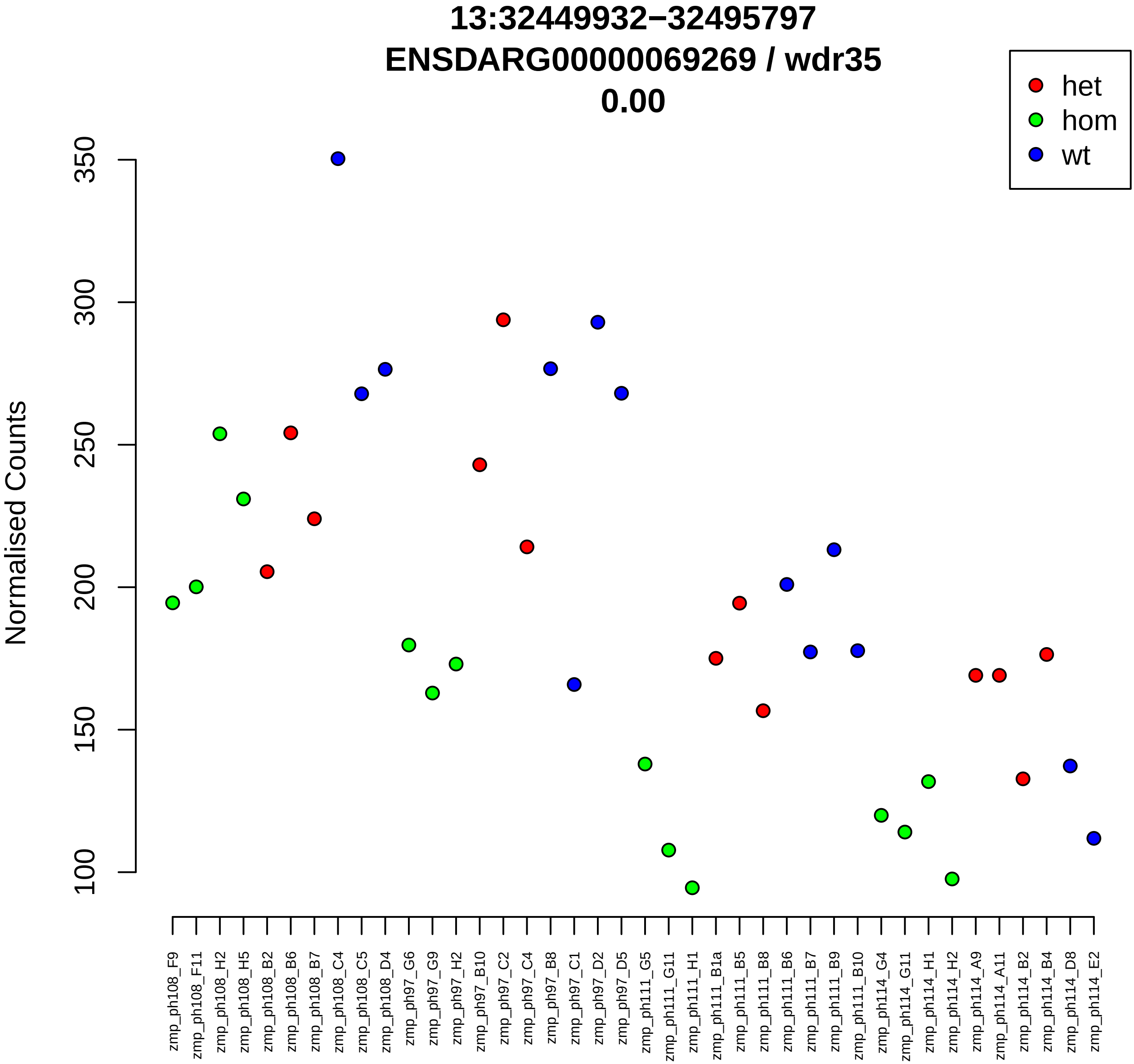
13:32449932−32495797
ENSDARG00000069269 / wdr35
0.00
het
hom
wt
Normalised Counts
100
150
200
250
300
350
zmp_ph108_F9
zmp_ph108_F11
zmp_ph108_H2
zmp_ph108_H5
zmp_ph108_B2
zmp_ph108_B6
zmp_ph108_B7
zmp_ph108_C4
zmp_ph108_C5
zmp_ph108_D4
zmp_ph97_G6
zmp_ph97_G9
zmp_ph97_H2
zmp_ph97_B10
zmp_ph97_C2
zmp_ph97_C4
zmp_ph97_B8
zmp_ph97_C1
zmp_ph97_D2
zmp_ph97_D5
zmp_ph111_G5
zmp_ph111_G11
zmp_ph111_H1
zmp_ph111_B1a
zmp_ph111_B5
zmp_ph111_B8
zmp_ph111_B6
zmp_ph111_B7
zmp_ph111_B9
zmp_ph111_B10
zmp_ph114_G4
zmp_ph114_G11
zmp_ph114_H1
zmp_ph114_H2
zmp_ph114_A9
zmp_ph114_A11
zmp_ph114_B2
zmp_ph114_B4
zmp_ph114_D8
zmp_ph114_E2

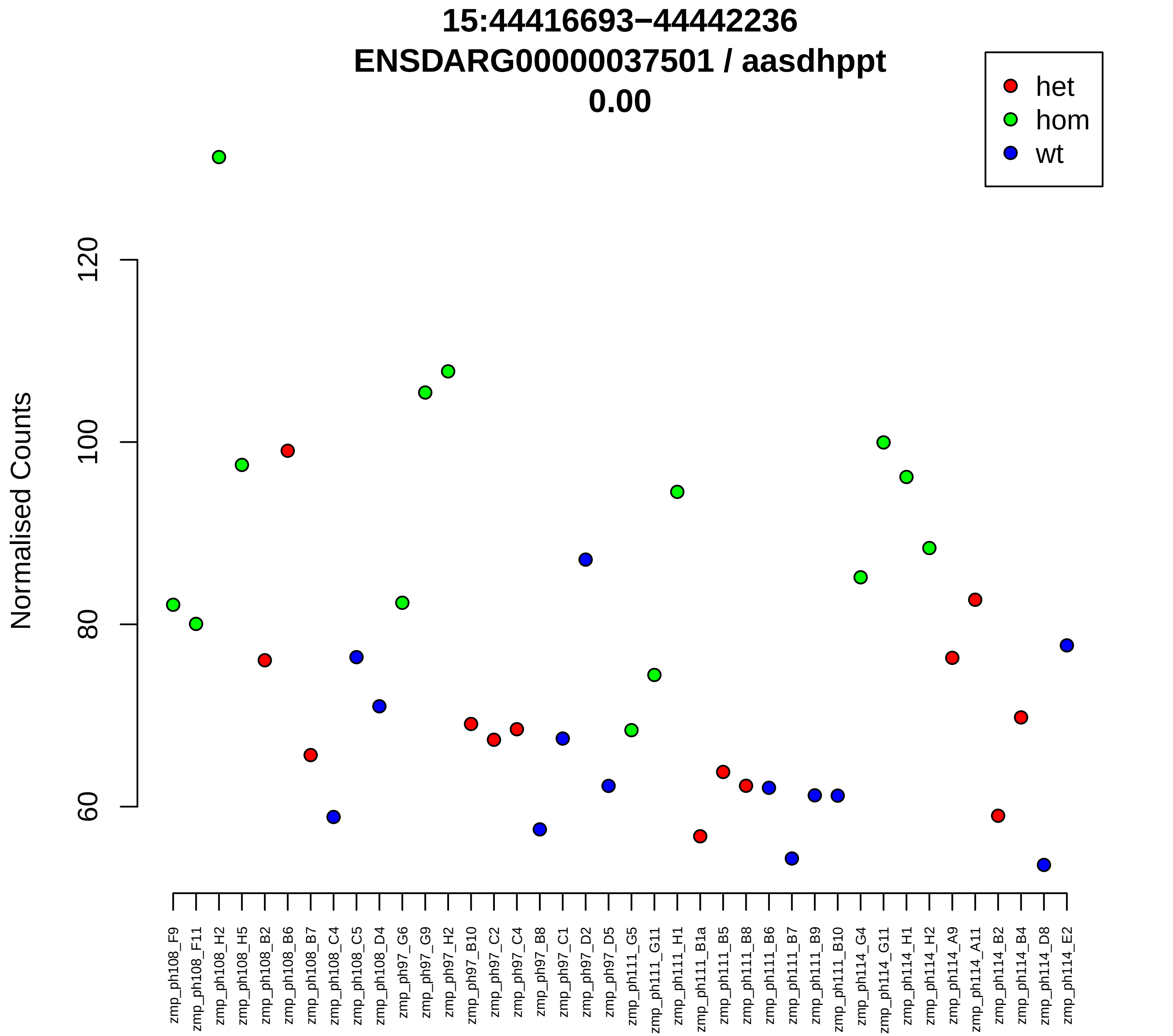

15:44416693−44442236
ENSDARG00000037501 / aasdhppt
0.00
het
hom
wt
Normalised Counts
60
80
100
120
zmp_ph108_F9
zmp_ph108_F11
zmp_ph108_H2
zmp_ph108_H5
zmp_ph108_B2
zmp_ph108_B6
zmp_ph108_B7
zmp_ph108_C4
zmp_ph108_C5
zmp_ph108_D4
zmp_ph97_G6
zmp_ph97_G9
zmp_ph97_H2
zmp_ph97_B10
zmp_ph97_C2
zmp_ph97_C4
zmp_ph97_B8
zmp_ph97_C1
zmp_ph97_D2
zmp_ph97_D5
zmp_ph111_G5
zmp_ph111_G11
zmp_ph111_H1
zmp_ph111_B1a
zmp_ph111_B5
zmp_ph111_B8
zmp_ph111_B6
zmp_ph111_B7
zmp_ph111_B9
zmp_ph111_B10
zmp_ph114_G4
zmp_ph114_G11
zmp_ph114_H1
zmp_ph114_H2
zmp_ph114_A9
zmp_ph114_A11
zmp_ph114_B2
zmp_ph114_B4
zmp_ph114_D8
zmp_ph114_E2

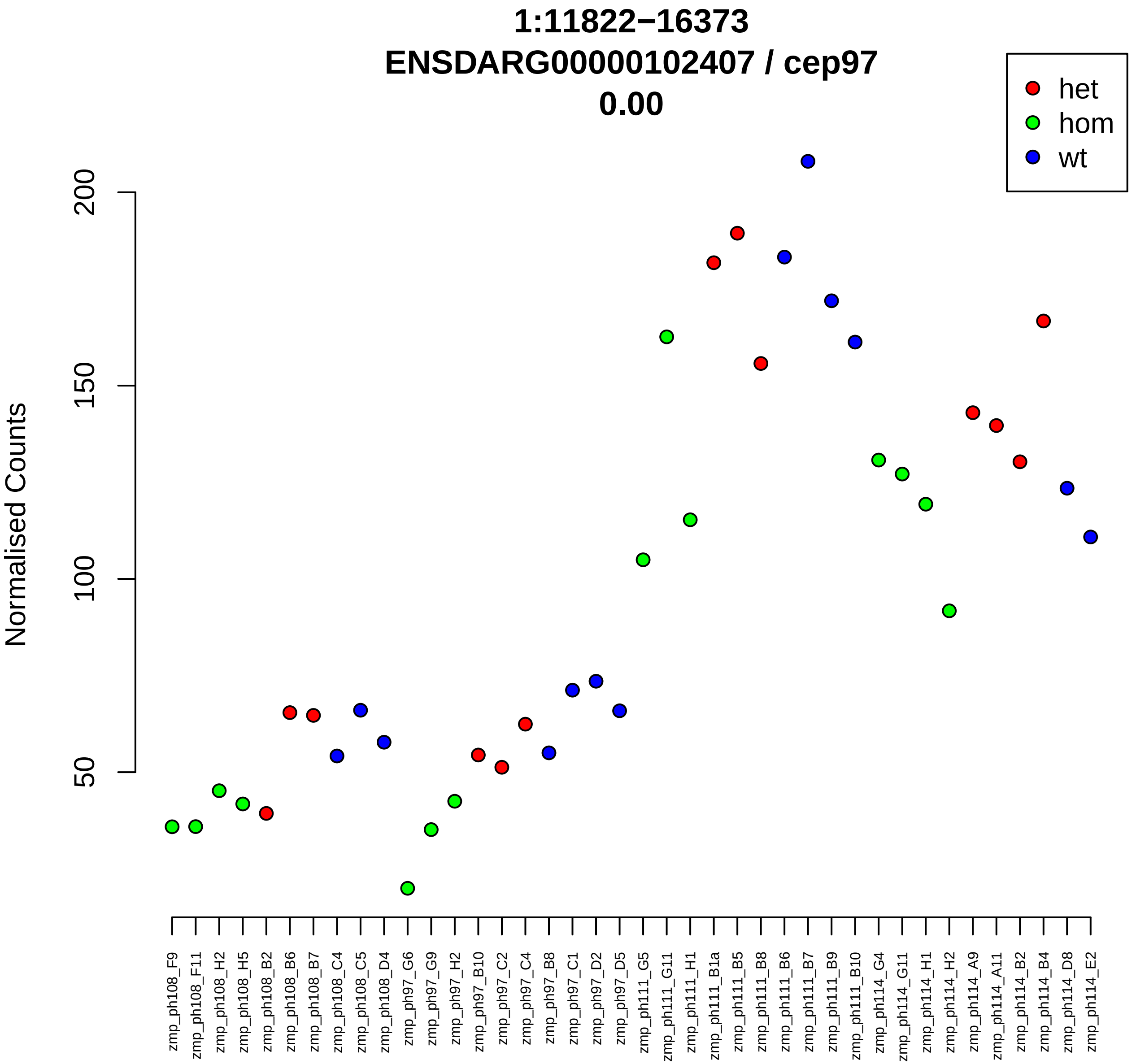
1:11822−16373
ENSDARG00000102407 / cep97
0.00
het
hom
wt
Normalised Counts
50
100
150
200
zmp_ph108_F9
zmp_ph108_F11
zmp_ph108_H2
zmp_ph108_H5
zmp_ph108_B2
zmp_ph108_B6
zmp_ph108_B7
zmp_ph108_C4
zmp_ph108_C5
zmp_ph108_D4
zmp_ph97_G6
zmp_ph97_G9
zmp_ph97_H2
zmp_ph97_B10
zmp_ph97_C2
zmp_ph97_C4
zmp_ph97_B8
zmp_ph97_C1
zmp_ph97_D2
zmp_ph97_D5
zmp_ph111_G5
zmp_ph111_G11
zmp_ph111_H1
zmp_ph111_B1a
zmp_ph111_B5
zmp_ph111_B8
zmp_ph111_B6
zmp_ph111_B7
zmp_ph111_B9
zmp_ph111_B10
zmp_ph114_G4
zmp_ph114_G11
zmp_ph114_H1
zmp_ph114_H2
zmp_ph114_A9
zmp_ph114_A11
zmp_ph114_B2
zmp_ph114_B4
zmp_ph114_D8
zmp_ph114_E2

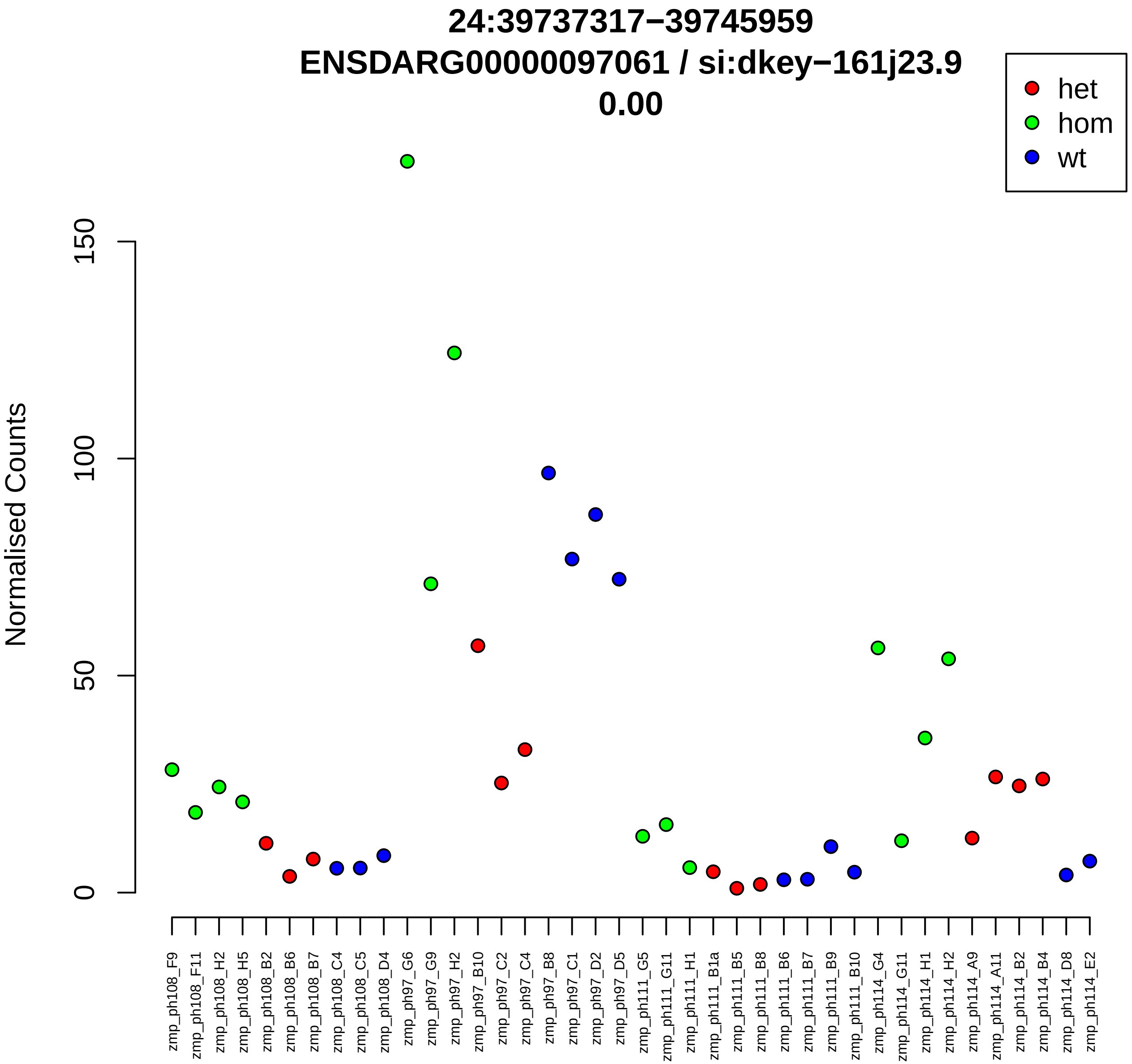
24:39737317−39745959
ENSDARG00000097061 / si:dkey−161j23.9
0.00
het
hom
wt
Normalised Counts
0
50
100
150
zmp_ph108_F9
zmp_ph108_F11
zmp_ph108_H2
zmp_ph108_H5
zmp_ph108_B2
zmp_ph108_B6
zmp_ph108_B7
zmp_ph108_C4
zmp_ph108_C5
zmp_ph108_D4
zmp_ph97_G6
zmp_ph97_G9
zmp_ph97_H2
zmp_ph97_B10
zmp_ph97_C2
zmp_ph97_C4
zmp_ph97_B8
zmp_ph97_C1
zmp_ph97_D2
zmp_ph97_D5
zmp_ph111_G5
zmp_ph111_G11
zmp_ph111_H1
zmp_ph111_B1a
zmp_ph111_B5
zmp_ph111_B8
zmp_ph111_B6
zmp_ph111_B7
zmp_ph111_B9
zmp_ph111_B10
zmp_ph114_G4
zmp_ph114_G11
zmp_ph114_H1
zmp_ph114_H2
zmp_ph114_A9
zmp_ph114_A11
zmp_ph114_B2
zmp_ph114_B4
zmp_ph114_D8
zmp_ph114_E2

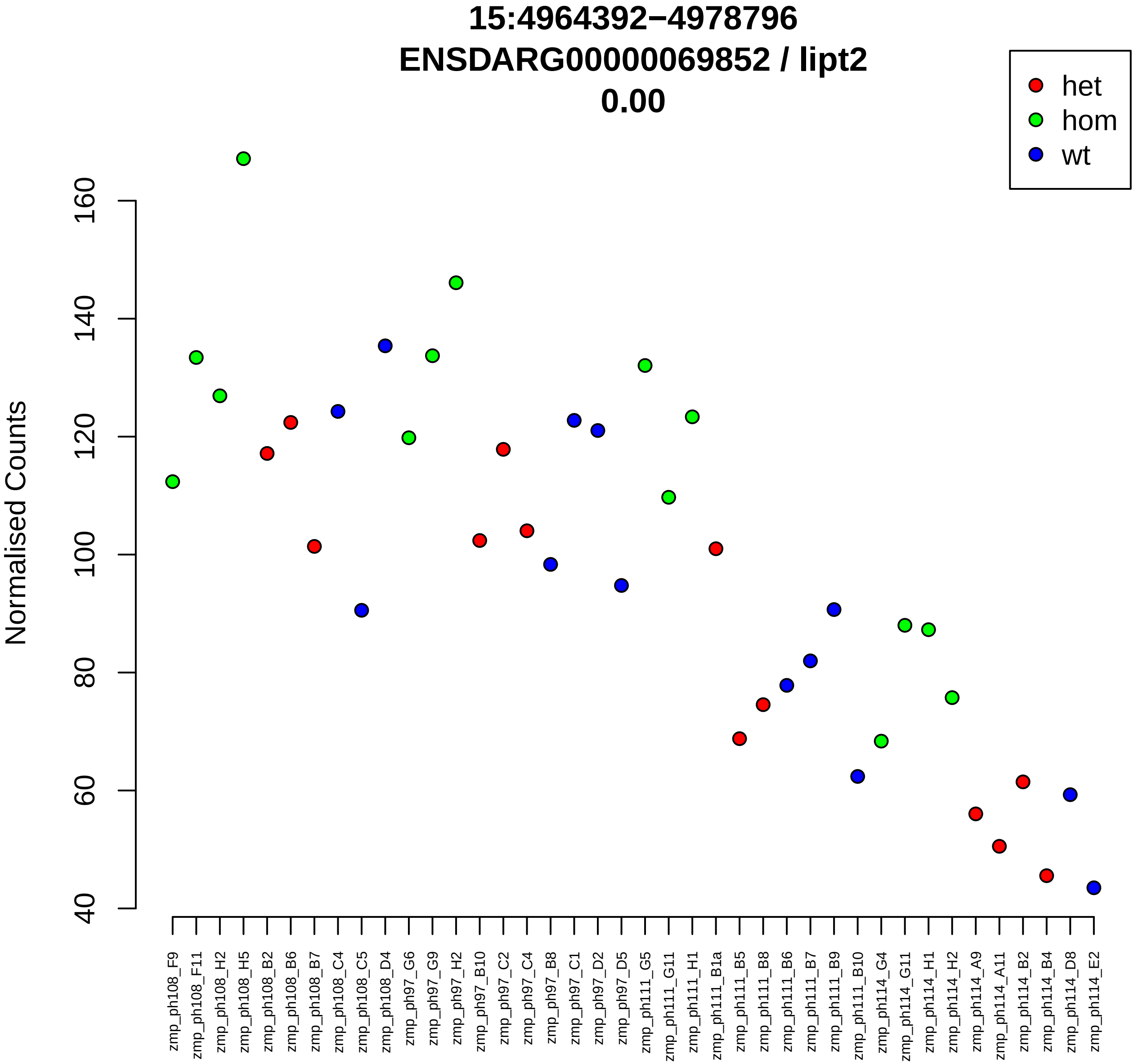
15:4964392−4978796
ENSDARG00000069852 / lipt2
0.00
het
hom
wt
Normalised Counts
40
60
80
100
120
140
160
zmp_ph108_F9
zmp_ph108_F11
zmp_ph108_H2
zmp_ph108_H5
zmp_ph108_B2
zmp_ph108_B6
zmp_ph108_B7
zmp_ph108_C4
zmp_ph108_C5
zmp_ph108_D4
zmp_ph97_G6
zmp_ph97_G9
zmp_ph97_H2
zmp_ph97_B10
zmp_ph97_C2
zmp_ph97_C4
zmp_ph97_B8
zmp_ph97_C1
zmp_ph97_D2
zmp_ph97_D5
zmp_ph111_G5
zmp_ph111_G11
zmp_ph111_H1
zmp_ph111_B1a
zmp_ph111_B5
zmp_ph111_B8
zmp_ph111_B6
zmp_ph111_B7
zmp_ph111_B9
zmp_ph111_B10
zmp_ph114_G4
zmp_ph114_G11
zmp_ph114_H1
zmp_ph114_H2
zmp_ph114_A9
zmp_ph114_A11
zmp_ph114_B2
zmp_ph114_B4
zmp_ph114_D8
zmp_ph114_E2

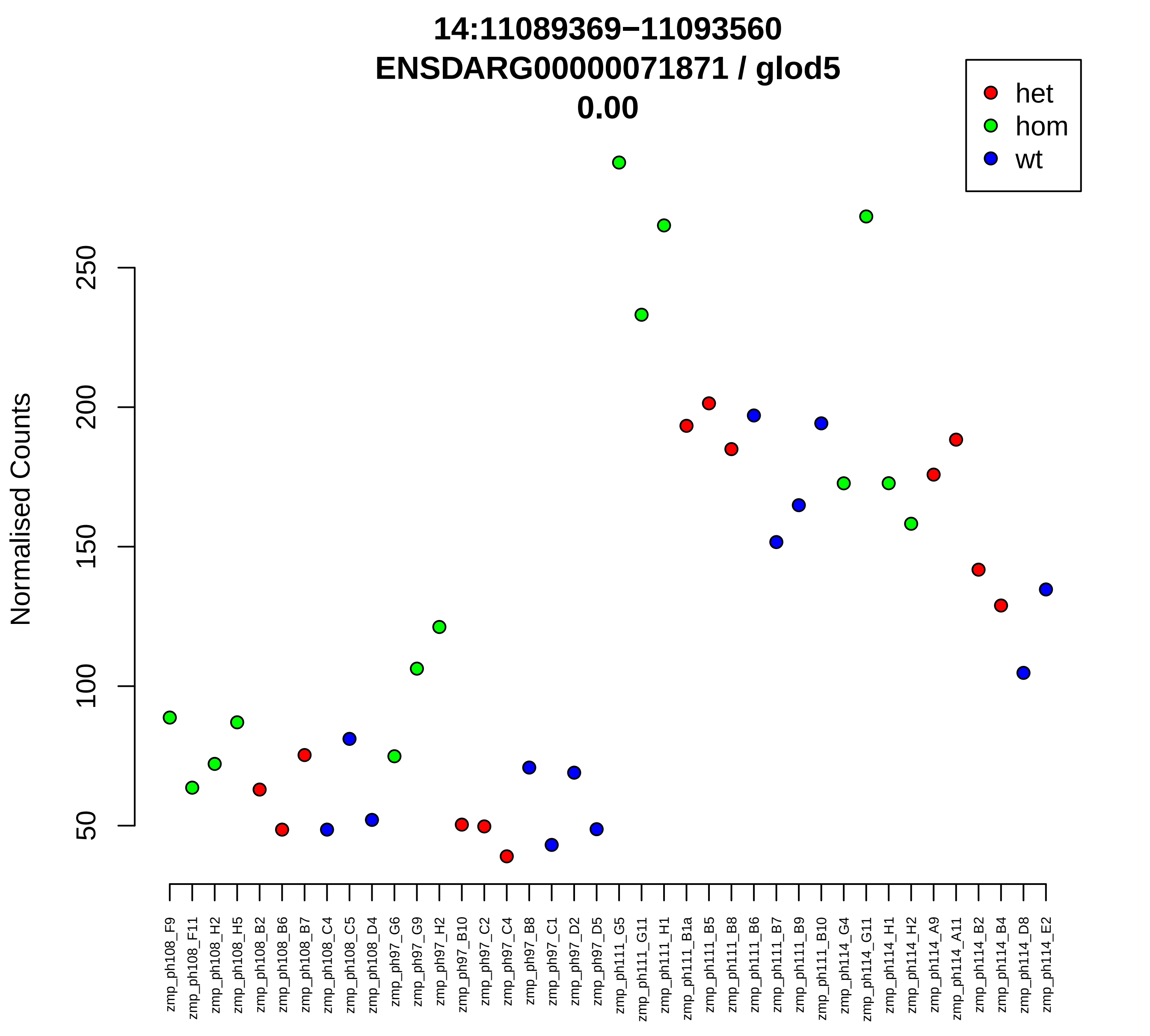

14:11089369−11093560
ENSDARG00000071871 / glod5
0.00
het
hom
wt
Normalised Counts
50
100
150
200
250
zmp_ph108_F9
zmp_ph108_F11
zmp_ph108_H2
zmp_ph108_H5
zmp_ph108_B2
zmp_ph108_B6
zmp_ph108_B7
zmp_ph108_C4
zmp_ph108_C5
zmp_ph108_D4
zmp_ph97_G6
zmp_ph97_G9
zmp_ph97_H2
zmp_ph97_B10
zmp_ph97_C2
zmp_ph97_C4
zmp_ph97_B8
zmp_ph97_C1
zmp_ph97_D2
zmp_ph97_D5
zmp_ph111_G5
zmp_ph111_G11
zmp_ph111_H1
zmp_ph111_B1a
zmp_ph111_B5
zmp_ph111_B8
zmp_ph111_B6
zmp_ph111_B7
zmp_ph111_B9
zmp_ph111_B10
zmp_ph114_G4
zmp_ph114_G11
zmp_ph114_H1
zmp_ph114_H2
zmp_ph114_A9
zmp_ph114_A11
zmp_ph114_B2
zmp_ph114_B4
zmp_ph114_D8
zmp_ph114_E2

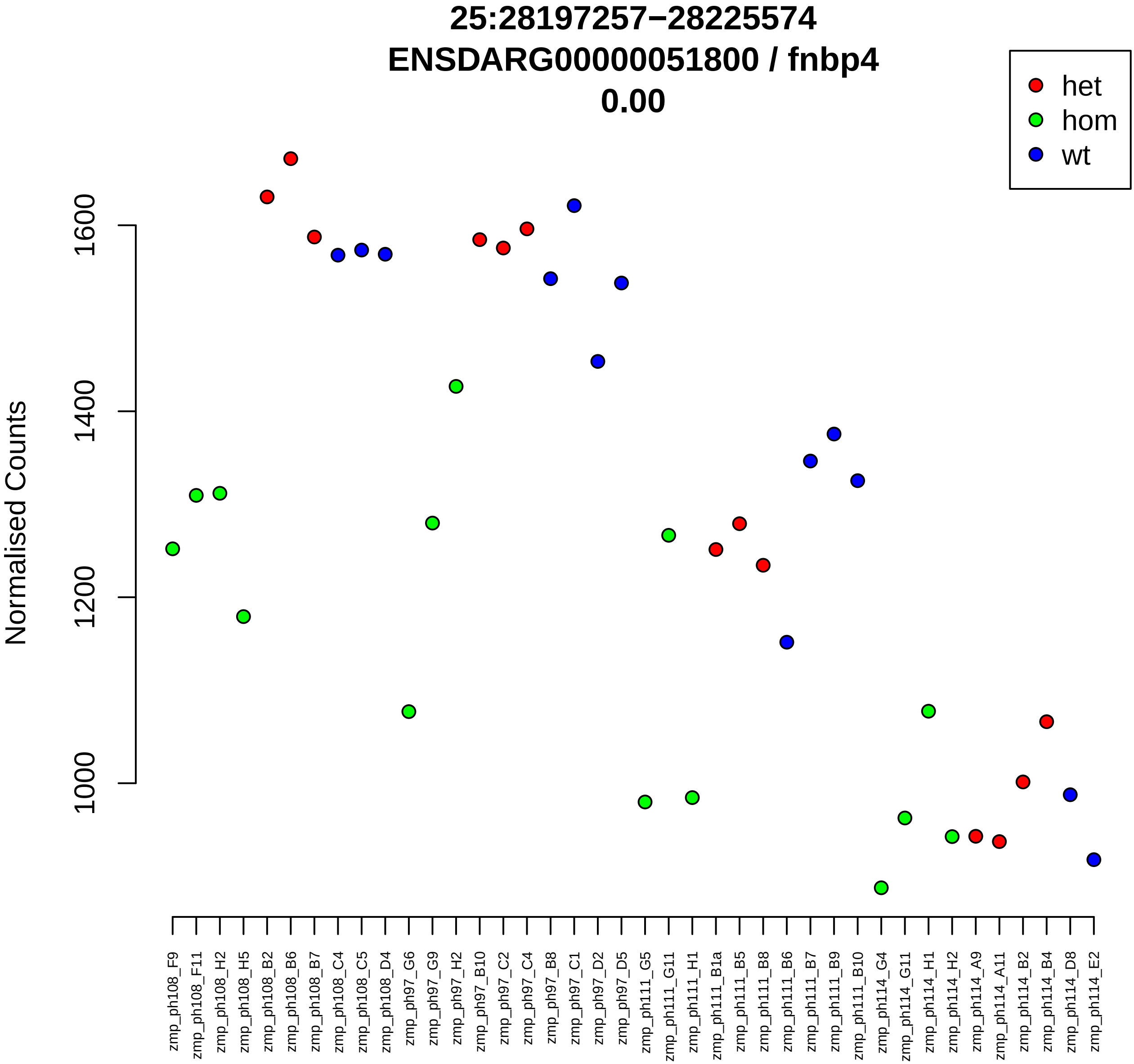

25:28197257−28225574
ENSDARG00000051800 / fnbp4
0.00
het
hom
wt
Normalised Counts
1000
1200
1400
1600
zmp_ph108_F9
zmp_ph108_F11
zmp_ph108_H2
zmp_ph108_H5
zmp_ph108_B2
zmp_ph108_B6
zmp_ph108_B7
zmp_ph108_C4
zmp_ph108_C5
zmp_ph108_D4
zmp_ph97_G6
zmp_ph97_G9
zmp_ph97_H2
zmp_ph97_B10
zmp_ph97_C2
zmp_ph97_C4
zmp_ph97_B8
zmp_ph97_C1
zmp_ph97_D2
zmp_ph97_D5
zmp_ph111_G5
zmp_ph111_G11
zmp_ph111_H1
zmp_ph111_B1a
zmp_ph111_B5
zmp_ph111_B8
zmp_ph111_B6
zmp_ph111_B7
zmp_ph111_B9
zmp_ph111_B10
zmp_ph114_G4
zmp_ph114_G11
zmp_ph114_H1
zmp_ph114_H2
zmp_ph114_A9
zmp_ph114_A11
zmp_ph114_B2
zmp_ph114_B4
zmp_ph114_D8
zmp_ph114_E2

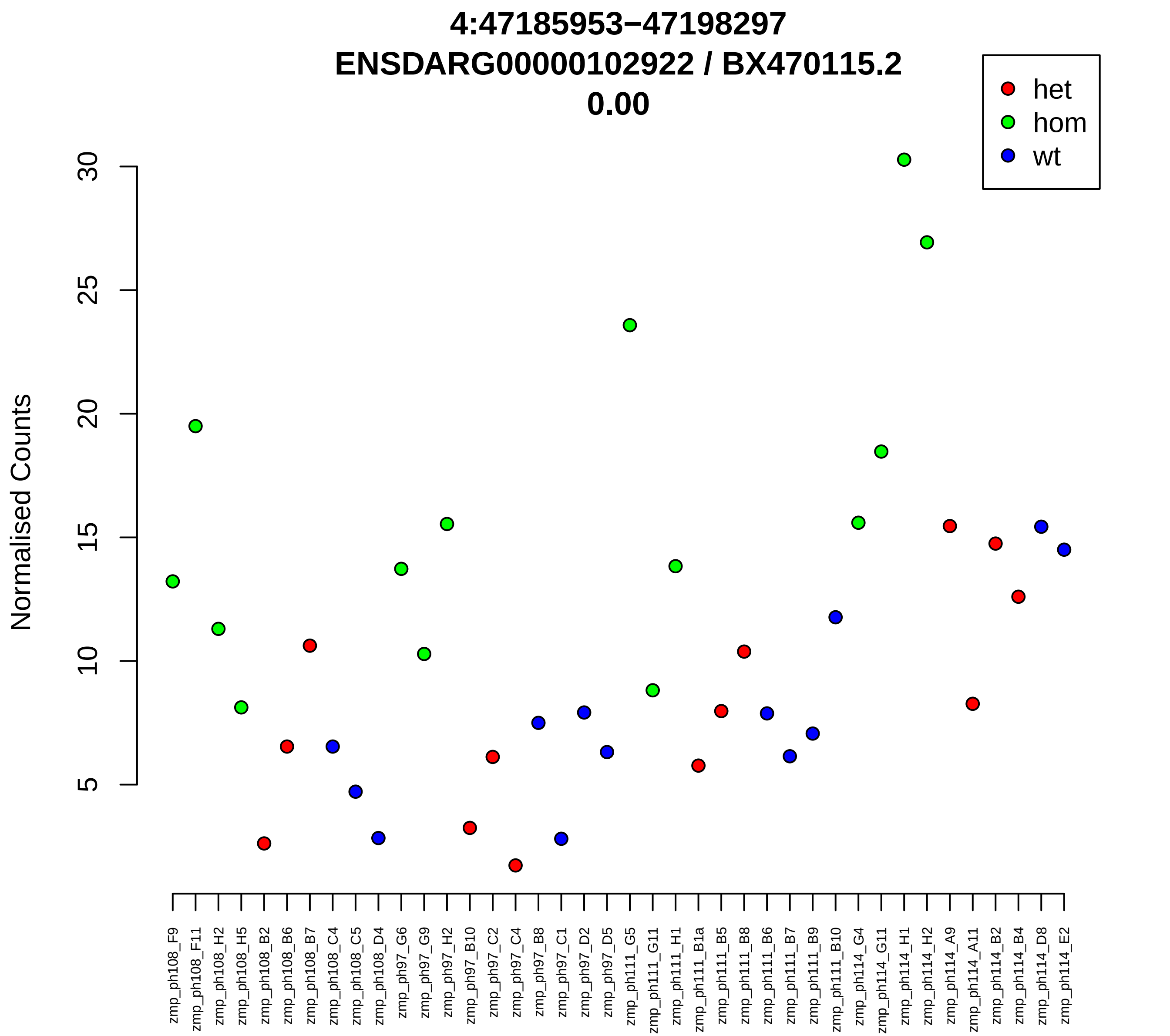
4:47185953−47198297
ENSDARG00000102922 / BX470115.2
0.00
het
hom
wt
Normalised Counts
5
10
15
20
25
30
zmp_ph108_F9
zmp_ph108_F11
zmp_ph108_H2
zmp_ph108_H5
zmp_ph108_B2
zmp_ph108_B6
zmp_ph108_B7
zmp_ph108_C4
zmp_ph108_C5
zmp_ph108_D4
zmp_ph97_G6
zmp_ph97_G9
zmp_ph97_H2
zmp_ph97_B10
zmp_ph97_C2
zmp_ph97_C4
zmp_ph97_B8
zmp_ph97_C1
zmp_ph97_D2
zmp_ph97_D5
zmp_ph111_G5
zmp_ph111_G11
zmp_ph111_H1
zmp_ph111_B1a
zmp_ph111_B5
zmp_ph111_B8
zmp_ph111_B6
zmp_ph111_B7
zmp_ph111_B9
zmp_ph111_B10
zmp_ph114_G4
zmp_ph114_G11
zmp_ph114_H1
zmp_ph114_H2
zmp_ph114_A9
zmp_ph114_A11
zmp_ph114_B2
zmp_ph114_B4
zmp_ph114_D8
zmp_ph114_E2

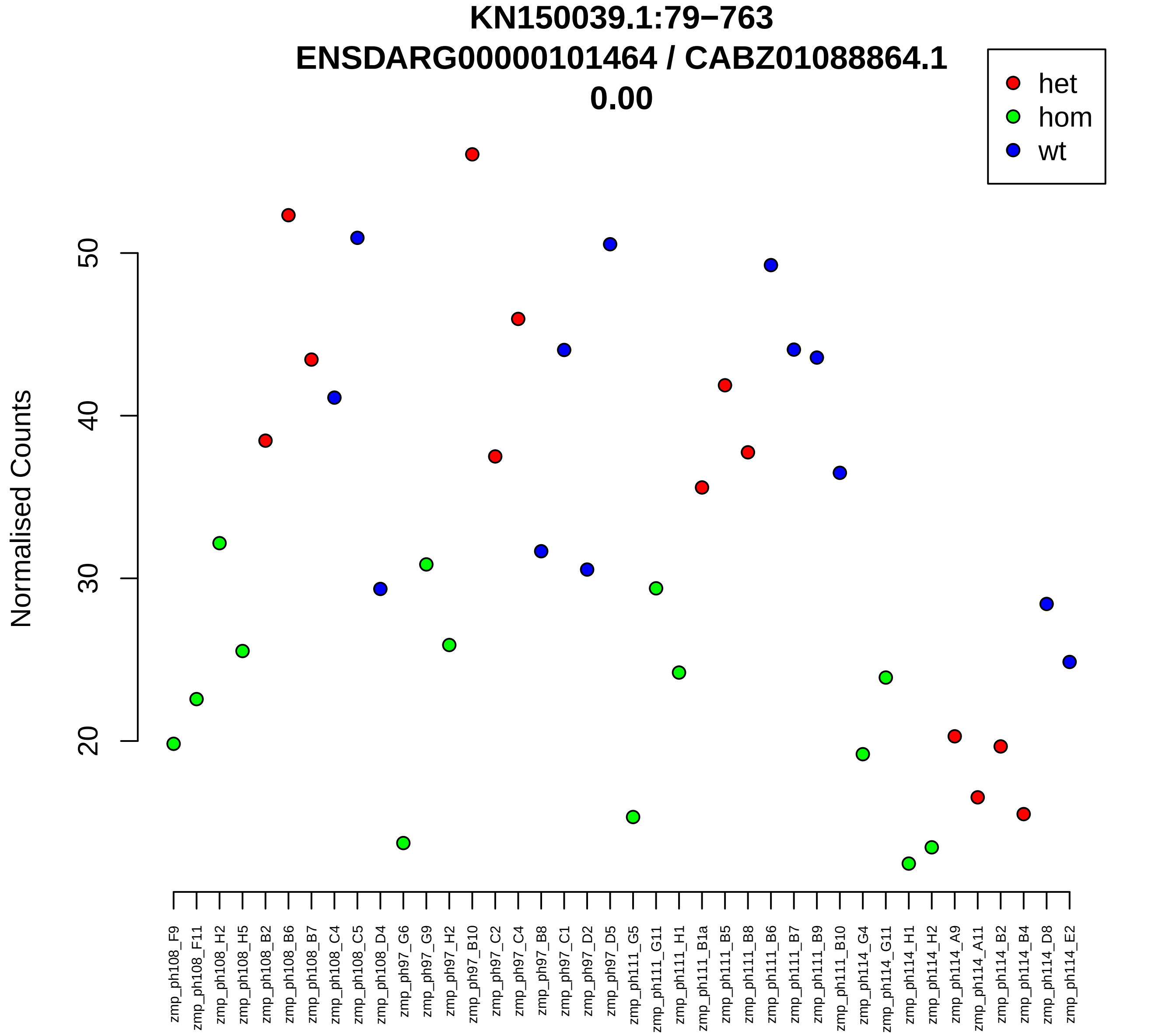
KN150039.1:79−763
ENSDARG00000101464 / CABZ01088864.1
0.00
het
hom
wt
Normalised Counts
20
30
40
50
zmp_ph108_F9
zmp_ph108_F11
zmp_ph108_H2
zmp_ph108_H5
zmp_ph108_B2
zmp_ph108_B6
zmp_ph108_B7
zmp_ph108_C4
zmp_ph108_C5
zmp_ph108_D4
zmp_ph97_G6
zmp_ph97_G9
zmp_ph97_H2
zmp_ph97_B10
zmp_ph97_C2
zmp_ph97_C4
zmp_ph97_B8
zmp_ph97_C1
zmp_ph97_D2
zmp_ph97_D5
zmp_ph111_G5
zmp_ph111_G11
zmp_ph111_H1
zmp_ph111_B1a
zmp_ph111_B5
zmp_ph111_B8
zmp_ph111_B6
zmp_ph111_B7
zmp_ph111_B9
zmp_ph111_B10
zmp_ph114_G4
zmp_ph114_G11
zmp_ph114_H1
zmp_ph114_H2
zmp_ph114_A9
zmp_ph114_A11
zmp_ph114_B2
zmp_ph114_B4
zmp_ph114_D8
zmp_ph114_E2

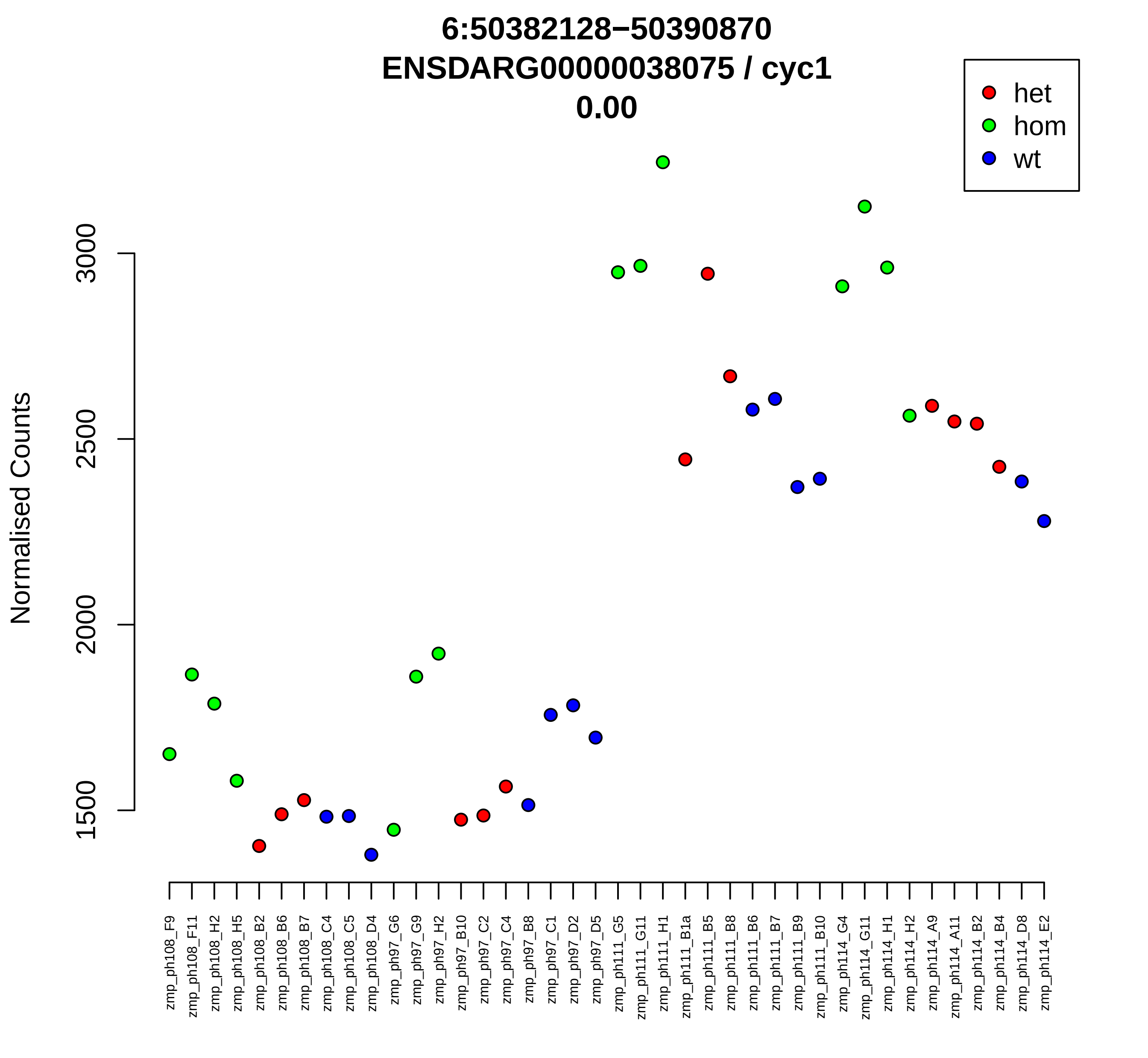

6:50382128−50390870
ENSDARG00000038075 / cyc1
0.00
het
hom
wt
Normalised Counts
1500
2000
2500
3000
zmp_ph108_F9
zmp_ph108_F11
zmp_ph108_H2
zmp_ph108_H5
zmp_ph108_B2
zmp_ph108_B6
zmp_ph108_B7
zmp_ph108_C4
zmp_ph108_C5
zmp_ph108_D4
zmp_ph97_G6
zmp_ph97_G9
zmp_ph97_H2
zmp_ph97_B10
zmp_ph97_C2
zmp_ph97_C4
zmp_ph97_B8
zmp_ph97_C1
zmp_ph97_D2
zmp_ph97_D5
zmp_ph111_G5
zmp_ph111_G11
zmp_ph111_H1
zmp_ph111_B1a
zmp_ph111_B5
zmp_ph111_B8
zmp_ph111_B6
zmp_ph111_B7
zmp_ph111_B9
zmp_ph111_B10
zmp_ph114_G4
zmp_ph114_G11
zmp_ph114_H1
zmp_ph114_H2
zmp_ph114_A9
zmp_ph114_A11
zmp_ph114_B2
zmp_ph114_B4
zmp_ph114_D8
zmp_ph114_E2

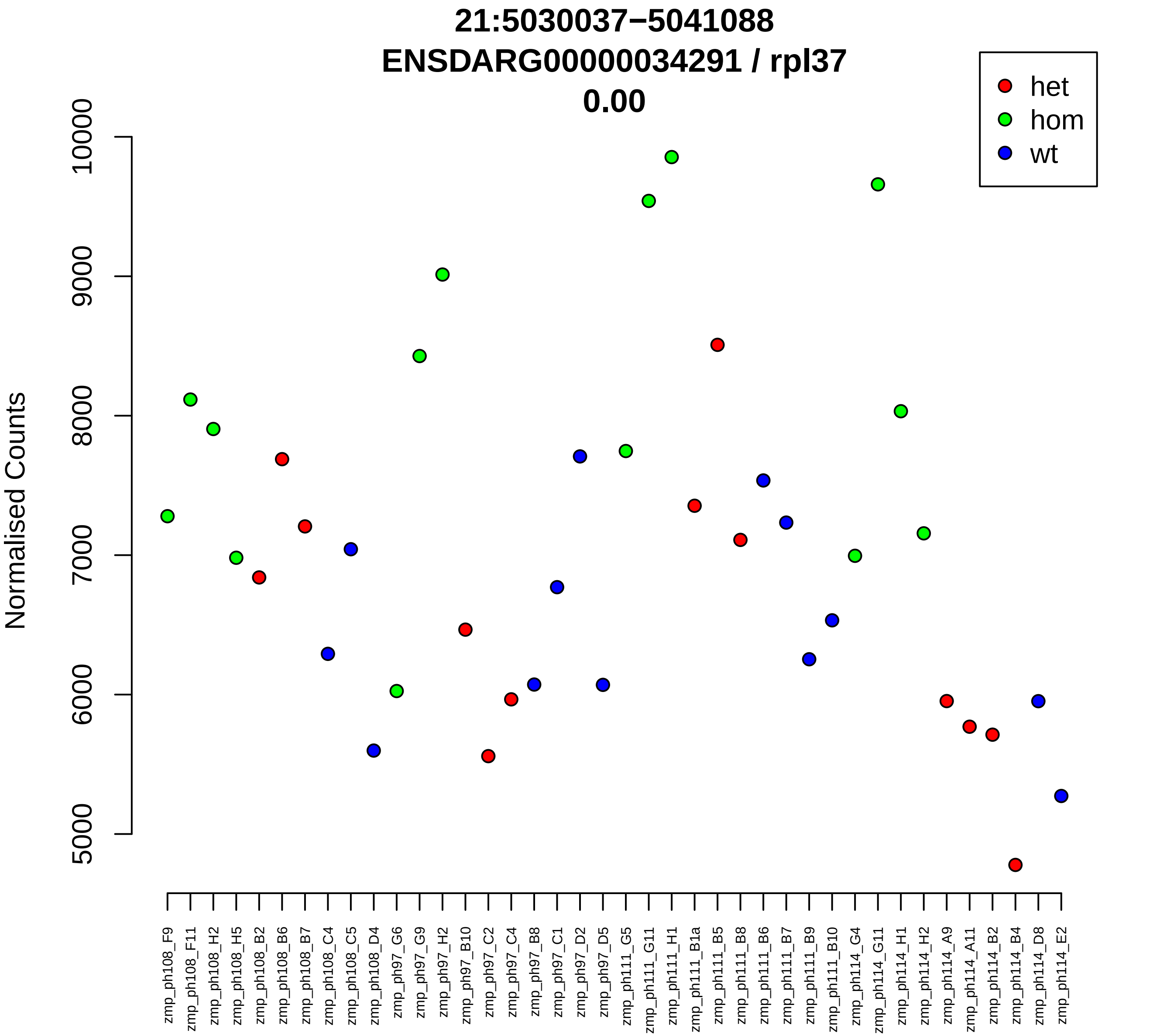

21:5030037−5041088
ENSDARG00000034291 / rpl37
0.00
het
hom
wt
Normalised Counts
5000
6000
7000
8000
9000
10000
zmp_ph108_F9
zmp_ph108_F11
zmp_ph108_H2
zmp_ph108_H5
zmp_ph108_B2
zmp_ph108_B6
zmp_ph108_B7
zmp_ph108_C4
zmp_ph108_C5
zmp_ph108_D4
zmp_ph97_G6
zmp_ph97_G9
zmp_ph97_H2
zmp_ph97_B10
zmp_ph97_C2
zmp_ph97_C4
zmp_ph97_B8
zmp_ph97_C1
zmp_ph97_D2
zmp_ph97_D5
zmp_ph111_G5
zmp_ph111_G11
zmp_ph111_H1
zmp_ph111_B1a
zmp_ph111_B5
zmp_ph111_B8
zmp_ph111_B6
zmp_ph111_B7
zmp_ph111_B9
zmp_ph111_B10
zmp_ph114_G4
zmp_ph114_G11
zmp_ph114_H1
zmp_ph114_H2
zmp_ph114_A9
zmp_ph114_A11
zmp_ph114_B2
zmp_ph114_B4
zmp_ph114_D8
zmp_ph114_E2

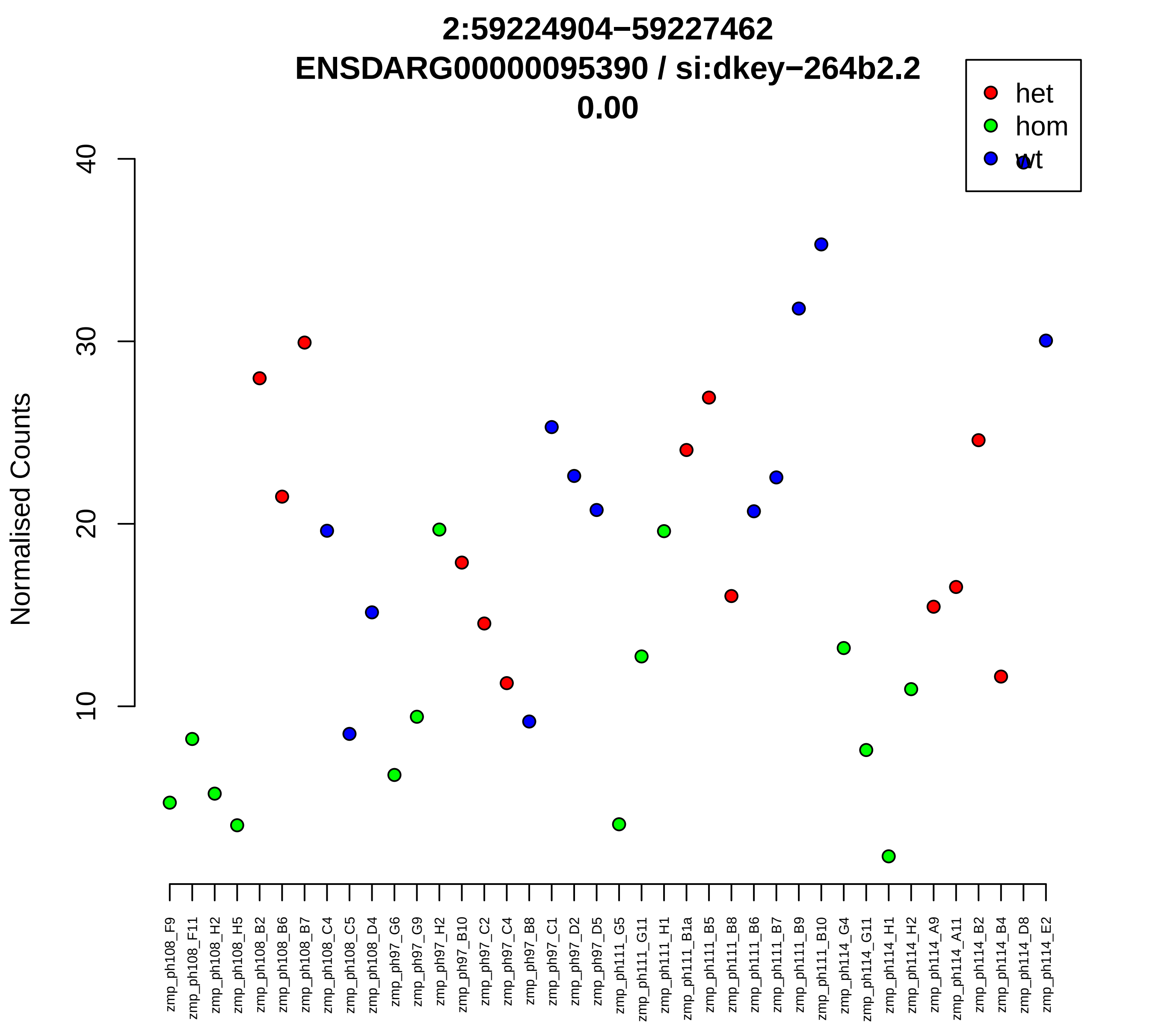
2:59224904−59227462
ENSDARG00000095390 / si:dkey−264b2.2
0.00
het
hom
wt
Normalised Counts
10
20
30
40
zmp_ph108_F9
zmp_ph108_F11
zmp_ph108_H2
zmp_ph108_H5
zmp_ph108_B2
zmp_ph108_B6
zmp_ph108_B7
zmp_ph108_C4
zmp_ph108_C5
zmp_ph108_D4
zmp_ph97_G6
zmp_ph97_G9
zmp_ph97_H2
zmp_ph97_B10
zmp_ph97_C2
zmp_ph97_C4
zmp_ph97_B8
zmp_ph97_C1
zmp_ph97_D2
zmp_ph97_D5
zmp_ph111_G5
zmp_ph111_G11
zmp_ph111_H1
zmp_ph111_B1a
zmp_ph111_B5
zmp_ph111_B8
zmp_ph111_B6
zmp_ph111_B7
zmp_ph111_B9
zmp_ph111_B10
zmp_ph114_G4
zmp_ph114_G11
zmp_ph114_H1
zmp_ph114_H2
zmp_ph114_A9
zmp_ph114_A11
zmp_ph114_B2
zmp_ph114_B4
zmp_ph114_D8
zmp_ph114_E2

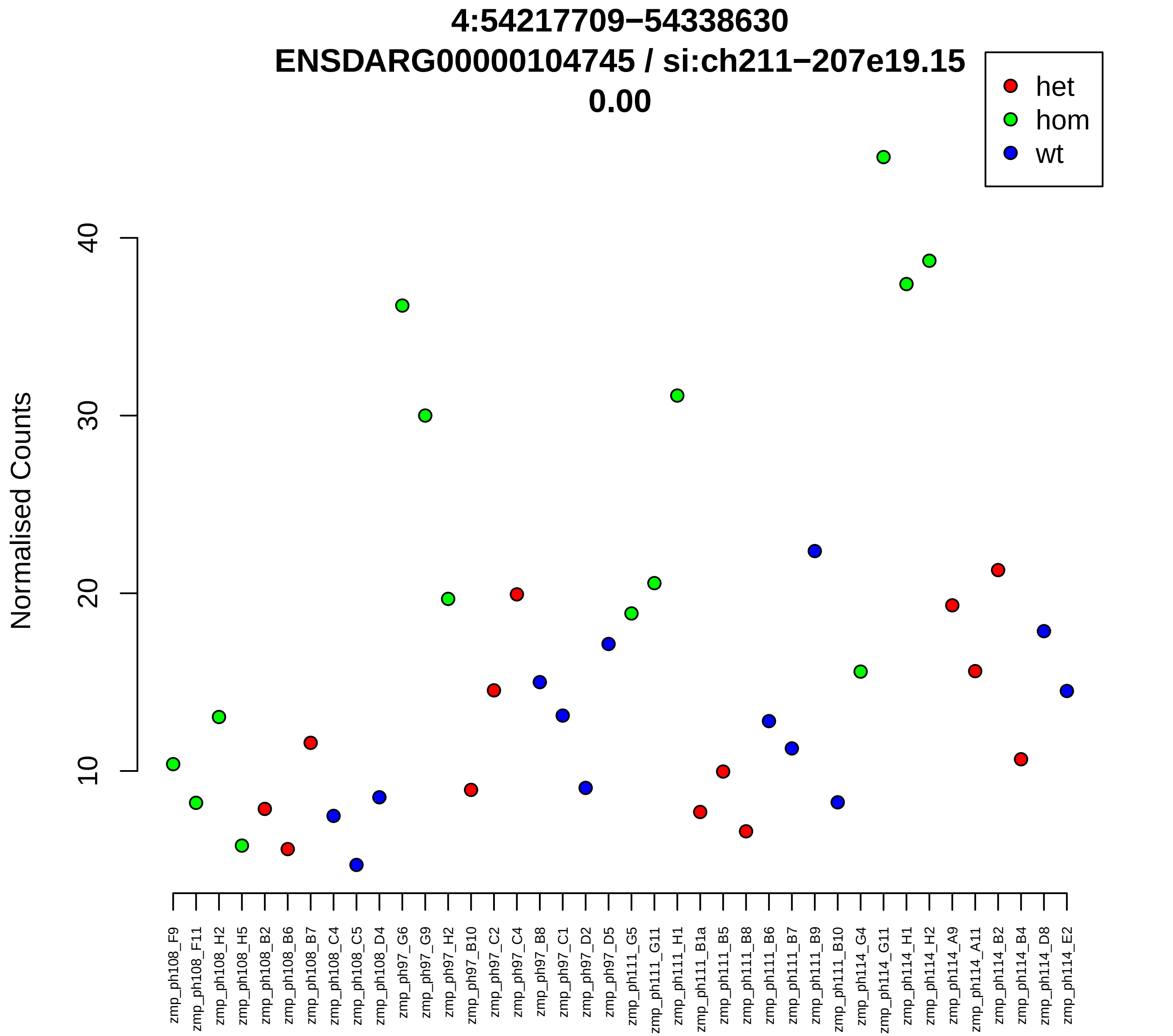

4:54217709−54338630
ENSDARG00000104745 / si:ch211−207e19.15
0.00
het
hom
wt
Normalised Counts
10
20
30
40
zmp_ph108_F9
zmp_ph108_F11
zmp_ph108_H2
zmp_ph108_H5
zmp_ph108_B2
zmp_ph108_B6
zmp_ph108_B7
zmp_ph108_C4
zmp_ph108_C5
zmp_ph108_D4
zmp_ph97_G6
zmp_ph97_G9
zmp_ph97_H2
zmp_ph97_B10
zmp_ph97_C2
zmp_ph97_C4
zmp_ph97_B8
zmp_ph97_C1
zmp_ph97_D2
zmp_ph97_D5
zmp_ph111_G5
zmp_ph111_G11
zmp_ph111_H1
zmp_ph111_B1a
zmp_ph111_B5
zmp_ph111_B8
zmp_ph111_B6
zmp_ph111_B7
zmp_ph111_B9
zmp_ph111_B10
zmp_ph114_G4
zmp_ph114_G11
zmp_ph114_H1
zmp_ph114_H2
zmp_ph114_A9
zmp_ph114_A11
zmp_ph114_B2
zmp_ph114_B4
zmp_ph114_D8
zmp_ph114_E2

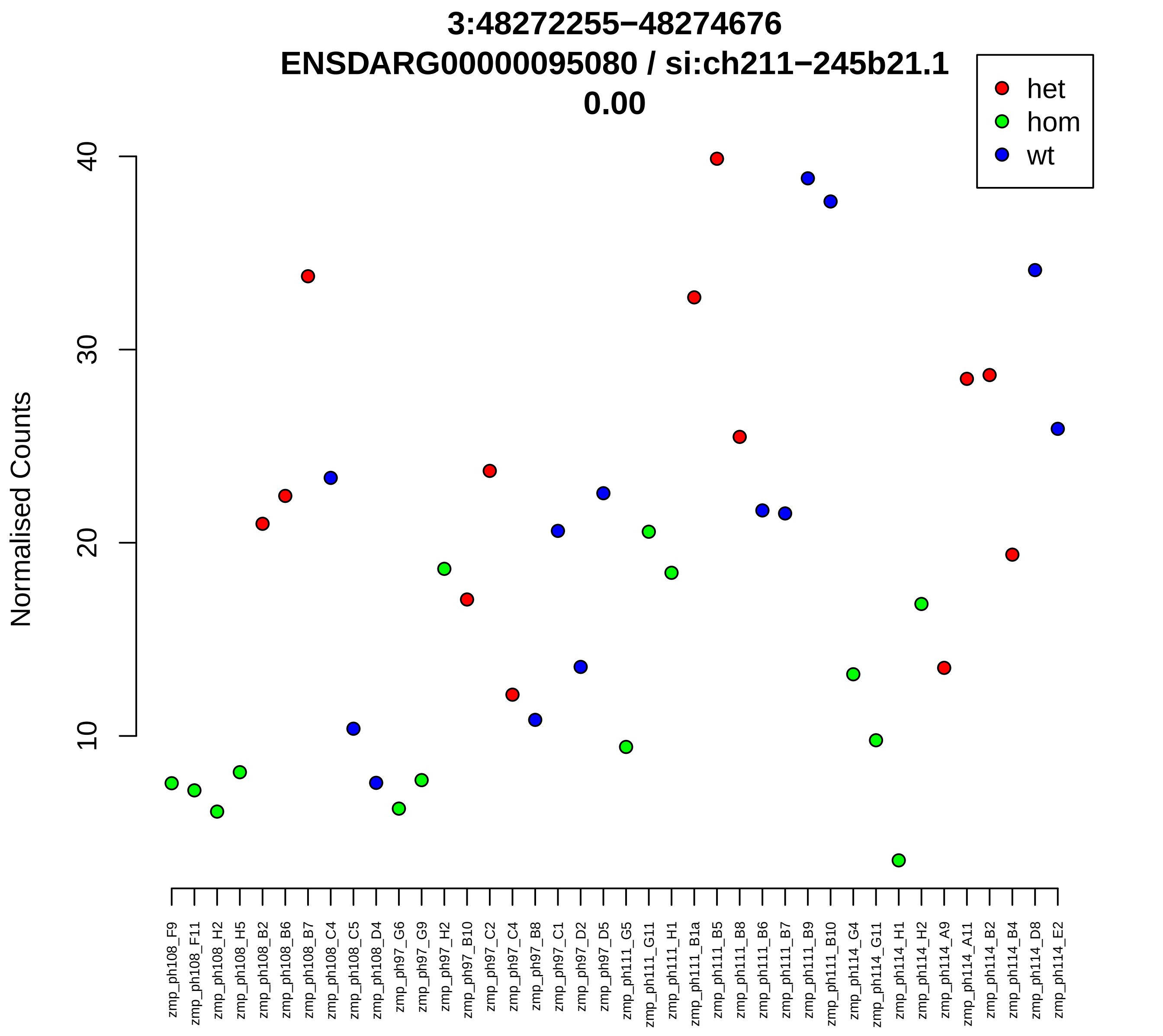

3:48272255−48274676
ENSDARG00000095080 / si:ch211−245b21.1
0.00
het
hom
wt
Normalised Counts
10
20
30
40
zmp_ph108_F9
zmp_ph108_F11
zmp_ph108_H2
zmp_ph108_H5
zmp_ph108_B2
zmp_ph108_B6
zmp_ph108_B7
zmp_ph108_C4
zmp_ph108_C5
zmp_ph108_D4
zmp_ph97_G6
zmp_ph97_G9
zmp_ph97_H2
zmp_ph97_B10
zmp_ph97_C2
zmp_ph97_C4
zmp_ph97_B8
zmp_ph97_C1
zmp_ph97_D2
zmp_ph97_D5
zmp_ph111_G5
zmp_ph111_G11
zmp_ph111_H1
zmp_ph111_B1a
zmp_ph111_B5
zmp_ph111_B8
zmp_ph111_B6
zmp_ph111_B7
zmp_ph111_B9
zmp_ph111_B10
zmp_ph114_G4
zmp_ph114_G11
zmp_ph114_H1
zmp_ph114_H2
zmp_ph114_A9
zmp_ph114_A11
zmp_ph114_B2
zmp_ph114_B4
zmp_ph114_D8
zmp_ph114_E2

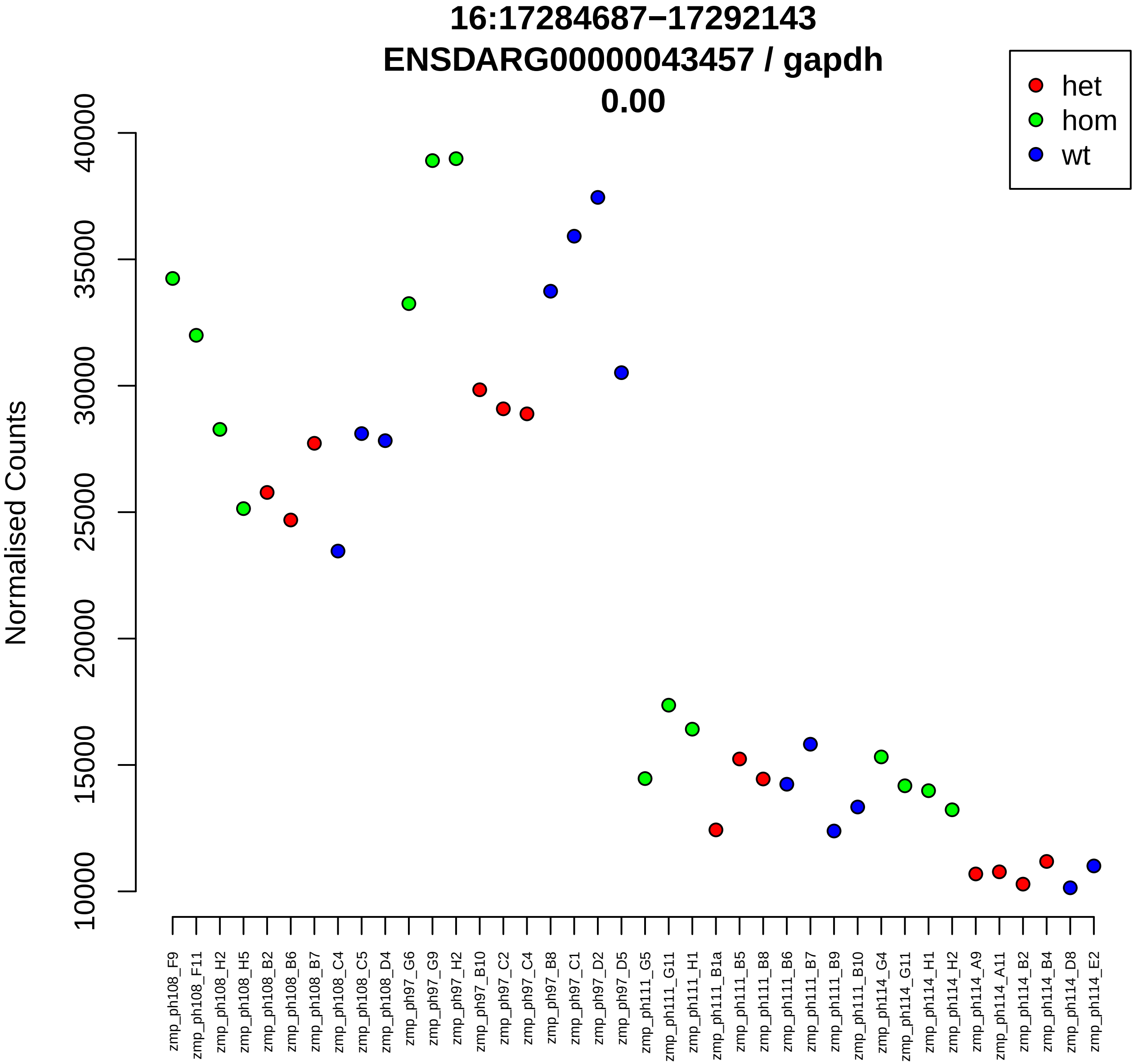

16:17284687−17292143
ENSDARG00000043457 / gapdh
0.00
het
hom
wt
Normalised Counts
10000
15000
20000
25000
30000
35000
40000
zmp_ph108_F9
zmp_ph108_F11
zmp_ph108_H2
zmp_ph108_H5
zmp_ph108_B2
zmp_ph108_B6
zmp_ph108_B7
zmp_ph108_C4
zmp_ph108_C5
zmp_ph108_D4
zmp_ph97_G6
zmp_ph97_G9
zmp_ph97_H2
zmp_ph97_B10
zmp_ph97_C2
zmp_ph97_C4
zmp_ph97_B8
zmp_ph97_C1
zmp_ph97_D2
zmp_ph97_D5
zmp_ph111_G5
zmp_ph111_G11
zmp_ph111_H1
zmp_ph111_B1a
zmp_ph111_B5
zmp_ph111_B8
zmp_ph111_B6
zmp_ph111_B7
zmp_ph111_B9
zmp_ph111_B10
zmp_ph114_G4
zmp_ph114_G11
zmp_ph114_H1
zmp_ph114_H2
zmp_ph114_A9
zmp_ph114_A11
zmp_ph114_B2
zmp_ph114_B4
zmp_ph114_D8
zmp_ph114_E2

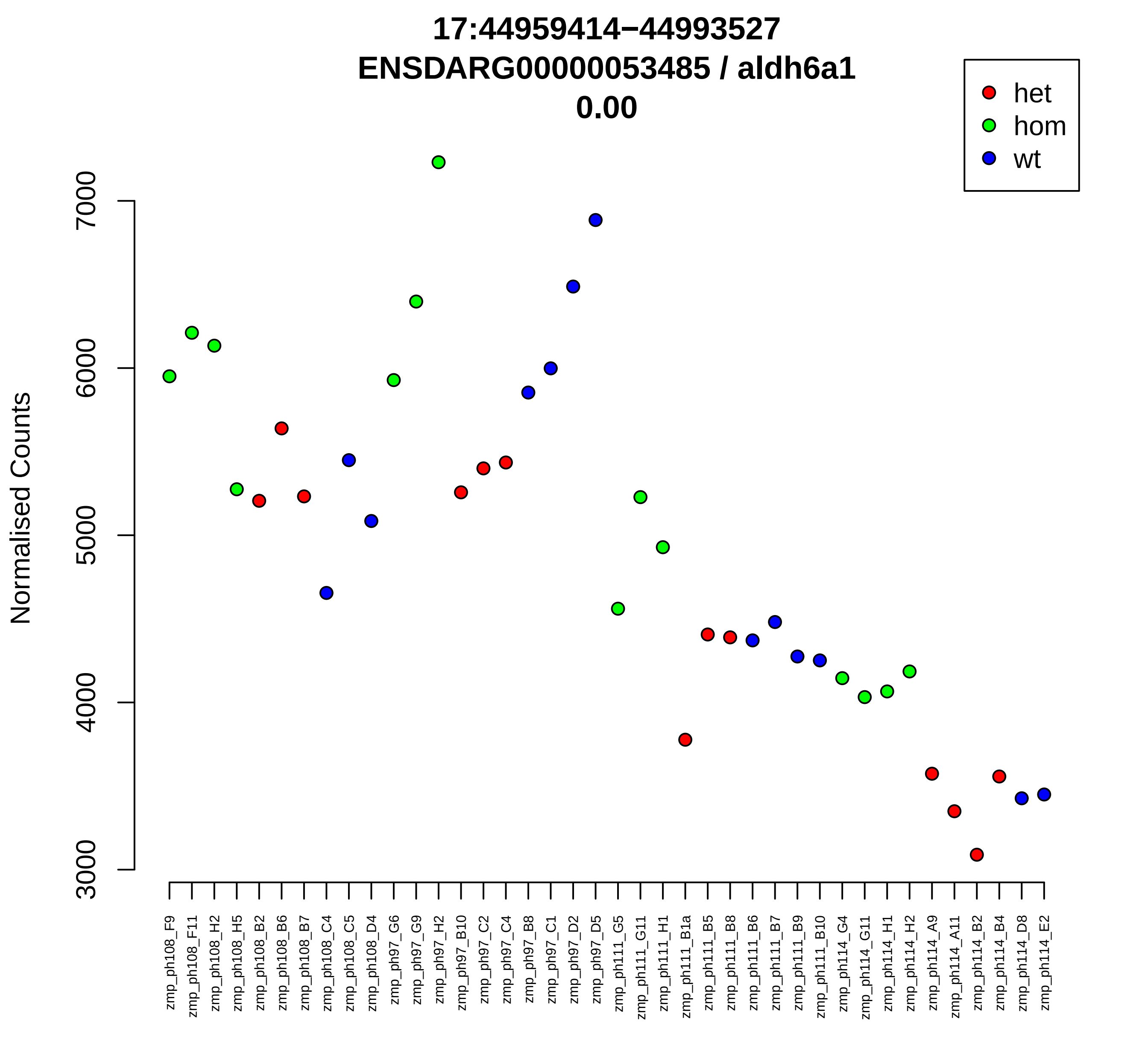

17:44959414−44993527
ENSDARG00000053485 / aldh6a1
0.00
het
hom
wt
Normalised Counts
3000
4000
5000
6000
7000
zmp_ph108_F9
zmp_ph108_F11
zmp_ph108_H2
zmp_ph108_H5
zmp_ph108_B2
zmp_ph108_B6
zmp_ph108_B7
zmp_ph108_C4
zmp_ph108_C5
zmp_ph108_D4
zmp_ph97_G6
zmp_ph97_G9
zmp_ph97_H2
zmp_ph97_B10
zmp_ph97_C2
zmp_ph97_C4
zmp_ph97_B8
zmp_ph97_C1
zmp_ph97_D2
zmp_ph97_D5
zmp_ph111_G5
zmp_ph111_G11
zmp_ph111_H1
zmp_ph111_B1a
zmp_ph111_B5
zmp_ph111_B8
zmp_ph111_B6
zmp_ph111_B7
zmp_ph111_B9
zmp_ph111_B10
zmp_ph114_G4
zmp_ph114_G11
zmp_ph114_H1
zmp_ph114_H2
zmp_ph114_A9
zmp_ph114_A11
zmp_ph114_B2
zmp_ph114_B4
zmp_ph114_D8
zmp_ph114_E2

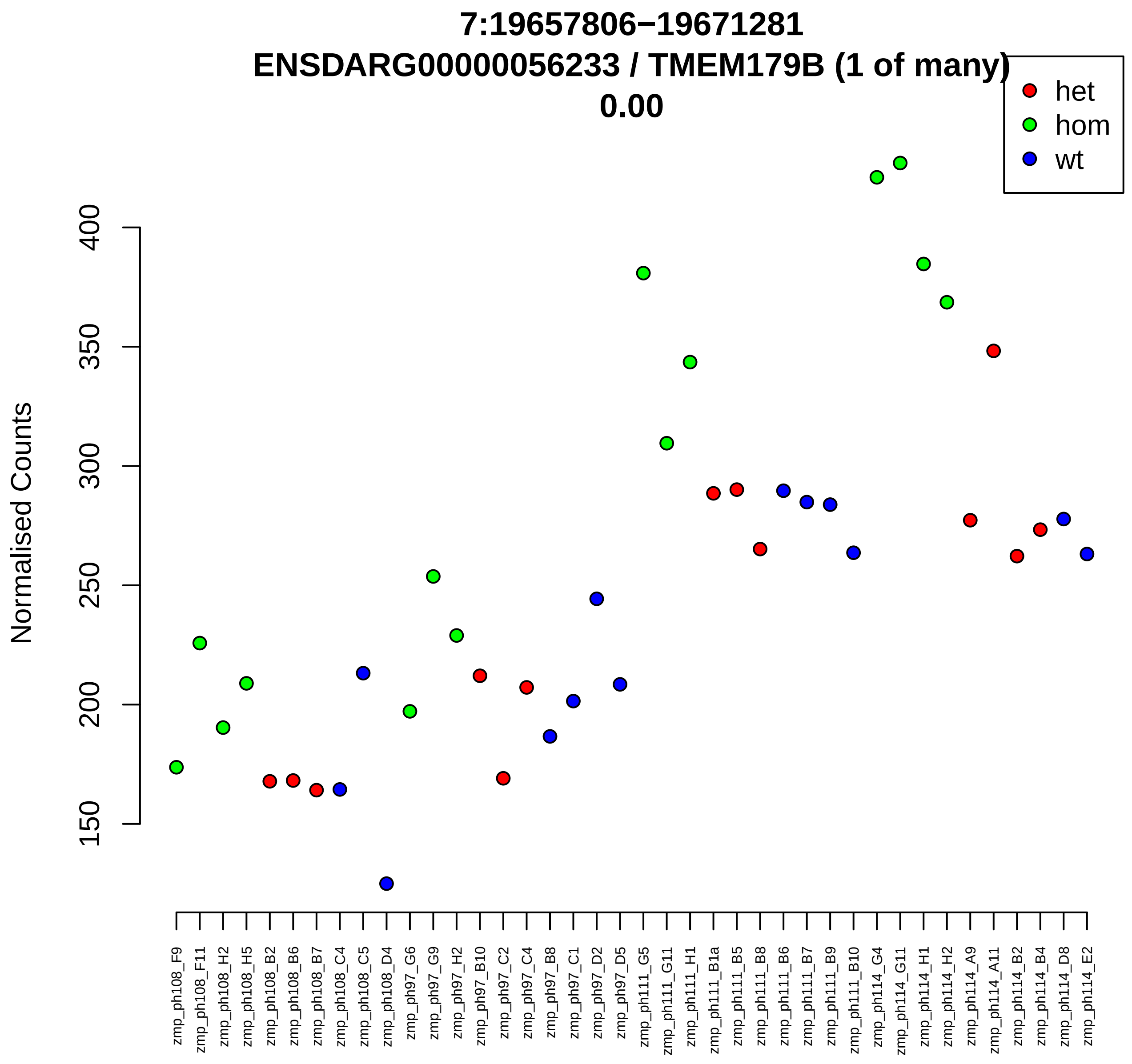

7:19657806−19671281
ENSDARG00000056233 / TMEM179B (1 of many)
0.00
het
hom
wt
Normalised Counts
150
200
250
300
350
400
zmp_ph108_F9
zmp_ph108_F11
zmp_ph108_H2
zmp_ph108_H5
zmp_ph108_B2
zmp_ph108_B6
zmp_ph108_B7
zmp_ph108_C4
zmp_ph108_C5
zmp_ph108_D4
zmp_ph97_G6
zmp_ph97_G9
zmp_ph97_H2
zmp_ph97_B10
zmp_ph97_C2
zmp_ph97_C4
zmp_ph97_B8
zmp_ph97_C1
zmp_ph97_D2
zmp_ph97_D5
zmp_ph111_G5
zmp_ph111_G11
zmp_ph111_H1
zmp_ph111_B1a
zmp_ph111_B5
zmp_ph111_B8
zmp_ph111_B6
zmp_ph111_B7
zmp_ph111_B9
zmp_ph111_B10
zmp_ph114_G4
zmp_ph114_G11
zmp_ph114_H1
zmp_ph114_H2
zmp_ph114_A9
zmp_ph114_A11
zmp_ph114_B2
zmp_ph114_B4
zmp_ph114_D8
zmp_ph114_E2

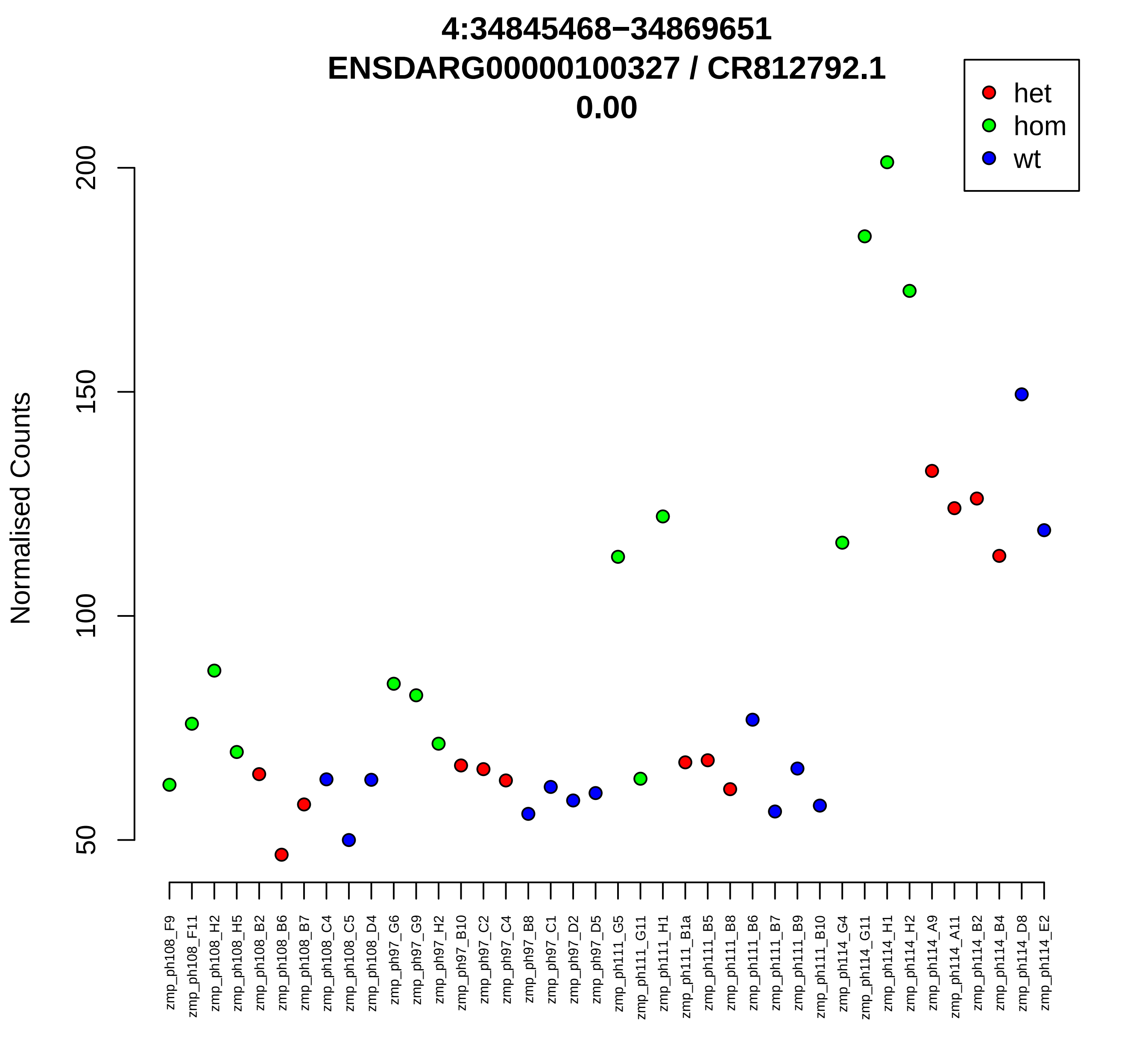

4:34845468−34869651
ENSDARG00000100327 / CR812792.1
0.00
het
hom
wt
Normalised Counts
50
100
150
200
zmp_ph108_F9
zmp_ph108_F11
zmp_ph108_H2
zmp_ph108_H5
zmp_ph108_B2
zmp_ph108_B6
zmp_ph108_B7
zmp_ph108_C4
zmp_ph108_C5
zmp_ph108_D4
zmp_ph97_G6
zmp_ph97_G9
zmp_ph97_H2
zmp_ph97_B10
zmp_ph97_C2
zmp_ph97_C4
zmp_ph97_B8
zmp_ph97_C1
zmp_ph97_D2
zmp_ph97_D5
zmp_ph111_G5
zmp_ph111_G11
zmp_ph111_H1
zmp_ph111_B1a
zmp_ph111_B5
zmp_ph111_B8
zmp_ph111_B6
zmp_ph111_B7
zmp_ph111_B9
zmp_ph111_B10
zmp_ph114_G4
zmp_ph114_G11
zmp_ph114_H1
zmp_ph114_H2
zmp_ph114_A9
zmp_ph114_A11
zmp_ph114_B2
zmp_ph114_B4
zmp_ph114_D8
zmp_ph114_E2

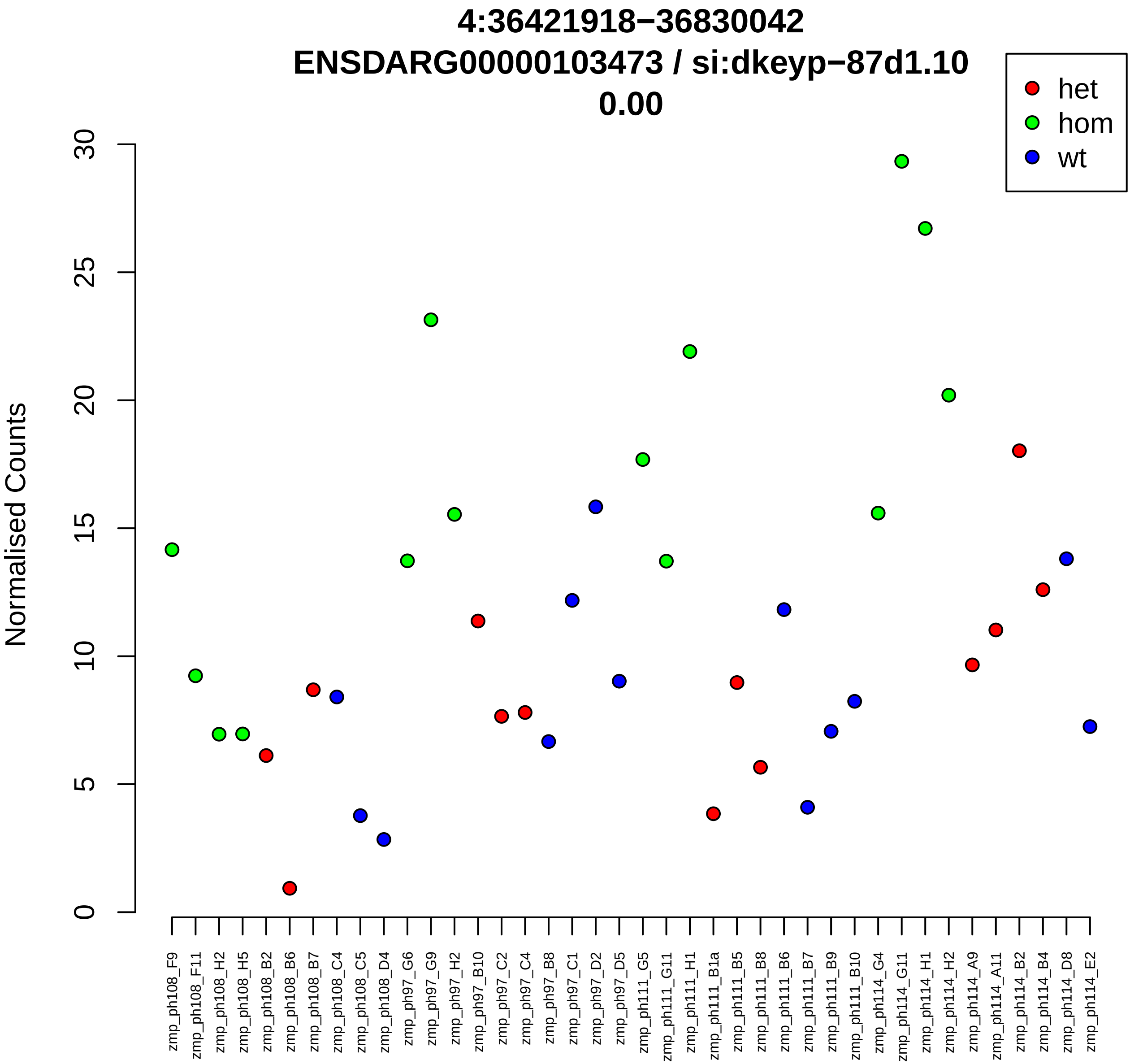

4:36421918−36830042
ENSDARG00000103473 / si:dkeyp−87d1.10
0.00
het
hom
wt
Normalised Counts
0
5
10
15
20
25
30
zmp_ph108_F9
zmp_ph108_F11
zmp_ph108_H2
zmp_ph108_H5
zmp_ph108_B2
zmp_ph108_B6
zmp_ph108_B7
zmp_ph108_C4
zmp_ph108_C5
zmp_ph108_D4
zmp_ph97_G6
zmp_ph97_G9
zmp_ph97_H2
zmp_ph97_B10
zmp_ph97_C2
zmp_ph97_C4
zmp_ph97_B8
zmp_ph97_C1
zmp_ph97_D2
zmp_ph97_D5
zmp_ph111_G5
zmp_ph111_G11
zmp_ph111_H1
zmp_ph111_B1a
zmp_ph111_B5
zmp_ph111_B8
zmp_ph111_B6
zmp_ph111_B7
zmp_ph111_B9
zmp_ph111_B10
zmp_ph114_G4
zmp_ph114_G11
zmp_ph114_H1
zmp_ph114_H2
zmp_ph114_A9
zmp_ph114_A11
zmp_ph114_B2
zmp_ph114_B4
zmp_ph114_D8
zmp_ph114_E2

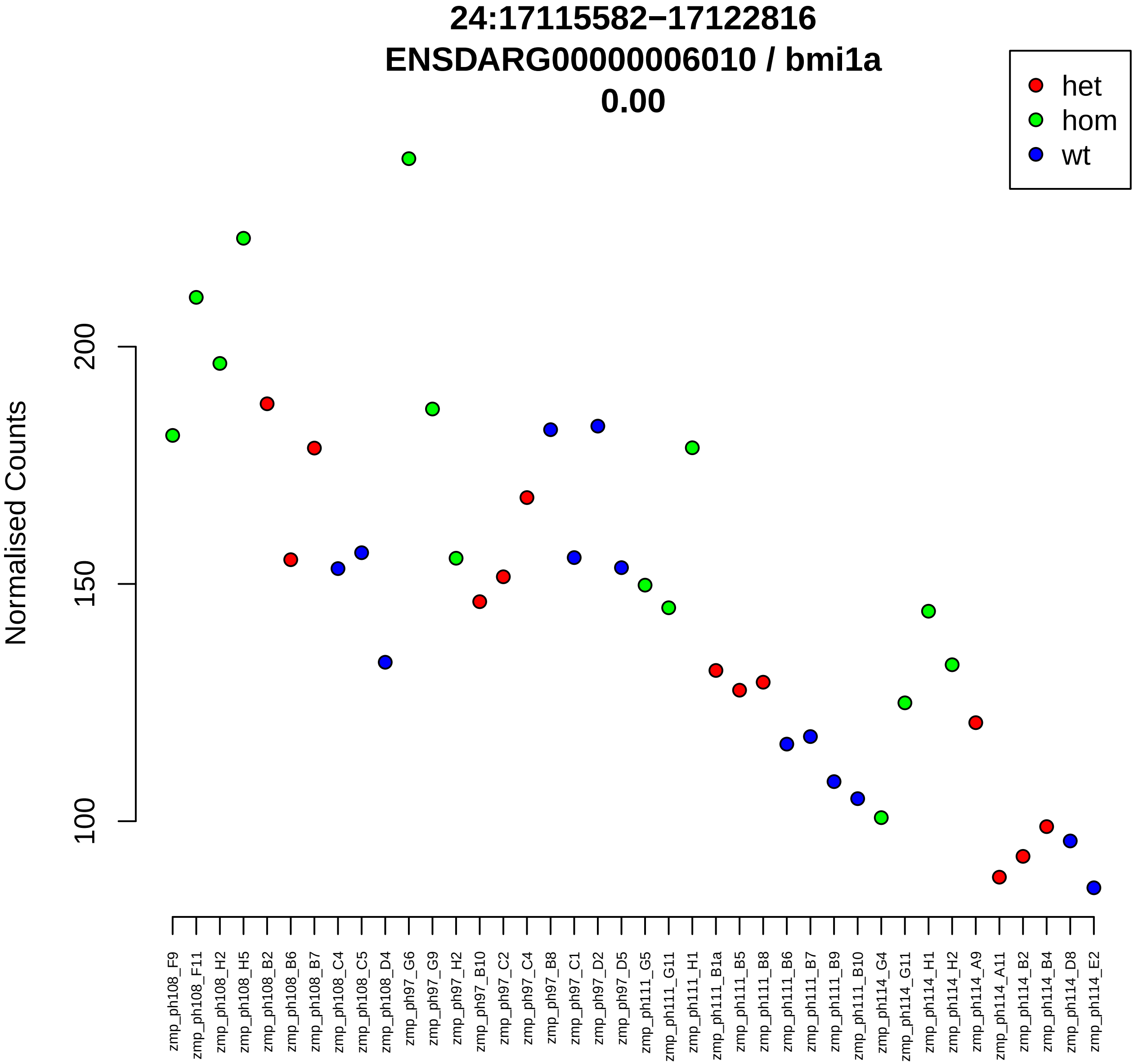
24:17115582−17122816
ENSDARG00000006010 / bmi1a
0.00
het
hom
wt
Normalised Counts
100
150
200
zmp_ph108_F9
zmp_ph108_F11
zmp_ph108_H2
zmp_ph108_H5
zmp_ph108_B2
zmp_ph108_B6
zmp_ph108_B7
zmp_ph108_C4
zmp_ph108_C5
zmp_ph108_D4
zmp_ph97_G6
zmp_ph97_G9
zmp_ph97_H2
zmp_ph97_B10
zmp_ph97_C2
zmp_ph97_C4
zmp_ph97_B8
zmp_ph97_C1
zmp_ph97_D2
zmp_ph97_D5
zmp_ph111_G5
zmp_ph111_G11
zmp_ph111_H1
zmp_ph111_B1a
zmp_ph111_B5
zmp_ph111_B8
zmp_ph111_B6
zmp_ph111_B7
zmp_ph111_B9
zmp_ph111_B10
zmp_ph114_G4
zmp_ph114_G11
zmp_ph114_H1
zmp_ph114_H2
zmp_ph114_A9
zmp_ph114_A11
zmp_ph114_B2
zmp_ph114_B4
zmp_ph114_D8
zmp_ph114_E2

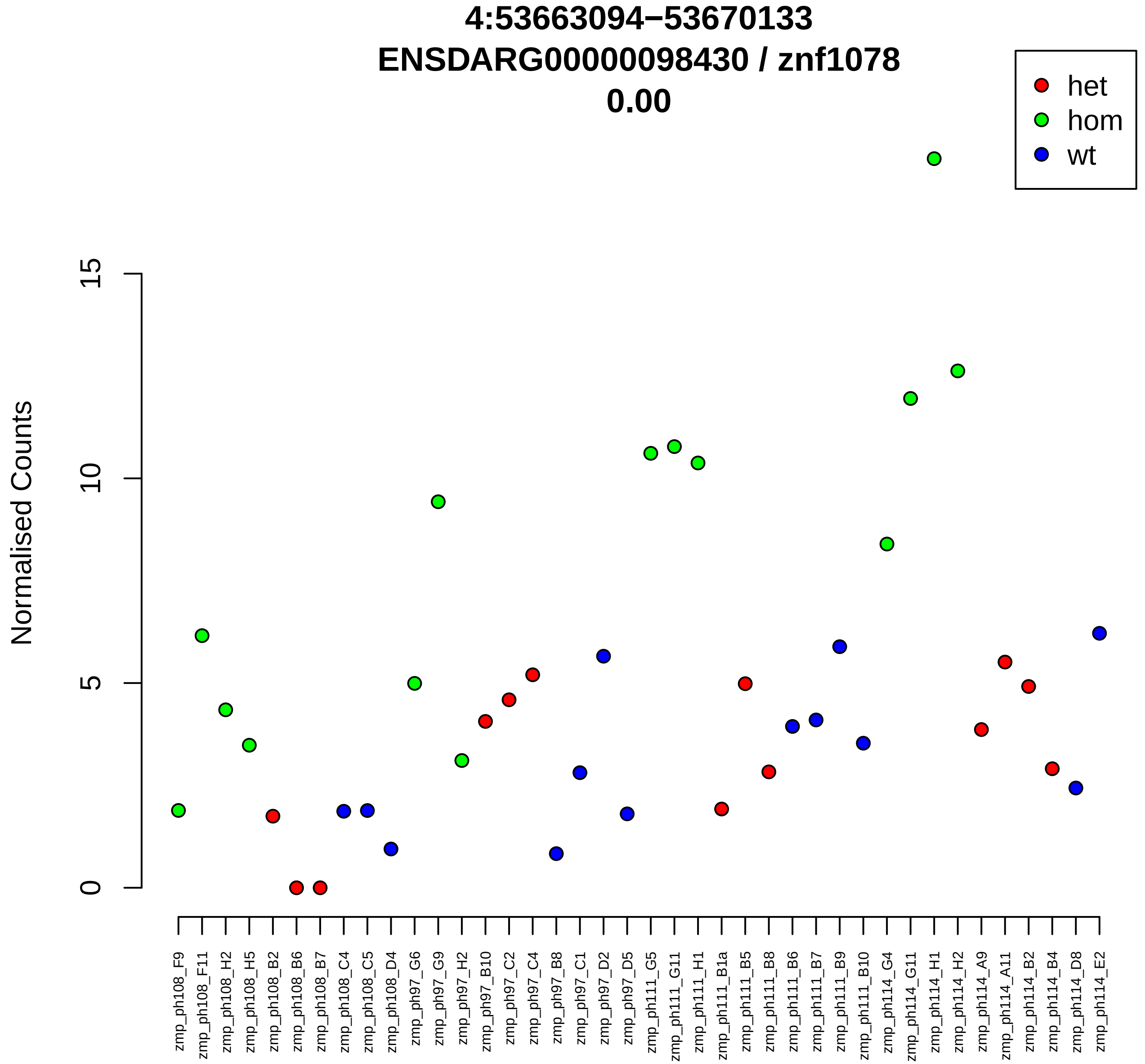
4:53663094−53670133
ENSDARG00000098430 / znf1078
0.00
het
hom
wt
Normalised Counts
0
5
10
15
zmp_ph108_F9
zmp_ph108_F11
zmp_ph108_H2
zmp_ph108_H5
zmp_ph108_B2
zmp_ph108_B6
zmp_ph108_B7
zmp_ph108_C4
zmp_ph108_C5
zmp_ph108_D4
zmp_ph97_G6
zmp_ph97_G9
zmp_ph97_H2
zmp_ph97_B10
zmp_ph97_C2
zmp_ph97_C4
zmp_ph97_B8
zmp_ph97_C1
zmp_ph97_D2
zmp_ph97_D5
zmp_ph111_G5
zmp_ph111_G11
zmp_ph111_H1
zmp_ph111_B1a
zmp_ph111_B5
zmp_ph111_B8
zmp_ph111_B6
zmp_ph111_B7
zmp_ph111_B9
zmp_ph111_B10
zmp_ph114_G4
zmp_ph114_G11
zmp_ph114_H1
zmp_ph114_H2
zmp_ph114_A9
zmp_ph114_A11
zmp_ph114_B2
zmp_ph114_B4
zmp_ph114_D8
zmp_ph114_E2

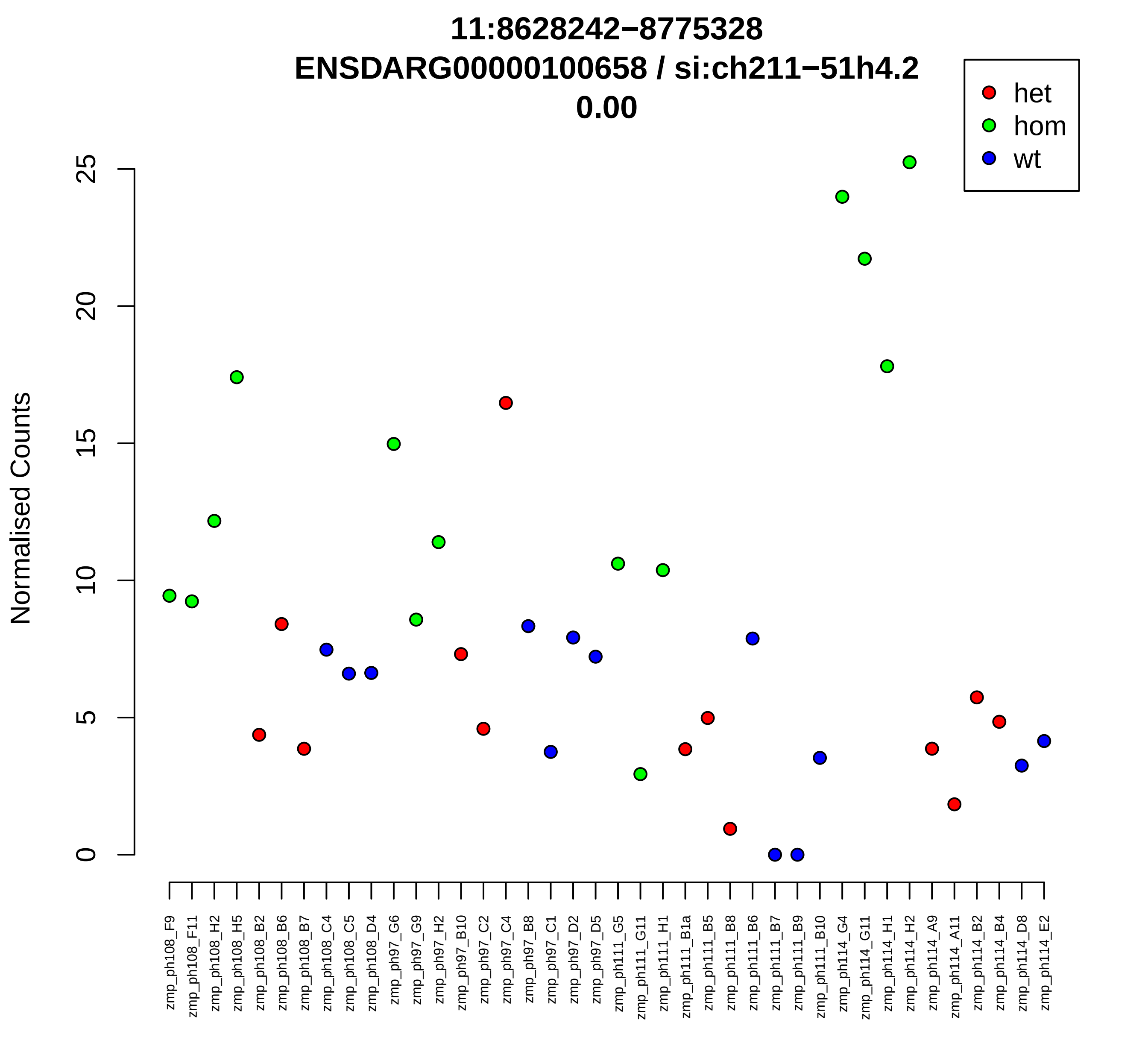
11:8628242−8775328
ENSDARG00000100658 / si:ch211−51h4.2
0.00
het
hom
wt
Normalised Counts
0
5
10
15
20
25
zmp_ph108_F9
zmp_ph108_F11
zmp_ph108_H2
zmp_ph108_H5
zmp_ph108_B2
zmp_ph108_B6
zmp_ph108_B7
zmp_ph108_C4
zmp_ph108_C5
zmp_ph108_D4
zmp_ph97_G6
zmp_ph97_G9
zmp_ph97_H2
zmp_ph97_B10
zmp_ph97_C2
zmp_ph97_C4
zmp_ph97_B8
zmp_ph97_C1
zmp_ph97_D2
zmp_ph97_D5
zmp_ph111_G5
zmp_ph111_G11
zmp_ph111_H1
zmp_ph111_B1a
zmp_ph111_B5
zmp_ph111_B8
zmp_ph111_B6
zmp_ph111_B7
zmp_ph111_B9
zmp_ph111_B10
zmp_ph114_G4
zmp_ph114_G11
zmp_ph114_H1
zmp_ph114_H2
zmp_ph114_A9
zmp_ph114_A11
zmp_ph114_B2
zmp_ph114_B4
zmp_ph114_D8
zmp_ph114_E2

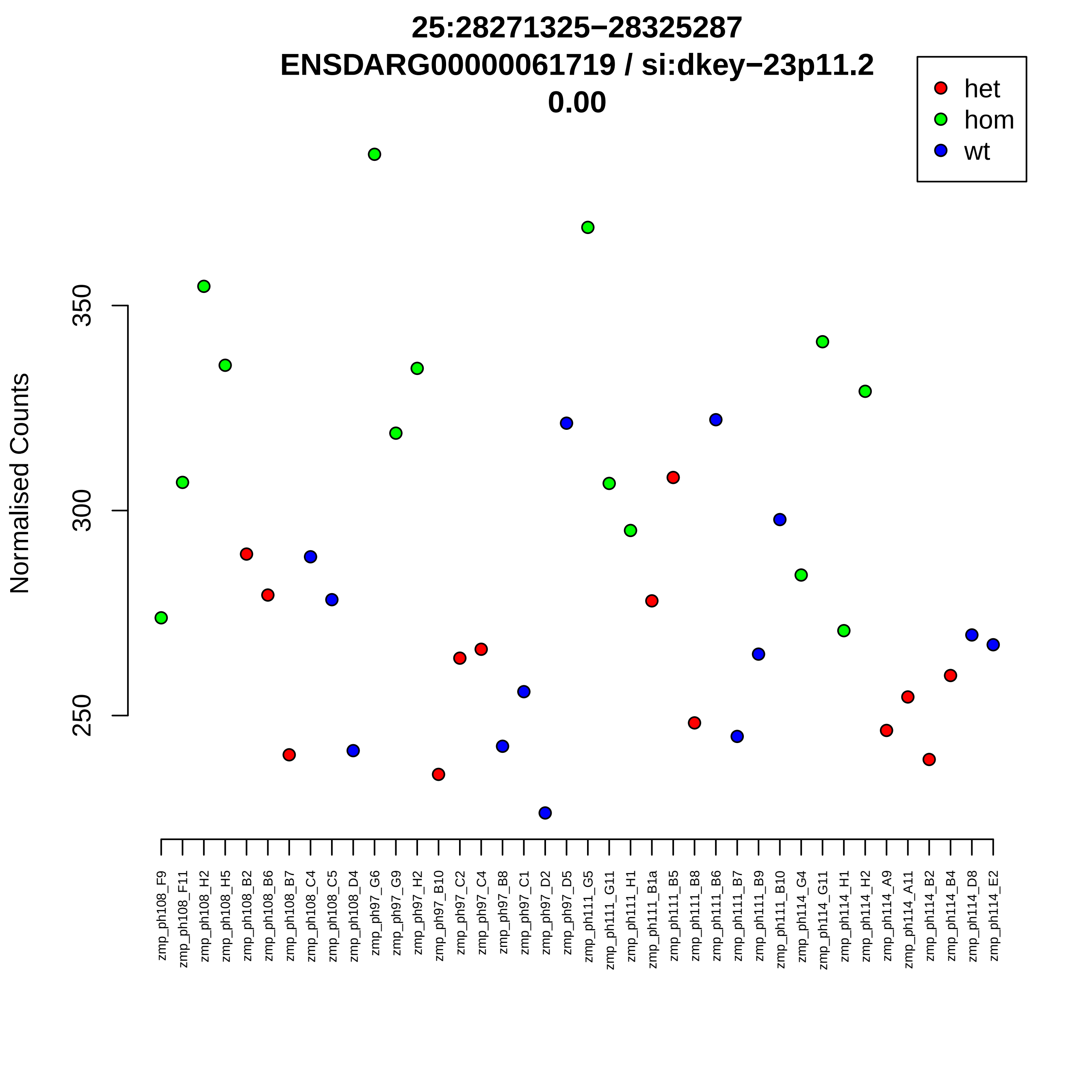
25:28271325−28325287
ENSDARG00000061719 / si:dkey−23p11.2
0.00
het
hom
wt
Normalised Counts
250
300
350
zmp_ph108_F9
zmp_ph108_F11
zmp_ph108_H2
zmp_ph108_H5
zmp_ph108_B2
zmp_ph108_B6
zmp_ph108_B7
zmp_ph108_C4
zmp_ph108_C5
zmp_ph108_D4
zmp_ph97_G6
zmp_ph97_G9
zmp_ph97_H2
zmp_ph97_B10
zmp_ph97_C2
zmp_ph97_C4
zmp_ph97_B8
zmp_ph97_C1
zmp_ph97_D2
zmp_ph97_D5
zmp_ph111_G5
zmp_ph111_G11
zmp_ph111_H1
zmp_ph111_B1a
zmp_ph111_B5
zmp_ph111_B8
zmp_ph111_B6
zmp_ph111_B7
zmp_ph111_B9
zmp_ph111_B10
zmp_ph114_G4
zmp_ph114_G11
zmp_ph114_H1
zmp_ph114_H2
zmp_ph114_A9
zmp_ph114_A11
zmp_ph114_B2
zmp_ph114_B4
zmp_ph114_D8
zmp_ph114_E2

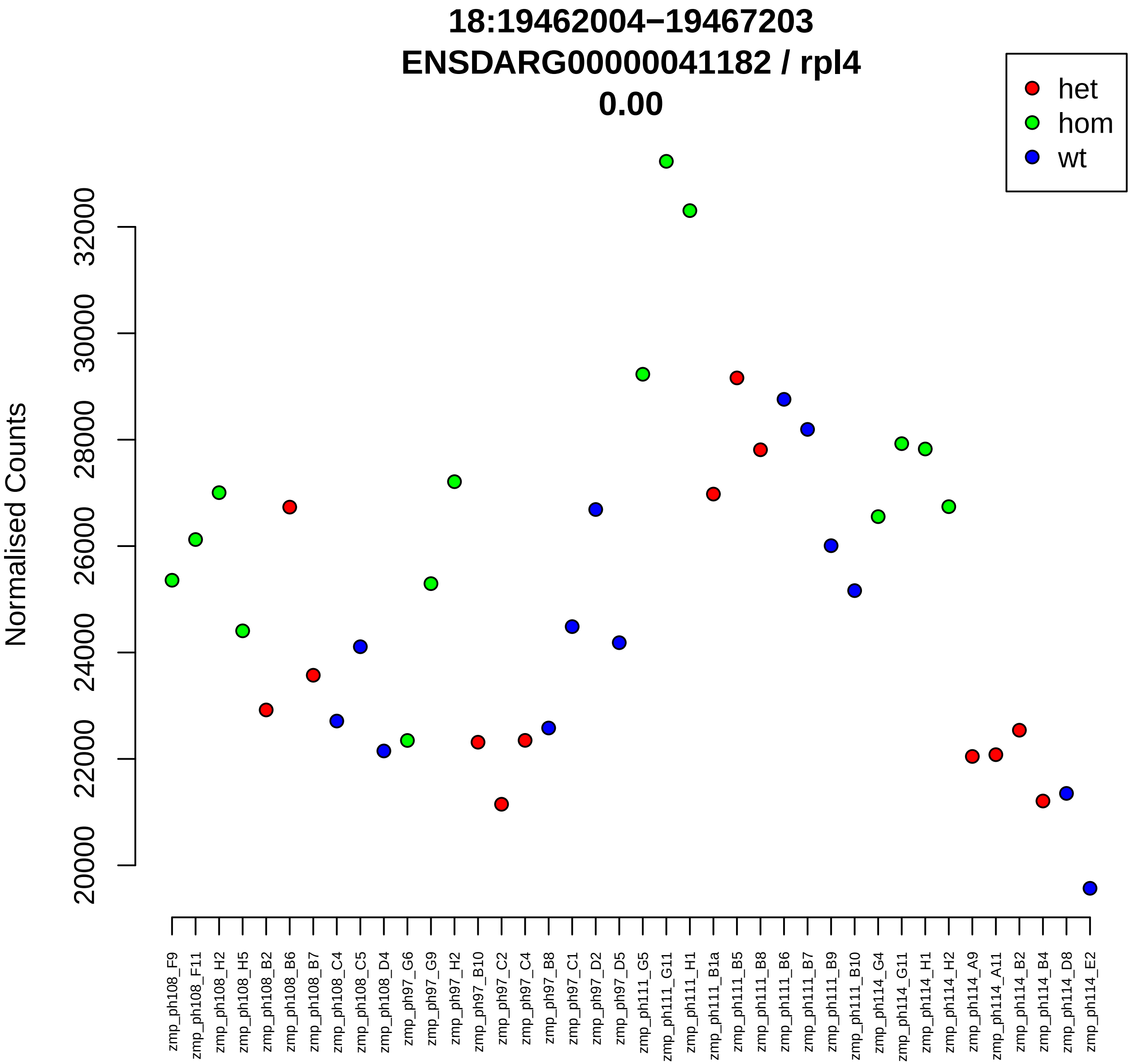
18:19462004−19467203
ENSDARG00000041182 / rpl4
0.00
het
hom
wt
Normalised Counts
20000
22000
24000
26000
28000
30000
32000
zmp_ph108_F9
zmp_ph108_F11
zmp_ph108_H2
zmp_ph108_H5
zmp_ph108_B2
zmp_ph108_B6
zmp_ph108_B7
zmp_ph108_C4
zmp_ph108_C5
zmp_ph108_D4
zmp_ph97_G6
zmp_ph97_G9
zmp_ph97_H2
zmp_ph97_B10
zmp_ph97_C2
zmp_ph97_C4
zmp_ph97_B8
zmp_ph97_C1
zmp_ph97_D2
zmp_ph97_D5
zmp_ph111_G5
zmp_ph111_G11
zmp_ph111_H1
zmp_ph111_B1a
zmp_ph111_B5
zmp_ph111_B8
zmp_ph111_B6
zmp_ph111_B7
zmp_ph111_B9
zmp_ph111_B10
zmp_ph114_G4
zmp_ph114_G11
zmp_ph114_H1
zmp_ph114_H2
zmp_ph114_A9
zmp_ph114_A11
zmp_ph114_B2
zmp_ph114_B4
zmp_ph114_D8
zmp_ph114_E2

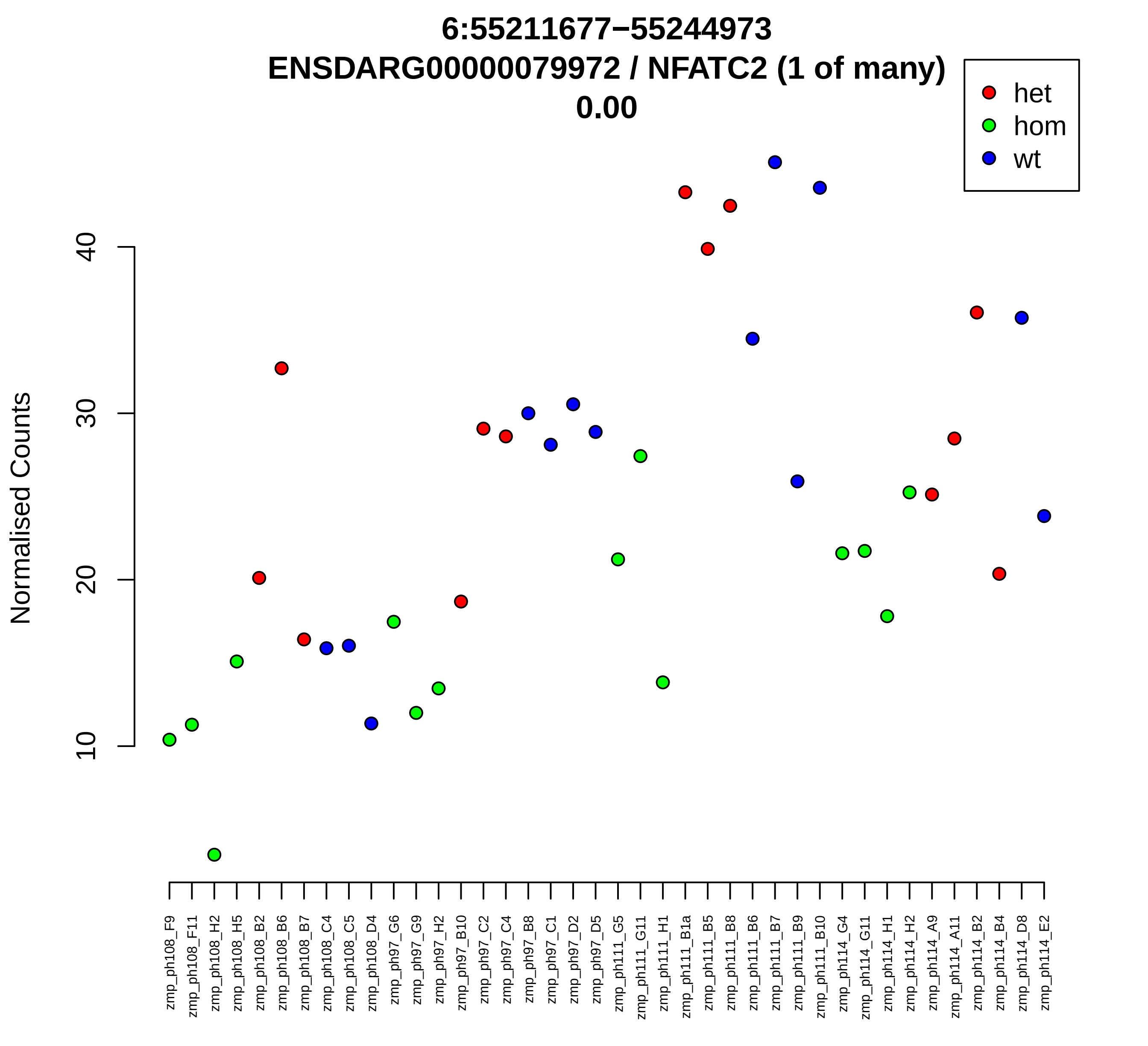

6:55211677−55244973
ENSDARG00000079972 / NFATC2 (1 of many)
0.00
het
hom
wt
Normalised Counts
10
20
30
40
zmp_ph108_F9
zmp_ph108_F11
zmp_ph108_H2
zmp_ph108_H5
zmp_ph108_B2
zmp_ph108_B6
zmp_ph108_B7
zmp_ph108_C4
zmp_ph108_C5
zmp_ph108_D4
zmp_ph97_G6
zmp_ph97_G9
zmp_ph97_H2
zmp_ph97_B10
zmp_ph97_C2
zmp_ph97_C4
zmp_ph97_B8
zmp_ph97_C1
zmp_ph97_D2
zmp_ph97_D5
zmp_ph111_G5
zmp_ph111_G11
zmp_ph111_H1
zmp_ph111_B1a
zmp_ph111_B5
zmp_ph111_B8
zmp_ph111_B6
zmp_ph111_B7
zmp_ph111_B9
zmp_ph111_B10
zmp_ph114_G4
zmp_ph114_G11
zmp_ph114_H1
zmp_ph114_H2
zmp_ph114_A9
zmp_ph114_A11
zmp_ph114_B2
zmp_ph114_B4
zmp_ph114_D8
zmp_ph114_E2

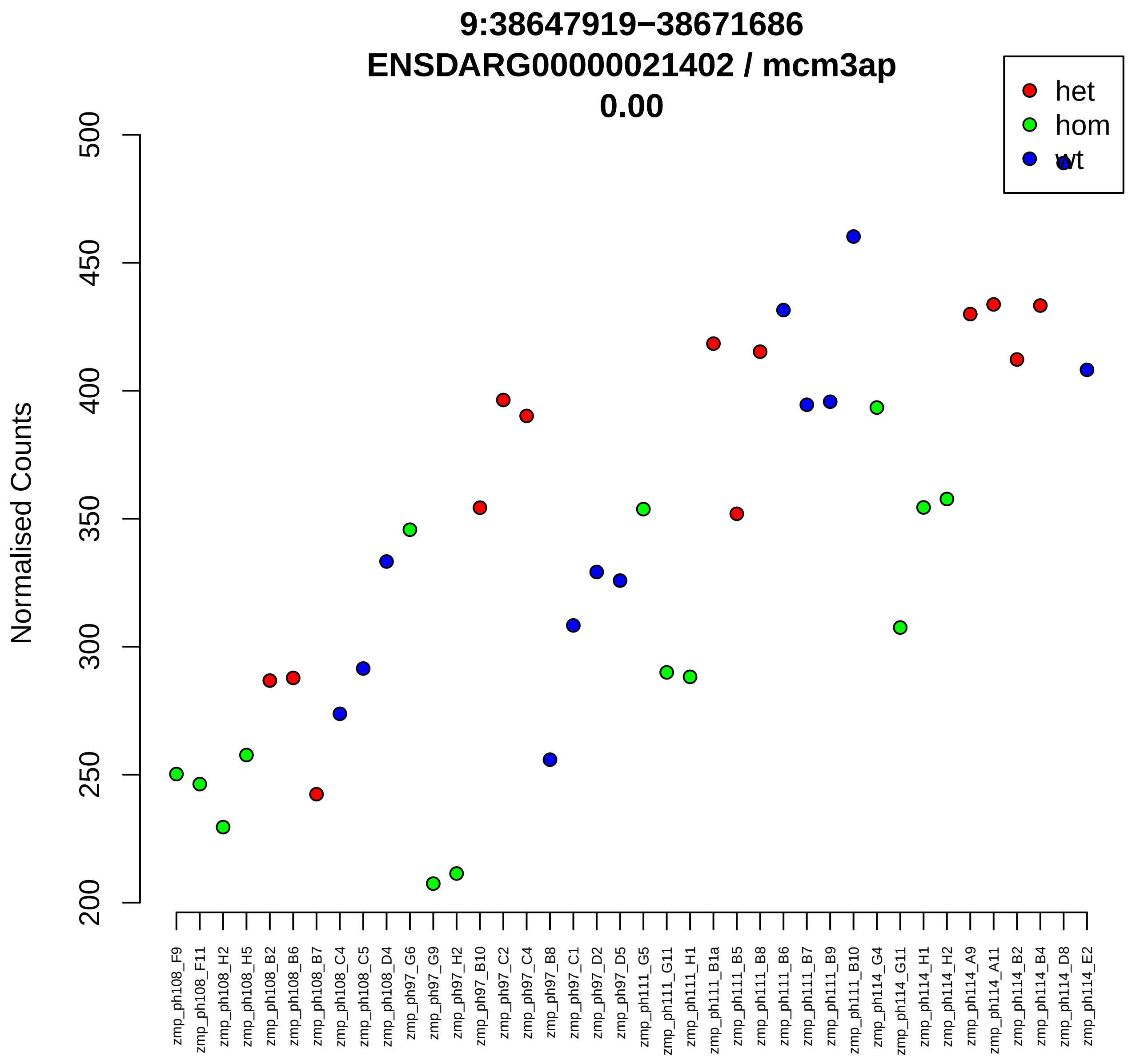

9:38647919−38671686
ENSDARG00000021402 / mcm3ap
0.00
Normalised Counts
het
hom
wt
200
250
300
350
400
450
500
zmp_ph108_F9
zmp_ph108_F11
zmp_ph108_H2
zmp_ph108_H5
zmp_ph108_B2
zmp_ph108_B6
zmp_ph108_B7
zmp_ph108_C4
zmp_ph108_C5
zmp_ph108_D4
zmp_ph97_G6
zmp_ph97_G9
zmp_ph97_H2
zmp_ph97_B10
zmp_ph97_C2
zmp_ph97_C4
zmp_ph97_B8
zmp_ph97_C1
zmp_ph97_D2
zmp_ph97_D5
zmp_ph111_G5
zmp_ph111_G11
zmp_ph111_H1
zmp_ph111_B1a
zmp_ph111_B5
zmp_ph111_B8
zmp_ph111_B6
zmp_ph111_B7
zmp_ph111_B9
zmp_ph111_B10
zmp_ph114_G4
zmp_ph114_G11
zmp_ph114_H1
zmp_ph114_H2
zmp_ph114_A9
zmp_ph114_A11
zmp_ph114_B2
zmp_ph114_B4
zmp_ph114_D8
zmp_ph114_E2

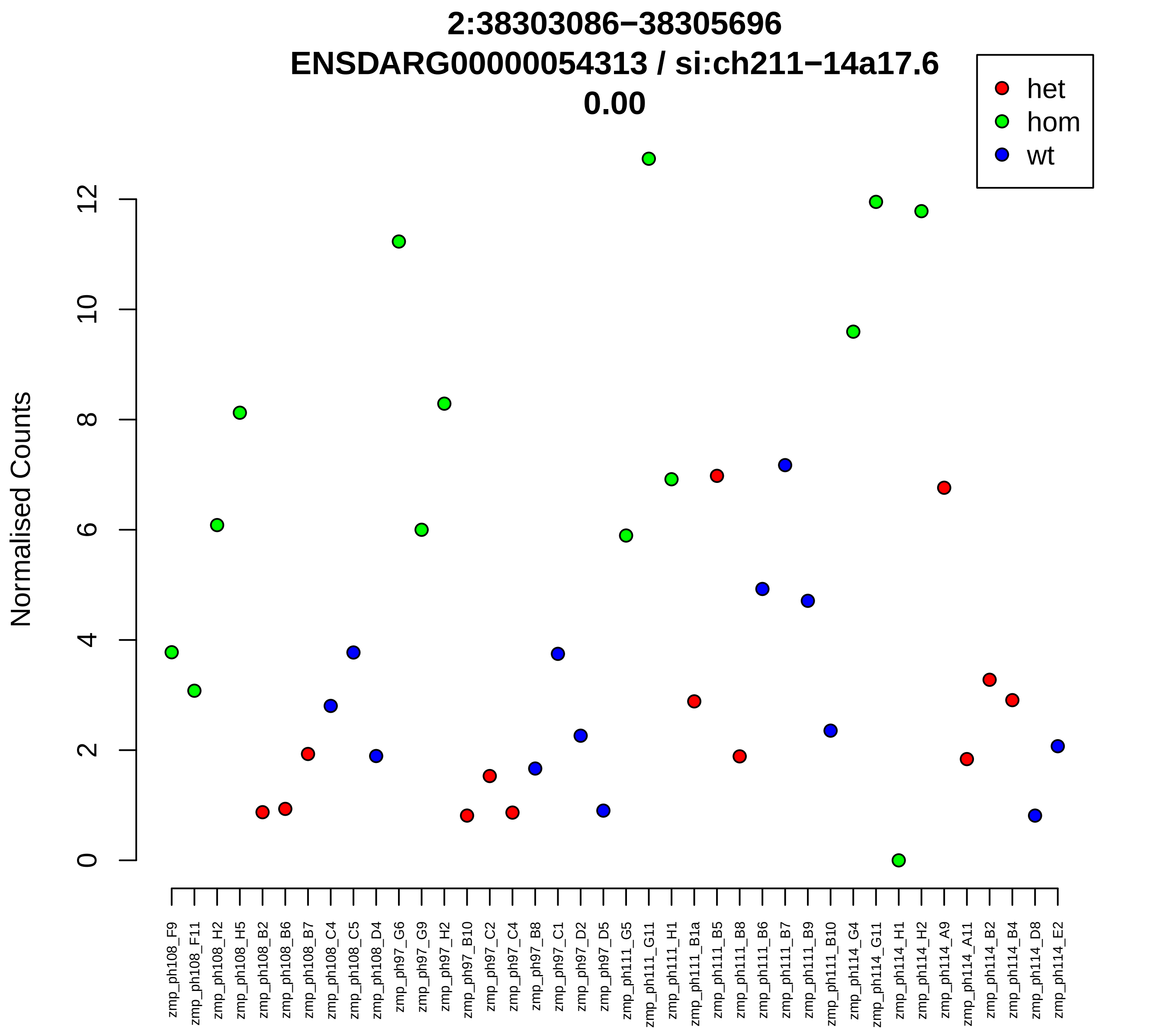

2:38303086−38305696
ENSDARG00000054313 / si:ch211−14a17.6
0.00
het
hom
wt
Normalised Counts
0
2
4
6
8
10
12
zmp_ph108_F9
zmp_ph108_F11
zmp_ph108_H2
zmp_ph108_H5
zmp_ph108_B2
zmp_ph108_B6
zmp_ph108_B7
zmp_ph108_C4
zmp_ph108_C5
zmp_ph108_D4
zmp_ph97_G6
zmp_ph97_G9
zmp_ph97_H2
zmp_ph97_B10
zmp_ph97_C2
zmp_ph97_C4
zmp_ph97_B8
zmp_ph97_C1
zmp_ph97_D2
zmp_ph97_D5
zmp_ph111_G5
zmp_ph111_G11
zmp_ph111_H1
zmp_ph111_B1a
zmp_ph111_B5
zmp_ph111_B8
zmp_ph111_B6
zmp_ph111_B7
zmp_ph111_B9
zmp_ph111_B10
zmp_ph114_G4
zmp_ph114_G11
zmp_ph114_H1
zmp_ph114_H2
zmp_ph114_A9
zmp_ph114_A11
zmp_ph114_B2
zmp_ph114_B4
zmp_ph114_D8
zmp_ph114_E2

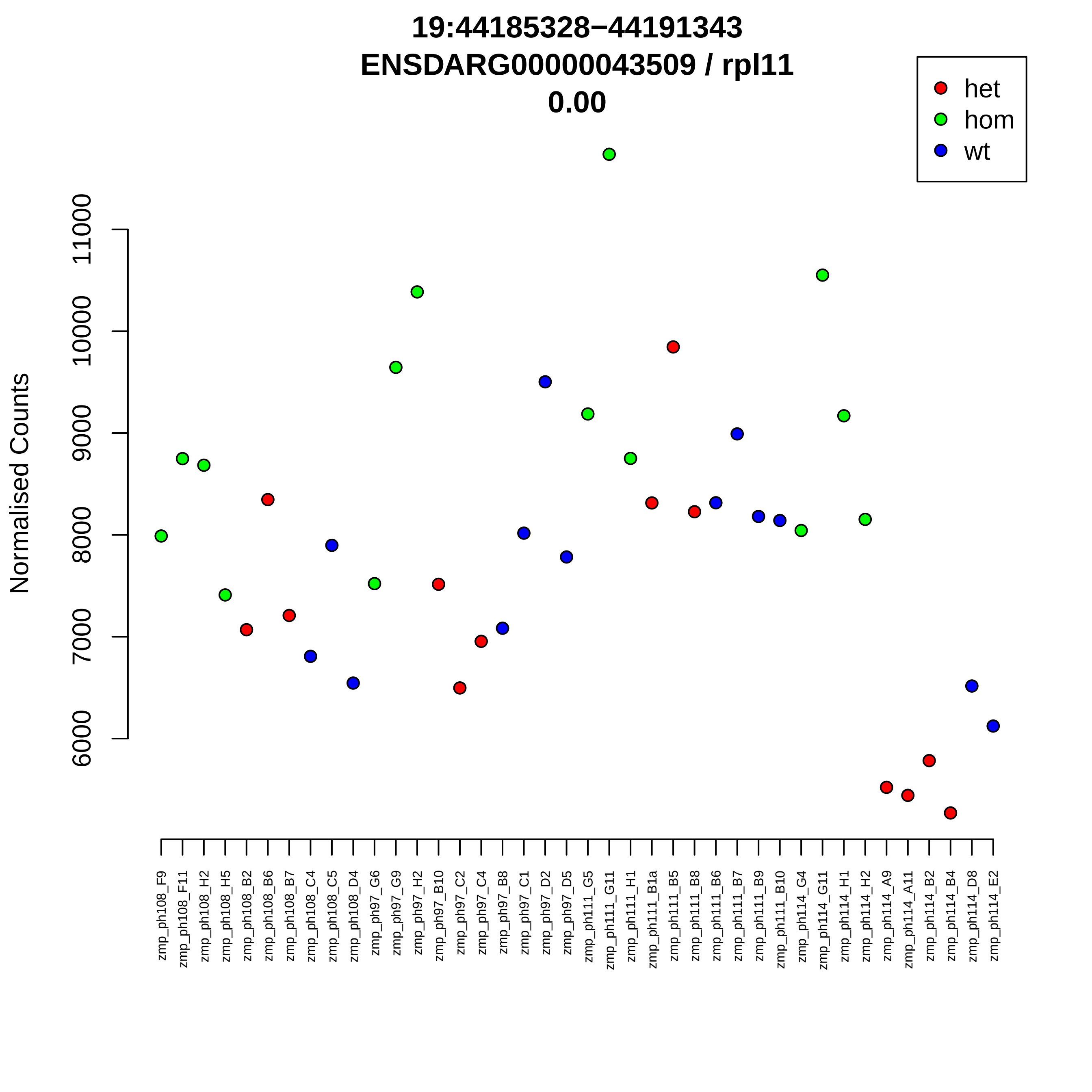
19:44185328−44191343
ENSDARG00000043509 / rpl11
0.00
het
hom
wt
Normalised Counts
6000
7000
8000
9000
10000
11000
zmp_ph108_F9
zmp_ph108_F11
zmp_ph108_H2
zmp_ph108_H5
zmp_ph108_B2
zmp_ph108_B6
zmp_ph108_B7
zmp_ph108_C4
zmp_ph108_C5
zmp_ph108_D4
zmp_ph97_G6
zmp_ph97_G9
zmp_ph97_H2
zmp_ph97_B10
zmp_ph97_C2
zmp_ph97_C4
zmp_ph97_B8
zmp_ph97_C1
zmp_ph97_D2
zmp_ph97_D5
zmp_ph111_G5
zmp_ph111_G11
zmp_ph111_H1
zmp_ph111_B1a
zmp_ph111_B5
zmp_ph111_B8
zmp_ph111_B6
zmp_ph111_B7
zmp_ph111_B9
zmp_ph111_B10
zmp_ph114_G4
zmp_ph114_G11
zmp_ph114_H1
zmp_ph114_H2
zmp_ph114_A9
zmp_ph114_A11
zmp_ph114_B2
zmp_ph114_B4
zmp_ph114_D8
zmp_ph114_E2

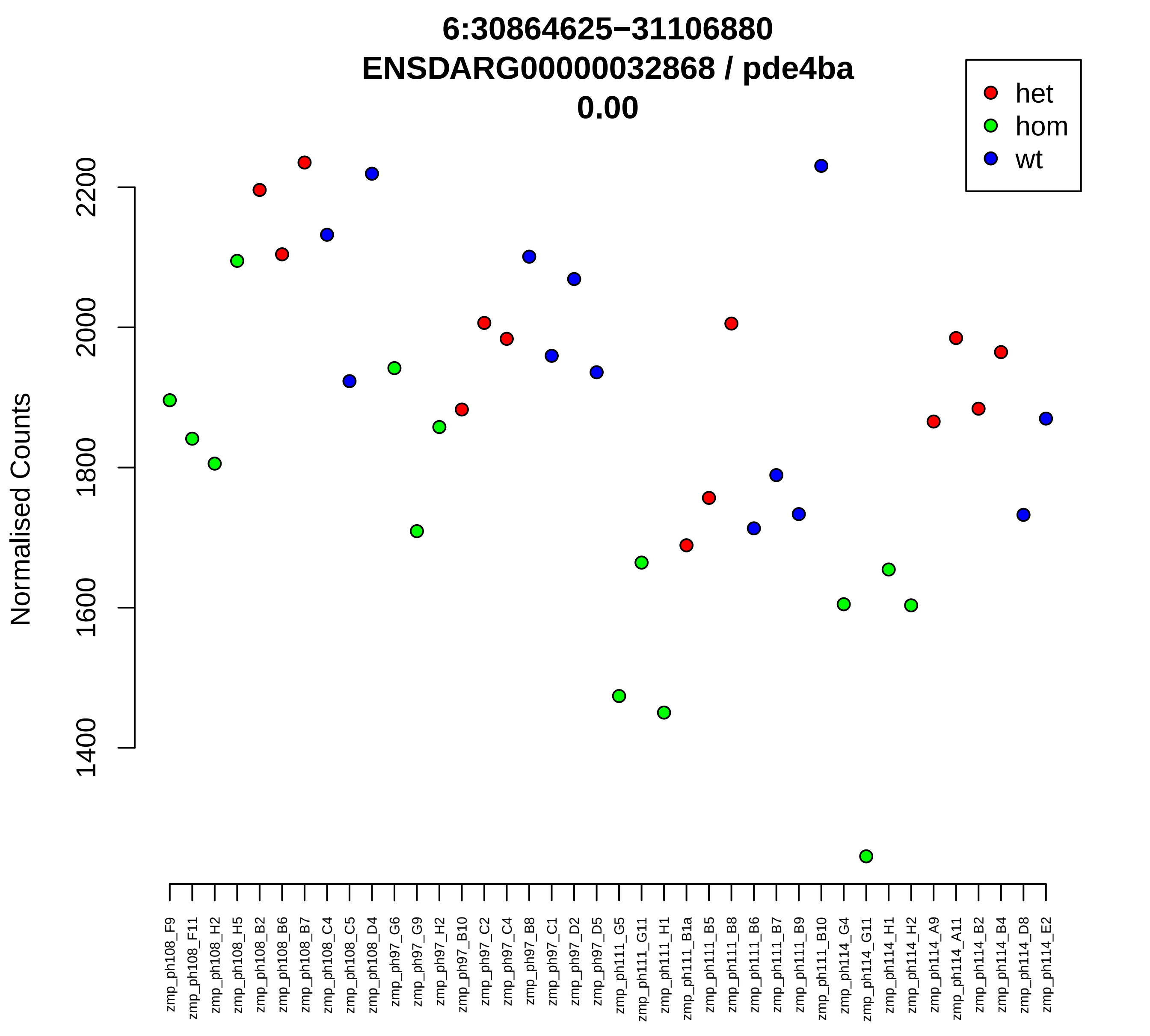
6:30864625−31106880
ENSDARG00000032868 / pde4ba
0.00
het
hom
wt
Normalised Counts
1400
1600
1800
2000
2200
zmp_ph108_F9
zmp_ph108_F11
zmp_ph108_H2
zmp_ph108_H5
zmp_ph108_B2
zmp_ph108_B6
zmp_ph108_B7
zmp_ph108_C4
zmp_ph108_C5
zmp_ph108_D4
zmp_ph97_G6
zmp_ph97_G9
zmp_ph97_H2
zmp_ph97_B10
zmp_ph97_C2
zmp_ph97_C4
zmp_ph97_B8
zmp_ph97_C1
zmp_ph97_D2
zmp_ph97_D5
zmp_ph111_G5
zmp_ph111_G11
zmp_ph111_H1
zmp_ph111_B1a
zmp_ph111_B5
zmp_ph111_B8
zmp_ph111_B6
zmp_ph111_B7
zmp_ph111_B9
zmp_ph111_B10
zmp_ph114_G4
zmp_ph114_G11
zmp_ph114_H1
zmp_ph114_H2
zmp_ph114_A9
zmp_ph114_A11
zmp_ph114_B2
zmp_ph114_B4
zmp_ph114_D8
zmp_ph114_E2

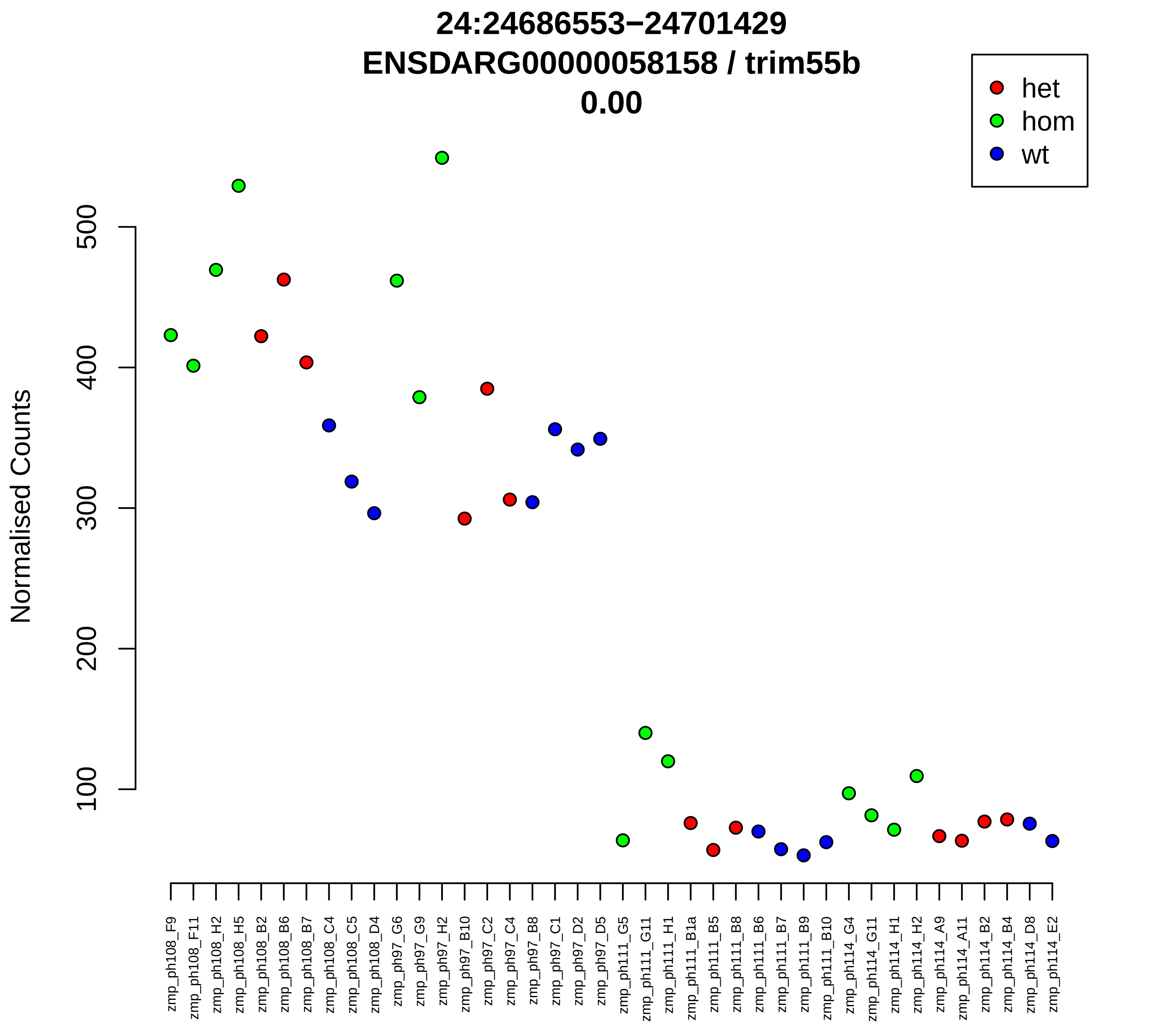

24:24686553−24701429
ENSDARG00000058158 / trim55b
0.00
het
hom
wt
Normalised Counts
100
200
300
400
500
zmp_ph108_F9
zmp_ph108_F11
zmp_ph108_H2
zmp_ph108_H5
zmp_ph108_B2
zmp_ph108_B6
zmp_ph108_B7
zmp_ph108_C4
zmp_ph108_C5
zmp_ph108_D4
zmp_ph97_G6
zmp_ph97_G9
zmp_ph97_H2
zmp_ph97_B10
zmp_ph97_C2
zmp_ph97_C4
zmp_ph97_B8
zmp_ph97_C1
zmp_ph97_D2
zmp_ph97_D5
zmp_ph111_G5
zmp_ph111_G11
zmp_ph111_H1
zmp_ph111_B1a
zmp_ph111_B5
zmp_ph111_B8
zmp_ph111_B6
zmp_ph111_B7
zmp_ph111_B9
zmp_ph111_B10
zmp_ph114_G4
zmp_ph114_G11
zmp_ph114_H1
zmp_ph114_H2
zmp_ph114_A9
zmp_ph114_A11
zmp_ph114_B2
zmp_ph114_B4
zmp_ph114_D8
zmp_ph114_E2

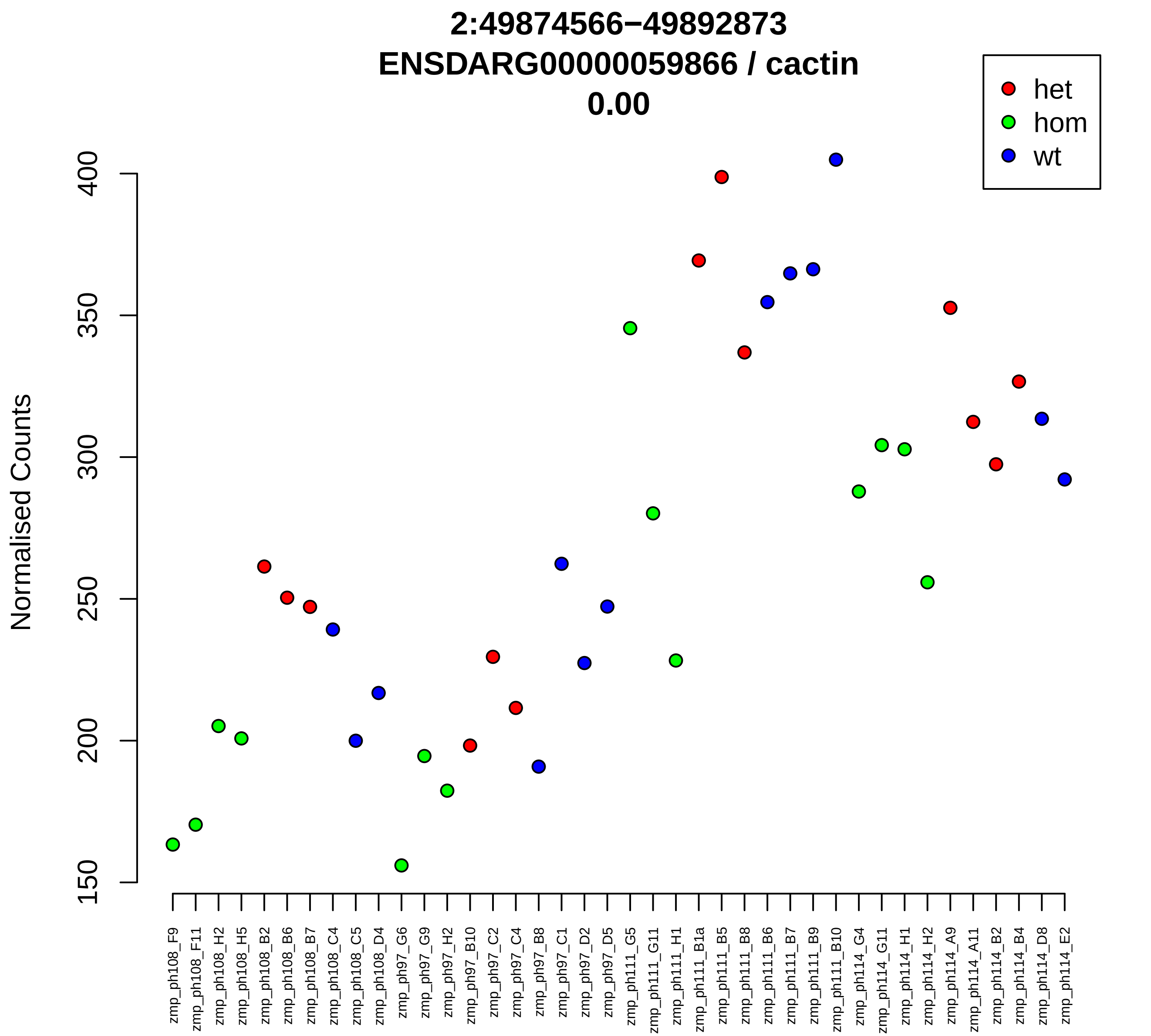
2:49874566−49892873
ENSDARG00000059866 / cactin
0.00
het
hom
wt
Normalised Counts
150
200
250
300
350
400
zmp_ph108_F9
zmp_ph108_F11
zmp_ph108_H2
zmp_ph108_H5
zmp_ph108_B2
zmp_ph108_B6
zmp_ph108_B7
zmp_ph108_C4
zmp_ph108_C5
zmp_ph108_D4
zmp_ph97_G6
zmp_ph97_G9
zmp_ph97_H2
zmp_ph97_B10
zmp_ph97_C2
zmp_ph97_C4
zmp_ph97_B8
zmp_ph97_C1
zmp_ph97_D2
zmp_ph97_D5
zmp_ph111_G5
zmp_ph111_G11
zmp_ph111_H1
zmp_ph111_B1a
zmp_ph111_B5
zmp_ph111_B8
zmp_ph111_B6
zmp_ph111_B7
zmp_ph111_B9
zmp_ph111_B10
zmp_ph114_G4
zmp_ph114_G11
zmp_ph114_H1
zmp_ph114_H2
zmp_ph114_A9
zmp_ph114_A11
zmp_ph114_B2
zmp_ph114_B4
zmp_ph114_D8
zmp_ph114_E2

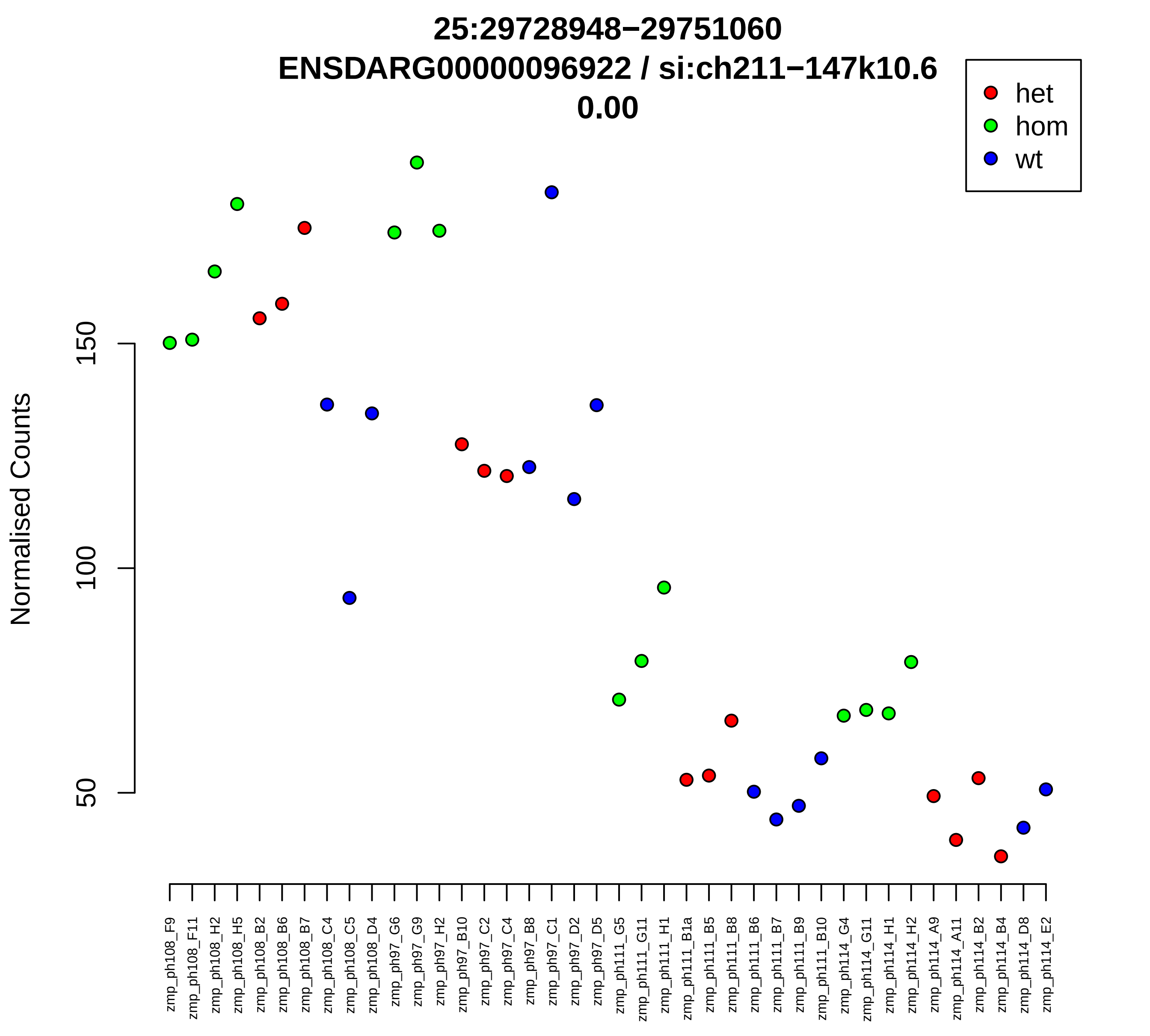

25:29728948−29751060
ENSDARG00000096922 / si:ch211−147k10.6
0.00
het
hom
wt
Normalised Counts
50
100
150
zmp_ph108_F9
zmp_ph108_F11
zmp_ph108_H2
zmp_ph108_H5
zmp_ph108_B2
zmp_ph108_B6
zmp_ph108_B7
zmp_ph108_C4
zmp_ph108_C5
zmp_ph108_D4
zmp_ph97_G6
zmp_ph97_G9
zmp_ph97_H2
zmp_ph97_B10
zmp_ph97_C2
zmp_ph97_C4
zmp_ph97_B8
zmp_ph97_C1
zmp_ph97_D2
zmp_ph97_D5
zmp_ph111_G5
zmp_ph111_G11
zmp_ph111_H1
zmp_ph111_B1a
zmp_ph111_B5
zmp_ph111_B8
zmp_ph111_B6
zmp_ph111_B7
zmp_ph111_B9
zmp_ph111_B10
zmp_ph114_G4
zmp_ph114_G11
zmp_ph114_H1
zmp_ph114_H2
zmp_ph114_A9
zmp_ph114_A11
zmp_ph114_B2
zmp_ph114_B4
zmp_ph114_D8
zmp_ph114_E2

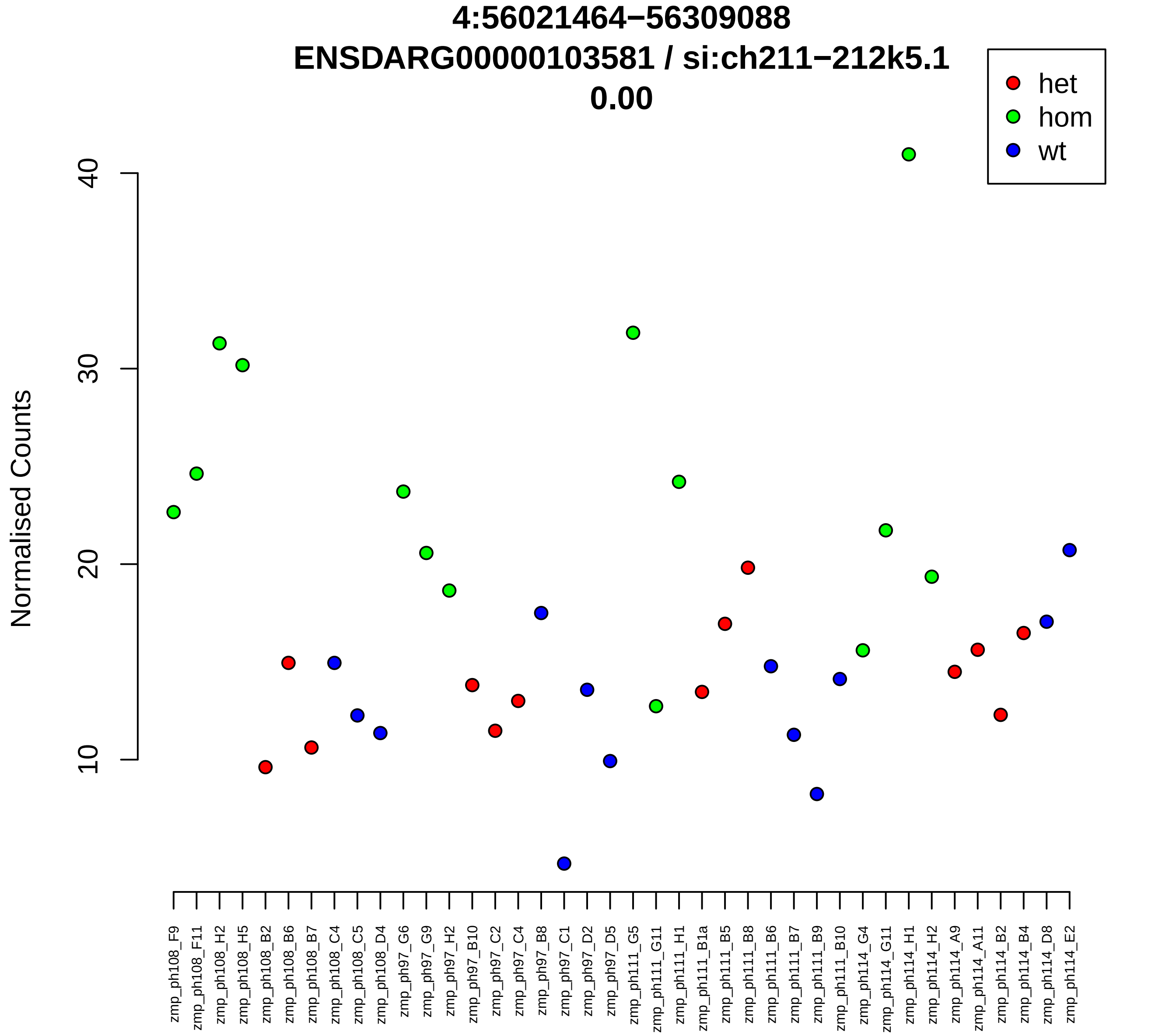

4:56021464−56309088
ENSDARG00000103581 / si:ch211−212k5.1
0.00
het
hom
wt
Normalised Counts
10
20
30
40
zmp_ph108_F9
zmp_ph108_F11
zmp_ph108_H2
zmp_ph108_H5
zmp_ph108_B2
zmp_ph108_B6
zmp_ph108_B7
zmp_ph108_C4
zmp_ph108_C5
zmp_ph108_D4
zmp_ph97_G6
zmp_ph97_G9
zmp_ph97_H2
zmp_ph97_B10
zmp_ph97_C2
zmp_ph97_C4
zmp_ph97_B8
zmp_ph97_C1
zmp_ph97_D2
zmp_ph97_D5
zmp_ph111_G5
zmp_ph111_G11
zmp_ph111_H1
zmp_ph111_B1a
zmp_ph111_B5
zmp_ph111_B8
zmp_ph111_B6
zmp_ph111_B7
zmp_ph111_B9
zmp_ph111_B10
zmp_ph114_G4
zmp_ph114_G11
zmp_ph114_H1
zmp_ph114_H2
zmp_ph114_A9
zmp_ph114_A11
zmp_ph114_B2
zmp_ph114_B4
zmp_ph114_D8
zmp_ph114_E2

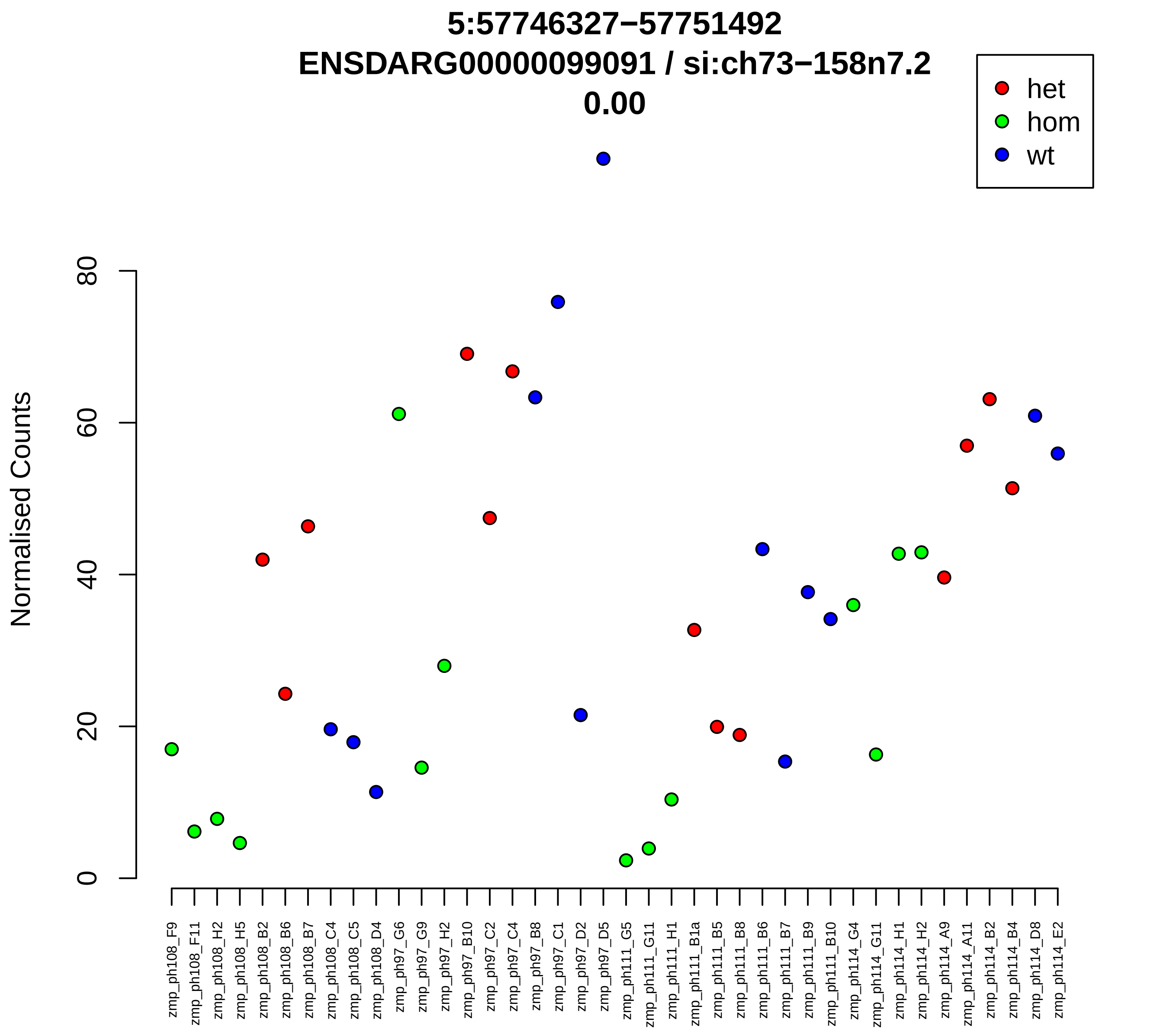

5:57746327−57751492
ENSDARG00000099091 / si:ch73−158n7.2
0.00
het
hom
wt
Normalised Counts
0
20
40
60
80
zmp_ph108_F9
zmp_ph108_F11
zmp_ph108_H2
zmp_ph108_H5
zmp_ph108_B2
zmp_ph108_B6
zmp_ph108_B7
zmp_ph108_C4
zmp_ph108_C5
zmp_ph108_D4
zmp_ph97_G6
zmp_ph97_G9
zmp_ph97_H2
zmp_ph97_B10
zmp_ph97_C2
zmp_ph97_C4
zmp_ph97_B8
zmp_ph97_C1
zmp_ph97_D2
zmp_ph97_D5
zmp_ph111_G5
zmp_ph111_G11
zmp_ph111_H1
zmp_ph111_B1a
zmp_ph111_B5
zmp_ph111_B8
zmp_ph111_B6
zmp_ph111_B7
zmp_ph111_B9
zmp_ph111_B10
zmp_ph114_G4
zmp_ph114_G11
zmp_ph114_H1
zmp_ph114_H2
zmp_ph114_A9
zmp_ph114_A11
zmp_ph114_B2
zmp_ph114_B4
zmp_ph114_D8
zmp_ph114_E2

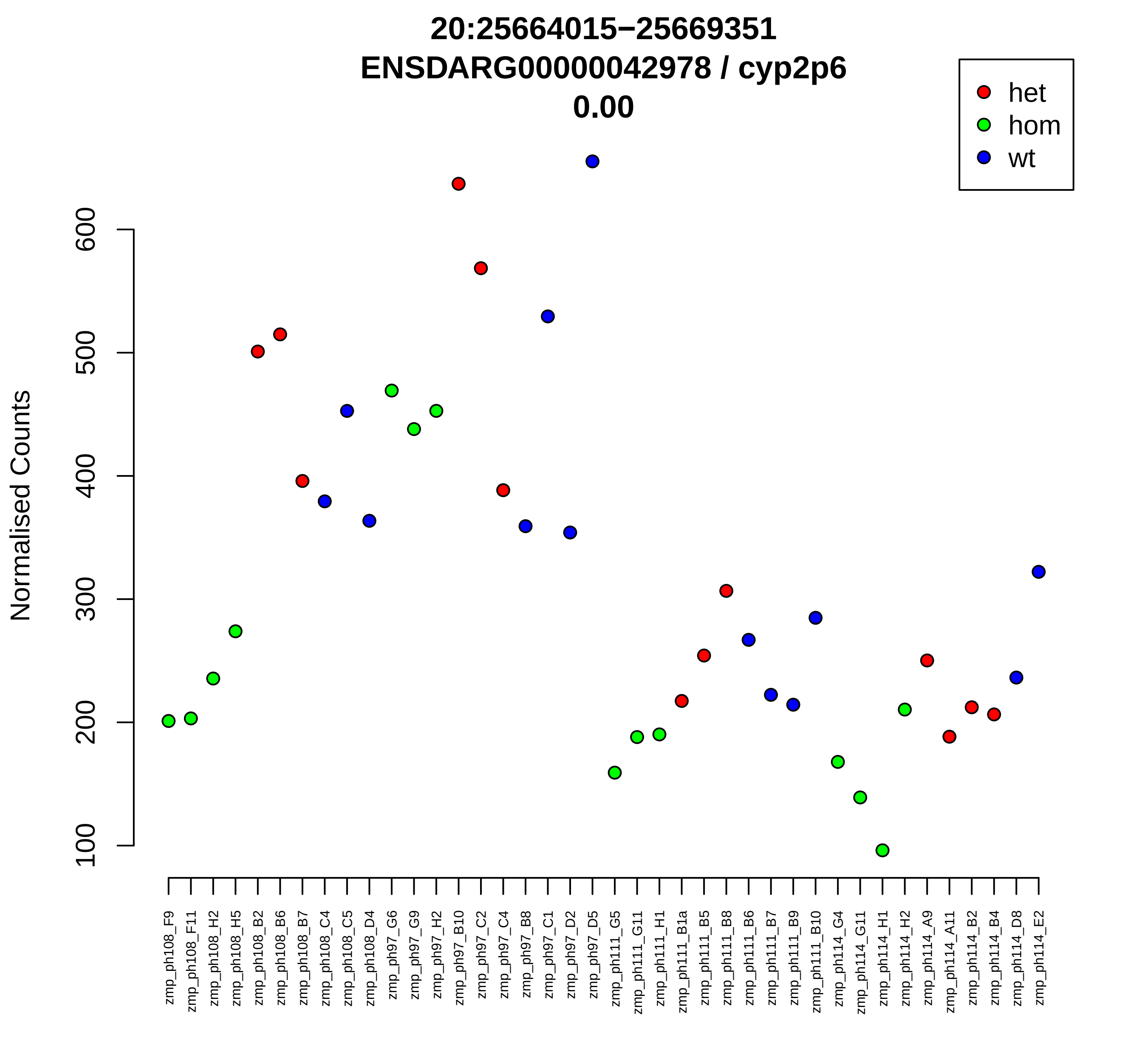

20:25664015−25669351
ENSDARG00000042978 / cyp2p6
0.00
het
hom
wt
Normalised Counts
100
200
300
400
500
600
zmp_ph108_F9
zmp_ph108_F11
zmp_ph108_H2
zmp_ph108_H5
zmp_ph108_B2
zmp_ph108_B6
zmp_ph108_B7
zmp_ph108_C4
zmp_ph108_C5
zmp_ph108_D4
zmp_ph97_G6
zmp_ph97_G9
zmp_ph97_H2
zmp_ph97_B10
zmp_ph97_C2
zmp_ph97_C4
zmp_ph97_B8
zmp_ph97_C1
zmp_ph97_D2
zmp_ph97_D5
zmp_ph111_G5
zmp_ph111_G11
zmp_ph111_H1
zmp_ph111_B1a
zmp_ph111_B5
zmp_ph111_B8
zmp_ph111_B6
zmp_ph111_B7
zmp_ph111_B9
zmp_ph111_B10
zmp_ph114_G4
zmp_ph114_G11
zmp_ph114_H1
zmp_ph114_H2
zmp_ph114_A9
zmp_ph114_A11
zmp_ph114_B2
zmp_ph114_B4
zmp_ph114_D8
zmp_ph114_E2

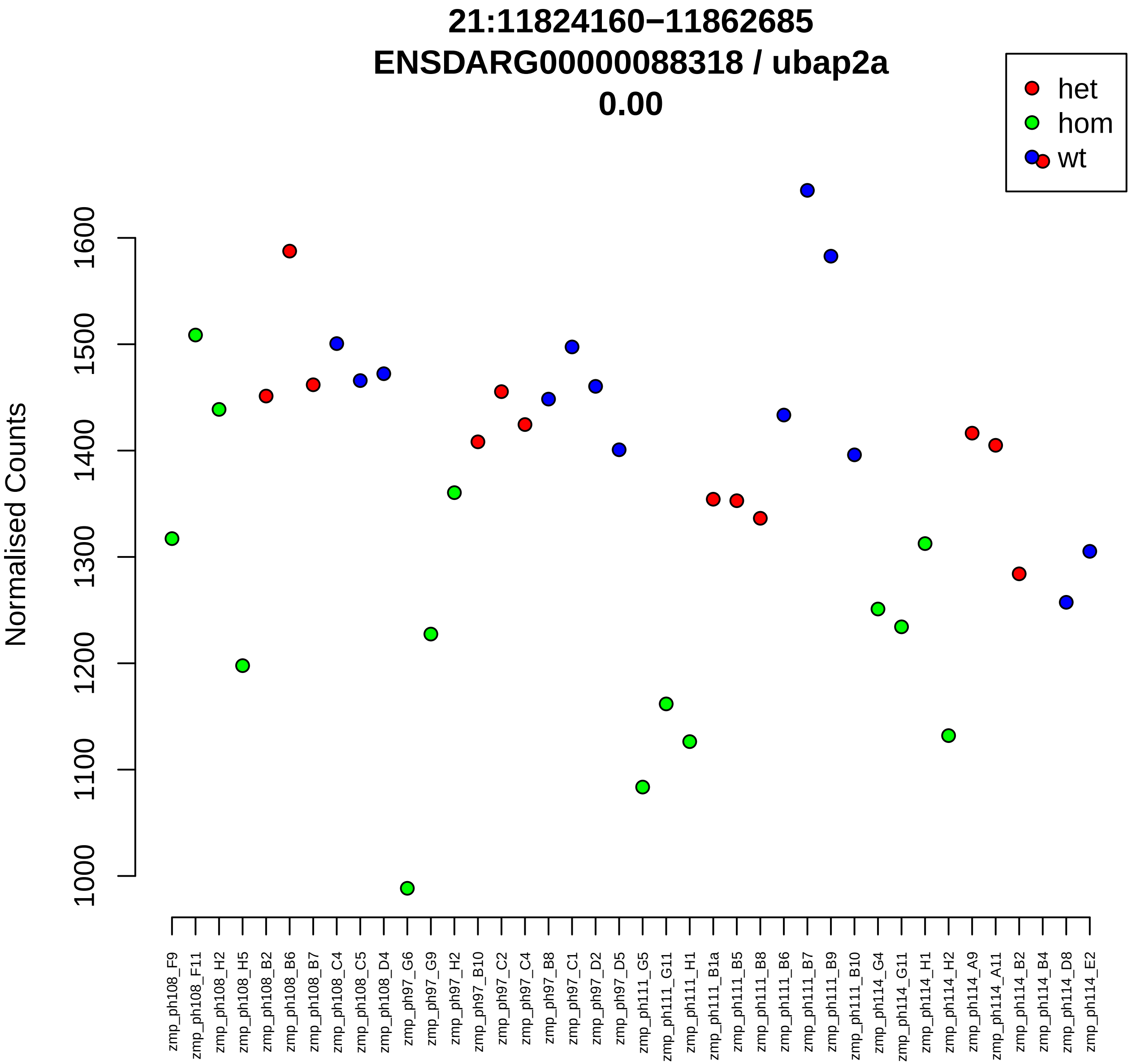

21:11824160−11862685
ENSDARG00000088318 / ubap2a
0.00
het
hom
wt
Normalised Counts
1000
1100
1200
1300
1400
1500
1600
zmp_ph108_F9
zmp_ph108_F11
zmp_ph108_H2
zmp_ph108_H5
zmp_ph108_B2
zmp_ph108_B6
zmp_ph108_B7
zmp_ph108_C4
zmp_ph108_C5
zmp_ph108_D4
zmp_ph97_G6
zmp_ph97_G9
zmp_ph97_H2
zmp_ph97_B10
zmp_ph97_C2
zmp_ph97_C4
zmp_ph97_B8
zmp_ph97_C1
zmp_ph97_D2
zmp_ph97_D5
zmp_ph111_G5
zmp_ph111_G11
zmp_ph111_H1
zmp_ph111_B1a
zmp_ph111_B5
zmp_ph111_B8
zmp_ph111_B6
zmp_ph111_B7
zmp_ph111_B9
zmp_ph111_B10
zmp_ph114_G4
zmp_ph114_G11
zmp_ph114_H1
zmp_ph114_H2
zmp_ph114_A9
zmp_ph114_A11
zmp_ph114_B2
zmp_ph114_B4
zmp_ph114_D8
zmp_ph114_E2

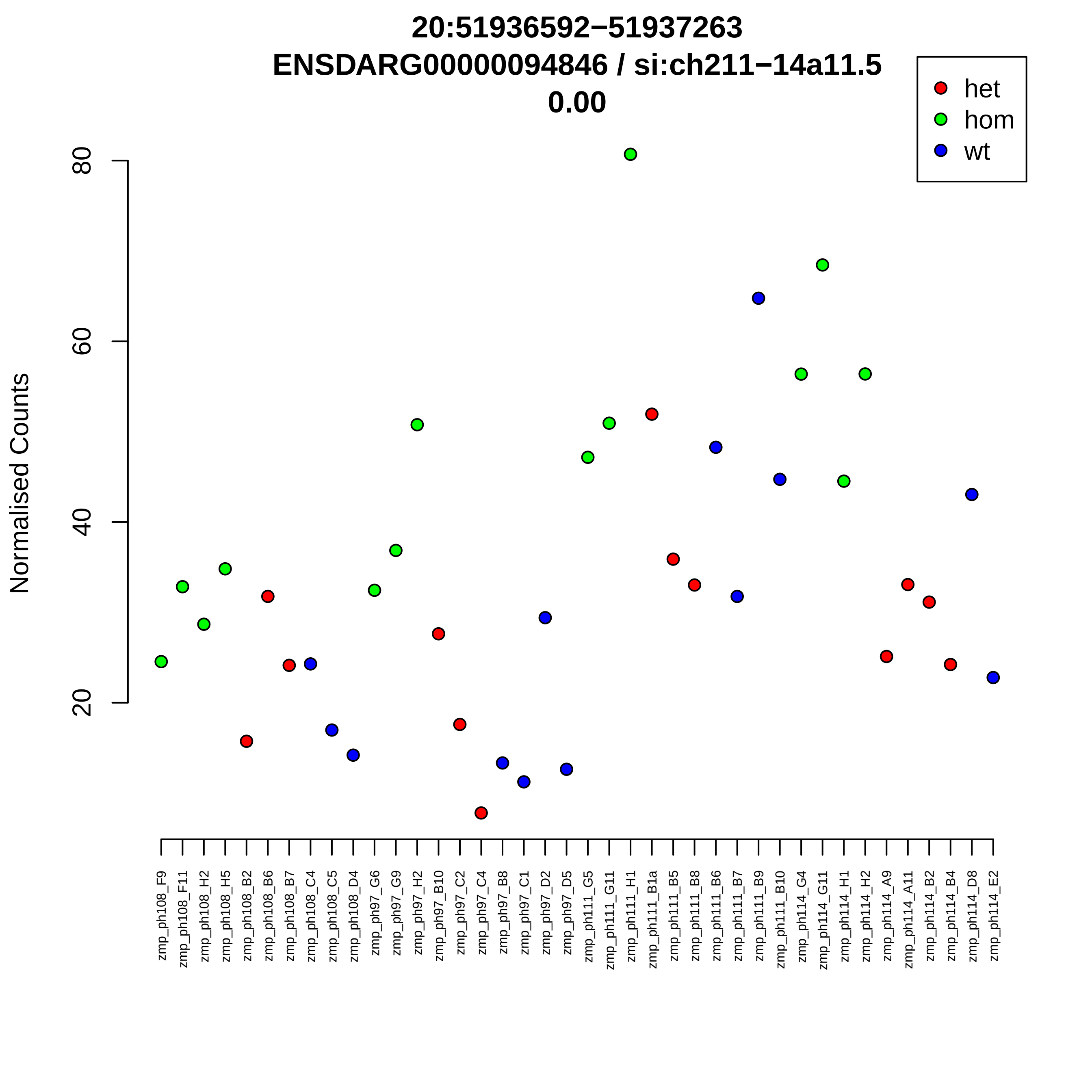

20:51936592−51937263
ENSDARG00000094846 / si:ch211−14a11.5
0.00
het
hom
wt
Normalised Counts
20
40
60
80
zmp_ph108_F9
zmp_ph108_F11
zmp_ph108_H2
zmp_ph108_H5
zmp_ph108_B2
zmp_ph108_B6
zmp_ph108_B7
zmp_ph108_C4
zmp_ph108_C5
zmp_ph108_D4
zmp_ph97_G6
zmp_ph97_G9
zmp_ph97_H2
zmp_ph97_B10
zmp_ph97_C2
zmp_ph97_C4
zmp_ph97_B8
zmp_ph97_C1
zmp_ph97_D2
zmp_ph97_D5
zmp_ph111_G5
zmp_ph111_G11
zmp_ph111_H1
zmp_ph111_B1a
zmp_ph111_B5
zmp_ph111_B8
zmp_ph111_B6
zmp_ph111_B7
zmp_ph111_B9
zmp_ph111_B10
zmp_ph114_G4
zmp_ph114_G11
zmp_ph114_H1
zmp_ph114_H2
zmp_ph114_A9
zmp_ph114_A11
zmp_ph114_B2
zmp_ph114_B4
zmp_ph114_D8
zmp_ph114_E2

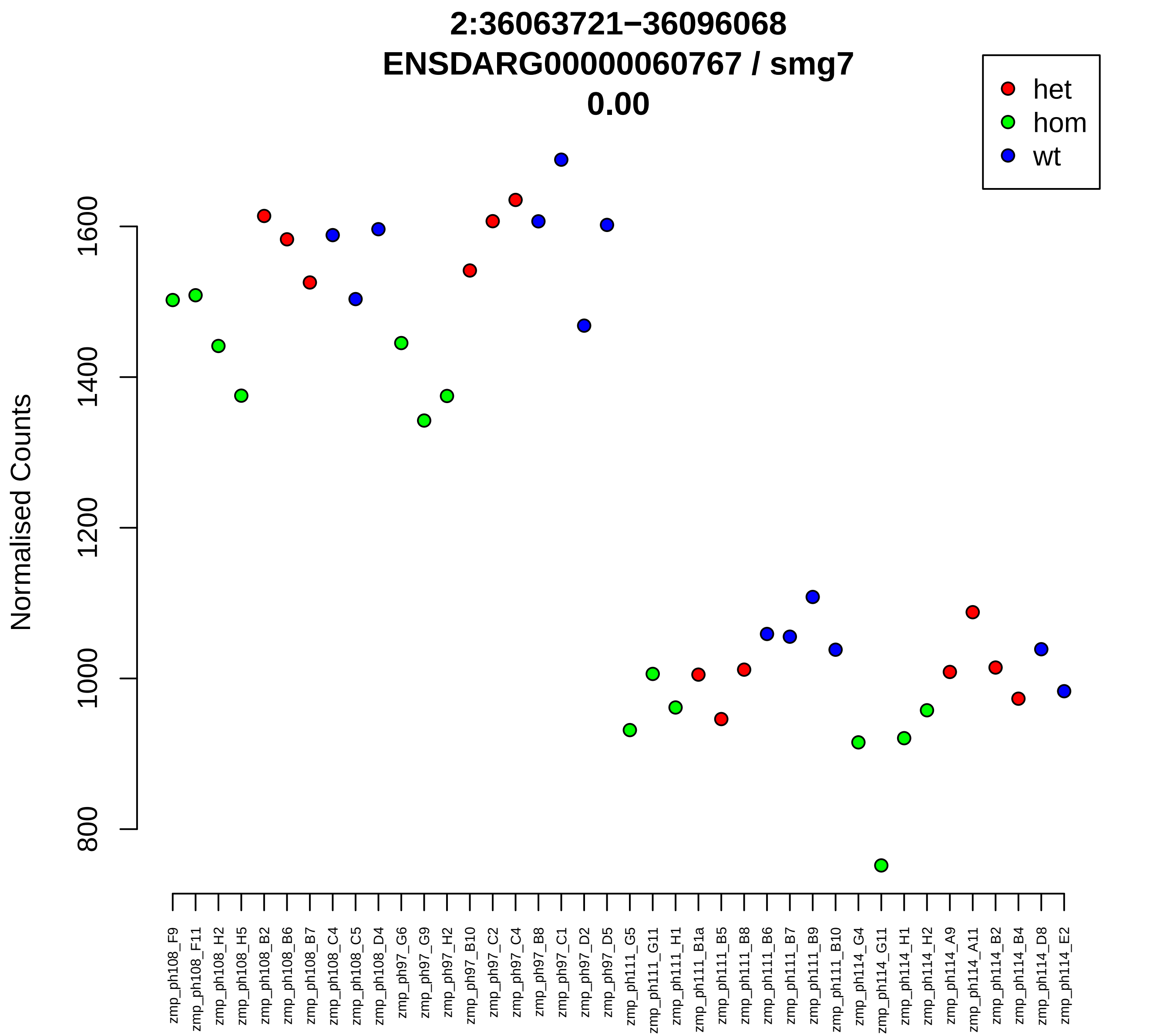

2:36063721−36096068
ENSDARG00000060767 / smg7
0.00
het
hom
wt
Normalised Counts
800
1000
1200
1400
1600
zmp_ph108_F9
zmp_ph108_F11
zmp_ph108_H2
zmp_ph108_H5
zmp_ph108_B2
zmp_ph108_B6
zmp_ph108_B7
zmp_ph108_C4
zmp_ph108_C5
zmp_ph108_D4
zmp_ph97_G6
zmp_ph97_G9
zmp_ph97_H2
zmp_ph97_B10
zmp_ph97_C2
zmp_ph97_C4
zmp_ph97_B8
zmp_ph97_C1
zmp_ph97_D2
zmp_ph97_D5
zmp_ph111_G5
zmp_ph111_G11
zmp_ph111_H1
zmp_ph111_B1a
zmp_ph111_B5
zmp_ph111_B8
zmp_ph111_B6
zmp_ph111_B7
zmp_ph111_B9
zmp_ph111_B10
zmp_ph114_G4
zmp_ph114_G11
zmp_ph114_H1
zmp_ph114_H2
zmp_ph114_A9
zmp_ph114_A11
zmp_ph114_B2
zmp_ph114_B4
zmp_ph114_D8
zmp_ph114_E2

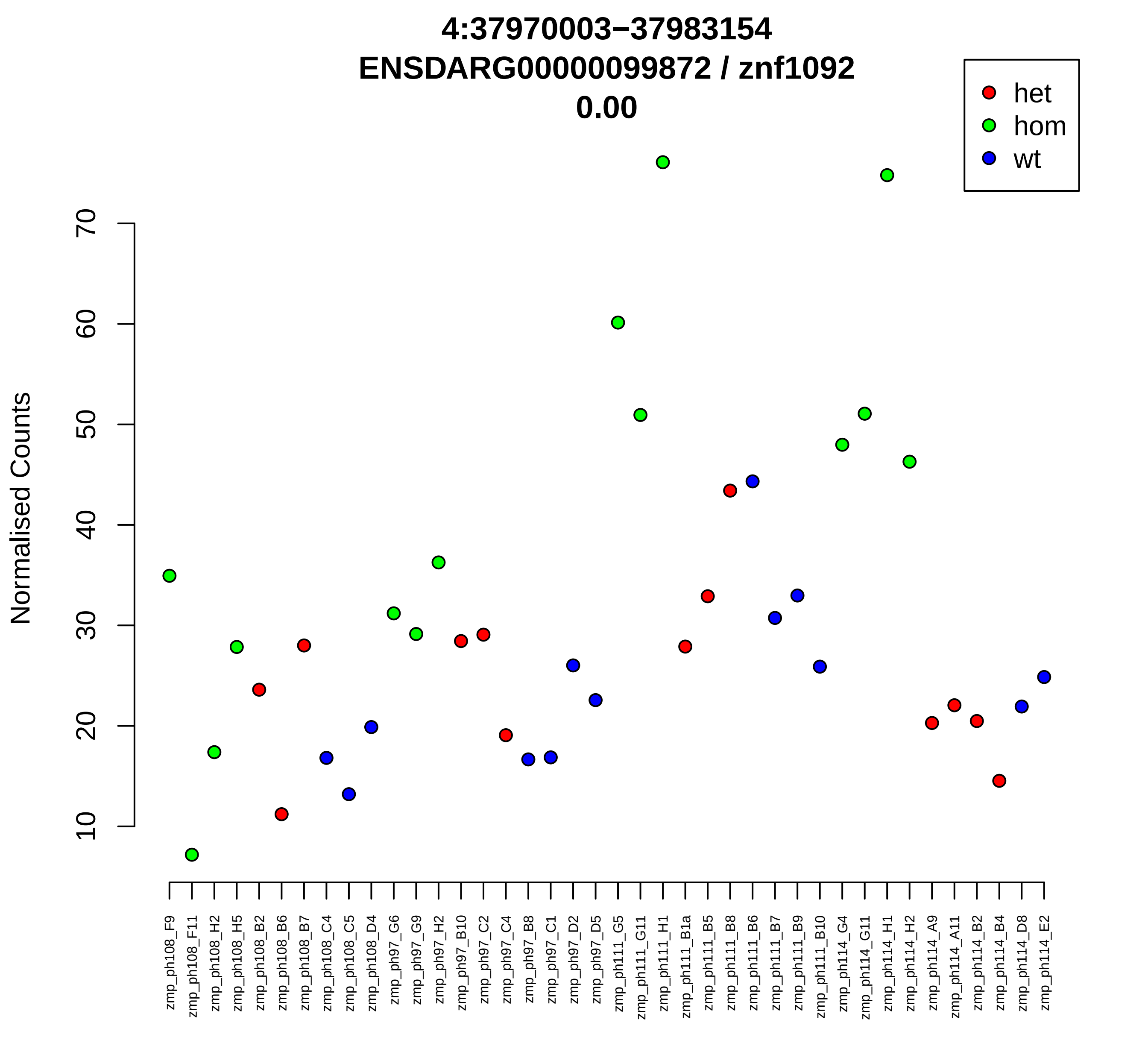

4:37970003−37983154
ENSDARG00000099872 / znf1092
0.00
het
hom
wt
Normalised Counts
10
20
30
40
50
60
70
zmp_ph108_F9
zmp_ph108_F11
zmp_ph108_H2
zmp_ph108_H5
zmp_ph108_B2
zmp_ph108_B6
zmp_ph108_B7
zmp_ph108_C4
zmp_ph108_C5
zmp_ph108_D4
zmp_ph97_G6
zmp_ph97_G9
zmp_ph97_H2
zmp_ph97_B10
zmp_ph97_C2
zmp_ph97_C4
zmp_ph97_B8
zmp_ph97_C1
zmp_ph97_D2
zmp_ph97_D5
zmp_ph111_G5
zmp_ph111_G11
zmp_ph111_H1
zmp_ph111_B1a
zmp_ph111_B5
zmp_ph111_B8
zmp_ph111_B6
zmp_ph111_B7
zmp_ph111_B9
zmp_ph111_B10
zmp_ph114_G4
zmp_ph114_G11
zmp_ph114_H1
zmp_ph114_H2
zmp_ph114_A9
zmp_ph114_A11
zmp_ph114_B2
zmp_ph114_B4
zmp_ph114_D8
zmp_ph114_E2

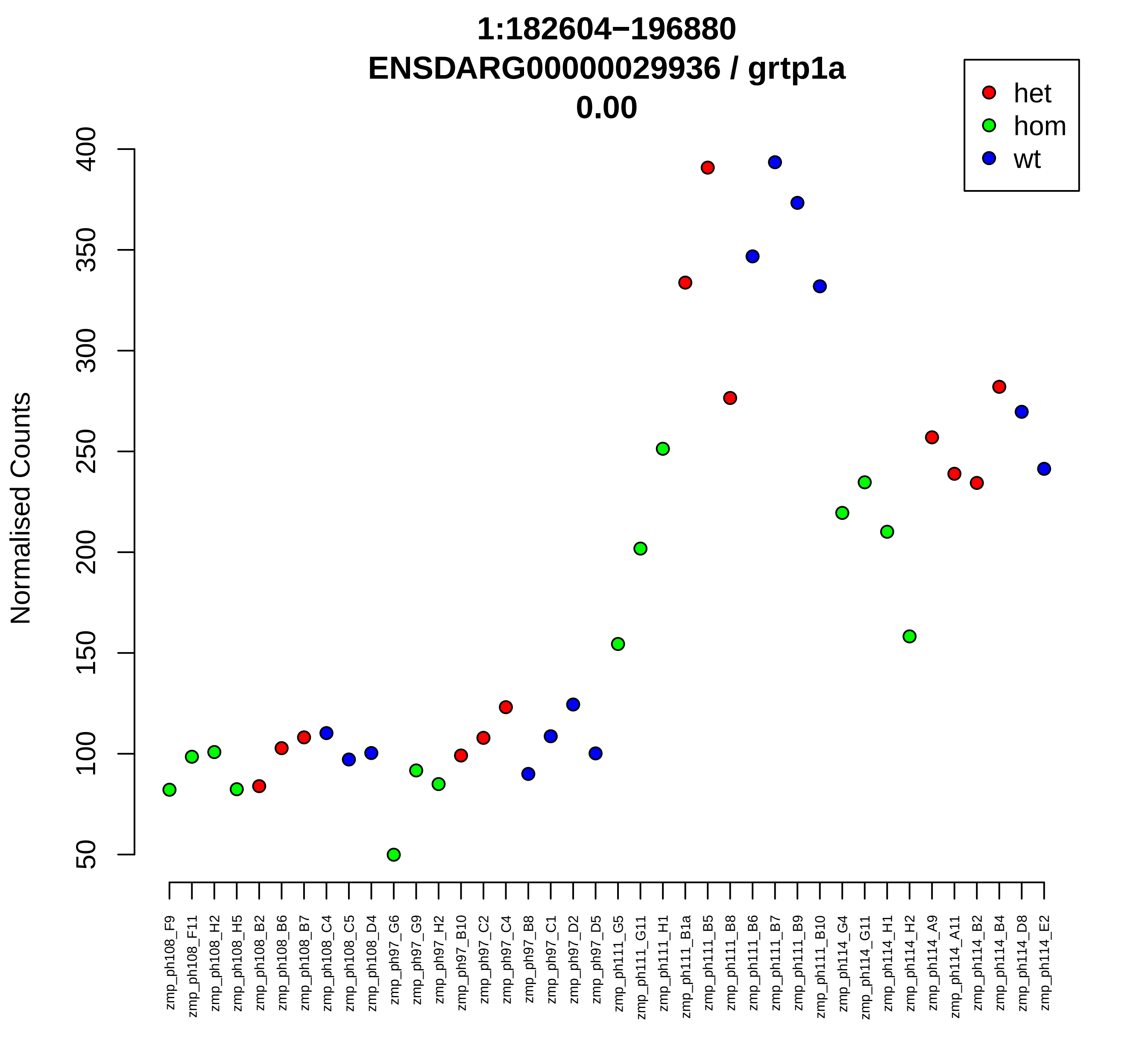

1:182604−196880
ENSDARG00000029936 / grtp1a
0.00
het
hom
wt
Normalised Counts
50
100
150
200
250
300
350
400
zmp_ph108_F9
zmp_ph108_F11
zmp_ph108_H2
zmp_ph108_H5
zmp_ph108_B2
zmp_ph108_B6
zmp_ph108_B7
zmp_ph108_C4
zmp_ph108_C5
zmp_ph108_D4
zmp_ph97_G6
zmp_ph97_G9
zmp_ph97_H2
zmp_ph97_B10
zmp_ph97_C2
zmp_ph97_C4
zmp_ph97_B8
zmp_ph97_C1
zmp_ph97_D2
zmp_ph97_D5
zmp_ph111_G5
zmp_ph111_G11
zmp_ph111_H1
zmp_ph111_B1a
zmp_ph111_B5
zmp_ph111_B8
zmp_ph111_B6
zmp_ph111_B7
zmp_ph111_B9
zmp_ph111_B10
zmp_ph114_G4
zmp_ph114_G11
zmp_ph114_H1
zmp_ph114_H2
zmp_ph114_A9
zmp_ph114_A11
zmp_ph114_B2
zmp_ph114_B4
zmp_ph114_D8
zmp_ph114_E2

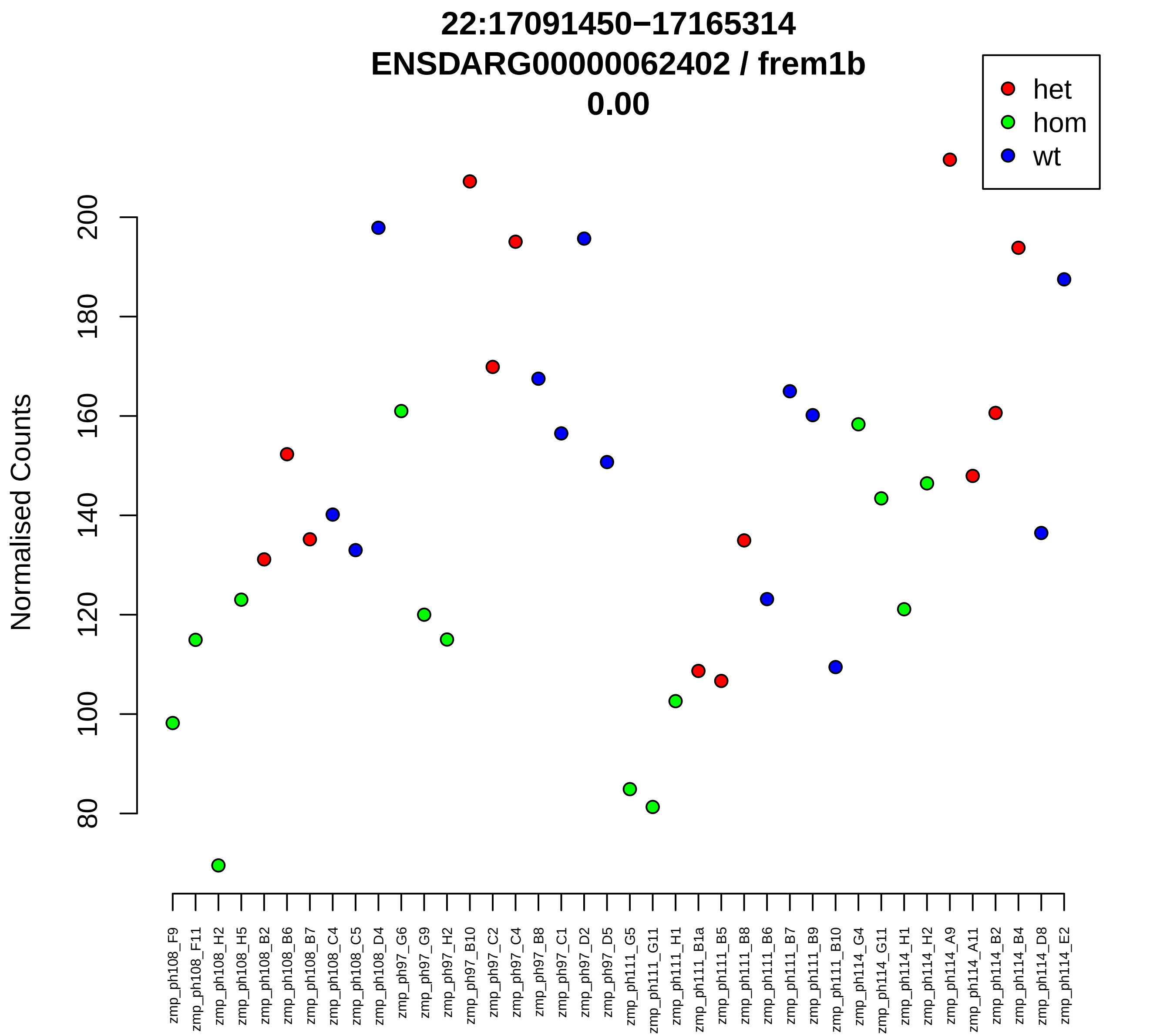
22:17091450−17165314
ENSDARG00000062402 / frem1b
0.00
het
hom
wt
Normalised Counts
80
100
120
140
160
180
200
zmp_ph108_F9
zmp_ph108_F11
zmp_ph108_H2
zmp_ph108_H5
zmp_ph108_B2
zmp_ph108_B6
zmp_ph108_B7
zmp_ph108_C4
zmp_ph108_C5
zmp_ph108_D4
zmp_ph97_G6
zmp_ph97_G9
zmp_ph97_H2
zmp_ph97_B10
zmp_ph97_C2
zmp_ph97_C4
zmp_ph97_B8
zmp_ph97_C1
zmp_ph97_D2
zmp_ph97_D5
zmp_ph111_G5
zmp_ph111_G11
zmp_ph111_H1
zmp_ph111_B1a
zmp_ph111_B5
zmp_ph111_B8
zmp_ph111_B6
zmp_ph111_B7
zmp_ph111_B9
zmp_ph111_B10
zmp_ph114_G4
zmp_ph114_G11
zmp_ph114_H1
zmp_ph114_H2
zmp_ph114_A9
zmp_ph114_A11
zmp_ph114_B2
zmp_ph114_B4
zmp_ph114_D8
zmp_ph114_E2

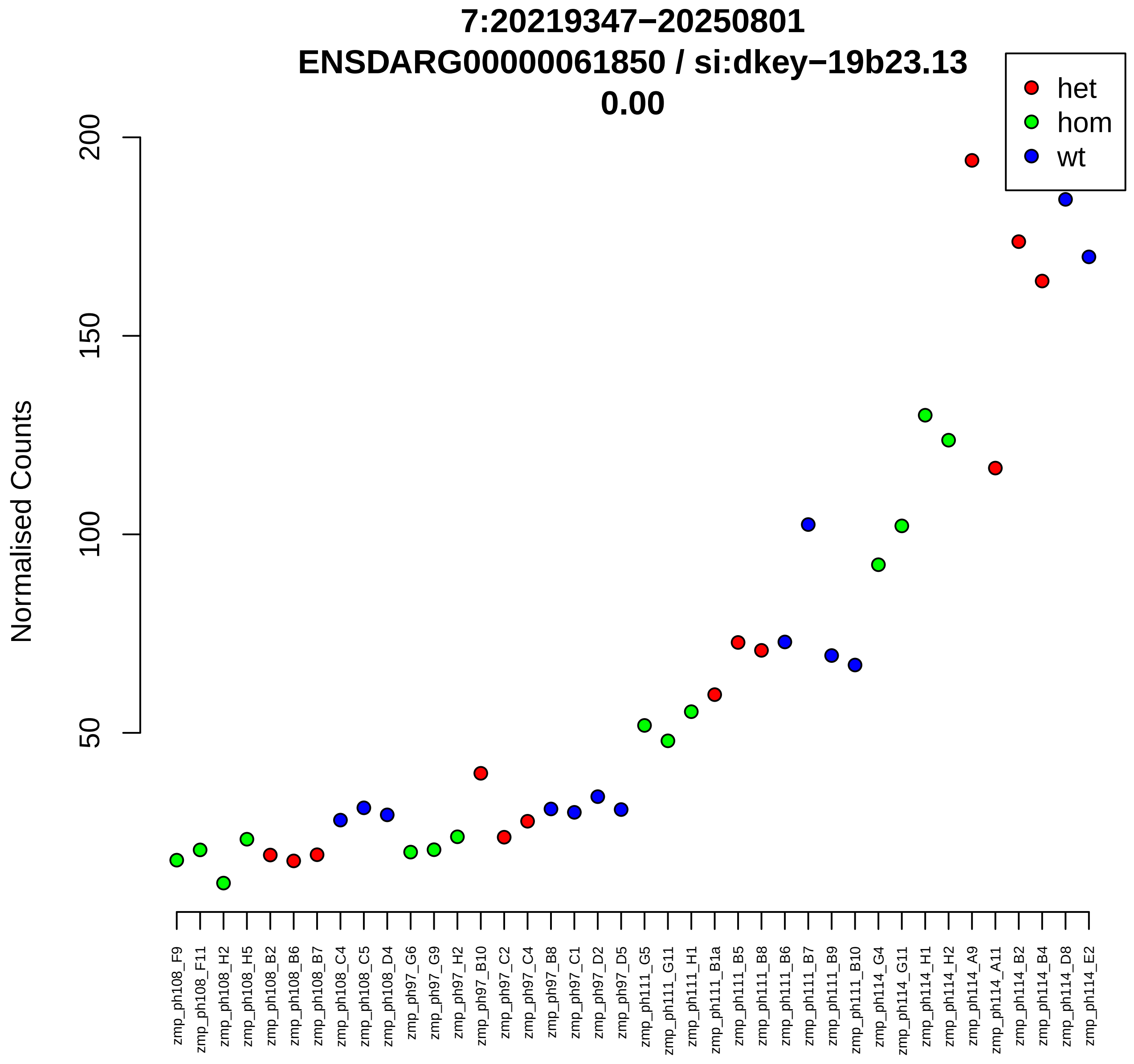
7:20219347−20250801
ENSDARG00000061850 / si:dkey−19b23.13
0.00
Normalised Counts
50
100
150
200
het
hom
wt
zmp_ph108_F9
zmp_ph108_F11
zmp_ph108_H2
zmp_ph108_H5
zmp_ph108_B2
zmp_ph108_B6
zmp_ph108_B7
zmp_ph108_C4
zmp_ph108_C5
zmp_ph108_D4
zmp_ph97_G6
zmp_ph97_G9
zmp_ph97_H2
zmp_ph97_B10
zmp_ph97_C2
zmp_ph97_C4
zmp_ph97_B8
zmp_ph97_C1
zmp_ph97_D2
zmp_ph97_D5
zmp_ph111_G5
zmp_ph111_G11
zmp_ph111_H1
zmp_ph111_B1a
zmp_ph111_B5
zmp_ph111_B8
zmp_ph111_B6
zmp_ph111_B7
zmp_ph111_B9
zmp_ph111_B10
zmp_ph114_G4
zmp_ph114_G11
zmp_ph114_H1
zmp_ph114_H2
zmp_ph114_A9
zmp_ph114_A11
zmp_ph114_B2
zmp_ph114_B4
zmp_ph114_D8
zmp_ph114_E2

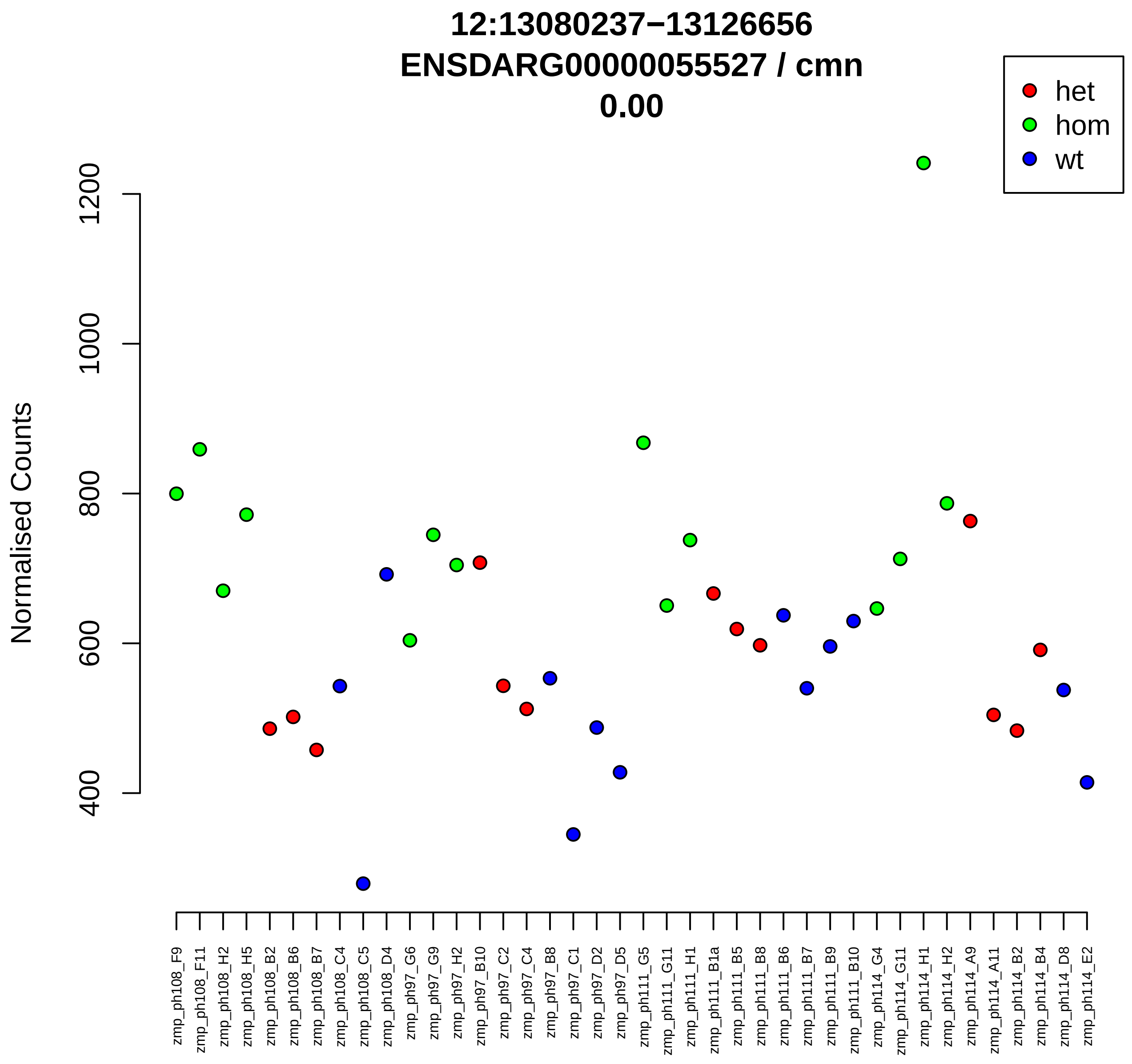

12:13080237−13126656
ENSDARG00000055527 / cmn
0.00
het
hom
wt
Normalised Counts
400
600
800
1000
1200
zmp_ph108_F9
zmp_ph108_F11
zmp_ph108_H2
zmp_ph108_H5
zmp_ph108_B2
zmp_ph108_B6
zmp_ph108_B7
zmp_ph108_C4
zmp_ph108_C5
zmp_ph108_D4
zmp_ph97_G6
zmp_ph97_G9
zmp_ph97_H2
zmp_ph97_B10
zmp_ph97_C2
zmp_ph97_C4
zmp_ph97_B8
zmp_ph97_C1
zmp_ph97_D2
zmp_ph97_D5
zmp_ph111_G5
zmp_ph111_G11
zmp_ph111_H1
zmp_ph111_B1a
zmp_ph111_B5
zmp_ph111_B8
zmp_ph111_B6
zmp_ph111_B7
zmp_ph111_B9
zmp_ph111_B10
zmp_ph114_G4
zmp_ph114_G11
zmp_ph114_H1
zmp_ph114_H2
zmp_ph114_A9
zmp_ph114_A11
zmp_ph114_B2
zmp_ph114_B4
zmp_ph114_D8
zmp_ph114_E2

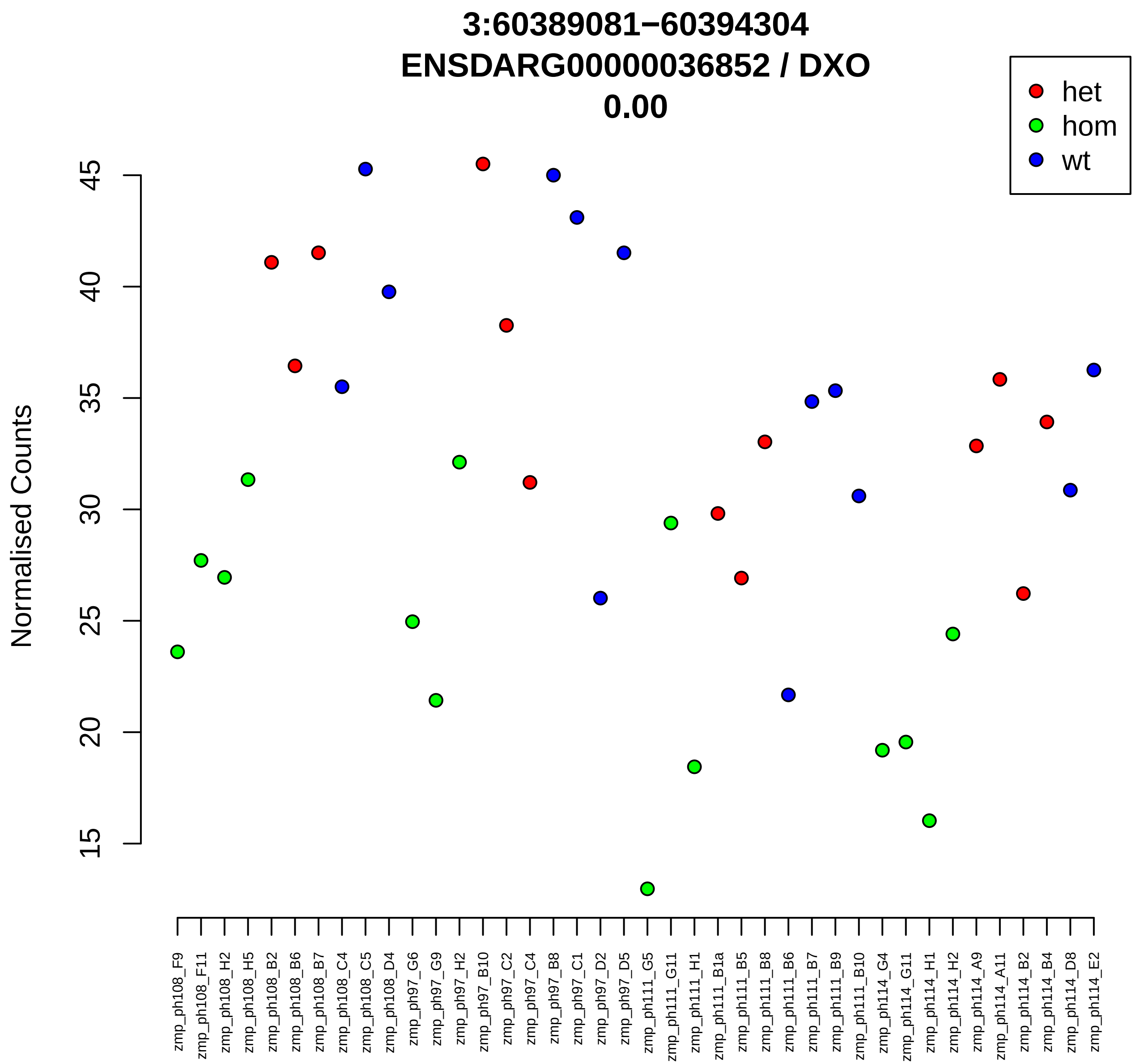
3:60389081−60394304
ENSDARG00000036852 / DXO
0.00
het
hom
wt
Normalised Counts
15
20
25
30
35
40
45
zmp_ph108_F9
zmp_ph108_F11
zmp_ph108_H2
zmp_ph108_H5
zmp_ph108_B2
zmp_ph108_B6
zmp_ph108_B7
zmp_ph108_C4
zmp_ph108_C5
zmp_ph108_D4
zmp_ph97_G6
zmp_ph97_G9
zmp_ph97_H2
zmp_ph97_B10
zmp_ph97_C2
zmp_ph97_C4
zmp_ph97_B8
zmp_ph97_C1
zmp_ph97_D2
zmp_ph97_D5
zmp_ph111_G5
zmp_ph111_G11
zmp_ph111_H1
zmp_ph111_B1a
zmp_ph111_B5
zmp_ph111_B8
zmp_ph111_B6
zmp_ph111_B7
zmp_ph111_B9
zmp_ph111_B10
zmp_ph114_G4
zmp_ph114_G11
zmp_ph114_H1
zmp_ph114_H2
zmp_ph114_A9
zmp_ph114_A11
zmp_ph114_B2
zmp_ph114_B4
zmp_ph114_D8
zmp_ph114_E2

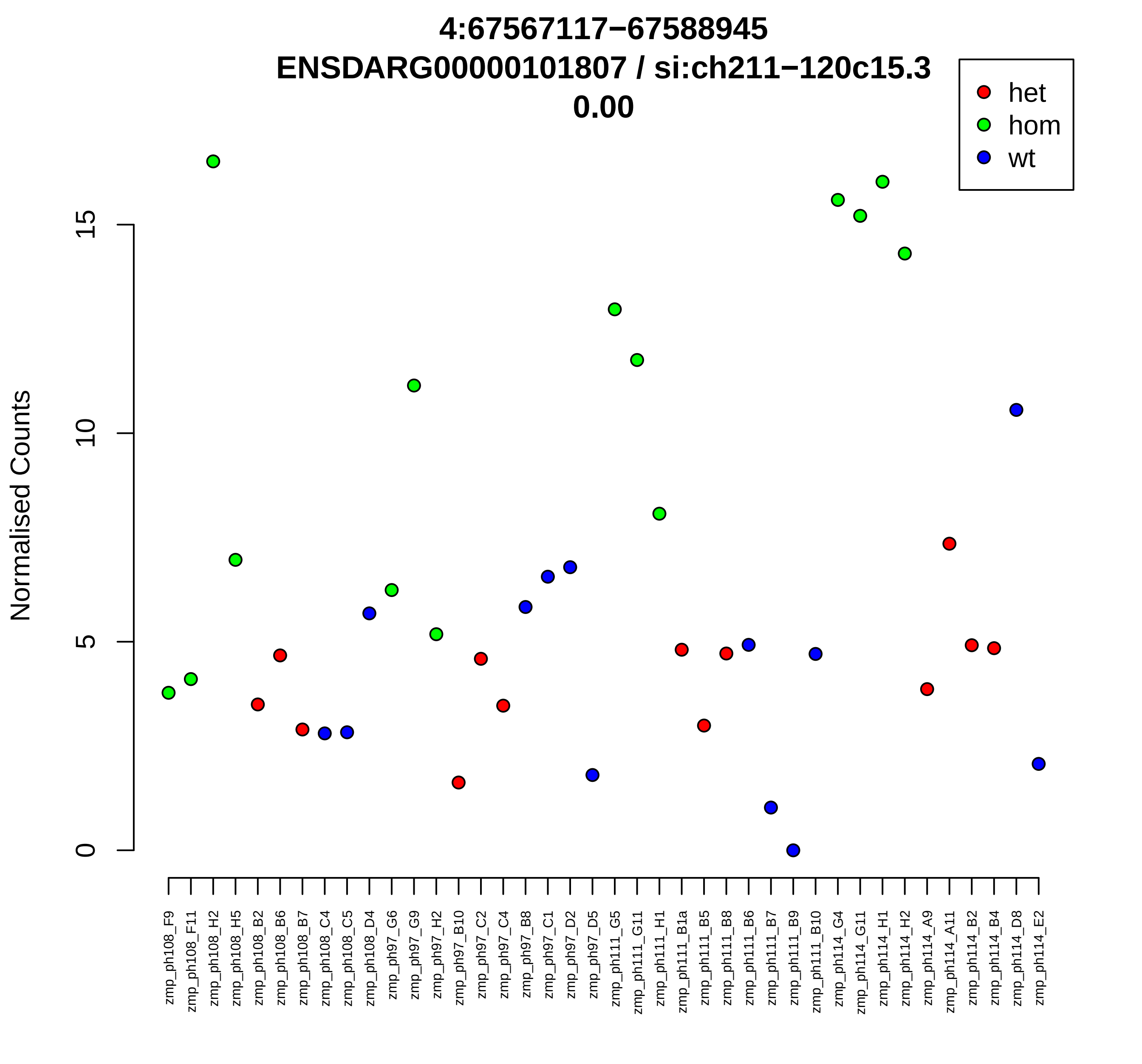

4:67567117−67588945
ENSDARG00000101807 / si:ch211−120c15.3
0.00
het
hom
wt
Normalised Counts
0
5
10
15
zmp_ph108_F9
zmp_ph108_F11
zmp_ph108_H2
zmp_ph108_H5
zmp_ph108_B2
zmp_ph108_B6
zmp_ph108_B7
zmp_ph108_C4
zmp_ph108_C5
zmp_ph108_D4
zmp_ph97_G6
zmp_ph97_G9
zmp_ph97_H2
zmp_ph97_B10
zmp_ph97_C2
zmp_ph97_C4
zmp_ph97_B8
zmp_ph97_C1
zmp_ph97_D2
zmp_ph97_D5
zmp_ph111_G5
zmp_ph111_G11
zmp_ph111_H1
zmp_ph111_B1a
zmp_ph111_B5
zmp_ph111_B8
zmp_ph111_B6
zmp_ph111_B7
zmp_ph111_B9
zmp_ph111_B10
zmp_ph114_G4
zmp_ph114_G11
zmp_ph114_H1
zmp_ph114_H2
zmp_ph114_A9
zmp_ph114_A11
zmp_ph114_B2
zmp_ph114_B4
zmp_ph114_D8
zmp_ph114_E2

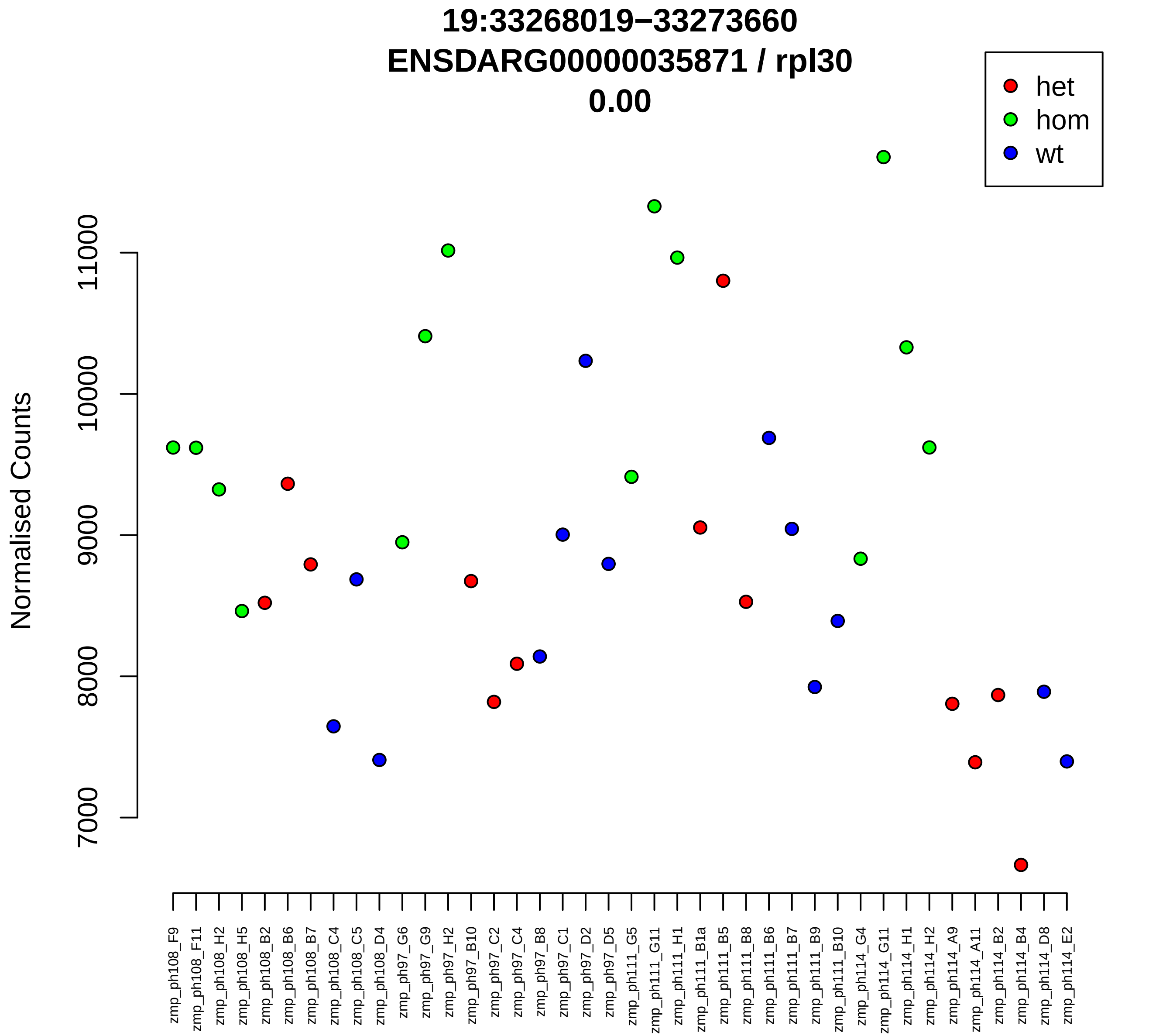
19:33268019−33273660
ENSDARG00000035871 / rpl30
0.00
het
hom
wt
Normalised Counts
7000
8000
9000
10000
11000
zmp_ph108_F9
zmp_ph108_F11
zmp_ph108_H2
zmp_ph108_H5
zmp_ph108_B2
zmp_ph108_B6
zmp_ph108_B7
zmp_ph108_C4
zmp_ph108_C5
zmp_ph108_D4
zmp_ph97_G6
zmp_ph97_G9
zmp_ph97_H2
zmp_ph97_B10
zmp_ph97_C2
zmp_ph97_C4
zmp_ph97_B8
zmp_ph97_C1
zmp_ph97_D2
zmp_ph97_D5
zmp_ph111_G5
zmp_ph111_G11
zmp_ph111_H1
zmp_ph111_B1a
zmp_ph111_B5
zmp_ph111_B8
zmp_ph111_B6
zmp_ph111_B7
zmp_ph111_B9
zmp_ph111_B10
zmp_ph114_G4
zmp_ph114_G11
zmp_ph114_H1
zmp_ph114_H2
zmp_ph114_A9
zmp_ph114_A11
zmp_ph114_B2
zmp_ph114_B4
zmp_ph114_D8
zmp_ph114_E2

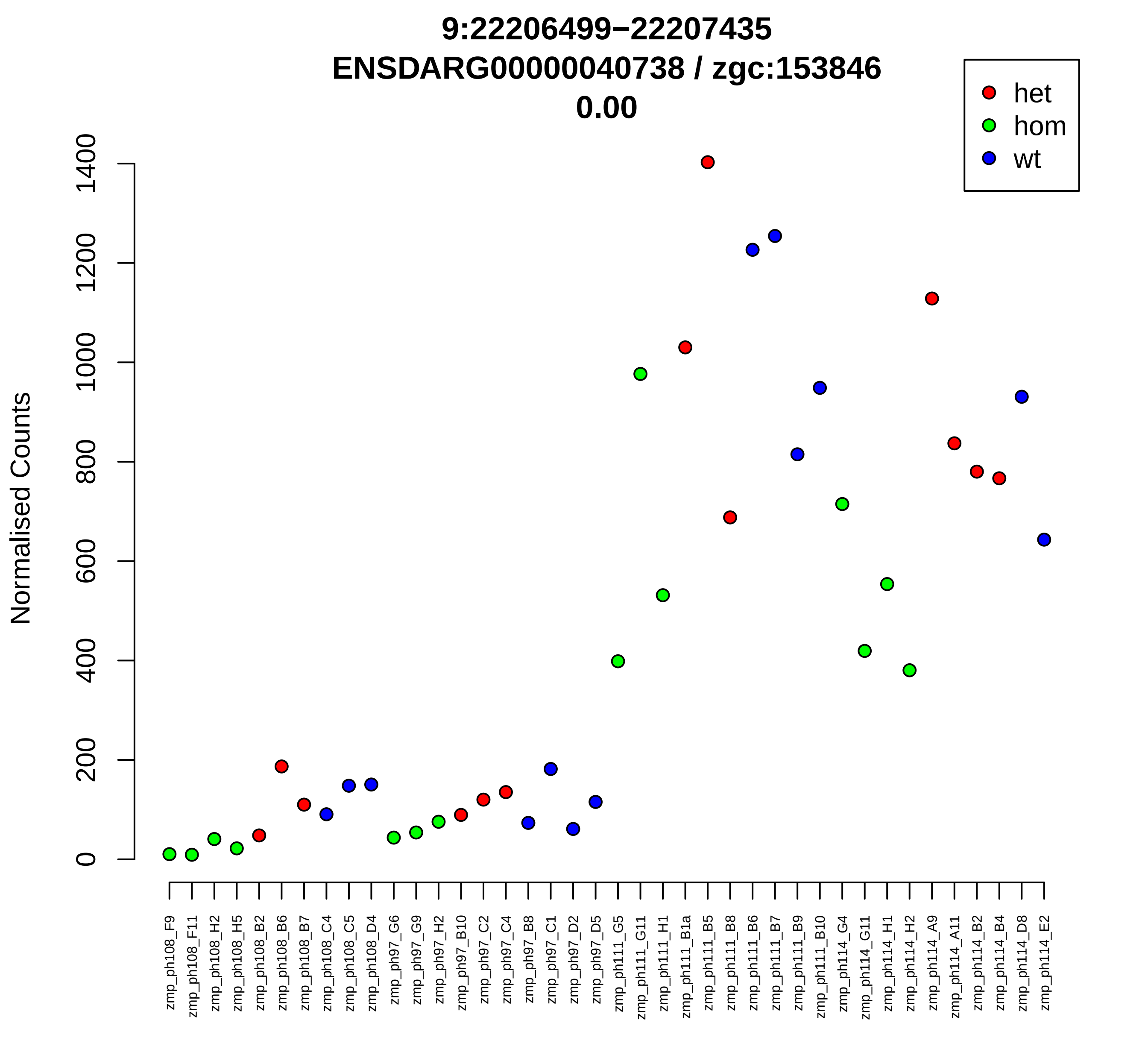

9:22206499−22207435
ENSDARG00000040738 / zgc:153846
0.00
het
hom
wt
Normalised Counts
0
200
400
600
800
1000
1200
1400
zmp_ph108_F9
zmp_ph108_F11
zmp_ph108_H2
zmp_ph108_H5
zmp_ph108_B2
zmp_ph108_B6
zmp_ph108_B7
zmp_ph108_C4
zmp_ph108_C5
zmp_ph108_D4
zmp_ph97_G6
zmp_ph97_G9
zmp_ph97_H2
zmp_ph97_B10
zmp_ph97_C2
zmp_ph97_C4
zmp_ph97_B8
zmp_ph97_C1
zmp_ph97_D2
zmp_ph97_D5
zmp_ph111_G5
zmp_ph111_G11
zmp_ph111_H1
zmp_ph111_B1a
zmp_ph111_B5
zmp_ph111_B8
zmp_ph111_B6
zmp_ph111_B7
zmp_ph111_B9
zmp_ph111_B10
zmp_ph114_G4
zmp_ph114_G11
zmp_ph114_H1
zmp_ph114_H2
zmp_ph114_A9
zmp_ph114_A11
zmp_ph114_B2
zmp_ph114_B4
zmp_ph114_D8
zmp_ph114_E2

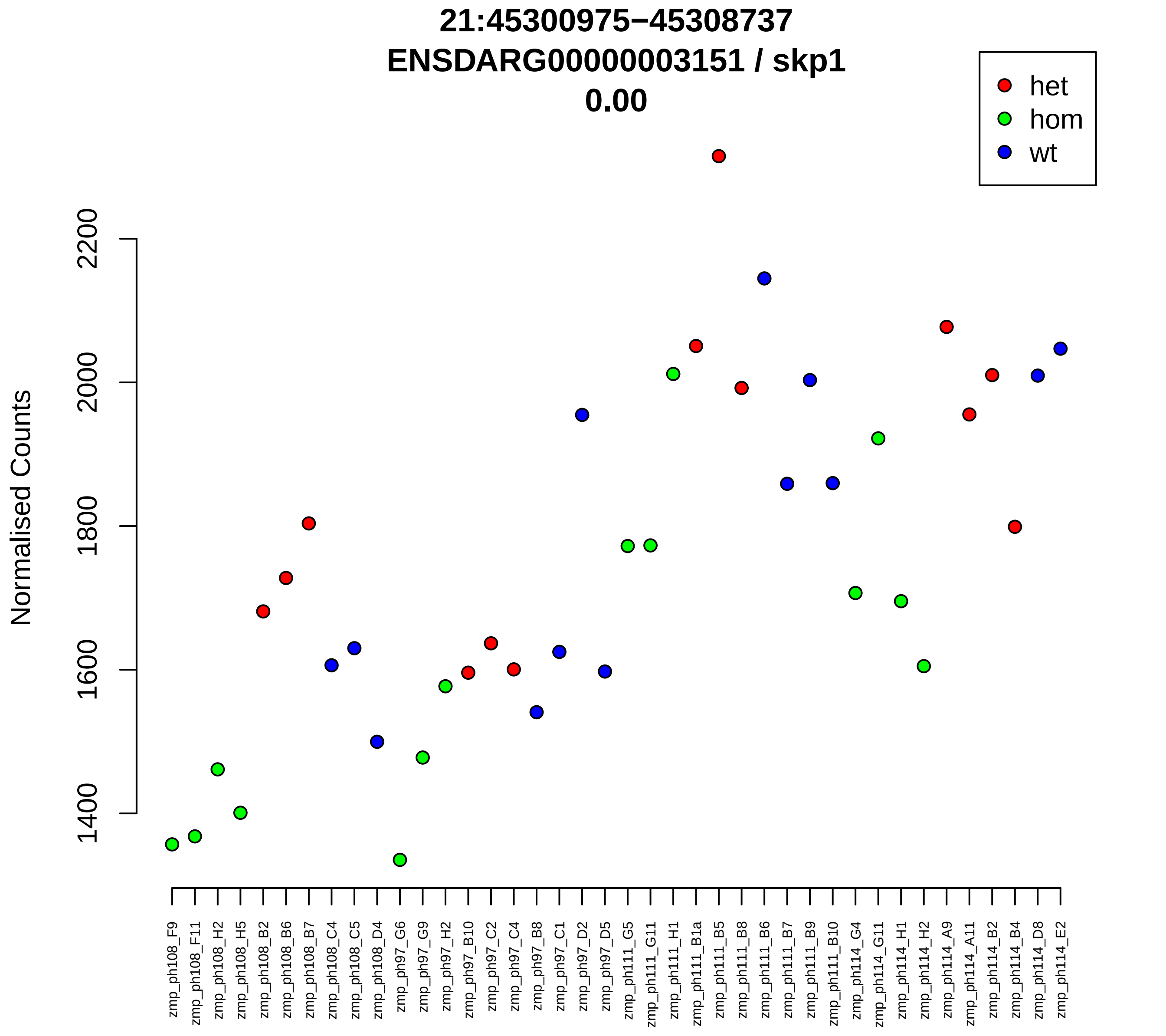

21:45300975−45308737
ENSDARG00000003151 / skp1
0.00
het
hom
wt
Normalised Counts
1400
1600
1800
2000
2200
zmp_ph108_F9
zmp_ph108_F11
zmp_ph108_H2
zmp_ph108_H5
zmp_ph108_B2
zmp_ph108_B6
zmp_ph108_B7
zmp_ph108_C4
zmp_ph108_C5
zmp_ph108_D4
zmp_ph97_G6
zmp_ph97_G9
zmp_ph97_H2
zmp_ph97_B10
zmp_ph97_C2
zmp_ph97_C4
zmp_ph97_B8
zmp_ph97_C1
zmp_ph97_D2
zmp_ph97_D5
zmp_ph111_G5
zmp_ph111_G11
zmp_ph111_H1
zmp_ph111_B1a
zmp_ph111_B5
zmp_ph111_B8
zmp_ph111_B6
zmp_ph111_B7
zmp_ph111_B9
zmp_ph111_B10
zmp_ph114_G4
zmp_ph114_G11
zmp_ph114_H1
zmp_ph114_H2
zmp_ph114_A9
zmp_ph114_A11
zmp_ph114_B2
zmp_ph114_B4
zmp_ph114_D8
zmp_ph114_E2

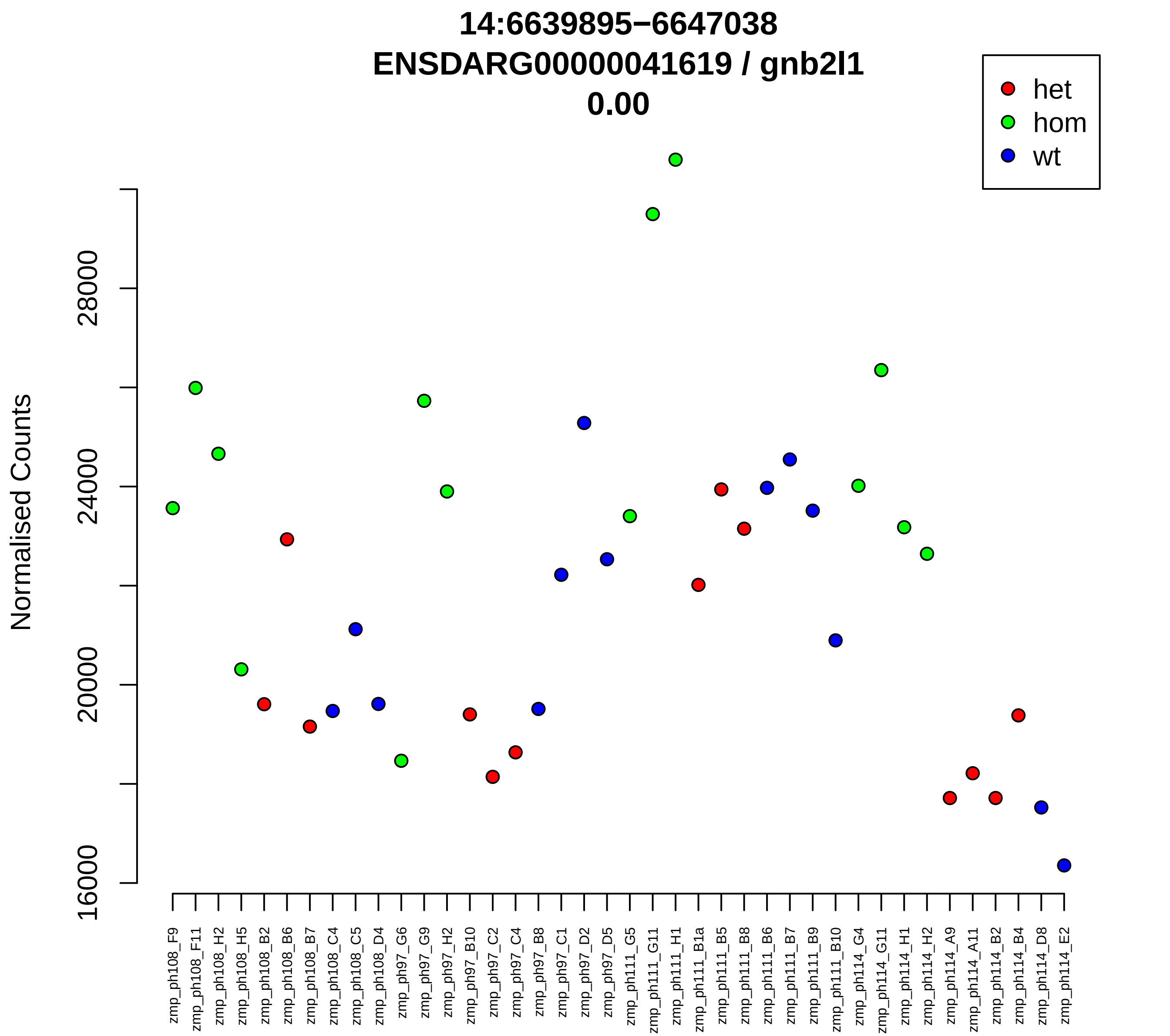
14:6639895−6647038
ENSDARG00000041619 / gnb2l1
0.00
het
hom
wt
Normalised Counts
16000
20000
24000
28000
zmp_ph108_F9
zmp_ph108_F11
zmp_ph108_H2
zmp_ph108_H5
zmp_ph108_B2
zmp_ph108_B6
zmp_ph108_B7
zmp_ph108_C4
zmp_ph108_C5
zmp_ph108_D4
zmp_ph97_G6
zmp_ph97_G9
zmp_ph97_H2
zmp_ph97_B10
zmp_ph97_C2
zmp_ph97_C4
zmp_ph97_B8
zmp_ph97_C1
zmp_ph97_D2
zmp_ph97_D5
zmp_ph111_G5
zmp_ph111_G11
zmp_ph111_H1
zmp_ph111_B1a
zmp_ph111_B5
zmp_ph111_B8
zmp_ph111_B6
zmp_ph111_B7
zmp_ph111_B9
zmp_ph111_B10
zmp_ph114_G4
zmp_ph114_G11
zmp_ph114_H1
zmp_ph114_H2
zmp_ph114_A9
zmp_ph114_A11
zmp_ph114_B2
zmp_ph114_B4
zmp_ph114_D8
zmp_ph114_E2

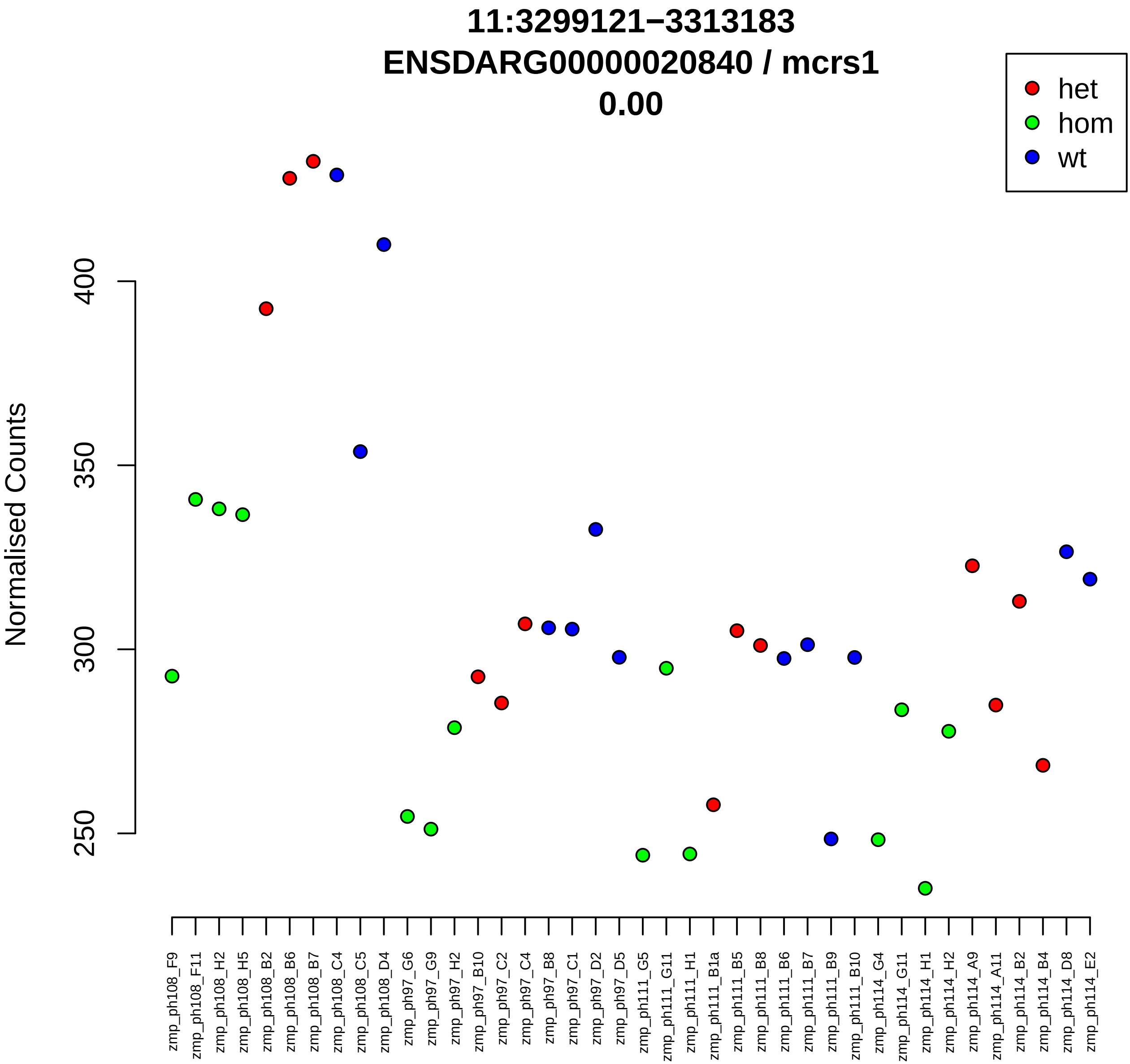
11:3299121−3313183
ENSDARG00000020840 / mcrs1
0.00
het
hom
wt
Normalised Counts
400
350
300
250
zmp_ph108_F9
zmp_ph108_F11
zmp_ph108_H2
zmp_ph108_H5
zmp_ph108_B2
zmp_ph108_B6
zmp_ph108_B7
zmp_ph108_C4
zmp_ph108_C5
zmp_ph108_D4
zmp_ph97_G6
zmp_ph97_G9
zmp_ph97_H2
zmp_ph97_B10
zmp_ph97_C2
zmp_ph97_C4
zmp_ph97_B8
zmp_ph97_C1
zmp_ph97_D2
zmp_ph97_D5
zmp_ph111_G5
zmp_ph111_G11
zmp_ph111_H1
zmp_ph111_B1a
zmp_ph111_B5
zmp_ph111_B8
zmp_ph111_B6
zmp_ph111_B7
zmp_ph111_B9
zmp_ph111_B10
zmp_ph114_G4
zmp_ph114_G11
zmp_ph114_H1
zmp_ph114_H2
zmp_ph114_A9
zmp_ph114_A11
zmp_ph114_B2
zmp_ph114_B4
zmp_ph114_D8
zmp_ph114_E2

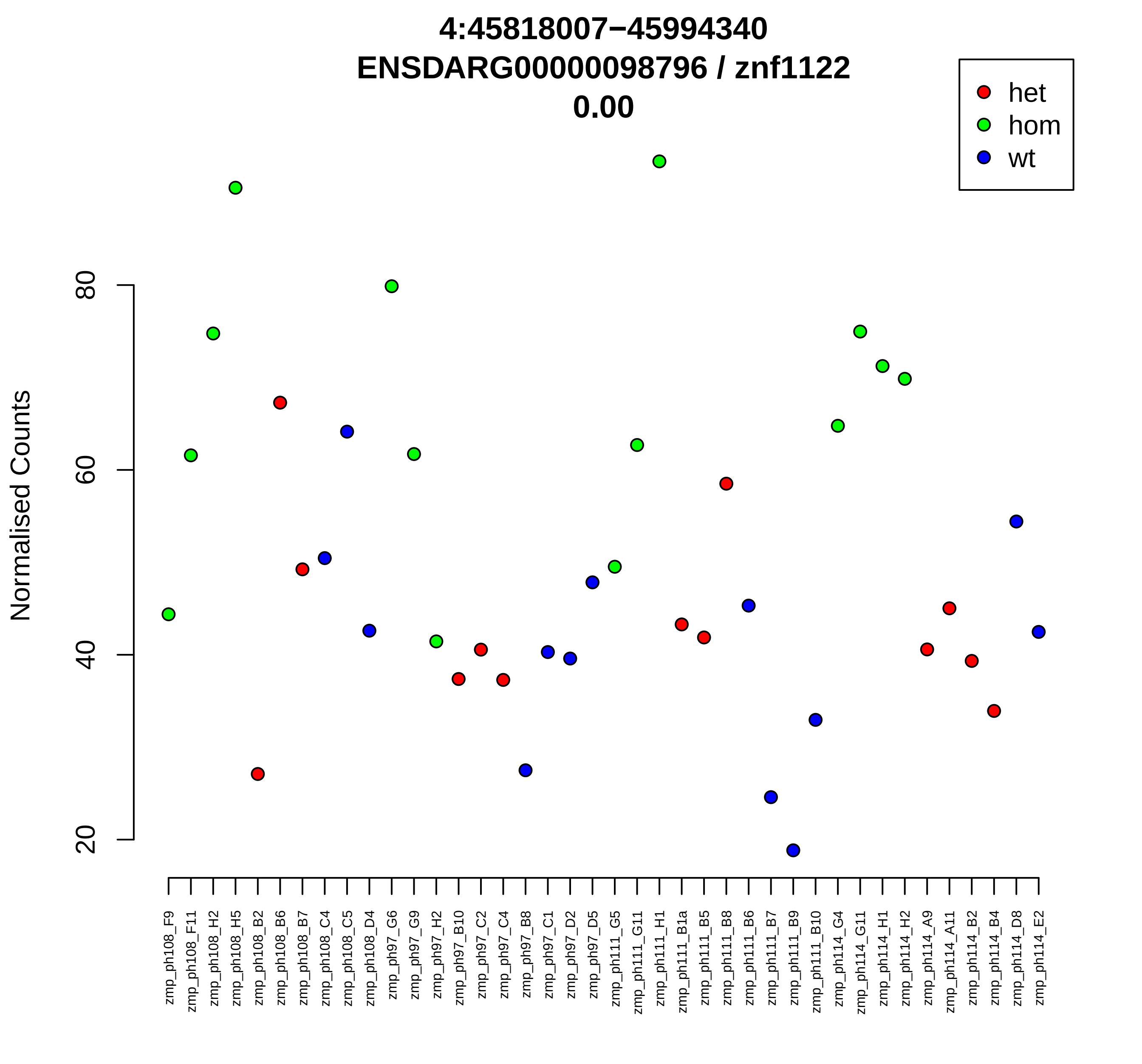
4:45818007−45994340
ENSDARG00000098796 / znf1122
0.00
het
hom
wt
Normalised Counts
20
40
60
80
zmp_ph108_F9
zmp_ph108_F11
zmp_ph108_H2
zmp_ph108_H5
zmp_ph108_B2
zmp_ph108_B6
zmp_ph108_B7
zmp_ph108_C4
zmp_ph108_C5
zmp_ph108_D4
zmp_ph97_G6
zmp_ph97_G9
zmp_ph97_H2
zmp_ph97_B10
zmp_ph97_C2
zmp_ph97_C4
zmp_ph97_B8
zmp_ph97_C1
zmp_ph97_D2
zmp_ph97_D5
zmp_ph111_G5
zmp_ph111_G11
zmp_ph111_H1
zmp_ph111_B1a
zmp_ph111_B5
zmp_ph111_B8
zmp_ph111_B6
zmp_ph111_B7
zmp_ph111_B9
zmp_ph111_B10
zmp_ph114_G4
zmp_ph114_G11
zmp_ph114_H1
zmp_ph114_H2
zmp_ph114_A9
zmp_ph114_A11
zmp_ph114_B2
zmp_ph114_B4
zmp_ph114_D8
zmp_ph114_E2

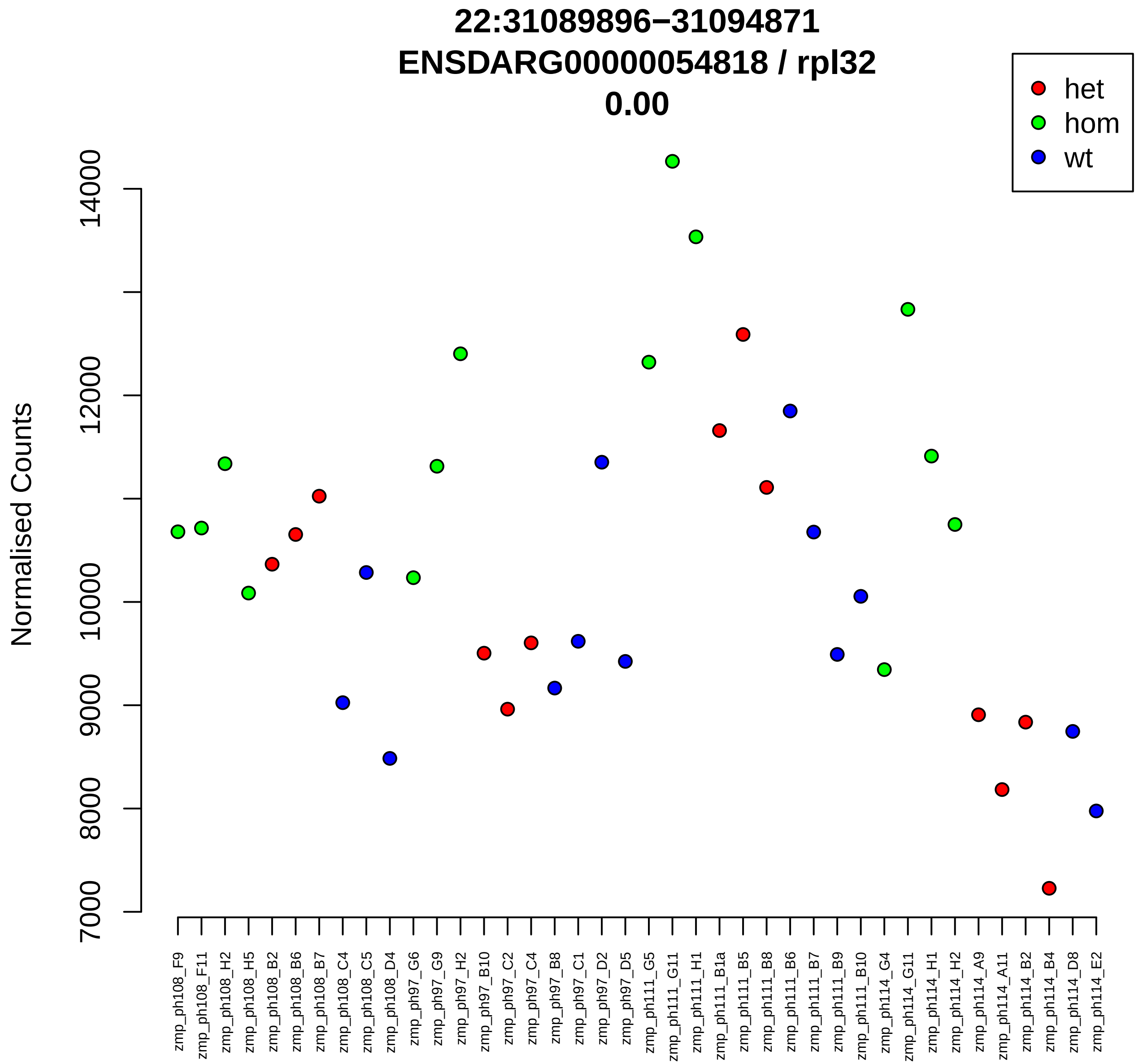

22:31089896−31094871
ENSDARG00000054818 / rpl32
0.00
het
hom
wt
Normalised Counts
14000
12000
10000
9000
8000
7000
zmp_ph108_F9
zmp_ph108_F11
zmp_ph108_H2
zmp_ph108_H5
zmp_ph108_B2
zmp_ph108_B6
zmp_ph108_B7
zmp_ph108_C4
zmp_ph108_C5
zmp_ph108_D4
zmp_ph97_G6
zmp_ph97_G9
zmp_ph97_H2
zmp_ph97_B10
zmp_ph97_C2
zmp_ph97_C4
zmp_ph97_B8
zmp_ph97_C1
zmp_ph97_D2
zmp_ph97_D5
zmp_ph111_G5
zmp_ph111_G11
zmp_ph111_H1
zmp_ph111_B1a
zmp_ph111_B5
zmp_ph111_B8
zmp_ph111_B6
zmp_ph111_B7
zmp_ph111_B9
zmp_ph111_B10
zmp_ph114_G4
zmp_ph114_G11
zmp_ph114_H1
zmp_ph114_H2
zmp_ph114_A9
zmp_ph114_A11
zmp_ph114_B2
zmp_ph114_B4
zmp_ph114_D8
zmp_ph114_E2

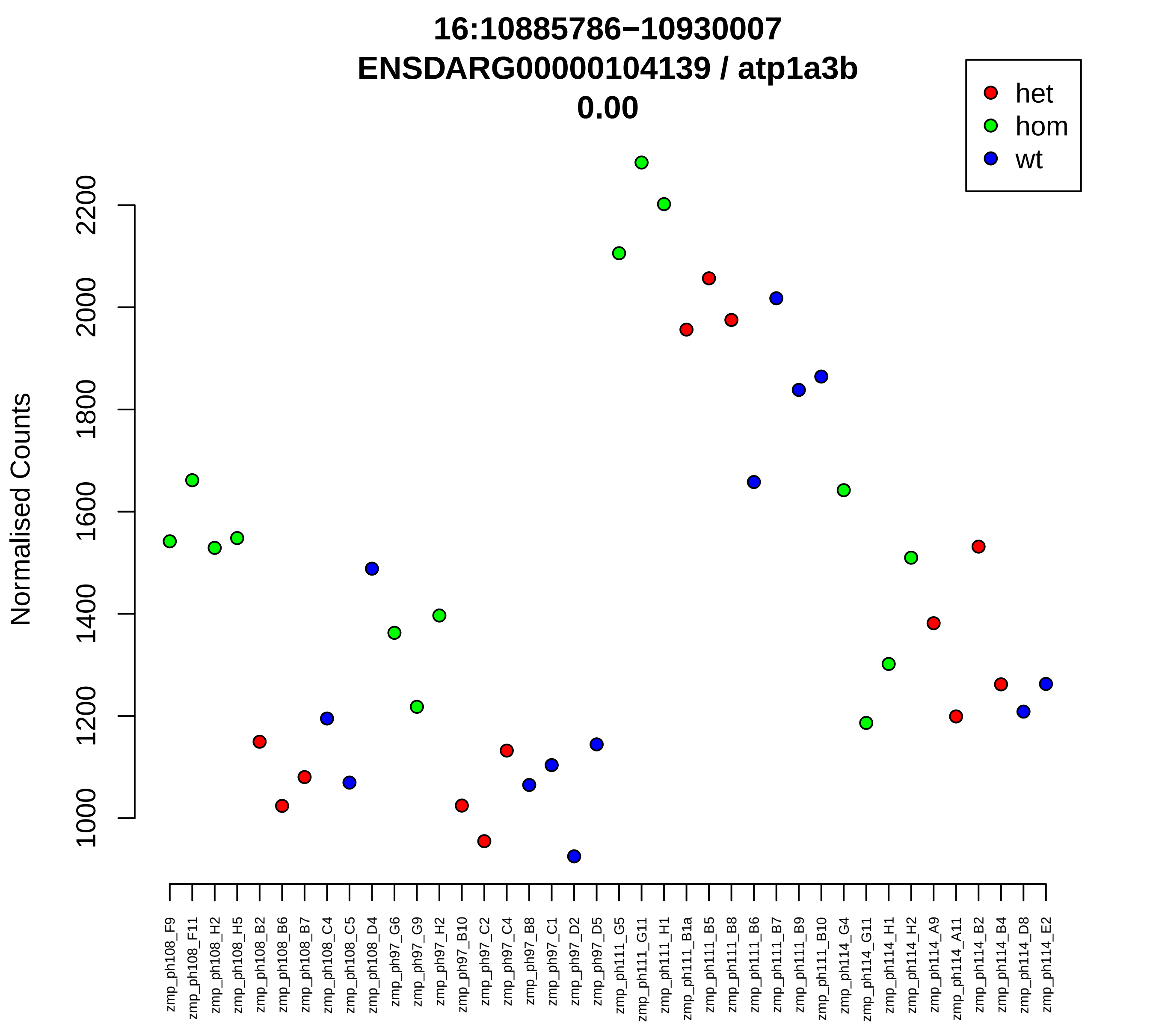
16:10885786−10930007
ENSDARG00000104139 / atp1a3b
0.00
het
hom
wt
Normalised Counts
1000
1200
1400
1600
1800
2000
2200
zmp_ph108_F9
zmp_ph108_F11
zmp_ph108_H2
zmp_ph108_H5
zmp_ph108_B2
zmp_ph108_B6
zmp_ph108_B7
zmp_ph108_C4
zmp_ph108_C5
zmp_ph108_D4
zmp_ph97_G6
zmp_ph97_G9
zmp_ph97_H2
zmp_ph97_B10
zmp_ph97_C2
zmp_ph97_C4
zmp_ph97_B8
zmp_ph97_C1
zmp_ph97_D2
zmp_ph97_D5
zmp_ph111_G5
zmp_ph111_G11
zmp_ph111_H1
zmp_ph111_B1a
zmp_ph111_B5
zmp_ph111_B8
zmp_ph111_B6
zmp_ph111_B7
zmp_ph111_B9
zmp_ph111_B10
zmp_ph114_G4
zmp_ph114_G11
zmp_ph114_H1
zmp_ph114_H2
zmp_ph114_A9
zmp_ph114_A11
zmp_ph114_B2
zmp_ph114_B4
zmp_ph114_D8
zmp_ph114_E2

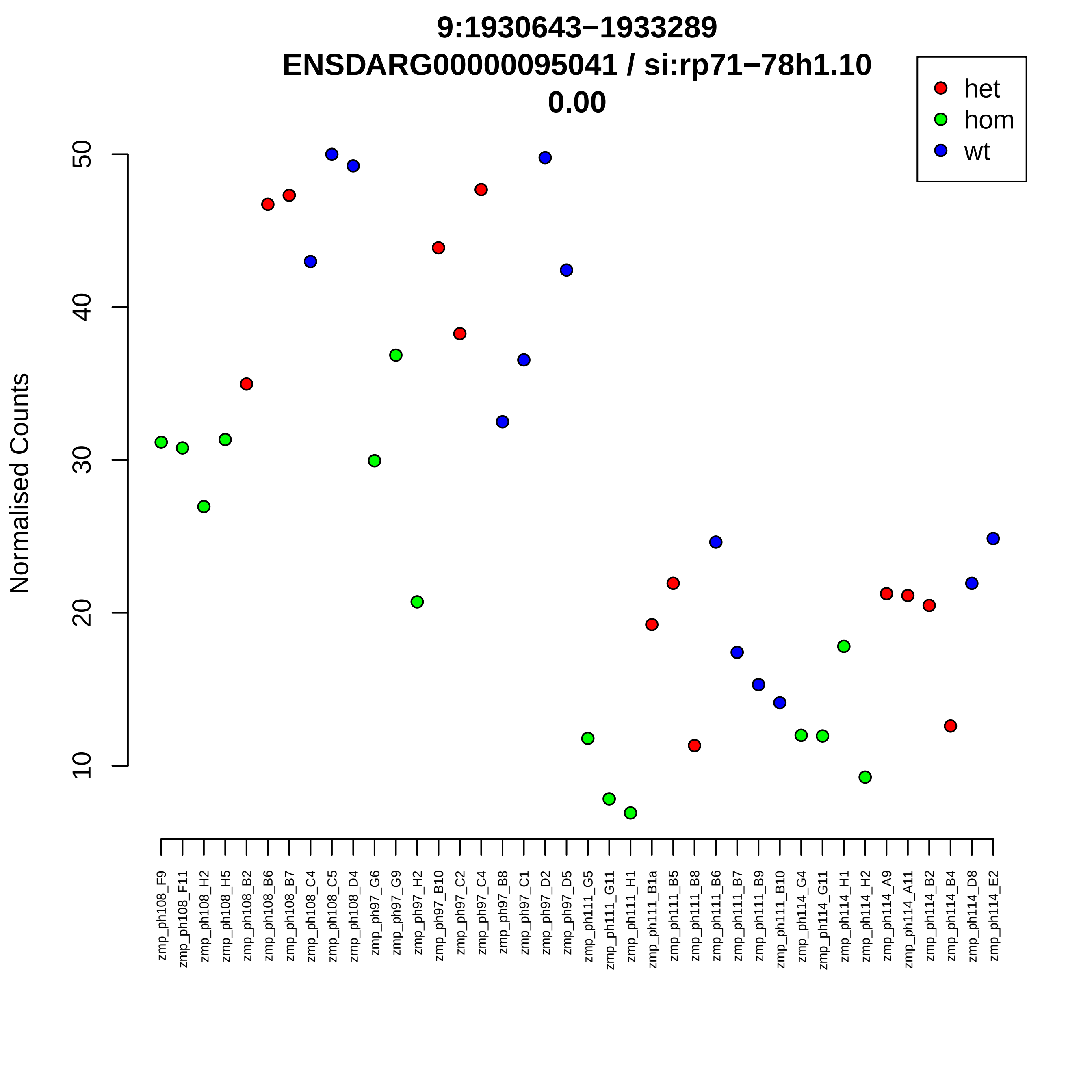

9:1930643−1933289
ENSDARG00000095041 / si:rp71−78h1.10
0.00
het
hom
wt
Normalised Counts
10
20
30
40
50
zmp_ph108_F9
zmp_ph108_F11
zmp_ph108_H2
zmp_ph108_H5
zmp_ph108_B2
zmp_ph108_B6
zmp_ph108_B7
zmp_ph108_C4
zmp_ph108_C5
zmp_ph108_D4
zmp_ph97_G6
zmp_ph97_G9
zmp_ph97_H2
zmp_ph97_B10
zmp_ph97_C2
zmp_ph97_C4
zmp_ph97_B8
zmp_ph97_C1
zmp_ph97_D2
zmp_ph97_D5
zmp_ph111_G5
zmp_ph111_G11
zmp_ph111_H1
zmp_ph111_B1a
zmp_ph111_B5
zmp_ph111_B8
zmp_ph111_B6
zmp_ph111_B7
zmp_ph111_B9
zmp_ph111_B10
zmp_ph114_G4
zmp_ph114_G11
zmp_ph114_H1
zmp_ph114_H2
zmp_ph114_A9
zmp_ph114_A11
zmp_ph114_B2
zmp_ph114_B4
zmp_ph114_D8
zmp_ph114_E2

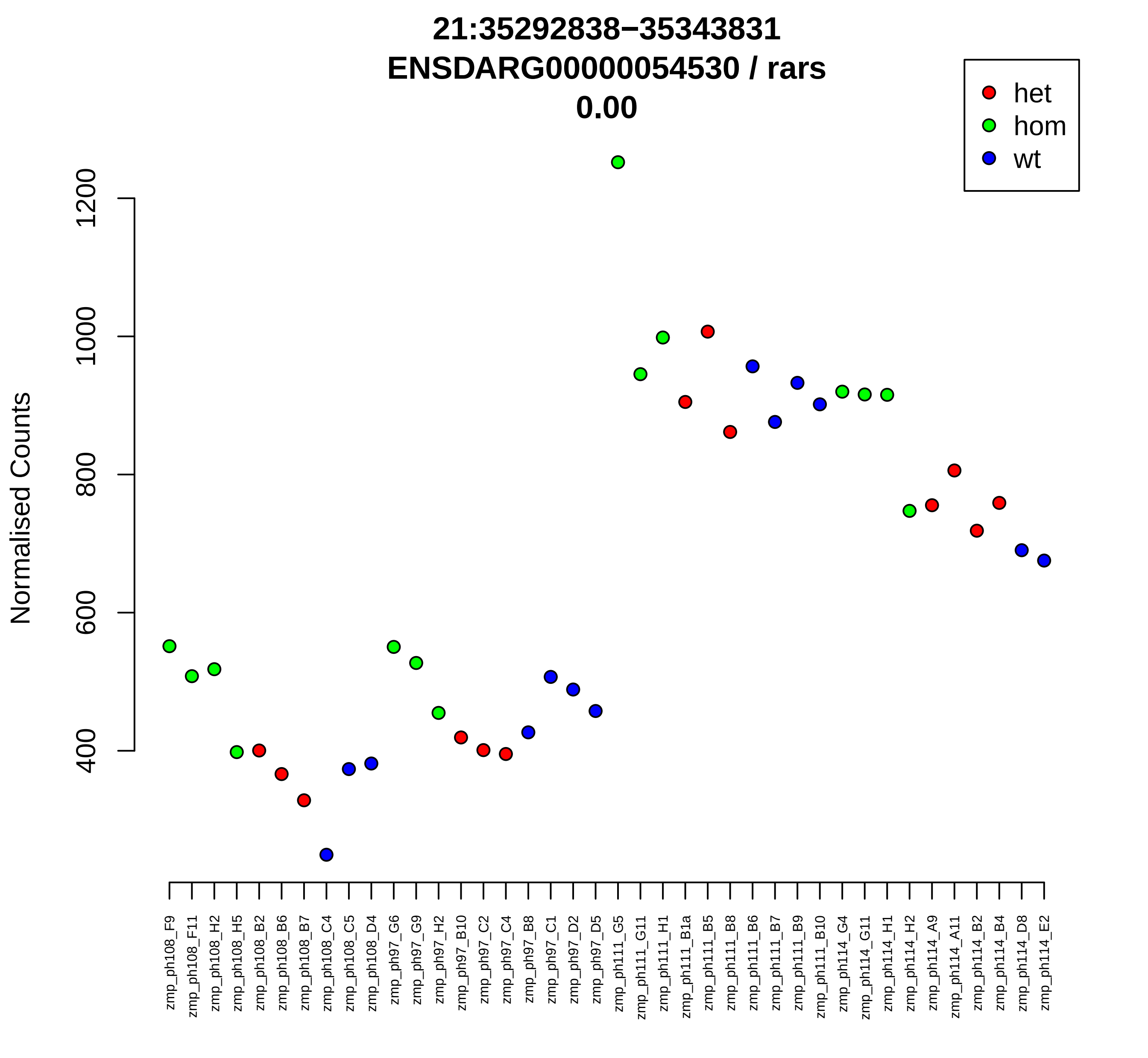

21:35292838−35343831
ENSDARG00000054530 / rars
0.00
het
hom
wt
Normalised Counts
400
600
800
1000
1200
zmp_ph108_F9
zmp_ph108_F11
zmp_ph108_H2
zmp_ph108_H5
zmp_ph108_B2
zmp_ph108_B6
zmp_ph108_B7
zmp_ph108_C4
zmp_ph108_C5
zmp_ph108_D4
zmp_ph97_G6
zmp_ph97_G9
zmp_ph97_H2
zmp_ph97_B10
zmp_ph97_C2
zmp_ph97_C4
zmp_ph97_B8
zmp_ph97_C1
zmp_ph97_D2
zmp_ph97_D5
zmp_ph111_G5
zmp_ph111_G11
zmp_ph111_H1
zmp_ph111_B1a
zmp_ph111_B5
zmp_ph111_B8
zmp_ph111_B6
zmp_ph111_B7
zmp_ph111_B9
zmp_ph111_B10
zmp_ph114_G4
zmp_ph114_G11
zmp_ph114_H1
zmp_ph114_H2
zmp_ph114_A9
zmp_ph114_A11
zmp_ph114_B2
zmp_ph114_B4
zmp_ph114_D8
zmp_ph114_E2

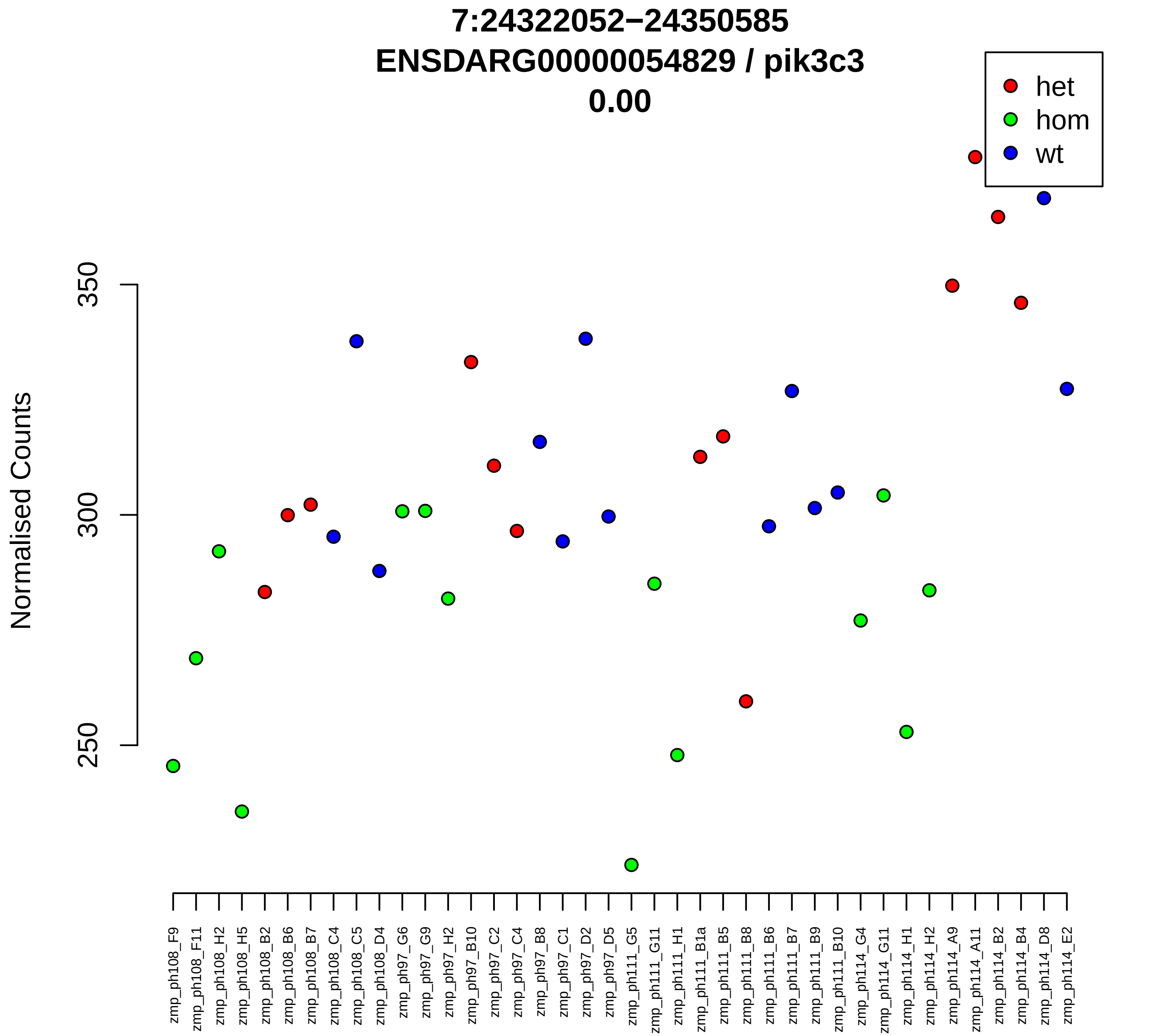
7:24322052−24350585
ENSDARG00000054829 / pik3c3
0.00
het
hom
wt
Normalised Counts
250
300
350
zmp_ph108_F9
zmp_ph108_F11
zmp_ph108_H2
zmp_ph108_H5
zmp_ph108_B2
zmp_ph108_B6
zmp_ph108_B7
zmp_ph108_C4
zmp_ph108_C5
zmp_ph108_D4
zmp_ph97_G6
zmp_ph97_G9
zmp_ph97_H2
zmp_ph97_B10
zmp_ph97_C2
zmp_ph97_C4
zmp_ph97_B8
zmp_ph97_C1
zmp_ph97_D2
zmp_ph97_D5
zmp_ph111_G5
zmp_ph111_G11
zmp_ph111_H1
zmp_ph111_B1a
zmp_ph111_B5
zmp_ph111_B8
zmp_ph111_B6
zmp_ph111_B7
zmp_ph111_B9
zmp_ph111_B10
zmp_ph114_G4
zmp_ph114_G11
zmp_ph114_H1
zmp_ph114_H2
zmp_ph114_A9
zmp_ph114_A11
zmp_ph114_B2
zmp_ph114_B4
zmp_ph114_D8
zmp_ph114_E2

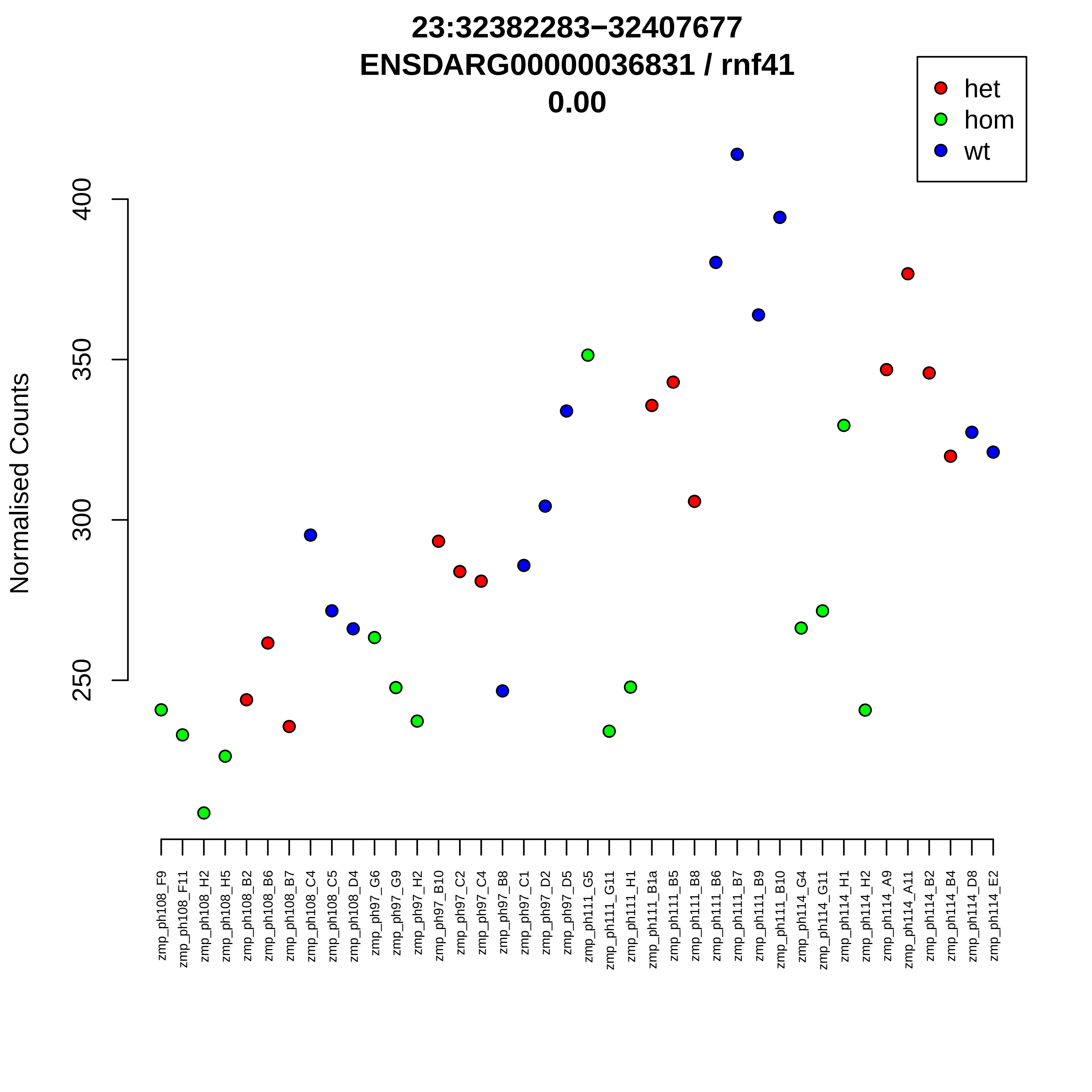

23:32382283−32407677
ENSDARG00000036831 / rnf41
0.00
het
hom
wt
Normalised Counts
250
300
350
400
zmp_ph108_F9
zmp_ph108_F11
zmp_ph108_H2
zmp_ph108_H5
zmp_ph108_B2
zmp_ph108_B6
zmp_ph108_B7
zmp_ph108_C4
zmp_ph108_C5
zmp_ph108_D4
zmp_ph97_G6
zmp_ph97_G9
zmp_ph97_H2
zmp_ph97_B10
zmp_ph97_C2
zmp_ph97_C4
zmp_ph97_B8
zmp_ph97_C1
zmp_ph97_D2
zmp_ph97_D5
zmp_ph111_G5
zmp_ph111_G11
zmp_ph111_H1
zmp_ph111_B1a
zmp_ph111_B5
zmp_ph111_B8
zmp_ph111_B6
zmp_ph111_B7
zmp_ph111_B9
zmp_ph111_B10
zmp_ph114_G4
zmp_ph114_G11
zmp_ph114_H1
zmp_ph114_H2
zmp_ph114_A9
zmp_ph114_A11
zmp_ph114_B2
zmp_ph114_B4
zmp_ph114_D8
zmp_ph114_E2

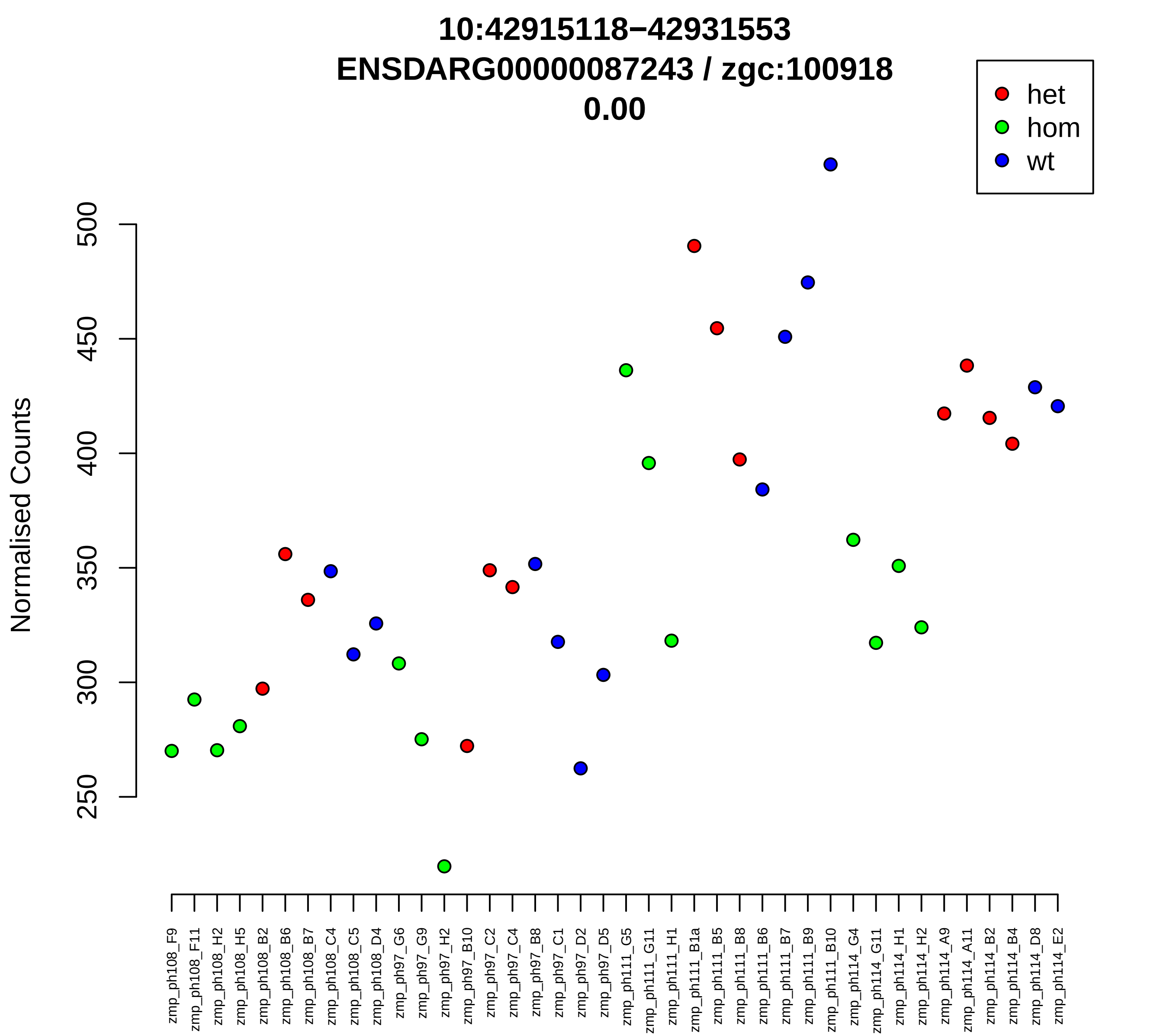
10:42915118−42931553
ENSDARG00000087243 / zgc:100918
0.00
het
hom
wt
Normalised Counts
250
300
350
400
450
500
zmp_ph108_F9
zmp_ph108_F11
zmp_ph108_H2
zmp_ph108_H5
zmp_ph108_B2
zmp_ph108_B6
zmp_ph108_B7
zmp_ph108_C4
zmp_ph108_C5
zmp_ph108_D4
zmp_ph97_G6
zmp_ph97_G9
zmp_ph97_H2
zmp_ph97_B10
zmp_ph97_C2
zmp_ph97_C4
zmp_ph97_B8
zmp_ph97_C1
zmp_ph97_D2
zmp_ph97_D5
zmp_ph111_G5
zmp_ph111_G11
zmp_ph111_H1
zmp_ph111_B1a
zmp_ph111_B5
zmp_ph111_B8
zmp_ph111_B6
zmp_ph111_B7
zmp_ph111_B9
zmp_ph111_B10
zmp_ph114_G4
zmp_ph114_G11
zmp_ph114_H1
zmp_ph114_H2
zmp_ph114_A9
zmp_ph114_A11
zmp_ph114_B2
zmp_ph114_B4
zmp_ph114_D8
zmp_ph114_E2

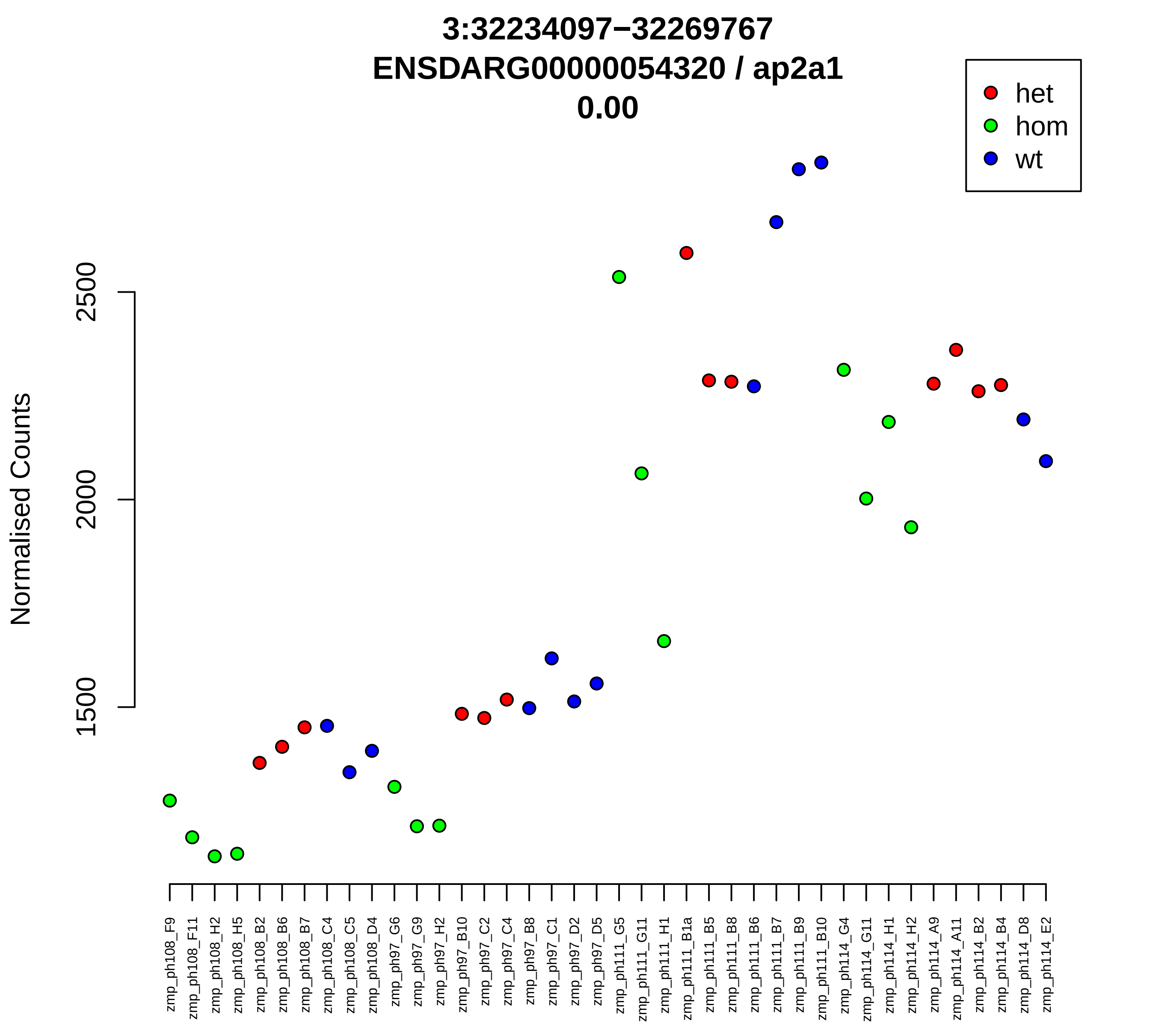
3:32234097−32269767
ENSDARG00000054320 / ap2a1
0.00
het
hom
wt
Normalised Counts
1500
2000
2500
zmp_ph108_F9
zmp_ph108_F11
zmp_ph108_H2
zmp_ph108_H5
zmp_ph108_B2
zmp_ph108_B6
zmp_ph108_B7
zmp_ph108_C4
zmp_ph108_C5
zmp_ph108_D4
zmp_ph97_G6
zmp_ph97_G9
zmp_ph97_H2
zmp_ph97_B10
zmp_ph97_C2
zmp_ph97_C4
zmp_ph97_B8
zmp_ph97_C1
zmp_ph97_D2
zmp_ph97_D5
zmp_ph111_G5
zmp_ph111_G11
zmp_ph111_H1
zmp_ph111_B1a
zmp_ph111_B5
zmp_ph111_B8
zmp_ph111_B6
zmp_ph111_B7
zmp_ph111_B9
zmp_ph111_B10
zmp_ph114_G4
zmp_ph114_G11
zmp_ph114_H1
zmp_ph114_H2
zmp_ph114_A9
zmp_ph114_A11
zmp_ph114_B2
zmp_ph114_B4
zmp_ph114_D8
zmp_ph114_E2

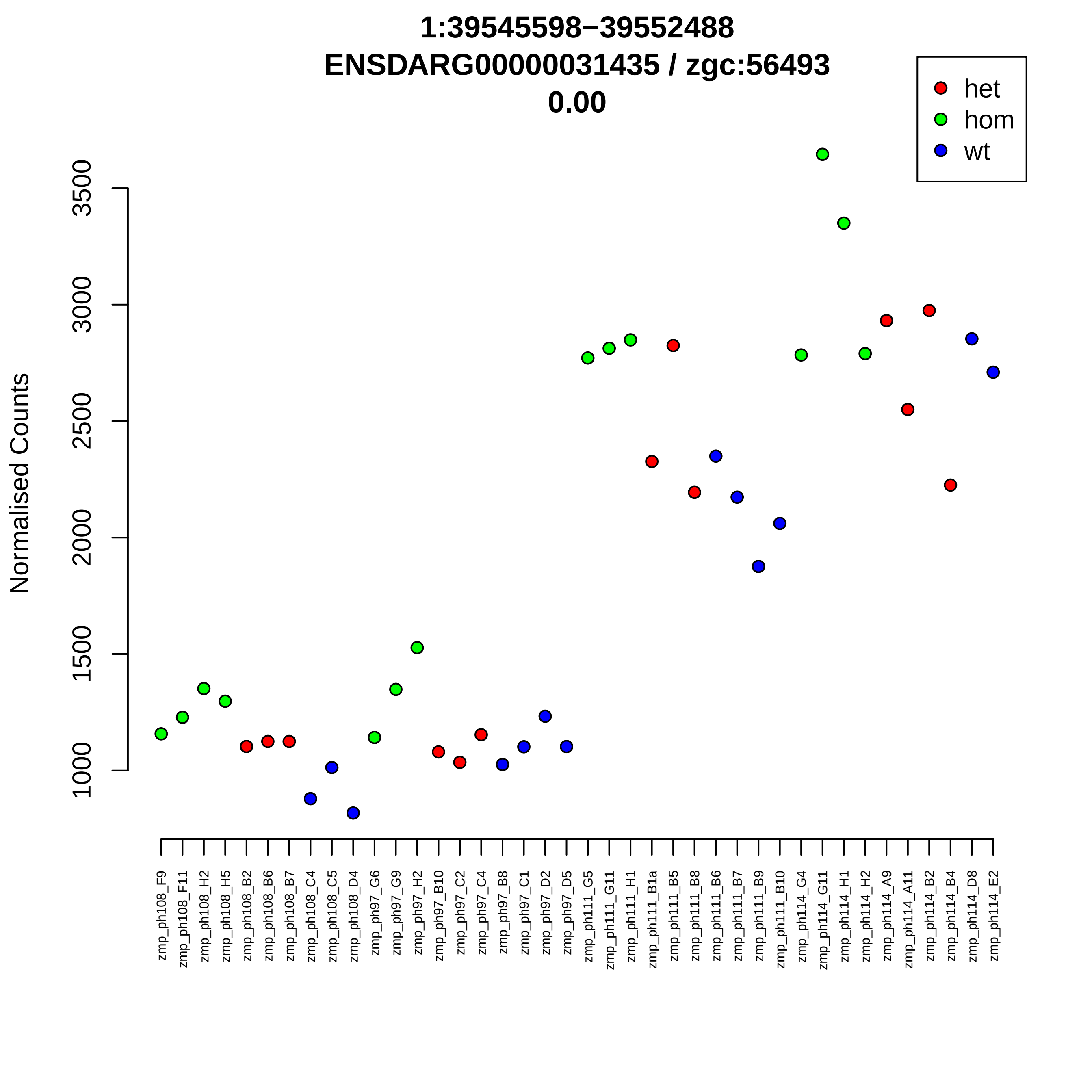

1:39545598−39552488
ENSDARG00000031435 / zgc:56493
0.00
het
hom
wt
Normalised Counts
1000
1500
2000
2500
3000
3500
zmp_ph108_F9
zmp_ph108_F11
zmp_ph108_H2
zmp_ph108_H5
zmp_ph108_B2
zmp_ph108_B6
zmp_ph108_B7
zmp_ph108_C4
zmp_ph108_C5
zmp_ph108_D4
zmp_ph97_G6
zmp_ph97_G9
zmp_ph97_H2
zmp_ph97_B10
zmp_ph97_C2
zmp_ph97_C4
zmp_ph97_B8
zmp_ph97_C1
zmp_ph97_D2
zmp_ph97_D5
zmp_ph111_G5
zmp_ph111_G11
zmp_ph111_H1
zmp_ph111_B1a
zmp_ph111_B5
zmp_ph111_B8
zmp_ph111_B6
zmp_ph111_B7
zmp_ph111_B9
zmp_ph111_B10
zmp_ph114_G4
zmp_ph114_G11
zmp_ph114_H1
zmp_ph114_H2
zmp_ph114_A9
zmp_ph114_A11
zmp_ph114_B2
zmp_ph114_B4
zmp_ph114_D8
zmp_ph114_E2

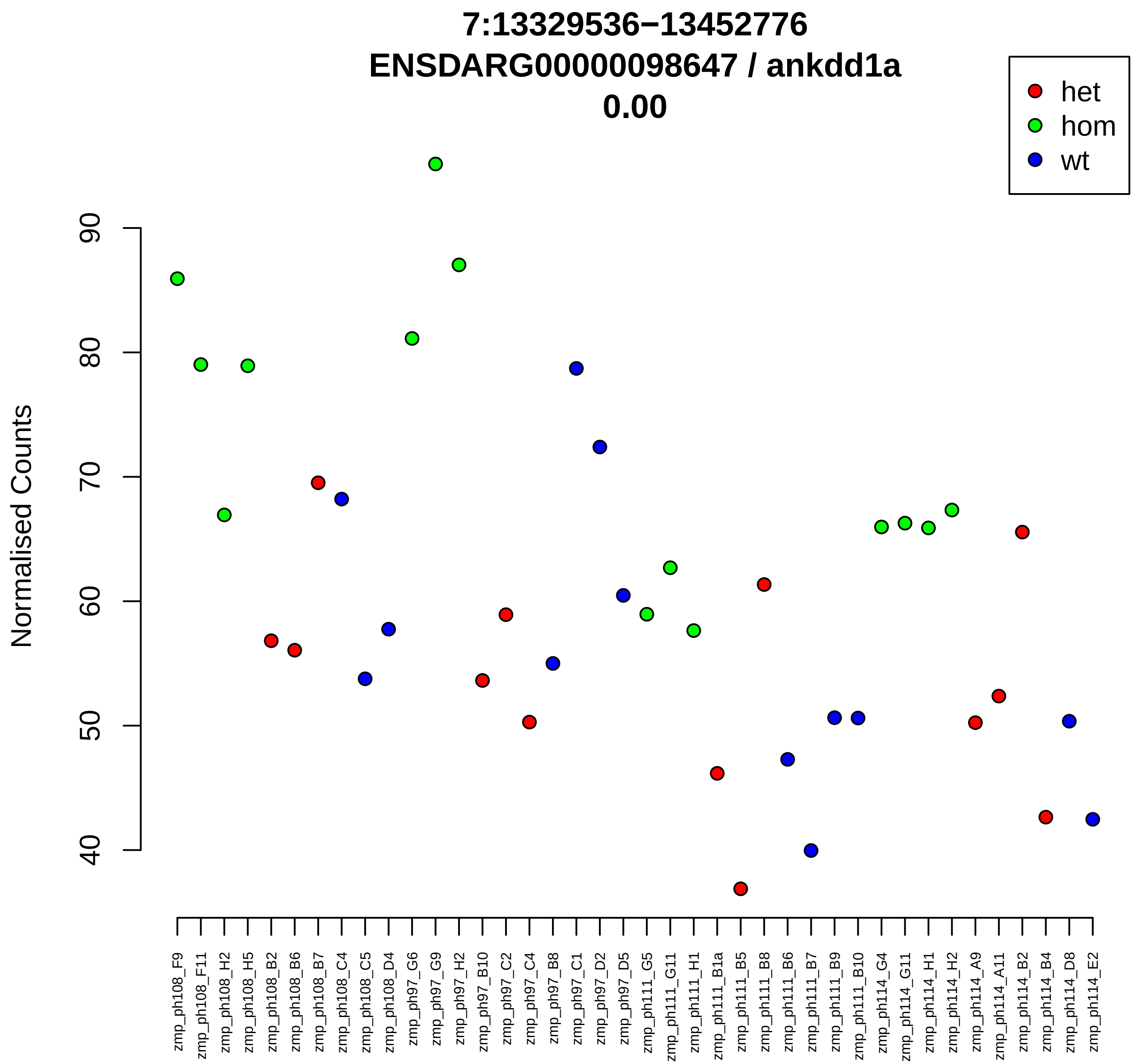
7:13329536−13452776
ENSDARG00000098647 / ankdd1a
0.00
het
hom
wt
Normalised Counts
40
50
60
70
80
90
zmp_ph108_F9
zmp_ph108_F11
zmp_ph108_H2
zmp_ph108_H5
zmp_ph108_B2
zmp_ph108_B6
zmp_ph108_B7
zmp_ph108_C4
zmp_ph108_C5
zmp_ph108_D4
zmp_ph97_G6
zmp_ph97_G9
zmp_ph97_H2
zmp_ph97_B10
zmp_ph97_C2
zmp_ph97_C4
zmp_ph97_B8
zmp_ph97_C1
zmp_ph97_D2
zmp_ph97_D5
zmp_ph111_G5
zmp_ph111_G11
zmp_ph111_H1
zmp_ph111_B1a
zmp_ph111_B5
zmp_ph111_B8
zmp_ph111_B6
zmp_ph111_B7
zmp_ph111_B9
zmp_ph111_B10
zmp_ph114_G4
zmp_ph114_G11
zmp_ph114_H1
zmp_ph114_H2
zmp_ph114_A9
zmp_ph114_A11
zmp_ph114_B2
zmp_ph114_B4
zmp_ph114_D8
zmp_ph114_E2

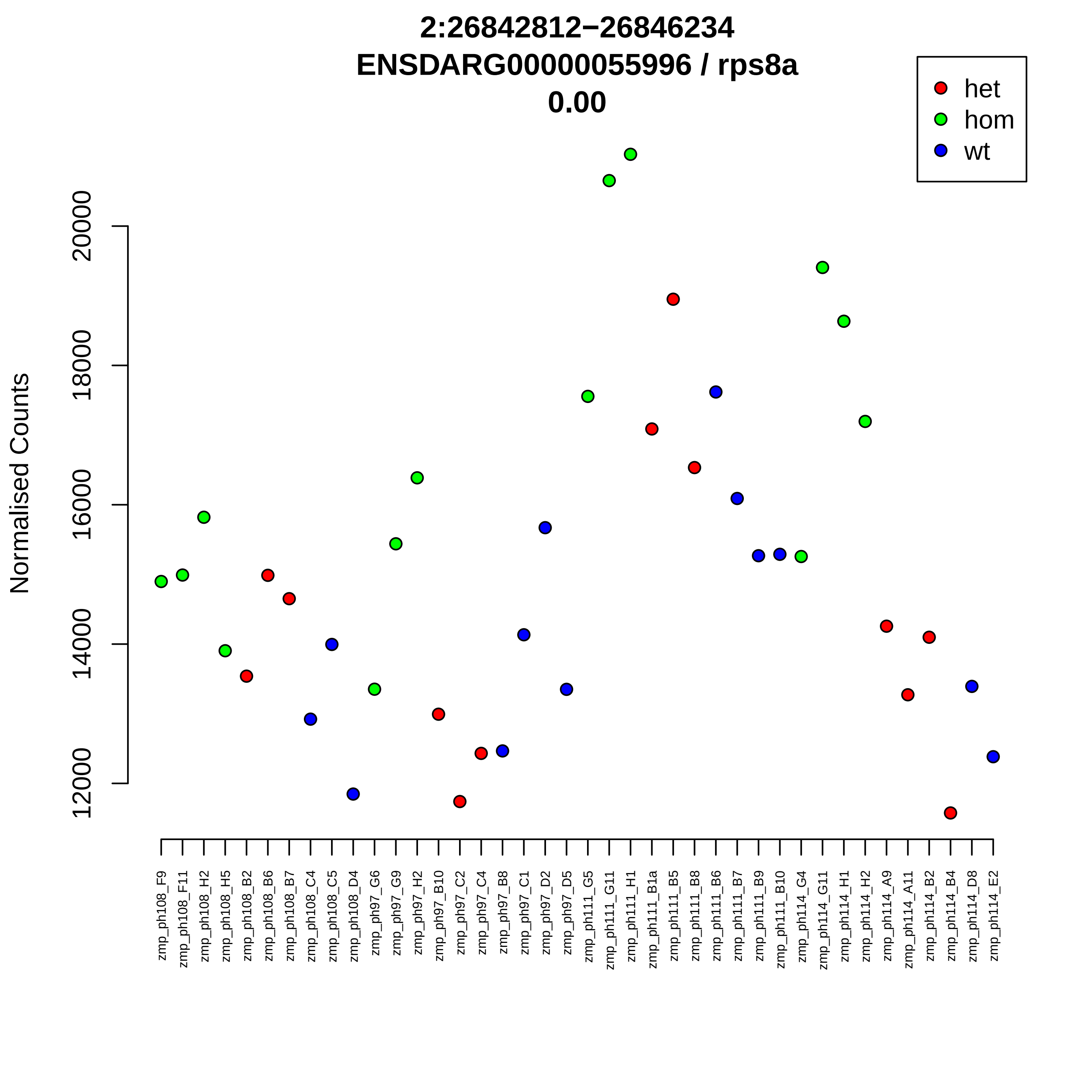

2:26842812−26846234
ENSDARG00000055996 / rps8a
0.00
het
hom
wt
Normalised Counts
12000
14000
16000
18000
20000
zmp_ph108_F9
zmp_ph108_F11
zmp_ph108_H2
zmp_ph108_H5
zmp_ph108_B2
zmp_ph108_B6
zmp_ph108_B7
zmp_ph108_C4
zmp_ph108_C5
zmp_ph108_D4
zmp_ph97_G6
zmp_ph97_G9
zmp_ph97_H2
zmp_ph97_B10
zmp_ph97_C2
zmp_ph97_C4
zmp_ph97_B8
zmp_ph97_C1
zmp_ph97_D2
zmp_ph97_D5
zmp_ph111_G5
zmp_ph111_G11
zmp_ph111_H1
zmp_ph111_B1a
zmp_ph111_B5
zmp_ph111_B8
zmp_ph111_B6
zmp_ph111_B7
zmp_ph111_B9
zmp_ph111_B10
zmp_ph114_G4
zmp_ph114_G11
zmp_ph114_H1
zmp_ph114_H2
zmp_ph114_A9
zmp_ph114_A11
zmp_ph114_B2
zmp_ph114_B4
zmp_ph114_D8
zmp_ph114_E2

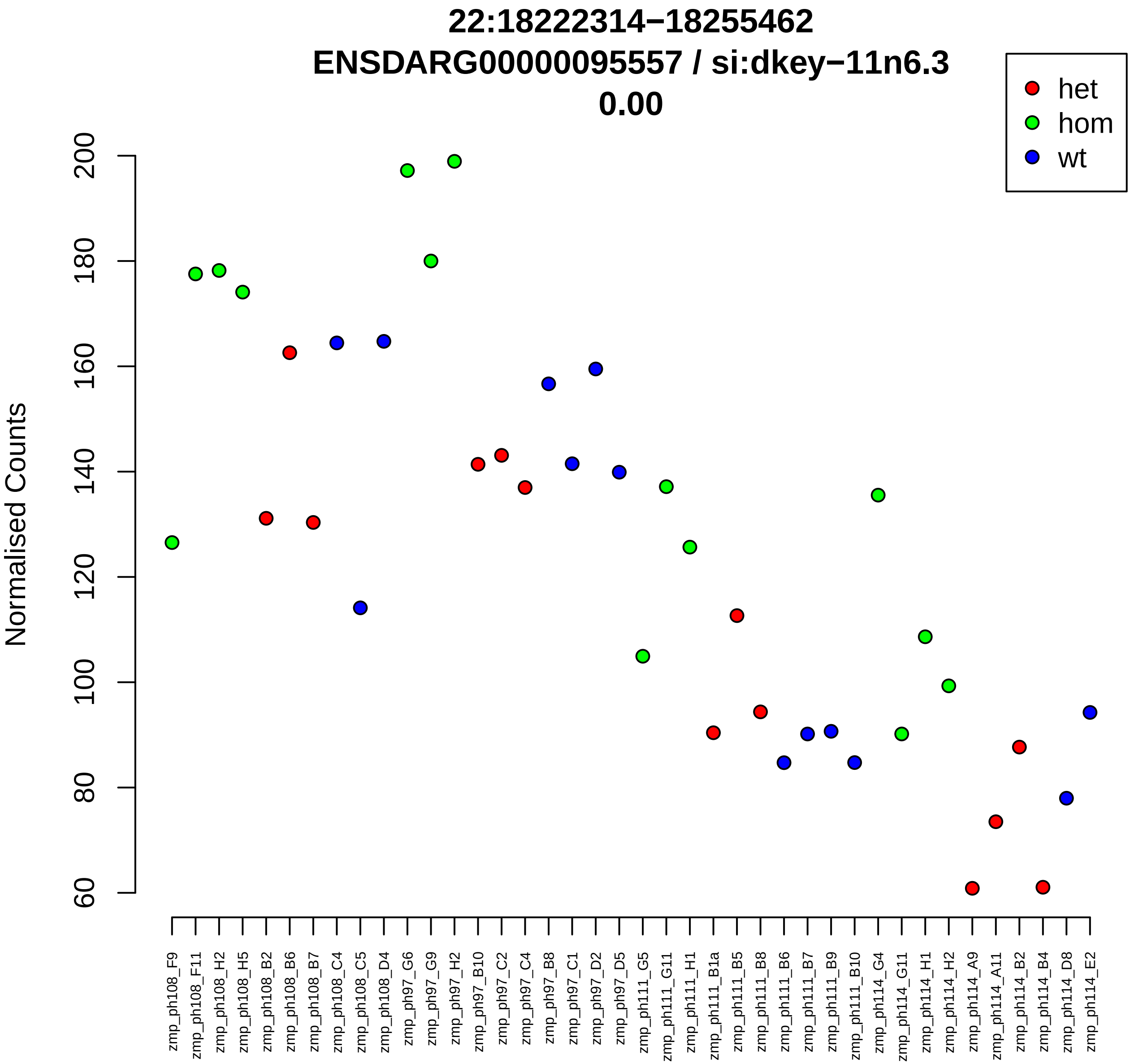

22:18222314−18255462
ENSDARG00000095557 / si:dkey−11n6.3
0.00
het
hom
wt
Normalised Counts
200
180
160
140
120
100
80
60
zmp_ph108_F9
zmp_ph108_F11
zmp_ph108_H2
zmp_ph108_H5
zmp_ph108_B2
zmp_ph108_B6
zmp_ph108_B7
zmp_ph108_C4
zmp_ph108_C5
zmp_ph108_D4
zmp_ph97_G6
zmp_ph97_G9
zmp_ph97_H2
zmp_ph97_B10
zmp_ph97_C2
zmp_ph97_C4
zmp_ph97_B8
zmp_ph97_C1
zmp_ph97_D2
zmp_ph97_D5
zmp_ph111_G5
zmp_ph111_G11
zmp_ph111_H1
zmp_ph111_B1a
zmp_ph111_B5
zmp_ph111_B8
zmp_ph111_B6
zmp_ph111_B7
zmp_ph111_B9
zmp_ph111_B10
zmp_ph114_G4
zmp_ph114_G11
zmp_ph114_H1
zmp_ph114_H2
zmp_ph114_A9
zmp_ph114_A11
zmp_ph114_B2
zmp_ph114_B4
zmp_ph114_D8
zmp_ph114_E2

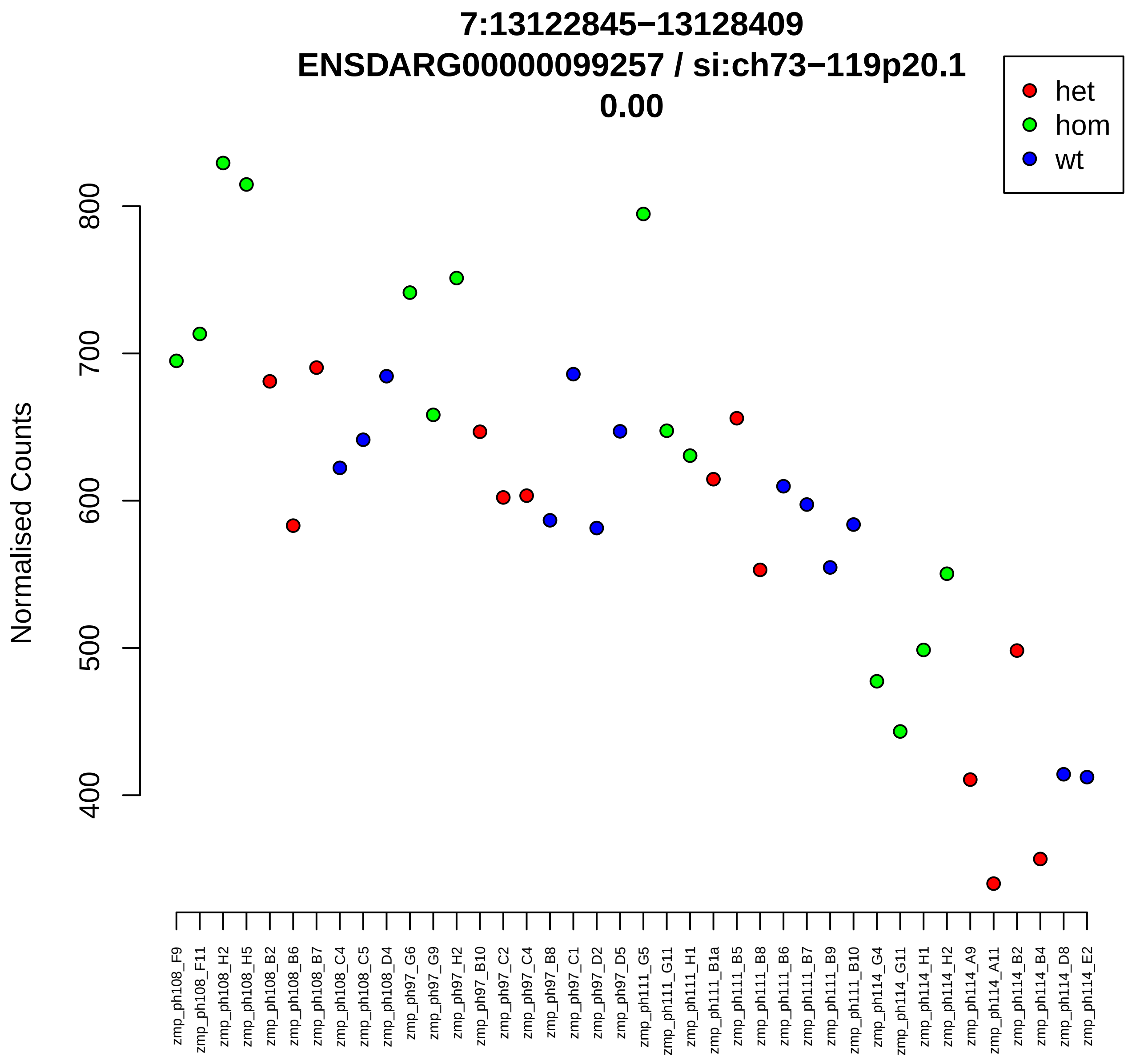

7:13122845−13128409
ENSDARG00000099257 / si:ch73−119p20.1
0.00
het
hom
wt
Normalised Counts
400
500
600
700
800
zmp_ph108_F9
zmp_ph108_F11
zmp_ph108_H2
zmp_ph108_H5
zmp_ph108_B2
zmp_ph108_B6
zmp_ph108_B7
zmp_ph108_C4
zmp_ph108_C5
zmp_ph108_D4
zmp_ph97_G6
zmp_ph97_G9
zmp_ph97_H2
zmp_ph97_B10
zmp_ph97_C2
zmp_ph97_C4
zmp_ph97_B8
zmp_ph97_C1
zmp_ph97_D2
zmp_ph97_D5
zmp_ph111_G5
zmp_ph111_G11
zmp_ph111_H1
zmp_ph111_B1a
zmp_ph111_B5
zmp_ph111_B8
zmp_ph111_B6
zmp_ph111_B7
zmp_ph111_B9
zmp_ph111_B10
zmp_ph114_G4
zmp_ph114_G11
zmp_ph114_H1
zmp_ph114_H2
zmp_ph114_A9
zmp_ph114_A11
zmp_ph114_B2
zmp_ph114_B4
zmp_ph114_D8
zmp_ph114_E2

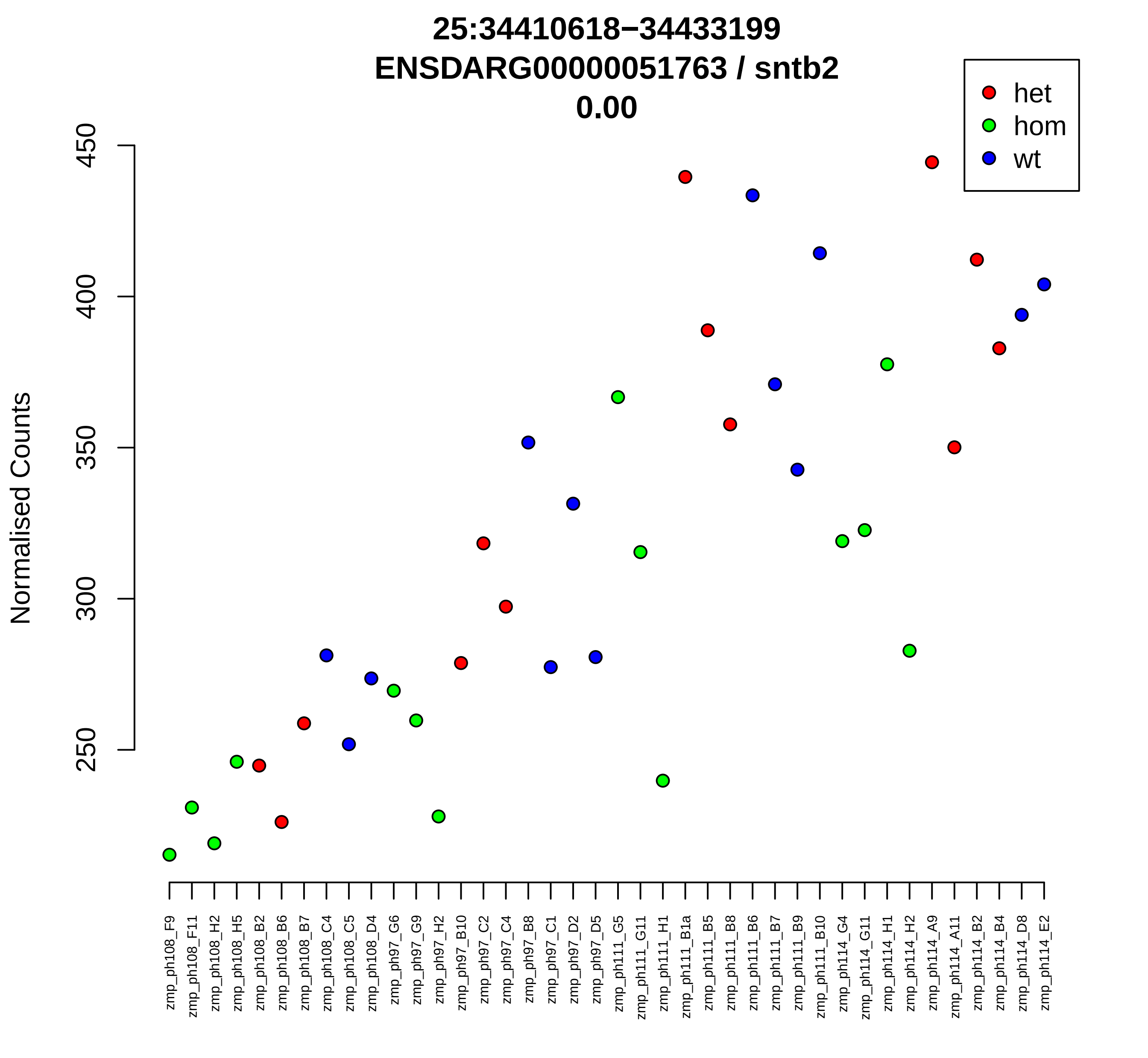

25:34410618−34433199
ENSDARG00000051763 / sntb2
0.00
het
hom
wt
Normalised Counts
250
300
350
400
450
zmp_ph108_F9
zmp_ph108_F11
zmp_ph108_H2
zmp_ph108_H5
zmp_ph108_B2
zmp_ph108_B6
zmp_ph108_B7
zmp_ph108_C4
zmp_ph108_C5
zmp_ph108_D4
zmp_ph97_G6
zmp_ph97_G9
zmp_ph97_H2
zmp_ph97_B10
zmp_ph97_C2
zmp_ph97_C4
zmp_ph97_B8
zmp_ph97_C1
zmp_ph97_D2
zmp_ph97_D5
zmp_ph111_G5
zmp_ph111_G11
zmp_ph111_H1
zmp_ph111_B1a
zmp_ph111_B5
zmp_ph111_B8
zmp_ph111_B6
zmp_ph111_B7
zmp_ph111_B9
zmp_ph111_B10
zmp_ph114_G4
zmp_ph114_G11
zmp_ph114_H1
zmp_ph114_H2
zmp_ph114_A9
zmp_ph114_A11
zmp_ph114_B2
zmp_ph114_B4
zmp_ph114_D8
zmp_ph114_E2

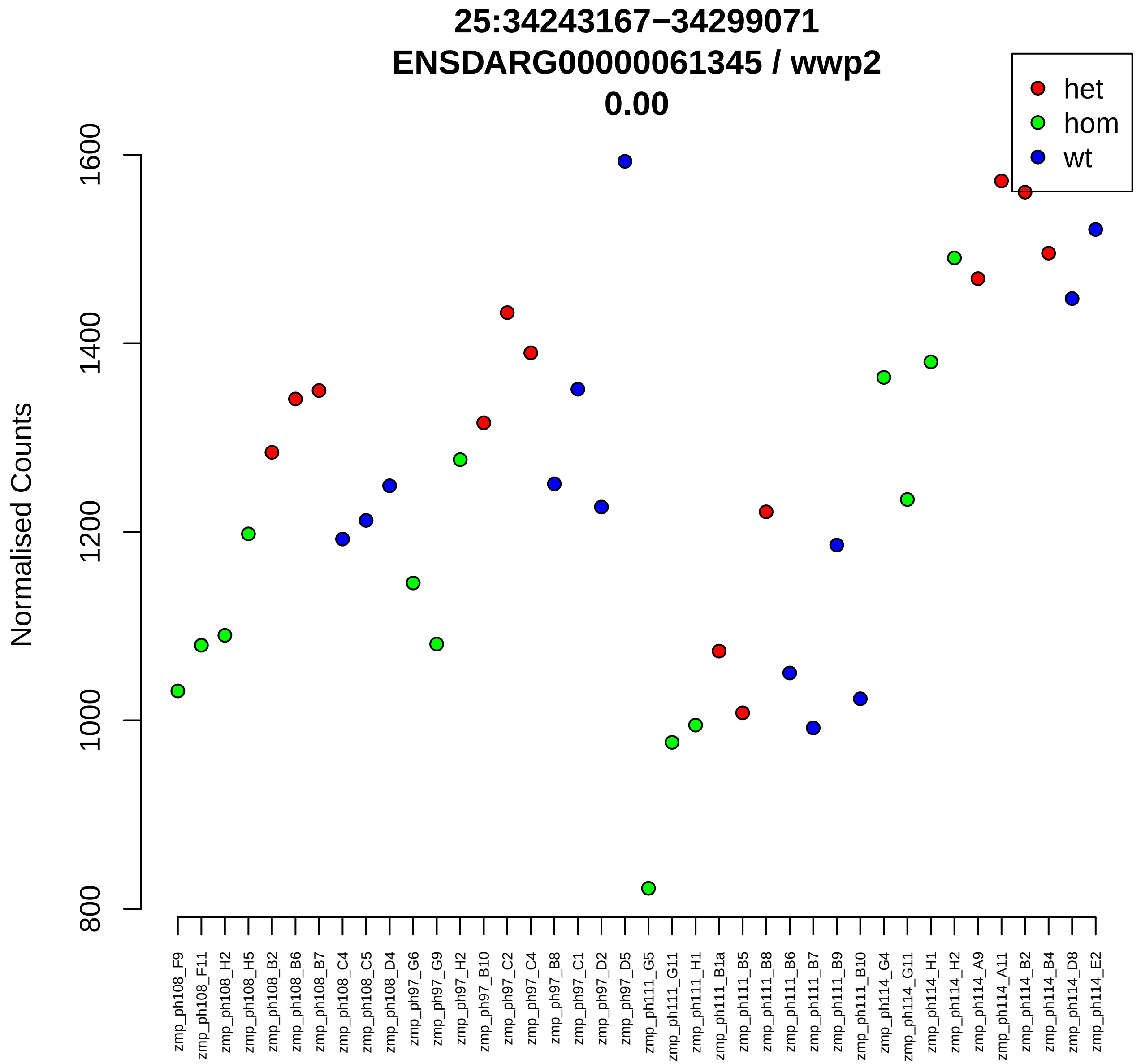
25:34243167−34299071
ENSDARG00000061345 / wwp2
0.00
Normalised Counts
800
1000
1200
1400
1600
het
hom
wt
zmp_ph108_F9
zmp_ph108_F11
zmp_ph108_H2
zmp_ph108_H5
zmp_ph108_B2
zmp_ph108_B6
zmp_ph108_B7
zmp_ph108_C4
zmp_ph108_C5
zmp_ph108_D4
zmp_ph97_G6
zmp_ph97_G9
zmp_ph97_H2
zmp_ph97_B10
zmp_ph97_C2
zmp_ph97_C4
zmp_ph97_B8
zmp_ph97_C1
zmp_ph97_D2
zmp_ph97_D5
zmp_ph111_G5
zmp_ph111_G11
zmp_ph111_H1
zmp_ph111_B1a
zmp_ph111_B5
zmp_ph111_B8
zmp_ph111_B6
zmp_ph111_B7
zmp_ph111_B9
zmp_ph111_B10
zmp_ph114_G4
zmp_ph114_G11
zmp_ph114_H1
zmp_ph114_H2
zmp_ph114_A9
zmp_ph114_A11
zmp_ph114_B2
zmp_ph114_B4
zmp_ph114_D8
zmp_ph114_E2

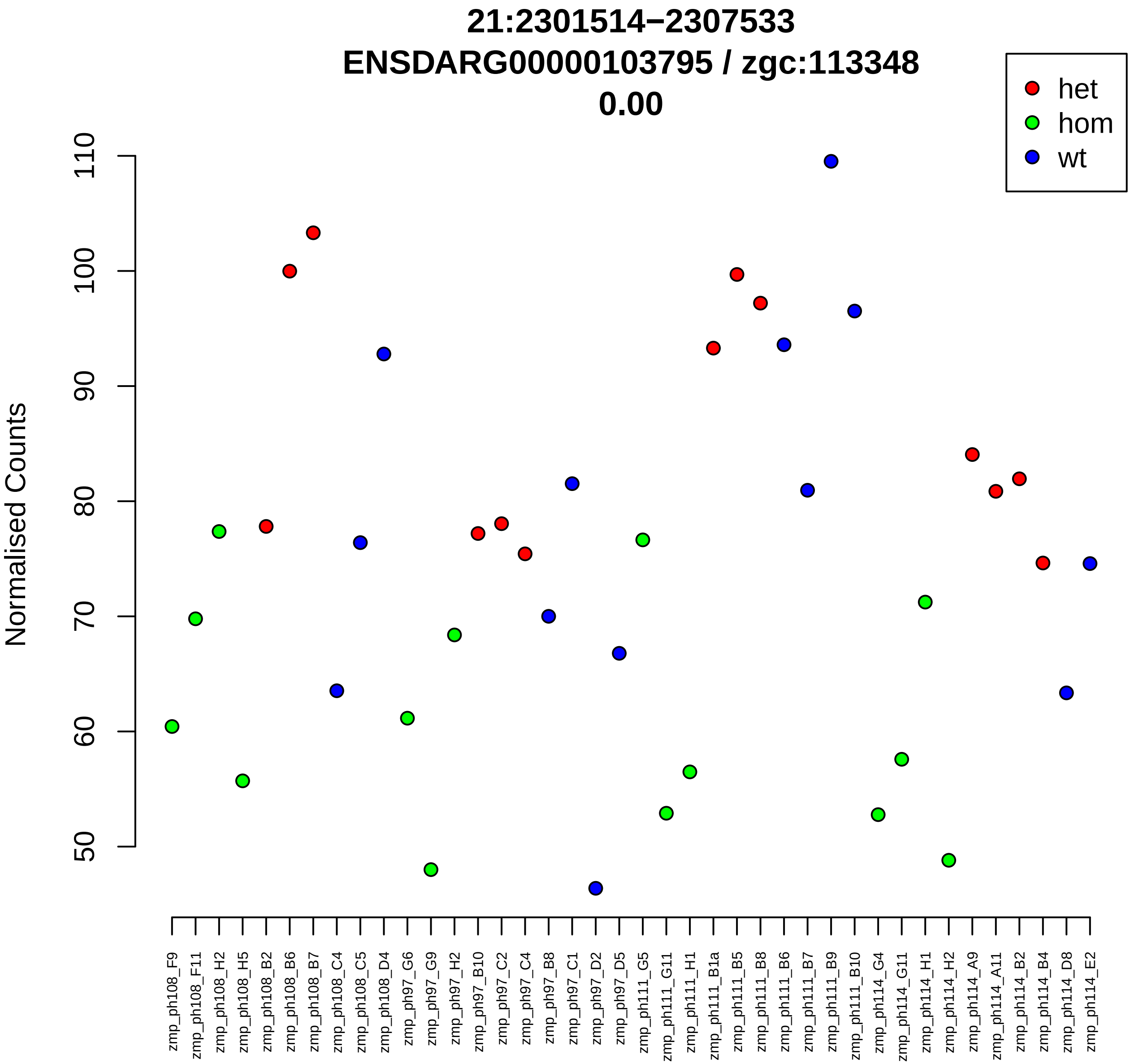

21:2301514−2307533
ENSDARG00000103795 / zgc:113348
0.00
Normalised Counts
het
hom
wt
50
60
70
80
90
100
110
zmp_ph108_F9
zmp_ph108_F11
zmp_ph108_H2
zmp_ph108_H5
zmp_ph108_B2
zmp_ph108_B6
zmp_ph108_B7
zmp_ph108_C4
zmp_ph108_C5
zmp_ph108_D4
zmp_ph97_G6
zmp_ph97_G9
zmp_ph97_H2
zmp_ph97_B10
zmp_ph97_C2
zmp_ph97_C4
zmp_ph97_B8
zmp_ph97_C1
zmp_ph97_D2
zmp_ph97_D5
zmp_ph111_G5
zmp_ph111_G11
zmp_ph111_H1
zmp_ph111_B1a
zmp_ph111_B5
zmp_ph111_B8
zmp_ph111_B6
zmp_ph111_B7
zmp_ph111_B9
zmp_ph111_B10
zmp_ph114_G4
zmp_ph114_G11
zmp_ph114_H1
zmp_ph114_H2
zmp_ph114_A9
zmp_ph114_A11
zmp_ph114_B2
zmp_ph114_B4
zmp_ph114_D8
zmp_ph114_E2

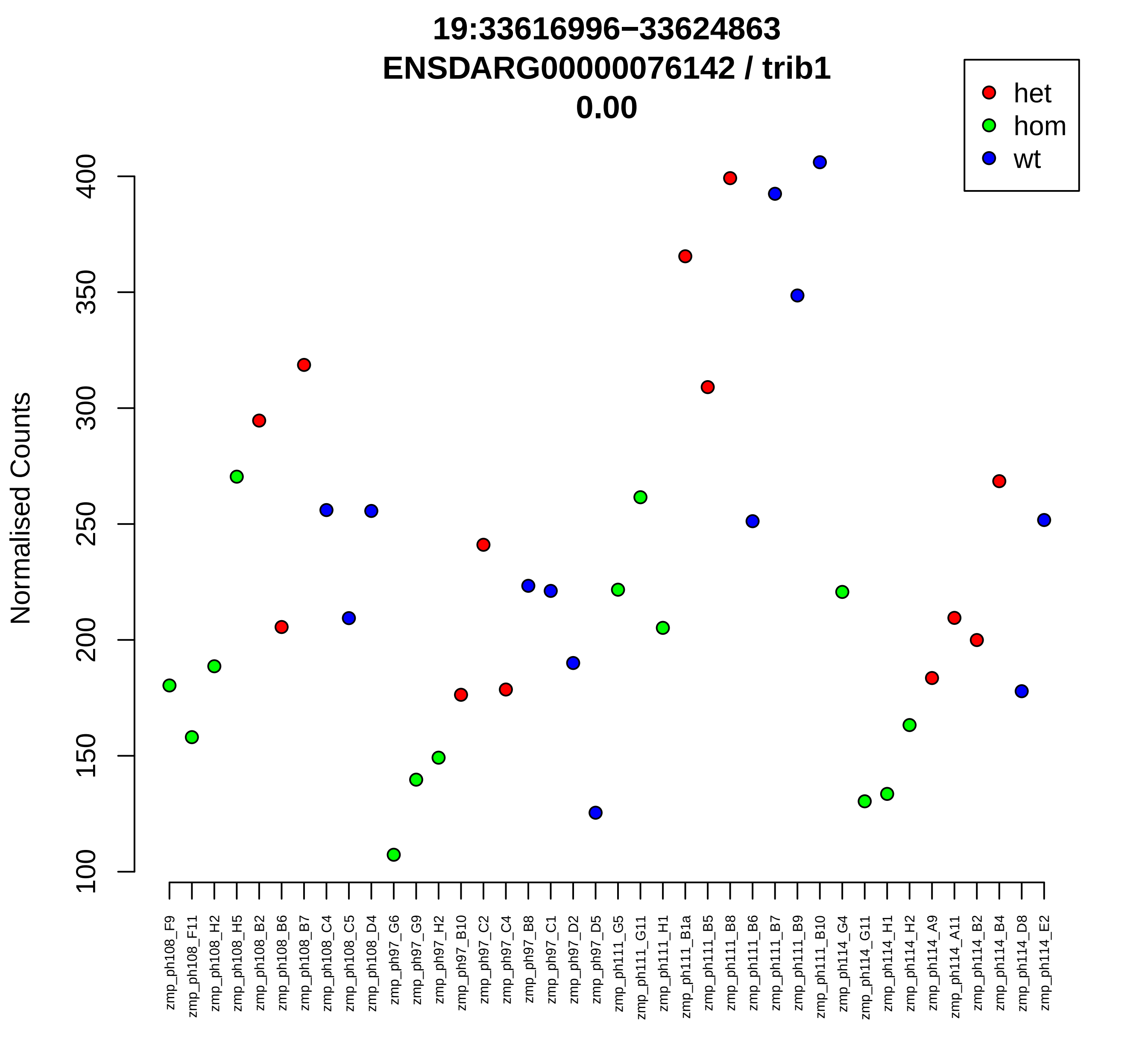

19:33616996−33624863
ENSDARG00000076142 / trib1
0.00
het
hom
wt
Normalised Counts
100
150
200
250
300
350
400
zmp_ph108_F9
zmp_ph108_F11
zmp_ph108_H2
zmp_ph108_H5
zmp_ph108_B2
zmp_ph108_B6
zmp_ph108_B7
zmp_ph108_C4
zmp_ph108_C5
zmp_ph108_D4
zmp_ph97_G6
zmp_ph97_G9
zmp_ph97_H2
zmp_ph97_B10
zmp_ph97_C2
zmp_ph97_C4
zmp_ph97_B8
zmp_ph97_C1
zmp_ph97_D2
zmp_ph97_D5
zmp_ph111_G5
zmp_ph111_G11
zmp_ph111_H1
zmp_ph111_B1a
zmp_ph111_B5
zmp_ph111_B8
zmp_ph111_B6
zmp_ph111_B7
zmp_ph111_B9
zmp_ph111_B10
zmp_ph114_G4
zmp_ph114_G11
zmp_ph114_H1
zmp_ph114_H2
zmp_ph114_A9
zmp_ph114_A11
zmp_ph114_B2
zmp_ph114_B4
zmp_ph114_D8
zmp_ph114_E2

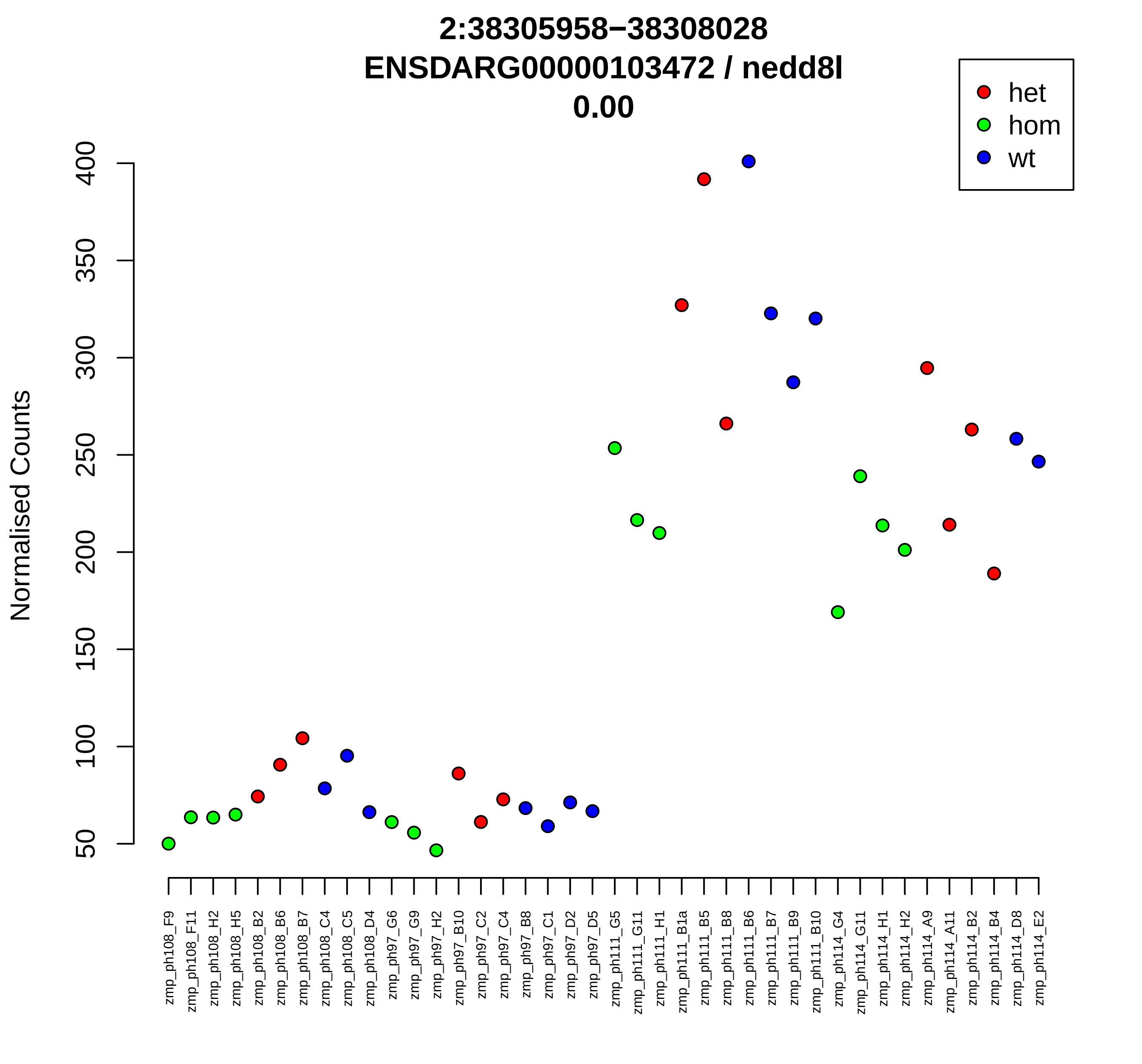

2:38305958−38308028
ENSDARG00000103472 / nedd8l
0.00
het
hom
wt
Normalised Counts
50
100
150
200
250
300
350
400
zmp_ph108_F9
zmp_ph108_F11
zmp_ph108_H2
zmp_ph108_H5
zmp_ph108_B2
zmp_ph108_B6
zmp_ph108_B7
zmp_ph108_C4
zmp_ph108_C5
zmp_ph108_D4
zmp_ph97_G6
zmp_ph97_G9
zmp_ph97_H2
zmp_ph97_B10
zmp_ph97_C2
zmp_ph97_C4
zmp_ph97_B8
zmp_ph97_C1
zmp_ph97_D2
zmp_ph97_D5
zmp_ph111_G5
zmp_ph111_G11
zmp_ph111_H1
zmp_ph111_B1a
zmp_ph111_B5
zmp_ph111_B8
zmp_ph111_B6
zmp_ph111_B7
zmp_ph111_B9
zmp_ph111_B10
zmp_ph114_G4
zmp_ph114_G11
zmp_ph114_H1
zmp_ph114_H2
zmp_ph114_A9
zmp_ph114_A11
zmp_ph114_B2
zmp_ph114_B4
zmp_ph114_D8
zmp_ph114_E2

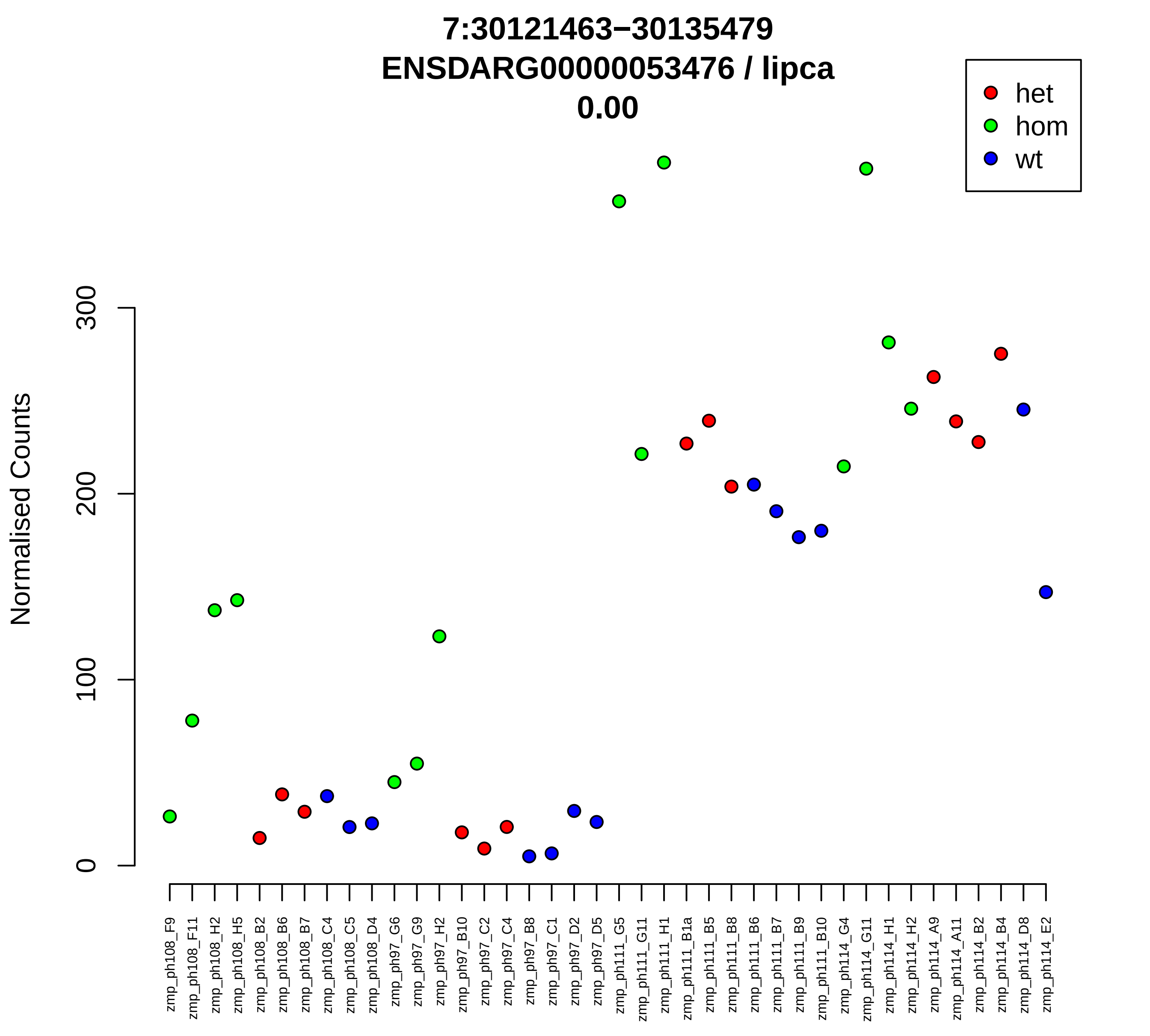
7:30121463−30135479
ENSDARG00000053476 / lipca
0.00
het
hom
wt
Normalised Counts
0
100
200
300
zmp_ph108_F9
zmp_ph108_F11
zmp_ph108_H2
zmp_ph108_H5
zmp_ph108_B2
zmp_ph108_B6
zmp_ph108_B7
zmp_ph108_C4
zmp_ph108_C5
zmp_ph108_D4
zmp_ph97_G6
zmp_ph97_G9
zmp_ph97_H2
zmp_ph97_B10
zmp_ph97_C2
zmp_ph97_C4
zmp_ph97_B8
zmp_ph97_C1
zmp_ph97_D2
zmp_ph97_D5
zmp_ph111_G5
zmp_ph111_G11
zmp_ph111_H1
zmp_ph111_B1a
zmp_ph111_B5
zmp_ph111_B8
zmp_ph111_B6
zmp_ph111_B7
zmp_ph111_B9
zmp_ph111_B10
zmp_ph114_G4
zmp_ph114_G11
zmp_ph114_H1
zmp_ph114_H2
zmp_ph114_A9
zmp_ph114_A11
zmp_ph114_B2
zmp_ph114_B4
zmp_ph114_D8
zmp_ph114_E2

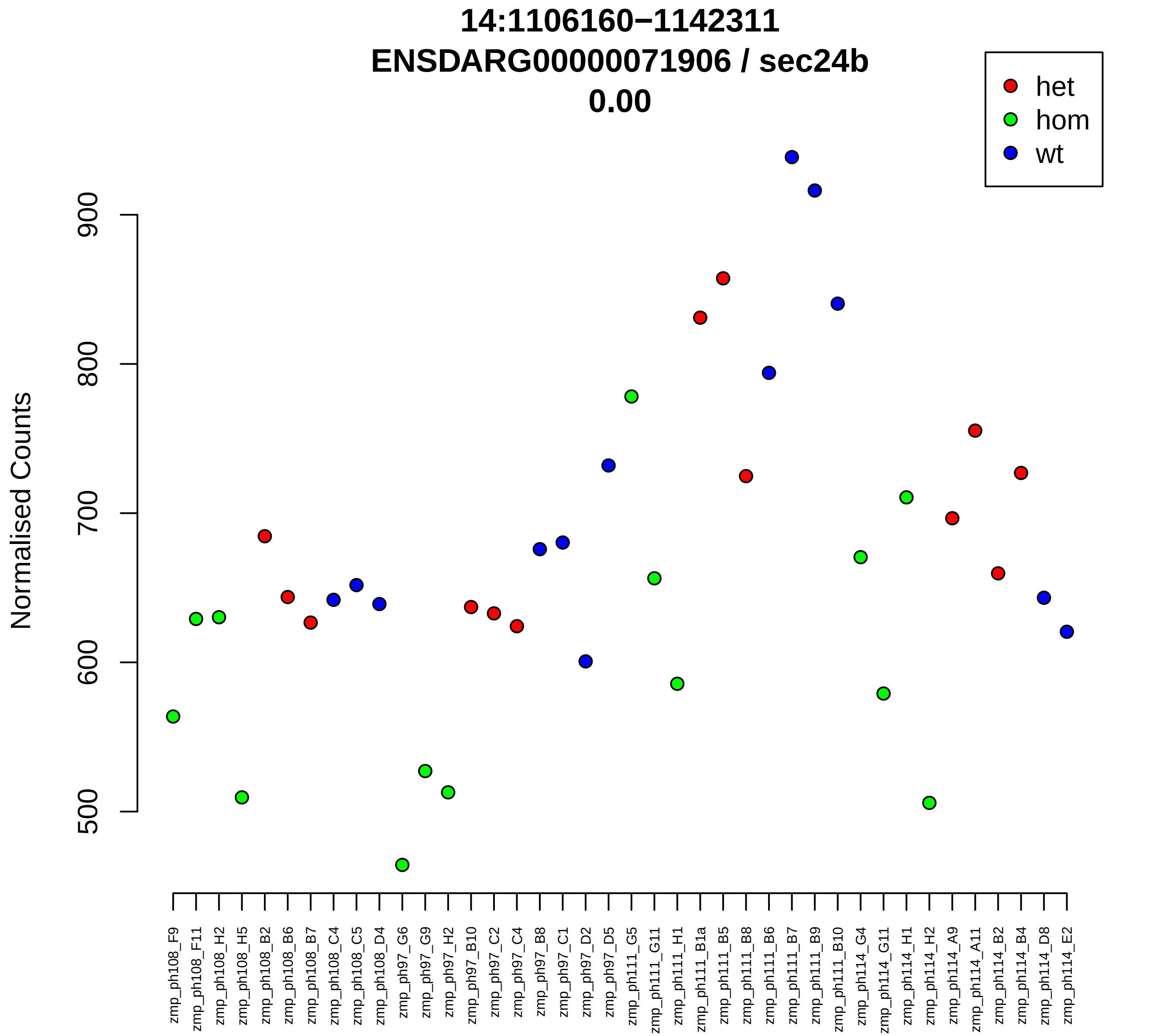
14:1106160−1142311
ENSDARG00000071906 / sec24b
0.00
het
hom
wt
Normalised Counts
500
600
700
800
900
zmp_ph108_F9
zmp_ph108_F11
zmp_ph108_H2
zmp_ph108_H5
zmp_ph108_B2
zmp_ph108_B6
zmp_ph108_B7
zmp_ph108_C4
zmp_ph108_C5
zmp_ph108_D4
zmp_ph97_G6
zmp_ph97_G9
zmp_ph97_H2
zmp_ph97_B10
zmp_ph97_C2
zmp_ph97_C4
zmp_ph97_B8
zmp_ph97_C1
zmp_ph97_D2
zmp_ph97_D5
zmp_ph111_G5
zmp_ph111_G11
zmp_ph111_H1
zmp_ph111_B1a
zmp_ph111_B5
zmp_ph111_B8
zmp_ph111_B6
zmp_ph111_B7
zmp_ph111_B9
zmp_ph111_B10
zmp_ph114_G4
zmp_ph114_G11
zmp_ph114_H1
zmp_ph114_H2
zmp_ph114_A9
zmp_ph114_A11
zmp_ph114_B2
zmp_ph114_B4
zmp_ph114_D8
zmp_ph114_E2

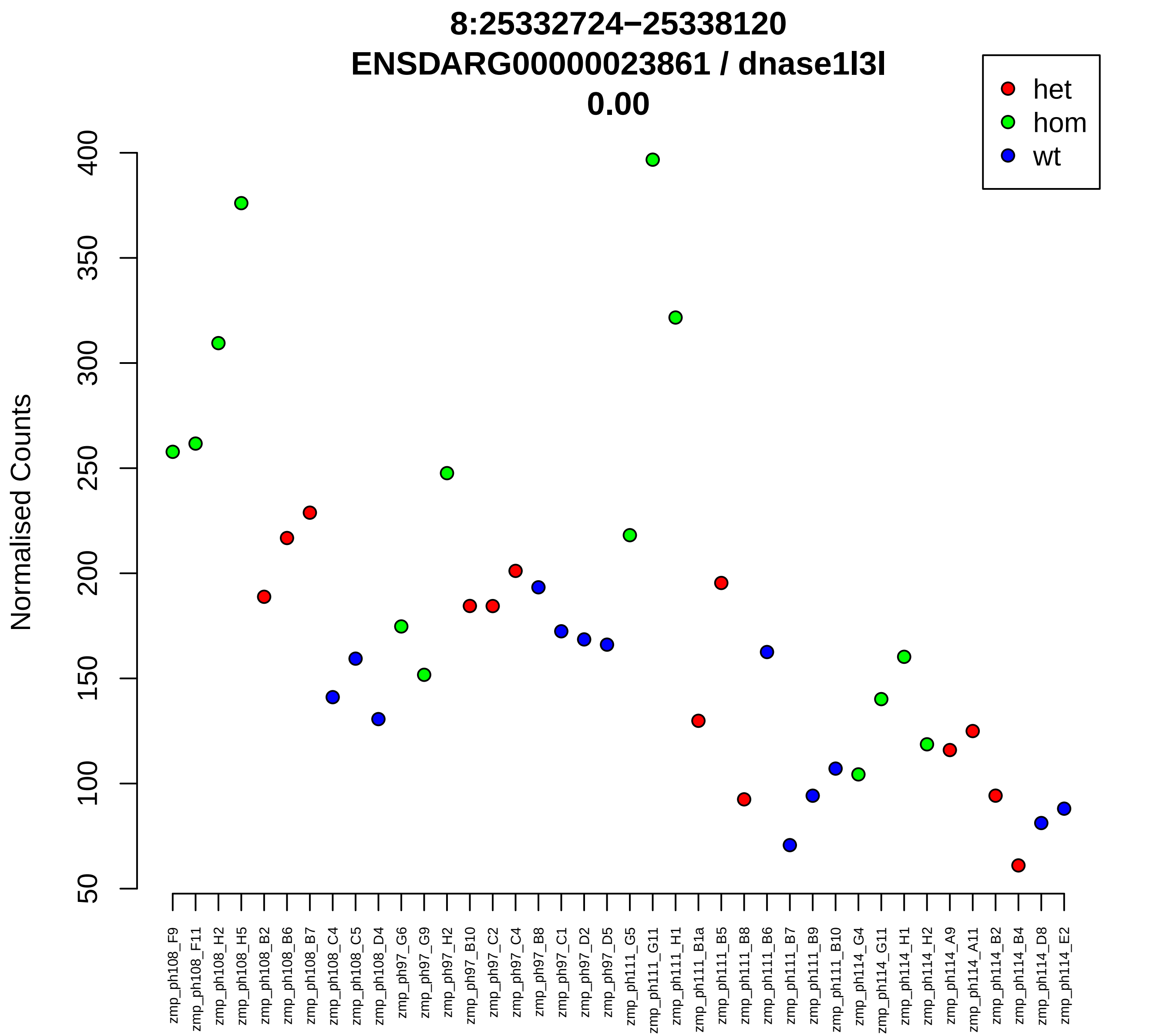

8:25332724−25338120
ENSDARG00000023861 / dnase1l3l
0.00
het
hom
wt
Normalised Counts
50
100
150
200
250
300
350
400
zmp_ph108_F9
zmp_ph108_F11
zmp_ph108_H2
zmp_ph108_H5
zmp_ph108_B2
zmp_ph108_B6
zmp_ph108_B7
zmp_ph108_C4
zmp_ph108_C5
zmp_ph108_D4
zmp_ph97_G6
zmp_ph97_G9
zmp_ph97_H2
zmp_ph97_B10
zmp_ph97_C2
zmp_ph97_C4
zmp_ph97_B8
zmp_ph97_C1
zmp_ph97_D2
zmp_ph97_D5
zmp_ph111_G5
zmp_ph111_G11
zmp_ph111_H1
zmp_ph111_B1a
zmp_ph111_B5
zmp_ph111_B8
zmp_ph111_B6
zmp_ph111_B7
zmp_ph111_B9
zmp_ph111_B10
zmp_ph114_G4
zmp_ph114_G11
zmp_ph114_H1
zmp_ph114_H2
zmp_ph114_A9
zmp_ph114_A11
zmp_ph114_B2
zmp_ph114_B4
zmp_ph114_D8
zmp_ph114_E2

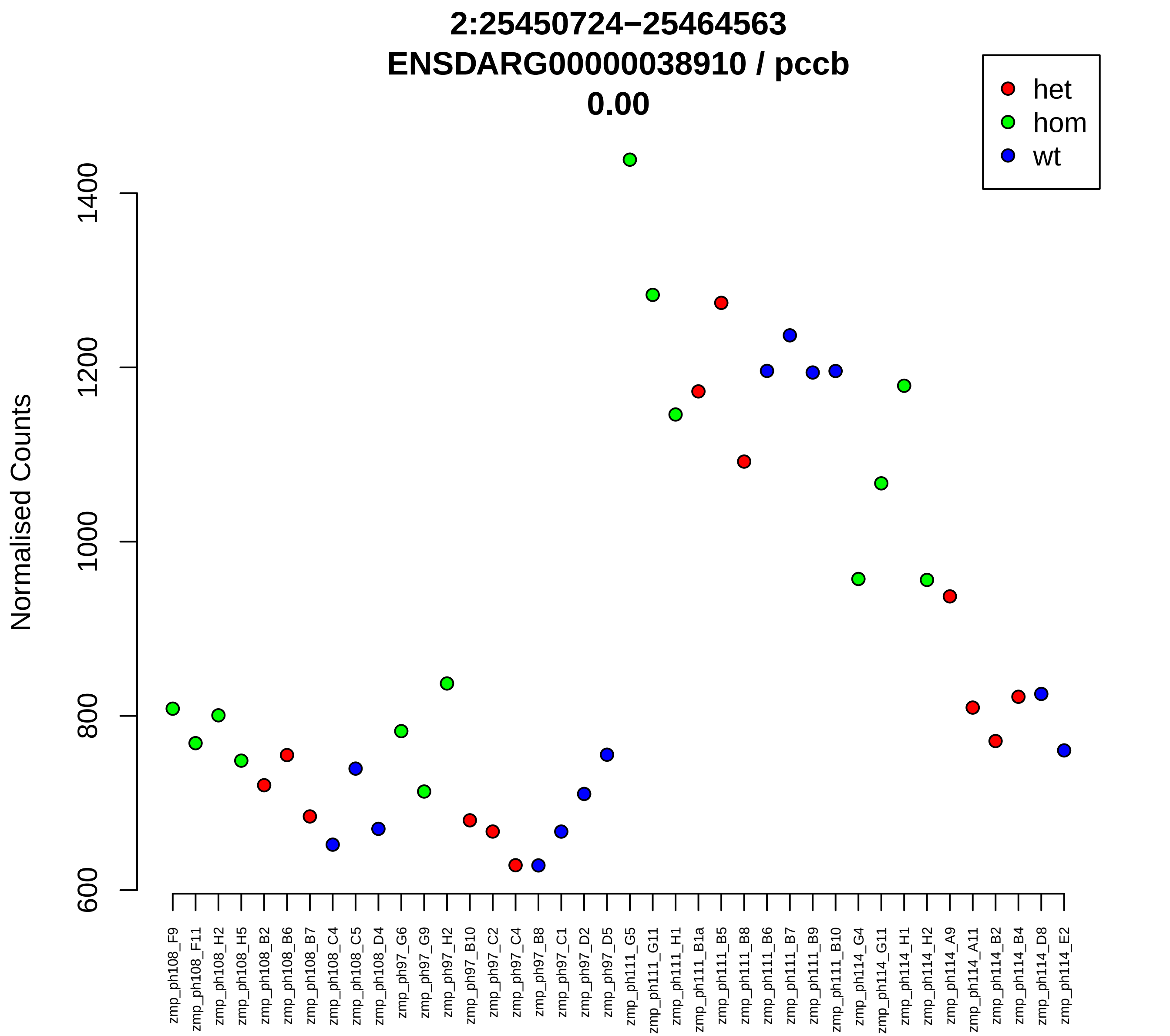
2:25450724−25464563
ENSDARG00000038910 / pccb
0.00
het
hom
wt
Normalised Counts
600
800
1000
1200
1400
zmp_ph108_F9
zmp_ph108_F11
zmp_ph108_H2
zmp_ph108_H5
zmp_ph108_B2
zmp_ph108_B6
zmp_ph108_B7
zmp_ph108_C4
zmp_ph108_C5
zmp_ph108_D4
zmp_ph97_G6
zmp_ph97_G9
zmp_ph97_H2
zmp_ph97_B10
zmp_ph97_C2
zmp_ph97_C4
zmp_ph97_B8
zmp_ph97_C1
zmp_ph97_D2
zmp_ph97_D5
zmp_ph111_G5
zmp_ph111_G11
zmp_ph111_H1
zmp_ph111_B1a
zmp_ph111_B5
zmp_ph111_B8
zmp_ph111_B6
zmp_ph111_B7
zmp_ph111_B9
zmp_ph111_B10
zmp_ph114_G4
zmp_ph114_G11
zmp_ph114_H1
zmp_ph114_H2
zmp_ph114_A9
zmp_ph114_A11
zmp_ph114_B2
zmp_ph114_B4
zmp_ph114_D8
zmp_ph114_E2

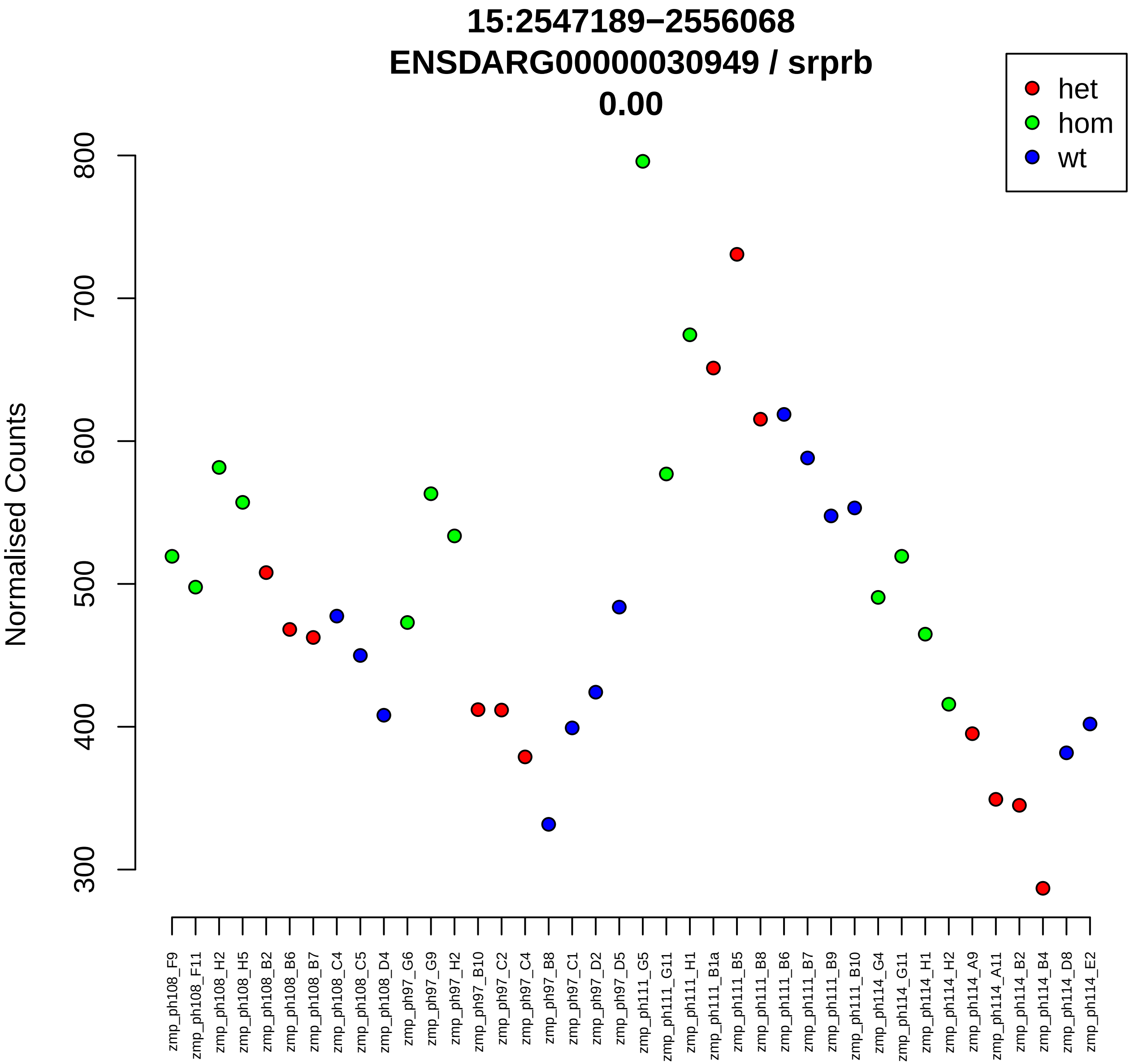

15:2547189−2556068
ENSDARG00000030949 / srprb
0.00
het
hom
wt
Normalised Counts
300
400
500
600
700
800
zmp_ph108_F9
zmp_ph108_F11
zmp_ph108_H2
zmp_ph108_H5
zmp_ph108_B2
zmp_ph108_B6
zmp_ph108_B7
zmp_ph108_C4
zmp_ph108_C5
zmp_ph108_D4
zmp_ph97_G6
zmp_ph97_G9
zmp_ph97_H2
zmp_ph97_B10
zmp_ph97_C2
zmp_ph97_C4
zmp_ph97_B8
zmp_ph97_C1
zmp_ph97_D2
zmp_ph97_D5
zmp_ph111_G5
zmp_ph111_G11
zmp_ph111_H1
zmp_ph111_B1a
zmp_ph111_B5
zmp_ph111_B8
zmp_ph111_B6
zmp_ph111_B7
zmp_ph111_B9
zmp_ph111_B10
zmp_ph114_G4
zmp_ph114_G11
zmp_ph114_H1
zmp_ph114_H2
zmp_ph114_A9
zmp_ph114_A11
zmp_ph114_B2
zmp_ph114_B4
zmp_ph114_D8
zmp_ph114_E2

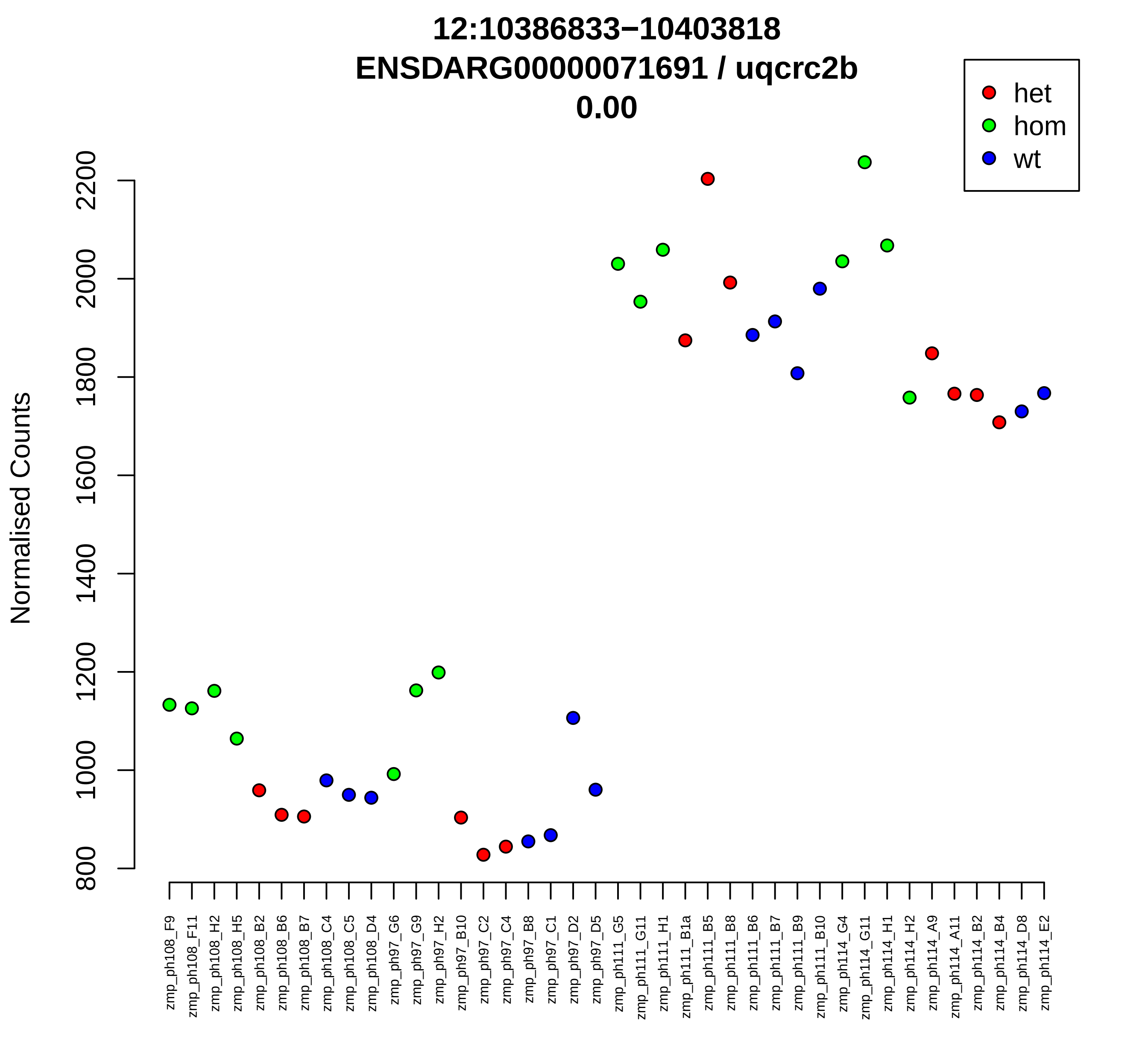
12:10386833−10403818
ENSDARG00000071691 / uqcrc2b
0.00
het
hom
wt
Normalised Counts
800
1000
1200
1400
1600
1800
2000
2200
zmp_ph108_F9
zmp_ph108_F11
zmp_ph108_H2
zmp_ph108_H5
zmp_ph108_B2
zmp_ph108_B6
zmp_ph108_B7
zmp_ph108_C4
zmp_ph108_C5
zmp_ph108_D4
zmp_ph97_G6
zmp_ph97_G9
zmp_ph97_H2
zmp_ph97_B10
zmp_ph97_C2
zmp_ph97_C4
zmp_ph97_B8
zmp_ph97_C1
zmp_ph97_D2
zmp_ph97_D5
zmp_ph111_G5
zmp_ph111_G11
zmp_ph111_H1
zmp_ph111_B1a
zmp_ph111_B5
zmp_ph111_B8
zmp_ph111_B6
zmp_ph111_B7
zmp_ph111_B9
zmp_ph111_B10
zmp_ph114_G4
zmp_ph114_G11
zmp_ph114_H1
zmp_ph114_H2
zmp_ph114_A9
zmp_ph114_A11
zmp_ph114_B2
zmp_ph114_B4
zmp_ph114_D8
zmp_ph114_E2

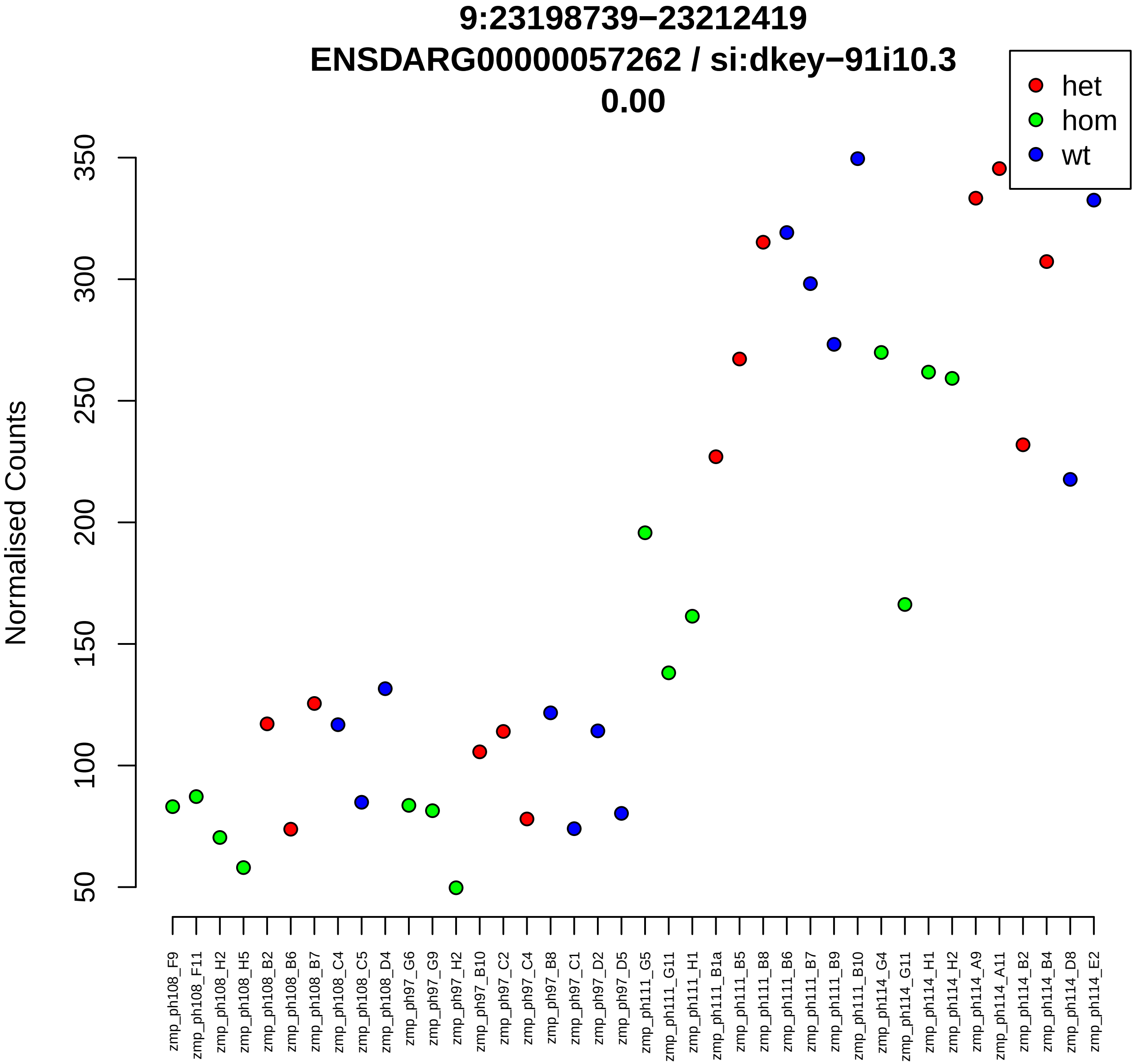
9:23198739−23212419
ENSDARG00000057262 / si:dkey−91i10.3
0.00
het
hom
wt
Normalised Counts
50
100
150
200
250
300
350
zmp_ph108_F9
zmp_ph108_F11
zmp_ph108_H2
zmp_ph108_H5
zmp_ph108_B2
zmp_ph108_B6
zmp_ph108_B7
zmp_ph108_C4
zmp_ph108_C5
zmp_ph108_D4
zmp_ph97_G6
zmp_ph97_G9
zmp_ph97_H2
zmp_ph97_B10
zmp_ph97_C2
zmp_ph97_C4
zmp_ph97_B8
zmp_ph97_C1
zmp_ph97_D2
zmp_ph97_D5
zmp_ph111_G5
zmp_ph111_G11
zmp_ph111_H1
zmp_ph111_B1a
zmp_ph111_B5
zmp_ph111_B8
zmp_ph111_B6
zmp_ph111_B7
zmp_ph111_B9
zmp_ph111_B10
zmp_ph114_G4
zmp_ph114_G11
zmp_ph114_H1
zmp_ph114_H2
zmp_ph114_A9
zmp_ph114_A11
zmp_ph114_B2
zmp_ph114_B4
zmp_ph114_D8
zmp_ph114_E2

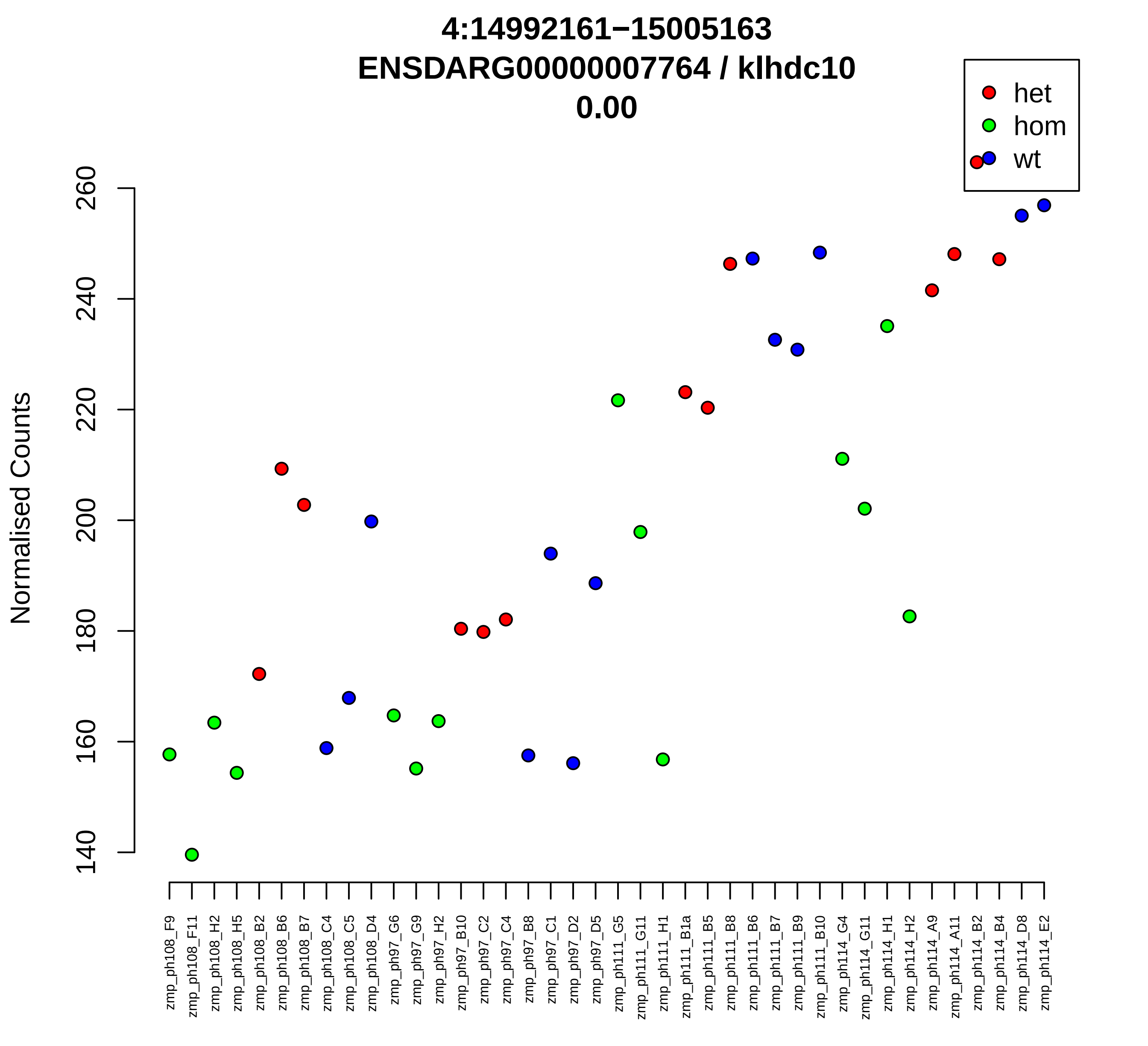
4:14992161−15005163
ENSDARG00000007764 / klhdc10
0.00
het
hom
wt
Normalised Counts
140
160
180
200
220
240
260
zmp_ph108_F9
zmp_ph108_F11
zmp_ph108_H2
zmp_ph108_H5
zmp_ph108_B2
zmp_ph108_B6
zmp_ph108_B7
zmp_ph108_C4
zmp_ph108_C5
zmp_ph108_D4
zmp_ph97_G6
zmp_ph97_G9
zmp_ph97_H2
zmp_ph97_B10
zmp_ph97_C2
zmp_ph97_C4
zmp_ph97_B8
zmp_ph97_C1
zmp_ph97_D2
zmp_ph97_D5
zmp_ph111_G5
zmp_ph111_G11
zmp_ph111_H1
zmp_ph111_B1a
zmp_ph111_B5
zmp_ph111_B8
zmp_ph111_B6
zmp_ph111_B7
zmp_ph111_B9
zmp_ph111_B10
zmp_ph114_G4
zmp_ph114_G11
zmp_ph114_H1
zmp_ph114_H2
zmp_ph114_A9
zmp_ph114_A11
zmp_ph114_B2
zmp_ph114_B4
zmp_ph114_D8
zmp_ph114_E2

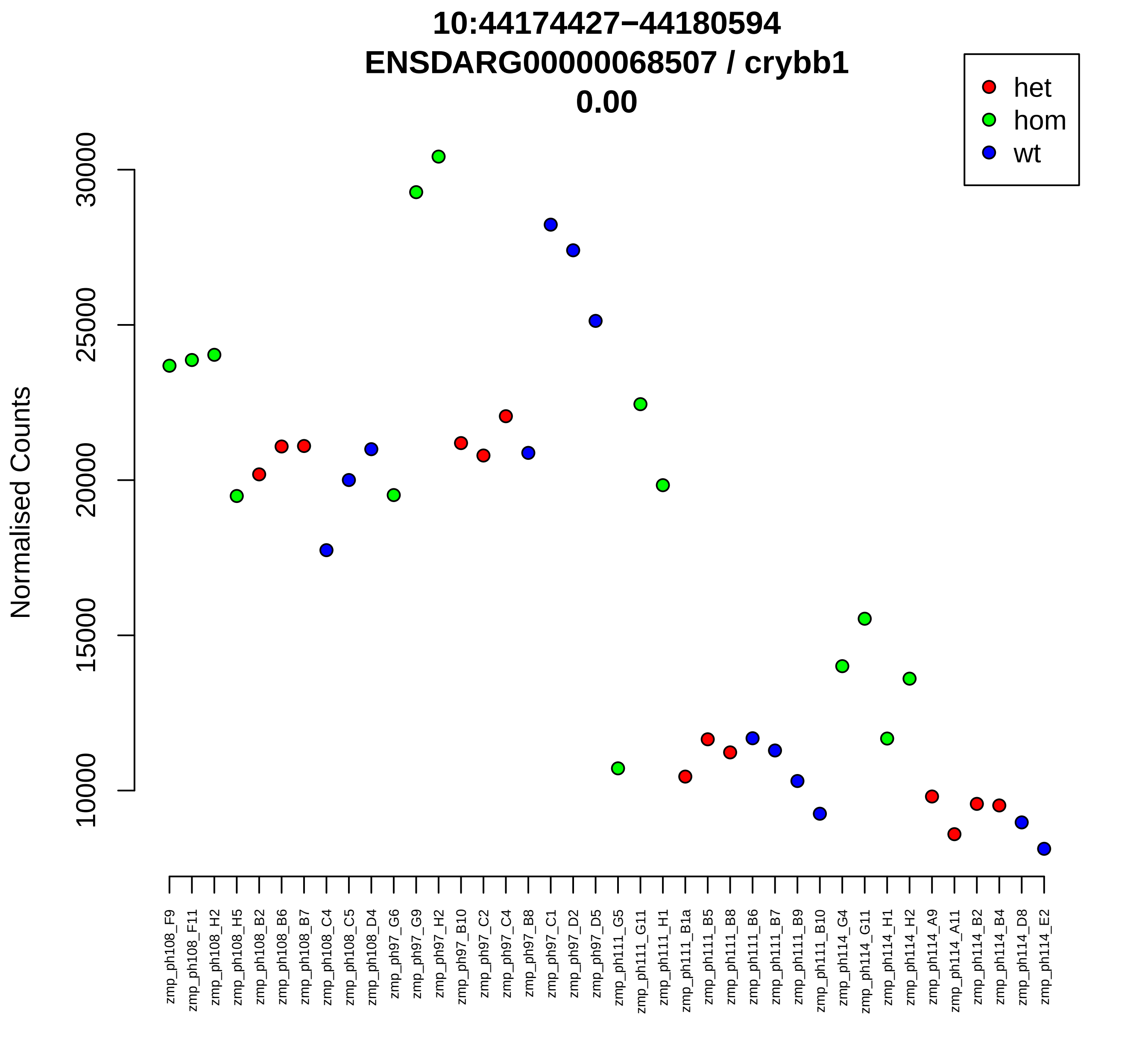
10:44174427−44180594
ENSDARG00000068507 / crybb1
0.00
het
hom
wt
Normalised Counts
10000
15000
20000
25000
30000
zmp_ph108_F9
zmp_ph108_F11
zmp_ph108_H2
zmp_ph108_H5
zmp_ph108_B2
zmp_ph108_B6
zmp_ph108_B7
zmp_ph108_C4
zmp_ph108_C5
zmp_ph108_D4
zmp_ph97_G6
zmp_ph97_G9
zmp_ph97_H2
zmp_ph97_B10
zmp_ph97_C2
zmp_ph97_C4
zmp_ph97_B8
zmp_ph97_C1
zmp_ph97_D2
zmp_ph97_D5
zmp_ph111_G5
zmp_ph111_G11
zmp_ph111_H1
zmp_ph111_B1a
zmp_ph111_B5
zmp_ph111_B8
zmp_ph111_B6
zmp_ph111_B7
zmp_ph111_B9
zmp_ph111_B10
zmp_ph114_G4
zmp_ph114_G11
zmp_ph114_H1
zmp_ph114_H2
zmp_ph114_A9
zmp_ph114_A11
zmp_ph114_B2
zmp_ph114_B4
zmp_ph114_D8
zmp_ph114_E2

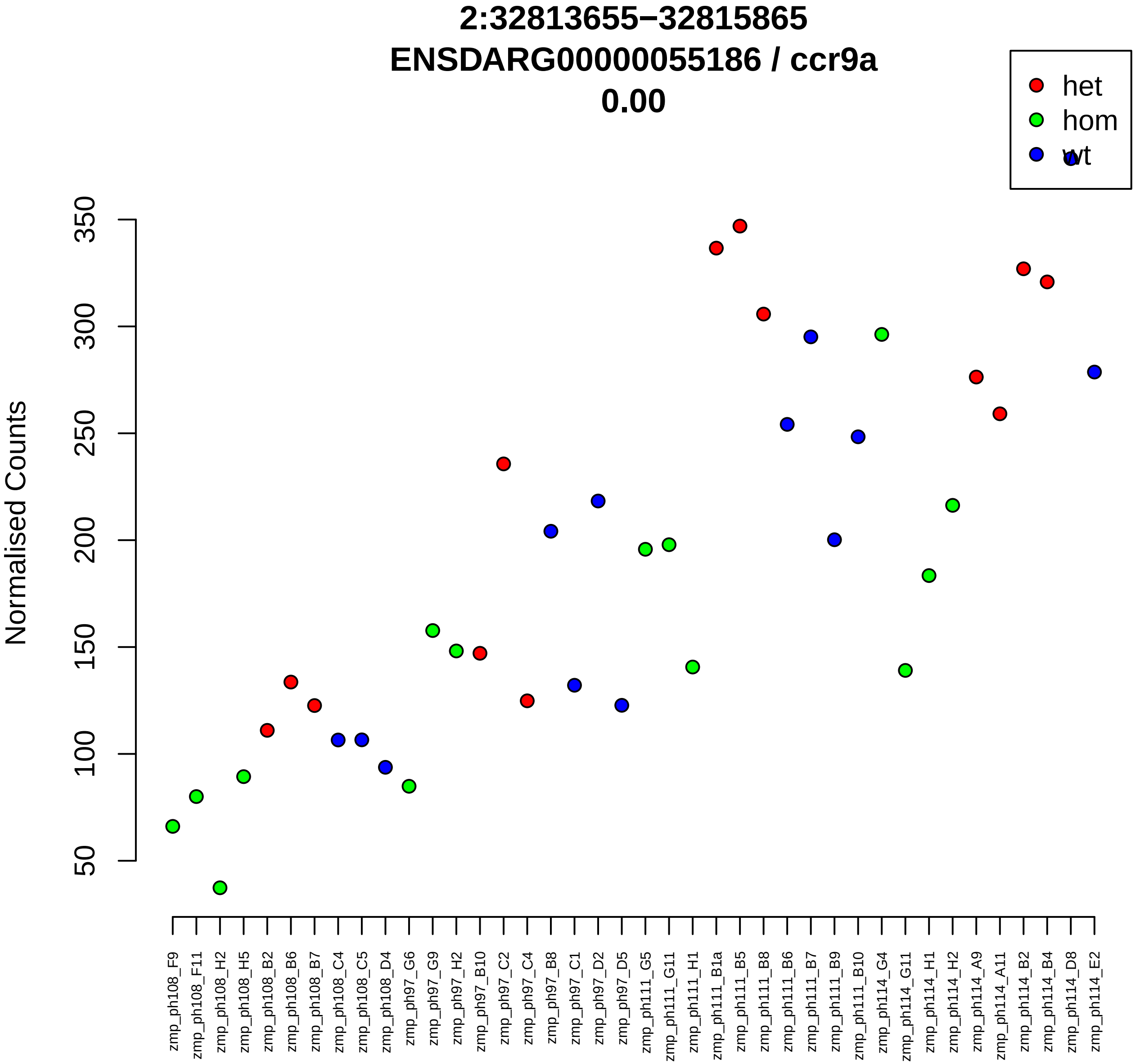
2:32813655−32815865
ENSDARG00000055186 / ccr9a
0.00
het
hom
wt
Normalised Counts
50
100
150
200
250
300
350
zmp_ph108_F9
zmp_ph108_F11
zmp_ph108_H2
zmp_ph108_H5
zmp_ph108_B2
zmp_ph108_B6
zmp_ph108_B7
zmp_ph108_C4
zmp_ph108_C5
zmp_ph108_D4
zmp_ph97_G6
zmp_ph97_G9
zmp_ph97_H2
zmp_ph97_B10
zmp_ph97_C2
zmp_ph97_C4
zmp_ph97_B8
zmp_ph97_C1
zmp_ph97_D2
zmp_ph97_D5
zmp_ph111_G5
zmp_ph111_G11
zmp_ph111_H1
zmp_ph111_B1a
zmp_ph111_B5
zmp_ph111_B8
zmp_ph111_B6
zmp_ph111_B7
zmp_ph111_B9
zmp_ph111_B10
zmp_ph114_G4
zmp_ph114_G11
zmp_ph114_H1
zmp_ph114_H2
zmp_ph114_A9
zmp_ph114_A11
zmp_ph114_B2
zmp_ph114_B4
zmp_ph114_D8
zmp_ph114_E2

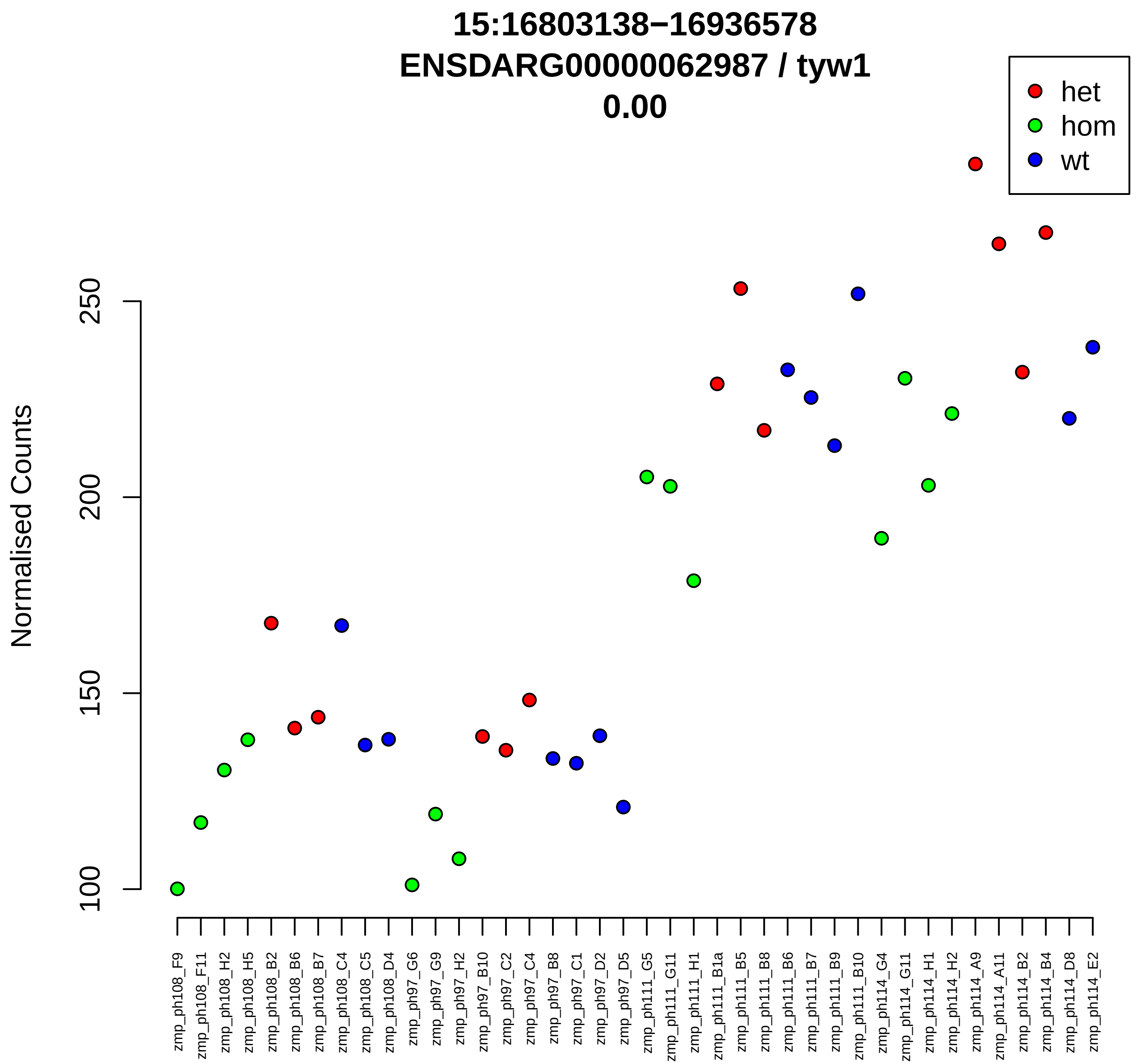

15:16803138−16936578
ENSDARG00000062987 / tyw1
0.00
het
hom
wt
Normalised Counts
100
150
200
250
zmp_ph108_F9
zmp_ph108_F11
zmp_ph108_H2
zmp_ph108_H5
zmp_ph108_B2
zmp_ph108_B6
zmp_ph108_B7
zmp_ph108_C4
zmp_ph108_C5
zmp_ph108_D4
zmp_ph97_G6
zmp_ph97_G9
zmp_ph97_H2
zmp_ph97_B10
zmp_ph97_C2
zmp_ph97_C4
zmp_ph97_B8
zmp_ph97_C1
zmp_ph97_D2
zmp_ph97_D5
zmp_ph111_G5
zmp_ph111_G11
zmp_ph111_H1
zmp_ph111_B1a
zmp_ph111_B5
zmp_ph111_B8
zmp_ph111_B6
zmp_ph111_B7
zmp_ph111_B9
zmp_ph111_B10
zmp_ph114_G4
zmp_ph114_G11
zmp_ph114_H1
zmp_ph114_H2
zmp_ph114_A9
zmp_ph114_A11
zmp_ph114_B2
zmp_ph114_B4
zmp_ph114_D8
zmp_ph114_E2

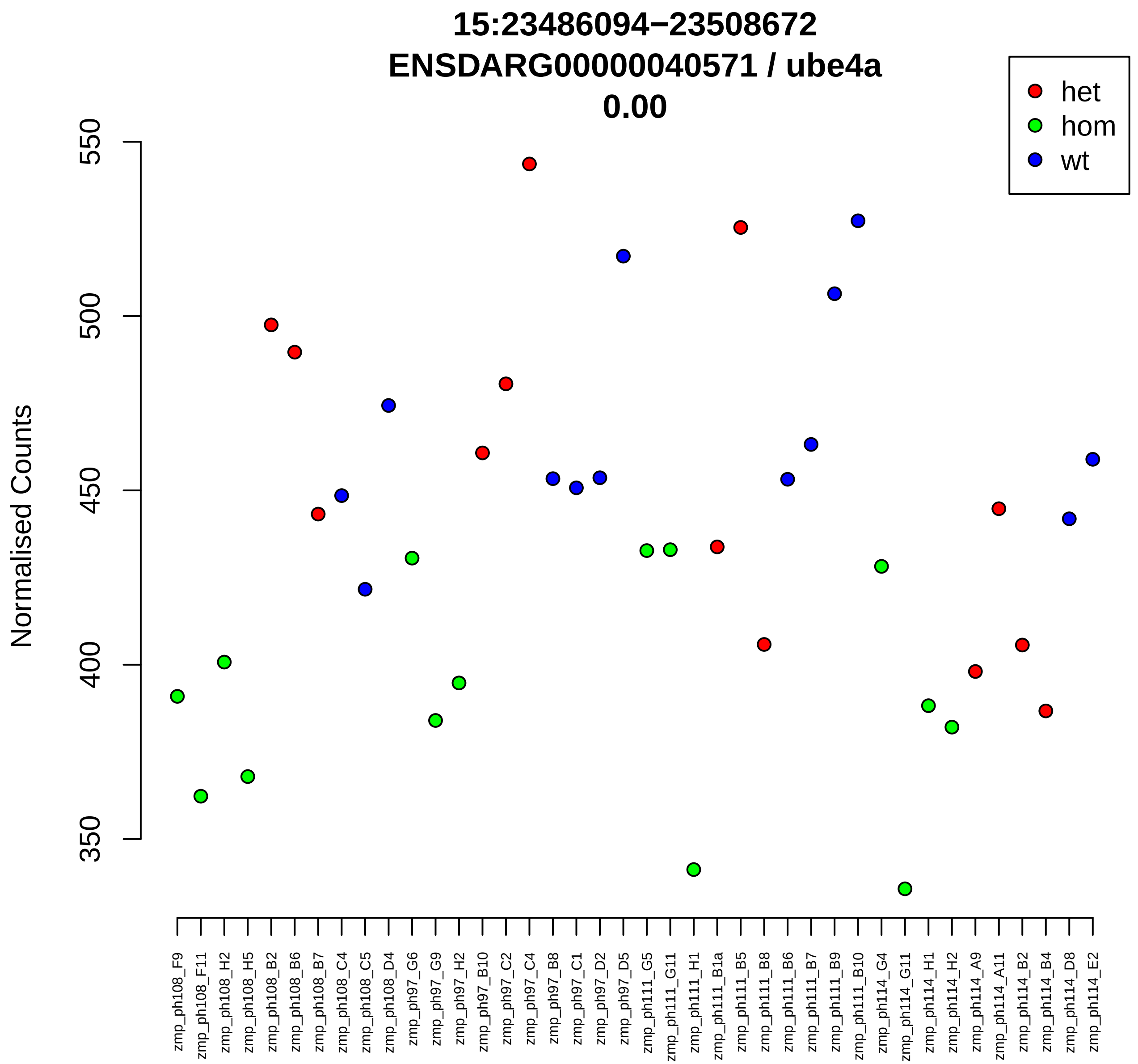

15:23486094−23508672
ENSDARG00000040571 / ube4a
0.00
het
hom
wt
Normalised Counts
350
400
450
500
550
zmp_ph108_F9
zmp_ph108_F11
zmp_ph108_H2
zmp_ph108_H5
zmp_ph108_B2
zmp_ph108_B6
zmp_ph108_B7
zmp_ph108_C4
zmp_ph108_C5
zmp_ph108_D4
zmp_ph97_G6
zmp_ph97_G9
zmp_ph97_H2
zmp_ph97_B10
zmp_ph97_C2
zmp_ph97_C4
zmp_ph97_B8
zmp_ph97_C1
zmp_ph97_D2
zmp_ph97_D5
zmp_ph111_G5
zmp_ph111_G11
zmp_ph111_H1
zmp_ph111_B1a
zmp_ph111_B5
zmp_ph111_B8
zmp_ph111_B6
zmp_ph111_B7
zmp_ph111_B9
zmp_ph111_B10
zmp_ph114_G4
zmp_ph114_G11
zmp_ph114_H1
zmp_ph114_H2
zmp_ph114_A9
zmp_ph114_A11
zmp_ph114_B2
zmp_ph114_B4
zmp_ph114_D8
zmp_ph114_E2

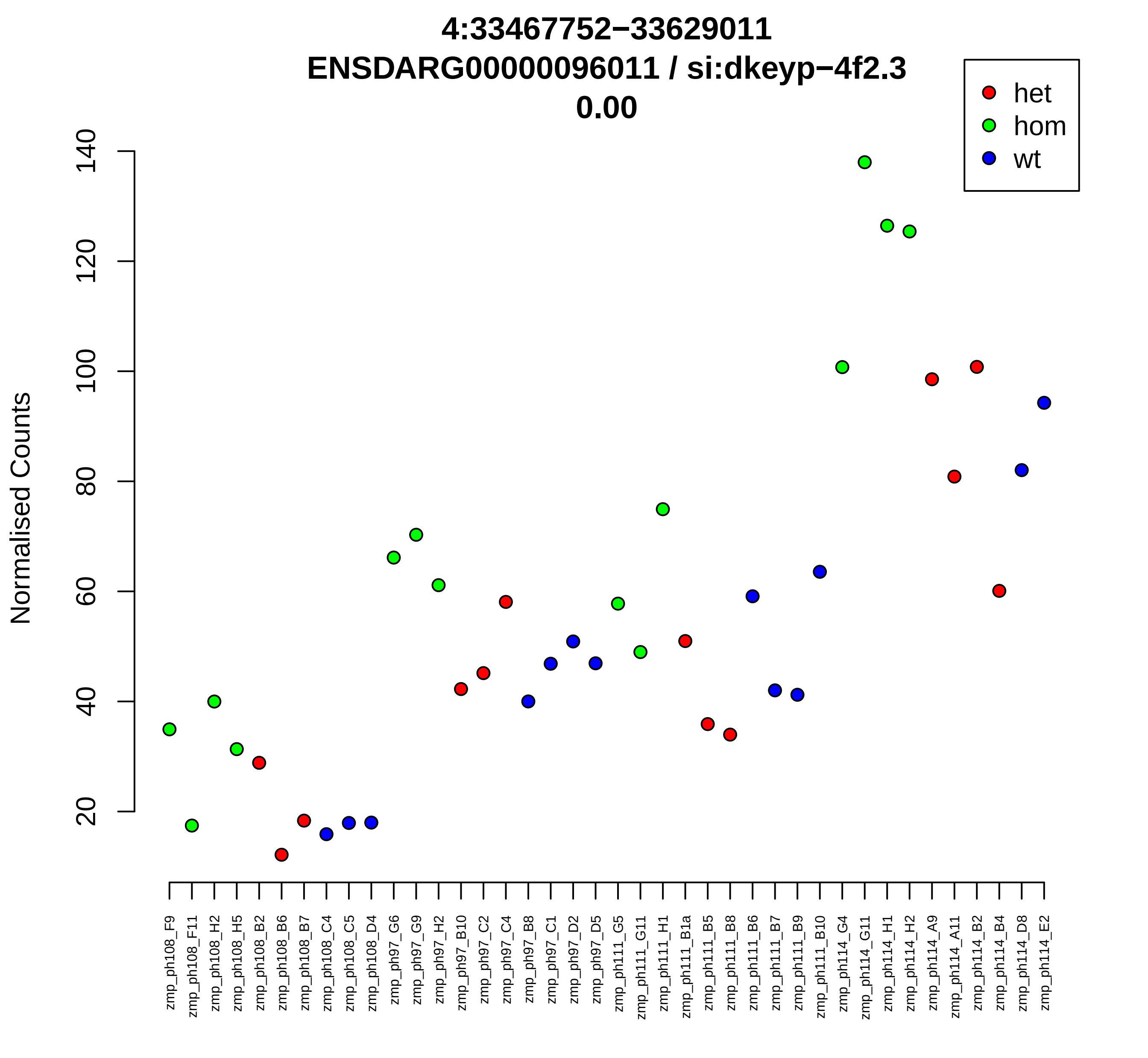

4:33467752−33629011
ENSDARG00000096011 / si:dkeyp−4f2.3
0.00
het
hom
wt
Normalised Counts
20
40
60
80
100
120
140
zmp_ph108_F9
zmp_ph108_F11
zmp_ph108_H2
zmp_ph108_H5
zmp_ph108_B2
zmp_ph108_B6
zmp_ph108_B7
zmp_ph108_C4
zmp_ph108_C5
zmp_ph108_D4
zmp_ph97_G6
zmp_ph97_G9
zmp_ph97_H2
zmp_ph97_B10
zmp_ph97_C2
zmp_ph97_C4
zmp_ph97_B8
zmp_ph97_C1
zmp_ph97_D2
zmp_ph97_D5
zmp_ph111_G5
zmp_ph111_G11
zmp_ph111_H1
zmp_ph111_B1a
zmp_ph111_B5
zmp_ph111_B8
zmp_ph111_B6
zmp_ph111_B7
zmp_ph111_B9
zmp_ph111_B10
zmp_ph114_G4
zmp_ph114_G11
zmp_ph114_H1
zmp_ph114_H2
zmp_ph114_A9
zmp_ph114_A11
zmp_ph114_B2
zmp_ph114_B4
zmp_ph114_D8
zmp_ph114_E2

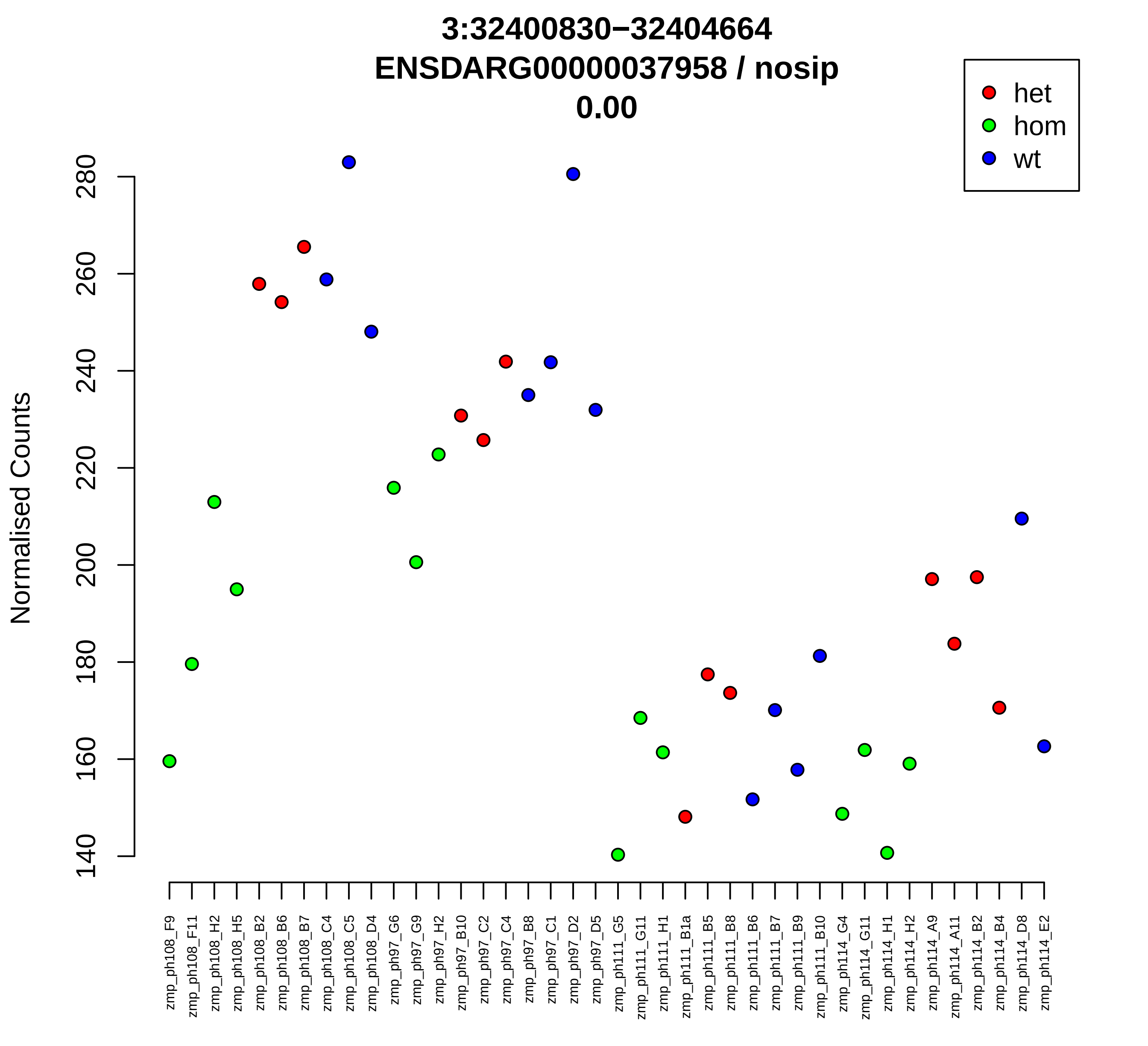
3:32400830−32404664
ENSDARG00000037958 / nosip
0.00
het
hom
wt
Normalised Counts
140
160
180
200
220
240
260
280
zmp_ph108_F9
zmp_ph108_F11
zmp_ph108_H2
zmp_ph108_H5
zmp_ph108_B2
zmp_ph108_B6
zmp_ph108_B7
zmp_ph108_C4
zmp_ph108_C5
zmp_ph108_D4
zmp_ph97_G6
zmp_ph97_G9
zmp_ph97_H2
zmp_ph97_B10
zmp_ph97_C2
zmp_ph97_C4
zmp_ph97_B8
zmp_ph97_C1
zmp_ph97_D2
zmp_ph97_D5
zmp_ph111_G5
zmp_ph111_G11
zmp_ph111_H1
zmp_ph111_B1a
zmp_ph111_B5
zmp_ph111_B8
zmp_ph111_B6
zmp_ph111_B7
zmp_ph111_B9
zmp_ph111_B10
zmp_ph114_G4
zmp_ph114_G11
zmp_ph114_H1
zmp_ph114_H2
zmp_ph114_A9
zmp_ph114_A11
zmp_ph114_B2
zmp_ph114_B4
zmp_ph114_D8
zmp_ph114_E2

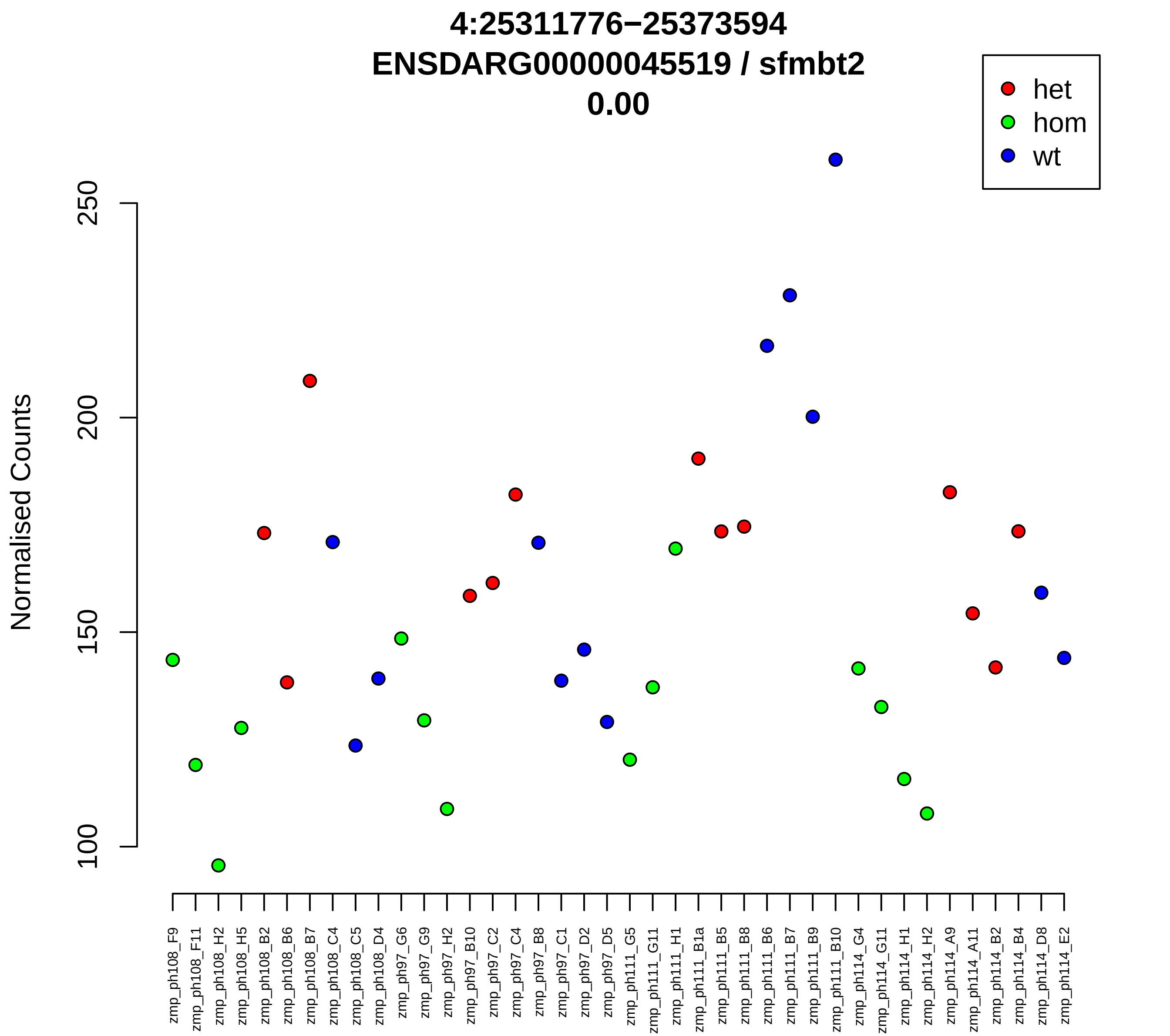
4:25311776−25373594
ENSDARG00000045519 / sfmbt2
0.00
het
hom
wt
Normalised Counts
100
150
200
250
zmp_ph108_F9
zmp_ph108_F11
zmp_ph108_H2
zmp_ph108_H5
zmp_ph108_B2
zmp_ph108_B6
zmp_ph108_B7
zmp_ph108_C4
zmp_ph108_C5
zmp_ph108_D4
zmp_ph97_G6
zmp_ph97_G9
zmp_ph97_H2
zmp_ph97_B10
zmp_ph97_C2
zmp_ph97_C4
zmp_ph97_B8
zmp_ph97_C1
zmp_ph97_D2
zmp_ph97_D5
zmp_ph111_G5
zmp_ph111_G11
zmp_ph111_H1
zmp_ph111_B1a
zmp_ph111_B5
zmp_ph111_B8
zmp_ph111_B6
zmp_ph111_B7
zmp_ph111_B9
zmp_ph111_B10
zmp_ph114_G4
zmp_ph114_G11
zmp_ph114_H1
zmp_ph114_H2
zmp_ph114_A9
zmp_ph114_A11
zmp_ph114_B2
zmp_ph114_B4
zmp_ph114_D8
zmp_ph114_E2

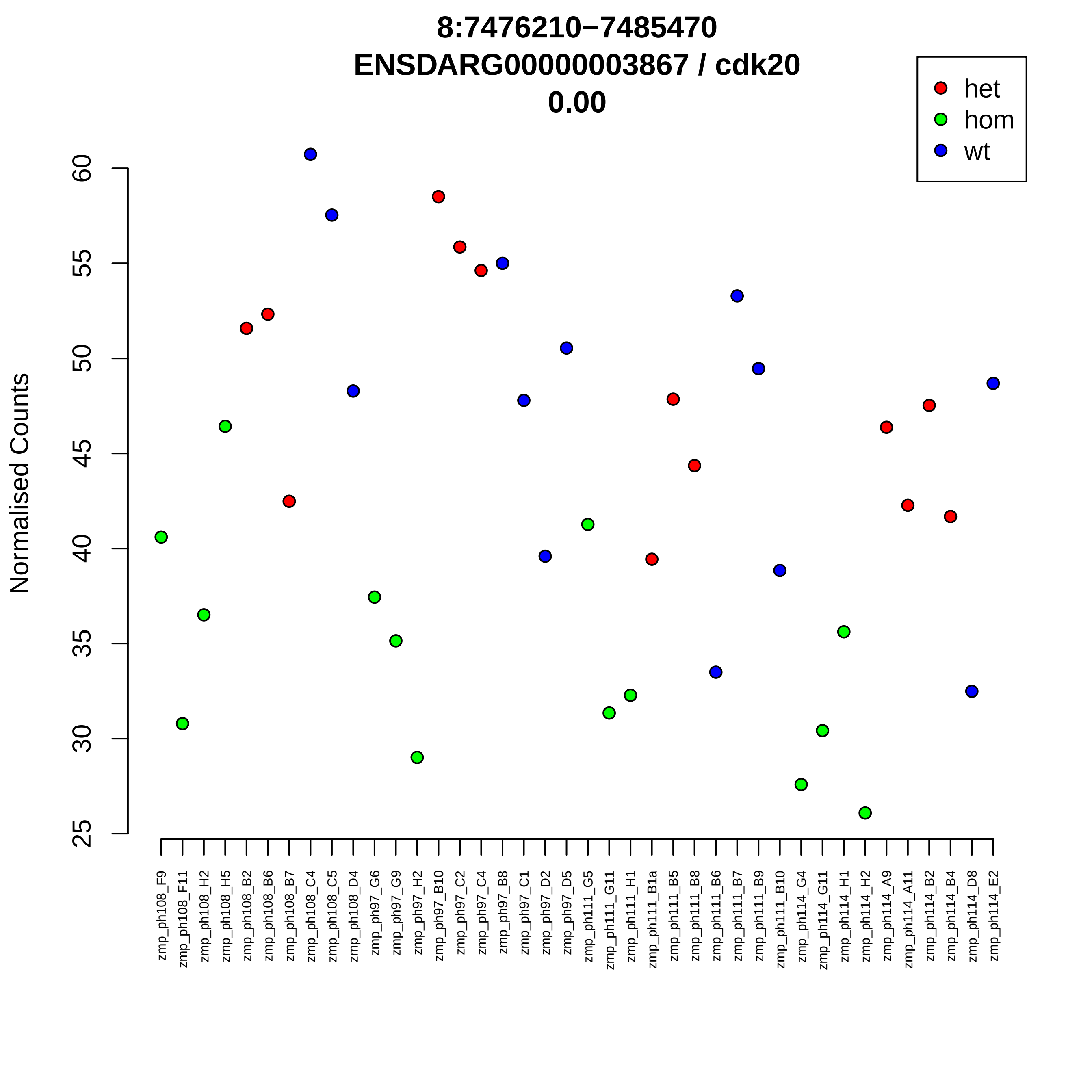
8:7476210−7485470
ENSDARG00000003867 / cdk20
0.00
het
hom
wt
Normalised Counts
25
30
35
40
45
50
55
60
zmp_ph108_F9
zmp_ph108_F11
zmp_ph108_H2
zmp_ph108_H5
zmp_ph108_B2
zmp_ph108_B6
zmp_ph108_B7
zmp_ph108_C4
zmp_ph108_C5
zmp_ph108_D4
zmp_ph97_G6
zmp_ph97_G9
zmp_ph97_H2
zmp_ph97_B10
zmp_ph97_C2
zmp_ph97_C4
zmp_ph97_B8
zmp_ph97_C1
zmp_ph97_D2
zmp_ph97_D5
zmp_ph111_G5
zmp_ph111_G11
zmp_ph111_H1
zmp_ph111_B1a
zmp_ph111_B5
zmp_ph111_B8
zmp_ph111_B6
zmp_ph111_B7
zmp_ph111_B9
zmp_ph111_B10
zmp_ph114_G4
zmp_ph114_G11
zmp_ph114_H1
zmp_ph114_H2
zmp_ph114_A9
zmp_ph114_A11
zmp_ph114_B2
zmp_ph114_B4
zmp_ph114_D8
zmp_ph114_E2

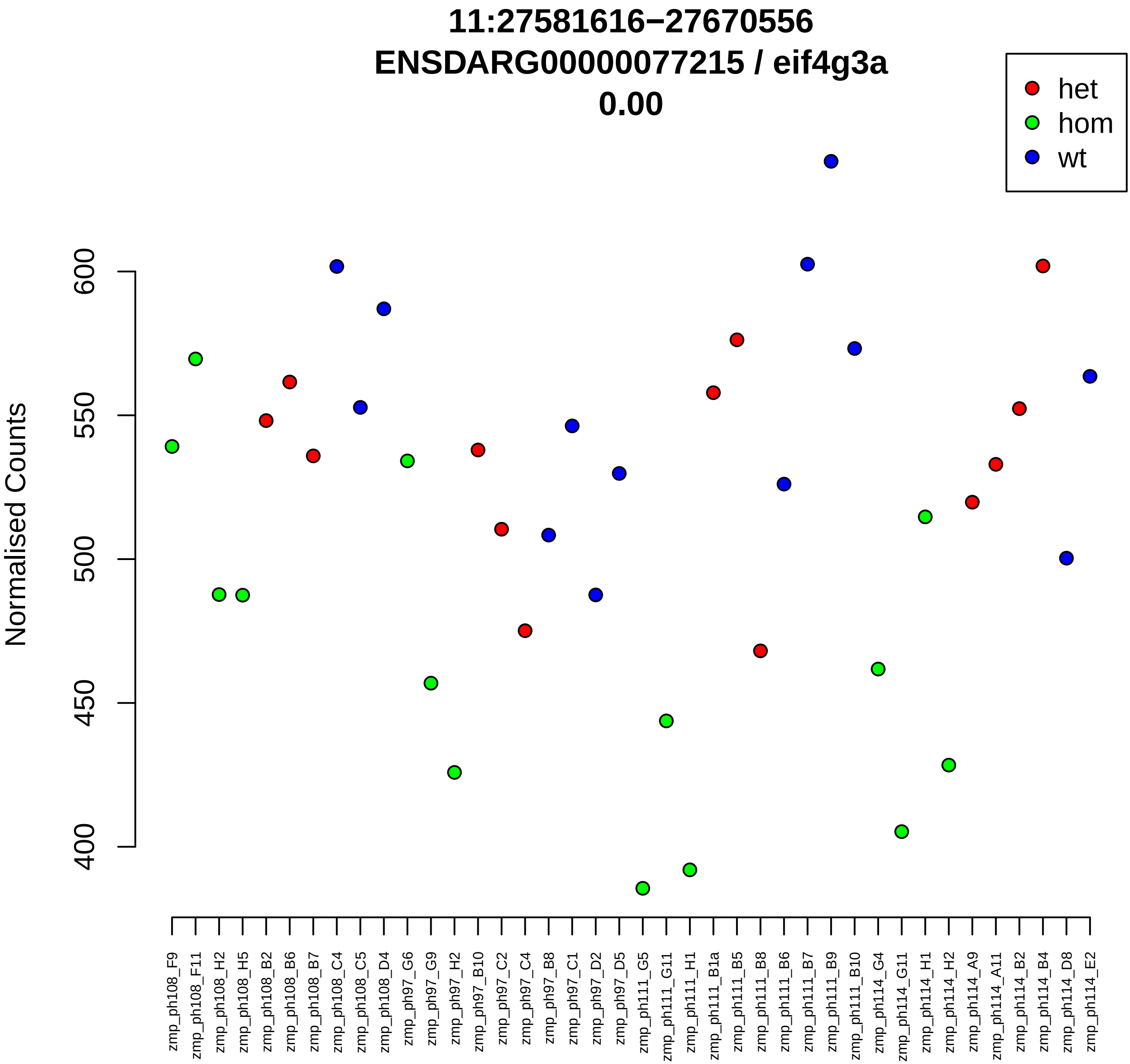

11:27581616−27670556
ENSDARG00000077215 / eif4g3a
0.00
het
hom
wt
Normalised Counts
400
450
500
550
600
zmp_ph108_F9
zmp_ph108_F11
zmp_ph108_H2
zmp_ph108_H5
zmp_ph108_B2
zmp_ph108_B6
zmp_ph108_B7
zmp_ph108_C4
zmp_ph108_C5
zmp_ph108_D4
zmp_ph97_G6
zmp_ph97_G9
zmp_ph97_H2
zmp_ph97_B10
zmp_ph97_C2
zmp_ph97_C4
zmp_ph97_B8
zmp_ph97_C1
zmp_ph97_D2
zmp_ph97_D5
zmp_ph111_G5
zmp_ph111_G11
zmp_ph111_H1
zmp_ph111_B1a
zmp_ph111_B5
zmp_ph111_B8
zmp_ph111_B6
zmp_ph111_B7
zmp_ph111_B9
zmp_ph111_B10
zmp_ph114_G4
zmp_ph114_G11
zmp_ph114_H1
zmp_ph114_H2
zmp_ph114_A9
zmp_ph114_A11
zmp_ph114_B2
zmp_ph114_B4
zmp_ph114_D8
zmp_ph114_E2

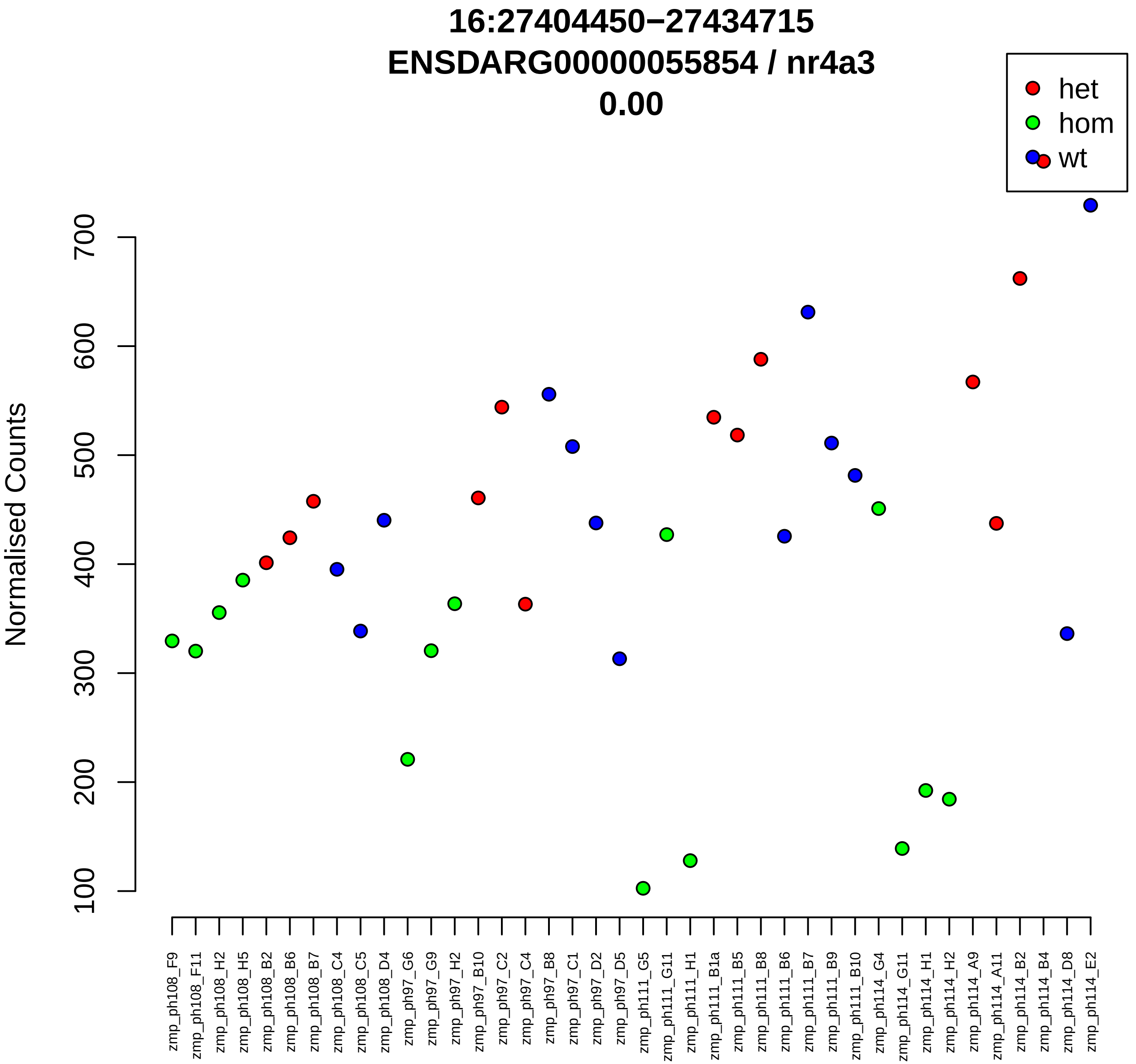
16:27404450−27434715
ENSDARG00000055854 / nr4a3
0.00
het
hom
wt
Normalised Counts
100
200
300
400
500
600
700
zmp_ph108_F9
zmp_ph108_F11
zmp_ph108_H2
zmp_ph108_H5
zmp_ph108_B2
zmp_ph108_B6
zmp_ph108_B7
zmp_ph108_C4
zmp_ph108_C5
zmp_ph108_D4
zmp_ph97_G6
zmp_ph97_G9
zmp_ph97_H2
zmp_ph97_B10
zmp_ph97_C2
zmp_ph97_C4
zmp_ph97_B8
zmp_ph97_C1
zmp_ph97_D2
zmp_ph97_D5
zmp_ph111_G5
zmp_ph111_G11
zmp_ph111_H1
zmp_ph111_B1a
zmp_ph111_B5
zmp_ph111_B8
zmp_ph111_B6
zmp_ph111_B7
zmp_ph111_B9
zmp_ph111_B10
zmp_ph114_G4
zmp_ph114_G11
zmp_ph114_H1
zmp_ph114_H2
zmp_ph114_A9
zmp_ph114_A11
zmp_ph114_B2
zmp_ph114_B4
zmp_ph114_D8
zmp_ph114_E2

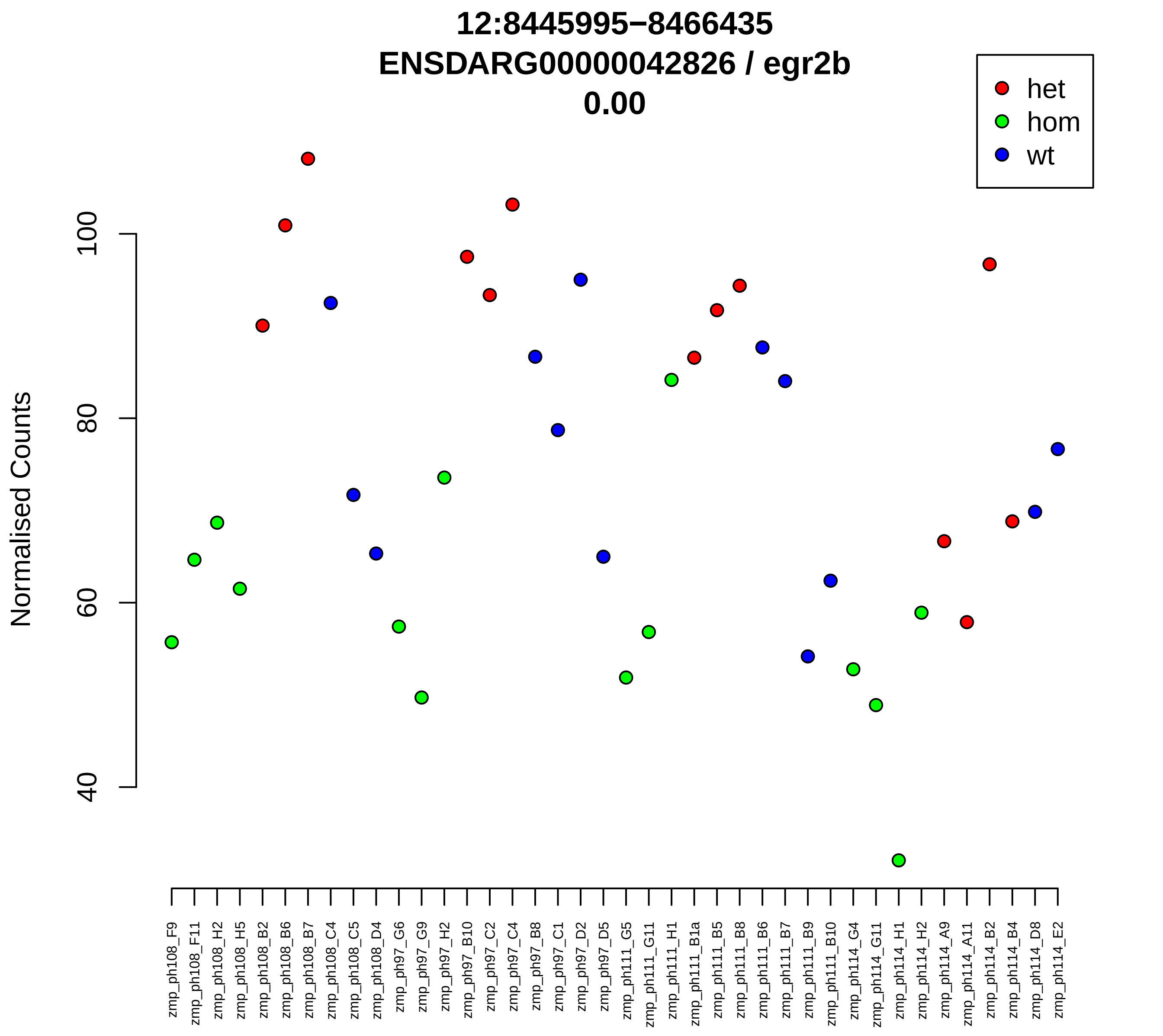
12:8445995−8466435
ENSDARG00000042826 / egr2b
0.00
het
hom
wt
Normalised Counts
40
60
80
100
zmp_ph108_F9
zmp_ph108_F11
zmp_ph108_H2
zmp_ph108_H5
zmp_ph108_B2
zmp_ph108_B6
zmp_ph108_B7
zmp_ph108_C4
zmp_ph108_C5
zmp_ph108_D4
zmp_ph97_G6
zmp_ph97_G9
zmp_ph97_H2
zmp_ph97_B10
zmp_ph97_C2
zmp_ph97_C4
zmp_ph97_B8
zmp_ph97_C1
zmp_ph97_D2
zmp_ph97_D5
zmp_ph111_G5
zmp_ph111_G11
zmp_ph111_H1
zmp_ph111_B1a
zmp_ph111_B5
zmp_ph111_B8
zmp_ph111_B6
zmp_ph111_B7
zmp_ph111_B9
zmp_ph111_B10
zmp_ph114_G4
zmp_ph114_G11
zmp_ph114_H1
zmp_ph114_H2
zmp_ph114_A9
zmp_ph114_A11
zmp_ph114_B2
zmp_ph114_B4
zmp_ph114_D8
zmp_ph114_E2

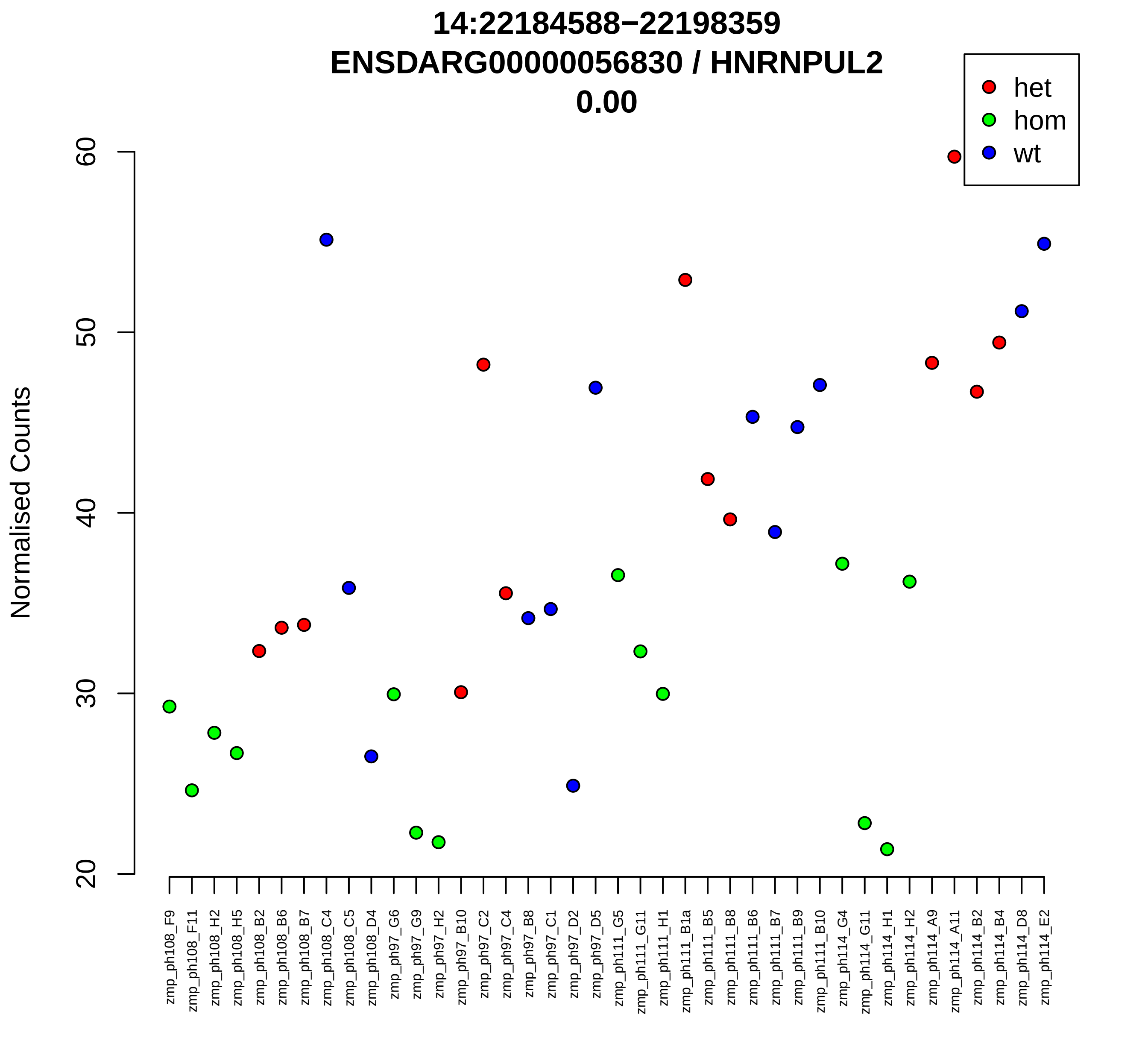
14:22184588−22198359
ENSDARG00000056830 / HNRNPUL2
0.00
het
hom
wt
Normalised Counts
20
30
40
50
60
zmp_ph108_F9
zmp_ph108_F11
zmp_ph108_H2
zmp_ph108_H5
zmp_ph108_B2
zmp_ph108_B6
zmp_ph108_B7
zmp_ph108_C4
zmp_ph108_C5
zmp_ph108_D4
zmp_ph97_G6
zmp_ph97_G9
zmp_ph97_H2
zmp_ph97_B10
zmp_ph97_C2
zmp_ph97_C4
zmp_ph97_B8
zmp_ph97_C1
zmp_ph97_D2
zmp_ph97_D5
zmp_ph111_G5
zmp_ph111_G11
zmp_ph111_H1
zmp_ph111_B1a
zmp_ph111_B5
zmp_ph111_B8
zmp_ph111_B6
zmp_ph111_B7
zmp_ph111_B9
zmp_ph111_B10
zmp_ph114_G4
zmp_ph114_G11
zmp_ph114_H1
zmp_ph114_H2
zmp_ph114_A9
zmp_ph114_A11
zmp_ph114_B2
zmp_ph114_B4
zmp_ph114_D8
zmp_ph114_E2

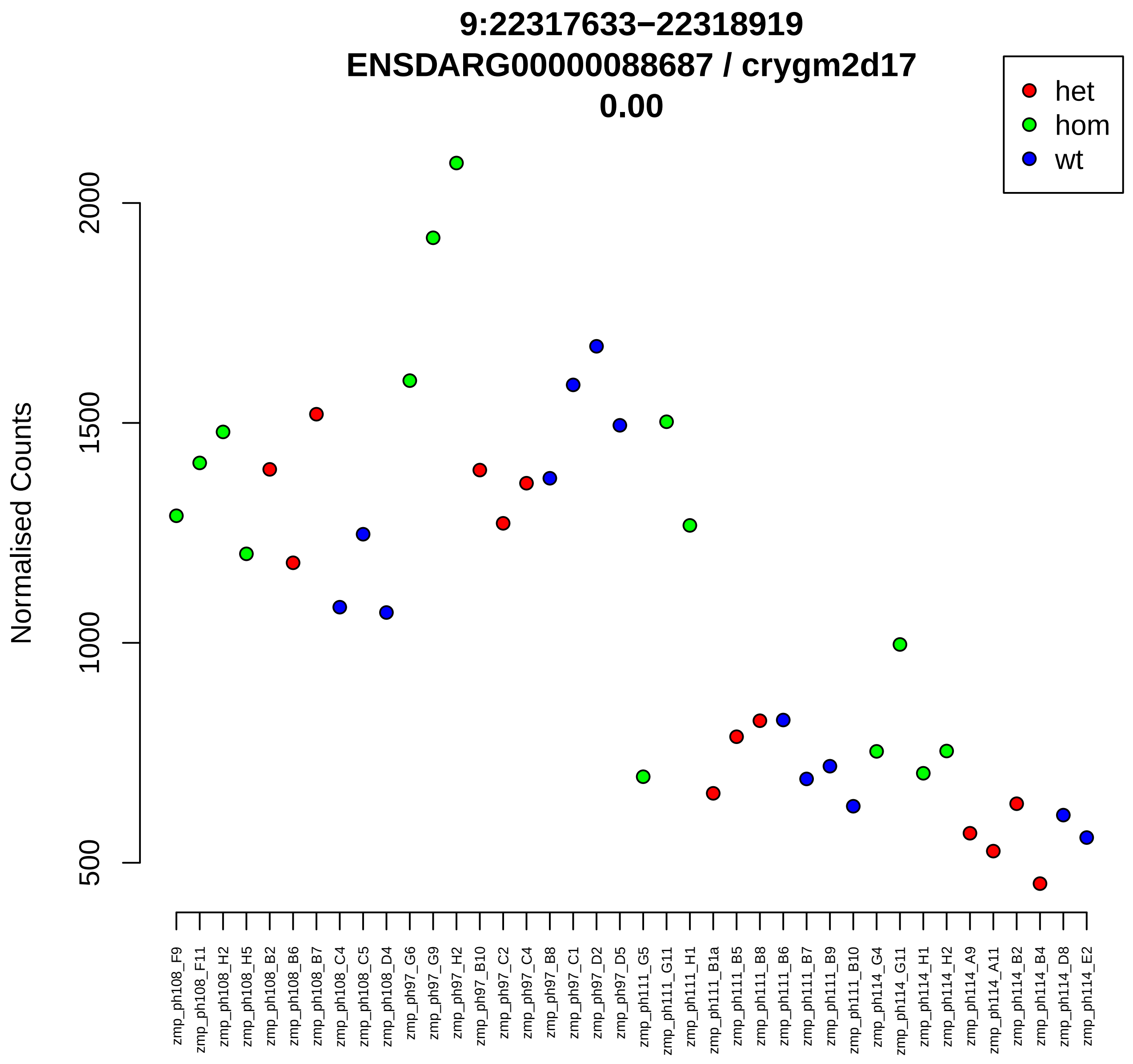

9:22317633−22318919
ENSDARG00000088687 / crygm2d17
0.00
het
hom
wt
Normalised Counts
500
1000
1500
2000
zmp_ph108_F9
zmp_ph108_F11
zmp_ph108_H2
zmp_ph108_H5
zmp_ph108_B2
zmp_ph108_B6
zmp_ph108_B7
zmp_ph108_C4
zmp_ph108_C5
zmp_ph108_D4
zmp_ph97_G6
zmp_ph97_G9
zmp_ph97_H2
zmp_ph97_B10
zmp_ph97_C2
zmp_ph97_C4
zmp_ph97_B8
zmp_ph97_C1
zmp_ph97_D2
zmp_ph97_D5
zmp_ph111_G5
zmp_ph111_G11
zmp_ph111_H1
zmp_ph111_B1a
zmp_ph111_B5
zmp_ph111_B8
zmp_ph111_B6
zmp_ph111_B7
zmp_ph111_B9
zmp_ph111_B10
zmp_ph114_G4
zmp_ph114_G11
zmp_ph114_H1
zmp_ph114_H2
zmp_ph114_A9
zmp_ph114_A11
zmp_ph114_B2
zmp_ph114_B4
zmp_ph114_D8
zmp_ph114_E2

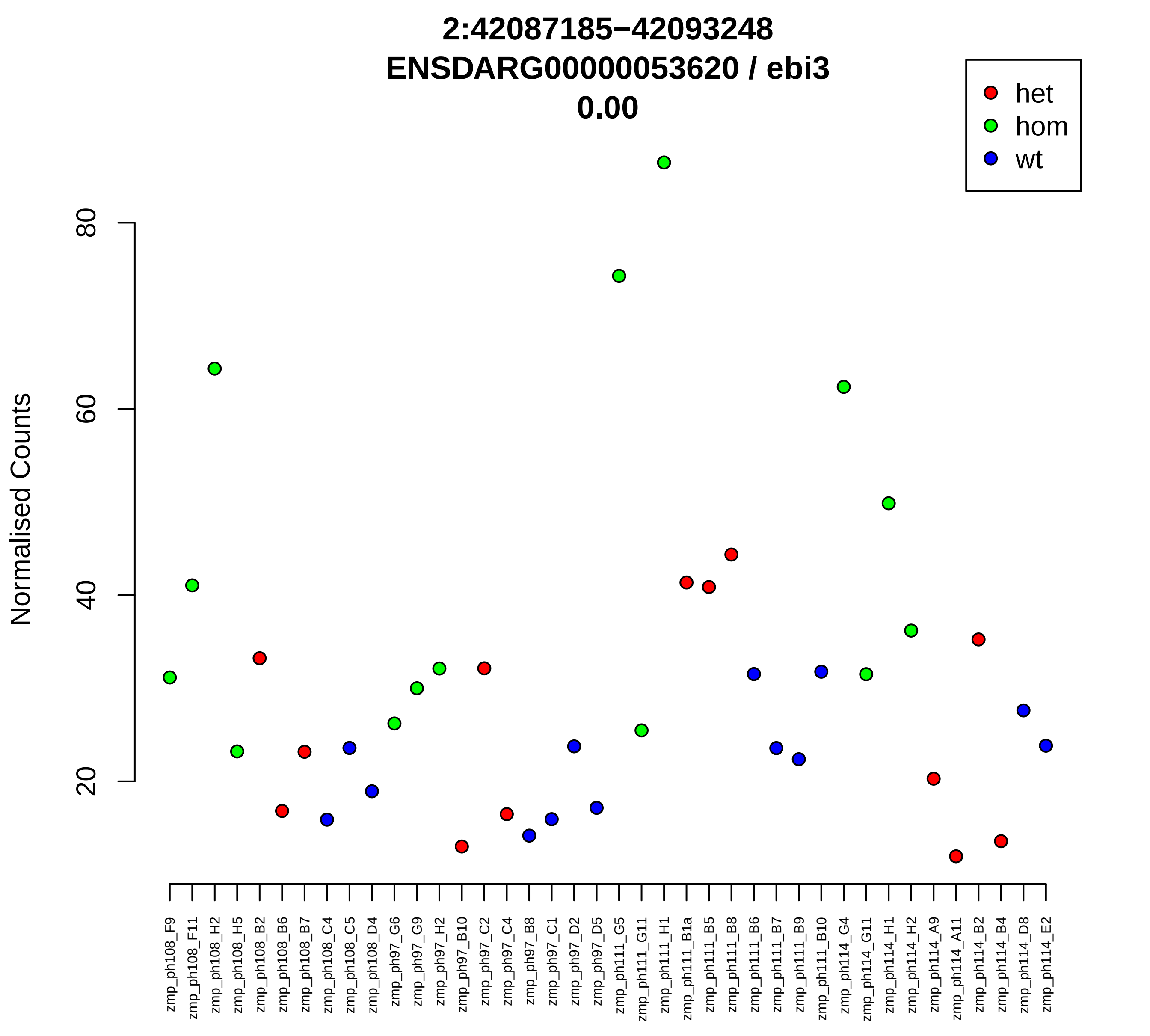

2:42087185−42093248
ENSDARG00000053620 / ebi3
0.00
het
hom
wt
Normalised Counts
20
40
60
80
zmp_ph108_F9
zmp_ph108_F11
zmp_ph108_H2
zmp_ph108_H5
zmp_ph108_B2
zmp_ph108_B6
zmp_ph108_B7
zmp_ph108_C4
zmp_ph108_C5
zmp_ph108_D4
zmp_ph97_G6
zmp_ph97_G9
zmp_ph97_H2
zmp_ph97_B10
zmp_ph97_C2
zmp_ph97_C4
zmp_ph97_B8
zmp_ph97_C1
zmp_ph97_D2
zmp_ph97_D5
zmp_ph111_G5
zmp_ph111_G11
zmp_ph111_H1
zmp_ph111_B1a
zmp_ph111_B5
zmp_ph111_B8
zmp_ph111_B6
zmp_ph111_B7
zmp_ph111_B9
zmp_ph111_B10
zmp_ph114_G4
zmp_ph114_G11
zmp_ph114_H1
zmp_ph114_H2
zmp_ph114_A9
zmp_ph114_A11
zmp_ph114_B2
zmp_ph114_B4
zmp_ph114_D8
zmp_ph114_E2

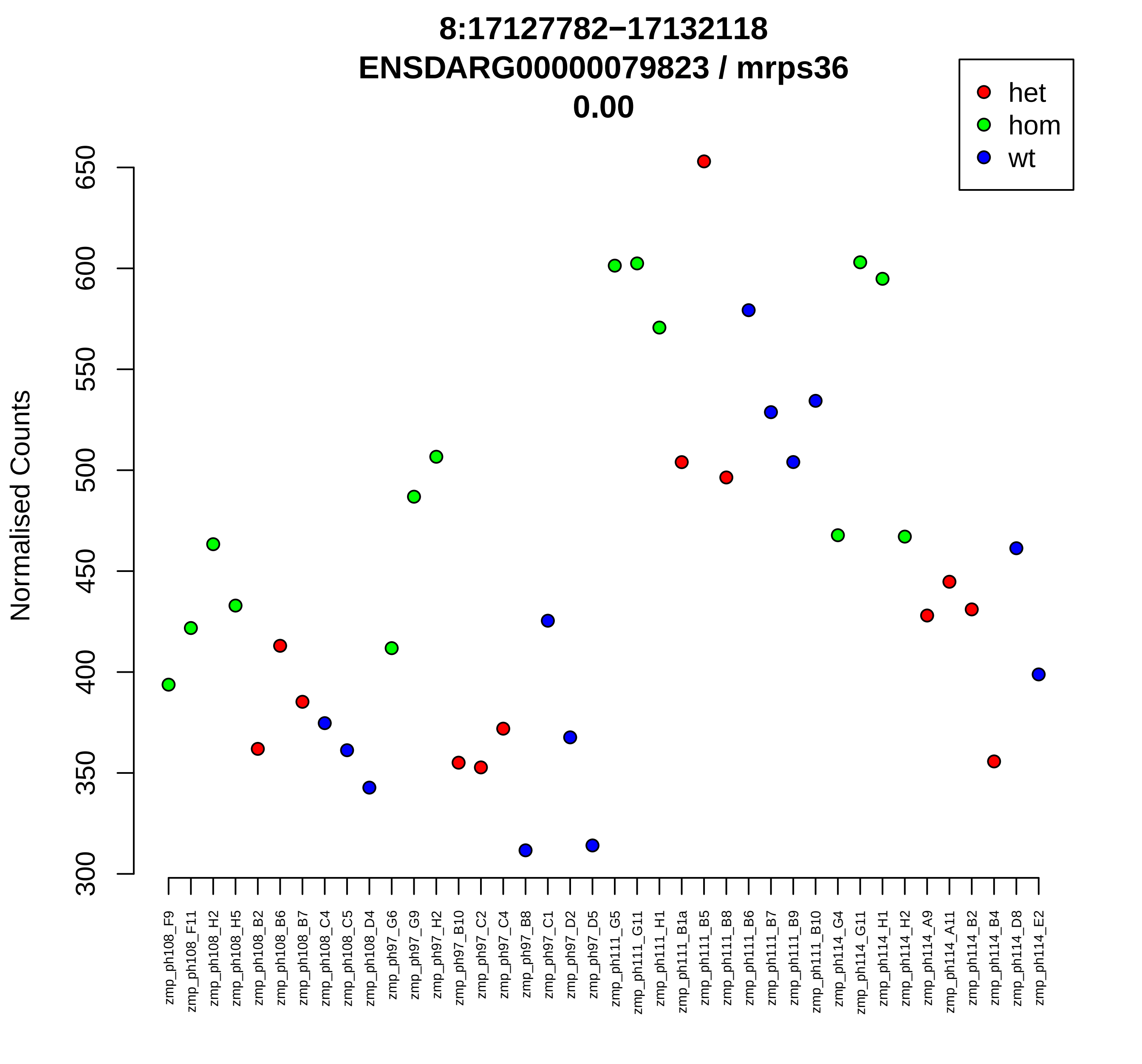
8:17127782−17132118
ENSDARG00000079823 / mrps36
0.00
het
hom
wt
Normalised Counts
300
350
400
450
500
550
600
650
zmp_ph108_F9
zmp_ph108_F11
zmp_ph108_H2
zmp_ph108_H5
zmp_ph108_B2
zmp_ph108_B6
zmp_ph108_B7
zmp_ph108_C4
zmp_ph108_C5
zmp_ph108_D4
zmp_ph97_G6
zmp_ph97_G9
zmp_ph97_H2
zmp_ph97_B10
zmp_ph97_C2
zmp_ph97_C4
zmp_ph97_B8
zmp_ph97_C1
zmp_ph97_D2
zmp_ph97_D5
zmp_ph111_G5
zmp_ph111_G11
zmp_ph111_H1
zmp_ph111_B1a
zmp_ph111_B5
zmp_ph111_B8
zmp_ph111_B6
zmp_ph111_B7
zmp_ph111_B9
zmp_ph111_B10
zmp_ph114_G4
zmp_ph114_G11
zmp_ph114_H1
zmp_ph114_H2
zmp_ph114_A9
zmp_ph114_A11
zmp_ph114_B2
zmp_ph114_B4
zmp_ph114_D8
zmp_ph114_E2

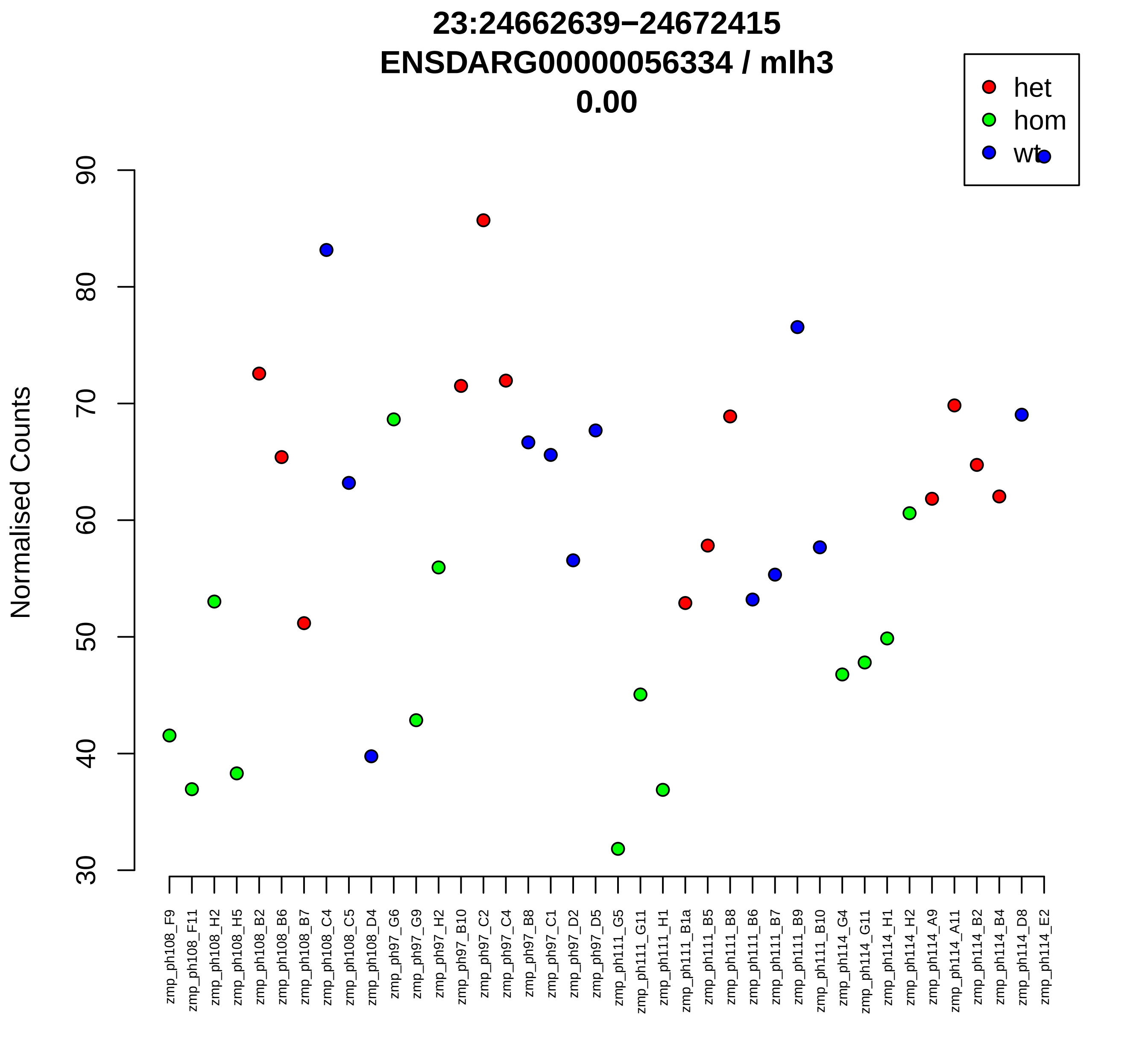

23:24662639−24672415
ENSDARG00000056334 / mlh3
0.00
het
hom
wt
Normalised Counts
30
40
50
60
70
80
90
zmp_ph108_F9
zmp_ph108_F11
zmp_ph108_H2
zmp_ph108_H5
zmp_ph108_B2
zmp_ph108_B6
zmp_ph108_B7
zmp_ph108_C4
zmp_ph108_C5
zmp_ph108_D4
zmp_ph97_G6
zmp_ph97_G9
zmp_ph97_H2
zmp_ph97_B10
zmp_ph97_C2
zmp_ph97_C4
zmp_ph97_B8
zmp_ph97_C1
zmp_ph97_D2
zmp_ph97_D5
zmp_ph111_G5
zmp_ph111_G11
zmp_ph111_H1
zmp_ph111_B1a
zmp_ph111_B5
zmp_ph111_B8
zmp_ph111_B6
zmp_ph111_B7
zmp_ph111_B9
zmp_ph111_B10
zmp_ph114_G4
zmp_ph114_G11
zmp_ph114_H1
zmp_ph114_H2
zmp_ph114_A9
zmp_ph114_A11
zmp_ph114_B2
zmp_ph114_B4
zmp_ph114_D8
zmp_ph114_E2

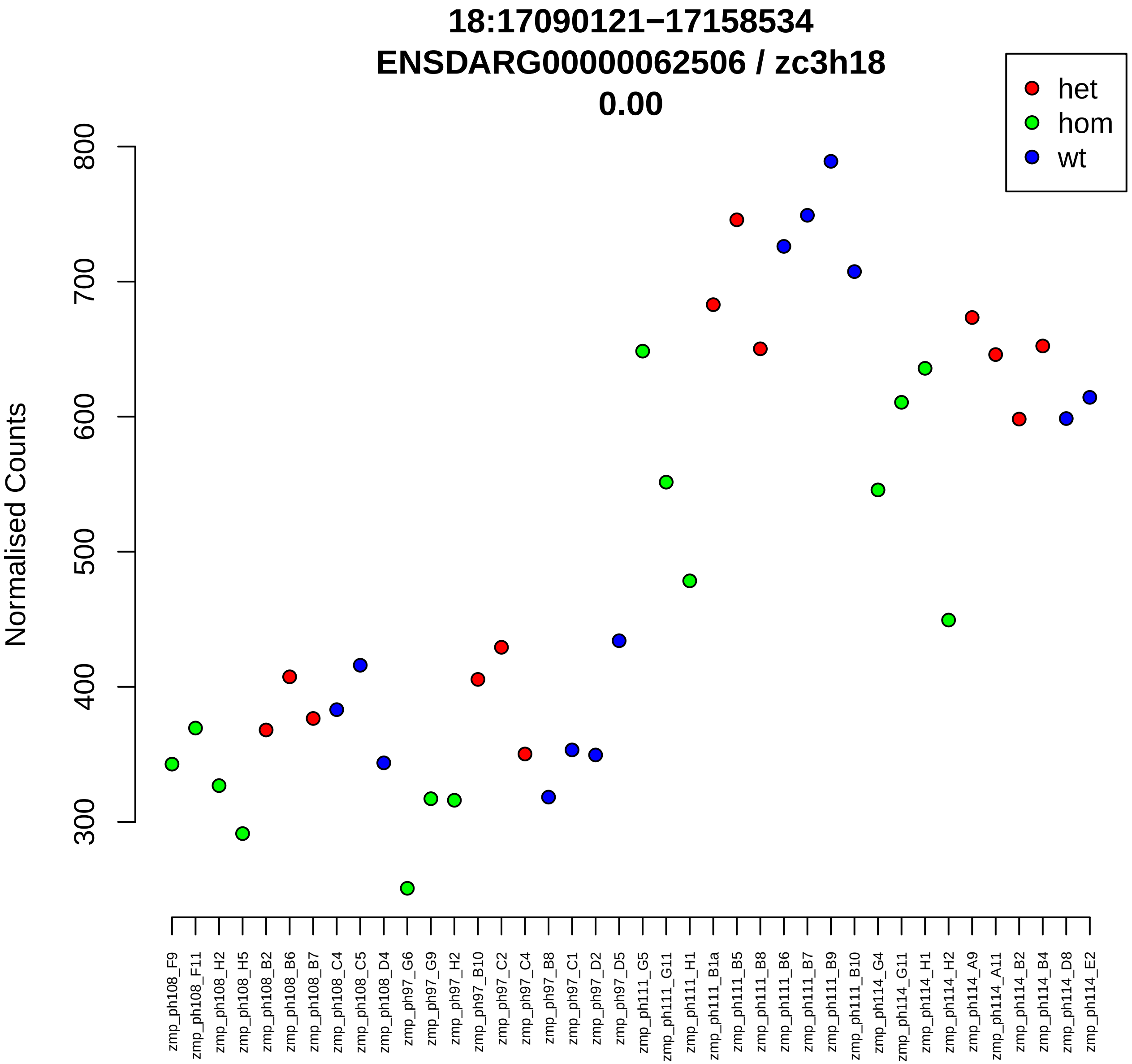
18:17090121−17158534
ENSDARG00000062506 / zc3h18
0.00
het
hom
wt
Normalised Counts
300
400
500
600
700
800
zmp_ph108_F9
zmp_ph108_F11
zmp_ph108_H2
zmp_ph108_H5
zmp_ph108_B2
zmp_ph108_B6
zmp_ph108_B7
zmp_ph108_C4
zmp_ph108_C5
zmp_ph108_D4
zmp_ph97_G6
zmp_ph97_G9
zmp_ph97_H2
zmp_ph97_B10
zmp_ph97_C2
zmp_ph97_C4
zmp_ph97_B8
zmp_ph97_C1
zmp_ph97_D2
zmp_ph97_D5
zmp_ph111_G5
zmp_ph111_G11
zmp_ph111_H1
zmp_ph111_B1a
zmp_ph111_B5
zmp_ph111_B8
zmp_ph111_B6
zmp_ph111_B7
zmp_ph111_B9
zmp_ph111_B10
zmp_ph114_G4
zmp_ph114_G11
zmp_ph114_H1
zmp_ph114_H2
zmp_ph114_A9
zmp_ph114_A11
zmp_ph114_B2
zmp_ph114_B4
zmp_ph114_D8
zmp_ph114_E2

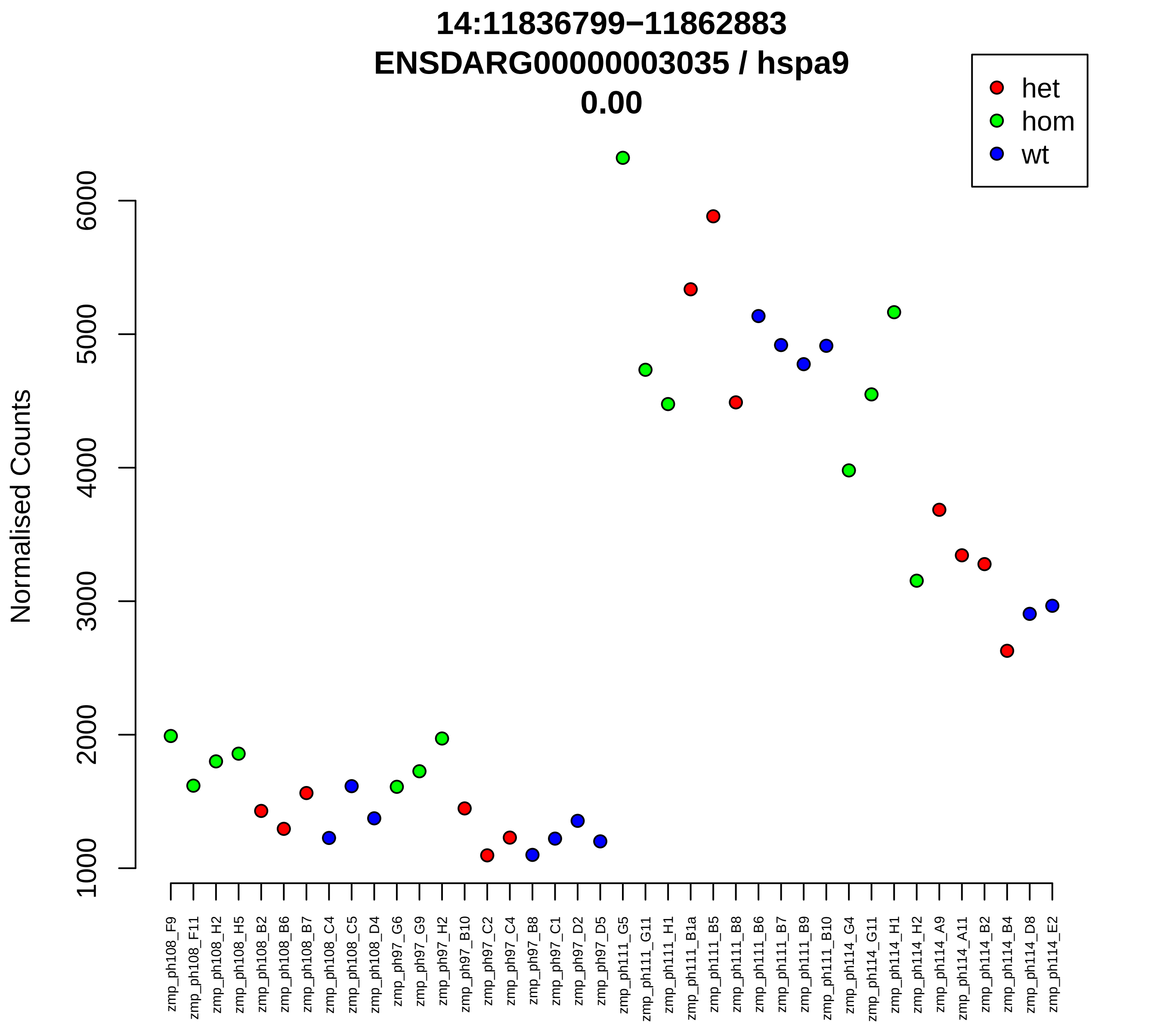
14:11836799−11862883
ENSDARG00000003035 / hspa9
0.00
het
hom
wt
Normalised Counts
1000
2000
3000
4000
5000
6000
zmp_ph108_F9
zmp_ph108_F11
zmp_ph108_H2
zmp_ph108_H5
zmp_ph108_B2
zmp_ph108_B6
zmp_ph108_B7
zmp_ph108_C4
zmp_ph108_C5
zmp_ph108_D4
zmp_ph97_G6
zmp_ph97_G9
zmp_ph97_H2
zmp_ph97_B10
zmp_ph97_C2
zmp_ph97_C4
zmp_ph97_B8
zmp_ph97_C1
zmp_ph97_D2
zmp_ph97_D5
zmp_ph111_G5
zmp_ph111_G11
zmp_ph111_H1
zmp_ph111_B1a
zmp_ph111_B5
zmp_ph111_B8
zmp_ph111_B6
zmp_ph111_B7
zmp_ph111_B9
zmp_ph111_B10
zmp_ph114_G4
zmp_ph114_G11
zmp_ph114_H1
zmp_ph114_H2
zmp_ph114_A9
zmp_ph114_A11
zmp_ph114_B2
zmp_ph114_B4
zmp_ph114_D8
zmp_ph114_E2

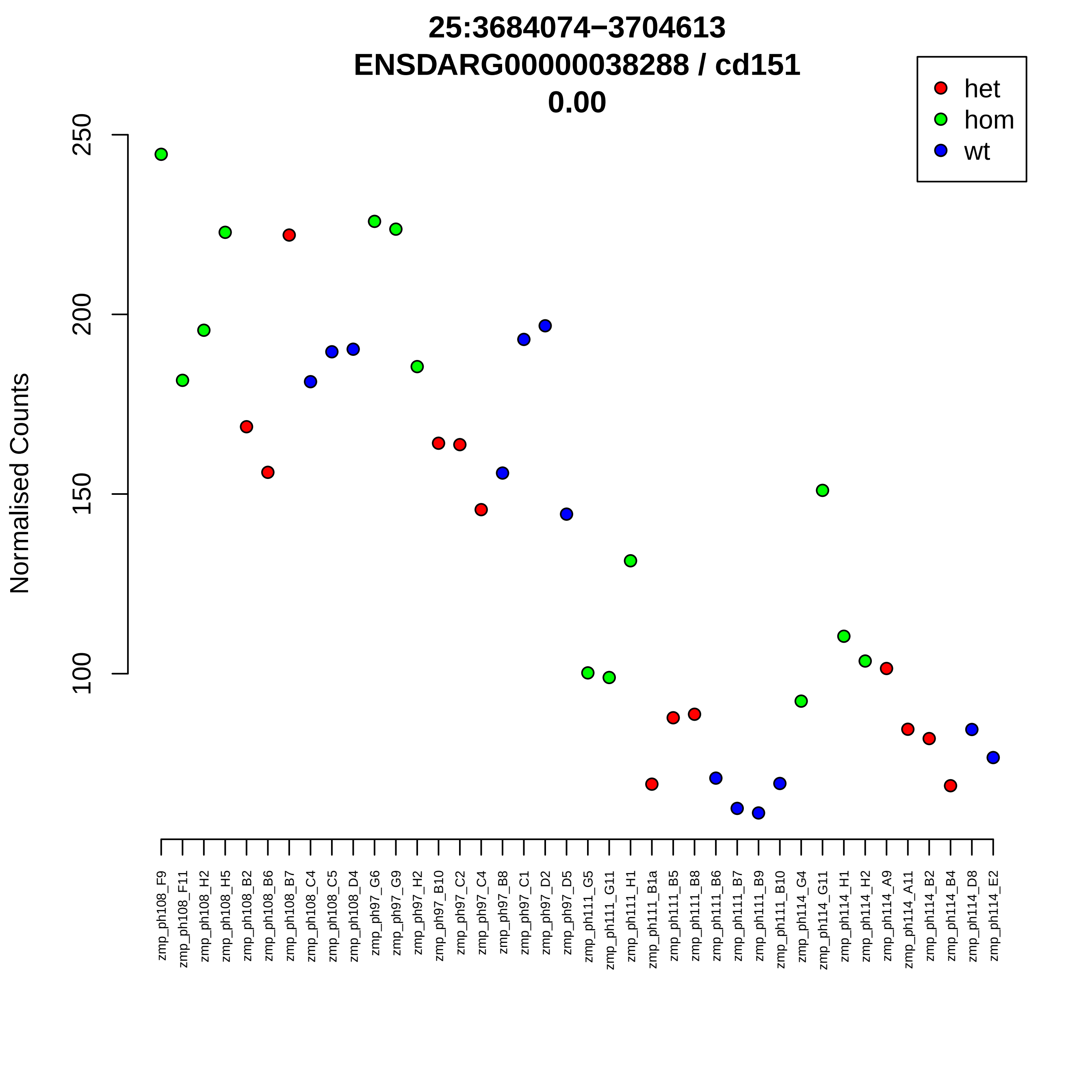
25:3684074−3704613
ENSDARG00000038288 / cd151
0.00
het
hom
wt
Normalised Counts
100
150
200
250
zmp_ph108_F9
zmp_ph108_F11
zmp_ph108_H2
zmp_ph108_H5
zmp_ph108_B2
zmp_ph108_B6
zmp_ph108_B7
zmp_ph108_C4
zmp_ph108_C5
zmp_ph108_D4
zmp_ph97_G6
zmp_ph97_G9
zmp_ph97_H2
zmp_ph97_B10
zmp_ph97_C2
zmp_ph97_C4
zmp_ph97_B8
zmp_ph97_C1
zmp_ph97_D2
zmp_ph97_D5
zmp_ph111_G5
zmp_ph111_G11
zmp_ph111_H1
zmp_ph111_B1a
zmp_ph111_B5
zmp_ph111_B8
zmp_ph111_B6
zmp_ph111_B7
zmp_ph111_B9
zmp_ph111_B10
zmp_ph114_G4
zmp_ph114_G11
zmp_ph114_H1
zmp_ph114_H2
zmp_ph114_A9
zmp_ph114_A11
zmp_ph114_B2
zmp_ph114_B4
zmp_ph114_D8
zmp_ph114_E2

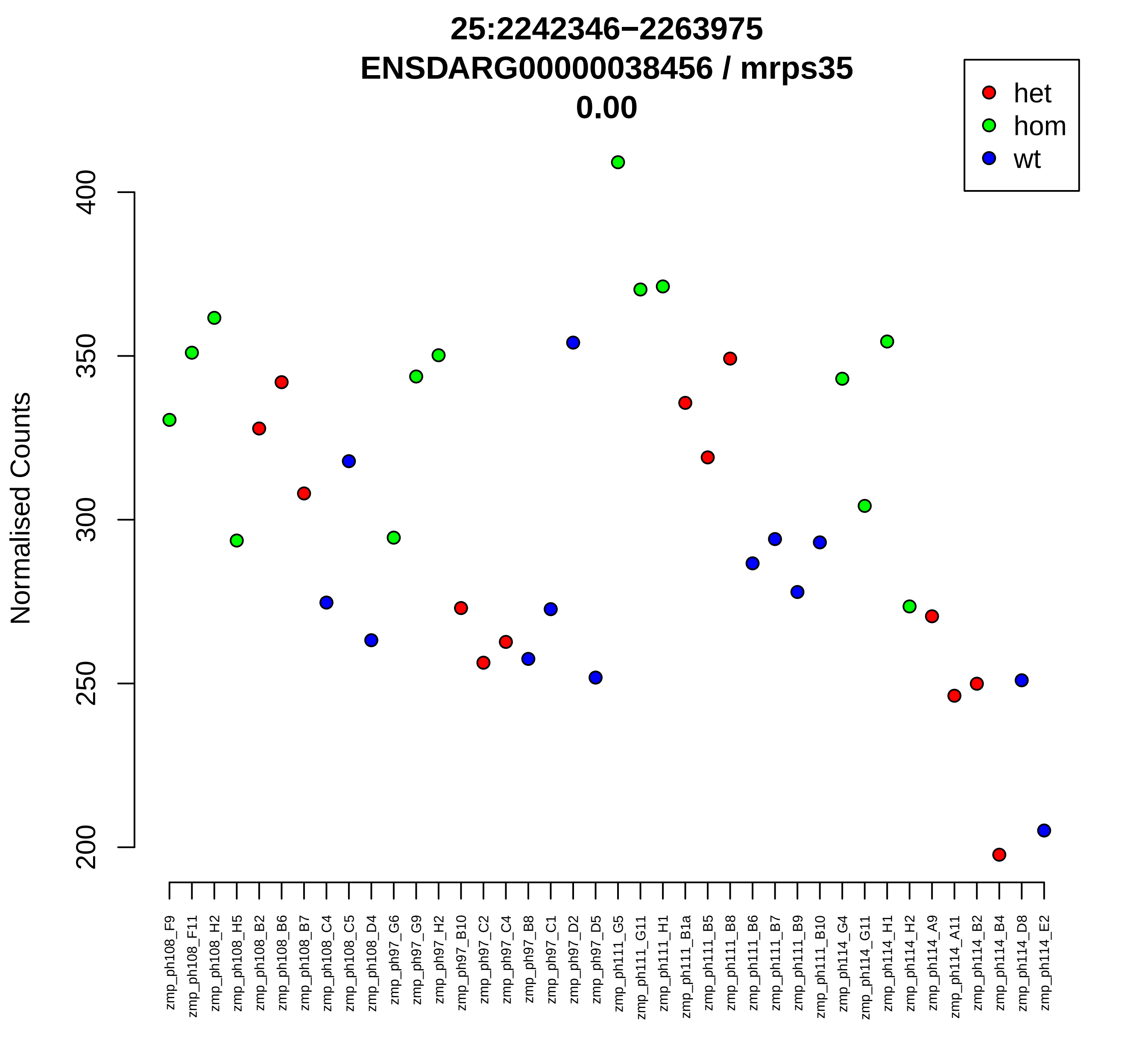

25:2242346−2263975
ENSDARG00000038456 / mrps35
0.00
het
hom
wt
Normalised Counts
400
350
300
250
200
zmp_ph108_F9
zmp_ph108_F11
zmp_ph108_H2
zmp_ph108_H5
zmp_ph108_B2
zmp_ph108_B6
zmp_ph108_B7
zmp_ph108_C4
zmp_ph108_C5
zmp_ph108_D4
zmp_ph97_G6
zmp_ph97_G9
zmp_ph97_H2
zmp_ph97_B10
zmp_ph97_C2
zmp_ph97_C4
zmp_ph97_B8
zmp_ph97_C1
zmp_ph97_D2
zmp_ph97_D5
zmp_ph111_G5
zmp_ph111_G11
zmp_ph111_H1
zmp_ph111_B1a
zmp_ph111_B5
zmp_ph111_B8
zmp_ph111_B6
zmp_ph111_B7
zmp_ph111_B9
zmp_ph111_B10
zmp_ph114_G4
zmp_ph114_G11
zmp_ph114_H1
zmp_ph114_H2
zmp_ph114_A9
zmp_ph114_A11
zmp_ph114_B2
zmp_ph114_B4
zmp_ph114_D8
zmp_ph114_E2

9:22335634−22336698
ENSDARG00000087301 / crygm2d14
0.00

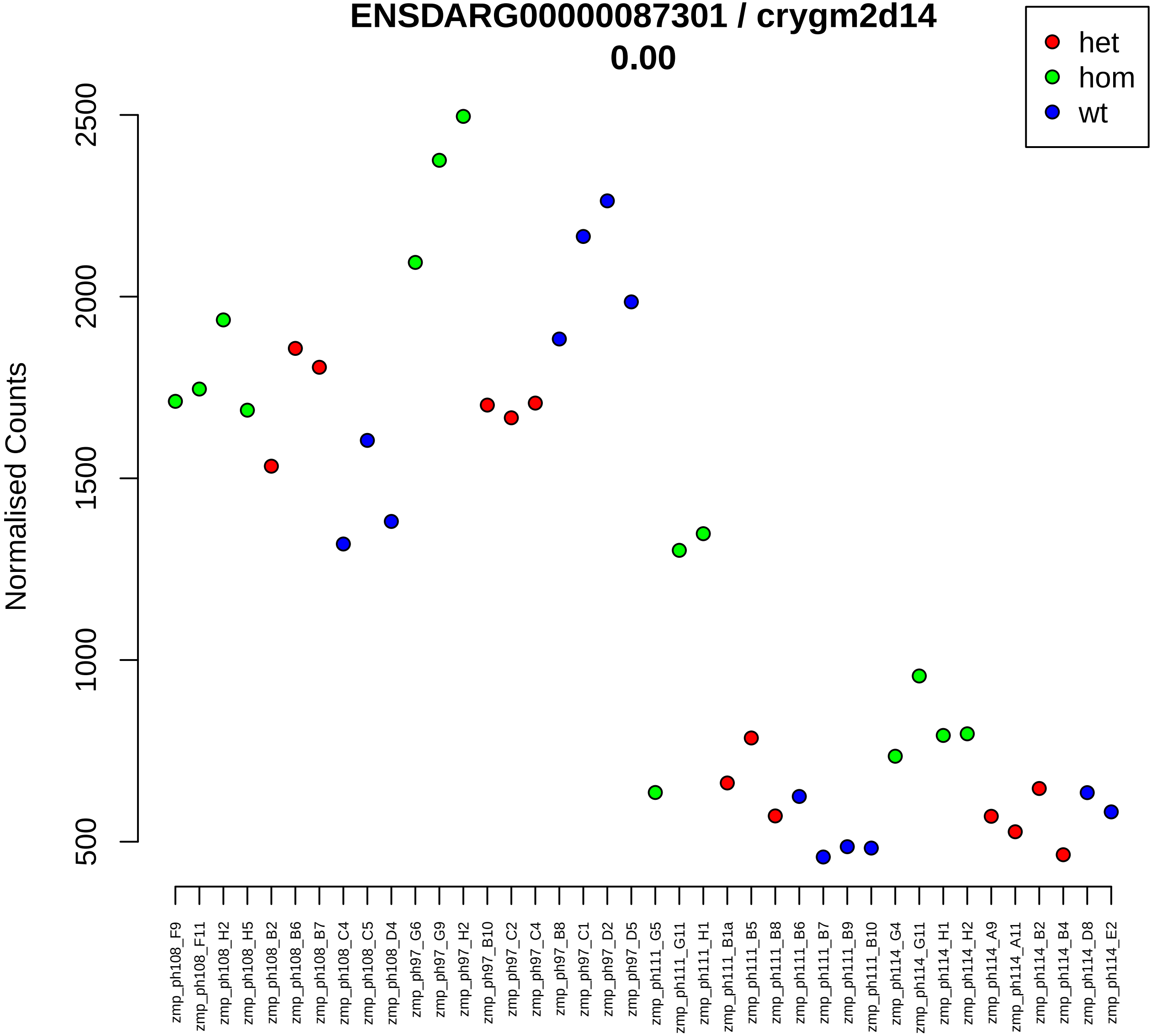

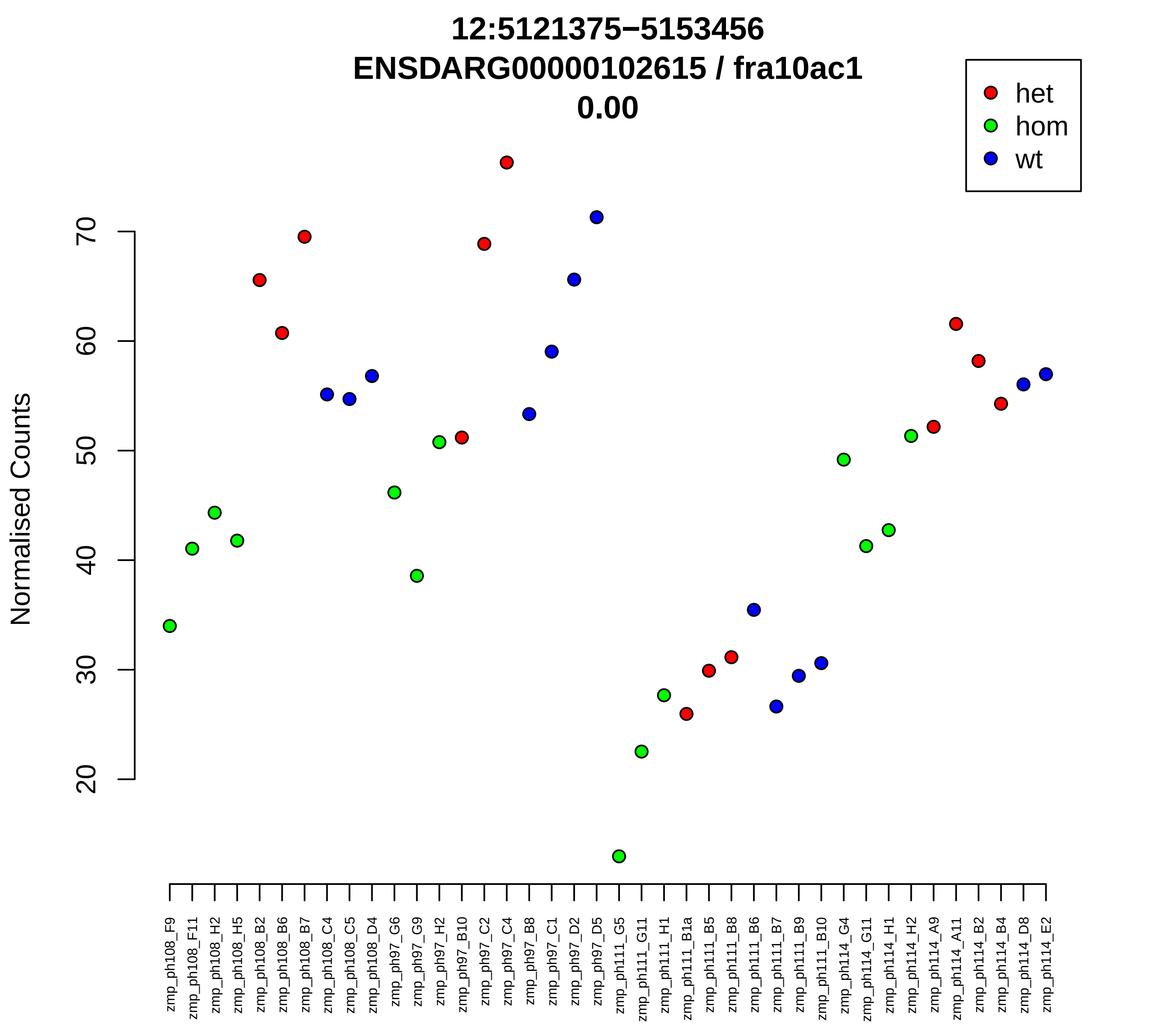
12:5121375−5153456
ENSDARG00000102615 / fra10ac1
0.00
het
hom
wt
Normalised Counts
20
30
40
50
60
70
zmp_ph108_F9
zmp_ph108_F11
zmp_ph108_H2
zmp_ph108_H5
zmp_ph108_B2
zmp_ph108_B6
zmp_ph108_B7
zmp_ph108_C4
zmp_ph108_C5
zmp_ph108_D4
zmp_ph97_G6
zmp_ph97_G9
zmp_ph97_H2
zmp_ph97_B10
zmp_ph97_C2
zmp_ph97_C4
zmp_ph97_B8
zmp_ph97_C1
zmp_ph97_D2
zmp_ph97_D5
zmp_ph111_G5
zmp_ph111_G11
zmp_ph111_H1
zmp_ph111_B1a
zmp_ph111_B5
zmp_ph111_B8
zmp_ph111_B6
zmp_ph111_B7
zmp_ph111_B9
zmp_ph111_B10
zmp_ph114_G4
zmp_ph114_G11
zmp_ph114_H1
zmp_ph114_H2
zmp_ph114_A9
zmp_ph114_A11
zmp_ph114_B2
zmp_ph114_B4
zmp_ph114_D8
zmp_ph114_E2

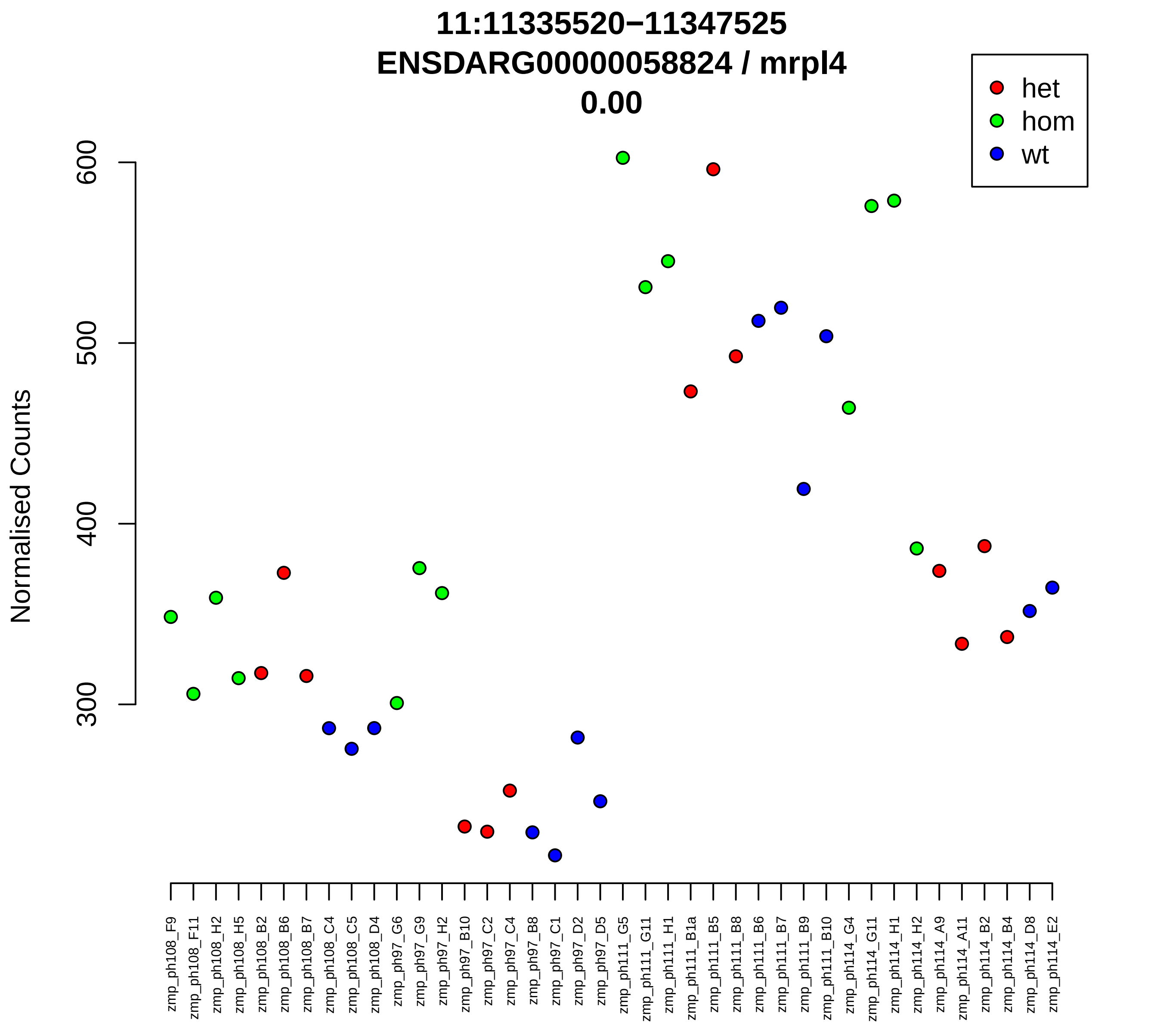
11:11335520−11347525
ENSDARG00000058824 / mrpl4
0.00
het
hom
wt
Normalised Counts
300
400
500
600
zmp_ph108_F9
zmp_ph108_F11
zmp_ph108_H2
zmp_ph108_H5
zmp_ph108_B2
zmp_ph108_B6
zmp_ph108_B7
zmp_ph108_C4
zmp_ph108_C5
zmp_ph108_D4
zmp_ph97_G6
zmp_ph97_G9
zmp_ph97_H2
zmp_ph97_B10
zmp_ph97_C2
zmp_ph97_C4
zmp_ph97_B8
zmp_ph97_C1
zmp_ph97_D2
zmp_ph97_D5
zmp_ph111_G5
zmp_ph111_G11
zmp_ph111_H1
zmp_ph111_B1a
zmp_ph111_B5
zmp_ph111_B8
zmp_ph111_B6
zmp_ph111_B7
zmp_ph111_B9
zmp_ph111_B10
zmp_ph114_G4
zmp_ph114_G11
zmp_ph114_H1
zmp_ph114_H2
zmp_ph114_A9
zmp_ph114_A11
zmp_ph114_B2
zmp_ph114_B4
zmp_ph114_D8
zmp_ph114_E2

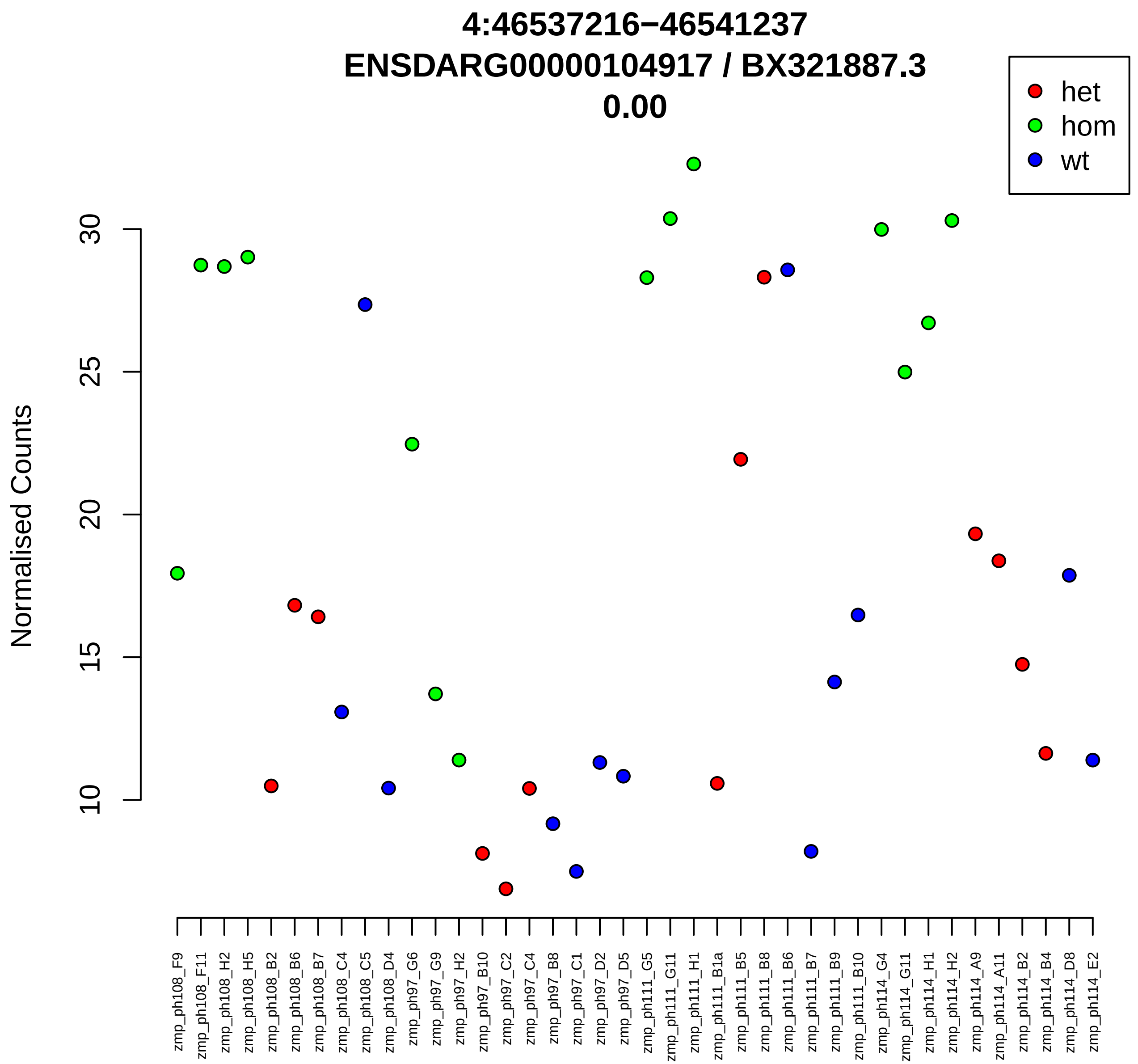

4:46537216−46541237
ENSDARG00000104917 / BX321887.3
0.00
het
hom
wt
Normalised Counts
10
15
20
25
30
zmp_ph108_F9
zmp_ph108_F11
zmp_ph108_H2
zmp_ph108_H5
zmp_ph108_B2
zmp_ph108_B6
zmp_ph108_B7
zmp_ph108_C4
zmp_ph108_C5
zmp_ph108_D4
zmp_ph97_G6
zmp_ph97_G9
zmp_ph97_H2
zmp_ph97_B10
zmp_ph97_C2
zmp_ph97_C4
zmp_ph97_B8
zmp_ph97_C1
zmp_ph97_D2
zmp_ph97_D5
zmp_ph111_G5
zmp_ph111_G11
zmp_ph111_H1
zmp_ph111_B1a
zmp_ph111_B5
zmp_ph111_B8
zmp_ph111_B6
zmp_ph111_B7
zmp_ph111_B9
zmp_ph111_B10
zmp_ph114_G4
zmp_ph114_G11
zmp_ph114_H1
zmp_ph114_H2
zmp_ph114_A9
zmp_ph114_A11
zmp_ph114_B2
zmp_ph114_B4
zmp_ph114_D8
zmp_ph114_E2

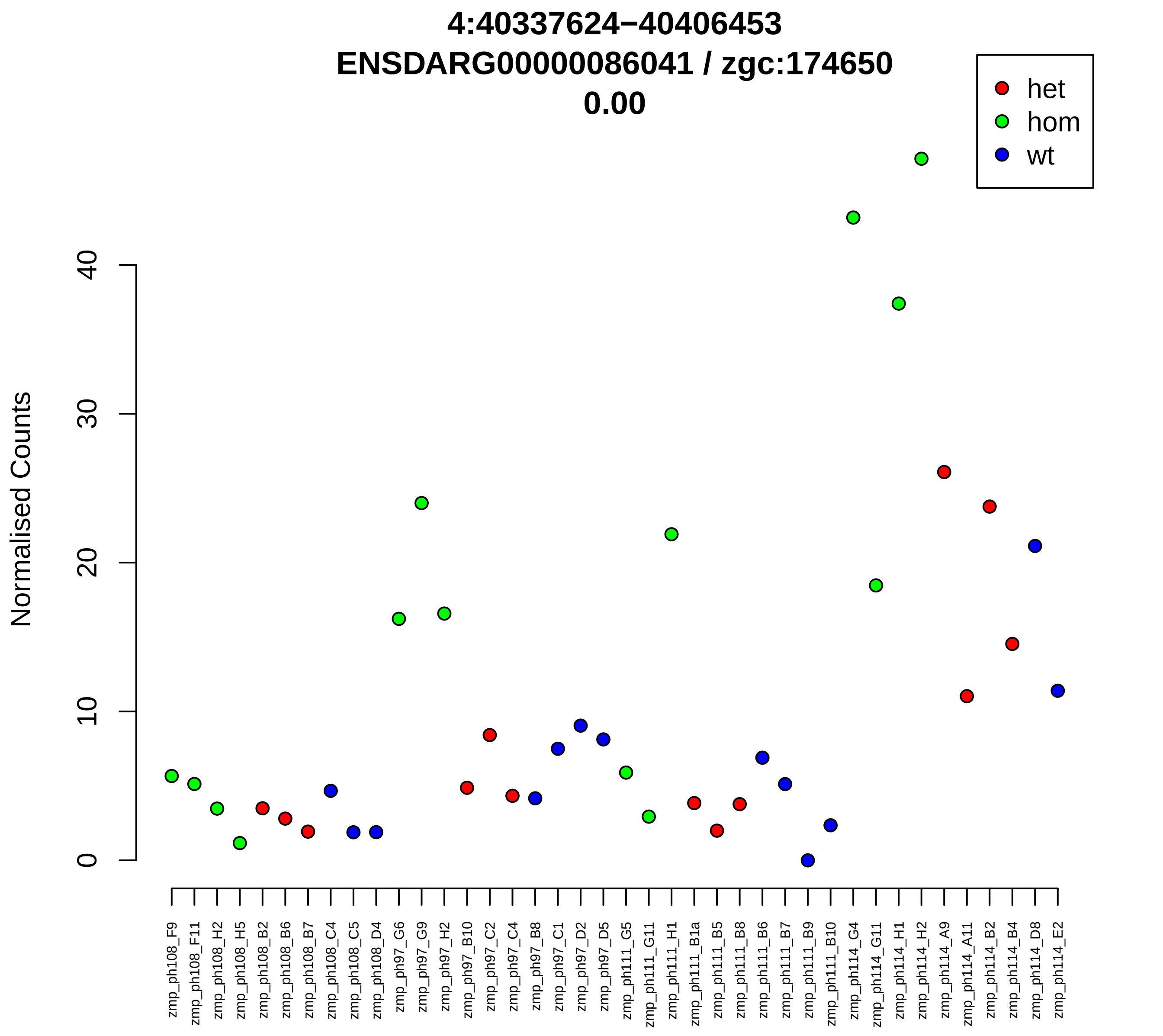
4:40337624−40406453
ENSDARG00000086041 / zgc:174650
0.00
het
hom
wt
Normalised Counts
0
10
20
30
40
zmp_ph108_F9
zmp_ph108_F11
zmp_ph108_H2
zmp_ph108_H5
zmp_ph108_B2
zmp_ph108_B6
zmp_ph108_B7
zmp_ph108_C4
zmp_ph108_C5
zmp_ph108_D4
zmp_ph97_G6
zmp_ph97_G9
zmp_ph97_H2
zmp_ph97_B10
zmp_ph97_C2
zmp_ph97_C4
zmp_ph97_B8
zmp_ph97_C1
zmp_ph97_D2
zmp_ph97_D5
zmp_ph111_G5
zmp_ph111_G11
zmp_ph111_H1
zmp_ph111_B1a
zmp_ph111_B5
zmp_ph111_B8
zmp_ph111_B6
zmp_ph111_B7
zmp_ph111_B9
zmp_ph111_B10
zmp_ph114_G4
zmp_ph114_G11
zmp_ph114_H1
zmp_ph114_H2
zmp_ph114_A9
zmp_ph114_A11
zmp_ph114_B2
zmp_ph114_B4
zmp_ph114_D8
zmp_ph114_E2

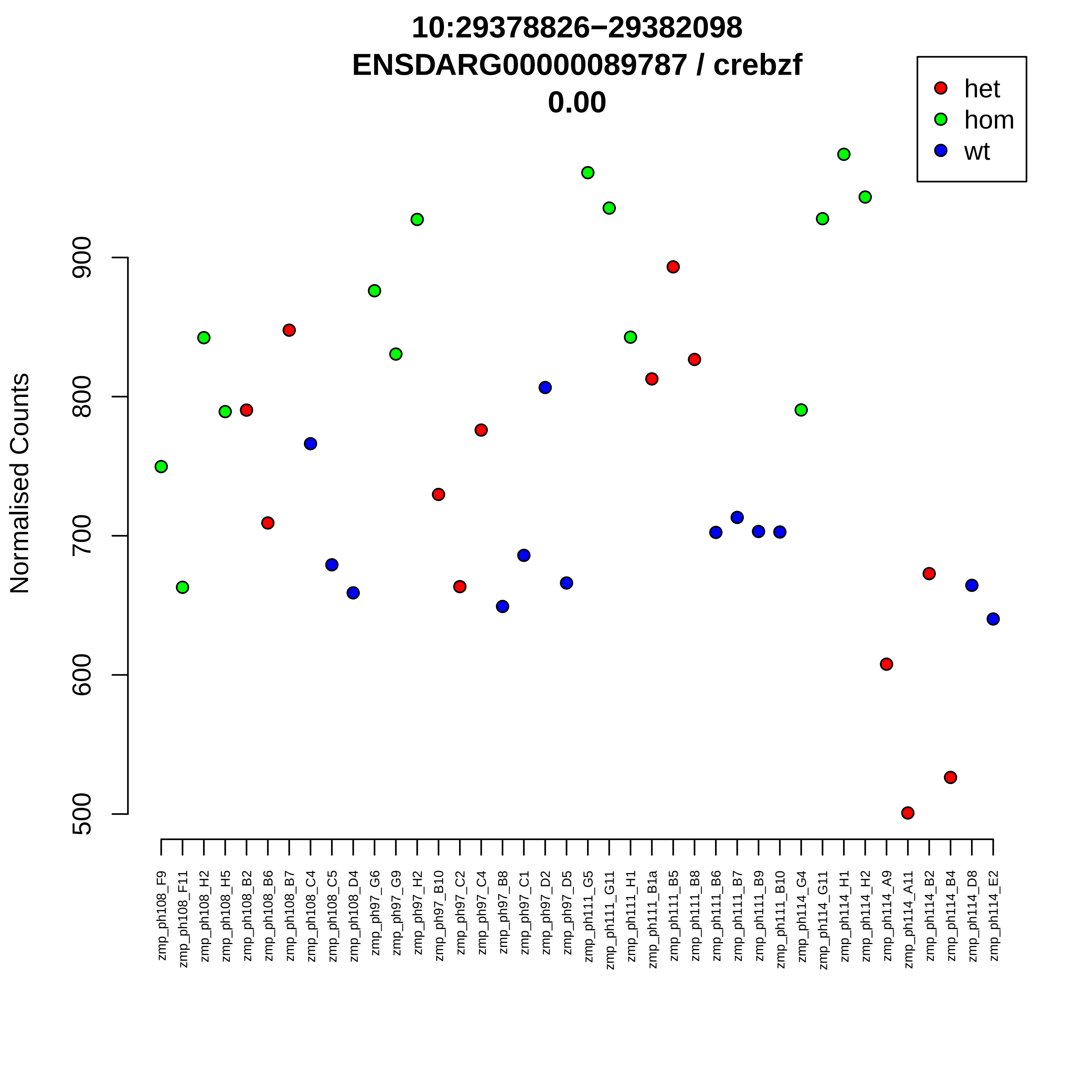
10:29378826−29382098
ENSDARG00000089787 / crebzf
0.00
het
hom
wt
Normalised Counts
500
600
700
800
900
zmp_ph108_F9
zmp_ph108_F11
zmp_ph108_H2
zmp_ph108_H5
zmp_ph108_B2
zmp_ph108_B6
zmp_ph108_B7
zmp_ph108_C4
zmp_ph108_C5
zmp_ph108_D4
zmp_ph97_G6
zmp_ph97_G9
zmp_ph97_H2
zmp_ph97_B10
zmp_ph97_C2
zmp_ph97_C4
zmp_ph97_B8
zmp_ph97_C1
zmp_ph97_D2
zmp_ph97_D5
zmp_ph111_G5
zmp_ph111_G11
zmp_ph111_H1
zmp_ph111_B1a
zmp_ph111_B5
zmp_ph111_B8
zmp_ph111_B6
zmp_ph111_B7
zmp_ph111_B9
zmp_ph111_B10
zmp_ph114_G4
zmp_ph114_G11
zmp_ph114_H1
zmp_ph114_H2
zmp_ph114_A9
zmp_ph114_A11
zmp_ph114_B2
zmp_ph114_B4
zmp_ph114_D8
zmp_ph114_E2

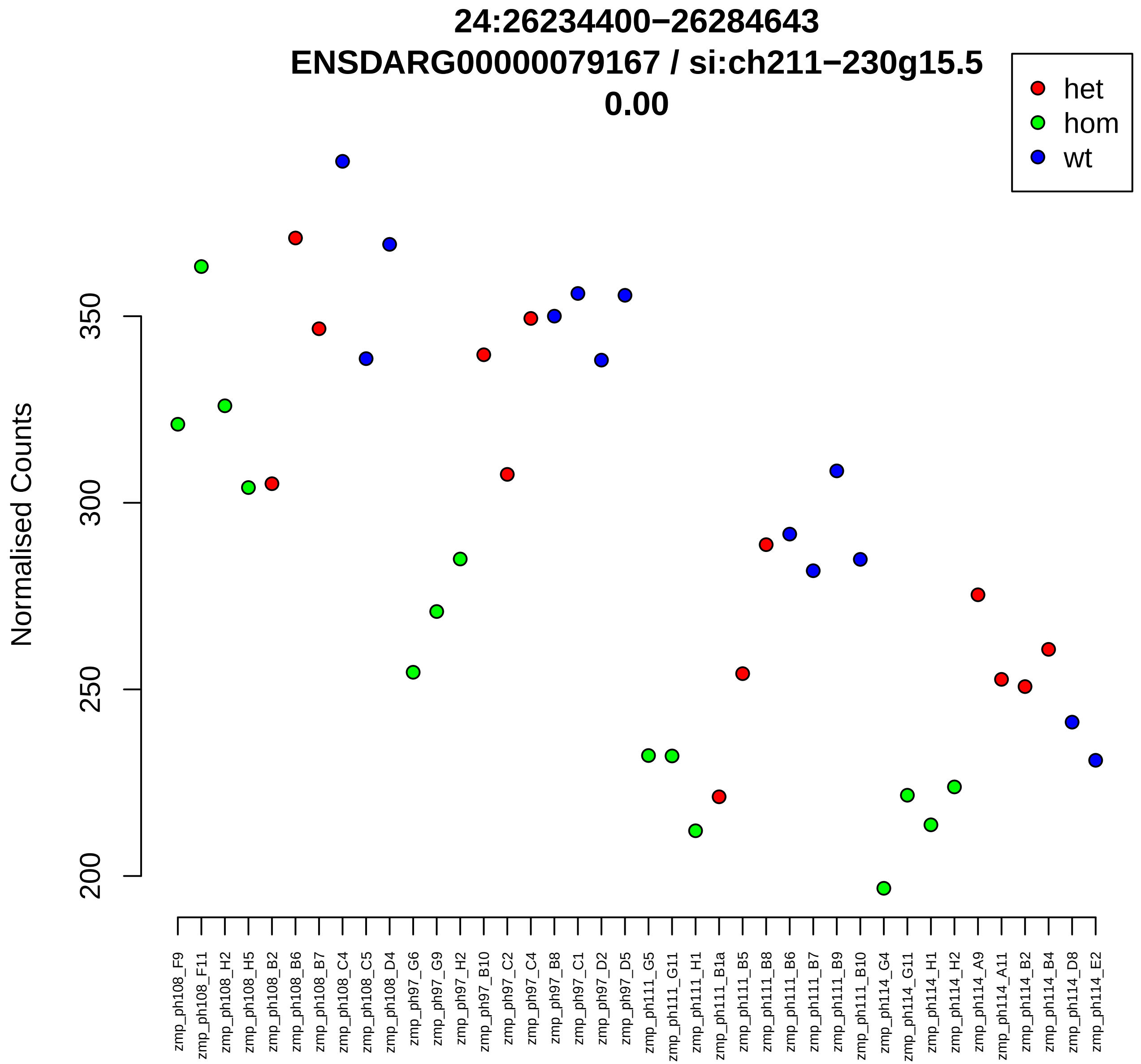

24:26234400−26284643
ENSDARG00000079167 / si:ch211−230g15.5
0.00
het
hom
wt
Normalised Counts
200
250
300
350
zmp_ph108_F9
zmp_ph108_F11
zmp_ph108_H2
zmp_ph108_H5
zmp_ph108_B2
zmp_ph108_B6
zmp_ph108_B7
zmp_ph108_C4
zmp_ph108_C5
zmp_ph108_D4
zmp_ph97_G6
zmp_ph97_G9
zmp_ph97_H2
zmp_ph97_B10
zmp_ph97_C2
zmp_ph97_C4
zmp_ph97_B8
zmp_ph97_C1
zmp_ph97_D2
zmp_ph97_D5
zmp_ph111_G5
zmp_ph111_G11
zmp_ph111_H1
zmp_ph111_B1a
zmp_ph111_B5
zmp_ph111_B8
zmp_ph111_B6
zmp_ph111_B7
zmp_ph111_B9
zmp_ph111_B10
zmp_ph114_G4
zmp_ph114_G11
zmp_ph114_H1
zmp_ph114_H2
zmp_ph114_A9
zmp_ph114_A11
zmp_ph114_B2
zmp_ph114_B4
zmp_ph114_D8
zmp_ph114_E2

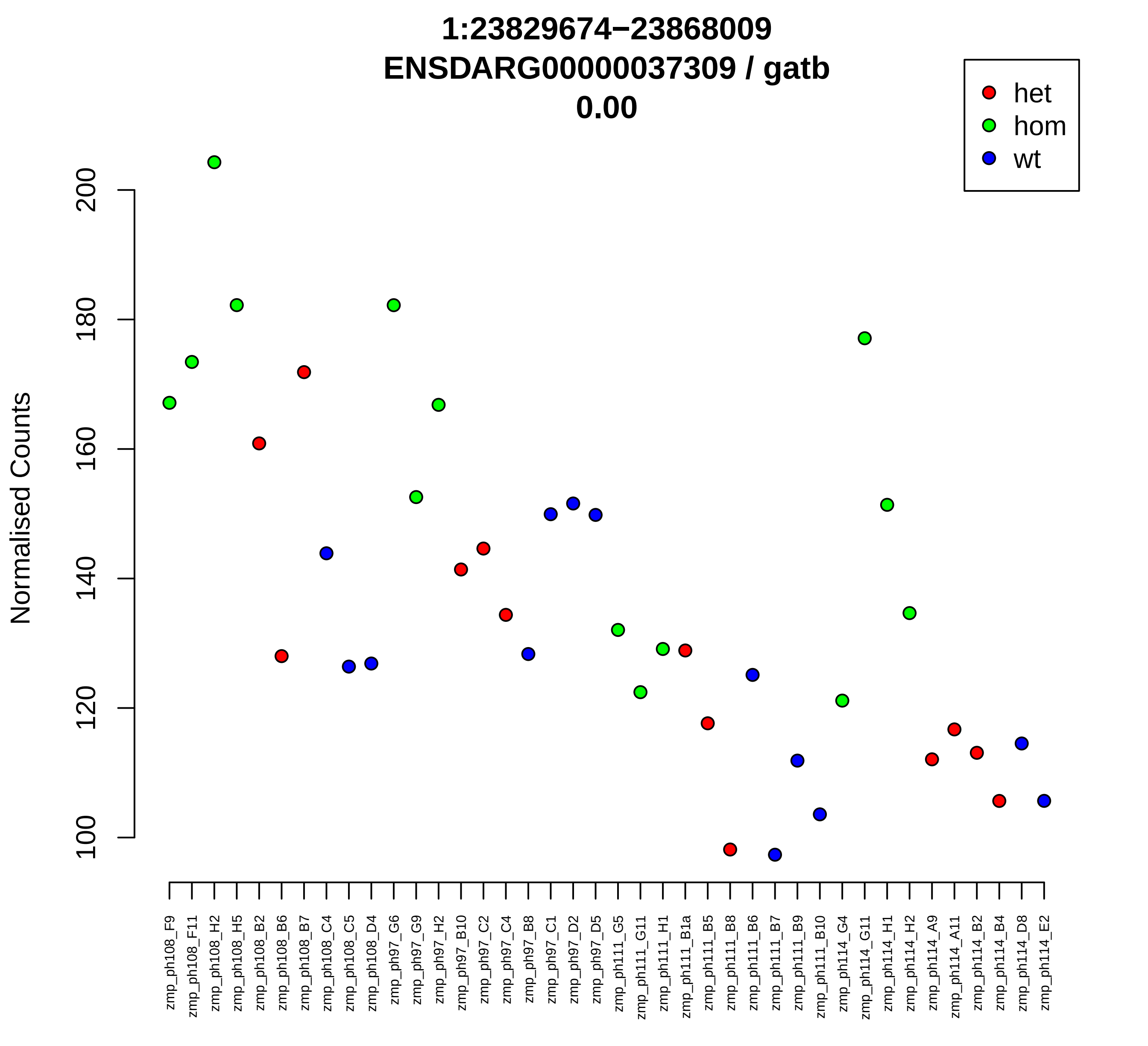
1:23829674−23868009
ENSDARG00000037309 / gatb
0.00
het
hom
wt
Normalised Counts
100
120
140
160
180
200
zmp_ph108_F9
zmp_ph108_F11
zmp_ph108_H2
zmp_ph108_H5
zmp_ph108_B2
zmp_ph108_B6
zmp_ph108_B7
zmp_ph108_C4
zmp_ph108_C5
zmp_ph108_D4
zmp_ph97_G6
zmp_ph97_G9
zmp_ph97_H2
zmp_ph97_B10
zmp_ph97_C2
zmp_ph97_C4
zmp_ph97_B8
zmp_ph97_C1
zmp_ph97_D2
zmp_ph97_D5
zmp_ph111_G5
zmp_ph111_G11
zmp_ph111_H1
zmp_ph111_B1a
zmp_ph111_B5
zmp_ph111_B8
zmp_ph111_B6
zmp_ph111_B7
zmp_ph111_B9
zmp_ph111_B10
zmp_ph114_G4
zmp_ph114_G11
zmp_ph114_H1
zmp_ph114_H2
zmp_ph114_A9
zmp_ph114_A11
zmp_ph114_B2
zmp_ph114_B4
zmp_ph114_D8
zmp_ph114_E2

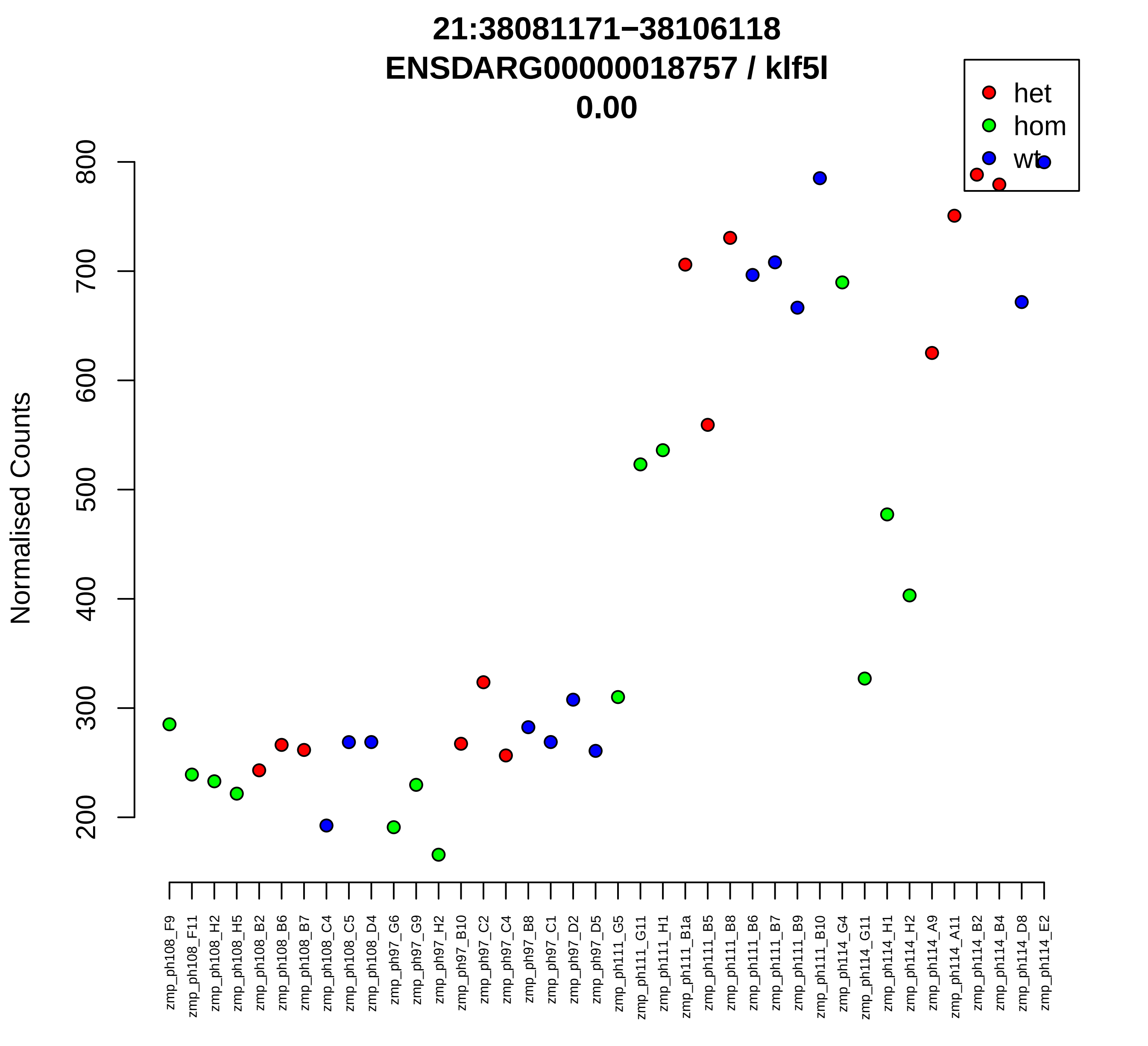

21:38081171−38106118
ENSDARG00000018757 / klf5l
0.00
het
hom
wt
Normalised Counts
200
300
400
500
600
700
800
zmp_ph108_F9
zmp_ph108_F11
zmp_ph108_H2
zmp_ph108_H5
zmp_ph108_B2
zmp_ph108_B6
zmp_ph108_B7
zmp_ph108_C4
zmp_ph108_C5
zmp_ph108_D4
zmp_ph97_G6
zmp_ph97_G9
zmp_ph97_H2
zmp_ph97_B10
zmp_ph97_C2
zmp_ph97_C4
zmp_ph97_B8
zmp_ph97_C1
zmp_ph97_D2
zmp_ph97_D5
zmp_ph111_G5
zmp_ph111_G11
zmp_ph111_H1
zmp_ph111_B1a
zmp_ph111_B5
zmp_ph111_B8
zmp_ph111_B6
zmp_ph111_B7
zmp_ph111_B9
zmp_ph111_B10
zmp_ph114_G4
zmp_ph114_G11
zmp_ph114_H1
zmp_ph114_H2
zmp_ph114_A9
zmp_ph114_A11
zmp_ph114_B2
zmp_ph114_B4
zmp_ph114_D8
zmp_ph114_E2

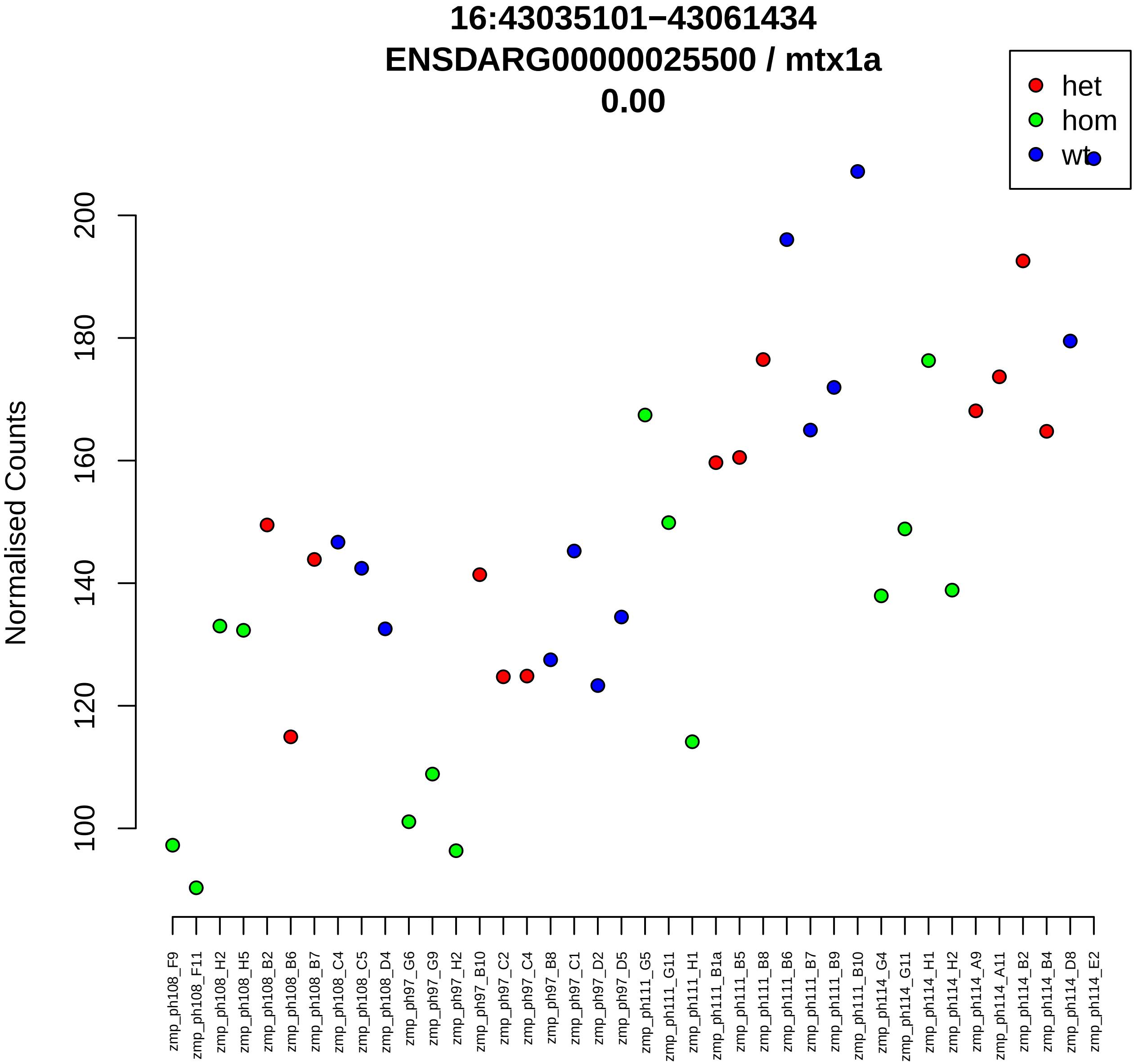

16:43035101−43061434
ENSDARG00000025500 / mtx1a
0.00
het
hom
wt
Normalised Counts
100
120
140
160
180
200
zmp_ph108_F9
zmp_ph108_F11
zmp_ph108_H2
zmp_ph108_H5
zmp_ph108_B2
zmp_ph108_B6
zmp_ph108_B7
zmp_ph108_C4
zmp_ph108_C5
zmp_ph108_D4
zmp_ph97_G6
zmp_ph97_G9
zmp_ph97_H2
zmp_ph97_B10
zmp_ph97_C2
zmp_ph97_C4
zmp_ph97_B8
zmp_ph97_C1
zmp_ph97_D2
zmp_ph97_D5
zmp_ph111_G5
zmp_ph111_G11
zmp_ph111_H1
zmp_ph111_B1a
zmp_ph111_B5
zmp_ph111_B8
zmp_ph111_B6
zmp_ph111_B7
zmp_ph111_B9
zmp_ph111_B10
zmp_ph114_G4
zmp_ph114_G11
zmp_ph114_H1
zmp_ph114_H2
zmp_ph114_A9
zmp_ph114_A11
zmp_ph114_B2
zmp_ph114_B4
zmp_ph114_D8
zmp_ph114_E2

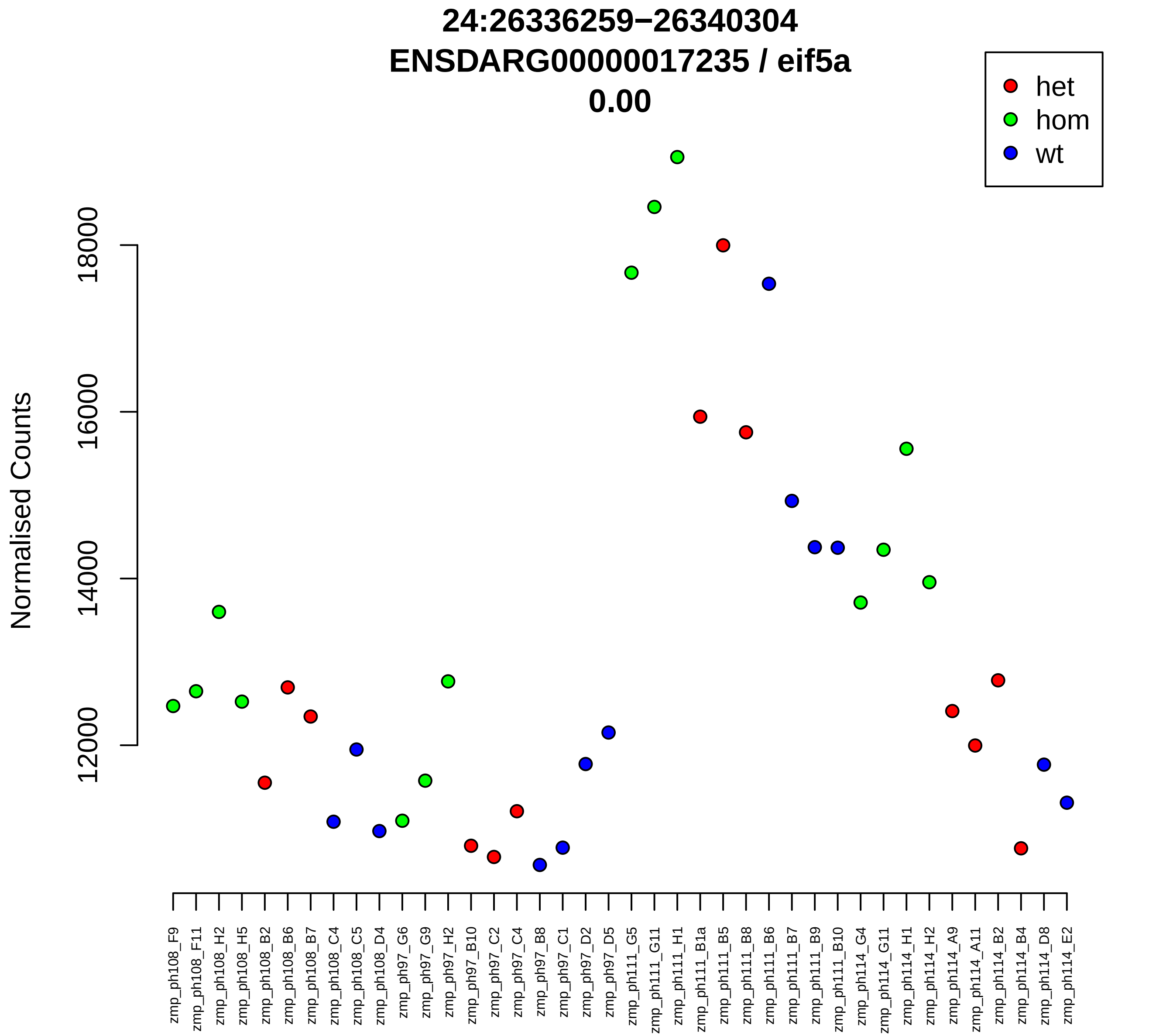
24:26336259−26340304
ENSDARG00000017235 / eif5a
0.00
het
hom
wt
Normalised Counts
12000
14000
16000
18000
zmp_ph108_F9
zmp_ph108_F11
zmp_ph108_H2
zmp_ph108_H5
zmp_ph108_B2
zmp_ph108_B6
zmp_ph108_B7
zmp_ph108_C4
zmp_ph108_C5
zmp_ph108_D4
zmp_ph97_G6
zmp_ph97_G9
zmp_ph97_H2
zmp_ph97_B10
zmp_ph97_C2
zmp_ph97_C4
zmp_ph97_B8
zmp_ph97_C1
zmp_ph97_D2
zmp_ph97_D5
zmp_ph111_G5
zmp_ph111_G11
zmp_ph111_H1
zmp_ph111_B1a
zmp_ph111_B5
zmp_ph111_B8
zmp_ph111_B6
zmp_ph111_B7
zmp_ph111_B9
zmp_ph111_B10
zmp_ph114_G4
zmp_ph114_G11
zmp_ph114_H1
zmp_ph114_H2
zmp_ph114_A9
zmp_ph114_A11
zmp_ph114_B2
zmp_ph114_B4
zmp_ph114_D8
zmp_ph114_E2

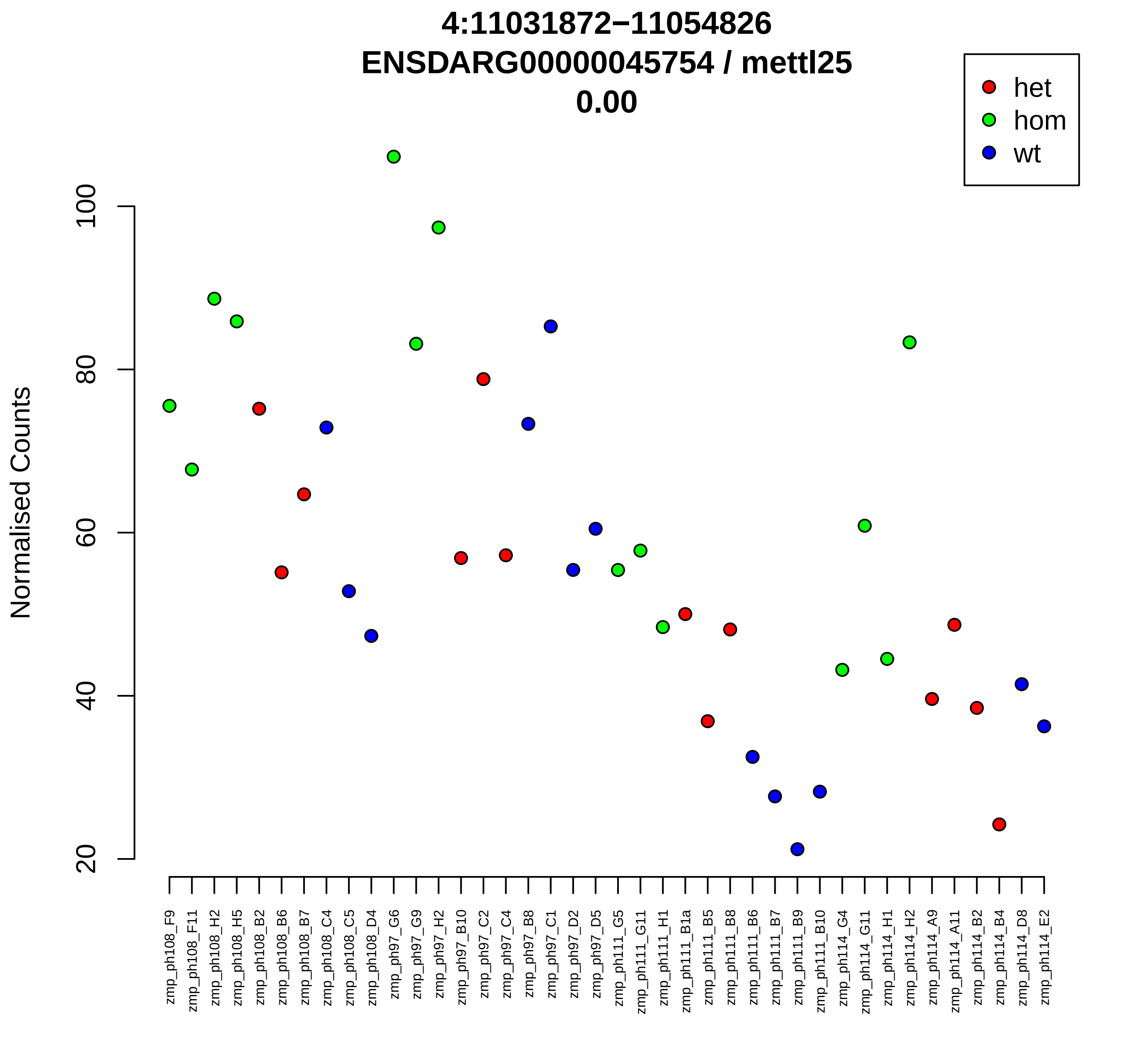

4:11031872−11054826
ENSDARG00000045754 / mettl25
0.00
het
hom
wt
Normalised Counts
20
40
60
80
100
zmp_ph108_F9
zmp_ph108_F11
zmp_ph108_H2
zmp_ph108_H5
zmp_ph108_B2
zmp_ph108_B6
zmp_ph108_B7
zmp_ph108_C4
zmp_ph108_C5
zmp_ph108_D4
zmp_ph97_G6
zmp_ph97_G9
zmp_ph97_H2
zmp_ph97_B10
zmp_ph97_C2
zmp_ph97_C4
zmp_ph97_B8
zmp_ph97_C1
zmp_ph97_D2
zmp_ph97_D5
zmp_ph111_G5
zmp_ph111_G11
zmp_ph111_H1
zmp_ph111_B1a
zmp_ph111_B5
zmp_ph111_B8
zmp_ph111_B6
zmp_ph111_B7
zmp_ph111_B9
zmp_ph111_B10
zmp_ph114_G4
zmp_ph114_G11
zmp_ph114_H1
zmp_ph114_H2
zmp_ph114_A9
zmp_ph114_A11
zmp_ph114_B2
zmp_ph114_B4
zmp_ph114_D8
zmp_ph114_E2

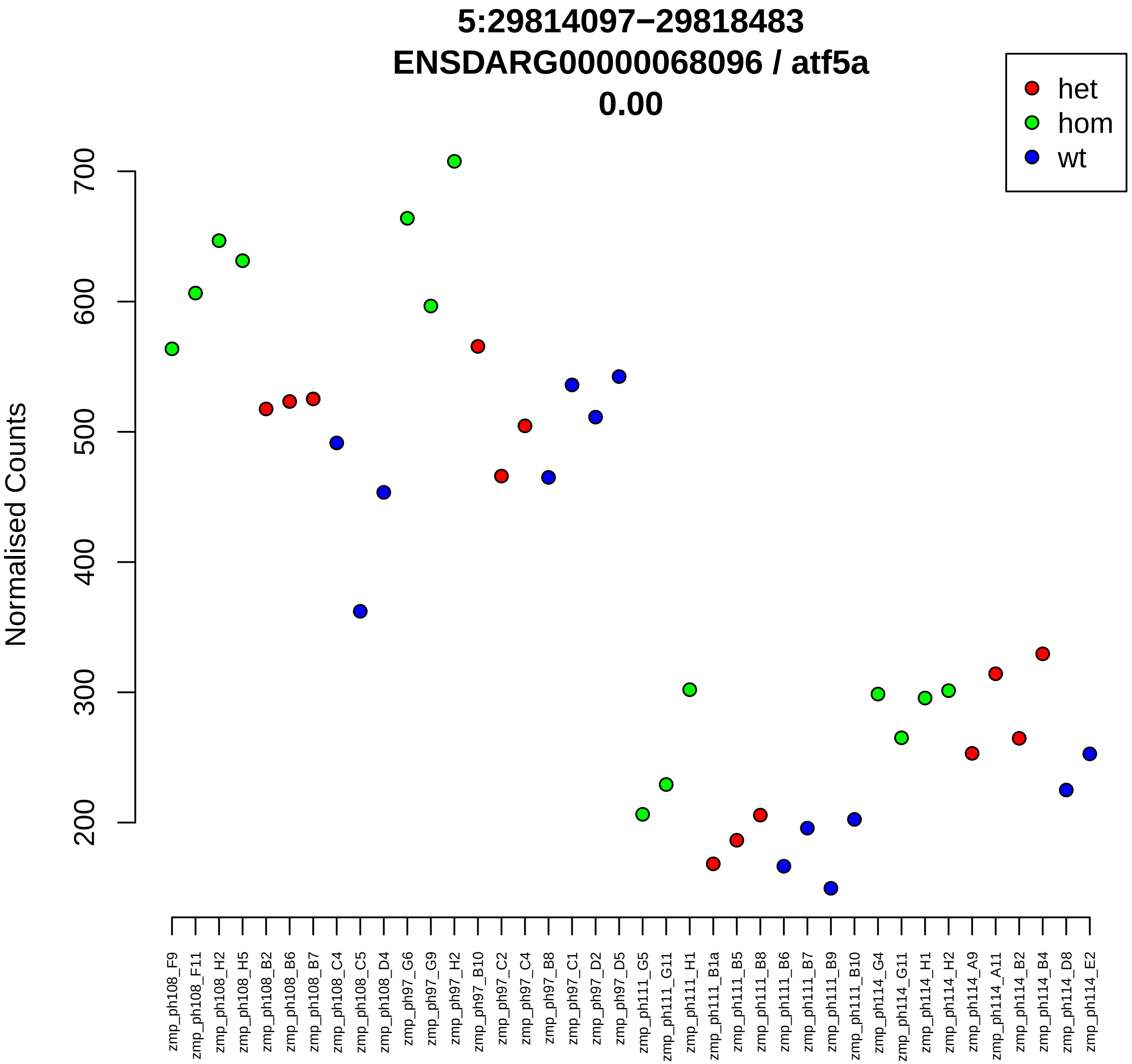

5:29814097−29818483
ENSDARG00000068096 / atf5a
0.00
het
hom
wt
Normalised Counts
200
300
400
500
600
700
zmp_ph108_F9
zmp_ph108_F11
zmp_ph108_H2
zmp_ph108_H5
zmp_ph108_B2
zmp_ph108_B6
zmp_ph108_B7
zmp_ph108_C4
zmp_ph108_C5
zmp_ph108_D4
zmp_ph97_G6
zmp_ph97_G9
zmp_ph97_H2
zmp_ph97_B10
zmp_ph97_C2
zmp_ph97_C4
zmp_ph97_B8
zmp_ph97_C1
zmp_ph97_D2
zmp_ph97_D5
zmp_ph111_G5
zmp_ph111_G11
zmp_ph111_H1
zmp_ph111_B1a
zmp_ph111_B5
zmp_ph111_B8
zmp_ph111_B6
zmp_ph111_B7
zmp_ph111_B9
zmp_ph111_B10
zmp_ph114_G4
zmp_ph114_G11
zmp_ph114_H1
zmp_ph114_H2
zmp_ph114_A9
zmp_ph114_A11
zmp_ph114_B2
zmp_ph114_B4
zmp_ph114_D8
zmp_ph114_E2

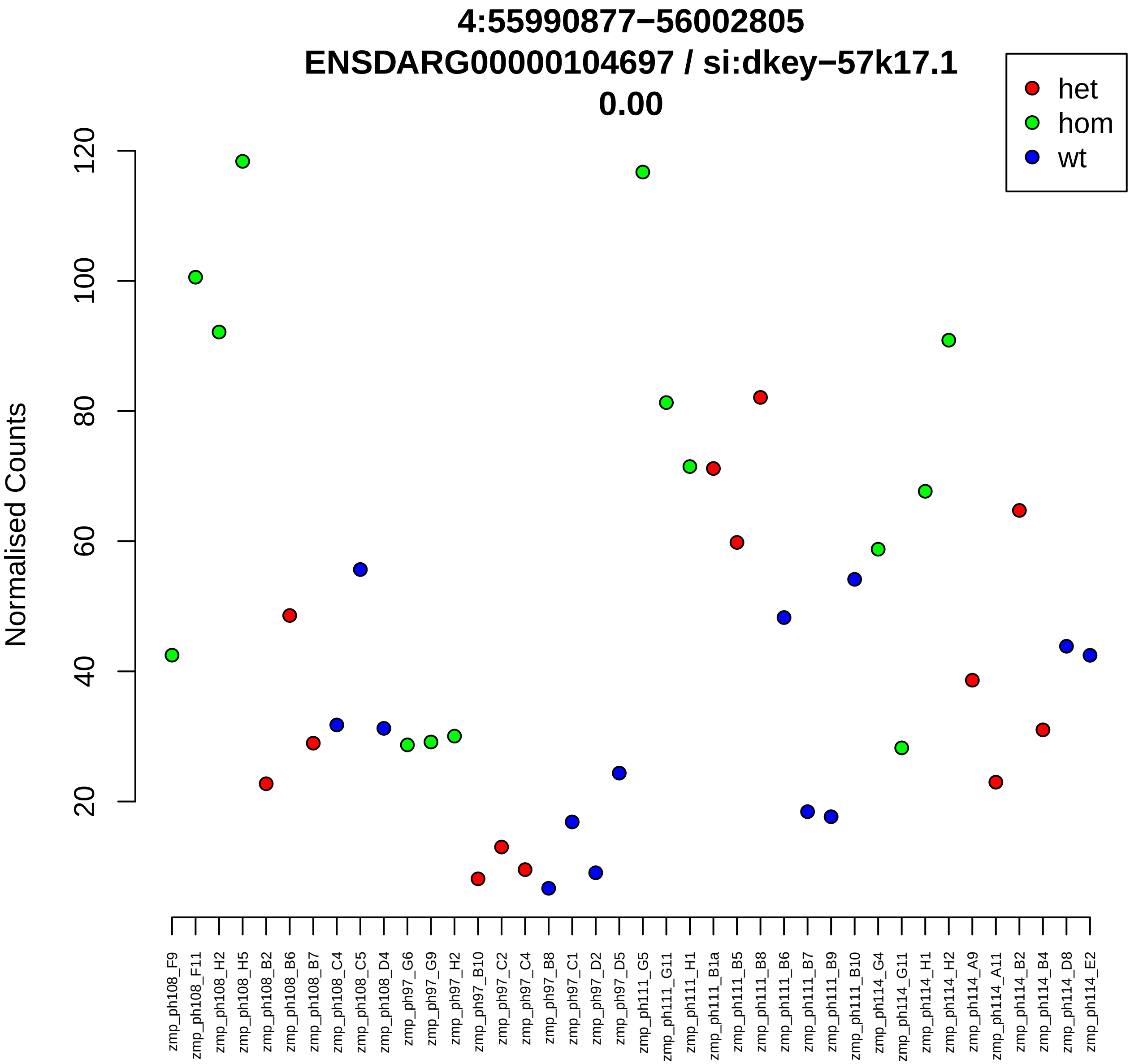

4:55990877−56002805
ENSDARG00000104697 / si:dkey−57k17.1
0.00
het
hom
wt
Normalised Counts
20
40
60
80
100
120
zmp_ph108_F9
zmp_ph108_F11
zmp_ph108_H2
zmp_ph108_H5
zmp_ph108_B2
zmp_ph108_B6
zmp_ph108_B7
zmp_ph108_C4
zmp_ph108_C5
zmp_ph108_D4
zmp_ph97_G6
zmp_ph97_G9
zmp_ph97_H2
zmp_ph97_B10
zmp_ph97_C2
zmp_ph97_C4
zmp_ph97_B8
zmp_ph97_C1
zmp_ph97_D2
zmp_ph97_D5
zmp_ph111_G5
zmp_ph111_G11
zmp_ph111_H1
zmp_ph111_B1a
zmp_ph111_B5
zmp_ph111_B8
zmp_ph111_B6
zmp_ph111_B7
zmp_ph111_B9
zmp_ph111_B10
zmp_ph114_G4
zmp_ph114_G11
zmp_ph114_H1
zmp_ph114_H2
zmp_ph114_A9
zmp_ph114_A11
zmp_ph114_B2
zmp_ph114_B4
zmp_ph114_D8
zmp_ph114_E2

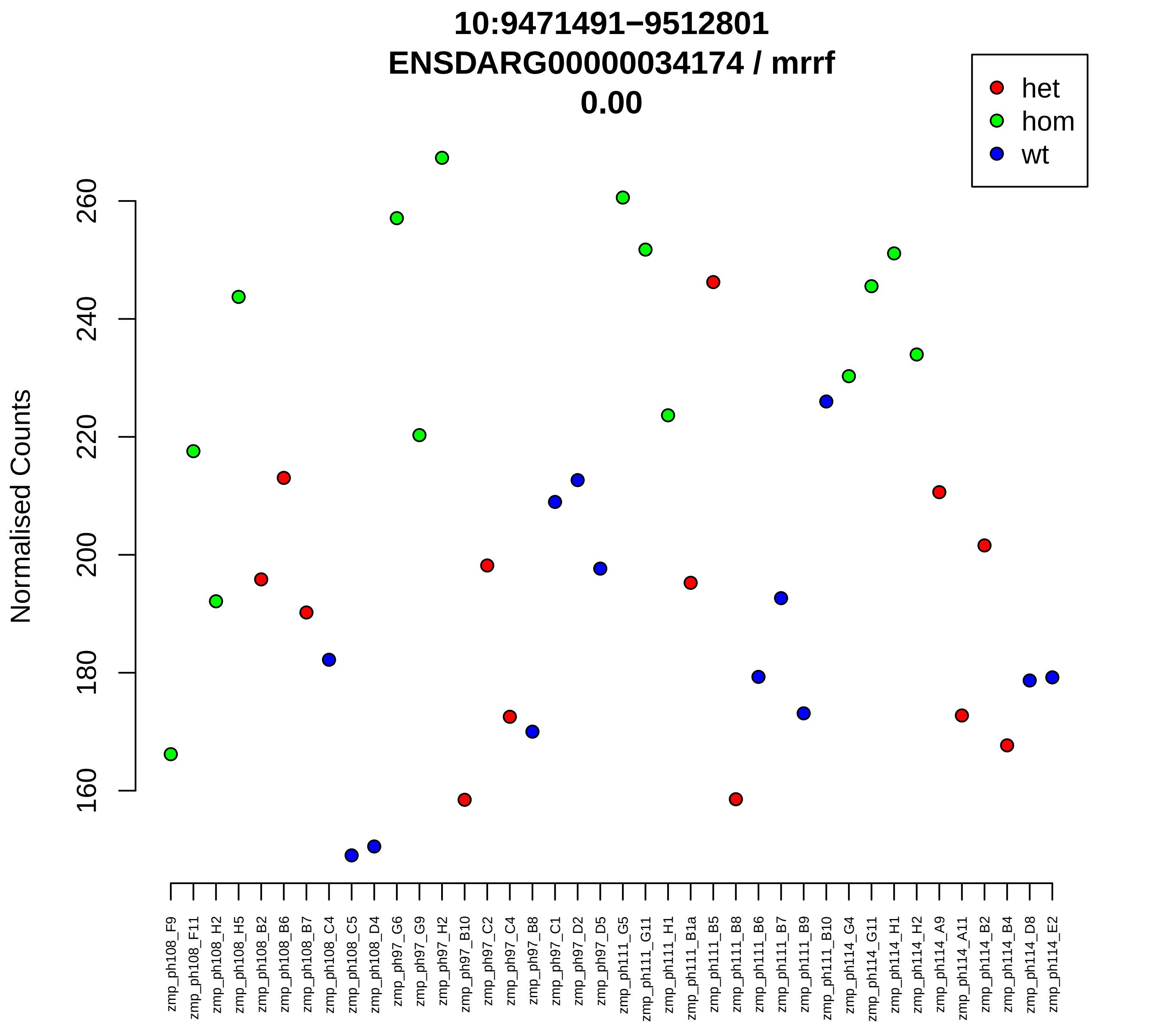

10:9471491−9512801
ENSDARG00000034174 / mrrf
0.00
het
hom
wt
Normalised Counts
160
180
200
220
240
260
zmp_ph108_F9
zmp_ph108_F11
zmp_ph108_H2
zmp_ph108_H5
zmp_ph108_B2
zmp_ph108_B6
zmp_ph108_B7
zmp_ph108_C4
zmp_ph108_C5
zmp_ph108_D4
zmp_ph97_G6
zmp_ph97_G9
zmp_ph97_H2
zmp_ph97_B10
zmp_ph97_C2
zmp_ph97_C4
zmp_ph97_B8
zmp_ph97_C1
zmp_ph97_D2
zmp_ph97_D5
zmp_ph111_G5
zmp_ph111_G11
zmp_ph111_H1
zmp_ph111_B1a
zmp_ph111_B5
zmp_ph111_B8
zmp_ph111_B6
zmp_ph111_B7
zmp_ph111_B9
zmp_ph111_B10
zmp_ph114_G4
zmp_ph114_G11
zmp_ph114_H1
zmp_ph114_H2
zmp_ph114_A9
zmp_ph114_A11
zmp_ph114_B2
zmp_ph114_B4
zmp_ph114_D8
zmp_ph114_E2

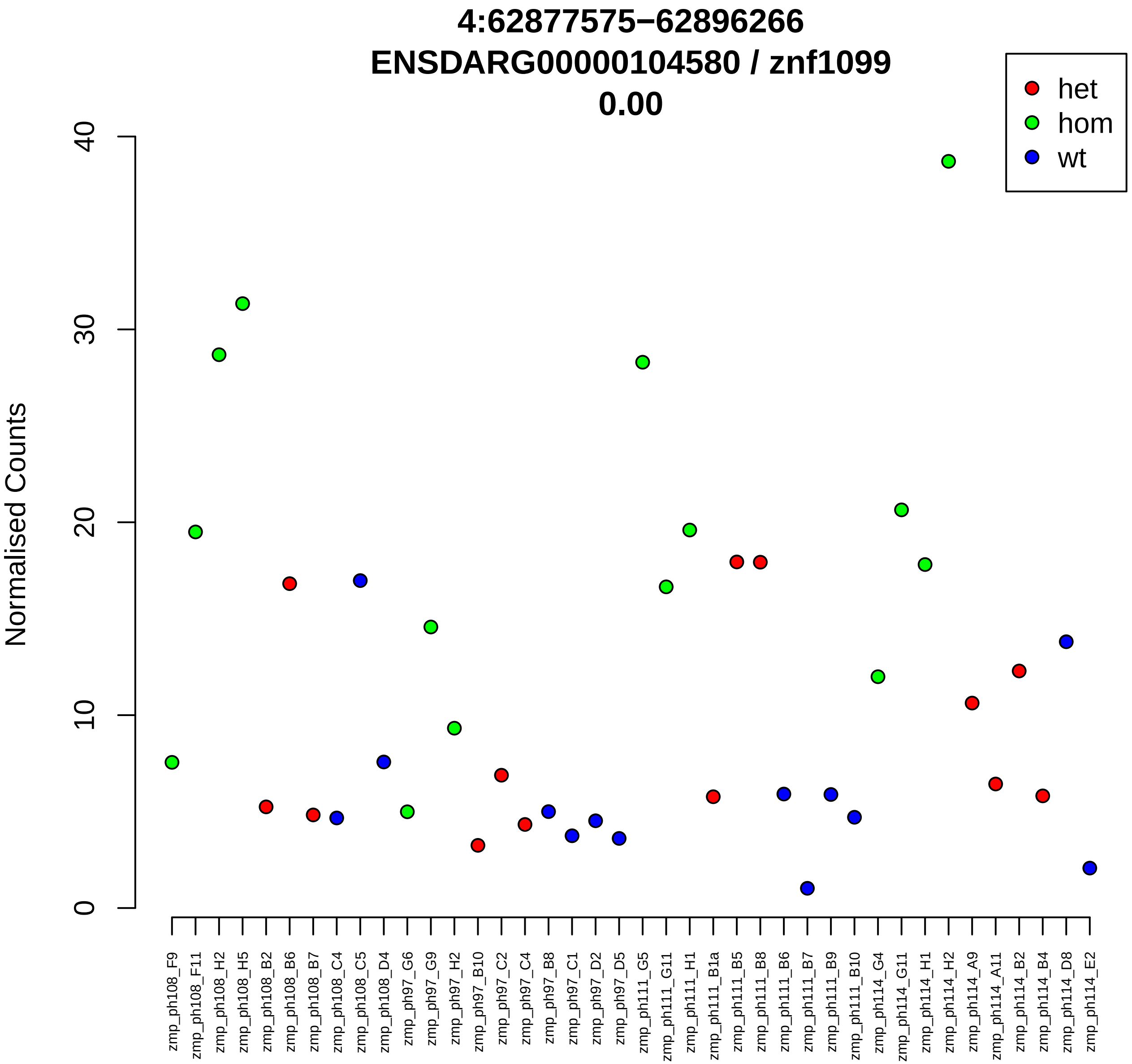
4:62877575−62896266
ENSDARG00000104580 / znf1099
0.00
het
hom
wt
Normalised Counts
0
10
20
30
40
zmp_ph108_F9
zmp_ph108_F11
zmp_ph108_H2
zmp_ph108_H5
zmp_ph108_B2
zmp_ph108_B6
zmp_ph108_B7
zmp_ph108_C4
zmp_ph108_C5
zmp_ph108_D4
zmp_ph97_G6
zmp_ph97_G9
zmp_ph97_H2
zmp_ph97_B10
zmp_ph97_C2
zmp_ph97_C4
zmp_ph97_B8
zmp_ph97_C1
zmp_ph97_D2
zmp_ph97_D5
zmp_ph111_G5
zmp_ph111_G11
zmp_ph111_H1
zmp_ph111_B1a
zmp_ph111_B5
zmp_ph111_B8
zmp_ph111_B6
zmp_ph111_B7
zmp_ph111_B9
zmp_ph111_B10
zmp_ph114_G4
zmp_ph114_G11
zmp_ph114_H1
zmp_ph114_H2
zmp_ph114_A9
zmp_ph114_A11
zmp_ph114_B2
zmp_ph114_B4
zmp_ph114_D8
zmp_ph114_E2

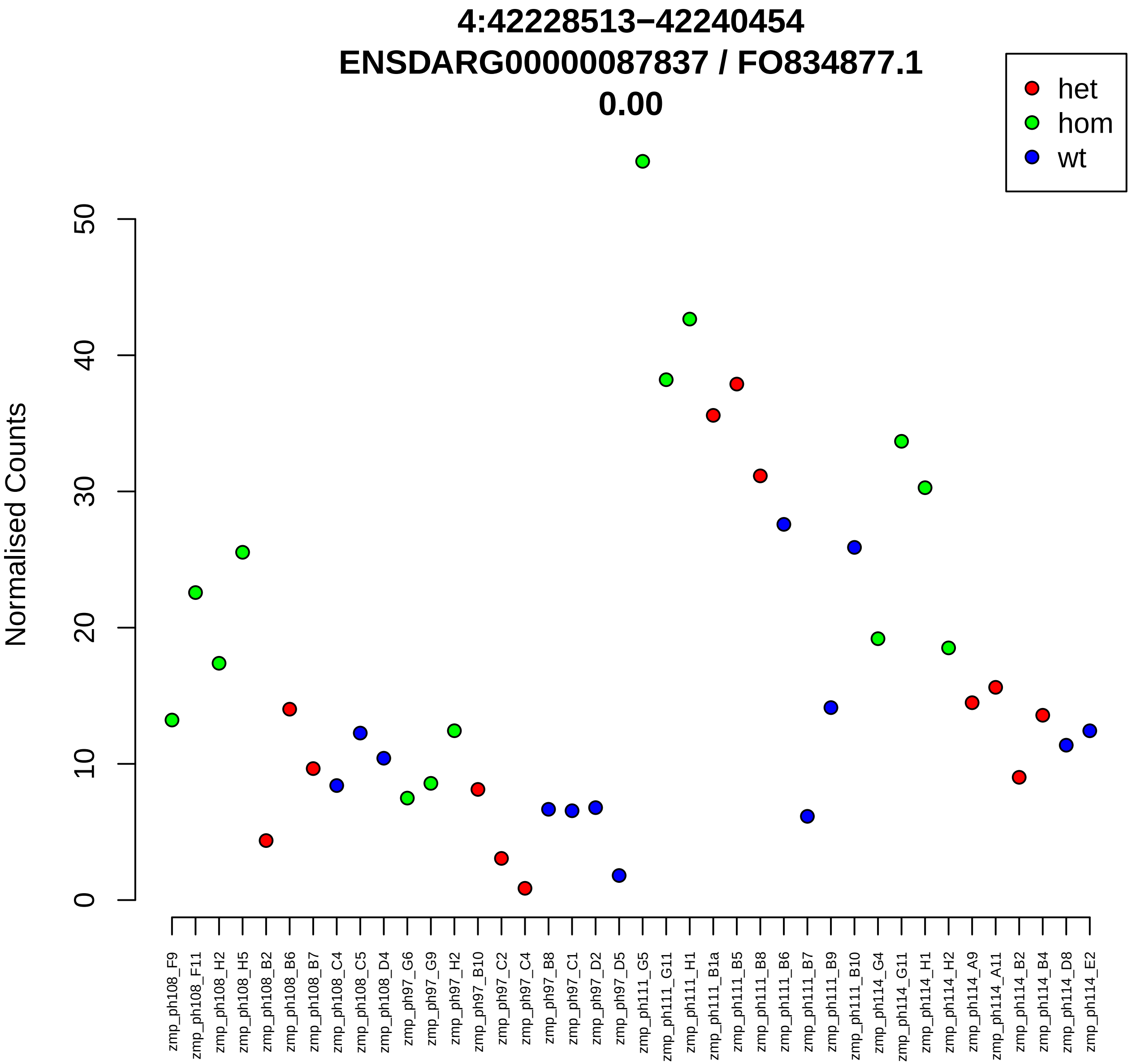
4:42228513−42240454
ENSDARG00000087837 / FO834877.1
0.00
het
hom
wt
Normalised Counts
0
10
20
30
40
50
zmp_ph108_F9
zmp_ph108_F11
zmp_ph108_H2
zmp_ph108_H5
zmp_ph108_B2
zmp_ph108_B6
zmp_ph108_B7
zmp_ph108_C4
zmp_ph108_C5
zmp_ph108_D4
zmp_ph97_G6
zmp_ph97_G9
zmp_ph97_H2
zmp_ph97_B10
zmp_ph97_C2
zmp_ph97_C4
zmp_ph97_B8
zmp_ph97_C1
zmp_ph97_D2
zmp_ph97_D5
zmp_ph111_G5
zmp_ph111_G11
zmp_ph111_H1
zmp_ph111_B1a
zmp_ph111_B5
zmp_ph111_B8
zmp_ph111_B6
zmp_ph111_B7
zmp_ph111_B9
zmp_ph111_B10
zmp_ph114_G4
zmp_ph114_G11
zmp_ph114_H1
zmp_ph114_H2
zmp_ph114_A9
zmp_ph114_A11
zmp_ph114_B2
zmp_ph114_B4
zmp_ph114_D8
zmp_ph114_E2

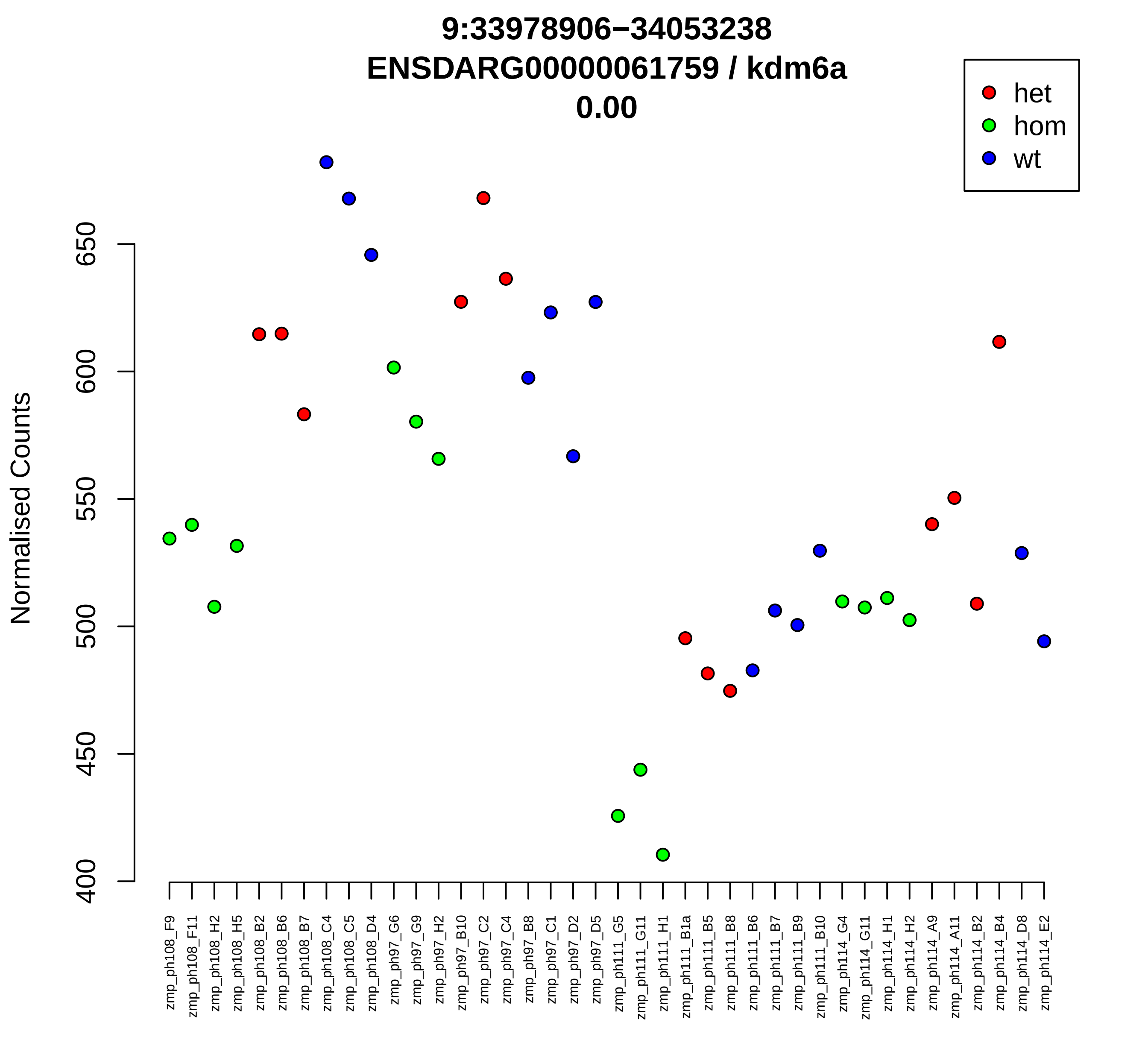

9:33978906−34053238
ENSDARG00000061759 / kdm6a
0.00
het
hom
wt
Normalised Counts
400
450
500
550
600
650
zmp_ph108_F9
zmp_ph108_F11
zmp_ph108_H2
zmp_ph108_H5
zmp_ph108_B2
zmp_ph108_B6
zmp_ph108_B7
zmp_ph108_C4
zmp_ph108_C5
zmp_ph108_D4
zmp_ph97_G6
zmp_ph97_G9
zmp_ph97_H2
zmp_ph97_B10
zmp_ph97_C2
zmp_ph97_C4
zmp_ph97_B8
zmp_ph97_C1
zmp_ph97_D2
zmp_ph97_D5
zmp_ph111_G5
zmp_ph111_G11
zmp_ph111_H1
zmp_ph111_B1a
zmp_ph111_B5
zmp_ph111_B8
zmp_ph111_B6
zmp_ph111_B7
zmp_ph111_B9
zmp_ph111_B10
zmp_ph114_G4
zmp_ph114_G11
zmp_ph114_H1
zmp_ph114_H2
zmp_ph114_A9
zmp_ph114_A11
zmp_ph114_B2
zmp_ph114_B4
zmp_ph114_D8
zmp_ph114_E2

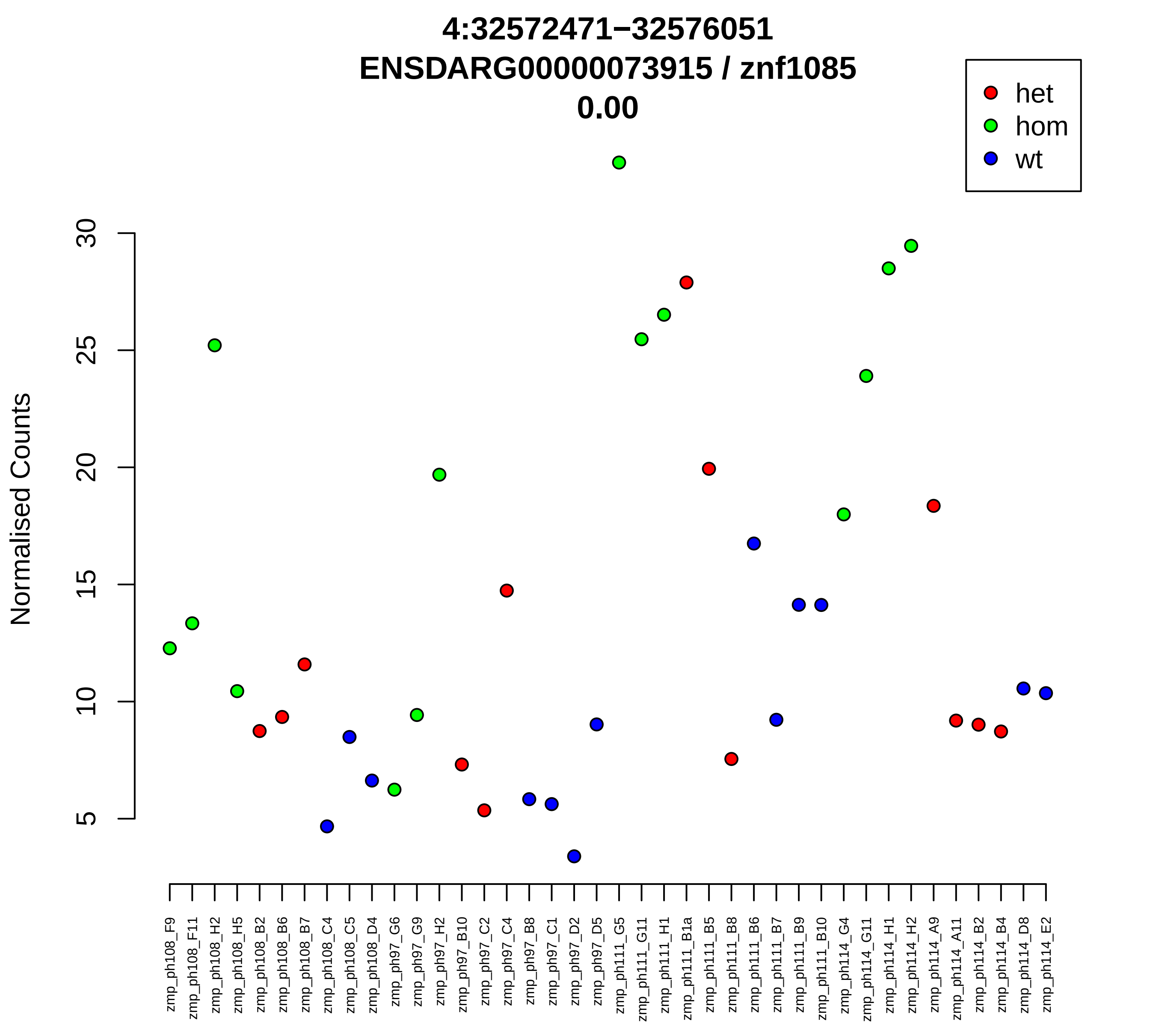
4:32572471−32576051
ENSDARG00000073915 / znf1085
0.00
het
hom
wt
Normalised Counts
5
10
15
20
25
30
zmp_ph108_F9
zmp_ph108_F11
zmp_ph108_H2
zmp_ph108_H5
zmp_ph108_B2
zmp_ph108_B6
zmp_ph108_B7
zmp_ph108_C4
zmp_ph108_C5
zmp_ph108_D4
zmp_ph97_G6
zmp_ph97_G9
zmp_ph97_H2
zmp_ph97_B10
zmp_ph97_C2
zmp_ph97_C4
zmp_ph97_B8
zmp_ph97_C1
zmp_ph97_D2
zmp_ph97_D5
zmp_ph111_G5
zmp_ph111_G11
zmp_ph111_H1
zmp_ph111_B1a
zmp_ph111_B5
zmp_ph111_B8
zmp_ph111_B6
zmp_ph111_B7
zmp_ph111_B9
zmp_ph111_B10
zmp_ph114_G4
zmp_ph114_G11
zmp_ph114_H1
zmp_ph114_H2
zmp_ph114_A9
zmp_ph114_A11
zmp_ph114_B2
zmp_ph114_B4
zmp_ph114_D8
zmp_ph114_E2

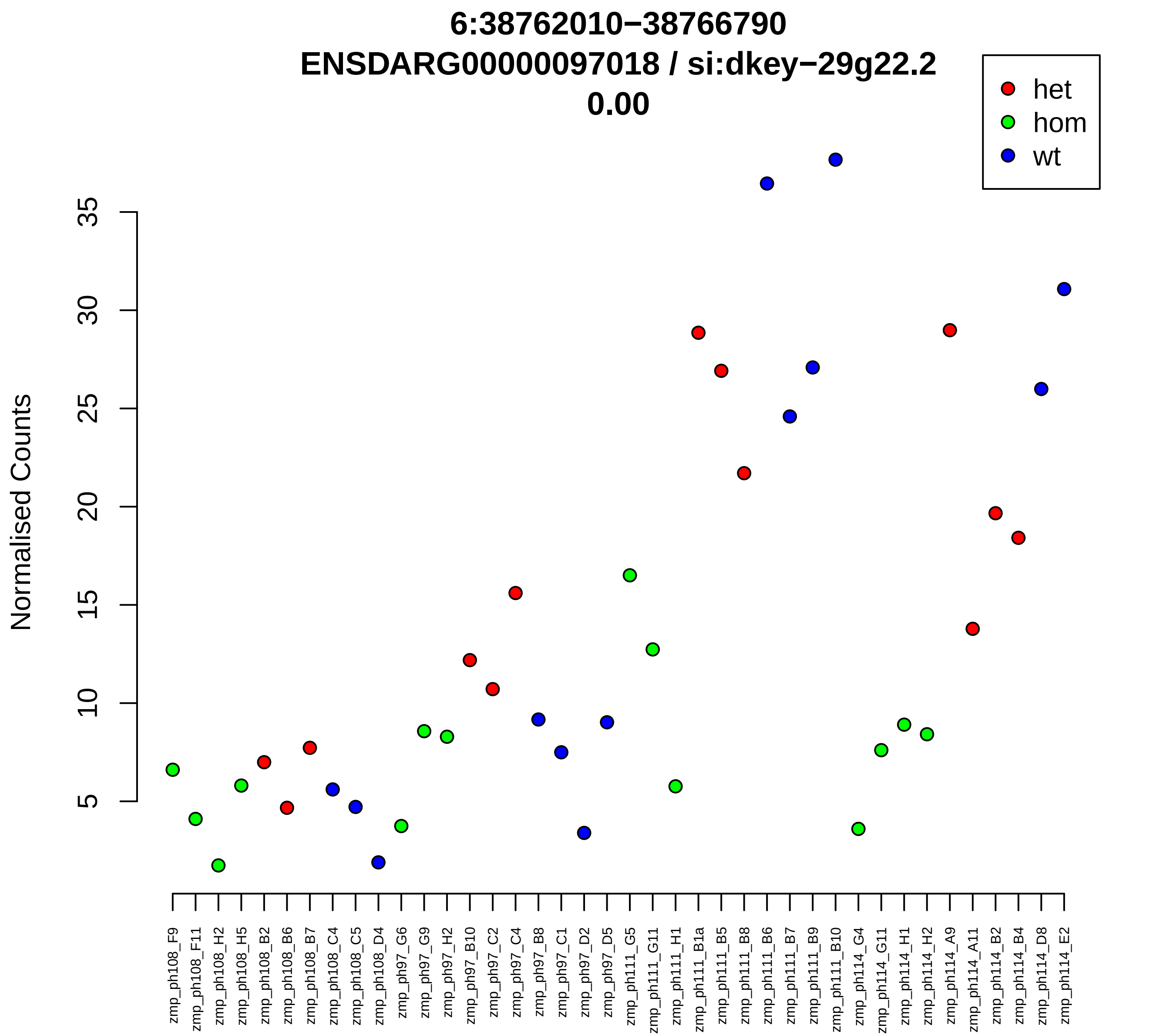

6:38762010−38766790
ENSDARG00000097018 / si:dkey−29g22.2
0.00
het
hom
wt
Normalised Counts
5
10
15
20
25
30
35
zmp_ph108_F9
zmp_ph108_F11
zmp_ph108_H2
zmp_ph108_H5
zmp_ph108_B2
zmp_ph108_B6
zmp_ph108_B7
zmp_ph108_C4
zmp_ph108_C5
zmp_ph108_D4
zmp_ph97_G6
zmp_ph97_G9
zmp_ph97_H2
zmp_ph97_B10
zmp_ph97_C2
zmp_ph97_C4
zmp_ph97_B8
zmp_ph97_C1
zmp_ph97_D2
zmp_ph97_D5
zmp_ph111_G5
zmp_ph111_G11
zmp_ph111_H1
zmp_ph111_B1a
zmp_ph111_B5
zmp_ph111_B8
zmp_ph111_B6
zmp_ph111_B7
zmp_ph111_B9
zmp_ph111_B10
zmp_ph114_G4
zmp_ph114_G11
zmp_ph114_H1
zmp_ph114_H2
zmp_ph114_A9
zmp_ph114_A11
zmp_ph114_B2
zmp_ph114_B4
zmp_ph114_D8
zmp_ph114_E2

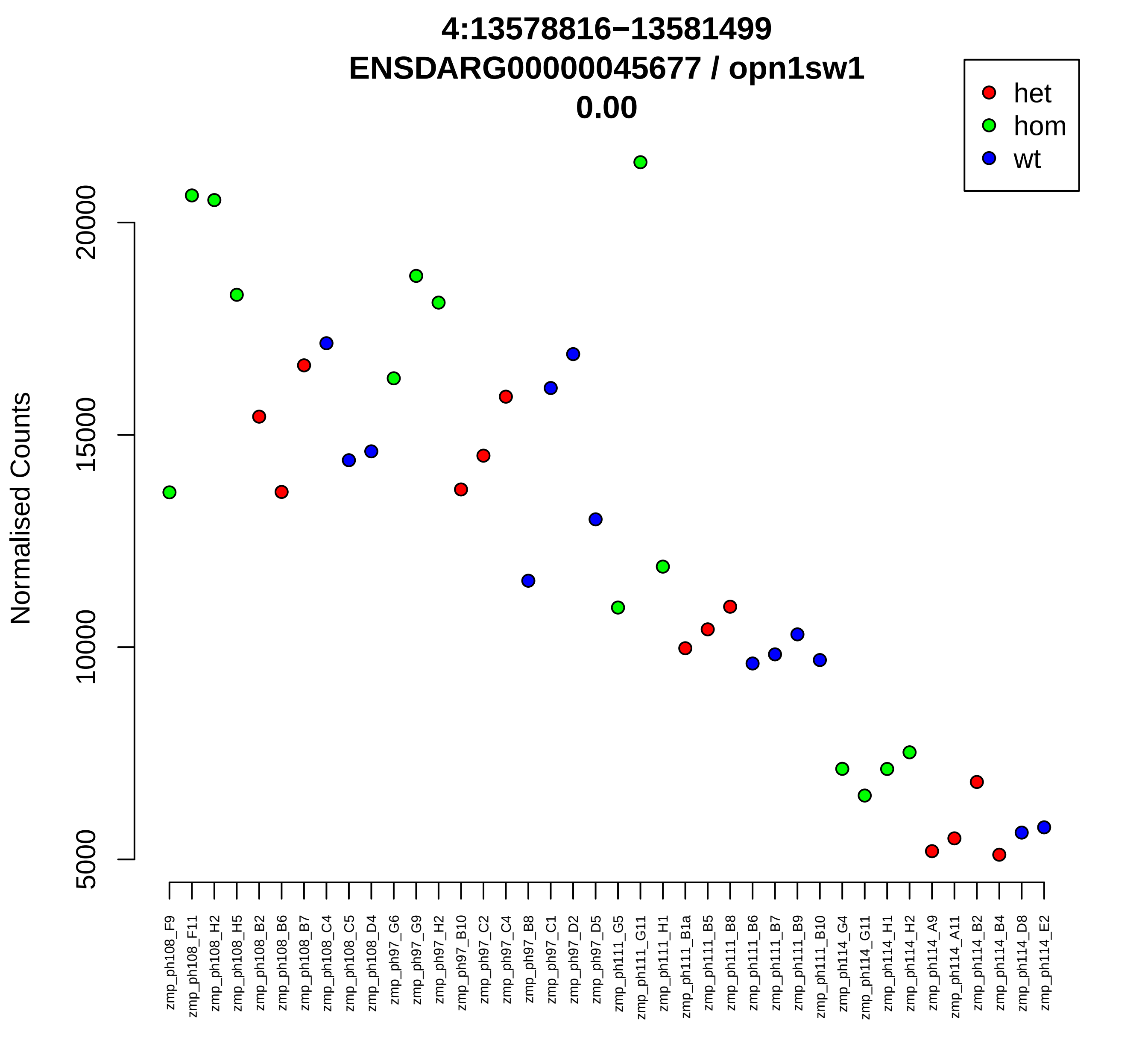
4:13578816−13581499
ENSDARG00000045677 / opn1sw1
0.00
het
hom
wt
Normalised Counts
5000
10000
15000
20000
zmp_ph108_F9
zmp_ph108_F11
zmp_ph108_H2
zmp_ph108_H5
zmp_ph108_B2
zmp_ph108_B6
zmp_ph108_B7
zmp_ph108_C4
zmp_ph108_C5
zmp_ph108_D4
zmp_ph97_G6
zmp_ph97_G9
zmp_ph97_H2
zmp_ph97_B10
zmp_ph97_C2
zmp_ph97_C4
zmp_ph97_B8
zmp_ph97_C1
zmp_ph97_D2
zmp_ph97_D5
zmp_ph111_G5
zmp_ph111_G11
zmp_ph111_H1
zmp_ph111_B1a
zmp_ph111_B5
zmp_ph111_B8
zmp_ph111_B6
zmp_ph111_B7
zmp_ph111_B9
zmp_ph111_B10
zmp_ph114_G4
zmp_ph114_G11
zmp_ph114_H1
zmp_ph114_H2
zmp_ph114_A9
zmp_ph114_A11
zmp_ph114_B2
zmp_ph114_B4
zmp_ph114_D8
zmp_ph114_E2

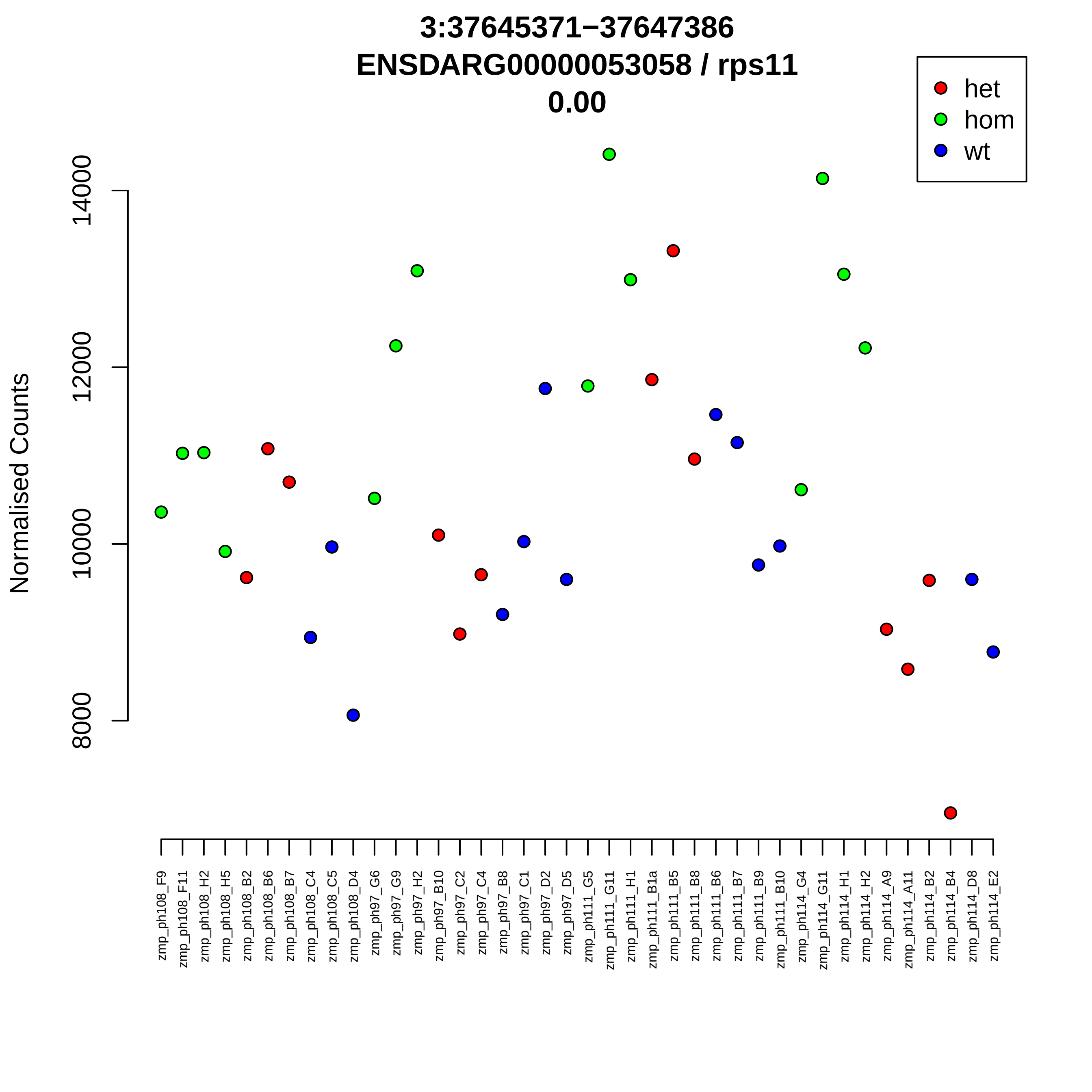

3:37645371−37647386
ENSDARG00000053058 / rps11
0.00
het
hom
wt
Normalised Counts
8000
10000
12000
14000
zmp_ph108_F9
zmp_ph108_F11
zmp_ph108_H2
zmp_ph108_H5
zmp_ph108_B2
zmp_ph108_B6
zmp_ph108_B7
zmp_ph108_C4
zmp_ph108_C5
zmp_ph108_D4
zmp_ph97_G6
zmp_ph97_G9
zmp_ph97_H2
zmp_ph97_B10
zmp_ph97_C2
zmp_ph97_C4
zmp_ph97_B8
zmp_ph97_C1
zmp_ph97_D2
zmp_ph97_D5
zmp_ph111_G5
zmp_ph111_G11
zmp_ph111_H1
zmp_ph111_B1a
zmp_ph111_B5
zmp_ph111_B8
zmp_ph111_B6
zmp_ph111_B7
zmp_ph111_B9
zmp_ph111_B10
zmp_ph114_G4
zmp_ph114_G11
zmp_ph114_H1
zmp_ph114_H2
zmp_ph114_A9
zmp_ph114_A11
zmp_ph114_B2
zmp_ph114_B4
zmp_ph114_D8
zmp_ph114_E2

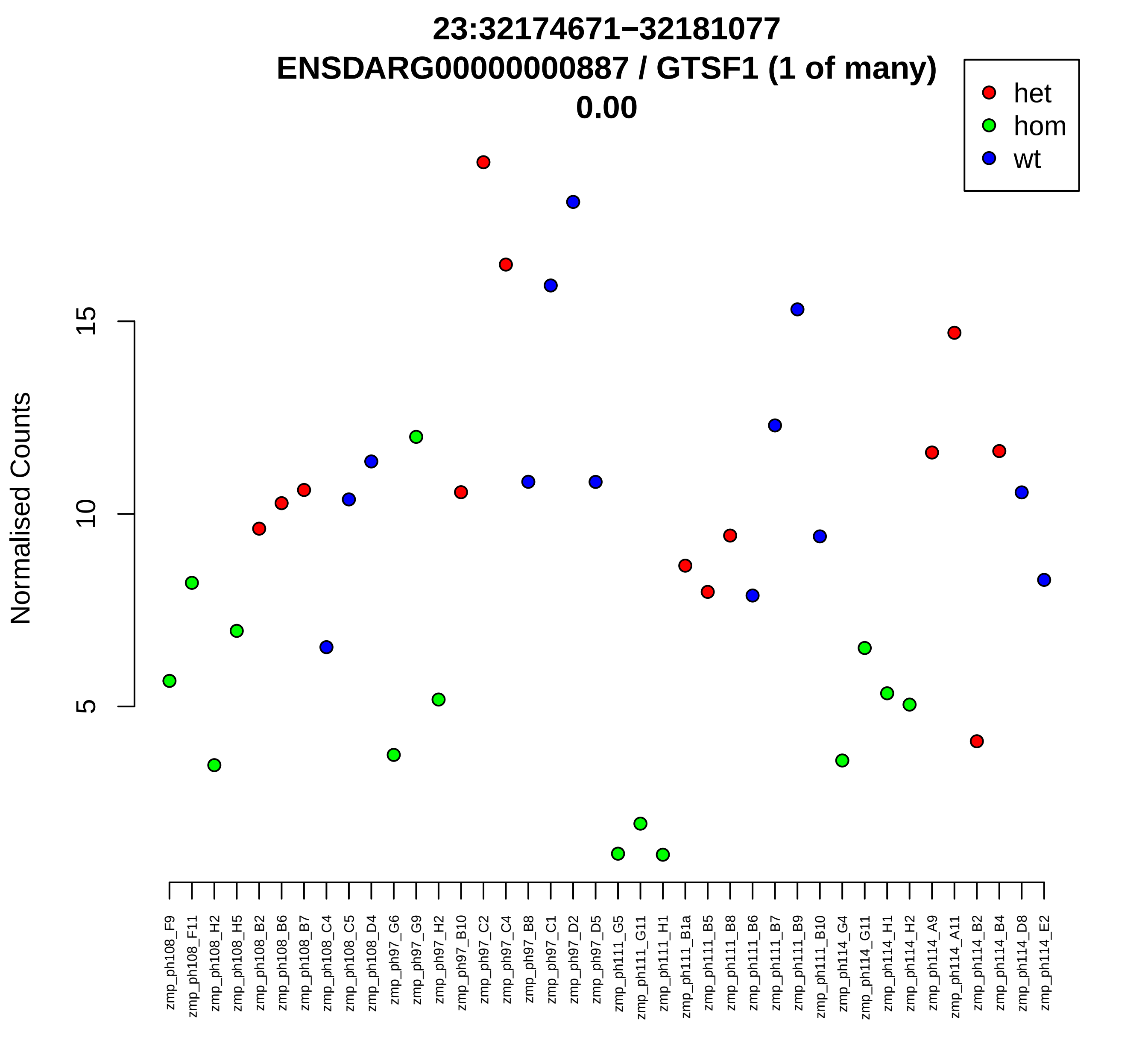
23:32174671−32181077
ENSDARG00000000887 / GTSF1 (1 of many)
0.00
het
hom
wt
Normalised Counts
5
10
15
zmp_ph108_F9
zmp_ph108_F11
zmp_ph108_H2
zmp_ph108_H5
zmp_ph108_B2
zmp_ph108_B6
zmp_ph108_B7
zmp_ph108_C4
zmp_ph108_C5
zmp_ph108_D4
zmp_ph97_G6
zmp_ph97_G9
zmp_ph97_H2
zmp_ph97_B10
zmp_ph97_C2
zmp_ph97_C4
zmp_ph97_B8
zmp_ph97_C1
zmp_ph97_D2
zmp_ph97_D5
zmp_ph111_G5
zmp_ph111_G11
zmp_ph111_H1
zmp_ph111_B1a
zmp_ph111_B5
zmp_ph111_B8
zmp_ph111_B6
zmp_ph111_B7
zmp_ph111_B9
zmp_ph111_B10
zmp_ph114_G4
zmp_ph114_G11
zmp_ph114_H1
zmp_ph114_H2
zmp_ph114_A9
zmp_ph114_A11
zmp_ph114_B2
zmp_ph114_B4
zmp_ph114_D8
zmp_ph114_E2

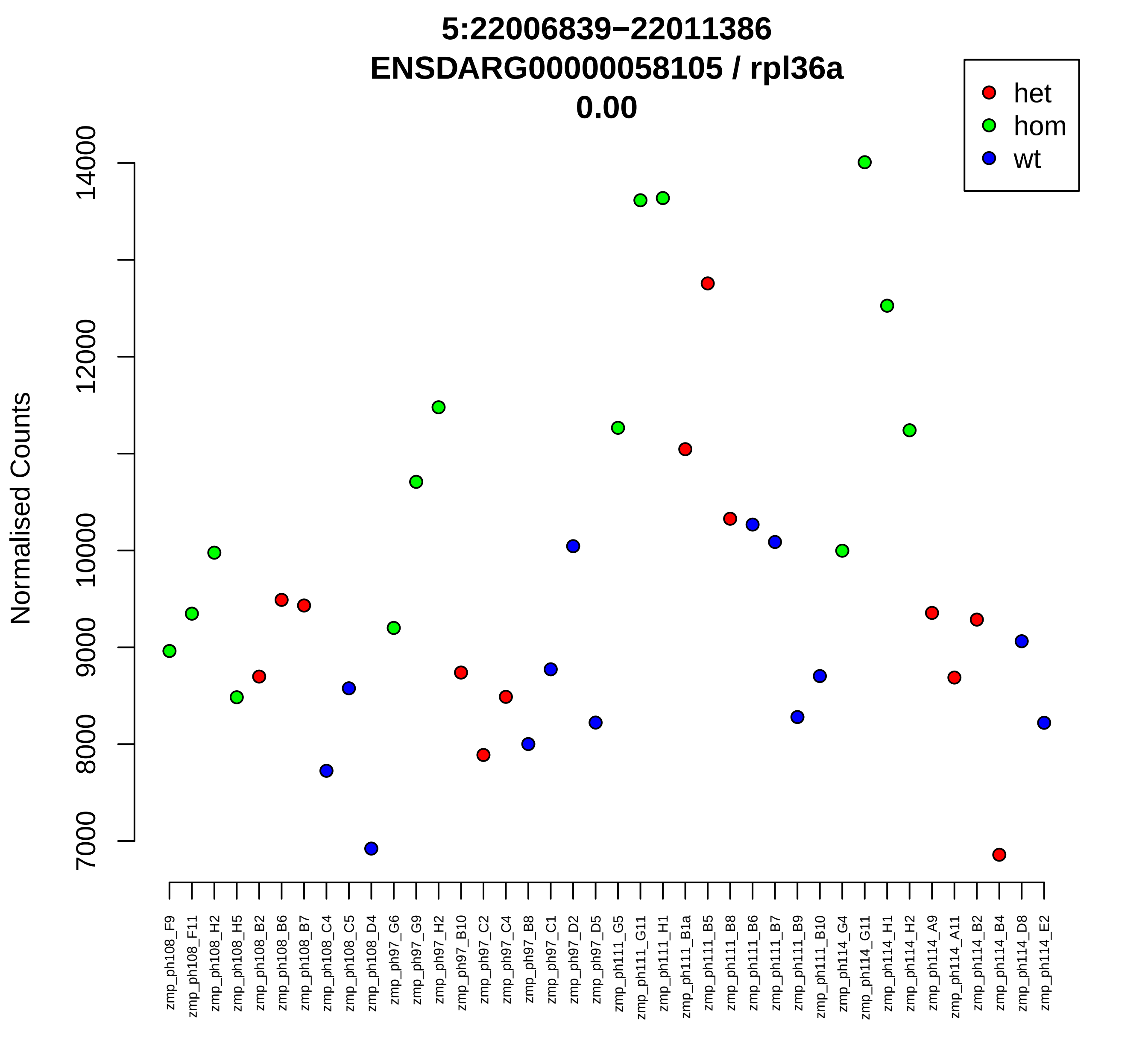

5:22006839−22011386
ENSDARG00000058105 / rpl36a
0.00
het
hom
wt
Normalised Counts
7000
8000
9000
10000
12000
14000
zmp_ph108_F9
zmp_ph108_F11
zmp_ph108_H2
zmp_ph108_H5
zmp_ph108_B2
zmp_ph108_B6
zmp_ph108_B7
zmp_ph108_C4
zmp_ph108_C5
zmp_ph108_D4
zmp_ph97_G6
zmp_ph97_G9
zmp_ph97_H2
zmp_ph97_B10
zmp_ph97_C2
zmp_ph97_C4
zmp_ph97_B8
zmp_ph97_C1
zmp_ph97_D2
zmp_ph97_D5
zmp_ph111_G5
zmp_ph111_G11
zmp_ph111_H1
zmp_ph111_B1a
zmp_ph111_B5
zmp_ph111_B8
zmp_ph111_B6
zmp_ph111_B7
zmp_ph111_B9
zmp_ph111_B10
zmp_ph114_G4
zmp_ph114_G11
zmp_ph114_H1
zmp_ph114_H2
zmp_ph114_A9
zmp_ph114_A11
zmp_ph114_B2
zmp_ph114_B4
zmp_ph114_D8
zmp_ph114_E2

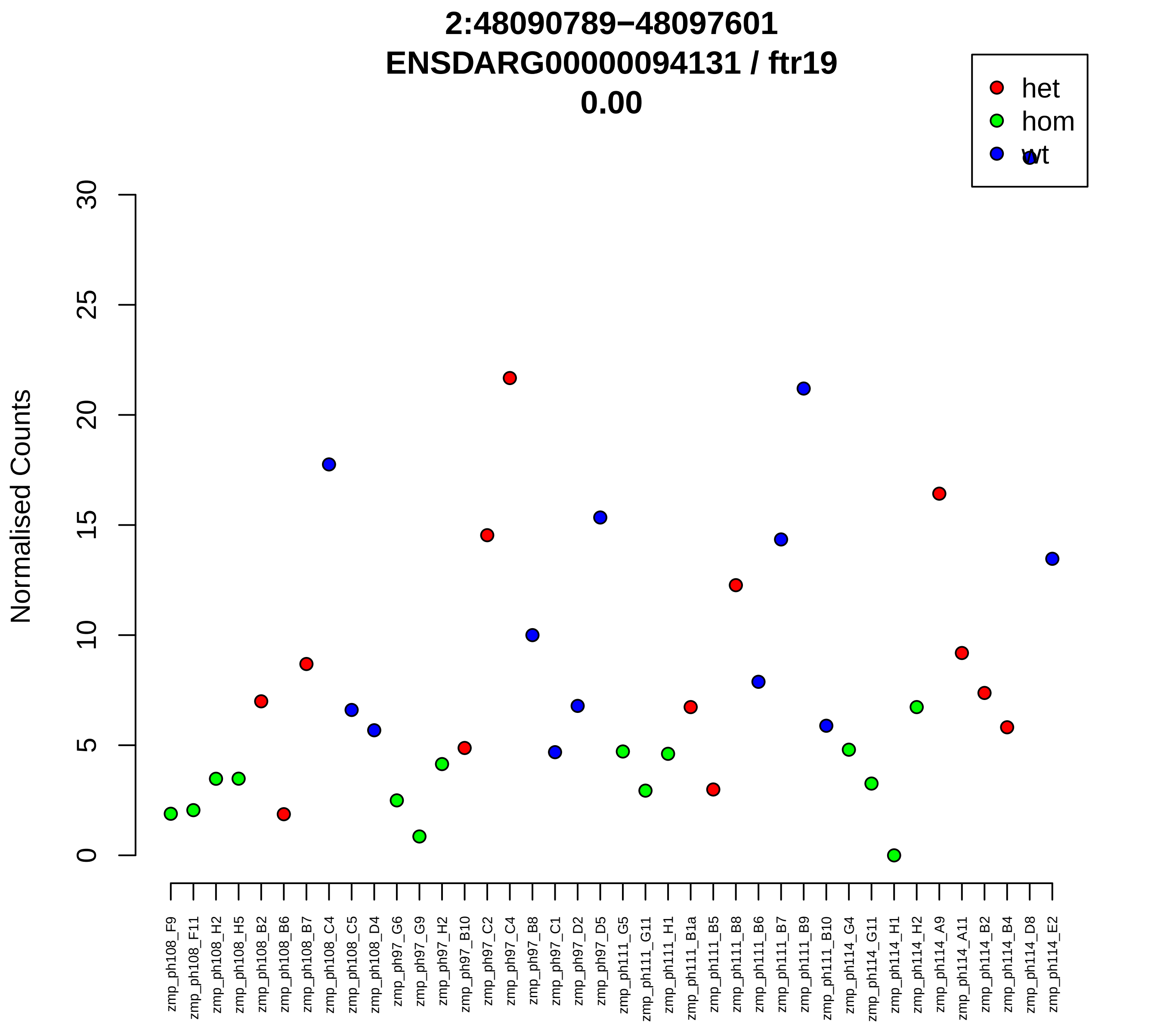
2:48090789−48097601
ENSDARG00000094131 / ftr19
0.00
het
hom
wt
Normalised Counts
0
5
10
15
20
25
30
zmp_ph108_F9
zmp_ph108_F11
zmp_ph108_H2
zmp_ph108_H5
zmp_ph108_B2
zmp_ph108_B6
zmp_ph108_B7
zmp_ph108_C4
zmp_ph108_C5
zmp_ph108_D4
zmp_ph97_G6
zmp_ph97_G9
zmp_ph97_H2
zmp_ph97_B10
zmp_ph97_C2
zmp_ph97_C4
zmp_ph97_B8
zmp_ph97_C1
zmp_ph97_D2
zmp_ph97_D5
zmp_ph111_G5
zmp_ph111_G11
zmp_ph111_H1
zmp_ph111_B1a
zmp_ph111_B5
zmp_ph111_B8
zmp_ph111_B6
zmp_ph111_B7
zmp_ph111_B9
zmp_ph111_B10
zmp_ph114_G4
zmp_ph114_G11
zmp_ph114_H1
zmp_ph114_H2
zmp_ph114_A9
zmp_ph114_A11
zmp_ph114_B2
zmp_ph114_B4
zmp_ph114_D8
zmp_ph114_E2

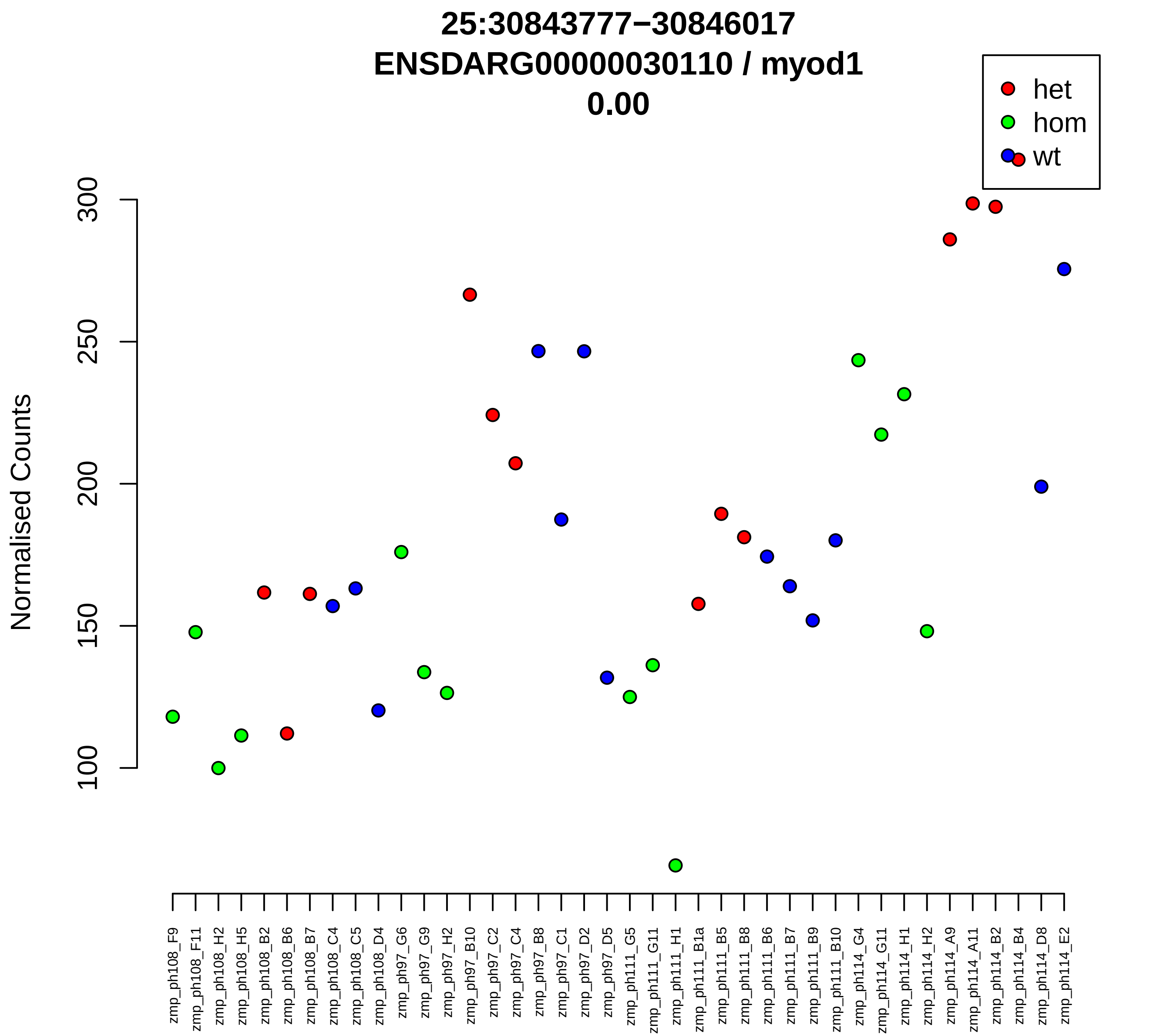
25:30843777−30846017
ENSDARG00000030110 / myod1
0.00
het
hom
wt
Normalised Counts
100
150
200
250
300
zmp_ph108_F9
zmp_ph108_F11
zmp_ph108_H2
zmp_ph108_H5
zmp_ph108_B2
zmp_ph108_B6
zmp_ph108_B7
zmp_ph108_C4
zmp_ph108_C5
zmp_ph108_D4
zmp_ph97_G6
zmp_ph97_G9
zmp_ph97_H2
zmp_ph97_B10
zmp_ph97_C2
zmp_ph97_C4
zmp_ph97_B8
zmp_ph97_C1
zmp_ph97_D2
zmp_ph97_D5
zmp_ph111_G5
zmp_ph111_G11
zmp_ph111_H1
zmp_ph111_B1a
zmp_ph111_B5
zmp_ph111_B8
zmp_ph111_B6
zmp_ph111_B7
zmp_ph111_B9
zmp_ph111_B10
zmp_ph114_G4
zmp_ph114_G11
zmp_ph114_H1
zmp_ph114_H2
zmp_ph114_A9
zmp_ph114_A11
zmp_ph114_B2
zmp_ph114_B4
zmp_ph114_D8
zmp_ph114_E2

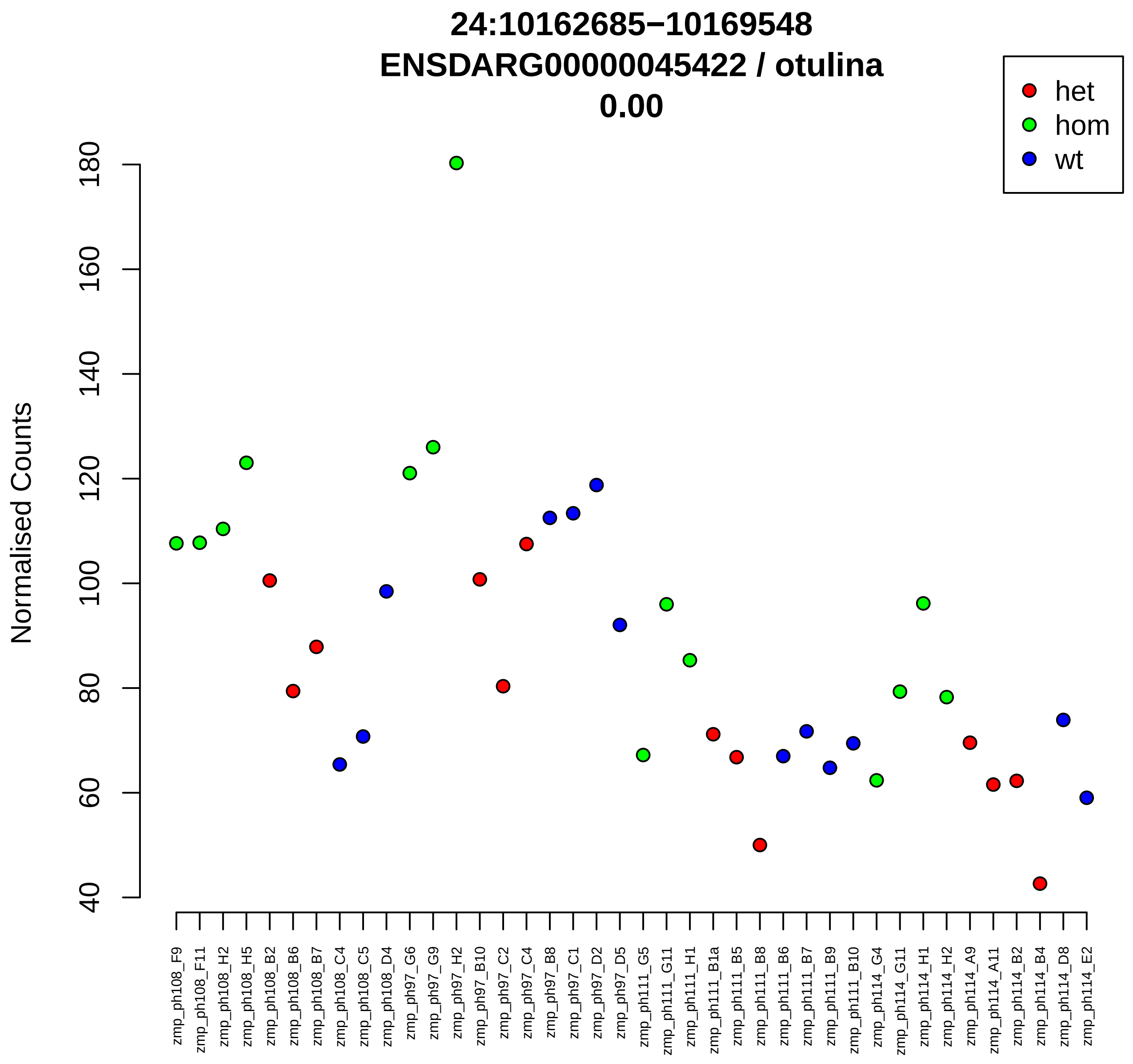
24:10162685−10169548
ENSDARG00000045422 / otulina
0.00
het
hom
wt
Normalised Counts
40
60
80
100
120
140
160
180
zmp_ph108_F9
zmp_ph108_F11
zmp_ph108_H2
zmp_ph108_H5
zmp_ph108_B2
zmp_ph108_B6
zmp_ph108_B7
zmp_ph108_C4
zmp_ph108_C5
zmp_ph108_D4
zmp_ph97_G6
zmp_ph97_G9
zmp_ph97_H2
zmp_ph97_B10
zmp_ph97_C2
zmp_ph97_C4
zmp_ph97_B8
zmp_ph97_C1
zmp_ph97_D2
zmp_ph97_D5
zmp_ph111_G5
zmp_ph111_G11
zmp_ph111_H1
zmp_ph111_B1a
zmp_ph111_B5
zmp_ph111_B8
zmp_ph111_B6
zmp_ph111_B7
zmp_ph111_B9
zmp_ph111_B10
zmp_ph114_G4
zmp_ph114_G11
zmp_ph114_H1
zmp_ph114_H2
zmp_ph114_A9
zmp_ph114_A11
zmp_ph114_B2
zmp_ph114_B4
zmp_ph114_D8
zmp_ph114_E2

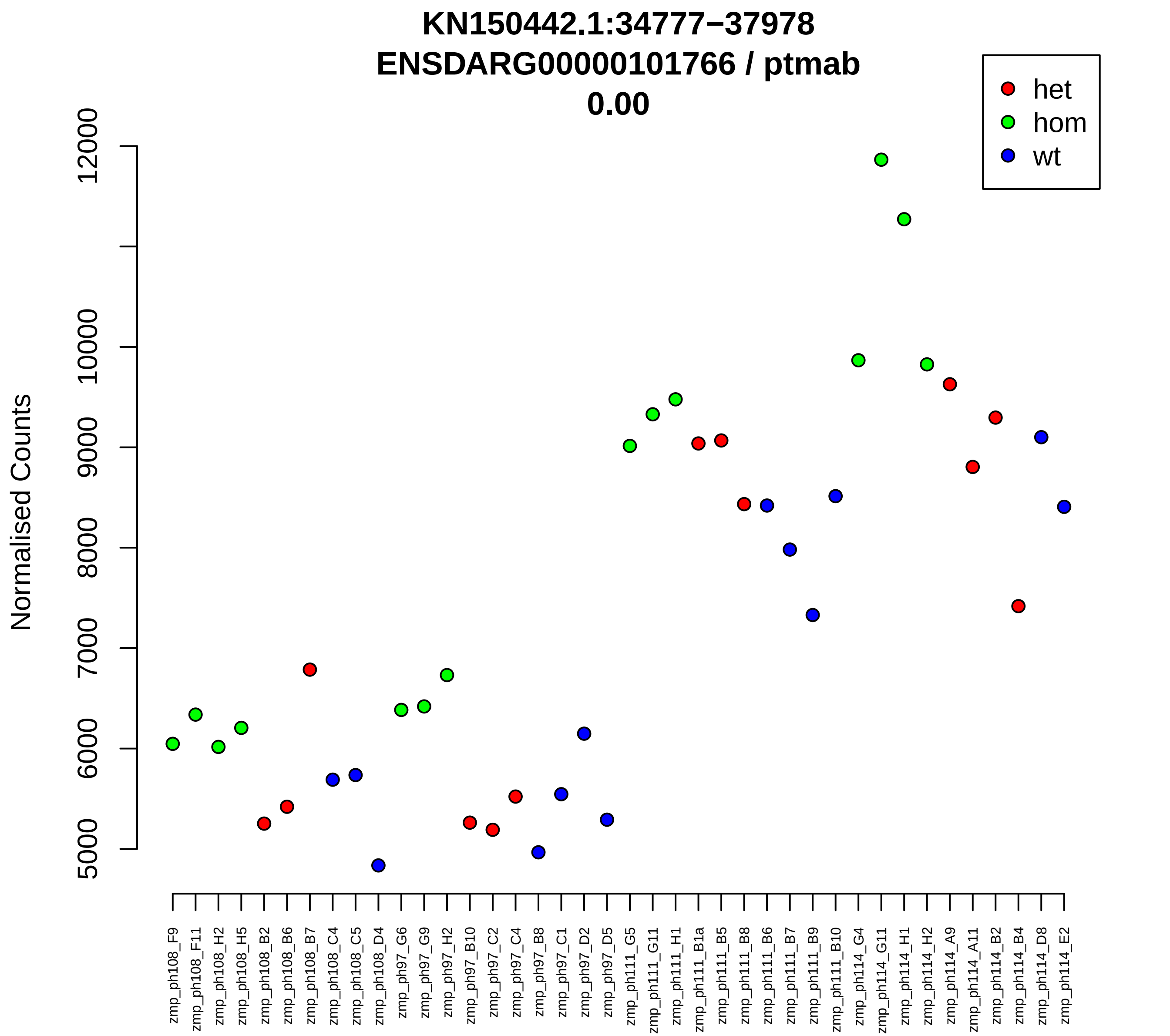
KN150442.1:34777−37978
ENSDARG00000101766 / ptmab
0.00
het
hom
wt
Normalised Counts
5000
6000
7000
8000
9000
10000
12000
zmp_ph108_F9
zmp_ph108_F11
zmp_ph108_H2
zmp_ph108_H5
zmp_ph108_B2
zmp_ph108_B6
zmp_ph108_B7
zmp_ph108_C4
zmp_ph108_C5
zmp_ph108_D4
zmp_ph97_G6
zmp_ph97_G9
zmp_ph97_H2
zmp_ph97_B10
zmp_ph97_C2
zmp_ph97_C4
zmp_ph97_B8
zmp_ph97_C1
zmp_ph97_D2
zmp_ph97_D5
zmp_ph111_G5
zmp_ph111_G11
zmp_ph111_H1
zmp_ph111_B1a
zmp_ph111_B5
zmp_ph111_B8
zmp_ph111_B6
zmp_ph111_B7
zmp_ph111_B9
zmp_ph111_B10
zmp_ph114_G4
zmp_ph114_G11
zmp_ph114_H1
zmp_ph114_H2
zmp_ph114_A9
zmp_ph114_A11
zmp_ph114_B2
zmp_ph114_B4
zmp_ph114_D8
zmp_ph114_E2

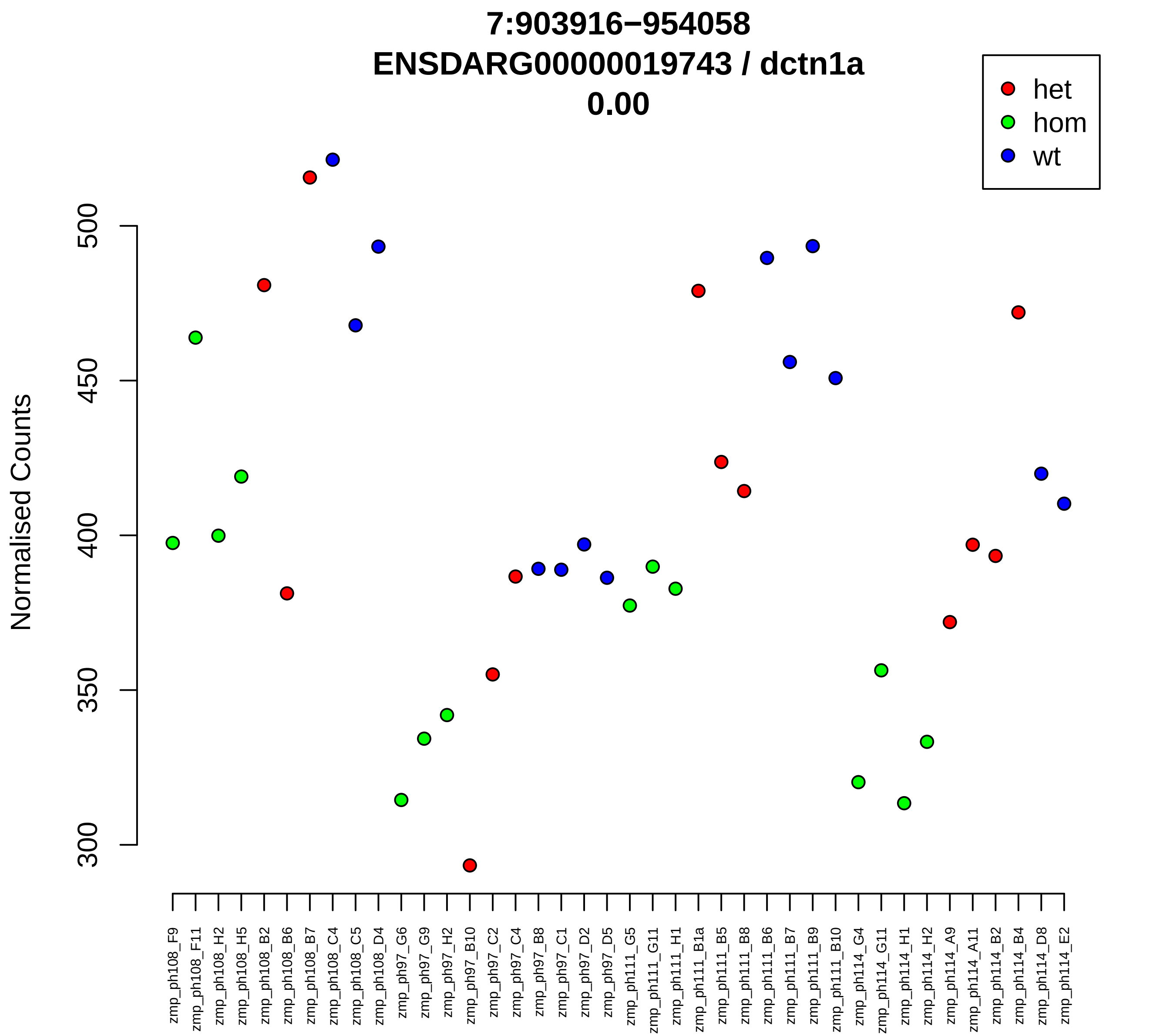

7:903916−954058
ENSDARG00000019743 / dctn1a
0.00
het
hom
wt
Normalised Counts
300
350
400
450
500
zmp_ph108_F9
zmp_ph108_F11
zmp_ph108_H2
zmp_ph108_H5
zmp_ph108_B2
zmp_ph108_B6
zmp_ph108_B7
zmp_ph108_C4
zmp_ph108_C5
zmp_ph108_D4
zmp_ph97_G6
zmp_ph97_G9
zmp_ph97_H2
zmp_ph97_B10
zmp_ph97_C2
zmp_ph97_C4
zmp_ph97_B8
zmp_ph97_C1
zmp_ph97_D2
zmp_ph97_D5
zmp_ph111_G5
zmp_ph111_G11
zmp_ph111_H1
zmp_ph111_B1a
zmp_ph111_B5
zmp_ph111_B8
zmp_ph111_B6
zmp_ph111_B7
zmp_ph111_B9
zmp_ph111_B10
zmp_ph114_G4
zmp_ph114_G11
zmp_ph114_H1
zmp_ph114_H2
zmp_ph114_A9
zmp_ph114_A11
zmp_ph114_B2
zmp_ph114_B4
zmp_ph114_D8
zmp_ph114_E2

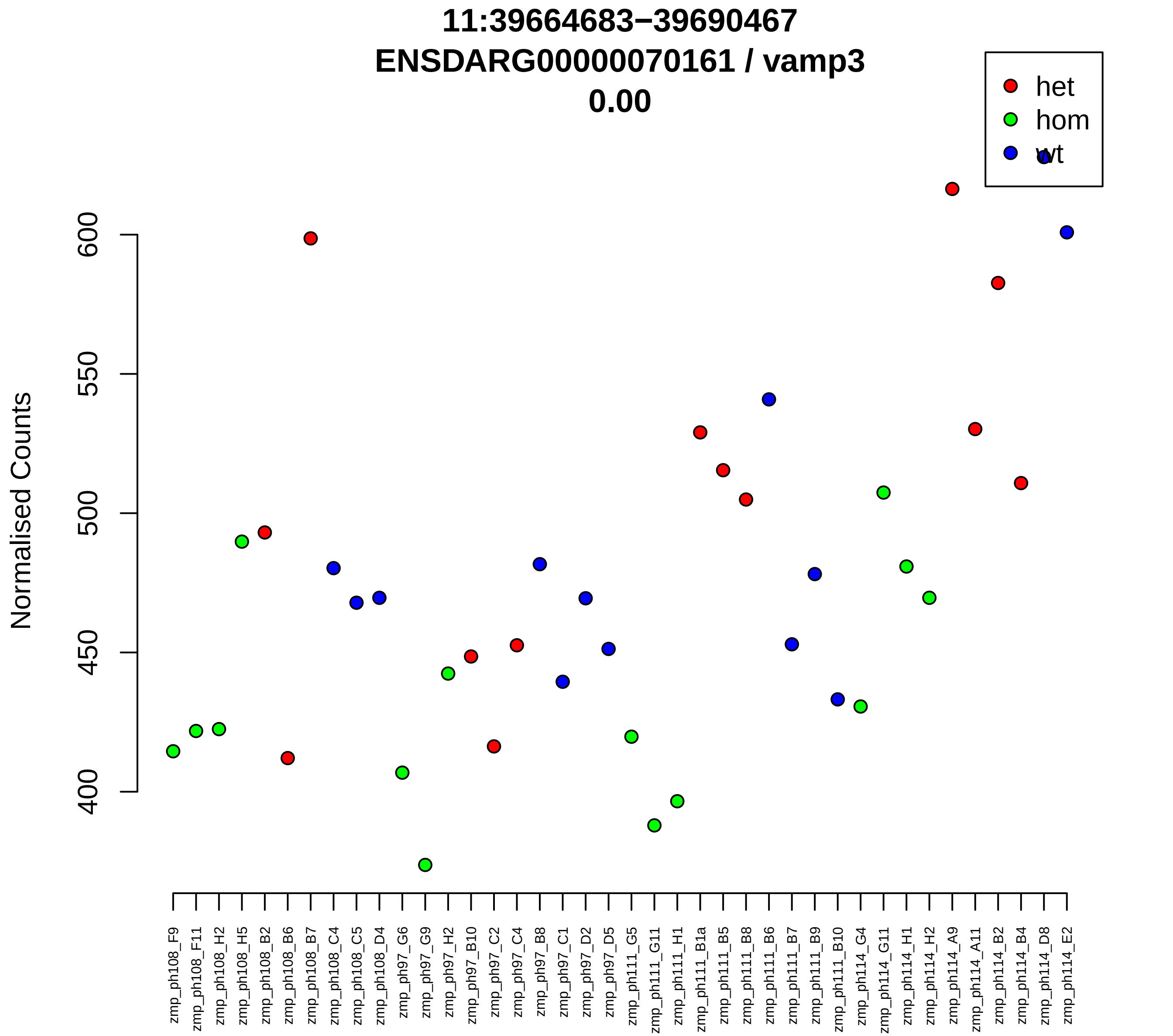

11:39664683−39690467
ENSDARG00000070161 / vamp3
0.00
het
hom
wt
Normalised Counts
400
450
500
550
600
zmp_ph108_F9
zmp_ph108_F11
zmp_ph108_H2
zmp_ph108_H5
zmp_ph108_B2
zmp_ph108_B6
zmp_ph108_B7
zmp_ph108_C4
zmp_ph108_C5
zmp_ph108_D4
zmp_ph97_G6
zmp_ph97_G9
zmp_ph97_H2
zmp_ph97_B10
zmp_ph97_C2
zmp_ph97_C4
zmp_ph97_B8
zmp_ph97_C1
zmp_ph97_D2
zmp_ph97_D5
zmp_ph111_G5
zmp_ph111_G11
zmp_ph111_H1
zmp_ph111_B1a
zmp_ph111_B5
zmp_ph111_B8
zmp_ph111_B6
zmp_ph111_B7
zmp_ph111_B9
zmp_ph111_B10
zmp_ph114_G4
zmp_ph114_G11
zmp_ph114_H1
zmp_ph114_H2
zmp_ph114_A9
zmp_ph114_A11
zmp_ph114_B2
zmp_ph114_B4
zmp_ph114_D8
zmp_ph114_E2

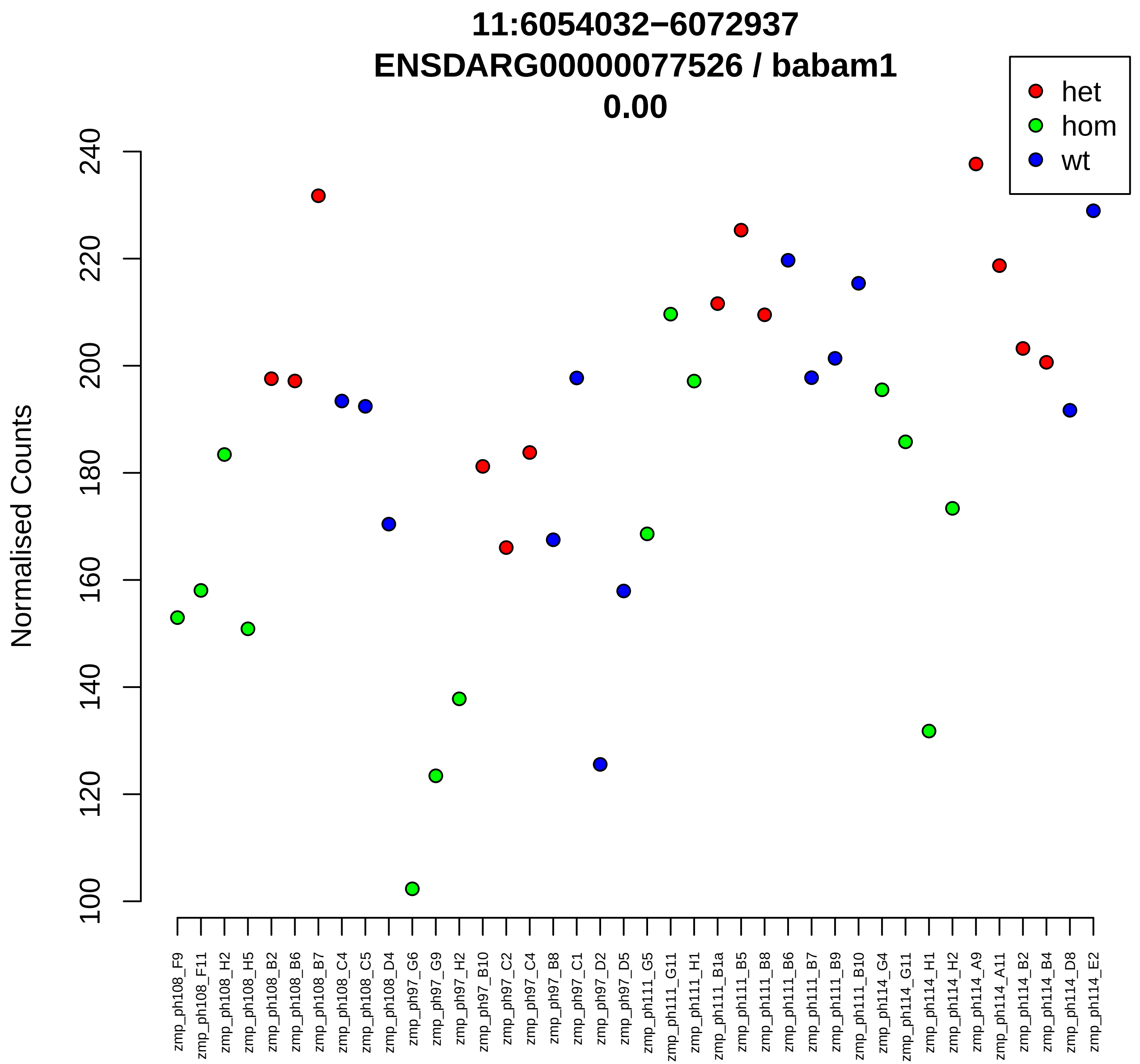

11:6054032−6072937
ENSDARG00000077526 / babam1
0.00
het
hom
wt
Normalised Counts
100
120
140
160
180
200
220
240
zmp_ph108_F9
zmp_ph108_F11
zmp_ph108_H2
zmp_ph108_H5
zmp_ph108_B2
zmp_ph108_B6
zmp_ph108_B7
zmp_ph108_C4
zmp_ph108_C5
zmp_ph108_D4
zmp_ph97_G6
zmp_ph97_G9
zmp_ph97_H2
zmp_ph97_B10
zmp_ph97_C2
zmp_ph97_C4
zmp_ph97_B8
zmp_ph97_C1
zmp_ph97_D2
zmp_ph97_D5
zmp_ph111_G5
zmp_ph111_G11
zmp_ph111_H1
zmp_ph111_B1a
zmp_ph111_B5
zmp_ph111_B8
zmp_ph111_B6
zmp_ph111_B7
zmp_ph111_B9
zmp_ph111_B10
zmp_ph114_G4
zmp_ph114_G11
zmp_ph114_H1
zmp_ph114_H2
zmp_ph114_A9
zmp_ph114_A11
zmp_ph114_B2
zmp_ph114_B4
zmp_ph114_D8
zmp_ph114_E2

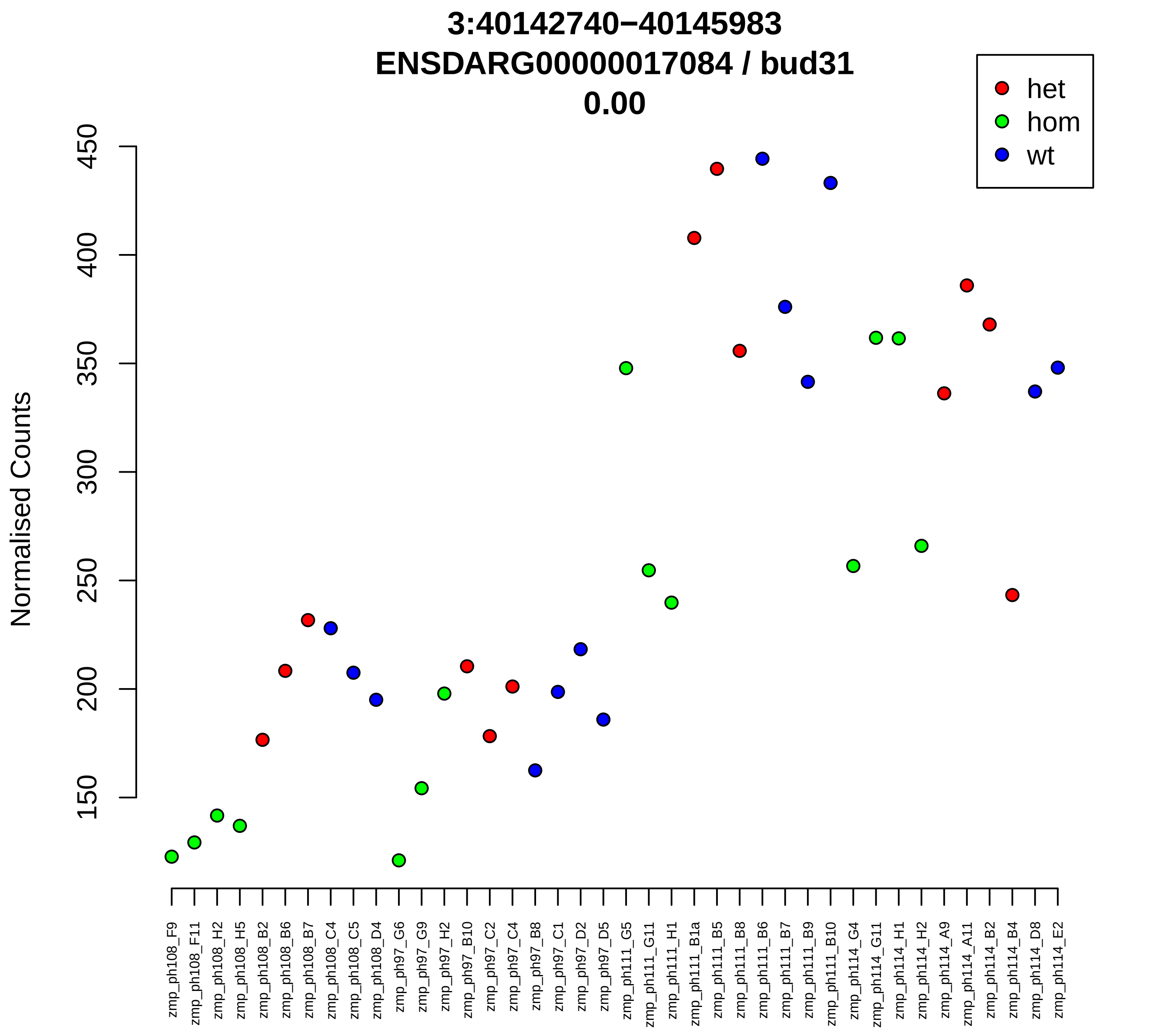
3:40142740−40145983
ENSDARG00000017084 / bud31
0.00
het
hom
wt
Normalised Counts
150
200
250
300
350
400
450
zmp_ph108_F9
zmp_ph108_F11
zmp_ph108_H2
zmp_ph108_H5
zmp_ph108_B2
zmp_ph108_B6
zmp_ph108_B7
zmp_ph108_C4
zmp_ph108_C5
zmp_ph108_D4
zmp_ph97_G6
zmp_ph97_G9
zmp_ph97_H2
zmp_ph97_B10
zmp_ph97_C2
zmp_ph97_C4
zmp_ph97_B8
zmp_ph97_C1
zmp_ph97_D2
zmp_ph97_D5
zmp_ph111_G5
zmp_ph111_G11
zmp_ph111_H1
zmp_ph111_B1a
zmp_ph111_B5
zmp_ph111_B8
zmp_ph111_B6
zmp_ph111_B7
zmp_ph111_B9
zmp_ph111_B10
zmp_ph114_G4
zmp_ph114_G11
zmp_ph114_H1
zmp_ph114_H2
zmp_ph114_A9
zmp_ph114_A11
zmp_ph114_B2
zmp_ph114_B4
zmp_ph114_D8
zmp_ph114_E2

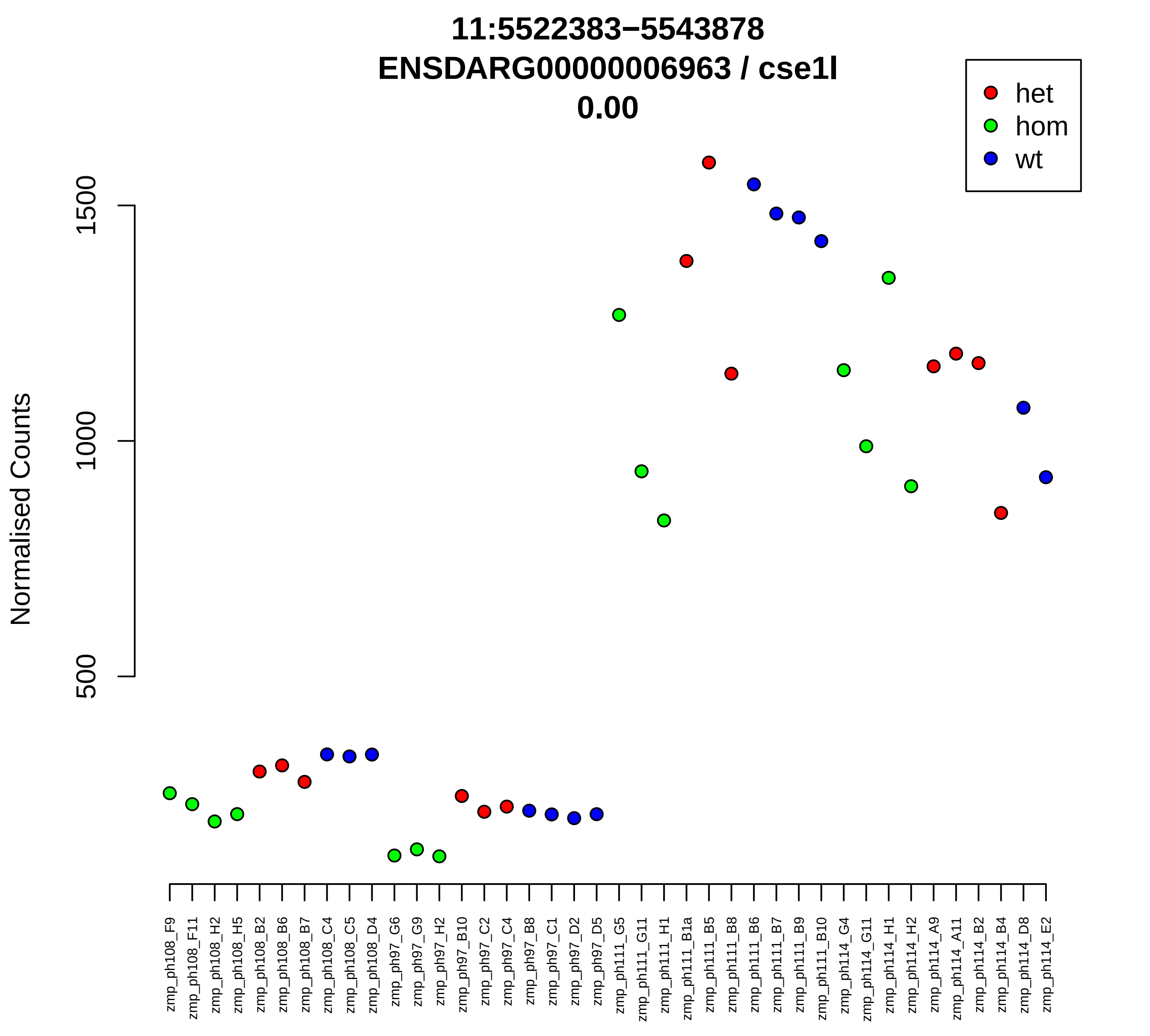

11:5522383−5543878
ENSDARG00000006963 / cse1l
0.00
het
hom
wt
Normalised Counts
500
1000
1500
zmp_ph108_F9
zmp_ph108_F11
zmp_ph108_H2
zmp_ph108_H5
zmp_ph108_B2
zmp_ph108_B6
zmp_ph108_B7
zmp_ph108_C4
zmp_ph108_C5
zmp_ph108_D4
zmp_ph97_G6
zmp_ph97_G9
zmp_ph97_H2
zmp_ph97_B10
zmp_ph97_C2
zmp_ph97_C4
zmp_ph97_B8
zmp_ph97_C1
zmp_ph97_D2
zmp_ph97_D5
zmp_ph111_G5
zmp_ph111_G11
zmp_ph111_H1
zmp_ph111_B1a
zmp_ph111_B5
zmp_ph111_B8
zmp_ph111_B6
zmp_ph111_B7
zmp_ph111_B9
zmp_ph111_B10
zmp_ph114_G4
zmp_ph114_G11
zmp_ph114_H1
zmp_ph114_H2
zmp_ph114_A9
zmp_ph114_A11
zmp_ph114_B2
zmp_ph114_B4
zmp_ph114_D8
zmp_ph114_E2

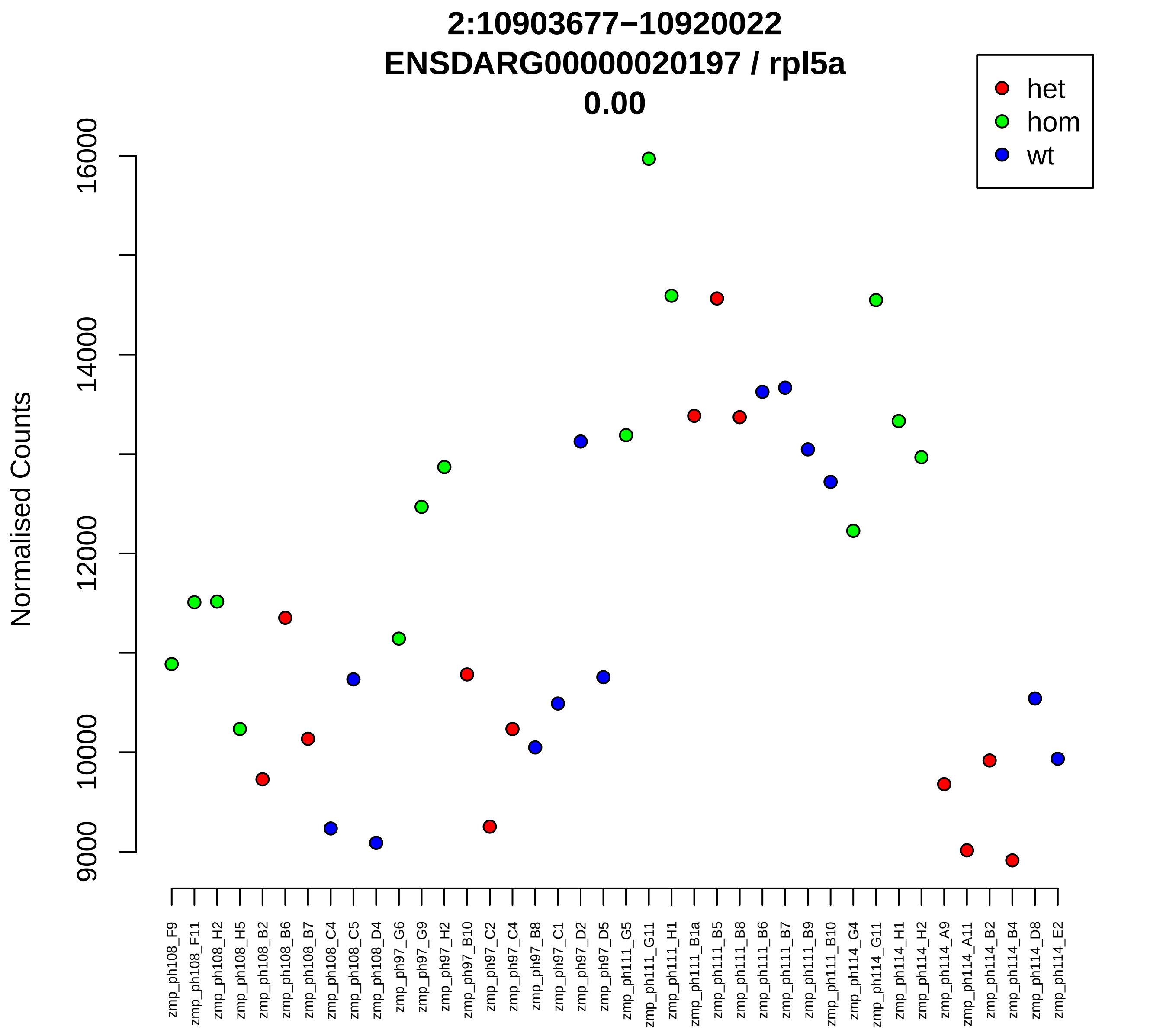
2:10903677−10920022
ENSDARG00000020197 / rpl5a
0.00
het
hom
wt
Normalised Counts
16000
14000
12000
10000
9000
zmp_ph108_F9
zmp_ph108_F11
zmp_ph108_H2
zmp_ph108_H5
zmp_ph108_B2
zmp_ph108_B6
zmp_ph108_B7
zmp_ph108_C4
zmp_ph108_C5
zmp_ph108_D4
zmp_ph97_G6
zmp_ph97_G9
zmp_ph97_H2
zmp_ph97_B10
zmp_ph97_C2
zmp_ph97_C4
zmp_ph97_B8
zmp_ph97_C1
zmp_ph97_D2
zmp_ph97_D5
zmp_ph111_G5
zmp_ph111_G11
zmp_ph111_H1
zmp_ph111_B1a
zmp_ph111_B5
zmp_ph111_B8
zmp_ph111_B6
zmp_ph111_B7
zmp_ph111_B9
zmp_ph111_B10
zmp_ph114_G4
zmp_ph114_G11
zmp_ph114_H1
zmp_ph114_H2
zmp_ph114_A9
zmp_ph114_A11
zmp_ph114_B2
zmp_ph114_B4
zmp_ph114_D8
zmp_ph114_E2

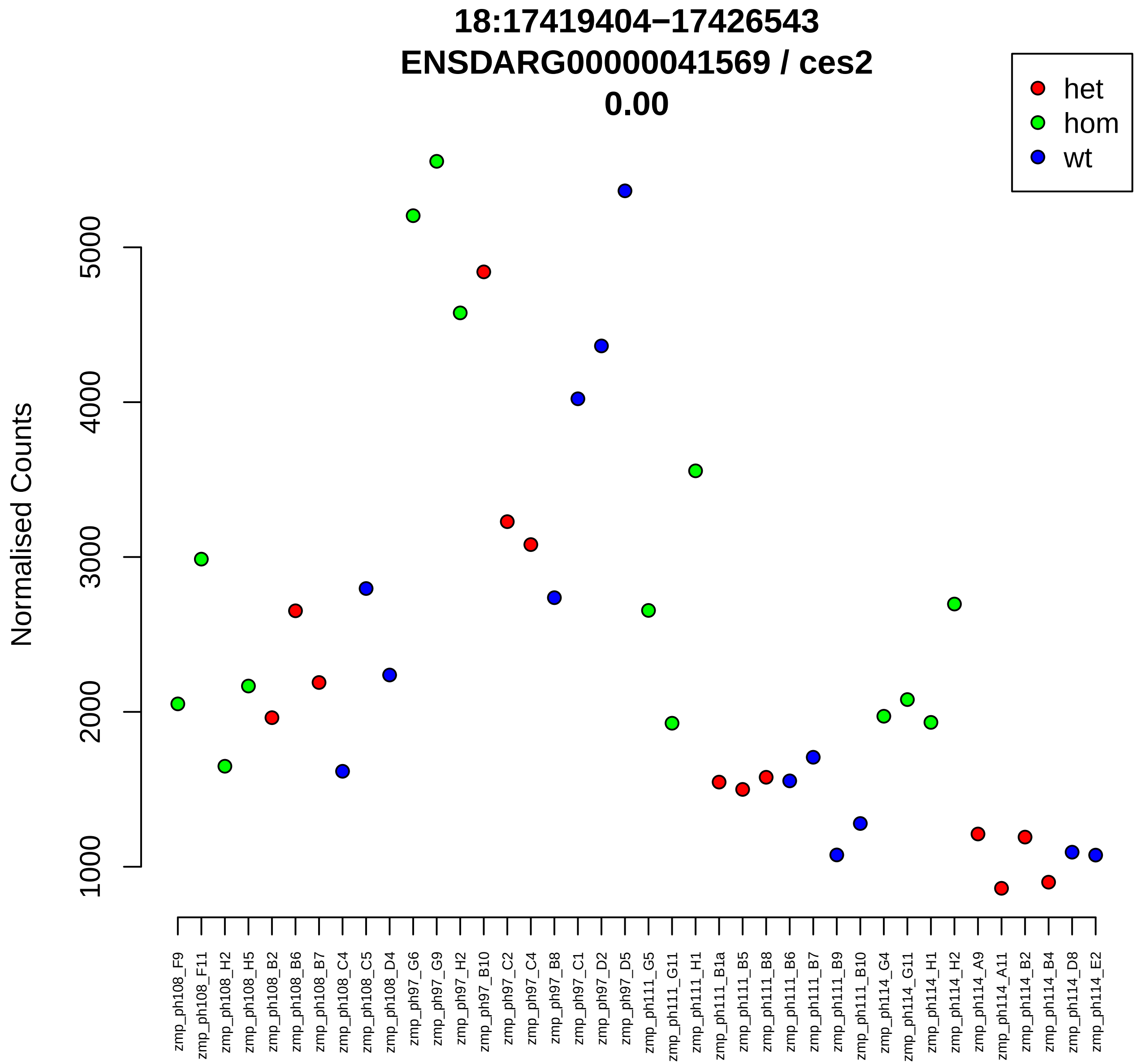

18:17419404−17426543
ENSDARG00000041569 / ces2
0.00
het
hom
wt
Normalised Counts
1000
2000
3000
4000
5000
zmp_ph108_F9
zmp_ph108_F11
zmp_ph108_H2
zmp_ph108_H5
zmp_ph108_B2
zmp_ph108_B6
zmp_ph108_B7
zmp_ph108_C4
zmp_ph108_C5
zmp_ph108_D4
zmp_ph97_G6
zmp_ph97_G9
zmp_ph97_H2
zmp_ph97_B10
zmp_ph97_C2
zmp_ph97_C4
zmp_ph97_B8
zmp_ph97_C1
zmp_ph97_D2
zmp_ph97_D5
zmp_ph111_G5
zmp_ph111_G11
zmp_ph111_H1
zmp_ph111_B1a
zmp_ph111_B5
zmp_ph111_B8
zmp_ph111_B6
zmp_ph111_B7
zmp_ph111_B9
zmp_ph111_B10
zmp_ph114_G4
zmp_ph114_G11
zmp_ph114_H1
zmp_ph114_H2
zmp_ph114_A9
zmp_ph114_A11
zmp_ph114_B2
zmp_ph114_B4
zmp_ph114_D8
zmp_ph114_E2

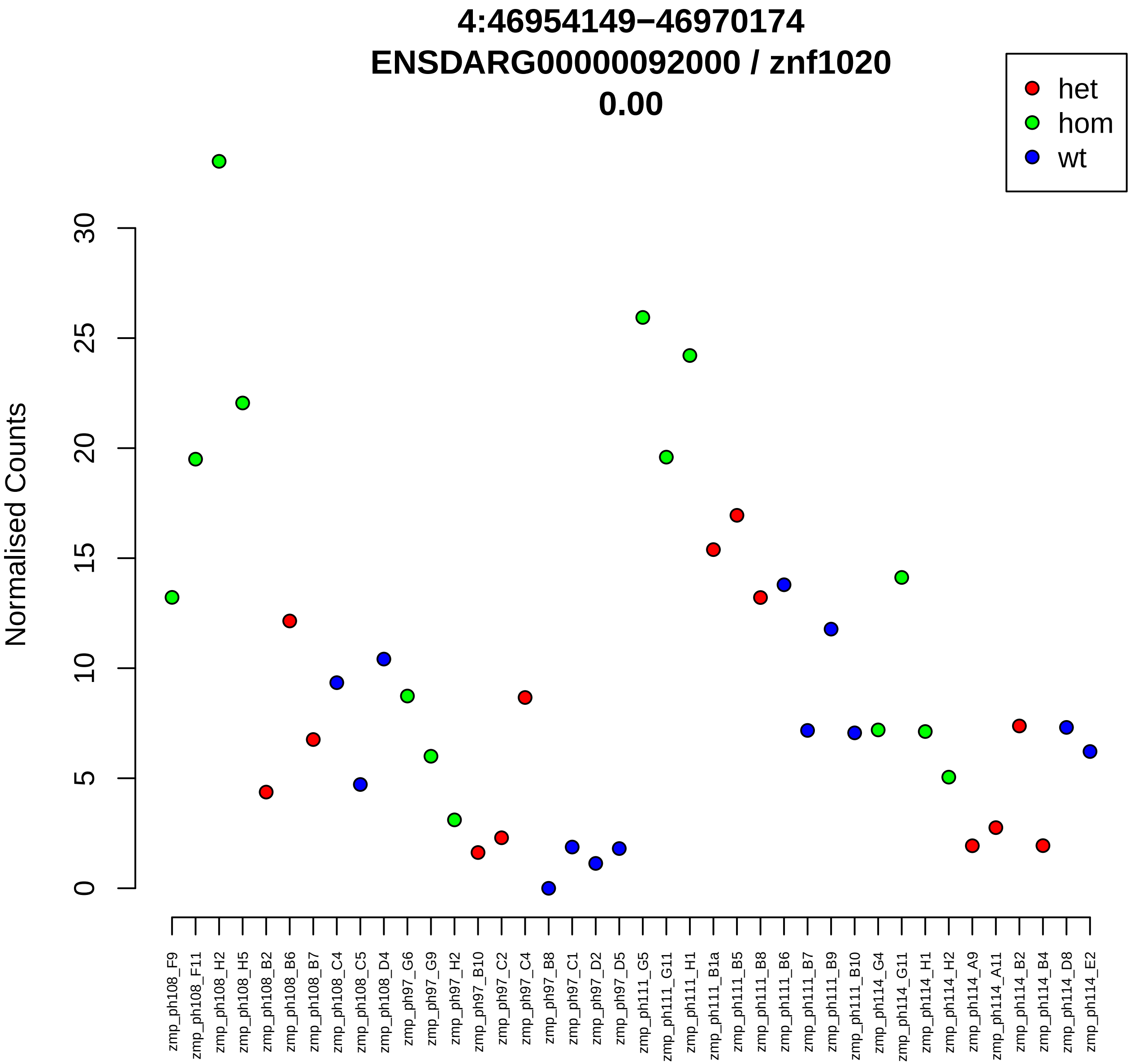

4:46954149−46970174
ENSDARG00000092000 / znf1020
0.00
het
hom
wt
Normalised Counts
0
5
10
15
20
25
30
zmp_ph108_F9
zmp_ph108_F11
zmp_ph108_H2
zmp_ph108_H5
zmp_ph108_B2
zmp_ph108_B6
zmp_ph108_B7
zmp_ph108_C4
zmp_ph108_C5
zmp_ph108_D4
zmp_ph97_G6
zmp_ph97_G9
zmp_ph97_H2
zmp_ph97_B10
zmp_ph97_C2
zmp_ph97_C4
zmp_ph97_B8
zmp_ph97_C1
zmp_ph97_D2
zmp_ph97_D5
zmp_ph111_G5
zmp_ph111_G11
zmp_ph111_H1
zmp_ph111_B1a
zmp_ph111_B5
zmp_ph111_B8
zmp_ph111_B6
zmp_ph111_B7
zmp_ph111_B9
zmp_ph111_B10
zmp_ph114_G4
zmp_ph114_G11
zmp_ph114_H1
zmp_ph114_H2
zmp_ph114_A9
zmp_ph114_A11
zmp_ph114_B2
zmp_ph114_B4
zmp_ph114_D8
zmp_ph114_E2

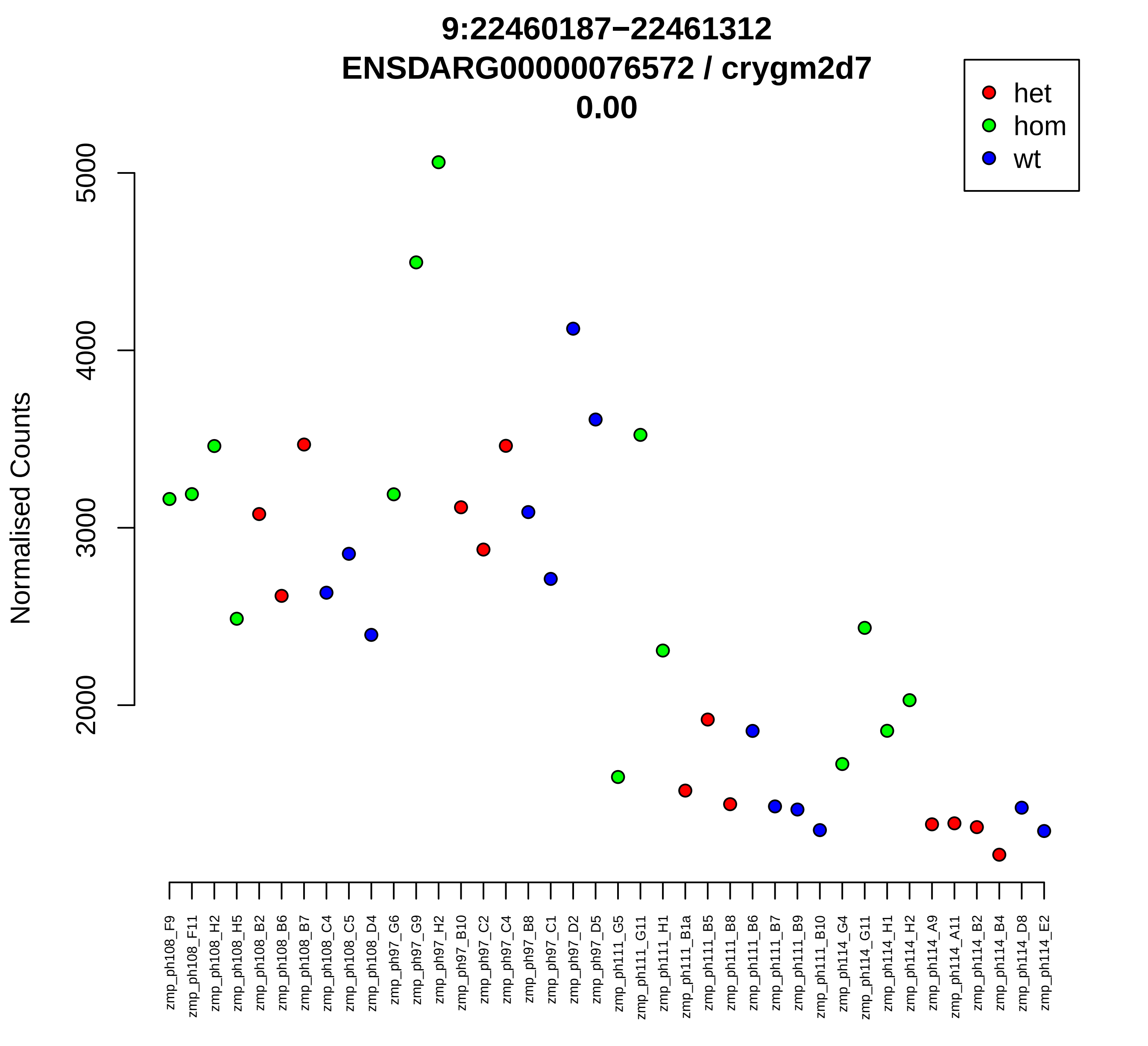

9:22460187−22461312
ENSDARG00000076572 / crygm2d7
0.00
het
hom
wt
Normalised Counts
2000
3000
4000
5000
zmp_ph108_F9
zmp_ph108_F11
zmp_ph108_H2
zmp_ph108_H5
zmp_ph108_B2
zmp_ph108_B6
zmp_ph108_B7
zmp_ph108_C4
zmp_ph108_C5
zmp_ph108_D4
zmp_ph97_G6
zmp_ph97_G9
zmp_ph97_H2
zmp_ph97_B10
zmp_ph97_C2
zmp_ph97_C4
zmp_ph97_B8
zmp_ph97_C1
zmp_ph97_D2
zmp_ph97_D5
zmp_ph111_G5
zmp_ph111_G11
zmp_ph111_H1
zmp_ph111_B1a
zmp_ph111_B5
zmp_ph111_B8
zmp_ph111_B6
zmp_ph111_B7
zmp_ph111_B9
zmp_ph111_B10
zmp_ph114_G4
zmp_ph114_G11
zmp_ph114_H1
zmp_ph114_H2
zmp_ph114_A9
zmp_ph114_A11
zmp_ph114_B2
zmp_ph114_B4
zmp_ph114_D8
zmp_ph114_E2

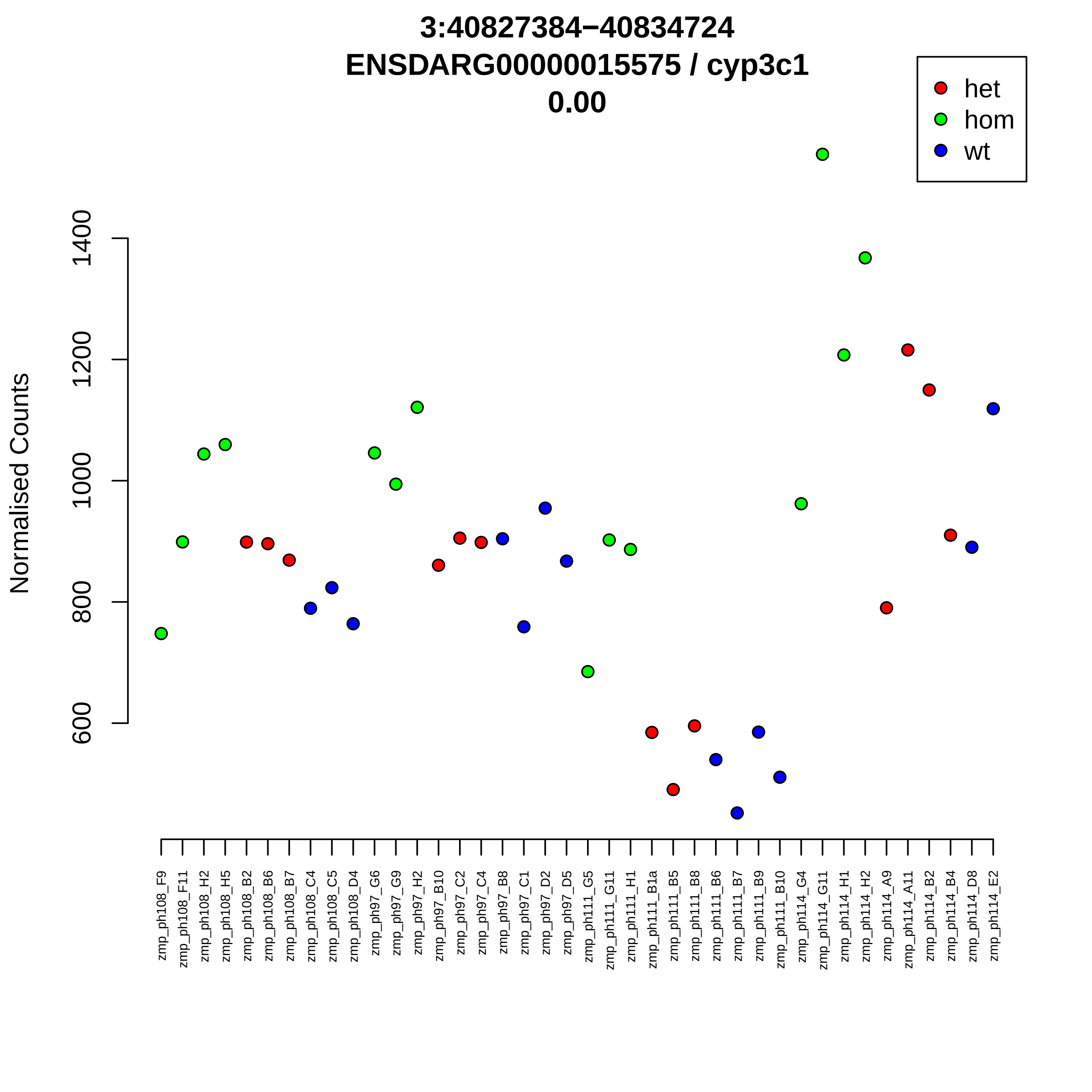

3:40827384−40834724
ENSDARG00000015575 / cyp3c1
0.00
het
hom
wt
Normalised Counts
600
800
1000
1200
1400
zmp_ph108_F9
zmp_ph108_F11
zmp_ph108_H2
zmp_ph108_H5
zmp_ph108_B2
zmp_ph108_B6
zmp_ph108_B7
zmp_ph108_C4
zmp_ph108_C5
zmp_ph108_D4
zmp_ph97_G6
zmp_ph97_G9
zmp_ph97_H2
zmp_ph97_B10
zmp_ph97_C2
zmp_ph97_C4
zmp_ph97_B8
zmp_ph97_C1
zmp_ph97_D2
zmp_ph97_D5
zmp_ph111_G5
zmp_ph111_G11
zmp_ph111_H1
zmp_ph111_B1a
zmp_ph111_B5
zmp_ph111_B8
zmp_ph111_B6
zmp_ph111_B7
zmp_ph111_B9
zmp_ph111_B10
zmp_ph114_G4
zmp_ph114_G11
zmp_ph114_H1
zmp_ph114_H2
zmp_ph114_A9
zmp_ph114_A11
zmp_ph114_B2
zmp_ph114_B4
zmp_ph114_D8
zmp_ph114_E2

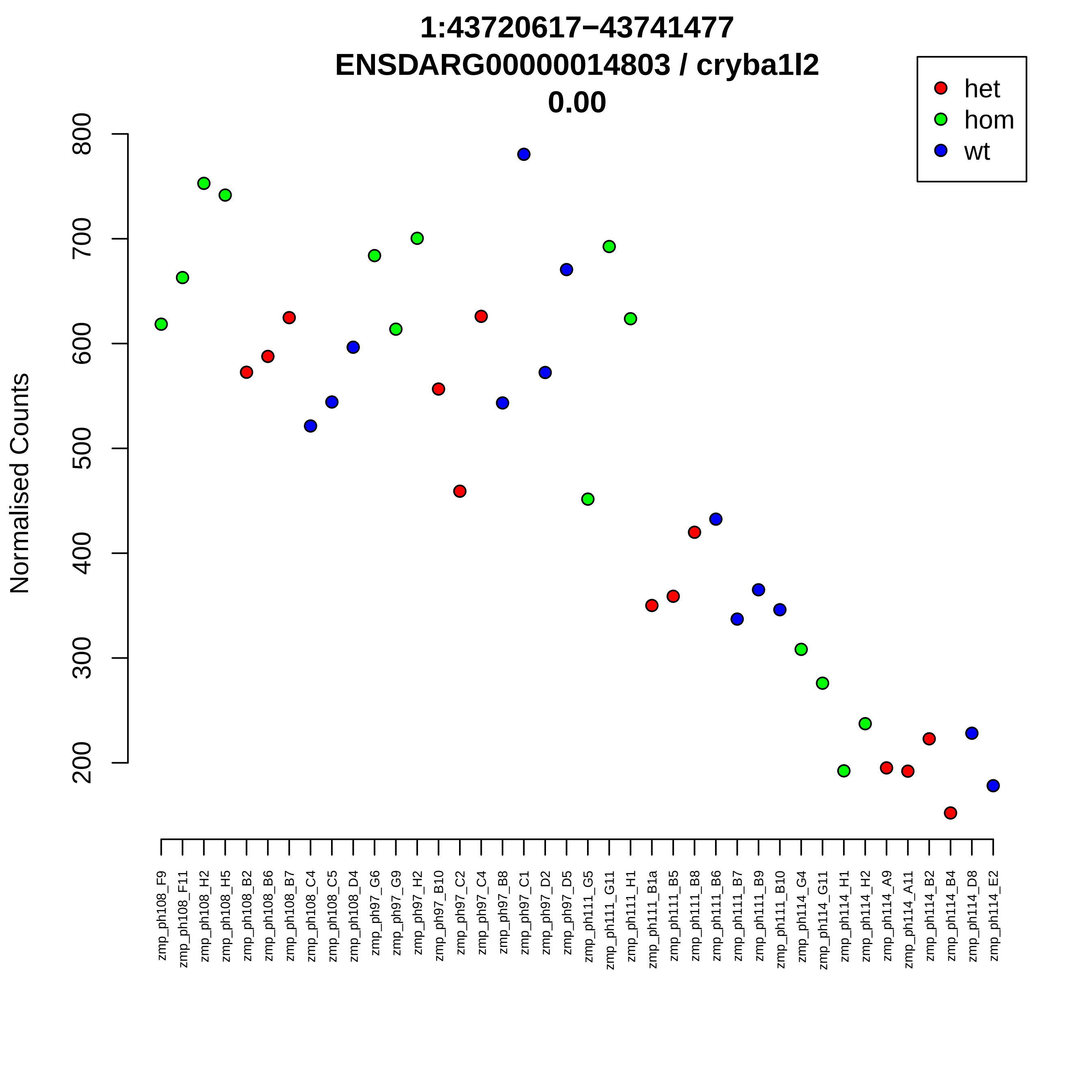
1:43720617−43741477
ENSDARG00000014803 / cryba1l2
0.00
Normalised Counts
200
300
400
500
600
700
800
het
hom
wt
zmp_ph108_F9
zmp_ph108_F11
zmp_ph108_H2
zmp_ph108_H5
zmp_ph108_B2
zmp_ph108_B6
zmp_ph108_B7
zmp_ph108_C4
zmp_ph108_C5
zmp_ph108_D4
zmp_ph97_G6
zmp_ph97_G9
zmp_ph97_H2
zmp_ph97_B10
zmp_ph97_C2
zmp_ph97_C4
zmp_ph97_B8
zmp_ph97_C1
zmp_ph97_D2
zmp_ph97_D5
zmp_ph111_G5
zmp_ph111_G11
zmp_ph111_H1
zmp_ph111_B1a
zmp_ph111_B5
zmp_ph111_B8
zmp_ph111_B6
zmp_ph111_B7
zmp_ph111_B9
zmp_ph111_B10
zmp_ph114_G4
zmp_ph114_G11
zmp_ph114_H1
zmp_ph114_H2
zmp_ph114_A9
zmp_ph114_A11
zmp_ph114_B2
zmp_ph114_B4
zmp_ph114_D8
zmp_ph114_E2

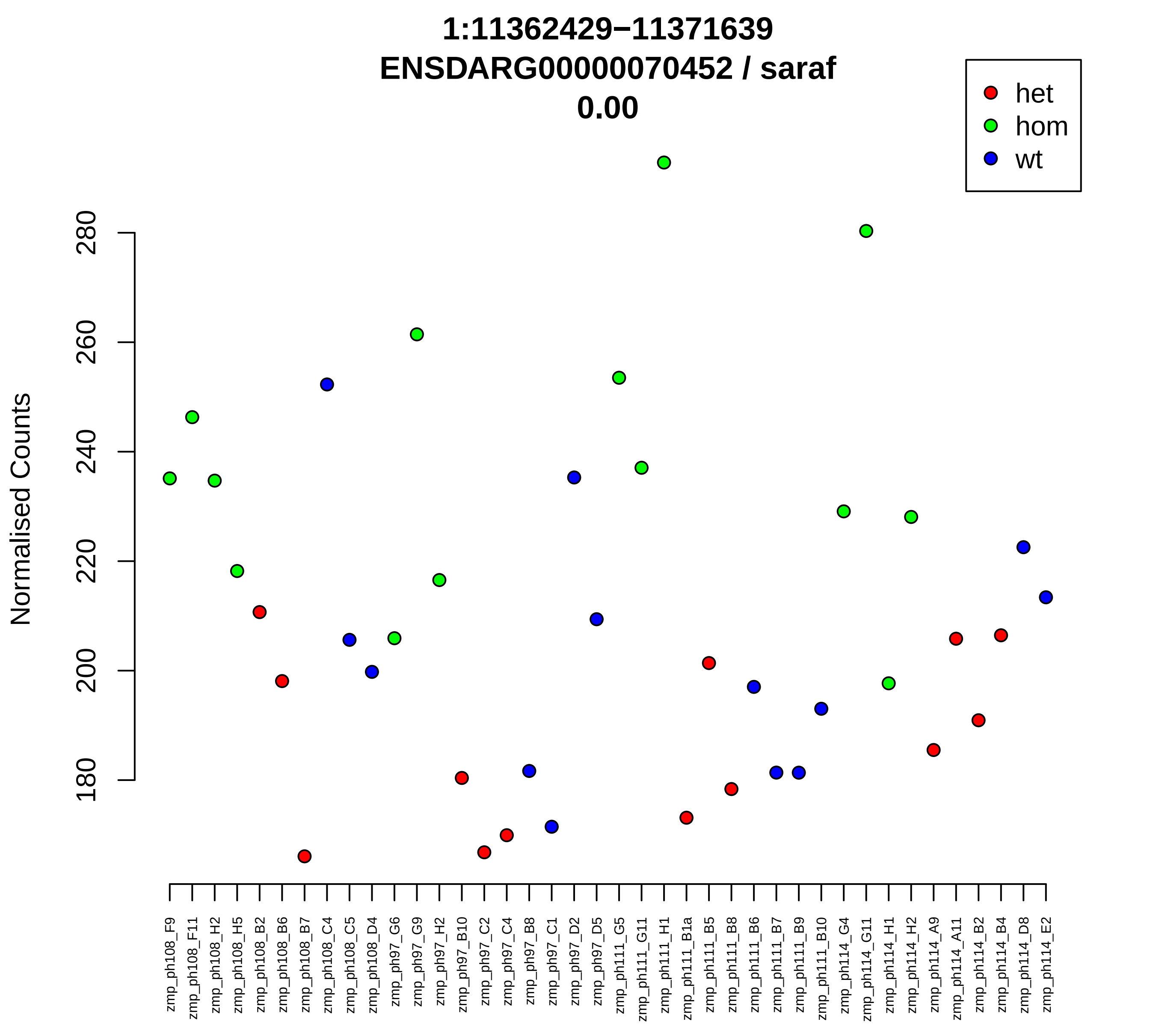

1:11362429−11371639
ENSDARG00000070452 / saraf
0.00
het
hom
wt
Normalised Counts
180
200
220
240
260
280
zmp_ph108_F9
zmp_ph108_F11
zmp_ph108_H2
zmp_ph108_H5
zmp_ph108_B2
zmp_ph108_B6
zmp_ph108_B7
zmp_ph108_C4
zmp_ph108_C5
zmp_ph108_D4
zmp_ph97_G6
zmp_ph97_G9
zmp_ph97_H2
zmp_ph97_B10
zmp_ph97_C2
zmp_ph97_C4
zmp_ph97_B8
zmp_ph97_C1
zmp_ph97_D2
zmp_ph97_D5
zmp_ph111_G5
zmp_ph111_G11
zmp_ph111_H1
zmp_ph111_B1a
zmp_ph111_B5
zmp_ph111_B8
zmp_ph111_B6
zmp_ph111_B7
zmp_ph111_B9
zmp_ph111_B10
zmp_ph114_G4
zmp_ph114_G11
zmp_ph114_H1
zmp_ph114_H2
zmp_ph114_A9
zmp_ph114_A11
zmp_ph114_B2
zmp_ph114_B4
zmp_ph114_D8
zmp_ph114_E2

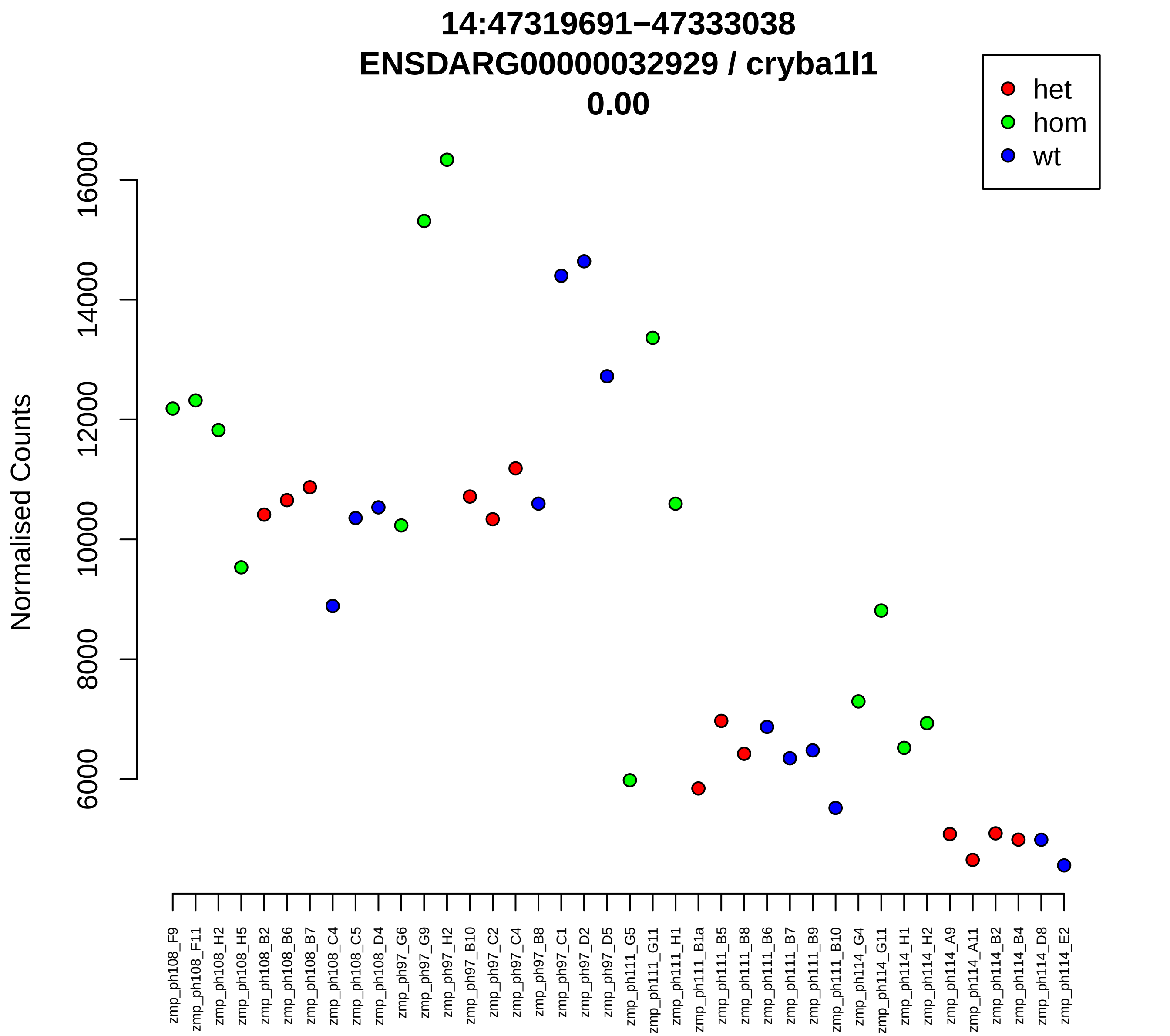

14:47319691−47333038
ENSDARG00000032929 / cryba1l1
0.00
het
hom
wt
Normalised Counts
6000
8000
10000
12000
14000
16000
zmp_ph108_F9
zmp_ph108_F11
zmp_ph108_H2
zmp_ph108_H5
zmp_ph108_B2
zmp_ph108_B6
zmp_ph108_B7
zmp_ph108_C4
zmp_ph108_C5
zmp_ph108_D4
zmp_ph97_G6
zmp_ph97_G9
zmp_ph97_H2
zmp_ph97_B10
zmp_ph97_C2
zmp_ph97_C4
zmp_ph97_B8
zmp_ph97_C1
zmp_ph97_D2
zmp_ph97_D5
zmp_ph111_G5
zmp_ph111_G11
zmp_ph111_H1
zmp_ph111_B1a
zmp_ph111_B5
zmp_ph111_B8
zmp_ph111_B6
zmp_ph111_B7
zmp_ph111_B9
zmp_ph111_B10
zmp_ph114_G4
zmp_ph114_G11
zmp_ph114_H1
zmp_ph114_H2
zmp_ph114_A9
zmp_ph114_A11
zmp_ph114_B2
zmp_ph114_B4
zmp_ph114_D8
zmp_ph114_E2

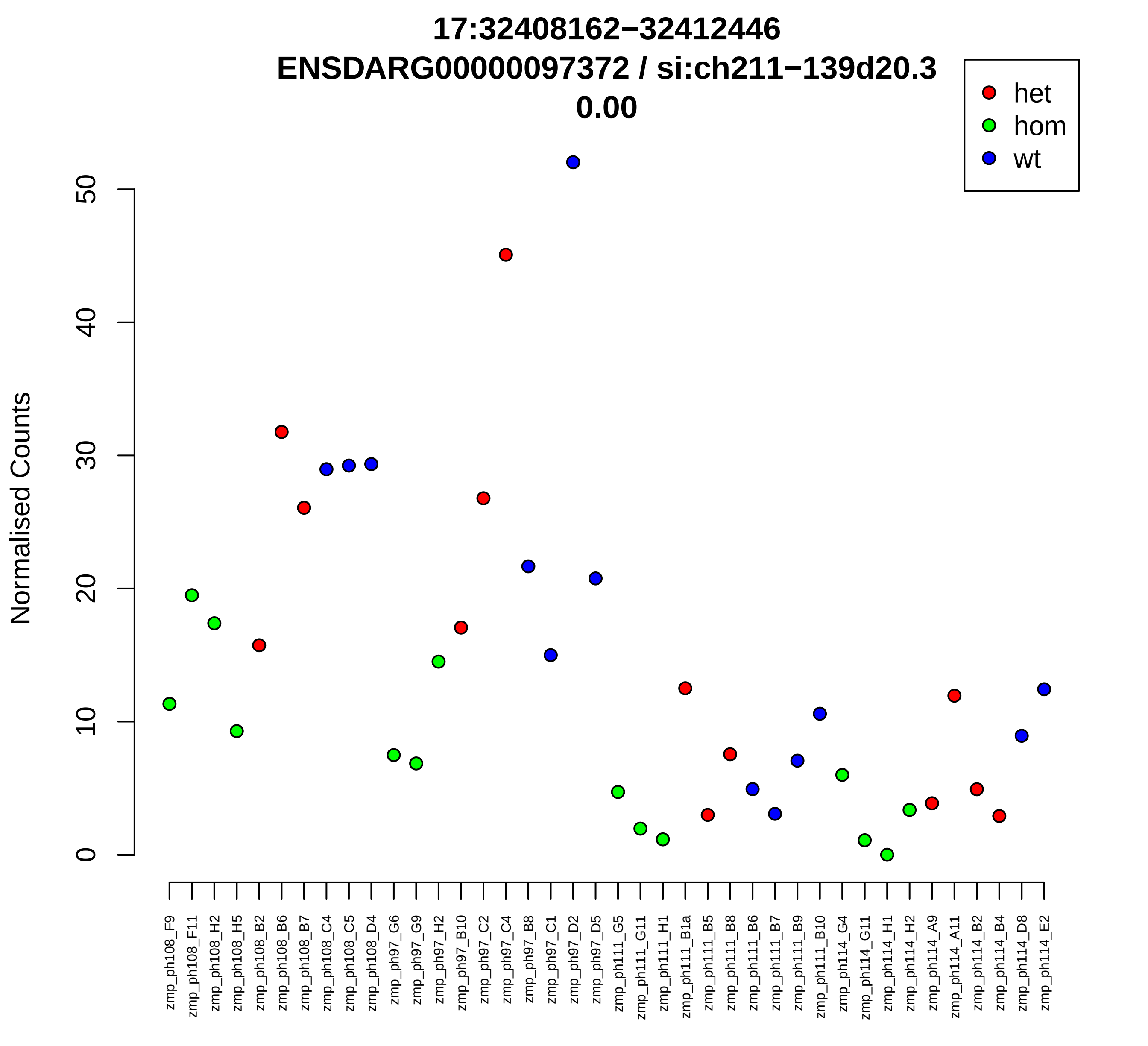

17:32408162−32412446
ENSDARG00000097372 / si:ch211−139d20.3
0.00
het
hom
wt
Normalised Counts
0
10
20
30
40
50
zmp_ph108_F9
zmp_ph108_F11
zmp_ph108_H2
zmp_ph108_H5
zmp_ph108_B2
zmp_ph108_B6
zmp_ph108_B7
zmp_ph108_C4
zmp_ph108_C5
zmp_ph108_D4
zmp_ph97_G6
zmp_ph97_G9
zmp_ph97_H2
zmp_ph97_B10
zmp_ph97_C2
zmp_ph97_C4
zmp_ph97_B8
zmp_ph97_C1
zmp_ph97_D2
zmp_ph97_D5
zmp_ph111_G5
zmp_ph111_G11
zmp_ph111_H1
zmp_ph111_B1a
zmp_ph111_B5
zmp_ph111_B8
zmp_ph111_B6
zmp_ph111_B7
zmp_ph111_B9
zmp_ph111_B10
zmp_ph114_G4
zmp_ph114_G11
zmp_ph114_H1
zmp_ph114_H2
zmp_ph114_A9
zmp_ph114_A11
zmp_ph114_B2
zmp_ph114_B4
zmp_ph114_D8
zmp_ph114_E2

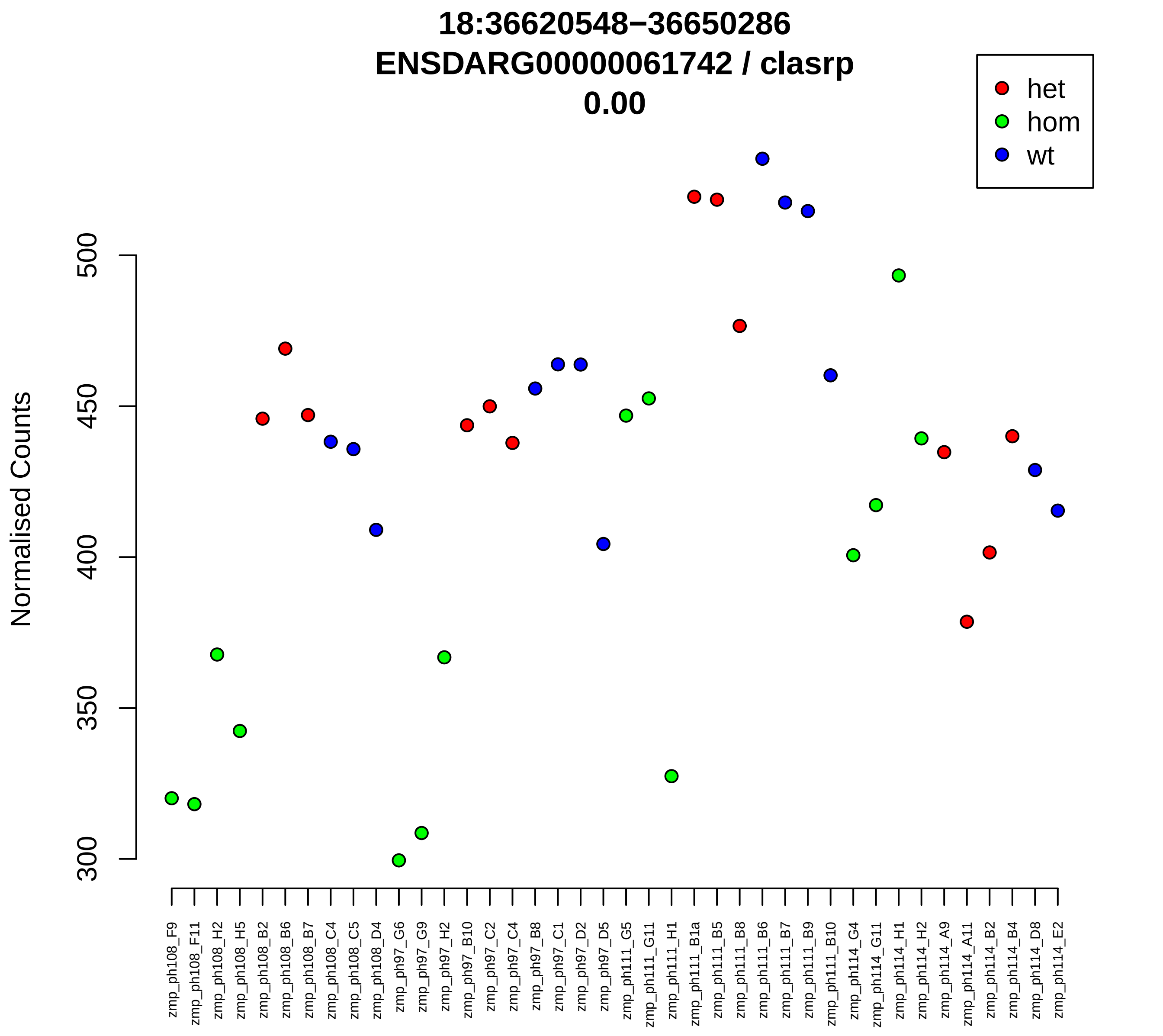
18:36620548−36650286
ENSDARG00000061742 / clasrp
0.00
het
hom
wt
Normalised Counts
300
350
400
450
500
zmp_ph108_F9
zmp_ph108_F11
zmp_ph108_H2
zmp_ph108_H5
zmp_ph108_B2
zmp_ph108_B6
zmp_ph108_B7
zmp_ph108_C4
zmp_ph108_C5
zmp_ph108_D4
zmp_ph97_G6
zmp_ph97_G9
zmp_ph97_H2
zmp_ph97_B10
zmp_ph97_C2
zmp_ph97_C4
zmp_ph97_B8
zmp_ph97_C1
zmp_ph97_D2
zmp_ph97_D5
zmp_ph111_G5
zmp_ph111_G11
zmp_ph111_H1
zmp_ph111_B1a
zmp_ph111_B5
zmp_ph111_B8
zmp_ph111_B6
zmp_ph111_B7
zmp_ph111_B9
zmp_ph111_B10
zmp_ph114_G4
zmp_ph114_G11
zmp_ph114_H1
zmp_ph114_H2
zmp_ph114_A9
zmp_ph114_A11
zmp_ph114_B2
zmp_ph114_B4
zmp_ph114_D8
zmp_ph114_E2

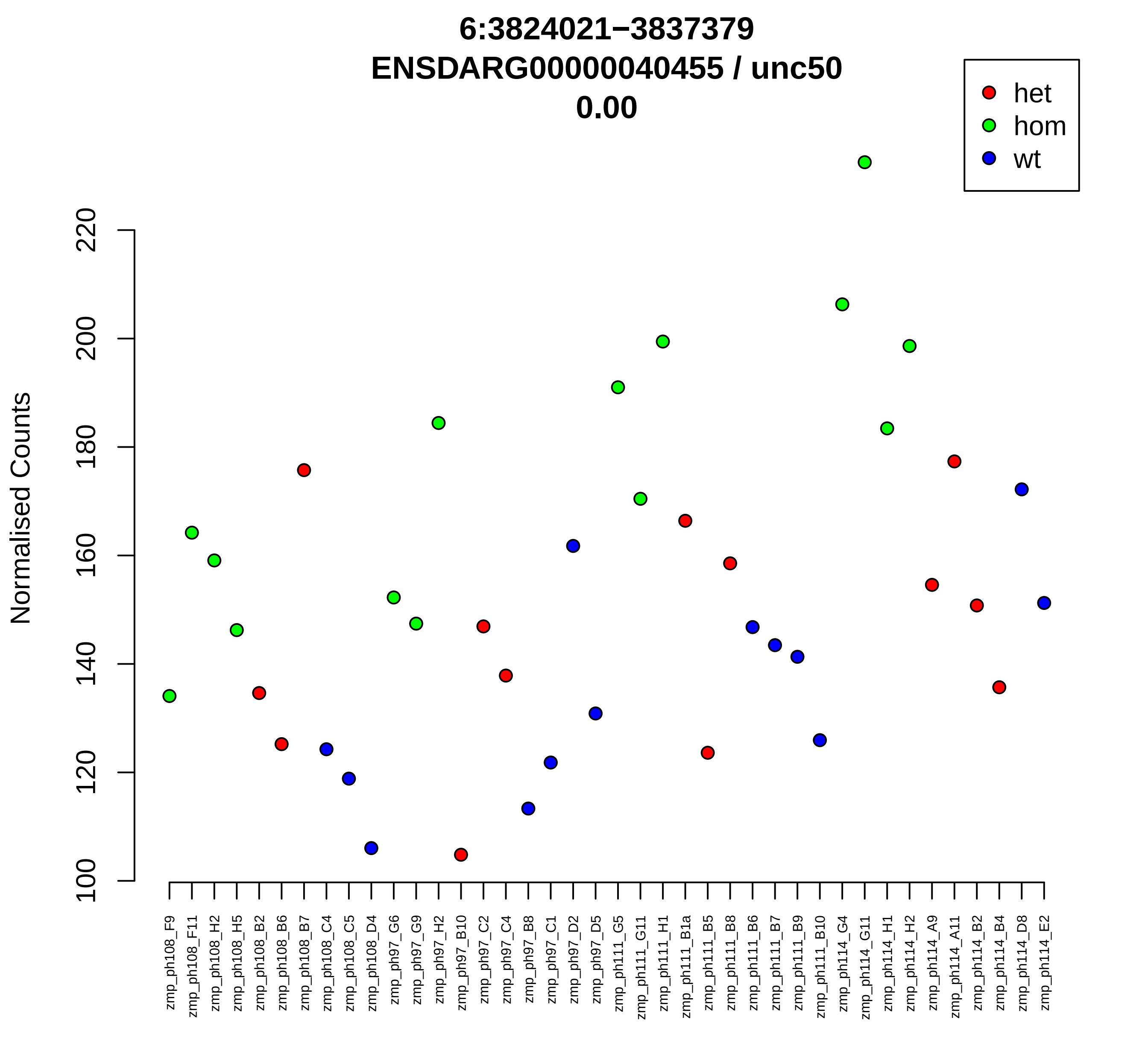
6:3824021−3837379
ENSDARG00000040455 / unc50
0.00
het
hom
wt
Normalised Counts
100
120
140
160
180
200
220
zmp_ph108_F9
zmp_ph108_F11
zmp_ph108_H2
zmp_ph108_H5
zmp_ph108_B2
zmp_ph108_B6
zmp_ph108_B7
zmp_ph108_C4
zmp_ph108_C5
zmp_ph108_D4
zmp_ph97_G6
zmp_ph97_G9
zmp_ph97_H2
zmp_ph97_B10
zmp_ph97_C2
zmp_ph97_C4
zmp_ph97_B8
zmp_ph97_C1
zmp_ph97_D2
zmp_ph97_D5
zmp_ph111_G5
zmp_ph111_G11
zmp_ph111_H1
zmp_ph111_B1a
zmp_ph111_B5
zmp_ph111_B8
zmp_ph111_B6
zmp_ph111_B7
zmp_ph111_B9
zmp_ph111_B10
zmp_ph114_G4
zmp_ph114_G11
zmp_ph114_H1
zmp_ph114_H2
zmp_ph114_A9
zmp_ph114_A11
zmp_ph114_B2
zmp_ph114_B4
zmp_ph114_D8
zmp_ph114_E2

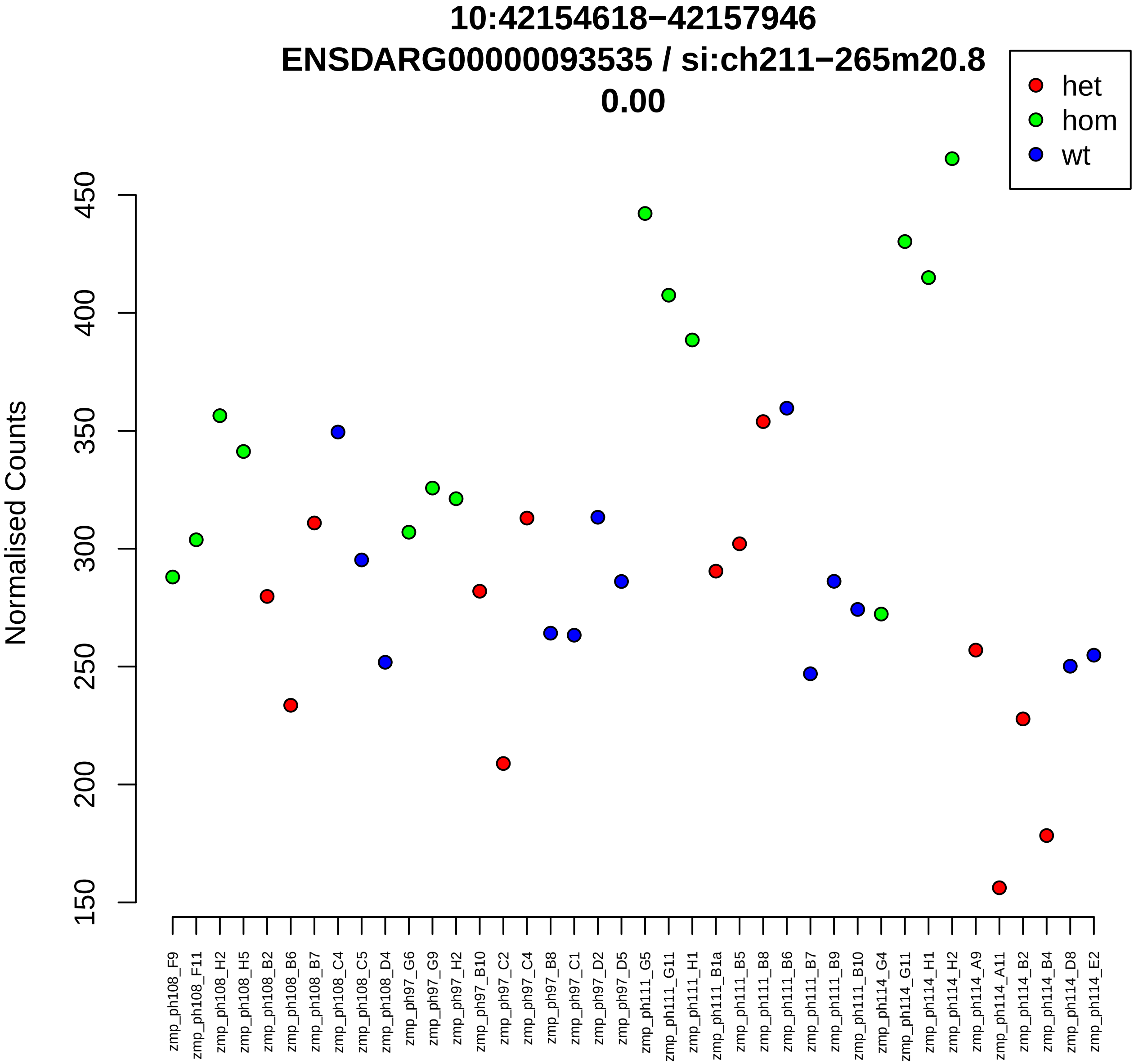

10:42154618−42157946
ENSDARG00000093535 / si:ch211−265m20.8
0.00
het
hom
wt
Normalised Counts
150
200
250
300
350
400
450
zmp_ph108_F9
zmp_ph108_F11
zmp_ph108_H2
zmp_ph108_H5
zmp_ph108_B2
zmp_ph108_B6
zmp_ph108_B7
zmp_ph108_C4
zmp_ph108_C5
zmp_ph108_D4
zmp_ph97_G6
zmp_ph97_G9
zmp_ph97_H2
zmp_ph97_B10
zmp_ph97_C2
zmp_ph97_C4
zmp_ph97_B8
zmp_ph97_C1
zmp_ph97_D2
zmp_ph97_D5
zmp_ph111_G5
zmp_ph111_G11
zmp_ph111_H1
zmp_ph111_B1a
zmp_ph111_B5
zmp_ph111_B8
zmp_ph111_B6
zmp_ph111_B7
zmp_ph111_B9
zmp_ph111_B10
zmp_ph114_G4
zmp_ph114_G11
zmp_ph114_H1
zmp_ph114_H2
zmp_ph114_A9
zmp_ph114_A11
zmp_ph114_B2
zmp_ph114_B4
zmp_ph114_D8
zmp_ph114_E2

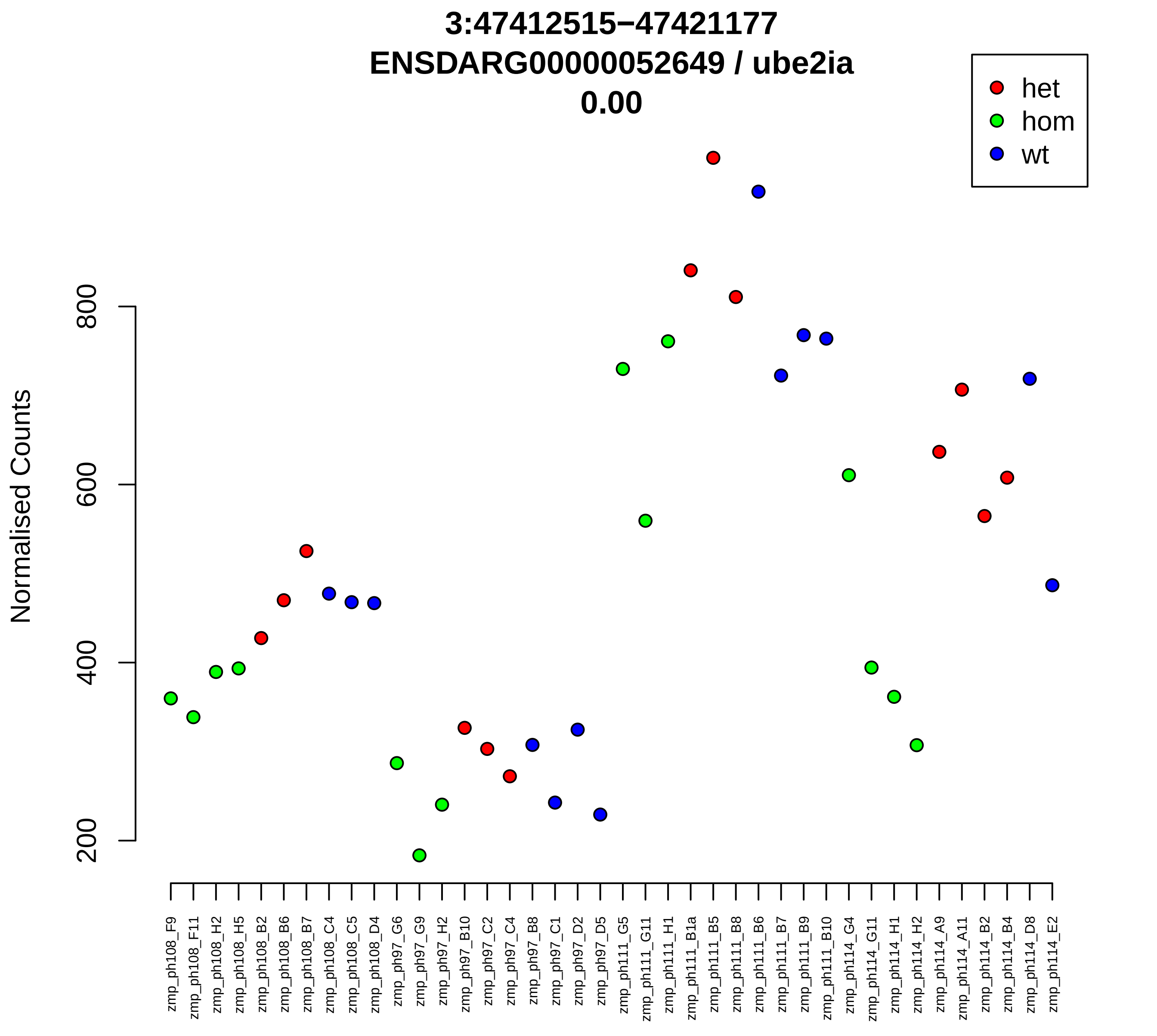

3:47412515−47421177
ENSDARG00000052649 / ube2ia
0.00
het
hom
wt
Normalised Counts
200
400
600
800
zmp_ph108_F9
zmp_ph108_F11
zmp_ph108_H2
zmp_ph108_H5
zmp_ph108_B2
zmp_ph108_B6
zmp_ph108_B7
zmp_ph108_C4
zmp_ph108_C5
zmp_ph108_D4
zmp_ph97_G6
zmp_ph97_G9
zmp_ph97_H2
zmp_ph97_B10
zmp_ph97_C2
zmp_ph97_C4
zmp_ph97_B8
zmp_ph97_C1
zmp_ph97_D2
zmp_ph97_D5
zmp_ph111_G5
zmp_ph111_G11
zmp_ph111_H1
zmp_ph111_B1a
zmp_ph111_B5
zmp_ph111_B8
zmp_ph111_B6
zmp_ph111_B7
zmp_ph111_B9
zmp_ph111_B10
zmp_ph114_G4
zmp_ph114_G11
zmp_ph114_H1
zmp_ph114_H2
zmp_ph114_A9
zmp_ph114_A11
zmp_ph114_B2
zmp_ph114_B4
zmp_ph114_D8
zmp_ph114_E2

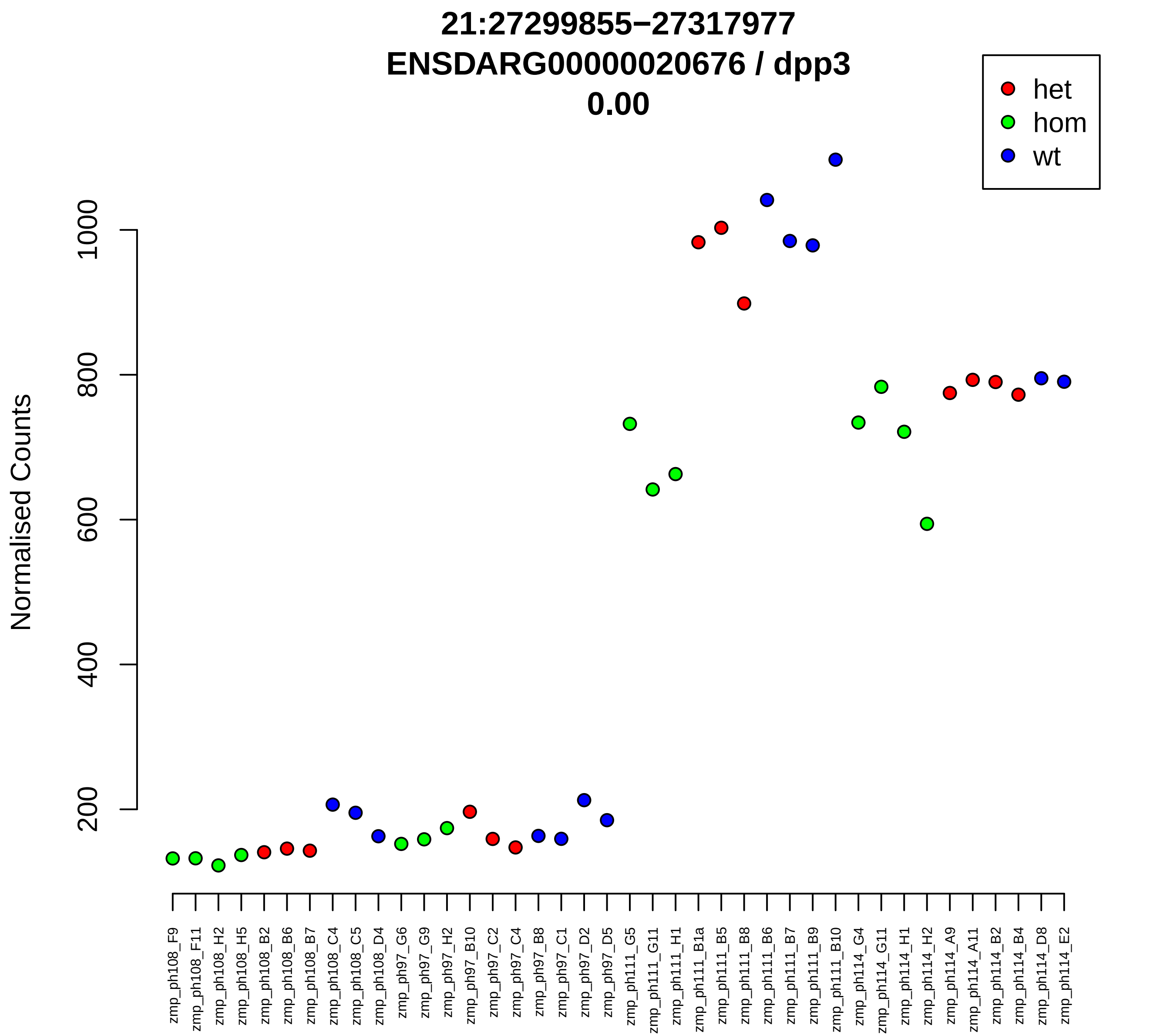

21:27299855−27317977
ENSDARG00000020676 / dpp3
0.00
het
hom
wt
Normalised Counts
200
400
600
800
1000
zmp_ph108_F9
zmp_ph108_F11
zmp_ph108_H2
zmp_ph108_H5
zmp_ph108_B2
zmp_ph108_B6
zmp_ph108_B7
zmp_ph108_C4
zmp_ph108_C5
zmp_ph108_D4
zmp_ph97_G6
zmp_ph97_G9
zmp_ph97_H2
zmp_ph97_B10
zmp_ph97_C2
zmp_ph97_C4
zmp_ph97_B8
zmp_ph97_C1
zmp_ph97_D2
zmp_ph97_D5
zmp_ph111_G5
zmp_ph111_G11
zmp_ph111_H1
zmp_ph111_B1a
zmp_ph111_B5
zmp_ph111_B8
zmp_ph111_B6
zmp_ph111_B7
zmp_ph111_B9
zmp_ph111_B10
zmp_ph114_G4
zmp_ph114_G11
zmp_ph114_H1
zmp_ph114_H2
zmp_ph114_A9
zmp_ph114_A11
zmp_ph114_B2
zmp_ph114_B4
zmp_ph114_D8
zmp_ph114_E2

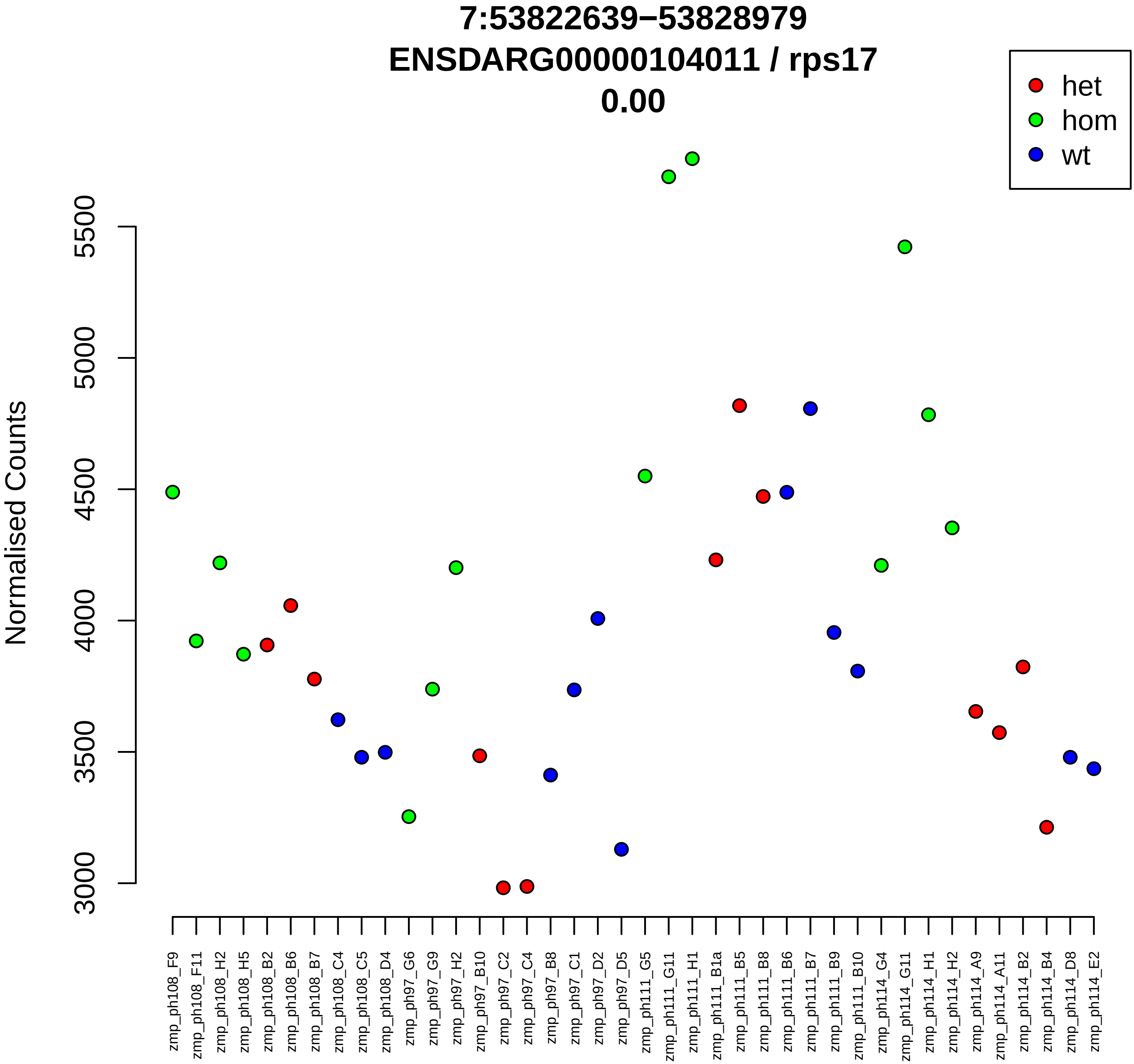

7:53822639−53828979
ENSDARG00000104011 / rps17
0.00
het
hom
wt
Normalised Counts
3000
3500
4000
4500
5000
5500
zmp_ph108_F9
zmp_ph108_F11
zmp_ph108_H2
zmp_ph108_H5
zmp_ph108_B2
zmp_ph108_B6
zmp_ph108_B7
zmp_ph108_C4
zmp_ph108_C5
zmp_ph108_D4
zmp_ph97_G6
zmp_ph97_G9
zmp_ph97_H2
zmp_ph97_B10
zmp_ph97_C2
zmp_ph97_C4
zmp_ph97_B8
zmp_ph97_C1
zmp_ph97_D2
zmp_ph97_D5
zmp_ph111_G5
zmp_ph111_G11
zmp_ph111_H1
zmp_ph111_B1a
zmp_ph111_B5
zmp_ph111_B8
zmp_ph111_B6
zmp_ph111_B7
zmp_ph111_B9
zmp_ph111_B10
zmp_ph114_G4
zmp_ph114_G11
zmp_ph114_H1
zmp_ph114_H2
zmp_ph114_A9
zmp_ph114_A11
zmp_ph114_B2
zmp_ph114_B4
zmp_ph114_D8
zmp_ph114_E2

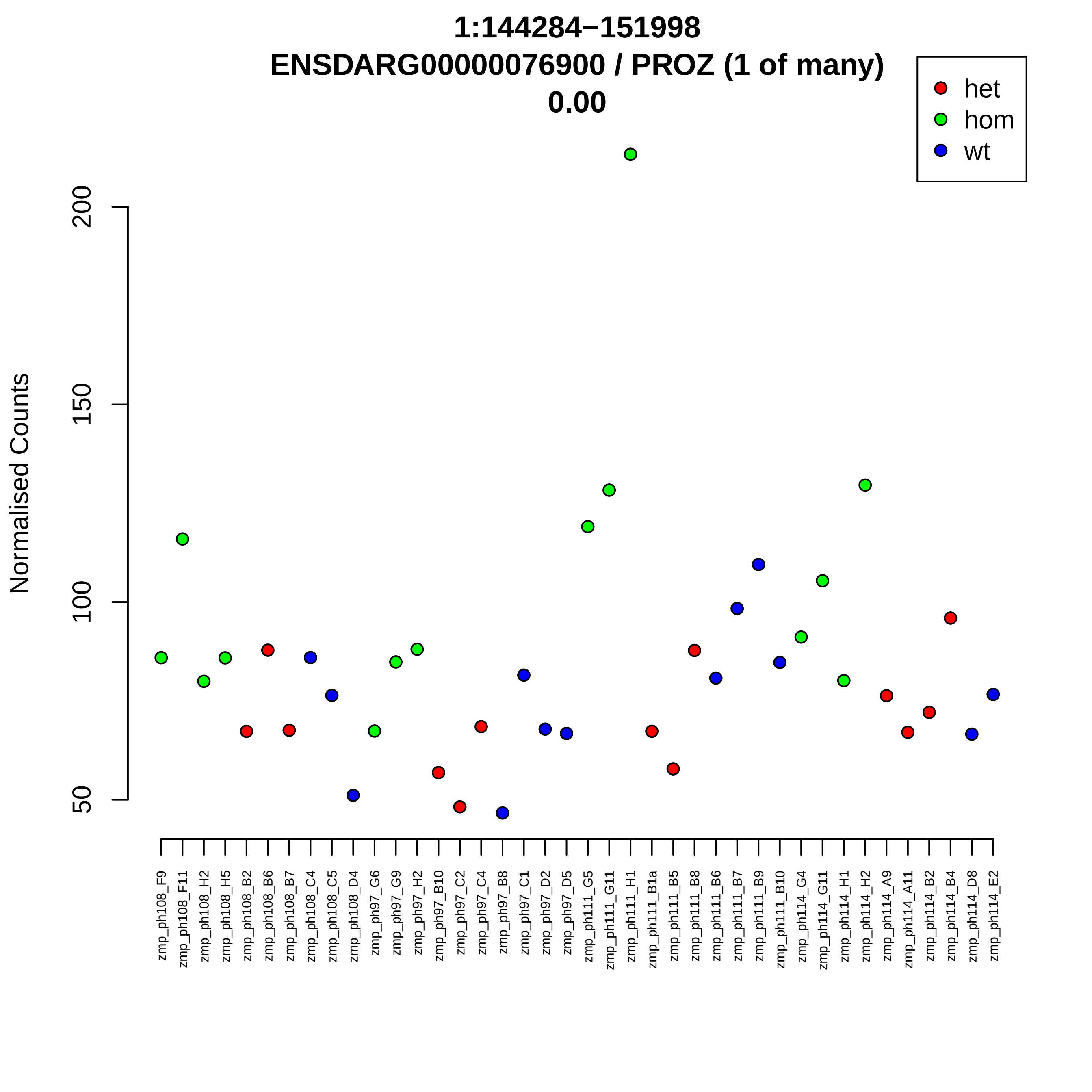
1:144284−151998
ENSDARG00000076900 / PROZ (1 of many)
0.00
het
hom
wt
Normalised Counts
200
150
100
50
zmp_ph108_F9
zmp_ph108_F11
zmp_ph108_H2
zmp_ph108_H5
zmp_ph108_B2
zmp_ph108_B6
zmp_ph108_B7
zmp_ph108_C4
zmp_ph108_C5
zmp_ph108_D4
zmp_ph97_G6
zmp_ph97_G9
zmp_ph97_H2
zmp_ph97_B10
zmp_ph97_C2
zmp_ph97_C4
zmp_ph97_B8
zmp_ph97_C1
zmp_ph97_D2
zmp_ph97_D5
zmp_ph111_G5
zmp_ph111_G11
zmp_ph111_H1
zmp_ph111_B1a
zmp_ph111_B5
zmp_ph111_B8
zmp_ph111_B6
zmp_ph111_B7
zmp_ph111_B9
zmp_ph111_B10
zmp_ph114_G4
zmp_ph114_G11
zmp_ph114_H1
zmp_ph114_H2
zmp_ph114_A9
zmp_ph114_A11
zmp_ph114_B2
zmp_ph114_B4
zmp_ph114_D8
zmp_ph114_E2

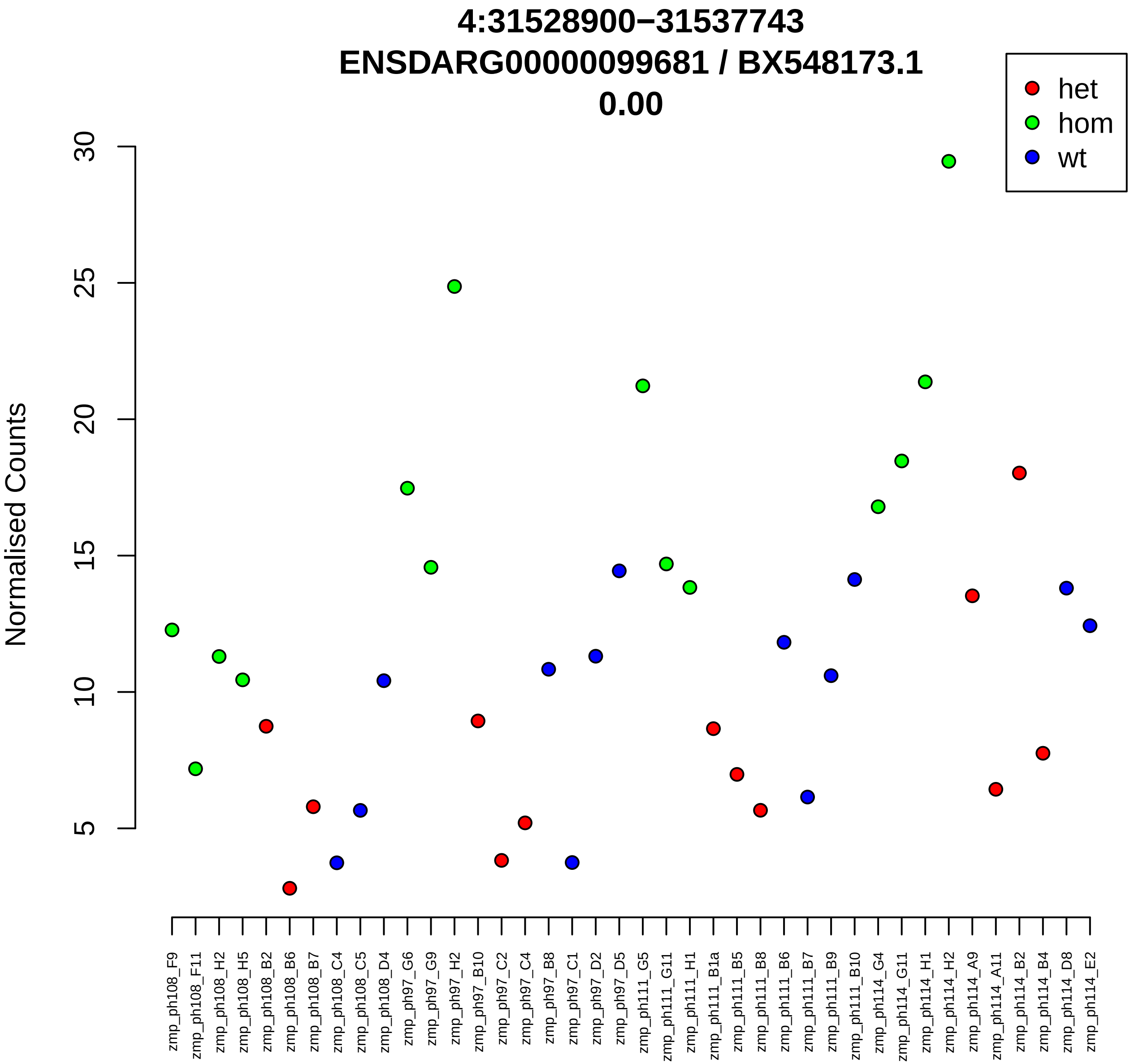
4:31528900−31537743
ENSDARG00000099681 / BX548173.1
0.00
het
hom
wt
Normalised Counts
30
25
20
15
10
5
zmp_ph108_F9
zmp_ph108_F11
zmp_ph108_H2
zmp_ph108_H5
zmp_ph108_B2
zmp_ph108_B6
zmp_ph108_B7
zmp_ph108_C4
zmp_ph108_C5
zmp_ph108_D4
zmp_ph97_G6
zmp_ph97_G9
zmp_ph97_H2
zmp_ph97_B10
zmp_ph97_C2
zmp_ph97_C4
zmp_ph97_B8
zmp_ph97_C1
zmp_ph97_D2
zmp_ph97_D5
zmp_ph111_G5
zmp_ph111_G11
zmp_ph111_H1
zmp_ph111_B1a
zmp_ph111_B5
zmp_ph111_B8
zmp_ph111_B6
zmp_ph111_B7
zmp_ph111_B9
zmp_ph111_B10
zmp_ph114_G4
zmp_ph114_G11
zmp_ph114_H1
zmp_ph114_H2
zmp_ph114_A9
zmp_ph114_A11
zmp_ph114_B2
zmp_ph114_B4
zmp_ph114_D8
zmp_ph114_E2

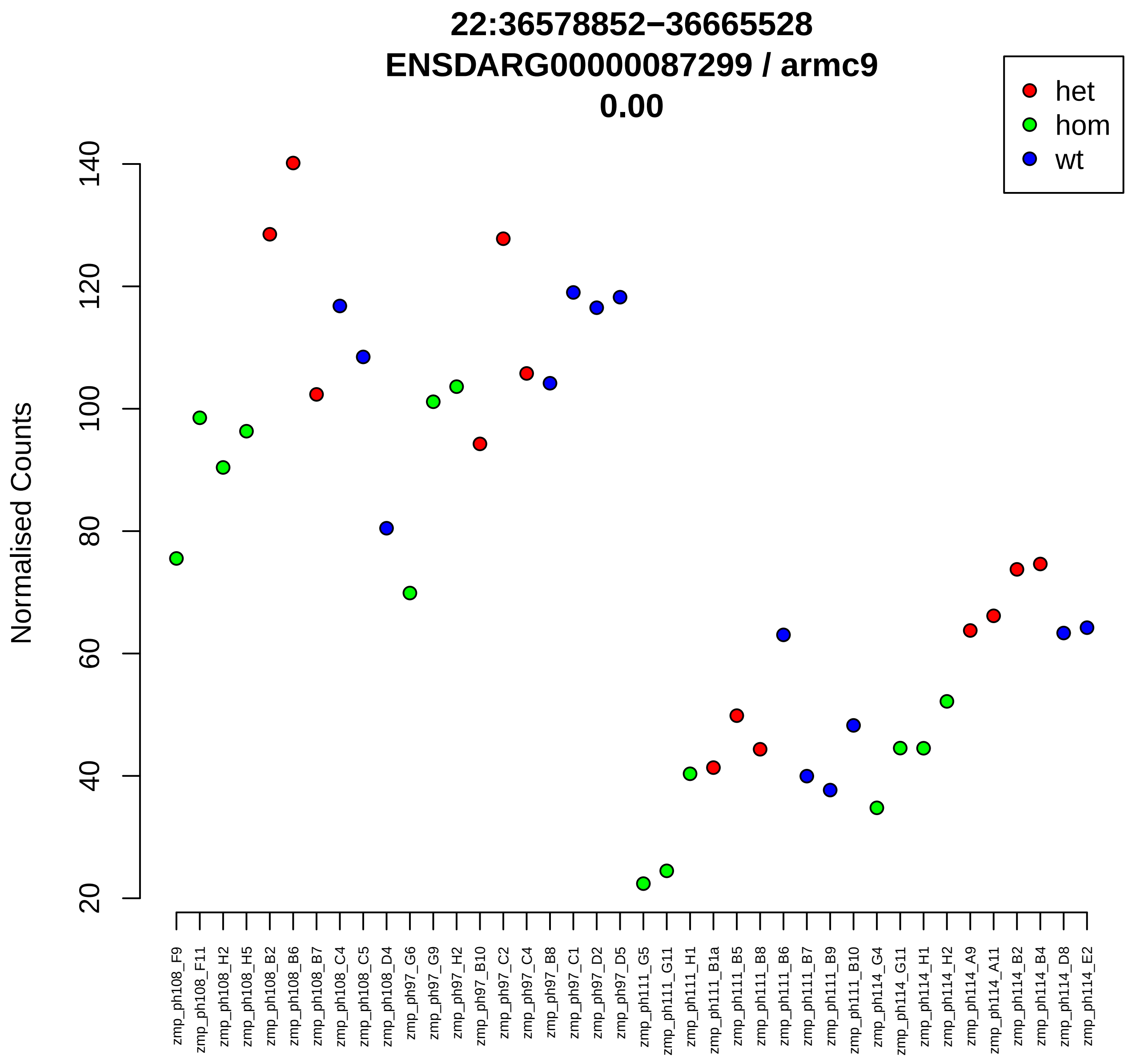

22:36578852−36665528
ENSDARG00000087299 / armc9
0.00
het
hom
wt
Normalised Counts
20
40
60
80
100
120
140
zmp_ph108_F9
zmp_ph108_F11
zmp_ph108_H2
zmp_ph108_H5
zmp_ph108_B2
zmp_ph108_B6
zmp_ph108_B7
zmp_ph108_C4
zmp_ph108_C5
zmp_ph108_D4
zmp_ph97_G6
zmp_ph97_G9
zmp_ph97_H2
zmp_ph97_B10
zmp_ph97_C2
zmp_ph97_C4
zmp_ph97_B8
zmp_ph97_C1
zmp_ph97_D2
zmp_ph97_D5
zmp_ph111_G5
zmp_ph111_G11
zmp_ph111_H1
zmp_ph111_B1a
zmp_ph111_B5
zmp_ph111_B8
zmp_ph111_B6
zmp_ph111_B7
zmp_ph111_B9
zmp_ph111_B10
zmp_ph114_G4
zmp_ph114_G11
zmp_ph114_H1
zmp_ph114_H2
zmp_ph114_A9
zmp_ph114_A11
zmp_ph114_B2
zmp_ph114_B4
zmp_ph114_D8
zmp_ph114_E2

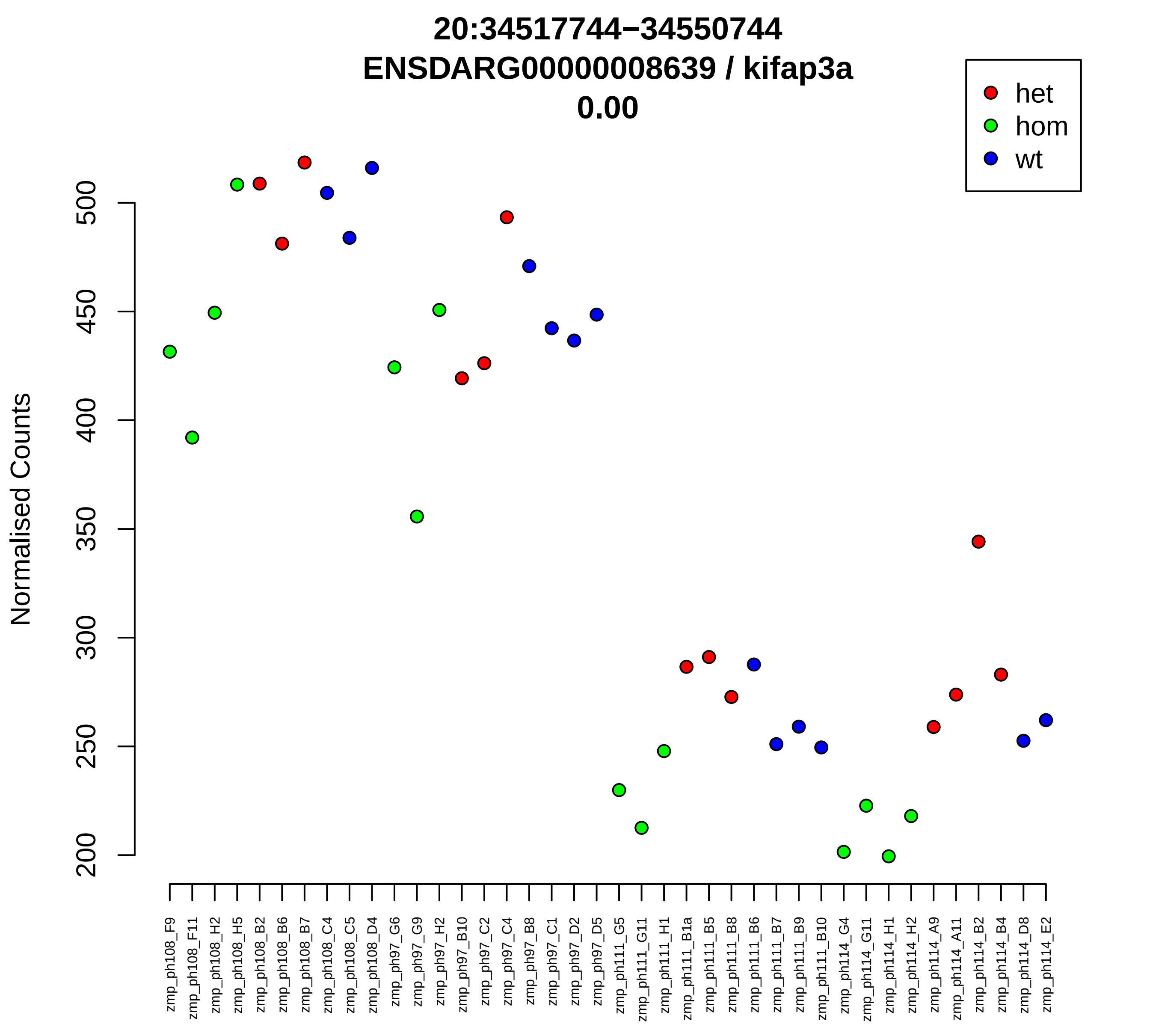

20:34517744−34550744
ENSDARG00000008639 / kifap3a
0.00
het
hom
wt
Normalised Counts
200
250
300
350
400
450
500
zmp_ph108_F9
zmp_ph108_F11
zmp_ph108_H2
zmp_ph108_H5
zmp_ph108_B2
zmp_ph108_B6
zmp_ph108_B7
zmp_ph108_C4
zmp_ph108_C5
zmp_ph108_D4
zmp_ph97_G6
zmp_ph97_G9
zmp_ph97_H2
zmp_ph97_B10
zmp_ph97_C2
zmp_ph97_C4
zmp_ph97_B8
zmp_ph97_C1
zmp_ph97_D2
zmp_ph97_D5
zmp_ph111_G5
zmp_ph111_G11
zmp_ph111_H1
zmp_ph111_B1a
zmp_ph111_B5
zmp_ph111_B8
zmp_ph111_B6
zmp_ph111_B7
zmp_ph111_B9
zmp_ph111_B10
zmp_ph114_G4
zmp_ph114_G11
zmp_ph114_H1
zmp_ph114_H2
zmp_ph114_A9
zmp_ph114_A11
zmp_ph114_B2
zmp_ph114_B4
zmp_ph114_D8
zmp_ph114_E2

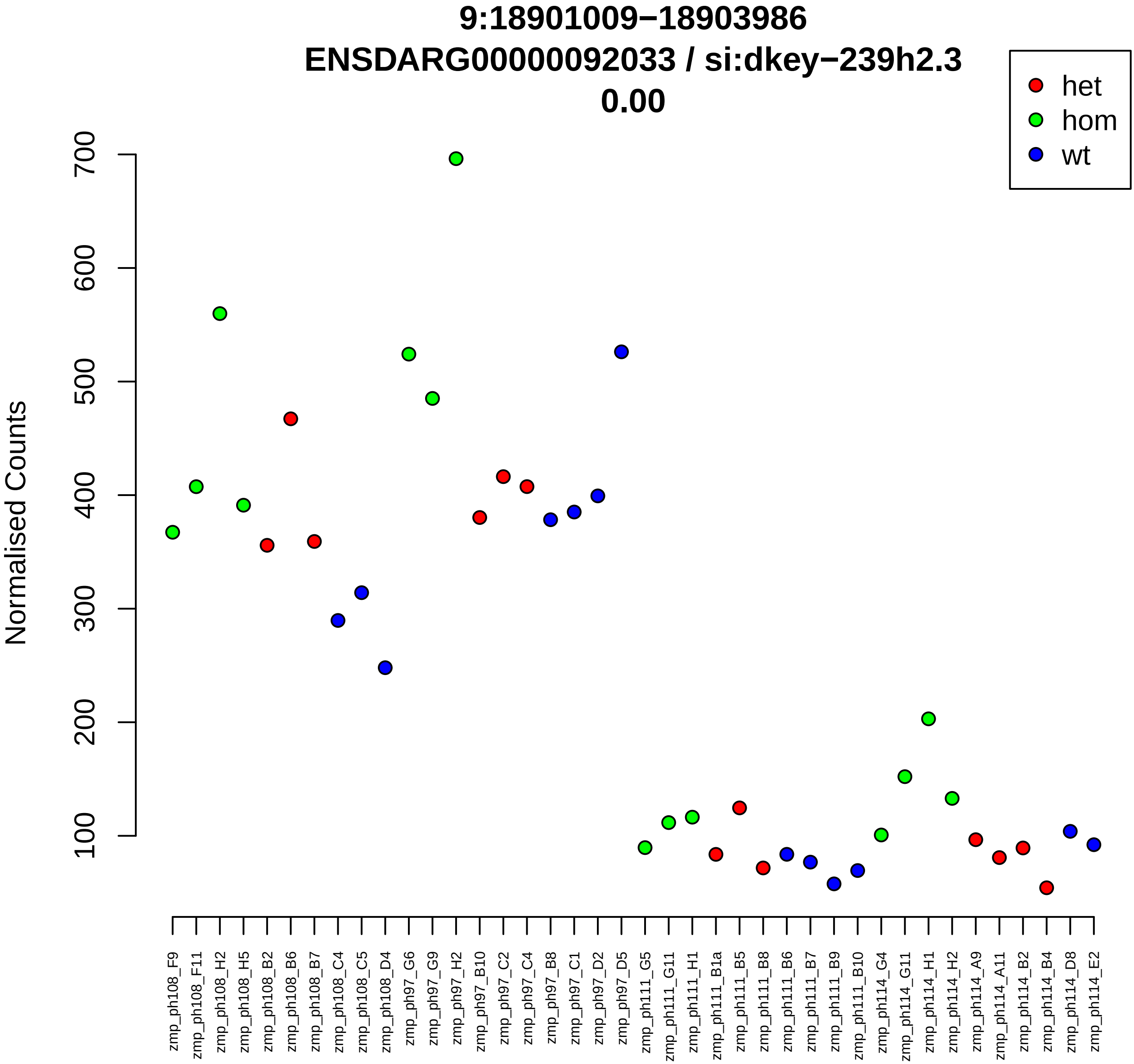
9:18901009−18903986
ENSDARG00000092033 / si:dkey−239h2.3
0.00
het
hom
wt
Normalised Counts
100
200
300
400
500
600
700
zmp_ph108_F9
zmp_ph108_F11
zmp_ph108_H2
zmp_ph108_H5
zmp_ph108_B2
zmp_ph108_B6
zmp_ph108_B7
zmp_ph108_C4
zmp_ph108_C5
zmp_ph108_D4
zmp_ph97_G6
zmp_ph97_G9
zmp_ph97_H2
zmp_ph97_B10
zmp_ph97_C2
zmp_ph97_C4
zmp_ph97_B8
zmp_ph97_C1
zmp_ph97_D2
zmp_ph97_D5
zmp_ph111_G5
zmp_ph111_G11
zmp_ph111_H1
zmp_ph111_B1a
zmp_ph111_B5
zmp_ph111_B8
zmp_ph111_B6
zmp_ph111_B7
zmp_ph111_B9
zmp_ph111_B10
zmp_ph114_G4
zmp_ph114_G11
zmp_ph114_H1
zmp_ph114_H2
zmp_ph114_A9
zmp_ph114_A11
zmp_ph114_B2
zmp_ph114_B4
zmp_ph114_D8
zmp_ph114_E2

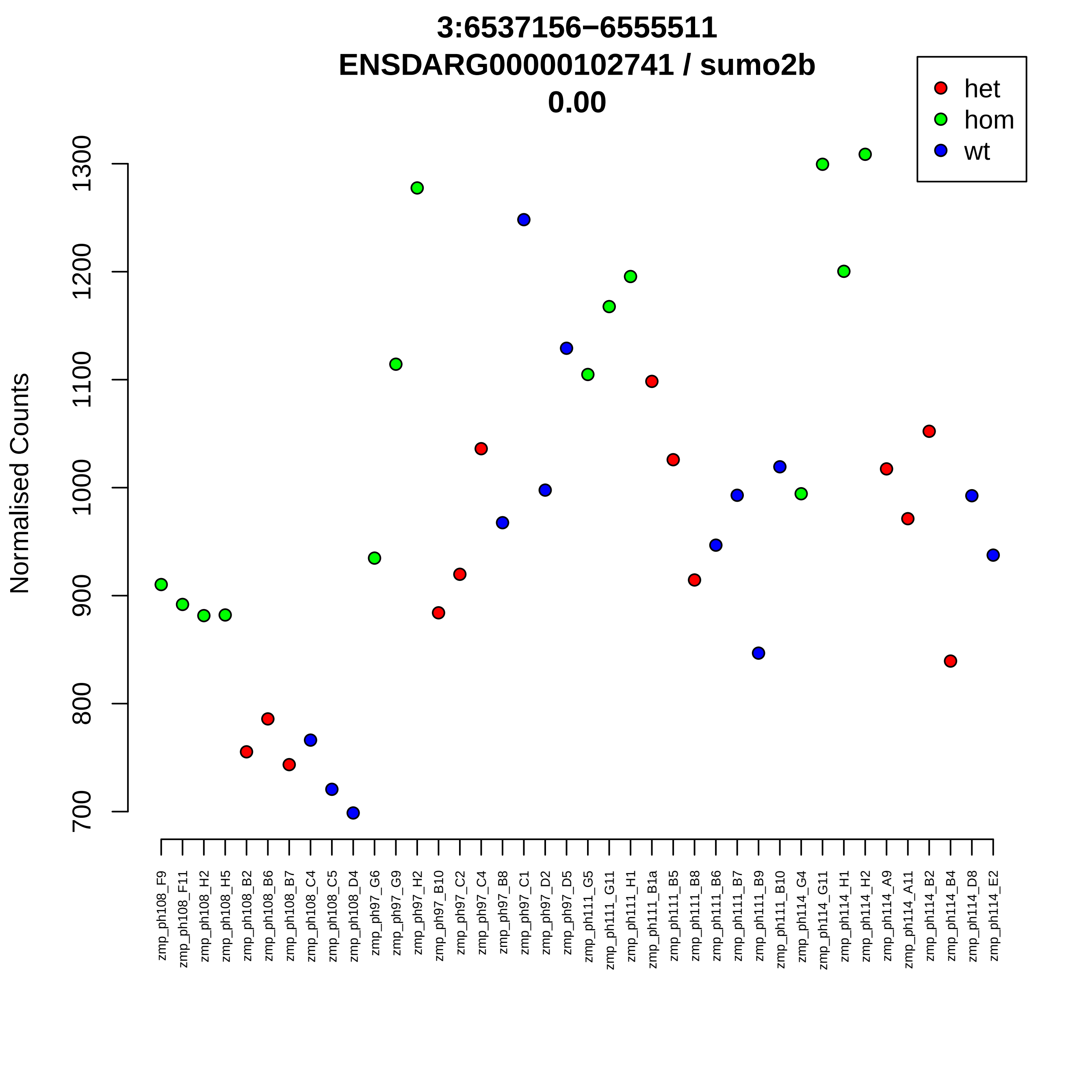

3:6537156−6555511
ENSDARG00000102741 / sumo2b
0.00
het
hom
wt
Normalised Counts
700
800
900
1000
1100
1200
1300
zmp_ph108_F9
zmp_ph108_F11
zmp_ph108_H2
zmp_ph108_H5
zmp_ph108_B2
zmp_ph108_B6
zmp_ph108_B7
zmp_ph108_C4
zmp_ph108_C5
zmp_ph108_D4
zmp_ph97_G6
zmp_ph97_G9
zmp_ph97_H2
zmp_ph97_B10
zmp_ph97_C2
zmp_ph97_C4
zmp_ph97_B8
zmp_ph97_C1
zmp_ph97_D2
zmp_ph97_D5
zmp_ph111_G5
zmp_ph111_G11
zmp_ph111_H1
zmp_ph111_B1a
zmp_ph111_B5
zmp_ph111_B8
zmp_ph111_B6
zmp_ph111_B7
zmp_ph111_B9
zmp_ph111_B10
zmp_ph114_G4
zmp_ph114_G11
zmp_ph114_H1
zmp_ph114_H2
zmp_ph114_A9
zmp_ph114_A11
zmp_ph114_B2
zmp_ph114_B4
zmp_ph114_D8
zmp_ph114_E2

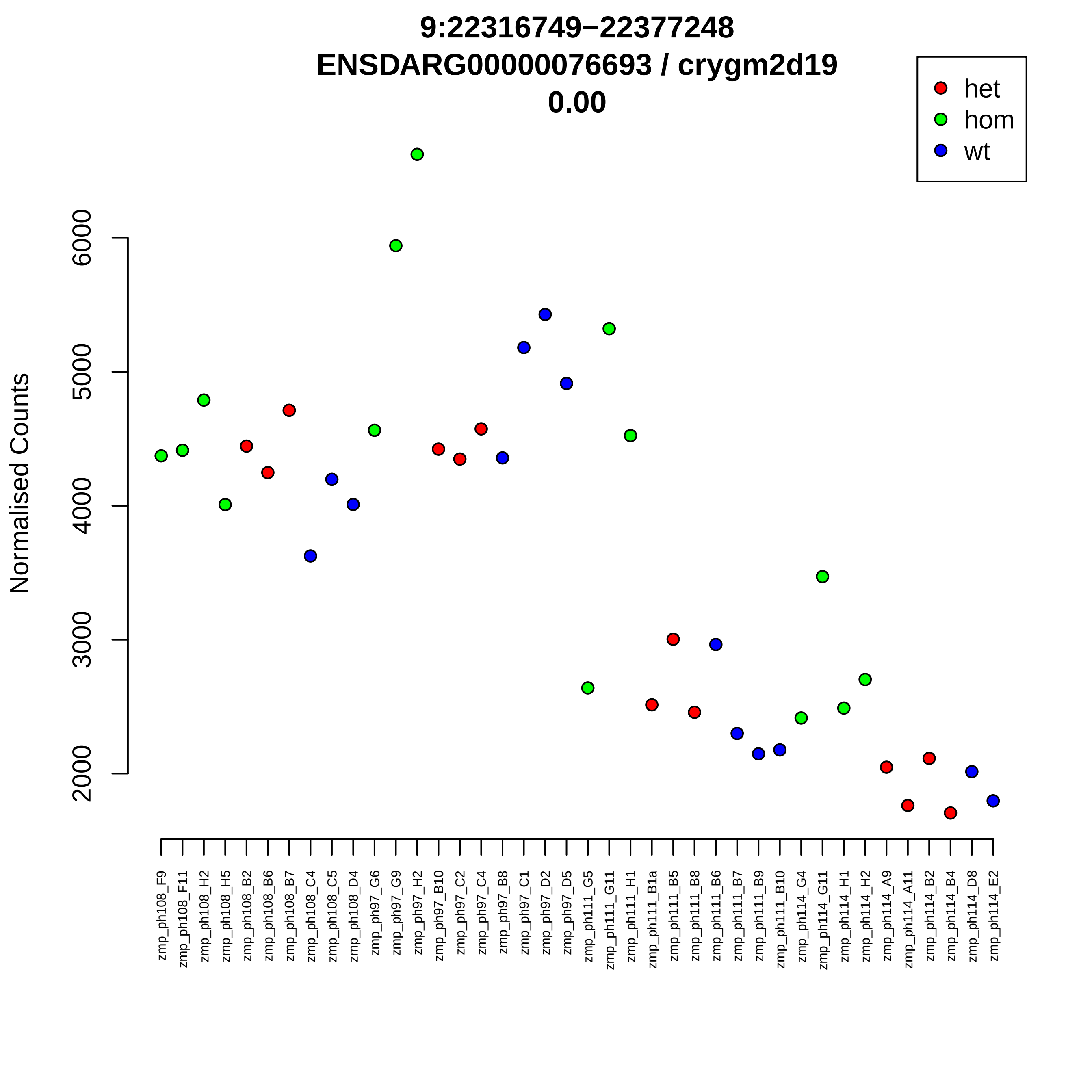
9:22316749−22377248
ENSDARG00000076693 / crygm2d19
0.00
het
hom
wt
Normalised Counts
2000
3000
4000
5000
6000
zmp_ph108_F9
zmp_ph108_F11
zmp_ph108_H2
zmp_ph108_H5
zmp_ph108_B2
zmp_ph108_B6
zmp_ph108_B7
zmp_ph108_C4
zmp_ph108_C5
zmp_ph108_D4
zmp_ph97_G6
zmp_ph97_G9
zmp_ph97_H2
zmp_ph97_B10
zmp_ph97_C2
zmp_ph97_C4
zmp_ph97_B8
zmp_ph97_C1
zmp_ph97_D2
zmp_ph97_D5
zmp_ph111_G5
zmp_ph111_G11
zmp_ph111_H1
zmp_ph111_B1a
zmp_ph111_B5
zmp_ph111_B8
zmp_ph111_B6
zmp_ph111_B7
zmp_ph111_B9
zmp_ph111_B10
zmp_ph114_G4
zmp_ph114_G11
zmp_ph114_H1
zmp_ph114_H2
zmp_ph114_A9
zmp_ph114_A11
zmp_ph114_B2
zmp_ph114_B4
zmp_ph114_D8
zmp_ph114_E2

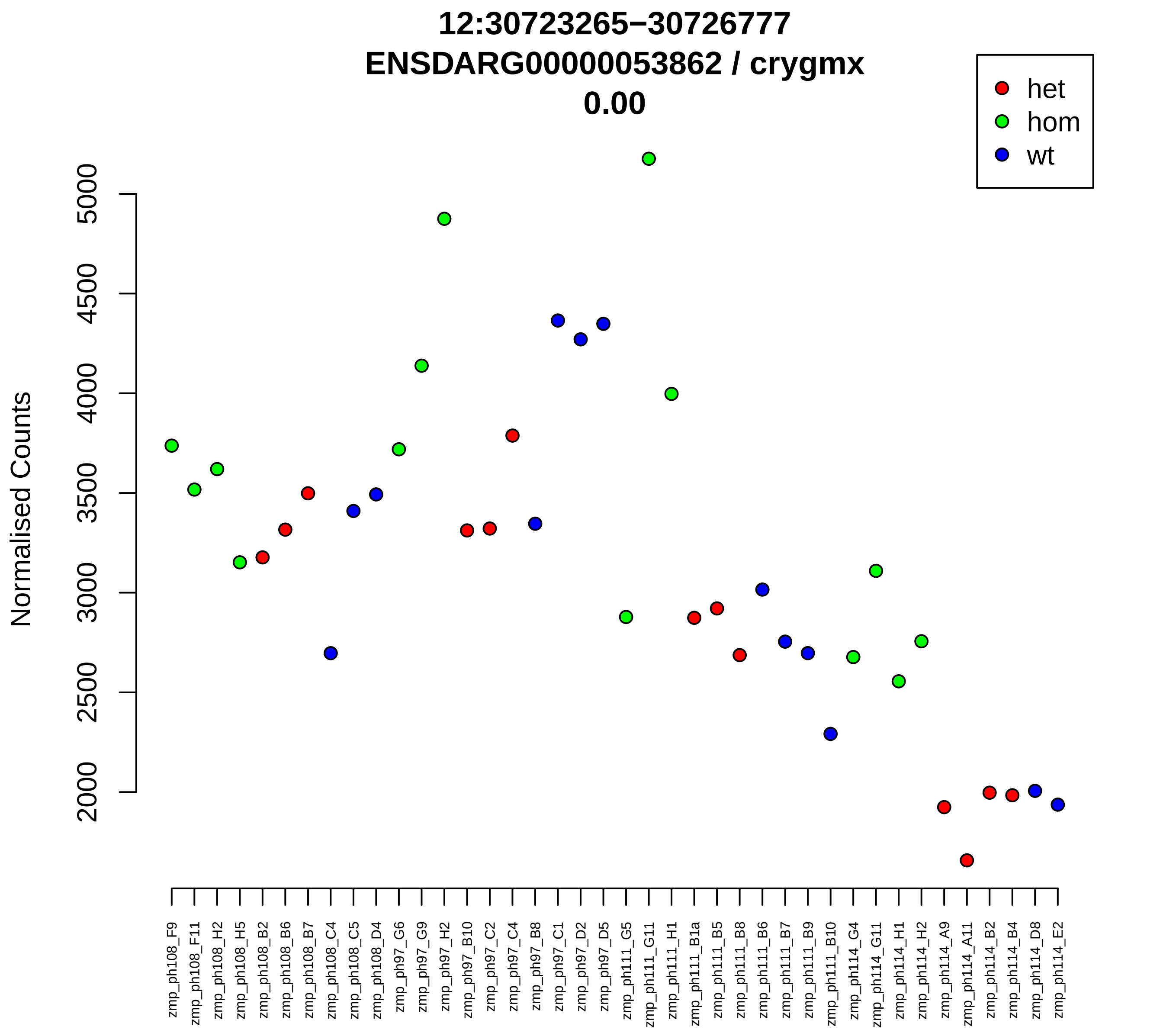
12:30723265−30726777
ENSDARG00000053862 / crygmx
0.00
het
hom
wt
Normalised Counts
5000
4500
4000
3500
3000
2500
2000
zmp_ph108_F9
zmp_ph108_F11
zmp_ph108_H2
zmp_ph108_H5
zmp_ph108_B2
zmp_ph108_B6
zmp_ph108_B7
zmp_ph108_C4
zmp_ph108_C5
zmp_ph108_D4
zmp_ph97_G6
zmp_ph97_G9
zmp_ph97_H2
zmp_ph97_B10
zmp_ph97_C2
zmp_ph97_C4
zmp_ph97_B8
zmp_ph97_C1
zmp_ph97_D2
zmp_ph97_D5
zmp_ph111_G5
zmp_ph111_G11
zmp_ph111_H1
zmp_ph111_B1a
zmp_ph111_B5
zmp_ph111_B8
zmp_ph111_B6
zmp_ph111_B7
zmp_ph111_B9
zmp_ph111_B10
zmp_ph114_G4
zmp_ph114_G11
zmp_ph114_H1
zmp_ph114_H2
zmp_ph114_A9
zmp_ph114_A11
zmp_ph114_B2
zmp_ph114_B4
zmp_ph114_D8
zmp_ph114_E2

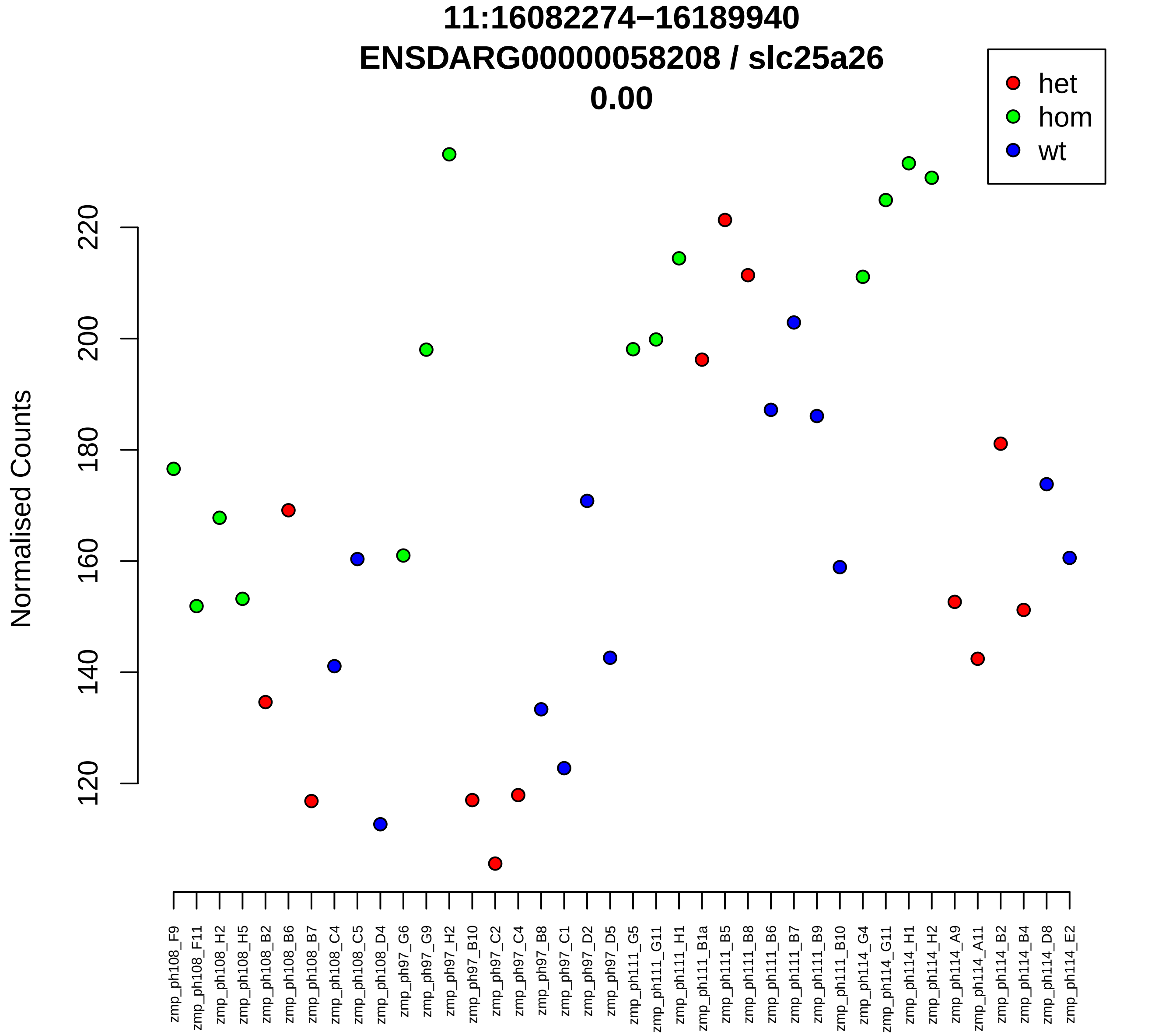

11:16082274−16189940
ENSDARG00000058208 / slc25a26
0.00
het
hom
wt
Normalised Counts
120
140
160
180
200
220
zmp_ph108_F9
zmp_ph108_F11
zmp_ph108_H2
zmp_ph108_H5
zmp_ph108_B2
zmp_ph108_B6
zmp_ph108_B7
zmp_ph108_C4
zmp_ph108_C5
zmp_ph108_D4
zmp_ph97_G6
zmp_ph97_G9
zmp_ph97_H2
zmp_ph97_B10
zmp_ph97_C2
zmp_ph97_C4
zmp_ph97_B8
zmp_ph97_C1
zmp_ph97_D2
zmp_ph97_D5
zmp_ph111_G5
zmp_ph111_G11
zmp_ph111_H1
zmp_ph111_B1a
zmp_ph111_B5
zmp_ph111_B8
zmp_ph111_B6
zmp_ph111_B7
zmp_ph111_B9
zmp_ph111_B10
zmp_ph114_G4
zmp_ph114_G11
zmp_ph114_H1
zmp_ph114_H2
zmp_ph114_A9
zmp_ph114_A11
zmp_ph114_B2
zmp_ph114_B4
zmp_ph114_D8
zmp_ph114_E2

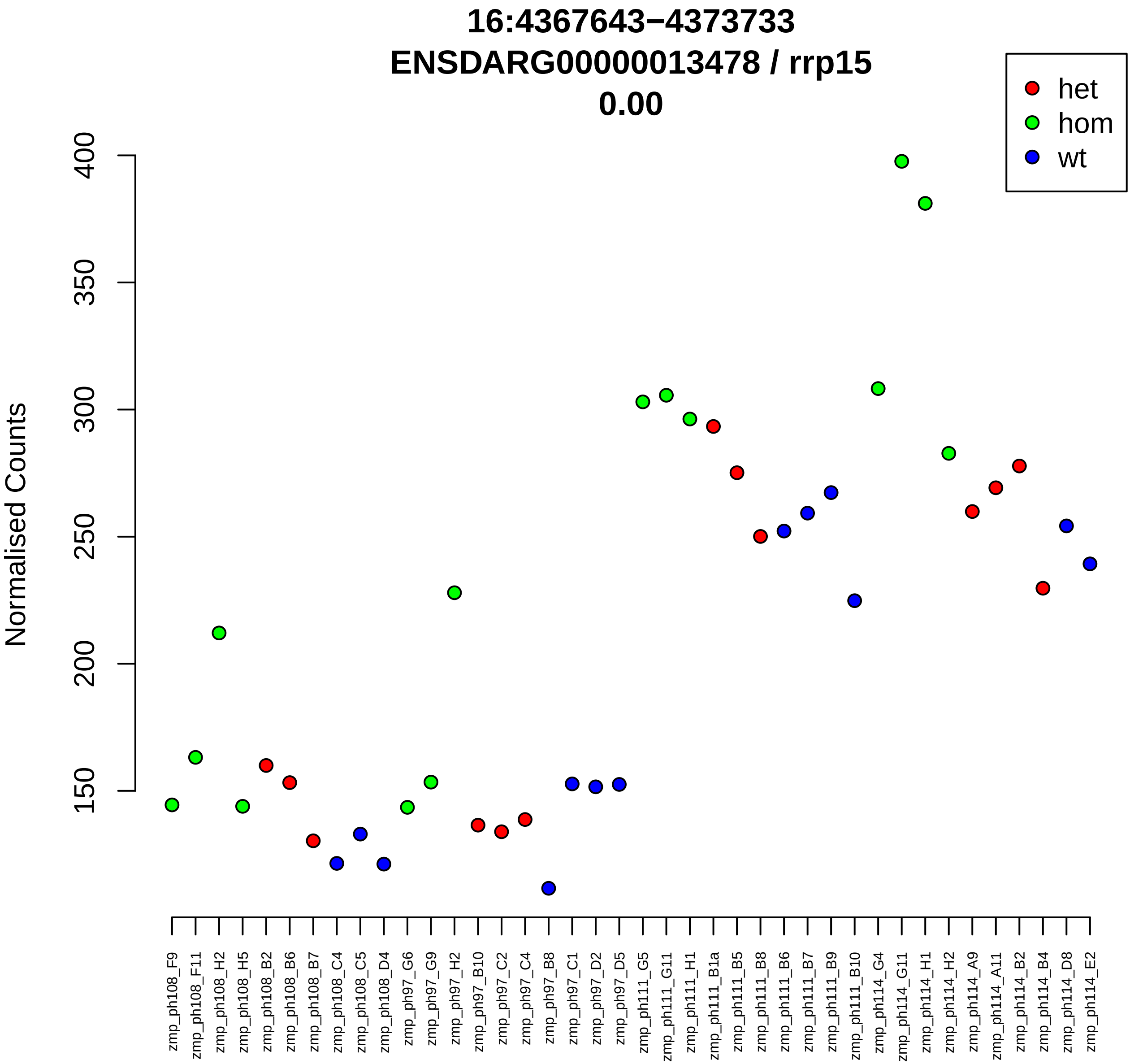
16:4367643−4373733
ENSDARG00000013478 / rrp15
0.00
het
hom
wt
Normalised Counts
150
200
250
300
350
400
zmp_ph108_F9
zmp_ph108_F11
zmp_ph108_H2
zmp_ph108_H5
zmp_ph108_B2
zmp_ph108_B6
zmp_ph108_B7
zmp_ph108_C4
zmp_ph108_C5
zmp_ph108_D4
zmp_ph97_G6
zmp_ph97_G9
zmp_ph97_H2
zmp_ph97_B10
zmp_ph97_C2
zmp_ph97_C4
zmp_ph97_B8
zmp_ph97_C1
zmp_ph97_D2
zmp_ph97_D5
zmp_ph111_G5
zmp_ph111_G11
zmp_ph111_H1
zmp_ph111_B1a
zmp_ph111_B5
zmp_ph111_B8
zmp_ph111_B6
zmp_ph111_B7
zmp_ph111_B9
zmp_ph111_B10
zmp_ph114_G4
zmp_ph114_G11
zmp_ph114_H1
zmp_ph114_H2
zmp_ph114_A9
zmp_ph114_A11
zmp_ph114_B2
zmp_ph114_B4
zmp_ph114_D8
zmp_ph114_E2

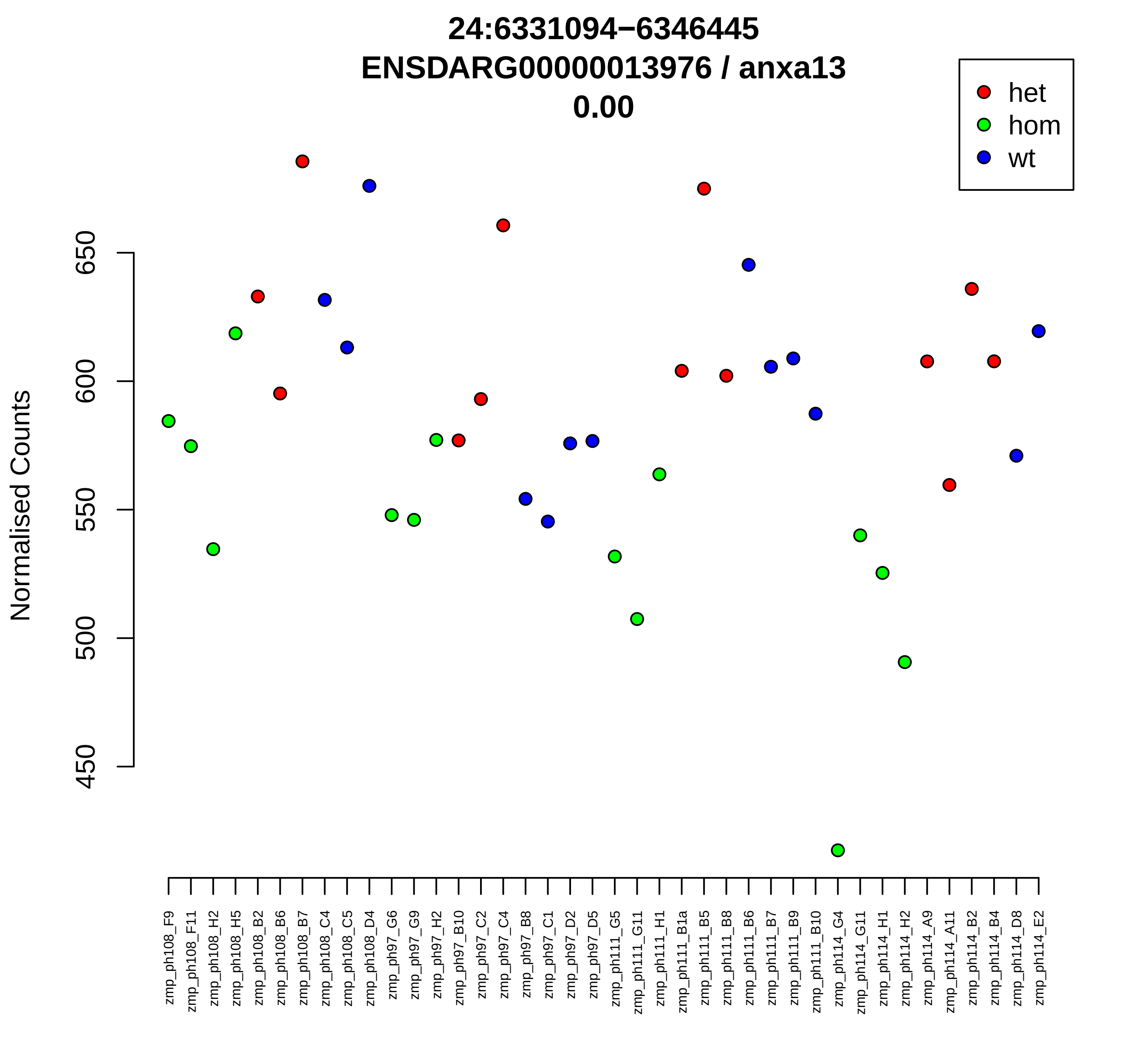

24:6331094−6346445
ENSDARG00000013976 / anxa13
0.00
het
hom
wt
Normalised Counts
450
500
550
600
650
zmp_ph108_F9
zmp_ph108_F11
zmp_ph108_H2
zmp_ph108_H5
zmp_ph108_B2
zmp_ph108_B6
zmp_ph108_B7
zmp_ph108_C4
zmp_ph108_C5
zmp_ph108_D4
zmp_ph97_G6
zmp_ph97_G9
zmp_ph97_H2
zmp_ph97_B10
zmp_ph97_C2
zmp_ph97_C4
zmp_ph97_B8
zmp_ph97_C1
zmp_ph97_D2
zmp_ph97_D5
zmp_ph111_G5
zmp_ph111_G11
zmp_ph111_H1
zmp_ph111_B1a
zmp_ph111_B5
zmp_ph111_B8
zmp_ph111_B6
zmp_ph111_B7
zmp_ph111_B9
zmp_ph111_B10
zmp_ph114_G4
zmp_ph114_G11
zmp_ph114_H1
zmp_ph114_H2
zmp_ph114_A9
zmp_ph114_A11
zmp_ph114_B2
zmp_ph114_B4
zmp_ph114_D8
zmp_ph114_E2

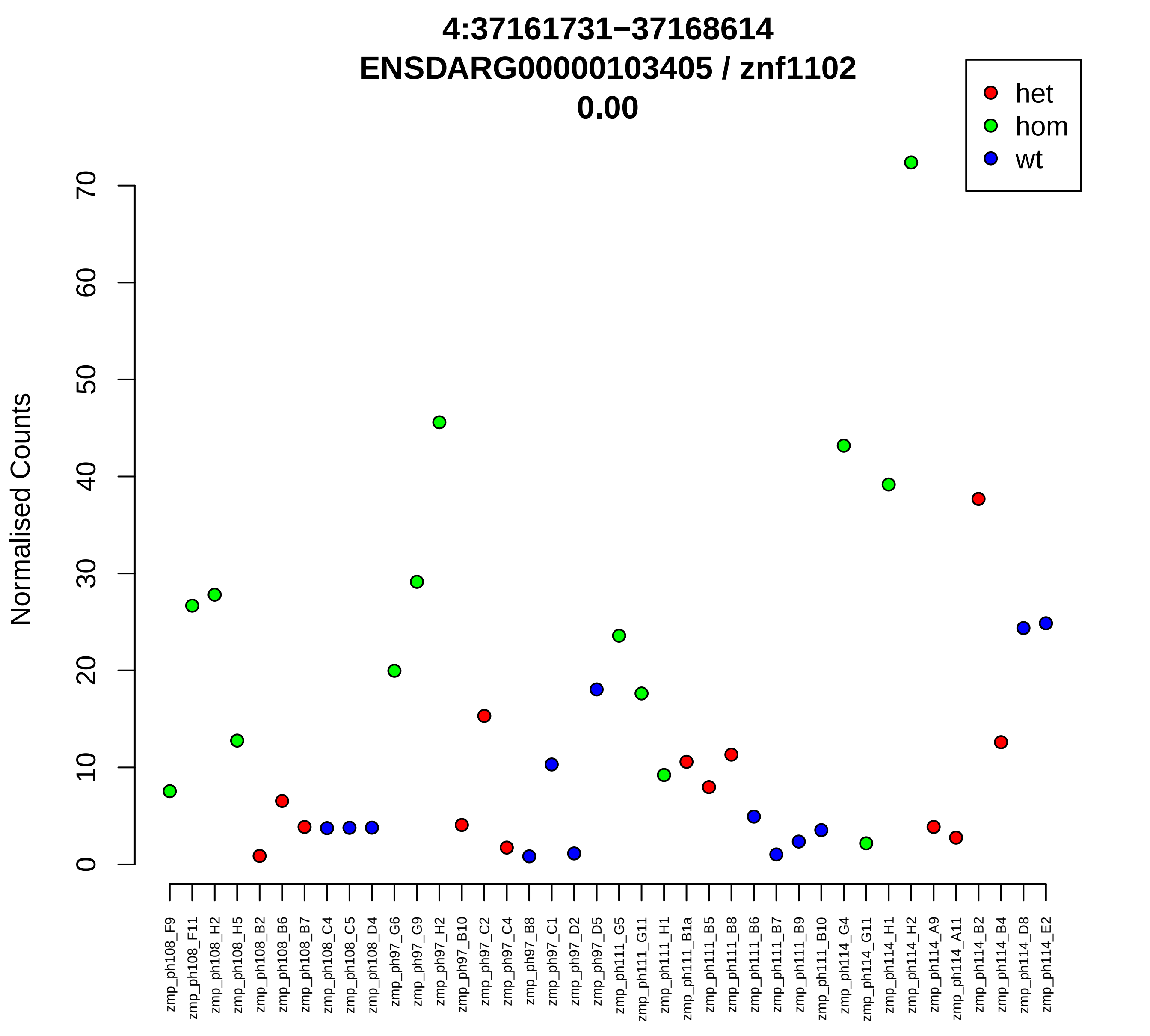
4:37161731−37168614
ENSDARG00000103405 / znf1102
0.00
het
hom
wt
Normalised Counts
0
10
20
30
40
50
60
70
zmp_ph108_F9
zmp_ph108_F11
zmp_ph108_H2
zmp_ph108_H5
zmp_ph108_B2
zmp_ph108_B6
zmp_ph108_B7
zmp_ph108_C4
zmp_ph108_C5
zmp_ph108_D4
zmp_ph97_G6
zmp_ph97_G9
zmp_ph97_H2
zmp_ph97_B10
zmp_ph97_C2
zmp_ph97_C4
zmp_ph97_B8
zmp_ph97_C1
zmp_ph97_D2
zmp_ph97_D5
zmp_ph111_G5
zmp_ph111_G11
zmp_ph111_H1
zmp_ph111_B1a
zmp_ph111_B5
zmp_ph111_B8
zmp_ph111_B6
zmp_ph111_B7
zmp_ph111_B9
zmp_ph111_B10
zmp_ph114_G4
zmp_ph114_G11
zmp_ph114_H1
zmp_ph114_H2
zmp_ph114_A9
zmp_ph114_A11
zmp_ph114_B2
zmp_ph114_B4
zmp_ph114_D8
zmp_ph114_E2

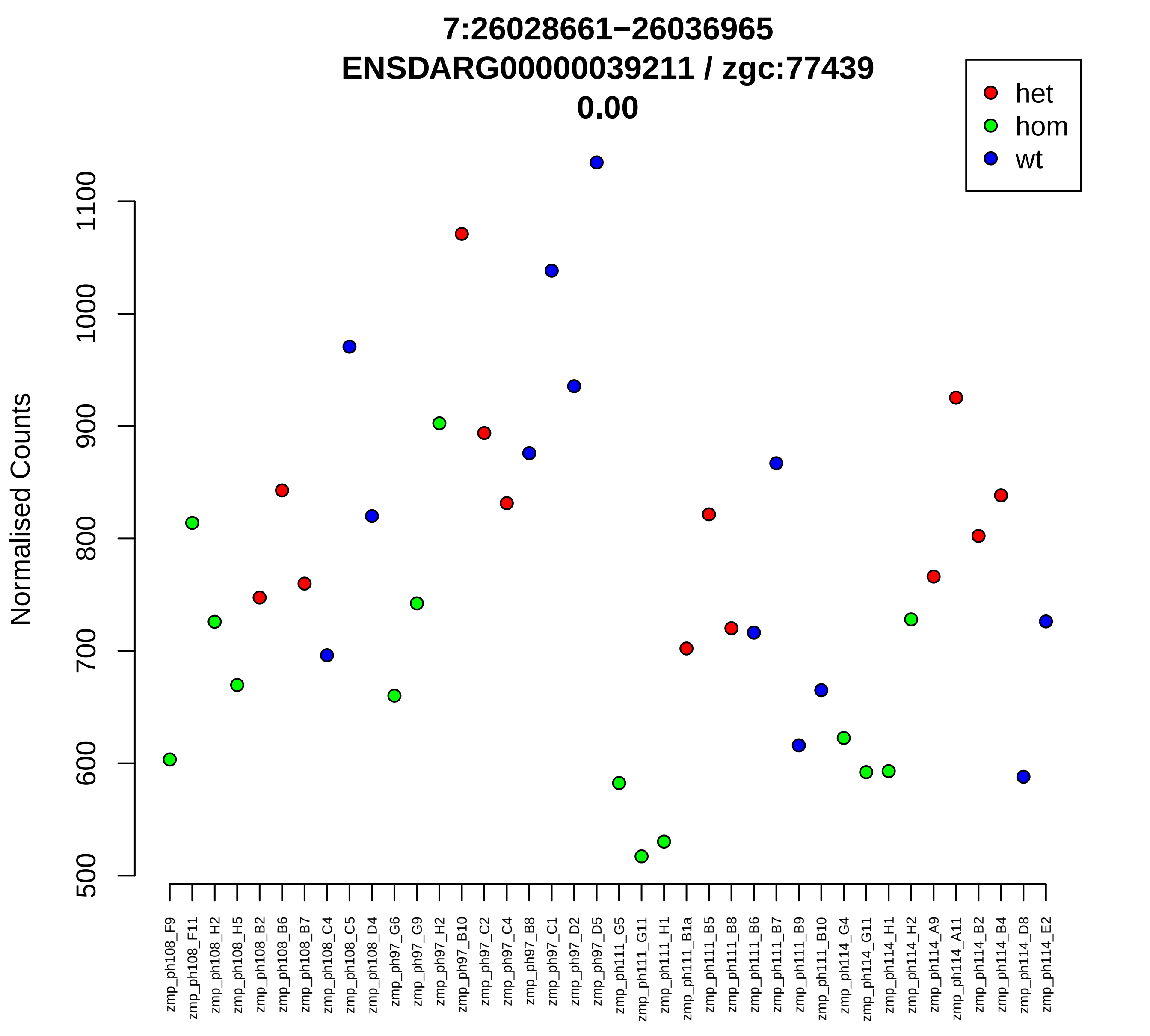
7:26028661−26036965
ENSDARG00000039211 / zgc:77439
0.00
het
hom
wt
Normalised Counts
500
600
700
800
900
1000
1100
zmp_ph108_F9
zmp_ph108_F11
zmp_ph108_H2
zmp_ph108_H5
zmp_ph108_B2
zmp_ph108_B6
zmp_ph108_B7
zmp_ph108_C4
zmp_ph108_C5
zmp_ph108_D4
zmp_ph97_G6
zmp_ph97_G9
zmp_ph97_H2
zmp_ph97_B10
zmp_ph97_C2
zmp_ph97_C4
zmp_ph97_B8
zmp_ph97_C1
zmp_ph97_D2
zmp_ph97_D5
zmp_ph111_G5
zmp_ph111_G11
zmp_ph111_H1
zmp_ph111_B1a
zmp_ph111_B5
zmp_ph111_B8
zmp_ph111_B6
zmp_ph111_B7
zmp_ph111_B9
zmp_ph111_B10
zmp_ph114_G4
zmp_ph114_G11
zmp_ph114_H1
zmp_ph114_H2
zmp_ph114_A9
zmp_ph114_A11
zmp_ph114_B2
zmp_ph114_B4
zmp_ph114_D8
zmp_ph114_E2

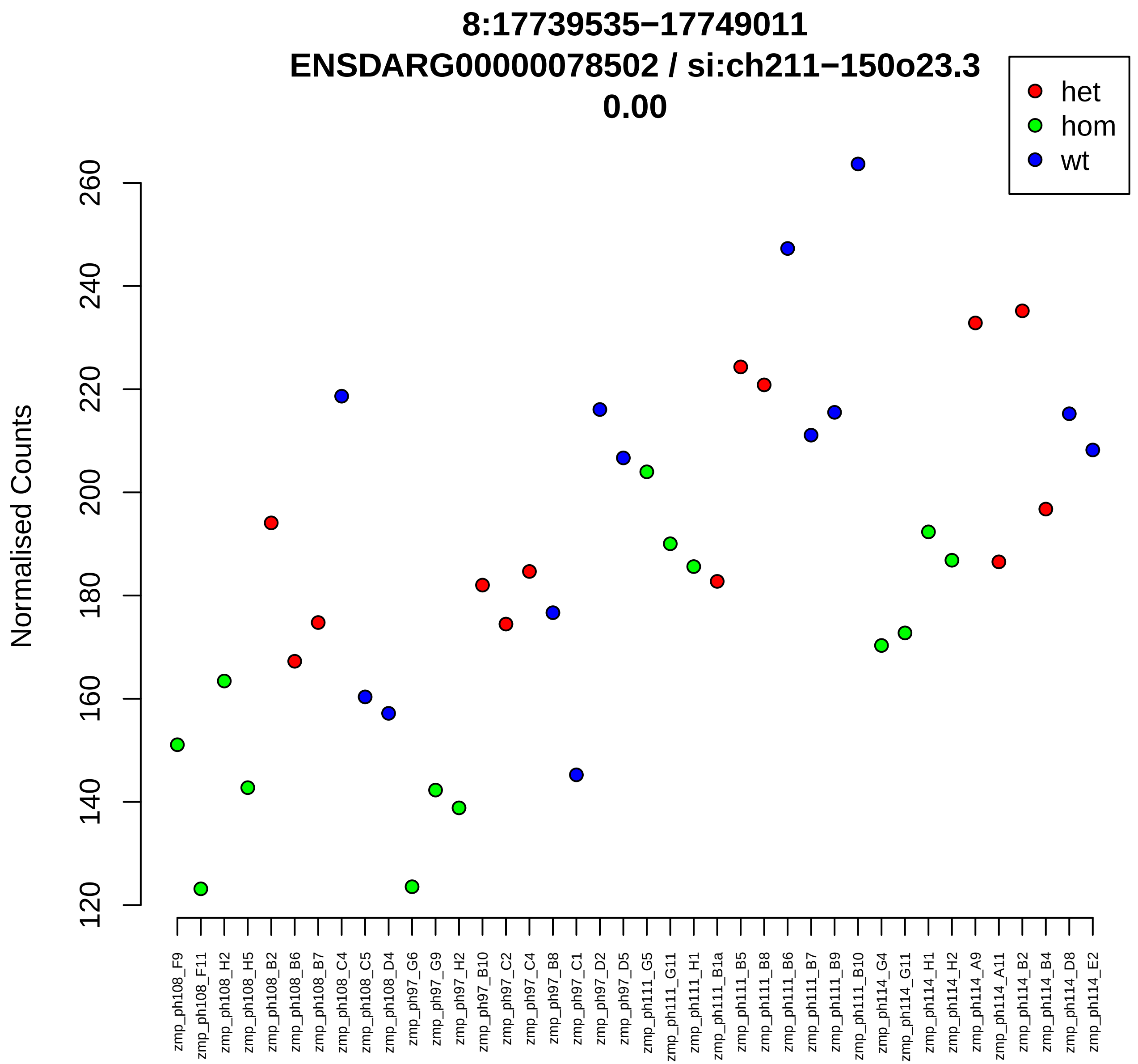
8:17739535−17749011
ENSDARG00000078502 / si:ch211−150o23.3
0.00
het
hom
wt
Normalised Counts
120
140
160
180
200
220
240
260
zmp_ph108_F9
zmp_ph108_F11
zmp_ph108_H2
zmp_ph108_H5
zmp_ph108_B2
zmp_ph108_B6
zmp_ph108_B7
zmp_ph108_C4
zmp_ph108_C5
zmp_ph108_D4
zmp_ph97_G6
zmp_ph97_G9
zmp_ph97_H2
zmp_ph97_B10
zmp_ph97_C2
zmp_ph97_C4
zmp_ph97_B8
zmp_ph97_C1
zmp_ph97_D2
zmp_ph97_D5
zmp_ph111_G5
zmp_ph111_G11
zmp_ph111_H1
zmp_ph111_B1a
zmp_ph111_B5
zmp_ph111_B8
zmp_ph111_B6
zmp_ph111_B7
zmp_ph111_B9
zmp_ph111_B10
zmp_ph114_G4
zmp_ph114_G11
zmp_ph114_H1
zmp_ph114_H2
zmp_ph114_A9
zmp_ph114_A11
zmp_ph114_B2
zmp_ph114_B4
zmp_ph114_D8
zmp_ph114_E2

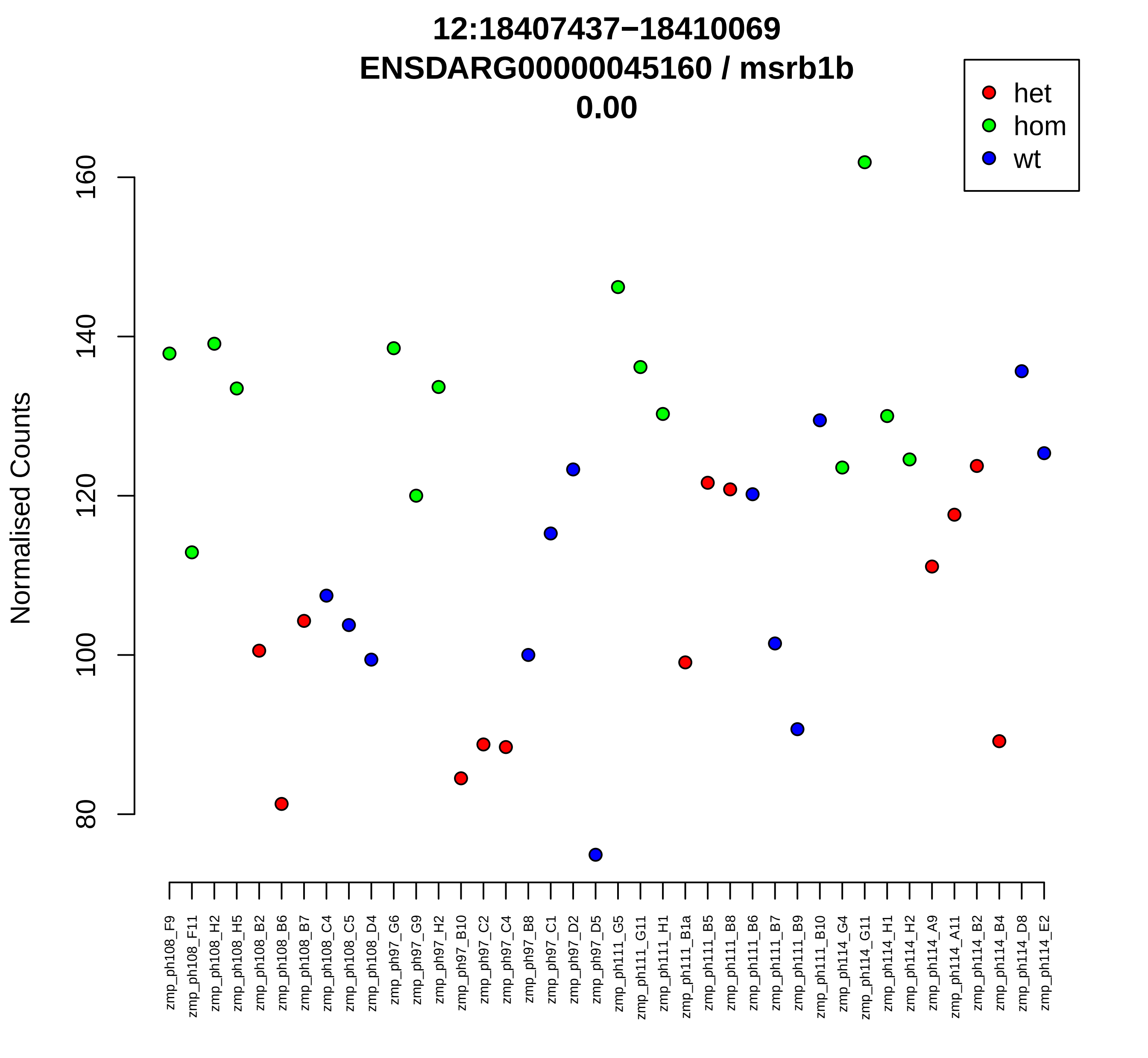
12:18407437−18410069
ENSDARG00000045160 / msrb1b
0.00
het
hom
wt
Normalised Counts
80
100
120
140
160
zmp_ph108_F9
zmp_ph108_F11
zmp_ph108_H2
zmp_ph108_H5
zmp_ph108_B2
zmp_ph108_B6
zmp_ph108_B7
zmp_ph108_C4
zmp_ph108_C5
zmp_ph108_D4
zmp_ph97_G6
zmp_ph97_G9
zmp_ph97_H2
zmp_ph97_B10
zmp_ph97_C2
zmp_ph97_C4
zmp_ph97_B8
zmp_ph97_C1
zmp_ph97_D2
zmp_ph97_D5
zmp_ph111_G5
zmp_ph111_G11
zmp_ph111_H1
zmp_ph111_B1a
zmp_ph111_B5
zmp_ph111_B8
zmp_ph111_B6
zmp_ph111_B7
zmp_ph111_B9
zmp_ph111_B10
zmp_ph114_G4
zmp_ph114_G11
zmp_ph114_H1
zmp_ph114_H2
zmp_ph114_A9
zmp_ph114_A11
zmp_ph114_B2
zmp_ph114_B4
zmp_ph114_D8
zmp_ph114_E2

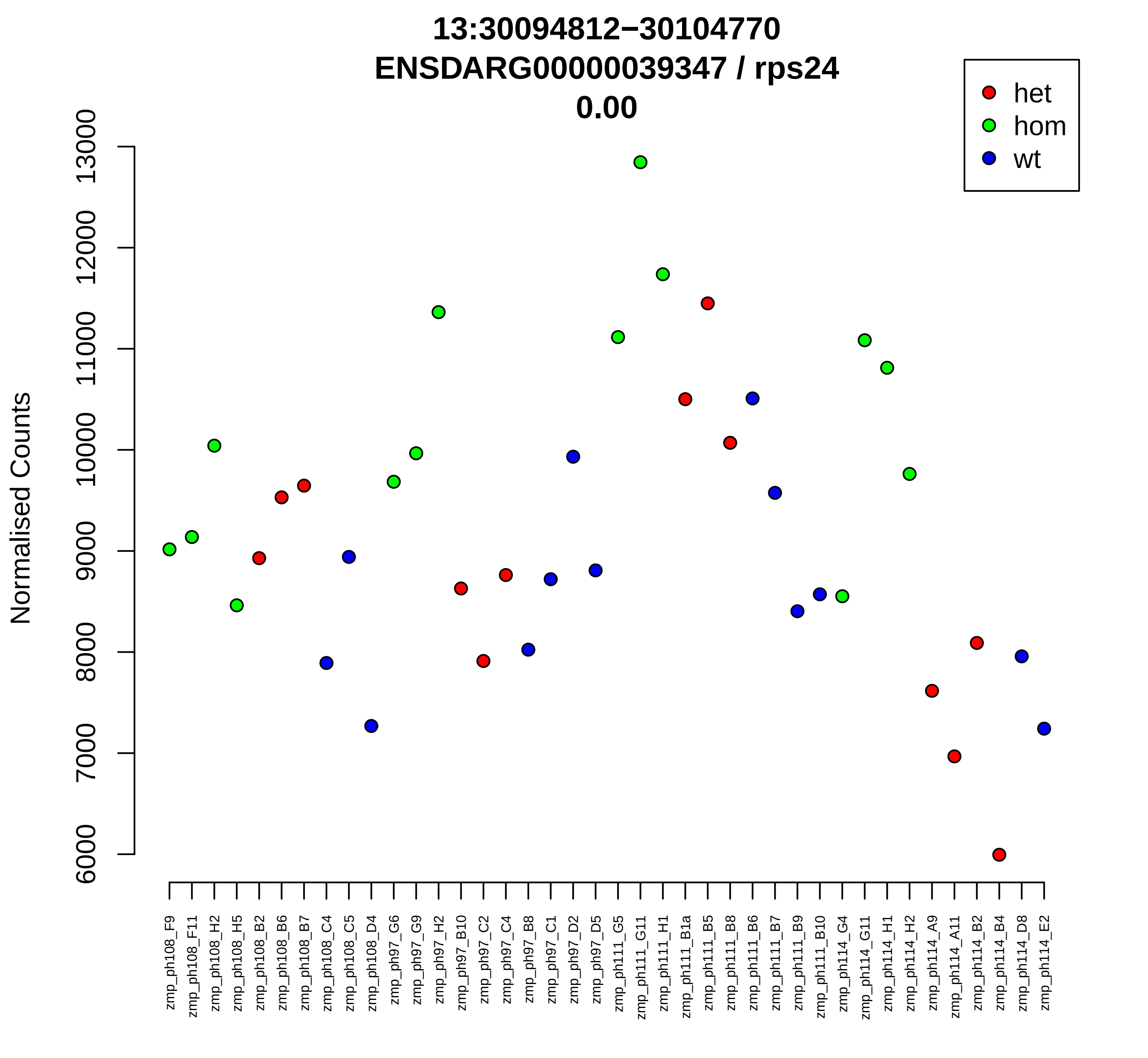

13:30094812−30104770
ENSDARG00000039347 / rps24
0.00
het
hom
wt
Normalised Counts
6000
7000
8000
9000
10000
11000
12000
13000
zmp_ph108_F9
zmp_ph108_F11
zmp_ph108_H2
zmp_ph108_H5
zmp_ph108_B2
zmp_ph108_B6
zmp_ph108_B7
zmp_ph108_C4
zmp_ph108_C5
zmp_ph108_D4
zmp_ph97_G6
zmp_ph97_G9
zmp_ph97_H2
zmp_ph97_B10
zmp_ph97_C2
zmp_ph97_C4
zmp_ph97_B8
zmp_ph97_C1
zmp_ph97_D2
zmp_ph97_D5
zmp_ph111_G5
zmp_ph111_G11
zmp_ph111_H1
zmp_ph111_B1a
zmp_ph111_B5
zmp_ph111_B8
zmp_ph111_B6
zmp_ph111_B7
zmp_ph111_B9
zmp_ph111_B10
zmp_ph114_G4
zmp_ph114_G11
zmp_ph114_H1
zmp_ph114_H2
zmp_ph114_A9
zmp_ph114_A11
zmp_ph114_B2
zmp_ph114_B4
zmp_ph114_D8
zmp_ph114_E2

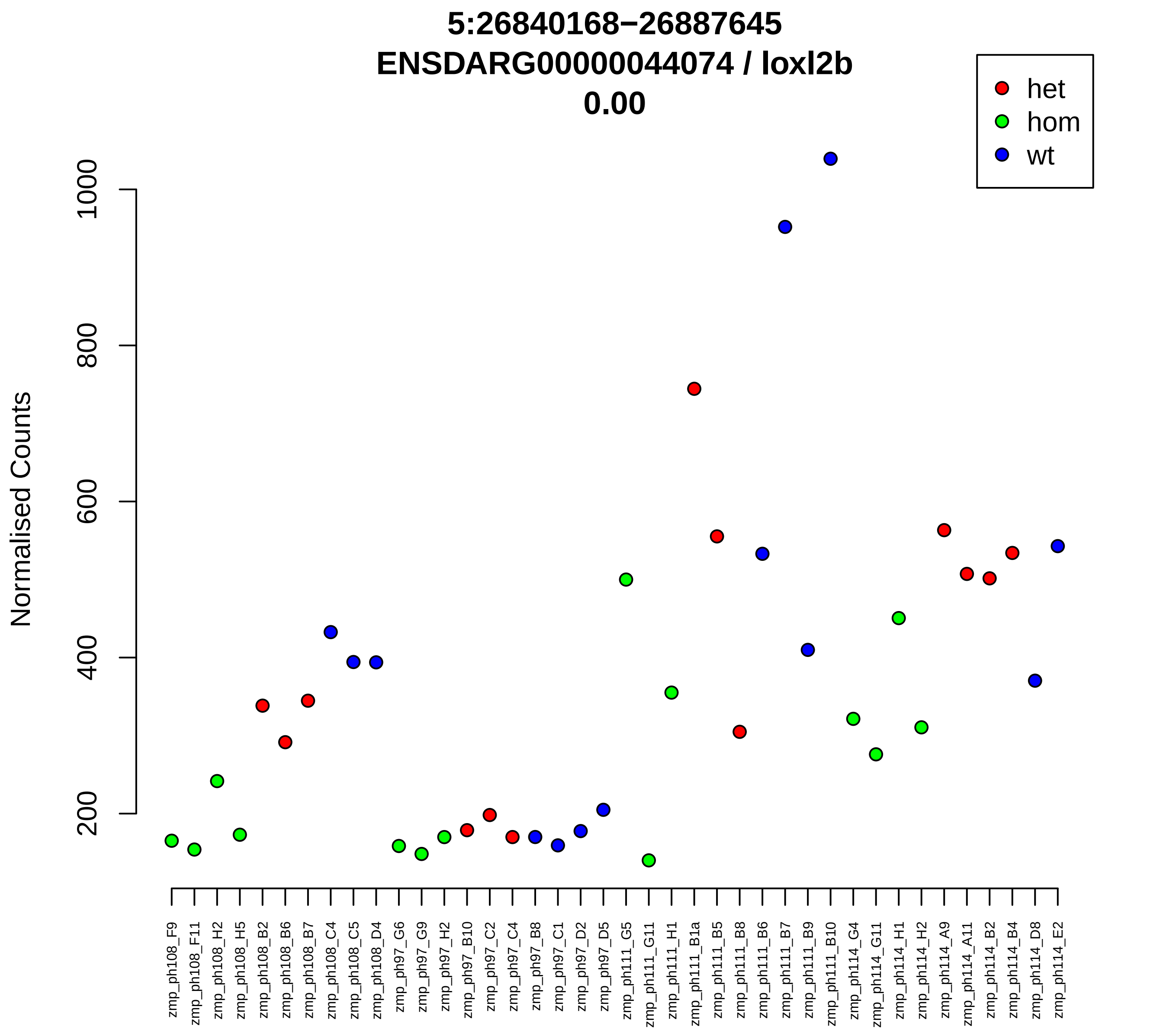

5:26840168−26887645
ENSDARG00000044074 / loxl2b
0.00
het
hom
wt
Normalised Counts
200
400
600
800
1000
zmp_ph108_F9
zmp_ph108_F11
zmp_ph108_H2
zmp_ph108_H5
zmp_ph108_B2
zmp_ph108_B6
zmp_ph108_B7
zmp_ph108_C4
zmp_ph108_C5
zmp_ph108_D4
zmp_ph97_G6
zmp_ph97_G9
zmp_ph97_H2
zmp_ph97_B10
zmp_ph97_C2
zmp_ph97_C4
zmp_ph97_B8
zmp_ph97_C1
zmp_ph97_D2
zmp_ph97_D5
zmp_ph111_G5
zmp_ph111_G11
zmp_ph111_H1
zmp_ph111_B1a
zmp_ph111_B5
zmp_ph111_B8
zmp_ph111_B6
zmp_ph111_B7
zmp_ph111_B9
zmp_ph111_B10
zmp_ph114_G4
zmp_ph114_G11
zmp_ph114_H1
zmp_ph114_H2
zmp_ph114_A9
zmp_ph114_A11
zmp_ph114_B2
zmp_ph114_B4
zmp_ph114_D8
zmp_ph114_E2

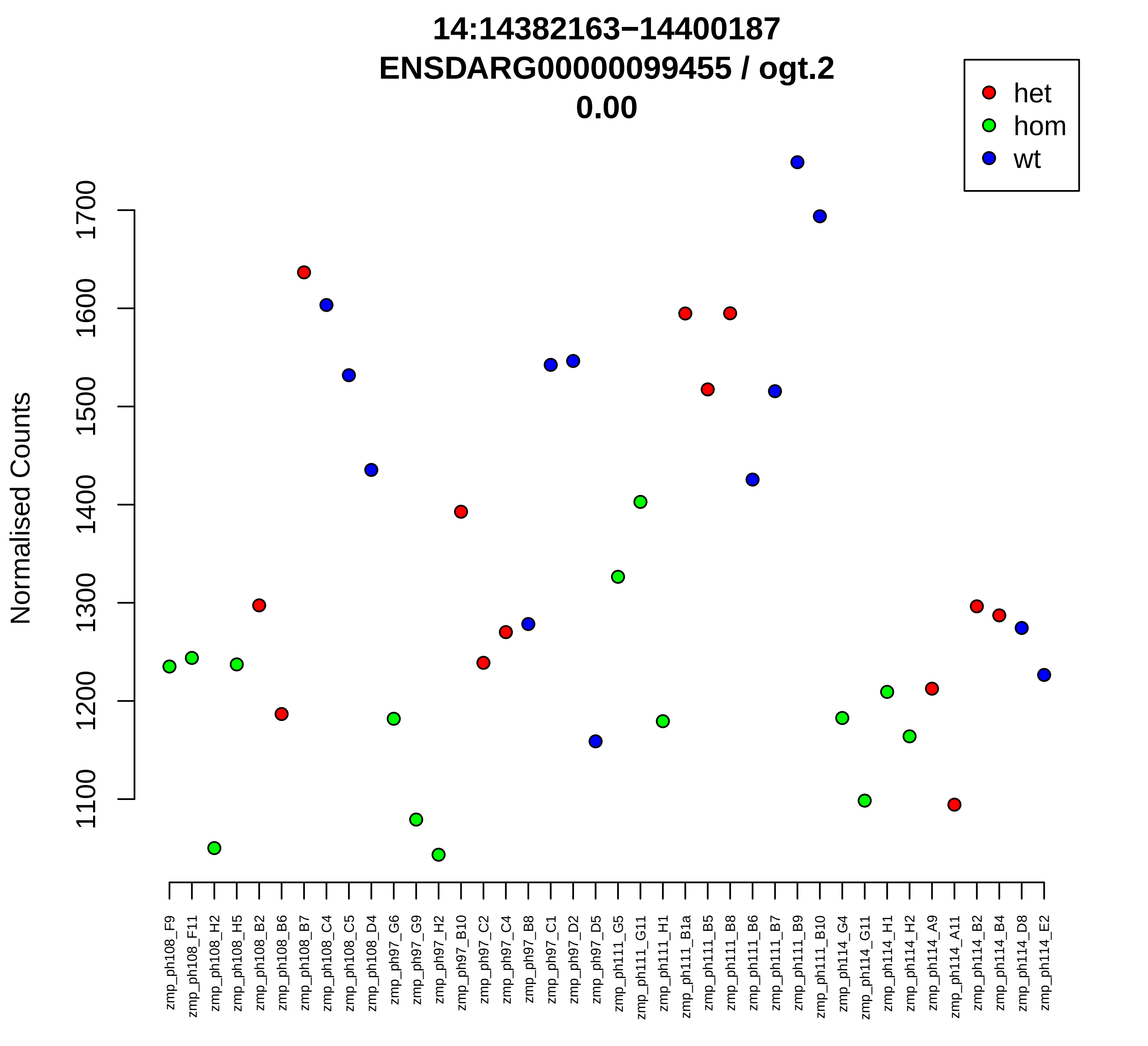

14:14382163−14400187
ENSDARG00000099455 / ogt.2
0.00
het
hom
wt
Normalised Counts
1100
1200
1300
1400
1500
1600
1700
zmp_ph108_F9
zmp_ph108_F11
zmp_ph108_H2
zmp_ph108_H5
zmp_ph108_B2
zmp_ph108_B6
zmp_ph108_B7
zmp_ph108_C4
zmp_ph108_C5
zmp_ph108_D4
zmp_ph97_G6
zmp_ph97_G9
zmp_ph97_H2
zmp_ph97_B10
zmp_ph97_C2
zmp_ph97_C4
zmp_ph97_B8
zmp_ph97_C1
zmp_ph97_D2
zmp_ph97_D5
zmp_ph111_G5
zmp_ph111_G11
zmp_ph111_H1
zmp_ph111_B1a
zmp_ph111_B5
zmp_ph111_B8
zmp_ph111_B6
zmp_ph111_B7
zmp_ph111_B9
zmp_ph111_B10
zmp_ph114_G4
zmp_ph114_G11
zmp_ph114_H1
zmp_ph114_H2
zmp_ph114_A9
zmp_ph114_A11
zmp_ph114_B2
zmp_ph114_B4
zmp_ph114_D8
zmp_ph114_E2

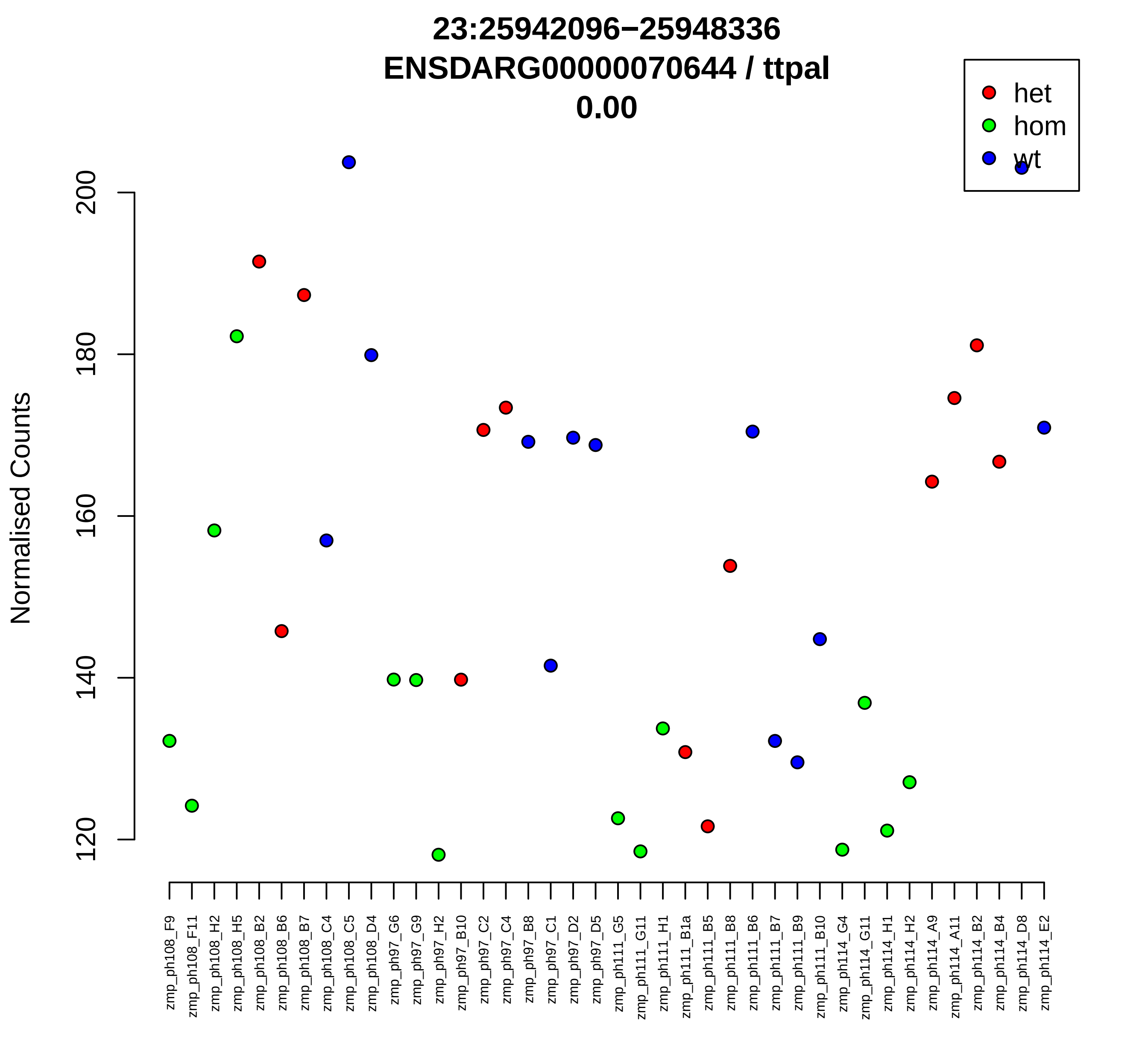

23:25942096−25948336
ENSDARG00000070644 / ttpal
0.00
het
hom
wt
Normalised Counts
120
140
160
180
200
zmp_ph108_F9
zmp_ph108_F11
zmp_ph108_H2
zmp_ph108_H5
zmp_ph108_B2
zmp_ph108_B6
zmp_ph108_B7
zmp_ph108_C4
zmp_ph108_C5
zmp_ph108_D4
zmp_ph97_G6
zmp_ph97_G9
zmp_ph97_H2
zmp_ph97_B10
zmp_ph97_C2
zmp_ph97_C4
zmp_ph97_B8
zmp_ph97_C1
zmp_ph97_D2
zmp_ph97_D5
zmp_ph111_G5
zmp_ph111_G11
zmp_ph111_H1
zmp_ph111_B1a
zmp_ph111_B5
zmp_ph111_B8
zmp_ph111_B6
zmp_ph111_B7
zmp_ph111_B9
zmp_ph111_B10
zmp_ph114_G4
zmp_ph114_G11
zmp_ph114_H1
zmp_ph114_H2
zmp_ph114_A9
zmp_ph114_A11
zmp_ph114_B2
zmp_ph114_B4
zmp_ph114_D8
zmp_ph114_E2

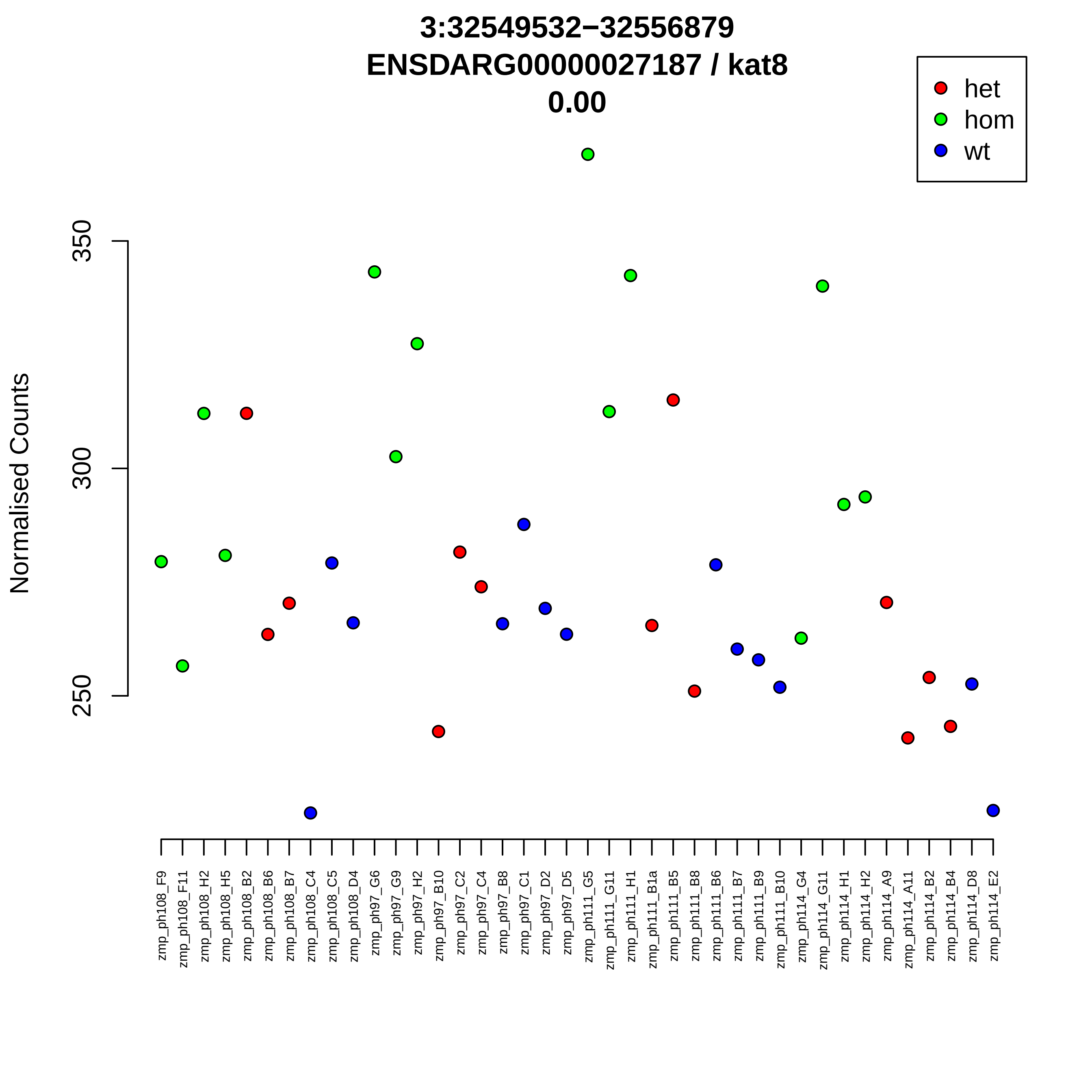
3:32549532−32556879
ENSDARG00000027187 / kat8
0.00
het
hom
wt
Normalised Counts
250
300
350
zmp_ph108_F9
zmp_ph108_F11
zmp_ph108_H2
zmp_ph108_H5
zmp_ph108_B2
zmp_ph108_B6
zmp_ph108_B7
zmp_ph108_C4
zmp_ph108_C5
zmp_ph108_D4
zmp_ph97_G6
zmp_ph97_G9
zmp_ph97_H2
zmp_ph97_B10
zmp_ph97_C2
zmp_ph97_C4
zmp_ph97_B8
zmp_ph97_C1
zmp_ph97_D2
zmp_ph97_D5
zmp_ph111_G5
zmp_ph111_G11
zmp_ph111_H1
zmp_ph111_B1a
zmp_ph111_B5
zmp_ph111_B8
zmp_ph111_B6
zmp_ph111_B7
zmp_ph111_B9
zmp_ph111_B10
zmp_ph114_G4
zmp_ph114_G11
zmp_ph114_H1
zmp_ph114_H2
zmp_ph114_A9
zmp_ph114_A11
zmp_ph114_B2
zmp_ph114_B4
zmp_ph114_D8
zmp_ph114_E2

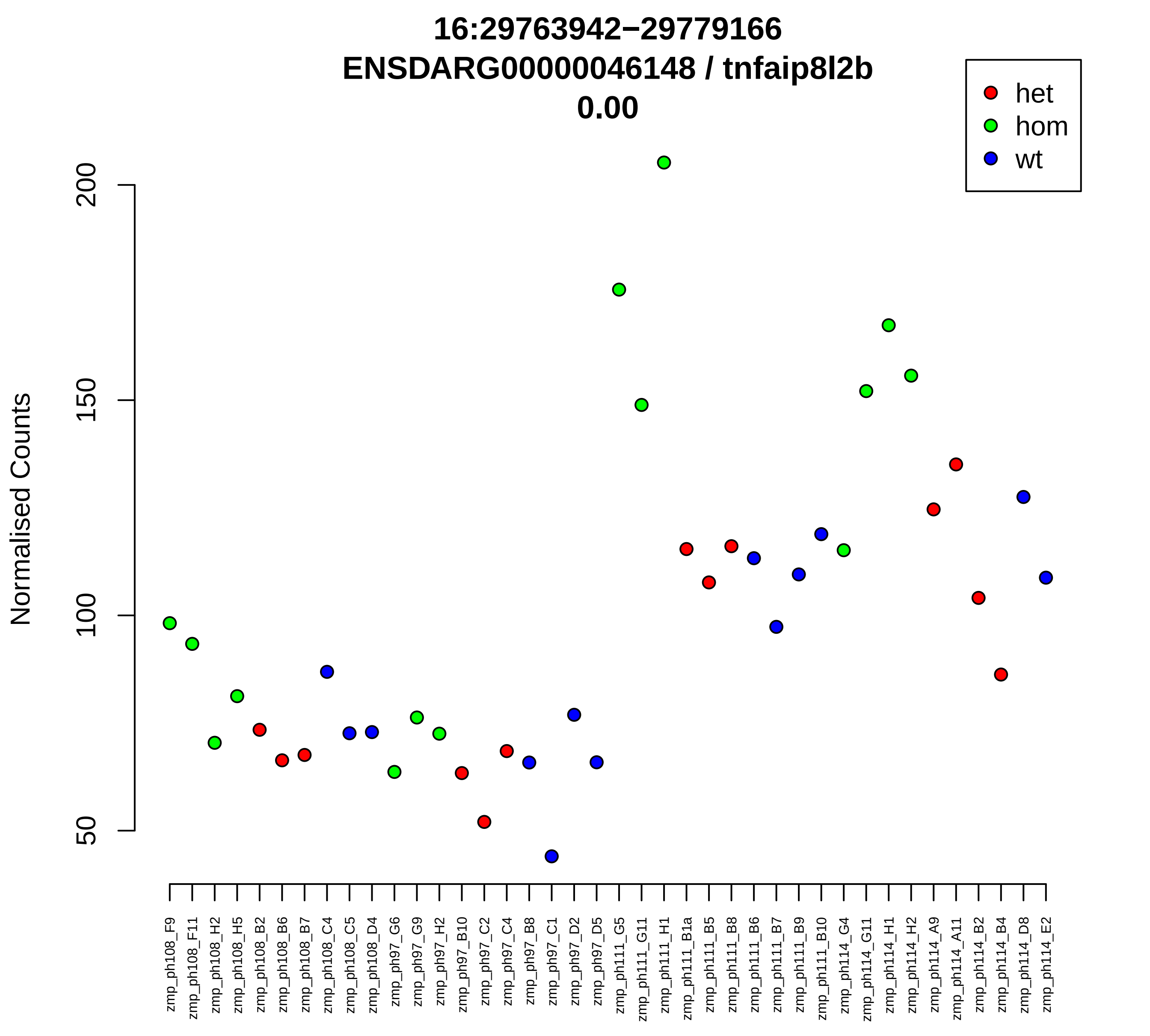
16:29763942−29779166
ENSDARG00000046148 / tnfaip8l2b
0.00
het
hom
wt
Normalised Counts
50
100
150
200
zmp_ph108_F9
zmp_ph108_F11
zmp_ph108_H2
zmp_ph108_H5
zmp_ph108_B2
zmp_ph108_B6
zmp_ph108_B7
zmp_ph108_C4
zmp_ph108_C5
zmp_ph108_D4
zmp_ph97_G6
zmp_ph97_G9
zmp_ph97_H2
zmp_ph97_B10
zmp_ph97_C2
zmp_ph97_C4
zmp_ph97_B8
zmp_ph97_C1
zmp_ph97_D2
zmp_ph97_D5
zmp_ph111_G5
zmp_ph111_G11
zmp_ph111_H1
zmp_ph111_B1a
zmp_ph111_B5
zmp_ph111_B8
zmp_ph111_B6
zmp_ph111_B7
zmp_ph111_B9
zmp_ph111_B10
zmp_ph114_G4
zmp_ph114_G11
zmp_ph114_H1
zmp_ph114_H2
zmp_ph114_A9
zmp_ph114_A11
zmp_ph114_B2
zmp_ph114_B4
zmp_ph114_D8
zmp_ph114_E2

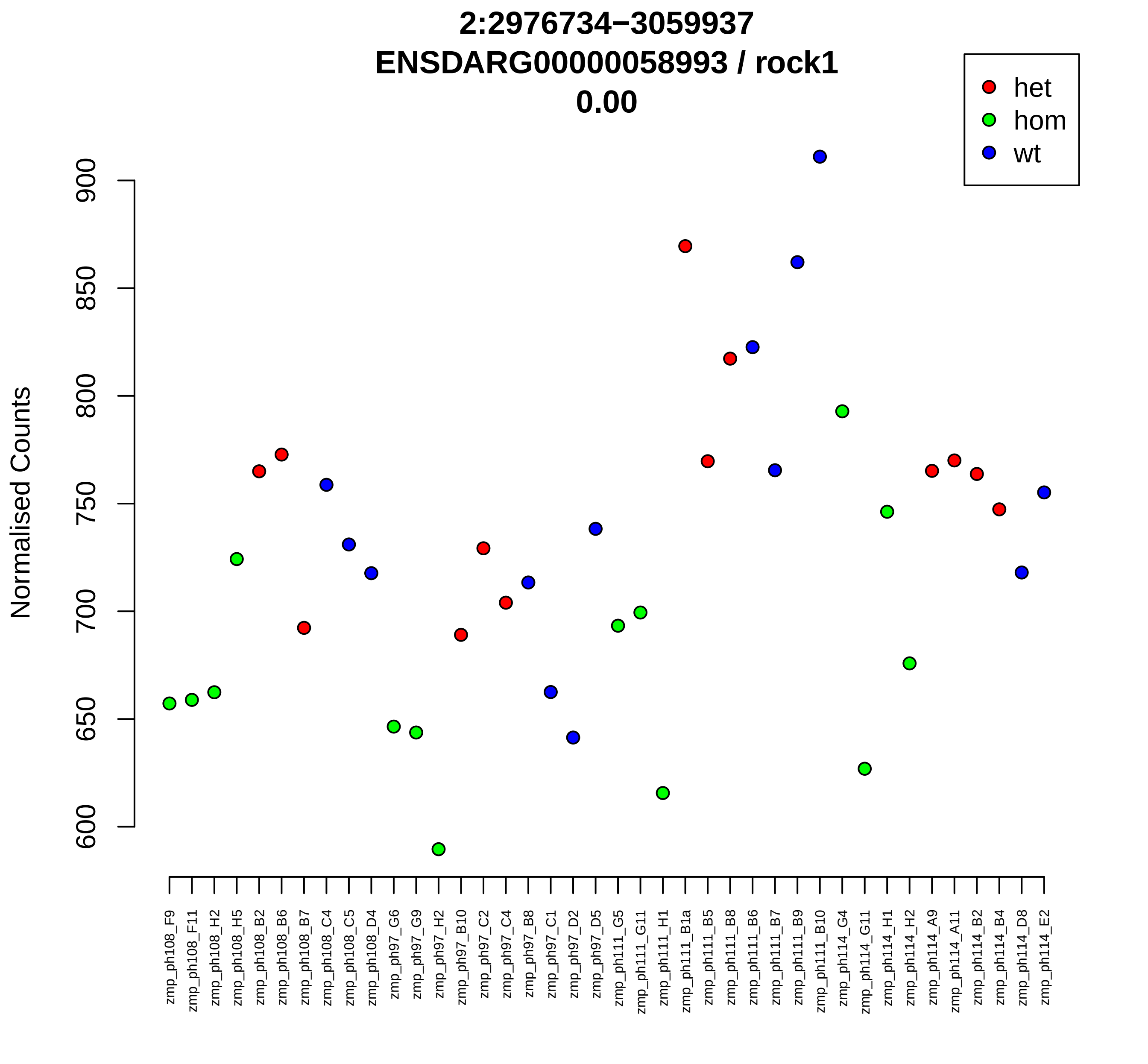
2:2976734−3059937
ENSDARG00000058993 / rock1
0.00
het
hom
wt
Normalised Counts
600
650
700
750
800
850
900
zmp_ph108_F9
zmp_ph108_F11
zmp_ph108_H2
zmp_ph108_H5
zmp_ph108_B2
zmp_ph108_B6
zmp_ph108_B7
zmp_ph108_C4
zmp_ph108_C5
zmp_ph108_D4
zmp_ph97_G6
zmp_ph97_G9
zmp_ph97_H2
zmp_ph97_B10
zmp_ph97_C2
zmp_ph97_C4
zmp_ph97_B8
zmp_ph97_C1
zmp_ph97_D2
zmp_ph97_D5
zmp_ph111_G5
zmp_ph111_G11
zmp_ph111_H1
zmp_ph111_B1a
zmp_ph111_B5
zmp_ph111_B8
zmp_ph111_B6
zmp_ph111_B7
zmp_ph111_B9
zmp_ph111_B10
zmp_ph114_G4
zmp_ph114_G11
zmp_ph114_H1
zmp_ph114_H2
zmp_ph114_A9
zmp_ph114_A11
zmp_ph114_B2
zmp_ph114_B4
zmp_ph114_D8
zmp_ph114_E2

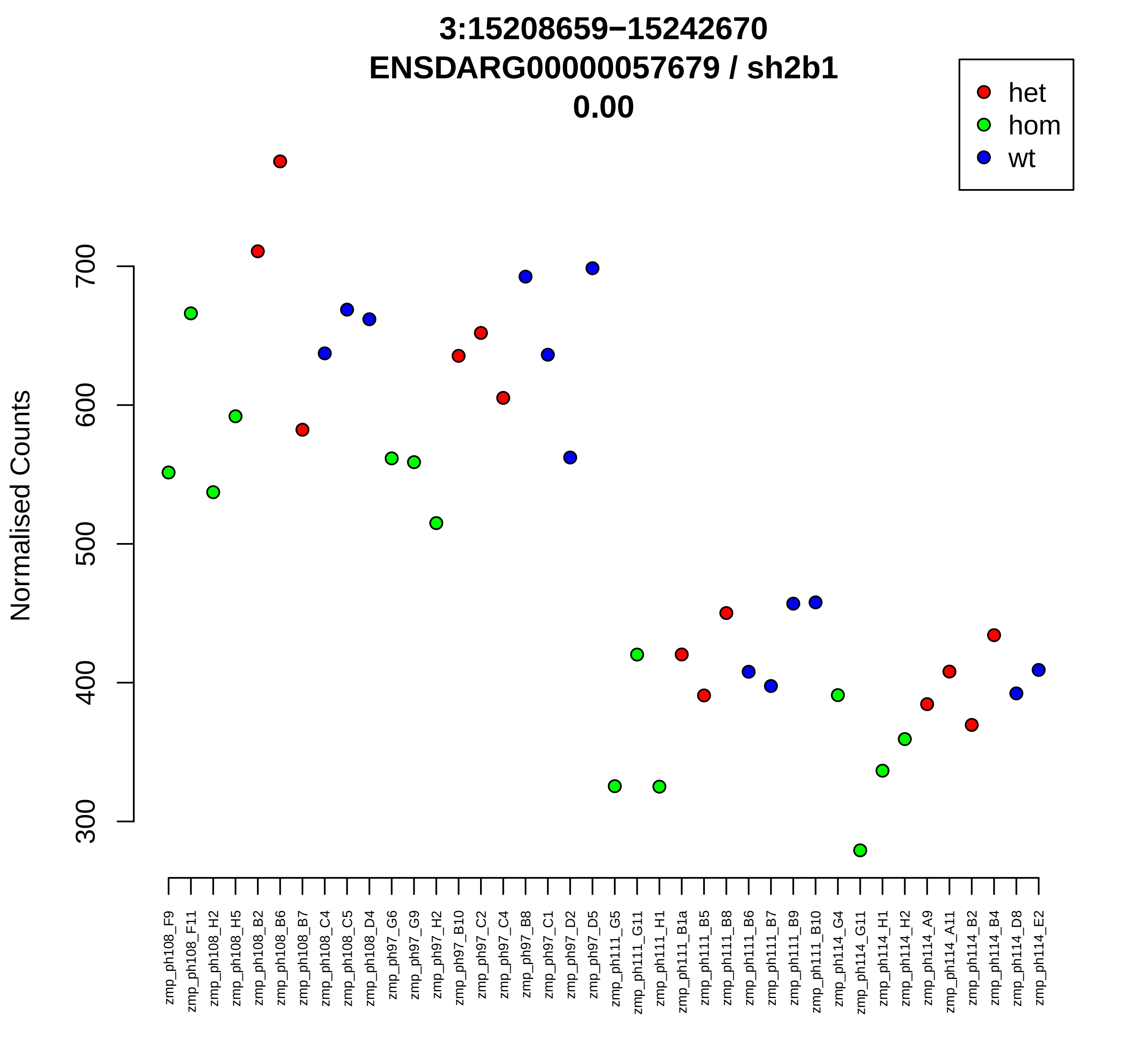

3:15208659−15242670
ENSDARG00000057679 / sh2b1
0.00
het
hom
wt
Normalised Counts
300
400
500
600
700
zmp_ph108_F9
zmp_ph108_F11
zmp_ph108_H2
zmp_ph108_H5
zmp_ph108_B2
zmp_ph108_B6
zmp_ph108_B7
zmp_ph108_C4
zmp_ph108_C5
zmp_ph108_D4
zmp_ph97_G6
zmp_ph97_G9
zmp_ph97_H2
zmp_ph97_B10
zmp_ph97_C2
zmp_ph97_C4
zmp_ph97_B8
zmp_ph97_C1
zmp_ph97_D2
zmp_ph97_D5
zmp_ph111_G5
zmp_ph111_G11
zmp_ph111_H1
zmp_ph111_B1a
zmp_ph111_B5
zmp_ph111_B8
zmp_ph111_B6
zmp_ph111_B7
zmp_ph111_B9
zmp_ph111_B10
zmp_ph114_G4
zmp_ph114_G11
zmp_ph114_H1
zmp_ph114_H2
zmp_ph114_A9
zmp_ph114_A11
zmp_ph114_B2
zmp_ph114_B4
zmp_ph114_D8
zmp_ph114_E2

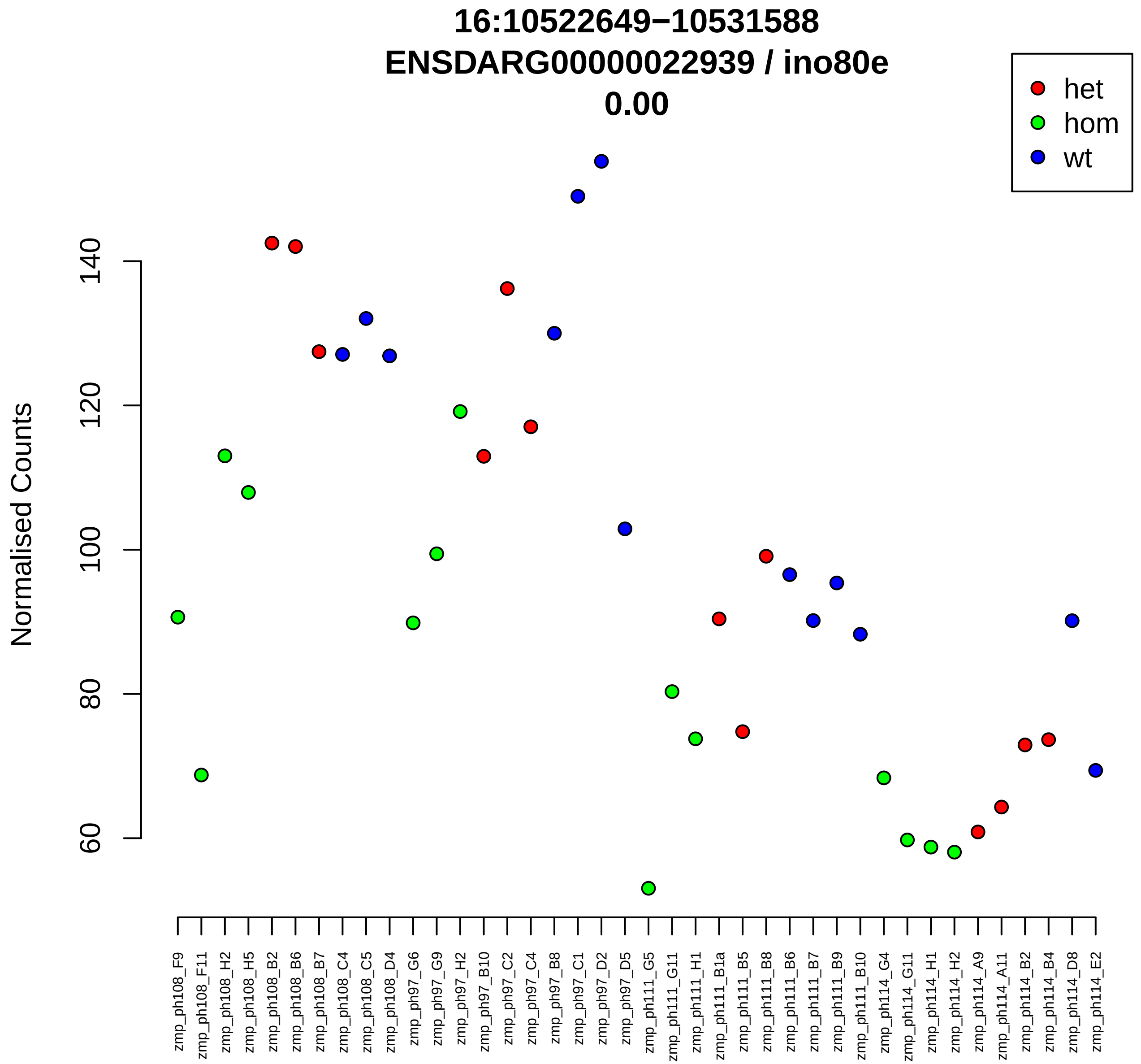
16:10522649−10531588
ENSDARG00000022939 / ino80e
0.00
het
hom
wt
Normalised Counts
60
80
100
120
140
zmp_ph108_F9
zmp_ph108_F11
zmp_ph108_H2
zmp_ph108_H5
zmp_ph108_B2
zmp_ph108_B6
zmp_ph108_B7
zmp_ph108_C4
zmp_ph108_C5
zmp_ph108_D4
zmp_ph97_G6
zmp_ph97_G9
zmp_ph97_H2
zmp_ph97_B10
zmp_ph97_C2
zmp_ph97_C4
zmp_ph97_B8
zmp_ph97_C1
zmp_ph97_D2
zmp_ph97_D5
zmp_ph111_G5
zmp_ph111_G11
zmp_ph111_H1
zmp_ph111_B1a
zmp_ph111_B5
zmp_ph111_B8
zmp_ph111_B6
zmp_ph111_B7
zmp_ph111_B9
zmp_ph111_B10
zmp_ph114_G4
zmp_ph114_G11
zmp_ph114_H1
zmp_ph114_H2
zmp_ph114_A9
zmp_ph114_A11
zmp_ph114_B2
zmp_ph114_B4
zmp_ph114_D8
zmp_ph114_E2

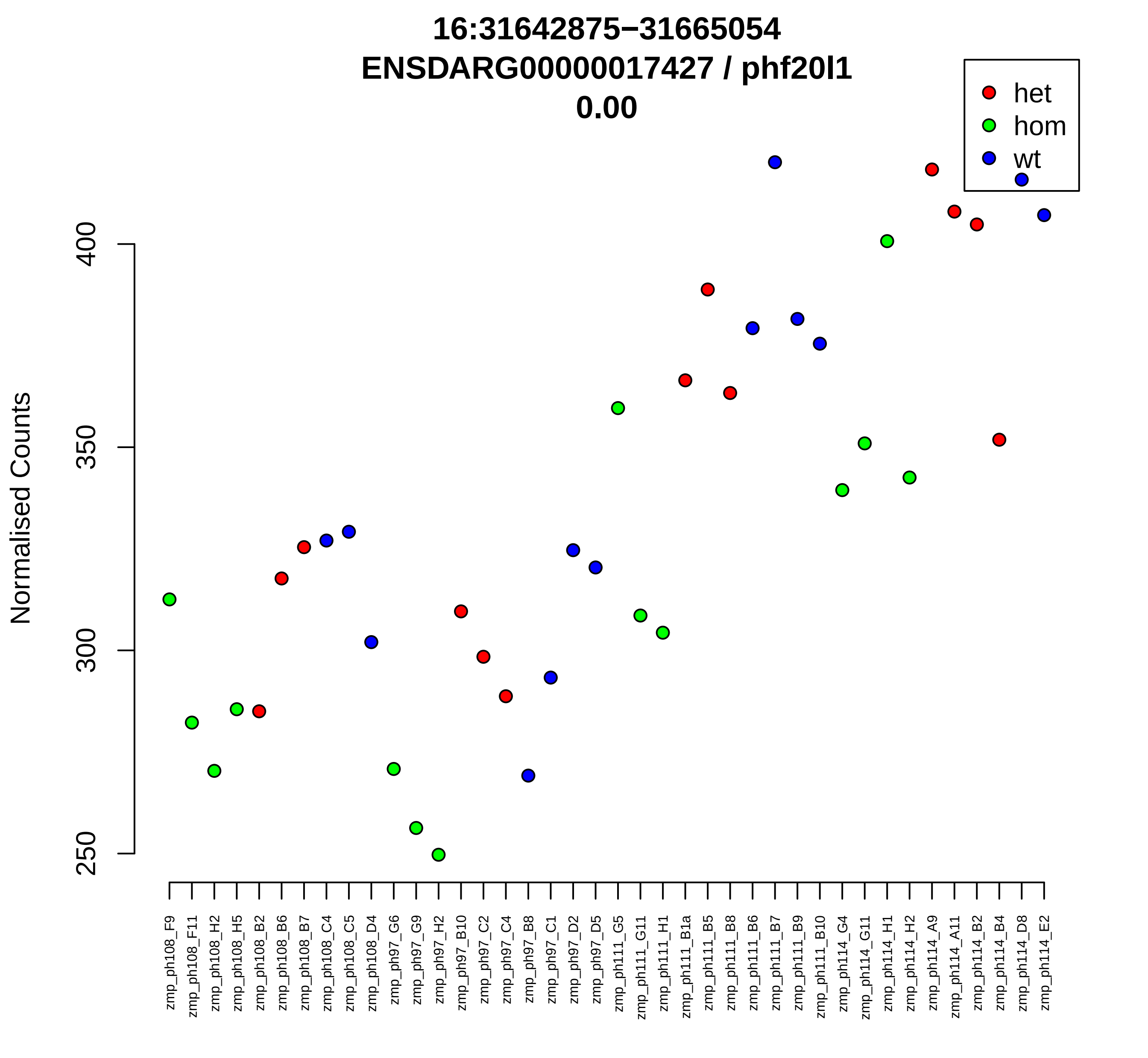

16:31642875−31665054
ENSDARG00000017427 / phf20l1
0.00
het
hom
wt
Normalised Counts
250
300
350
400
zmp_ph108_F9
zmp_ph108_F11
zmp_ph108_H2
zmp_ph108_H5
zmp_ph108_B2
zmp_ph108_B6
zmp_ph108_B7
zmp_ph108_C4
zmp_ph108_C5
zmp_ph108_D4
zmp_ph97_G6
zmp_ph97_G9
zmp_ph97_H2
zmp_ph97_B10
zmp_ph97_C2
zmp_ph97_C4
zmp_ph97_B8
zmp_ph97_C1
zmp_ph97_D2
zmp_ph97_D5
zmp_ph111_G5
zmp_ph111_G11
zmp_ph111_H1
zmp_ph111_B1a
zmp_ph111_B5
zmp_ph111_B8
zmp_ph111_B6
zmp_ph111_B7
zmp_ph111_B9
zmp_ph111_B10
zmp_ph114_G4
zmp_ph114_G11
zmp_ph114_H1
zmp_ph114_H2
zmp_ph114_A9
zmp_ph114_A11
zmp_ph114_B2
zmp_ph114_B4
zmp_ph114_D8
zmp_ph114_E2

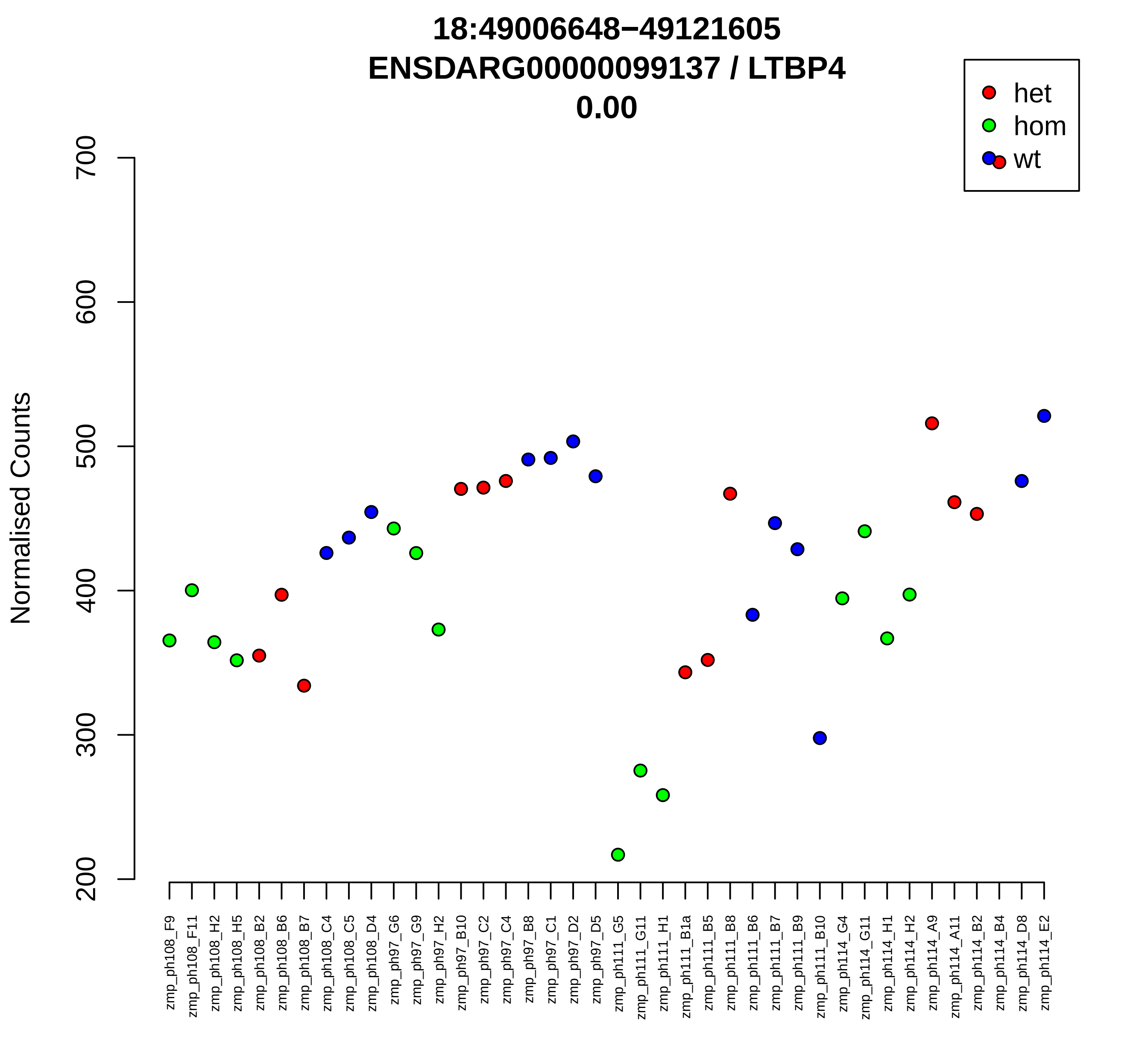
18:49006648−49121605
ENSDARG00000099137 / LTBP4
0.00
het
hom
wt
Normalised Counts
200
300
400
500
600
700
zmp_ph108_F9
zmp_ph108_F11
zmp_ph108_H2
zmp_ph108_H5
zmp_ph108_B2
zmp_ph108_B6
zmp_ph108_B7
zmp_ph108_C4
zmp_ph108_C5
zmp_ph108_D4
zmp_ph97_G6
zmp_ph97_G9
zmp_ph97_H2
zmp_ph97_B10
zmp_ph97_C2
zmp_ph97_C4
zmp_ph97_B8
zmp_ph97_C1
zmp_ph97_D2
zmp_ph97_D5
zmp_ph111_G5
zmp_ph111_G11
zmp_ph111_H1
zmp_ph111_B1a
zmp_ph111_B5
zmp_ph111_B8
zmp_ph111_B6
zmp_ph111_B7
zmp_ph111_B9
zmp_ph111_B10
zmp_ph114_G4
zmp_ph114_G11
zmp_ph114_H1
zmp_ph114_H2
zmp_ph114_A9
zmp_ph114_A11
zmp_ph114_B2
zmp_ph114_B4
zmp_ph114_D8
zmp_ph114_E2

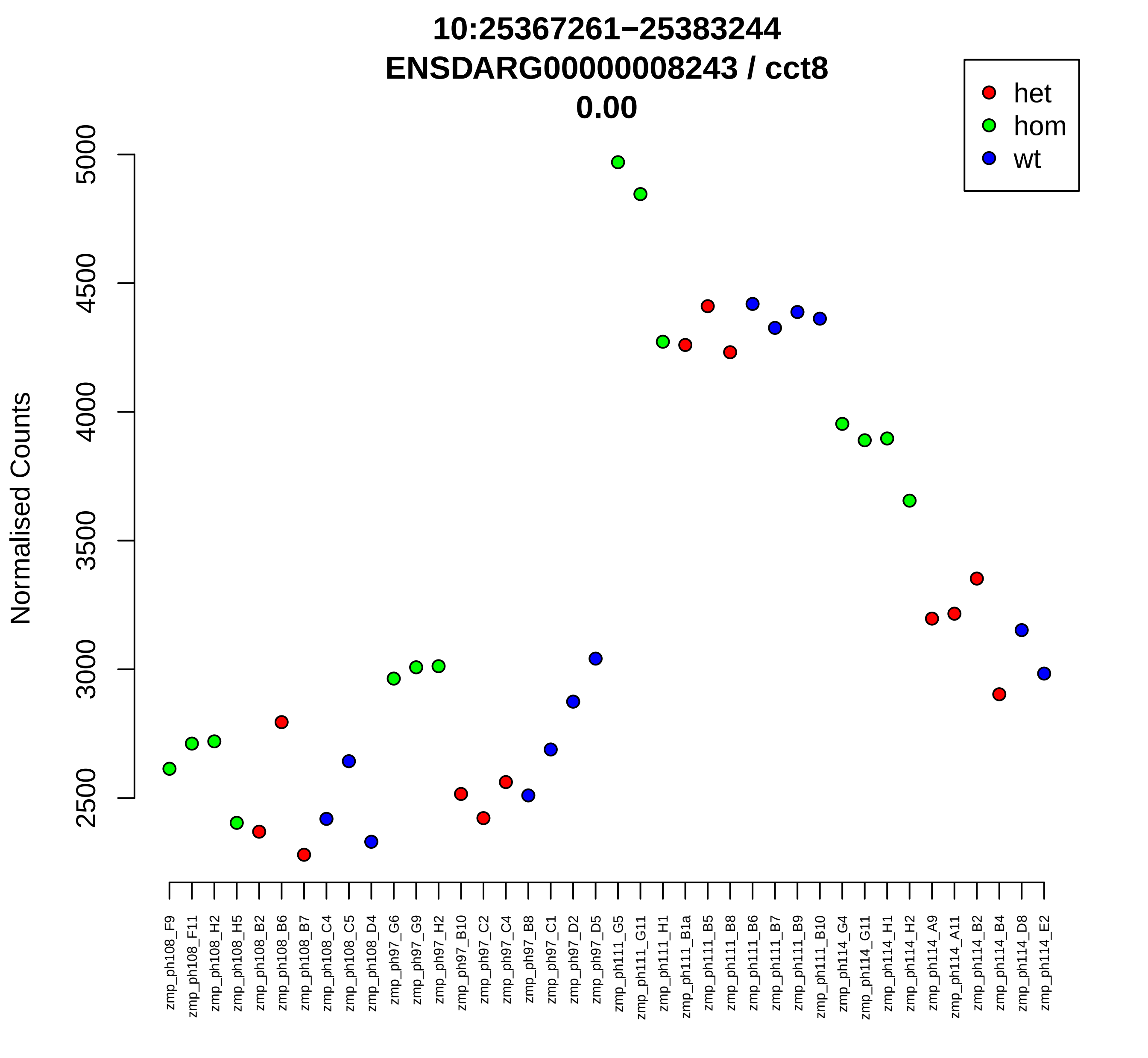
10:25367261−25383244
ENSDARG00000008243 / cct8
0.00
het
hom
wt
Normalised Counts
2500
3000
3500
4000
4500
5000
zmp_ph108_F9
zmp_ph108_F11
zmp_ph108_H2
zmp_ph108_H5
zmp_ph108_B2
zmp_ph108_B6
zmp_ph108_B7
zmp_ph108_C4
zmp_ph108_C5
zmp_ph108_D4
zmp_ph97_G6
zmp_ph97_G9
zmp_ph97_H2
zmp_ph97_B10
zmp_ph97_C2
zmp_ph97_C4
zmp_ph97_B8
zmp_ph97_C1
zmp_ph97_D2
zmp_ph97_D5
zmp_ph111_G5
zmp_ph111_G11
zmp_ph111_H1
zmp_ph111_B1a
zmp_ph111_B5
zmp_ph111_B8
zmp_ph111_B6
zmp_ph111_B7
zmp_ph111_B9
zmp_ph111_B10
zmp_ph114_G4
zmp_ph114_G11
zmp_ph114_H1
zmp_ph114_H2
zmp_ph114_A9
zmp_ph114_A11
zmp_ph114_B2
zmp_ph114_B4
zmp_ph114_D8
zmp_ph114_E2

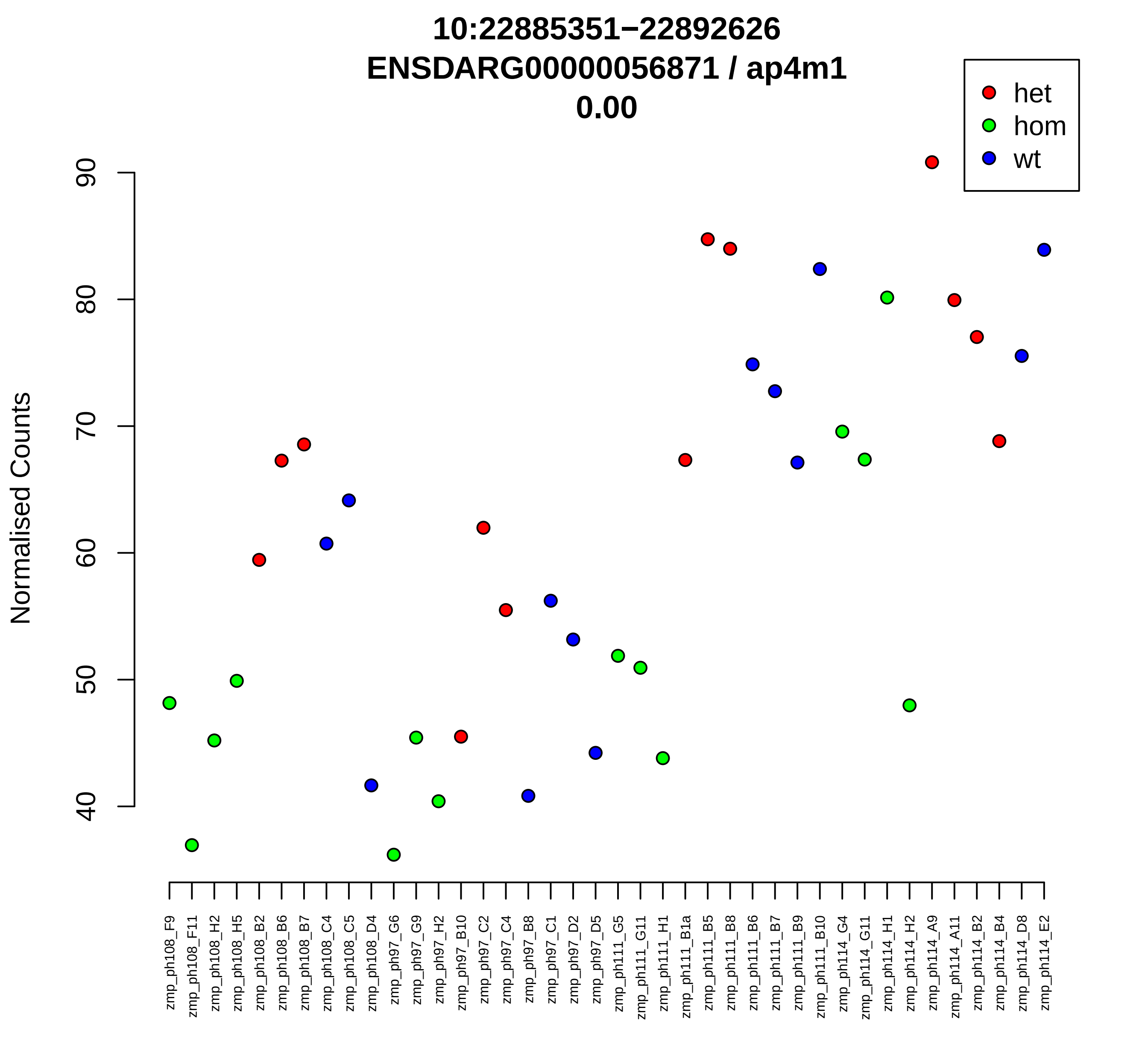

10:22885351−22892626
ENSDARG00000056871 / ap4m1
0.00
het
hom
wt
Normalised Counts
40
50
60
70
80
90
zmp_ph108_F9
zmp_ph108_F11
zmp_ph108_H2
zmp_ph108_H5
zmp_ph108_B2
zmp_ph108_B6
zmp_ph108_B7
zmp_ph108_C4
zmp_ph108_C5
zmp_ph108_D4
zmp_ph97_G6
zmp_ph97_G9
zmp_ph97_H2
zmp_ph97_B10
zmp_ph97_C2
zmp_ph97_C4
zmp_ph97_B8
zmp_ph97_C1
zmp_ph97_D2
zmp_ph97_D5
zmp_ph111_G5
zmp_ph111_G11
zmp_ph111_H1
zmp_ph111_B1a
zmp_ph111_B5
zmp_ph111_B8
zmp_ph111_B6
zmp_ph111_B7
zmp_ph111_B9
zmp_ph111_B10
zmp_ph114_G4
zmp_ph114_G11
zmp_ph114_H1
zmp_ph114_H2
zmp_ph114_A9
zmp_ph114_A11
zmp_ph114_B2
zmp_ph114_B4
zmp_ph114_D8
zmp_ph114_E2

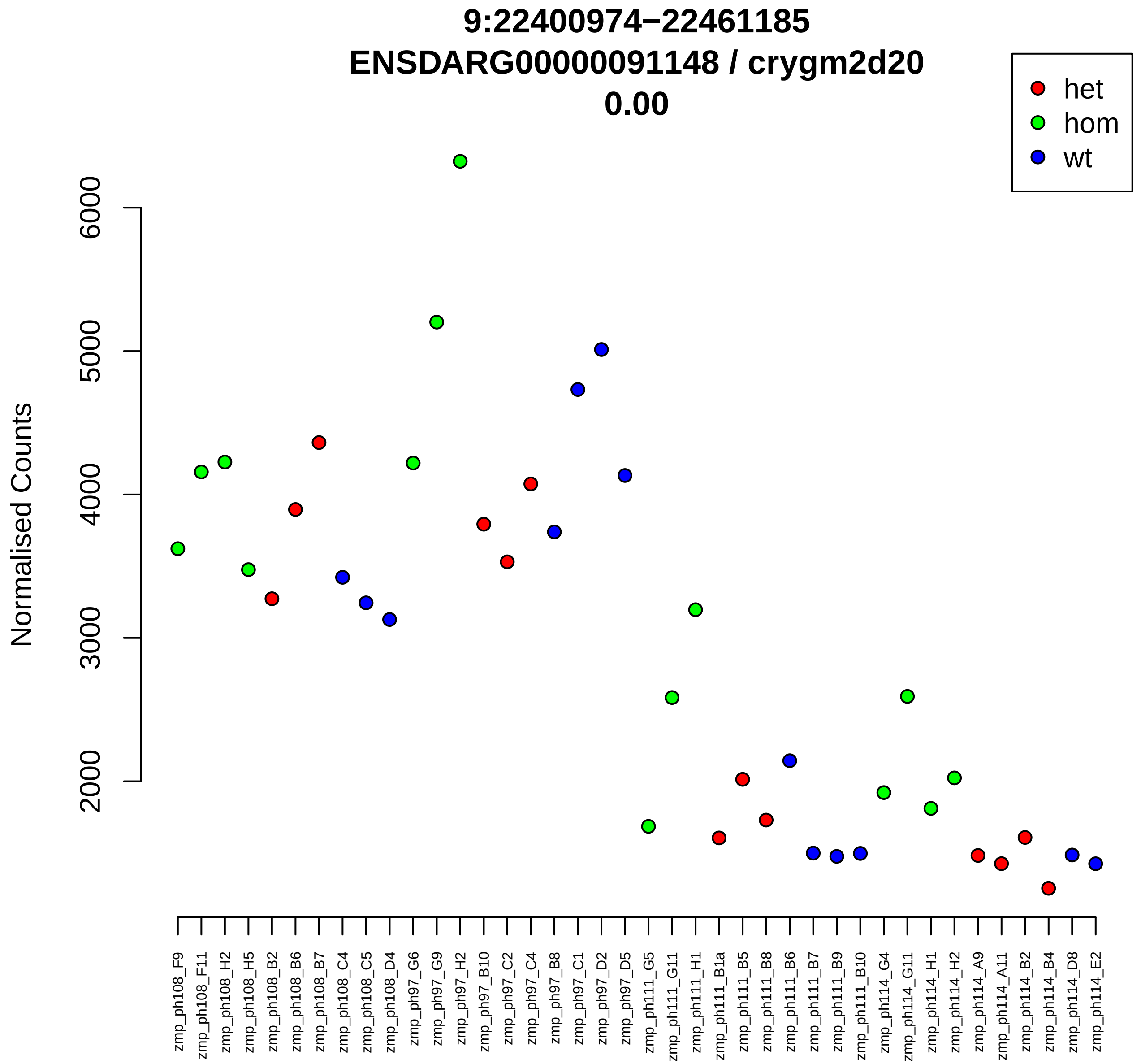
9:22400974−22461185
ENSDARG00000091148 / crygm2d20
0.00
het
hom
wt
Normalised Counts
2000
3000
4000
5000
6000
zmp_ph108_F9
zmp_ph108_F11
zmp_ph108_H2
zmp_ph108_H5
zmp_ph108_B2
zmp_ph108_B6
zmp_ph108_B7
zmp_ph108_C4
zmp_ph108_C5
zmp_ph108_D4
zmp_ph97_G6
zmp_ph97_G9
zmp_ph97_H2
zmp_ph97_B10
zmp_ph97_C2
zmp_ph97_C4
zmp_ph97_B8
zmp_ph97_C1
zmp_ph97_D2
zmp_ph97_D5
zmp_ph111_G5
zmp_ph111_G11
zmp_ph111_H1
zmp_ph111_B1a
zmp_ph111_B5
zmp_ph111_B8
zmp_ph111_B6
zmp_ph111_B7
zmp_ph111_B9
zmp_ph111_B10
zmp_ph114_G4
zmp_ph114_G11
zmp_ph114_H1
zmp_ph114_H2
zmp_ph114_A9
zmp_ph114_A11
zmp_ph114_B2
zmp_ph114_B4
zmp_ph114_D8
zmp_ph114_E2

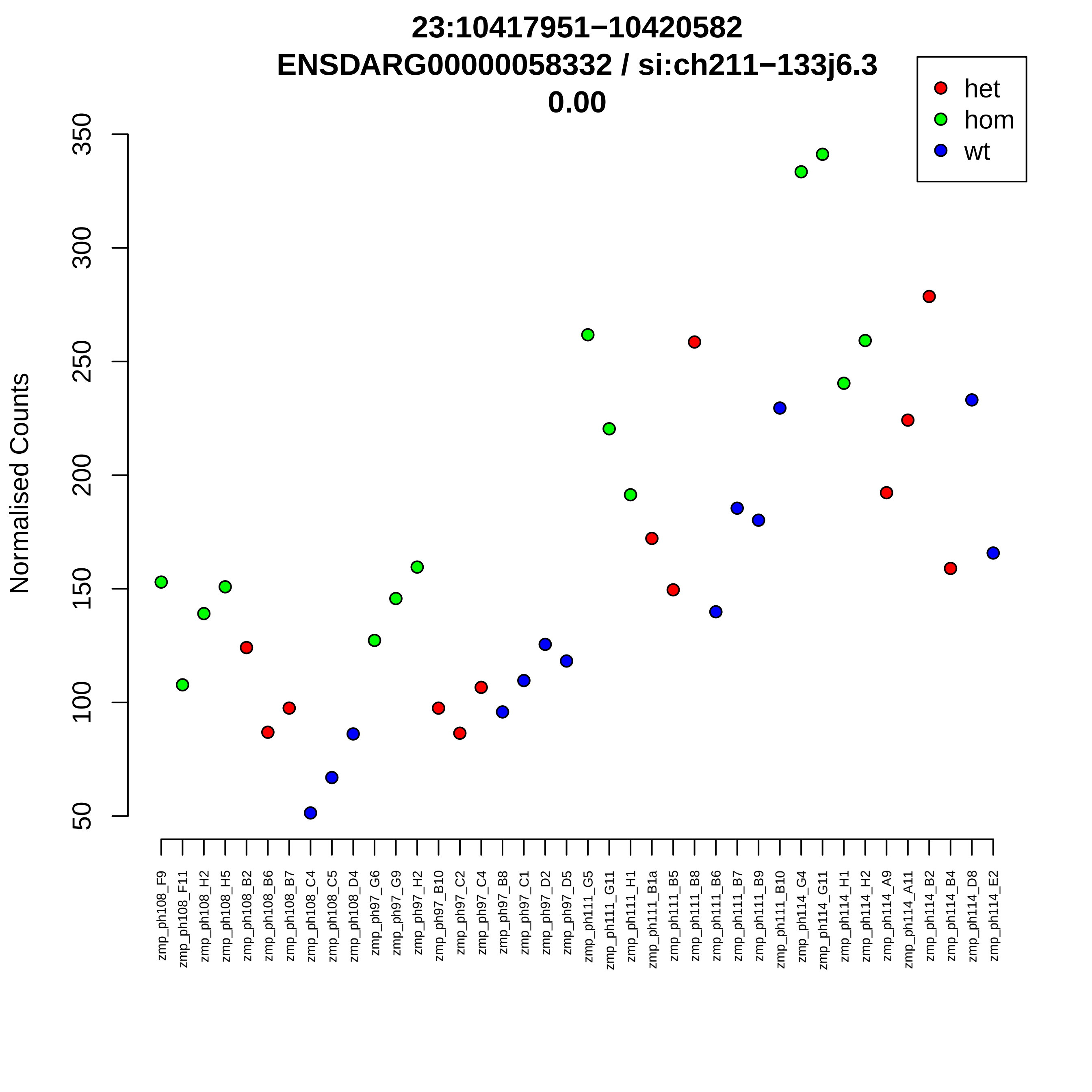

23:10417951−10420582
ENSDARG00000058332 / si:ch211−133j6.3
0.00
het
hom
wt
Normalised Counts
50
100
150
200
250
300
350
zmp_ph108_F9
zmp_ph108_F11
zmp_ph108_H2
zmp_ph108_H5
zmp_ph108_B2
zmp_ph108_B6
zmp_ph108_B7
zmp_ph108_C4
zmp_ph108_C5
zmp_ph108_D4
zmp_ph97_G6
zmp_ph97_G9
zmp_ph97_H2
zmp_ph97_B10
zmp_ph97_C2
zmp_ph97_C4
zmp_ph97_B8
zmp_ph97_C1
zmp_ph97_D2
zmp_ph97_D5
zmp_ph111_G5
zmp_ph111_G11
zmp_ph111_H1
zmp_ph111_B1a
zmp_ph111_B5
zmp_ph111_B8
zmp_ph111_B6
zmp_ph111_B7
zmp_ph111_B9
zmp_ph111_B10
zmp_ph114_G4
zmp_ph114_G11
zmp_ph114_H1
zmp_ph114_H2
zmp_ph114_A9
zmp_ph114_A11
zmp_ph114_B2
zmp_ph114_B4
zmp_ph114_D8
zmp_ph114_E2

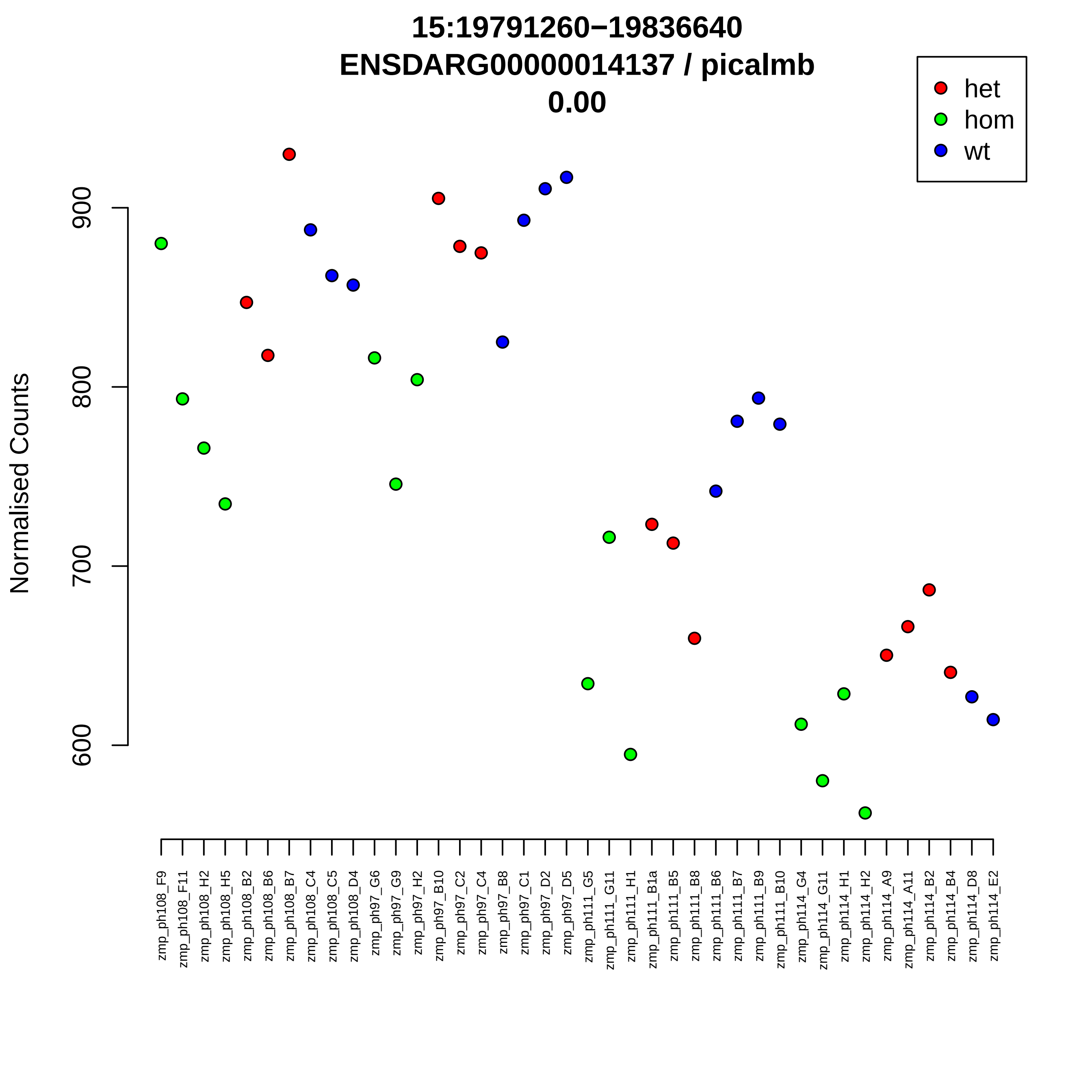

15:19791260−19836640
ENSDARG00000014137 / picalmb
0.00
het
hom
wt
Normalised Counts
600
700
800
900
zmp_ph108_F9
zmp_ph108_F11
zmp_ph108_H2
zmp_ph108_H5
zmp_ph108_B2
zmp_ph108_B6
zmp_ph108_B7
zmp_ph108_C4
zmp_ph108_C5
zmp_ph108_D4
zmp_ph97_G6
zmp_ph97_G9
zmp_ph97_H2
zmp_ph97_B10
zmp_ph97_C2
zmp_ph97_C4
zmp_ph97_B8
zmp_ph97_C1
zmp_ph97_D2
zmp_ph97_D5
zmp_ph111_G5
zmp_ph111_G11
zmp_ph111_H1
zmp_ph111_B1a
zmp_ph111_B5
zmp_ph111_B8
zmp_ph111_B6
zmp_ph111_B7
zmp_ph111_B9
zmp_ph111_B10
zmp_ph114_G4
zmp_ph114_G11
zmp_ph114_H1
zmp_ph114_H2
zmp_ph114_A9
zmp_ph114_A11
zmp_ph114_B2
zmp_ph114_B4
zmp_ph114_D8
zmp_ph114_E2

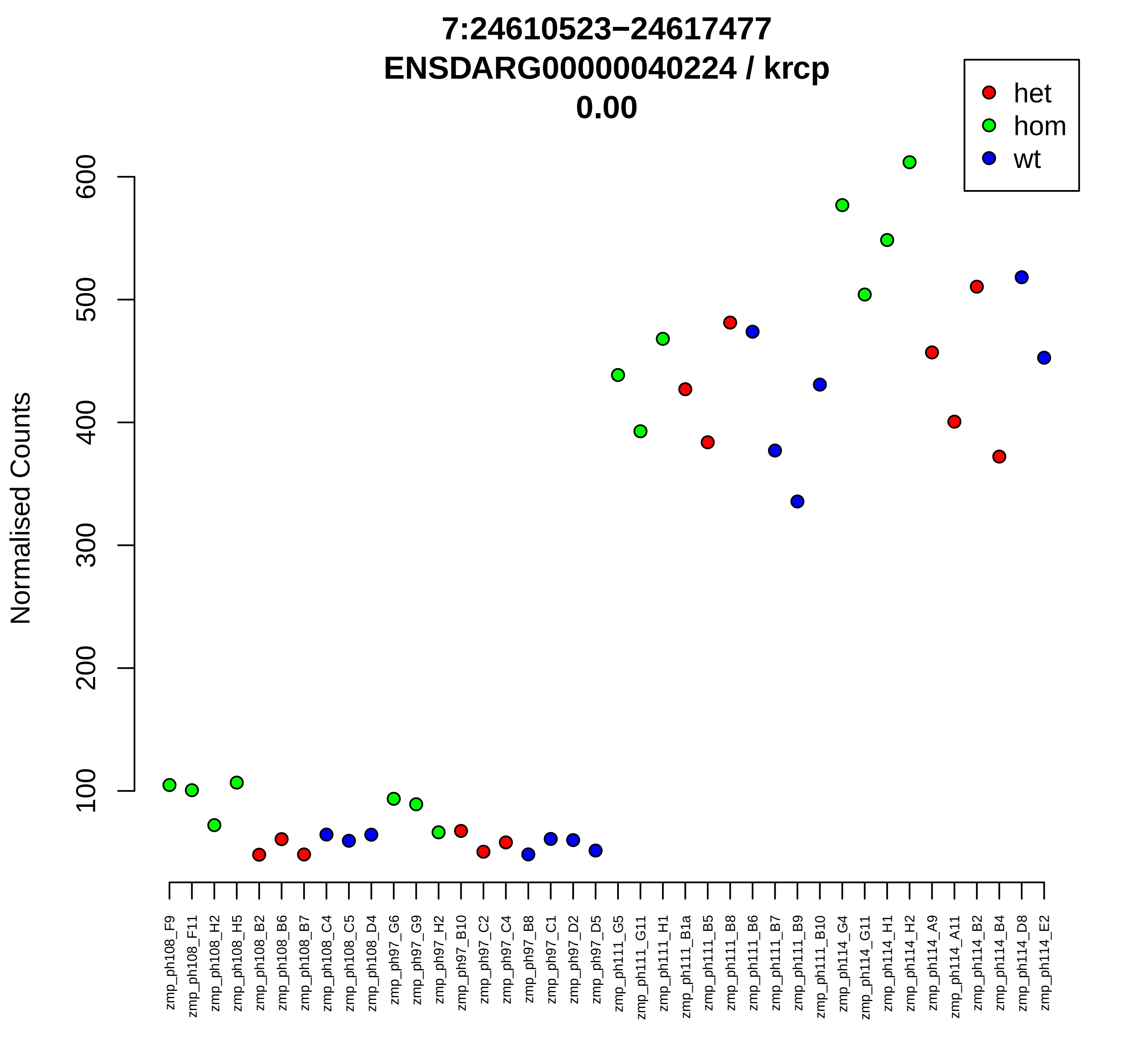

7:24610523−24617477
ENSDARG00000040224 / krcp
0.00
het
hom
wt
Normalised Counts
100
200
300
400
500
600
zmp_ph108_F9
zmp_ph108_F11
zmp_ph108_H2
zmp_ph108_H5
zmp_ph108_B2
zmp_ph108_B6
zmp_ph108_B7
zmp_ph108_C4
zmp_ph108_C5
zmp_ph108_D4
zmp_ph97_G6
zmp_ph97_G9
zmp_ph97_H2
zmp_ph97_B10
zmp_ph97_C2
zmp_ph97_C4
zmp_ph97_B8
zmp_ph97_C1
zmp_ph97_D2
zmp_ph97_D5
zmp_ph111_G5
zmp_ph111_G11
zmp_ph111_H1
zmp_ph111_B1a
zmp_ph111_B5
zmp_ph111_B8
zmp_ph111_B6
zmp_ph111_B7
zmp_ph111_B9
zmp_ph111_B10
zmp_ph114_G4
zmp_ph114_G11
zmp_ph114_H1
zmp_ph114_H2
zmp_ph114_A9
zmp_ph114_A11
zmp_ph114_B2
zmp_ph114_B4
zmp_ph114_D8
zmp_ph114_E2

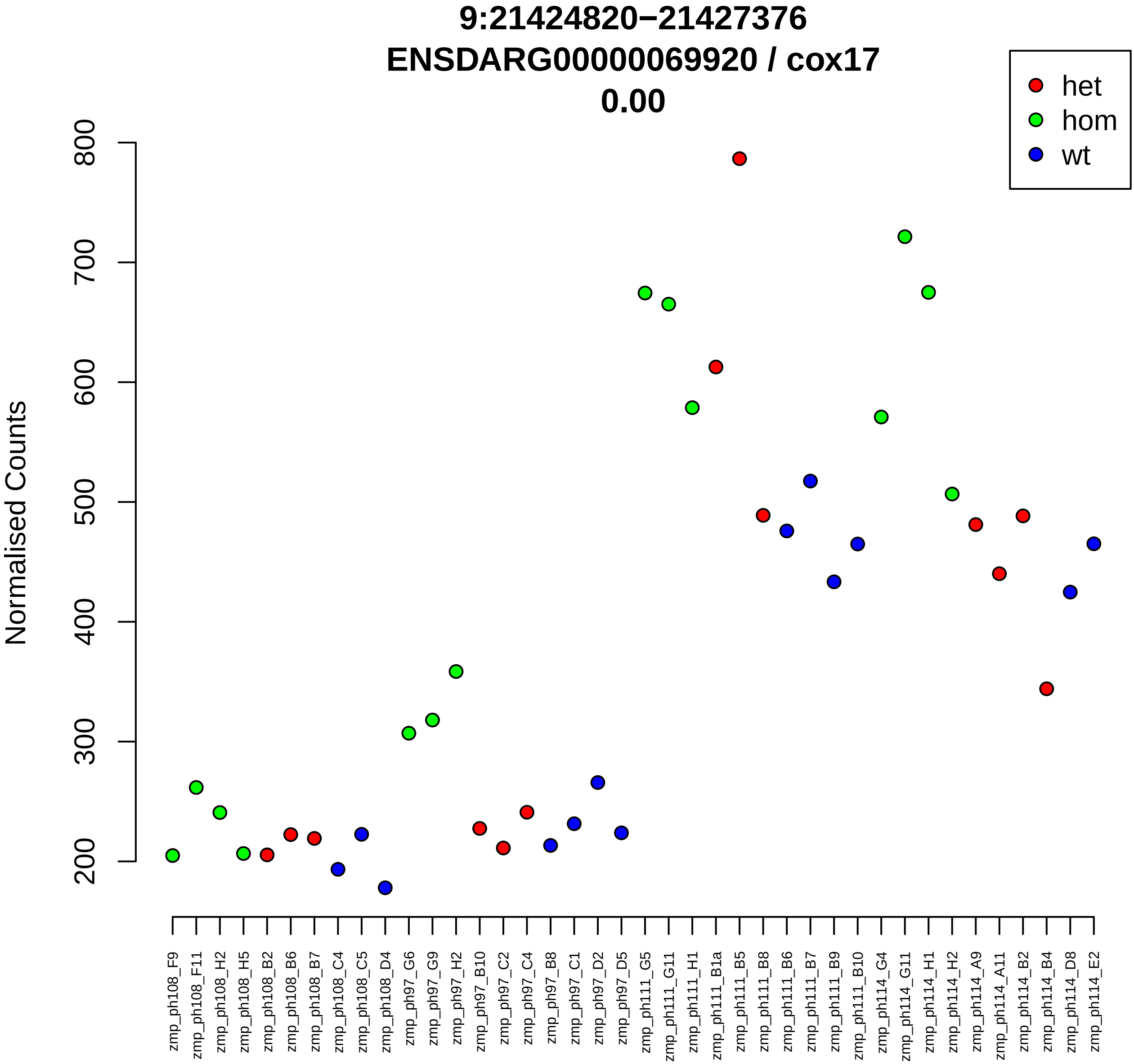

9:21424820−21427376
ENSDARG00000069920 / cox17
0.00
het
hom
wt
Normalised Counts
200
300
400
500
600
700
800
zmp_ph108_F9
zmp_ph108_F11
zmp_ph108_H2
zmp_ph108_H5
zmp_ph108_B2
zmp_ph108_B6
zmp_ph108_B7
zmp_ph108_C4
zmp_ph108_C5
zmp_ph108_D4
zmp_ph97_G6
zmp_ph97_G9
zmp_ph97_H2
zmp_ph97_B10
zmp_ph97_C2
zmp_ph97_C4
zmp_ph97_B8
zmp_ph97_C1
zmp_ph97_D2
zmp_ph97_D5
zmp_ph111_G5
zmp_ph111_G11
zmp_ph111_H1
zmp_ph111_B1a
zmp_ph111_B5
zmp_ph111_B8
zmp_ph111_B6
zmp_ph111_B7
zmp_ph111_B9
zmp_ph111_B10
zmp_ph114_G4
zmp_ph114_G11
zmp_ph114_H1
zmp_ph114_H2
zmp_ph114_A9
zmp_ph114_A11
zmp_ph114_B2
zmp_ph114_B4
zmp_ph114_D8
zmp_ph114_E2

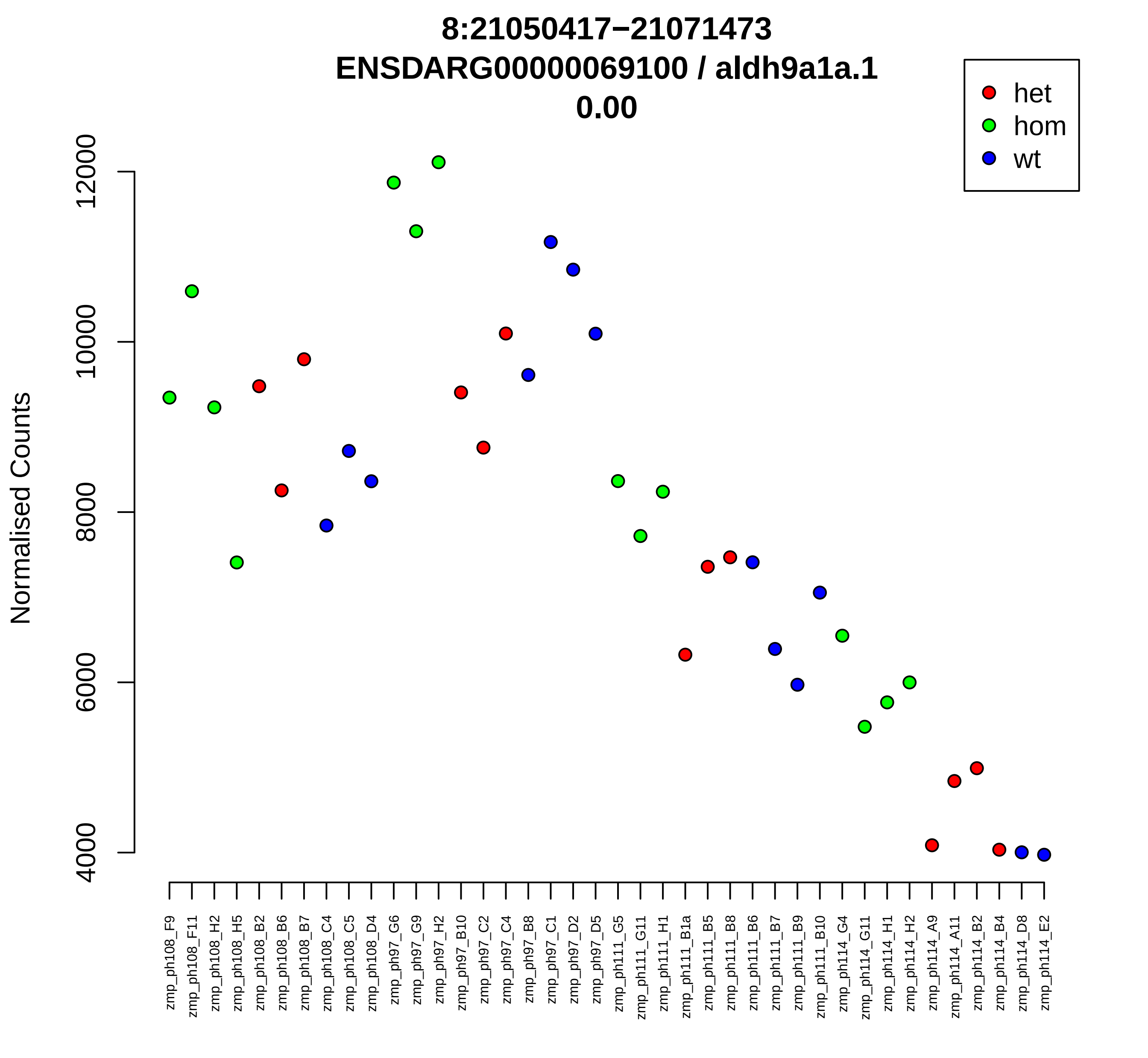

8:21050417−21071473
ENSDARG00000069100 / aldh9a1a.1
0.00
het
hom
wt
Normalised Counts
4000
6000
8000
10000
12000
zmp_ph108_F9
zmp_ph108_F11
zmp_ph108_H2
zmp_ph108_H5
zmp_ph108_B2
zmp_ph108_B6
zmp_ph108_B7
zmp_ph108_C4
zmp_ph108_C5
zmp_ph108_D4
zmp_ph97_G6
zmp_ph97_G9
zmp_ph97_H2
zmp_ph97_B10
zmp_ph97_C2
zmp_ph97_C4
zmp_ph97_B8
zmp_ph97_C1
zmp_ph97_D2
zmp_ph97_D5
zmp_ph111_G5
zmp_ph111_G11
zmp_ph111_H1
zmp_ph111_B1a
zmp_ph111_B5
zmp_ph111_B8
zmp_ph111_B6
zmp_ph111_B7
zmp_ph111_B9
zmp_ph111_B10
zmp_ph114_G4
zmp_ph114_G11
zmp_ph114_H1
zmp_ph114_H2
zmp_ph114_A9
zmp_ph114_A11
zmp_ph114_B2
zmp_ph114_B4
zmp_ph114_D8
zmp_ph114_E2

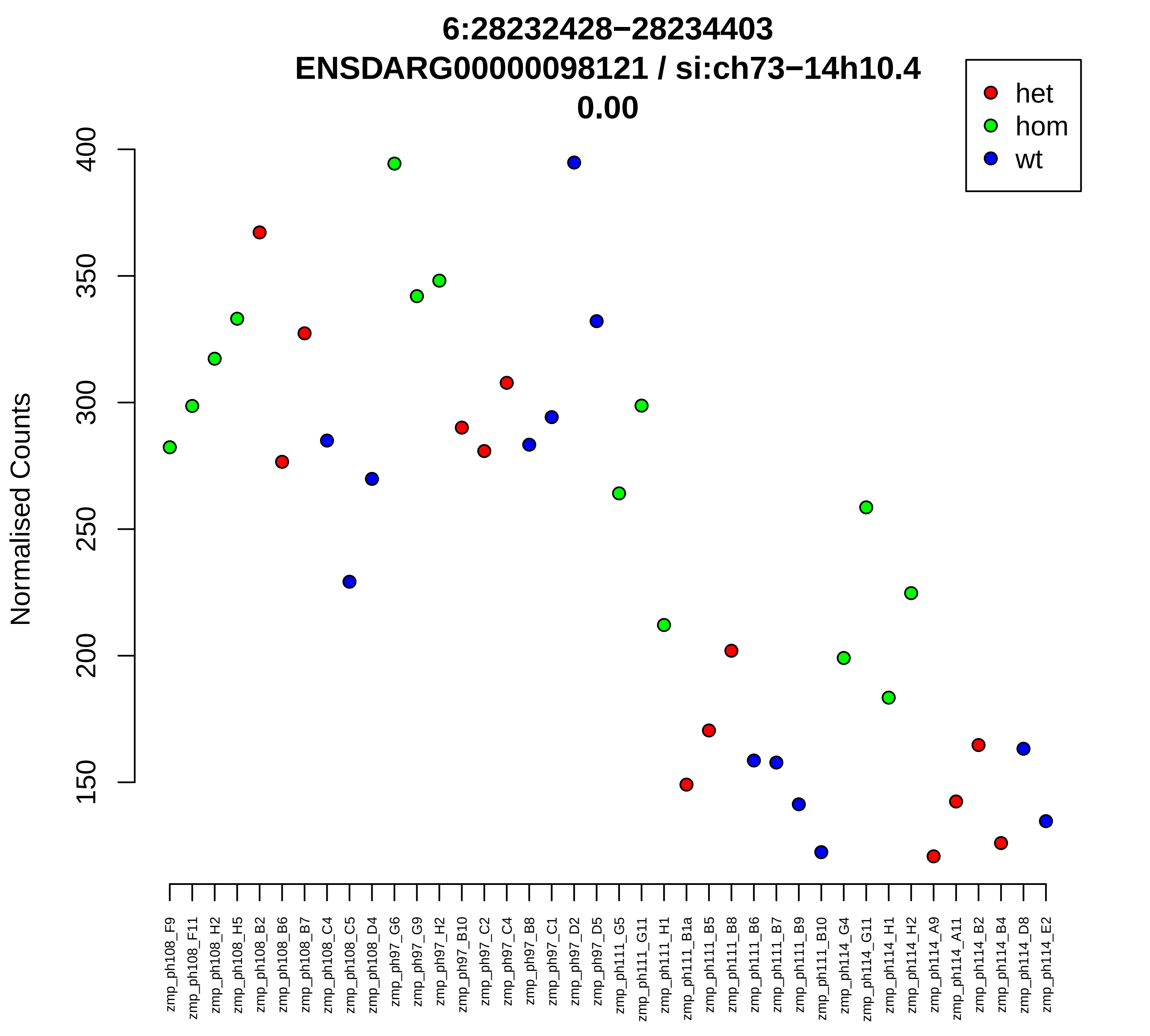
6:28232428−28234403
ENSDARG00000098121 / si:ch73−14h10.4
0.00
het
hom
wt
Normalised Counts
150
200
250
300
350
400
zmp_ph108_F9
zmp_ph108_F11
zmp_ph108_H2
zmp_ph108_H5
zmp_ph108_B2
zmp_ph108_B6
zmp_ph108_B7
zmp_ph108_C4
zmp_ph108_C5
zmp_ph108_D4
zmp_ph97_G6
zmp_ph97_G9
zmp_ph97_H2
zmp_ph97_B10
zmp_ph97_C2
zmp_ph97_C4
zmp_ph97_B8
zmp_ph97_C1
zmp_ph97_D2
zmp_ph97_D5
zmp_ph111_G5
zmp_ph111_G11
zmp_ph111_H1
zmp_ph111_B1a
zmp_ph111_B5
zmp_ph111_B8
zmp_ph111_B6
zmp_ph111_B7
zmp_ph111_B9
zmp_ph111_B10
zmp_ph114_G4
zmp_ph114_G11
zmp_ph114_H1
zmp_ph114_H2
zmp_ph114_A9
zmp_ph114_A11
zmp_ph114_B2
zmp_ph114_B4
zmp_ph114_D8
zmp_ph114_E2

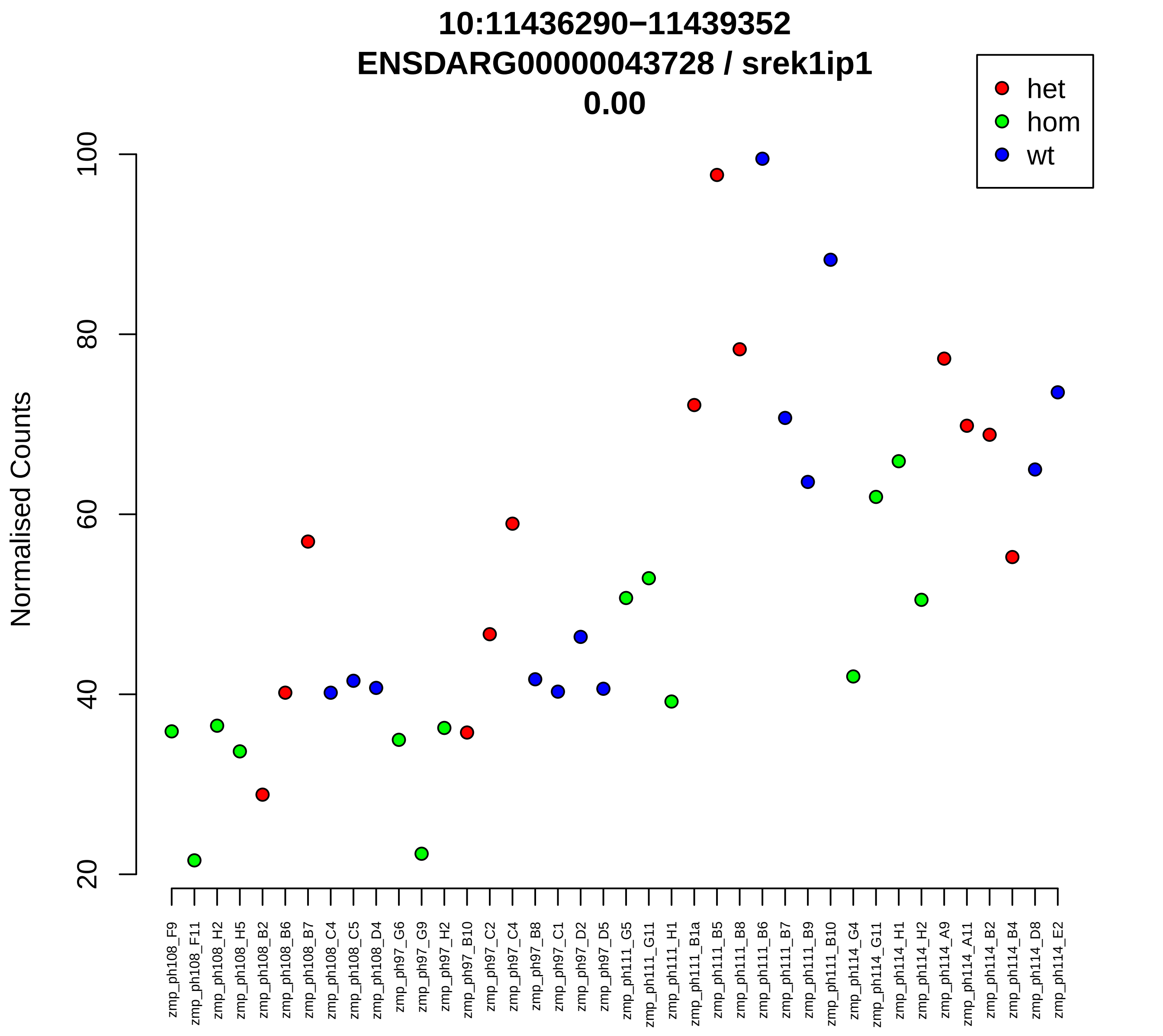

10:11436290−11439352
ENSDARG00000043728 / srek1ip1
0.00
het
hom
wt
Normalised Counts
20
40
60
80
100
zmp_ph108_F9
zmp_ph108_F11
zmp_ph108_H2
zmp_ph108_H5
zmp_ph108_B2
zmp_ph108_B6
zmp_ph108_B7
zmp_ph108_C4
zmp_ph108_C5
zmp_ph108_D4
zmp_ph97_G6
zmp_ph97_G9
zmp_ph97_H2
zmp_ph97_B10
zmp_ph97_C2
zmp_ph97_C4
zmp_ph97_B8
zmp_ph97_C1
zmp_ph97_D2
zmp_ph97_D5
zmp_ph111_G5
zmp_ph111_G11
zmp_ph111_H1
zmp_ph111_B1a
zmp_ph111_B5
zmp_ph111_B8
zmp_ph111_B6
zmp_ph111_B7
zmp_ph111_B9
zmp_ph111_B10
zmp_ph114_G4
zmp_ph114_G11
zmp_ph114_H1
zmp_ph114_H2
zmp_ph114_A9
zmp_ph114_A11
zmp_ph114_B2
zmp_ph114_B4
zmp_ph114_D8
zmp_ph114_E2

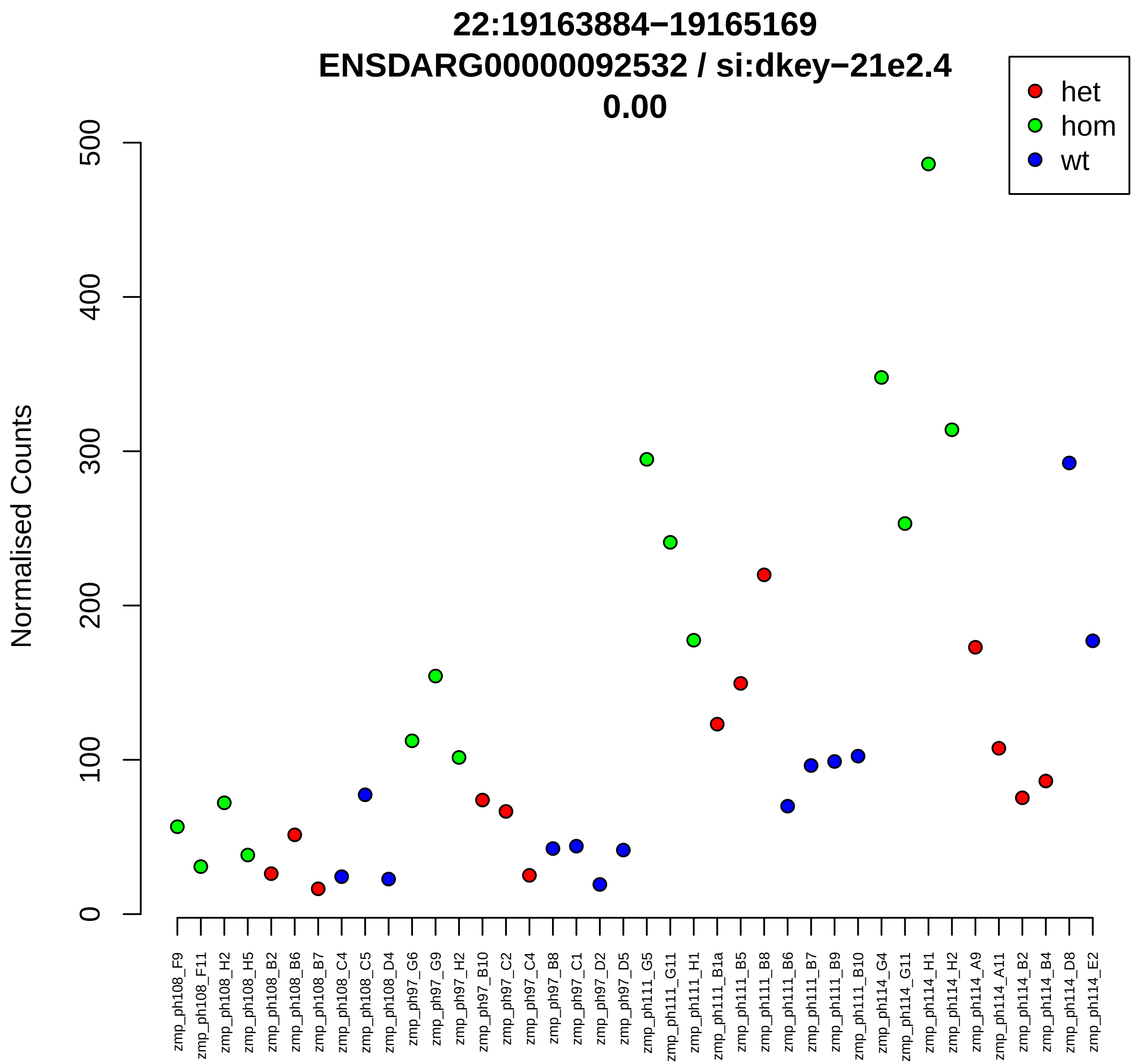

22:19163884−19165169
ENSDARG00000092532 / si:dkey−21e2.4
0.00
het
hom
wt
Normalised Counts
0
100
200
300
400
500
zmp_ph108_F9
zmp_ph108_F11
zmp_ph108_H2
zmp_ph108_H5
zmp_ph108_B2
zmp_ph108_B6
zmp_ph108_B7
zmp_ph108_C4
zmp_ph108_C5
zmp_ph108_D4
zmp_ph97_G6
zmp_ph97_G9
zmp_ph97_H2
zmp_ph97_B10
zmp_ph97_C2
zmp_ph97_C4
zmp_ph97_B8
zmp_ph97_C1
zmp_ph97_D2
zmp_ph97_D5
zmp_ph111_G5
zmp_ph111_G11
zmp_ph111_H1
zmp_ph111_B1a
zmp_ph111_B5
zmp_ph111_B8
zmp_ph111_B6
zmp_ph111_B7
zmp_ph111_B9
zmp_ph111_B10
zmp_ph114_G4
zmp_ph114_G11
zmp_ph114_H1
zmp_ph114_H2
zmp_ph114_A9
zmp_ph114_A11
zmp_ph114_B2
zmp_ph114_B4
zmp_ph114_D8
zmp_ph114_E2

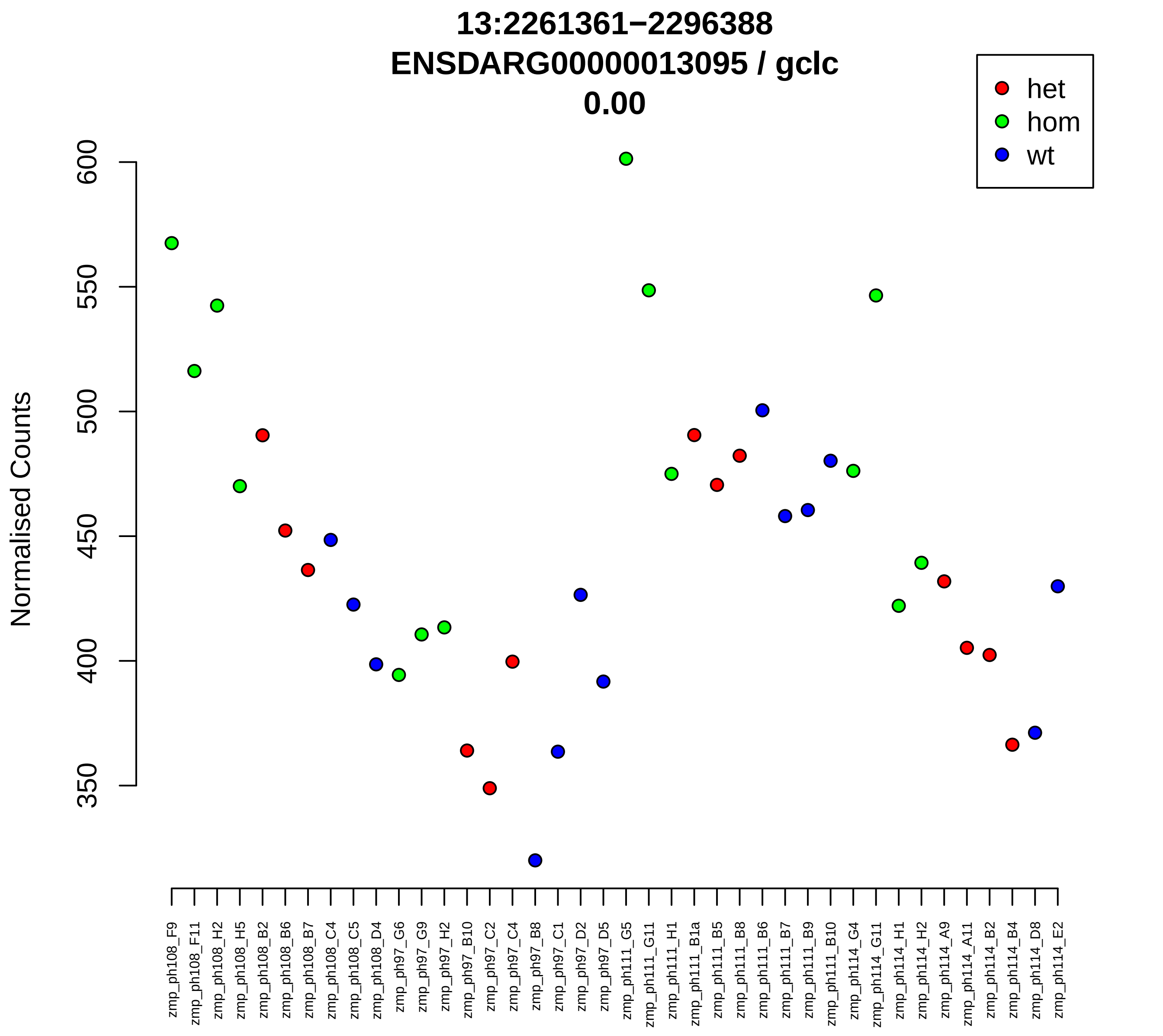
13:2261361−2296388
ENSDARG00000013095 / gclc
0.00
het
hom
wt
Normalised Counts
350
400
450
500
550
600
zmp_ph108_F9
zmp_ph108_F11
zmp_ph108_H2
zmp_ph108_H5
zmp_ph108_B2
zmp_ph108_B6
zmp_ph108_B7
zmp_ph108_C4
zmp_ph108_C5
zmp_ph108_D4
zmp_ph97_G6
zmp_ph97_G9
zmp_ph97_H2
zmp_ph97_B10
zmp_ph97_C2
zmp_ph97_C4
zmp_ph97_B8
zmp_ph97_C1
zmp_ph97_D2
zmp_ph97_D5
zmp_ph111_G5
zmp_ph111_G11
zmp_ph111_H1
zmp_ph111_B1a
zmp_ph111_B5
zmp_ph111_B8
zmp_ph111_B6
zmp_ph111_B7
zmp_ph111_B9
zmp_ph111_B10
zmp_ph114_G4
zmp_ph114_G11
zmp_ph114_H1
zmp_ph114_H2
zmp_ph114_A9
zmp_ph114_A11
zmp_ph114_B2
zmp_ph114_B4
zmp_ph114_D8
zmp_ph114_E2

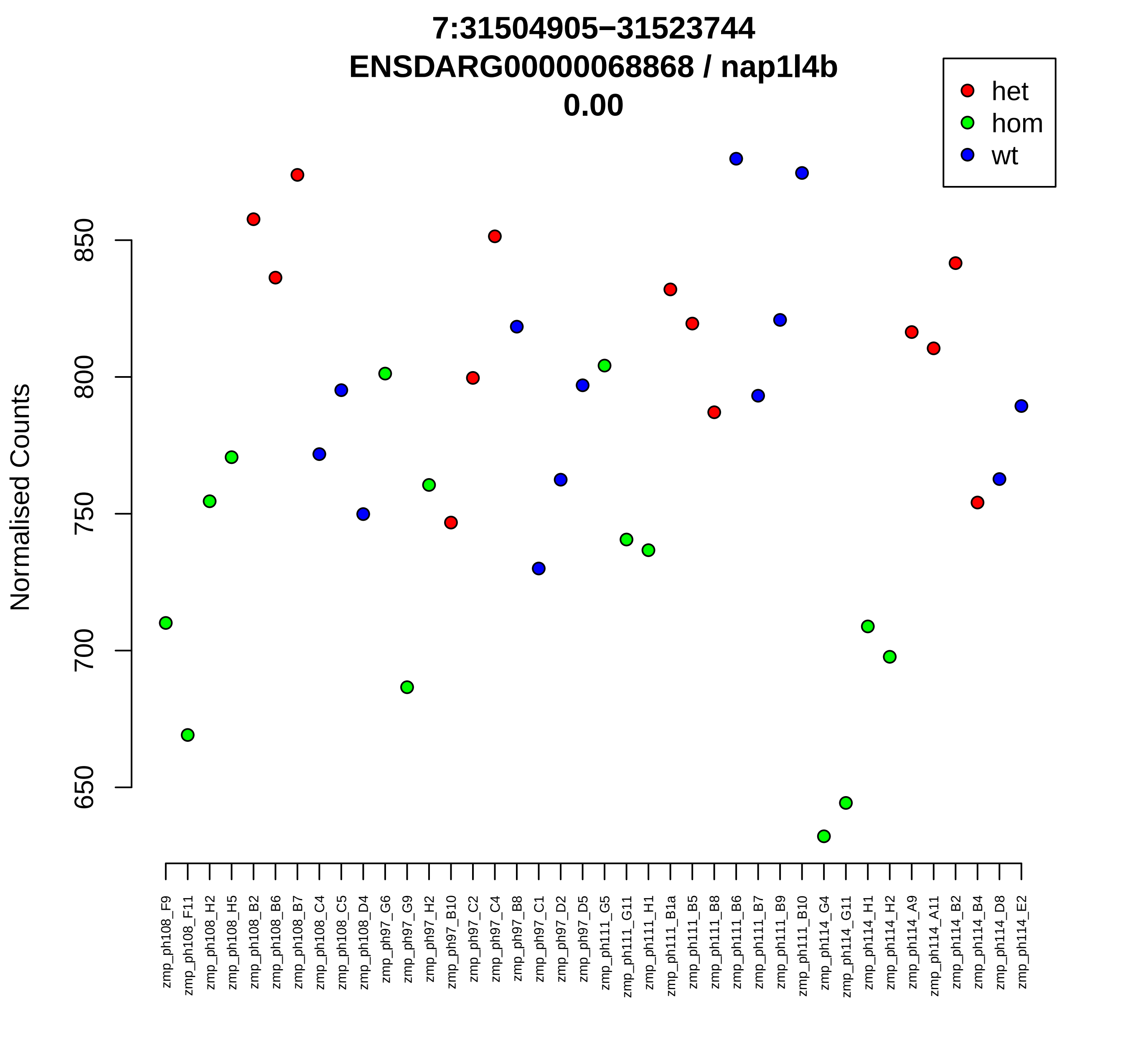

7:31504905−31523744
ENSDARG00000068868 / nap1l4b
0.00
het
hom
wt
Normalised Counts
650
700
750
800
850
zmp_ph108_F9
zmp_ph108_F11
zmp_ph108_H2
zmp_ph108_H5
zmp_ph108_B2
zmp_ph108_B6
zmp_ph108_B7
zmp_ph108_C4
zmp_ph108_C5
zmp_ph108_D4
zmp_ph97_G6
zmp_ph97_G9
zmp_ph97_H2
zmp_ph97_B10
zmp_ph97_C2
zmp_ph97_C4
zmp_ph97_B8
zmp_ph97_C1
zmp_ph97_D2
zmp_ph97_D5
zmp_ph111_G5
zmp_ph111_G11
zmp_ph111_H1
zmp_ph111_B1a
zmp_ph111_B5
zmp_ph111_B8
zmp_ph111_B6
zmp_ph111_B7
zmp_ph111_B9
zmp_ph111_B10
zmp_ph114_G4
zmp_ph114_G11
zmp_ph114_H1
zmp_ph114_H2
zmp_ph114_A9
zmp_ph114_A11
zmp_ph114_B2
zmp_ph114_B4
zmp_ph114_D8
zmp_ph114_E2

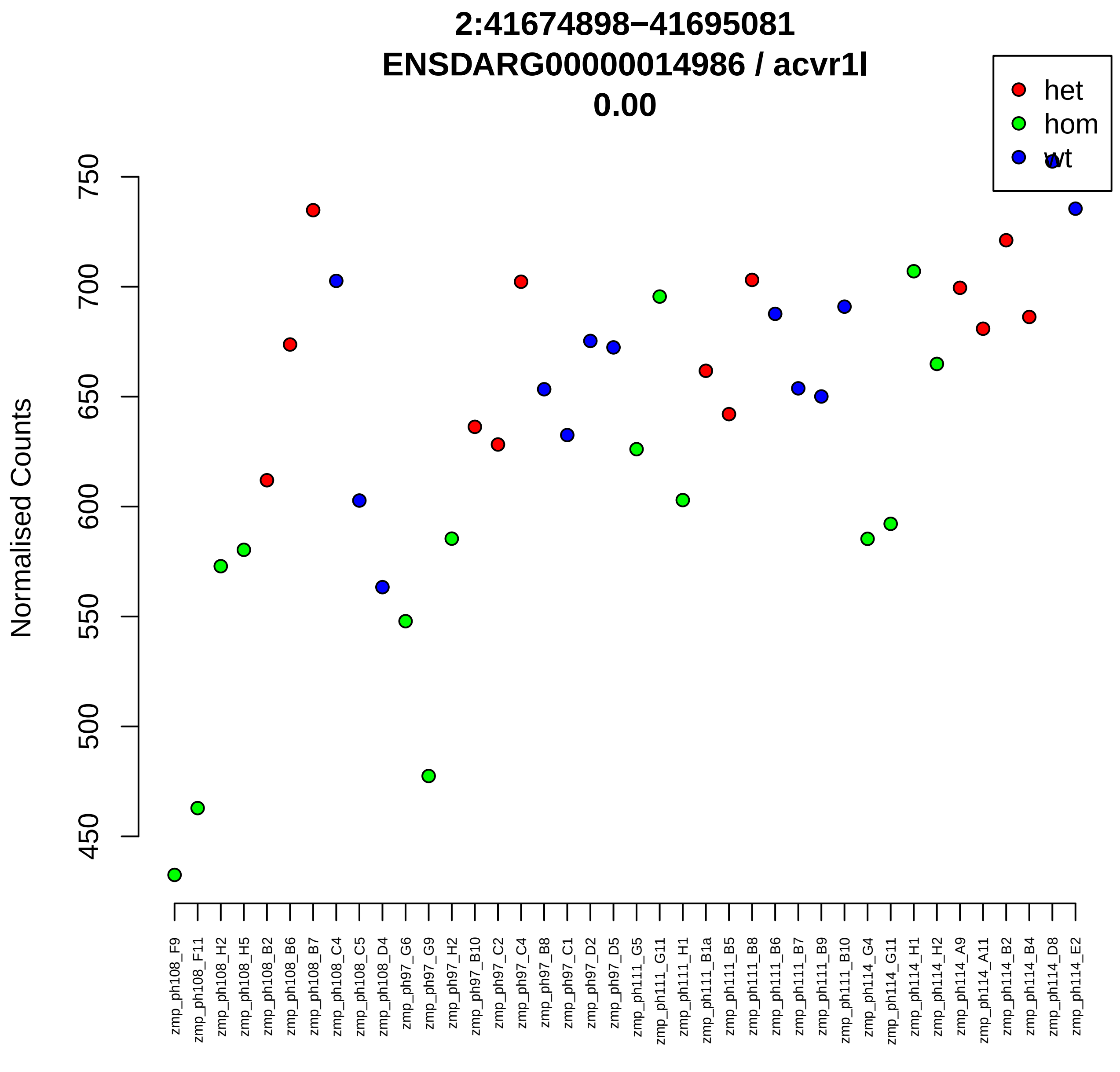

2:41674898−41695081
ENSDARG00000014986 / acvr1l
0.00
het
hom
wt
Normalised Counts
750
700
650
600
550
500
450
zmp_ph108_F9
zmp_ph108_F11
zmp_ph108_H2
zmp_ph108_H5
zmp_ph108_B2
zmp_ph108_B6
zmp_ph108_B7
zmp_ph108_C4
zmp_ph108_C5
zmp_ph108_D4
zmp_ph97_G6
zmp_ph97_G9
zmp_ph97_H2
zmp_ph97_B10
zmp_ph97_C2
zmp_ph97_C4
zmp_ph97_B8
zmp_ph97_C1
zmp_ph97_D2
zmp_ph97_D5
zmp_ph111_G5
zmp_ph111_G11
zmp_ph111_H1
zmp_ph111_B1a
zmp_ph111_B5
zmp_ph111_B8
zmp_ph111_B6
zmp_ph111_B7
zmp_ph111_B9
zmp_ph111_B10
zmp_ph114_G4
zmp_ph114_G11
zmp_ph114_H1
zmp_ph114_H2
zmp_ph114_A9
zmp_ph114_A11
zmp_ph114_B2
zmp_ph114_B4
zmp_ph114_D8
zmp_ph114_E2

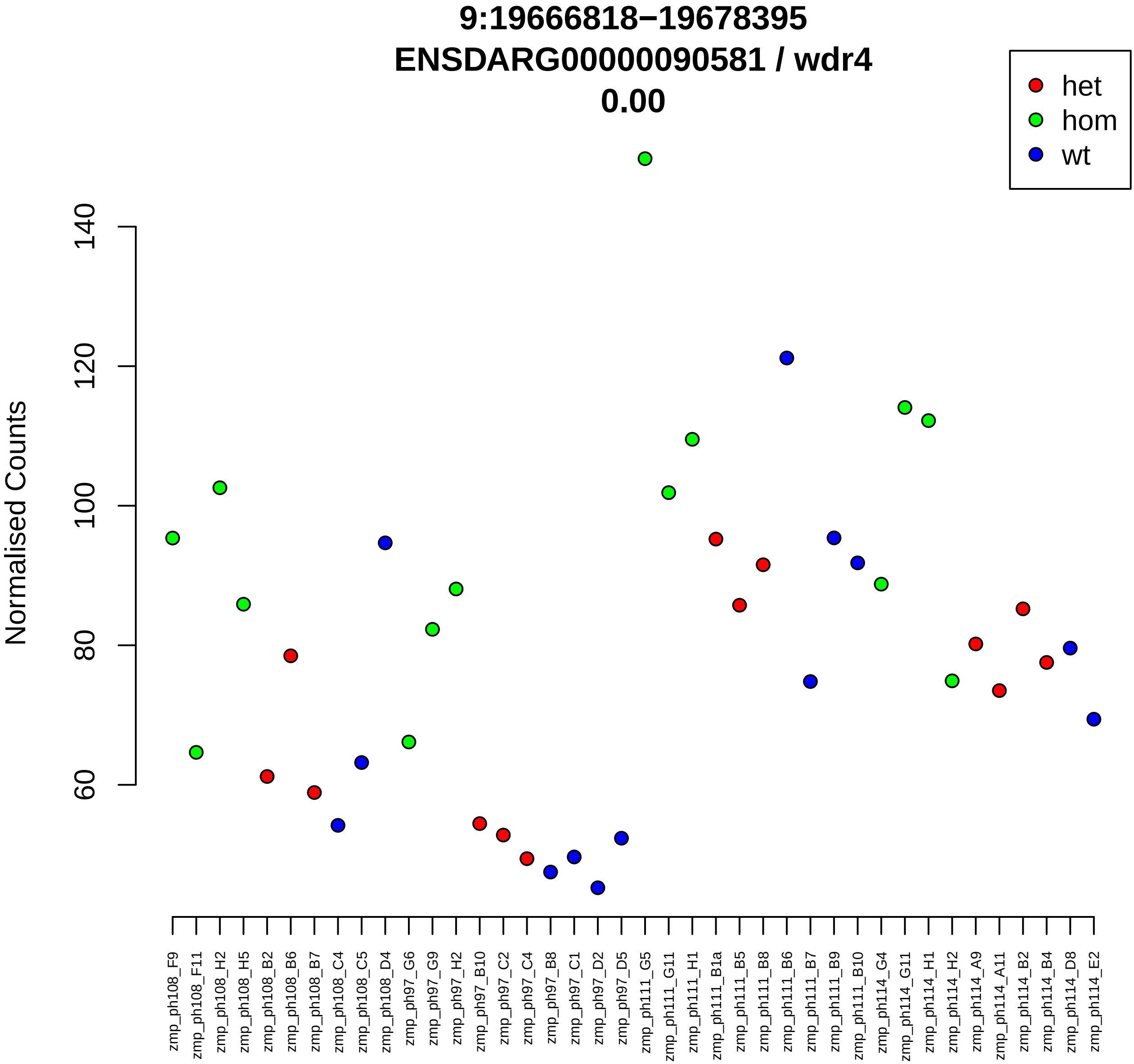

9:19666818−19678395
ENSDARG00000090581 / wdr4
0.00
het
hom
wt
Normalised Counts
60
80
100
120
140
zmp_ph108_F9
zmp_ph108_F11
zmp_ph108_H2
zmp_ph108_H5
zmp_ph108_B2
zmp_ph108_B6
zmp_ph108_B7
zmp_ph108_C4
zmp_ph108_C5
zmp_ph108_D4
zmp_ph97_G6
zmp_ph97_G9
zmp_ph97_H2
zmp_ph97_B10
zmp_ph97_C2
zmp_ph97_C4
zmp_ph97_B8
zmp_ph97_C1
zmp_ph97_D2
zmp_ph97_D5
zmp_ph111_G5
zmp_ph111_G11
zmp_ph111_H1
zmp_ph111_B1a
zmp_ph111_B5
zmp_ph111_B8
zmp_ph111_B6
zmp_ph111_B7
zmp_ph111_B9
zmp_ph111_B10
zmp_ph114_G4
zmp_ph114_G11
zmp_ph114_H1
zmp_ph114_H2
zmp_ph114_A9
zmp_ph114_A11
zmp_ph114_B2
zmp_ph114_B4
zmp_ph114_D8
zmp_ph114_E2

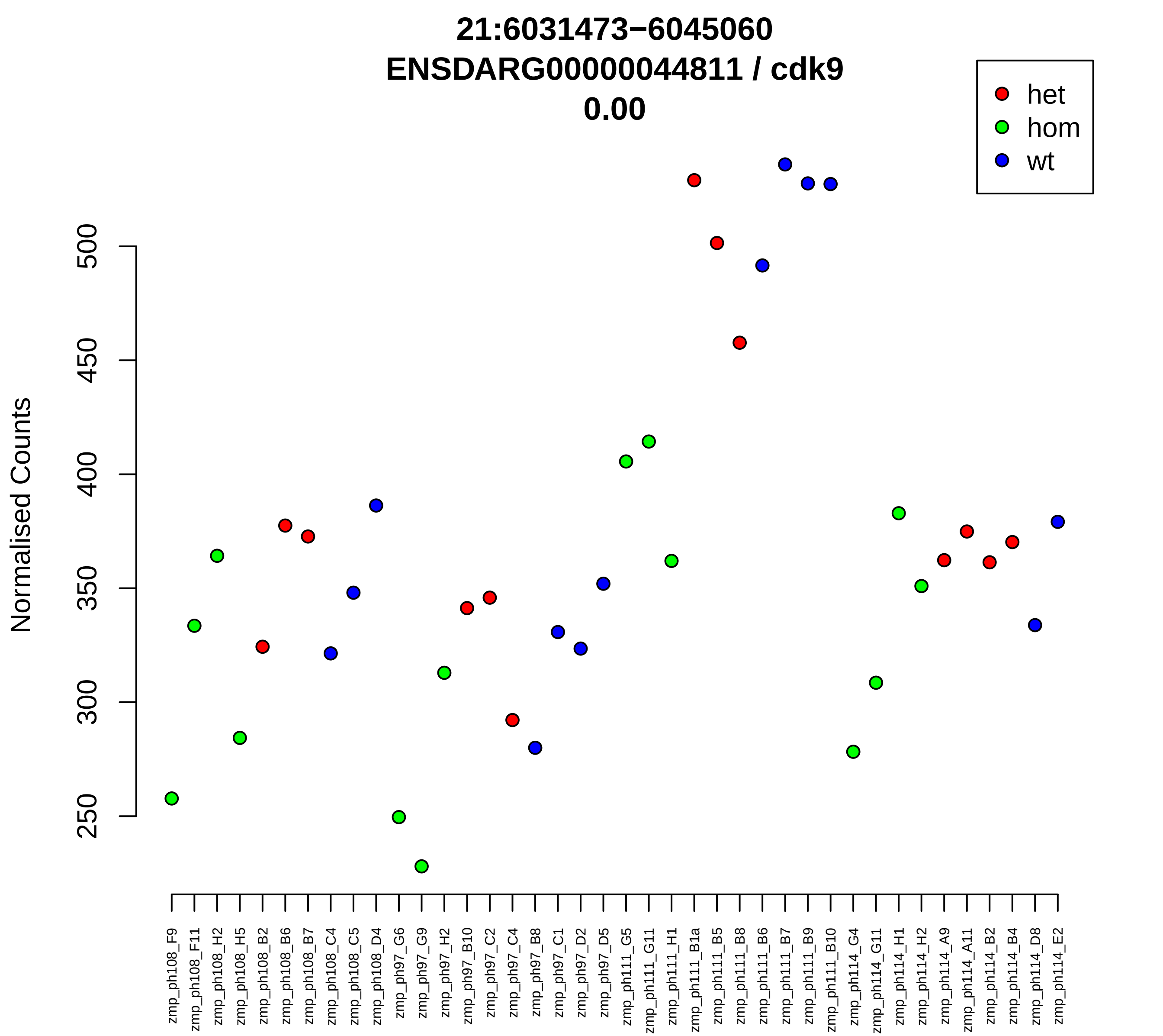

21:6031473−6045060
ENSDARG00000044811 / cdk9
0.00
het
hom
wt
Normalised Counts
250
300
350
400
450
500
zmp_ph108_F9
zmp_ph108_F11
zmp_ph108_H2
zmp_ph108_H5
zmp_ph108_B2
zmp_ph108_B6
zmp_ph108_B7
zmp_ph108_C4
zmp_ph108_C5
zmp_ph108_D4
zmp_ph97_G6
zmp_ph97_G9
zmp_ph97_H2
zmp_ph97_B10
zmp_ph97_C2
zmp_ph97_C4
zmp_ph97_B8
zmp_ph97_C1
zmp_ph97_D2
zmp_ph97_D5
zmp_ph111_G5
zmp_ph111_G11
zmp_ph111_H1
zmp_ph111_B1a
zmp_ph111_B5
zmp_ph111_B8
zmp_ph111_B6
zmp_ph111_B7
zmp_ph111_B9
zmp_ph111_B10
zmp_ph114_G4
zmp_ph114_G11
zmp_ph114_H1
zmp_ph114_H2
zmp_ph114_A9
zmp_ph114_A11
zmp_ph114_B2
zmp_ph114_B4
zmp_ph114_D8
zmp_ph114_E2

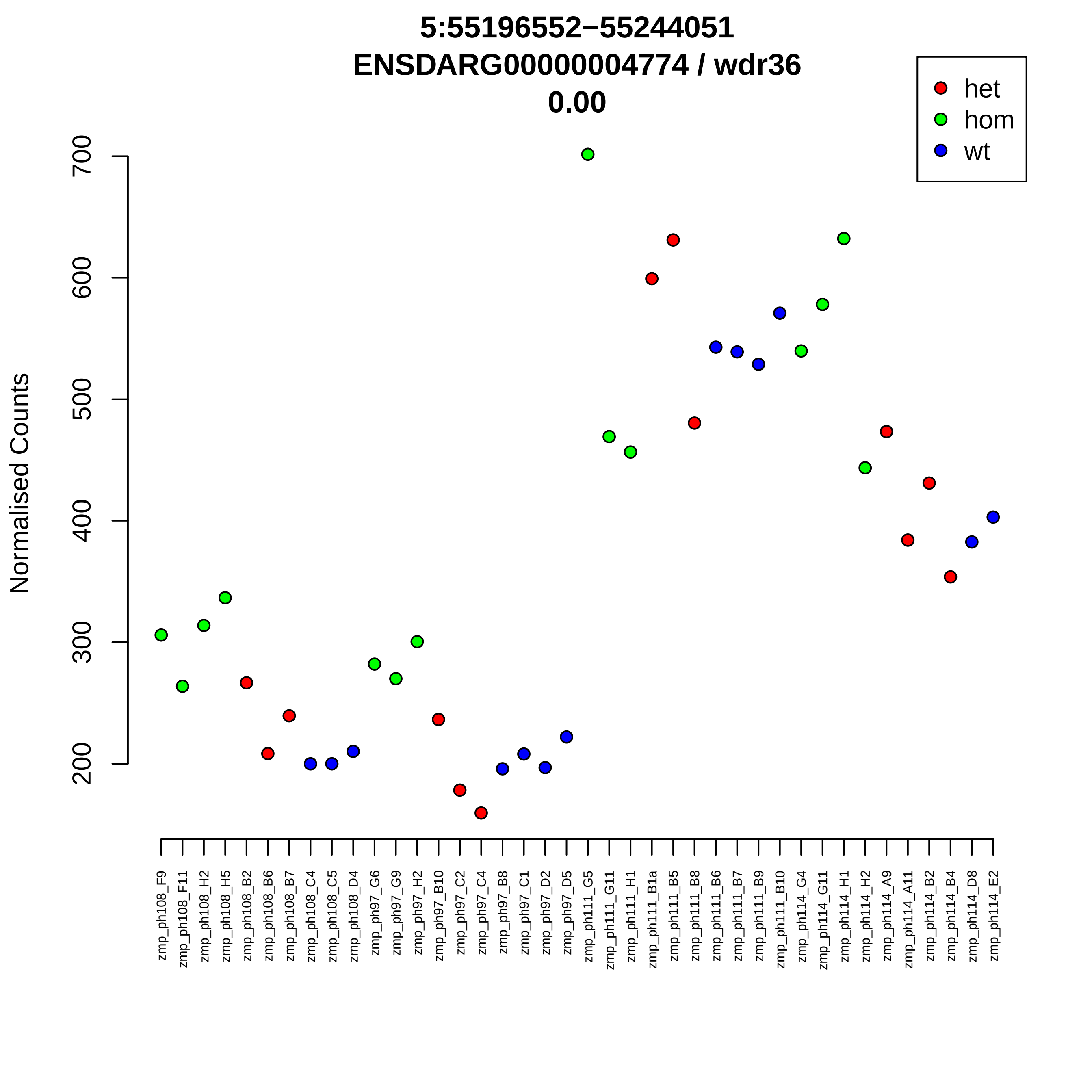

5:55196552−55244051
ENSDARG00000004774 / wdr36
0.00
het
hom
wt
Normalised Counts
200
300
400
500
600
700
zmp_ph108_F9
zmp_ph108_F11
zmp_ph108_H2
zmp_ph108_H5
zmp_ph108_B2
zmp_ph108_B6
zmp_ph108_B7
zmp_ph108_C4
zmp_ph108_C5
zmp_ph108_D4
zmp_ph97_G6
zmp_ph97_G9
zmp_ph97_H2
zmp_ph97_B10
zmp_ph97_C2
zmp_ph97_C4
zmp_ph97_B8
zmp_ph97_C1
zmp_ph97_D2
zmp_ph97_D5
zmp_ph111_G5
zmp_ph111_G11
zmp_ph111_H1
zmp_ph111_B1a
zmp_ph111_B5
zmp_ph111_B8
zmp_ph111_B6
zmp_ph111_B7
zmp_ph111_B9
zmp_ph111_B10
zmp_ph114_G4
zmp_ph114_G11
zmp_ph114_H1
zmp_ph114_H2
zmp_ph114_A9
zmp_ph114_A11
zmp_ph114_B2
zmp_ph114_B4
zmp_ph114_D8
zmp_ph114_E2

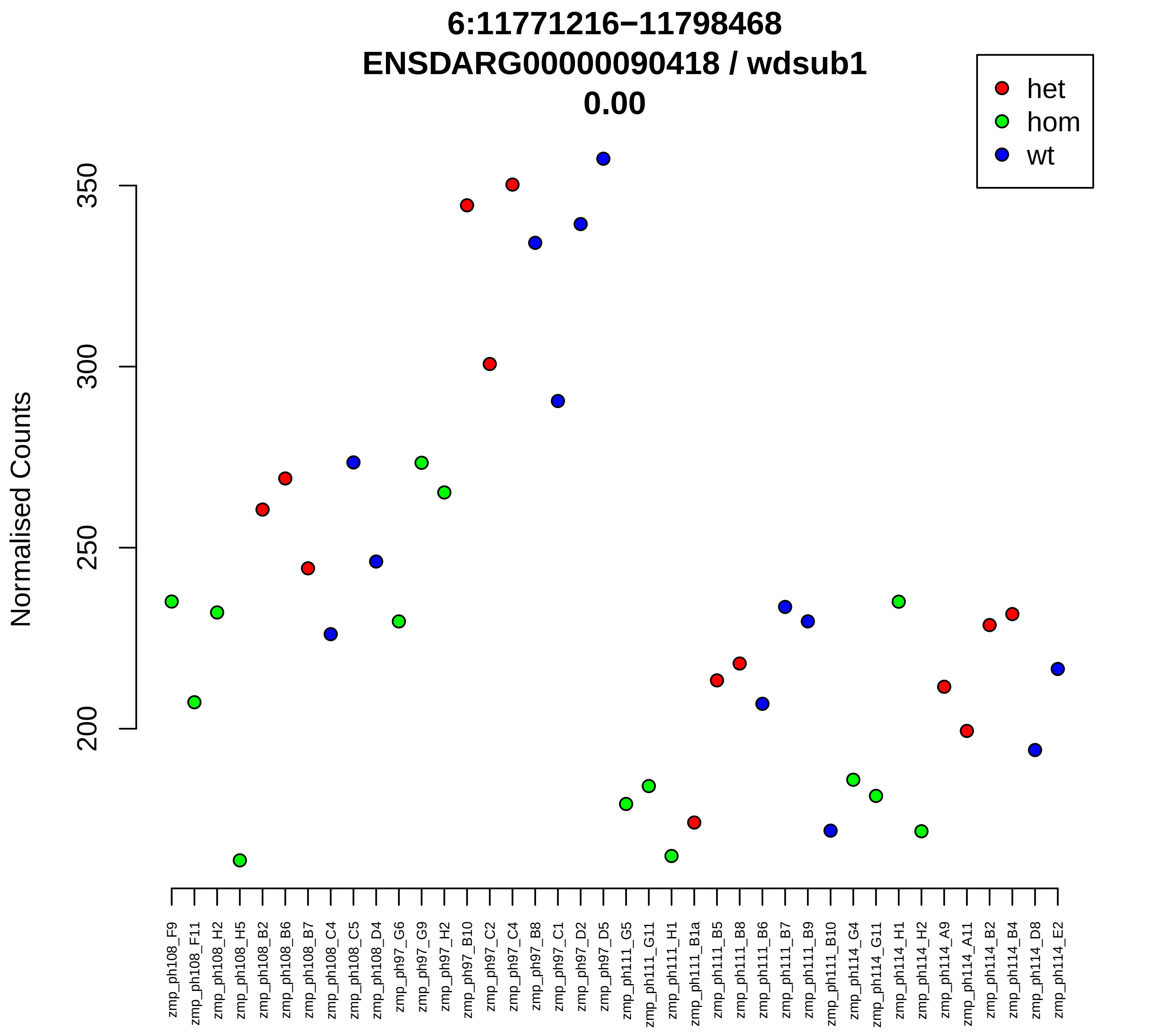

6:11771216−11798468
ENSDARG00000090418 / wdsub1
0.00
het
hom
wt
Normalised Counts
200
250
300
350
zmp_ph108_F9
zmp_ph108_F11
zmp_ph108_H2
zmp_ph108_H5
zmp_ph108_B2
zmp_ph108_B6
zmp_ph108_B7
zmp_ph108_C4
zmp_ph108_C5
zmp_ph108_D4
zmp_ph97_G6
zmp_ph97_G9
zmp_ph97_H2
zmp_ph97_B10
zmp_ph97_C2
zmp_ph97_C4
zmp_ph97_B8
zmp_ph97_C1
zmp_ph97_D2
zmp_ph97_D5
zmp_ph111_G5
zmp_ph111_G11
zmp_ph111_H1
zmp_ph111_B1a
zmp_ph111_B5
zmp_ph111_B8
zmp_ph111_B6
zmp_ph111_B7
zmp_ph111_B9
zmp_ph111_B10
zmp_ph114_G4
zmp_ph114_G11
zmp_ph114_H1
zmp_ph114_H2
zmp_ph114_A9
zmp_ph114_A11
zmp_ph114_B2
zmp_ph114_B4
zmp_ph114_D8
zmp_ph114_E2

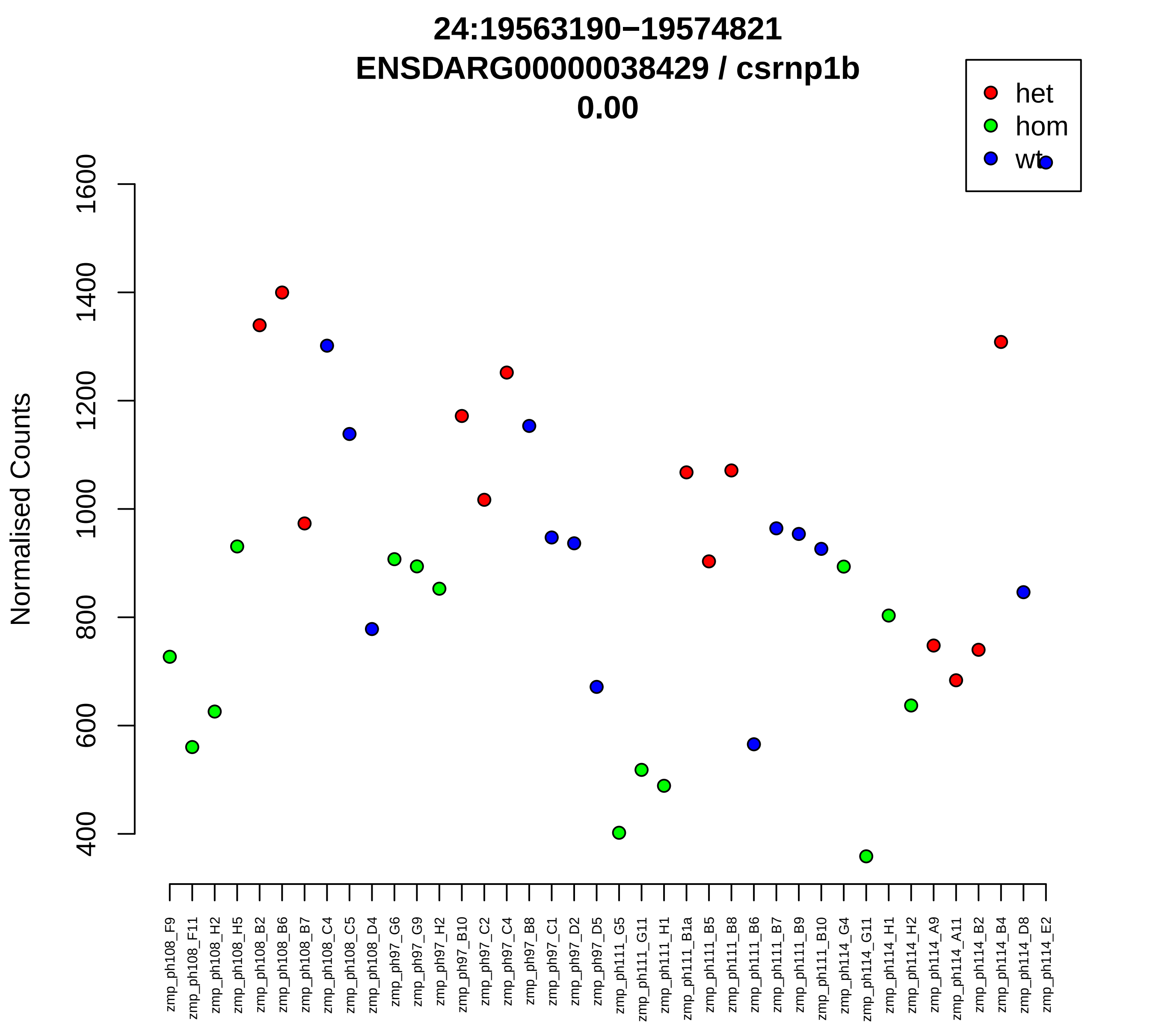
24:19563190−19574821
ENSDARG00000038429 / csrnp1b
0.00
het
hom
wt
Normalised Counts
400
600
800
1000
1200
1400
1600
zmp_ph108_F9
zmp_ph108_F11
zmp_ph108_H2
zmp_ph108_H5
zmp_ph108_B2
zmp_ph108_B6
zmp_ph108_B7
zmp_ph108_C4
zmp_ph108_C5
zmp_ph108_D4
zmp_ph97_G6
zmp_ph97_G9
zmp_ph97_H2
zmp_ph97_B10
zmp_ph97_C2
zmp_ph97_C4
zmp_ph97_B8
zmp_ph97_C1
zmp_ph97_D2
zmp_ph97_D5
zmp_ph111_G5
zmp_ph111_G11
zmp_ph111_H1
zmp_ph111_B1a
zmp_ph111_B5
zmp_ph111_B8
zmp_ph111_B6
zmp_ph111_B7
zmp_ph111_B9
zmp_ph111_B10
zmp_ph114_G4
zmp_ph114_G11
zmp_ph114_H1
zmp_ph114_H2
zmp_ph114_A9
zmp_ph114_A11
zmp_ph114_B2
zmp_ph114_B4
zmp_ph114_D8
zmp_ph114_E2

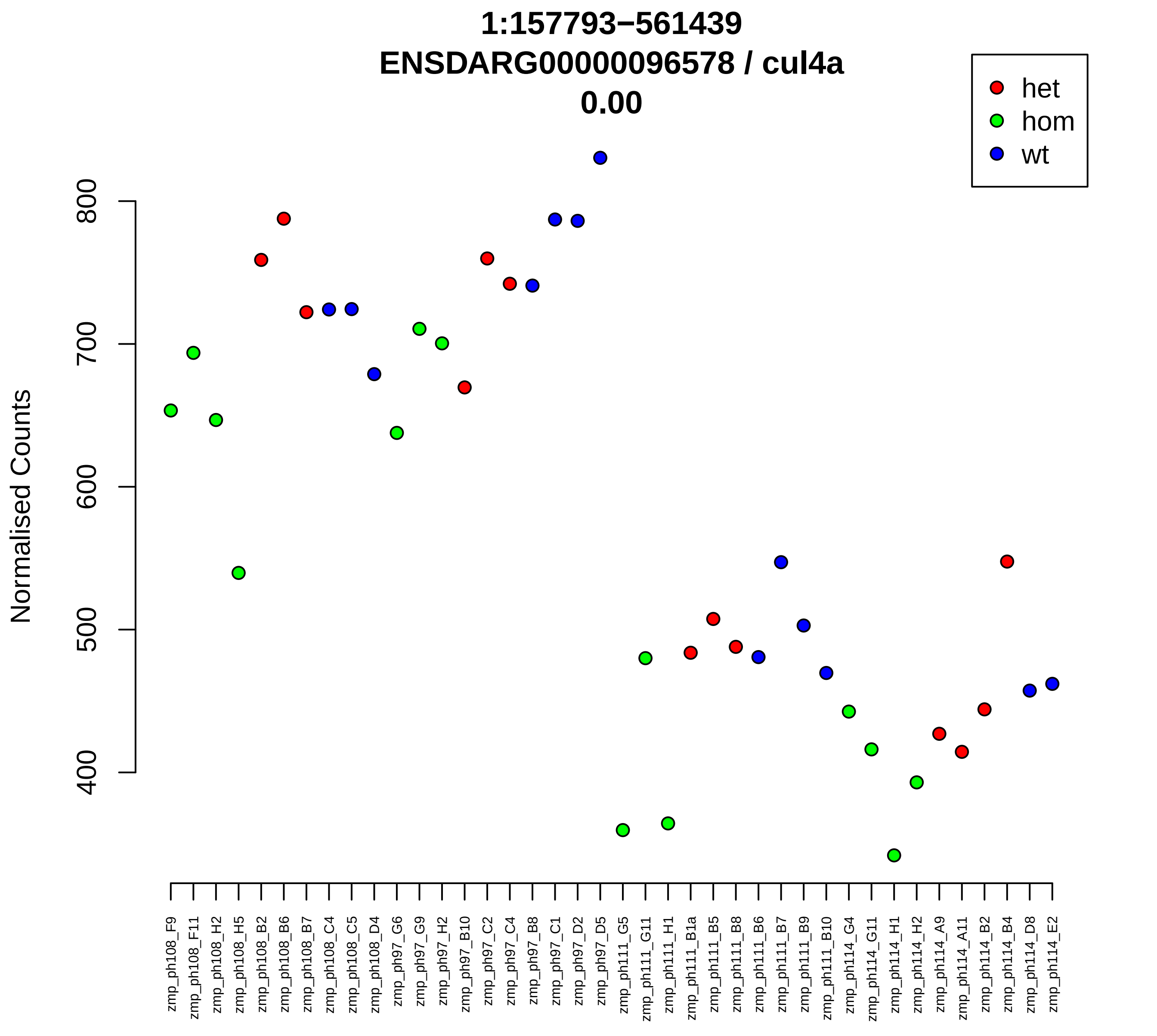
1:157793−561439
ENSDARG00000096578 / cul4a
0.00
het
hom
wt
Normalised Counts
400
500
600
700
800
zmp_ph108_F9
zmp_ph108_F11
zmp_ph108_H2
zmp_ph108_H5
zmp_ph108_B2
zmp_ph108_B6
zmp_ph108_B7
zmp_ph108_C4
zmp_ph108_C5
zmp_ph108_D4
zmp_ph97_G6
zmp_ph97_G9
zmp_ph97_H2
zmp_ph97_B10
zmp_ph97_C2
zmp_ph97_C4
zmp_ph97_B8
zmp_ph97_C1
zmp_ph97_D2
zmp_ph97_D5
zmp_ph111_G5
zmp_ph111_G11
zmp_ph111_H1
zmp_ph111_B1a
zmp_ph111_B5
zmp_ph111_B8
zmp_ph111_B6
zmp_ph111_B7
zmp_ph111_B9
zmp_ph111_B10
zmp_ph114_G4
zmp_ph114_G11
zmp_ph114_H1
zmp_ph114_H2
zmp_ph114_A9
zmp_ph114_A11
zmp_ph114_B2
zmp_ph114_B4
zmp_ph114_D8
zmp_ph114_E2

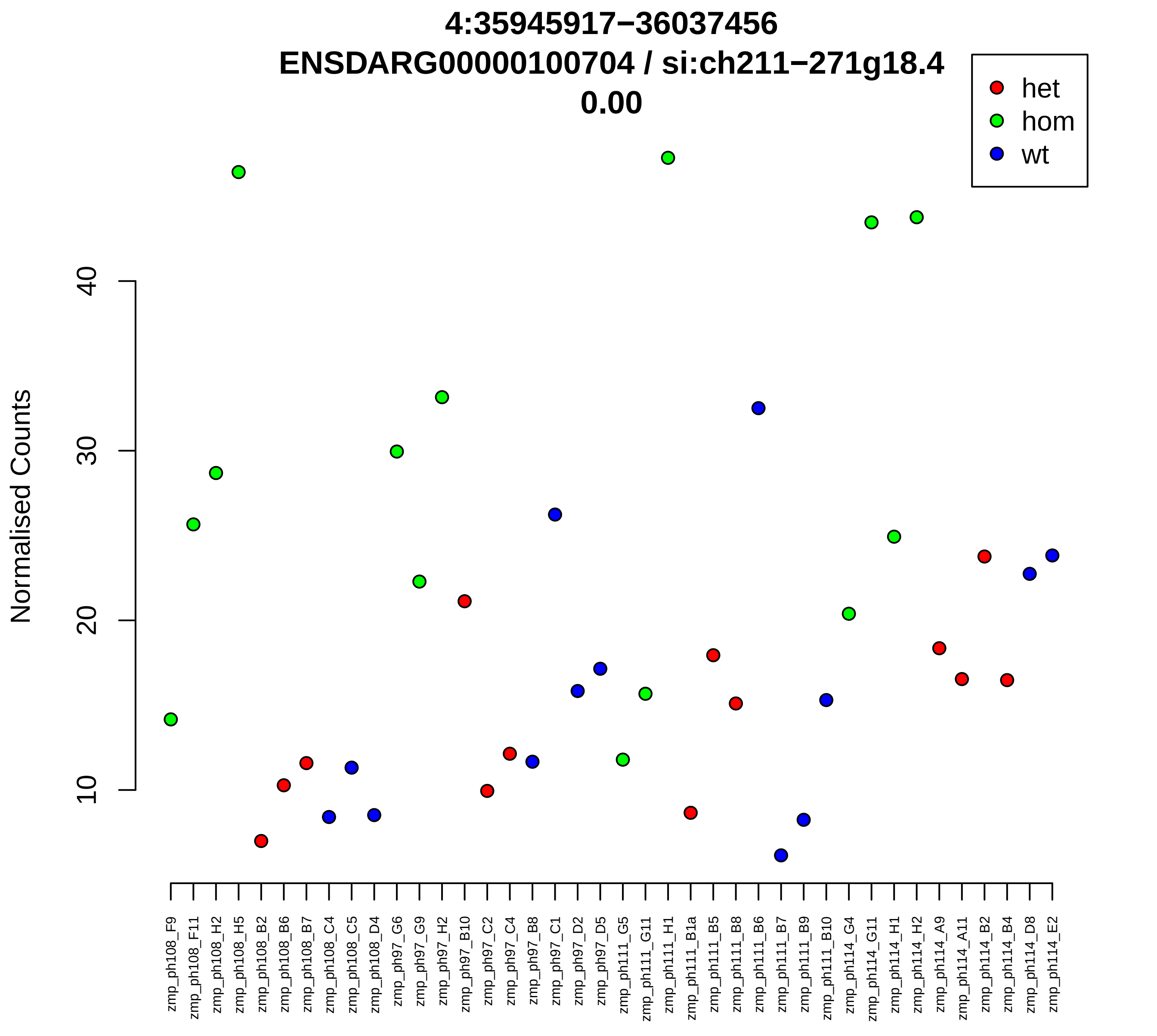
4:35945917−36037456
ENSDARG00000100704 / si:ch211−271g18.4
0.00
het
hom
wt
Normalised Counts
10
20
30
40
zmp_ph108_F9
zmp_ph108_F11
zmp_ph108_H2
zmp_ph108_H5
zmp_ph108_B2
zmp_ph108_B6
zmp_ph108_B7
zmp_ph108_C4
zmp_ph108_C5
zmp_ph108_D4
zmp_ph97_G6
zmp_ph97_G9
zmp_ph97_H2
zmp_ph97_B10
zmp_ph97_C2
zmp_ph97_C4
zmp_ph97_B8
zmp_ph97_C1
zmp_ph97_D2
zmp_ph97_D5
zmp_ph111_G5
zmp_ph111_G11
zmp_ph111_H1
zmp_ph111_B1a
zmp_ph111_B5
zmp_ph111_B8
zmp_ph111_B6
zmp_ph111_B7
zmp_ph111_B9
zmp_ph111_B10
zmp_ph114_G4
zmp_ph114_G11
zmp_ph114_H1
zmp_ph114_H2
zmp_ph114_A9
zmp_ph114_A11
zmp_ph114_B2
zmp_ph114_B4
zmp_ph114_D8
zmp_ph114_E2

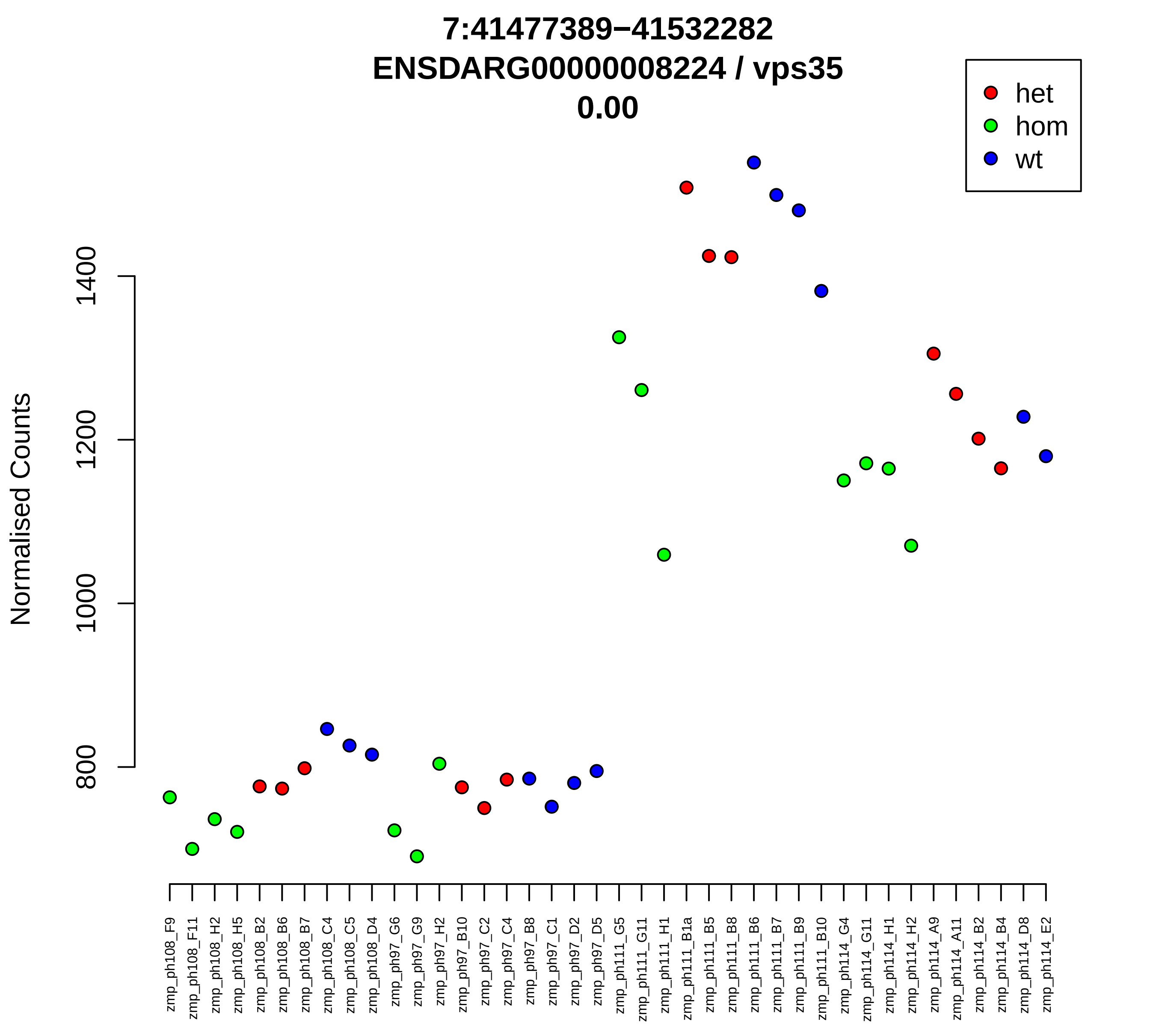
7:41477389−41532282
ENSDARG00000008224 / vps35
0.00
het
hom
wt
Normalised Counts
800
1000
1200
1400
zmp_ph108_F9
zmp_ph108_F11
zmp_ph108_H2
zmp_ph108_H5
zmp_ph108_B2
zmp_ph108_B6
zmp_ph108_B7
zmp_ph108_C4
zmp_ph108_C5
zmp_ph108_D4
zmp_ph97_G6
zmp_ph97_G9
zmp_ph97_H2
zmp_ph97_B10
zmp_ph97_C2
zmp_ph97_C4
zmp_ph97_B8
zmp_ph97_C1
zmp_ph97_D2
zmp_ph97_D5
zmp_ph111_G5
zmp_ph111_G11
zmp_ph111_H1
zmp_ph111_B1a
zmp_ph111_B5
zmp_ph111_B8
zmp_ph111_B6
zmp_ph111_B7
zmp_ph111_B9
zmp_ph111_B10
zmp_ph114_G4
zmp_ph114_G11
zmp_ph114_H1
zmp_ph114_H2
zmp_ph114_A9
zmp_ph114_A11
zmp_ph114_B2
zmp_ph114_B4
zmp_ph114_D8
zmp_ph114_E2

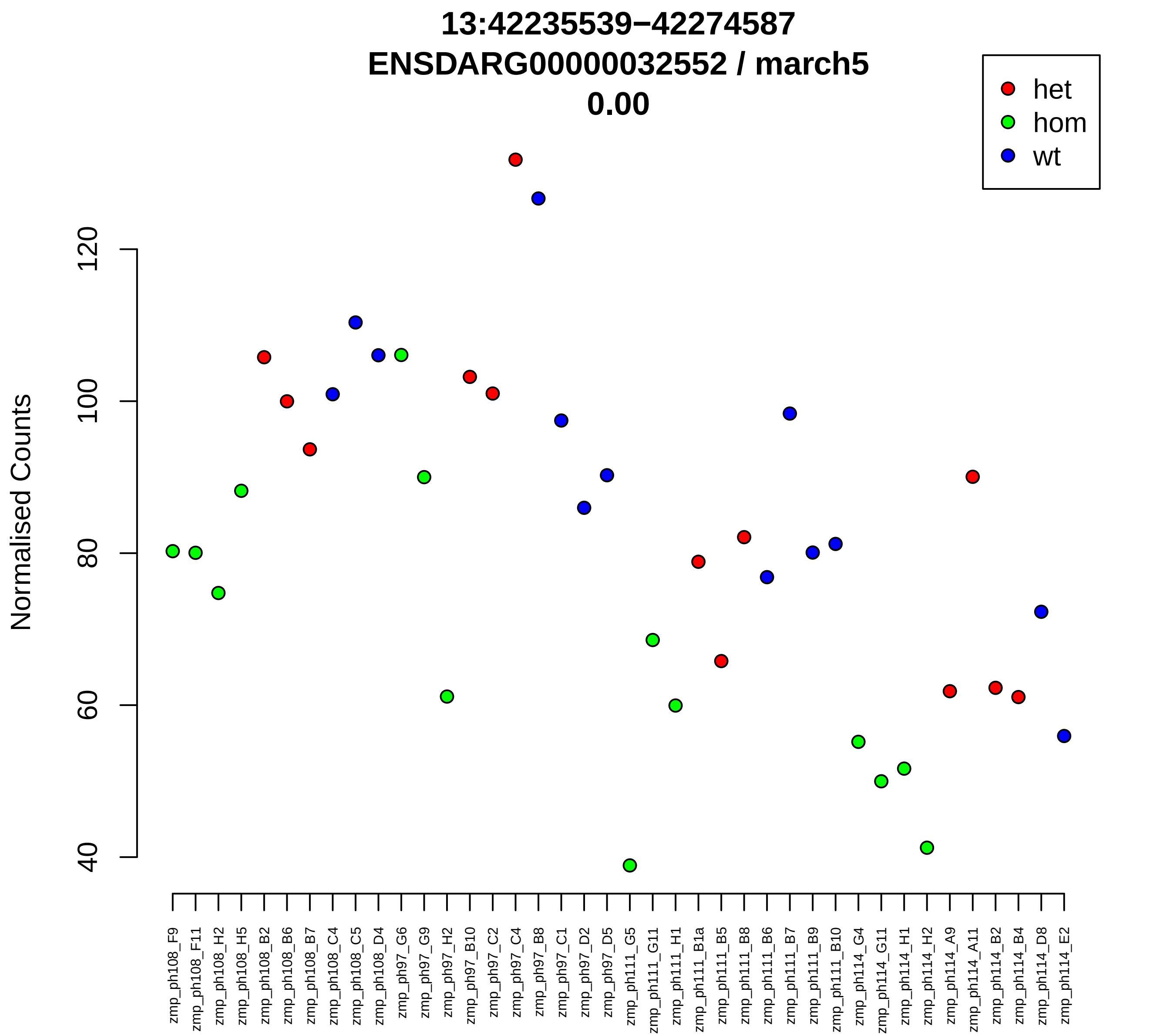

13:42235539−42274587
ENSDARG00000032552 / march5
0.00
het
hom
wt
Normalised Counts
40
60
80
100
120
zmp_ph108_F9
zmp_ph108_F11
zmp_ph108_H2
zmp_ph108_H5
zmp_ph108_B2
zmp_ph108_B6
zmp_ph108_B7
zmp_ph108_C4
zmp_ph108_C5
zmp_ph108_D4
zmp_ph97_G6
zmp_ph97_G9
zmp_ph97_H2
zmp_ph97_B10
zmp_ph97_C2
zmp_ph97_C4
zmp_ph97_B8
zmp_ph97_C1
zmp_ph97_D2
zmp_ph97_D5
zmp_ph111_G5
zmp_ph111_G11
zmp_ph111_H1
zmp_ph111_B1a
zmp_ph111_B5
zmp_ph111_B8
zmp_ph111_B6
zmp_ph111_B7
zmp_ph111_B9
zmp_ph111_B10
zmp_ph114_G4
zmp_ph114_G11
zmp_ph114_H1
zmp_ph114_H2
zmp_ph114_A9
zmp_ph114_A11
zmp_ph114_B2
zmp_ph114_B4
zmp_ph114_D8
zmp_ph114_E2

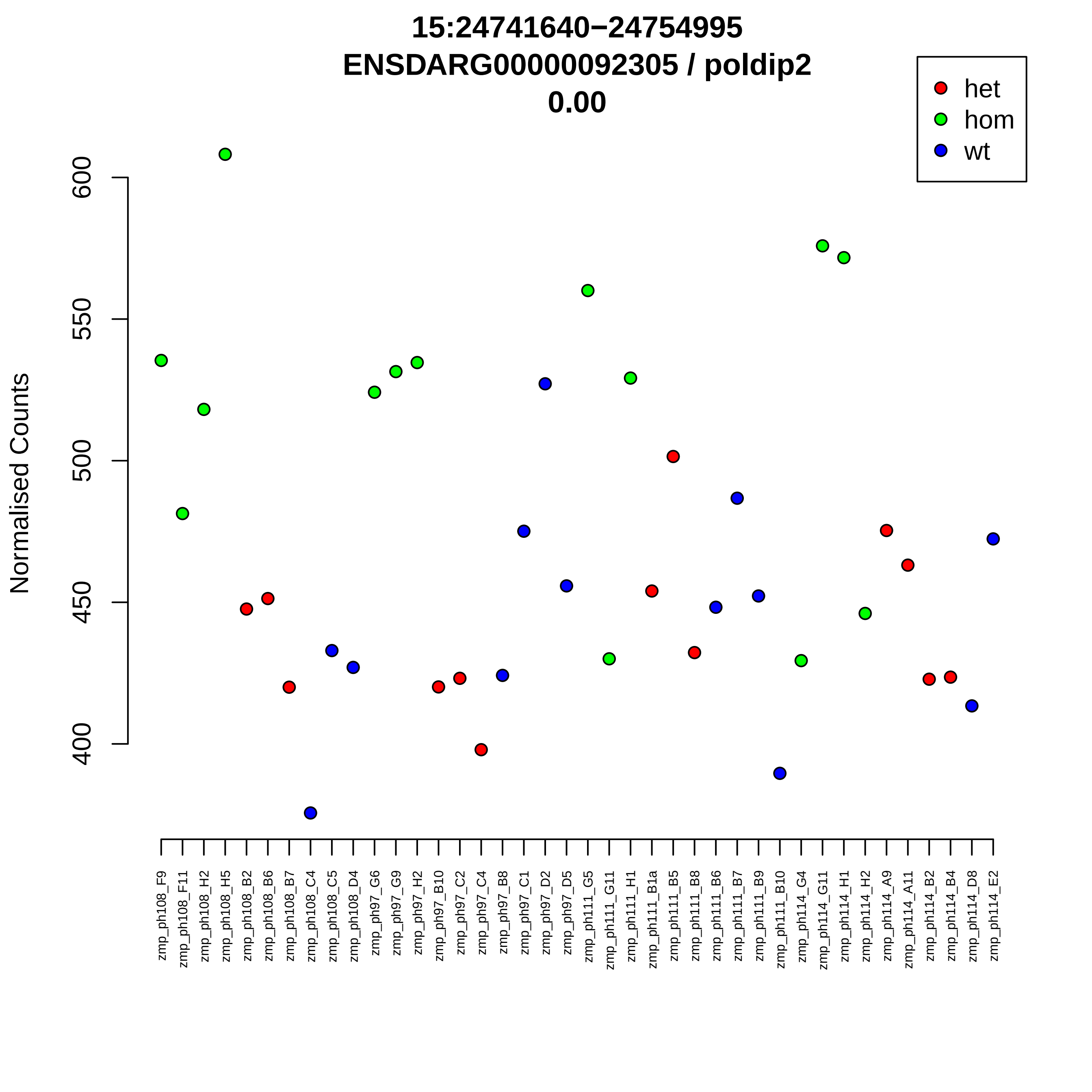
15:24741640−24754995
ENSDARG00000092305 / poldip2
0.00
het
hom
wt
Normalised Counts
400
450
500
550
600
zmp_ph108_F9
zmp_ph108_F11
zmp_ph108_H2
zmp_ph108_H5
zmp_ph108_B2
zmp_ph108_B6
zmp_ph108_B7
zmp_ph108_C4
zmp_ph108_C5
zmp_ph108_D4
zmp_ph97_G6
zmp_ph97_G9
zmp_ph97_H2
zmp_ph97_B10
zmp_ph97_C2
zmp_ph97_C4
zmp_ph97_B8
zmp_ph97_C1
zmp_ph97_D2
zmp_ph97_D5
zmp_ph111_G5
zmp_ph111_G11
zmp_ph111_H1
zmp_ph111_B1a
zmp_ph111_B5
zmp_ph111_B8
zmp_ph111_B6
zmp_ph111_B7
zmp_ph111_B9
zmp_ph111_B10
zmp_ph114_G4
zmp_ph114_G11
zmp_ph114_H1
zmp_ph114_H2
zmp_ph114_A9
zmp_ph114_A11
zmp_ph114_B2
zmp_ph114_B4
zmp_ph114_D8
zmp_ph114_E2

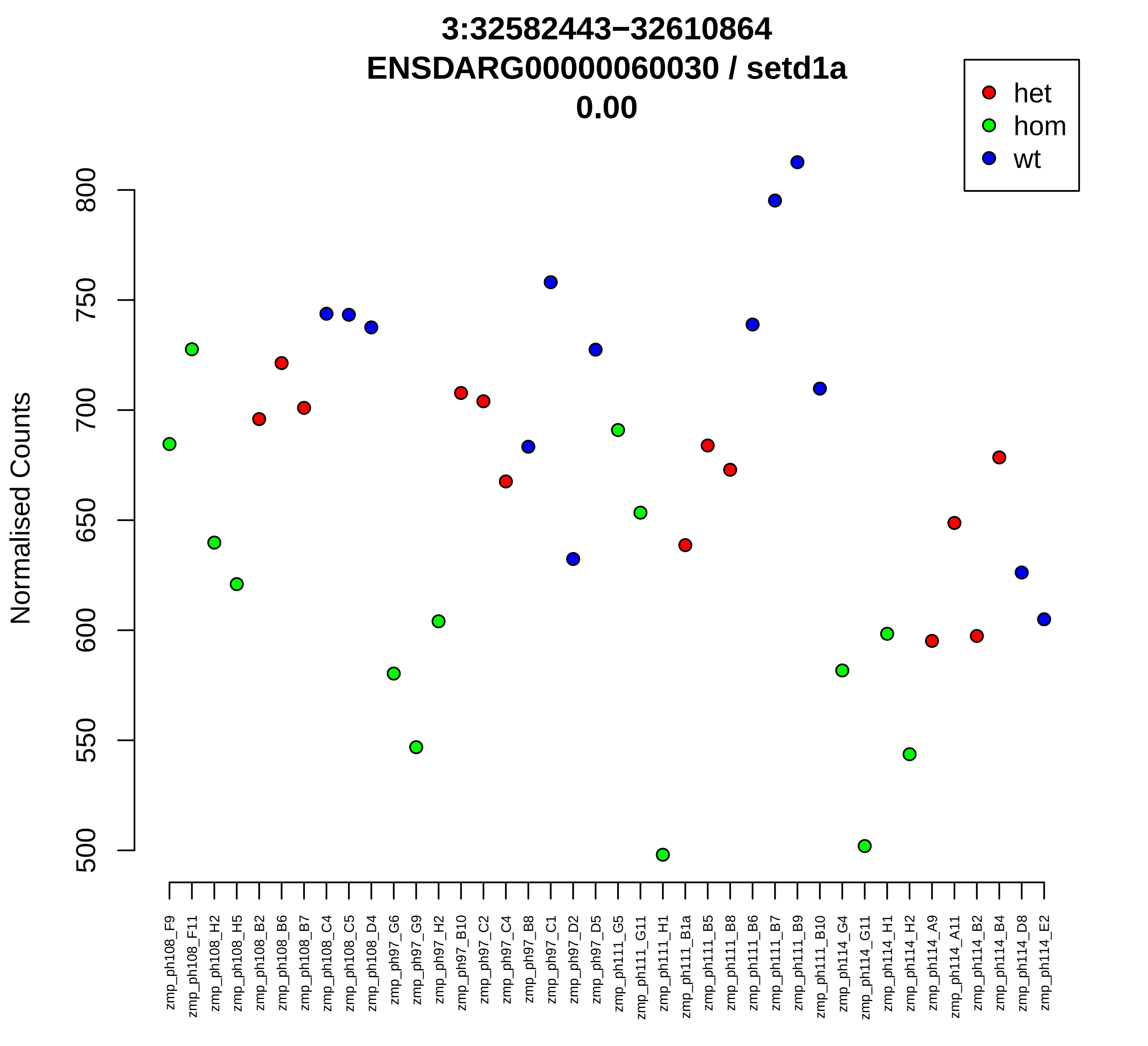
3:32582443−32610864
ENSDARG00000060030 / setd1a
0.00
het
hom
wt
Normalised Counts
500
550
600
650
700
750
800
zmp_ph108_F9
zmp_ph108_F11
zmp_ph108_H2
zmp_ph108_H5
zmp_ph108_B2
zmp_ph108_B6
zmp_ph108_B7
zmp_ph108_C4
zmp_ph108_C5
zmp_ph108_D4
zmp_ph97_G6
zmp_ph97_G9
zmp_ph97_H2
zmp_ph97_B10
zmp_ph97_C2
zmp_ph97_C4
zmp_ph97_B8
zmp_ph97_C1
zmp_ph97_D2
zmp_ph97_D5
zmp_ph111_G5
zmp_ph111_G11
zmp_ph111_H1
zmp_ph111_B1a
zmp_ph111_B5
zmp_ph111_B8
zmp_ph111_B6
zmp_ph111_B7
zmp_ph111_B9
zmp_ph111_B10
zmp_ph114_G4
zmp_ph114_G11
zmp_ph114_H1
zmp_ph114_H2
zmp_ph114_A9
zmp_ph114_A11
zmp_ph114_B2
zmp_ph114_B4
zmp_ph114_D8
zmp_ph114_E2

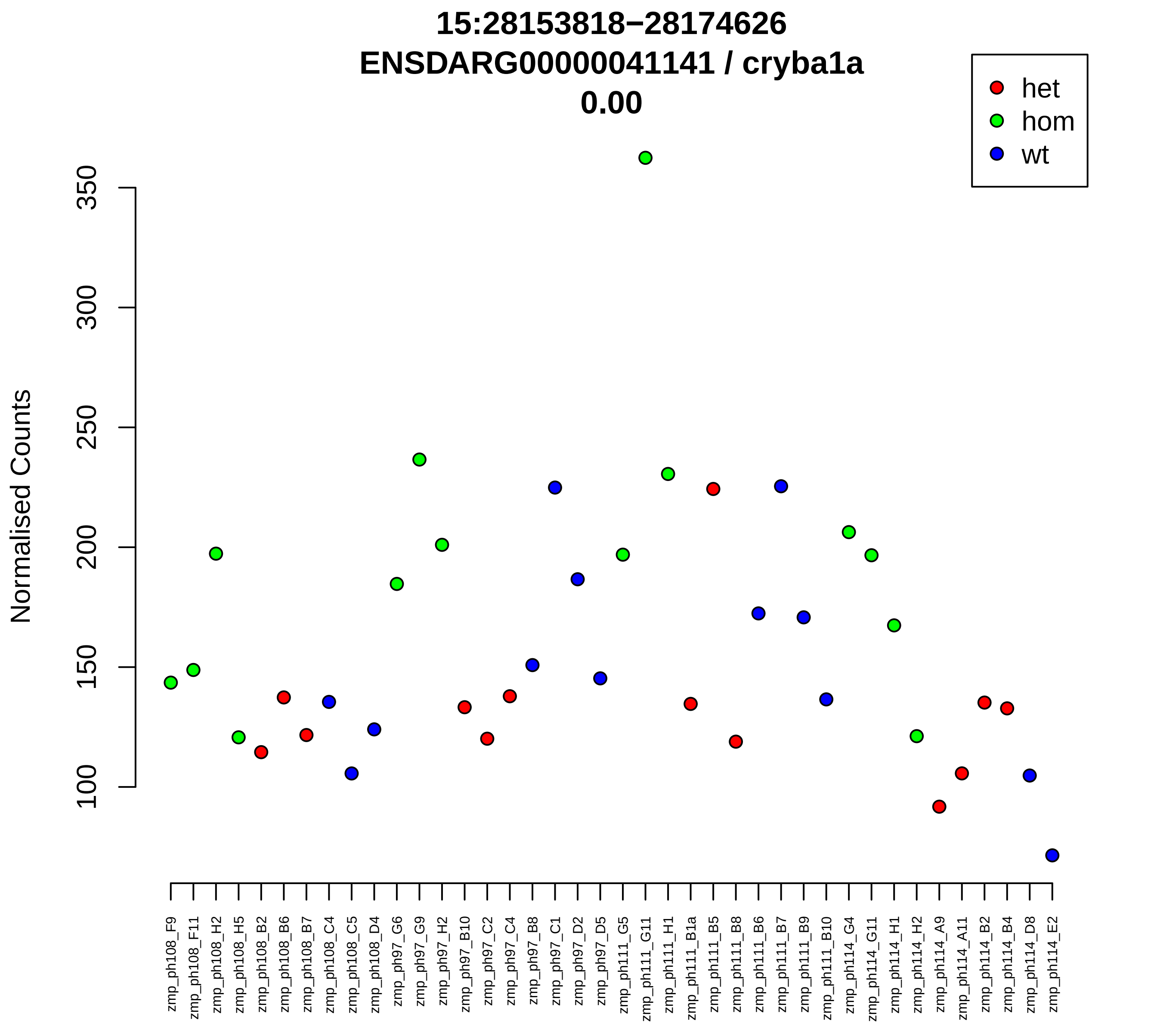

15:28153818−28174626
ENSDARG00000041141 / cryba1a
0.00
het
hom
wt
Normalised Counts
100
150
200
250
300
350
zmp_ph108_F9
zmp_ph108_F11
zmp_ph108_H2
zmp_ph108_H5
zmp_ph108_B2
zmp_ph108_B6
zmp_ph108_B7
zmp_ph108_C4
zmp_ph108_C5
zmp_ph108_D4
zmp_ph97_G6
zmp_ph97_G9
zmp_ph97_H2
zmp_ph97_B10
zmp_ph97_C2
zmp_ph97_C4
zmp_ph97_B8
zmp_ph97_C1
zmp_ph97_D2
zmp_ph97_D5
zmp_ph111_G5
zmp_ph111_G11
zmp_ph111_H1
zmp_ph111_B1a
zmp_ph111_B5
zmp_ph111_B8
zmp_ph111_B6
zmp_ph111_B7
zmp_ph111_B9
zmp_ph111_B10
zmp_ph114_G4
zmp_ph114_G11
zmp_ph114_H1
zmp_ph114_H2
zmp_ph114_A9
zmp_ph114_A11
zmp_ph114_B2
zmp_ph114_B4
zmp_ph114_D8
zmp_ph114_E2

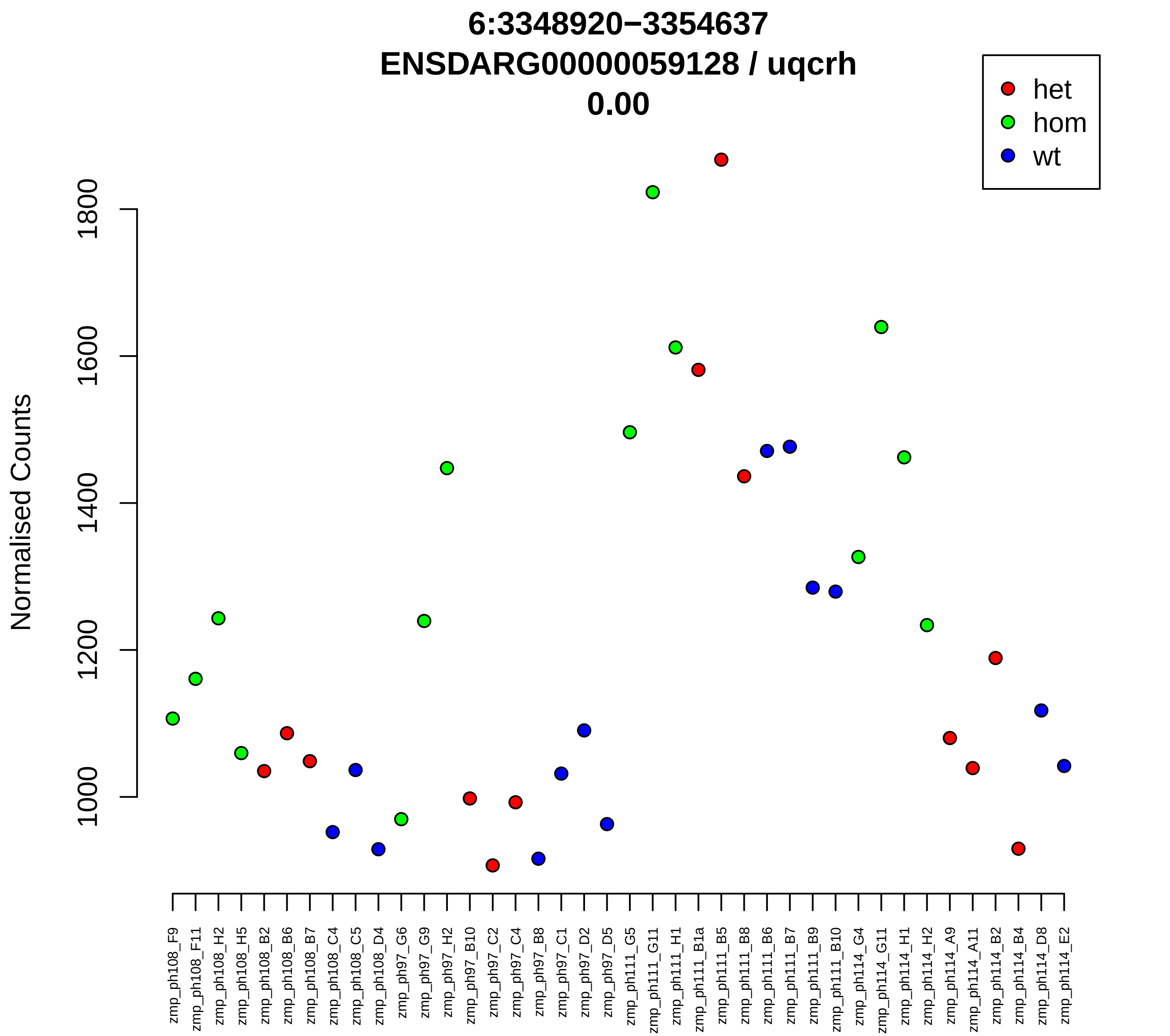

6:3348920−3354637
ENSDARG00000059128 / uqcrh
0.00
het
hom
wt
Normalised Counts
1000
1200
1400
1600
1800
zmp_ph108_F9
zmp_ph108_F11
zmp_ph108_H2
zmp_ph108_H5
zmp_ph108_B2
zmp_ph108_B6
zmp_ph108_B7
zmp_ph108_C4
zmp_ph108_C5
zmp_ph108_D4
zmp_ph97_G6
zmp_ph97_G9
zmp_ph97_H2
zmp_ph97_B10
zmp_ph97_C2
zmp_ph97_C4
zmp_ph97_B8
zmp_ph97_C1
zmp_ph97_D2
zmp_ph97_D5
zmp_ph111_G5
zmp_ph111_G11
zmp_ph111_H1
zmp_ph111_B1a
zmp_ph111_B5
zmp_ph111_B8
zmp_ph111_B6
zmp_ph111_B7
zmp_ph111_B9
zmp_ph111_B10
zmp_ph114_G4
zmp_ph114_G11
zmp_ph114_H1
zmp_ph114_H2
zmp_ph114_A9
zmp_ph114_A11
zmp_ph114_B2
zmp_ph114_B4
zmp_ph114_D8
zmp_ph114_E2

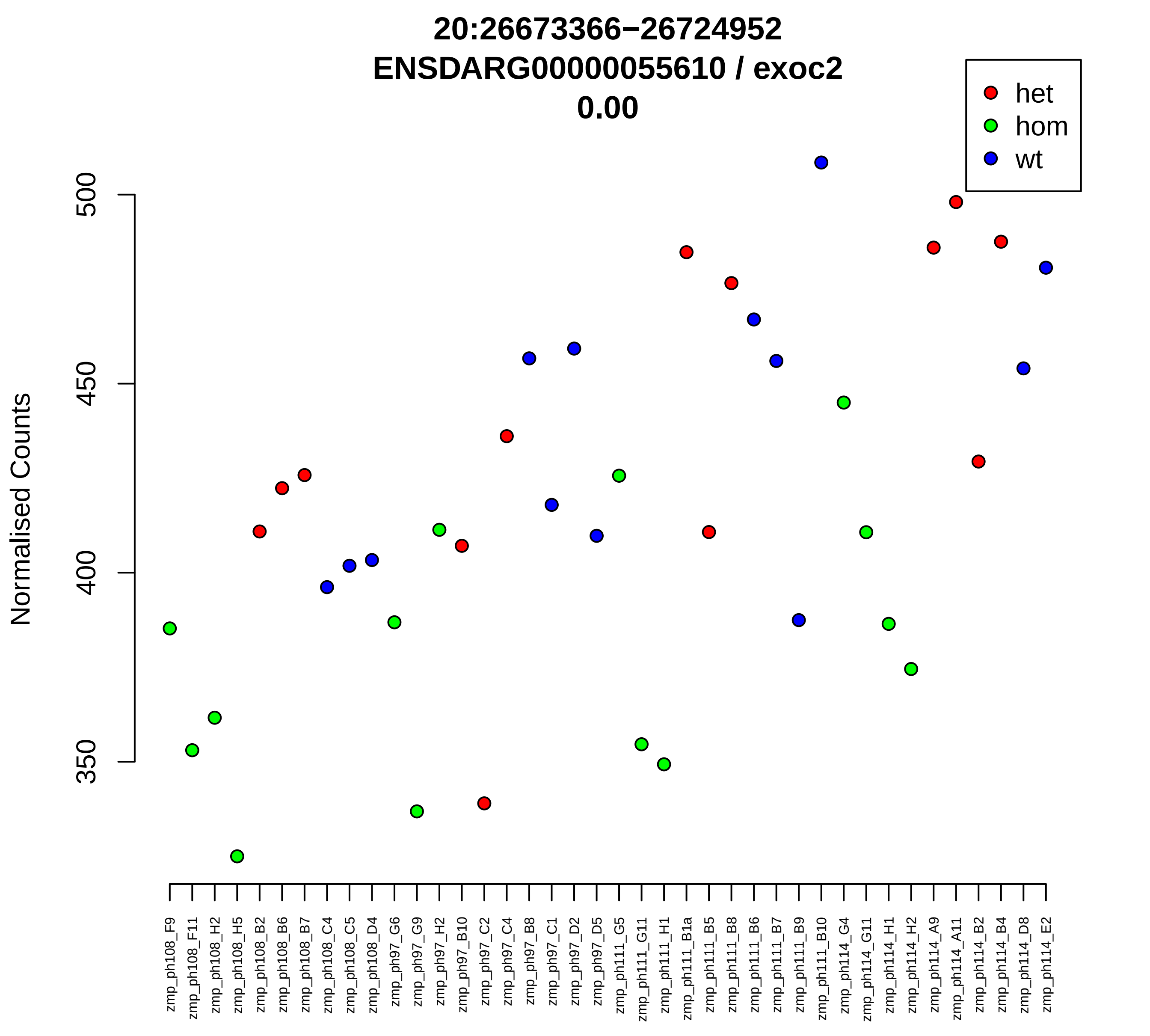

20:26673366−26724952
ENSDARG00000055610 / exoc2
0.00
het
hom
wt
Normalised Counts
500
450
400
350
zmp_ph108_F9
zmp_ph108_F11
zmp_ph108_H2
zmp_ph108_H5
zmp_ph108_B2
zmp_ph108_B6
zmp_ph108_B7
zmp_ph108_C4
zmp_ph108_C5
zmp_ph108_D4
zmp_ph97_G6
zmp_ph97_G9
zmp_ph97_H2
zmp_ph97_B10
zmp_ph97_C2
zmp_ph97_C4
zmp_ph97_B8
zmp_ph97_C1
zmp_ph97_D2
zmp_ph97_D5
zmp_ph111_G5
zmp_ph111_G11
zmp_ph111_H1
zmp_ph111_B1a
zmp_ph111_B5
zmp_ph111_B8
zmp_ph111_B6
zmp_ph111_B7
zmp_ph111_B9
zmp_ph111_B10
zmp_ph114_G4
zmp_ph114_G11
zmp_ph114_H1
zmp_ph114_H2
zmp_ph114_A9
zmp_ph114_A11
zmp_ph114_B2
zmp_ph114_B4
zmp_ph114_D8
zmp_ph114_E2

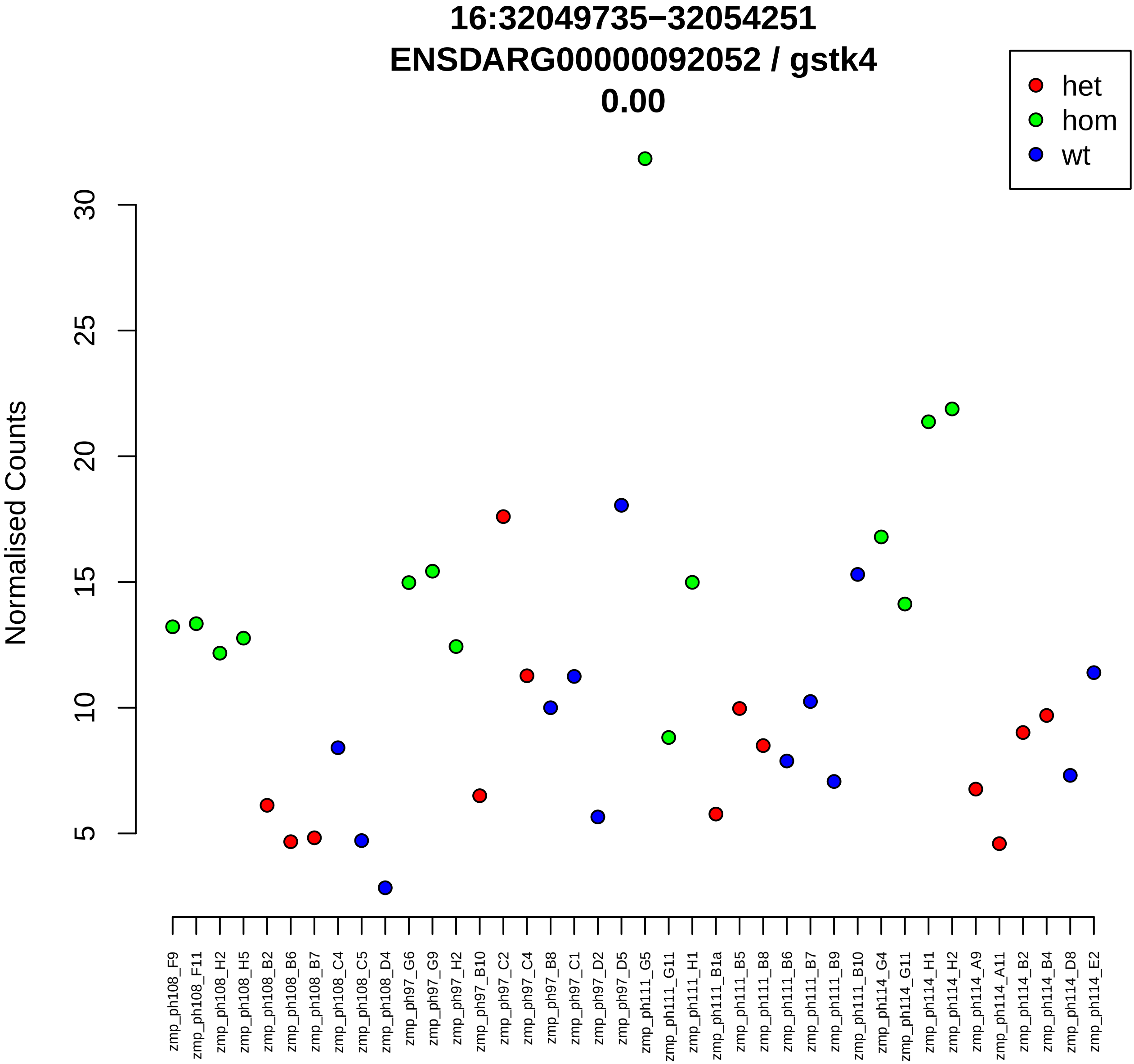
16:32049735−32054251
ENSDARG00000092052 / gstk4
0.00
het
hom
wt
Normalised Counts
5
10
15
20
25
30
zmp_ph108_F9
zmp_ph108_F11
zmp_ph108_H2
zmp_ph108_H5
zmp_ph108_B2
zmp_ph108_B6
zmp_ph108_B7
zmp_ph108_C4
zmp_ph108_C5
zmp_ph108_D4
zmp_ph97_G6
zmp_ph97_G9
zmp_ph97_H2
zmp_ph97_B10
zmp_ph97_C2
zmp_ph97_C4
zmp_ph97_B8
zmp_ph97_C1
zmp_ph97_D2
zmp_ph97_D5
zmp_ph111_G5
zmp_ph111_G11
zmp_ph111_H1
zmp_ph111_B1a
zmp_ph111_B5
zmp_ph111_B8
zmp_ph111_B6
zmp_ph111_B7
zmp_ph111_B9
zmp_ph111_B10
zmp_ph114_G4
zmp_ph114_G11
zmp_ph114_H1
zmp_ph114_H2
zmp_ph114_A9
zmp_ph114_A11
zmp_ph114_B2
zmp_ph114_B4
zmp_ph114_D8
zmp_ph114_E2

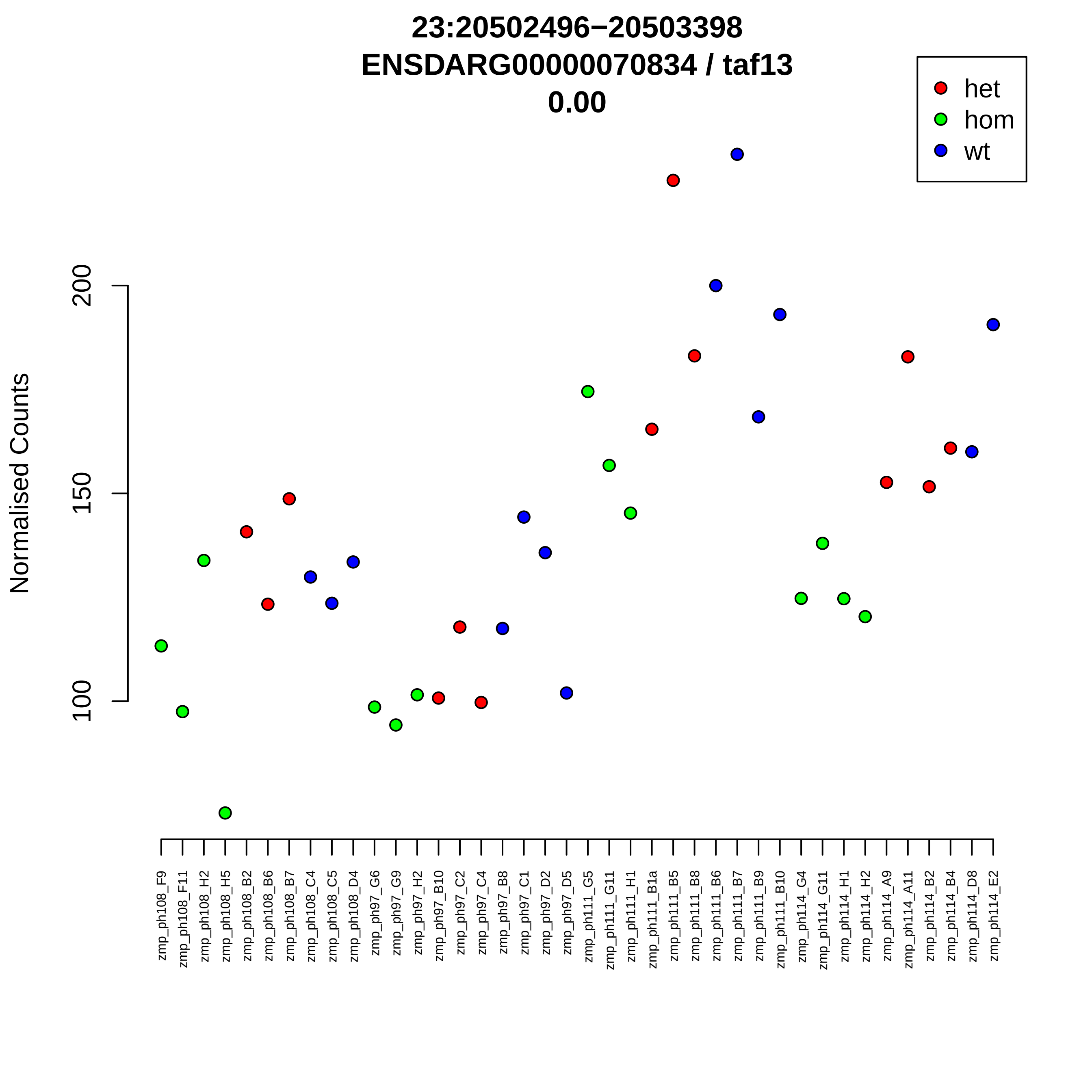

23:20502496−20503398
ENSDARG00000070834 / taf13
0.00
het
hom
wt
Normalised Counts
100
150
200
zmp_ph108_F9
zmp_ph108_F11
zmp_ph108_H2
zmp_ph108_H5
zmp_ph108_B2
zmp_ph108_B6
zmp_ph108_B7
zmp_ph108_C4
zmp_ph108_C5
zmp_ph108_D4
zmp_ph97_G6
zmp_ph97_G9
zmp_ph97_H2
zmp_ph97_B10
zmp_ph97_C2
zmp_ph97_C4
zmp_ph97_B8
zmp_ph97_C1
zmp_ph97_D2
zmp_ph97_D5
zmp_ph111_G5
zmp_ph111_G11
zmp_ph111_H1
zmp_ph111_B1a
zmp_ph111_B5
zmp_ph111_B8
zmp_ph111_B6
zmp_ph111_B7
zmp_ph111_B9
zmp_ph111_B10
zmp_ph114_G4
zmp_ph114_G11
zmp_ph114_H1
zmp_ph114_H2
zmp_ph114_A9
zmp_ph114_A11
zmp_ph114_B2
zmp_ph114_B4
zmp_ph114_D8
zmp_ph114_E2

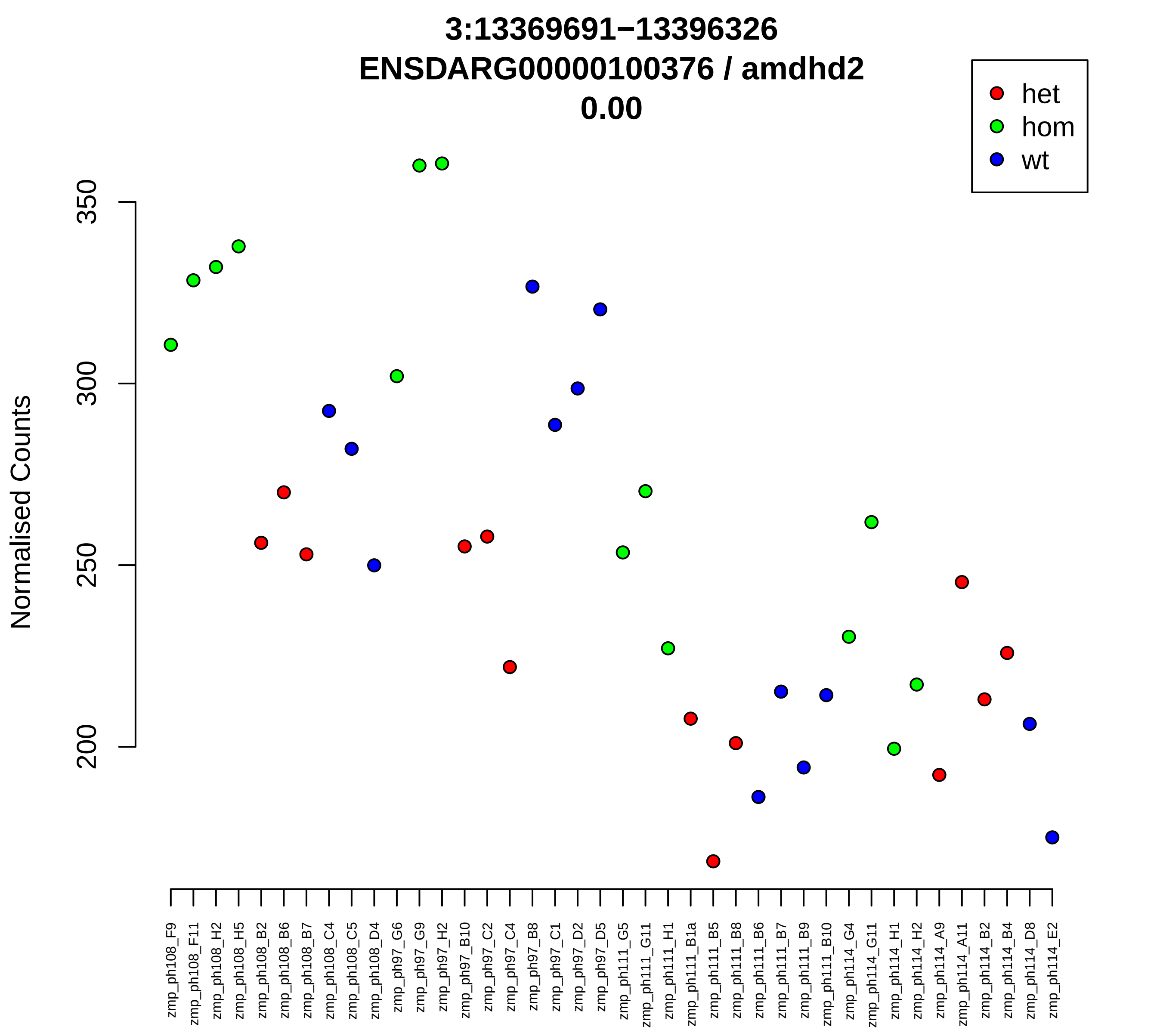

3:13369691−13396326
ENSDARG00000100376 / amdhd2
0.00
het
hom
wt
Normalised Counts
350
300
250
200
zmp_ph108_F9
zmp_ph108_F11
zmp_ph108_H2
zmp_ph108_H5
zmp_ph108_B2
zmp_ph108_B6
zmp_ph108_B7
zmp_ph108_C4
zmp_ph108_C5
zmp_ph108_D4
zmp_ph97_G6
zmp_ph97_G9
zmp_ph97_H2
zmp_ph97_B10
zmp_ph97_C2
zmp_ph97_C4
zmp_ph97_B8
zmp_ph97_C1
zmp_ph97_D2
zmp_ph97_D5
zmp_ph111_G5
zmp_ph111_G11
zmp_ph111_H1
zmp_ph111_B1a
zmp_ph111_B5
zmp_ph111_B8
zmp_ph111_B6
zmp_ph111_B7
zmp_ph111_B9
zmp_ph111_B10
zmp_ph114_G4
zmp_ph114_G11
zmp_ph114_H1
zmp_ph114_H2
zmp_ph114_A9
zmp_ph114_A11
zmp_ph114_B2
zmp_ph114_B4
zmp_ph114_D8
zmp_ph114_E2

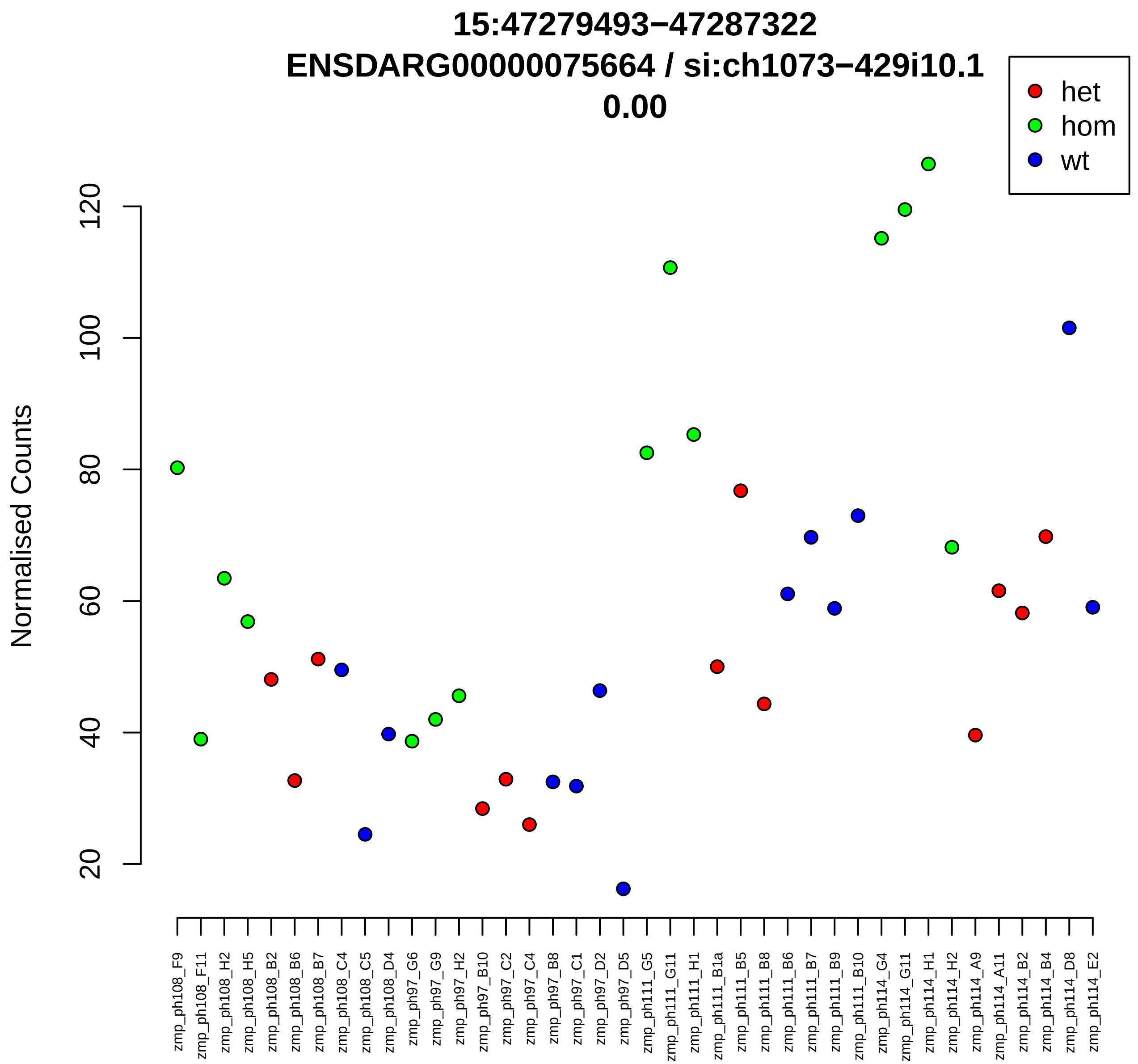

15:47279493−47287322
ENSDARG00000075664 / si:ch1073−429i10.1
0.00
het
hom
wt
Normalised Counts
20
40
60
80
100
120
zmp_ph108_F9
zmp_ph108_F11
zmp_ph108_H2
zmp_ph108_H5
zmp_ph108_B2
zmp_ph108_B6
zmp_ph108_B7
zmp_ph108_C4
zmp_ph108_C5
zmp_ph108_D4
zmp_ph97_G6
zmp_ph97_G9
zmp_ph97_H2
zmp_ph97_B10
zmp_ph97_C2
zmp_ph97_C4
zmp_ph97_B8
zmp_ph97_C1
zmp_ph97_D2
zmp_ph97_D5
zmp_ph111_G5
zmp_ph111_G11
zmp_ph111_H1
zmp_ph111_B1a
zmp_ph111_B5
zmp_ph111_B8
zmp_ph111_B6
zmp_ph111_B7
zmp_ph111_B9
zmp_ph111_B10
zmp_ph114_G4
zmp_ph114_G11
zmp_ph114_H1
zmp_ph114_H2
zmp_ph114_A9
zmp_ph114_A11
zmp_ph114_B2
zmp_ph114_B4
zmp_ph114_D8
zmp_ph114_E2

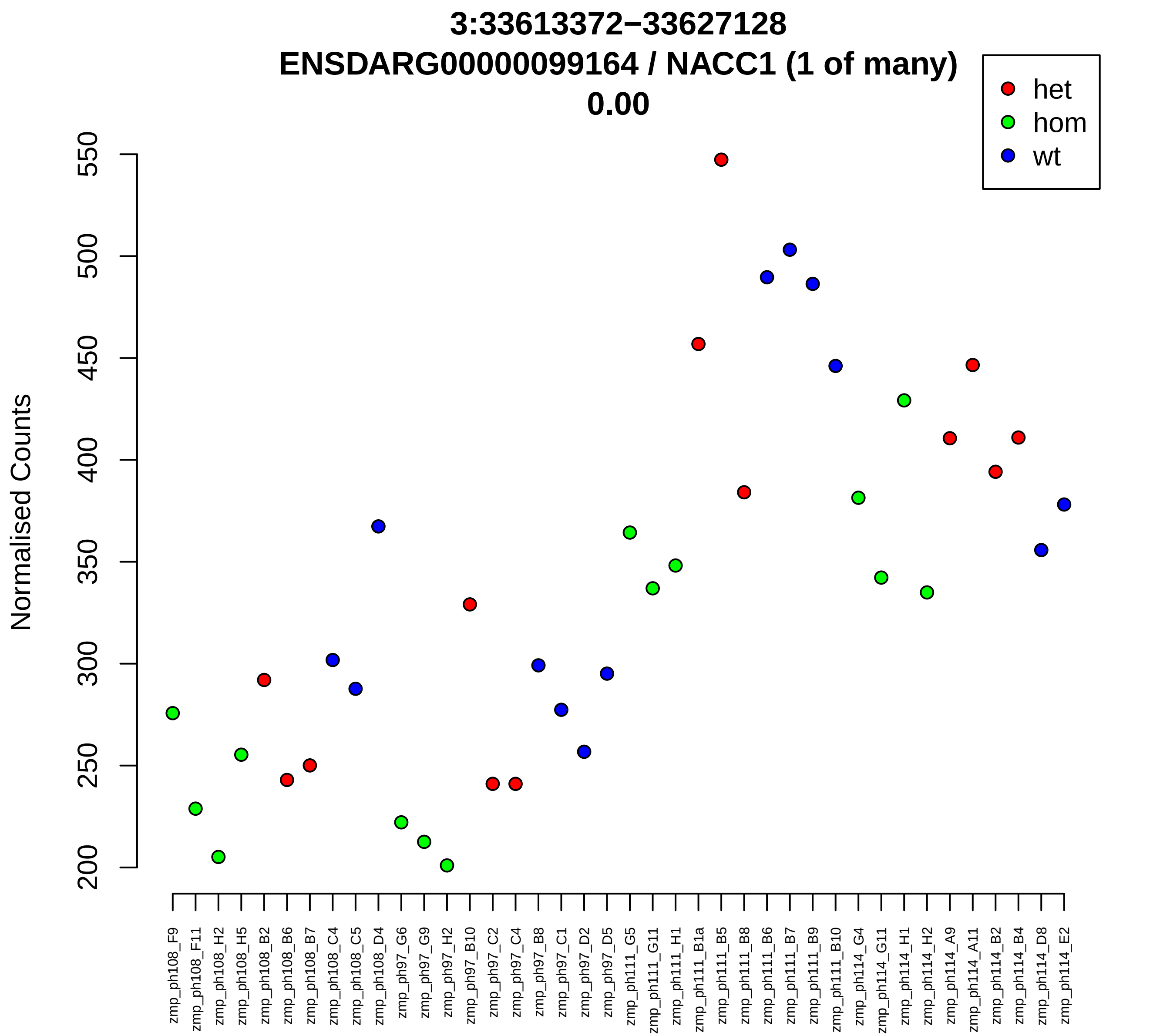
3:33613372−33627128
ENSDARG00000099164 / NACC1 (1 of many)
0.00
het
hom
wt
Normalised Counts
200
250
300
350
400
450
500
550
zmp_ph108_F9
zmp_ph108_F11
zmp_ph108_H2
zmp_ph108_H5
zmp_ph108_B2
zmp_ph108_B6
zmp_ph108_B7
zmp_ph108_C4
zmp_ph108_C5
zmp_ph108_D4
zmp_ph97_G6
zmp_ph97_G9
zmp_ph97_H2
zmp_ph97_B10
zmp_ph97_C2
zmp_ph97_C4
zmp_ph97_B8
zmp_ph97_C1
zmp_ph97_D2
zmp_ph97_D5
zmp_ph111_G5
zmp_ph111_G11
zmp_ph111_H1
zmp_ph111_B1a
zmp_ph111_B5
zmp_ph111_B8
zmp_ph111_B6
zmp_ph111_B7
zmp_ph111_B9
zmp_ph111_B10
zmp_ph114_G4
zmp_ph114_G11
zmp_ph114_H1
zmp_ph114_H2
zmp_ph114_A9
zmp_ph114_A11
zmp_ph114_B2
zmp_ph114_B4
zmp_ph114_D8
zmp_ph114_E2

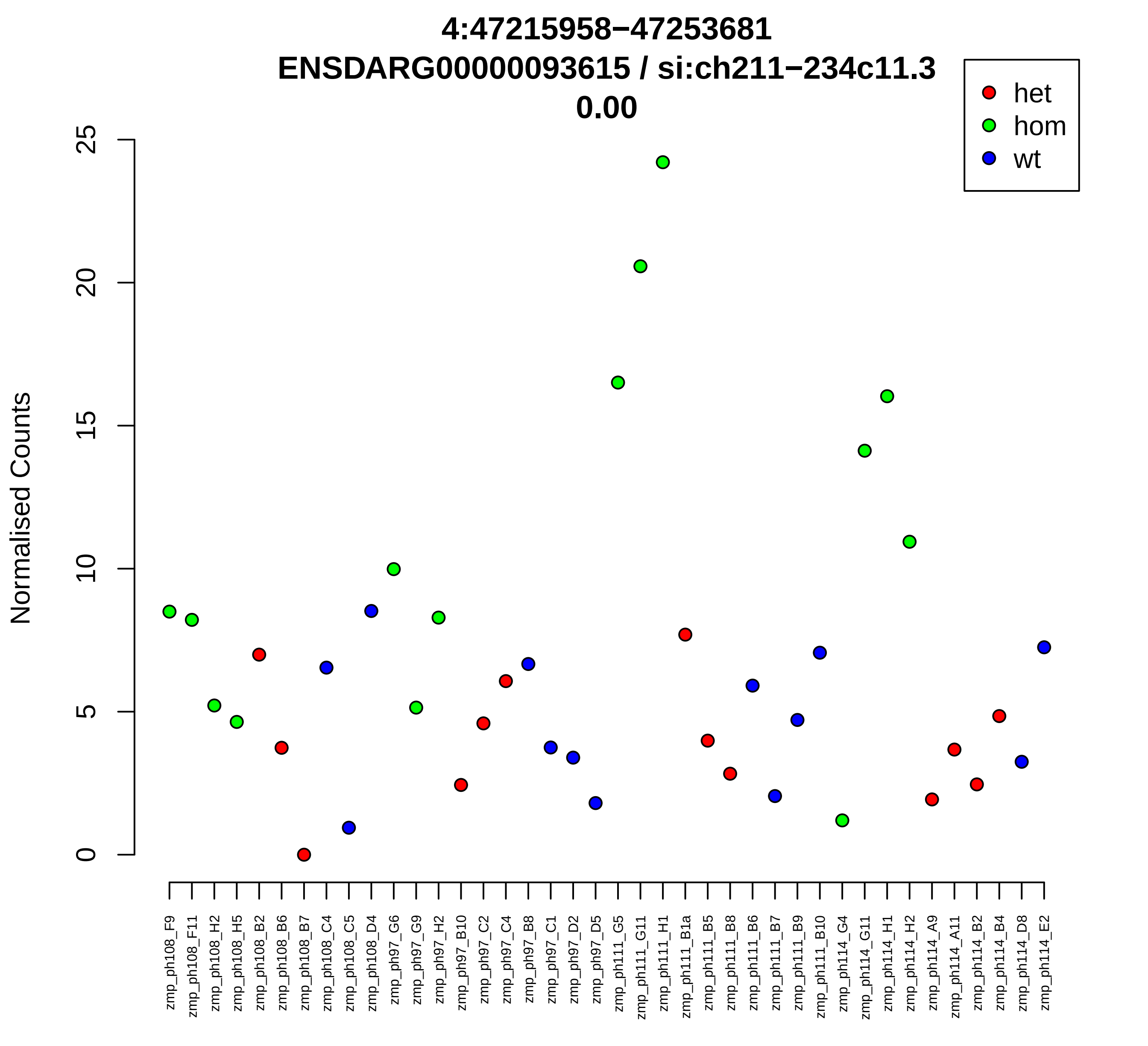
4:47215958−47253681
ENSDARG00000093615 / si:ch211−234c11.3
0.00
het
hom
wt
Normalised Counts
0
5
10
15
20
25
zmp_ph108_F9
zmp_ph108_F11
zmp_ph108_H2
zmp_ph108_H5
zmp_ph108_B2
zmp_ph108_B6
zmp_ph108_B7
zmp_ph108_C4
zmp_ph108_C5
zmp_ph108_D4
zmp_ph97_G6
zmp_ph97_G9
zmp_ph97_H2
zmp_ph97_B10
zmp_ph97_C2
zmp_ph97_C4
zmp_ph97_B8
zmp_ph97_C1
zmp_ph97_D2
zmp_ph97_D5
zmp_ph111_G5
zmp_ph111_G11
zmp_ph111_H1
zmp_ph111_B1a
zmp_ph111_B5
zmp_ph111_B8
zmp_ph111_B6
zmp_ph111_B7
zmp_ph111_B9
zmp_ph111_B10
zmp_ph114_G4
zmp_ph114_G11
zmp_ph114_H1
zmp_ph114_H2
zmp_ph114_A9
zmp_ph114_A11
zmp_ph114_B2
zmp_ph114_B4
zmp_ph114_D8
zmp_ph114_E2

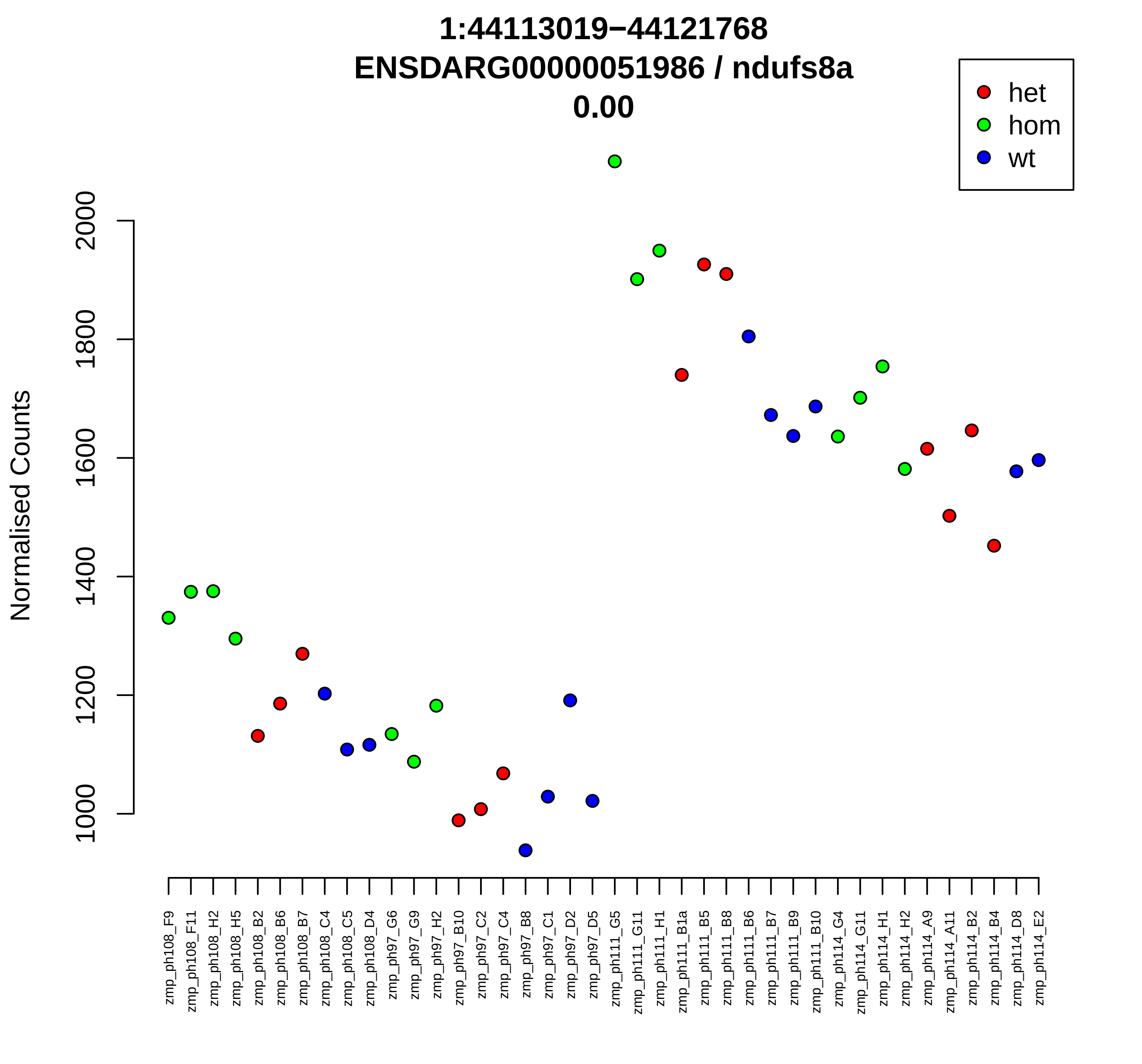

1:44113019−44121768
ENSDARG00000051986 / ndufs8a
0.00
het
hom
wt
Normalised Counts
1000
1200
1400
1600
1800
2000
zmp_ph108_F9
zmp_ph108_F11
zmp_ph108_H2
zmp_ph108_H5
zmp_ph108_B2
zmp_ph108_B6
zmp_ph108_B7
zmp_ph108_C4
zmp_ph108_C5
zmp_ph108_D4
zmp_ph97_G6
zmp_ph97_G9
zmp_ph97_H2
zmp_ph97_B10
zmp_ph97_C2
zmp_ph97_C4
zmp_ph97_B8
zmp_ph97_C1
zmp_ph97_D2
zmp_ph97_D5
zmp_ph111_G5
zmp_ph111_G11
zmp_ph111_H1
zmp_ph111_B1a
zmp_ph111_B5
zmp_ph111_B8
zmp_ph111_B6
zmp_ph111_B7
zmp_ph111_B9
zmp_ph111_B10
zmp_ph114_G4
zmp_ph114_G11
zmp_ph114_H1
zmp_ph114_H2
zmp_ph114_A9
zmp_ph114_A11
zmp_ph114_B2
zmp_ph114_B4
zmp_ph114_D8
zmp_ph114_E2

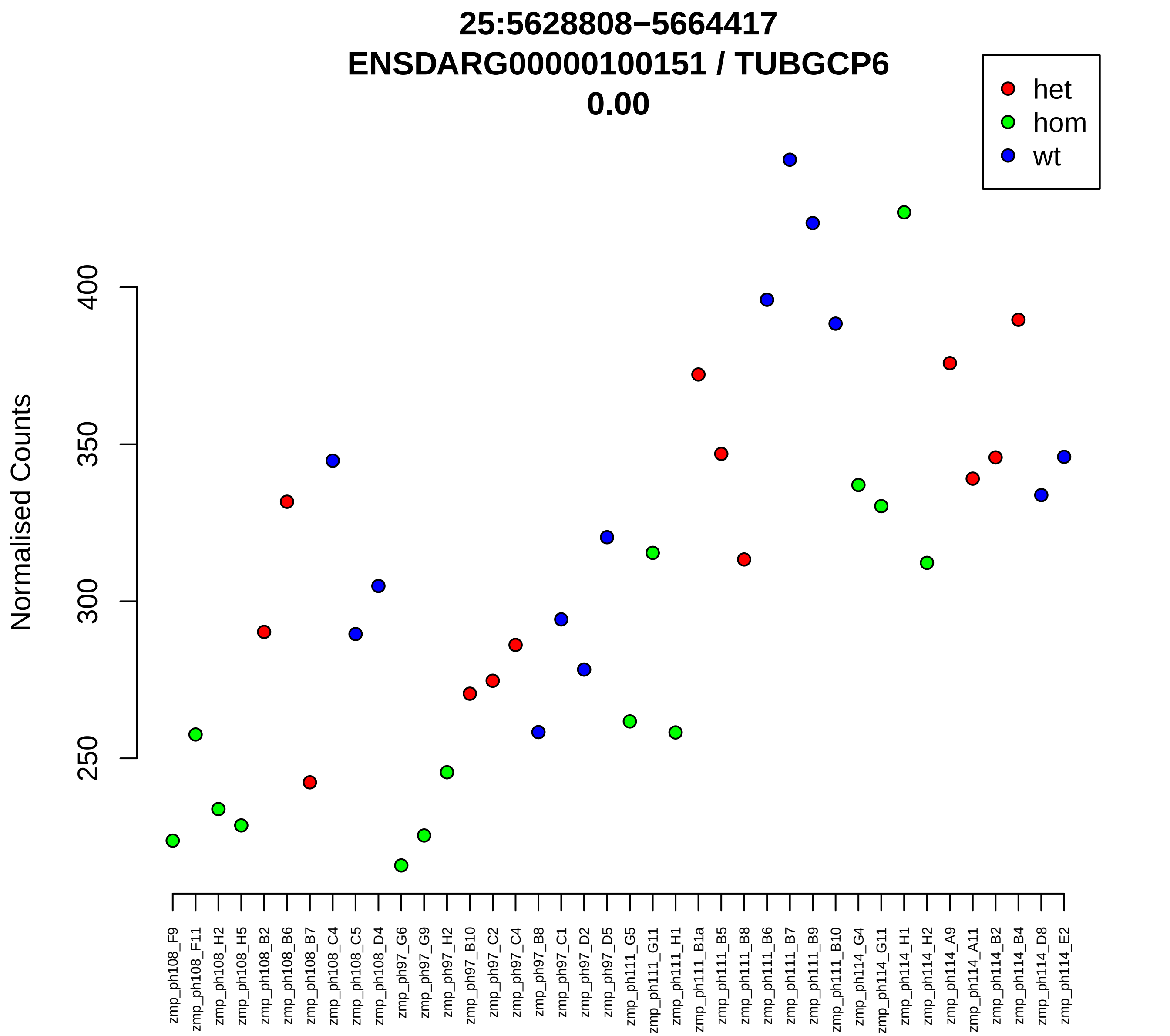
25:5628808−5664417
ENSDARG00000100151 / TUBGCP6
0.00
het
hom
wt
Normalised Counts
250
300
350
400
zmp_ph108_F9
zmp_ph108_F11
zmp_ph108_H2
zmp_ph108_H5
zmp_ph108_B2
zmp_ph108_B6
zmp_ph108_B7
zmp_ph108_C4
zmp_ph108_C5
zmp_ph108_D4
zmp_ph97_G6
zmp_ph97_G9
zmp_ph97_H2
zmp_ph97_B10
zmp_ph97_C2
zmp_ph97_C4
zmp_ph97_B8
zmp_ph97_C1
zmp_ph97_D2
zmp_ph97_D5
zmp_ph111_G5
zmp_ph111_G11
zmp_ph111_H1
zmp_ph111_B1a
zmp_ph111_B5
zmp_ph111_B8
zmp_ph111_B6
zmp_ph111_B7
zmp_ph111_B9
zmp_ph111_B10
zmp_ph114_G4
zmp_ph114_G11
zmp_ph114_H1
zmp_ph114_H2
zmp_ph114_A9
zmp_ph114_A11
zmp_ph114_B2
zmp_ph114_B4
zmp_ph114_D8
zmp_ph114_E2

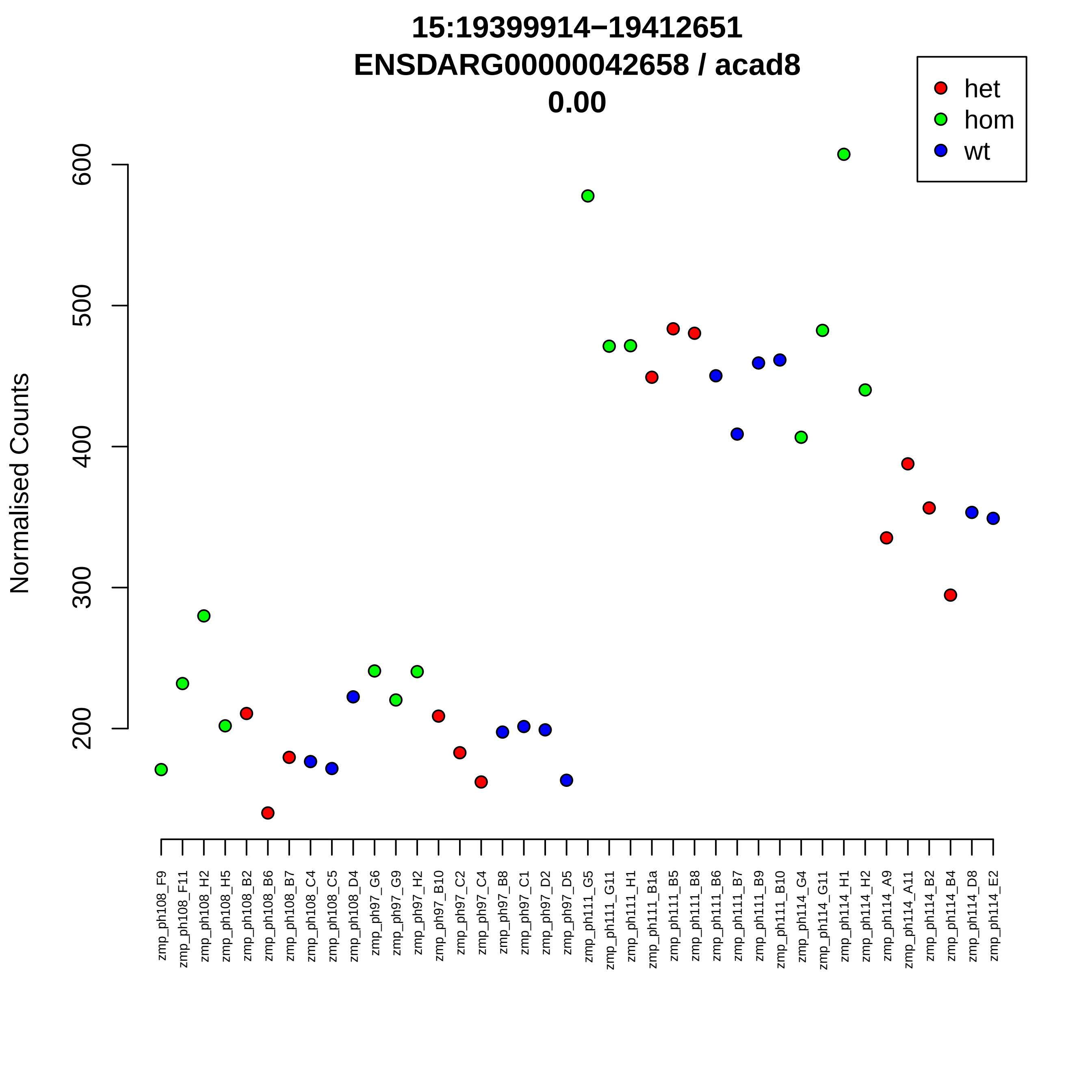

15:19399914−19412651
ENSDARG00000042658 / acad8
0.00
Normalised Counts
200
300
400
500
600
het
hom
wt
zmp_ph108_F9
zmp_ph108_F11
zmp_ph108_H2
zmp_ph108_H5
zmp_ph108_B2
zmp_ph108_B6
zmp_ph108_B7
zmp_ph108_C4
zmp_ph108_C5
zmp_ph108_D4
zmp_ph97_G6
zmp_ph97_G9
zmp_ph97_H2
zmp_ph97_B10
zmp_ph97_C2
zmp_ph97_C4
zmp_ph97_B8
zmp_ph97_C1
zmp_ph97_D2
zmp_ph97_D5
zmp_ph111_G5
zmp_ph111_G11
zmp_ph111_H1
zmp_ph111_B1a
zmp_ph111_B5
zmp_ph111_B8
zmp_ph111_B6
zmp_ph111_B7
zmp_ph111_B9
zmp_ph111_B10
zmp_ph114_G4
zmp_ph114_G11
zmp_ph114_H1
zmp_ph114_H2
zmp_ph114_A9
zmp_ph114_A11
zmp_ph114_B2
zmp_ph114_B4
zmp_ph114_D8
zmp_ph114_E2

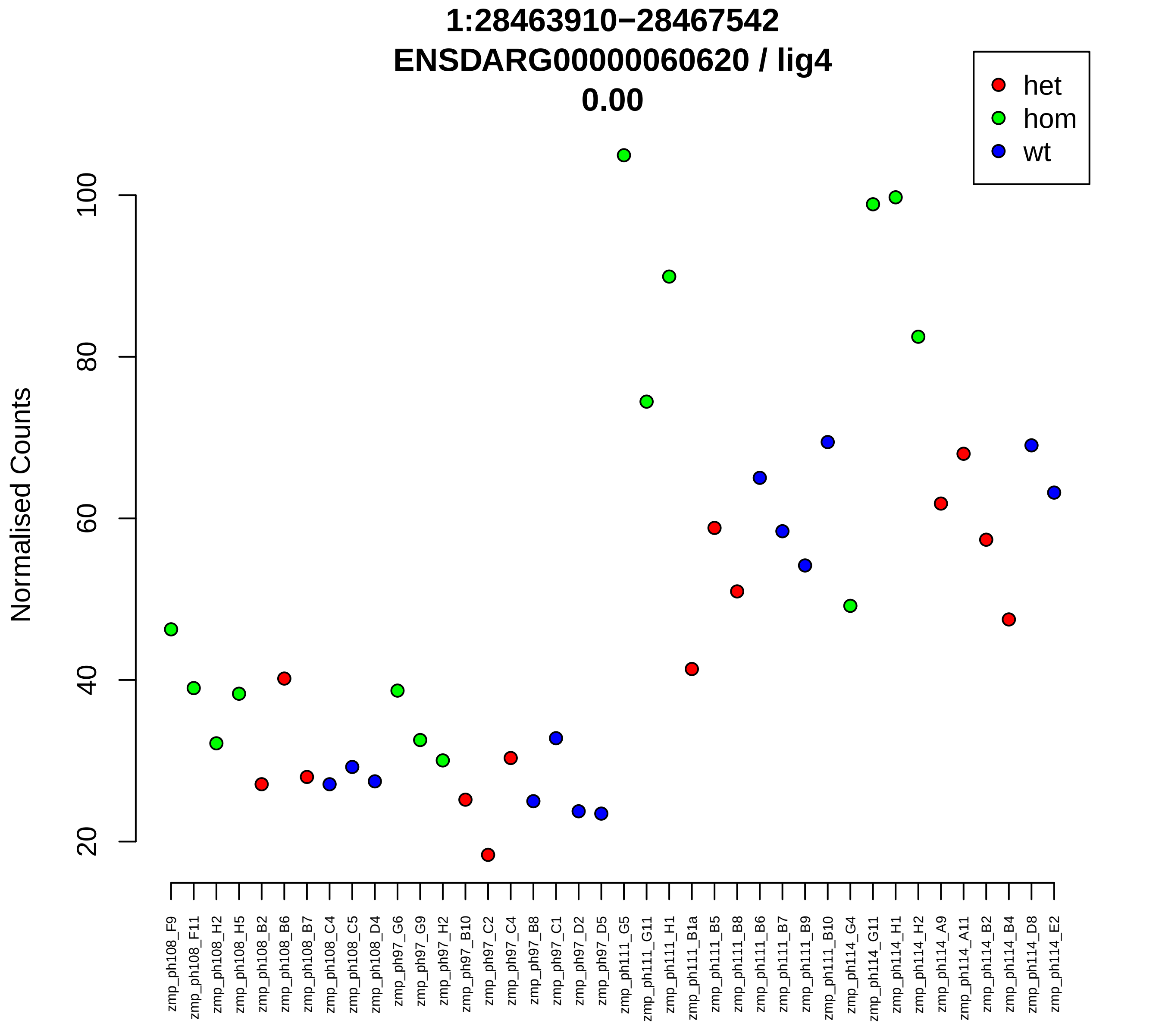

1:28463910−28467542
ENSDARG00000060620 / lig4
0.00
het
hom
wt
Normalised Counts
20
40
60
80
100
zmp_ph108_F9
zmp_ph108_F11
zmp_ph108_H2
zmp_ph108_H5
zmp_ph108_B2
zmp_ph108_B6
zmp_ph108_B7
zmp_ph108_C4
zmp_ph108_C5
zmp_ph108_D4
zmp_ph97_G6
zmp_ph97_G9
zmp_ph97_H2
zmp_ph97_B10
zmp_ph97_C2
zmp_ph97_C4
zmp_ph97_B8
zmp_ph97_C1
zmp_ph97_D2
zmp_ph97_D5
zmp_ph111_G5
zmp_ph111_G11
zmp_ph111_H1
zmp_ph111_B1a
zmp_ph111_B5
zmp_ph111_B8
zmp_ph111_B6
zmp_ph111_B7
zmp_ph111_B9
zmp_ph111_B10
zmp_ph114_G4
zmp_ph114_G11
zmp_ph114_H1
zmp_ph114_H2
zmp_ph114_A9
zmp_ph114_A11
zmp_ph114_B2
zmp_ph114_B4
zmp_ph114_D8
zmp_ph114_E2

6:55347375−55383496
ENSDARG00000104495 / pltp
0.00

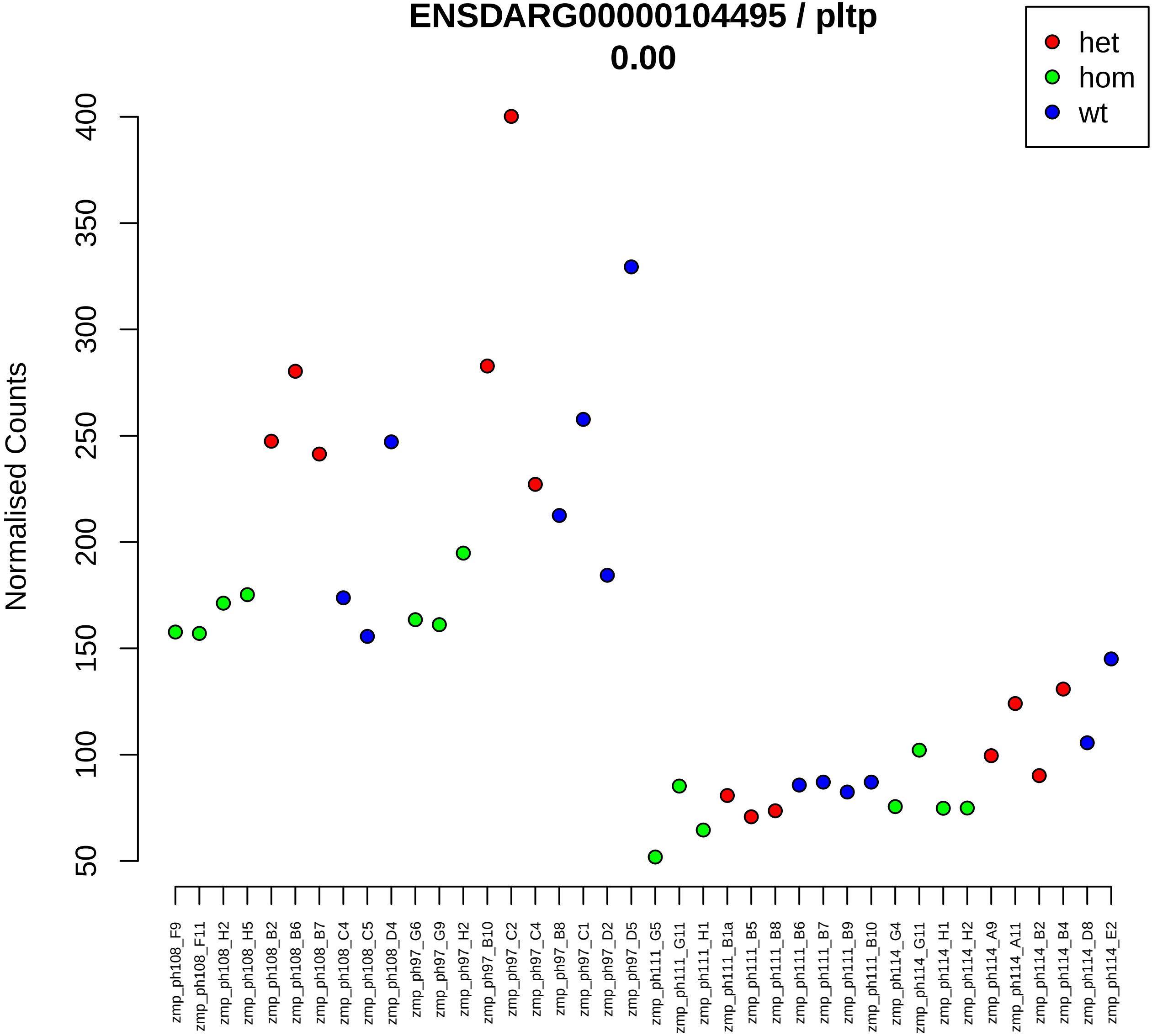

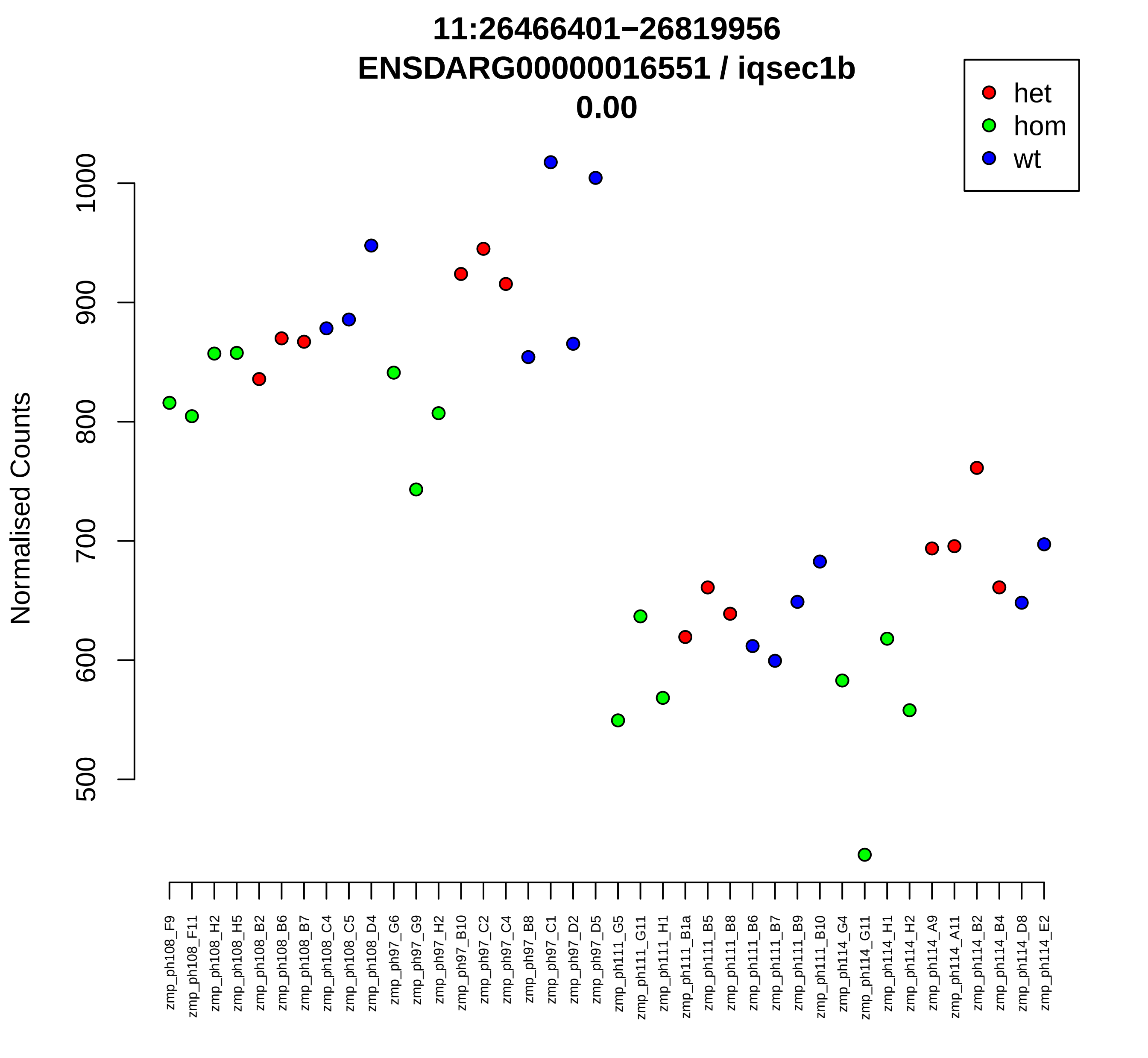
11:26466401−26819956
ENSDARG00000016551 / iqsec1b
0.00
het
hom
wt
Normalised Counts
500
600
700
800
900
1000
zmp_ph108_F9
zmp_ph108_F11
zmp_ph108_H2
zmp_ph108_H5
zmp_ph108_B2
zmp_ph108_B6
zmp_ph108_B7
zmp_ph108_C4
zmp_ph108_C5
zmp_ph108_D4
zmp_ph97_G6
zmp_ph97_G9
zmp_ph97_H2
zmp_ph97_B10
zmp_ph97_C2
zmp_ph97_C4
zmp_ph97_B8
zmp_ph97_C1
zmp_ph97_D2
zmp_ph97_D5
zmp_ph111_G5
zmp_ph111_G11
zmp_ph111_H1
zmp_ph111_B1a
zmp_ph111_B5
zmp_ph111_B8
zmp_ph111_B6
zmp_ph111_B7
zmp_ph111_B9
zmp_ph111_B10
zmp_ph114_G4
zmp_ph114_G11
zmp_ph114_H1
zmp_ph114_H2
zmp_ph114_A9
zmp_ph114_A11
zmp_ph114_B2
zmp_ph114_B4
zmp_ph114_D8
zmp_ph114_E2

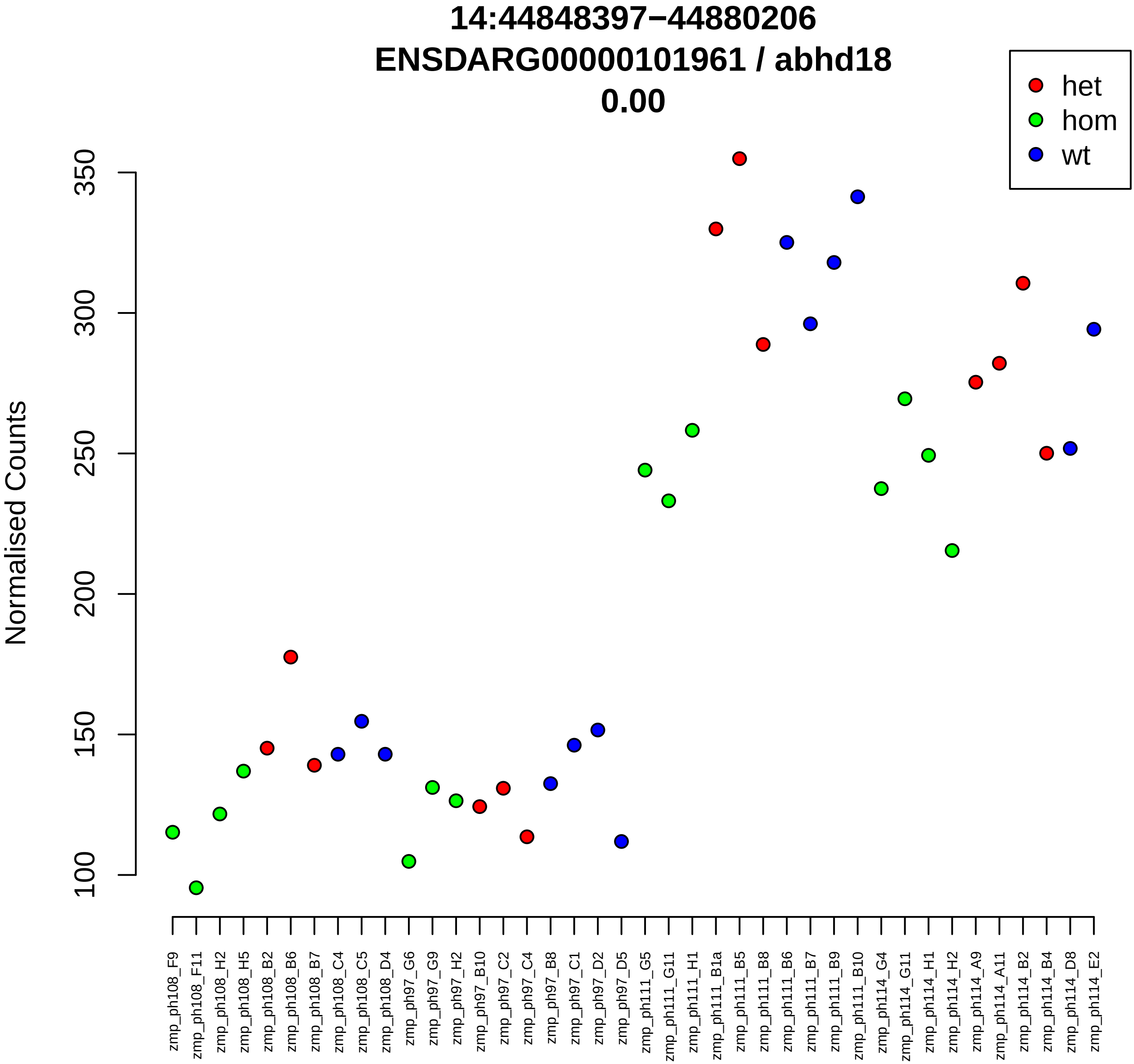
14:44848397−44880206
ENSDARG00000101961 / abhd18
0.00
het
hom
wt
Normalised Counts
100
150
200
250
300
350
zmp_ph108_F9
zmp_ph108_F11
zmp_ph108_H2
zmp_ph108_H5
zmp_ph108_B2
zmp_ph108_B6
zmp_ph108_B7
zmp_ph108_C4
zmp_ph108_C5
zmp_ph108_D4
zmp_ph97_G6
zmp_ph97_G9
zmp_ph97_H2
zmp_ph97_B10
zmp_ph97_C2
zmp_ph97_C4
zmp_ph97_B8
zmp_ph97_C1
zmp_ph97_D2
zmp_ph97_D5
zmp_ph111_G5
zmp_ph111_G11
zmp_ph111_H1
zmp_ph111_B1a
zmp_ph111_B5
zmp_ph111_B8
zmp_ph111_B6
zmp_ph111_B7
zmp_ph111_B9
zmp_ph111_B10
zmp_ph114_G4
zmp_ph114_G11
zmp_ph114_H1
zmp_ph114_H2
zmp_ph114_A9
zmp_ph114_A11
zmp_ph114_B2
zmp_ph114_B4
zmp_ph114_D8
zmp_ph114_E2

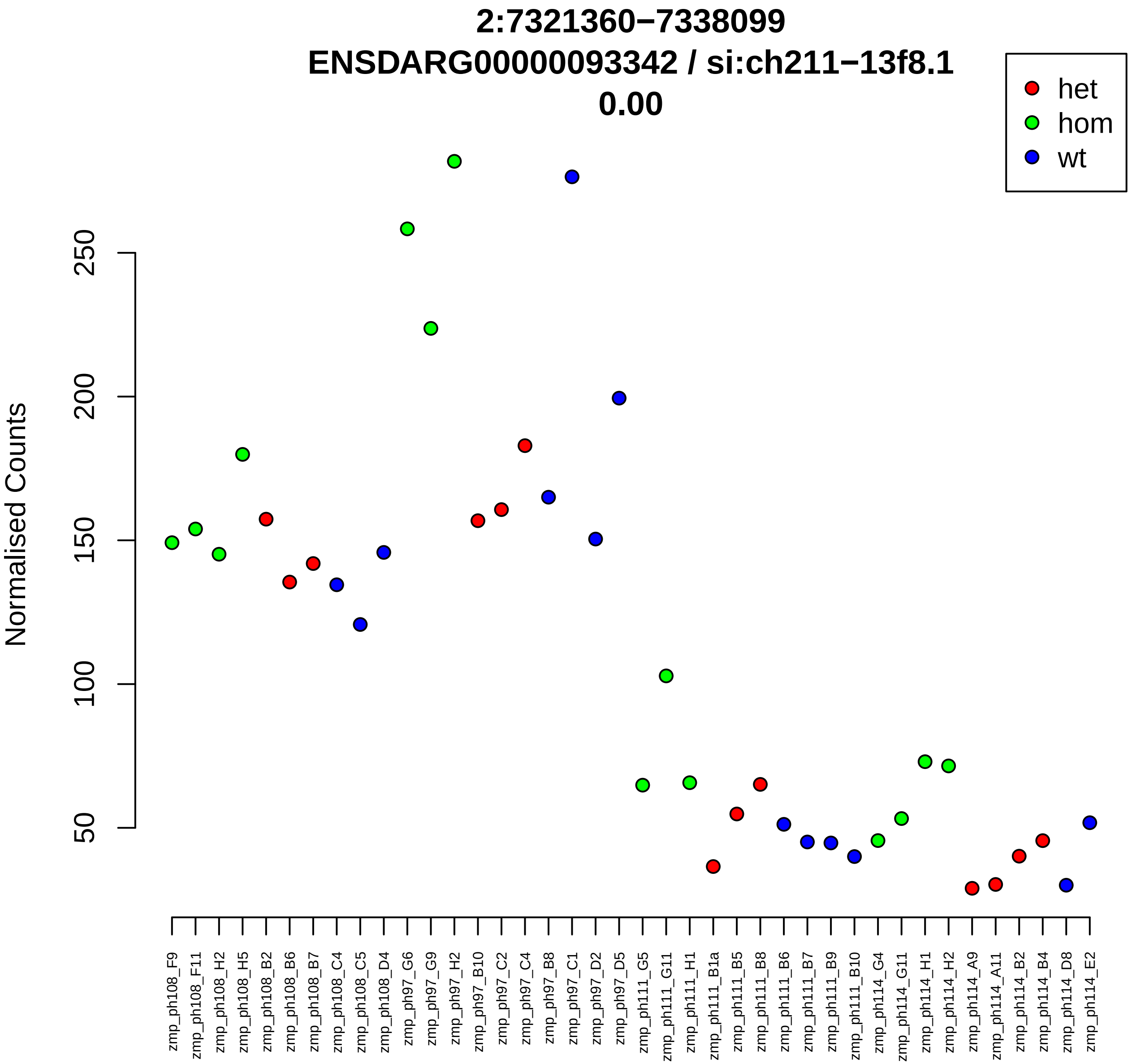

2:7321360−7338099
ENSDARG00000093342 / si:ch211−13f8.1
0.00
het
hom
wt
Normalised Counts
50
100
150
200
250
zmp_ph108_F9
zmp_ph108_F11
zmp_ph108_H2
zmp_ph108_H5
zmp_ph108_B2
zmp_ph108_B6
zmp_ph108_B7
zmp_ph108_C4
zmp_ph108_C5
zmp_ph108_D4
zmp_ph97_G6
zmp_ph97_G9
zmp_ph97_H2
zmp_ph97_B10
zmp_ph97_C2
zmp_ph97_C4
zmp_ph97_B8
zmp_ph97_C1
zmp_ph97_D2
zmp_ph97_D5
zmp_ph111_G5
zmp_ph111_G11
zmp_ph111_H1
zmp_ph111_B1a
zmp_ph111_B5
zmp_ph111_B8
zmp_ph111_B6
zmp_ph111_B7
zmp_ph111_B9
zmp_ph111_B10
zmp_ph114_G4
zmp_ph114_G11
zmp_ph114_H1
zmp_ph114_H2
zmp_ph114_A9
zmp_ph114_A11
zmp_ph114_B2
zmp_ph114_B4
zmp_ph114_D8
zmp_ph114_E2

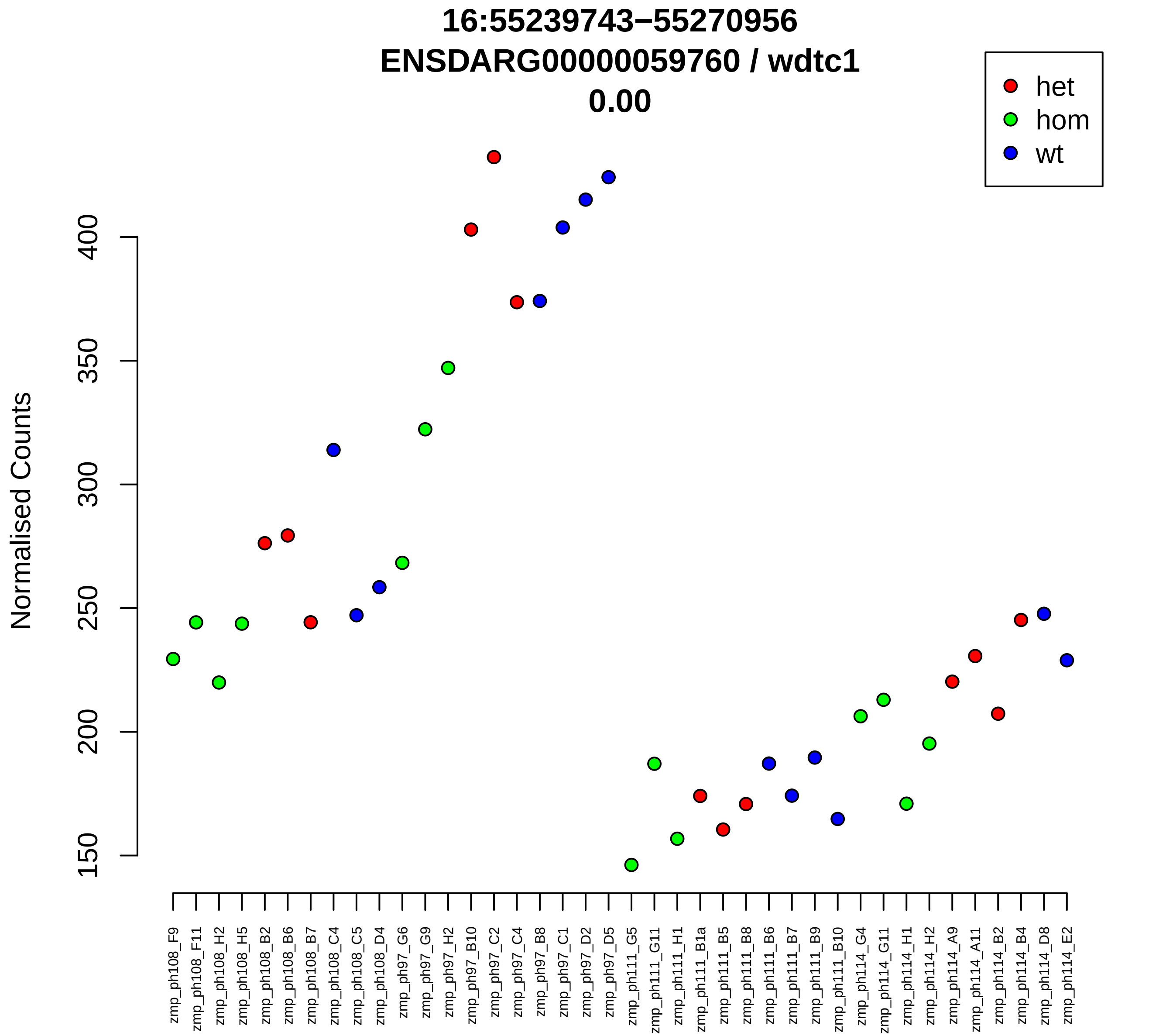

16:55239743−55270956
ENSDARG00000059760 / wdtc1
0.00
het
hom
wt
Normalised Counts
150
200
250
300
350
400
zmp_ph108_F9
zmp_ph108_F11
zmp_ph108_H2
zmp_ph108_H5
zmp_ph108_B2
zmp_ph108_B6
zmp_ph108_B7
zmp_ph108_C4
zmp_ph108_C5
zmp_ph108_D4
zmp_ph97_G6
zmp_ph97_G9
zmp_ph97_H2
zmp_ph97_B10
zmp_ph97_C2
zmp_ph97_C4
zmp_ph97_B8
zmp_ph97_C1
zmp_ph97_D2
zmp_ph97_D5
zmp_ph111_G5
zmp_ph111_G11
zmp_ph111_H1
zmp_ph111_B1a
zmp_ph111_B5
zmp_ph111_B8
zmp_ph111_B6
zmp_ph111_B7
zmp_ph111_B9
zmp_ph111_B10
zmp_ph114_G4
zmp_ph114_G11
zmp_ph114_H1
zmp_ph114_H2
zmp_ph114_A9
zmp_ph114_A11
zmp_ph114_B2
zmp_ph114_B4
zmp_ph114_D8
zmp_ph114_E2

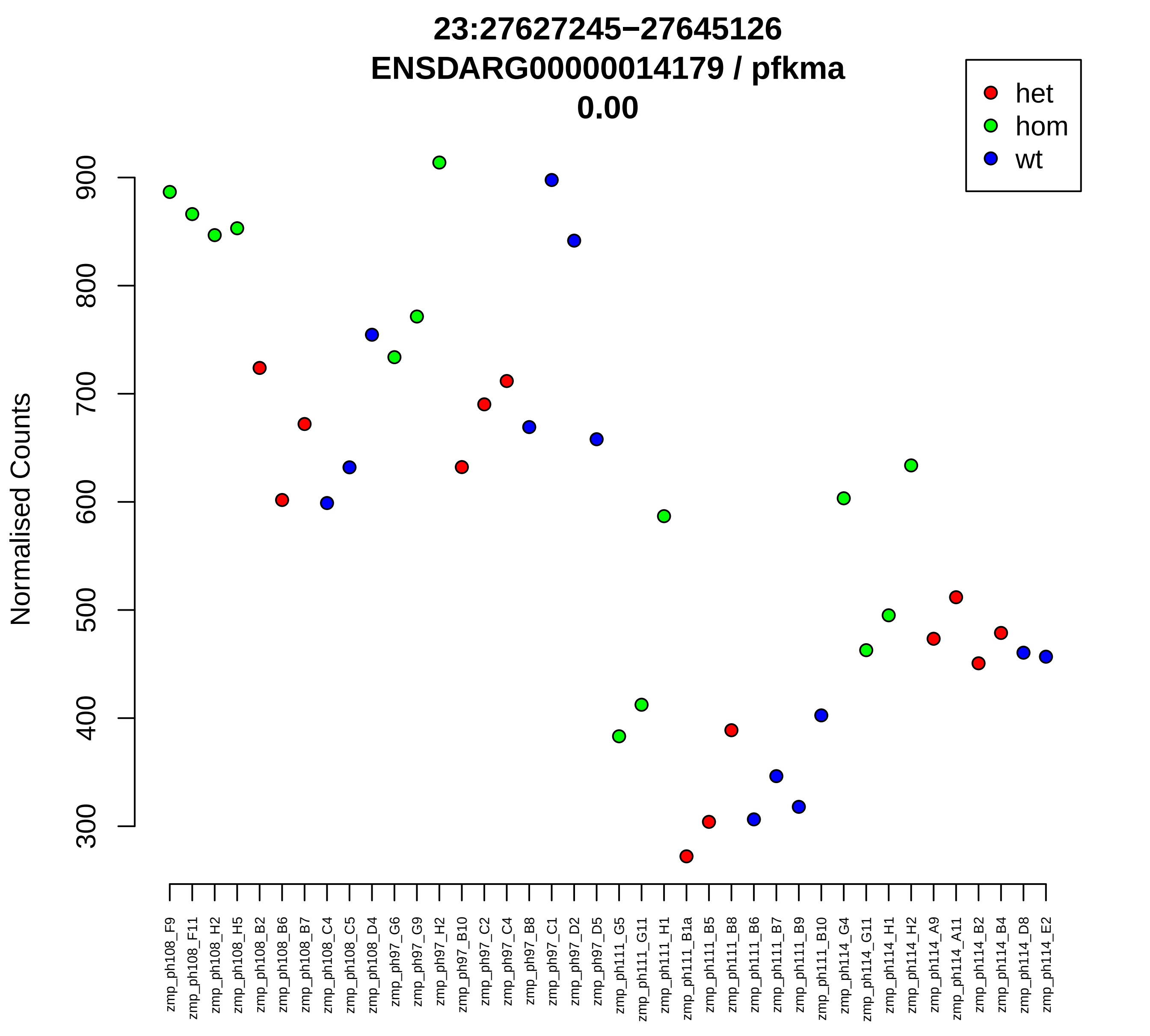

23:27627245−27645126
ENSDARG00000014179 / pfkma
0.00
het
hom
wt
Normalised Counts
300
400
500
600
700
800
900
zmp_ph108_F9
zmp_ph108_F11
zmp_ph108_H2
zmp_ph108_H5
zmp_ph108_B2
zmp_ph108_B6
zmp_ph108_B7
zmp_ph108_C4
zmp_ph108_C5
zmp_ph108_D4
zmp_ph97_G6
zmp_ph97_G9
zmp_ph97_H2
zmp_ph97_B10
zmp_ph97_C2
zmp_ph97_C4
zmp_ph97_B8
zmp_ph97_C1
zmp_ph97_D2
zmp_ph97_D5
zmp_ph111_G5
zmp_ph111_G11
zmp_ph111_H1
zmp_ph111_B1a
zmp_ph111_B5
zmp_ph111_B8
zmp_ph111_B6
zmp_ph111_B7
zmp_ph111_B9
zmp_ph111_B10
zmp_ph114_G4
zmp_ph114_G11
zmp_ph114_H1
zmp_ph114_H2
zmp_ph114_A9
zmp_ph114_A11
zmp_ph114_B2
zmp_ph114_B4
zmp_ph114_D8
zmp_ph114_E2

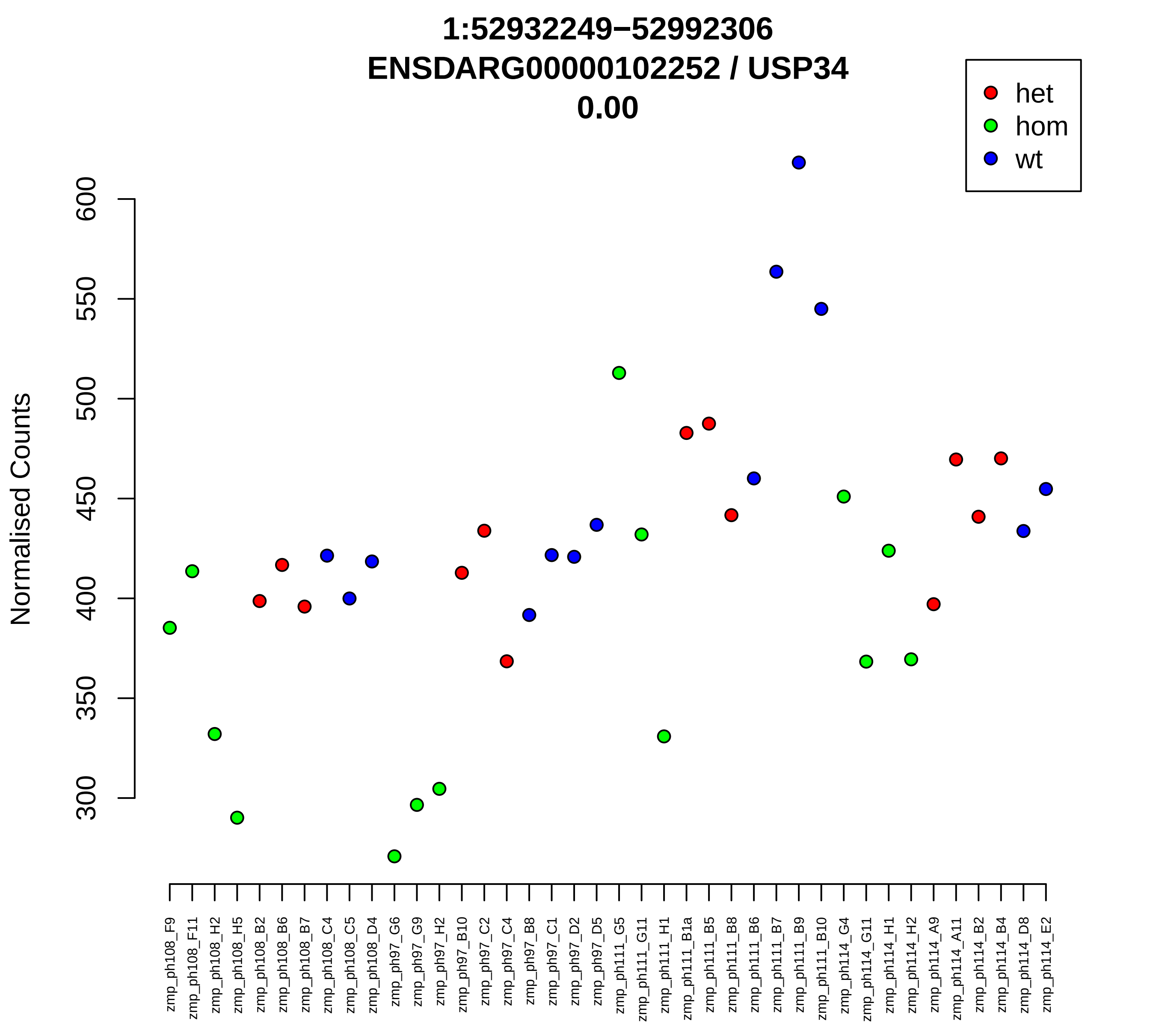
1:52932249−52992306
ENSDARG00000102252 / USP34
0.00
het
hom
wt
Normalised Counts
300
350
400
450
500
550
600
zmp_ph108_F9
zmp_ph108_F11
zmp_ph108_H2
zmp_ph108_H5
zmp_ph108_B2
zmp_ph108_B6
zmp_ph108_B7
zmp_ph108_C4
zmp_ph108_C5
zmp_ph108_D4
zmp_ph97_G6
zmp_ph97_G9
zmp_ph97_H2
zmp_ph97_B10
zmp_ph97_C2
zmp_ph97_C4
zmp_ph97_B8
zmp_ph97_C1
zmp_ph97_D2
zmp_ph97_D5
zmp_ph111_G5
zmp_ph111_G11
zmp_ph111_H1
zmp_ph111_B1a
zmp_ph111_B5
zmp_ph111_B8
zmp_ph111_B6
zmp_ph111_B7
zmp_ph111_B9
zmp_ph111_B10
zmp_ph114_G4
zmp_ph114_G11
zmp_ph114_H1
zmp_ph114_H2
zmp_ph114_A9
zmp_ph114_A11
zmp_ph114_B2
zmp_ph114_B4
zmp_ph114_D8
zmp_ph114_E2

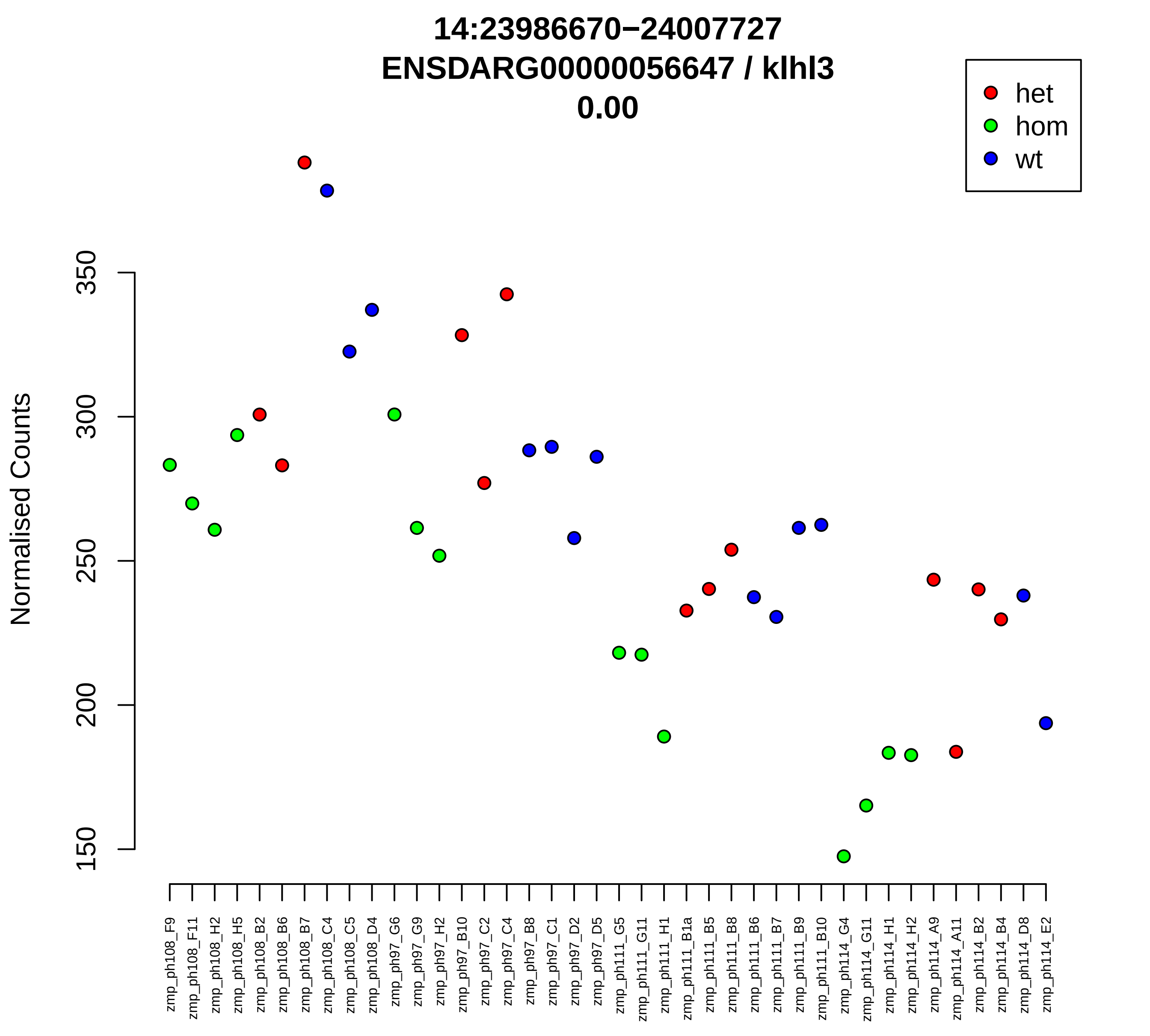

14:23986670−24007727
ENSDARG00000056647 / klhl3
0.00
het
hom
wt
Normalised Counts
150
200
250
300
350
zmp_ph108_F9
zmp_ph108_F11
zmp_ph108_H2
zmp_ph108_H5
zmp_ph108_B2
zmp_ph108_B6
zmp_ph108_B7
zmp_ph108_C4
zmp_ph108_C5
zmp_ph108_D4
zmp_ph97_G6
zmp_ph97_G9
zmp_ph97_H2
zmp_ph97_B10
zmp_ph97_C2
zmp_ph97_C4
zmp_ph97_B8
zmp_ph97_C1
zmp_ph97_D2
zmp_ph97_D5
zmp_ph111_G5
zmp_ph111_G11
zmp_ph111_H1
zmp_ph111_B1a
zmp_ph111_B5
zmp_ph111_B8
zmp_ph111_B6
zmp_ph111_B7
zmp_ph111_B9
zmp_ph111_B10
zmp_ph114_G4
zmp_ph114_G11
zmp_ph114_H1
zmp_ph114_H2
zmp_ph114_A9
zmp_ph114_A11
zmp_ph114_B2
zmp_ph114_B4
zmp_ph114_D8
zmp_ph114_E2

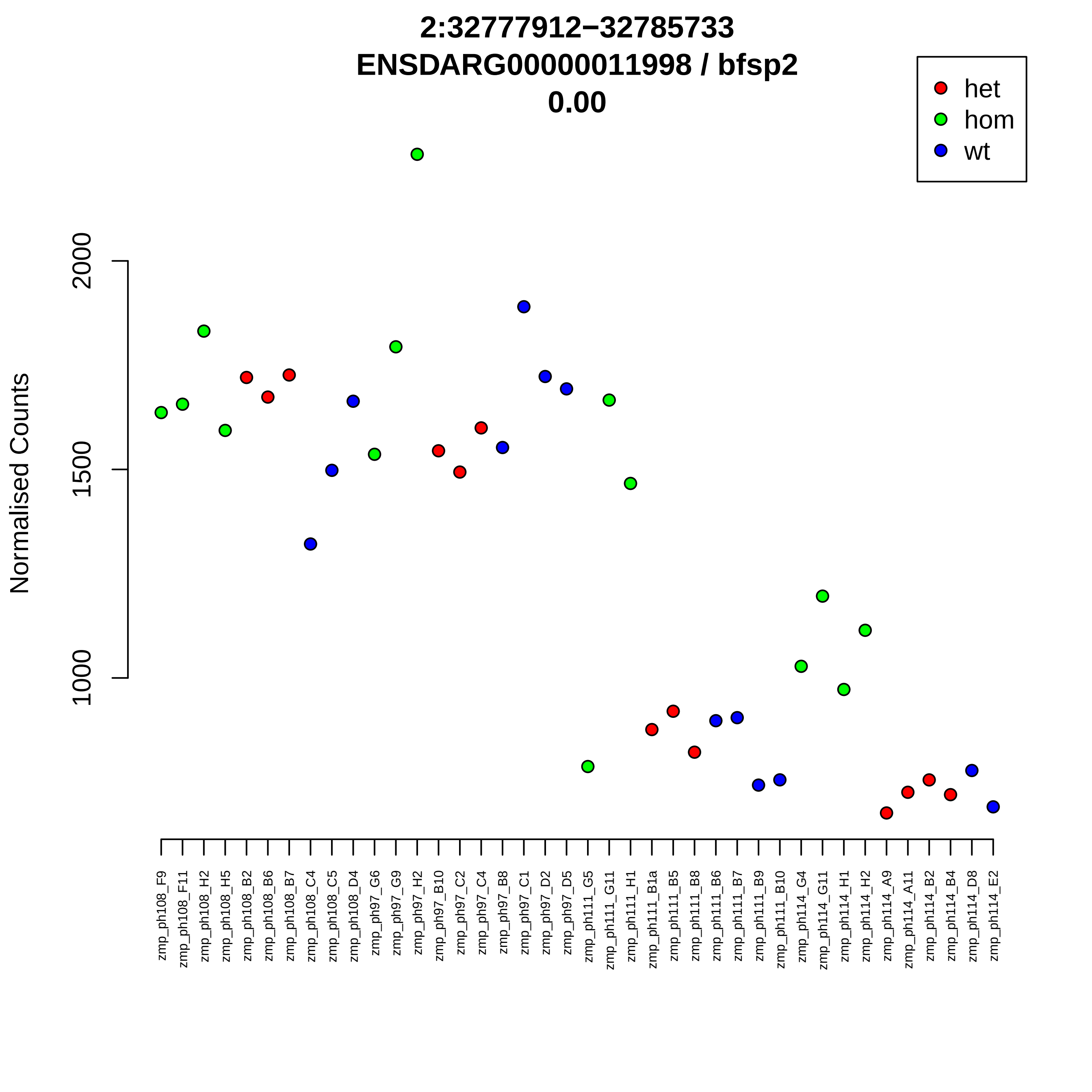

2:32777912−32785733
ENSDARG00000011998 / bfsp2
0.00
het
hom
wt
Normalised Counts
2000
1500
1000
zmp_ph108_F9
zmp_ph108_F11
zmp_ph108_H2
zmp_ph108_H5
zmp_ph108_B2
zmp_ph108_B6
zmp_ph108_B7
zmp_ph108_C4
zmp_ph108_C5
zmp_ph108_D4
zmp_ph97_G6
zmp_ph97_G9
zmp_ph97_H2
zmp_ph97_B10
zmp_ph97_C2
zmp_ph97_C4
zmp_ph97_B8
zmp_ph97_C1
zmp_ph97_D2
zmp_ph97_D5
zmp_ph111_G5
zmp_ph111_G11
zmp_ph111_H1
zmp_ph111_B1a
zmp_ph111_B5
zmp_ph111_B8
zmp_ph111_B6
zmp_ph111_B7
zmp_ph111_B9
zmp_ph111_B10
zmp_ph114_G4
zmp_ph114_G11
zmp_ph114_H1
zmp_ph114_H2
zmp_ph114_A9
zmp_ph114_A11
zmp_ph114_B2
zmp_ph114_B4
zmp_ph114_D8
zmp_ph114_E2

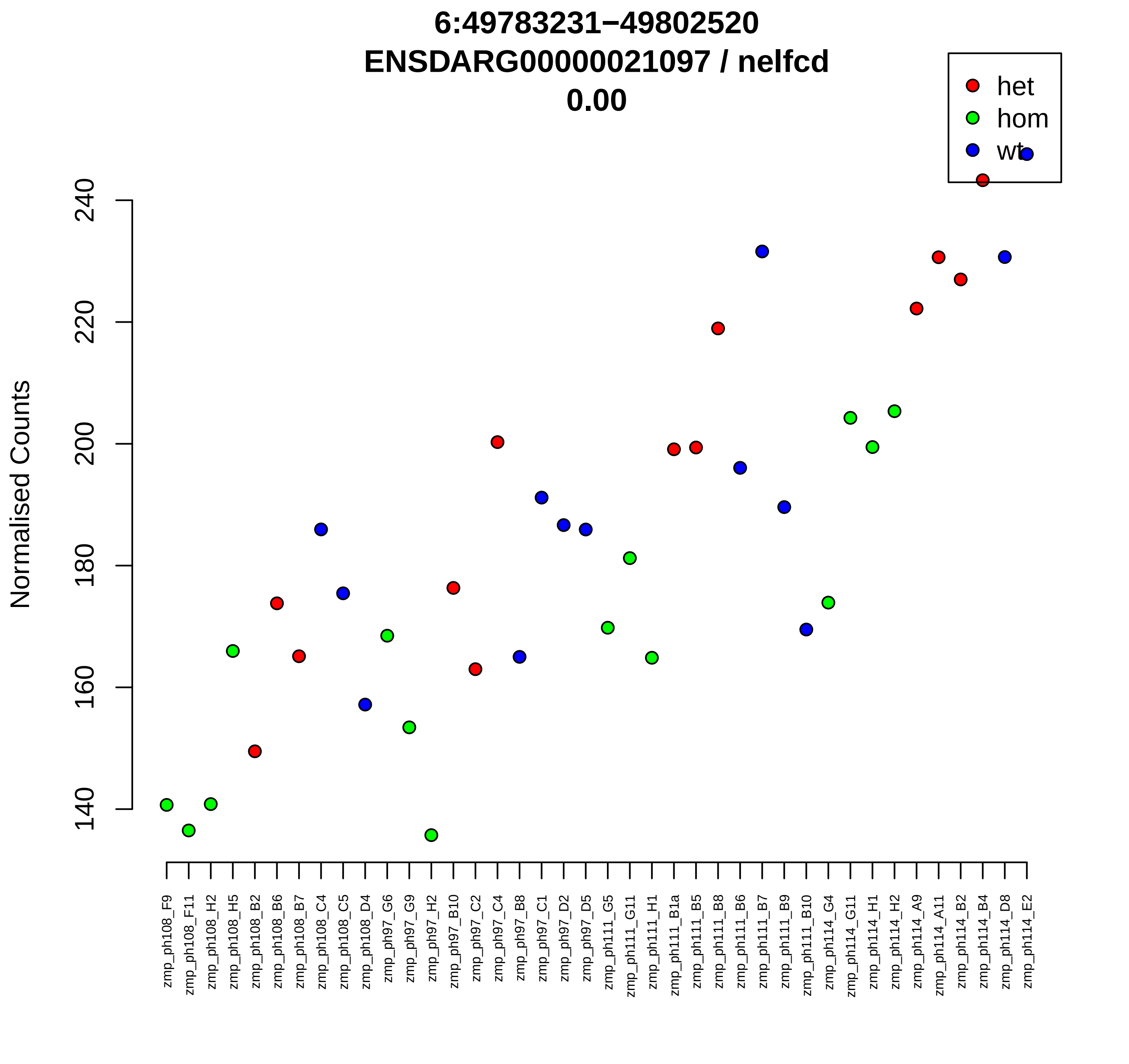
6:49783231−49802520
ENSDARG00000021097 / nelfcd
0.00
het
hom
wt
Normalised Counts
140
160
180
200
220
240
zmp_ph108_F9
zmp_ph108_F11
zmp_ph108_H2
zmp_ph108_H5
zmp_ph108_B2
zmp_ph108_B6
zmp_ph108_B7
zmp_ph108_C4
zmp_ph108_C5
zmp_ph108_D4
zmp_ph97_G6
zmp_ph97_G9
zmp_ph97_H2
zmp_ph97_B10
zmp_ph97_C2
zmp_ph97_C4
zmp_ph97_B8
zmp_ph97_C1
zmp_ph97_D2
zmp_ph97_D5
zmp_ph111_G5
zmp_ph111_G11
zmp_ph111_H1
zmp_ph111_B1a
zmp_ph111_B5
zmp_ph111_B8
zmp_ph111_B6
zmp_ph111_B7
zmp_ph111_B9
zmp_ph111_B10
zmp_ph114_G4
zmp_ph114_G11
zmp_ph114_H1
zmp_ph114_H2
zmp_ph114_A9
zmp_ph114_A11
zmp_ph114_B2
zmp_ph114_B4
zmp_ph114_D8
zmp_ph114_E2

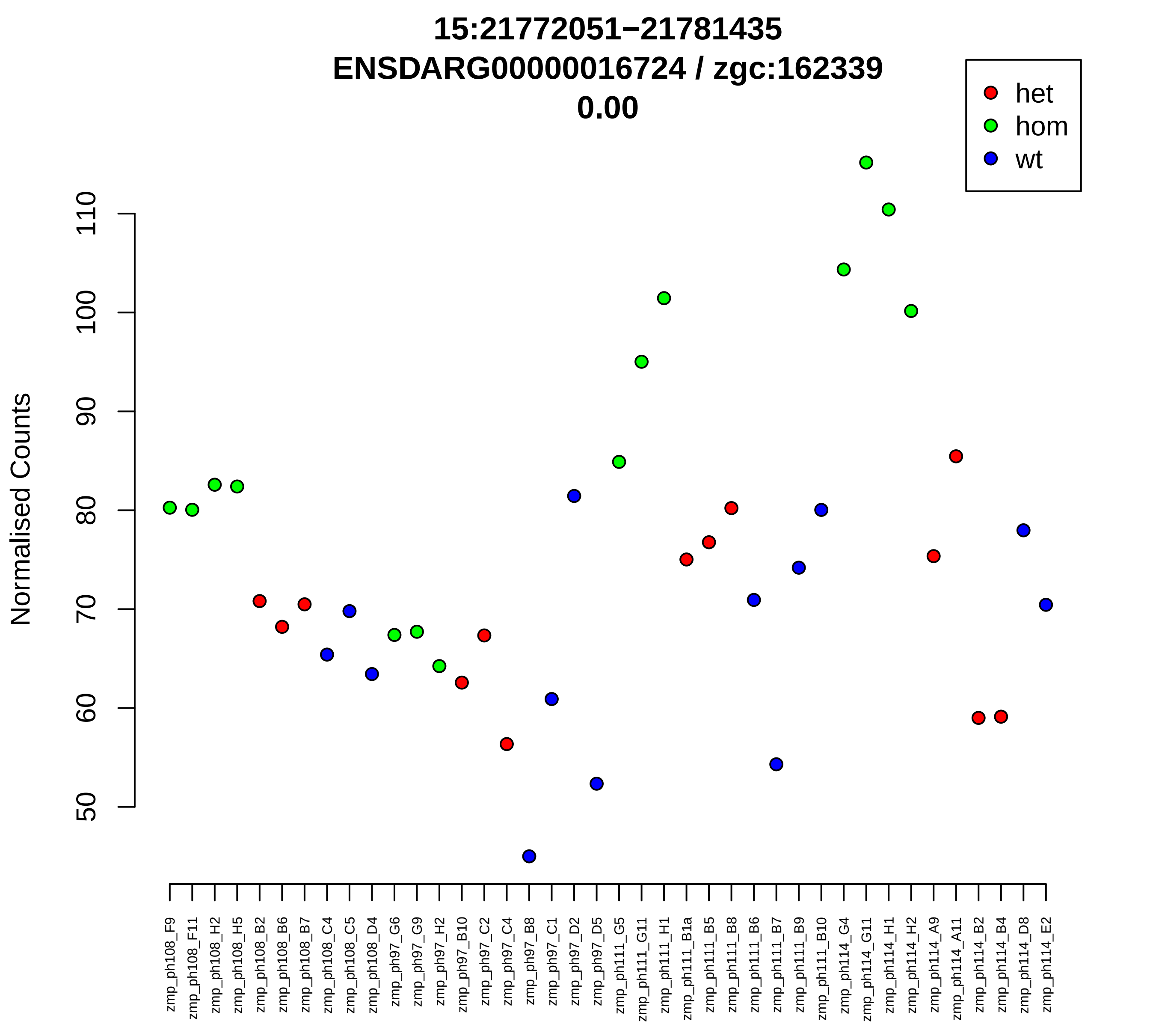

15:21772051−21781435
ENSDARG00000016724 / zgc:162339
0.00
het
hom
wt
Normalised Counts
50
60
70
80
90
100
110
zmp_ph108_F9
zmp_ph108_F11
zmp_ph108_H2
zmp_ph108_H5
zmp_ph108_B2
zmp_ph108_B6
zmp_ph108_B7
zmp_ph108_C4
zmp_ph108_C5
zmp_ph108_D4
zmp_ph97_G6
zmp_ph97_G9
zmp_ph97_H2
zmp_ph97_B10
zmp_ph97_C2
zmp_ph97_C4
zmp_ph97_B8
zmp_ph97_C1
zmp_ph97_D2
zmp_ph97_D5
zmp_ph111_G5
zmp_ph111_G11
zmp_ph111_H1
zmp_ph111_B1a
zmp_ph111_B5
zmp_ph111_B8
zmp_ph111_B6
zmp_ph111_B7
zmp_ph111_B9
zmp_ph111_B10
zmp_ph114_G4
zmp_ph114_G11
zmp_ph114_H1
zmp_ph114_H2
zmp_ph114_A9
zmp_ph114_A11
zmp_ph114_B2
zmp_ph114_B4
zmp_ph114_D8
zmp_ph114_E2

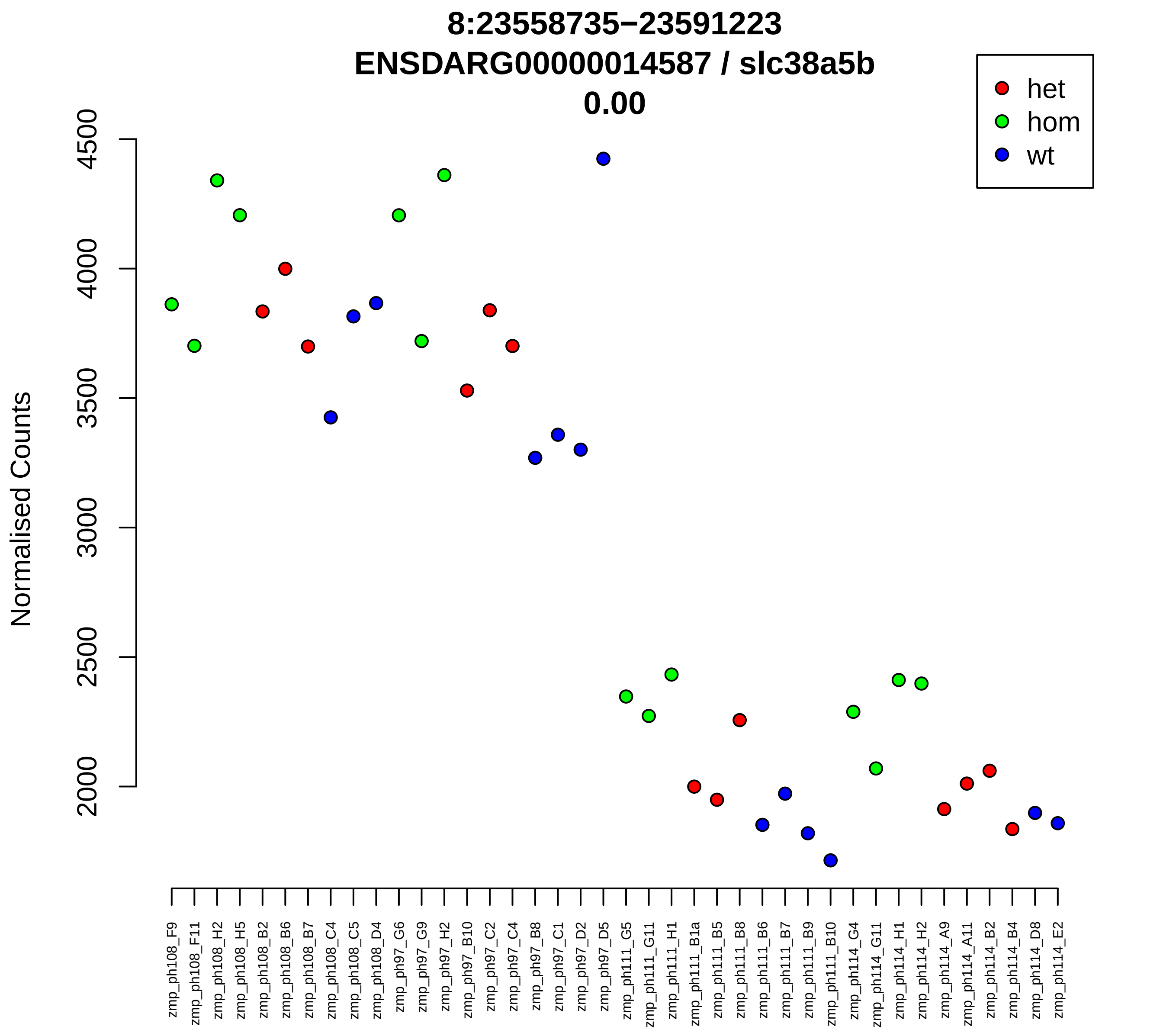
8:23558735−23591223
ENSDARG00000014587 / slc38a5b
0.00
het
hom
wt
Normalised Counts
2000
2500
3000
3500
4000
4500
zmp_ph108_F9
zmp_ph108_F11
zmp_ph108_H2
zmp_ph108_H5
zmp_ph108_B2
zmp_ph108_B6
zmp_ph108_B7
zmp_ph108_C4
zmp_ph108_C5
zmp_ph108_D4
zmp_ph97_G6
zmp_ph97_G9
zmp_ph97_H2
zmp_ph97_B10
zmp_ph97_C2
zmp_ph97_C4
zmp_ph97_B8
zmp_ph97_C1
zmp_ph97_D2
zmp_ph97_D5
zmp_ph111_G5
zmp_ph111_G11
zmp_ph111_H1
zmp_ph111_B1a
zmp_ph111_B5
zmp_ph111_B8
zmp_ph111_B6
zmp_ph111_B7
zmp_ph111_B9
zmp_ph111_B10
zmp_ph114_G4
zmp_ph114_G11
zmp_ph114_H1
zmp_ph114_H2
zmp_ph114_A9
zmp_ph114_A11
zmp_ph114_B2
zmp_ph114_B4
zmp_ph114_D8
zmp_ph114_E2

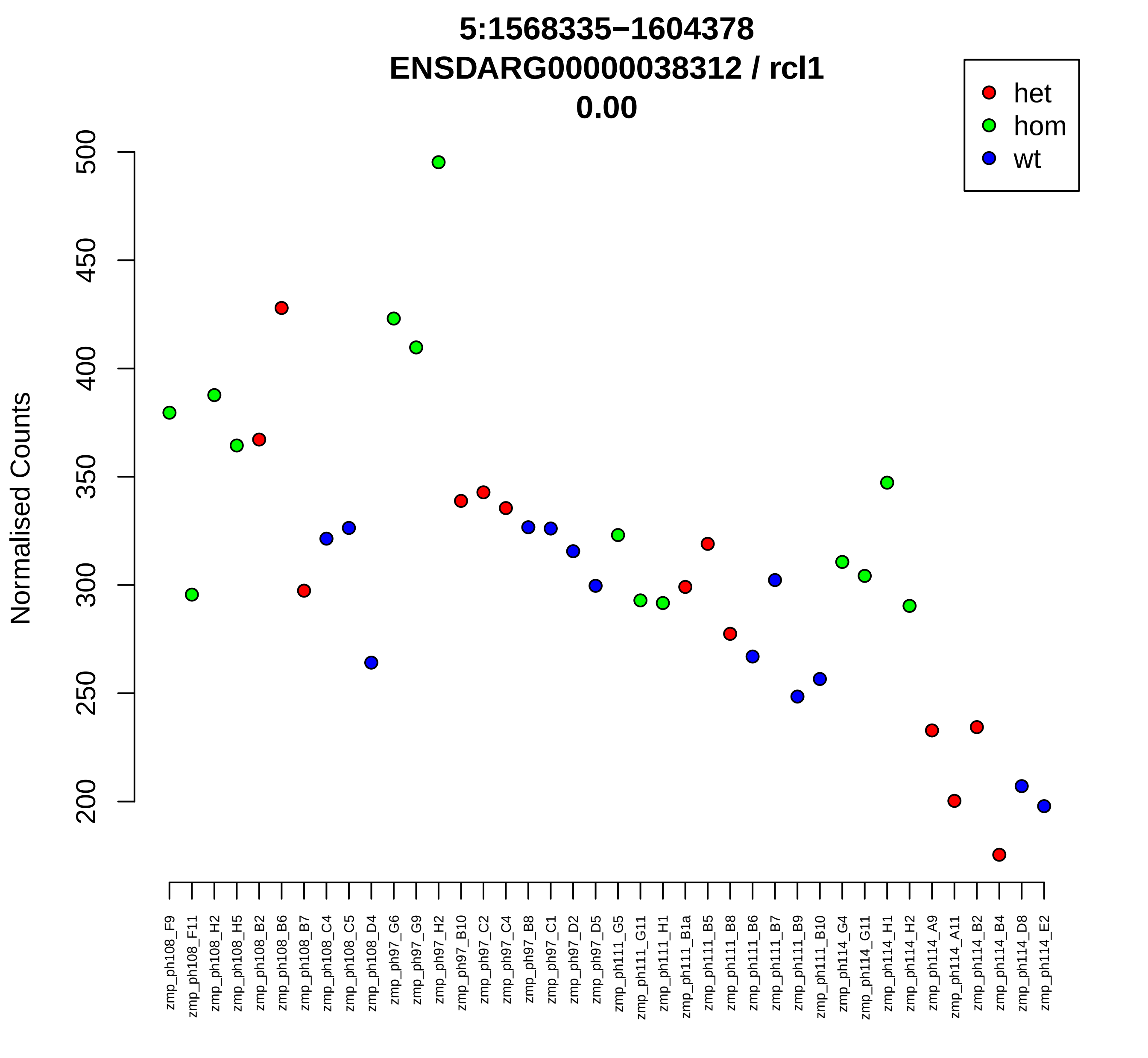

5:1568335−1604378
ENSDARG00000038312 / rcl1
0.00
het
hom
wt
Normalised Counts
500
450
400
350
300
250
200
zmp_ph108_F9
zmp_ph108_F11
zmp_ph108_H2
zmp_ph108_H5
zmp_ph108_B2
zmp_ph108_B6
zmp_ph108_B7
zmp_ph108_C4
zmp_ph108_C5
zmp_ph108_D4
zmp_ph97_G6
zmp_ph97_G9
zmp_ph97_H2
zmp_ph97_B10
zmp_ph97_C2
zmp_ph97_C4
zmp_ph97_B8
zmp_ph97_C1
zmp_ph97_D2
zmp_ph97_D5
zmp_ph111_G5
zmp_ph111_G11
zmp_ph111_H1
zmp_ph111_B1a
zmp_ph111_B5
zmp_ph111_B8
zmp_ph111_B6
zmp_ph111_B7
zmp_ph111_B9
zmp_ph111_B10
zmp_ph114_G4
zmp_ph114_G11
zmp_ph114_H1
zmp_ph114_H2
zmp_ph114_A9
zmp_ph114_A11
zmp_ph114_B2
zmp_ph114_B4
zmp_ph114_D8
zmp_ph114_E2

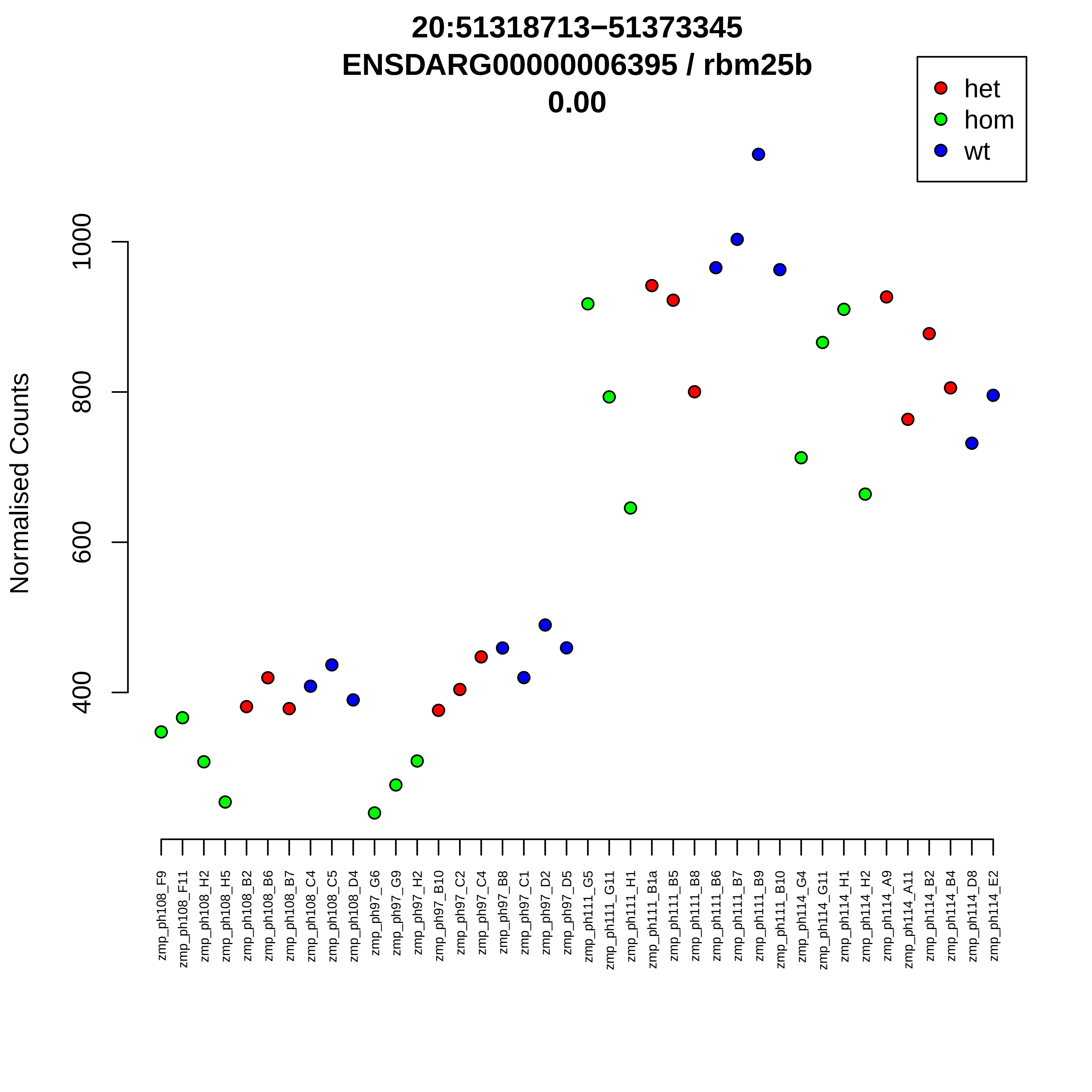

20:51318713−51373345
ENSDARG00000006395 / rbm25b
0.00
het
hom
wt
Normalised Counts
1000
800
600
400
zmp_ph108_F9
zmp_ph108_F11
zmp_ph108_H2
zmp_ph108_H5
zmp_ph108_B2
zmp_ph108_B6
zmp_ph108_B7
zmp_ph108_C4
zmp_ph108_C5
zmp_ph108_D4
zmp_ph97_G6
zmp_ph97_G9
zmp_ph97_H2
zmp_ph97_B10
zmp_ph97_C2
zmp_ph97_C4
zmp_ph97_B8
zmp_ph97_C1
zmp_ph97_D2
zmp_ph97_D5
zmp_ph111_G5
zmp_ph111_G11
zmp_ph111_H1
zmp_ph111_B1a
zmp_ph111_B5
zmp_ph111_B8
zmp_ph111_B6
zmp_ph111_B7
zmp_ph111_B9
zmp_ph111_B10
zmp_ph114_G4
zmp_ph114_G11
zmp_ph114_H1
zmp_ph114_H2
zmp_ph114_A9
zmp_ph114_A11
zmp_ph114_B2
zmp_ph114_B4
zmp_ph114_D8
zmp_ph114_E2

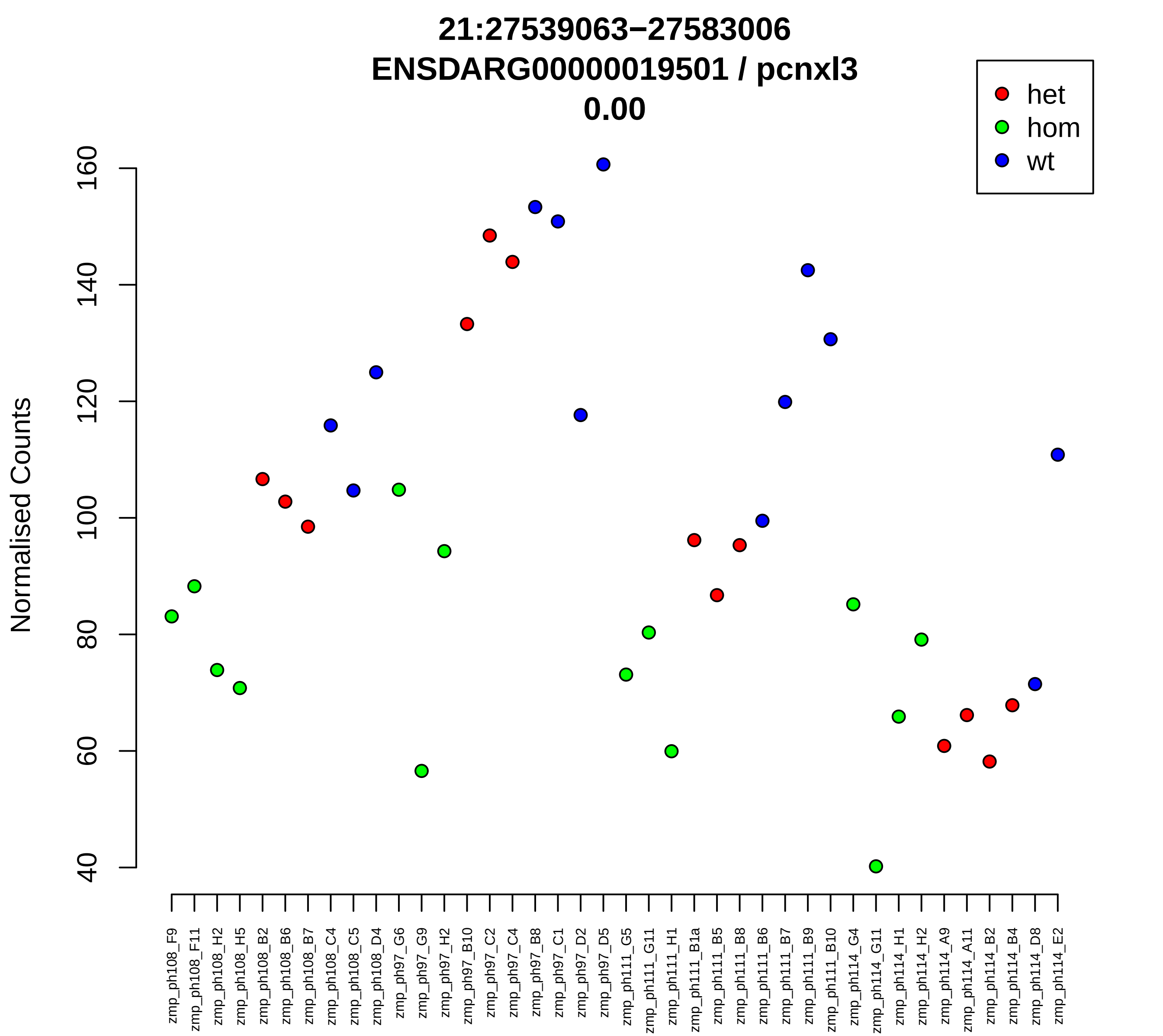
21:27539063−27583006
ENSDARG00000019501 / pcnxl3
0.00
het
hom
wt
Normalised Counts
40
60
80
100
120
140
160
zmp_ph108_F9
zmp_ph108_F11
zmp_ph108_H2
zmp_ph108_H5
zmp_ph108_B2
zmp_ph108_B6
zmp_ph108_B7
zmp_ph108_C4
zmp_ph108_C5
zmp_ph108_D4
zmp_ph97_G6
zmp_ph97_G9
zmp_ph97_H2
zmp_ph97_B10
zmp_ph97_C2
zmp_ph97_C4
zmp_ph97_B8
zmp_ph97_C1
zmp_ph97_D2
zmp_ph97_D5
zmp_ph111_G5
zmp_ph111_G11
zmp_ph111_H1
zmp_ph111_B1a
zmp_ph111_B5
zmp_ph111_B8
zmp_ph111_B6
zmp_ph111_B7
zmp_ph111_B9
zmp_ph111_B10
zmp_ph114_G4
zmp_ph114_G11
zmp_ph114_H1
zmp_ph114_H2
zmp_ph114_A9
zmp_ph114_A11
zmp_ph114_B2
zmp_ph114_B4
zmp_ph114_D8
zmp_ph114_E2

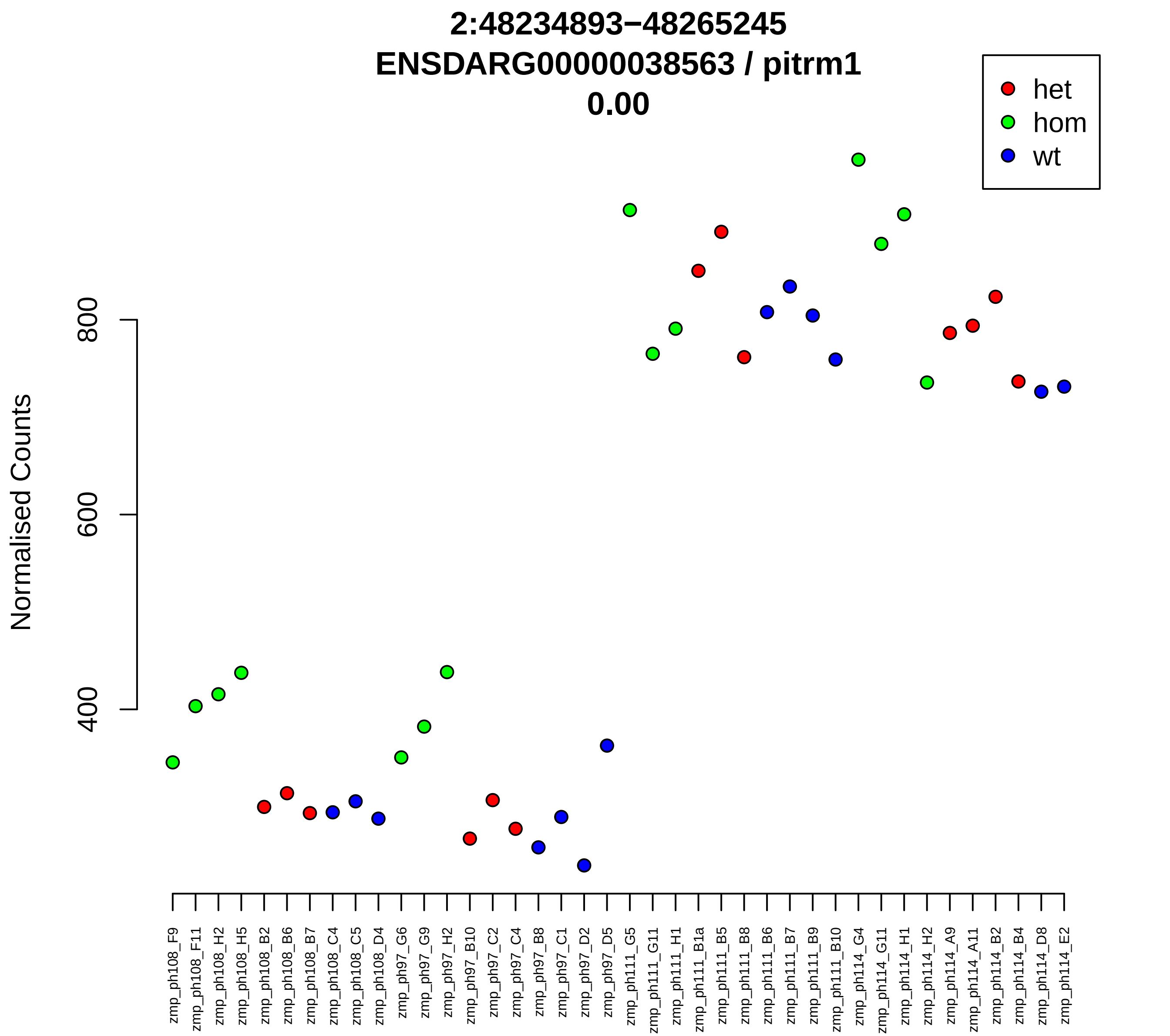
2:48234893−48265245
ENSDARG00000038563 / pitrm1
0.00
het
hom
wt
Normalised Counts
400
600
800
zmp_ph108_F9
zmp_ph108_F11
zmp_ph108_H2
zmp_ph108_H5
zmp_ph108_B2
zmp_ph108_B6
zmp_ph108_B7
zmp_ph108_C4
zmp_ph108_C5
zmp_ph108_D4
zmp_ph97_G6
zmp_ph97_G9
zmp_ph97_H2
zmp_ph97_B10
zmp_ph97_C2
zmp_ph97_C4
zmp_ph97_B8
zmp_ph97_C1
zmp_ph97_D2
zmp_ph97_D5
zmp_ph111_G5
zmp_ph111_G11
zmp_ph111_H1
zmp_ph111_B1a
zmp_ph111_B5
zmp_ph111_B8
zmp_ph111_B6
zmp_ph111_B7
zmp_ph111_B9
zmp_ph111_B10
zmp_ph114_G4
zmp_ph114_G11
zmp_ph114_H1
zmp_ph114_H2
zmp_ph114_A9
zmp_ph114_A11
zmp_ph114_B2
zmp_ph114_B4
zmp_ph114_D8
zmp_ph114_E2

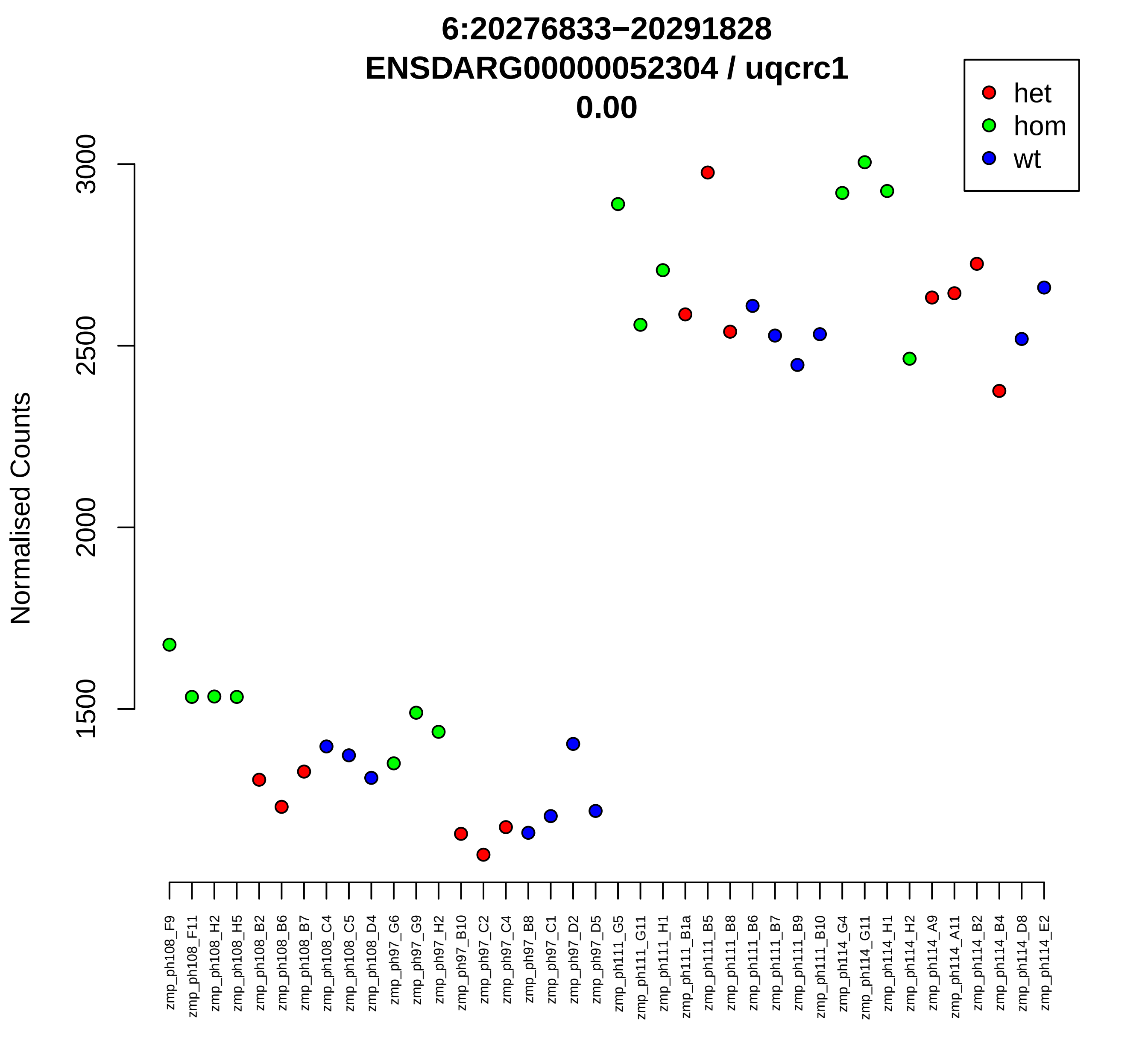

6:20276833−20291828
ENSDARG00000052304 / uqcrc1
0.00
het
hom
wt
Normalised Counts
1500
2000
2500
3000
zmp_ph108_F9
zmp_ph108_F11
zmp_ph108_H2
zmp_ph108_H5
zmp_ph108_B2
zmp_ph108_B6
zmp_ph108_B7
zmp_ph108_C4
zmp_ph108_C5
zmp_ph108_D4
zmp_ph97_G6
zmp_ph97_G9
zmp_ph97_H2
zmp_ph97_B10
zmp_ph97_C2
zmp_ph97_C4
zmp_ph97_B8
zmp_ph97_C1
zmp_ph97_D2
zmp_ph97_D5
zmp_ph111_G5
zmp_ph111_G11
zmp_ph111_H1
zmp_ph111_B1a
zmp_ph111_B5
zmp_ph111_B8
zmp_ph111_B6
zmp_ph111_B7
zmp_ph111_B9
zmp_ph111_B10
zmp_ph114_G4
zmp_ph114_G11
zmp_ph114_H1
zmp_ph114_H2
zmp_ph114_A9
zmp_ph114_A11
zmp_ph114_B2
zmp_ph114_B4
zmp_ph114_D8
zmp_ph114_E2

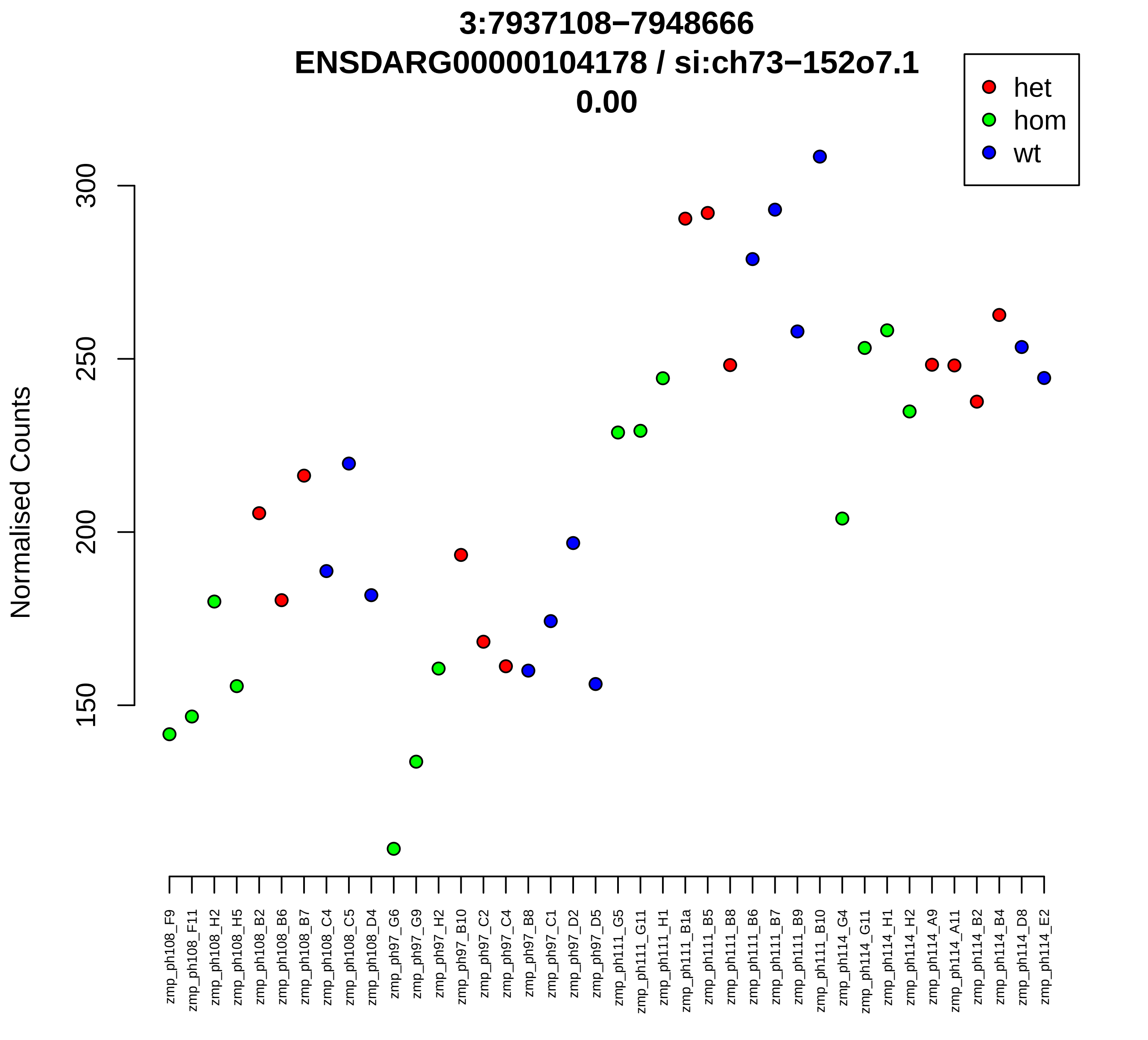

3:7937108−7948666
ENSDARG00000104178 / si:ch73−152o7.1
0.00
het
hom
wt
Normalised Counts
150
200
250
300
zmp_ph108_F9
zmp_ph108_F11
zmp_ph108_H2
zmp_ph108_H5
zmp_ph108_B2
zmp_ph108_B6
zmp_ph108_B7
zmp_ph108_C4
zmp_ph108_C5
zmp_ph108_D4
zmp_ph97_G6
zmp_ph97_G9
zmp_ph97_H2
zmp_ph97_B10
zmp_ph97_C2
zmp_ph97_C4
zmp_ph97_B8
zmp_ph97_C1
zmp_ph97_D2
zmp_ph97_D5
zmp_ph111_G5
zmp_ph111_G11
zmp_ph111_H1
zmp_ph111_B1a
zmp_ph111_B5
zmp_ph111_B8
zmp_ph111_B6
zmp_ph111_B7
zmp_ph111_B9
zmp_ph111_B10
zmp_ph114_G4
zmp_ph114_G11
zmp_ph114_H1
zmp_ph114_H2
zmp_ph114_A9
zmp_ph114_A11
zmp_ph114_B2
zmp_ph114_B4
zmp_ph114_D8
zmp_ph114_E2

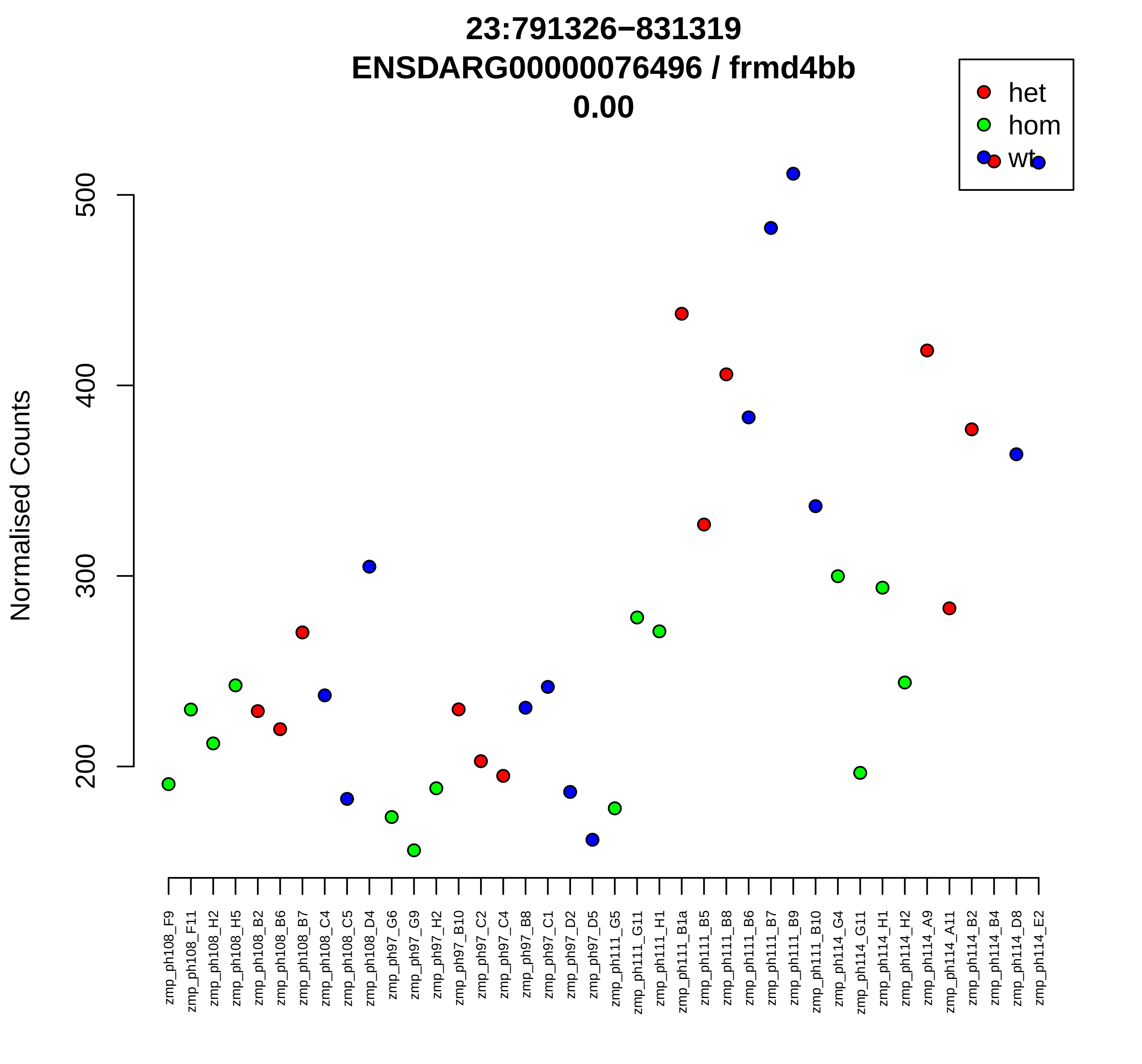
23:791326−831319
ENSDARG00000076496 / frmd4bb
0.00
het
hom
wt
Normalised Counts
200
300
400
500
zmp_ph108_F9
zmp_ph108_F11
zmp_ph108_H2
zmp_ph108_H5
zmp_ph108_B2
zmp_ph108_B6
zmp_ph108_B7
zmp_ph108_C4
zmp_ph108_C5
zmp_ph108_D4
zmp_ph97_G6
zmp_ph97_G9
zmp_ph97_H2
zmp_ph97_B10
zmp_ph97_C2
zmp_ph97_C4
zmp_ph97_B8
zmp_ph97_C1
zmp_ph97_D2
zmp_ph97_D5
zmp_ph111_G5
zmp_ph111_G11
zmp_ph111_H1
zmp_ph111_B1a
zmp_ph111_B5
zmp_ph111_B8
zmp_ph111_B6
zmp_ph111_B7
zmp_ph111_B9
zmp_ph111_B10
zmp_ph114_G4
zmp_ph114_G11
zmp_ph114_H1
zmp_ph114_H2
zmp_ph114_A9
zmp_ph114_A11
zmp_ph114_B2
zmp_ph114_B4
zmp_ph114_D8
zmp_ph114_E2

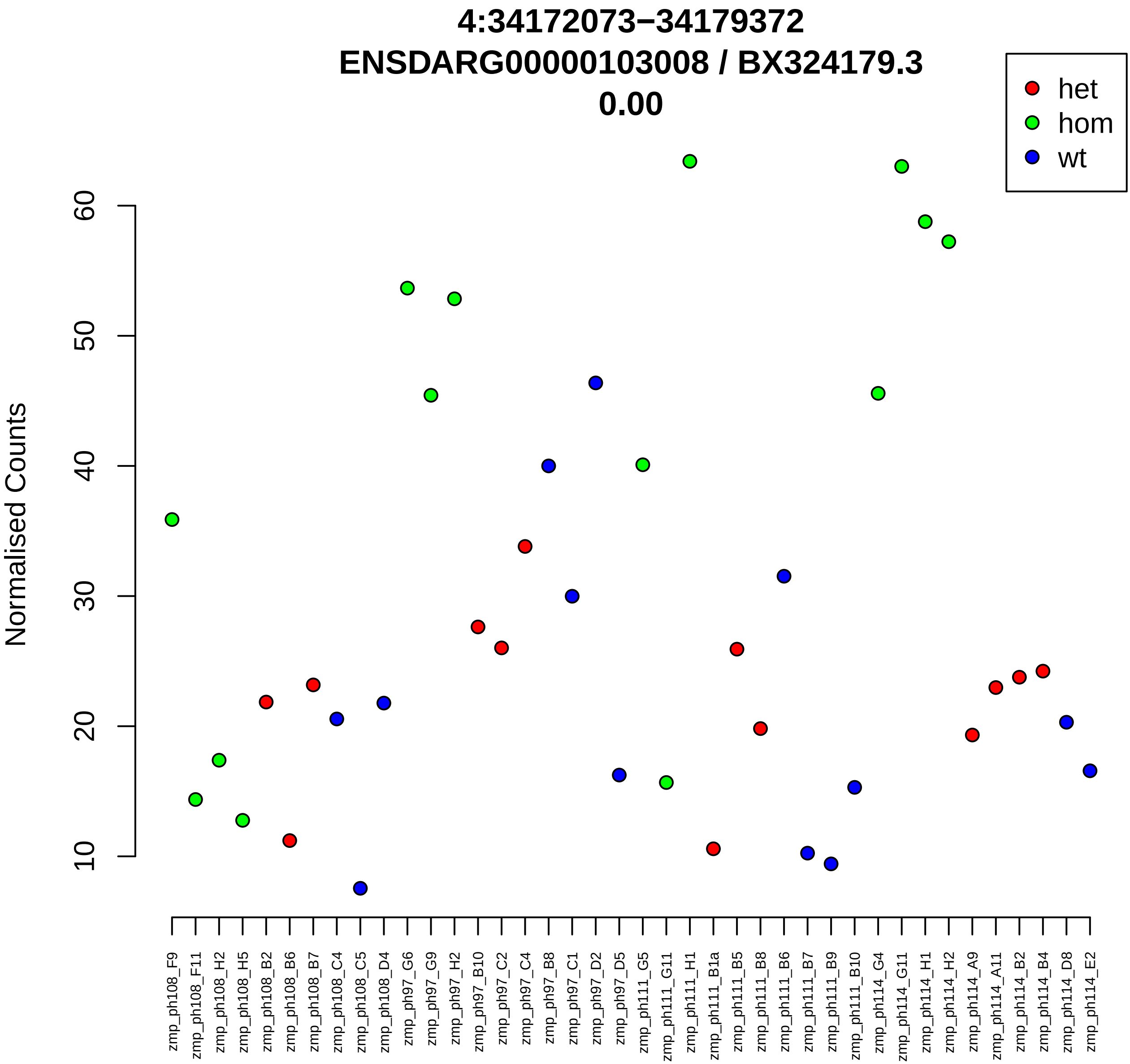
4:34172073−34179372
ENSDARG00000103008 / BX324179.3
0.00
het
hom
wt
Normalised Counts
10
20
30
40
50
60
zmp_ph108_F9
zmp_ph108_F11
zmp_ph108_H2
zmp_ph108_H5
zmp_ph108_B2
zmp_ph108_B6
zmp_ph108_B7
zmp_ph108_C4
zmp_ph108_C5
zmp_ph108_D4
zmp_ph97_G6
zmp_ph97_G9
zmp_ph97_H2
zmp_ph97_B10
zmp_ph97_C2
zmp_ph97_C4
zmp_ph97_B8
zmp_ph97_C1
zmp_ph97_D2
zmp_ph97_D5
zmp_ph111_G5
zmp_ph111_G11
zmp_ph111_H1
zmp_ph111_B1a
zmp_ph111_B5
zmp_ph111_B8
zmp_ph111_B6
zmp_ph111_B7
zmp_ph111_B9
zmp_ph111_B10
zmp_ph114_G4
zmp_ph114_G11
zmp_ph114_H1
zmp_ph114_H2
zmp_ph114_A9
zmp_ph114_A11
zmp_ph114_B2
zmp_ph114_B4
zmp_ph114_D8
zmp_ph114_E2

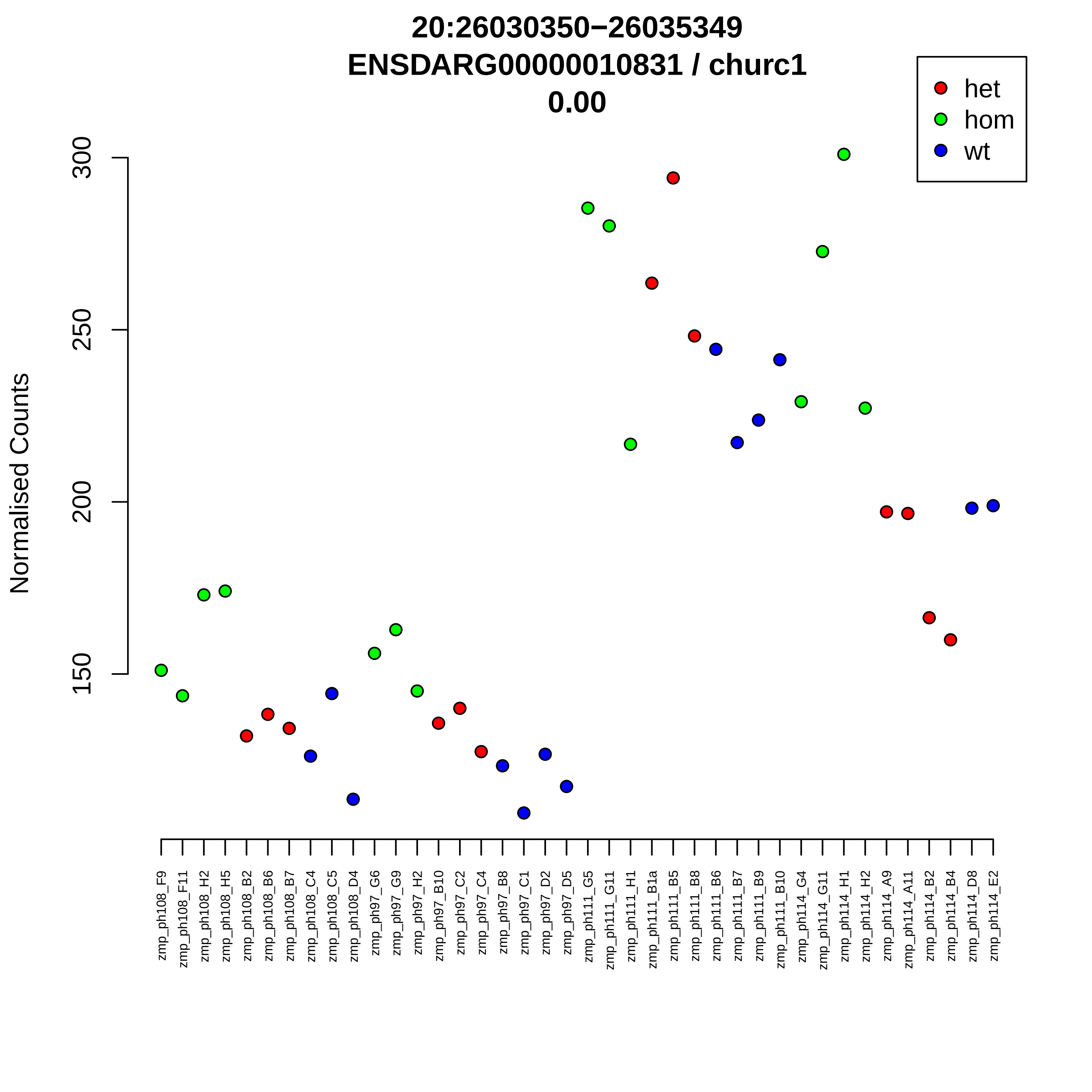

20:26030350−26035349
ENSDARG00000010831 / churc1
0.00
het
hom
wt
Normalised Counts
300
250
200
150
zmp_ph108_F9
zmp_ph108_F11
zmp_ph108_H2
zmp_ph108_H5
zmp_ph108_B2
zmp_ph108_B6
zmp_ph108_B7
zmp_ph108_C4
zmp_ph108_C5
zmp_ph108_D4
zmp_ph97_G6
zmp_ph97_G9
zmp_ph97_H2
zmp_ph97_B10
zmp_ph97_C2
zmp_ph97_C4
zmp_ph97_B8
zmp_ph97_C1
zmp_ph97_D2
zmp_ph97_D5
zmp_ph111_G5
zmp_ph111_G11
zmp_ph111_H1
zmp_ph111_B1a
zmp_ph111_B5
zmp_ph111_B8
zmp_ph111_B6
zmp_ph111_B7
zmp_ph111_B9
zmp_ph111_B10
zmp_ph114_G4
zmp_ph114_G11
zmp_ph114_H1
zmp_ph114_H2
zmp_ph114_A9
zmp_ph114_A11
zmp_ph114_B2
zmp_ph114_B4
zmp_ph114_D8
zmp_ph114_E2

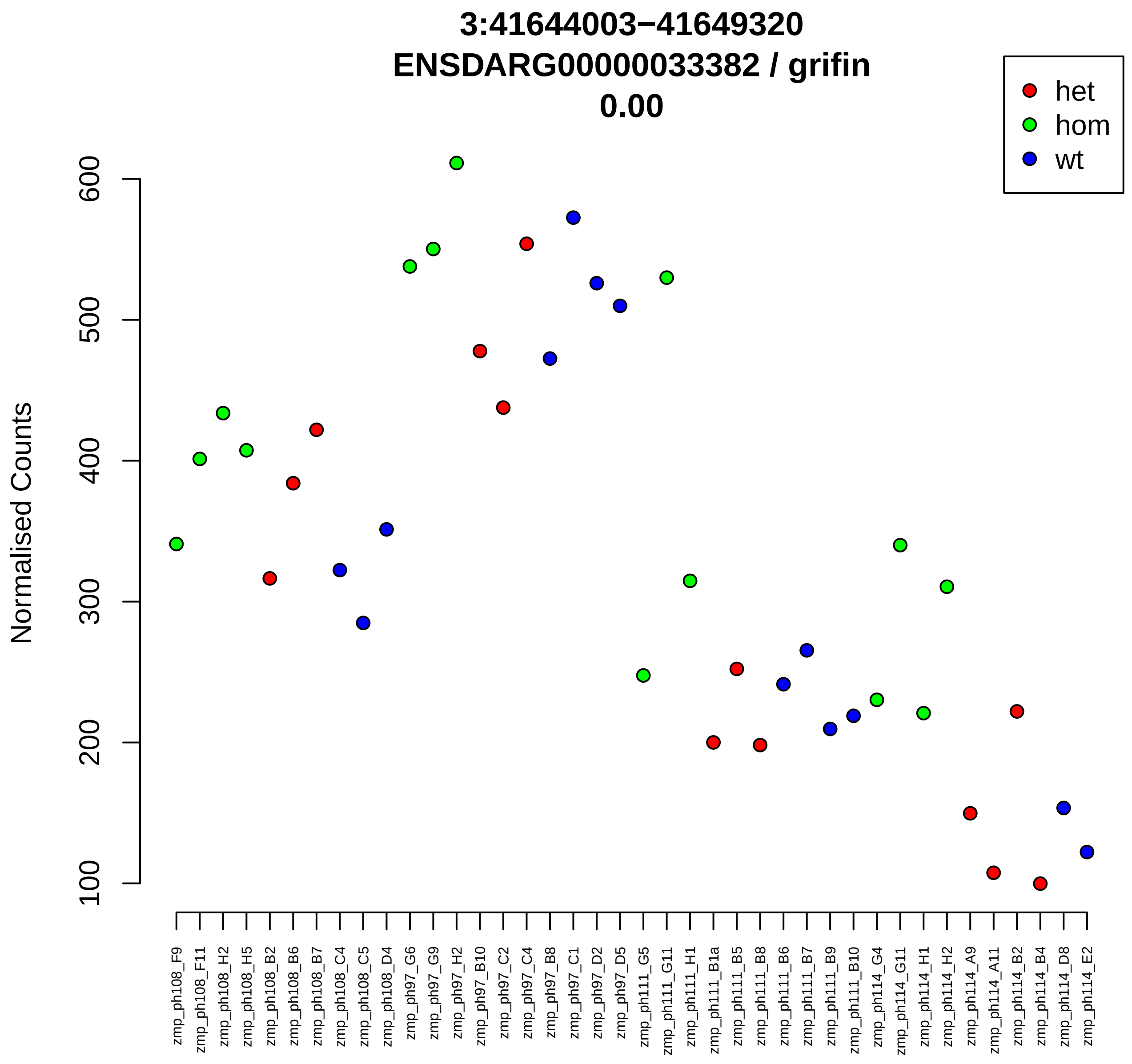

3:41644003−41649320
ENSDARG00000033382 / grifin
0.00
het
hom
wt
Normalised Counts
100
200
300
400
500
600
zmp_ph108_F9
zmp_ph108_F11
zmp_ph108_H2
zmp_ph108_H5
zmp_ph108_B2
zmp_ph108_B6
zmp_ph108_B7
zmp_ph108_C4
zmp_ph108_C5
zmp_ph108_D4
zmp_ph97_G6
zmp_ph97_G9
zmp_ph97_H2
zmp_ph97_B10
zmp_ph97_C2
zmp_ph97_C4
zmp_ph97_B8
zmp_ph97_C1
zmp_ph97_D2
zmp_ph97_D5
zmp_ph111_G5
zmp_ph111_G11
zmp_ph111_H1
zmp_ph111_B1a
zmp_ph111_B5
zmp_ph111_B8
zmp_ph111_B6
zmp_ph111_B7
zmp_ph111_B9
zmp_ph111_B10
zmp_ph114_G4
zmp_ph114_G11
zmp_ph114_H1
zmp_ph114_H2
zmp_ph114_A9
zmp_ph114_A11
zmp_ph114_B2
zmp_ph114_B4
zmp_ph114_D8
zmp_ph114_E2

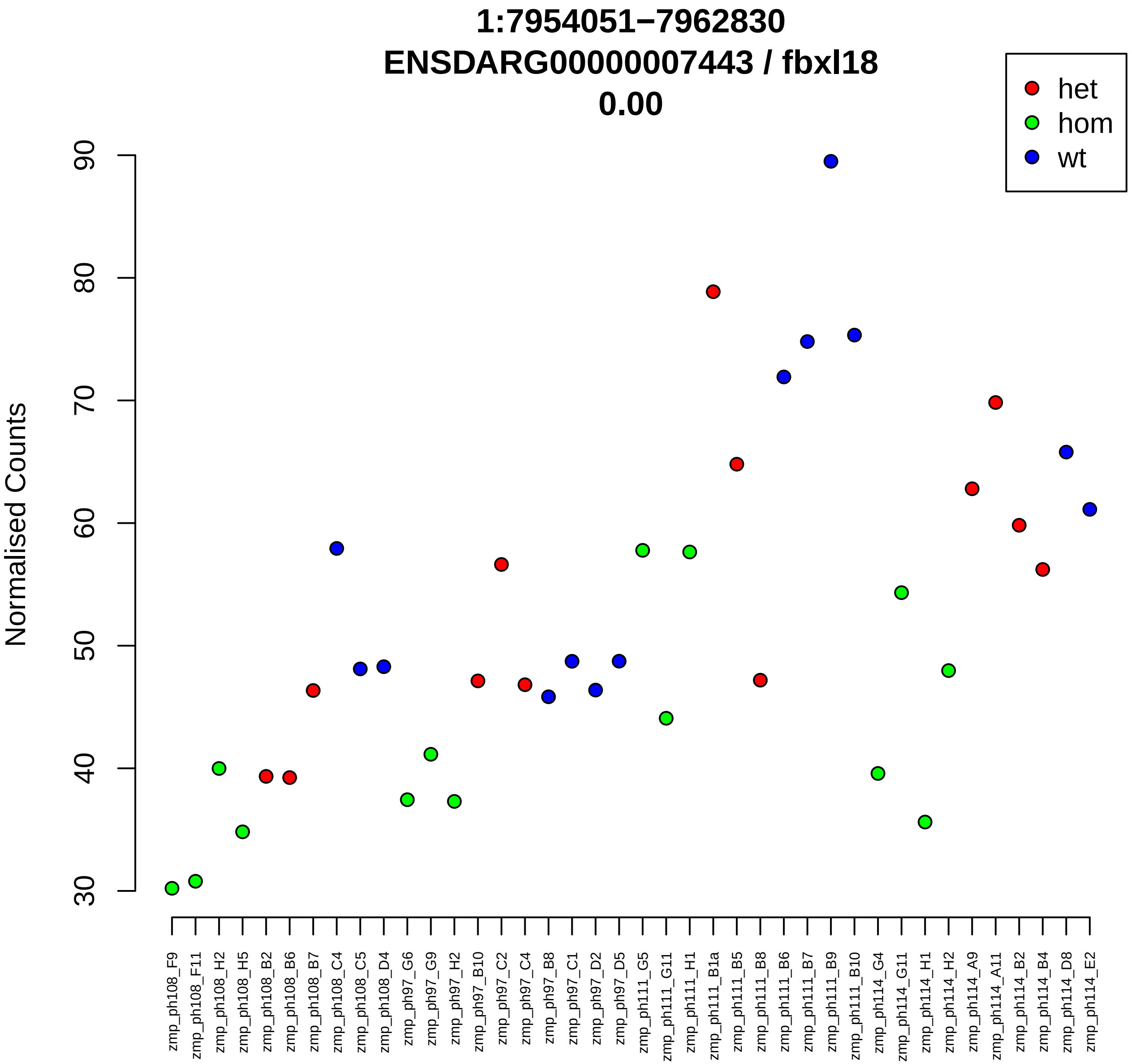

1:7954051−7962830
ENSDARG00000007443 / fbxl18
0.00
het
hom
wt
Normalised Counts
30
40
50
60
70
80
90
zmp_ph108_F9
zmp_ph108_F11
zmp_ph108_H2
zmp_ph108_H5
zmp_ph108_B2
zmp_ph108_B6
zmp_ph108_B7
zmp_ph108_C4
zmp_ph108_C5
zmp_ph108_D4
zmp_ph97_G6
zmp_ph97_G9
zmp_ph97_H2
zmp_ph97_B10
zmp_ph97_C2
zmp_ph97_C4
zmp_ph97_B8
zmp_ph97_C1
zmp_ph97_D2
zmp_ph97_D5
zmp_ph111_G5
zmp_ph111_G11
zmp_ph111_H1
zmp_ph111_B1a
zmp_ph111_B5
zmp_ph111_B8
zmp_ph111_B6
zmp_ph111_B7
zmp_ph111_B9
zmp_ph111_B10
zmp_ph114_G4
zmp_ph114_G11
zmp_ph114_H1
zmp_ph114_H2
zmp_ph114_A9
zmp_ph114_A11
zmp_ph114_B2
zmp_ph114_B4
zmp_ph114_D8
zmp_ph114_E2

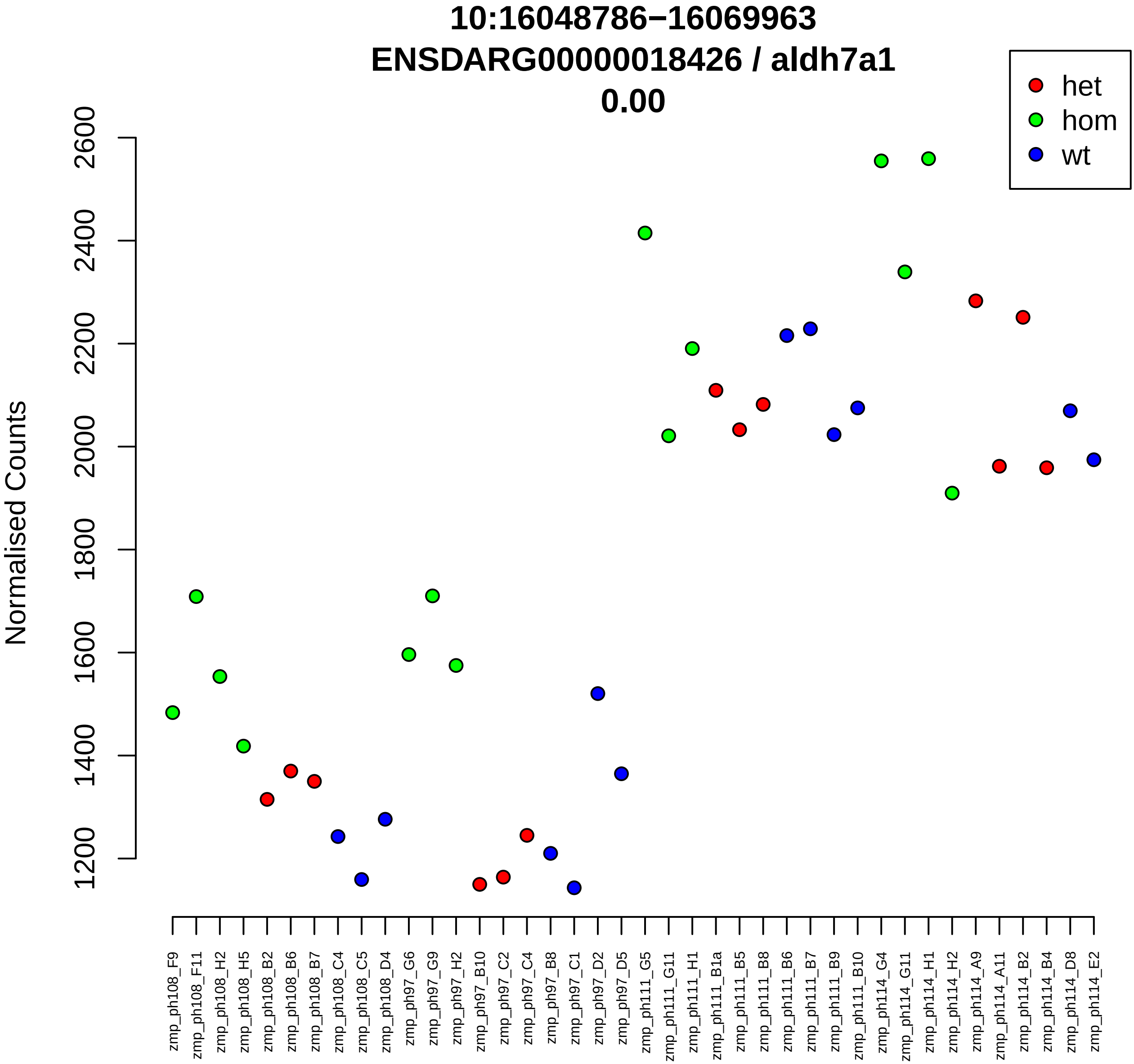

10:16048786−16069963
ENSDARG00000018426 / aldh7a1
0.00
Normalised Counts
het
hom
wt
1200
1400
1600
1800
2000
2200
2400
2600
zmp_ph108_F9
zmp_ph108_F11
zmp_ph108_H2
zmp_ph108_H5
zmp_ph108_B2
zmp_ph108_B6
zmp_ph108_B7
zmp_ph108_C4
zmp_ph108_C5
zmp_ph108_D4
zmp_ph97_G6
zmp_ph97_G9
zmp_ph97_H2
zmp_ph97_B10
zmp_ph97_C2
zmp_ph97_C4
zmp_ph97_B8
zmp_ph97_C1
zmp_ph97_D2
zmp_ph97_D5
zmp_ph111_G5
zmp_ph111_G11
zmp_ph111_H1
zmp_ph111_B1a
zmp_ph111_B5
zmp_ph111_B8
zmp_ph111_B6
zmp_ph111_B7
zmp_ph111_B9
zmp_ph111_B10
zmp_ph114_G4
zmp_ph114_G11
zmp_ph114_H1
zmp_ph114_H2
zmp_ph114_A9
zmp_ph114_A11
zmp_ph114_B2
zmp_ph114_B4
zmp_ph114_D8
zmp_ph114_E2

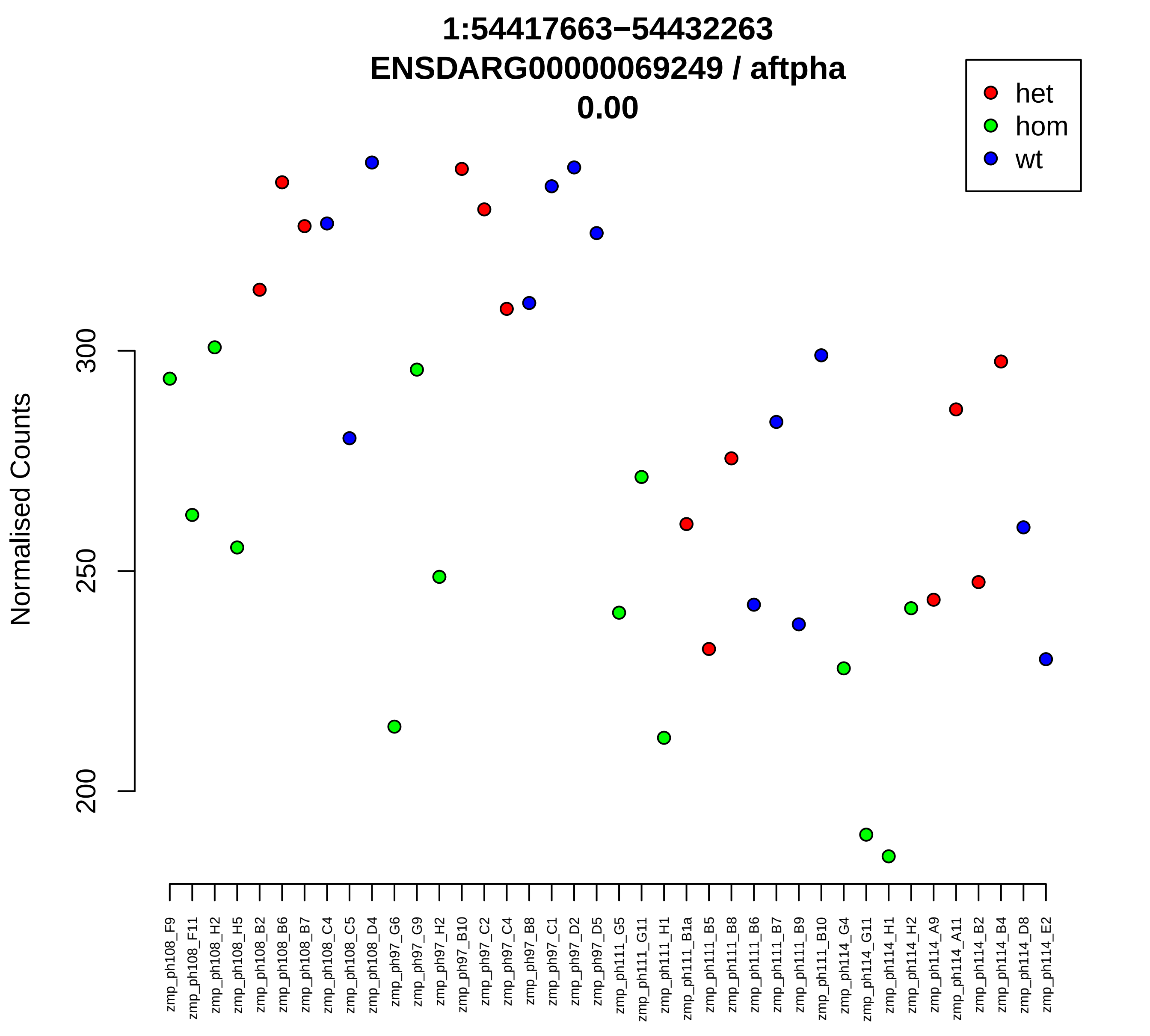

1:54417663−54432263
ENSDARG00000069249 / aftpha
0.00
het
hom
wt
Normalised Counts
300
250
200
zmp_ph108_F9
zmp_ph108_F11
zmp_ph108_H2
zmp_ph108_H5
zmp_ph108_B2
zmp_ph108_B6
zmp_ph108_B7
zmp_ph108_C4
zmp_ph108_C5
zmp_ph108_D4
zmp_ph97_G6
zmp_ph97_G9
zmp_ph97_H2
zmp_ph97_B10
zmp_ph97_C2
zmp_ph97_C4
zmp_ph97_B8
zmp_ph97_C1
zmp_ph97_D2
zmp_ph97_D5
zmp_ph111_G5
zmp_ph111_G11
zmp_ph111_H1
zmp_ph111_B1a
zmp_ph111_B5
zmp_ph111_B8
zmp_ph111_B6
zmp_ph111_B7
zmp_ph111_B9
zmp_ph111_B10
zmp_ph114_G4
zmp_ph114_G11
zmp_ph114_H1
zmp_ph114_H2
zmp_ph114_A9
zmp_ph114_A11
zmp_ph114_B2
zmp_ph114_B4
zmp_ph114_D8
zmp_ph114_E2

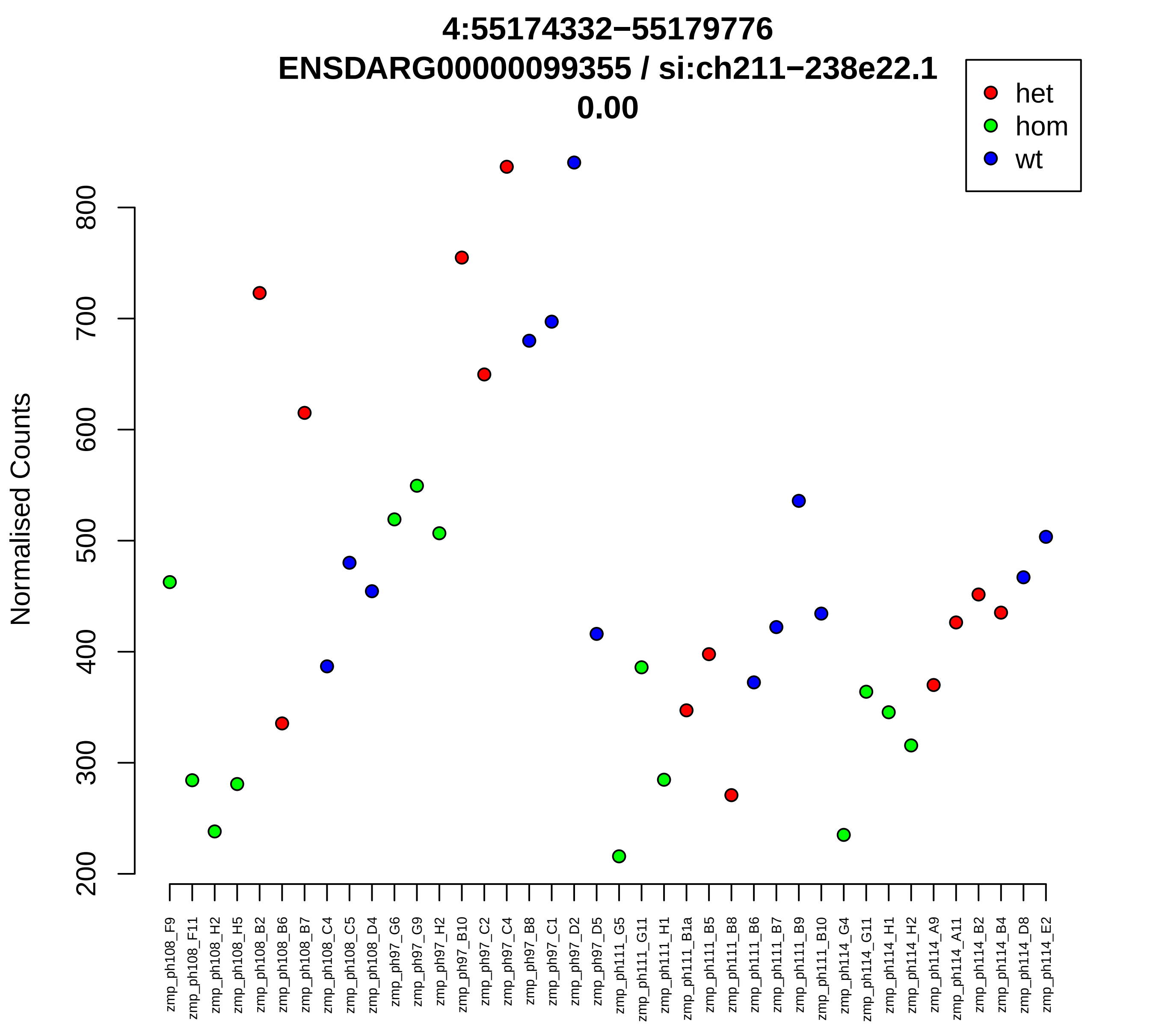
4:55174332−55179776
ENSDARG00000099355 / si:ch211−238e22.1
0.00
het
hom
wt
Normalised Counts
200
300
400
500
600
700
800
zmp_ph108_F9
zmp_ph108_F11
zmp_ph108_H2
zmp_ph108_H5
zmp_ph108_B2
zmp_ph108_B6
zmp_ph108_B7
zmp_ph108_C4
zmp_ph108_C5
zmp_ph108_D4
zmp_ph97_G6
zmp_ph97_G9
zmp_ph97_H2
zmp_ph97_B10
zmp_ph97_C2
zmp_ph97_C4
zmp_ph97_B8
zmp_ph97_C1
zmp_ph97_D2
zmp_ph97_D5
zmp_ph111_G5
zmp_ph111_G11
zmp_ph111_H1
zmp_ph111_B1a
zmp_ph111_B5
zmp_ph111_B8
zmp_ph111_B6
zmp_ph111_B7
zmp_ph111_B9
zmp_ph111_B10
zmp_ph114_G4
zmp_ph114_G11
zmp_ph114_H1
zmp_ph114_H2
zmp_ph114_A9
zmp_ph114_A11
zmp_ph114_B2
zmp_ph114_B4
zmp_ph114_D8
zmp_ph114_E2

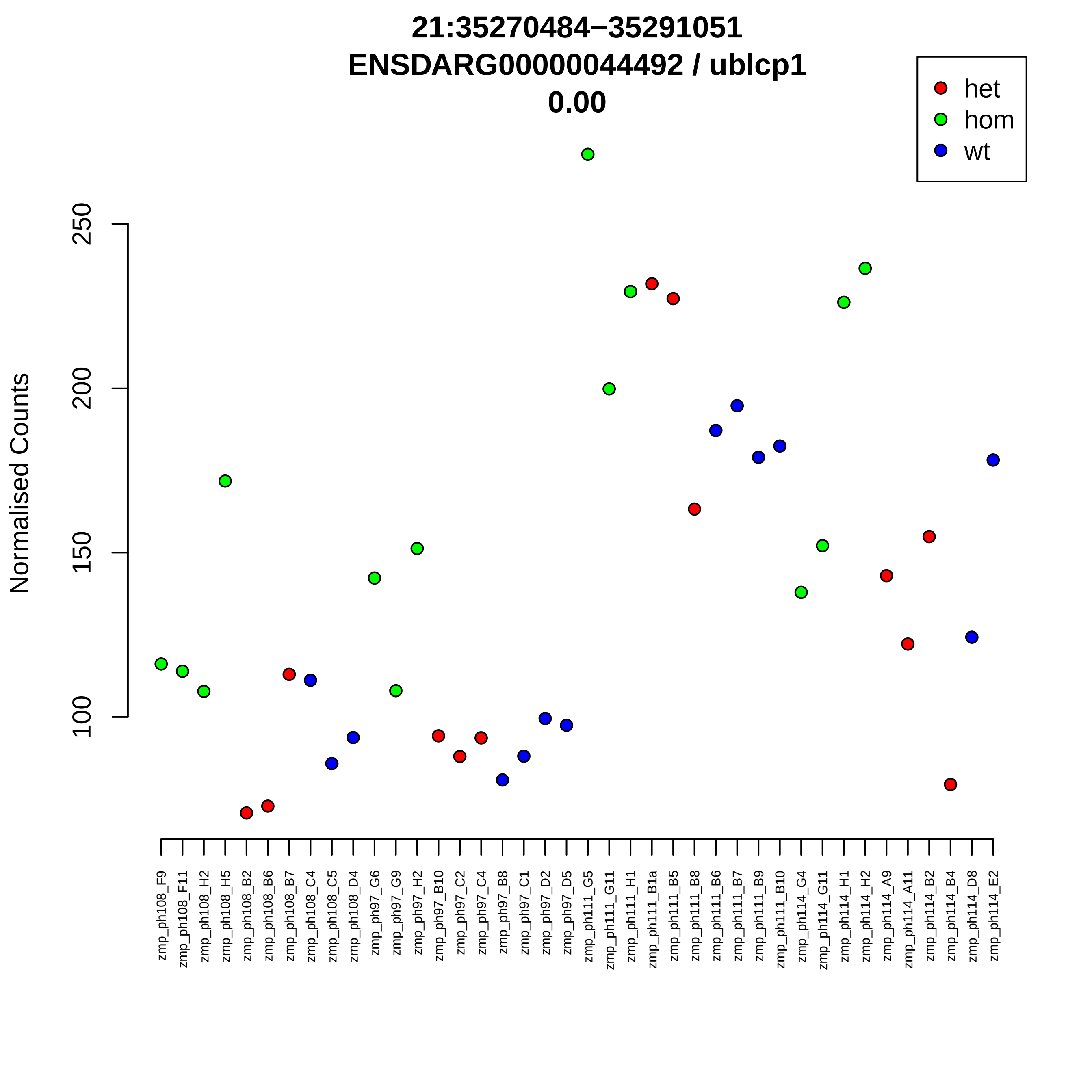
21:35270484−35291051
ENSDARG00000044492 / ublcp1
0.00
het
hom
wt
Normalised Counts
100
150
200
250
zmp_ph108_F9
zmp_ph108_F11
zmp_ph108_H2
zmp_ph108_H5
zmp_ph108_B2
zmp_ph108_B6
zmp_ph108_B7
zmp_ph108_C4
zmp_ph108_C5
zmp_ph108_D4
zmp_ph97_G6
zmp_ph97_G9
zmp_ph97_H2
zmp_ph97_B10
zmp_ph97_C2
zmp_ph97_C4
zmp_ph97_B8
zmp_ph97_C1
zmp_ph97_D2
zmp_ph97_D5
zmp_ph111_G5
zmp_ph111_G11
zmp_ph111_H1
zmp_ph111_B1a
zmp_ph111_B5
zmp_ph111_B8
zmp_ph111_B6
zmp_ph111_B7
zmp_ph111_B9
zmp_ph111_B10
zmp_ph114_G4
zmp_ph114_G11
zmp_ph114_H1
zmp_ph114_H2
zmp_ph114_A9
zmp_ph114_A11
zmp_ph114_B2
zmp_ph114_B4
zmp_ph114_D8
zmp_ph114_E2

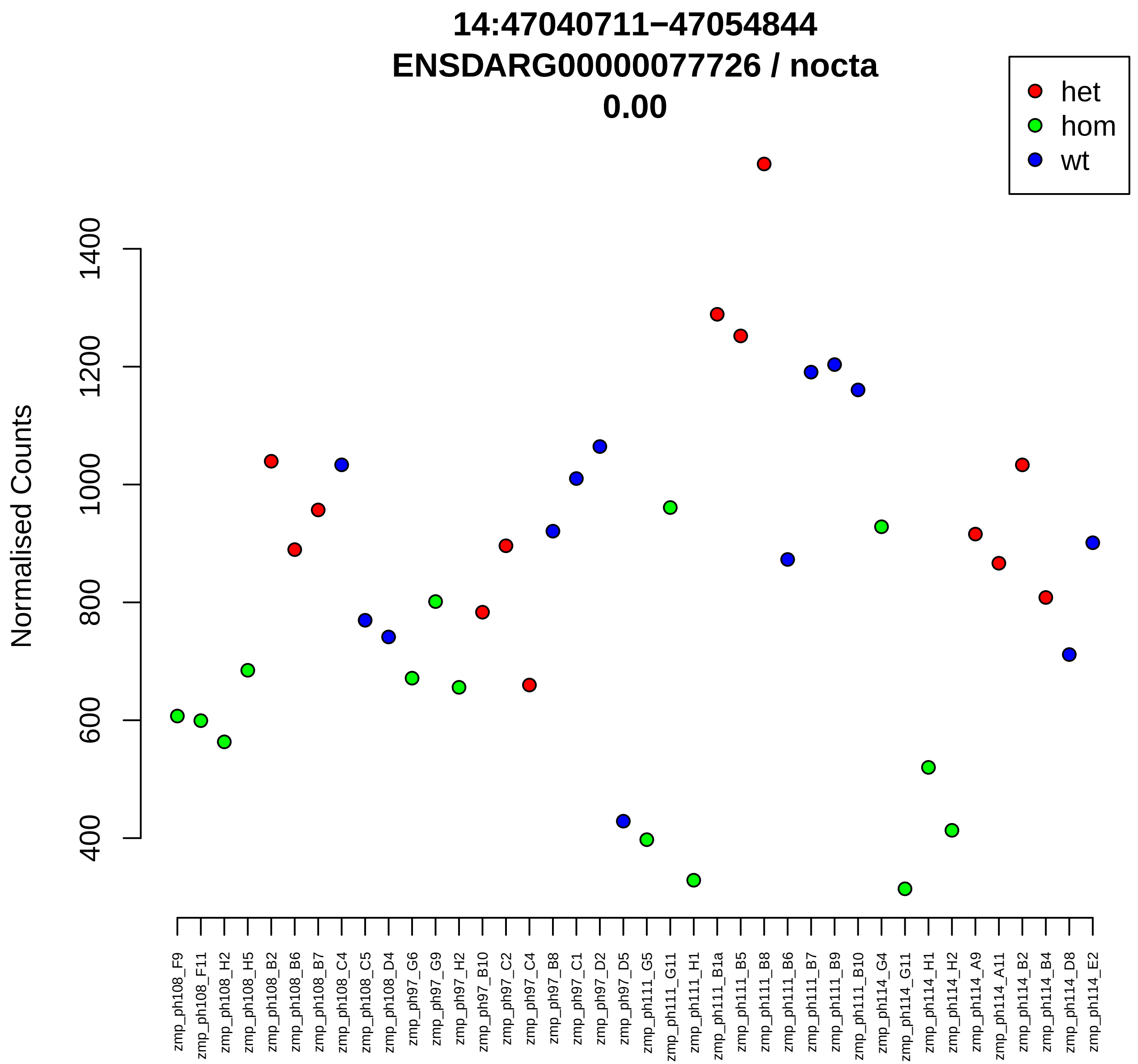

14:47040711−47054844
ENSDARG00000077726 / nocta
0.00
het
hom
wt
Normalised Counts
400
600
800
1000
1200
1400
zmp_ph108_F9
zmp_ph108_F11
zmp_ph108_H2
zmp_ph108_H5
zmp_ph108_B2
zmp_ph108_B6
zmp_ph108_B7
zmp_ph108_C4
zmp_ph108_C5
zmp_ph108_D4
zmp_ph97_G6
zmp_ph97_G9
zmp_ph97_H2
zmp_ph97_B10
zmp_ph97_C2
zmp_ph97_C4
zmp_ph97_B8
zmp_ph97_C1
zmp_ph97_D2
zmp_ph97_D5
zmp_ph111_G5
zmp_ph111_G11
zmp_ph111_H1
zmp_ph111_B1a
zmp_ph111_B5
zmp_ph111_B8
zmp_ph111_B6
zmp_ph111_B7
zmp_ph111_B9
zmp_ph111_B10
zmp_ph114_G4
zmp_ph114_G11
zmp_ph114_H1
zmp_ph114_H2
zmp_ph114_A9
zmp_ph114_A11
zmp_ph114_B2
zmp_ph114_B4
zmp_ph114_D8
zmp_ph114_E2

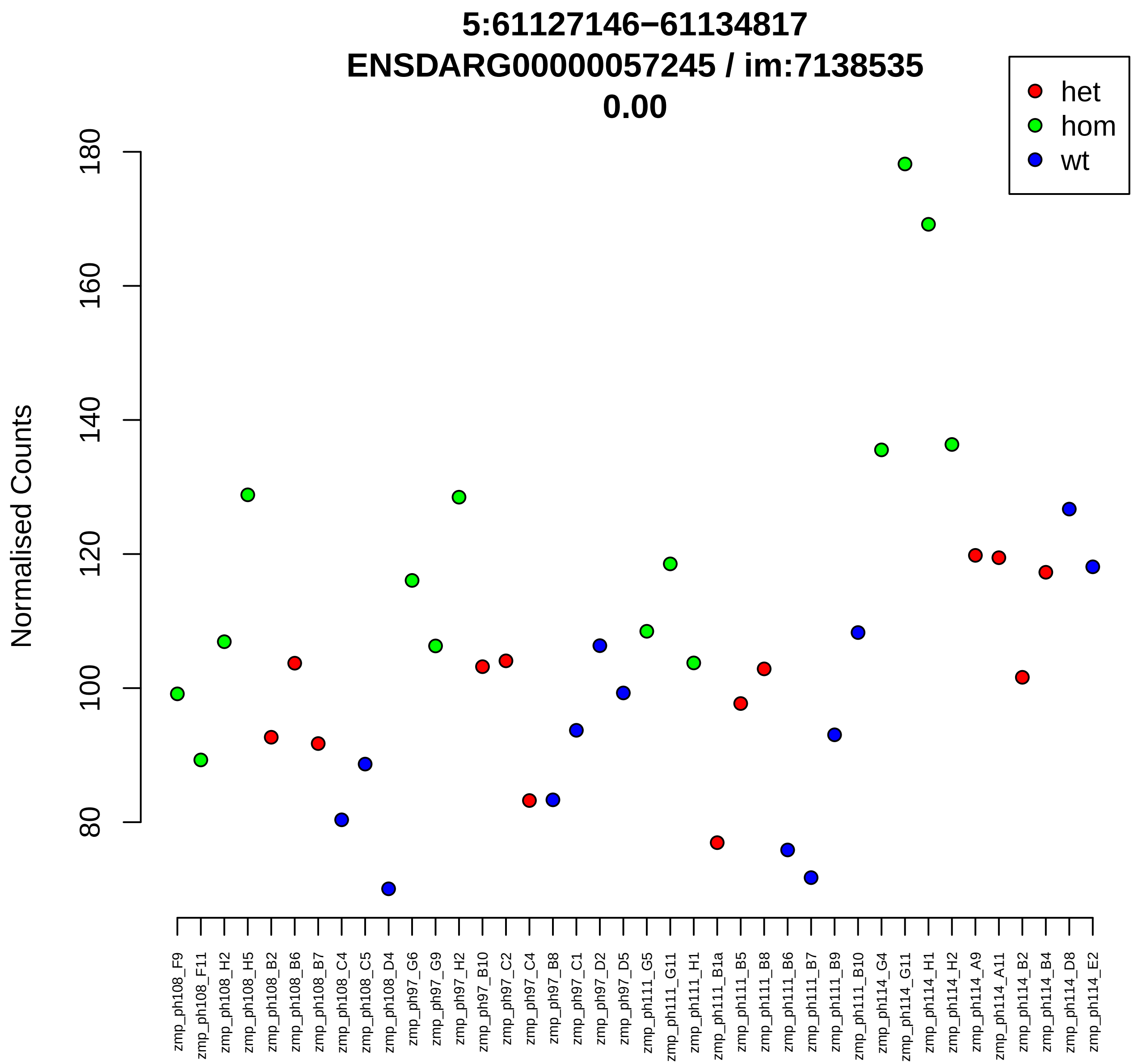
5:61127146−61134817
ENSDARG00000057245 / im:7138535
0.00
het
hom
wt
Normalised Counts
80
100
120
140
160
180
zmp_ph108_F9
zmp_ph108_F11
zmp_ph108_H2
zmp_ph108_H5
zmp_ph108_B2
zmp_ph108_B6
zmp_ph108_B7
zmp_ph108_C4
zmp_ph108_C5
zmp_ph108_D4
zmp_ph97_G6
zmp_ph97_G9
zmp_ph97_H2
zmp_ph97_B10
zmp_ph97_C2
zmp_ph97_C4
zmp_ph97_B8
zmp_ph97_C1
zmp_ph97_D2
zmp_ph97_D5
zmp_ph111_G5
zmp_ph111_G11
zmp_ph111_H1
zmp_ph111_B1a
zmp_ph111_B5
zmp_ph111_B8
zmp_ph111_B6
zmp_ph111_B7
zmp_ph111_B9
zmp_ph111_B10
zmp_ph114_G4
zmp_ph114_G11
zmp_ph114_H1
zmp_ph114_H2
zmp_ph114_A9
zmp_ph114_A11
zmp_ph114_B2
zmp_ph114_B4
zmp_ph114_D8
zmp_ph114_E2

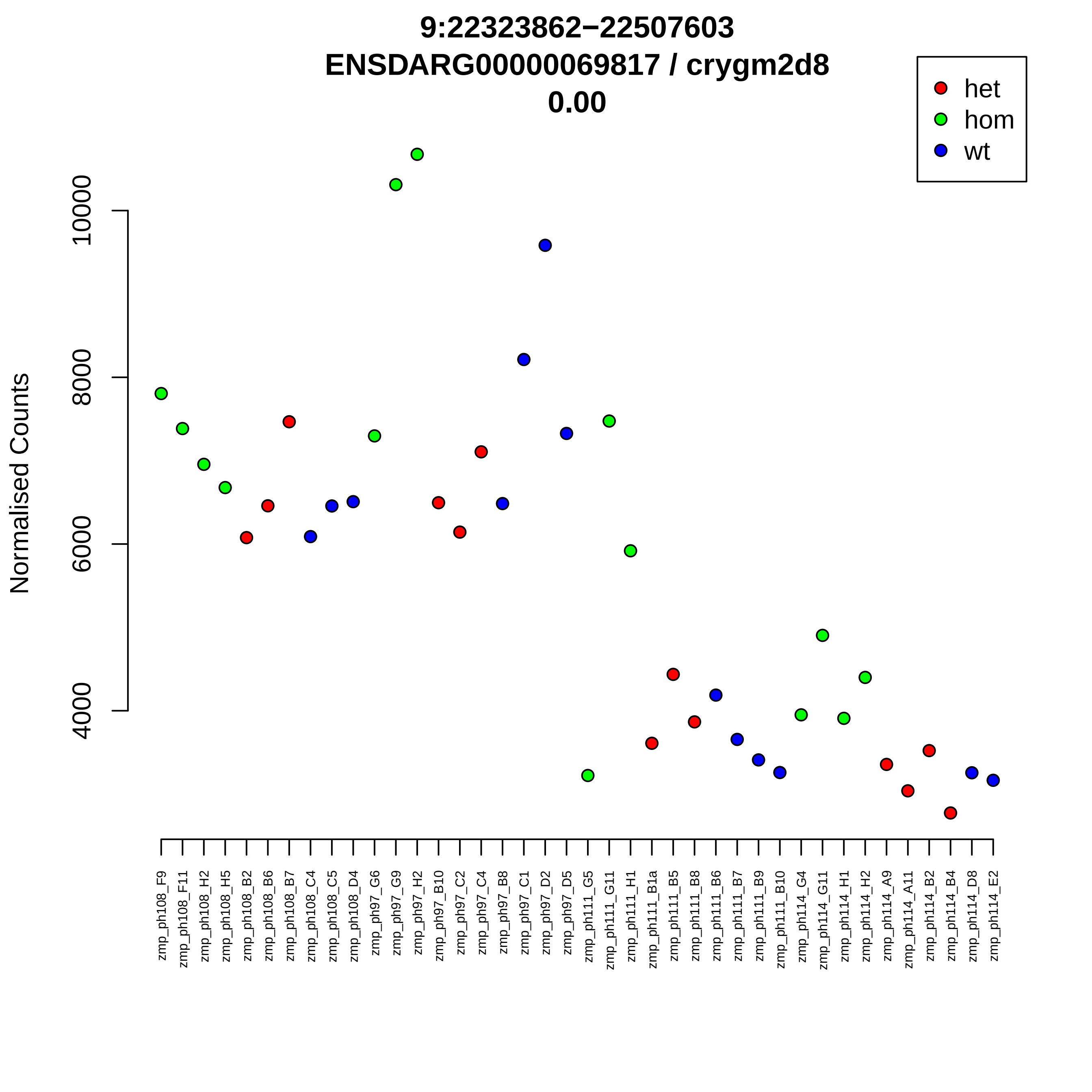

9:22323862−22507603
ENSDARG00000069817 / crygm2d8
0.00
het
hom
wt
Normalised Counts
4000
6000
8000
10000
zmp_ph108_F9
zmp_ph108_F11
zmp_ph108_H2
zmp_ph108_H5
zmp_ph108_B2
zmp_ph108_B6
zmp_ph108_B7
zmp_ph108_C4
zmp_ph108_C5
zmp_ph108_D4
zmp_ph97_G6
zmp_ph97_G9
zmp_ph97_H2
zmp_ph97_B10
zmp_ph97_C2
zmp_ph97_C4
zmp_ph97_B8
zmp_ph97_C1
zmp_ph97_D2
zmp_ph97_D5
zmp_ph111_G5
zmp_ph111_G11
zmp_ph111_H1
zmp_ph111_B1a
zmp_ph111_B5
zmp_ph111_B8
zmp_ph111_B6
zmp_ph111_B7
zmp_ph111_B9
zmp_ph111_B10
zmp_ph114_G4
zmp_ph114_G11
zmp_ph114_H1
zmp_ph114_H2
zmp_ph114_A9
zmp_ph114_A11
zmp_ph114_B2
zmp_ph114_B4
zmp_ph114_D8
zmp_ph114_E2

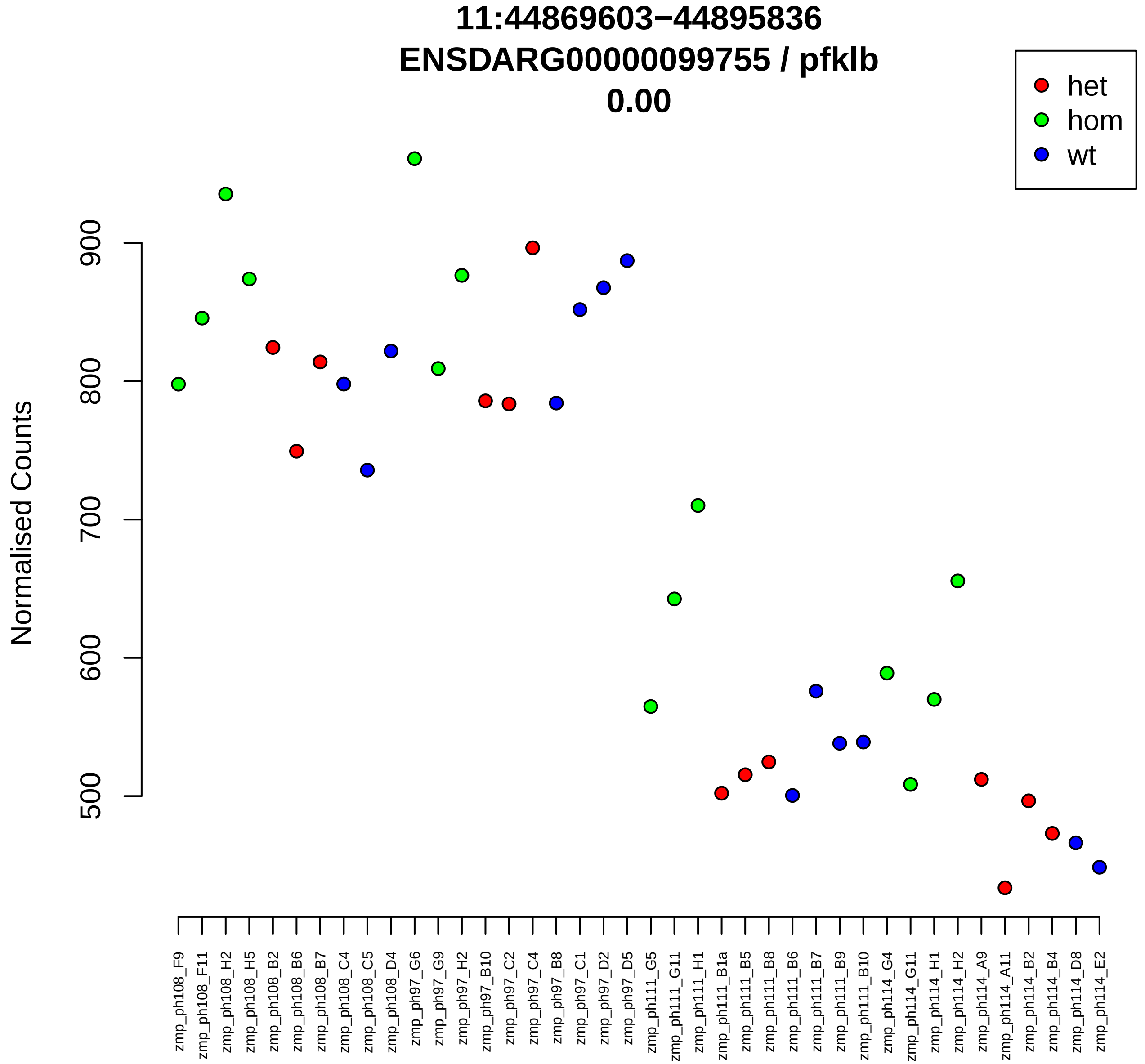

11:44869603−44895836
ENSDARG00000099755 / pfklb
0.00
het
hom
wt
Normalised Counts
900
800
700
600
500
zmp_ph108_F9
zmp_ph108_F11
zmp_ph108_H2
zmp_ph108_H5
zmp_ph108_B2
zmp_ph108_B6
zmp_ph108_B7
zmp_ph108_C4
zmp_ph108_C5
zmp_ph108_D4
zmp_ph97_G6
zmp_ph97_G9
zmp_ph97_H2
zmp_ph97_B10
zmp_ph97_C2
zmp_ph97_C4
zmp_ph97_B8
zmp_ph97_C1
zmp_ph97_D2
zmp_ph97_D5
zmp_ph111_G5
zmp_ph111_G11
zmp_ph111_H1
zmp_ph111_B1a
zmp_ph111_B5
zmp_ph111_B8
zmp_ph111_B6
zmp_ph111_B7
zmp_ph111_B9
zmp_ph111_B10
zmp_ph114_G4
zmp_ph114_G11
zmp_ph114_H1
zmp_ph114_H2
zmp_ph114_A9
zmp_ph114_A11
zmp_ph114_B2
zmp_ph114_B4
zmp_ph114_D8
zmp_ph114_E2

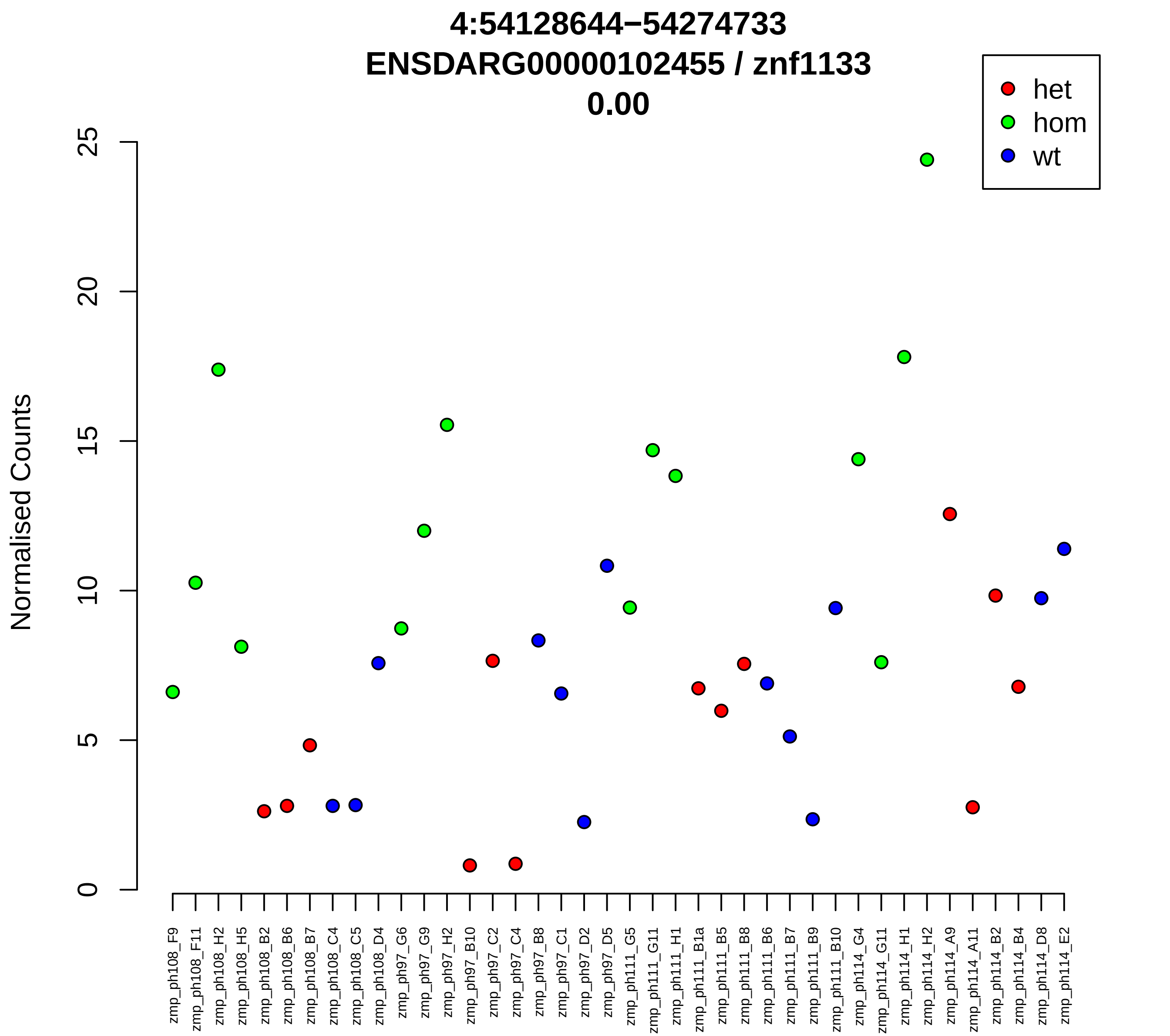

4:54128644−54274733
ENSDARG00000102455 / znf1133
0.00
het
hom
wt
Normalised Counts
0
5
10
15
20
25
zmp_ph108_F9
zmp_ph108_F11
zmp_ph108_H2
zmp_ph108_H5
zmp_ph108_B2
zmp_ph108_B6
zmp_ph108_B7
zmp_ph108_C4
zmp_ph108_C5
zmp_ph108_D4
zmp_ph97_G6
zmp_ph97_G9
zmp_ph97_H2
zmp_ph97_B10
zmp_ph97_C2
zmp_ph97_C4
zmp_ph97_B8
zmp_ph97_C1
zmp_ph97_D2
zmp_ph97_D5
zmp_ph111_G5
zmp_ph111_G11
zmp_ph111_H1
zmp_ph111_B1a
zmp_ph111_B5
zmp_ph111_B8
zmp_ph111_B6
zmp_ph111_B7
zmp_ph111_B9
zmp_ph111_B10
zmp_ph114_G4
zmp_ph114_G11
zmp_ph114_H1
zmp_ph114_H2
zmp_ph114_A9
zmp_ph114_A11
zmp_ph114_B2
zmp_ph114_B4
zmp_ph114_D8
zmp_ph114_E2

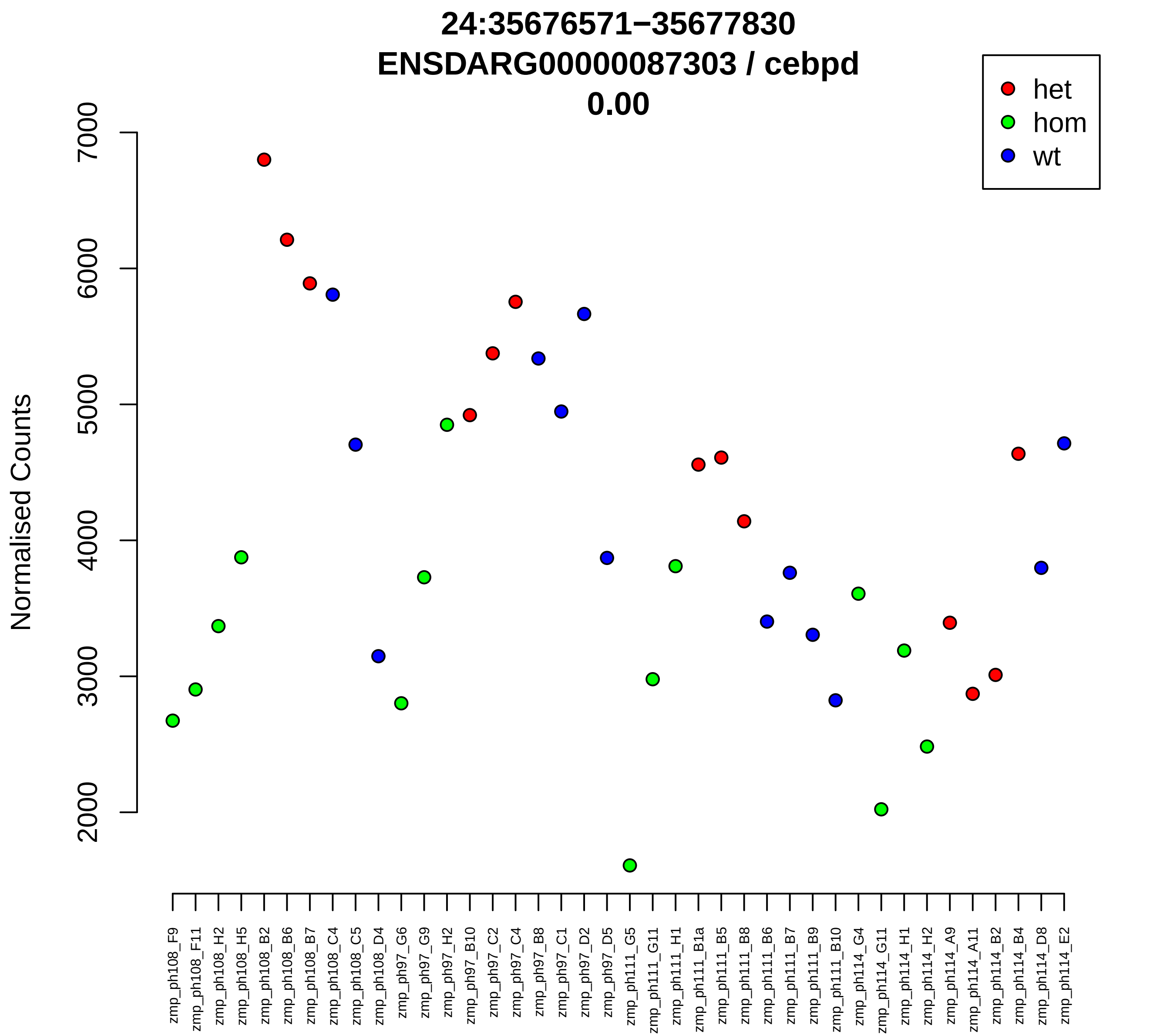

24:35676571−35677830
ENSDARG00000087303 / cebpd
0.00
het
hom
wt
Normalised Counts
2000
3000
4000
5000
6000
7000
zmp_ph108_F9
zmp_ph108_F11
zmp_ph108_H2
zmp_ph108_H5
zmp_ph108_B2
zmp_ph108_B6
zmp_ph108_B7
zmp_ph108_C4
zmp_ph108_C5
zmp_ph108_D4
zmp_ph97_G6
zmp_ph97_G9
zmp_ph97_H2
zmp_ph97_B10
zmp_ph97_C2
zmp_ph97_C4
zmp_ph97_B8
zmp_ph97_C1
zmp_ph97_D2
zmp_ph97_D5
zmp_ph111_G5
zmp_ph111_G11
zmp_ph111_H1
zmp_ph111_B1a
zmp_ph111_B5
zmp_ph111_B8
zmp_ph111_B6
zmp_ph111_B7
zmp_ph111_B9
zmp_ph111_B10
zmp_ph114_G4
zmp_ph114_G11
zmp_ph114_H1
zmp_ph114_H2
zmp_ph114_A9
zmp_ph114_A11
zmp_ph114_B2
zmp_ph114_B4
zmp_ph114_D8
zmp_ph114_E2

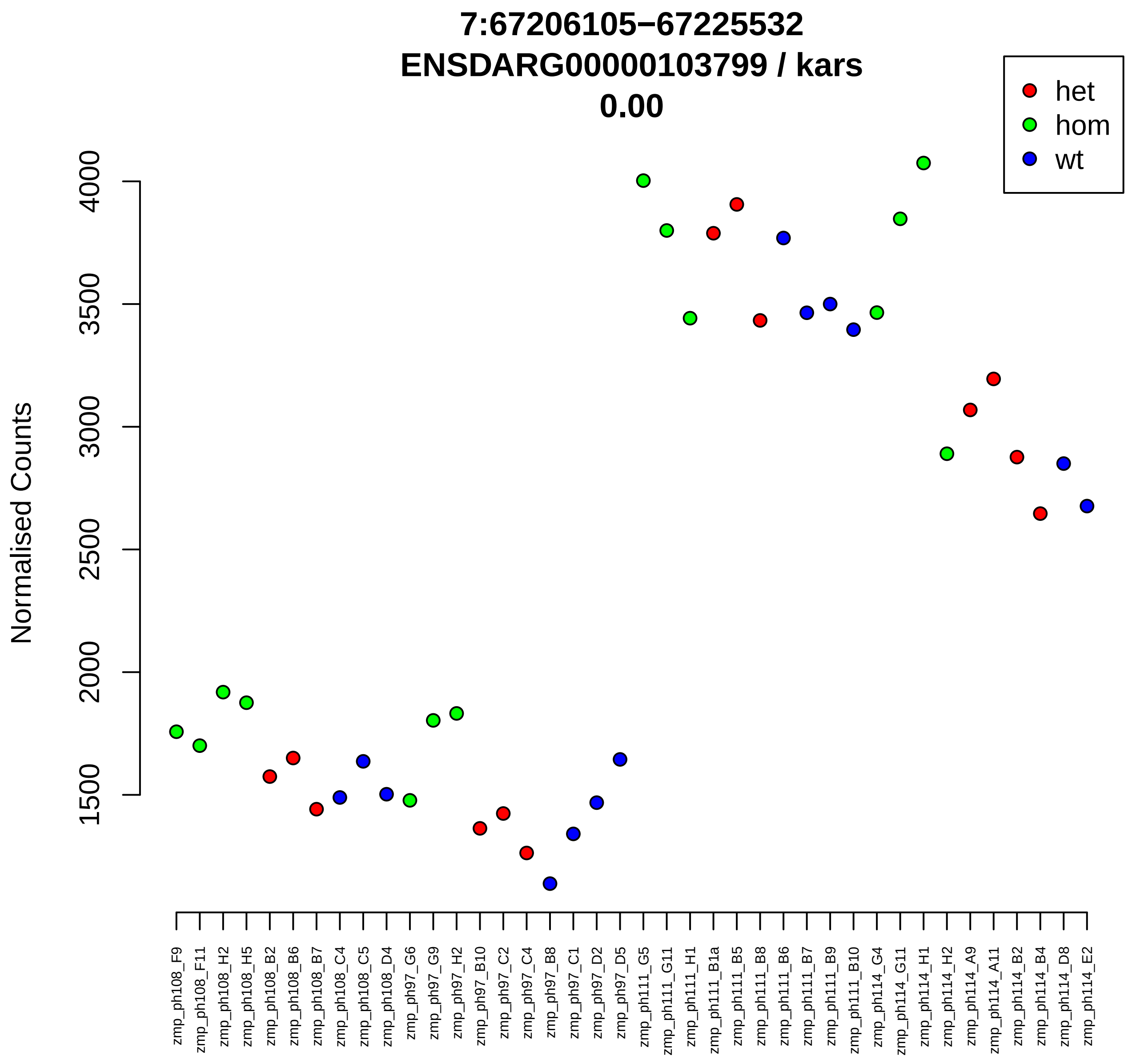
7:67206105−67225532
ENSDARG00000103799 / kars
0.00
het
hom
wt
Normalised Counts
4000
3500
3000
2500
2000
1500
zmp_ph108_F9
zmp_ph108_F11
zmp_ph108_H2
zmp_ph108_H5
zmp_ph108_B2
zmp_ph108_B6
zmp_ph108_B7
zmp_ph108_C4
zmp_ph108_C5
zmp_ph108_D4
zmp_ph97_G6
zmp_ph97_G9
zmp_ph97_H2
zmp_ph97_B10
zmp_ph97_C2
zmp_ph97_C4
zmp_ph97_B8
zmp_ph97_C1
zmp_ph97_D2
zmp_ph97_D5
zmp_ph111_G5
zmp_ph111_G11
zmp_ph111_H1
zmp_ph111_B1a
zmp_ph111_B5
zmp_ph111_B8
zmp_ph111_B6
zmp_ph111_B7
zmp_ph111_B9
zmp_ph111_B10
zmp_ph114_G4
zmp_ph114_G11
zmp_ph114_H1
zmp_ph114_H2
zmp_ph114_A9
zmp_ph114_A11
zmp_ph114_B2
zmp_ph114_B4
zmp_ph114_D8
zmp_ph114_E2

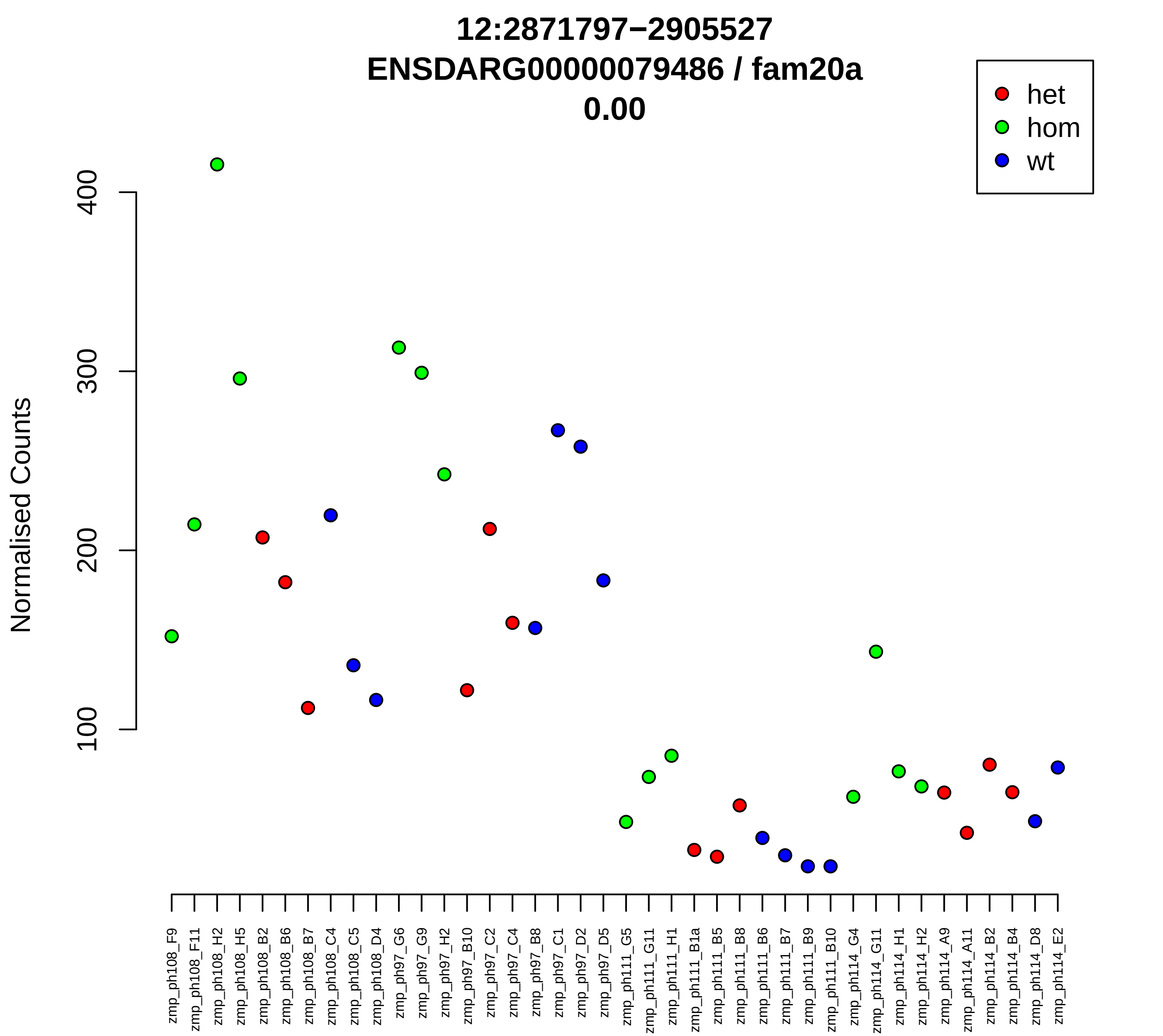

12:2871797−2905527
ENSDARG00000079486 / fam20a
0.00
het
hom
wt
Normalised Counts
100
200
300
400
zmp_ph108_F9
zmp_ph108_F11
zmp_ph108_H2
zmp_ph108_H5
zmp_ph108_B2
zmp_ph108_B6
zmp_ph108_B7
zmp_ph108_C4
zmp_ph108_C5
zmp_ph108_D4
zmp_ph97_G6
zmp_ph97_G9
zmp_ph97_H2
zmp_ph97_B10
zmp_ph97_C2
zmp_ph97_C4
zmp_ph97_B8
zmp_ph97_C1
zmp_ph97_D2
zmp_ph97_D5
zmp_ph111_G5
zmp_ph111_G11
zmp_ph111_H1
zmp_ph111_B1a
zmp_ph111_B5
zmp_ph111_B8
zmp_ph111_B6
zmp_ph111_B7
zmp_ph111_B9
zmp_ph111_B10
zmp_ph114_G4
zmp_ph114_G11
zmp_ph114_H1
zmp_ph114_H2
zmp_ph114_A9
zmp_ph114_A11
zmp_ph114_B2
zmp_ph114_B4
zmp_ph114_D8
zmp_ph114_E2

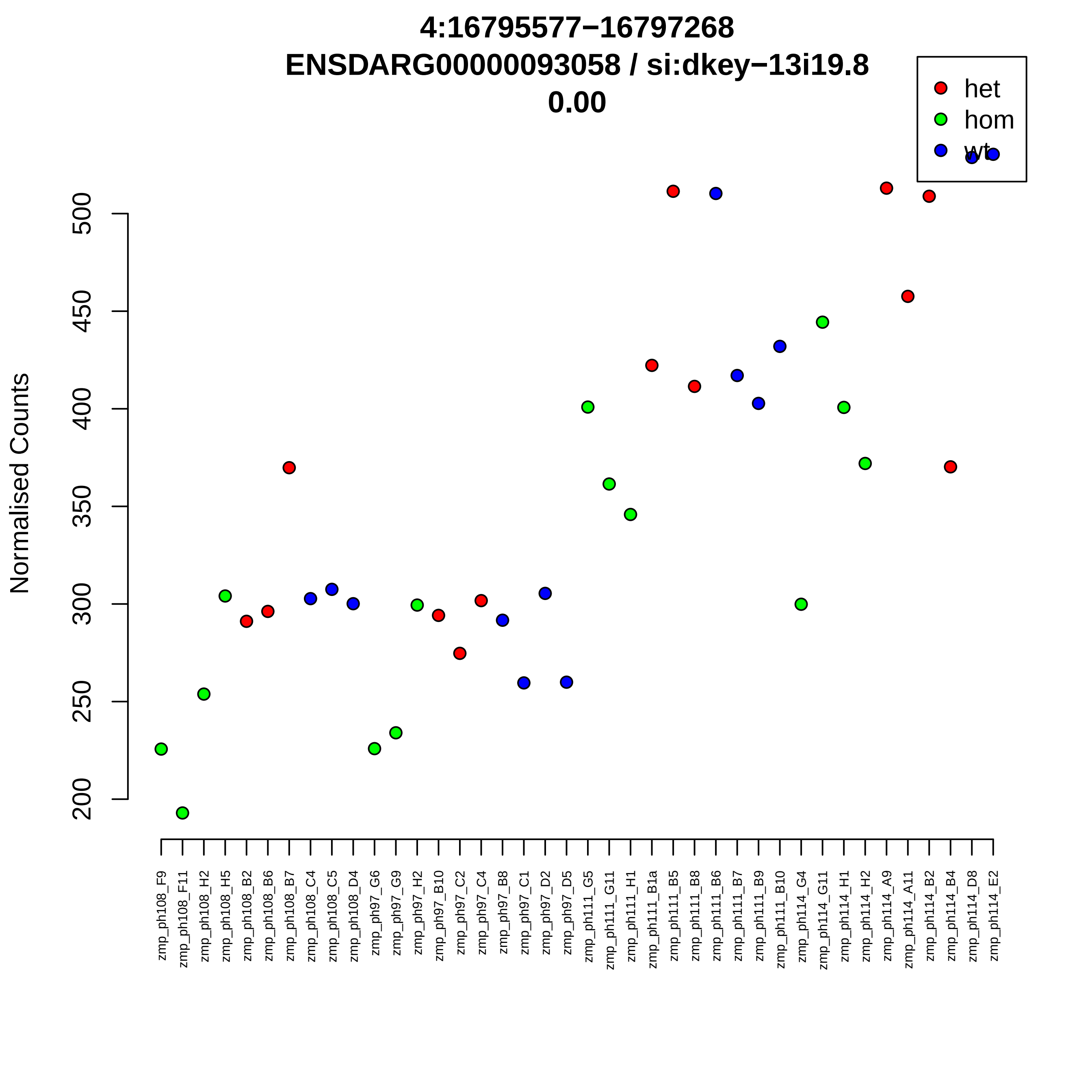

4:16795577−16797268
ENSDARG00000093058 / si:dkey−13i19.8
0.00
het
hom
wt
Normalised Counts
200
250
300
350
400
450
500
zmp_ph108_F9
zmp_ph108_F11
zmp_ph108_H2
zmp_ph108_H5
zmp_ph108_B2
zmp_ph108_B6
zmp_ph108_B7
zmp_ph108_C4
zmp_ph108_C5
zmp_ph108_D4
zmp_ph97_G6
zmp_ph97_G9
zmp_ph97_H2
zmp_ph97_B10
zmp_ph97_C2
zmp_ph97_C4
zmp_ph97_B8
zmp_ph97_C1
zmp_ph97_D2
zmp_ph97_D5
zmp_ph111_G5
zmp_ph111_G11
zmp_ph111_H1
zmp_ph111_B1a
zmp_ph111_B5
zmp_ph111_B8
zmp_ph111_B6
zmp_ph111_B7
zmp_ph111_B9
zmp_ph111_B10
zmp_ph114_G4
zmp_ph114_G11
zmp_ph114_H1
zmp_ph114_H2
zmp_ph114_A9
zmp_ph114_A11
zmp_ph114_B2
zmp_ph114_B4
zmp_ph114_D8
zmp_ph114_E2

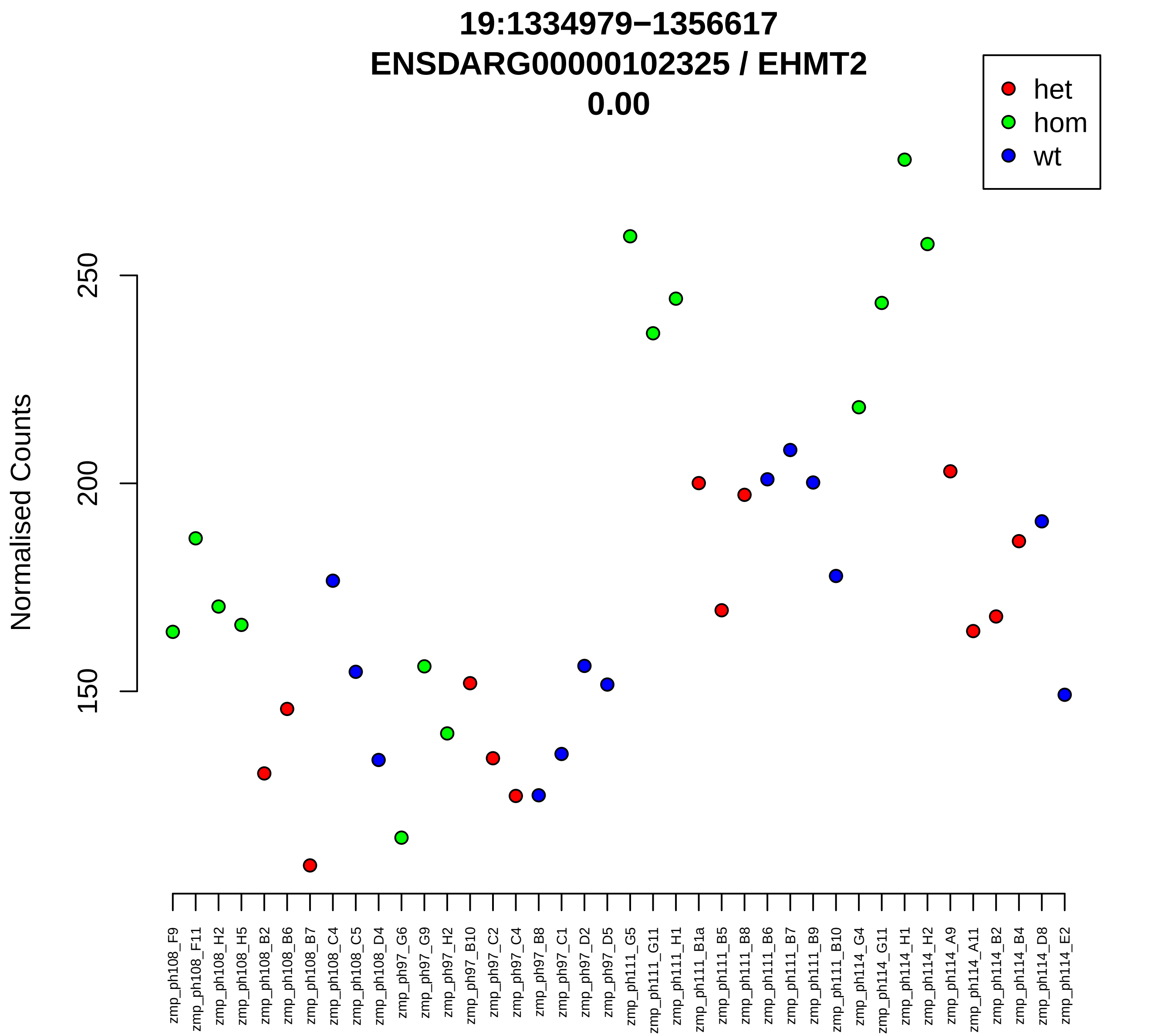

19:1334979−1356617
ENSDARG00000102325 / EHMT2
0.00
het
hom
wt
Normalised Counts
150
200
250
zmp_ph108_F9
zmp_ph108_F11
zmp_ph108_H2
zmp_ph108_H5
zmp_ph108_B2
zmp_ph108_B6
zmp_ph108_B7
zmp_ph108_C4
zmp_ph108_C5
zmp_ph108_D4
zmp_ph97_G6
zmp_ph97_G9
zmp_ph97_H2
zmp_ph97_B10
zmp_ph97_C2
zmp_ph97_C4
zmp_ph97_B8
zmp_ph97_C1
zmp_ph97_D2
zmp_ph97_D5
zmp_ph111_G5
zmp_ph111_G11
zmp_ph111_H1
zmp_ph111_B1a
zmp_ph111_B5
zmp_ph111_B8
zmp_ph111_B6
zmp_ph111_B7
zmp_ph111_B9
zmp_ph111_B10
zmp_ph114_G4
zmp_ph114_G11
zmp_ph114_H1
zmp_ph114_H2
zmp_ph114_A9
zmp_ph114_A11
zmp_ph114_B2
zmp_ph114_B4
zmp_ph114_D8
zmp_ph114_E2

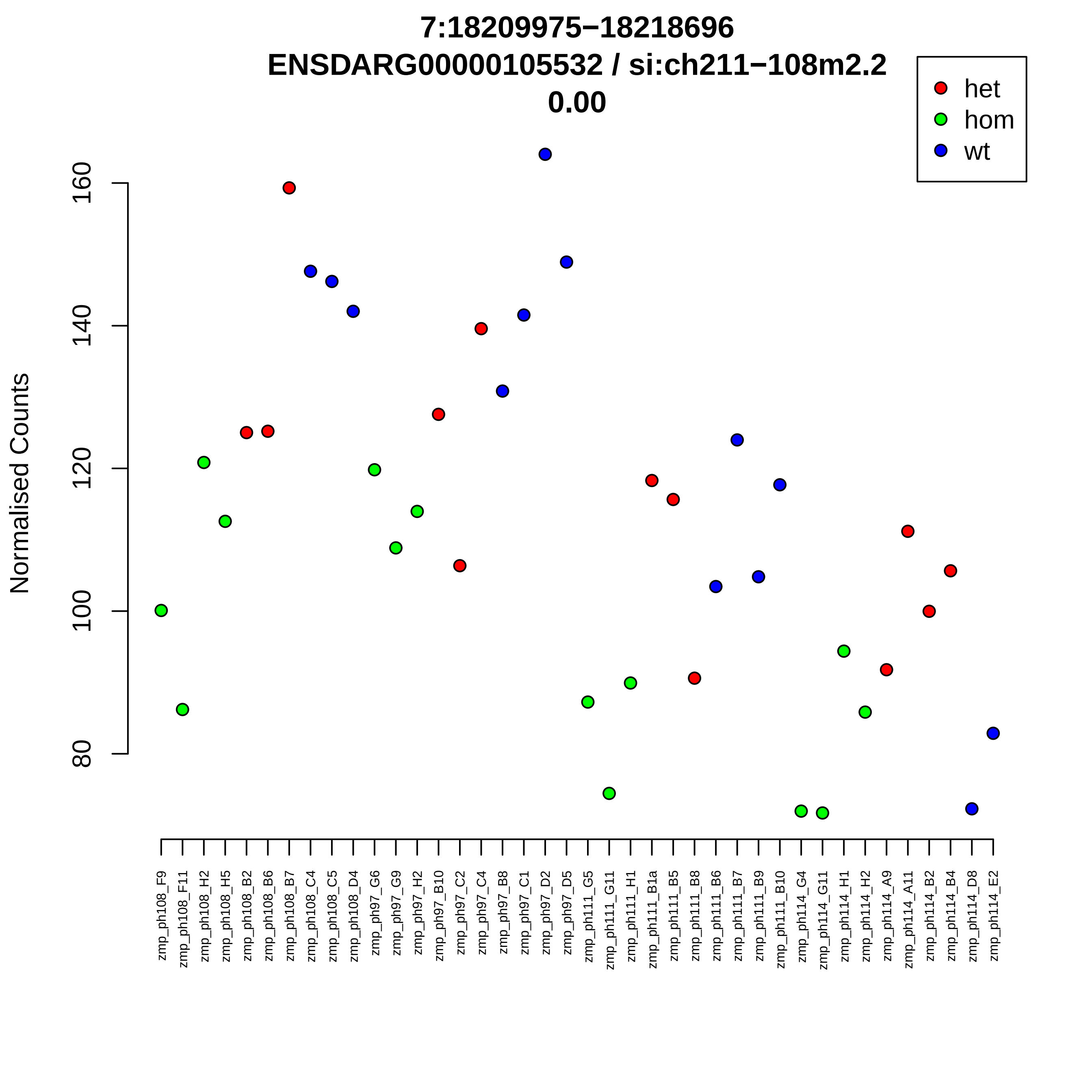

7:18209975−18218696
ENSDARG00000105532 / si:ch211−108m2.2
0.00
het
hom
wt
Normalised Counts
80
100
120
140
160
zmp_ph108_F9
zmp_ph108_F11
zmp_ph108_H2
zmp_ph108_H5
zmp_ph108_B2
zmp_ph108_B6
zmp_ph108_B7
zmp_ph108_C4
zmp_ph108_C5
zmp_ph108_D4
zmp_ph97_G6
zmp_ph97_G9
zmp_ph97_H2
zmp_ph97_B10
zmp_ph97_C2
zmp_ph97_C4
zmp_ph97_B8
zmp_ph97_C1
zmp_ph97_D2
zmp_ph97_D5
zmp_ph111_G5
zmp_ph111_G11
zmp_ph111_H1
zmp_ph111_B1a
zmp_ph111_B5
zmp_ph111_B8
zmp_ph111_B6
zmp_ph111_B7
zmp_ph111_B9
zmp_ph111_B10
zmp_ph114_G4
zmp_ph114_G11
zmp_ph114_H1
zmp_ph114_H2
zmp_ph114_A9
zmp_ph114_A11
zmp_ph114_B2
zmp_ph114_B4
zmp_ph114_D8
zmp_ph114_E2

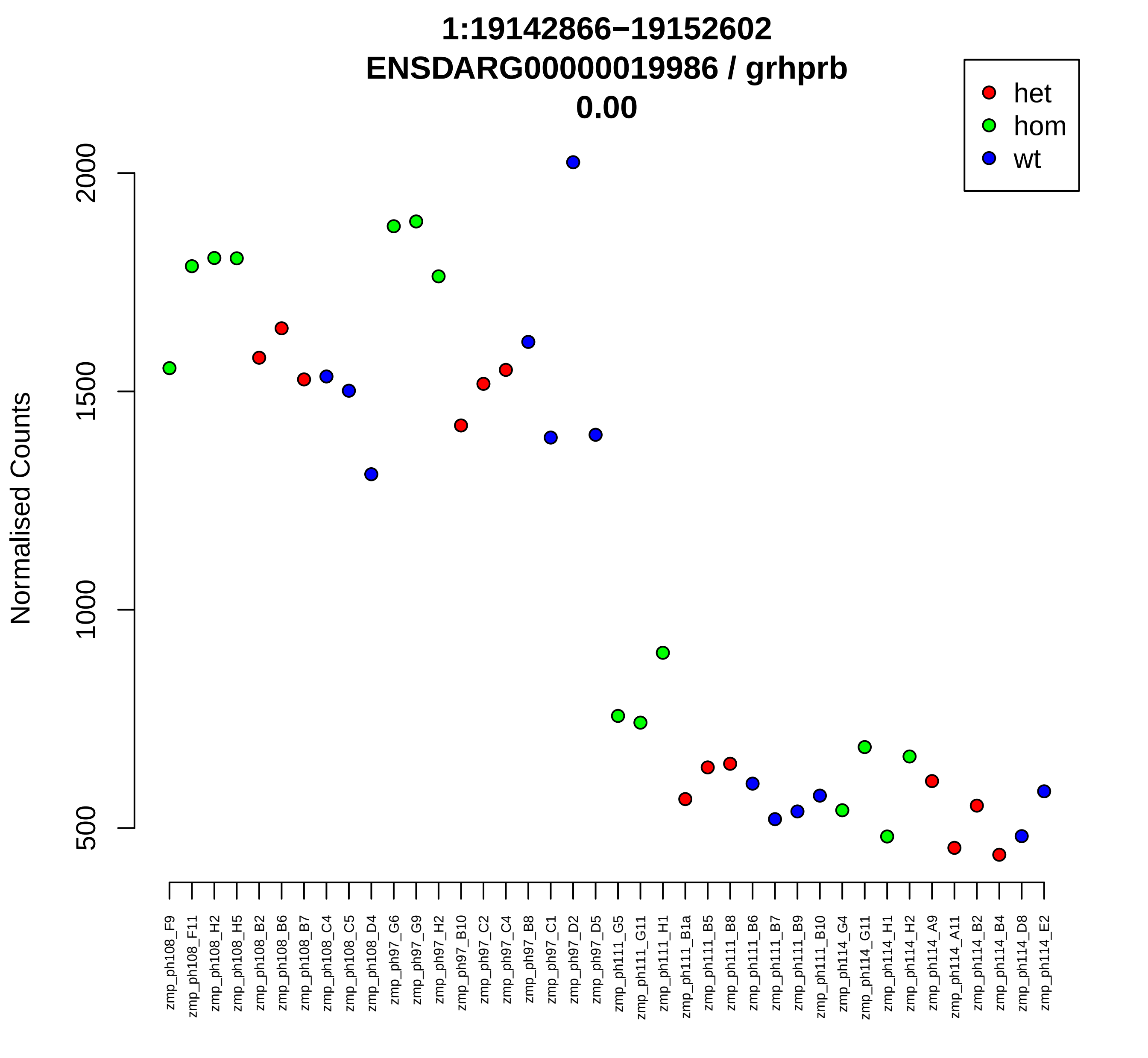

1:19142866−19152602
ENSDARG00000019986 / grhprb
0.00
het
hom
wt
Normalised Counts
500
1000
1500
2000
zmp_ph108_F9
zmp_ph108_F11
zmp_ph108_H2
zmp_ph108_H5
zmp_ph108_B2
zmp_ph108_B6
zmp_ph108_B7
zmp_ph108_C4
zmp_ph108_C5
zmp_ph108_D4
zmp_ph97_G6
zmp_ph97_G9
zmp_ph97_H2
zmp_ph97_B10
zmp_ph97_C2
zmp_ph97_C4
zmp_ph97_B8
zmp_ph97_C1
zmp_ph97_D2
zmp_ph97_D5
zmp_ph111_G5
zmp_ph111_G11
zmp_ph111_H1
zmp_ph111_B1a
zmp_ph111_B5
zmp_ph111_B8
zmp_ph111_B6
zmp_ph111_B7
zmp_ph111_B9
zmp_ph111_B10
zmp_ph114_G4
zmp_ph114_G11
zmp_ph114_H1
zmp_ph114_H2
zmp_ph114_A9
zmp_ph114_A11
zmp_ph114_B2
zmp_ph114_B4
zmp_ph114_D8
zmp_ph114_E2

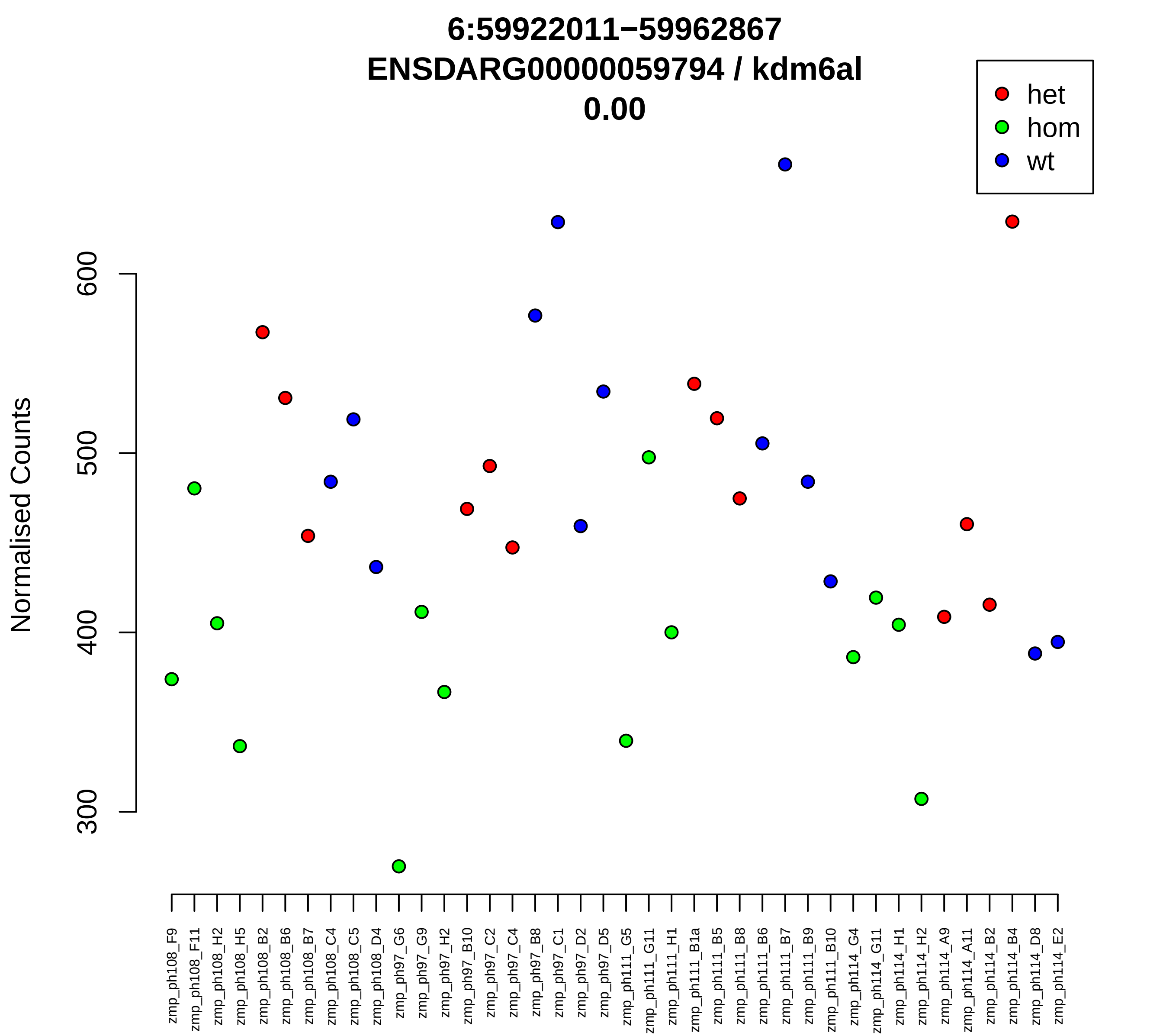

6:59922011−59962867
ENSDARG00000059794 / kdm6al
0.00
het
hom
wt
Normalised Counts
300
400
500
600
zmp_ph108_F9
zmp_ph108_F11
zmp_ph108_H2
zmp_ph108_H5
zmp_ph108_B2
zmp_ph108_B6
zmp_ph108_B7
zmp_ph108_C4
zmp_ph108_C5
zmp_ph108_D4
zmp_ph97_G6
zmp_ph97_G9
zmp_ph97_H2
zmp_ph97_B10
zmp_ph97_C2
zmp_ph97_C4
zmp_ph97_B8
zmp_ph97_C1
zmp_ph97_D2
zmp_ph97_D5
zmp_ph111_G5
zmp_ph111_G11
zmp_ph111_H1
zmp_ph111_B1a
zmp_ph111_B5
zmp_ph111_B8
zmp_ph111_B6
zmp_ph111_B7
zmp_ph111_B9
zmp_ph111_B10
zmp_ph114_G4
zmp_ph114_G11
zmp_ph114_H1
zmp_ph114_H2
zmp_ph114_A9
zmp_ph114_A11
zmp_ph114_B2
zmp_ph114_B4
zmp_ph114_D8
zmp_ph114_E2

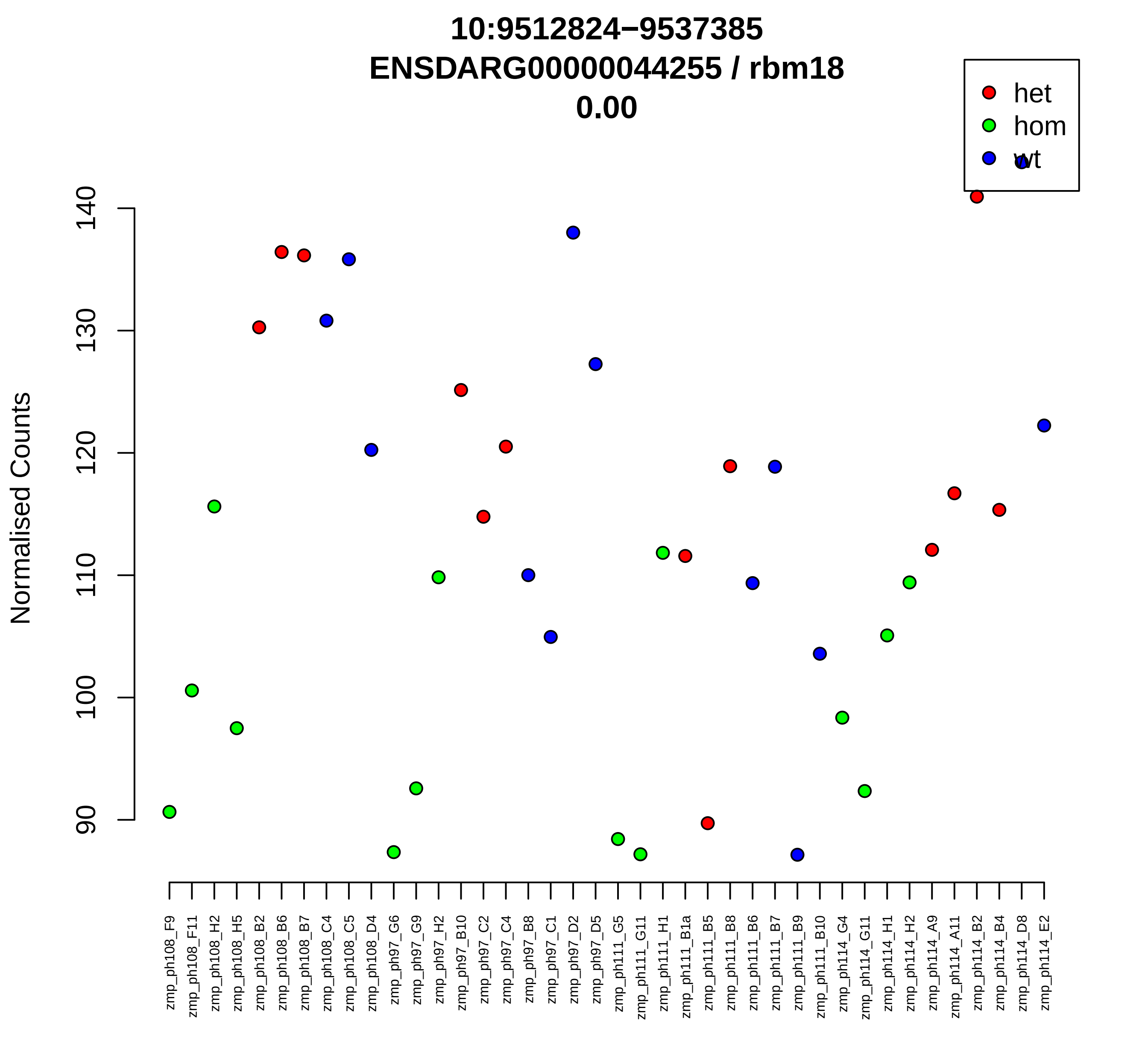
10:9512824−9537385
ENSDARG00000044255 / rbm18
0.00
het
hom
wt
Normalised Counts
90
100
110
120
130
140
zmp_ph108_F9
zmp_ph108_F11
zmp_ph108_H2
zmp_ph108_H5
zmp_ph108_B2
zmp_ph108_B6
zmp_ph108_B7
zmp_ph108_C4
zmp_ph108_C5
zmp_ph108_D4
zmp_ph97_G6
zmp_ph97_G9
zmp_ph97_H2
zmp_ph97_B10
zmp_ph97_C2
zmp_ph97_C4
zmp_ph97_B8
zmp_ph97_C1
zmp_ph97_D2
zmp_ph97_D5
zmp_ph111_G5
zmp_ph111_G11
zmp_ph111_H1
zmp_ph111_B1a
zmp_ph111_B5
zmp_ph111_B8
zmp_ph111_B6
zmp_ph111_B7
zmp_ph111_B9
zmp_ph111_B10
zmp_ph114_G4
zmp_ph114_G11
zmp_ph114_H1
zmp_ph114_H2
zmp_ph114_A9
zmp_ph114_A11
zmp_ph114_B2
zmp_ph114_B4
zmp_ph114_D8
zmp_ph114_E2

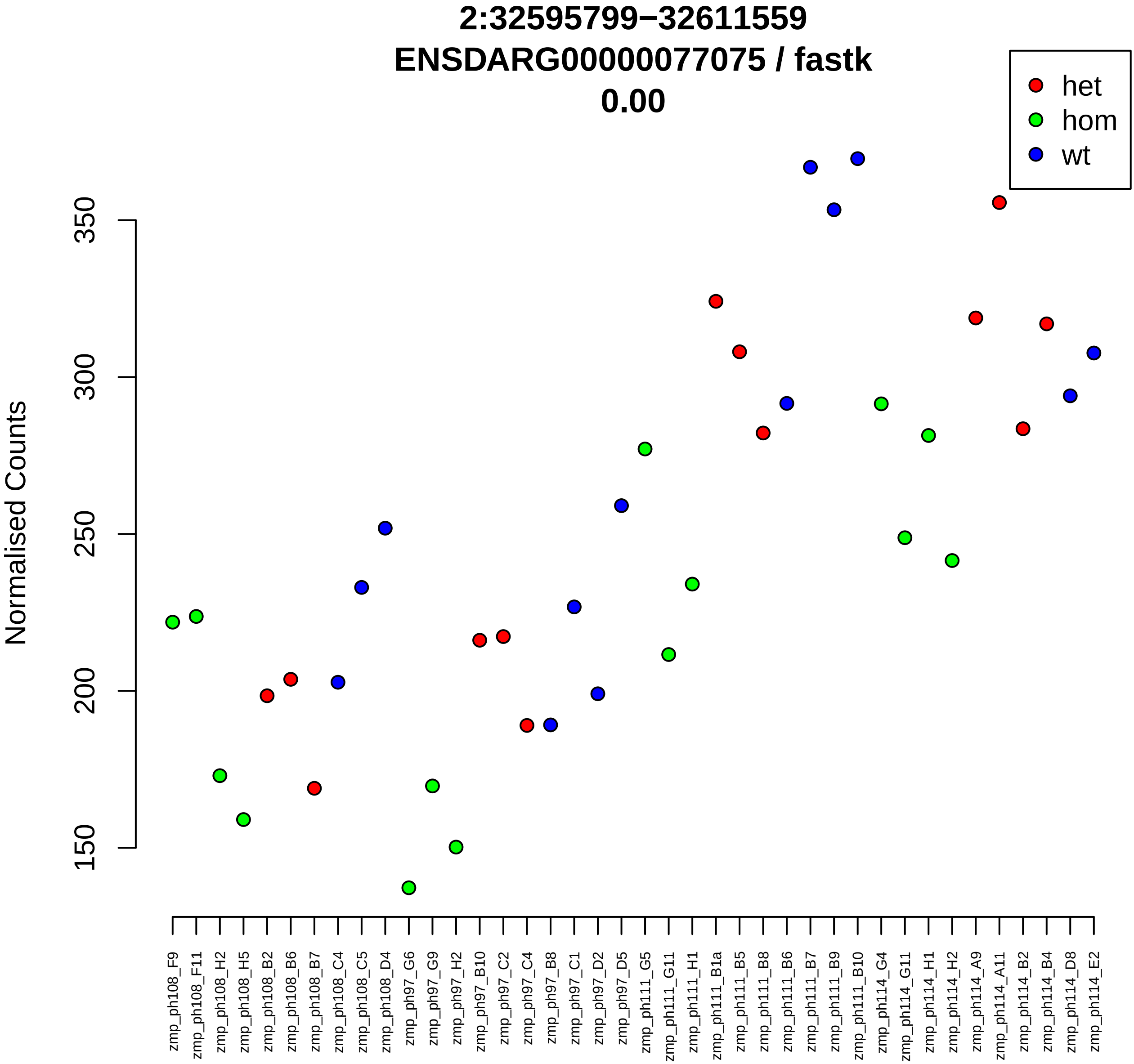

2:32595799−32611559
ENSDARG00000077075 / fastk
0.00
het
hom
wt
Normalised Counts
150
200
250
300
350
zmp_ph108_F9
zmp_ph108_F11
zmp_ph108_H2
zmp_ph108_H5
zmp_ph108_B2
zmp_ph108_B6
zmp_ph108_B7
zmp_ph108_C4
zmp_ph108_C5
zmp_ph108_D4
zmp_ph97_G6
zmp_ph97_G9
zmp_ph97_H2
zmp_ph97_B10
zmp_ph97_C2
zmp_ph97_C4
zmp_ph97_B8
zmp_ph97_C1
zmp_ph97_D2
zmp_ph97_D5
zmp_ph111_G5
zmp_ph111_G11
zmp_ph111_H1
zmp_ph111_B1a
zmp_ph111_B5
zmp_ph111_B8
zmp_ph111_B6
zmp_ph111_B7
zmp_ph111_B9
zmp_ph111_B10
zmp_ph114_G4
zmp_ph114_G11
zmp_ph114_H1
zmp_ph114_H2
zmp_ph114_A9
zmp_ph114_A11
zmp_ph114_B2
zmp_ph114_B4
zmp_ph114_D8
zmp_ph114_E2

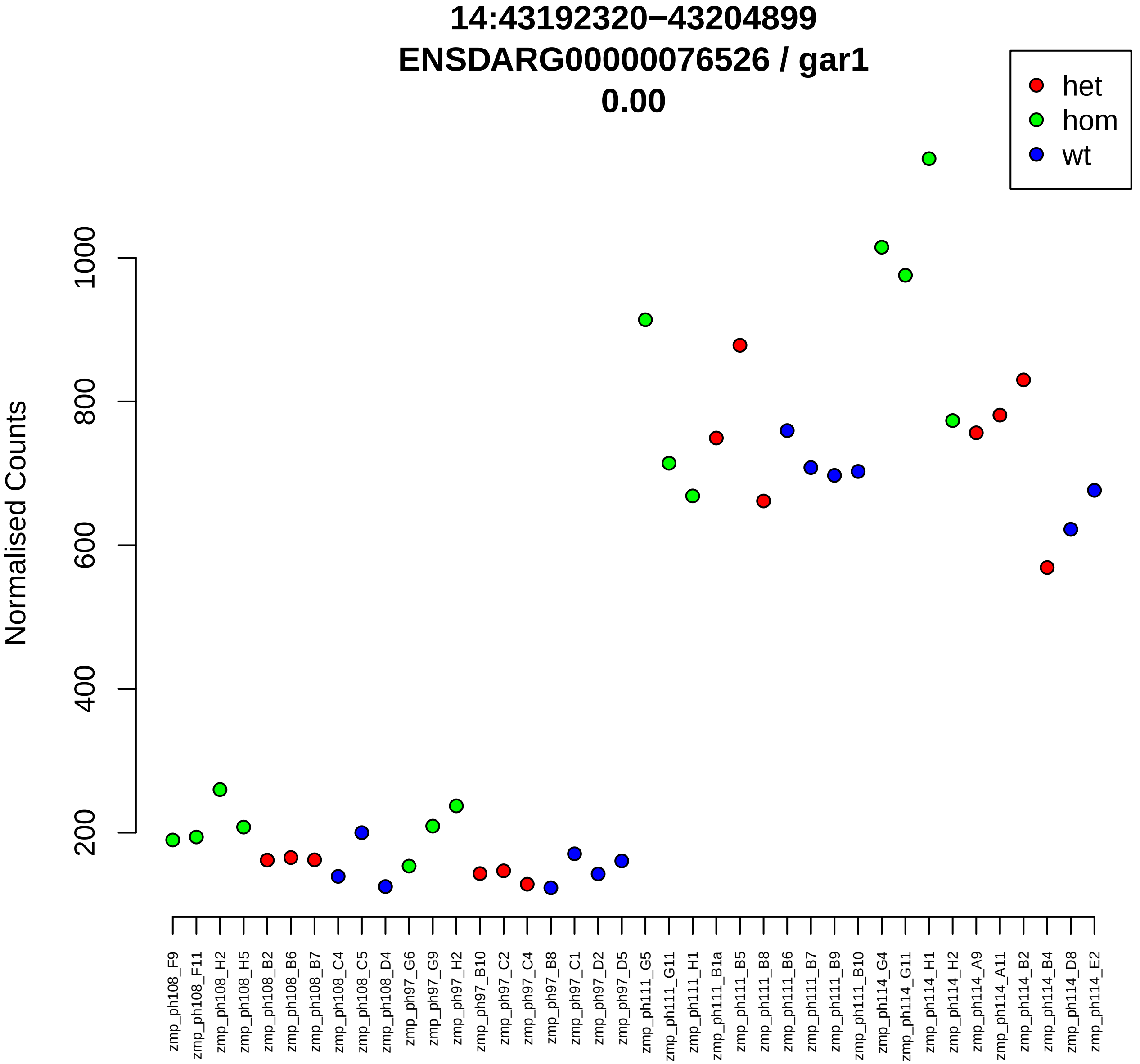

14:43192320−43204899
ENSDARG00000076526 / gar1
0.00
het
hom
wt
Normalised Counts
200
400
600
800
1000
zmp_ph108_F9
zmp_ph108_F11
zmp_ph108_H2
zmp_ph108_H5
zmp_ph108_B2
zmp_ph108_B6
zmp_ph108_B7
zmp_ph108_C4
zmp_ph108_C5
zmp_ph108_D4
zmp_ph97_G6
zmp_ph97_G9
zmp_ph97_H2
zmp_ph97_B10
zmp_ph97_C2
zmp_ph97_C4
zmp_ph97_B8
zmp_ph97_C1
zmp_ph97_D2
zmp_ph97_D5
zmp_ph111_G5
zmp_ph111_G11
zmp_ph111_H1
zmp_ph111_B1a
zmp_ph111_B5
zmp_ph111_B8
zmp_ph111_B6
zmp_ph111_B7
zmp_ph111_B9
zmp_ph111_B10
zmp_ph114_G4
zmp_ph114_G11
zmp_ph114_H1
zmp_ph114_H2
zmp_ph114_A9
zmp_ph114_A11
zmp_ph114_B2
zmp_ph114_B4
zmp_ph114_D8
zmp_ph114_E2

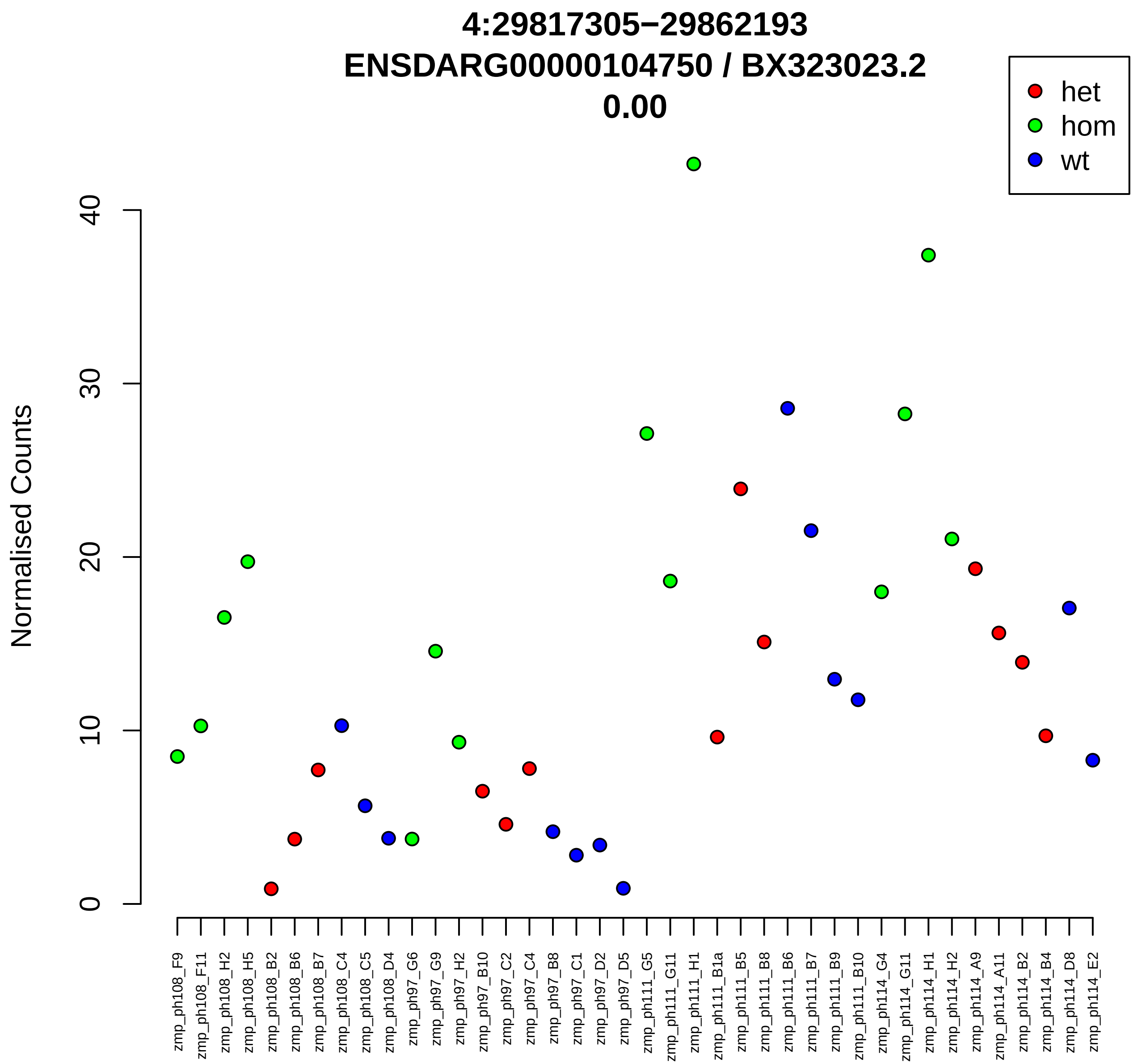
4:29817305−29862193
ENSDARG00000104750 / BX323023.2
0.00
het
hom
wt
Normalised Counts
0
10
20
30
40
zmp_ph108_F9
zmp_ph108_F11
zmp_ph108_H2
zmp_ph108_H5
zmp_ph108_B2
zmp_ph108_B6
zmp_ph108_B7
zmp_ph108_C4
zmp_ph108_C5
zmp_ph108_D4
zmp_ph97_G6
zmp_ph97_G9
zmp_ph97_H2
zmp_ph97_B10
zmp_ph97_C2
zmp_ph97_C4
zmp_ph97_B8
zmp_ph97_C1
zmp_ph97_D2
zmp_ph97_D5
zmp_ph111_G5
zmp_ph111_G11
zmp_ph111_H1
zmp_ph111_B1a
zmp_ph111_B5
zmp_ph111_B8
zmp_ph111_B6
zmp_ph111_B7
zmp_ph111_B9
zmp_ph111_B10
zmp_ph114_G4
zmp_ph114_G11
zmp_ph114_H1
zmp_ph114_H2
zmp_ph114_A9
zmp_ph114_A11
zmp_ph114_B2
zmp_ph114_B4
zmp_ph114_D8
zmp_ph114_E2

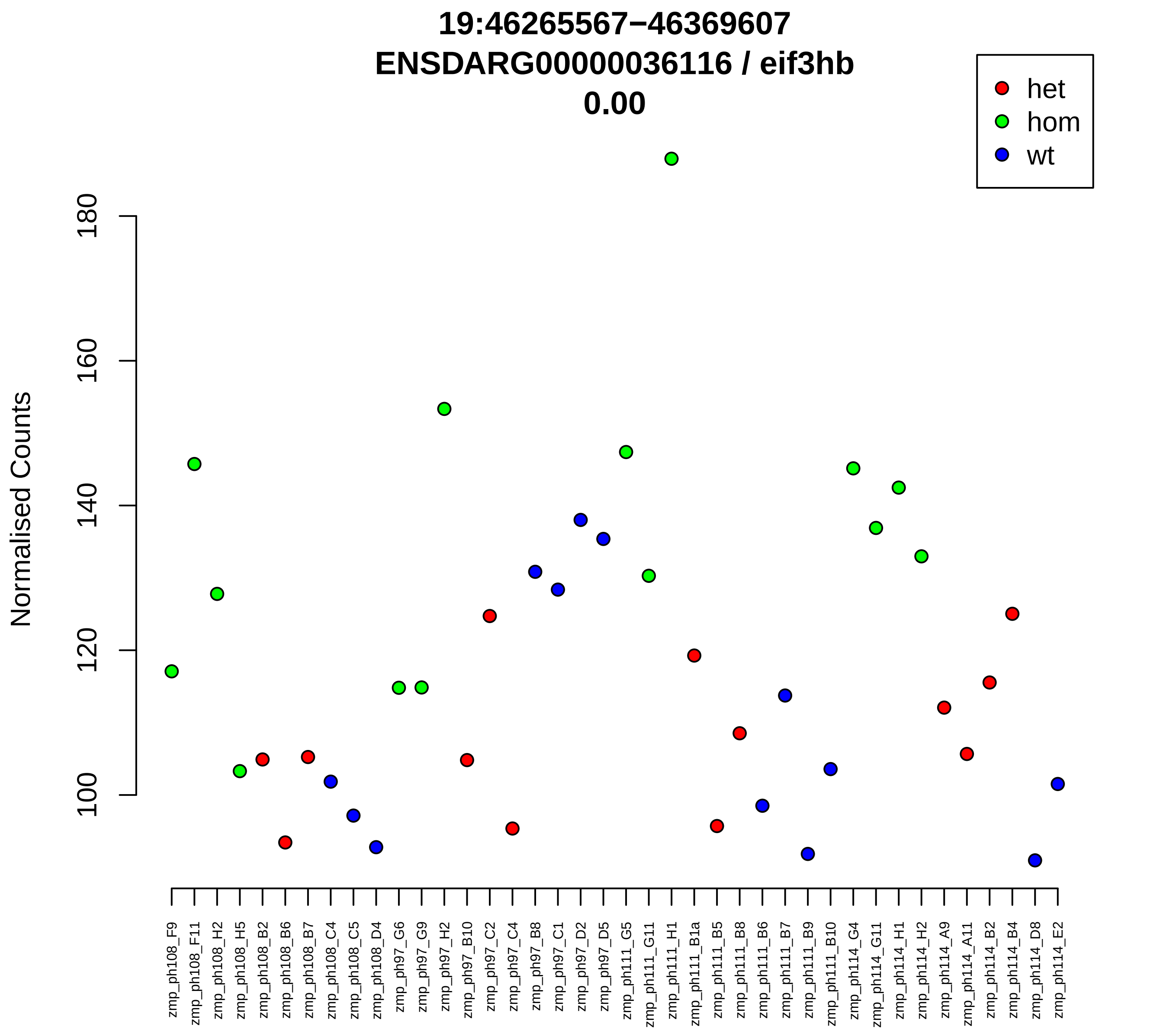

19:46265567−46369607
ENSDARG00000036116 / eif3hb
0.00
het
hom
wt
Normalised Counts
180
160
140
120
100
zmp_ph108_F9
zmp_ph108_F11
zmp_ph108_H2
zmp_ph108_H5
zmp_ph108_B2
zmp_ph108_B6
zmp_ph108_B7
zmp_ph108_C4
zmp_ph108_C5
zmp_ph108_D4
zmp_ph97_G6
zmp_ph97_G9
zmp_ph97_H2
zmp_ph97_B10
zmp_ph97_C2
zmp_ph97_C4
zmp_ph97_B8
zmp_ph97_C1
zmp_ph97_D2
zmp_ph97_D5
zmp_ph111_G5
zmp_ph111_G11
zmp_ph111_H1
zmp_ph111_B1a
zmp_ph111_B5
zmp_ph111_B8
zmp_ph111_B6
zmp_ph111_B7
zmp_ph111_B9
zmp_ph111_B10
zmp_ph114_G4
zmp_ph114_G11
zmp_ph114_H1
zmp_ph114_H2
zmp_ph114_A9
zmp_ph114_A11
zmp_ph114_B2
zmp_ph114_B4
zmp_ph114_D8
zmp_ph114_E2

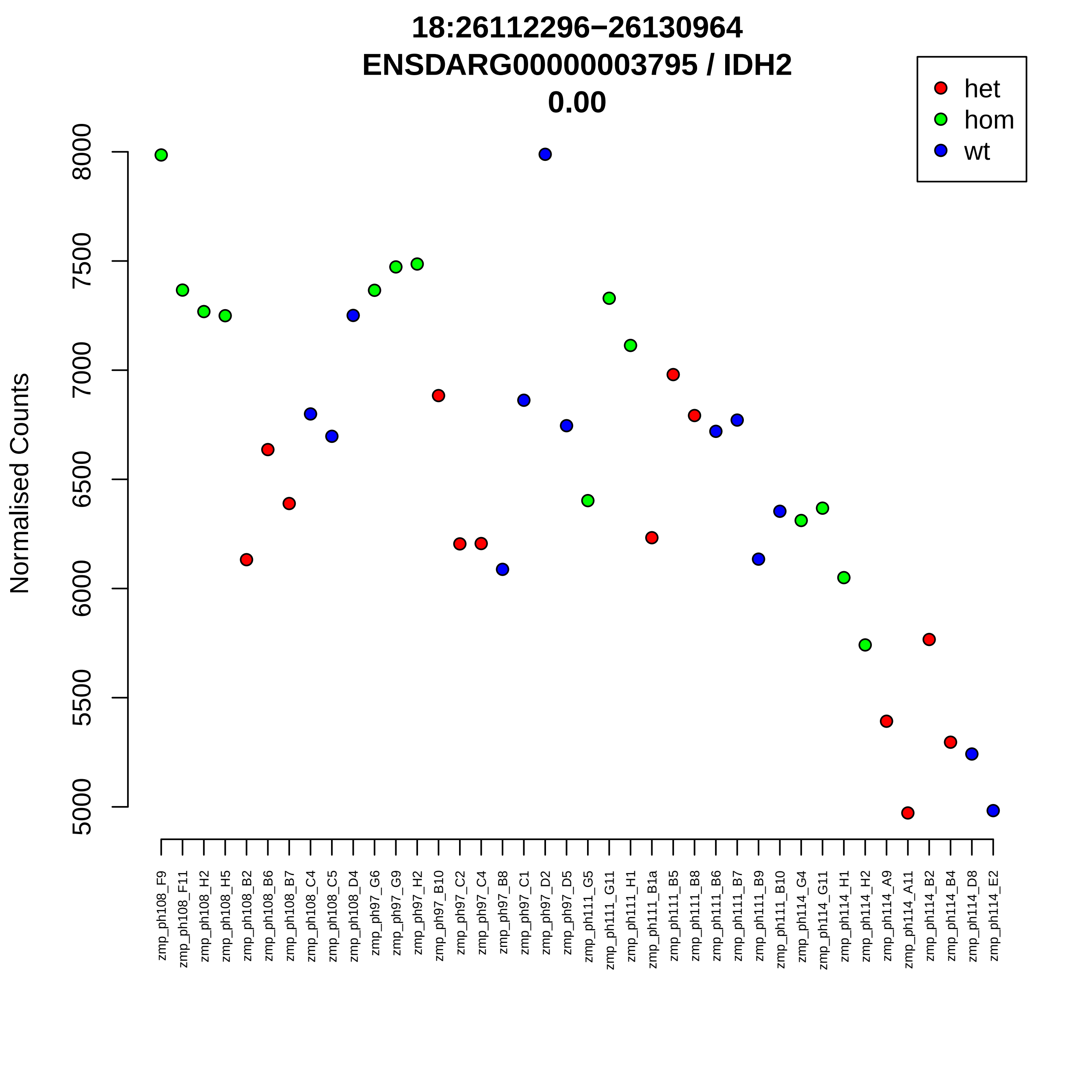

18:26112296−26130964
ENSDARG00000003795 / IDH2
0.00
het
hom
wt
Normalised Counts
8000
7500
7000
6500
6000
5500
5000
zmp_ph108_F9
zmp_ph108_F11
zmp_ph108_H2
zmp_ph108_H5
zmp_ph108_B2
zmp_ph108_B6
zmp_ph108_B7
zmp_ph108_C4
zmp_ph108_C5
zmp_ph108_D4
zmp_ph97_G6
zmp_ph97_G9
zmp_ph97_H2
zmp_ph97_B10
zmp_ph97_C2
zmp_ph97_C4
zmp_ph97_B8
zmp_ph97_C1
zmp_ph97_D2
zmp_ph97_D5
zmp_ph111_G5
zmp_ph111_G11
zmp_ph111_H1
zmp_ph111_B1a
zmp_ph111_B5
zmp_ph111_B8
zmp_ph111_B6
zmp_ph111_B7
zmp_ph111_B9
zmp_ph111_B10
zmp_ph114_G4
zmp_ph114_G11
zmp_ph114_H1
zmp_ph114_H2
zmp_ph114_A9
zmp_ph114_A11
zmp_ph114_B2
zmp_ph114_B4
zmp_ph114_D8
zmp_ph114_E2

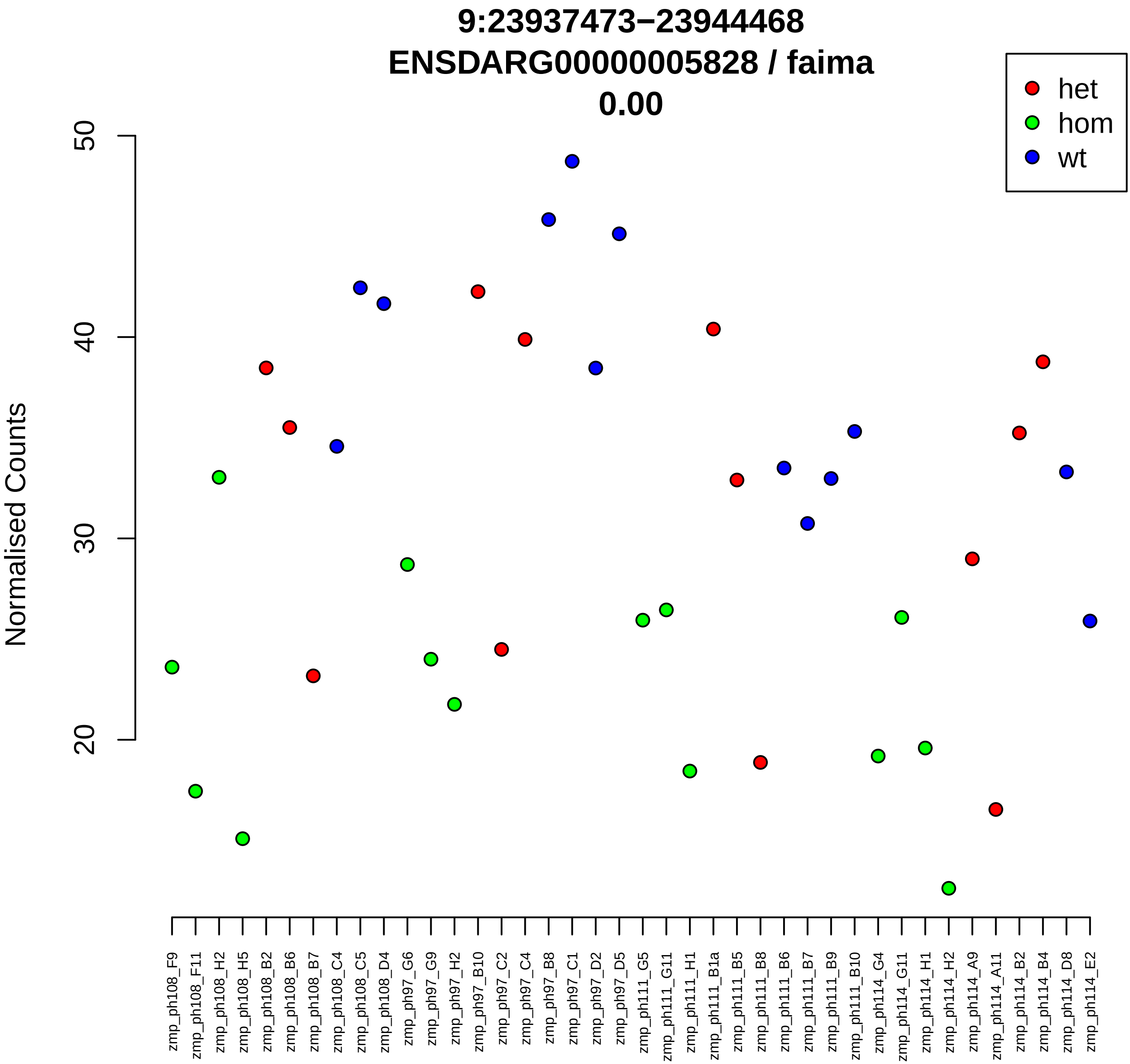
9:23937473−23944468
ENSDARG00000005828 / faima
0.00
Normalised Counts
20
30
40
50
het
hom
wt
zmp_ph108_F9
zmp_ph108_F11
zmp_ph108_H2
zmp_ph108_H5
zmp_ph108_B2
zmp_ph108_B6
zmp_ph108_B7
zmp_ph108_C4
zmp_ph108_C5
zmp_ph108_D4
zmp_ph97_G6
zmp_ph97_G9
zmp_ph97_H2
zmp_ph97_B10
zmp_ph97_C2
zmp_ph97_C4
zmp_ph97_B8
zmp_ph97_C1
zmp_ph97_D2
zmp_ph97_D5
zmp_ph111_G5
zmp_ph111_G11
zmp_ph111_H1
zmp_ph111_B1a
zmp_ph111_B5
zmp_ph111_B8
zmp_ph111_B6
zmp_ph111_B7
zmp_ph111_B9
zmp_ph111_B10
zmp_ph114_G4
zmp_ph114_G11
zmp_ph114_H1
zmp_ph114_H2
zmp_ph114_A9
zmp_ph114_A11
zmp_ph114_B2
zmp_ph114_B4
zmp_ph114_D8
zmp_ph114_E2

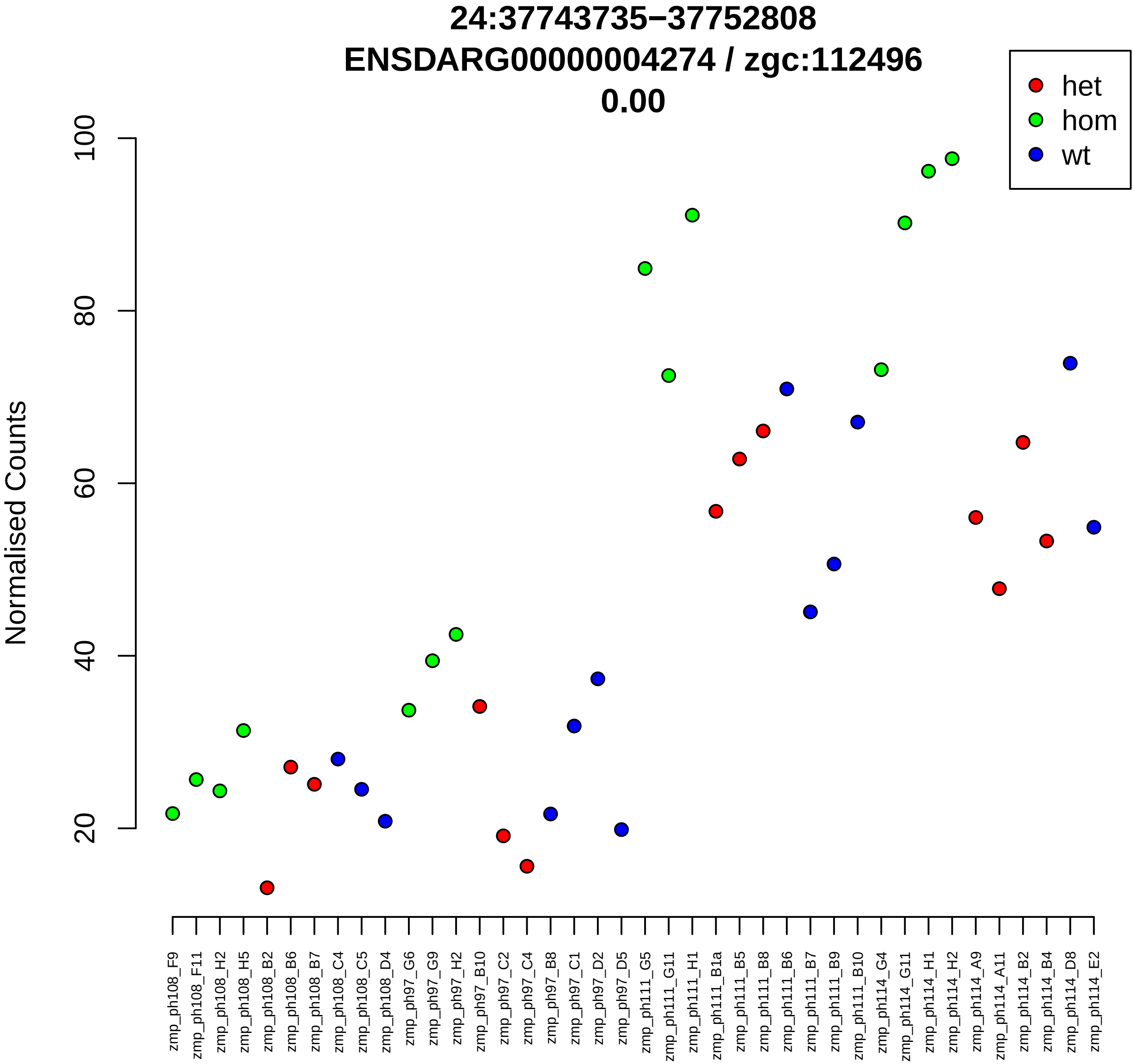

24:37743735−37752808
ENSDARG00000004274 / zgc:112496
0.00
Normalised Counts
100
80
60
40
20
het
hom
wt
zmp_ph108_F9
zmp_ph108_F11
zmp_ph108_H2
zmp_ph108_H5
zmp_ph108_B2
zmp_ph108_B6
zmp_ph108_B7
zmp_ph108_C4
zmp_ph108_C5
zmp_ph108_D4
zmp_ph97_G6
zmp_ph97_G9
zmp_ph97_H2
zmp_ph97_B10
zmp_ph97_C2
zmp_ph97_C4
zmp_ph97_B8
zmp_ph97_C1
zmp_ph97_D2
zmp_ph97_D5
zmp_ph111_G5
zmp_ph111_G11
zmp_ph111_H1
zmp_ph111_B1a
zmp_ph111_B5
zmp_ph111_B8
zmp_ph111_B6
zmp_ph111_B7
zmp_ph111_B9
zmp_ph111_B10
zmp_ph114_G4
zmp_ph114_G11
zmp_ph114_H1
zmp_ph114_H2
zmp_ph114_A9
zmp_ph114_A11
zmp_ph114_B2
zmp_ph114_B4
zmp_ph114_D8
zmp_ph114_E2

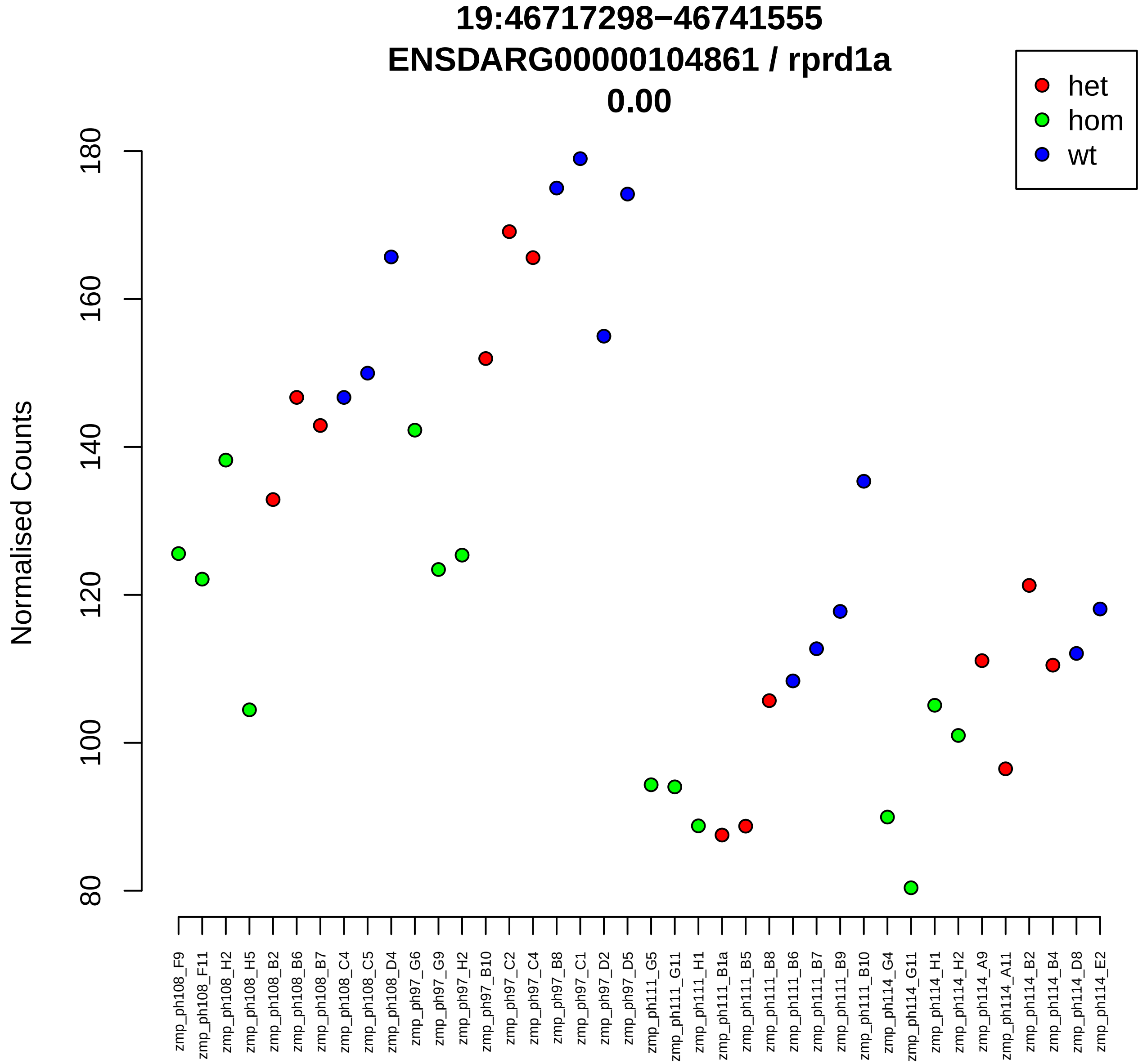

19:46717298−46741555
ENSDARG00000104861 / rprd1a
0.00
het
hom
wt
Normalised Counts
80
100
120
140
160
180
zmp_ph108_F9
zmp_ph108_F11
zmp_ph108_H2
zmp_ph108_H5
zmp_ph108_B2
zmp_ph108_B6
zmp_ph108_B7
zmp_ph108_C4
zmp_ph108_C5
zmp_ph108_D4
zmp_ph97_G6
zmp_ph97_G9
zmp_ph97_H2
zmp_ph97_B10
zmp_ph97_C2
zmp_ph97_C4
zmp_ph97_B8
zmp_ph97_C1
zmp_ph97_D2
zmp_ph97_D5
zmp_ph111_G5
zmp_ph111_G11
zmp_ph111_H1
zmp_ph111_B1a
zmp_ph111_B5
zmp_ph111_B8
zmp_ph111_B6
zmp_ph111_B7
zmp_ph111_B9
zmp_ph111_B10
zmp_ph114_G4
zmp_ph114_G11
zmp_ph114_H1
zmp_ph114_H2
zmp_ph114_A9
zmp_ph114_A11
zmp_ph114_B2
zmp_ph114_B4
zmp_ph114_D8
zmp_ph114_E2

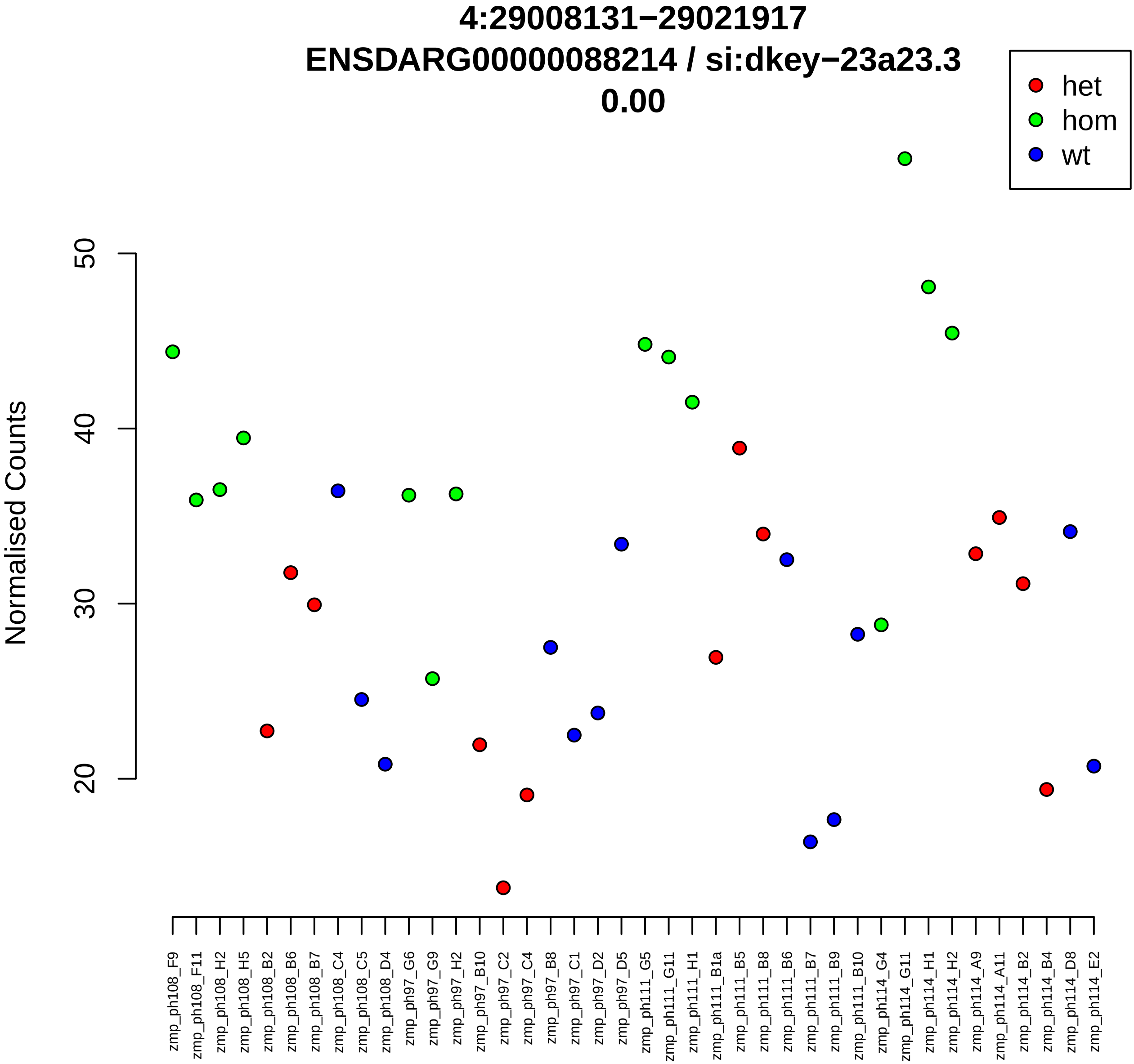
4:29008131−29021917
ENSDARG00000088214 / si:dkey−23a23.3
0.00
het
hom
wt
Normalised Counts
20
30
40
50
zmp_ph108_F9
zmp_ph108_F11
zmp_ph108_H2
zmp_ph108_H5
zmp_ph108_B2
zmp_ph108_B6
zmp_ph108_B7
zmp_ph108_C4
zmp_ph108_C5
zmp_ph108_D4
zmp_ph97_G6
zmp_ph97_G9
zmp_ph97_H2
zmp_ph97_B10
zmp_ph97_C2
zmp_ph97_C4
zmp_ph97_B8
zmp_ph97_C1
zmp_ph97_D2
zmp_ph97_D5
zmp_ph111_G5
zmp_ph111_G11
zmp_ph111_H1
zmp_ph111_B1a
zmp_ph111_B5
zmp_ph111_B8
zmp_ph111_B6
zmp_ph111_B7
zmp_ph111_B9
zmp_ph111_B10
zmp_ph114_G4
zmp_ph114_G11
zmp_ph114_H1
zmp_ph114_H2
zmp_ph114_A9
zmp_ph114_A11
zmp_ph114_B2
zmp_ph114_B4
zmp_ph114_D8
zmp_ph114_E2

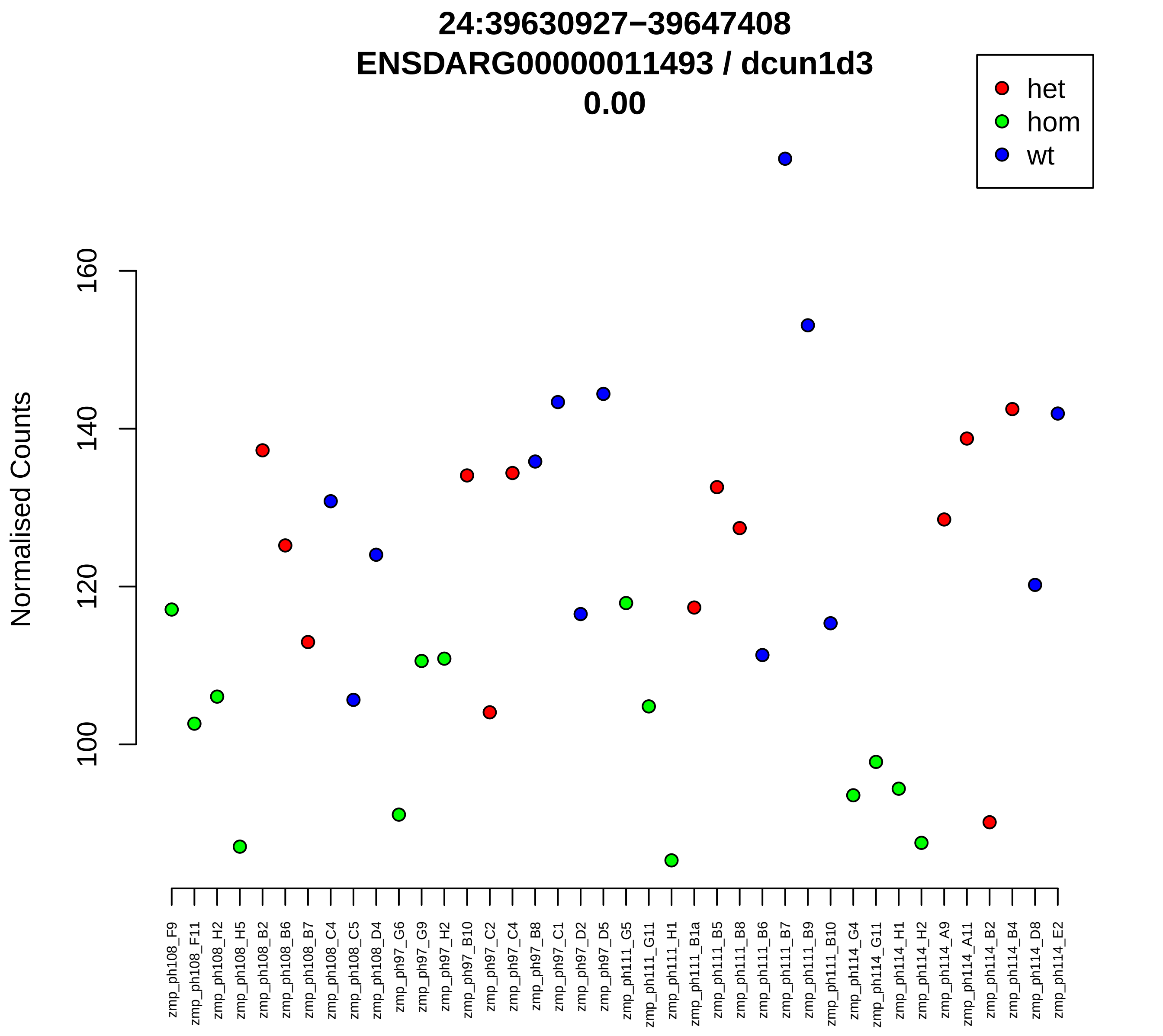

24:39630927−39647408
ENSDARG00000011493 / dcun1d3
0.00
het
hom
wt
Normalised Counts
100
120
140
160
zmp_ph108_F9
zmp_ph108_F11
zmp_ph108_H2
zmp_ph108_H5
zmp_ph108_B2
zmp_ph108_B6
zmp_ph108_B7
zmp_ph108_C4
zmp_ph108_C5
zmp_ph108_D4
zmp_ph97_G6
zmp_ph97_G9
zmp_ph97_H2
zmp_ph97_B10
zmp_ph97_C2
zmp_ph97_C4
zmp_ph97_B8
zmp_ph97_C1
zmp_ph97_D2
zmp_ph97_D5
zmp_ph111_G5
zmp_ph111_G11
zmp_ph111_H1
zmp_ph111_B1a
zmp_ph111_B5
zmp_ph111_B8
zmp_ph111_B6
zmp_ph111_B7
zmp_ph111_B9
zmp_ph111_B10
zmp_ph114_G4
zmp_ph114_G11
zmp_ph114_H1
zmp_ph114_H2
zmp_ph114_A9
zmp_ph114_A11
zmp_ph114_B2
zmp_ph114_B4
zmp_ph114_D8
zmp_ph114_E2

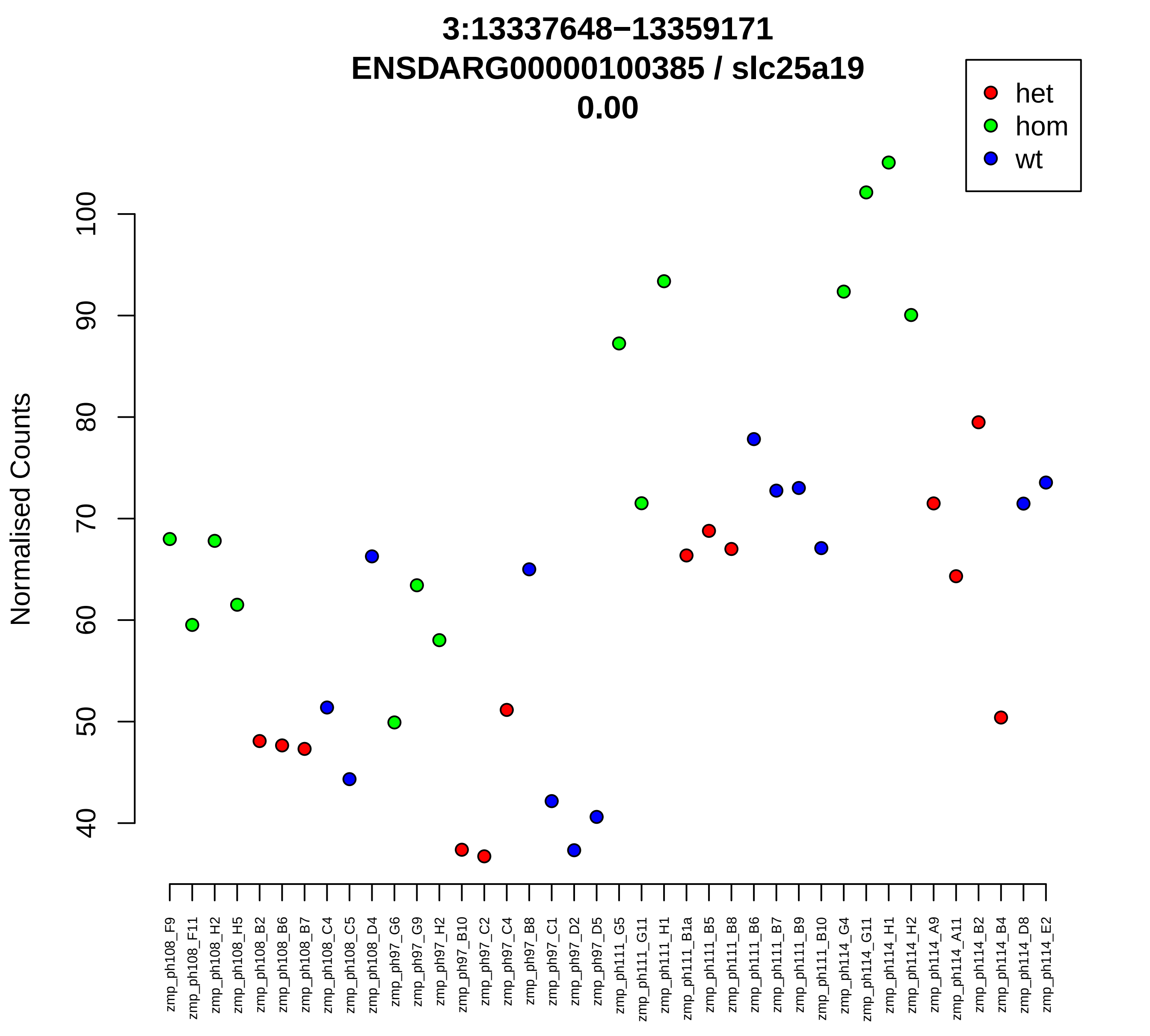
3:13337648−13359171
ENSDARG00000100385 / slc25a19
0.00
het
hom
wt
Normalised Counts
40
50
60
70
80
90
100
zmp_ph108_F9
zmp_ph108_F11
zmp_ph108_H2
zmp_ph108_H5
zmp_ph108_B2
zmp_ph108_B6
zmp_ph108_B7
zmp_ph108_C4
zmp_ph108_C5
zmp_ph108_D4
zmp_ph97_G6
zmp_ph97_G9
zmp_ph97_H2
zmp_ph97_B10
zmp_ph97_C2
zmp_ph97_C4
zmp_ph97_B8
zmp_ph97_C1
zmp_ph97_D2
zmp_ph97_D5
zmp_ph111_G5
zmp_ph111_G11
zmp_ph111_H1
zmp_ph111_B1a
zmp_ph111_B5
zmp_ph111_B8
zmp_ph111_B6
zmp_ph111_B7
zmp_ph111_B9
zmp_ph111_B10
zmp_ph114_G4
zmp_ph114_G11
zmp_ph114_H1
zmp_ph114_H2
zmp_ph114_A9
zmp_ph114_A11
zmp_ph114_B2
zmp_ph114_B4
zmp_ph114_D8
zmp_ph114_E2

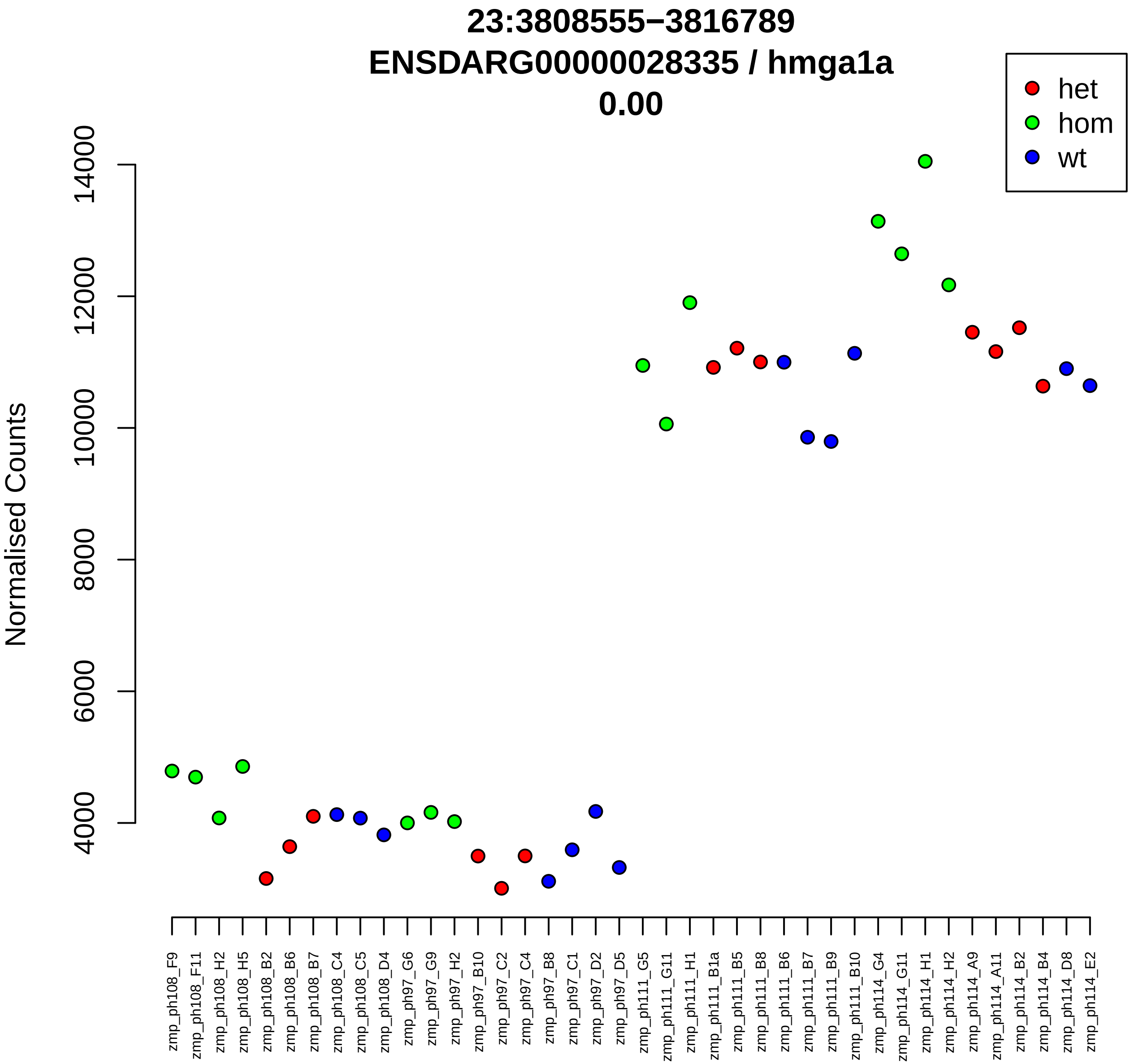

23:3808555−3816789
ENSDARG00000028335 / hmga1a
0.00
het
hom
wt
Normalised Counts
4000
6000
8000
10000
12000
14000
zmp_ph108_F9
zmp_ph108_F11
zmp_ph108_H2
zmp_ph108_H5
zmp_ph108_B2
zmp_ph108_B6
zmp_ph108_B7
zmp_ph108_C4
zmp_ph108_C5
zmp_ph108_D4
zmp_ph97_G6
zmp_ph97_G9
zmp_ph97_H2
zmp_ph97_B10
zmp_ph97_C2
zmp_ph97_C4
zmp_ph97_B8
zmp_ph97_C1
zmp_ph97_D2
zmp_ph97_D5
zmp_ph111_G5
zmp_ph111_G11
zmp_ph111_H1
zmp_ph111_B1a
zmp_ph111_B5
zmp_ph111_B8
zmp_ph111_B6
zmp_ph111_B7
zmp_ph111_B9
zmp_ph111_B10
zmp_ph114_G4
zmp_ph114_G11
zmp_ph114_H1
zmp_ph114_H2
zmp_ph114_A9
zmp_ph114_A11
zmp_ph114_B2
zmp_ph114_B4
zmp_ph114_D8
zmp_ph114_E2

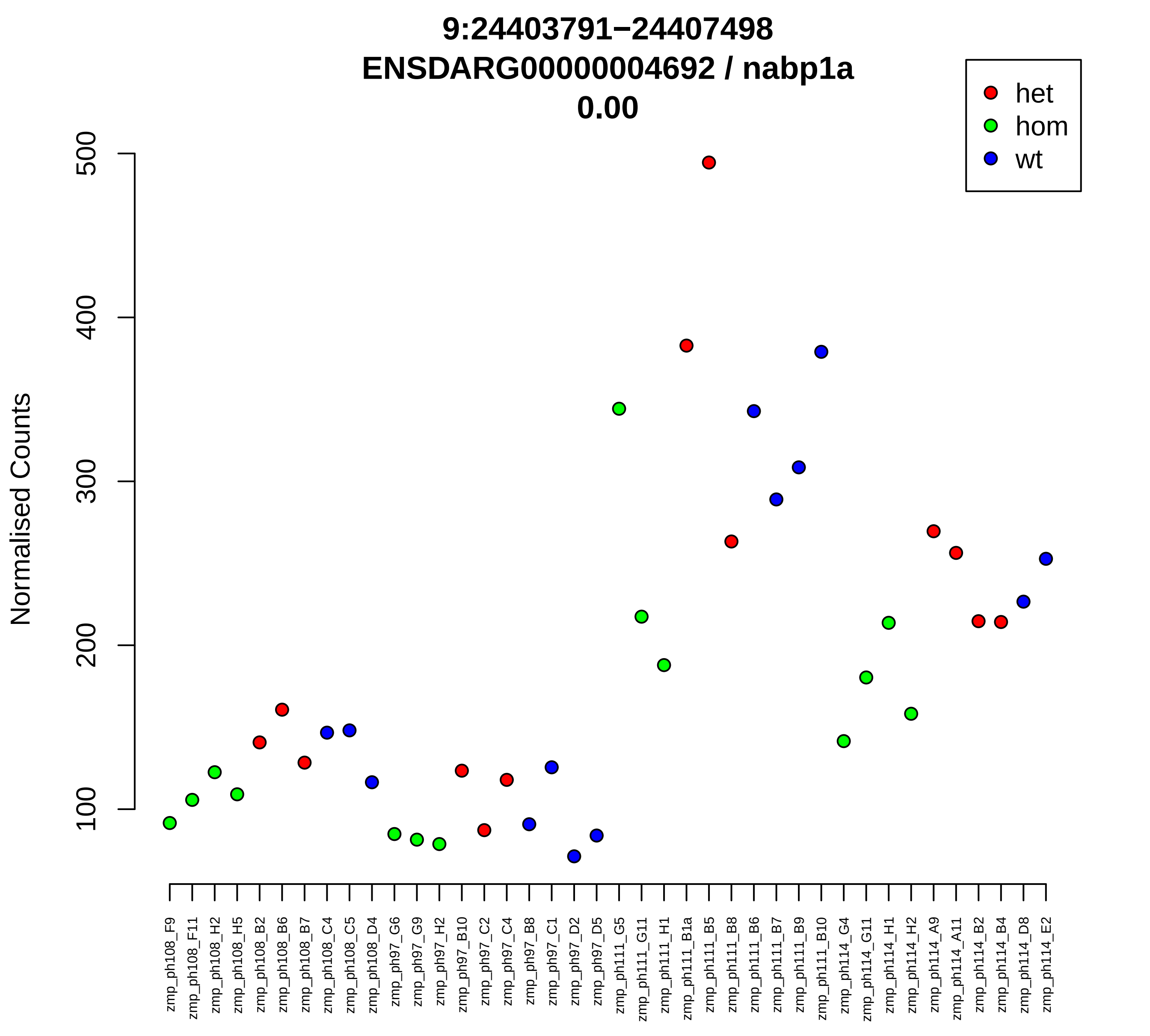
9:24403791−24407498
ENSDARG00000004692 / nabp1a
0.00
het
hom
wt
Normalised Counts
100
200
300
400
500
zmp_ph108_F9
zmp_ph108_F11
zmp_ph108_H2
zmp_ph108_H5
zmp_ph108_B2
zmp_ph108_B6
zmp_ph108_B7
zmp_ph108_C4
zmp_ph108_C5
zmp_ph108_D4
zmp_ph97_G6
zmp_ph97_G9
zmp_ph97_H2
zmp_ph97_B10
zmp_ph97_C2
zmp_ph97_C4
zmp_ph97_B8
zmp_ph97_C1
zmp_ph97_D2
zmp_ph97_D5
zmp_ph111_G5
zmp_ph111_G11
zmp_ph111_H1
zmp_ph111_B1a
zmp_ph111_B5
zmp_ph111_B8
zmp_ph111_B6
zmp_ph111_B7
zmp_ph111_B9
zmp_ph111_B10
zmp_ph114_G4
zmp_ph114_G11
zmp_ph114_H1
zmp_ph114_H2
zmp_ph114_A9
zmp_ph114_A11
zmp_ph114_B2
zmp_ph114_B4
zmp_ph114_D8
zmp_ph114_E2

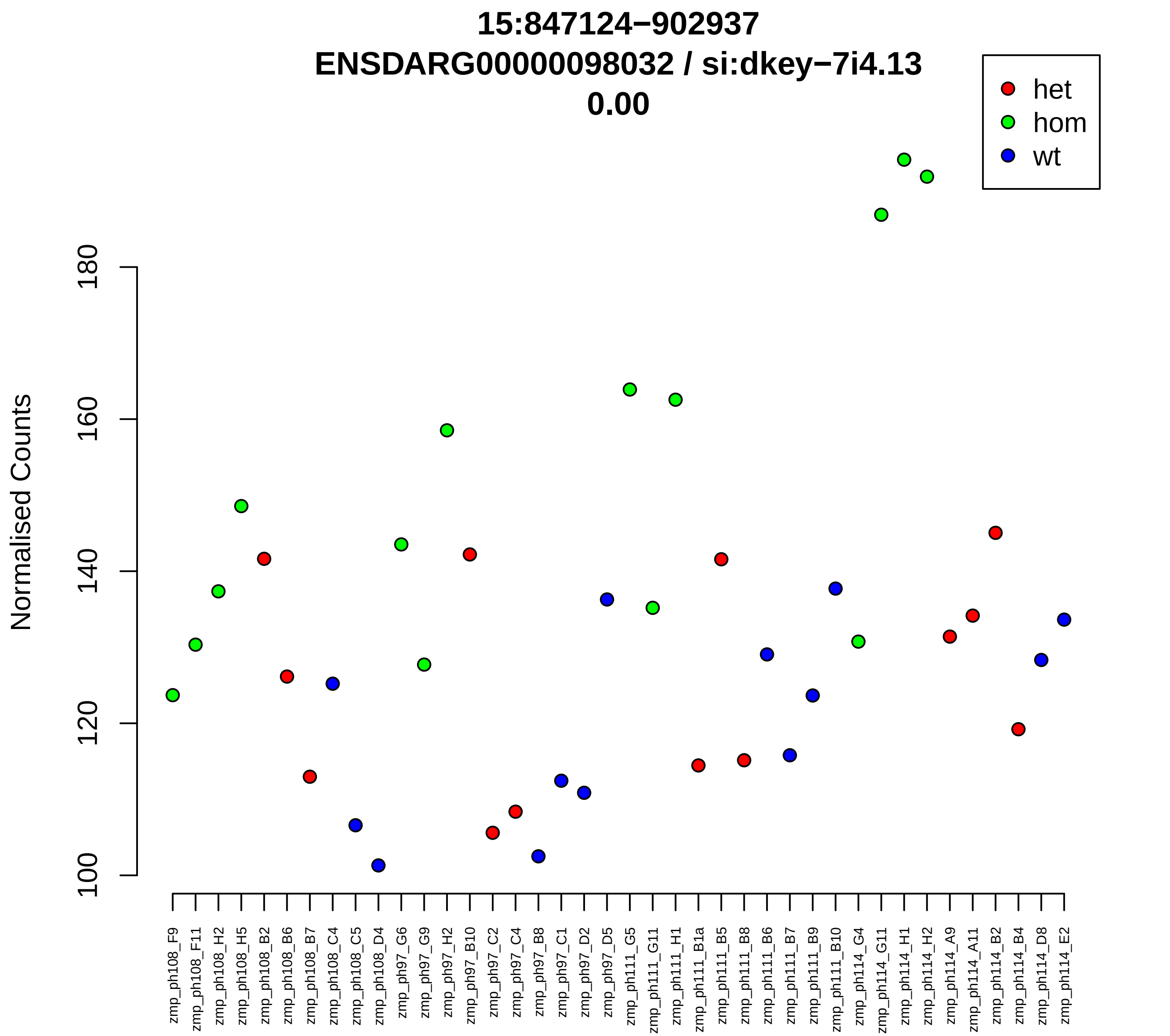
15:847124−902937
ENSDARG00000098032 / si:dkey−7i4.13
0.00
het
hom
wt
Normalised Counts
100
120
140
160
180
zmp_ph108_F9
zmp_ph108_F11
zmp_ph108_H2
zmp_ph108_H5
zmp_ph108_B2
zmp_ph108_B6
zmp_ph108_B7
zmp_ph108_C4
zmp_ph108_C5
zmp_ph108_D4
zmp_ph97_G6
zmp_ph97_G9
zmp_ph97_H2
zmp_ph97_B10
zmp_ph97_C2
zmp_ph97_C4
zmp_ph97_B8
zmp_ph97_C1
zmp_ph97_D2
zmp_ph97_D5
zmp_ph111_G5
zmp_ph111_G11
zmp_ph111_H1
zmp_ph111_B1a
zmp_ph111_B5
zmp_ph111_B8
zmp_ph111_B6
zmp_ph111_B7
zmp_ph111_B9
zmp_ph111_B10
zmp_ph114_G4
zmp_ph114_G11
zmp_ph114_H1
zmp_ph114_H2
zmp_ph114_A9
zmp_ph114_A11
zmp_ph114_B2
zmp_ph114_B4
zmp_ph114_D8
zmp_ph114_E2

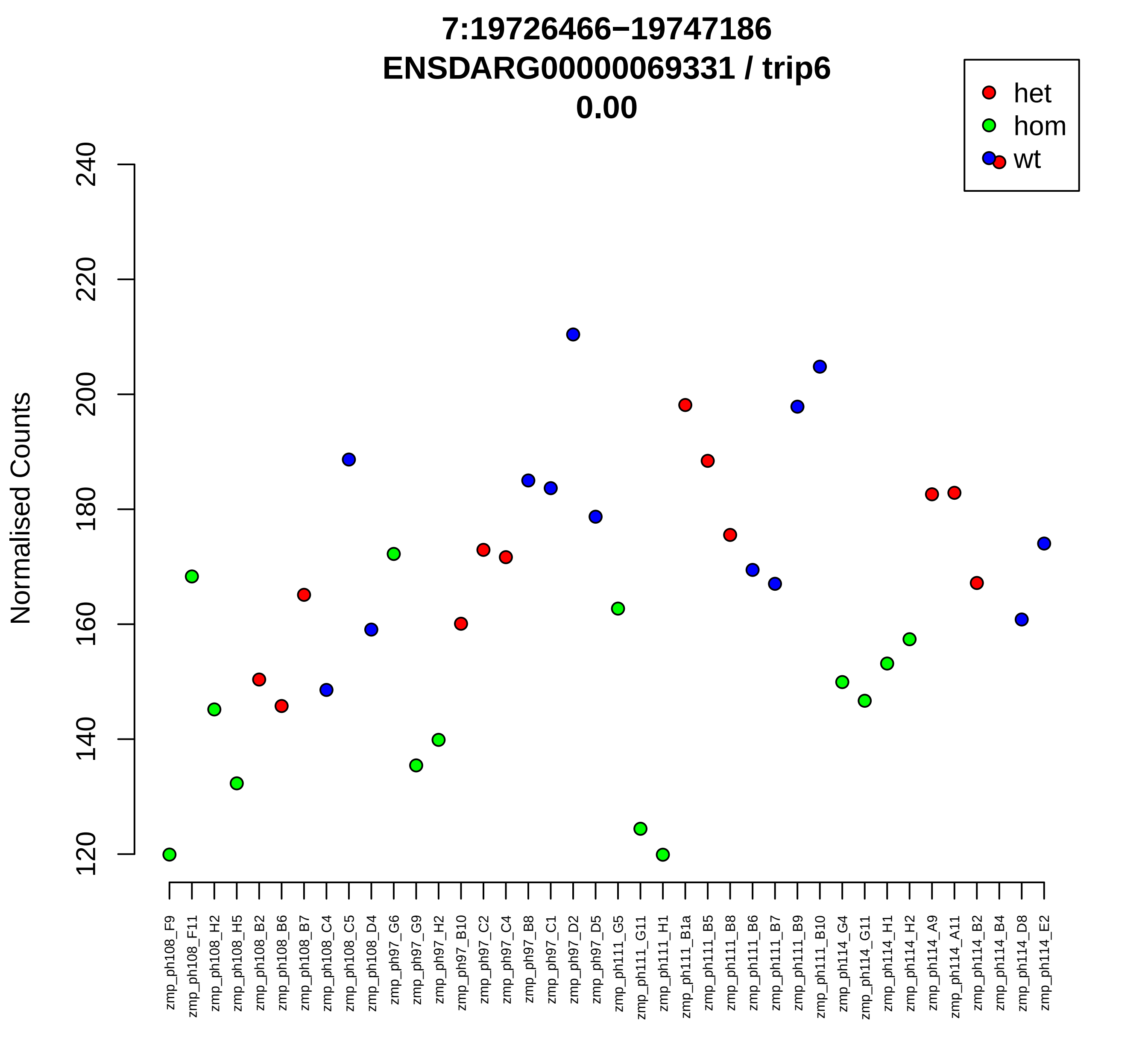

7:19726466−19747186
ENSDARG00000069331 / trip6
0.00
het
hom
wt
Normalised Counts
120
140
160
180
200
220
240
zmp_ph108_F9
zmp_ph108_F11
zmp_ph108_H2
zmp_ph108_H5
zmp_ph108_B2
zmp_ph108_B6
zmp_ph108_B7
zmp_ph108_C4
zmp_ph108_C5
zmp_ph108_D4
zmp_ph97_G6
zmp_ph97_G9
zmp_ph97_H2
zmp_ph97_B10
zmp_ph97_C2
zmp_ph97_C4
zmp_ph97_B8
zmp_ph97_C1
zmp_ph97_D2
zmp_ph97_D5
zmp_ph111_G5
zmp_ph111_G11
zmp_ph111_H1
zmp_ph111_B1a
zmp_ph111_B5
zmp_ph111_B8
zmp_ph111_B6
zmp_ph111_B7
zmp_ph111_B9
zmp_ph111_B10
zmp_ph114_G4
zmp_ph114_G11
zmp_ph114_H1
zmp_ph114_H2
zmp_ph114_A9
zmp_ph114_A11
zmp_ph114_B2
zmp_ph114_B4
zmp_ph114_D8
zmp_ph114_E2

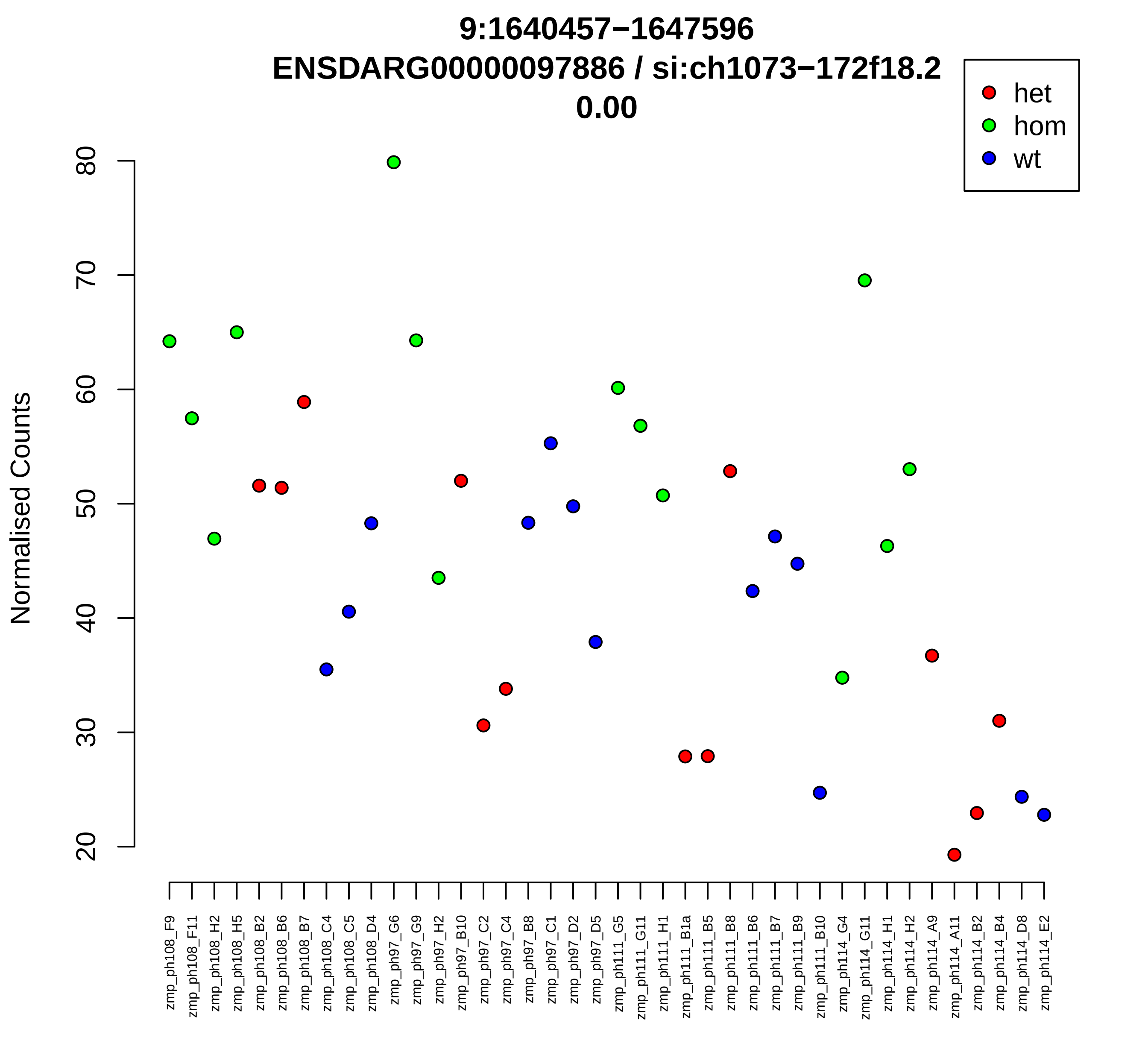

9:1640457−1647596
ENSDARG00000097886 / si:ch1073−172f18.2
0.00
het
hom
wt
Normalised Counts
20
30
40
50
60
70
80
zmp_ph108_F9
zmp_ph108_F11
zmp_ph108_H2
zmp_ph108_H5
zmp_ph108_B2
zmp_ph108_B6
zmp_ph108_B7
zmp_ph108_C4
zmp_ph108_C5
zmp_ph108_D4
zmp_ph97_G6
zmp_ph97_G9
zmp_ph97_H2
zmp_ph97_B10
zmp_ph97_C2
zmp_ph97_C4
zmp_ph97_B8
zmp_ph97_C1
zmp_ph97_D2
zmp_ph97_D5
zmp_ph111_G5
zmp_ph111_G11
zmp_ph111_H1
zmp_ph111_B1a
zmp_ph111_B5
zmp_ph111_B8
zmp_ph111_B6
zmp_ph111_B7
zmp_ph111_B9
zmp_ph111_B10
zmp_ph114_G4
zmp_ph114_G11
zmp_ph114_H1
zmp_ph114_H2
zmp_ph114_A9
zmp_ph114_A11
zmp_ph114_B2
zmp_ph114_B4
zmp_ph114_D8
zmp_ph114_E2

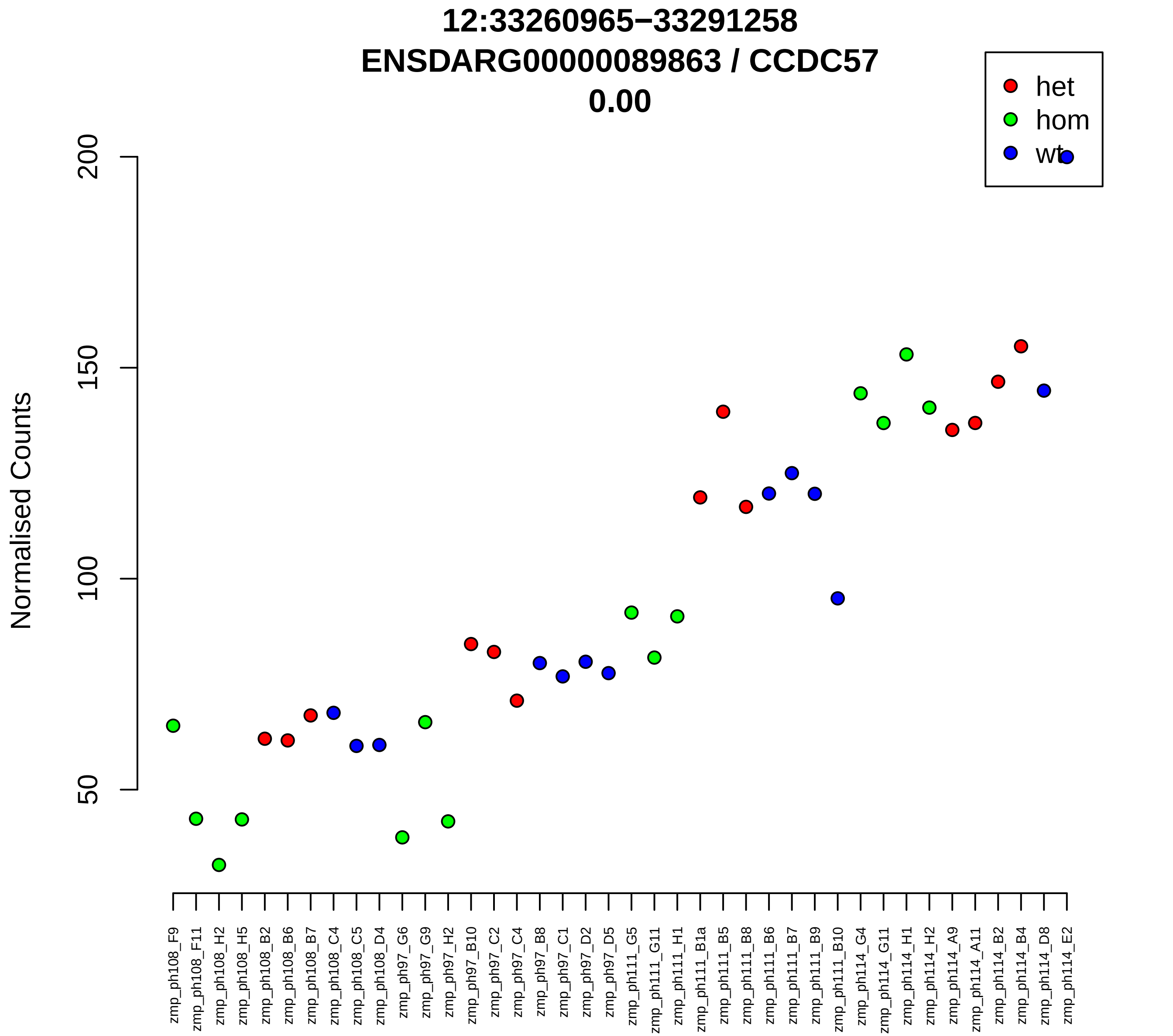

12:33260965−33291258
ENSDARG00000089863 / CCDC57
0.00
het
hom
wt
Normalised Counts
50
100
150
200
zmp_ph108_F9
zmp_ph108_F11
zmp_ph108_H2
zmp_ph108_H5
zmp_ph108_B2
zmp_ph108_B6
zmp_ph108_B7
zmp_ph108_C4
zmp_ph108_C5
zmp_ph108_D4
zmp_ph97_G6
zmp_ph97_G9
zmp_ph97_H2
zmp_ph97_B10
zmp_ph97_C2
zmp_ph97_C4
zmp_ph97_B8
zmp_ph97_C1
zmp_ph97_D2
zmp_ph97_D5
zmp_ph111_G5
zmp_ph111_G11
zmp_ph111_H1
zmp_ph111_B1a
zmp_ph111_B5
zmp_ph111_B8
zmp_ph111_B6
zmp_ph111_B7
zmp_ph111_B9
zmp_ph111_B10
zmp_ph114_G4
zmp_ph114_G11
zmp_ph114_H1
zmp_ph114_H2
zmp_ph114_A9
zmp_ph114_A11
zmp_ph114_B2
zmp_ph114_B4
zmp_ph114_D8
zmp_ph114_E2

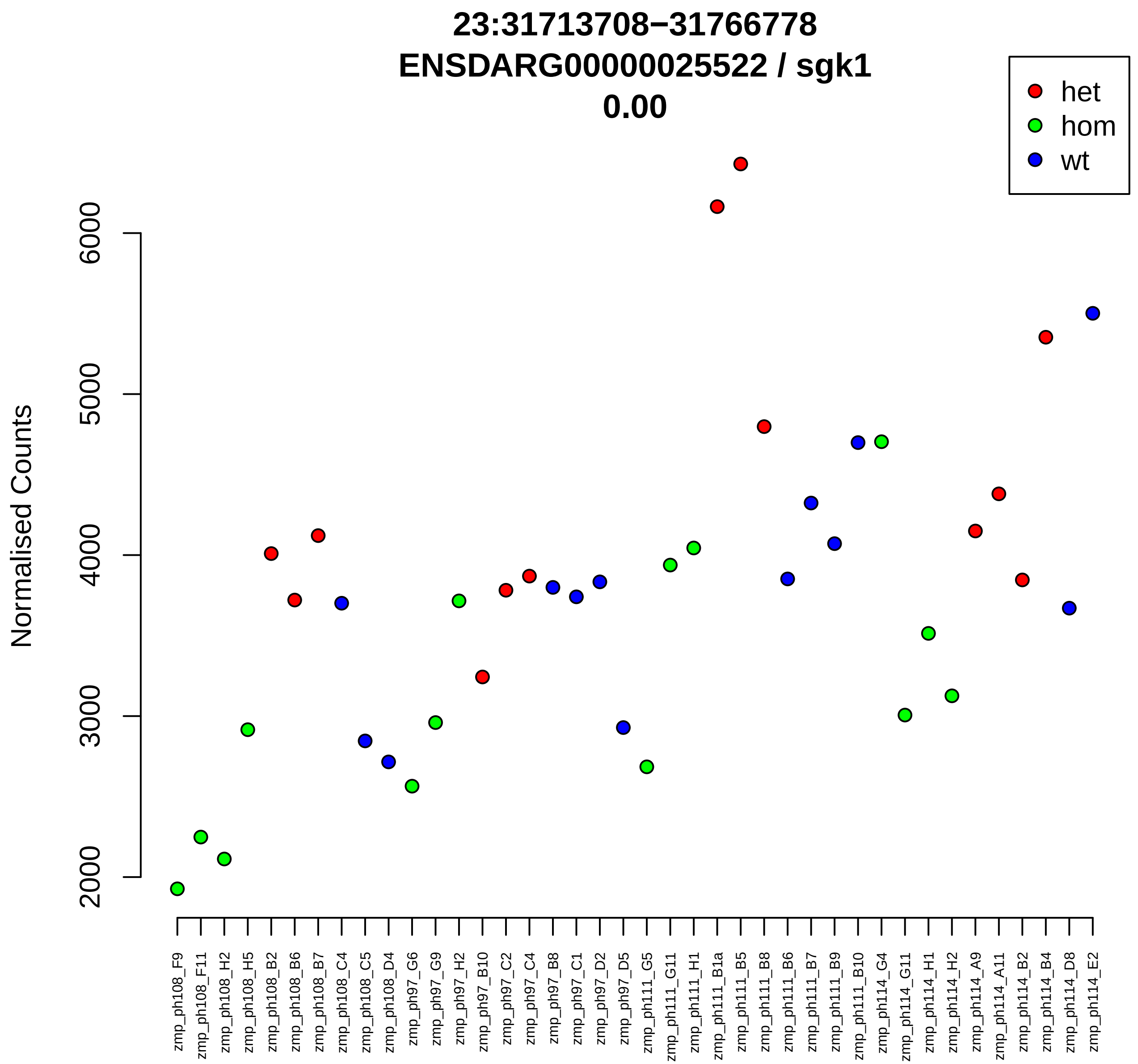
23:31713708−31766778
ENSDARG00000025522 / sgk1
0.00
het
hom
wt
Normalised Counts
2000
3000
4000
5000
6000
zmp_ph108_F9
zmp_ph108_F11
zmp_ph108_H2
zmp_ph108_H5
zmp_ph108_B2
zmp_ph108_B6
zmp_ph108_B7
zmp_ph108_C4
zmp_ph108_C5
zmp_ph108_D4
zmp_ph97_G6
zmp_ph97_G9
zmp_ph97_H2
zmp_ph97_B10
zmp_ph97_C2
zmp_ph97_C4
zmp_ph97_B8
zmp_ph97_C1
zmp_ph97_D2
zmp_ph97_D5
zmp_ph111_G5
zmp_ph111_G11
zmp_ph111_H1
zmp_ph111_B1a
zmp_ph111_B5
zmp_ph111_B8
zmp_ph111_B6
zmp_ph111_B7
zmp_ph111_B9
zmp_ph111_B10
zmp_ph114_G4
zmp_ph114_G11
zmp_ph114_H1
zmp_ph114_H2
zmp_ph114_A9
zmp_ph114_A11
zmp_ph114_B2
zmp_ph114_B4
zmp_ph114_D8
zmp_ph114_E2

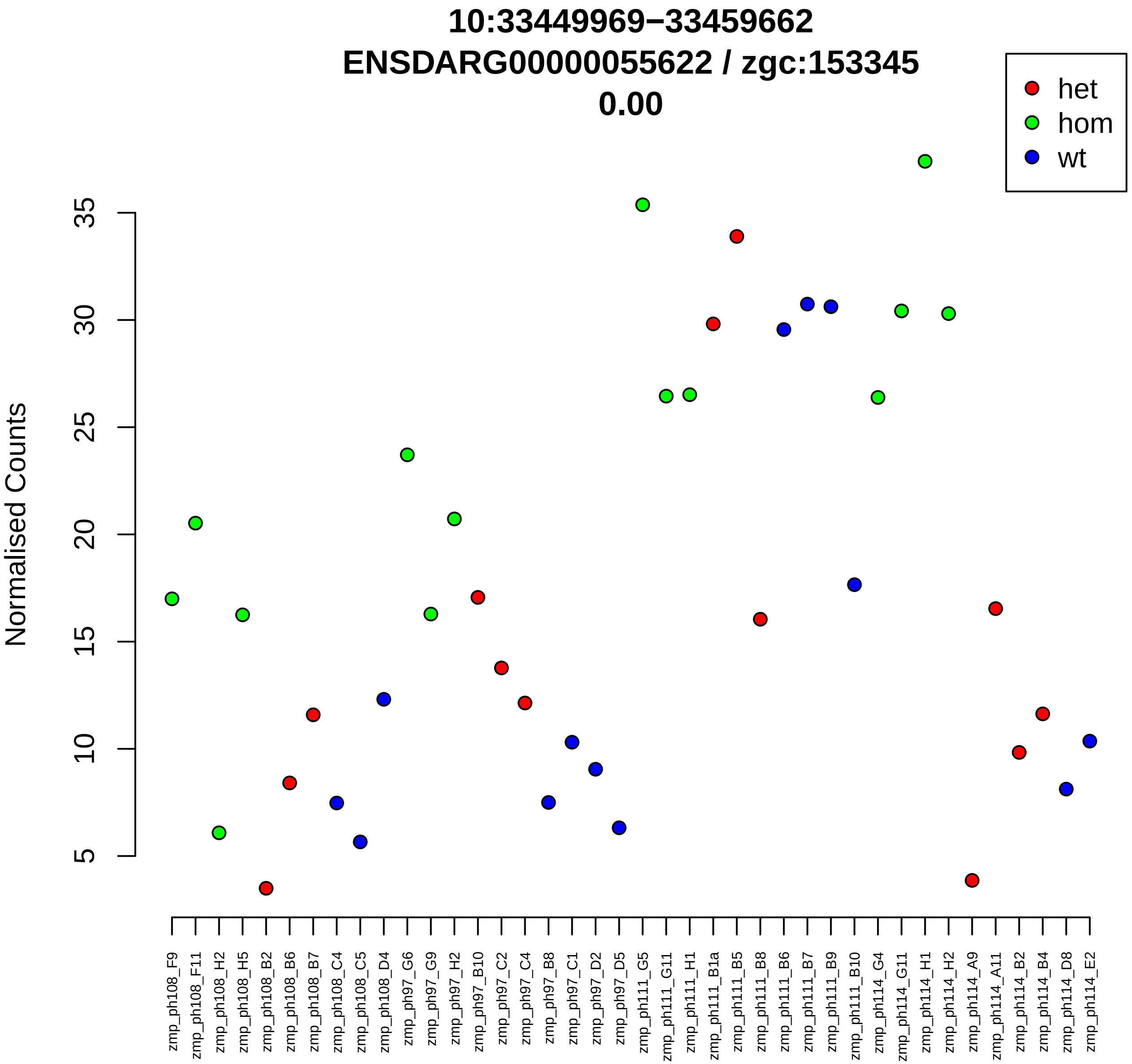
10:33449969−33459662
ENSDARG00000055622 / zgc:153345
0.00
het
hom
wt
Normalised Counts
5
10
15
20
25
30
35
zmp_ph108_F9
zmp_ph108_F11
zmp_ph108_H2
zmp_ph108_H5
zmp_ph108_B2
zmp_ph108_B6
zmp_ph108_B7
zmp_ph108_C4
zmp_ph108_C5
zmp_ph108_D4
zmp_ph97_G6
zmp_ph97_G9
zmp_ph97_H2
zmp_ph97_B10
zmp_ph97_C2
zmp_ph97_C4
zmp_ph97_B8
zmp_ph97_C1
zmp_ph97_D2
zmp_ph97_D5
zmp_ph111_G5
zmp_ph111_G11
zmp_ph111_H1
zmp_ph111_B1a
zmp_ph111_B5
zmp_ph111_B8
zmp_ph111_B6
zmp_ph111_B7
zmp_ph111_B9
zmp_ph111_B10
zmp_ph114_G4
zmp_ph114_G11
zmp_ph114_H1
zmp_ph114_H2
zmp_ph114_A9
zmp_ph114_A11
zmp_ph114_B2
zmp_ph114_B4
zmp_ph114_D8
zmp_ph114_E2

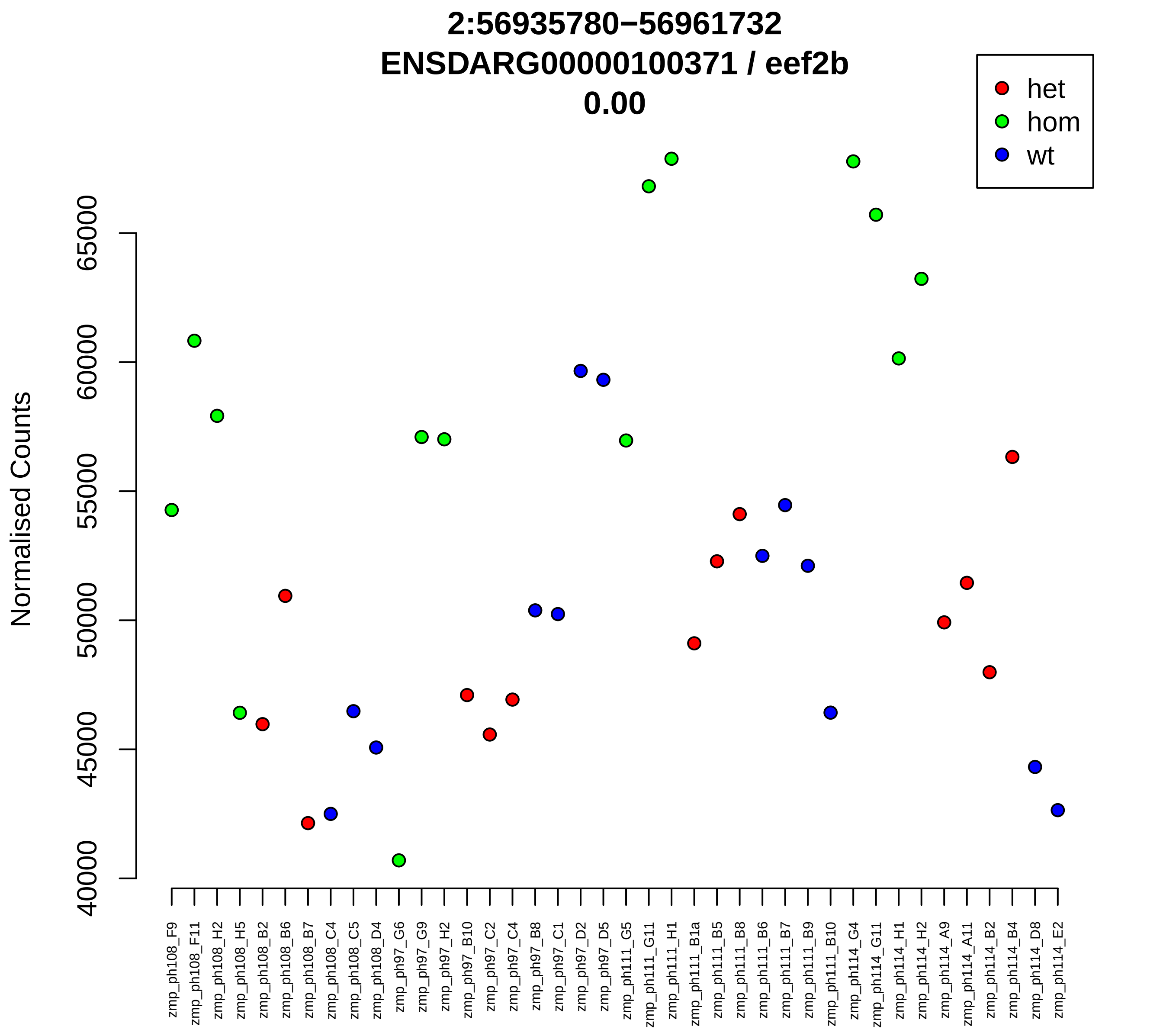
2:56935780−56961732
ENSDARG00000100371 / eef2b
0.00
het
hom
wt
Normalised Counts
40000
45000
50000
55000
60000
65000
zmp_ph108_F9
zmp_ph108_F11
zmp_ph108_H2
zmp_ph108_H5
zmp_ph108_B2
zmp_ph108_B6
zmp_ph108_B7
zmp_ph108_C4
zmp_ph108_C5
zmp_ph108_D4
zmp_ph97_G6
zmp_ph97_G9
zmp_ph97_H2
zmp_ph97_B10
zmp_ph97_C2
zmp_ph97_C4
zmp_ph97_B8
zmp_ph97_C1
zmp_ph97_D2
zmp_ph97_D5
zmp_ph111_G5
zmp_ph111_G11
zmp_ph111_H1
zmp_ph111_B1a
zmp_ph111_B5
zmp_ph111_B8
zmp_ph111_B6
zmp_ph111_B7
zmp_ph111_B9
zmp_ph111_B10
zmp_ph114_G4
zmp_ph114_G11
zmp_ph114_H1
zmp_ph114_H2
zmp_ph114_A9
zmp_ph114_A11
zmp_ph114_B2
zmp_ph114_B4
zmp_ph114_D8
zmp_ph114_E2

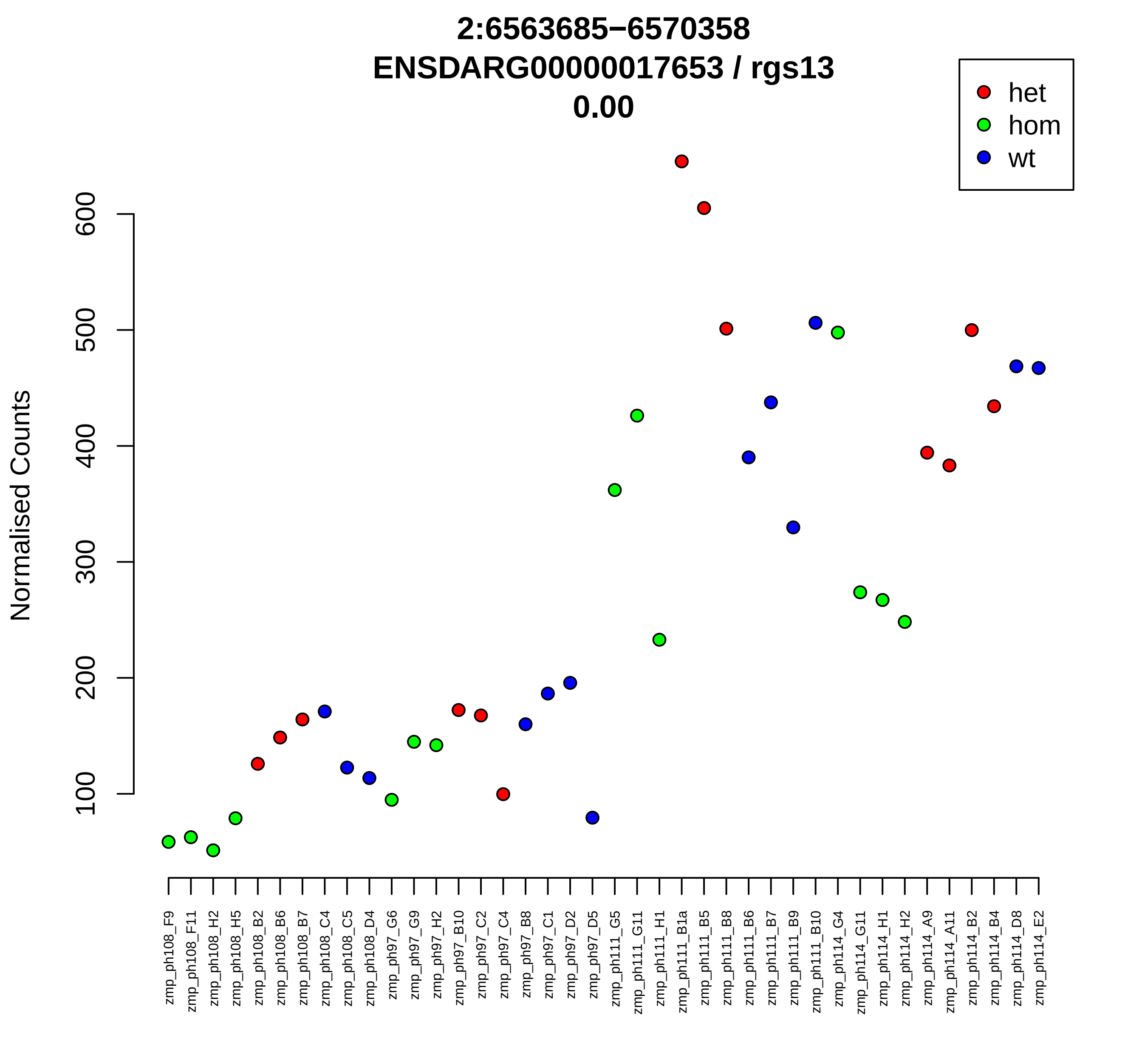

2:6563685−6570358
ENSDARG00000017653 / rgs13
0.00
het
hom
wt
Normalised Counts
100
200
300
400
500
600
zmp_ph108_F9
zmp_ph108_F11
zmp_ph108_H2
zmp_ph108_H5
zmp_ph108_B2
zmp_ph108_B6
zmp_ph108_B7
zmp_ph108_C4
zmp_ph108_C5
zmp_ph108_D4
zmp_ph97_G6
zmp_ph97_G9
zmp_ph97_H2
zmp_ph97_B10
zmp_ph97_C2
zmp_ph97_C4
zmp_ph97_B8
zmp_ph97_C1
zmp_ph97_D2
zmp_ph97_D5
zmp_ph111_G5
zmp_ph111_G11
zmp_ph111_H1
zmp_ph111_B1a
zmp_ph111_B5
zmp_ph111_B8
zmp_ph111_B6
zmp_ph111_B7
zmp_ph111_B9
zmp_ph111_B10
zmp_ph114_G4
zmp_ph114_G11
zmp_ph114_H1
zmp_ph114_H2
zmp_ph114_A9
zmp_ph114_A11
zmp_ph114_B2
zmp_ph114_B4
zmp_ph114_D8
zmp_ph114_E2

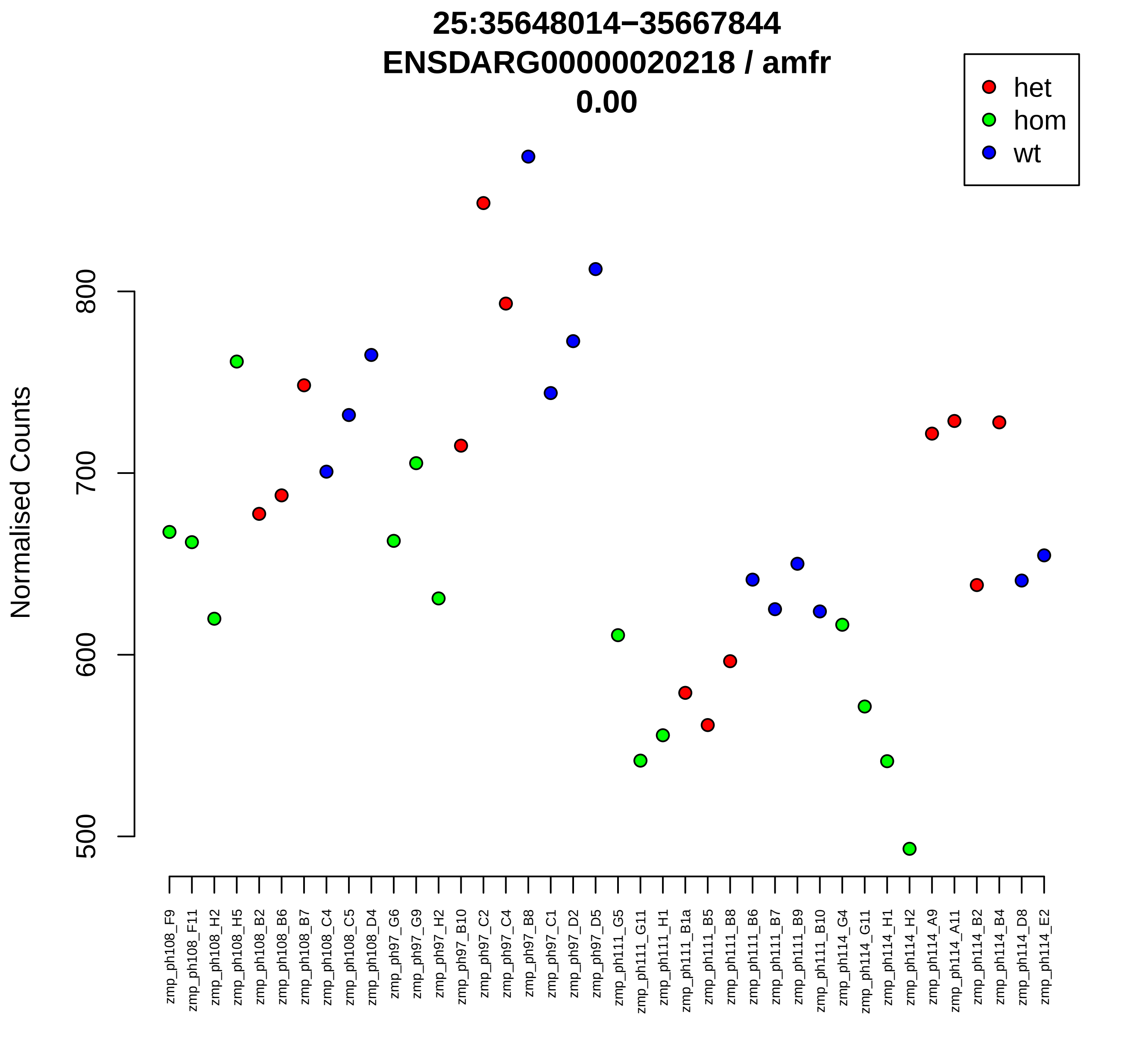

25:35648014−35667844
ENSDARG00000020218 / amfr
0.00
het
hom
wt
Normalised Counts
500
600
700
800
zmp_ph108_F9
zmp_ph108_F11
zmp_ph108_H2
zmp_ph108_H5
zmp_ph108_B2
zmp_ph108_B6
zmp_ph108_B7
zmp_ph108_C4
zmp_ph108_C5
zmp_ph108_D4
zmp_ph97_G6
zmp_ph97_G9
zmp_ph97_H2
zmp_ph97_B10
zmp_ph97_C2
zmp_ph97_C4
zmp_ph97_B8
zmp_ph97_C1
zmp_ph97_D2
zmp_ph97_D5
zmp_ph111_G5
zmp_ph111_G11
zmp_ph111_H1
zmp_ph111_B1a
zmp_ph111_B5
zmp_ph111_B8
zmp_ph111_B6
zmp_ph111_B7
zmp_ph111_B9
zmp_ph111_B10
zmp_ph114_G4
zmp_ph114_G11
zmp_ph114_H1
zmp_ph114_H2
zmp_ph114_A9
zmp_ph114_A11
zmp_ph114_B2
zmp_ph114_B4
zmp_ph114_D8
zmp_ph114_E2

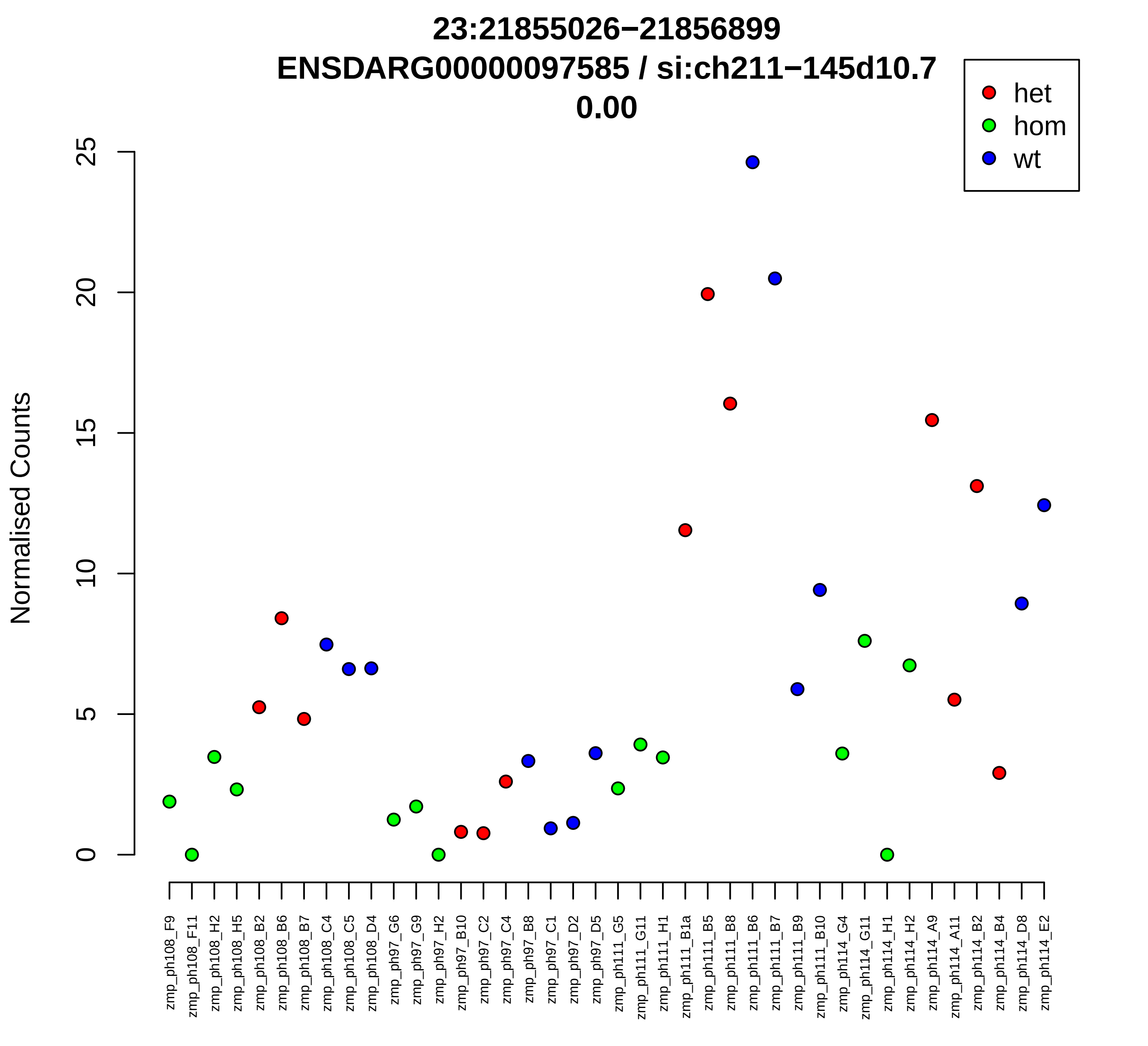

23:21855026−21856899
ENSDARG00000097585 / si:ch211−145d10.7
0.00
het
hom
wt
Normalised Counts
0
5
10
15
20
25
zmp_ph108_F9
zmp_ph108_F11
zmp_ph108_H2
zmp_ph108_H5
zmp_ph108_B2
zmp_ph108_B6
zmp_ph108_B7
zmp_ph108_C4
zmp_ph108_C5
zmp_ph108_D4
zmp_ph97_G6
zmp_ph97_G9
zmp_ph97_H2
zmp_ph97_B10
zmp_ph97_C2
zmp_ph97_C4
zmp_ph97_B8
zmp_ph97_C1
zmp_ph97_D2
zmp_ph97_D5
zmp_ph111_G5
zmp_ph111_G11
zmp_ph111_H1
zmp_ph111_B1a
zmp_ph111_B5
zmp_ph111_B8
zmp_ph111_B6
zmp_ph111_B7
zmp_ph111_B9
zmp_ph111_B10
zmp_ph114_G4
zmp_ph114_G11
zmp_ph114_H1
zmp_ph114_H2
zmp_ph114_A9
zmp_ph114_A11
zmp_ph114_B2
zmp_ph114_B4
zmp_ph114_D8
zmp_ph114_E2

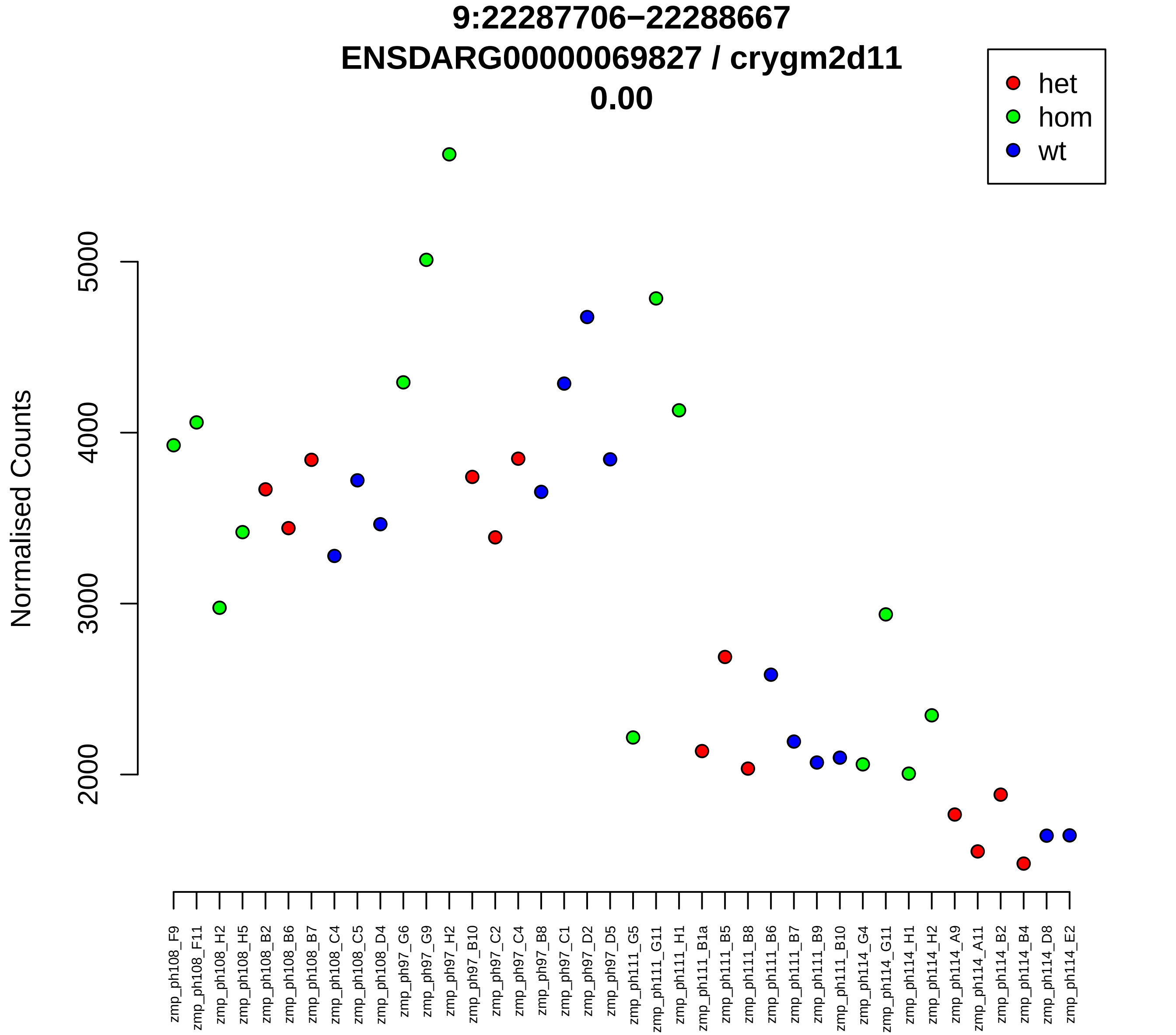
9:22287706−22288667
ENSDARG00000069827 / crygm2d11
0.00
het
hom
wt
Normalised Counts
2000
3000
4000
5000
zmp_ph108_F9
zmp_ph108_F11
zmp_ph108_H2
zmp_ph108_H5
zmp_ph108_B2
zmp_ph108_B6
zmp_ph108_B7
zmp_ph108_C4
zmp_ph108_C5
zmp_ph108_D4
zmp_ph97_G6
zmp_ph97_G9
zmp_ph97_H2
zmp_ph97_B10
zmp_ph97_C2
zmp_ph97_C4
zmp_ph97_B8
zmp_ph97_C1
zmp_ph97_D2
zmp_ph97_D5
zmp_ph111_G5
zmp_ph111_G11
zmp_ph111_H1
zmp_ph111_B1a
zmp_ph111_B5
zmp_ph111_B8
zmp_ph111_B6
zmp_ph111_B7
zmp_ph111_B9
zmp_ph111_B10
zmp_ph114_G4
zmp_ph114_G11
zmp_ph114_H1
zmp_ph114_H2
zmp_ph114_A9
zmp_ph114_A11
zmp_ph114_B2
zmp_ph114_B4
zmp_ph114_D8
zmp_ph114_E2

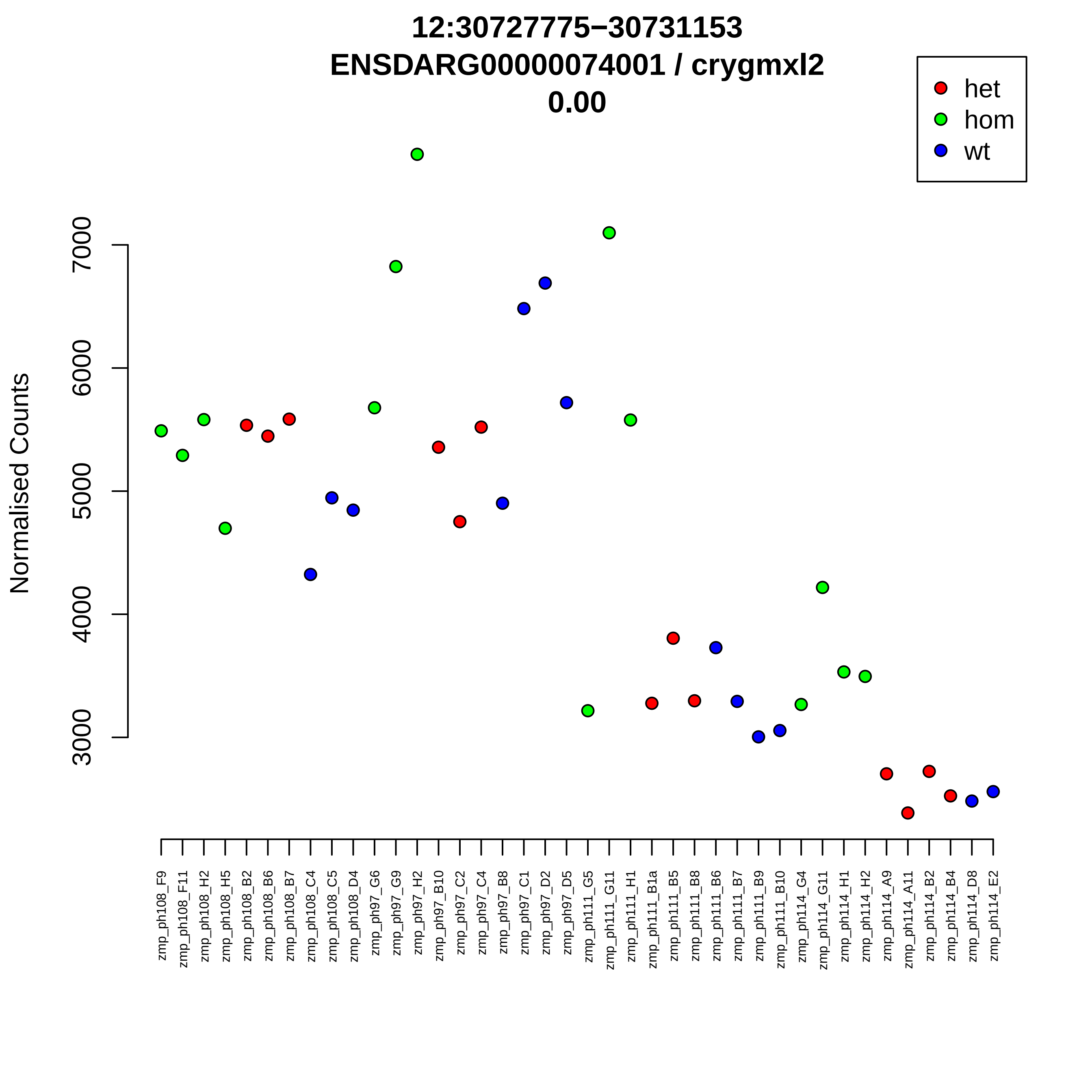

12:30727775−30731153
ENSDARG00000074001 / crygmxl2
0.00
het
hom
wt
Normalised Counts
3000
4000
5000
6000
7000
zmp_ph108_F9
zmp_ph108_F11
zmp_ph108_H2
zmp_ph108_H5
zmp_ph108_B2
zmp_ph108_B6
zmp_ph108_B7
zmp_ph108_C4
zmp_ph108_C5
zmp_ph108_D4
zmp_ph97_G6
zmp_ph97_G9
zmp_ph97_H2
zmp_ph97_B10
zmp_ph97_C2
zmp_ph97_C4
zmp_ph97_B8
zmp_ph97_C1
zmp_ph97_D2
zmp_ph97_D5
zmp_ph111_G5
zmp_ph111_G11
zmp_ph111_H1
zmp_ph111_B1a
zmp_ph111_B5
zmp_ph111_B8
zmp_ph111_B6
zmp_ph111_B7
zmp_ph111_B9
zmp_ph111_B10
zmp_ph114_G4
zmp_ph114_G11
zmp_ph114_H1
zmp_ph114_H2
zmp_ph114_A9
zmp_ph114_A11
zmp_ph114_B2
zmp_ph114_B4
zmp_ph114_D8
zmp_ph114_E2

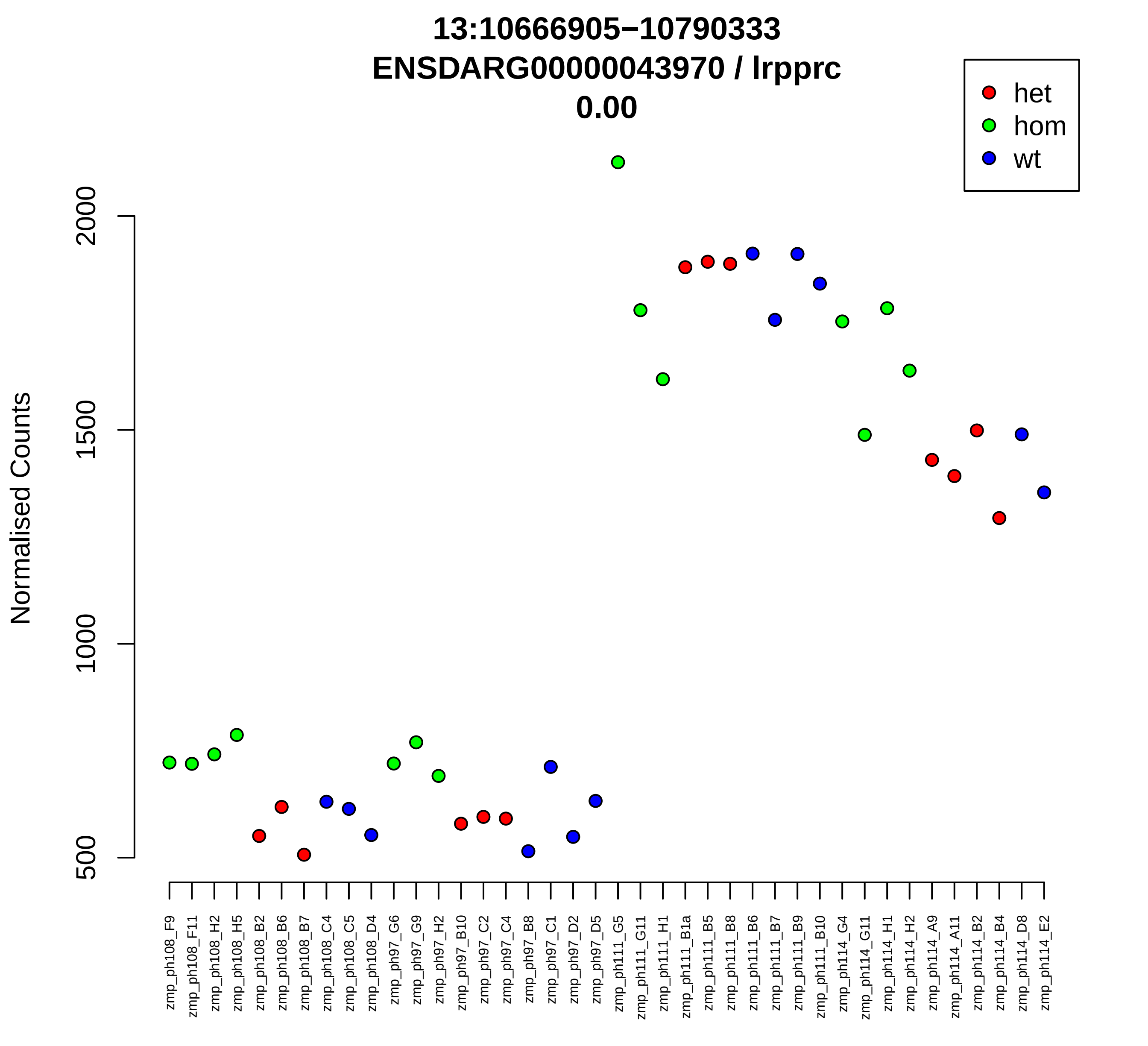

13:10666905−10790333
ENSDARG00000043970 / lrpprc
0.00
het
hom
wt
Normalised Counts
500
1000
1500
2000
zmp_ph108_F9
zmp_ph108_F11
zmp_ph108_H2
zmp_ph108_H5
zmp_ph108_B2
zmp_ph108_B6
zmp_ph108_B7
zmp_ph108_C4
zmp_ph108_C5
zmp_ph108_D4
zmp_ph97_G6
zmp_ph97_G9
zmp_ph97_H2
zmp_ph97_B10
zmp_ph97_C2
zmp_ph97_C4
zmp_ph97_B8
zmp_ph97_C1
zmp_ph97_D2
zmp_ph97_D5
zmp_ph111_G5
zmp_ph111_G11
zmp_ph111_H1
zmp_ph111_B1a
zmp_ph111_B5
zmp_ph111_B8
zmp_ph111_B6
zmp_ph111_B7
zmp_ph111_B9
zmp_ph111_B10
zmp_ph114_G4
zmp_ph114_G11
zmp_ph114_H1
zmp_ph114_H2
zmp_ph114_A9
zmp_ph114_A11
zmp_ph114_B2
zmp_ph114_B4
zmp_ph114_D8
zmp_ph114_E2

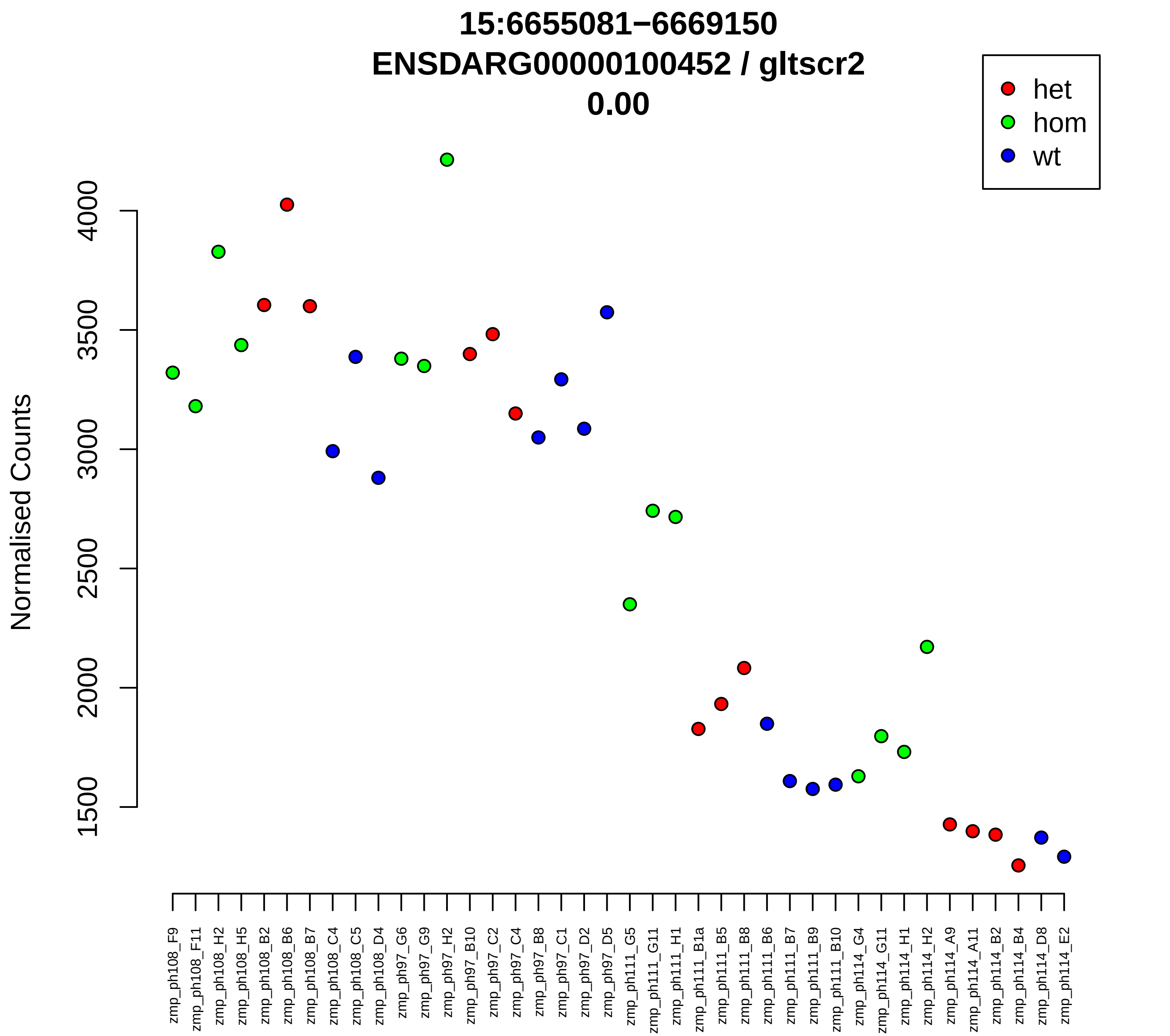

15:6655081−6669150
ENSDARG00000100452 / gltscr2
0.00
het
hom
wt
Normalised Counts
1500
2000
2500
3000
3500
4000
zmp_ph108_F9
zmp_ph108_F11
zmp_ph108_H2
zmp_ph108_H5
zmp_ph108_B2
zmp_ph108_B6
zmp_ph108_B7
zmp_ph108_C4
zmp_ph108_C5
zmp_ph108_D4
zmp_ph97_G6
zmp_ph97_G9
zmp_ph97_H2
zmp_ph97_B10
zmp_ph97_C2
zmp_ph97_C4
zmp_ph97_B8
zmp_ph97_C1
zmp_ph97_D2
zmp_ph97_D5
zmp_ph111_G5
zmp_ph111_G11
zmp_ph111_H1
zmp_ph111_B1a
zmp_ph111_B5
zmp_ph111_B8
zmp_ph111_B6
zmp_ph111_B7
zmp_ph111_B9
zmp_ph111_B10
zmp_ph114_G4
zmp_ph114_G11
zmp_ph114_H1
zmp_ph114_H2
zmp_ph114_A9
zmp_ph114_A11
zmp_ph114_B2
zmp_ph114_B4
zmp_ph114_D8
zmp_ph114_E2

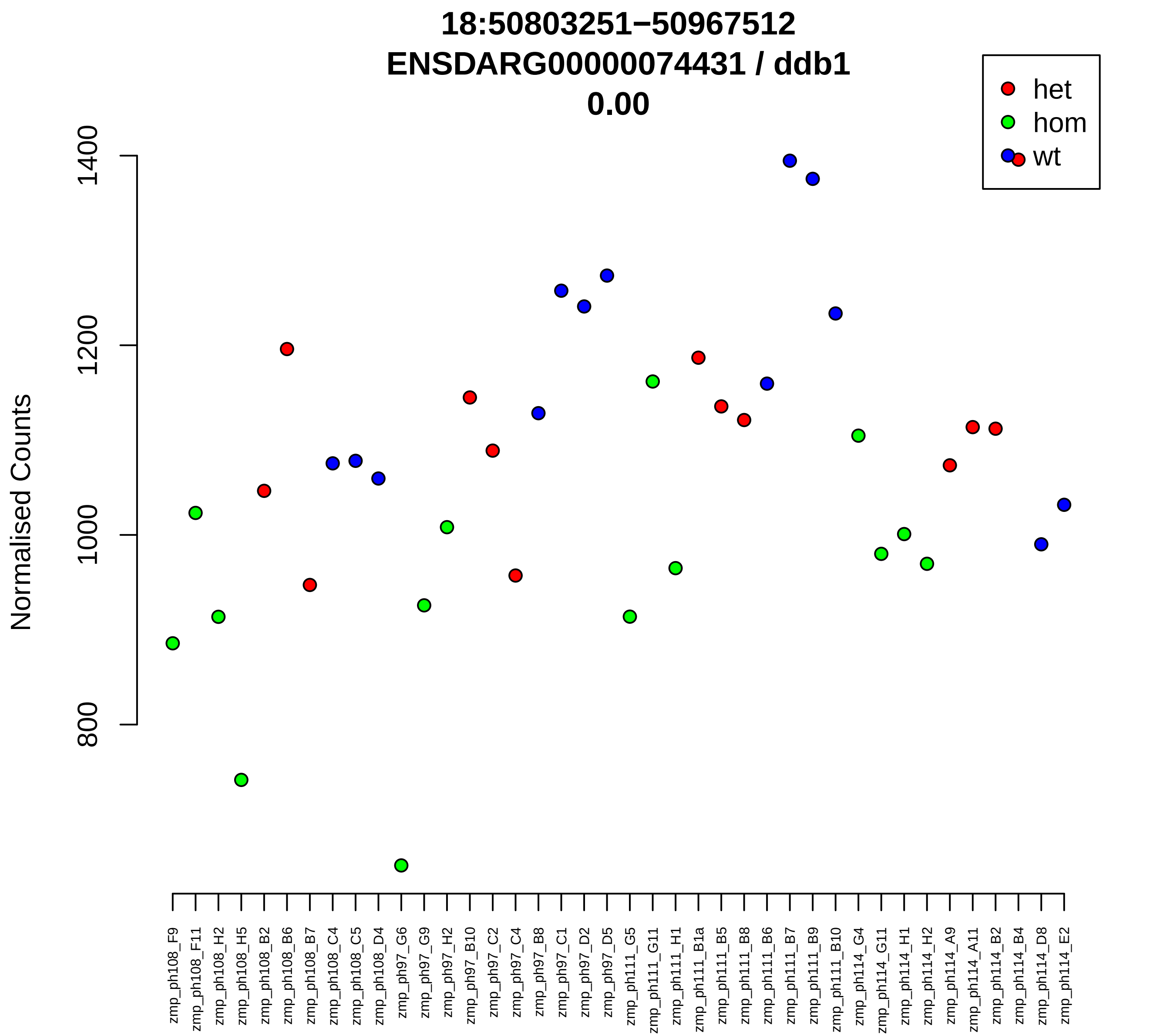

18:50803251−50967512
ENSDARG00000074431 / ddb1
0.00
het
hom
wt
Normalised Counts
1400
1200
1000
800
zmp_ph108_F9
zmp_ph108_F11
zmp_ph108_H2
zmp_ph108_H5
zmp_ph108_B2
zmp_ph108_B6
zmp_ph108_B7
zmp_ph108_C4
zmp_ph108_C5
zmp_ph108_D4
zmp_ph97_G6
zmp_ph97_G9
zmp_ph97_H2
zmp_ph97_B10
zmp_ph97_C2
zmp_ph97_C4
zmp_ph97_B8
zmp_ph97_C1
zmp_ph97_D2
zmp_ph97_D5
zmp_ph111_G5
zmp_ph111_G11
zmp_ph111_H1
zmp_ph111_B1a
zmp_ph111_B5
zmp_ph111_B8
zmp_ph111_B6
zmp_ph111_B7
zmp_ph111_B9
zmp_ph111_B10
zmp_ph114_G4
zmp_ph114_G11
zmp_ph114_H1
zmp_ph114_H2
zmp_ph114_A9
zmp_ph114_A11
zmp_ph114_B2
zmp_ph114_B4
zmp_ph114_D8
zmp_ph114_E2

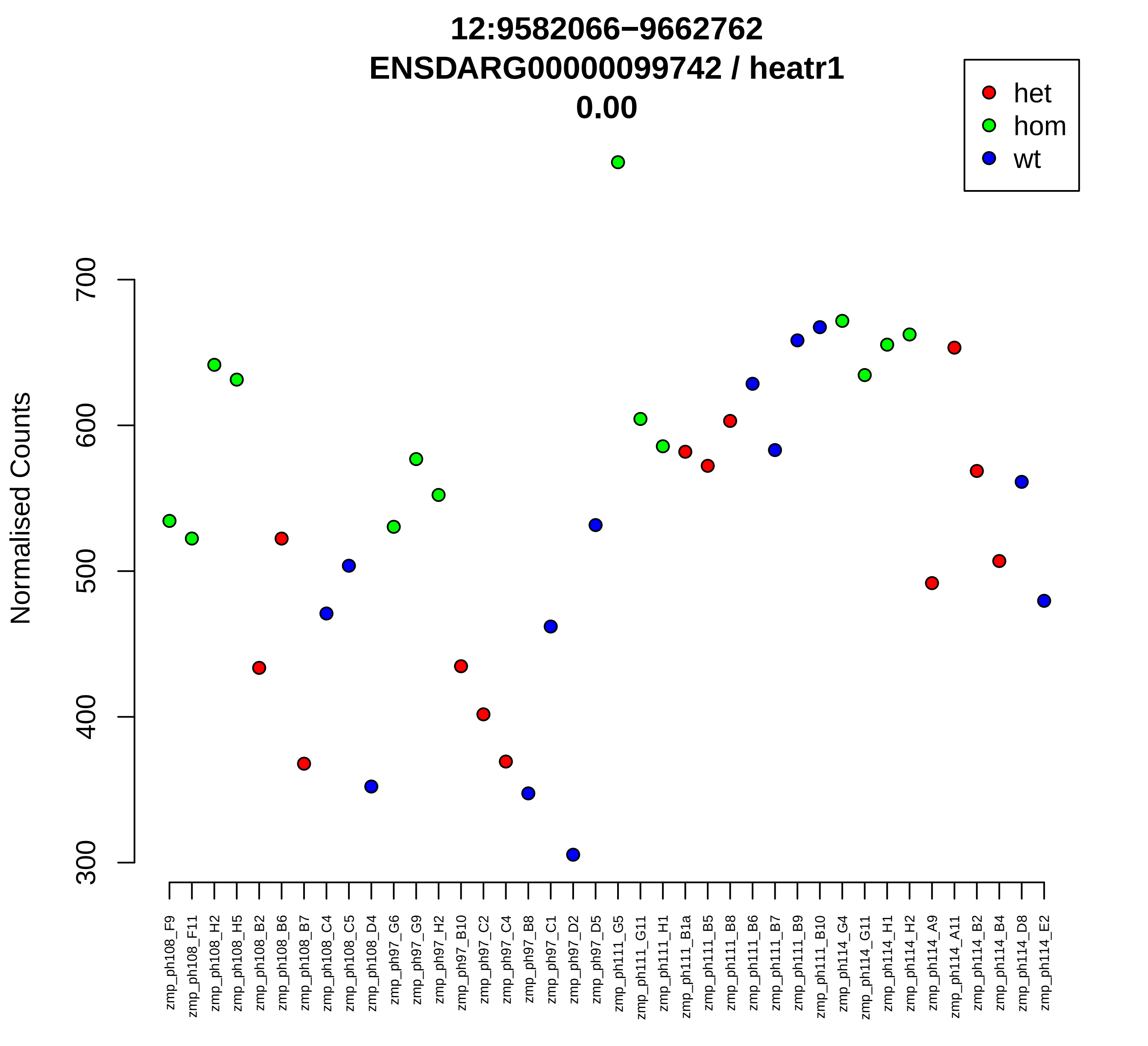
12:9582066−9662762
ENSDARG00000099742 / heatr1
0.00
het
hom
wt
Normalised Counts
300
400
500
600
700
zmp_ph108_F9
zmp_ph108_F11
zmp_ph108_H2
zmp_ph108_H5
zmp_ph108_B2
zmp_ph108_B6
zmp_ph108_B7
zmp_ph108_C4
zmp_ph108_C5
zmp_ph108_D4
zmp_ph97_G6
zmp_ph97_G9
zmp_ph97_H2
zmp_ph97_B10
zmp_ph97_C2
zmp_ph97_C4
zmp_ph97_B8
zmp_ph97_C1
zmp_ph97_D2
zmp_ph97_D5
zmp_ph111_G5
zmp_ph111_G11
zmp_ph111_H1
zmp_ph111_B1a
zmp_ph111_B5
zmp_ph111_B8
zmp_ph111_B6
zmp_ph111_B7
zmp_ph111_B9
zmp_ph111_B10
zmp_ph114_G4
zmp_ph114_G11
zmp_ph114_H1
zmp_ph114_H2
zmp_ph114_A9
zmp_ph114_A11
zmp_ph114_B2
zmp_ph114_B4
zmp_ph114_D8
zmp_ph114_E2

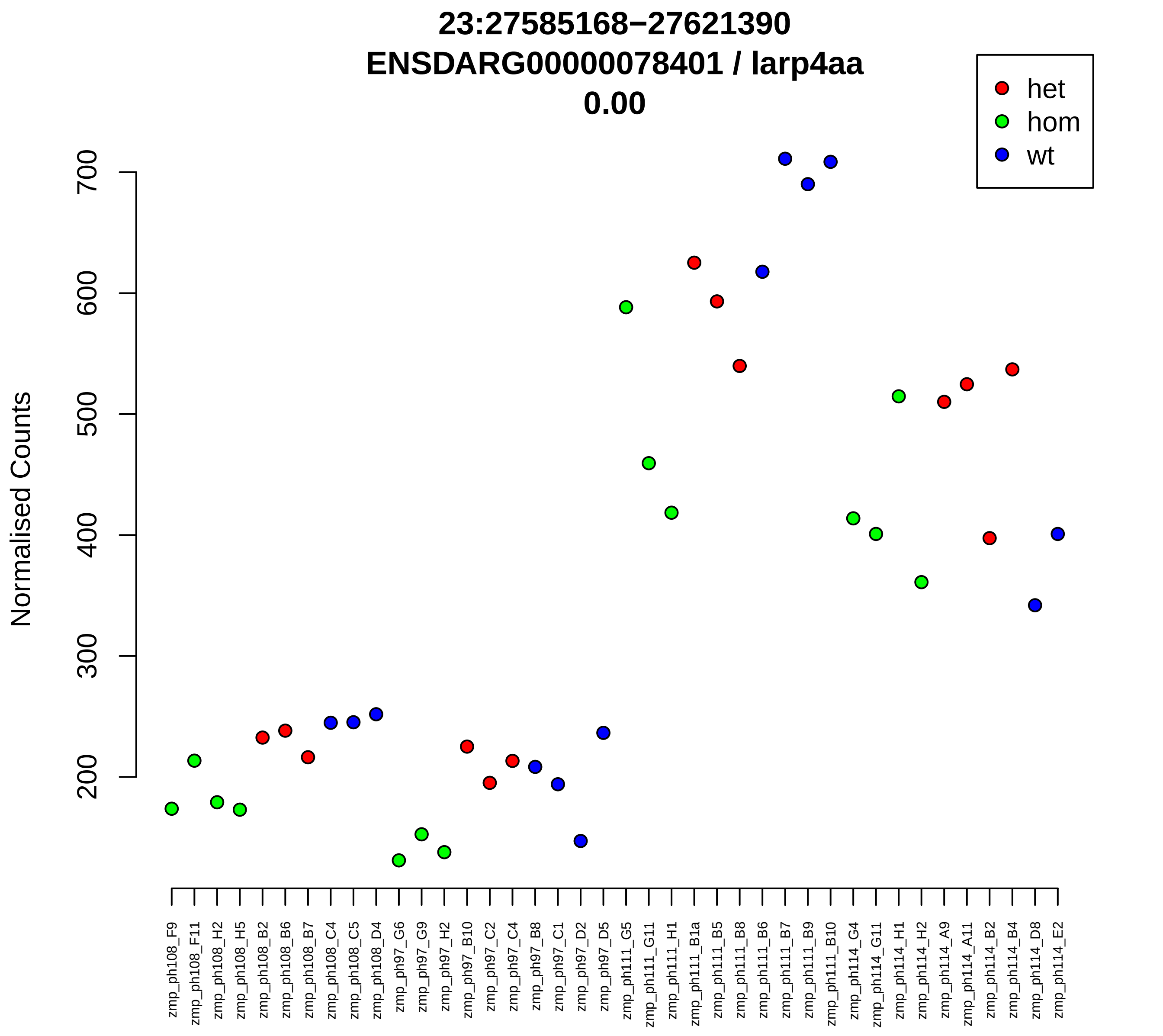

23:27585168−27621390
ENSDARG00000078401 / larp4aa
0.00
het
hom
wt
Normalised Counts
200
300
400
500
600
700
zmp_ph108_F9
zmp_ph108_F11
zmp_ph108_H2
zmp_ph108_H5
zmp_ph108_B2
zmp_ph108_B6
zmp_ph108_B7
zmp_ph108_C4
zmp_ph108_C5
zmp_ph108_D4
zmp_ph97_G6
zmp_ph97_G9
zmp_ph97_H2
zmp_ph97_B10
zmp_ph97_C2
zmp_ph97_C4
zmp_ph97_B8
zmp_ph97_C1
zmp_ph97_D2
zmp_ph97_D5
zmp_ph111_G5
zmp_ph111_G11
zmp_ph111_H1
zmp_ph111_B1a
zmp_ph111_B5
zmp_ph111_B8
zmp_ph111_B6
zmp_ph111_B7
zmp_ph111_B9
zmp_ph111_B10
zmp_ph114_G4
zmp_ph114_G11
zmp_ph114_H1
zmp_ph114_H2
zmp_ph114_A9
zmp_ph114_A11
zmp_ph114_B2
zmp_ph114_B4
zmp_ph114_D8
zmp_ph114_E2

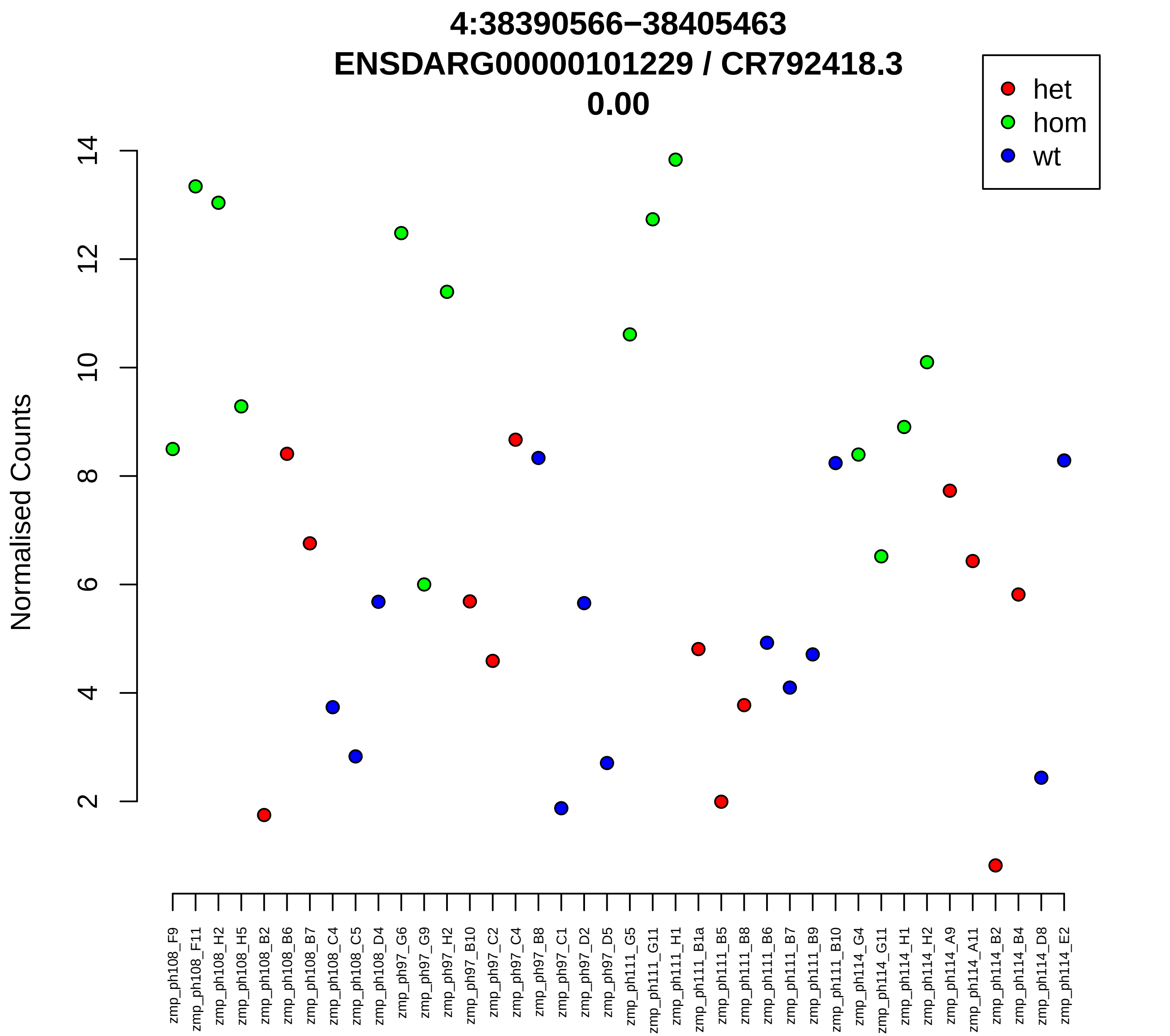

4:38390566−38405463
ENSDARG00000101229 / CR792418.3
0.00
het
hom
wt
Normalised Counts
2
4
6
8
10
12
14
zmp_ph108_F9
zmp_ph108_F11
zmp_ph108_H2
zmp_ph108_H5
zmp_ph108_B2
zmp_ph108_B6
zmp_ph108_B7
zmp_ph108_C4
zmp_ph108_C5
zmp_ph108_D4
zmp_ph97_G6
zmp_ph97_G9
zmp_ph97_H2
zmp_ph97_B10
zmp_ph97_C2
zmp_ph97_C4
zmp_ph97_B8
zmp_ph97_C1
zmp_ph97_D2
zmp_ph97_D5
zmp_ph111_G5
zmp_ph111_G11
zmp_ph111_H1
zmp_ph111_B1a
zmp_ph111_B5
zmp_ph111_B8
zmp_ph111_B6
zmp_ph111_B7
zmp_ph111_B9
zmp_ph111_B10
zmp_ph114_G4
zmp_ph114_G11
zmp_ph114_H1
zmp_ph114_H2
zmp_ph114_A9
zmp_ph114_A11
zmp_ph114_B2
zmp_ph114_B4
zmp_ph114_D8
zmp_ph114_E2

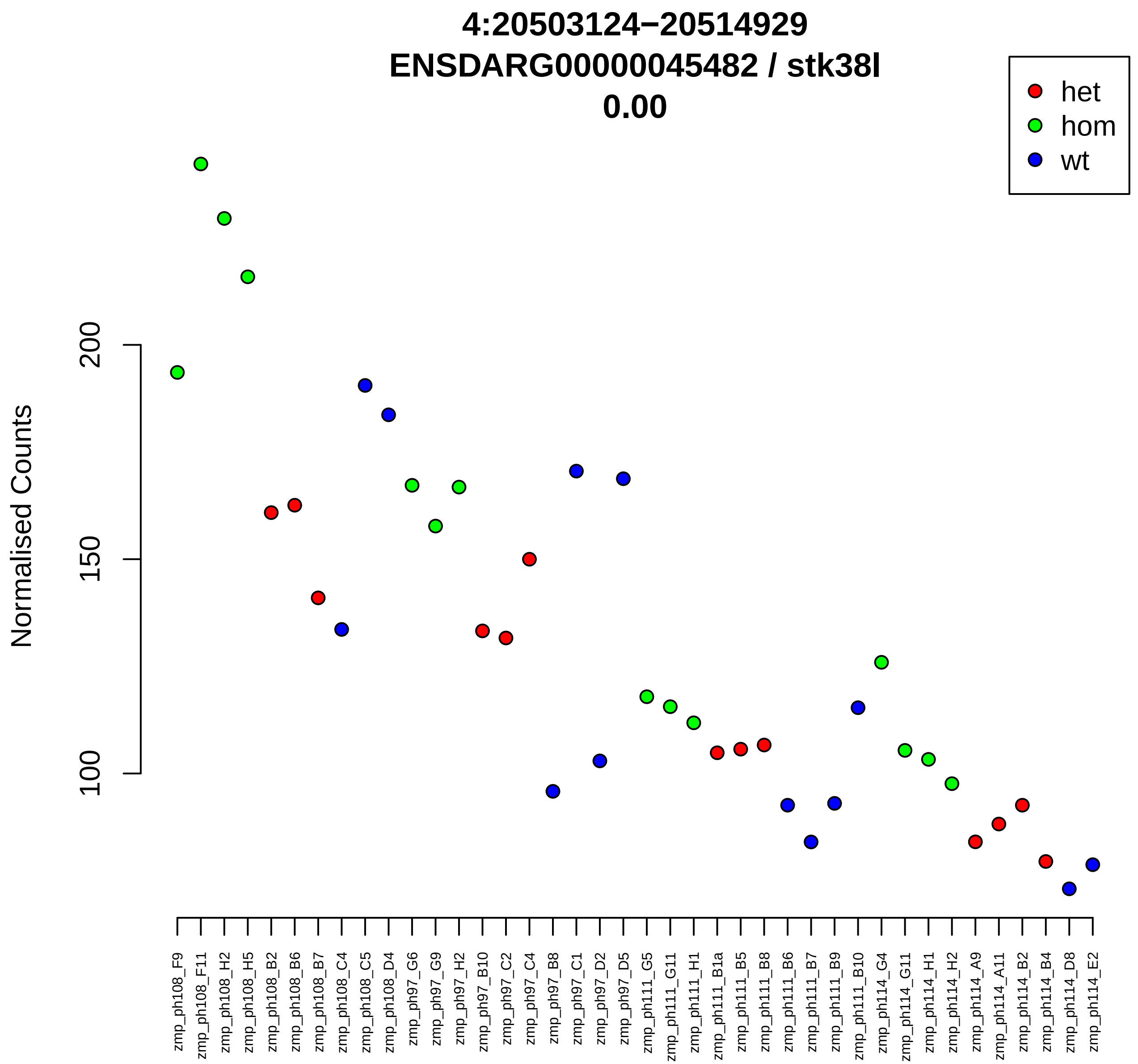

4:20503124−20514929
ENSDARG00000045482 / stk38l
0.00
het
hom
wt
Normalised Counts
100
150
200
zmp_ph108_F9
zmp_ph108_F11
zmp_ph108_H2
zmp_ph108_H5
zmp_ph108_B2
zmp_ph108_B6
zmp_ph108_B7
zmp_ph108_C4
zmp_ph108_C5
zmp_ph108_D4
zmp_ph97_G6
zmp_ph97_G9
zmp_ph97_H2
zmp_ph97_B10
zmp_ph97_C2
zmp_ph97_C4
zmp_ph97_B8
zmp_ph97_C1
zmp_ph97_D2
zmp_ph97_D5
zmp_ph111_G5
zmp_ph111_G11
zmp_ph111_H1
zmp_ph111_B1a
zmp_ph111_B5
zmp_ph111_B8
zmp_ph111_B6
zmp_ph111_B7
zmp_ph111_B9
zmp_ph111_B10
zmp_ph114_G4
zmp_ph114_G11
zmp_ph114_H1
zmp_ph114_H2
zmp_ph114_A9
zmp_ph114_A11
zmp_ph114_B2
zmp_ph114_B4
zmp_ph114_D8
zmp_ph114_E2

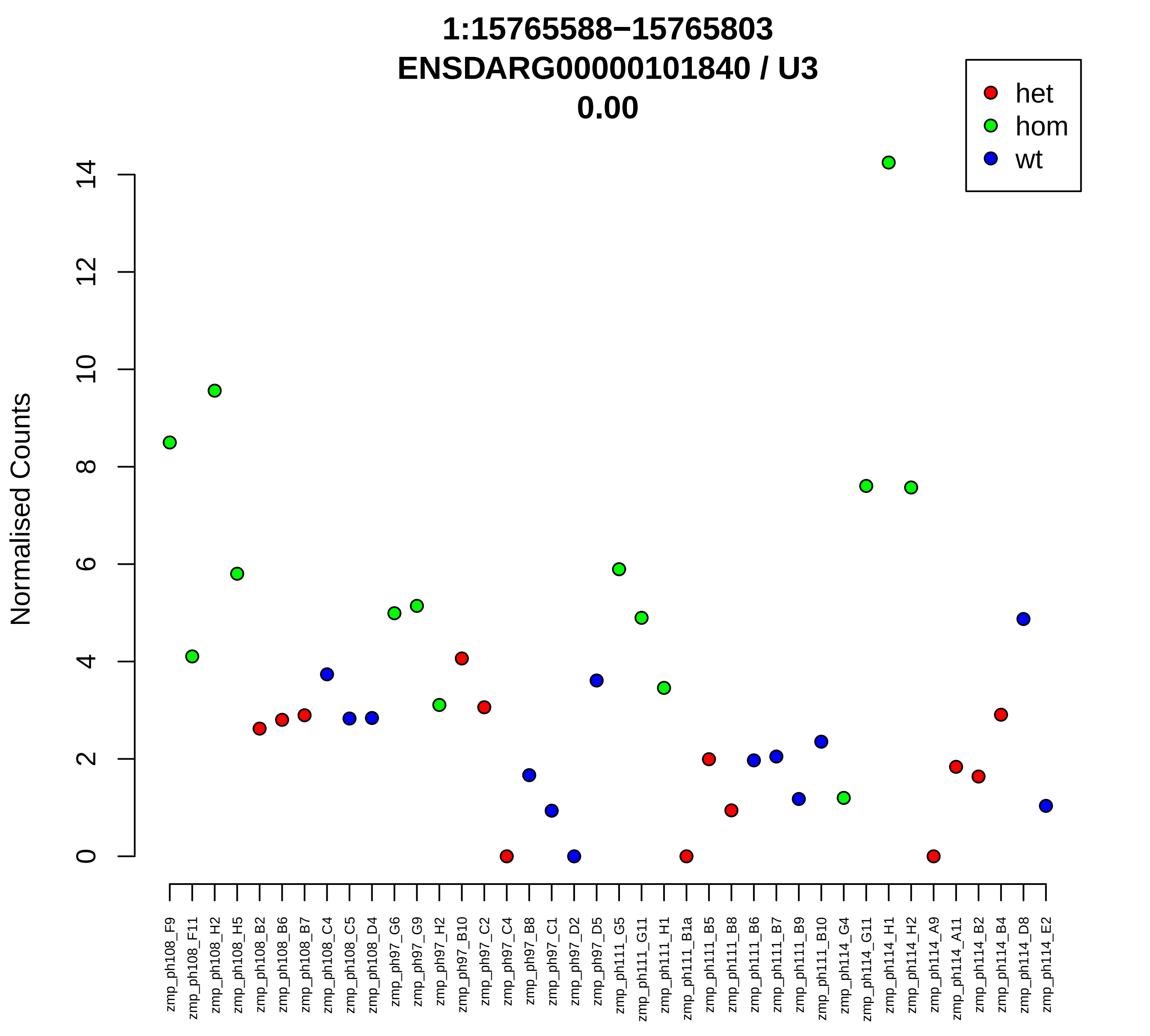

1:15765588−15765803
ENSDARG00000101840 / U3
0.00
het
hom
wt
Normalised Counts
0
2
4
6
8
10
12
14
zmp_ph108_F9
zmp_ph108_F11
zmp_ph108_H2
zmp_ph108_H5
zmp_ph108_B2
zmp_ph108_B6
zmp_ph108_B7
zmp_ph108_C4
zmp_ph108_C5
zmp_ph108_D4
zmp_ph97_G6
zmp_ph97_G9
zmp_ph97_H2
zmp_ph97_B10
zmp_ph97_C2
zmp_ph97_C4
zmp_ph97_B8
zmp_ph97_C1
zmp_ph97_D2
zmp_ph97_D5
zmp_ph111_G5
zmp_ph111_G11
zmp_ph111_H1
zmp_ph111_B1a
zmp_ph111_B5
zmp_ph111_B8
zmp_ph111_B6
zmp_ph111_B7
zmp_ph111_B9
zmp_ph111_B10
zmp_ph114_G4
zmp_ph114_G11
zmp_ph114_H1
zmp_ph114_H2
zmp_ph114_A9
zmp_ph114_A11
zmp_ph114_B2
zmp_ph114_B4
zmp_ph114_D8
zmp_ph114_E2

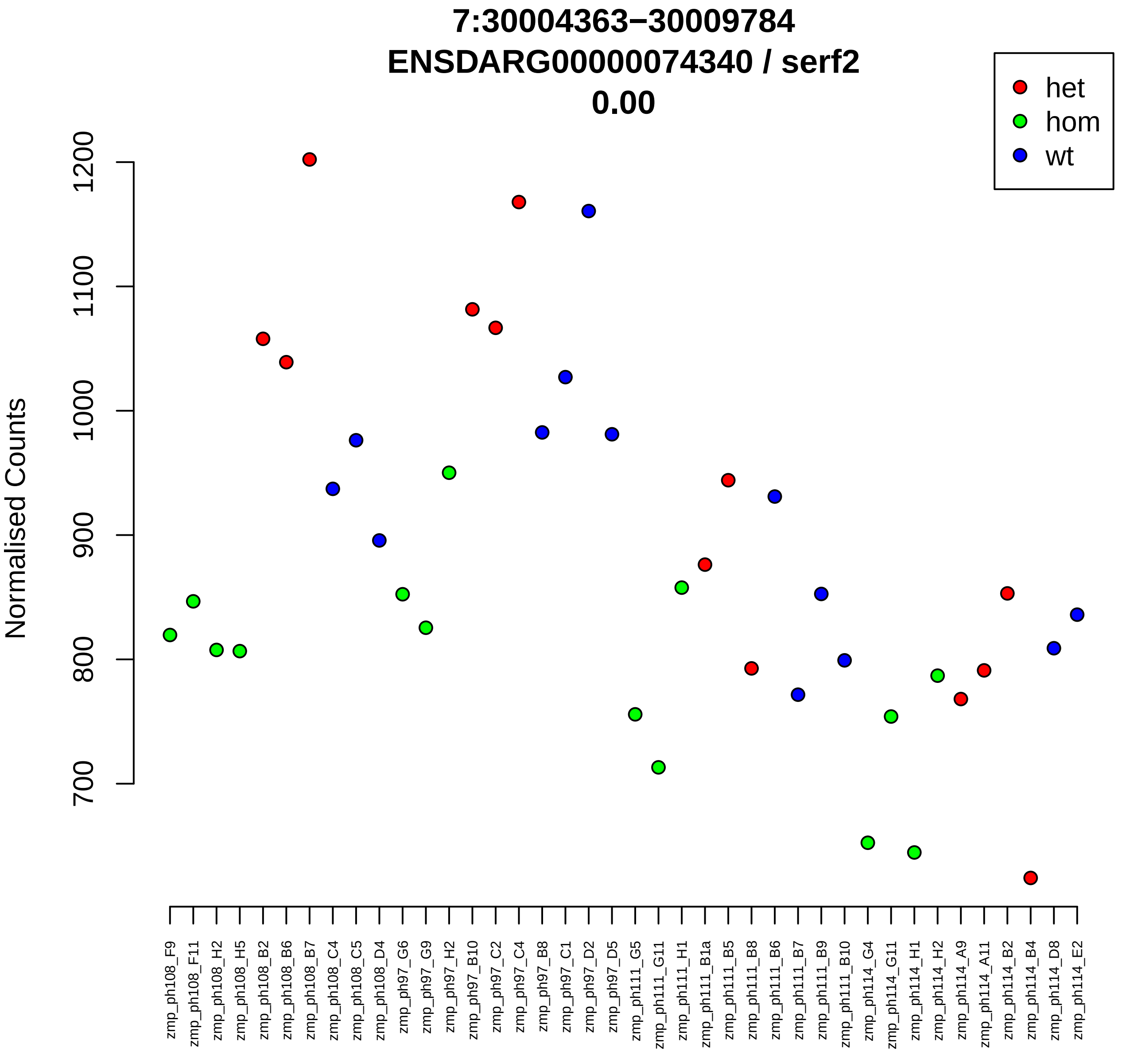

7:30004363−30009784
ENSDARG00000074340 / serf2
0.00
het
hom
wt
Normalised Counts
700
800
900
1000
1100
1200
zmp_ph108_F9
zmp_ph108_F11
zmp_ph108_H2
zmp_ph108_H5
zmp_ph108_B2
zmp_ph108_B6
zmp_ph108_B7
zmp_ph108_C4
zmp_ph108_C5
zmp_ph108_D4
zmp_ph97_G6
zmp_ph97_G9
zmp_ph97_H2
zmp_ph97_B10
zmp_ph97_C2
zmp_ph97_C4
zmp_ph97_B8
zmp_ph97_C1
zmp_ph97_D2
zmp_ph97_D5
zmp_ph111_G5
zmp_ph111_G11
zmp_ph111_H1
zmp_ph111_B1a
zmp_ph111_B5
zmp_ph111_B8
zmp_ph111_B6
zmp_ph111_B7
zmp_ph111_B9
zmp_ph111_B10
zmp_ph114_G4
zmp_ph114_G11
zmp_ph114_H1
zmp_ph114_H2
zmp_ph114_A9
zmp_ph114_A11
zmp_ph114_B2
zmp_ph114_B4
zmp_ph114_D8
zmp_ph114_E2

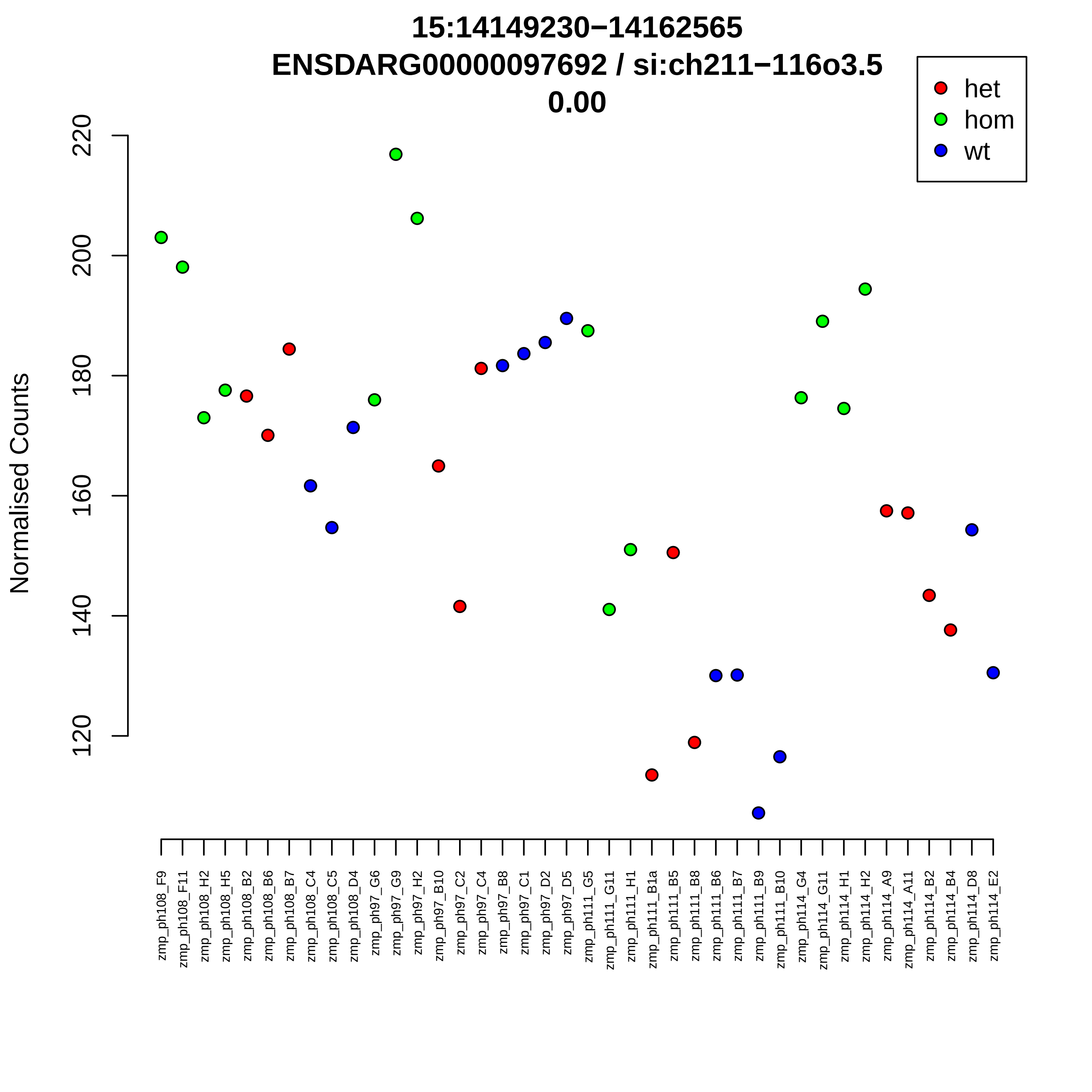
15:14149230−14162565
ENSDARG00000097692 / si:ch211−116o3.5
0.00
Normalised Counts
het
hom
wt
120
140
160
180
200
220
zmp_ph108_F9
zmp_ph108_F11
zmp_ph108_H2
zmp_ph108_H5
zmp_ph108_B2
zmp_ph108_B6
zmp_ph108_B7
zmp_ph108_C4
zmp_ph108_C5
zmp_ph108_D4
zmp_ph97_G6
zmp_ph97_G9
zmp_ph97_H2
zmp_ph97_B10
zmp_ph97_C2
zmp_ph97_C4
zmp_ph97_B8
zmp_ph97_C1
zmp_ph97_D2
zmp_ph97_D5
zmp_ph111_G5
zmp_ph111_G11
zmp_ph111_H1
zmp_ph111_B1a
zmp_ph111_B5
zmp_ph111_B8
zmp_ph111_B6
zmp_ph111_B7
zmp_ph111_B9
zmp_ph111_B10
zmp_ph114_G4
zmp_ph114_G11
zmp_ph114_H1
zmp_ph114_H2
zmp_ph114_A9
zmp_ph114_A11
zmp_ph114_B2
zmp_ph114_B4
zmp_ph114_D8
zmp_ph114_E2

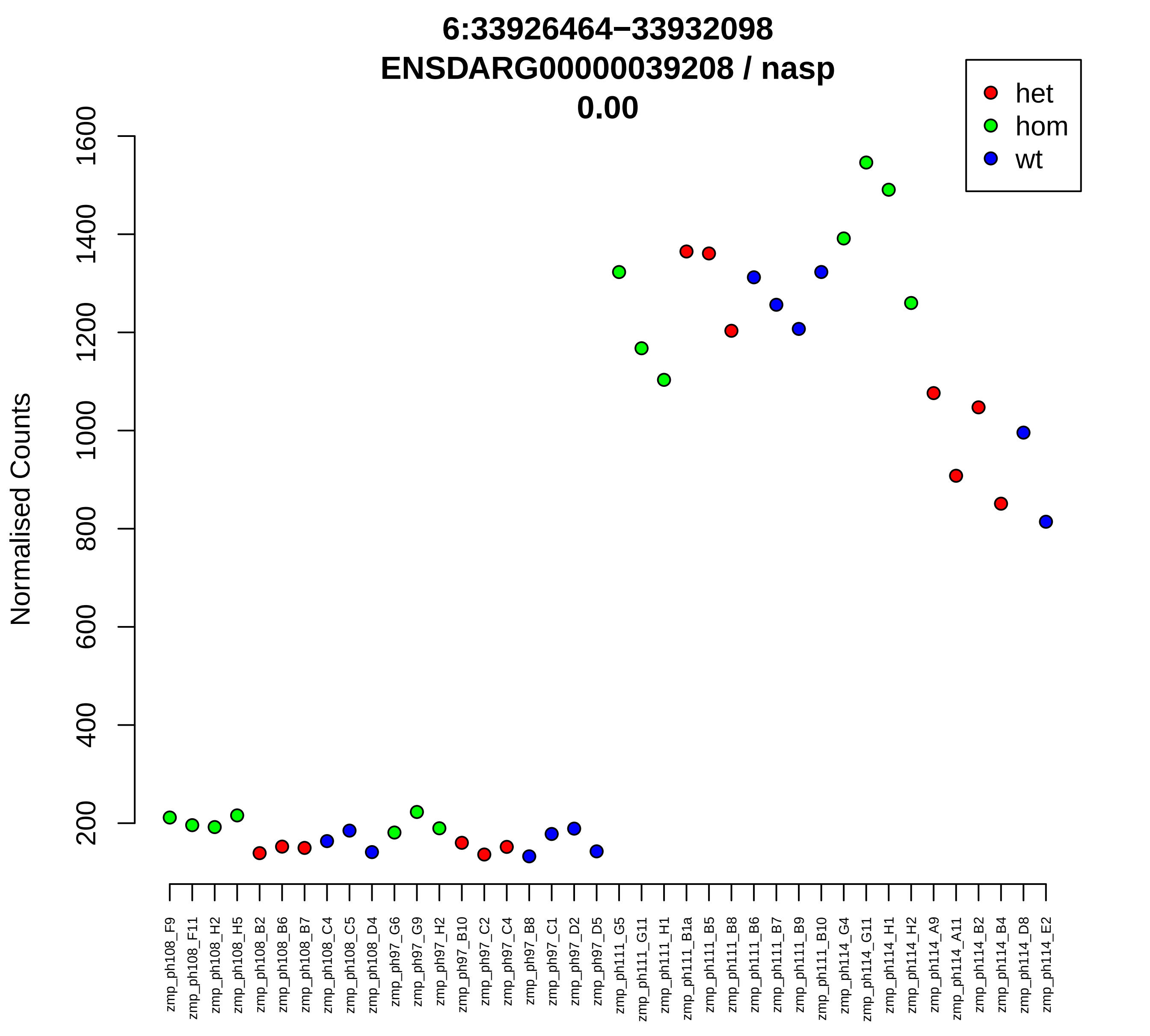
6:33926464−33932098
ENSDARG00000039208 / nasp
0.00
Normalised Counts
200
400
600
800
1000
1200
1400
1600
het
hom
wt
zmp_ph108_F9
zmp_ph108_F11
zmp_ph108_H2
zmp_ph108_H5
zmp_ph108_B2
zmp_ph108_B6
zmp_ph108_B7
zmp_ph108_C4
zmp_ph108_C5
zmp_ph108_D4
zmp_ph97_G6
zmp_ph97_G9
zmp_ph97_H2
zmp_ph97_B10
zmp_ph97_C2
zmp_ph97_C4
zmp_ph97_B8
zmp_ph97_C1
zmp_ph97_D2
zmp_ph97_D5
zmp_ph111_G5
zmp_ph111_G11
zmp_ph111_H1
zmp_ph111_B1a
zmp_ph111_B5
zmp_ph111_B8
zmp_ph111_B6
zmp_ph111_B7
zmp_ph111_B9
zmp_ph111_B10
zmp_ph114_G4
zmp_ph114_G11
zmp_ph114_H1
zmp_ph114_H2
zmp_ph114_A9
zmp_ph114_A11
zmp_ph114_B2
zmp_ph114_B4
zmp_ph114_D8
zmp_ph114_E2

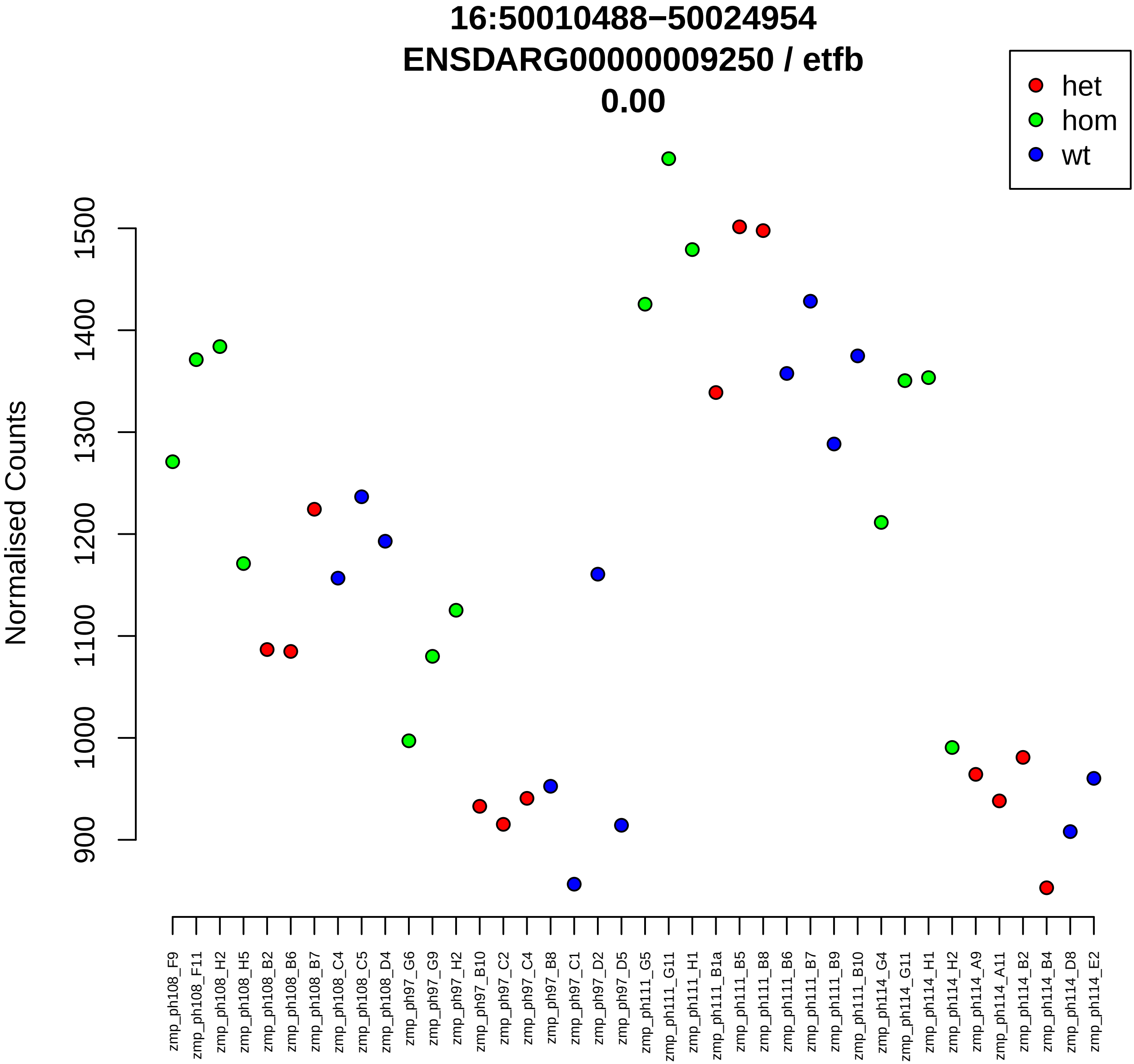

16:50010488−50024954
ENSDARG00000009250 / etfb
0.00
het
hom
wt
Normalised Counts
900
1000
1100
1200
1300
1400
1500
zmp_ph108_F9
zmp_ph108_F11
zmp_ph108_H2
zmp_ph108_H5
zmp_ph108_B2
zmp_ph108_B6
zmp_ph108_B7
zmp_ph108_C4
zmp_ph108_C5
zmp_ph108_D4
zmp_ph97_G6
zmp_ph97_G9
zmp_ph97_H2
zmp_ph97_B10
zmp_ph97_C2
zmp_ph97_C4
zmp_ph97_B8
zmp_ph97_C1
zmp_ph97_D2
zmp_ph97_D5
zmp_ph111_G5
zmp_ph111_G11
zmp_ph111_H1
zmp_ph111_B1a
zmp_ph111_B5
zmp_ph111_B8
zmp_ph111_B6
zmp_ph111_B7
zmp_ph111_B9
zmp_ph111_B10
zmp_ph114_G4
zmp_ph114_G11
zmp_ph114_H1
zmp_ph114_H2
zmp_ph114_A9
zmp_ph114_A11
zmp_ph114_B2
zmp_ph114_B4
zmp_ph114_D8
zmp_ph114_E2

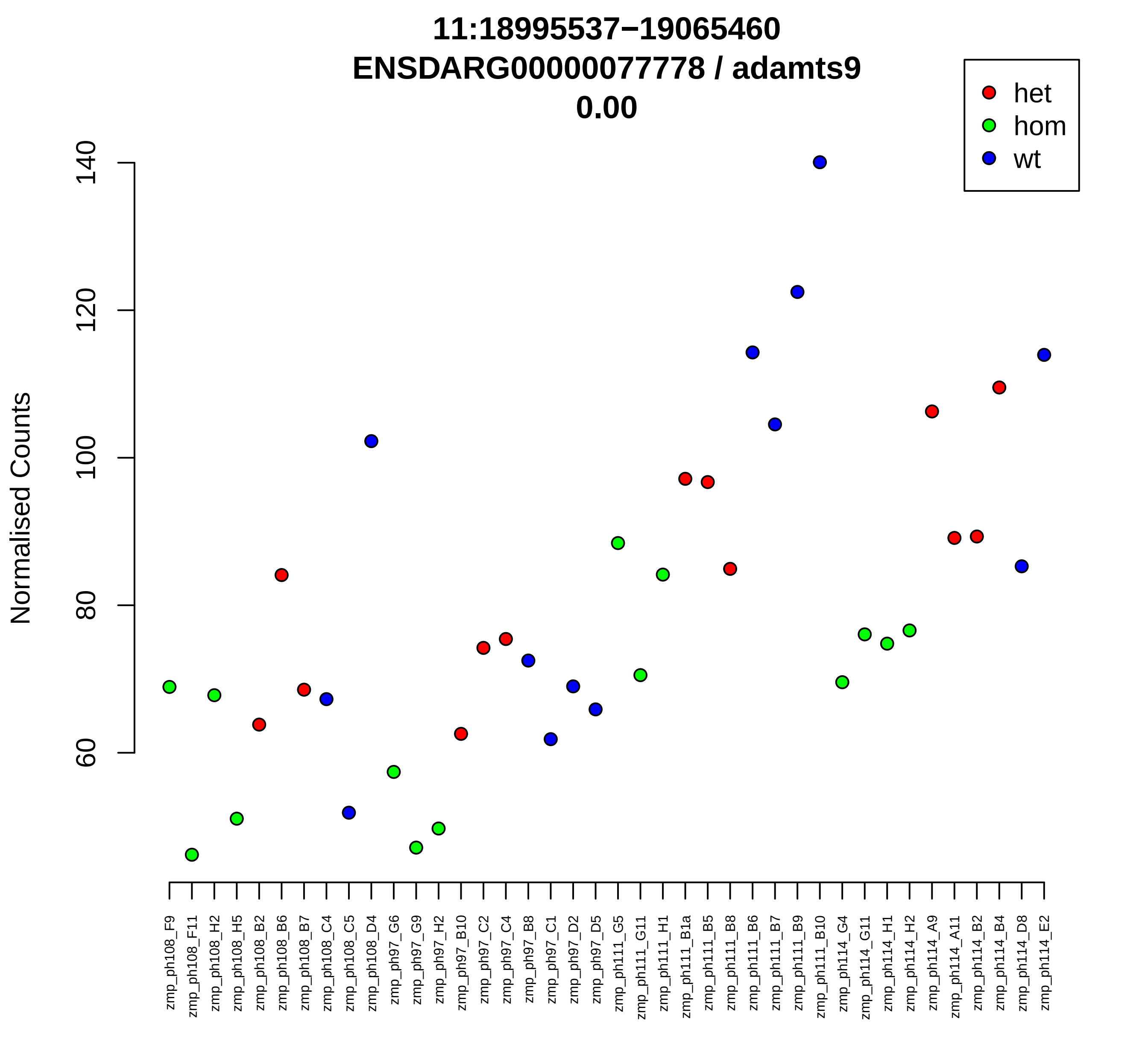

11:18995537−19065460
ENSDARG00000077778 / adamts9
0.00
het
hom
wt
Normalised Counts
60
80
100
120
140
zmp_ph108_F9
zmp_ph108_F11
zmp_ph108_H2
zmp_ph108_H5
zmp_ph108_B2
zmp_ph108_B6
zmp_ph108_B7
zmp_ph108_C4
zmp_ph108_C5
zmp_ph108_D4
zmp_ph97_G6
zmp_ph97_G9
zmp_ph97_H2
zmp_ph97_B10
zmp_ph97_C2
zmp_ph97_C4
zmp_ph97_B8
zmp_ph97_C1
zmp_ph97_D2
zmp_ph97_D5
zmp_ph111_G5
zmp_ph111_G11
zmp_ph111_H1
zmp_ph111_B1a
zmp_ph111_B5
zmp_ph111_B8
zmp_ph111_B6
zmp_ph111_B7
zmp_ph111_B9
zmp_ph111_B10
zmp_ph114_G4
zmp_ph114_G11
zmp_ph114_H1
zmp_ph114_H2
zmp_ph114_A9
zmp_ph114_A11
zmp_ph114_B2
zmp_ph114_B4
zmp_ph114_D8
zmp_ph114_E2

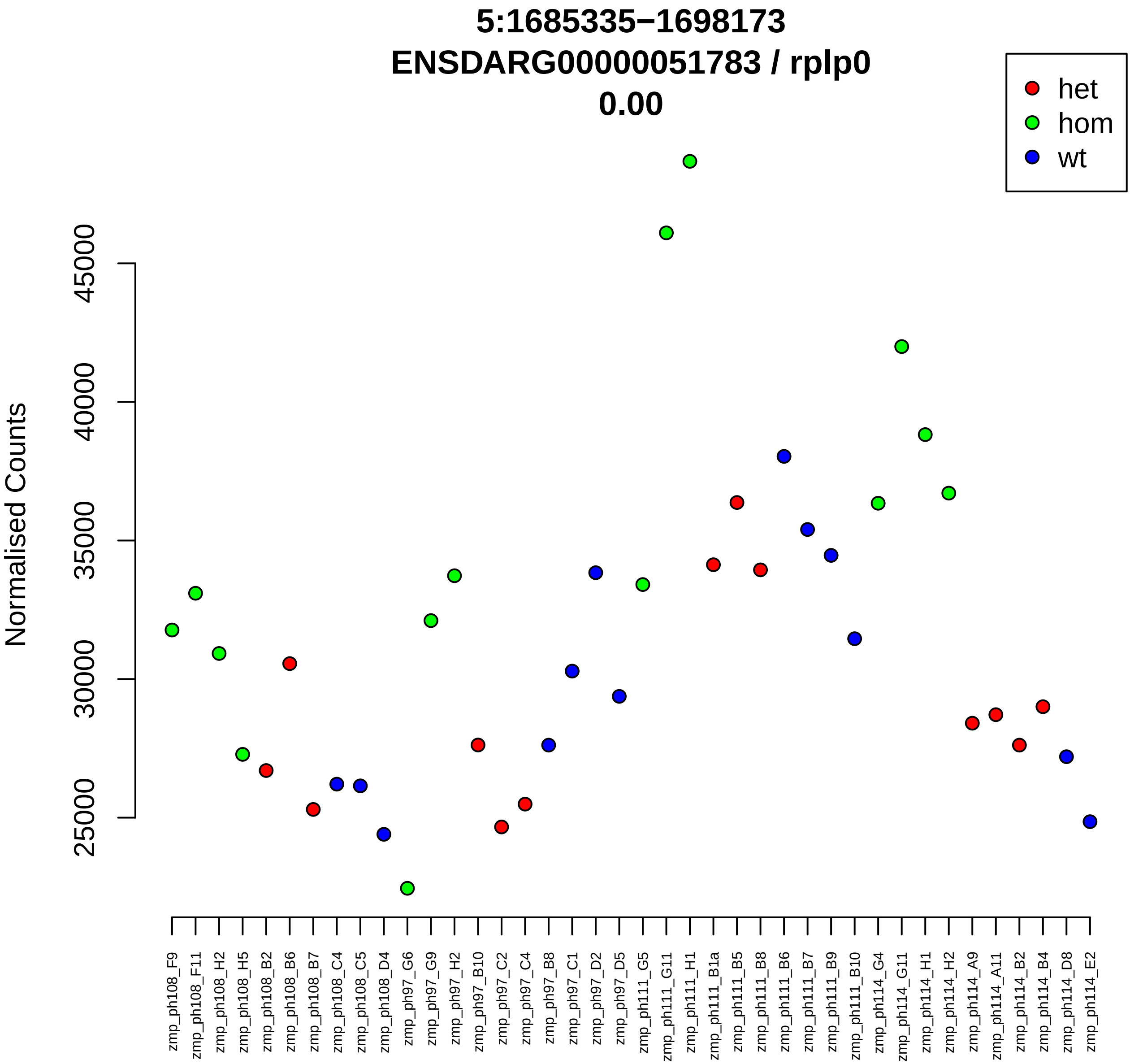

5:1685335−1698173
ENSDARG00000051783 / rplp0
0.00
het
hom
wt
Normalised Counts
25000
30000
35000
40000
45000
zmp_ph108_F9
zmp_ph108_F11
zmp_ph108_H2
zmp_ph108_H5
zmp_ph108_B2
zmp_ph108_B6
zmp_ph108_B7
zmp_ph108_C4
zmp_ph108_C5
zmp_ph108_D4
zmp_ph97_G6
zmp_ph97_G9
zmp_ph97_H2
zmp_ph97_B10
zmp_ph97_C2
zmp_ph97_C4
zmp_ph97_B8
zmp_ph97_C1
zmp_ph97_D2
zmp_ph97_D5
zmp_ph111_G5
zmp_ph111_G11
zmp_ph111_H1
zmp_ph111_B1a
zmp_ph111_B5
zmp_ph111_B8
zmp_ph111_B6
zmp_ph111_B7
zmp_ph111_B9
zmp_ph111_B10
zmp_ph114_G4
zmp_ph114_G11
zmp_ph114_H1
zmp_ph114_H2
zmp_ph114_A9
zmp_ph114_A11
zmp_ph114_B2
zmp_ph114_B4
zmp_ph114_D8
zmp_ph114_E2

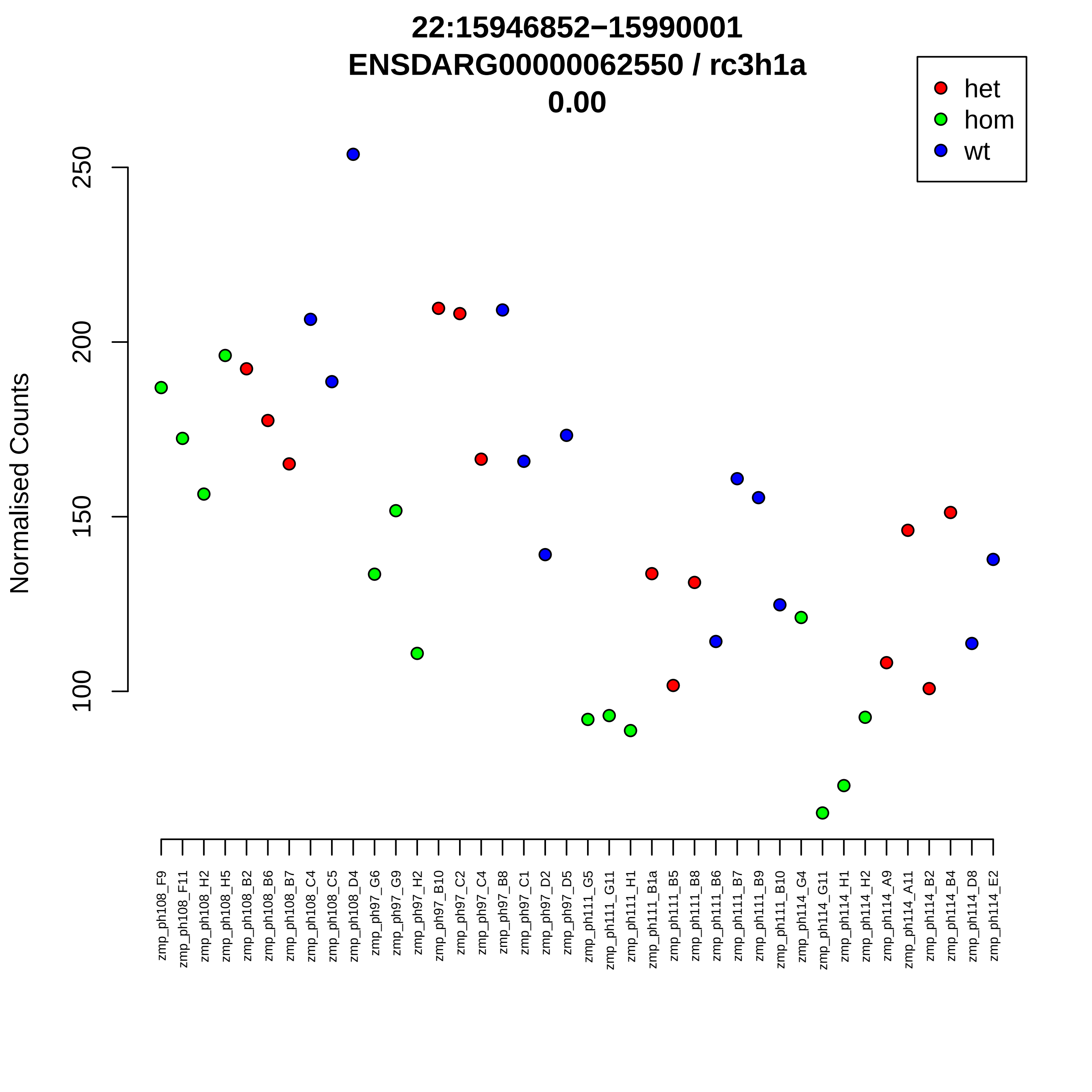
22:15946852−15990001
ENSDARG00000062550 / rc3h1a
0.00
het
hom
wt
Normalised Counts
250
200
150
100
zmp_ph108_F9
zmp_ph108_F11
zmp_ph108_H2
zmp_ph108_H5
zmp_ph108_B2
zmp_ph108_B6
zmp_ph108_B7
zmp_ph108_C4
zmp_ph108_C5
zmp_ph108_D4
zmp_ph97_G6
zmp_ph97_G9
zmp_ph97_H2
zmp_ph97_B10
zmp_ph97_C2
zmp_ph97_C4
zmp_ph97_B8
zmp_ph97_C1
zmp_ph97_D2
zmp_ph97_D5
zmp_ph111_G5
zmp_ph111_G11
zmp_ph111_H1
zmp_ph111_B1a
zmp_ph111_B5
zmp_ph111_B8
zmp_ph111_B6
zmp_ph111_B7
zmp_ph111_B9
zmp_ph111_B10
zmp_ph114_G4
zmp_ph114_G11
zmp_ph114_H1
zmp_ph114_H2
zmp_ph114_A9
zmp_ph114_A11
zmp_ph114_B2
zmp_ph114_B4
zmp_ph114_D8
zmp_ph114_E2

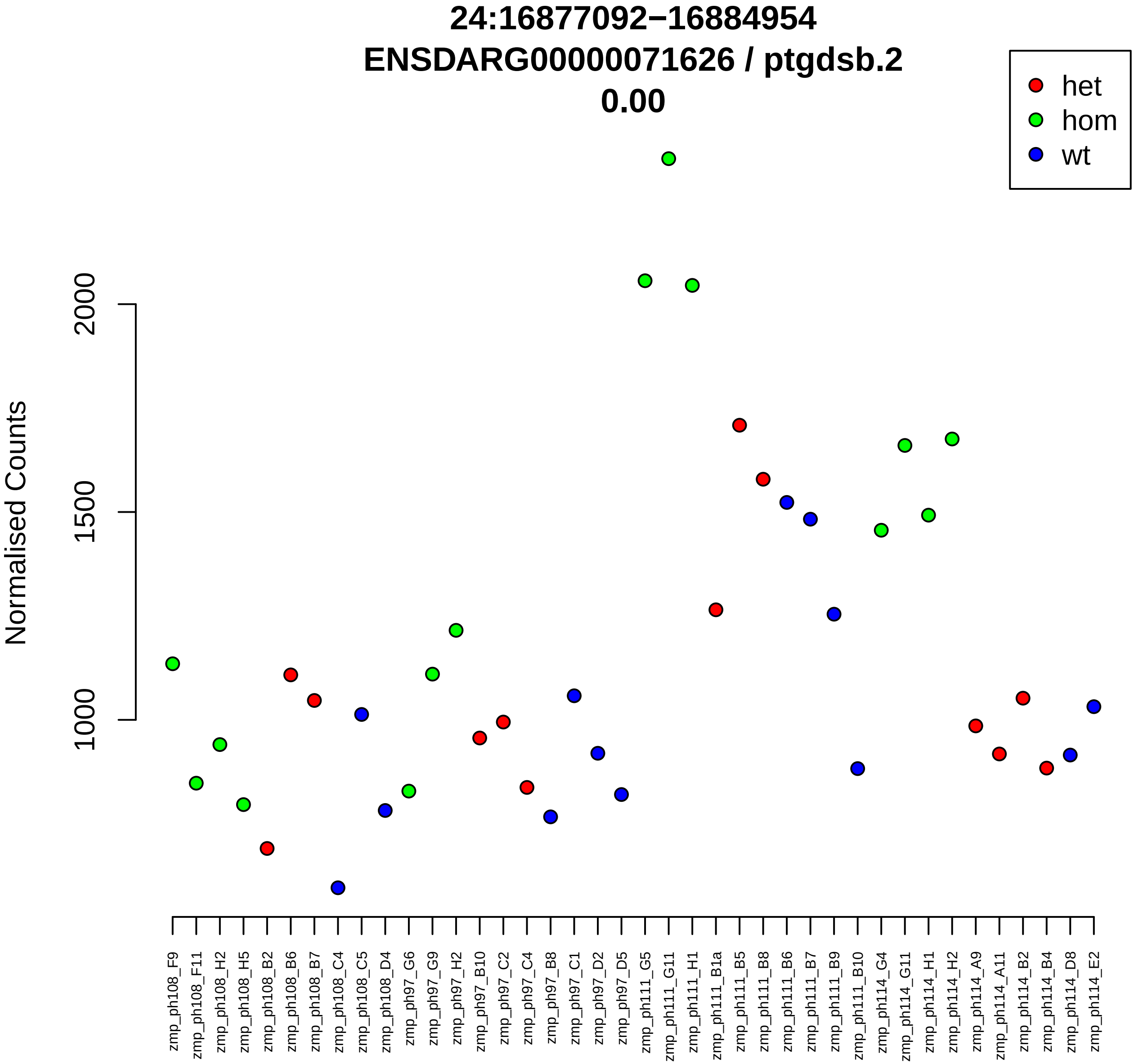

24:16877092−16884954
ENSDARG00000071626 / ptgdsb.2
0.00
het
hom
wt
Normalised Counts
1000
1500
2000
zmp_ph108_F9
zmp_ph108_F11
zmp_ph108_H2
zmp_ph108_H5
zmp_ph108_B2
zmp_ph108_B6
zmp_ph108_B7
zmp_ph108_C4
zmp_ph108_C5
zmp_ph108_D4
zmp_ph97_G6
zmp_ph97_G9
zmp_ph97_H2
zmp_ph97_B10
zmp_ph97_C2
zmp_ph97_C4
zmp_ph97_B8
zmp_ph97_C1
zmp_ph97_D2
zmp_ph97_D5
zmp_ph111_G5
zmp_ph111_G11
zmp_ph111_H1
zmp_ph111_B1a
zmp_ph111_B5
zmp_ph111_B8
zmp_ph111_B6
zmp_ph111_B7
zmp_ph111_B9
zmp_ph111_B10
zmp_ph114_G4
zmp_ph114_G11
zmp_ph114_H1
zmp_ph114_H2
zmp_ph114_A9
zmp_ph114_A11
zmp_ph114_B2
zmp_ph114_B4
zmp_ph114_D8
zmp_ph114_E2

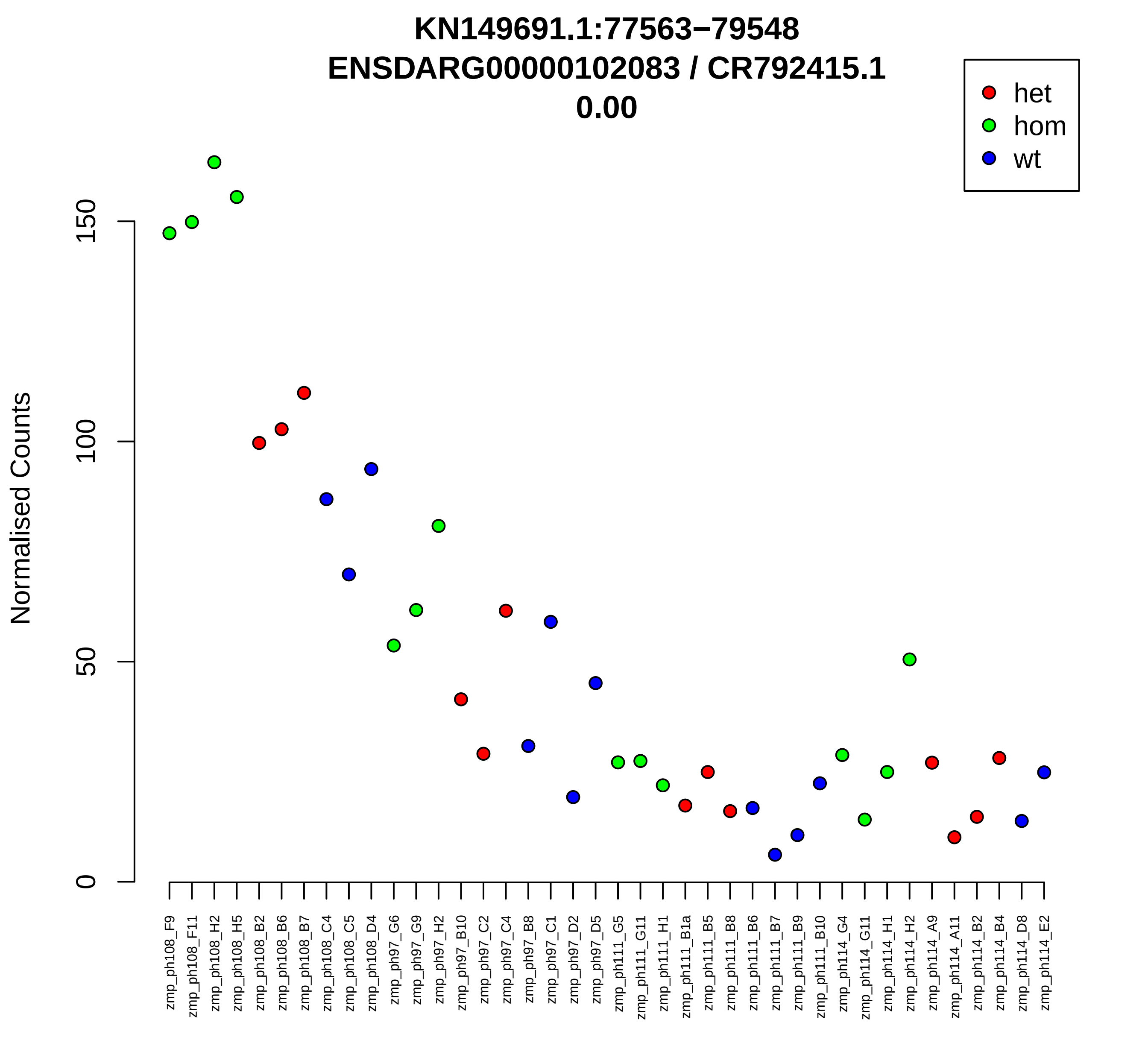
KN149691.1:77563−79548
ENSDARG00000102083 / CR792415.1
0.00
het
hom
wt
Normalised Counts
0
50
100
150
zmp_ph108_F9
zmp_ph108_F11
zmp_ph108_H2
zmp_ph108_H5
zmp_ph108_B2
zmp_ph108_B6
zmp_ph108_B7
zmp_ph108_C4
zmp_ph108_C5
zmp_ph108_D4
zmp_ph97_G6
zmp_ph97_G9
zmp_ph97_H2
zmp_ph97_B10
zmp_ph97_C2
zmp_ph97_C4
zmp_ph97_B8
zmp_ph97_C1
zmp_ph97_D2
zmp_ph97_D5
zmp_ph111_G5
zmp_ph111_G11
zmp_ph111_H1
zmp_ph111_B1a
zmp_ph111_B5
zmp_ph111_B8
zmp_ph111_B6
zmp_ph111_B7
zmp_ph111_B9
zmp_ph111_B10
zmp_ph114_G4
zmp_ph114_G11
zmp_ph114_H1
zmp_ph114_H2
zmp_ph114_A9
zmp_ph114_A11
zmp_ph114_B2
zmp_ph114_B4
zmp_ph114_D8
zmp_ph114_E2

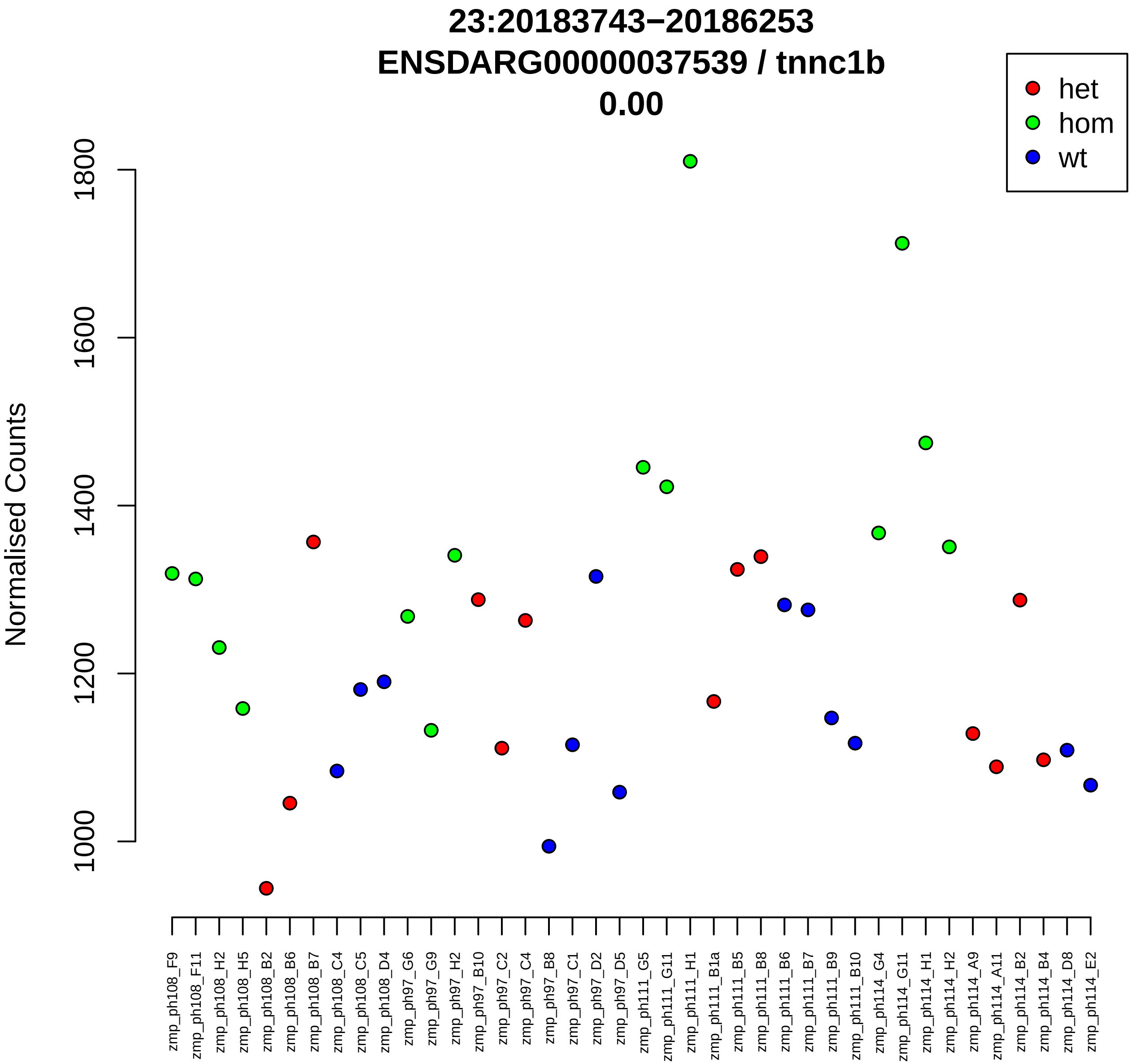
23:20183743−20186253
ENSDARG00000037539 / tnnc1b
0.00
het
hom
wt
Normalised Counts
1000
1200
1400
1600
1800
zmp_ph108_F9
zmp_ph108_F11
zmp_ph108_H2
zmp_ph108_H5
zmp_ph108_B2
zmp_ph108_B6
zmp_ph108_B7
zmp_ph108_C4
zmp_ph108_C5
zmp_ph108_D4
zmp_ph97_G6
zmp_ph97_G9
zmp_ph97_H2
zmp_ph97_B10
zmp_ph97_C2
zmp_ph97_C4
zmp_ph97_B8
zmp_ph97_C1
zmp_ph97_D2
zmp_ph97_D5
zmp_ph111_G5
zmp_ph111_G11
zmp_ph111_H1
zmp_ph111_B1a
zmp_ph111_B5
zmp_ph111_B8
zmp_ph111_B6
zmp_ph111_B7
zmp_ph111_B9
zmp_ph111_B10
zmp_ph114_G4
zmp_ph114_G11
zmp_ph114_H1
zmp_ph114_H2
zmp_ph114_A9
zmp_ph114_A11
zmp_ph114_B2
zmp_ph114_B4
zmp_ph114_D8
zmp_ph114_E2

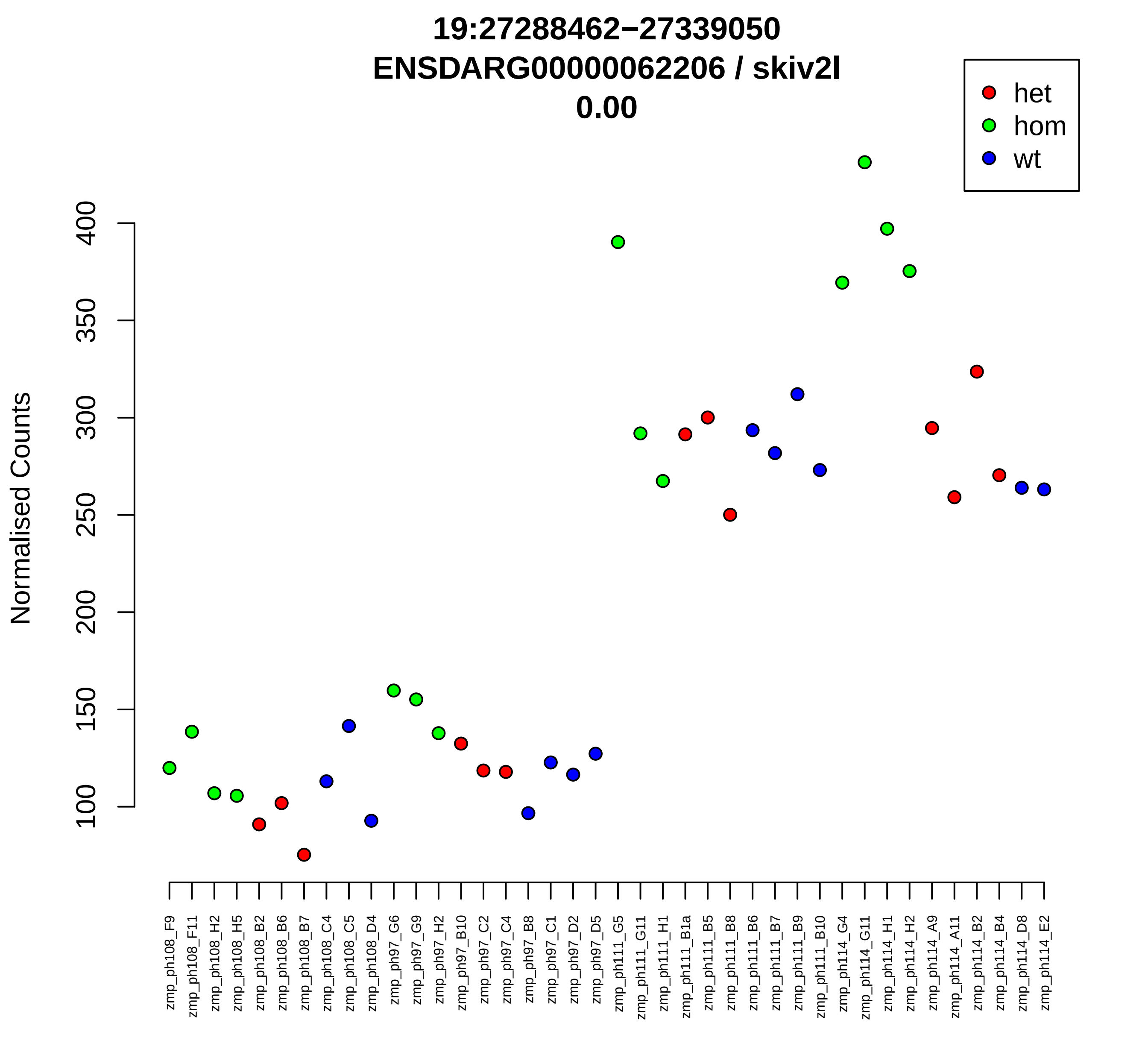
19:27288462−27339050
ENSDARG00000062206 / skiv2l
0.00
het
hom
wt
Normalised Counts
100
150
200
250
300
350
400
zmp_ph108_F9
zmp_ph108_F11
zmp_ph108_H2
zmp_ph108_H5
zmp_ph108_B2
zmp_ph108_B6
zmp_ph108_B7
zmp_ph108_C4
zmp_ph108_C5
zmp_ph108_D4
zmp_ph97_G6
zmp_ph97_G9
zmp_ph97_H2
zmp_ph97_B10
zmp_ph97_C2
zmp_ph97_C4
zmp_ph97_B8
zmp_ph97_C1
zmp_ph97_D2
zmp_ph97_D5
zmp_ph111_G5
zmp_ph111_G11
zmp_ph111_H1
zmp_ph111_B1a
zmp_ph111_B5
zmp_ph111_B8
zmp_ph111_B6
zmp_ph111_B7
zmp_ph111_B9
zmp_ph111_B10
zmp_ph114_G4
zmp_ph114_G11
zmp_ph114_H1
zmp_ph114_H2
zmp_ph114_A9
zmp_ph114_A11
zmp_ph114_B2
zmp_ph114_B4
zmp_ph114_D8
zmp_ph114_E2

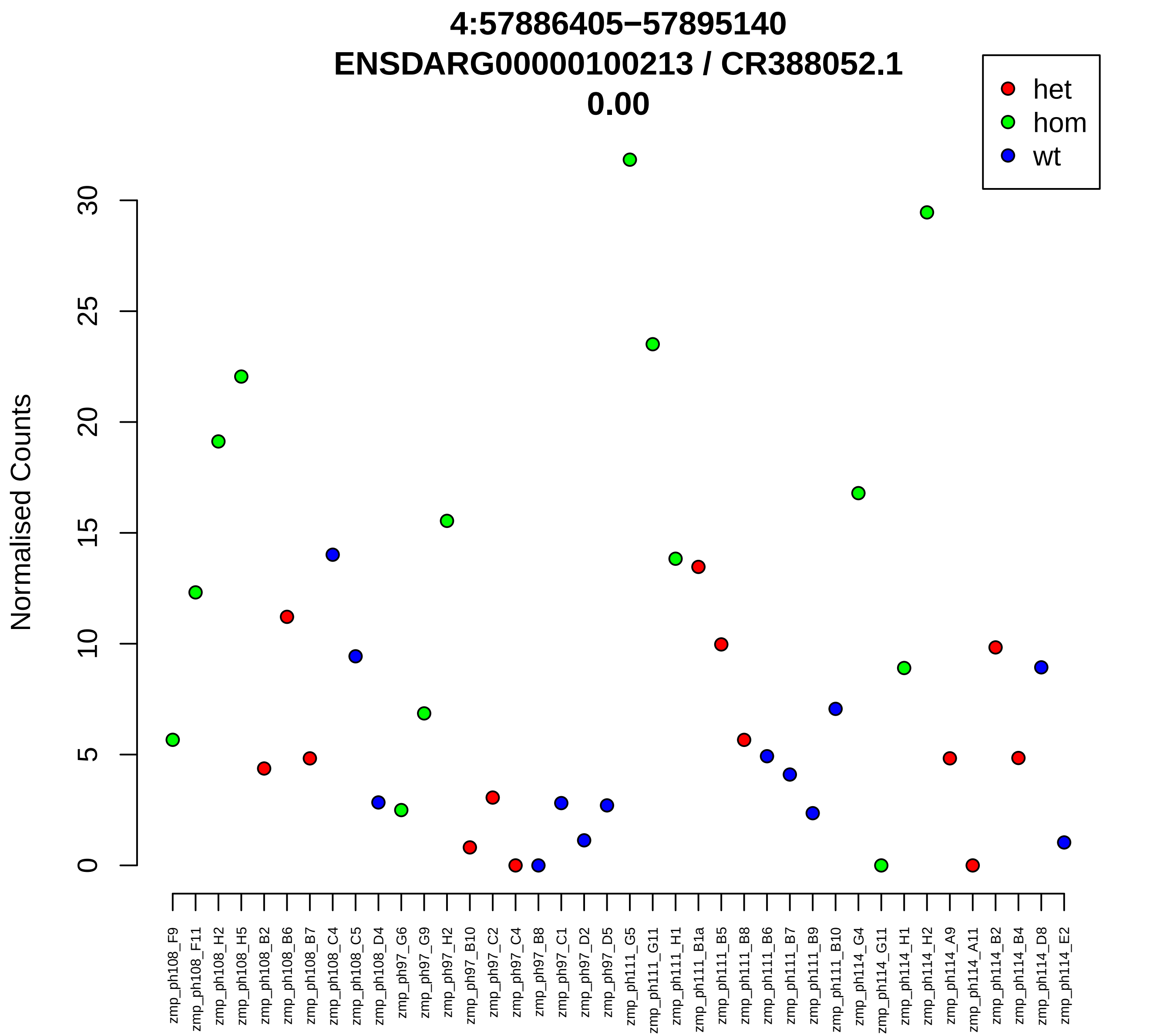

4:57886405−57895140
ENSDARG00000100213 / CR388052.1
0.00
het
hom
wt
Normalised Counts
0
5
10
15
20
25
30
zmp_ph108_F9
zmp_ph108_F11
zmp_ph108_H2
zmp_ph108_H5
zmp_ph108_B2
zmp_ph108_B6
zmp_ph108_B7
zmp_ph108_C4
zmp_ph108_C5
zmp_ph108_D4
zmp_ph97_G6
zmp_ph97_G9
zmp_ph97_H2
zmp_ph97_B10
zmp_ph97_C2
zmp_ph97_C4
zmp_ph97_B8
zmp_ph97_C1
zmp_ph97_D2
zmp_ph97_D5
zmp_ph111_G5
zmp_ph111_G11
zmp_ph111_H1
zmp_ph111_B1a
zmp_ph111_B5
zmp_ph111_B8
zmp_ph111_B6
zmp_ph111_B7
zmp_ph111_B9
zmp_ph111_B10
zmp_ph114_G4
zmp_ph114_G11
zmp_ph114_H1
zmp_ph114_H2
zmp_ph114_A9
zmp_ph114_A11
zmp_ph114_B2
zmp_ph114_B4
zmp_ph114_D8
zmp_ph114_E2

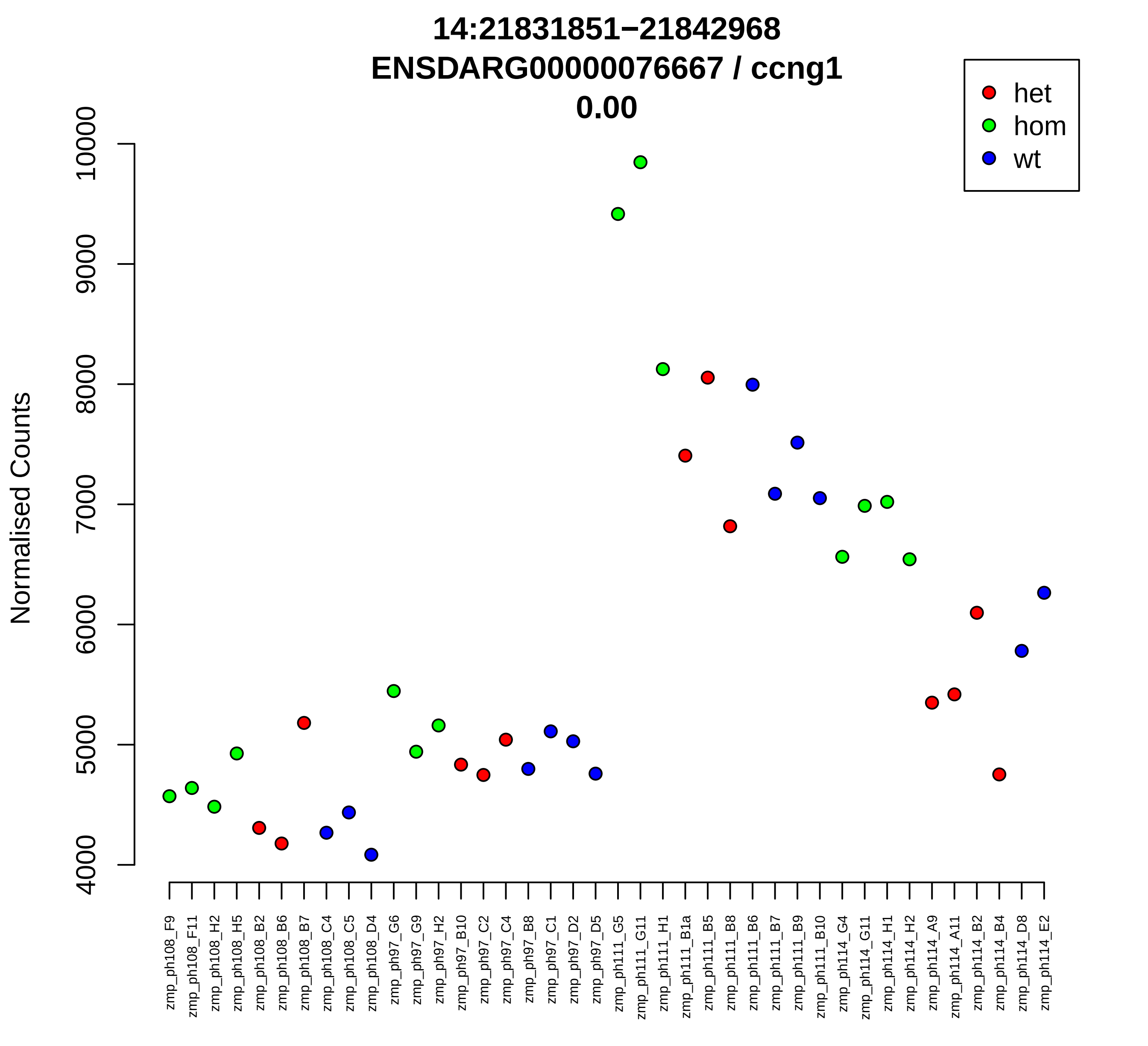

14:21831851−21842968
ENSDARG00000076667 / ccng1
0.00
het
hom
wt
Normalised Counts
4000
5000
6000
7000
8000
9000
10000
zmp_ph108_F9
zmp_ph108_F11
zmp_ph108_H2
zmp_ph108_H5
zmp_ph108_B2
zmp_ph108_B6
zmp_ph108_B7
zmp_ph108_C4
zmp_ph108_C5
zmp_ph108_D4
zmp_ph97_G6
zmp_ph97_G9
zmp_ph97_H2
zmp_ph97_B10
zmp_ph97_C2
zmp_ph97_C4
zmp_ph97_B8
zmp_ph97_C1
zmp_ph97_D2
zmp_ph97_D5
zmp_ph111_G5
zmp_ph111_G11
zmp_ph111_H1
zmp_ph111_B1a
zmp_ph111_B5
zmp_ph111_B8
zmp_ph111_B6
zmp_ph111_B7
zmp_ph111_B9
zmp_ph111_B10
zmp_ph114_G4
zmp_ph114_G11
zmp_ph114_H1
zmp_ph114_H2
zmp_ph114_A9
zmp_ph114_A11
zmp_ph114_B2
zmp_ph114_B4
zmp_ph114_D8
zmp_ph114_E2

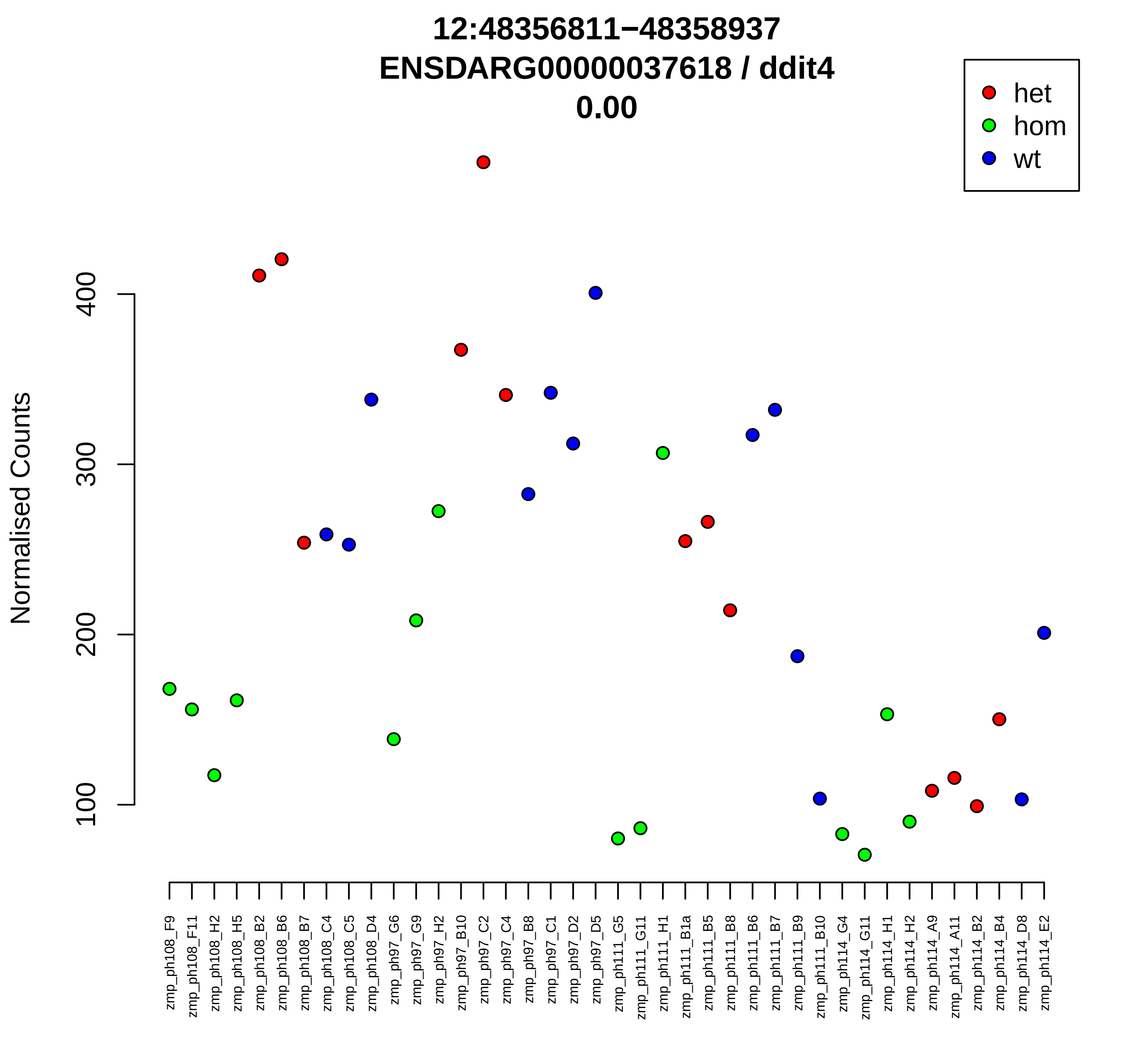
12:48356811−48358937
ENSDARG00000037618 / ddit4
0.00
het
hom
wt
Normalised Counts
100
200
300
400
zmp_ph108_F9
zmp_ph108_F11
zmp_ph108_H2
zmp_ph108_H5
zmp_ph108_B2
zmp_ph108_B6
zmp_ph108_B7
zmp_ph108_C4
zmp_ph108_C5
zmp_ph108_D4
zmp_ph97_G6
zmp_ph97_G9
zmp_ph97_H2
zmp_ph97_B10
zmp_ph97_C2
zmp_ph97_C4
zmp_ph97_B8
zmp_ph97_C1
zmp_ph97_D2
zmp_ph97_D5
zmp_ph111_G5
zmp_ph111_G11
zmp_ph111_H1
zmp_ph111_B1a
zmp_ph111_B5
zmp_ph111_B8
zmp_ph111_B6
zmp_ph111_B7
zmp_ph111_B9
zmp_ph111_B10
zmp_ph114_G4
zmp_ph114_G11
zmp_ph114_H1
zmp_ph114_H2
zmp_ph114_A9
zmp_ph114_A11
zmp_ph114_B2
zmp_ph114_B4
zmp_ph114_D8
zmp_ph114_E2

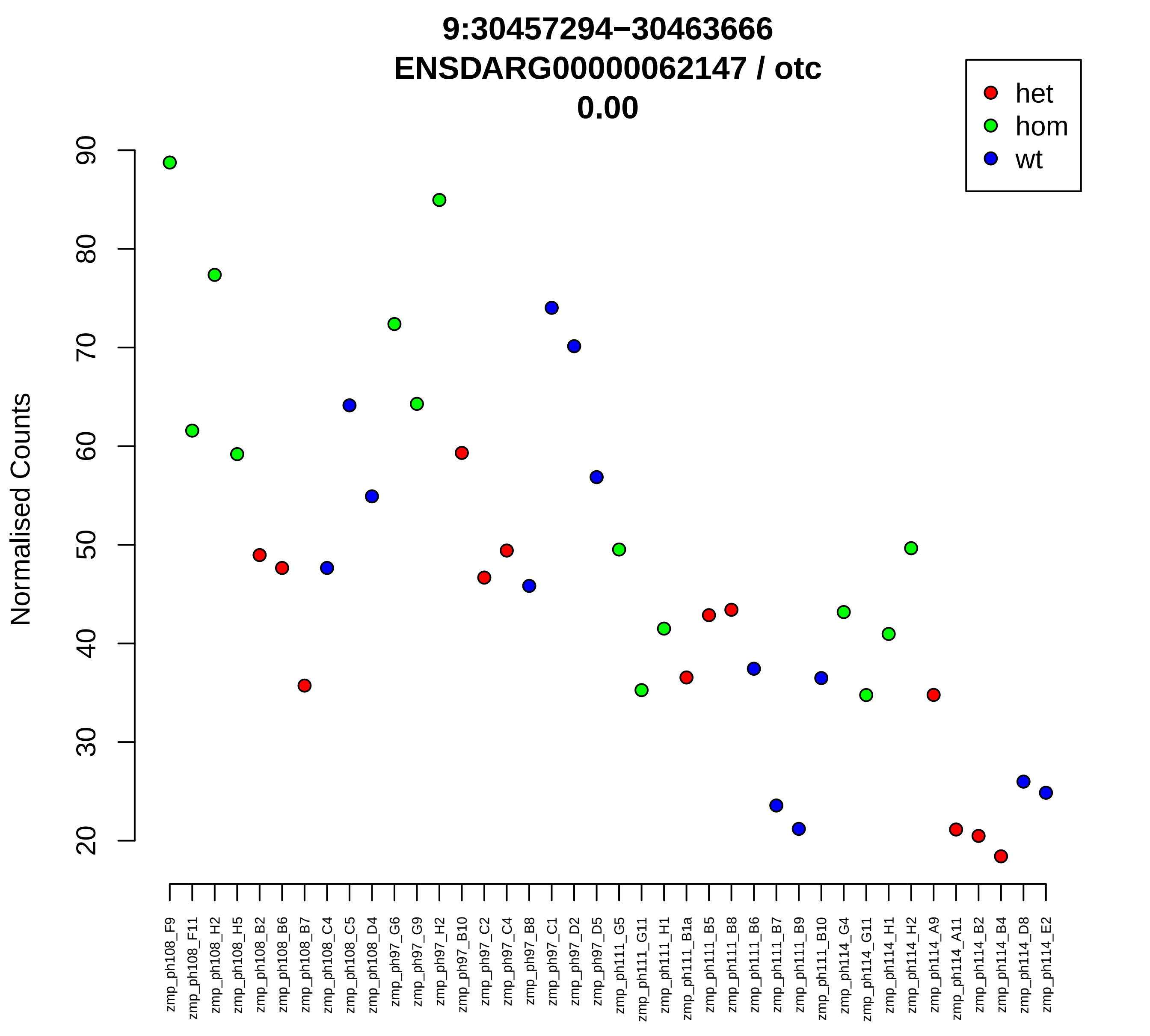
9:30457294−30463666
ENSDARG00000062147 / otc
0.00
het
hom
wt
Normalised Counts
20
30
40
50
60
70
80
90
zmp_ph108_F9
zmp_ph108_F11
zmp_ph108_H2
zmp_ph108_H5
zmp_ph108_B2
zmp_ph108_B6
zmp_ph108_B7
zmp_ph108_C4
zmp_ph108_C5
zmp_ph108_D4
zmp_ph97_G6
zmp_ph97_G9
zmp_ph97_H2
zmp_ph97_B10
zmp_ph97_C2
zmp_ph97_C4
zmp_ph97_B8
zmp_ph97_C1
zmp_ph97_D2
zmp_ph97_D5
zmp_ph111_G5
zmp_ph111_G11
zmp_ph111_H1
zmp_ph111_B1a
zmp_ph111_B5
zmp_ph111_B8
zmp_ph111_B6
zmp_ph111_B7
zmp_ph111_B9
zmp_ph111_B10
zmp_ph114_G4
zmp_ph114_G11
zmp_ph114_H1
zmp_ph114_H2
zmp_ph114_A9
zmp_ph114_A11
zmp_ph114_B2
zmp_ph114_B4
zmp_ph114_D8
zmp_ph114_E2

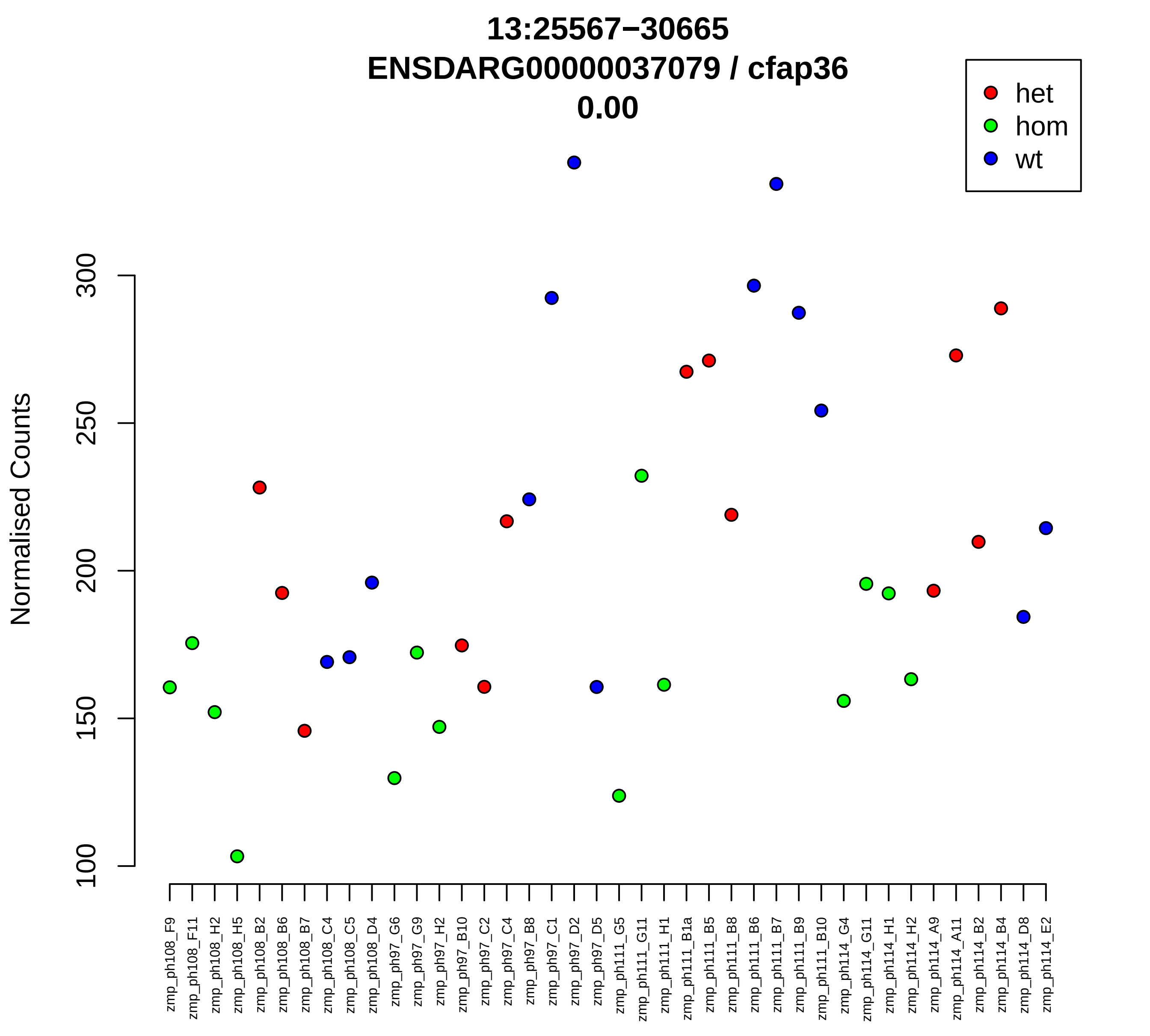
13:25567−30665
ENSDARG00000037079 / cfap36
0.00
het
hom
wt
Normalised Counts
100
150
200
250
300
zmp_ph108_F9
zmp_ph108_F11
zmp_ph108_H2
zmp_ph108_H5
zmp_ph108_B2
zmp_ph108_B6
zmp_ph108_B7
zmp_ph108_C4
zmp_ph108_C5
zmp_ph108_D4
zmp_ph97_G6
zmp_ph97_G9
zmp_ph97_H2
zmp_ph97_B10
zmp_ph97_C2
zmp_ph97_C4
zmp_ph97_B8
zmp_ph97_C1
zmp_ph97_D2
zmp_ph97_D5
zmp_ph111_G5
zmp_ph111_G11
zmp_ph111_H1
zmp_ph111_B1a
zmp_ph111_B5
zmp_ph111_B8
zmp_ph111_B6
zmp_ph111_B7
zmp_ph111_B9
zmp_ph111_B10
zmp_ph114_G4
zmp_ph114_G11
zmp_ph114_H1
zmp_ph114_H2
zmp_ph114_A9
zmp_ph114_A11
zmp_ph114_B2
zmp_ph114_B4
zmp_ph114_D8
zmp_ph114_E2

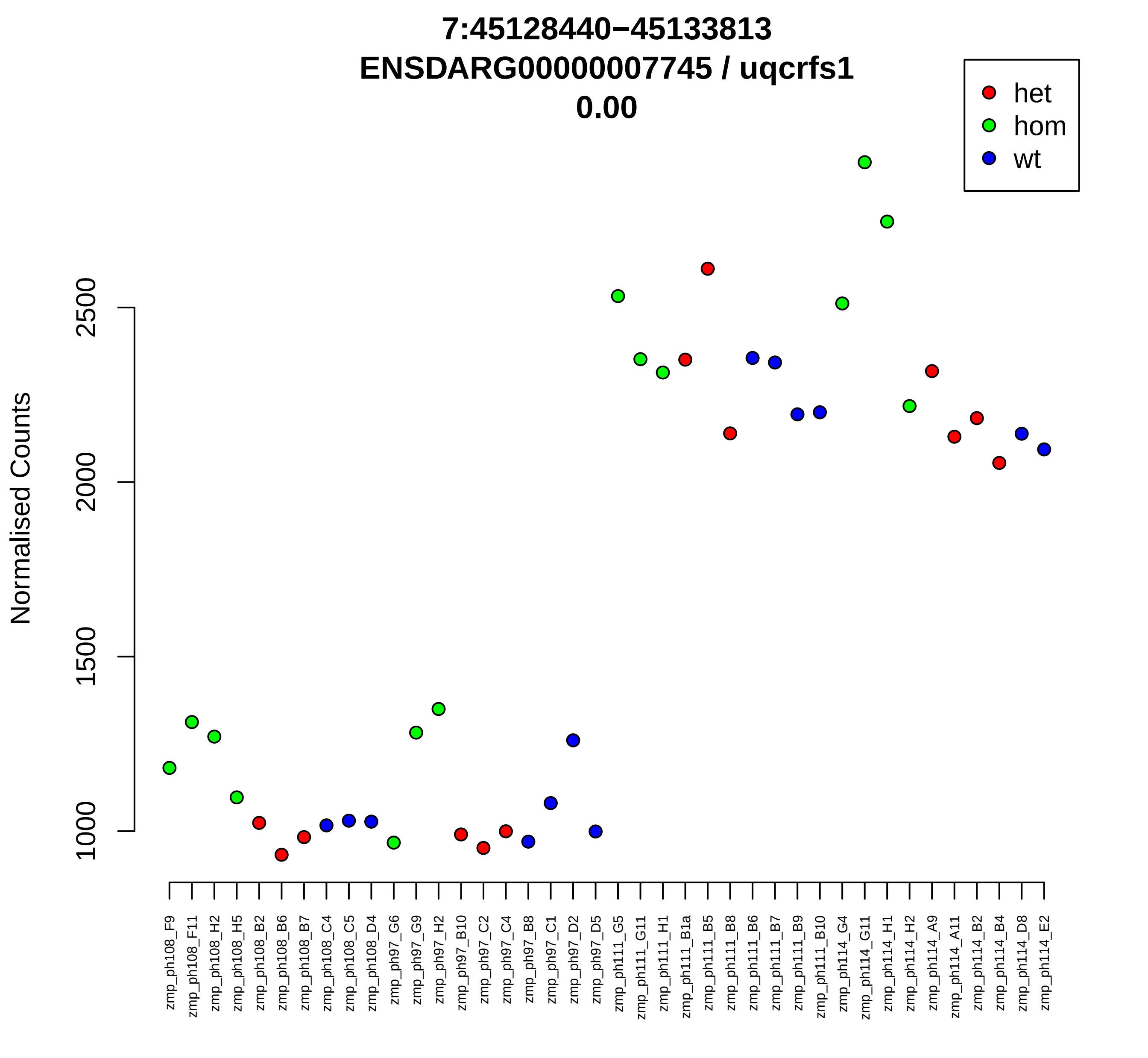
7:45128440−45133813
ENSDARG00000007745 / uqcrfs1
0.00
het
hom
wt
Normalised Counts
1000
1500
2000
2500
zmp_ph108_F9
zmp_ph108_F11
zmp_ph108_H2
zmp_ph108_H5
zmp_ph108_B2
zmp_ph108_B6
zmp_ph108_B7
zmp_ph108_C4
zmp_ph108_C5
zmp_ph108_D4
zmp_ph97_G6
zmp_ph97_G9
zmp_ph97_H2
zmp_ph97_B10
zmp_ph97_C2
zmp_ph97_C4
zmp_ph97_B8
zmp_ph97_C1
zmp_ph97_D2
zmp_ph97_D5
zmp_ph111_G5
zmp_ph111_G11
zmp_ph111_H1
zmp_ph111_B1a
zmp_ph111_B5
zmp_ph111_B8
zmp_ph111_B6
zmp_ph111_B7
zmp_ph111_B9
zmp_ph111_B10
zmp_ph114_G4
zmp_ph114_G11
zmp_ph114_H1
zmp_ph114_H2
zmp_ph114_A9
zmp_ph114_A11
zmp_ph114_B2
zmp_ph114_B4
zmp_ph114_D8
zmp_ph114_E2

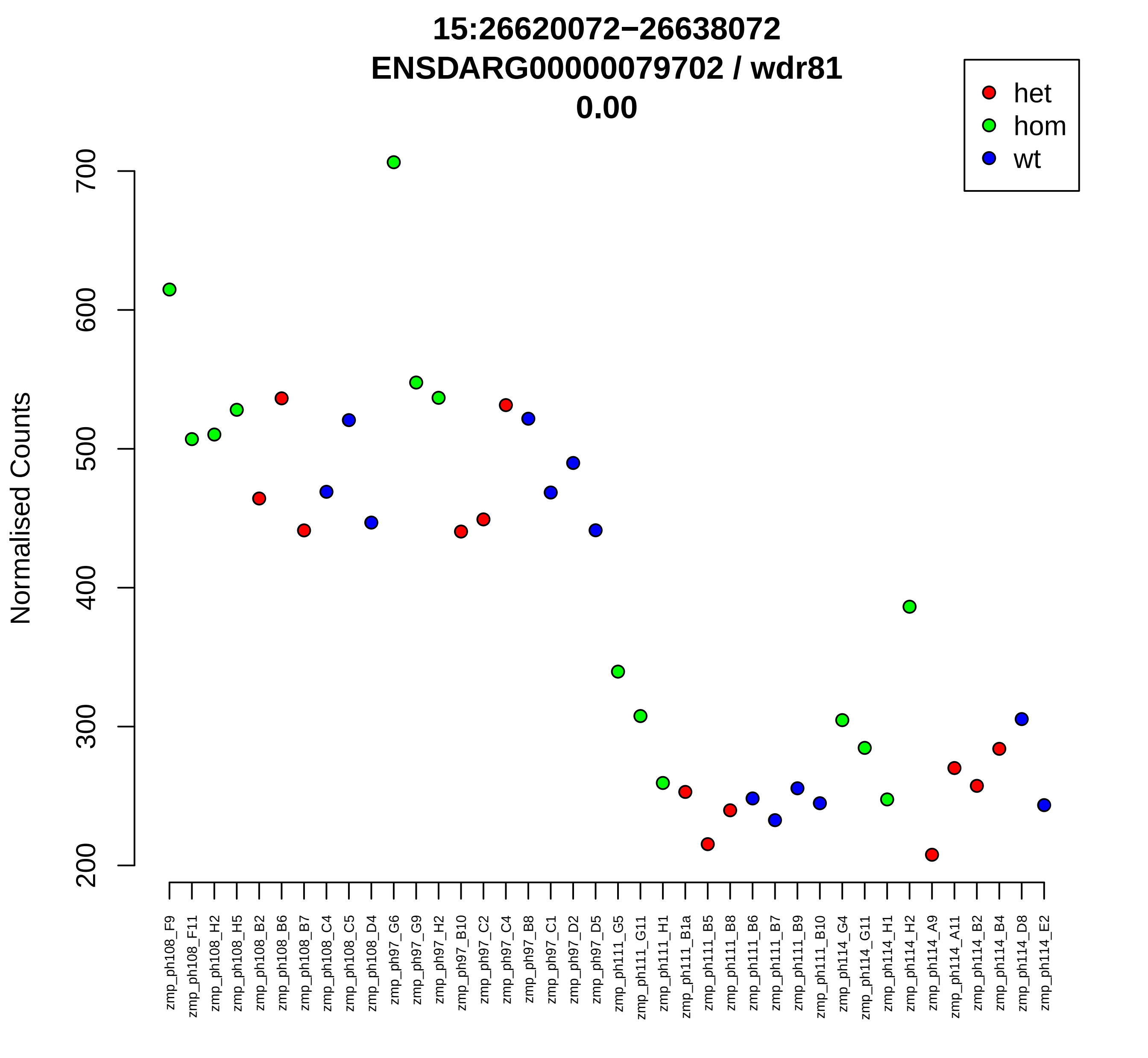

15:26620072−26638072
ENSDARG00000079702 / wdr81
0.00
het
hom
wt
Normalised Counts
200
300
400
500
600
700
zmp_ph108_F9
zmp_ph108_F11
zmp_ph108_H2
zmp_ph108_H5
zmp_ph108_B2
zmp_ph108_B6
zmp_ph108_B7
zmp_ph108_C4
zmp_ph108_C5
zmp_ph108_D4
zmp_ph97_G6
zmp_ph97_G9
zmp_ph97_H2
zmp_ph97_B10
zmp_ph97_C2
zmp_ph97_C4
zmp_ph97_B8
zmp_ph97_C1
zmp_ph97_D2
zmp_ph97_D5
zmp_ph111_G5
zmp_ph111_G11
zmp_ph111_H1
zmp_ph111_B1a
zmp_ph111_B5
zmp_ph111_B8
zmp_ph111_B6
zmp_ph111_B7
zmp_ph111_B9
zmp_ph111_B10
zmp_ph114_G4
zmp_ph114_G11
zmp_ph114_H1
zmp_ph114_H2
zmp_ph114_A9
zmp_ph114_A11
zmp_ph114_B2
zmp_ph114_B4
zmp_ph114_D8
zmp_ph114_E2

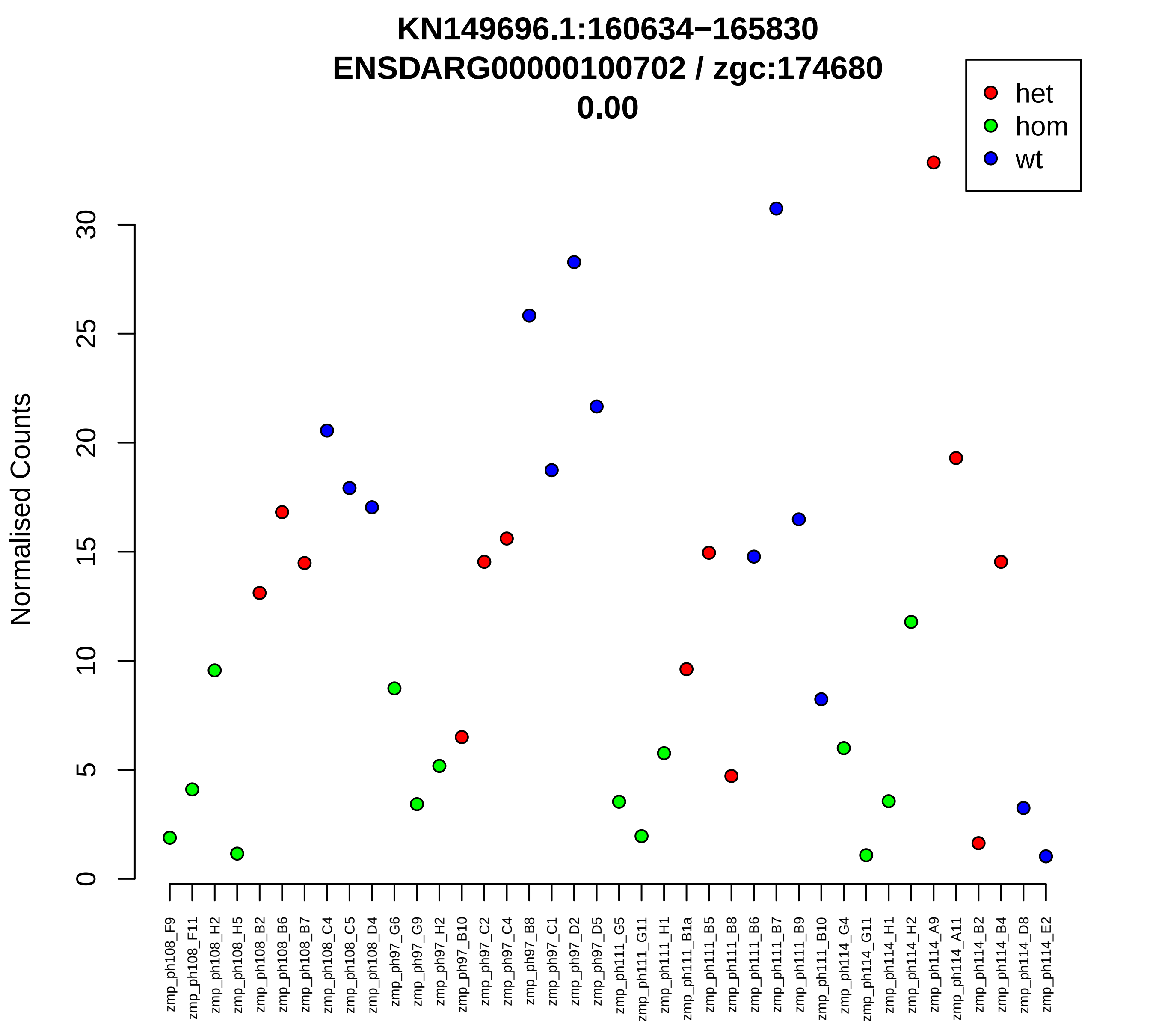

KN149696.1:160634−165830
ENSDARG00000100702 / zgc:174680
0.00
het
hom
wt
Normalised Counts
0
5
10
15
20
25
30
zmp_ph108_F9
zmp_ph108_F11
zmp_ph108_H2
zmp_ph108_H5
zmp_ph108_B2
zmp_ph108_B6
zmp_ph108_B7
zmp_ph108_C4
zmp_ph108_C5
zmp_ph108_D4
zmp_ph97_G6
zmp_ph97_G9
zmp_ph97_H2
zmp_ph97_B10
zmp_ph97_C2
zmp_ph97_C4
zmp_ph97_B8
zmp_ph97_C1
zmp_ph97_D2
zmp_ph97_D5
zmp_ph111_G5
zmp_ph111_G11
zmp_ph111_H1
zmp_ph111_B1a
zmp_ph111_B5
zmp_ph111_B8
zmp_ph111_B6
zmp_ph111_B7
zmp_ph111_B9
zmp_ph111_B10
zmp_ph114_G4
zmp_ph114_G11
zmp_ph114_H1
zmp_ph114_H2
zmp_ph114_A9
zmp_ph114_A11
zmp_ph114_B2
zmp_ph114_B4
zmp_ph114_D8
zmp_ph114_E2

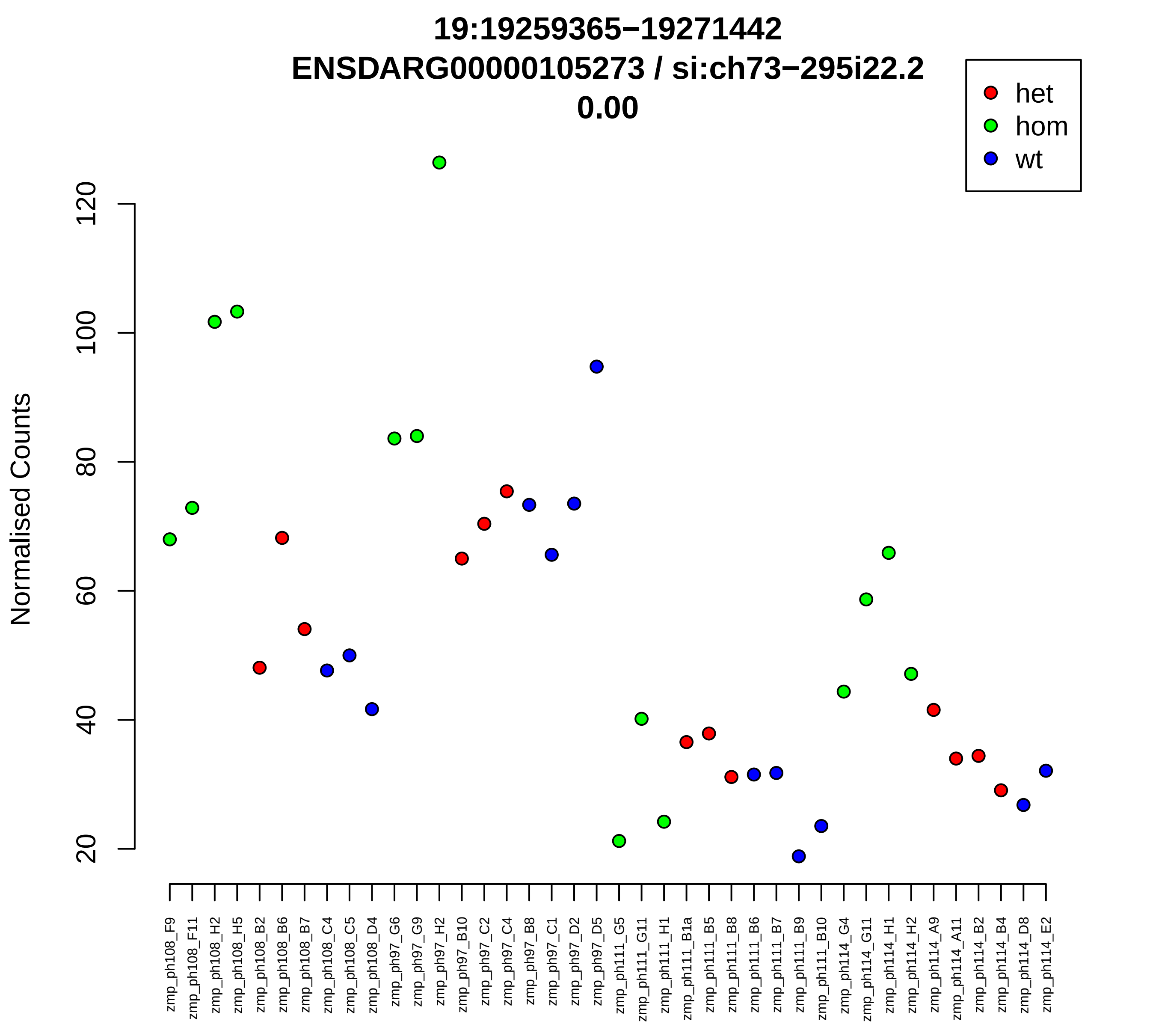

19:19259365−19271442
ENSDARG00000105273 / si:ch73−295i22.2
0.00
het
hom
wt
Normalised Counts
20
40
60
80
100
120
zmp_ph108_F9
zmp_ph108_F11
zmp_ph108_H2
zmp_ph108_H5
zmp_ph108_B2
zmp_ph108_B6
zmp_ph108_B7
zmp_ph108_C4
zmp_ph108_C5
zmp_ph108_D4
zmp_ph97_G6
zmp_ph97_G9
zmp_ph97_H2
zmp_ph97_B10
zmp_ph97_C2
zmp_ph97_C4
zmp_ph97_B8
zmp_ph97_C1
zmp_ph97_D2
zmp_ph97_D5
zmp_ph111_G5
zmp_ph111_G11
zmp_ph111_H1
zmp_ph111_B1a
zmp_ph111_B5
zmp_ph111_B8
zmp_ph111_B6
zmp_ph111_B7
zmp_ph111_B9
zmp_ph111_B10
zmp_ph114_G4
zmp_ph114_G11
zmp_ph114_H1
zmp_ph114_H2
zmp_ph114_A9
zmp_ph114_A11
zmp_ph114_B2
zmp_ph114_B4
zmp_ph114_D8
zmp_ph114_E2

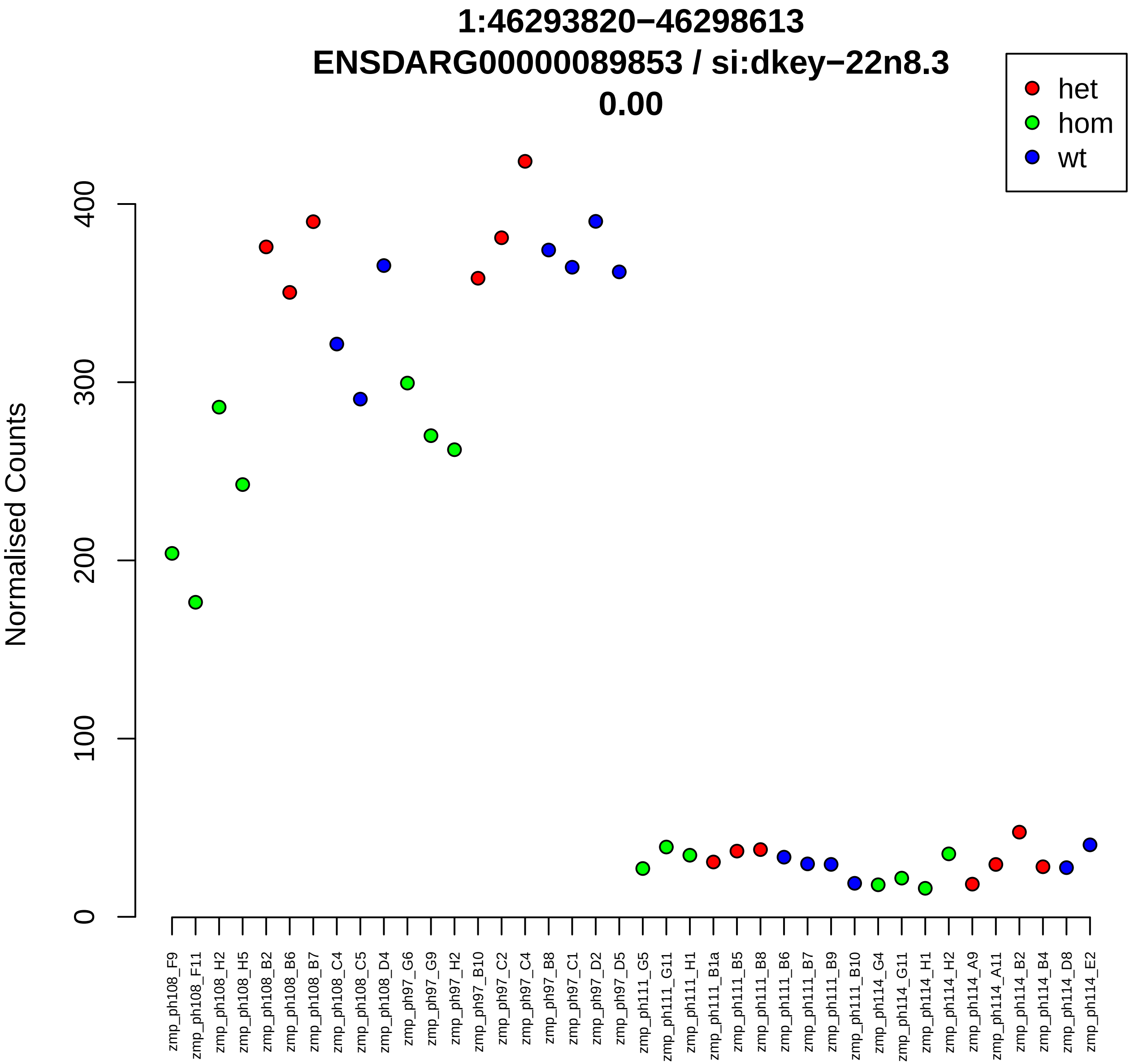

1:46293820−46298613
ENSDARG00000089853 / si:dkey−22n8.3
0.00
het
hom
wt
Normalised Counts
0
100
200
300
400
zmp_ph108_F9
zmp_ph108_F11
zmp_ph108_H2
zmp_ph108_H5
zmp_ph108_B2
zmp_ph108_B6
zmp_ph108_B7
zmp_ph108_C4
zmp_ph108_C5
zmp_ph108_D4
zmp_ph97_G6
zmp_ph97_G9
zmp_ph97_H2
zmp_ph97_B10
zmp_ph97_C2
zmp_ph97_C4
zmp_ph97_B8
zmp_ph97_C1
zmp_ph97_D2
zmp_ph97_D5
zmp_ph111_G5
zmp_ph111_G11
zmp_ph111_H1
zmp_ph111_B1a
zmp_ph111_B5
zmp_ph111_B8
zmp_ph111_B6
zmp_ph111_B7
zmp_ph111_B9
zmp_ph111_B10
zmp_ph114_G4
zmp_ph114_G11
zmp_ph114_H1
zmp_ph114_H2
zmp_ph114_A9
zmp_ph114_A11
zmp_ph114_B2
zmp_ph114_B4
zmp_ph114_D8
zmp_ph114_E2

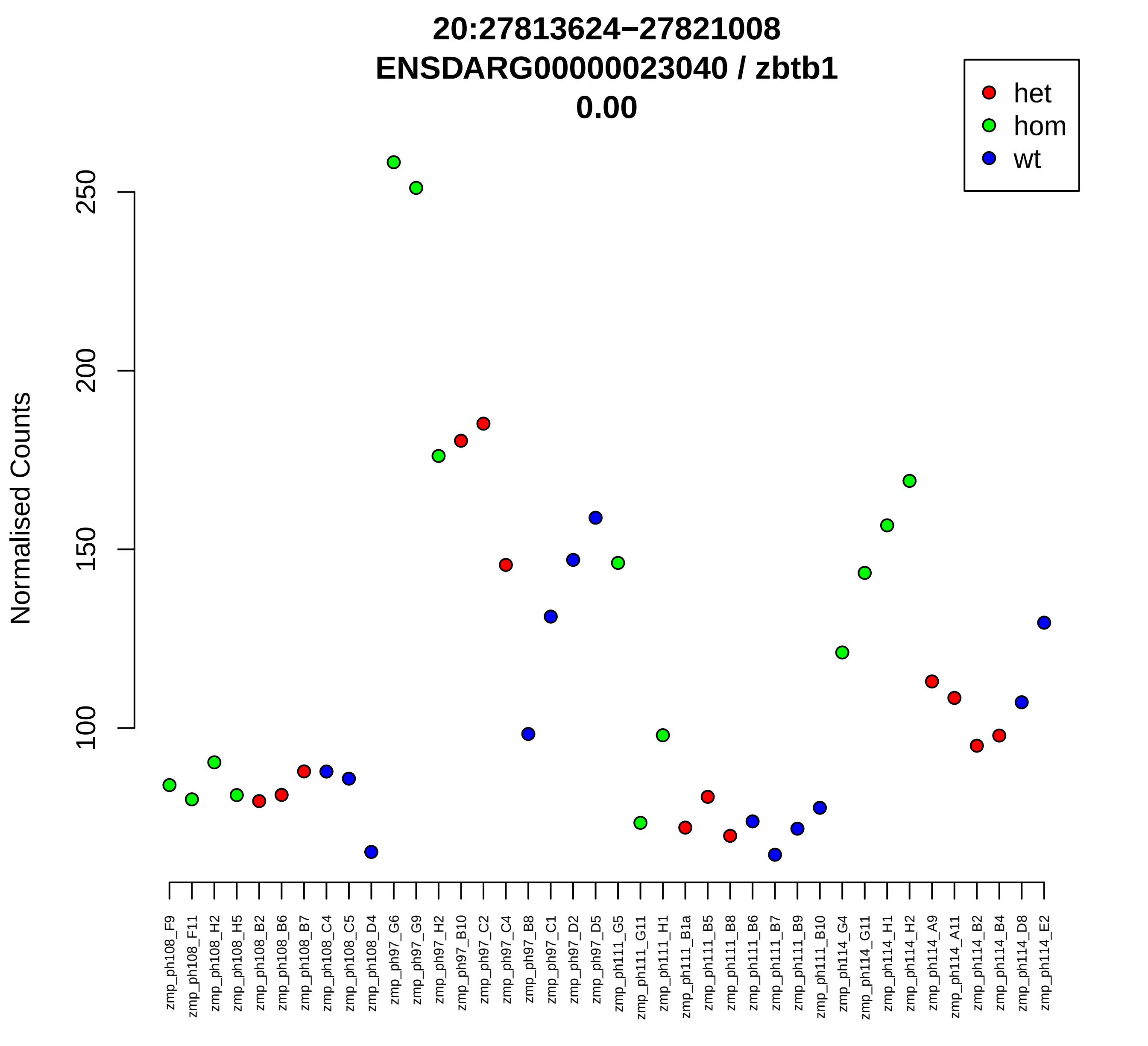
20:27813624−27821008
ENSDARG00000023040 / zbtb1
0.00
het
hom
wt
Normalised Counts
250
200
150
100
zmp_ph108_F9
zmp_ph108_F11
zmp_ph108_H2
zmp_ph108_H5
zmp_ph108_B2
zmp_ph108_B6
zmp_ph108_B7
zmp_ph108_C4
zmp_ph108_C5
zmp_ph108_D4
zmp_ph97_G6
zmp_ph97_G9
zmp_ph97_H2
zmp_ph97_B10
zmp_ph97_C2
zmp_ph97_C4
zmp_ph97_B8
zmp_ph97_C1
zmp_ph97_D2
zmp_ph97_D5
zmp_ph111_G5
zmp_ph111_G11
zmp_ph111_H1
zmp_ph111_B1a
zmp_ph111_B5
zmp_ph111_B8
zmp_ph111_B6
zmp_ph111_B7
zmp_ph111_B9
zmp_ph111_B10
zmp_ph114_G4
zmp_ph114_G11
zmp_ph114_H1
zmp_ph114_H2
zmp_ph114_A9
zmp_ph114_A11
zmp_ph114_B2
zmp_ph114_B4
zmp_ph114_D8
zmp_ph114_E2

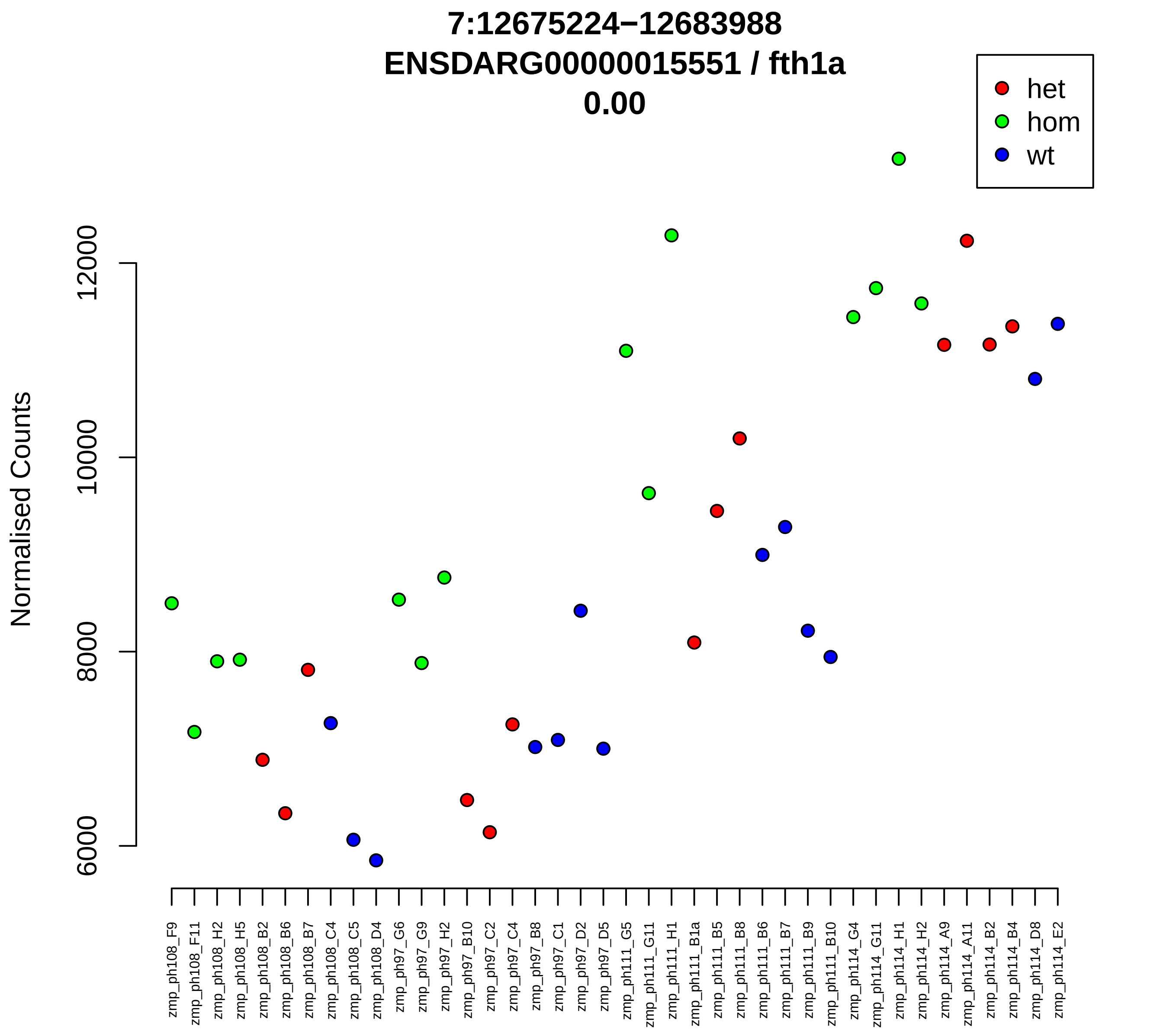

7:12675224−12683988
ENSDARG00000015551 / fth1a
0.00
het
hom
wt
Normalised Counts
6000
8000
10000
12000
zmp_ph108_F9
zmp_ph108_F11
zmp_ph108_H2
zmp_ph108_H5
zmp_ph108_B2
zmp_ph108_B6
zmp_ph108_B7
zmp_ph108_C4
zmp_ph108_C5
zmp_ph108_D4
zmp_ph97_G6
zmp_ph97_G9
zmp_ph97_H2
zmp_ph97_B10
zmp_ph97_C2
zmp_ph97_C4
zmp_ph97_B8
zmp_ph97_C1
zmp_ph97_D2
zmp_ph97_D5
zmp_ph111_G5
zmp_ph111_G11
zmp_ph111_H1
zmp_ph111_B1a
zmp_ph111_B5
zmp_ph111_B8
zmp_ph111_B6
zmp_ph111_B7
zmp_ph111_B9
zmp_ph111_B10
zmp_ph114_G4
zmp_ph114_G11
zmp_ph114_H1
zmp_ph114_H2
zmp_ph114_A9
zmp_ph114_A11
zmp_ph114_B2
zmp_ph114_B4
zmp_ph114_D8
zmp_ph114_E2

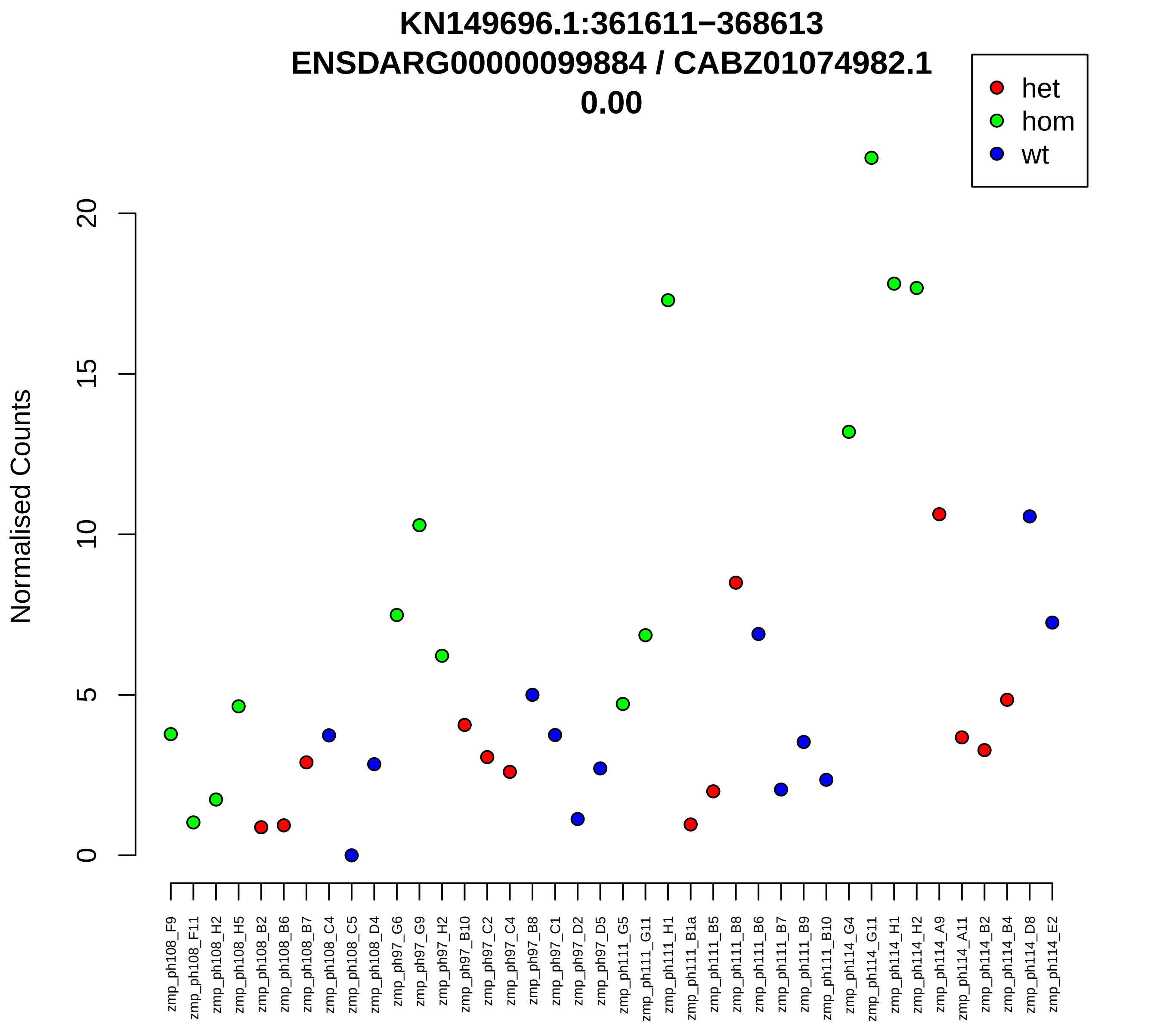

KN149696.1:361611−368613
ENSDARG00000099884 / CABZ01074982.1
0.00
het
hom
wt
Normalised Counts
0
5
10
15
20
zmp_ph108_F9
zmp_ph108_F11
zmp_ph108_H2
zmp_ph108_H5
zmp_ph108_B2
zmp_ph108_B6
zmp_ph108_B7
zmp_ph108_C4
zmp_ph108_C5
zmp_ph108_D4
zmp_ph97_G6
zmp_ph97_G9
zmp_ph97_H2
zmp_ph97_B10
zmp_ph97_C2
zmp_ph97_C4
zmp_ph97_B8
zmp_ph97_C1
zmp_ph97_D2
zmp_ph97_D5
zmp_ph111_G5
zmp_ph111_G11
zmp_ph111_H1
zmp_ph111_B1a
zmp_ph111_B5
zmp_ph111_B8
zmp_ph111_B6
zmp_ph111_B7
zmp_ph111_B9
zmp_ph111_B10
zmp_ph114_G4
zmp_ph114_G11
zmp_ph114_H1
zmp_ph114_H2
zmp_ph114_A9
zmp_ph114_A11
zmp_ph114_B2
zmp_ph114_B4
zmp_ph114_D8
zmp_ph114_E2

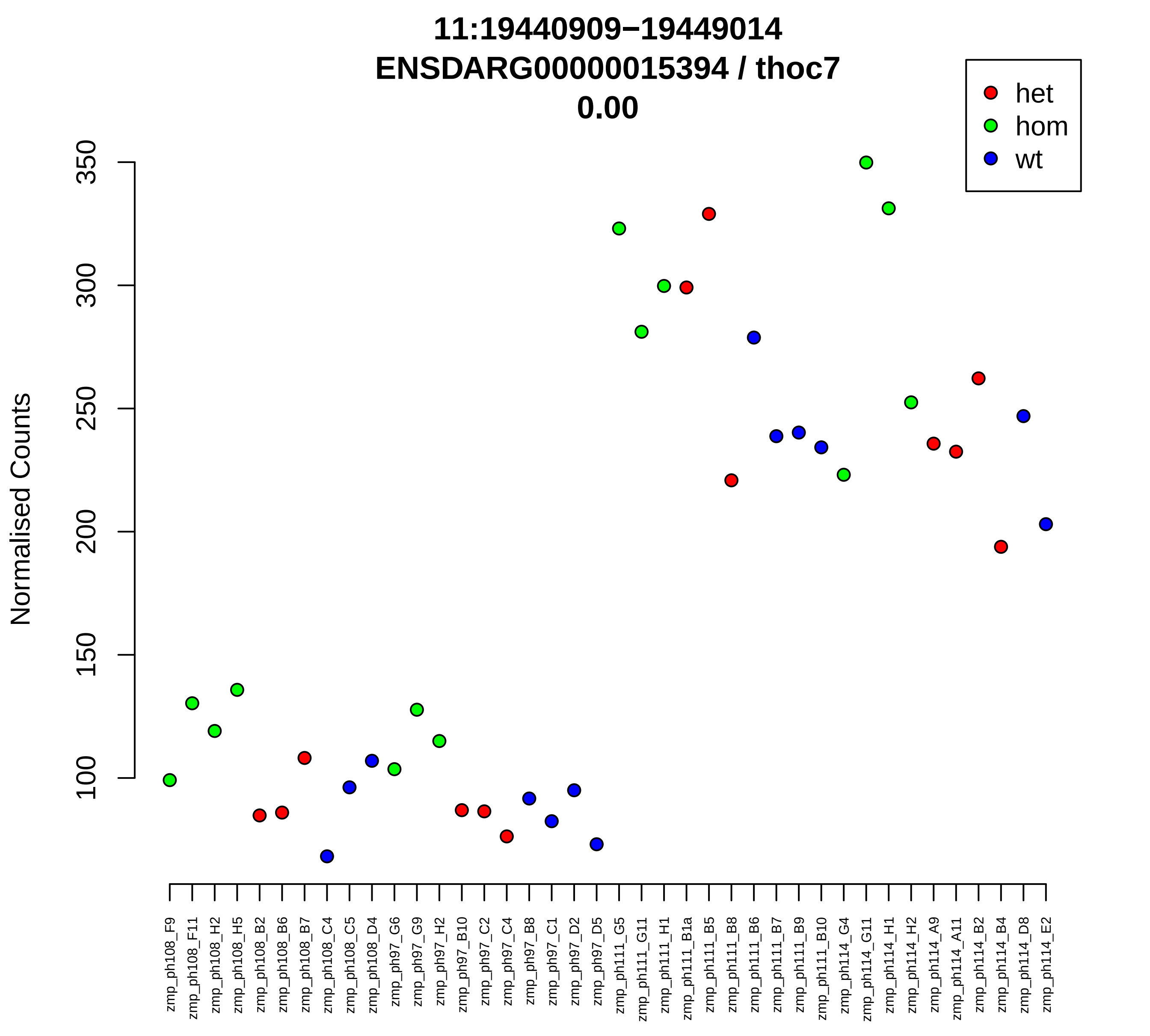

11:19440909−19449014
ENSDARG00000015394 / thoc7
0.00
het
hom
wt
Normalised Counts
350
300
250
200
150
100
zmp_ph108_F9
zmp_ph108_F11
zmp_ph108_H2
zmp_ph108_H5
zmp_ph108_B2
zmp_ph108_B6
zmp_ph108_B7
zmp_ph108_C4
zmp_ph108_C5
zmp_ph108_D4
zmp_ph97_G6
zmp_ph97_G9
zmp_ph97_H2
zmp_ph97_B10
zmp_ph97_C2
zmp_ph97_C4
zmp_ph97_B8
zmp_ph97_C1
zmp_ph97_D2
zmp_ph97_D5
zmp_ph111_G5
zmp_ph111_G11
zmp_ph111_H1
zmp_ph111_B1a
zmp_ph111_B5
zmp_ph111_B8
zmp_ph111_B6
zmp_ph111_B7
zmp_ph111_B9
zmp_ph111_B10
zmp_ph114_G4
zmp_ph114_G11
zmp_ph114_H1
zmp_ph114_H2
zmp_ph114_A9
zmp_ph114_A11
zmp_ph114_B2
zmp_ph114_B4
zmp_ph114_D8
zmp_ph114_E2

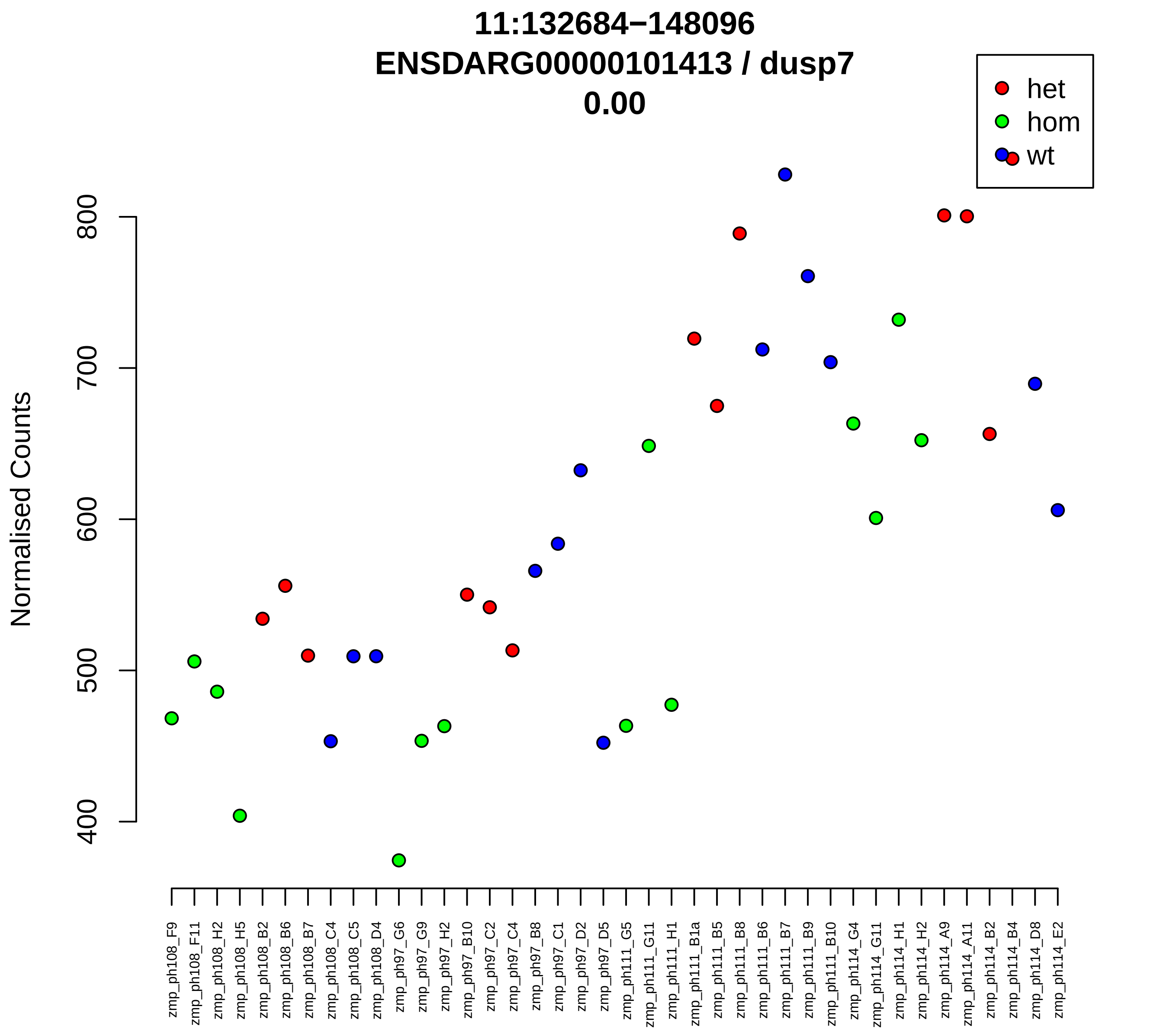

11:132684−148096
ENSDARG00000101413 / dusp7
0.00
het
hom
wt
Normalised Counts
400
500
600
700
800
zmp_ph108_F9
zmp_ph108_F11
zmp_ph108_H2
zmp_ph108_H5
zmp_ph108_B2
zmp_ph108_B6
zmp_ph108_B7
zmp_ph108_C4
zmp_ph108_C5
zmp_ph108_D4
zmp_ph97_G6
zmp_ph97_G9
zmp_ph97_H2
zmp_ph97_B10
zmp_ph97_C2
zmp_ph97_C4
zmp_ph97_B8
zmp_ph97_C1
zmp_ph97_D2
zmp_ph97_D5
zmp_ph111_G5
zmp_ph111_G11
zmp_ph111_H1
zmp_ph111_B1a
zmp_ph111_B5
zmp_ph111_B8
zmp_ph111_B6
zmp_ph111_B7
zmp_ph111_B9
zmp_ph111_B10
zmp_ph114_G4
zmp_ph114_G11
zmp_ph114_H1
zmp_ph114_H2
zmp_ph114_A9
zmp_ph114_A11
zmp_ph114_B2
zmp_ph114_B4
zmp_ph114_D8
zmp_ph114_E2

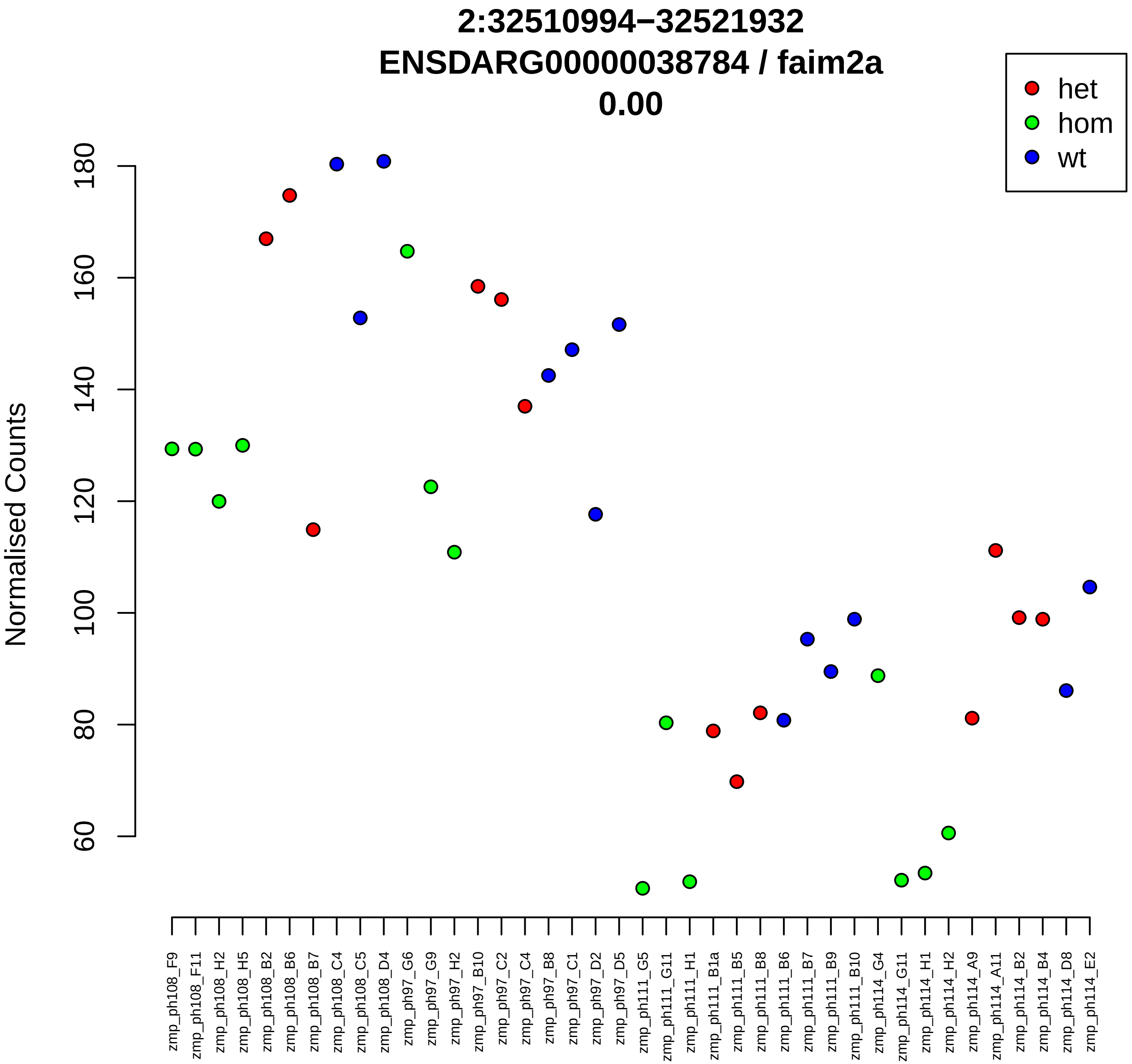
2:32510994−32521932
ENSDARG00000038784 / faim2a
0.00
het
hom
wt
Normalised Counts
60
80
100
120
140
160
180
zmp_ph108_F9
zmp_ph108_F11
zmp_ph108_H2
zmp_ph108_H5
zmp_ph108_B2
zmp_ph108_B6
zmp_ph108_B7
zmp_ph108_C4
zmp_ph108_C5
zmp_ph108_D4
zmp_ph97_G6
zmp_ph97_G9
zmp_ph97_H2
zmp_ph97_B10
zmp_ph97_C2
zmp_ph97_C4
zmp_ph97_B8
zmp_ph97_C1
zmp_ph97_D2
zmp_ph97_D5
zmp_ph111_G5
zmp_ph111_G11
zmp_ph111_H1
zmp_ph111_B1a
zmp_ph111_B5
zmp_ph111_B8
zmp_ph111_B6
zmp_ph111_B7
zmp_ph111_B9
zmp_ph111_B10
zmp_ph114_G4
zmp_ph114_G11
zmp_ph114_H1
zmp_ph114_H2
zmp_ph114_A9
zmp_ph114_A11
zmp_ph114_B2
zmp_ph114_B4
zmp_ph114_D8
zmp_ph114_E2

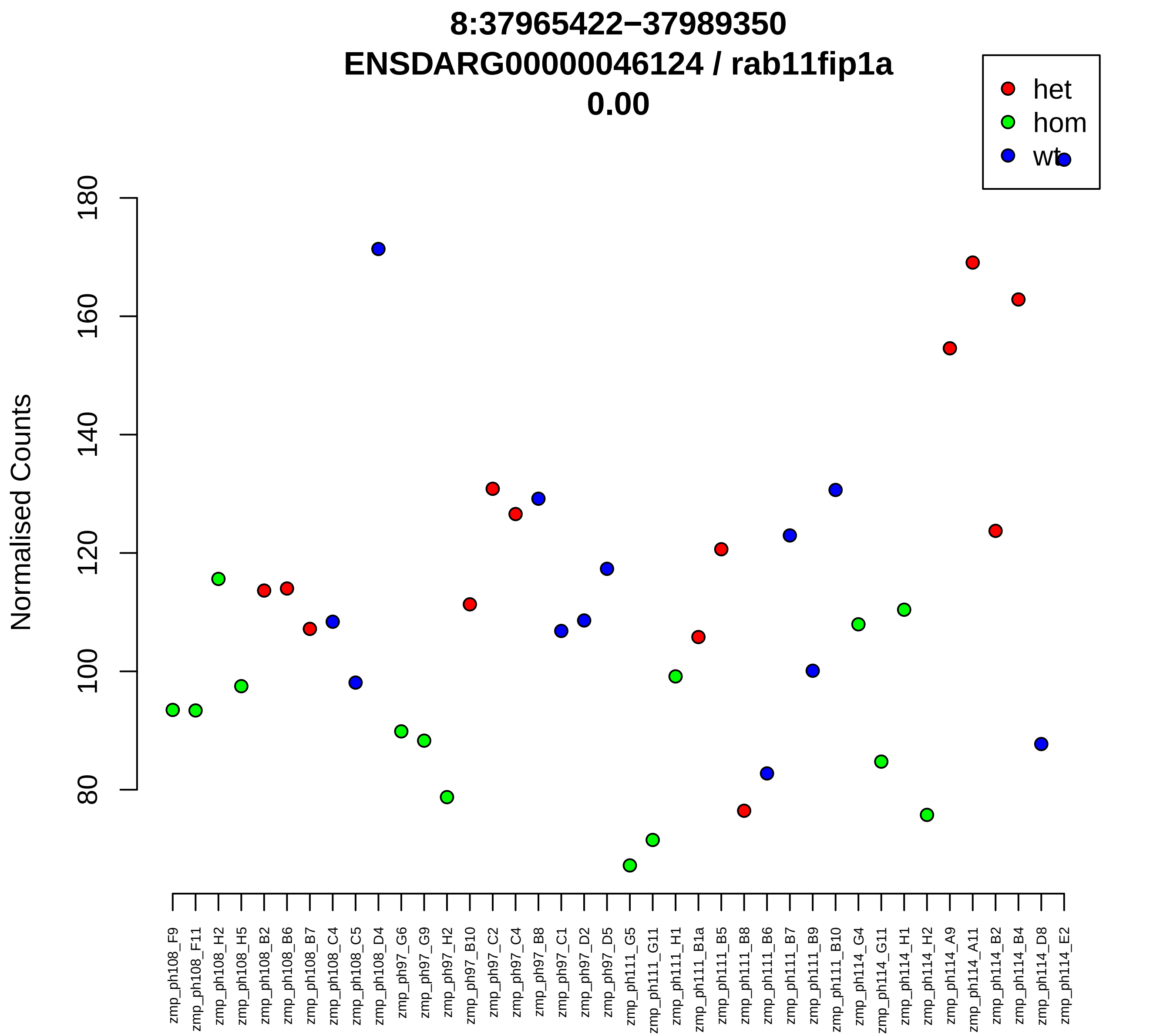

8:37965422−37989350
ENSDARG00000046124 / rab11fip1a
0.00
het
hom
wt
Normalised Counts
80
100
120
140
160
180
zmp_ph108_F9
zmp_ph108_F11
zmp_ph108_H2
zmp_ph108_H5
zmp_ph108_B2
zmp_ph108_B6
zmp_ph108_B7
zmp_ph108_C4
zmp_ph108_C5
zmp_ph108_D4
zmp_ph97_G6
zmp_ph97_G9
zmp_ph97_H2
zmp_ph97_B10
zmp_ph97_C2
zmp_ph97_C4
zmp_ph97_B8
zmp_ph97_C1
zmp_ph97_D2
zmp_ph97_D5
zmp_ph111_G5
zmp_ph111_G11
zmp_ph111_H1
zmp_ph111_B1a
zmp_ph111_B5
zmp_ph111_B8
zmp_ph111_B6
zmp_ph111_B7
zmp_ph111_B9
zmp_ph111_B10
zmp_ph114_G4
zmp_ph114_G11
zmp_ph114_H1
zmp_ph114_H2
zmp_ph114_A9
zmp_ph114_A11
zmp_ph114_B2
zmp_ph114_B4
zmp_ph114_D8
zmp_ph114_E2

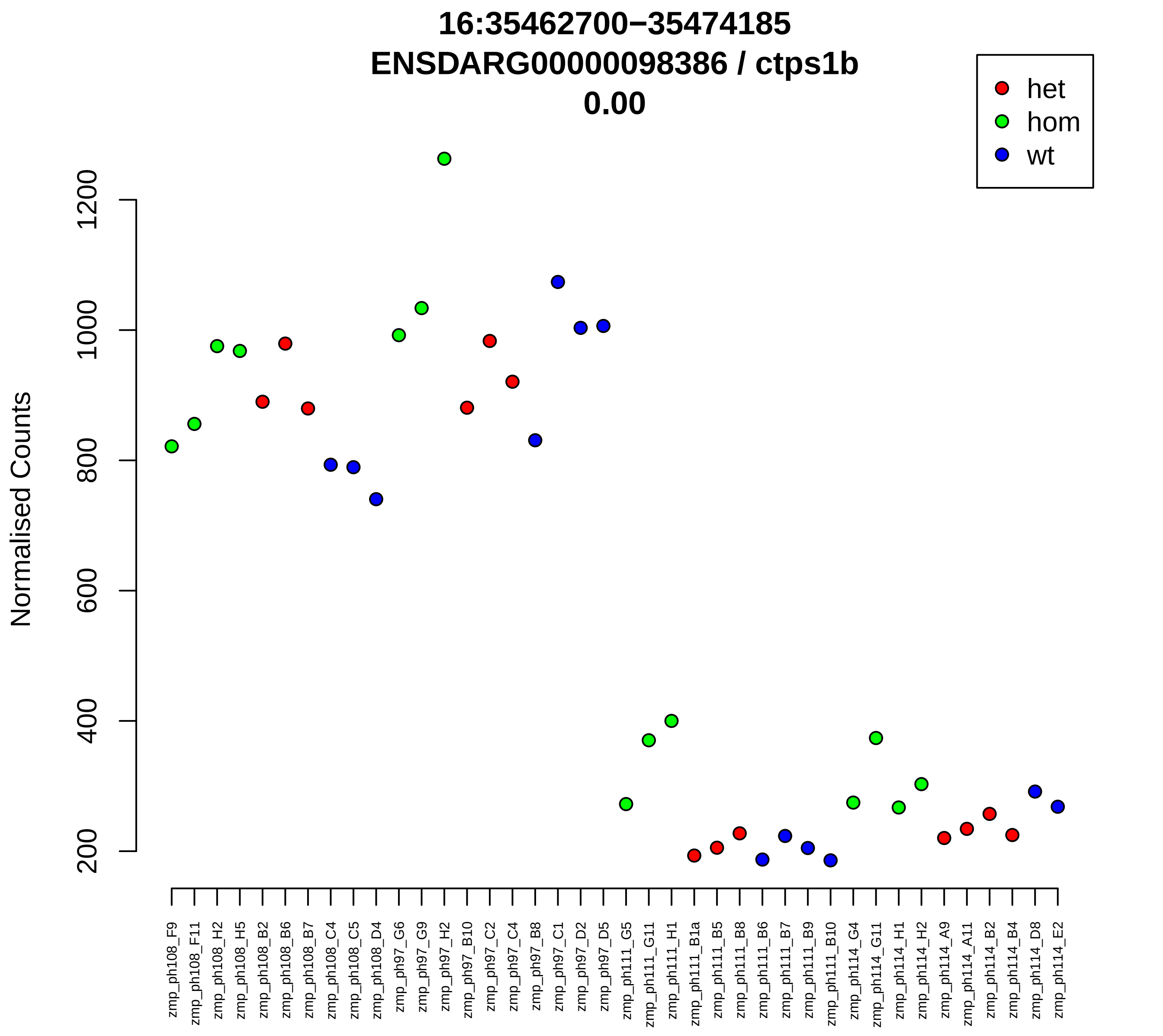

16:35462700−35474185
ENSDARG00000098386 / ctps1b
0.00
het
hom
wt
Normalised Counts
200
400
600
800
1000
1200
zmp_ph108_F9
zmp_ph108_F11
zmp_ph108_H2
zmp_ph108_H5
zmp_ph108_B2
zmp_ph108_B6
zmp_ph108_B7
zmp_ph108_C4
zmp_ph108_C5
zmp_ph108_D4
zmp_ph97_G6
zmp_ph97_G9
zmp_ph97_H2
zmp_ph97_B10
zmp_ph97_C2
zmp_ph97_C4
zmp_ph97_B8
zmp_ph97_C1
zmp_ph97_D2
zmp_ph97_D5
zmp_ph111_G5
zmp_ph111_G11
zmp_ph111_H1
zmp_ph111_B1a
zmp_ph111_B5
zmp_ph111_B8
zmp_ph111_B6
zmp_ph111_B7
zmp_ph111_B9
zmp_ph111_B10
zmp_ph114_G4
zmp_ph114_G11
zmp_ph114_H1
zmp_ph114_H2
zmp_ph114_A9
zmp_ph114_A11
zmp_ph114_B2
zmp_ph114_B4
zmp_ph114_D8
zmp_ph114_E2

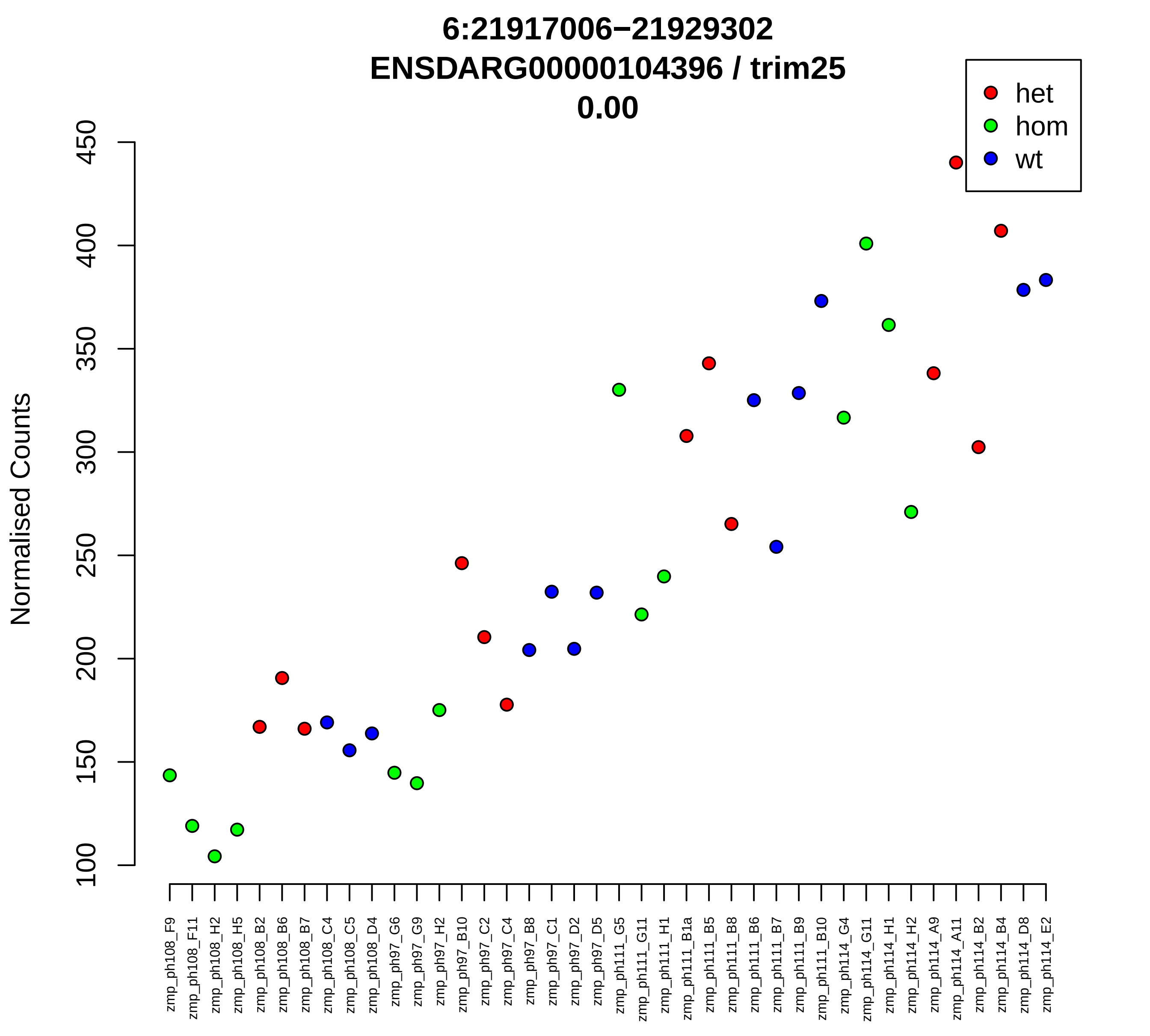
6:21917006−21929302
ENSDARG00000104396 / trim25
0.00
het
hom
wt
Normalised Counts
100
150
200
250
300
350
400
450
zmp_ph108_F9
zmp_ph108_F11
zmp_ph108_H2
zmp_ph108_H5
zmp_ph108_B2
zmp_ph108_B6
zmp_ph108_B7
zmp_ph108_C4
zmp_ph108_C5
zmp_ph108_D4
zmp_ph97_G6
zmp_ph97_G9
zmp_ph97_H2
zmp_ph97_B10
zmp_ph97_C2
zmp_ph97_C4
zmp_ph97_B8
zmp_ph97_C1
zmp_ph97_D2
zmp_ph97_D5
zmp_ph111_G5
zmp_ph111_G11
zmp_ph111_H1
zmp_ph111_B1a
zmp_ph111_B5
zmp_ph111_B8
zmp_ph111_B6
zmp_ph111_B7
zmp_ph111_B9
zmp_ph111_B10
zmp_ph114_G4
zmp_ph114_G11
zmp_ph114_H1
zmp_ph114_H2
zmp_ph114_A9
zmp_ph114_A11
zmp_ph114_B2
zmp_ph114_B4
zmp_ph114_D8
zmp_ph114_E2

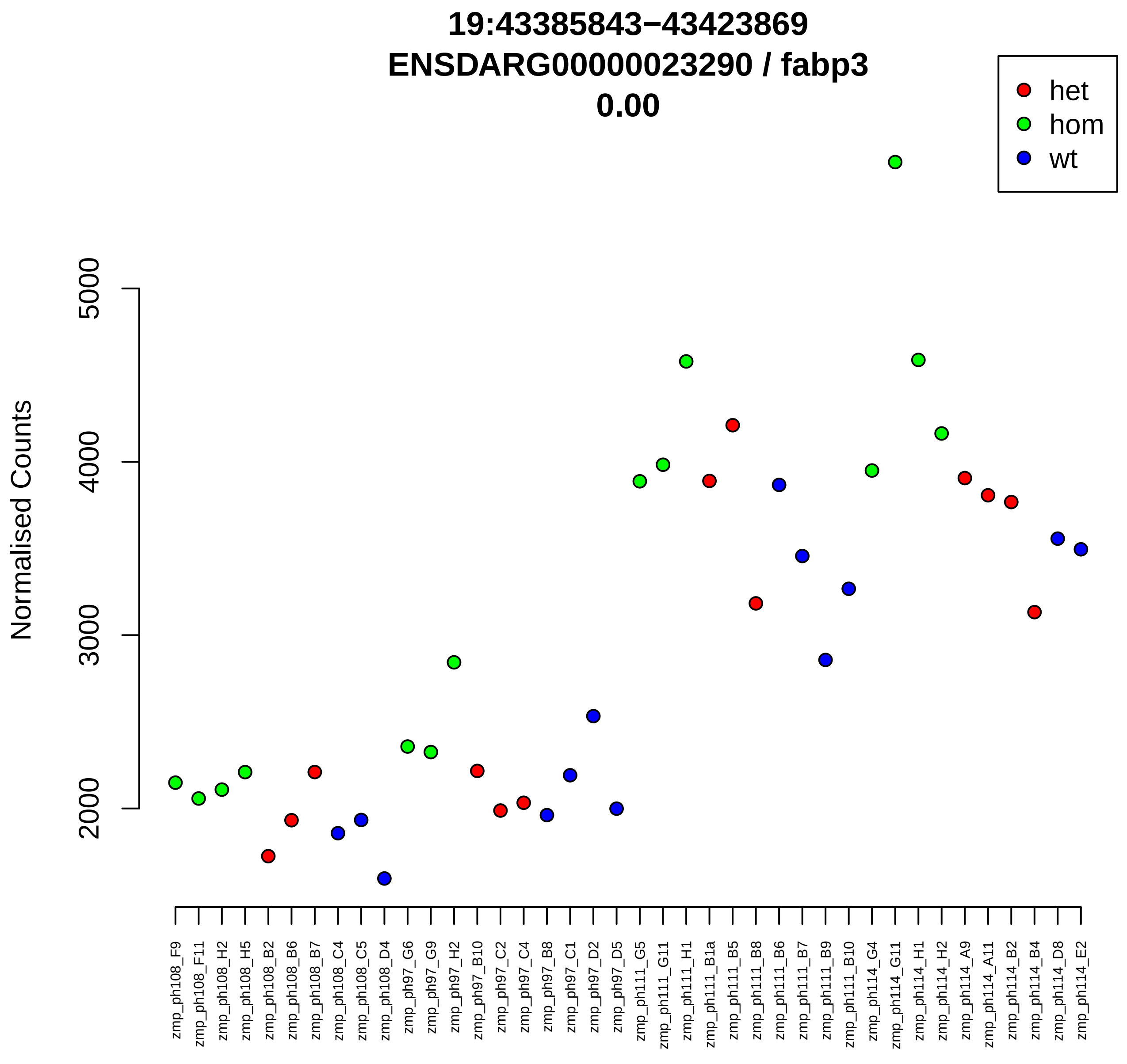
19:43385843−43423869
ENSDARG00000023290 / fabp3
0.00
het
hom
wt
Normalised Counts
2000
3000
4000
5000
zmp_ph108_F9
zmp_ph108_F11
zmp_ph108_H2
zmp_ph108_H5
zmp_ph108_B2
zmp_ph108_B6
zmp_ph108_B7
zmp_ph108_C4
zmp_ph108_C5
zmp_ph108_D4
zmp_ph97_G6
zmp_ph97_G9
zmp_ph97_H2
zmp_ph97_B10
zmp_ph97_C2
zmp_ph97_C4
zmp_ph97_B8
zmp_ph97_C1
zmp_ph97_D2
zmp_ph97_D5
zmp_ph111_G5
zmp_ph111_G11
zmp_ph111_H1
zmp_ph111_B1a
zmp_ph111_B5
zmp_ph111_B8
zmp_ph111_B6
zmp_ph111_B7
zmp_ph111_B9
zmp_ph111_B10
zmp_ph114_G4
zmp_ph114_G11
zmp_ph114_H1
zmp_ph114_H2
zmp_ph114_A9
zmp_ph114_A11
zmp_ph114_B2
zmp_ph114_B4
zmp_ph114_D8
zmp_ph114_E2

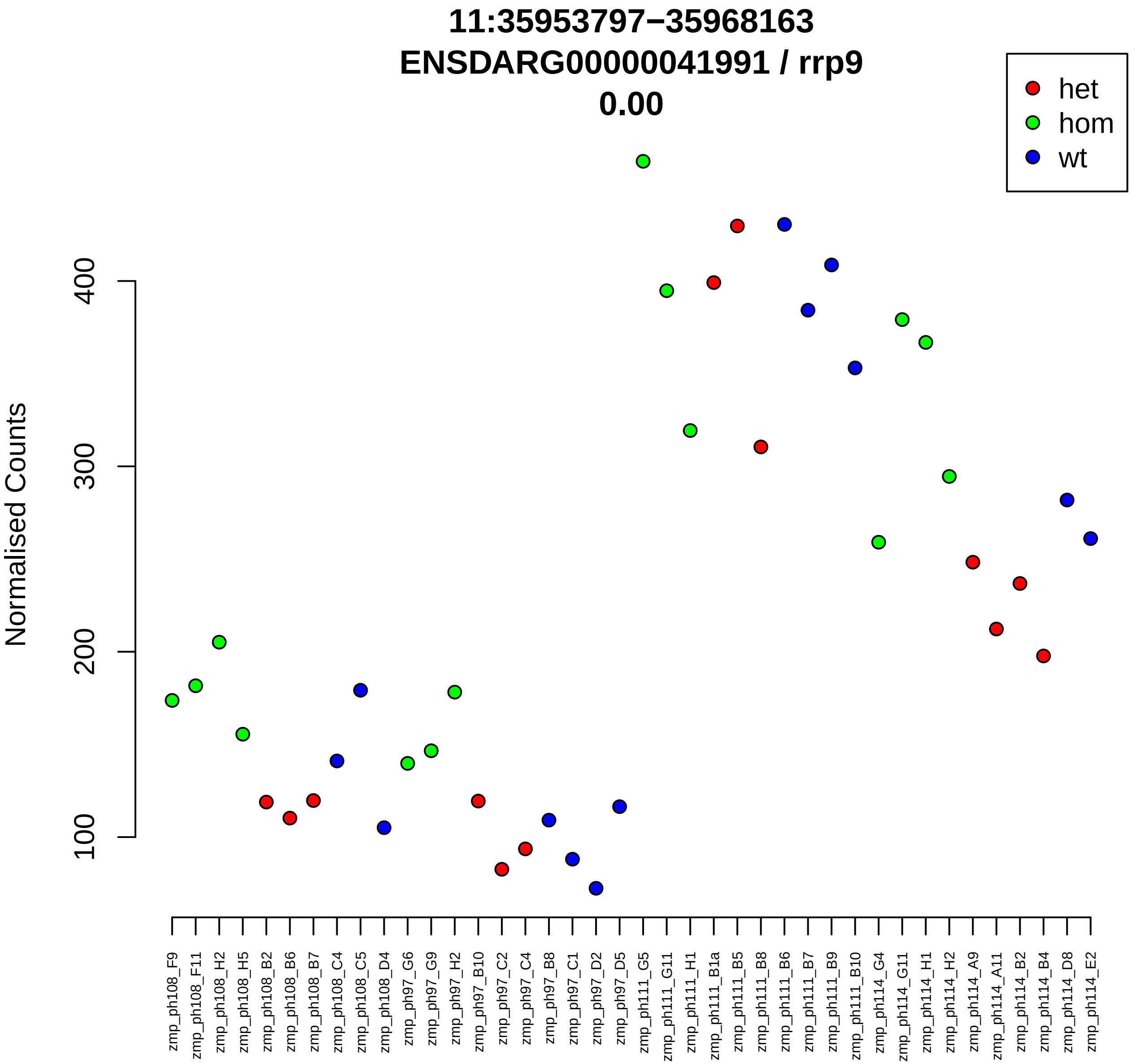

11:35953797−35968163
ENSDARG00000041991 / rrp9
0.00
het
hom
wt
Normalised Counts
100
200
300
400
zmp_ph108_F9
zmp_ph108_F11
zmp_ph108_H2
zmp_ph108_H5
zmp_ph108_B2
zmp_ph108_B6
zmp_ph108_B7
zmp_ph108_C4
zmp_ph108_C5
zmp_ph108_D4
zmp_ph97_G6
zmp_ph97_G9
zmp_ph97_H2
zmp_ph97_B10
zmp_ph97_C2
zmp_ph97_C4
zmp_ph97_B8
zmp_ph97_C1
zmp_ph97_D2
zmp_ph97_D5
zmp_ph111_G5
zmp_ph111_G11
zmp_ph111_H1
zmp_ph111_B1a
zmp_ph111_B5
zmp_ph111_B8
zmp_ph111_B6
zmp_ph111_B7
zmp_ph111_B9
zmp_ph111_B10
zmp_ph114_G4
zmp_ph114_G11
zmp_ph114_H1
zmp_ph114_H2
zmp_ph114_A9
zmp_ph114_A11
zmp_ph114_B2
zmp_ph114_B4
zmp_ph114_D8
zmp_ph114_E2

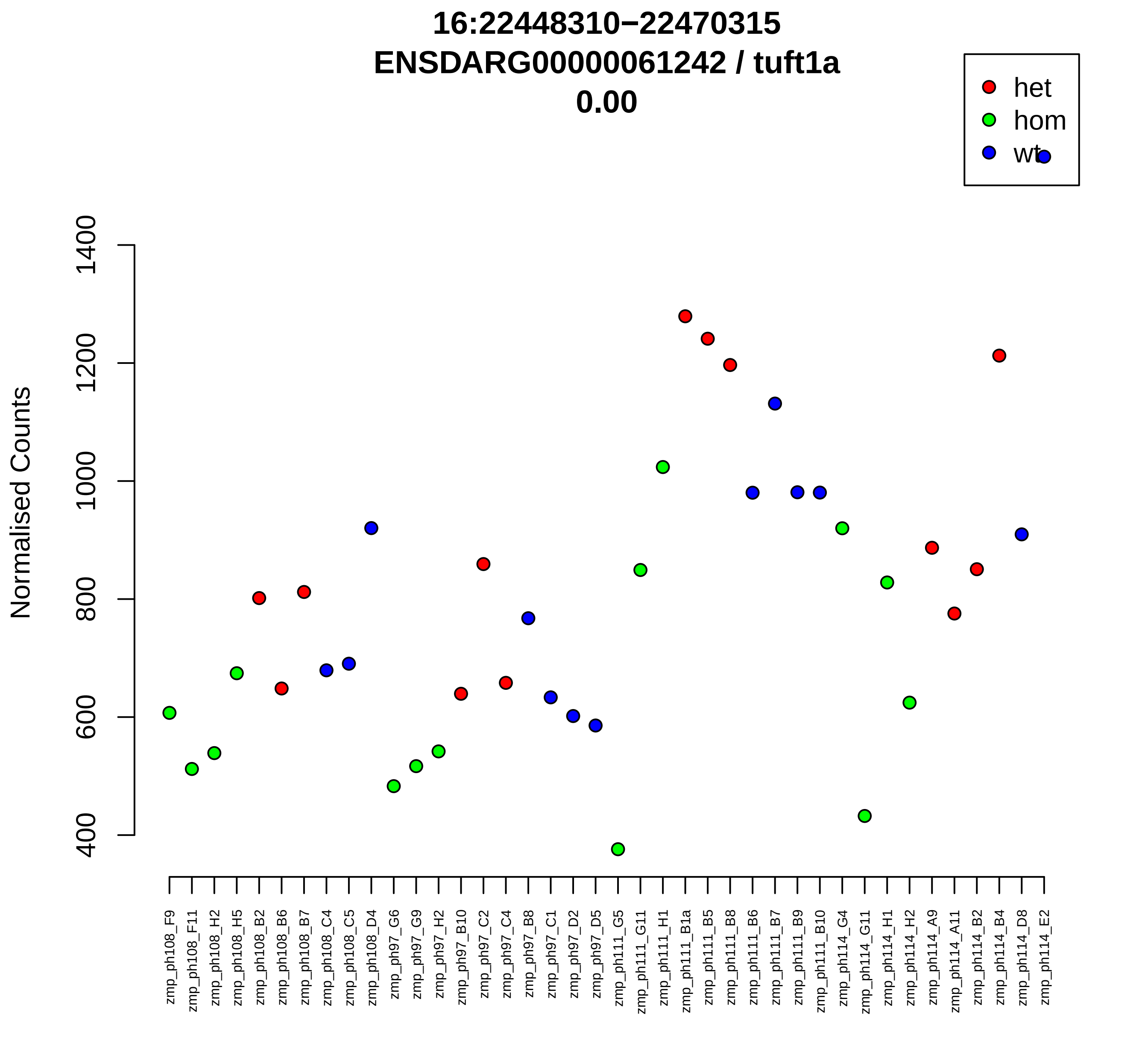

16:22448310−22470315
ENSDARG00000061242 / tuft1a
0.00
het
hom
wt
Normalised Counts
400
600
800
1000
1200
1400
zmp_ph108_F9
zmp_ph108_F11
zmp_ph108_H2
zmp_ph108_H5
zmp_ph108_B2
zmp_ph108_B6
zmp_ph108_B7
zmp_ph108_C4
zmp_ph108_C5
zmp_ph108_D4
zmp_ph97_G6
zmp_ph97_G9
zmp_ph97_H2
zmp_ph97_B10
zmp_ph97_C2
zmp_ph97_C4
zmp_ph97_B8
zmp_ph97_C1
zmp_ph97_D2
zmp_ph97_D5
zmp_ph111_G5
zmp_ph111_G11
zmp_ph111_H1
zmp_ph111_B1a
zmp_ph111_B5
zmp_ph111_B8
zmp_ph111_B6
zmp_ph111_B7
zmp_ph111_B9
zmp_ph111_B10
zmp_ph114_G4
zmp_ph114_G11
zmp_ph114_H1
zmp_ph114_H2
zmp_ph114_A9
zmp_ph114_A11
zmp_ph114_B2
zmp_ph114_B4
zmp_ph114_D8
zmp_ph114_E2

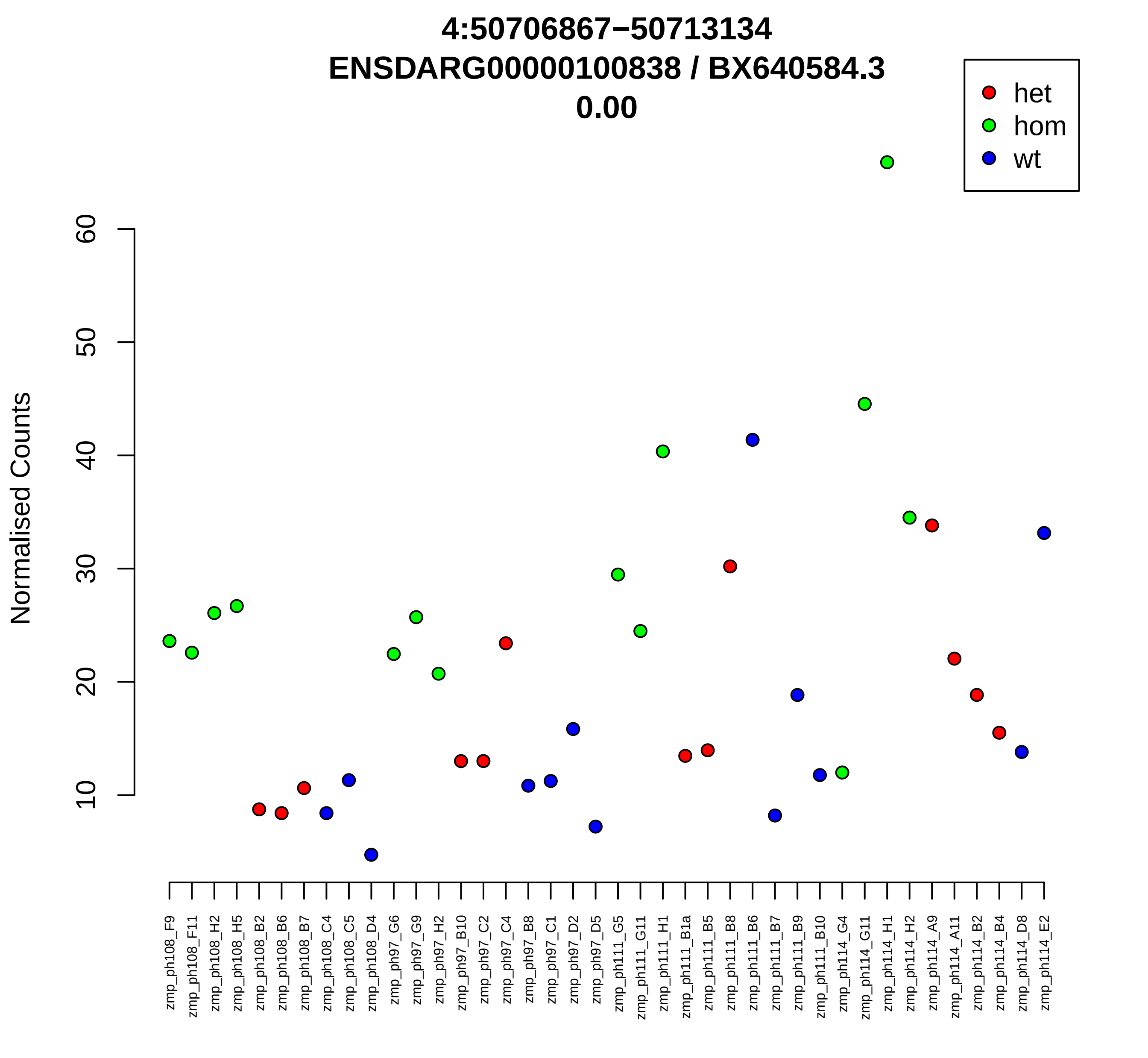
4:50706867−50713134
ENSDARG00000100838 / BX640584.3
0.00
het
hom
wt
Normalised Counts
10
20
30
40
50
60
zmp_ph108_F9
zmp_ph108_F11
zmp_ph108_H2
zmp_ph108_H5
zmp_ph108_B2
zmp_ph108_B6
zmp_ph108_B7
zmp_ph108_C4
zmp_ph108_C5
zmp_ph108_D4
zmp_ph97_G6
zmp_ph97_G9
zmp_ph97_H2
zmp_ph97_B10
zmp_ph97_C2
zmp_ph97_C4
zmp_ph97_B8
zmp_ph97_C1
zmp_ph97_D2
zmp_ph97_D5
zmp_ph111_G5
zmp_ph111_G11
zmp_ph111_H1
zmp_ph111_B1a
zmp_ph111_B5
zmp_ph111_B8
zmp_ph111_B6
zmp_ph111_B7
zmp_ph111_B9
zmp_ph111_B10
zmp_ph114_G4
zmp_ph114_G11
zmp_ph114_H1
zmp_ph114_H2
zmp_ph114_A9
zmp_ph114_A11
zmp_ph114_B2
zmp_ph114_B4
zmp_ph114_D8
zmp_ph114_E2

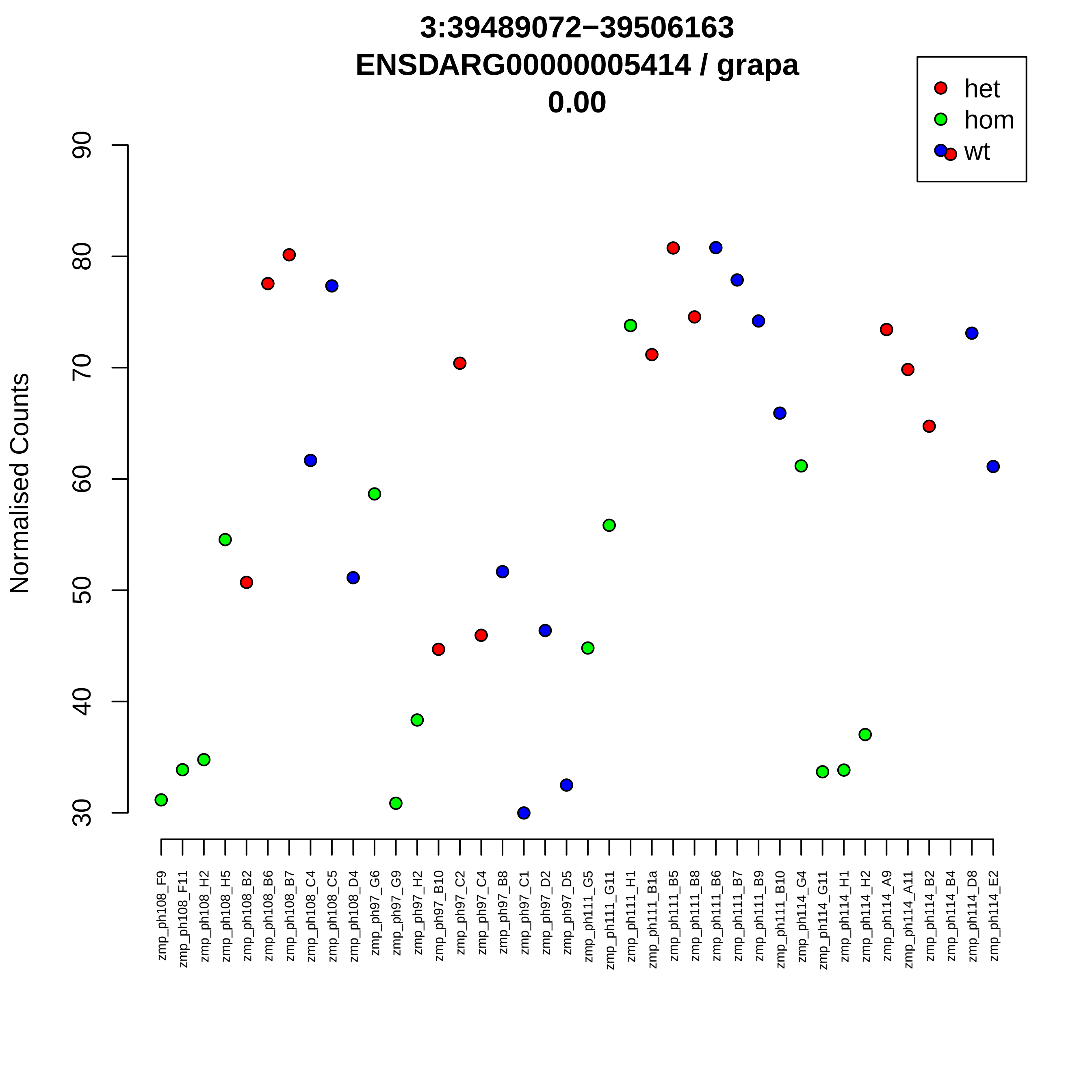
3:39489072−39506163
ENSDARG00000005414 / grapa
0.00
Normalised Counts
het
hom
wt
30
40
50
60
70
80
90
zmp_ph108_F9
zmp_ph108_F11
zmp_ph108_H2
zmp_ph108_H5
zmp_ph108_B2
zmp_ph108_B6
zmp_ph108_B7
zmp_ph108_C4
zmp_ph108_C5
zmp_ph108_D4
zmp_ph97_G6
zmp_ph97_G9
zmp_ph97_H2
zmp_ph97_B10
zmp_ph97_C2
zmp_ph97_C4
zmp_ph97_B8
zmp_ph97_C1
zmp_ph97_D2
zmp_ph97_D5
zmp_ph111_G5
zmp_ph111_G11
zmp_ph111_H1
zmp_ph111_B1a
zmp_ph111_B5
zmp_ph111_B8
zmp_ph111_B6
zmp_ph111_B7
zmp_ph111_B9
zmp_ph111_B10
zmp_ph114_G4
zmp_ph114_G11
zmp_ph114_H1
zmp_ph114_H2
zmp_ph114_A9
zmp_ph114_A11
zmp_ph114_B2
zmp_ph114_B4
zmp_ph114_D8
zmp_ph114_E2

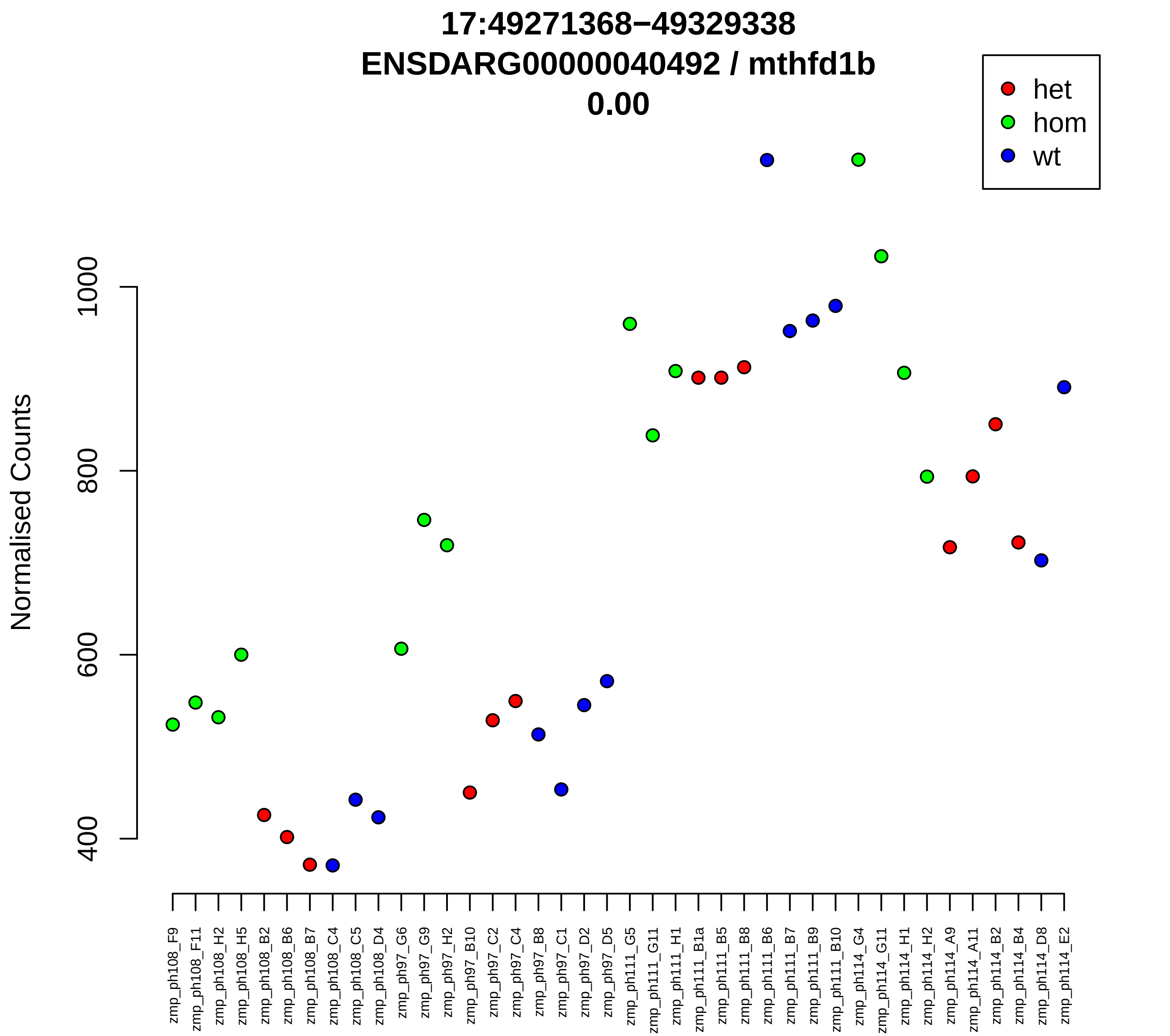
17:49271368−49329338
ENSDARG00000040492 / mthfd1b
0.00
het
hom
wt
Normalised Counts
400
600
800
1000
zmp_ph108_F9
zmp_ph108_F11
zmp_ph108_H2
zmp_ph108_H5
zmp_ph108_B2
zmp_ph108_B6
zmp_ph108_B7
zmp_ph108_C4
zmp_ph108_C5
zmp_ph108_D4
zmp_ph97_G6
zmp_ph97_G9
zmp_ph97_H2
zmp_ph97_B10
zmp_ph97_C2
zmp_ph97_C4
zmp_ph97_B8
zmp_ph97_C1
zmp_ph97_D2
zmp_ph97_D5
zmp_ph111_G5
zmp_ph111_G11
zmp_ph111_H1
zmp_ph111_B1a
zmp_ph111_B5
zmp_ph111_B8
zmp_ph111_B6
zmp_ph111_B7
zmp_ph111_B9
zmp_ph111_B10
zmp_ph114_G4
zmp_ph114_G11
zmp_ph114_H1
zmp_ph114_H2
zmp_ph114_A9
zmp_ph114_A11
zmp_ph114_B2
zmp_ph114_B4
zmp_ph114_D8
zmp_ph114_E2

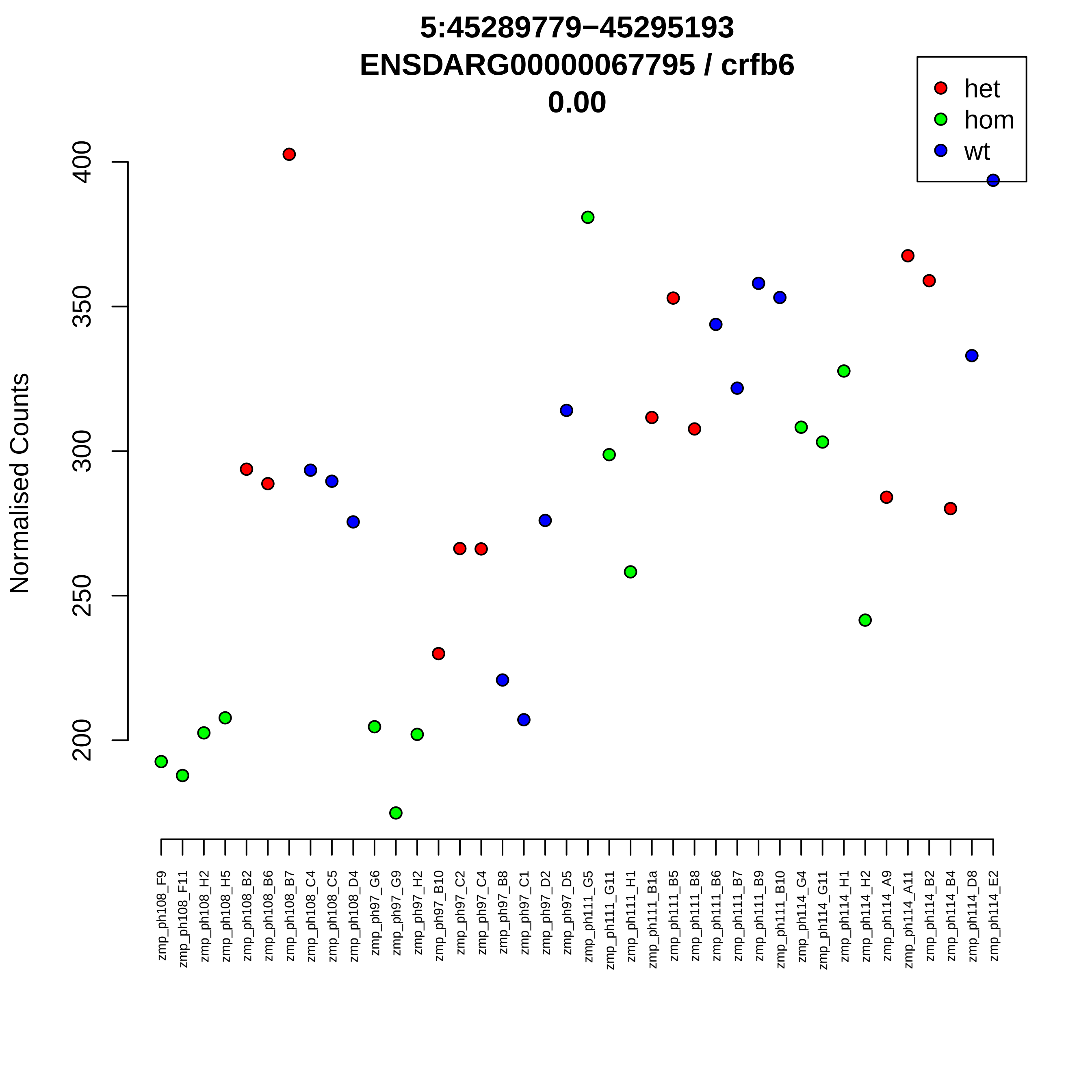
5:45289779−45295193
ENSDARG00000067795 / crfb6
0.00
het
hom
wt
Normalised Counts
200
250
300
350
400
zmp_ph108_F9
zmp_ph108_F11
zmp_ph108_H2
zmp_ph108_H5
zmp_ph108_B2
zmp_ph108_B6
zmp_ph108_B7
zmp_ph108_C4
zmp_ph108_C5
zmp_ph108_D4
zmp_ph97_G6
zmp_ph97_G9
zmp_ph97_H2
zmp_ph97_B10
zmp_ph97_C2
zmp_ph97_C4
zmp_ph97_B8
zmp_ph97_C1
zmp_ph97_D2
zmp_ph97_D5
zmp_ph111_G5
zmp_ph111_G11
zmp_ph111_H1
zmp_ph111_B1a
zmp_ph111_B5
zmp_ph111_B8
zmp_ph111_B6
zmp_ph111_B7
zmp_ph111_B9
zmp_ph111_B10
zmp_ph114_G4
zmp_ph114_G11
zmp_ph114_H1
zmp_ph114_H2
zmp_ph114_A9
zmp_ph114_A11
zmp_ph114_B2
zmp_ph114_B4
zmp_ph114_D8
zmp_ph114_E2

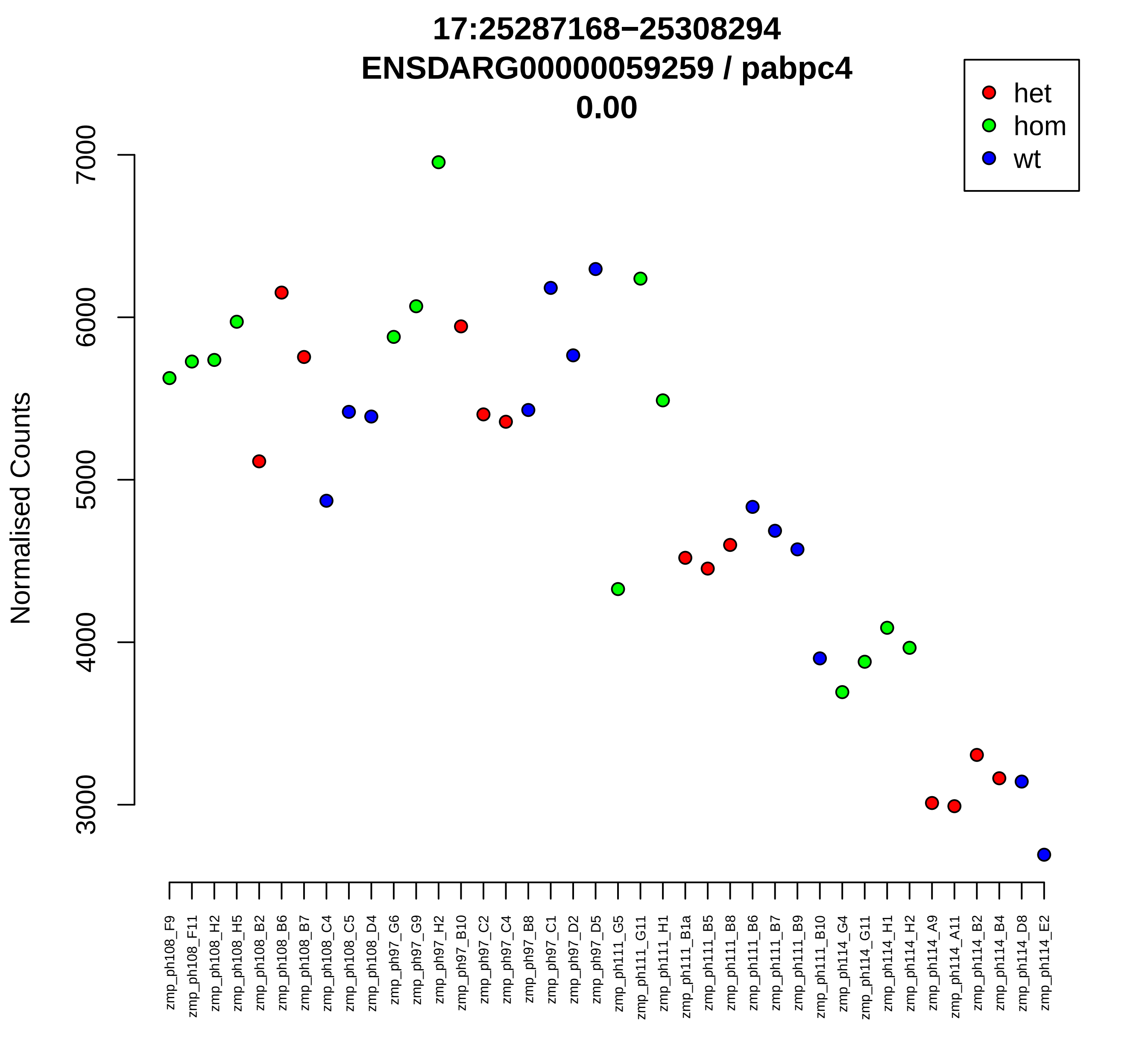

17:25287168−25308294
ENSDARG00000059259 / pabpc4
0.00
het
hom
wt
Normalised Counts
7000
6000
5000
4000
3000
zmp_ph108_F9
zmp_ph108_F11
zmp_ph108_H2
zmp_ph108_H5
zmp_ph108_B2
zmp_ph108_B6
zmp_ph108_B7
zmp_ph108_C4
zmp_ph108_C5
zmp_ph108_D4
zmp_ph97_G6
zmp_ph97_G9
zmp_ph97_H2
zmp_ph97_B10
zmp_ph97_C2
zmp_ph97_C4
zmp_ph97_B8
zmp_ph97_C1
zmp_ph97_D2
zmp_ph97_D5
zmp_ph111_G5
zmp_ph111_G11
zmp_ph111_H1
zmp_ph111_B1a
zmp_ph111_B5
zmp_ph111_B8
zmp_ph111_B6
zmp_ph111_B7
zmp_ph111_B9
zmp_ph111_B10
zmp_ph114_G4
zmp_ph114_G11
zmp_ph114_H1
zmp_ph114_H2
zmp_ph114_A9
zmp_ph114_A11
zmp_ph114_B2
zmp_ph114_B4
zmp_ph114_D8
zmp_ph114_E2

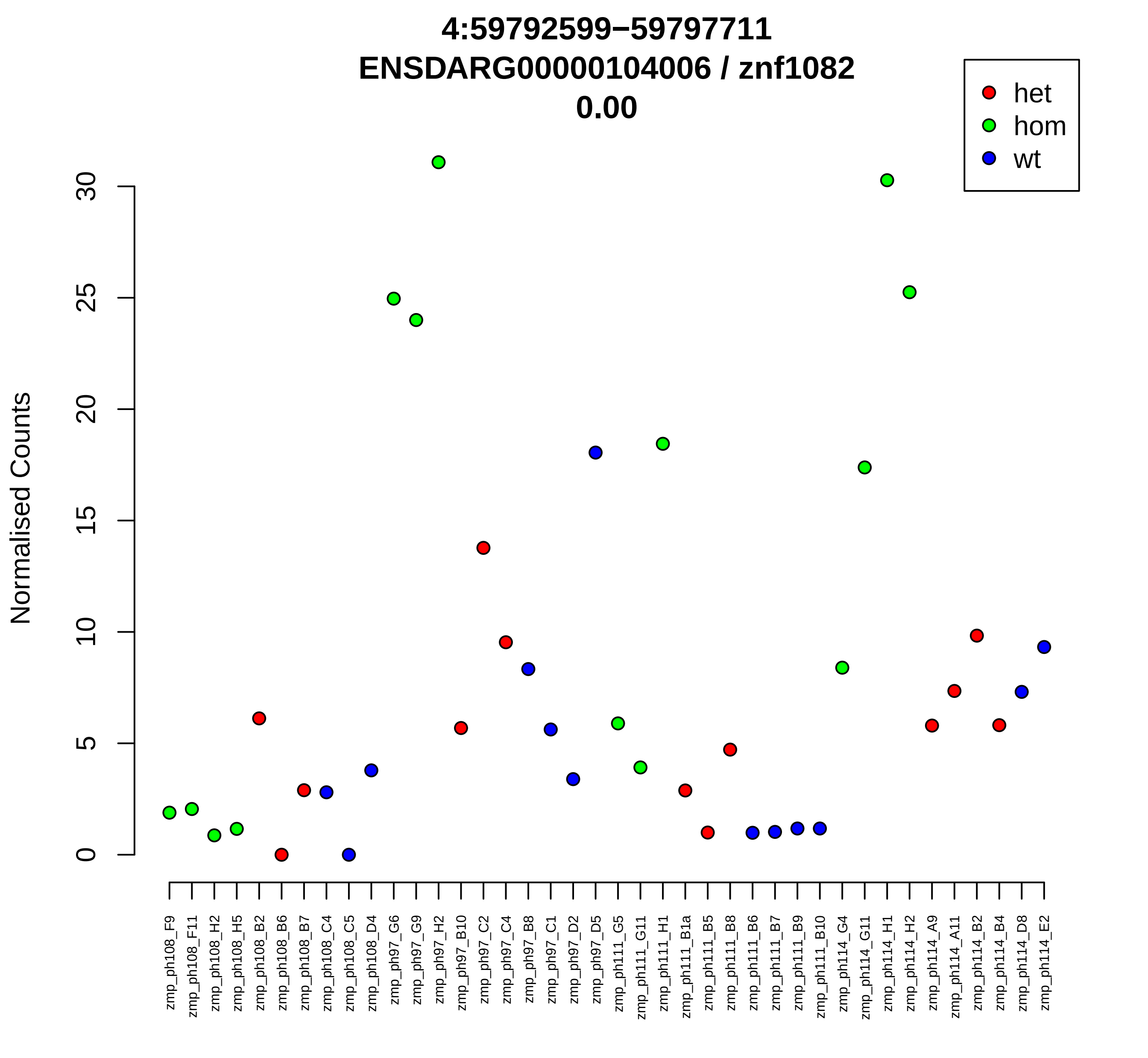

4:59792599−59797711
ENSDARG00000104006 / znf1082
0.00
het
hom
wt
Normalised Counts
0
5
10
15
20
25
30
zmp_ph108_F9
zmp_ph108_F11
zmp_ph108_H2
zmp_ph108_H5
zmp_ph108_B2
zmp_ph108_B6
zmp_ph108_B7
zmp_ph108_C4
zmp_ph108_C5
zmp_ph108_D4
zmp_ph97_G6
zmp_ph97_G9
zmp_ph97_H2
zmp_ph97_B10
zmp_ph97_C2
zmp_ph97_C4
zmp_ph97_B8
zmp_ph97_C1
zmp_ph97_D2
zmp_ph97_D5
zmp_ph111_G5
zmp_ph111_G11
zmp_ph111_H1
zmp_ph111_B1a
zmp_ph111_B5
zmp_ph111_B8
zmp_ph111_B6
zmp_ph111_B7
zmp_ph111_B9
zmp_ph111_B10
zmp_ph114_G4
zmp_ph114_G11
zmp_ph114_H1
zmp_ph114_H2
zmp_ph114_A9
zmp_ph114_A11
zmp_ph114_B2
zmp_ph114_B4
zmp_ph114_D8
zmp_ph114_E2

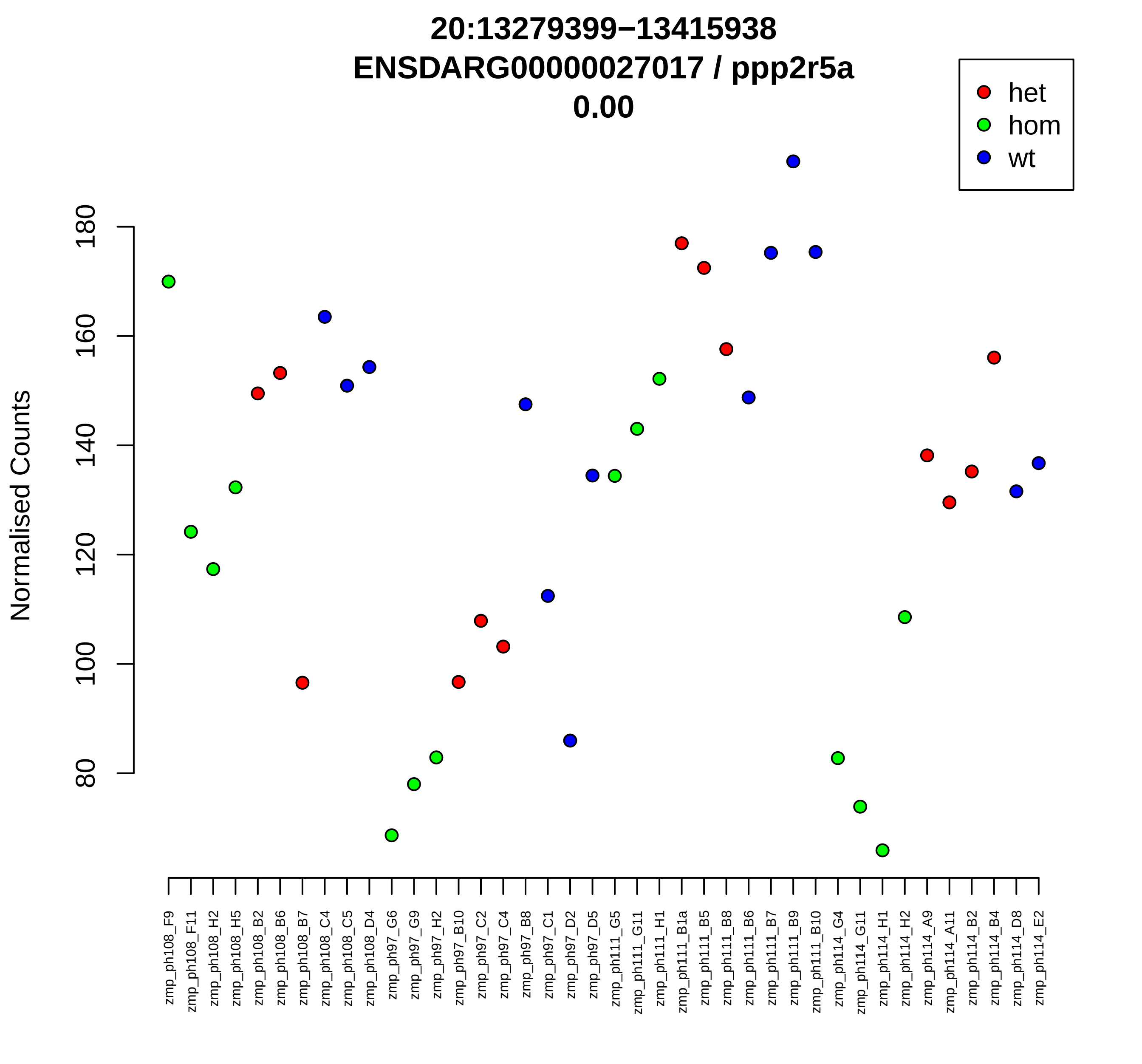

20:13279399−13415938
ENSDARG00000027017 / ppp2r5a
0.00
het
hom
wt
Normalised Counts
80
100
120
140
160
180
zmp_ph108_F9
zmp_ph108_F11
zmp_ph108_H2
zmp_ph108_H5
zmp_ph108_B2
zmp_ph108_B6
zmp_ph108_B7
zmp_ph108_C4
zmp_ph108_C5
zmp_ph108_D4
zmp_ph97_G6
zmp_ph97_G9
zmp_ph97_H2
zmp_ph97_B10
zmp_ph97_C2
zmp_ph97_C4
zmp_ph97_B8
zmp_ph97_C1
zmp_ph97_D2
zmp_ph97_D5
zmp_ph111_G5
zmp_ph111_G11
zmp_ph111_H1
zmp_ph111_B1a
zmp_ph111_B5
zmp_ph111_B8
zmp_ph111_B6
zmp_ph111_B7
zmp_ph111_B9
zmp_ph111_B10
zmp_ph114_G4
zmp_ph114_G11
zmp_ph114_H1
zmp_ph114_H2
zmp_ph114_A9
zmp_ph114_A11
zmp_ph114_B2
zmp_ph114_B4
zmp_ph114_D8
zmp_ph114_E2

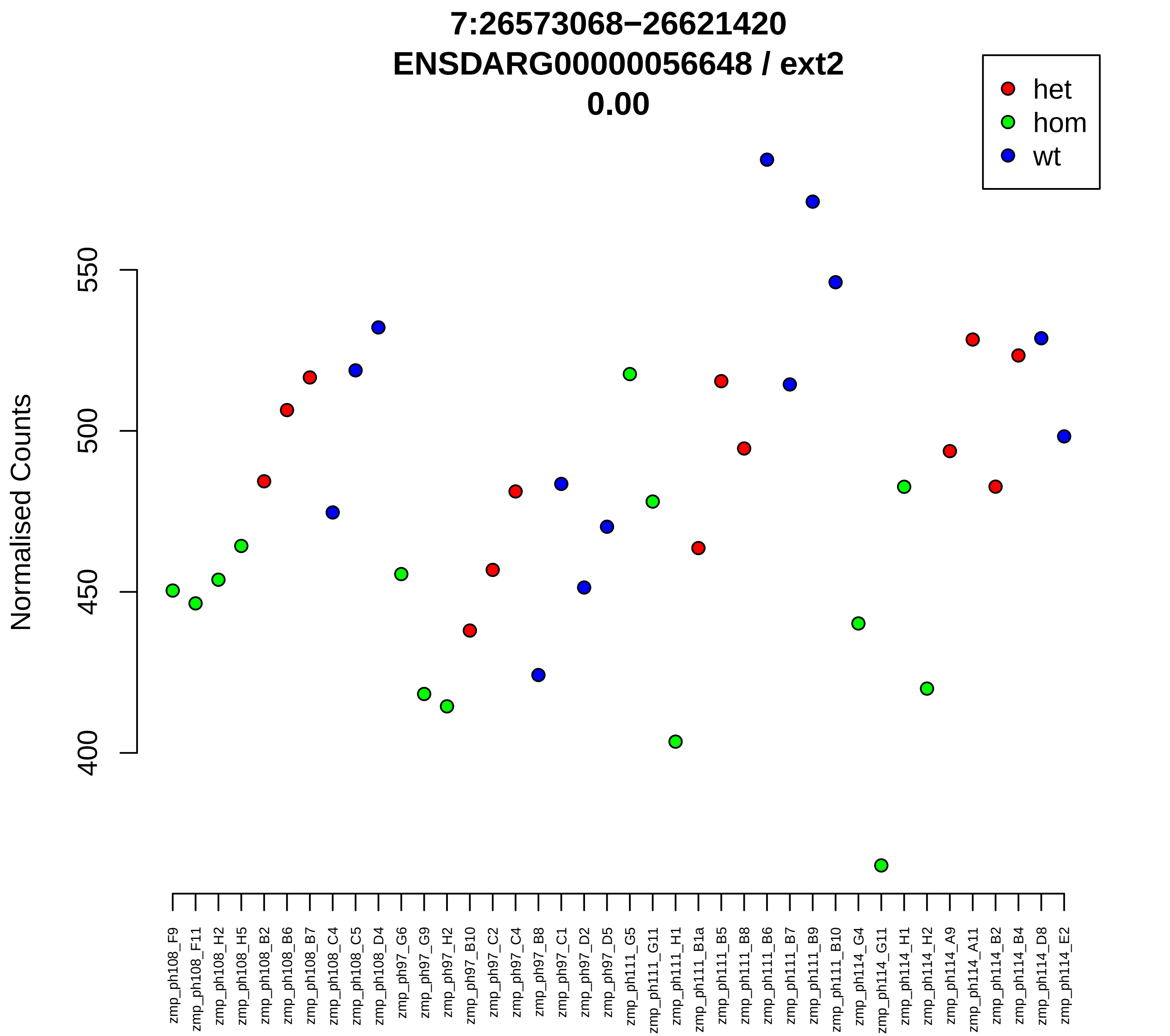
7:26573068−26621420
ENSDARG00000056648 / ext2
0.00
het
hom
wt
Normalised Counts
400
450
500
550
zmp_ph108_F9
zmp_ph108_F11
zmp_ph108_H2
zmp_ph108_H5
zmp_ph108_B2
zmp_ph108_B6
zmp_ph108_B7
zmp_ph108_C4
zmp_ph108_C5
zmp_ph108_D4
zmp_ph97_G6
zmp_ph97_G9
zmp_ph97_H2
zmp_ph97_B10
zmp_ph97_C2
zmp_ph97_C4
zmp_ph97_B8
zmp_ph97_C1
zmp_ph97_D2
zmp_ph97_D5
zmp_ph111_G5
zmp_ph111_G11
zmp_ph111_H1
zmp_ph111_B1a
zmp_ph111_B5
zmp_ph111_B8
zmp_ph111_B6
zmp_ph111_B7
zmp_ph111_B9
zmp_ph111_B10
zmp_ph114_G4
zmp_ph114_G11
zmp_ph114_H1
zmp_ph114_H2
zmp_ph114_A9
zmp_ph114_A11
zmp_ph114_B2
zmp_ph114_B4
zmp_ph114_D8
zmp_ph114_E2

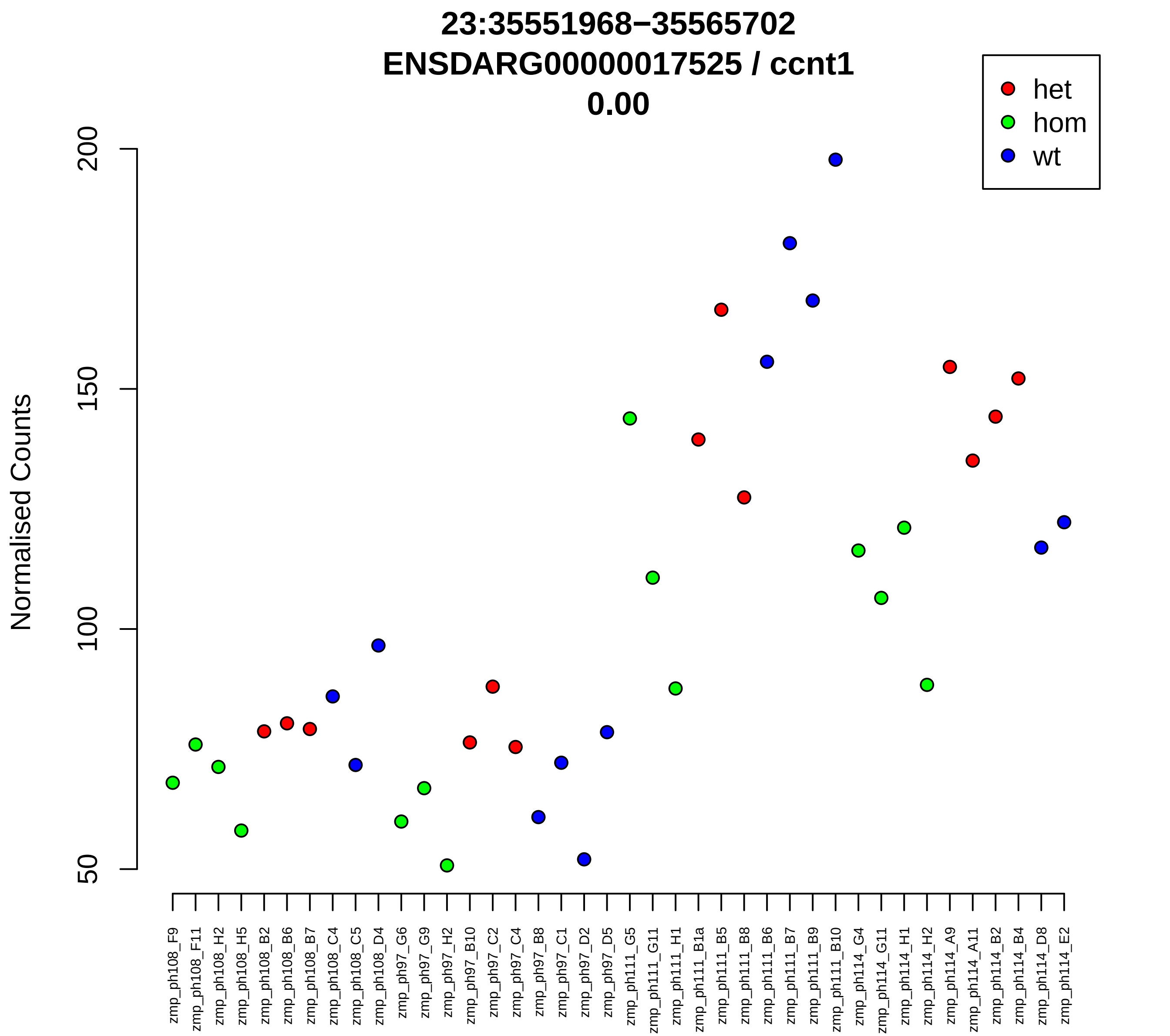

23:35551968−35565702
ENSDARG00000017525 / ccnt1
0.00
het
hom
wt
Normalised Counts
200
150
100
50
zmp_ph108_F9
zmp_ph108_F11
zmp_ph108_H2
zmp_ph108_H5
zmp_ph108_B2
zmp_ph108_B6
zmp_ph108_B7
zmp_ph108_C4
zmp_ph108_C5
zmp_ph108_D4
zmp_ph97_G6
zmp_ph97_G9
zmp_ph97_H2
zmp_ph97_B10
zmp_ph97_C2
zmp_ph97_C4
zmp_ph97_B8
zmp_ph97_C1
zmp_ph97_D2
zmp_ph97_D5
zmp_ph111_G5
zmp_ph111_G11
zmp_ph111_H1
zmp_ph111_B1a
zmp_ph111_B5
zmp_ph111_B8
zmp_ph111_B6
zmp_ph111_B7
zmp_ph111_B9
zmp_ph111_B10
zmp_ph114_G4
zmp_ph114_G11
zmp_ph114_H1
zmp_ph114_H2
zmp_ph114_A9
zmp_ph114_A11
zmp_ph114_B2
zmp_ph114_B4
zmp_ph114_D8
zmp_ph114_E2

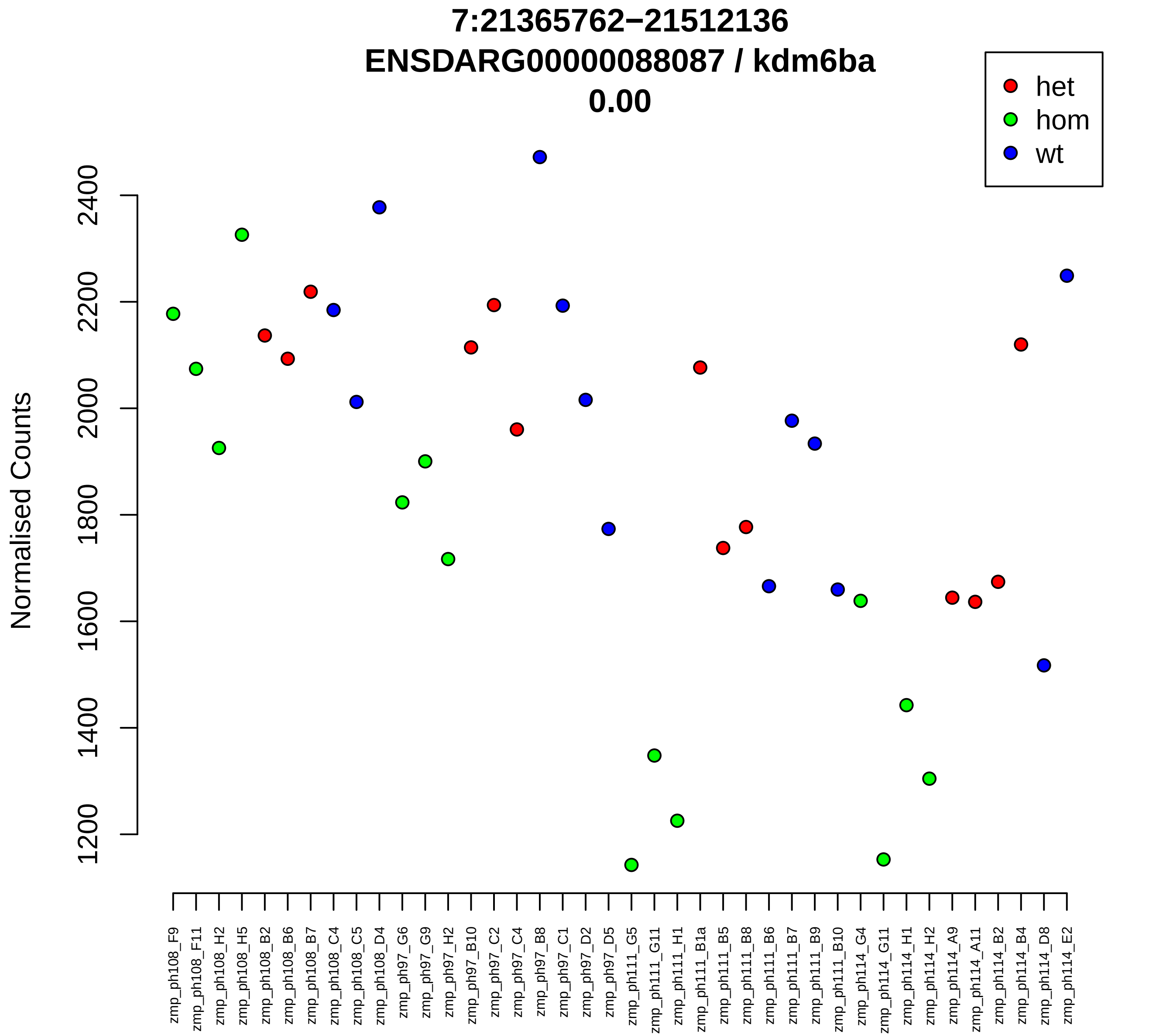

7:21365762−21512136
ENSDARG00000088087 / kdm6ba
0.00
het
hom
wt
Normalised Counts
1200
1400
1600
1800
2000
2200
2400
zmp_ph108_F9
zmp_ph108_F11
zmp_ph108_H2
zmp_ph108_H5
zmp_ph108_B2
zmp_ph108_B6
zmp_ph108_B7
zmp_ph108_C4
zmp_ph108_C5
zmp_ph108_D4
zmp_ph97_G6
zmp_ph97_G9
zmp_ph97_H2
zmp_ph97_B10
zmp_ph97_C2
zmp_ph97_C4
zmp_ph97_B8
zmp_ph97_C1
zmp_ph97_D2
zmp_ph97_D5
zmp_ph111_G5
zmp_ph111_G11
zmp_ph111_H1
zmp_ph111_B1a
zmp_ph111_B5
zmp_ph111_B8
zmp_ph111_B6
zmp_ph111_B7
zmp_ph111_B9
zmp_ph111_B10
zmp_ph114_G4
zmp_ph114_G11
zmp_ph114_H1
zmp_ph114_H2
zmp_ph114_A9
zmp_ph114_A11
zmp_ph114_B2
zmp_ph114_B4
zmp_ph114_D8
zmp_ph114_E2

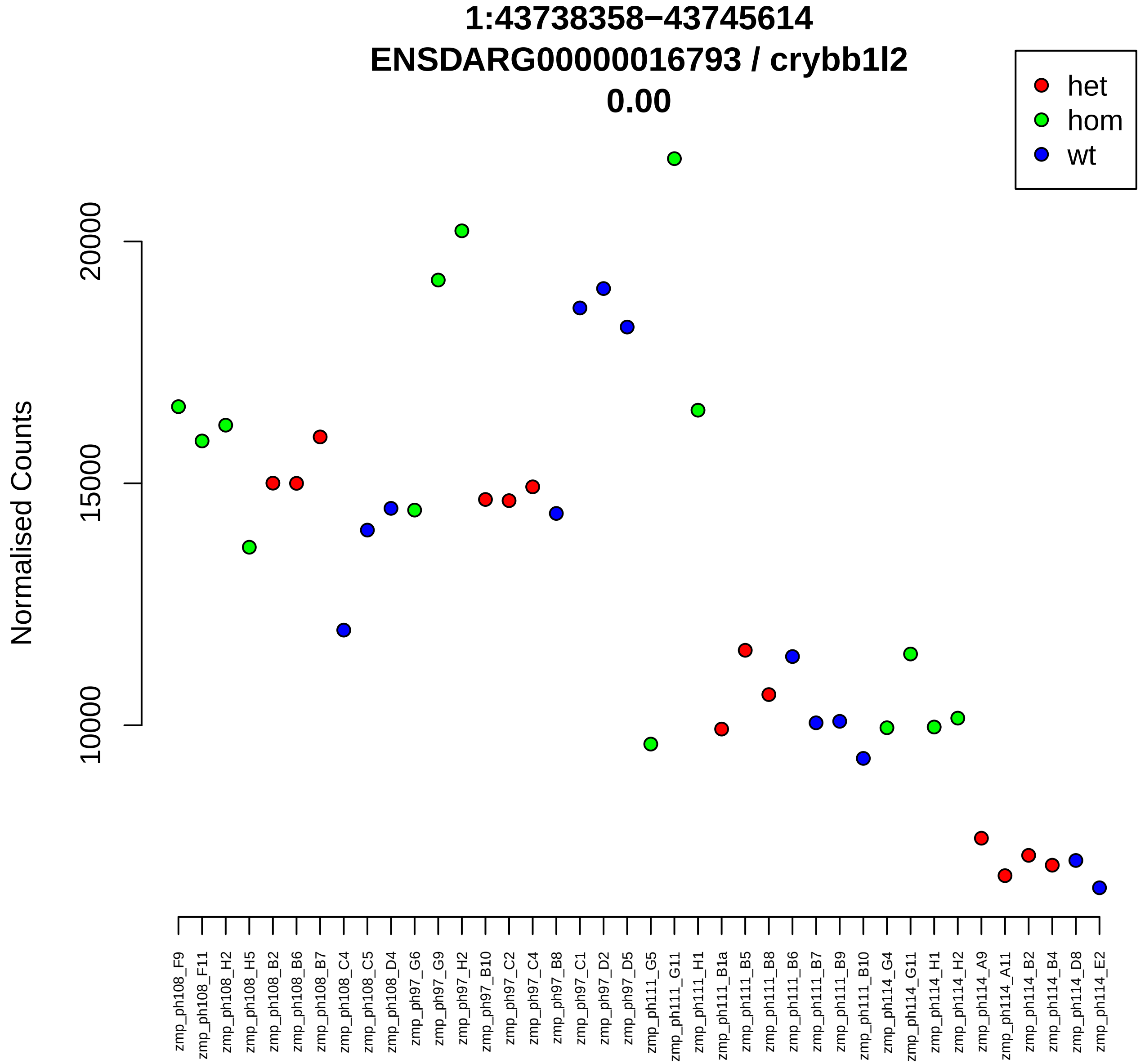

1:43738358−43745614
ENSDARG00000016793 / crybb1l2
0.00
het
hom
wt
Normalised Counts
10000
15000
20000
zmp_ph108_F9
zmp_ph108_F11
zmp_ph108_H2
zmp_ph108_H5
zmp_ph108_B2
zmp_ph108_B6
zmp_ph108_B7
zmp_ph108_C4
zmp_ph108_C5
zmp_ph108_D4
zmp_ph97_G6
zmp_ph97_G9
zmp_ph97_H2
zmp_ph97_B10
zmp_ph97_C2
zmp_ph97_C4
zmp_ph97_B8
zmp_ph97_C1
zmp_ph97_D2
zmp_ph97_D5
zmp_ph111_G5
zmp_ph111_G11
zmp_ph111_H1
zmp_ph111_B1a
zmp_ph111_B5
zmp_ph111_B8
zmp_ph111_B6
zmp_ph111_B7
zmp_ph111_B9
zmp_ph111_B10
zmp_ph114_G4
zmp_ph114_G11
zmp_ph114_H1
zmp_ph114_H2
zmp_ph114_A9
zmp_ph114_A11
zmp_ph114_B2
zmp_ph114_B4
zmp_ph114_D8
zmp_ph114_E2

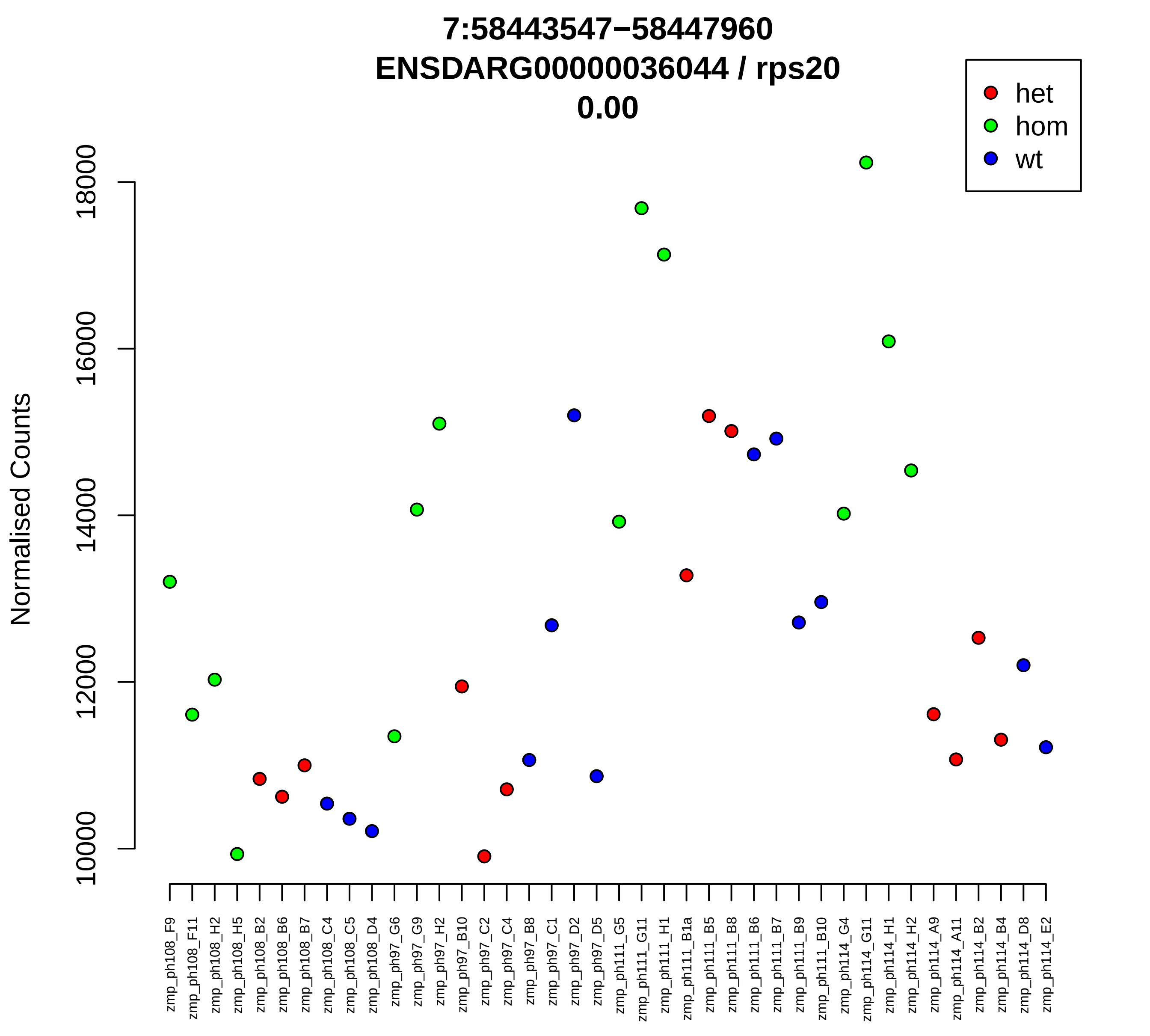
7:58443547−58447960
ENSDARG00000036044 / rps20
0.00
het
hom
wt
Normalised Counts
10000
12000
14000
16000
18000
zmp_ph108_F9
zmp_ph108_F11
zmp_ph108_H2
zmp_ph108_H5
zmp_ph108_B2
zmp_ph108_B6
zmp_ph108_B7
zmp_ph108_C4
zmp_ph108_C5
zmp_ph108_D4
zmp_ph97_G6
zmp_ph97_G9
zmp_ph97_H2
zmp_ph97_B10
zmp_ph97_C2
zmp_ph97_C4
zmp_ph97_B8
zmp_ph97_C1
zmp_ph97_D2
zmp_ph97_D5
zmp_ph111_G5
zmp_ph111_G11
zmp_ph111_H1
zmp_ph111_B1a
zmp_ph111_B5
zmp_ph111_B8
zmp_ph111_B6
zmp_ph111_B7
zmp_ph111_B9
zmp_ph111_B10
zmp_ph114_G4
zmp_ph114_G11
zmp_ph114_H1
zmp_ph114_H2
zmp_ph114_A9
zmp_ph114_A11
zmp_ph114_B2
zmp_ph114_B4
zmp_ph114_D8
zmp_ph114_E2

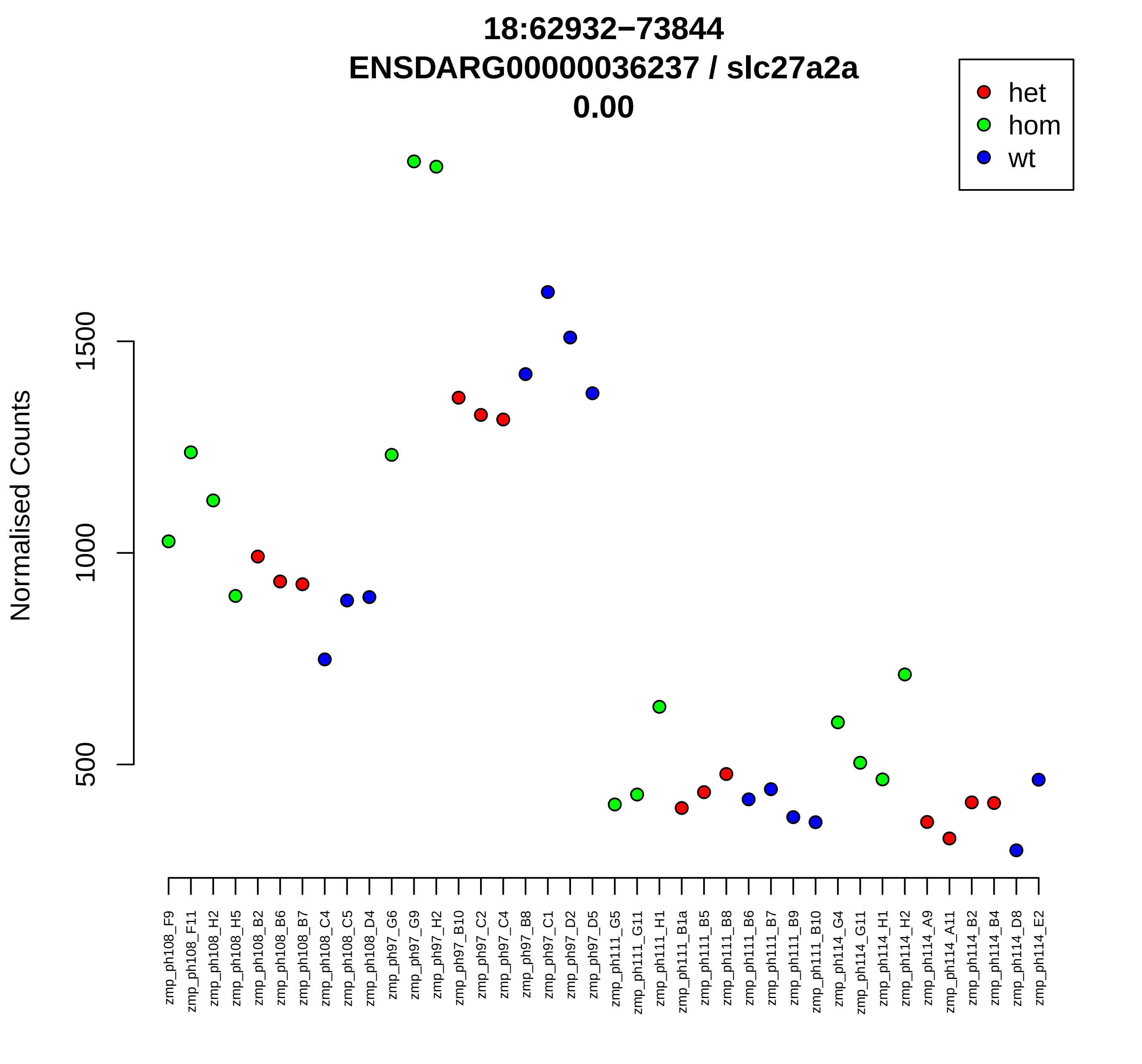

18:62932−73844
ENSDARG00000036237 / slc27a2a
0.00
het
hom
wt
Normalised Counts
500
1000
1500
zmp_ph108_F9
zmp_ph108_F11
zmp_ph108_H2
zmp_ph108_H5
zmp_ph108_B2
zmp_ph108_B6
zmp_ph108_B7
zmp_ph108_C4
zmp_ph108_C5
zmp_ph108_D4
zmp_ph97_G6
zmp_ph97_G9
zmp_ph97_H2
zmp_ph97_B10
zmp_ph97_C2
zmp_ph97_C4
zmp_ph97_B8
zmp_ph97_C1
zmp_ph97_D2
zmp_ph97_D5
zmp_ph111_G5
zmp_ph111_G11
zmp_ph111_H1
zmp_ph111_B1a
zmp_ph111_B5
zmp_ph111_B8
zmp_ph111_B6
zmp_ph111_B7
zmp_ph111_B9
zmp_ph111_B10
zmp_ph114_G4
zmp_ph114_G11
zmp_ph114_H1
zmp_ph114_H2
zmp_ph114_A9
zmp_ph114_A11
zmp_ph114_B2
zmp_ph114_B4
zmp_ph114_D8
zmp_ph114_E2

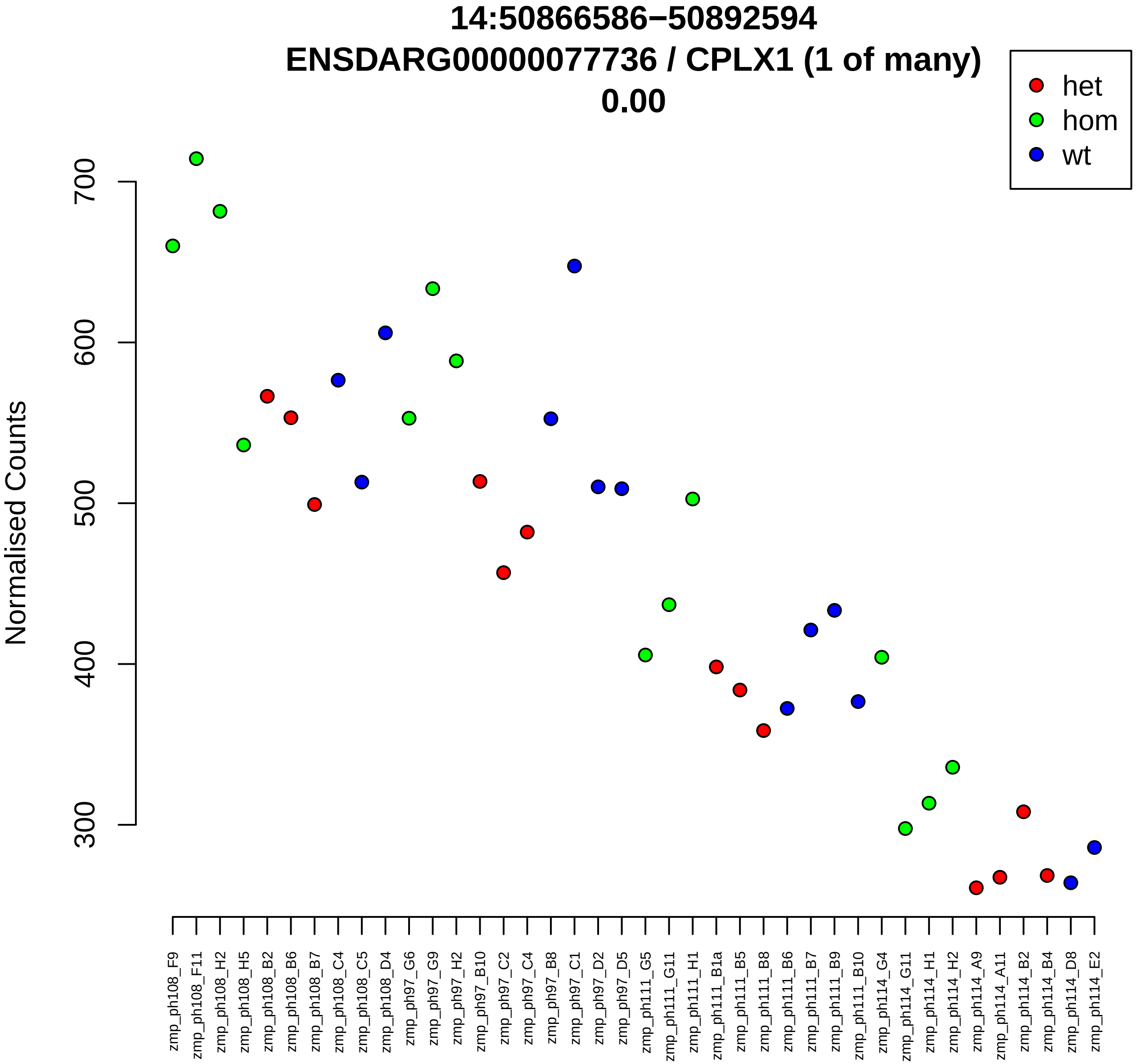
14:50866586−50892594
ENSDARG00000077736 / CPLX1 (1 of many)
0.00
het
hom
wt
Normalised Counts
700
600
500
400
300
zmp_ph108_F9
zmp_ph108_F11
zmp_ph108_H2
zmp_ph108_H5
zmp_ph108_B2
zmp_ph108_B6
zmp_ph108_B7
zmp_ph108_C4
zmp_ph108_C5
zmp_ph108_D4
zmp_ph97_G6
zmp_ph97_G9
zmp_ph97_H2
zmp_ph97_B10
zmp_ph97_C2
zmp_ph97_C4
zmp_ph97_B8
zmp_ph97_C1
zmp_ph97_D2
zmp_ph97_D5
zmp_ph111_G5
zmp_ph111_G11
zmp_ph111_H1
zmp_ph111_B1a
zmp_ph111_B5
zmp_ph111_B8
zmp_ph111_B6
zmp_ph111_B7
zmp_ph111_B9
zmp_ph111_B10
zmp_ph114_G4
zmp_ph114_G11
zmp_ph114_H1
zmp_ph114_H2
zmp_ph114_A9
zmp_ph114_A11
zmp_ph114_B2
zmp_ph114_B4
zmp_ph114_D8
zmp_ph114_E2

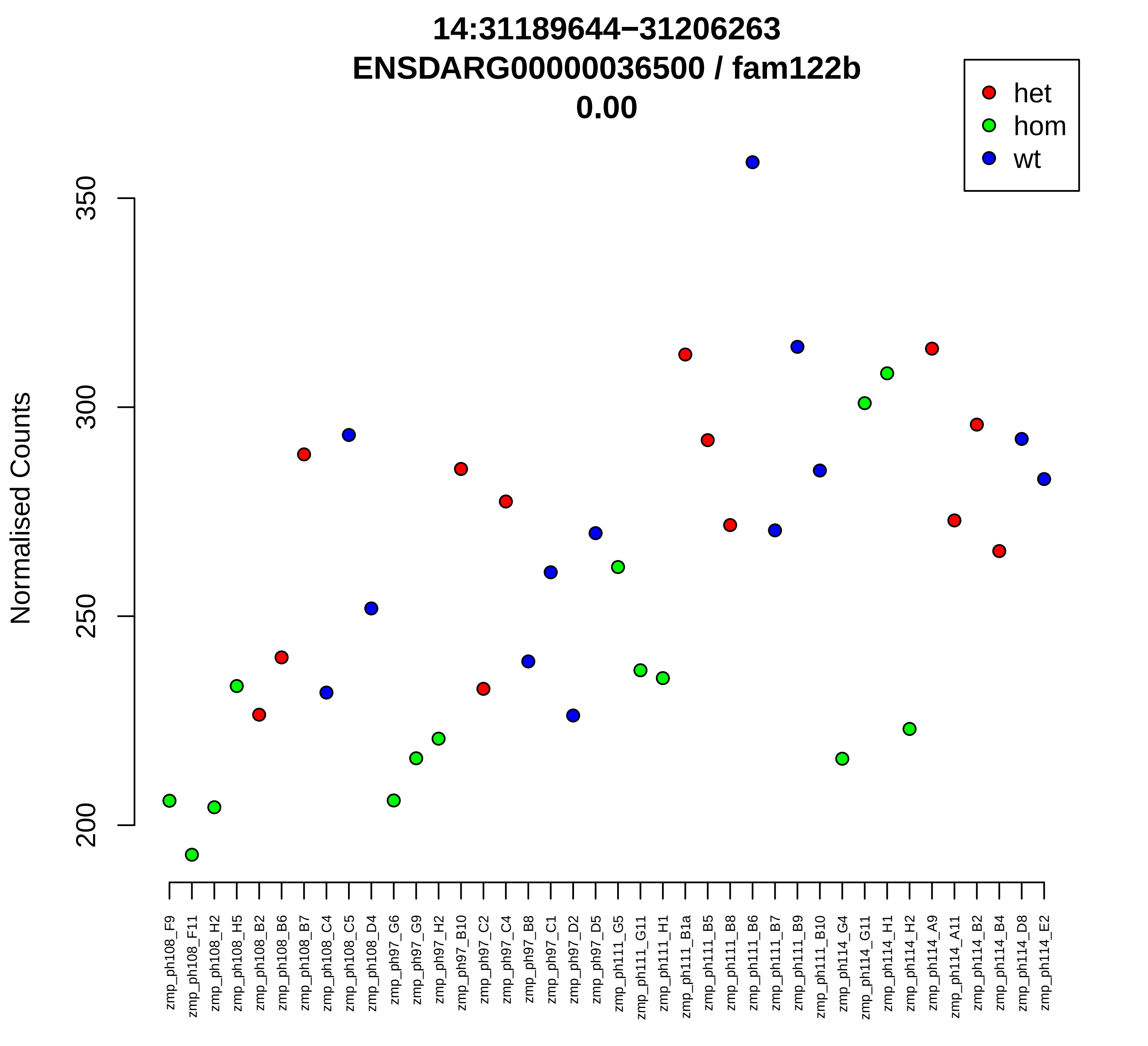

14:31189644−31206263
ENSDARG00000036500 / fam122b
0.00
het
hom
wt
Normalised Counts
200
250
300
350
zmp_ph108_F9
zmp_ph108_F11
zmp_ph108_H2
zmp_ph108_H5
zmp_ph108_B2
zmp_ph108_B6
zmp_ph108_B7
zmp_ph108_C4
zmp_ph108_C5
zmp_ph108_D4
zmp_ph97_G6
zmp_ph97_G9
zmp_ph97_H2
zmp_ph97_B10
zmp_ph97_C2
zmp_ph97_C4
zmp_ph97_B8
zmp_ph97_C1
zmp_ph97_D2
zmp_ph97_D5
zmp_ph111_G5
zmp_ph111_G11
zmp_ph111_H1
zmp_ph111_B1a
zmp_ph111_B5
zmp_ph111_B8
zmp_ph111_B6
zmp_ph111_B7
zmp_ph111_B9
zmp_ph111_B10
zmp_ph114_G4
zmp_ph114_G11
zmp_ph114_H1
zmp_ph114_H2
zmp_ph114_A9
zmp_ph114_A11
zmp_ph114_B2
zmp_ph114_B4
zmp_ph114_D8
zmp_ph114_E2

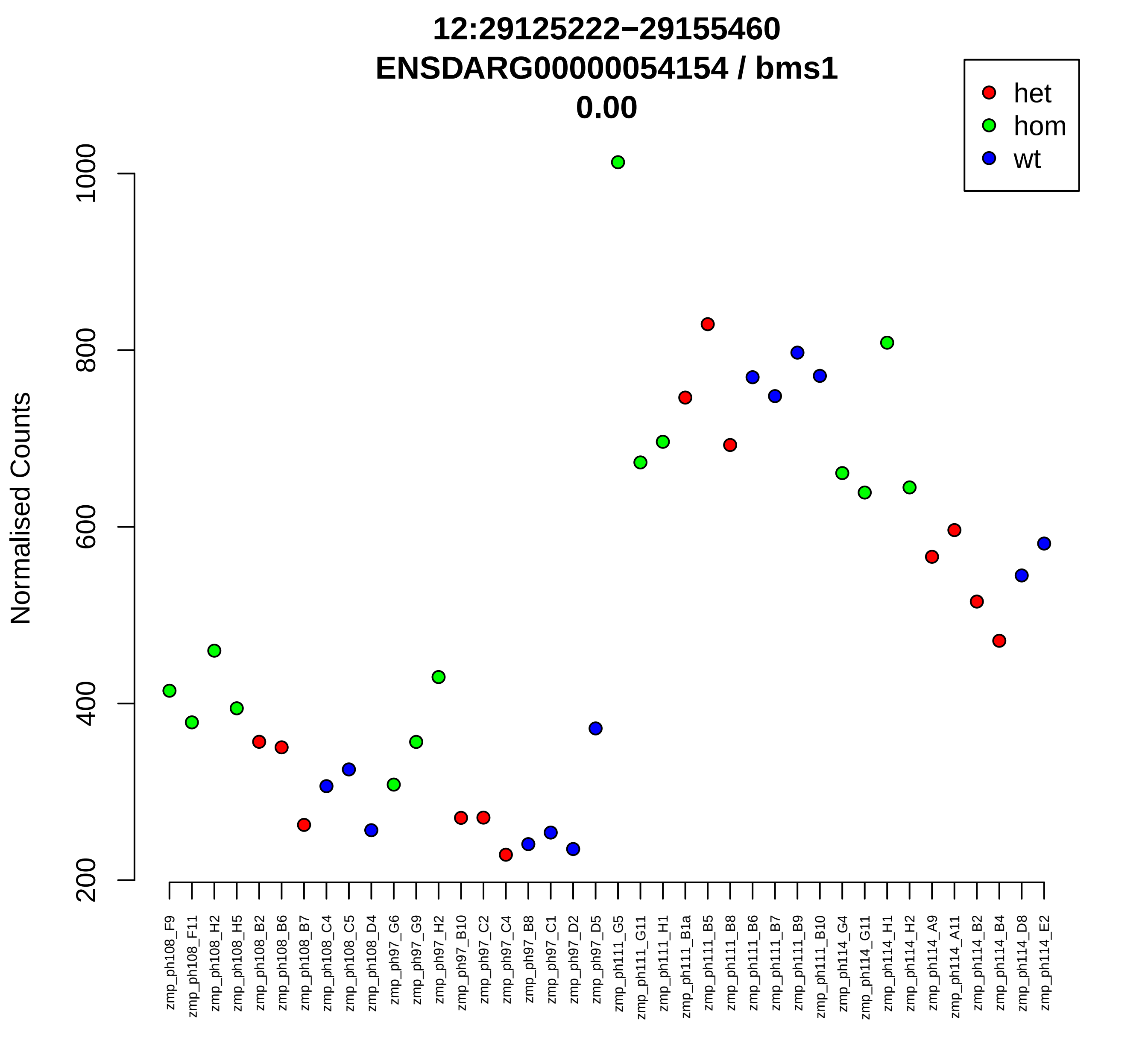

12:29125222−29155460
ENSDARG00000054154 / bms1
0.00
het
hom
wt
Normalised Counts
1000
800
600
400
200
zmp_ph108_F9
zmp_ph108_F11
zmp_ph108_H2
zmp_ph108_H5
zmp_ph108_B2
zmp_ph108_B6
zmp_ph108_B7
zmp_ph108_C4
zmp_ph108_C5
zmp_ph108_D4
zmp_ph97_G6
zmp_ph97_G9
zmp_ph97_H2
zmp_ph97_B10
zmp_ph97_C2
zmp_ph97_C4
zmp_ph97_B8
zmp_ph97_C1
zmp_ph97_D2
zmp_ph97_D5
zmp_ph111_G5
zmp_ph111_G11
zmp_ph111_H1
zmp_ph111_B1a
zmp_ph111_B5
zmp_ph111_B8
zmp_ph111_B6
zmp_ph111_B7
zmp_ph111_B9
zmp_ph111_B10
zmp_ph114_G4
zmp_ph114_G11
zmp_ph114_H1
zmp_ph114_H2
zmp_ph114_A9
zmp_ph114_A11
zmp_ph114_B2
zmp_ph114_B4
zmp_ph114_D8
zmp_ph114_E2

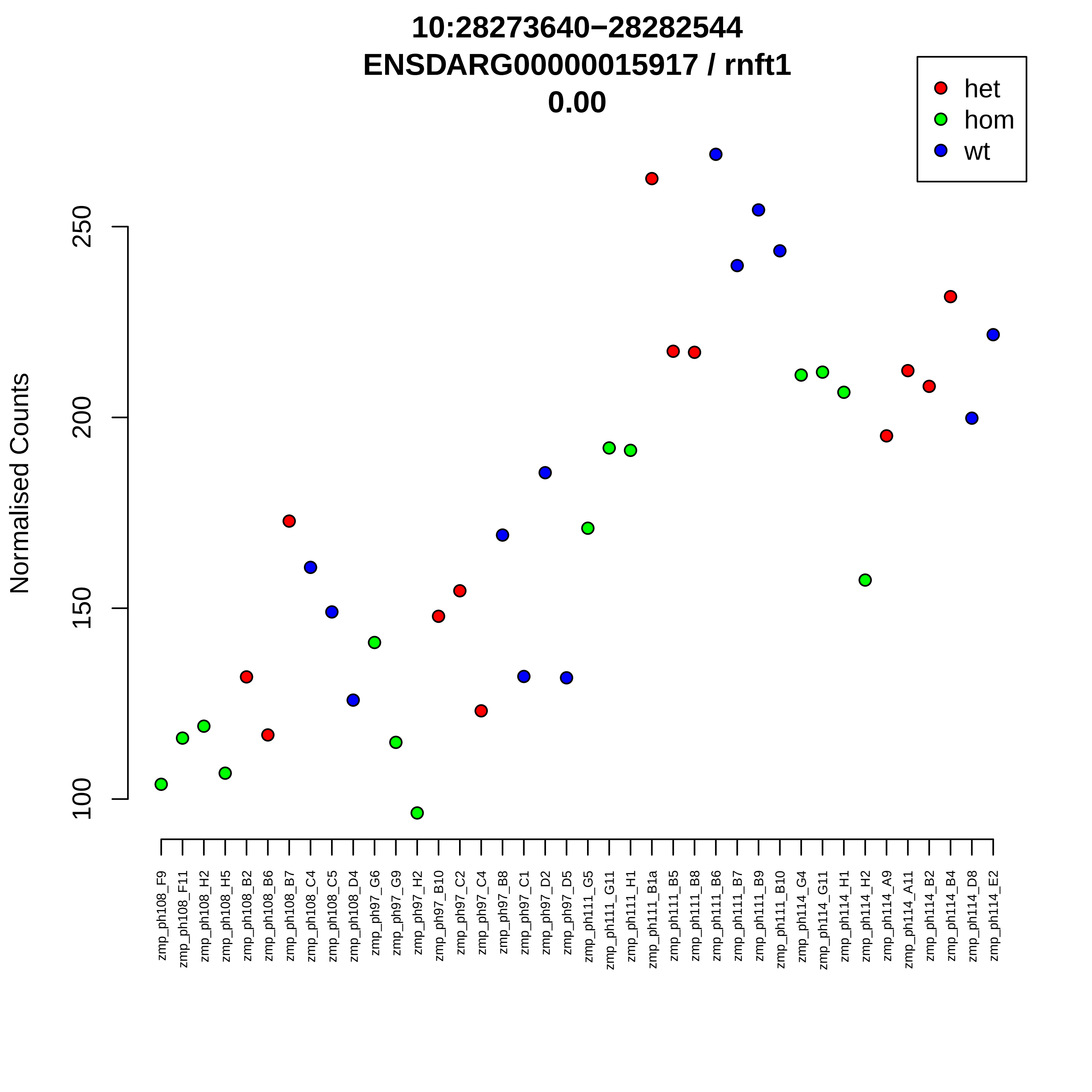

10:28273640−28282544
ENSDARG00000015917 / rnft1
0.00
het
hom
wt
Normalised Counts
100
150
200
250
zmp_ph108_F9
zmp_ph108_F11
zmp_ph108_H2
zmp_ph108_H5
zmp_ph108_B2
zmp_ph108_B6
zmp_ph108_B7
zmp_ph108_C4
zmp_ph108_C5
zmp_ph108_D4
zmp_ph97_G6
zmp_ph97_G9
zmp_ph97_H2
zmp_ph97_B10
zmp_ph97_C2
zmp_ph97_C4
zmp_ph97_B8
zmp_ph97_C1
zmp_ph97_D2
zmp_ph97_D5
zmp_ph111_G5
zmp_ph111_G11
zmp_ph111_H1
zmp_ph111_B1a
zmp_ph111_B5
zmp_ph111_B8
zmp_ph111_B6
zmp_ph111_B7
zmp_ph111_B9
zmp_ph111_B10
zmp_ph114_G4
zmp_ph114_G11
zmp_ph114_H1
zmp_ph114_H2
zmp_ph114_A9
zmp_ph114_A11
zmp_ph114_B2
zmp_ph114_B4
zmp_ph114_D8
zmp_ph114_E2

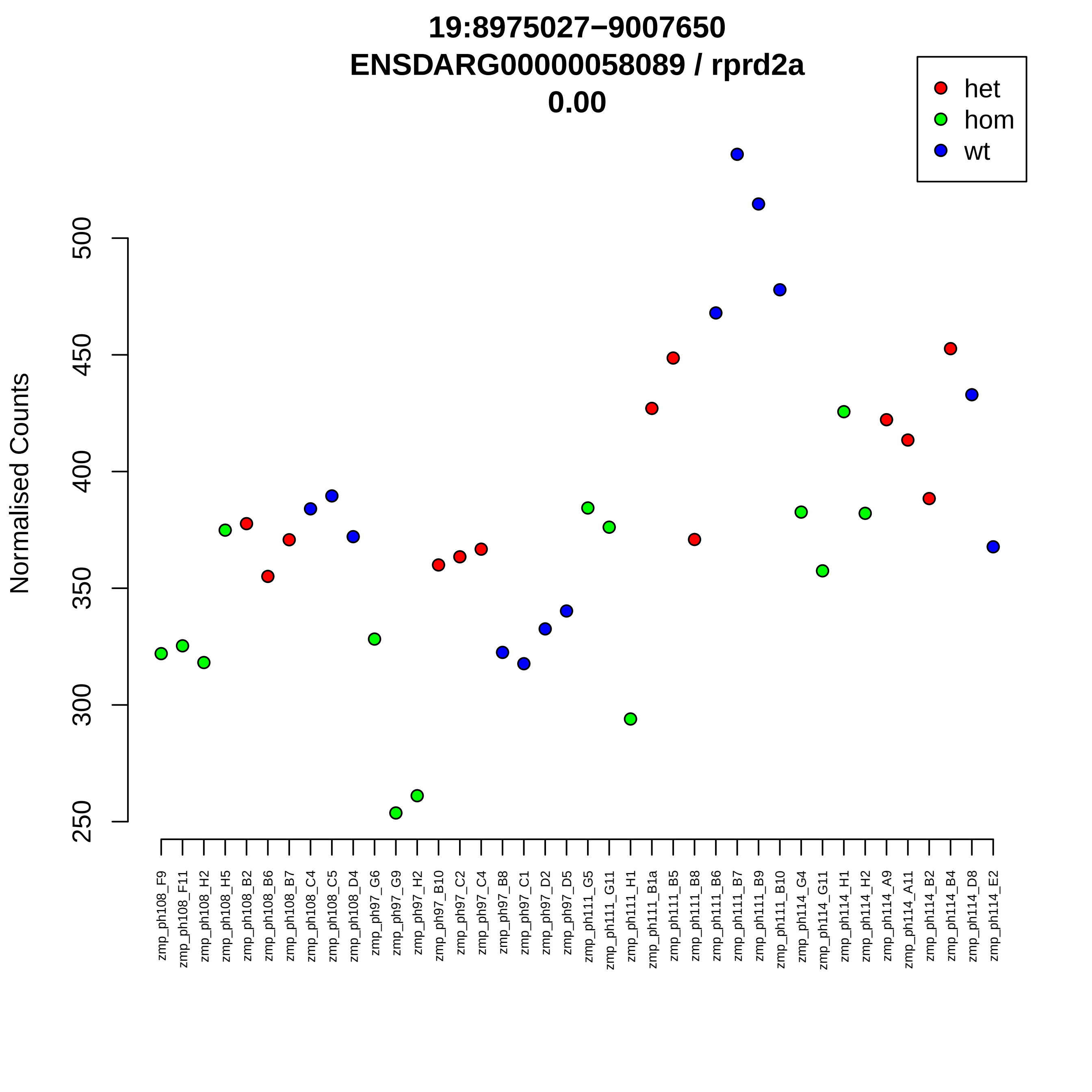
19:8975027−9007650
ENSDARG00000058089 / rprd2a
0.00
het
hom
wt
Normalised Counts
250
300
350
400
450
500
zmp_ph108_F9
zmp_ph108_F11
zmp_ph108_H2
zmp_ph108_H5
zmp_ph108_B2
zmp_ph108_B6
zmp_ph108_B7
zmp_ph108_C4
zmp_ph108_C5
zmp_ph108_D4
zmp_ph97_G6
zmp_ph97_G9
zmp_ph97_H2
zmp_ph97_B10
zmp_ph97_C2
zmp_ph97_C4
zmp_ph97_B8
zmp_ph97_C1
zmp_ph97_D2
zmp_ph97_D5
zmp_ph111_G5
zmp_ph111_G11
zmp_ph111_H1
zmp_ph111_B1a
zmp_ph111_B5
zmp_ph111_B8
zmp_ph111_B6
zmp_ph111_B7
zmp_ph111_B9
zmp_ph111_B10
zmp_ph114_G4
zmp_ph114_G11
zmp_ph114_H1
zmp_ph114_H2
zmp_ph114_A9
zmp_ph114_A11
zmp_ph114_B2
zmp_ph114_B4
zmp_ph114_D8
zmp_ph114_E2

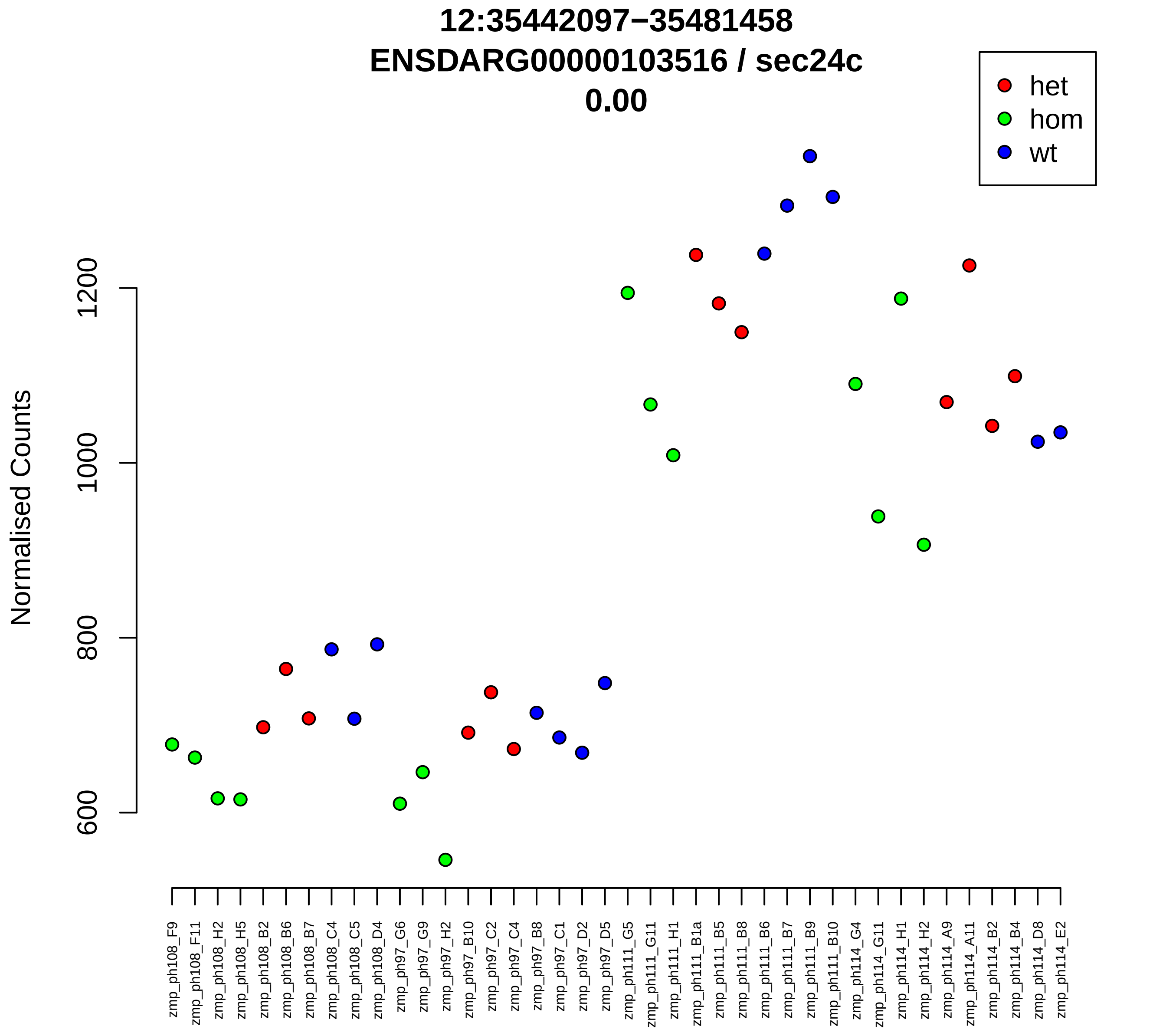

12:35442097−35481458
ENSDARG00000103516 / sec24c
0.00
het
hom
wt
Normalised Counts
600
800
1000
1200
zmp_ph108_F9
zmp_ph108_F11
zmp_ph108_H2
zmp_ph108_H5
zmp_ph108_B2
zmp_ph108_B6
zmp_ph108_B7
zmp_ph108_C4
zmp_ph108_C5
zmp_ph108_D4
zmp_ph97_G6
zmp_ph97_G9
zmp_ph97_H2
zmp_ph97_B10
zmp_ph97_C2
zmp_ph97_C4
zmp_ph97_B8
zmp_ph97_C1
zmp_ph97_D2
zmp_ph97_D5
zmp_ph111_G5
zmp_ph111_G11
zmp_ph111_H1
zmp_ph111_B1a
zmp_ph111_B5
zmp_ph111_B8
zmp_ph111_B6
zmp_ph111_B7
zmp_ph111_B9
zmp_ph111_B10
zmp_ph114_G4
zmp_ph114_G11
zmp_ph114_H1
zmp_ph114_H2
zmp_ph114_A9
zmp_ph114_A11
zmp_ph114_B2
zmp_ph114_B4
zmp_ph114_D8
zmp_ph114_E2

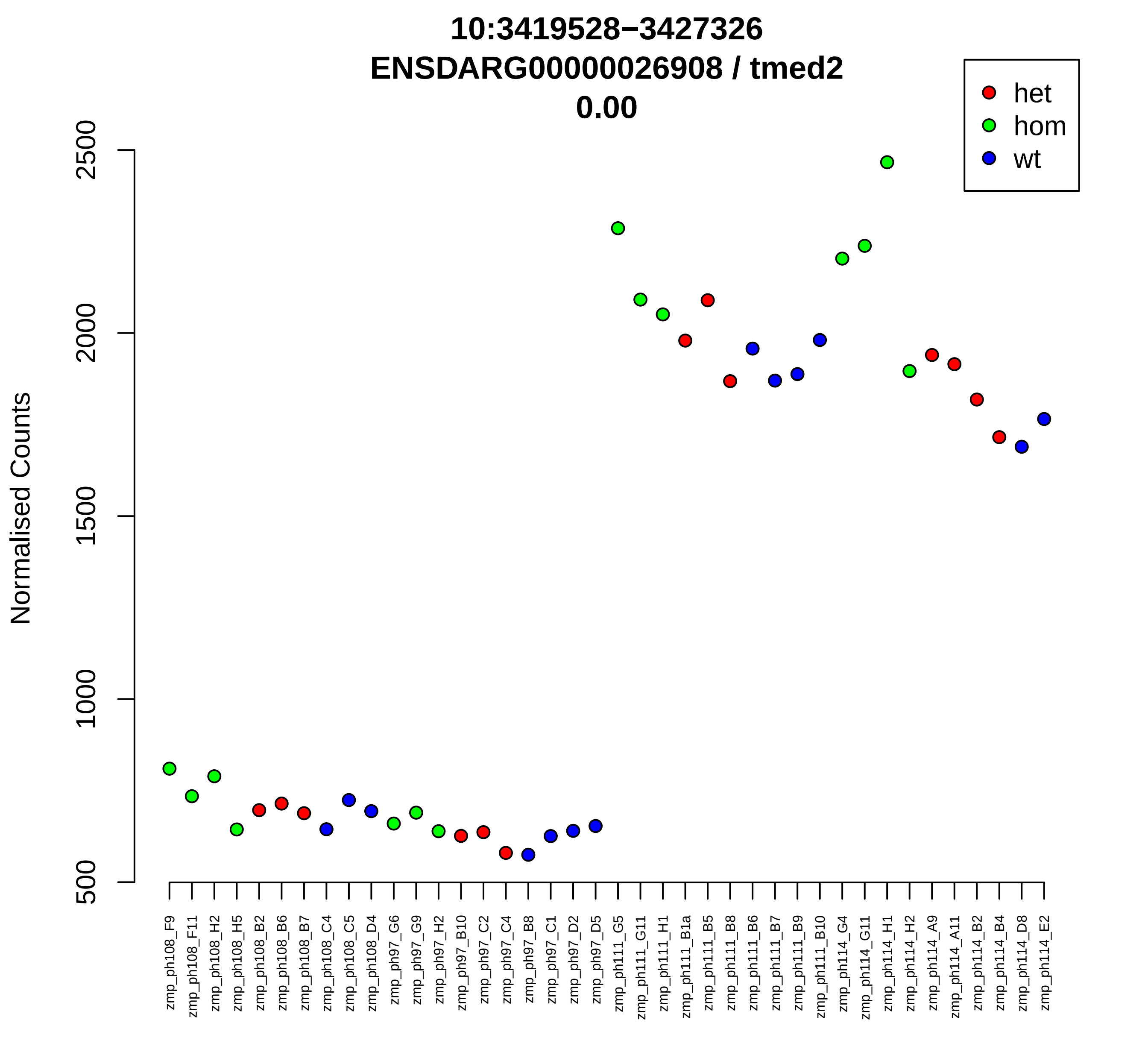

10:3419528−3427326
ENSDARG00000026908 / tmed2
0.00
het
hom
wt
Normalised Counts
500
1000
1500
2000
2500
zmp_ph108_F9
zmp_ph108_F11
zmp_ph108_H2
zmp_ph108_H5
zmp_ph108_B2
zmp_ph108_B6
zmp_ph108_B7
zmp_ph108_C4
zmp_ph108_C5
zmp_ph108_D4
zmp_ph97_G6
zmp_ph97_G9
zmp_ph97_H2
zmp_ph97_B10
zmp_ph97_C2
zmp_ph97_C4
zmp_ph97_B8
zmp_ph97_C1
zmp_ph97_D2
zmp_ph97_D5
zmp_ph111_G5
zmp_ph111_G11
zmp_ph111_H1
zmp_ph111_B1a
zmp_ph111_B5
zmp_ph111_B8
zmp_ph111_B6
zmp_ph111_B7
zmp_ph111_B9
zmp_ph111_B10
zmp_ph114_G4
zmp_ph114_G11
zmp_ph114_H1
zmp_ph114_H2
zmp_ph114_A9
zmp_ph114_A11
zmp_ph114_B2
zmp_ph114_B4
zmp_ph114_D8
zmp_ph114_E2

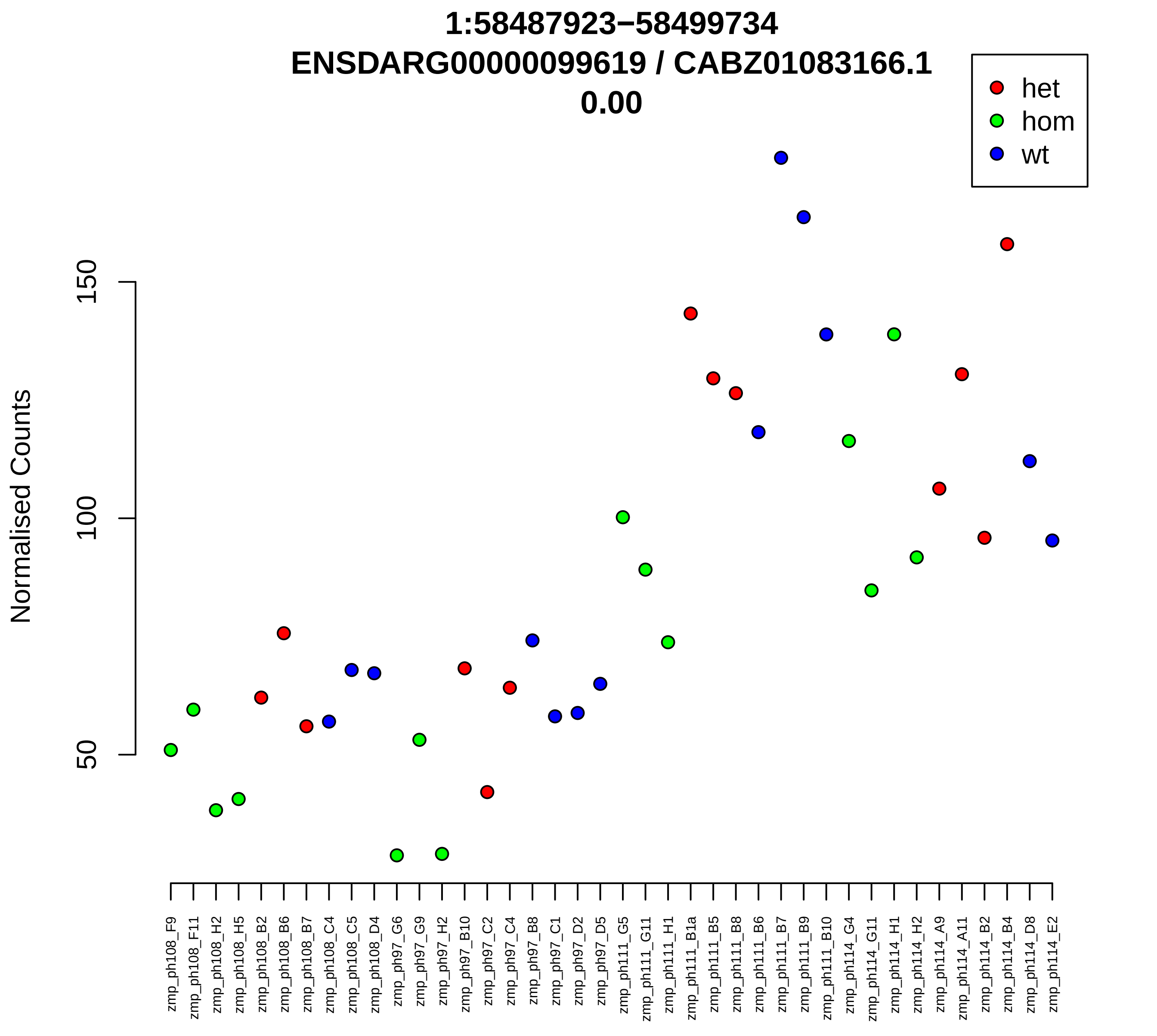

1:58487923−58499734
ENSDARG00000099619 / CABZ01083166.1
0.00
het
hom
wt
Normalised Counts
50
100
150
zmp_ph108_F9
zmp_ph108_F11
zmp_ph108_H2
zmp_ph108_H5
zmp_ph108_B2
zmp_ph108_B6
zmp_ph108_B7
zmp_ph108_C4
zmp_ph108_C5
zmp_ph108_D4
zmp_ph97_G6
zmp_ph97_G9
zmp_ph97_H2
zmp_ph97_B10
zmp_ph97_C2
zmp_ph97_C4
zmp_ph97_B8
zmp_ph97_C1
zmp_ph97_D2
zmp_ph97_D5
zmp_ph111_G5
zmp_ph111_G11
zmp_ph111_H1
zmp_ph111_B1a
zmp_ph111_B5
zmp_ph111_B8
zmp_ph111_B6
zmp_ph111_B7
zmp_ph111_B9
zmp_ph111_B10
zmp_ph114_G4
zmp_ph114_G11
zmp_ph114_H1
zmp_ph114_H2
zmp_ph114_A9
zmp_ph114_A11
zmp_ph114_B2
zmp_ph114_B4
zmp_ph114_D8
zmp_ph114_E2

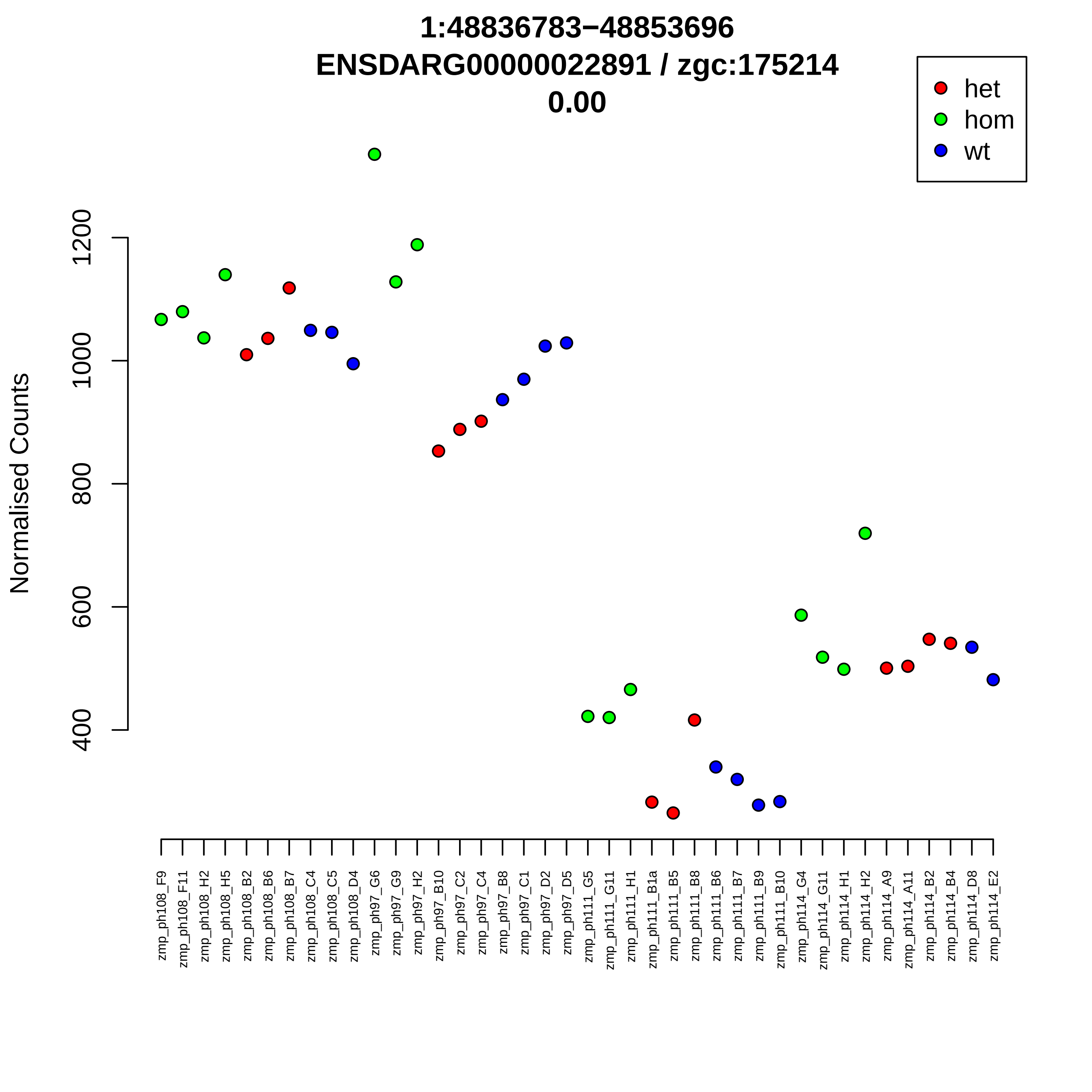

1:48836783−48853696
ENSDARG00000022891 / zgc:175214
0.00
het
hom
wt
Normalised Counts
400
600
800
1000
1200
zmp_ph108_F9
zmp_ph108_F11
zmp_ph108_H2
zmp_ph108_H5
zmp_ph108_B2
zmp_ph108_B6
zmp_ph108_B7
zmp_ph108_C4
zmp_ph108_C5
zmp_ph108_D4
zmp_ph97_G6
zmp_ph97_G9
zmp_ph97_H2
zmp_ph97_B10
zmp_ph97_C2
zmp_ph97_C4
zmp_ph97_B8
zmp_ph97_C1
zmp_ph97_D2
zmp_ph97_D5
zmp_ph111_G5
zmp_ph111_G11
zmp_ph111_H1
zmp_ph111_B1a
zmp_ph111_B5
zmp_ph111_B8
zmp_ph111_B6
zmp_ph111_B7
zmp_ph111_B9
zmp_ph111_B10
zmp_ph114_G4
zmp_ph114_G11
zmp_ph114_H1
zmp_ph114_H2
zmp_ph114_A9
zmp_ph114_A11
zmp_ph114_B2
zmp_ph114_B4
zmp_ph114_D8
zmp_ph114_E2

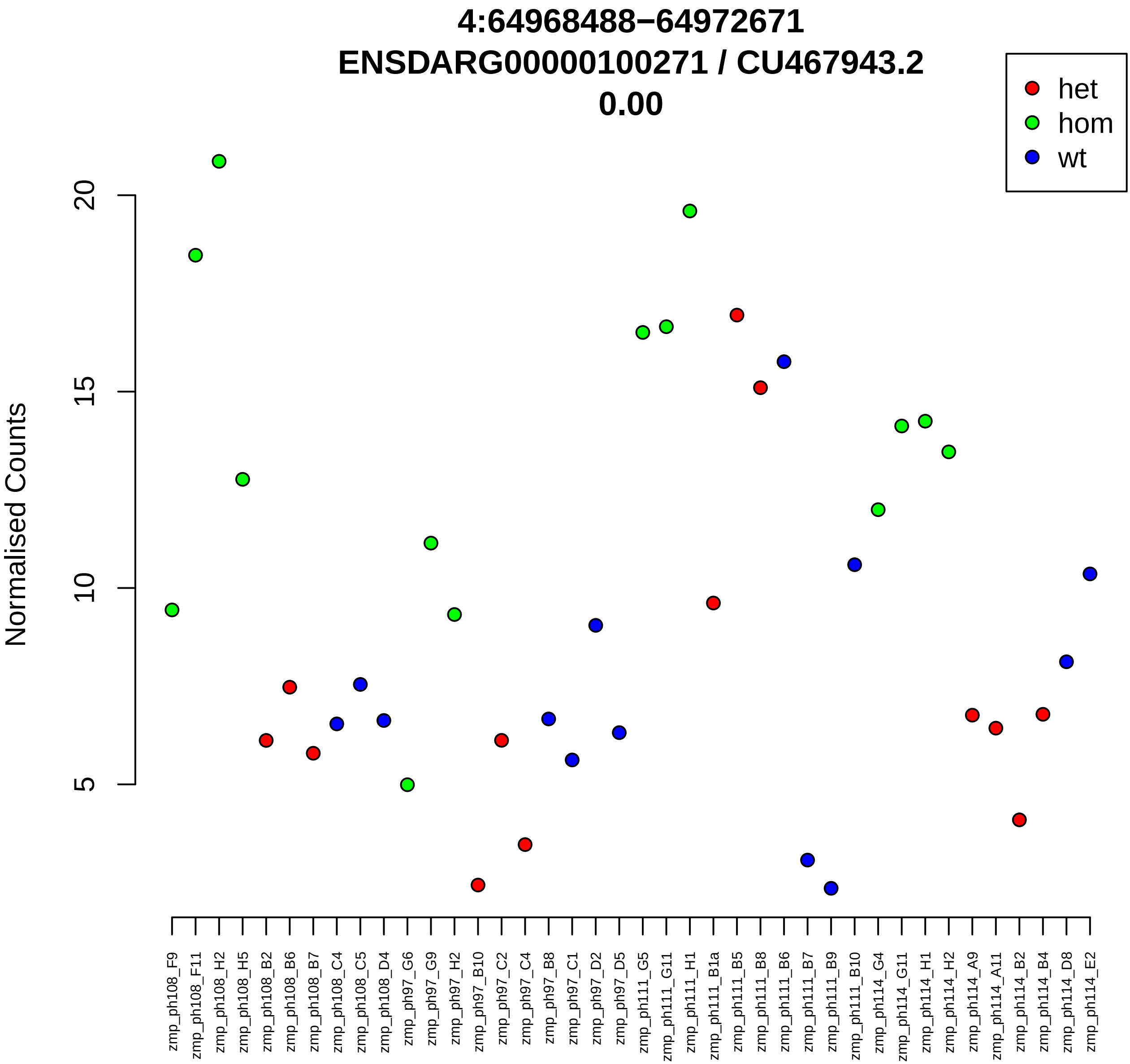

4:64968488−64972671
ENSDARG00000100271 / CU467943.2
0.00
het
hom
wt
Normalised Counts
20
15
10
5
zmp_ph108_F9
zmp_ph108_F11
zmp_ph108_H2
zmp_ph108_H5
zmp_ph108_B2
zmp_ph108_B6
zmp_ph108_B7
zmp_ph108_C4
zmp_ph108_C5
zmp_ph108_D4
zmp_ph97_G6
zmp_ph97_G9
zmp_ph97_H2
zmp_ph97_B10
zmp_ph97_C2
zmp_ph97_C4
zmp_ph97_B8
zmp_ph97_C1
zmp_ph97_D2
zmp_ph97_D5
zmp_ph111_G5
zmp_ph111_G11
zmp_ph111_H1
zmp_ph111_B1a
zmp_ph111_B5
zmp_ph111_B8
zmp_ph111_B6
zmp_ph111_B7
zmp_ph111_B9
zmp_ph111_B10
zmp_ph114_G4
zmp_ph114_G11
zmp_ph114_H1
zmp_ph114_H2
zmp_ph114_A9
zmp_ph114_A11
zmp_ph114_B2
zmp_ph114_B4
zmp_ph114_D8
zmp_ph114_E2

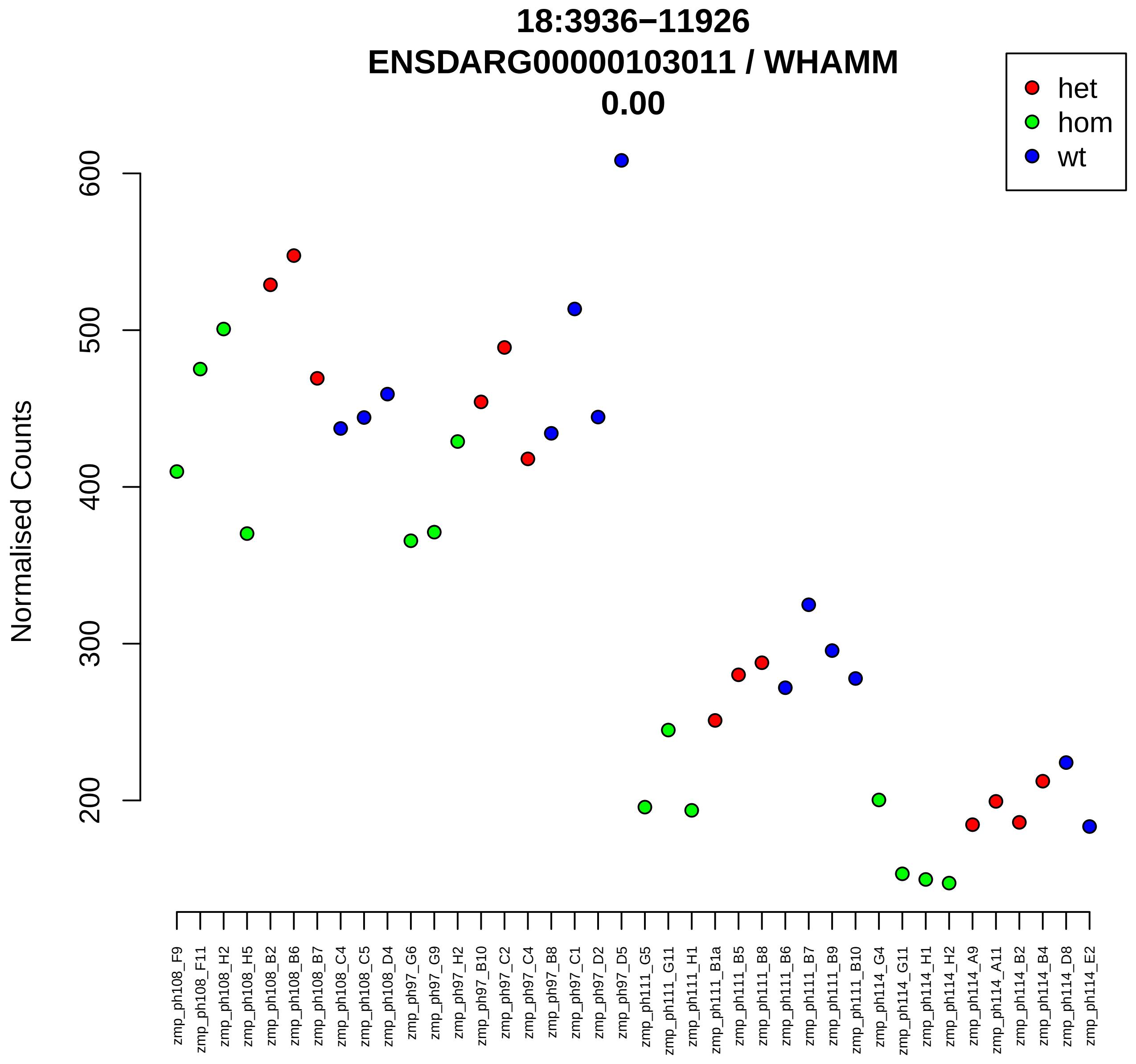
18:3936−11926
ENSDARG00000103011 / WHAMM
0.00
het
hom
wt
Normalised Counts
200
300
400
500
600
zmp_ph108_F9
zmp_ph108_F11
zmp_ph108_H2
zmp_ph108_H5
zmp_ph108_B2
zmp_ph108_B6
zmp_ph108_B7
zmp_ph108_C4
zmp_ph108_C5
zmp_ph108_D4
zmp_ph97_G6
zmp_ph97_G9
zmp_ph97_H2
zmp_ph97_B10
zmp_ph97_C2
zmp_ph97_C4
zmp_ph97_B8
zmp_ph97_C1
zmp_ph97_D2
zmp_ph97_D5
zmp_ph111_G5
zmp_ph111_G11
zmp_ph111_H1
zmp_ph111_B1a
zmp_ph111_B5
zmp_ph111_B8
zmp_ph111_B6
zmp_ph111_B7
zmp_ph111_B9
zmp_ph111_B10
zmp_ph114_G4
zmp_ph114_G11
zmp_ph114_H1
zmp_ph114_H2
zmp_ph114_A9
zmp_ph114_A11
zmp_ph114_B2
zmp_ph114_B4
zmp_ph114_D8
zmp_ph114_E2

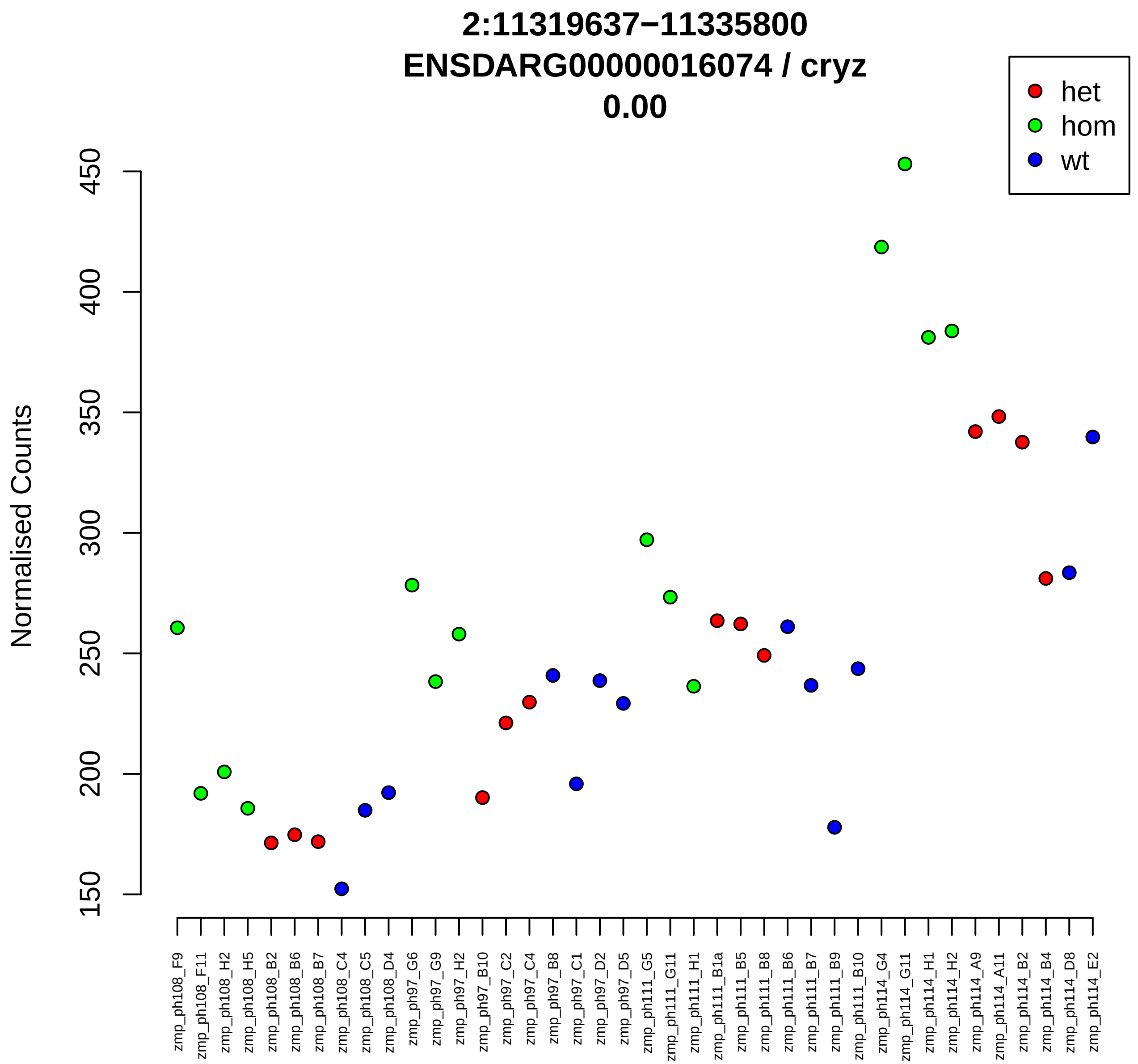

2:11319637−11335800
ENSDARG00000016074 / cryz
0.00
het
hom
wt
Normalised Counts
150
200
250
300
350
400
450
zmp_ph108_F9
zmp_ph108_F11
zmp_ph108_H2
zmp_ph108_H5
zmp_ph108_B2
zmp_ph108_B6
zmp_ph108_B7
zmp_ph108_C4
zmp_ph108_C5
zmp_ph108_D4
zmp_ph97_G6
zmp_ph97_G9
zmp_ph97_H2
zmp_ph97_B10
zmp_ph97_C2
zmp_ph97_C4
zmp_ph97_B8
zmp_ph97_C1
zmp_ph97_D2
zmp_ph97_D5
zmp_ph111_G5
zmp_ph111_G11
zmp_ph111_H1
zmp_ph111_B1a
zmp_ph111_B5
zmp_ph111_B8
zmp_ph111_B6
zmp_ph111_B7
zmp_ph111_B9
zmp_ph111_B10
zmp_ph114_G4
zmp_ph114_G11
zmp_ph114_H1
zmp_ph114_H2
zmp_ph114_A9
zmp_ph114_A11
zmp_ph114_B2
zmp_ph114_B4
zmp_ph114_D8
zmp_ph114_E2

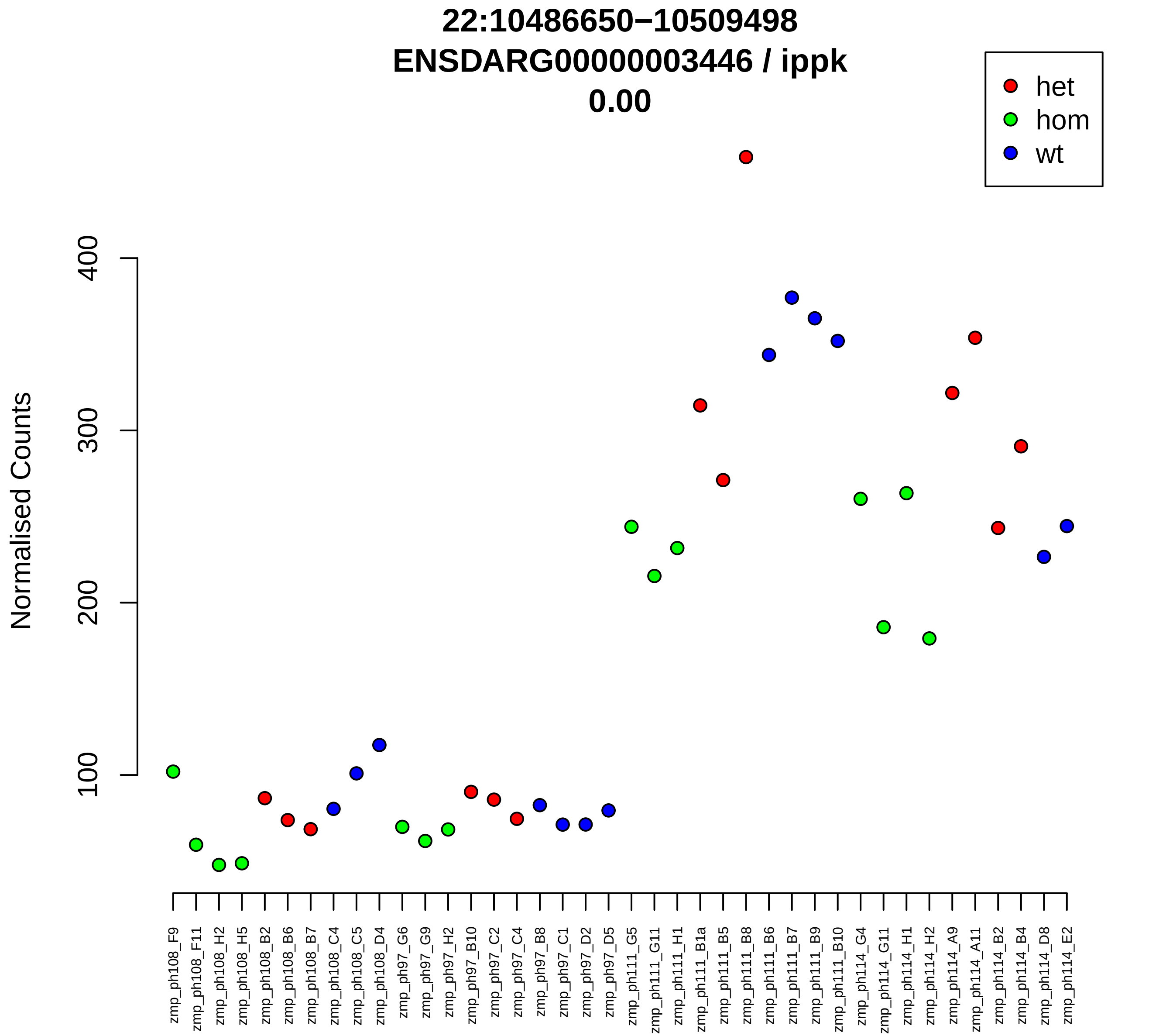

22:10486650−10509498
ENSDARG00000003446 / ippk
0.00
het
hom
wt
Normalised Counts
100
200
300
400
zmp_ph108_F9
zmp_ph108_F11
zmp_ph108_H2
zmp_ph108_H5
zmp_ph108_B2
zmp_ph108_B6
zmp_ph108_B7
zmp_ph108_C4
zmp_ph108_C5
zmp_ph108_D4
zmp_ph97_G6
zmp_ph97_G9
zmp_ph97_H2
zmp_ph97_B10
zmp_ph97_C2
zmp_ph97_C4
zmp_ph97_B8
zmp_ph97_C1
zmp_ph97_D2
zmp_ph97_D5
zmp_ph111_G5
zmp_ph111_G11
zmp_ph111_H1
zmp_ph111_B1a
zmp_ph111_B5
zmp_ph111_B8
zmp_ph111_B6
zmp_ph111_B7
zmp_ph111_B9
zmp_ph111_B10
zmp_ph114_G4
zmp_ph114_G11
zmp_ph114_H1
zmp_ph114_H2
zmp_ph114_A9
zmp_ph114_A11
zmp_ph114_B2
zmp_ph114_B4
zmp_ph114_D8
zmp_ph114_E2

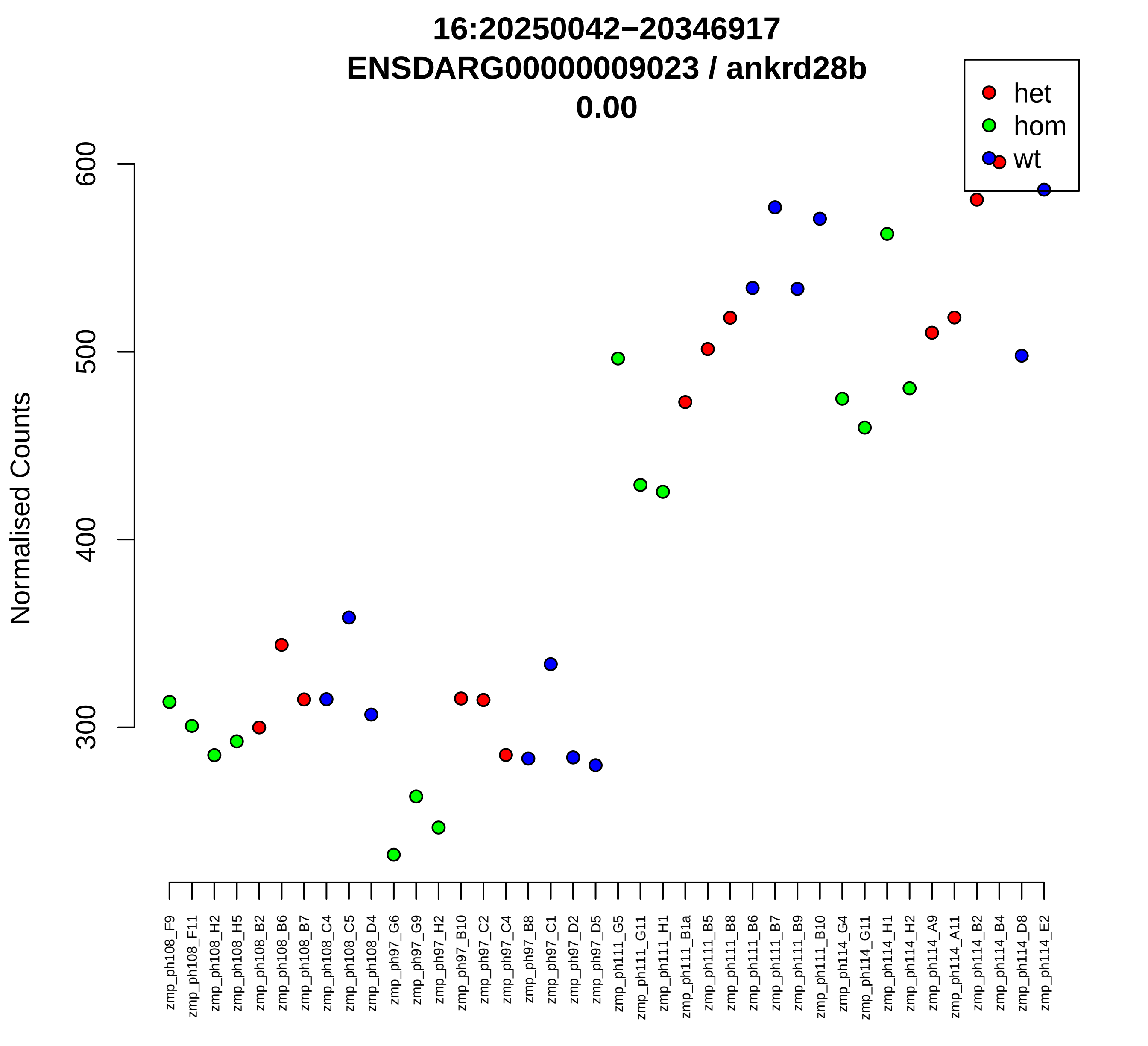
16:20250042−20346917
ENSDARG00000009023 / ankrd28b
0.00
het
hom
wt
Normalised Counts
300
400
500
600
zmp_ph108_F9
zmp_ph108_F11
zmp_ph108_H2
zmp_ph108_H5
zmp_ph108_B2
zmp_ph108_B6
zmp_ph108_B7
zmp_ph108_C4
zmp_ph108_C5
zmp_ph108_D4
zmp_ph97_G6
zmp_ph97_G9
zmp_ph97_H2
zmp_ph97_B10
zmp_ph97_C2
zmp_ph97_C4
zmp_ph97_B8
zmp_ph97_C1
zmp_ph97_D2
zmp_ph97_D5
zmp_ph111_G5
zmp_ph111_G11
zmp_ph111_H1
zmp_ph111_B1a
zmp_ph111_B5
zmp_ph111_B8
zmp_ph111_B6
zmp_ph111_B7
zmp_ph111_B9
zmp_ph111_B10
zmp_ph114_G4
zmp_ph114_G11
zmp_ph114_H1
zmp_ph114_H2
zmp_ph114_A9
zmp_ph114_A11
zmp_ph114_B2
zmp_ph114_B4
zmp_ph114_D8
zmp_ph114_E2

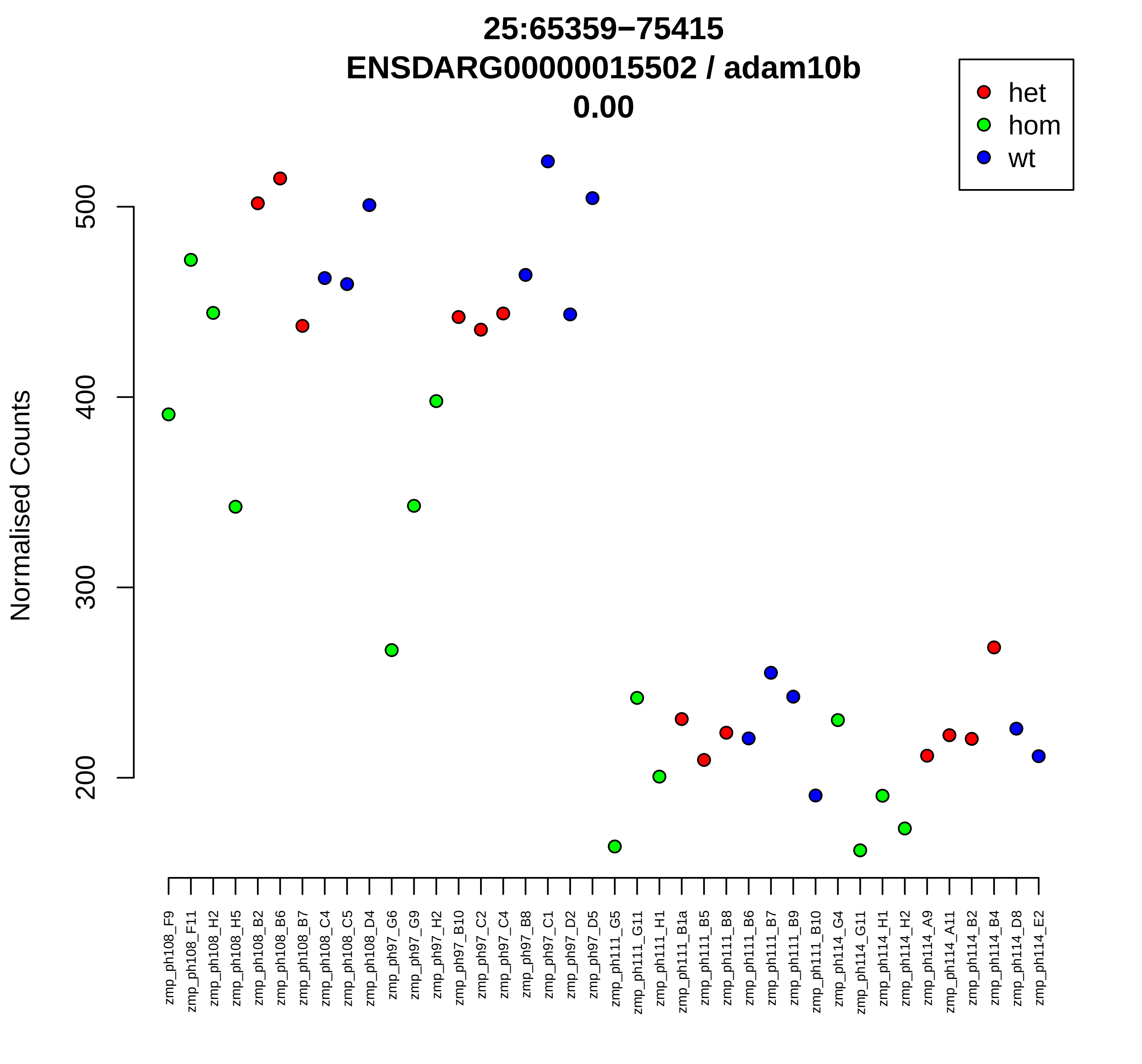
25:65359−75415
ENSDARG00000015502 / adam10b
0.00
het
hom
wt
Normalised Counts
200
300
400
500
zmp_ph108_F9
zmp_ph108_F11
zmp_ph108_H2
zmp_ph108_H5
zmp_ph108_B2
zmp_ph108_B6
zmp_ph108_B7
zmp_ph108_C4
zmp_ph108_C5
zmp_ph108_D4
zmp_ph97_G6
zmp_ph97_G9
zmp_ph97_H2
zmp_ph97_B10
zmp_ph97_C2
zmp_ph97_C4
zmp_ph97_B8
zmp_ph97_C1
zmp_ph97_D2
zmp_ph97_D5
zmp_ph111_G5
zmp_ph111_G11
zmp_ph111_H1
zmp_ph111_B1a
zmp_ph111_B5
zmp_ph111_B8
zmp_ph111_B6
zmp_ph111_B7
zmp_ph111_B9
zmp_ph111_B10
zmp_ph114_G4
zmp_ph114_G11
zmp_ph114_H1
zmp_ph114_H2
zmp_ph114_A9
zmp_ph114_A11
zmp_ph114_B2
zmp_ph114_B4
zmp_ph114_D8
zmp_ph114_E2

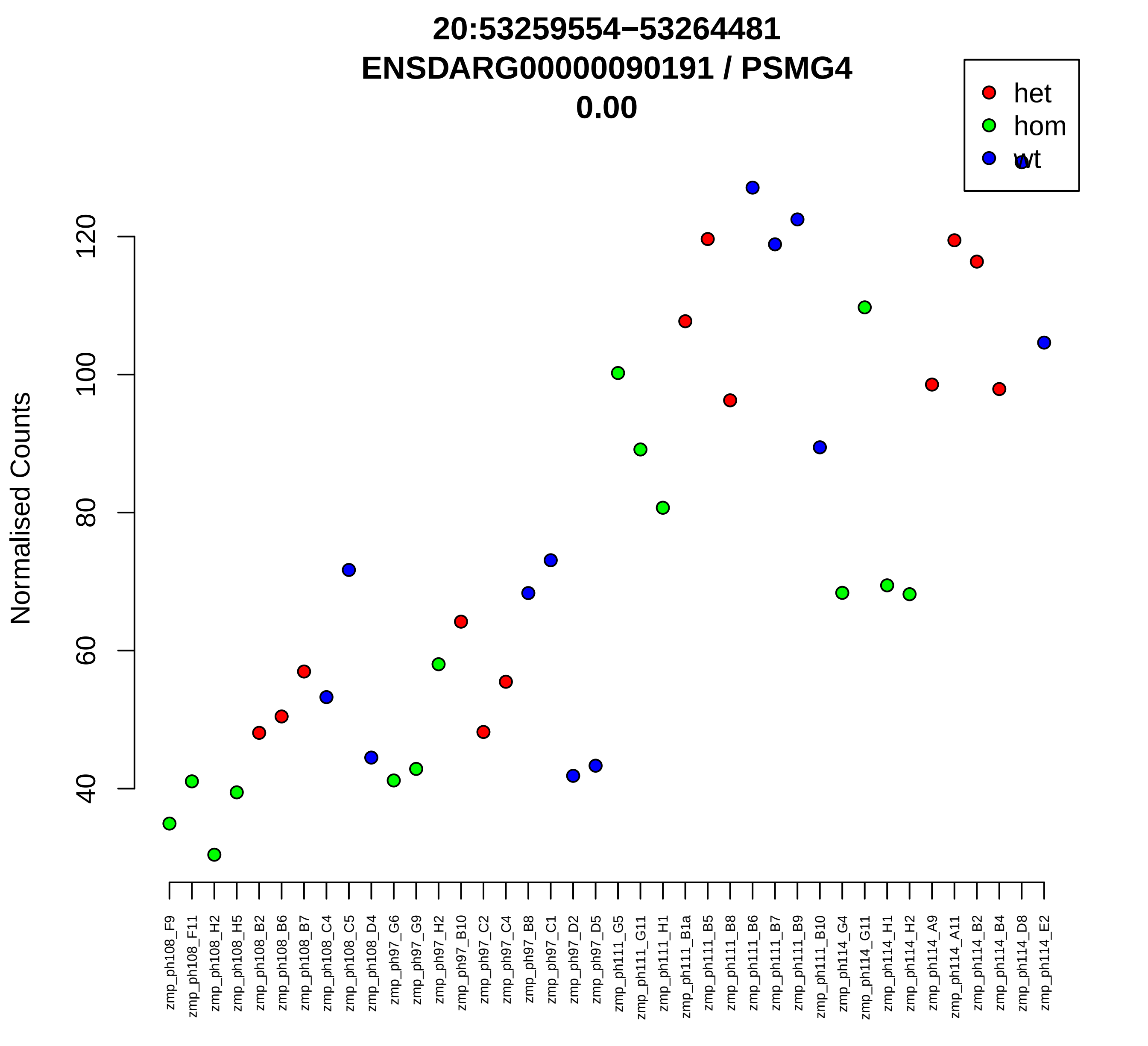

20:53259554−53264481
ENSDARG00000090191 / PSMG4
0.00
het
hom
wt
Normalised Counts
40
60
80
100
120
zmp_ph108_F9
zmp_ph108_F11
zmp_ph108_H2
zmp_ph108_H5
zmp_ph108_B2
zmp_ph108_B6
zmp_ph108_B7
zmp_ph108_C4
zmp_ph108_C5
zmp_ph108_D4
zmp_ph97_G6
zmp_ph97_G9
zmp_ph97_H2
zmp_ph97_B10
zmp_ph97_C2
zmp_ph97_C4
zmp_ph97_B8
zmp_ph97_C1
zmp_ph97_D2
zmp_ph97_D5
zmp_ph111_G5
zmp_ph111_G11
zmp_ph111_H1
zmp_ph111_B1a
zmp_ph111_B5
zmp_ph111_B8
zmp_ph111_B6
zmp_ph111_B7
zmp_ph111_B9
zmp_ph111_B10
zmp_ph114_G4
zmp_ph114_G11
zmp_ph114_H1
zmp_ph114_H2
zmp_ph114_A9
zmp_ph114_A11
zmp_ph114_B2
zmp_ph114_B4
zmp_ph114_D8
zmp_ph114_E2

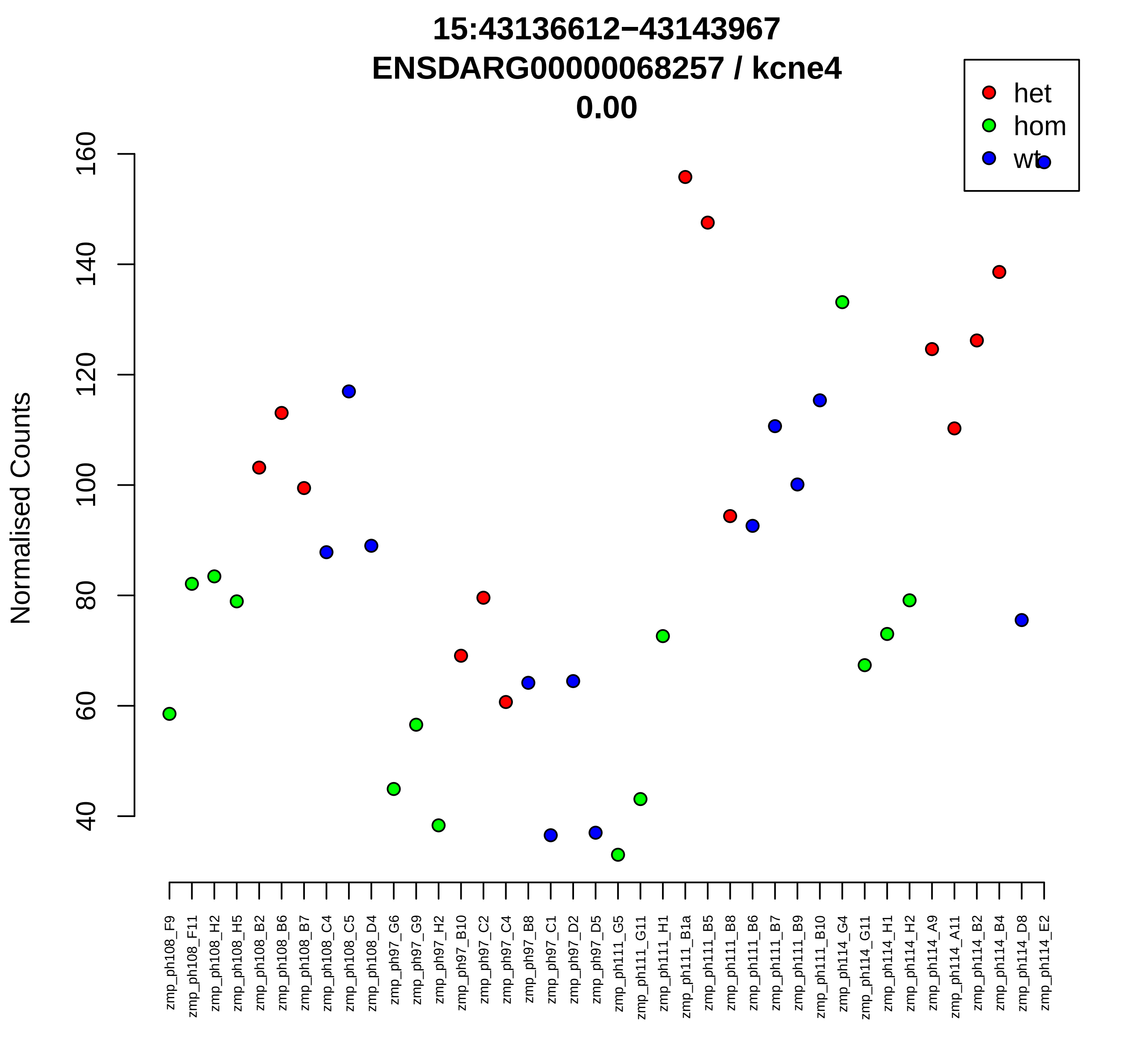

15:43136612−43143967
ENSDARG00000068257 / kcne4
0.00
Normalised Counts
40
60
80
100
120
140
160
het
hom
wt
zmp_ph108_F9
zmp_ph108_F11
zmp_ph108_H2
zmp_ph108_H5
zmp_ph108_B2
zmp_ph108_B6
zmp_ph108_B7
zmp_ph108_C4
zmp_ph108_C5
zmp_ph108_D4
zmp_ph97_G6
zmp_ph97_G9
zmp_ph97_H2
zmp_ph97_B10
zmp_ph97_C2
zmp_ph97_C4
zmp_ph97_B8
zmp_ph97_C1
zmp_ph97_D2
zmp_ph97_D5
zmp_ph111_G5
zmp_ph111_G11
zmp_ph111_H1
zmp_ph111_B1a
zmp_ph111_B5
zmp_ph111_B8
zmp_ph111_B6
zmp_ph111_B7
zmp_ph111_B9
zmp_ph111_B10
zmp_ph114_G4
zmp_ph114_G11
zmp_ph114_H1
zmp_ph114_H2
zmp_ph114_A9
zmp_ph114_A11
zmp_ph114_B2
zmp_ph114_B4
zmp_ph114_D8
zmp_ph114_E2

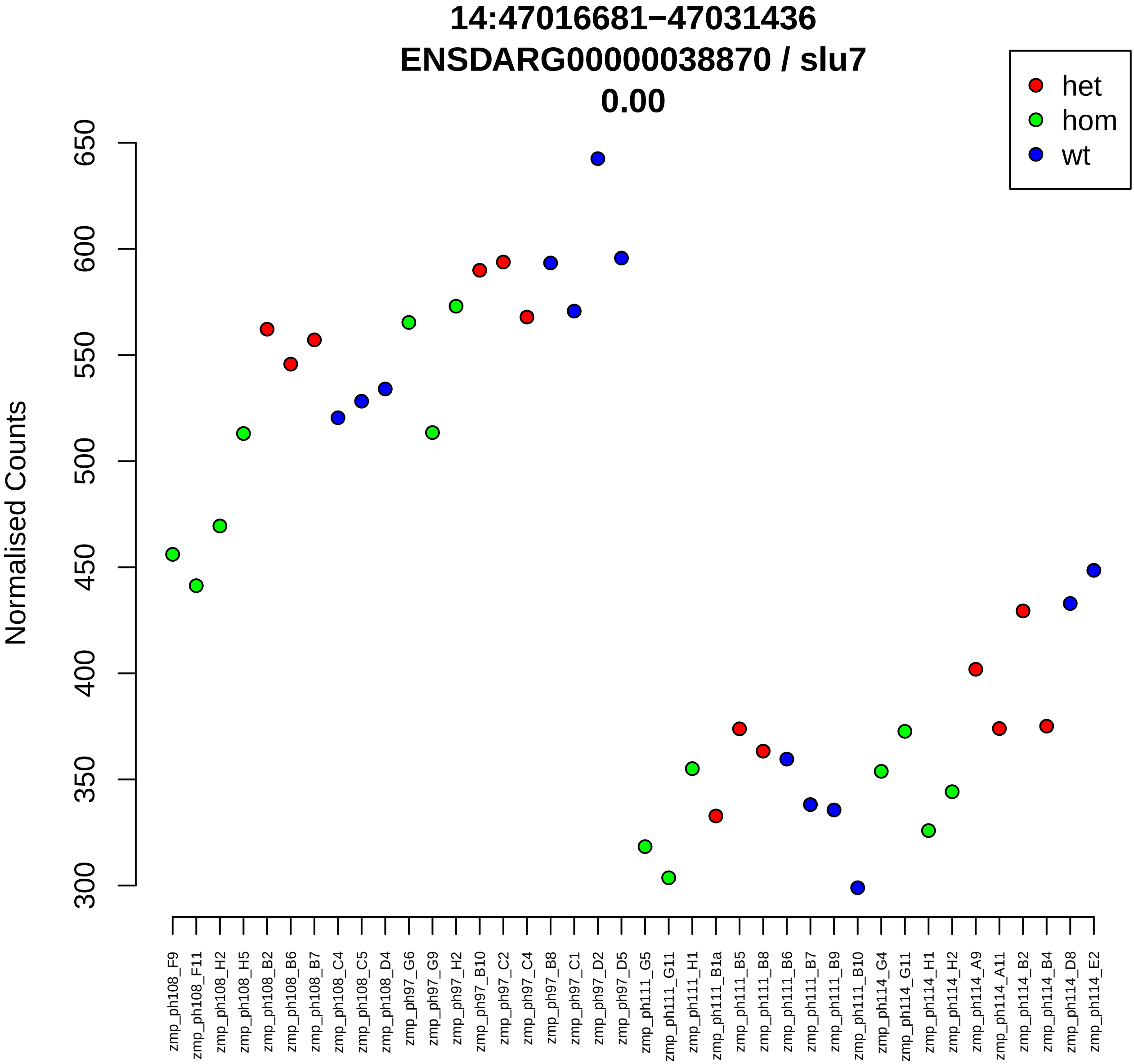

14:47016681−47031436
ENSDARG00000038870 / slu7
0.00
het
hom
wt
Normalised Counts
650
600
550
500
450
400
350
300
zmp_ph108_F9
zmp_ph108_F11
zmp_ph108_H2
zmp_ph108_H5
zmp_ph108_B2
zmp_ph108_B6
zmp_ph108_B7
zmp_ph108_C4
zmp_ph108_C5
zmp_ph108_D4
zmp_ph97_G6
zmp_ph97_G9
zmp_ph97_H2
zmp_ph97_B10
zmp_ph97_C2
zmp_ph97_C4
zmp_ph97_B8
zmp_ph97_C1
zmp_ph97_D2
zmp_ph97_D5
zmp_ph111_G5
zmp_ph111_G11
zmp_ph111_H1
zmp_ph111_B1a
zmp_ph111_B5
zmp_ph111_B8
zmp_ph111_B6
zmp_ph111_B7
zmp_ph111_B9
zmp_ph111_B10
zmp_ph114_G4
zmp_ph114_G11
zmp_ph114_H1
zmp_ph114_H2
zmp_ph114_A9
zmp_ph114_A11
zmp_ph114_B2
zmp_ph114_B4
zmp_ph114_D8
zmp_ph114_E2

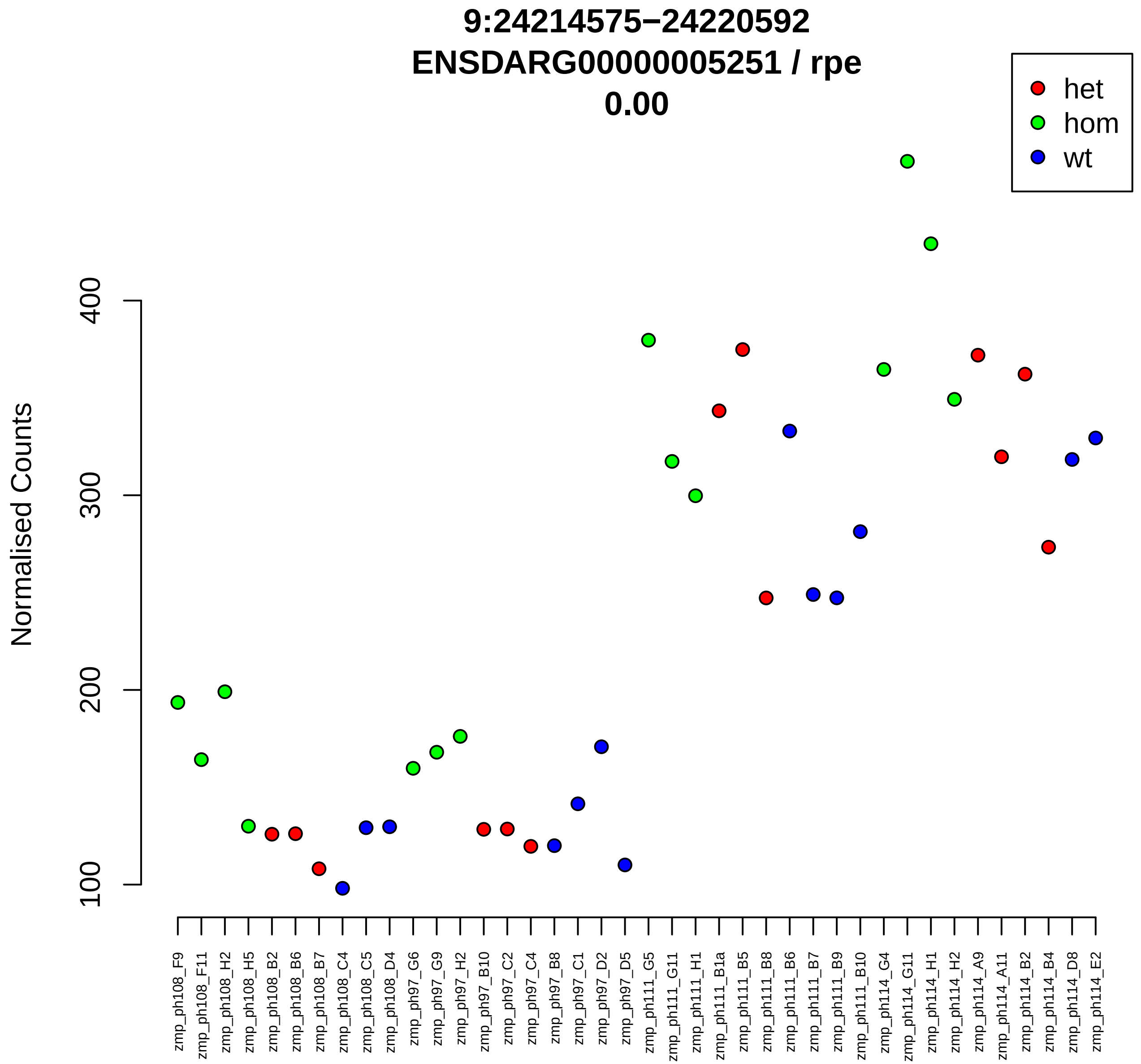

9:24214575−24220592
ENSDARG00000005251 / rpe
0.00
het
hom
wt
Normalised Counts
100
200
300
400
zmp_ph108_F9
zmp_ph108_F11
zmp_ph108_H2
zmp_ph108_H5
zmp_ph108_B2
zmp_ph108_B6
zmp_ph108_B7
zmp_ph108_C4
zmp_ph108_C5
zmp_ph108_D4
zmp_ph97_G6
zmp_ph97_G9
zmp_ph97_H2
zmp_ph97_B10
zmp_ph97_C2
zmp_ph97_C4
zmp_ph97_B8
zmp_ph97_C1
zmp_ph97_D2
zmp_ph97_D5
zmp_ph111_G5
zmp_ph111_G11
zmp_ph111_H1
zmp_ph111_B1a
zmp_ph111_B5
zmp_ph111_B8
zmp_ph111_B6
zmp_ph111_B7
zmp_ph111_B9
zmp_ph111_B10
zmp_ph114_G4
zmp_ph114_G11
zmp_ph114_H1
zmp_ph114_H2
zmp_ph114_A9
zmp_ph114_A11
zmp_ph114_B2
zmp_ph114_B4
zmp_ph114_D8
zmp_ph114_E2

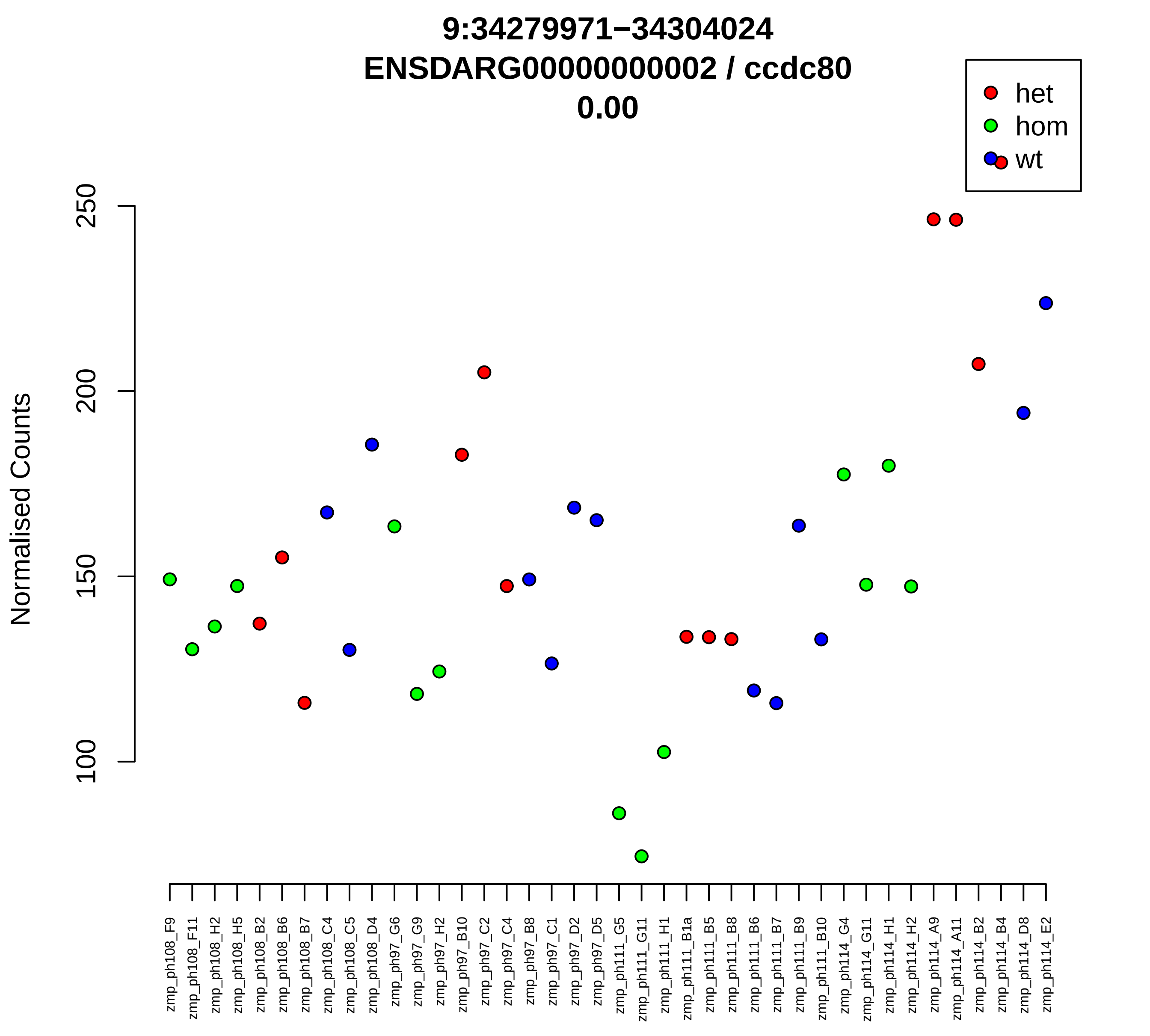
9:34279971−34304024
ENSDARG00000000002 / ccdc80
0.00
het
hom
wt
Normalised Counts
100
150
200
250
zmp_ph108_F9
zmp_ph108_F11
zmp_ph108_H2
zmp_ph108_H5
zmp_ph108_B2
zmp_ph108_B6
zmp_ph108_B7
zmp_ph108_C4
zmp_ph108_C5
zmp_ph108_D4
zmp_ph97_G6
zmp_ph97_G9
zmp_ph97_H2
zmp_ph97_B10
zmp_ph97_C2
zmp_ph97_C4
zmp_ph97_B8
zmp_ph97_C1
zmp_ph97_D2
zmp_ph97_D5
zmp_ph111_G5
zmp_ph111_G11
zmp_ph111_H1
zmp_ph111_B1a
zmp_ph111_B5
zmp_ph111_B8
zmp_ph111_B6
zmp_ph111_B7
zmp_ph111_B9
zmp_ph111_B10
zmp_ph114_G4
zmp_ph114_G11
zmp_ph114_H1
zmp_ph114_H2
zmp_ph114_A9
zmp_ph114_A11
zmp_ph114_B2
zmp_ph114_B4
zmp_ph114_D8
zmp_ph114_E2

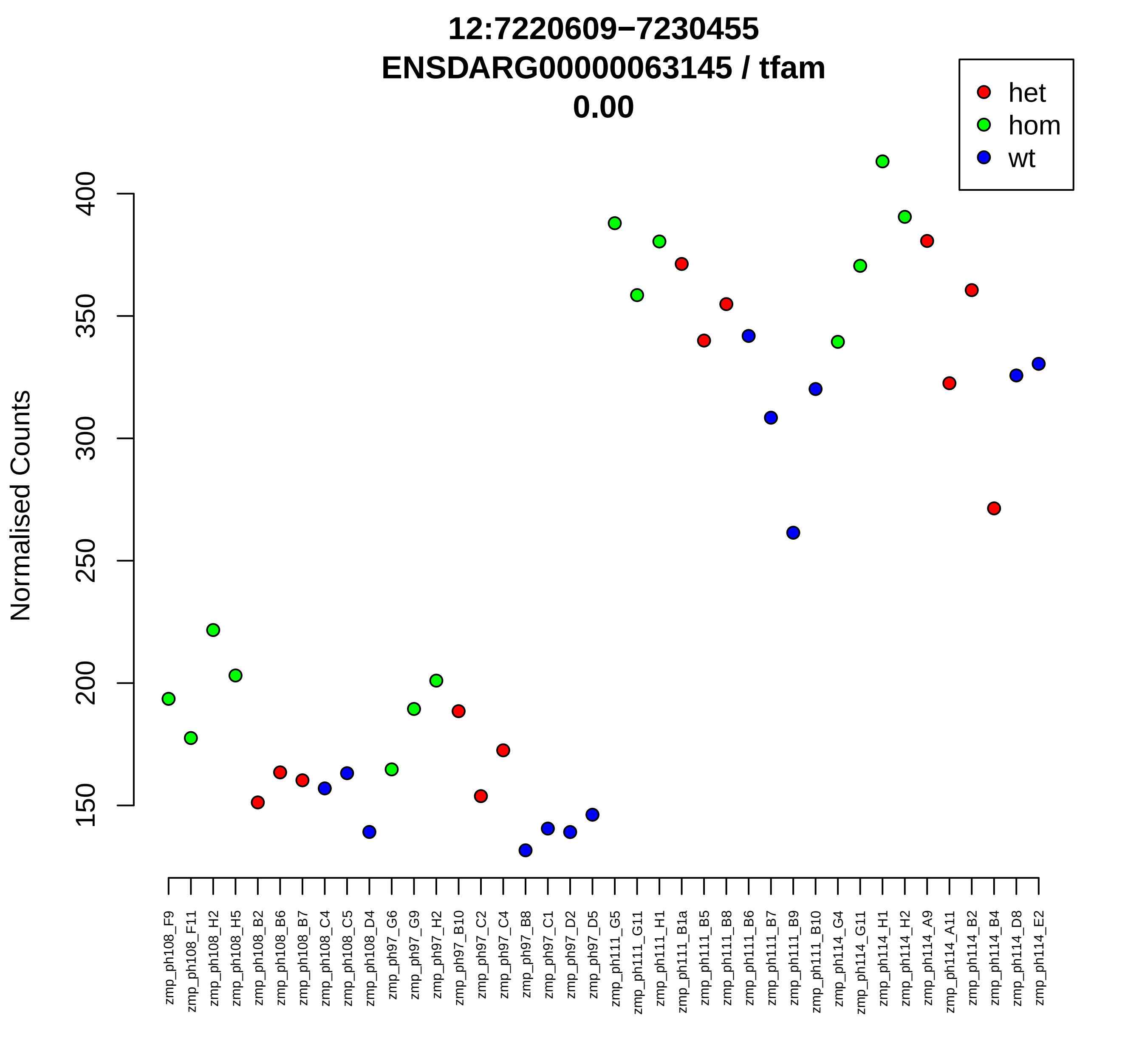

12:7220609−7230455
ENSDARG00000063145 / tfam
0.00
het
hom
wt
Normalised Counts
150
200
250
300
350
400
zmp_ph108_F9
zmp_ph108_F11
zmp_ph108_H2
zmp_ph108_H5
zmp_ph108_B2
zmp_ph108_B6
zmp_ph108_B7
zmp_ph108_C4
zmp_ph108_C5
zmp_ph108_D4
zmp_ph97_G6
zmp_ph97_G9
zmp_ph97_H2
zmp_ph97_B10
zmp_ph97_C2
zmp_ph97_C4
zmp_ph97_B8
zmp_ph97_C1
zmp_ph97_D2
zmp_ph97_D5
zmp_ph111_G5
zmp_ph111_G11
zmp_ph111_H1
zmp_ph111_B1a
zmp_ph111_B5
zmp_ph111_B8
zmp_ph111_B6
zmp_ph111_B7
zmp_ph111_B9
zmp_ph111_B10
zmp_ph114_G4
zmp_ph114_G11
zmp_ph114_H1
zmp_ph114_H2
zmp_ph114_A9
zmp_ph114_A11
zmp_ph114_B2
zmp_ph114_B4
zmp_ph114_D8
zmp_ph114_E2

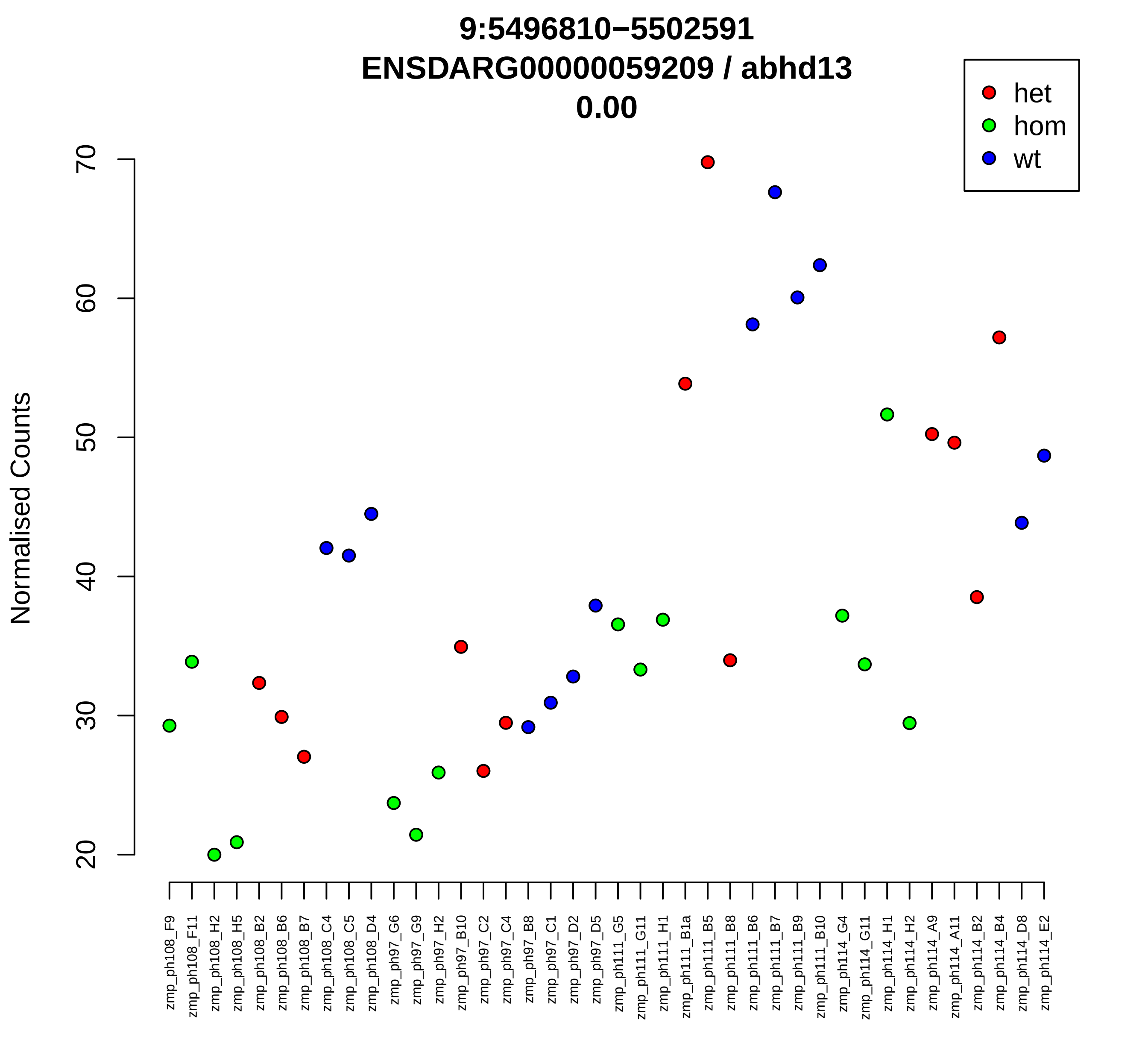
9:5496810−5502591
ENSDARG00000059209 / abhd13
0.00
het
hom
wt
Normalised Counts
20
30
40
50
60
70
zmp_ph108_F9
zmp_ph108_F11
zmp_ph108_H2
zmp_ph108_H5
zmp_ph108_B2
zmp_ph108_B6
zmp_ph108_B7
zmp_ph108_C4
zmp_ph108_C5
zmp_ph108_D4
zmp_ph97_G6
zmp_ph97_G9
zmp_ph97_H2
zmp_ph97_B10
zmp_ph97_C2
zmp_ph97_C4
zmp_ph97_B8
zmp_ph97_C1
zmp_ph97_D2
zmp_ph97_D5
zmp_ph111_G5
zmp_ph111_G11
zmp_ph111_H1
zmp_ph111_B1a
zmp_ph111_B5
zmp_ph111_B8
zmp_ph111_B6
zmp_ph111_B7
zmp_ph111_B9
zmp_ph111_B10
zmp_ph114_G4
zmp_ph114_G11
zmp_ph114_H1
zmp_ph114_H2
zmp_ph114_A9
zmp_ph114_A11
zmp_ph114_B2
zmp_ph114_B4
zmp_ph114_D8
zmp_ph114_E2

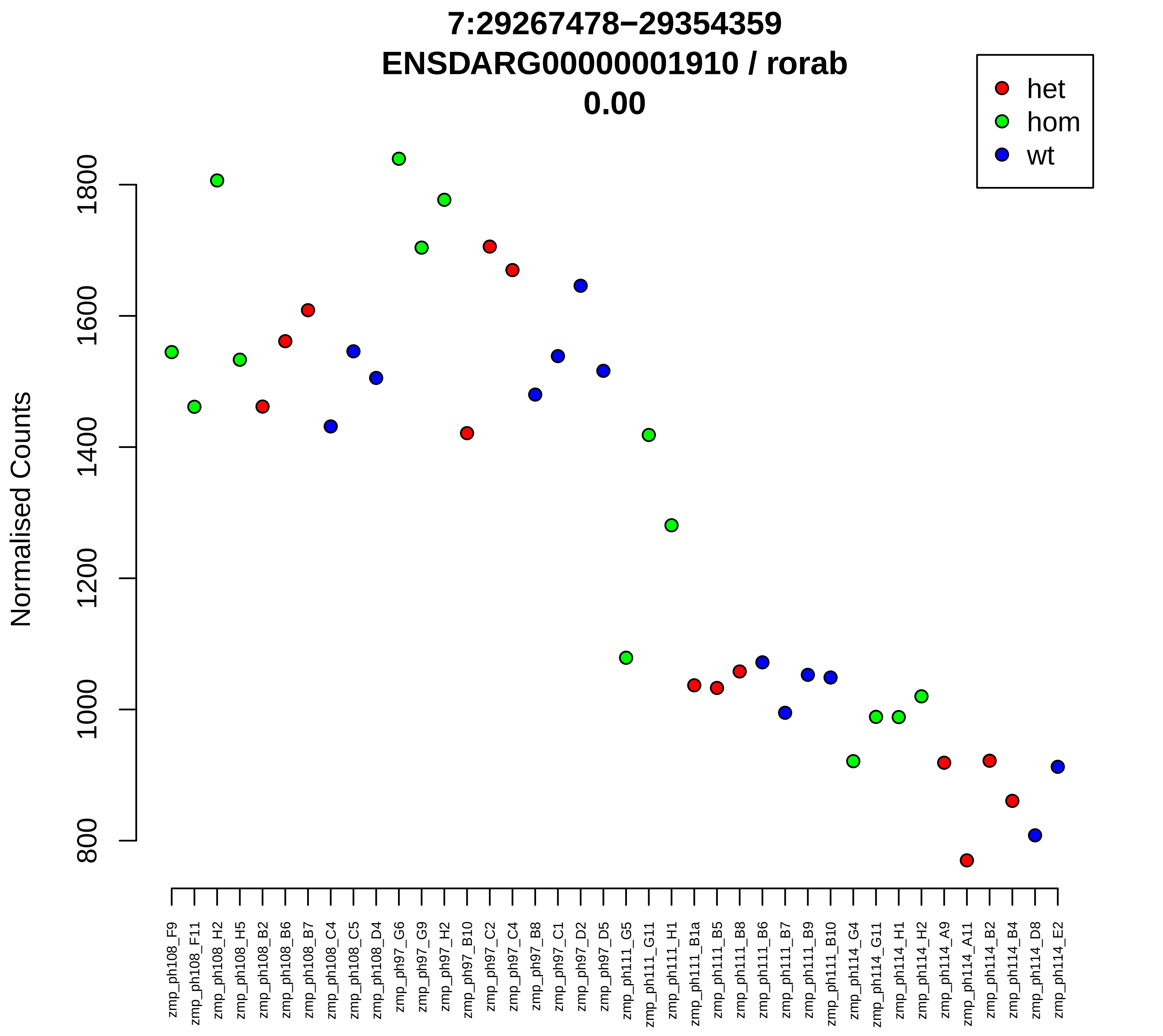
7:29267478−29354359
ENSDARG00000001910 / rorab
0.00
het
hom
wt
Normalised Counts
800
1000
1200
1400
1600
1800
zmp_ph108_F9
zmp_ph108_F11
zmp_ph108_H2
zmp_ph108_H5
zmp_ph108_B2
zmp_ph108_B6
zmp_ph108_B7
zmp_ph108_C4
zmp_ph108_C5
zmp_ph108_D4
zmp_ph97_G6
zmp_ph97_G9
zmp_ph97_H2
zmp_ph97_B10
zmp_ph97_C2
zmp_ph97_C4
zmp_ph97_B8
zmp_ph97_C1
zmp_ph97_D2
zmp_ph97_D5
zmp_ph111_G5
zmp_ph111_G11
zmp_ph111_H1
zmp_ph111_B1a
zmp_ph111_B5
zmp_ph111_B8
zmp_ph111_B6
zmp_ph111_B7
zmp_ph111_B9
zmp_ph111_B10
zmp_ph114_G4
zmp_ph114_G11
zmp_ph114_H1
zmp_ph114_H2
zmp_ph114_A9
zmp_ph114_A11
zmp_ph114_B2
zmp_ph114_B4
zmp_ph114_D8
zmp_ph114_E2

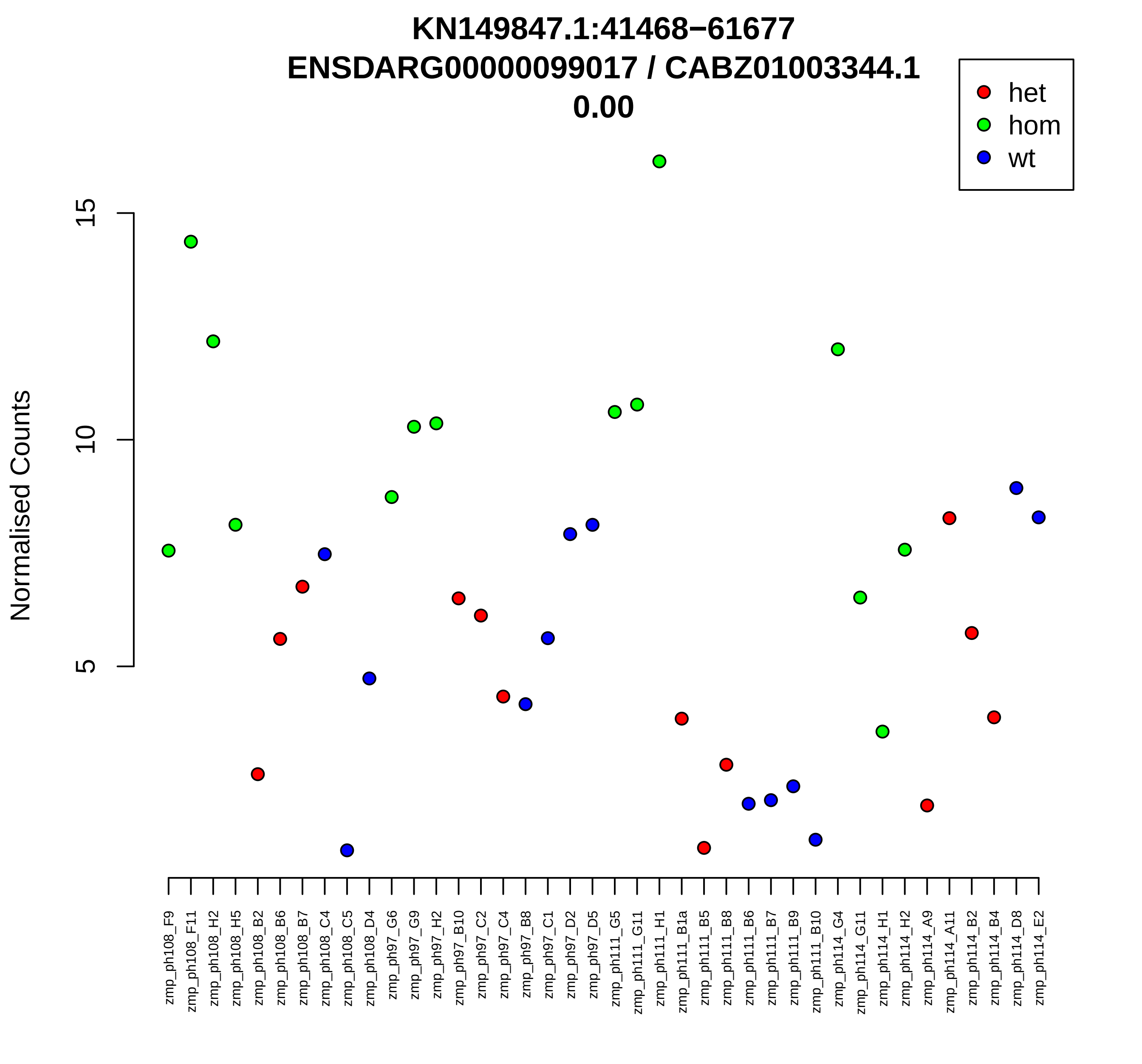
KN149847.1:41468−61677
ENSDARG00000099017 / CABZ01003344.1
0.00
het
hom
wt
Normalised Counts
5
10
15
zmp_ph108_F9
zmp_ph108_F11
zmp_ph108_H2
zmp_ph108_H5
zmp_ph108_B2
zmp_ph108_B6
zmp_ph108_B7
zmp_ph108_C4
zmp_ph108_C5
zmp_ph108_D4
zmp_ph97_G6
zmp_ph97_G9
zmp_ph97_H2
zmp_ph97_B10
zmp_ph97_C2
zmp_ph97_C4
zmp_ph97_B8
zmp_ph97_C1
zmp_ph97_D2
zmp_ph97_D5
zmp_ph111_G5
zmp_ph111_G11
zmp_ph111_H1
zmp_ph111_B1a
zmp_ph111_B5
zmp_ph111_B8
zmp_ph111_B6
zmp_ph111_B7
zmp_ph111_B9
zmp_ph111_B10
zmp_ph114_G4
zmp_ph114_G11
zmp_ph114_H1
zmp_ph114_H2
zmp_ph114_A9
zmp_ph114_A11
zmp_ph114_B2
zmp_ph114_B4
zmp_ph114_D8
zmp_ph114_E2

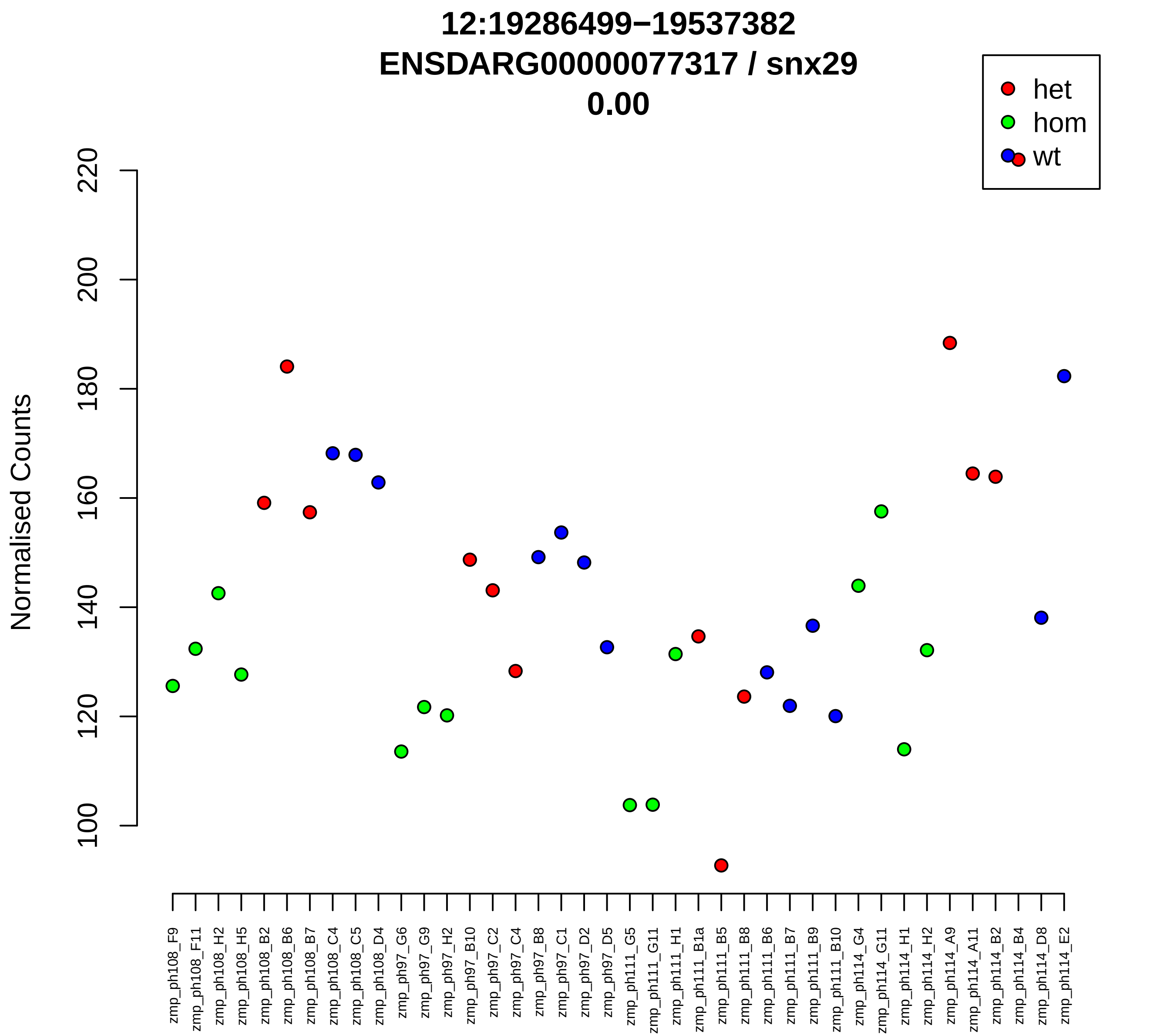
12:19286499−19537382
ENSDARG00000077317 / snx29
0.00
het
hom
wt
Normalised Counts
100
120
140
160
180
200
220
zmp_ph108_F9
zmp_ph108_F11
zmp_ph108_H2
zmp_ph108_H5
zmp_ph108_B2
zmp_ph108_B6
zmp_ph108_B7
zmp_ph108_C4
zmp_ph108_C5
zmp_ph108_D4
zmp_ph97_G6
zmp_ph97_G9
zmp_ph97_H2
zmp_ph97_B10
zmp_ph97_C2
zmp_ph97_C4
zmp_ph97_B8
zmp_ph97_C1
zmp_ph97_D2
zmp_ph97_D5
zmp_ph111_G5
zmp_ph111_G11
zmp_ph111_H1
zmp_ph111_B1a
zmp_ph111_B5
zmp_ph111_B8
zmp_ph111_B6
zmp_ph111_B7
zmp_ph111_B9
zmp_ph111_B10
zmp_ph114_G4
zmp_ph114_G11
zmp_ph114_H1
zmp_ph114_H2
zmp_ph114_A9
zmp_ph114_A11
zmp_ph114_B2
zmp_ph114_B4
zmp_ph114_D8
zmp_ph114_E2

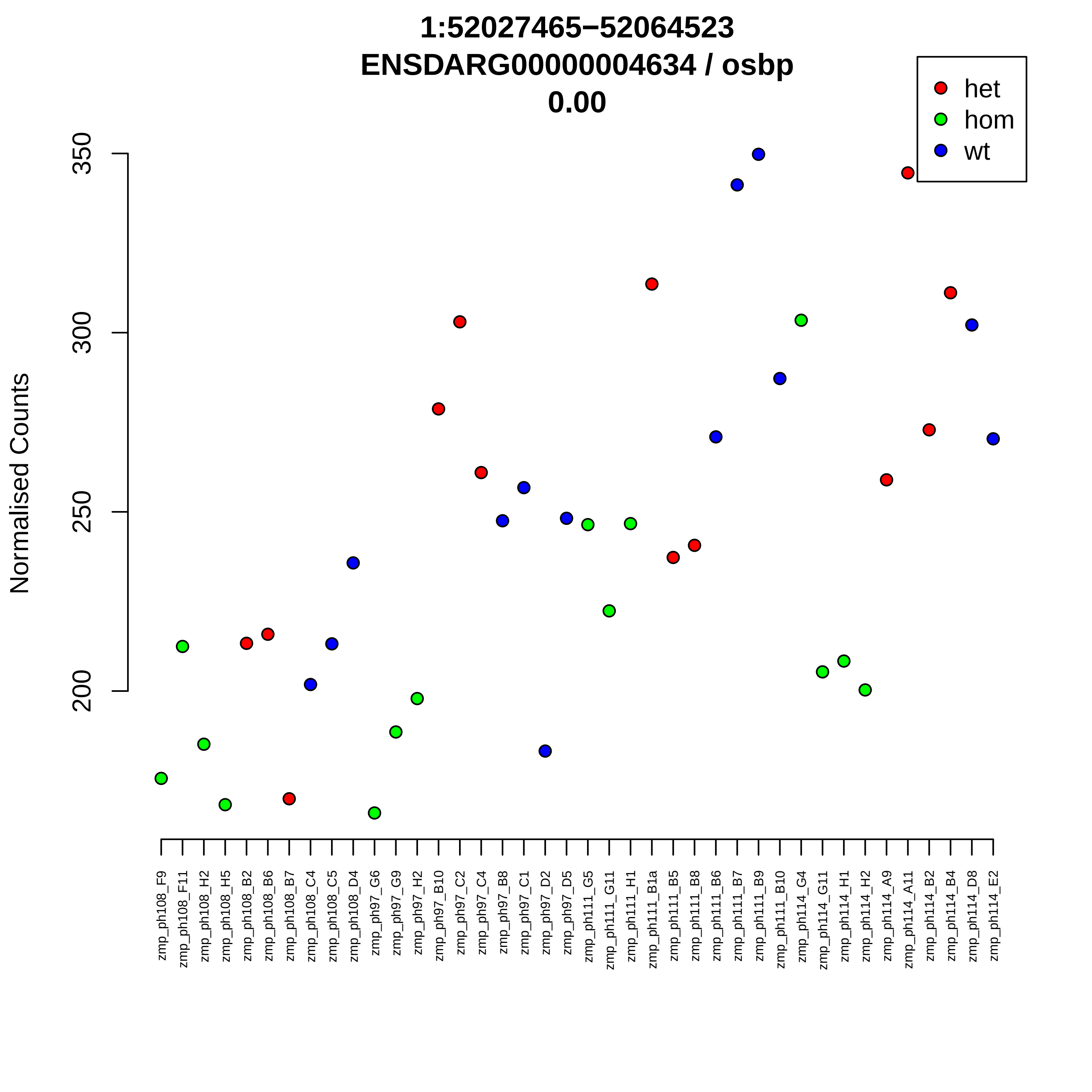

1:52027465−52064523
ENSDARG00000004634 / osbp
0.00
het
hom
wt
Normalised Counts
200
250
300
350
zmp_ph108_F9
zmp_ph108_F11
zmp_ph108_H2
zmp_ph108_H5
zmp_ph108_B2
zmp_ph108_B6
zmp_ph108_B7
zmp_ph108_C4
zmp_ph108_C5
zmp_ph108_D4
zmp_ph97_G6
zmp_ph97_G9
zmp_ph97_H2
zmp_ph97_B10
zmp_ph97_C2
zmp_ph97_C4
zmp_ph97_B8
zmp_ph97_C1
zmp_ph97_D2
zmp_ph97_D5
zmp_ph111_G5
zmp_ph111_G11
zmp_ph111_H1
zmp_ph111_B1a
zmp_ph111_B5
zmp_ph111_B8
zmp_ph111_B6
zmp_ph111_B7
zmp_ph111_B9
zmp_ph111_B10
zmp_ph114_G4
zmp_ph114_G11
zmp_ph114_H1
zmp_ph114_H2
zmp_ph114_A9
zmp_ph114_A11
zmp_ph114_B2
zmp_ph114_B4
zmp_ph114_D8
zmp_ph114_E2

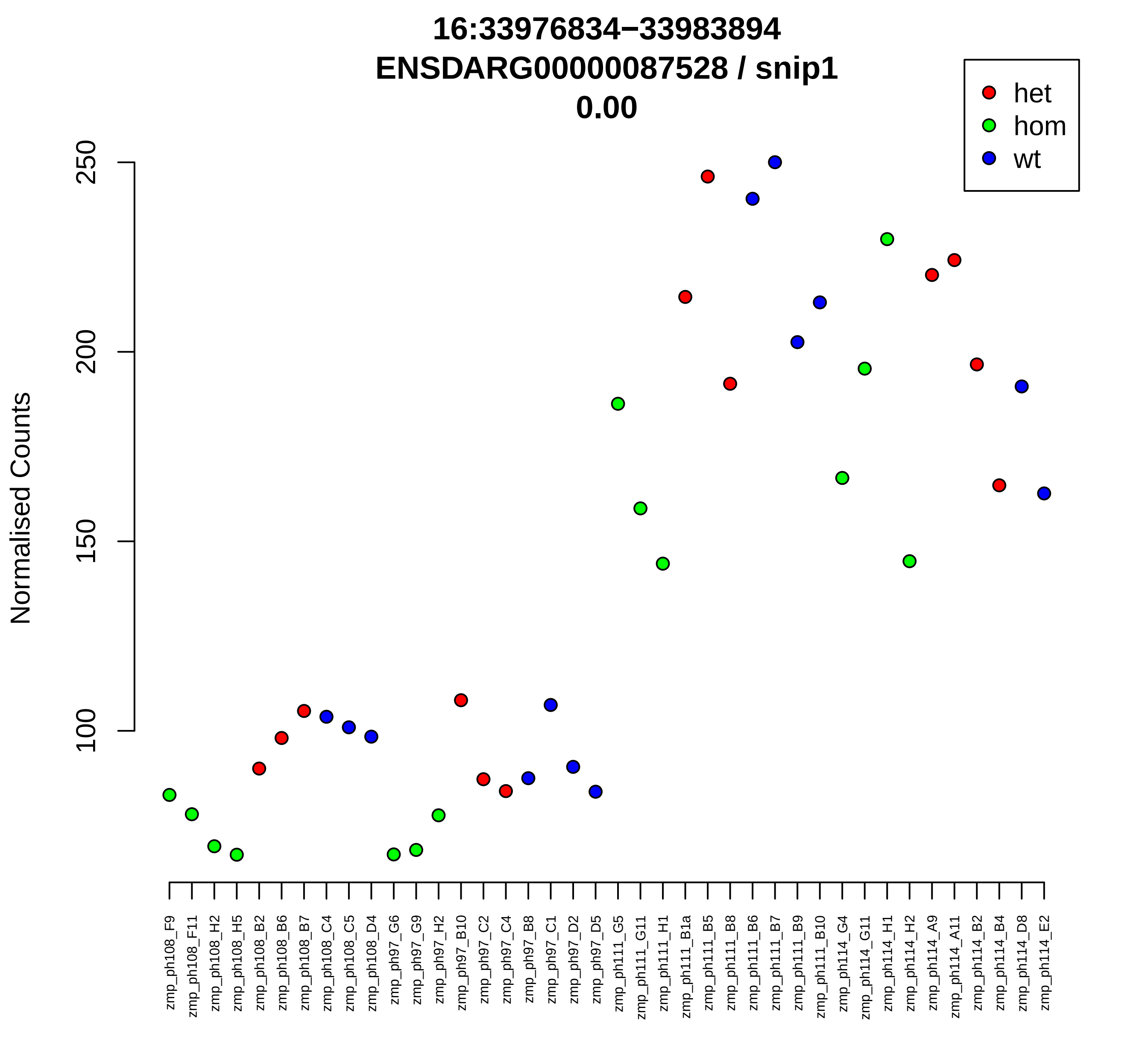

16:33976834−33983894
ENSDARG00000087528 / snip1
0.00
het
hom
wt
Normalised Counts
100
150
200
250
zmp_ph108_F9
zmp_ph108_F11
zmp_ph108_H2
zmp_ph108_H5
zmp_ph108_B2
zmp_ph108_B6
zmp_ph108_B7
zmp_ph108_C4
zmp_ph108_C5
zmp_ph108_D4
zmp_ph97_G6
zmp_ph97_G9
zmp_ph97_H2
zmp_ph97_B10
zmp_ph97_C2
zmp_ph97_C4
zmp_ph97_B8
zmp_ph97_C1
zmp_ph97_D2
zmp_ph97_D5
zmp_ph111_G5
zmp_ph111_G11
zmp_ph111_H1
zmp_ph111_B1a
zmp_ph111_B5
zmp_ph111_B8
zmp_ph111_B6
zmp_ph111_B7
zmp_ph111_B9
zmp_ph111_B10
zmp_ph114_G4
zmp_ph114_G11
zmp_ph114_H1
zmp_ph114_H2
zmp_ph114_A9
zmp_ph114_A11
zmp_ph114_B2
zmp_ph114_B4
zmp_ph114_D8
zmp_ph114_E2

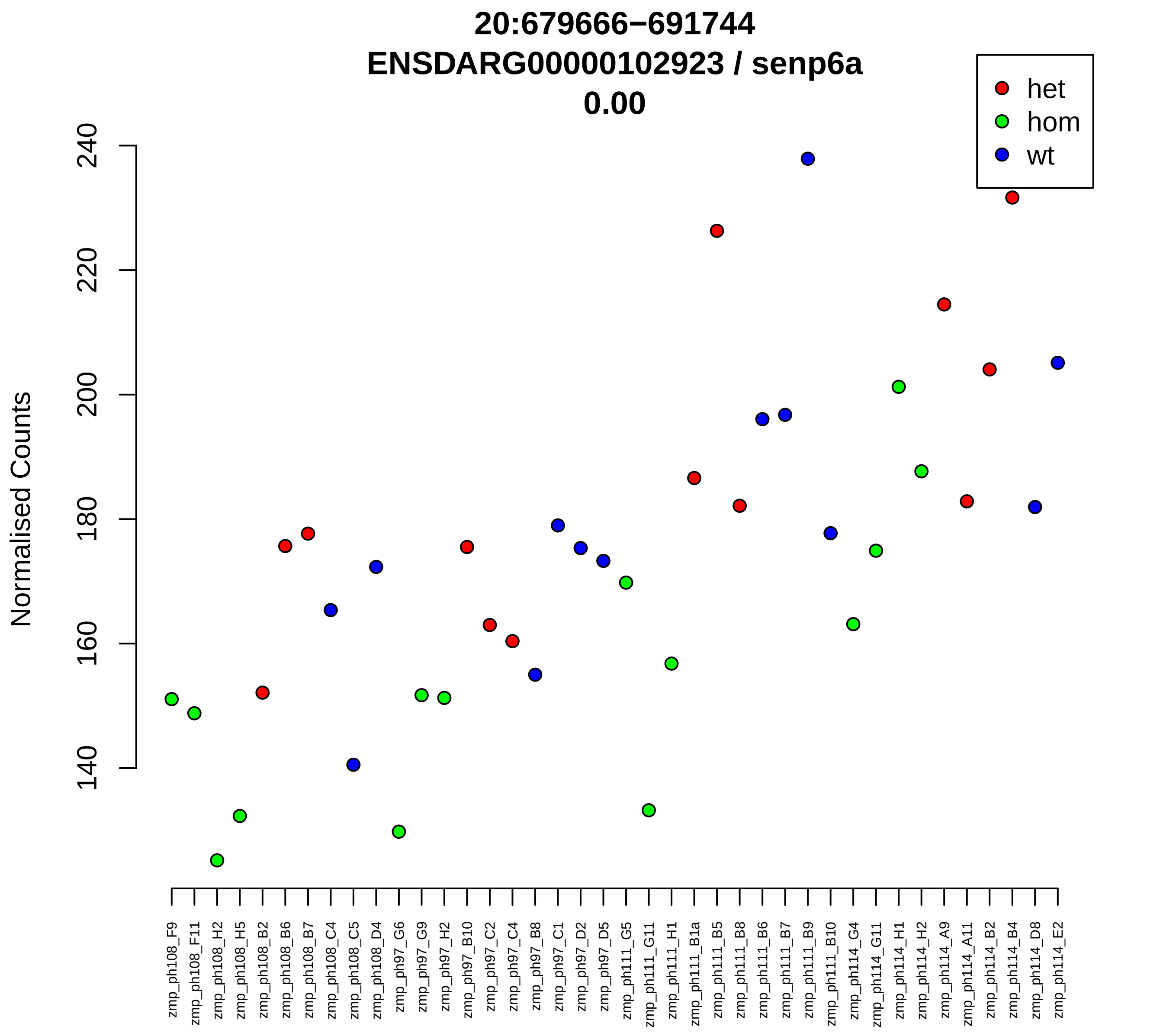

20:679666−691744
ENSDARG00000102923 / senp6a
0.00
het
hom
wt
Normalised Counts
140
160
180
200
220
240
zmp_ph108_F9
zmp_ph108_F11
zmp_ph108_H2
zmp_ph108_H5
zmp_ph108_B2
zmp_ph108_B6
zmp_ph108_B7
zmp_ph108_C4
zmp_ph108_C5
zmp_ph108_D4
zmp_ph97_G6
zmp_ph97_G9
zmp_ph97_H2
zmp_ph97_B10
zmp_ph97_C2
zmp_ph97_C4
zmp_ph97_B8
zmp_ph97_C1
zmp_ph97_D2
zmp_ph97_D5
zmp_ph111_G5
zmp_ph111_G11
zmp_ph111_H1
zmp_ph111_B1a
zmp_ph111_B5
zmp_ph111_B8
zmp_ph111_B6
zmp_ph111_B7
zmp_ph111_B9
zmp_ph111_B10
zmp_ph114_G4
zmp_ph114_G11
zmp_ph114_H1
zmp_ph114_H2
zmp_ph114_A9
zmp_ph114_A11
zmp_ph114_B2
zmp_ph114_B4
zmp_ph114_D8
zmp_ph114_E2

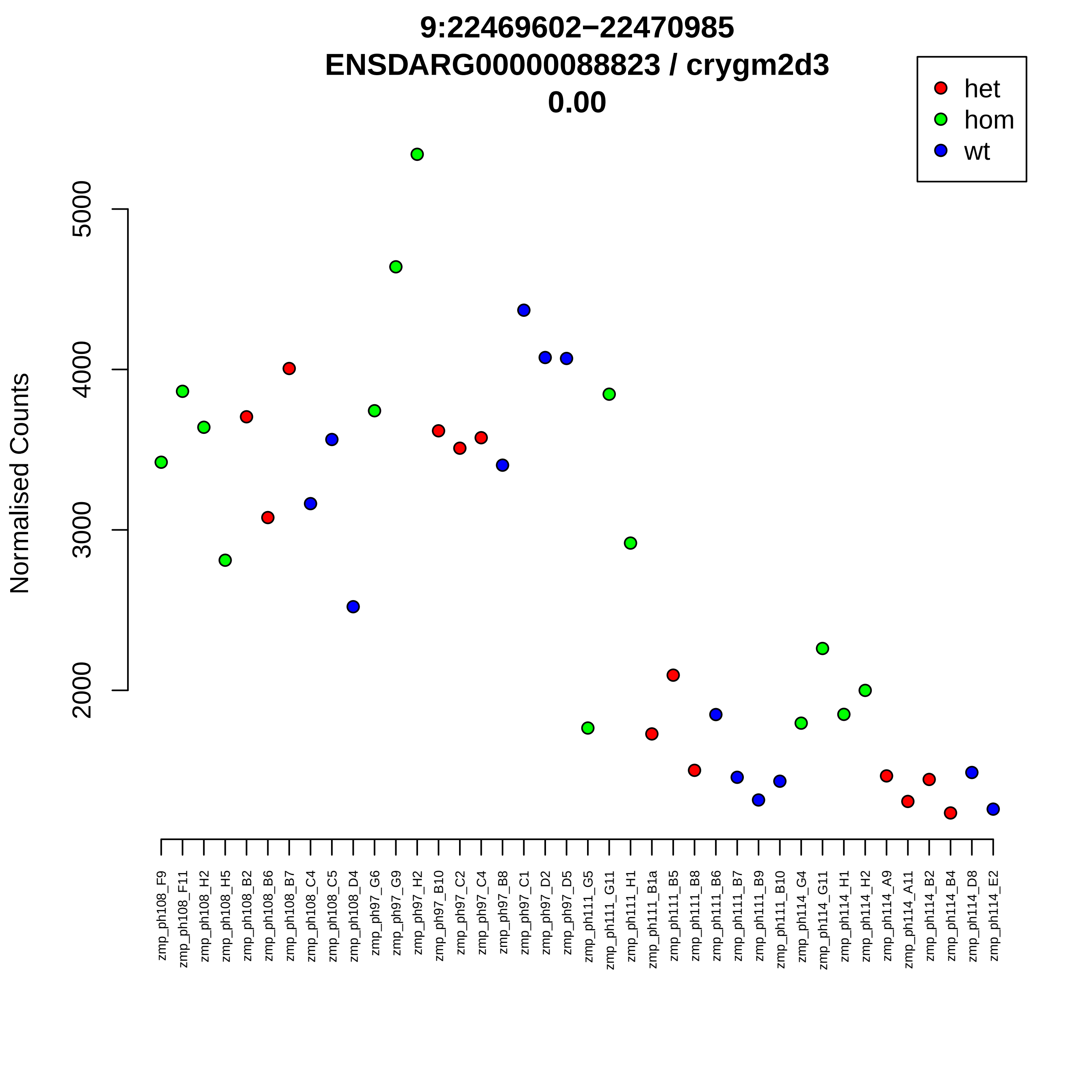

9:22469602−22470985
ENSDARG00000088823 / crygm2d3
0.00
het
hom
wt
Normalised Counts
2000
3000
4000
5000
zmp_ph108_F9
zmp_ph108_F11
zmp_ph108_H2
zmp_ph108_H5
zmp_ph108_B2
zmp_ph108_B6
zmp_ph108_B7
zmp_ph108_C4
zmp_ph108_C5
zmp_ph108_D4
zmp_ph97_G6
zmp_ph97_G9
zmp_ph97_H2
zmp_ph97_B10
zmp_ph97_C2
zmp_ph97_C4
zmp_ph97_B8
zmp_ph97_C1
zmp_ph97_D2
zmp_ph97_D5
zmp_ph111_G5
zmp_ph111_G11
zmp_ph111_H1
zmp_ph111_B1a
zmp_ph111_B5
zmp_ph111_B8
zmp_ph111_B6
zmp_ph111_B7
zmp_ph111_B9
zmp_ph111_B10
zmp_ph114_G4
zmp_ph114_G11
zmp_ph114_H1
zmp_ph114_H2
zmp_ph114_A9
zmp_ph114_A11
zmp_ph114_B2
zmp_ph114_B4
zmp_ph114_D8
zmp_ph114_E2

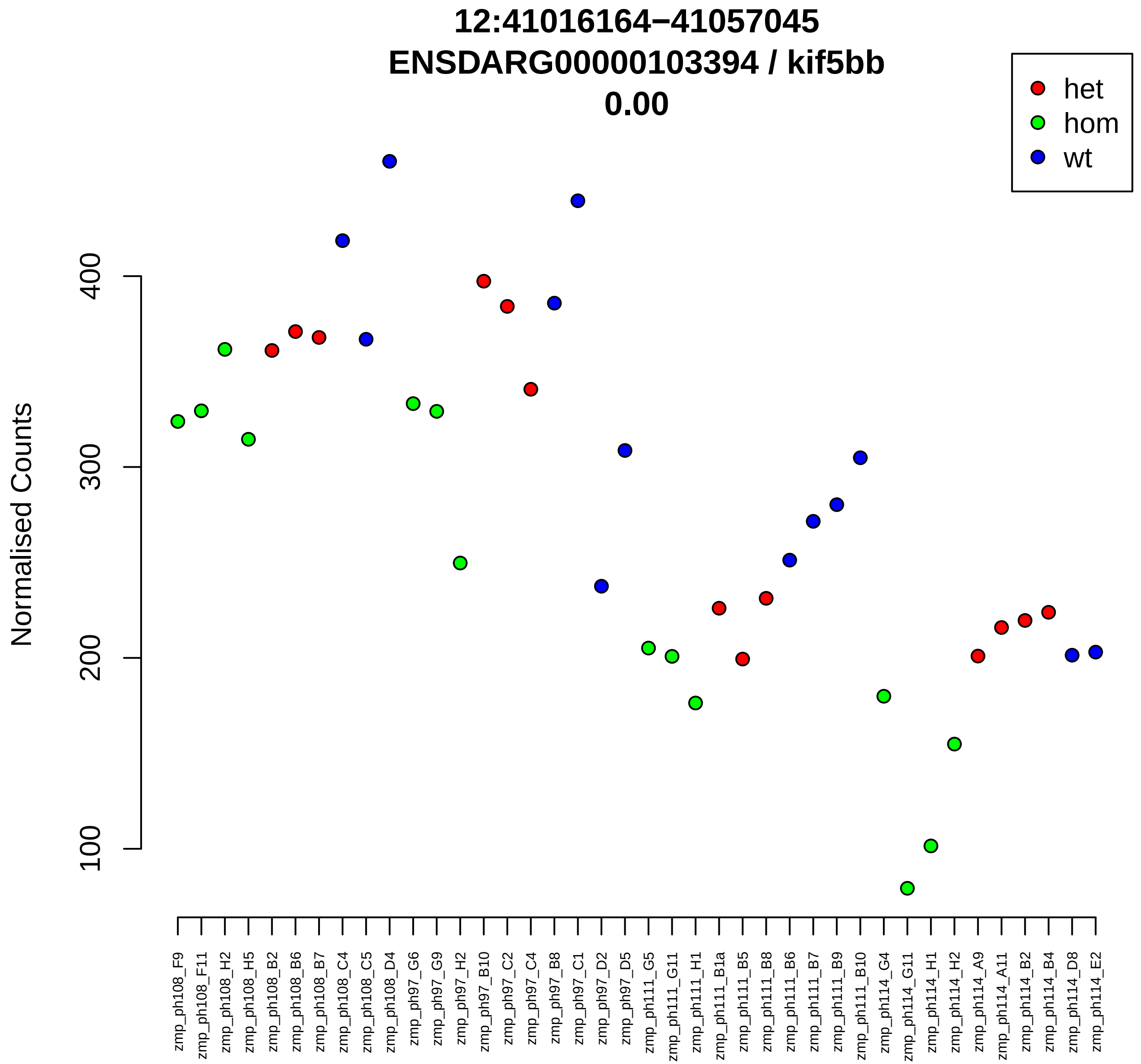
12:41016164−41057045
ENSDARG00000103394 / kif5bb
0.00
het
hom
wt
Normalised Counts
100
200
300
400
zmp_ph108_F9
zmp_ph108_F11
zmp_ph108_H2
zmp_ph108_H5
zmp_ph108_B2
zmp_ph108_B6
zmp_ph108_B7
zmp_ph108_C4
zmp_ph108_C5
zmp_ph108_D4
zmp_ph97_G6
zmp_ph97_G9
zmp_ph97_H2
zmp_ph97_B10
zmp_ph97_C2
zmp_ph97_C4
zmp_ph97_B8
zmp_ph97_C1
zmp_ph97_D2
zmp_ph97_D5
zmp_ph111_G5
zmp_ph111_G11
zmp_ph111_H1
zmp_ph111_B1a
zmp_ph111_B5
zmp_ph111_B8
zmp_ph111_B6
zmp_ph111_B7
zmp_ph111_B9
zmp_ph111_B10
zmp_ph114_G4
zmp_ph114_G11
zmp_ph114_H1
zmp_ph114_H2
zmp_ph114_A9
zmp_ph114_A11
zmp_ph114_B2
zmp_ph114_B4
zmp_ph114_D8
zmp_ph114_E2

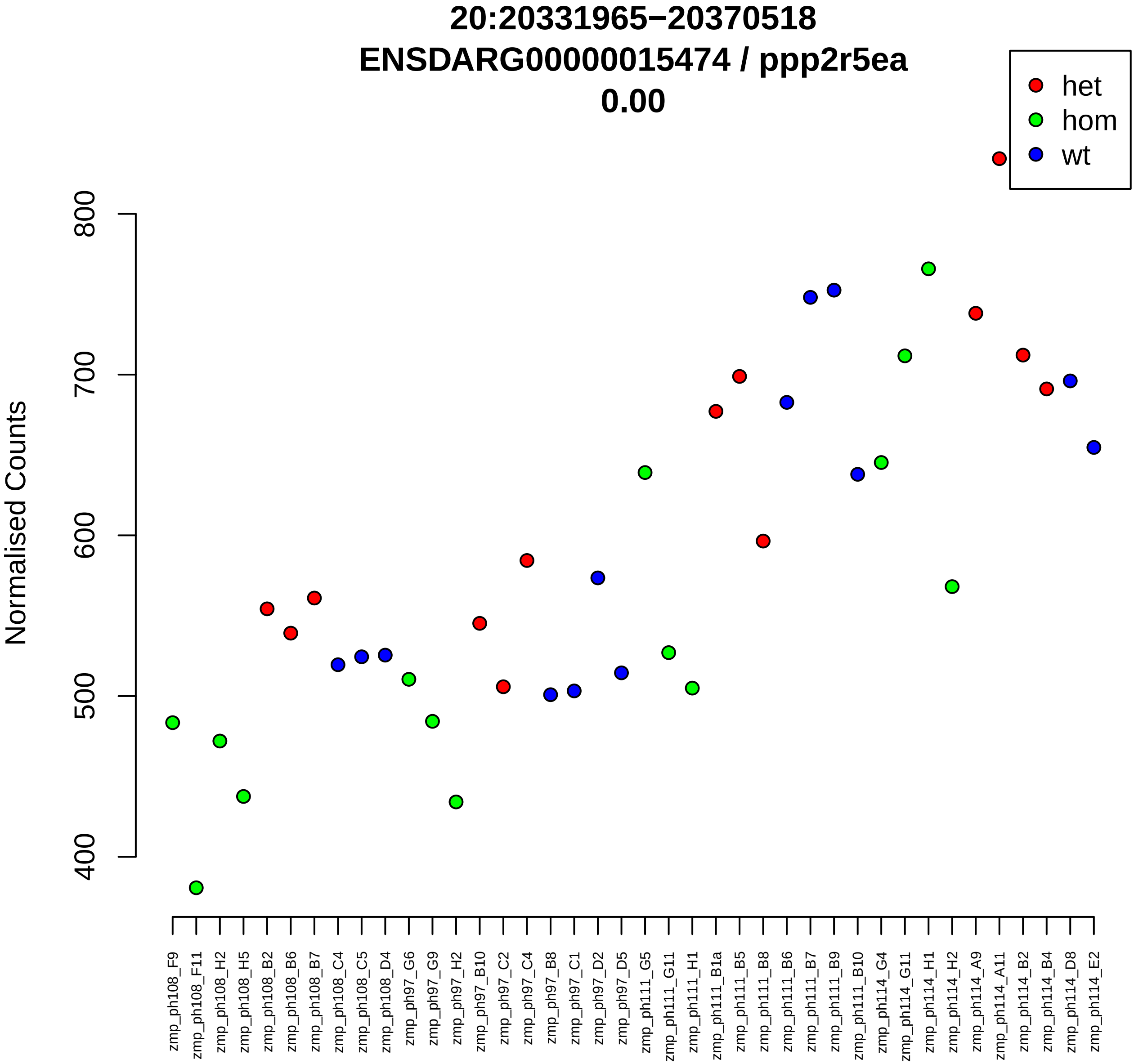
20:20331965−20370518
ENSDARG00000015474 / ppp2r5ea
0.00
het
hom
wt
Normalised Counts
400
500
600
700
800
zmp_ph108_F9
zmp_ph108_F11
zmp_ph108_H2
zmp_ph108_H5
zmp_ph108_B2
zmp_ph108_B6
zmp_ph108_B7
zmp_ph108_C4
zmp_ph108_C5
zmp_ph108_D4
zmp_ph97_G6
zmp_ph97_G9
zmp_ph97_H2
zmp_ph97_B10
zmp_ph97_C2
zmp_ph97_C4
zmp_ph97_B8
zmp_ph97_C1
zmp_ph97_D2
zmp_ph97_D5
zmp_ph111_G5
zmp_ph111_G11
zmp_ph111_H1
zmp_ph111_B1a
zmp_ph111_B5
zmp_ph111_B8
zmp_ph111_B6
zmp_ph111_B7
zmp_ph111_B9
zmp_ph111_B10
zmp_ph114_G4
zmp_ph114_G11
zmp_ph114_H1
zmp_ph114_H2
zmp_ph114_A9
zmp_ph114_A11
zmp_ph114_B2
zmp_ph114_B4
zmp_ph114_D8
zmp_ph114_E2

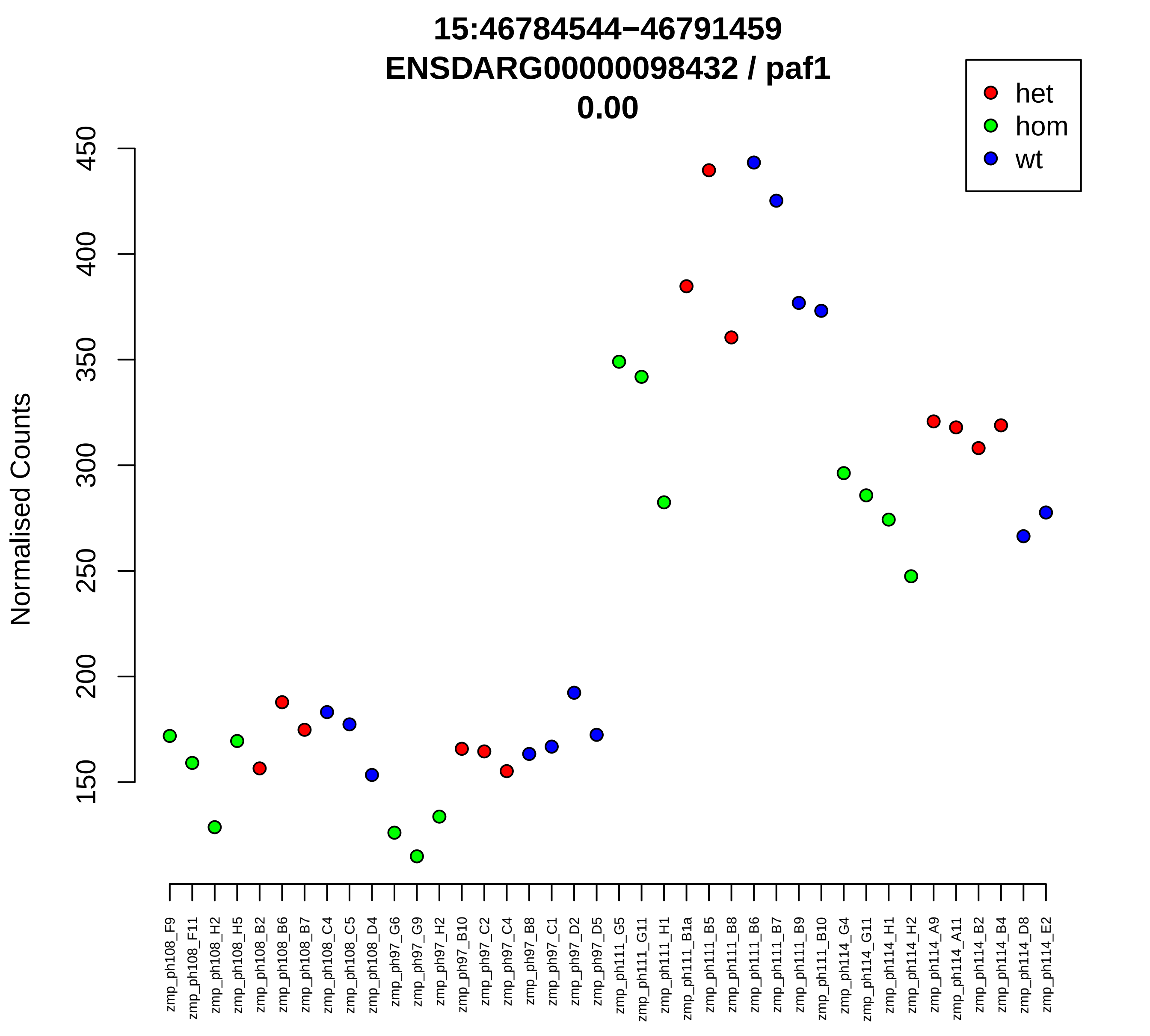

15:46784544−46791459
ENSDARG00000098432 / paf1
0.00
het
hom
wt
Normalised Counts
150
200
250
300
350
400
450
zmp_ph108_F9
zmp_ph108_F11
zmp_ph108_H2
zmp_ph108_H5
zmp_ph108_B2
zmp_ph108_B6
zmp_ph108_B7
zmp_ph108_C4
zmp_ph108_C5
zmp_ph108_D4
zmp_ph97_G6
zmp_ph97_G9
zmp_ph97_H2
zmp_ph97_B10
zmp_ph97_C2
zmp_ph97_C4
zmp_ph97_B8
zmp_ph97_C1
zmp_ph97_D2
zmp_ph97_D5
zmp_ph111_G5
zmp_ph111_G11
zmp_ph111_H1
zmp_ph111_B1a
zmp_ph111_B5
zmp_ph111_B8
zmp_ph111_B6
zmp_ph111_B7
zmp_ph111_B9
zmp_ph111_B10
zmp_ph114_G4
zmp_ph114_G11
zmp_ph114_H1
zmp_ph114_H2
zmp_ph114_A9
zmp_ph114_A11
zmp_ph114_B2
zmp_ph114_B4
zmp_ph114_D8
zmp_ph114_E2

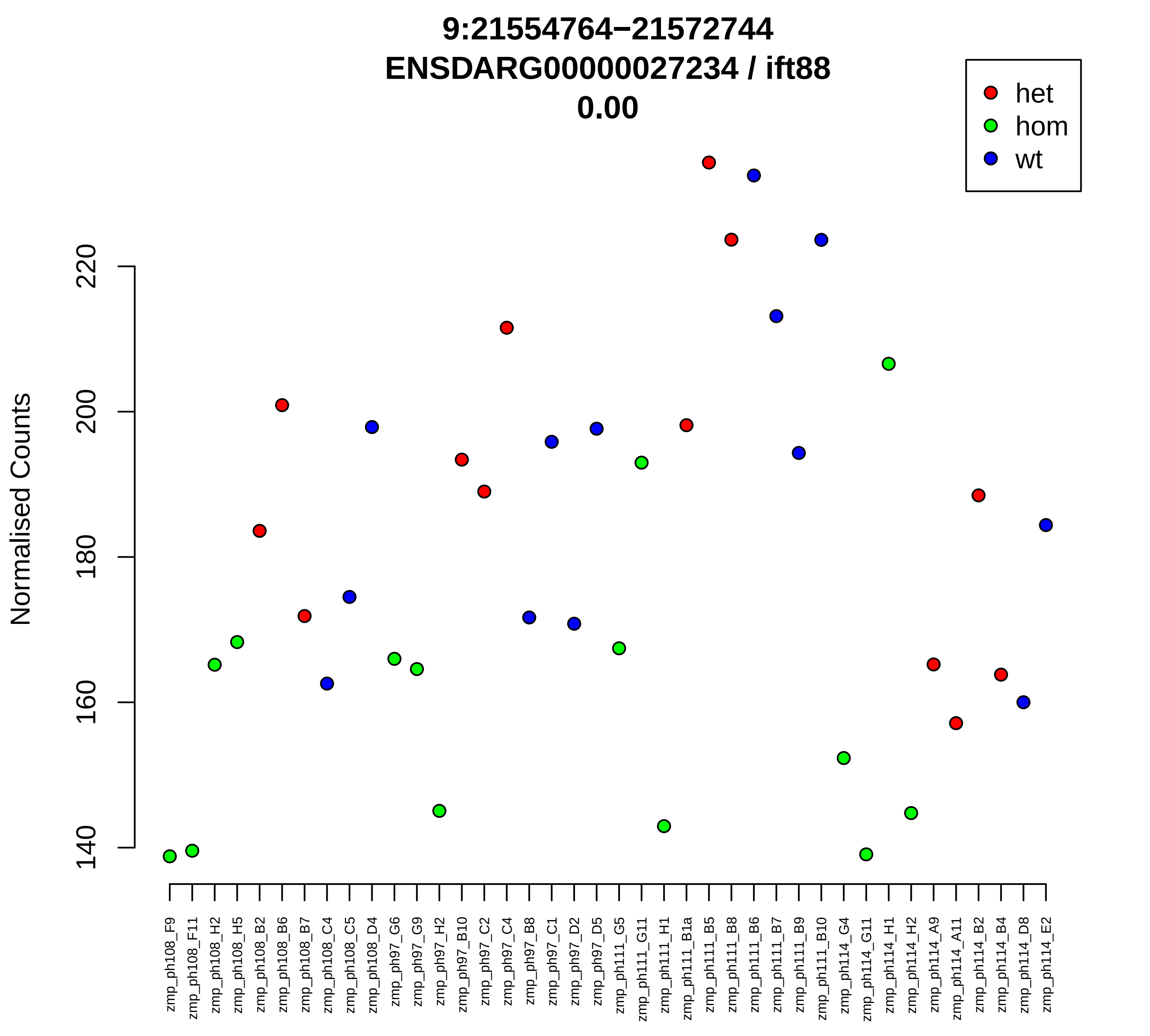
9:21554764−21572744
ENSDARG00000027234 / ift88
0.00
het
hom
wt
Normalised Counts
140
160
180
200
220
zmp_ph108_F9
zmp_ph108_F11
zmp_ph108_H2
zmp_ph108_H5
zmp_ph108_B2
zmp_ph108_B6
zmp_ph108_B7
zmp_ph108_C4
zmp_ph108_C5
zmp_ph108_D4
zmp_ph97_G6
zmp_ph97_G9
zmp_ph97_H2
zmp_ph97_B10
zmp_ph97_C2
zmp_ph97_C4
zmp_ph97_B8
zmp_ph97_C1
zmp_ph97_D2
zmp_ph97_D5
zmp_ph111_G5
zmp_ph111_G11
zmp_ph111_H1
zmp_ph111_B1a
zmp_ph111_B5
zmp_ph111_B8
zmp_ph111_B6
zmp_ph111_B7
zmp_ph111_B9
zmp_ph111_B10
zmp_ph114_G4
zmp_ph114_G11
zmp_ph114_H1
zmp_ph114_H2
zmp_ph114_A9
zmp_ph114_A11
zmp_ph114_B2
zmp_ph114_B4
zmp_ph114_D8
zmp_ph114_E2

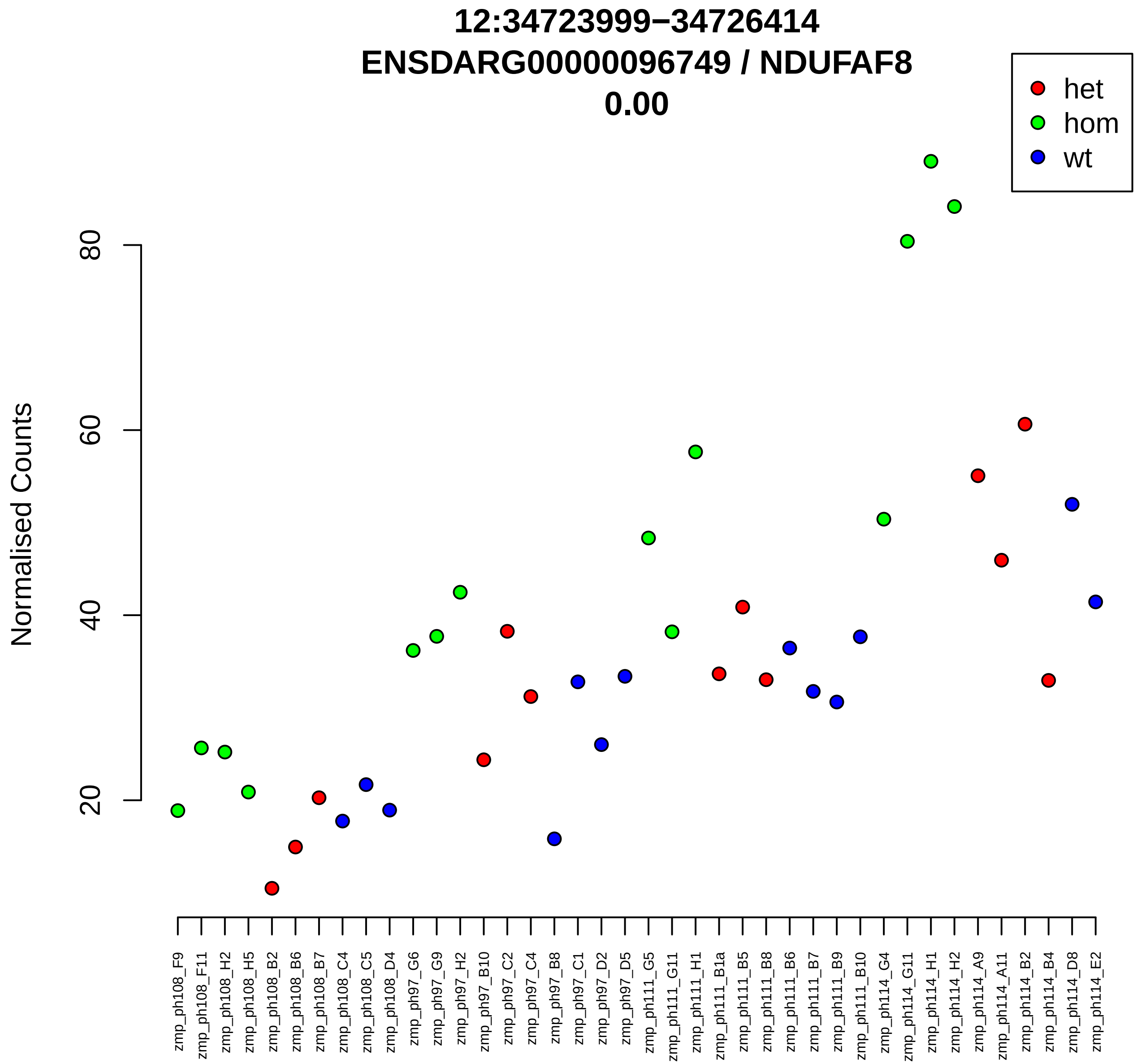
12:34723999−34726414
ENSDARG00000096749 / NDUFAF8
0.00
het
hom
wt
Normalised Counts
20
40
60
80
zmp_ph108_F9
zmp_ph108_F11
zmp_ph108_H2
zmp_ph108_H5
zmp_ph108_B2
zmp_ph108_B6
zmp_ph108_B7
zmp_ph108_C4
zmp_ph108_C5
zmp_ph108_D4
zmp_ph97_G6
zmp_ph97_G9
zmp_ph97_H2
zmp_ph97_B10
zmp_ph97_C2
zmp_ph97_C4
zmp_ph97_B8
zmp_ph97_C1
zmp_ph97_D2
zmp_ph97_D5
zmp_ph111_G5
zmp_ph111_G11
zmp_ph111_H1
zmp_ph111_B1a
zmp_ph111_B5
zmp_ph111_B8
zmp_ph111_B6
zmp_ph111_B7
zmp_ph111_B9
zmp_ph111_B10
zmp_ph114_G4
zmp_ph114_G11
zmp_ph114_H1
zmp_ph114_H2
zmp_ph114_A9
zmp_ph114_A11
zmp_ph114_B2
zmp_ph114_B4
zmp_ph114_D8
zmp_ph114_E2

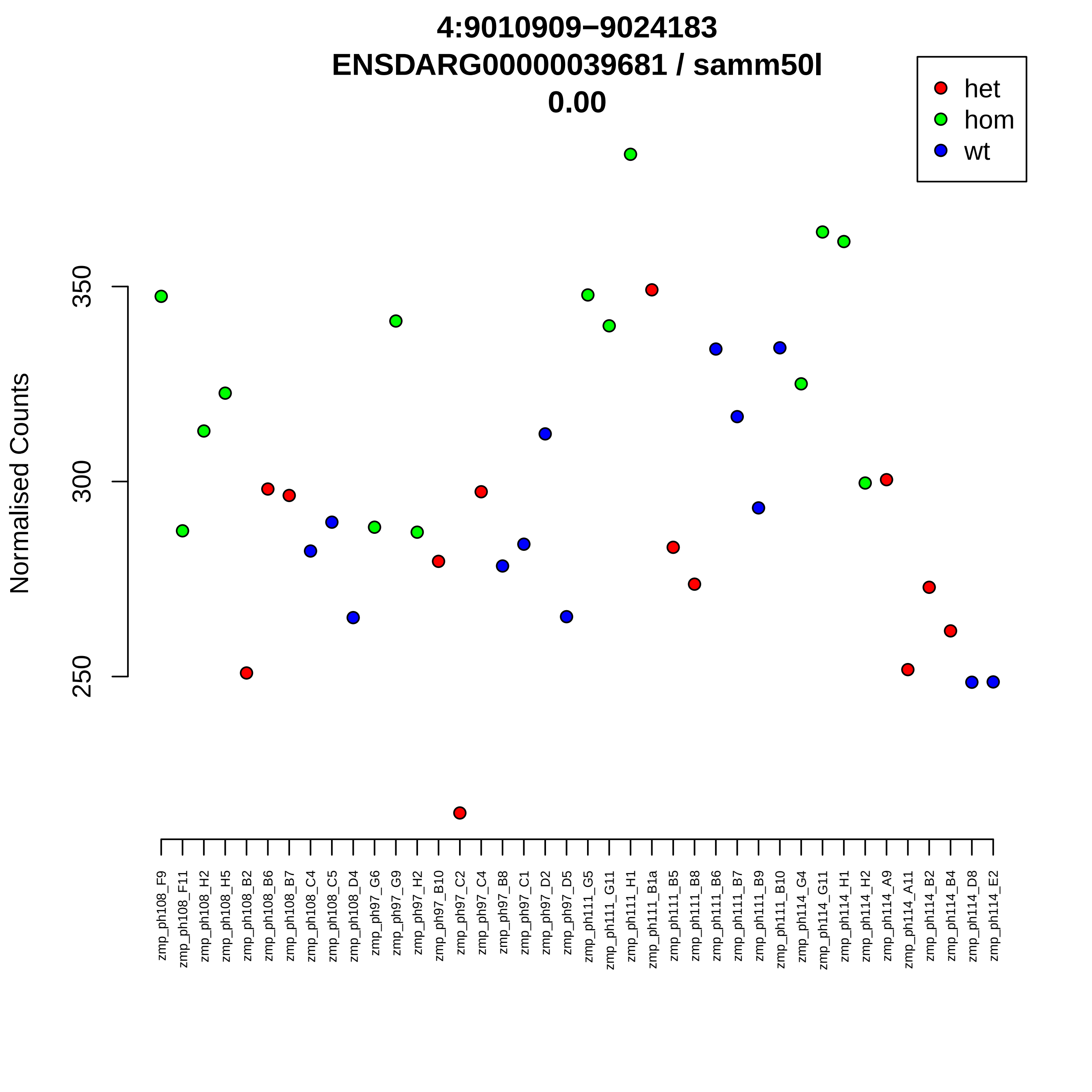
4:9010909−9024183
ENSDARG00000039681 / samm50l
0.00
het
hom
wt
Normalised Counts
250
300
350
zmp_ph108_F9
zmp_ph108_F11
zmp_ph108_H2
zmp_ph108_H5
zmp_ph108_B2
zmp_ph108_B6
zmp_ph108_B7
zmp_ph108_C4
zmp_ph108_C5
zmp_ph108_D4
zmp_ph97_G6
zmp_ph97_G9
zmp_ph97_H2
zmp_ph97_B10
zmp_ph97_C2
zmp_ph97_C4
zmp_ph97_B8
zmp_ph97_C1
zmp_ph97_D2
zmp_ph97_D5
zmp_ph111_G5
zmp_ph111_G11
zmp_ph111_H1
zmp_ph111_B1a
zmp_ph111_B5
zmp_ph111_B8
zmp_ph111_B6
zmp_ph111_B7
zmp_ph111_B9
zmp_ph111_B10
zmp_ph114_G4
zmp_ph114_G11
zmp_ph114_H1
zmp_ph114_H2
zmp_ph114_A9
zmp_ph114_A11
zmp_ph114_B2
zmp_ph114_B4
zmp_ph114_D8
zmp_ph114_E2

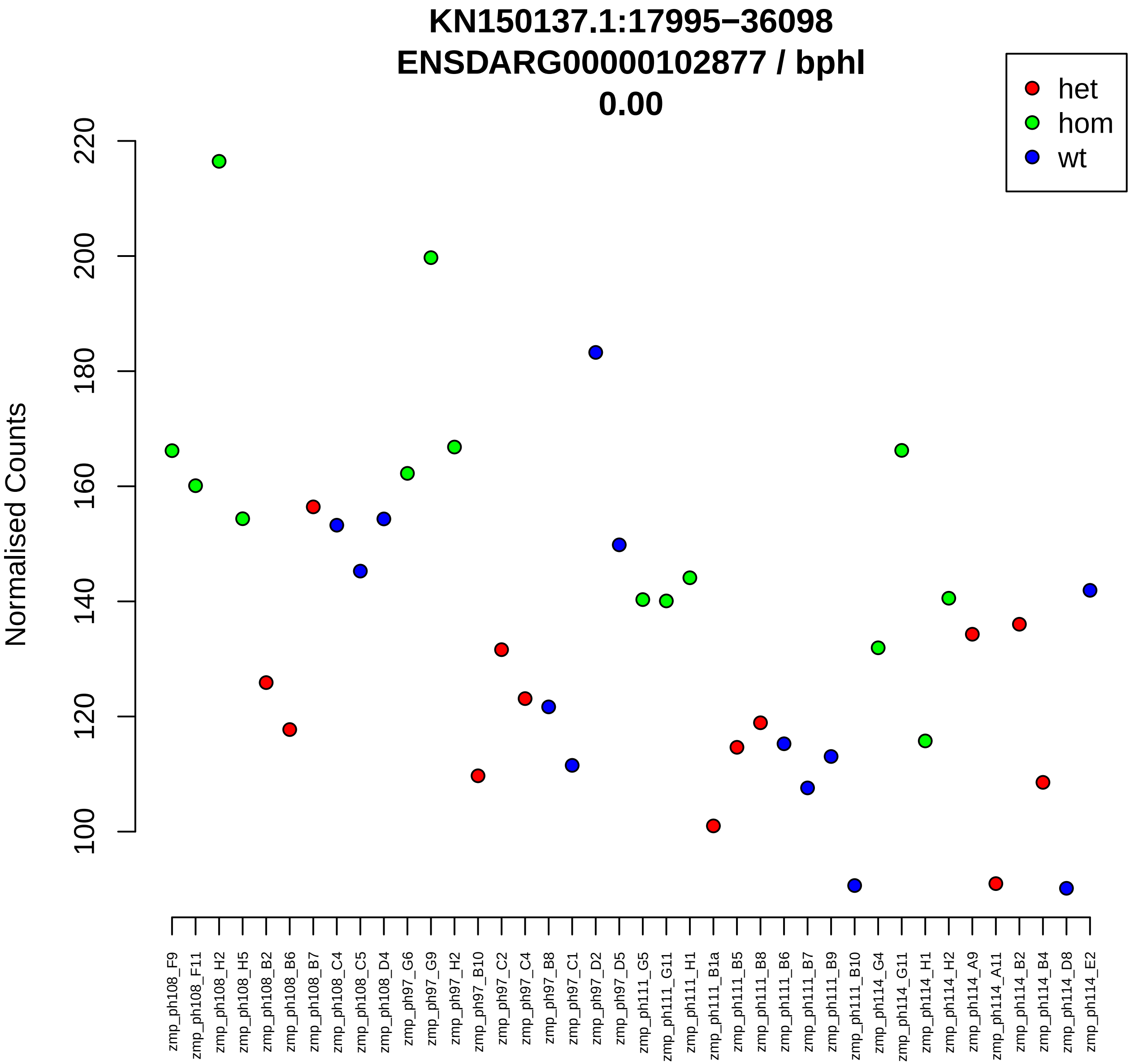

KN150137.1:17995−36098
ENSDARG00000102877 / bphl
0.00
het
hom
wt
Normalised Counts
100
120
140
160
180
200
220
zmp_ph108_F9
zmp_ph108_F11
zmp_ph108_H2
zmp_ph108_H5
zmp_ph108_B2
zmp_ph108_B6
zmp_ph108_B7
zmp_ph108_C4
zmp_ph108_C5
zmp_ph108_D4
zmp_ph97_G6
zmp_ph97_G9
zmp_ph97_H2
zmp_ph97_B10
zmp_ph97_C2
zmp_ph97_C4
zmp_ph97_B8
zmp_ph97_C1
zmp_ph97_D2
zmp_ph97_D5
zmp_ph111_G5
zmp_ph111_G11
zmp_ph111_H1
zmp_ph111_B1a
zmp_ph111_B5
zmp_ph111_B8
zmp_ph111_B6
zmp_ph111_B7
zmp_ph111_B9
zmp_ph111_B10
zmp_ph114_G4
zmp_ph114_G11
zmp_ph114_H1
zmp_ph114_H2
zmp_ph114_A9
zmp_ph114_A11
zmp_ph114_B2
zmp_ph114_B4
zmp_ph114_D8
zmp_ph114_E2

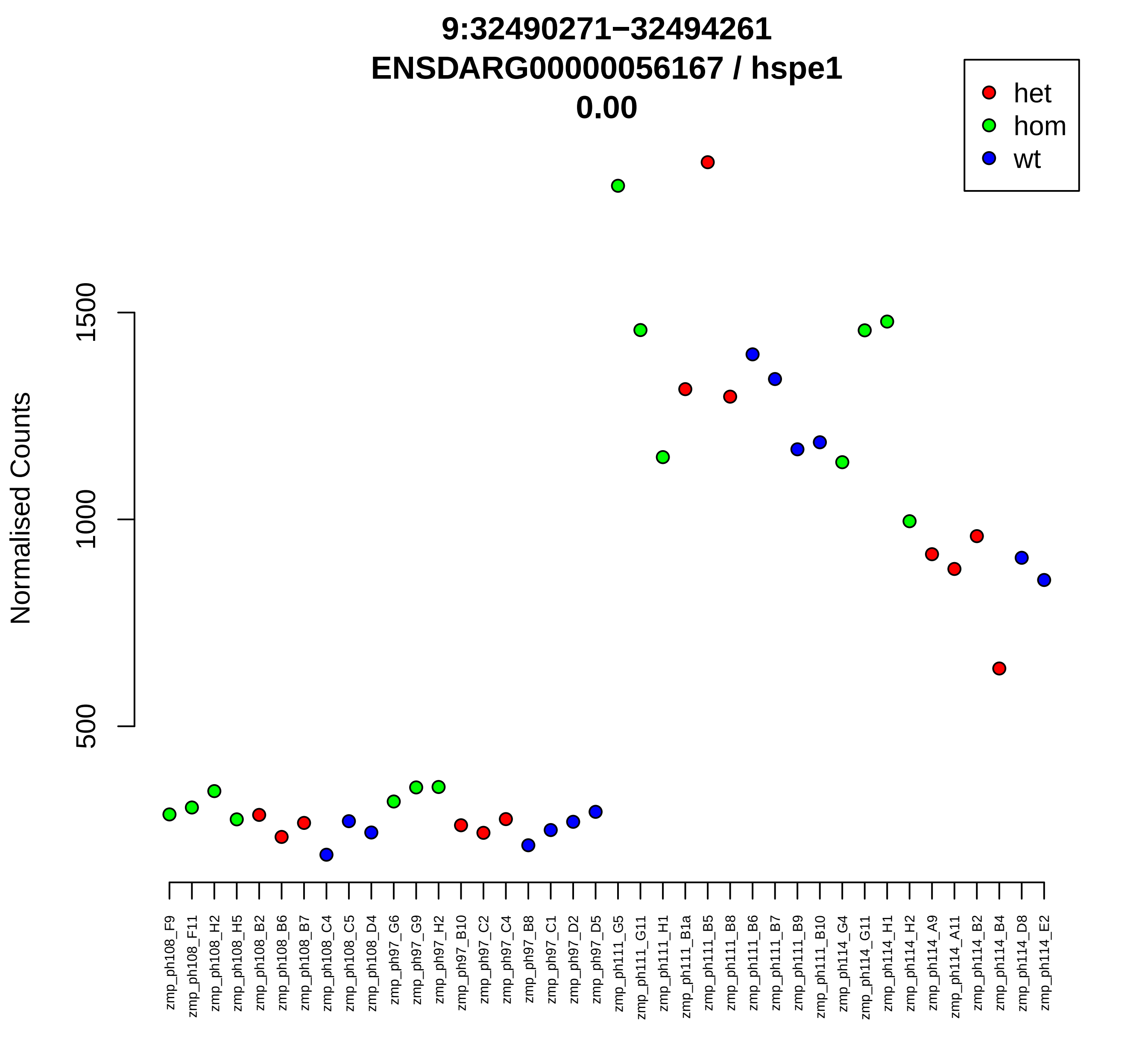

9:32490271−32494261
ENSDARG00000056167 / hspe1
0.00
het
hom
wt
Normalised Counts
500
1000
1500
zmp_ph108_F9
zmp_ph108_F11
zmp_ph108_H2
zmp_ph108_H5
zmp_ph108_B2
zmp_ph108_B6
zmp_ph108_B7
zmp_ph108_C4
zmp_ph108_C5
zmp_ph108_D4
zmp_ph97_G6
zmp_ph97_G9
zmp_ph97_H2
zmp_ph97_B10
zmp_ph97_C2
zmp_ph97_C4
zmp_ph97_B8
zmp_ph97_C1
zmp_ph97_D2
zmp_ph97_D5
zmp_ph111_G5
zmp_ph111_G11
zmp_ph111_H1
zmp_ph111_B1a
zmp_ph111_B5
zmp_ph111_B8
zmp_ph111_B6
zmp_ph111_B7
zmp_ph111_B9
zmp_ph111_B10
zmp_ph114_G4
zmp_ph114_G11
zmp_ph114_H1
zmp_ph114_H2
zmp_ph114_A9
zmp_ph114_A11
zmp_ph114_B2
zmp_ph114_B4
zmp_ph114_D8
zmp_ph114_E2

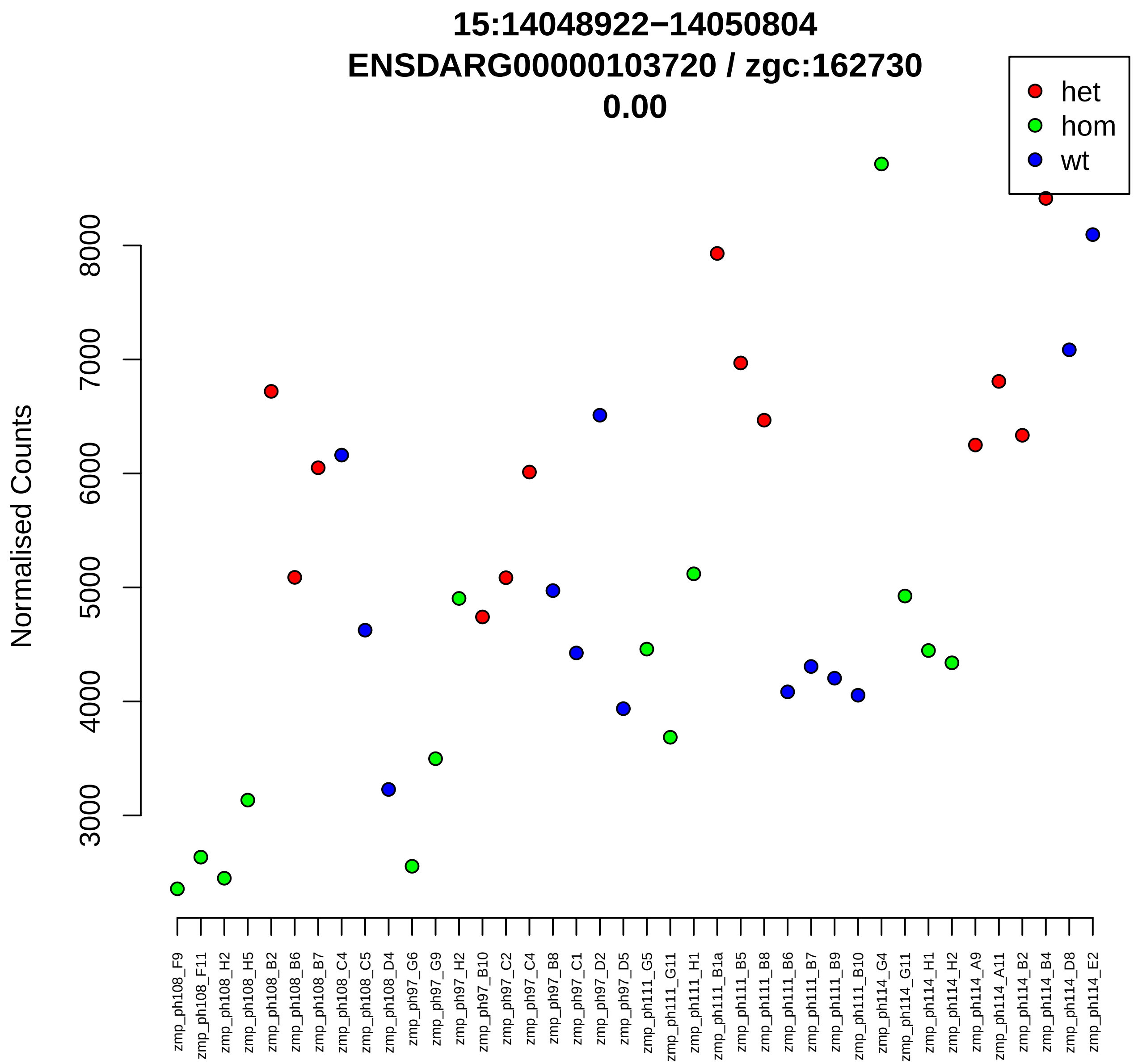
15:14048922−14050804
ENSDARG00000103720 / zgc:162730
0.00
het
hom
wt
Normalised Counts
3000
4000
5000
6000
7000
8000
zmp_ph108_F9
zmp_ph108_F11
zmp_ph108_H2
zmp_ph108_H5
zmp_ph108_B2
zmp_ph108_B6
zmp_ph108_B7
zmp_ph108_C4
zmp_ph108_C5
zmp_ph108_D4
zmp_ph97_G6
zmp_ph97_G9
zmp_ph97_H2
zmp_ph97_B10
zmp_ph97_C2
zmp_ph97_C4
zmp_ph97_B8
zmp_ph97_C1
zmp_ph97_D2
zmp_ph97_D5
zmp_ph111_G5
zmp_ph111_G11
zmp_ph111_H1
zmp_ph111_B1a
zmp_ph111_B5
zmp_ph111_B8
zmp_ph111_B6
zmp_ph111_B7
zmp_ph111_B9
zmp_ph111_B10
zmp_ph114_G4
zmp_ph114_G11
zmp_ph114_H1
zmp_ph114_H2
zmp_ph114_A9
zmp_ph114_A11
zmp_ph114_B2
zmp_ph114_B4
zmp_ph114_D8
zmp_ph114_E2

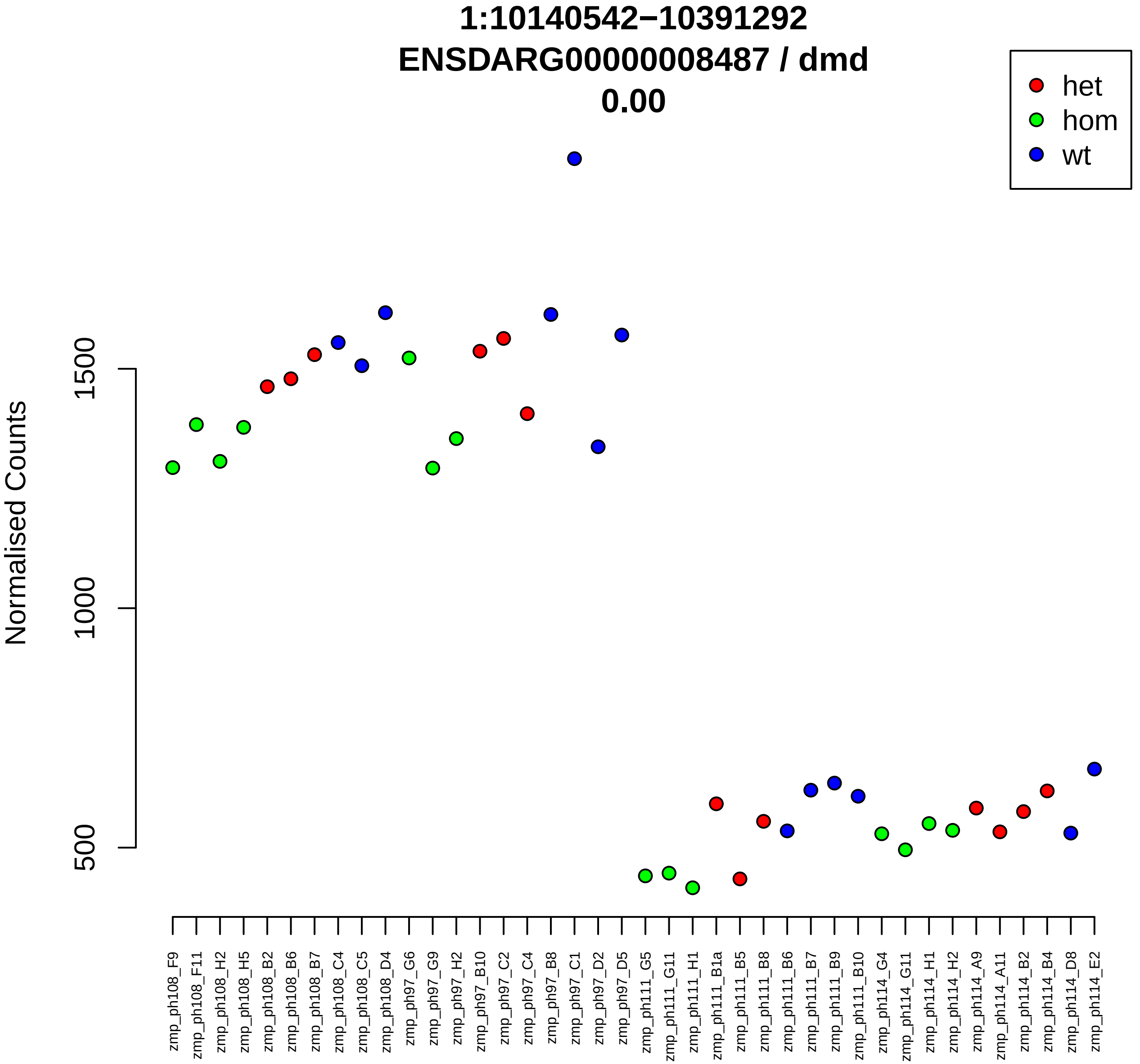

1:10140542−10391292
ENSDARG00000008487 / dmd
0.00
het
hom
wt
Normalised Counts
500
1000
1500
zmp_ph108_F9
zmp_ph108_F11
zmp_ph108_H2
zmp_ph108_H5
zmp_ph108_B2
zmp_ph108_B6
zmp_ph108_B7
zmp_ph108_C4
zmp_ph108_C5
zmp_ph108_D4
zmp_ph97_G6
zmp_ph97_G9
zmp_ph97_H2
zmp_ph97_B10
zmp_ph97_C2
zmp_ph97_C4
zmp_ph97_B8
zmp_ph97_C1
zmp_ph97_D2
zmp_ph97_D5
zmp_ph111_G5
zmp_ph111_G11
zmp_ph111_H1
zmp_ph111_B1a
zmp_ph111_B5
zmp_ph111_B8
zmp_ph111_B6
zmp_ph111_B7
zmp_ph111_B9
zmp_ph111_B10
zmp_ph114_G4
zmp_ph114_G11
zmp_ph114_H1
zmp_ph114_H2
zmp_ph114_A9
zmp_ph114_A11
zmp_ph114_B2
zmp_ph114_B4
zmp_ph114_D8
zmp_ph114_E2

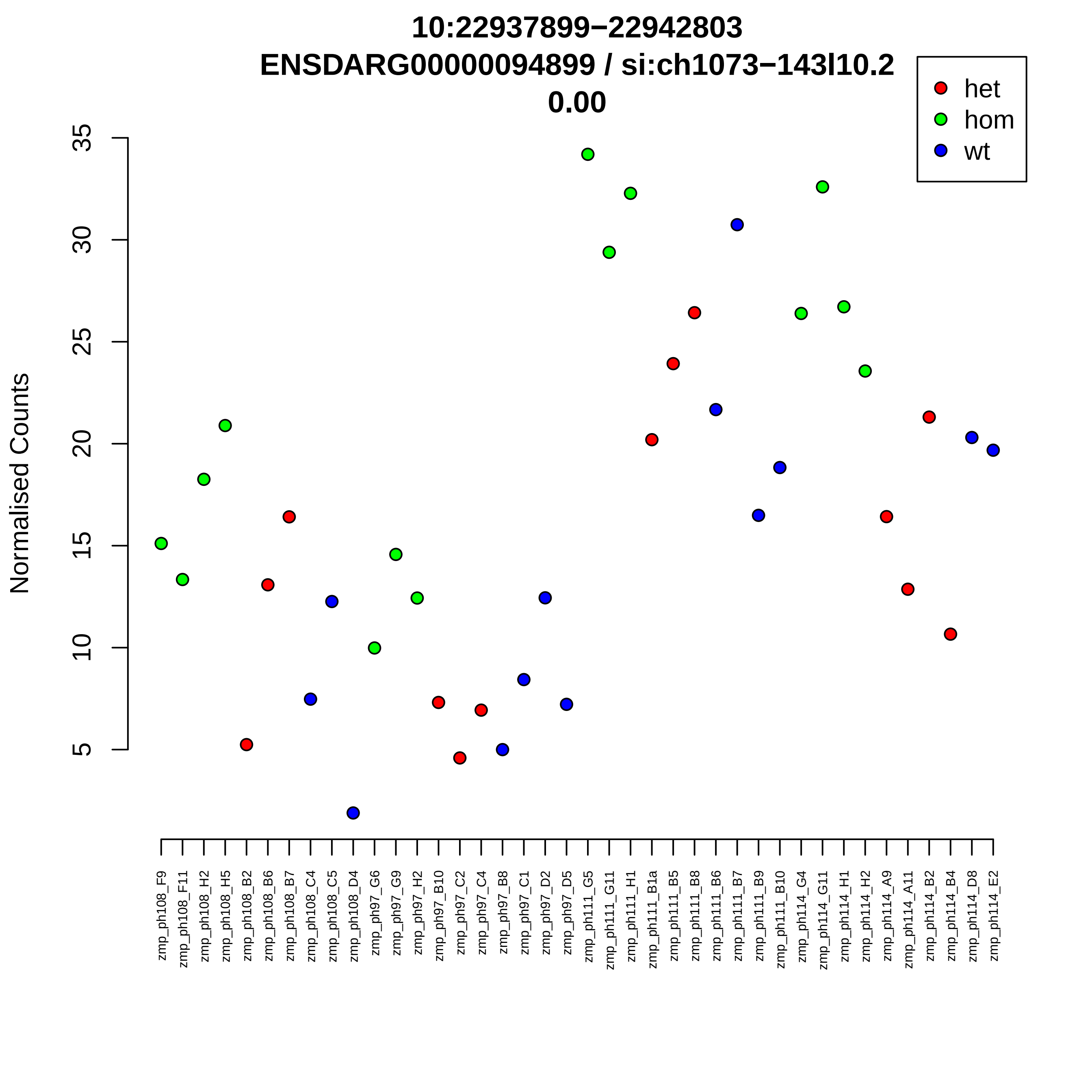
10:22937899−22942803
ENSDARG00000094899 / si:ch1073−143l10.2
0.00
het
hom
wt
Normalised Counts
35
30
25
20
15
10
5
zmp_ph108_F9
zmp_ph108_F11
zmp_ph108_H2
zmp_ph108_H5
zmp_ph108_B2
zmp_ph108_B6
zmp_ph108_B7
zmp_ph108_C4
zmp_ph108_C5
zmp_ph108_D4
zmp_ph97_G6
zmp_ph97_G9
zmp_ph97_H2
zmp_ph97_B10
zmp_ph97_C2
zmp_ph97_C4
zmp_ph97_B8
zmp_ph97_C1
zmp_ph97_D2
zmp_ph97_D5
zmp_ph111_G5
zmp_ph111_G11
zmp_ph111_H1
zmp_ph111_B1a
zmp_ph111_B5
zmp_ph111_B8
zmp_ph111_B6
zmp_ph111_B7
zmp_ph111_B9
zmp_ph111_B10
zmp_ph114_G4
zmp_ph114_G11
zmp_ph114_H1
zmp_ph114_H2
zmp_ph114_A9
zmp_ph114_A11
zmp_ph114_B2
zmp_ph114_B4
zmp_ph114_D8
zmp_ph114_E2

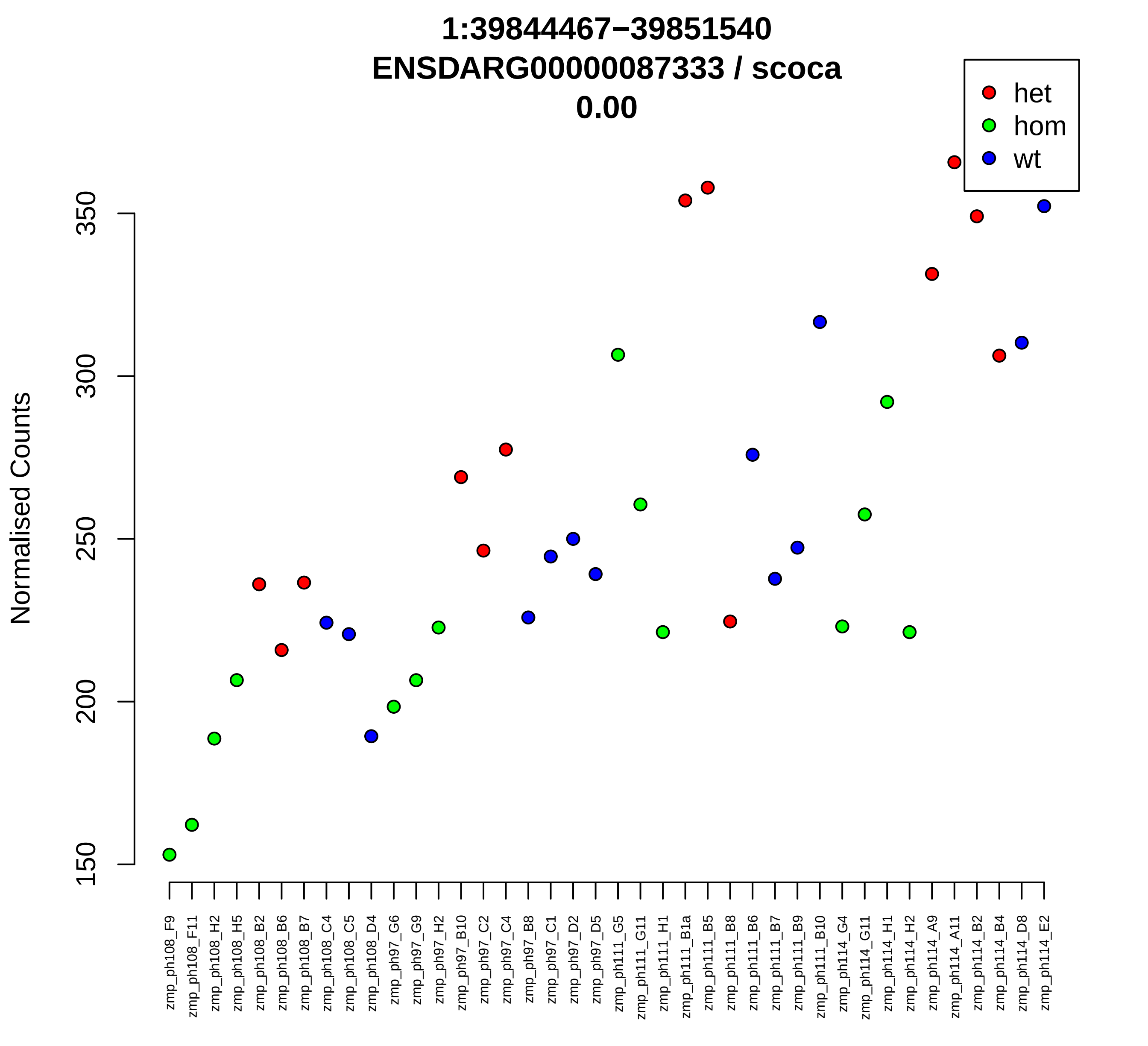
1:39844467−39851540
ENSDARG00000087333 / scoca
0.00
het
hom
wt
Normalised Counts
150
200
250
300
350
zmp_ph108_F9
zmp_ph108_F11
zmp_ph108_H2
zmp_ph108_H5
zmp_ph108_B2
zmp_ph108_B6
zmp_ph108_B7
zmp_ph108_C4
zmp_ph108_C5
zmp_ph108_D4
zmp_ph97_G6
zmp_ph97_G9
zmp_ph97_H2
zmp_ph97_B10
zmp_ph97_C2
zmp_ph97_C4
zmp_ph97_B8
zmp_ph97_C1
zmp_ph97_D2
zmp_ph97_D5
zmp_ph111_G5
zmp_ph111_G11
zmp_ph111_H1
zmp_ph111_B1a
zmp_ph111_B5
zmp_ph111_B8
zmp_ph111_B6
zmp_ph111_B7
zmp_ph111_B9
zmp_ph111_B10
zmp_ph114_G4
zmp_ph114_G11
zmp_ph114_H1
zmp_ph114_H2
zmp_ph114_A9
zmp_ph114_A11
zmp_ph114_B2
zmp_ph114_B4
zmp_ph114_D8
zmp_ph114_E2

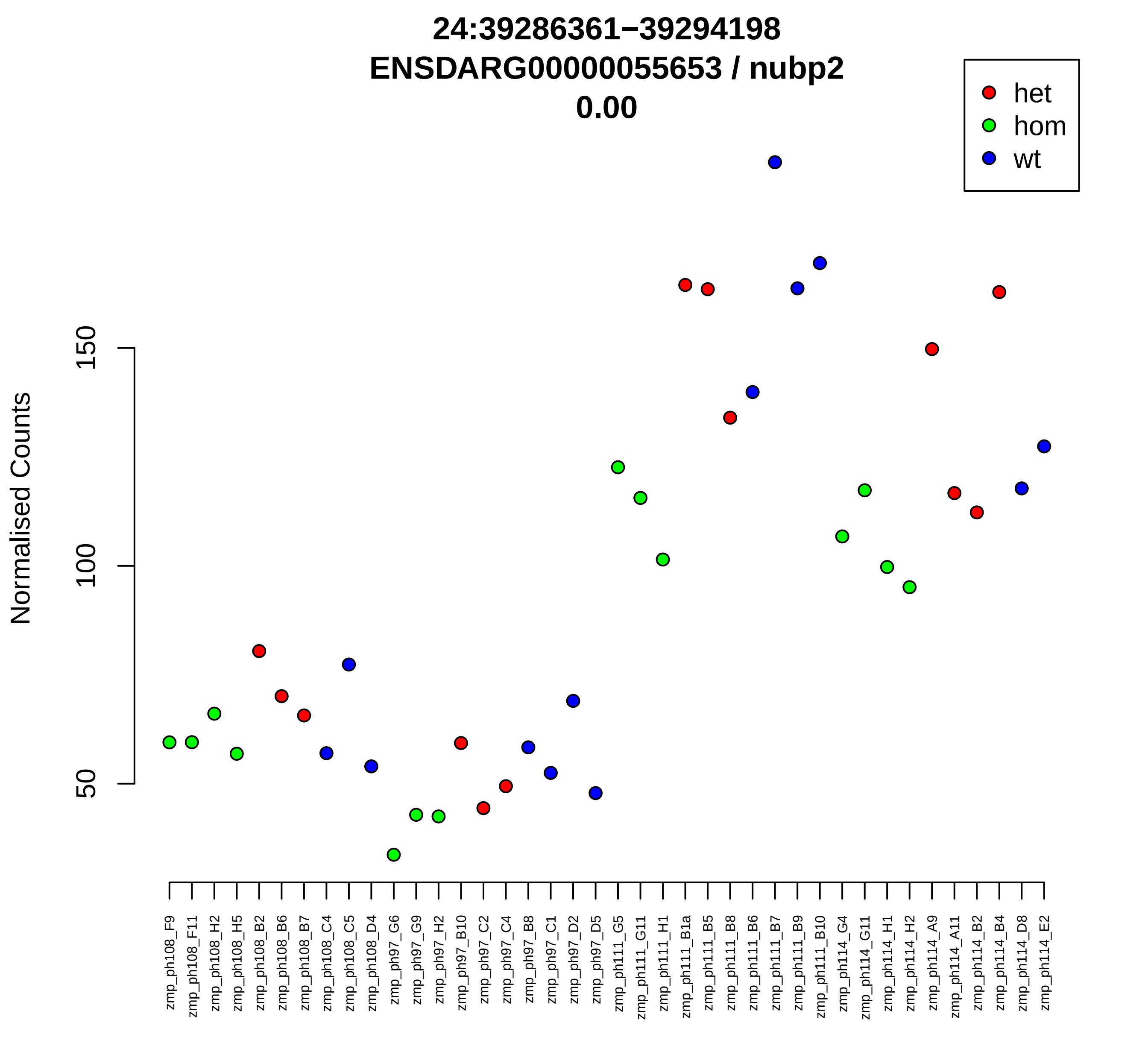
24:39286361−39294198
ENSDARG00000055653 / nubp2
0.00
het
hom
wt
Normalised Counts
50
100
150
zmp_ph108_F9
zmp_ph108_F11
zmp_ph108_H2
zmp_ph108_H5
zmp_ph108_B2
zmp_ph108_B6
zmp_ph108_B7
zmp_ph108_C4
zmp_ph108_C5
zmp_ph108_D4
zmp_ph97_G6
zmp_ph97_G9
zmp_ph97_H2
zmp_ph97_B10
zmp_ph97_C2
zmp_ph97_C4
zmp_ph97_B8
zmp_ph97_C1
zmp_ph97_D2
zmp_ph97_D5
zmp_ph111_G5
zmp_ph111_G11
zmp_ph111_H1
zmp_ph111_B1a
zmp_ph111_B5
zmp_ph111_B8
zmp_ph111_B6
zmp_ph111_B7
zmp_ph111_B9
zmp_ph111_B10
zmp_ph114_G4
zmp_ph114_G11
zmp_ph114_H1
zmp_ph114_H2
zmp_ph114_A9
zmp_ph114_A11
zmp_ph114_B2
zmp_ph114_B4
zmp_ph114_D8
zmp_ph114_E2

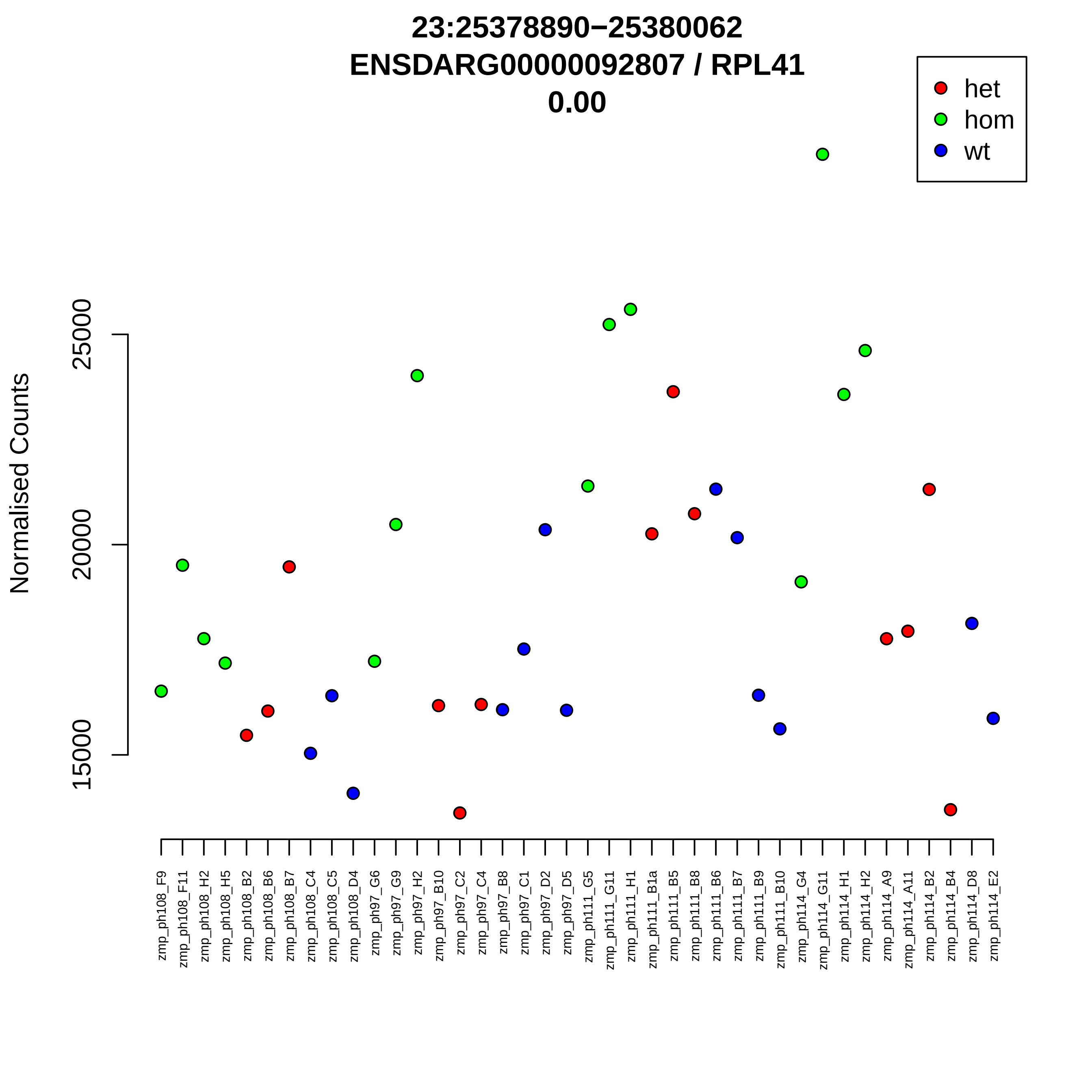

23:25378890−25380062
ENSDARG00000092807 / RPL41
0.00
het
hom
wt
Normalised Counts
15000
20000
25000
zmp_ph108_F9
zmp_ph108_F11
zmp_ph108_H2
zmp_ph108_H5
zmp_ph108_B2
zmp_ph108_B6
zmp_ph108_B7
zmp_ph108_C4
zmp_ph108_C5
zmp_ph108_D4
zmp_ph97_G6
zmp_ph97_G9
zmp_ph97_H2
zmp_ph97_B10
zmp_ph97_C2
zmp_ph97_C4
zmp_ph97_B8
zmp_ph97_C1
zmp_ph97_D2
zmp_ph97_D5
zmp_ph111_G5
zmp_ph111_G11
zmp_ph111_H1
zmp_ph111_B1a
zmp_ph111_B5
zmp_ph111_B8
zmp_ph111_B6
zmp_ph111_B7
zmp_ph111_B9
zmp_ph111_B10
zmp_ph114_G4
zmp_ph114_G11
zmp_ph114_H1
zmp_ph114_H2
zmp_ph114_A9
zmp_ph114_A11
zmp_ph114_B2
zmp_ph114_B4
zmp_ph114_D8
zmp_ph114_E2

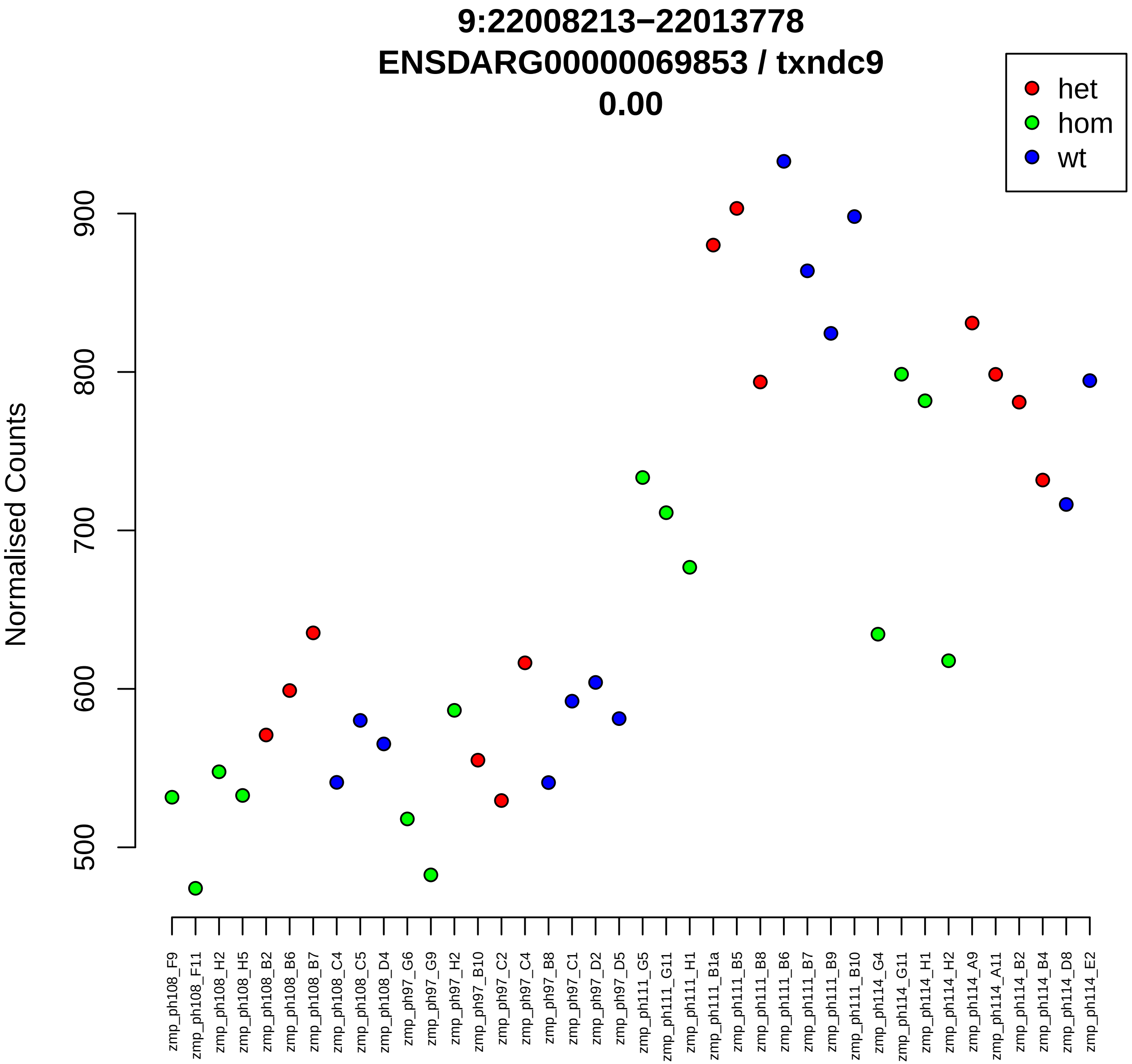
9:22008213−22013778
ENSDARG00000069853 / txndc9
0.00
het
hom
wt
Normalised Counts
500
600
700
800
900
zmp_ph108_F9
zmp_ph108_F11
zmp_ph108_H2
zmp_ph108_H5
zmp_ph108_B2
zmp_ph108_B6
zmp_ph108_B7
zmp_ph108_C4
zmp_ph108_C5
zmp_ph108_D4
zmp_ph97_G6
zmp_ph97_G9
zmp_ph97_H2
zmp_ph97_B10
zmp_ph97_C2
zmp_ph97_C4
zmp_ph97_B8
zmp_ph97_C1
zmp_ph97_D2
zmp_ph97_D5
zmp_ph111_G5
zmp_ph111_G11
zmp_ph111_H1
zmp_ph111_B1a
zmp_ph111_B5
zmp_ph111_B8
zmp_ph111_B6
zmp_ph111_B7
zmp_ph111_B9
zmp_ph111_B10
zmp_ph114_G4
zmp_ph114_G11
zmp_ph114_H1
zmp_ph114_H2
zmp_ph114_A9
zmp_ph114_A11
zmp_ph114_B2
zmp_ph114_B4
zmp_ph114_D8
zmp_ph114_E2

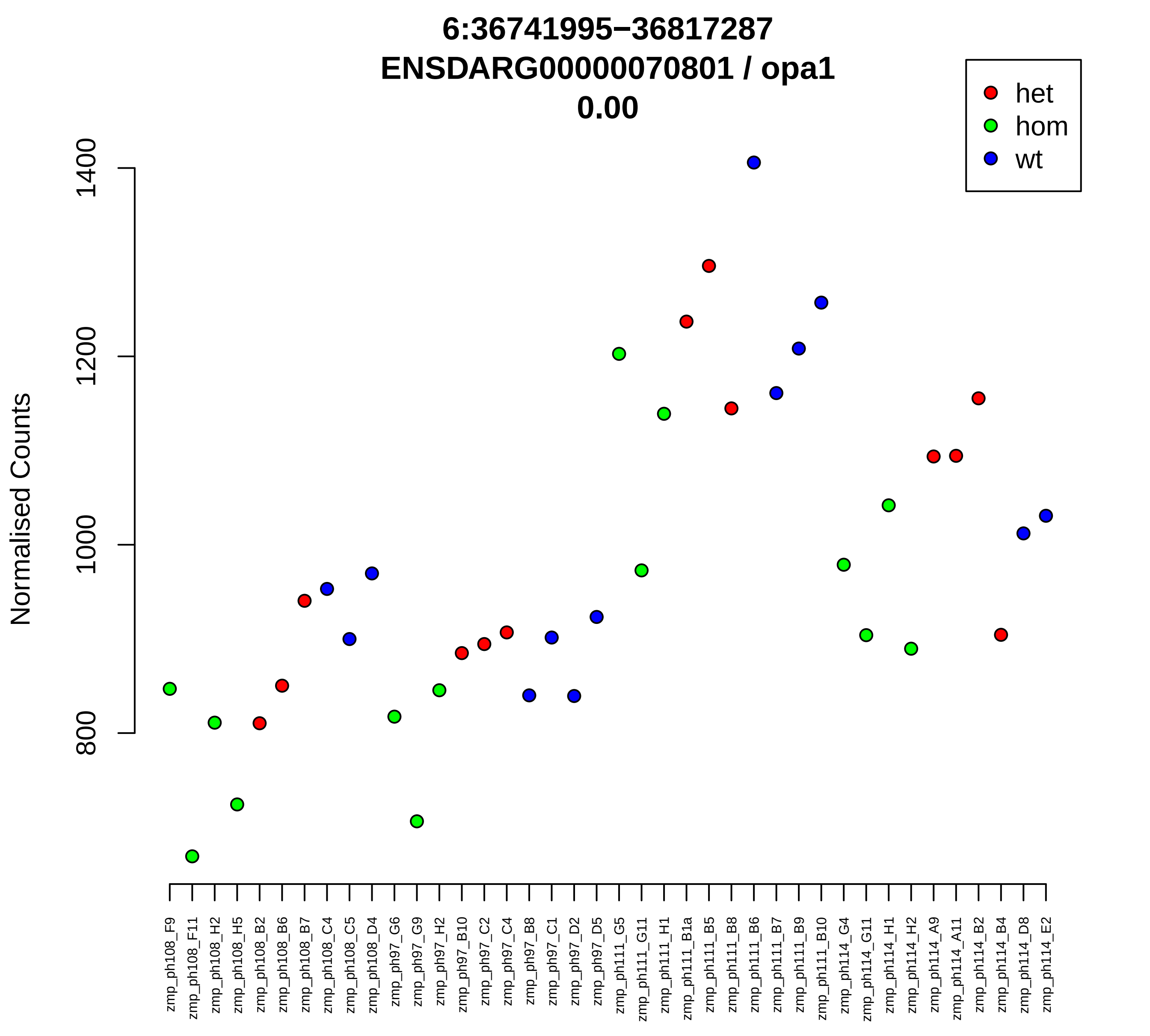
6:36741995−36817287
ENSDARG00000070801 / opa1
0.00
het
hom
wt
Normalised Counts
800
1000
1200
1400
zmp_ph108_F9
zmp_ph108_F11
zmp_ph108_H2
zmp_ph108_H5
zmp_ph108_B2
zmp_ph108_B6
zmp_ph108_B7
zmp_ph108_C4
zmp_ph108_C5
zmp_ph108_D4
zmp_ph97_G6
zmp_ph97_G9
zmp_ph97_H2
zmp_ph97_B10
zmp_ph97_C2
zmp_ph97_C4
zmp_ph97_B8
zmp_ph97_C1
zmp_ph97_D2
zmp_ph97_D5
zmp_ph111_G5
zmp_ph111_G11
zmp_ph111_H1
zmp_ph111_B1a
zmp_ph111_B5
zmp_ph111_B8
zmp_ph111_B6
zmp_ph111_B7
zmp_ph111_B9
zmp_ph111_B10
zmp_ph114_G4
zmp_ph114_G11
zmp_ph114_H1
zmp_ph114_H2
zmp_ph114_A9
zmp_ph114_A11
zmp_ph114_B2
zmp_ph114_B4
zmp_ph114_D8
zmp_ph114_E2

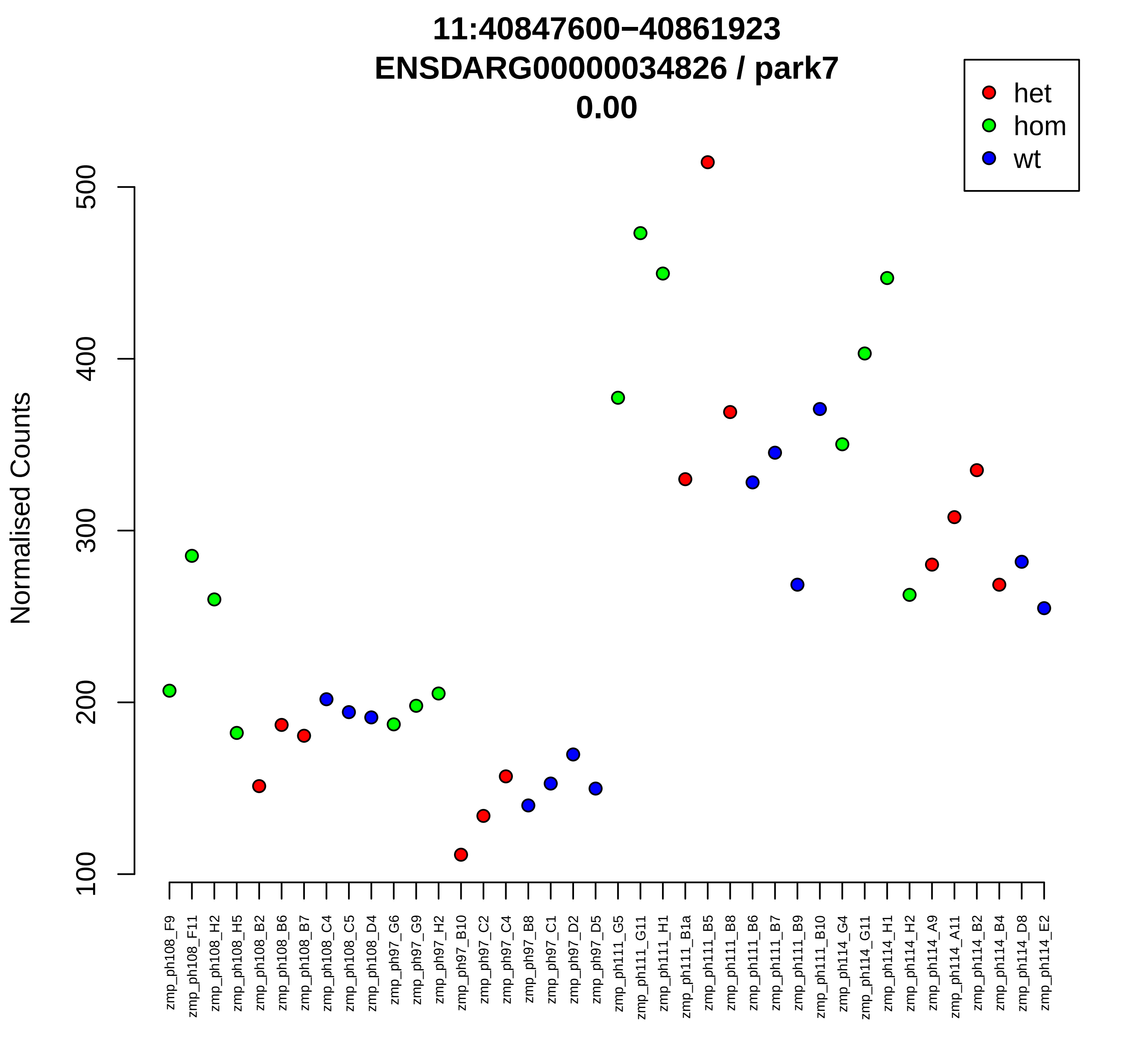

11:40847600−40861923
ENSDARG00000034826 / park7
0.00
het
hom
wt
Normalised Counts
100
200
300
400
500
zmp_ph108_F9
zmp_ph108_F11
zmp_ph108_H2
zmp_ph108_H5
zmp_ph108_B2
zmp_ph108_B6
zmp_ph108_B7
zmp_ph108_C4
zmp_ph108_C5
zmp_ph108_D4
zmp_ph97_G6
zmp_ph97_G9
zmp_ph97_H2
zmp_ph97_B10
zmp_ph97_C2
zmp_ph97_C4
zmp_ph97_B8
zmp_ph97_C1
zmp_ph97_D2
zmp_ph97_D5
zmp_ph111_G5
zmp_ph111_G11
zmp_ph111_H1
zmp_ph111_B1a
zmp_ph111_B5
zmp_ph111_B8
zmp_ph111_B6
zmp_ph111_B7
zmp_ph111_B9
zmp_ph111_B10
zmp_ph114_G4
zmp_ph114_G11
zmp_ph114_H1
zmp_ph114_H2
zmp_ph114_A9
zmp_ph114_A11
zmp_ph114_B2
zmp_ph114_B4
zmp_ph114_D8
zmp_ph114_E2

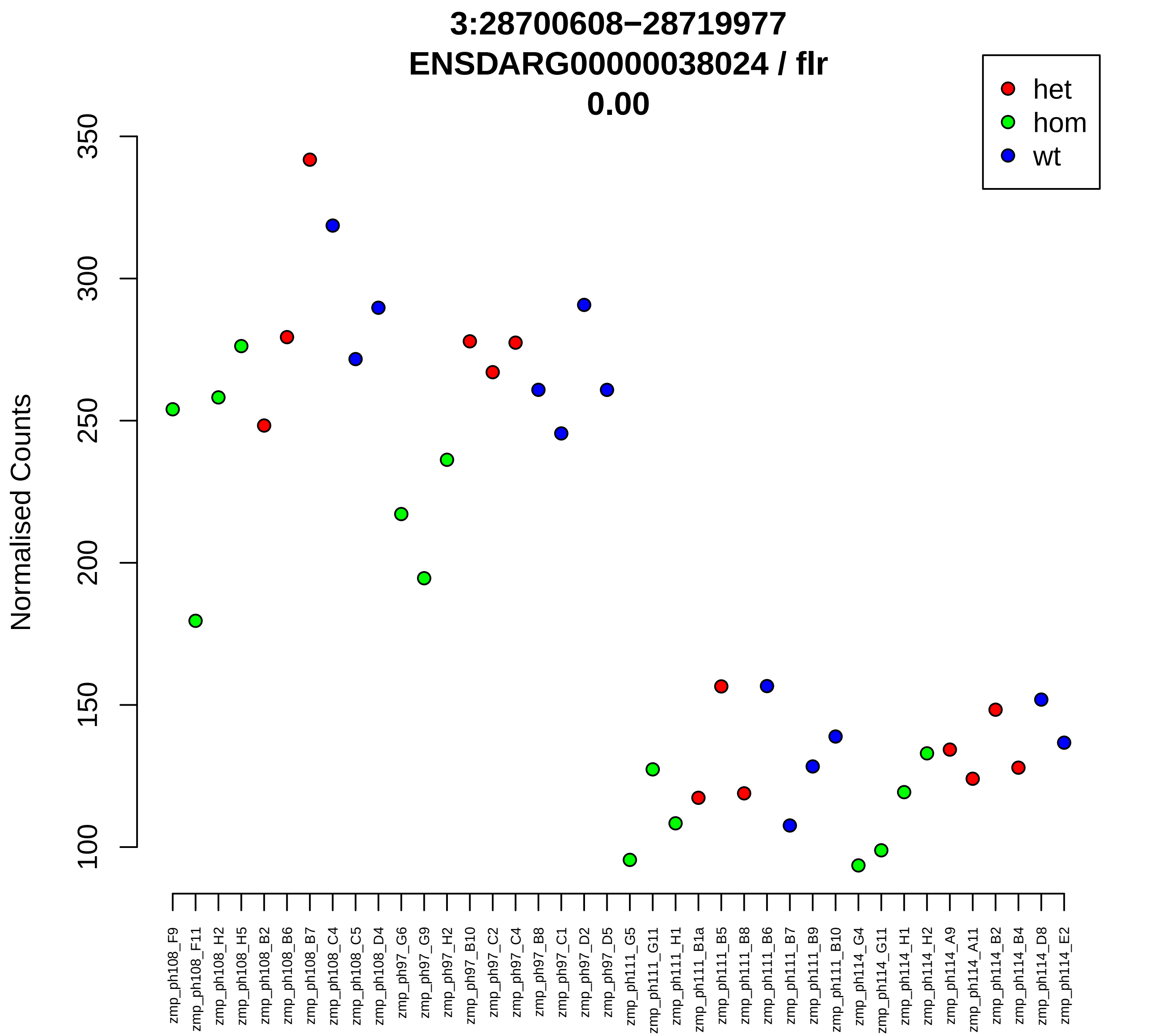

3:28700608−28719977
ENSDARG00000038024 / flr
0.00
het
hom
wt
Normalised Counts
100
150
200
250
300
350
zmp_ph108_F9
zmp_ph108_F11
zmp_ph108_H2
zmp_ph108_H5
zmp_ph108_B2
zmp_ph108_B6
zmp_ph108_B7
zmp_ph108_C4
zmp_ph108_C5
zmp_ph108_D4
zmp_ph97_G6
zmp_ph97_G9
zmp_ph97_H2
zmp_ph97_B10
zmp_ph97_C2
zmp_ph97_C4
zmp_ph97_B8
zmp_ph97_C1
zmp_ph97_D2
zmp_ph97_D5
zmp_ph111_G5
zmp_ph111_G11
zmp_ph111_H1
zmp_ph111_B1a
zmp_ph111_B5
zmp_ph111_B8
zmp_ph111_B6
zmp_ph111_B7
zmp_ph111_B9
zmp_ph111_B10
zmp_ph114_G4
zmp_ph114_G11
zmp_ph114_H1
zmp_ph114_H2
zmp_ph114_A9
zmp_ph114_A11
zmp_ph114_B2
zmp_ph114_B4
zmp_ph114_D8
zmp_ph114_E2

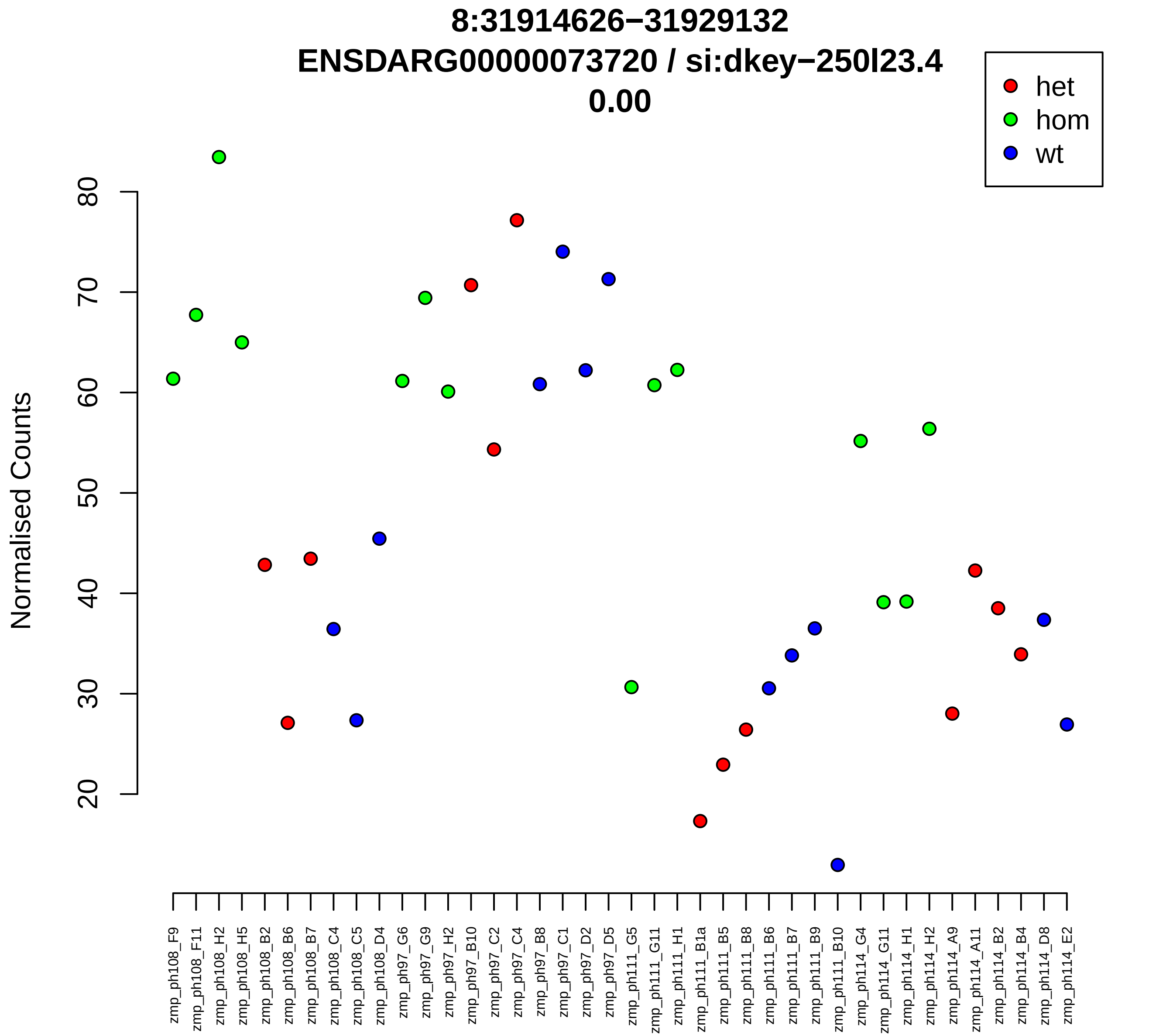

8:31914626−31929132
ENSDARG00000073720 / si:dkey−250l23.4
0.00
het
hom
wt
Normalised Counts
20
30
40
50
60
70
80
zmp_ph108_F9
zmp_ph108_F11
zmp_ph108_H2
zmp_ph108_H5
zmp_ph108_B2
zmp_ph108_B6
zmp_ph108_B7
zmp_ph108_C4
zmp_ph108_C5
zmp_ph108_D4
zmp_ph97_G6
zmp_ph97_G9
zmp_ph97_H2
zmp_ph97_B10
zmp_ph97_C2
zmp_ph97_C4
zmp_ph97_B8
zmp_ph97_C1
zmp_ph97_D2
zmp_ph97_D5
zmp_ph111_G5
zmp_ph111_G11
zmp_ph111_H1
zmp_ph111_B1a
zmp_ph111_B5
zmp_ph111_B8
zmp_ph111_B6
zmp_ph111_B7
zmp_ph111_B9
zmp_ph111_B10
zmp_ph114_G4
zmp_ph114_G11
zmp_ph114_H1
zmp_ph114_H2
zmp_ph114_A9
zmp_ph114_A11
zmp_ph114_B2
zmp_ph114_B4
zmp_ph114_D8
zmp_ph114_E2

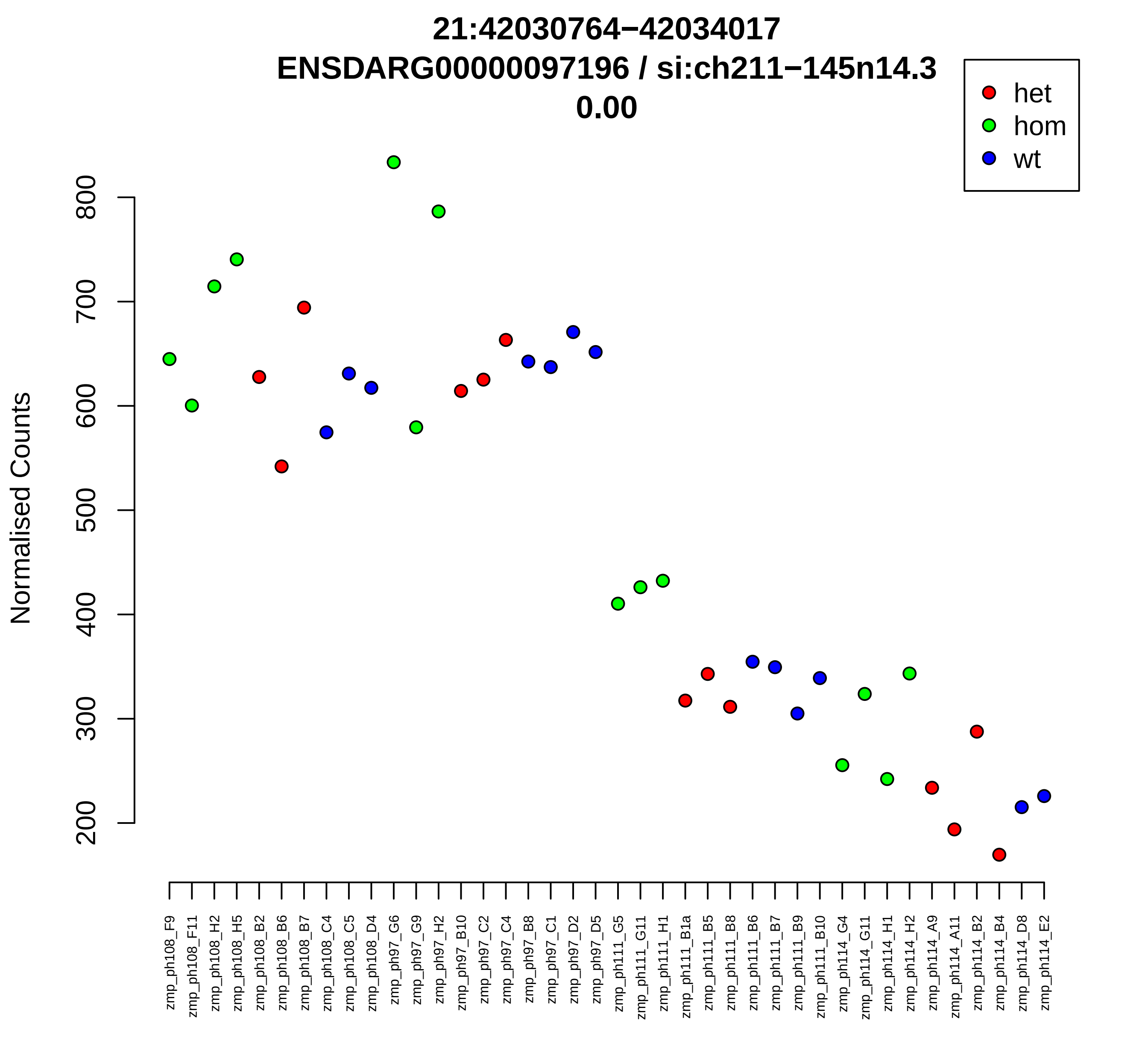

21:42030764−42034017
ENSDARG00000097196 / si:ch211−145n14.3
0.00
het
hom
wt
Normalised Counts
800
700
600
500
400
300
200
zmp_ph108_F9
zmp_ph108_F11
zmp_ph108_H2
zmp_ph108_H5
zmp_ph108_B2
zmp_ph108_B6
zmp_ph108_B7
zmp_ph108_C4
zmp_ph108_C5
zmp_ph108_D4
zmp_ph97_G6
zmp_ph97_G9
zmp_ph97_H2
zmp_ph97_B10
zmp_ph97_C2
zmp_ph97_C4
zmp_ph97_B8
zmp_ph97_C1
zmp_ph97_D2
zmp_ph97_D5
zmp_ph111_G5
zmp_ph111_G11
zmp_ph111_H1
zmp_ph111_B1a
zmp_ph111_B5
zmp_ph111_B8
zmp_ph111_B6
zmp_ph111_B7
zmp_ph111_B9
zmp_ph111_B10
zmp_ph114_G4
zmp_ph114_G11
zmp_ph114_H1
zmp_ph114_H2
zmp_ph114_A9
zmp_ph114_A11
zmp_ph114_B2
zmp_ph114_B4
zmp_ph114_D8
zmp_ph114_E2

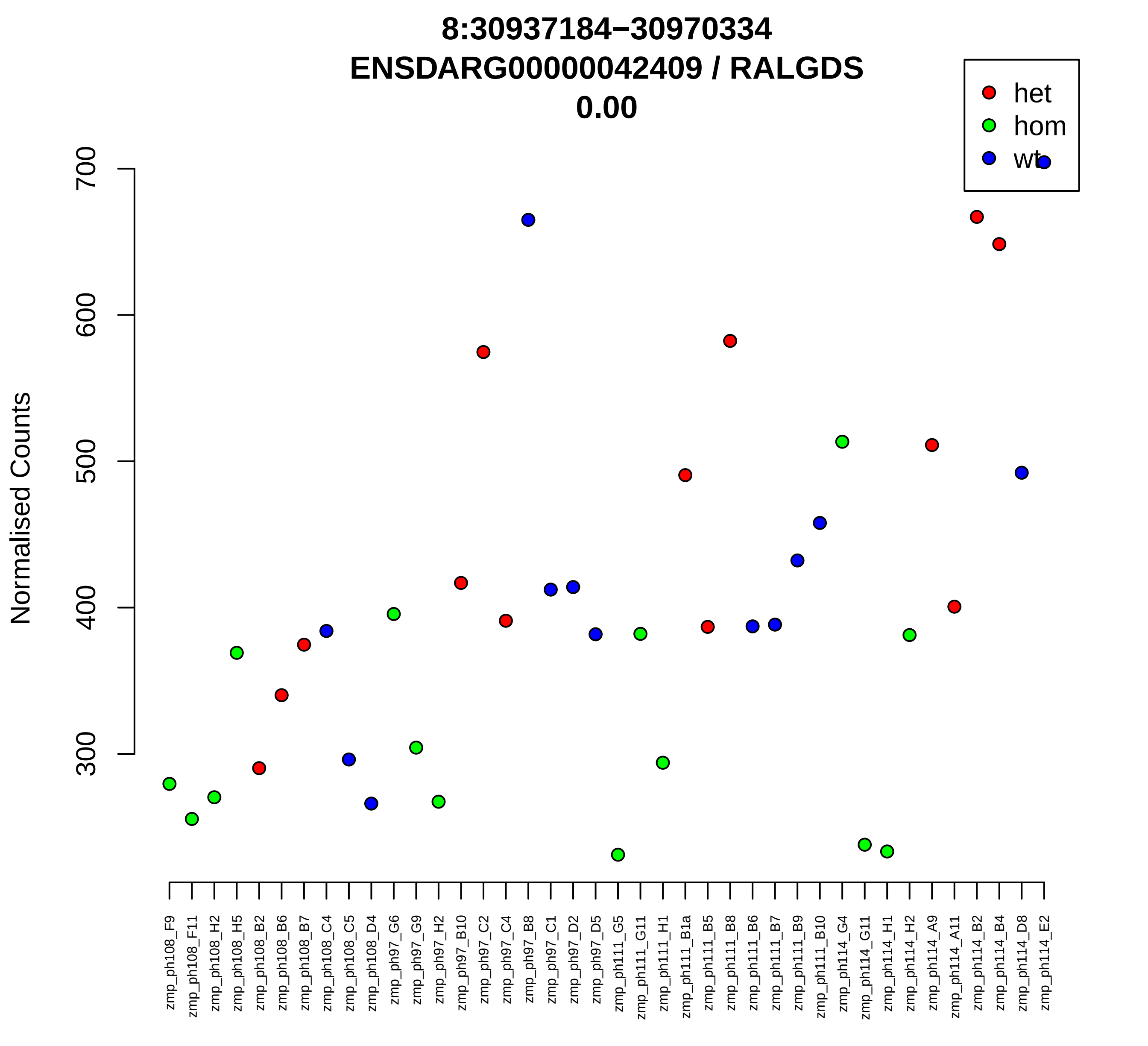
8:30937184−30970334
ENSDARG00000042409 / RALGDS
0.00
het
hom
wt
Normalised Counts
300
400
500
600
700
zmp_ph108_F9
zmp_ph108_F11
zmp_ph108_H2
zmp_ph108_H5
zmp_ph108_B2
zmp_ph108_B6
zmp_ph108_B7
zmp_ph108_C4
zmp_ph108_C5
zmp_ph108_D4
zmp_ph97_G6
zmp_ph97_G9
zmp_ph97_H2
zmp_ph97_B10
zmp_ph97_C2
zmp_ph97_C4
zmp_ph97_B8
zmp_ph97_C1
zmp_ph97_D2
zmp_ph97_D5
zmp_ph111_G5
zmp_ph111_G11
zmp_ph111_H1
zmp_ph111_B1a
zmp_ph111_B5
zmp_ph111_B8
zmp_ph111_B6
zmp_ph111_B7
zmp_ph111_B9
zmp_ph111_B10
zmp_ph114_G4
zmp_ph114_G11
zmp_ph114_H1
zmp_ph114_H2
zmp_ph114_A9
zmp_ph114_A11
zmp_ph114_B2
zmp_ph114_B4
zmp_ph114_D8
zmp_ph114_E2

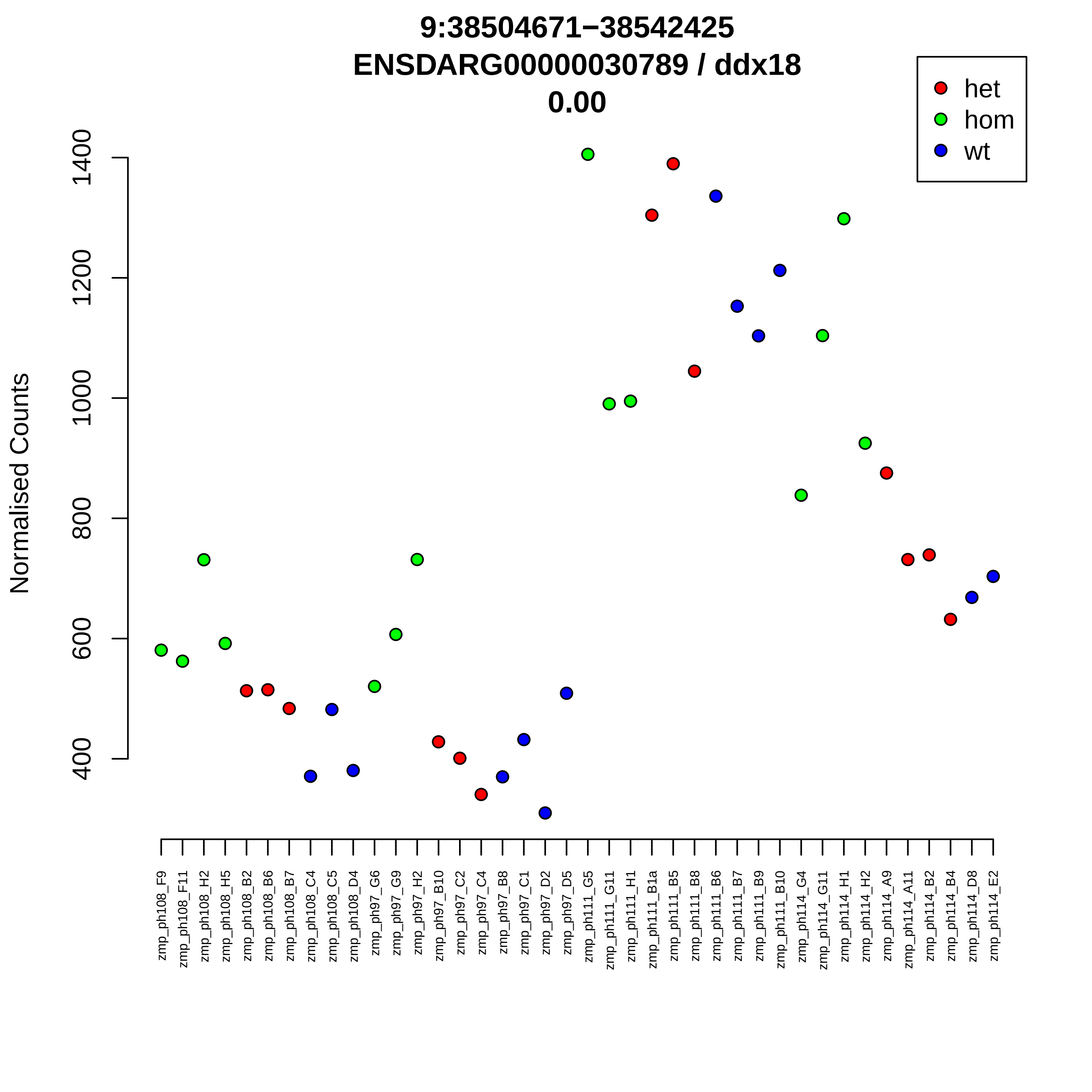

9:38504671−38542425
ENSDARG00000030789 / ddx18
0.00
het
hom
wt
Normalised Counts
400
600
800
1000
1200
1400
zmp_ph108_F9
zmp_ph108_F11
zmp_ph108_H2
zmp_ph108_H5
zmp_ph108_B2
zmp_ph108_B6
zmp_ph108_B7
zmp_ph108_C4
zmp_ph108_C5
zmp_ph108_D4
zmp_ph97_G6
zmp_ph97_G9
zmp_ph97_H2
zmp_ph97_B10
zmp_ph97_C2
zmp_ph97_C4
zmp_ph97_B8
zmp_ph97_C1
zmp_ph97_D2
zmp_ph97_D5
zmp_ph111_G5
zmp_ph111_G11
zmp_ph111_H1
zmp_ph111_B1a
zmp_ph111_B5
zmp_ph111_B8
zmp_ph111_B6
zmp_ph111_B7
zmp_ph111_B9
zmp_ph111_B10
zmp_ph114_G4
zmp_ph114_G11
zmp_ph114_H1
zmp_ph114_H2
zmp_ph114_A9
zmp_ph114_A11
zmp_ph114_B2
zmp_ph114_B4
zmp_ph114_D8
zmp_ph114_E2

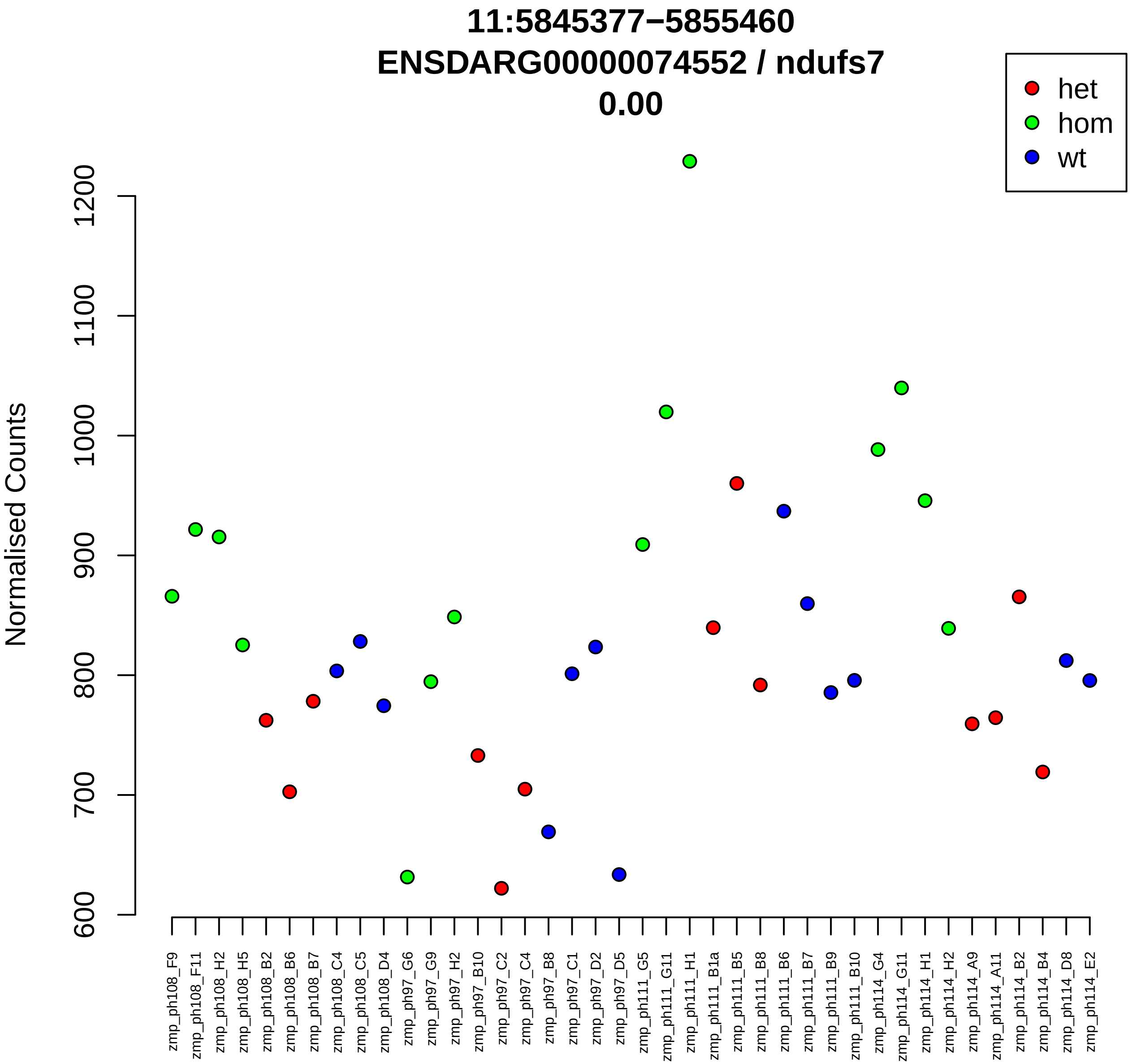

11:5845377−5855460
ENSDARG00000074552 / ndufs7
0.00
het
hom
wt
Normalised Counts
600
700
800
900
1000
1100
1200
zmp_ph108_F9
zmp_ph108_F11
zmp_ph108_H2
zmp_ph108_H5
zmp_ph108_B2
zmp_ph108_B6
zmp_ph108_B7
zmp_ph108_C4
zmp_ph108_C5
zmp_ph108_D4
zmp_ph97_G6
zmp_ph97_G9
zmp_ph97_H2
zmp_ph97_B10
zmp_ph97_C2
zmp_ph97_C4
zmp_ph97_B8
zmp_ph97_C1
zmp_ph97_D2
zmp_ph97_D5
zmp_ph111_G5
zmp_ph111_G11
zmp_ph111_H1
zmp_ph111_B1a
zmp_ph111_B5
zmp_ph111_B8
zmp_ph111_B6
zmp_ph111_B7
zmp_ph111_B9
zmp_ph111_B10
zmp_ph114_G4
zmp_ph114_G11
zmp_ph114_H1
zmp_ph114_H2
zmp_ph114_A9
zmp_ph114_A11
zmp_ph114_B2
zmp_ph114_B4
zmp_ph114_D8
zmp_ph114_E2

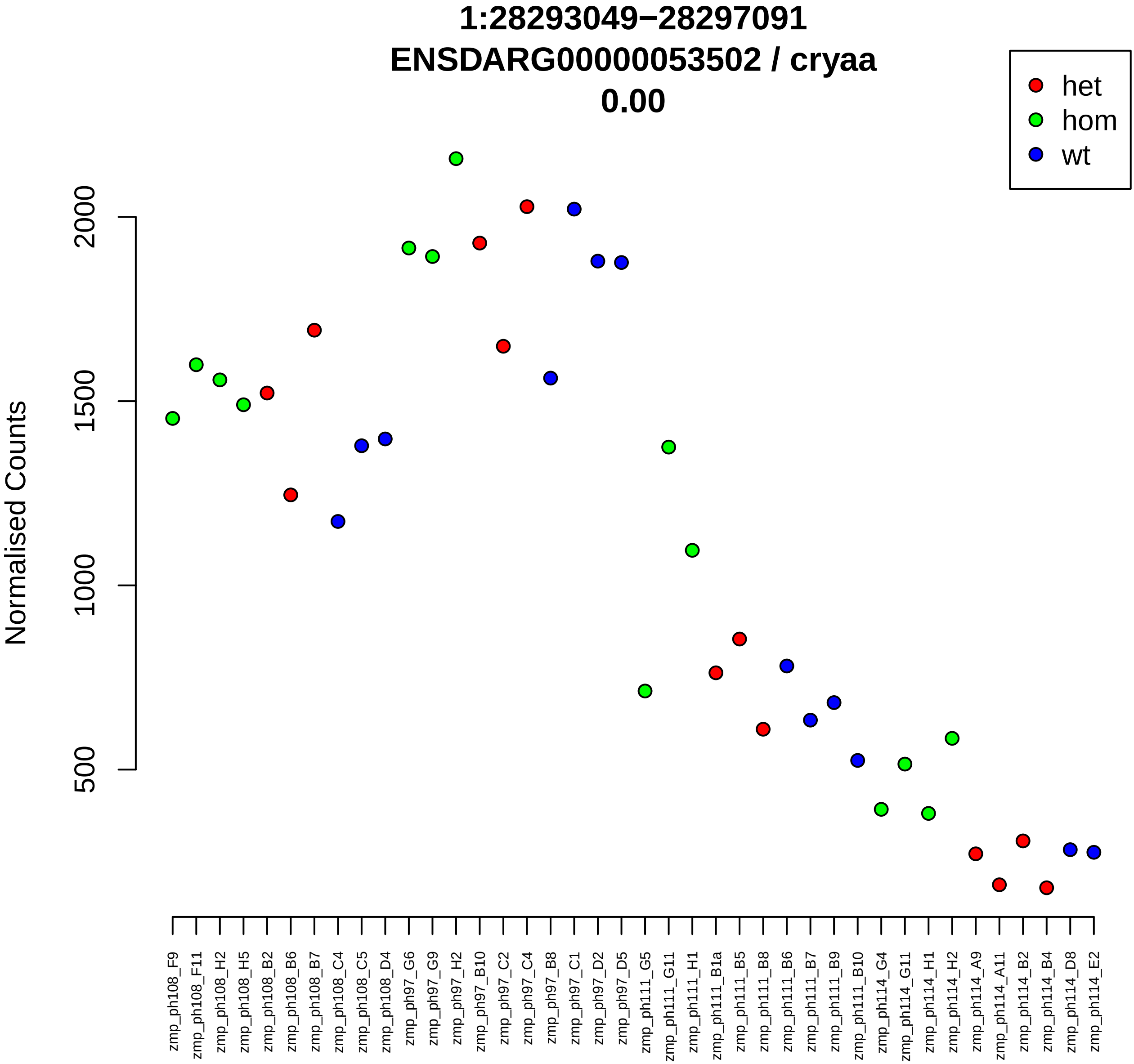

1:28293049−28297091
ENSDARG00000053502 / cryaa
0.00
het
hom
wt
Normalised Counts
500
1000
1500
2000
zmp_ph108_F9
zmp_ph108_F11
zmp_ph108_H2
zmp_ph108_H5
zmp_ph108_B2
zmp_ph108_B6
zmp_ph108_B7
zmp_ph108_C4
zmp_ph108_C5
zmp_ph108_D4
zmp_ph97_G6
zmp_ph97_G9
zmp_ph97_H2
zmp_ph97_B10
zmp_ph97_C2
zmp_ph97_C4
zmp_ph97_B8
zmp_ph97_C1
zmp_ph97_D2
zmp_ph97_D5
zmp_ph111_G5
zmp_ph111_G11
zmp_ph111_H1
zmp_ph111_B1a
zmp_ph111_B5
zmp_ph111_B8
zmp_ph111_B6
zmp_ph111_B7
zmp_ph111_B9
zmp_ph111_B10
zmp_ph114_G4
zmp_ph114_G11
zmp_ph114_H1
zmp_ph114_H2
zmp_ph114_A9
zmp_ph114_A11
zmp_ph114_B2
zmp_ph114_B4
zmp_ph114_D8
zmp_ph114_E2

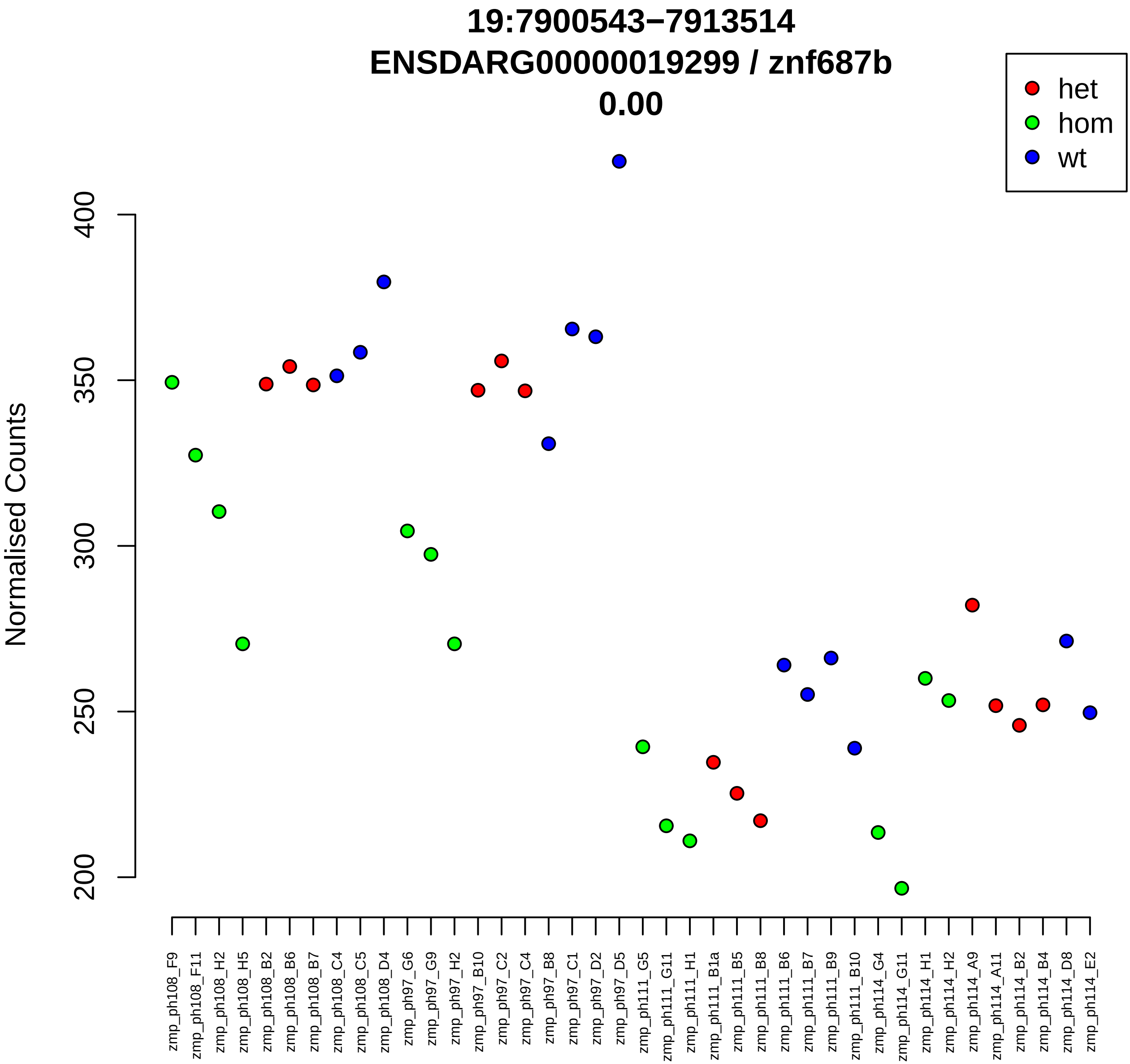

19:7900543−7913514
ENSDARG00000019299 / znf687b
0.00
het
hom
wt
Normalised Counts
200
250
300
350
400
zmp_ph108_F9
zmp_ph108_F11
zmp_ph108_H2
zmp_ph108_H5
zmp_ph108_B2
zmp_ph108_B6
zmp_ph108_B7
zmp_ph108_C4
zmp_ph108_C5
zmp_ph108_D4
zmp_ph97_G6
zmp_ph97_G9
zmp_ph97_H2
zmp_ph97_B10
zmp_ph97_C2
zmp_ph97_C4
zmp_ph97_B8
zmp_ph97_C1
zmp_ph97_D2
zmp_ph97_D5
zmp_ph111_G5
zmp_ph111_G11
zmp_ph111_H1
zmp_ph111_B1a
zmp_ph111_B5
zmp_ph111_B8
zmp_ph111_B6
zmp_ph111_B7
zmp_ph111_B9
zmp_ph111_B10
zmp_ph114_G4
zmp_ph114_G11
zmp_ph114_H1
zmp_ph114_H2
zmp_ph114_A9
zmp_ph114_A11
zmp_ph114_B2
zmp_ph114_B4
zmp_ph114_D8
zmp_ph114_E2

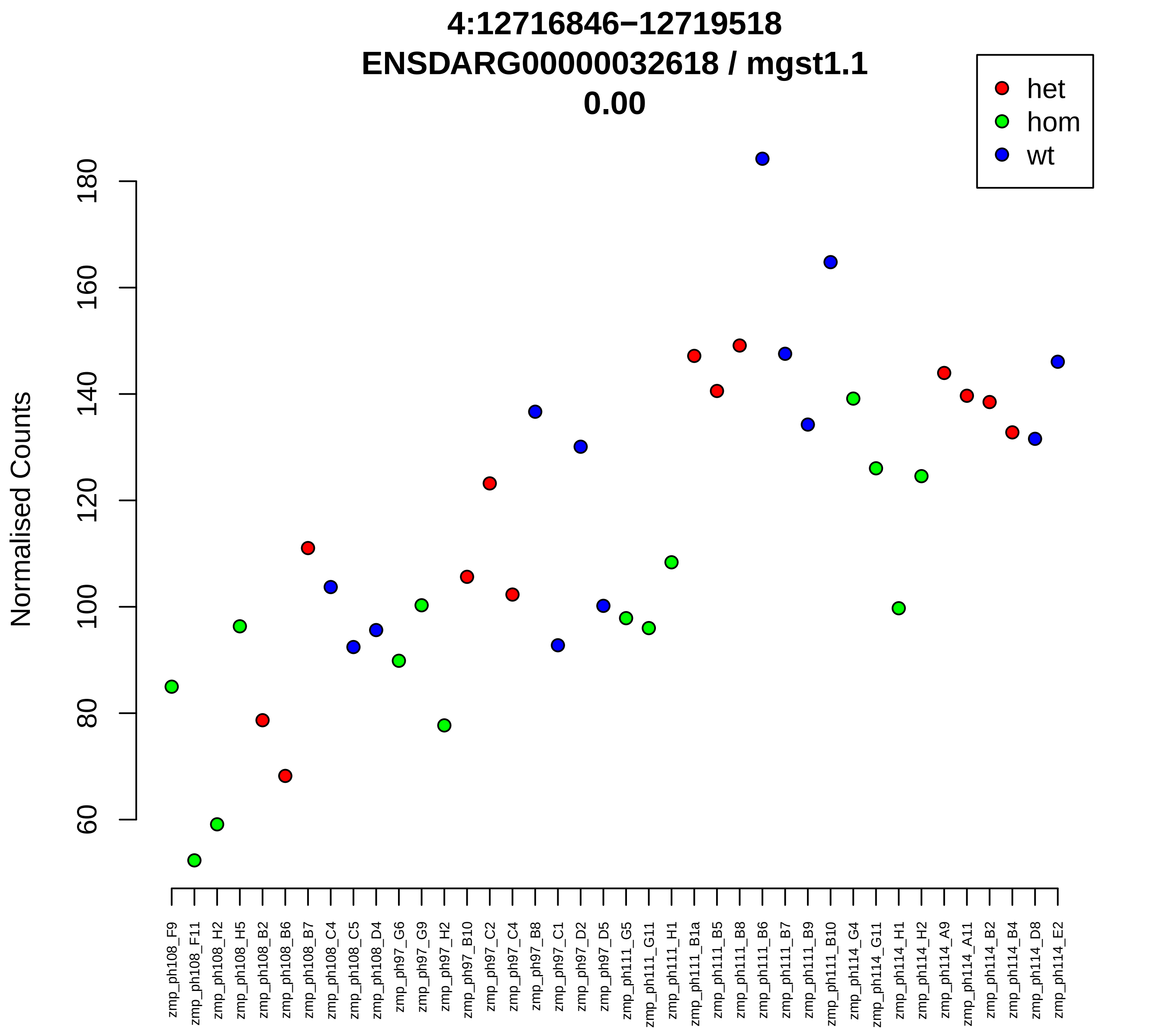

4:12716846−12719518
ENSDARG00000032618 / mgst1.1
0.00
het
hom
wt
Normalised Counts
60
80
100
120
140
160
180
zmp_ph108_F9
zmp_ph108_F11
zmp_ph108_H2
zmp_ph108_H5
zmp_ph108_B2
zmp_ph108_B6
zmp_ph108_B7
zmp_ph108_C4
zmp_ph108_C5
zmp_ph108_D4
zmp_ph97_G6
zmp_ph97_G9
zmp_ph97_H2
zmp_ph97_B10
zmp_ph97_C2
zmp_ph97_C4
zmp_ph97_B8
zmp_ph97_C1
zmp_ph97_D2
zmp_ph97_D5
zmp_ph111_G5
zmp_ph111_G11
zmp_ph111_H1
zmp_ph111_B1a
zmp_ph111_B5
zmp_ph111_B8
zmp_ph111_B6
zmp_ph111_B7
zmp_ph111_B9
zmp_ph111_B10
zmp_ph114_G4
zmp_ph114_G11
zmp_ph114_H1
zmp_ph114_H2
zmp_ph114_A9
zmp_ph114_A11
zmp_ph114_B2
zmp_ph114_B4
zmp_ph114_D8
zmp_ph114_E2

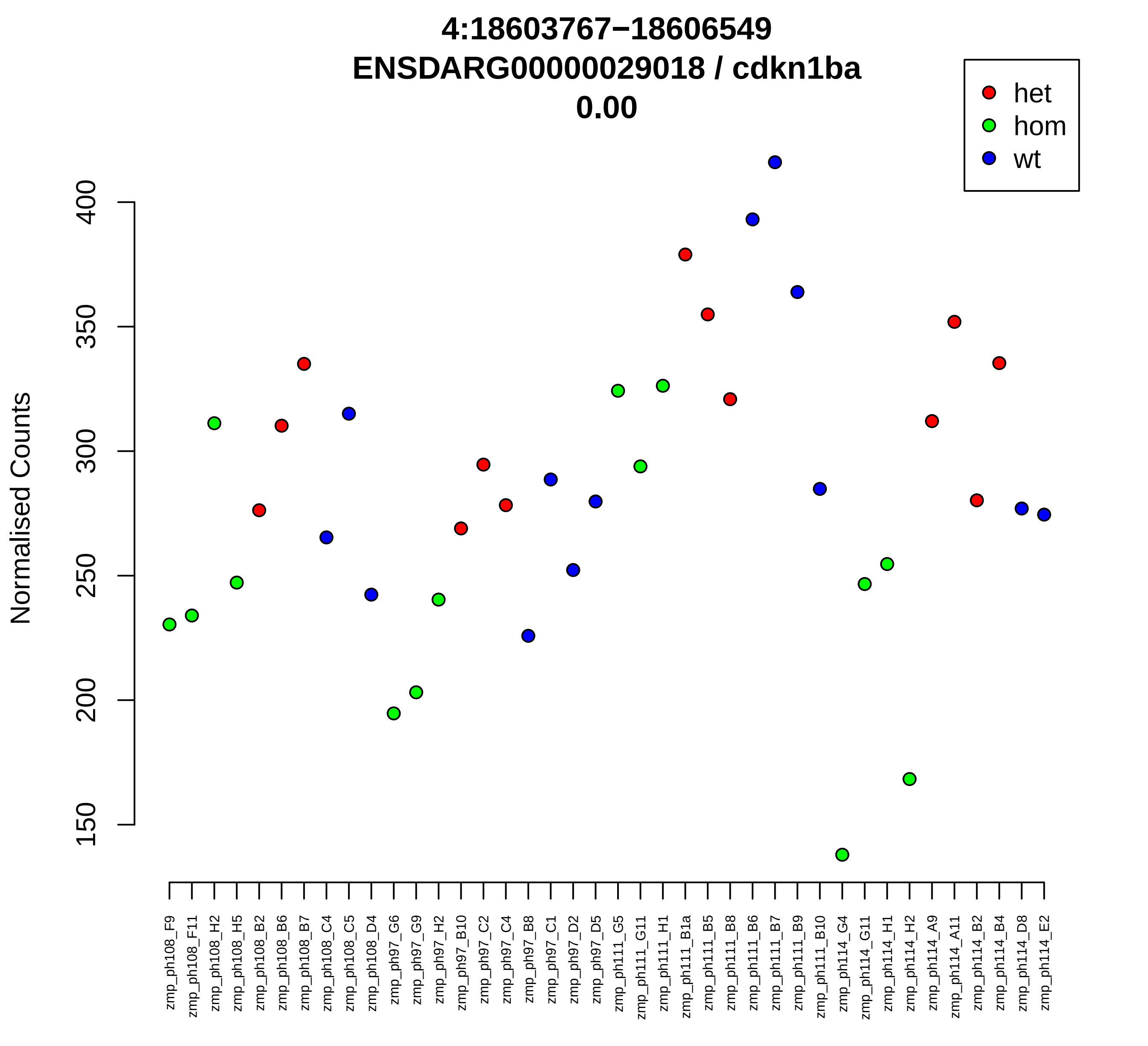
4:18603767−18606549
ENSDARG00000029018 / cdkn1ba
0.00
het
hom
wt
Normalised Counts
150
200
250
300
350
400
zmp_ph108_F9
zmp_ph108_F11
zmp_ph108_H2
zmp_ph108_H5
zmp_ph108_B2
zmp_ph108_B6
zmp_ph108_B7
zmp_ph108_C4
zmp_ph108_C5
zmp_ph108_D4
zmp_ph97_G6
zmp_ph97_G9
zmp_ph97_H2
zmp_ph97_B10
zmp_ph97_C2
zmp_ph97_C4
zmp_ph97_B8
zmp_ph97_C1
zmp_ph97_D2
zmp_ph97_D5
zmp_ph111_G5
zmp_ph111_G11
zmp_ph111_H1
zmp_ph111_B1a
zmp_ph111_B5
zmp_ph111_B8
zmp_ph111_B6
zmp_ph111_B7
zmp_ph111_B9
zmp_ph111_B10
zmp_ph114_G4
zmp_ph114_G11
zmp_ph114_H1
zmp_ph114_H2
zmp_ph114_A9
zmp_ph114_A11
zmp_ph114_B2
zmp_ph114_B4
zmp_ph114_D8
zmp_ph114_E2

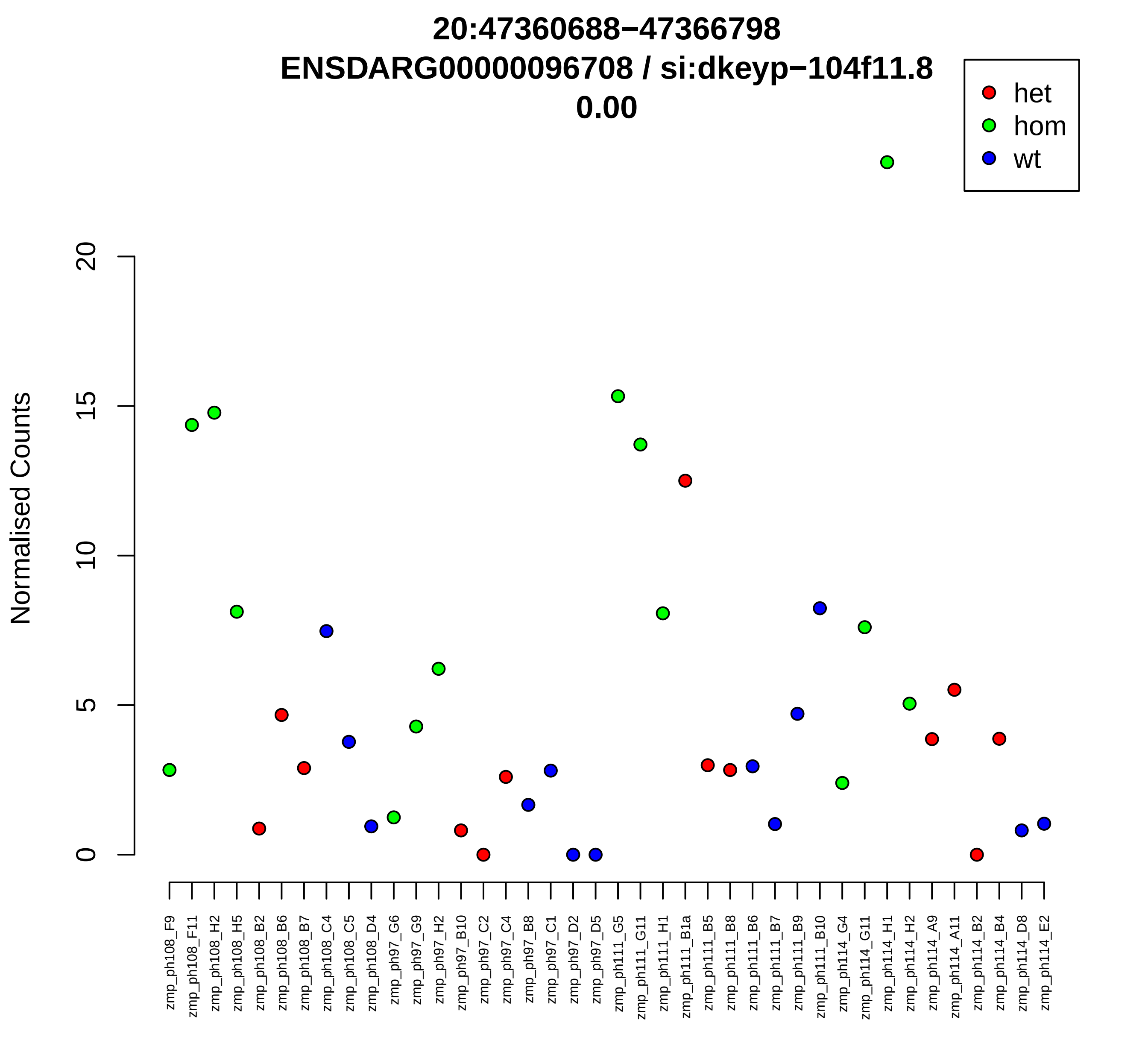

20:47360688−47366798
ENSDARG00000096708 / si:dkeyp−104f11.8
0.00
het
hom
wt
Normalised Counts
0
5
10
15
20
zmp_ph108_F9
zmp_ph108_F11
zmp_ph108_H2
zmp_ph108_H5
zmp_ph108_B2
zmp_ph108_B6
zmp_ph108_B7
zmp_ph108_C4
zmp_ph108_C5
zmp_ph108_D4
zmp_ph97_G6
zmp_ph97_G9
zmp_ph97_H2
zmp_ph97_B10
zmp_ph97_C2
zmp_ph97_C4
zmp_ph97_B8
zmp_ph97_C1
zmp_ph97_D2
zmp_ph97_D5
zmp_ph111_G5
zmp_ph111_G11
zmp_ph111_H1
zmp_ph111_B1a
zmp_ph111_B5
zmp_ph111_B8
zmp_ph111_B6
zmp_ph111_B7
zmp_ph111_B9
zmp_ph111_B10
zmp_ph114_G4
zmp_ph114_G11
zmp_ph114_H1
zmp_ph114_H2
zmp_ph114_A9
zmp_ph114_A11
zmp_ph114_B2
zmp_ph114_B4
zmp_ph114_D8
zmp_ph114_E2

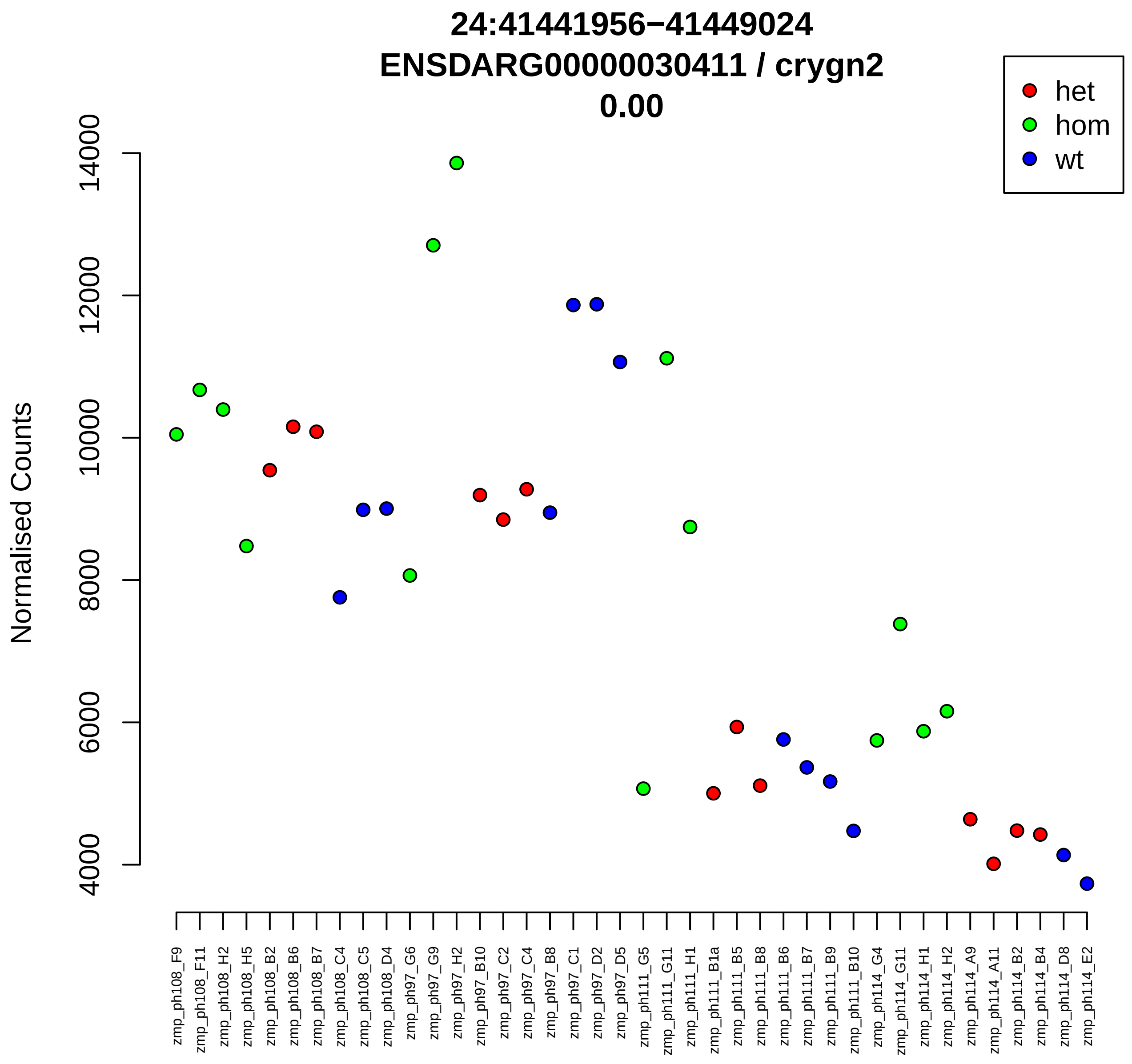

24:41441956−41449024
ENSDARG00000030411 / crygn2
0.00
het
hom
wt
Normalised Counts
4000
6000
8000
10000
12000
14000
zmp_ph108_F9
zmp_ph108_F11
zmp_ph108_H2
zmp_ph108_H5
zmp_ph108_B2
zmp_ph108_B6
zmp_ph108_B7
zmp_ph108_C4
zmp_ph108_C5
zmp_ph108_D4
zmp_ph97_G6
zmp_ph97_G9
zmp_ph97_H2
zmp_ph97_B10
zmp_ph97_C2
zmp_ph97_C4
zmp_ph97_B8
zmp_ph97_C1
zmp_ph97_D2
zmp_ph97_D5
zmp_ph111_G5
zmp_ph111_G11
zmp_ph111_H1
zmp_ph111_B1a
zmp_ph111_B5
zmp_ph111_B8
zmp_ph111_B6
zmp_ph111_B7
zmp_ph111_B9
zmp_ph111_B10
zmp_ph114_G4
zmp_ph114_G11
zmp_ph114_H1
zmp_ph114_H2
zmp_ph114_A9
zmp_ph114_A11
zmp_ph114_B2
zmp_ph114_B4
zmp_ph114_D8
zmp_ph114_E2

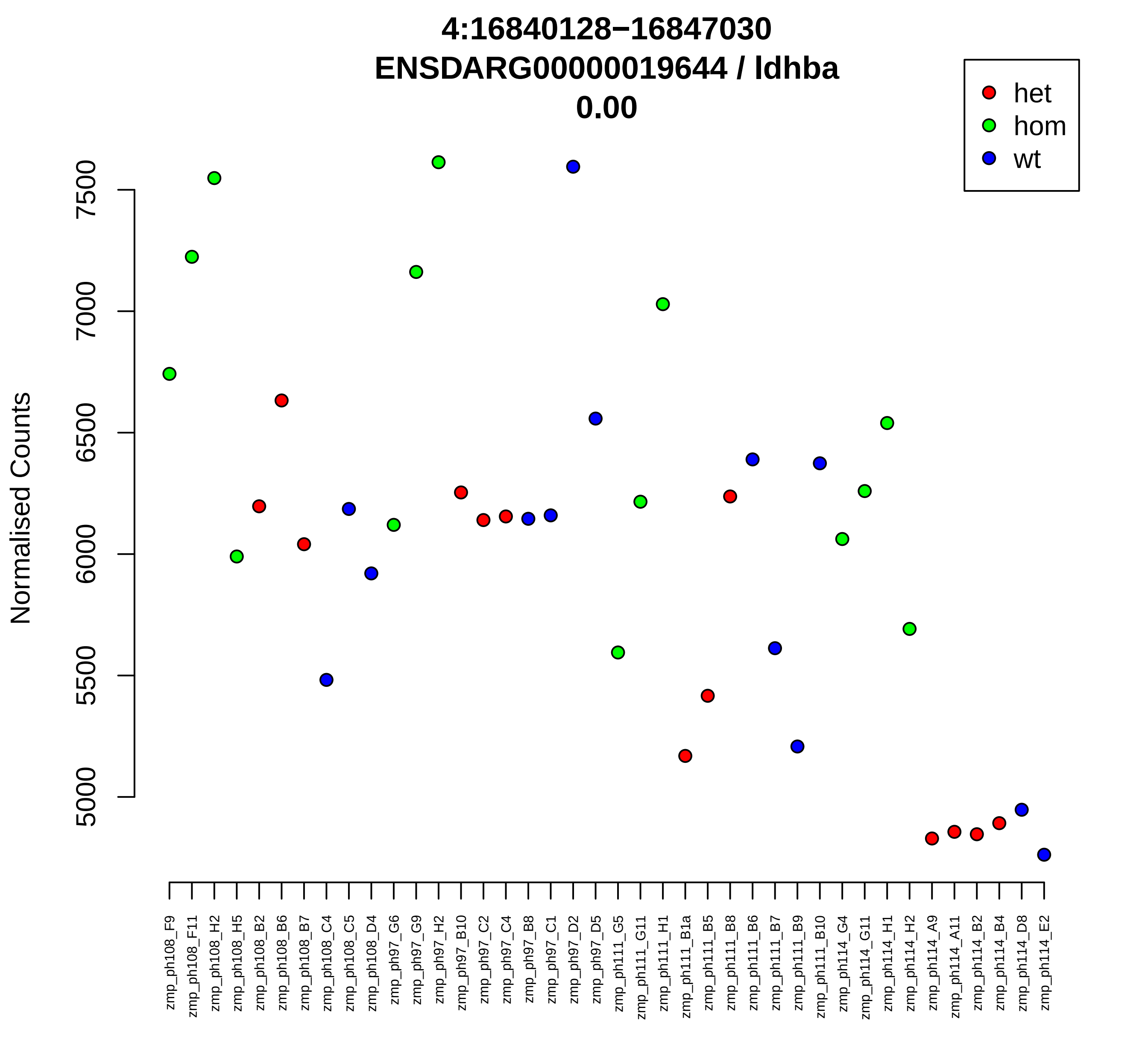

4:16840128−16847030
ENSDARG00000019644 / ldhba
0.00
het
hom
wt
Normalised Counts
5000
5500
6000
6500
7000
7500
zmp_ph108_F9
zmp_ph108_F11
zmp_ph108_H2
zmp_ph108_H5
zmp_ph108_B2
zmp_ph108_B6
zmp_ph108_B7
zmp_ph108_C4
zmp_ph108_C5
zmp_ph108_D4
zmp_ph97_G6
zmp_ph97_G9
zmp_ph97_H2
zmp_ph97_B10
zmp_ph97_C2
zmp_ph97_C4
zmp_ph97_B8
zmp_ph97_C1
zmp_ph97_D2
zmp_ph97_D5
zmp_ph111_G5
zmp_ph111_G11
zmp_ph111_H1
zmp_ph111_B1a
zmp_ph111_B5
zmp_ph111_B8
zmp_ph111_B6
zmp_ph111_B7
zmp_ph111_B9
zmp_ph111_B10
zmp_ph114_G4
zmp_ph114_G11
zmp_ph114_H1
zmp_ph114_H2
zmp_ph114_A9
zmp_ph114_A11
zmp_ph114_B2
zmp_ph114_B4
zmp_ph114_D8
zmp_ph114_E2

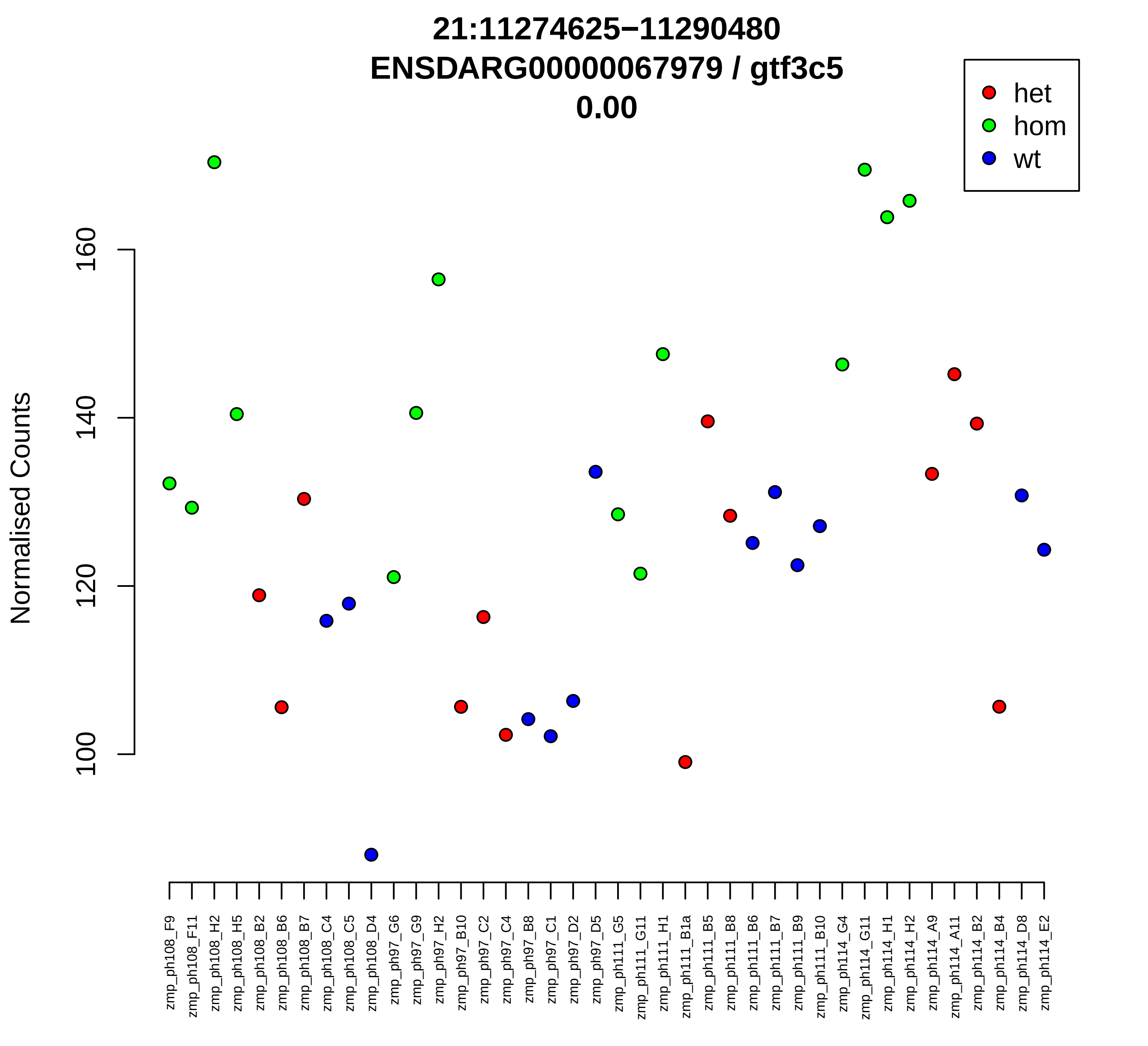

21:11274625−11290480
ENSDARG00000067979 / gtf3c5
0.00
het
hom
wt
Normalised Counts
100
120
140
160
zmp_ph108_F9
zmp_ph108_F11
zmp_ph108_H2
zmp_ph108_H5
zmp_ph108_B2
zmp_ph108_B6
zmp_ph108_B7
zmp_ph108_C4
zmp_ph108_C5
zmp_ph108_D4
zmp_ph97_G6
zmp_ph97_G9
zmp_ph97_H2
zmp_ph97_B10
zmp_ph97_C2
zmp_ph97_C4
zmp_ph97_B8
zmp_ph97_C1
zmp_ph97_D2
zmp_ph97_D5
zmp_ph111_G5
zmp_ph111_G11
zmp_ph111_H1
zmp_ph111_B1a
zmp_ph111_B5
zmp_ph111_B8
zmp_ph111_B6
zmp_ph111_B7
zmp_ph111_B9
zmp_ph111_B10
zmp_ph114_G4
zmp_ph114_G11
zmp_ph114_H1
zmp_ph114_H2
zmp_ph114_A9
zmp_ph114_A11
zmp_ph114_B2
zmp_ph114_B4
zmp_ph114_D8
zmp_ph114_E2

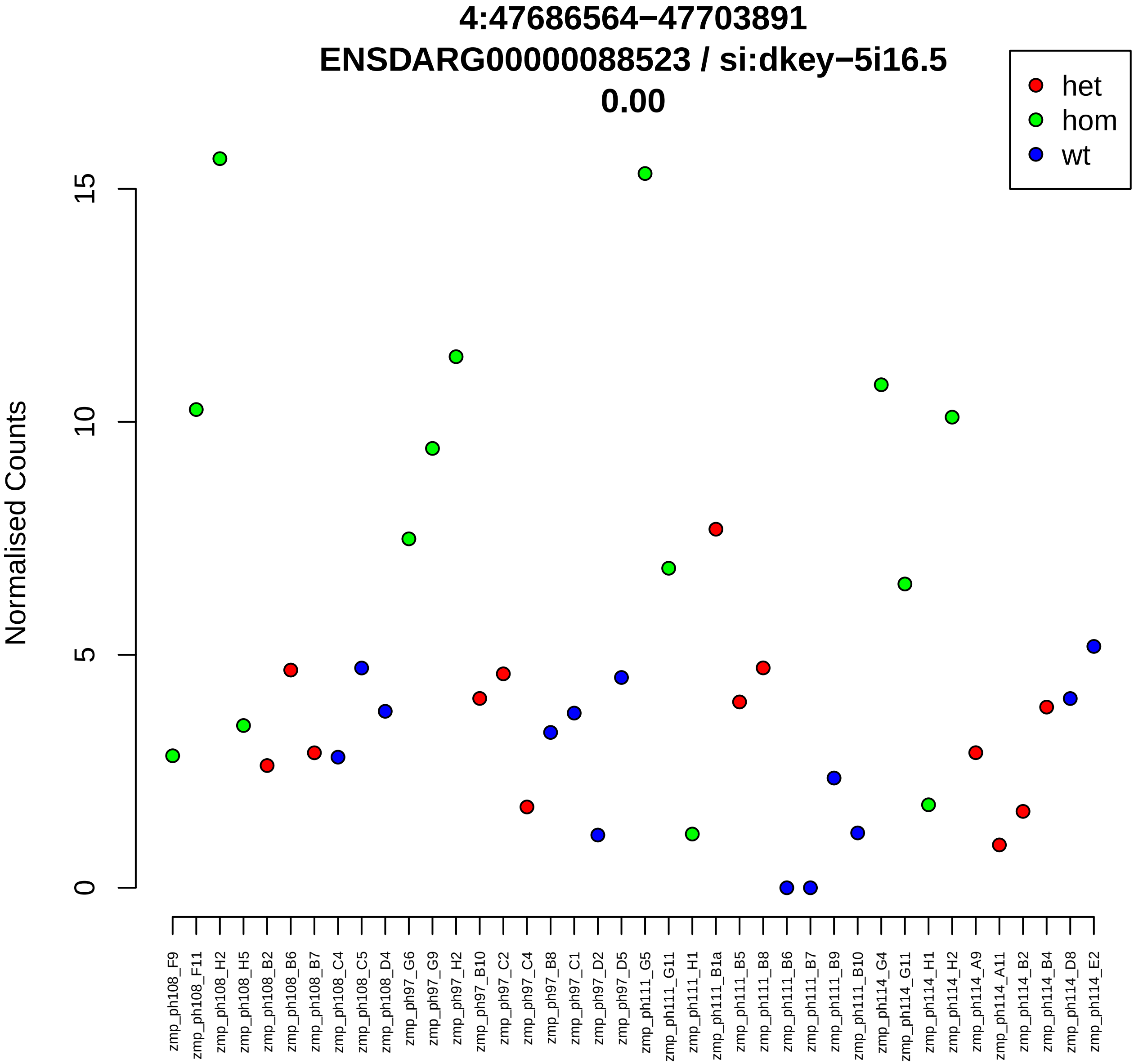

4:47686564−47703891
ENSDARG00000088523 / si:dkey−5i16.5
0.00
het
hom
wt
Normalised Counts
0
5
10
15
zmp_ph108_F9
zmp_ph108_F11
zmp_ph108_H2
zmp_ph108_H5
zmp_ph108_B2
zmp_ph108_B6
zmp_ph108_B7
zmp_ph108_C4
zmp_ph108_C5
zmp_ph108_D4
zmp_ph97_G6
zmp_ph97_G9
zmp_ph97_H2
zmp_ph97_B10
zmp_ph97_C2
zmp_ph97_C4
zmp_ph97_B8
zmp_ph97_C1
zmp_ph97_D2
zmp_ph97_D5
zmp_ph111_G5
zmp_ph111_G11
zmp_ph111_H1
zmp_ph111_B1a
zmp_ph111_B5
zmp_ph111_B8
zmp_ph111_B6
zmp_ph111_B7
zmp_ph111_B9
zmp_ph111_B10
zmp_ph114_G4
zmp_ph114_G11
zmp_ph114_H1
zmp_ph114_H2
zmp_ph114_A9
zmp_ph114_A11
zmp_ph114_B2
zmp_ph114_B4
zmp_ph114_D8
zmp_ph114_E2

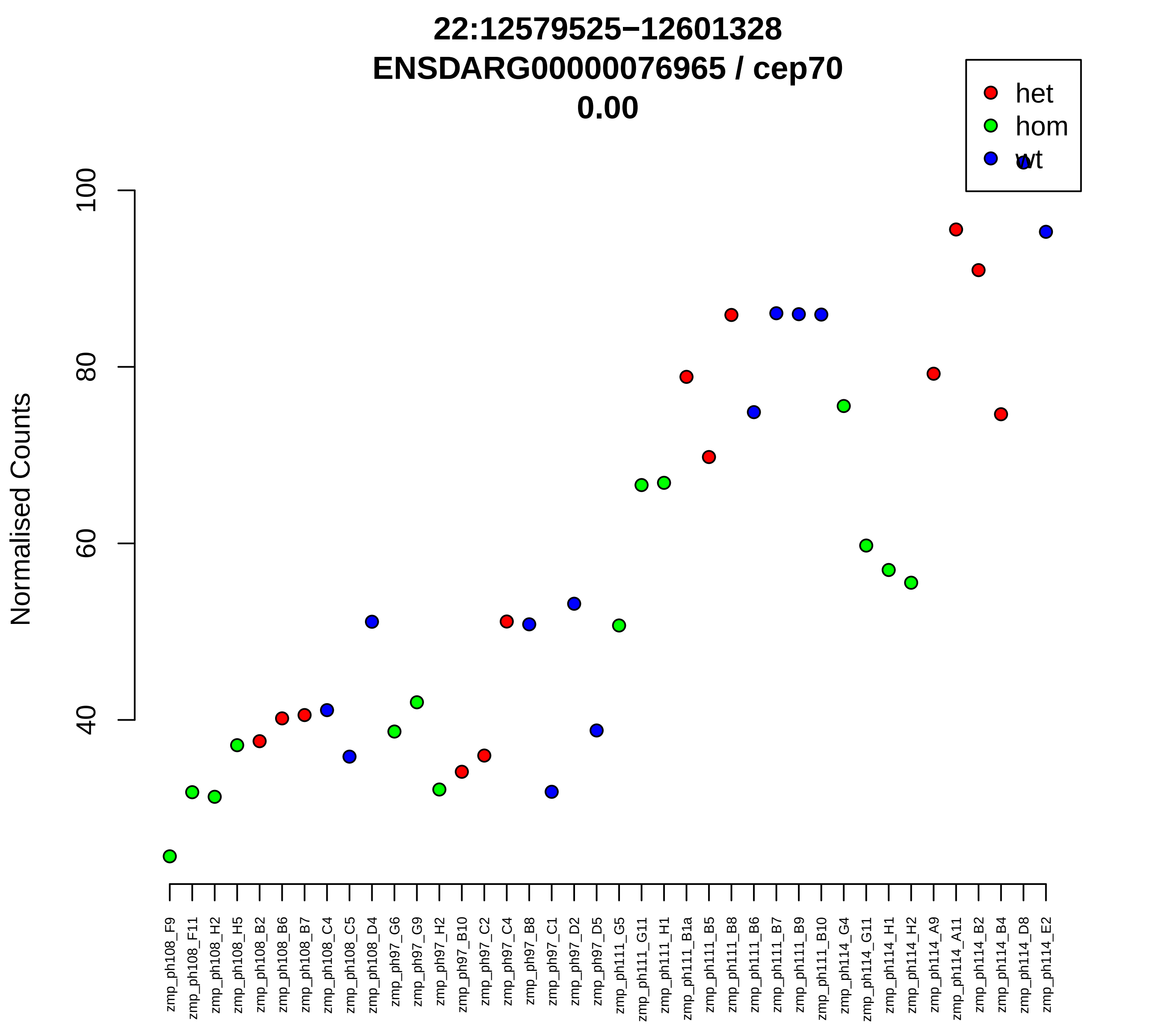

22:12579525−12601328
ENSDARG00000076965 / cep70
0.00
het
hom
wt
Normalised Counts
100
80
60
40
zmp_ph108_F9
zmp_ph108_F11
zmp_ph108_H2
zmp_ph108_H5
zmp_ph108_B2
zmp_ph108_B6
zmp_ph108_B7
zmp_ph108_C4
zmp_ph108_C5
zmp_ph108_D4
zmp_ph97_G6
zmp_ph97_G9
zmp_ph97_H2
zmp_ph97_B10
zmp_ph97_C2
zmp_ph97_C4
zmp_ph97_B8
zmp_ph97_C1
zmp_ph97_D2
zmp_ph97_D5
zmp_ph111_G5
zmp_ph111_G11
zmp_ph111_H1
zmp_ph111_B1a
zmp_ph111_B5
zmp_ph111_B8
zmp_ph111_B6
zmp_ph111_B7
zmp_ph111_B9
zmp_ph111_B10
zmp_ph114_G4
zmp_ph114_G11
zmp_ph114_H1
zmp_ph114_H2
zmp_ph114_A9
zmp_ph114_A11
zmp_ph114_B2
zmp_ph114_B4
zmp_ph114_D8
zmp_ph114_E2

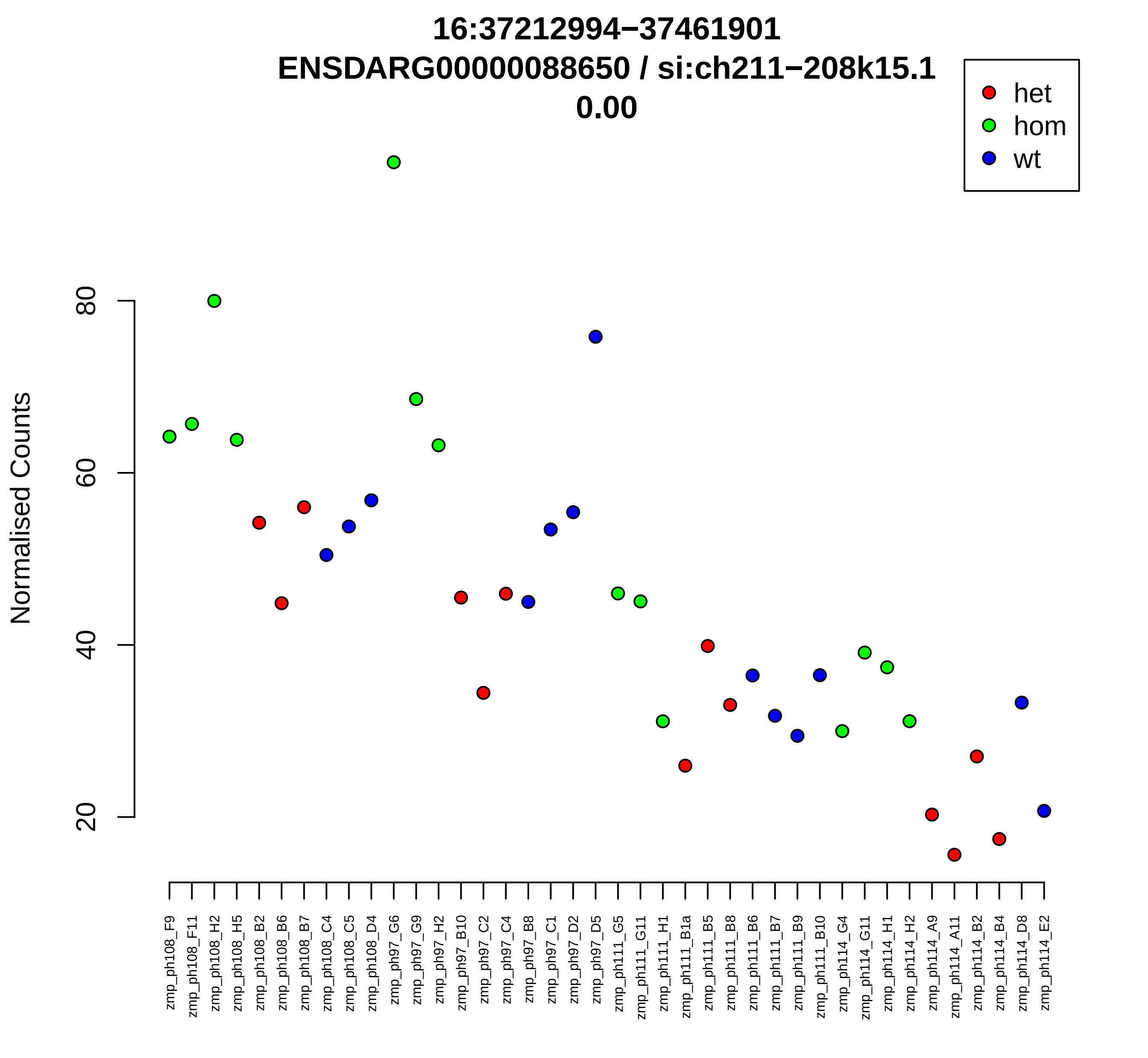
16:37212994−37461901
ENSDARG00000088650 / si:ch211−208k15.1
0.00
het
hom
wt
Normalised Counts
20
40
60
80
zmp_ph108_F9
zmp_ph108_F11
zmp_ph108_H2
zmp_ph108_H5
zmp_ph108_B2
zmp_ph108_B6
zmp_ph108_B7
zmp_ph108_C4
zmp_ph108_C5
zmp_ph108_D4
zmp_ph97_G6
zmp_ph97_G9
zmp_ph97_H2
zmp_ph97_B10
zmp_ph97_C2
zmp_ph97_C4
zmp_ph97_B8
zmp_ph97_C1
zmp_ph97_D2
zmp_ph97_D5
zmp_ph111_G5
zmp_ph111_G11
zmp_ph111_H1
zmp_ph111_B1a
zmp_ph111_B5
zmp_ph111_B8
zmp_ph111_B6
zmp_ph111_B7
zmp_ph111_B9
zmp_ph111_B10
zmp_ph114_G4
zmp_ph114_G11
zmp_ph114_H1
zmp_ph114_H2
zmp_ph114_A9
zmp_ph114_A11
zmp_ph114_B2
zmp_ph114_B4
zmp_ph114_D8
zmp_ph114_E2

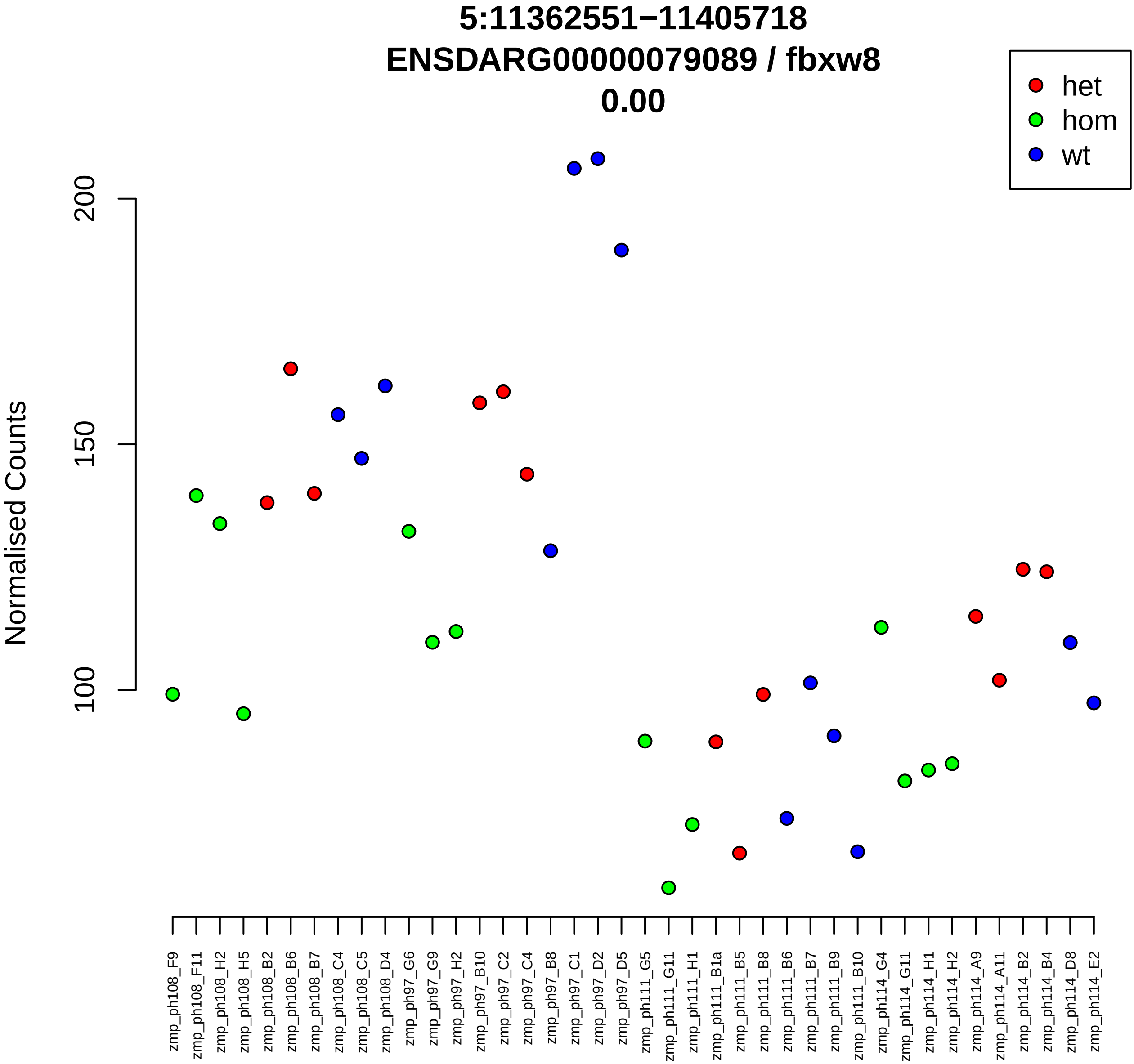

5:11362551−11405718
ENSDARG00000079089 / fbxw8
0.00
het
hom
wt
Normalised Counts
100
150
200
zmp_ph108_F9
zmp_ph108_F11
zmp_ph108_H2
zmp_ph108_H5
zmp_ph108_B2
zmp_ph108_B6
zmp_ph108_B7
zmp_ph108_C4
zmp_ph108_C5
zmp_ph108_D4
zmp_ph97_G6
zmp_ph97_G9
zmp_ph97_H2
zmp_ph97_B10
zmp_ph97_C2
zmp_ph97_C4
zmp_ph97_B8
zmp_ph97_C1
zmp_ph97_D2
zmp_ph97_D5
zmp_ph111_G5
zmp_ph111_G11
zmp_ph111_H1
zmp_ph111_B1a
zmp_ph111_B5
zmp_ph111_B8
zmp_ph111_B6
zmp_ph111_B7
zmp_ph111_B9
zmp_ph111_B10
zmp_ph114_G4
zmp_ph114_G11
zmp_ph114_H1
zmp_ph114_H2
zmp_ph114_A9
zmp_ph114_A11
zmp_ph114_B2
zmp_ph114_B4
zmp_ph114_D8
zmp_ph114_E2

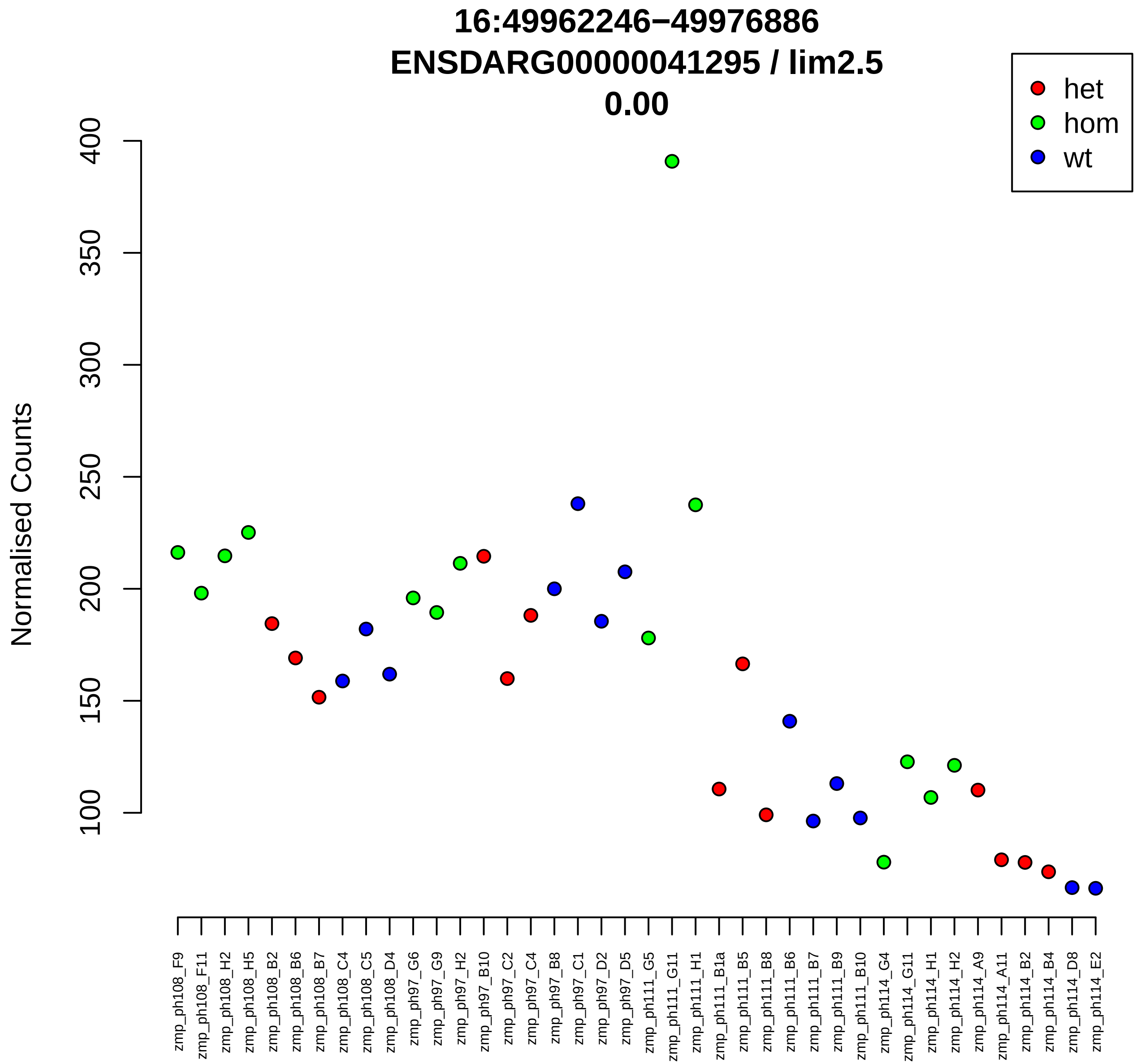
16:49962246−49976886
ENSDARG00000041295 / lim2.5
0.00
het
hom
wt
Normalised Counts
100
150
200
250
300
350
400
zmp_ph108_F9
zmp_ph108_F11
zmp_ph108_H2
zmp_ph108_H5
zmp_ph108_B2
zmp_ph108_B6
zmp_ph108_B7
zmp_ph108_C4
zmp_ph108_C5
zmp_ph108_D4
zmp_ph97_G6
zmp_ph97_G9
zmp_ph97_H2
zmp_ph97_B10
zmp_ph97_C2
zmp_ph97_C4
zmp_ph97_B8
zmp_ph97_C1
zmp_ph97_D2
zmp_ph97_D5
zmp_ph111_G5
zmp_ph111_G11
zmp_ph111_H1
zmp_ph111_B1a
zmp_ph111_B5
zmp_ph111_B8
zmp_ph111_B6
zmp_ph111_B7
zmp_ph111_B9
zmp_ph111_B10
zmp_ph114_G4
zmp_ph114_G11
zmp_ph114_H1
zmp_ph114_H2
zmp_ph114_A9
zmp_ph114_A11
zmp_ph114_B2
zmp_ph114_B4
zmp_ph114_D8
zmp_ph114_E2

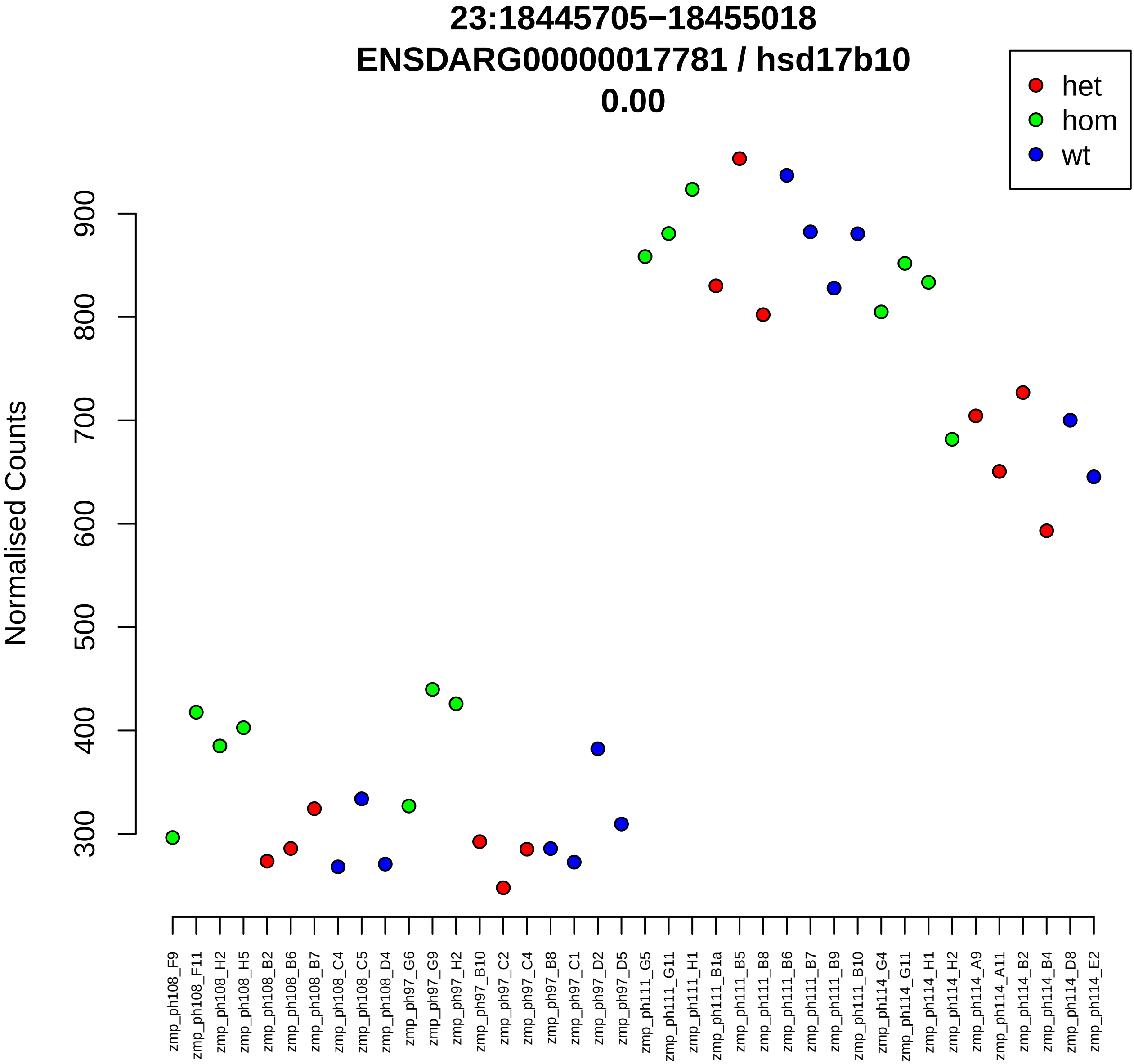
23:18445705−18455018
ENSDARG00000017781 / hsd17b10
0.00
het
hom
wt
Normalised Counts
300
400
500
600
700
800
900
zmp_ph108_F9
zmp_ph108_F11
zmp_ph108_H2
zmp_ph108_H5
zmp_ph108_B2
zmp_ph108_B6
zmp_ph108_B7
zmp_ph108_C4
zmp_ph108_C5
zmp_ph108_D4
zmp_ph97_G6
zmp_ph97_G9
zmp_ph97_H2
zmp_ph97_B10
zmp_ph97_C2
zmp_ph97_C4
zmp_ph97_B8
zmp_ph97_C1
zmp_ph97_D2
zmp_ph97_D5
zmp_ph111_G5
zmp_ph111_G11
zmp_ph111_H1
zmp_ph111_B1a
zmp_ph111_B5
zmp_ph111_B8
zmp_ph111_B6
zmp_ph111_B7
zmp_ph111_B9
zmp_ph111_B10
zmp_ph114_G4
zmp_ph114_G11
zmp_ph114_H1
zmp_ph114_H2
zmp_ph114_A9
zmp_ph114_A11
zmp_ph114_B2
zmp_ph114_B4
zmp_ph114_D8
zmp_ph114_E2

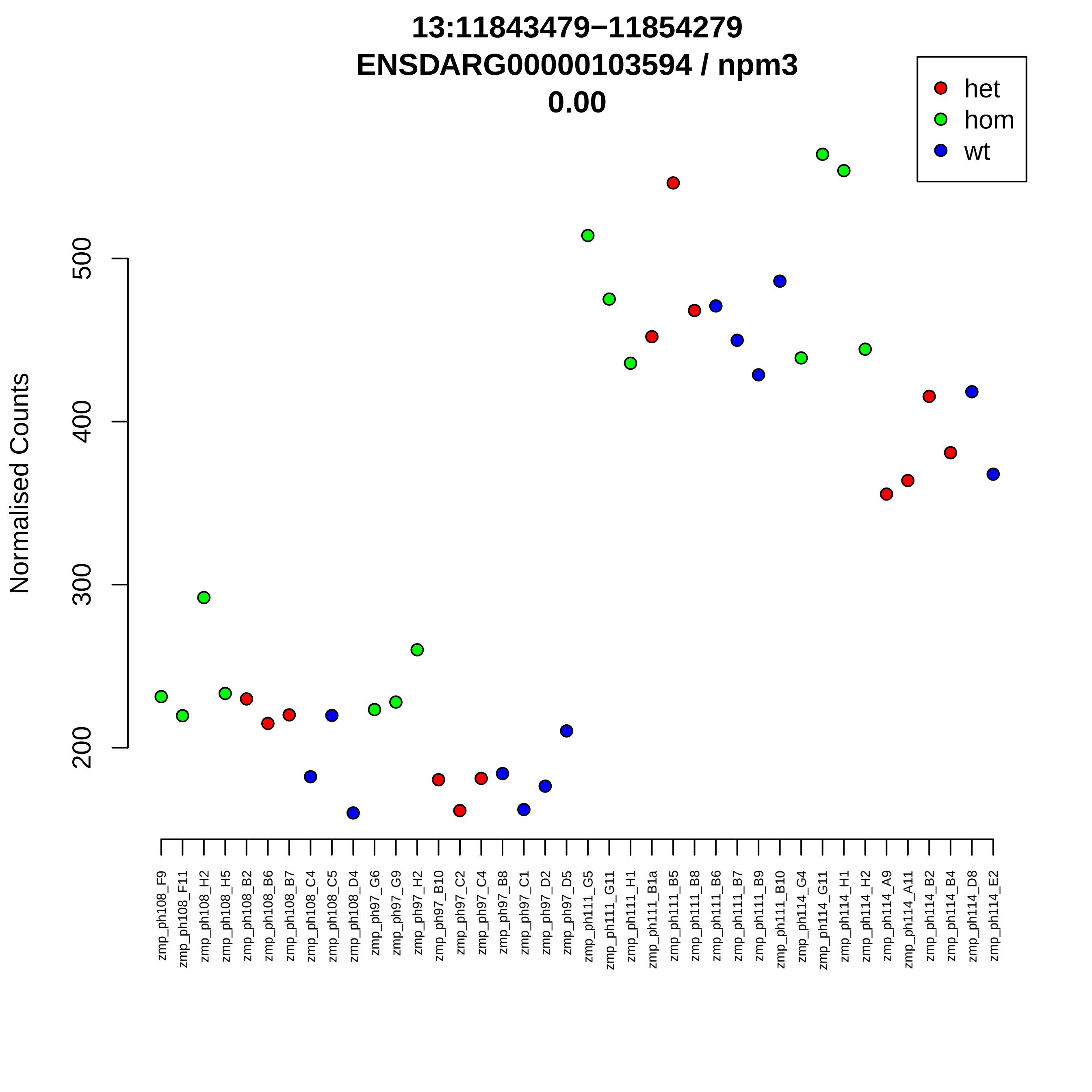

13:11843479−11854279
ENSDARG00000103594 / npm3
0.00
het
hom
wt
Normalised Counts
200
300
400
500
zmp_ph108_F9
zmp_ph108_F11
zmp_ph108_H2
zmp_ph108_H5
zmp_ph108_B2
zmp_ph108_B6
zmp_ph108_B7
zmp_ph108_C4
zmp_ph108_C5
zmp_ph108_D4
zmp_ph97_G6
zmp_ph97_G9
zmp_ph97_H2
zmp_ph97_B10
zmp_ph97_C2
zmp_ph97_C4
zmp_ph97_B8
zmp_ph97_C1
zmp_ph97_D2
zmp_ph97_D5
zmp_ph111_G5
zmp_ph111_G11
zmp_ph111_H1
zmp_ph111_B1a
zmp_ph111_B5
zmp_ph111_B8
zmp_ph111_B6
zmp_ph111_B7
zmp_ph111_B9
zmp_ph111_B10
zmp_ph114_G4
zmp_ph114_G11
zmp_ph114_H1
zmp_ph114_H2
zmp_ph114_A9
zmp_ph114_A11
zmp_ph114_B2
zmp_ph114_B4
zmp_ph114_D8
zmp_ph114_E2

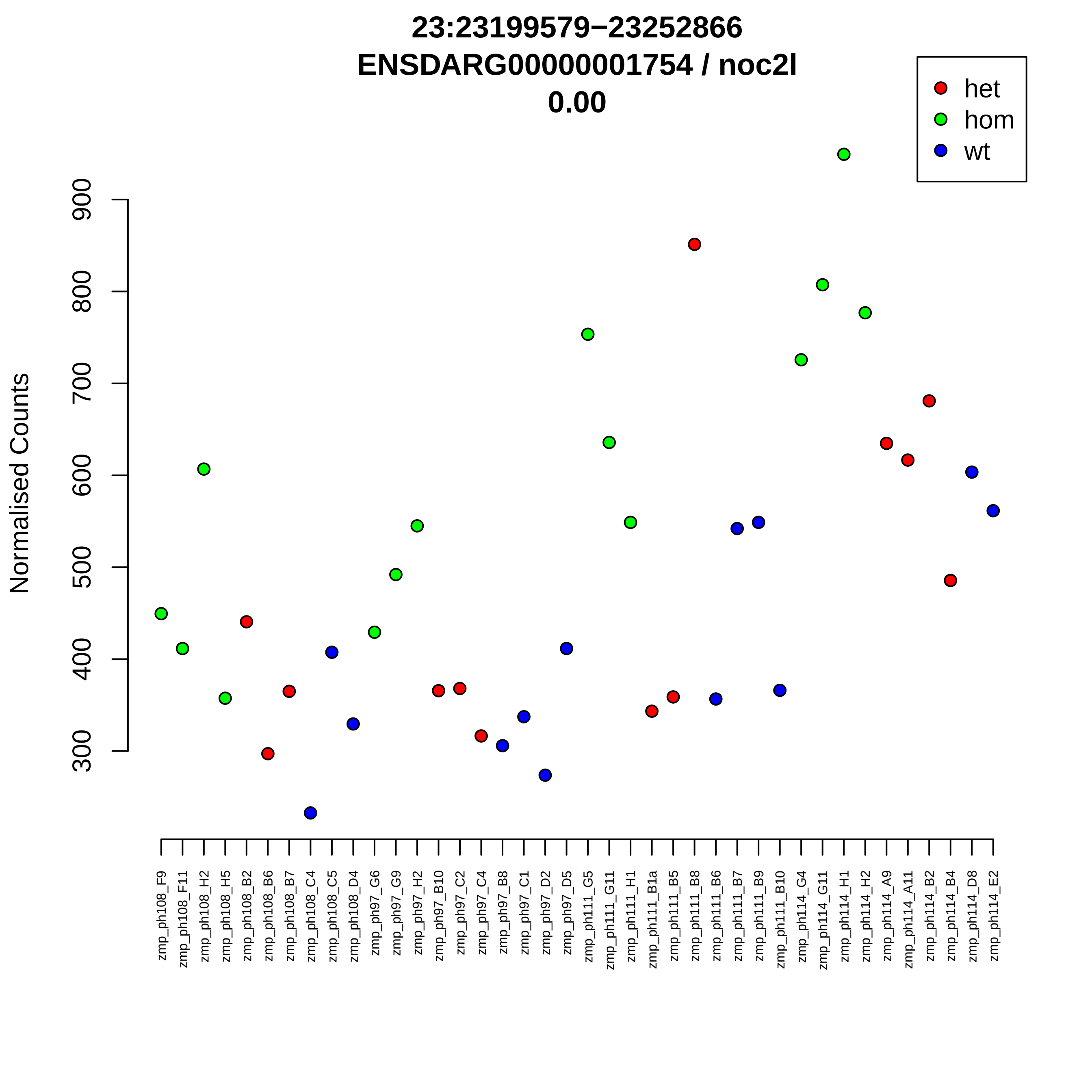

23:23199579−23252866
ENSDARG00000001754 / noc2l
0.00
het
hom
wt
Normalised Counts
300
400
500
600
700
800
900
zmp_ph108_F9
zmp_ph108_F11
zmp_ph108_H2
zmp_ph108_H5
zmp_ph108_B2
zmp_ph108_B6
zmp_ph108_B7
zmp_ph108_C4
zmp_ph108_C5
zmp_ph108_D4
zmp_ph97_G6
zmp_ph97_G9
zmp_ph97_H2
zmp_ph97_B10
zmp_ph97_C2
zmp_ph97_C4
zmp_ph97_B8
zmp_ph97_C1
zmp_ph97_D2
zmp_ph97_D5
zmp_ph111_G5
zmp_ph111_G11
zmp_ph111_H1
zmp_ph111_B1a
zmp_ph111_B5
zmp_ph111_B8
zmp_ph111_B6
zmp_ph111_B7
zmp_ph111_B9
zmp_ph111_B10
zmp_ph114_G4
zmp_ph114_G11
zmp_ph114_H1
zmp_ph114_H2
zmp_ph114_A9
zmp_ph114_A11
zmp_ph114_B2
zmp_ph114_B4
zmp_ph114_D8
zmp_ph114_E2

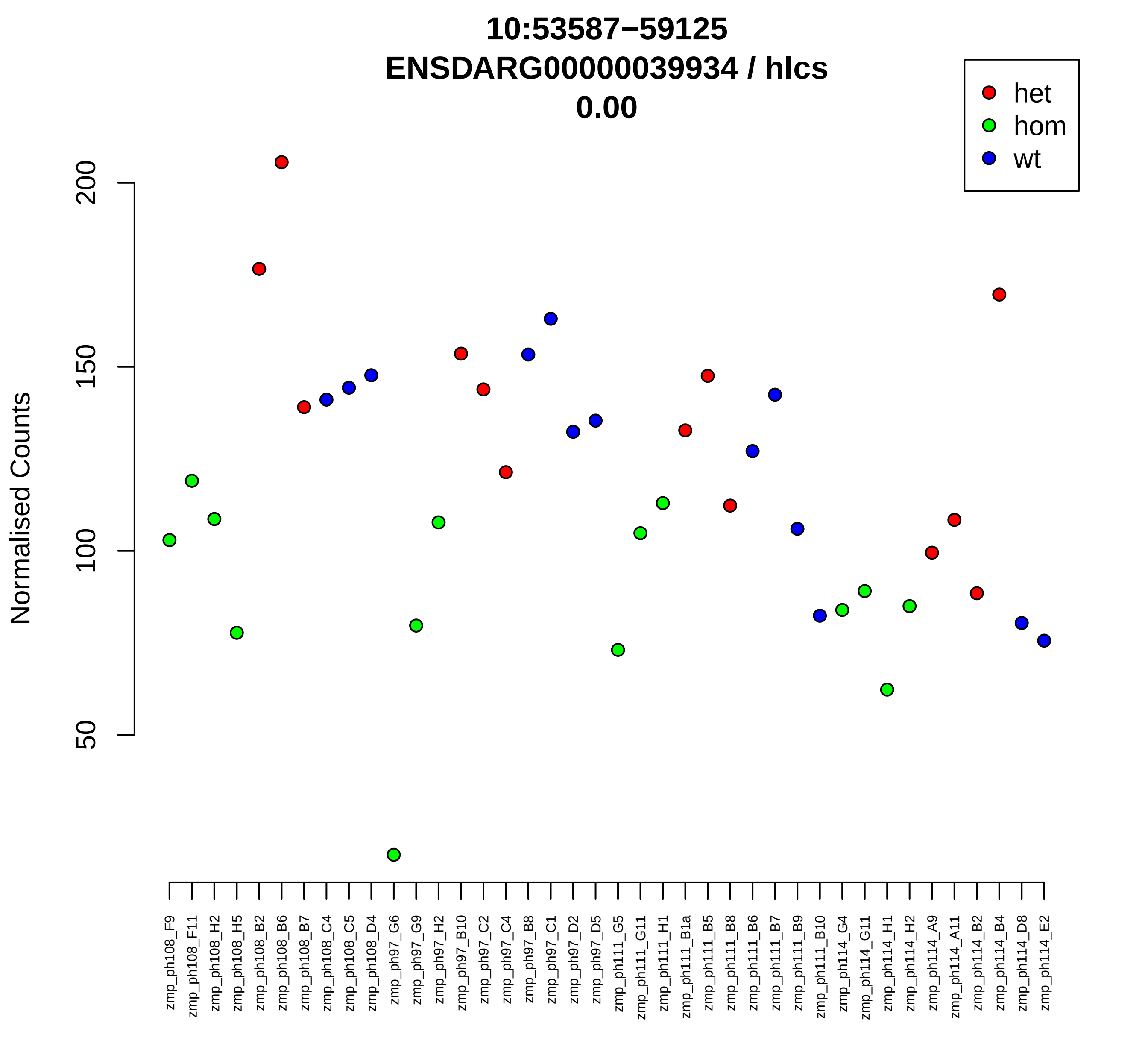
10:53587−59125
ENSDARG00000039934 / hlcs
0.00
het
hom
wt
Normalised Counts
50
100
150
200
zmp_ph108_F9
zmp_ph108_F11
zmp_ph108_H2
zmp_ph108_H5
zmp_ph108_B2
zmp_ph108_B6
zmp_ph108_B7
zmp_ph108_C4
zmp_ph108_C5
zmp_ph108_D4
zmp_ph97_G6
zmp_ph97_G9
zmp_ph97_H2
zmp_ph97_B10
zmp_ph97_C2
zmp_ph97_C4
zmp_ph97_B8
zmp_ph97_C1
zmp_ph97_D2
zmp_ph97_D5
zmp_ph111_G5
zmp_ph111_G11
zmp_ph111_H1
zmp_ph111_B1a
zmp_ph111_B5
zmp_ph111_B8
zmp_ph111_B6
zmp_ph111_B7
zmp_ph111_B9
zmp_ph111_B10
zmp_ph114_G4
zmp_ph114_G11
zmp_ph114_H1
zmp_ph114_H2
zmp_ph114_A9
zmp_ph114_A11
zmp_ph114_B2
zmp_ph114_B4
zmp_ph114_D8
zmp_ph114_E2

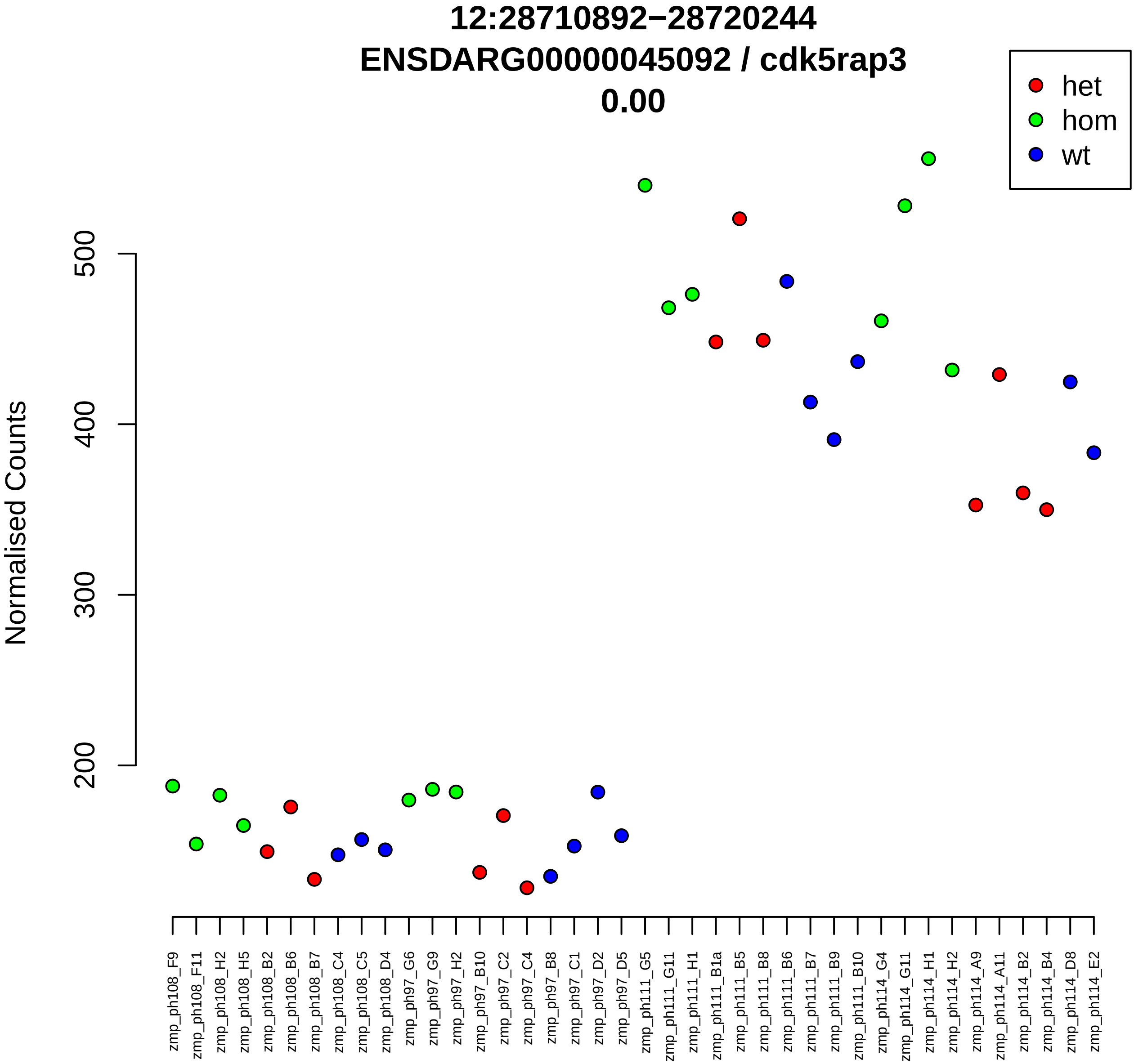
12:28710892−28720244
ENSDARG00000045092 / cdk5rap3
0.00
het
hom
wt
Normalised Counts
200
300
400
500
zmp_ph108_F9
zmp_ph108_F11
zmp_ph108_H2
zmp_ph108_H5
zmp_ph108_B2
zmp_ph108_B6
zmp_ph108_B7
zmp_ph108_C4
zmp_ph108_C5
zmp_ph108_D4
zmp_ph97_G6
zmp_ph97_G9
zmp_ph97_H2
zmp_ph97_B10
zmp_ph97_C2
zmp_ph97_C4
zmp_ph97_B8
zmp_ph97_C1
zmp_ph97_D2
zmp_ph97_D5
zmp_ph111_G5
zmp_ph111_G11
zmp_ph111_H1
zmp_ph111_B1a
zmp_ph111_B5
zmp_ph111_B8
zmp_ph111_B6
zmp_ph111_B7
zmp_ph111_B9
zmp_ph111_B10
zmp_ph114_G4
zmp_ph114_G11
zmp_ph114_H1
zmp_ph114_H2
zmp_ph114_A9
zmp_ph114_A11
zmp_ph114_B2
zmp_ph114_B4
zmp_ph114_D8
zmp_ph114_E2

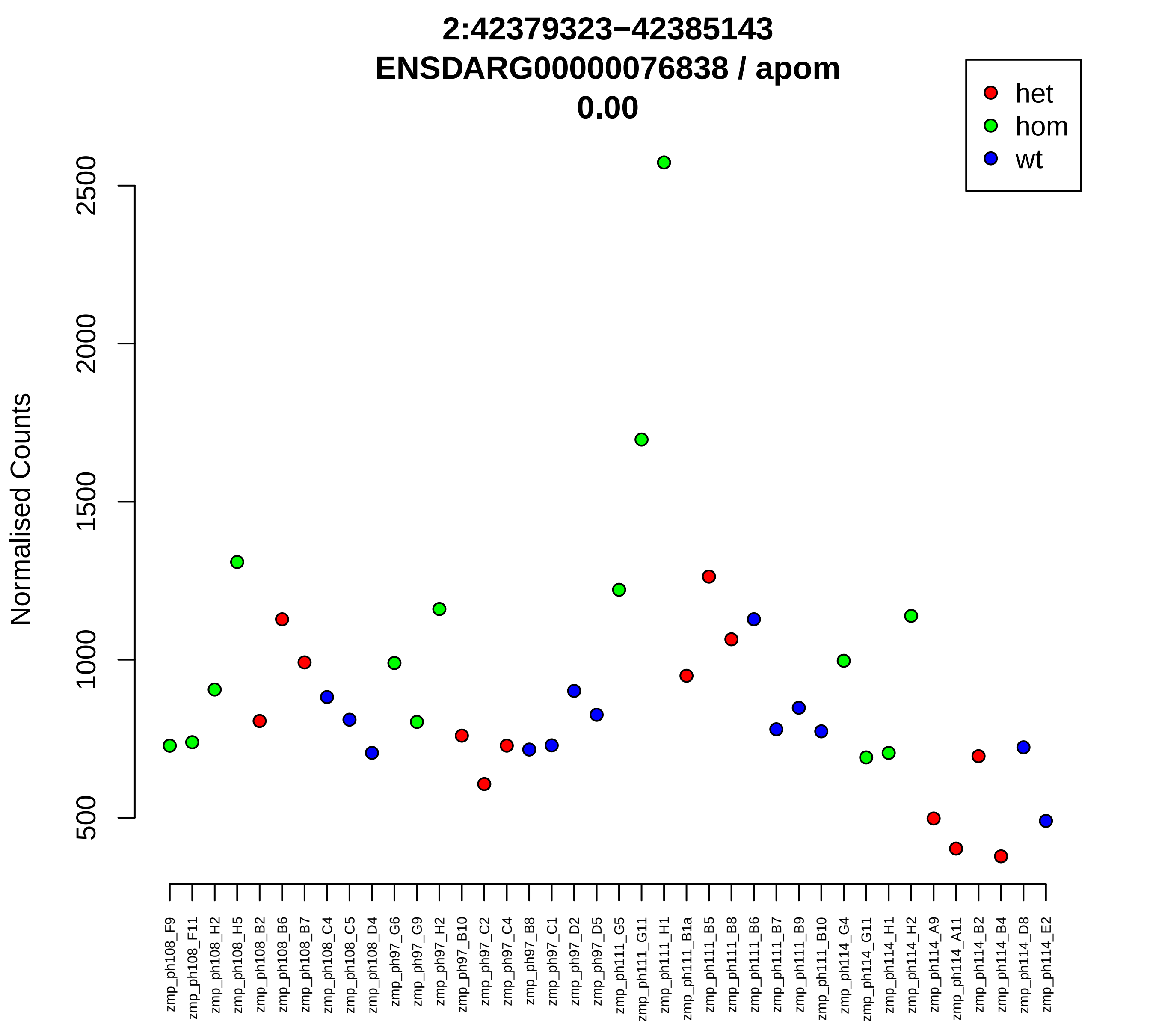

2:42379323−42385143
ENSDARG00000076838 / apom
0.00
het
hom
wt
Normalised Counts
500
1000
1500
2000
2500
zmp_ph108_F9
zmp_ph108_F11
zmp_ph108_H2
zmp_ph108_H5
zmp_ph108_B2
zmp_ph108_B6
zmp_ph108_B7
zmp_ph108_C4
zmp_ph108_C5
zmp_ph108_D4
zmp_ph97_G6
zmp_ph97_G9
zmp_ph97_H2
zmp_ph97_B10
zmp_ph97_C2
zmp_ph97_C4
zmp_ph97_B8
zmp_ph97_C1
zmp_ph97_D2
zmp_ph97_D5
zmp_ph111_G5
zmp_ph111_G11
zmp_ph111_H1
zmp_ph111_B1a
zmp_ph111_B5
zmp_ph111_B8
zmp_ph111_B6
zmp_ph111_B7
zmp_ph111_B9
zmp_ph111_B10
zmp_ph114_G4
zmp_ph114_G11
zmp_ph114_H1
zmp_ph114_H2
zmp_ph114_A9
zmp_ph114_A11
zmp_ph114_B2
zmp_ph114_B4
zmp_ph114_D8
zmp_ph114_E2

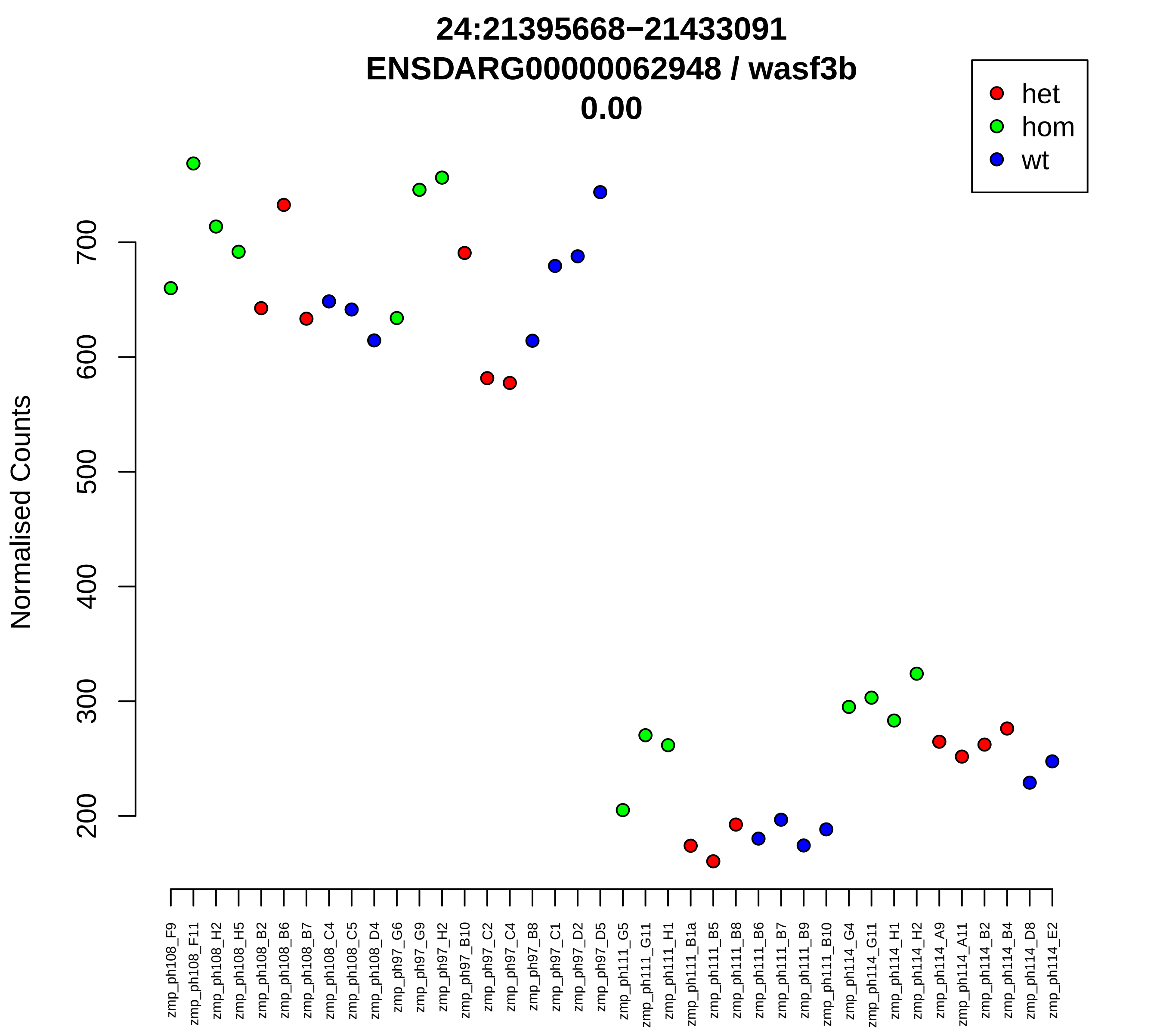

24:21395668−21433091
ENSDARG00000062948 / wasf3b
0.00
het
hom
wt
Normalised Counts
200
300
400
500
600
700
zmp_ph108_F9
zmp_ph108_F11
zmp_ph108_H2
zmp_ph108_H5
zmp_ph108_B2
zmp_ph108_B6
zmp_ph108_B7
zmp_ph108_C4
zmp_ph108_C5
zmp_ph108_D4
zmp_ph97_G6
zmp_ph97_G9
zmp_ph97_H2
zmp_ph97_B10
zmp_ph97_C2
zmp_ph97_C4
zmp_ph97_B8
zmp_ph97_C1
zmp_ph97_D2
zmp_ph97_D5
zmp_ph111_G5
zmp_ph111_G11
zmp_ph111_H1
zmp_ph111_B1a
zmp_ph111_B5
zmp_ph111_B8
zmp_ph111_B6
zmp_ph111_B7
zmp_ph111_B9
zmp_ph111_B10
zmp_ph114_G4
zmp_ph114_G11
zmp_ph114_H1
zmp_ph114_H2
zmp_ph114_A9
zmp_ph114_A11
zmp_ph114_B2
zmp_ph114_B4
zmp_ph114_D8
zmp_ph114_E2

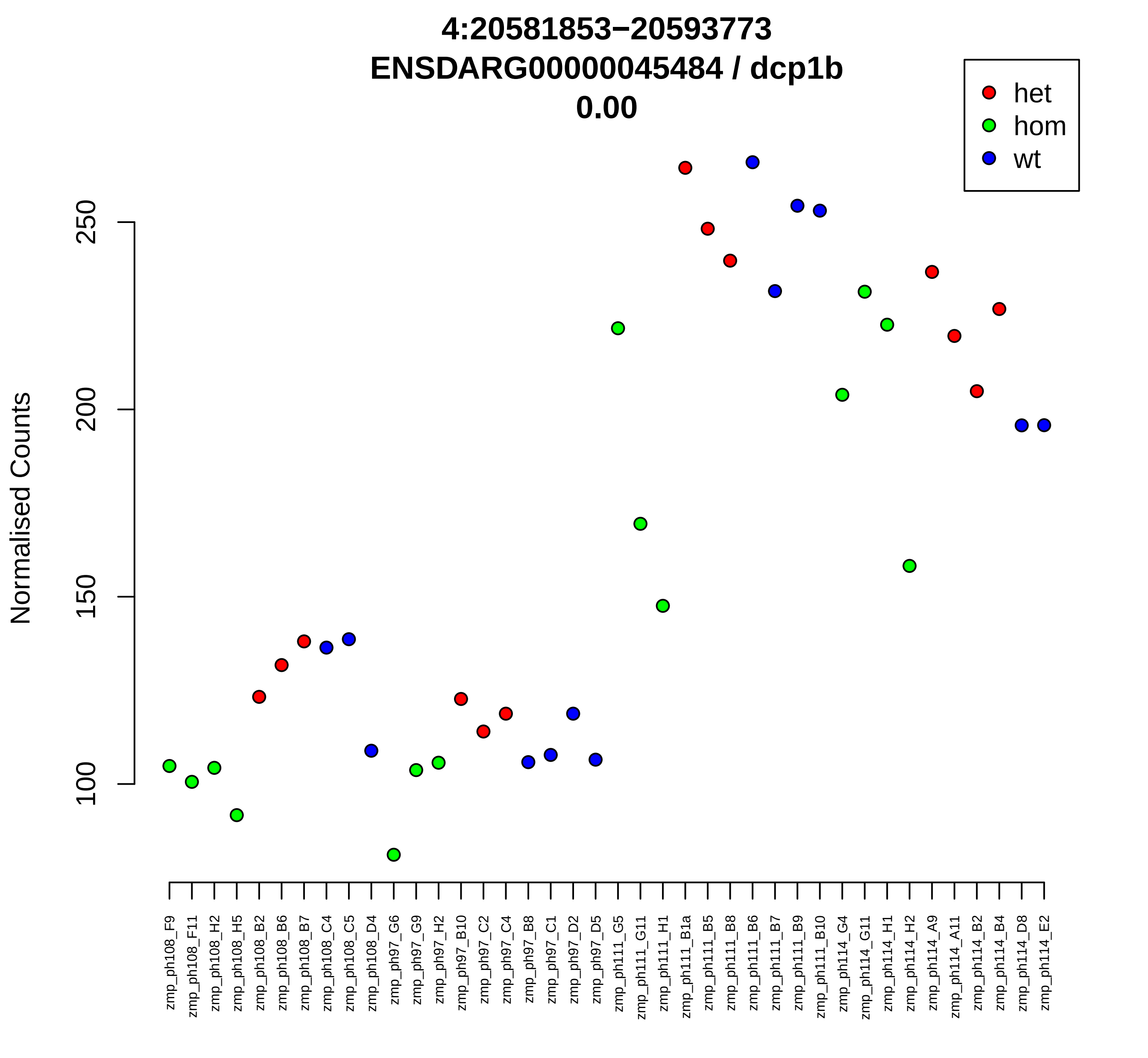
4:20581853−20593773
ENSDARG00000045484 / dcp1b
0.00
het
hom
wt
Normalised Counts
100
150
200
250
zmp_ph108_F9
zmp_ph108_F11
zmp_ph108_H2
zmp_ph108_H5
zmp_ph108_B2
zmp_ph108_B6
zmp_ph108_B7
zmp_ph108_C4
zmp_ph108_C5
zmp_ph108_D4
zmp_ph97_G6
zmp_ph97_G9
zmp_ph97_H2
zmp_ph97_B10
zmp_ph97_C2
zmp_ph97_C4
zmp_ph97_B8
zmp_ph97_C1
zmp_ph97_D2
zmp_ph97_D5
zmp_ph111_G5
zmp_ph111_G11
zmp_ph111_H1
zmp_ph111_B1a
zmp_ph111_B5
zmp_ph111_B8
zmp_ph111_B6
zmp_ph111_B7
zmp_ph111_B9
zmp_ph111_B10
zmp_ph114_G4
zmp_ph114_G11
zmp_ph114_H1
zmp_ph114_H2
zmp_ph114_A9
zmp_ph114_A11
zmp_ph114_B2
zmp_ph114_B4
zmp_ph114_D8
zmp_ph114_E2

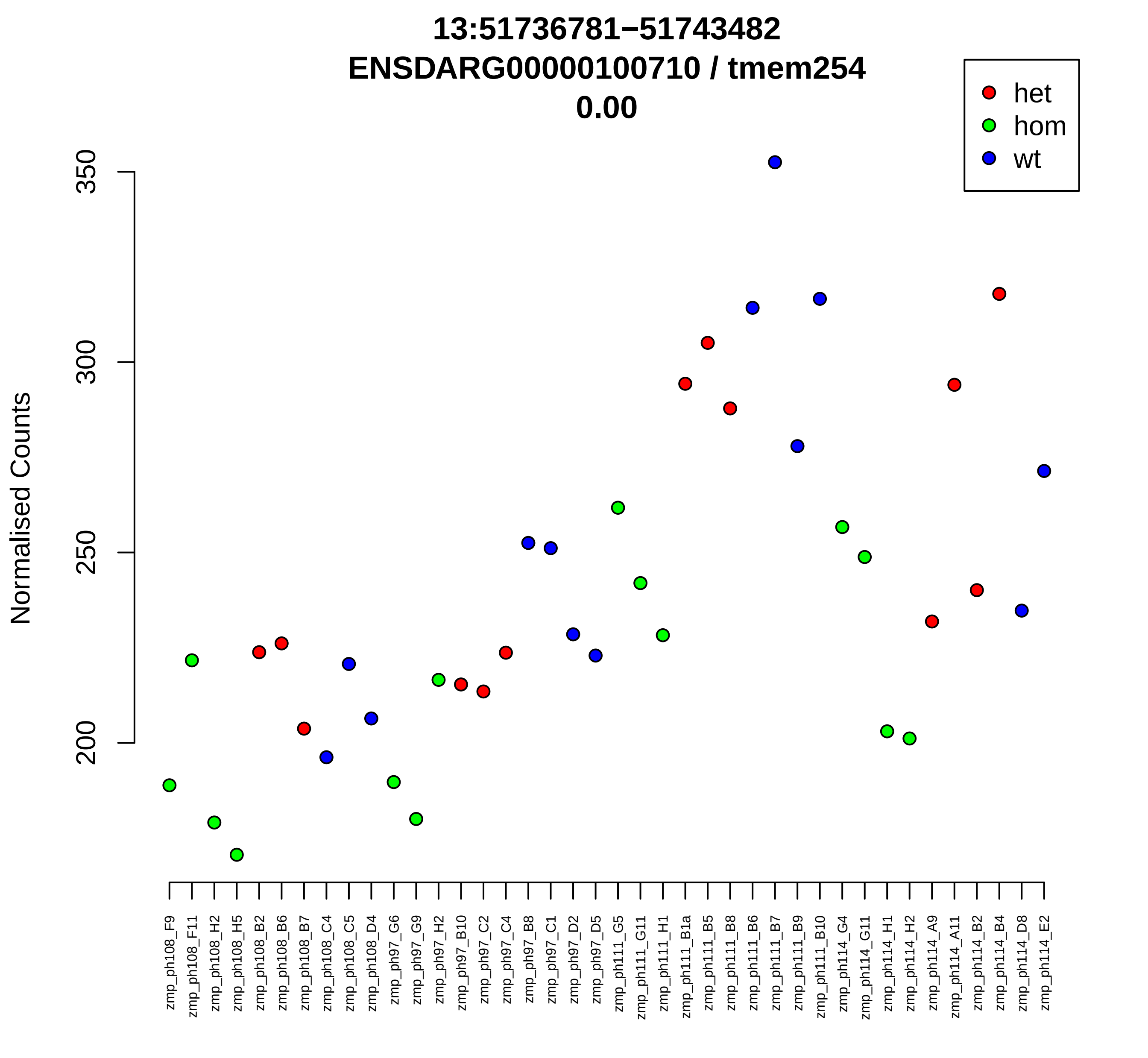
13:51736781−51743482
ENSDARG00000100710 / tmem254
0.00
het
hom
wt
Normalised Counts
200
250
300
350
zmp_ph108_F9
zmp_ph108_F11
zmp_ph108_H2
zmp_ph108_H5
zmp_ph108_B2
zmp_ph108_B6
zmp_ph108_B7
zmp_ph108_C4
zmp_ph108_C5
zmp_ph108_D4
zmp_ph97_G6
zmp_ph97_G9
zmp_ph97_H2
zmp_ph97_B10
zmp_ph97_C2
zmp_ph97_C4
zmp_ph97_B8
zmp_ph97_C1
zmp_ph97_D2
zmp_ph97_D5
zmp_ph111_G5
zmp_ph111_G11
zmp_ph111_H1
zmp_ph111_B1a
zmp_ph111_B5
zmp_ph111_B8
zmp_ph111_B6
zmp_ph111_B7
zmp_ph111_B9
zmp_ph111_B10
zmp_ph114_G4
zmp_ph114_G11
zmp_ph114_H1
zmp_ph114_H2
zmp_ph114_A9
zmp_ph114_A11
zmp_ph114_B2
zmp_ph114_B4
zmp_ph114_D8
zmp_ph114_E2

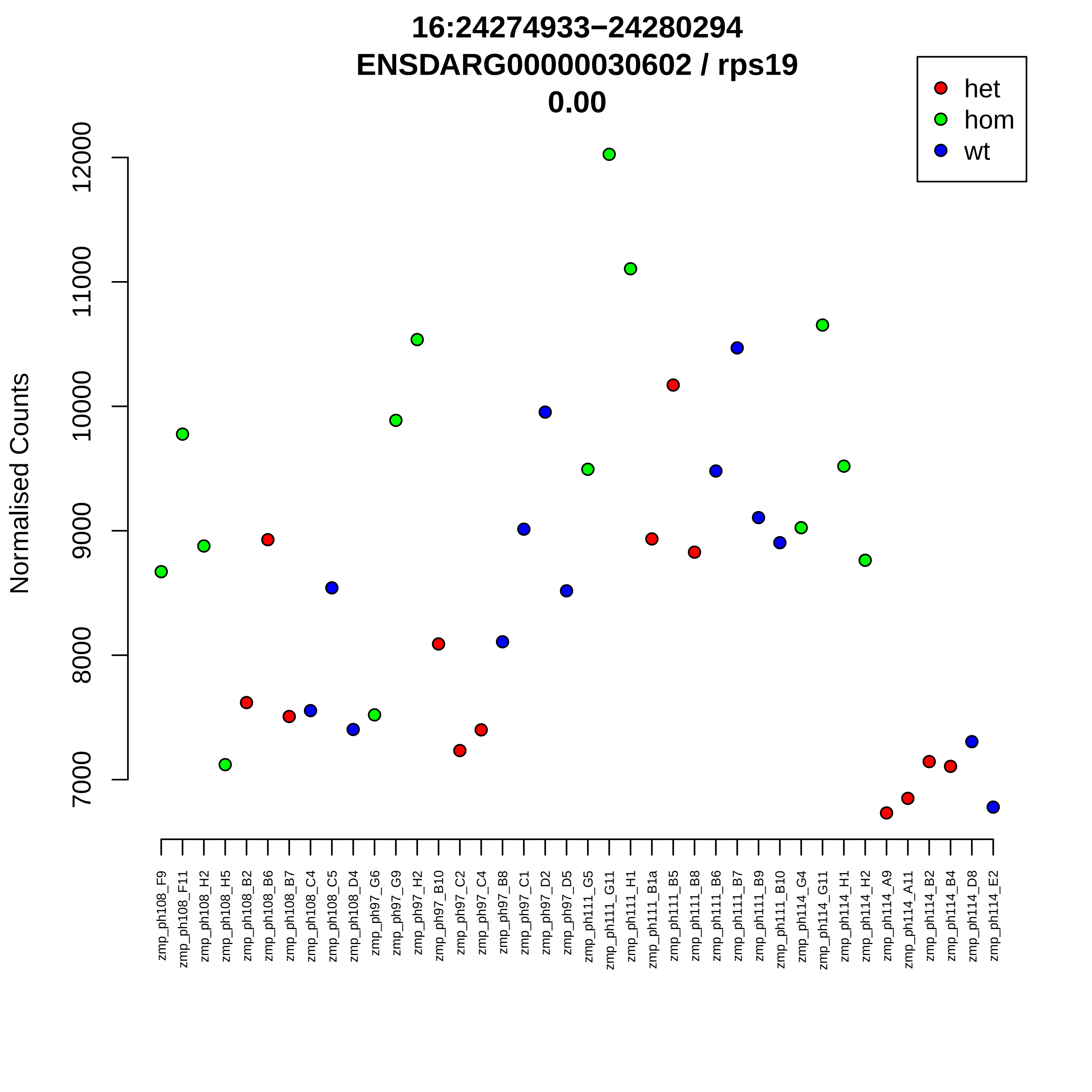

16:24274933−24280294
ENSDARG00000030602 / rps19
0.00
het
hom
wt
Normalised Counts
7000
8000
9000
10000
11000
12000
zmp_ph108_F9
zmp_ph108_F11
zmp_ph108_H2
zmp_ph108_H5
zmp_ph108_B2
zmp_ph108_B6
zmp_ph108_B7
zmp_ph108_C4
zmp_ph108_C5
zmp_ph108_D4
zmp_ph97_G6
zmp_ph97_G9
zmp_ph97_H2
zmp_ph97_B10
zmp_ph97_C2
zmp_ph97_C4
zmp_ph97_B8
zmp_ph97_C1
zmp_ph97_D2
zmp_ph97_D5
zmp_ph111_G5
zmp_ph111_G11
zmp_ph111_H1
zmp_ph111_B1a
zmp_ph111_B5
zmp_ph111_B8
zmp_ph111_B6
zmp_ph111_B7
zmp_ph111_B9
zmp_ph111_B10
zmp_ph114_G4
zmp_ph114_G11
zmp_ph114_H1
zmp_ph114_H2
zmp_ph114_A9
zmp_ph114_A11
zmp_ph114_B2
zmp_ph114_B4
zmp_ph114_D8
zmp_ph114_E2

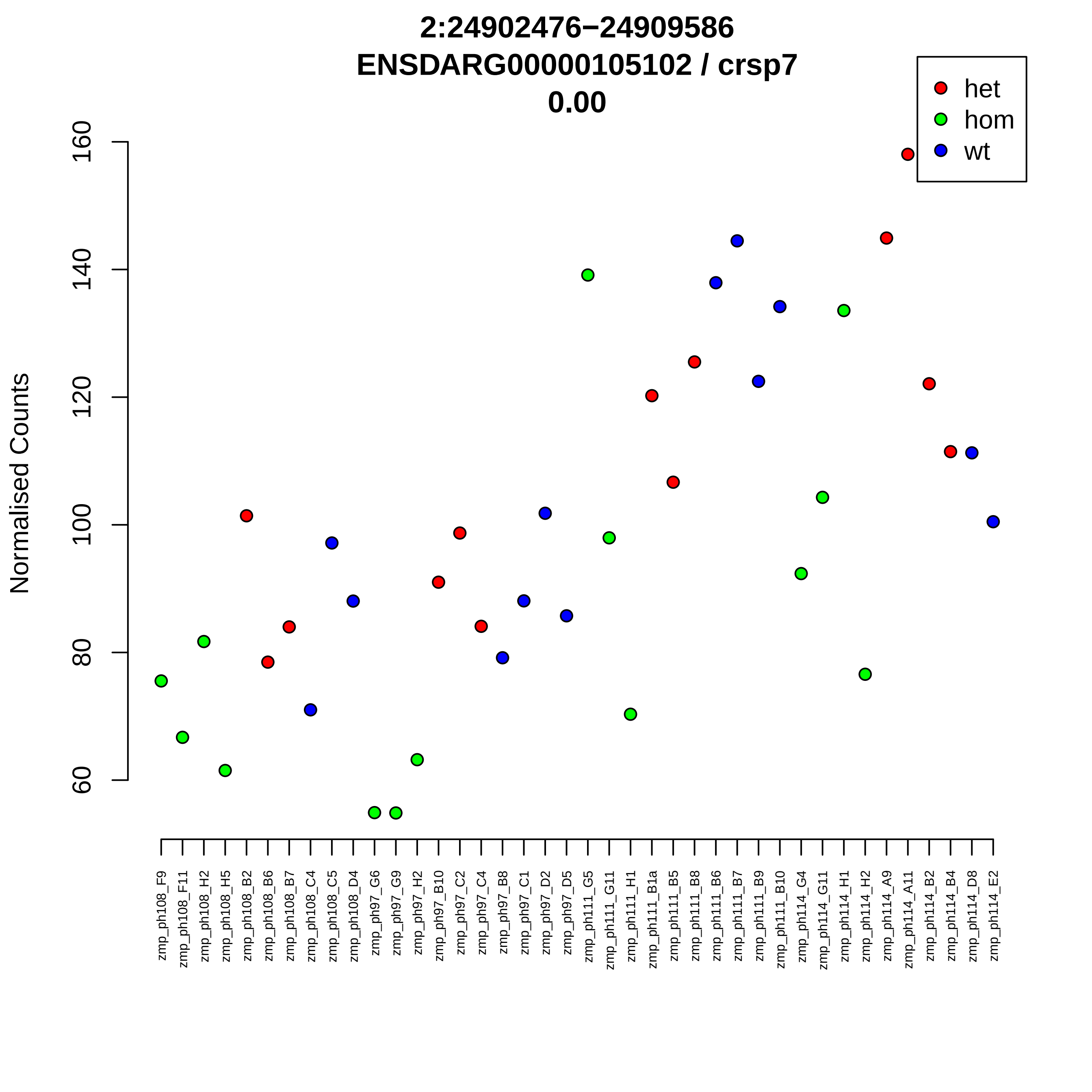
2:24902476−24909586
ENSDARG00000105102 / crsp7
0.00
het
hom
wt
Normalised Counts
60
80
100
120
140
160
zmp_ph108_F9
zmp_ph108_F11
zmp_ph108_H2
zmp_ph108_H5
zmp_ph108_B2
zmp_ph108_B6
zmp_ph108_B7
zmp_ph108_C4
zmp_ph108_C5
zmp_ph108_D4
zmp_ph97_G6
zmp_ph97_G9
zmp_ph97_H2
zmp_ph97_B10
zmp_ph97_C2
zmp_ph97_C4
zmp_ph97_B8
zmp_ph97_C1
zmp_ph97_D2
zmp_ph97_D5
zmp_ph111_G5
zmp_ph111_G11
zmp_ph111_H1
zmp_ph111_B1a
zmp_ph111_B5
zmp_ph111_B8
zmp_ph111_B6
zmp_ph111_B7
zmp_ph111_B9
zmp_ph111_B10
zmp_ph114_G4
zmp_ph114_G11
zmp_ph114_H1
zmp_ph114_H2
zmp_ph114_A9
zmp_ph114_A11
zmp_ph114_B2
zmp_ph114_B4
zmp_ph114_D8
zmp_ph114_E2

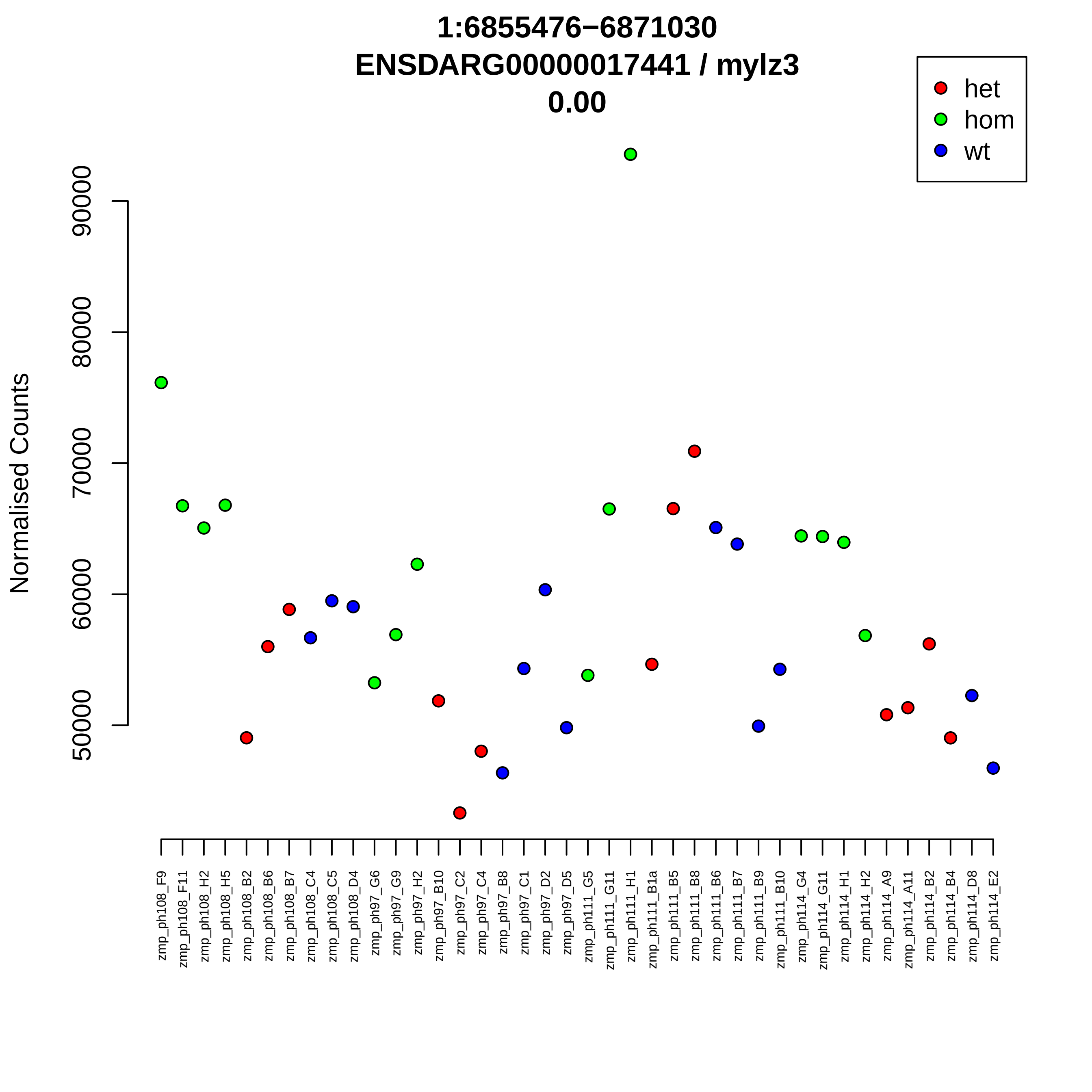
1:6855476−6871030
ENSDARG00000017441 / mylz3
0.00
het
hom
wt
Normalised Counts
50000
60000
70000
80000
90000
zmp_ph108_F9
zmp_ph108_F11
zmp_ph108_H2
zmp_ph108_H5
zmp_ph108_B2
zmp_ph108_B6
zmp_ph108_B7
zmp_ph108_C4
zmp_ph108_C5
zmp_ph108_D4
zmp_ph97_G6
zmp_ph97_G9
zmp_ph97_H2
zmp_ph97_B10
zmp_ph97_C2
zmp_ph97_C4
zmp_ph97_B8
zmp_ph97_C1
zmp_ph97_D2
zmp_ph97_D5
zmp_ph111_G5
zmp_ph111_G11
zmp_ph111_H1
zmp_ph111_B1a
zmp_ph111_B5
zmp_ph111_B8
zmp_ph111_B6
zmp_ph111_B7
zmp_ph111_B9
zmp_ph111_B10
zmp_ph114_G4
zmp_ph114_G11
zmp_ph114_H1
zmp_ph114_H2
zmp_ph114_A9
zmp_ph114_A11
zmp_ph114_B2
zmp_ph114_B4
zmp_ph114_D8
zmp_ph114_E2

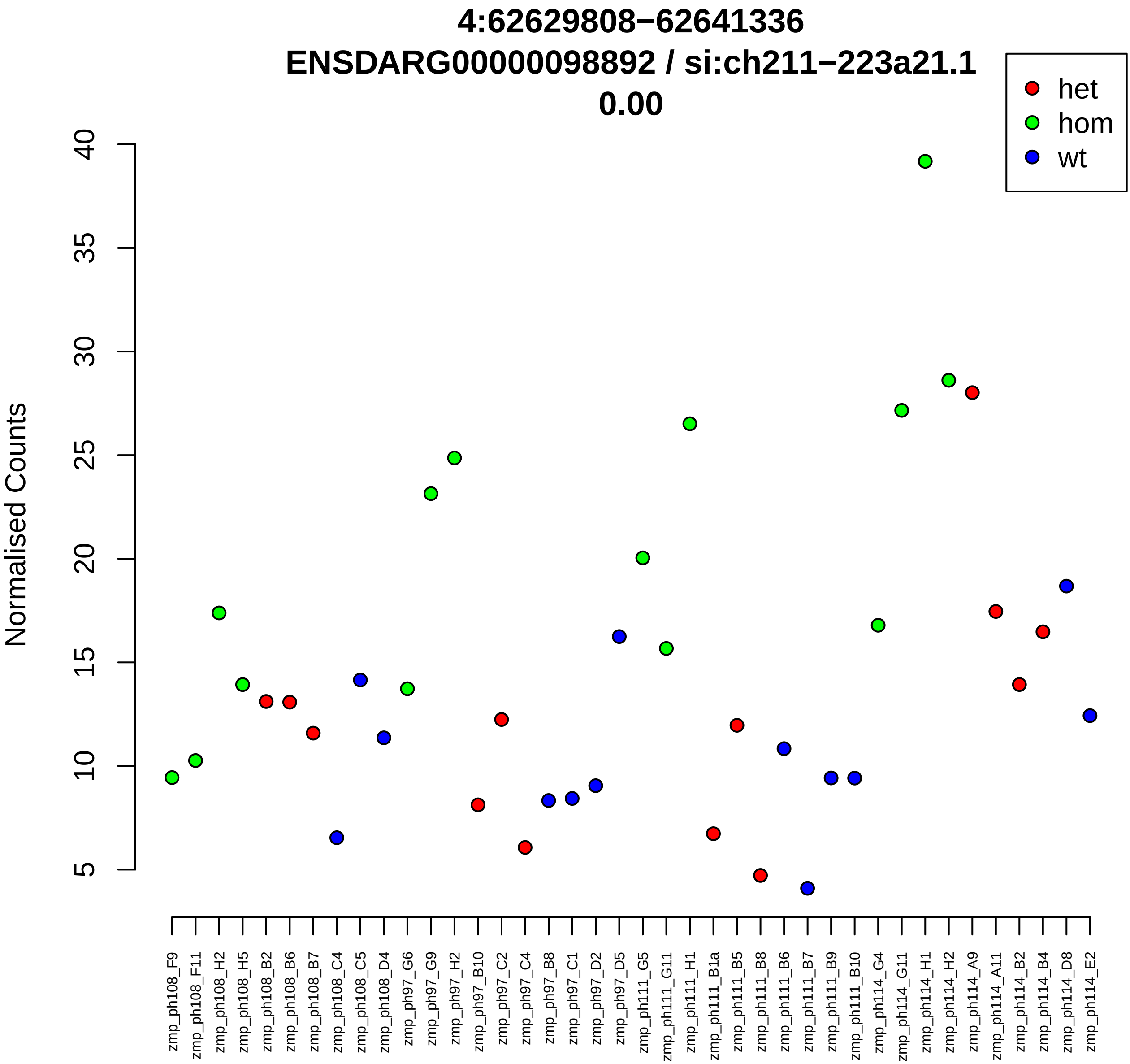

4:62629808−62641336
ENSDARG00000098892 / si:ch211−223a21.1
0.00
het
hom
wt
Normalised Counts
5
10
15
20
25
30
35
40
zmp_ph108_F9
zmp_ph108_F11
zmp_ph108_H2
zmp_ph108_H5
zmp_ph108_B2
zmp_ph108_B6
zmp_ph108_B7
zmp_ph108_C4
zmp_ph108_C5
zmp_ph108_D4
zmp_ph97_G6
zmp_ph97_G9
zmp_ph97_H2
zmp_ph97_B10
zmp_ph97_C2
zmp_ph97_C4
zmp_ph97_B8
zmp_ph97_C1
zmp_ph97_D2
zmp_ph97_D5
zmp_ph111_G5
zmp_ph111_G11
zmp_ph111_H1
zmp_ph111_B1a
zmp_ph111_B5
zmp_ph111_B8
zmp_ph111_B6
zmp_ph111_B7
zmp_ph111_B9
zmp_ph111_B10
zmp_ph114_G4
zmp_ph114_G11
zmp_ph114_H1
zmp_ph114_H2
zmp_ph114_A9
zmp_ph114_A11
zmp_ph114_B2
zmp_ph114_B4
zmp_ph114_D8
zmp_ph114_E2

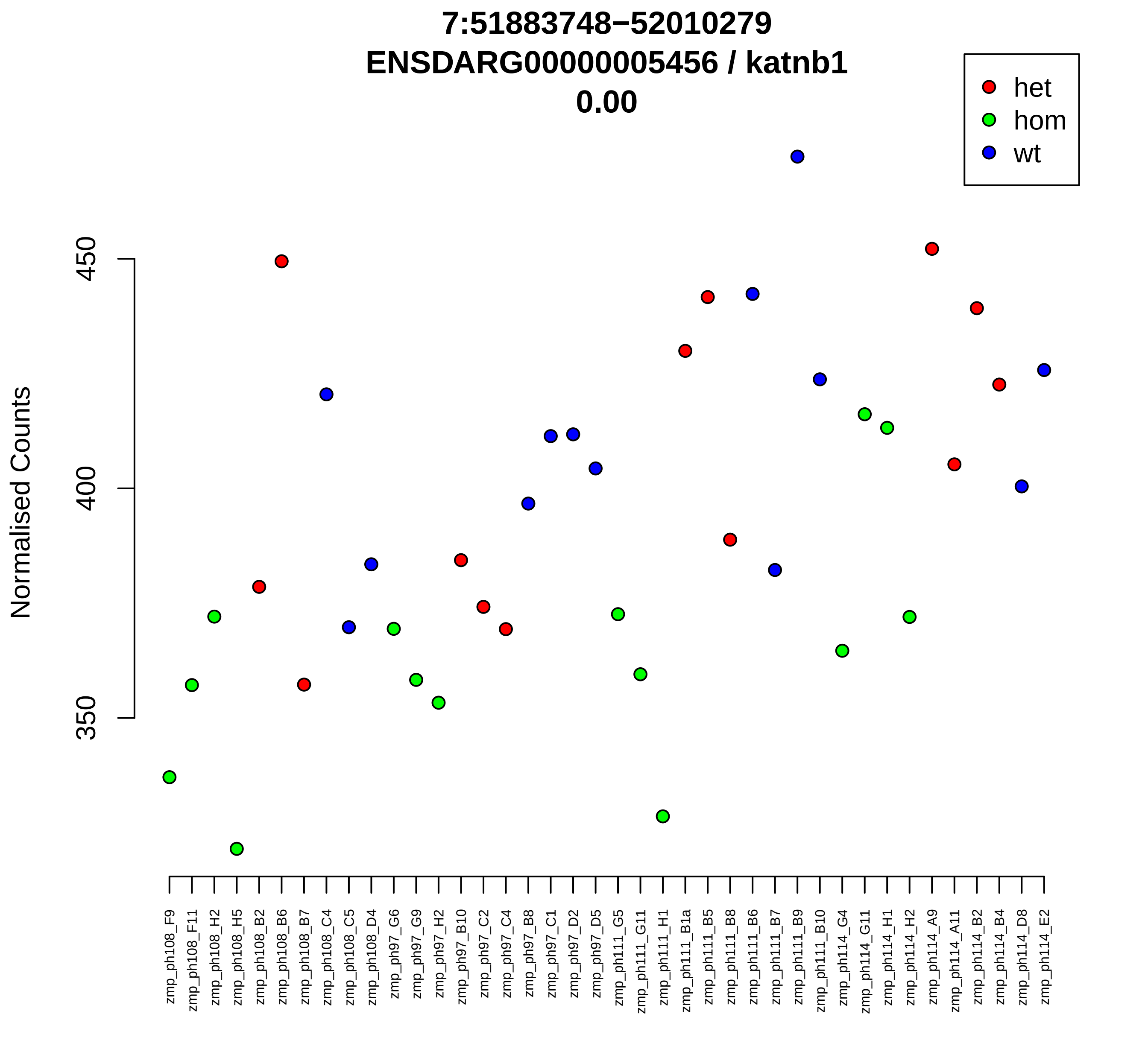

7:51883748−52010279
ENSDARG00000005456 / katnb1
0.00
het
hom
wt
Normalised Counts
450
400
350
zmp_ph108_F9
zmp_ph108_F11
zmp_ph108_H2
zmp_ph108_H5
zmp_ph108_B2
zmp_ph108_B6
zmp_ph108_B7
zmp_ph108_C4
zmp_ph108_C5
zmp_ph108_D4
zmp_ph97_G6
zmp_ph97_G9
zmp_ph97_H2
zmp_ph97_B10
zmp_ph97_C2
zmp_ph97_C4
zmp_ph97_B8
zmp_ph97_C1
zmp_ph97_D2
zmp_ph97_D5
zmp_ph111_G5
zmp_ph111_G11
zmp_ph111_H1
zmp_ph111_B1a
zmp_ph111_B5
zmp_ph111_B8
zmp_ph111_B6
zmp_ph111_B7
zmp_ph111_B9
zmp_ph111_B10
zmp_ph114_G4
zmp_ph114_G11
zmp_ph114_H1
zmp_ph114_H2
zmp_ph114_A9
zmp_ph114_A11
zmp_ph114_B2
zmp_ph114_B4
zmp_ph114_D8
zmp_ph114_E2

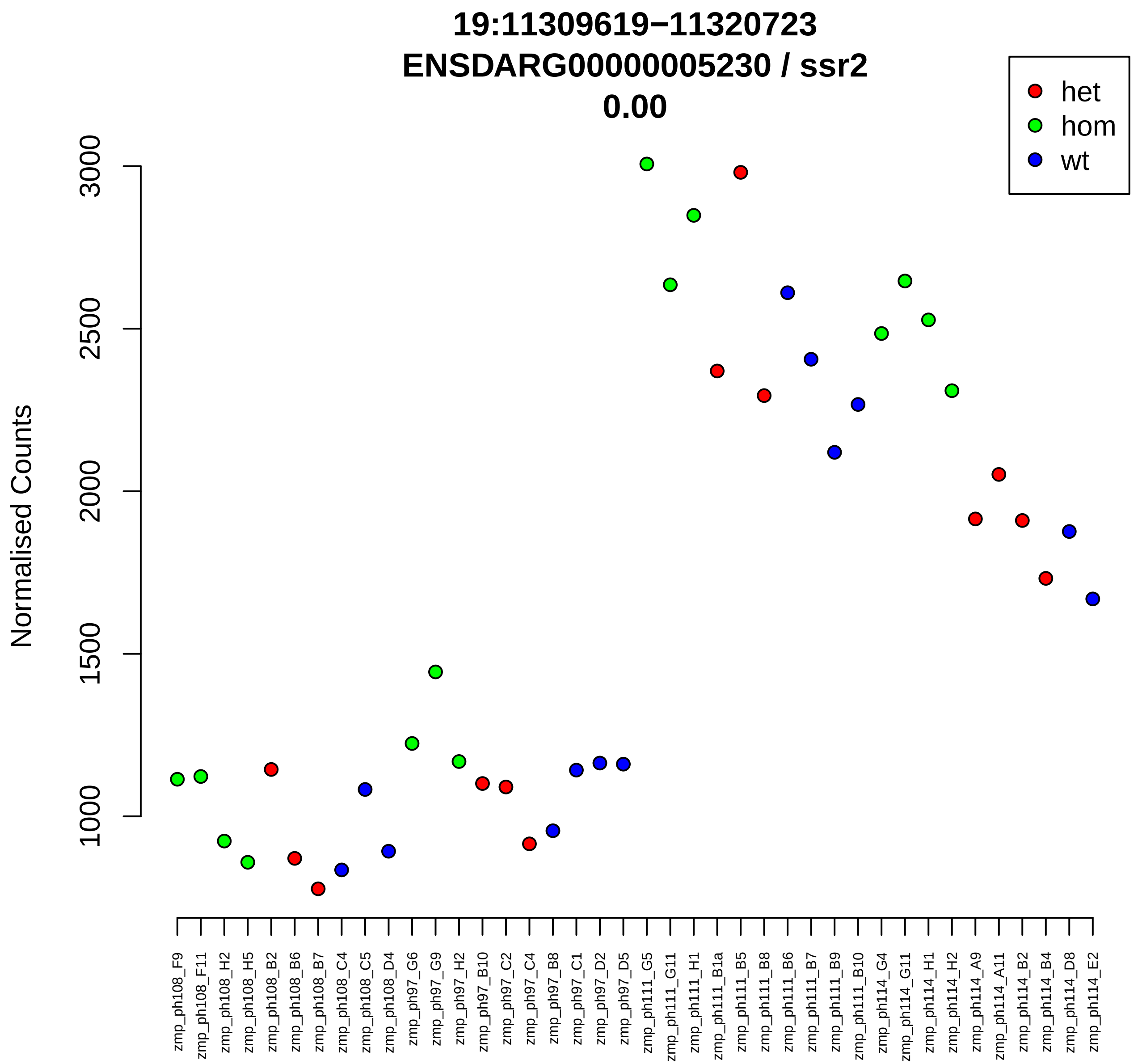

19:11309619−11320723
ENSDARG00000005230 / ssr2
0.00
het
hom
wt
Normalised Counts
1000
1500
2000
2500
3000
zmp_ph108_F9
zmp_ph108_F11
zmp_ph108_H2
zmp_ph108_H5
zmp_ph108_B2
zmp_ph108_B6
zmp_ph108_B7
zmp_ph108_C4
zmp_ph108_C5
zmp_ph108_D4
zmp_ph97_G6
zmp_ph97_G9
zmp_ph97_H2
zmp_ph97_B10
zmp_ph97_C2
zmp_ph97_C4
zmp_ph97_B8
zmp_ph97_C1
zmp_ph97_D2
zmp_ph97_D5
zmp_ph111_G5
zmp_ph111_G11
zmp_ph111_H1
zmp_ph111_B1a
zmp_ph111_B5
zmp_ph111_B8
zmp_ph111_B6
zmp_ph111_B7
zmp_ph111_B9
zmp_ph111_B10
zmp_ph114_G4
zmp_ph114_G11
zmp_ph114_H1
zmp_ph114_H2
zmp_ph114_A9
zmp_ph114_A11
zmp_ph114_B2
zmp_ph114_B4
zmp_ph114_D8
zmp_ph114_E2

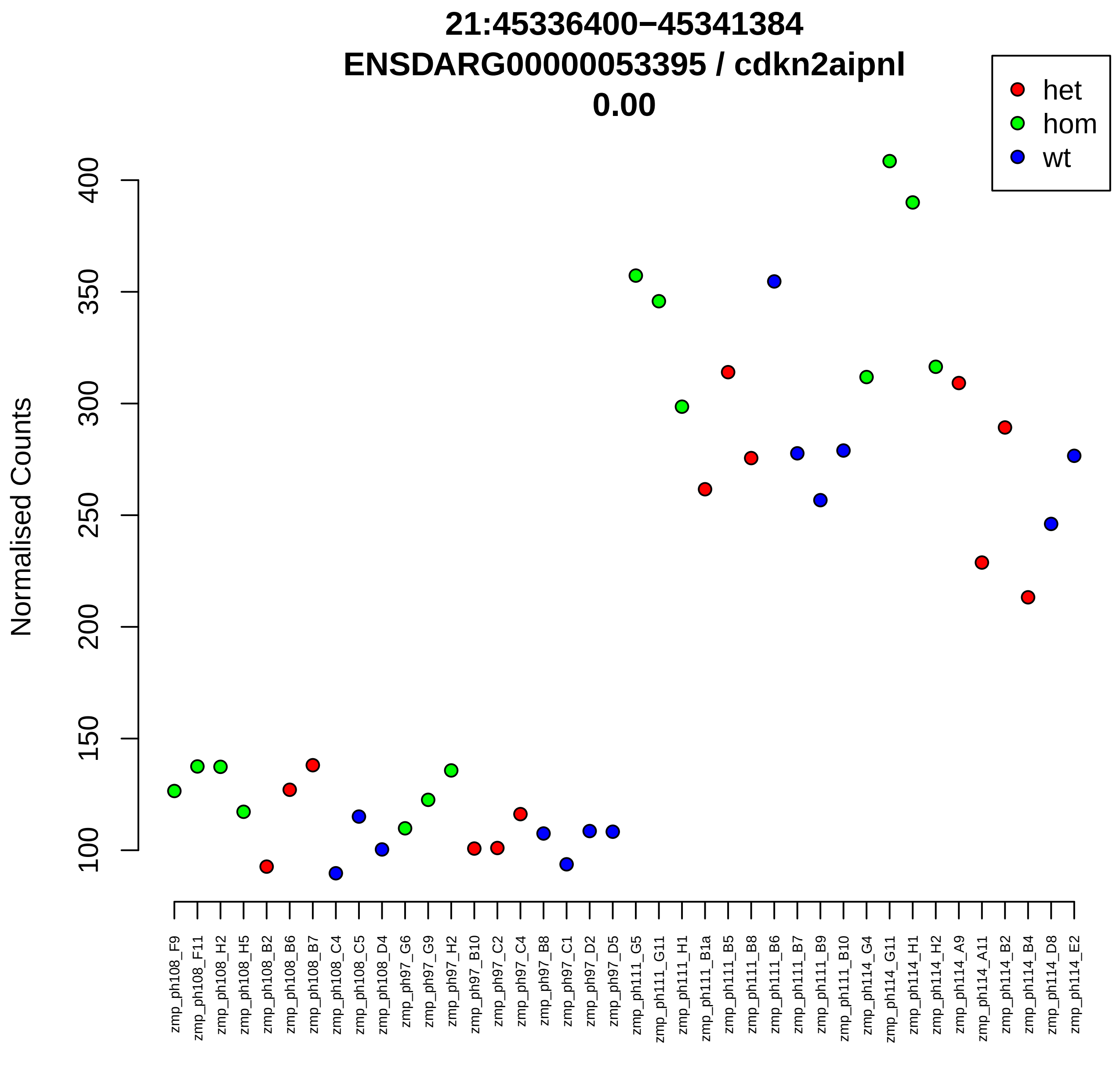

21:45336400−45341384
ENSDARG00000053395 / cdkn2aipnl
0.00
het
hom
wt
Normalised Counts
100
150
200
250
300
350
400
zmp_ph108_F9
zmp_ph108_F11
zmp_ph108_H2
zmp_ph108_H5
zmp_ph108_B2
zmp_ph108_B6
zmp_ph108_B7
zmp_ph108_C4
zmp_ph108_C5
zmp_ph108_D4
zmp_ph97_G6
zmp_ph97_G9
zmp_ph97_H2
zmp_ph97_B10
zmp_ph97_C2
zmp_ph97_C4
zmp_ph97_B8
zmp_ph97_C1
zmp_ph97_D2
zmp_ph97_D5
zmp_ph111_G5
zmp_ph111_G11
zmp_ph111_H1
zmp_ph111_B1a
zmp_ph111_B5
zmp_ph111_B8
zmp_ph111_B6
zmp_ph111_B7
zmp_ph111_B9
zmp_ph111_B10
zmp_ph114_G4
zmp_ph114_G11
zmp_ph114_H1
zmp_ph114_H2
zmp_ph114_A9
zmp_ph114_A11
zmp_ph114_B2
zmp_ph114_B4
zmp_ph114_D8
zmp_ph114_E2

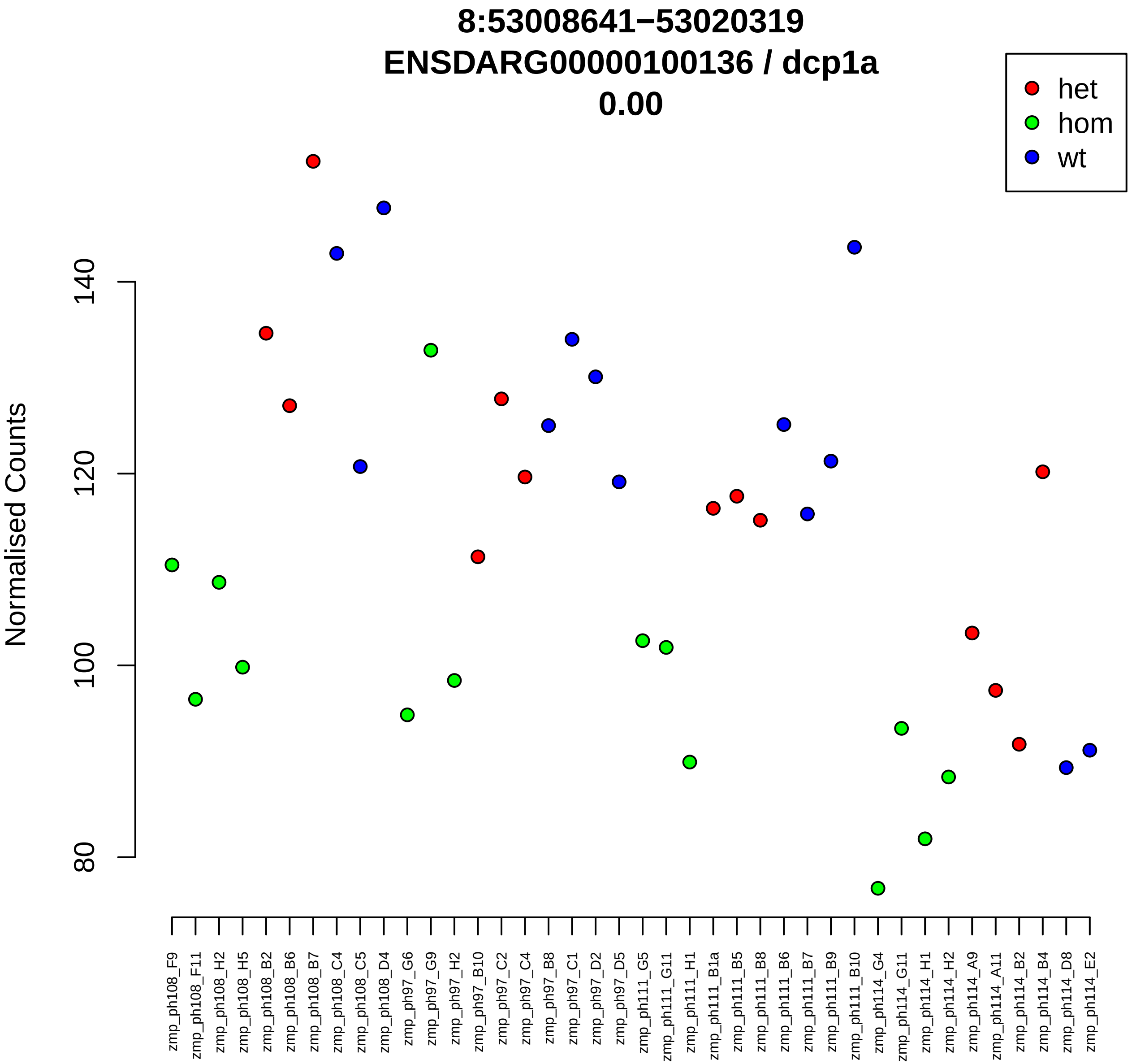

8:53008641−53020319
ENSDARG00000100136 / dcp1a
0.00
het
hom
wt
Normalised Counts
80
100
120
140
zmp_ph108_F9
zmp_ph108_F11
zmp_ph108_H2
zmp_ph108_H5
zmp_ph108_B2
zmp_ph108_B6
zmp_ph108_B7
zmp_ph108_C4
zmp_ph108_C5
zmp_ph108_D4
zmp_ph97_G6
zmp_ph97_G9
zmp_ph97_H2
zmp_ph97_B10
zmp_ph97_C2
zmp_ph97_C4
zmp_ph97_B8
zmp_ph97_C1
zmp_ph97_D2
zmp_ph97_D5
zmp_ph111_G5
zmp_ph111_G11
zmp_ph111_H1
zmp_ph111_B1a
zmp_ph111_B5
zmp_ph111_B8
zmp_ph111_B6
zmp_ph111_B7
zmp_ph111_B9
zmp_ph111_B10
zmp_ph114_G4
zmp_ph114_G11
zmp_ph114_H1
zmp_ph114_H2
zmp_ph114_A9
zmp_ph114_A11
zmp_ph114_B2
zmp_ph114_B4
zmp_ph114_D8
zmp_ph114_E2

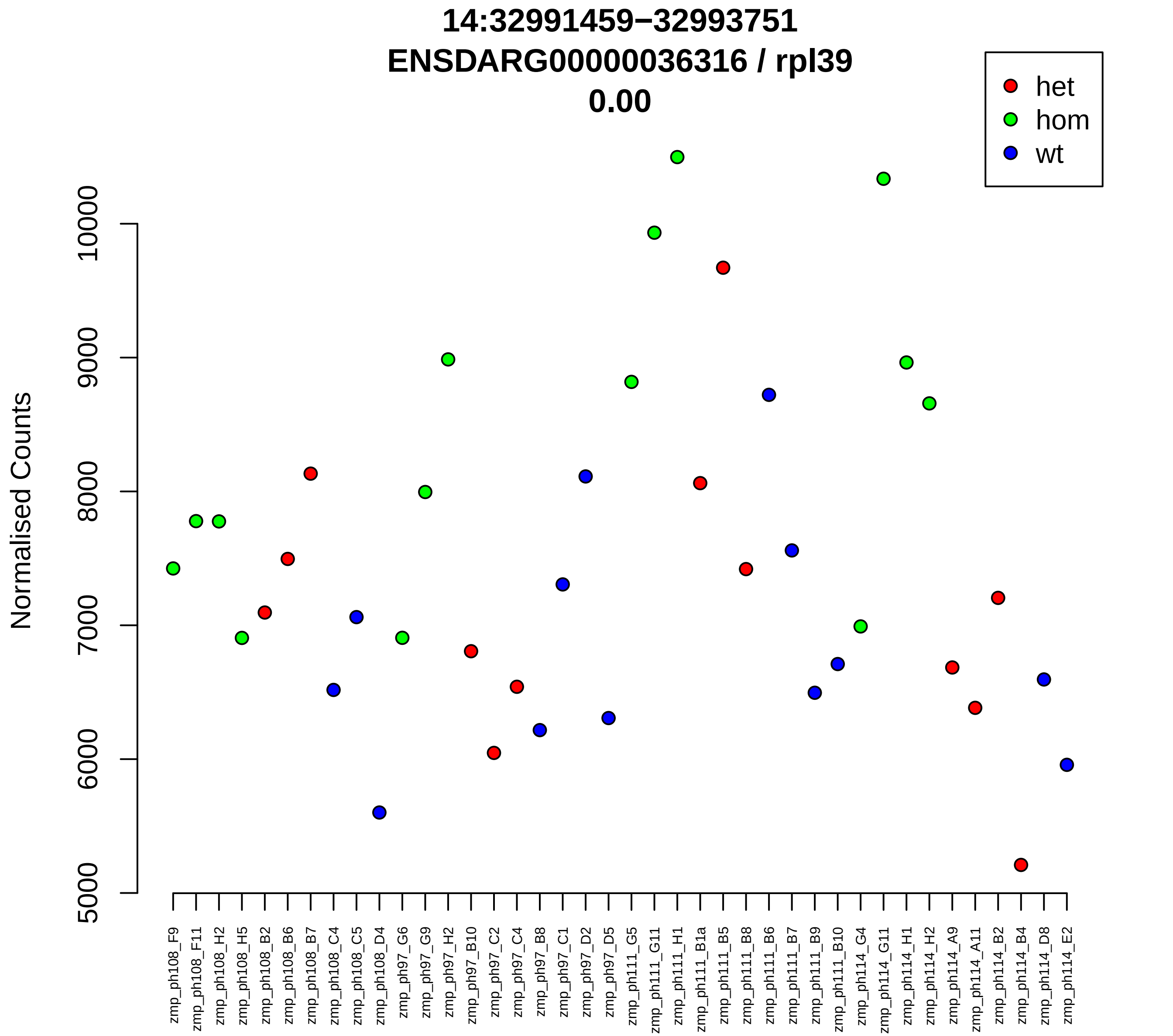

14:32991459−32993751
ENSDARG00000036316 / rpl39
0.00
het
hom
wt
Normalised Counts
5000
6000
7000
8000
9000
10000
zmp_ph108_F9
zmp_ph108_F11
zmp_ph108_H2
zmp_ph108_H5
zmp_ph108_B2
zmp_ph108_B6
zmp_ph108_B7
zmp_ph108_C4
zmp_ph108_C5
zmp_ph108_D4
zmp_ph97_G6
zmp_ph97_G9
zmp_ph97_H2
zmp_ph97_B10
zmp_ph97_C2
zmp_ph97_C4
zmp_ph97_B8
zmp_ph97_C1
zmp_ph97_D2
zmp_ph97_D5
zmp_ph111_G5
zmp_ph111_G11
zmp_ph111_H1
zmp_ph111_B1a
zmp_ph111_B5
zmp_ph111_B8
zmp_ph111_B6
zmp_ph111_B7
zmp_ph111_B9
zmp_ph111_B10
zmp_ph114_G4
zmp_ph114_G11
zmp_ph114_H1
zmp_ph114_H2
zmp_ph114_A9
zmp_ph114_A11
zmp_ph114_B2
zmp_ph114_B4
zmp_ph114_D8
zmp_ph114_E2

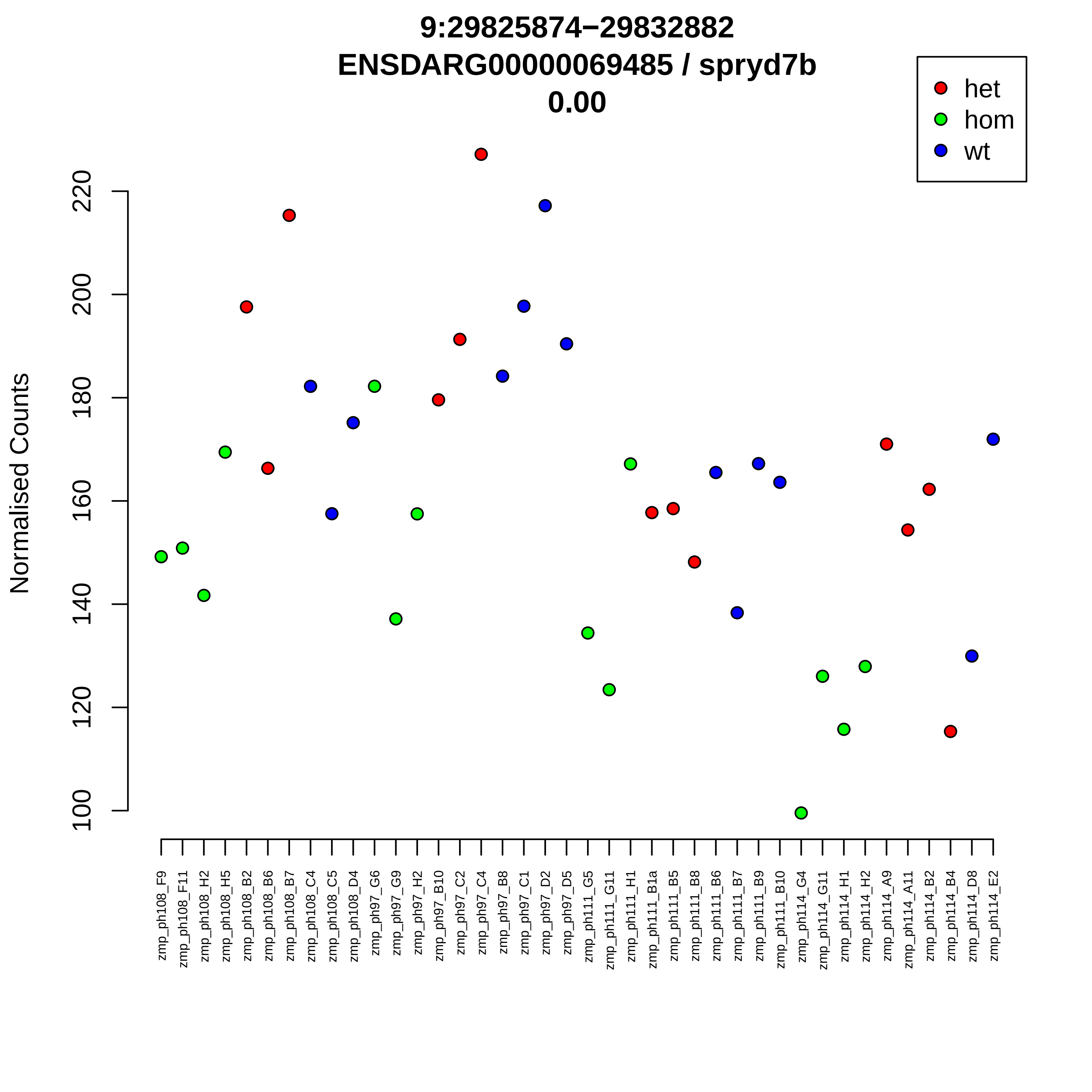
9:29825874−29832882
ENSDARG00000069485 / spryd7b
0.00
het
hom
wt
Normalised Counts
100
120
140
160
180
200
220
zmp_ph108_F9
zmp_ph108_F11
zmp_ph108_H2
zmp_ph108_H5
zmp_ph108_B2
zmp_ph108_B6
zmp_ph108_B7
zmp_ph108_C4
zmp_ph108_C5
zmp_ph108_D4
zmp_ph97_G6
zmp_ph97_G9
zmp_ph97_H2
zmp_ph97_B10
zmp_ph97_C2
zmp_ph97_C4
zmp_ph97_B8
zmp_ph97_C1
zmp_ph97_D2
zmp_ph97_D5
zmp_ph111_G5
zmp_ph111_G11
zmp_ph111_H1
zmp_ph111_B1a
zmp_ph111_B5
zmp_ph111_B8
zmp_ph111_B6
zmp_ph111_B7
zmp_ph111_B9
zmp_ph111_B10
zmp_ph114_G4
zmp_ph114_G11
zmp_ph114_H1
zmp_ph114_H2
zmp_ph114_A9
zmp_ph114_A11
zmp_ph114_B2
zmp_ph114_B4
zmp_ph114_D8
zmp_ph114_E2

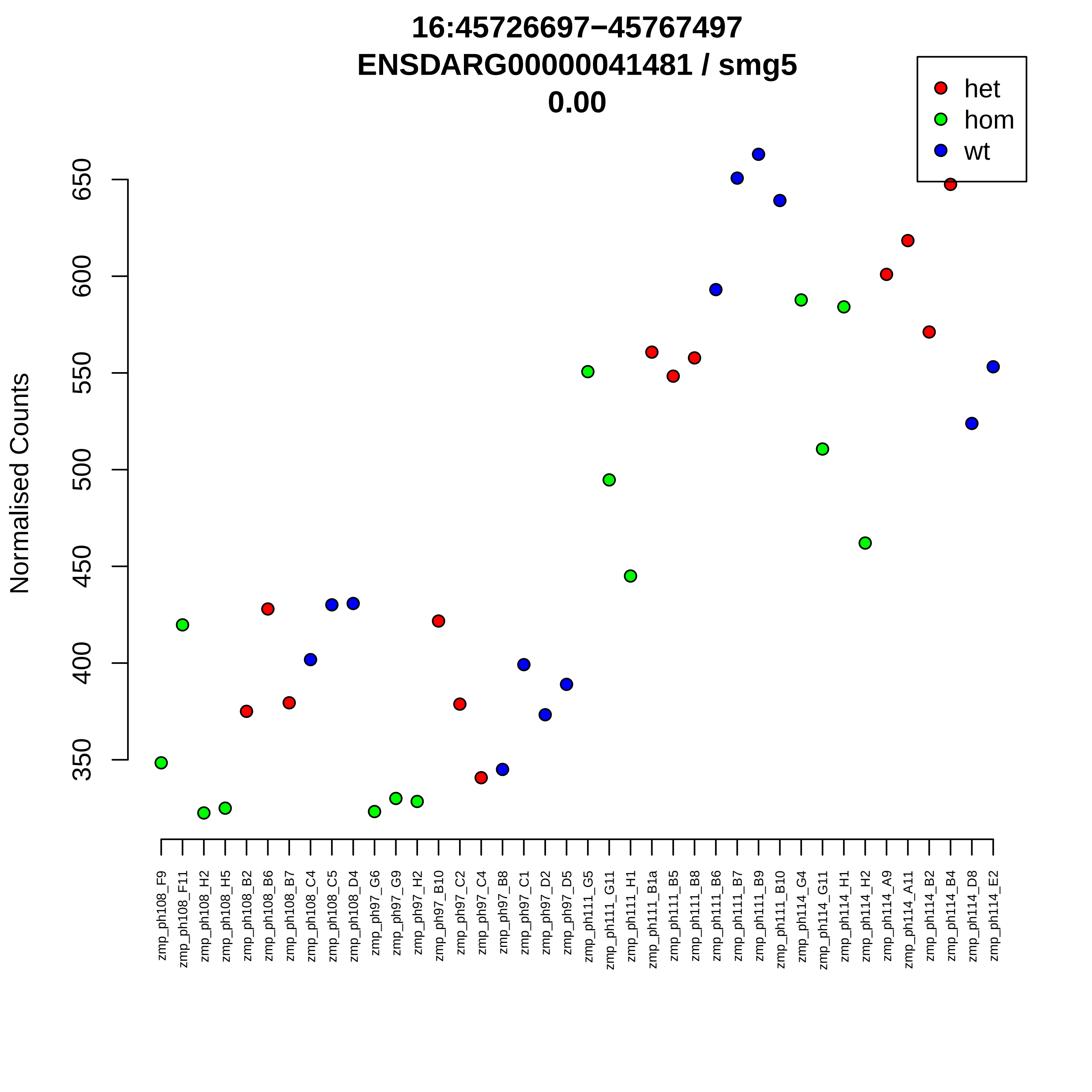

16:45726697−45767497
ENSDARG00000041481 / smg5
0.00
het
hom
wt
Normalised Counts
350
400
450
500
550
600
650
zmp_ph108_F9
zmp_ph108_F11
zmp_ph108_H2
zmp_ph108_H5
zmp_ph108_B2
zmp_ph108_B6
zmp_ph108_B7
zmp_ph108_C4
zmp_ph108_C5
zmp_ph108_D4
zmp_ph97_G6
zmp_ph97_G9
zmp_ph97_H2
zmp_ph97_B10
zmp_ph97_C2
zmp_ph97_C4
zmp_ph97_B8
zmp_ph97_C1
zmp_ph97_D2
zmp_ph97_D5
zmp_ph111_G5
zmp_ph111_G11
zmp_ph111_H1
zmp_ph111_B1a
zmp_ph111_B5
zmp_ph111_B8
zmp_ph111_B6
zmp_ph111_B7
zmp_ph111_B9
zmp_ph111_B10
zmp_ph114_G4
zmp_ph114_G11
zmp_ph114_H1
zmp_ph114_H2
zmp_ph114_A9
zmp_ph114_A11
zmp_ph114_B2
zmp_ph114_B4
zmp_ph114_D8
zmp_ph114_E2

14:13738230−13788843
ENSDARG00000104149 / rraga
0.00

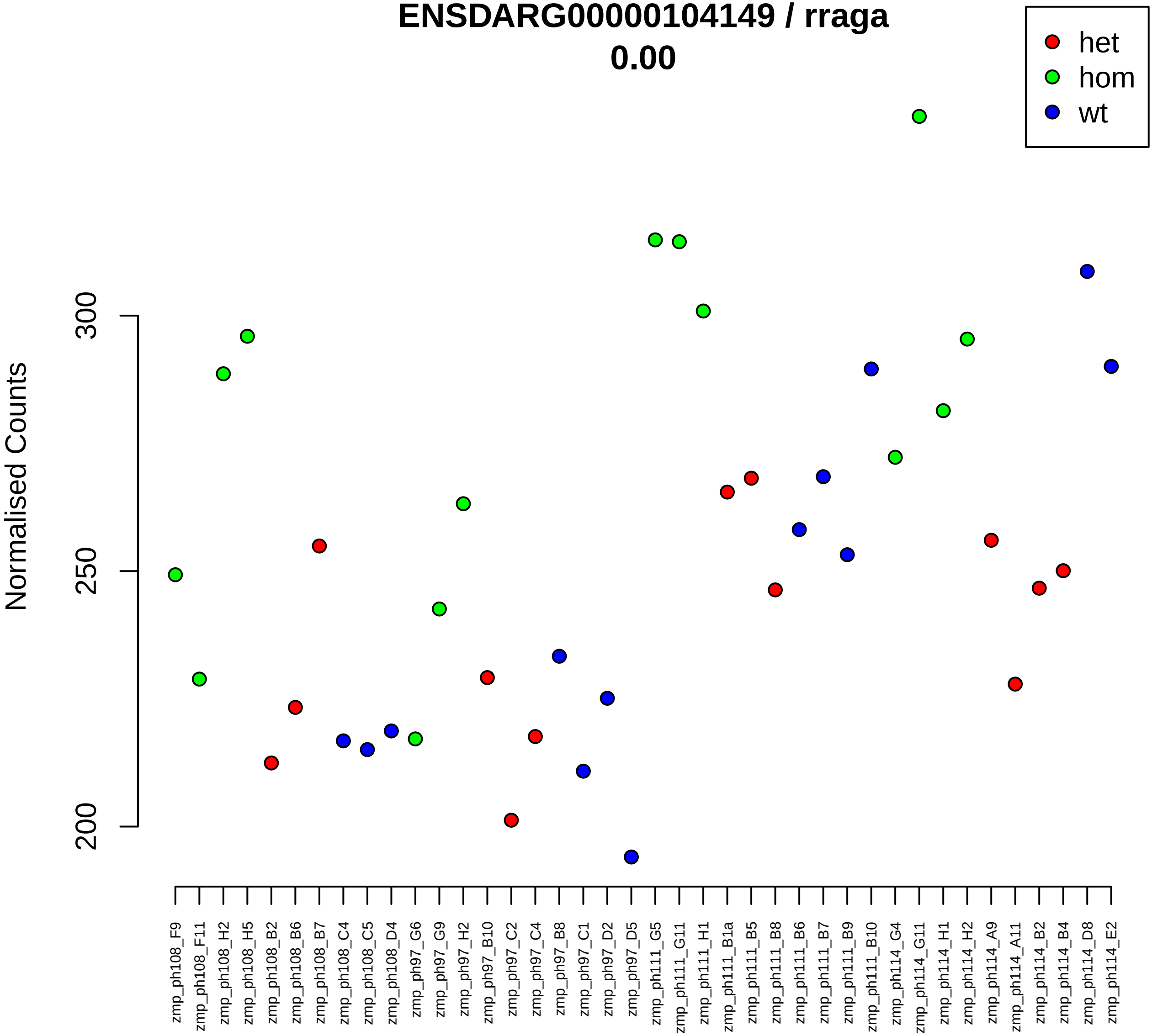

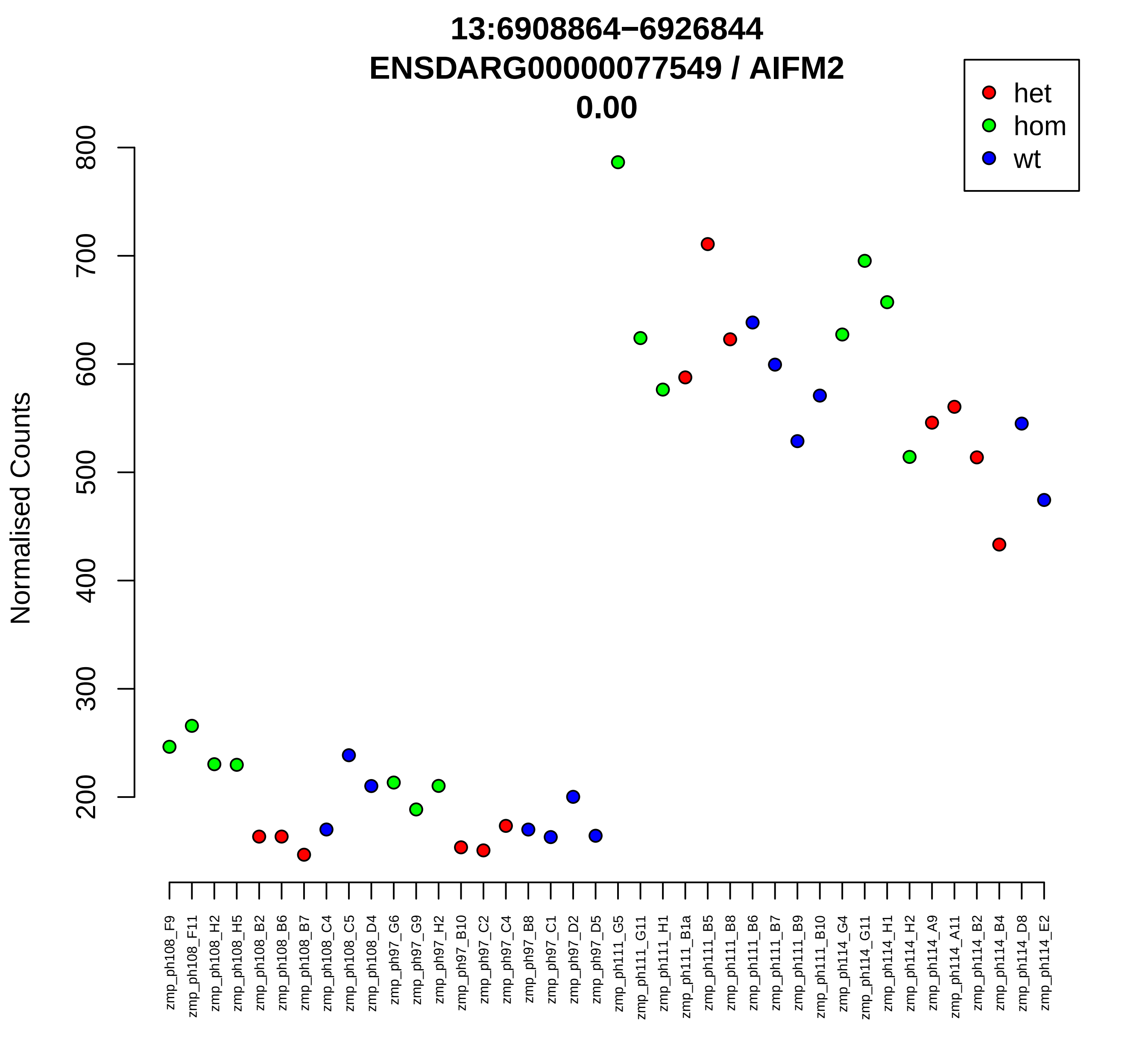

13:6908864−6926844
ENSDARG00000077549 / AIFM2
0.00
het
hom
wt
Normalised Counts
800
700
600
500
400
300
200
zmp_ph108_F9
zmp_ph108_F11
zmp_ph108_H2
zmp_ph108_H5
zmp_ph108_B2
zmp_ph108_B6
zmp_ph108_B7
zmp_ph108_C4
zmp_ph108_C5
zmp_ph108_D4
zmp_ph97_G6
zmp_ph97_G9
zmp_ph97_H2
zmp_ph97_B10
zmp_ph97_C2
zmp_ph97_C4
zmp_ph97_B8
zmp_ph97_C1
zmp_ph97_D2
zmp_ph97_D5
zmp_ph111_G5
zmp_ph111_G11
zmp_ph111_H1
zmp_ph111_B1a
zmp_ph111_B5
zmp_ph111_B8
zmp_ph111_B6
zmp_ph111_B7
zmp_ph111_B9
zmp_ph111_B10
zmp_ph114_G4
zmp_ph114_G11
zmp_ph114_H1
zmp_ph114_H2
zmp_ph114_A9
zmp_ph114_A11
zmp_ph114_B2
zmp_ph114_B4
zmp_ph114_D8
zmp_ph114_E2

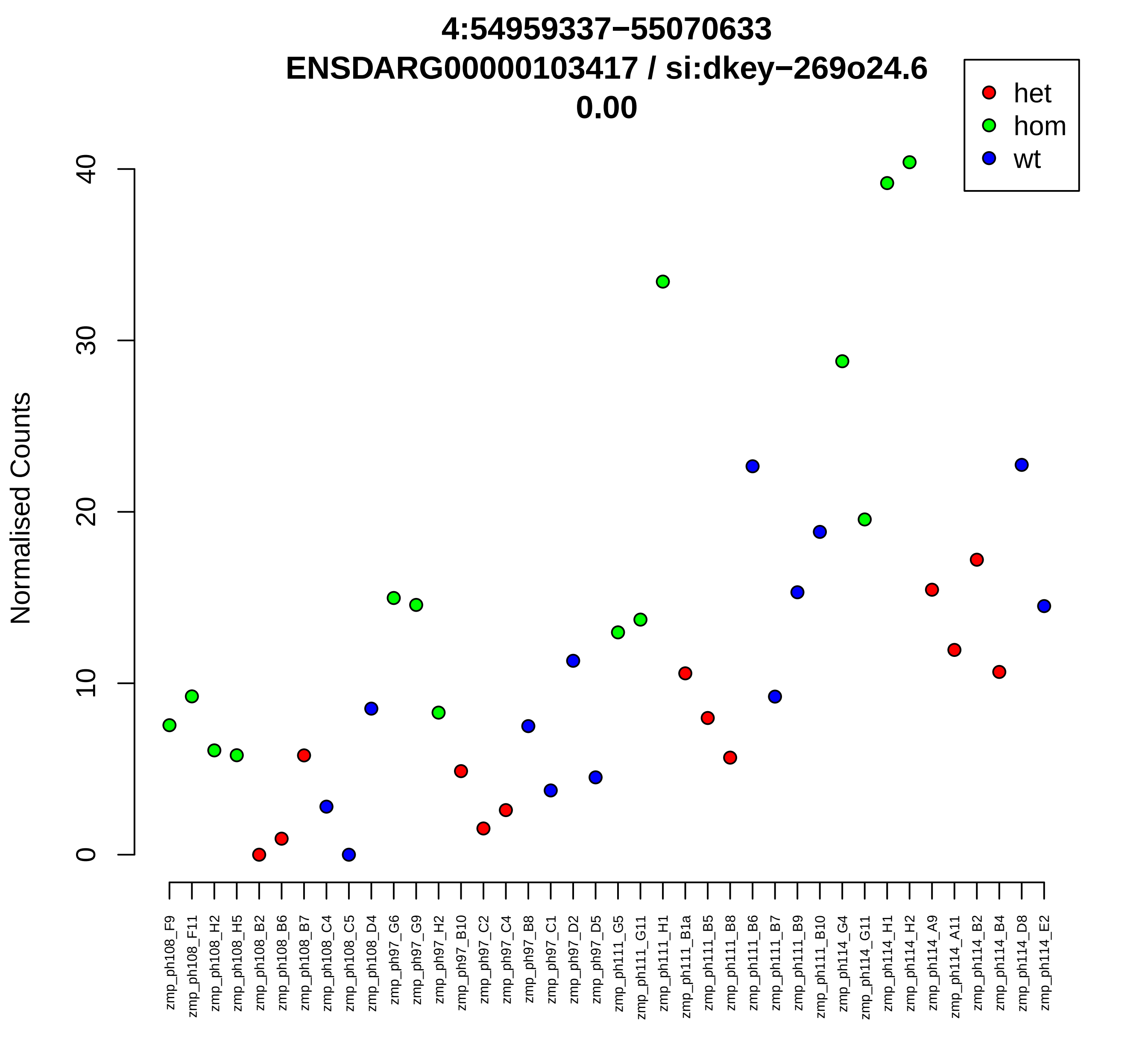

4:54959337−55070633
ENSDARG00000103417 / si:dkey−269o24.6
0.00
Normalised Counts
0
10
20
30
40
het
hom
wt
zmp_ph108_F9
zmp_ph108_F11
zmp_ph108_H2
zmp_ph108_H5
zmp_ph108_B2
zmp_ph108_B6
zmp_ph108_B7
zmp_ph108_C4
zmp_ph108_C5
zmp_ph108_D4
zmp_ph97_G6
zmp_ph97_G9
zmp_ph97_H2
zmp_ph97_B10
zmp_ph97_C2
zmp_ph97_C4
zmp_ph97_B8
zmp_ph97_C1
zmp_ph97_D2
zmp_ph97_D5
zmp_ph111_G5
zmp_ph111_G11
zmp_ph111_H1
zmp_ph111_B1a
zmp_ph111_B5
zmp_ph111_B8
zmp_ph111_B6
zmp_ph111_B7
zmp_ph111_B9
zmp_ph111_B10
zmp_ph114_G4
zmp_ph114_G11
zmp_ph114_H1
zmp_ph114_H2
zmp_ph114_A9
zmp_ph114_A11
zmp_ph114_B2
zmp_ph114_B4
zmp_ph114_D8
zmp_ph114_E2

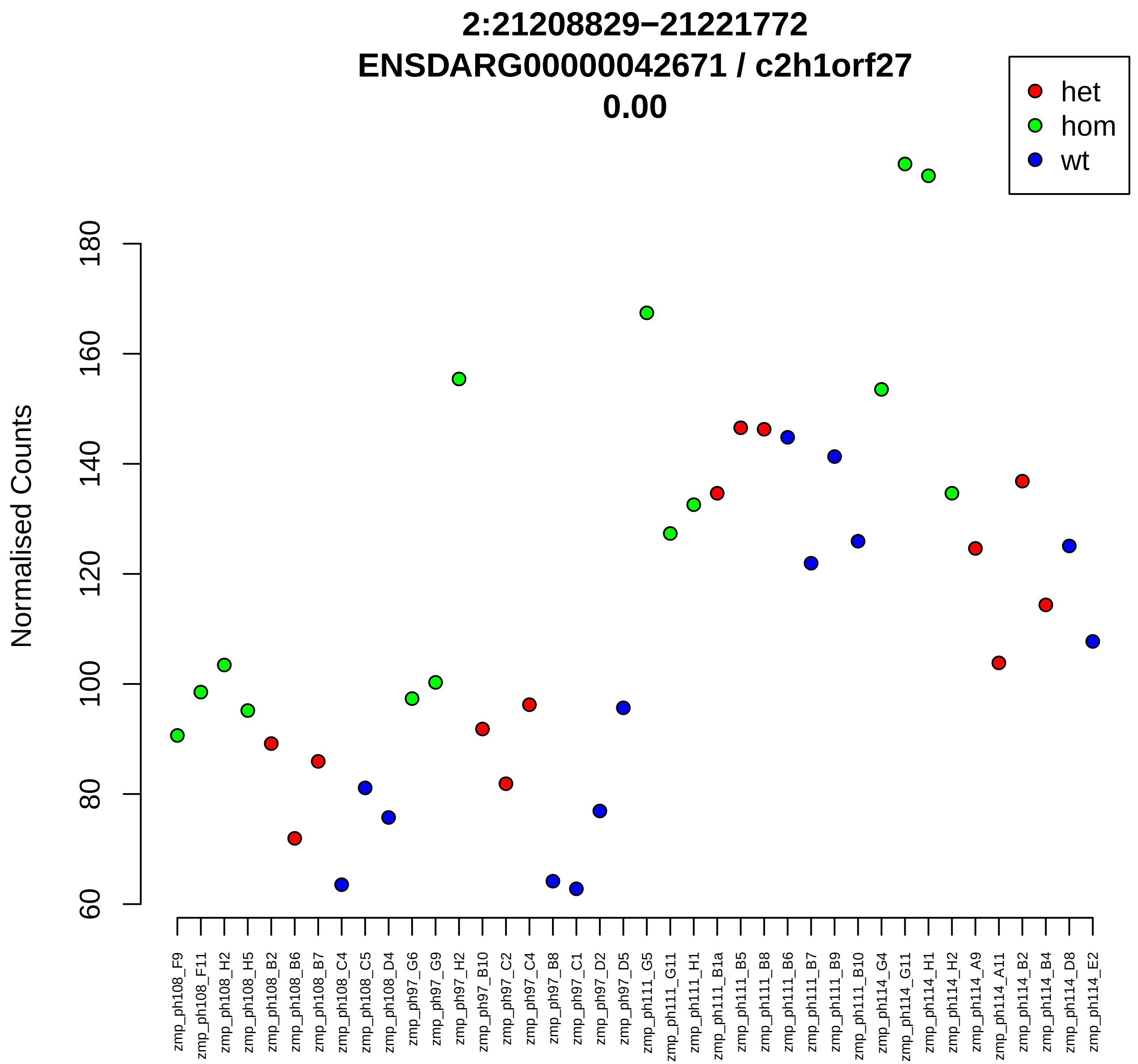

2:21208829−21221772
ENSDARG00000042671 / c2h1orf27
0.00
het
hom
wt
Normalised Counts
60
80
100
120
140
160
180
zmp_ph108_F9
zmp_ph108_F11
zmp_ph108_H2
zmp_ph108_H5
zmp_ph108_B2
zmp_ph108_B6
zmp_ph108_B7
zmp_ph108_C4
zmp_ph108_C5
zmp_ph108_D4
zmp_ph97_G6
zmp_ph97_G9
zmp_ph97_H2
zmp_ph97_B10
zmp_ph97_C2
zmp_ph97_C4
zmp_ph97_B8
zmp_ph97_C1
zmp_ph97_D2
zmp_ph97_D5
zmp_ph111_G5
zmp_ph111_G11
zmp_ph111_H1
zmp_ph111_B1a
zmp_ph111_B5
zmp_ph111_B8
zmp_ph111_B6
zmp_ph111_B7
zmp_ph111_B9
zmp_ph111_B10
zmp_ph114_G4
zmp_ph114_G11
zmp_ph114_H1
zmp_ph114_H2
zmp_ph114_A9
zmp_ph114_A11
zmp_ph114_B2
zmp_ph114_B4
zmp_ph114_D8
zmp_ph114_E2

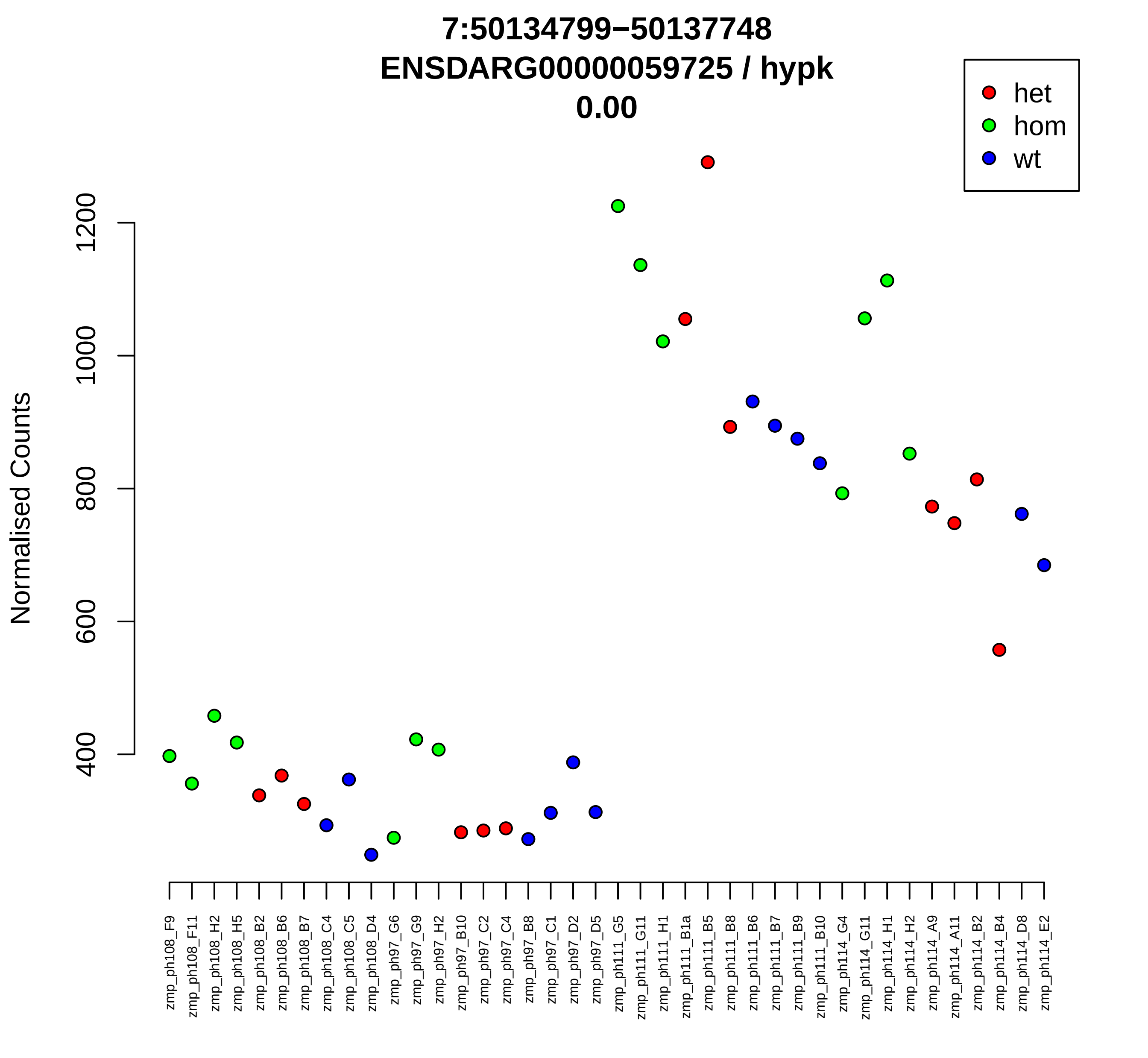
7:50134799−50137748
ENSDARG00000059725 / hypk
0.00
het
hom
wt
Normalised Counts
400
600
800
1000
1200
zmp_ph108_F9
zmp_ph108_F11
zmp_ph108_H2
zmp_ph108_H5
zmp_ph108_B2
zmp_ph108_B6
zmp_ph108_B7
zmp_ph108_C4
zmp_ph108_C5
zmp_ph108_D4
zmp_ph97_G6
zmp_ph97_G9
zmp_ph97_H2
zmp_ph97_B10
zmp_ph97_C2
zmp_ph97_C4
zmp_ph97_B8
zmp_ph97_C1
zmp_ph97_D2
zmp_ph97_D5
zmp_ph111_G5
zmp_ph111_G11
zmp_ph111_H1
zmp_ph111_B1a
zmp_ph111_B5
zmp_ph111_B8
zmp_ph111_B6
zmp_ph111_B7
zmp_ph111_B9
zmp_ph111_B10
zmp_ph114_G4
zmp_ph114_G11
zmp_ph114_H1
zmp_ph114_H2
zmp_ph114_A9
zmp_ph114_A11
zmp_ph114_B2
zmp_ph114_B4
zmp_ph114_D8
zmp_ph114_E2

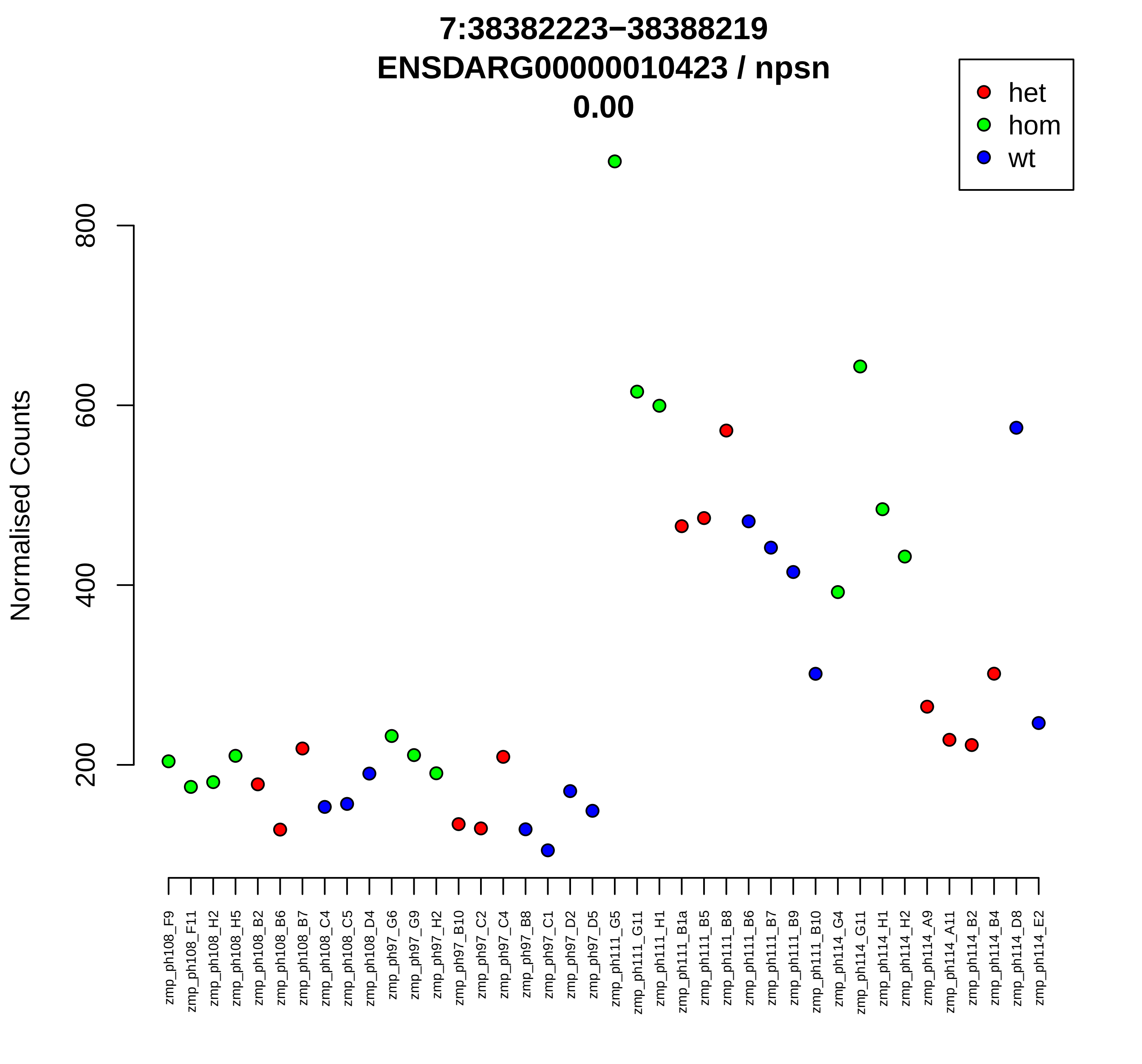

7:38382223−38388219
ENSDARG00000010423 / npsn
0.00
het
hom
wt
Normalised Counts
200
400
600
800
zmp_ph108_F9
zmp_ph108_F11
zmp_ph108_H2
zmp_ph108_H5
zmp_ph108_B2
zmp_ph108_B6
zmp_ph108_B7
zmp_ph108_C4
zmp_ph108_C5
zmp_ph108_D4
zmp_ph97_G6
zmp_ph97_G9
zmp_ph97_H2
zmp_ph97_B10
zmp_ph97_C2
zmp_ph97_C4
zmp_ph97_B8
zmp_ph97_C1
zmp_ph97_D2
zmp_ph97_D5
zmp_ph111_G5
zmp_ph111_G11
zmp_ph111_H1
zmp_ph111_B1a
zmp_ph111_B5
zmp_ph111_B8
zmp_ph111_B6
zmp_ph111_B7
zmp_ph111_B9
zmp_ph111_B10
zmp_ph114_G4
zmp_ph114_G11
zmp_ph114_H1
zmp_ph114_H2
zmp_ph114_A9
zmp_ph114_A11
zmp_ph114_B2
zmp_ph114_B4
zmp_ph114_D8
zmp_ph114_E2

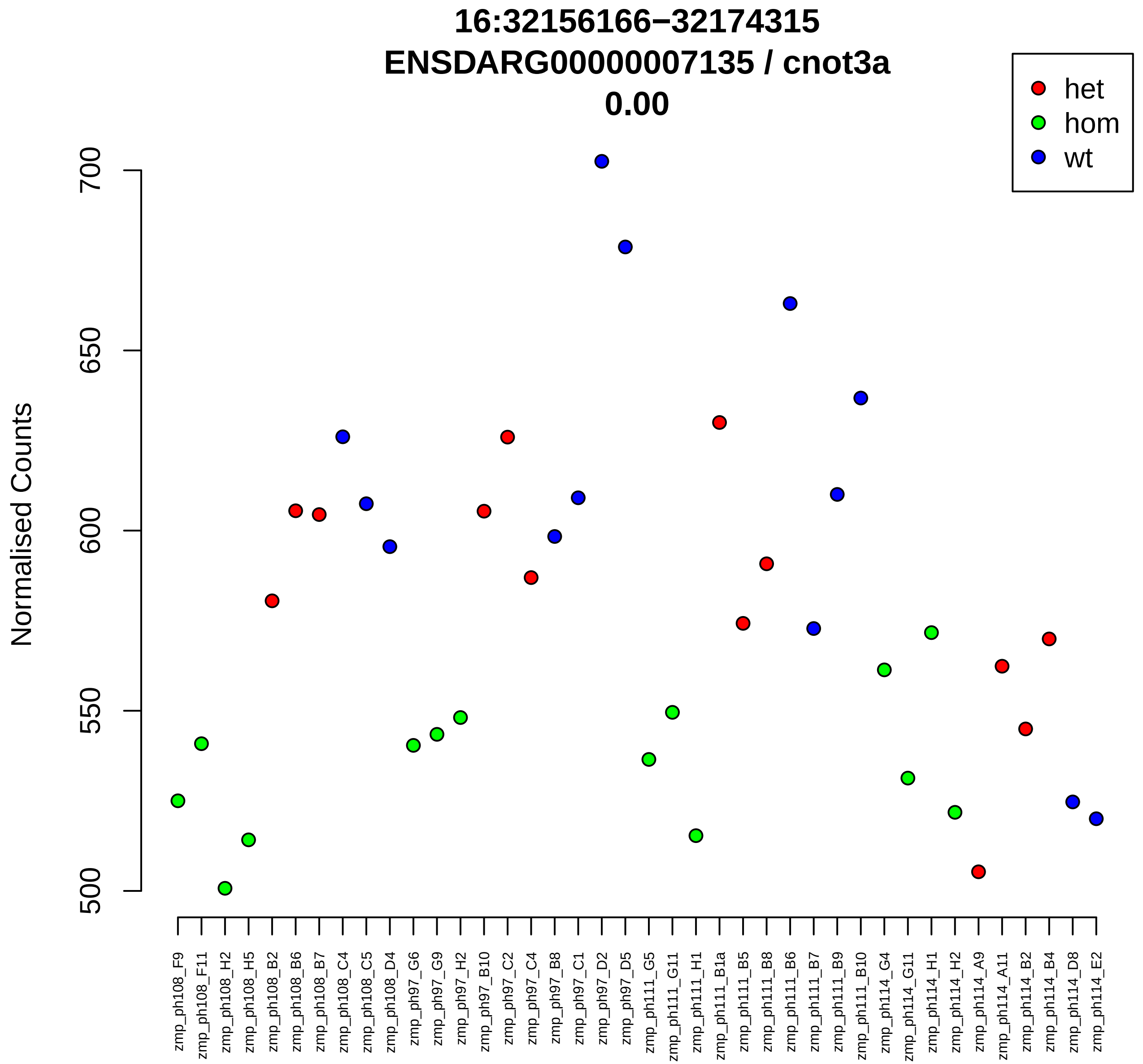

16:32156166−32174315
ENSDARG00000007135 / cnot3a
0.00
het
hom
wt
Normalised Counts
500
550
600
650
700
zmp_ph108_F9
zmp_ph108_F11
zmp_ph108_H2
zmp_ph108_H5
zmp_ph108_B2
zmp_ph108_B6
zmp_ph108_B7
zmp_ph108_C4
zmp_ph108_C5
zmp_ph108_D4
zmp_ph97_G6
zmp_ph97_G9
zmp_ph97_H2
zmp_ph97_B10
zmp_ph97_C2
zmp_ph97_C4
zmp_ph97_B8
zmp_ph97_C1
zmp_ph97_D2
zmp_ph97_D5
zmp_ph111_G5
zmp_ph111_G11
zmp_ph111_H1
zmp_ph111_B1a
zmp_ph111_B5
zmp_ph111_B8
zmp_ph111_B6
zmp_ph111_B7
zmp_ph111_B9
zmp_ph111_B10
zmp_ph114_G4
zmp_ph114_G11
zmp_ph114_H1
zmp_ph114_H2
zmp_ph114_A9
zmp_ph114_A11
zmp_ph114_B2
zmp_ph114_B4
zmp_ph114_D8
zmp_ph114_E2

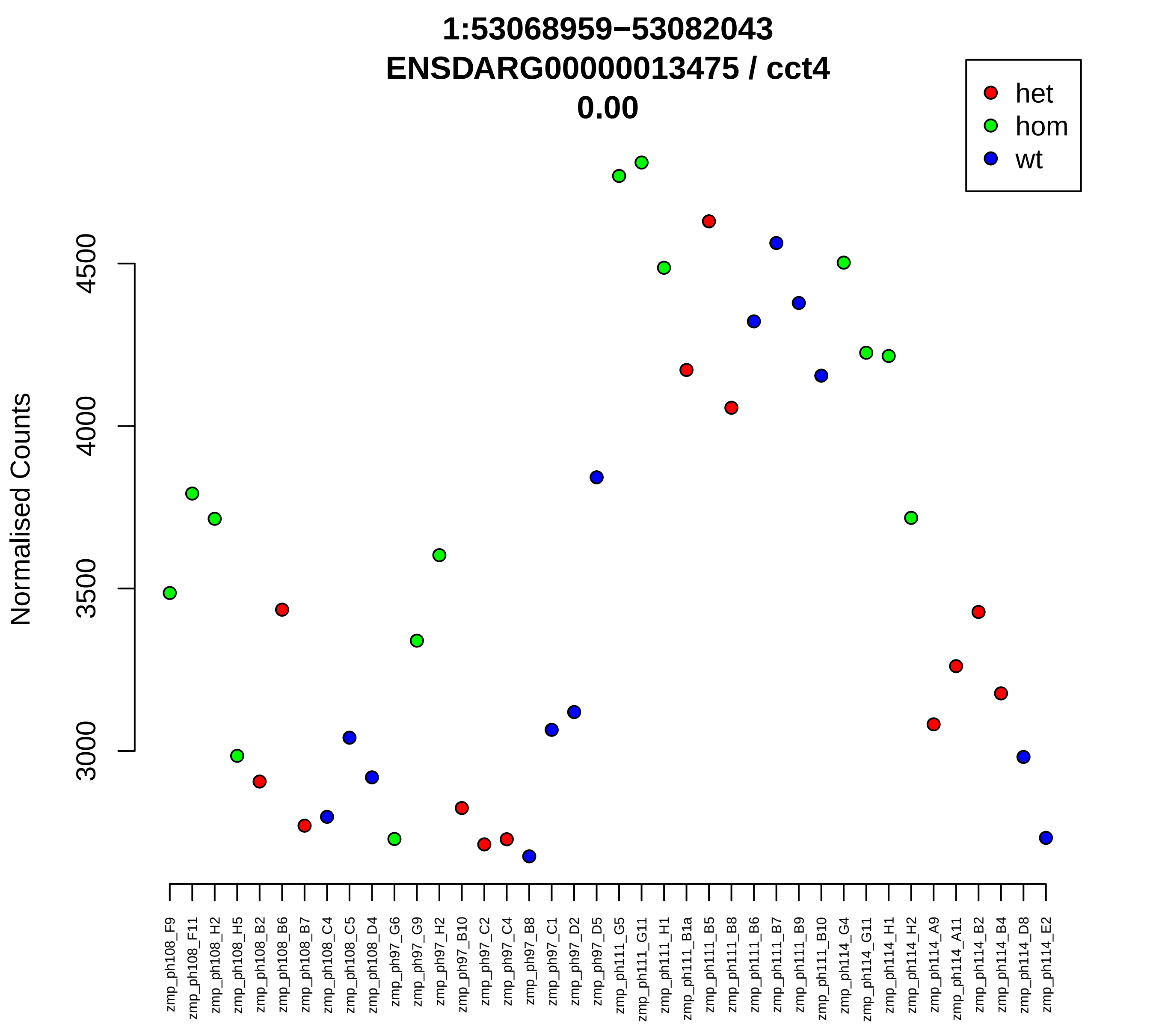

1:53068959−53082043
ENSDARG00000013475 / cct4
0.00
het
hom
wt
Normalised Counts
3000
3500
4000
4500
zmp_ph108_F9
zmp_ph108_F11
zmp_ph108_H2
zmp_ph108_H5
zmp_ph108_B2
zmp_ph108_B6
zmp_ph108_B7
zmp_ph108_C4
zmp_ph108_C5
zmp_ph108_D4
zmp_ph97_G6
zmp_ph97_G9
zmp_ph97_H2
zmp_ph97_B10
zmp_ph97_C2
zmp_ph97_C4
zmp_ph97_B8
zmp_ph97_C1
zmp_ph97_D2
zmp_ph97_D5
zmp_ph111_G5
zmp_ph111_G11
zmp_ph111_H1
zmp_ph111_B1a
zmp_ph111_B5
zmp_ph111_B8
zmp_ph111_B6
zmp_ph111_B7
zmp_ph111_B9
zmp_ph111_B10
zmp_ph114_G4
zmp_ph114_G11
zmp_ph114_H1
zmp_ph114_H2
zmp_ph114_A9
zmp_ph114_A11
zmp_ph114_B2
zmp_ph114_B4
zmp_ph114_D8
zmp_ph114_E2

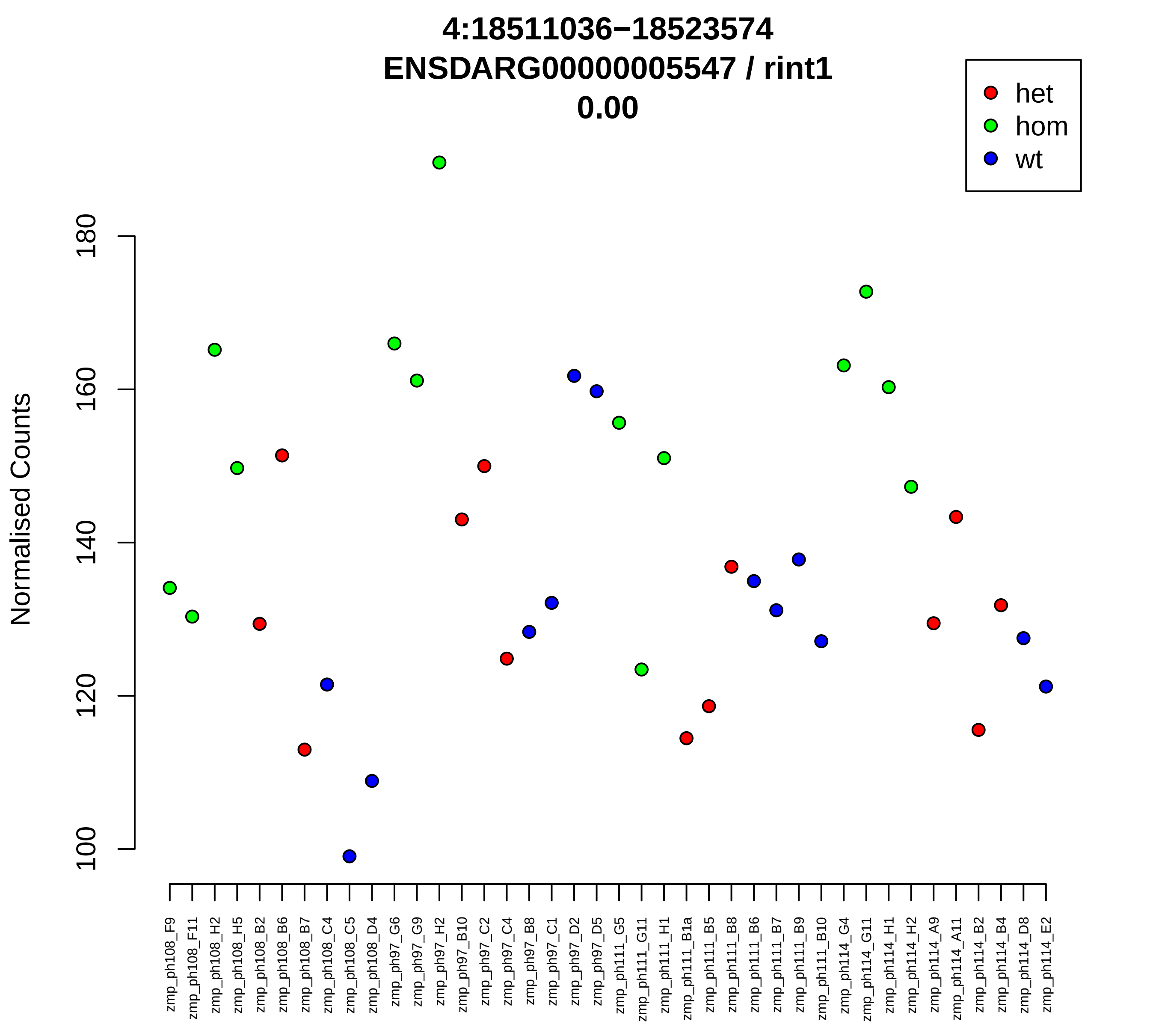

4:18511036−18523574
ENSDARG00000005547 / rint1
0.00
het
hom
wt
Normalised Counts
100
120
140
160
180
zmp_ph108_F9
zmp_ph108_F11
zmp_ph108_H2
zmp_ph108_H5
zmp_ph108_B2
zmp_ph108_B6
zmp_ph108_B7
zmp_ph108_C4
zmp_ph108_C5
zmp_ph108_D4
zmp_ph97_G6
zmp_ph97_G9
zmp_ph97_H2
zmp_ph97_B10
zmp_ph97_C2
zmp_ph97_C4
zmp_ph97_B8
zmp_ph97_C1
zmp_ph97_D2
zmp_ph97_D5
zmp_ph111_G5
zmp_ph111_G11
zmp_ph111_H1
zmp_ph111_B1a
zmp_ph111_B5
zmp_ph111_B8
zmp_ph111_B6
zmp_ph111_B7
zmp_ph111_B9
zmp_ph111_B10
zmp_ph114_G4
zmp_ph114_G11
zmp_ph114_H1
zmp_ph114_H2
zmp_ph114_A9
zmp_ph114_A11
zmp_ph114_B2
zmp_ph114_B4
zmp_ph114_D8
zmp_ph114_E2

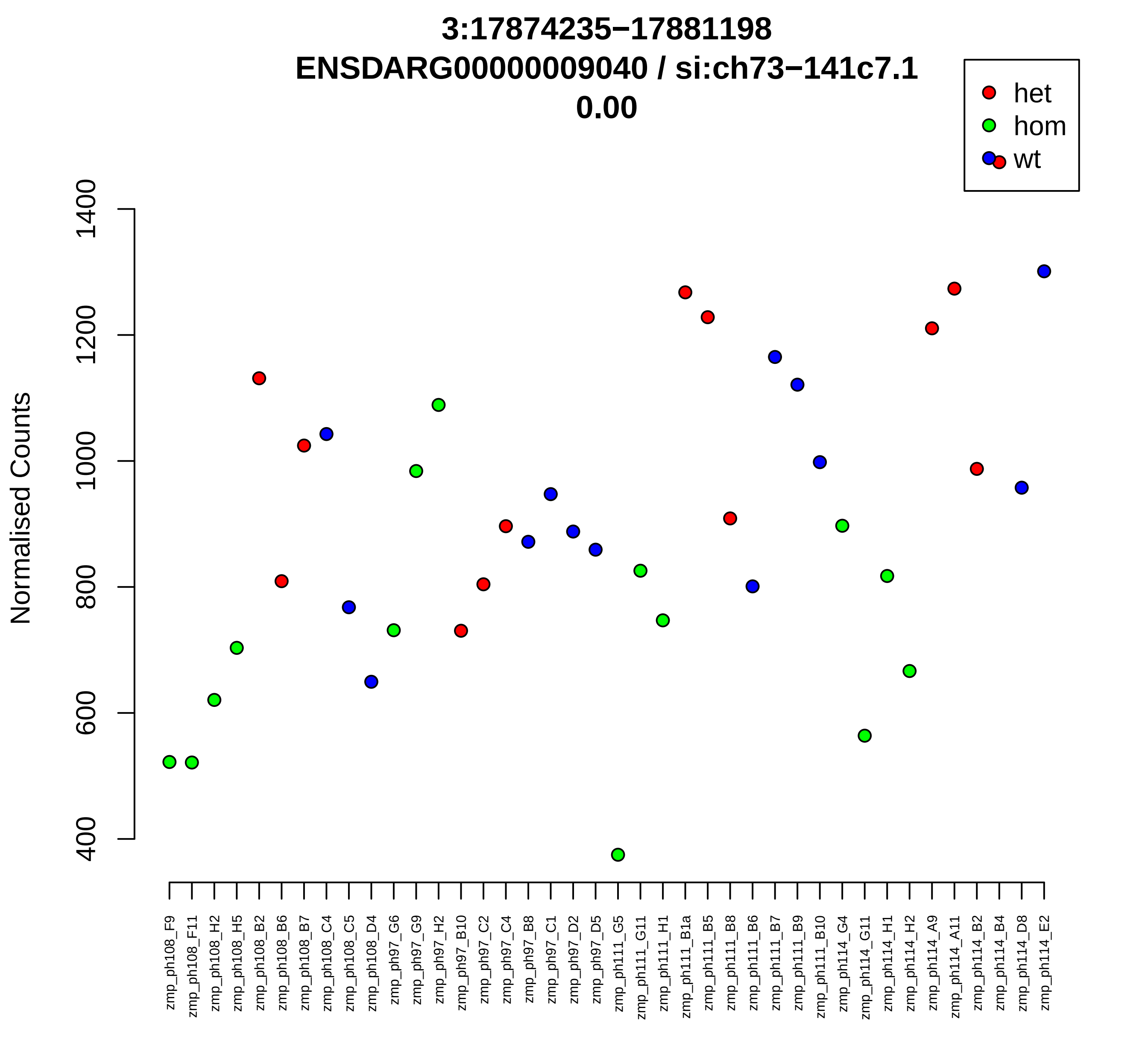

3:17874235−17881198
ENSDARG00000009040 / si:ch73−141c7.1
0.00
het
hom
wt
Normalised Counts
400
600
800
1000
1200
1400
zmp_ph108_F9
zmp_ph108_F11
zmp_ph108_H2
zmp_ph108_H5
zmp_ph108_B2
zmp_ph108_B6
zmp_ph108_B7
zmp_ph108_C4
zmp_ph108_C5
zmp_ph108_D4
zmp_ph97_G6
zmp_ph97_G9
zmp_ph97_H2
zmp_ph97_B10
zmp_ph97_C2
zmp_ph97_C4
zmp_ph97_B8
zmp_ph97_C1
zmp_ph97_D2
zmp_ph97_D5
zmp_ph111_G5
zmp_ph111_G11
zmp_ph111_H1
zmp_ph111_B1a
zmp_ph111_B5
zmp_ph111_B8
zmp_ph111_B6
zmp_ph111_B7
zmp_ph111_B9
zmp_ph111_B10
zmp_ph114_G4
zmp_ph114_G11
zmp_ph114_H1
zmp_ph114_H2
zmp_ph114_A9
zmp_ph114_A11
zmp_ph114_B2
zmp_ph114_B4
zmp_ph114_D8
zmp_ph114_E2

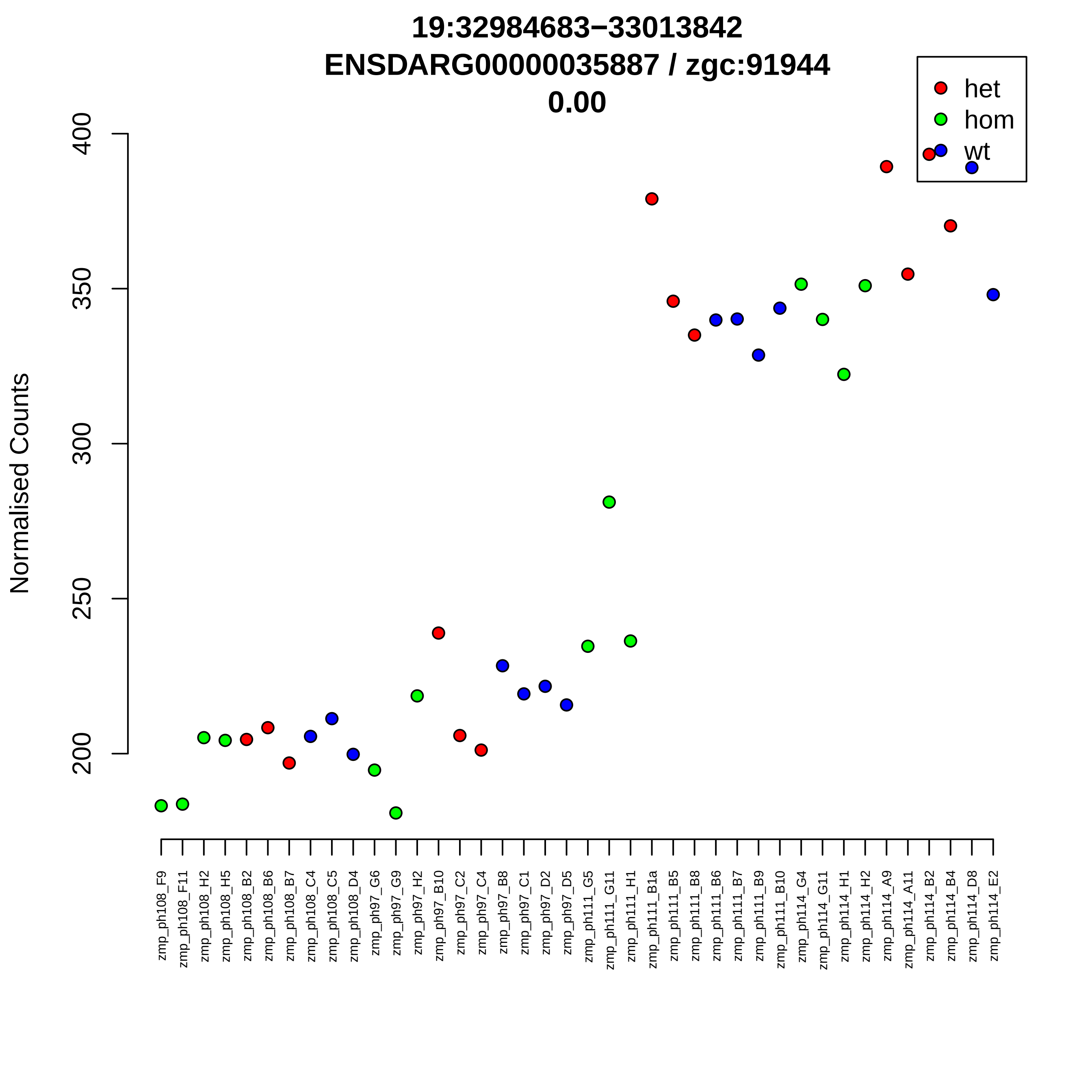

19:32984683−33013842
ENSDARG00000035887 / zgc:91944
0.00
het
hom
wt
Normalised Counts
200
250
300
350
400
zmp_ph108_F9
zmp_ph108_F11
zmp_ph108_H2
zmp_ph108_H5
zmp_ph108_B2
zmp_ph108_B6
zmp_ph108_B7
zmp_ph108_C4
zmp_ph108_C5
zmp_ph108_D4
zmp_ph97_G6
zmp_ph97_G9
zmp_ph97_H2
zmp_ph97_B10
zmp_ph97_C2
zmp_ph97_C4
zmp_ph97_B8
zmp_ph97_C1
zmp_ph97_D2
zmp_ph97_D5
zmp_ph111_G5
zmp_ph111_G11
zmp_ph111_H1
zmp_ph111_B1a
zmp_ph111_B5
zmp_ph111_B8
zmp_ph111_B6
zmp_ph111_B7
zmp_ph111_B9
zmp_ph111_B10
zmp_ph114_G4
zmp_ph114_G11
zmp_ph114_H1
zmp_ph114_H2
zmp_ph114_A9
zmp_ph114_A11
zmp_ph114_B2
zmp_ph114_B4
zmp_ph114_D8
zmp_ph114_E2

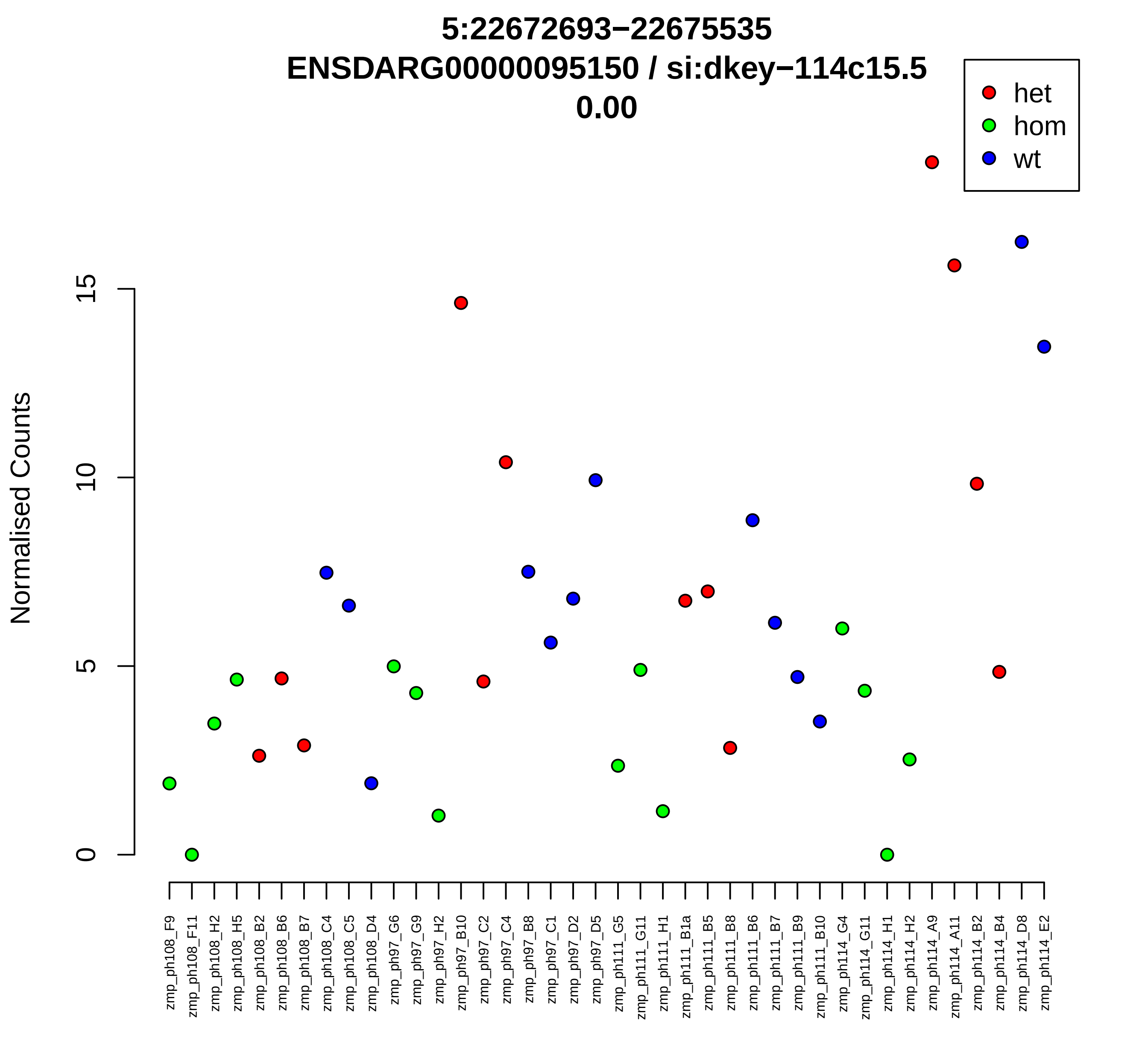

5:22672693−22675535
ENSDARG00000095150 / si:dkey−114c15.5
0.00
het
hom
wt
Normalised Counts
0
5
10
15
zmp_ph108_F9
zmp_ph108_F11
zmp_ph108_H2
zmp_ph108_H5
zmp_ph108_B2
zmp_ph108_B6
zmp_ph108_B7
zmp_ph108_C4
zmp_ph108_C5
zmp_ph108_D4
zmp_ph97_G6
zmp_ph97_G9
zmp_ph97_H2
zmp_ph97_B10
zmp_ph97_C2
zmp_ph97_C4
zmp_ph97_B8
zmp_ph97_C1
zmp_ph97_D2
zmp_ph97_D5
zmp_ph111_G5
zmp_ph111_G11
zmp_ph111_H1
zmp_ph111_B1a
zmp_ph111_B5
zmp_ph111_B8
zmp_ph111_B6
zmp_ph111_B7
zmp_ph111_B9
zmp_ph111_B10
zmp_ph114_G4
zmp_ph114_G11
zmp_ph114_H1
zmp_ph114_H2
zmp_ph114_A9
zmp_ph114_A11
zmp_ph114_B2
zmp_ph114_B4
zmp_ph114_D8
zmp_ph114_E2

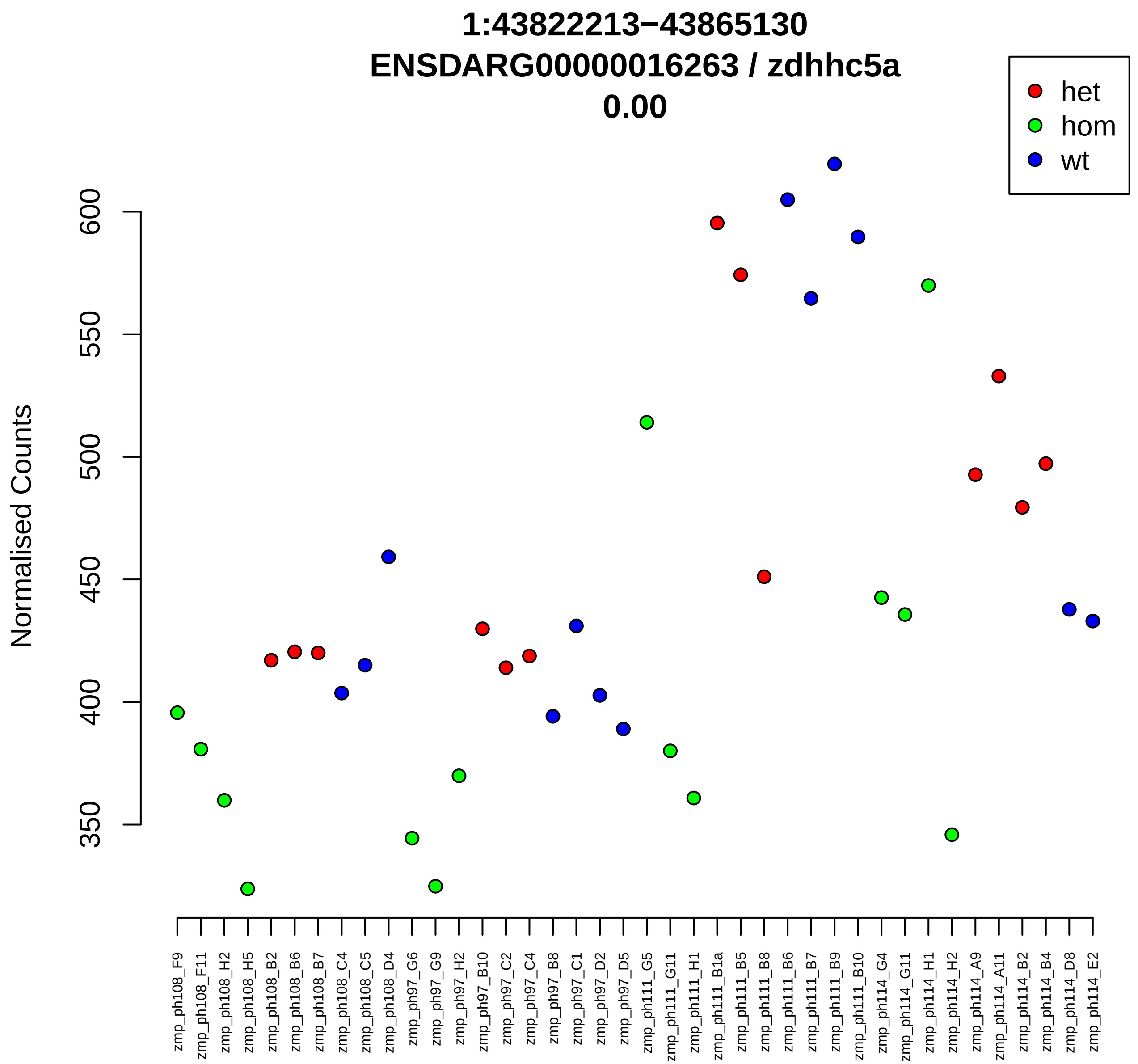
1:43822213−43865130
ENSDARG00000016263 / zdhhc5a
0.00
het
hom
wt
Normalised Counts
350
400
450
500
550
600
zmp_ph108_F9
zmp_ph108_F11
zmp_ph108_H2
zmp_ph108_H5
zmp_ph108_B2
zmp_ph108_B6
zmp_ph108_B7
zmp_ph108_C4
zmp_ph108_C5
zmp_ph108_D4
zmp_ph97_G6
zmp_ph97_G9
zmp_ph97_H2
zmp_ph97_B10
zmp_ph97_C2
zmp_ph97_C4
zmp_ph97_B8
zmp_ph97_C1
zmp_ph97_D2
zmp_ph97_D5
zmp_ph111_G5
zmp_ph111_G11
zmp_ph111_H1
zmp_ph111_B1a
zmp_ph111_B5
zmp_ph111_B8
zmp_ph111_B6
zmp_ph111_B7
zmp_ph111_B9
zmp_ph111_B10
zmp_ph114_G4
zmp_ph114_G11
zmp_ph114_H1
zmp_ph114_H2
zmp_ph114_A9
zmp_ph114_A11
zmp_ph114_B2
zmp_ph114_B4
zmp_ph114_D8
zmp_ph114_E2

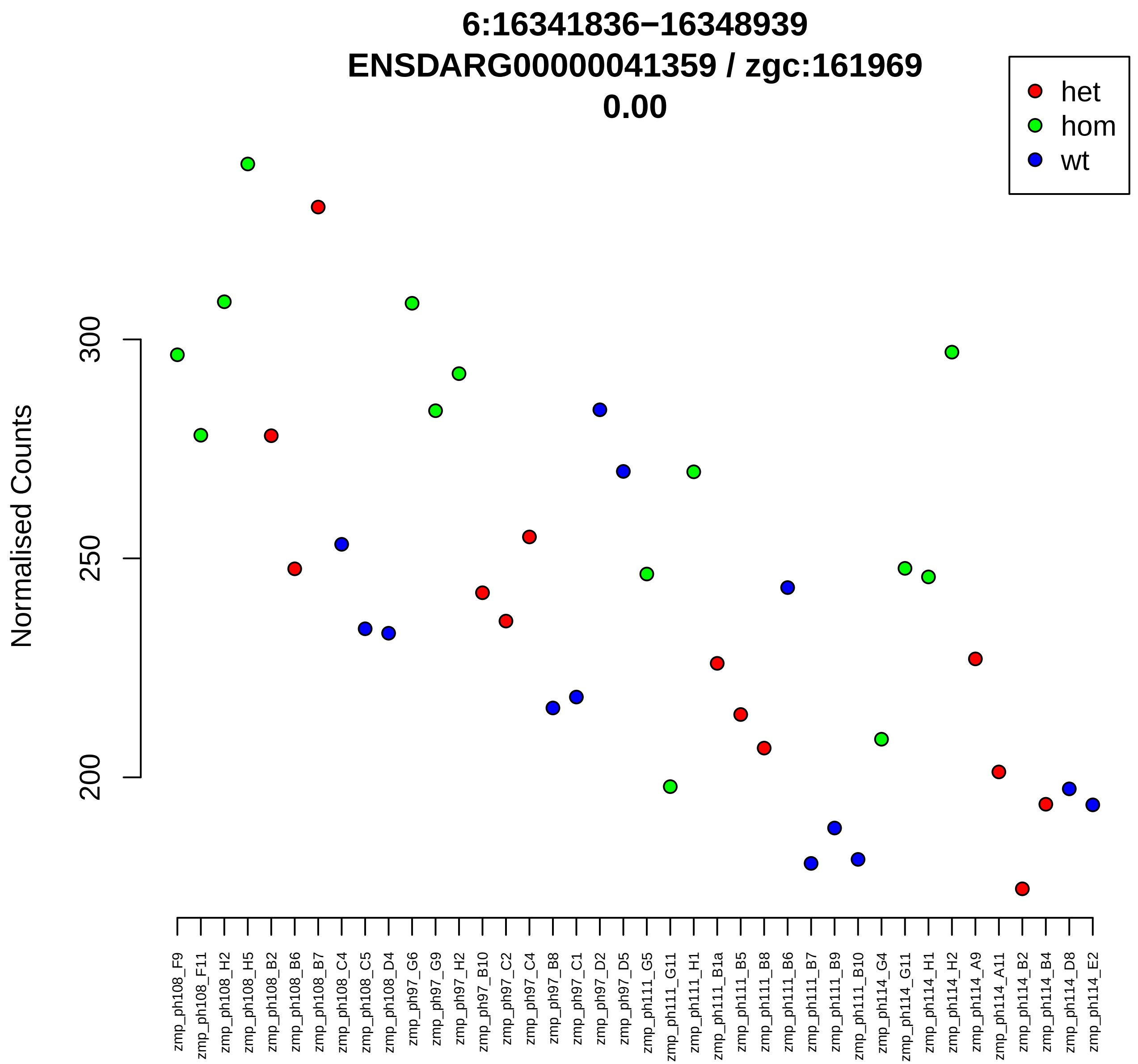
6:16341836−16348939
ENSDARG00000041359 / zgc:161969
0.00
het
hom
wt
Normalised Counts
300
250
200
zmp_ph108_F9
zmp_ph108_F11
zmp_ph108_H2
zmp_ph108_H5
zmp_ph108_B2
zmp_ph108_B6
zmp_ph108_B7
zmp_ph108_C4
zmp_ph108_C5
zmp_ph108_D4
zmp_ph97_G6
zmp_ph97_G9
zmp_ph97_H2
zmp_ph97_B10
zmp_ph97_C2
zmp_ph97_C4
zmp_ph97_B8
zmp_ph97_C1
zmp_ph97_D2
zmp_ph97_D5
zmp_ph111_G5
zmp_ph111_G11
zmp_ph111_H1
zmp_ph111_B1a
zmp_ph111_B5
zmp_ph111_B8
zmp_ph111_B6
zmp_ph111_B7
zmp_ph111_B9
zmp_ph111_B10
zmp_ph114_G4
zmp_ph114_G11
zmp_ph114_H1
zmp_ph114_H2
zmp_ph114_A9
zmp_ph114_A11
zmp_ph114_B2
zmp_ph114_B4
zmp_ph114_D8
zmp_ph114_E2

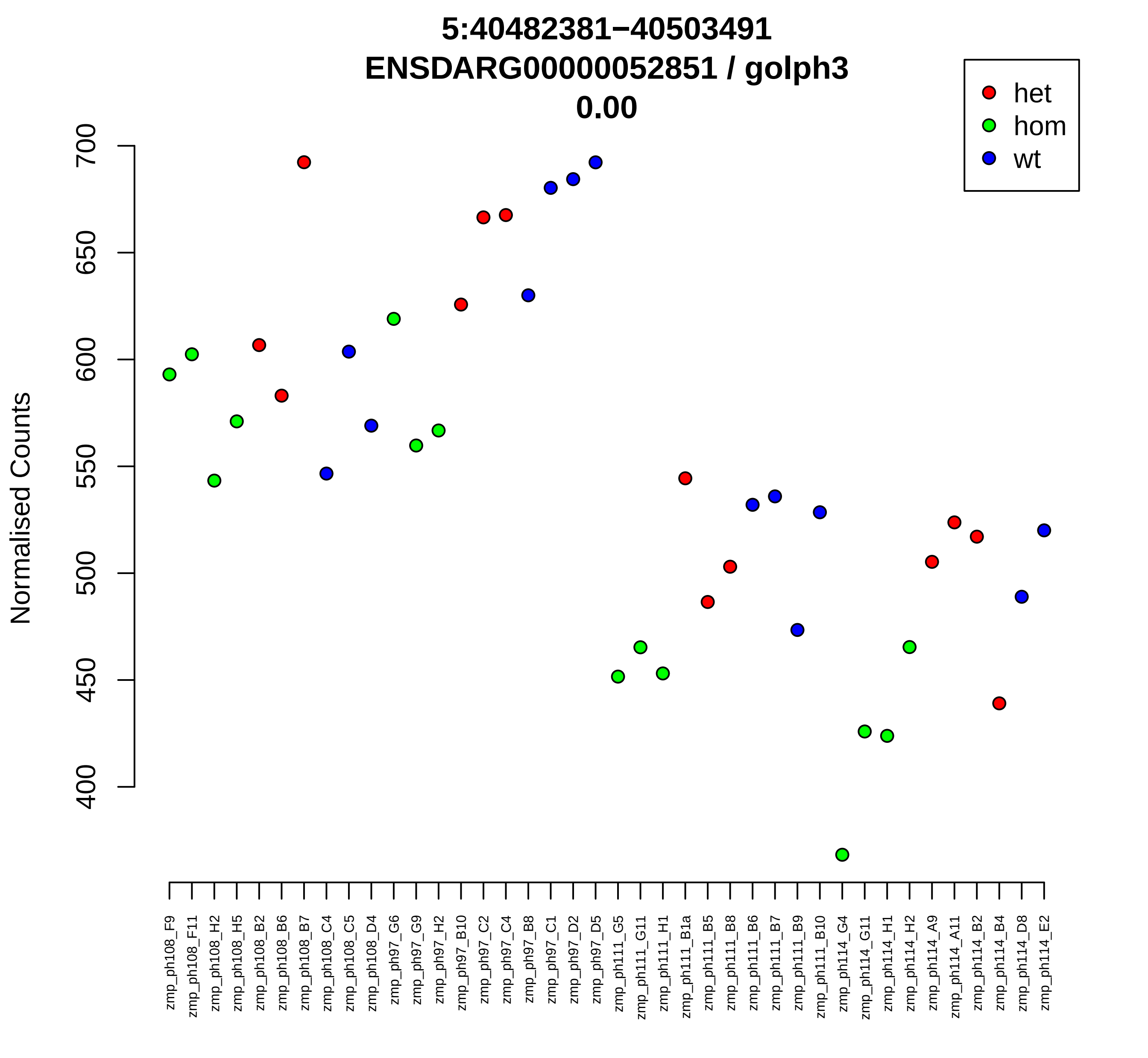
5:40482381−40503491
ENSDARG00000052851 / golph3
0.00
het
hom
wt
Normalised Counts
400
450
500
550
600
650
700
zmp_ph108_F9
zmp_ph108_F11
zmp_ph108_H2
zmp_ph108_H5
zmp_ph108_B2
zmp_ph108_B6
zmp_ph108_B7
zmp_ph108_C4
zmp_ph108_C5
zmp_ph108_D4
zmp_ph97_G6
zmp_ph97_G9
zmp_ph97_H2
zmp_ph97_B10
zmp_ph97_C2
zmp_ph97_C4
zmp_ph97_B8
zmp_ph97_C1
zmp_ph97_D2
zmp_ph97_D5
zmp_ph111_G5
zmp_ph111_G11
zmp_ph111_H1
zmp_ph111_B1a
zmp_ph111_B5
zmp_ph111_B8
zmp_ph111_B6
zmp_ph111_B7
zmp_ph111_B9
zmp_ph111_B10
zmp_ph114_G4
zmp_ph114_G11
zmp_ph114_H1
zmp_ph114_H2
zmp_ph114_A9
zmp_ph114_A11
zmp_ph114_B2
zmp_ph114_B4
zmp_ph114_D8
zmp_ph114_E2

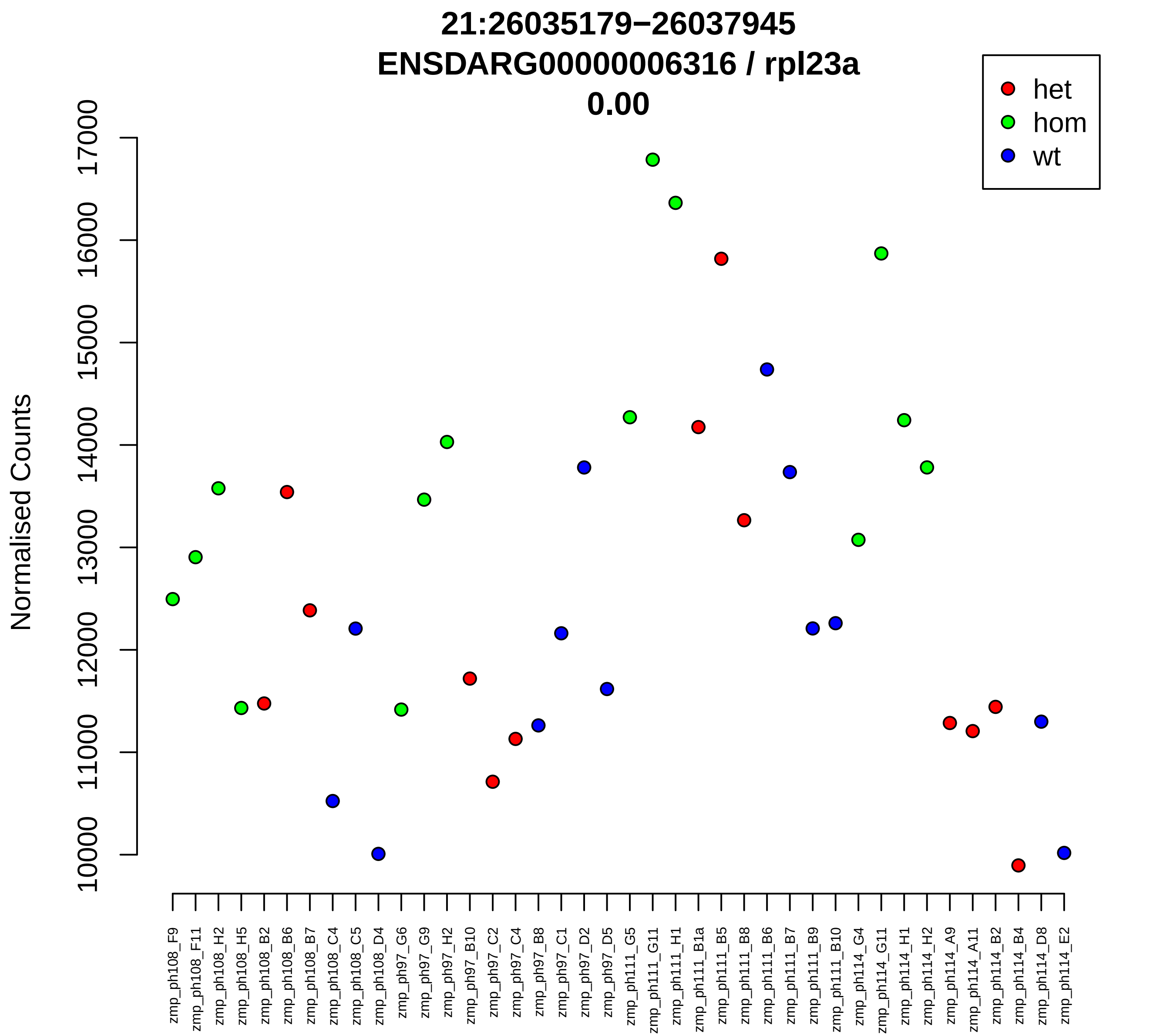

21:26035179−26037945
ENSDARG00000006316 / rpl23a
0.00
het
hom
wt
Normalised Counts
10000
11000
12000
13000
14000
15000
16000
17000
zmp_ph108_F9
zmp_ph108_F11
zmp_ph108_H2
zmp_ph108_H5
zmp_ph108_B2
zmp_ph108_B6
zmp_ph108_B7
zmp_ph108_C4
zmp_ph108_C5
zmp_ph108_D4
zmp_ph97_G6
zmp_ph97_G9
zmp_ph97_H2
zmp_ph97_B10
zmp_ph97_C2
zmp_ph97_C4
zmp_ph97_B8
zmp_ph97_C1
zmp_ph97_D2
zmp_ph97_D5
zmp_ph111_G5
zmp_ph111_G11
zmp_ph111_H1
zmp_ph111_B1a
zmp_ph111_B5
zmp_ph111_B8
zmp_ph111_B6
zmp_ph111_B7
zmp_ph111_B9
zmp_ph111_B10
zmp_ph114_G4
zmp_ph114_G11
zmp_ph114_H1
zmp_ph114_H2
zmp_ph114_A9
zmp_ph114_A11
zmp_ph114_B2
zmp_ph114_B4
zmp_ph114_D8
zmp_ph114_E2

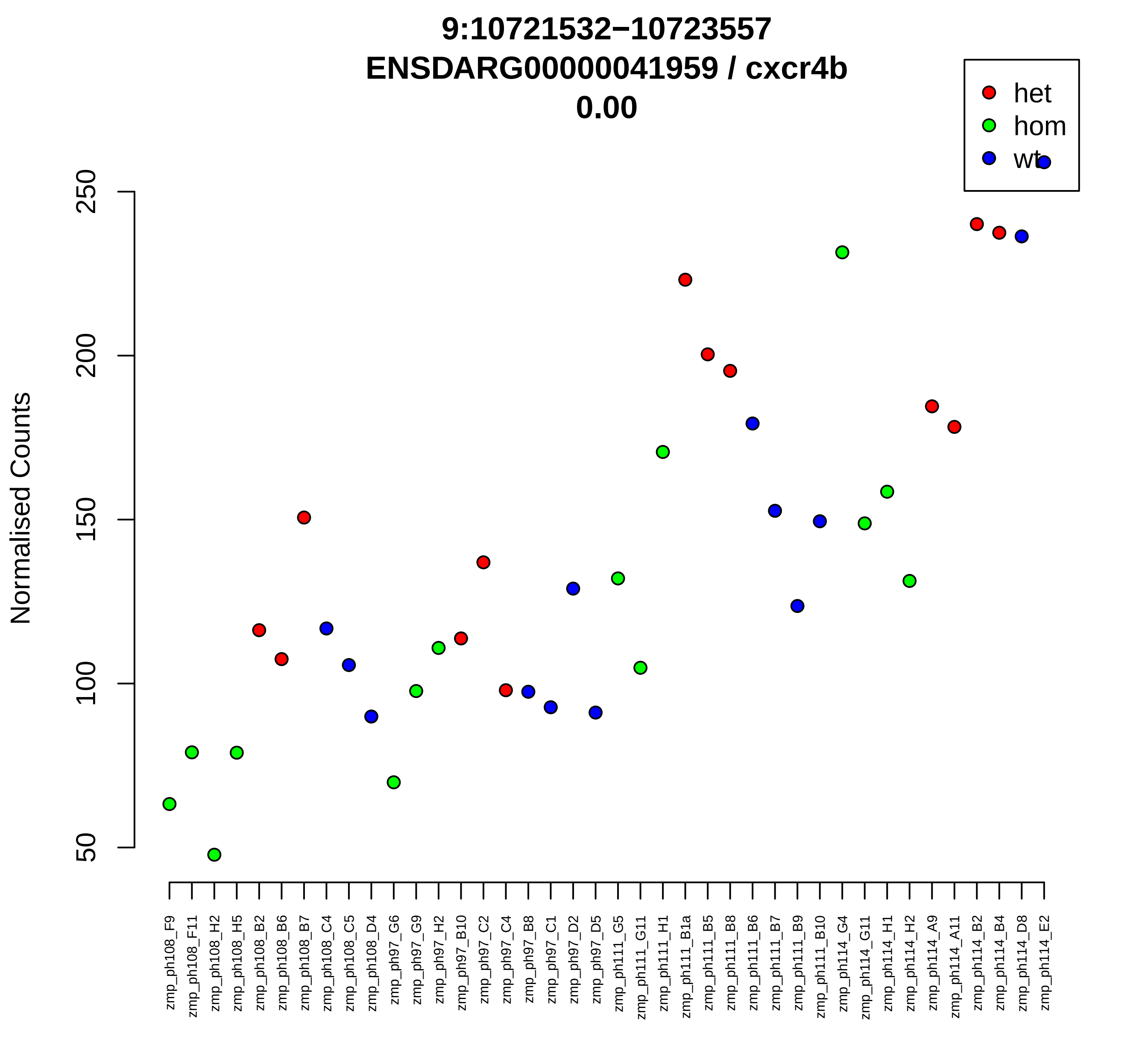

9:10721532−10723557
ENSDARG00000041959 / cxcr4b
0.00
het
hom
wt
Normalised Counts
50
100
150
200
250
zmp_ph108_F9
zmp_ph108_F11
zmp_ph108_H2
zmp_ph108_H5
zmp_ph108_B2
zmp_ph108_B6
zmp_ph108_B7
zmp_ph108_C4
zmp_ph108_C5
zmp_ph108_D4
zmp_ph97_G6
zmp_ph97_G9
zmp_ph97_H2
zmp_ph97_B10
zmp_ph97_C2
zmp_ph97_C4
zmp_ph97_B8
zmp_ph97_C1
zmp_ph97_D2
zmp_ph97_D5
zmp_ph111_G5
zmp_ph111_G11
zmp_ph111_H1
zmp_ph111_B1a
zmp_ph111_B5
zmp_ph111_B8
zmp_ph111_B6
zmp_ph111_B7
zmp_ph111_B9
zmp_ph111_B10
zmp_ph114_G4
zmp_ph114_G11
zmp_ph114_H1
zmp_ph114_H2
zmp_ph114_A9
zmp_ph114_A11
zmp_ph114_B2
zmp_ph114_B4
zmp_ph114_D8
zmp_ph114_E2

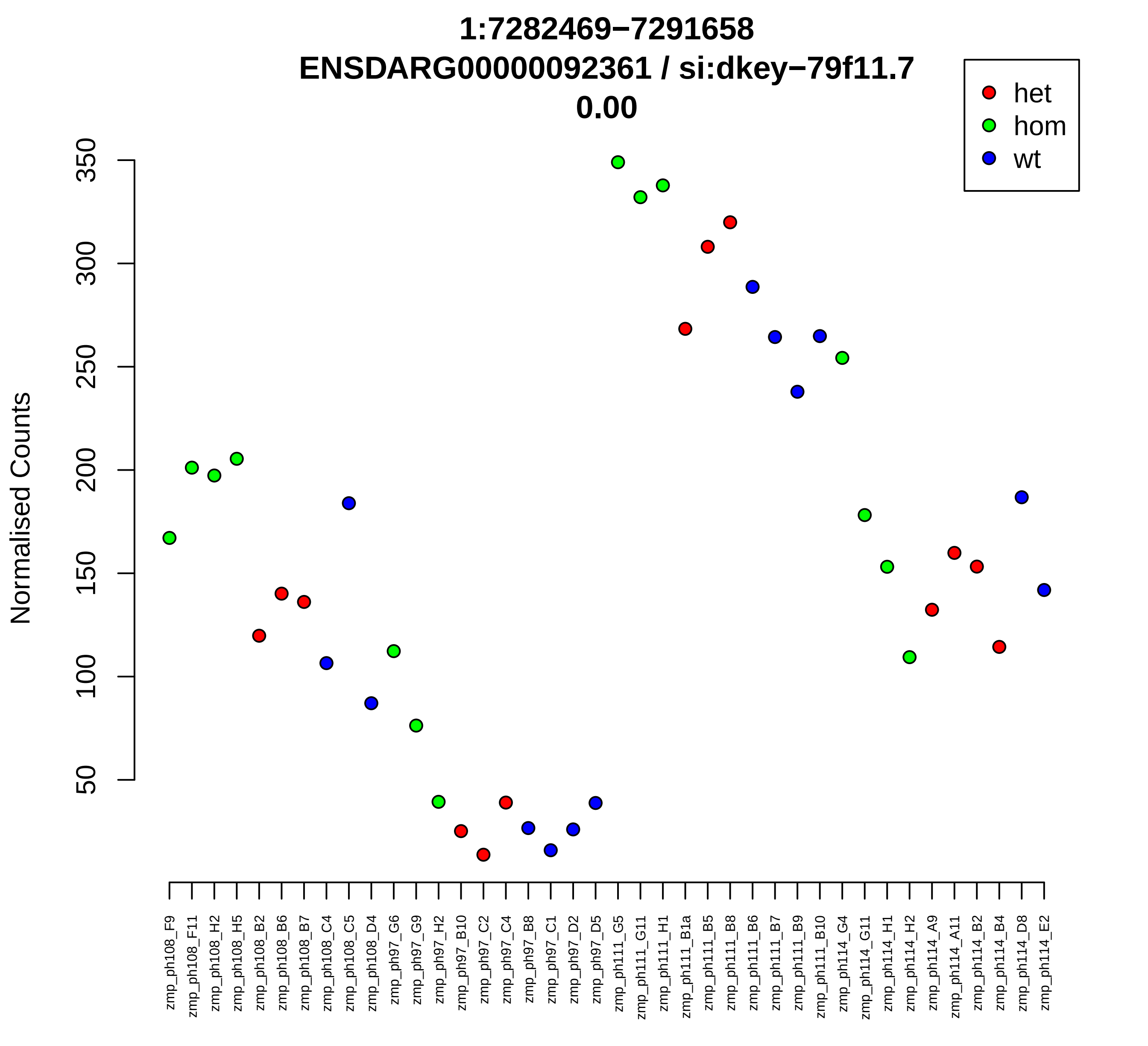
1:7282469−7291658
ENSDARG00000092361 / si:dkey−79f11.7
0.00
het
hom
wt
Normalised Counts
50
100
150
200
250
300
350
zmp_ph108_F9
zmp_ph108_F11
zmp_ph108_H2
zmp_ph108_H5
zmp_ph108_B2
zmp_ph108_B6
zmp_ph108_B7
zmp_ph108_C4
zmp_ph108_C5
zmp_ph108_D4
zmp_ph97_G6
zmp_ph97_G9
zmp_ph97_H2
zmp_ph97_B10
zmp_ph97_C2
zmp_ph97_C4
zmp_ph97_B8
zmp_ph97_C1
zmp_ph97_D2
zmp_ph97_D5
zmp_ph111_G5
zmp_ph111_G11
zmp_ph111_H1
zmp_ph111_B1a
zmp_ph111_B5
zmp_ph111_B8
zmp_ph111_B6
zmp_ph111_B7
zmp_ph111_B9
zmp_ph111_B10
zmp_ph114_G4
zmp_ph114_G11
zmp_ph114_H1
zmp_ph114_H2
zmp_ph114_A9
zmp_ph114_A11
zmp_ph114_B2
zmp_ph114_B4
zmp_ph114_D8
zmp_ph114_E2

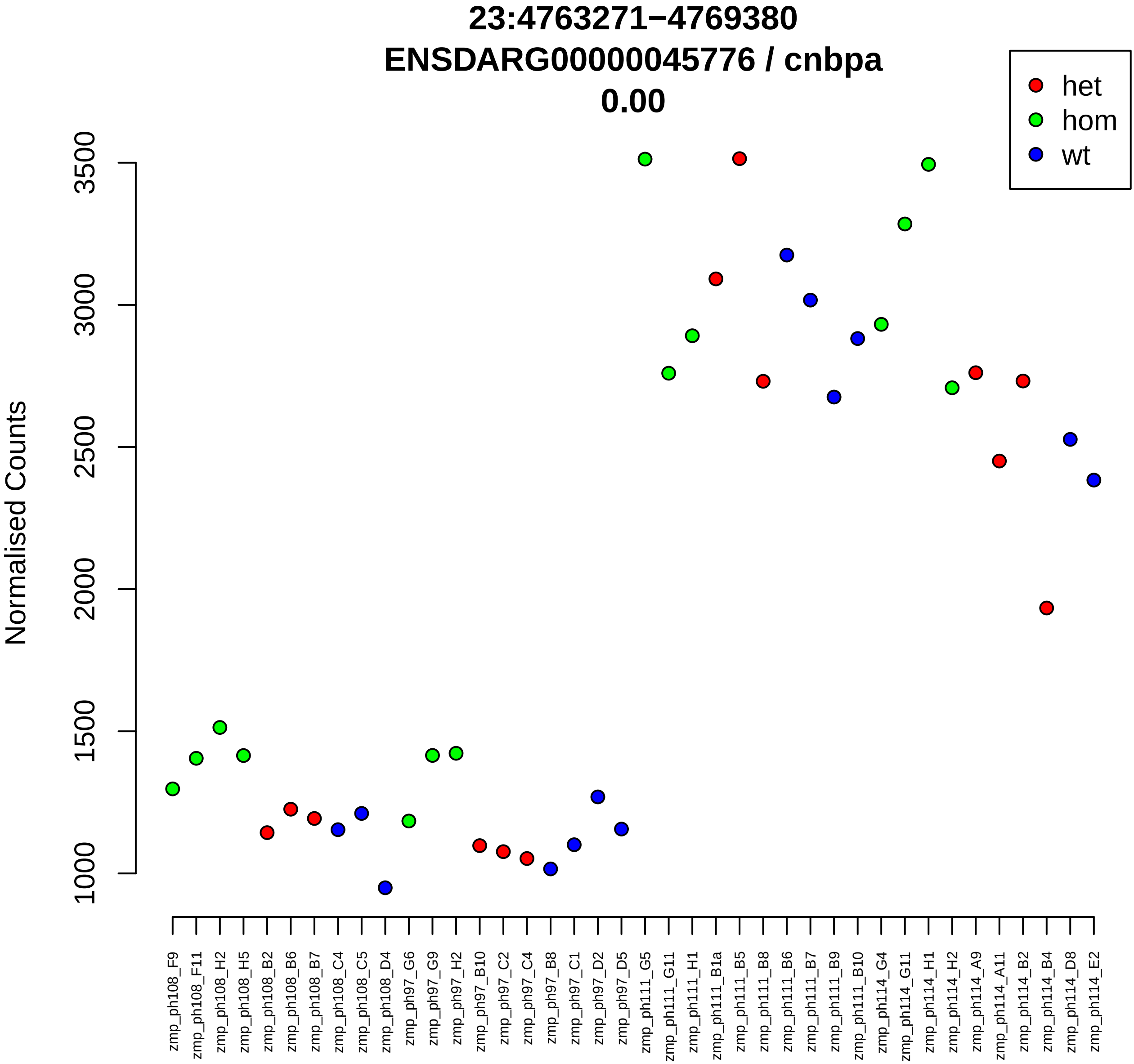

23:4763271−4769380
ENSDARG00000045776 / cnbpa
0.00
het
hom
wt
Normalised Counts
1000
1500
2000
2500
3000
3500
zmp_ph108_F9
zmp_ph108_F11
zmp_ph108_H2
zmp_ph108_H5
zmp_ph108_B2
zmp_ph108_B6
zmp_ph108_B7
zmp_ph108_C4
zmp_ph108_C5
zmp_ph108_D4
zmp_ph97_G6
zmp_ph97_G9
zmp_ph97_H2
zmp_ph97_B10
zmp_ph97_C2
zmp_ph97_C4
zmp_ph97_B8
zmp_ph97_C1
zmp_ph97_D2
zmp_ph97_D5
zmp_ph111_G5
zmp_ph111_G11
zmp_ph111_H1
zmp_ph111_B1a
zmp_ph111_B5
zmp_ph111_B8
zmp_ph111_B6
zmp_ph111_B7
zmp_ph111_B9
zmp_ph111_B10
zmp_ph114_G4
zmp_ph114_G11
zmp_ph114_H1
zmp_ph114_H2
zmp_ph114_A9
zmp_ph114_A11
zmp_ph114_B2
zmp_ph114_B4
zmp_ph114_D8
zmp_ph114_E2

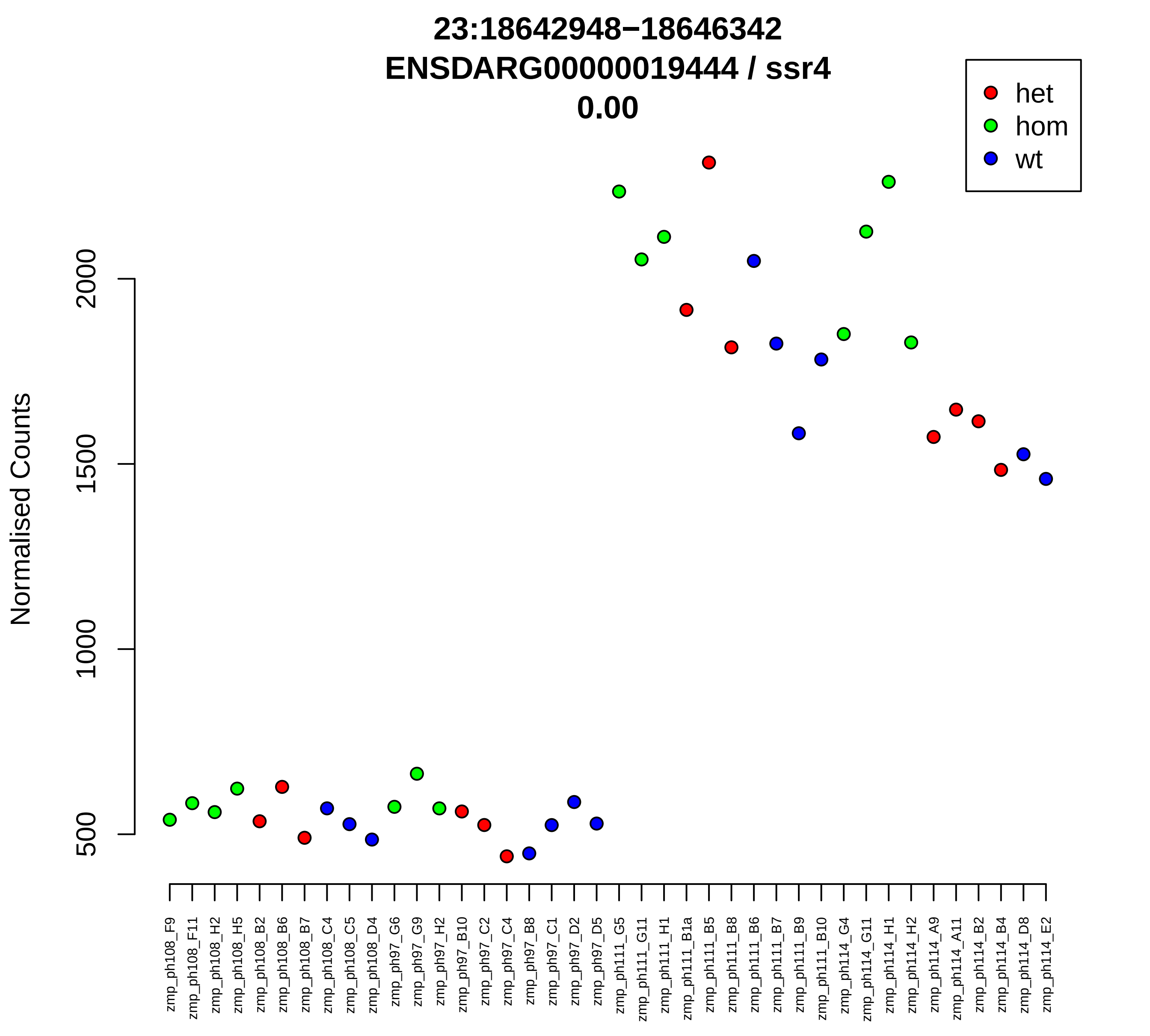

23:18642948−18646342
ENSDARG00000019444 / ssr4
0.00
het
hom
wt
Normalised Counts
500
1000
1500
2000
zmp_ph108_F9
zmp_ph108_F11
zmp_ph108_H2
zmp_ph108_H5
zmp_ph108_B2
zmp_ph108_B6
zmp_ph108_B7
zmp_ph108_C4
zmp_ph108_C5
zmp_ph108_D4
zmp_ph97_G6
zmp_ph97_G9
zmp_ph97_H2
zmp_ph97_B10
zmp_ph97_C2
zmp_ph97_C4
zmp_ph97_B8
zmp_ph97_C1
zmp_ph97_D2
zmp_ph97_D5
zmp_ph111_G5
zmp_ph111_G11
zmp_ph111_H1
zmp_ph111_B1a
zmp_ph111_B5
zmp_ph111_B8
zmp_ph111_B6
zmp_ph111_B7
zmp_ph111_B9
zmp_ph111_B10
zmp_ph114_G4
zmp_ph114_G11
zmp_ph114_H1
zmp_ph114_H2
zmp_ph114_A9
zmp_ph114_A11
zmp_ph114_B2
zmp_ph114_B4
zmp_ph114_D8
zmp_ph114_E2

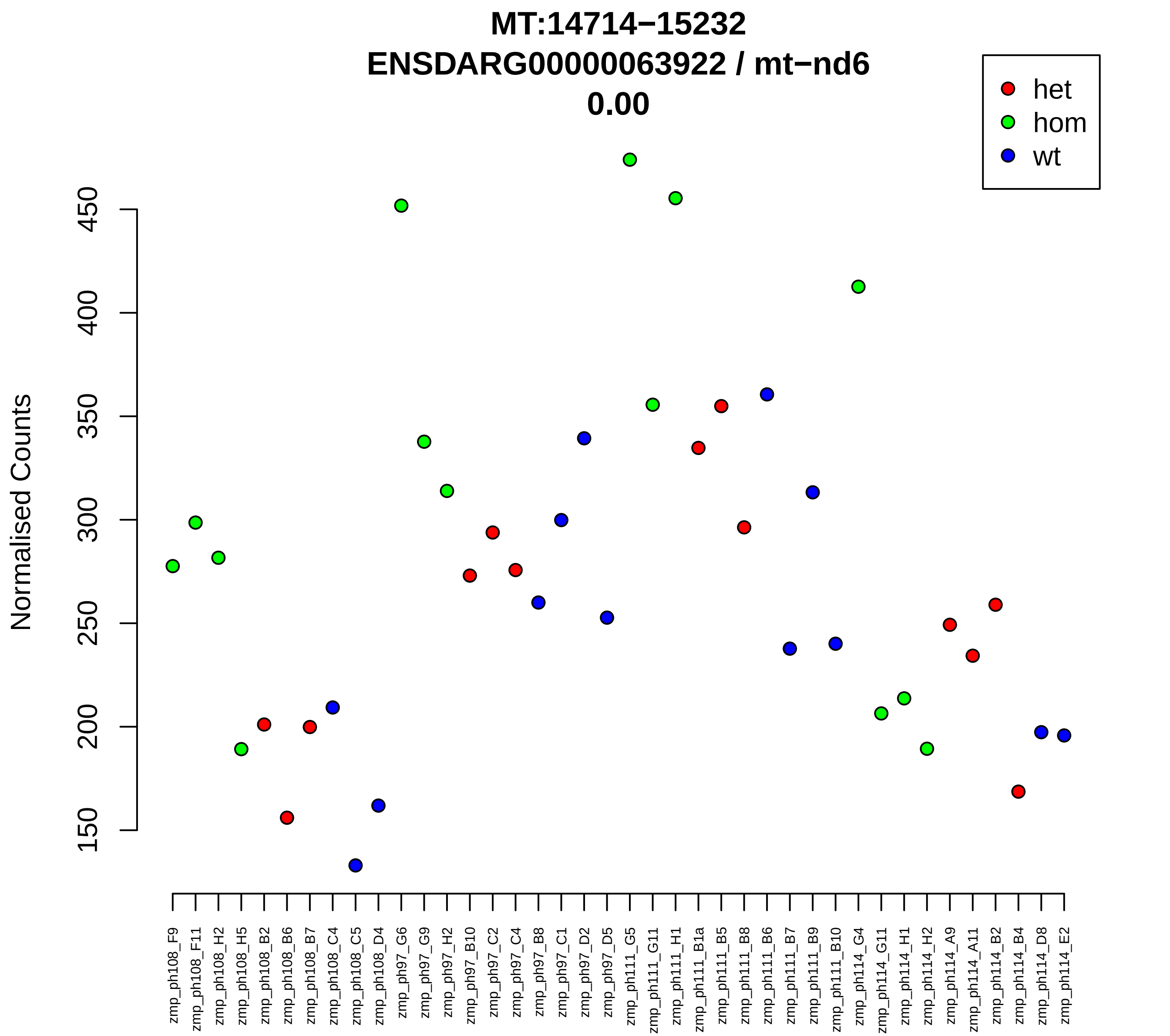

MT:14714−15232
ENSDARG00000063922 / mt−nd6
0.00
het
hom
wt
Normalised Counts
150
200
250
300
350
400
450
zmp_ph108_F9
zmp_ph108_F11
zmp_ph108_H2
zmp_ph108_H5
zmp_ph108_B2
zmp_ph108_B6
zmp_ph108_B7
zmp_ph108_C4
zmp_ph108_C5
zmp_ph108_D4
zmp_ph97_G6
zmp_ph97_G9
zmp_ph97_H2
zmp_ph97_B10
zmp_ph97_C2
zmp_ph97_C4
zmp_ph97_B8
zmp_ph97_C1
zmp_ph97_D2
zmp_ph97_D5
zmp_ph111_G5
zmp_ph111_G11
zmp_ph111_H1
zmp_ph111_B1a
zmp_ph111_B5
zmp_ph111_B8
zmp_ph111_B6
zmp_ph111_B7
zmp_ph111_B9
zmp_ph111_B10
zmp_ph114_G4
zmp_ph114_G11
zmp_ph114_H1
zmp_ph114_H2
zmp_ph114_A9
zmp_ph114_A11
zmp_ph114_B2
zmp_ph114_B4
zmp_ph114_D8
zmp_ph114_E2

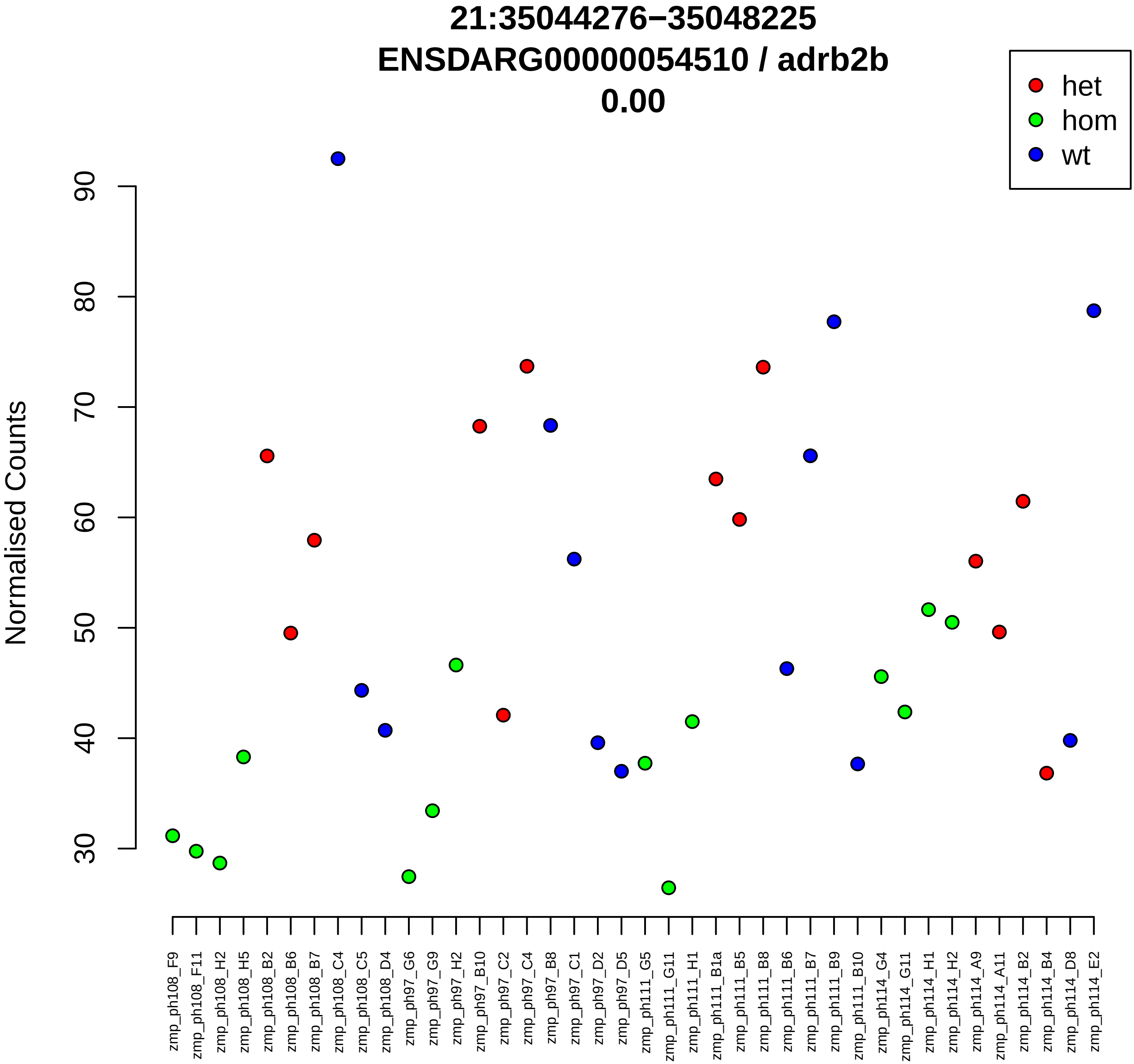
21:35044276−35048225
ENSDARG00000054510 / adrb2b
0.00
het
hom
wt
Normalised Counts
30
40
50
60
70
80
90
zmp_ph108_F9
zmp_ph108_F11
zmp_ph108_H2
zmp_ph108_H5
zmp_ph108_B2
zmp_ph108_B6
zmp_ph108_B7
zmp_ph108_C4
zmp_ph108_C5
zmp_ph108_D4
zmp_ph97_G6
zmp_ph97_G9
zmp_ph97_H2
zmp_ph97_B10
zmp_ph97_C2
zmp_ph97_C4
zmp_ph97_B8
zmp_ph97_C1
zmp_ph97_D2
zmp_ph97_D5
zmp_ph111_G5
zmp_ph111_G11
zmp_ph111_H1
zmp_ph111_B1a
zmp_ph111_B5
zmp_ph111_B8
zmp_ph111_B6
zmp_ph111_B7
zmp_ph111_B9
zmp_ph111_B10
zmp_ph114_G4
zmp_ph114_G11
zmp_ph114_H1
zmp_ph114_H2
zmp_ph114_A9
zmp_ph114_A11
zmp_ph114_B2
zmp_ph114_B4
zmp_ph114_D8
zmp_ph114_E2

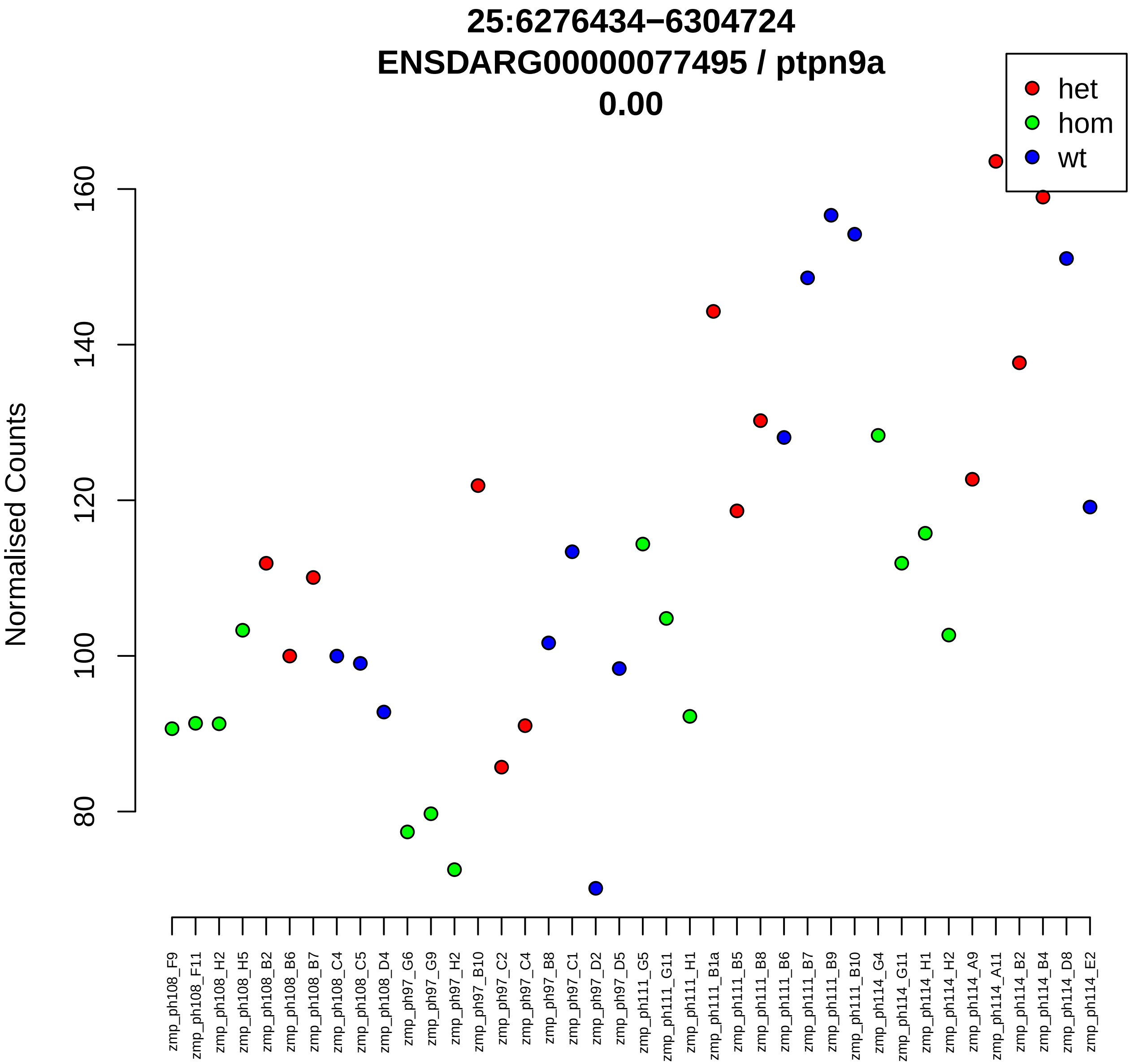

25:6276434−6304724
ENSDARG00000077495 / ptpn9a
0.00
het
hom
wt
Normalised Counts
80
100
120
140
160
zmp_ph108_F9
zmp_ph108_F11
zmp_ph108_H2
zmp_ph108_H5
zmp_ph108_B2
zmp_ph108_B6
zmp_ph108_B7
zmp_ph108_C4
zmp_ph108_C5
zmp_ph108_D4
zmp_ph97_G6
zmp_ph97_G9
zmp_ph97_H2
zmp_ph97_B10
zmp_ph97_C2
zmp_ph97_C4
zmp_ph97_B8
zmp_ph97_C1
zmp_ph97_D2
zmp_ph97_D5
zmp_ph111_G5
zmp_ph111_G11
zmp_ph111_H1
zmp_ph111_B1a
zmp_ph111_B5
zmp_ph111_B8
zmp_ph111_B6
zmp_ph111_B7
zmp_ph111_B9
zmp_ph111_B10
zmp_ph114_G4
zmp_ph114_G11
zmp_ph114_H1
zmp_ph114_H2
zmp_ph114_A9
zmp_ph114_A11
zmp_ph114_B2
zmp_ph114_B4
zmp_ph114_D8
zmp_ph114_E2

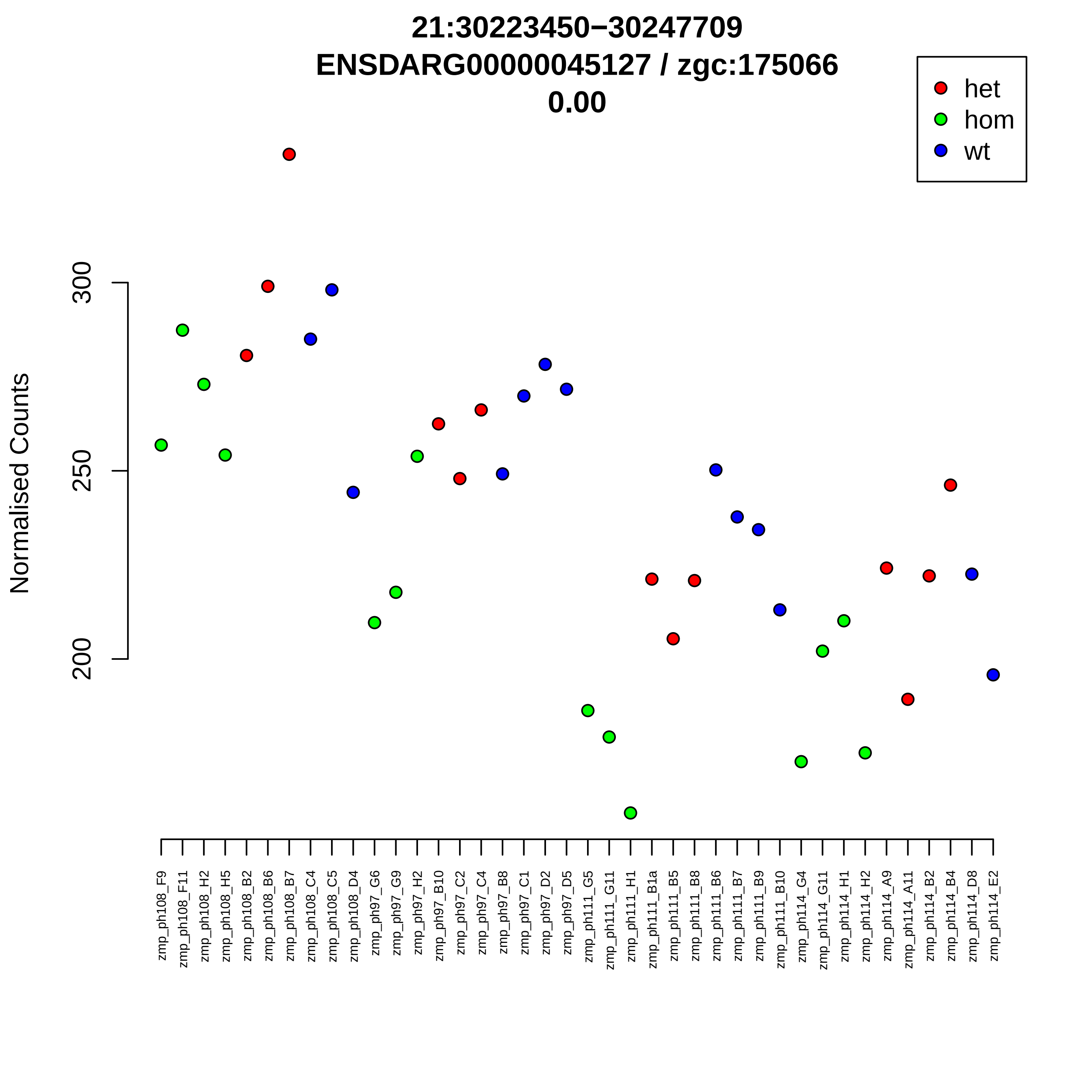

21:30223450−30247709
ENSDARG00000045127 / zgc:175066
0.00
het
hom
wt
Normalised Counts
300
250
200
zmp_ph108_F9
zmp_ph108_F11
zmp_ph108_H2
zmp_ph108_H5
zmp_ph108_B2
zmp_ph108_B6
zmp_ph108_B7
zmp_ph108_C4
zmp_ph108_C5
zmp_ph108_D4
zmp_ph97_G6
zmp_ph97_G9
zmp_ph97_H2
zmp_ph97_B10
zmp_ph97_C2
zmp_ph97_C4
zmp_ph97_B8
zmp_ph97_C1
zmp_ph97_D2
zmp_ph97_D5
zmp_ph111_G5
zmp_ph111_G11
zmp_ph111_H1
zmp_ph111_B1a
zmp_ph111_B5
zmp_ph111_B8
zmp_ph111_B6
zmp_ph111_B7
zmp_ph111_B9
zmp_ph111_B10
zmp_ph114_G4
zmp_ph114_G11
zmp_ph114_H1
zmp_ph114_H2
zmp_ph114_A9
zmp_ph114_A11
zmp_ph114_B2
zmp_ph114_B4
zmp_ph114_D8
zmp_ph114_E2

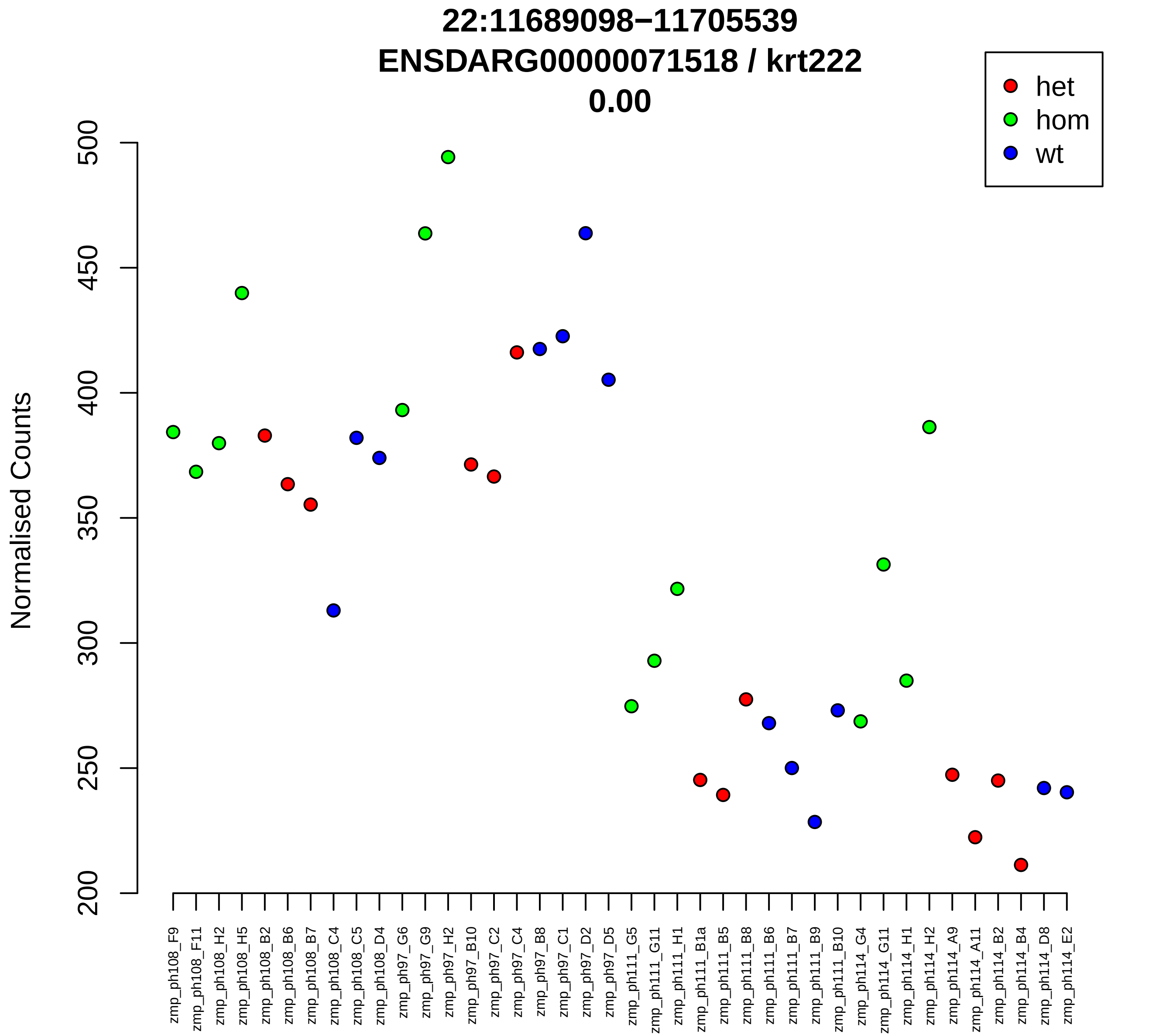
22:11689098−11705539
ENSDARG00000071518 / krt222
0.00
Normalised Counts
200
250
300
350
400
450
500
het
hom
wt
zmp_ph108_F9
zmp_ph108_F11
zmp_ph108_H2
zmp_ph108_H5
zmp_ph108_B2
zmp_ph108_B6
zmp_ph108_B7
zmp_ph108_C4
zmp_ph108_C5
zmp_ph108_D4
zmp_ph97_G6
zmp_ph97_G9
zmp_ph97_H2
zmp_ph97_B10
zmp_ph97_C2
zmp_ph97_C4
zmp_ph97_B8
zmp_ph97_C1
zmp_ph97_D2
zmp_ph97_D5
zmp_ph111_G5
zmp_ph111_G11
zmp_ph111_H1
zmp_ph111_B1a
zmp_ph111_B5
zmp_ph111_B8
zmp_ph111_B6
zmp_ph111_B7
zmp_ph111_B9
zmp_ph111_B10
zmp_ph114_G4
zmp_ph114_G11
zmp_ph114_H1
zmp_ph114_H2
zmp_ph114_A9
zmp_ph114_A11
zmp_ph114_B2
zmp_ph114_B4
zmp_ph114_D8
zmp_ph114_E2

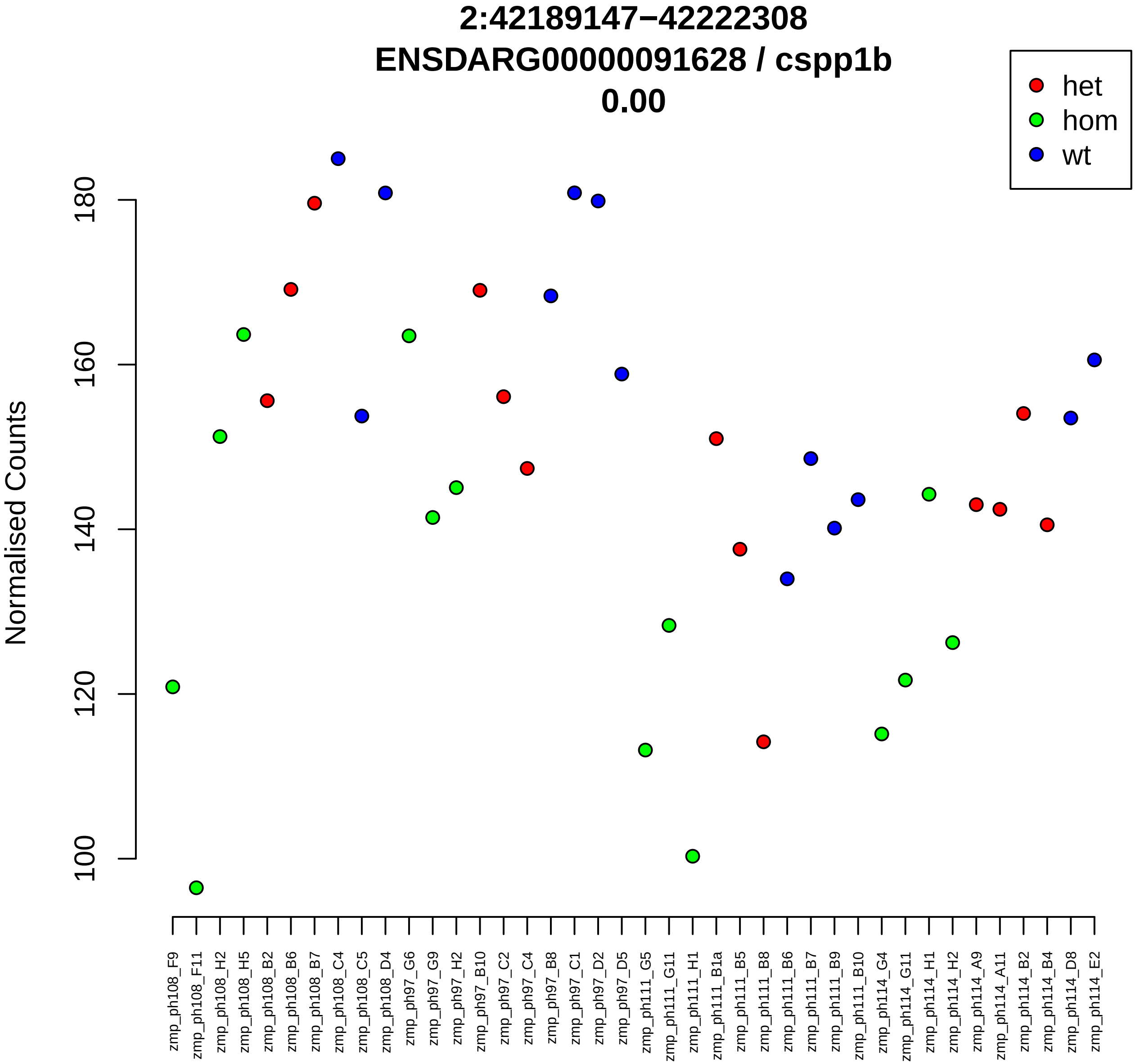
2:42189147−42222308
ENSDARG00000091628 / cspp1b
0.00
het
hom
wt
Normalised Counts
100
120
140
160
180
zmp_ph108_F9
zmp_ph108_F11
zmp_ph108_H2
zmp_ph108_H5
zmp_ph108_B2
zmp_ph108_B6
zmp_ph108_B7
zmp_ph108_C4
zmp_ph108_C5
zmp_ph108_D4
zmp_ph97_G6
zmp_ph97_G9
zmp_ph97_H2
zmp_ph97_B10
zmp_ph97_C2
zmp_ph97_C4
zmp_ph97_B8
zmp_ph97_C1
zmp_ph97_D2
zmp_ph97_D5
zmp_ph111_G5
zmp_ph111_G11
zmp_ph111_H1
zmp_ph111_B1a
zmp_ph111_B5
zmp_ph111_B8
zmp_ph111_B6
zmp_ph111_B7
zmp_ph111_B9
zmp_ph111_B10
zmp_ph114_G4
zmp_ph114_G11
zmp_ph114_H1
zmp_ph114_H2
zmp_ph114_A9
zmp_ph114_A11
zmp_ph114_B2
zmp_ph114_B4
zmp_ph114_D8
zmp_ph114_E2

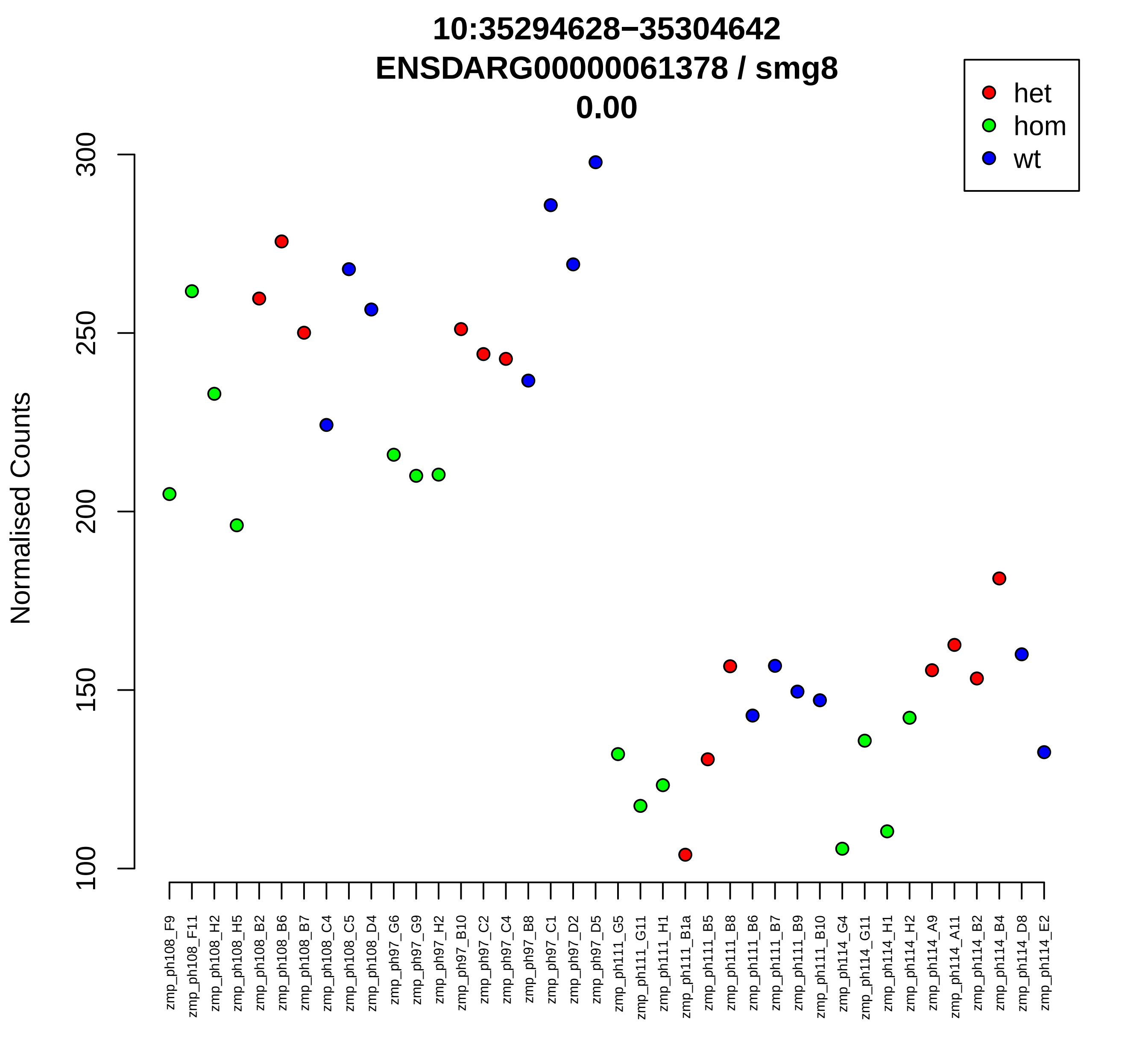

10:35294628−35304642
ENSDARG00000061378 / smg8
0.00
het
hom
wt
Normalised Counts
100
150
200
250
300
zmp_ph108_F9
zmp_ph108_F11
zmp_ph108_H2
zmp_ph108_H5
zmp_ph108_B2
zmp_ph108_B6
zmp_ph108_B7
zmp_ph108_C4
zmp_ph108_C5
zmp_ph108_D4
zmp_ph97_G6
zmp_ph97_G9
zmp_ph97_H2
zmp_ph97_B10
zmp_ph97_C2
zmp_ph97_C4
zmp_ph97_B8
zmp_ph97_C1
zmp_ph97_D2
zmp_ph97_D5
zmp_ph111_G5
zmp_ph111_G11
zmp_ph111_H1
zmp_ph111_B1a
zmp_ph111_B5
zmp_ph111_B8
zmp_ph111_B6
zmp_ph111_B7
zmp_ph111_B9
zmp_ph111_B10
zmp_ph114_G4
zmp_ph114_G11
zmp_ph114_H1
zmp_ph114_H2
zmp_ph114_A9
zmp_ph114_A11
zmp_ph114_B2
zmp_ph114_B4
zmp_ph114_D8
zmp_ph114_E2

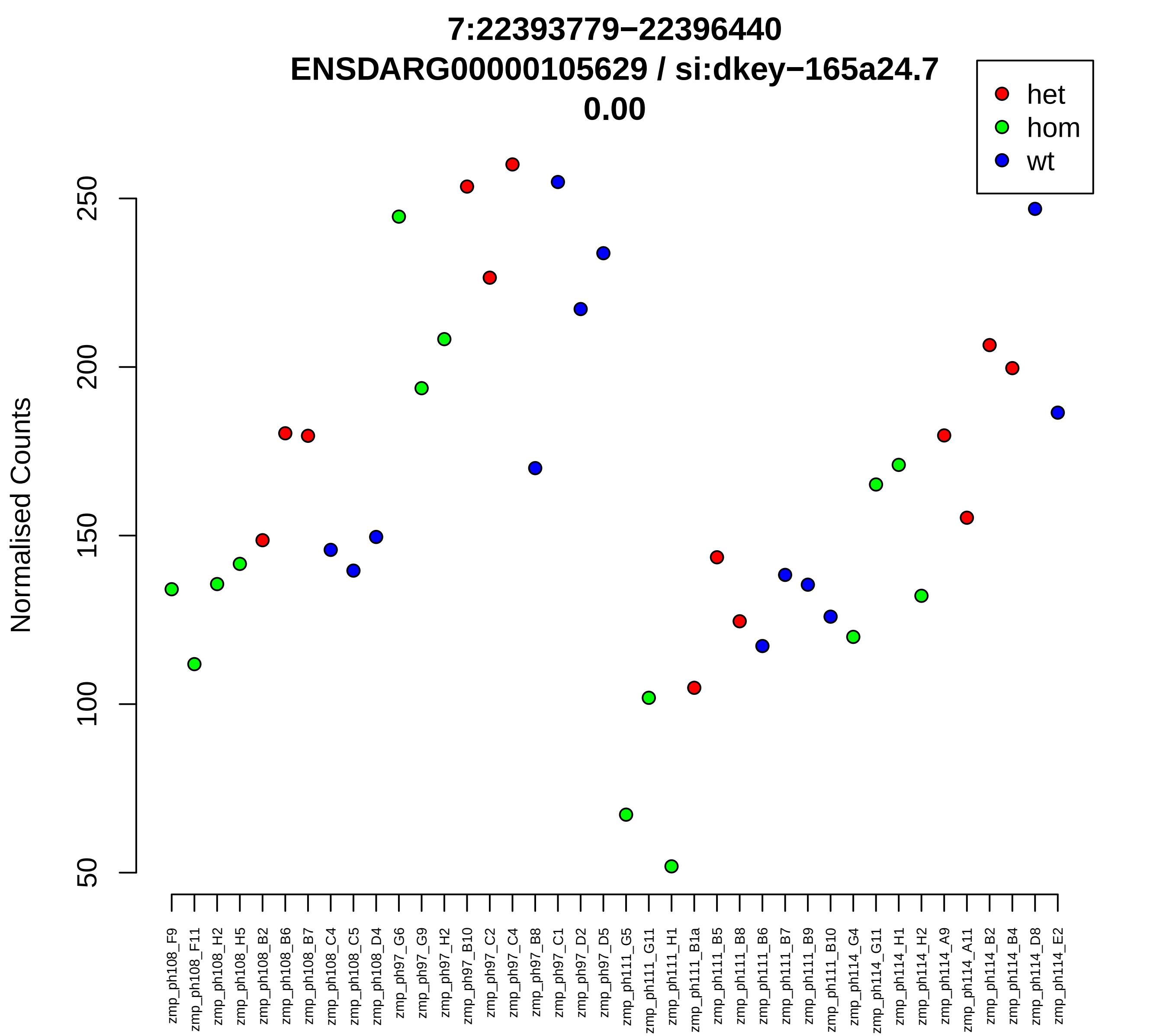

7:22393779−22396440
ENSDARG00000105629 / si:dkey−165a24.7
0.00
het
hom
wt
Normalised Counts
50
100
150
200
250
zmp_ph108_F9
zmp_ph108_F11
zmp_ph108_H2
zmp_ph108_H5
zmp_ph108_B2
zmp_ph108_B6
zmp_ph108_B7
zmp_ph108_C4
zmp_ph108_C5
zmp_ph108_D4
zmp_ph97_G6
zmp_ph97_G9
zmp_ph97_H2
zmp_ph97_B10
zmp_ph97_C2
zmp_ph97_C4
zmp_ph97_B8
zmp_ph97_C1
zmp_ph97_D2
zmp_ph97_D5
zmp_ph111_G5
zmp_ph111_G11
zmp_ph111_H1
zmp_ph111_B1a
zmp_ph111_B5
zmp_ph111_B8
zmp_ph111_B6
zmp_ph111_B7
zmp_ph111_B9
zmp_ph111_B10
zmp_ph114_G4
zmp_ph114_G11
zmp_ph114_H1
zmp_ph114_H2
zmp_ph114_A9
zmp_ph114_A11
zmp_ph114_B2
zmp_ph114_B4
zmp_ph114_D8
zmp_ph114_E2

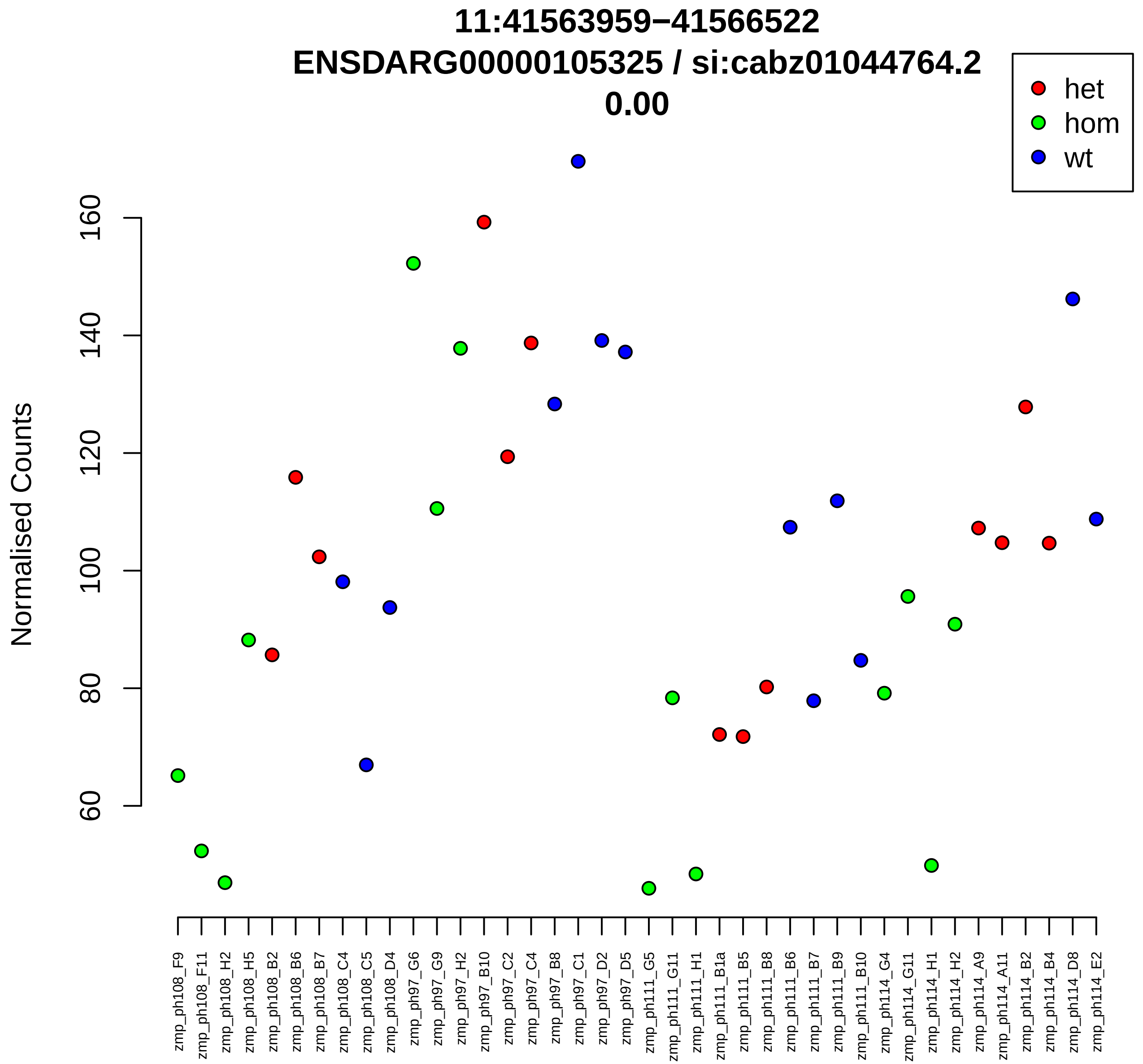

11:41563959−41566522
ENSDARG00000105325 / si:cabz01044764.2
0.00
het
hom
wt
Normalised Counts
60
80
100
120
140
160
zmp_ph108_F9
zmp_ph108_F11
zmp_ph108_H2
zmp_ph108_H5
zmp_ph108_B2
zmp_ph108_B6
zmp_ph108_B7
zmp_ph108_C4
zmp_ph108_C5
zmp_ph108_D4
zmp_ph97_G6
zmp_ph97_G9
zmp_ph97_H2
zmp_ph97_B10
zmp_ph97_C2
zmp_ph97_C4
zmp_ph97_B8
zmp_ph97_C1
zmp_ph97_D2
zmp_ph97_D5
zmp_ph111_G5
zmp_ph111_G11
zmp_ph111_H1
zmp_ph111_B1a
zmp_ph111_B5
zmp_ph111_B8
zmp_ph111_B6
zmp_ph111_B7
zmp_ph111_B9
zmp_ph111_B10
zmp_ph114_G4
zmp_ph114_G11
zmp_ph114_H1
zmp_ph114_H2
zmp_ph114_A9
zmp_ph114_A11
zmp_ph114_B2
zmp_ph114_B4
zmp_ph114_D8
zmp_ph114_E2

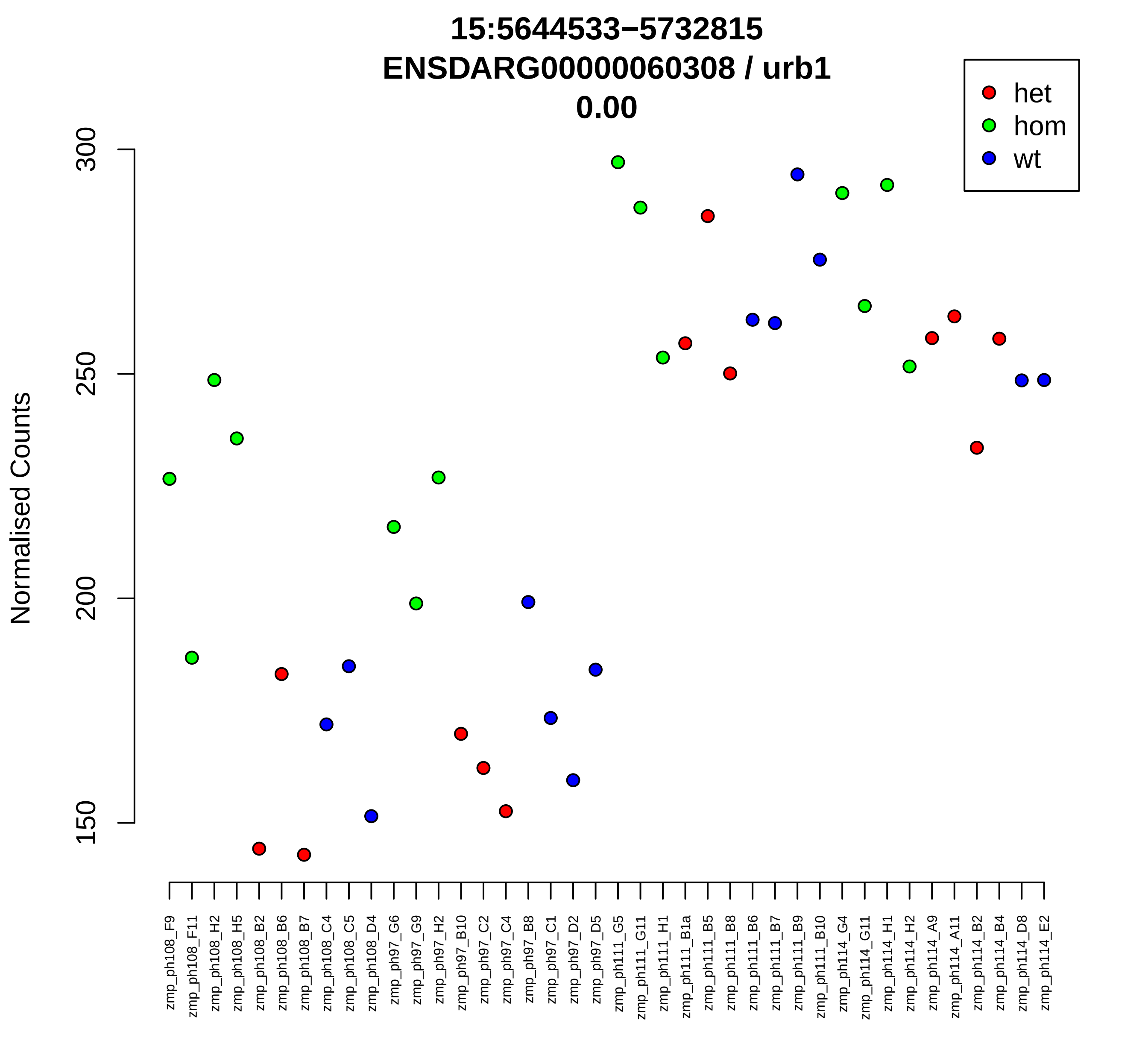

15:5644533−5732815
ENSDARG00000060308 / urb1
0.00
het
hom
wt
Normalised Counts
300
250
200
150
zmp_ph108_F9
zmp_ph108_F11
zmp_ph108_H2
zmp_ph108_H5
zmp_ph108_B2
zmp_ph108_B6
zmp_ph108_B7
zmp_ph108_C4
zmp_ph108_C5
zmp_ph108_D4
zmp_ph97_G6
zmp_ph97_G9
zmp_ph97_H2
zmp_ph97_B10
zmp_ph97_C2
zmp_ph97_C4
zmp_ph97_B8
zmp_ph97_C1
zmp_ph97_D2
zmp_ph97_D5
zmp_ph111_G5
zmp_ph111_G11
zmp_ph111_H1
zmp_ph111_B1a
zmp_ph111_B5
zmp_ph111_B8
zmp_ph111_B6
zmp_ph111_B7
zmp_ph111_B9
zmp_ph111_B10
zmp_ph114_G4
zmp_ph114_G11
zmp_ph114_H1
zmp_ph114_H2
zmp_ph114_A9
zmp_ph114_A11
zmp_ph114_B2
zmp_ph114_B4
zmp_ph114_D8
zmp_ph114_E2

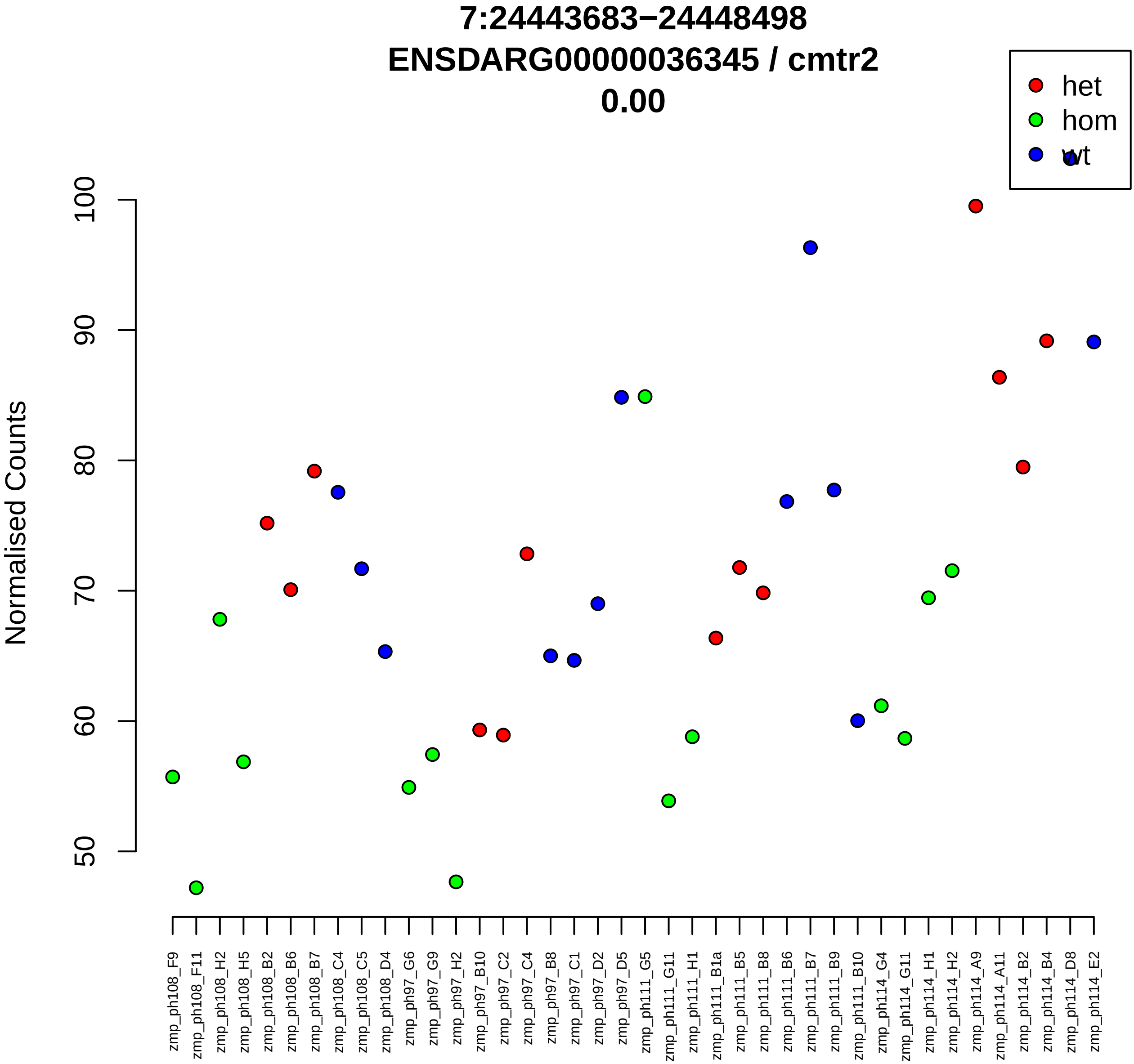

7:24443683−24448498
ENSDARG00000036345 / cmtr2
0.00
het
hom
wt
Normalised Counts
100
90
80
70
60
50
zmp_ph108_F9
zmp_ph108_F11
zmp_ph108_H2
zmp_ph108_H5
zmp_ph108_B2
zmp_ph108_B6
zmp_ph108_B7
zmp_ph108_C4
zmp_ph108_C5
zmp_ph108_D4
zmp_ph97_G6
zmp_ph97_G9
zmp_ph97_H2
zmp_ph97_B10
zmp_ph97_C2
zmp_ph97_C4
zmp_ph97_B8
zmp_ph97_C1
zmp_ph97_D2
zmp_ph97_D5
zmp_ph111_G5
zmp_ph111_G11
zmp_ph111_H1
zmp_ph111_B1a
zmp_ph111_B5
zmp_ph111_B8
zmp_ph111_B6
zmp_ph111_B7
zmp_ph111_B9
zmp_ph111_B10
zmp_ph114_G4
zmp_ph114_G11
zmp_ph114_H1
zmp_ph114_H2
zmp_ph114_A9
zmp_ph114_A11
zmp_ph114_B2
zmp_ph114_B4
zmp_ph114_D8
zmp_ph114_E2

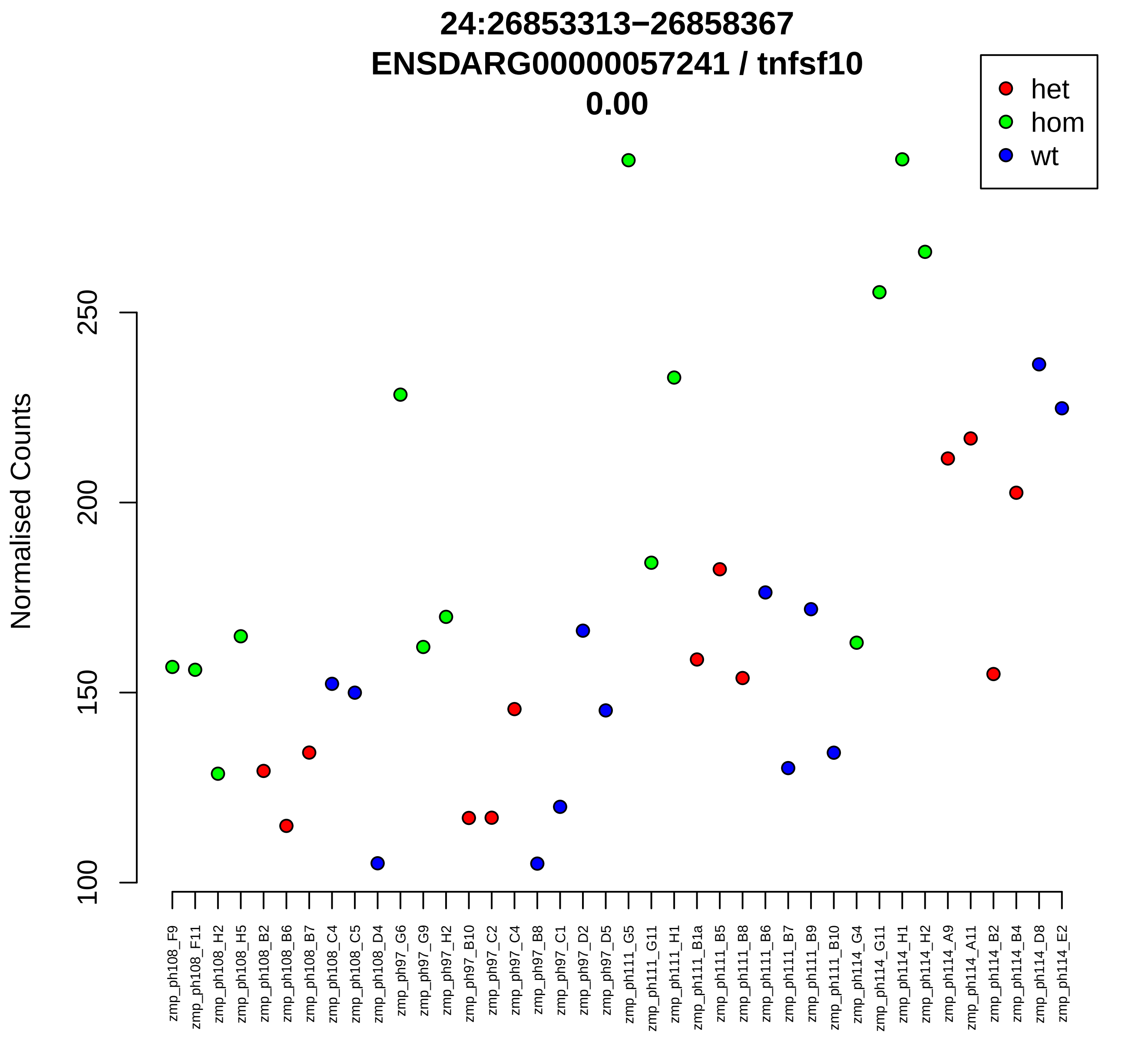

24:26853313−26858367
ENSDARG00000057241 / tnfsf10
0.00
het
hom
wt
Normalised Counts
100
150
200
250
zmp_ph108_F9
zmp_ph108_F11
zmp_ph108_H2
zmp_ph108_H5
zmp_ph108_B2
zmp_ph108_B6
zmp_ph108_B7
zmp_ph108_C4
zmp_ph108_C5
zmp_ph108_D4
zmp_ph97_G6
zmp_ph97_G9
zmp_ph97_H2
zmp_ph97_B10
zmp_ph97_C2
zmp_ph97_C4
zmp_ph97_B8
zmp_ph97_C1
zmp_ph97_D2
zmp_ph97_D5
zmp_ph111_G5
zmp_ph111_G11
zmp_ph111_H1
zmp_ph111_B1a
zmp_ph111_B5
zmp_ph111_B8
zmp_ph111_B6
zmp_ph111_B7
zmp_ph111_B9
zmp_ph111_B10
zmp_ph114_G4
zmp_ph114_G11
zmp_ph114_H1
zmp_ph114_H2
zmp_ph114_A9
zmp_ph114_A11
zmp_ph114_B2
zmp_ph114_B4
zmp_ph114_D8
zmp_ph114_E2

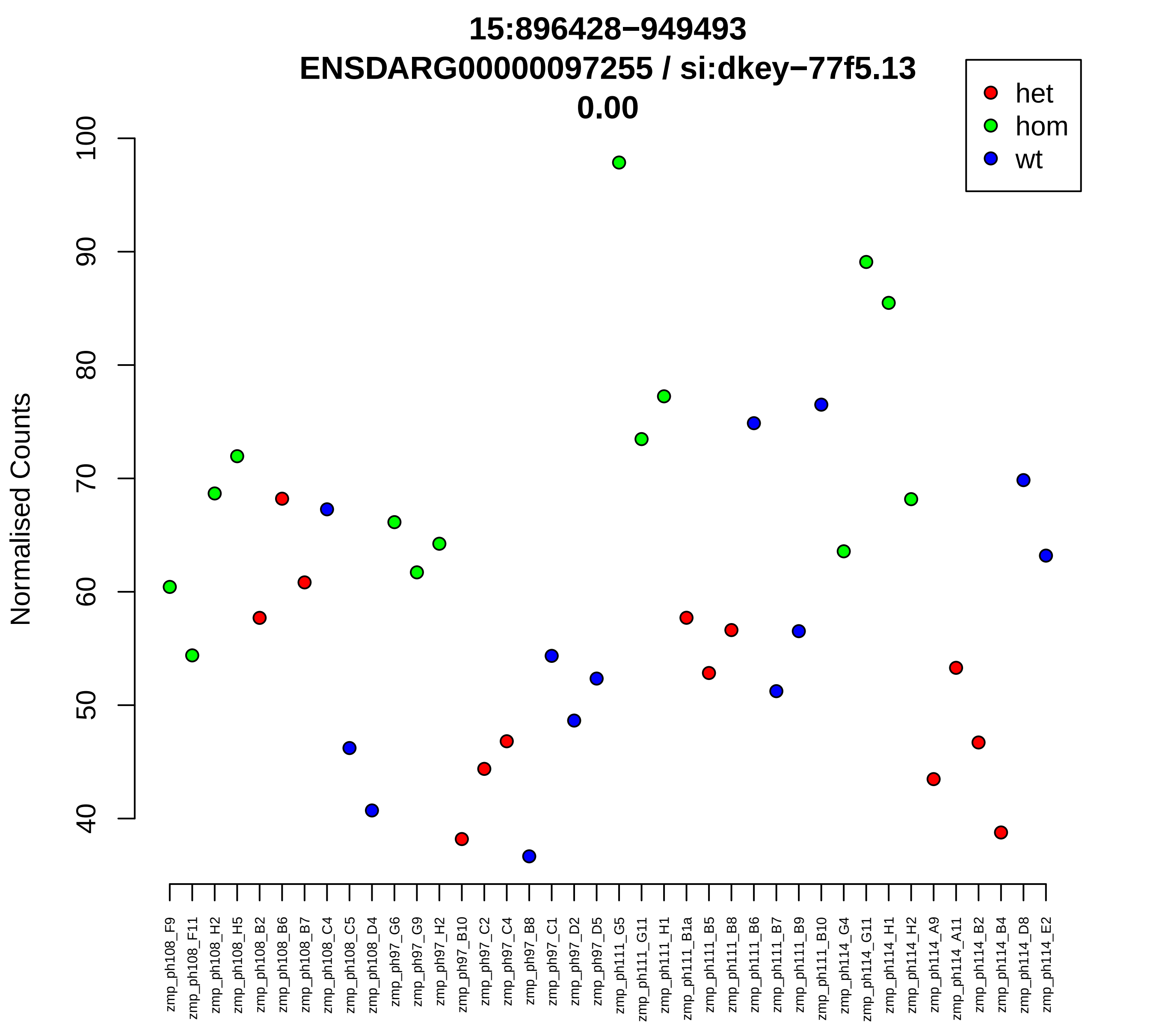

15:896428−949493
ENSDARG00000097255 / si:dkey−77f5.13
0.00
het
hom
wt
Normalised Counts
100
90
80
70
60
50
40
zmp_ph108_F9
zmp_ph108_F11
zmp_ph108_H2
zmp_ph108_H5
zmp_ph108_B2
zmp_ph108_B6
zmp_ph108_B7
zmp_ph108_C4
zmp_ph108_C5
zmp_ph108_D4
zmp_ph97_G6
zmp_ph97_G9
zmp_ph97_H2
zmp_ph97_B10
zmp_ph97_C2
zmp_ph97_C4
zmp_ph97_B8
zmp_ph97_C1
zmp_ph97_D2
zmp_ph97_D5
zmp_ph111_G5
zmp_ph111_G11
zmp_ph111_H1
zmp_ph111_B1a
zmp_ph111_B5
zmp_ph111_B8
zmp_ph111_B6
zmp_ph111_B7
zmp_ph111_B9
zmp_ph111_B10
zmp_ph114_G4
zmp_ph114_G11
zmp_ph114_H1
zmp_ph114_H2
zmp_ph114_A9
zmp_ph114_A11
zmp_ph114_B2
zmp_ph114_B4
zmp_ph114_D8
zmp_ph114_E2

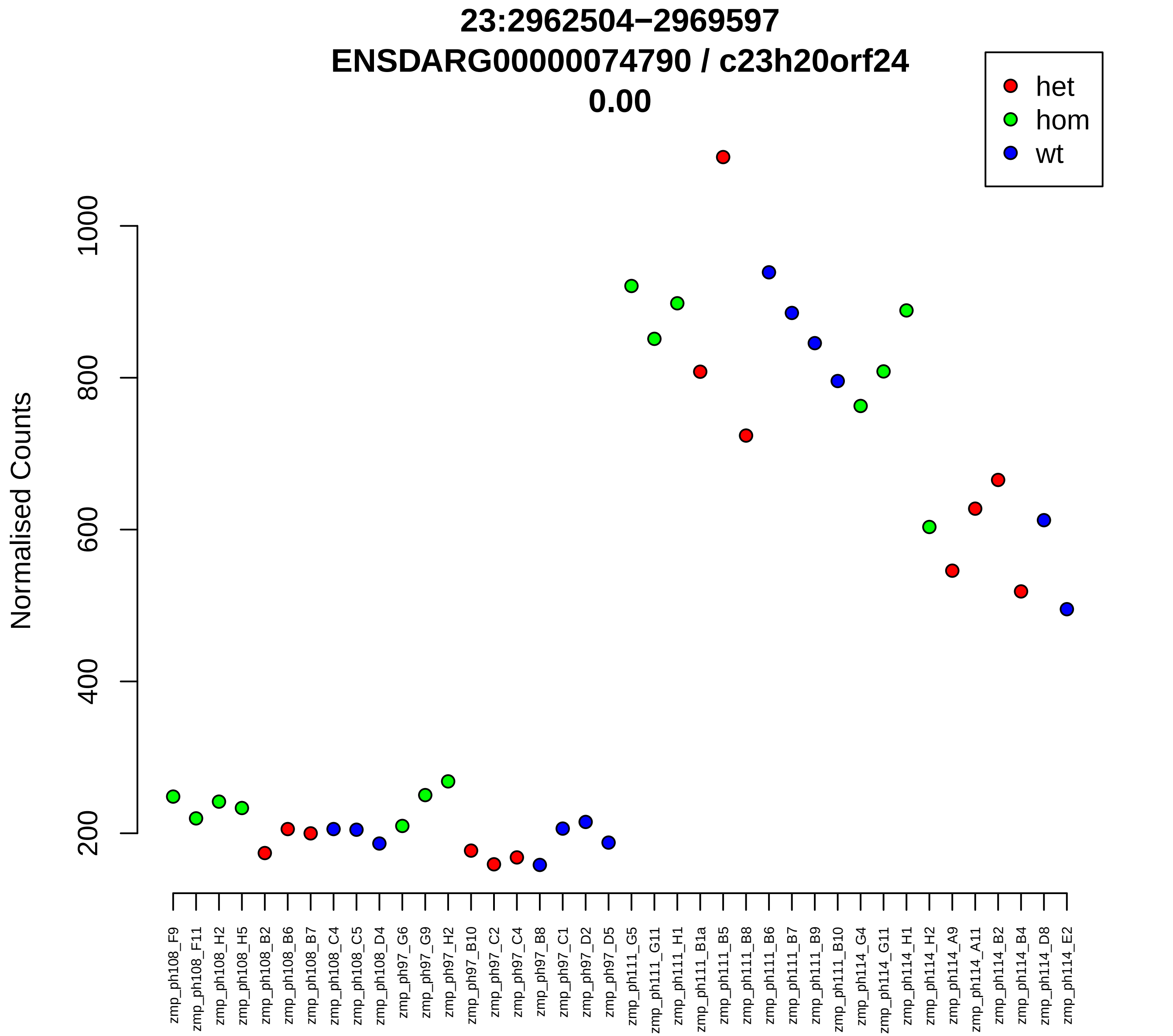
23:2962504−2969597
ENSDARG00000074790 / c23h20orf24
0.00
het
hom
wt
Normalised Counts
200
400
600
800
1000
zmp_ph108_F9
zmp_ph108_F11
zmp_ph108_H2
zmp_ph108_H5
zmp_ph108_B2
zmp_ph108_B6
zmp_ph108_B7
zmp_ph108_C4
zmp_ph108_C5
zmp_ph108_D4
zmp_ph97_G6
zmp_ph97_G9
zmp_ph97_H2
zmp_ph97_B10
zmp_ph97_C2
zmp_ph97_C4
zmp_ph97_B8
zmp_ph97_C1
zmp_ph97_D2
zmp_ph97_D5
zmp_ph111_G5
zmp_ph111_G11
zmp_ph111_H1
zmp_ph111_B1a
zmp_ph111_B5
zmp_ph111_B8
zmp_ph111_B6
zmp_ph111_B7
zmp_ph111_B9
zmp_ph111_B10
zmp_ph114_G4
zmp_ph114_G11
zmp_ph114_H1
zmp_ph114_H2
zmp_ph114_A9
zmp_ph114_A11
zmp_ph114_B2
zmp_ph114_B4
zmp_ph114_D8
zmp_ph114_E2

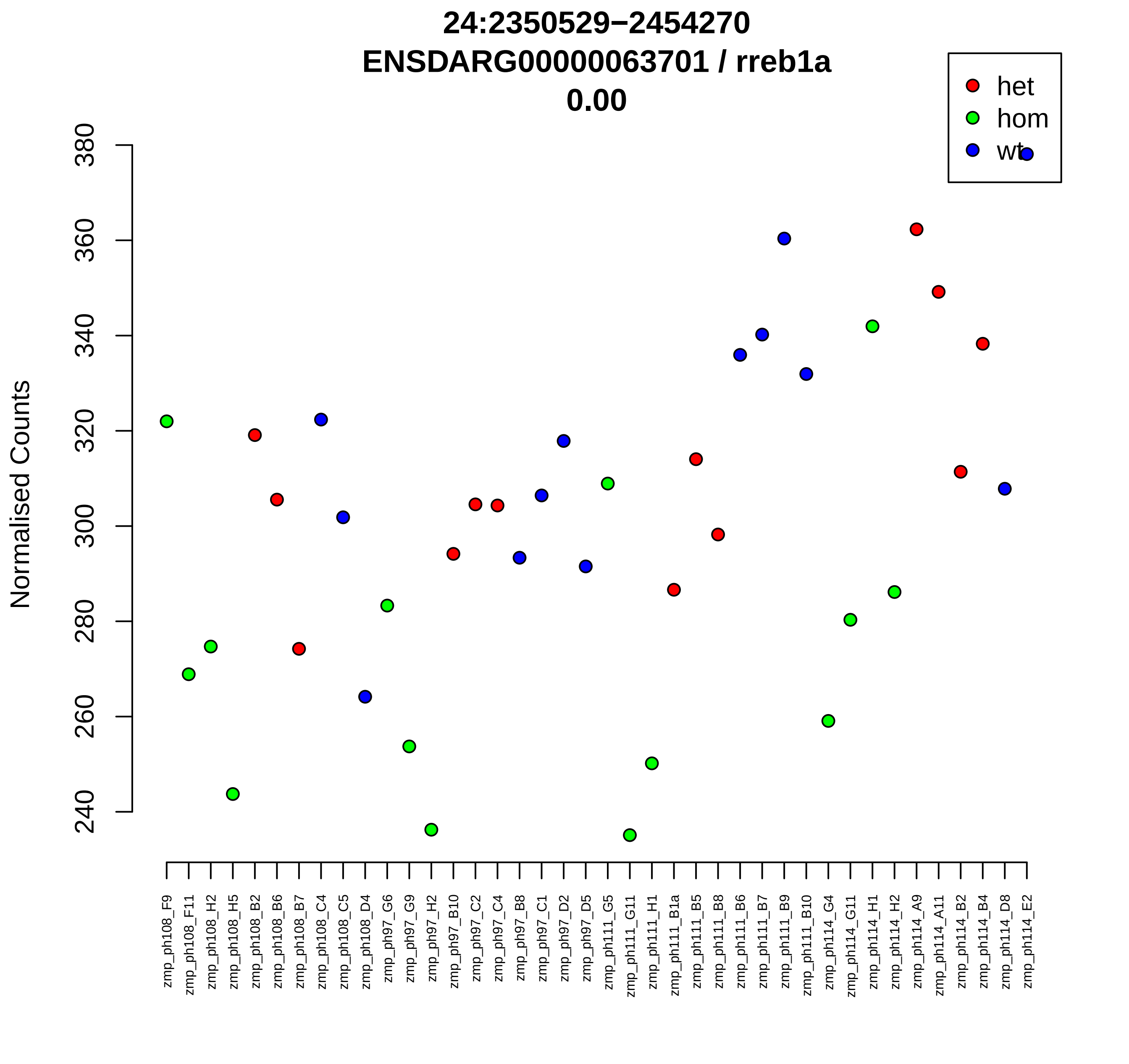

24:2350529−2454270
ENSDARG00000063701 / rreb1a
0.00
het
hom
wt
Normalised Counts
240
260
280
300
320
340
360
380
zmp_ph108_F9
zmp_ph108_F11
zmp_ph108_H2
zmp_ph108_H5
zmp_ph108_B2
zmp_ph108_B6
zmp_ph108_B7
zmp_ph108_C4
zmp_ph108_C5
zmp_ph108_D4
zmp_ph97_G6
zmp_ph97_G9
zmp_ph97_H2
zmp_ph97_B10
zmp_ph97_C2
zmp_ph97_C4
zmp_ph97_B8
zmp_ph97_C1
zmp_ph97_D2
zmp_ph97_D5
zmp_ph111_G5
zmp_ph111_G11
zmp_ph111_H1
zmp_ph111_B1a
zmp_ph111_B5
zmp_ph111_B8
zmp_ph111_B6
zmp_ph111_B7
zmp_ph111_B9
zmp_ph111_B10
zmp_ph114_G4
zmp_ph114_G11
zmp_ph114_H1
zmp_ph114_H2
zmp_ph114_A9
zmp_ph114_A11
zmp_ph114_B2
zmp_ph114_B4
zmp_ph114_D8
zmp_ph114_E2

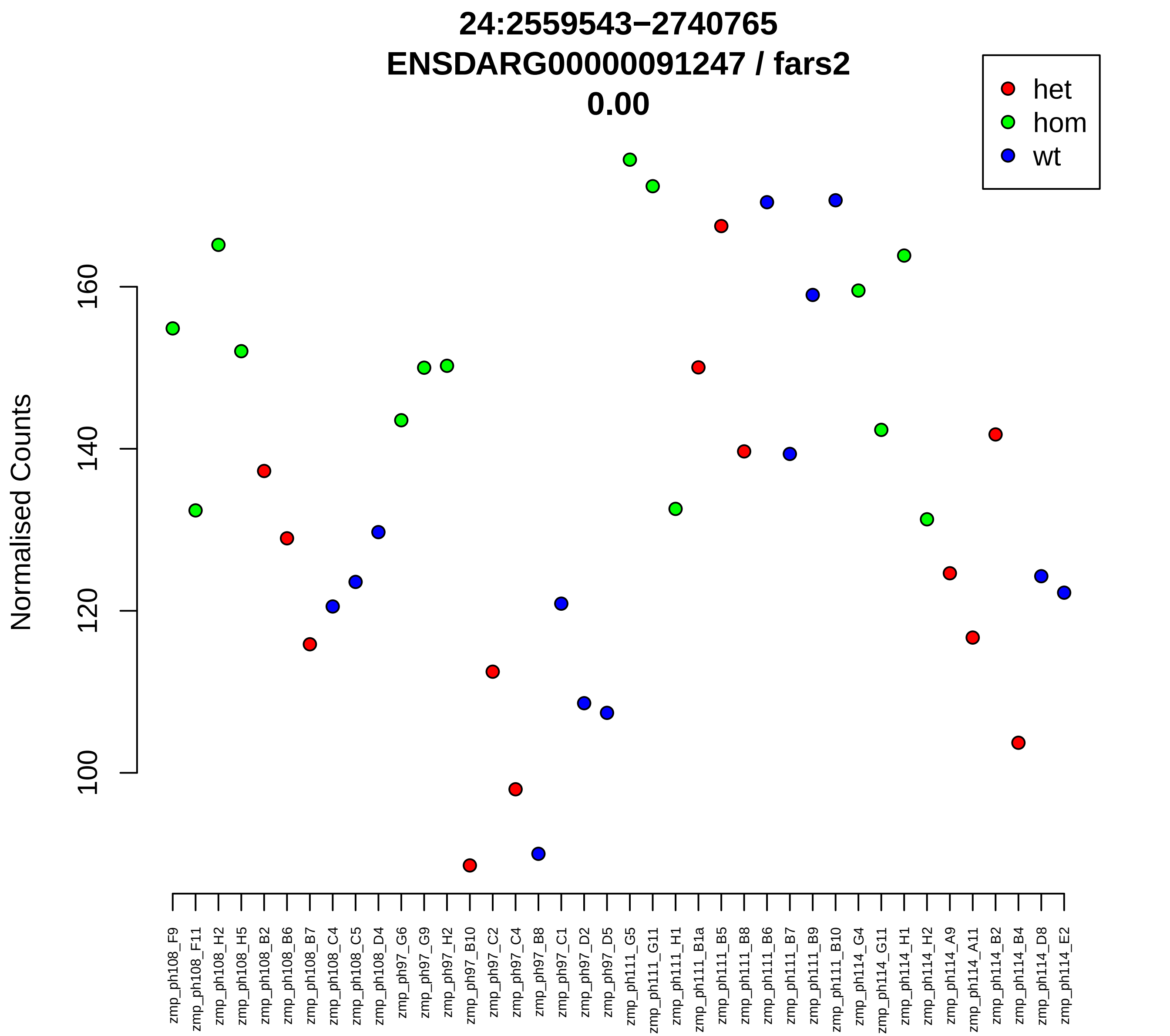

24:2559543−2740765
ENSDARG00000091247 / fars2
0.00
het
hom
wt
Normalised Counts
100
120
140
160
zmp_ph108_F9
zmp_ph108_F11
zmp_ph108_H2
zmp_ph108_H5
zmp_ph108_B2
zmp_ph108_B6
zmp_ph108_B7
zmp_ph108_C4
zmp_ph108_C5
zmp_ph108_D4
zmp_ph97_G6
zmp_ph97_G9
zmp_ph97_H2
zmp_ph97_B10
zmp_ph97_C2
zmp_ph97_C4
zmp_ph97_B8
zmp_ph97_C1
zmp_ph97_D2
zmp_ph97_D5
zmp_ph111_G5
zmp_ph111_G11
zmp_ph111_H1
zmp_ph111_B1a
zmp_ph111_B5
zmp_ph111_B8
zmp_ph111_B6
zmp_ph111_B7
zmp_ph111_B9
zmp_ph111_B10
zmp_ph114_G4
zmp_ph114_G11
zmp_ph114_H1
zmp_ph114_H2
zmp_ph114_A9
zmp_ph114_A11
zmp_ph114_B2
zmp_ph114_B4
zmp_ph114_D8
zmp_ph114_E2

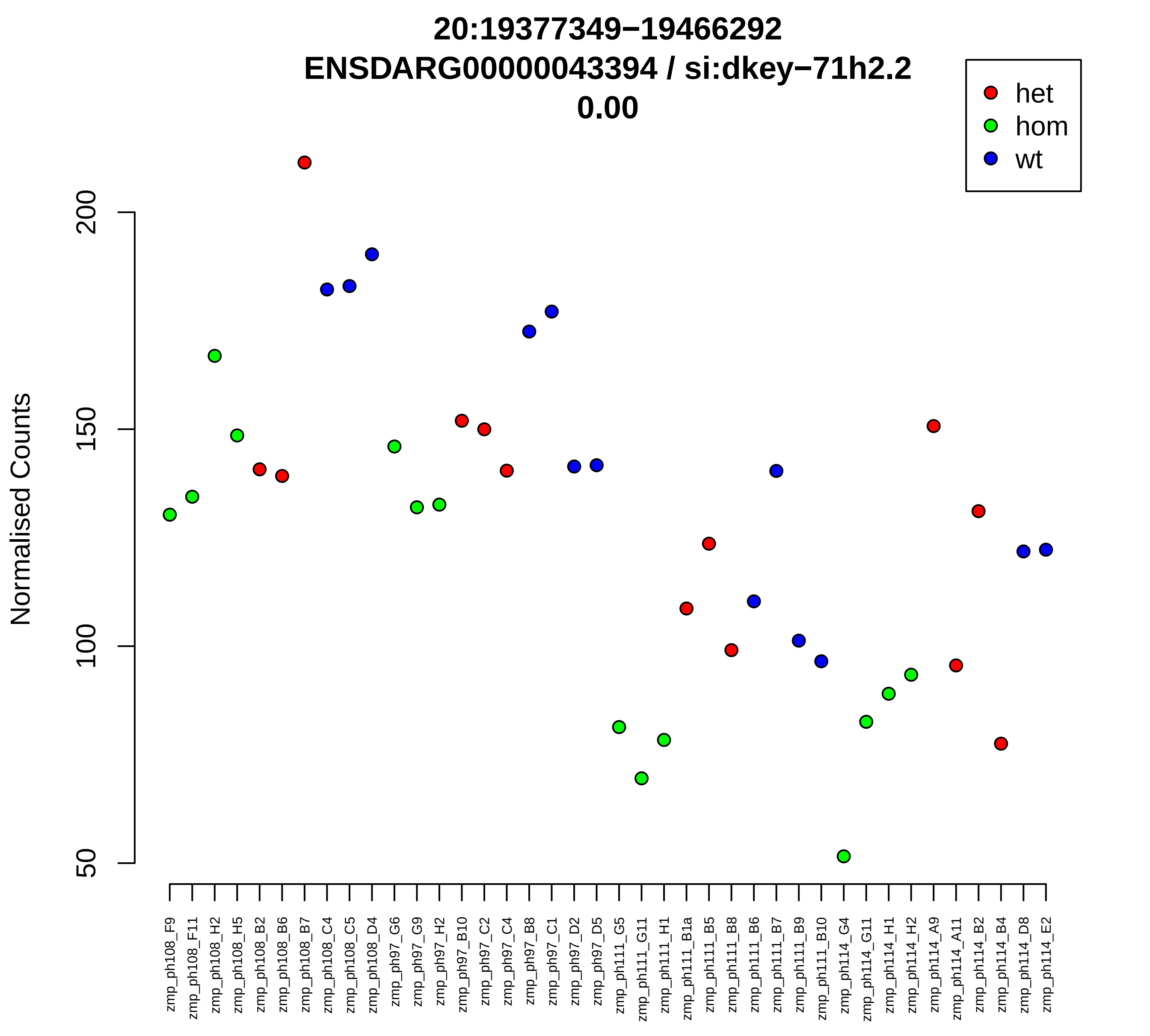
20:19377349−19466292
ENSDARG00000043394 / si:dkey−71h2.2
0.00
het
hom
wt
Normalised Counts
50
100
150
200
zmp_ph108_F9
zmp_ph108_F11
zmp_ph108_H2
zmp_ph108_H5
zmp_ph108_B2
zmp_ph108_B6
zmp_ph108_B7
zmp_ph108_C4
zmp_ph108_C5
zmp_ph108_D4
zmp_ph97_G6
zmp_ph97_G9
zmp_ph97_H2
zmp_ph97_B10
zmp_ph97_C2
zmp_ph97_C4
zmp_ph97_B8
zmp_ph97_C1
zmp_ph97_D2
zmp_ph97_D5
zmp_ph111_G5
zmp_ph111_G11
zmp_ph111_H1
zmp_ph111_B1a
zmp_ph111_B5
zmp_ph111_B8
zmp_ph111_B6
zmp_ph111_B7
zmp_ph111_B9
zmp_ph111_B10
zmp_ph114_G4
zmp_ph114_G11
zmp_ph114_H1
zmp_ph114_H2
zmp_ph114_A9
zmp_ph114_A11
zmp_ph114_B2
zmp_ph114_B4
zmp_ph114_D8
zmp_ph114_E2

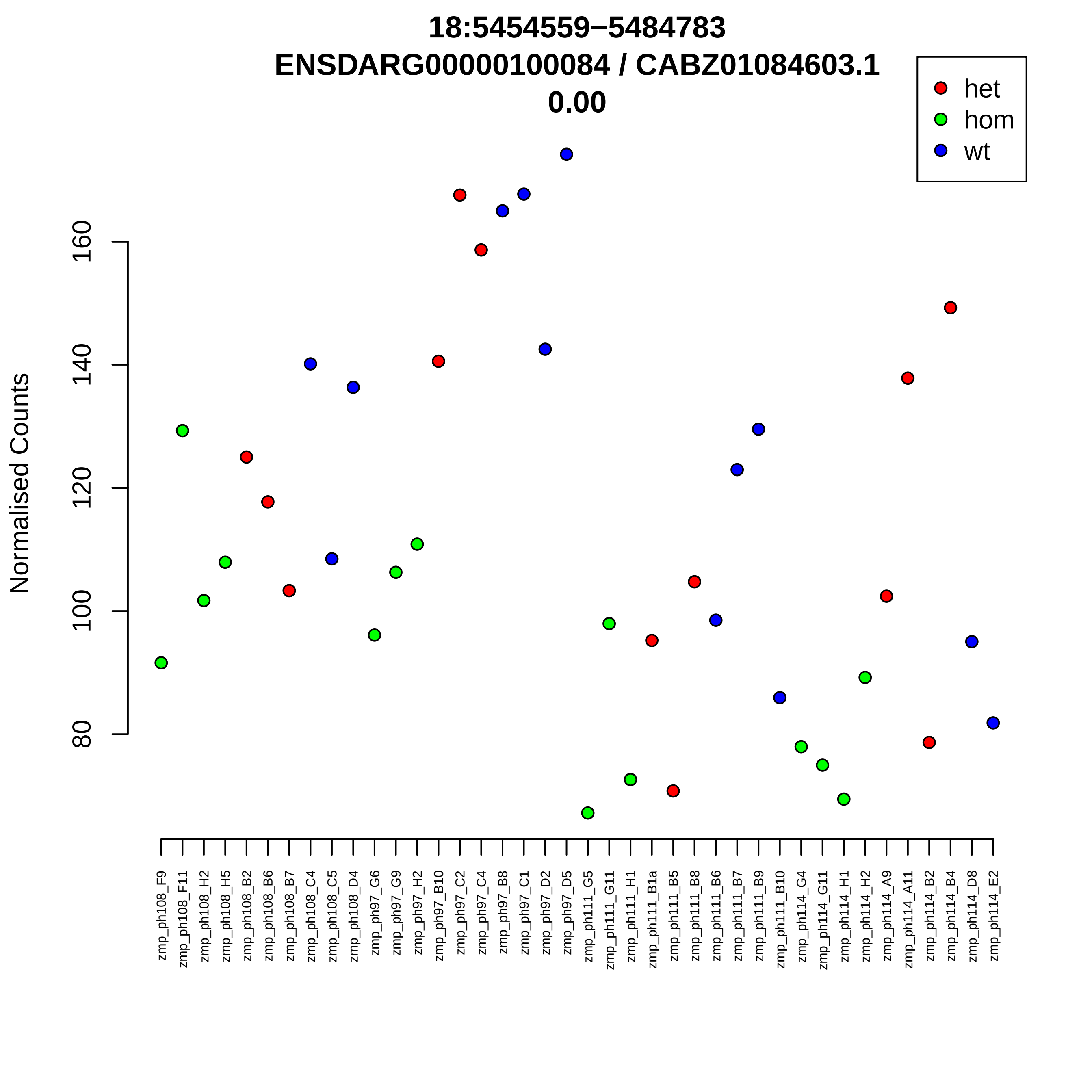

18:5454559−5484783
ENSDARG00000100084 / CABZ01084603.1
0.00
het
hom
wt
Normalised Counts
80
100
120
140
160
zmp_ph108_F9
zmp_ph108_F11
zmp_ph108_H2
zmp_ph108_H5
zmp_ph108_B2
zmp_ph108_B6
zmp_ph108_B7
zmp_ph108_C4
zmp_ph108_C5
zmp_ph108_D4
zmp_ph97_G6
zmp_ph97_G9
zmp_ph97_H2
zmp_ph97_B10
zmp_ph97_C2
zmp_ph97_C4
zmp_ph97_B8
zmp_ph97_C1
zmp_ph97_D2
zmp_ph97_D5
zmp_ph111_G5
zmp_ph111_G11
zmp_ph111_H1
zmp_ph111_B1a
zmp_ph111_B5
zmp_ph111_B8
zmp_ph111_B6
zmp_ph111_B7
zmp_ph111_B9
zmp_ph111_B10
zmp_ph114_G4
zmp_ph114_G11
zmp_ph114_H1
zmp_ph114_H2
zmp_ph114_A9
zmp_ph114_A11
zmp_ph114_B2
zmp_ph114_B4
zmp_ph114_D8
zmp_ph114_E2

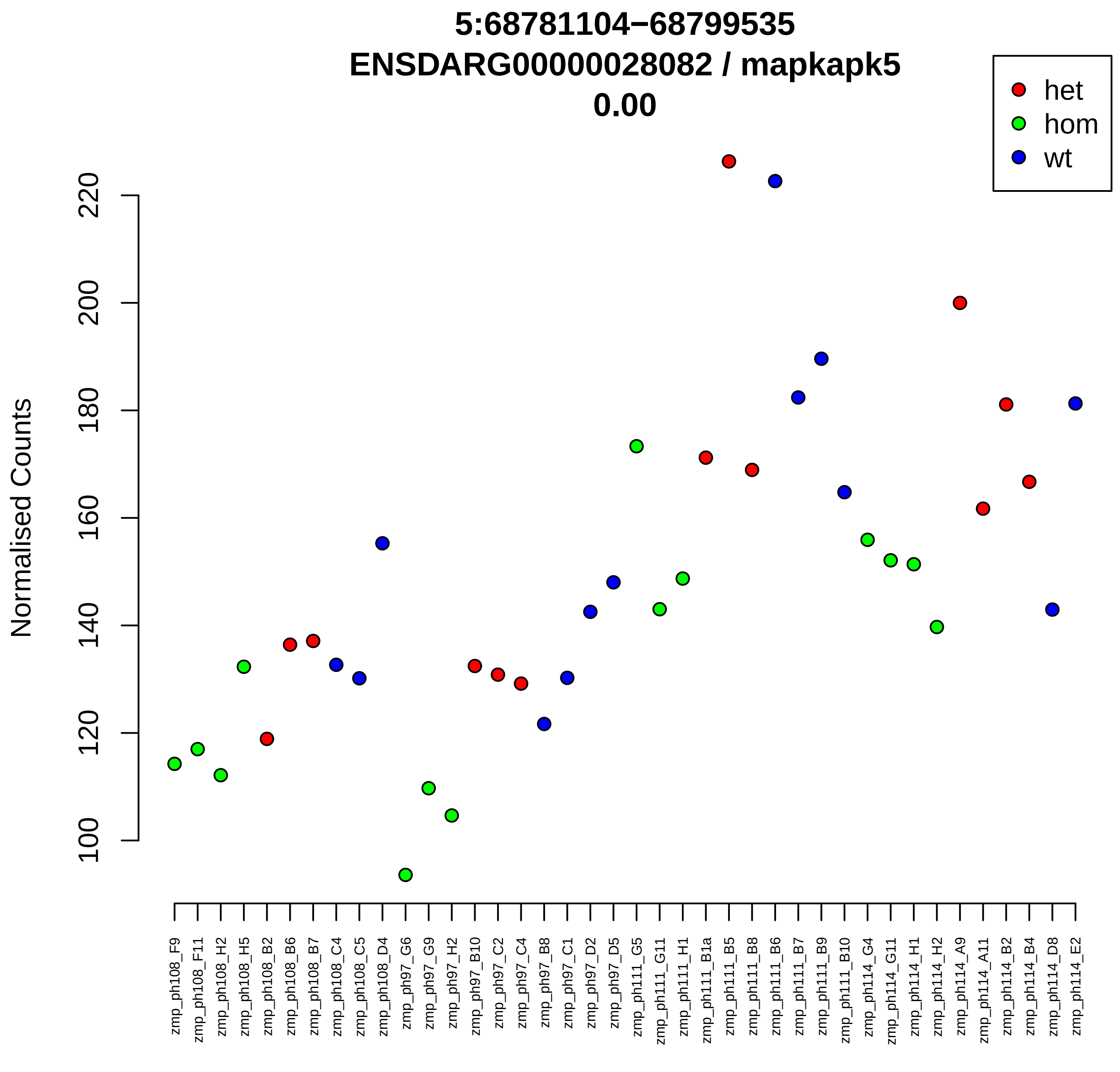
5:68781104−68799535
ENSDARG00000028082 / mapkapk5
0.00
het
hom
wt
Normalised Counts
100
120
140
160
180
200
220
zmp_ph108_F9
zmp_ph108_F11
zmp_ph108_H2
zmp_ph108_H5
zmp_ph108_B2
zmp_ph108_B6
zmp_ph108_B7
zmp_ph108_C4
zmp_ph108_C5
zmp_ph108_D4
zmp_ph97_G6
zmp_ph97_G9
zmp_ph97_H2
zmp_ph97_B10
zmp_ph97_C2
zmp_ph97_C4
zmp_ph97_B8
zmp_ph97_C1
zmp_ph97_D2
zmp_ph97_D5
zmp_ph111_G5
zmp_ph111_G11
zmp_ph111_H1
zmp_ph111_B1a
zmp_ph111_B5
zmp_ph111_B8
zmp_ph111_B6
zmp_ph111_B7
zmp_ph111_B9
zmp_ph111_B10
zmp_ph114_G4
zmp_ph114_G11
zmp_ph114_H1
zmp_ph114_H2
zmp_ph114_A9
zmp_ph114_A11
zmp_ph114_B2
zmp_ph114_B4
zmp_ph114_D8
zmp_ph114_E2

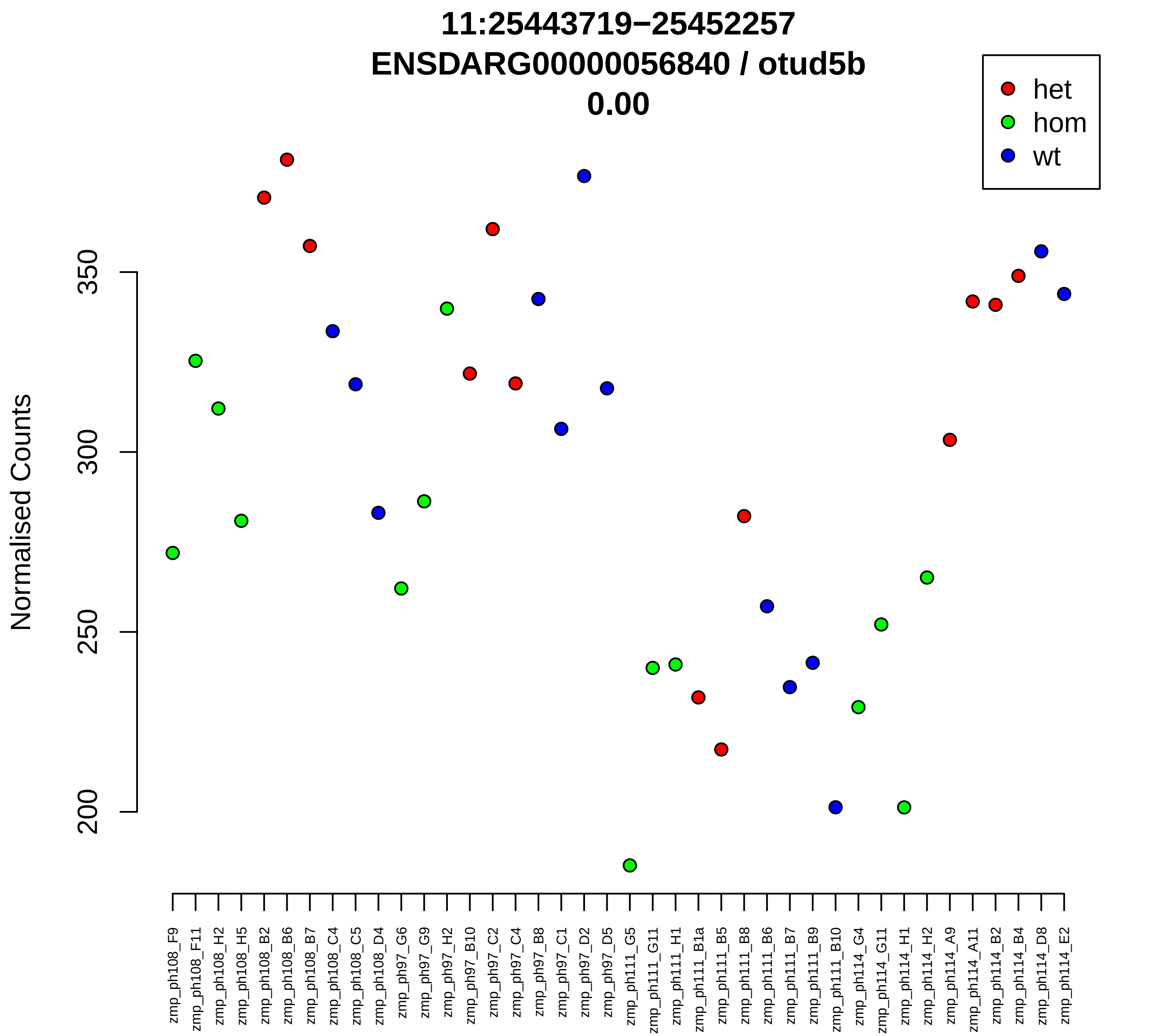
11:25443719−25452257
ENSDARG00000056840 / otud5b
0.00
het
hom
wt
Normalised Counts
200
250
300
350
zmp_ph108_F9
zmp_ph108_F11
zmp_ph108_H2
zmp_ph108_H5
zmp_ph108_B2
zmp_ph108_B6
zmp_ph108_B7
zmp_ph108_C4
zmp_ph108_C5
zmp_ph108_D4
zmp_ph97_G6
zmp_ph97_G9
zmp_ph97_H2
zmp_ph97_B10
zmp_ph97_C2
zmp_ph97_C4
zmp_ph97_B8
zmp_ph97_C1
zmp_ph97_D2
zmp_ph97_D5
zmp_ph111_G5
zmp_ph111_G11
zmp_ph111_H1
zmp_ph111_B1a
zmp_ph111_B5
zmp_ph111_B8
zmp_ph111_B6
zmp_ph111_B7
zmp_ph111_B9
zmp_ph111_B10
zmp_ph114_G4
zmp_ph114_G11
zmp_ph114_H1
zmp_ph114_H2
zmp_ph114_A9
zmp_ph114_A11
zmp_ph114_B2
zmp_ph114_B4
zmp_ph114_D8
zmp_ph114_E2

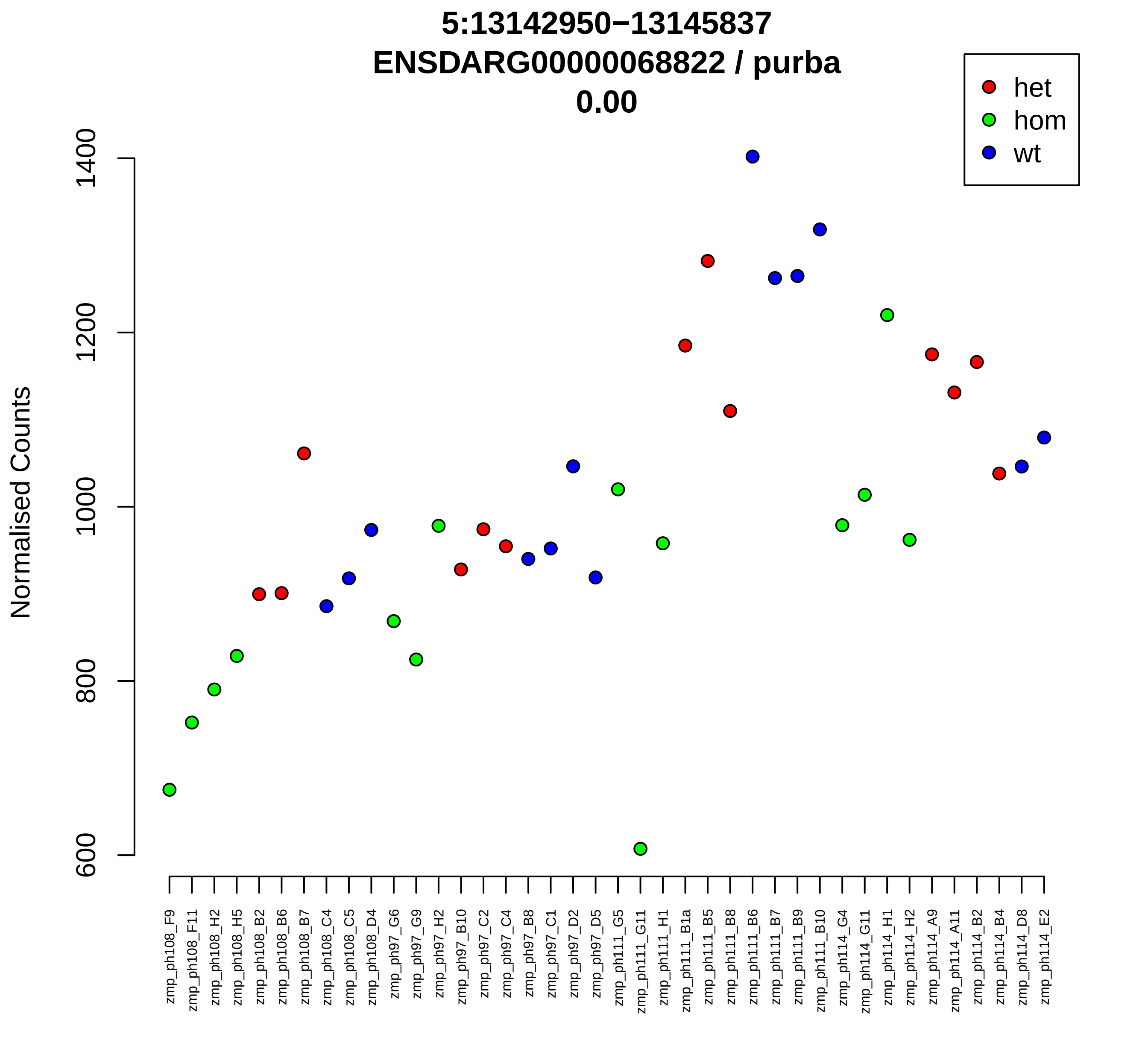

5:13142950−13145837
ENSDARG00000068822 / purba
0.00
het
hom
wt
Normalised Counts
600
800
1000
1200
1400
zmp_ph108_F9
zmp_ph108_F11
zmp_ph108_H2
zmp_ph108_H5
zmp_ph108_B2
zmp_ph108_B6
zmp_ph108_B7
zmp_ph108_C4
zmp_ph108_C5
zmp_ph108_D4
zmp_ph97_G6
zmp_ph97_G9
zmp_ph97_H2
zmp_ph97_B10
zmp_ph97_C2
zmp_ph97_C4
zmp_ph97_B8
zmp_ph97_C1
zmp_ph97_D2
zmp_ph97_D5
zmp_ph111_G5
zmp_ph111_G11
zmp_ph111_H1
zmp_ph111_B1a
zmp_ph111_B5
zmp_ph111_B8
zmp_ph111_B6
zmp_ph111_B7
zmp_ph111_B9
zmp_ph111_B10
zmp_ph114_G4
zmp_ph114_G11
zmp_ph114_H1
zmp_ph114_H2
zmp_ph114_A9
zmp_ph114_A11
zmp_ph114_B2
zmp_ph114_B4
zmp_ph114_D8
zmp_ph114_E2

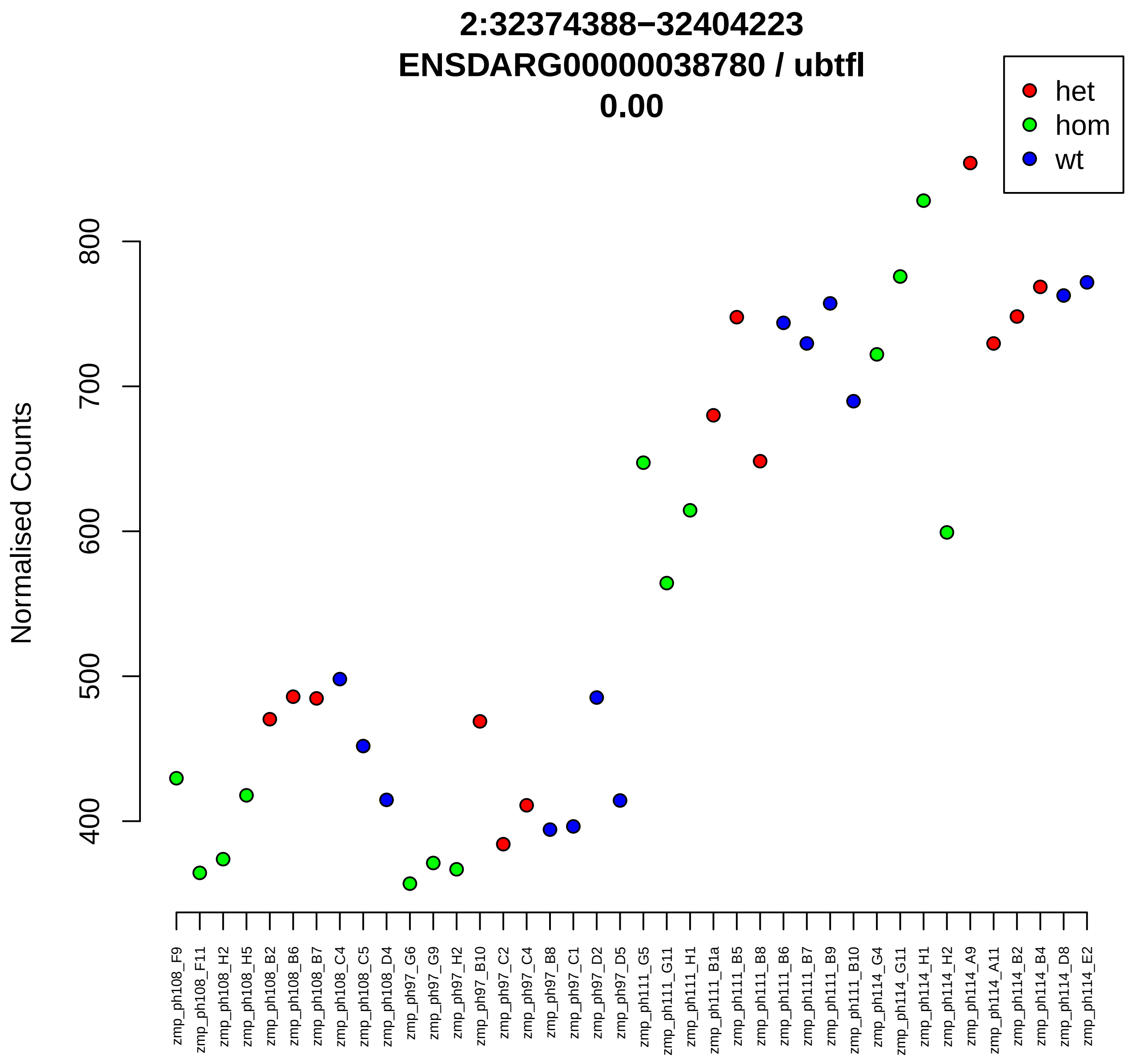

2:32374388−32404223
ENSDARG00000038780 / ubtfl
0.00
het
hom
wt
Normalised Counts
400
500
600
700
800
zmp_ph108_F9
zmp_ph108_F11
zmp_ph108_H2
zmp_ph108_H5
zmp_ph108_B2
zmp_ph108_B6
zmp_ph108_B7
zmp_ph108_C4
zmp_ph108_C5
zmp_ph108_D4
zmp_ph97_G6
zmp_ph97_G9
zmp_ph97_H2
zmp_ph97_B10
zmp_ph97_C2
zmp_ph97_C4
zmp_ph97_B8
zmp_ph97_C1
zmp_ph97_D2
zmp_ph97_D5
zmp_ph111_G5
zmp_ph111_G11
zmp_ph111_H1
zmp_ph111_B1a
zmp_ph111_B5
zmp_ph111_B8
zmp_ph111_B6
zmp_ph111_B7
zmp_ph111_B9
zmp_ph111_B10
zmp_ph114_G4
zmp_ph114_G11
zmp_ph114_H1
zmp_ph114_H2
zmp_ph114_A9
zmp_ph114_A11
zmp_ph114_B2
zmp_ph114_B4
zmp_ph114_D8
zmp_ph114_E2

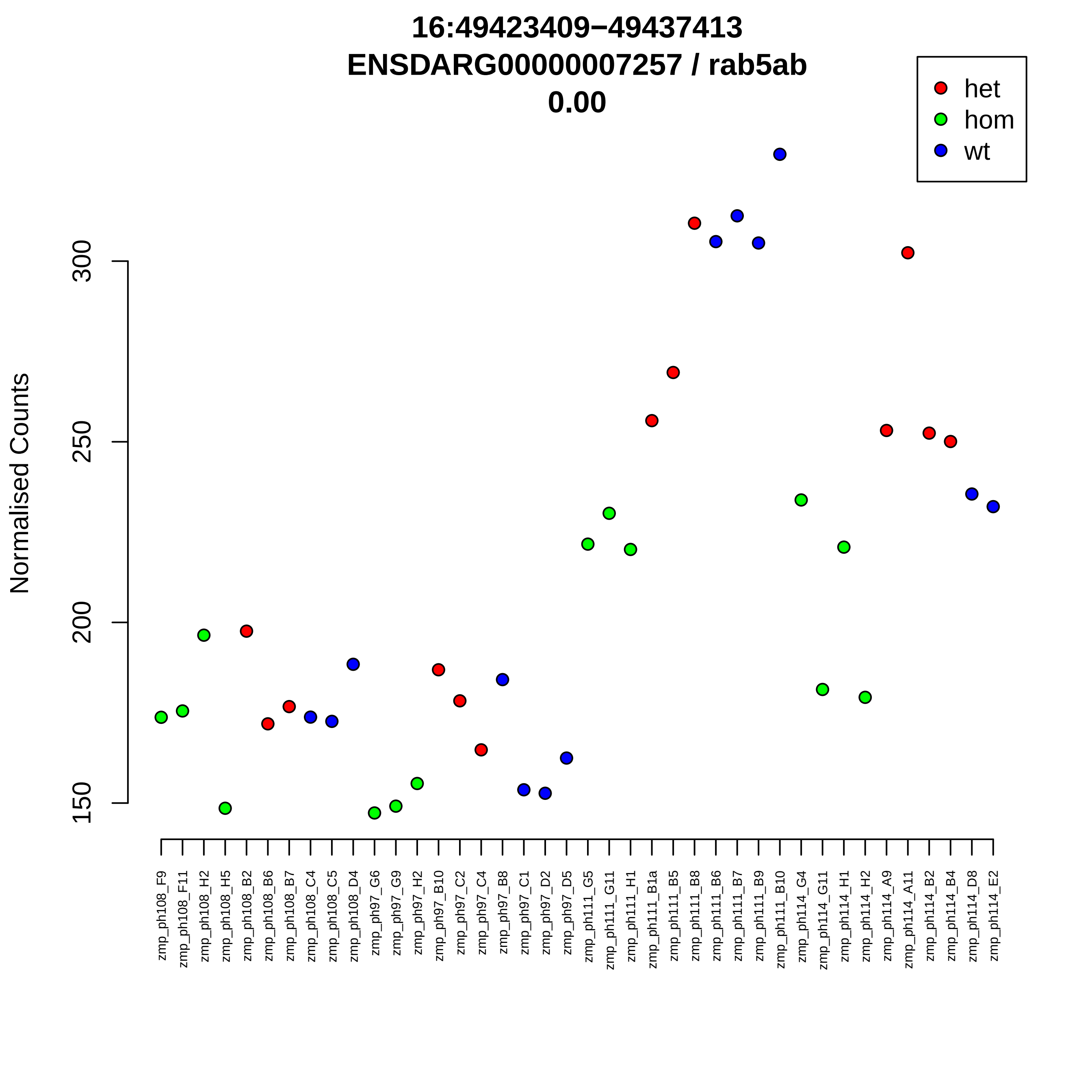

16:49423409−49437413
ENSDARG00000007257 / rab5ab
0.00
het
hom
wt
Normalised Counts
150
200
250
300
zmp_ph108_F9
zmp_ph108_F11
zmp_ph108_H2
zmp_ph108_H5
zmp_ph108_B2
zmp_ph108_B6
zmp_ph108_B7
zmp_ph108_C4
zmp_ph108_C5
zmp_ph108_D4
zmp_ph97_G6
zmp_ph97_G9
zmp_ph97_H2
zmp_ph97_B10
zmp_ph97_C2
zmp_ph97_C4
zmp_ph97_B8
zmp_ph97_C1
zmp_ph97_D2
zmp_ph97_D5
zmp_ph111_G5
zmp_ph111_G11
zmp_ph111_H1
zmp_ph111_B1a
zmp_ph111_B5
zmp_ph111_B8
zmp_ph111_B6
zmp_ph111_B7
zmp_ph111_B9
zmp_ph111_B10
zmp_ph114_G4
zmp_ph114_G11
zmp_ph114_H1
zmp_ph114_H2
zmp_ph114_A9
zmp_ph114_A11
zmp_ph114_B2
zmp_ph114_B4
zmp_ph114_D8
zmp_ph114_E2

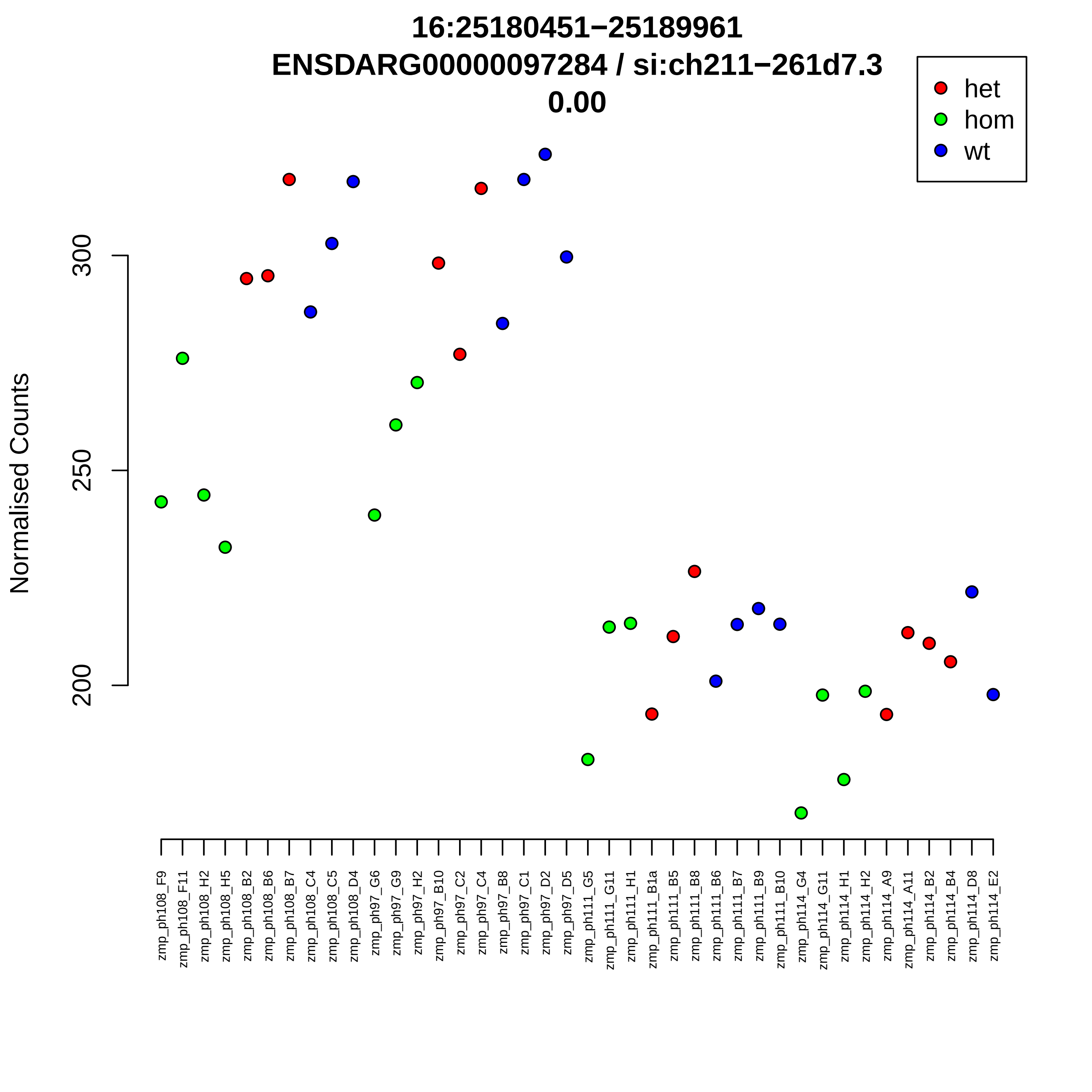
16:25180451−25189961
ENSDARG00000097284 / si:ch211−261d7.3
0.00
het
hom
wt
Normalised Counts
200
250
300
zmp_ph108_F9
zmp_ph108_F11
zmp_ph108_H2
zmp_ph108_H5
zmp_ph108_B2
zmp_ph108_B6
zmp_ph108_B7
zmp_ph108_C4
zmp_ph108_C5
zmp_ph108_D4
zmp_ph97_G6
zmp_ph97_G9
zmp_ph97_H2
zmp_ph97_B10
zmp_ph97_C2
zmp_ph97_C4
zmp_ph97_B8
zmp_ph97_C1
zmp_ph97_D2
zmp_ph97_D5
zmp_ph111_G5
zmp_ph111_G11
zmp_ph111_H1
zmp_ph111_B1a
zmp_ph111_B5
zmp_ph111_B8
zmp_ph111_B6
zmp_ph111_B7
zmp_ph111_B9
zmp_ph111_B10
zmp_ph114_G4
zmp_ph114_G11
zmp_ph114_H1
zmp_ph114_H2
zmp_ph114_A9
zmp_ph114_A11
zmp_ph114_B2
zmp_ph114_B4
zmp_ph114_D8
zmp_ph114_E2

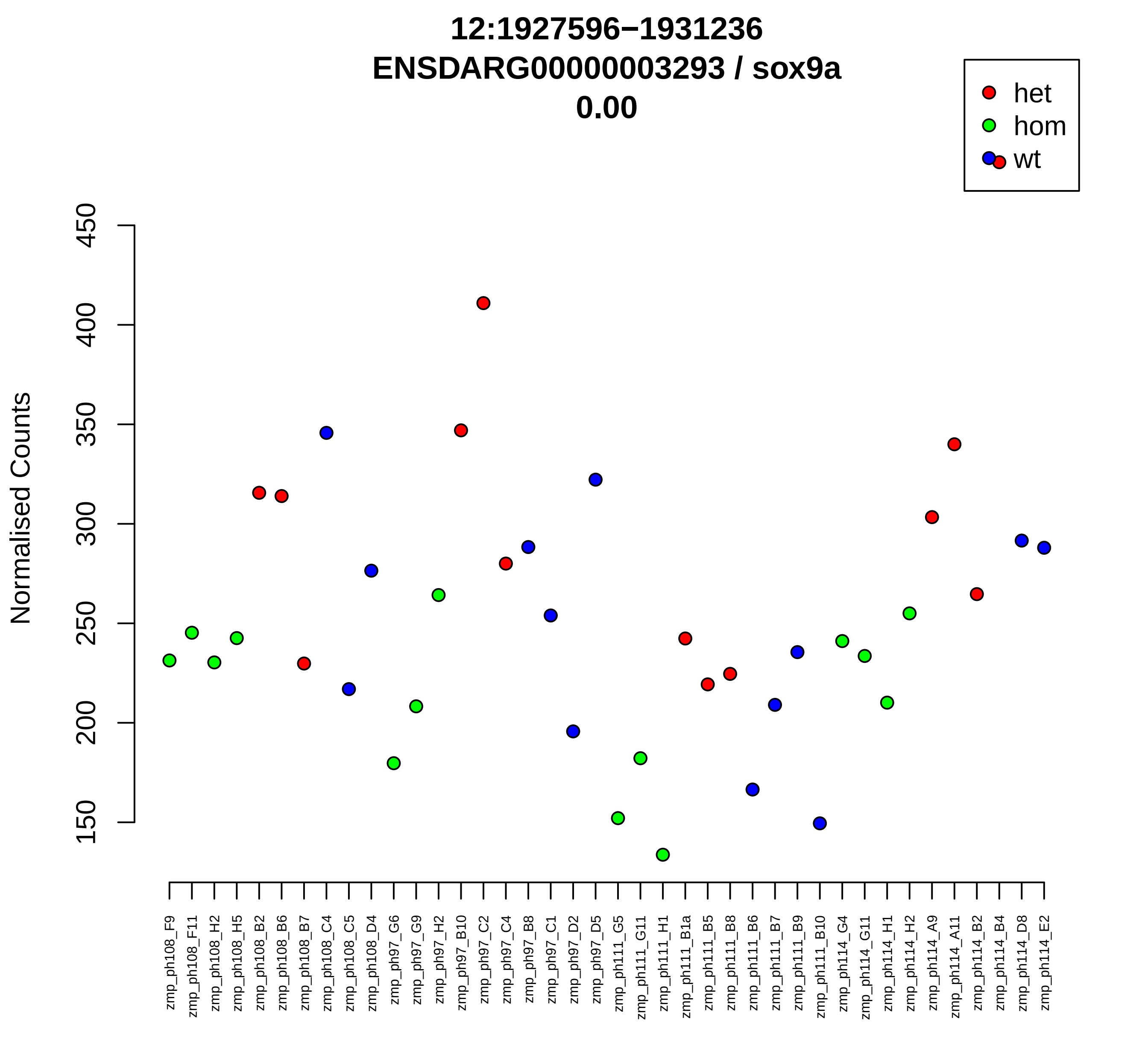
12:1927596−1931236
ENSDARG00000003293 / sox9a
0.00
het
hom
wt
Normalised Counts
450
400
350
300
250
200
150
zmp_ph108_F9
zmp_ph108_F11
zmp_ph108_H2
zmp_ph108_H5
zmp_ph108_B2
zmp_ph108_B6
zmp_ph108_B7
zmp_ph108_C4
zmp_ph108_C5
zmp_ph108_D4
zmp_ph97_G6
zmp_ph97_G9
zmp_ph97_H2
zmp_ph97_B10
zmp_ph97_C2
zmp_ph97_C4
zmp_ph97_B8
zmp_ph97_C1
zmp_ph97_D2
zmp_ph97_D5
zmp_ph111_G5
zmp_ph111_G11
zmp_ph111_H1
zmp_ph111_B1a
zmp_ph111_B5
zmp_ph111_B8
zmp_ph111_B6
zmp_ph111_B7
zmp_ph111_B9
zmp_ph111_B10
zmp_ph114_G4
zmp_ph114_G11
zmp_ph114_H1
zmp_ph114_H2
zmp_ph114_A9
zmp_ph114_A11
zmp_ph114_B2
zmp_ph114_B4
zmp_ph114_D8
zmp_ph114_E2

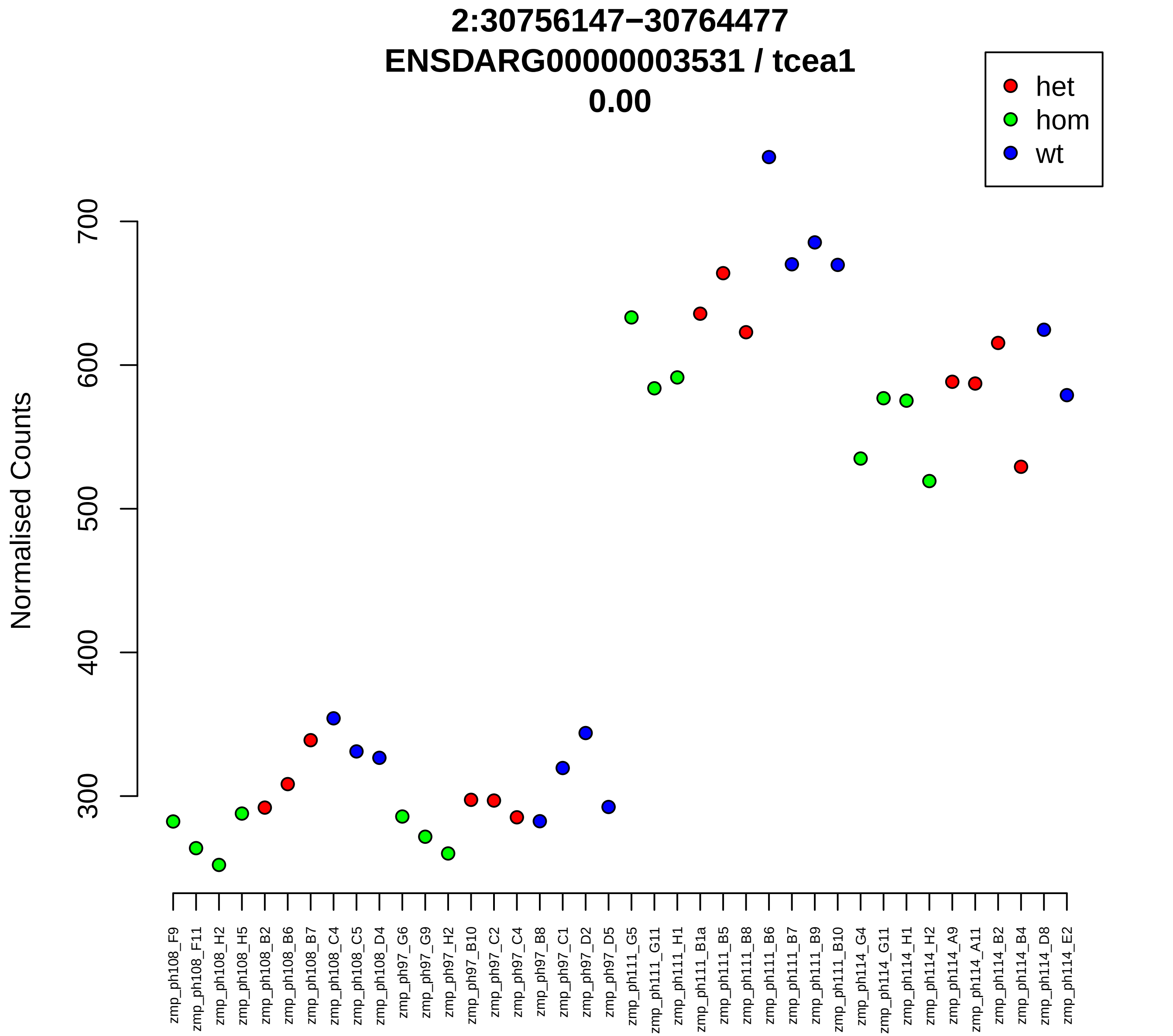

2:30756147−30764477
ENSDARG00000003531 / tcea1
0.00
het
hom
wt
Normalised Counts
300
400
500
600
700
zmp_ph108_F9
zmp_ph108_F11
zmp_ph108_H2
zmp_ph108_H5
zmp_ph108_B2
zmp_ph108_B6
zmp_ph108_B7
zmp_ph108_C4
zmp_ph108_C5
zmp_ph108_D4
zmp_ph97_G6
zmp_ph97_G9
zmp_ph97_H2
zmp_ph97_B10
zmp_ph97_C2
zmp_ph97_C4
zmp_ph97_B8
zmp_ph97_C1
zmp_ph97_D2
zmp_ph97_D5
zmp_ph111_G5
zmp_ph111_G11
zmp_ph111_H1
zmp_ph111_B1a
zmp_ph111_B5
zmp_ph111_B8
zmp_ph111_B6
zmp_ph111_B7
zmp_ph111_B9
zmp_ph111_B10
zmp_ph114_G4
zmp_ph114_G11
zmp_ph114_H1
zmp_ph114_H2
zmp_ph114_A9
zmp_ph114_A11
zmp_ph114_B2
zmp_ph114_B4
zmp_ph114_D8
zmp_ph114_E2

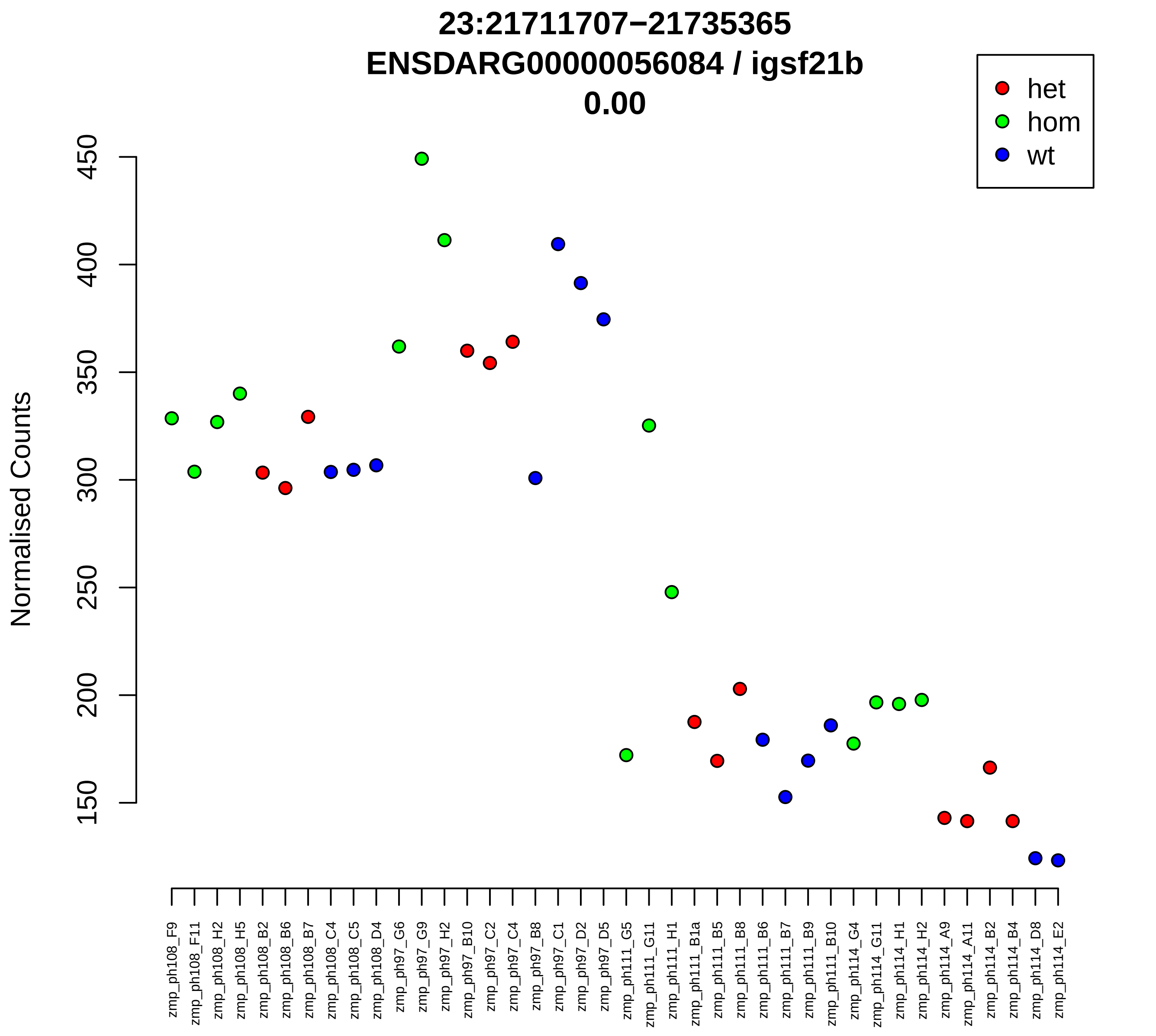

23:21711707−21735365
ENSDARG00000056084 / igsf21b
0.00
het
hom
wt
Normalised Counts
150
200
250
300
350
400
450
zmp_ph108_F9
zmp_ph108_F11
zmp_ph108_H2
zmp_ph108_H5
zmp_ph108_B2
zmp_ph108_B6
zmp_ph108_B7
zmp_ph108_C4
zmp_ph108_C5
zmp_ph108_D4
zmp_ph97_G6
zmp_ph97_G9
zmp_ph97_H2
zmp_ph97_B10
zmp_ph97_C2
zmp_ph97_C4
zmp_ph97_B8
zmp_ph97_C1
zmp_ph97_D2
zmp_ph97_D5
zmp_ph111_G5
zmp_ph111_G11
zmp_ph111_H1
zmp_ph111_B1a
zmp_ph111_B5
zmp_ph111_B8
zmp_ph111_B6
zmp_ph111_B7
zmp_ph111_B9
zmp_ph111_B10
zmp_ph114_G4
zmp_ph114_G11
zmp_ph114_H1
zmp_ph114_H2
zmp_ph114_A9
zmp_ph114_A11
zmp_ph114_B2
zmp_ph114_B4
zmp_ph114_D8
zmp_ph114_E2

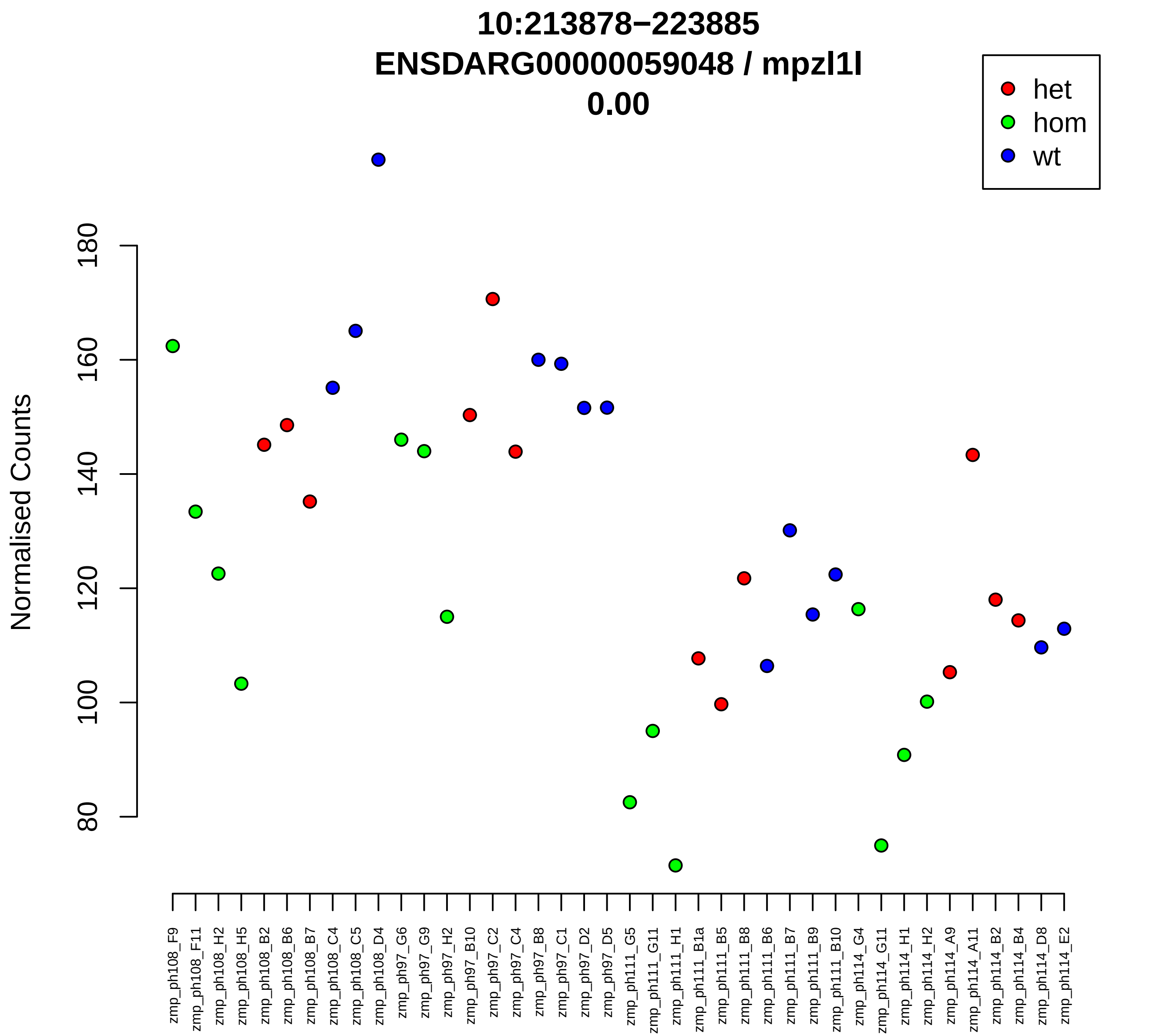

10:213878−223885
ENSDARG00000059048 / mpzl1l
0.00
het
hom
wt
Normalised Counts
80
100
120
140
160
180
zmp_ph108_F9
zmp_ph108_F11
zmp_ph108_H2
zmp_ph108_H5
zmp_ph108_B2
zmp_ph108_B6
zmp_ph108_B7
zmp_ph108_C4
zmp_ph108_C5
zmp_ph108_D4
zmp_ph97_G6
zmp_ph97_G9
zmp_ph97_H2
zmp_ph97_B10
zmp_ph97_C2
zmp_ph97_C4
zmp_ph97_B8
zmp_ph97_C1
zmp_ph97_D2
zmp_ph97_D5
zmp_ph111_G5
zmp_ph111_G11
zmp_ph111_H1
zmp_ph111_B1a
zmp_ph111_B5
zmp_ph111_B8
zmp_ph111_B6
zmp_ph111_B7
zmp_ph111_B9
zmp_ph111_B10
zmp_ph114_G4
zmp_ph114_G11
zmp_ph114_H1
zmp_ph114_H2
zmp_ph114_A9
zmp_ph114_A11
zmp_ph114_B2
zmp_ph114_B4
zmp_ph114_D8
zmp_ph114_E2

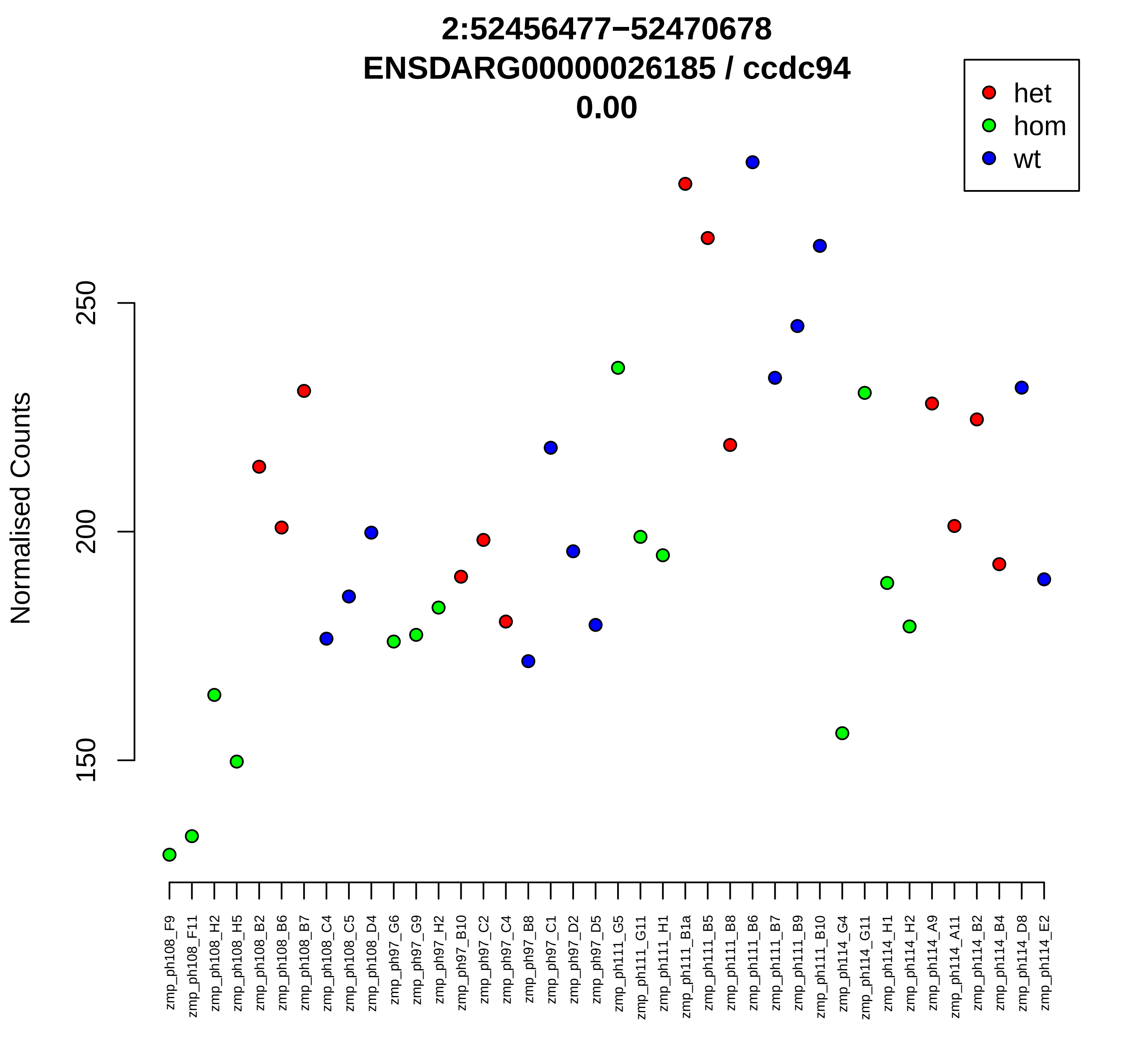

2:52456477−52470678
ENSDARG00000026185 / ccdc94
0.00
het
hom
wt
Normalised Counts
150
200
250
zmp_ph108_F9
zmp_ph108_F11
zmp_ph108_H2
zmp_ph108_H5
zmp_ph108_B2
zmp_ph108_B6
zmp_ph108_B7
zmp_ph108_C4
zmp_ph108_C5
zmp_ph108_D4
zmp_ph97_G6
zmp_ph97_G9
zmp_ph97_H2
zmp_ph97_B10
zmp_ph97_C2
zmp_ph97_C4
zmp_ph97_B8
zmp_ph97_C1
zmp_ph97_D2
zmp_ph97_D5
zmp_ph111_G5
zmp_ph111_G11
zmp_ph111_H1
zmp_ph111_B1a
zmp_ph111_B5
zmp_ph111_B8
zmp_ph111_B6
zmp_ph111_B7
zmp_ph111_B9
zmp_ph111_B10
zmp_ph114_G4
zmp_ph114_G11
zmp_ph114_H1
zmp_ph114_H2
zmp_ph114_A9
zmp_ph114_A11
zmp_ph114_B2
zmp_ph114_B4
zmp_ph114_D8
zmp_ph114_E2

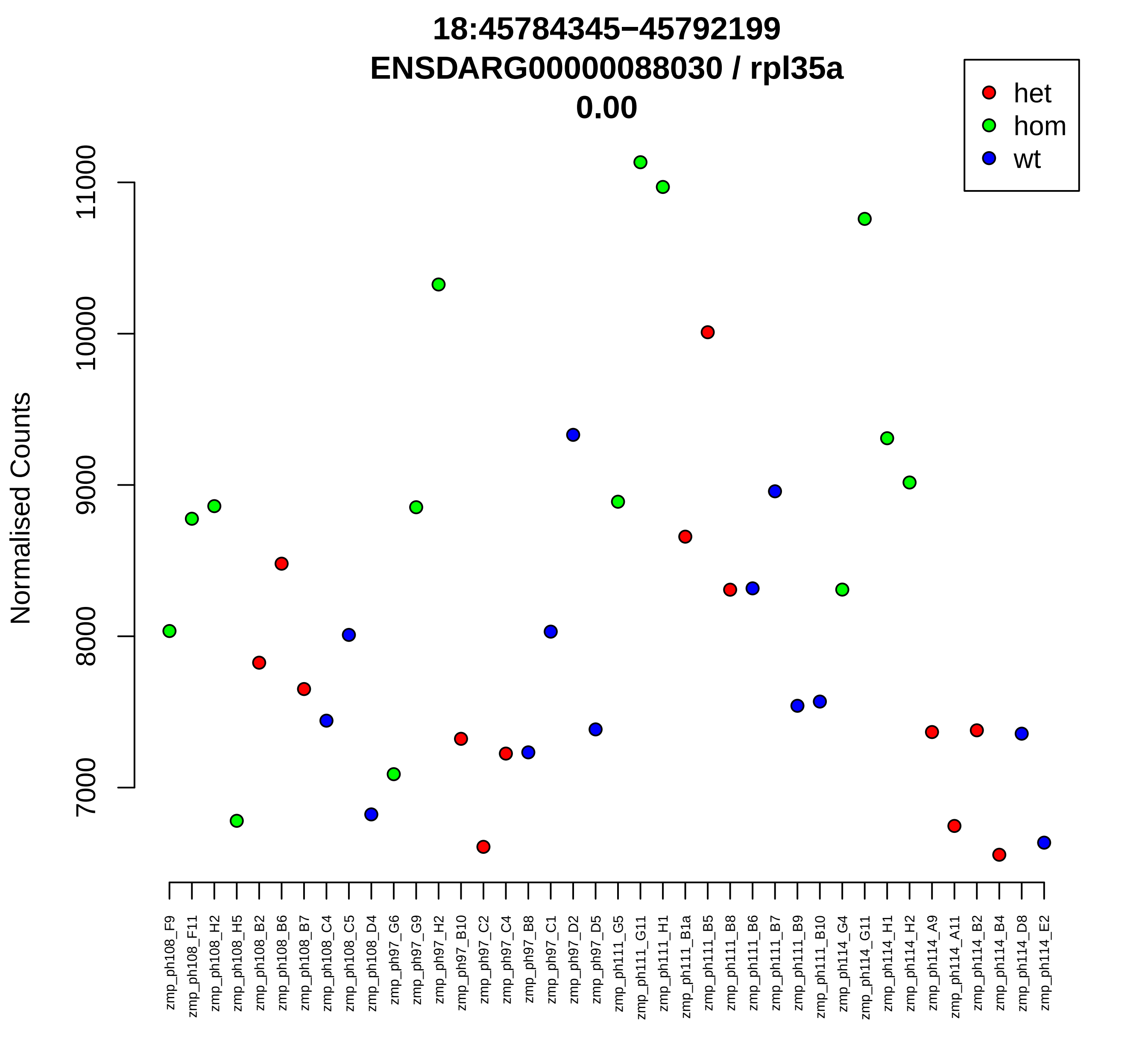
18:45784345−45792199
ENSDARG00000088030 / rpl35a
0.00
het
hom
wt
Normalised Counts
7000
8000
9000
10000
11000
zmp_ph108_F9
zmp_ph108_F11
zmp_ph108_H2
zmp_ph108_H5
zmp_ph108_B2
zmp_ph108_B6
zmp_ph108_B7
zmp_ph108_C4
zmp_ph108_C5
zmp_ph108_D4
zmp_ph97_G6
zmp_ph97_G9
zmp_ph97_H2
zmp_ph97_B10
zmp_ph97_C2
zmp_ph97_C4
zmp_ph97_B8
zmp_ph97_C1
zmp_ph97_D2
zmp_ph97_D5
zmp_ph111_G5
zmp_ph111_G11
zmp_ph111_H1
zmp_ph111_B1a
zmp_ph111_B5
zmp_ph111_B8
zmp_ph111_B6
zmp_ph111_B7
zmp_ph111_B9
zmp_ph111_B10
zmp_ph114_G4
zmp_ph114_G11
zmp_ph114_H1
zmp_ph114_H2
zmp_ph114_A9
zmp_ph114_A11
zmp_ph114_B2
zmp_ph114_B4
zmp_ph114_D8
zmp_ph114_E2

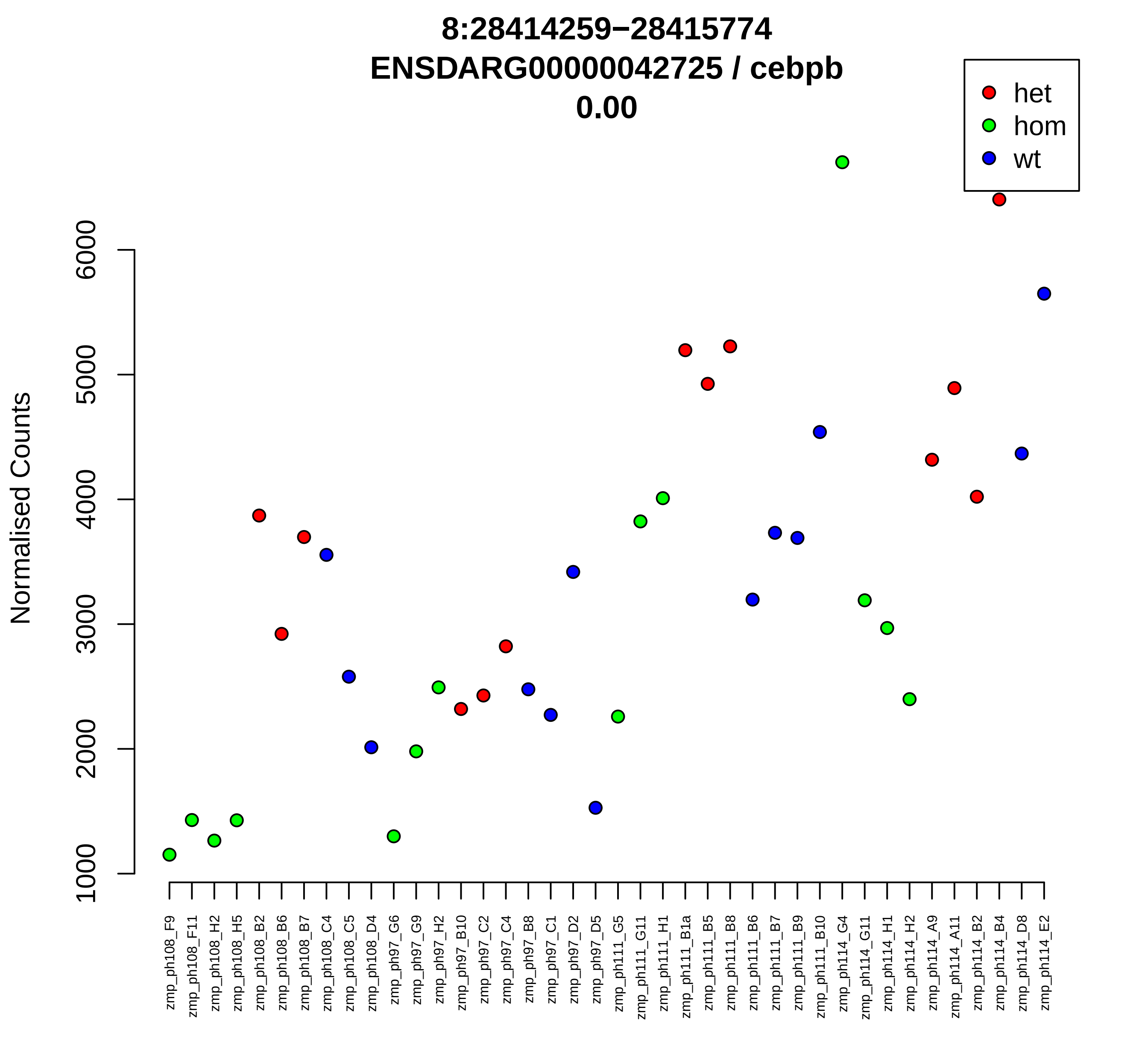

8:28414259−28415774
ENSDARG00000042725 / cebpb
0.00
het
hom
wt
Normalised Counts
1000
2000
3000
4000
5000
6000
zmp_ph108_F9
zmp_ph108_F11
zmp_ph108_H2
zmp_ph108_H5
zmp_ph108_B2
zmp_ph108_B6
zmp_ph108_B7
zmp_ph108_C4
zmp_ph108_C5
zmp_ph108_D4
zmp_ph97_G6
zmp_ph97_G9
zmp_ph97_H2
zmp_ph97_B10
zmp_ph97_C2
zmp_ph97_C4
zmp_ph97_B8
zmp_ph97_C1
zmp_ph97_D2
zmp_ph97_D5
zmp_ph111_G5
zmp_ph111_G11
zmp_ph111_H1
zmp_ph111_B1a
zmp_ph111_B5
zmp_ph111_B8
zmp_ph111_B6
zmp_ph111_B7
zmp_ph111_B9
zmp_ph111_B10
zmp_ph114_G4
zmp_ph114_G11
zmp_ph114_H1
zmp_ph114_H2
zmp_ph114_A9
zmp_ph114_A11
zmp_ph114_B2
zmp_ph114_B4
zmp_ph114_D8
zmp_ph114_E2

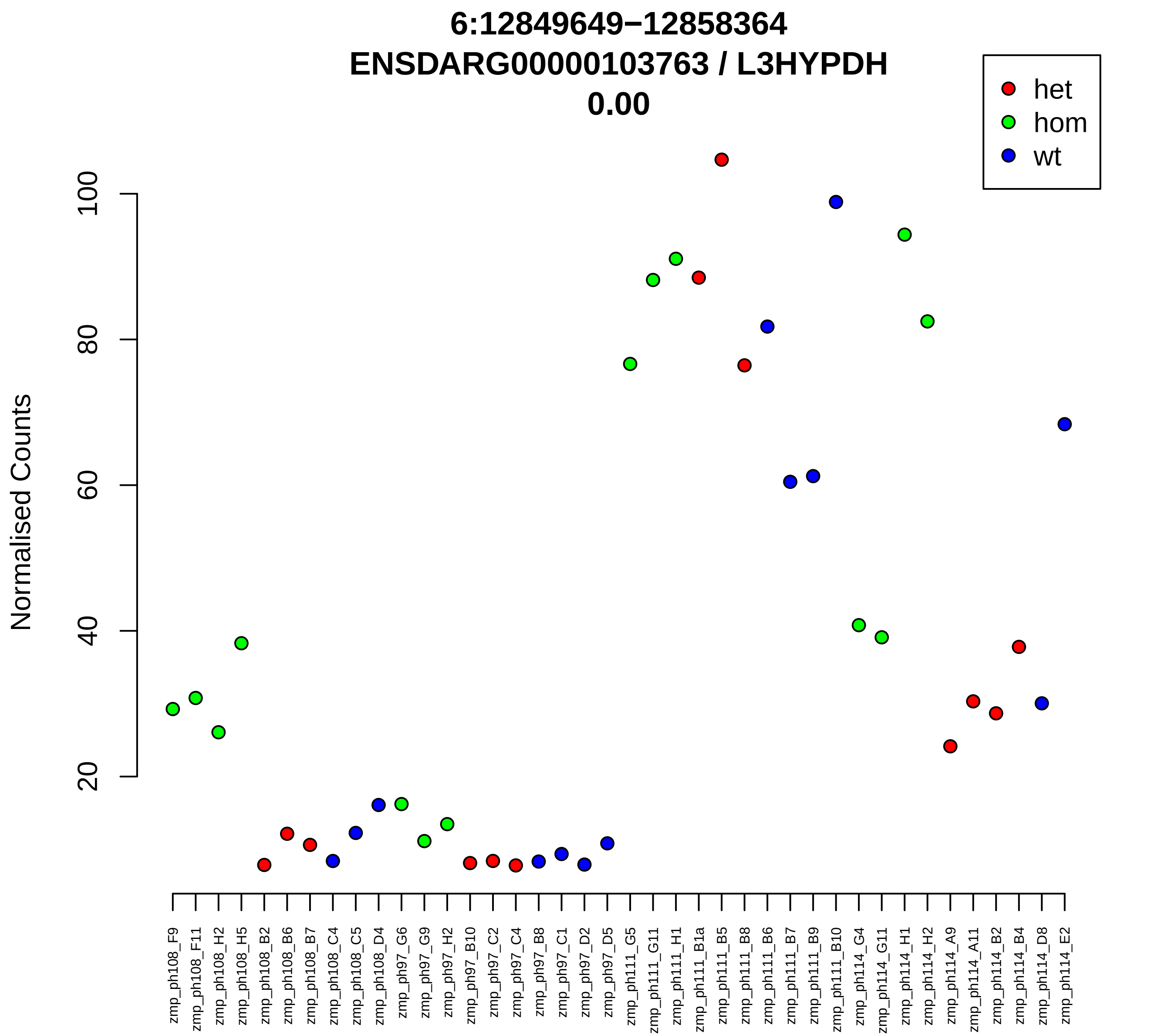

6:12849649−12858364
ENSDARG00000103763 / L3HYPDH
0.00
het
hom
wt
Normalised Counts
20
40
60
80
100
zmp_ph108_F9
zmp_ph108_F11
zmp_ph108_H2
zmp_ph108_H5
zmp_ph108_B2
zmp_ph108_B6
zmp_ph108_B7
zmp_ph108_C4
zmp_ph108_C5
zmp_ph108_D4
zmp_ph97_G6
zmp_ph97_G9
zmp_ph97_H2
zmp_ph97_B10
zmp_ph97_C2
zmp_ph97_C4
zmp_ph97_B8
zmp_ph97_C1
zmp_ph97_D2
zmp_ph97_D5
zmp_ph111_G5
zmp_ph111_G11
zmp_ph111_H1
zmp_ph111_B1a
zmp_ph111_B5
zmp_ph111_B8
zmp_ph111_B6
zmp_ph111_B7
zmp_ph111_B9
zmp_ph111_B10
zmp_ph114_G4
zmp_ph114_G11
zmp_ph114_H1
zmp_ph114_H2
zmp_ph114_A9
zmp_ph114_A11
zmp_ph114_B2
zmp_ph114_B4
zmp_ph114_D8
zmp_ph114_E2

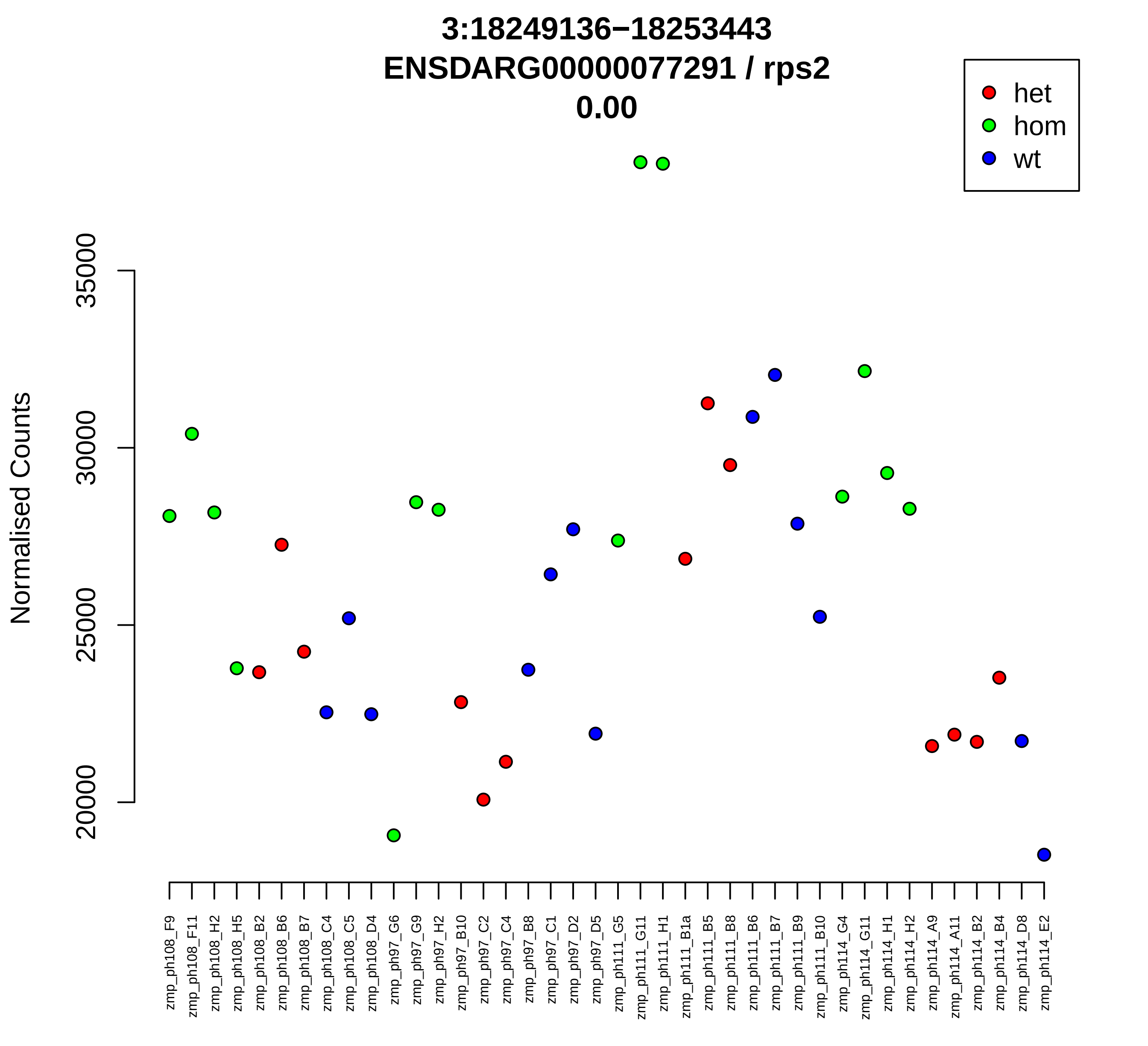

3:18249136−18253443
ENSDARG00000077291 / rps2
0.00
het
hom
wt
Normalised Counts
20000
25000
30000
35000
zmp_ph108_F9
zmp_ph108_F11
zmp_ph108_H2
zmp_ph108_H5
zmp_ph108_B2
zmp_ph108_B6
zmp_ph108_B7
zmp_ph108_C4
zmp_ph108_C5
zmp_ph108_D4
zmp_ph97_G6
zmp_ph97_G9
zmp_ph97_H2
zmp_ph97_B10
zmp_ph97_C2
zmp_ph97_C4
zmp_ph97_B8
zmp_ph97_C1
zmp_ph97_D2
zmp_ph97_D5
zmp_ph111_G5
zmp_ph111_G11
zmp_ph111_H1
zmp_ph111_B1a
zmp_ph111_B5
zmp_ph111_B8
zmp_ph111_B6
zmp_ph111_B7
zmp_ph111_B9
zmp_ph111_B10
zmp_ph114_G4
zmp_ph114_G11
zmp_ph114_H1
zmp_ph114_H2
zmp_ph114_A9
zmp_ph114_A11
zmp_ph114_B2
zmp_ph114_B4
zmp_ph114_D8
zmp_ph114_E2

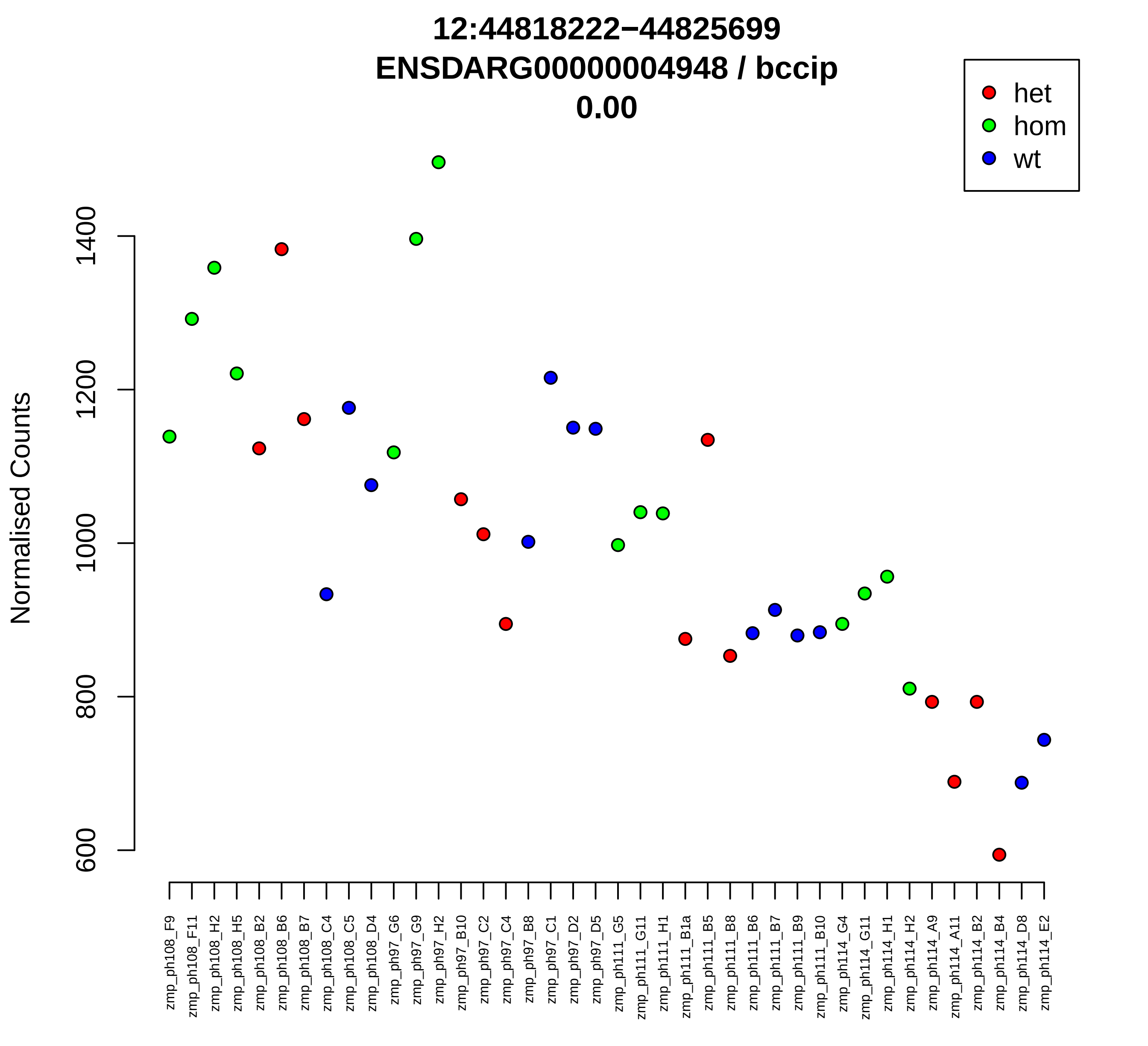

12:44818222−44825699
ENSDARG00000004948 / bccip
0.00
het
hom
wt
Normalised Counts
600
800
1000
1200
1400
zmp_ph108_F9
zmp_ph108_F11
zmp_ph108_H2
zmp_ph108_H5
zmp_ph108_B2
zmp_ph108_B6
zmp_ph108_B7
zmp_ph108_C4
zmp_ph108_C5
zmp_ph108_D4
zmp_ph97_G6
zmp_ph97_G9
zmp_ph97_H2
zmp_ph97_B10
zmp_ph97_C2
zmp_ph97_C4
zmp_ph97_B8
zmp_ph97_C1
zmp_ph97_D2
zmp_ph97_D5
zmp_ph111_G5
zmp_ph111_G11
zmp_ph111_H1
zmp_ph111_B1a
zmp_ph111_B5
zmp_ph111_B8
zmp_ph111_B6
zmp_ph111_B7
zmp_ph111_B9
zmp_ph111_B10
zmp_ph114_G4
zmp_ph114_G11
zmp_ph114_H1
zmp_ph114_H2
zmp_ph114_A9
zmp_ph114_A11
zmp_ph114_B2
zmp_ph114_B4
zmp_ph114_D8
zmp_ph114_E2

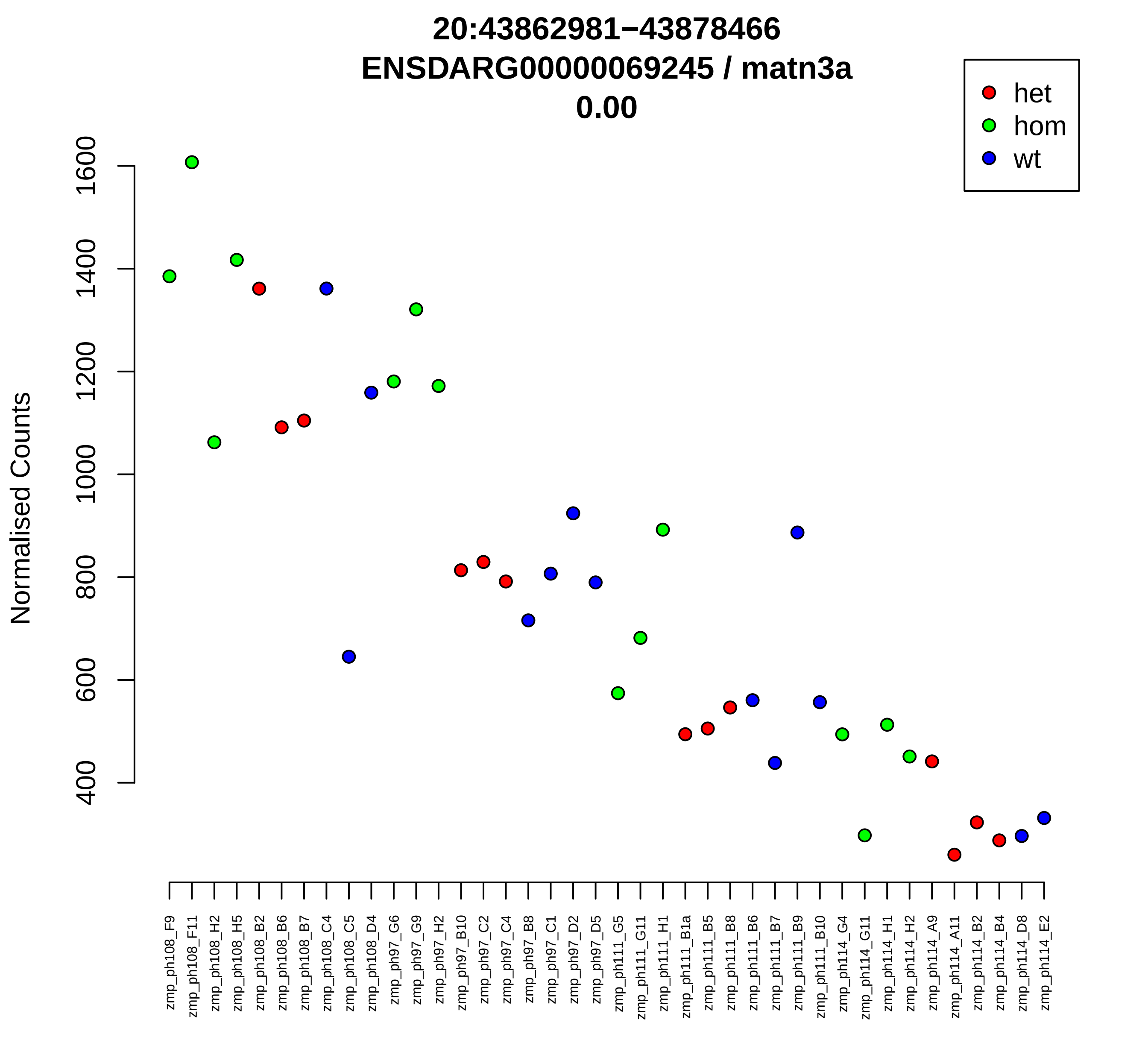

20:43862981−43878466
ENSDARG00000069245 / matn3a
0.00
het
hom
wt
Normalised Counts
400
600
800
1000
1200
1400
1600
zmp_ph108_F9
zmp_ph108_F11
zmp_ph108_H2
zmp_ph108_H5
zmp_ph108_B2
zmp_ph108_B6
zmp_ph108_B7
zmp_ph108_C4
zmp_ph108_C5
zmp_ph108_D4
zmp_ph97_G6
zmp_ph97_G9
zmp_ph97_H2
zmp_ph97_B10
zmp_ph97_C2
zmp_ph97_C4
zmp_ph97_B8
zmp_ph97_C1
zmp_ph97_D2
zmp_ph97_D5
zmp_ph111_G5
zmp_ph111_G11
zmp_ph111_H1
zmp_ph111_B1a
zmp_ph111_B5
zmp_ph111_B8
zmp_ph111_B6
zmp_ph111_B7
zmp_ph111_B9
zmp_ph111_B10
zmp_ph114_G4
zmp_ph114_G11
zmp_ph114_H1
zmp_ph114_H2
zmp_ph114_A9
zmp_ph114_A11
zmp_ph114_B2
zmp_ph114_B4
zmp_ph114_D8
zmp_ph114_E2

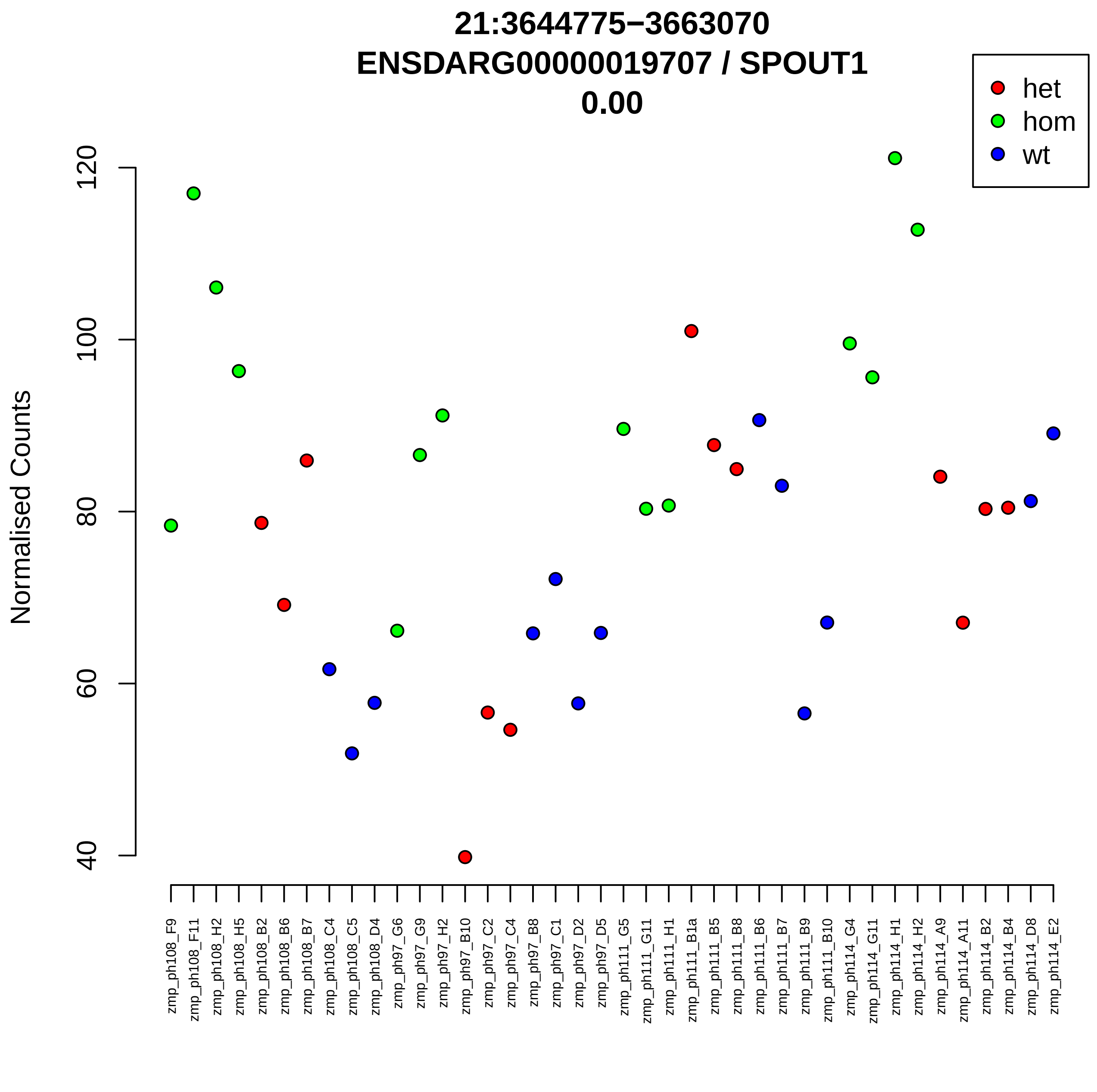

21:3644775−3663070
ENSDARG00000019707 / SPOUT1
0.00
het
hom
wt
Normalised Counts
40
60
80
100
120
zmp_ph108_F9
zmp_ph108_F11
zmp_ph108_H2
zmp_ph108_H5
zmp_ph108_B2
zmp_ph108_B6
zmp_ph108_B7
zmp_ph108_C4
zmp_ph108_C5
zmp_ph108_D4
zmp_ph97_G6
zmp_ph97_G9
zmp_ph97_H2
zmp_ph97_B10
zmp_ph97_C2
zmp_ph97_C4
zmp_ph97_B8
zmp_ph97_C1
zmp_ph97_D2
zmp_ph97_D5
zmp_ph111_G5
zmp_ph111_G11
zmp_ph111_H1
zmp_ph111_B1a
zmp_ph111_B5
zmp_ph111_B8
zmp_ph111_B6
zmp_ph111_B7
zmp_ph111_B9
zmp_ph111_B10
zmp_ph114_G4
zmp_ph114_G11
zmp_ph114_H1
zmp_ph114_H2
zmp_ph114_A9
zmp_ph114_A11
zmp_ph114_B2
zmp_ph114_B4
zmp_ph114_D8
zmp_ph114_E2

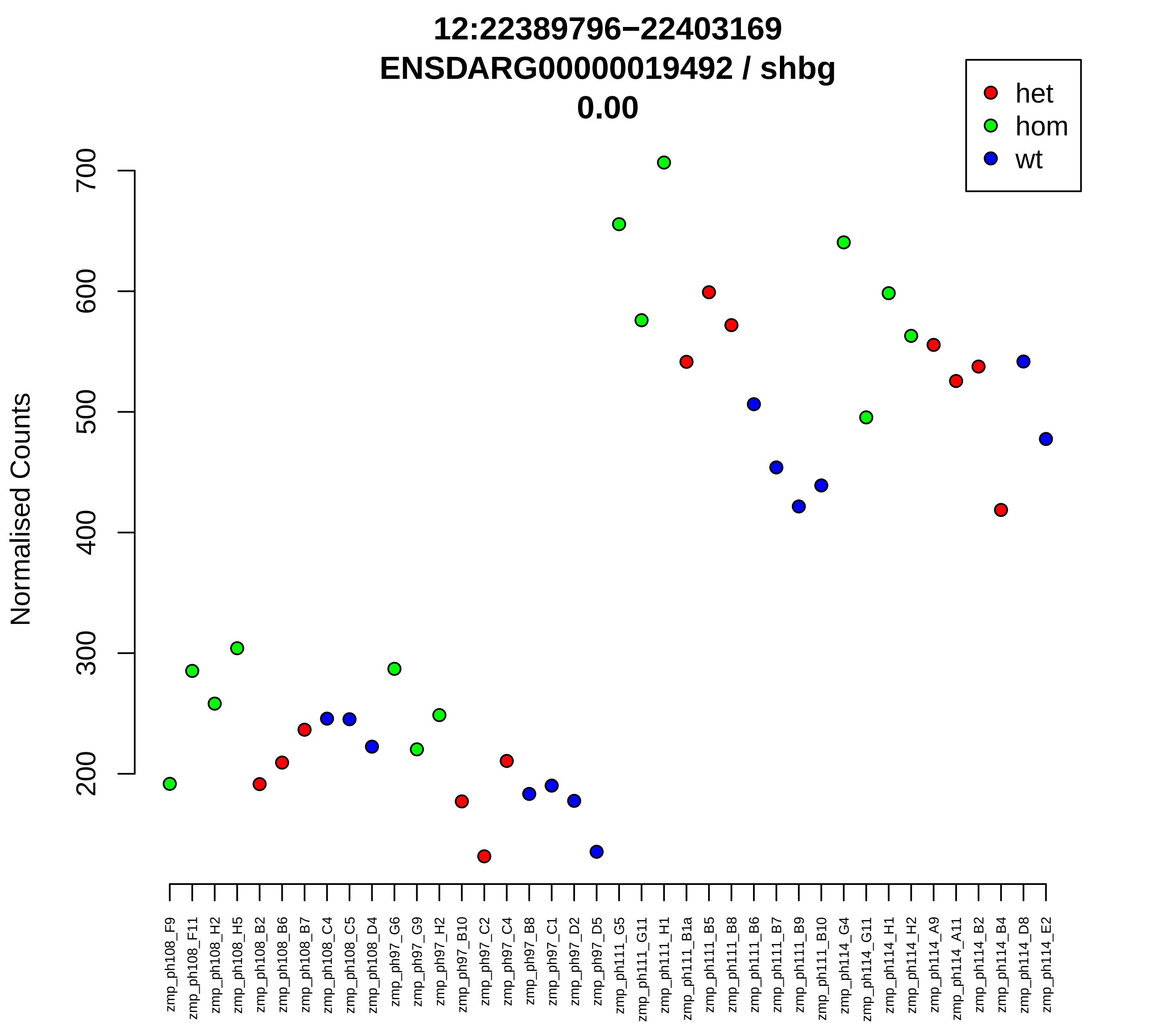

12:22389796−22403169
ENSDARG00000019492 / shbg
0.00
het
hom
wt
Normalised Counts
200
300
400
500
600
700
zmp_ph108_F9
zmp_ph108_F11
zmp_ph108_H2
zmp_ph108_H5
zmp_ph108_B2
zmp_ph108_B6
zmp_ph108_B7
zmp_ph108_C4
zmp_ph108_C5
zmp_ph108_D4
zmp_ph97_G6
zmp_ph97_G9
zmp_ph97_H2
zmp_ph97_B10
zmp_ph97_C2
zmp_ph97_C4
zmp_ph97_B8
zmp_ph97_C1
zmp_ph97_D2
zmp_ph97_D5
zmp_ph111_G5
zmp_ph111_G11
zmp_ph111_H1
zmp_ph111_B1a
zmp_ph111_B5
zmp_ph111_B8
zmp_ph111_B6
zmp_ph111_B7
zmp_ph111_B9
zmp_ph111_B10
zmp_ph114_G4
zmp_ph114_G11
zmp_ph114_H1
zmp_ph114_H2
zmp_ph114_A9
zmp_ph114_A11
zmp_ph114_B2
zmp_ph114_B4
zmp_ph114_D8
zmp_ph114_E2

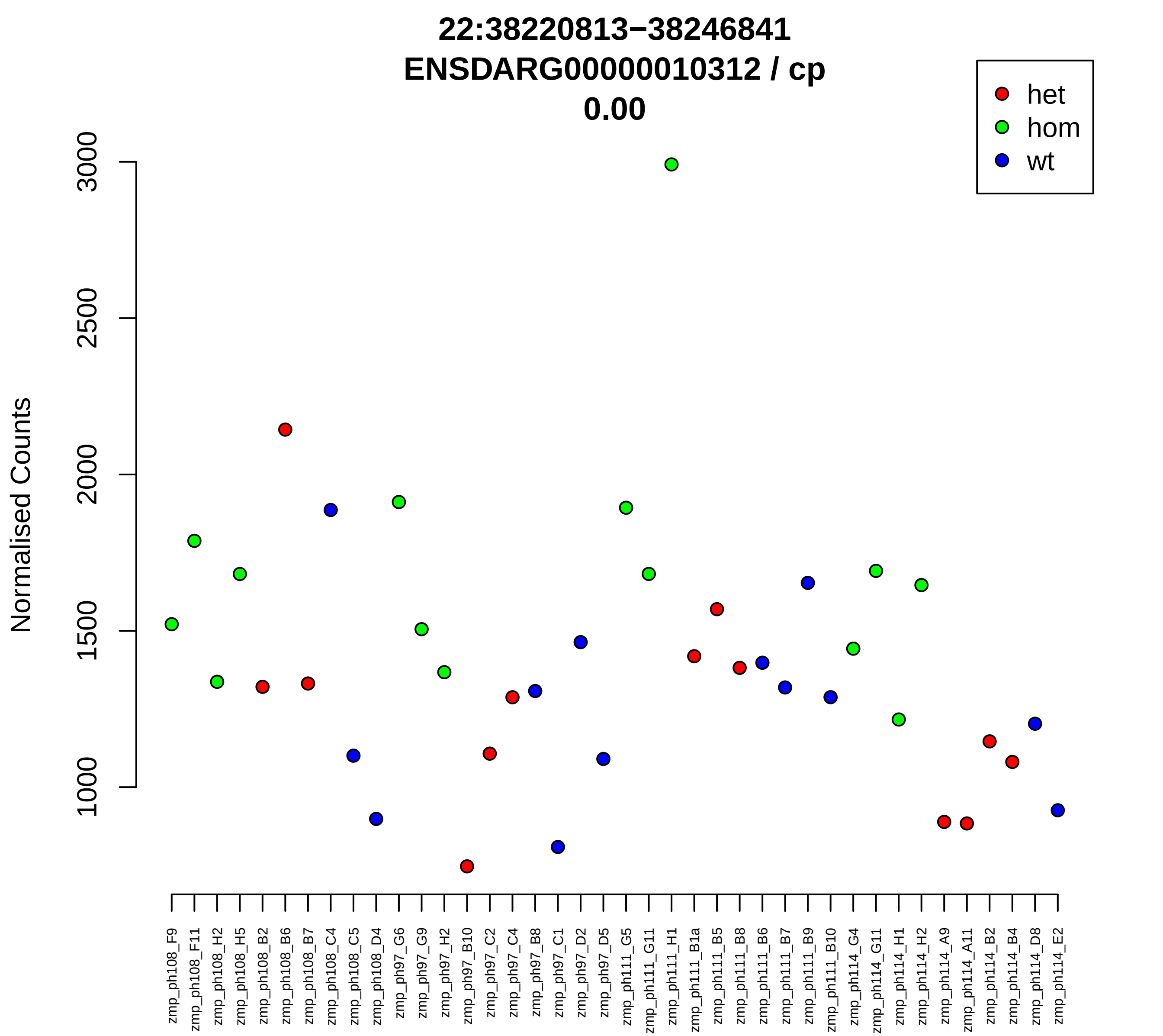

22:38220813−38246841
ENSDARG00000010312 / cp
0.00
het
hom
wt
Normalised Counts
1000
1500
2000
2500
3000
zmp_ph108_F9
zmp_ph108_F11
zmp_ph108_H2
zmp_ph108_H5
zmp_ph108_B2
zmp_ph108_B6
zmp_ph108_B7
zmp_ph108_C4
zmp_ph108_C5
zmp_ph108_D4
zmp_ph97_G6
zmp_ph97_G9
zmp_ph97_H2
zmp_ph97_B10
zmp_ph97_C2
zmp_ph97_C4
zmp_ph97_B8
zmp_ph97_C1
zmp_ph97_D2
zmp_ph97_D5
zmp_ph111_G5
zmp_ph111_G11
zmp_ph111_H1
zmp_ph111_B1a
zmp_ph111_B5
zmp_ph111_B8
zmp_ph111_B6
zmp_ph111_B7
zmp_ph111_B9
zmp_ph111_B10
zmp_ph114_G4
zmp_ph114_G11
zmp_ph114_H1
zmp_ph114_H2
zmp_ph114_A9
zmp_ph114_A11
zmp_ph114_B2
zmp_ph114_B4
zmp_ph114_D8
zmp_ph114_E2

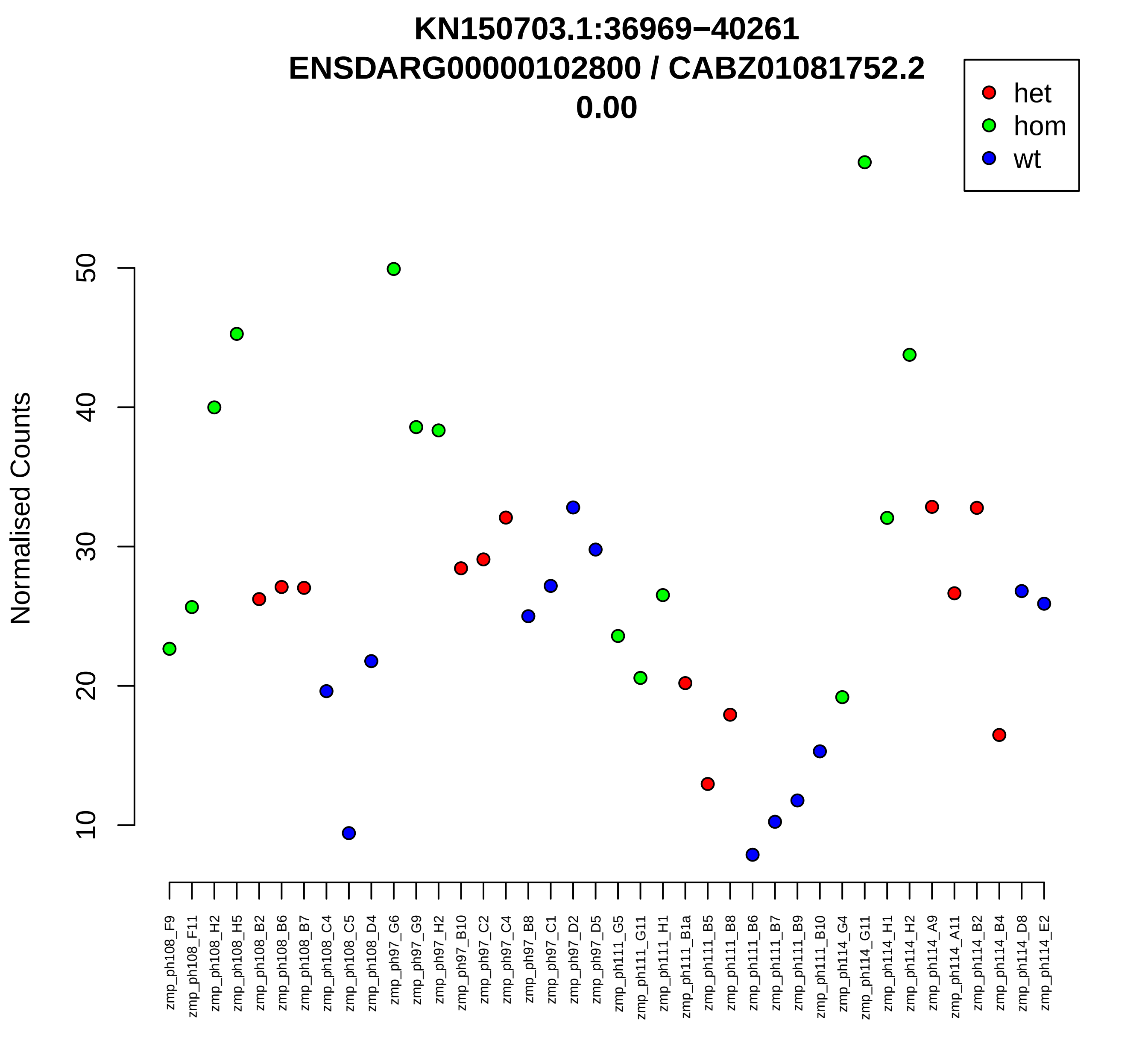
KN150703.1:36969−40261
ENSDARG00000102800 / CABZ01081752.2
0.00
het
hom
wt
Normalised Counts
10
20
30
40
50
zmp_ph108_F9
zmp_ph108_F11
zmp_ph108_H2
zmp_ph108_H5
zmp_ph108_B2
zmp_ph108_B6
zmp_ph108_B7
zmp_ph108_C4
zmp_ph108_C5
zmp_ph108_D4
zmp_ph97_G6
zmp_ph97_G9
zmp_ph97_H2
zmp_ph97_B10
zmp_ph97_C2
zmp_ph97_C4
zmp_ph97_B8
zmp_ph97_C1
zmp_ph97_D2
zmp_ph97_D5
zmp_ph111_G5
zmp_ph111_G11
zmp_ph111_H1
zmp_ph111_B1a
zmp_ph111_B5
zmp_ph111_B8
zmp_ph111_B6
zmp_ph111_B7
zmp_ph111_B9
zmp_ph111_B10
zmp_ph114_G4
zmp_ph114_G11
zmp_ph114_H1
zmp_ph114_H2
zmp_ph114_A9
zmp_ph114_A11
zmp_ph114_B2
zmp_ph114_B4
zmp_ph114_D8
zmp_ph114_E2

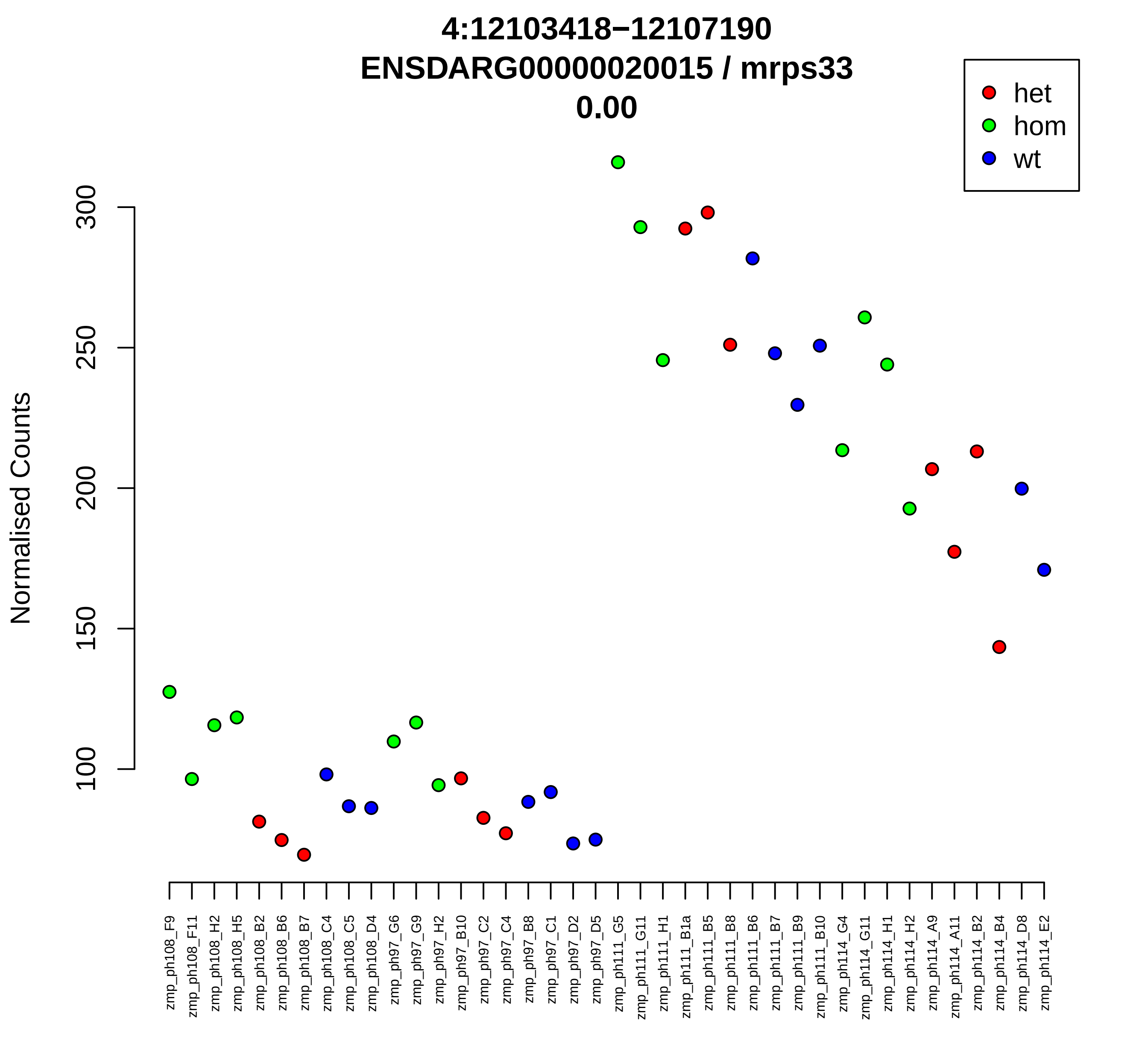
4:12103418−12107190
ENSDARG00000020015 / mrps33
0.00
het
hom
wt
Normalised Counts
100
150
200
250
300
zmp_ph108_F9
zmp_ph108_F11
zmp_ph108_H2
zmp_ph108_H5
zmp_ph108_B2
zmp_ph108_B6
zmp_ph108_B7
zmp_ph108_C4
zmp_ph108_C5
zmp_ph108_D4
zmp_ph97_G6
zmp_ph97_G9
zmp_ph97_H2
zmp_ph97_B10
zmp_ph97_C2
zmp_ph97_C4
zmp_ph97_B8
zmp_ph97_C1
zmp_ph97_D2
zmp_ph97_D5
zmp_ph111_G5
zmp_ph111_G11
zmp_ph111_H1
zmp_ph111_B1a
zmp_ph111_B5
zmp_ph111_B8
zmp_ph111_B6
zmp_ph111_B7
zmp_ph111_B9
zmp_ph111_B10
zmp_ph114_G4
zmp_ph114_G11
zmp_ph114_H1
zmp_ph114_H2
zmp_ph114_A9
zmp_ph114_A11
zmp_ph114_B2
zmp_ph114_B4
zmp_ph114_D8
zmp_ph114_E2

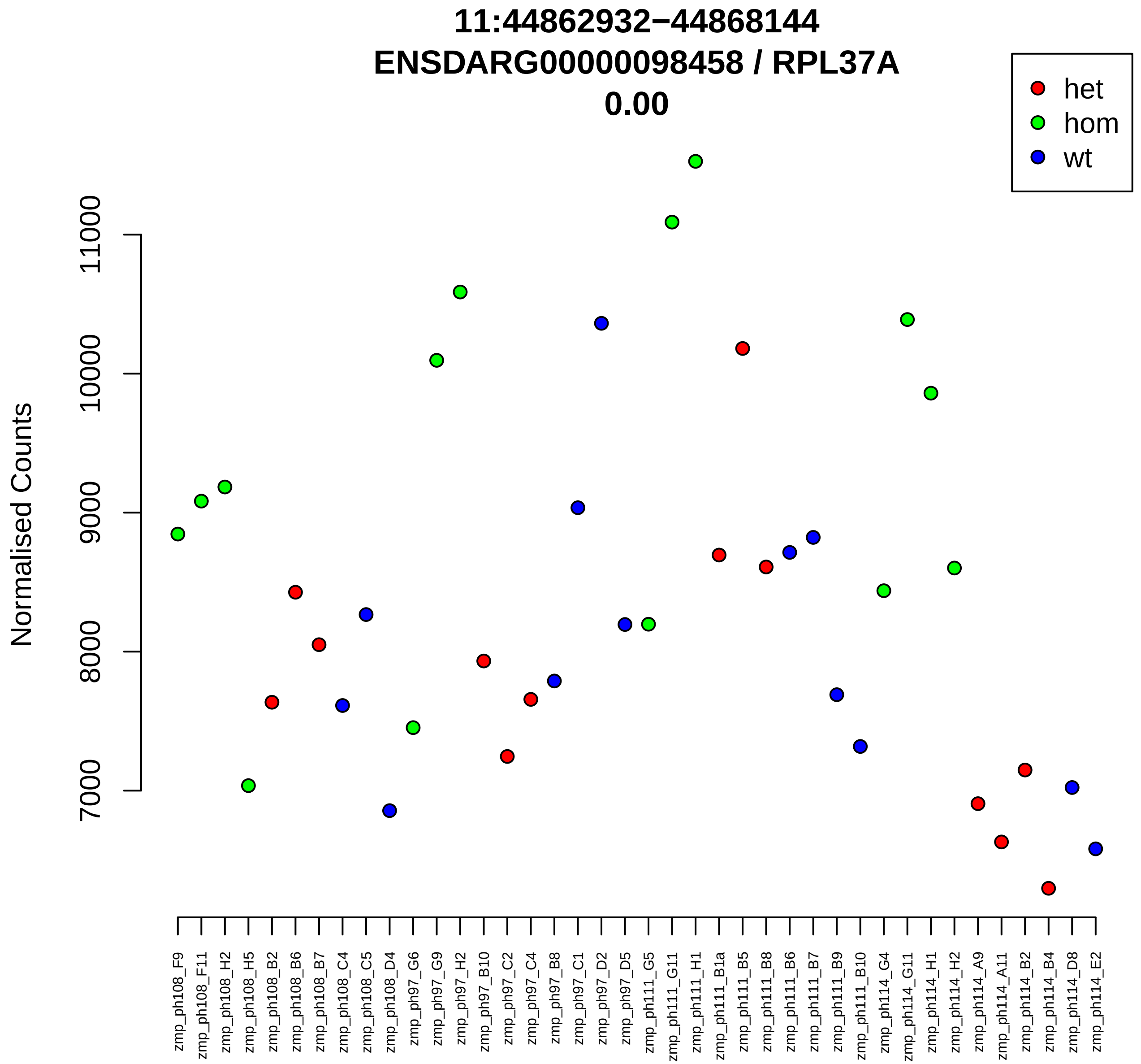

11:44862932−44868144
ENSDARG00000098458 / RPL37A
0.00
het
hom
wt
Normalised Counts
7000
8000
9000
10000
11000
zmp_ph108_F9
zmp_ph108_F11
zmp_ph108_H2
zmp_ph108_H5
zmp_ph108_B2
zmp_ph108_B6
zmp_ph108_B7
zmp_ph108_C4
zmp_ph108_C5
zmp_ph108_D4
zmp_ph97_G6
zmp_ph97_G9
zmp_ph97_H2
zmp_ph97_B10
zmp_ph97_C2
zmp_ph97_C4
zmp_ph97_B8
zmp_ph97_C1
zmp_ph97_D2
zmp_ph97_D5
zmp_ph111_G5
zmp_ph111_G11
zmp_ph111_H1
zmp_ph111_B1a
zmp_ph111_B5
zmp_ph111_B8
zmp_ph111_B6
zmp_ph111_B7
zmp_ph111_B9
zmp_ph111_B10
zmp_ph114_G4
zmp_ph114_G11
zmp_ph114_H1
zmp_ph114_H2
zmp_ph114_A9
zmp_ph114_A11
zmp_ph114_B2
zmp_ph114_B4
zmp_ph114_D8
zmp_ph114_E2

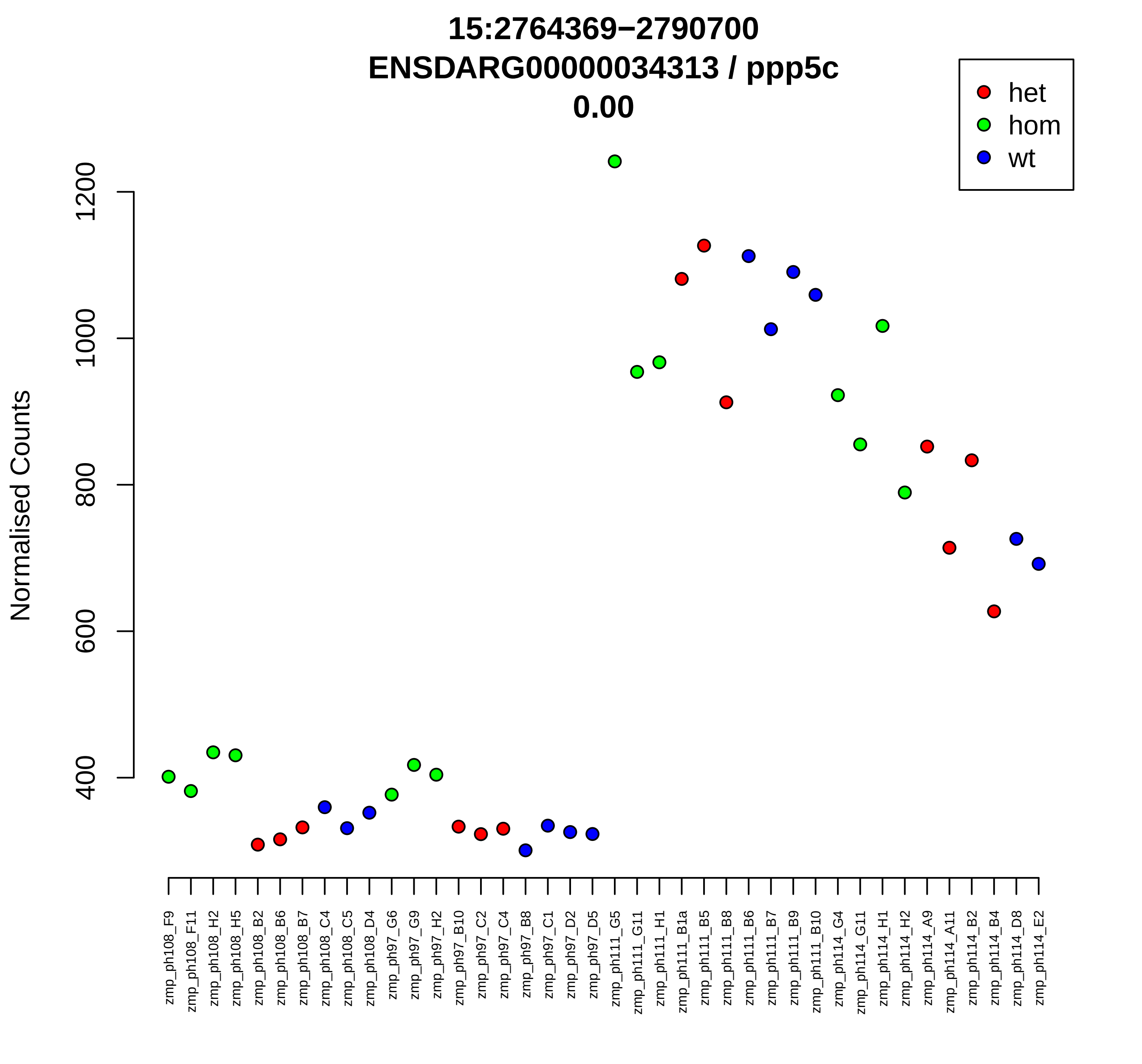

15:2764369−2790700
ENSDARG00000034313 / ppp5c
0.00
het
hom
wt
Normalised Counts
400
600
800
1000
1200
zmp_ph108_F9
zmp_ph108_F11
zmp_ph108_H2
zmp_ph108_H5
zmp_ph108_B2
zmp_ph108_B6
zmp_ph108_B7
zmp_ph108_C4
zmp_ph108_C5
zmp_ph108_D4
zmp_ph97_G6
zmp_ph97_G9
zmp_ph97_H2
zmp_ph97_B10
zmp_ph97_C2
zmp_ph97_C4
zmp_ph97_B8
zmp_ph97_C1
zmp_ph97_D2
zmp_ph97_D5
zmp_ph111_G5
zmp_ph111_G11
zmp_ph111_H1
zmp_ph111_B1a
zmp_ph111_B5
zmp_ph111_B8
zmp_ph111_B6
zmp_ph111_B7
zmp_ph111_B9
zmp_ph111_B10
zmp_ph114_G4
zmp_ph114_G11
zmp_ph114_H1
zmp_ph114_H2
zmp_ph114_A9
zmp_ph114_A11
zmp_ph114_B2
zmp_ph114_B4
zmp_ph114_D8
zmp_ph114_E2

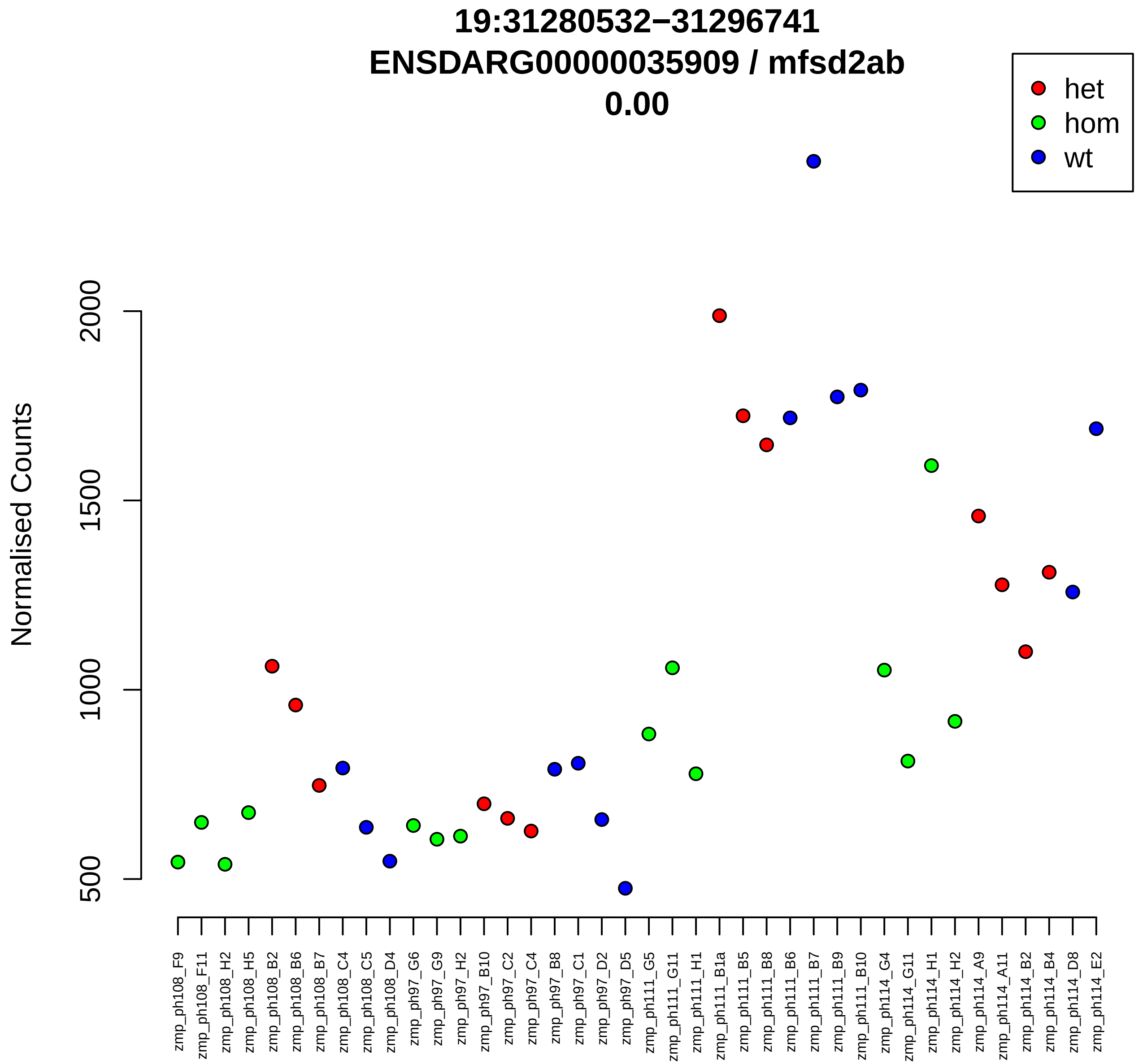

19:31280532−31296741
ENSDARG00000035909 / mfsd2ab
0.00
het
hom
wt
Normalised Counts
500
1000
1500
2000
zmp_ph108_F9
zmp_ph108_F11
zmp_ph108_H2
zmp_ph108_H5
zmp_ph108_B2
zmp_ph108_B6
zmp_ph108_B7
zmp_ph108_C4
zmp_ph108_C5
zmp_ph108_D4
zmp_ph97_G6
zmp_ph97_G9
zmp_ph97_H2
zmp_ph97_B10
zmp_ph97_C2
zmp_ph97_C4
zmp_ph97_B8
zmp_ph97_C1
zmp_ph97_D2
zmp_ph97_D5
zmp_ph111_G5
zmp_ph111_G11
zmp_ph111_H1
zmp_ph111_B1a
zmp_ph111_B5
zmp_ph111_B8
zmp_ph111_B6
zmp_ph111_B7
zmp_ph111_B9
zmp_ph111_B10
zmp_ph114_G4
zmp_ph114_G11
zmp_ph114_H1
zmp_ph114_H2
zmp_ph114_A9
zmp_ph114_A11
zmp_ph114_B2
zmp_ph114_B4
zmp_ph114_D8
zmp_ph114_E2

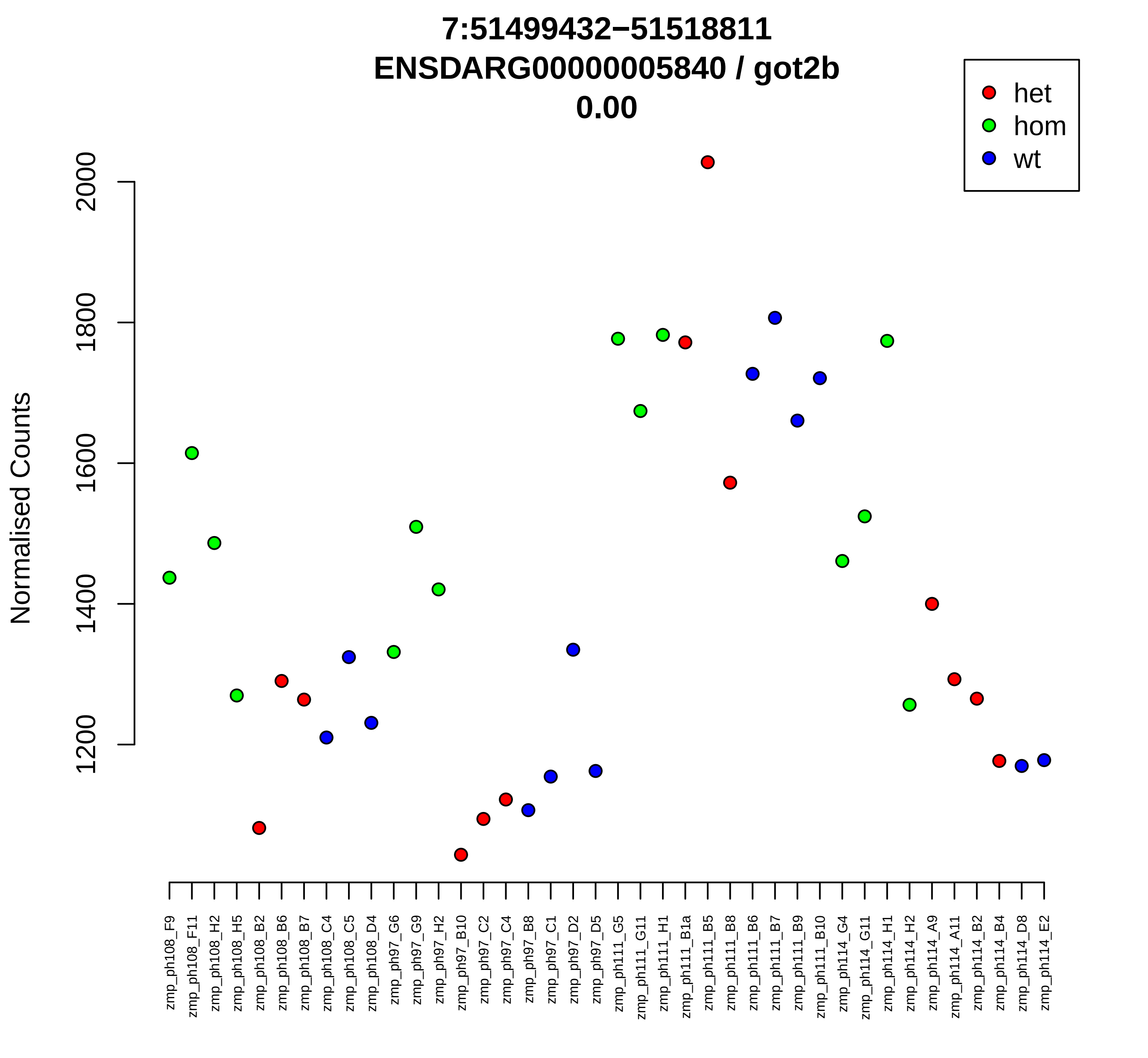

7:51499432−51518811
ENSDARG00000005840 / got2b
0.00
het
hom
wt
Normalised Counts
2000
1800
1600
1400
1200
zmp_ph108_F9
zmp_ph108_F11
zmp_ph108_H2
zmp_ph108_H5
zmp_ph108_B2
zmp_ph108_B6
zmp_ph108_B7
zmp_ph108_C4
zmp_ph108_C5
zmp_ph108_D4
zmp_ph97_G6
zmp_ph97_G9
zmp_ph97_H2
zmp_ph97_B10
zmp_ph97_C2
zmp_ph97_C4
zmp_ph97_B8
zmp_ph97_C1
zmp_ph97_D2
zmp_ph97_D5
zmp_ph111_G5
zmp_ph111_G11
zmp_ph111_H1
zmp_ph111_B1a
zmp_ph111_B5
zmp_ph111_B8
zmp_ph111_B6
zmp_ph111_B7
zmp_ph111_B9
zmp_ph111_B10
zmp_ph114_G4
zmp_ph114_G11
zmp_ph114_H1
zmp_ph114_H2
zmp_ph114_A9
zmp_ph114_A11
zmp_ph114_B2
zmp_ph114_B4
zmp_ph114_D8
zmp_ph114_E2

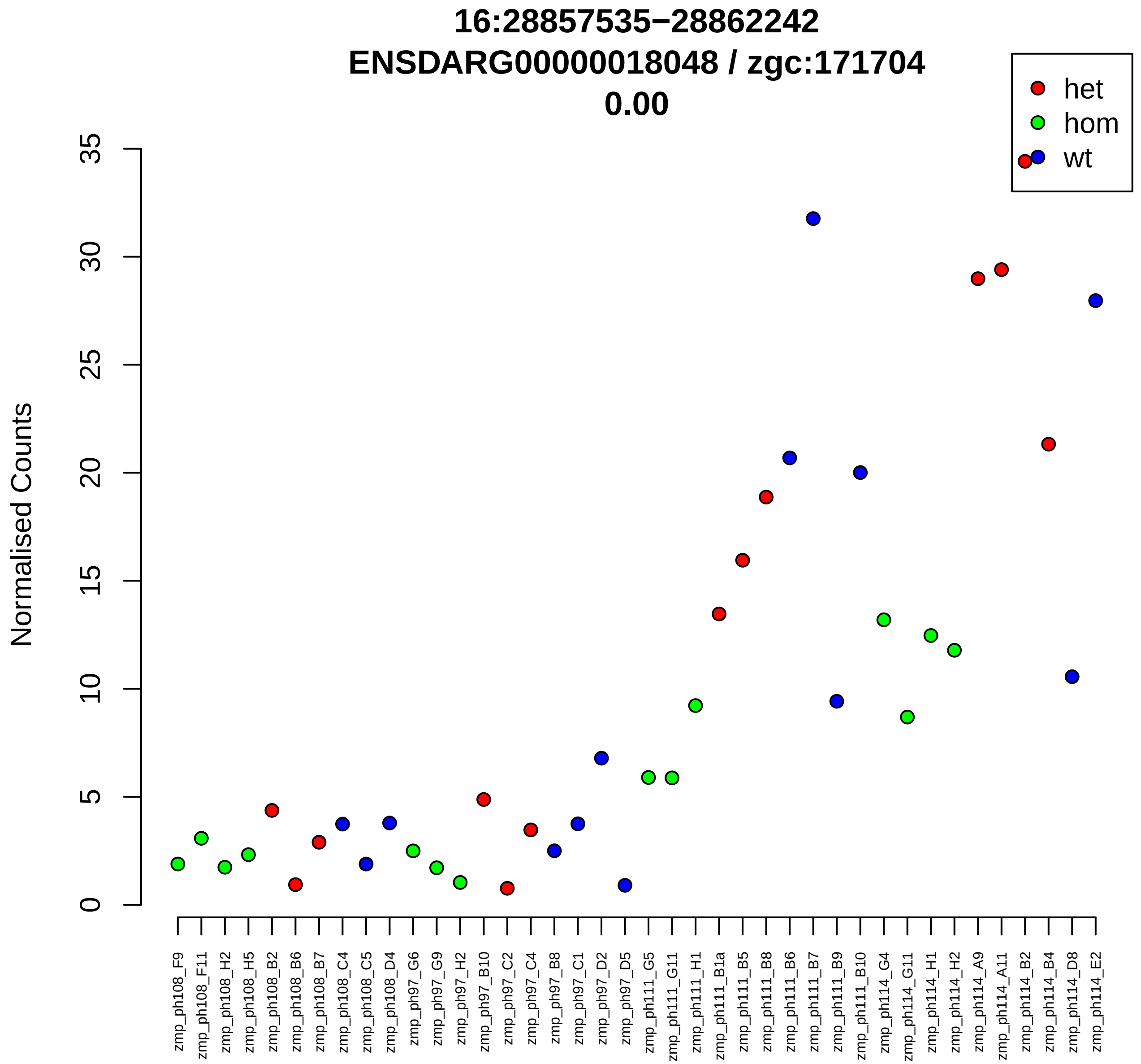

16:28857535−28862242
ENSDARG00000018048 / zgc:171704
0.00
het
hom
wt
Normalised Counts
0
5
10
15
20
25
30
35
zmp_ph108_F9
zmp_ph108_F11
zmp_ph108_H2
zmp_ph108_H5
zmp_ph108_B2
zmp_ph108_B6
zmp_ph108_B7
zmp_ph108_C4
zmp_ph108_C5
zmp_ph108_D4
zmp_ph97_G6
zmp_ph97_G9
zmp_ph97_H2
zmp_ph97_B10
zmp_ph97_C2
zmp_ph97_C4
zmp_ph97_B8
zmp_ph97_C1
zmp_ph97_D2
zmp_ph97_D5
zmp_ph111_G5
zmp_ph111_G11
zmp_ph111_H1
zmp_ph111_B1a
zmp_ph111_B5
zmp_ph111_B8
zmp_ph111_B6
zmp_ph111_B7
zmp_ph111_B9
zmp_ph111_B10
zmp_ph114_G4
zmp_ph114_G11
zmp_ph114_H1
zmp_ph114_H2
zmp_ph114_A9
zmp_ph114_A11
zmp_ph114_B2
zmp_ph114_B4
zmp_ph114_D8
zmp_ph114_E2

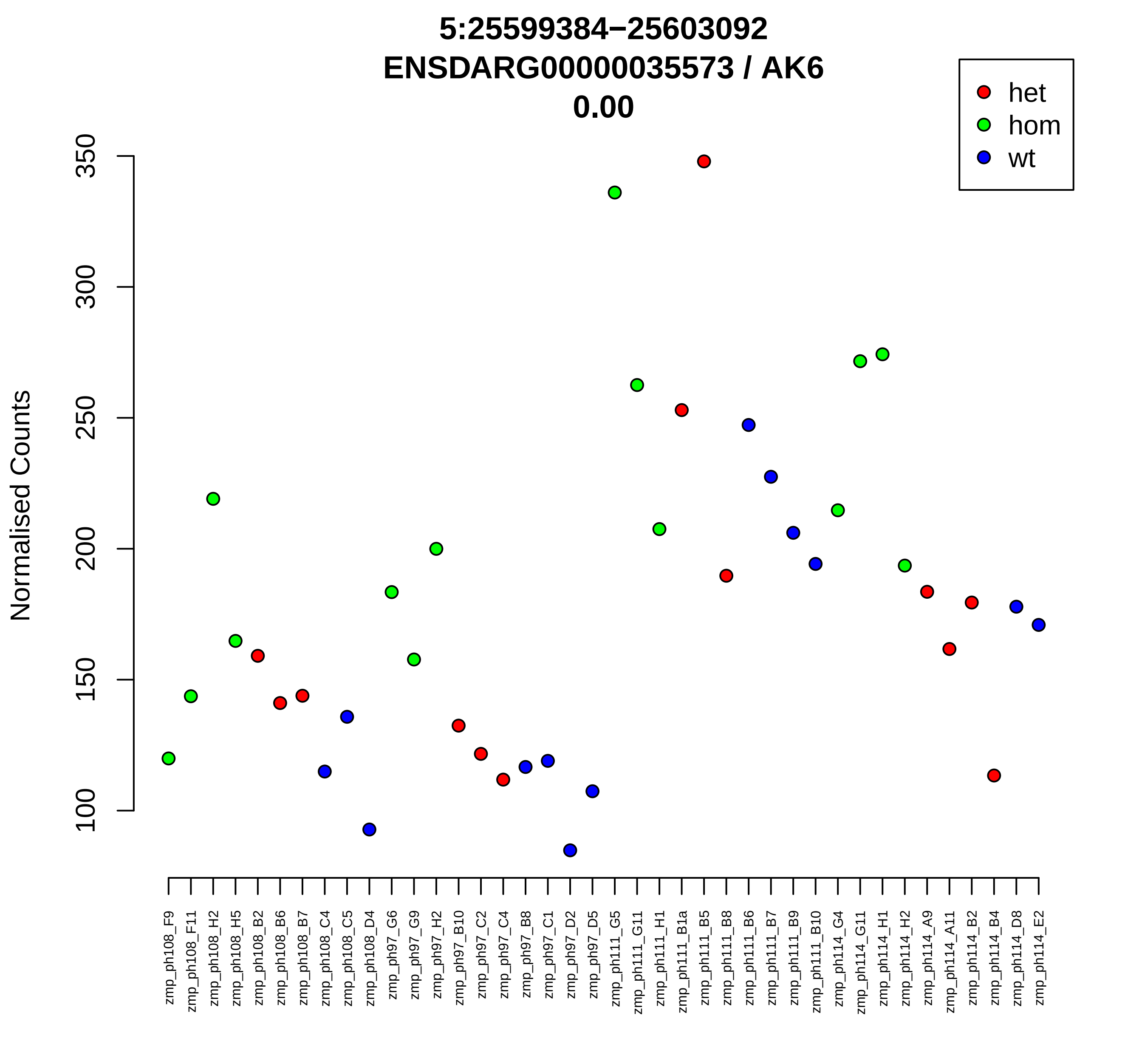
5:25599384−25603092
ENSDARG00000035573 / AK6
0.00
het
hom
wt
Normalised Counts
100
150
200
250
300
350
zmp_ph108_F9
zmp_ph108_F11
zmp_ph108_H2
zmp_ph108_H5
zmp_ph108_B2
zmp_ph108_B6
zmp_ph108_B7
zmp_ph108_C4
zmp_ph108_C5
zmp_ph108_D4
zmp_ph97_G6
zmp_ph97_G9
zmp_ph97_H2
zmp_ph97_B10
zmp_ph97_C2
zmp_ph97_C4
zmp_ph97_B8
zmp_ph97_C1
zmp_ph97_D2
zmp_ph97_D5
zmp_ph111_G5
zmp_ph111_G11
zmp_ph111_H1
zmp_ph111_B1a
zmp_ph111_B5
zmp_ph111_B8
zmp_ph111_B6
zmp_ph111_B7
zmp_ph111_B9
zmp_ph111_B10
zmp_ph114_G4
zmp_ph114_G11
zmp_ph114_H1
zmp_ph114_H2
zmp_ph114_A9
zmp_ph114_A11
zmp_ph114_B2
zmp_ph114_B4
zmp_ph114_D8
zmp_ph114_E2

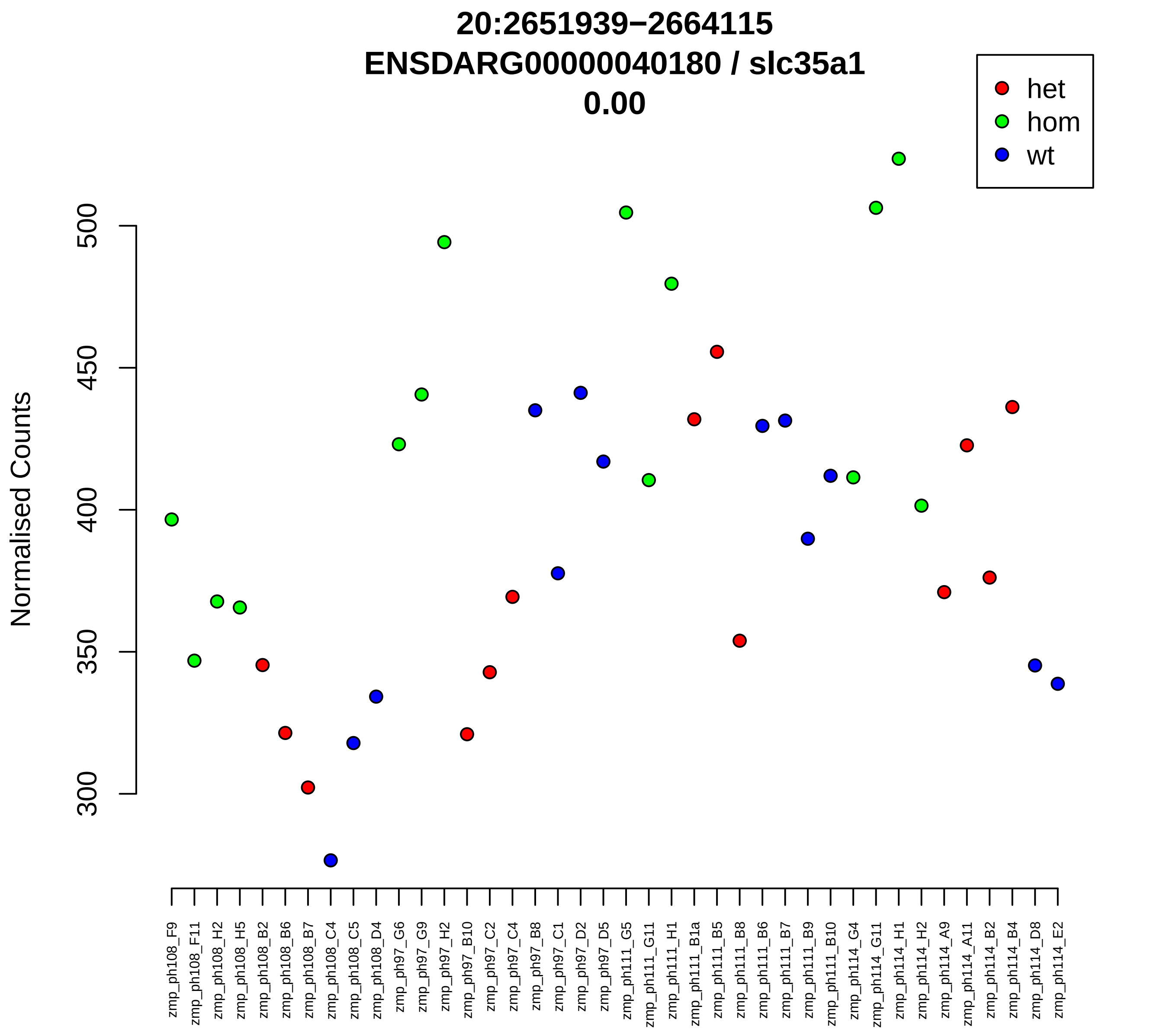

20:2651939−2664115
ENSDARG00000040180 / slc35a1
0.00
het
hom
wt
Normalised Counts
300
350
400
450
500
zmp_ph108_F9
zmp_ph108_F11
zmp_ph108_H2
zmp_ph108_H5
zmp_ph108_B2
zmp_ph108_B6
zmp_ph108_B7
zmp_ph108_C4
zmp_ph108_C5
zmp_ph108_D4
zmp_ph97_G6
zmp_ph97_G9
zmp_ph97_H2
zmp_ph97_B10
zmp_ph97_C2
zmp_ph97_C4
zmp_ph97_B8
zmp_ph97_C1
zmp_ph97_D2
zmp_ph97_D5
zmp_ph111_G5
zmp_ph111_G11
zmp_ph111_H1
zmp_ph111_B1a
zmp_ph111_B5
zmp_ph111_B8
zmp_ph111_B6
zmp_ph111_B7
zmp_ph111_B9
zmp_ph111_B10
zmp_ph114_G4
zmp_ph114_G11
zmp_ph114_H1
zmp_ph114_H2
zmp_ph114_A9
zmp_ph114_A11
zmp_ph114_B2
zmp_ph114_B4
zmp_ph114_D8
zmp_ph114_E2

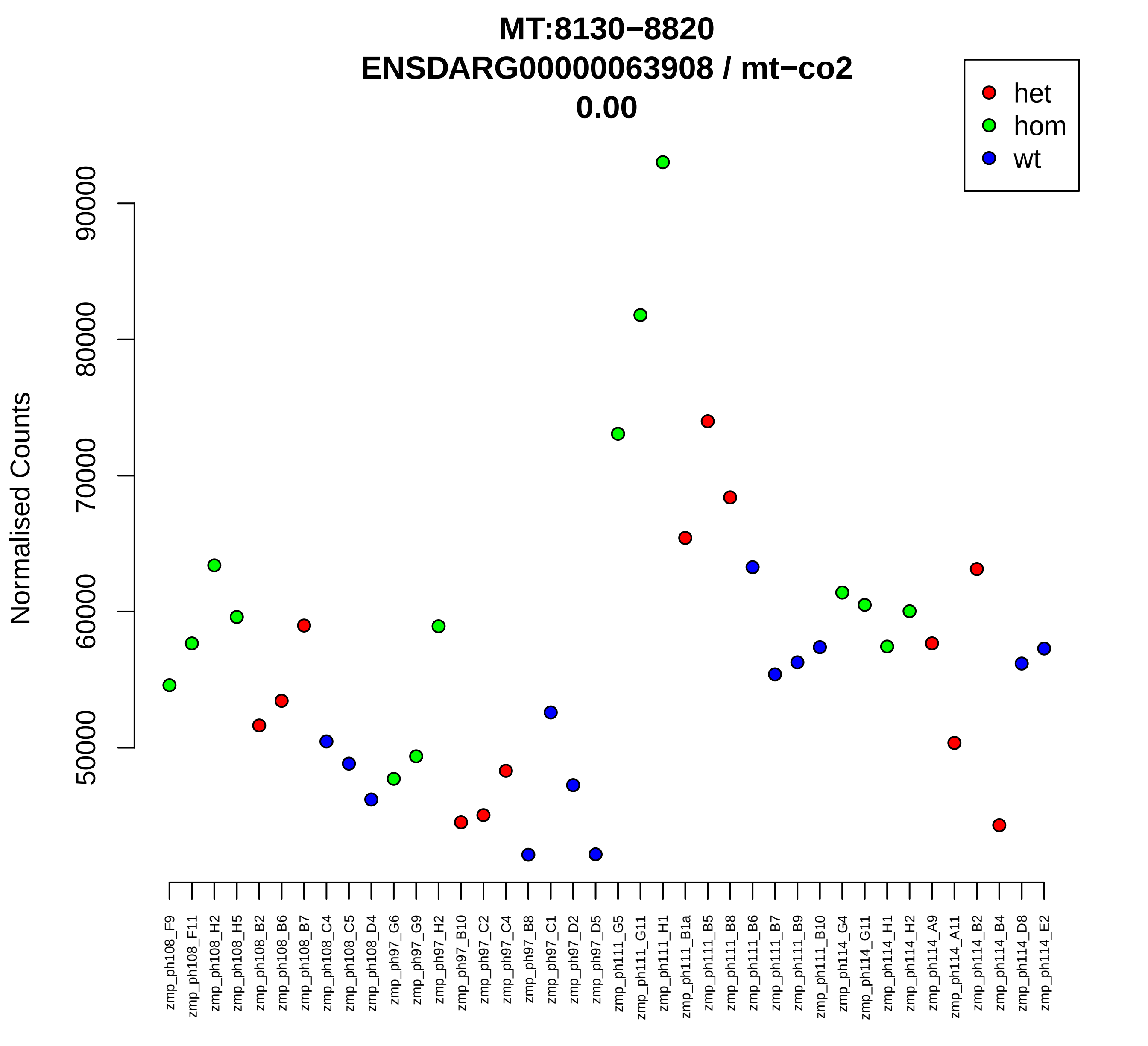
MT:8130−8820
ENSDARG00000063908 / mt−co2
0.00
het
hom
wt
Normalised Counts
50000
60000
70000
80000
90000
zmp_ph108_F9
zmp_ph108_F11
zmp_ph108_H2
zmp_ph108_H5
zmp_ph108_B2
zmp_ph108_B6
zmp_ph108_B7
zmp_ph108_C4
zmp_ph108_C5
zmp_ph108_D4
zmp_ph97_G6
zmp_ph97_G9
zmp_ph97_H2
zmp_ph97_B10
zmp_ph97_C2
zmp_ph97_C4
zmp_ph97_B8
zmp_ph97_C1
zmp_ph97_D2
zmp_ph97_D5
zmp_ph111_G5
zmp_ph111_G11
zmp_ph111_H1
zmp_ph111_B1a
zmp_ph111_B5
zmp_ph111_B8
zmp_ph111_B6
zmp_ph111_B7
zmp_ph111_B9
zmp_ph111_B10
zmp_ph114_G4
zmp_ph114_G11
zmp_ph114_H1
zmp_ph114_H2
zmp_ph114_A9
zmp_ph114_A11
zmp_ph114_B2
zmp_ph114_B4
zmp_ph114_D8
zmp_ph114_E2

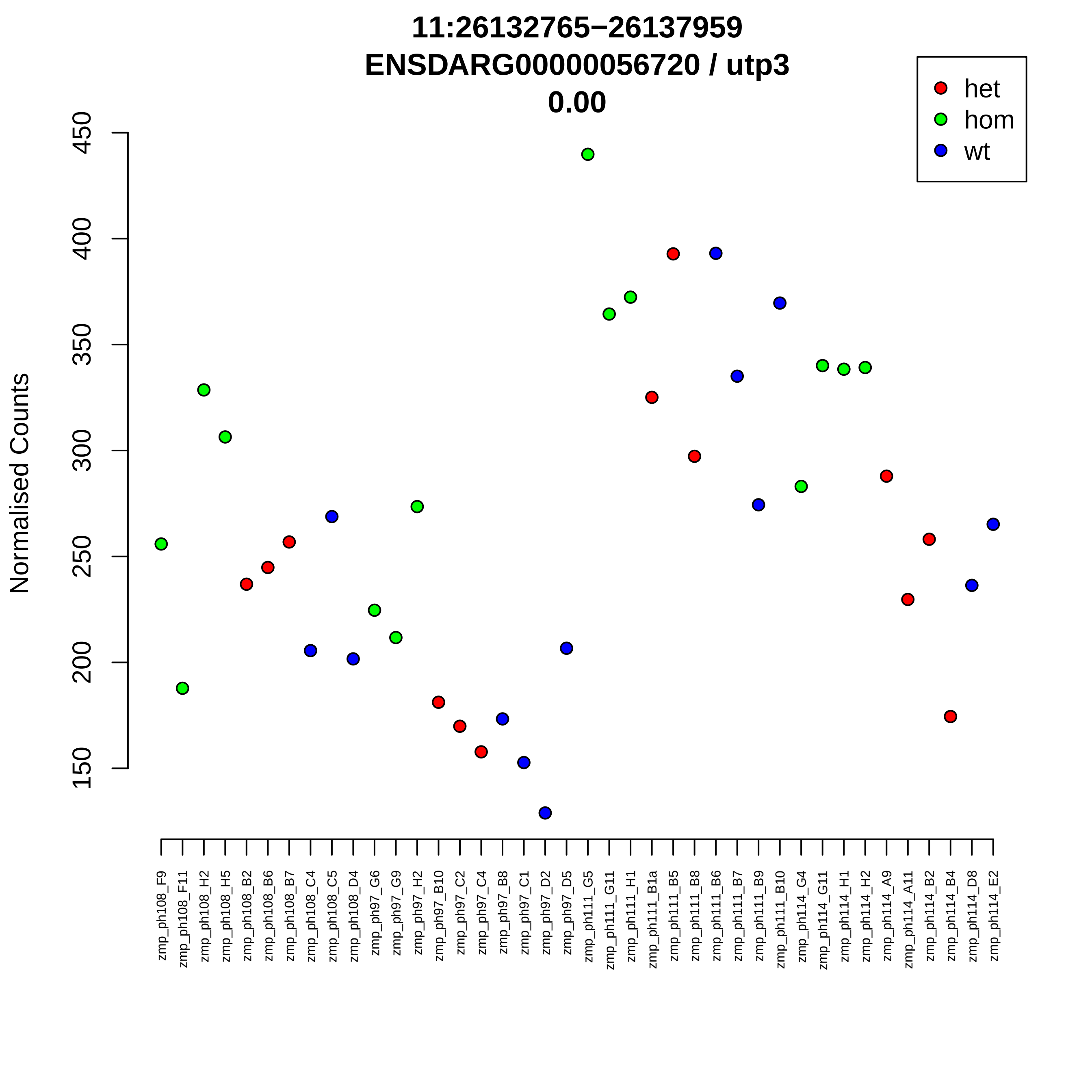
11:26132765−26137959
ENSDARG00000056720 / utp3
0.00
Normalised Counts
150
200
250
300
350
400
450
het
hom
wt
zmp_ph108_F9
zmp_ph108_F11
zmp_ph108_H2
zmp_ph108_H5
zmp_ph108_B2
zmp_ph108_B6
zmp_ph108_B7
zmp_ph108_C4
zmp_ph108_C5
zmp_ph108_D4
zmp_ph97_G6
zmp_ph97_G9
zmp_ph97_H2
zmp_ph97_B10
zmp_ph97_C2
zmp_ph97_C4
zmp_ph97_B8
zmp_ph97_C1
zmp_ph97_D2
zmp_ph97_D5
zmp_ph111_G5
zmp_ph111_G11
zmp_ph111_H1
zmp_ph111_B1a
zmp_ph111_B5
zmp_ph111_B8
zmp_ph111_B6
zmp_ph111_B7
zmp_ph111_B9
zmp_ph111_B10
zmp_ph114_G4
zmp_ph114_G11
zmp_ph114_H1
zmp_ph114_H2
zmp_ph114_A9
zmp_ph114_A11
zmp_ph114_B2
zmp_ph114_B4
zmp_ph114_D8
zmp_ph114_E2

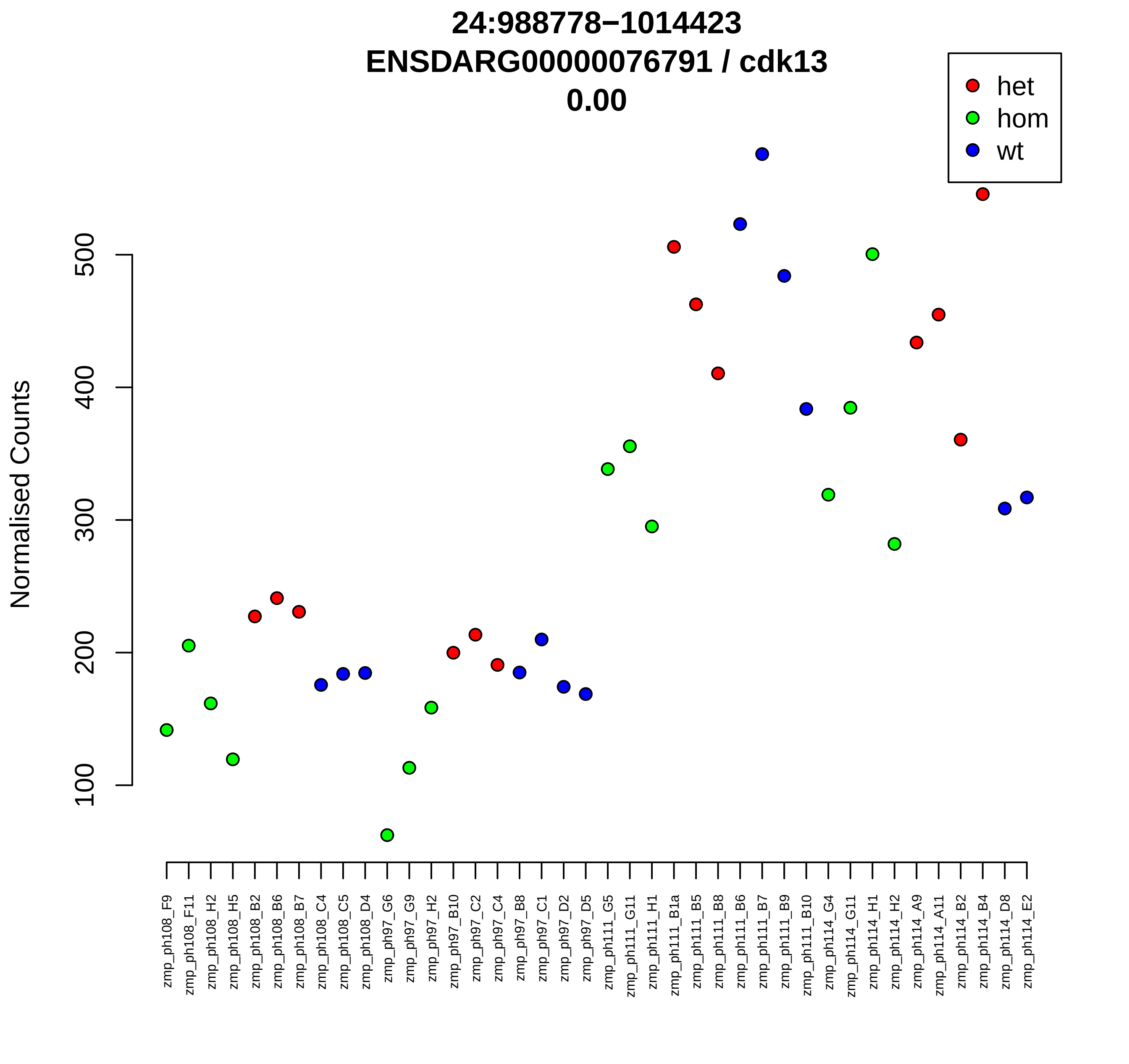
24:988778−1014423
ENSDARG00000076791 / cdk13
0.00
het
hom
wt
Normalised Counts
100
200
300
400
500
zmp_ph108_F9
zmp_ph108_F11
zmp_ph108_H2
zmp_ph108_H5
zmp_ph108_B2
zmp_ph108_B6
zmp_ph108_B7
zmp_ph108_C4
zmp_ph108_C5
zmp_ph108_D4
zmp_ph97_G6
zmp_ph97_G9
zmp_ph97_H2
zmp_ph97_B10
zmp_ph97_C2
zmp_ph97_C4
zmp_ph97_B8
zmp_ph97_C1
zmp_ph97_D2
zmp_ph97_D5
zmp_ph111_G5
zmp_ph111_G11
zmp_ph111_H1
zmp_ph111_B1a
zmp_ph111_B5
zmp_ph111_B8
zmp_ph111_B6
zmp_ph111_B7
zmp_ph111_B9
zmp_ph111_B10
zmp_ph114_G4
zmp_ph114_G11
zmp_ph114_H1
zmp_ph114_H2
zmp_ph114_A9
zmp_ph114_A11
zmp_ph114_B2
zmp_ph114_B4
zmp_ph114_D8
zmp_ph114_E2

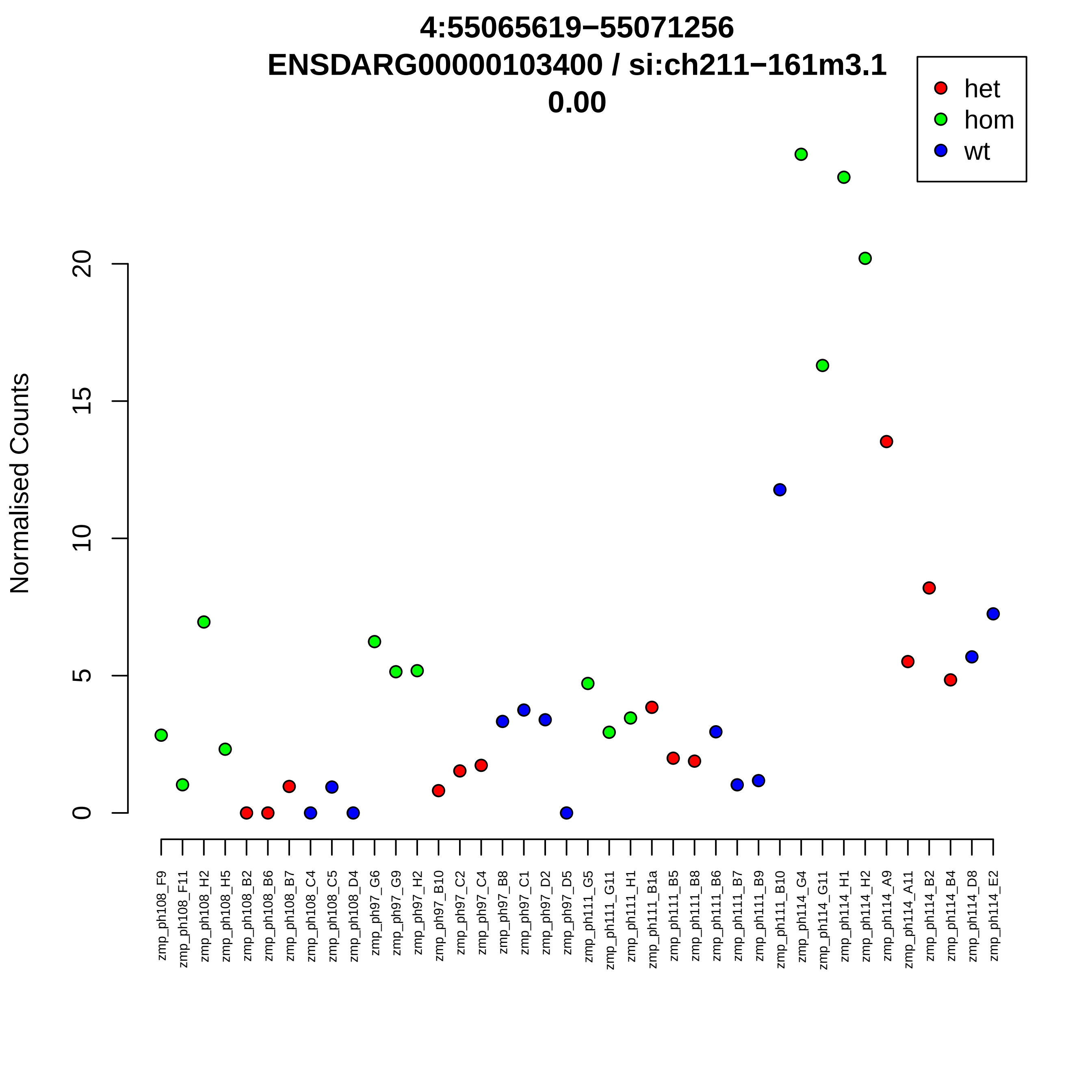

4:55065619−55071256
ENSDARG00000103400 / si:ch211−161m3.1
0.00
het
hom
wt
Normalised Counts
0
5
10
15
20
zmp_ph108_F9
zmp_ph108_F11
zmp_ph108_H2
zmp_ph108_H5
zmp_ph108_B2
zmp_ph108_B6
zmp_ph108_B7
zmp_ph108_C4
zmp_ph108_C5
zmp_ph108_D4
zmp_ph97_G6
zmp_ph97_G9
zmp_ph97_H2
zmp_ph97_B10
zmp_ph97_C2
zmp_ph97_C4
zmp_ph97_B8
zmp_ph97_C1
zmp_ph97_D2
zmp_ph97_D5
zmp_ph111_G5
zmp_ph111_G11
zmp_ph111_H1
zmp_ph111_B1a
zmp_ph111_B5
zmp_ph111_B8
zmp_ph111_B6
zmp_ph111_B7
zmp_ph111_B9
zmp_ph111_B10
zmp_ph114_G4
zmp_ph114_G11
zmp_ph114_H1
zmp_ph114_H2
zmp_ph114_A9
zmp_ph114_A11
zmp_ph114_B2
zmp_ph114_B4
zmp_ph114_D8
zmp_ph114_E2

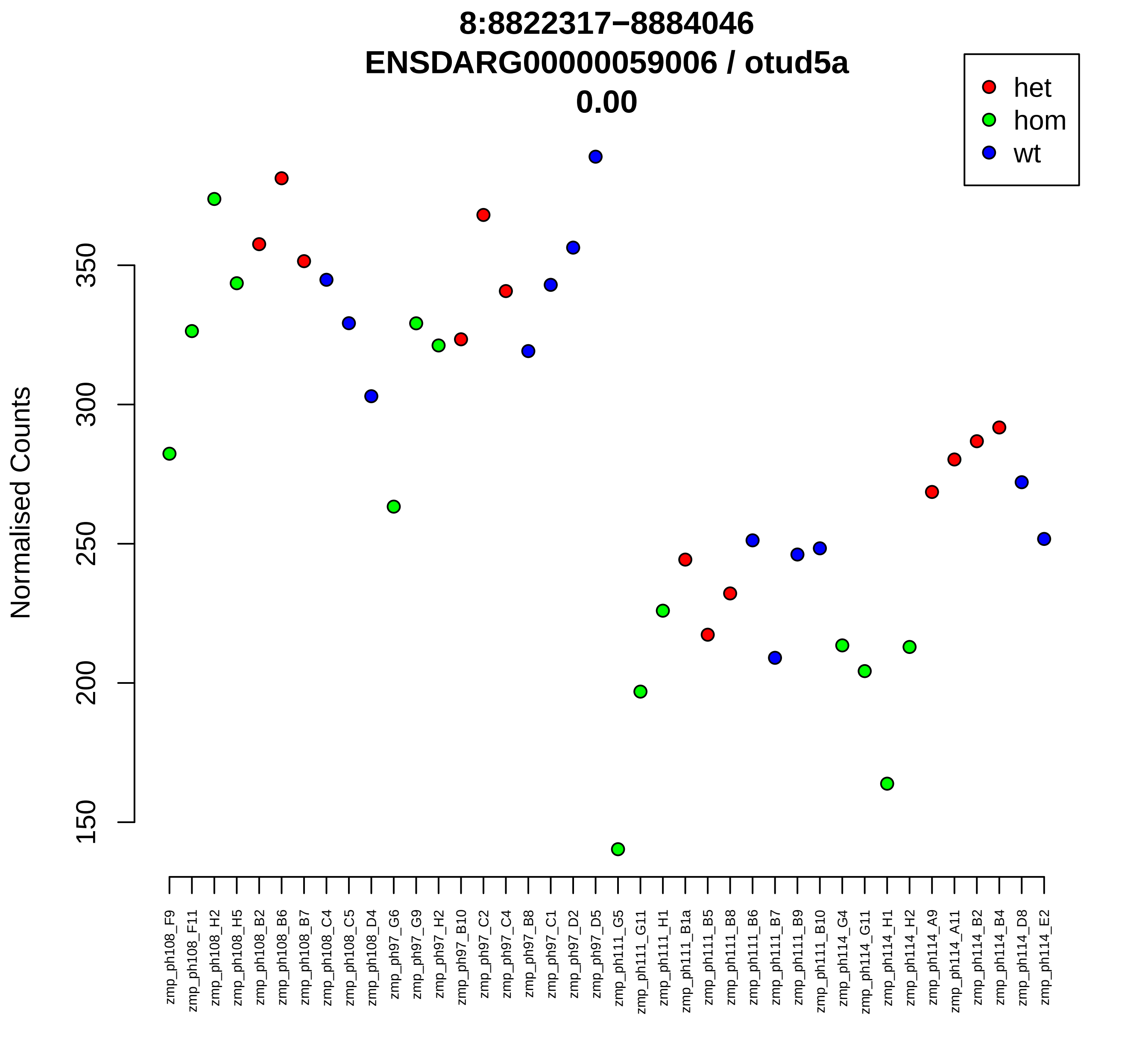

8:8822317−8884046
ENSDARG00000059006 / otud5a
0.00
het
hom
wt
Normalised Counts
150
200
250
300
350
zmp_ph108_F9
zmp_ph108_F11
zmp_ph108_H2
zmp_ph108_H5
zmp_ph108_B2
zmp_ph108_B6
zmp_ph108_B7
zmp_ph108_C4
zmp_ph108_C5
zmp_ph108_D4
zmp_ph97_G6
zmp_ph97_G9
zmp_ph97_H2
zmp_ph97_B10
zmp_ph97_C2
zmp_ph97_C4
zmp_ph97_B8
zmp_ph97_C1
zmp_ph97_D2
zmp_ph97_D5
zmp_ph111_G5
zmp_ph111_G11
zmp_ph111_H1
zmp_ph111_B1a
zmp_ph111_B5
zmp_ph111_B8
zmp_ph111_B6
zmp_ph111_B7
zmp_ph111_B9
zmp_ph111_B10
zmp_ph114_G4
zmp_ph114_G11
zmp_ph114_H1
zmp_ph114_H2
zmp_ph114_A9
zmp_ph114_A11
zmp_ph114_B2
zmp_ph114_B4
zmp_ph114_D8
zmp_ph114_E2

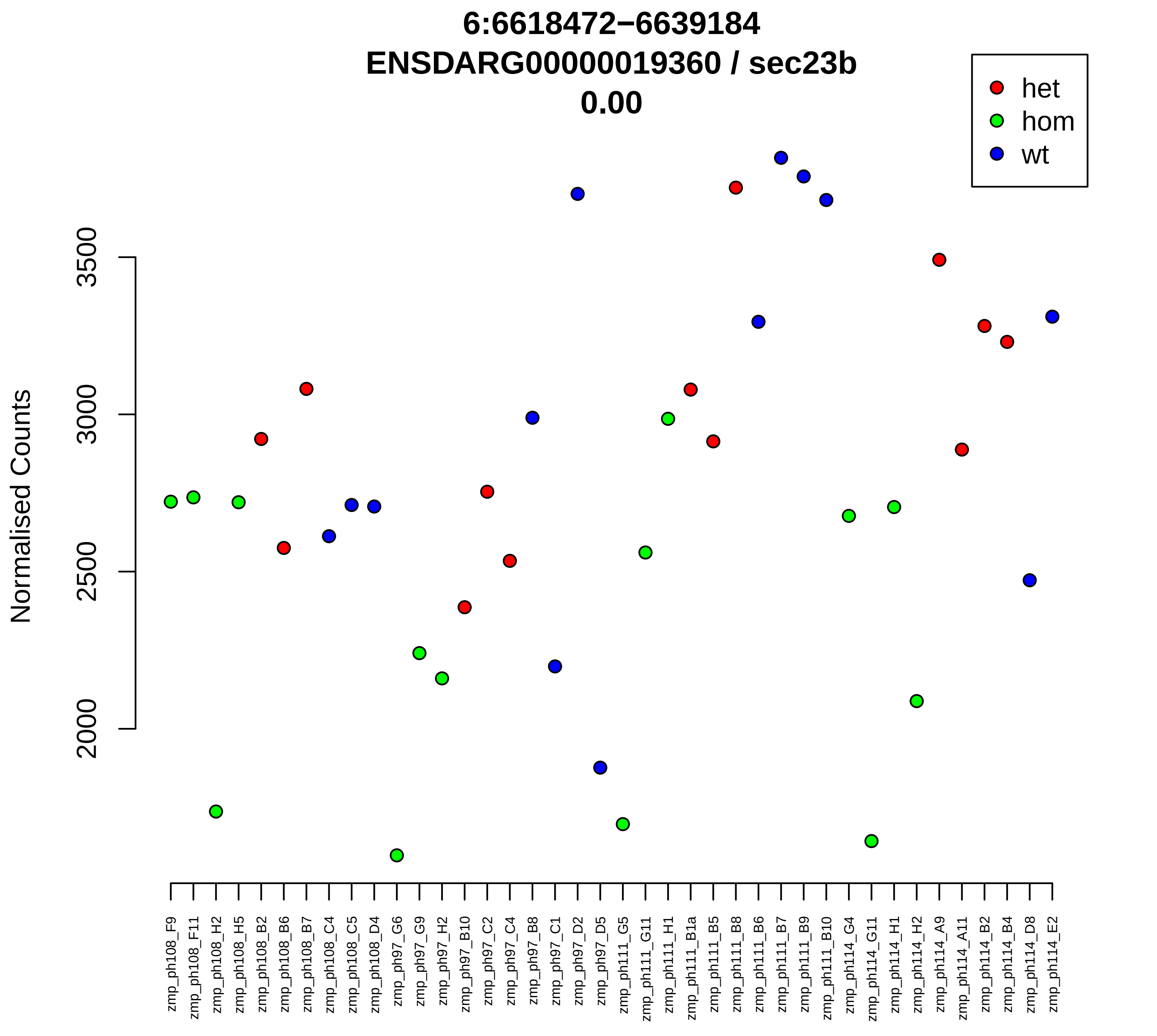
6:6618472−6639184
ENSDARG00000019360 / sec23b
0.00
het
hom
wt
Normalised Counts
2000
2500
3000
3500
zmp_ph108_F9
zmp_ph108_F11
zmp_ph108_H2
zmp_ph108_H5
zmp_ph108_B2
zmp_ph108_B6
zmp_ph108_B7
zmp_ph108_C4
zmp_ph108_C5
zmp_ph108_D4
zmp_ph97_G6
zmp_ph97_G9
zmp_ph97_H2
zmp_ph97_B10
zmp_ph97_C2
zmp_ph97_C4
zmp_ph97_B8
zmp_ph97_C1
zmp_ph97_D2
zmp_ph97_D5
zmp_ph111_G5
zmp_ph111_G11
zmp_ph111_H1
zmp_ph111_B1a
zmp_ph111_B5
zmp_ph111_B8
zmp_ph111_B6
zmp_ph111_B7
zmp_ph111_B9
zmp_ph111_B10
zmp_ph114_G4
zmp_ph114_G11
zmp_ph114_H1
zmp_ph114_H2
zmp_ph114_A9
zmp_ph114_A11
zmp_ph114_B2
zmp_ph114_B4
zmp_ph114_D8
zmp_ph114_E2

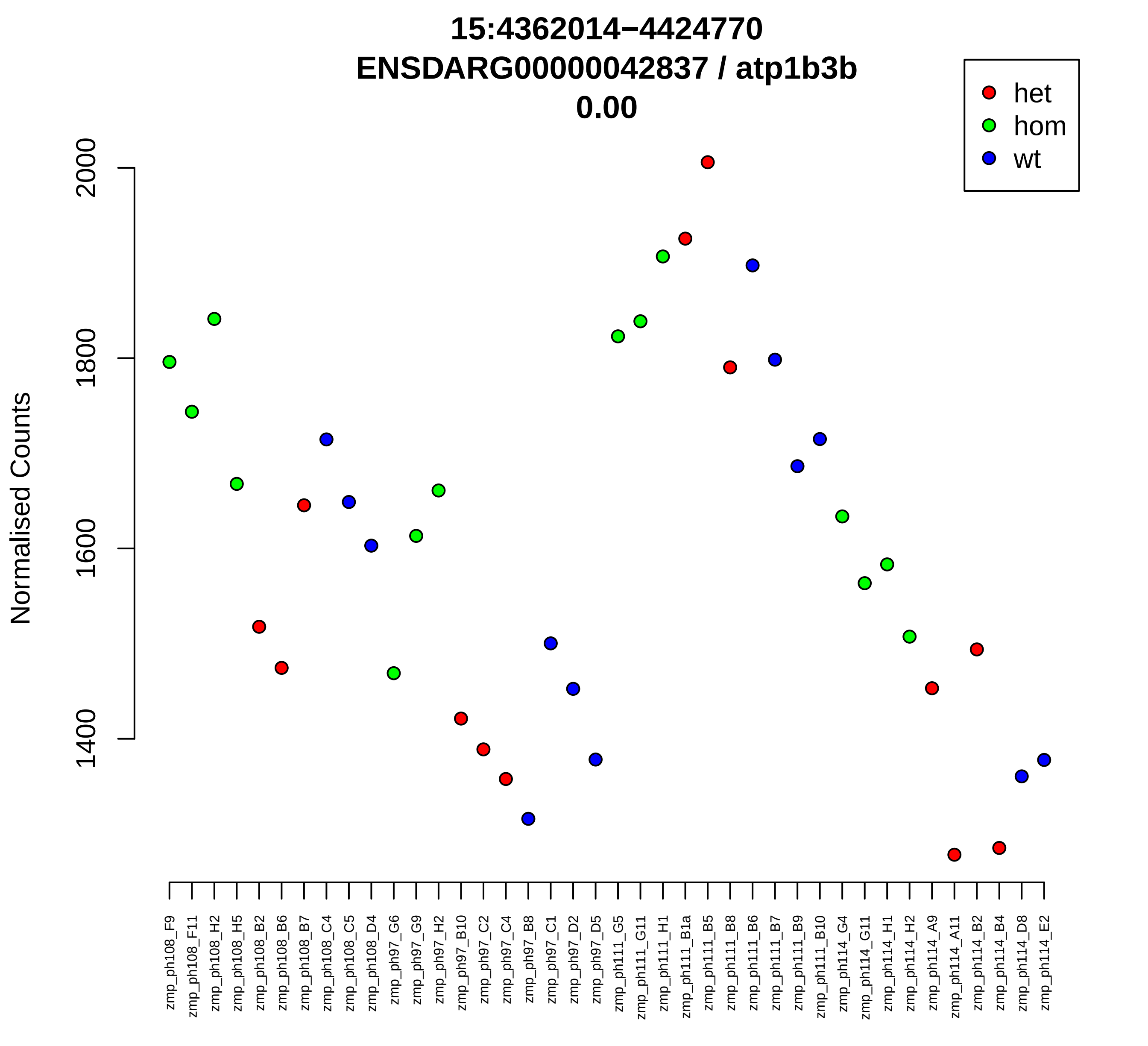
15:4362014−4424770
ENSDARG00000042837 / atp1b3b
0.00
het
hom
wt
Normalised Counts
2000
1800
1600
1400
zmp_ph108_F9
zmp_ph108_F11
zmp_ph108_H2
zmp_ph108_H5
zmp_ph108_B2
zmp_ph108_B6
zmp_ph108_B7
zmp_ph108_C4
zmp_ph108_C5
zmp_ph108_D4
zmp_ph97_G6
zmp_ph97_G9
zmp_ph97_H2
zmp_ph97_B10
zmp_ph97_C2
zmp_ph97_C4
zmp_ph97_B8
zmp_ph97_C1
zmp_ph97_D2
zmp_ph97_D5
zmp_ph111_G5
zmp_ph111_G11
zmp_ph111_H1
zmp_ph111_B1a
zmp_ph111_B5
zmp_ph111_B8
zmp_ph111_B6
zmp_ph111_B7
zmp_ph111_B9
zmp_ph111_B10
zmp_ph114_G4
zmp_ph114_G11
zmp_ph114_H1
zmp_ph114_H2
zmp_ph114_A9
zmp_ph114_A11
zmp_ph114_B2
zmp_ph114_B4
zmp_ph114_D8
zmp_ph114_E2

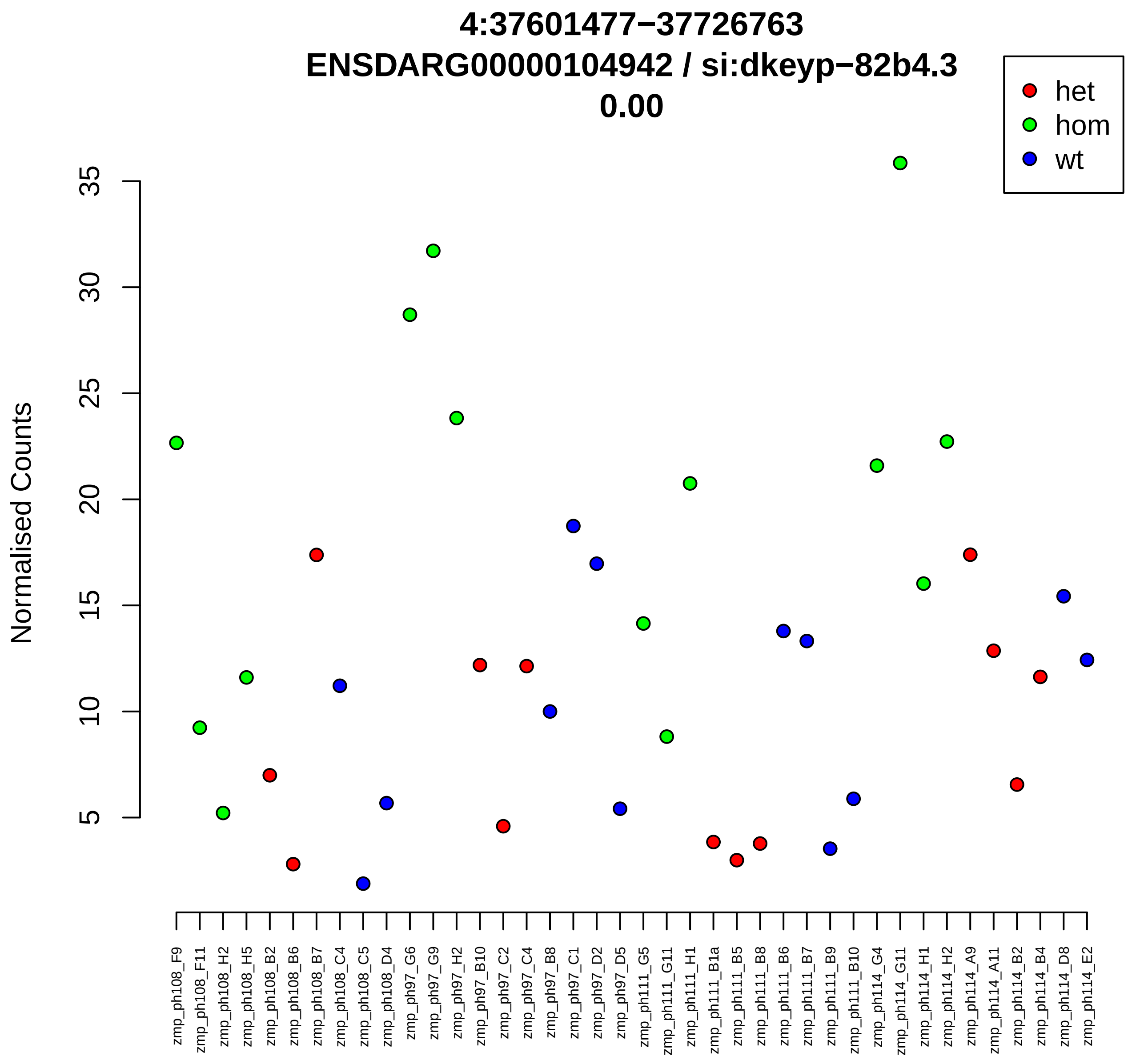
4:37601477−37726763
ENSDARG00000104942 / si:dkeyp−82b4.3
0.00
het
hom
wt
Normalised Counts
5
10
15
20
25
30
35
zmp_ph108_F9
zmp_ph108_F11
zmp_ph108_H2
zmp_ph108_H5
zmp_ph108_B2
zmp_ph108_B6
zmp_ph108_B7
zmp_ph108_C4
zmp_ph108_C5
zmp_ph108_D4
zmp_ph97_G6
zmp_ph97_G9
zmp_ph97_H2
zmp_ph97_B10
zmp_ph97_C2
zmp_ph97_C4
zmp_ph97_B8
zmp_ph97_C1
zmp_ph97_D2
zmp_ph97_D5
zmp_ph111_G5
zmp_ph111_G11
zmp_ph111_H1
zmp_ph111_B1a
zmp_ph111_B5
zmp_ph111_B8
zmp_ph111_B6
zmp_ph111_B7
zmp_ph111_B9
zmp_ph111_B10
zmp_ph114_G4
zmp_ph114_G11
zmp_ph114_H1
zmp_ph114_H2
zmp_ph114_A9
zmp_ph114_A11
zmp_ph114_B2
zmp_ph114_B4
zmp_ph114_D8
zmp_ph114_E2

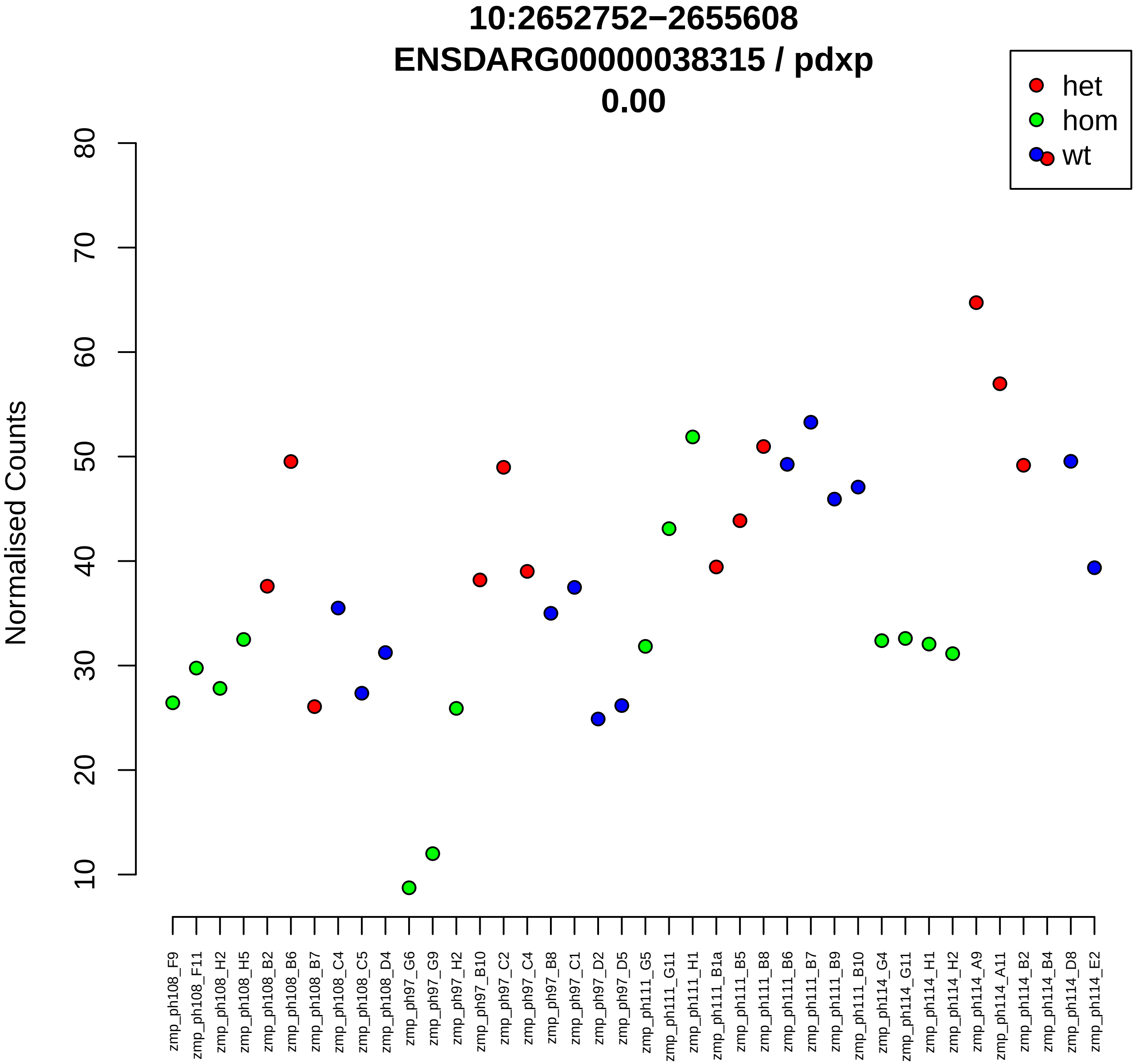

10:2652752−2655608
ENSDARG00000038315 / pdxp
0.00
het
hom
wt
Normalised Counts
10
20
30
40
50
60
70
80
zmp_ph108_F9
zmp_ph108_F11
zmp_ph108_H2
zmp_ph108_H5
zmp_ph108_B2
zmp_ph108_B6
zmp_ph108_B7
zmp_ph108_C4
zmp_ph108_C5
zmp_ph108_D4
zmp_ph97_G6
zmp_ph97_G9
zmp_ph97_H2
zmp_ph97_B10
zmp_ph97_C2
zmp_ph97_C4
zmp_ph97_B8
zmp_ph97_C1
zmp_ph97_D2
zmp_ph97_D5
zmp_ph111_G5
zmp_ph111_G11
zmp_ph111_H1
zmp_ph111_B1a
zmp_ph111_B5
zmp_ph111_B8
zmp_ph111_B6
zmp_ph111_B7
zmp_ph111_B9
zmp_ph111_B10
zmp_ph114_G4
zmp_ph114_G11
zmp_ph114_H1
zmp_ph114_H2
zmp_ph114_A9
zmp_ph114_A11
zmp_ph114_B2
zmp_ph114_B4
zmp_ph114_D8
zmp_ph114_E2

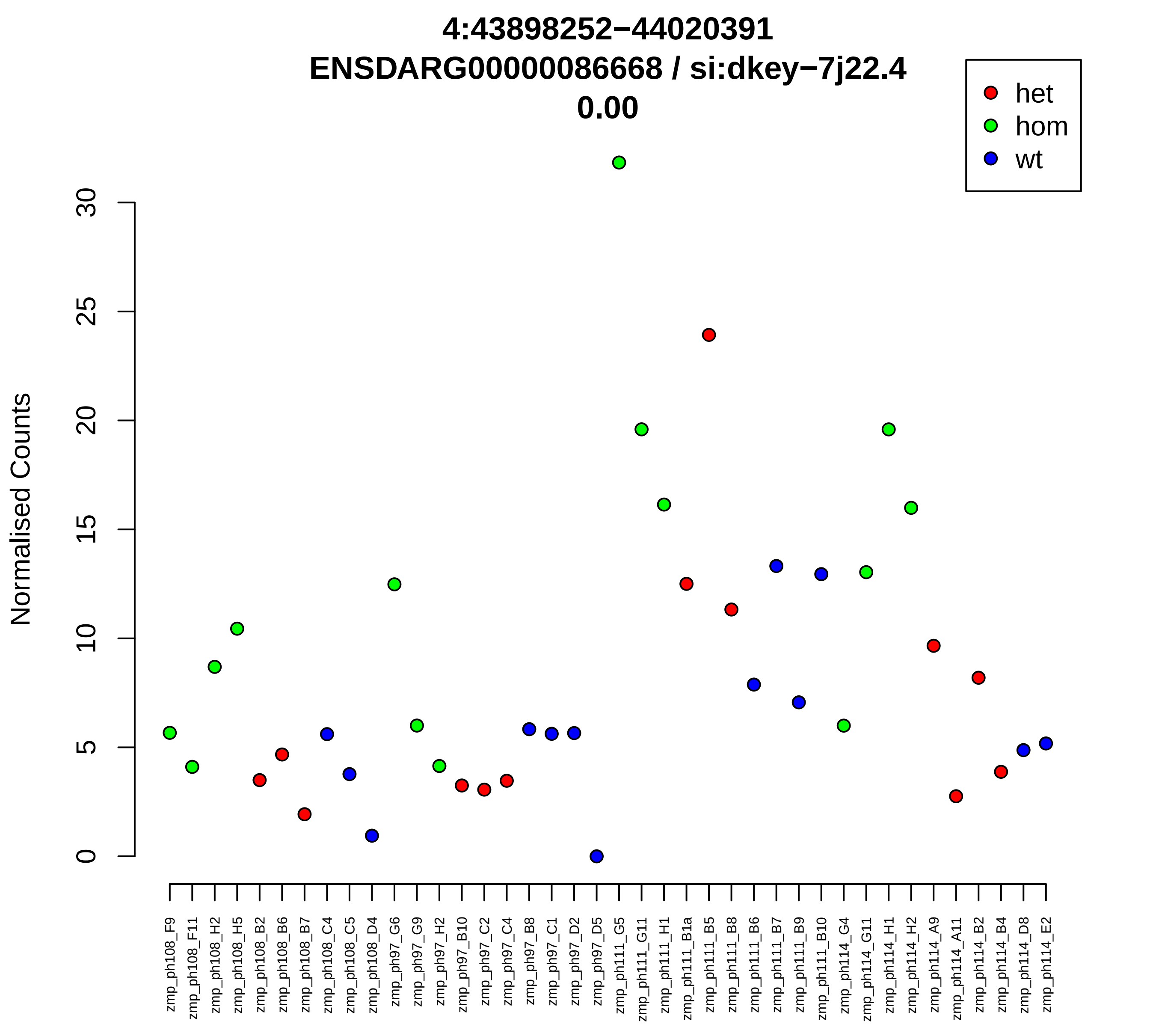

4:43898252−44020391
ENSDARG00000086668 / si:dkey−7j22.4
0.00
het
hom
wt
Normalised Counts
0
5
10
15
20
25
30
zmp_ph108_F9
zmp_ph108_F11
zmp_ph108_H2
zmp_ph108_H5
zmp_ph108_B2
zmp_ph108_B6
zmp_ph108_B7
zmp_ph108_C4
zmp_ph108_C5
zmp_ph108_D4
zmp_ph97_G6
zmp_ph97_G9
zmp_ph97_H2
zmp_ph97_B10
zmp_ph97_C2
zmp_ph97_C4
zmp_ph97_B8
zmp_ph97_C1
zmp_ph97_D2
zmp_ph97_D5
zmp_ph111_G5
zmp_ph111_G11
zmp_ph111_H1
zmp_ph111_B1a
zmp_ph111_B5
zmp_ph111_B8
zmp_ph111_B6
zmp_ph111_B7
zmp_ph111_B9
zmp_ph111_B10
zmp_ph114_G4
zmp_ph114_G11
zmp_ph114_H1
zmp_ph114_H2
zmp_ph114_A9
zmp_ph114_A11
zmp_ph114_B2
zmp_ph114_B4
zmp_ph114_D8
zmp_ph114_E2

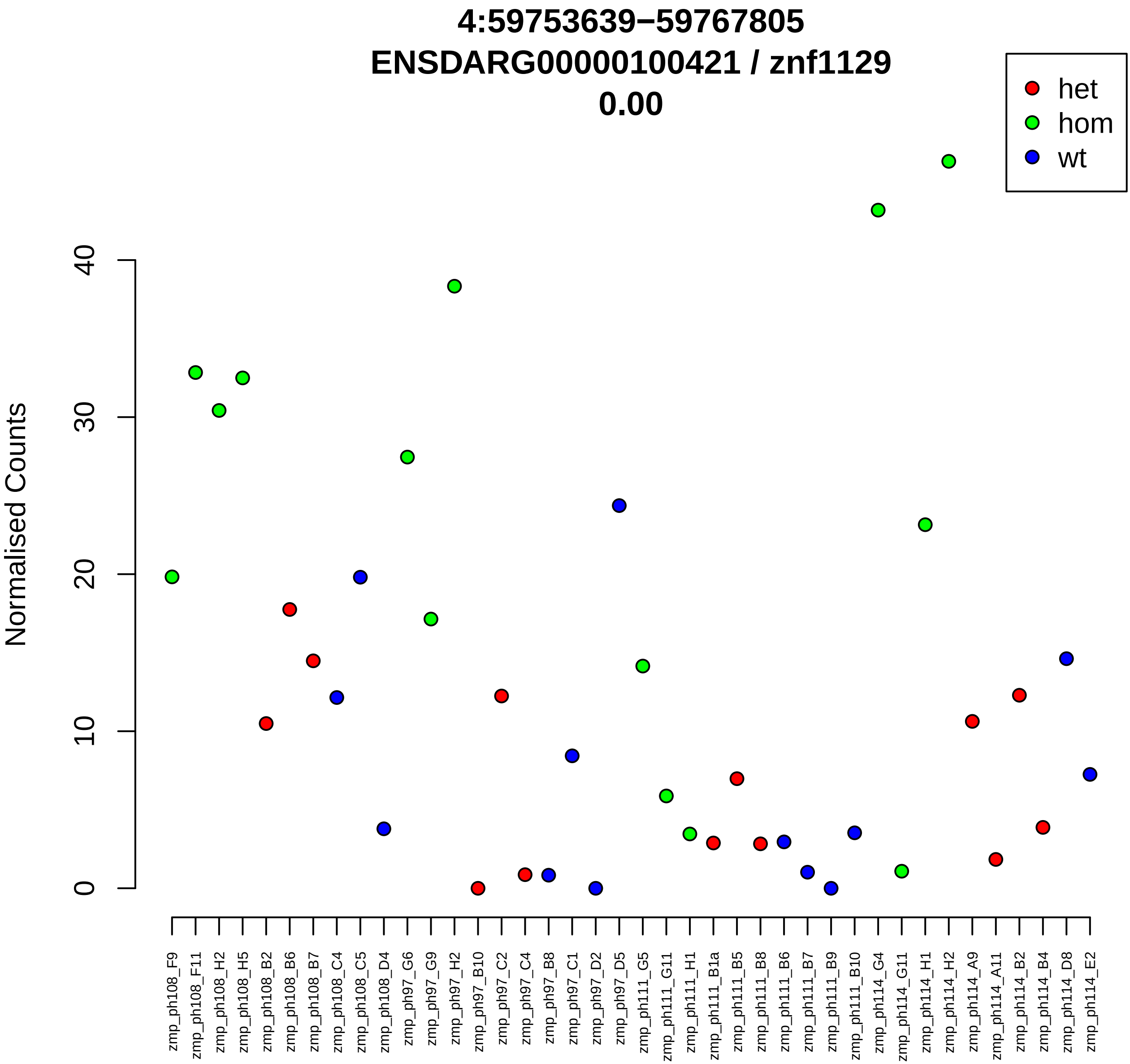
4:59753639−59767805
ENSDARG00000100421 / znf1129
0.00
het
hom
wt
Normalised Counts
0
10
20
30
40
zmp_ph108_F9
zmp_ph108_F11
zmp_ph108_H2
zmp_ph108_H5
zmp_ph108_B2
zmp_ph108_B6
zmp_ph108_B7
zmp_ph108_C4
zmp_ph108_C5
zmp_ph108_D4
zmp_ph97_G6
zmp_ph97_G9
zmp_ph97_H2
zmp_ph97_B10
zmp_ph97_C2
zmp_ph97_C4
zmp_ph97_B8
zmp_ph97_C1
zmp_ph97_D2
zmp_ph97_D5
zmp_ph111_G5
zmp_ph111_G11
zmp_ph111_H1
zmp_ph111_B1a
zmp_ph111_B5
zmp_ph111_B8
zmp_ph111_B6
zmp_ph111_B7
zmp_ph111_B9
zmp_ph111_B10
zmp_ph114_G4
zmp_ph114_G11
zmp_ph114_H1
zmp_ph114_H2
zmp_ph114_A9
zmp_ph114_A11
zmp_ph114_B2
zmp_ph114_B4
zmp_ph114_D8
zmp_ph114_E2

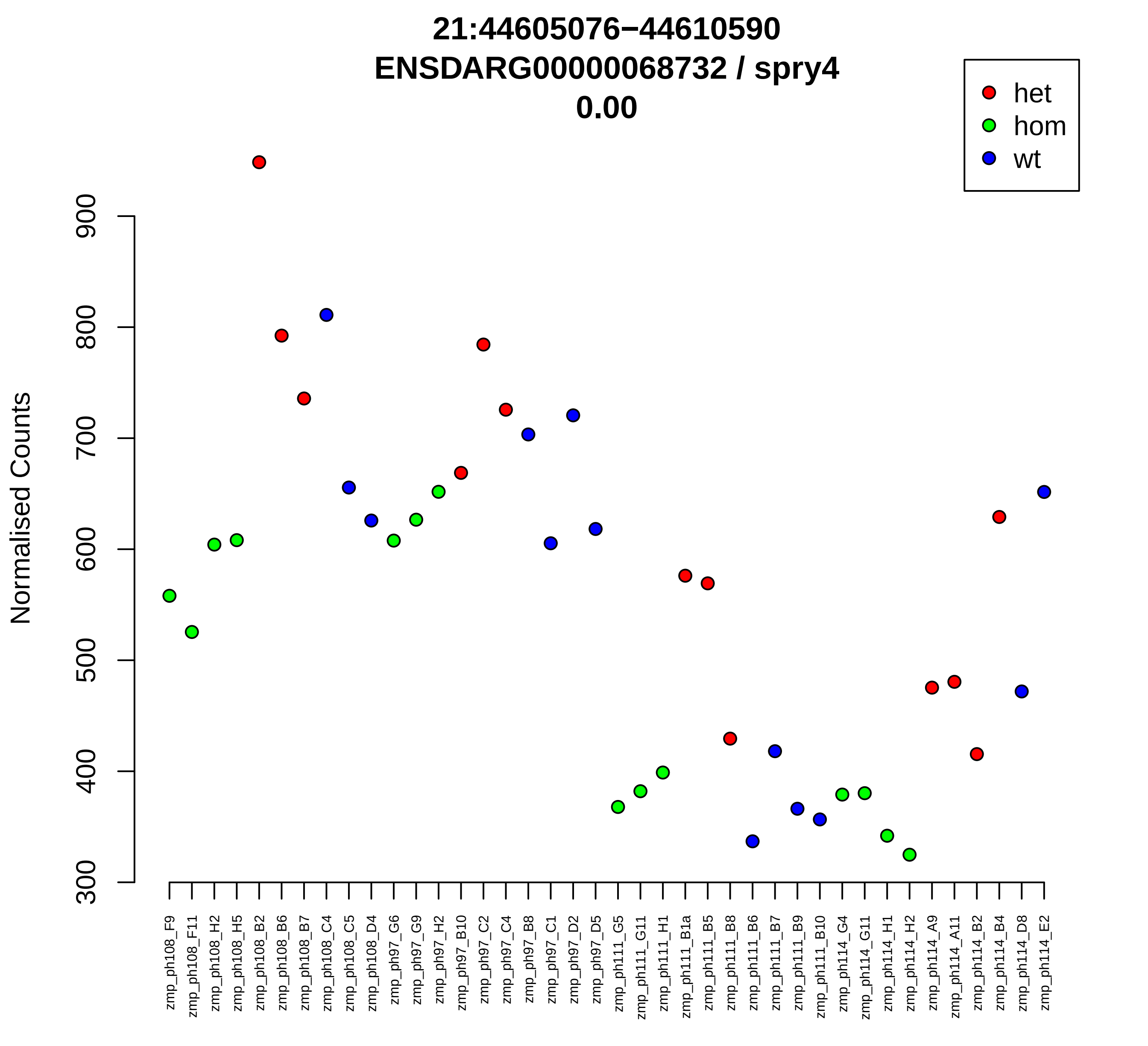

21:44605076−44610590
ENSDARG00000068732 / spry4
0.00
het
hom
wt
Normalised Counts
300
400
500
600
700
800
900
zmp_ph108_F9
zmp_ph108_F11
zmp_ph108_H2
zmp_ph108_H5
zmp_ph108_B2
zmp_ph108_B6
zmp_ph108_B7
zmp_ph108_C4
zmp_ph108_C5
zmp_ph108_D4
zmp_ph97_G6
zmp_ph97_G9
zmp_ph97_H2
zmp_ph97_B10
zmp_ph97_C2
zmp_ph97_C4
zmp_ph97_B8
zmp_ph97_C1
zmp_ph97_D2
zmp_ph97_D5
zmp_ph111_G5
zmp_ph111_G11
zmp_ph111_H1
zmp_ph111_B1a
zmp_ph111_B5
zmp_ph111_B8
zmp_ph111_B6
zmp_ph111_B7
zmp_ph111_B9
zmp_ph111_B10
zmp_ph114_G4
zmp_ph114_G11
zmp_ph114_H1
zmp_ph114_H2
zmp_ph114_A9
zmp_ph114_A11
zmp_ph114_B2
zmp_ph114_B4
zmp_ph114_D8
zmp_ph114_E2

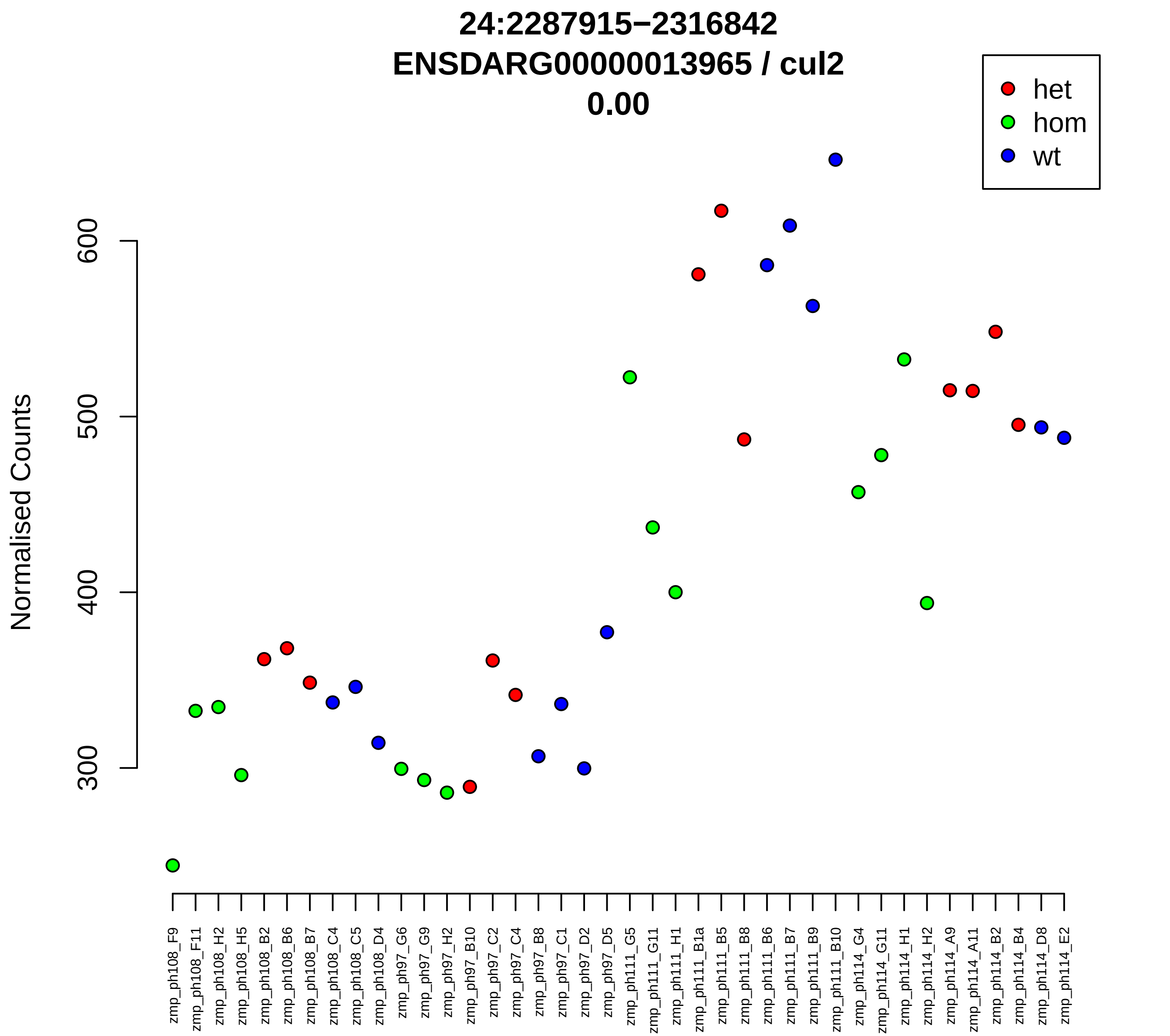

24:2287915−2316842
ENSDARG00000013965 / cul2
0.00
het
hom
wt
Normalised Counts
300
400
500
600
zmp_ph108_F9
zmp_ph108_F11
zmp_ph108_H2
zmp_ph108_H5
zmp_ph108_B2
zmp_ph108_B6
zmp_ph108_B7
zmp_ph108_C4
zmp_ph108_C5
zmp_ph108_D4
zmp_ph97_G6
zmp_ph97_G9
zmp_ph97_H2
zmp_ph97_B10
zmp_ph97_C2
zmp_ph97_C4
zmp_ph97_B8
zmp_ph97_C1
zmp_ph97_D2
zmp_ph97_D5
zmp_ph111_G5
zmp_ph111_G11
zmp_ph111_H1
zmp_ph111_B1a
zmp_ph111_B5
zmp_ph111_B8
zmp_ph111_B6
zmp_ph111_B7
zmp_ph111_B9
zmp_ph111_B10
zmp_ph114_G4
zmp_ph114_G11
zmp_ph114_H1
zmp_ph114_H2
zmp_ph114_A9
zmp_ph114_A11
zmp_ph114_B2
zmp_ph114_B4
zmp_ph114_D8
zmp_ph114_E2

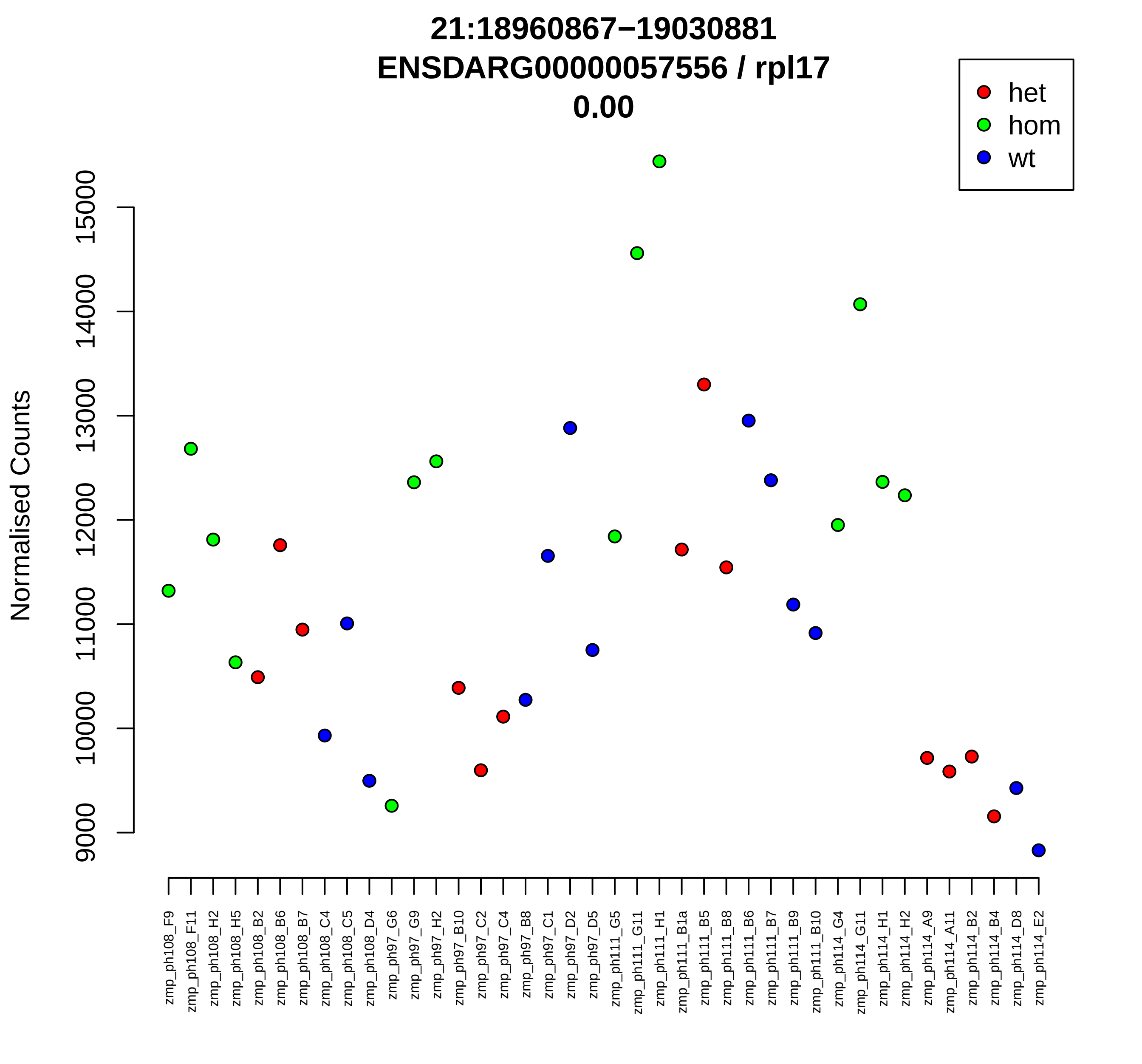
21:18960867−19030881
ENSDARG00000057556 / rpl17
0.00
het
hom
wt
Normalised Counts
9000
10000
11000
12000
13000
14000
15000
zmp_ph108_F9
zmp_ph108_F11
zmp_ph108_H2
zmp_ph108_H5
zmp_ph108_B2
zmp_ph108_B6
zmp_ph108_B7
zmp_ph108_C4
zmp_ph108_C5
zmp_ph108_D4
zmp_ph97_G6
zmp_ph97_G9
zmp_ph97_H2
zmp_ph97_B10
zmp_ph97_C2
zmp_ph97_C4
zmp_ph97_B8
zmp_ph97_C1
zmp_ph97_D2
zmp_ph97_D5
zmp_ph111_G5
zmp_ph111_G11
zmp_ph111_H1
zmp_ph111_B1a
zmp_ph111_B5
zmp_ph111_B8
zmp_ph111_B6
zmp_ph111_B7
zmp_ph111_B9
zmp_ph111_B10
zmp_ph114_G4
zmp_ph114_G11
zmp_ph114_H1
zmp_ph114_H2
zmp_ph114_A9
zmp_ph114_A11
zmp_ph114_B2
zmp_ph114_B4
zmp_ph114_D8
zmp_ph114_E2

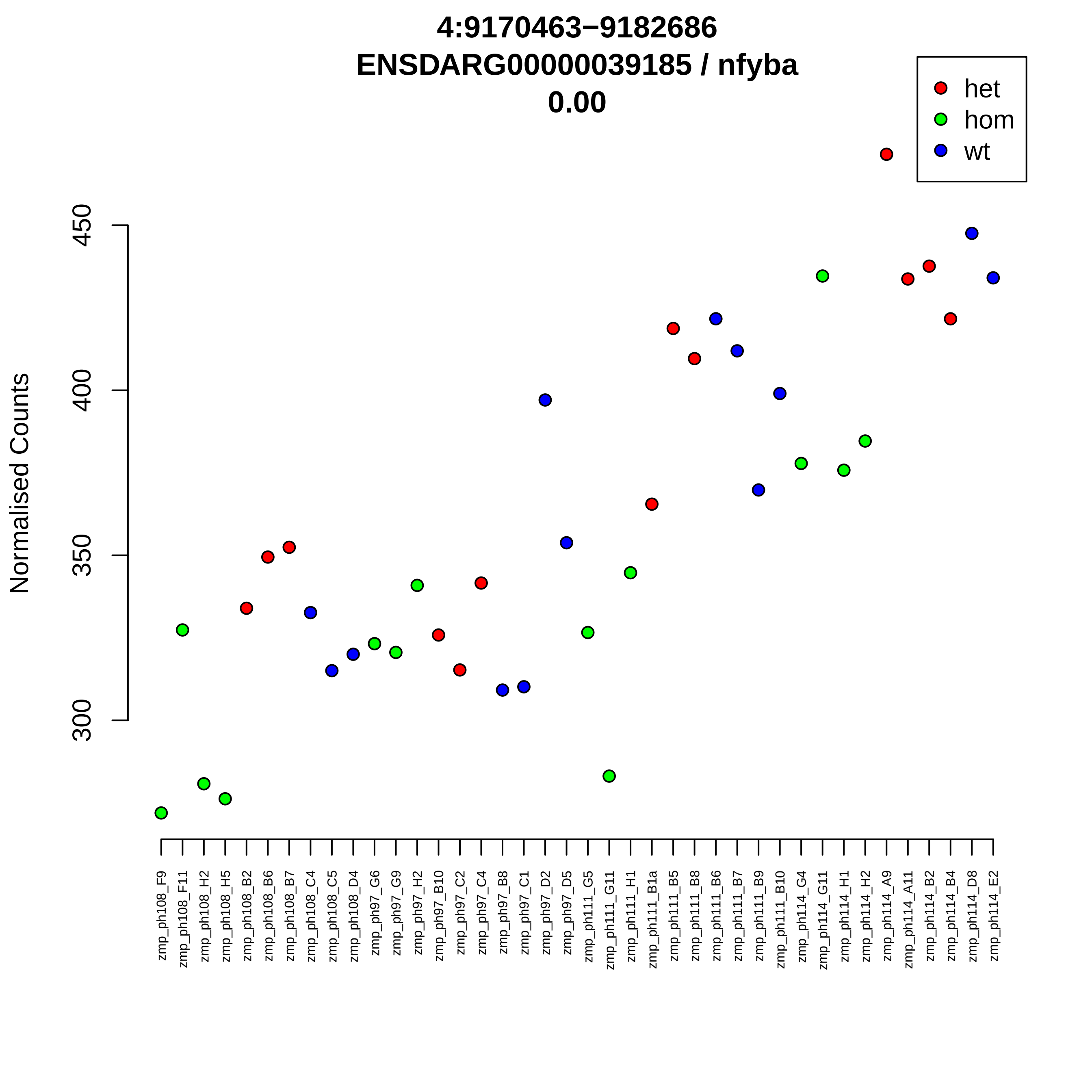

4:9170463−9182686
ENSDARG00000039185 / nfyba
0.00
het
hom
wt
Normalised Counts
300
350
400
450
zmp_ph108_F9
zmp_ph108_F11
zmp_ph108_H2
zmp_ph108_H5
zmp_ph108_B2
zmp_ph108_B6
zmp_ph108_B7
zmp_ph108_C4
zmp_ph108_C5
zmp_ph108_D4
zmp_ph97_G6
zmp_ph97_G9
zmp_ph97_H2
zmp_ph97_B10
zmp_ph97_C2
zmp_ph97_C4
zmp_ph97_B8
zmp_ph97_C1
zmp_ph97_D2
zmp_ph97_D5
zmp_ph111_G5
zmp_ph111_G11
zmp_ph111_H1
zmp_ph111_B1a
zmp_ph111_B5
zmp_ph111_B8
zmp_ph111_B6
zmp_ph111_B7
zmp_ph111_B9
zmp_ph111_B10
zmp_ph114_G4
zmp_ph114_G11
zmp_ph114_H1
zmp_ph114_H2
zmp_ph114_A9
zmp_ph114_A11
zmp_ph114_B2
zmp_ph114_B4
zmp_ph114_D8
zmp_ph114_E2

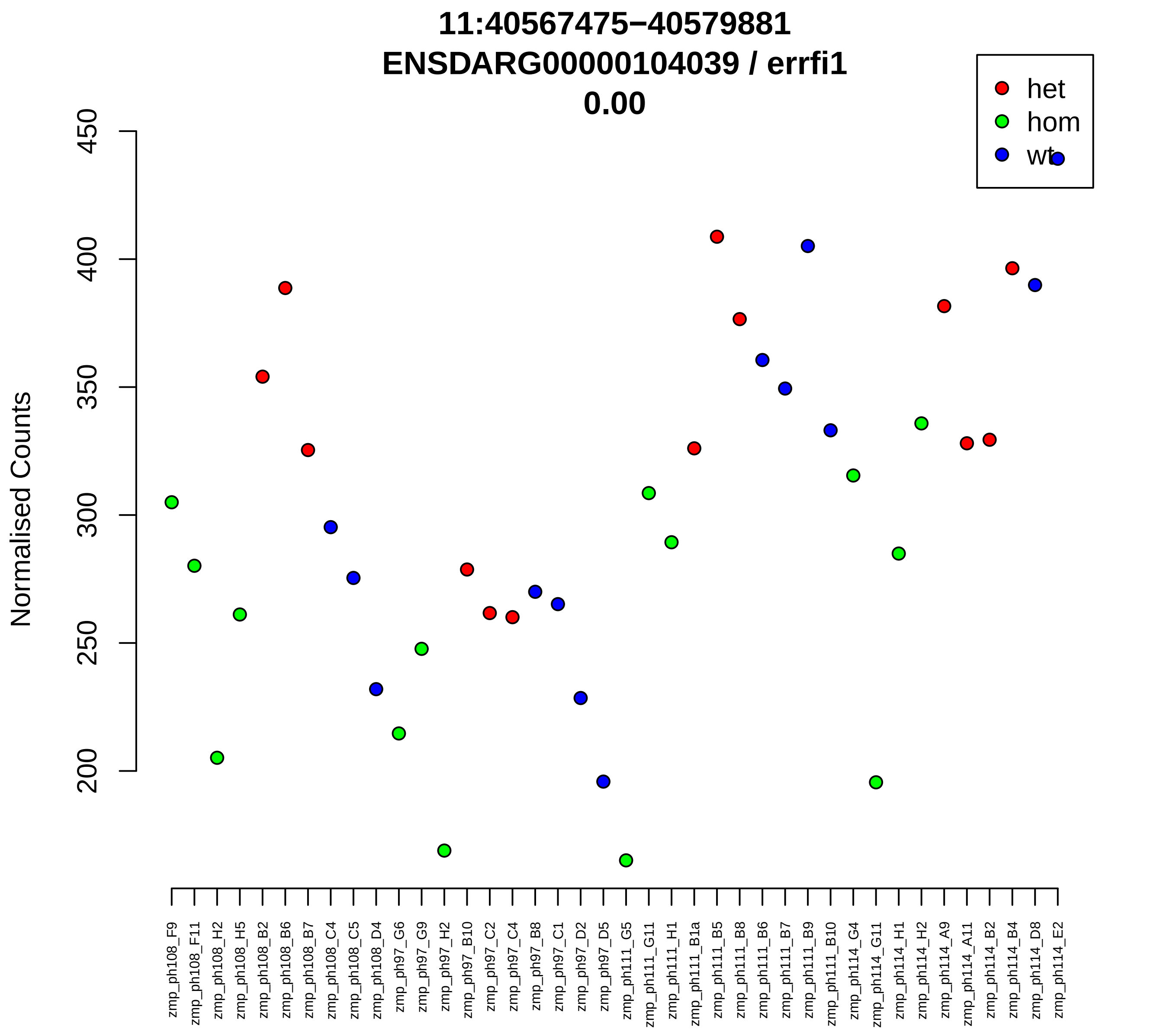

11:40567475−40579881
ENSDARG00000104039 / errfi1
0.00
het
hom
wt
Normalised Counts
200
250
300
350
400
450
zmp_ph108_F9
zmp_ph108_F11
zmp_ph108_H2
zmp_ph108_H5
zmp_ph108_B2
zmp_ph108_B6
zmp_ph108_B7
zmp_ph108_C4
zmp_ph108_C5
zmp_ph108_D4
zmp_ph97_G6
zmp_ph97_G9
zmp_ph97_H2
zmp_ph97_B10
zmp_ph97_C2
zmp_ph97_C4
zmp_ph97_B8
zmp_ph97_C1
zmp_ph97_D2
zmp_ph97_D5
zmp_ph111_G5
zmp_ph111_G11
zmp_ph111_H1
zmp_ph111_B1a
zmp_ph111_B5
zmp_ph111_B8
zmp_ph111_B6
zmp_ph111_B7
zmp_ph111_B9
zmp_ph111_B10
zmp_ph114_G4
zmp_ph114_G11
zmp_ph114_H1
zmp_ph114_H2
zmp_ph114_A9
zmp_ph114_A11
zmp_ph114_B2
zmp_ph114_B4
zmp_ph114_D8
zmp_ph114_E2

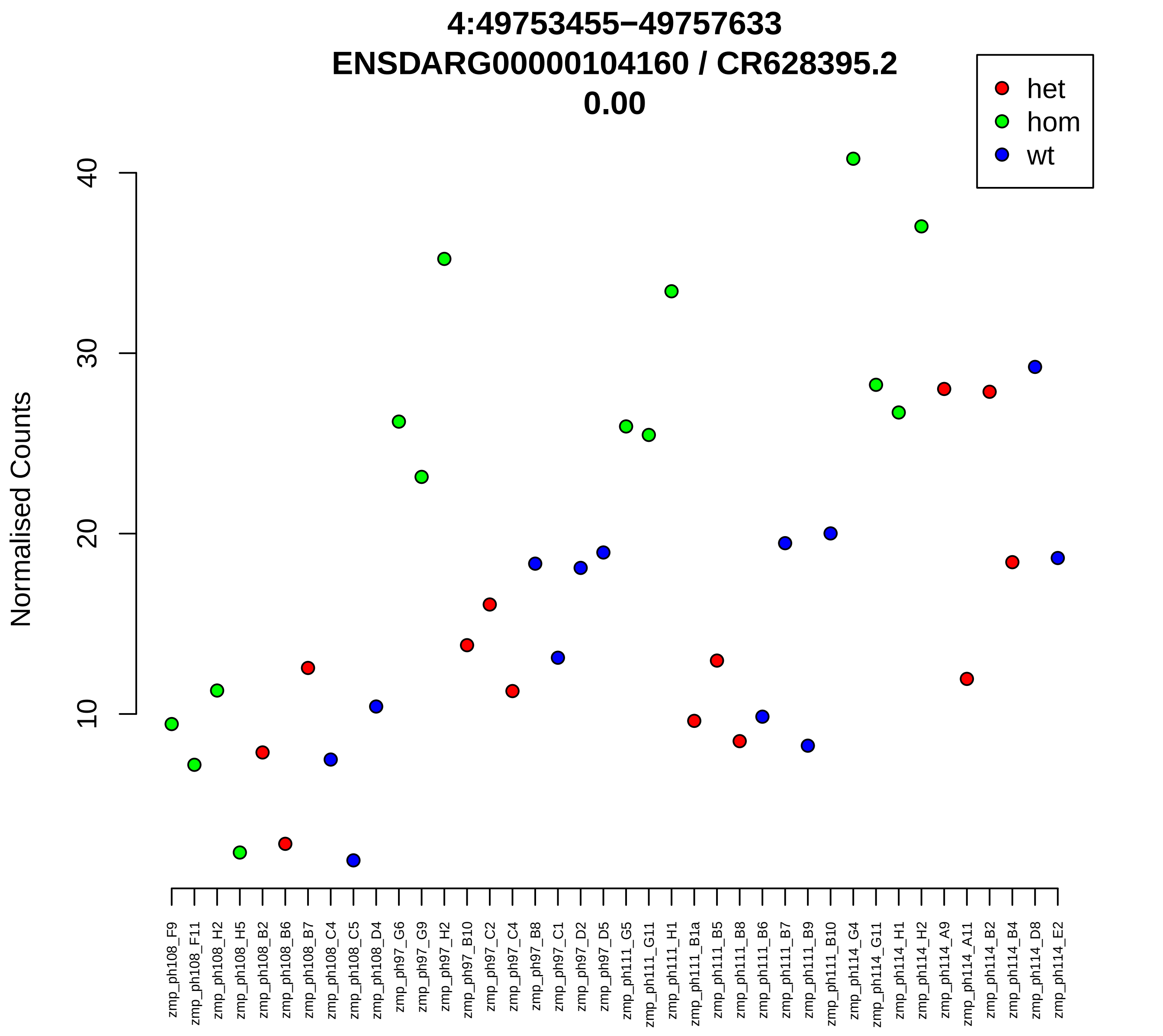

4:49753455−49757633
ENSDARG00000104160 / CR628395.2
0.00
het
hom
wt
Normalised Counts
10
20
30
40
zmp_ph108_F9
zmp_ph108_F11
zmp_ph108_H2
zmp_ph108_H5
zmp_ph108_B2
zmp_ph108_B6
zmp_ph108_B7
zmp_ph108_C4
zmp_ph108_C5
zmp_ph108_D4
zmp_ph97_G6
zmp_ph97_G9
zmp_ph97_H2
zmp_ph97_B10
zmp_ph97_C2
zmp_ph97_C4
zmp_ph97_B8
zmp_ph97_C1
zmp_ph97_D2
zmp_ph97_D5
zmp_ph111_G5
zmp_ph111_G11
zmp_ph111_H1
zmp_ph111_B1a
zmp_ph111_B5
zmp_ph111_B8
zmp_ph111_B6
zmp_ph111_B7
zmp_ph111_B9
zmp_ph111_B10
zmp_ph114_G4
zmp_ph114_G11
zmp_ph114_H1
zmp_ph114_H2
zmp_ph114_A9
zmp_ph114_A11
zmp_ph114_B2
zmp_ph114_B4
zmp_ph114_D8
zmp_ph114_E2

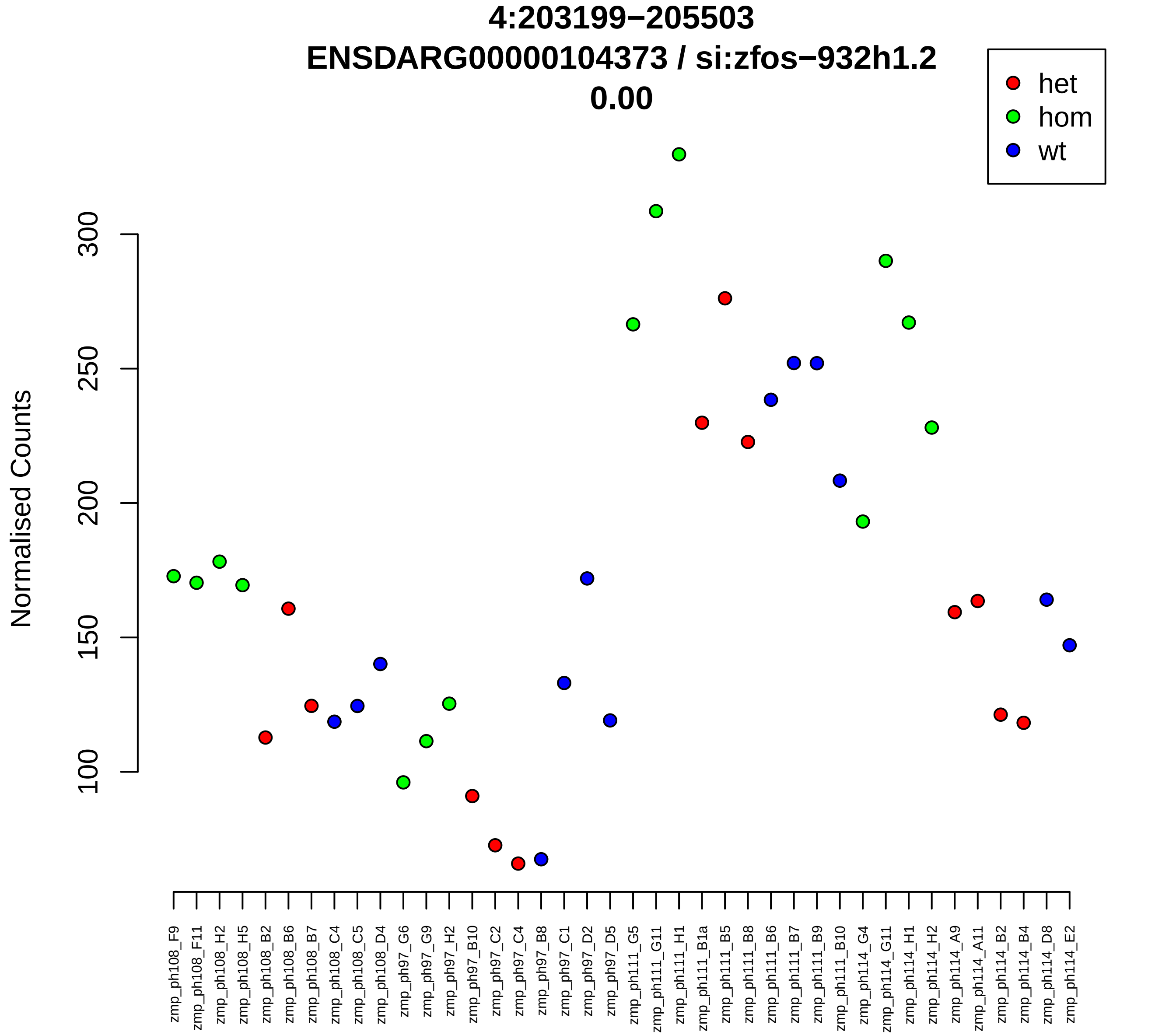
4:203199−205503
ENSDARG00000104373 / si:zfos−932h1.2
0.00
het
hom
wt
Normalised Counts
100
150
200
250
300
zmp_ph108_F9
zmp_ph108_F11
zmp_ph108_H2
zmp_ph108_H5
zmp_ph108_B2
zmp_ph108_B6
zmp_ph108_B7
zmp_ph108_C4
zmp_ph108_C5
zmp_ph108_D4
zmp_ph97_G6
zmp_ph97_G9
zmp_ph97_H2
zmp_ph97_B10
zmp_ph97_C2
zmp_ph97_C4
zmp_ph97_B8
zmp_ph97_C1
zmp_ph97_D2
zmp_ph97_D5
zmp_ph111_G5
zmp_ph111_G11
zmp_ph111_H1
zmp_ph111_B1a
zmp_ph111_B5
zmp_ph111_B8
zmp_ph111_B6
zmp_ph111_B7
zmp_ph111_B9
zmp_ph111_B10
zmp_ph114_G4
zmp_ph114_G11
zmp_ph114_H1
zmp_ph114_H2
zmp_ph114_A9
zmp_ph114_A11
zmp_ph114_B2
zmp_ph114_B4
zmp_ph114_D8
zmp_ph114_E2

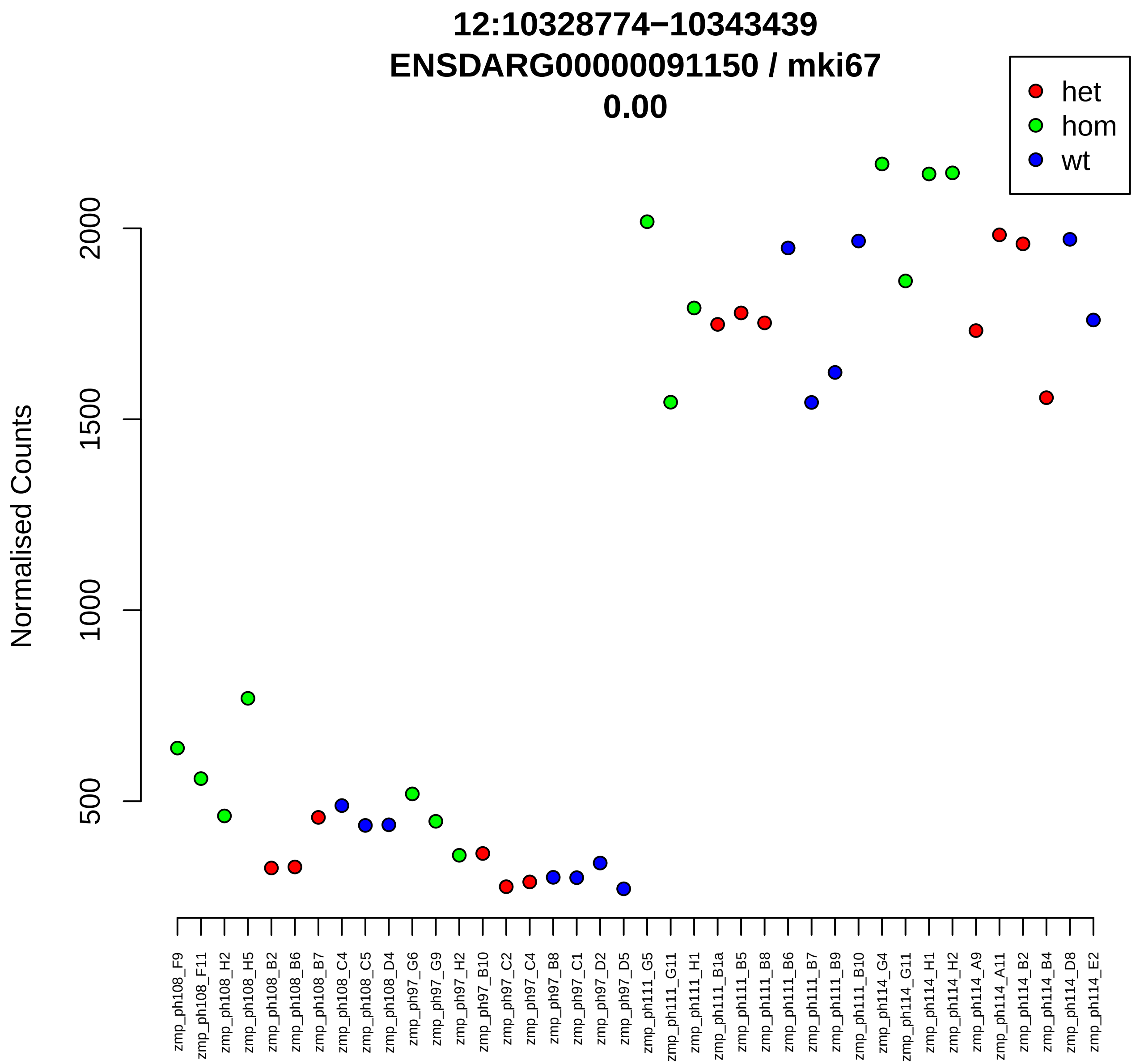

12:10328774−10343439
ENSDARG00000091150 / mki67
0.00
het
hom
wt
Normalised Counts
500
1000
1500
2000
zmp_ph108_F9
zmp_ph108_F11
zmp_ph108_H2
zmp_ph108_H5
zmp_ph108_B2
zmp_ph108_B6
zmp_ph108_B7
zmp_ph108_C4
zmp_ph108_C5
zmp_ph108_D4
zmp_ph97_G6
zmp_ph97_G9
zmp_ph97_H2
zmp_ph97_B10
zmp_ph97_C2
zmp_ph97_C4
zmp_ph97_B8
zmp_ph97_C1
zmp_ph97_D2
zmp_ph97_D5
zmp_ph111_G5
zmp_ph111_G11
zmp_ph111_H1
zmp_ph111_B1a
zmp_ph111_B5
zmp_ph111_B8
zmp_ph111_B6
zmp_ph111_B7
zmp_ph111_B9
zmp_ph111_B10
zmp_ph114_G4
zmp_ph114_G11
zmp_ph114_H1
zmp_ph114_H2
zmp_ph114_A9
zmp_ph114_A11
zmp_ph114_B2
zmp_ph114_B4
zmp_ph114_D8
zmp_ph114_E2

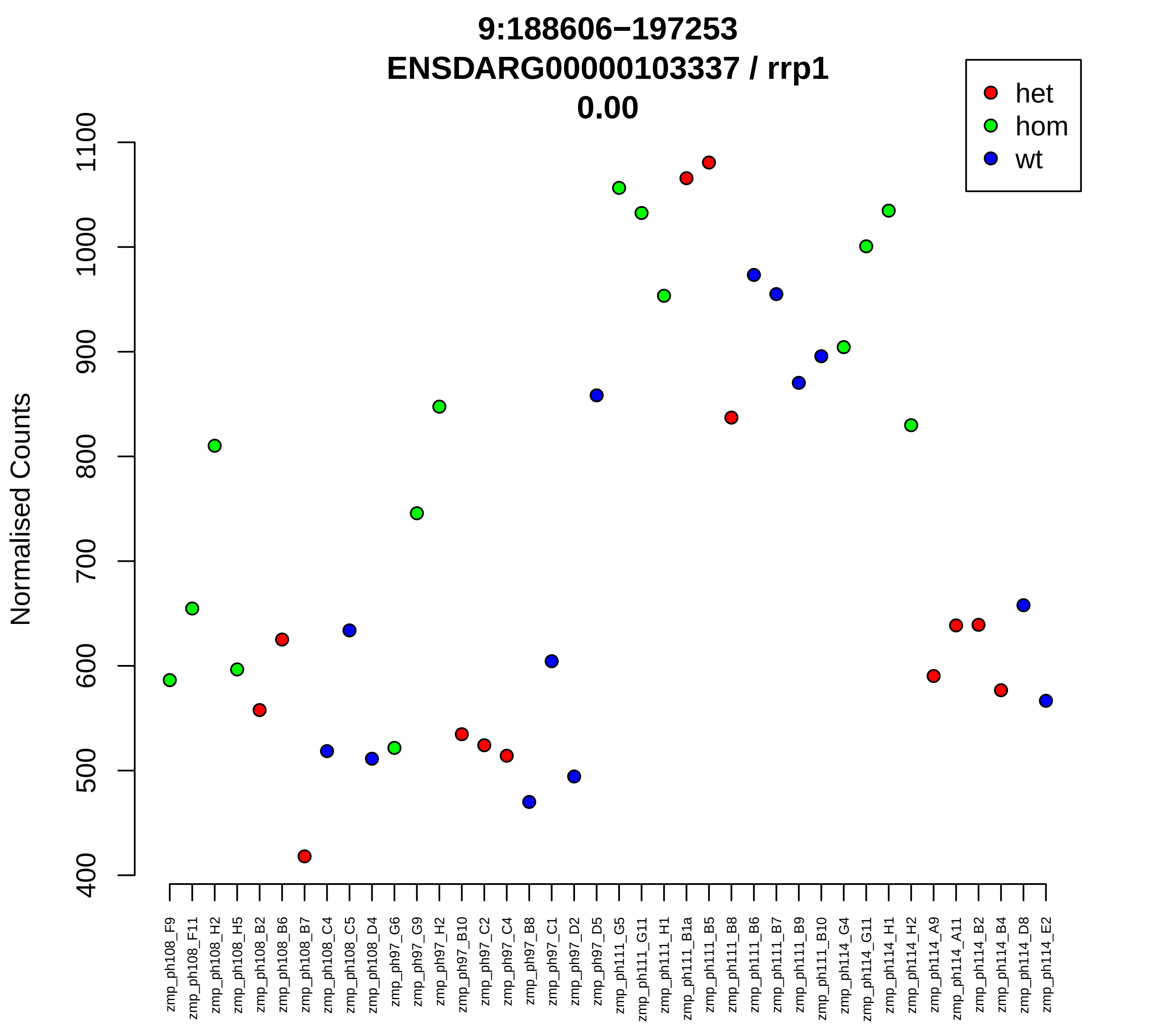

9:188606−197253
ENSDARG00000103337 / rrp1
0.00
het
hom
wt
Normalised Counts
400
500
600
700
800
900
1000
1100
zmp_ph108_F9
zmp_ph108_F11
zmp_ph108_H2
zmp_ph108_H5
zmp_ph108_B2
zmp_ph108_B6
zmp_ph108_B7
zmp_ph108_C4
zmp_ph108_C5
zmp_ph108_D4
zmp_ph97_G6
zmp_ph97_G9
zmp_ph97_H2
zmp_ph97_B10
zmp_ph97_C2
zmp_ph97_C4
zmp_ph97_B8
zmp_ph97_C1
zmp_ph97_D2
zmp_ph97_D5
zmp_ph111_G5
zmp_ph111_G11
zmp_ph111_H1
zmp_ph111_B1a
zmp_ph111_B5
zmp_ph111_B8
zmp_ph111_B6
zmp_ph111_B7
zmp_ph111_B9
zmp_ph111_B10
zmp_ph114_G4
zmp_ph114_G11
zmp_ph114_H1
zmp_ph114_H2
zmp_ph114_A9
zmp_ph114_A11
zmp_ph114_B2
zmp_ph114_B4
zmp_ph114_D8
zmp_ph114_E2

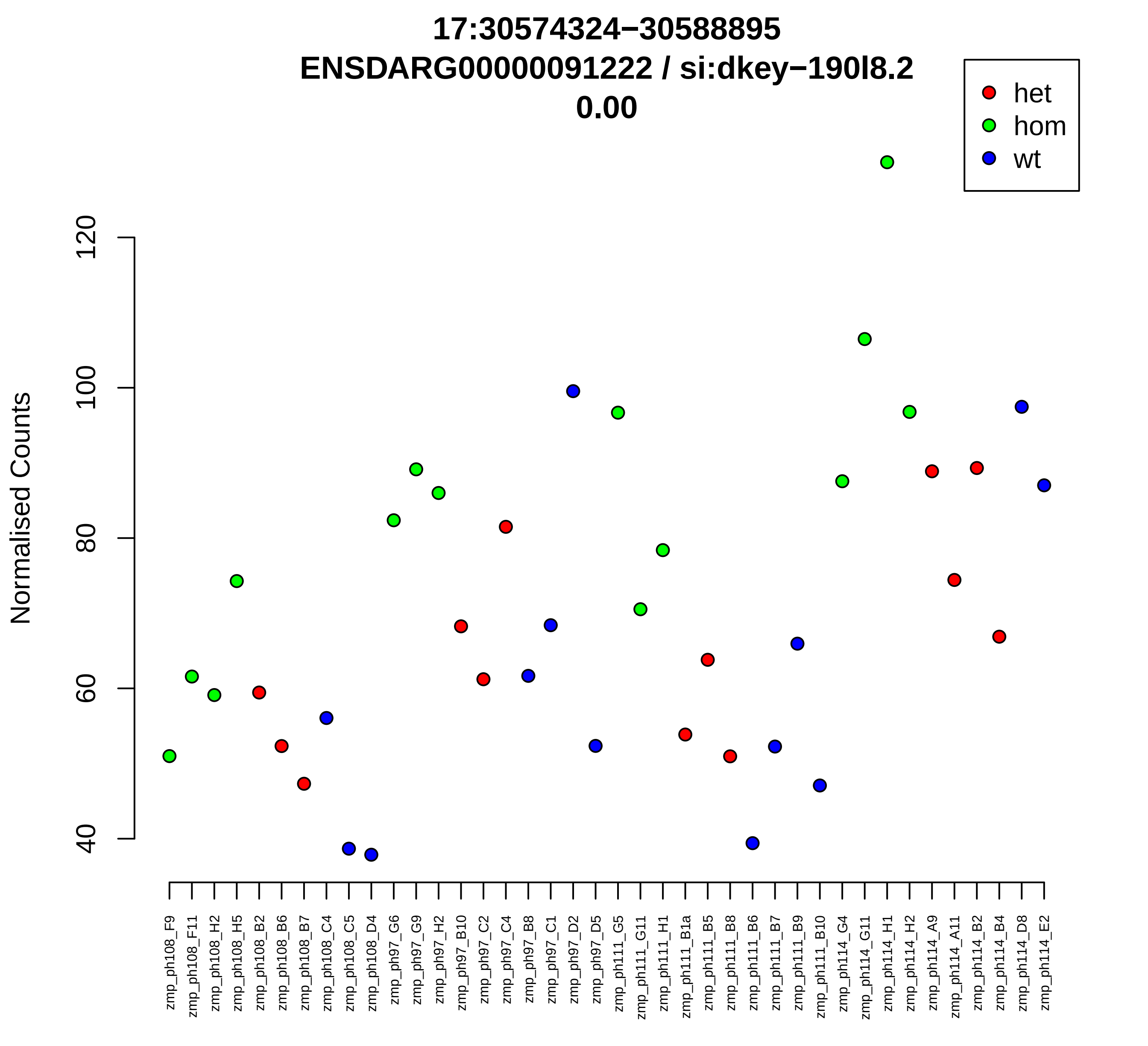
17:30574324−30588895
ENSDARG00000091222 / si:dkey−190l8.2
0.00
het
hom
wt
Normalised Counts
40
60
80
100
120
zmp_ph108_F9
zmp_ph108_F11
zmp_ph108_H2
zmp_ph108_H5
zmp_ph108_B2
zmp_ph108_B6
zmp_ph108_B7
zmp_ph108_C4
zmp_ph108_C5
zmp_ph108_D4
zmp_ph97_G6
zmp_ph97_G9
zmp_ph97_H2
zmp_ph97_B10
zmp_ph97_C2
zmp_ph97_C4
zmp_ph97_B8
zmp_ph97_C1
zmp_ph97_D2
zmp_ph97_D5
zmp_ph111_G5
zmp_ph111_G11
zmp_ph111_H1
zmp_ph111_B1a
zmp_ph111_B5
zmp_ph111_B8
zmp_ph111_B6
zmp_ph111_B7
zmp_ph111_B9
zmp_ph111_B10
zmp_ph114_G4
zmp_ph114_G11
zmp_ph114_H1
zmp_ph114_H2
zmp_ph114_A9
zmp_ph114_A11
zmp_ph114_B2
zmp_ph114_B4
zmp_ph114_D8
zmp_ph114_E2

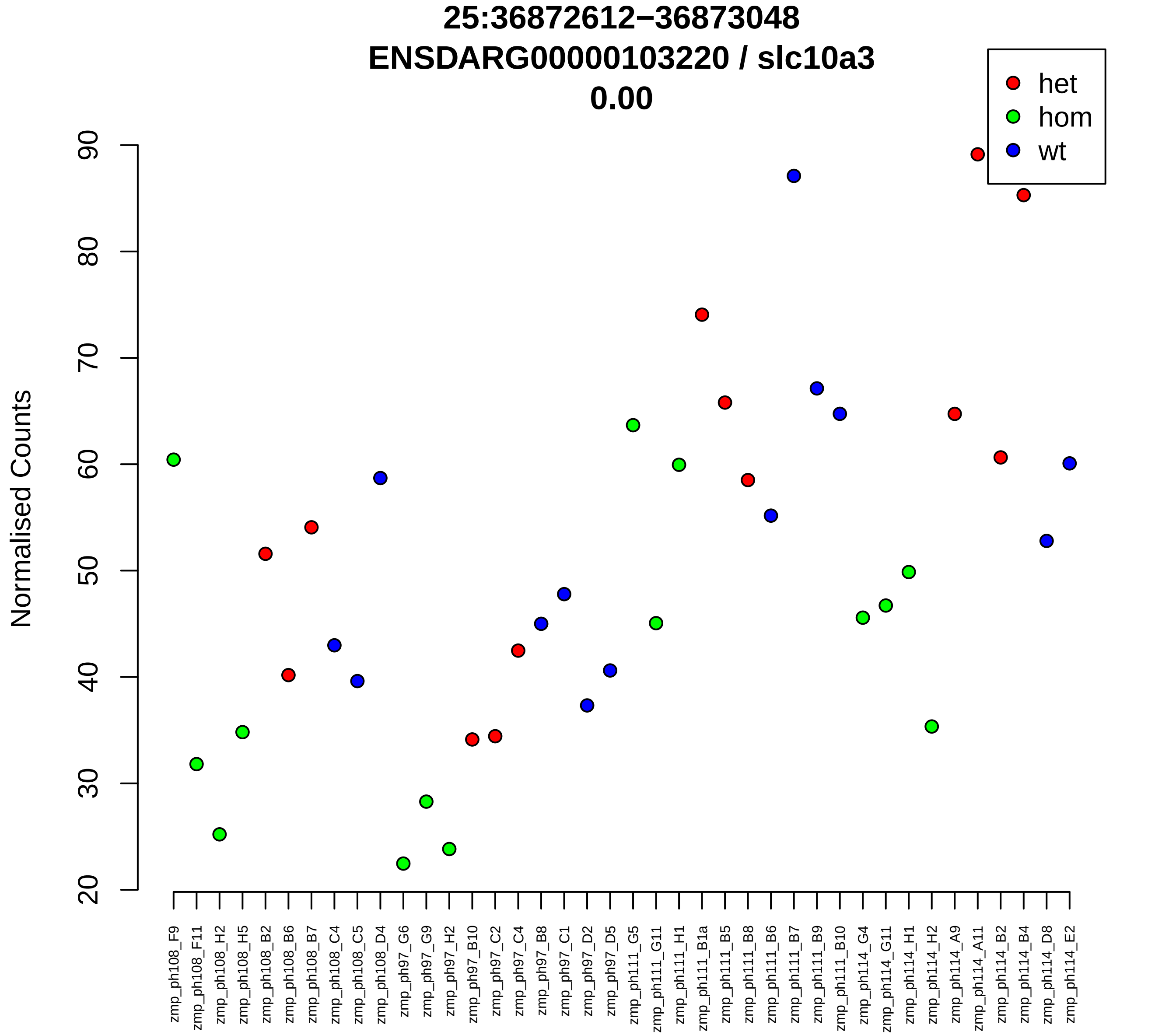
25:36872612−36873048
ENSDARG00000103220 / slc10a3
0.00
het
hom
wt
Normalised Counts
20
30
40
50
60
70
80
90
zmp_ph108_F9
zmp_ph108_F11
zmp_ph108_H2
zmp_ph108_H5
zmp_ph108_B2
zmp_ph108_B6
zmp_ph108_B7
zmp_ph108_C4
zmp_ph108_C5
zmp_ph108_D4
zmp_ph97_G6
zmp_ph97_G9
zmp_ph97_H2
zmp_ph97_B10
zmp_ph97_C2
zmp_ph97_C4
zmp_ph97_B8
zmp_ph97_C1
zmp_ph97_D2
zmp_ph97_D5
zmp_ph111_G5
zmp_ph111_G11
zmp_ph111_H1
zmp_ph111_B1a
zmp_ph111_B5
zmp_ph111_B8
zmp_ph111_B6
zmp_ph111_B7
zmp_ph111_B9
zmp_ph111_B10
zmp_ph114_G4
zmp_ph114_G11
zmp_ph114_H1
zmp_ph114_H2
zmp_ph114_A9
zmp_ph114_A11
zmp_ph114_B2
zmp_ph114_B4
zmp_ph114_D8
zmp_ph114_E2

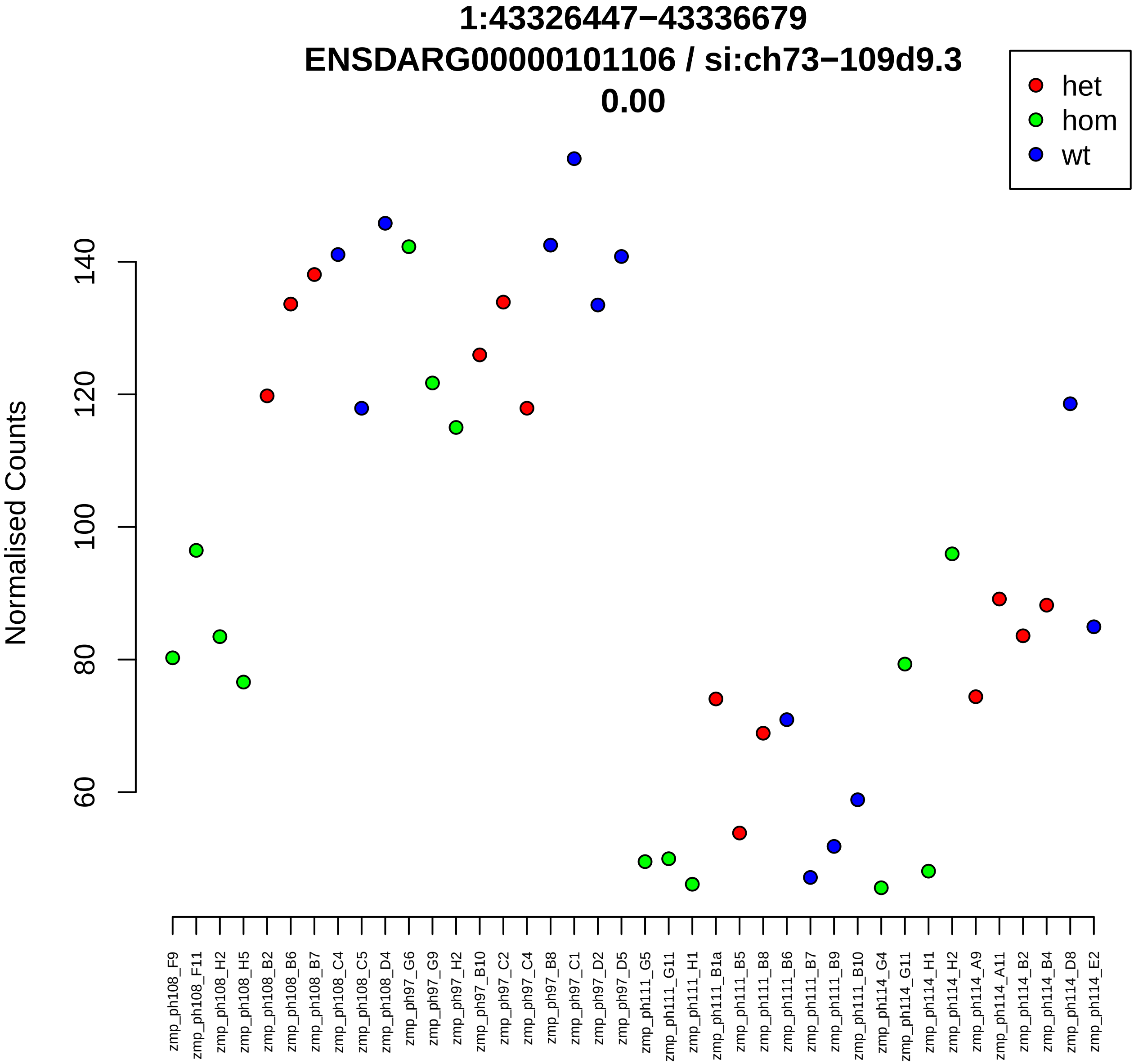

1:43326447−43336679
ENSDARG00000101106 / si:ch73−109d9.3
0.00
het
hom
wt
Normalised Counts
60
80
100
120
140
zmp_ph108_F9
zmp_ph108_F11
zmp_ph108_H2
zmp_ph108_H5
zmp_ph108_B2
zmp_ph108_B6
zmp_ph108_B7
zmp_ph108_C4
zmp_ph108_C5
zmp_ph108_D4
zmp_ph97_G6
zmp_ph97_G9
zmp_ph97_H2
zmp_ph97_B10
zmp_ph97_C2
zmp_ph97_C4
zmp_ph97_B8
zmp_ph97_C1
zmp_ph97_D2
zmp_ph97_D5
zmp_ph111_G5
zmp_ph111_G11
zmp_ph111_H1
zmp_ph111_B1a
zmp_ph111_B5
zmp_ph111_B8
zmp_ph111_B6
zmp_ph111_B7
zmp_ph111_B9
zmp_ph111_B10
zmp_ph114_G4
zmp_ph114_G11
zmp_ph114_H1
zmp_ph114_H2
zmp_ph114_A9
zmp_ph114_A11
zmp_ph114_B2
zmp_ph114_B4
zmp_ph114_D8
zmp_ph114_E2

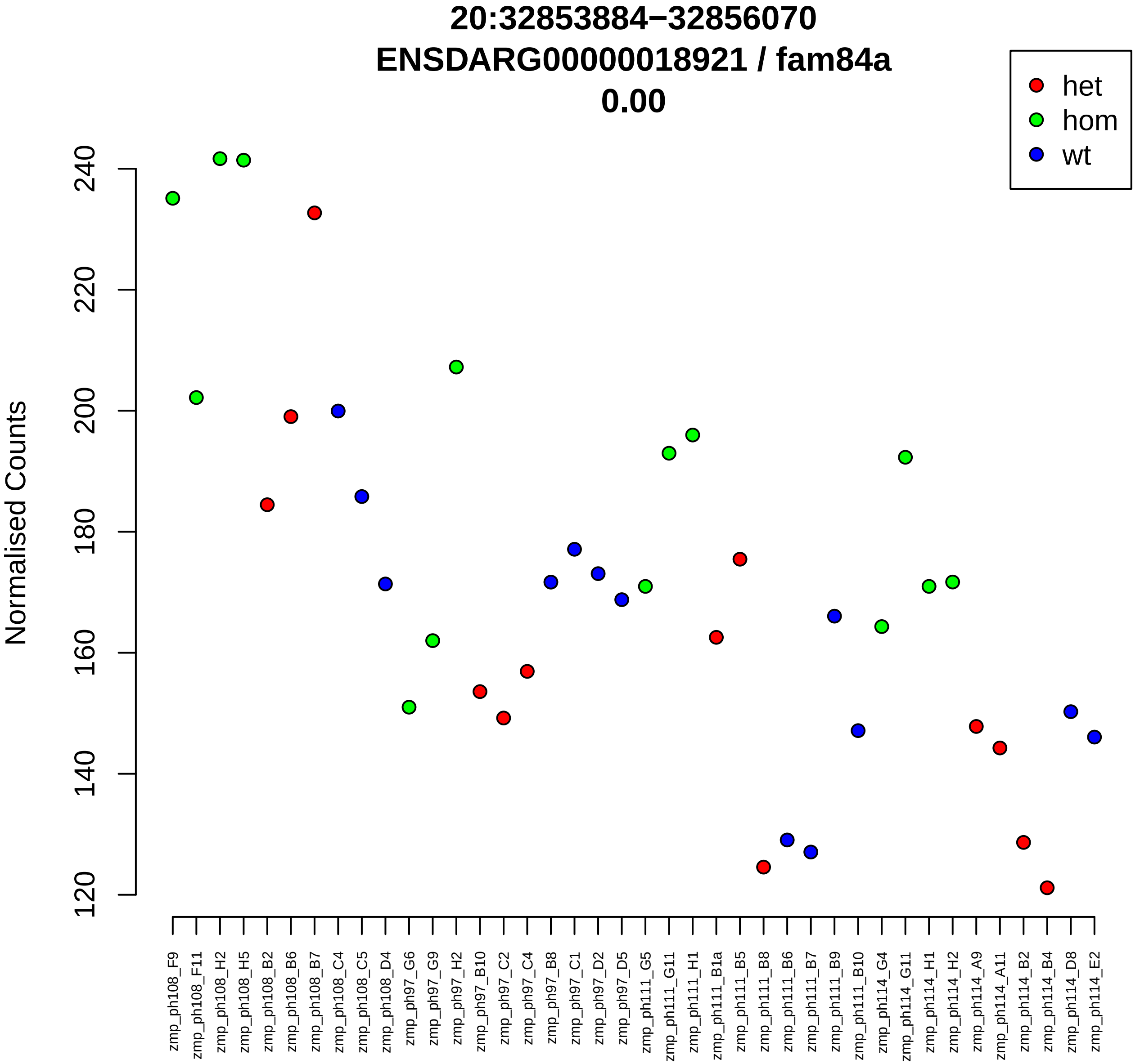

20:32853884−32856070
ENSDARG00000018921 / fam84a
0.00
het
hom
wt
Normalised Counts
120
140
160
180
200
220
240
zmp_ph108_F9
zmp_ph108_F11
zmp_ph108_H2
zmp_ph108_H5
zmp_ph108_B2
zmp_ph108_B6
zmp_ph108_B7
zmp_ph108_C4
zmp_ph108_C5
zmp_ph108_D4
zmp_ph97_G6
zmp_ph97_G9
zmp_ph97_H2
zmp_ph97_B10
zmp_ph97_C2
zmp_ph97_C4
zmp_ph97_B8
zmp_ph97_C1
zmp_ph97_D2
zmp_ph97_D5
zmp_ph111_G5
zmp_ph111_G11
zmp_ph111_H1
zmp_ph111_B1a
zmp_ph111_B5
zmp_ph111_B8
zmp_ph111_B6
zmp_ph111_B7
zmp_ph111_B9
zmp_ph111_B10
zmp_ph114_G4
zmp_ph114_G11
zmp_ph114_H1
zmp_ph114_H2
zmp_ph114_A9
zmp_ph114_A11
zmp_ph114_B2
zmp_ph114_B4
zmp_ph114_D8
zmp_ph114_E2

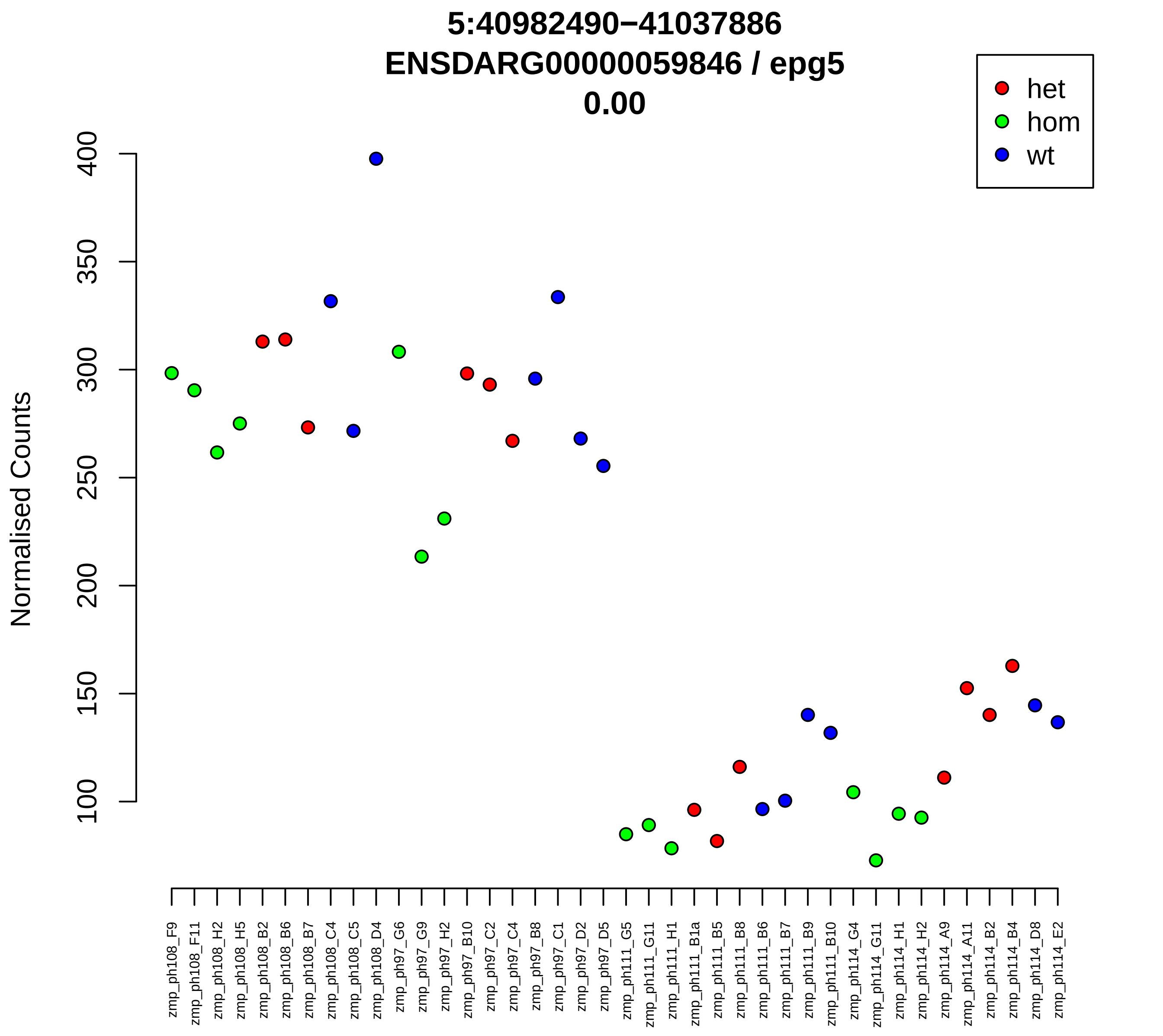

5:40982490−41037886
ENSDARG00000059846 / epg5
0.00
het
hom
wt
Normalised Counts
100
150
200
250
300
350
400
zmp_ph108_F9
zmp_ph108_F11
zmp_ph108_H2
zmp_ph108_H5
zmp_ph108_B2
zmp_ph108_B6
zmp_ph108_B7
zmp_ph108_C4
zmp_ph108_C5
zmp_ph108_D4
zmp_ph97_G6
zmp_ph97_G9
zmp_ph97_H2
zmp_ph97_B10
zmp_ph97_C2
zmp_ph97_C4
zmp_ph97_B8
zmp_ph97_C1
zmp_ph97_D2
zmp_ph97_D5
zmp_ph111_G5
zmp_ph111_G11
zmp_ph111_H1
zmp_ph111_B1a
zmp_ph111_B5
zmp_ph111_B8
zmp_ph111_B6
zmp_ph111_B7
zmp_ph111_B9
zmp_ph111_B10
zmp_ph114_G4
zmp_ph114_G11
zmp_ph114_H1
zmp_ph114_H2
zmp_ph114_A9
zmp_ph114_A11
zmp_ph114_B2
zmp_ph114_B4
zmp_ph114_D8
zmp_ph114_E2

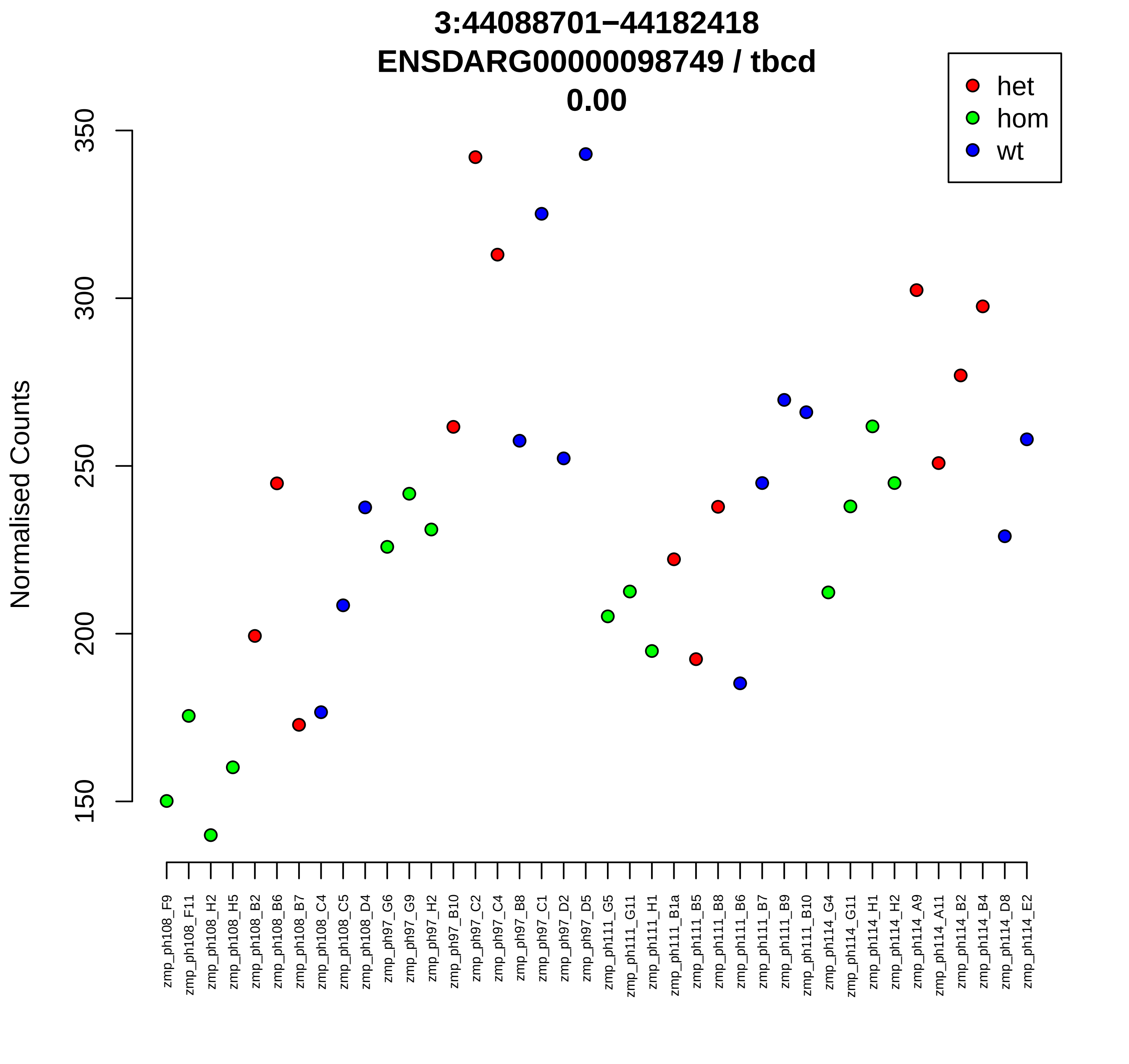

3:44088701−44182418
ENSDARG00000098749 / tbcd
0.00
Normalised Counts
150
200
250
300
350
het
hom
wt
zmp_ph108_F9
zmp_ph108_F11
zmp_ph108_H2
zmp_ph108_H5
zmp_ph108_B2
zmp_ph108_B6
zmp_ph108_B7
zmp_ph108_C4
zmp_ph108_C5
zmp_ph108_D4
zmp_ph97_G6
zmp_ph97_G9
zmp_ph97_H2
zmp_ph97_B10
zmp_ph97_C2
zmp_ph97_C4
zmp_ph97_B8
zmp_ph97_C1
zmp_ph97_D2
zmp_ph97_D5
zmp_ph111_G5
zmp_ph111_G11
zmp_ph111_H1
zmp_ph111_B1a
zmp_ph111_B5
zmp_ph111_B8
zmp_ph111_B6
zmp_ph111_B7
zmp_ph111_B9
zmp_ph111_B10
zmp_ph114_G4
zmp_ph114_G11
zmp_ph114_H1
zmp_ph114_H2
zmp_ph114_A9
zmp_ph114_A11
zmp_ph114_B2
zmp_ph114_B4
zmp_ph114_D8
zmp_ph114_E2

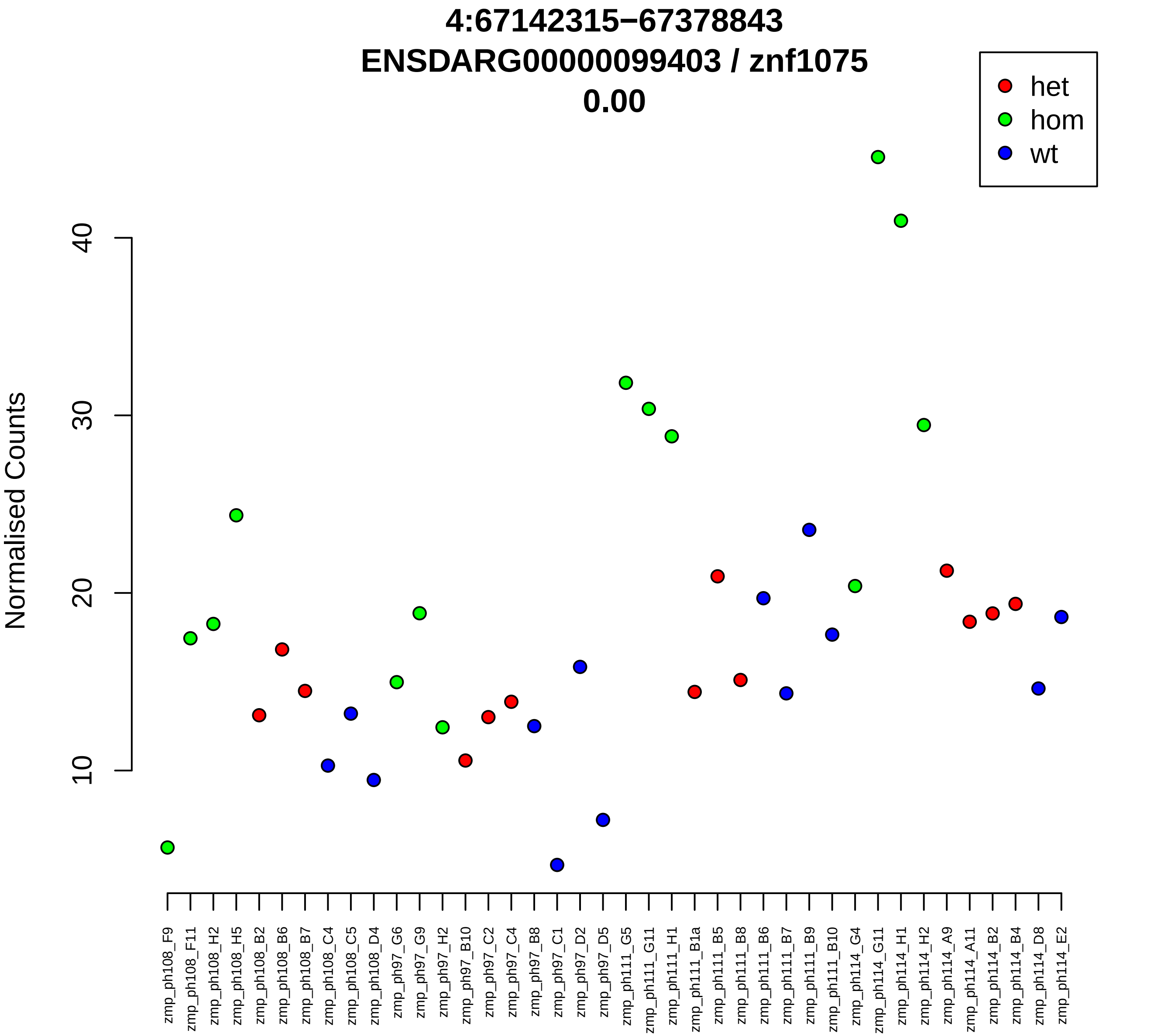
4:67142315−67378843
ENSDARG00000099403 / znf1075
0.00
het
hom
wt
Normalised Counts
10
20
30
40
zmp_ph108_F9
zmp_ph108_F11
zmp_ph108_H2
zmp_ph108_H5
zmp_ph108_B2
zmp_ph108_B6
zmp_ph108_B7
zmp_ph108_C4
zmp_ph108_C5
zmp_ph108_D4
zmp_ph97_G6
zmp_ph97_G9
zmp_ph97_H2
zmp_ph97_B10
zmp_ph97_C2
zmp_ph97_C4
zmp_ph97_B8
zmp_ph97_C1
zmp_ph97_D2
zmp_ph97_D5
zmp_ph111_G5
zmp_ph111_G11
zmp_ph111_H1
zmp_ph111_B1a
zmp_ph111_B5
zmp_ph111_B8
zmp_ph111_B6
zmp_ph111_B7
zmp_ph111_B9
zmp_ph111_B10
zmp_ph114_G4
zmp_ph114_G11
zmp_ph114_H1
zmp_ph114_H2
zmp_ph114_A9
zmp_ph114_A11
zmp_ph114_B2
zmp_ph114_B4
zmp_ph114_D8
zmp_ph114_E2

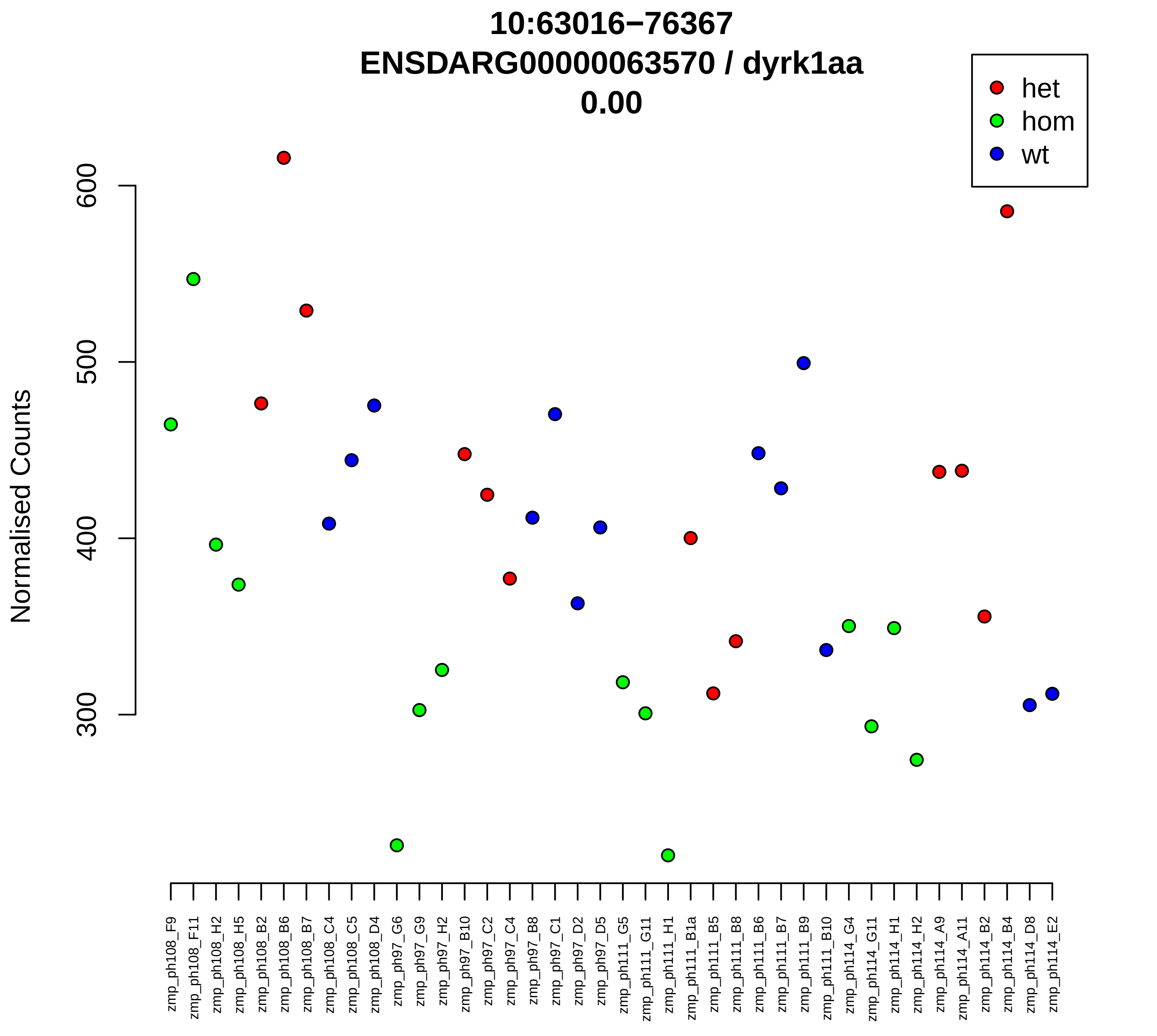

10:63016−76367
ENSDARG00000063570 / dyrk1aa
0.00
het
hom
wt
Normalised Counts
300
400
500
600
zmp_ph108_F9
zmp_ph108_F11
zmp_ph108_H2
zmp_ph108_H5
zmp_ph108_B2
zmp_ph108_B6
zmp_ph108_B7
zmp_ph108_C4
zmp_ph108_C5
zmp_ph108_D4
zmp_ph97_G6
zmp_ph97_G9
zmp_ph97_H2
zmp_ph97_B10
zmp_ph97_C2
zmp_ph97_C4
zmp_ph97_B8
zmp_ph97_C1
zmp_ph97_D2
zmp_ph97_D5
zmp_ph111_G5
zmp_ph111_G11
zmp_ph111_H1
zmp_ph111_B1a
zmp_ph111_B5
zmp_ph111_B8
zmp_ph111_B6
zmp_ph111_B7
zmp_ph111_B9
zmp_ph111_B10
zmp_ph114_G4
zmp_ph114_G11
zmp_ph114_H1
zmp_ph114_H2
zmp_ph114_A9
zmp_ph114_A11
zmp_ph114_B2
zmp_ph114_B4
zmp_ph114_D8
zmp_ph114_E2

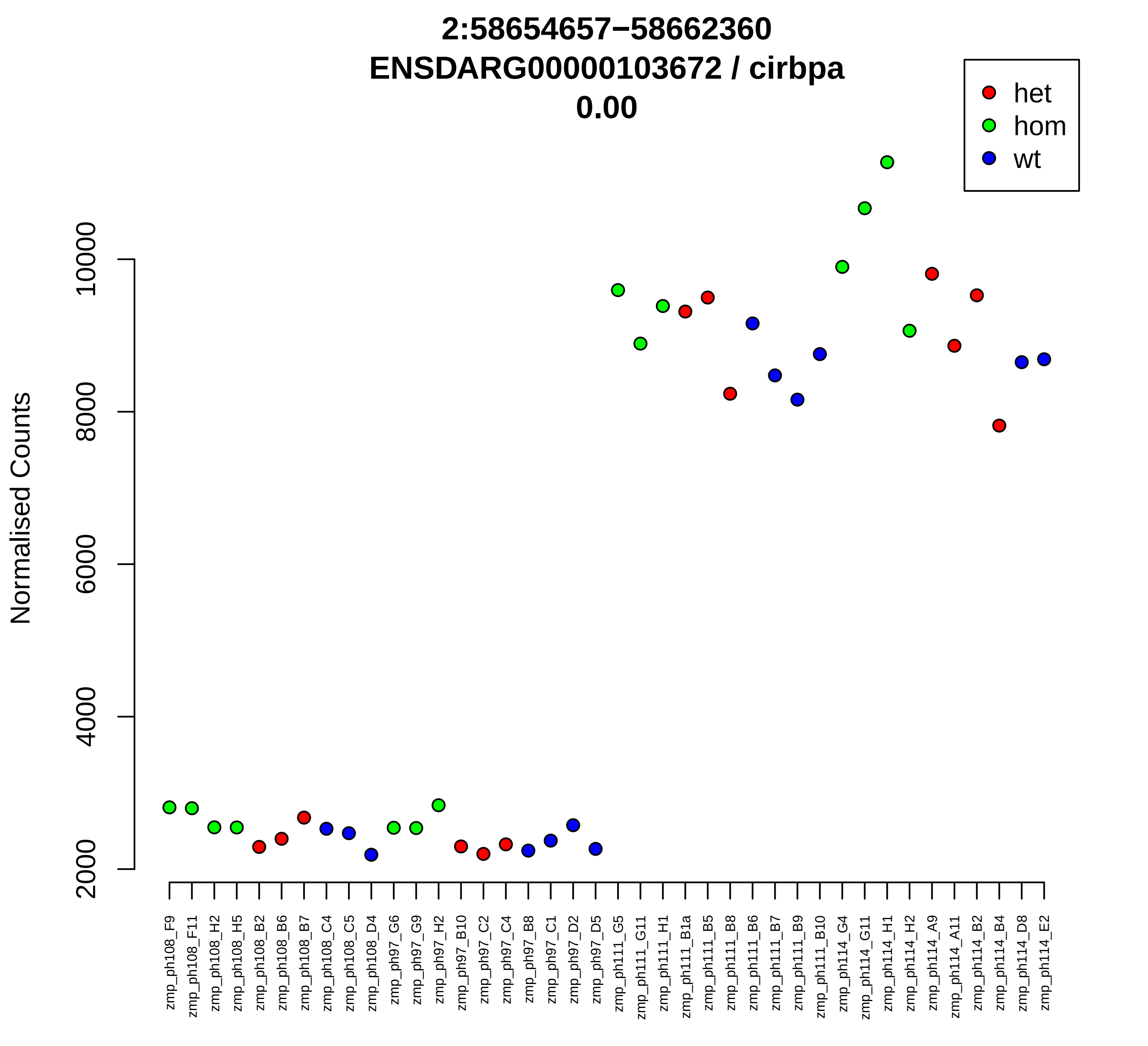

2:58654657−58662360
ENSDARG00000103672 / cirbpa
0.00
het
hom
wt
Normalised Counts
2000
4000
6000
8000
10000
zmp_ph108_F9
zmp_ph108_F11
zmp_ph108_H2
zmp_ph108_H5
zmp_ph108_B2
zmp_ph108_B6
zmp_ph108_B7
zmp_ph108_C4
zmp_ph108_C5
zmp_ph108_D4
zmp_ph97_G6
zmp_ph97_G9
zmp_ph97_H2
zmp_ph97_B10
zmp_ph97_C2
zmp_ph97_C4
zmp_ph97_B8
zmp_ph97_C1
zmp_ph97_D2
zmp_ph97_D5
zmp_ph111_G5
zmp_ph111_G11
zmp_ph111_H1
zmp_ph111_B1a
zmp_ph111_B5
zmp_ph111_B8
zmp_ph111_B6
zmp_ph111_B7
zmp_ph111_B9
zmp_ph111_B10
zmp_ph114_G4
zmp_ph114_G11
zmp_ph114_H1
zmp_ph114_H2
zmp_ph114_A9
zmp_ph114_A11
zmp_ph114_B2
zmp_ph114_B4
zmp_ph114_D8
zmp_ph114_E2

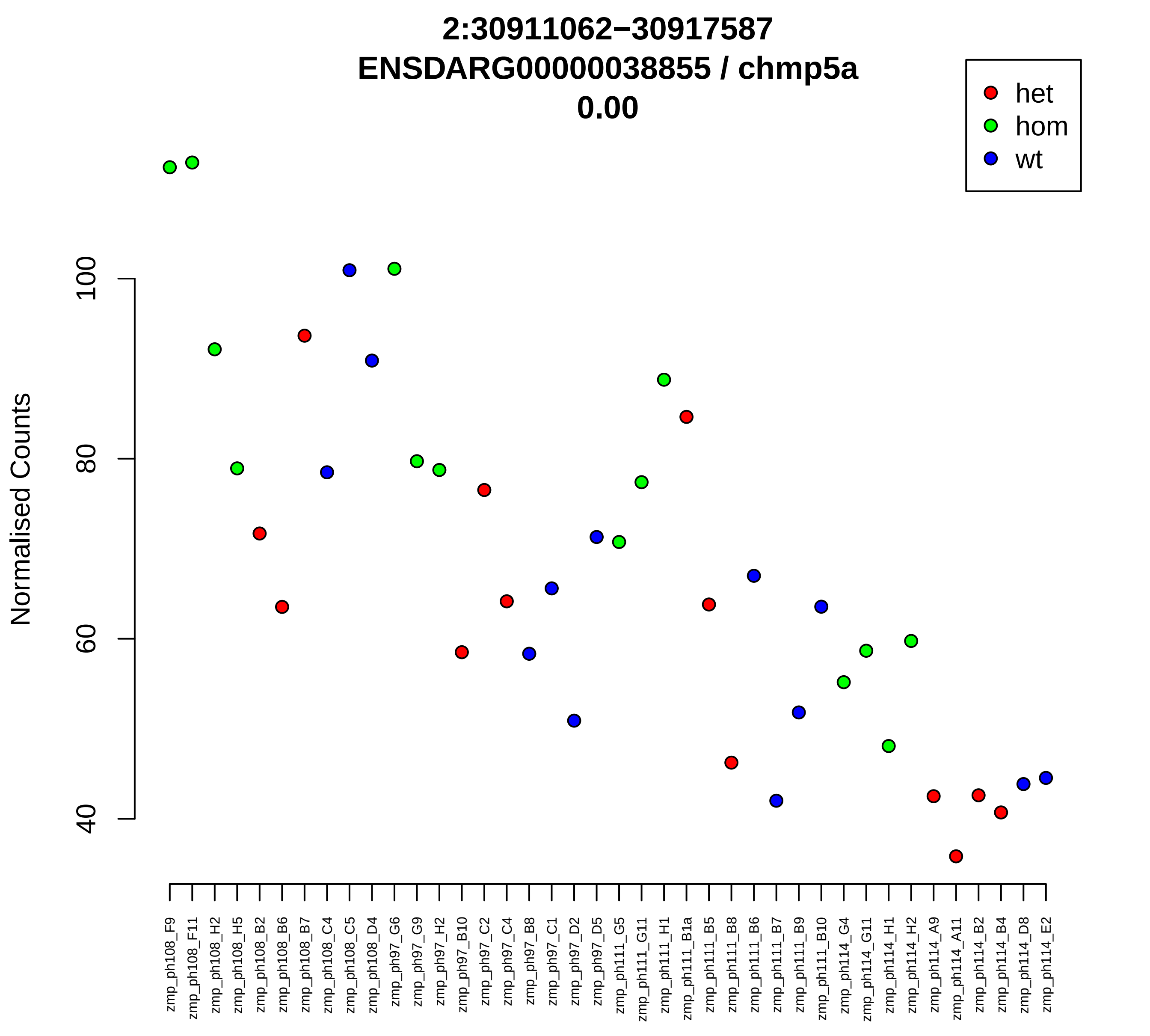

2:30911062−30917587
ENSDARG00000038855 / chmp5a
0.00
het
hom
wt
Normalised Counts
40
60
80
100
zmp_ph108_F9
zmp_ph108_F11
zmp_ph108_H2
zmp_ph108_H5
zmp_ph108_B2
zmp_ph108_B6
zmp_ph108_B7
zmp_ph108_C4
zmp_ph108_C5
zmp_ph108_D4
zmp_ph97_G6
zmp_ph97_G9
zmp_ph97_H2
zmp_ph97_B10
zmp_ph97_C2
zmp_ph97_C4
zmp_ph97_B8
zmp_ph97_C1
zmp_ph97_D2
zmp_ph97_D5
zmp_ph111_G5
zmp_ph111_G11
zmp_ph111_H1
zmp_ph111_B1a
zmp_ph111_B5
zmp_ph111_B8
zmp_ph111_B6
zmp_ph111_B7
zmp_ph111_B9
zmp_ph111_B10
zmp_ph114_G4
zmp_ph114_G11
zmp_ph114_H1
zmp_ph114_H2
zmp_ph114_A9
zmp_ph114_A11
zmp_ph114_B2
zmp_ph114_B4
zmp_ph114_D8
zmp_ph114_E2

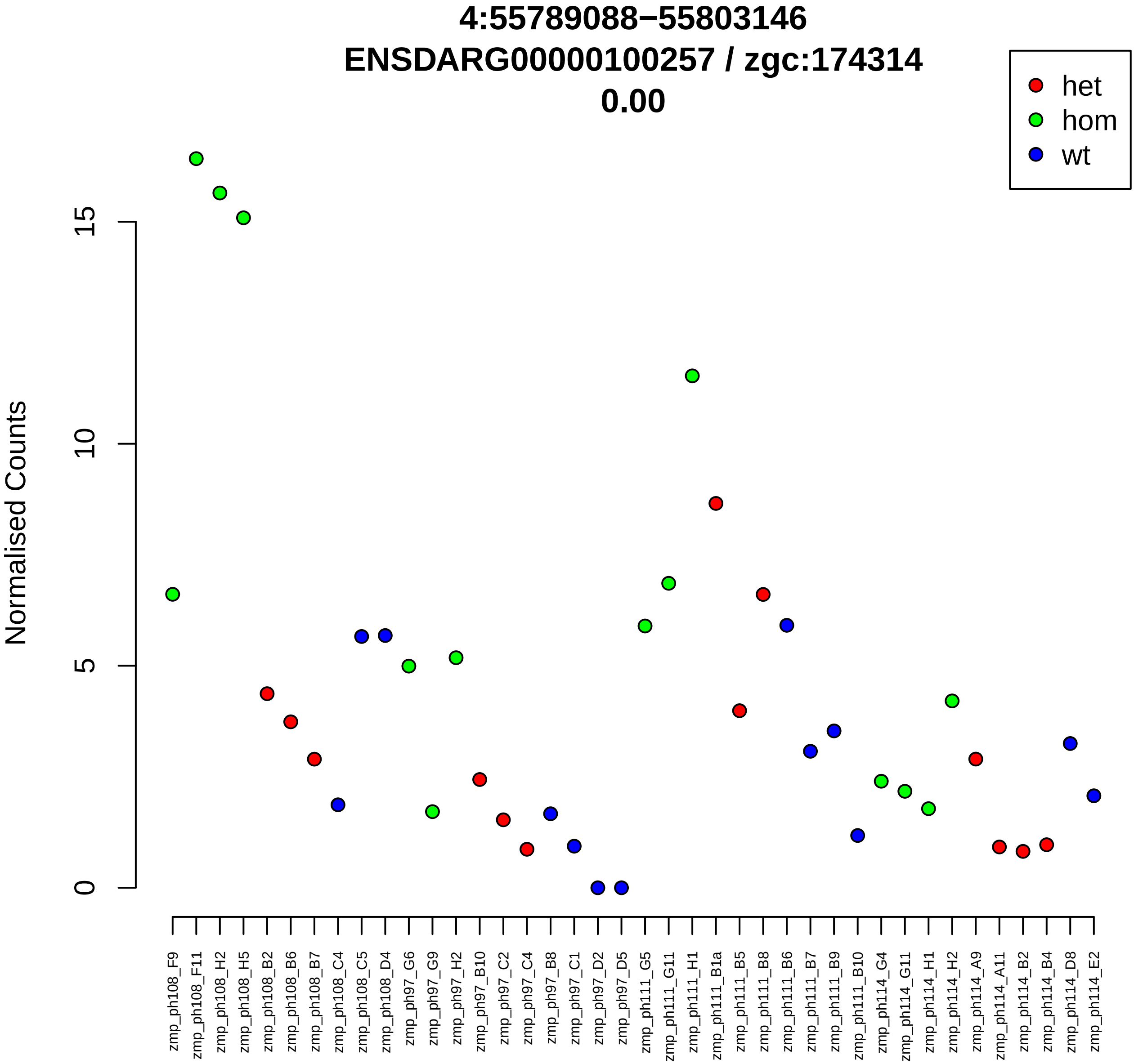

4:55789088−55803146
ENSDARG00000100257 / zgc:174314
0.00
het
hom
wt
Normalised Counts
0
5
10
15
zmp_ph108_F9
zmp_ph108_F11
zmp_ph108_H2
zmp_ph108_H5
zmp_ph108_B2
zmp_ph108_B6
zmp_ph108_B7
zmp_ph108_C4
zmp_ph108_C5
zmp_ph108_D4
zmp_ph97_G6
zmp_ph97_G9
zmp_ph97_H2
zmp_ph97_B10
zmp_ph97_C2
zmp_ph97_C4
zmp_ph97_B8
zmp_ph97_C1
zmp_ph97_D2
zmp_ph97_D5
zmp_ph111_G5
zmp_ph111_G11
zmp_ph111_H1
zmp_ph111_B1a
zmp_ph111_B5
zmp_ph111_B8
zmp_ph111_B6
zmp_ph111_B7
zmp_ph111_B9
zmp_ph111_B10
zmp_ph114_G4
zmp_ph114_G11
zmp_ph114_H1
zmp_ph114_H2
zmp_ph114_A9
zmp_ph114_A11
zmp_ph114_B2
zmp_ph114_B4
zmp_ph114_D8
zmp_ph114_E2

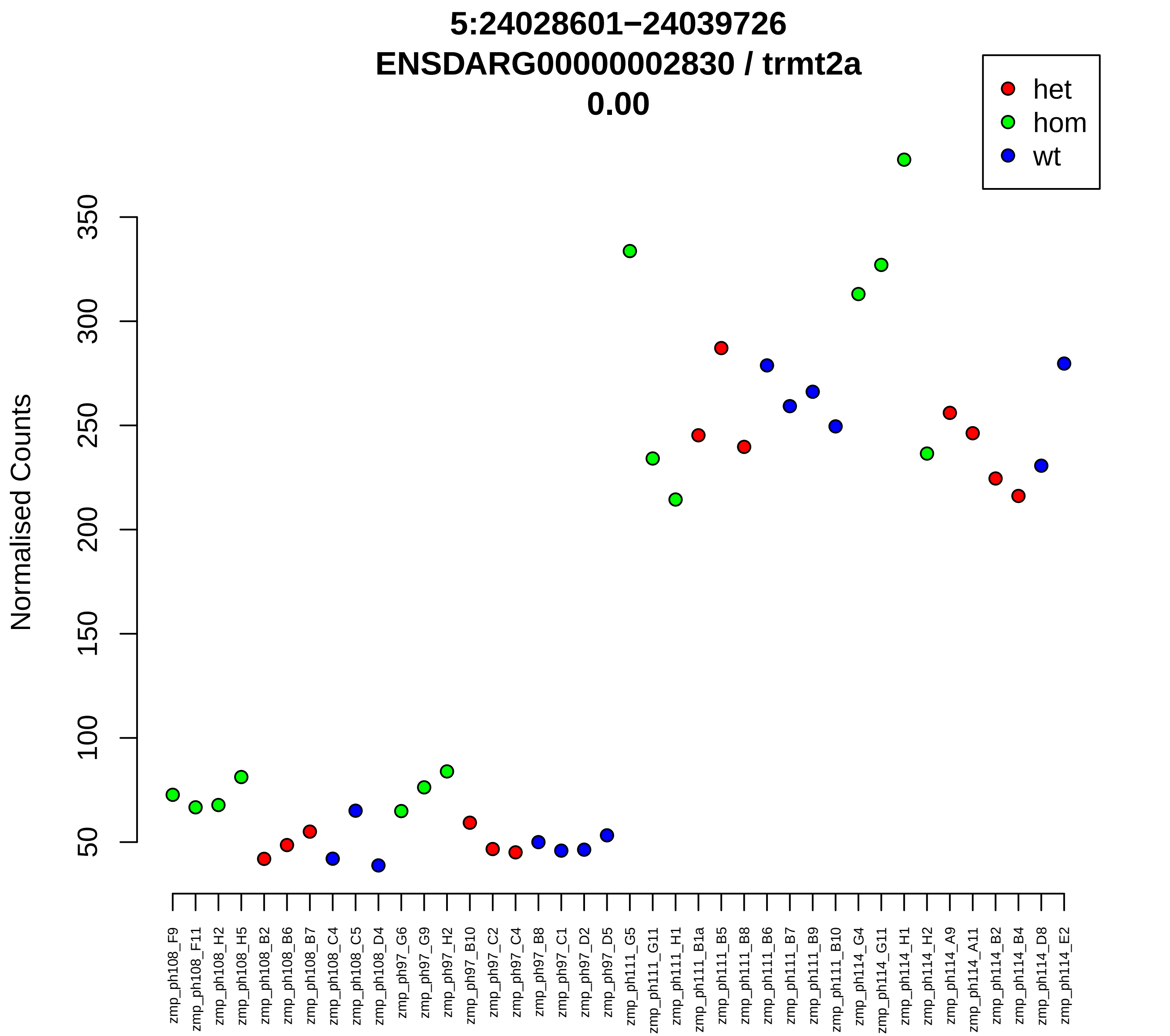

5:24028601−24039726
ENSDARG00000002830 / trmt2a
0.00
het
hom
wt
Normalised Counts
50
100
150
200
250
300
350
zmp_ph108_F9
zmp_ph108_F11
zmp_ph108_H2
zmp_ph108_H5
zmp_ph108_B2
zmp_ph108_B6
zmp_ph108_B7
zmp_ph108_C4
zmp_ph108_C5
zmp_ph108_D4
zmp_ph97_G6
zmp_ph97_G9
zmp_ph97_H2
zmp_ph97_B10
zmp_ph97_C2
zmp_ph97_C4
zmp_ph97_B8
zmp_ph97_C1
zmp_ph97_D2
zmp_ph97_D5
zmp_ph111_G5
zmp_ph111_G11
zmp_ph111_H1
zmp_ph111_B1a
zmp_ph111_B5
zmp_ph111_B8
zmp_ph111_B6
zmp_ph111_B7
zmp_ph111_B9
zmp_ph111_B10
zmp_ph114_G4
zmp_ph114_G11
zmp_ph114_H1
zmp_ph114_H2
zmp_ph114_A9
zmp_ph114_A11
zmp_ph114_B2
zmp_ph114_B4
zmp_ph114_D8
zmp_ph114_E2

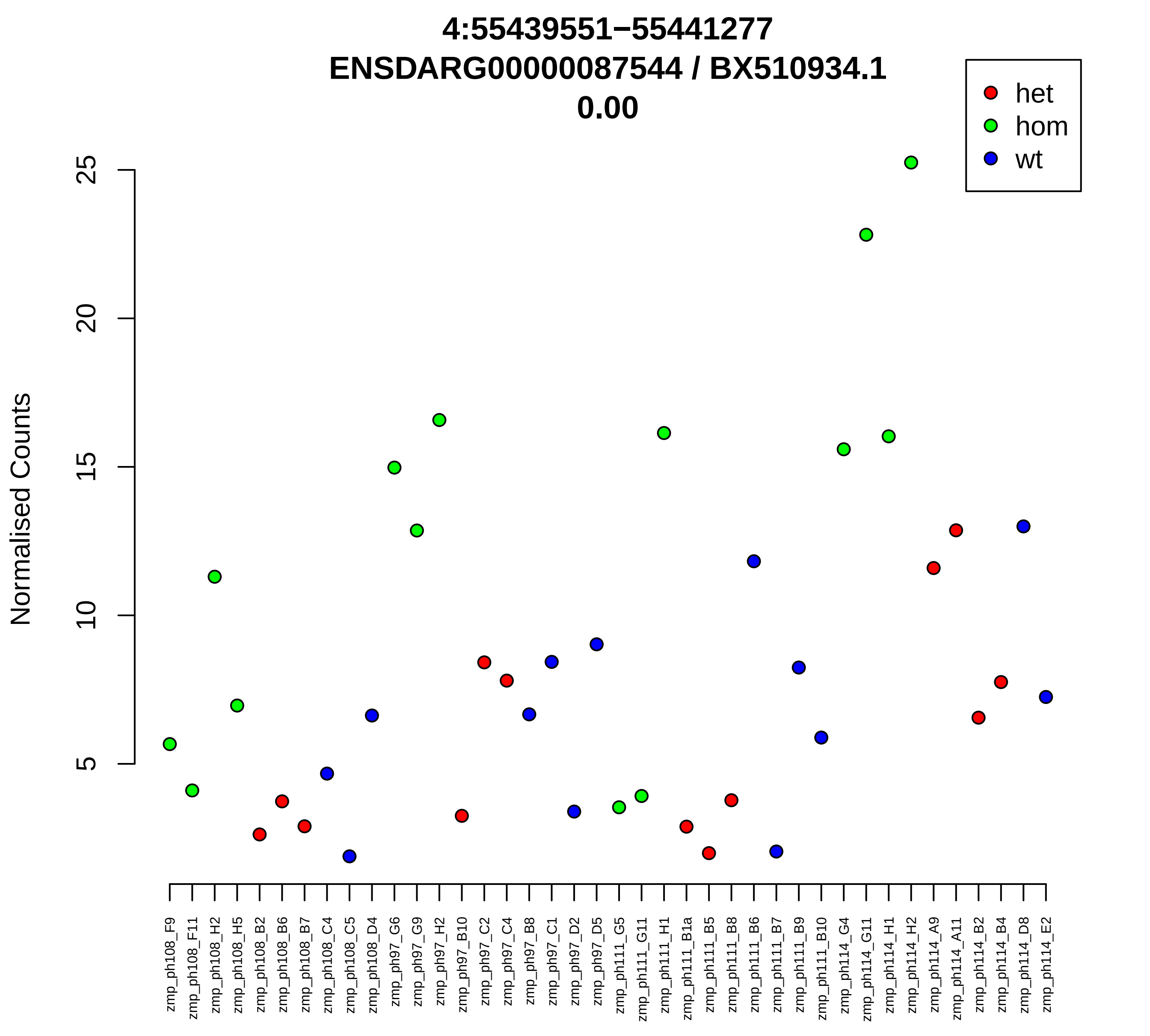

4:55439551−55441277
ENSDARG00000087544 / BX510934.1
0.00
het
hom
wt
Normalised Counts
5
10
15
20
25
zmp_ph108_F9
zmp_ph108_F11
zmp_ph108_H2
zmp_ph108_H5
zmp_ph108_B2
zmp_ph108_B6
zmp_ph108_B7
zmp_ph108_C4
zmp_ph108_C5
zmp_ph108_D4
zmp_ph97_G6
zmp_ph97_G9
zmp_ph97_H2
zmp_ph97_B10
zmp_ph97_C2
zmp_ph97_C4
zmp_ph97_B8
zmp_ph97_C1
zmp_ph97_D2
zmp_ph97_D5
zmp_ph111_G5
zmp_ph111_G11
zmp_ph111_H1
zmp_ph111_B1a
zmp_ph111_B5
zmp_ph111_B8
zmp_ph111_B6
zmp_ph111_B7
zmp_ph111_B9
zmp_ph111_B10
zmp_ph114_G4
zmp_ph114_G11
zmp_ph114_H1
zmp_ph114_H2
zmp_ph114_A9
zmp_ph114_A11
zmp_ph114_B2
zmp_ph114_B4
zmp_ph114_D8
zmp_ph114_E2

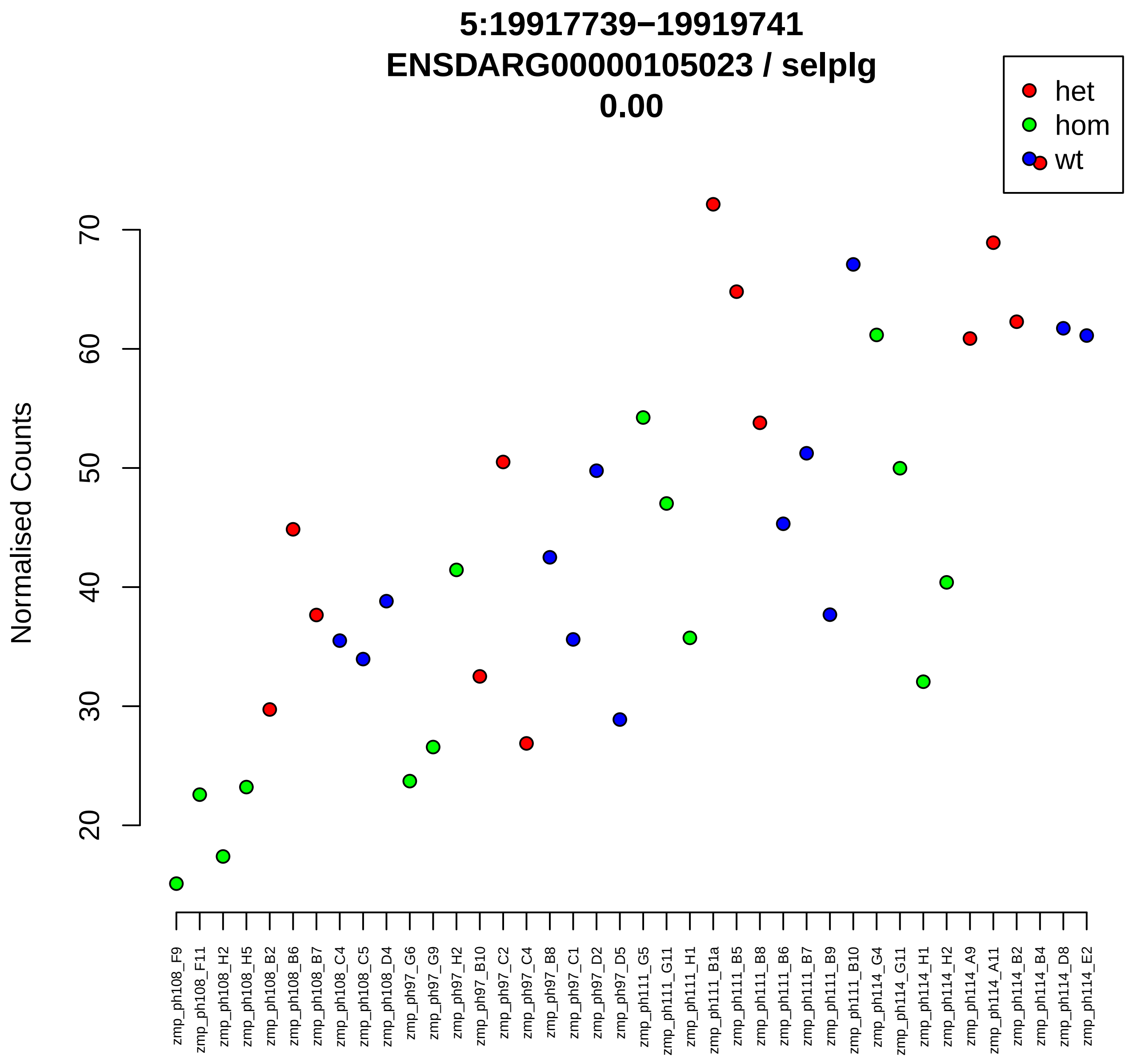

5:19917739−19919741
ENSDARG00000105023 / selplg
0.00
het
hom
wt
Normalised Counts
20
30
40
50
60
70
zmp_ph108_F9
zmp_ph108_F11
zmp_ph108_H2
zmp_ph108_H5
zmp_ph108_B2
zmp_ph108_B6
zmp_ph108_B7
zmp_ph108_C4
zmp_ph108_C5
zmp_ph108_D4
zmp_ph97_G6
zmp_ph97_G9
zmp_ph97_H2
zmp_ph97_B10
zmp_ph97_C2
zmp_ph97_C4
zmp_ph97_B8
zmp_ph97_C1
zmp_ph97_D2
zmp_ph97_D5
zmp_ph111_G5
zmp_ph111_G11
zmp_ph111_H1
zmp_ph111_B1a
zmp_ph111_B5
zmp_ph111_B8
zmp_ph111_B6
zmp_ph111_B7
zmp_ph111_B9
zmp_ph111_B10
zmp_ph114_G4
zmp_ph114_G11
zmp_ph114_H1
zmp_ph114_H2
zmp_ph114_A9
zmp_ph114_A11
zmp_ph114_B2
zmp_ph114_B4
zmp_ph114_D8
zmp_ph114_E2

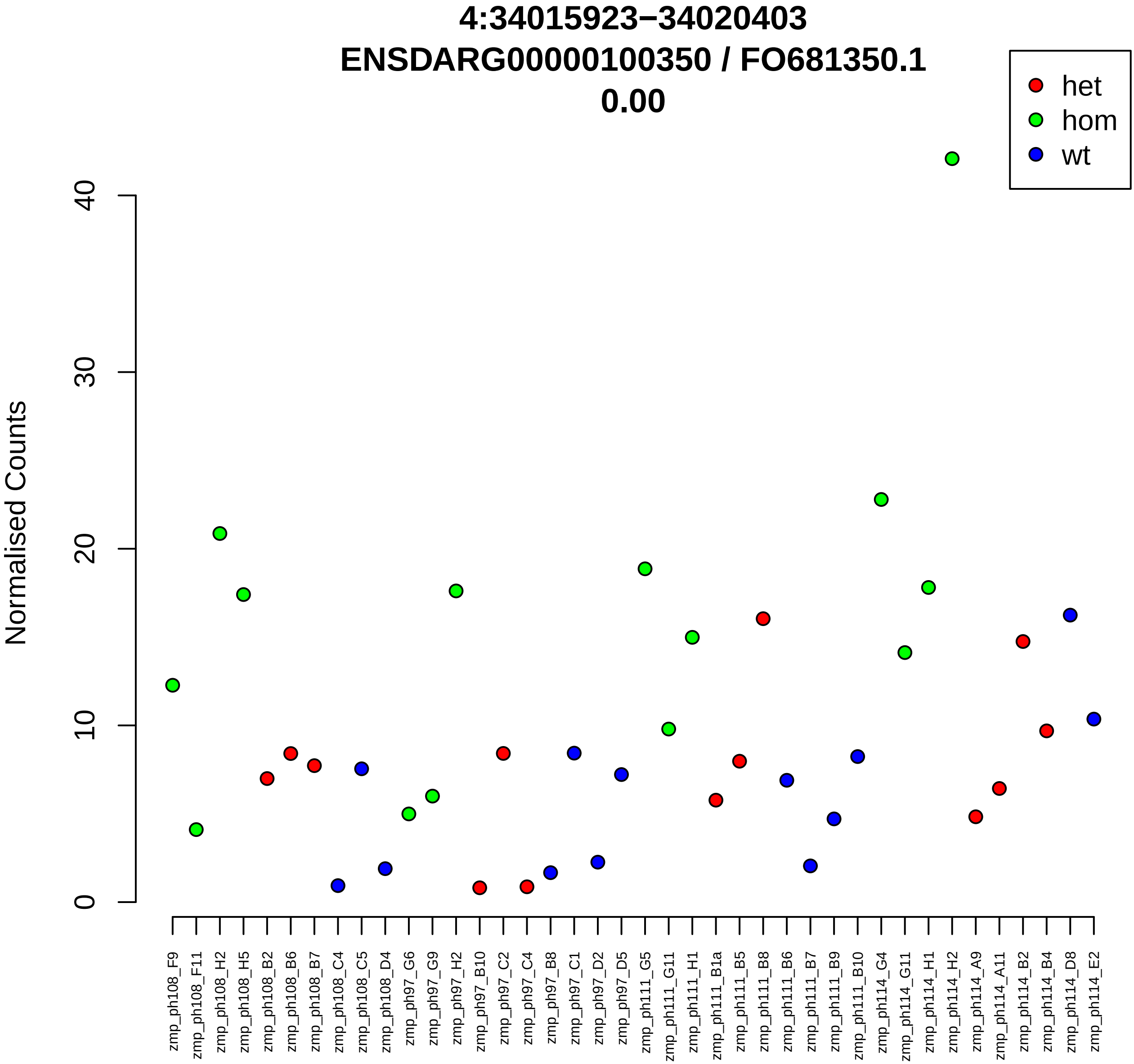
4:34015923−34020403
ENSDARG00000100350 / FO681350.1
0.00
het
hom
wt
Normalised Counts
0
10
20
30
40
zmp_ph108_F9
zmp_ph108_F11
zmp_ph108_H2
zmp_ph108_H5
zmp_ph108_B2
zmp_ph108_B6
zmp_ph108_B7
zmp_ph108_C4
zmp_ph108_C5
zmp_ph108_D4
zmp_ph97_G6
zmp_ph97_G9
zmp_ph97_H2
zmp_ph97_B10
zmp_ph97_C2
zmp_ph97_C4
zmp_ph97_B8
zmp_ph97_C1
zmp_ph97_D2
zmp_ph97_D5
zmp_ph111_G5
zmp_ph111_G11
zmp_ph111_H1
zmp_ph111_B1a
zmp_ph111_B5
zmp_ph111_B8
zmp_ph111_B6
zmp_ph111_B7
zmp_ph111_B9
zmp_ph111_B10
zmp_ph114_G4
zmp_ph114_G11
zmp_ph114_H1
zmp_ph114_H2
zmp_ph114_A9
zmp_ph114_A11
zmp_ph114_B2
zmp_ph114_B4
zmp_ph114_D8
zmp_ph114_E2

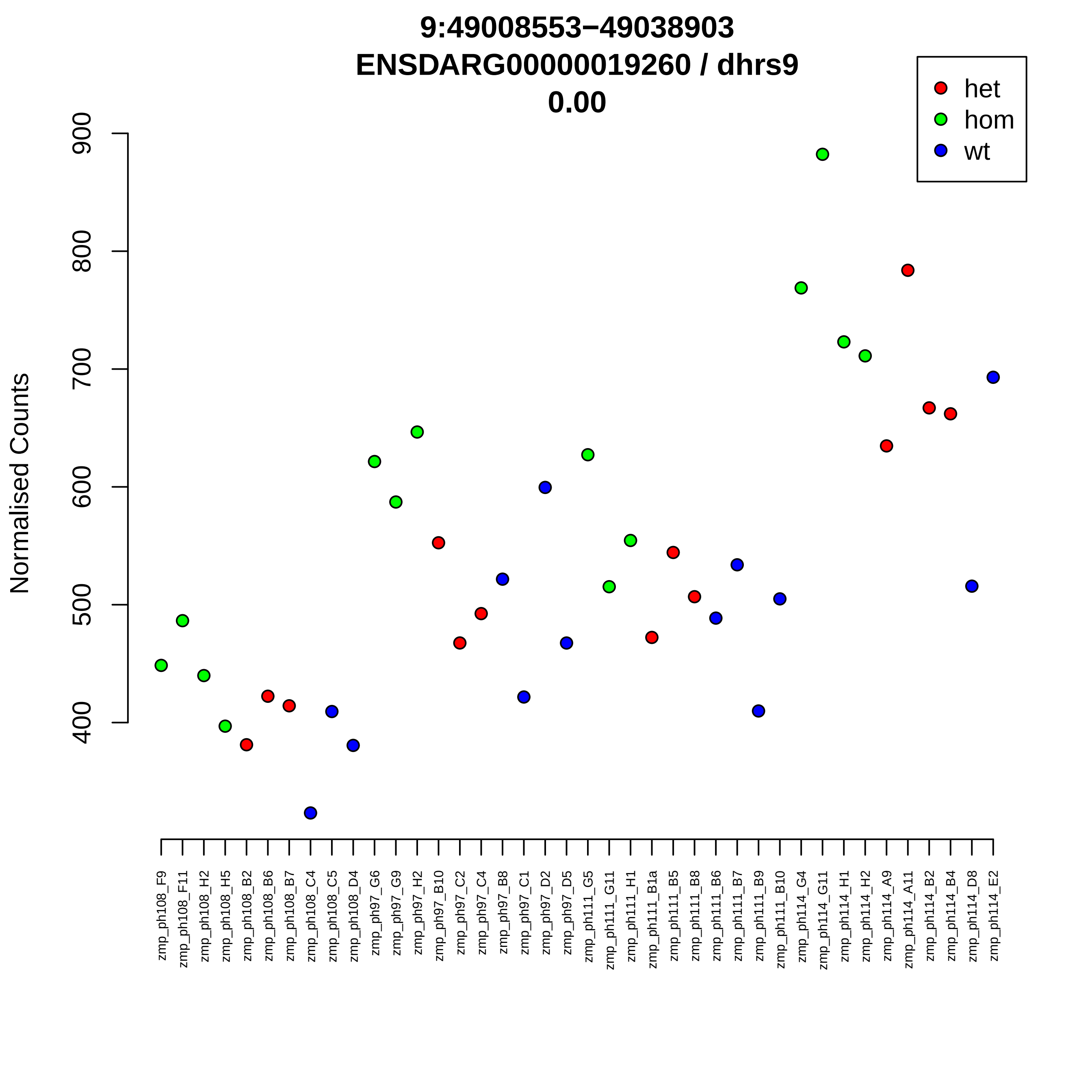

9:49008553−49038903
ENSDARG00000019260 / dhrs9
0.00
het
hom
wt
Normalised Counts
400
500
600
700
800
900
zmp_ph108_F9
zmp_ph108_F11
zmp_ph108_H2
zmp_ph108_H5
zmp_ph108_B2
zmp_ph108_B6
zmp_ph108_B7
zmp_ph108_C4
zmp_ph108_C5
zmp_ph108_D4
zmp_ph97_G6
zmp_ph97_G9
zmp_ph97_H2
zmp_ph97_B10
zmp_ph97_C2
zmp_ph97_C4
zmp_ph97_B8
zmp_ph97_C1
zmp_ph97_D2
zmp_ph97_D5
zmp_ph111_G5
zmp_ph111_G11
zmp_ph111_H1
zmp_ph111_B1a
zmp_ph111_B5
zmp_ph111_B8
zmp_ph111_B6
zmp_ph111_B7
zmp_ph111_B9
zmp_ph111_B10
zmp_ph114_G4
zmp_ph114_G11
zmp_ph114_H1
zmp_ph114_H2
zmp_ph114_A9
zmp_ph114_A11
zmp_ph114_B2
zmp_ph114_B4
zmp_ph114_D8
zmp_ph114_E2

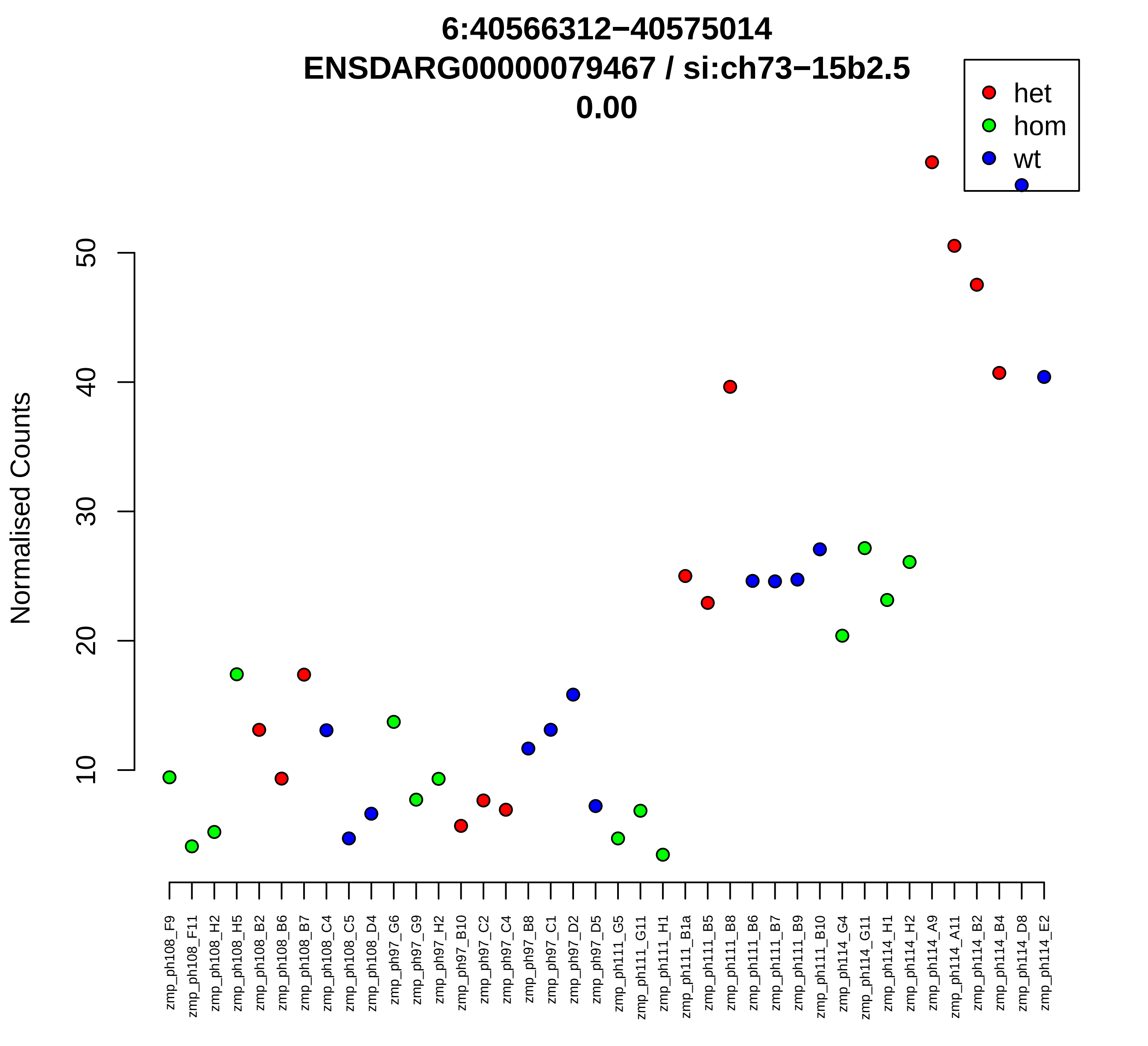

6:40566312−40575014
ENSDARG00000079467 / si:ch73−15b2.5
0.00
het
hom
wt
Normalised Counts
10
20
30
40
50
zmp_ph108_F9
zmp_ph108_F11
zmp_ph108_H2
zmp_ph108_H5
zmp_ph108_B2
zmp_ph108_B6
zmp_ph108_B7
zmp_ph108_C4
zmp_ph108_C5
zmp_ph108_D4
zmp_ph97_G6
zmp_ph97_G9
zmp_ph97_H2
zmp_ph97_B10
zmp_ph97_C2
zmp_ph97_C4
zmp_ph97_B8
zmp_ph97_C1
zmp_ph97_D2
zmp_ph97_D5
zmp_ph111_G5
zmp_ph111_G11
zmp_ph111_H1
zmp_ph111_B1a
zmp_ph111_B5
zmp_ph111_B8
zmp_ph111_B6
zmp_ph111_B7
zmp_ph111_B9
zmp_ph111_B10
zmp_ph114_G4
zmp_ph114_G11
zmp_ph114_H1
zmp_ph114_H2
zmp_ph114_A9
zmp_ph114_A11
zmp_ph114_B2
zmp_ph114_B4
zmp_ph114_D8
zmp_ph114_E2

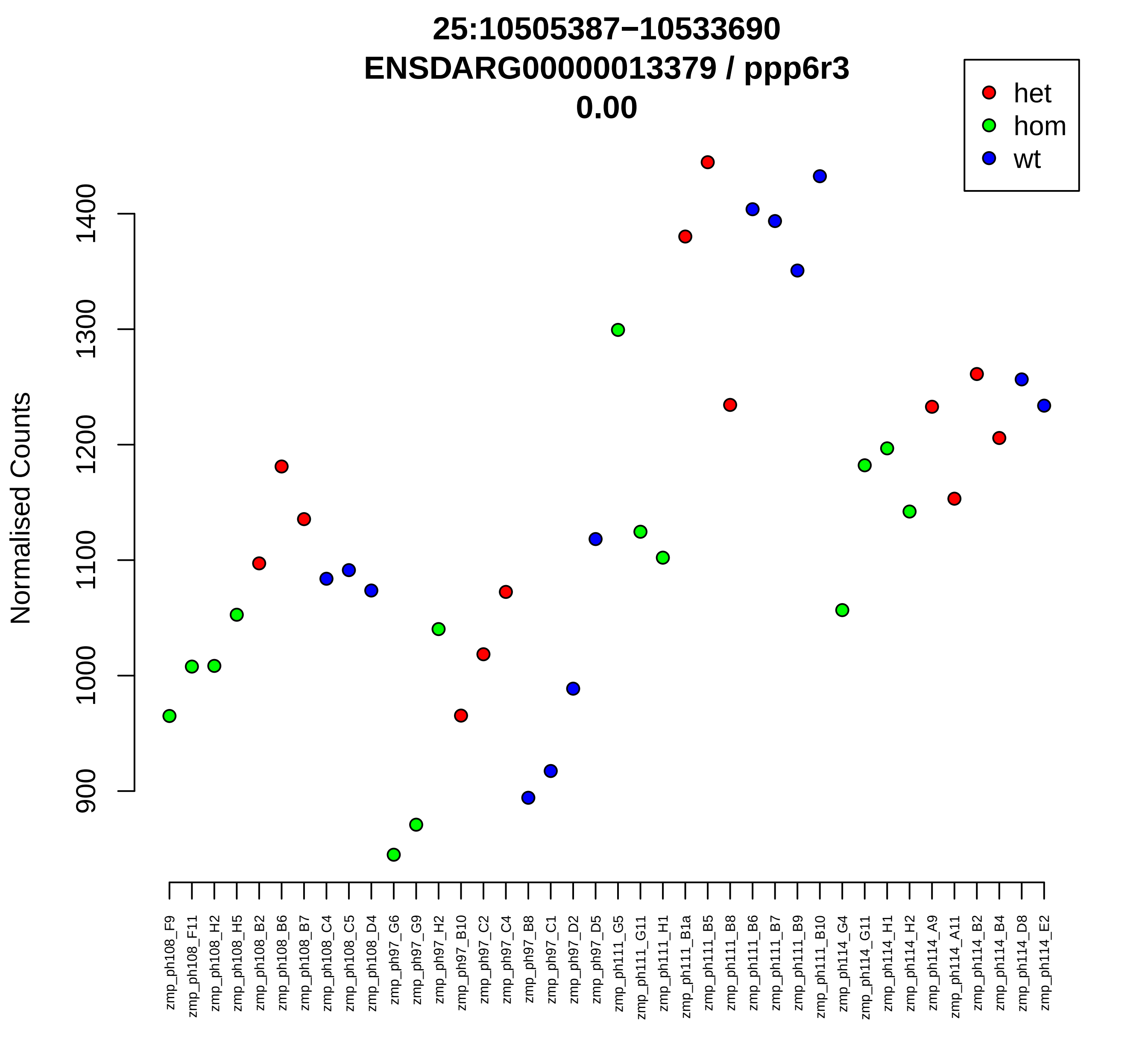

25:10505387−10533690
ENSDARG00000013379 / ppp6r3
0.00
het
hom
wt
Normalised Counts
900
1000
1100
1200
1300
1400
zmp_ph108_F9
zmp_ph108_F11
zmp_ph108_H2
zmp_ph108_H5
zmp_ph108_B2
zmp_ph108_B6
zmp_ph108_B7
zmp_ph108_C4
zmp_ph108_C5
zmp_ph108_D4
zmp_ph97_G6
zmp_ph97_G9
zmp_ph97_H2
zmp_ph97_B10
zmp_ph97_C2
zmp_ph97_C4
zmp_ph97_B8
zmp_ph97_C1
zmp_ph97_D2
zmp_ph97_D5
zmp_ph111_G5
zmp_ph111_G11
zmp_ph111_H1
zmp_ph111_B1a
zmp_ph111_B5
zmp_ph111_B8
zmp_ph111_B6
zmp_ph111_B7
zmp_ph111_B9
zmp_ph111_B10
zmp_ph114_G4
zmp_ph114_G11
zmp_ph114_H1
zmp_ph114_H2
zmp_ph114_A9
zmp_ph114_A11
zmp_ph114_B2
zmp_ph114_B4
zmp_ph114_D8
zmp_ph114_E2

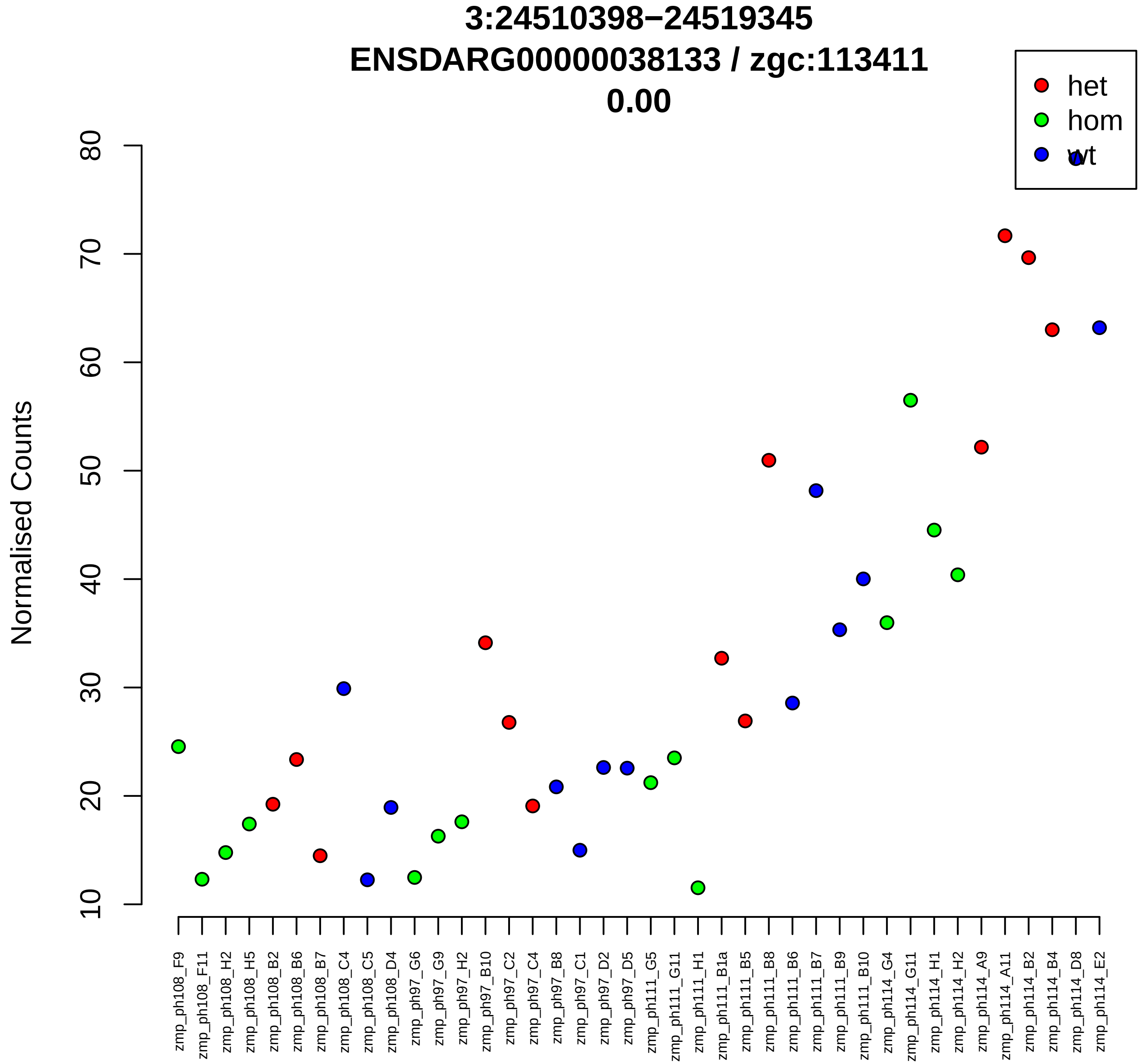
3:24510398−24519345
ENSDARG00000038133 / zgc:113411
0.00
Normalised Counts
10
20
30
40
50
60
70
80
het
hom
wt
zmp_ph108_F9
zmp_ph108_F11
zmp_ph108_H2
zmp_ph108_H5
zmp_ph108_B2
zmp_ph108_B6
zmp_ph108_B7
zmp_ph108_C4
zmp_ph108_C5
zmp_ph108_D4
zmp_ph97_G6
zmp_ph97_G9
zmp_ph97_H2
zmp_ph97_B10
zmp_ph97_C2
zmp_ph97_C4
zmp_ph97_B8
zmp_ph97_C1
zmp_ph97_D2
zmp_ph97_D5
zmp_ph111_G5
zmp_ph111_G11
zmp_ph111_H1
zmp_ph111_B1a
zmp_ph111_B5
zmp_ph111_B8
zmp_ph111_B6
zmp_ph111_B7
zmp_ph111_B9
zmp_ph111_B10
zmp_ph114_G4
zmp_ph114_G11
zmp_ph114_H1
zmp_ph114_H2
zmp_ph114_A9
zmp_ph114_A11
zmp_ph114_B2
zmp_ph114_B4
zmp_ph114_D8
zmp_ph114_E2

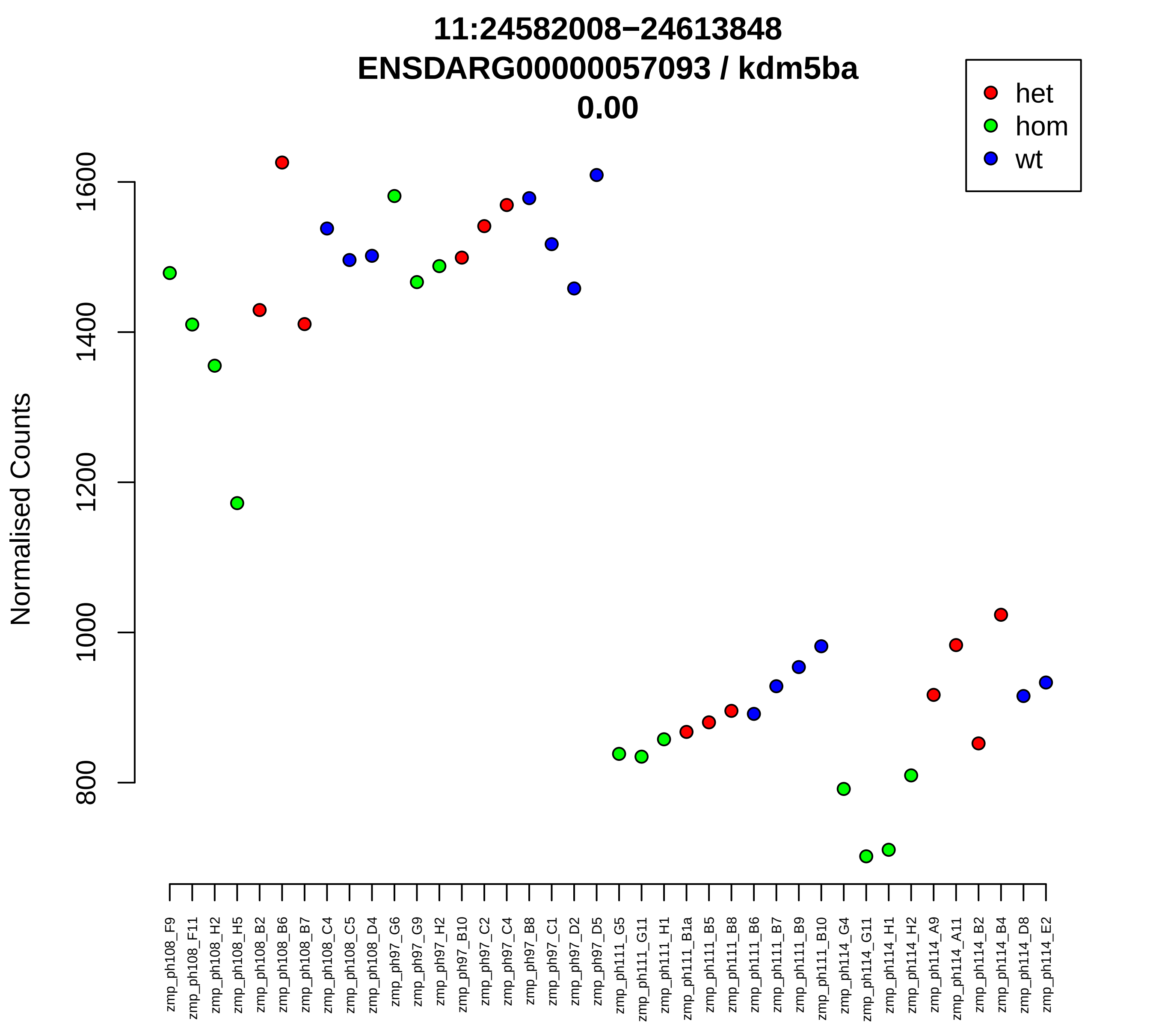

11:24582008−24613848
ENSDARG00000057093 / kdm5ba
0.00
het
hom
wt
Normalised Counts
800
1000
1200
1400
1600
zmp_ph108_F9
zmp_ph108_F11
zmp_ph108_H2
zmp_ph108_H5
zmp_ph108_B2
zmp_ph108_B6
zmp_ph108_B7
zmp_ph108_C4
zmp_ph108_C5
zmp_ph108_D4
zmp_ph97_G6
zmp_ph97_G9
zmp_ph97_H2
zmp_ph97_B10
zmp_ph97_C2
zmp_ph97_C4
zmp_ph97_B8
zmp_ph97_C1
zmp_ph97_D2
zmp_ph97_D5
zmp_ph111_G5
zmp_ph111_G11
zmp_ph111_H1
zmp_ph111_B1a
zmp_ph111_B5
zmp_ph111_B8
zmp_ph111_B6
zmp_ph111_B7
zmp_ph111_B9
zmp_ph111_B10
zmp_ph114_G4
zmp_ph114_G11
zmp_ph114_H1
zmp_ph114_H2
zmp_ph114_A9
zmp_ph114_A11
zmp_ph114_B2
zmp_ph114_B4
zmp_ph114_D8
zmp_ph114_E2

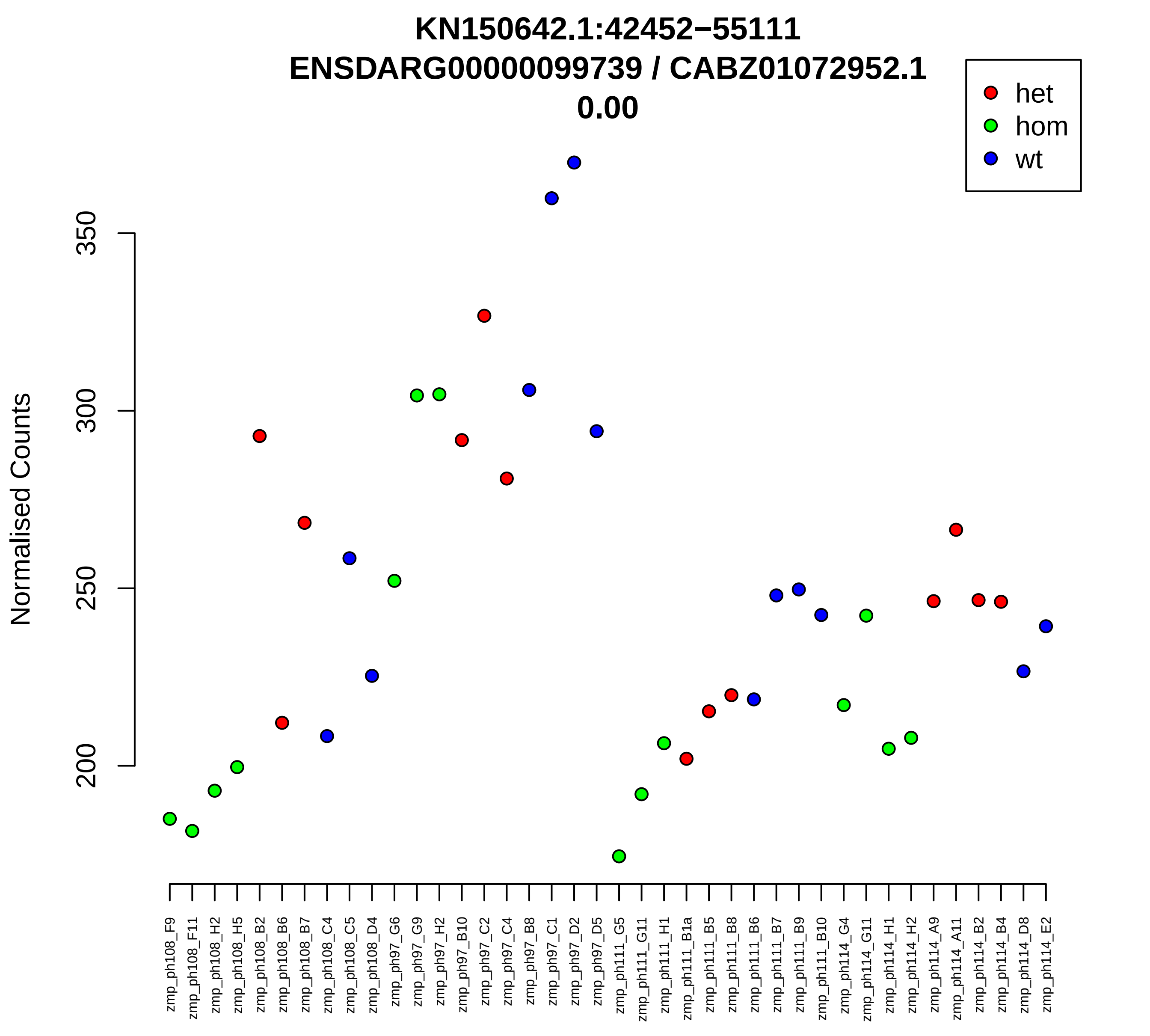

KN150642.1:42452−55111
ENSDARG00000099739 / CABZ01072952.1
0.00
het
hom
wt
Normalised Counts
200
250
300
350
zmp_ph108_F9
zmp_ph108_F11
zmp_ph108_H2
zmp_ph108_H5
zmp_ph108_B2
zmp_ph108_B6
zmp_ph108_B7
zmp_ph108_C4
zmp_ph108_C5
zmp_ph108_D4
zmp_ph97_G6
zmp_ph97_G9
zmp_ph97_H2
zmp_ph97_B10
zmp_ph97_C2
zmp_ph97_C4
zmp_ph97_B8
zmp_ph97_C1
zmp_ph97_D2
zmp_ph97_D5
zmp_ph111_G5
zmp_ph111_G11
zmp_ph111_H1
zmp_ph111_B1a
zmp_ph111_B5
zmp_ph111_B8
zmp_ph111_B6
zmp_ph111_B7
zmp_ph111_B9
zmp_ph111_B10
zmp_ph114_G4
zmp_ph114_G11
zmp_ph114_H1
zmp_ph114_H2
zmp_ph114_A9
zmp_ph114_A11
zmp_ph114_B2
zmp_ph114_B4
zmp_ph114_D8
zmp_ph114_E2

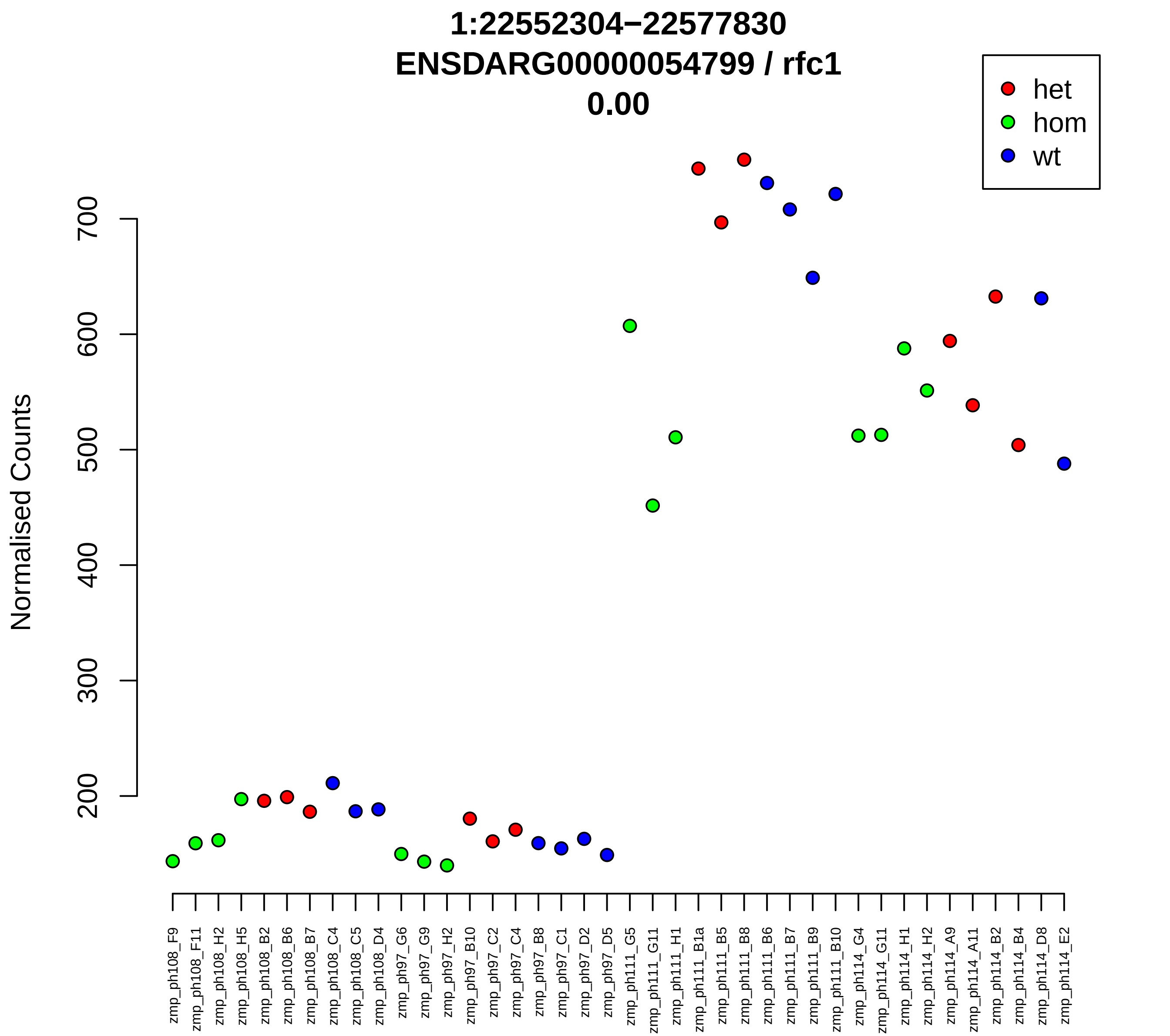

1:22552304−22577830
ENSDARG00000054799 / rfc1
0.00
het
hom
wt
Normalised Counts
200
300
400
500
600
700
zmp_ph108_F9
zmp_ph108_F11
zmp_ph108_H2
zmp_ph108_H5
zmp_ph108_B2
zmp_ph108_B6
zmp_ph108_B7
zmp_ph108_C4
zmp_ph108_C5
zmp_ph108_D4
zmp_ph97_G6
zmp_ph97_G9
zmp_ph97_H2
zmp_ph97_B10
zmp_ph97_C2
zmp_ph97_C4
zmp_ph97_B8
zmp_ph97_C1
zmp_ph97_D2
zmp_ph97_D5
zmp_ph111_G5
zmp_ph111_G11
zmp_ph111_H1
zmp_ph111_B1a
zmp_ph111_B5
zmp_ph111_B8
zmp_ph111_B6
zmp_ph111_B7
zmp_ph111_B9
zmp_ph111_B10
zmp_ph114_G4
zmp_ph114_G11
zmp_ph114_H1
zmp_ph114_H2
zmp_ph114_A9
zmp_ph114_A11
zmp_ph114_B2
zmp_ph114_B4
zmp_ph114_D8
zmp_ph114_E2

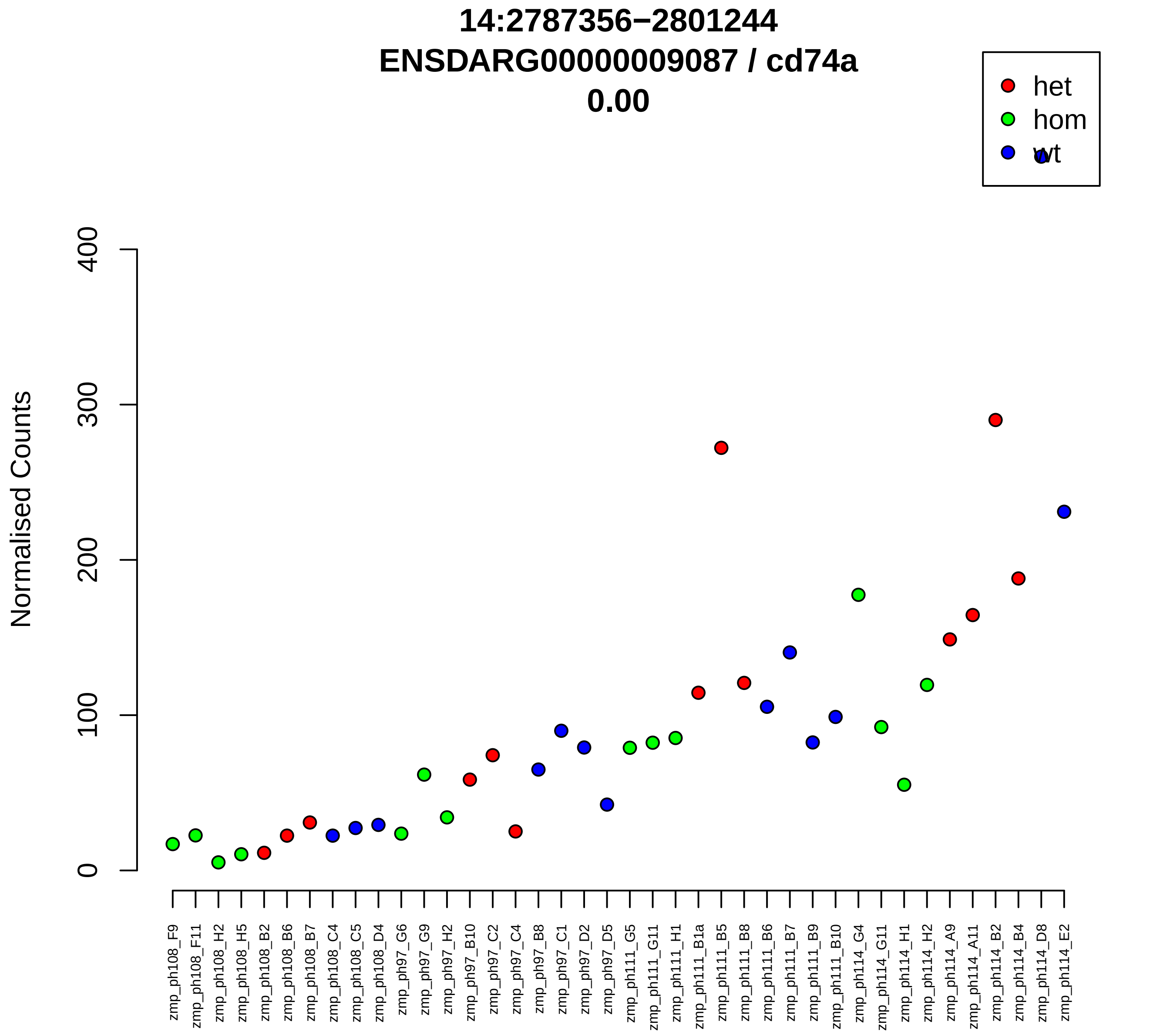
14:2787356−2801244
ENSDARG00000009087 / cd74a
0.00
het
hom
wt
Normalised Counts
0
100
200
300
400
zmp_ph108_F9
zmp_ph108_F11
zmp_ph108_H2
zmp_ph108_H5
zmp_ph108_B2
zmp_ph108_B6
zmp_ph108_B7
zmp_ph108_C4
zmp_ph108_C5
zmp_ph108_D4
zmp_ph97_G6
zmp_ph97_G9
zmp_ph97_H2
zmp_ph97_B10
zmp_ph97_C2
zmp_ph97_C4
zmp_ph97_B8
zmp_ph97_C1
zmp_ph97_D2
zmp_ph97_D5
zmp_ph111_G5
zmp_ph111_G11
zmp_ph111_H1
zmp_ph111_B1a
zmp_ph111_B5
zmp_ph111_B8
zmp_ph111_B6
zmp_ph111_B7
zmp_ph111_B9
zmp_ph111_B10
zmp_ph114_G4
zmp_ph114_G11
zmp_ph114_H1
zmp_ph114_H2
zmp_ph114_A9
zmp_ph114_A11
zmp_ph114_B2
zmp_ph114_B4
zmp_ph114_D8
zmp_ph114_E2

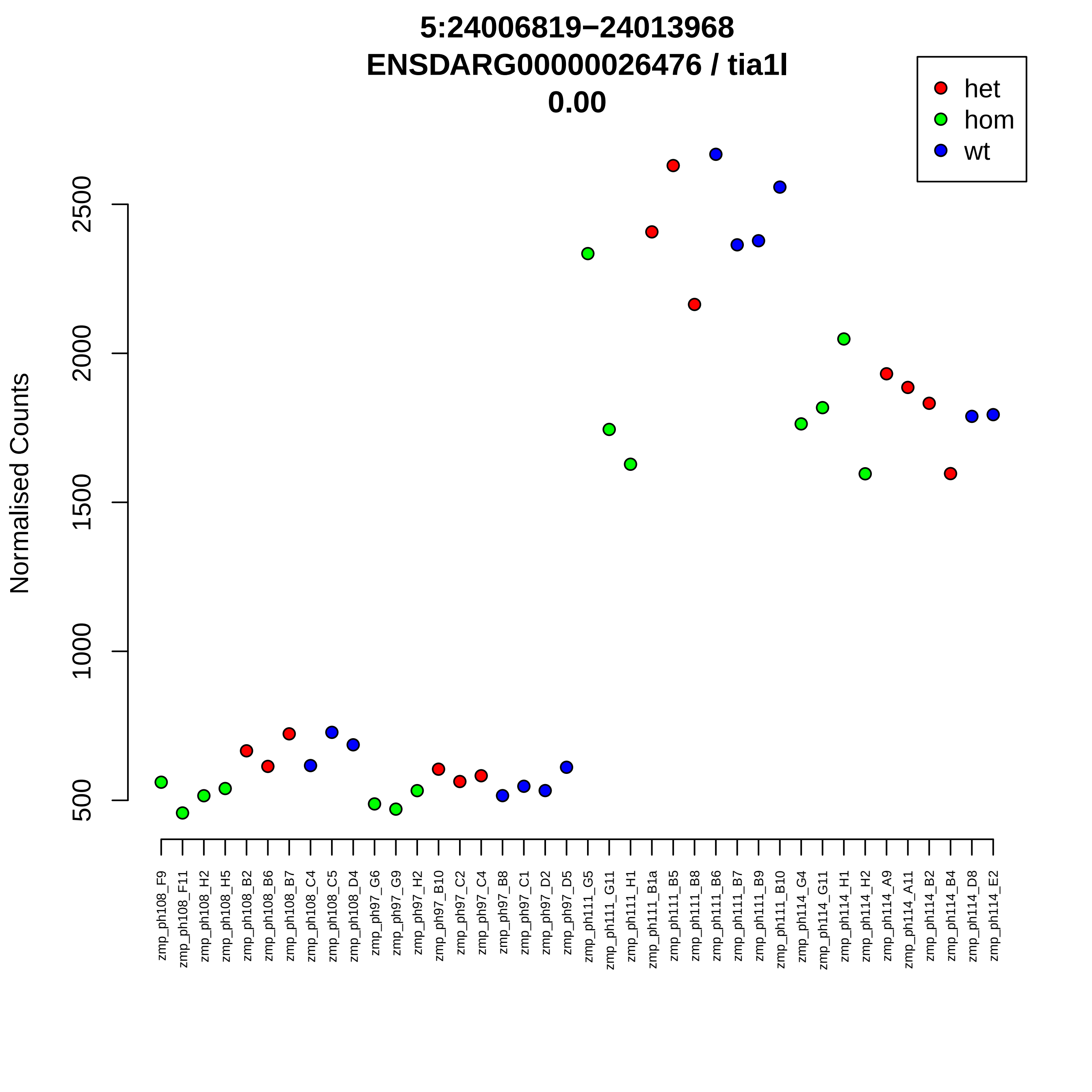

5:24006819−24013968
ENSDARG00000026476 / tia1l
0.00
het
hom
wt
Normalised Counts
500
1000
1500
2000
2500
zmp_ph108_F9
zmp_ph108_F11
zmp_ph108_H2
zmp_ph108_H5
zmp_ph108_B2
zmp_ph108_B6
zmp_ph108_B7
zmp_ph108_C4
zmp_ph108_C5
zmp_ph108_D4
zmp_ph97_G6
zmp_ph97_G9
zmp_ph97_H2
zmp_ph97_B10
zmp_ph97_C2
zmp_ph97_C4
zmp_ph97_B8
zmp_ph97_C1
zmp_ph97_D2
zmp_ph97_D5
zmp_ph111_G5
zmp_ph111_G11
zmp_ph111_H1
zmp_ph111_B1a
zmp_ph111_B5
zmp_ph111_B8
zmp_ph111_B6
zmp_ph111_B7
zmp_ph111_B9
zmp_ph111_B10
zmp_ph114_G4
zmp_ph114_G11
zmp_ph114_H1
zmp_ph114_H2
zmp_ph114_A9
zmp_ph114_A11
zmp_ph114_B2
zmp_ph114_B4
zmp_ph114_D8
zmp_ph114_E2

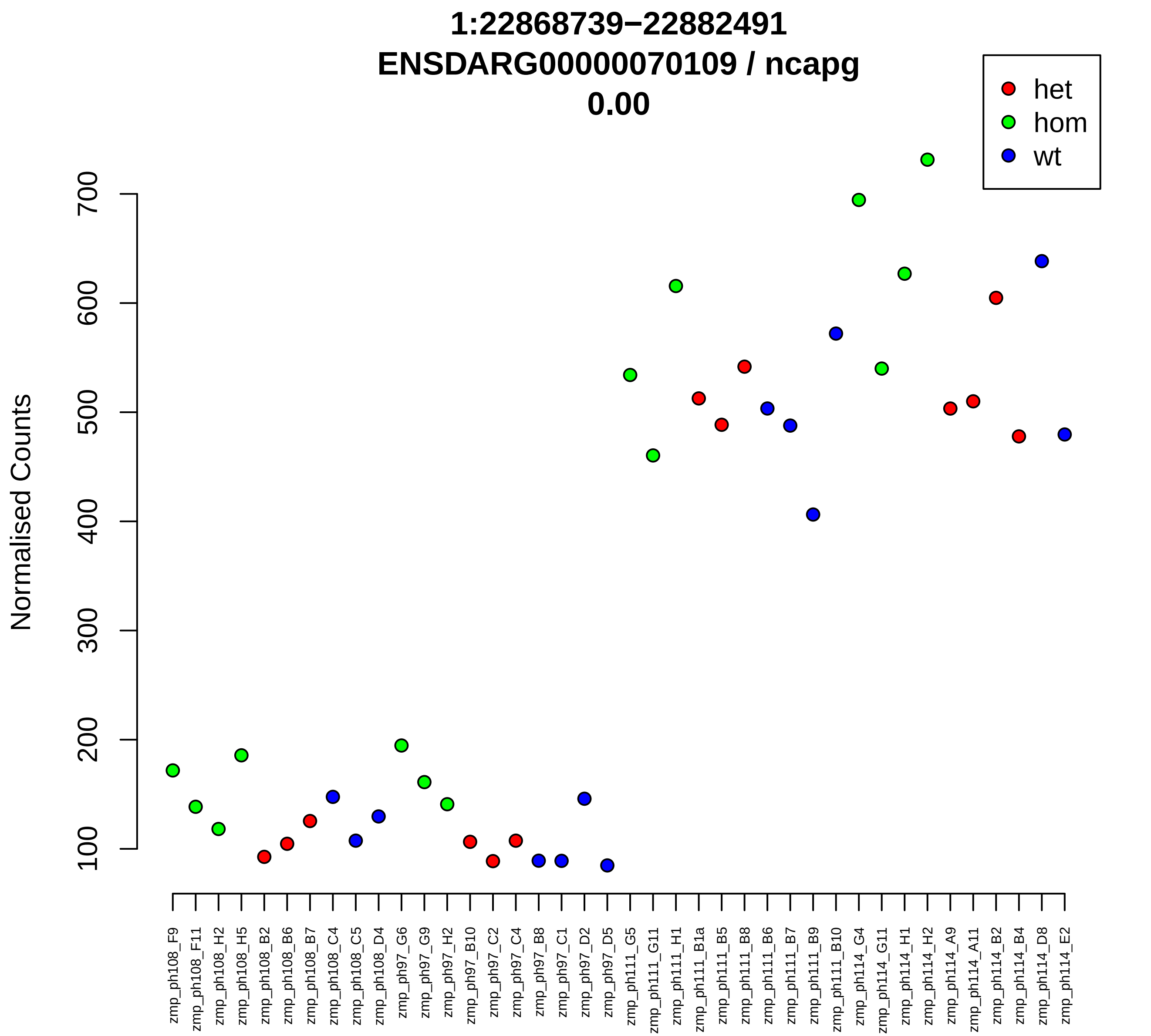
1:22868739−22882491
ENSDARG00000070109 / ncapg
0.00
het
hom
wt
Normalised Counts
100
200
300
400
500
600
700
zmp_ph108_F9
zmp_ph108_F11
zmp_ph108_H2
zmp_ph108_H5
zmp_ph108_B2
zmp_ph108_B6
zmp_ph108_B7
zmp_ph108_C4
zmp_ph108_C5
zmp_ph108_D4
zmp_ph97_G6
zmp_ph97_G9
zmp_ph97_H2
zmp_ph97_B10
zmp_ph97_C2
zmp_ph97_C4
zmp_ph97_B8
zmp_ph97_C1
zmp_ph97_D2
zmp_ph97_D5
zmp_ph111_G5
zmp_ph111_G11
zmp_ph111_H1
zmp_ph111_B1a
zmp_ph111_B5
zmp_ph111_B8
zmp_ph111_B6
zmp_ph111_B7
zmp_ph111_B9
zmp_ph111_B10
zmp_ph114_G4
zmp_ph114_G11
zmp_ph114_H1
zmp_ph114_H2
zmp_ph114_A9
zmp_ph114_A11
zmp_ph114_B2
zmp_ph114_B4
zmp_ph114_D8
zmp_ph114_E2

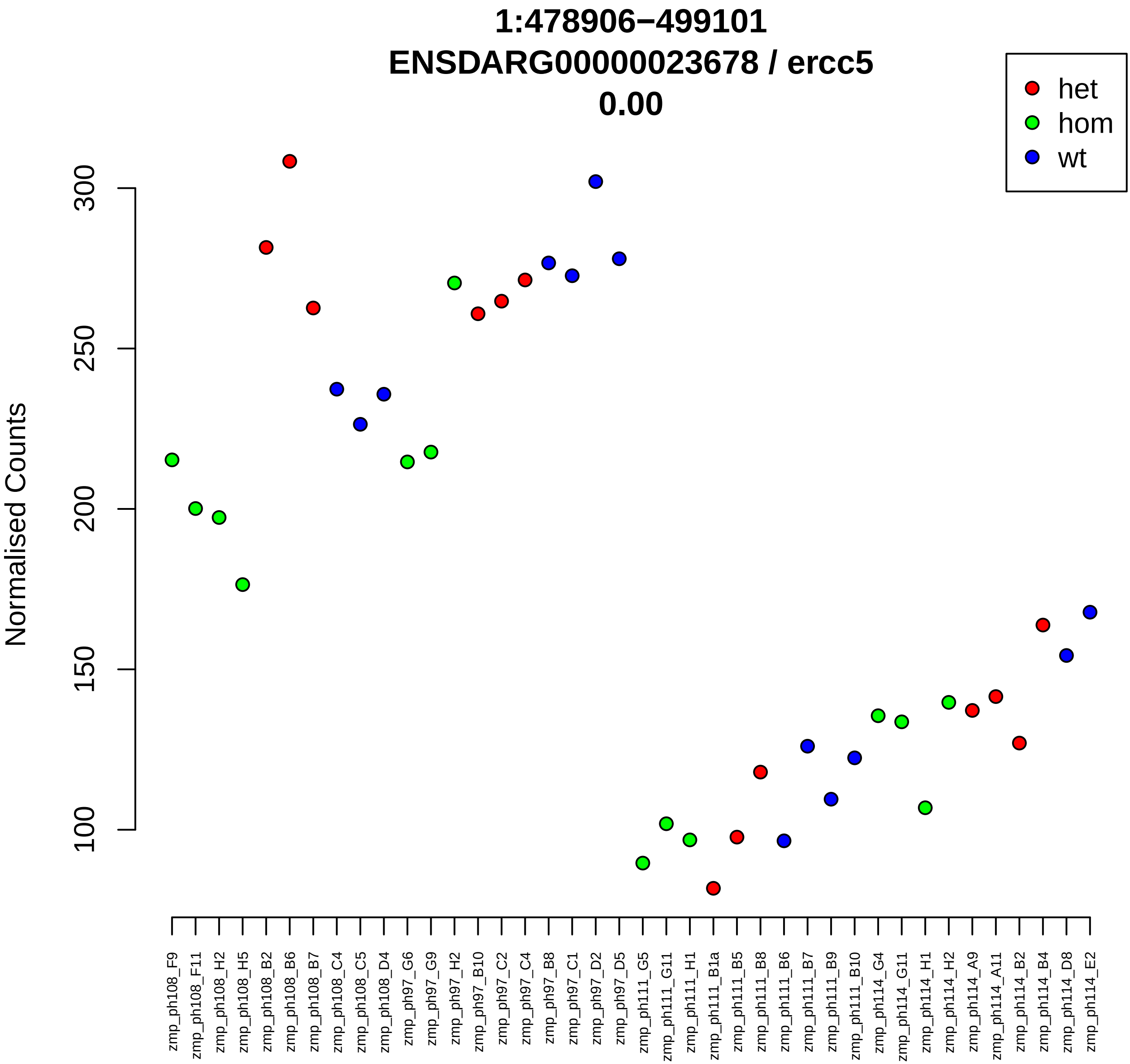
1:478906−499101
ENSDARG00000023678 / ercc5
0.00
het
hom
wt
Normalised Counts
100
150
200
250
300
zmp_ph108_F9
zmp_ph108_F11
zmp_ph108_H2
zmp_ph108_H5
zmp_ph108_B2
zmp_ph108_B6
zmp_ph108_B7
zmp_ph108_C4
zmp_ph108_C5
zmp_ph108_D4
zmp_ph97_G6
zmp_ph97_G9
zmp_ph97_H2
zmp_ph97_B10
zmp_ph97_C2
zmp_ph97_C4
zmp_ph97_B8
zmp_ph97_C1
zmp_ph97_D2
zmp_ph97_D5
zmp_ph111_G5
zmp_ph111_G11
zmp_ph111_H1
zmp_ph111_B1a
zmp_ph111_B5
zmp_ph111_B8
zmp_ph111_B6
zmp_ph111_B7
zmp_ph111_B9
zmp_ph111_B10
zmp_ph114_G4
zmp_ph114_G11
zmp_ph114_H1
zmp_ph114_H2
zmp_ph114_A9
zmp_ph114_A11
zmp_ph114_B2
zmp_ph114_B4
zmp_ph114_D8
zmp_ph114_E2

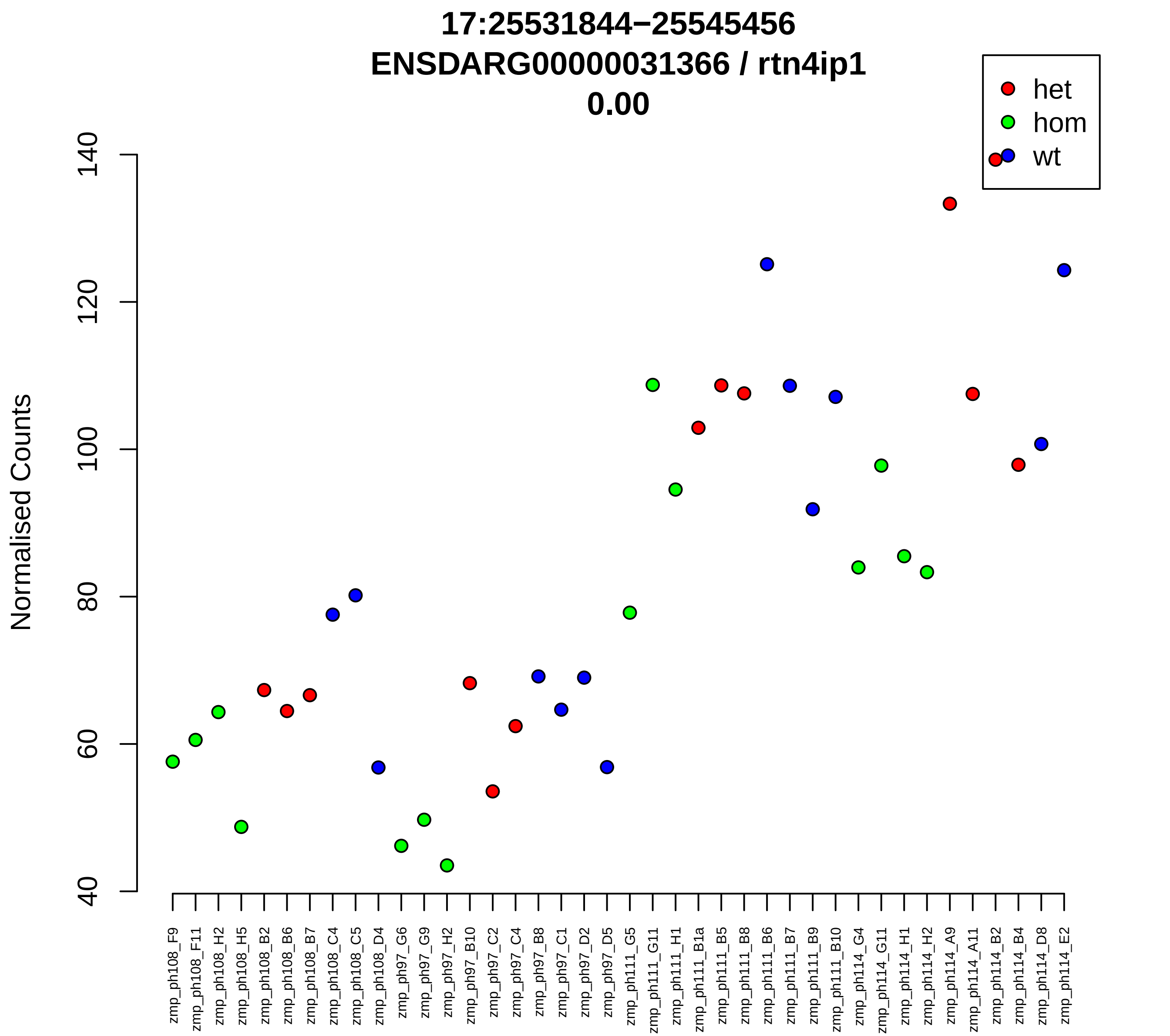

17:25531844−25545456
ENSDARG00000031366 / rtn4ip1
0.00
het
hom
wt
Normalised Counts
40
60
80
100
120
140
zmp_ph108_F9
zmp_ph108_F11
zmp_ph108_H2
zmp_ph108_H5
zmp_ph108_B2
zmp_ph108_B6
zmp_ph108_B7
zmp_ph108_C4
zmp_ph108_C5
zmp_ph108_D4
zmp_ph97_G6
zmp_ph97_G9
zmp_ph97_H2
zmp_ph97_B10
zmp_ph97_C2
zmp_ph97_C4
zmp_ph97_B8
zmp_ph97_C1
zmp_ph97_D2
zmp_ph97_D5
zmp_ph111_G5
zmp_ph111_G11
zmp_ph111_H1
zmp_ph111_B1a
zmp_ph111_B5
zmp_ph111_B8
zmp_ph111_B6
zmp_ph111_B7
zmp_ph111_B9
zmp_ph111_B10
zmp_ph114_G4
zmp_ph114_G11
zmp_ph114_H1
zmp_ph114_H2
zmp_ph114_A9
zmp_ph114_A11
zmp_ph114_B2
zmp_ph114_B4
zmp_ph114_D8
zmp_ph114_E2

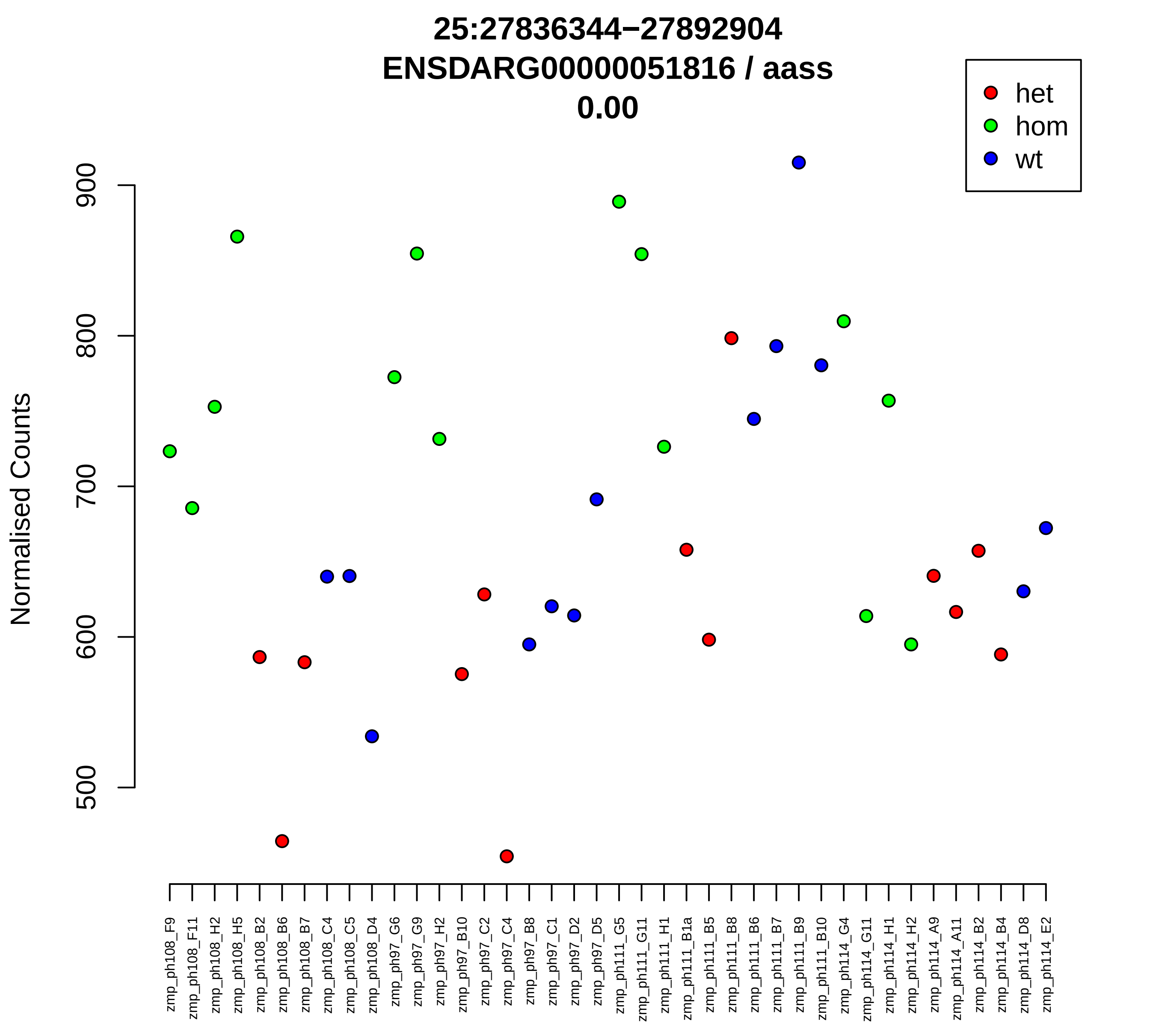

25:27836344−27892904
ENSDARG00000051816 / aass
0.00
het
hom
wt
Normalised Counts
500
600
700
800
900
zmp_ph108_F9
zmp_ph108_F11
zmp_ph108_H2
zmp_ph108_H5
zmp_ph108_B2
zmp_ph108_B6
zmp_ph108_B7
zmp_ph108_C4
zmp_ph108_C5
zmp_ph108_D4
zmp_ph97_G6
zmp_ph97_G9
zmp_ph97_H2
zmp_ph97_B10
zmp_ph97_C2
zmp_ph97_C4
zmp_ph97_B8
zmp_ph97_C1
zmp_ph97_D2
zmp_ph97_D5
zmp_ph111_G5
zmp_ph111_G11
zmp_ph111_H1
zmp_ph111_B1a
zmp_ph111_B5
zmp_ph111_B8
zmp_ph111_B6
zmp_ph111_B7
zmp_ph111_B9
zmp_ph111_B10
zmp_ph114_G4
zmp_ph114_G11
zmp_ph114_H1
zmp_ph114_H2
zmp_ph114_A9
zmp_ph114_A11
zmp_ph114_B2
zmp_ph114_B4
zmp_ph114_D8
zmp_ph114_E2

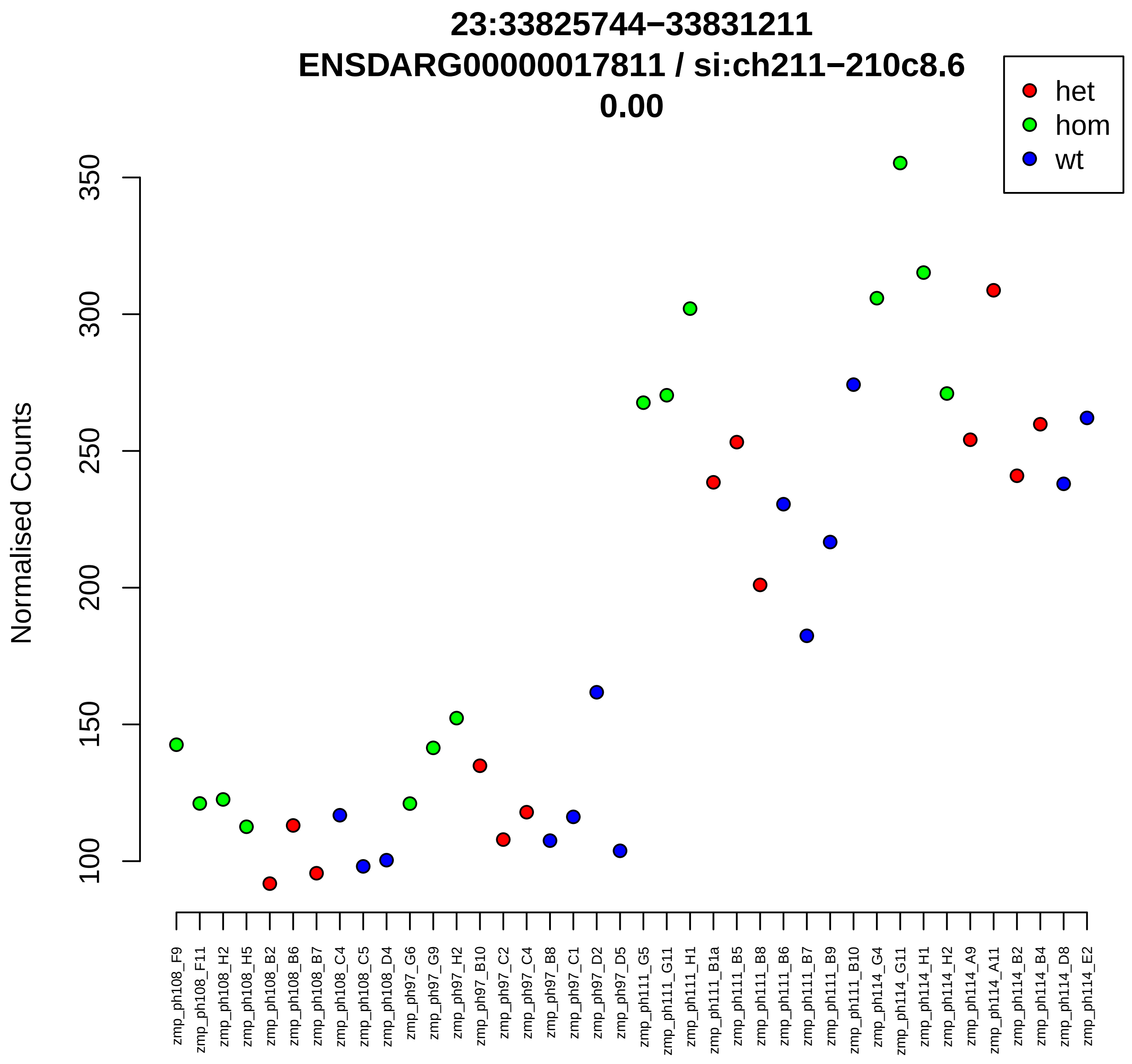

23:33825744−33831211
ENSDARG00000017811 / si:ch211−210c8.6
0.00
het
hom
wt
Normalised Counts
100
150
200
250
300
350
zmp_ph108_F9
zmp_ph108_F11
zmp_ph108_H2
zmp_ph108_H5
zmp_ph108_B2
zmp_ph108_B6
zmp_ph108_B7
zmp_ph108_C4
zmp_ph108_C5
zmp_ph108_D4
zmp_ph97_G6
zmp_ph97_G9
zmp_ph97_H2
zmp_ph97_B10
zmp_ph97_C2
zmp_ph97_C4
zmp_ph97_B8
zmp_ph97_C1
zmp_ph97_D2
zmp_ph97_D5
zmp_ph111_G5
zmp_ph111_G11
zmp_ph111_H1
zmp_ph111_B1a
zmp_ph111_B5
zmp_ph111_B8
zmp_ph111_B6
zmp_ph111_B7
zmp_ph111_B9
zmp_ph111_B10
zmp_ph114_G4
zmp_ph114_G11
zmp_ph114_H1
zmp_ph114_H2
zmp_ph114_A9
zmp_ph114_A11
zmp_ph114_B2
zmp_ph114_B4
zmp_ph114_D8
zmp_ph114_E2

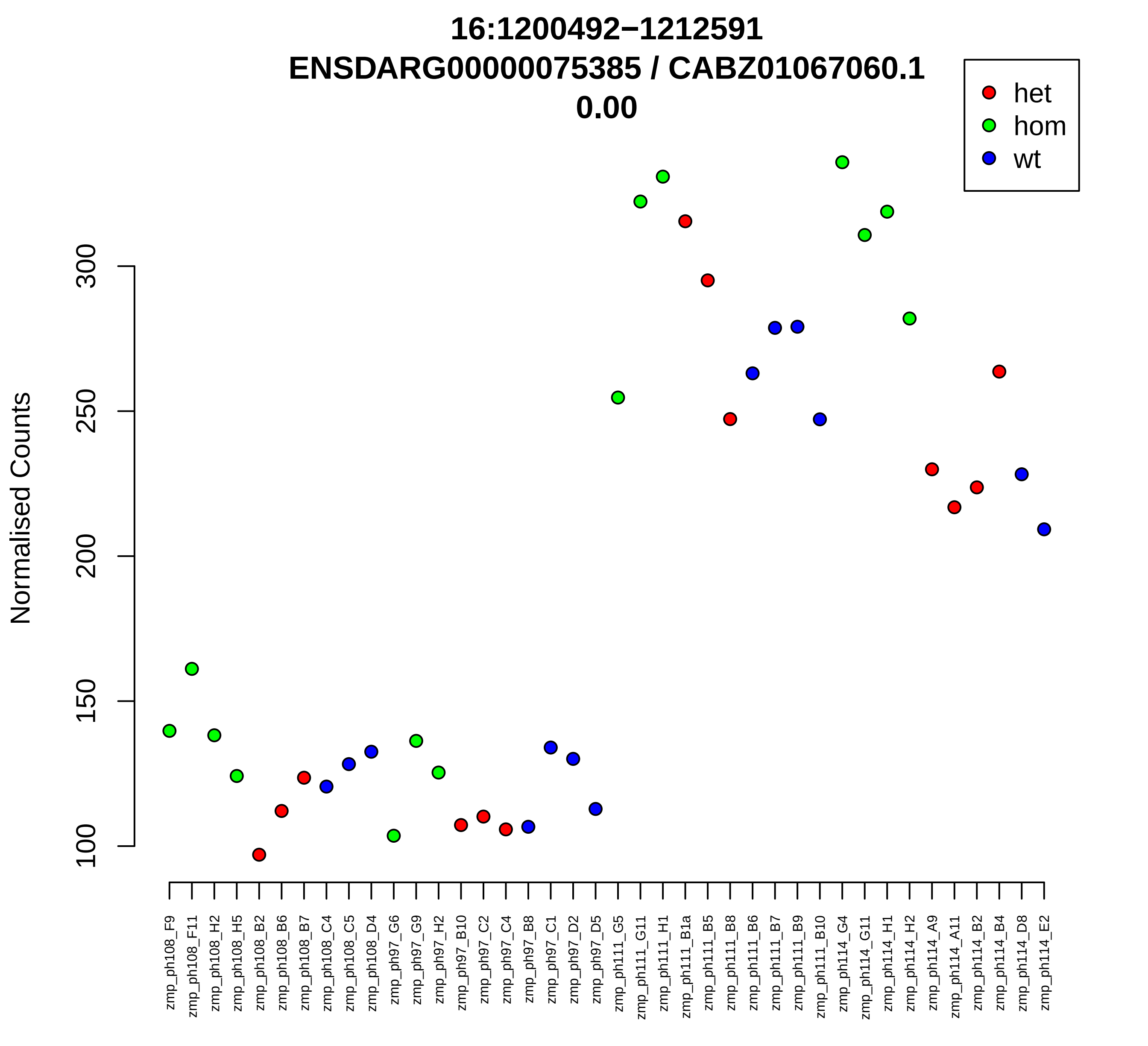
16:1200492−1212591
ENSDARG00000075385 / CABZ01067060.1
0.00
het
hom
wt
Normalised Counts
100
150
200
250
300
zmp_ph108_F9
zmp_ph108_F11
zmp_ph108_H2
zmp_ph108_H5
zmp_ph108_B2
zmp_ph108_B6
zmp_ph108_B7
zmp_ph108_C4
zmp_ph108_C5
zmp_ph108_D4
zmp_ph97_G6
zmp_ph97_G9
zmp_ph97_H2
zmp_ph97_B10
zmp_ph97_C2
zmp_ph97_C4
zmp_ph97_B8
zmp_ph97_C1
zmp_ph97_D2
zmp_ph97_D5
zmp_ph111_G5
zmp_ph111_G11
zmp_ph111_H1
zmp_ph111_B1a
zmp_ph111_B5
zmp_ph111_B8
zmp_ph111_B6
zmp_ph111_B7
zmp_ph111_B9
zmp_ph111_B10
zmp_ph114_G4
zmp_ph114_G11
zmp_ph114_H1
zmp_ph114_H2
zmp_ph114_A9
zmp_ph114_A11
zmp_ph114_B2
zmp_ph114_B4
zmp_ph114_D8
zmp_ph114_E2

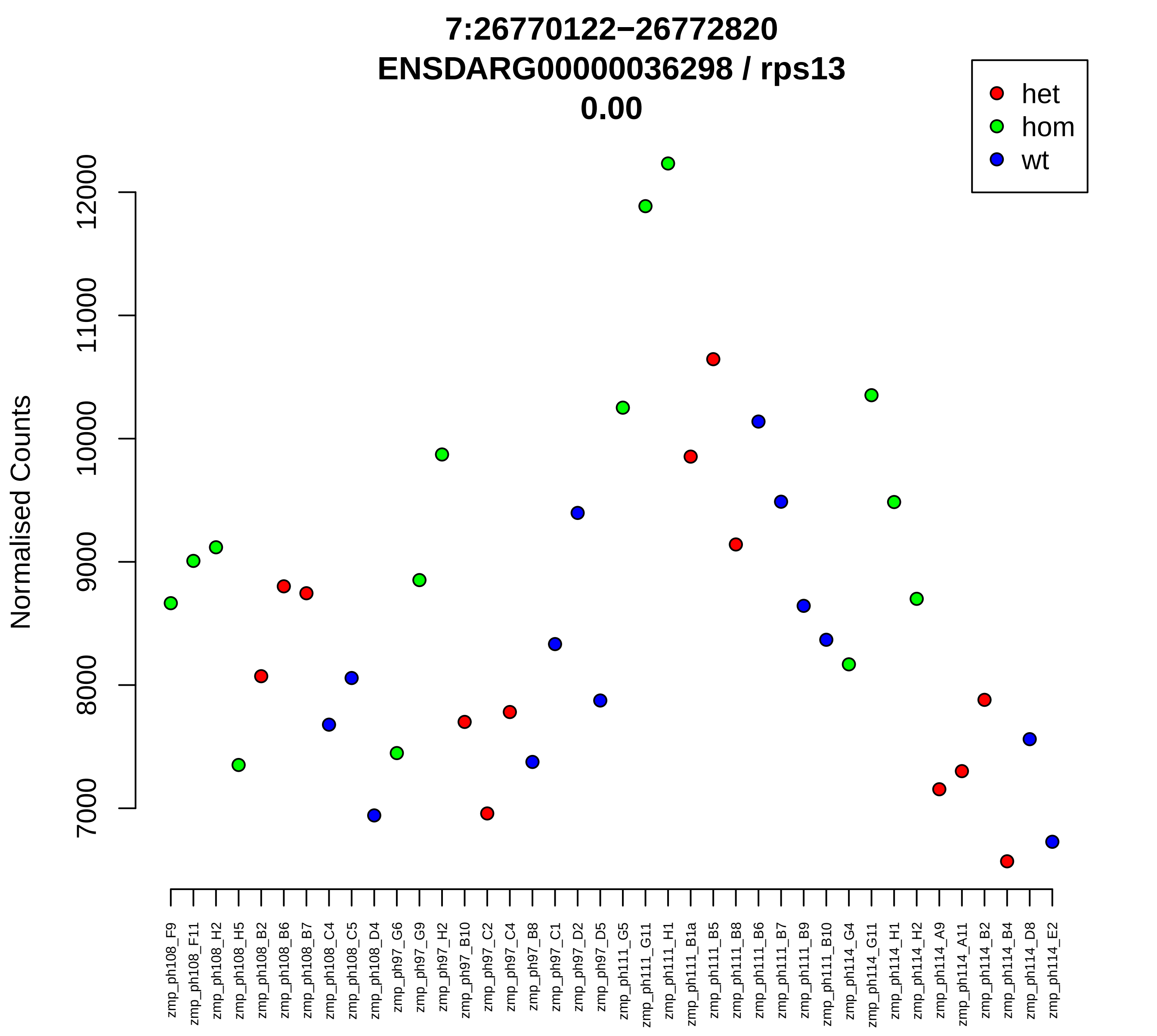
7:26770122−26772820
ENSDARG00000036298 / rps13
0.00
het
hom
wt
Normalised Counts
7000
8000
9000
10000
11000
12000
zmp_ph108_F9
zmp_ph108_F11
zmp_ph108_H2
zmp_ph108_H5
zmp_ph108_B2
zmp_ph108_B6
zmp_ph108_B7
zmp_ph108_C4
zmp_ph108_C5
zmp_ph108_D4
zmp_ph97_G6
zmp_ph97_G9
zmp_ph97_H2
zmp_ph97_B10
zmp_ph97_C2
zmp_ph97_C4
zmp_ph97_B8
zmp_ph97_C1
zmp_ph97_D2
zmp_ph97_D5
zmp_ph111_G5
zmp_ph111_G11
zmp_ph111_H1
zmp_ph111_B1a
zmp_ph111_B5
zmp_ph111_B8
zmp_ph111_B6
zmp_ph111_B7
zmp_ph111_B9
zmp_ph111_B10
zmp_ph114_G4
zmp_ph114_G11
zmp_ph114_H1
zmp_ph114_H2
zmp_ph114_A9
zmp_ph114_A11
zmp_ph114_B2
zmp_ph114_B4
zmp_ph114_D8
zmp_ph114_E2

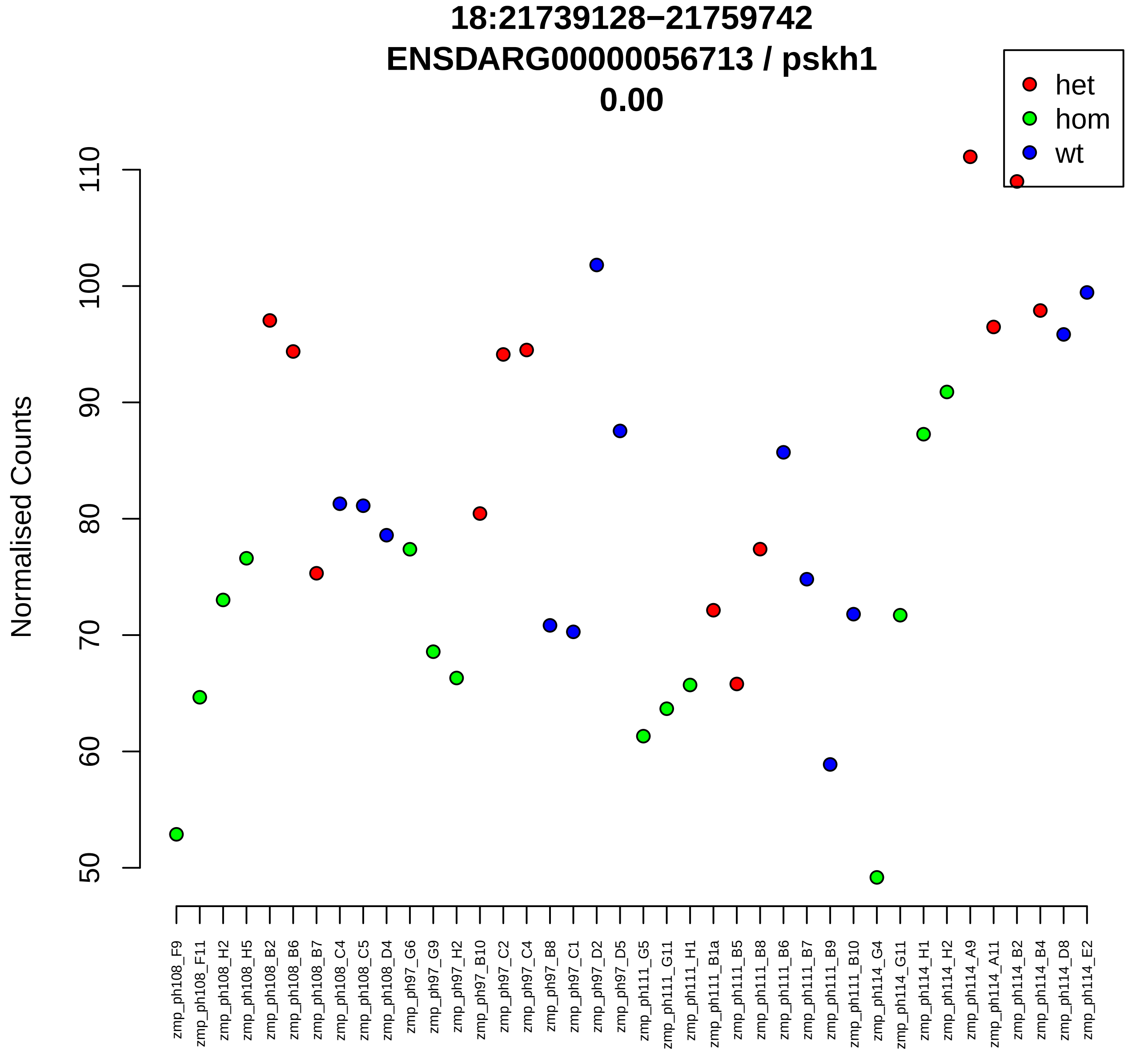

18:21739128−21759742
ENSDARG00000056713 / pskh1
0.00
het
hom
wt
Normalised Counts
50
60
70
80
90
100
110
zmp_ph108_F9
zmp_ph108_F11
zmp_ph108_H2
zmp_ph108_H5
zmp_ph108_B2
zmp_ph108_B6
zmp_ph108_B7
zmp_ph108_C4
zmp_ph108_C5
zmp_ph108_D4
zmp_ph97_G6
zmp_ph97_G9
zmp_ph97_H2
zmp_ph97_B10
zmp_ph97_C2
zmp_ph97_C4
zmp_ph97_B8
zmp_ph97_C1
zmp_ph97_D2
zmp_ph97_D5
zmp_ph111_G5
zmp_ph111_G11
zmp_ph111_H1
zmp_ph111_B1a
zmp_ph111_B5
zmp_ph111_B8
zmp_ph111_B6
zmp_ph111_B7
zmp_ph111_B9
zmp_ph111_B10
zmp_ph114_G4
zmp_ph114_G11
zmp_ph114_H1
zmp_ph114_H2
zmp_ph114_A9
zmp_ph114_A11
zmp_ph114_B2
zmp_ph114_B4
zmp_ph114_D8
zmp_ph114_E2

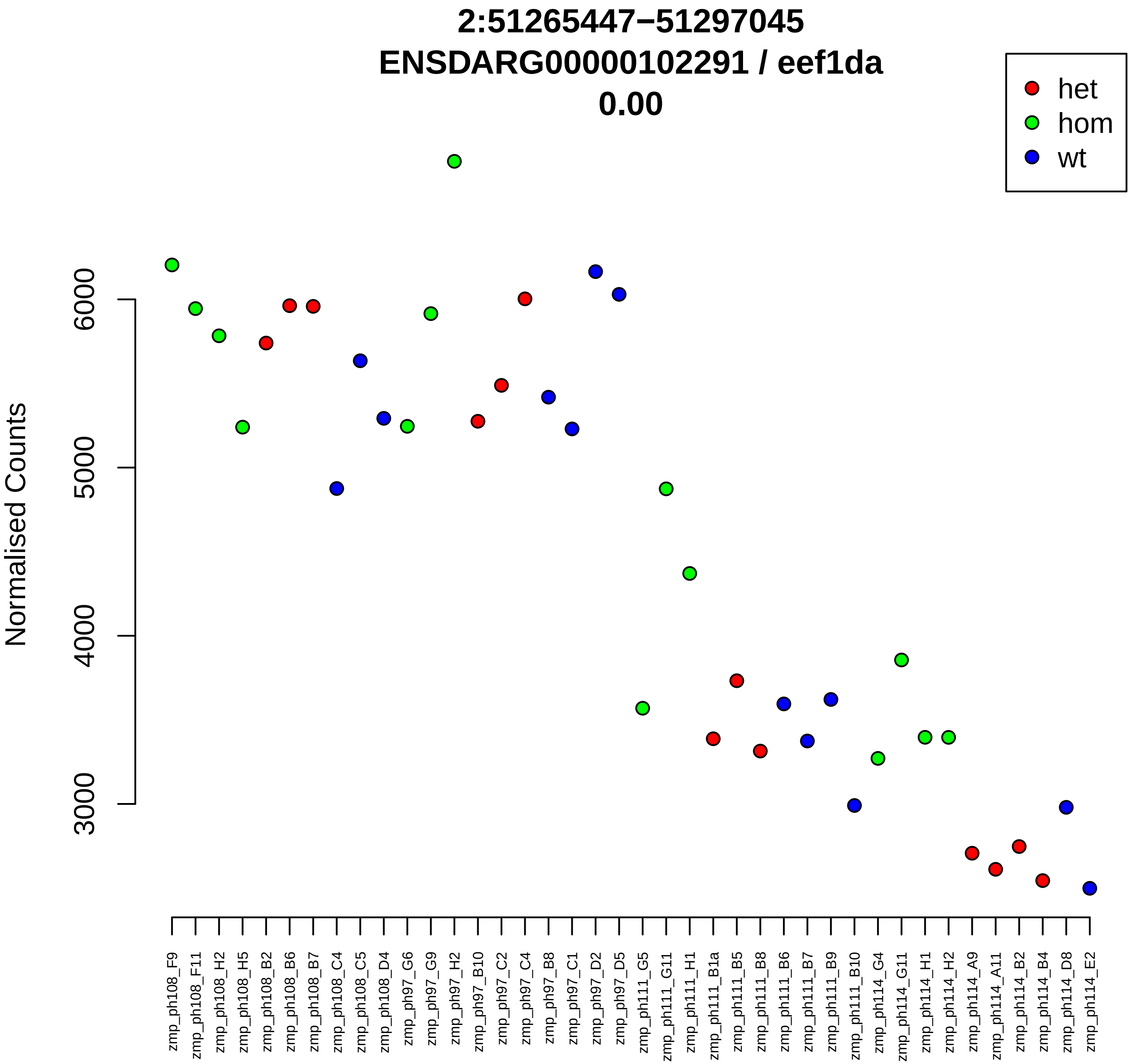
2:51265447−51297045
ENSDARG00000102291 / eef1da
0.00
het
hom
wt
Normalised Counts
3000
4000
5000
6000
zmp_ph108_F9
zmp_ph108_F11
zmp_ph108_H2
zmp_ph108_H5
zmp_ph108_B2
zmp_ph108_B6
zmp_ph108_B7
zmp_ph108_C4
zmp_ph108_C5
zmp_ph108_D4
zmp_ph97_G6
zmp_ph97_G9
zmp_ph97_H2
zmp_ph97_B10
zmp_ph97_C2
zmp_ph97_C4
zmp_ph97_B8
zmp_ph97_C1
zmp_ph97_D2
zmp_ph97_D5
zmp_ph111_G5
zmp_ph111_G11
zmp_ph111_H1
zmp_ph111_B1a
zmp_ph111_B5
zmp_ph111_B8
zmp_ph111_B6
zmp_ph111_B7
zmp_ph111_B9
zmp_ph111_B10
zmp_ph114_G4
zmp_ph114_G11
zmp_ph114_H1
zmp_ph114_H2
zmp_ph114_A9
zmp_ph114_A11
zmp_ph114_B2
zmp_ph114_B4
zmp_ph114_D8
zmp_ph114_E2

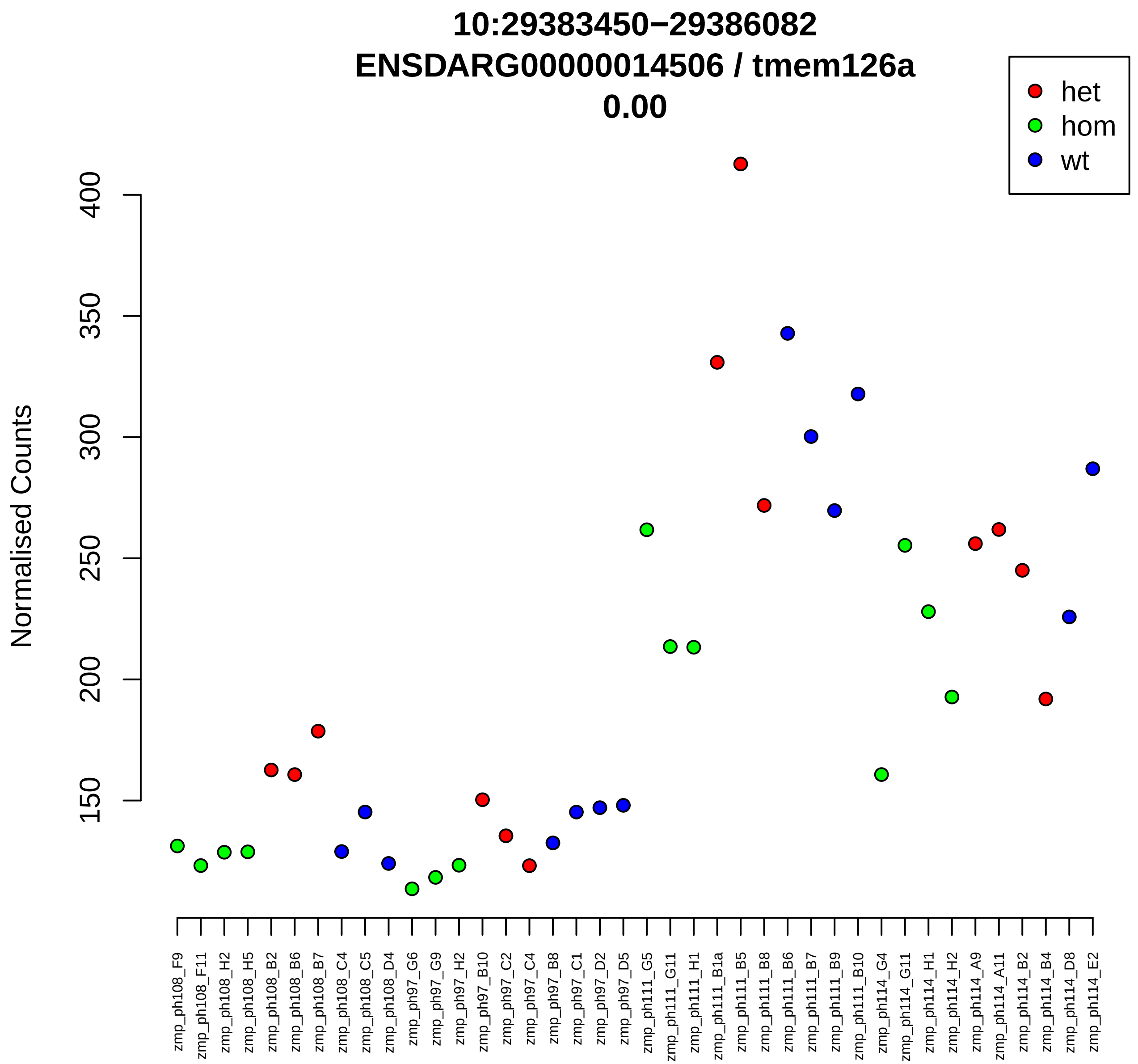
10:29383450−29386082
ENSDARG00000014506 / tmem126a
0.00
het
hom
wt
Normalised Counts
150
200
250
300
350
400
zmp_ph108_F9
zmp_ph108_F11
zmp_ph108_H2
zmp_ph108_H5
zmp_ph108_B2
zmp_ph108_B6
zmp_ph108_B7
zmp_ph108_C4
zmp_ph108_C5
zmp_ph108_D4
zmp_ph97_G6
zmp_ph97_G9
zmp_ph97_H2
zmp_ph97_B10
zmp_ph97_C2
zmp_ph97_C4
zmp_ph97_B8
zmp_ph97_C1
zmp_ph97_D2
zmp_ph97_D5
zmp_ph111_G5
zmp_ph111_G11
zmp_ph111_H1
zmp_ph111_B1a
zmp_ph111_B5
zmp_ph111_B8
zmp_ph111_B6
zmp_ph111_B7
zmp_ph111_B9
zmp_ph111_B10
zmp_ph114_G4
zmp_ph114_G11
zmp_ph114_H1
zmp_ph114_H2
zmp_ph114_A9
zmp_ph114_A11
zmp_ph114_B2
zmp_ph114_B4
zmp_ph114_D8
zmp_ph114_E2

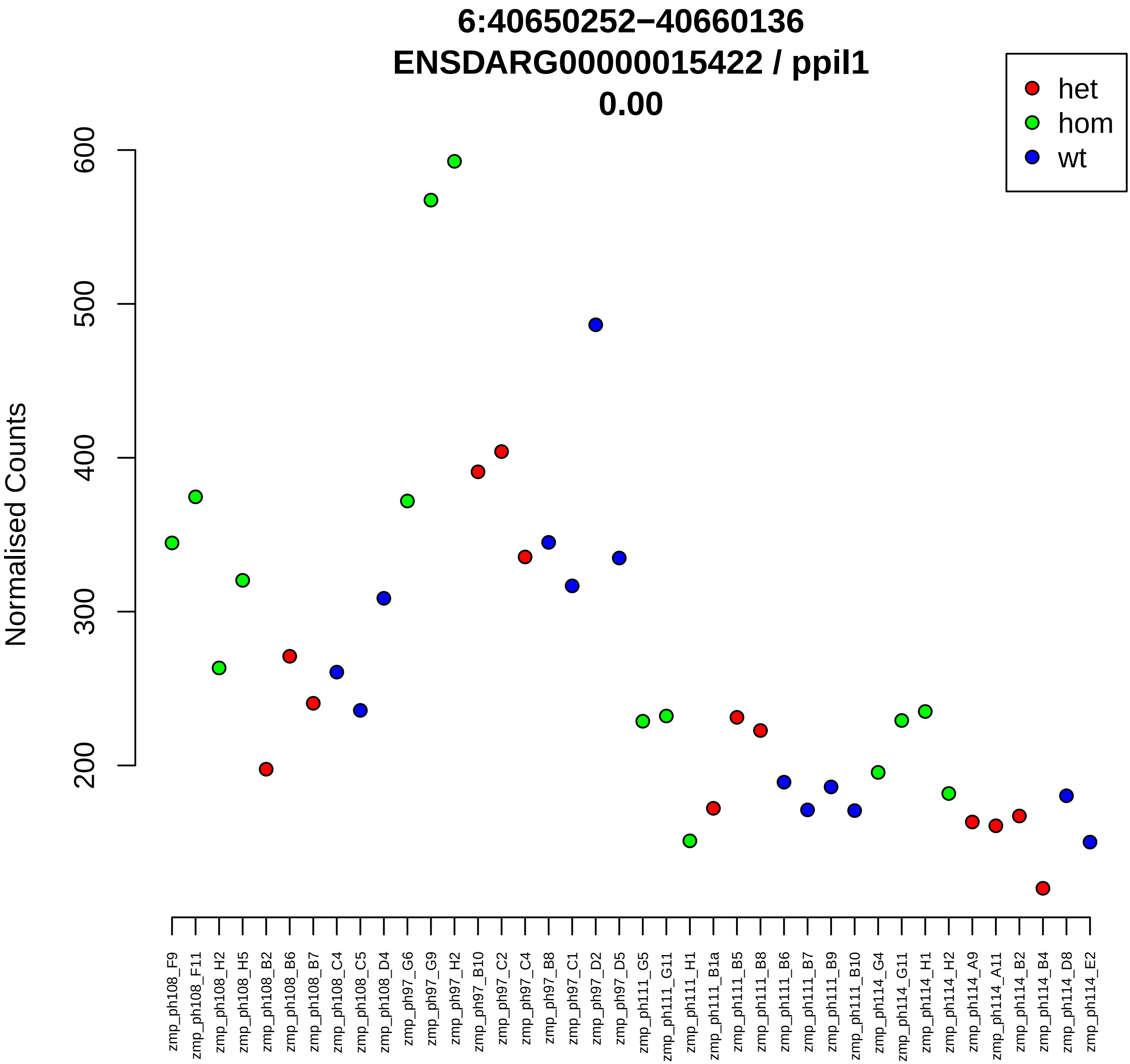

6:40650252−40660136
ENSDARG00000015422 / ppil1
0.00
het
hom
wt
Normalised Counts
200
300
400
500
600
zmp_ph108_F9
zmp_ph108_F11
zmp_ph108_H2
zmp_ph108_H5
zmp_ph108_B2
zmp_ph108_B6
zmp_ph108_B7
zmp_ph108_C4
zmp_ph108_C5
zmp_ph108_D4
zmp_ph97_G6
zmp_ph97_G9
zmp_ph97_H2
zmp_ph97_B10
zmp_ph97_C2
zmp_ph97_C4
zmp_ph97_B8
zmp_ph97_C1
zmp_ph97_D2
zmp_ph97_D5
zmp_ph111_G5
zmp_ph111_G11
zmp_ph111_H1
zmp_ph111_B1a
zmp_ph111_B5
zmp_ph111_B8
zmp_ph111_B6
zmp_ph111_B7
zmp_ph111_B9
zmp_ph111_B10
zmp_ph114_G4
zmp_ph114_G11
zmp_ph114_H1
zmp_ph114_H2
zmp_ph114_A9
zmp_ph114_A11
zmp_ph114_B2
zmp_ph114_B4
zmp_ph114_D8
zmp_ph114_E2

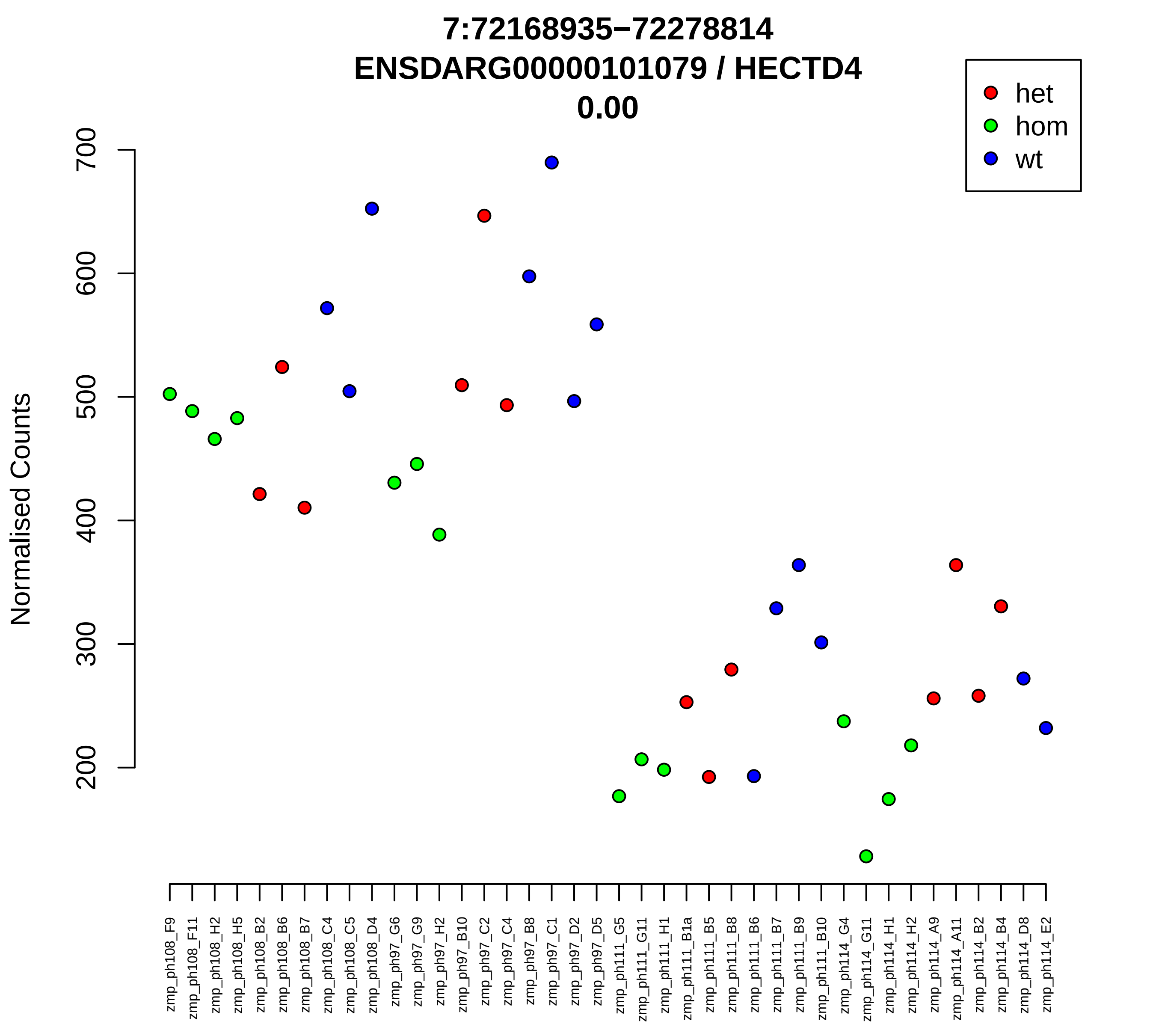
7:72168935−72278814
ENSDARG00000101079 / HECTD4
0.00
het
hom
wt
Normalised Counts
200
300
400
500
600
700
zmp_ph108_F9
zmp_ph108_F11
zmp_ph108_H2
zmp_ph108_H5
zmp_ph108_B2
zmp_ph108_B6
zmp_ph108_B7
zmp_ph108_C4
zmp_ph108_C5
zmp_ph108_D4
zmp_ph97_G6
zmp_ph97_G9
zmp_ph97_H2
zmp_ph97_B10
zmp_ph97_C2
zmp_ph97_C4
zmp_ph97_B8
zmp_ph97_C1
zmp_ph97_D2
zmp_ph97_D5
zmp_ph111_G5
zmp_ph111_G11
zmp_ph111_H1
zmp_ph111_B1a
zmp_ph111_B5
zmp_ph111_B8
zmp_ph111_B6
zmp_ph111_B7
zmp_ph111_B9
zmp_ph111_B10
zmp_ph114_G4
zmp_ph114_G11
zmp_ph114_H1
zmp_ph114_H2
zmp_ph114_A9
zmp_ph114_A11
zmp_ph114_B2
zmp_ph114_B4
zmp_ph114_D8
zmp_ph114_E2

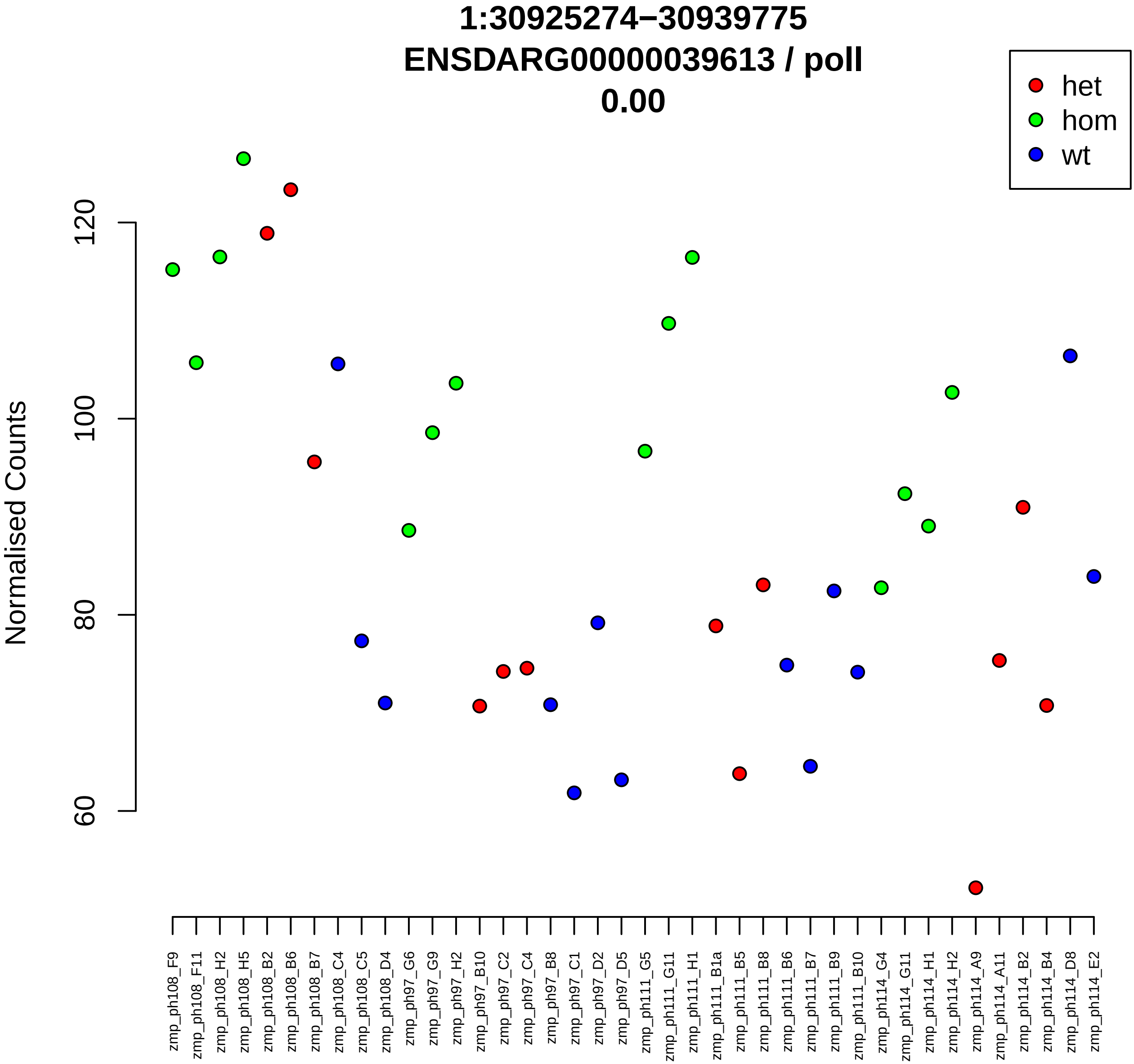

1:30925274−30939775
ENSDARG00000039613 / polI
0.00
het
hom
wt
Normalised Counts
60
80
100
120
zmp_ph108_F9
zmp_ph108_F11
zmp_ph108_H2
zmp_ph108_H5
zmp_ph108_B2
zmp_ph108_B6
zmp_ph108_B7
zmp_ph108_C4
zmp_ph108_C5
zmp_ph108_D4
zmp_ph97_G6
zmp_ph97_G9
zmp_ph97_H2
zmp_ph97_B10
zmp_ph97_C2
zmp_ph97_C4
zmp_ph97_B8
zmp_ph97_C1
zmp_ph97_D2
zmp_ph97_D5
zmp_ph111_G5
zmp_ph111_G11
zmp_ph111_H1
zmp_ph111_B1a
zmp_ph111_B5
zmp_ph111_B8
zmp_ph111_B6
zmp_ph111_B7
zmp_ph111_B9
zmp_ph111_B10
zmp_ph114_G4
zmp_ph114_G11
zmp_ph114_H1
zmp_ph114_H2
zmp_ph114_A9
zmp_ph114_A11
zmp_ph114_B2
zmp_ph114_B4
zmp_ph114_D8
zmp_ph114_E2

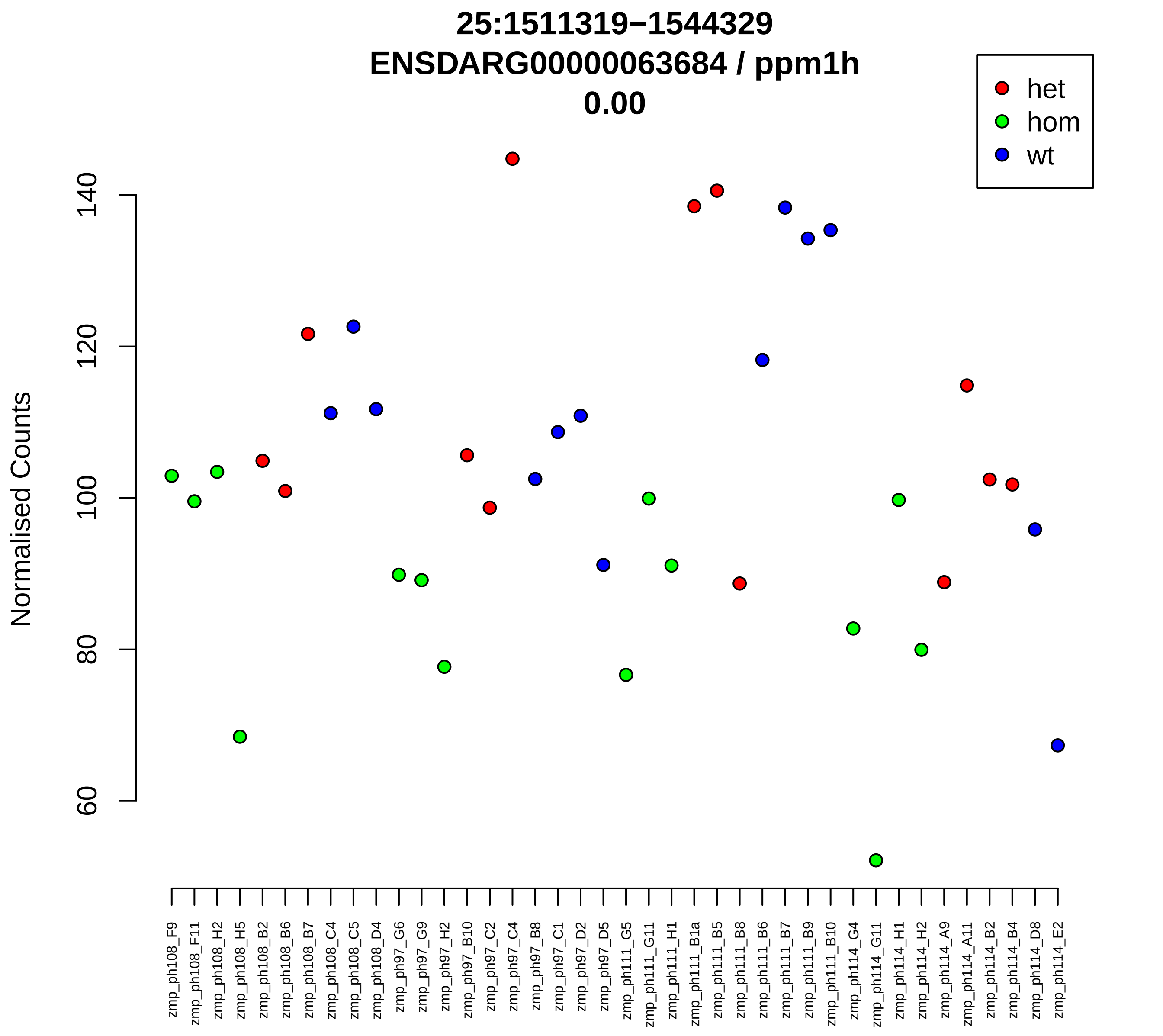

25:1511319−1544329
ENSDARG00000063684 / ppm1h
0.00
het
hom
wt
Normalised Counts
60
80
100
120
140
zmp_ph108_F9
zmp_ph108_F11
zmp_ph108_H2
zmp_ph108_H5
zmp_ph108_B2
zmp_ph108_B6
zmp_ph108_B7
zmp_ph108_C4
zmp_ph108_C5
zmp_ph108_D4
zmp_ph97_G6
zmp_ph97_G9
zmp_ph97_H2
zmp_ph97_B10
zmp_ph97_C2
zmp_ph97_C4
zmp_ph97_B8
zmp_ph97_C1
zmp_ph97_D2
zmp_ph97_D5
zmp_ph111_G5
zmp_ph111_G11
zmp_ph111_H1
zmp_ph111_B1a
zmp_ph111_B5
zmp_ph111_B8
zmp_ph111_B6
zmp_ph111_B7
zmp_ph111_B9
zmp_ph111_B10
zmp_ph114_G4
zmp_ph114_G11
zmp_ph114_H1
zmp_ph114_H2
zmp_ph114_A9
zmp_ph114_A11
zmp_ph114_B2
zmp_ph114_B4
zmp_ph114_D8
zmp_ph114_E2

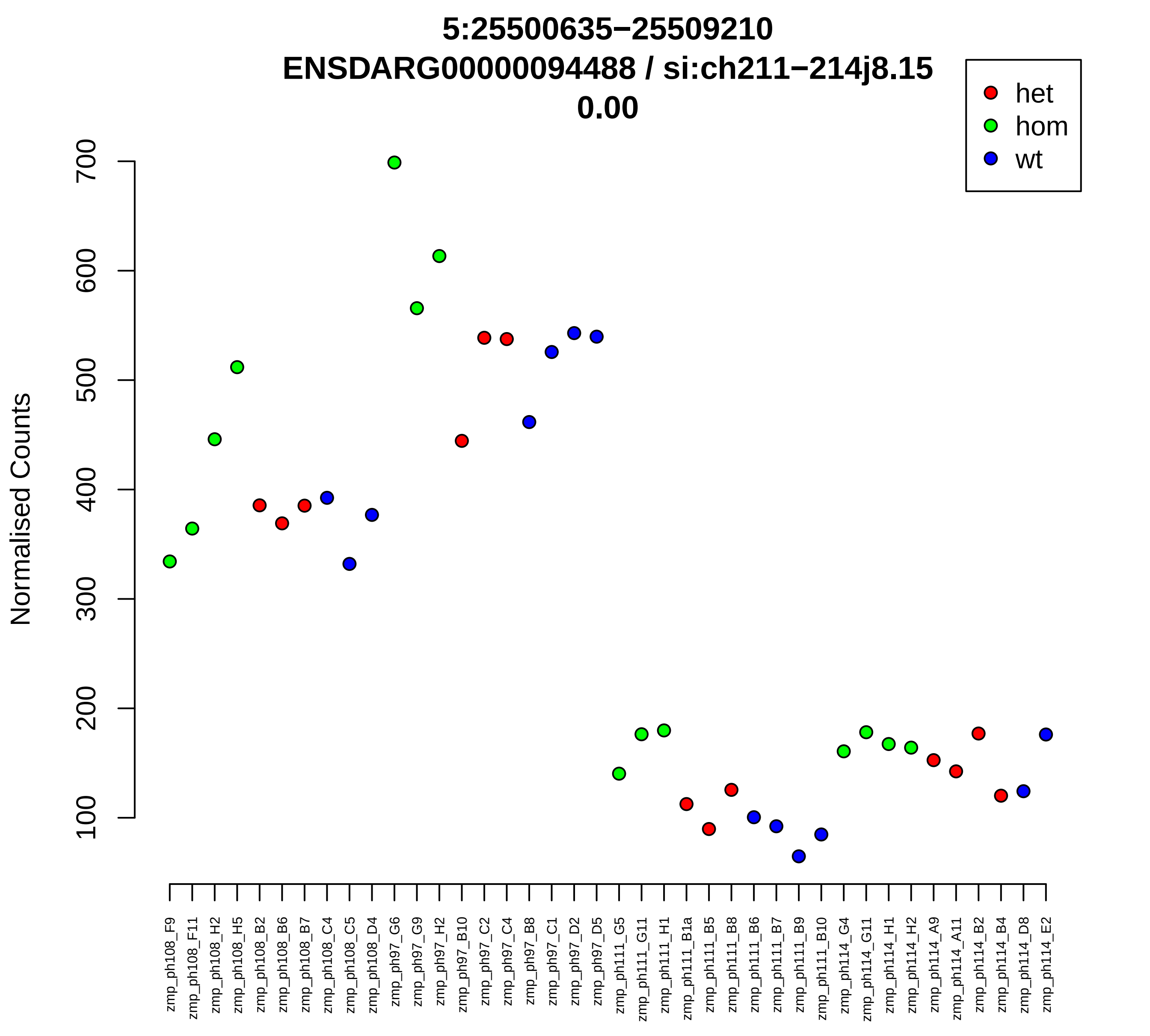
5:25500635−25509210
ENSDARG00000094488 / si:ch211−214j8.15
0.00
het
hom
wt
Normalised Counts
100
200
300
400
500
600
700
zmp_ph108_F9
zmp_ph108_F11
zmp_ph108_H2
zmp_ph108_H5
zmp_ph108_B2
zmp_ph108_B6
zmp_ph108_B7
zmp_ph108_C4
zmp_ph108_C5
zmp_ph108_D4
zmp_ph97_G6
zmp_ph97_G9
zmp_ph97_H2
zmp_ph97_B10
zmp_ph97_C2
zmp_ph97_C4
zmp_ph97_B8
zmp_ph97_C1
zmp_ph97_D2
zmp_ph97_D5
zmp_ph111_G5
zmp_ph111_G11
zmp_ph111_H1
zmp_ph111_B1a
zmp_ph111_B5
zmp_ph111_B8
zmp_ph111_B6
zmp_ph111_B7
zmp_ph111_B9
zmp_ph111_B10
zmp_ph114_G4
zmp_ph114_G11
zmp_ph114_H1
zmp_ph114_H2
zmp_ph114_A9
zmp_ph114_A11
zmp_ph114_B2
zmp_ph114_B4
zmp_ph114_D8
zmp_ph114_E2

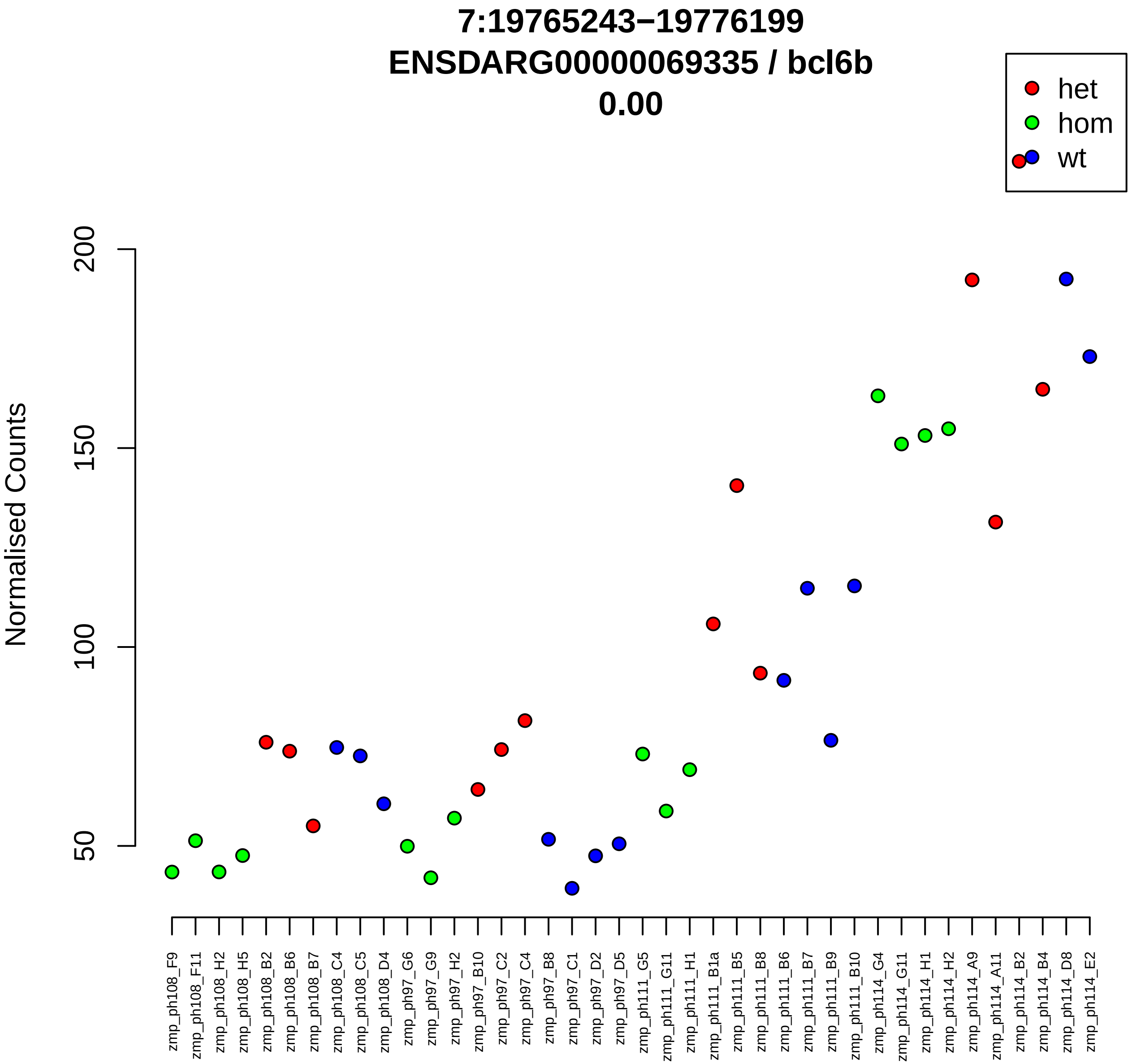
7:19765243−19776199
ENSDARG00000069335 / bcl6b
0.00
het
hom
wt
Normalised Counts
50
100
150
200
zmp_ph108_F9
zmp_ph108_F11
zmp_ph108_H2
zmp_ph108_H5
zmp_ph108_B2
zmp_ph108_B6
zmp_ph108_B7
zmp_ph108_C4
zmp_ph108_C5
zmp_ph108_D4
zmp_ph97_G6
zmp_ph97_G9
zmp_ph97_H2
zmp_ph97_B10
zmp_ph97_C2
zmp_ph97_C4
zmp_ph97_B8
zmp_ph97_C1
zmp_ph97_D2
zmp_ph97_D5
zmp_ph111_G5
zmp_ph111_G11
zmp_ph111_H1
zmp_ph111_B1a
zmp_ph111_B5
zmp_ph111_B8
zmp_ph111_B6
zmp_ph111_B7
zmp_ph111_B9
zmp_ph111_B10
zmp_ph114_G4
zmp_ph114_G11
zmp_ph114_H1
zmp_ph114_H2
zmp_ph114_A9
zmp_ph114_A11
zmp_ph114_B2
zmp_ph114_B4
zmp_ph114_D8
zmp_ph114_E2

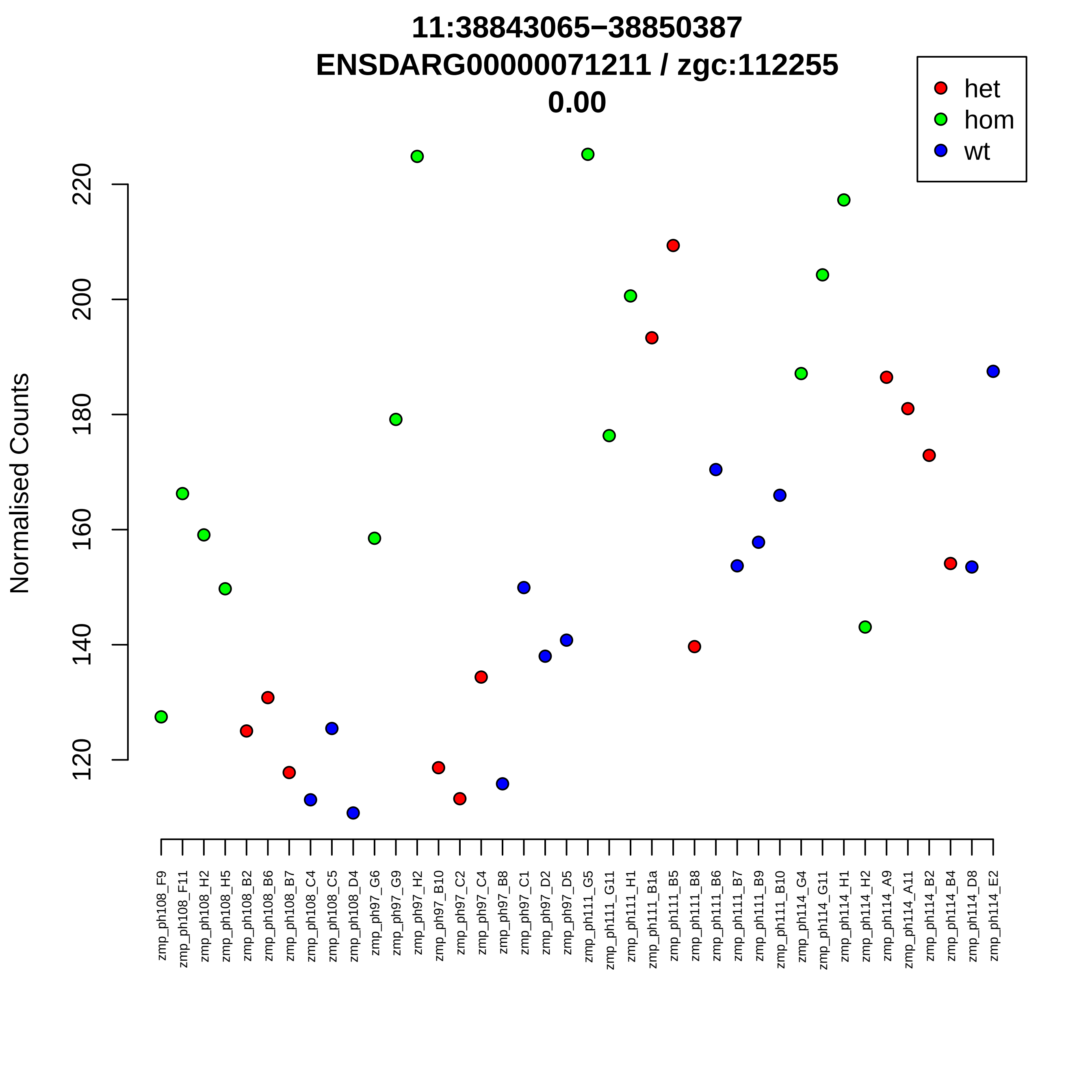

11:38843065−38850387
ENSDARG00000071211 / zgc:112255
0.00
het
hom
wt
Normalised Counts
120
140
160
180
200
220
zmp_ph108_F9
zmp_ph108_F11
zmp_ph108_H2
zmp_ph108_H5
zmp_ph108_B2
zmp_ph108_B6
zmp_ph108_B7
zmp_ph108_C4
zmp_ph108_C5
zmp_ph108_D4
zmp_ph97_G6
zmp_ph97_G9
zmp_ph97_H2
zmp_ph97_B10
zmp_ph97_C2
zmp_ph97_C4
zmp_ph97_B8
zmp_ph97_C1
zmp_ph97_D2
zmp_ph97_D5
zmp_ph111_G5
zmp_ph111_G11
zmp_ph111_H1
zmp_ph111_B1a
zmp_ph111_B5
zmp_ph111_B8
zmp_ph111_B6
zmp_ph111_B7
zmp_ph111_B9
zmp_ph111_B10
zmp_ph114_G4
zmp_ph114_G11
zmp_ph114_H1
zmp_ph114_H2
zmp_ph114_A9
zmp_ph114_A11
zmp_ph114_B2
zmp_ph114_B4
zmp_ph114_D8
zmp_ph114_E2

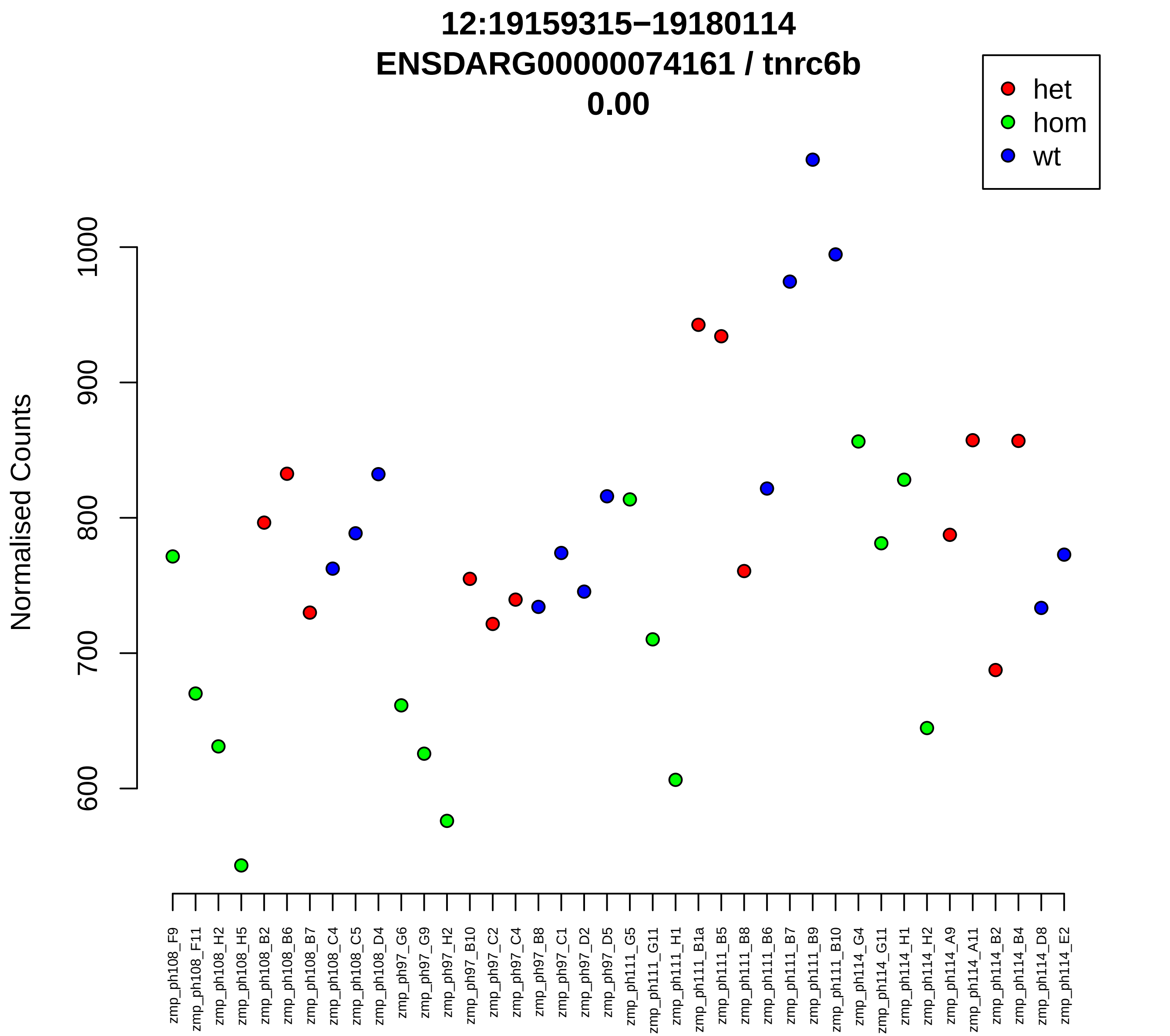

12:19159315−19180114
ENSDARG00000074161 / tnrc6b
0.00
het
hom
wt
Normalised Counts
600
700
800
900
1000
zmp_ph108_F9
zmp_ph108_F11
zmp_ph108_H2
zmp_ph108_H5
zmp_ph108_B2
zmp_ph108_B6
zmp_ph108_B7
zmp_ph108_C4
zmp_ph108_C5
zmp_ph108_D4
zmp_ph97_G6
zmp_ph97_G9
zmp_ph97_H2
zmp_ph97_B10
zmp_ph97_C2
zmp_ph97_C4
zmp_ph97_B8
zmp_ph97_C1
zmp_ph97_D2
zmp_ph97_D5
zmp_ph111_G5
zmp_ph111_G11
zmp_ph111_H1
zmp_ph111_B1a
zmp_ph111_B5
zmp_ph111_B8
zmp_ph111_B6
zmp_ph111_B7
zmp_ph111_B9
zmp_ph111_B10
zmp_ph114_G4
zmp_ph114_G11
zmp_ph114_H1
zmp_ph114_H2
zmp_ph114_A9
zmp_ph114_A11
zmp_ph114_B2
zmp_ph114_B4
zmp_ph114_D8
zmp_ph114_E2

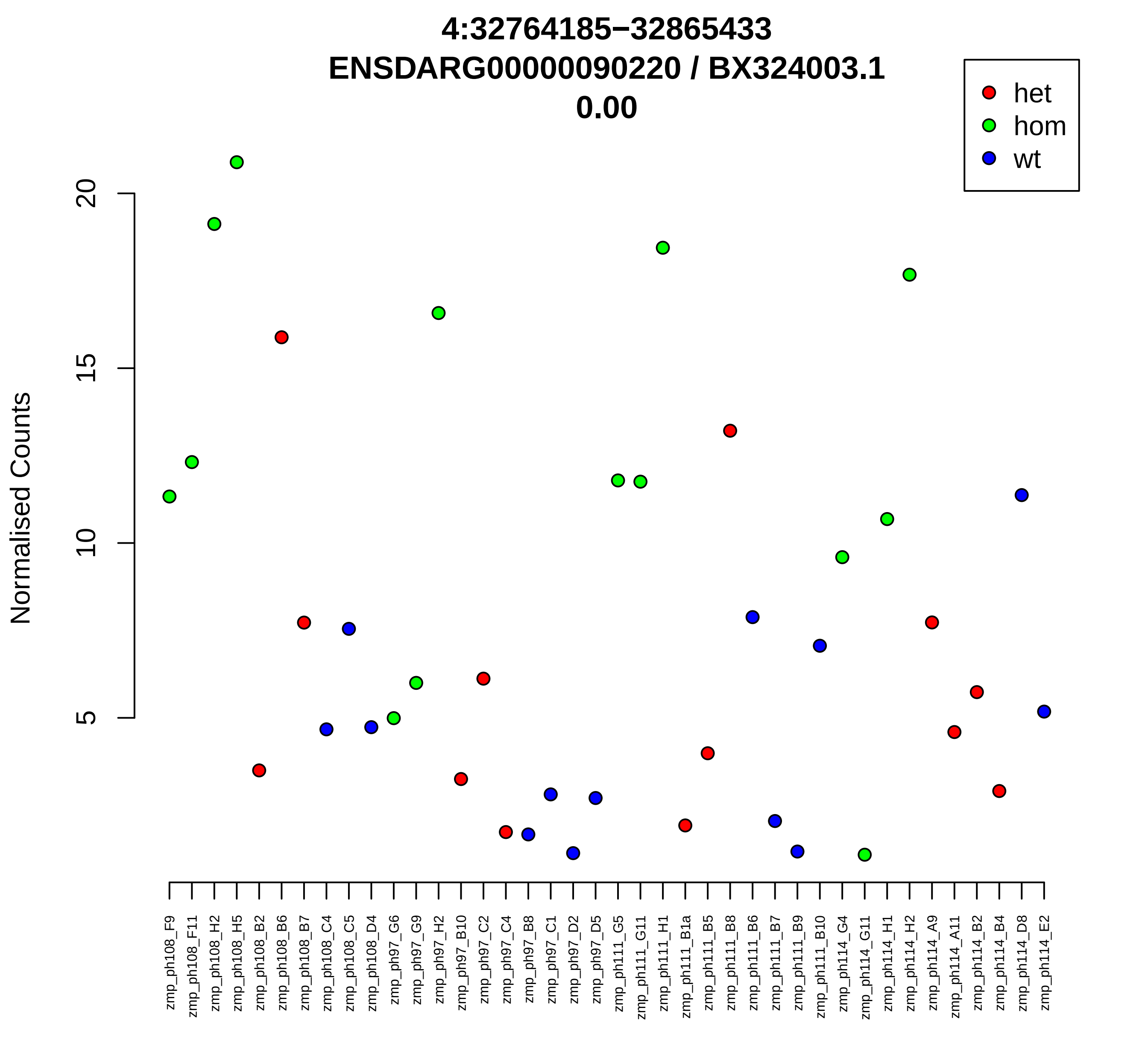

4:32764185−32865433
ENSDARG00000090220 / BX324003.1
0.00
het
hom
wt
Normalised Counts
5
10
15
20
zmp_ph108_F9
zmp_ph108_F11
zmp_ph108_H2
zmp_ph108_H5
zmp_ph108_B2
zmp_ph108_B6
zmp_ph108_B7
zmp_ph108_C4
zmp_ph108_C5
zmp_ph108_D4
zmp_ph97_G6
zmp_ph97_G9
zmp_ph97_H2
zmp_ph97_B10
zmp_ph97_C2
zmp_ph97_C4
zmp_ph97_B8
zmp_ph97_C1
zmp_ph97_D2
zmp_ph97_D5
zmp_ph111_G5
zmp_ph111_G11
zmp_ph111_H1
zmp_ph111_B1a
zmp_ph111_B5
zmp_ph111_B8
zmp_ph111_B6
zmp_ph111_B7
zmp_ph111_B9
zmp_ph111_B10
zmp_ph114_G4
zmp_ph114_G11
zmp_ph114_H1
zmp_ph114_H2
zmp_ph114_A9
zmp_ph114_A11
zmp_ph114_B2
zmp_ph114_B4
zmp_ph114_D8
zmp_ph114_E2

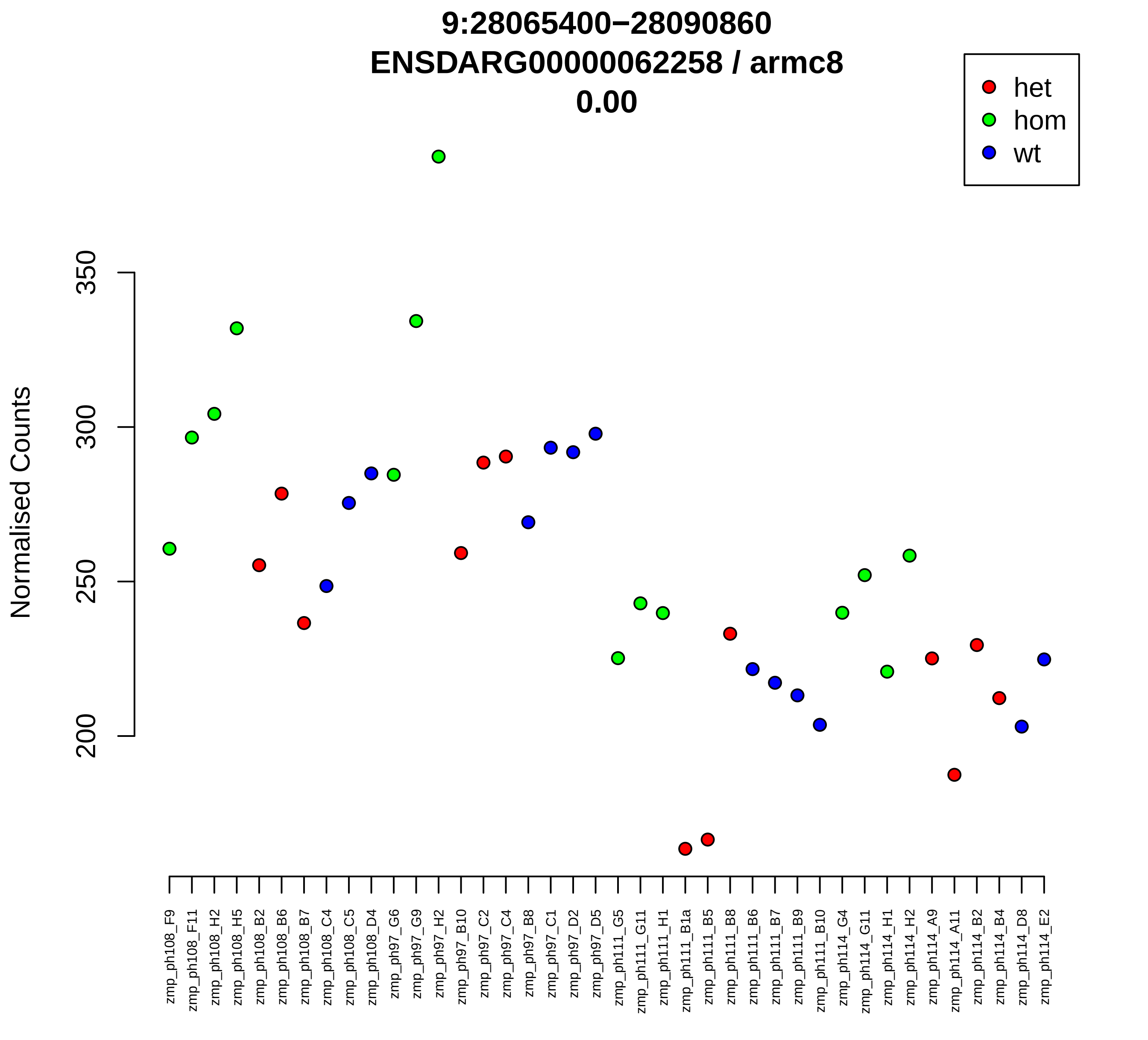
9:28065400−28090860
ENSDARG00000062258 / armc8
0.00
het
hom
wt
Normalised Counts
200
250
300
350
zmp_ph108_F9
zmp_ph108_F11
zmp_ph108_H2
zmp_ph108_H5
zmp_ph108_B2
zmp_ph108_B6
zmp_ph108_B7
zmp_ph108_C4
zmp_ph108_C5
zmp_ph108_D4
zmp_ph97_G6
zmp_ph97_G9
zmp_ph97_H2
zmp_ph97_B10
zmp_ph97_C2
zmp_ph97_C4
zmp_ph97_B8
zmp_ph97_C1
zmp_ph97_D2
zmp_ph97_D5
zmp_ph111_G5
zmp_ph111_G11
zmp_ph111_H1
zmp_ph111_B1a
zmp_ph111_B5
zmp_ph111_B8
zmp_ph111_B6
zmp_ph111_B7
zmp_ph111_B9
zmp_ph111_B10
zmp_ph114_G4
zmp_ph114_G11
zmp_ph114_H1
zmp_ph114_H2
zmp_ph114_A9
zmp_ph114_A11
zmp_ph114_B2
zmp_ph114_B4
zmp_ph114_D8
zmp_ph114_E2

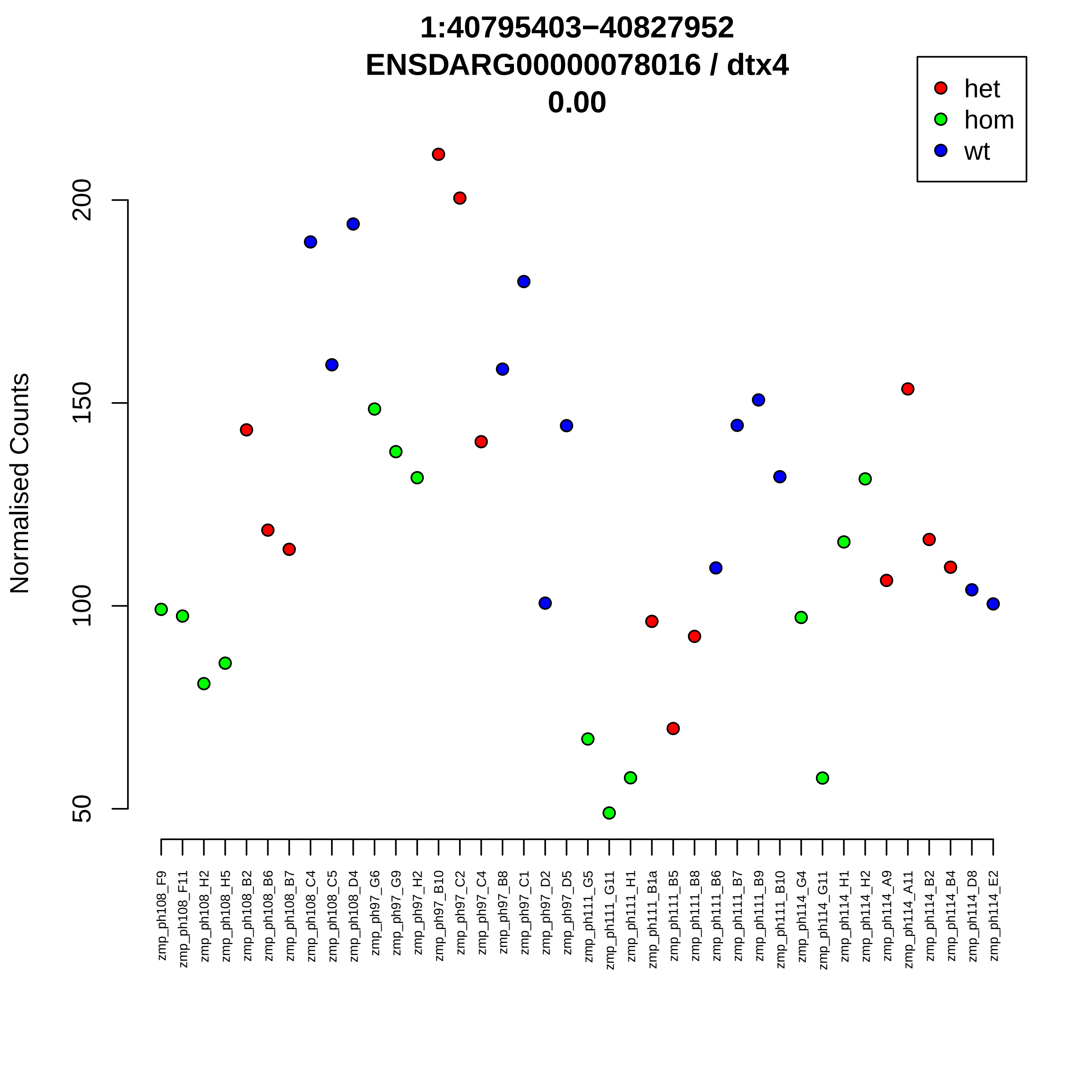

1:40795403−40827952
ENSDARG00000078016 / dtx4
0.00
het
hom
wt
Normalised Counts
50
100
150
200
zmp_ph108_F9
zmp_ph108_F11
zmp_ph108_H2
zmp_ph108_H5
zmp_ph108_B2
zmp_ph108_B6
zmp_ph108_B7
zmp_ph108_C4
zmp_ph108_C5
zmp_ph108_D4
zmp_ph97_G6
zmp_ph97_G9
zmp_ph97_H2
zmp_ph97_B10
zmp_ph97_C2
zmp_ph97_C4
zmp_ph97_B8
zmp_ph97_C1
zmp_ph97_D2
zmp_ph97_D5
zmp_ph111_G5
zmp_ph111_G11
zmp_ph111_H1
zmp_ph111_B1a
zmp_ph111_B5
zmp_ph111_B8
zmp_ph111_B6
zmp_ph111_B7
zmp_ph111_B9
zmp_ph111_B10
zmp_ph114_G4
zmp_ph114_G11
zmp_ph114_H1
zmp_ph114_H2
zmp_ph114_A9
zmp_ph114_A11
zmp_ph114_B2
zmp_ph114_B4
zmp_ph114_D8
zmp_ph114_E2

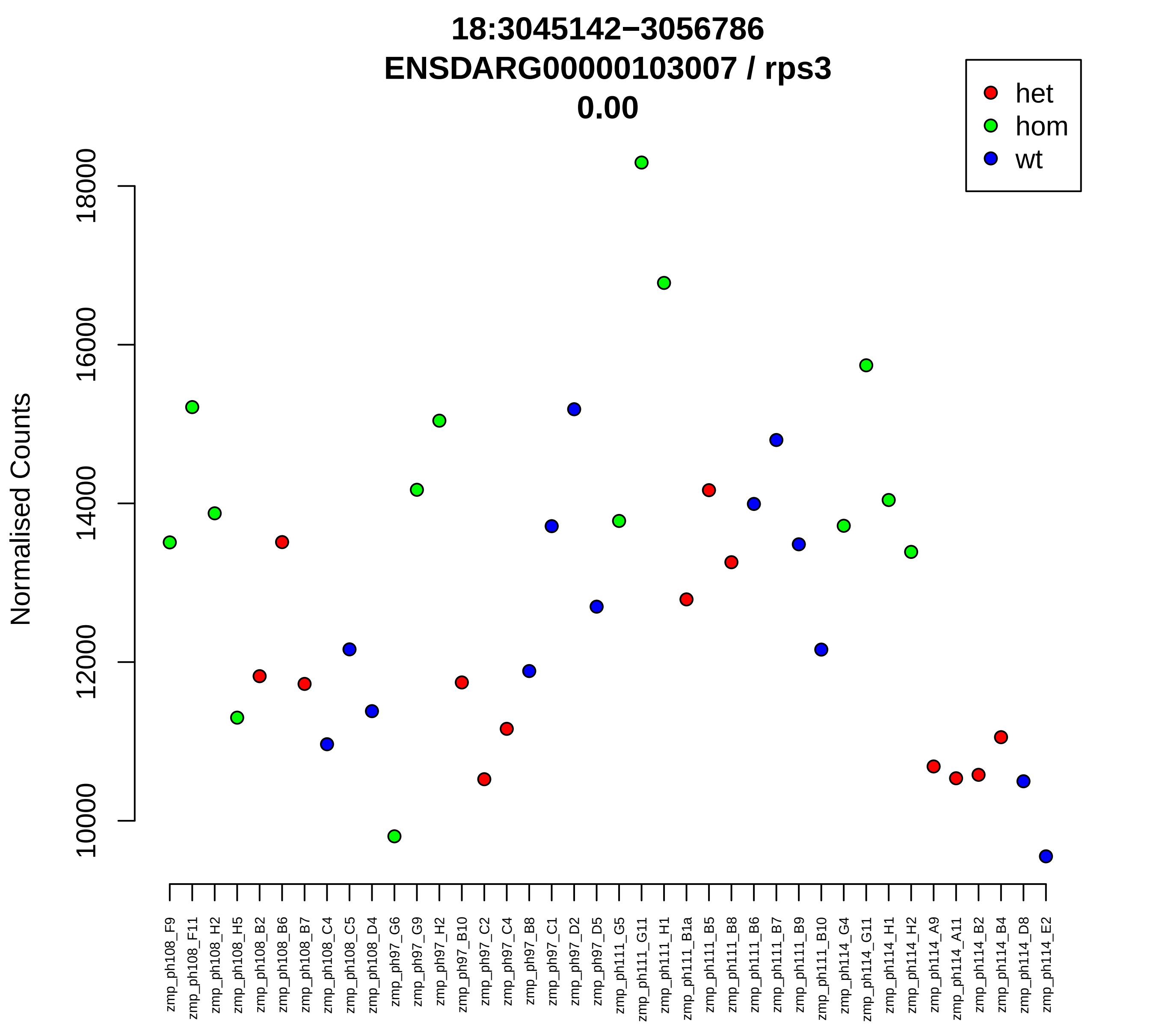

18:3045142−3056786
ENSDARG00000103007 / rps3
0.00
het
hom
wt
Normalised Counts
10000
12000
14000
16000
18000
zmp_ph108_F9
zmp_ph108_F11
zmp_ph108_H2
zmp_ph108_H5
zmp_ph108_B2
zmp_ph108_B6
zmp_ph108_B7
zmp_ph108_C4
zmp_ph108_C5
zmp_ph108_D4
zmp_ph97_G6
zmp_ph97_G9
zmp_ph97_H2
zmp_ph97_B10
zmp_ph97_C2
zmp_ph97_C4
zmp_ph97_B8
zmp_ph97_C1
zmp_ph97_D2
zmp_ph97_D5
zmp_ph111_G5
zmp_ph111_G11
zmp_ph111_H1
zmp_ph111_B1a
zmp_ph111_B5
zmp_ph111_B8
zmp_ph111_B6
zmp_ph111_B7
zmp_ph111_B9
zmp_ph111_B10
zmp_ph114_G4
zmp_ph114_G11
zmp_ph114_H1
zmp_ph114_H2
zmp_ph114_A9
zmp_ph114_A11
zmp_ph114_B2
zmp_ph114_B4
zmp_ph114_D8
zmp_ph114_E2

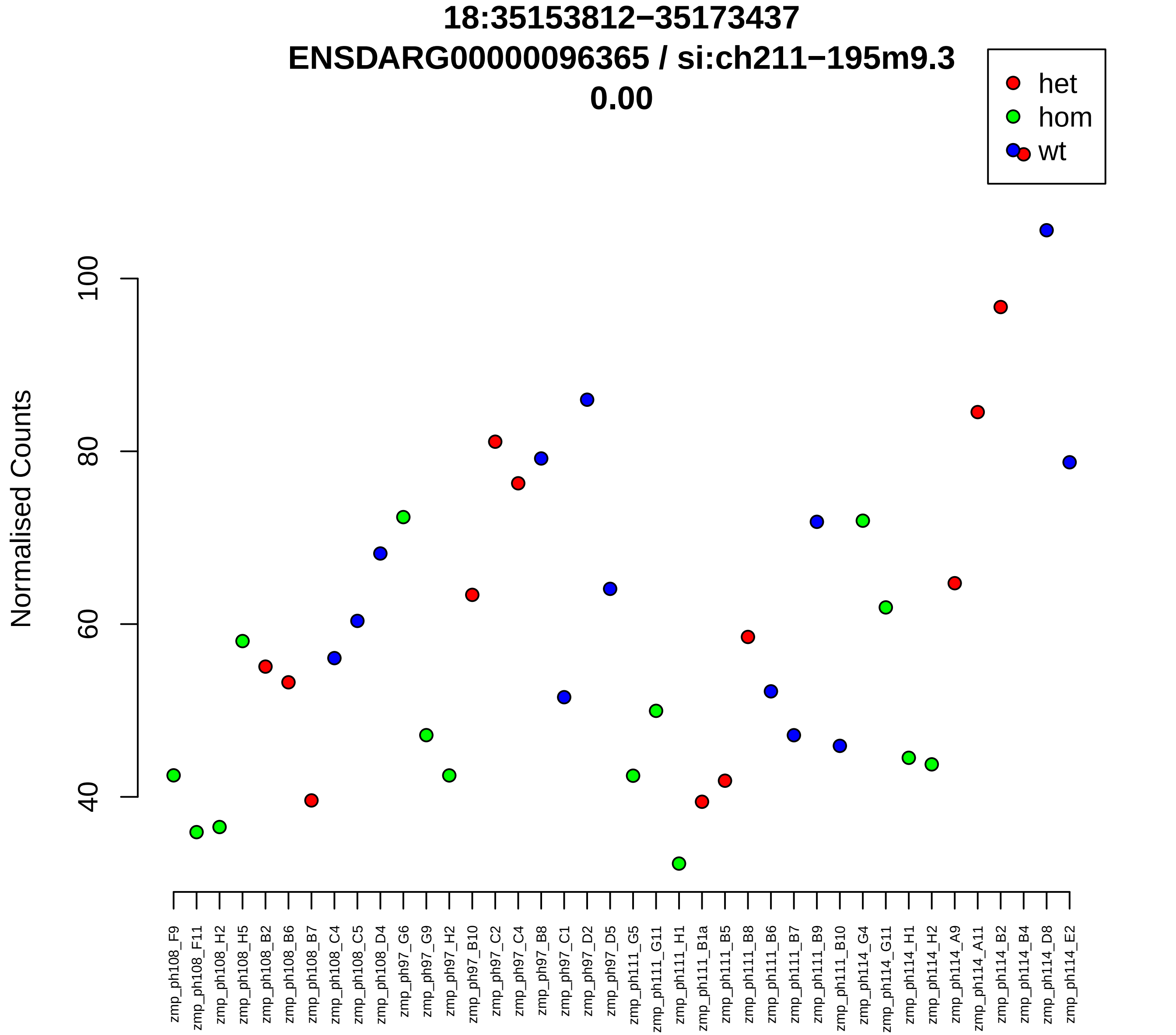

18:35153812−35173437
ENSDARG00000096365 / si:ch211−195m9.3
0.00
het
hom
wt
Normalised Counts
40
60
80
100
zmp_ph108_F9
zmp_ph108_F11
zmp_ph108_H2
zmp_ph108_H5
zmp_ph108_B2
zmp_ph108_B6
zmp_ph108_B7
zmp_ph108_C4
zmp_ph108_C5
zmp_ph108_D4
zmp_ph97_G6
zmp_ph97_G9
zmp_ph97_H2
zmp_ph97_B10
zmp_ph97_C2
zmp_ph97_C4
zmp_ph97_B8
zmp_ph97_C1
zmp_ph97_D2
zmp_ph97_D5
zmp_ph111_G5
zmp_ph111_G11
zmp_ph111_H1
zmp_ph111_B1a
zmp_ph111_B5
zmp_ph111_B8
zmp_ph111_B6
zmp_ph111_B7
zmp_ph111_B9
zmp_ph111_B10
zmp_ph114_G4
zmp_ph114_G11
zmp_ph114_H1
zmp_ph114_H2
zmp_ph114_A9
zmp_ph114_A11
zmp_ph114_B2
zmp_ph114_B4
zmp_ph114_D8
zmp_ph114_E2

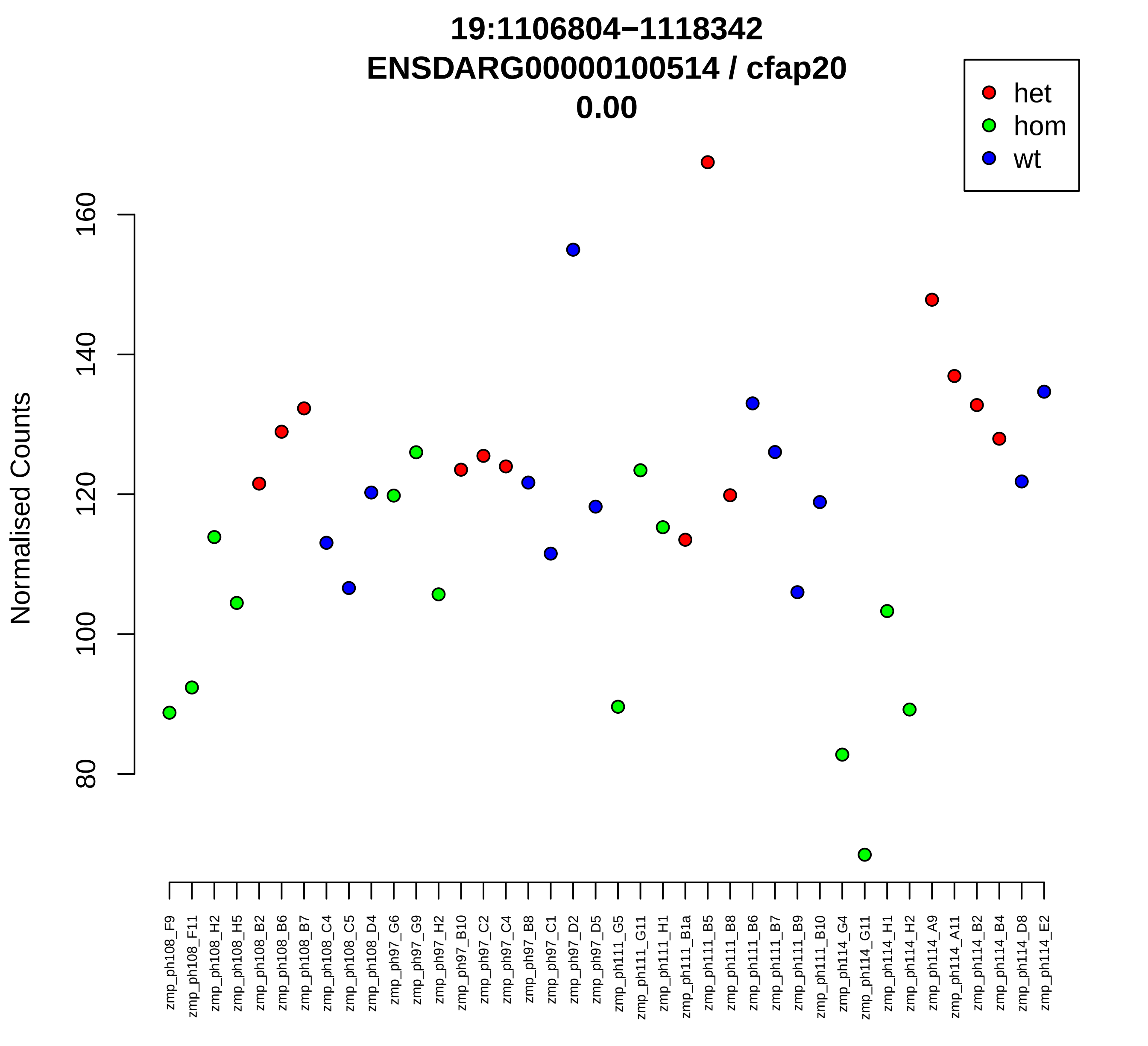
19:1106804−1118342
ENSDARG00000100514 / cfap20
0.00
het
hom
wt
Normalised Counts
80
100
120
140
160
zmp_ph108_F9
zmp_ph108_F11
zmp_ph108_H2
zmp_ph108_H5
zmp_ph108_B2
zmp_ph108_B6
zmp_ph108_B7
zmp_ph108_C4
zmp_ph108_C5
zmp_ph108_D4
zmp_ph97_G6
zmp_ph97_G9
zmp_ph97_H2
zmp_ph97_B10
zmp_ph97_C2
zmp_ph97_C4
zmp_ph97_B8
zmp_ph97_C1
zmp_ph97_D2
zmp_ph97_D5
zmp_ph111_G5
zmp_ph111_G11
zmp_ph111_H1
zmp_ph111_B1a
zmp_ph111_B5
zmp_ph111_B8
zmp_ph111_B6
zmp_ph111_B7
zmp_ph111_B9
zmp_ph111_B10
zmp_ph114_G4
zmp_ph114_G11
zmp_ph114_H1
zmp_ph114_H2
zmp_ph114_A9
zmp_ph114_A11
zmp_ph114_B2
zmp_ph114_B4
zmp_ph114_D8
zmp_ph114_E2

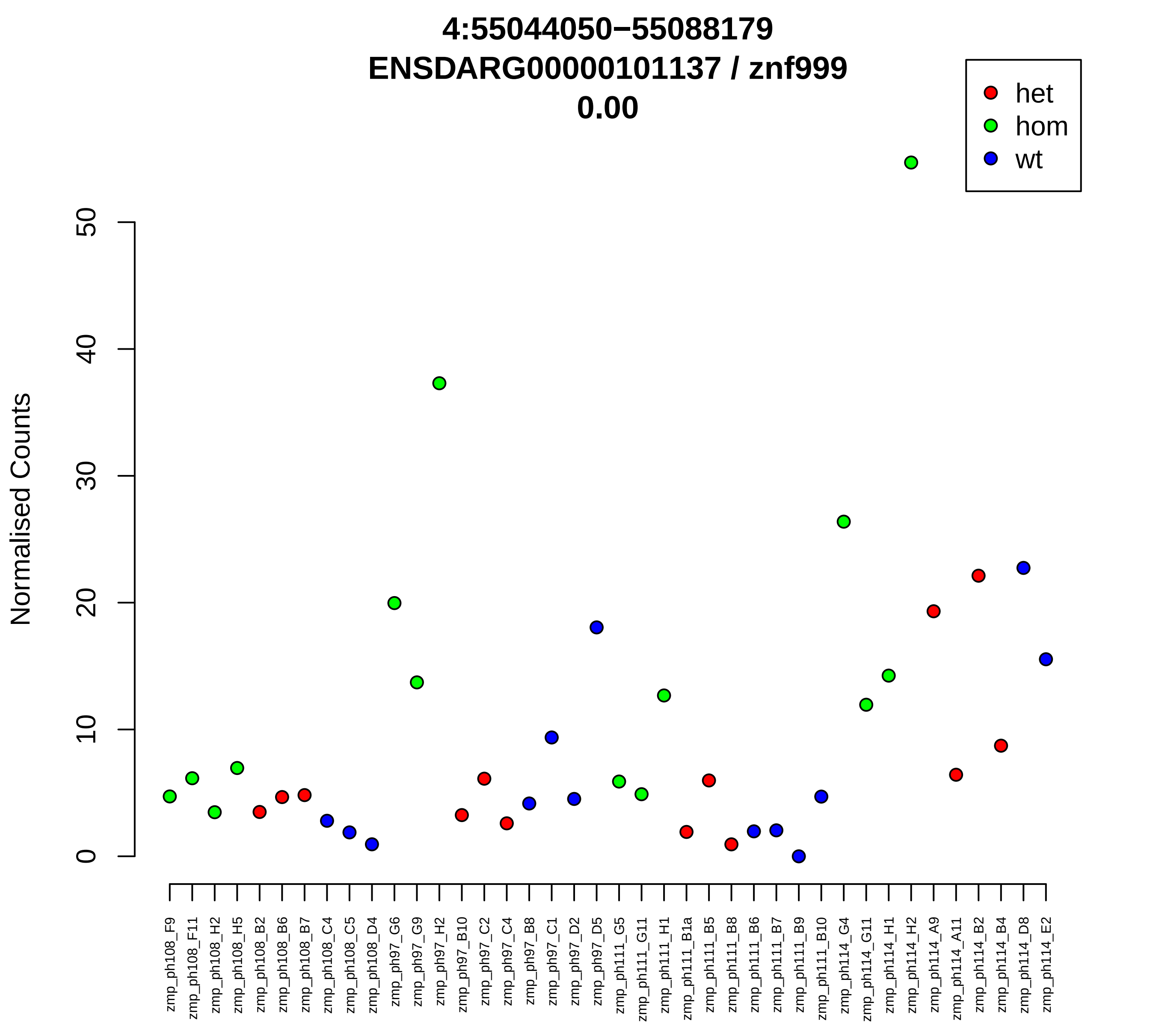
4:55044050−55088179
ENSDARG00000101137 / znf999
0.00
het
hom
wt
Normalised Counts
0
10
20
30
40
50
zmp_ph108_F9
zmp_ph108_F11
zmp_ph108_H2
zmp_ph108_H5
zmp_ph108_B2
zmp_ph108_B6
zmp_ph108_B7
zmp_ph108_C4
zmp_ph108_C5
zmp_ph108_D4
zmp_ph97_G6
zmp_ph97_G9
zmp_ph97_H2
zmp_ph97_B10
zmp_ph97_C2
zmp_ph97_C4
zmp_ph97_B8
zmp_ph97_C1
zmp_ph97_D2
zmp_ph97_D5
zmp_ph111_G5
zmp_ph111_G11
zmp_ph111_H1
zmp_ph111_B1a
zmp_ph111_B5
zmp_ph111_B8
zmp_ph111_B6
zmp_ph111_B7
zmp_ph111_B9
zmp_ph111_B10
zmp_ph114_G4
zmp_ph114_G11
zmp_ph114_H1
zmp_ph114_H2
zmp_ph114_A9
zmp_ph114_A11
zmp_ph114_B2
zmp_ph114_B4
zmp_ph114_D8
zmp_ph114_E2

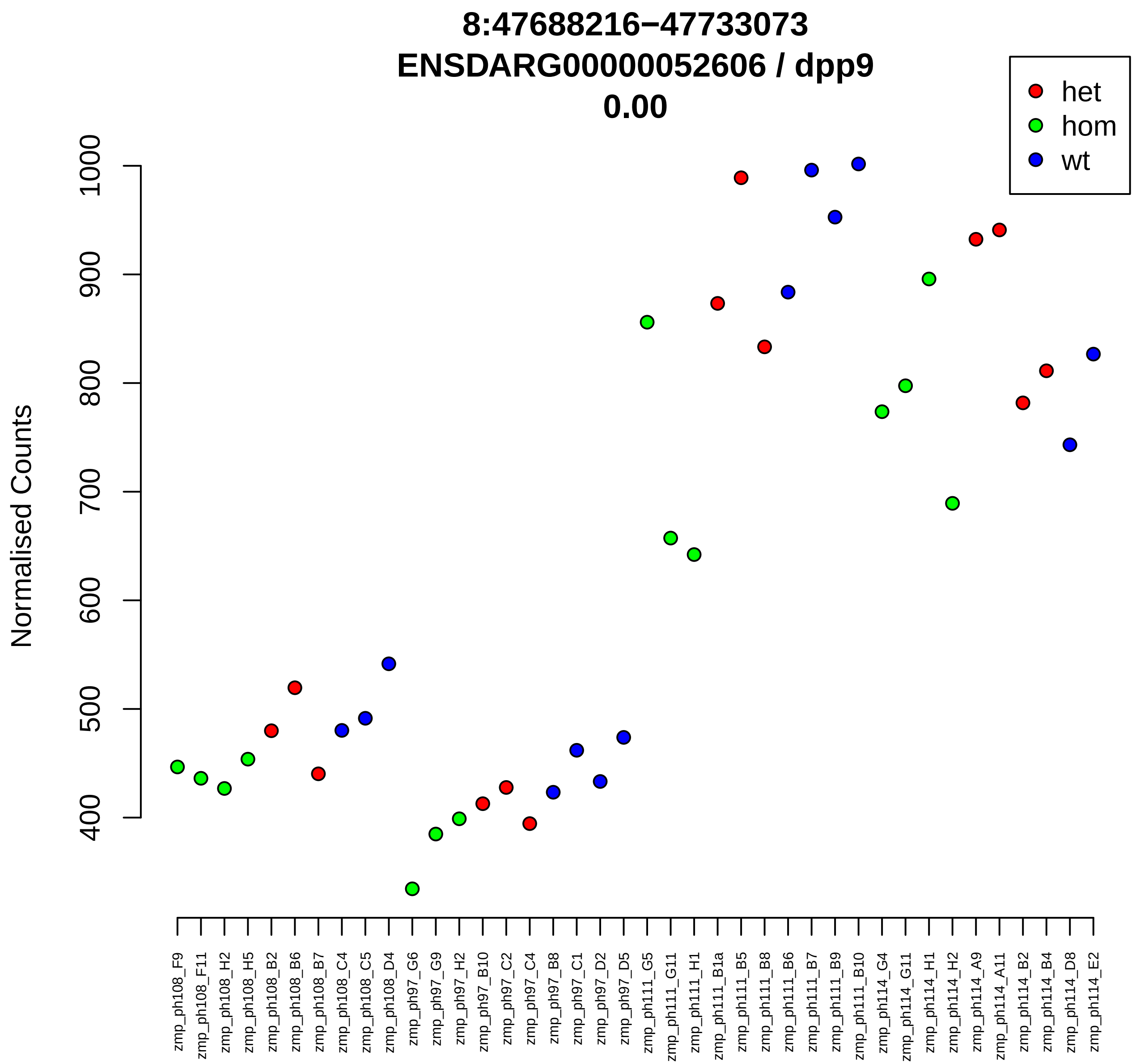

8:47688216−47733073
ENSDARG00000052606 / dpp9
0.00
het
hom
wt
Normalised Counts
400
500
600
700
800
900
1000
zmp_ph108_F9
zmp_ph108_F11
zmp_ph108_H2
zmp_ph108_H5
zmp_ph108_B2
zmp_ph108_B6
zmp_ph108_B7
zmp_ph108_C4
zmp_ph108_C5
zmp_ph108_D4
zmp_ph97_G6
zmp_ph97_G9
zmp_ph97_H2
zmp_ph97_B10
zmp_ph97_C2
zmp_ph97_C4
zmp_ph97_B8
zmp_ph97_C1
zmp_ph97_D2
zmp_ph97_D5
zmp_ph111_G5
zmp_ph111_G11
zmp_ph111_H1
zmp_ph111_B1a
zmp_ph111_B5
zmp_ph111_B8
zmp_ph111_B6
zmp_ph111_B7
zmp_ph111_B9
zmp_ph111_B10
zmp_ph114_G4
zmp_ph114_G11
zmp_ph114_H1
zmp_ph114_H2
zmp_ph114_A9
zmp_ph114_A11
zmp_ph114_B2
zmp_ph114_B4
zmp_ph114_D8
zmp_ph114_E2

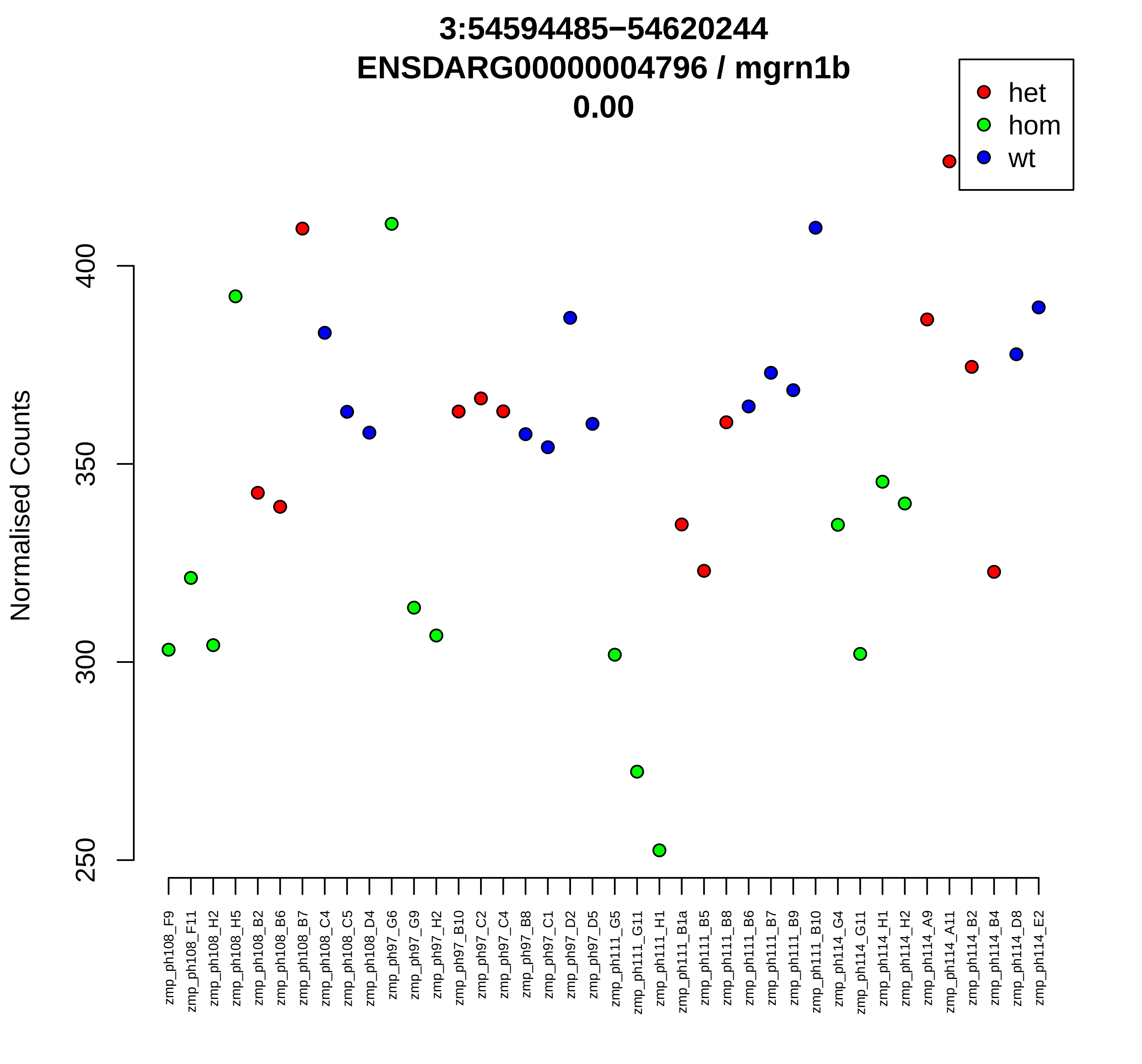
3:54594485−54620244
ENSDARG00000004796 / mgrn1b
0.00
het
hom
wt
Normalised Counts
250
300
350
400
zmp_ph108_F9
zmp_ph108_F11
zmp_ph108_H2
zmp_ph108_H5
zmp_ph108_B2
zmp_ph108_B6
zmp_ph108_B7
zmp_ph108_C4
zmp_ph108_C5
zmp_ph108_D4
zmp_ph97_G6
zmp_ph97_G9
zmp_ph97_H2
zmp_ph97_B10
zmp_ph97_C2
zmp_ph97_C4
zmp_ph97_B8
zmp_ph97_C1
zmp_ph97_D2
zmp_ph97_D5
zmp_ph111_G5
zmp_ph111_G11
zmp_ph111_H1
zmp_ph111_B1a
zmp_ph111_B5
zmp_ph111_B8
zmp_ph111_B6
zmp_ph111_B7
zmp_ph111_B9
zmp_ph111_B10
zmp_ph114_G4
zmp_ph114_G11
zmp_ph114_H1
zmp_ph114_H2
zmp_ph114_A9
zmp_ph114_A11
zmp_ph114_B2
zmp_ph114_B4
zmp_ph114_D8
zmp_ph114_E2

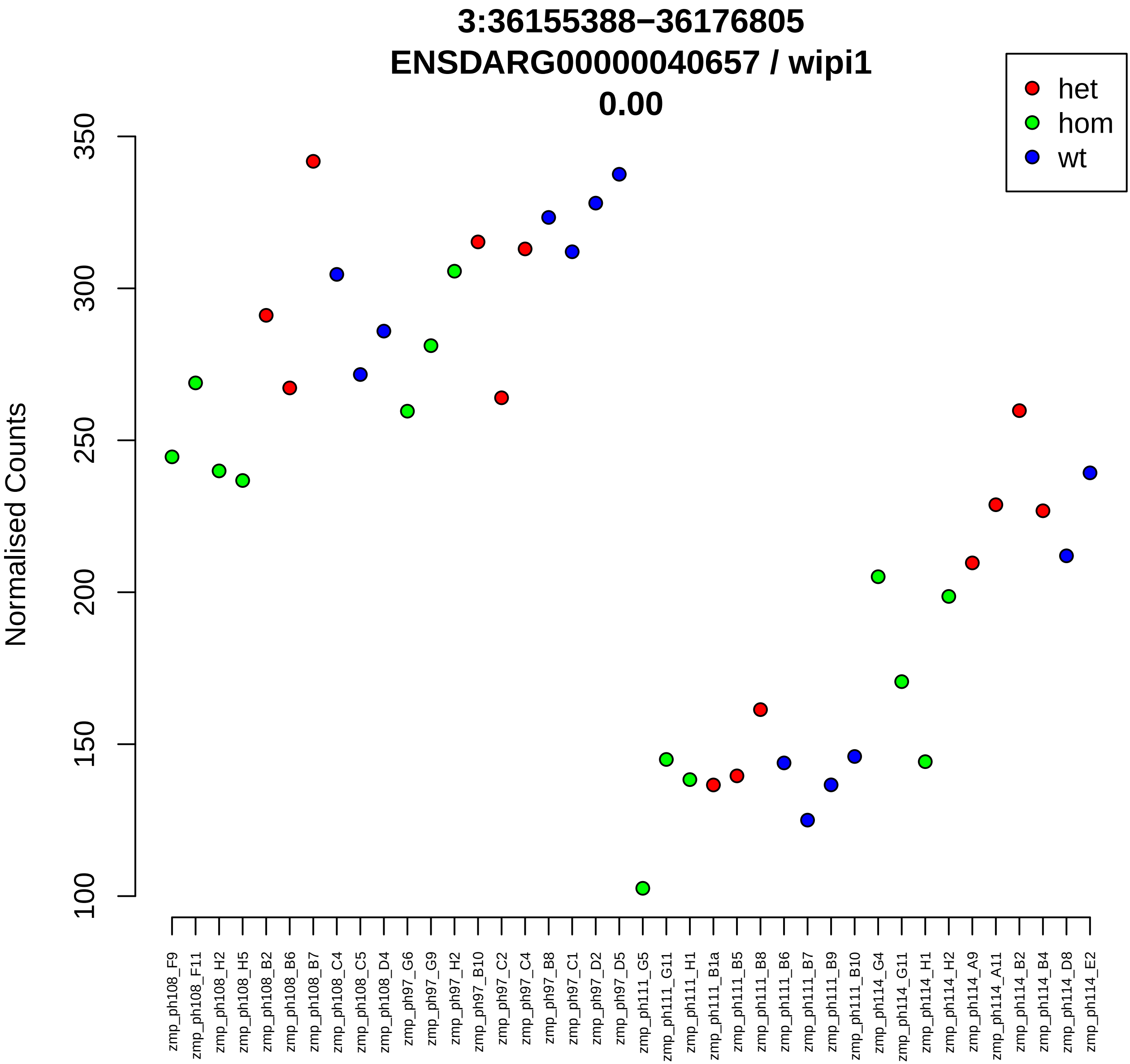

3:36155388−36176805
ENSDARG00000040657 / wipi1
0.00
het
hom
wt
Normalised Counts
100
150
200
250
300
350
zmp_ph108_F9
zmp_ph108_F11
zmp_ph108_H2
zmp_ph108_H5
zmp_ph108_B2
zmp_ph108_B6
zmp_ph108_B7
zmp_ph108_C4
zmp_ph108_C5
zmp_ph108_D4
zmp_ph97_G6
zmp_ph97_G9
zmp_ph97_H2
zmp_ph97_B10
zmp_ph97_C2
zmp_ph97_C4
zmp_ph97_B8
zmp_ph97_C1
zmp_ph97_D2
zmp_ph97_D5
zmp_ph111_G5
zmp_ph111_G11
zmp_ph111_H1
zmp_ph111_B1a
zmp_ph111_B5
zmp_ph111_B8
zmp_ph111_B6
zmp_ph111_B7
zmp_ph111_B9
zmp_ph111_B10
zmp_ph114_G4
zmp_ph114_G11
zmp_ph114_H1
zmp_ph114_H2
zmp_ph114_A9
zmp_ph114_A11
zmp_ph114_B2
zmp_ph114_B4
zmp_ph114_D8
zmp_ph114_E2

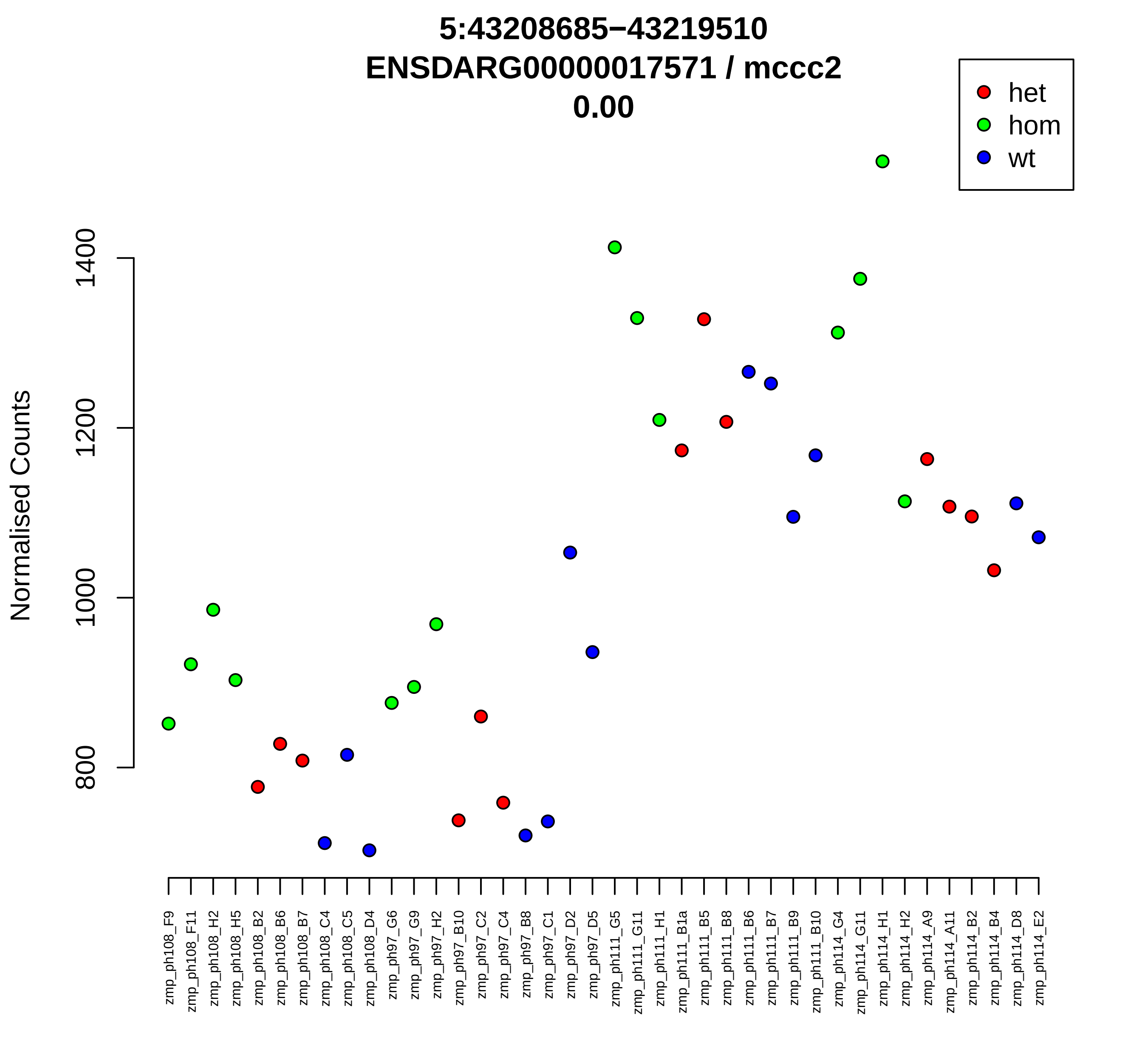
5:43208685−43219510
ENSDARG00000017571 / mccc2
0.00
het
hom
wt
Normalised Counts
800
1000
1200
1400
zmp_ph108_F9
zmp_ph108_F11
zmp_ph108_H2
zmp_ph108_H5
zmp_ph108_B2
zmp_ph108_B6
zmp_ph108_B7
zmp_ph108_C4
zmp_ph108_C5
zmp_ph108_D4
zmp_ph97_G6
zmp_ph97_G9
zmp_ph97_H2
zmp_ph97_B10
zmp_ph97_C2
zmp_ph97_C4
zmp_ph97_B8
zmp_ph97_C1
zmp_ph97_D2
zmp_ph97_D5
zmp_ph111_G5
zmp_ph111_G11
zmp_ph111_H1
zmp_ph111_B1a
zmp_ph111_B5
zmp_ph111_B8
zmp_ph111_B6
zmp_ph111_B7
zmp_ph111_B9
zmp_ph111_B10
zmp_ph114_G4
zmp_ph114_G11
zmp_ph114_H1
zmp_ph114_H2
zmp_ph114_A9
zmp_ph114_A11
zmp_ph114_B2
zmp_ph114_B4
zmp_ph114_D8
zmp_ph114_E2

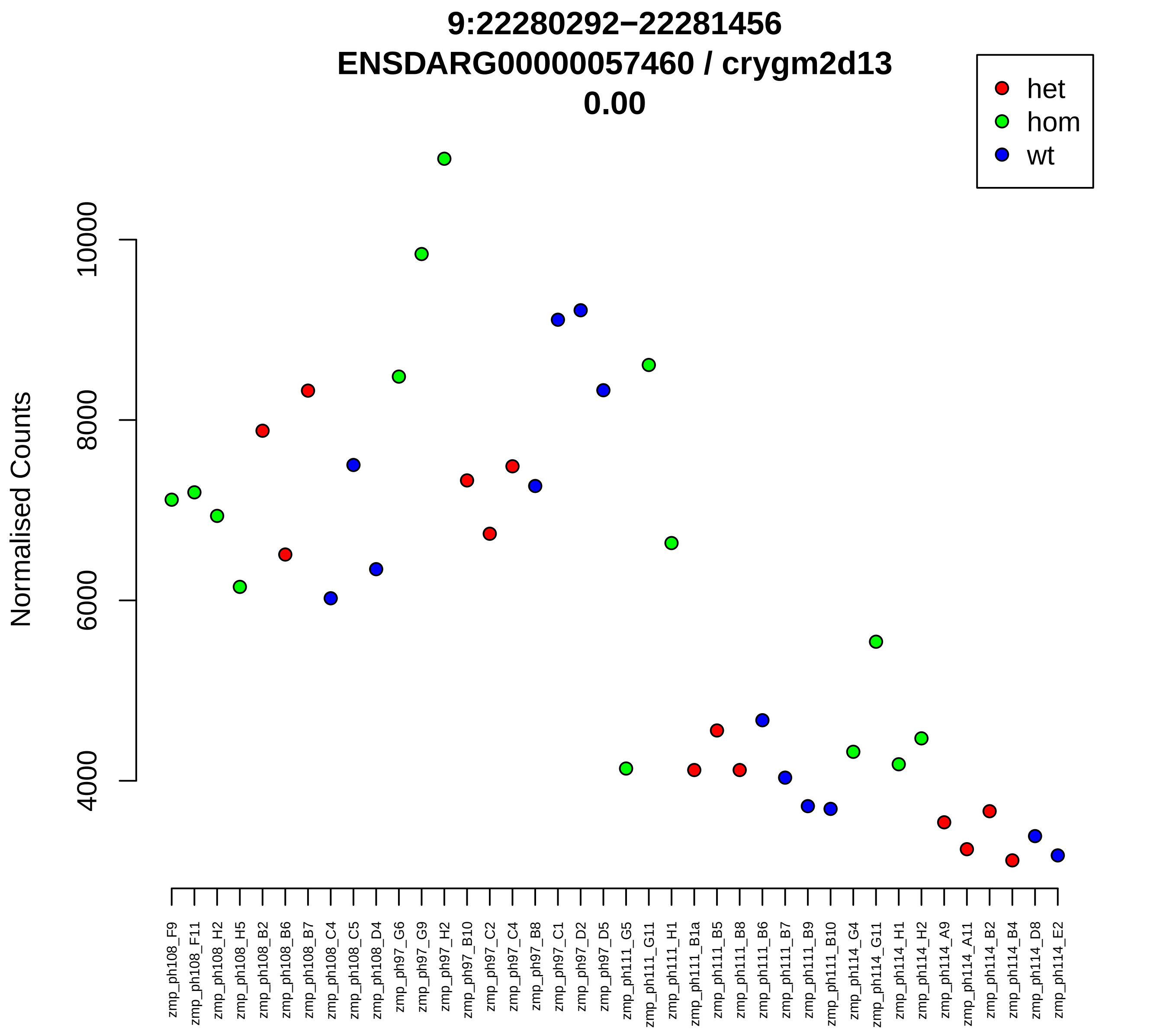
9:22280292−22281456
ENSDARG00000057460 / crygm2d13
0.00
het
hom
wt
Normalised Counts
4000
6000
8000
10000
zmp_ph108_F9
zmp_ph108_F11
zmp_ph108_H2
zmp_ph108_H5
zmp_ph108_B2
zmp_ph108_B6
zmp_ph108_B7
zmp_ph108_C4
zmp_ph108_C5
zmp_ph108_D4
zmp_ph97_G6
zmp_ph97_G9
zmp_ph97_H2
zmp_ph97_B10
zmp_ph97_C2
zmp_ph97_C4
zmp_ph97_B8
zmp_ph97_C1
zmp_ph97_D2
zmp_ph97_D5
zmp_ph111_G5
zmp_ph111_G11
zmp_ph111_H1
zmp_ph111_B1a
zmp_ph111_B5
zmp_ph111_B8
zmp_ph111_B6
zmp_ph111_B7
zmp_ph111_B9
zmp_ph111_B10
zmp_ph114_G4
zmp_ph114_G11
zmp_ph114_H1
zmp_ph114_H2
zmp_ph114_A9
zmp_ph114_A11
zmp_ph114_B2
zmp_ph114_B4
zmp_ph114_D8
zmp_ph114_E2

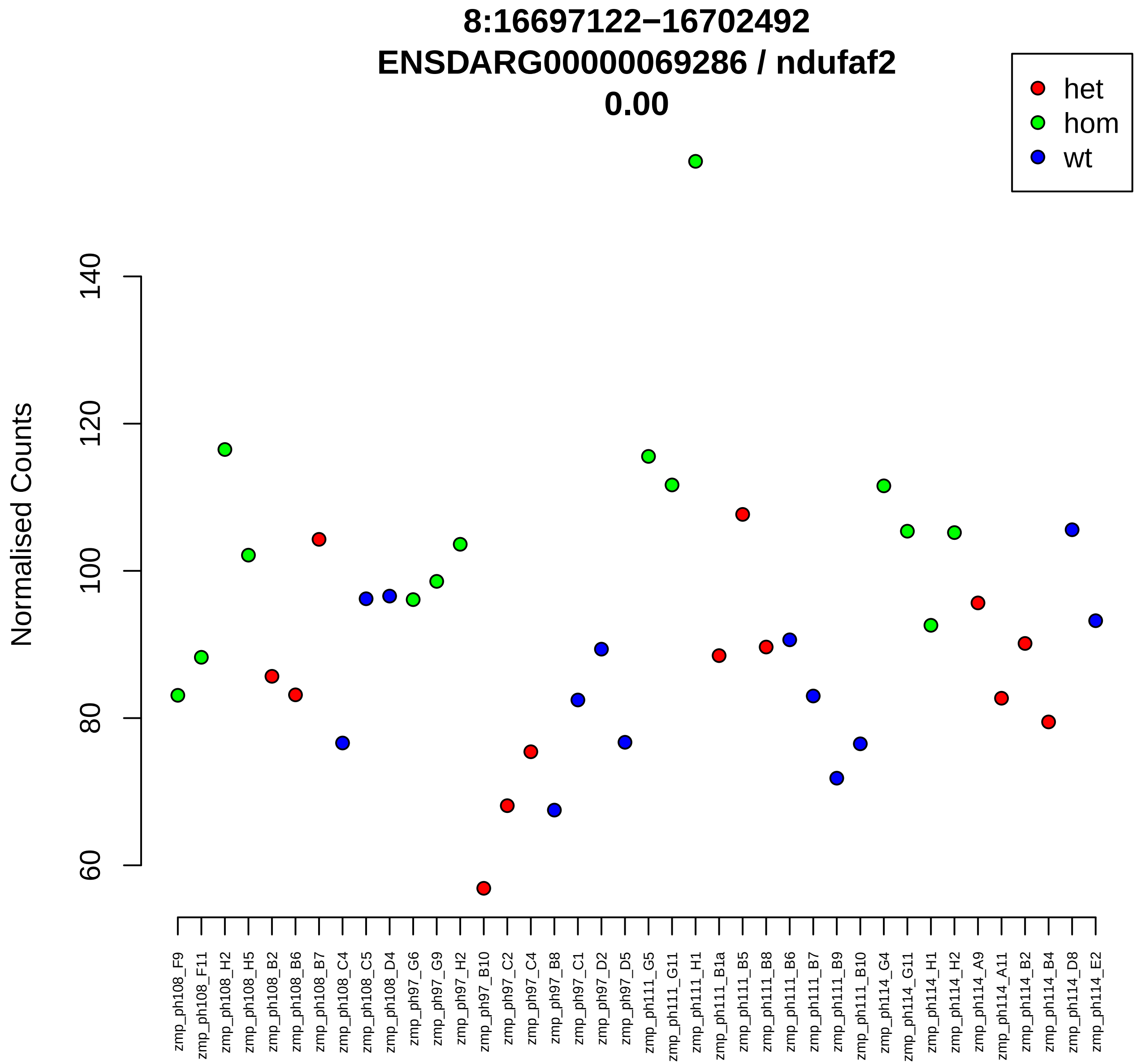
8:16697122−16702492
ENSDARG00000069286 / ndufaf2
0.00
het
hom
wt
Normalised Counts
60
80
100
120
140
zmp_ph108_F9
zmp_ph108_F11
zmp_ph108_H2
zmp_ph108_H5
zmp_ph108_B2
zmp_ph108_B6
zmp_ph108_B7
zmp_ph108_C4
zmp_ph108_C5
zmp_ph108_D4
zmp_ph97_G6
zmp_ph97_G9
zmp_ph97_H2
zmp_ph97_B10
zmp_ph97_C2
zmp_ph97_C4
zmp_ph97_B8
zmp_ph97_C1
zmp_ph97_D2
zmp_ph97_D5
zmp_ph111_G5
zmp_ph111_G11
zmp_ph111_H1
zmp_ph111_B1a
zmp_ph111_B5
zmp_ph111_B8
zmp_ph111_B6
zmp_ph111_B7
zmp_ph111_B9
zmp_ph111_B10
zmp_ph114_G4
zmp_ph114_G11
zmp_ph114_H1
zmp_ph114_H2
zmp_ph114_A9
zmp_ph114_A11
zmp_ph114_B2
zmp_ph114_B4
zmp_ph114_D8
zmp_ph114_E2

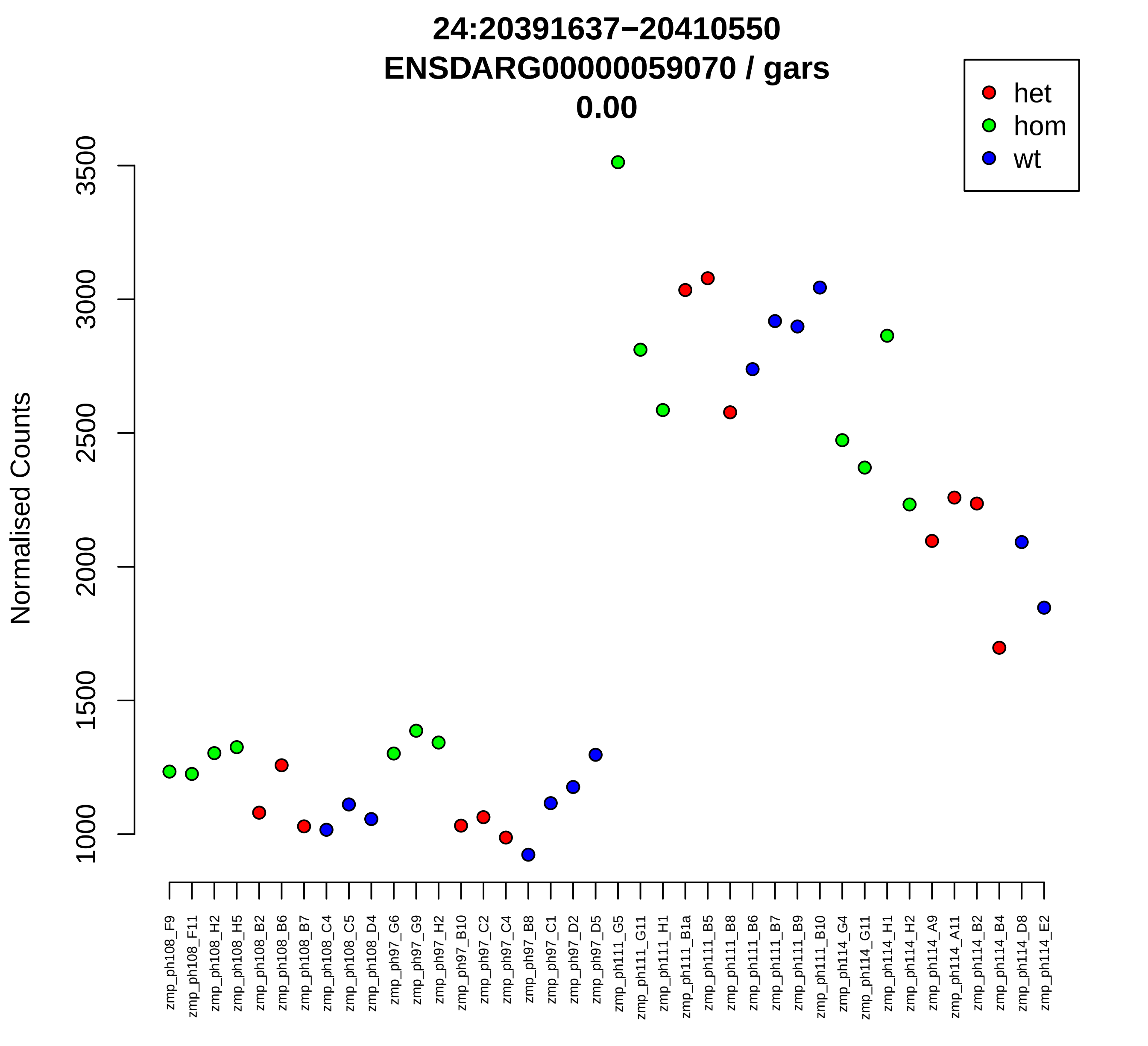

24:20391637−20410550
ENSDARG00000059070 / gars
0.00
het
hom
wt
Normalised Counts
1000
1500
2000
2500
3000
3500
zmp_ph108_F9
zmp_ph108_F11
zmp_ph108_H2
zmp_ph108_H5
zmp_ph108_B2
zmp_ph108_B6
zmp_ph108_B7
zmp_ph108_C4
zmp_ph108_C5
zmp_ph108_D4
zmp_ph97_G6
zmp_ph97_G9
zmp_ph97_H2
zmp_ph97_B10
zmp_ph97_C2
zmp_ph97_C4
zmp_ph97_B8
zmp_ph97_C1
zmp_ph97_D2
zmp_ph97_D5
zmp_ph111_G5
zmp_ph111_G11
zmp_ph111_H1
zmp_ph111_B1a
zmp_ph111_B5
zmp_ph111_B8
zmp_ph111_B6
zmp_ph111_B7
zmp_ph111_B9
zmp_ph111_B10
zmp_ph114_G4
zmp_ph114_G11
zmp_ph114_H1
zmp_ph114_H2
zmp_ph114_A9
zmp_ph114_A11
zmp_ph114_B2
zmp_ph114_B4
zmp_ph114_D8
zmp_ph114_E2

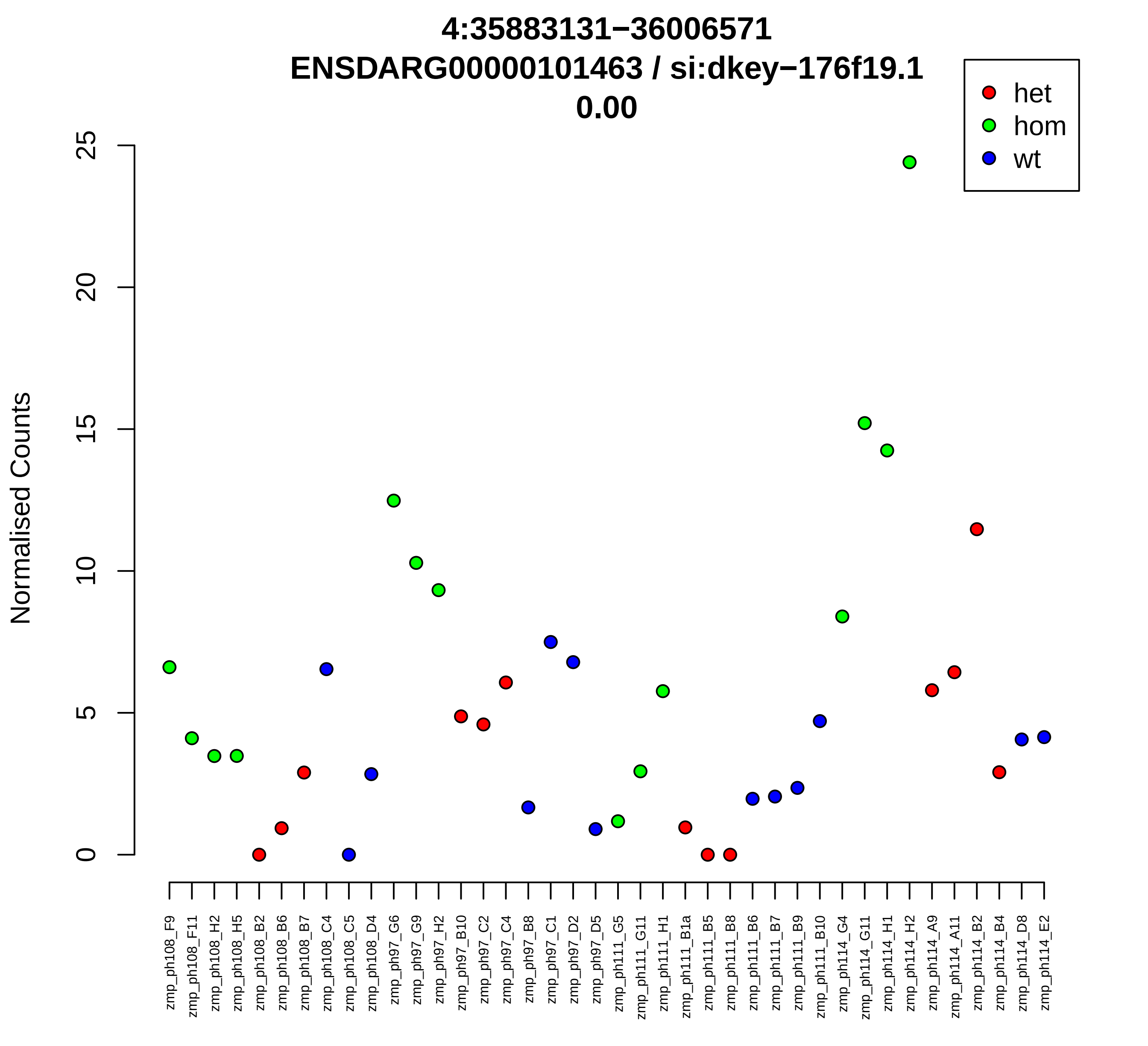

4:35883131−36006571
ENSDARG00000101463 / si:dkey−176f19.1
0.00
het
hom
wt
Normalised Counts
0
5
10
15
20
25
zmp_ph108_F9
zmp_ph108_F11
zmp_ph108_H2
zmp_ph108_H5
zmp_ph108_B2
zmp_ph108_B6
zmp_ph108_B7
zmp_ph108_C4
zmp_ph108_C5
zmp_ph108_D4
zmp_ph97_G6
zmp_ph97_G9
zmp_ph97_H2
zmp_ph97_B10
zmp_ph97_C2
zmp_ph97_C4
zmp_ph97_B8
zmp_ph97_C1
zmp_ph97_D2
zmp_ph97_D5
zmp_ph111_G5
zmp_ph111_G11
zmp_ph111_H1
zmp_ph111_B1a
zmp_ph111_B5
zmp_ph111_B8
zmp_ph111_B6
zmp_ph111_B7
zmp_ph111_B9
zmp_ph111_B10
zmp_ph114_G4
zmp_ph114_G11
zmp_ph114_H1
zmp_ph114_H2
zmp_ph114_A9
zmp_ph114_A11
zmp_ph114_B2
zmp_ph114_B4
zmp_ph114_D8
zmp_ph114_E2

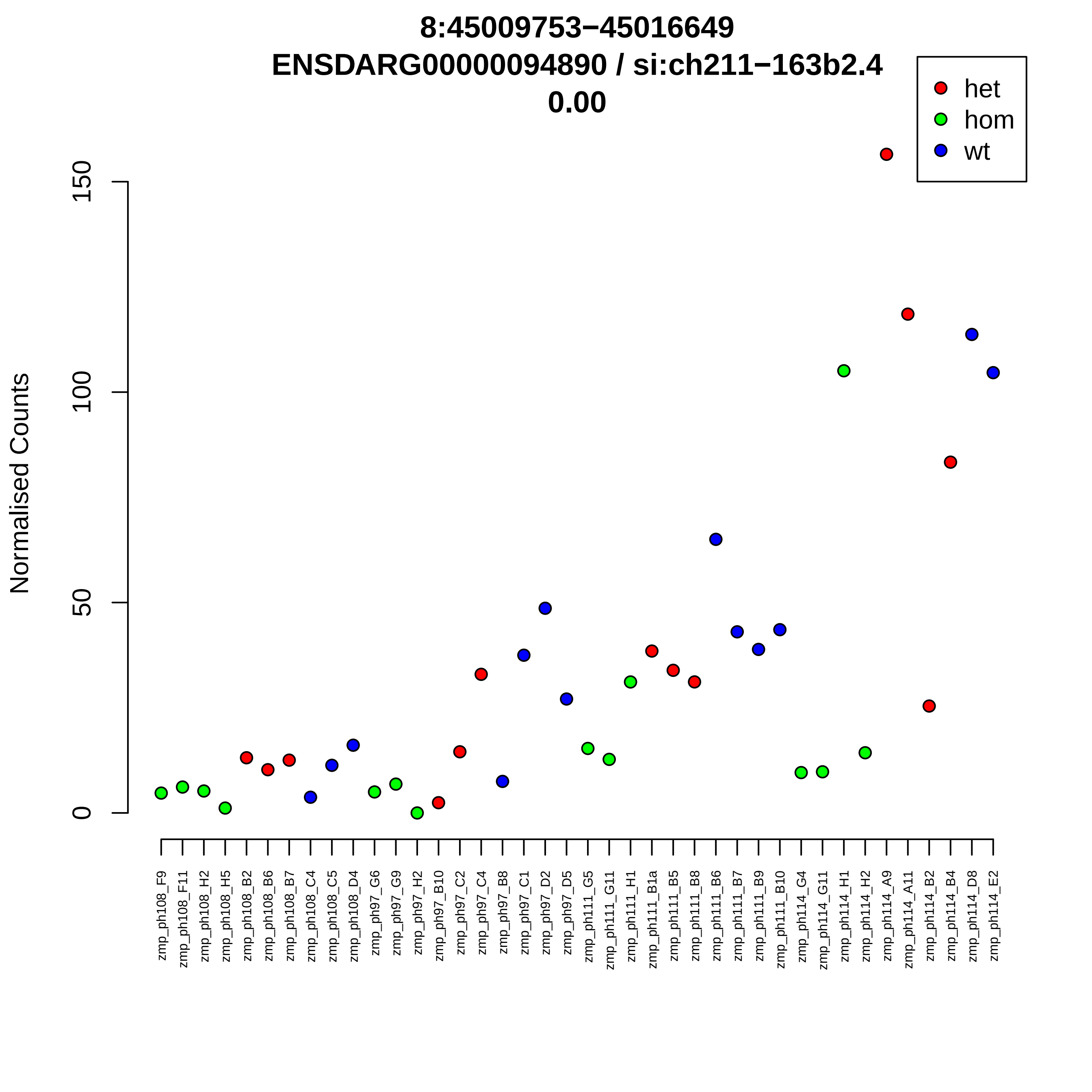

8:45009753−45016649
ENSDARG00000094890 / si:ch211−163b2.4
0.00
het
hom
wt
Normalised Counts
0
50
100
150
zmp_ph108_F9
zmp_ph108_F11
zmp_ph108_H2
zmp_ph108_H5
zmp_ph108_B2
zmp_ph108_B6
zmp_ph108_B7
zmp_ph108_C4
zmp_ph108_C5
zmp_ph108_D4
zmp_ph97_G6
zmp_ph97_G9
zmp_ph97_H2
zmp_ph97_B10
zmp_ph97_C2
zmp_ph97_C4
zmp_ph97_B8
zmp_ph97_C1
zmp_ph97_D2
zmp_ph97_D5
zmp_ph111_G5
zmp_ph111_G11
zmp_ph111_H1
zmp_ph111_B1a
zmp_ph111_B5
zmp_ph111_B8
zmp_ph111_B6
zmp_ph111_B7
zmp_ph111_B9
zmp_ph111_B10
zmp_ph114_G4
zmp_ph114_G11
zmp_ph114_H1
zmp_ph114_H2
zmp_ph114_A9
zmp_ph114_A11
zmp_ph114_B2
zmp_ph114_B4
zmp_ph114_D8
zmp_ph114_E2

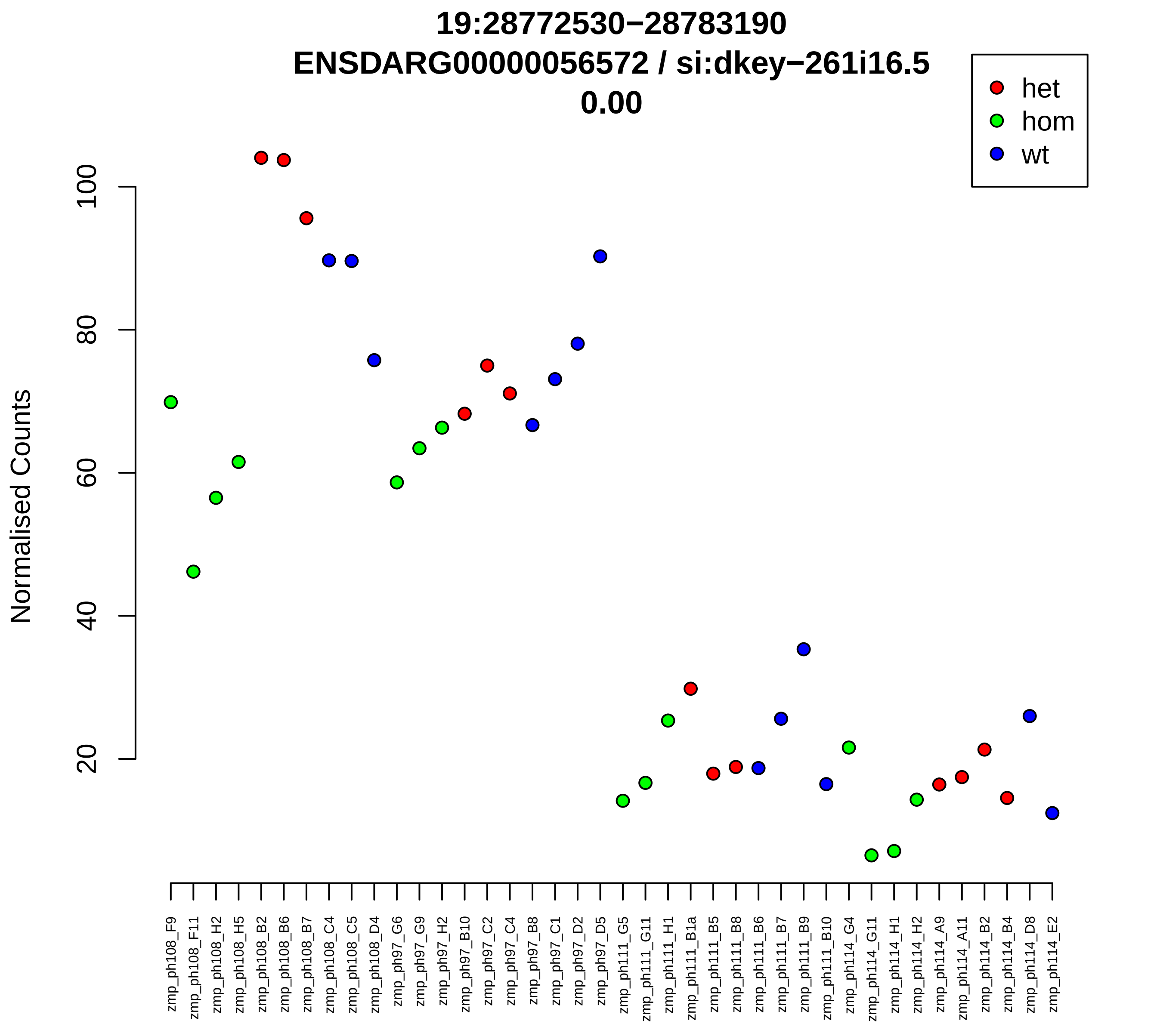

19:28772530−28783190
ENSDARG00000056572 / si:dkey−261i16.5
0.00
het
hom
wt
Normalised Counts
20
40
60
80
100
zmp_ph108_F9
zmp_ph108_F11
zmp_ph108_H2
zmp_ph108_H5
zmp_ph108_B2
zmp_ph108_B6
zmp_ph108_B7
zmp_ph108_C4
zmp_ph108_C5
zmp_ph108_D4
zmp_ph97_G6
zmp_ph97_G9
zmp_ph97_H2
zmp_ph97_B10
zmp_ph97_C2
zmp_ph97_C4
zmp_ph97_B8
zmp_ph97_C1
zmp_ph97_D2
zmp_ph97_D5
zmp_ph111_G5
zmp_ph111_G11
zmp_ph111_H1
zmp_ph111_B1a
zmp_ph111_B5
zmp_ph111_B8
zmp_ph111_B6
zmp_ph111_B7
zmp_ph111_B9
zmp_ph111_B10
zmp_ph114_G4
zmp_ph114_G11
zmp_ph114_H1
zmp_ph114_H2
zmp_ph114_A9
zmp_ph114_A11
zmp_ph114_B2
zmp_ph114_B4
zmp_ph114_D8
zmp_ph114_E2

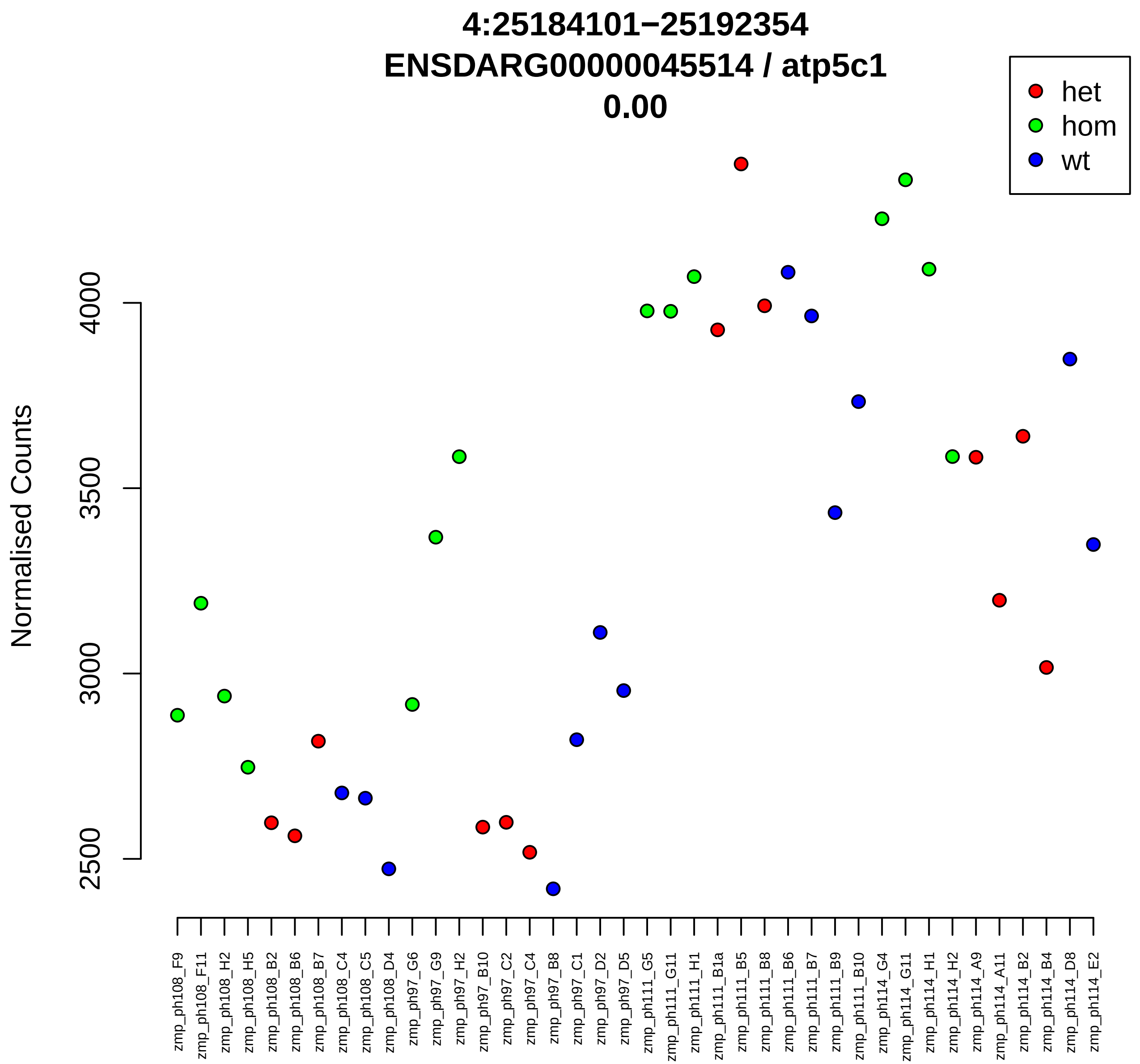

4:25184101−25192354
ENSDARG00000045514 / atp5c1
0.00
het
hom
wt
Normalised Counts
2500
3000
3500
4000
zmp_ph108_F9
zmp_ph108_F11
zmp_ph108_H2
zmp_ph108_H5
zmp_ph108_B2
zmp_ph108_B6
zmp_ph108_B7
zmp_ph108_C4
zmp_ph108_C5
zmp_ph108_D4
zmp_ph97_G6
zmp_ph97_G9
zmp_ph97_H2
zmp_ph97_B10
zmp_ph97_C2
zmp_ph97_C4
zmp_ph97_B8
zmp_ph97_C1
zmp_ph97_D2
zmp_ph97_D5
zmp_ph111_G5
zmp_ph111_G11
zmp_ph111_H1
zmp_ph111_B1a
zmp_ph111_B5
zmp_ph111_B8
zmp_ph111_B6
zmp_ph111_B7
zmp_ph111_B9
zmp_ph111_B10
zmp_ph114_G4
zmp_ph114_G11
zmp_ph114_H1
zmp_ph114_H2
zmp_ph114_A9
zmp_ph114_A11
zmp_ph114_B2
zmp_ph114_B4
zmp_ph114_D8
zmp_ph114_E2

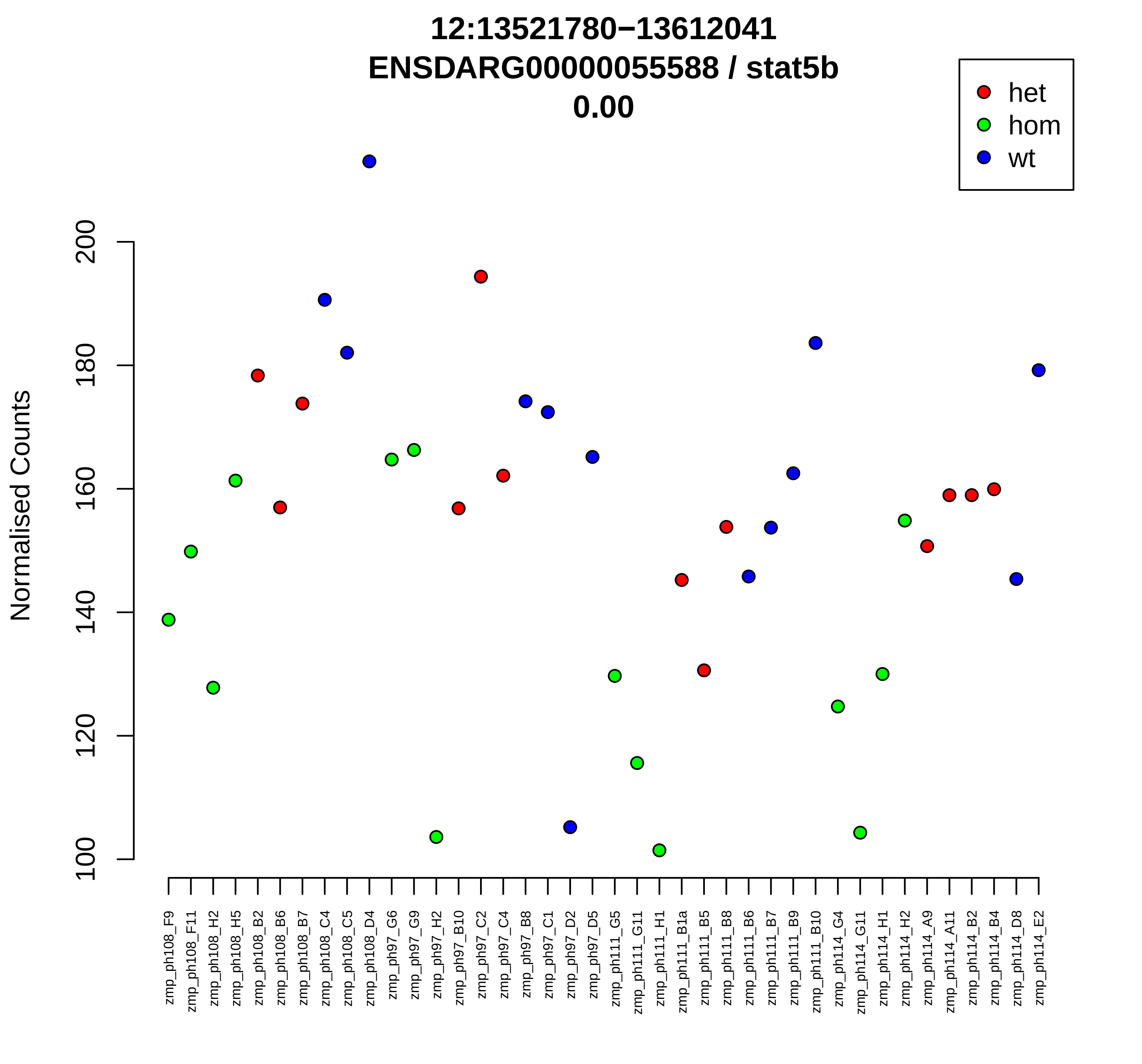
12:13521780−13612041
ENSDARG00000055588 / stat5b
0.00
het
hom
wt
Normalised Counts
100
120
140
160
180
200
zmp_ph108_F9
zmp_ph108_F11
zmp_ph108_H2
zmp_ph108_H5
zmp_ph108_B2
zmp_ph108_B6
zmp_ph108_B7
zmp_ph108_C4
zmp_ph108_C5
zmp_ph108_D4
zmp_ph97_G6
zmp_ph97_G9
zmp_ph97_H2
zmp_ph97_B10
zmp_ph97_C2
zmp_ph97_C4
zmp_ph97_B8
zmp_ph97_C1
zmp_ph97_D2
zmp_ph97_D5
zmp_ph111_G5
zmp_ph111_G11
zmp_ph111_H1
zmp_ph111_B1a
zmp_ph111_B5
zmp_ph111_B8
zmp_ph111_B6
zmp_ph111_B7
zmp_ph111_B9
zmp_ph111_B10
zmp_ph114_G4
zmp_ph114_G11
zmp_ph114_H1
zmp_ph114_H2
zmp_ph114_A9
zmp_ph114_A11
zmp_ph114_B2
zmp_ph114_B4
zmp_ph114_D8
zmp_ph114_E2

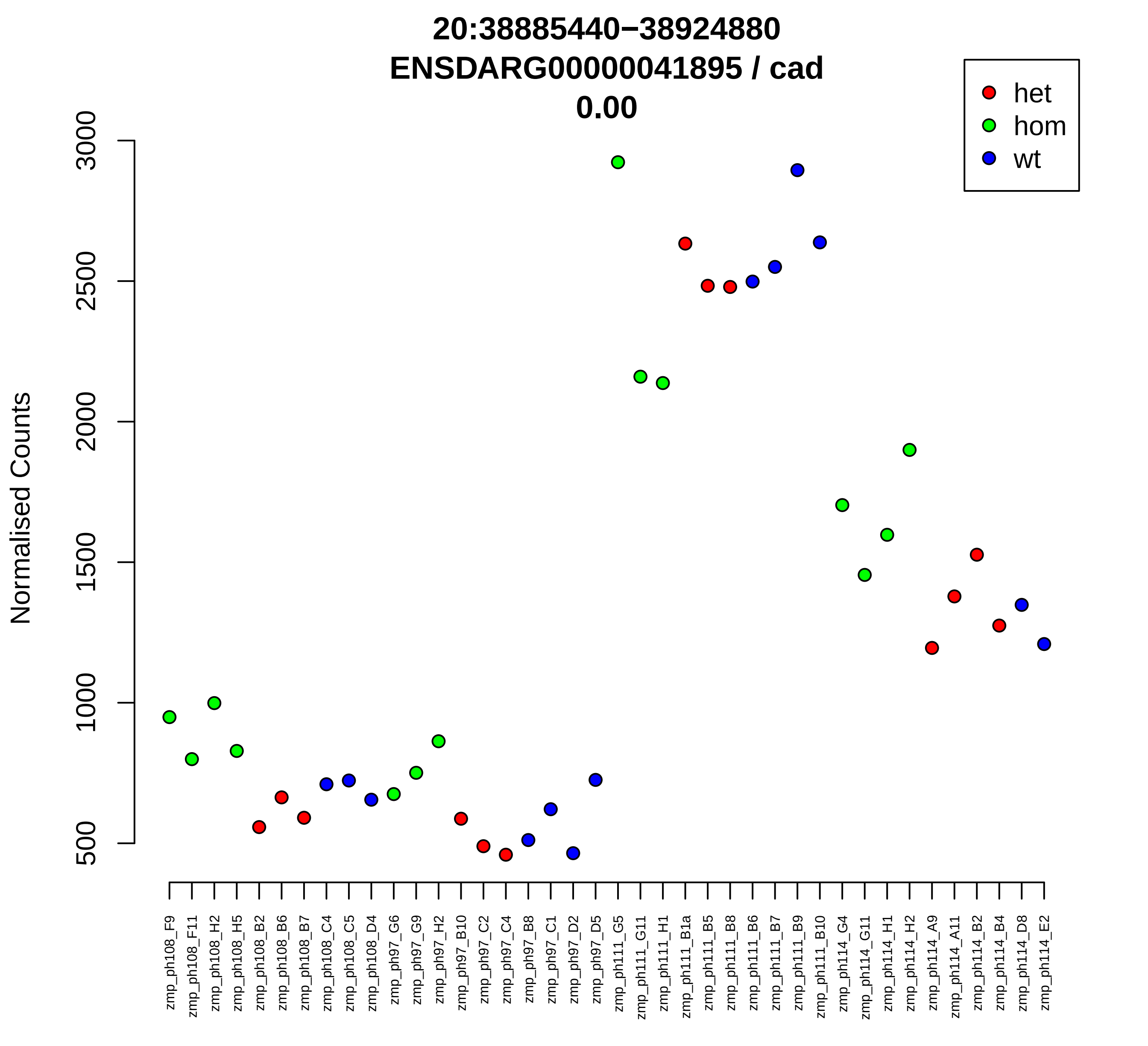

20:38885440−38924880
ENSDARG00000041895 / cad
0.00
het
hom
wt
Normalised Counts
500
1000
1500
2000
2500
3000
zmp_ph108_F9
zmp_ph108_F11
zmp_ph108_H2
zmp_ph108_H5
zmp_ph108_B2
zmp_ph108_B6
zmp_ph108_B7
zmp_ph108_C4
zmp_ph108_C5
zmp_ph108_D4
zmp_ph97_G6
zmp_ph97_G9
zmp_ph97_H2
zmp_ph97_B10
zmp_ph97_C2
zmp_ph97_C4
zmp_ph97_B8
zmp_ph97_C1
zmp_ph97_D2
zmp_ph97_D5
zmp_ph111_G5
zmp_ph111_G11
zmp_ph111_H1
zmp_ph111_B1a
zmp_ph111_B5
zmp_ph111_B8
zmp_ph111_B6
zmp_ph111_B7
zmp_ph111_B9
zmp_ph111_B10
zmp_ph114_G4
zmp_ph114_G11
zmp_ph114_H1
zmp_ph114_H2
zmp_ph114_A9
zmp_ph114_A11
zmp_ph114_B2
zmp_ph114_B4
zmp_ph114_D8
zmp_ph114_E2

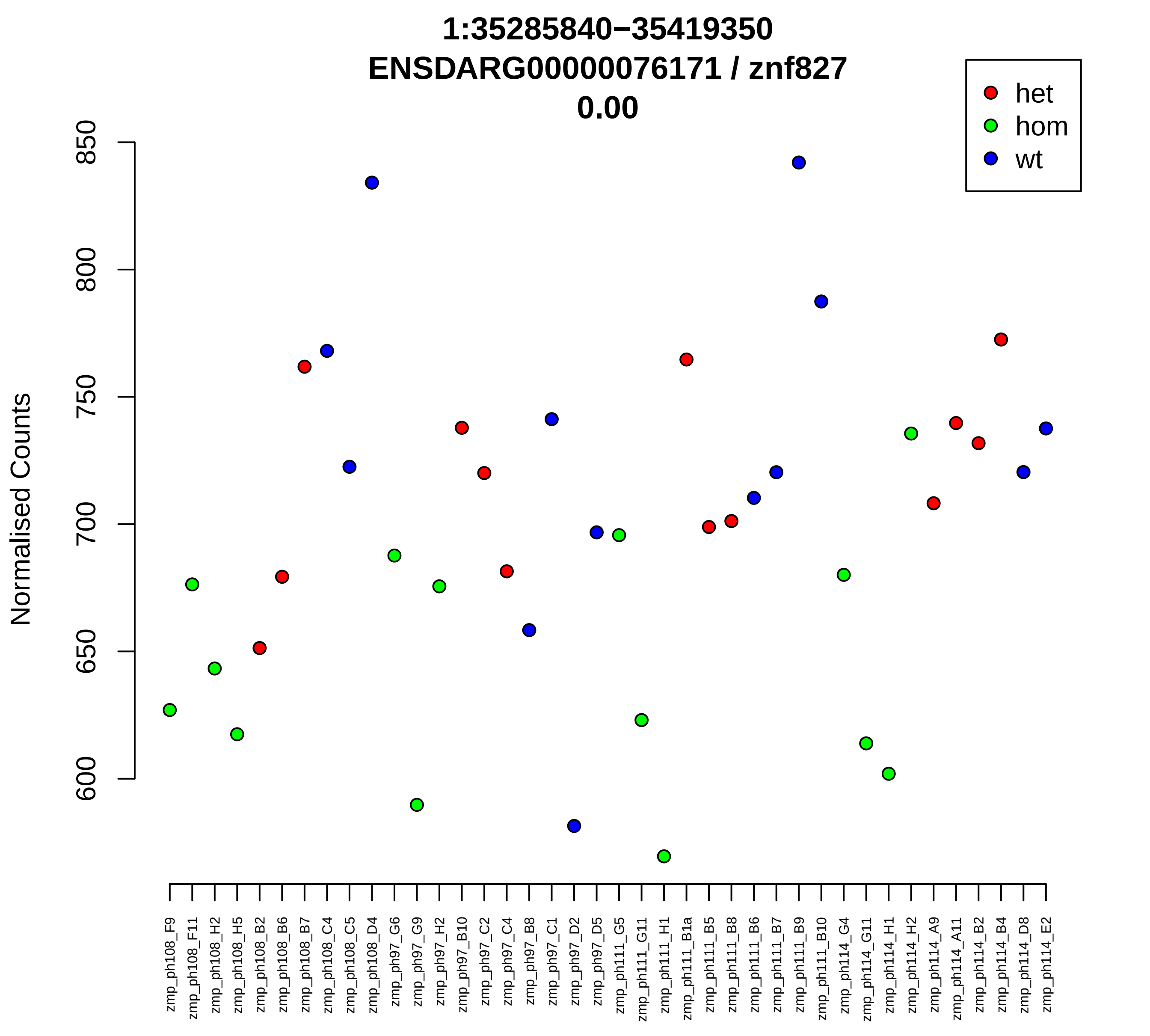

1:35285840−35419350
ENSDARG00000076171 / znf827
0.00
het
hom
wt
Normalised Counts
600
650
700
750
800
850
zmp_ph108_F9
zmp_ph108_F11
zmp_ph108_H2
zmp_ph108_H5
zmp_ph108_B2
zmp_ph108_B6
zmp_ph108_B7
zmp_ph108_C4
zmp_ph108_C5
zmp_ph108_D4
zmp_ph97_G6
zmp_ph97_G9
zmp_ph97_H2
zmp_ph97_B10
zmp_ph97_C2
zmp_ph97_C4
zmp_ph97_B8
zmp_ph97_C1
zmp_ph97_D2
zmp_ph97_D5
zmp_ph111_G5
zmp_ph111_G11
zmp_ph111_H1
zmp_ph111_B1a
zmp_ph111_B5
zmp_ph111_B8
zmp_ph111_B6
zmp_ph111_B7
zmp_ph111_B9
zmp_ph111_B10
zmp_ph114_G4
zmp_ph114_G11
zmp_ph114_H1
zmp_ph114_H2
zmp_ph114_A9
zmp_ph114_A11
zmp_ph114_B2
zmp_ph114_B4
zmp_ph114_D8
zmp_ph114_E2

16:24686108−24727834
ENSDARG00000097057 / fxyd6l
0.00

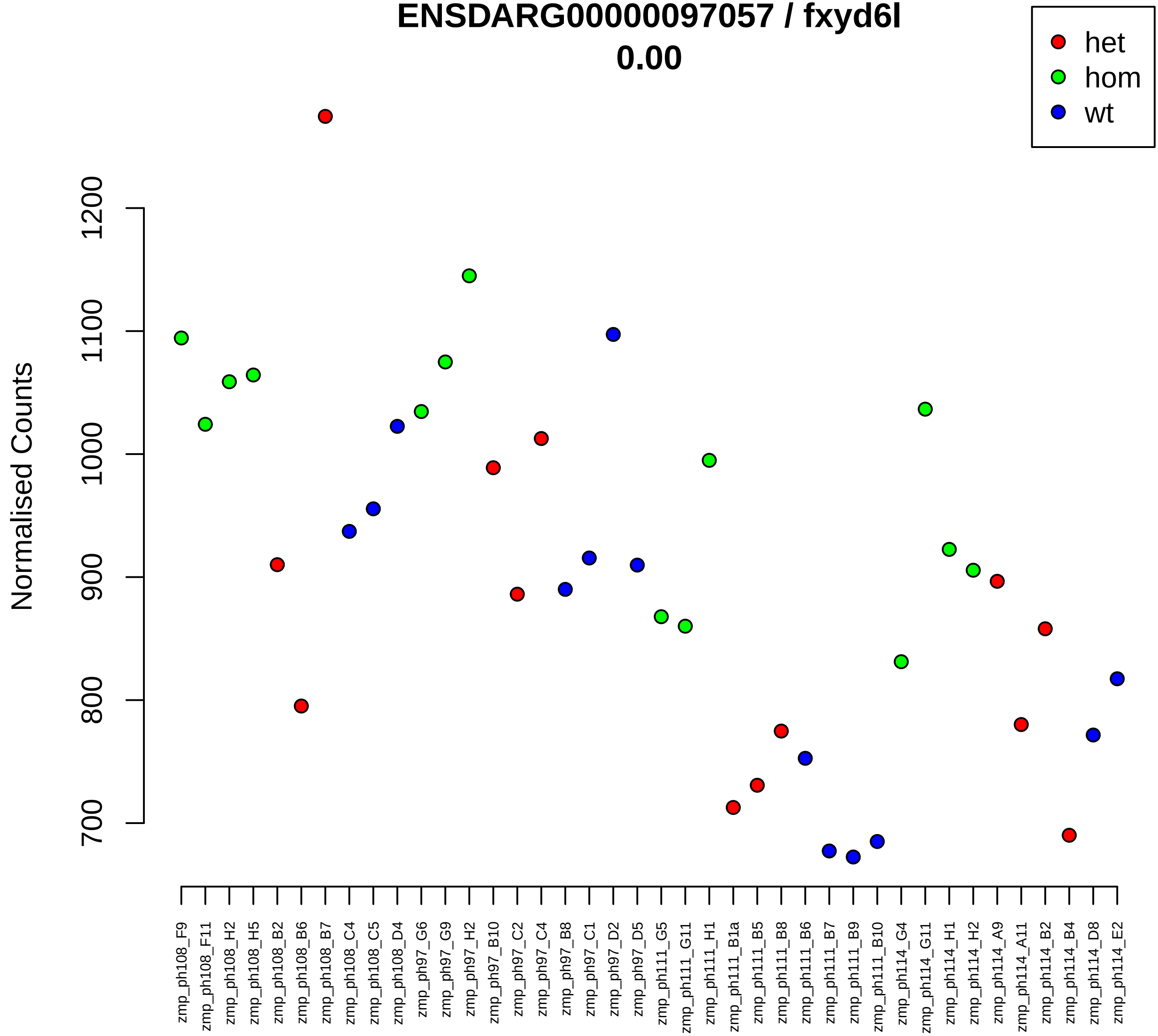

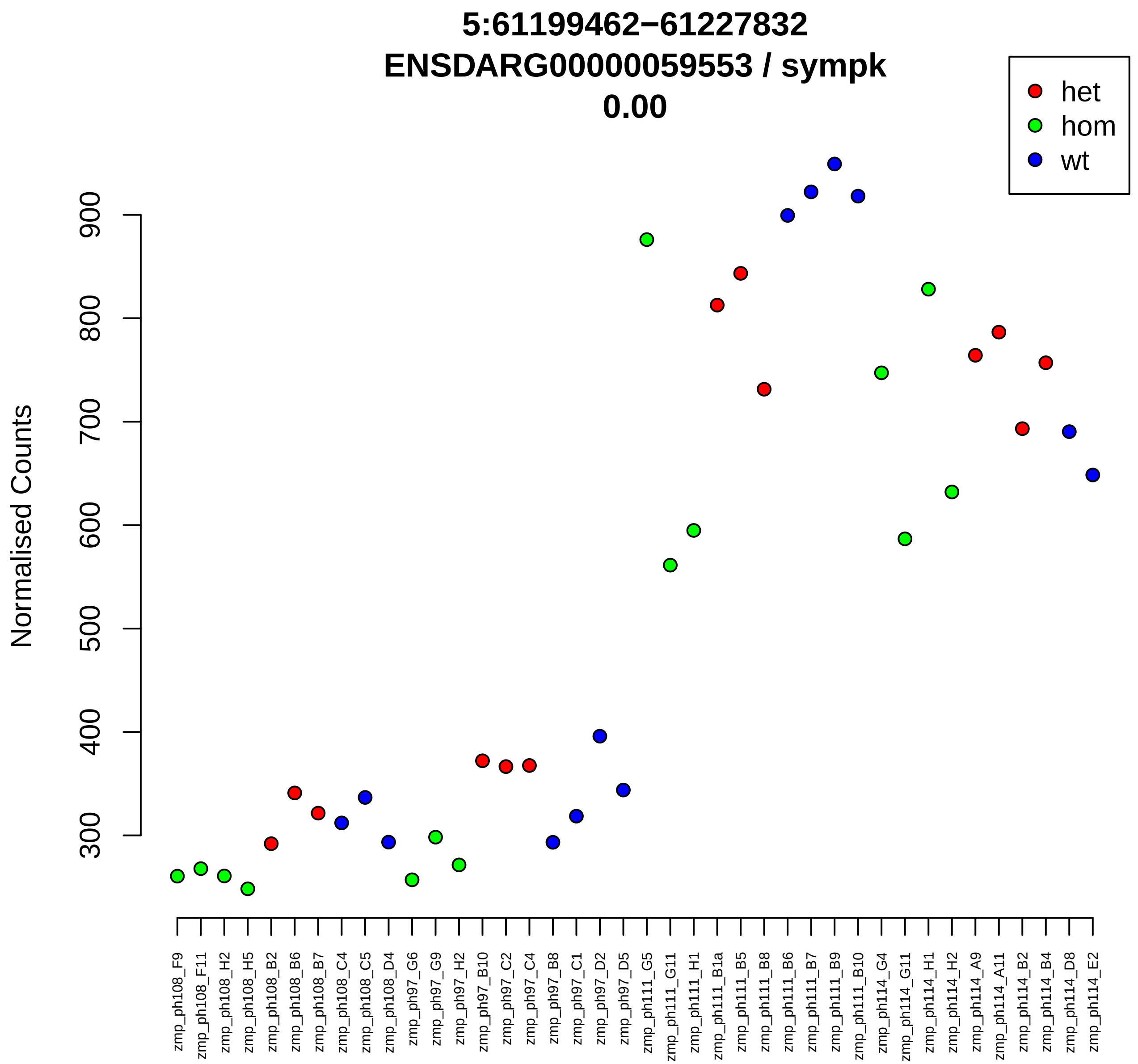

5:61199462−61227832
ENSDARG00000059553 / sympk
0.00
het
hom
wt
Normalised Counts
300
400
500
600
700
800
900
zmp_ph108_F9
zmp_ph108_F11
zmp_ph108_H2
zmp_ph108_H5
zmp_ph108_B2
zmp_ph108_B6
zmp_ph108_B7
zmp_ph108_C4
zmp_ph108_C5
zmp_ph108_D4
zmp_ph97_G6
zmp_ph97_G9
zmp_ph97_H2
zmp_ph97_B10
zmp_ph97_C2
zmp_ph97_C4
zmp_ph97_B8
zmp_ph97_C1
zmp_ph97_D2
zmp_ph97_D5
zmp_ph111_G5
zmp_ph111_G11
zmp_ph111_H1
zmp_ph111_B1a
zmp_ph111_B5
zmp_ph111_B8
zmp_ph111_B6
zmp_ph111_B7
zmp_ph111_B9
zmp_ph111_B10
zmp_ph114_G4
zmp_ph114_G11
zmp_ph114_H1
zmp_ph114_H2
zmp_ph114_A9
zmp_ph114_A11
zmp_ph114_B2
zmp_ph114_B4
zmp_ph114_D8
zmp_ph114_E2

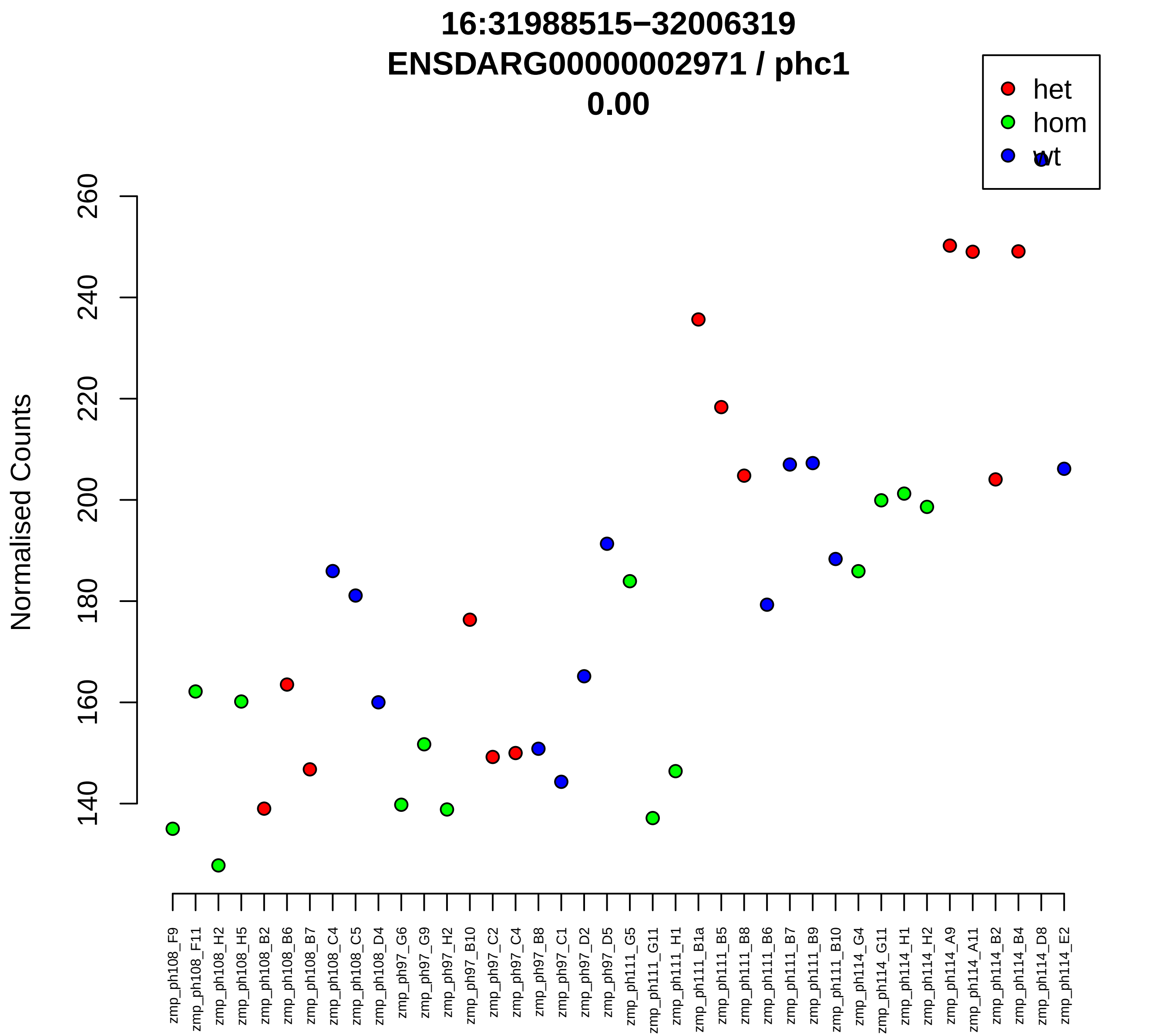

16:31988515−32006319
ENSDARG00000002971 / phc1
0.00
het
hom
wt
Normalised Counts
140
160
180
200
220
240
260
zmp_ph108_F9
zmp_ph108_F11
zmp_ph108_H2
zmp_ph108_H5
zmp_ph108_B2
zmp_ph108_B6
zmp_ph108_B7
zmp_ph108_C4
zmp_ph108_C5
zmp_ph108_D4
zmp_ph97_G6
zmp_ph97_G9
zmp_ph97_H2
zmp_ph97_B10
zmp_ph97_C2
zmp_ph97_C4
zmp_ph97_B8
zmp_ph97_C1
zmp_ph97_D2
zmp_ph97_D5
zmp_ph111_G5
zmp_ph111_G11
zmp_ph111_H1
zmp_ph111_B1a
zmp_ph111_B5
zmp_ph111_B8
zmp_ph111_B6
zmp_ph111_B7
zmp_ph111_B9
zmp_ph111_B10
zmp_ph114_G4
zmp_ph114_G11
zmp_ph114_H1
zmp_ph114_H2
zmp_ph114_A9
zmp_ph114_A11
zmp_ph114_B2
zmp_ph114_B4
zmp_ph114_D8
zmp_ph114_E2

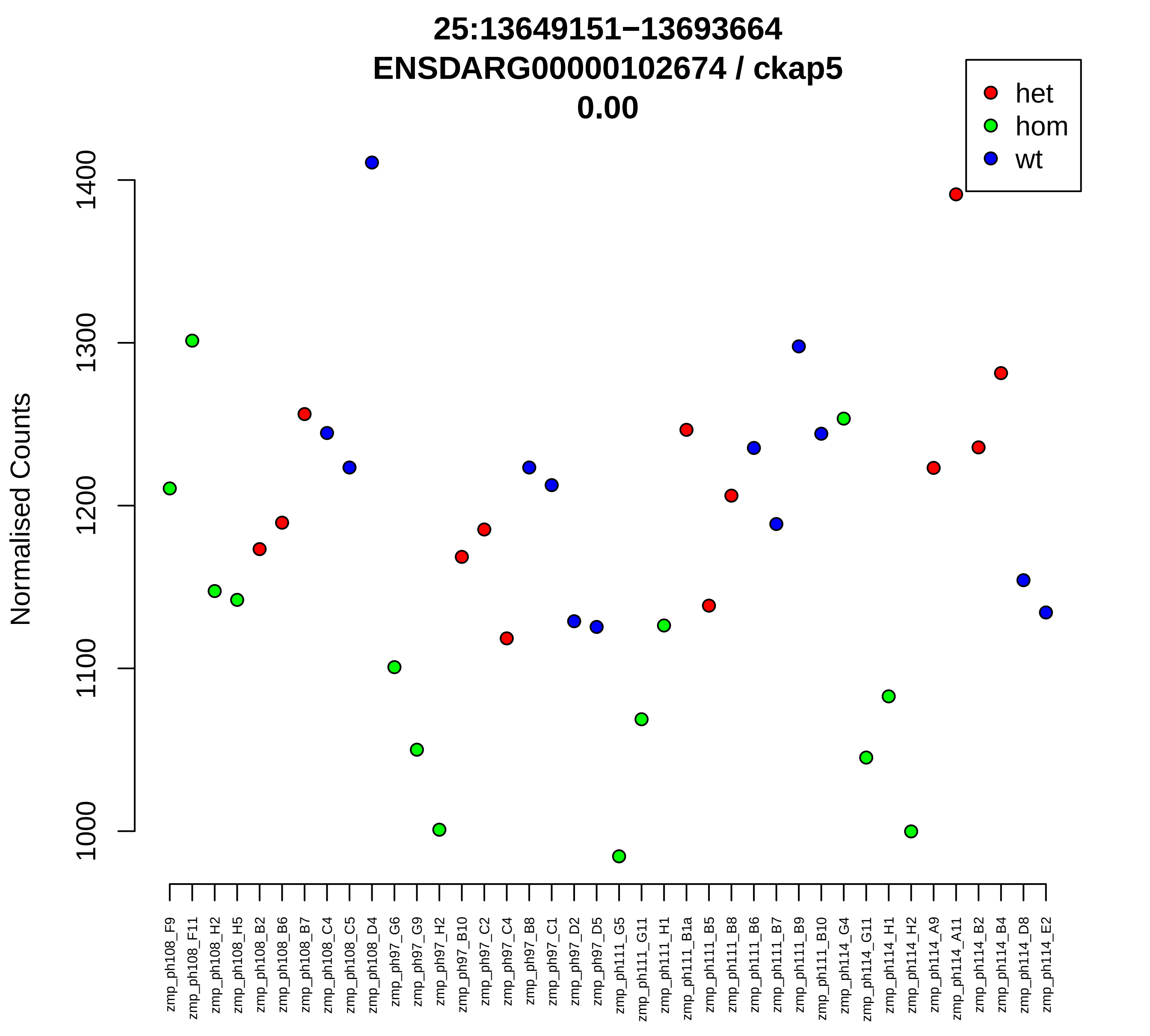

25:13649151−13693664
ENSDARG00000102674 / ckap5
0.00
het
hom
wt
Normalised Counts
1000
1100
1200
1300
1400
zmp_ph108_F9
zmp_ph108_F11
zmp_ph108_H2
zmp_ph108_H5
zmp_ph108_B2
zmp_ph108_B6
zmp_ph108_B7
zmp_ph108_C4
zmp_ph108_C5
zmp_ph108_D4
zmp_ph97_G6
zmp_ph97_G9
zmp_ph97_H2
zmp_ph97_B10
zmp_ph97_C2
zmp_ph97_C4
zmp_ph97_B8
zmp_ph97_C1
zmp_ph97_D2
zmp_ph97_D5
zmp_ph111_G5
zmp_ph111_G11
zmp_ph111_H1
zmp_ph111_B1a
zmp_ph111_B5
zmp_ph111_B8
zmp_ph111_B6
zmp_ph111_B7
zmp_ph111_B9
zmp_ph111_B10
zmp_ph114_G4
zmp_ph114_G11
zmp_ph114_H1
zmp_ph114_H2
zmp_ph114_A9
zmp_ph114_A11
zmp_ph114_B2
zmp_ph114_B4
zmp_ph114_D8
zmp_ph114_E2

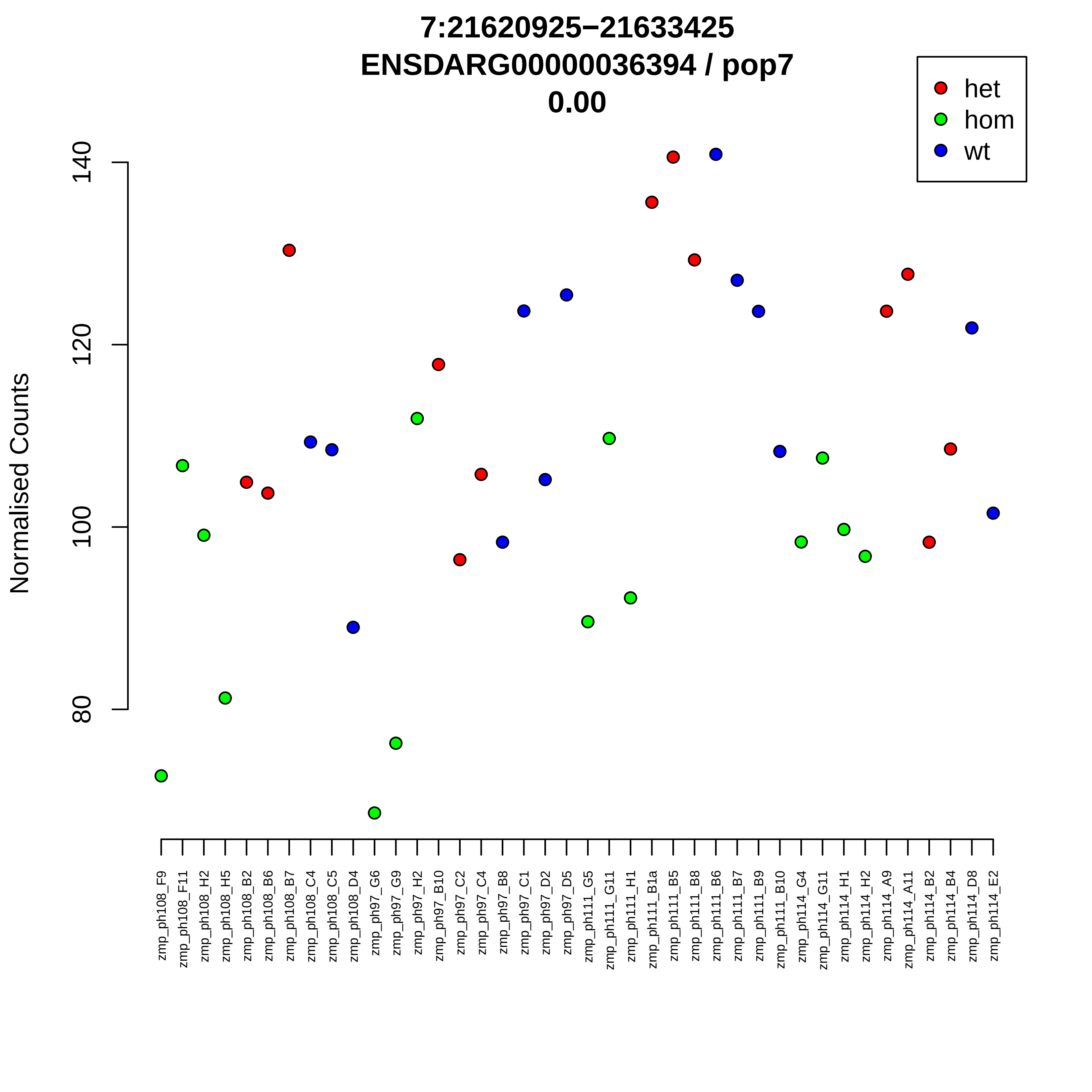

7:21620925−21633425
ENSDARG00000036394 / pop7
0.00
het
hom
wt
Normalised Counts
140
120
100
80
zmp_ph108_F9
zmp_ph108_F11
zmp_ph108_H2
zmp_ph108_H5
zmp_ph108_B2
zmp_ph108_B6
zmp_ph108_B7
zmp_ph108_C4
zmp_ph108_C5
zmp_ph108_D4
zmp_ph97_G6
zmp_ph97_G9
zmp_ph97_H2
zmp_ph97_B10
zmp_ph97_C2
zmp_ph97_C4
zmp_ph97_B8
zmp_ph97_C1
zmp_ph97_D2
zmp_ph97_D5
zmp_ph111_G5
zmp_ph111_G11
zmp_ph111_H1
zmp_ph111_B1a
zmp_ph111_B5
zmp_ph111_B8
zmp_ph111_B6
zmp_ph111_B7
zmp_ph111_B9
zmp_ph111_B10
zmp_ph114_G4
zmp_ph114_G11
zmp_ph114_H1
zmp_ph114_H2
zmp_ph114_A9
zmp_ph114_A11
zmp_ph114_B2
zmp_ph114_B4
zmp_ph114_D8
zmp_ph114_E2

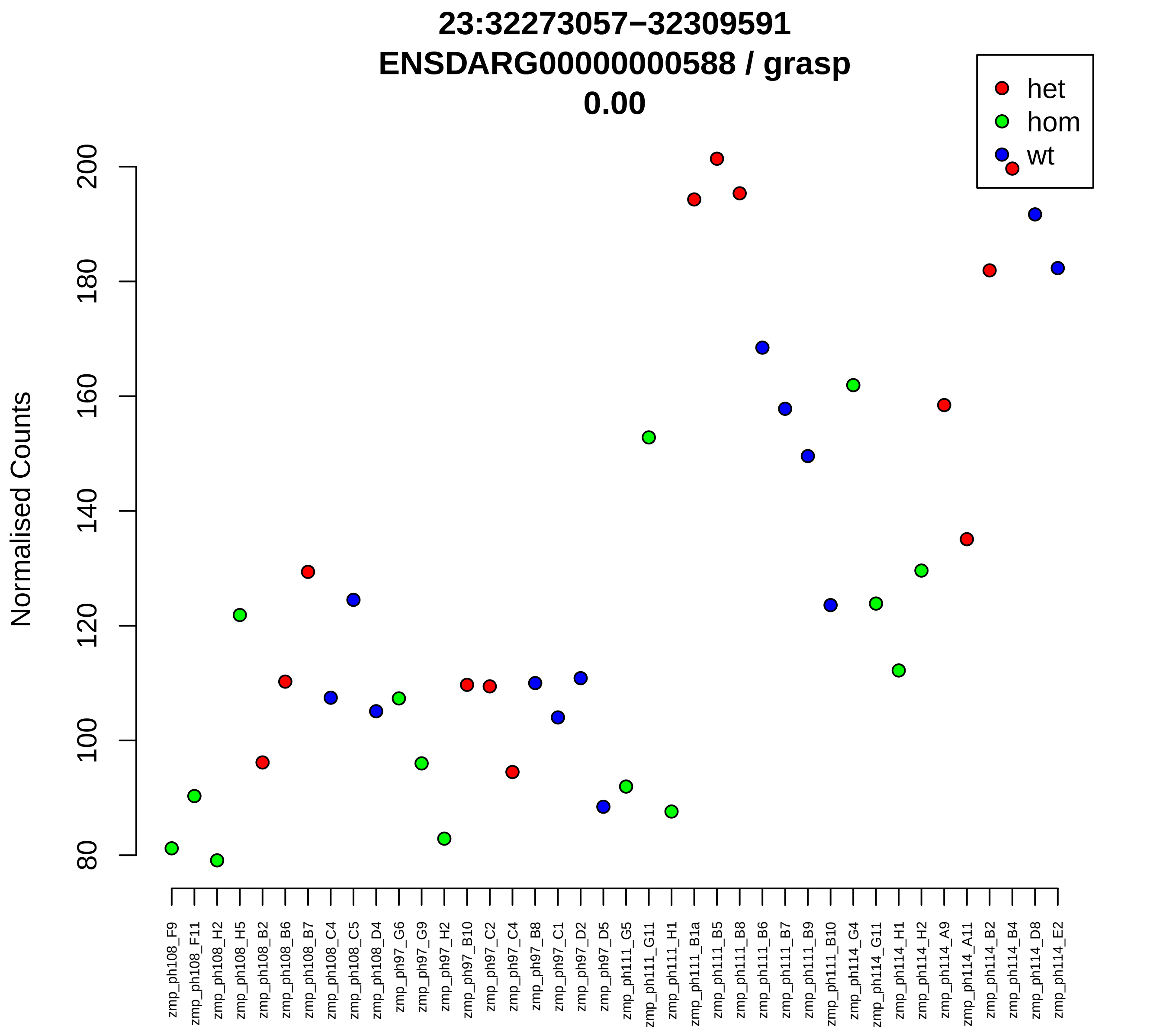
23:32273057−32309591
ENSDARG00000000588 / grasp
0.00
het
hom
wt
Normalised Counts
80
100
120
140
160
180
200
zmp_ph108_F9
zmp_ph108_F11
zmp_ph108_H2
zmp_ph108_H5
zmp_ph108_B2
zmp_ph108_B6
zmp_ph108_B7
zmp_ph108_C4
zmp_ph108_C5
zmp_ph108_D4
zmp_ph97_G6
zmp_ph97_G9
zmp_ph97_H2
zmp_ph97_B10
zmp_ph97_C2
zmp_ph97_C4
zmp_ph97_B8
zmp_ph97_C1
zmp_ph97_D2
zmp_ph97_D5
zmp_ph111_G5
zmp_ph111_G11
zmp_ph111_H1
zmp_ph111_B1a
zmp_ph111_B5
zmp_ph111_B8
zmp_ph111_B6
zmp_ph111_B7
zmp_ph111_B9
zmp_ph111_B10
zmp_ph114_G4
zmp_ph114_G11
zmp_ph114_H1
zmp_ph114_H2
zmp_ph114_A9
zmp_ph114_A11
zmp_ph114_B2
zmp_ph114_B4
zmp_ph114_D8
zmp_ph114_E2

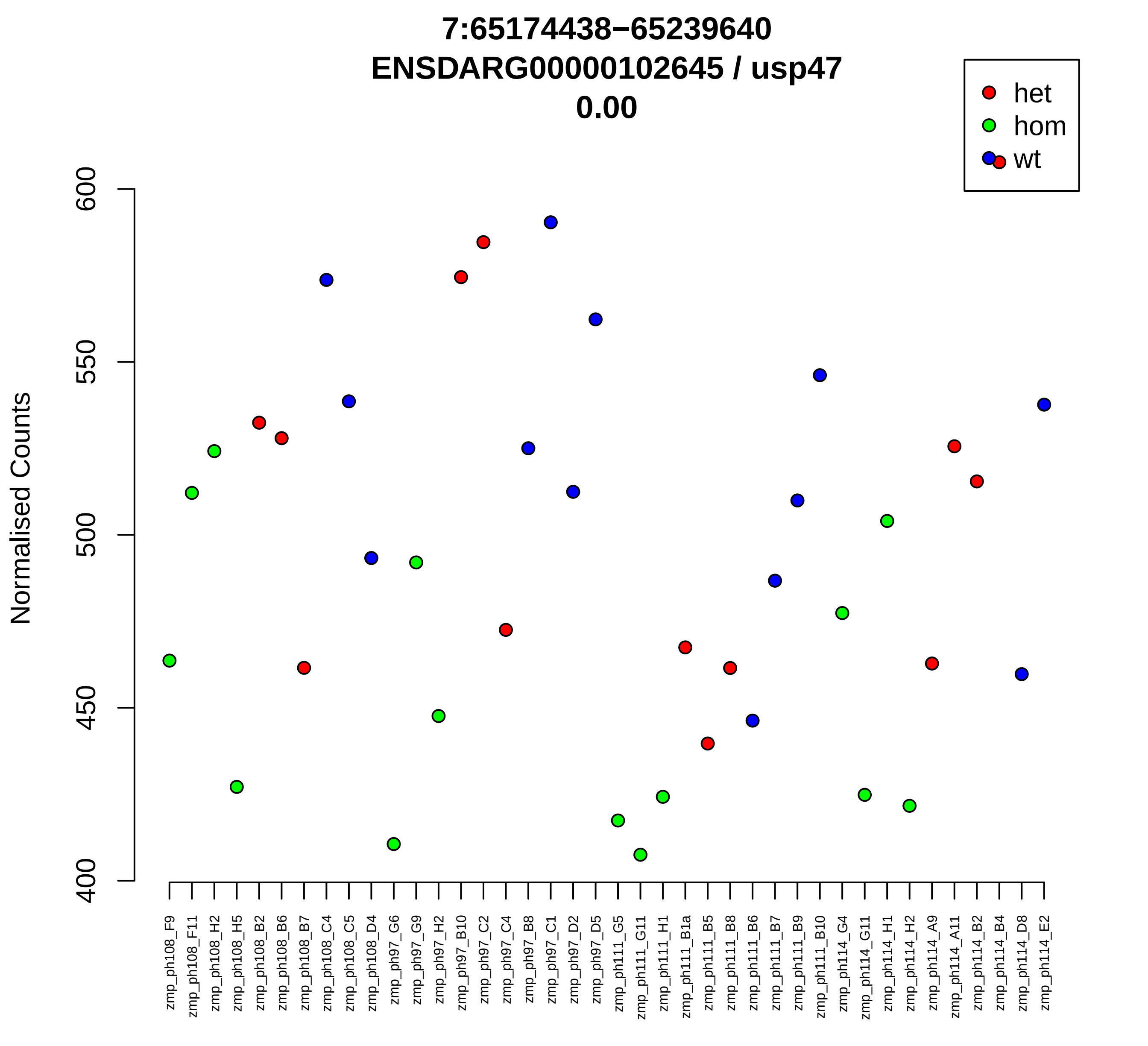
7:65174438−65239640
ENSDARG00000102645 / usp47
0.00
het
hom
wt
Normalised Counts
600
550
500
450
400
zmp_ph108_F9
zmp_ph108_F11
zmp_ph108_H2
zmp_ph108_H5
zmp_ph108_B2
zmp_ph108_B6
zmp_ph108_B7
zmp_ph108_C4
zmp_ph108_C5
zmp_ph108_D4
zmp_ph97_G6
zmp_ph97_G9
zmp_ph97_H2
zmp_ph97_B10
zmp_ph97_C2
zmp_ph97_C4
zmp_ph97_B8
zmp_ph97_C1
zmp_ph97_D2
zmp_ph97_D5
zmp_ph111_G5
zmp_ph111_G11
zmp_ph111_H1
zmp_ph111_B1a
zmp_ph111_B5
zmp_ph111_B8
zmp_ph111_B6
zmp_ph111_B7
zmp_ph111_B9
zmp_ph111_B10
zmp_ph114_G4
zmp_ph114_G11
zmp_ph114_H1
zmp_ph114_H2
zmp_ph114_A9
zmp_ph114_A11
zmp_ph114_B2
zmp_ph114_B4
zmp_ph114_D8
zmp_ph114_E2

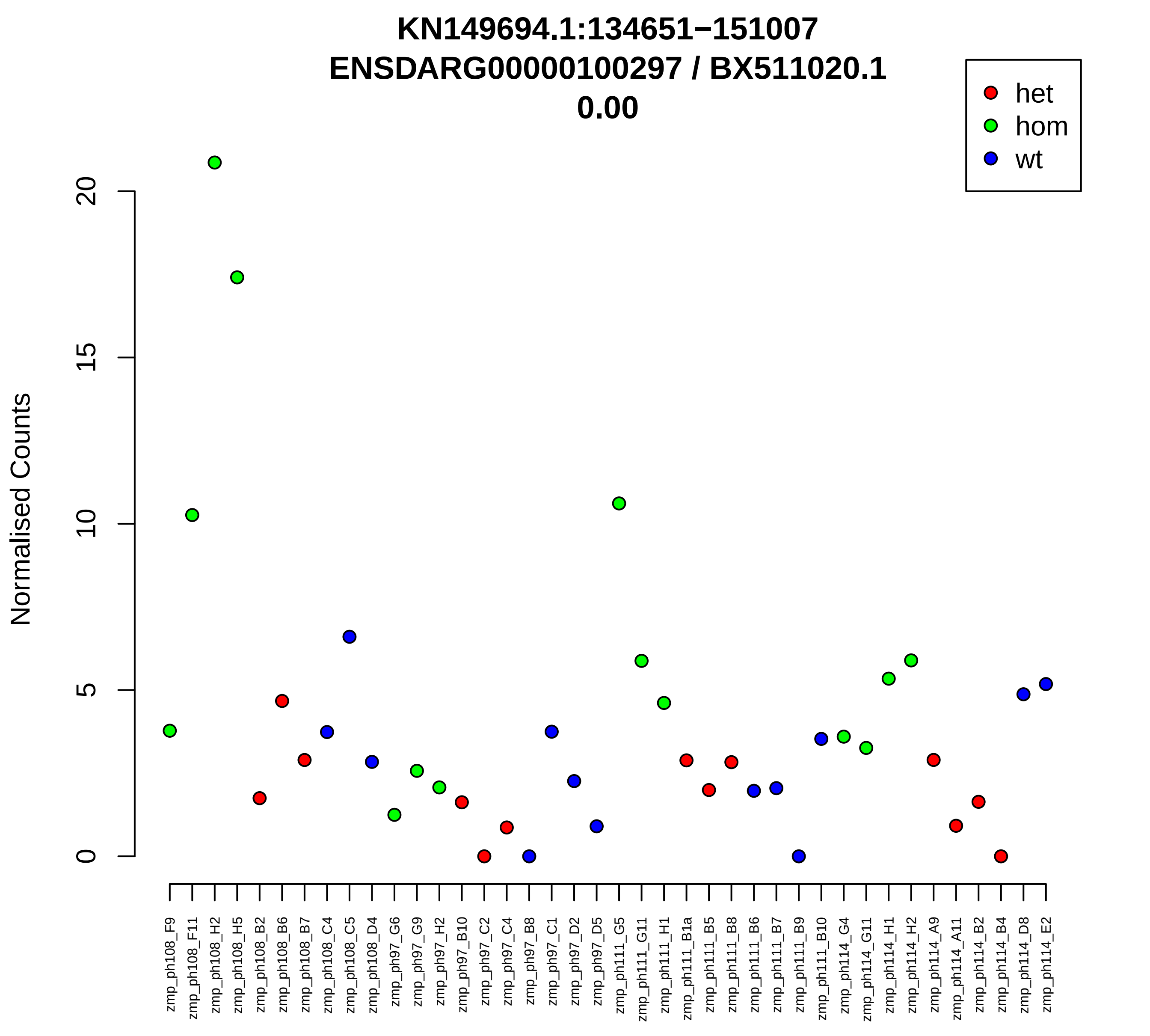

KN149694.1:134651−151007
ENSDARG00000100297 / BX511020.1
0.00
het
hom
wt
Normalised Counts
0
5
10
15
20
zmp_ph108_F9
zmp_ph108_F11
zmp_ph108_H2
zmp_ph108_H5
zmp_ph108_B2
zmp_ph108_B6
zmp_ph108_B7
zmp_ph108_C4
zmp_ph108_C5
zmp_ph108_D4
zmp_ph97_G6
zmp_ph97_G9
zmp_ph97_H2
zmp_ph97_B10
zmp_ph97_C2
zmp_ph97_C4
zmp_ph97_B8
zmp_ph97_C1
zmp_ph97_D2
zmp_ph97_D5
zmp_ph111_G5
zmp_ph111_G11
zmp_ph111_H1
zmp_ph111_B1a
zmp_ph111_B5
zmp_ph111_B8
zmp_ph111_B6
zmp_ph111_B7
zmp_ph111_B9
zmp_ph111_B10
zmp_ph114_G4
zmp_ph114_G11
zmp_ph114_H1
zmp_ph114_H2
zmp_ph114_A9
zmp_ph114_A11
zmp_ph114_B2
zmp_ph114_B4
zmp_ph114_D8
zmp_ph114_E2

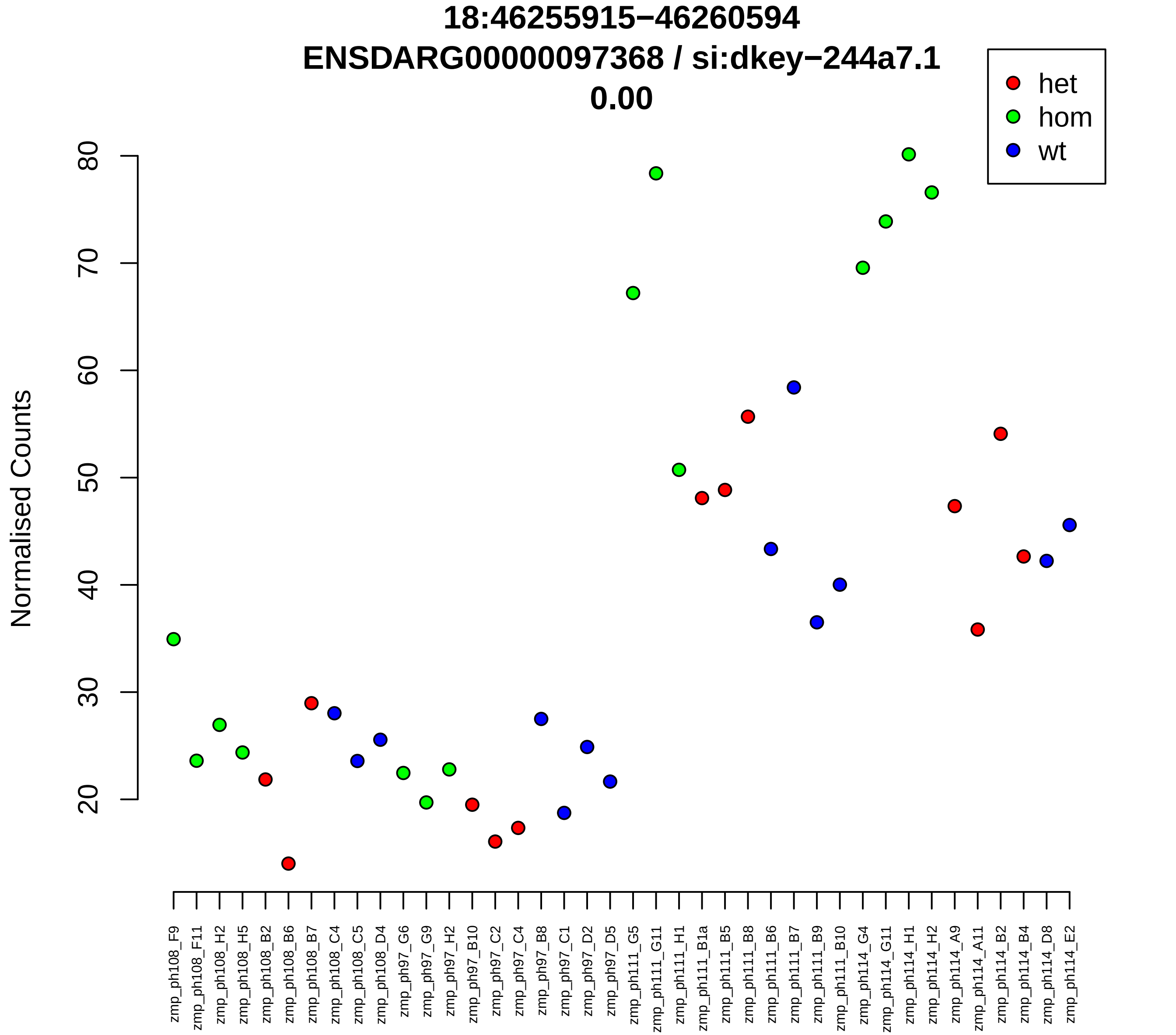

18:46255915−46260594
ENSDARG00000097368 / si:dkey−244a7.1
0.00
het
hom
wt
Normalised Counts
20
30
40
50
60
70
80
zmp_ph108_F9
zmp_ph108_F11
zmp_ph108_H2
zmp_ph108_H5
zmp_ph108_B2
zmp_ph108_B6
zmp_ph108_B7
zmp_ph108_C4
zmp_ph108_C5
zmp_ph108_D4
zmp_ph97_G6
zmp_ph97_G9
zmp_ph97_H2
zmp_ph97_B10
zmp_ph97_C2
zmp_ph97_C4
zmp_ph97_B8
zmp_ph97_C1
zmp_ph97_D2
zmp_ph97_D5
zmp_ph111_G5
zmp_ph111_G11
zmp_ph111_H1
zmp_ph111_B1a
zmp_ph111_B5
zmp_ph111_B8
zmp_ph111_B6
zmp_ph111_B7
zmp_ph111_B9
zmp_ph111_B10
zmp_ph114_G4
zmp_ph114_G11
zmp_ph114_H1
zmp_ph114_H2
zmp_ph114_A9
zmp_ph114_A11
zmp_ph114_B2
zmp_ph114_B4
zmp_ph114_D8
zmp_ph114_E2

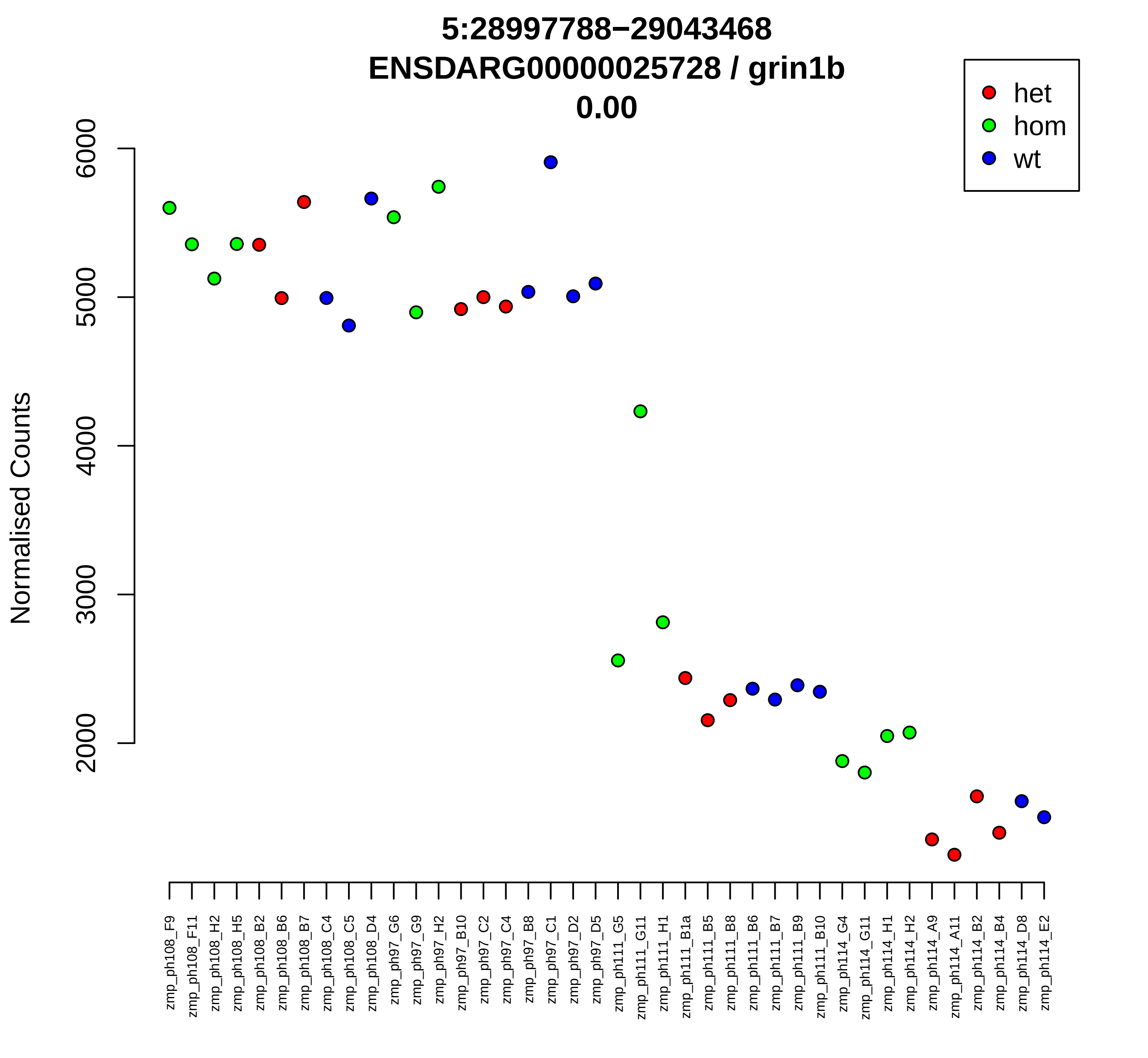

5:28997788−29043468
ENSDARG00000025728 / grin1b
0.00
het
hom
wt
Normalised Counts
6000
5000
4000
3000
2000
zmp_ph108_F9
zmp_ph108_F11
zmp_ph108_H2
zmp_ph108_H5
zmp_ph108_B2
zmp_ph108_B6
zmp_ph108_B7
zmp_ph108_C4
zmp_ph108_C5
zmp_ph108_D4
zmp_ph97_G6
zmp_ph97_G9
zmp_ph97_H2
zmp_ph97_B10
zmp_ph97_C2
zmp_ph97_C4
zmp_ph97_B8
zmp_ph97_C1
zmp_ph97_D2
zmp_ph97_D5
zmp_ph111_G5
zmp_ph111_G11
zmp_ph111_H1
zmp_ph111_B1a
zmp_ph111_B5
zmp_ph111_B8
zmp_ph111_B6
zmp_ph111_B7
zmp_ph111_B9
zmp_ph111_B10
zmp_ph114_G4
zmp_ph114_G11
zmp_ph114_H1
zmp_ph114_H2
zmp_ph114_A9
zmp_ph114_A11
zmp_ph114_B2
zmp_ph114_B4
zmp_ph114_D8
zmp_ph114_E2

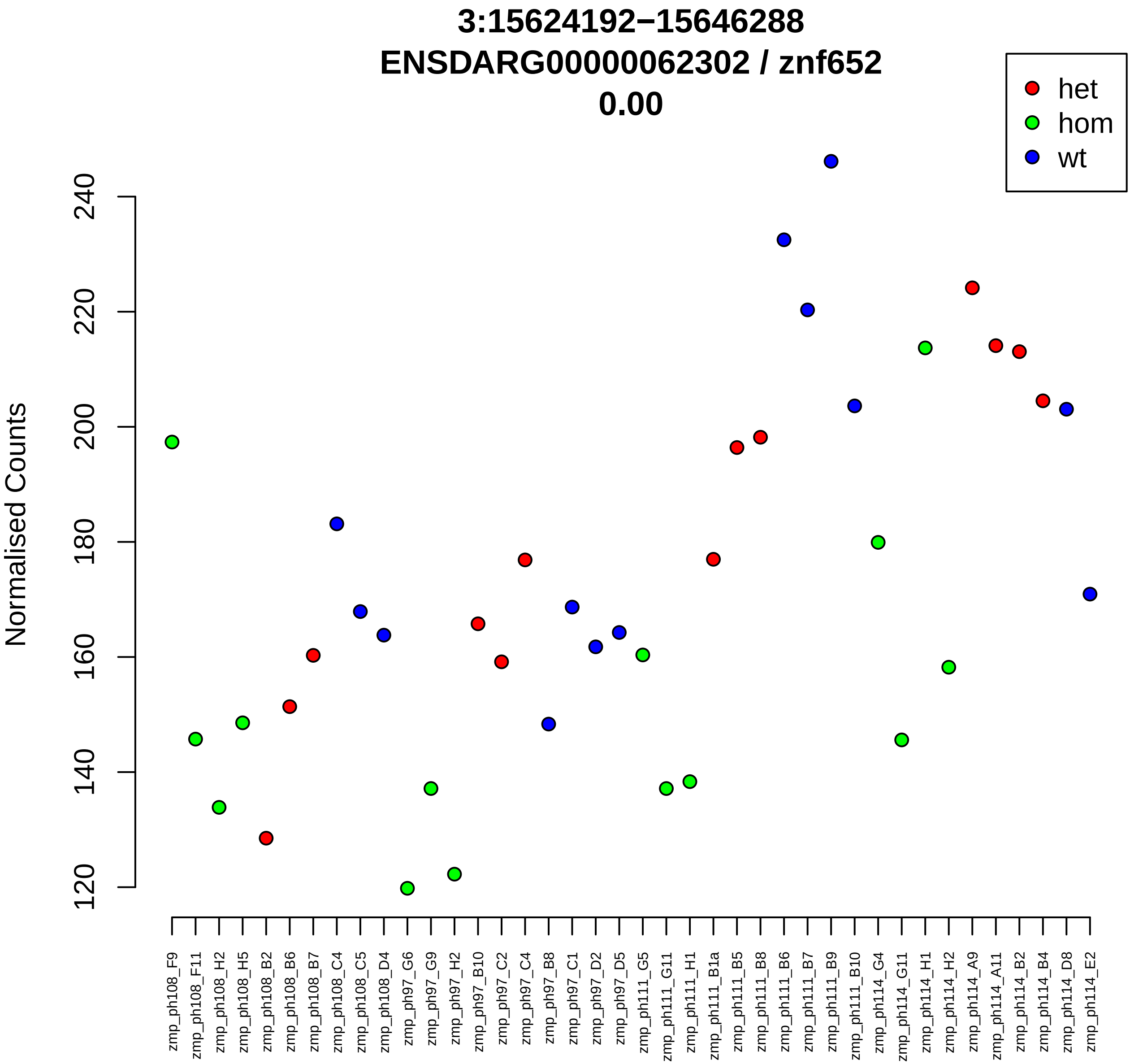
3:15624192−15646288
ENSDARG00000062302 / znf652
0.00
het
hom
wt
Normalised Counts
120
140
160
180
200
220
240
zmp_ph108_F9
zmp_ph108_F11
zmp_ph108_H2
zmp_ph108_H5
zmp_ph108_B2
zmp_ph108_B6
zmp_ph108_B7
zmp_ph108_C4
zmp_ph108_C5
zmp_ph108_D4
zmp_ph97_G6
zmp_ph97_G9
zmp_ph97_H2
zmp_ph97_B10
zmp_ph97_C2
zmp_ph97_C4
zmp_ph97_B8
zmp_ph97_C1
zmp_ph97_D2
zmp_ph97_D5
zmp_ph111_G5
zmp_ph111_G11
zmp_ph111_H1
zmp_ph111_B1a
zmp_ph111_B5
zmp_ph111_B8
zmp_ph111_B6
zmp_ph111_B7
zmp_ph111_B9
zmp_ph111_B10
zmp_ph114_G4
zmp_ph114_G11
zmp_ph114_H1
zmp_ph114_H2
zmp_ph114_A9
zmp_ph114_A11
zmp_ph114_B2
zmp_ph114_B4
zmp_ph114_D8
zmp_ph114_E2

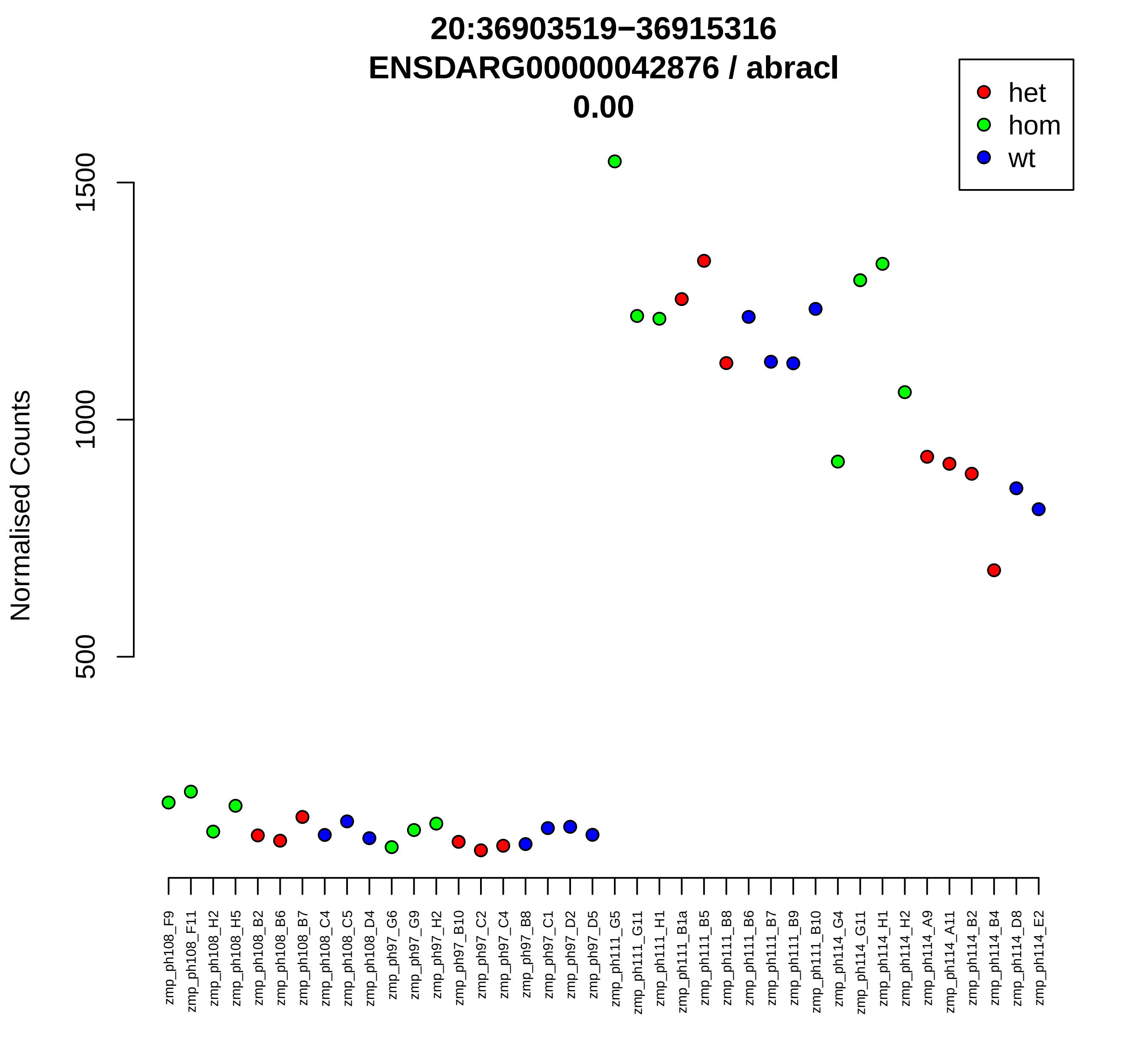
20:36903519−36915316
ENSDARG00000042876 / abracl
0.00
het
hom
wt
Normalised Counts
500
1000
1500
zmp_ph108_F9
zmp_ph108_F11
zmp_ph108_H2
zmp_ph108_H5
zmp_ph108_B2
zmp_ph108_B6
zmp_ph108_B7
zmp_ph108_C4
zmp_ph108_C5
zmp_ph108_D4
zmp_ph97_G6
zmp_ph97_G9
zmp_ph97_H2
zmp_ph97_B10
zmp_ph97_C2
zmp_ph97_C4
zmp_ph97_B8
zmp_ph97_C1
zmp_ph97_D2
zmp_ph97_D5
zmp_ph111_G5
zmp_ph111_G11
zmp_ph111_H1
zmp_ph111_B1a
zmp_ph111_B5
zmp_ph111_B8
zmp_ph111_B6
zmp_ph111_B7
zmp_ph111_B9
zmp_ph111_B10
zmp_ph114_G4
zmp_ph114_G11
zmp_ph114_H1
zmp_ph114_H2
zmp_ph114_A9
zmp_ph114_A11
zmp_ph114_B2
zmp_ph114_B4
zmp_ph114_D8
zmp_ph114_E2

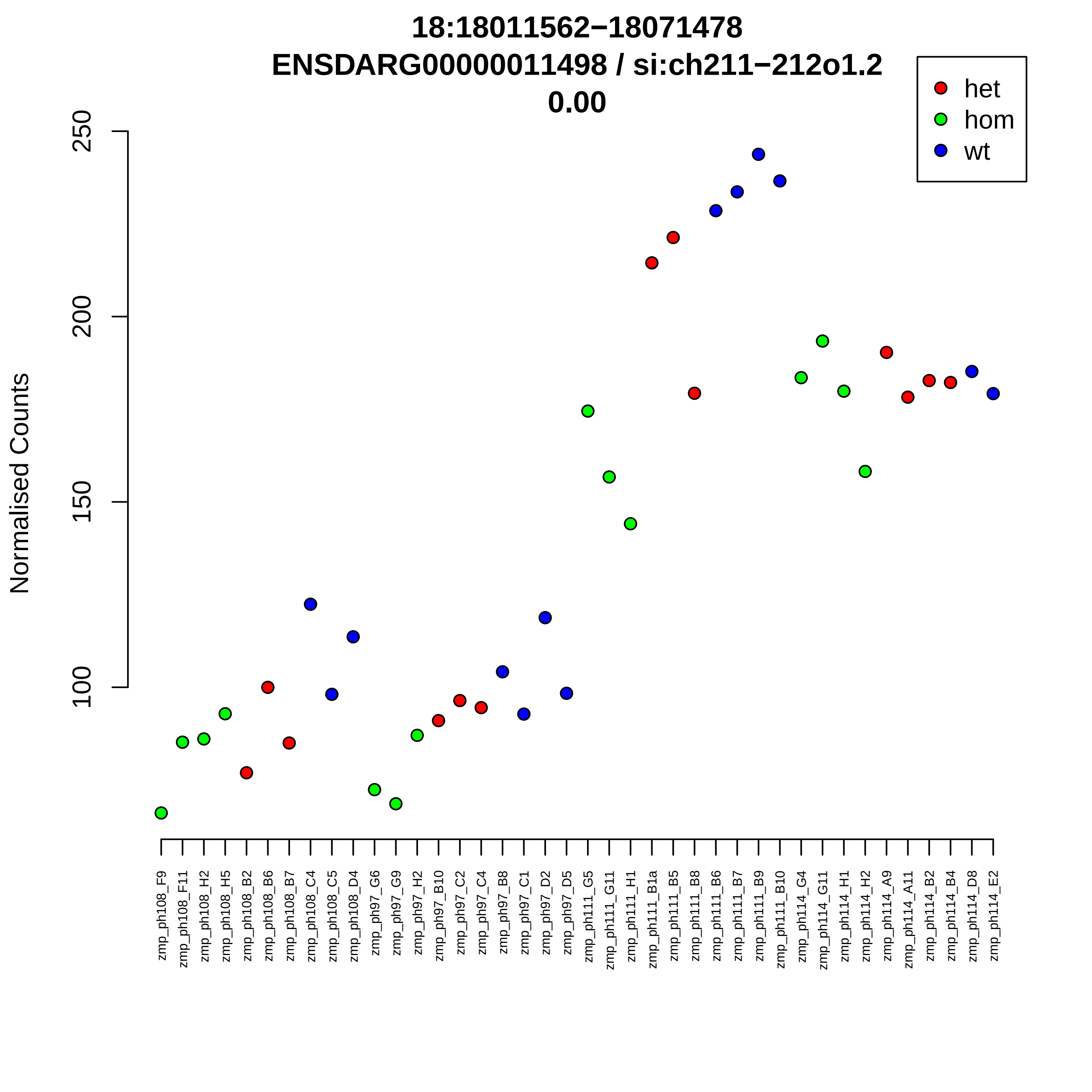

18:18011562−18071478
ENSDARG00000011498 / si:ch211−212o1.2
0.00
het
hom
wt
Normalised Counts
250
200
150
100
zmp_ph108_F9
zmp_ph108_F11
zmp_ph108_H2
zmp_ph108_H5
zmp_ph108_B2
zmp_ph108_B6
zmp_ph108_B7
zmp_ph108_C4
zmp_ph108_C5
zmp_ph108_D4
zmp_ph97_G6
zmp_ph97_G9
zmp_ph97_H2
zmp_ph97_B10
zmp_ph97_C2
zmp_ph97_C4
zmp_ph97_B8
zmp_ph97_C1
zmp_ph97_D2
zmp_ph97_D5
zmp_ph111_G5
zmp_ph111_G11
zmp_ph111_H1
zmp_ph111_B1a
zmp_ph111_B5
zmp_ph111_B8
zmp_ph111_B6
zmp_ph111_B7
zmp_ph111_B9
zmp_ph111_B10
zmp_ph114_G4
zmp_ph114_G11
zmp_ph114_H1
zmp_ph114_H2
zmp_ph114_A9
zmp_ph114_A11
zmp_ph114_B2
zmp_ph114_B4
zmp_ph114_D8
zmp_ph114_E2

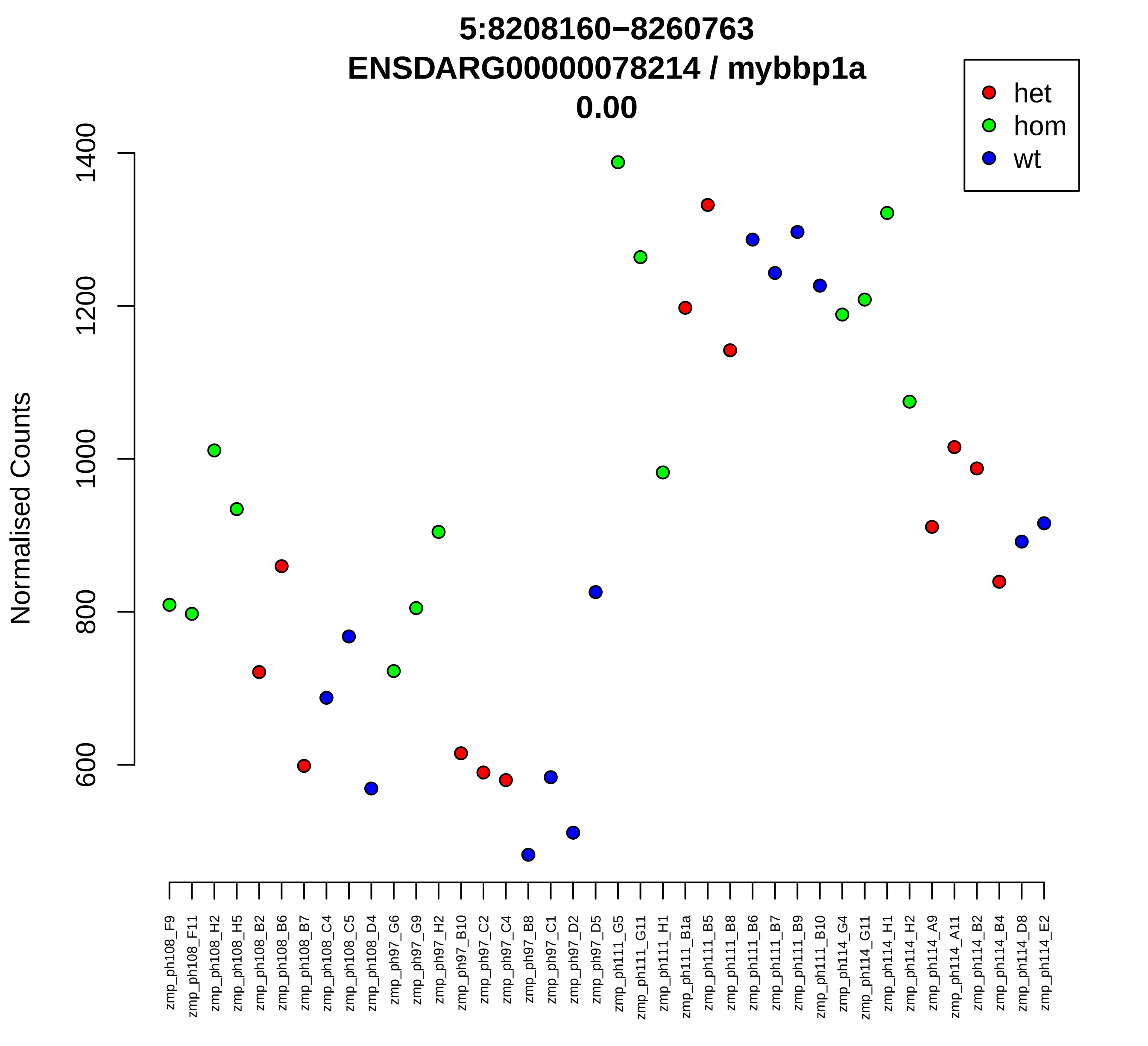
5:8208160−8260763
ENSDARG00000078214 / mybbp1a
0.00
het
hom
wt
Normalised Counts
1400
1200
1000
800
600
zmp_ph108_F9
zmp_ph108_F11
zmp_ph108_H2
zmp_ph108_H5
zmp_ph108_B2
zmp_ph108_B6
zmp_ph108_B7
zmp_ph108_C4
zmp_ph108_C5
zmp_ph108_D4
zmp_ph97_G6
zmp_ph97_G9
zmp_ph97_H2
zmp_ph97_B10
zmp_ph97_C2
zmp_ph97_C4
zmp_ph97_B8
zmp_ph97_C1
zmp_ph97_D2
zmp_ph97_D5
zmp_ph111_G5
zmp_ph111_G11
zmp_ph111_H1
zmp_ph111_B1a
zmp_ph111_B5
zmp_ph111_B8
zmp_ph111_B6
zmp_ph111_B7
zmp_ph111_B9
zmp_ph111_B10
zmp_ph114_G4
zmp_ph114_G11
zmp_ph114_H1
zmp_ph114_H2
zmp_ph114_A9
zmp_ph114_A11
zmp_ph114_B2
zmp_ph114_B4
zmp_ph114_D8
zmp_ph114_E2

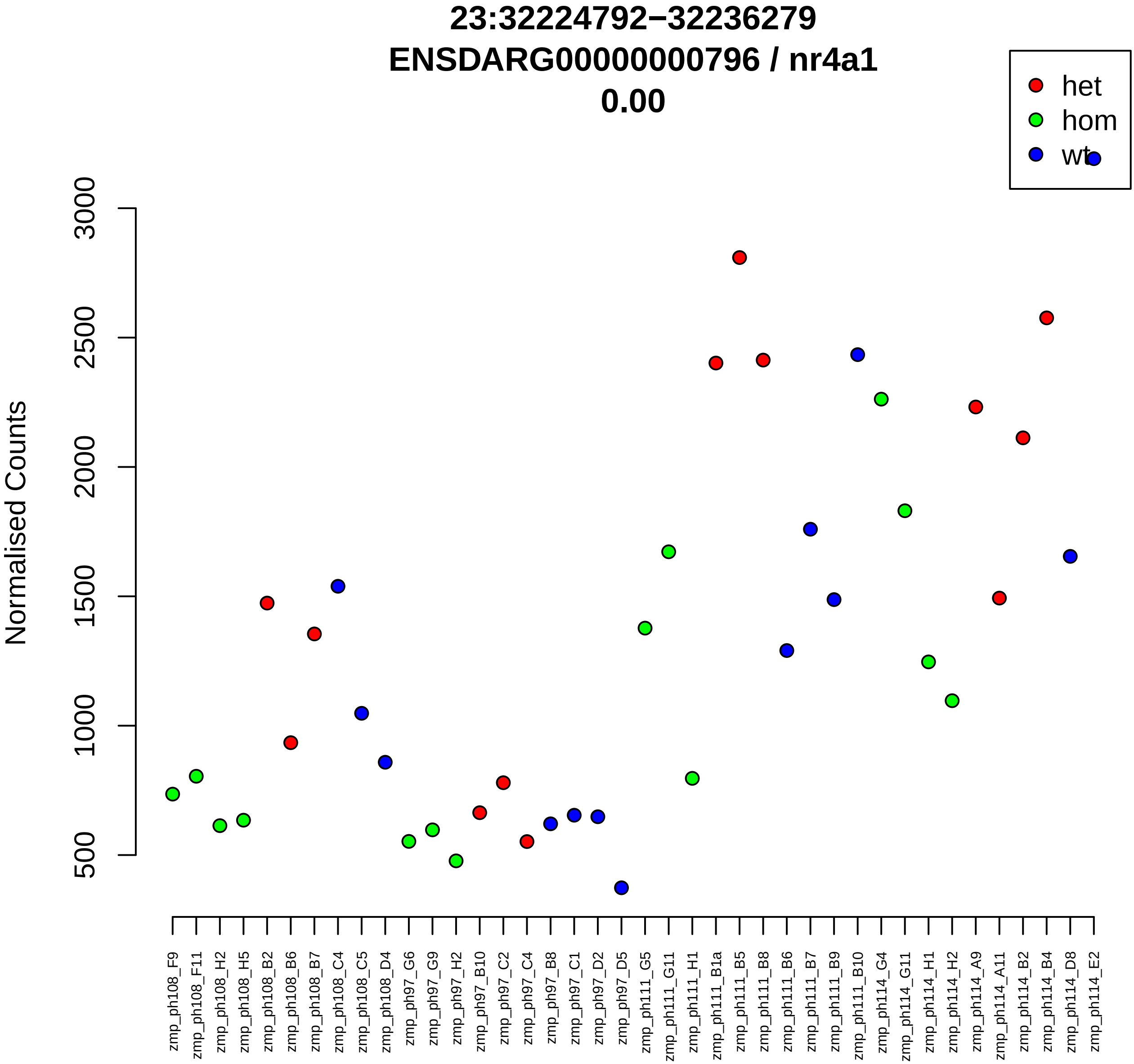

23:32224792−32236279
ENSDARG00000000796 / nr4a1
0.00
het
hom
wt
Normalised Counts
500
1000
1500
2000
2500
3000
zmp_ph108_F9
zmp_ph108_F11
zmp_ph108_H2
zmp_ph108_H5
zmp_ph108_B2
zmp_ph108_B6
zmp_ph108_B7
zmp_ph108_C4
zmp_ph108_C5
zmp_ph108_D4
zmp_ph97_G6
zmp_ph97_G9
zmp_ph97_H2
zmp_ph97_B10
zmp_ph97_C2
zmp_ph97_C4
zmp_ph97_B8
zmp_ph97_C1
zmp_ph97_D2
zmp_ph97_D5
zmp_ph111_G5
zmp_ph111_G11
zmp_ph111_H1
zmp_ph111_B1a
zmp_ph111_B5
zmp_ph111_B8
zmp_ph111_B6
zmp_ph111_B7
zmp_ph111_B9
zmp_ph111_B10
zmp_ph114_G4
zmp_ph114_G11
zmp_ph114_H1
zmp_ph114_H2
zmp_ph114_A9
zmp_ph114_A11
zmp_ph114_B2
zmp_ph114_B4
zmp_ph114_D8
zmp_ph114_E2

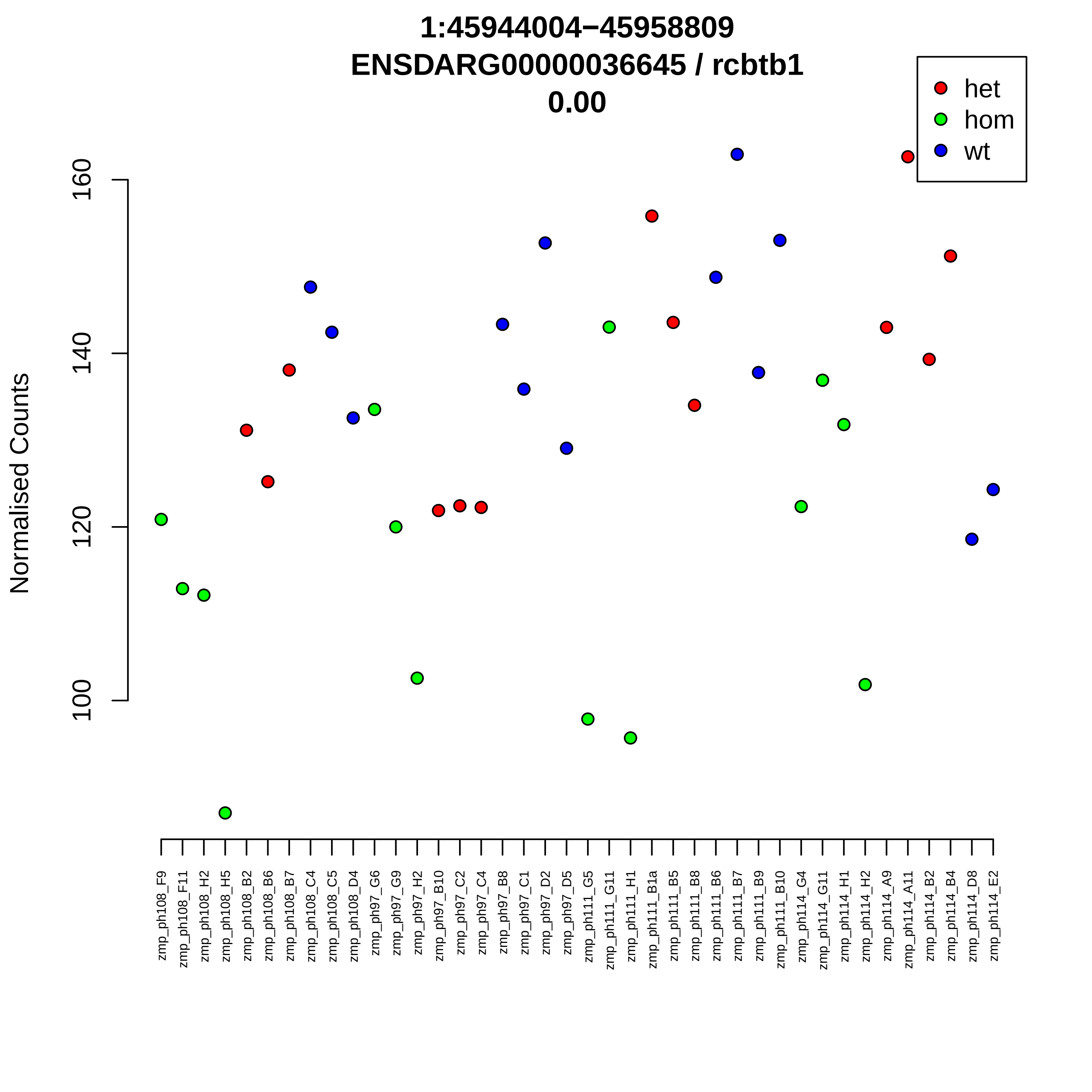

1:45944004−45958809
ENSDARG00000036645 / rcbtb1
0.00
het
hom
wt
Normalised Counts
100
120
140
160
zmp_ph108_F9
zmp_ph108_F11
zmp_ph108_H2
zmp_ph108_H5
zmp_ph108_B2
zmp_ph108_B6
zmp_ph108_B7
zmp_ph108_C4
zmp_ph108_C5
zmp_ph108_D4
zmp_ph97_G6
zmp_ph97_G9
zmp_ph97_H2
zmp_ph97_B10
zmp_ph97_C2
zmp_ph97_C4
zmp_ph97_B8
zmp_ph97_C1
zmp_ph97_D2
zmp_ph97_D5
zmp_ph111_G5
zmp_ph111_G11
zmp_ph111_H1
zmp_ph111_B1a
zmp_ph111_B5
zmp_ph111_B8
zmp_ph111_B6
zmp_ph111_B7
zmp_ph111_B9
zmp_ph111_B10
zmp_ph114_G4
zmp_ph114_G11
zmp_ph114_H1
zmp_ph114_H2
zmp_ph114_A9
zmp_ph114_A11
zmp_ph114_B2
zmp_ph114_B4
zmp_ph114_D8
zmp_ph114_E2

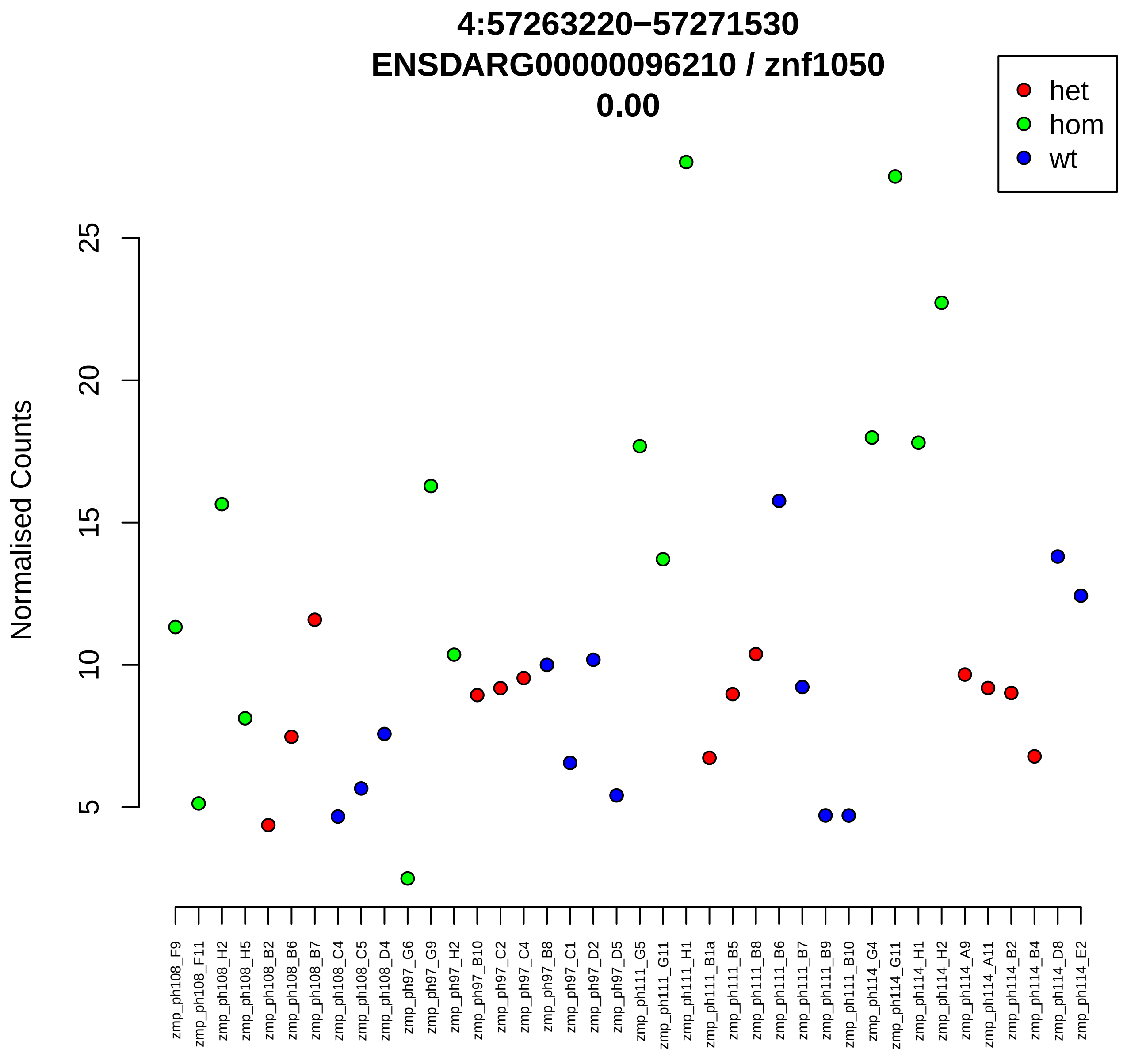

4:57263220−57271530
ENSDARG00000096210 / znf1050
0.00
het
hom
wt
Normalised Counts
5
10
15
20
25
zmp_ph108_F9
zmp_ph108_F11
zmp_ph108_H2
zmp_ph108_H5
zmp_ph108_B2
zmp_ph108_B6
zmp_ph108_B7
zmp_ph108_C4
zmp_ph108_C5
zmp_ph108_D4
zmp_ph97_G6
zmp_ph97_G9
zmp_ph97_H2
zmp_ph97_B10
zmp_ph97_C2
zmp_ph97_C4
zmp_ph97_B8
zmp_ph97_C1
zmp_ph97_D2
zmp_ph97_D5
zmp_ph111_G5
zmp_ph111_G11
zmp_ph111_H1
zmp_ph111_B1a
zmp_ph111_B5
zmp_ph111_B8
zmp_ph111_B6
zmp_ph111_B7
zmp_ph111_B9
zmp_ph111_B10
zmp_ph114_G4
zmp_ph114_G11
zmp_ph114_H1
zmp_ph114_H2
zmp_ph114_A9
zmp_ph114_A11
zmp_ph114_B2
zmp_ph114_B4
zmp_ph114_D8
zmp_ph114_E2

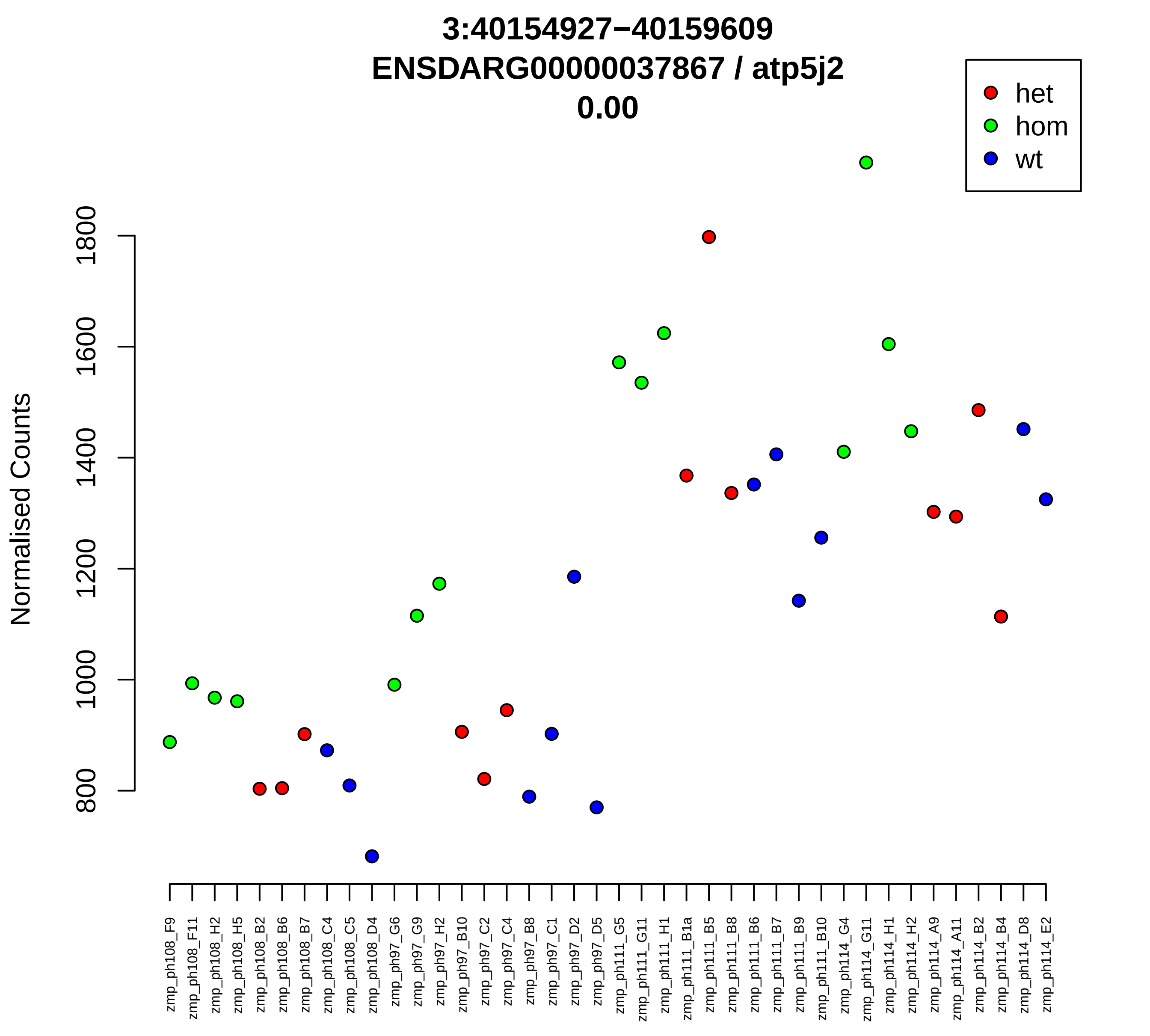

3:40154927−40159609
ENSDARG00000037867 / atp5j2
0.00
het
hom
wt
Normalised Counts
800
1000
1200
1400
1600
1800
zmp_ph108_F9
zmp_ph108_F11
zmp_ph108_H2
zmp_ph108_H5
zmp_ph108_B2
zmp_ph108_B6
zmp_ph108_B7
zmp_ph108_C4
zmp_ph108_C5
zmp_ph108_D4
zmp_ph97_G6
zmp_ph97_G9
zmp_ph97_H2
zmp_ph97_B10
zmp_ph97_C2
zmp_ph97_C4
zmp_ph97_B8
zmp_ph97_C1
zmp_ph97_D2
zmp_ph97_D5
zmp_ph111_G5
zmp_ph111_G11
zmp_ph111_H1
zmp_ph111_B1a
zmp_ph111_B5
zmp_ph111_B8
zmp_ph111_B6
zmp_ph111_B7
zmp_ph111_B9
zmp_ph111_B10
zmp_ph114_G4
zmp_ph114_G11
zmp_ph114_H1
zmp_ph114_H2
zmp_ph114_A9
zmp_ph114_A11
zmp_ph114_B2
zmp_ph114_B4
zmp_ph114_D8
zmp_ph114_E2

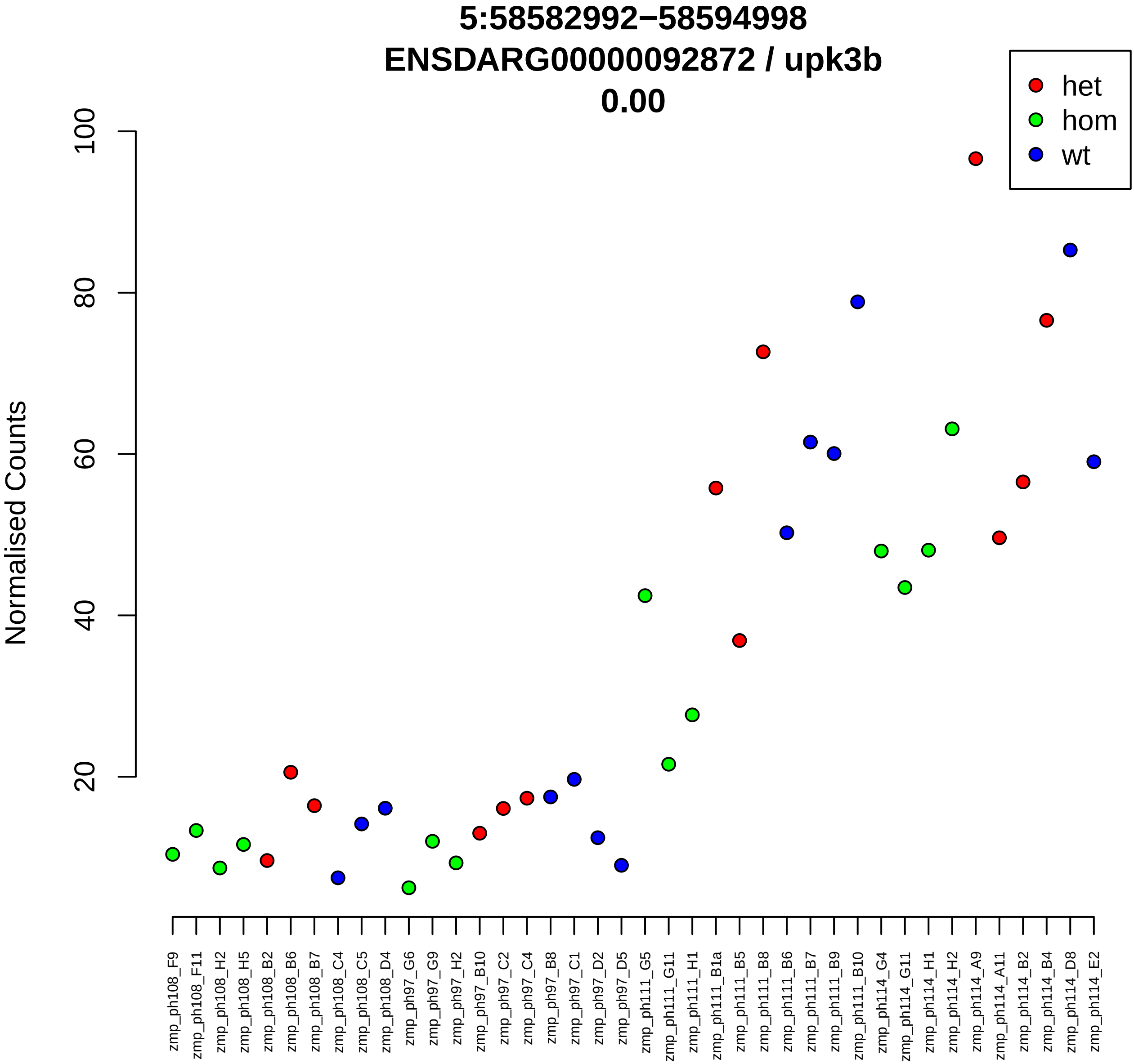

5:58582992−58594998
ENSDARG00000092872 / upk3b
0.00
Normalised Counts
20
40
60
80
100
het
hom
wt
zmp_ph108_F9
zmp_ph108_F11
zmp_ph108_H2
zmp_ph108_H5
zmp_ph108_B2
zmp_ph108_B6
zmp_ph108_B7
zmp_ph108_C4
zmp_ph108_C5
zmp_ph108_D4
zmp_ph97_G6
zmp_ph97_G9
zmp_ph97_H2
zmp_ph97_B10
zmp_ph97_C2
zmp_ph97_C4
zmp_ph97_B8
zmp_ph97_C1
zmp_ph97_D2
zmp_ph97_D5
zmp_ph111_G5
zmp_ph111_G11
zmp_ph111_H1
zmp_ph111_B1a
zmp_ph111_B5
zmp_ph111_B8
zmp_ph111_B6
zmp_ph111_B7
zmp_ph111_B9
zmp_ph111_B10
zmp_ph114_G4
zmp_ph114_G11
zmp_ph114_H1
zmp_ph114_H2
zmp_ph114_A9
zmp_ph114_A11
zmp_ph114_B2
zmp_ph114_B4
zmp_ph114_D8
zmp_ph114_E2

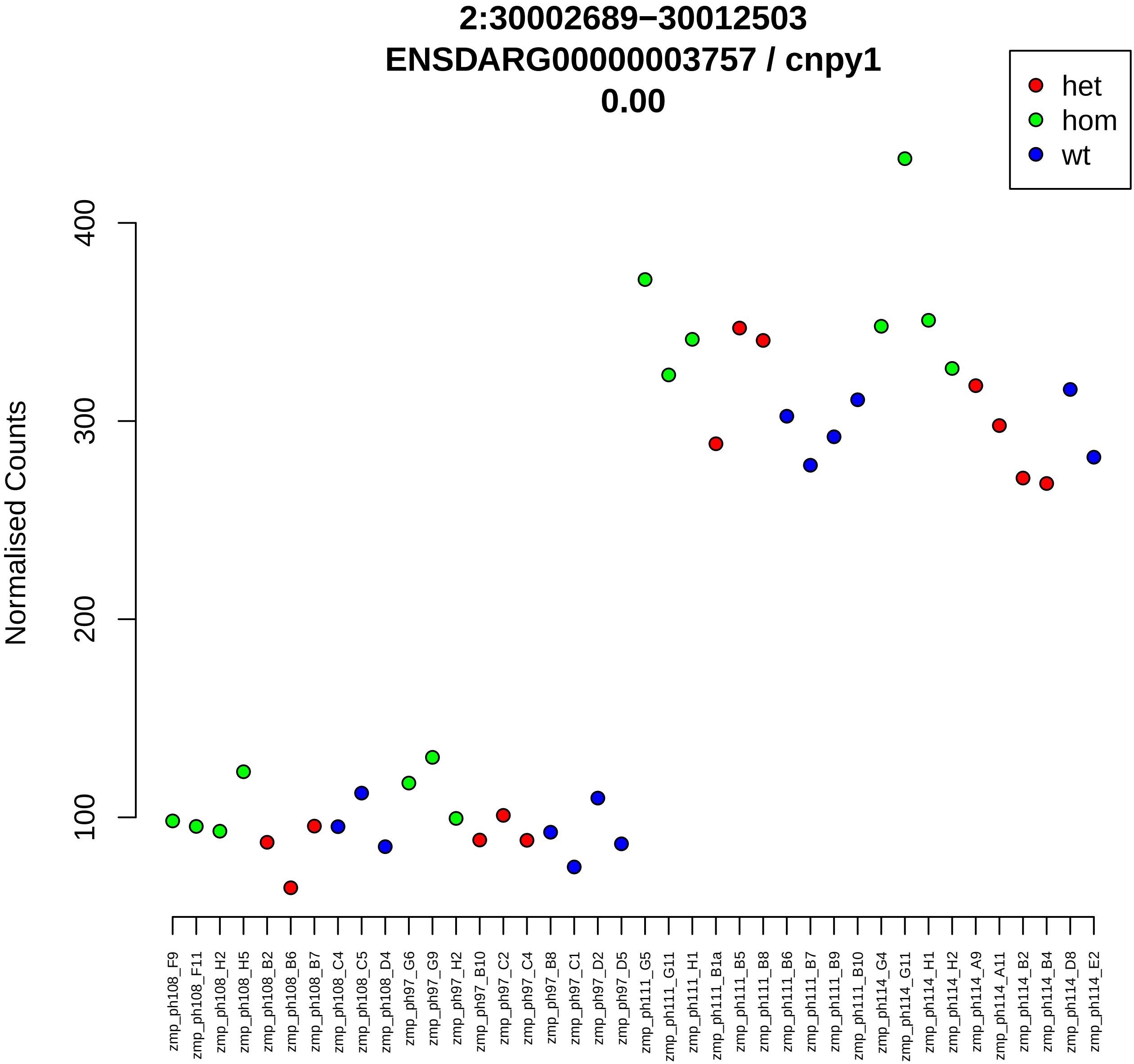

2:30002689−30012503
ENSDARG00000003757 / cnpy1
0.00
het
hom
wt
Normalised Counts
100
200
300
400
zmp_ph108_F9
zmp_ph108_F11
zmp_ph108_H2
zmp_ph108_H5
zmp_ph108_B2
zmp_ph108_B6
zmp_ph108_B7
zmp_ph108_C4
zmp_ph108_C5
zmp_ph108_D4
zmp_ph97_G6
zmp_ph97_G9
zmp_ph97_H2
zmp_ph97_B10
zmp_ph97_C2
zmp_ph97_C4
zmp_ph97_B8
zmp_ph97_C1
zmp_ph97_D2
zmp_ph97_D5
zmp_ph111_G5
zmp_ph111_G11
zmp_ph111_H1
zmp_ph111_B1a
zmp_ph111_B5
zmp_ph111_B8
zmp_ph111_B6
zmp_ph111_B7
zmp_ph111_B9
zmp_ph111_B10
zmp_ph114_G4
zmp_ph114_G11
zmp_ph114_H1
zmp_ph114_H2
zmp_ph114_A9
zmp_ph114_A11
zmp_ph114_B2
zmp_ph114_B4
zmp_ph114_D8
zmp_ph114_E2

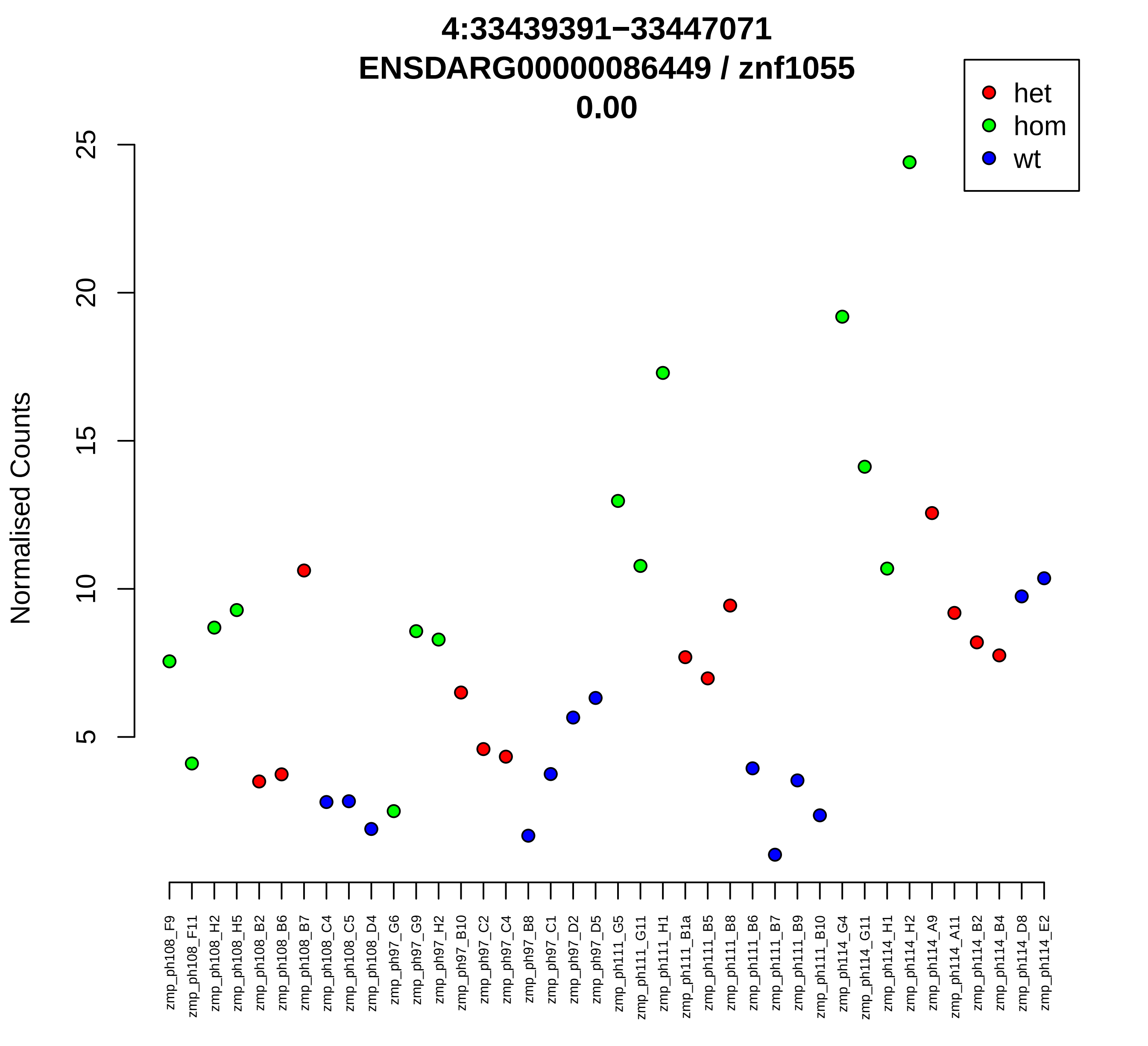
4:33439391−33447071
ENSDARG00000086449 / znf1055
0.00
het
hom
wt
Normalised Counts
5
10
15
20
25
zmp_ph108_F9
zmp_ph108_F11
zmp_ph108_H2
zmp_ph108_H5
zmp_ph108_B2
zmp_ph108_B6
zmp_ph108_B7
zmp_ph108_C4
zmp_ph108_C5
zmp_ph108_D4
zmp_ph97_G6
zmp_ph97_G9
zmp_ph97_H2
zmp_ph97_B10
zmp_ph97_C2
zmp_ph97_C4
zmp_ph97_B8
zmp_ph97_C1
zmp_ph97_D2
zmp_ph97_D5
zmp_ph111_G5
zmp_ph111_G11
zmp_ph111_H1
zmp_ph111_B1a
zmp_ph111_B5
zmp_ph111_B8
zmp_ph111_B6
zmp_ph111_B7
zmp_ph111_B9
zmp_ph111_B10
zmp_ph114_G4
zmp_ph114_G11
zmp_ph114_H1
zmp_ph114_H2
zmp_ph114_A9
zmp_ph114_A11
zmp_ph114_B2
zmp_ph114_B4
zmp_ph114_D8
zmp_ph114_E2

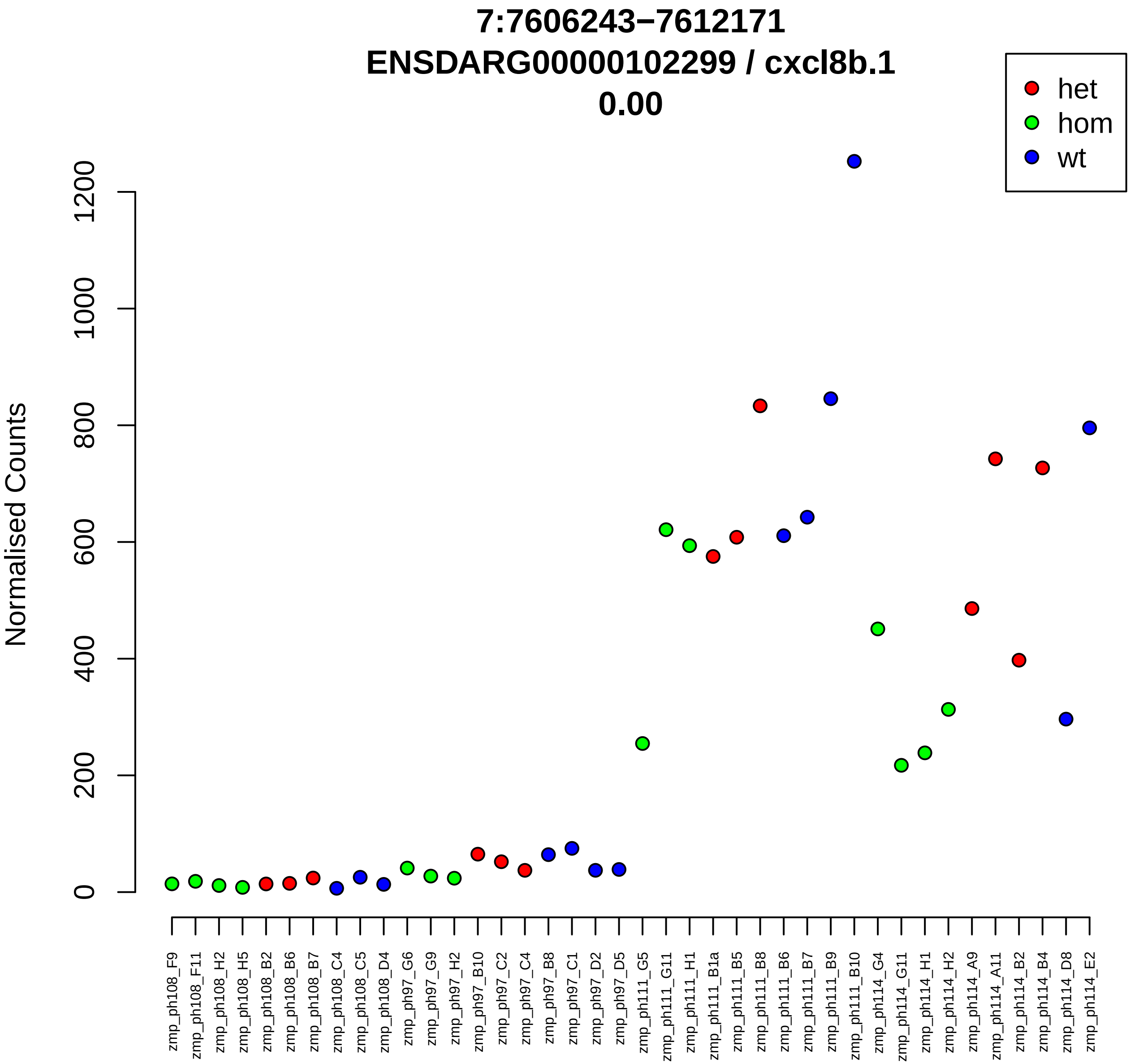

7:7606243−7612171
ENSDARG00000102299 / cxcl8b.1
0.00
het
hom
wt
Normalised Counts
0
200
400
600
800
1000
1200
zmp_ph108_F9
zmp_ph108_F11
zmp_ph108_H2
zmp_ph108_H5
zmp_ph108_B2
zmp_ph108_B6
zmp_ph108_B7
zmp_ph108_C4
zmp_ph108_C5
zmp_ph108_D4
zmp_ph97_G6
zmp_ph97_G9
zmp_ph97_H2
zmp_ph97_B10
zmp_ph97_C2
zmp_ph97_C4
zmp_ph97_B8
zmp_ph97_C1
zmp_ph97_D2
zmp_ph97_D5
zmp_ph111_G5
zmp_ph111_G11
zmp_ph111_H1
zmp_ph111_B1a
zmp_ph111_B5
zmp_ph111_B8
zmp_ph111_B6
zmp_ph111_B7
zmp_ph111_B9
zmp_ph111_B10
zmp_ph114_G4
zmp_ph114_G11
zmp_ph114_H1
zmp_ph114_H2
zmp_ph114_A9
zmp_ph114_A11
zmp_ph114_B2
zmp_ph114_B4
zmp_ph114_D8
zmp_ph114_E2

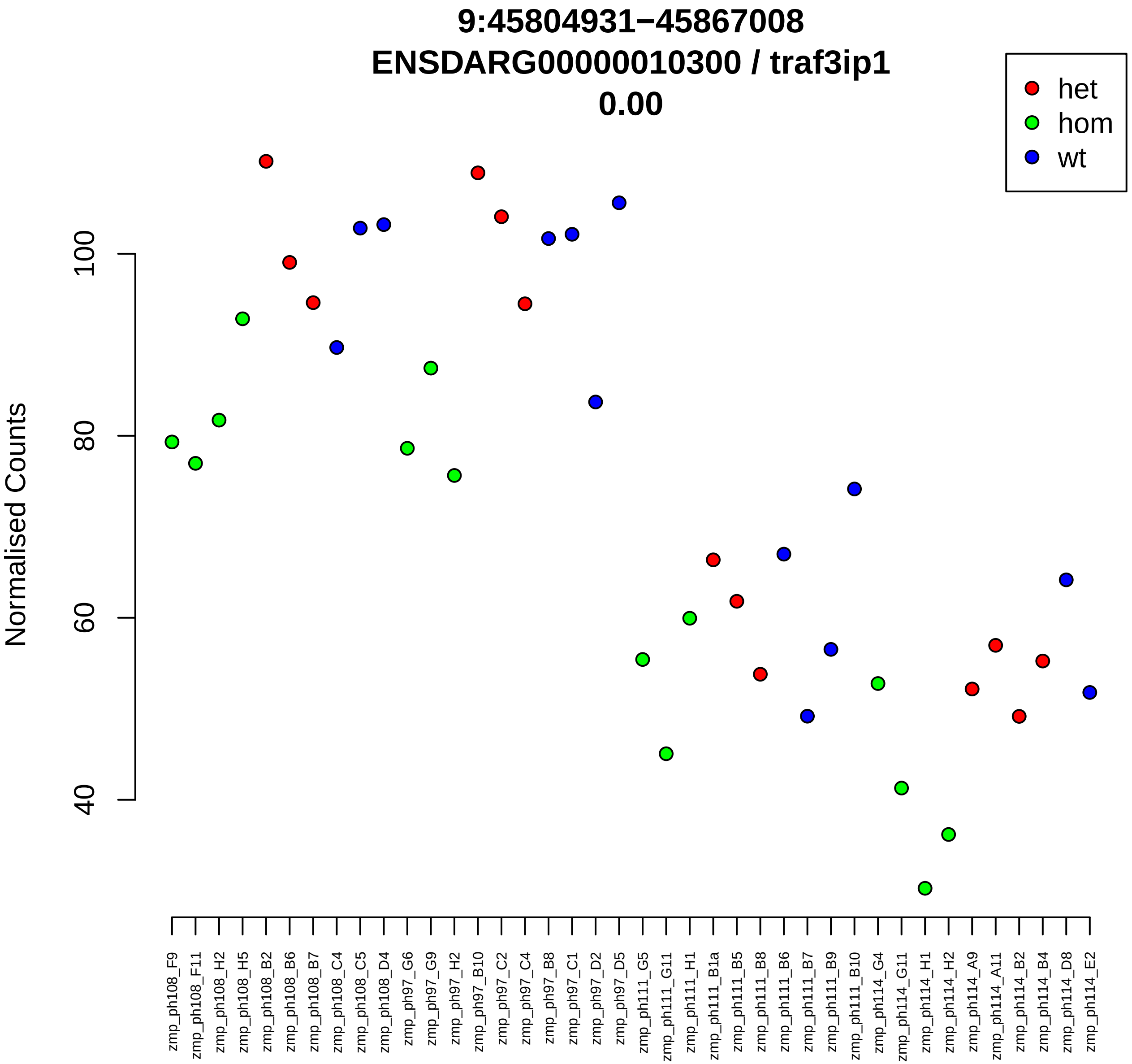

9:45804931−45867008
ENSDARG00000010300 / traf3ip1
0.00
het
hom
wt
Normalised Counts
40
60
80
100
zmp_ph108_F9
zmp_ph108_F11
zmp_ph108_H2
zmp_ph108_H5
zmp_ph108_B2
zmp_ph108_B6
zmp_ph108_B7
zmp_ph108_C4
zmp_ph108_C5
zmp_ph108_D4
zmp_ph97_G6
zmp_ph97_G9
zmp_ph97_H2
zmp_ph97_B10
zmp_ph97_C2
zmp_ph97_C4
zmp_ph97_B8
zmp_ph97_C1
zmp_ph97_D2
zmp_ph97_D5
zmp_ph111_G5
zmp_ph111_G11
zmp_ph111_H1
zmp_ph111_B1a
zmp_ph111_B5
zmp_ph111_B8
zmp_ph111_B6
zmp_ph111_B7
zmp_ph111_B9
zmp_ph111_B10
zmp_ph114_G4
zmp_ph114_G11
zmp_ph114_H1
zmp_ph114_H2
zmp_ph114_A9
zmp_ph114_A11
zmp_ph114_B2
zmp_ph114_B4
zmp_ph114_D8
zmp_ph114_E2

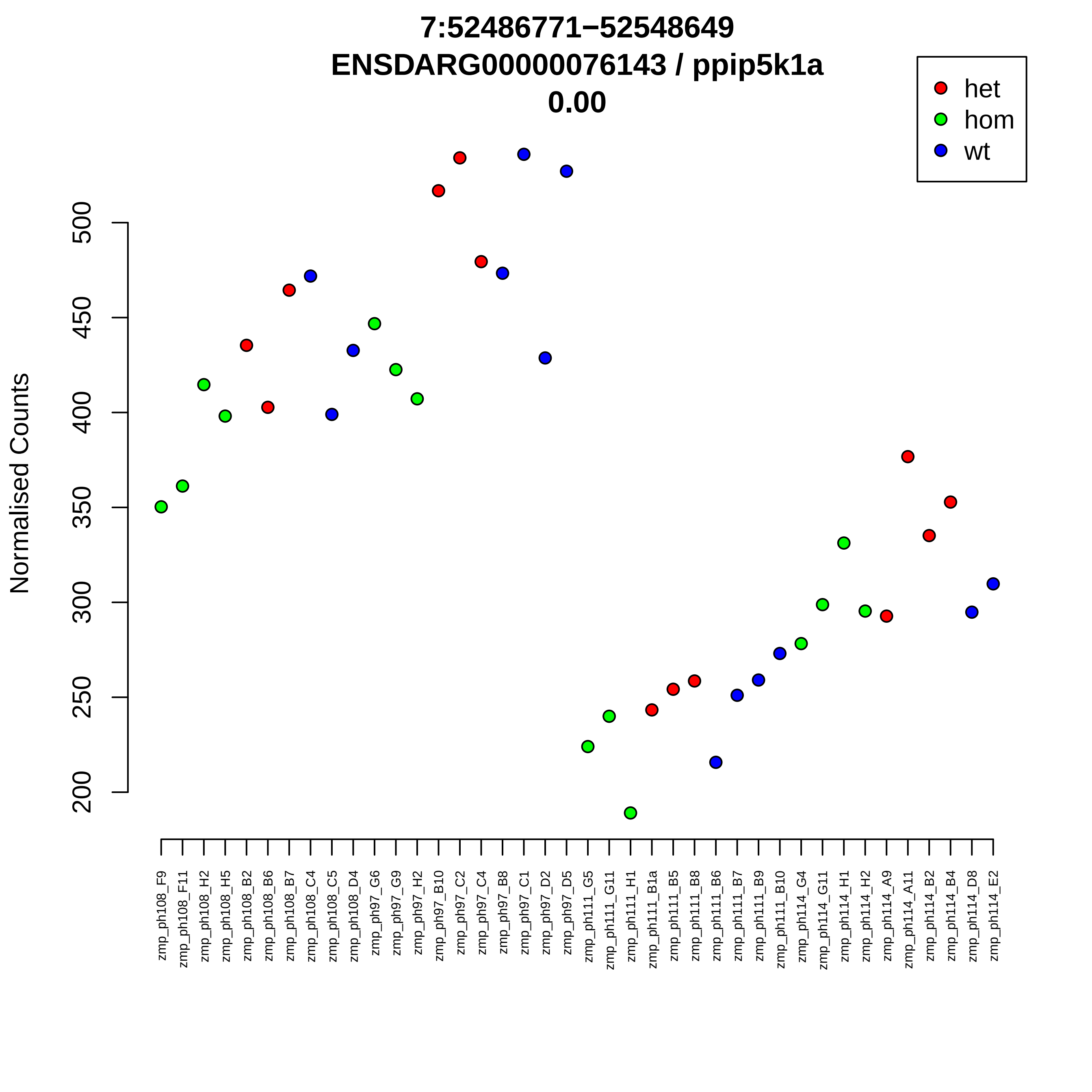

7:52486771−52548649
ENSDARG00000076143 / ppip5k1a
0.00
het
hom
wt
Normalised Counts
200
250
300
350
400
450
500
zmp_ph108_F9
zmp_ph108_F11
zmp_ph108_H2
zmp_ph108_H5
zmp_ph108_B2
zmp_ph108_B6
zmp_ph108_B7
zmp_ph108_C4
zmp_ph108_C5
zmp_ph108_D4
zmp_ph97_G6
zmp_ph97_G9
zmp_ph97_H2
zmp_ph97_B10
zmp_ph97_C2
zmp_ph97_C4
zmp_ph97_B8
zmp_ph97_C1
zmp_ph97_D2
zmp_ph97_D5
zmp_ph111_G5
zmp_ph111_G11
zmp_ph111_H1
zmp_ph111_B1a
zmp_ph111_B5
zmp_ph111_B8
zmp_ph111_B6
zmp_ph111_B7
zmp_ph111_B9
zmp_ph111_B10
zmp_ph114_G4
zmp_ph114_G11
zmp_ph114_H1
zmp_ph114_H2
zmp_ph114_A9
zmp_ph114_A11
zmp_ph114_B2
zmp_ph114_B4
zmp_ph114_D8
zmp_ph114_E2

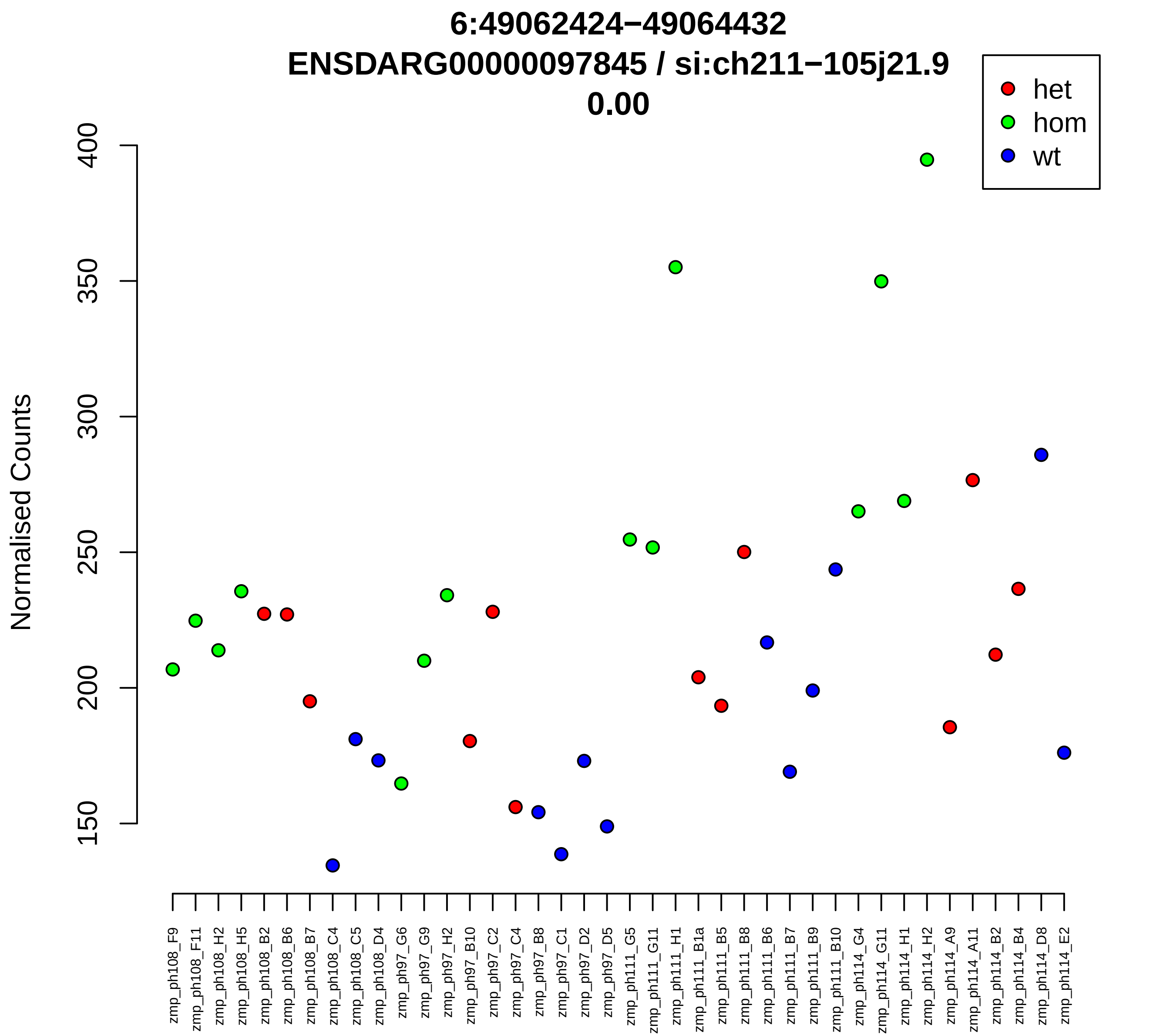

6:49062424−49064432
ENSDARG00000097845 / si:ch211−105j21.9
0.00
het
hom
wt
Normalised Counts
400
350
300
250
200
150
zmp_ph108_F9
zmp_ph108_F11
zmp_ph108_H2
zmp_ph108_H5
zmp_ph108_B2
zmp_ph108_B6
zmp_ph108_B7
zmp_ph108_C4
zmp_ph108_C5
zmp_ph108_D4
zmp_ph97_G6
zmp_ph97_G9
zmp_ph97_H2
zmp_ph97_B10
zmp_ph97_C2
zmp_ph97_C4
zmp_ph97_B8
zmp_ph97_C1
zmp_ph97_D2
zmp_ph97_D5
zmp_ph111_G5
zmp_ph111_G11
zmp_ph111_H1
zmp_ph111_B1a
zmp_ph111_B5
zmp_ph111_B8
zmp_ph111_B6
zmp_ph111_B7
zmp_ph111_B9
zmp_ph111_B10
zmp_ph114_G4
zmp_ph114_G11
zmp_ph114_H1
zmp_ph114_H2
zmp_ph114_A9
zmp_ph114_A11
zmp_ph114_B2
zmp_ph114_B4
zmp_ph114_D8
zmp_ph114_E2

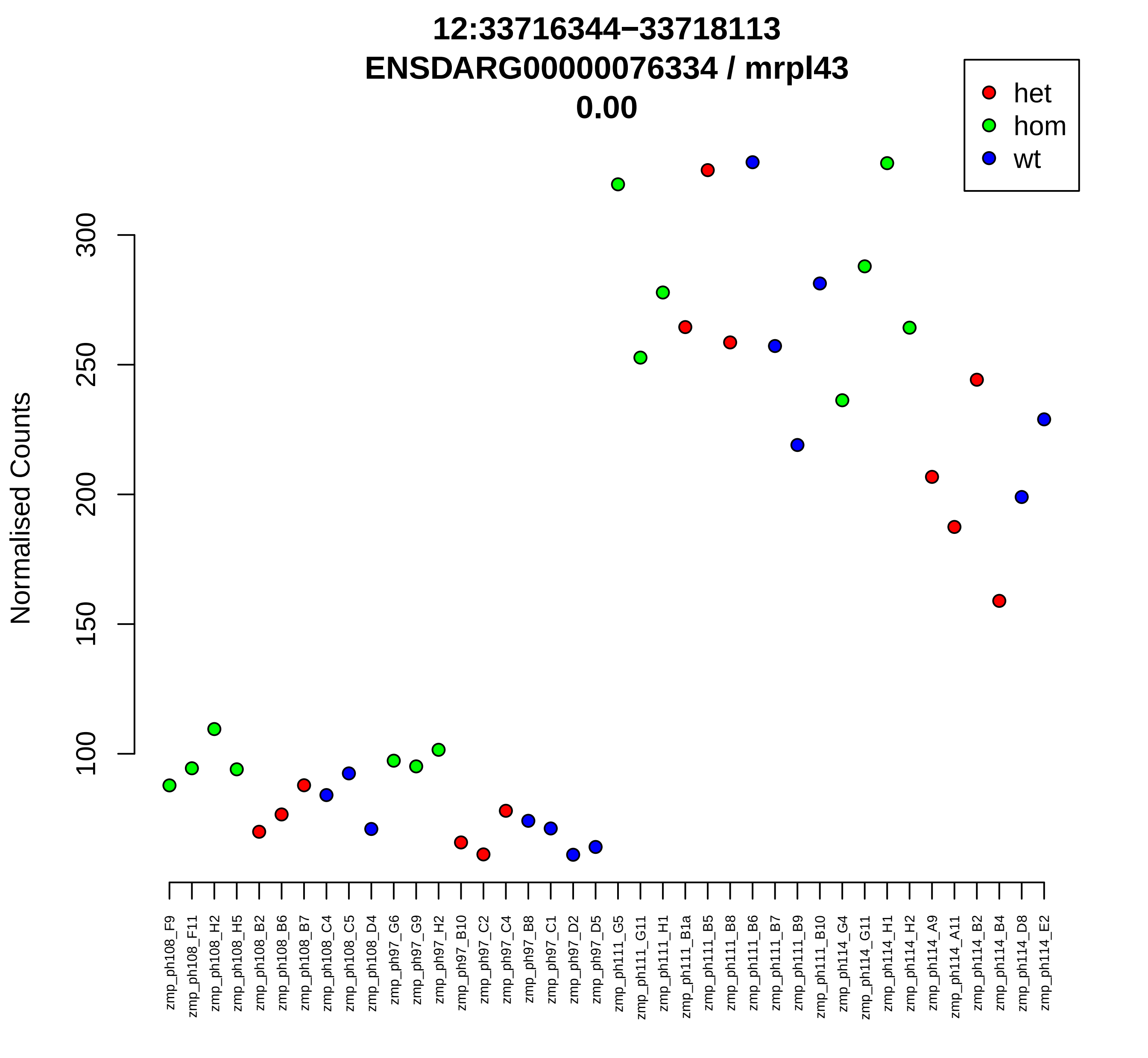

12:33716344−33718113
ENSDARG00000076334 / mrpl43
0.00
het
hom
wt
Normalised Counts
100
150
200
250
300
zmp_ph108_F9
zmp_ph108_F11
zmp_ph108_H2
zmp_ph108_H5
zmp_ph108_B2
zmp_ph108_B6
zmp_ph108_B7
zmp_ph108_C4
zmp_ph108_C5
zmp_ph108_D4
zmp_ph97_G6
zmp_ph97_G9
zmp_ph97_H2
zmp_ph97_B10
zmp_ph97_C2
zmp_ph97_C4
zmp_ph97_B8
zmp_ph97_C1
zmp_ph97_D2
zmp_ph97_D5
zmp_ph111_G5
zmp_ph111_G11
zmp_ph111_H1
zmp_ph111_B1a
zmp_ph111_B5
zmp_ph111_B8
zmp_ph111_B6
zmp_ph111_B7
zmp_ph111_B9
zmp_ph111_B10
zmp_ph114_G4
zmp_ph114_G11
zmp_ph114_H1
zmp_ph114_H2
zmp_ph114_A9
zmp_ph114_A11
zmp_ph114_B2
zmp_ph114_B4
zmp_ph114_D8
zmp_ph114_E2

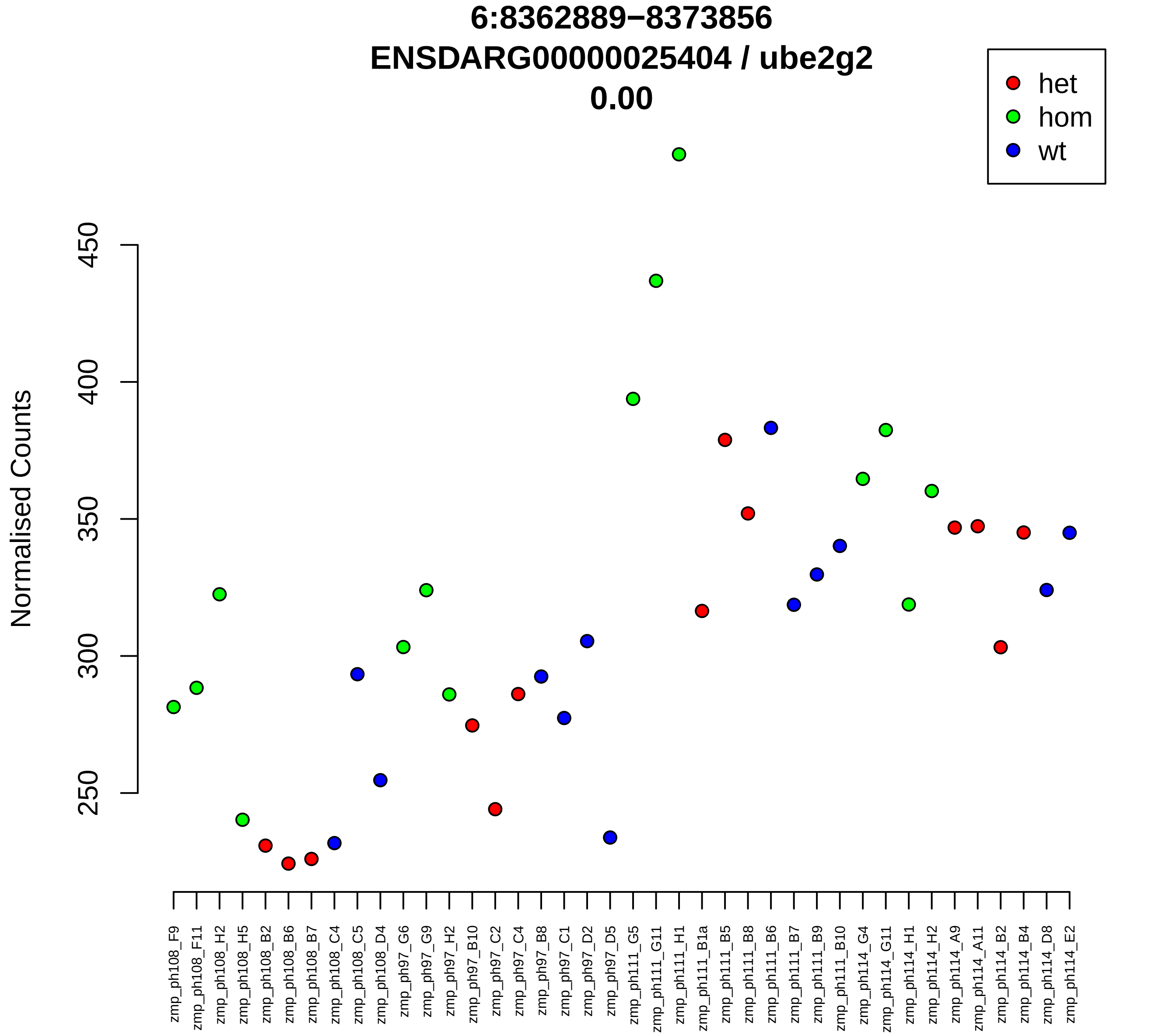
6:8362889−8373856
ENSDARG00000025404 / ube2g2
0.00
het
hom
wt
Normalised Counts
250
300
350
400
450
zmp_ph108_F9
zmp_ph108_F11
zmp_ph108_H2
zmp_ph108_H5
zmp_ph108_B2
zmp_ph108_B6
zmp_ph108_B7
zmp_ph108_C4
zmp_ph108_C5
zmp_ph108_D4
zmp_ph97_G6
zmp_ph97_G9
zmp_ph97_H2
zmp_ph97_B10
zmp_ph97_C2
zmp_ph97_C4
zmp_ph97_B8
zmp_ph97_C1
zmp_ph97_D2
zmp_ph97_D5
zmp_ph111_G5
zmp_ph111_G11
zmp_ph111_H1
zmp_ph111_B1a
zmp_ph111_B5
zmp_ph111_B8
zmp_ph111_B6
zmp_ph111_B7
zmp_ph111_B9
zmp_ph111_B10
zmp_ph114_G4
zmp_ph114_G11
zmp_ph114_H1
zmp_ph114_H2
zmp_ph114_A9
zmp_ph114_A11
zmp_ph114_B2
zmp_ph114_B4
zmp_ph114_D8
zmp_ph114_E2

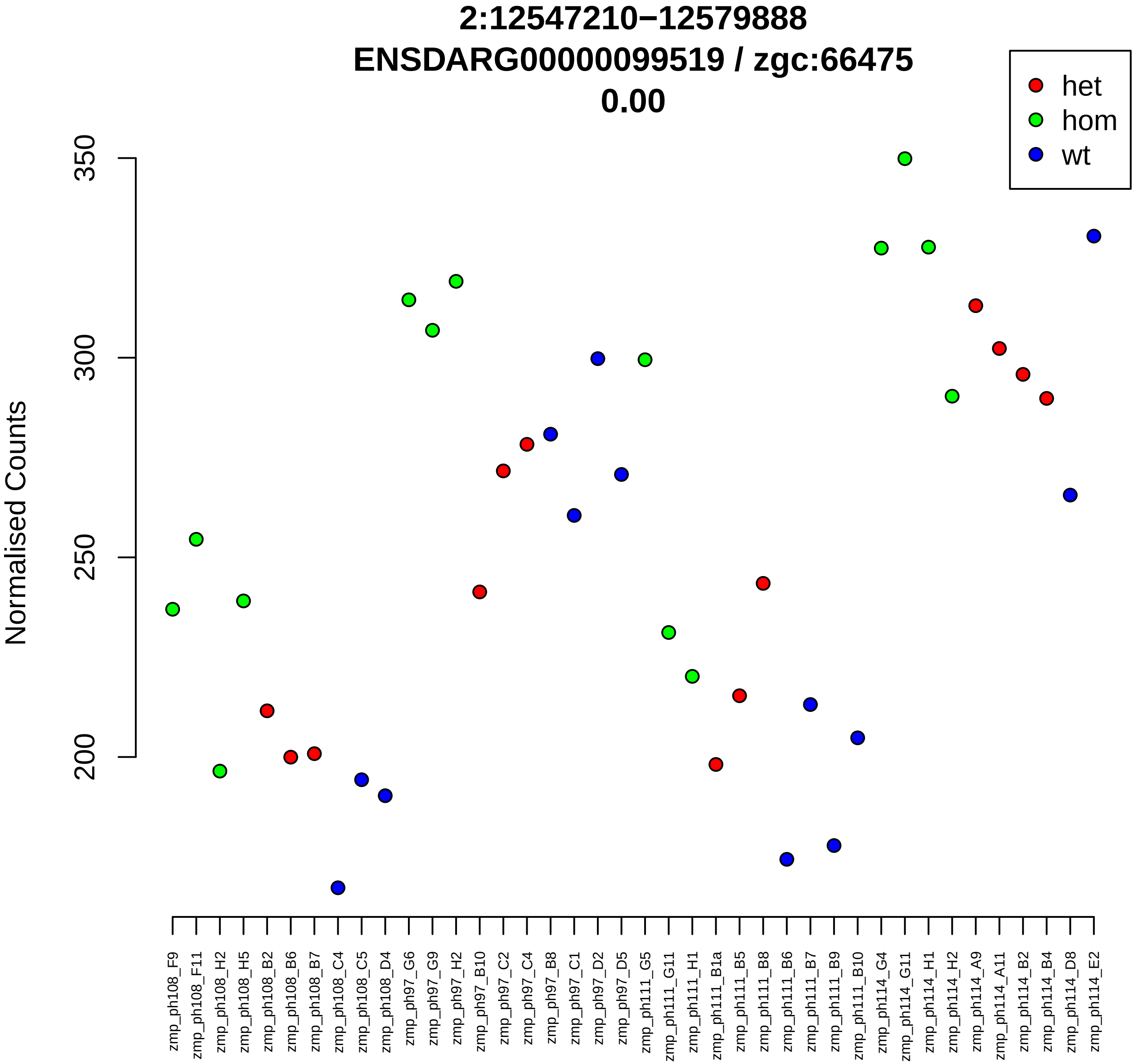
2:12547210−12579888
ENSDARG00000099519 / zgc:66475
0.00
het
hom
wt
Normalised Counts
350
300
250
200
zmp_ph108_F9
zmp_ph108_F11
zmp_ph108_H2
zmp_ph108_H5
zmp_ph108_B2
zmp_ph108_B6
zmp_ph108_B7
zmp_ph108_C4
zmp_ph108_C5
zmp_ph108_D4
zmp_ph97_G6
zmp_ph97_G9
zmp_ph97_H2
zmp_ph97_B10
zmp_ph97_C2
zmp_ph97_C4
zmp_ph97_B8
zmp_ph97_C1
zmp_ph97_D2
zmp_ph97_D5
zmp_ph111_G5
zmp_ph111_G11
zmp_ph111_H1
zmp_ph111_B1a
zmp_ph111_B5
zmp_ph111_B8
zmp_ph111_B6
zmp_ph111_B7
zmp_ph111_B9
zmp_ph111_B10
zmp_ph114_G4
zmp_ph114_G11
zmp_ph114_H1
zmp_ph114_H2
zmp_ph114_A9
zmp_ph114_A11
zmp_ph114_B2
zmp_ph114_B4
zmp_ph114_D8
zmp_ph114_E2

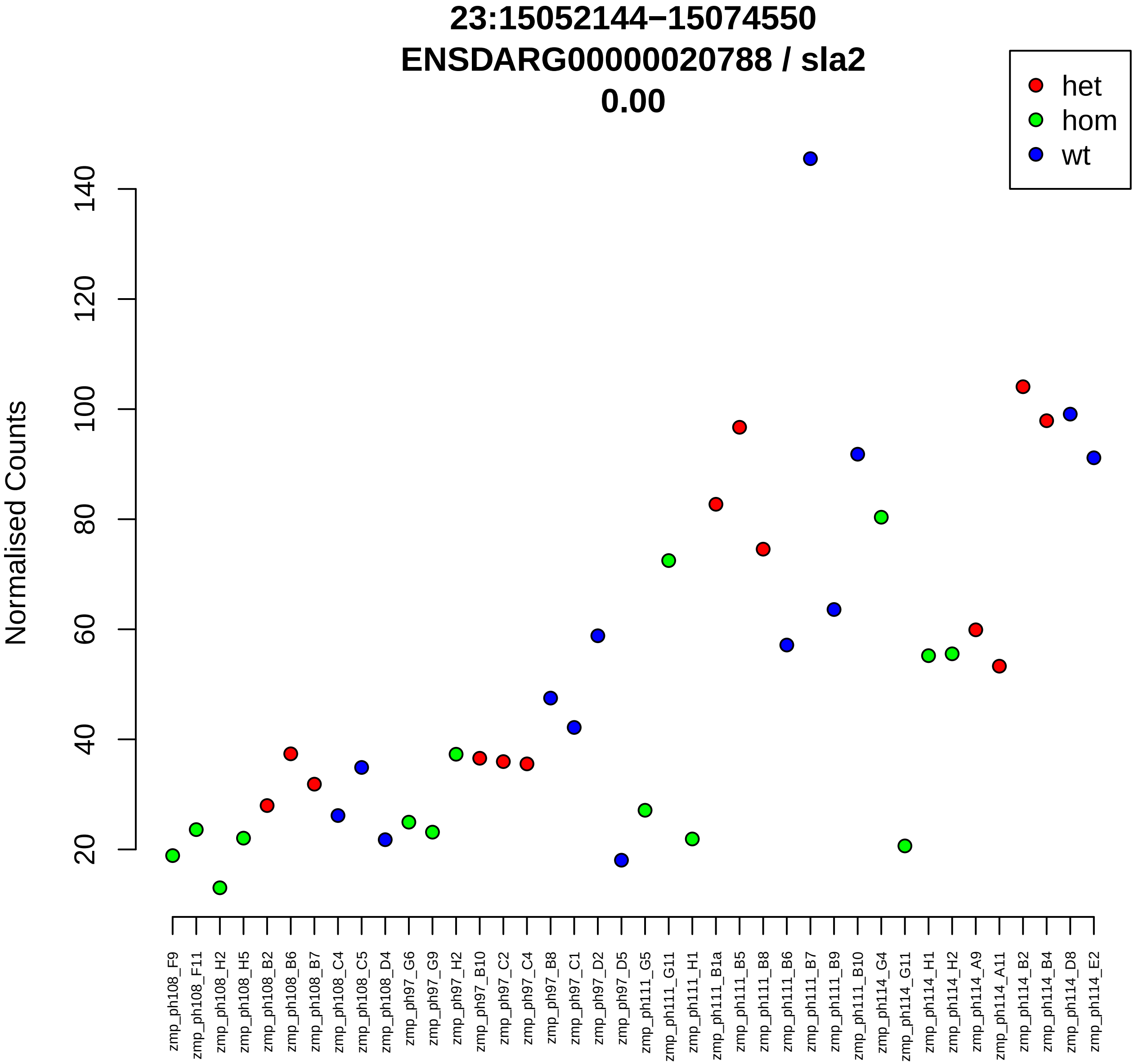
23:15052144−15074550
ENSDARG00000020788 / sla2
0.00
het
hom
wt
Normalised Counts
20
40
60
80
100
120
140
zmp_ph108_F9
zmp_ph108_F11
zmp_ph108_H2
zmp_ph108_H5
zmp_ph108_B2
zmp_ph108_B6
zmp_ph108_B7
zmp_ph108_C4
zmp_ph108_C5
zmp_ph108_D4
zmp_ph97_G6
zmp_ph97_G9
zmp_ph97_H2
zmp_ph97_B10
zmp_ph97_C2
zmp_ph97_C4
zmp_ph97_B8
zmp_ph97_C1
zmp_ph97_D2
zmp_ph97_D5
zmp_ph111_G5
zmp_ph111_G11
zmp_ph111_H1
zmp_ph111_B1a
zmp_ph111_B5
zmp_ph111_B8
zmp_ph111_B6
zmp_ph111_B7
zmp_ph111_B9
zmp_ph111_B10
zmp_ph114_G4
zmp_ph114_G11
zmp_ph114_H1
zmp_ph114_H2
zmp_ph114_A9
zmp_ph114_A11
zmp_ph114_B2
zmp_ph114_B4
zmp_ph114_D8
zmp_ph114_E2

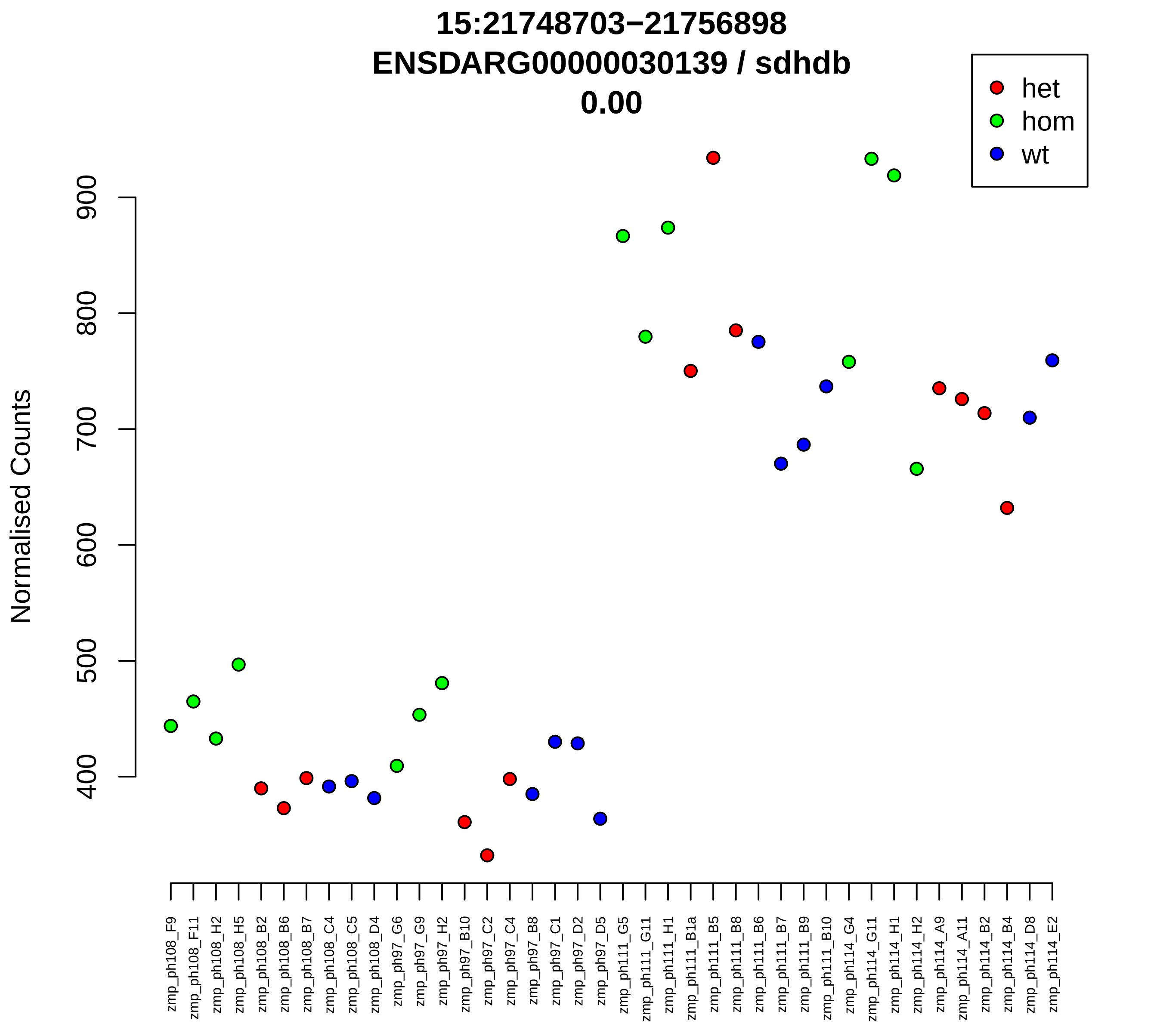
15:21748703−21756898
ENSDARG00000030139 / sdhdb
0.00
het
hom
wt
Normalised Counts
400
500
600
700
800
900
zmp_ph108_F9
zmp_ph108_F11
zmp_ph108_H2
zmp_ph108_H5
zmp_ph108_B2
zmp_ph108_B6
zmp_ph108_B7
zmp_ph108_C4
zmp_ph108_C5
zmp_ph108_D4
zmp_ph97_G6
zmp_ph97_G9
zmp_ph97_H2
zmp_ph97_B10
zmp_ph97_C2
zmp_ph97_C4
zmp_ph97_B8
zmp_ph97_C1
zmp_ph97_D2
zmp_ph97_D5
zmp_ph111_G5
zmp_ph111_G11
zmp_ph111_H1
zmp_ph111_B1a
zmp_ph111_B5
zmp_ph111_B8
zmp_ph111_B6
zmp_ph111_B7
zmp_ph111_B9
zmp_ph111_B10
zmp_ph114_G4
zmp_ph114_G11
zmp_ph114_H1
zmp_ph114_H2
zmp_ph114_A9
zmp_ph114_A11
zmp_ph114_B2
zmp_ph114_B4
zmp_ph114_D8
zmp_ph114_E2

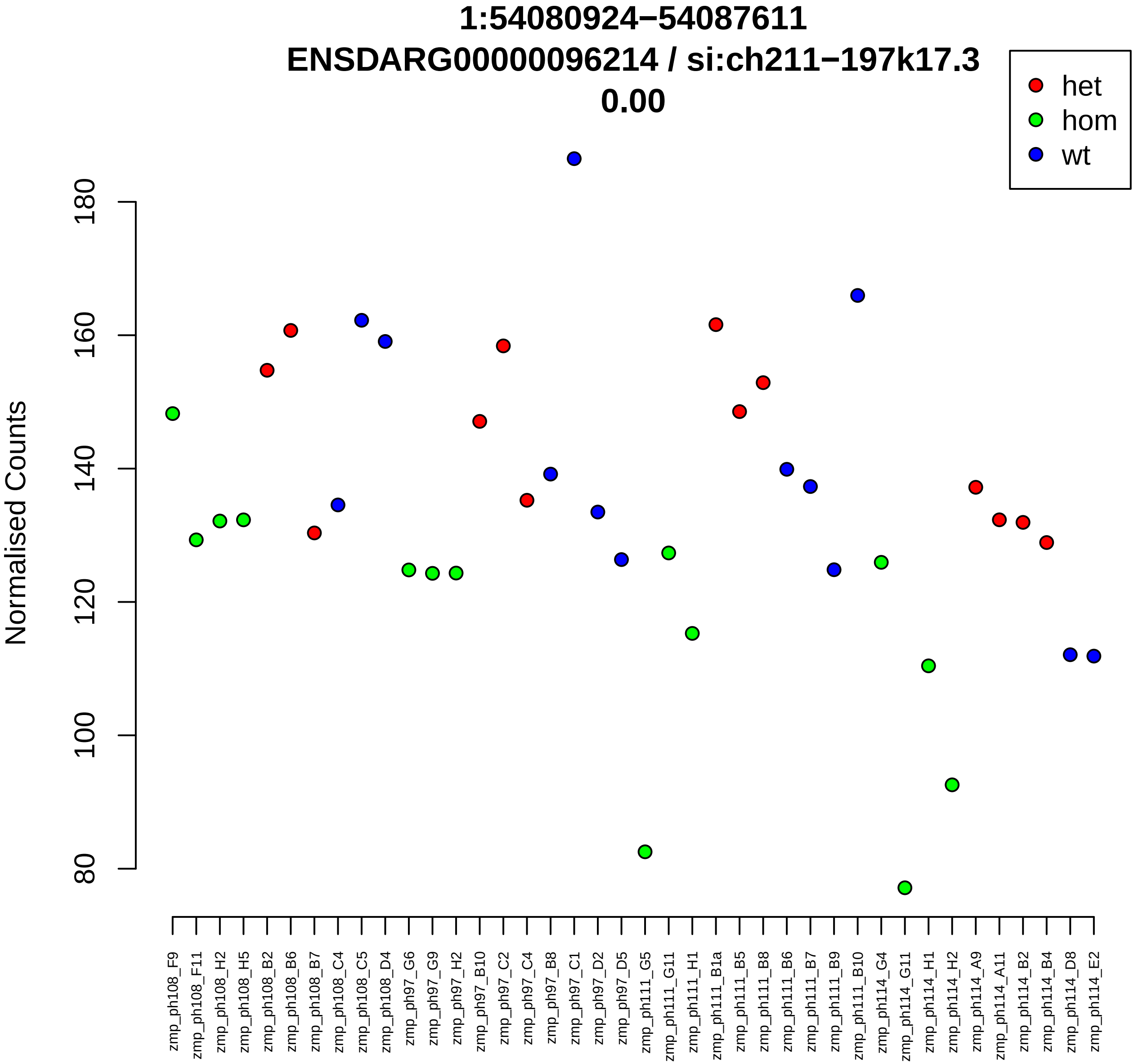
1:54080924−54087611
ENSDARG00000096214 / si:ch211−197k17.3
0.00
het
hom
wt
Normalised Counts
80
100
120
140
160
180
zmp_ph108_F9
zmp_ph108_F11
zmp_ph108_H2
zmp_ph108_H5
zmp_ph108_B2
zmp_ph108_B6
zmp_ph108_B7
zmp_ph108_C4
zmp_ph108_C5
zmp_ph108_D4
zmp_ph97_G6
zmp_ph97_G9
zmp_ph97_H2
zmp_ph97_B10
zmp_ph97_C2
zmp_ph97_C4
zmp_ph97_B8
zmp_ph97_C1
zmp_ph97_D2
zmp_ph97_D5
zmp_ph111_G5
zmp_ph111_G11
zmp_ph111_H1
zmp_ph111_B1a
zmp_ph111_B5
zmp_ph111_B8
zmp_ph111_B6
zmp_ph111_B7
zmp_ph111_B9
zmp_ph111_B10
zmp_ph114_G4
zmp_ph114_G11
zmp_ph114_H1
zmp_ph114_H2
zmp_ph114_A9
zmp_ph114_A11
zmp_ph114_B2
zmp_ph114_B4
zmp_ph114_D8
zmp_ph114_E2

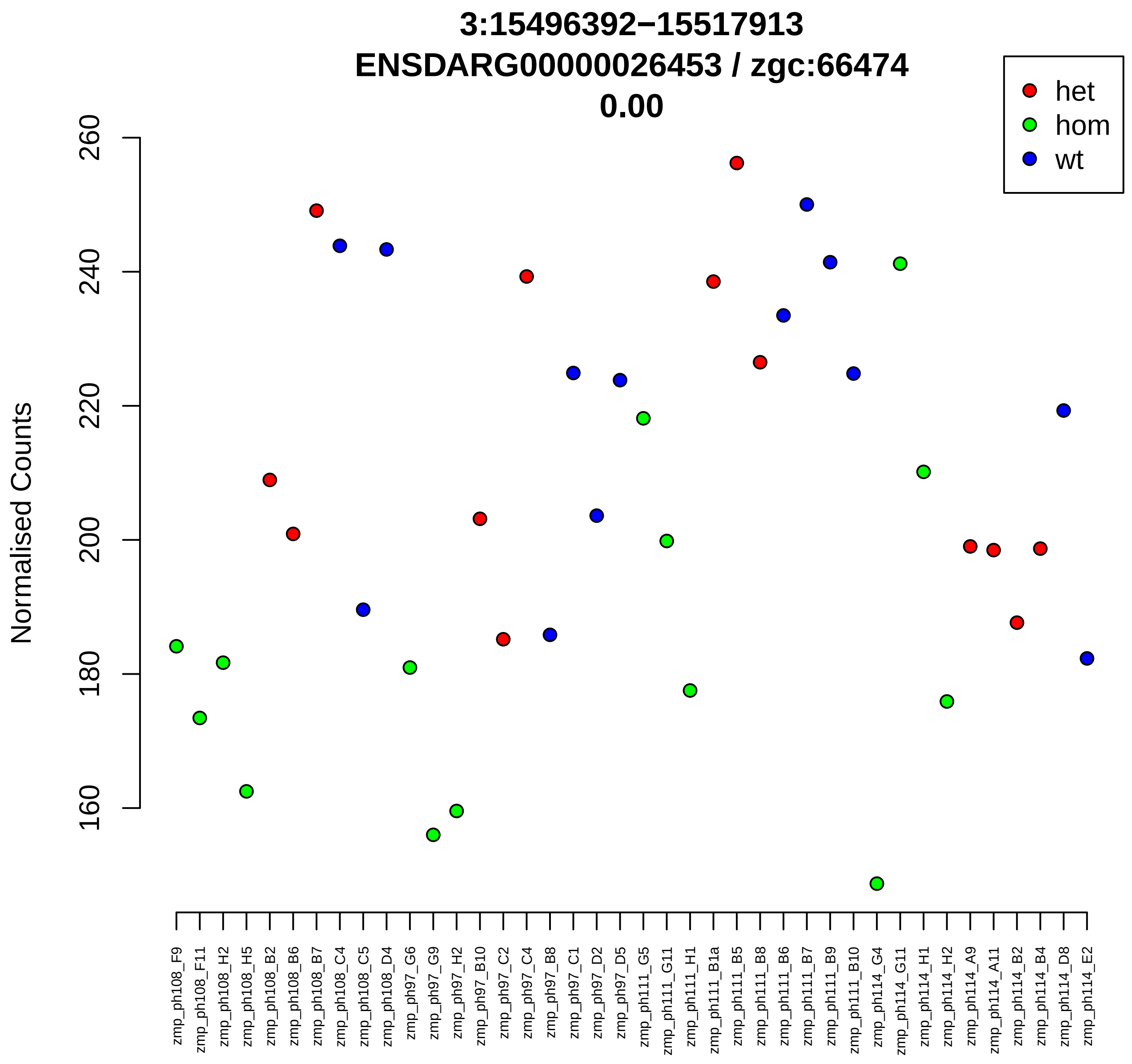
3:15496392−15517913
ENSDARG00000026453 / zgc:66474
0.00
het
hom
wt
Normalised Counts
260
240
220
200
180
160
zmp_ph108_F9
zmp_ph108_F11
zmp_ph108_H2
zmp_ph108_H5
zmp_ph108_B2
zmp_ph108_B6
zmp_ph108_B7
zmp_ph108_C4
zmp_ph108_C5
zmp_ph108_D4
zmp_ph97_G6
zmp_ph97_G9
zmp_ph97_H2
zmp_ph97_B10
zmp_ph97_C2
zmp_ph97_C4
zmp_ph97_B8
zmp_ph97_C1
zmp_ph97_D2
zmp_ph97_D5
zmp_ph111_G5
zmp_ph111_G11
zmp_ph111_H1
zmp_ph111_B1a
zmp_ph111_B5
zmp_ph111_B8
zmp_ph111_B6
zmp_ph111_B7
zmp_ph111_B9
zmp_ph111_B10
zmp_ph114_G4
zmp_ph114_G11
zmp_ph114_H1
zmp_ph114_H2
zmp_ph114_A9
zmp_ph114_A11
zmp_ph114_B2
zmp_ph114_B4
zmp_ph114_D8
zmp_ph114_E2

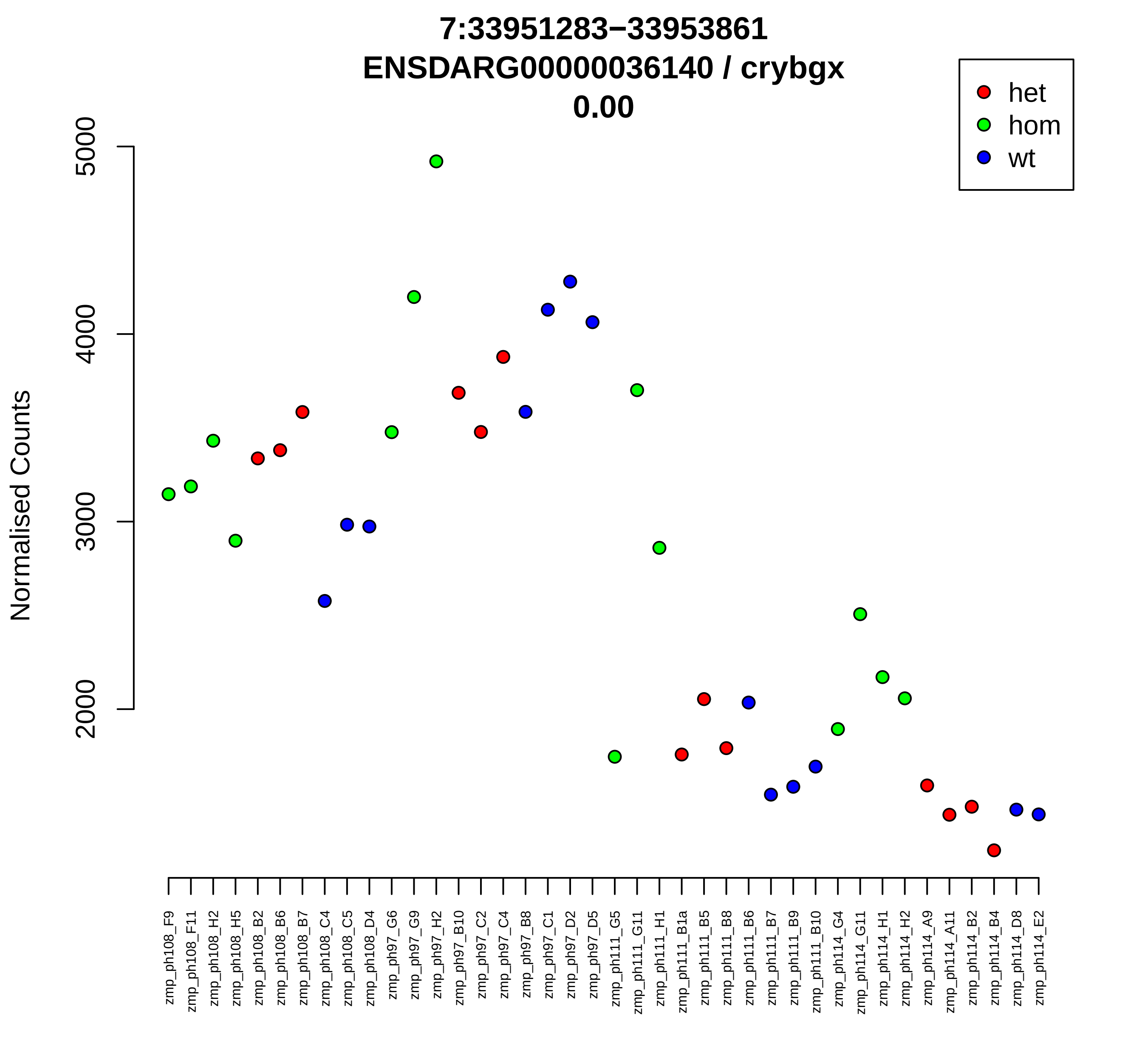

7:33951283−33953861
ENSDARG00000036140 / crybgx
0.00
het
hom
wt
Normalised Counts
5000
4000
3000
2000
zmp_ph108_F9
zmp_ph108_F11
zmp_ph108_H2
zmp_ph108_H5
zmp_ph108_B2
zmp_ph108_B6
zmp_ph108_B7
zmp_ph108_C4
zmp_ph108_C5
zmp_ph108_D4
zmp_ph97_G6
zmp_ph97_G9
zmp_ph97_H2
zmp_ph97_B10
zmp_ph97_C2
zmp_ph97_C4
zmp_ph97_B8
zmp_ph97_C1
zmp_ph97_D2
zmp_ph97_D5
zmp_ph111_G5
zmp_ph111_G11
zmp_ph111_H1
zmp_ph111_B1a
zmp_ph111_B5
zmp_ph111_B8
zmp_ph111_B6
zmp_ph111_B7
zmp_ph111_B9
zmp_ph111_B10
zmp_ph114_G4
zmp_ph114_G11
zmp_ph114_H1
zmp_ph114_H2
zmp_ph114_A9
zmp_ph114_A11
zmp_ph114_B2
zmp_ph114_B4
zmp_ph114_D8
zmp_ph114_E2

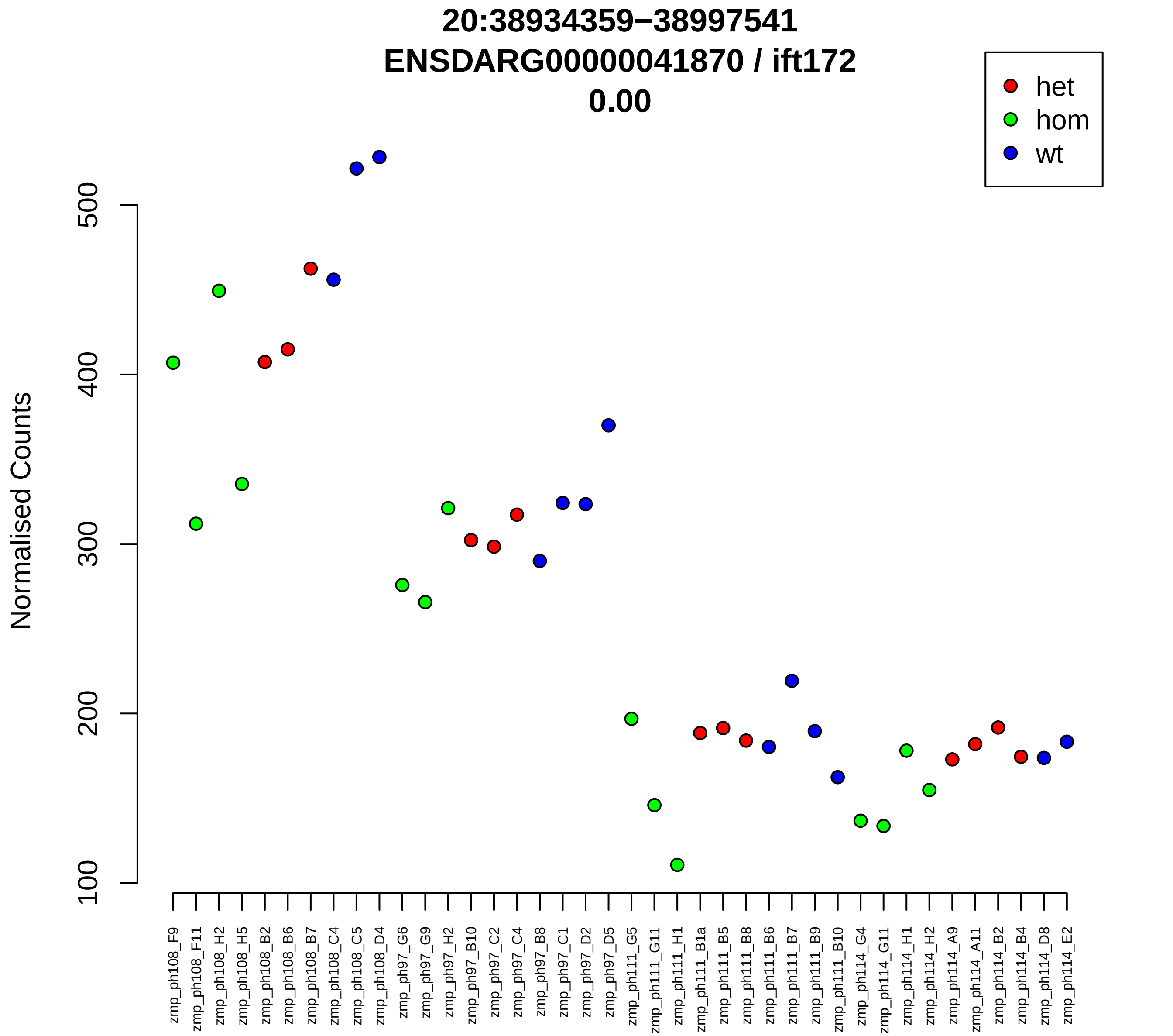

20:38934359−38997541
ENSDARG00000041870 / ift172
0.00
het
hom
wt
Normalised Counts
100
200
300
400
500
zmp_ph108_F9
zmp_ph108_F11
zmp_ph108_H2
zmp_ph108_H5
zmp_ph108_B2
zmp_ph108_B6
zmp_ph108_B7
zmp_ph108_C4
zmp_ph108_C5
zmp_ph108_D4
zmp_ph97_G6
zmp_ph97_G9
zmp_ph97_H2
zmp_ph97_B10
zmp_ph97_C2
zmp_ph97_C4
zmp_ph97_B8
zmp_ph97_C1
zmp_ph97_D2
zmp_ph97_D5
zmp_ph111_G5
zmp_ph111_G11
zmp_ph111_H1
zmp_ph111_B1a
zmp_ph111_B5
zmp_ph111_B8
zmp_ph111_B6
zmp_ph111_B7
zmp_ph111_B9
zmp_ph111_B10
zmp_ph114_G4
zmp_ph114_G11
zmp_ph114_H1
zmp_ph114_H2
zmp_ph114_A9
zmp_ph114_A11
zmp_ph114_B2
zmp_ph114_B4
zmp_ph114_D8
zmp_ph114_E2

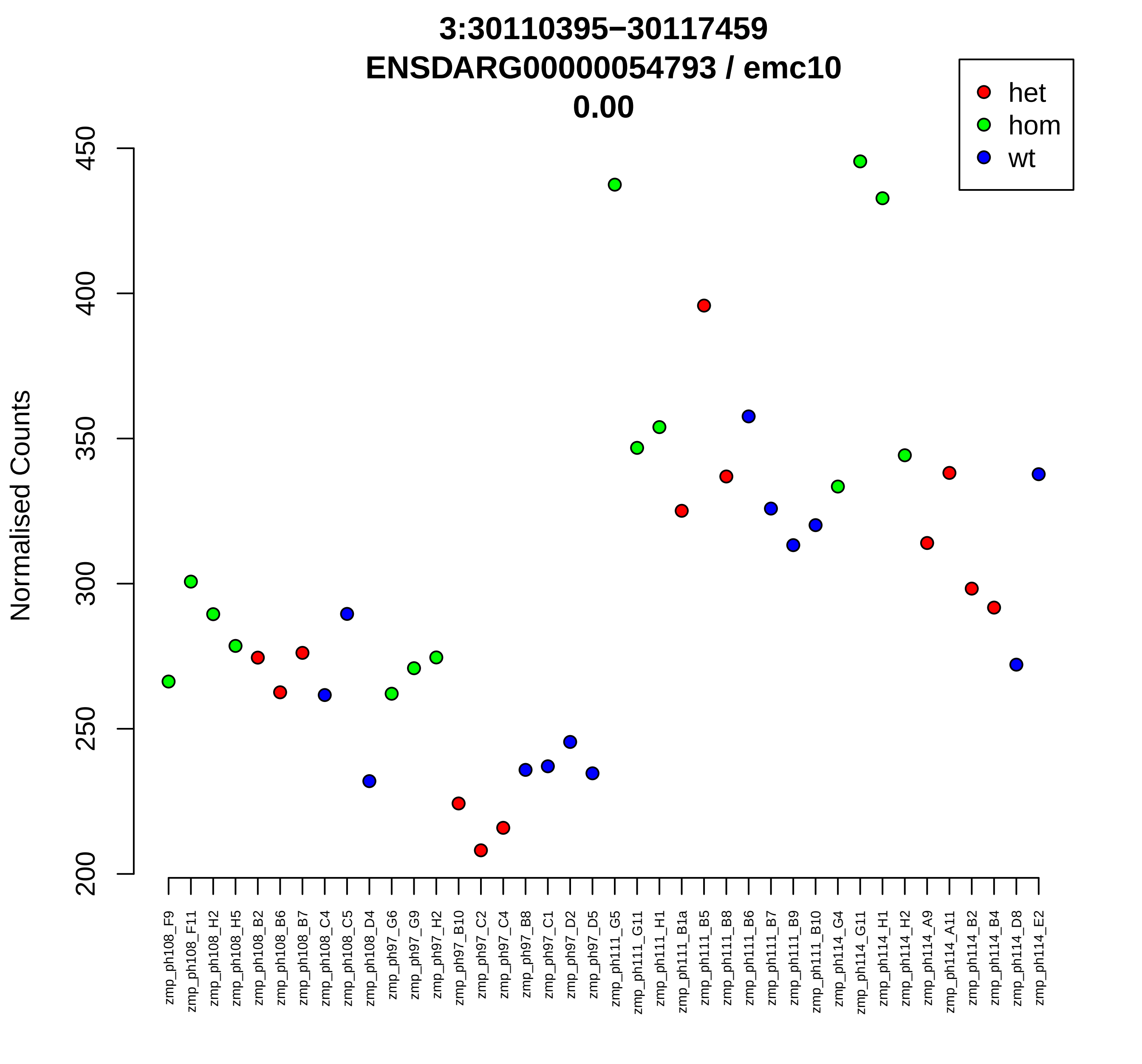
3:30110395−30117459
ENSDARG00000054793 / emc10
0.00
het
hom
wt
Normalised Counts
200
250
300
350
400
450
zmp_ph108_F9
zmp_ph108_F11
zmp_ph108_H2
zmp_ph108_H5
zmp_ph108_B2
zmp_ph108_B6
zmp_ph108_B7
zmp_ph108_C4
zmp_ph108_C5
zmp_ph108_D4
zmp_ph97_G6
zmp_ph97_G9
zmp_ph97_H2
zmp_ph97_B10
zmp_ph97_C2
zmp_ph97_C4
zmp_ph97_B8
zmp_ph97_C1
zmp_ph97_D2
zmp_ph97_D5
zmp_ph111_G5
zmp_ph111_G11
zmp_ph111_H1
zmp_ph111_B1a
zmp_ph111_B5
zmp_ph111_B8
zmp_ph111_B6
zmp_ph111_B7
zmp_ph111_B9
zmp_ph111_B10
zmp_ph114_G4
zmp_ph114_G11
zmp_ph114_H1
zmp_ph114_H2
zmp_ph114_A9
zmp_ph114_A11
zmp_ph114_B2
zmp_ph114_B4
zmp_ph114_D8
zmp_ph114_E2

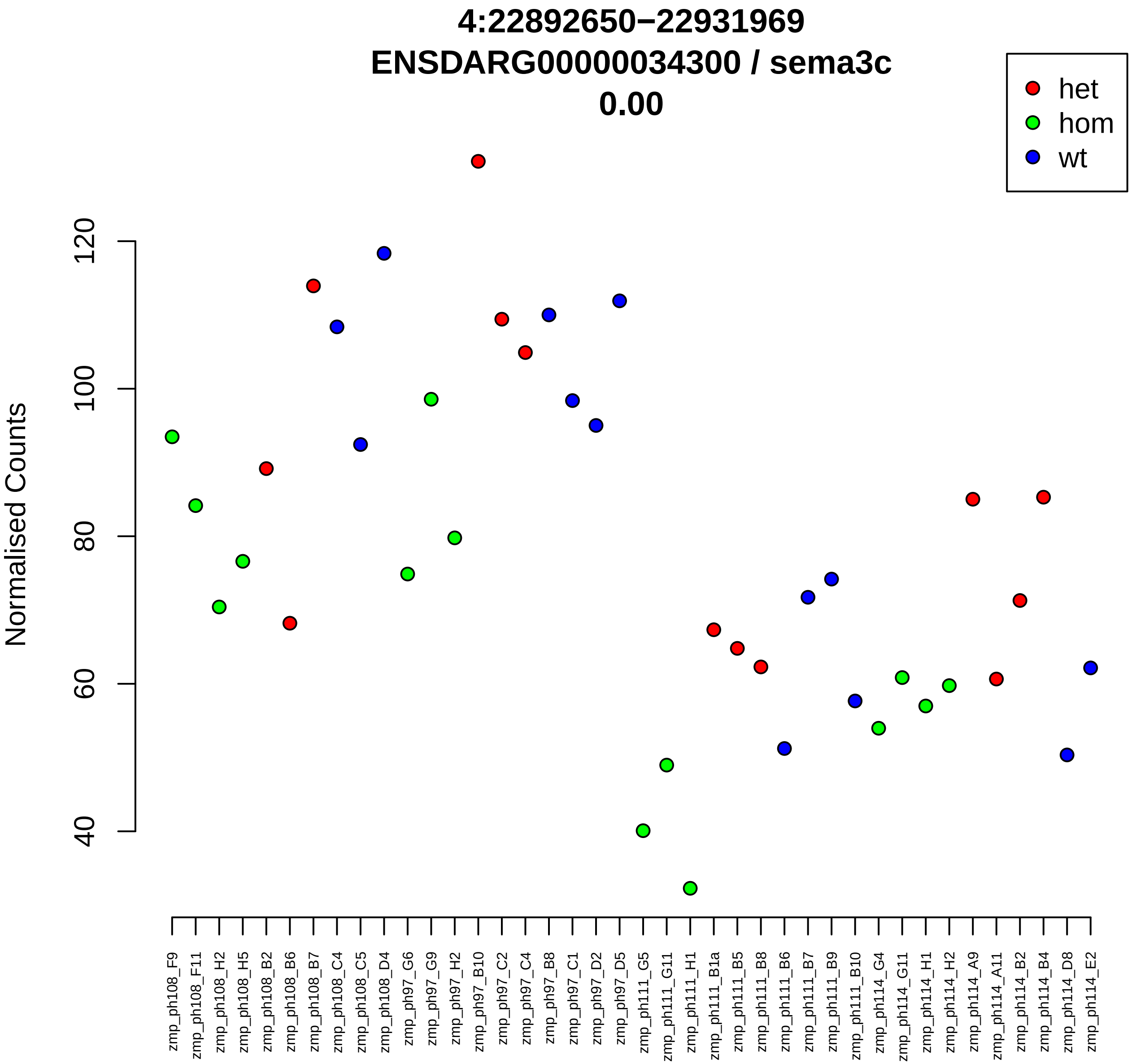
4:22892650−22931969
ENSDARG00000034300 / sema3c
0.00
het
hom
wt
Normalised Counts
40
60
80
100
120
zmp_ph108_F9
zmp_ph108_F11
zmp_ph108_H2
zmp_ph108_H5
zmp_ph108_B2
zmp_ph108_B6
zmp_ph108_B7
zmp_ph108_C4
zmp_ph108_C5
zmp_ph108_D4
zmp_ph97_G6
zmp_ph97_G9
zmp_ph97_H2
zmp_ph97_B10
zmp_ph97_C2
zmp_ph97_C4
zmp_ph97_B8
zmp_ph97_C1
zmp_ph97_D2
zmp_ph97_D5
zmp_ph111_G5
zmp_ph111_G11
zmp_ph111_H1
zmp_ph111_B1a
zmp_ph111_B5
zmp_ph111_B8
zmp_ph111_B6
zmp_ph111_B7
zmp_ph111_B9
zmp_ph111_B10
zmp_ph114_G4
zmp_ph114_G11
zmp_ph114_H1
zmp_ph114_H2
zmp_ph114_A9
zmp_ph114_A11
zmp_ph114_B2
zmp_ph114_B4
zmp_ph114_D8
zmp_ph114_E2

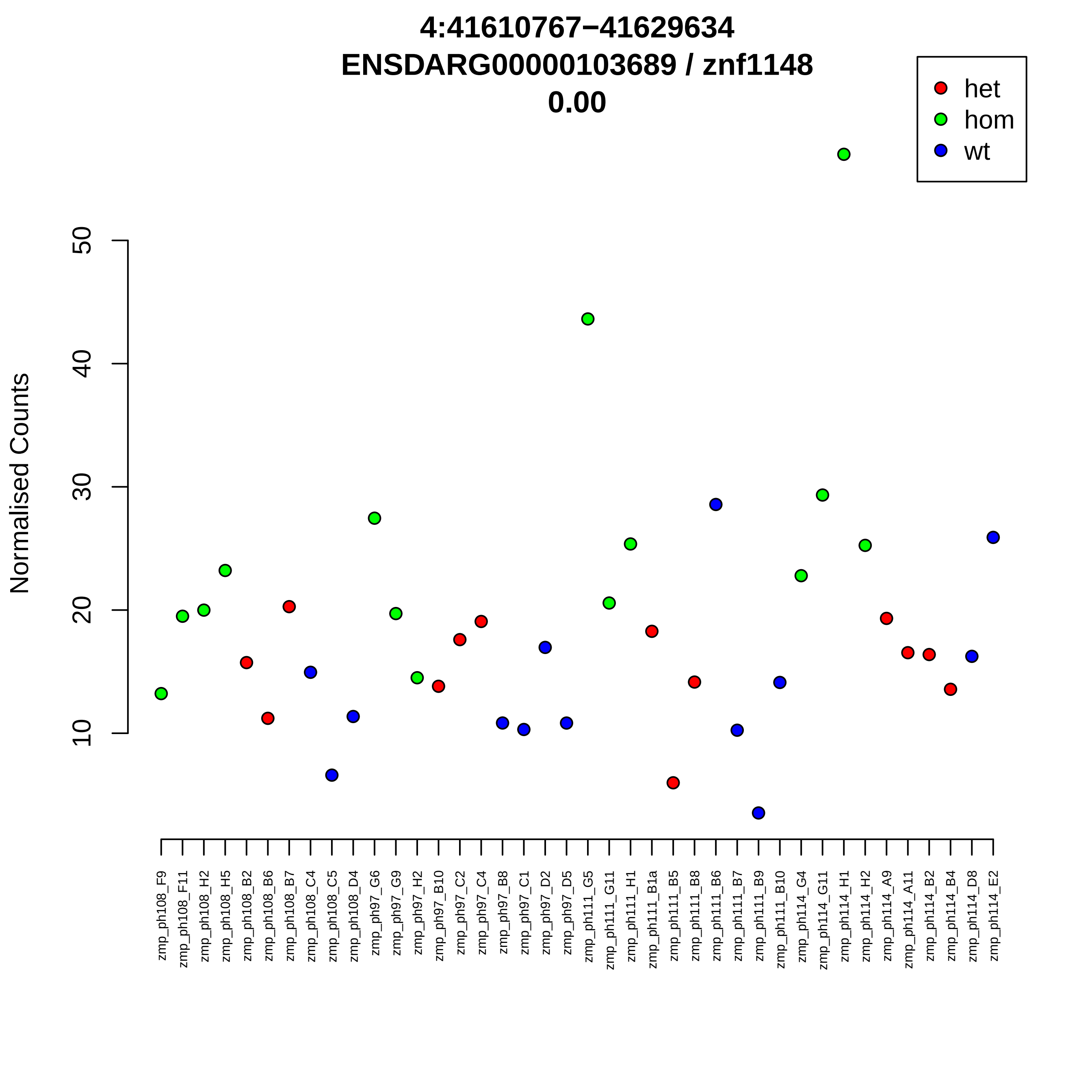

4:41610767−41629634
ENSDARG00000103689 / znf1148
0.00
het
hom
wt
Normalised Counts
10
20
30
40
50
zmp_ph108_F9
zmp_ph108_F11
zmp_ph108_H2
zmp_ph108_H5
zmp_ph108_B2
zmp_ph108_B6
zmp_ph108_B7
zmp_ph108_C4
zmp_ph108_C5
zmp_ph108_D4
zmp_ph97_G6
zmp_ph97_G9
zmp_ph97_H2
zmp_ph97_B10
zmp_ph97_C2
zmp_ph97_C4
zmp_ph97_B8
zmp_ph97_C1
zmp_ph97_D2
zmp_ph97_D5
zmp_ph111_G5
zmp_ph111_G11
zmp_ph111_H1
zmp_ph111_B1a
zmp_ph111_B5
zmp_ph111_B8
zmp_ph111_B6
zmp_ph111_B7
zmp_ph111_B9
zmp_ph111_B10
zmp_ph114_G4
zmp_ph114_G11
zmp_ph114_H1
zmp_ph114_H2
zmp_ph114_A9
zmp_ph114_A11
zmp_ph114_B2
zmp_ph114_B4
zmp_ph114_D8
zmp_ph114_E2

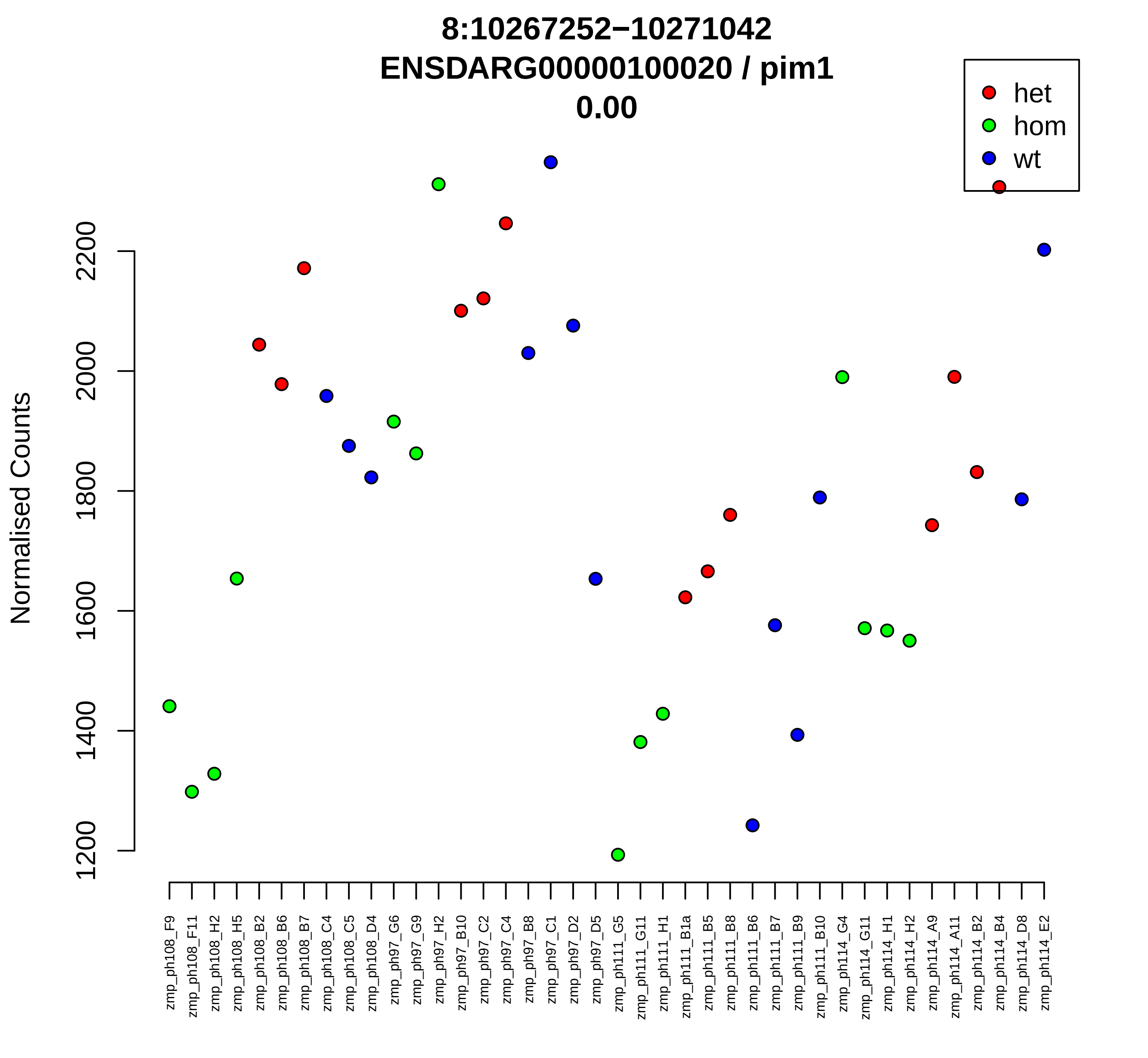

8:10267252−10271042
ENSDARG00000100020 / pim1
0.00
het
hom
wt
Normalised Counts
1200
1400
1600
1800
2000
2200
zmp_ph108_F9
zmp_ph108_F11
zmp_ph108_H2
zmp_ph108_H5
zmp_ph108_B2
zmp_ph108_B6
zmp_ph108_B7
zmp_ph108_C4
zmp_ph108_C5
zmp_ph108_D4
zmp_ph97_G6
zmp_ph97_G9
zmp_ph97_H2
zmp_ph97_B10
zmp_ph97_C2
zmp_ph97_C4
zmp_ph97_B8
zmp_ph97_C1
zmp_ph97_D2
zmp_ph97_D5
zmp_ph111_G5
zmp_ph111_G11
zmp_ph111_H1
zmp_ph111_B1a
zmp_ph111_B5
zmp_ph111_B8
zmp_ph111_B6
zmp_ph111_B7
zmp_ph111_B9
zmp_ph111_B10
zmp_ph114_G4
zmp_ph114_G11
zmp_ph114_H1
zmp_ph114_H2
zmp_ph114_A9
zmp_ph114_A11
zmp_ph114_B2
zmp_ph114_B4
zmp_ph114_D8
zmp_ph114_E2

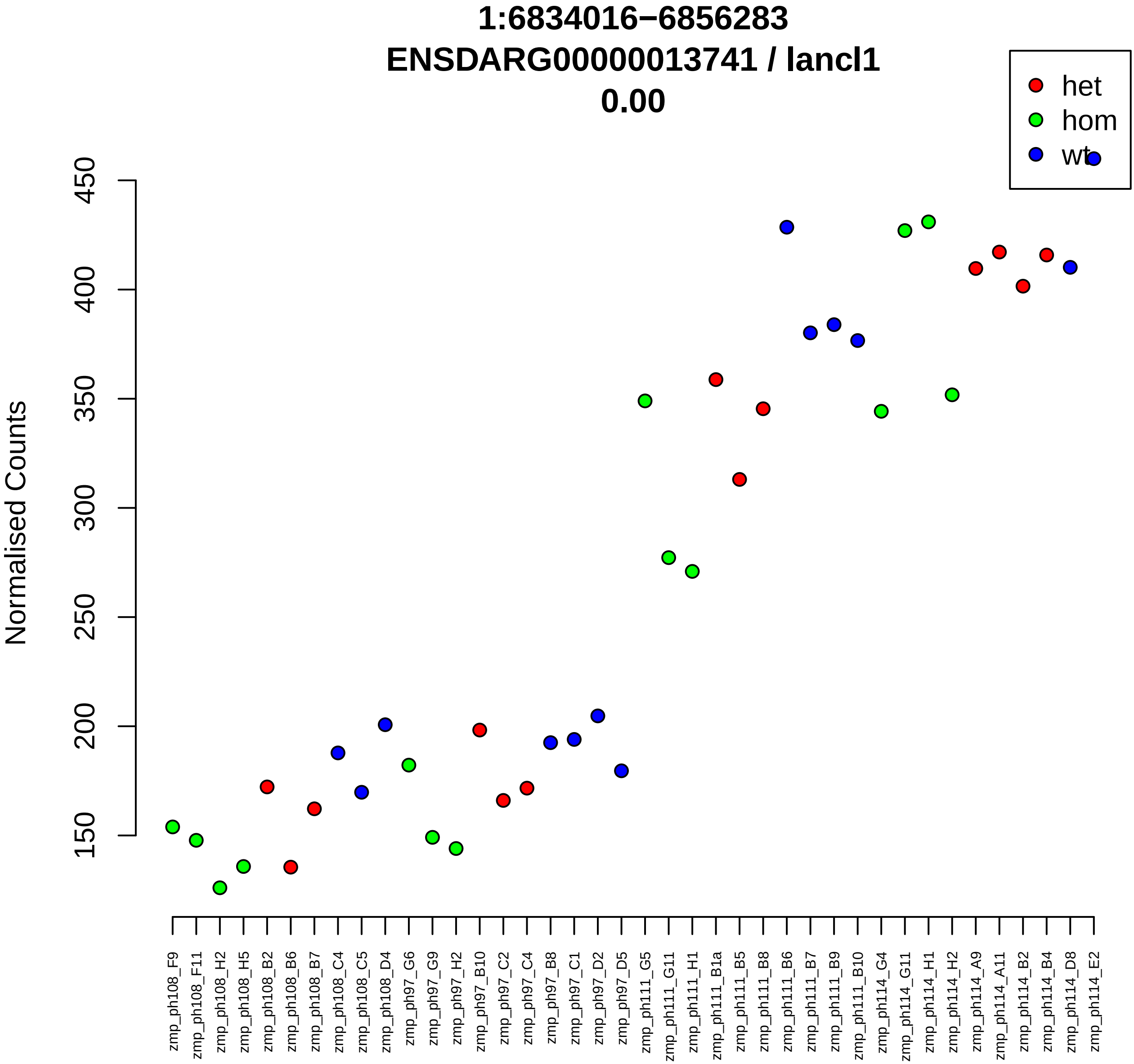

1:6834016−6856283
ENSDARG00000013741 / lancl1
0.00
het
hom
wt
Normalised Counts
150
200
250
300
350
400
450
zmp_ph108_F9
zmp_ph108_F11
zmp_ph108_H2
zmp_ph108_H5
zmp_ph108_B2
zmp_ph108_B6
zmp_ph108_B7
zmp_ph108_C4
zmp_ph108_C5
zmp_ph108_D4
zmp_ph97_G6
zmp_ph97_G9
zmp_ph97_H2
zmp_ph97_B10
zmp_ph97_C2
zmp_ph97_C4
zmp_ph97_B8
zmp_ph97_C1
zmp_ph97_D2
zmp_ph97_D5
zmp_ph111_G5
zmp_ph111_G11
zmp_ph111_H1
zmp_ph111_B1a
zmp_ph111_B5
zmp_ph111_B8
zmp_ph111_B6
zmp_ph111_B7
zmp_ph111_B9
zmp_ph111_B10
zmp_ph114_G4
zmp_ph114_G11
zmp_ph114_H1
zmp_ph114_H2
zmp_ph114_A9
zmp_ph114_A11
zmp_ph114_B2
zmp_ph114_B4
zmp_ph114_D8
zmp_ph114_E2

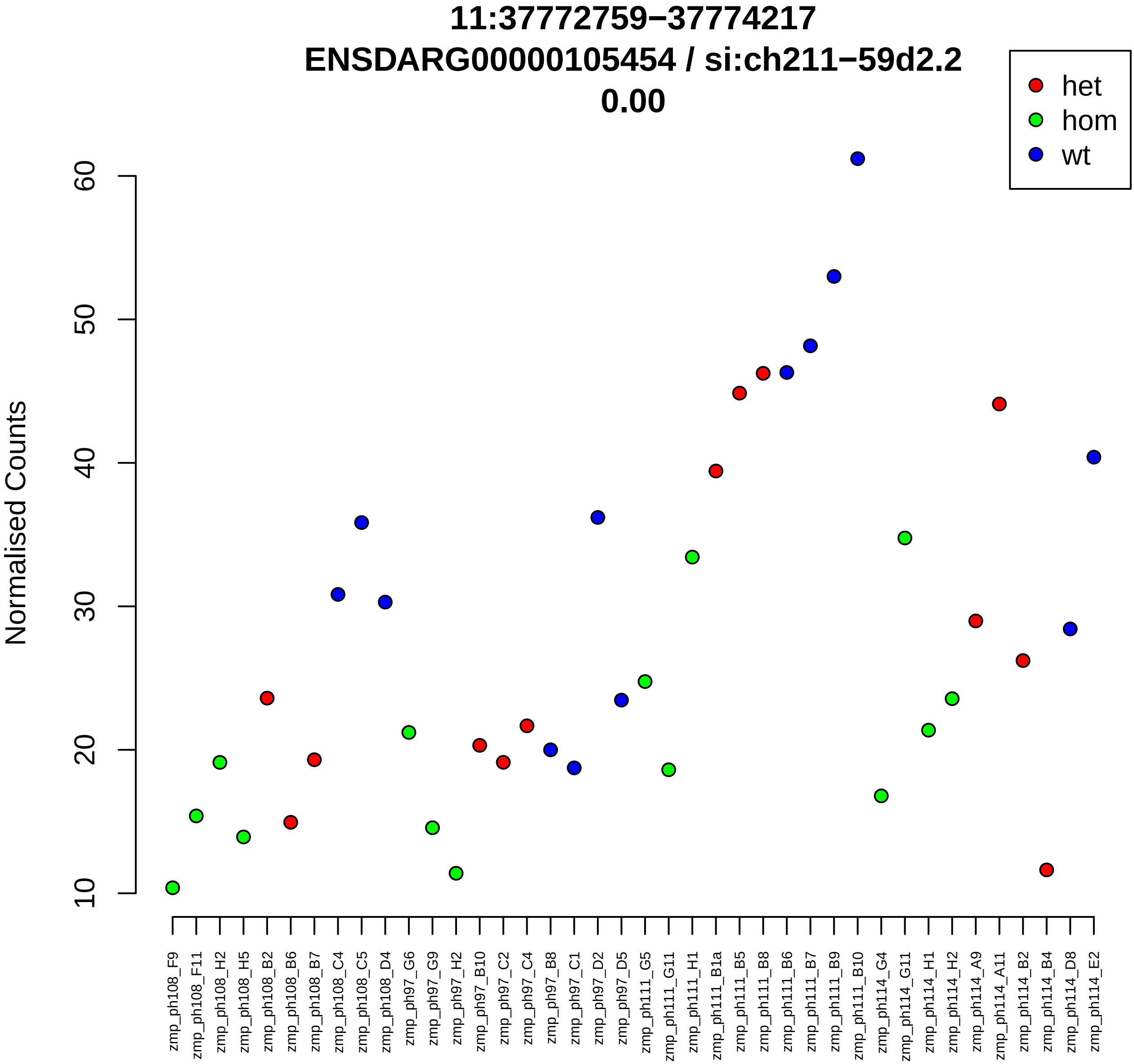
11:37772759−37774217
ENSDARG00000105454 / si:ch211−59d2.2
0.00
het
hom
wt
Normalised Counts
10
20
30
40
50
60
zmp_ph108_F9
zmp_ph108_F11
zmp_ph108_H2
zmp_ph108_H5
zmp_ph108_B2
zmp_ph108_B6
zmp_ph108_B7
zmp_ph108_C4
zmp_ph108_C5
zmp_ph108_D4
zmp_ph97_G6
zmp_ph97_G9
zmp_ph97_H2
zmp_ph97_B10
zmp_ph97_C2
zmp_ph97_C4
zmp_ph97_B8
zmp_ph97_C1
zmp_ph97_D2
zmp_ph97_D5
zmp_ph111_G5
zmp_ph111_G11
zmp_ph111_H1
zmp_ph111_B1a
zmp_ph111_B5
zmp_ph111_B8
zmp_ph111_B6
zmp_ph111_B7
zmp_ph111_B9
zmp_ph111_B10
zmp_ph114_G4
zmp_ph114_G11
zmp_ph114_H1
zmp_ph114_H2
zmp_ph114_A9
zmp_ph114_A11
zmp_ph114_B2
zmp_ph114_B4
zmp_ph114_D8
zmp_ph114_E2

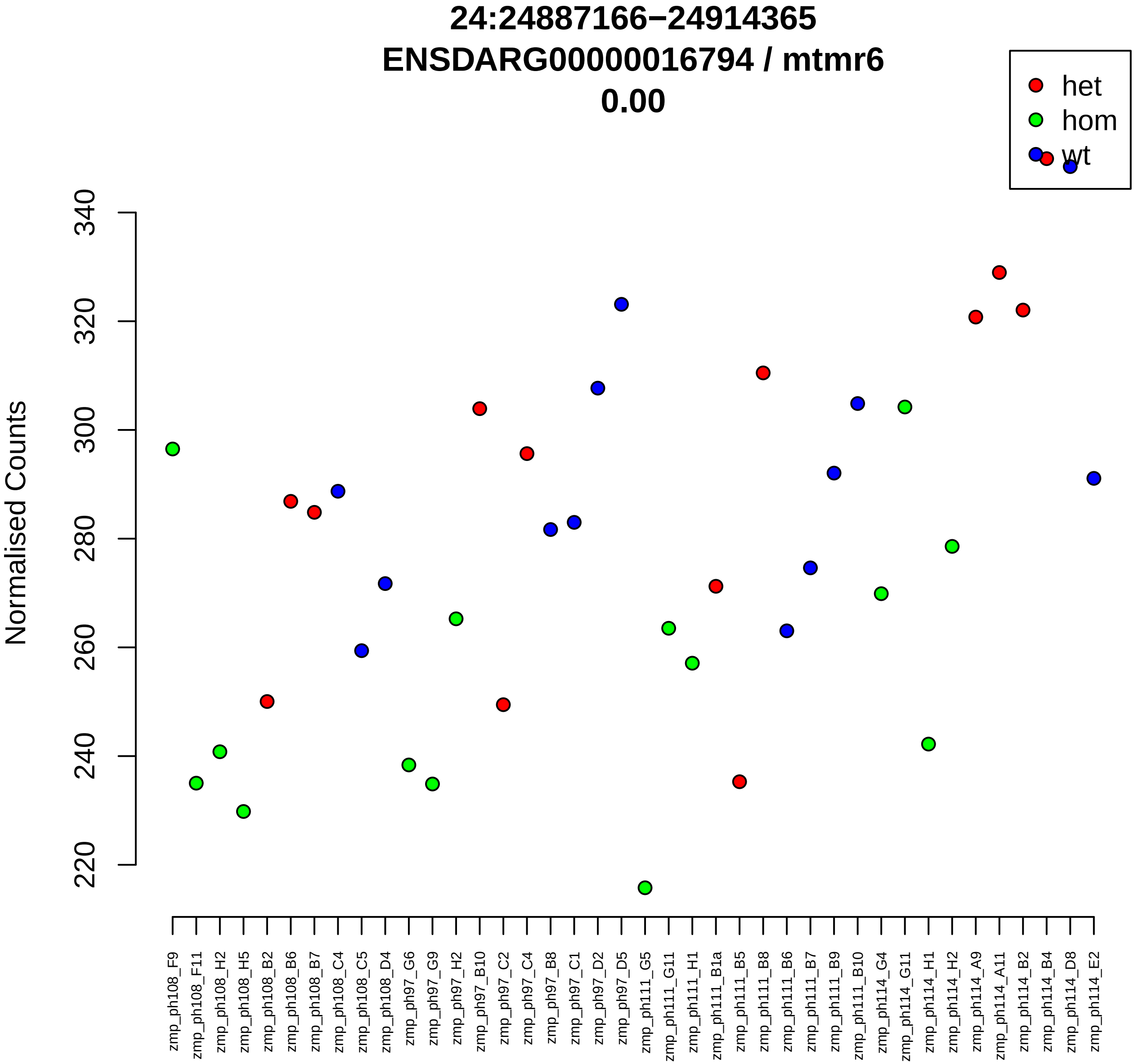

24:24887166−24914365
ENSDARG00000016794 / mtmr6
0.00
het
hom
wt
Normalised Counts
220
240
260
280
300
320
340
zmp_ph108_F9
zmp_ph108_F11
zmp_ph108_H2
zmp_ph108_H5
zmp_ph108_B2
zmp_ph108_B6
zmp_ph108_B7
zmp_ph108_C4
zmp_ph108_C5
zmp_ph108_D4
zmp_ph97_G6
zmp_ph97_G9
zmp_ph97_H2
zmp_ph97_B10
zmp_ph97_C2
zmp_ph97_C4
zmp_ph97_B8
zmp_ph97_C1
zmp_ph97_D2
zmp_ph97_D5
zmp_ph111_G5
zmp_ph111_G11
zmp_ph111_H1
zmp_ph111_B1a
zmp_ph111_B5
zmp_ph111_B8
zmp_ph111_B6
zmp_ph111_B7
zmp_ph111_B9
zmp_ph111_B10
zmp_ph114_G4
zmp_ph114_G11
zmp_ph114_H1
zmp_ph114_H2
zmp_ph114_A9
zmp_ph114_A11
zmp_ph114_B2
zmp_ph114_B4
zmp_ph114_D8
zmp_ph114_E2

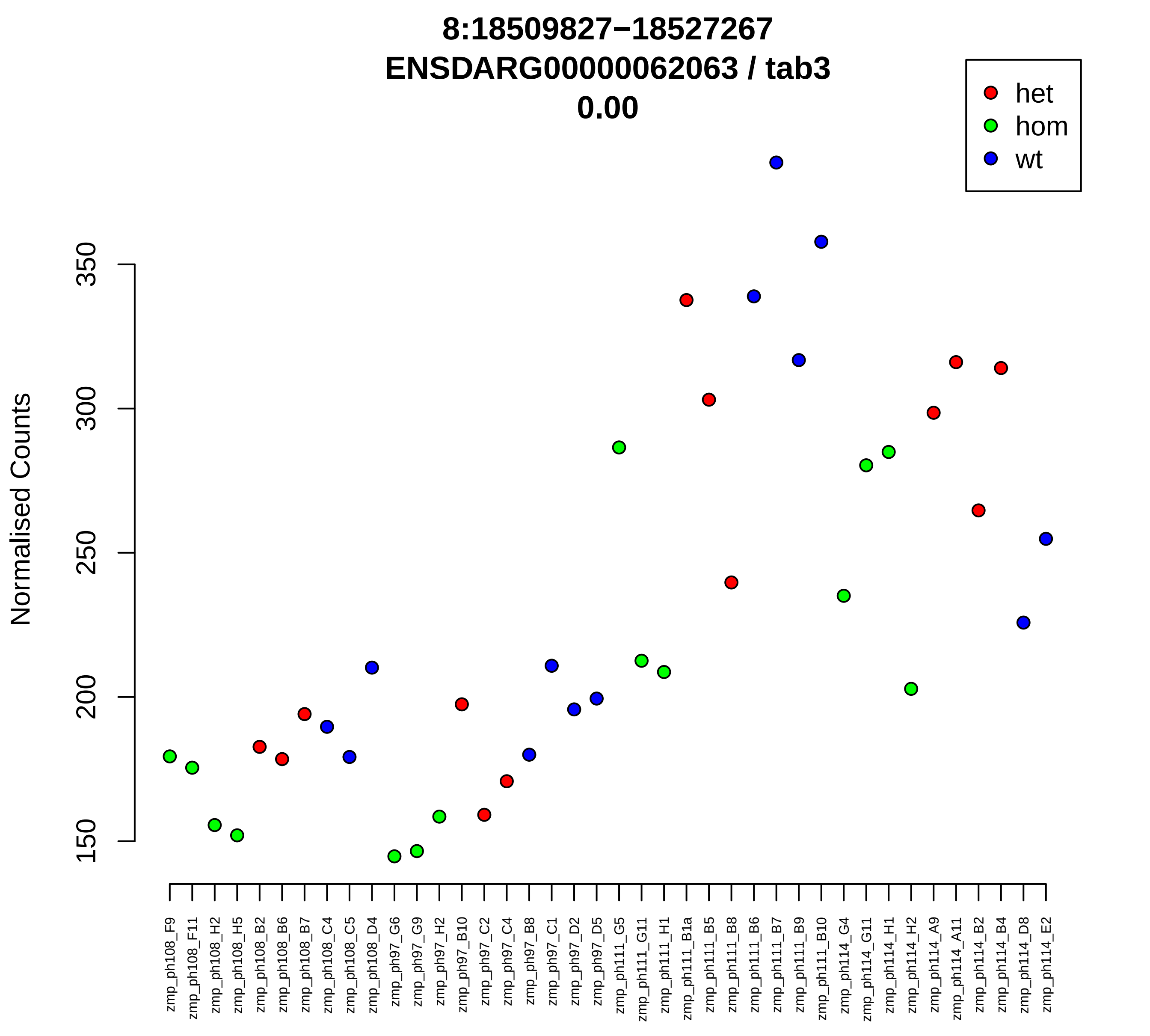
8:18509827−18527267
ENSDARG00000062063 / tab3
0.00
het
hom
wt
Normalised Counts
150
200
250
300
350
zmp_ph108_F9
zmp_ph108_F11
zmp_ph108_H2
zmp_ph108_H5
zmp_ph108_B2
zmp_ph108_B6
zmp_ph108_B7
zmp_ph108_C4
zmp_ph108_C5
zmp_ph108_D4
zmp_ph97_G6
zmp_ph97_G9
zmp_ph97_H2
zmp_ph97_B10
zmp_ph97_C2
zmp_ph97_C4
zmp_ph97_B8
zmp_ph97_C1
zmp_ph97_D2
zmp_ph97_D5
zmp_ph111_G5
zmp_ph111_G11
zmp_ph111_H1
zmp_ph111_B1a
zmp_ph111_B5
zmp_ph111_B8
zmp_ph111_B6
zmp_ph111_B7
zmp_ph111_B9
zmp_ph111_B10
zmp_ph114_G4
zmp_ph114_G11
zmp_ph114_H1
zmp_ph114_H2
zmp_ph114_A9
zmp_ph114_A11
zmp_ph114_B2
zmp_ph114_B4
zmp_ph114_D8
zmp_ph114_E2

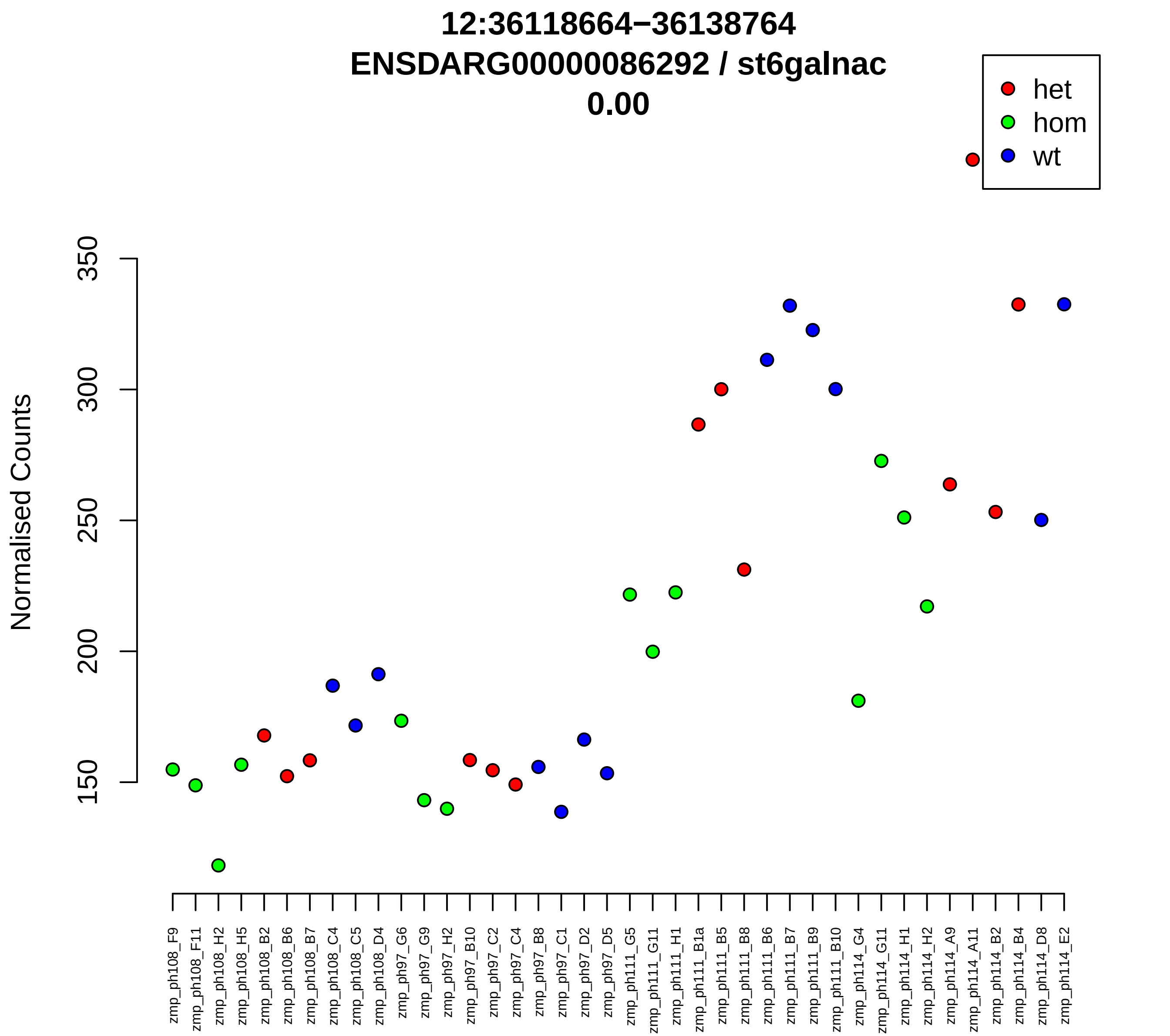
12:36118664−36138764
ENSDARG00000086292 / st6galnac
0.00
het
hom
wt
Normalised Counts
350
300
250
200
150
zmp_ph108_F9
zmp_ph108_F11
zmp_ph108_H2
zmp_ph108_H5
zmp_ph108_B2
zmp_ph108_B6
zmp_ph108_B7
zmp_ph108_C4
zmp_ph108_C5
zmp_ph108_D4
zmp_ph97_G6
zmp_ph97_G9
zmp_ph97_H2
zmp_ph97_B10
zmp_ph97_C2
zmp_ph97_C4
zmp_ph97_B8
zmp_ph97_C1
zmp_ph97_D2
zmp_ph97_D5
zmp_ph111_G5
zmp_ph111_G11
zmp_ph111_H1
zmp_ph111_B1a
zmp_ph111_B5
zmp_ph111_B8
zmp_ph111_B6
zmp_ph111_B7
zmp_ph111_B9
zmp_ph111_B10
zmp_ph114_G4
zmp_ph114_G11
zmp_ph114_H1
zmp_ph114_H2
zmp_ph114_A9
zmp_ph114_A11
zmp_ph114_B2
zmp_ph114_B4
zmp_ph114_D8
zmp_ph114_E2

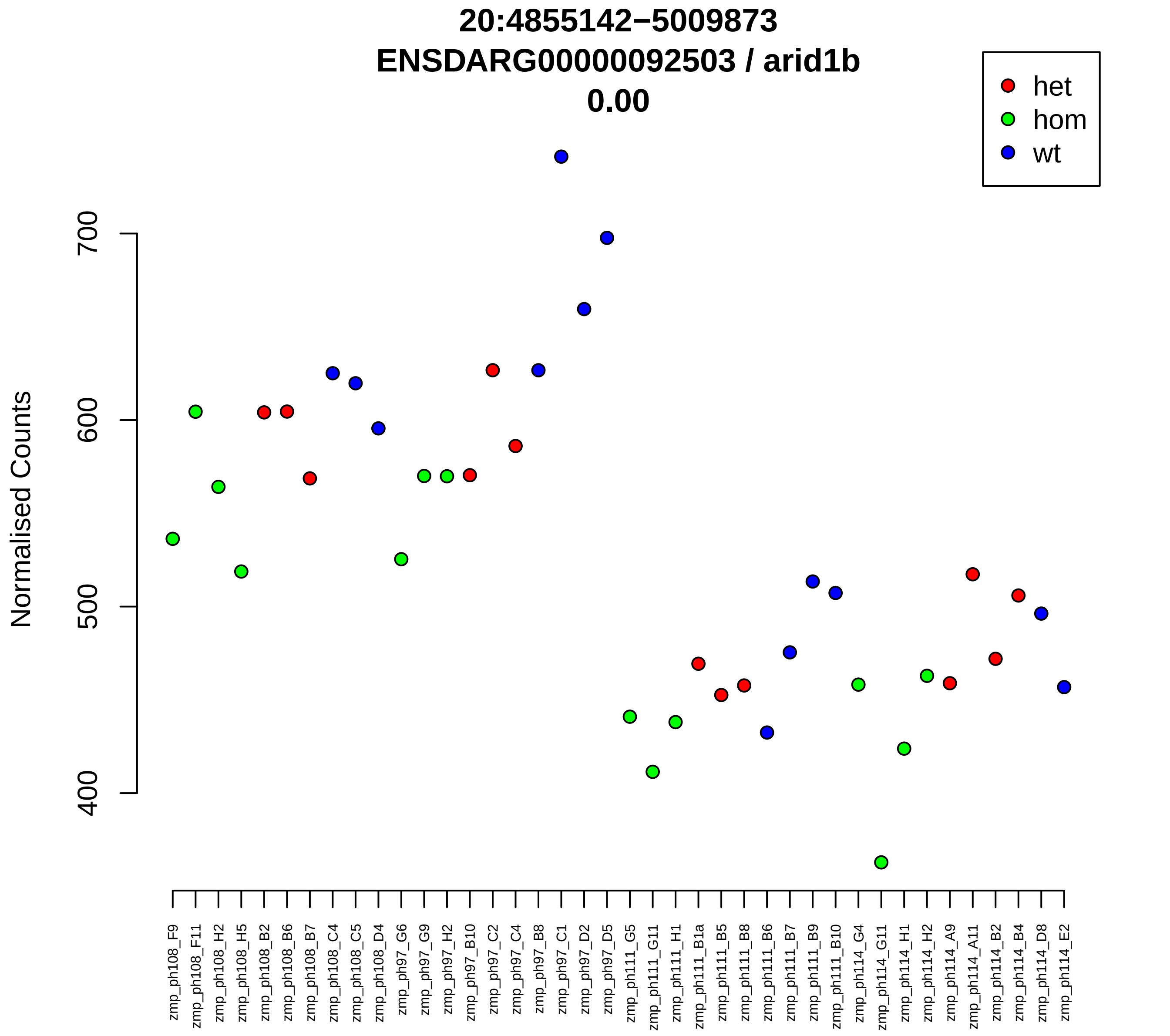

20:4855142−5009873
ENSDARG00000092503 / arid1b
0.00
het
hom
wt
Normalised Counts
400
500
600
700
zmp_ph108_F9
zmp_ph108_F11
zmp_ph108_H2
zmp_ph108_H5
zmp_ph108_B2
zmp_ph108_B6
zmp_ph108_B7
zmp_ph108_C4
zmp_ph108_C5
zmp_ph108_D4
zmp_ph97_G6
zmp_ph97_G9
zmp_ph97_H2
zmp_ph97_B10
zmp_ph97_C2
zmp_ph97_C4
zmp_ph97_B8
zmp_ph97_C1
zmp_ph97_D2
zmp_ph97_D5
zmp_ph111_G5
zmp_ph111_G11
zmp_ph111_H1
zmp_ph111_B1a
zmp_ph111_B5
zmp_ph111_B8
zmp_ph111_B6
zmp_ph111_B7
zmp_ph111_B9
zmp_ph111_B10
zmp_ph114_G4
zmp_ph114_G11
zmp_ph114_H1
zmp_ph114_H2
zmp_ph114_A9
zmp_ph114_A11
zmp_ph114_B2
zmp_ph114_B4
zmp_ph114_D8
zmp_ph114_E2

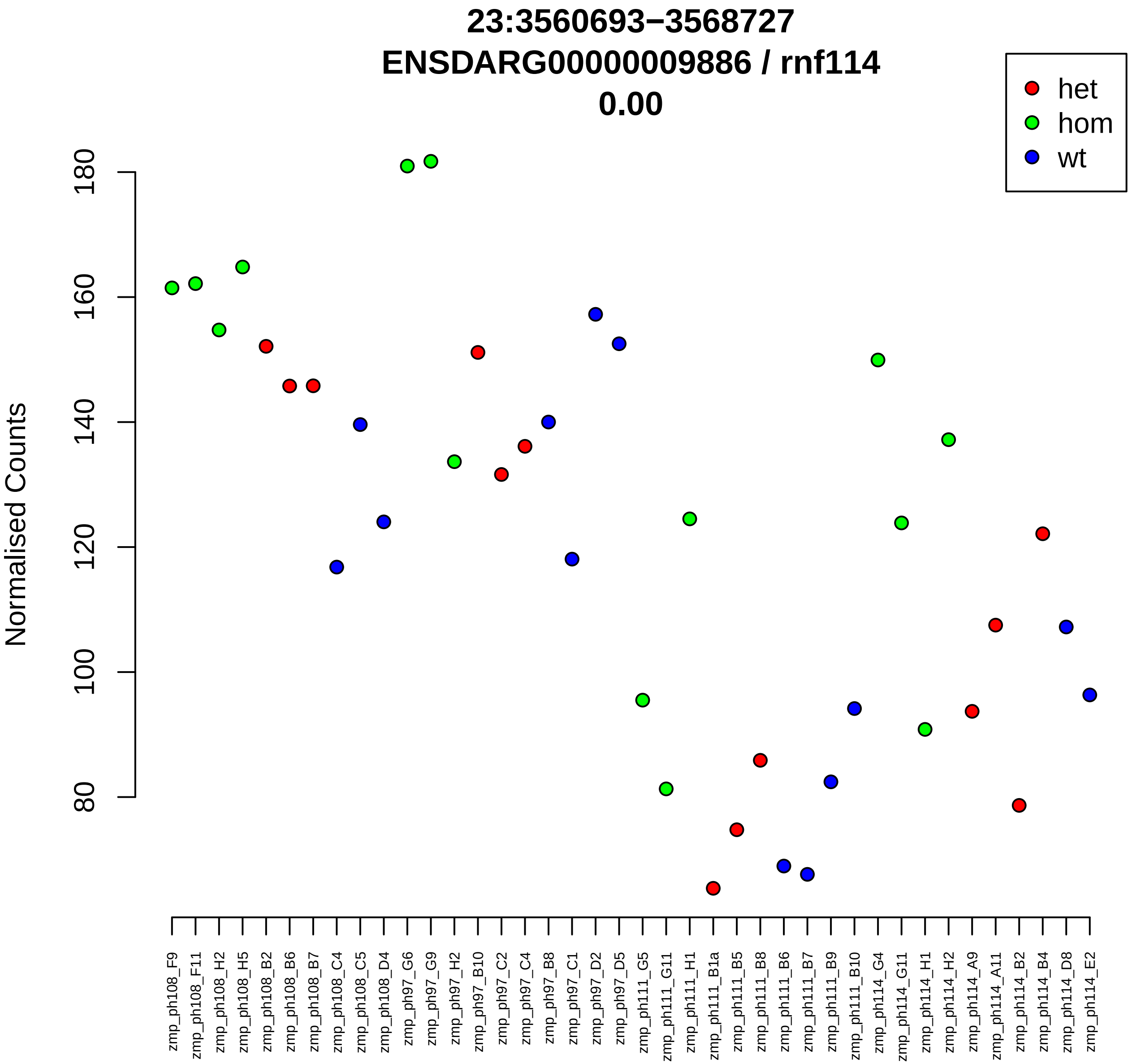
23:3560693−3568727
ENSDARG00000009886 / rnf114
0.00
het
hom
wt
Normalised Counts
80
100
120
140
160
180
zmp_ph108_F9
zmp_ph108_F11
zmp_ph108_H2
zmp_ph108_H5
zmp_ph108_B2
zmp_ph108_B6
zmp_ph108_B7
zmp_ph108_C4
zmp_ph108_C5
zmp_ph108_D4
zmp_ph97_G6
zmp_ph97_G9
zmp_ph97_H2
zmp_ph97_B10
zmp_ph97_C2
zmp_ph97_C4
zmp_ph97_B8
zmp_ph97_C1
zmp_ph97_D2
zmp_ph97_D5
zmp_ph111_G5
zmp_ph111_G11
zmp_ph111_H1
zmp_ph111_B1a
zmp_ph111_B5
zmp_ph111_B8
zmp_ph111_B6
zmp_ph111_B7
zmp_ph111_B9
zmp_ph111_B10
zmp_ph114_G4
zmp_ph114_G11
zmp_ph114_H1
zmp_ph114_H2
zmp_ph114_A9
zmp_ph114_A11
zmp_ph114_B2
zmp_ph114_B4
zmp_ph114_D8
zmp_ph114_E2

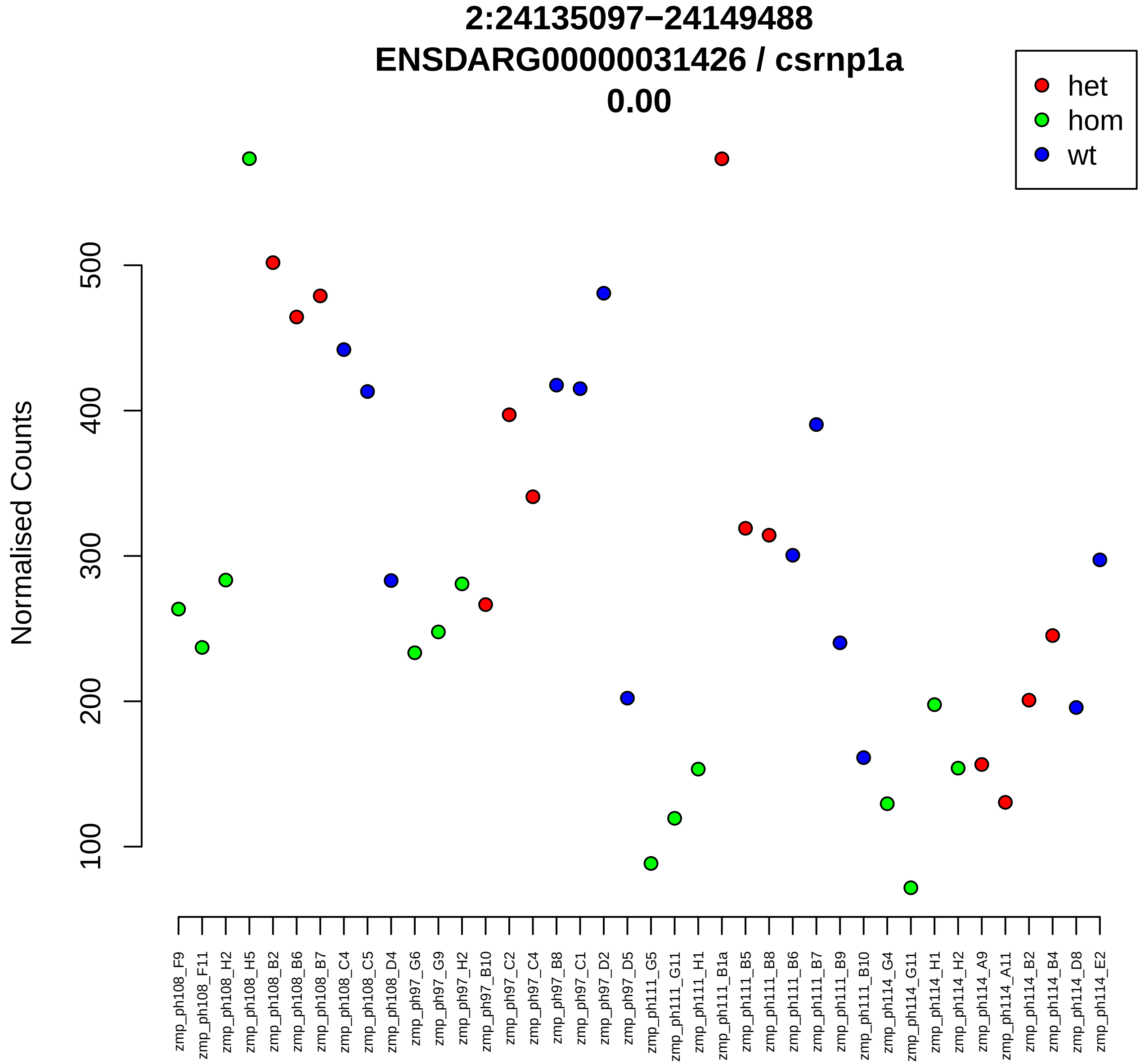

2:24135097−24149488
ENSDARG00000031426 / csrnp1a
0.00
het
hom
wt
Normalised Counts
100
200
300
400
500
zmp_ph108_F9
zmp_ph108_F11
zmp_ph108_H2
zmp_ph108_H5
zmp_ph108_B2
zmp_ph108_B6
zmp_ph108_B7
zmp_ph108_C4
zmp_ph108_C5
zmp_ph108_D4
zmp_ph97_G6
zmp_ph97_G9
zmp_ph97_H2
zmp_ph97_B10
zmp_ph97_C2
zmp_ph97_C4
zmp_ph97_B8
zmp_ph97_C1
zmp_ph97_D2
zmp_ph97_D5
zmp_ph111_G5
zmp_ph111_G11
zmp_ph111_H1
zmp_ph111_B1a
zmp_ph111_B5
zmp_ph111_B8
zmp_ph111_B6
zmp_ph111_B7
zmp_ph111_B9
zmp_ph111_B10
zmp_ph114_G4
zmp_ph114_G11
zmp_ph114_H1
zmp_ph114_H2
zmp_ph114_A9
zmp_ph114_A11
zmp_ph114_B2
zmp_ph114_B4
zmp_ph114_D8
zmp_ph114_E2

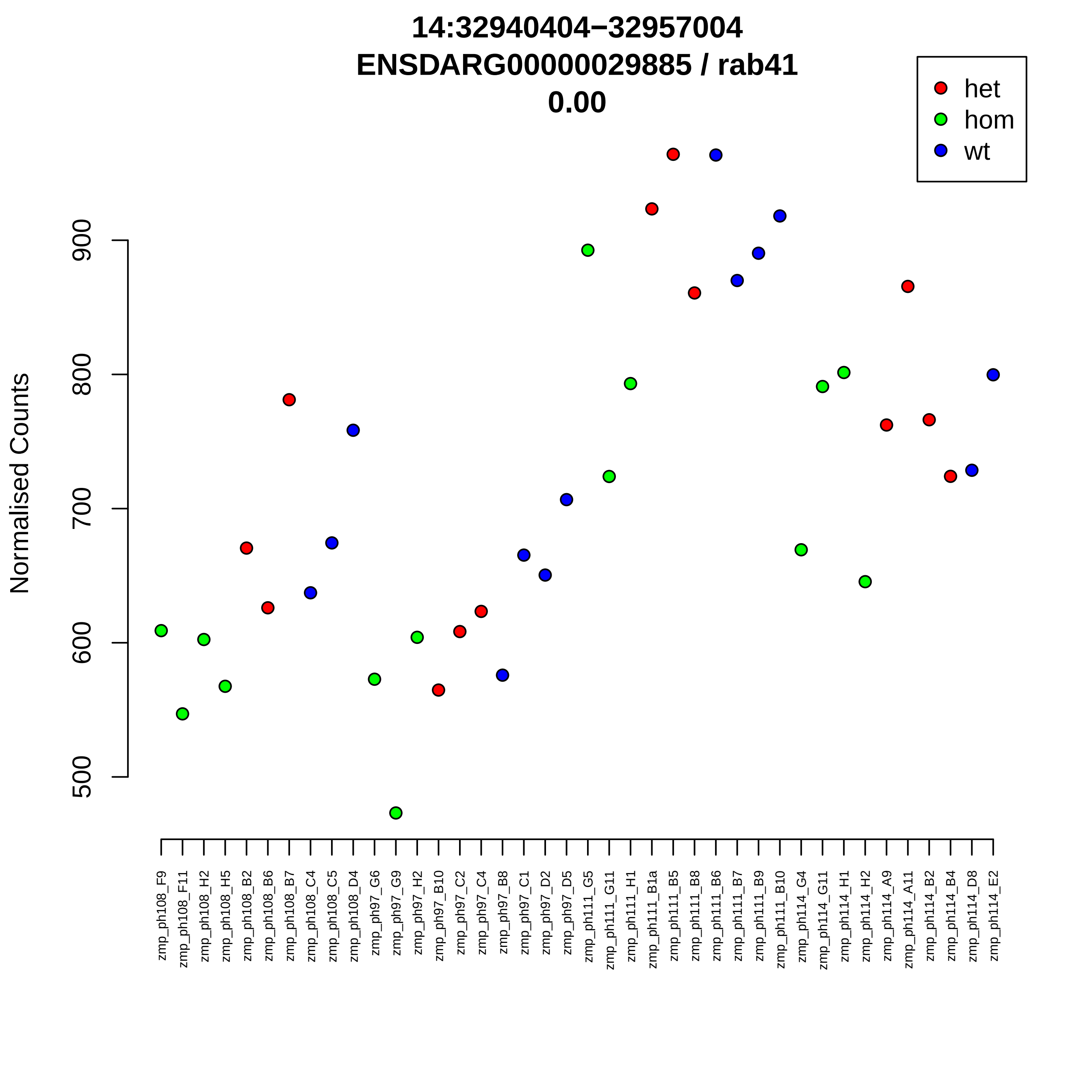

14:32940404−32957004
ENSDARG00000029885 / rab41
0.00
het
hom
wt
Normalised Counts
500
600
700
800
900
zmp_ph108_F9
zmp_ph108_F11
zmp_ph108_H2
zmp_ph108_H5
zmp_ph108_B2
zmp_ph108_B6
zmp_ph108_B7
zmp_ph108_C4
zmp_ph108_C5
zmp_ph108_D4
zmp_ph97_G6
zmp_ph97_G9
zmp_ph97_H2
zmp_ph97_B10
zmp_ph97_C2
zmp_ph97_C4
zmp_ph97_B8
zmp_ph97_C1
zmp_ph97_D2
zmp_ph97_D5
zmp_ph111_G5
zmp_ph111_G11
zmp_ph111_H1
zmp_ph111_B1a
zmp_ph111_B5
zmp_ph111_B8
zmp_ph111_B6
zmp_ph111_B7
zmp_ph111_B9
zmp_ph111_B10
zmp_ph114_G4
zmp_ph114_G11
zmp_ph114_H1
zmp_ph114_H2
zmp_ph114_A9
zmp_ph114_A11
zmp_ph114_B2
zmp_ph114_B4
zmp_ph114_D8
zmp_ph114_E2

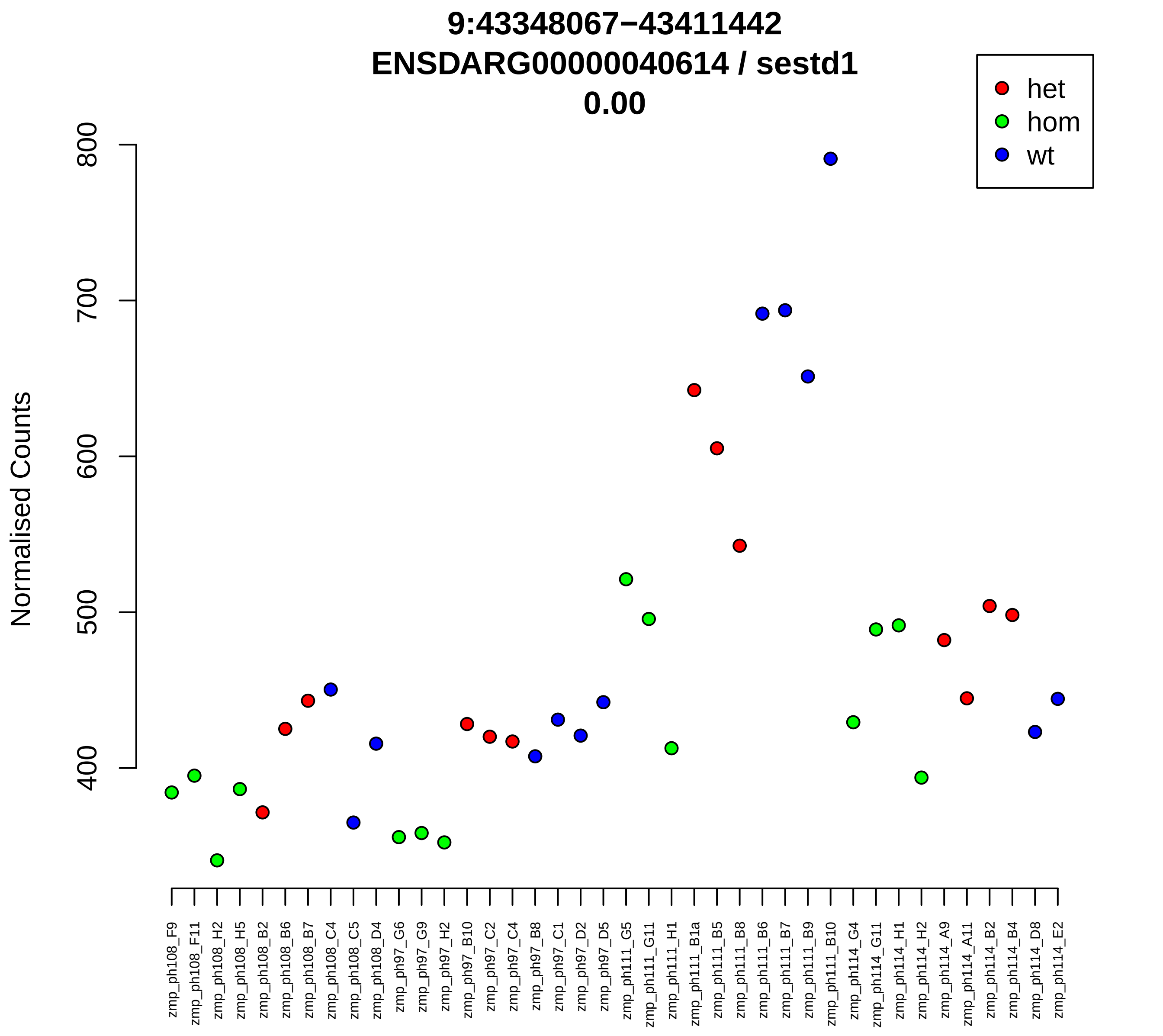
9:43348067−43411442
ENSDARG00000040614 / sestd1
0.00
het
hom
wt
Normalised Counts
400
500
600
700
800
zmp_ph108_F9
zmp_ph108_F11
zmp_ph108_H2
zmp_ph108_H5
zmp_ph108_B2
zmp_ph108_B6
zmp_ph108_B7
zmp_ph108_C4
zmp_ph108_C5
zmp_ph108_D4
zmp_ph97_G6
zmp_ph97_G9
zmp_ph97_H2
zmp_ph97_B10
zmp_ph97_C2
zmp_ph97_C4
zmp_ph97_B8
zmp_ph97_C1
zmp_ph97_D2
zmp_ph97_D5
zmp_ph111_G5
zmp_ph111_G11
zmp_ph111_H1
zmp_ph111_B1a
zmp_ph111_B5
zmp_ph111_B8
zmp_ph111_B6
zmp_ph111_B7
zmp_ph111_B9
zmp_ph111_B10
zmp_ph114_G4
zmp_ph114_G11
zmp_ph114_H1
zmp_ph114_H2
zmp_ph114_A9
zmp_ph114_A11
zmp_ph114_B2
zmp_ph114_B4
zmp_ph114_D8
zmp_ph114_E2

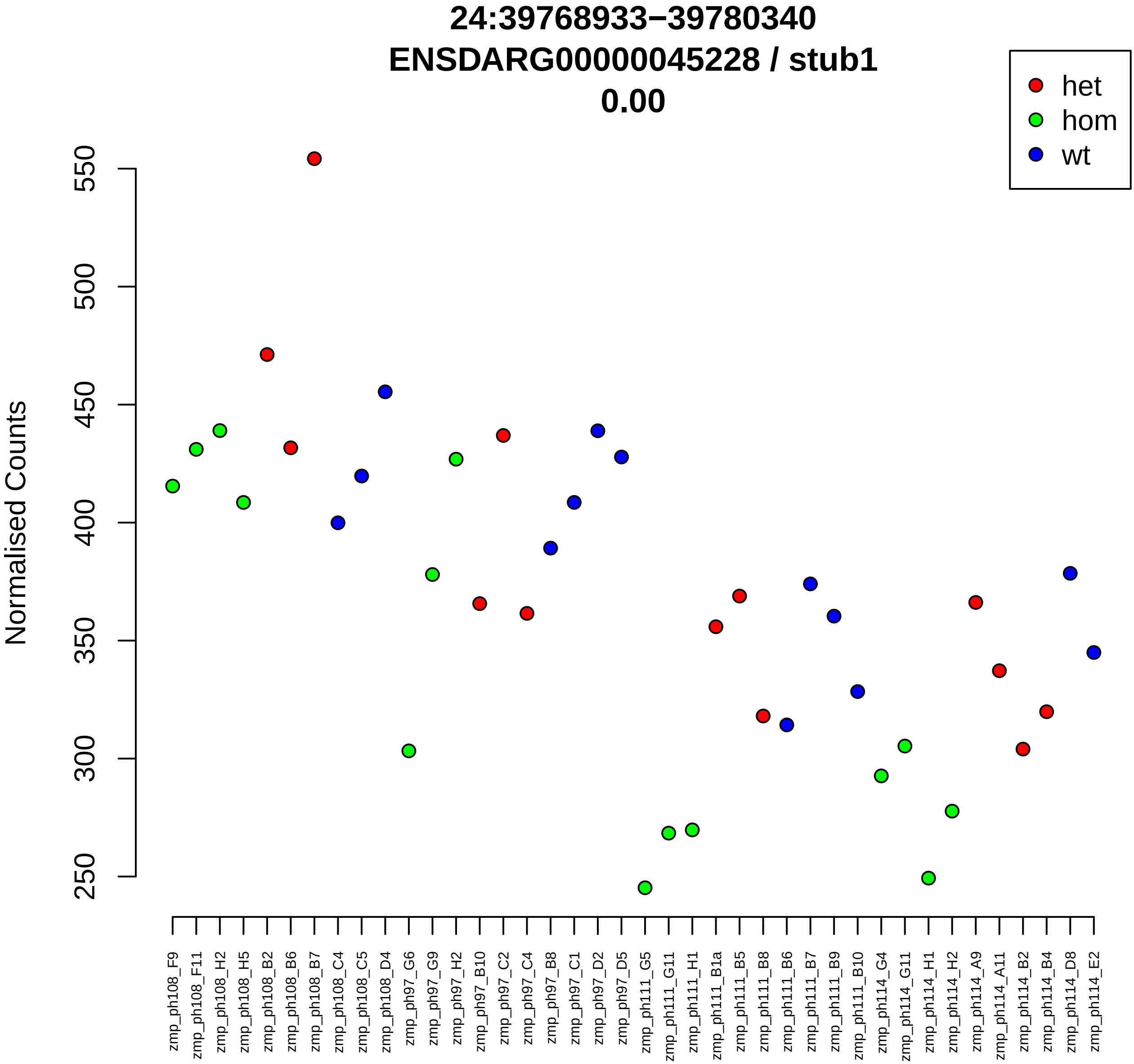
24:39768933−39780340
ENSDARG00000045228 / stub1
0.00
het
hom
wt
Normalised Counts
250
300
350
400
450
500
550
zmp_ph108_F9
zmp_ph108_F11
zmp_ph108_H2
zmp_ph108_H5
zmp_ph108_B2
zmp_ph108_B6
zmp_ph108_B7
zmp_ph108_C4
zmp_ph108_C5
zmp_ph108_D4
zmp_ph97_G6
zmp_ph97_G9
zmp_ph97_H2
zmp_ph97_B10
zmp_ph97_C2
zmp_ph97_C4
zmp_ph97_B8
zmp_ph97_C1
zmp_ph97_D2
zmp_ph97_D5
zmp_ph111_G5
zmp_ph111_G11
zmp_ph111_H1
zmp_ph111_B1a
zmp_ph111_B5
zmp_ph111_B8
zmp_ph111_B6
zmp_ph111_B7
zmp_ph111_B9
zmp_ph111_B10
zmp_ph114_G4
zmp_ph114_G11
zmp_ph114_H1
zmp_ph114_H2
zmp_ph114_A9
zmp_ph114_A11
zmp_ph114_B2
zmp_ph114_B4
zmp_ph114_D8
zmp_ph114_E2

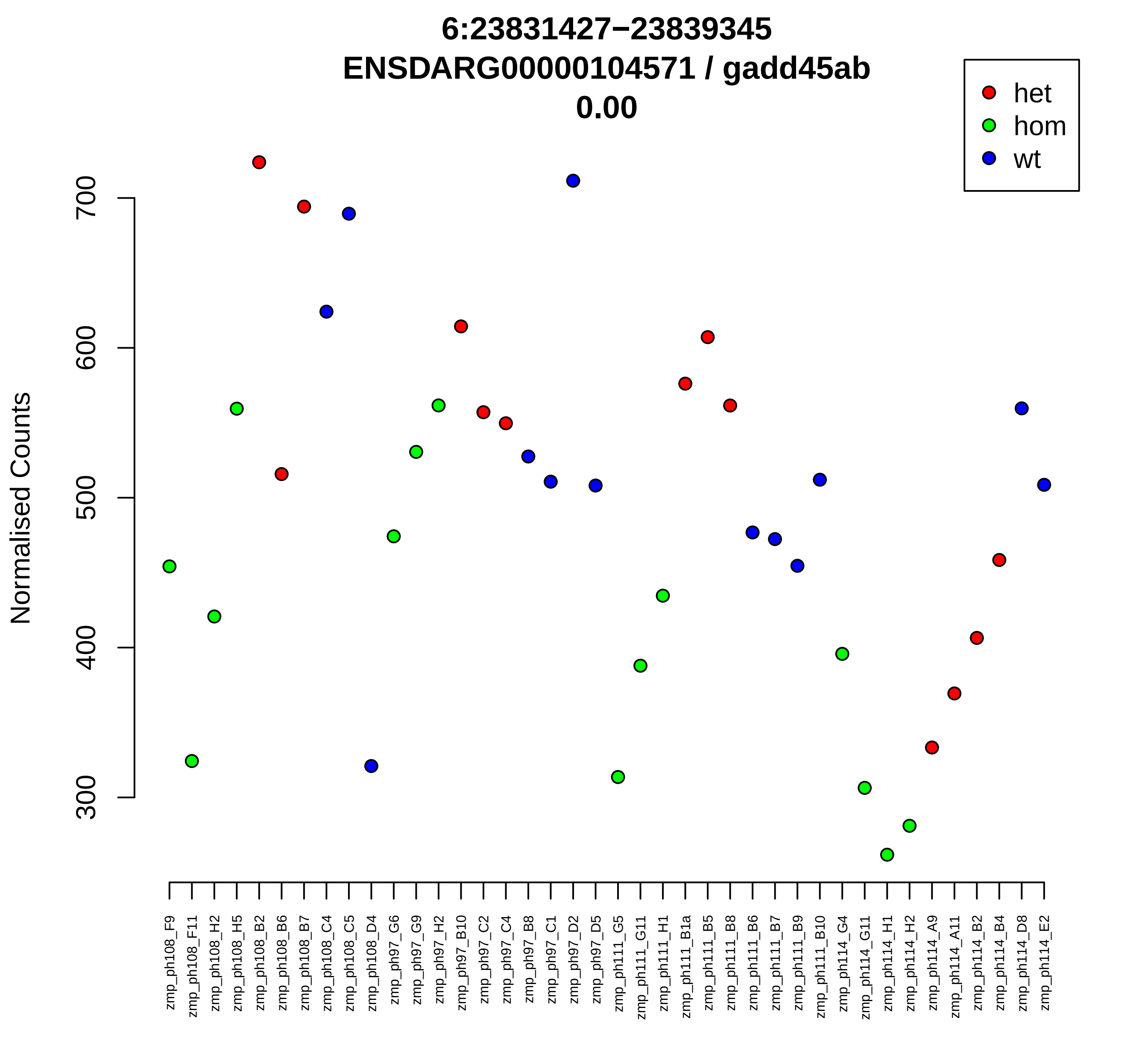
6:23831427−23839345
ENSDARG00000104571 / gadd45ab
0.00
het
hom
wt
Normalised Counts
300
400
500
600
700
zmp_ph108_F9
zmp_ph108_F11
zmp_ph108_H2
zmp_ph108_H5
zmp_ph108_B2
zmp_ph108_B6
zmp_ph108_B7
zmp_ph108_C4
zmp_ph108_C5
zmp_ph108_D4
zmp_ph97_G6
zmp_ph97_G9
zmp_ph97_H2
zmp_ph97_B10
zmp_ph97_C2
zmp_ph97_C4
zmp_ph97_B8
zmp_ph97_C1
zmp_ph97_D2
zmp_ph97_D5
zmp_ph111_G5
zmp_ph111_G11
zmp_ph111_H1
zmp_ph111_B1a
zmp_ph111_B5
zmp_ph111_B8
zmp_ph111_B6
zmp_ph111_B7
zmp_ph111_B9
zmp_ph111_B10
zmp_ph114_G4
zmp_ph114_G11
zmp_ph114_H1
zmp_ph114_H2
zmp_ph114_A9
zmp_ph114_A11
zmp_ph114_B2
zmp_ph114_B4
zmp_ph114_D8
zmp_ph114_E2

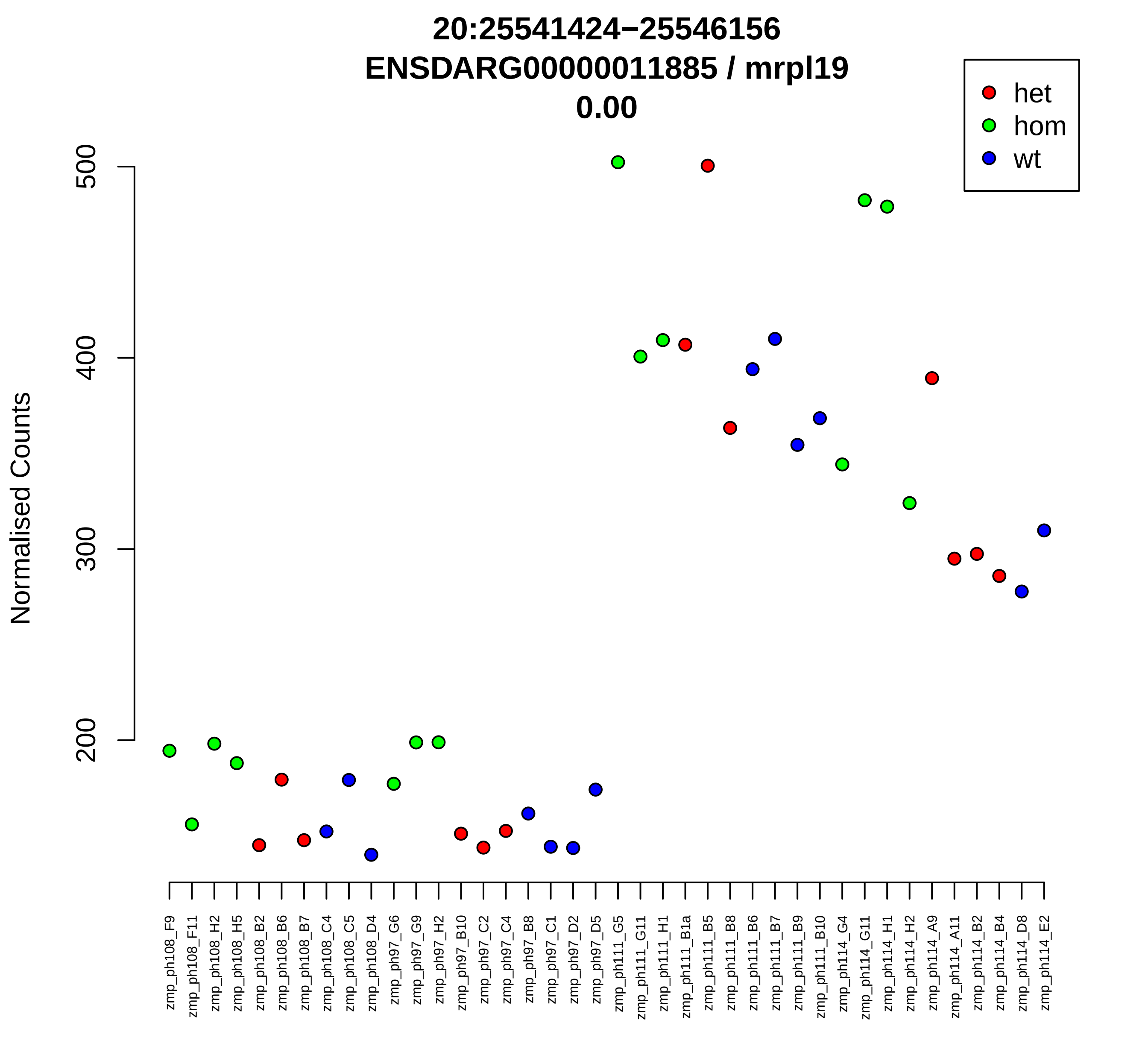

20:25541424−25546156
ENSDARG00000011885 / mrpl19
0.00
het
hom
wt
Normalised Counts
500
400
300
200
zmp_ph108_F9
zmp_ph108_F11
zmp_ph108_H2
zmp_ph108_H5
zmp_ph108_B2
zmp_ph108_B6
zmp_ph108_B7
zmp_ph108_C4
zmp_ph108_C5
zmp_ph108_D4
zmp_ph97_G6
zmp_ph97_G9
zmp_ph97_H2
zmp_ph97_B10
zmp_ph97_C2
zmp_ph97_C4
zmp_ph97_B8
zmp_ph97_C1
zmp_ph97_D2
zmp_ph97_D5
zmp_ph111_G5
zmp_ph111_G11
zmp_ph111_H1
zmp_ph111_B1a
zmp_ph111_B5
zmp_ph111_B8
zmp_ph111_B6
zmp_ph111_B7
zmp_ph111_B9
zmp_ph111_B10
zmp_ph114_G4
zmp_ph114_G11
zmp_ph114_H1
zmp_ph114_H2
zmp_ph114_A9
zmp_ph114_A11
zmp_ph114_B2
zmp_ph114_B4
zmp_ph114_D8
zmp_ph114_E2

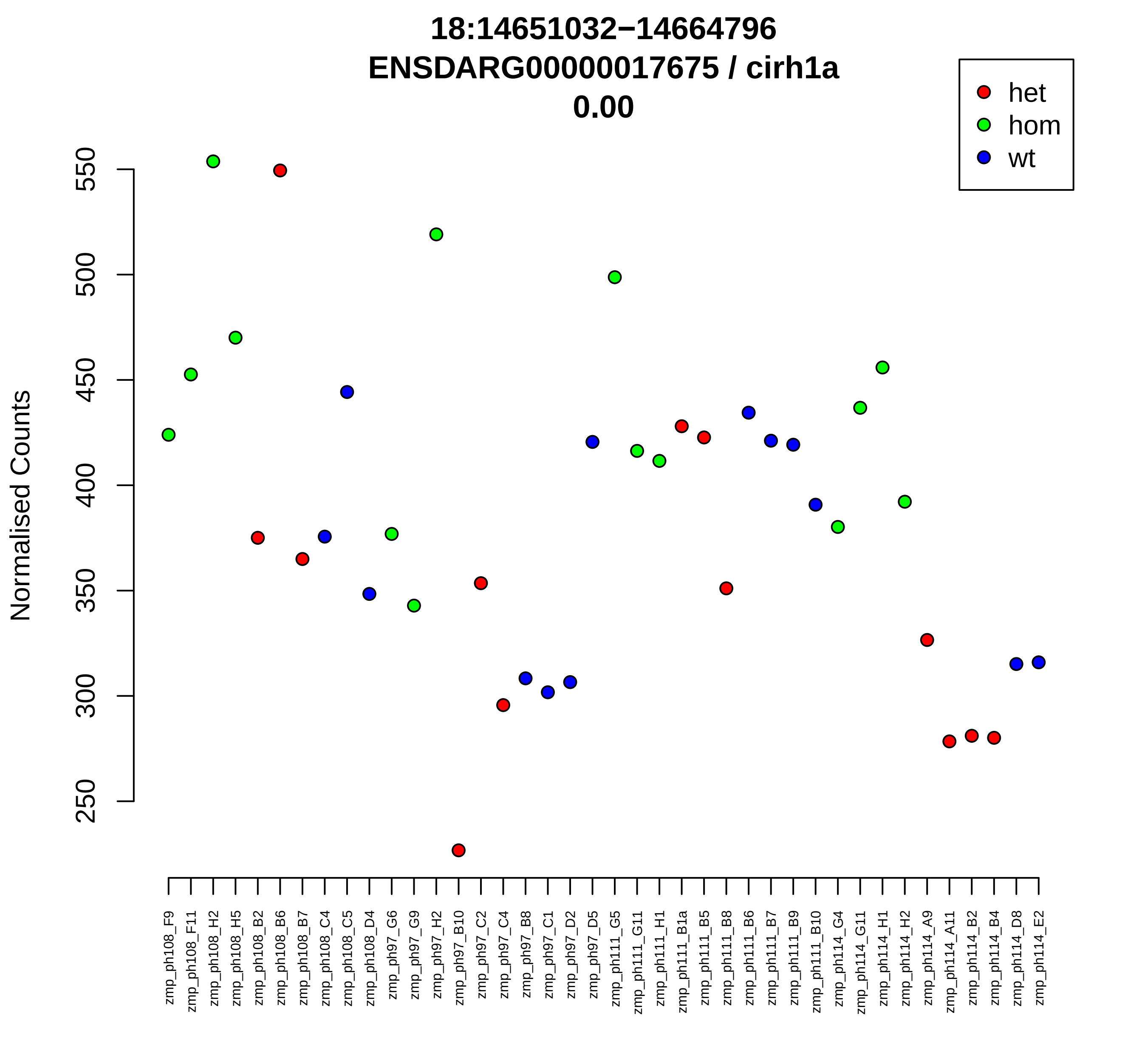

18:14651032−14664796
ENSDARG00000017675 / cirh1a
0.00
het
hom
wt
Normalised Counts
250
300
350
400
450
500
550
zmp_ph108_F9
zmp_ph108_F11
zmp_ph108_H2
zmp_ph108_H5
zmp_ph108_B2
zmp_ph108_B6
zmp_ph108_B7
zmp_ph108_C4
zmp_ph108_C5
zmp_ph108_D4
zmp_ph97_G6
zmp_ph97_G9
zmp_ph97_H2
zmp_ph97_B10
zmp_ph97_C2
zmp_ph97_C4
zmp_ph97_B8
zmp_ph97_C1
zmp_ph97_D2
zmp_ph97_D5
zmp_ph111_G5
zmp_ph111_G11
zmp_ph111_H1
zmp_ph111_B1a
zmp_ph111_B5
zmp_ph111_B8
zmp_ph111_B6
zmp_ph111_B7
zmp_ph111_B9
zmp_ph111_B10
zmp_ph114_G4
zmp_ph114_G11
zmp_ph114_H1
zmp_ph114_H2
zmp_ph114_A9
zmp_ph114_A11
zmp_ph114_B2
zmp_ph114_B4
zmp_ph114_D8
zmp_ph114_E2

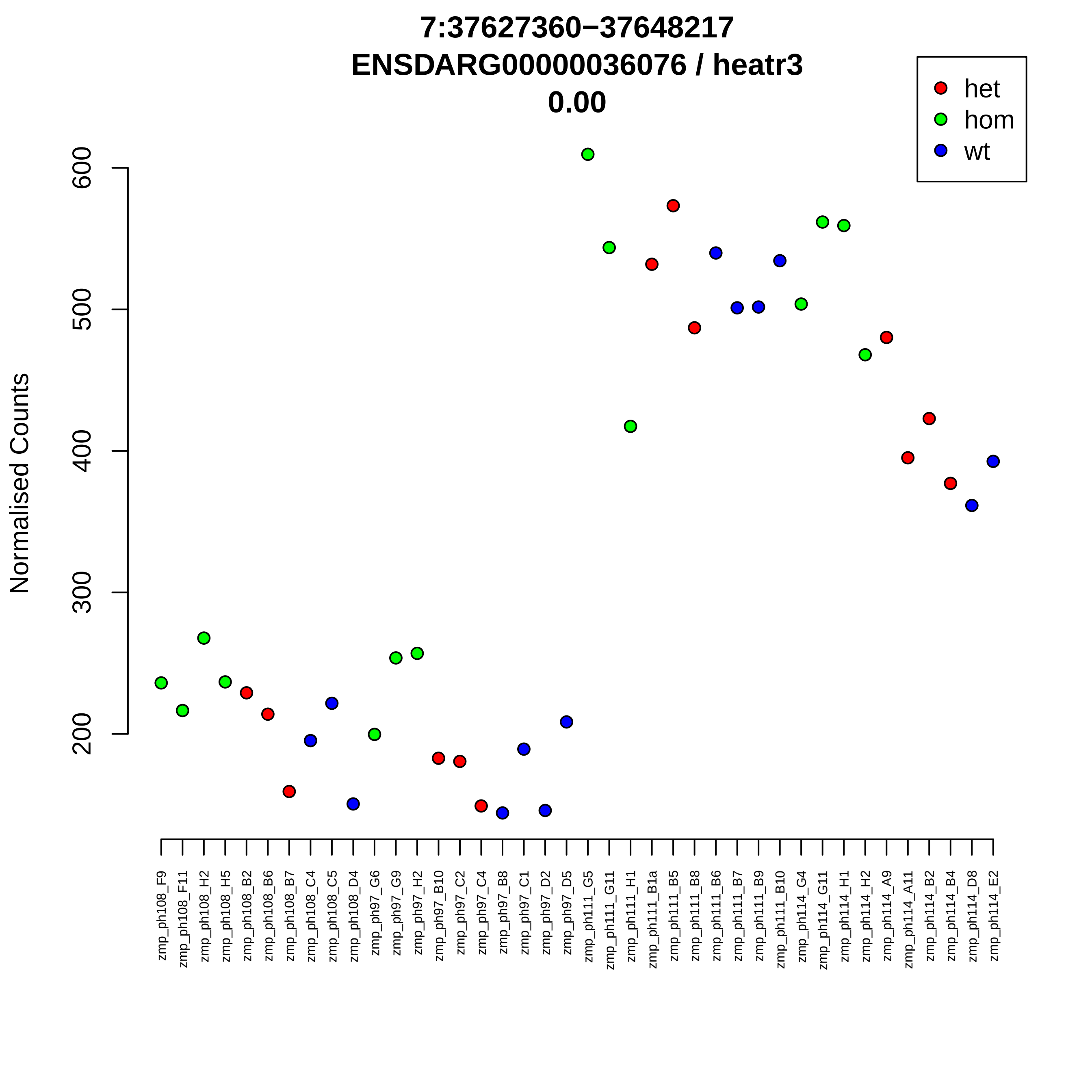

7:37627360−37648217
ENSDARG00000036076 / heatr3
0.00
het
hom
wt
Normalised Counts
200
300
400
500
600
zmp_ph108_F9
zmp_ph108_F11
zmp_ph108_H2
zmp_ph108_H5
zmp_ph108_B2
zmp_ph108_B6
zmp_ph108_B7
zmp_ph108_C4
zmp_ph108_C5
zmp_ph108_D4
zmp_ph97_G6
zmp_ph97_G9
zmp_ph97_H2
zmp_ph97_B10
zmp_ph97_C2
zmp_ph97_C4
zmp_ph97_B8
zmp_ph97_C1
zmp_ph97_D2
zmp_ph97_D5
zmp_ph111_G5
zmp_ph111_G11
zmp_ph111_H1
zmp_ph111_B1a
zmp_ph111_B5
zmp_ph111_B8
zmp_ph111_B6
zmp_ph111_B7
zmp_ph111_B9
zmp_ph111_B10
zmp_ph114_G4
zmp_ph114_G11
zmp_ph114_H1
zmp_ph114_H2
zmp_ph114_A9
zmp_ph114_A11
zmp_ph114_B2
zmp_ph114_B4
zmp_ph114_D8
zmp_ph114_E2

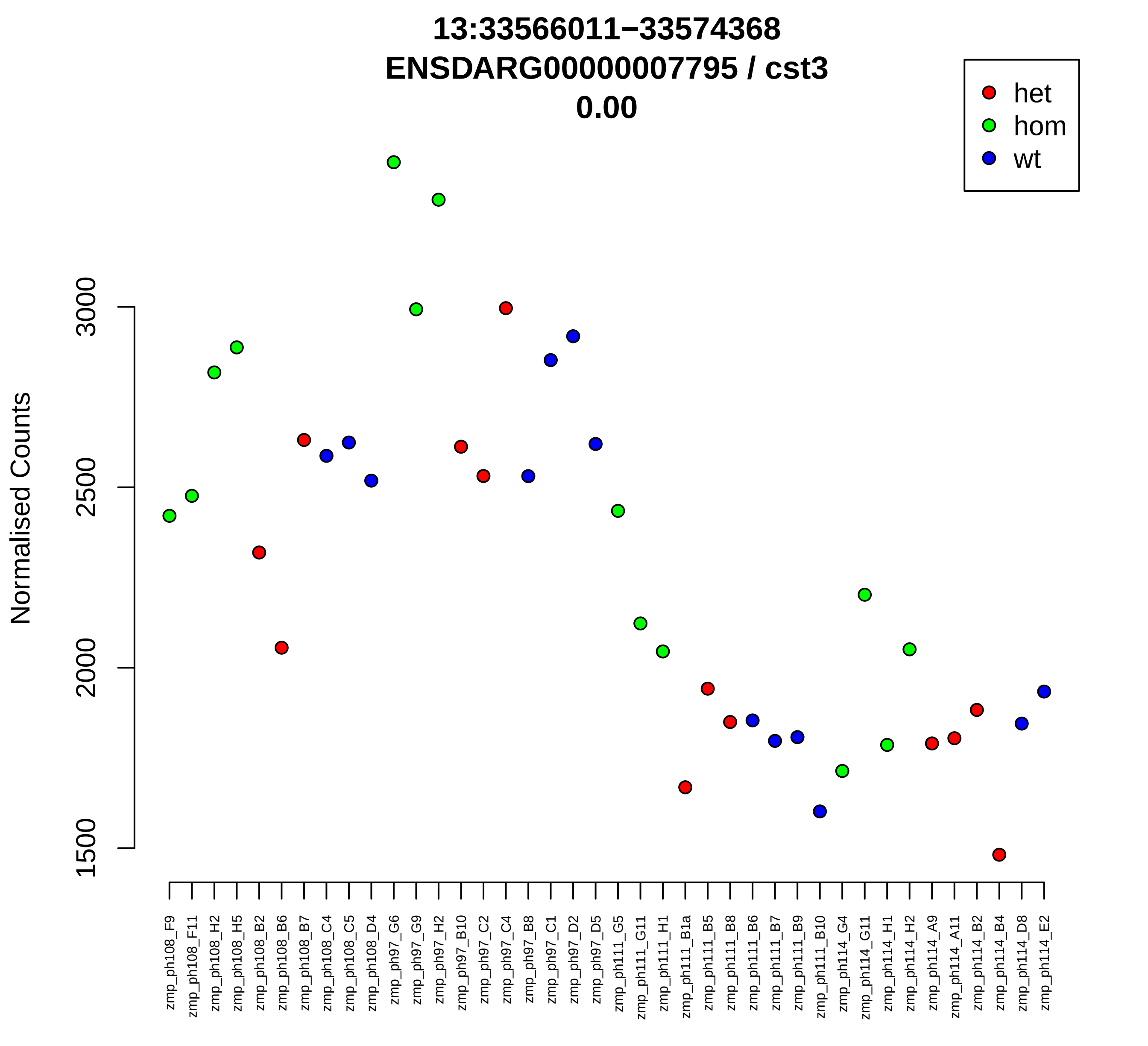

13:33566011−33574368
ENSDARG00000007795 / cst3
0.00
het
hom
wt
Normalised Counts
1500
2000
2500
3000
zmp_ph108_F9
zmp_ph108_F11
zmp_ph108_H2
zmp_ph108_H5
zmp_ph108_B2
zmp_ph108_B6
zmp_ph108_B7
zmp_ph108_C4
zmp_ph108_C5
zmp_ph108_D4
zmp_ph97_G6
zmp_ph97_G9
zmp_ph97_H2
zmp_ph97_B10
zmp_ph97_C2
zmp_ph97_C4
zmp_ph97_B8
zmp_ph97_C1
zmp_ph97_D2
zmp_ph97_D5
zmp_ph111_G5
zmp_ph111_G11
zmp_ph111_H1
zmp_ph111_B1a
zmp_ph111_B5
zmp_ph111_B8
zmp_ph111_B6
zmp_ph111_B7
zmp_ph111_B9
zmp_ph111_B10
zmp_ph114_G4
zmp_ph114_G11
zmp_ph114_H1
zmp_ph114_H2
zmp_ph114_A9
zmp_ph114_A11
zmp_ph114_B2
zmp_ph114_B4
zmp_ph114_D8
zmp_ph114_E2

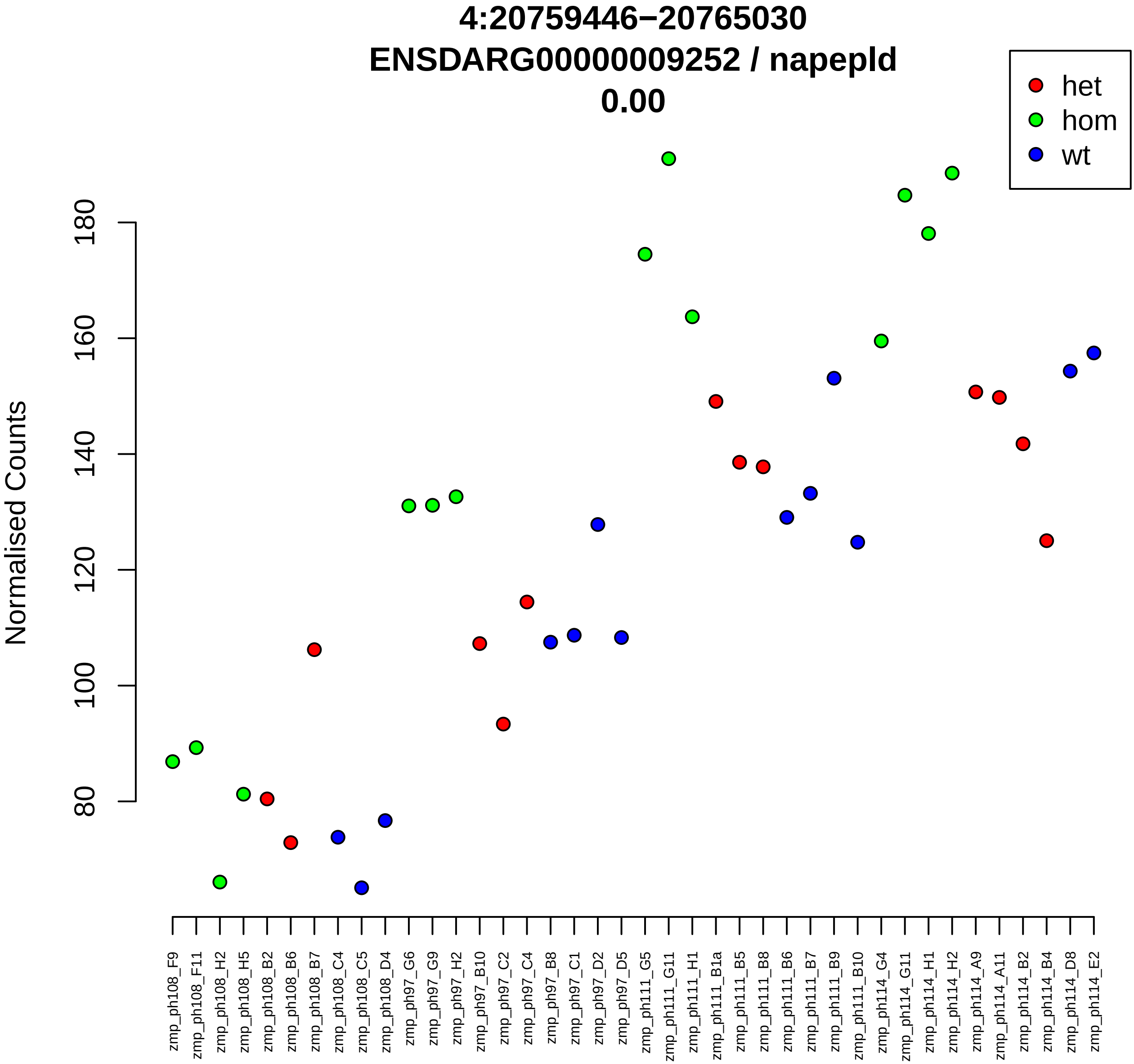
4:20759446−20765030
ENSDARG00000009252 / napepld
0.00
het
hom
wt
Normalised Counts
80
100
120
140
160
180
zmp_ph108_F9
zmp_ph108_F11
zmp_ph108_H2
zmp_ph108_H5
zmp_ph108_B2
zmp_ph108_B6
zmp_ph108_B7
zmp_ph108_C4
zmp_ph108_C5
zmp_ph108_D4
zmp_ph97_G6
zmp_ph97_G9
zmp_ph97_H2
zmp_ph97_B10
zmp_ph97_C2
zmp_ph97_C4
zmp_ph97_B8
zmp_ph97_C1
zmp_ph97_D2
zmp_ph97_D5
zmp_ph111_G5
zmp_ph111_G11
zmp_ph111_H1
zmp_ph111_B1a
zmp_ph111_B5
zmp_ph111_B8
zmp_ph111_B6
zmp_ph111_B7
zmp_ph111_B9
zmp_ph111_B10
zmp_ph114_G4
zmp_ph114_G11
zmp_ph114_H1
zmp_ph114_H2
zmp_ph114_A9
zmp_ph114_A11
zmp_ph114_B2
zmp_ph114_B4
zmp_ph114_D8
zmp_ph114_E2

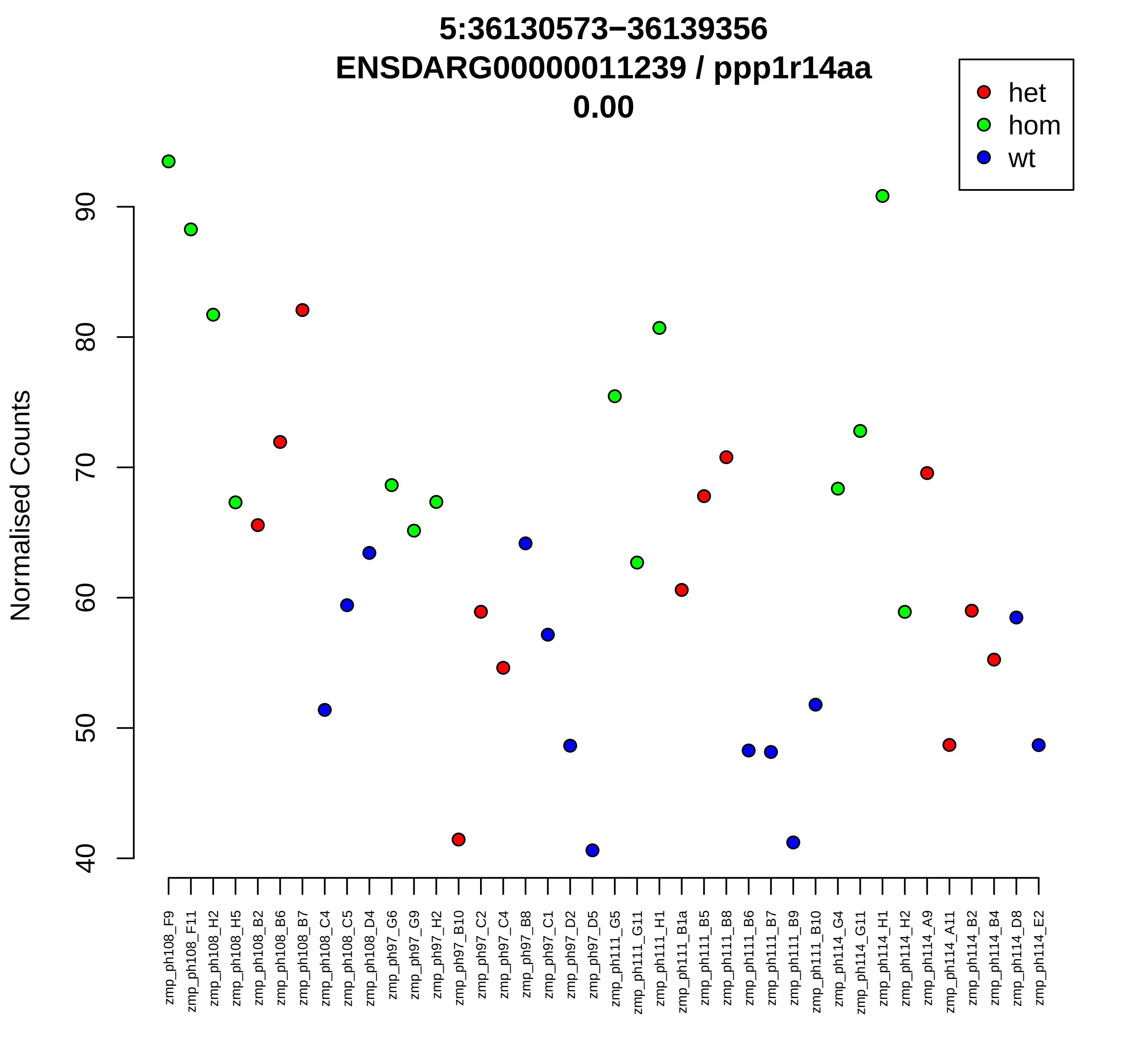
5:36130573−36139356
ENSDARG00000011239 / ppp1r14aa
0.00
het
hom
wt
Normalised Counts
40
50
60
70
80
90
zmp_ph108_F9
zmp_ph108_F11
zmp_ph108_H2
zmp_ph108_H5
zmp_ph108_B2
zmp_ph108_B6
zmp_ph108_B7
zmp_ph108_C4
zmp_ph108_C5
zmp_ph108_D4
zmp_ph97_G6
zmp_ph97_G9
zmp_ph97_H2
zmp_ph97_B10
zmp_ph97_C2
zmp_ph97_C4
zmp_ph97_B8
zmp_ph97_C1
zmp_ph97_D2
zmp_ph97_D5
zmp_ph111_G5
zmp_ph111_G11
zmp_ph111_H1
zmp_ph111_B1a
zmp_ph111_B5
zmp_ph111_B8
zmp_ph111_B6
zmp_ph111_B7
zmp_ph111_B9
zmp_ph111_B10
zmp_ph114_G4
zmp_ph114_G11
zmp_ph114_H1
zmp_ph114_H2
zmp_ph114_A9
zmp_ph114_A11
zmp_ph114_B2
zmp_ph114_B4
zmp_ph114_D8
zmp_ph114_E2

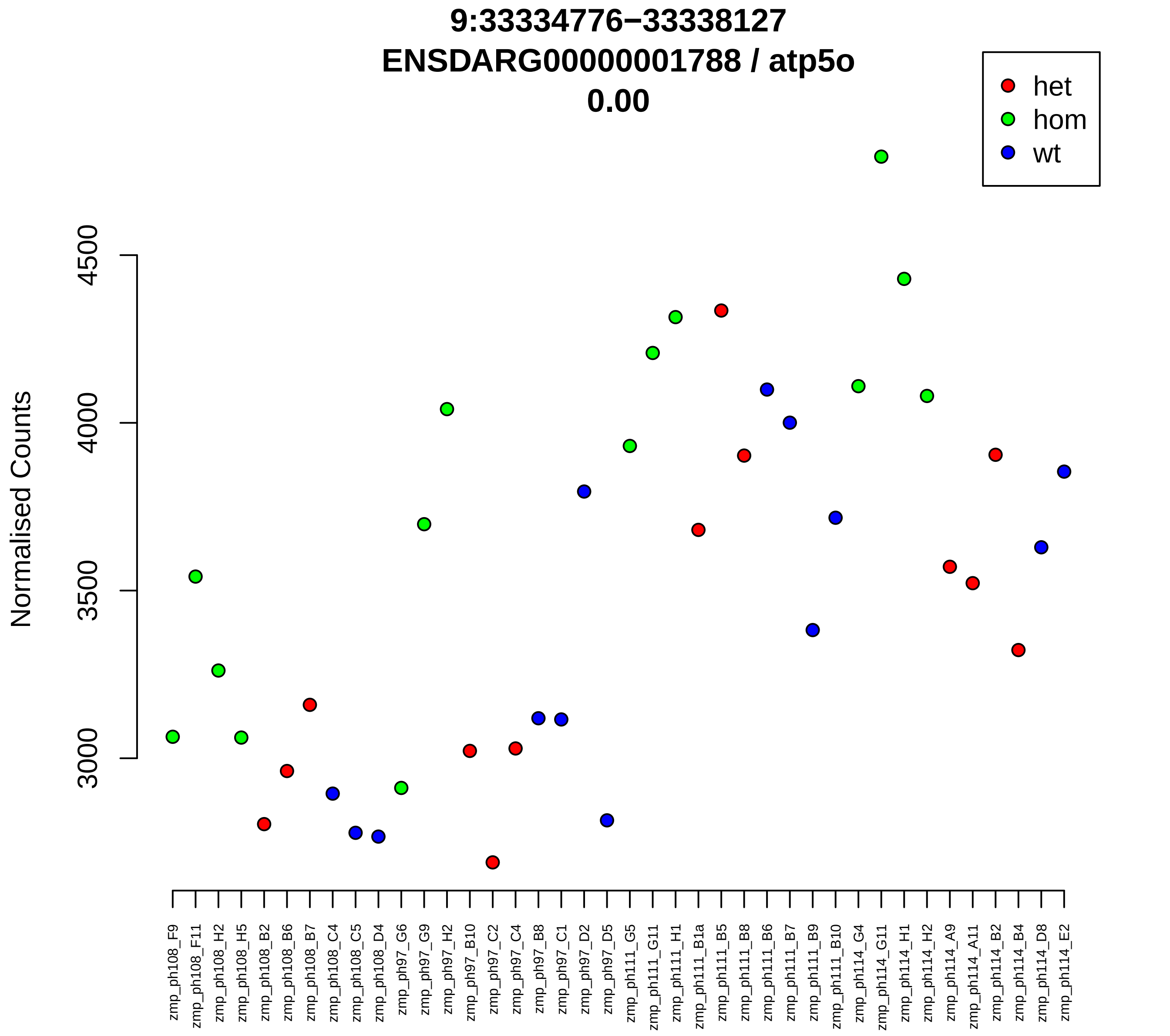

9:33334776−33338127
ENSDARG00000001788 / atp5o
0.00
het
hom
wt
Normalised Counts
3000
3500
4000
4500
zmp_ph108_F9
zmp_ph108_F11
zmp_ph108_H2
zmp_ph108_H5
zmp_ph108_B2
zmp_ph108_B6
zmp_ph108_B7
zmp_ph108_C4
zmp_ph108_C5
zmp_ph108_D4
zmp_ph97_G6
zmp_ph97_G9
zmp_ph97_H2
zmp_ph97_B10
zmp_ph97_C2
zmp_ph97_C4
zmp_ph97_B8
zmp_ph97_C1
zmp_ph97_D2
zmp_ph97_D5
zmp_ph111_G5
zmp_ph111_G11
zmp_ph111_H1
zmp_ph111_B1a
zmp_ph111_B5
zmp_ph111_B8
zmp_ph111_B6
zmp_ph111_B7
zmp_ph111_B9
zmp_ph111_B10
zmp_ph114_G4
zmp_ph114_G11
zmp_ph114_H1
zmp_ph114_H2
zmp_ph114_A9
zmp_ph114_A11
zmp_ph114_B2
zmp_ph114_B4
zmp_ph114_D8
zmp_ph114_E2

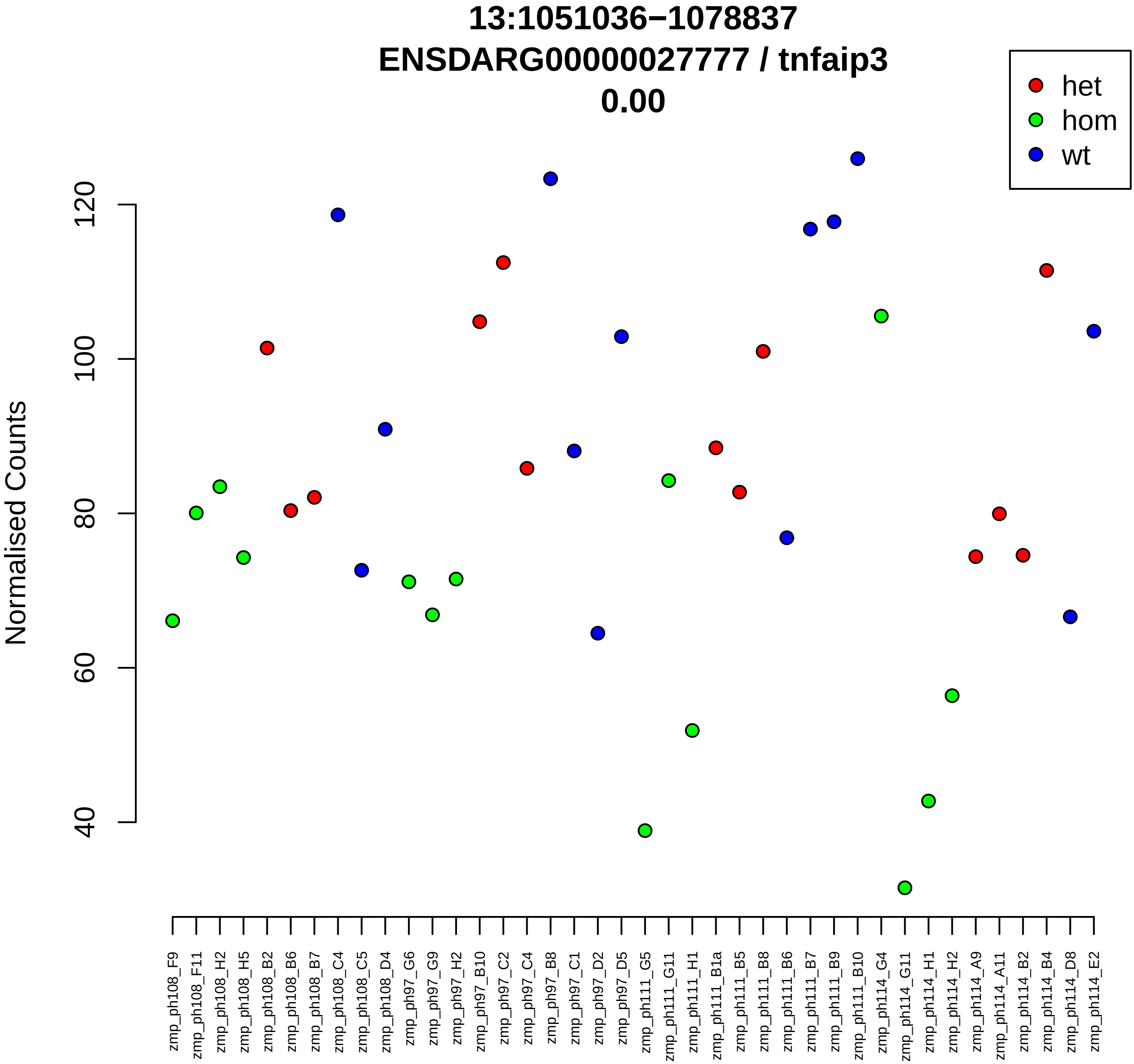

13:1051036−1078837
ENSDARG00000027777 / tnfaip3
0.00
het
hom
wt
Normalised Counts
40
60
80
100
120
zmp_ph108_F9
zmp_ph108_F11
zmp_ph108_H2
zmp_ph108_H5
zmp_ph108_B2
zmp_ph108_B6
zmp_ph108_B7
zmp_ph108_C4
zmp_ph108_C5
zmp_ph108_D4
zmp_ph97_G6
zmp_ph97_G9
zmp_ph97_H2
zmp_ph97_B10
zmp_ph97_C2
zmp_ph97_C4
zmp_ph97_B8
zmp_ph97_C1
zmp_ph97_D2
zmp_ph97_D5
zmp_ph111_G5
zmp_ph111_G11
zmp_ph111_H1
zmp_ph111_B1a
zmp_ph111_B5
zmp_ph111_B8
zmp_ph111_B6
zmp_ph111_B7
zmp_ph111_B9
zmp_ph111_B10
zmp_ph114_G4
zmp_ph114_G11
zmp_ph114_H1
zmp_ph114_H2
zmp_ph114_A9
zmp_ph114_A11
zmp_ph114_B2
zmp_ph114_B4
zmp_ph114_D8
zmp_ph114_E2

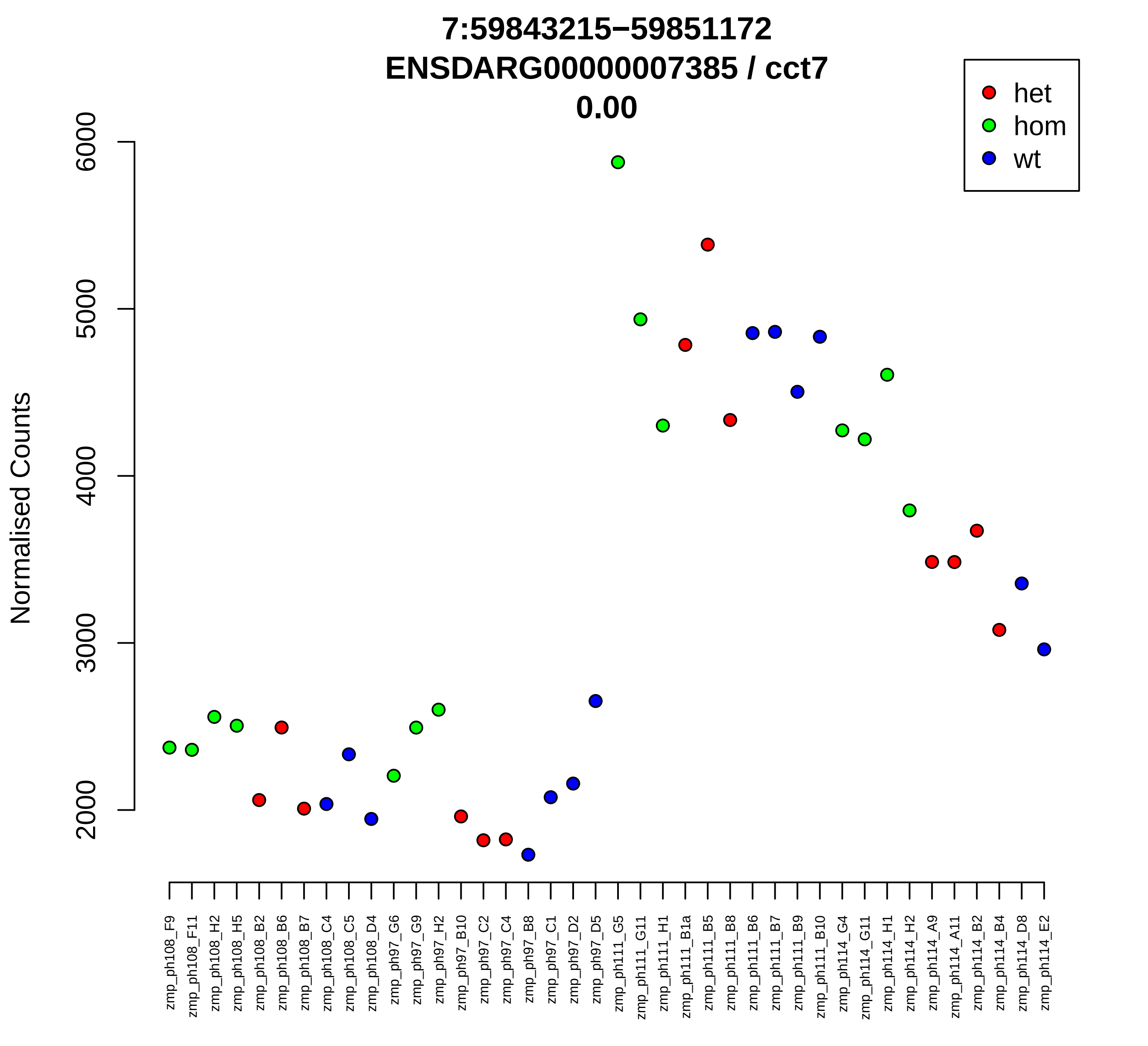
7:59843215−59851172
ENSDARG00000007385 / cct7
0.00
het
hom
wt
Normalised Counts
2000
3000
4000
5000
6000
zmp_ph108_F9
zmp_ph108_F11
zmp_ph108_H2
zmp_ph108_H5
zmp_ph108_B2
zmp_ph108_B6
zmp_ph108_B7
zmp_ph108_C4
zmp_ph108_C5
zmp_ph108_D4
zmp_ph97_G6
zmp_ph97_G9
zmp_ph97_H2
zmp_ph97_B10
zmp_ph97_C2
zmp_ph97_C4
zmp_ph97_B8
zmp_ph97_C1
zmp_ph97_D2
zmp_ph97_D5
zmp_ph111_G5
zmp_ph111_G11
zmp_ph111_H1
zmp_ph111_B1a
zmp_ph111_B5
zmp_ph111_B8
zmp_ph111_B6
zmp_ph111_B7
zmp_ph111_B9
zmp_ph111_B10
zmp_ph114_G4
zmp_ph114_G11
zmp_ph114_H1
zmp_ph114_H2
zmp_ph114_A9
zmp_ph114_A11
zmp_ph114_B2
zmp_ph114_B4
zmp_ph114_D8
zmp_ph114_E2

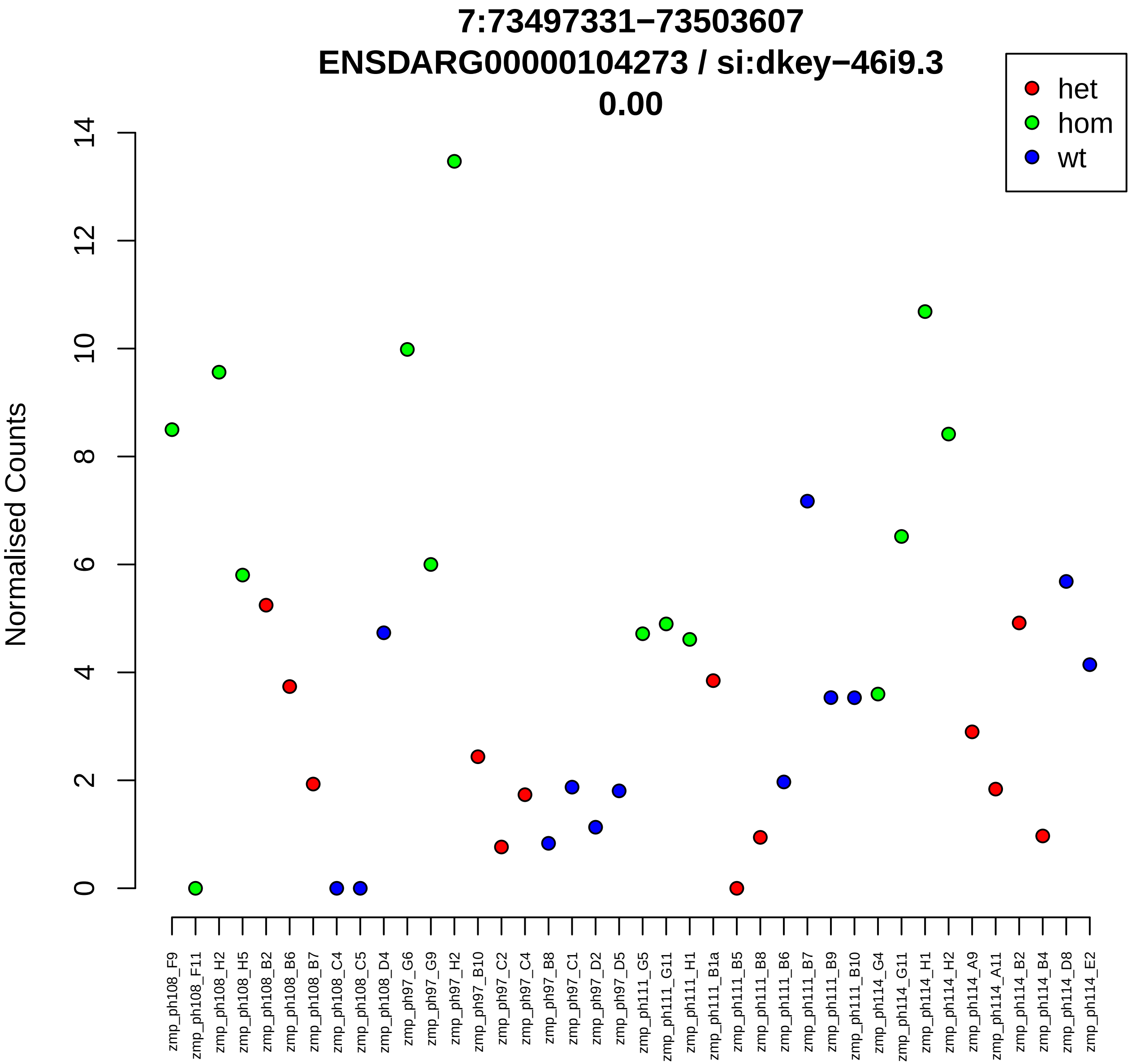
7:73497331−73503607
ENSDARG00000104273 / si:dkey−46i9.3
0.00
het
hom
wt
Normalised Counts
0
2
4
6
8
10
12
14
zmp_ph108_F9
zmp_ph108_F11
zmp_ph108_H2
zmp_ph108_H5
zmp_ph108_B2
zmp_ph108_B6
zmp_ph108_B7
zmp_ph108_C4
zmp_ph108_C5
zmp_ph108_D4
zmp_ph97_G6
zmp_ph97_G9
zmp_ph97_H2
zmp_ph97_B10
zmp_ph97_C2
zmp_ph97_C4
zmp_ph97_B8
zmp_ph97_C1
zmp_ph97_D2
zmp_ph97_D5
zmp_ph111_G5
zmp_ph111_G11
zmp_ph111_H1
zmp_ph111_B1a
zmp_ph111_B5
zmp_ph111_B8
zmp_ph111_B6
zmp_ph111_B7
zmp_ph111_B9
zmp_ph111_B10
zmp_ph114_G4
zmp_ph114_G11
zmp_ph114_H1
zmp_ph114_H2
zmp_ph114_A9
zmp_ph114_A11
zmp_ph114_B2
zmp_ph114_B4
zmp_ph114_D8
zmp_ph114_E2

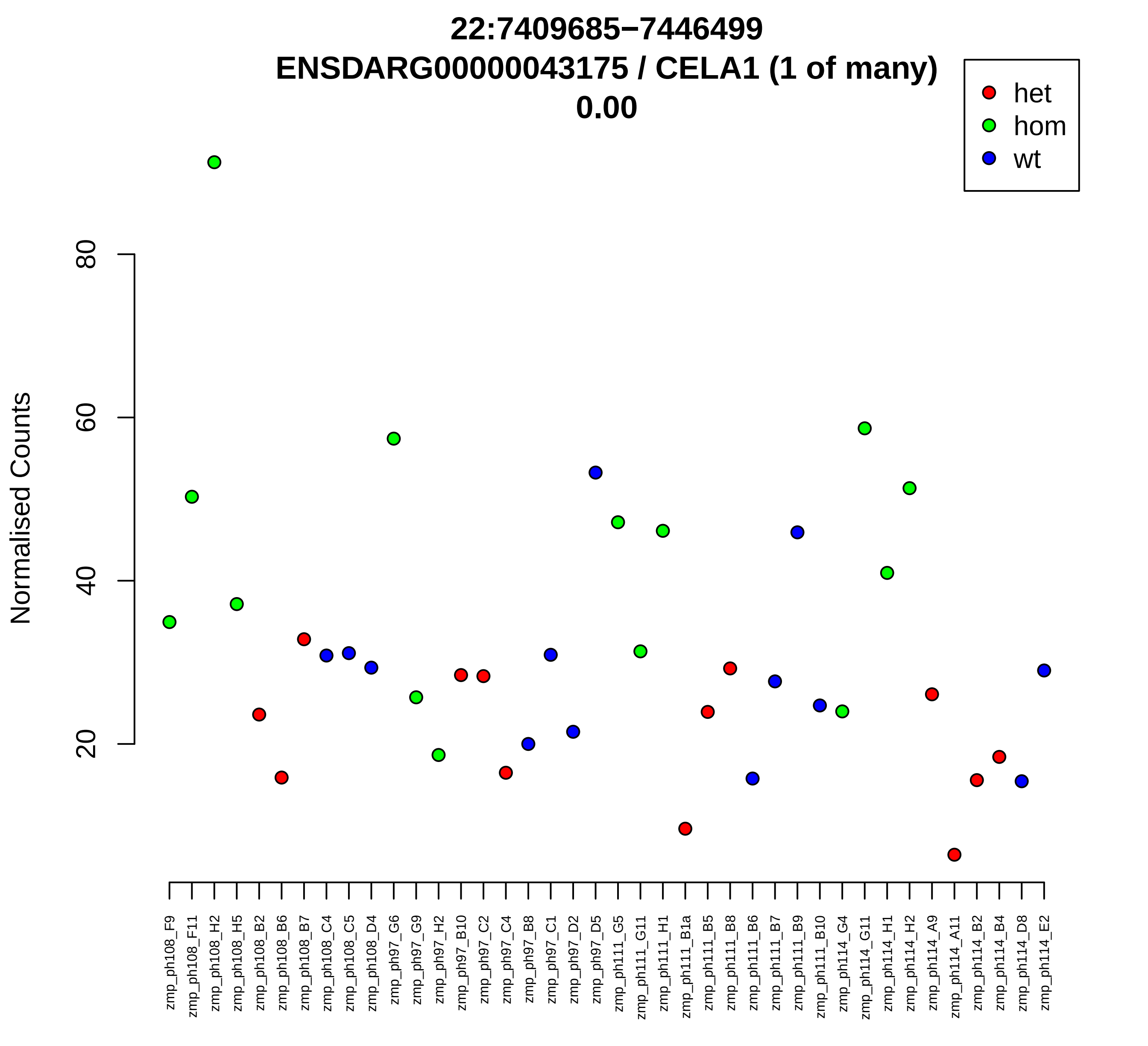

22:7409685−7446499
ENSDARG00000043175 / CELA1 (1 of many)
0.00
het
hom
wt
Normalised Counts
20
40
60
80
zmp_ph108_F9
zmp_ph108_F11
zmp_ph108_H2
zmp_ph108_H5
zmp_ph108_B2
zmp_ph108_B6
zmp_ph108_B7
zmp_ph108_C4
zmp_ph108_C5
zmp_ph108_D4
zmp_ph97_G6
zmp_ph97_G9
zmp_ph97_H2
zmp_ph97_B10
zmp_ph97_C2
zmp_ph97_C4
zmp_ph97_B8
zmp_ph97_C1
zmp_ph97_D2
zmp_ph97_D5
zmp_ph111_G5
zmp_ph111_G11
zmp_ph111_H1
zmp_ph111_B1a
zmp_ph111_B5
zmp_ph111_B8
zmp_ph111_B6
zmp_ph111_B7
zmp_ph111_B9
zmp_ph111_B10
zmp_ph114_G4
zmp_ph114_G11
zmp_ph114_H1
zmp_ph114_H2
zmp_ph114_A9
zmp_ph114_A11
zmp_ph114_B2
zmp_ph114_B4
zmp_ph114_D8
zmp_ph114_E2

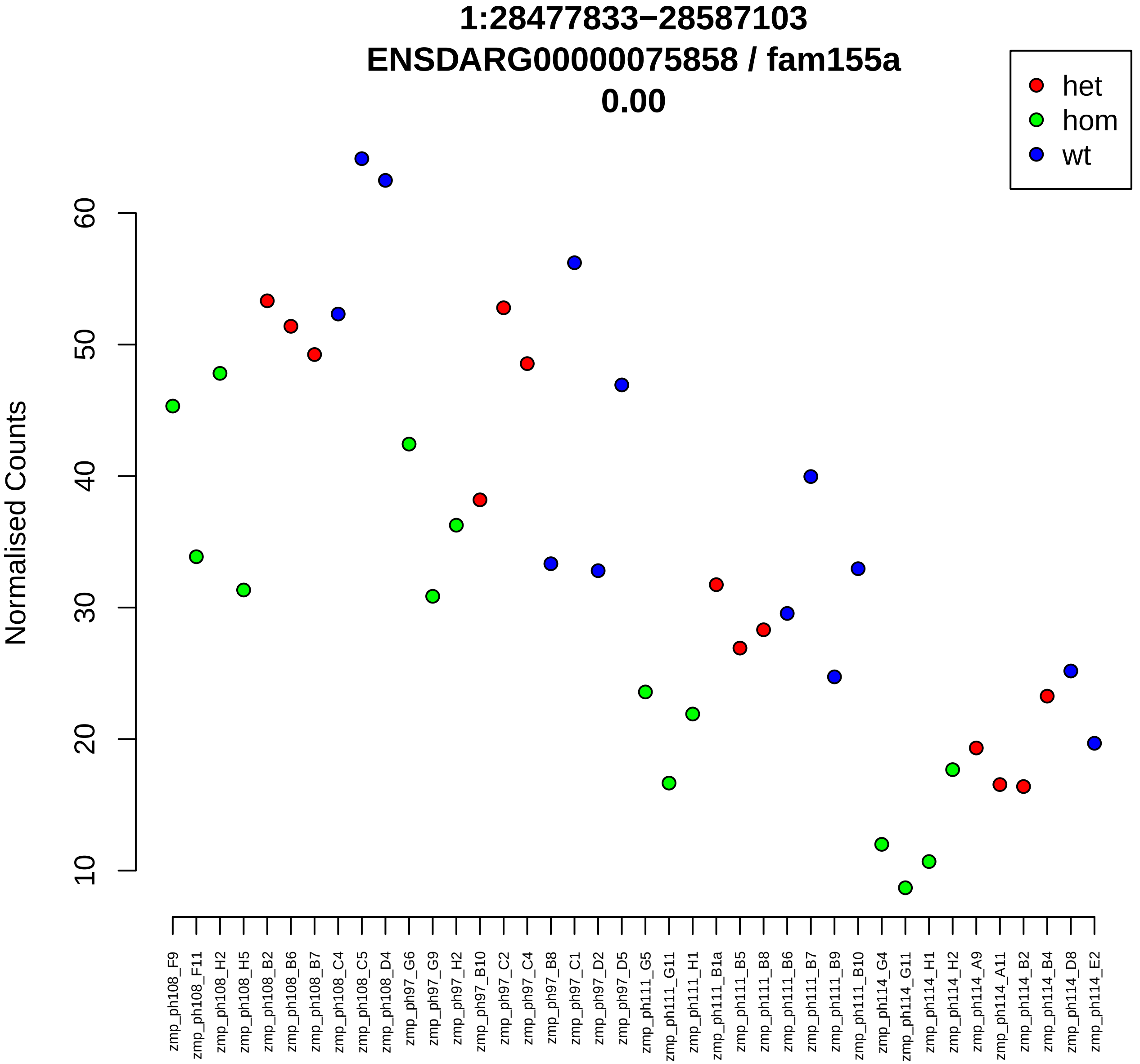

1:28477833−28587103
ENSDARG00000075858 / fam155a
0.00
het
hom
wt
Normalised Counts
10
20
30
40
50
60
zmp_ph108_F9
zmp_ph108_F11
zmp_ph108_H2
zmp_ph108_H5
zmp_ph108_B2
zmp_ph108_B6
zmp_ph108_B7
zmp_ph108_C4
zmp_ph108_C5
zmp_ph108_D4
zmp_ph97_G6
zmp_ph97_G9
zmp_ph97_H2
zmp_ph97_B10
zmp_ph97_C2
zmp_ph97_C4
zmp_ph97_B8
zmp_ph97_C1
zmp_ph97_D2
zmp_ph97_D5
zmp_ph111_G5
zmp_ph111_G11
zmp_ph111_H1
zmp_ph111_B1a
zmp_ph111_B5
zmp_ph111_B8
zmp_ph111_B6
zmp_ph111_B7
zmp_ph111_B9
zmp_ph111_B10
zmp_ph114_G4
zmp_ph114_G11
zmp_ph114_H1
zmp_ph114_H2
zmp_ph114_A9
zmp_ph114_A11
zmp_ph114_B2
zmp_ph114_B4
zmp_ph114_D8
zmp_ph114_E2

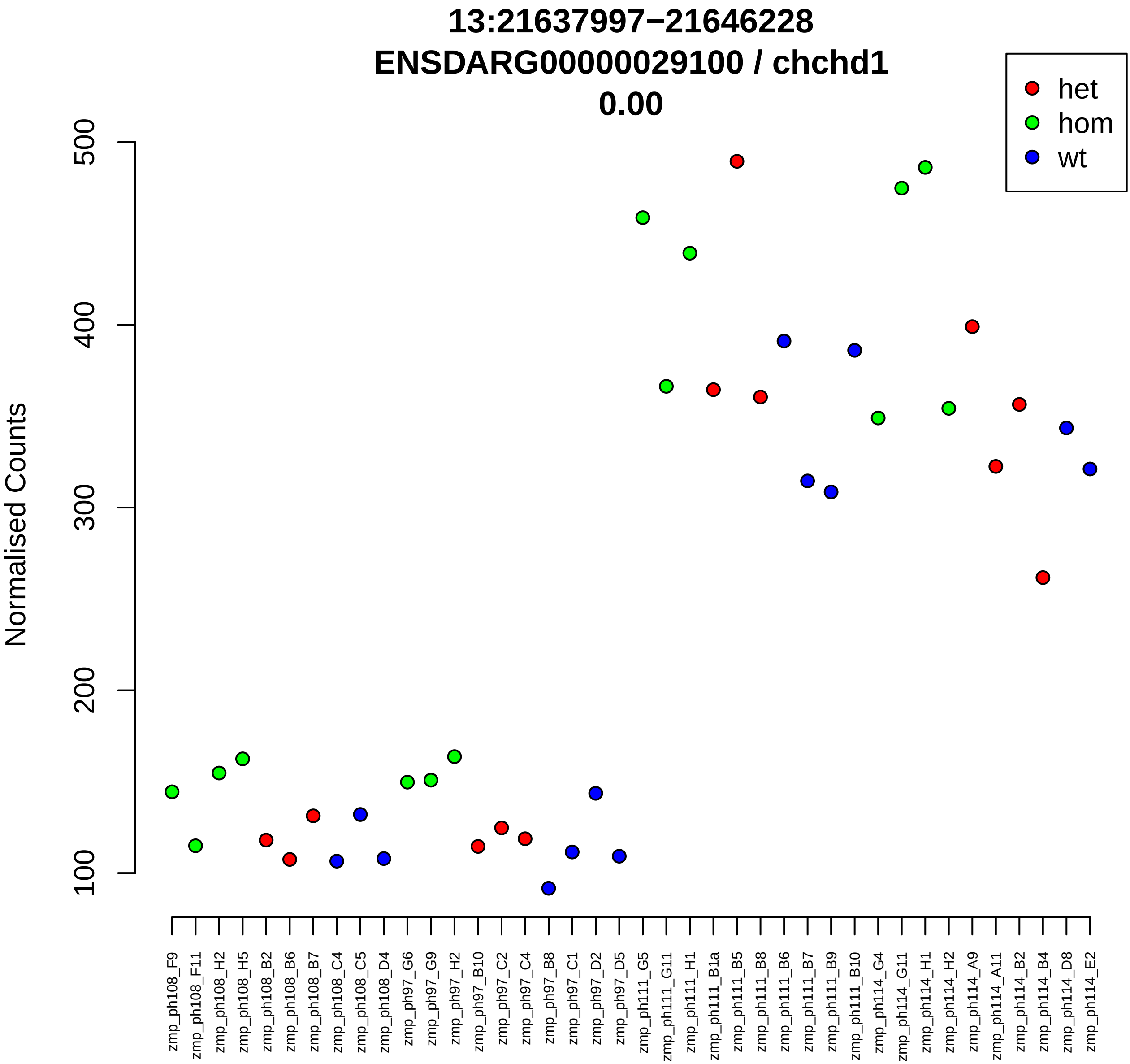

13:21637997−21646228
ENSDARG00000029100 / chchd1
0.00
het
hom
wt
Normalised Counts
100
200
300
400
500
zmp_ph108_F9
zmp_ph108_F11
zmp_ph108_H2
zmp_ph108_H5
zmp_ph108_B2
zmp_ph108_B6
zmp_ph108_B7
zmp_ph108_C4
zmp_ph108_C5
zmp_ph108_D4
zmp_ph97_G6
zmp_ph97_G9
zmp_ph97_H2
zmp_ph97_B10
zmp_ph97_C2
zmp_ph97_C4
zmp_ph97_B8
zmp_ph97_C1
zmp_ph97_D2
zmp_ph97_D5
zmp_ph111_G5
zmp_ph111_G11
zmp_ph111_H1
zmp_ph111_B1a
zmp_ph111_B5
zmp_ph111_B8
zmp_ph111_B6
zmp_ph111_B7
zmp_ph111_B9
zmp_ph111_B10
zmp_ph114_G4
zmp_ph114_G11
zmp_ph114_H1
zmp_ph114_H2
zmp_ph114_A9
zmp_ph114_A11
zmp_ph114_B2
zmp_ph114_B4
zmp_ph114_D8
zmp_ph114_E2

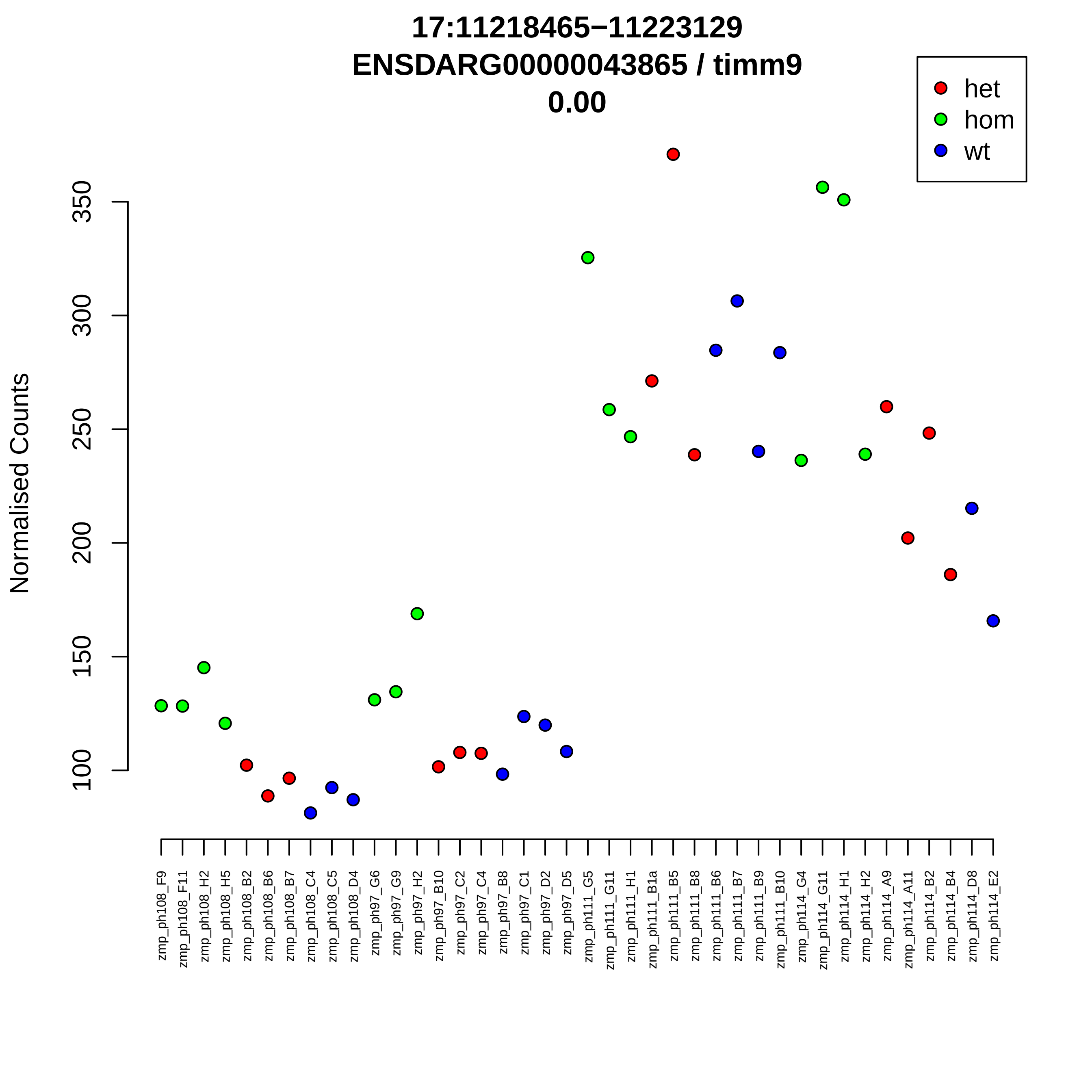
17:11218465−11223129
ENSDARG00000043865 / timm9
0.00
het
hom
wt
Normalised Counts
100
150
200
250
300
350
zmp_ph108_F9
zmp_ph108_F11
zmp_ph108_H2
zmp_ph108_H5
zmp_ph108_B2
zmp_ph108_B6
zmp_ph108_B7
zmp_ph108_C4
zmp_ph108_C5
zmp_ph108_D4
zmp_ph97_G6
zmp_ph97_G9
zmp_ph97_H2
zmp_ph97_B10
zmp_ph97_C2
zmp_ph97_C4
zmp_ph97_B8
zmp_ph97_C1
zmp_ph97_D2
zmp_ph97_D5
zmp_ph111_G5
zmp_ph111_G11
zmp_ph111_H1
zmp_ph111_B1a
zmp_ph111_B5
zmp_ph111_B8
zmp_ph111_B6
zmp_ph111_B7
zmp_ph111_B9
zmp_ph111_B10
zmp_ph114_G4
zmp_ph114_G11
zmp_ph114_H1
zmp_ph114_H2
zmp_ph114_A9
zmp_ph114_A11
zmp_ph114_B2
zmp_ph114_B4
zmp_ph114_D8
zmp_ph114_E2

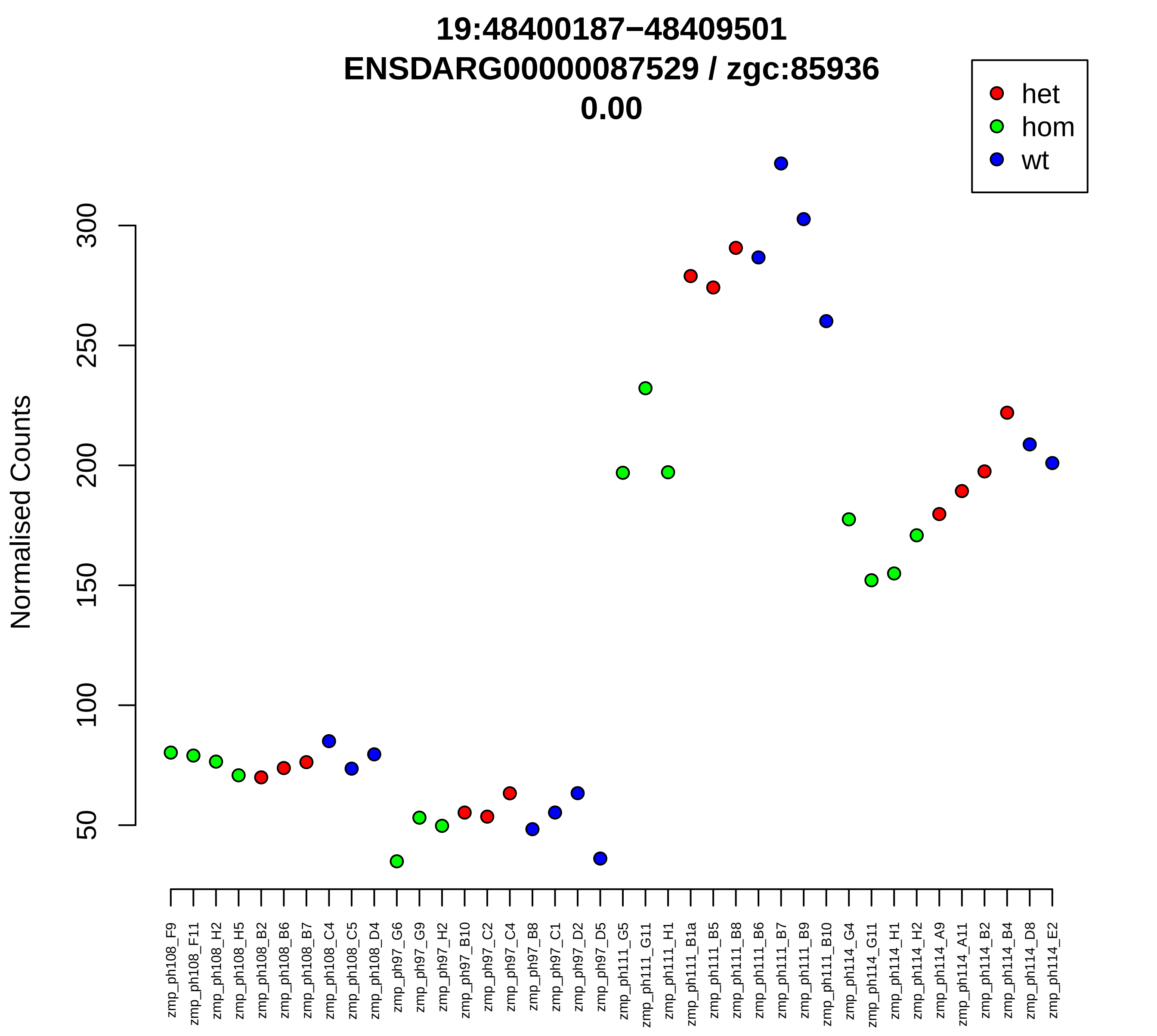

19:48400187−48409501
ENSDARG00000087529 / zgc:85936
0.00
het
hom
wt
Normalised Counts
50
100
150
200
250
300
zmp_ph108_F9
zmp_ph108_F11
zmp_ph108_H2
zmp_ph108_H5
zmp_ph108_B2
zmp_ph108_B6
zmp_ph108_B7
zmp_ph108_C4
zmp_ph108_C5
zmp_ph108_D4
zmp_ph97_G6
zmp_ph97_G9
zmp_ph97_H2
zmp_ph97_B10
zmp_ph97_C2
zmp_ph97_C4
zmp_ph97_B8
zmp_ph97_C1
zmp_ph97_D2
zmp_ph97_D5
zmp_ph111_G5
zmp_ph111_G11
zmp_ph111_H1
zmp_ph111_B1a
zmp_ph111_B5
zmp_ph111_B8
zmp_ph111_B6
zmp_ph111_B7
zmp_ph111_B9
zmp_ph111_B10
zmp_ph114_G4
zmp_ph114_G11
zmp_ph114_H1
zmp_ph114_H2
zmp_ph114_A9
zmp_ph114_A11
zmp_ph114_B2
zmp_ph114_B4
zmp_ph114_D8
zmp_ph114_E2

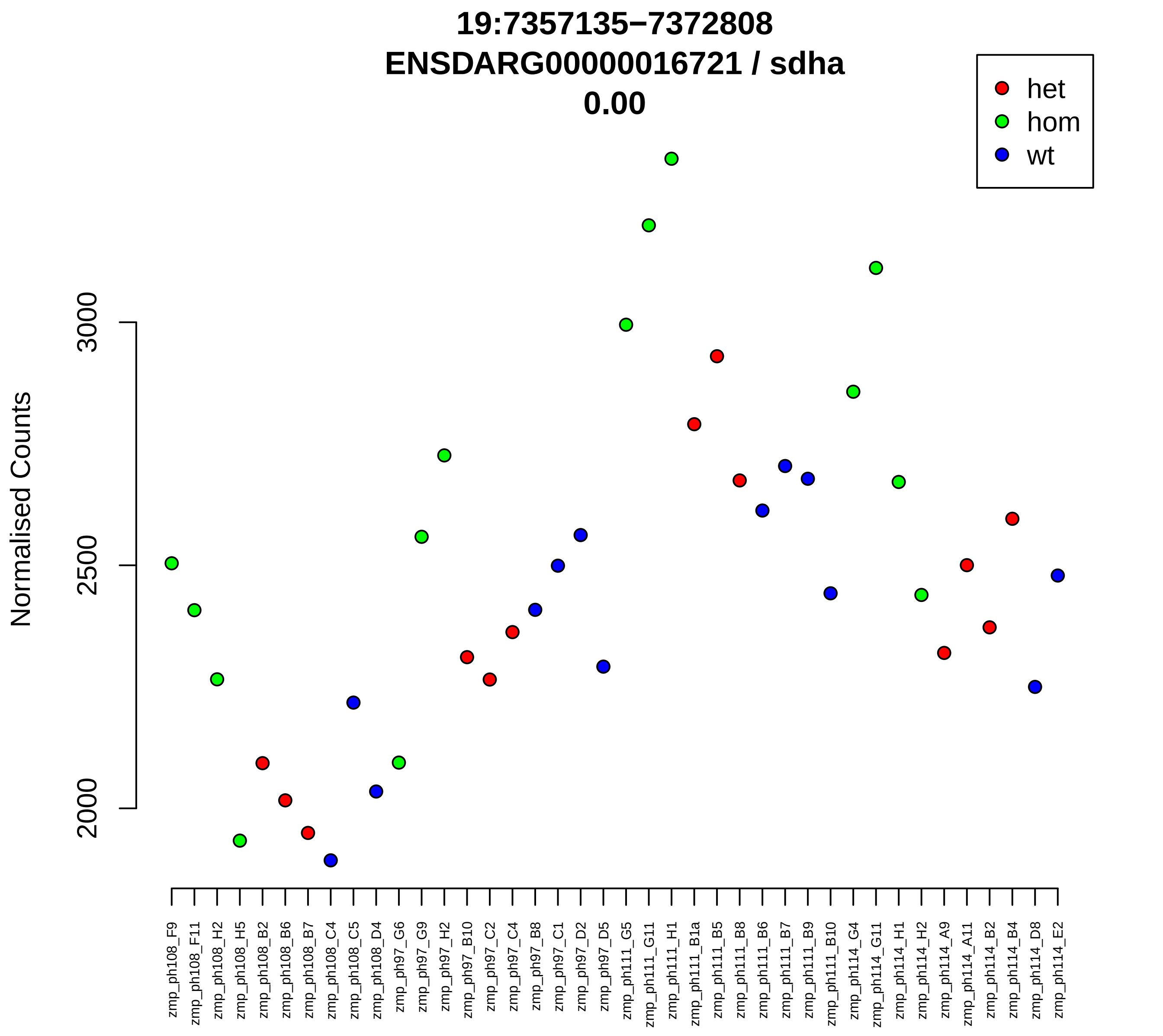

19:7357135−7372808
ENSDARG00000016721 / sdha
0.00
het
hom
wt
Normalised Counts
2000
2500
3000
zmp_ph108_F9
zmp_ph108_F11
zmp_ph108_H2
zmp_ph108_H5
zmp_ph108_B2
zmp_ph108_B6
zmp_ph108_B7
zmp_ph108_C4
zmp_ph108_C5
zmp_ph108_D4
zmp_ph97_G6
zmp_ph97_G9
zmp_ph97_H2
zmp_ph97_B10
zmp_ph97_C2
zmp_ph97_C4
zmp_ph97_B8
zmp_ph97_C1
zmp_ph97_D2
zmp_ph97_D5
zmp_ph111_G5
zmp_ph111_G11
zmp_ph111_H1
zmp_ph111_B1a
zmp_ph111_B5
zmp_ph111_B8
zmp_ph111_B6
zmp_ph111_B7
zmp_ph111_B9
zmp_ph111_B10
zmp_ph114_G4
zmp_ph114_G11
zmp_ph114_H1
zmp_ph114_H2
zmp_ph114_A9
zmp_ph114_A11
zmp_ph114_B2
zmp_ph114_B4
zmp_ph114_D8
zmp_ph114_E2

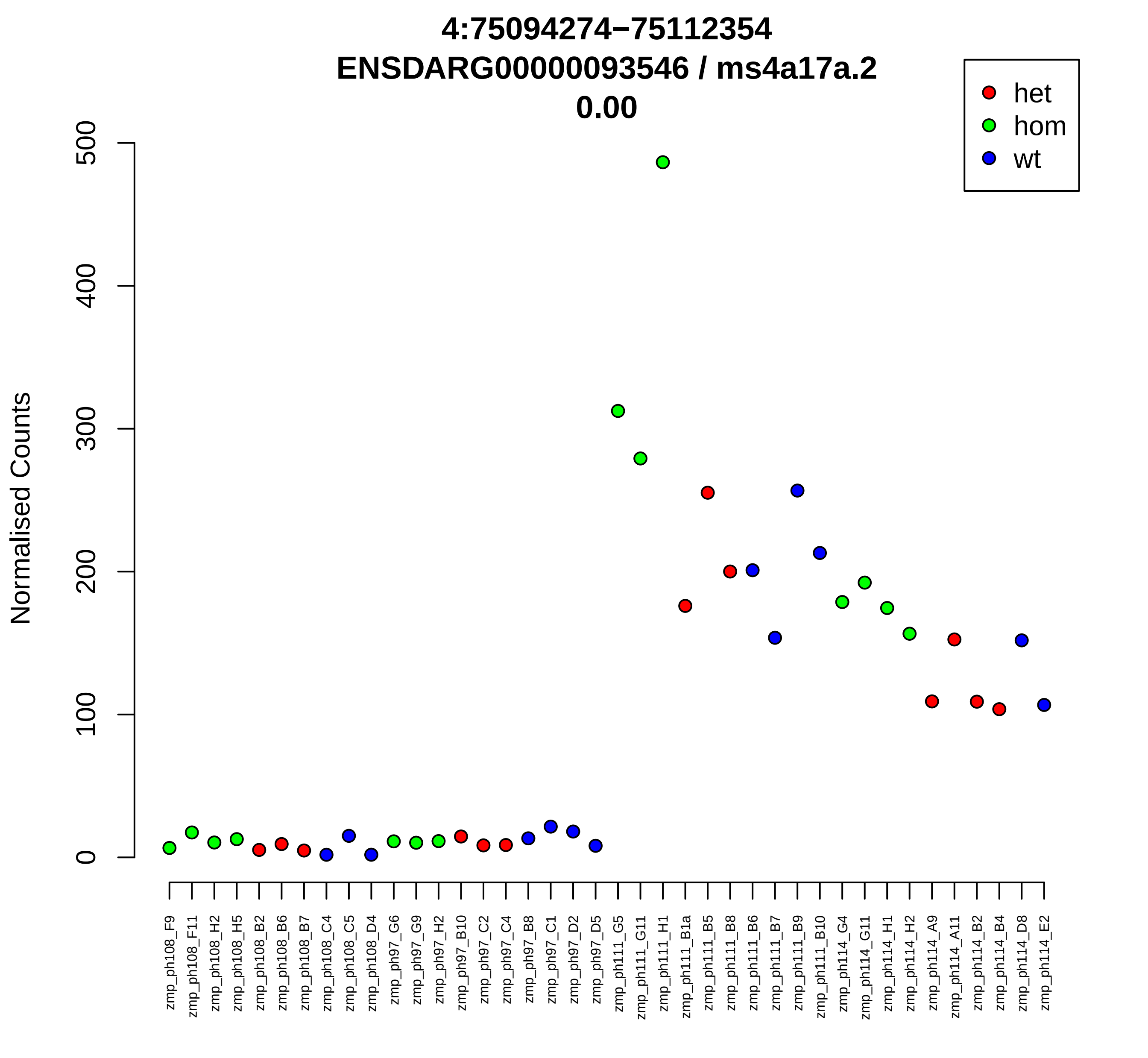

4:75094274−75112354
ENSDARG00000093546 / ms4a17a.2
0.00
het
hom
wt
Normalised Counts
0
100
200
300
400
500
zmp_ph108_F9
zmp_ph108_F11
zmp_ph108_H2
zmp_ph108_H5
zmp_ph108_B2
zmp_ph108_B6
zmp_ph108_B7
zmp_ph108_C4
zmp_ph108_C5
zmp_ph108_D4
zmp_ph97_G6
zmp_ph97_G9
zmp_ph97_H2
zmp_ph97_B10
zmp_ph97_C2
zmp_ph97_C4
zmp_ph97_B8
zmp_ph97_C1
zmp_ph97_D2
zmp_ph97_D5
zmp_ph111_G5
zmp_ph111_G11
zmp_ph111_H1
zmp_ph111_B1a
zmp_ph111_B5
zmp_ph111_B8
zmp_ph111_B6
zmp_ph111_B7
zmp_ph111_B9
zmp_ph111_B10
zmp_ph114_G4
zmp_ph114_G11
zmp_ph114_H1
zmp_ph114_H2
zmp_ph114_A9
zmp_ph114_A11
zmp_ph114_B2
zmp_ph114_B4
zmp_ph114_D8
zmp_ph114_E2

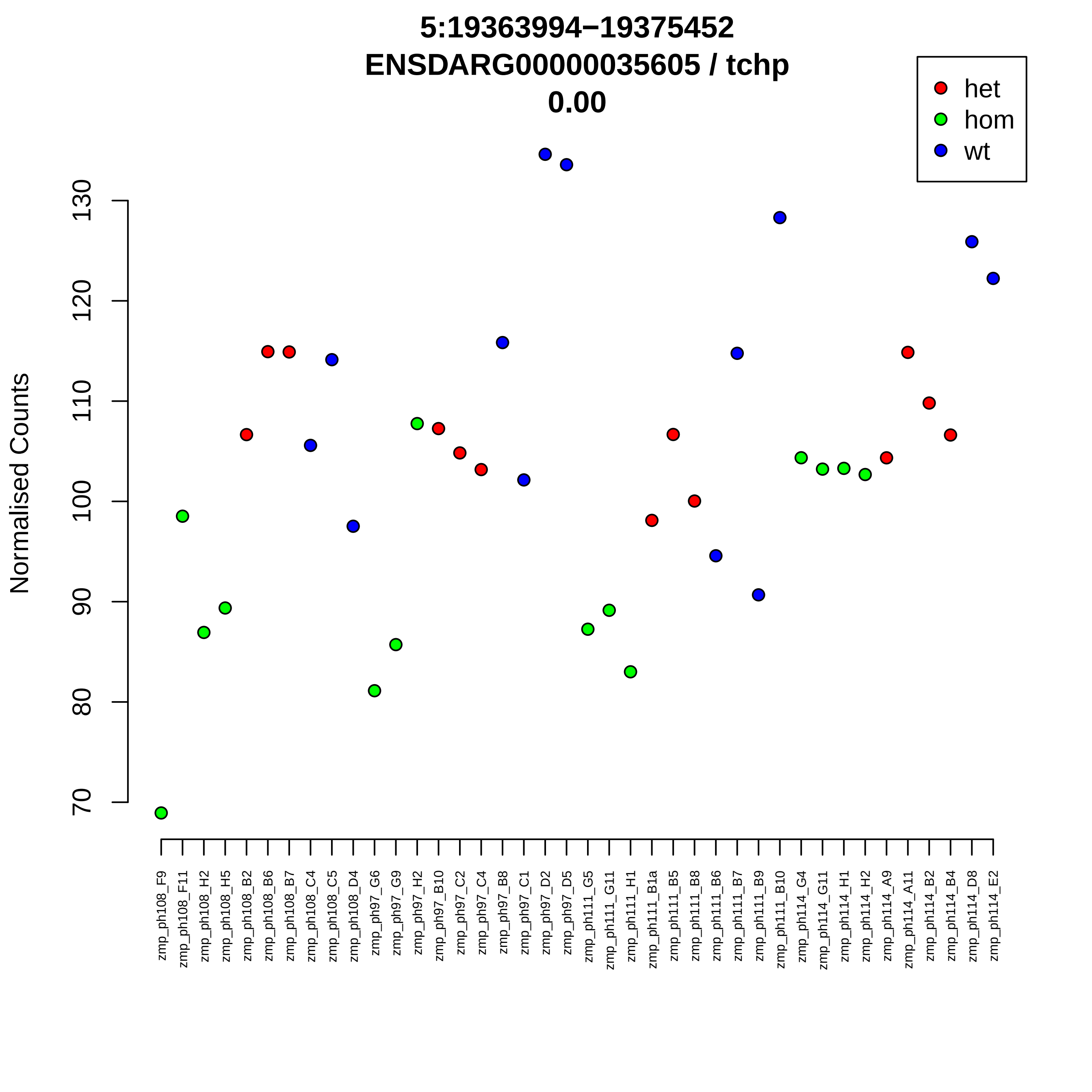

5:19363994−19375452
ENSDARG00000035605 / tchp
0.00
het
hom
wt
Normalised Counts
70
80
90
100
110
120
130
zmp_ph108_F9
zmp_ph108_F11
zmp_ph108_H2
zmp_ph108_H5
zmp_ph108_B2
zmp_ph108_B6
zmp_ph108_B7
zmp_ph108_C4
zmp_ph108_C5
zmp_ph108_D4
zmp_ph97_G6
zmp_ph97_G9
zmp_ph97_H2
zmp_ph97_B10
zmp_ph97_C2
zmp_ph97_C4
zmp_ph97_B8
zmp_ph97_C1
zmp_ph97_D2
zmp_ph97_D5
zmp_ph111_G5
zmp_ph111_G11
zmp_ph111_H1
zmp_ph111_B1a
zmp_ph111_B5
zmp_ph111_B8
zmp_ph111_B6
zmp_ph111_B7
zmp_ph111_B9
zmp_ph111_B10
zmp_ph114_G4
zmp_ph114_G11
zmp_ph114_H1
zmp_ph114_H2
zmp_ph114_A9
zmp_ph114_A11
zmp_ph114_B2
zmp_ph114_B4
zmp_ph114_D8
zmp_ph114_E2

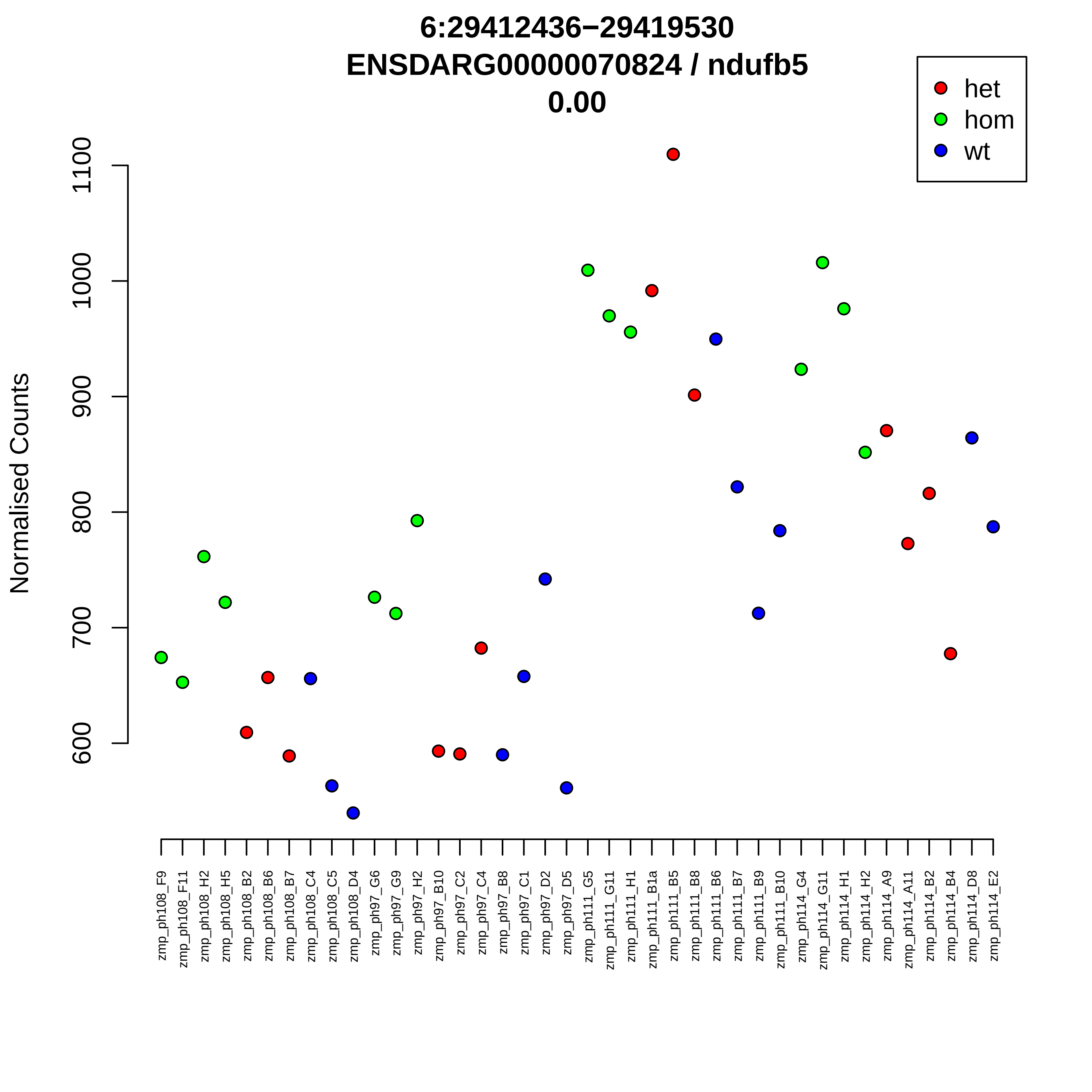

6:29412436−29419530
ENSDARG00000070824 / ndufb5
0.00
het
hom
wt
Normalised Counts
600
700
800
900
1000
1100
zmp_ph108_F9
zmp_ph108_F11
zmp_ph108_H2
zmp_ph108_H5
zmp_ph108_B2
zmp_ph108_B6
zmp_ph108_B7
zmp_ph108_C4
zmp_ph108_C5
zmp_ph108_D4
zmp_ph97_G6
zmp_ph97_G9
zmp_ph97_H2
zmp_ph97_B10
zmp_ph97_C2
zmp_ph97_C4
zmp_ph97_B8
zmp_ph97_C1
zmp_ph97_D2
zmp_ph97_D5
zmp_ph111_G5
zmp_ph111_G11
zmp_ph111_H1
zmp_ph111_B1a
zmp_ph111_B5
zmp_ph111_B8
zmp_ph111_B6
zmp_ph111_B7
zmp_ph111_B9
zmp_ph111_B10
zmp_ph114_G4
zmp_ph114_G11
zmp_ph114_H1
zmp_ph114_H2
zmp_ph114_A9
zmp_ph114_A11
zmp_ph114_B2
zmp_ph114_B4
zmp_ph114_D8
zmp_ph114_E2

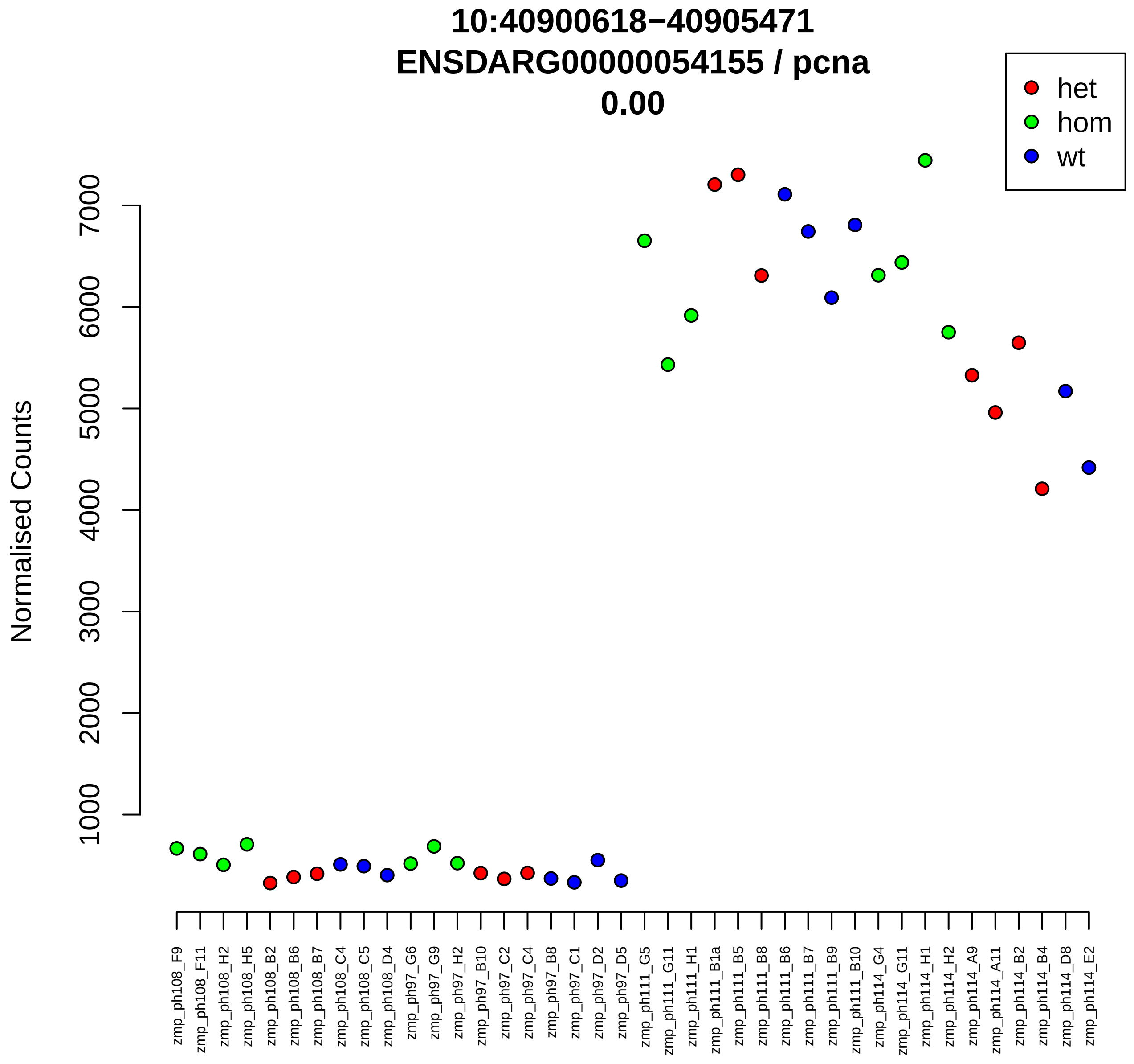

10:40900618−40905471
ENSDARG00000054155 / pcna
0.00
het
hom
wt
Normalised Counts
1000
2000
3000
4000
5000
6000
7000
zmp_ph108_F9
zmp_ph108_F11
zmp_ph108_H2
zmp_ph108_H5
zmp_ph108_B2
zmp_ph108_B6
zmp_ph108_B7
zmp_ph108_C4
zmp_ph108_C5
zmp_ph108_D4
zmp_ph97_G6
zmp_ph97_G9
zmp_ph97_H2
zmp_ph97_B10
zmp_ph97_C2
zmp_ph97_C4
zmp_ph97_B8
zmp_ph97_C1
zmp_ph97_D2
zmp_ph97_D5
zmp_ph111_G5
zmp_ph111_G11
zmp_ph111_H1
zmp_ph111_B1a
zmp_ph111_B5
zmp_ph111_B8
zmp_ph111_B6
zmp_ph111_B7
zmp_ph111_B9
zmp_ph111_B10
zmp_ph114_G4
zmp_ph114_G11
zmp_ph114_H1
zmp_ph114_H2
zmp_ph114_A9
zmp_ph114_A11
zmp_ph114_B2
zmp_ph114_B4
zmp_ph114_D8
zmp_ph114_E2

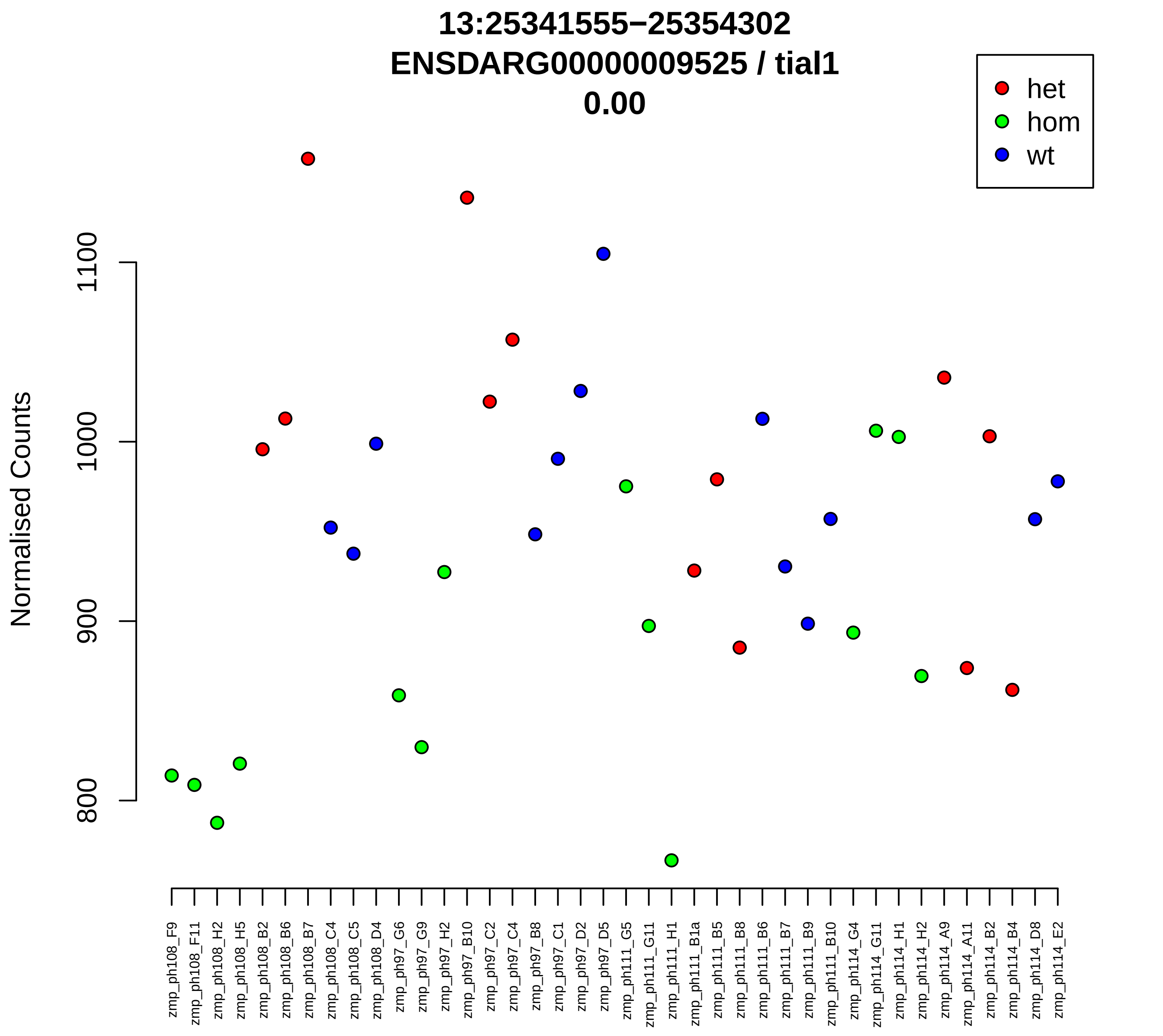

13:25341555−25354302
ENSDARG00000009525 / tial1
0.00
het
hom
wt
Normalised Counts
800
900
1000
1100
zmp_ph108_F9
zmp_ph108_F11
zmp_ph108_H2
zmp_ph108_H5
zmp_ph108_B2
zmp_ph108_B6
zmp_ph108_B7
zmp_ph108_C4
zmp_ph108_C5
zmp_ph108_D4
zmp_ph97_G6
zmp_ph97_G9
zmp_ph97_H2
zmp_ph97_B10
zmp_ph97_C2
zmp_ph97_C4
zmp_ph97_B8
zmp_ph97_C1
zmp_ph97_D2
zmp_ph97_D5
zmp_ph111_G5
zmp_ph111_G11
zmp_ph111_H1
zmp_ph111_B1a
zmp_ph111_B5
zmp_ph111_B8
zmp_ph111_B6
zmp_ph111_B7
zmp_ph111_B9
zmp_ph111_B10
zmp_ph114_G4
zmp_ph114_G11
zmp_ph114_H1
zmp_ph114_H2
zmp_ph114_A9
zmp_ph114_A11
zmp_ph114_B2
zmp_ph114_B4
zmp_ph114_D8
zmp_ph114_E2

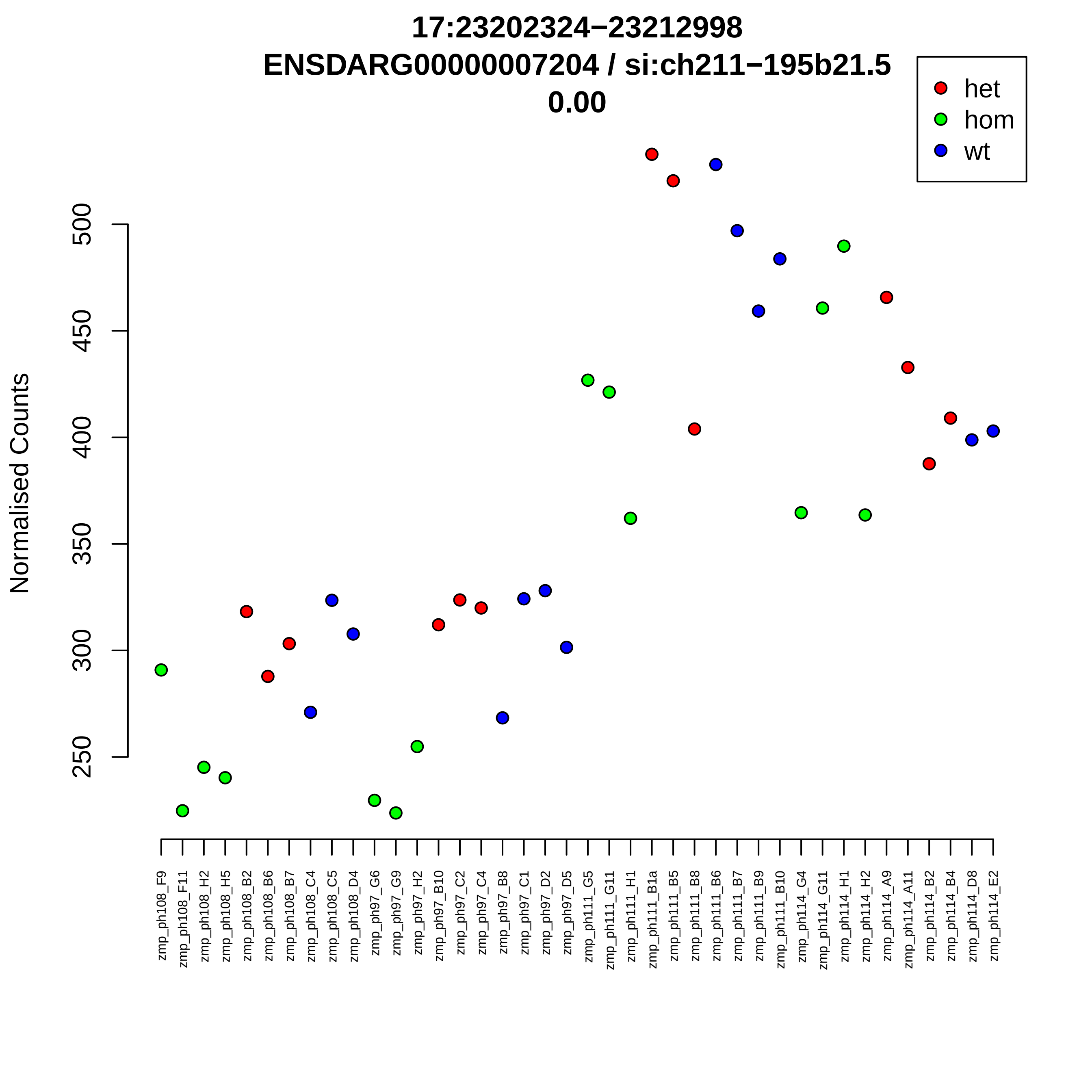

17:23202324−23212998
ENSDARG00000007204 / si:ch211−195b21.5
0.00
het
hom
wt
Normalised Counts
250
300
350
400
450
500
zmp_ph108_F9
zmp_ph108_F11
zmp_ph108_H2
zmp_ph108_H5
zmp_ph108_B2
zmp_ph108_B6
zmp_ph108_B7
zmp_ph108_C4
zmp_ph108_C5
zmp_ph108_D4
zmp_ph97_G6
zmp_ph97_G9
zmp_ph97_H2
zmp_ph97_B10
zmp_ph97_C2
zmp_ph97_C4
zmp_ph97_B8
zmp_ph97_C1
zmp_ph97_D2
zmp_ph97_D5
zmp_ph111_G5
zmp_ph111_G11
zmp_ph111_H1
zmp_ph111_B1a
zmp_ph111_B5
zmp_ph111_B8
zmp_ph111_B6
zmp_ph111_B7
zmp_ph111_B9
zmp_ph111_B10
zmp_ph114_G4
zmp_ph114_G11
zmp_ph114_H1
zmp_ph114_H2
zmp_ph114_A9
zmp_ph114_A11
zmp_ph114_B2
zmp_ph114_B4
zmp_ph114_D8
zmp_ph114_E2

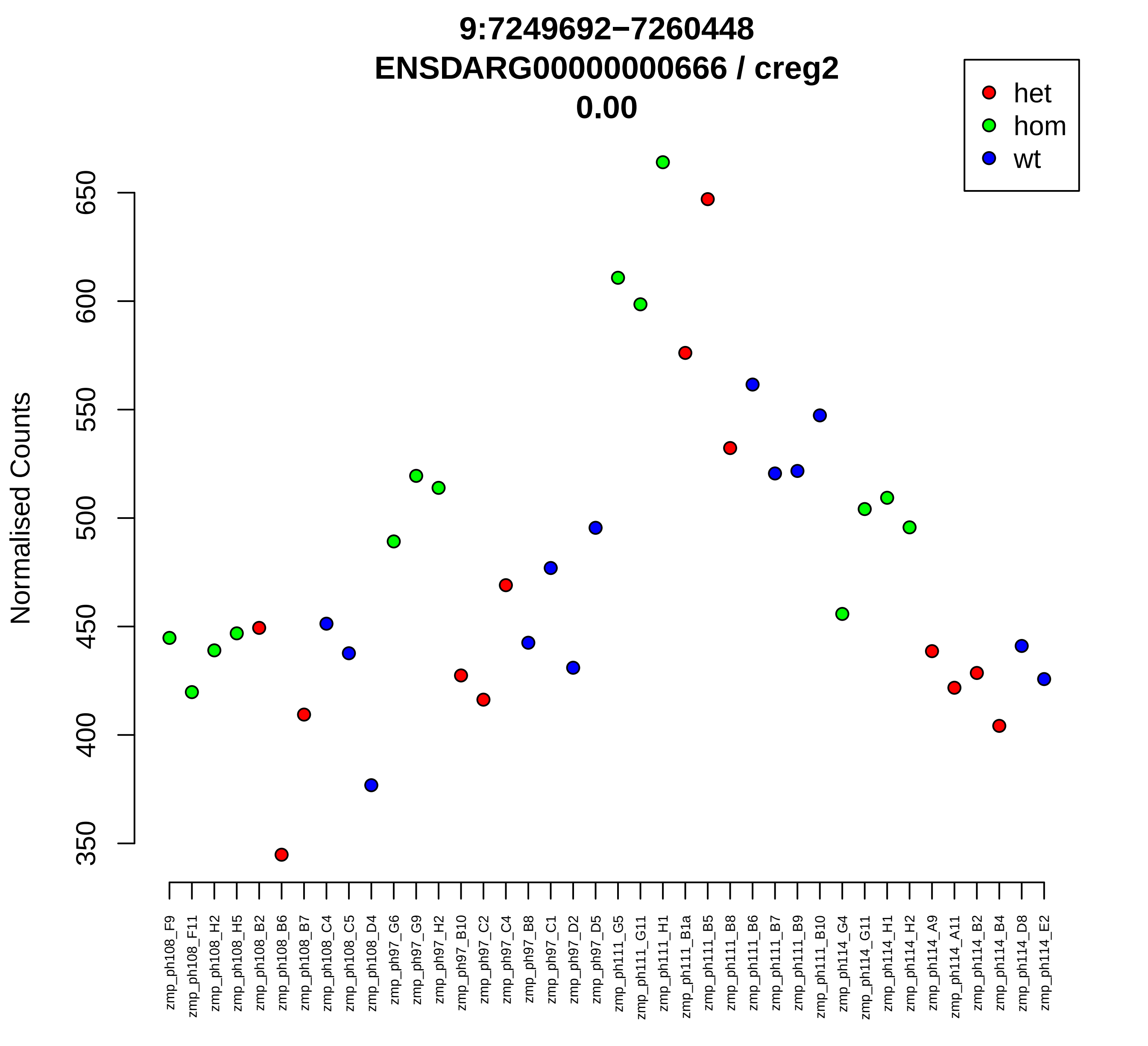

9:7249692−7260448
ENSDARG00000000666 / creg2
0.00
het
hom
wt
Normalised Counts
350
400
450
500
550
600
650
zmp_ph108_F9
zmp_ph108_F11
zmp_ph108_H2
zmp_ph108_H5
zmp_ph108_B2
zmp_ph108_B6
zmp_ph108_B7
zmp_ph108_C4
zmp_ph108_C5
zmp_ph108_D4
zmp_ph97_G6
zmp_ph97_G9
zmp_ph97_H2
zmp_ph97_B10
zmp_ph97_C2
zmp_ph97_C4
zmp_ph97_B8
zmp_ph97_C1
zmp_ph97_D2
zmp_ph97_D5
zmp_ph111_G5
zmp_ph111_G11
zmp_ph111_H1
zmp_ph111_B1a
zmp_ph111_B5
zmp_ph111_B8
zmp_ph111_B6
zmp_ph111_B7
zmp_ph111_B9
zmp_ph111_B10
zmp_ph114_G4
zmp_ph114_G11
zmp_ph114_H1
zmp_ph114_H2
zmp_ph114_A9
zmp_ph114_A11
zmp_ph114_B2
zmp_ph114_B4
zmp_ph114_D8
zmp_ph114_E2

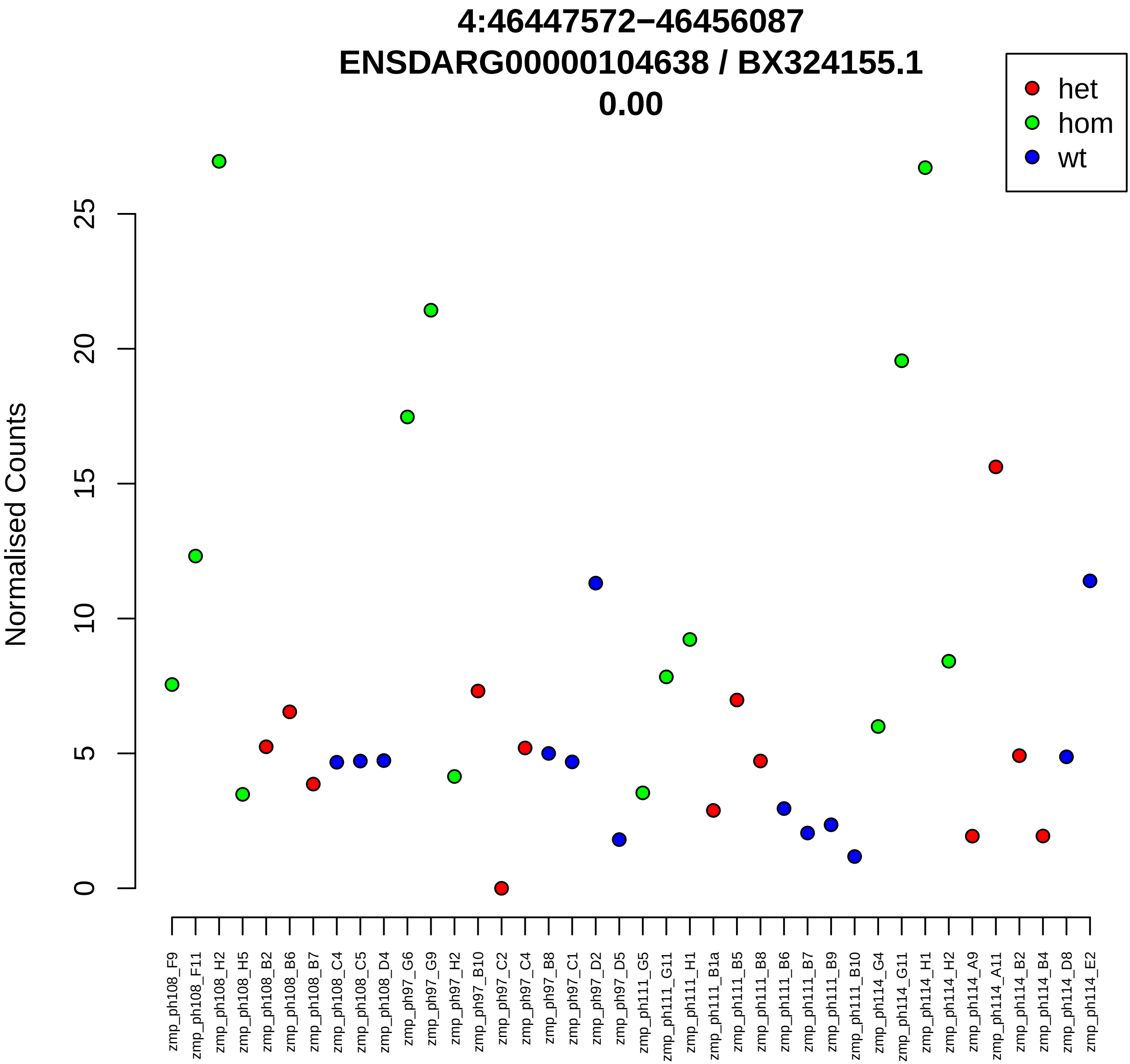

4:46447572−46456087
ENSDARG00000104638 / BX324155.1
0.00
het
hom
wt
Normalised Counts
0
5
10
15
20
25
zmp_ph108_F9
zmp_ph108_F11
zmp_ph108_H2
zmp_ph108_H5
zmp_ph108_B2
zmp_ph108_B6
zmp_ph108_B7
zmp_ph108_C4
zmp_ph108_C5
zmp_ph108_D4
zmp_ph97_G6
zmp_ph97_G9
zmp_ph97_H2
zmp_ph97_B10
zmp_ph97_C2
zmp_ph97_C4
zmp_ph97_B8
zmp_ph97_C1
zmp_ph97_D2
zmp_ph97_D5
zmp_ph111_G5
zmp_ph111_G11
zmp_ph111_H1
zmp_ph111_B1a
zmp_ph111_B5
zmp_ph111_B8
zmp_ph111_B6
zmp_ph111_B7
zmp_ph111_B9
zmp_ph111_B10
zmp_ph114_G4
zmp_ph114_G11
zmp_ph114_H1
zmp_ph114_H2
zmp_ph114_A9
zmp_ph114_A11
zmp_ph114_B2
zmp_ph114_B4
zmp_ph114_D8
zmp_ph114_E2

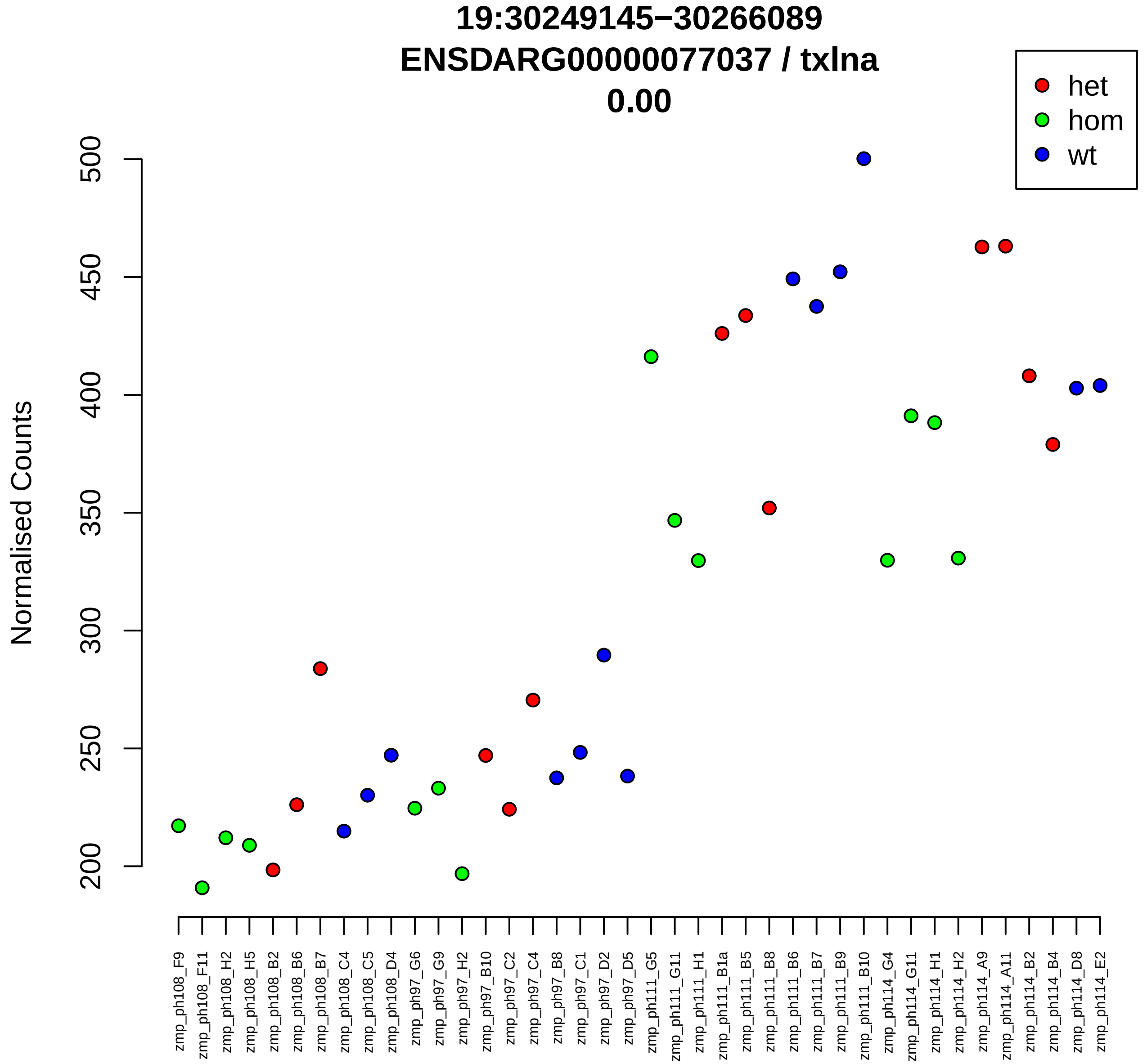

19:30249145−30266089
ENSDARG00000077037 / txlna
0.00
het
hom
wt
Normalised Counts
200
250
300
350
400
450
500
zmp_ph108_F9
zmp_ph108_F11
zmp_ph108_H2
zmp_ph108_H5
zmp_ph108_B2
zmp_ph108_B6
zmp_ph108_B7
zmp_ph108_C4
zmp_ph108_C5
zmp_ph108_D4
zmp_ph97_G6
zmp_ph97_G9
zmp_ph97_H2
zmp_ph97_B10
zmp_ph97_C2
zmp_ph97_C4
zmp_ph97_B8
zmp_ph97_C1
zmp_ph97_D2
zmp_ph97_D5
zmp_ph111_G5
zmp_ph111_G11
zmp_ph111_H1
zmp_ph111_B1a
zmp_ph111_B5
zmp_ph111_B8
zmp_ph111_B6
zmp_ph111_B7
zmp_ph111_B9
zmp_ph111_B10
zmp_ph114_G4
zmp_ph114_G11
zmp_ph114_H1
zmp_ph114_H2
zmp_ph114_A9
zmp_ph114_A11
zmp_ph114_B2
zmp_ph114_B4
zmp_ph114_D8
zmp_ph114_E2

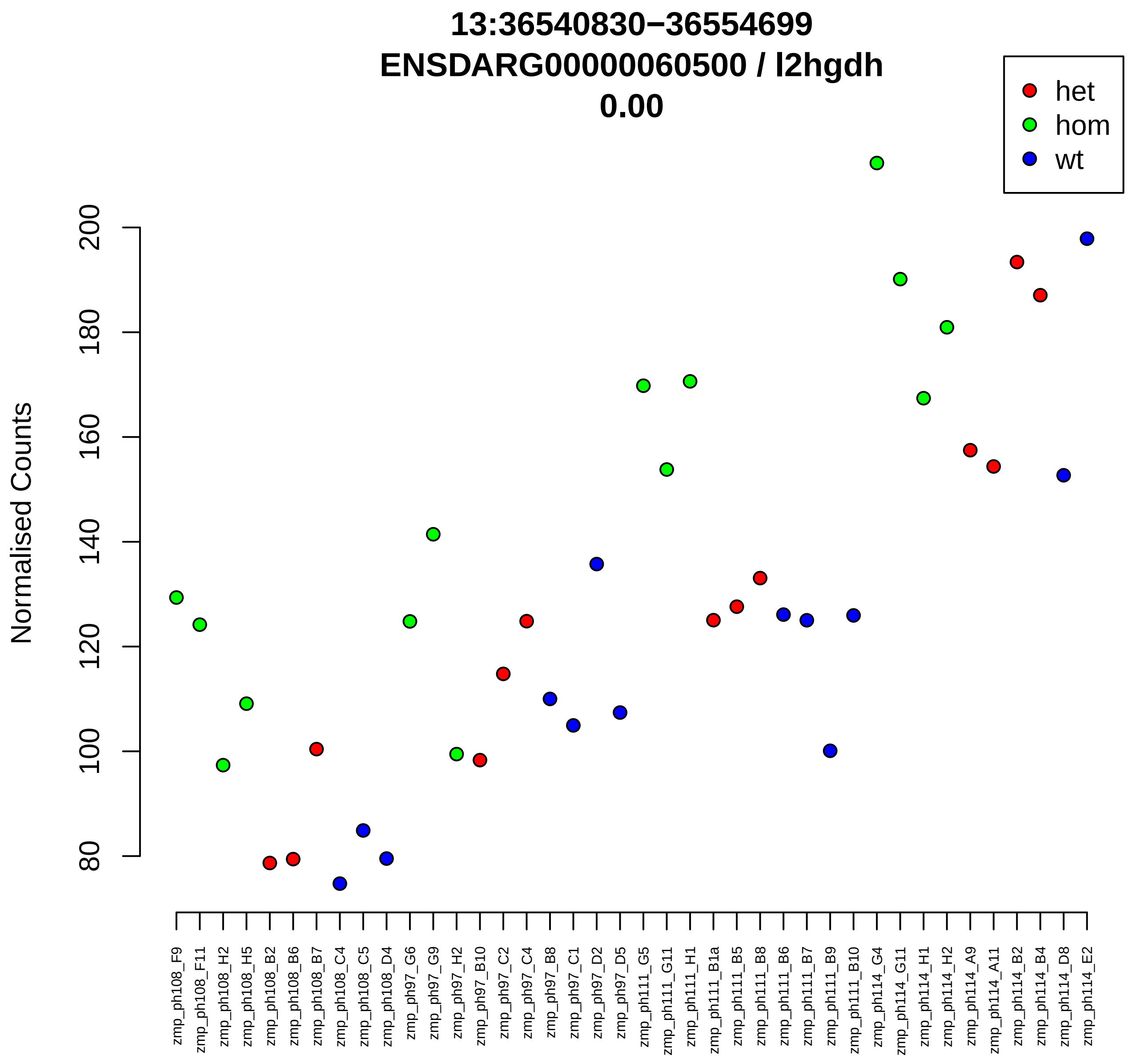
13:36540830−36554699
ENSDARG00000060500 / l2hgdh
0.00
het
hom
wt
Normalised Counts
80
100
120
140
160
180
200
zmp_ph108_F9
zmp_ph108_F11
zmp_ph108_H2
zmp_ph108_H5
zmp_ph108_B2
zmp_ph108_B6
zmp_ph108_B7
zmp_ph108_C4
zmp_ph108_C5
zmp_ph108_D4
zmp_ph97_G6
zmp_ph97_G9
zmp_ph97_H2
zmp_ph97_B10
zmp_ph97_C2
zmp_ph97_C4
zmp_ph97_B8
zmp_ph97_C1
zmp_ph97_D2
zmp_ph97_D5
zmp_ph111_G5
zmp_ph111_G11
zmp_ph111_H1
zmp_ph111_B1a
zmp_ph111_B5
zmp_ph111_B8
zmp_ph111_B6
zmp_ph111_B7
zmp_ph111_B9
zmp_ph111_B10
zmp_ph114_G4
zmp_ph114_G11
zmp_ph114_H1
zmp_ph114_H2
zmp_ph114_A9
zmp_ph114_A11
zmp_ph114_B2
zmp_ph114_B4
zmp_ph114_D8
zmp_ph114_E2

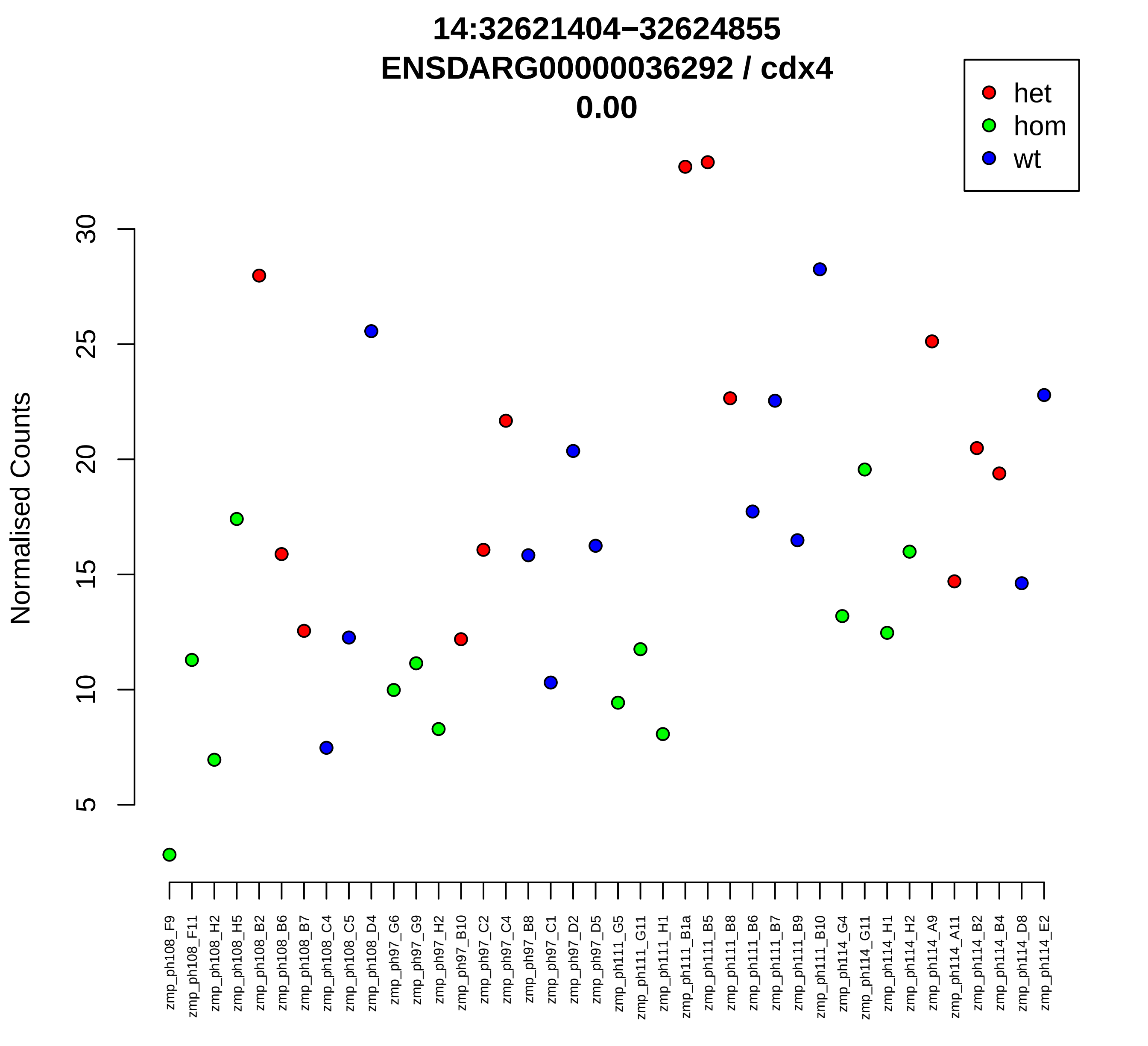
14:32621404−32624855
ENSDARG00000036292 / cdx4
0.00
het
hom
wt
Normalised Counts
5
10
15
20
25
30
zmp_ph108_F9
zmp_ph108_F11
zmp_ph108_H2
zmp_ph108_H5
zmp_ph108_B2
zmp_ph108_B6
zmp_ph108_B7
zmp_ph108_C4
zmp_ph108_C5
zmp_ph108_D4
zmp_ph97_G6
zmp_ph97_G9
zmp_ph97_H2
zmp_ph97_B10
zmp_ph97_C2
zmp_ph97_C4
zmp_ph97_B8
zmp_ph97_C1
zmp_ph97_D2
zmp_ph97_D5
zmp_ph111_G5
zmp_ph111_G11
zmp_ph111_H1
zmp_ph111_B1a
zmp_ph111_B5
zmp_ph111_B8
zmp_ph111_B6
zmp_ph111_B7
zmp_ph111_B9
zmp_ph111_B10
zmp_ph114_G4
zmp_ph114_G11
zmp_ph114_H1
zmp_ph114_H2
zmp_ph114_A9
zmp_ph114_A11
zmp_ph114_B2
zmp_ph114_B4
zmp_ph114_D8
zmp_ph114_E2

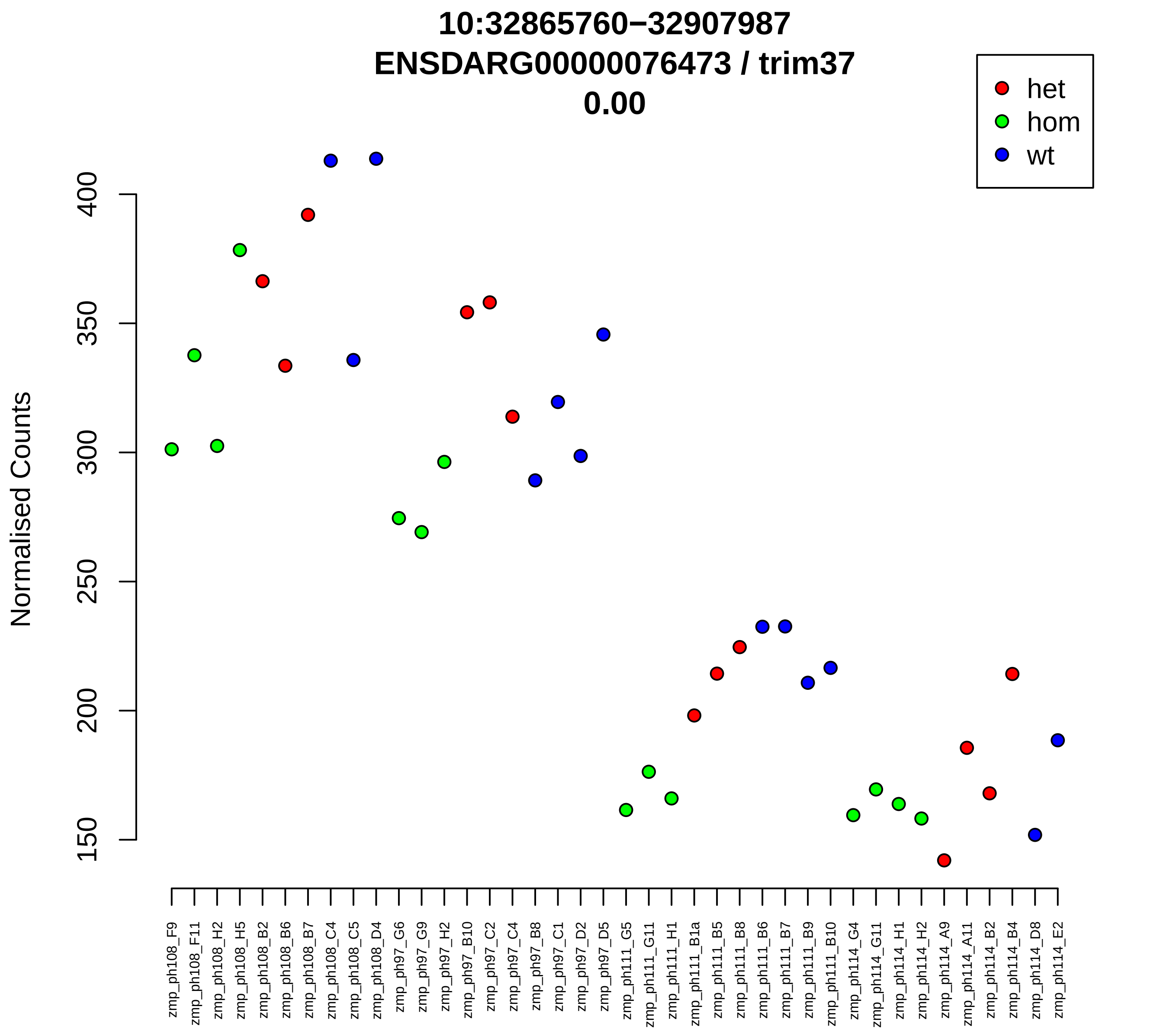
10:32865760−32907987
ENSDARG00000076473 / trim37
0.00
het
hom
wt
Normalised Counts
150
200
250
300
350
400
zmp_ph108_F9
zmp_ph108_F11
zmp_ph108_H2
zmp_ph108_H5
zmp_ph108_B2
zmp_ph108_B6
zmp_ph108_B7
zmp_ph108_C4
zmp_ph108_C5
zmp_ph108_D4
zmp_ph97_G6
zmp_ph97_G9
zmp_ph97_H2
zmp_ph97_B10
zmp_ph97_C2
zmp_ph97_C4
zmp_ph97_B8
zmp_ph97_C1
zmp_ph97_D2
zmp_ph97_D5
zmp_ph111_G5
zmp_ph111_G11
zmp_ph111_H1
zmp_ph111_B1a
zmp_ph111_B5
zmp_ph111_B8
zmp_ph111_B6
zmp_ph111_B7
zmp_ph111_B9
zmp_ph111_B10
zmp_ph114_G4
zmp_ph114_G11
zmp_ph114_H1
zmp_ph114_H2
zmp_ph114_A9
zmp_ph114_A11
zmp_ph114_B2
zmp_ph114_B4
zmp_ph114_D8
zmp_ph114_E2

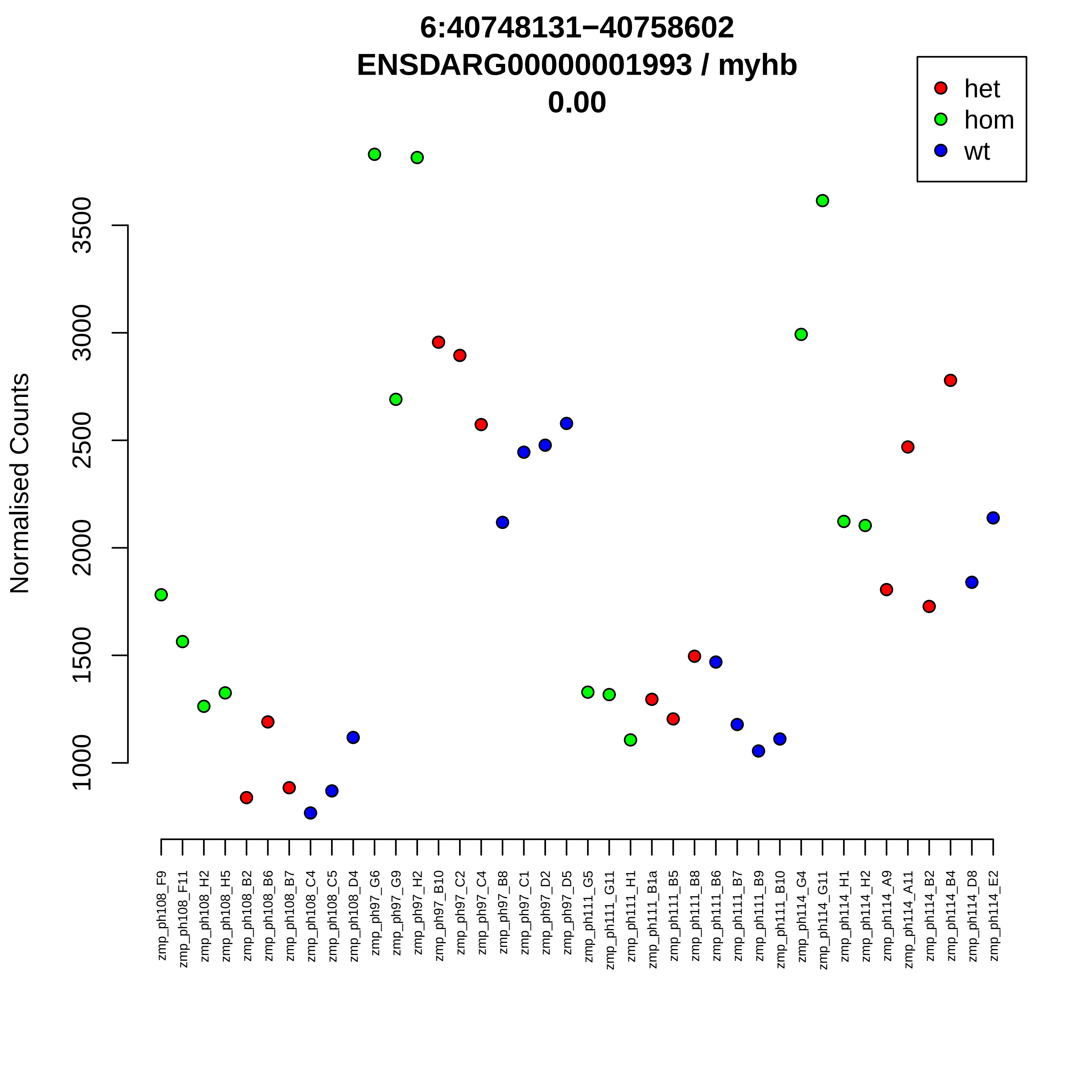

6:40748131−40758602
ENSDARG00000001993 / myhb
0.00
het
hom
wt
Normalised Counts
1000
1500
2000
2500
3000
3500
zmp_ph108_F9
zmp_ph108_F11
zmp_ph108_H2
zmp_ph108_H5
zmp_ph108_B2
zmp_ph108_B6
zmp_ph108_B7
zmp_ph108_C4
zmp_ph108_C5
zmp_ph108_D4
zmp_ph97_G6
zmp_ph97_G9
zmp_ph97_H2
zmp_ph97_B10
zmp_ph97_C2
zmp_ph97_C4
zmp_ph97_B8
zmp_ph97_C1
zmp_ph97_D2
zmp_ph97_D5
zmp_ph111_G5
zmp_ph111_G11
zmp_ph111_H1
zmp_ph111_B1a
zmp_ph111_B5
zmp_ph111_B8
zmp_ph111_B6
zmp_ph111_B7
zmp_ph111_B9
zmp_ph111_B10
zmp_ph114_G4
zmp_ph114_G11
zmp_ph114_H1
zmp_ph114_H2
zmp_ph114_A9
zmp_ph114_A11
zmp_ph114_B2
zmp_ph114_B4
zmp_ph114_D8
zmp_ph114_E2

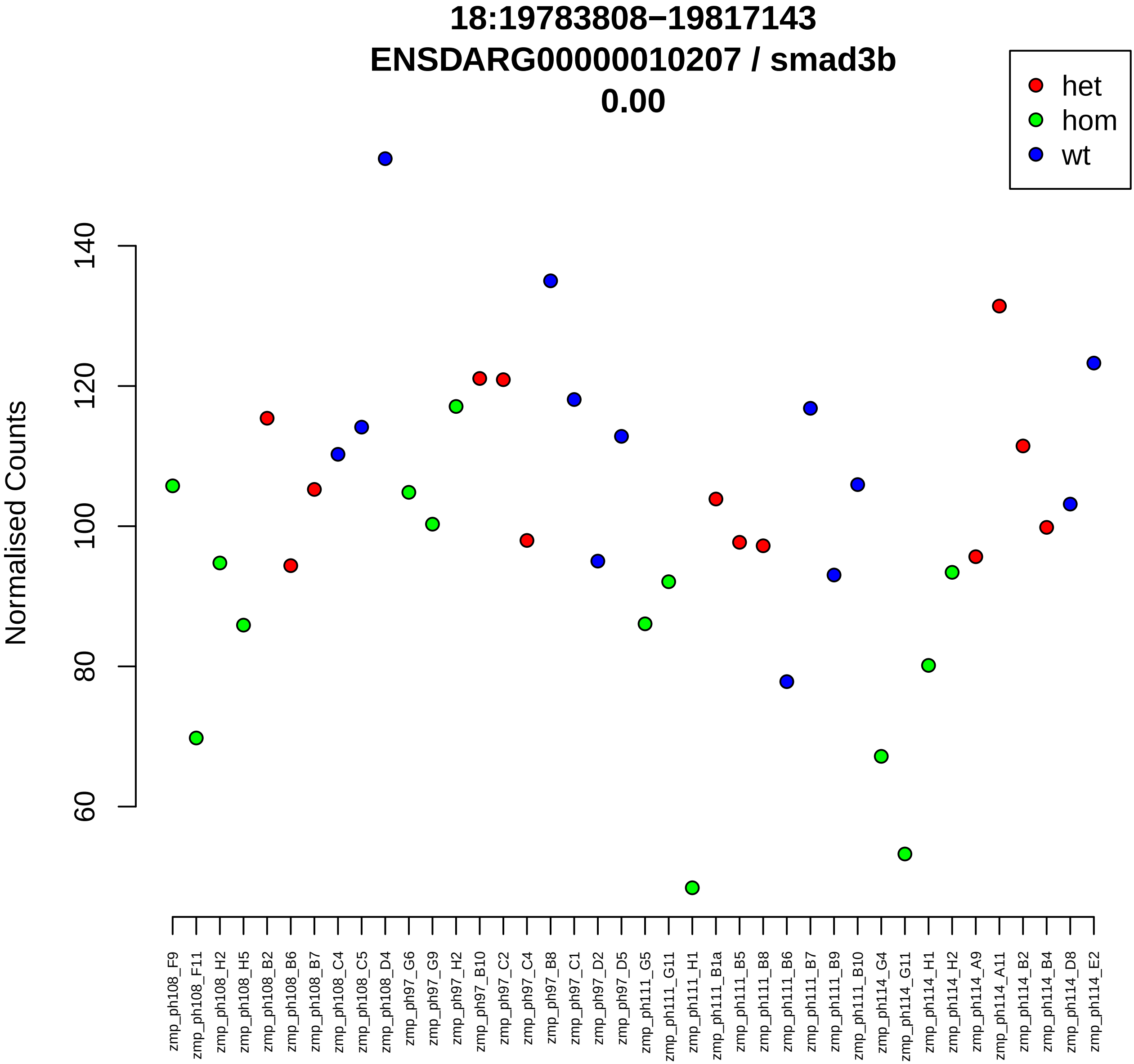

18:19783808−19817143
ENSDARG00000010207 / smad3b
0.00
het
hom
wt
Normalised Counts
60
80
100
120
140
zmp_ph108_F9
zmp_ph108_F11
zmp_ph108_H2
zmp_ph108_H5
zmp_ph108_B2
zmp_ph108_B6
zmp_ph108_B7
zmp_ph108_C4
zmp_ph108_C5
zmp_ph108_D4
zmp_ph97_G6
zmp_ph97_G9
zmp_ph97_H2
zmp_ph97_B10
zmp_ph97_C2
zmp_ph97_C4
zmp_ph97_B8
zmp_ph97_C1
zmp_ph97_D2
zmp_ph97_D5
zmp_ph111_G5
zmp_ph111_G11
zmp_ph111_H1
zmp_ph111_B1a
zmp_ph111_B5
zmp_ph111_B8
zmp_ph111_B6
zmp_ph111_B7
zmp_ph111_B9
zmp_ph111_B10
zmp_ph114_G4
zmp_ph114_G11
zmp_ph114_H1
zmp_ph114_H2
zmp_ph114_A9
zmp_ph114_A11
zmp_ph114_B2
zmp_ph114_B4
zmp_ph114_D8
zmp_ph114_E2

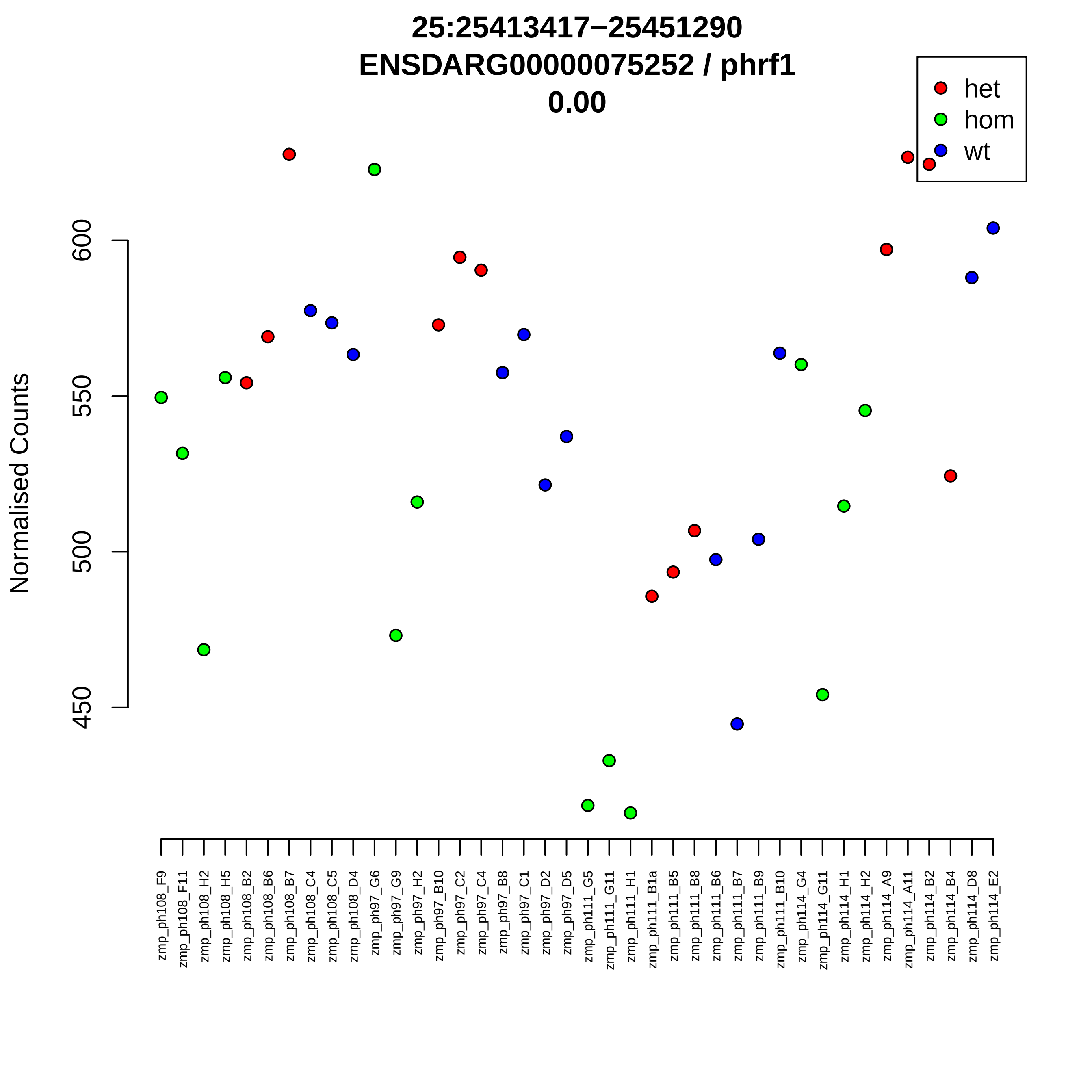

25:25413417−25451290
ENSDARG00000075252 / phrf1
0.00
het
hom
wt
Normalised Counts
600
550
500
450
zmp_ph108_F9
zmp_ph108_F11
zmp_ph108_H2
zmp_ph108_H5
zmp_ph108_B2
zmp_ph108_B6
zmp_ph108_B7
zmp_ph108_C4
zmp_ph108_C5
zmp_ph108_D4
zmp_ph97_G6
zmp_ph97_G9
zmp_ph97_H2
zmp_ph97_B10
zmp_ph97_C2
zmp_ph97_C4
zmp_ph97_B8
zmp_ph97_C1
zmp_ph97_D2
zmp_ph97_D5
zmp_ph111_G5
zmp_ph111_G11
zmp_ph111_H1
zmp_ph111_B1a
zmp_ph111_B5
zmp_ph111_B8
zmp_ph111_B6
zmp_ph111_B7
zmp_ph111_B9
zmp_ph111_B10
zmp_ph114_G4
zmp_ph114_G11
zmp_ph114_H1
zmp_ph114_H2
zmp_ph114_A9
zmp_ph114_A11
zmp_ph114_B2
zmp_ph114_B4
zmp_ph114_D8
zmp_ph114_E2

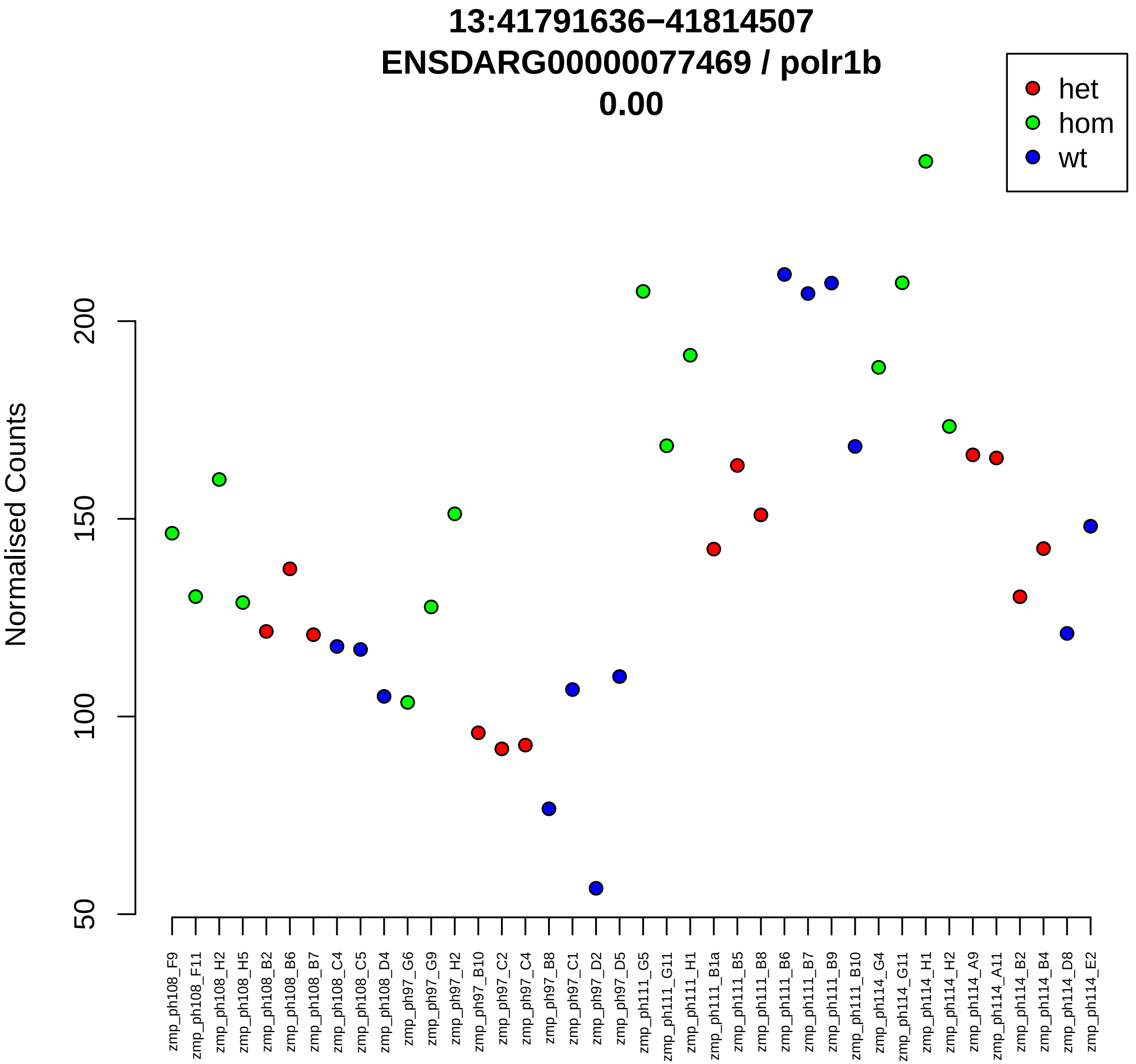
13:41791636−41814507
ENSDARG00000077469 / polr1b
0.00
het
hom
wt
Normalised Counts
50
100
150
200
zmp_ph108_F9
zmp_ph108_F11
zmp_ph108_H2
zmp_ph108_H5
zmp_ph108_B2
zmp_ph108_B6
zmp_ph108_B7
zmp_ph108_C4
zmp_ph108_C5
zmp_ph108_D4
zmp_ph97_G6
zmp_ph97_G9
zmp_ph97_H2
zmp_ph97_B10
zmp_ph97_C2
zmp_ph97_C4
zmp_ph97_B8
zmp_ph97_C1
zmp_ph97_D2
zmp_ph97_D5
zmp_ph111_G5
zmp_ph111_G11
zmp_ph111_H1
zmp_ph111_B1a
zmp_ph111_B5
zmp_ph111_B8
zmp_ph111_B6
zmp_ph111_B7
zmp_ph111_B9
zmp_ph111_B10
zmp_ph114_G4
zmp_ph114_G11
zmp_ph114_H1
zmp_ph114_H2
zmp_ph114_A9
zmp_ph114_A11
zmp_ph114_B2
zmp_ph114_B4
zmp_ph114_D8
zmp_ph114_E2

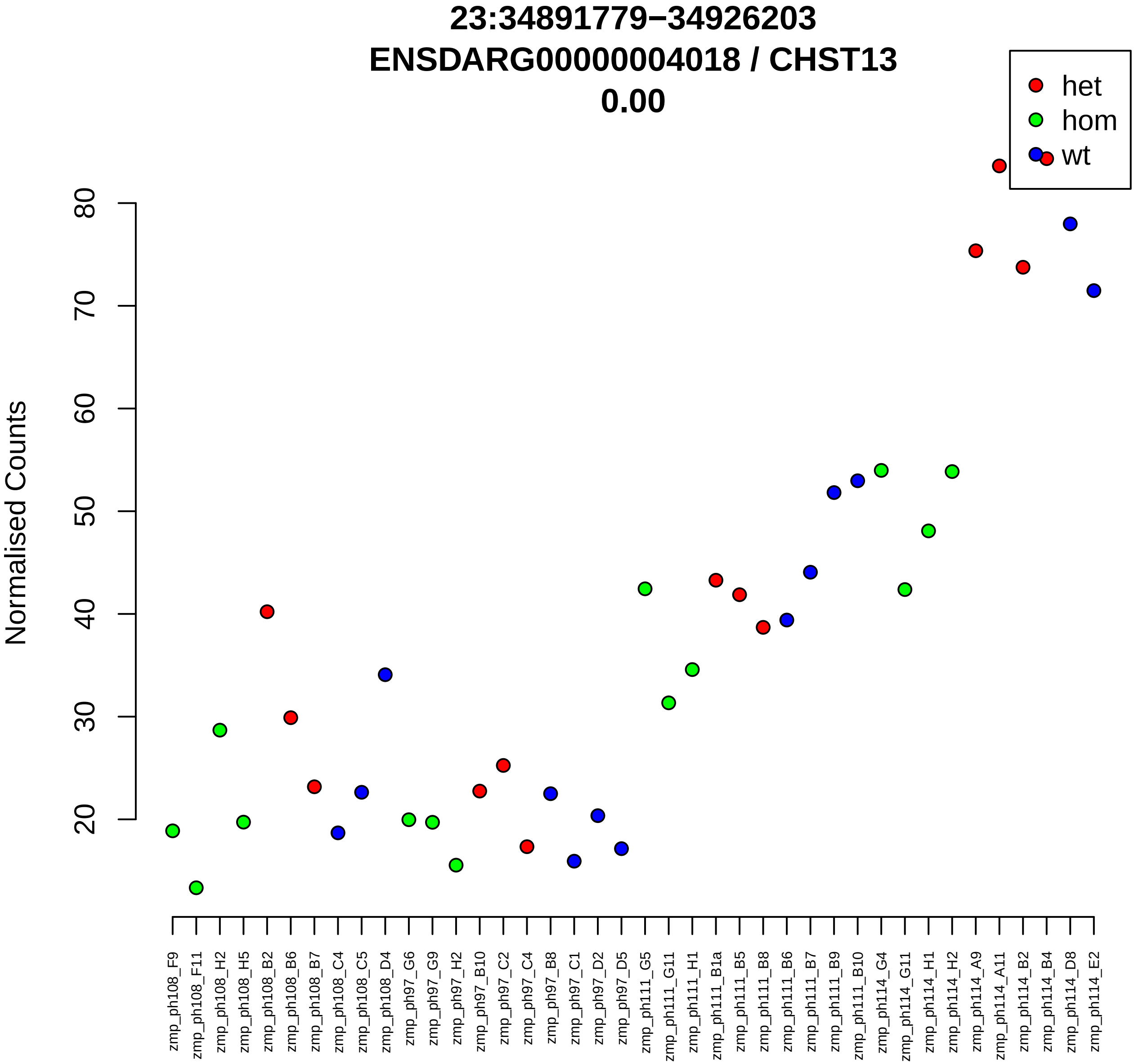

23:34891779−34926203
ENSDARG00000004018 / CHST13
0.00
het
hom
wt
Normalised Counts
20
30
40
50
60
70
80
zmp_ph108_F9
zmp_ph108_F11
zmp_ph108_H2
zmp_ph108_H5
zmp_ph108_B2
zmp_ph108_B6
zmp_ph108_B7
zmp_ph108_C4
zmp_ph108_C5
zmp_ph108_D4
zmp_ph97_G6
zmp_ph97_G9
zmp_ph97_H2
zmp_ph97_B10
zmp_ph97_C2
zmp_ph97_C4
zmp_ph97_B8
zmp_ph97_C1
zmp_ph97_D2
zmp_ph97_D5
zmp_ph111_G5
zmp_ph111_G11
zmp_ph111_H1
zmp_ph111_B1a
zmp_ph111_B5
zmp_ph111_B8
zmp_ph111_B6
zmp_ph111_B7
zmp_ph111_B9
zmp_ph111_B10
zmp_ph114_G4
zmp_ph114_G11
zmp_ph114_H1
zmp_ph114_H2
zmp_ph114_A9
zmp_ph114_A11
zmp_ph114_B2
zmp_ph114_B4
zmp_ph114_D8
zmp_ph114_E2

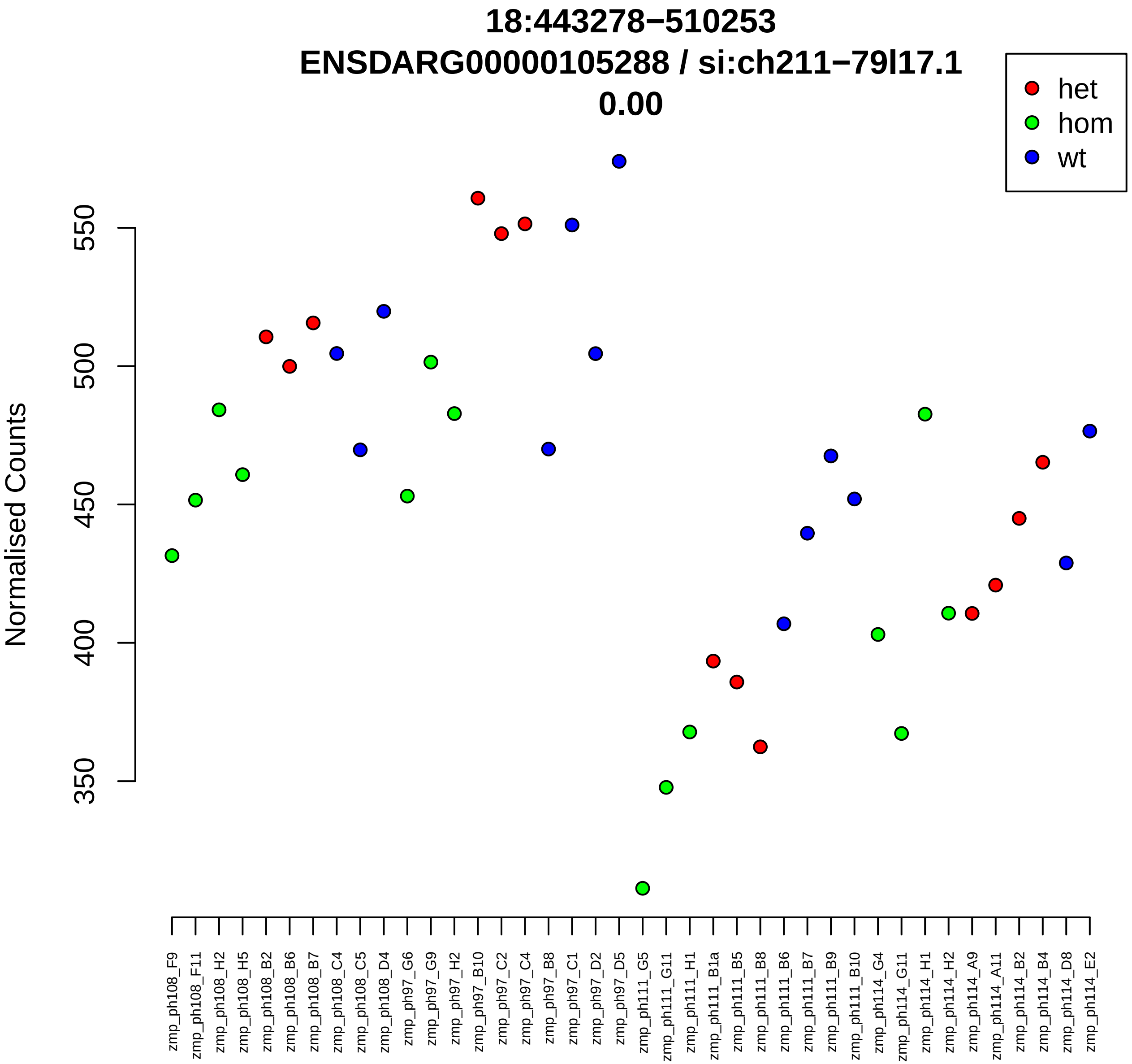
18:443278−510253
ENSDARG00000105288 / si:ch211−79l17.1
0.00
het
hom
wt
Normalised Counts
350
400
450
500
550
zmp_ph108_F9
zmp_ph108_F11
zmp_ph108_H2
zmp_ph108_H5
zmp_ph108_B2
zmp_ph108_B6
zmp_ph108_B7
zmp_ph108_C4
zmp_ph108_C5
zmp_ph108_D4
zmp_ph97_G6
zmp_ph97_G9
zmp_ph97_H2
zmp_ph97_B10
zmp_ph97_C2
zmp_ph97_C4
zmp_ph97_B8
zmp_ph97_C1
zmp_ph97_D2
zmp_ph97_D5
zmp_ph111_G5
zmp_ph111_G11
zmp_ph111_H1
zmp_ph111_B1a
zmp_ph111_B5
zmp_ph111_B8
zmp_ph111_B6
zmp_ph111_B7
zmp_ph111_B9
zmp_ph111_B10
zmp_ph114_G4
zmp_ph114_G11
zmp_ph114_H1
zmp_ph114_H2
zmp_ph114_A9
zmp_ph114_A11
zmp_ph114_B2
zmp_ph114_B4
zmp_ph114_D8
zmp_ph114_E2

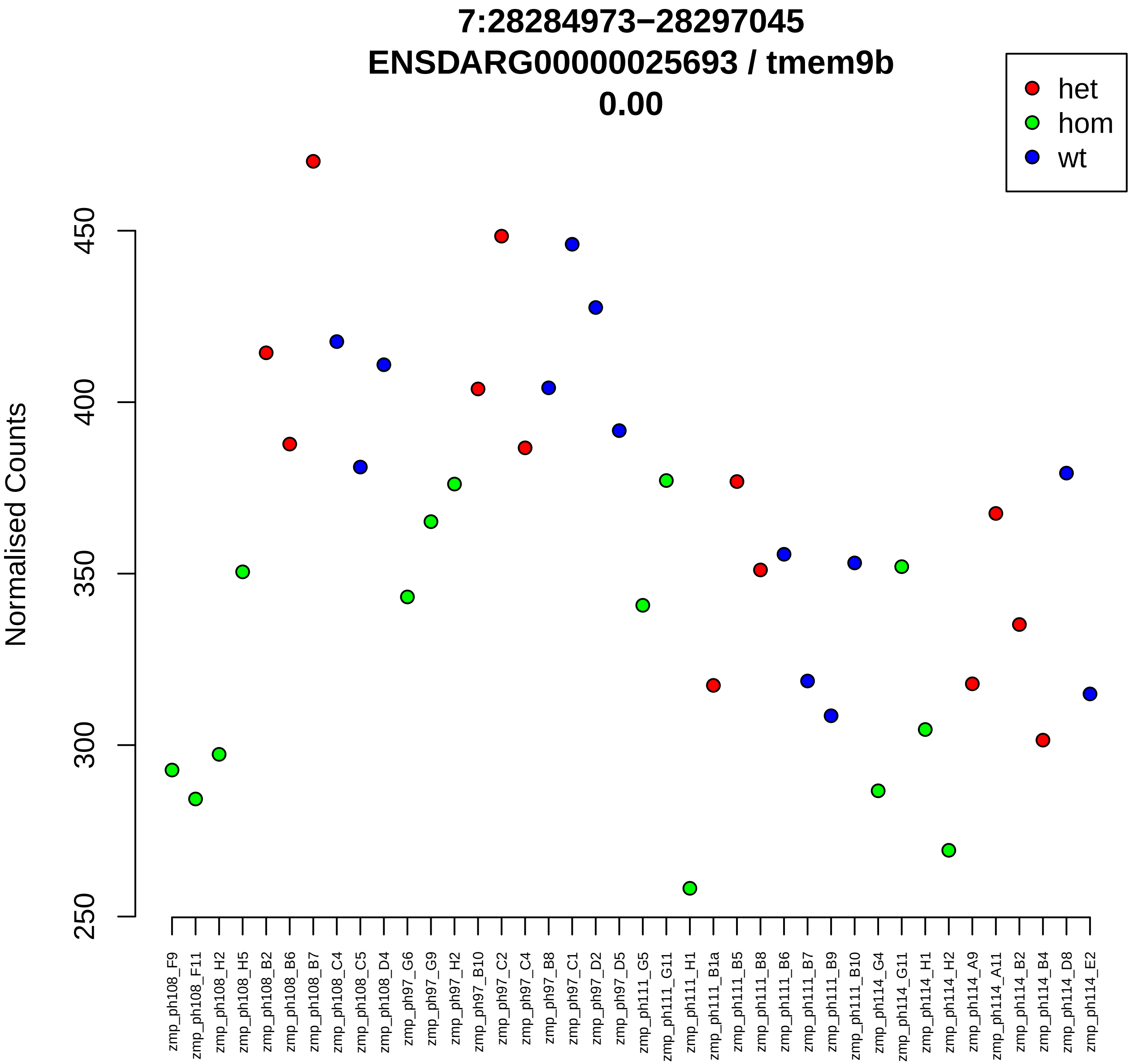

7:28284973−28297045
ENSDARG00000025693 / tmem9b
0.00
het
hom
wt
Normalised Counts
250
300
350
400
450
zmp_ph108_F9
zmp_ph108_F11
zmp_ph108_H2
zmp_ph108_H5
zmp_ph108_B2
zmp_ph108_B6
zmp_ph108_B7
zmp_ph108_C4
zmp_ph108_C5
zmp_ph108_D4
zmp_ph97_G6
zmp_ph97_G9
zmp_ph97_H2
zmp_ph97_B10
zmp_ph97_C2
zmp_ph97_C4
zmp_ph97_B8
zmp_ph97_C1
zmp_ph97_D2
zmp_ph97_D5
zmp_ph111_G5
zmp_ph111_G11
zmp_ph111_H1
zmp_ph111_B1a
zmp_ph111_B5
zmp_ph111_B8
zmp_ph111_B6
zmp_ph111_B7
zmp_ph111_B9
zmp_ph111_B10
zmp_ph114_G4
zmp_ph114_G11
zmp_ph114_H1
zmp_ph114_H2
zmp_ph114_A9
zmp_ph114_A11
zmp_ph114_B2
zmp_ph114_B4
zmp_ph114_D8
zmp_ph114_E2

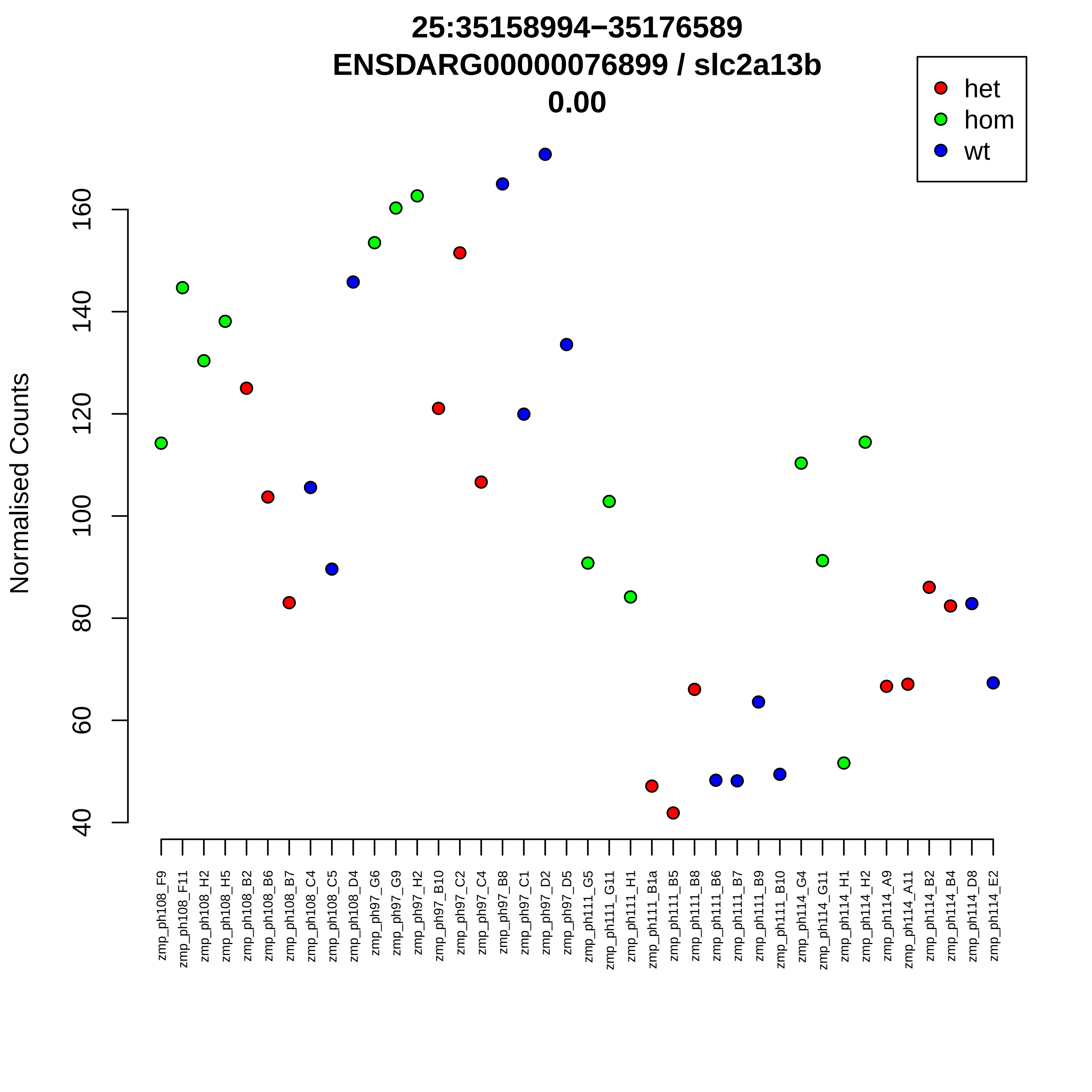
25:35158994−35176589
ENSDARG00000076899 / slc2a13b
0.00
het
hom
wt
Normalised Counts
40
60
80
100
120
140
160
zmp_ph108_F9
zmp_ph108_F11
zmp_ph108_H2
zmp_ph108_H5
zmp_ph108_B2
zmp_ph108_B6
zmp_ph108_B7
zmp_ph108_C4
zmp_ph108_C5
zmp_ph108_D4
zmp_ph97_G6
zmp_ph97_G9
zmp_ph97_H2
zmp_ph97_B10
zmp_ph97_C2
zmp_ph97_C4
zmp_ph97_B8
zmp_ph97_C1
zmp_ph97_D2
zmp_ph97_D5
zmp_ph111_G5
zmp_ph111_G11
zmp_ph111_H1
zmp_ph111_B1a
zmp_ph111_B5
zmp_ph111_B8
zmp_ph111_B6
zmp_ph111_B7
zmp_ph111_B9
zmp_ph111_B10
zmp_ph114_G4
zmp_ph114_G11
zmp_ph114_H1
zmp_ph114_H2
zmp_ph114_A9
zmp_ph114_A11
zmp_ph114_B2
zmp_ph114_B4
zmp_ph114_D8
zmp_ph114_E2

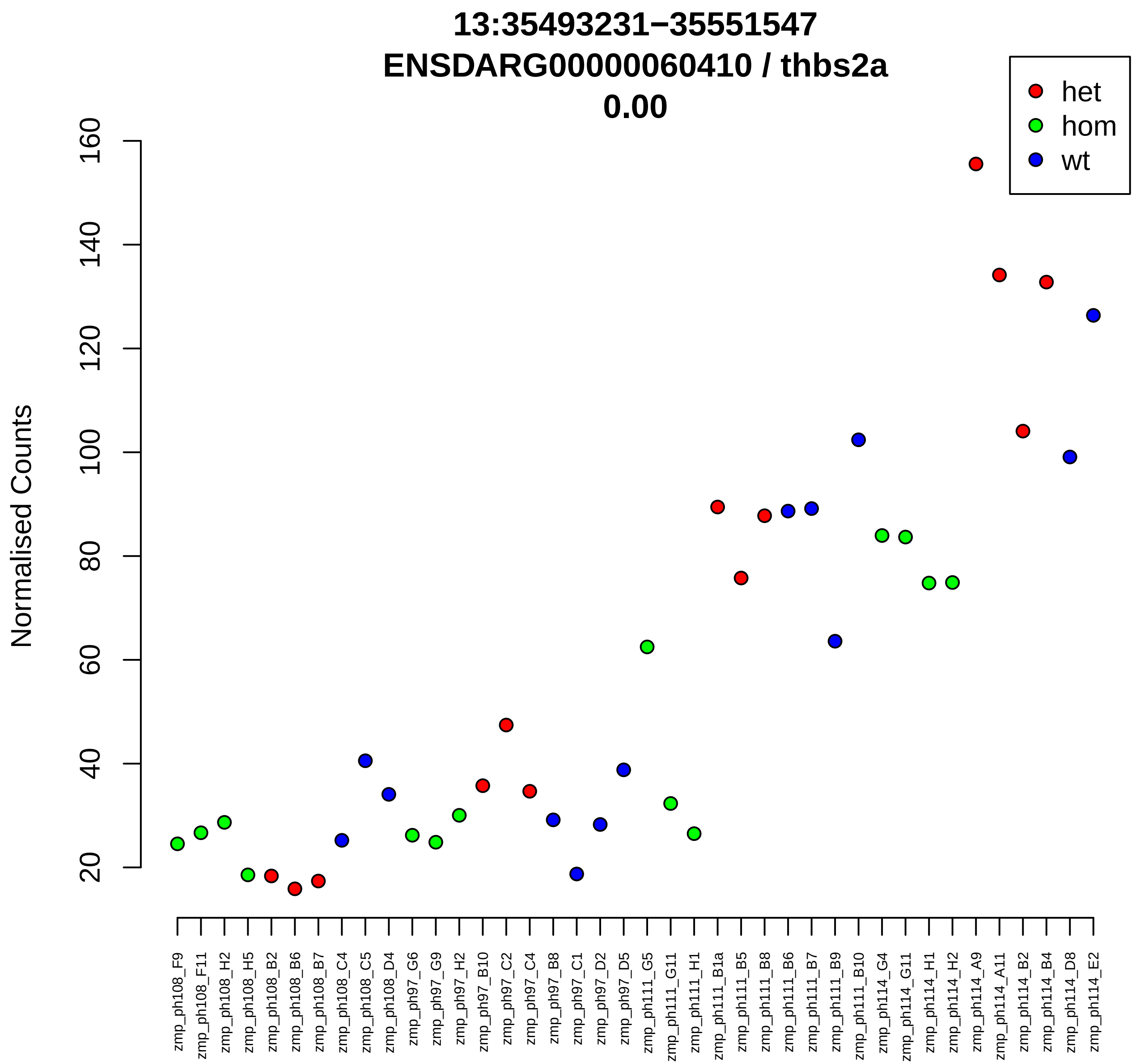

13:35493231−35551547
ENSDARG00000060410 / thbs2a
0.00
het
hom
wt
Normalised Counts
20
40
60
80
100
120
140
160
zmp_ph108_F9
zmp_ph108_F11
zmp_ph108_H2
zmp_ph108_H5
zmp_ph108_B2
zmp_ph108_B6
zmp_ph108_B7
zmp_ph108_C4
zmp_ph108_C5
zmp_ph108_D4
zmp_ph97_G6
zmp_ph97_G9
zmp_ph97_H2
zmp_ph97_B10
zmp_ph97_C2
zmp_ph97_C4
zmp_ph97_B8
zmp_ph97_C1
zmp_ph97_D2
zmp_ph97_D5
zmp_ph111_G5
zmp_ph111_G11
zmp_ph111_H1
zmp_ph111_B1a
zmp_ph111_B5
zmp_ph111_B8
zmp_ph111_B6
zmp_ph111_B7
zmp_ph111_B9
zmp_ph111_B10
zmp_ph114_G4
zmp_ph114_G11
zmp_ph114_H1
zmp_ph114_H2
zmp_ph114_A9
zmp_ph114_A11
zmp_ph114_B2
zmp_ph114_B4
zmp_ph114_D8
zmp_ph114_E2

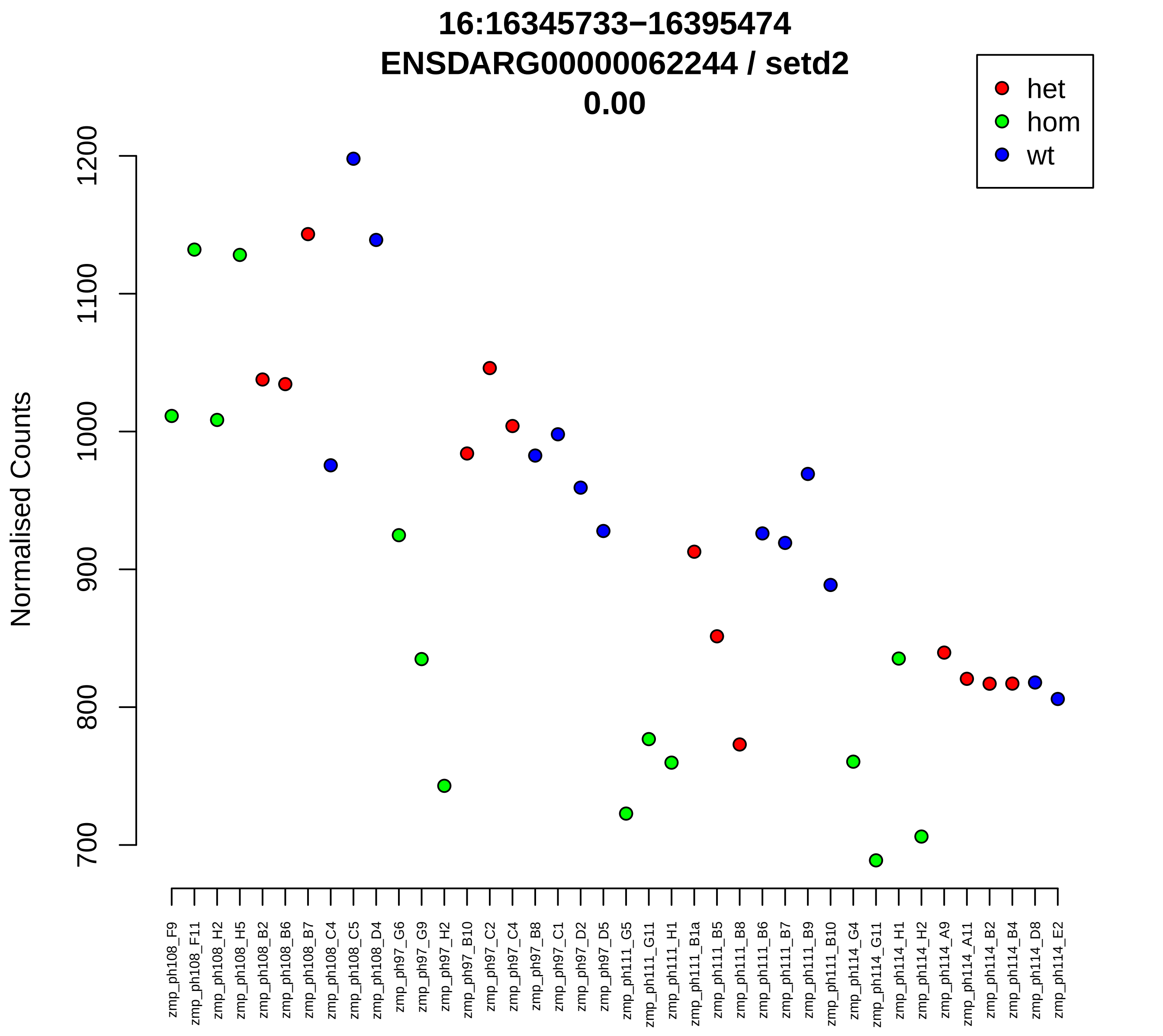

16:16345733−16395474
ENSDARG00000062244 / setd2
0.00
het
hom
wt
Normalised Counts
1200
1100
1000
900
800
700
zmp_ph108_F9
zmp_ph108_F11
zmp_ph108_H2
zmp_ph108_H5
zmp_ph108_B2
zmp_ph108_B6
zmp_ph108_B7
zmp_ph108_C4
zmp_ph108_C5
zmp_ph108_D4
zmp_ph97_G6
zmp_ph97_G9
zmp_ph97_H2
zmp_ph97_B10
zmp_ph97_C2
zmp_ph97_C4
zmp_ph97_B8
zmp_ph97_C1
zmp_ph97_D2
zmp_ph97_D5
zmp_ph111_G5
zmp_ph111_G11
zmp_ph111_H1
zmp_ph111_B1a
zmp_ph111_B5
zmp_ph111_B8
zmp_ph111_B6
zmp_ph111_B7
zmp_ph111_B9
zmp_ph111_B10
zmp_ph114_G4
zmp_ph114_G11
zmp_ph114_H1
zmp_ph114_H2
zmp_ph114_A9
zmp_ph114_A11
zmp_ph114_B2
zmp_ph114_B4
zmp_ph114_D8
zmp_ph114_E2

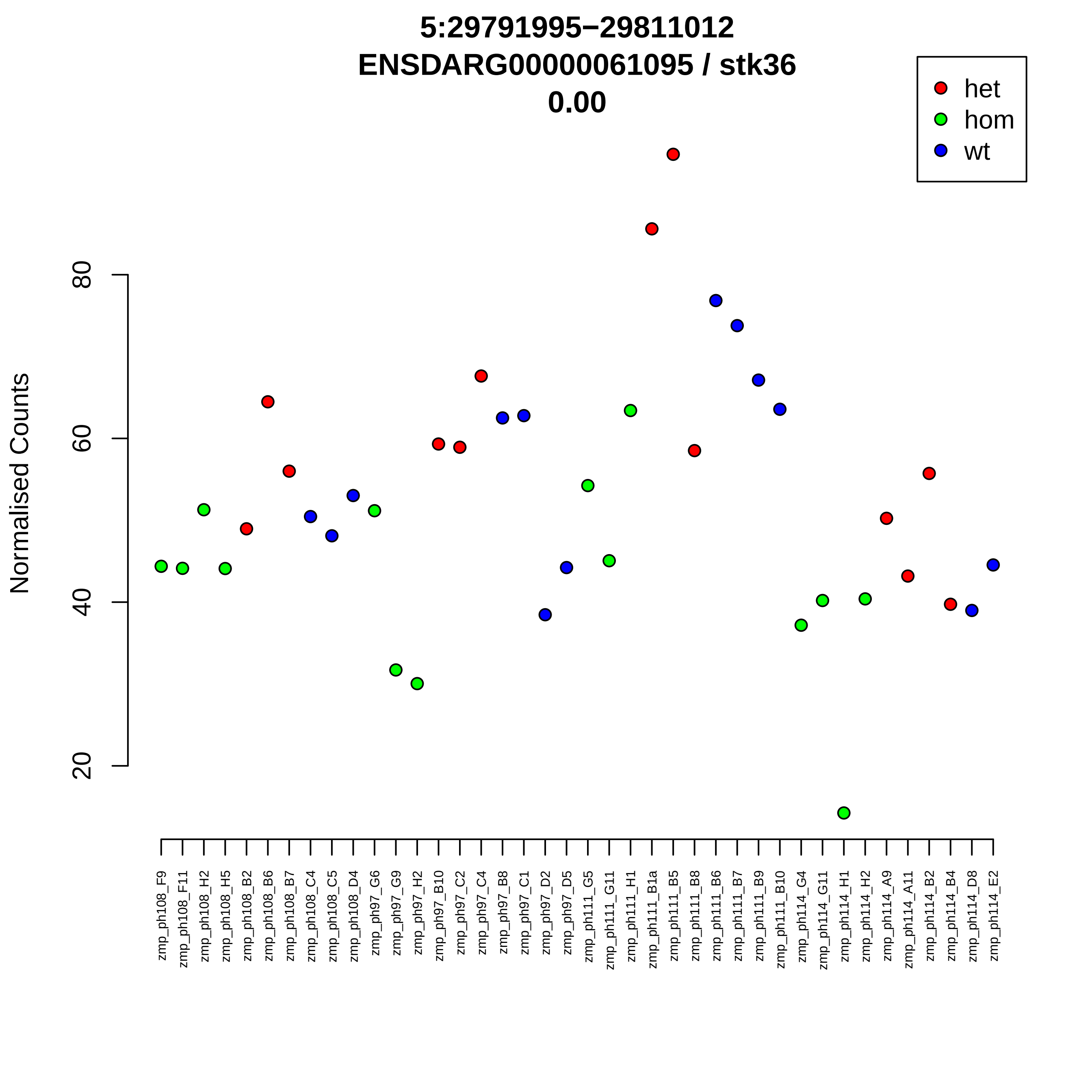
5:29791995−29811012
ENSDARG00000061095 / stk36
0.00
het
hom
wt
Normalised Counts
20
40
60
80
zmp_ph108_F9
zmp_ph108_F11
zmp_ph108_H2
zmp_ph108_H5
zmp_ph108_B2
zmp_ph108_B6
zmp_ph108_B7
zmp_ph108_C4
zmp_ph108_C5
zmp_ph108_D4
zmp_ph97_G6
zmp_ph97_G9
zmp_ph97_H2
zmp_ph97_B10
zmp_ph97_C2
zmp_ph97_C4
zmp_ph97_B8
zmp_ph97_C1
zmp_ph97_D2
zmp_ph97_D5
zmp_ph111_G5
zmp_ph111_G11
zmp_ph111_H1
zmp_ph111_B1a
zmp_ph111_B5
zmp_ph111_B8
zmp_ph111_B6
zmp_ph111_B7
zmp_ph111_B9
zmp_ph111_B10
zmp_ph114_G4
zmp_ph114_G11
zmp_ph114_H1
zmp_ph114_H2
zmp_ph114_A9
zmp_ph114_A11
zmp_ph114_B2
zmp_ph114_B4
zmp_ph114_D8
zmp_ph114_E2

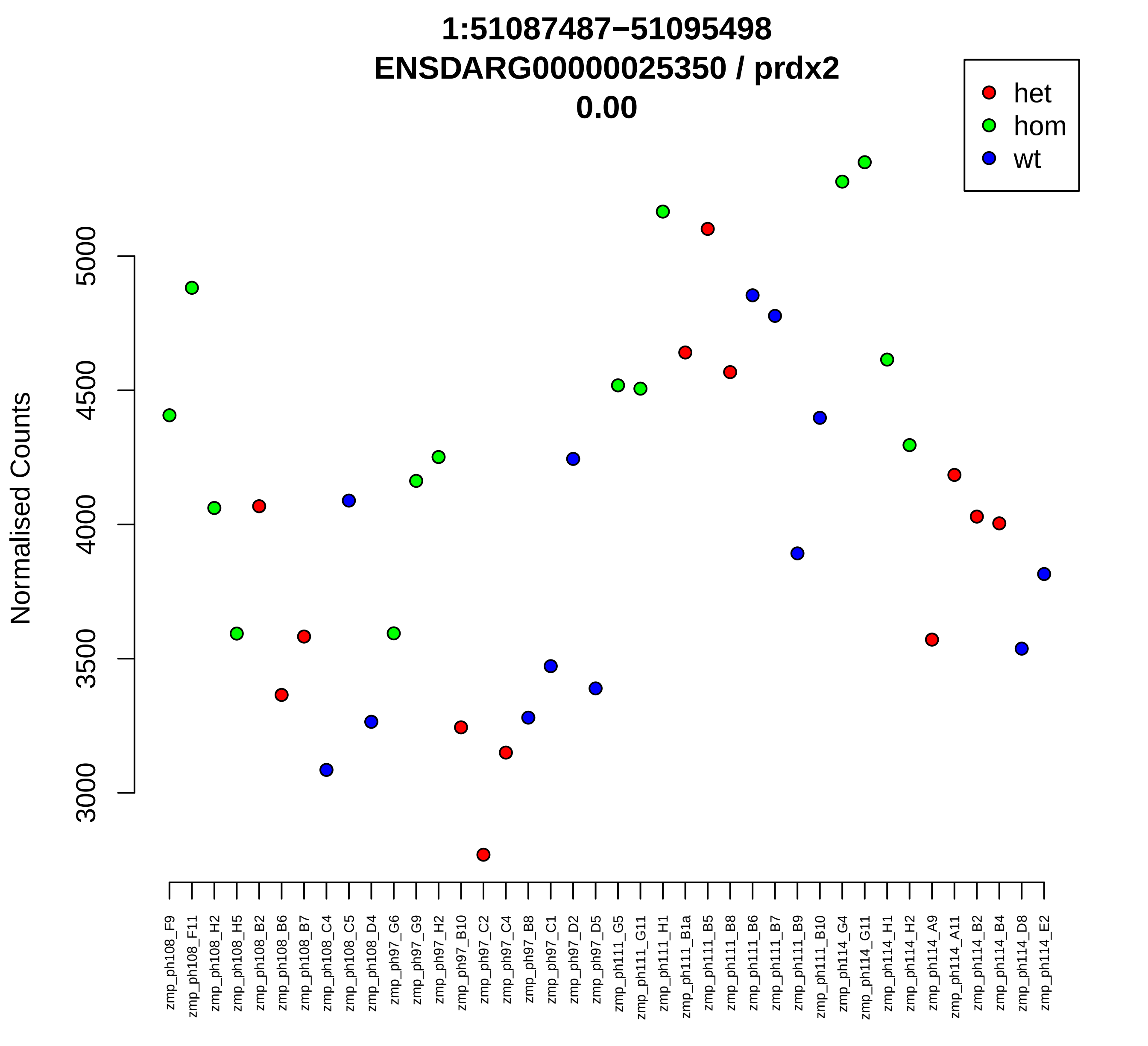
1:51087487−51095498
ENSDARG00000025350 / prdx2
0.00
het
hom
wt
Normalised Counts
3000
3500
4000
4500
5000
zmp_ph108_F9
zmp_ph108_F11
zmp_ph108_H2
zmp_ph108_H5
zmp_ph108_B2
zmp_ph108_B6
zmp_ph108_B7
zmp_ph108_C4
zmp_ph108_C5
zmp_ph108_D4
zmp_ph97_G6
zmp_ph97_G9
zmp_ph97_H2
zmp_ph97_B10
zmp_ph97_C2
zmp_ph97_C4
zmp_ph97_B8
zmp_ph97_C1
zmp_ph97_D2
zmp_ph97_D5
zmp_ph111_G5
zmp_ph111_G11
zmp_ph111_H1
zmp_ph111_B1a
zmp_ph111_B5
zmp_ph111_B8
zmp_ph111_B6
zmp_ph111_B7
zmp_ph111_B9
zmp_ph111_B10
zmp_ph114_G4
zmp_ph114_G11
zmp_ph114_H1
zmp_ph114_H2
zmp_ph114_A9
zmp_ph114_A11
zmp_ph114_B2
zmp_ph114_B4
zmp_ph114_D8
zmp_ph114_E2

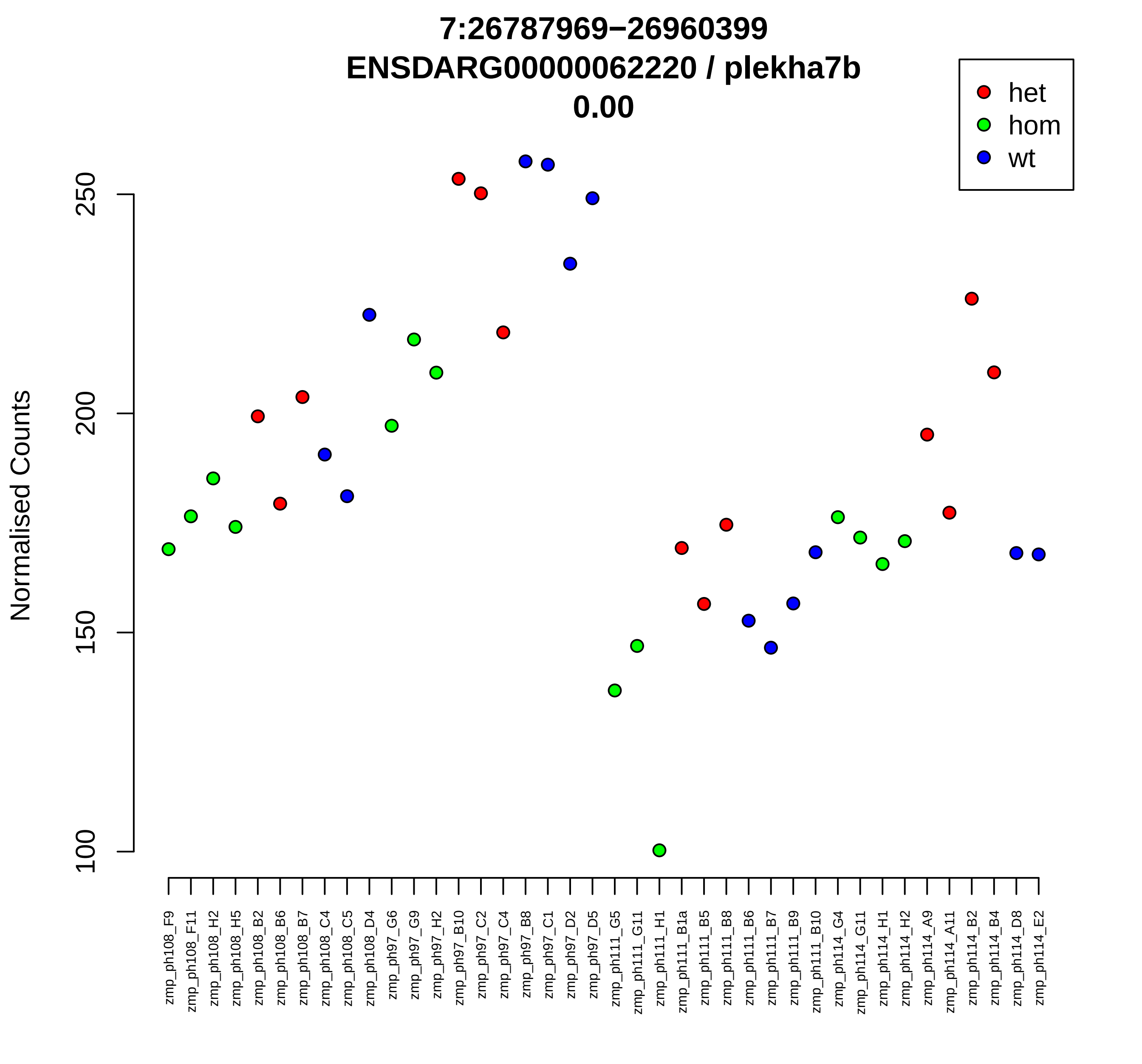
7:26787969−26960399
ENSDARG00000062220 / plekha7b
0.00
het
hom
wt
Normalised Counts
250
200
150
100
zmp_ph108_F9
zmp_ph108_F11
zmp_ph108_H2
zmp_ph108_H5
zmp_ph108_B2
zmp_ph108_B6
zmp_ph108_B7
zmp_ph108_C4
zmp_ph108_C5
zmp_ph108_D4
zmp_ph97_G6
zmp_ph97_G9
zmp_ph97_H2
zmp_ph97_B10
zmp_ph97_C2
zmp_ph97_C4
zmp_ph97_B8
zmp_ph97_C1
zmp_ph97_D2
zmp_ph97_D5
zmp_ph111_G5
zmp_ph111_G11
zmp_ph111_H1
zmp_ph111_B1a
zmp_ph111_B5
zmp_ph111_B8
zmp_ph111_B6
zmp_ph111_B7
zmp_ph111_B9
zmp_ph111_B10
zmp_ph114_G4
zmp_ph114_G11
zmp_ph114_H1
zmp_ph114_H2
zmp_ph114_A9
zmp_ph114_A11
zmp_ph114_B2
zmp_ph114_B4
zmp_ph114_D8
zmp_ph114_E2

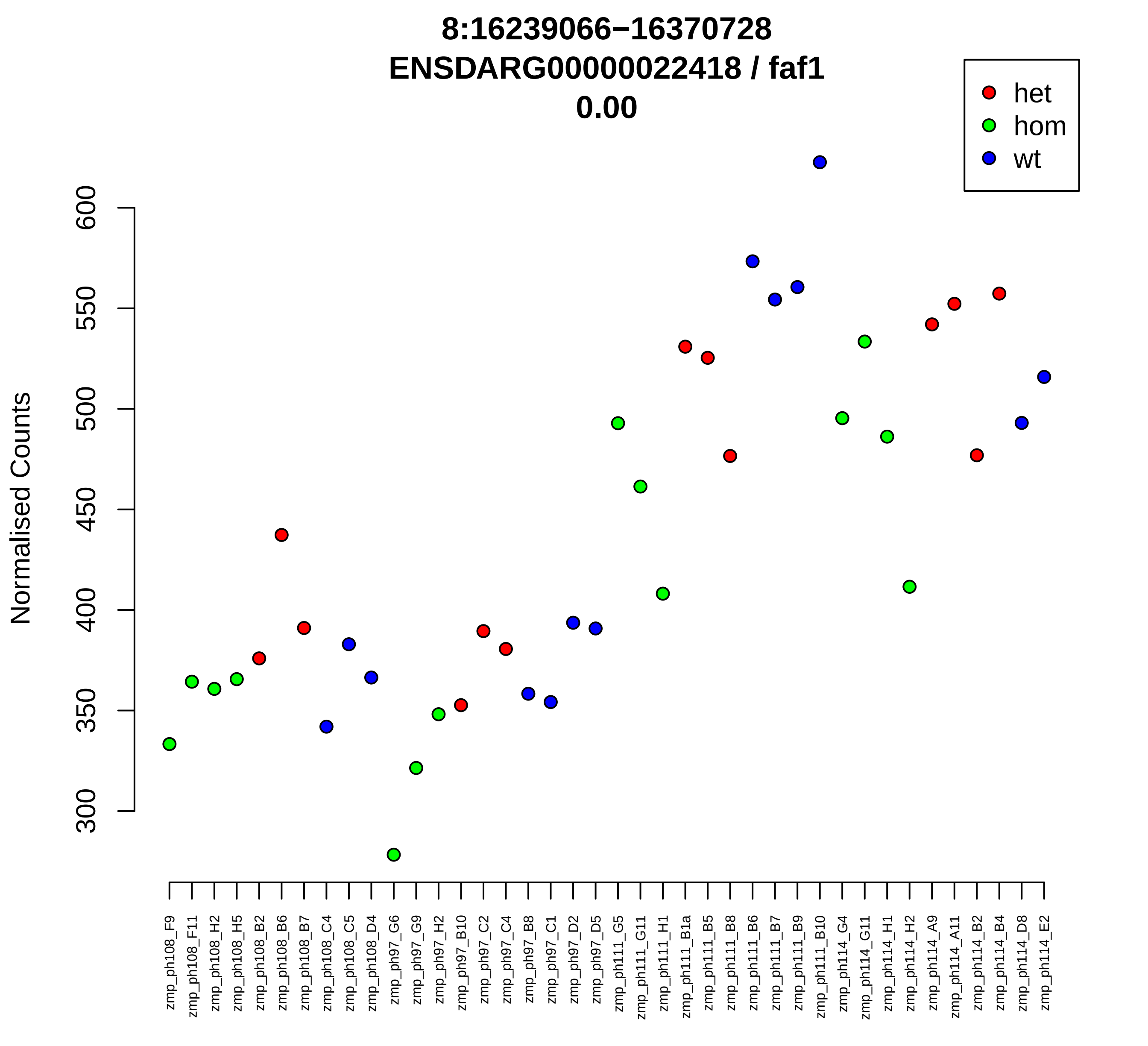

8:16239066−16370728
ENSDARG00000022418 / faf1
0.00
het
hom
wt
Normalised Counts
300
350
400
450
500
550
600
zmp_ph108_F9
zmp_ph108_F11
zmp_ph108_H2
zmp_ph108_H5
zmp_ph108_B2
zmp_ph108_B6
zmp_ph108_B7
zmp_ph108_C4
zmp_ph108_C5
zmp_ph108_D4
zmp_ph97_G6
zmp_ph97_G9
zmp_ph97_H2
zmp_ph97_B10
zmp_ph97_C2
zmp_ph97_C4
zmp_ph97_B8
zmp_ph97_C1
zmp_ph97_D2
zmp_ph97_D5
zmp_ph111_G5
zmp_ph111_G11
zmp_ph111_H1
zmp_ph111_B1a
zmp_ph111_B5
zmp_ph111_B8
zmp_ph111_B6
zmp_ph111_B7
zmp_ph111_B9
zmp_ph111_B10
zmp_ph114_G4
zmp_ph114_G11
zmp_ph114_H1
zmp_ph114_H2
zmp_ph114_A9
zmp_ph114_A11
zmp_ph114_B2
zmp_ph114_B4
zmp_ph114_D8
zmp_ph114_E2

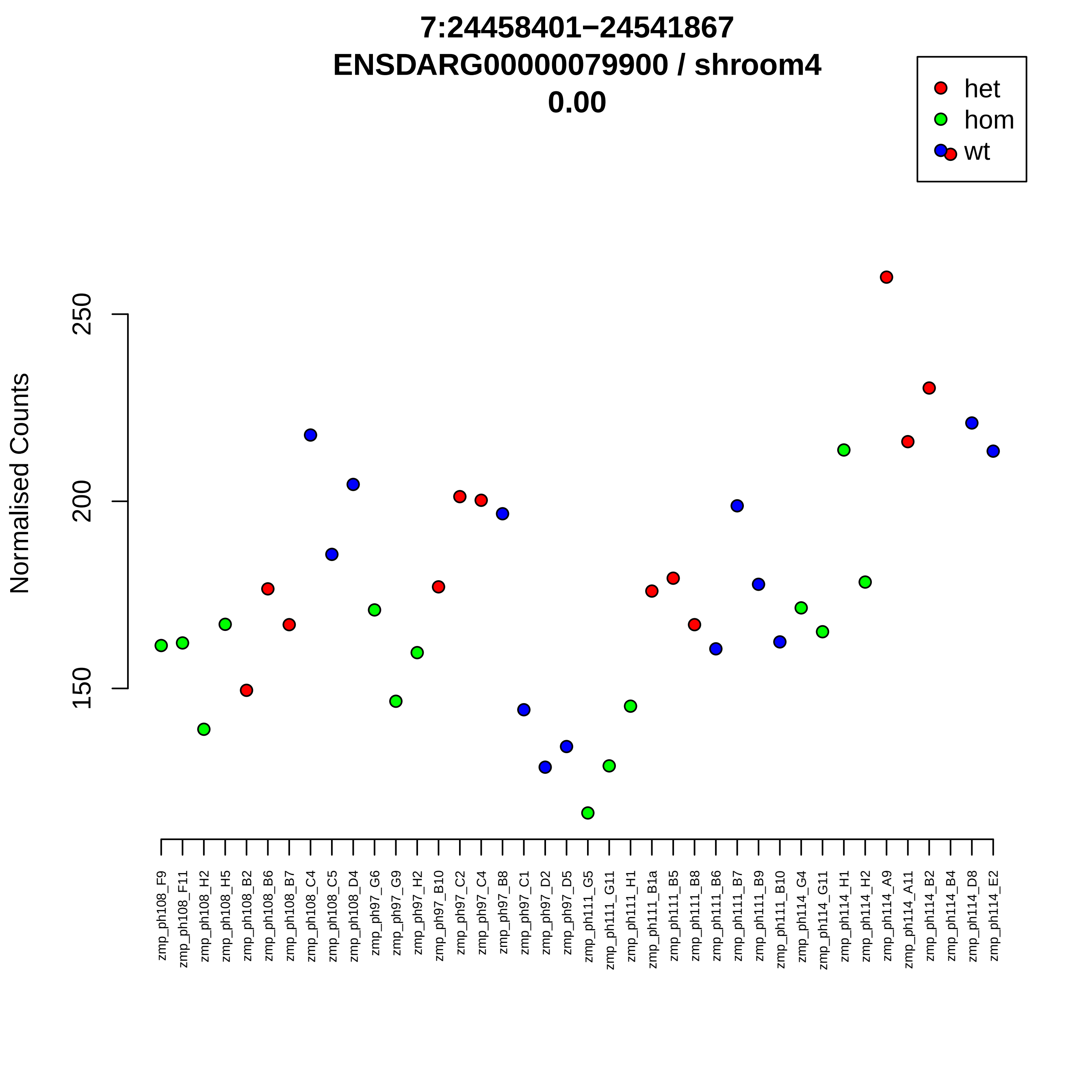
7:24458401−24541867
ENSDARG00000079900 / shroom4
0.00
het
hom
wt
Normalised Counts
150
200
250
zmp_ph108_F9
zmp_ph108_F11
zmp_ph108_H2
zmp_ph108_H5
zmp_ph108_B2
zmp_ph108_B6
zmp_ph108_B7
zmp_ph108_C4
zmp_ph108_C5
zmp_ph108_D4
zmp_ph97_G6
zmp_ph97_G9
zmp_ph97_H2
zmp_ph97_B10
zmp_ph97_C2
zmp_ph97_C4
zmp_ph97_B8
zmp_ph97_C1
zmp_ph97_D2
zmp_ph97_D5
zmp_ph111_G5
zmp_ph111_G11
zmp_ph111_H1
zmp_ph111_B1a
zmp_ph111_B5
zmp_ph111_B8
zmp_ph111_B6
zmp_ph111_B7
zmp_ph111_B9
zmp_ph111_B10
zmp_ph114_G4
zmp_ph114_G11
zmp_ph114_H1
zmp_ph114_H2
zmp_ph114_A9
zmp_ph114_A11
zmp_ph114_B2
zmp_ph114_B4
zmp_ph114_D8
zmp_ph114_E2

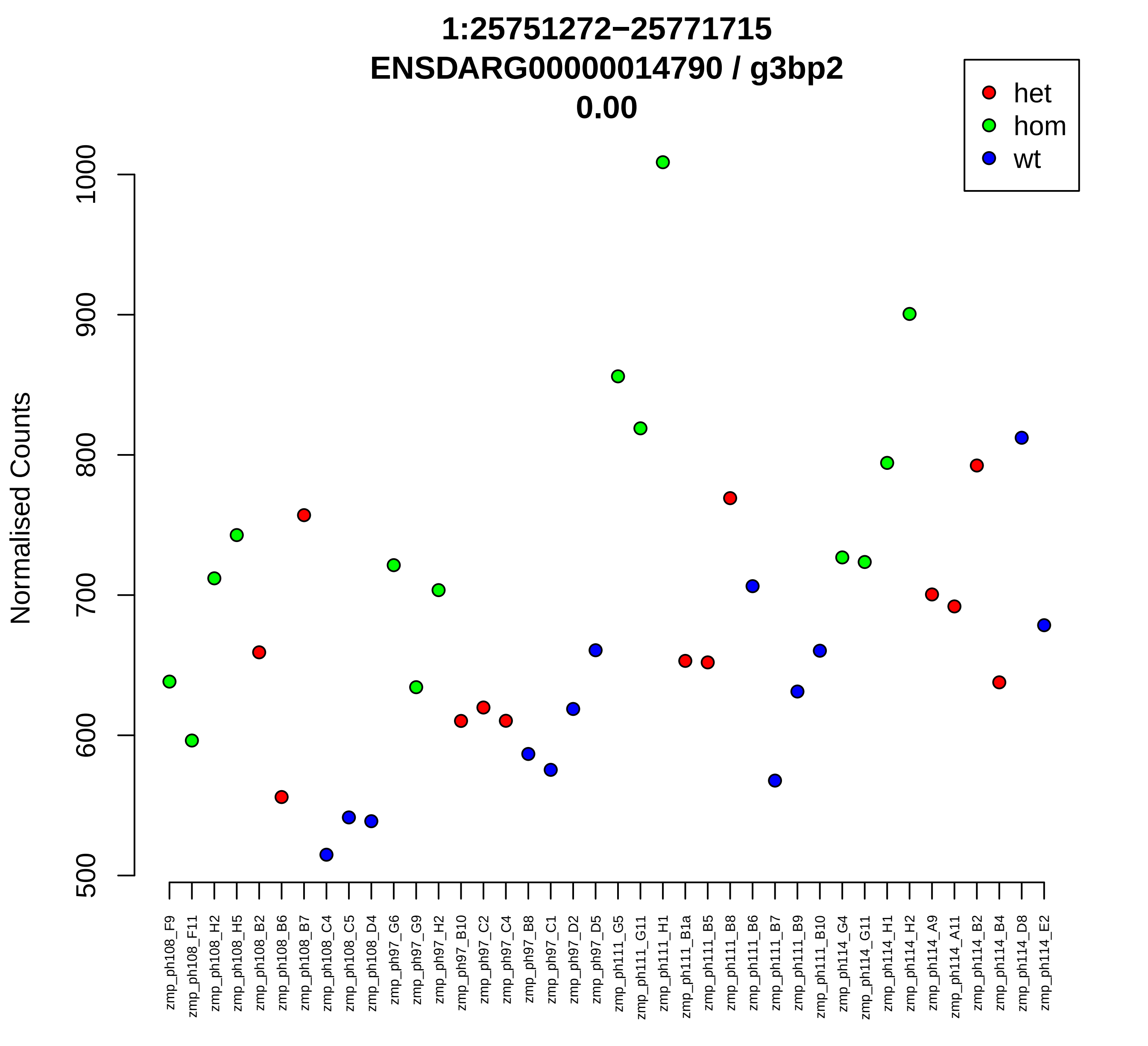
1:25751272−25771715
ENSDARG00000014790 / g3bp2
0.00
het
hom
wt
Normalised Counts
500
600
700
800
900
1000
zmp_ph108_F9
zmp_ph108_F11
zmp_ph108_H2
zmp_ph108_H5
zmp_ph108_B2
zmp_ph108_B6
zmp_ph108_B7
zmp_ph108_C4
zmp_ph108_C5
zmp_ph108_D4
zmp_ph97_G6
zmp_ph97_G9
zmp_ph97_H2
zmp_ph97_B10
zmp_ph97_C2
zmp_ph97_C4
zmp_ph97_B8
zmp_ph97_C1
zmp_ph97_D2
zmp_ph97_D5
zmp_ph111_G5
zmp_ph111_G11
zmp_ph111_H1
zmp_ph111_B1a
zmp_ph111_B5
zmp_ph111_B8
zmp_ph111_B6
zmp_ph111_B7
zmp_ph111_B9
zmp_ph111_B10
zmp_ph114_G4
zmp_ph114_G11
zmp_ph114_H1
zmp_ph114_H2
zmp_ph114_A9
zmp_ph114_A11
zmp_ph114_B2
zmp_ph114_B4
zmp_ph114_D8
zmp_ph114_E2

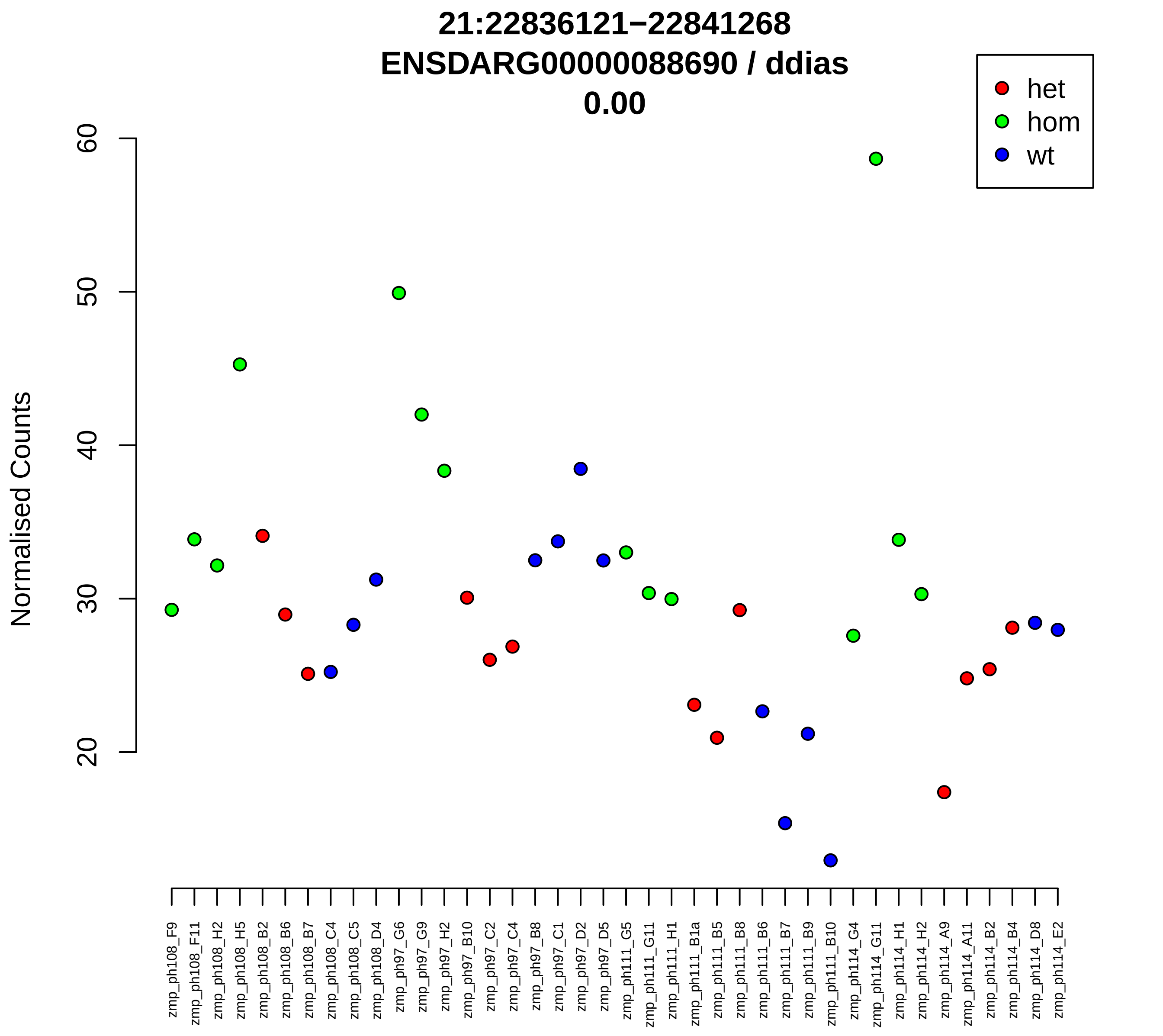
21:22836121−22841268
ENSDARG00000088690 / ddias
0.00
Normalised Counts
het
hom
wt
20
30
40
50
60
zmp_ph108_F9
zmp_ph108_F11
zmp_ph108_H2
zmp_ph108_H5
zmp_ph108_B2
zmp_ph108_B6
zmp_ph108_B7
zmp_ph108_C4
zmp_ph108_C5
zmp_ph108_D4
zmp_ph97_G6
zmp_ph97_G9
zmp_ph97_H2
zmp_ph97_B10
zmp_ph97_C2
zmp_ph97_C4
zmp_ph97_B8
zmp_ph97_C1
zmp_ph97_D2
zmp_ph97_D5
zmp_ph111_G5
zmp_ph111_G11
zmp_ph111_H1
zmp_ph111_B1a
zmp_ph111_B5
zmp_ph111_B8
zmp_ph111_B6
zmp_ph111_B7
zmp_ph111_B9
zmp_ph111_B10
zmp_ph114_G4
zmp_ph114_G11
zmp_ph114_H1
zmp_ph114_H2
zmp_ph114_A9
zmp_ph114_A11
zmp_ph114_B2
zmp_ph114_B4
zmp_ph114_D8
zmp_ph114_E2

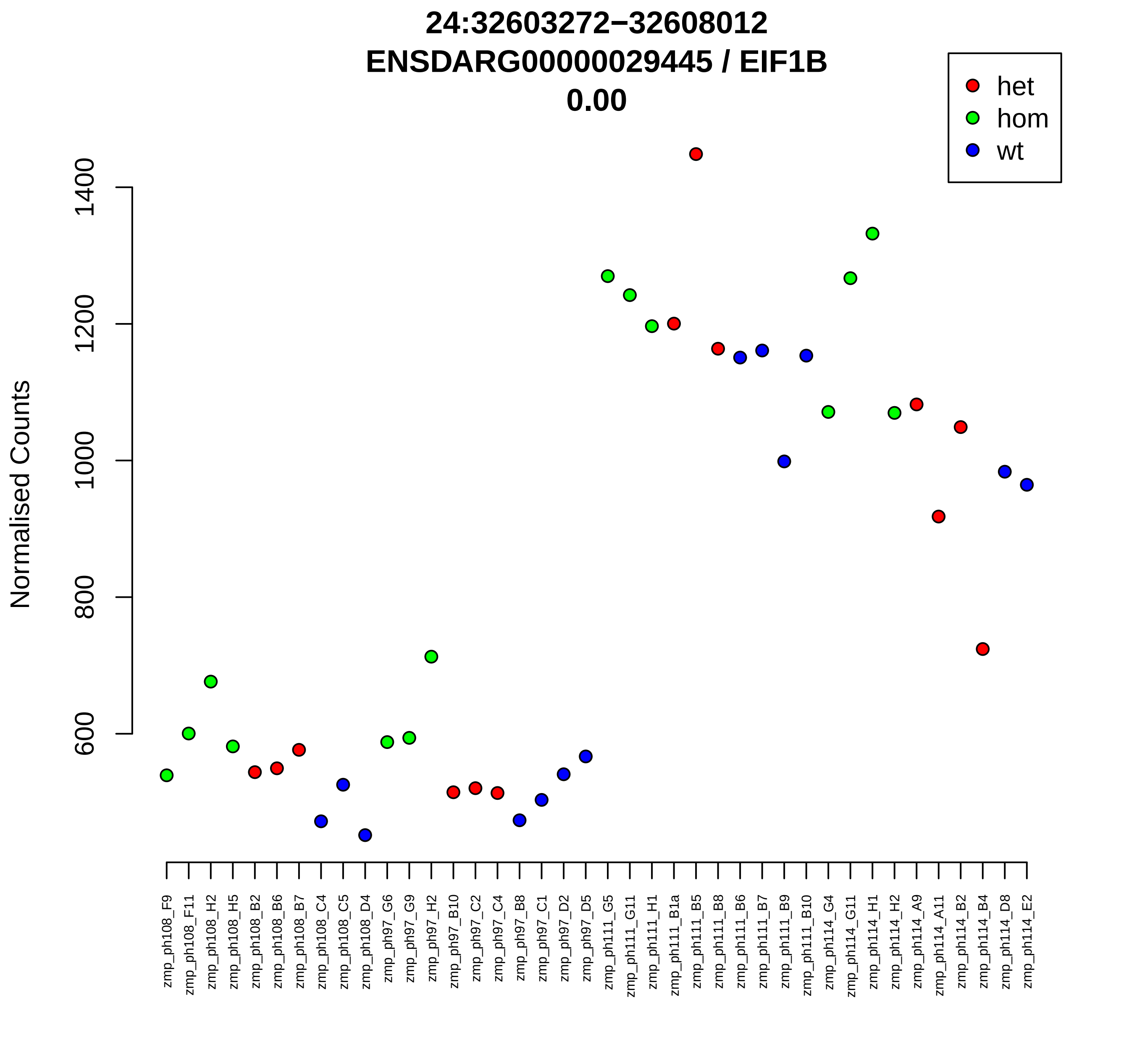

24:32603272−32608012
ENSDARG00000029445 / EIF1B
0.00
het
hom
wt
Normalised Counts
600
800
1000
1200
1400
zmp_ph108_F9
zmp_ph108_F11
zmp_ph108_H2
zmp_ph108_H5
zmp_ph108_B2
zmp_ph108_B6
zmp_ph108_B7
zmp_ph108_C4
zmp_ph108_C5
zmp_ph108_D4
zmp_ph97_G6
zmp_ph97_G9
zmp_ph97_H2
zmp_ph97_B10
zmp_ph97_C2
zmp_ph97_C4
zmp_ph97_B8
zmp_ph97_C1
zmp_ph97_D2
zmp_ph97_D5
zmp_ph111_G5
zmp_ph111_G11
zmp_ph111_H1
zmp_ph111_B1a
zmp_ph111_B5
zmp_ph111_B8
zmp_ph111_B6
zmp_ph111_B7
zmp_ph111_B9
zmp_ph111_B10
zmp_ph114_G4
zmp_ph114_G11
zmp_ph114_H1
zmp_ph114_H2
zmp_ph114_A9
zmp_ph114_A11
zmp_ph114_B2
zmp_ph114_B4
zmp_ph114_D8
zmp_ph114_E2

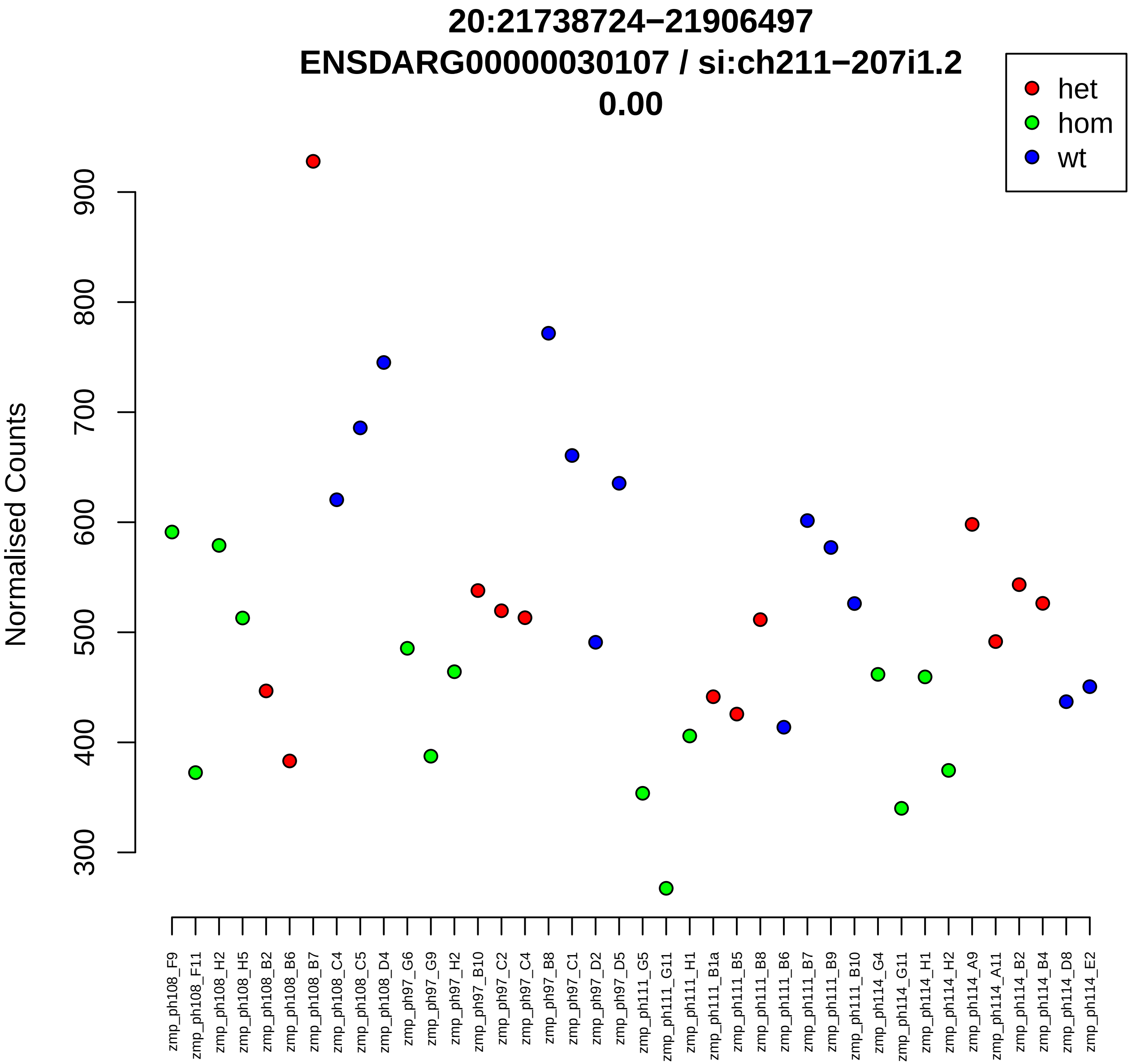

20:21738724−21906497
ENSDARG00000030107 / si:ch211−207i1.2
0.00
het
hom
wt
Normalised Counts
300
400
500
600
700
800
900
zmp_ph108_F9
zmp_ph108_F11
zmp_ph108_H2
zmp_ph108_H5
zmp_ph108_B2
zmp_ph108_B6
zmp_ph108_B7
zmp_ph108_C4
zmp_ph108_C5
zmp_ph108_D4
zmp_ph97_G6
zmp_ph97_G9
zmp_ph97_H2
zmp_ph97_B10
zmp_ph97_C2
zmp_ph97_C4
zmp_ph97_B8
zmp_ph97_C1
zmp_ph97_D2
zmp_ph97_D5
zmp_ph111_G5
zmp_ph111_G11
zmp_ph111_H1
zmp_ph111_B1a
zmp_ph111_B5
zmp_ph111_B8
zmp_ph111_B6
zmp_ph111_B7
zmp_ph111_B9
zmp_ph111_B10
zmp_ph114_G4
zmp_ph114_G11
zmp_ph114_H1
zmp_ph114_H2
zmp_ph114_A9
zmp_ph114_A11
zmp_ph114_B2
zmp_ph114_B4
zmp_ph114_D8
zmp_ph114_E2

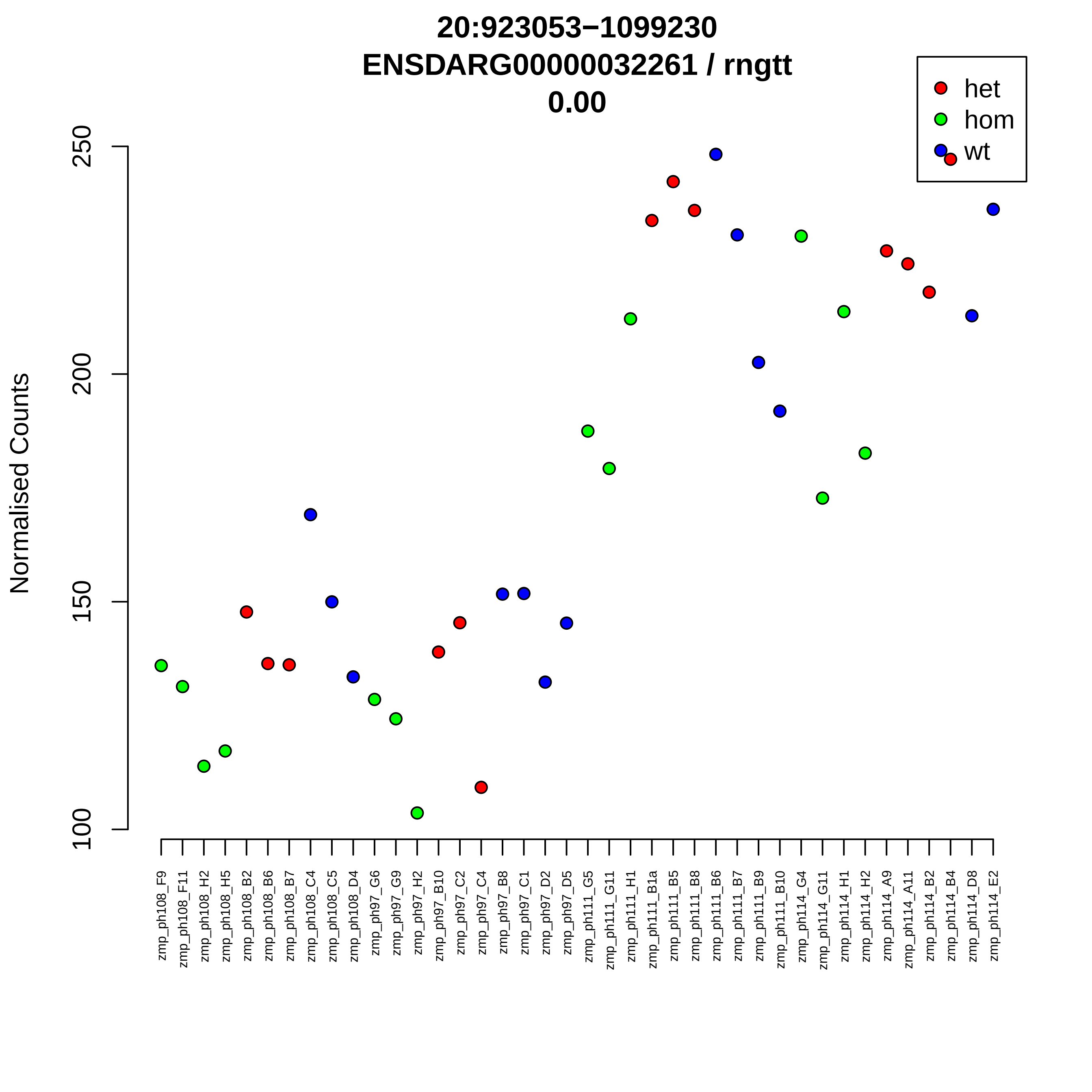

20:923053−1099230
ENSDARG00000032261 / rngtt
0.00
het
hom
wt
Normalised Counts
100
150
200
250
zmp_ph108_F9
zmp_ph108_F11
zmp_ph108_H2
zmp_ph108_H5
zmp_ph108_B2
zmp_ph108_B6
zmp_ph108_B7
zmp_ph108_C4
zmp_ph108_C5
zmp_ph108_D4
zmp_ph97_G6
zmp_ph97_G9
zmp_ph97_H2
zmp_ph97_B10
zmp_ph97_C2
zmp_ph97_C4
zmp_ph97_B8
zmp_ph97_C1
zmp_ph97_D2
zmp_ph97_D5
zmp_ph111_G5
zmp_ph111_G11
zmp_ph111_H1
zmp_ph111_B1a
zmp_ph111_B5
zmp_ph111_B8
zmp_ph111_B6
zmp_ph111_B7
zmp_ph111_B9
zmp_ph111_B10
zmp_ph114_G4
zmp_ph114_G11
zmp_ph114_H1
zmp_ph114_H2
zmp_ph114_A9
zmp_ph114_A11
zmp_ph114_B2
zmp_ph114_B4
zmp_ph114_D8
zmp_ph114_E2

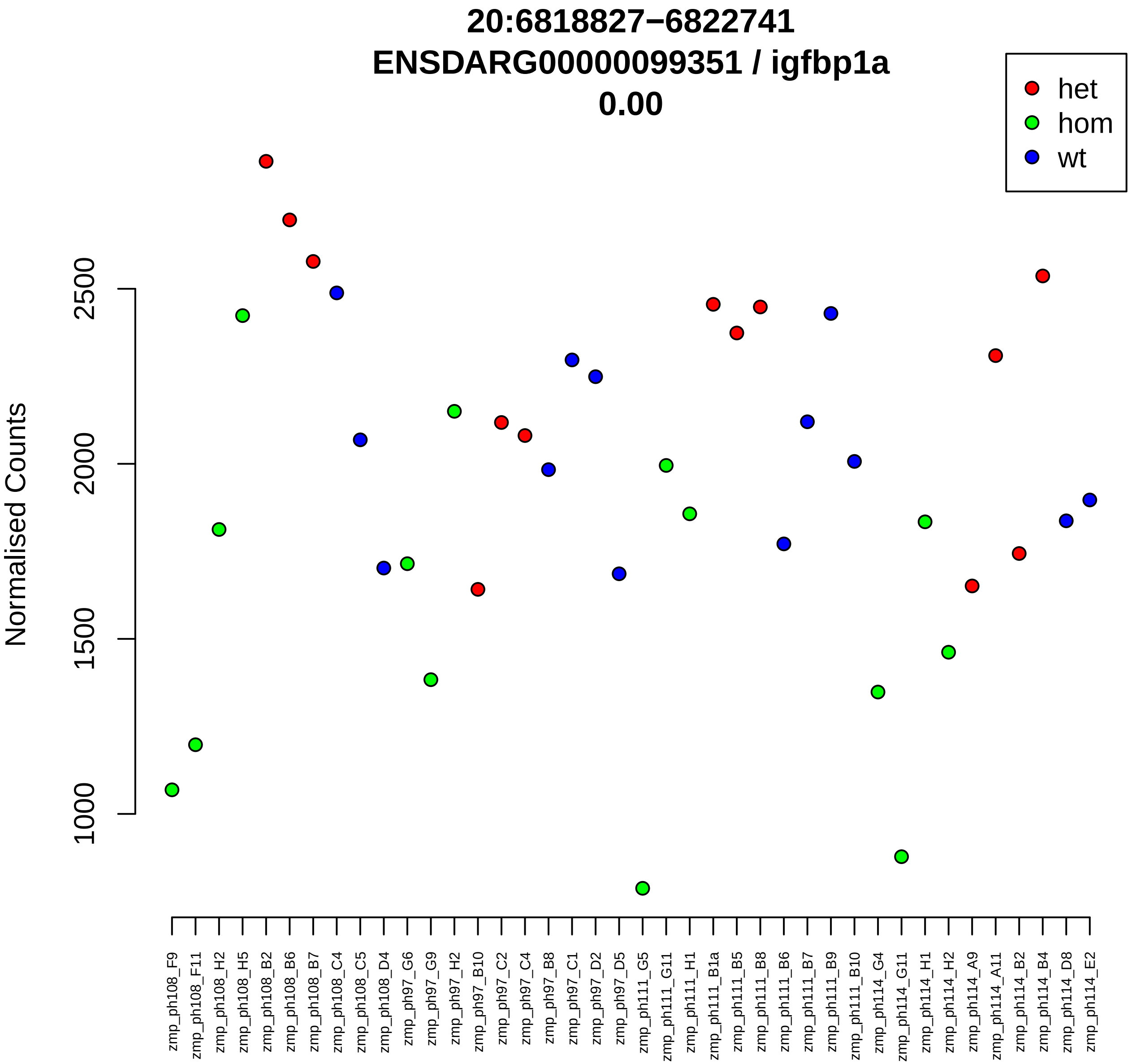
20:6818827−6822741
ENSDARG00000099351 / igfbp1a
0.00
het
hom
wt
Normalised Counts
1000
1500
2000
2500
zmp_ph108_F9
zmp_ph108_F11
zmp_ph108_H2
zmp_ph108_H5
zmp_ph108_B2
zmp_ph108_B6
zmp_ph108_B7
zmp_ph108_C4
zmp_ph108_C5
zmp_ph108_D4
zmp_ph97_G6
zmp_ph97_G9
zmp_ph97_H2
zmp_ph97_B10
zmp_ph97_C2
zmp_ph97_C4
zmp_ph97_B8
zmp_ph97_C1
zmp_ph97_D2
zmp_ph97_D5
zmp_ph111_G5
zmp_ph111_G11
zmp_ph111_H1
zmp_ph111_B1a
zmp_ph111_B5
zmp_ph111_B8
zmp_ph111_B6
zmp_ph111_B7
zmp_ph111_B9
zmp_ph111_B10
zmp_ph114_G4
zmp_ph114_G11
zmp_ph114_H1
zmp_ph114_H2
zmp_ph114_A9
zmp_ph114_A11
zmp_ph114_B2
zmp_ph114_B4
zmp_ph114_D8
zmp_ph114_E2

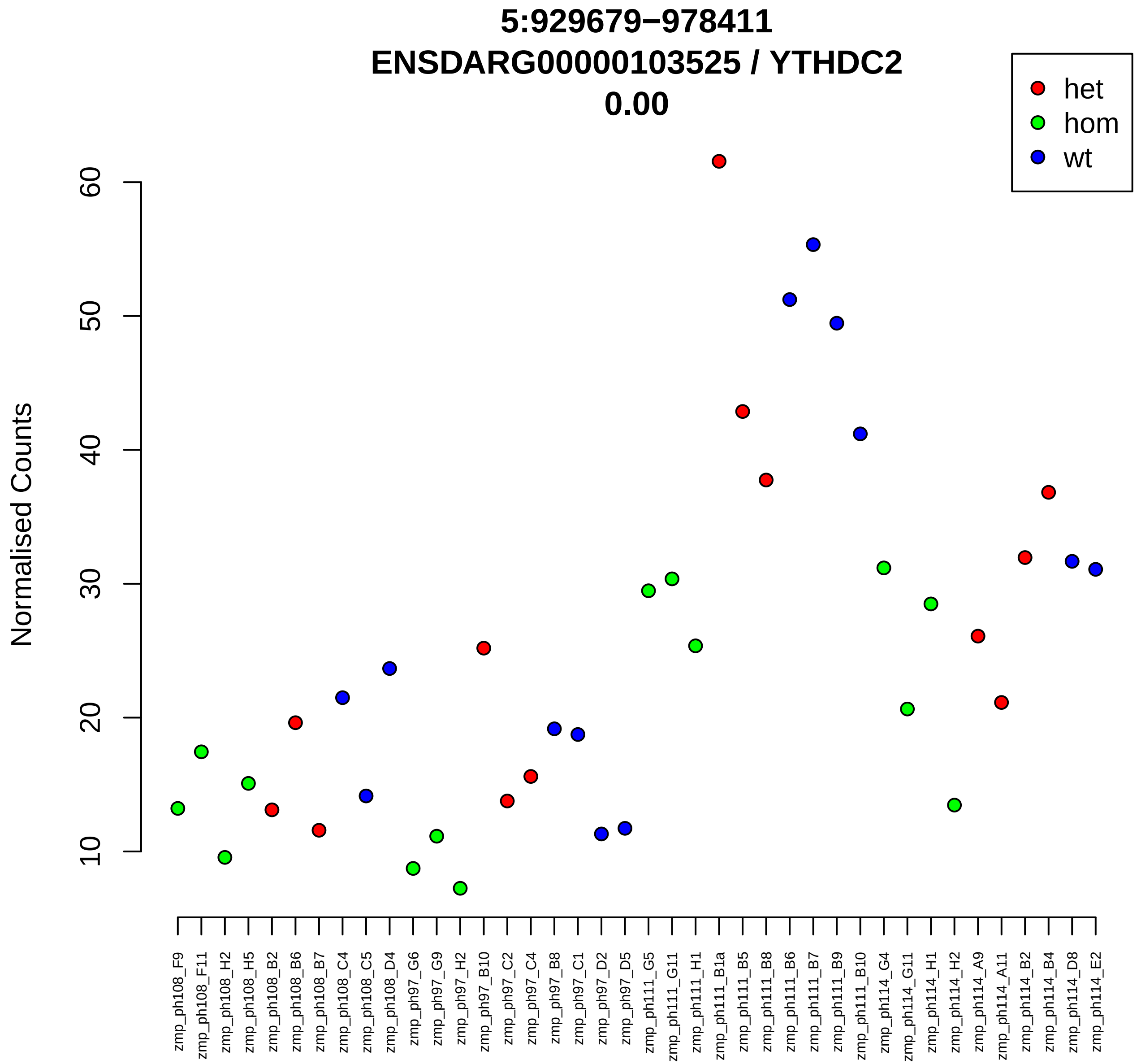
5:929679−978411
ENSDARG00000103525 / YTHDC2
0.00
het
hom
wt
Normalised Counts
10
20
30
40
50
60
zmp_ph108_F9
zmp_ph108_F11
zmp_ph108_H2
zmp_ph108_H5
zmp_ph108_B2
zmp_ph108_B6
zmp_ph108_B7
zmp_ph108_C4
zmp_ph108_C5
zmp_ph108_D4
zmp_ph97_G6
zmp_ph97_G9
zmp_ph97_H2
zmp_ph97_B10
zmp_ph97_C2
zmp_ph97_C4
zmp_ph97_B8
zmp_ph97_C1
zmp_ph97_D2
zmp_ph97_D5
zmp_ph111_G5
zmp_ph111_G11
zmp_ph111_H1
zmp_ph111_B1a
zmp_ph111_B5
zmp_ph111_B8
zmp_ph111_B6
zmp_ph111_B7
zmp_ph111_B9
zmp_ph111_B10
zmp_ph114_G4
zmp_ph114_G11
zmp_ph114_H1
zmp_ph114_H2
zmp_ph114_A9
zmp_ph114_A11
zmp_ph114_B2
zmp_ph114_B4
zmp_ph114_D8
zmp_ph114_E2

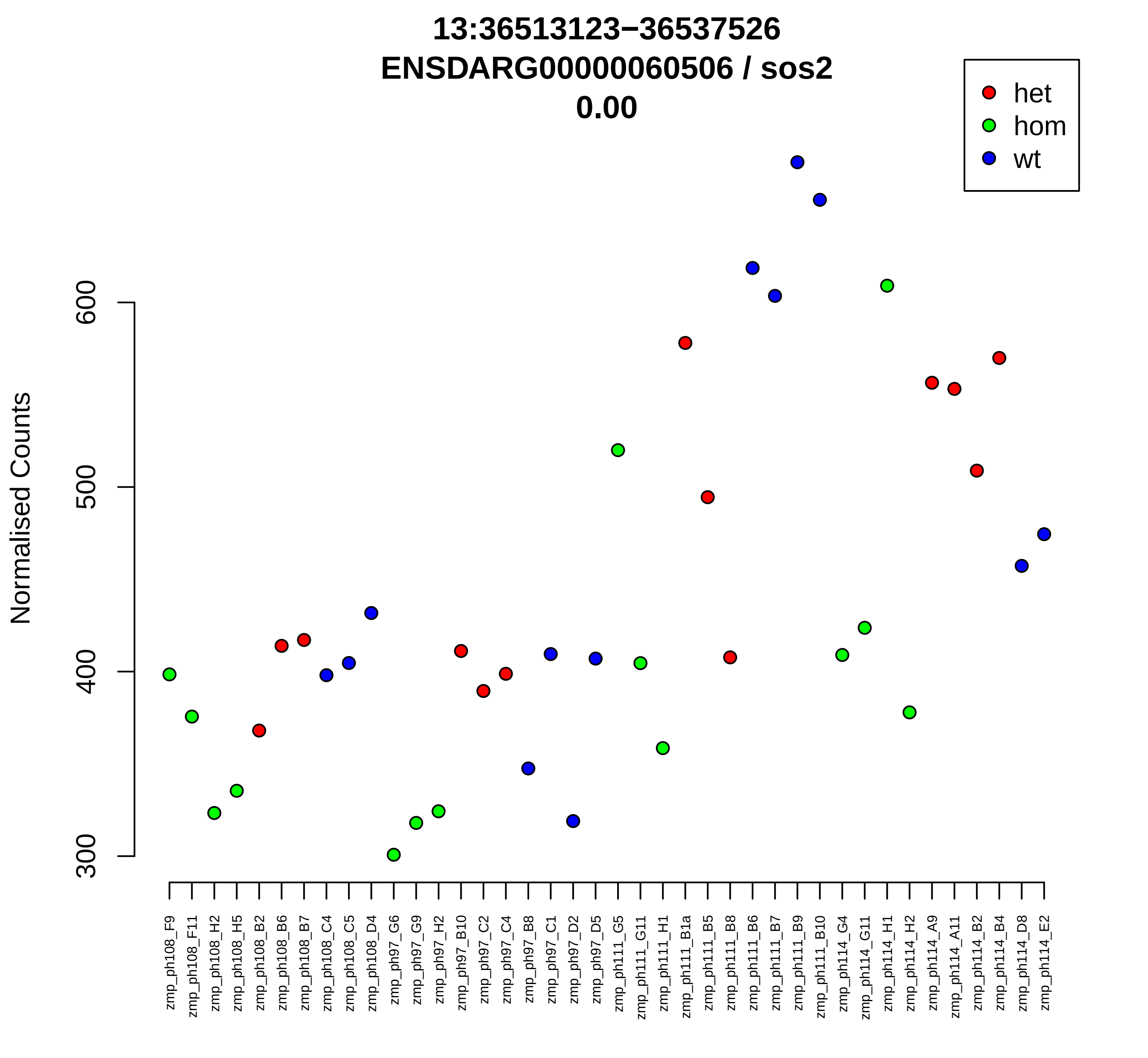

13:36513123−36537526
ENSDARG00000060506 / sos2
0.00
het
hom
wt
Normalised Counts
300
400
500
600
zmp_ph108_F9
zmp_ph108_F11
zmp_ph108_H2
zmp_ph108_H5
zmp_ph108_B2
zmp_ph108_B6
zmp_ph108_B7
zmp_ph108_C4
zmp_ph108_C5
zmp_ph108_D4
zmp_ph97_G6
zmp_ph97_G9
zmp_ph97_H2
zmp_ph97_B10
zmp_ph97_C2
zmp_ph97_C4
zmp_ph97_B8
zmp_ph97_C1
zmp_ph97_D2
zmp_ph97_D5
zmp_ph111_G5
zmp_ph111_G11
zmp_ph111_H1
zmp_ph111_B1a
zmp_ph111_B5
zmp_ph111_B8
zmp_ph111_B6
zmp_ph111_B7
zmp_ph111_B9
zmp_ph111_B10
zmp_ph114_G4
zmp_ph114_G11
zmp_ph114_H1
zmp_ph114_H2
zmp_ph114_A9
zmp_ph114_A11
zmp_ph114_B2
zmp_ph114_B4
zmp_ph114_D8
zmp_ph114_E2

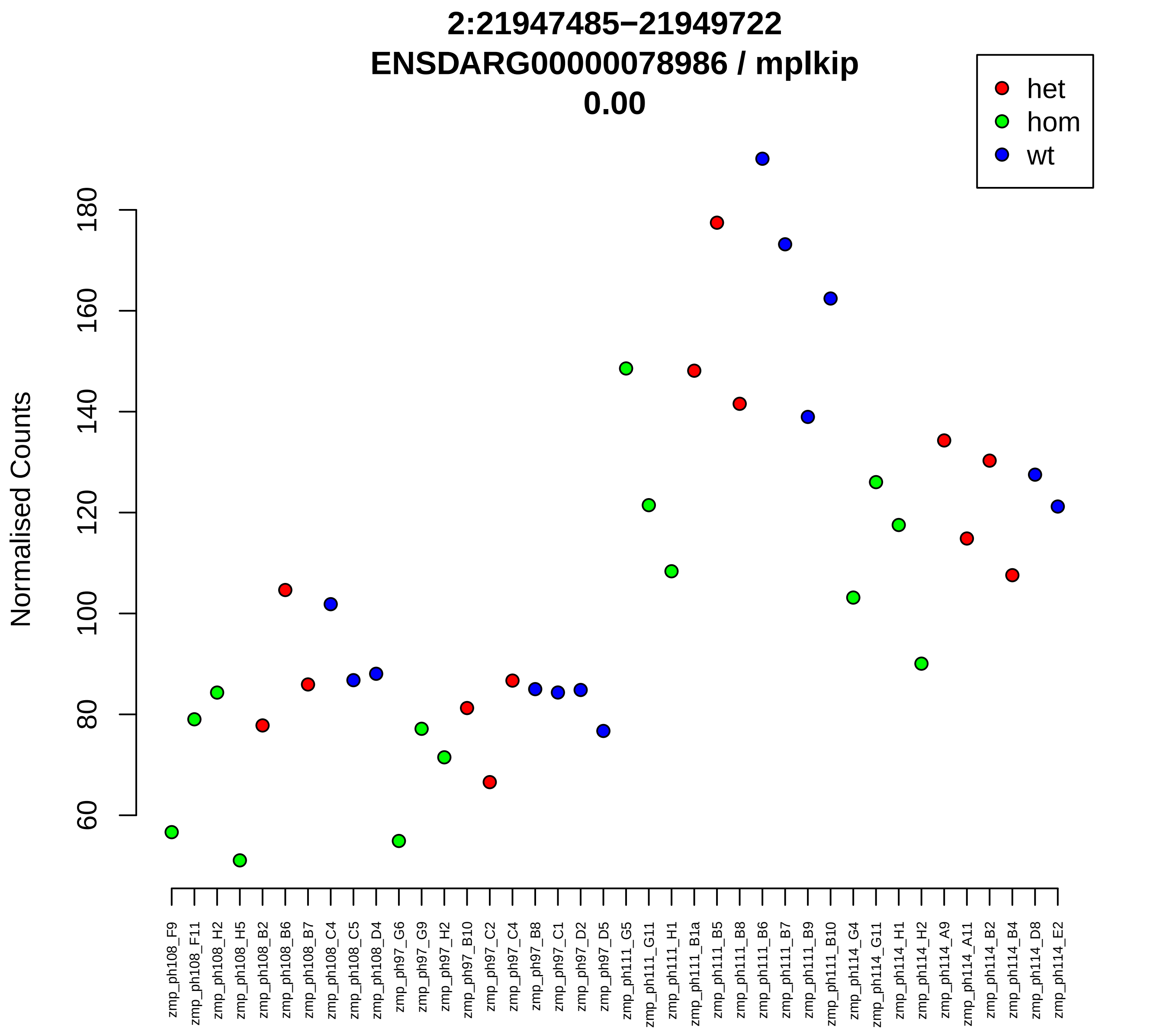

2:21947485−21949722
ENSDARG00000078986 / mplkip
0.00
het
hom
wt
Normalised Counts
60
80
100
120
140
160
180
zmp_ph108_F9
zmp_ph108_F11
zmp_ph108_H2
zmp_ph108_H5
zmp_ph108_B2
zmp_ph108_B6
zmp_ph108_B7
zmp_ph108_C4
zmp_ph108_C5
zmp_ph108_D4
zmp_ph97_G6
zmp_ph97_G9
zmp_ph97_H2
zmp_ph97_B10
zmp_ph97_C2
zmp_ph97_C4
zmp_ph97_B8
zmp_ph97_C1
zmp_ph97_D2
zmp_ph97_D5
zmp_ph111_G5
zmp_ph111_G11
zmp_ph111_H1
zmp_ph111_B1a
zmp_ph111_B5
zmp_ph111_B8
zmp_ph111_B6
zmp_ph111_B7
zmp_ph111_B9
zmp_ph111_B10
zmp_ph114_G4
zmp_ph114_G11
zmp_ph114_H1
zmp_ph114_H2
zmp_ph114_A9
zmp_ph114_A11
zmp_ph114_B2
zmp_ph114_B4
zmp_ph114_D8
zmp_ph114_E2

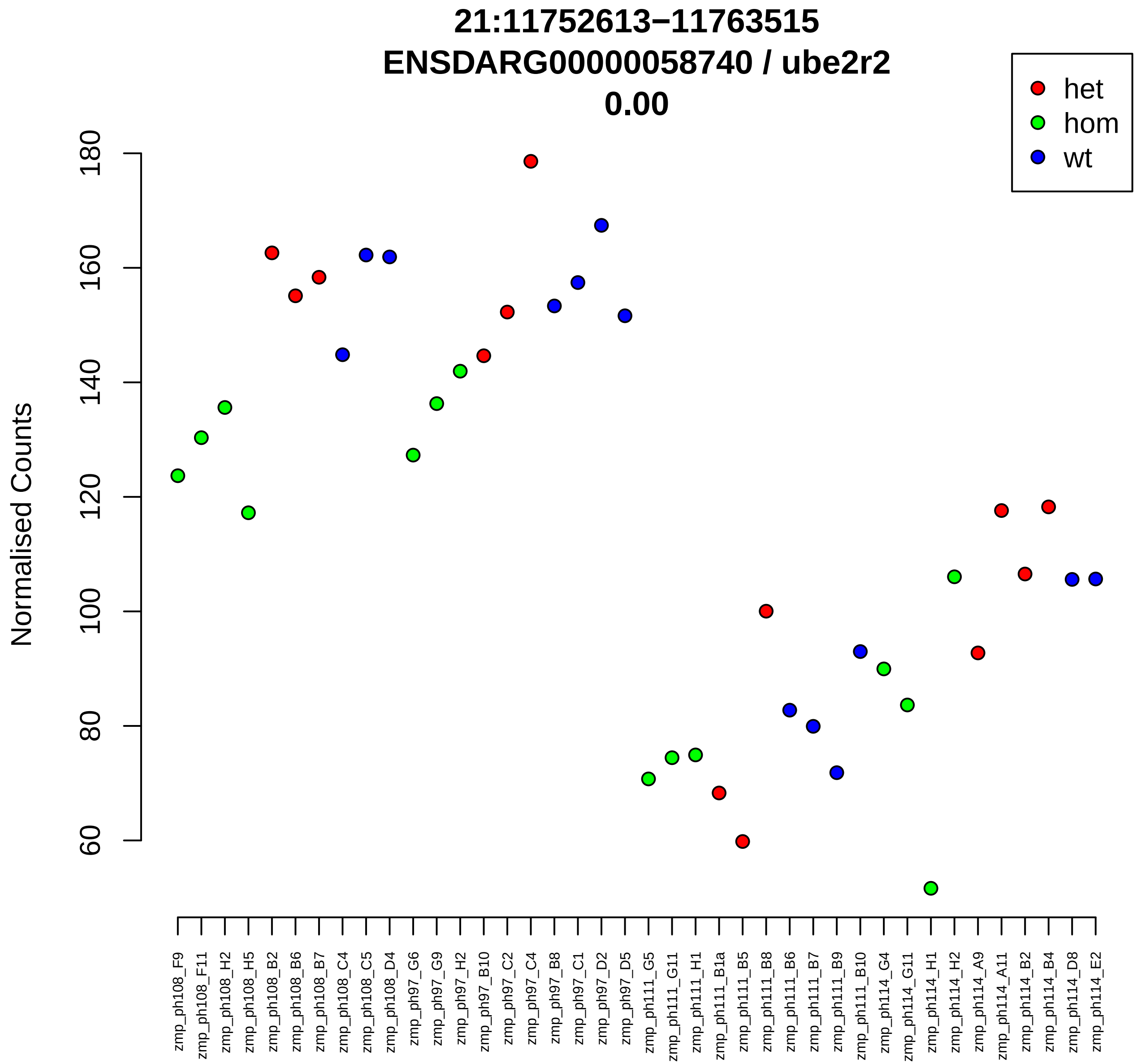

21:11752613−11763515
ENSDARG00000058740 / ube2r2
0.00
het
hom
wt
Normalised Counts
60
80
100
120
140
160
180
zmp_ph108_F9
zmp_ph108_F11
zmp_ph108_H2
zmp_ph108_H5
zmp_ph108_B2
zmp_ph108_B6
zmp_ph108_B7
zmp_ph108_C4
zmp_ph108_C5
zmp_ph108_D4
zmp_ph97_G6
zmp_ph97_G9
zmp_ph97_H2
zmp_ph97_B10
zmp_ph97_C2
zmp_ph97_C4
zmp_ph97_B8
zmp_ph97_C1
zmp_ph97_D2
zmp_ph97_D5
zmp_ph111_G5
zmp_ph111_G11
zmp_ph111_H1
zmp_ph111_B1a
zmp_ph111_B5
zmp_ph111_B8
zmp_ph111_B6
zmp_ph111_B7
zmp_ph111_B9
zmp_ph111_B10
zmp_ph114_G4
zmp_ph114_G11
zmp_ph114_H1
zmp_ph114_H2
zmp_ph114_A9
zmp_ph114_A11
zmp_ph114_B2
zmp_ph114_B4
zmp_ph114_D8
zmp_ph114_E2

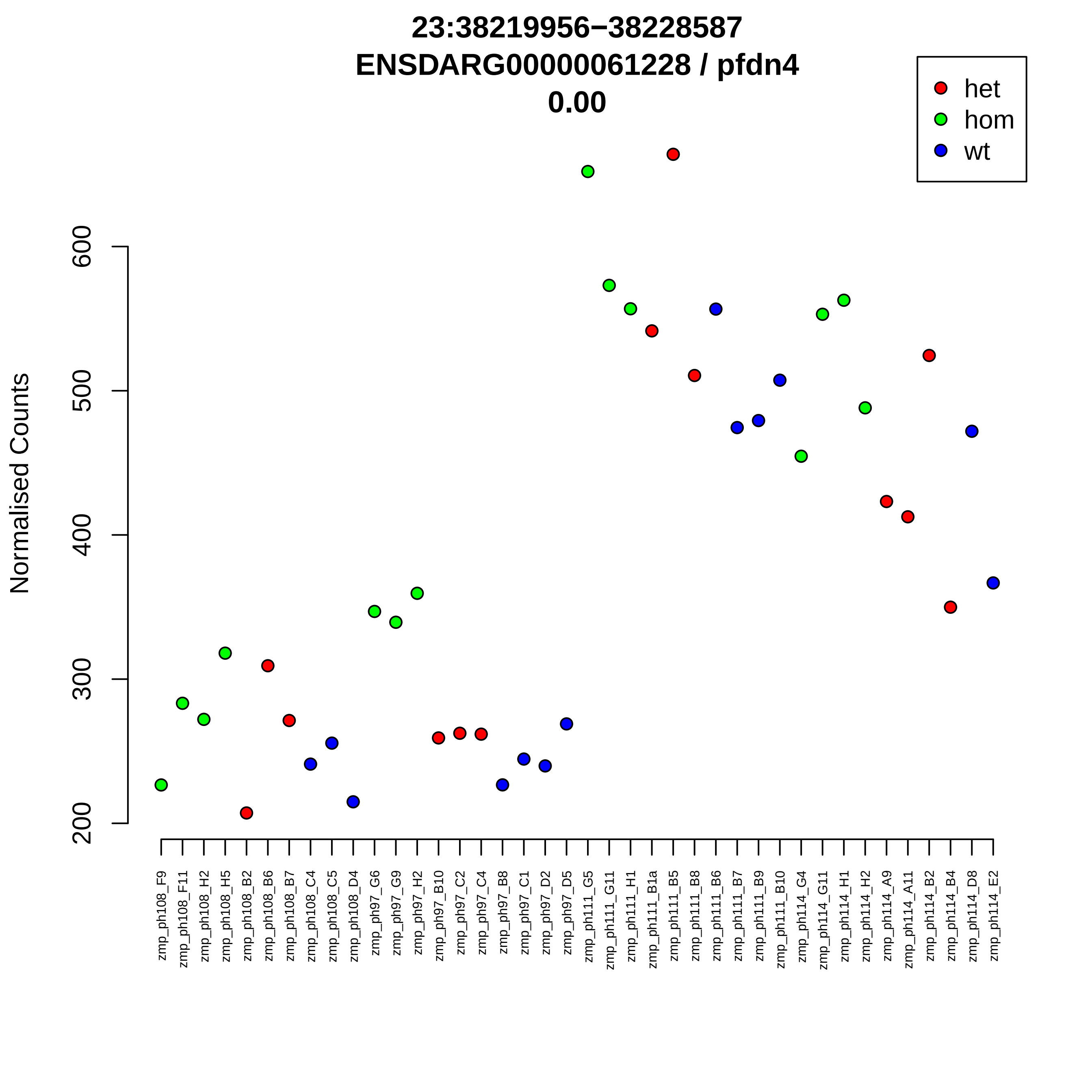

23:38219956−38228587
ENSDARG00000061228 / pfdn4
0.00
het
hom
wt
Normalised Counts
200
300
400
500
600
zmp_ph108_F9
zmp_ph108_F11
zmp_ph108_H2
zmp_ph108_H5
zmp_ph108_B2
zmp_ph108_B6
zmp_ph108_B7
zmp_ph108_C4
zmp_ph108_C5
zmp_ph108_D4
zmp_ph97_G6
zmp_ph97_G9
zmp_ph97_H2
zmp_ph97_B10
zmp_ph97_C2
zmp_ph97_C4
zmp_ph97_B8
zmp_ph97_C1
zmp_ph97_D2
zmp_ph97_D5
zmp_ph111_G5
zmp_ph111_G11
zmp_ph111_H1
zmp_ph111_B1a
zmp_ph111_B5
zmp_ph111_B8
zmp_ph111_B6
zmp_ph111_B7
zmp_ph111_B9
zmp_ph111_B10
zmp_ph114_G4
zmp_ph114_G11
zmp_ph114_H1
zmp_ph114_H2
zmp_ph114_A9
zmp_ph114_A11
zmp_ph114_B2
zmp_ph114_B4
zmp_ph114_D8
zmp_ph114_E2

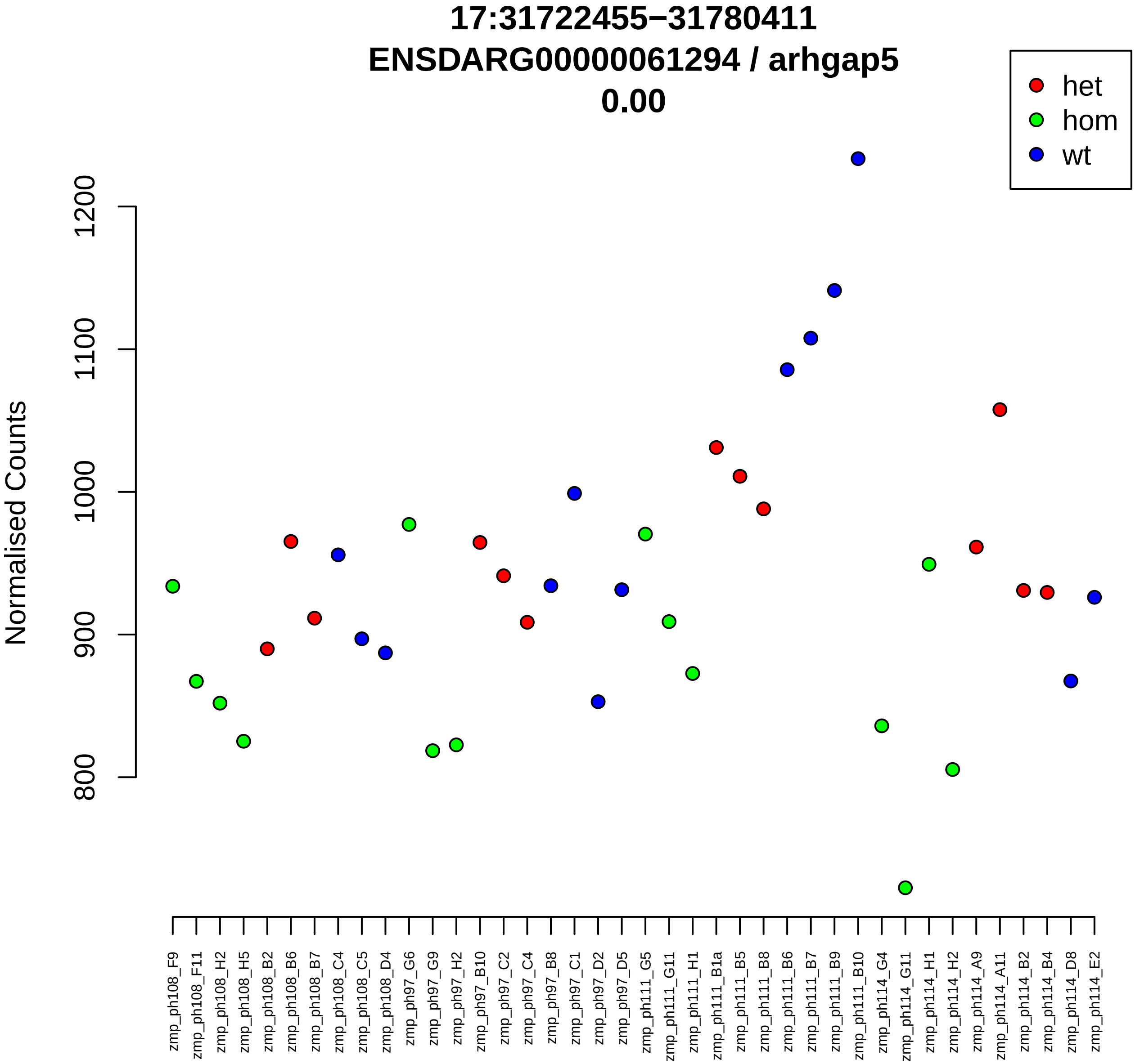

17:31722455−31780411
ENSDARG00000061294 / arhgap5
0.00
het
hom
wt
Normalised Counts
1200
1100
1000
900
800
zmp_ph108_F9
zmp_ph108_F11
zmp_ph108_H2
zmp_ph108_H5
zmp_ph108_B2
zmp_ph108_B6
zmp_ph108_B7
zmp_ph108_C4
zmp_ph108_C5
zmp_ph108_D4
zmp_ph97_G6
zmp_ph97_G9
zmp_ph97_H2
zmp_ph97_B10
zmp_ph97_C2
zmp_ph97_C4
zmp_ph97_B8
zmp_ph97_C1
zmp_ph97_D2
zmp_ph97_D5
zmp_ph111_G5
zmp_ph111_G11
zmp_ph111_H1
zmp_ph111_B1a
zmp_ph111_B5
zmp_ph111_B8
zmp_ph111_B6
zmp_ph111_B7
zmp_ph111_B9
zmp_ph111_B10
zmp_ph114_G4
zmp_ph114_G11
zmp_ph114_H1
zmp_ph114_H2
zmp_ph114_A9
zmp_ph114_A11
zmp_ph114_B2
zmp_ph114_B4
zmp_ph114_D8
zmp_ph114_E2

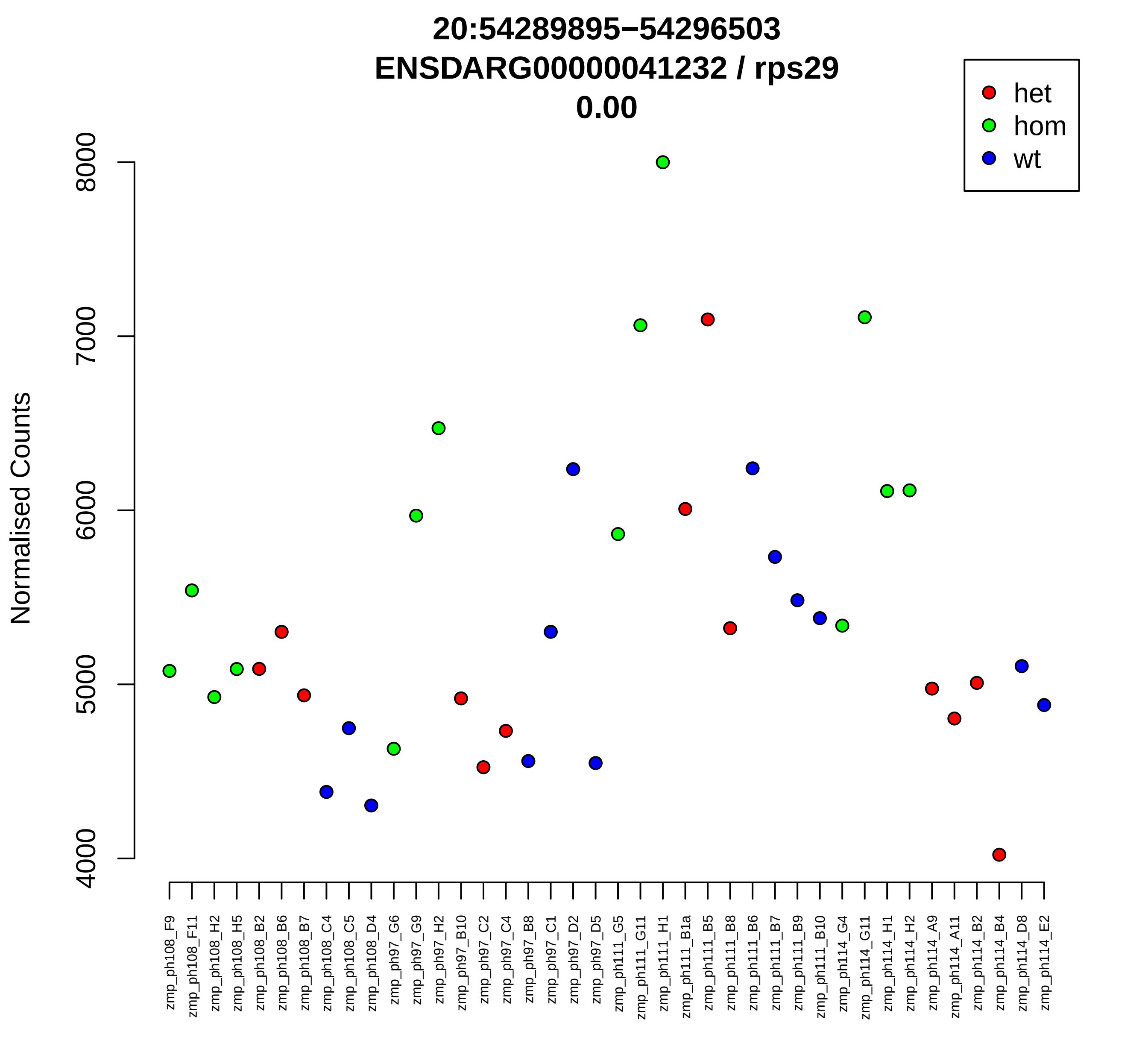
20:54289895−54296503
ENSDARG00000041232 / rps29
0.00
het
hom
wt
Normalised Counts
8000
7000
6000
5000
4000
zmp_ph108_F9
zmp_ph108_F11
zmp_ph108_H2
zmp_ph108_H5
zmp_ph108_B2
zmp_ph108_B6
zmp_ph108_B7
zmp_ph108_C4
zmp_ph108_C5
zmp_ph108_D4
zmp_ph97_G6
zmp_ph97_G9
zmp_ph97_H2
zmp_ph97_B10
zmp_ph97_C2
zmp_ph97_C4
zmp_ph97_B8
zmp_ph97_C1
zmp_ph97_D2
zmp_ph97_D5
zmp_ph111_G5
zmp_ph111_G11
zmp_ph111_H1
zmp_ph111_B1a
zmp_ph111_B5
zmp_ph111_B8
zmp_ph111_B6
zmp_ph111_B7
zmp_ph111_B9
zmp_ph111_B10
zmp_ph114_G4
zmp_ph114_G11
zmp_ph114_H1
zmp_ph114_H2
zmp_ph114_A9
zmp_ph114_A11
zmp_ph114_B2
zmp_ph114_B4
zmp_ph114_D8
zmp_ph114_E2

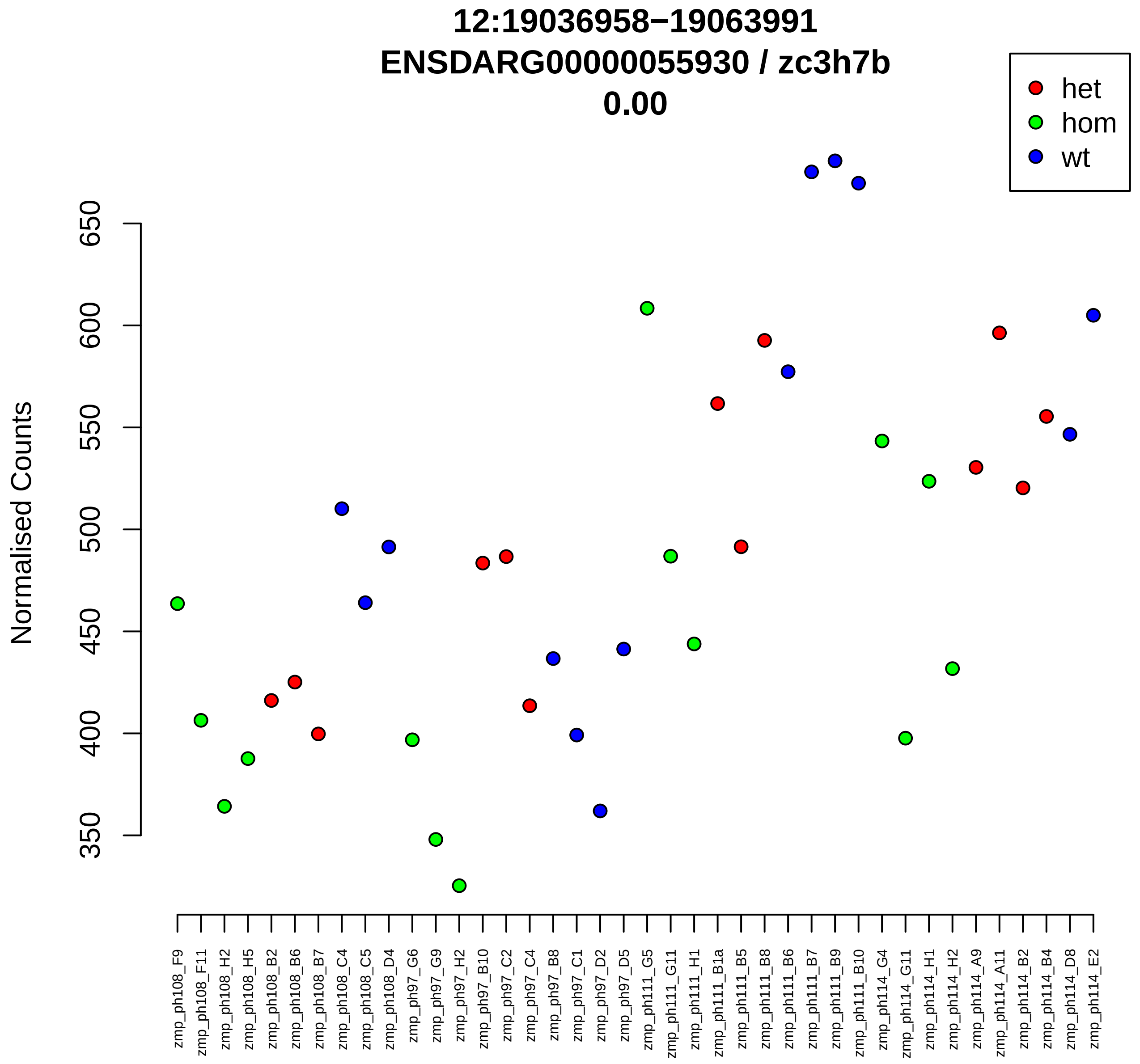

12:19036958−19063991
ENSDARG00000055930 / zc3h7b
0.00
het
hom
wt
Normalised Counts
350
400
450
500
550
600
650
zmp_ph108_F9
zmp_ph108_F11
zmp_ph108_H2
zmp_ph108_H5
zmp_ph108_B2
zmp_ph108_B6
zmp_ph108_B7
zmp_ph108_C4
zmp_ph108_C5
zmp_ph108_D4
zmp_ph97_G6
zmp_ph97_G9
zmp_ph97_H2
zmp_ph97_B10
zmp_ph97_C2
zmp_ph97_C4
zmp_ph97_B8
zmp_ph97_C1
zmp_ph97_D2
zmp_ph97_D5
zmp_ph111_G5
zmp_ph111_G11
zmp_ph111_H1
zmp_ph111_B1a
zmp_ph111_B5
zmp_ph111_B8
zmp_ph111_B6
zmp_ph111_B7
zmp_ph111_B9
zmp_ph111_B10
zmp_ph114_G4
zmp_ph114_G11
zmp_ph114_H1
zmp_ph114_H2
zmp_ph114_A9
zmp_ph114_A11
zmp_ph114_B2
zmp_ph114_B4
zmp_ph114_D8
zmp_ph114_E2

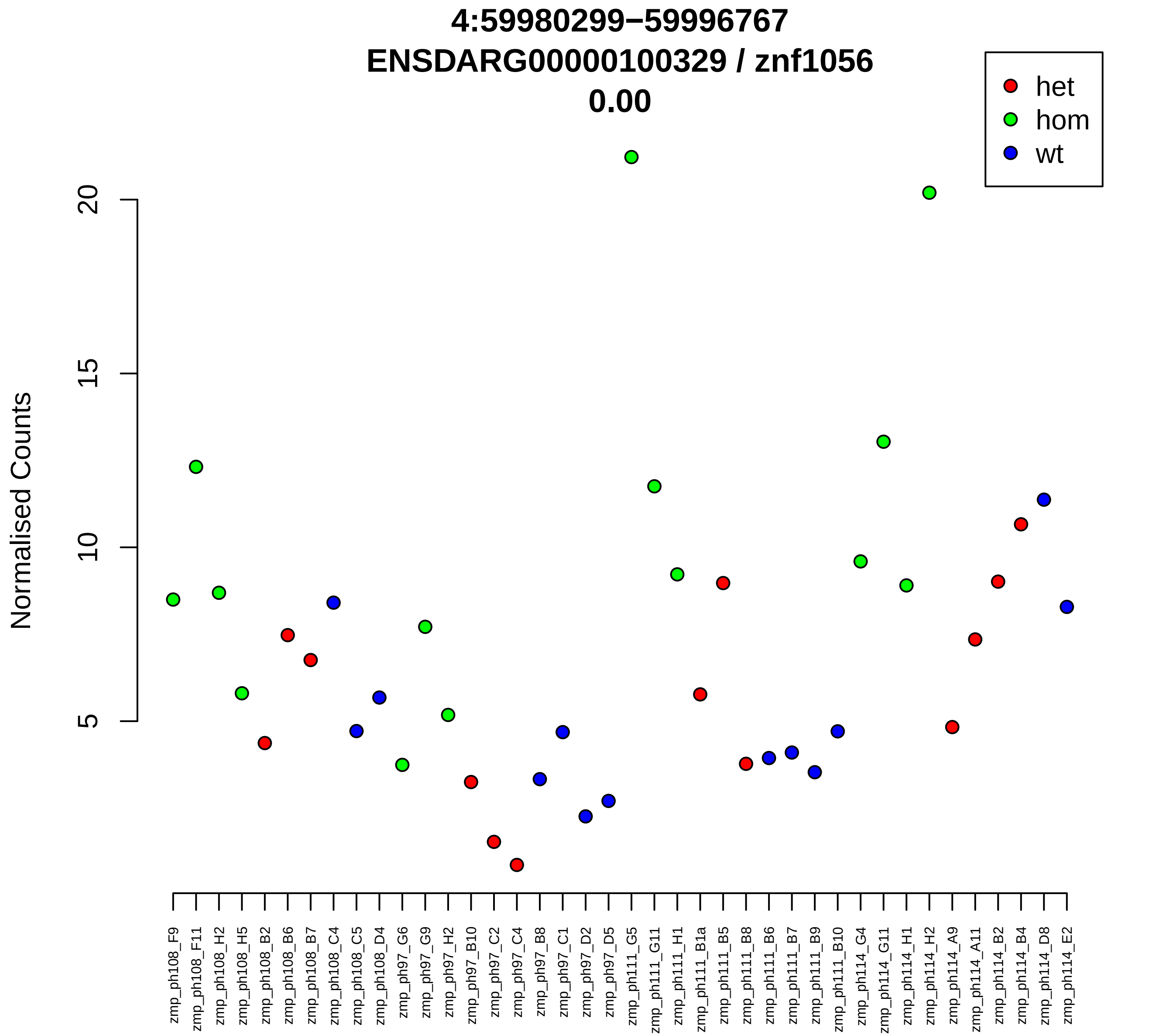

4:59980299−59996767
ENSDARG00000100329 / znf1056
0.00
het
hom
wt
Normalised Counts
5
10
15
20
zmp_ph108_F9
zmp_ph108_F11
zmp_ph108_H2
zmp_ph108_H5
zmp_ph108_B2
zmp_ph108_B6
zmp_ph108_B7
zmp_ph108_C4
zmp_ph108_C5
zmp_ph108_D4
zmp_ph97_G6
zmp_ph97_G9
zmp_ph97_H2
zmp_ph97_B10
zmp_ph97_C2
zmp_ph97_C4
zmp_ph97_B8
zmp_ph97_C1
zmp_ph97_D2
zmp_ph97_D5
zmp_ph111_G5
zmp_ph111_G11
zmp_ph111_H1
zmp_ph111_B1a
zmp_ph111_B5
zmp_ph111_B8
zmp_ph111_B6
zmp_ph111_B7
zmp_ph111_B9
zmp_ph111_B10
zmp_ph114_G4
zmp_ph114_G11
zmp_ph114_H1
zmp_ph114_H2
zmp_ph114_A9
zmp_ph114_A11
zmp_ph114_B2
zmp_ph114_B4
zmp_ph114_D8
zmp_ph114_E2

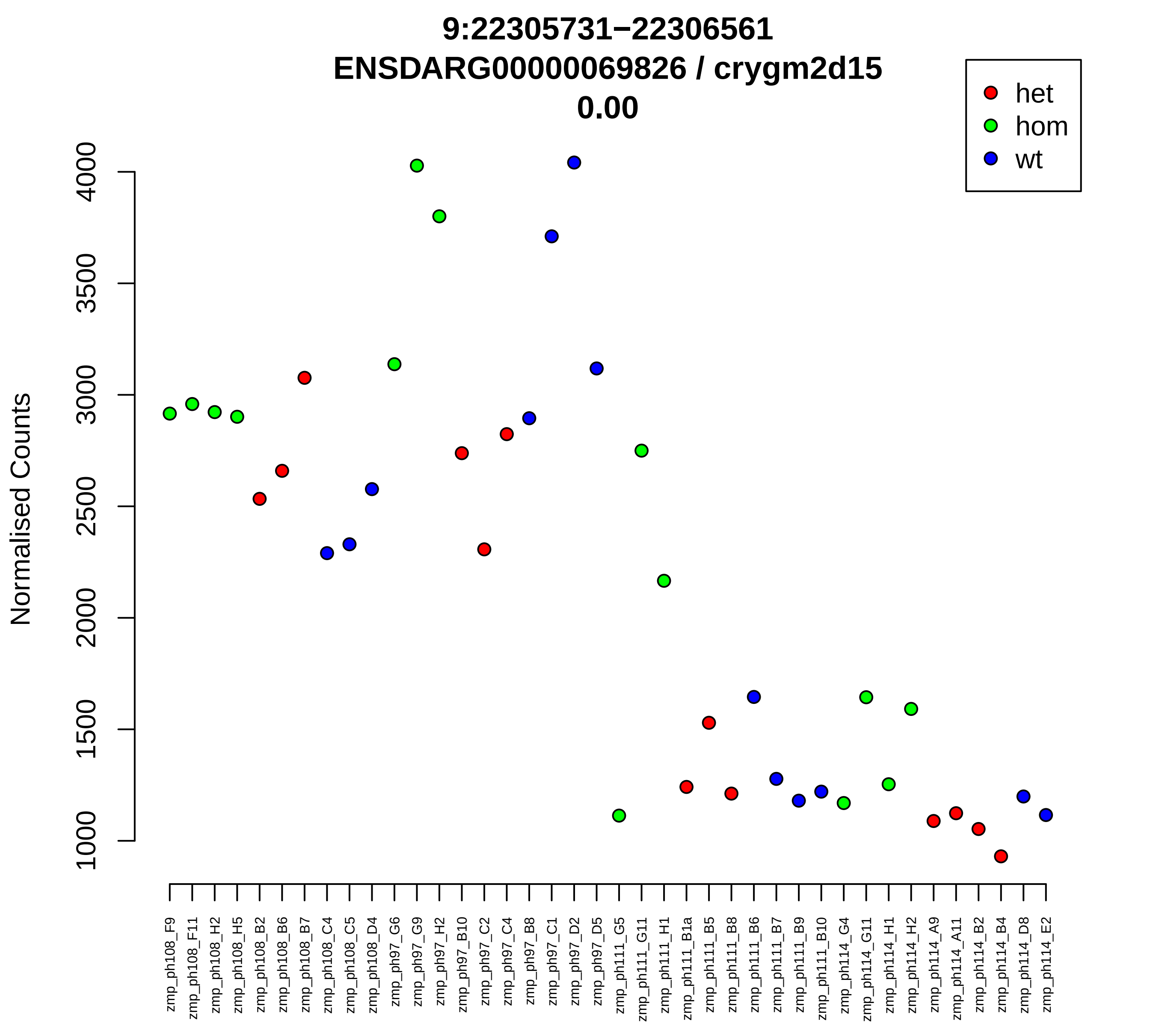
9:22305731−22306561
ENSDARG00000069826 / crygm2d15
0.00
het
hom
wt
Normalised Counts
1000
1500
2000
2500
3000
3500
4000
zmp_ph108_F9
zmp_ph108_F11
zmp_ph108_H2
zmp_ph108_H5
zmp_ph108_B2
zmp_ph108_B6
zmp_ph108_B7
zmp_ph108_C4
zmp_ph108_C5
zmp_ph108_D4
zmp_ph97_G6
zmp_ph97_G9
zmp_ph97_H2
zmp_ph97_B10
zmp_ph97_C2
zmp_ph97_C4
zmp_ph97_B8
zmp_ph97_C1
zmp_ph97_D2
zmp_ph97_D5
zmp_ph111_G5
zmp_ph111_G11
zmp_ph111_H1
zmp_ph111_B1a
zmp_ph111_B5
zmp_ph111_B8
zmp_ph111_B6
zmp_ph111_B7
zmp_ph111_B9
zmp_ph111_B10
zmp_ph114_G4
zmp_ph114_G11
zmp_ph114_H1
zmp_ph114_H2
zmp_ph114_A9
zmp_ph114_A11
zmp_ph114_B2
zmp_ph114_B4
zmp_ph114_D8
zmp_ph114_E2

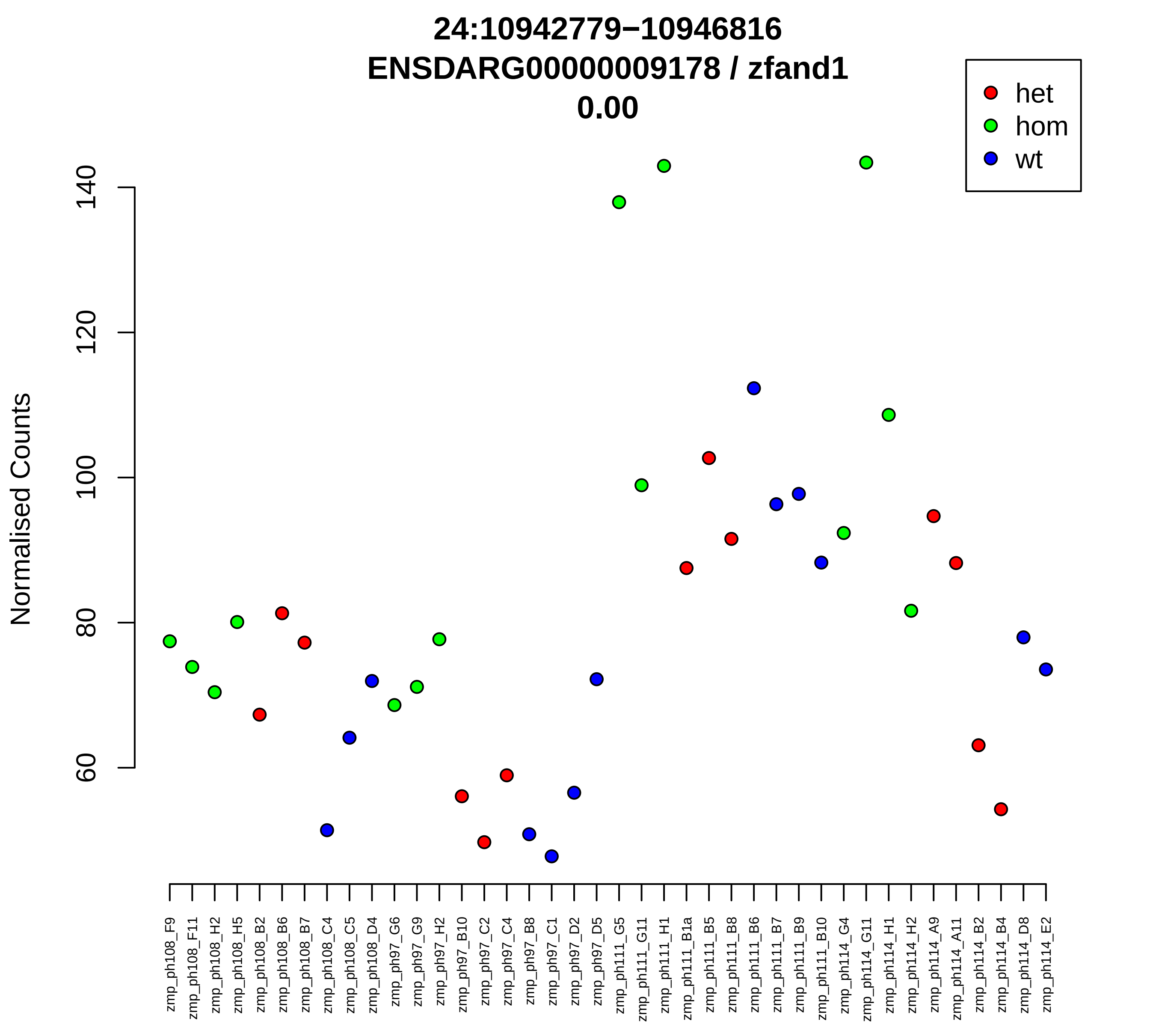

24:10942779−10946816
ENSDARG00000009178 / zfand1
0.00
het
hom
wt
Normalised Counts
60
80
100
120
140
zmp_ph108_F9
zmp_ph108_F11
zmp_ph108_H2
zmp_ph108_H5
zmp_ph108_B2
zmp_ph108_B6
zmp_ph108_B7
zmp_ph108_C4
zmp_ph108_C5
zmp_ph108_D4
zmp_ph97_G6
zmp_ph97_G9
zmp_ph97_H2
zmp_ph97_B10
zmp_ph97_C2
zmp_ph97_C4
zmp_ph97_B8
zmp_ph97_C1
zmp_ph97_D2
zmp_ph97_D5
zmp_ph111_G5
zmp_ph111_G11
zmp_ph111_H1
zmp_ph111_B1a
zmp_ph111_B5
zmp_ph111_B8
zmp_ph111_B6
zmp_ph111_B7
zmp_ph111_B9
zmp_ph111_B10
zmp_ph114_G4
zmp_ph114_G11
zmp_ph114_H1
zmp_ph114_H2
zmp_ph114_A9
zmp_ph114_A11
zmp_ph114_B2
zmp_ph114_B4
zmp_ph114_D8
zmp_ph114_E2

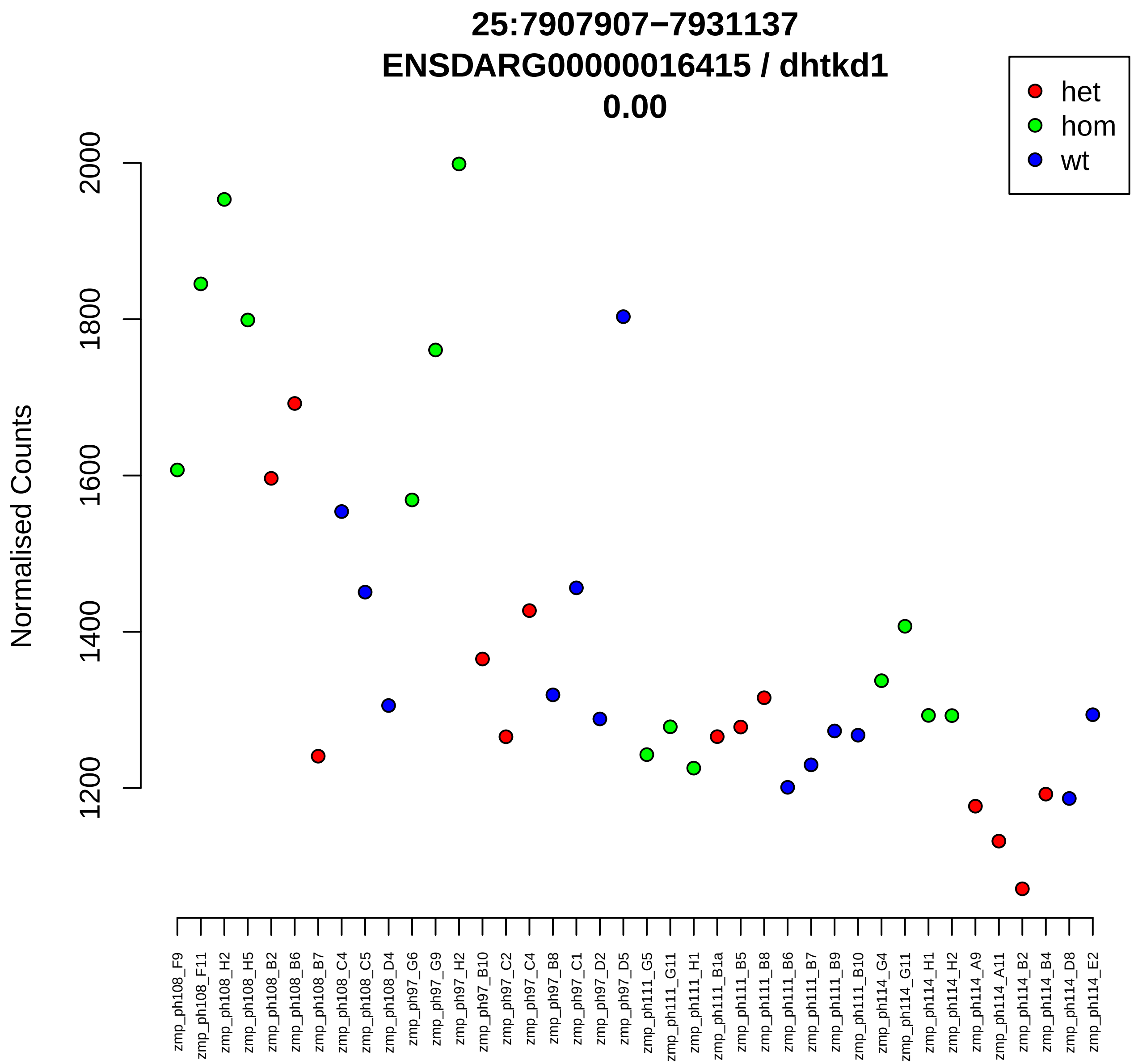
25:7907907−7931137
ENSDARG00000016415 / dhtkd1
0.00
het
hom
wt
Normalised Counts
2000
1800
1600
1400
1200
zmp_ph108_F9
zmp_ph108_F11
zmp_ph108_H2
zmp_ph108_H5
zmp_ph108_B2
zmp_ph108_B6
zmp_ph108_B7
zmp_ph108_C4
zmp_ph108_C5
zmp_ph108_D4
zmp_ph97_G6
zmp_ph97_G9
zmp_ph97_H2
zmp_ph97_B10
zmp_ph97_C2
zmp_ph97_C4
zmp_ph97_B8
zmp_ph97_C1
zmp_ph97_D2
zmp_ph97_D5
zmp_ph111_G5
zmp_ph111_G11
zmp_ph111_H1
zmp_ph111_B1a
zmp_ph111_B5
zmp_ph111_B8
zmp_ph111_B6
zmp_ph111_B7
zmp_ph111_B9
zmp_ph111_B10
zmp_ph114_G4
zmp_ph114_G11
zmp_ph114_H1
zmp_ph114_H2
zmp_ph114_A9
zmp_ph114_A11
zmp_ph114_B2
zmp_ph114_B4
zmp_ph114_D8
zmp_ph114_E2

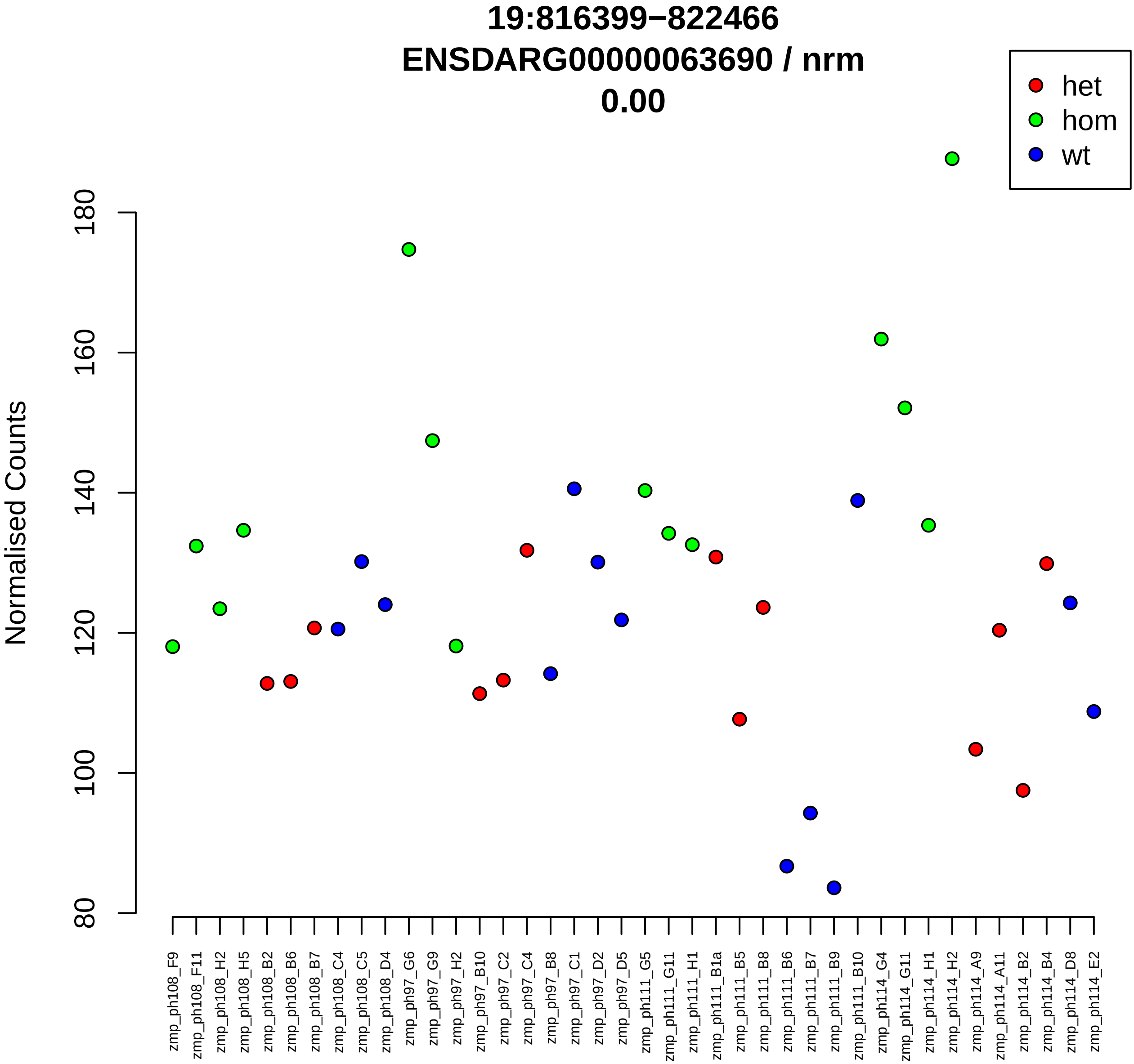

19:816399−822466
ENSDARG00000063690 / nrm
0.00
het
hom
wt
Normalised Counts
80
100
120
140
160
180
zmp_ph108_F9
zmp_ph108_F11
zmp_ph108_H2
zmp_ph108_H5
zmp_ph108_B2
zmp_ph108_B6
zmp_ph108_B7
zmp_ph108_C4
zmp_ph108_C5
zmp_ph108_D4
zmp_ph97_G6
zmp_ph97_G9
zmp_ph97_H2
zmp_ph97_B10
zmp_ph97_C2
zmp_ph97_C4
zmp_ph97_B8
zmp_ph97_C1
zmp_ph97_D2
zmp_ph97_D5
zmp_ph111_G5
zmp_ph111_G11
zmp_ph111_H1
zmp_ph111_B1a
zmp_ph111_B5
zmp_ph111_B8
zmp_ph111_B6
zmp_ph111_B7
zmp_ph111_B9
zmp_ph111_B10
zmp_ph114_G4
zmp_ph114_G11
zmp_ph114_H1
zmp_ph114_H2
zmp_ph114_A9
zmp_ph114_A11
zmp_ph114_B2
zmp_ph114_B4
zmp_ph114_D8
zmp_ph114_E2

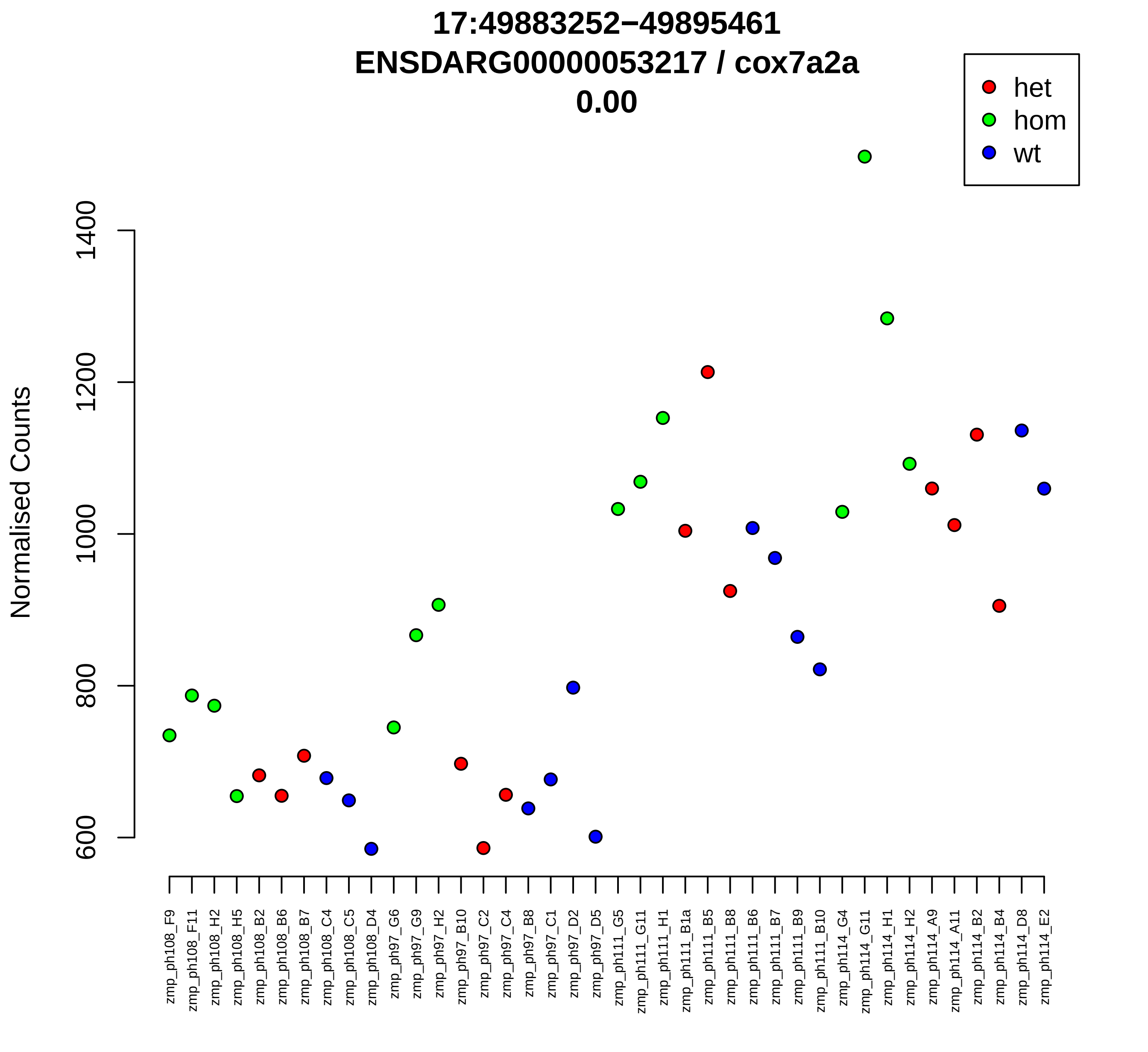
17:49883252−49895461
ENSDARG00000053217 / cox7a2a
0.00
het
hom
wt
Normalised Counts
600
800
1000
1200
1400
zmp_ph108_F9
zmp_ph108_F11
zmp_ph108_H2
zmp_ph108_H5
zmp_ph108_B2
zmp_ph108_B6
zmp_ph108_B7
zmp_ph108_C4
zmp_ph108_C5
zmp_ph108_D4
zmp_ph97_G6
zmp_ph97_G9
zmp_ph97_H2
zmp_ph97_B10
zmp_ph97_C2
zmp_ph97_C4
zmp_ph97_B8
zmp_ph97_C1
zmp_ph97_D2
zmp_ph97_D5
zmp_ph111_G5
zmp_ph111_G11
zmp_ph111_H1
zmp_ph111_B1a
zmp_ph111_B5
zmp_ph111_B8
zmp_ph111_B6
zmp_ph111_B7
zmp_ph111_B9
zmp_ph111_B10
zmp_ph114_G4
zmp_ph114_G11
zmp_ph114_H1
zmp_ph114_H2
zmp_ph114_A9
zmp_ph114_A11
zmp_ph114_B2
zmp_ph114_B4
zmp_ph114_D8
zmp_ph114_E2

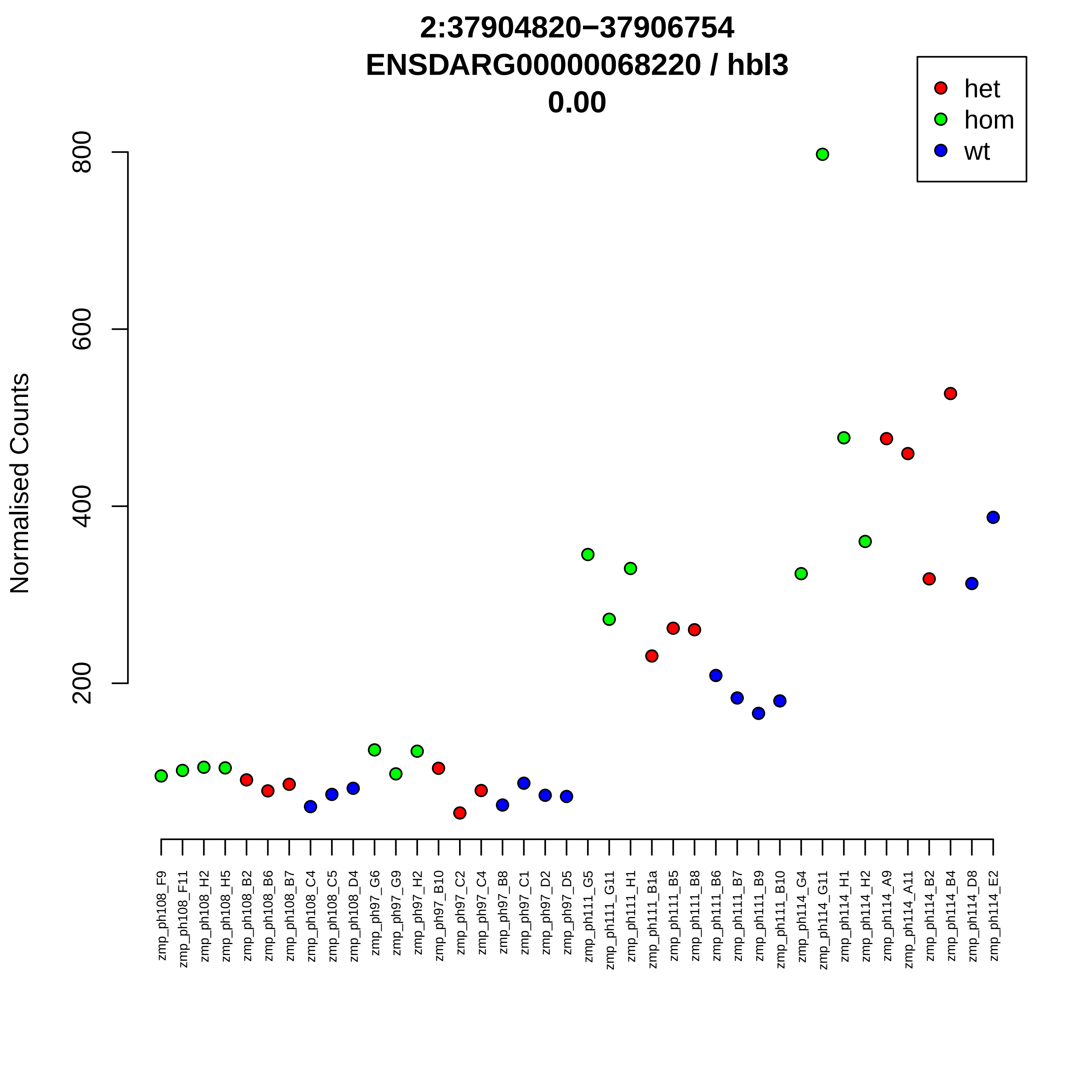

2:37904820−37906754
ENSDARG00000068220 / hbl3
0.00
het
hom
wt
Normalised Counts
200
400
600
800
zmp_ph108_F9
zmp_ph108_F11
zmp_ph108_H2
zmp_ph108_H5
zmp_ph108_B2
zmp_ph108_B6
zmp_ph108_B7
zmp_ph108_C4
zmp_ph108_C5
zmp_ph108_D4
zmp_ph97_G6
zmp_ph97_G9
zmp_ph97_H2
zmp_ph97_B10
zmp_ph97_C2
zmp_ph97_C4
zmp_ph97_B8
zmp_ph97_C1
zmp_ph97_D2
zmp_ph97_D5
zmp_ph111_G5
zmp_ph111_G11
zmp_ph111_H1
zmp_ph111_B1a
zmp_ph111_B5
zmp_ph111_B8
zmp_ph111_B6
zmp_ph111_B7
zmp_ph111_B9
zmp_ph111_B10
zmp_ph114_G4
zmp_ph114_G11
zmp_ph114_H1
zmp_ph114_H2
zmp_ph114_A9
zmp_ph114_A11
zmp_ph114_B2
zmp_ph114_B4
zmp_ph114_D8
zmp_ph114_E2

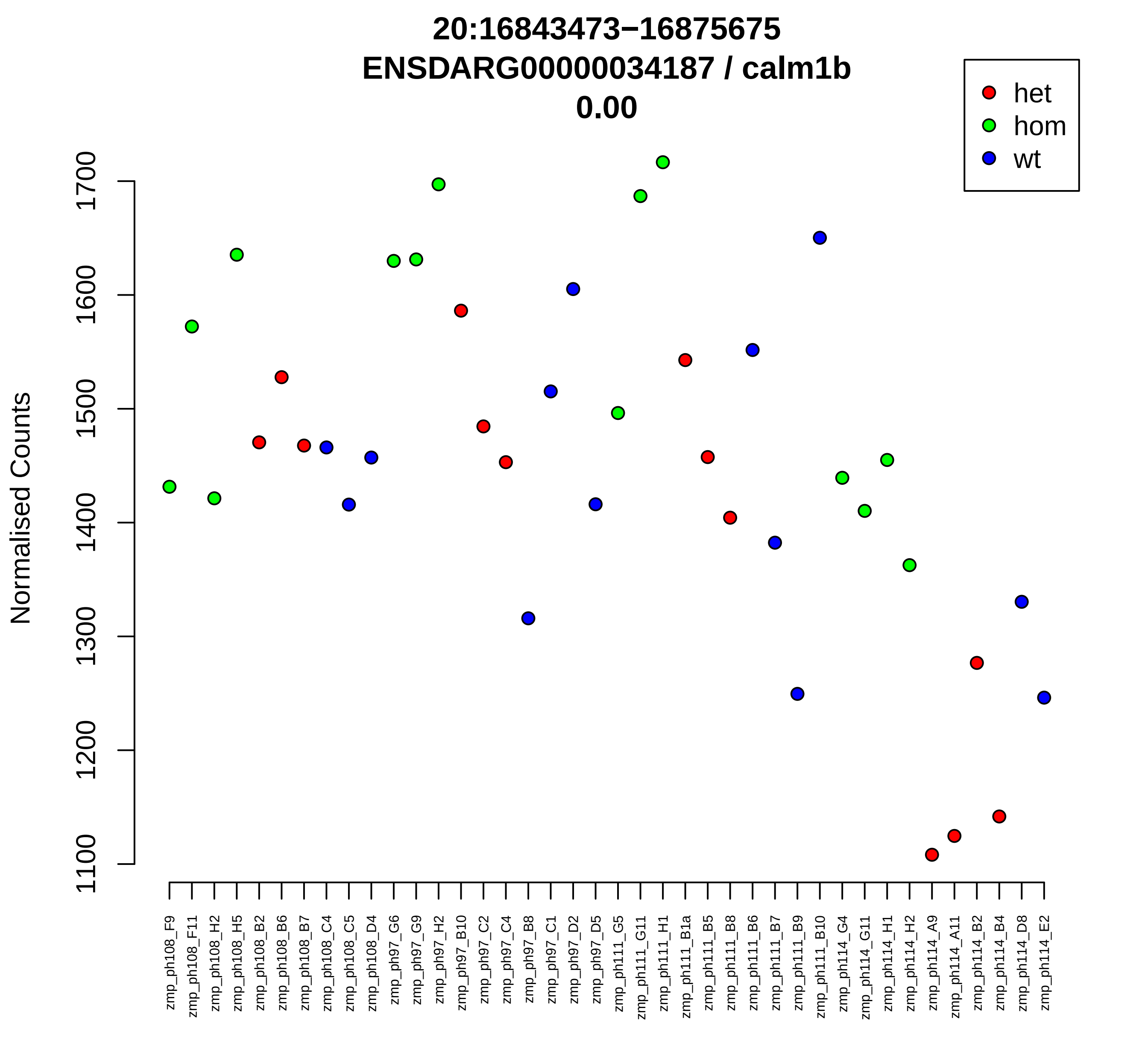

20:16843473−16875675
ENSDARG00000034187 / calm1b
0.00
het
hom
wt
Normalised Counts
1100
1200
1300
1400
1500
1600
1700
zmp_ph108_F9
zmp_ph108_F11
zmp_ph108_H2
zmp_ph108_H5
zmp_ph108_B2
zmp_ph108_B6
zmp_ph108_B7
zmp_ph108_C4
zmp_ph108_C5
zmp_ph108_D4
zmp_ph97_G6
zmp_ph97_G9
zmp_ph97_H2
zmp_ph97_B10
zmp_ph97_C2
zmp_ph97_C4
zmp_ph97_B8
zmp_ph97_C1
zmp_ph97_D2
zmp_ph97_D5
zmp_ph111_G5
zmp_ph111_G11
zmp_ph111_H1
zmp_ph111_B1a
zmp_ph111_B5
zmp_ph111_B8
zmp_ph111_B6
zmp_ph111_B7
zmp_ph111_B9
zmp_ph111_B10
zmp_ph114_G4
zmp_ph114_G11
zmp_ph114_H1
zmp_ph114_H2
zmp_ph114_A9
zmp_ph114_A11
zmp_ph114_B2
zmp_ph114_B4
zmp_ph114_D8
zmp_ph114_E2

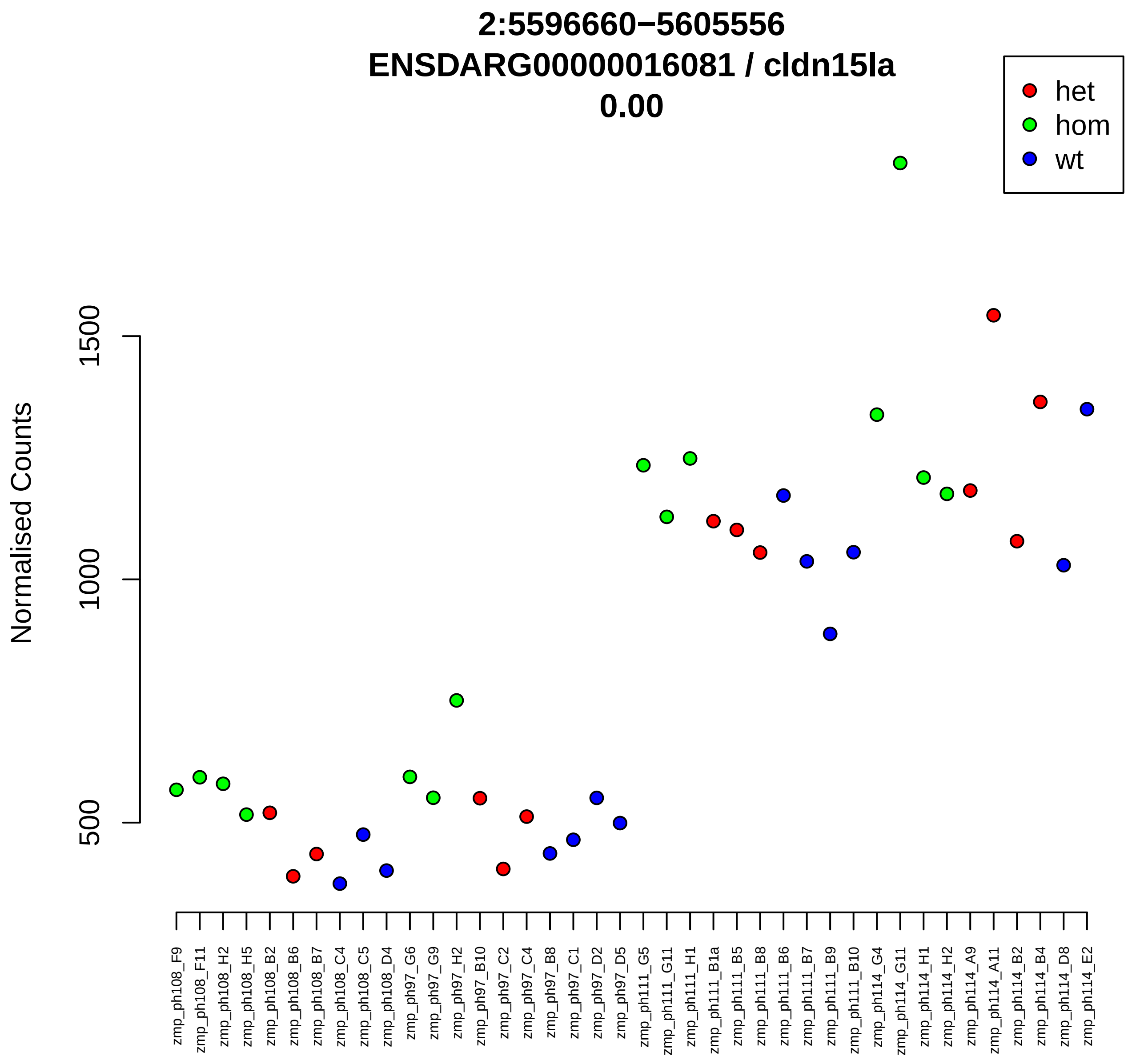

2:5596660−5605556
ENSDARG00000016081 / cldn15la
0.00
het
hom
wt
Normalised Counts
500
1000
1500
zmp_ph108_F9
zmp_ph108_F11
zmp_ph108_H2
zmp_ph108_H5
zmp_ph108_B2
zmp_ph108_B6
zmp_ph108_B7
zmp_ph108_C4
zmp_ph108_C5
zmp_ph108_D4
zmp_ph97_G6
zmp_ph97_G9
zmp_ph97_H2
zmp_ph97_B10
zmp_ph97_C2
zmp_ph97_C4
zmp_ph97_B8
zmp_ph97_C1
zmp_ph97_D2
zmp_ph97_D5
zmp_ph111_G5
zmp_ph111_G11
zmp_ph111_H1
zmp_ph111_B1a
zmp_ph111_B5
zmp_ph111_B8
zmp_ph111_B6
zmp_ph111_B7
zmp_ph111_B9
zmp_ph111_B10
zmp_ph114_G4
zmp_ph114_G11
zmp_ph114_H1
zmp_ph114_H2
zmp_ph114_A9
zmp_ph114_A11
zmp_ph114_B2
zmp_ph114_B4
zmp_ph114_D8
zmp_ph114_E2

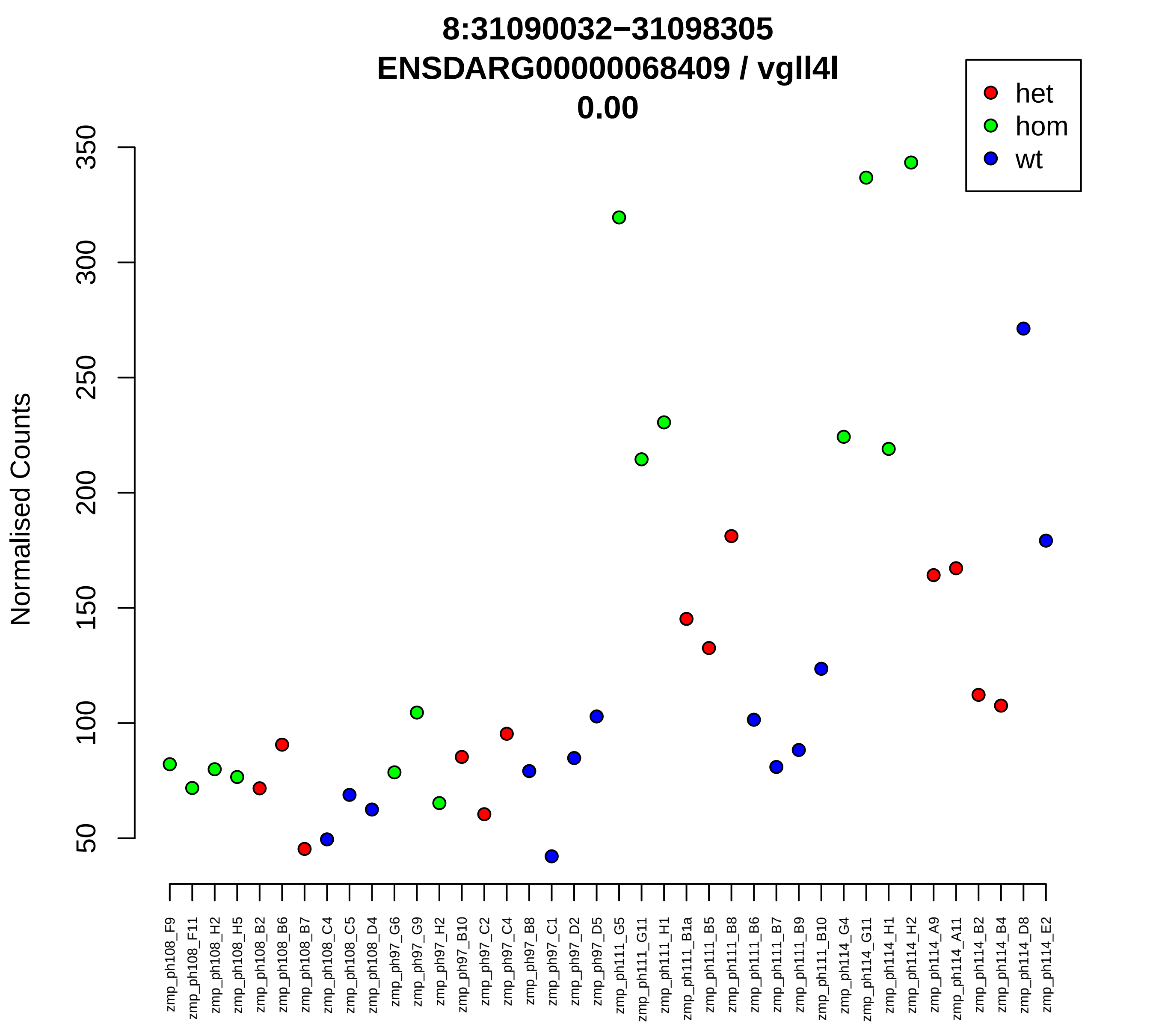

8:31090032−31098305
ENSDARG00000068409 / vgll4l
0.00
het
hom
wt
Normalised Counts
50
100
150
200
250
300
350
zmp_ph108_F9
zmp_ph108_F11
zmp_ph108_H2
zmp_ph108_H5
zmp_ph108_B2
zmp_ph108_B6
zmp_ph108_B7
zmp_ph108_C4
zmp_ph108_C5
zmp_ph108_D4
zmp_ph97_G6
zmp_ph97_G9
zmp_ph97_H2
zmp_ph97_B10
zmp_ph97_C2
zmp_ph97_C4
zmp_ph97_B8
zmp_ph97_C1
zmp_ph97_D2
zmp_ph97_D5
zmp_ph111_G5
zmp_ph111_G11
zmp_ph111_H1
zmp_ph111_B1a
zmp_ph111_B5
zmp_ph111_B8
zmp_ph111_B6
zmp_ph111_B7
zmp_ph111_B9
zmp_ph111_B10
zmp_ph114_G4
zmp_ph114_G11
zmp_ph114_H1
zmp_ph114_H2
zmp_ph114_A9
zmp_ph114_A11
zmp_ph114_B2
zmp_ph114_B4
zmp_ph114_D8
zmp_ph114_E2

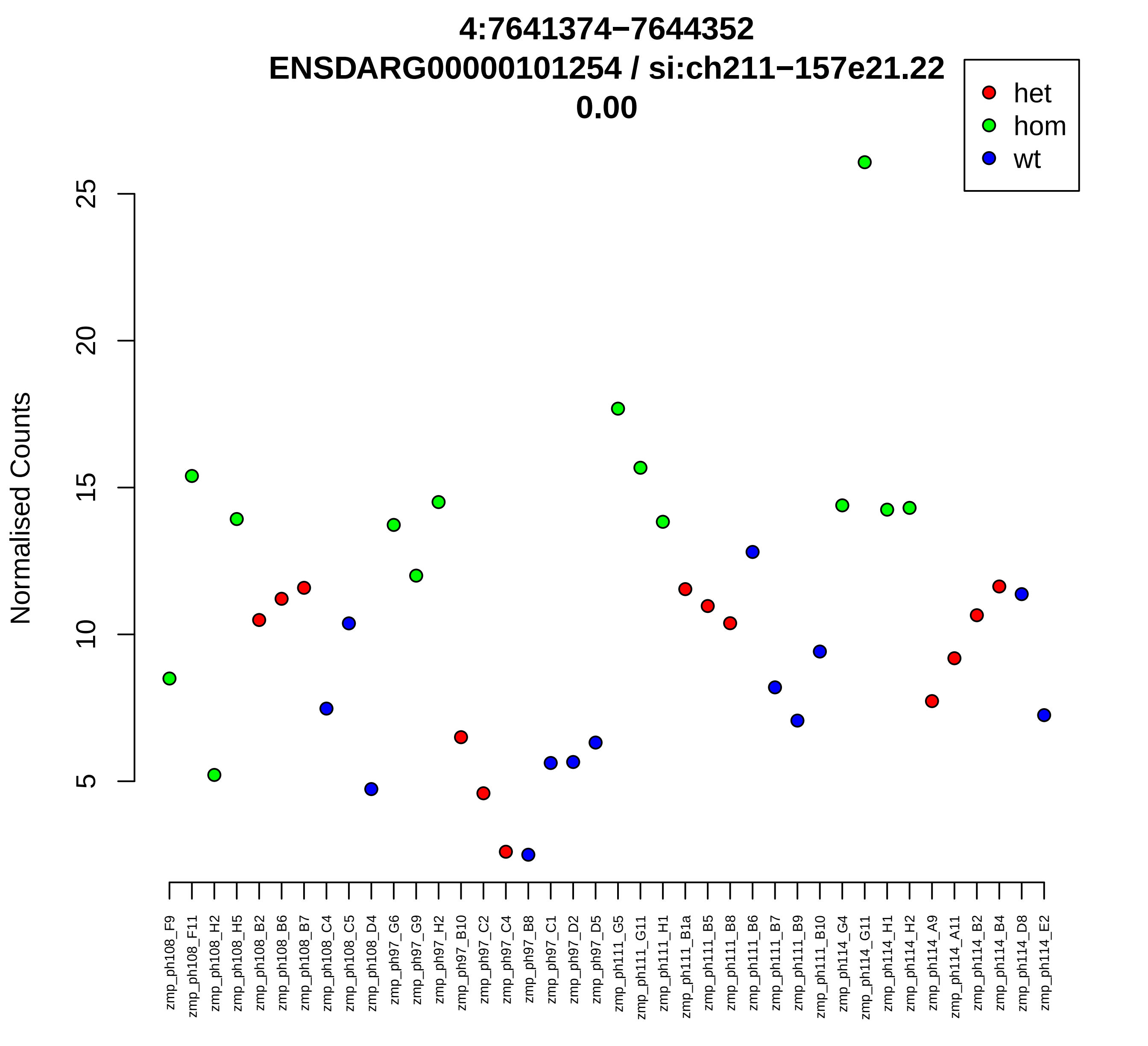
4:7641374−7644352
ENSDARG00000101254 / si:ch211−157e21.22
0.00
het
hom
wt
Normalised Counts
5
10
15
20
25
zmp_ph108_F9
zmp_ph108_F11
zmp_ph108_H2
zmp_ph108_H5
zmp_ph108_B2
zmp_ph108_B6
zmp_ph108_B7
zmp_ph108_C4
zmp_ph108_C5
zmp_ph108_D4
zmp_ph97_G6
zmp_ph97_G9
zmp_ph97_H2
zmp_ph97_B10
zmp_ph97_C2
zmp_ph97_C4
zmp_ph97_B8
zmp_ph97_C1
zmp_ph97_D2
zmp_ph97_D5
zmp_ph111_G5
zmp_ph111_G11
zmp_ph111_H1
zmp_ph111_B1a
zmp_ph111_B5
zmp_ph111_B8
zmp_ph111_B6
zmp_ph111_B7
zmp_ph111_B9
zmp_ph111_B10
zmp_ph114_G4
zmp_ph114_G11
zmp_ph114_H1
zmp_ph114_H2
zmp_ph114_A9
zmp_ph114_A11
zmp_ph114_B2
zmp_ph114_B4
zmp_ph114_D8
zmp_ph114_E2

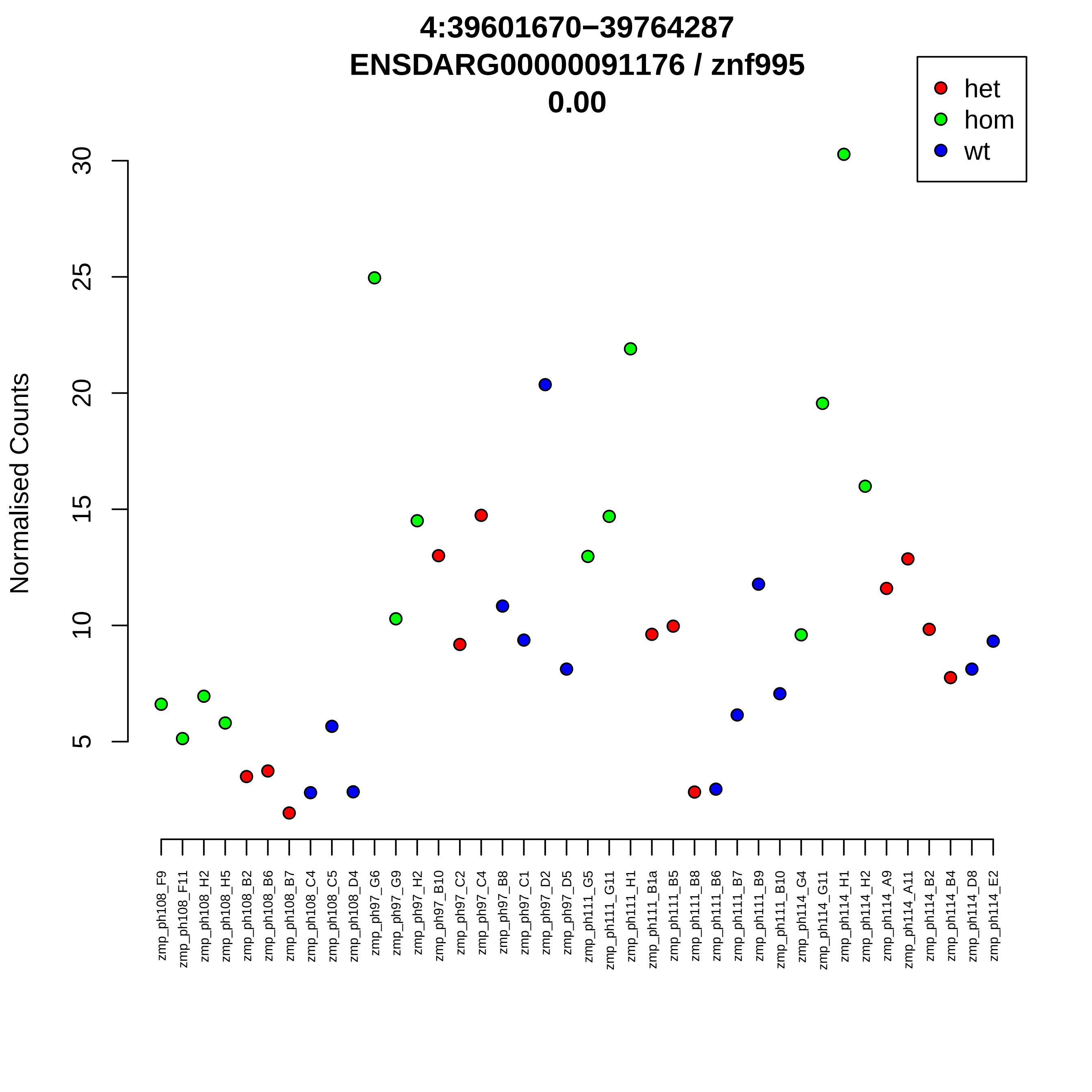
4:39601670−39764287
ENSDARG00000091176 / znf995
0.00
het
hom
wt
Normalised Counts
5
10
15
20
25
30
zmp_ph108_F9
zmp_ph108_F11
zmp_ph108_H2
zmp_ph108_H5
zmp_ph108_B2
zmp_ph108_B6
zmp_ph108_B7
zmp_ph108_C4
zmp_ph108_C5
zmp_ph108_D4
zmp_ph97_G6
zmp_ph97_G9
zmp_ph97_H2
zmp_ph97_B10
zmp_ph97_C2
zmp_ph97_C4
zmp_ph97_B8
zmp_ph97_C1
zmp_ph97_D2
zmp_ph97_D5
zmp_ph111_G5
zmp_ph111_G11
zmp_ph111_H1
zmp_ph111_B1a
zmp_ph111_B5
zmp_ph111_B8
zmp_ph111_B6
zmp_ph111_B7
zmp_ph111_B9
zmp_ph111_B10
zmp_ph114_G4
zmp_ph114_G11
zmp_ph114_H1
zmp_ph114_H2
zmp_ph114_A9
zmp_ph114_A11
zmp_ph114_B2
zmp_ph114_B4
zmp_ph114_D8
zmp_ph114_E2

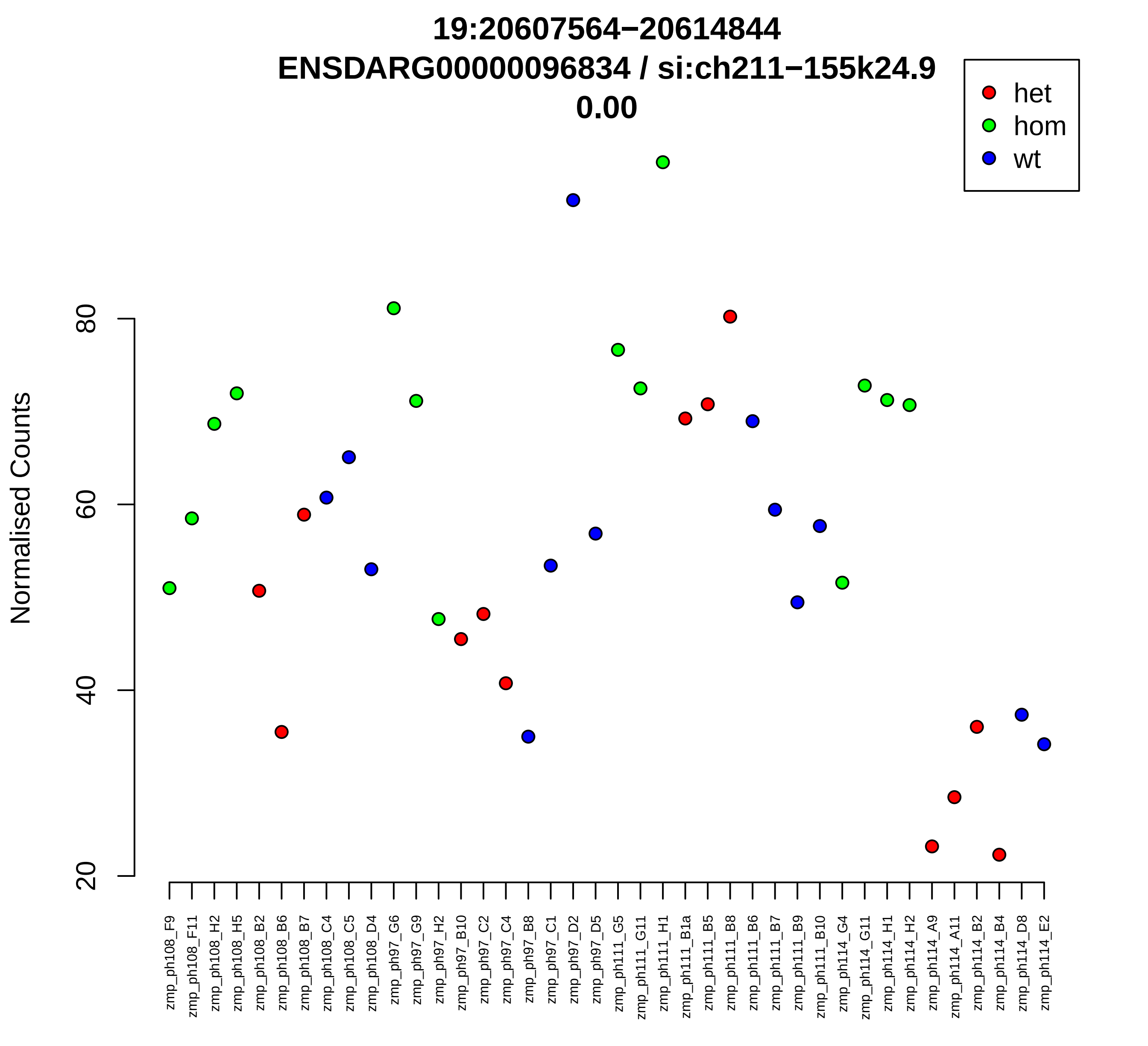

19:20607564−20614844
ENSDARG00000096834 / si:ch211−155k24.9
0.00
het
hom
wt
Normalised Counts
20
40
60
80
zmp_ph108_F9
zmp_ph108_F11
zmp_ph108_H2
zmp_ph108_H5
zmp_ph108_B2
zmp_ph108_B6
zmp_ph108_B7
zmp_ph108_C4
zmp_ph108_C5
zmp_ph108_D4
zmp_ph97_G6
zmp_ph97_G9
zmp_ph97_H2
zmp_ph97_B10
zmp_ph97_C2
zmp_ph97_C4
zmp_ph97_B8
zmp_ph97_C1
zmp_ph97_D2
zmp_ph97_D5
zmp_ph111_G5
zmp_ph111_G11
zmp_ph111_H1
zmp_ph111_B1a
zmp_ph111_B5
zmp_ph111_B8
zmp_ph111_B6
zmp_ph111_B7
zmp_ph111_B9
zmp_ph111_B10
zmp_ph114_G4
zmp_ph114_G11
zmp_ph114_H1
zmp_ph114_H2
zmp_ph114_A9
zmp_ph114_A11
zmp_ph114_B2
zmp_ph114_B4
zmp_ph114_D8
zmp_ph114_E2

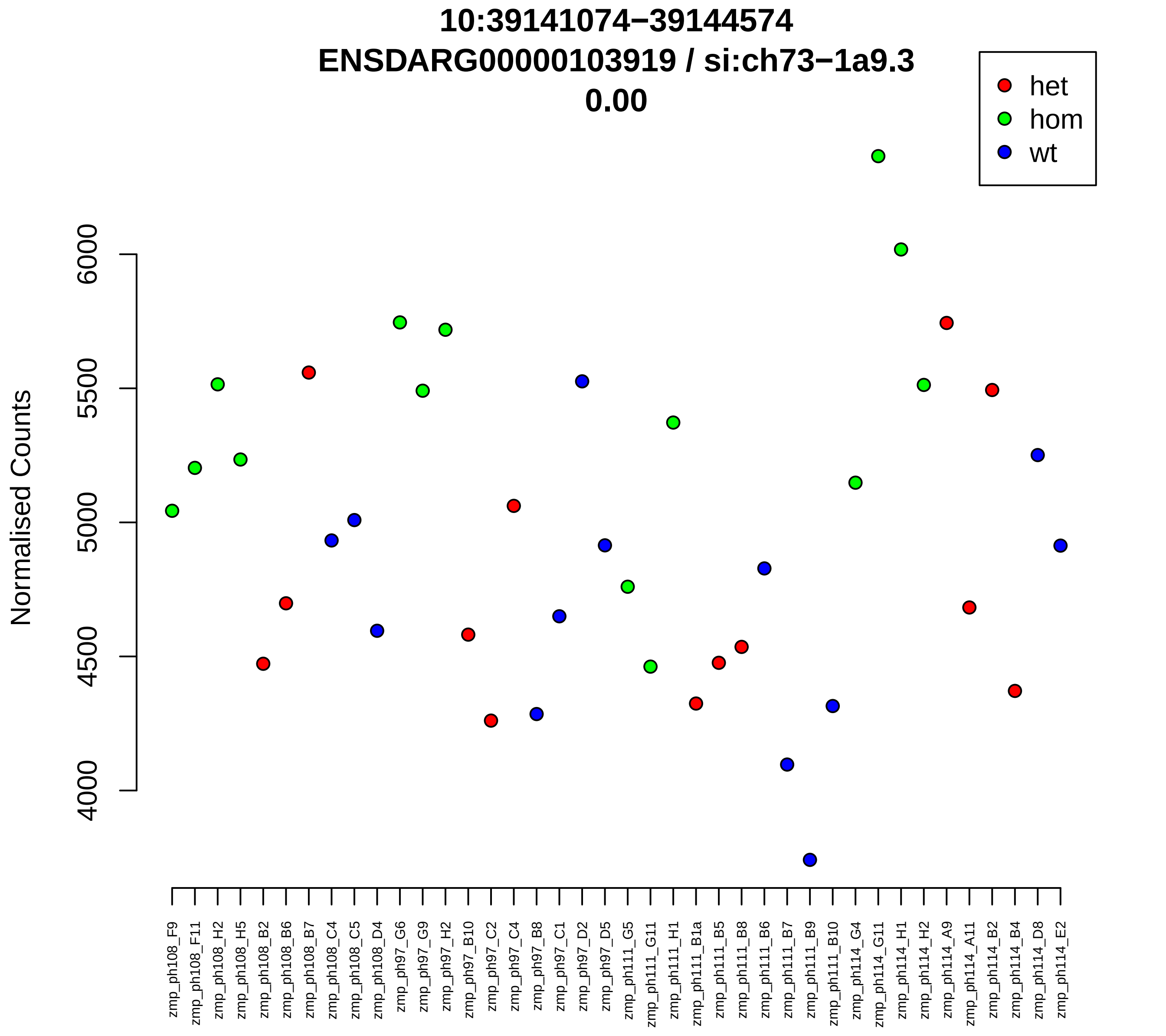

10:39141074−39144574
ENSDARG00000103919 / si:ch73−1a9.3
0.00
het
hom
wt
Normalised Counts
4000
4500
5000
5500
6000
zmp_ph108_F9
zmp_ph108_F11
zmp_ph108_H2
zmp_ph108_H5
zmp_ph108_B2
zmp_ph108_B6
zmp_ph108_B7
zmp_ph108_C4
zmp_ph108_C5
zmp_ph108_D4
zmp_ph97_G6
zmp_ph97_G9
zmp_ph97_H2
zmp_ph97_B10
zmp_ph97_C2
zmp_ph97_C4
zmp_ph97_B8
zmp_ph97_C1
zmp_ph97_D2
zmp_ph97_D5
zmp_ph111_G5
zmp_ph111_G11
zmp_ph111_H1
zmp_ph111_B1a
zmp_ph111_B5
zmp_ph111_B8
zmp_ph111_B6
zmp_ph111_B7
zmp_ph111_B9
zmp_ph111_B10
zmp_ph114_G4
zmp_ph114_G11
zmp_ph114_H1
zmp_ph114_H2
zmp_ph114_A9
zmp_ph114_A11
zmp_ph114_B2
zmp_ph114_B4
zmp_ph114_D8
zmp_ph114_E2

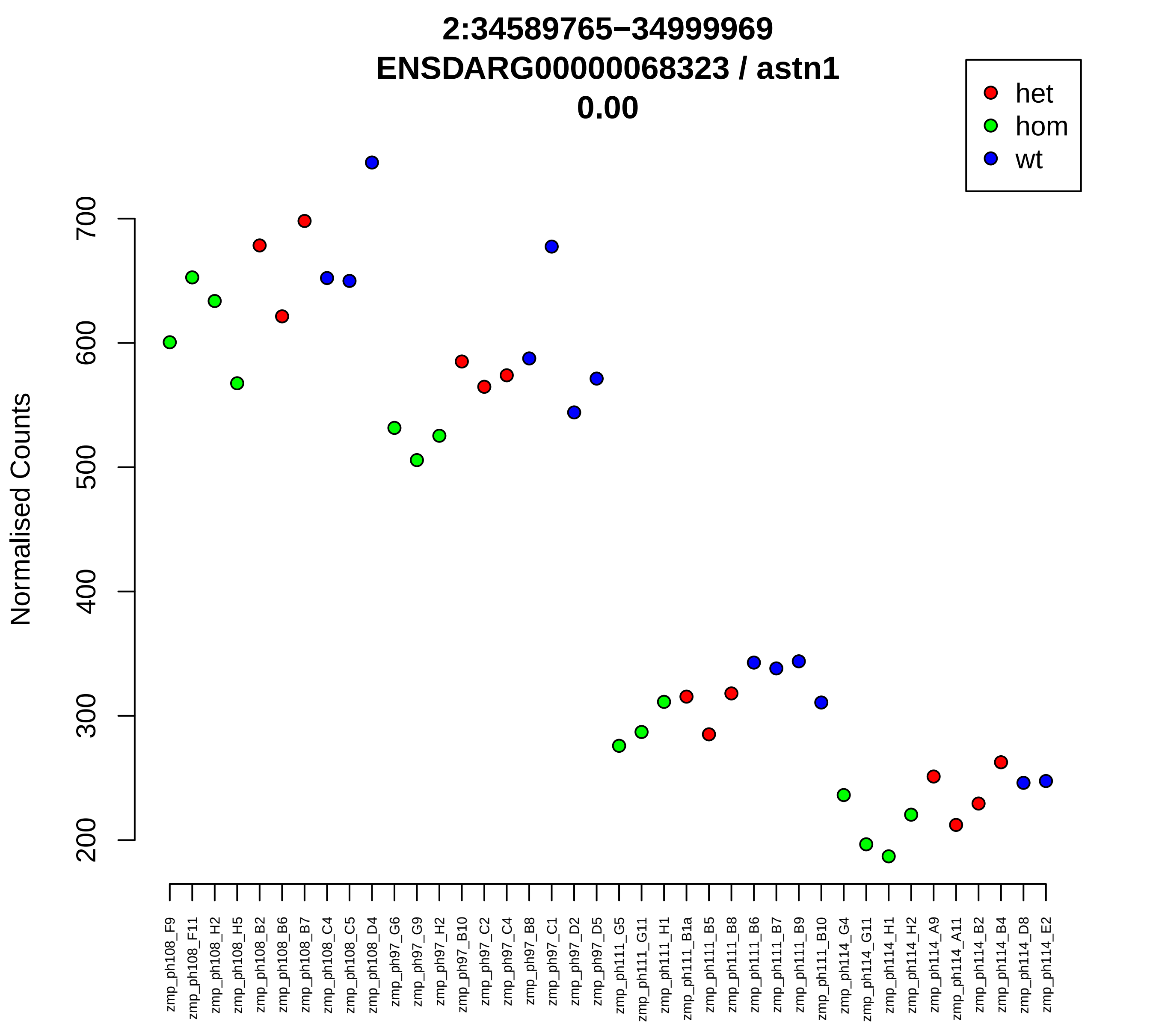

2:34589765−34999969
ENSDARG00000068323 / astn1
0.00
het
hom
wt
Normalised Counts
200
300
400
500
600
700
zmp_ph108_F9
zmp_ph108_F11
zmp_ph108_H2
zmp_ph108_H5
zmp_ph108_B2
zmp_ph108_B6
zmp_ph108_B7
zmp_ph108_C4
zmp_ph108_C5
zmp_ph108_D4
zmp_ph97_G6
zmp_ph97_G9
zmp_ph97_H2
zmp_ph97_B10
zmp_ph97_C2
zmp_ph97_C4
zmp_ph97_B8
zmp_ph97_C1
zmp_ph97_D2
zmp_ph97_D5
zmp_ph111_G5
zmp_ph111_G11
zmp_ph111_H1
zmp_ph111_B1a
zmp_ph111_B5
zmp_ph111_B8
zmp_ph111_B6
zmp_ph111_B7
zmp_ph111_B9
zmp_ph111_B10
zmp_ph114_G4
zmp_ph114_G11
zmp_ph114_H1
zmp_ph114_H2
zmp_ph114_A9
zmp_ph114_A11
zmp_ph114_B2
zmp_ph114_B4
zmp_ph114_D8
zmp_ph114_E2

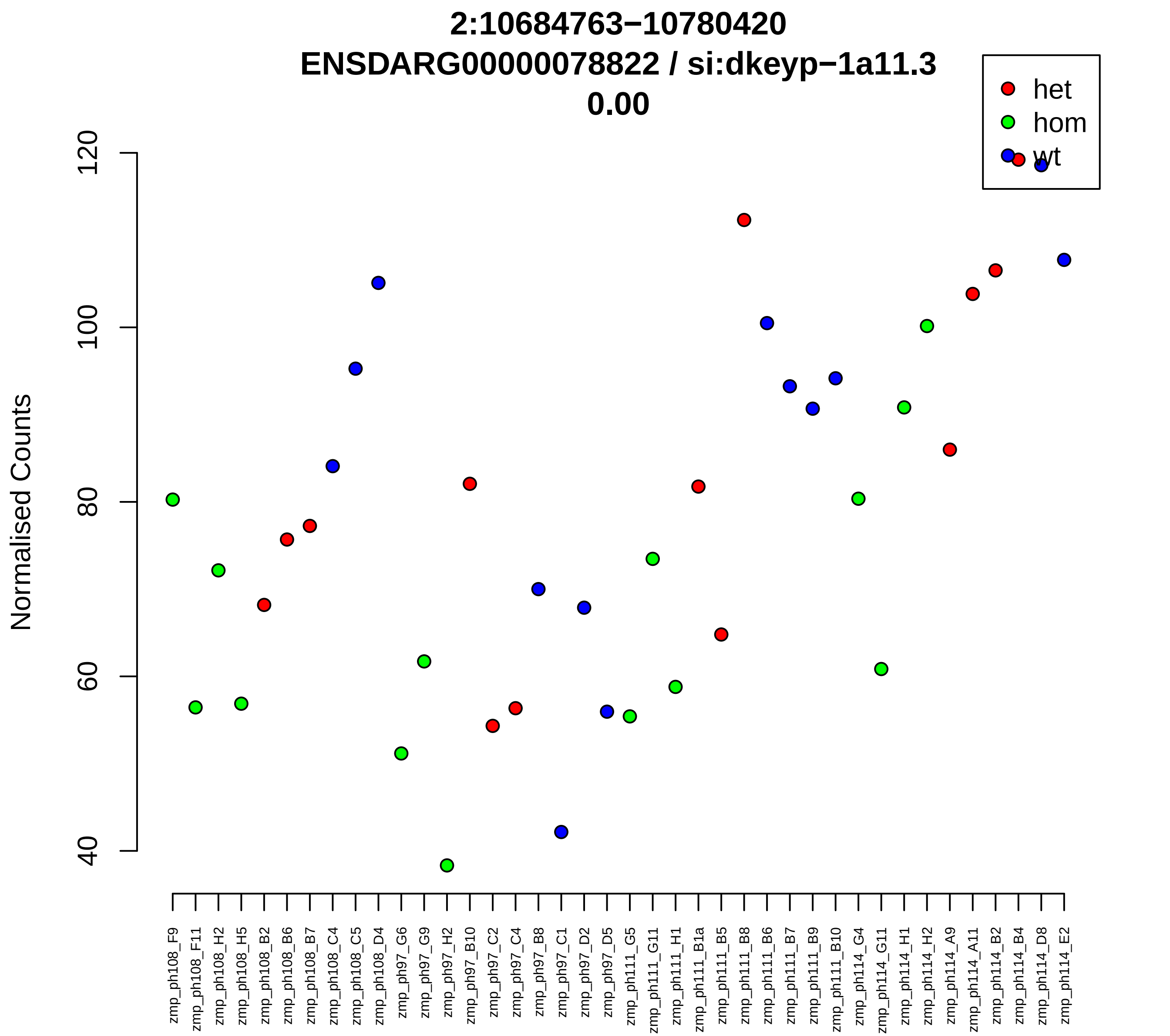

2:10684763−10780420
ENSDARG00000078822 / si:dkeyp−1a11.3
0.00
het
hom
wt
Normalised Counts
40
60
80
100
120
zmp_ph108_F9
zmp_ph108_F11
zmp_ph108_H2
zmp_ph108_H5
zmp_ph108_B2
zmp_ph108_B6
zmp_ph108_B7
zmp_ph108_C4
zmp_ph108_C5
zmp_ph108_D4
zmp_ph97_G6
zmp_ph97_G9
zmp_ph97_H2
zmp_ph97_B10
zmp_ph97_C2
zmp_ph97_C4
zmp_ph97_B8
zmp_ph97_C1
zmp_ph97_D2
zmp_ph97_D5
zmp_ph111_G5
zmp_ph111_G11
zmp_ph111_H1
zmp_ph111_B1a
zmp_ph111_B5
zmp_ph111_B8
zmp_ph111_B6
zmp_ph111_B7
zmp_ph111_B9
zmp_ph111_B10
zmp_ph114_G4
zmp_ph114_G11
zmp_ph114_H1
zmp_ph114_H2
zmp_ph114_A9
zmp_ph114_A11
zmp_ph114_B2
zmp_ph114_B4
zmp_ph114_D8
zmp_ph114_E2

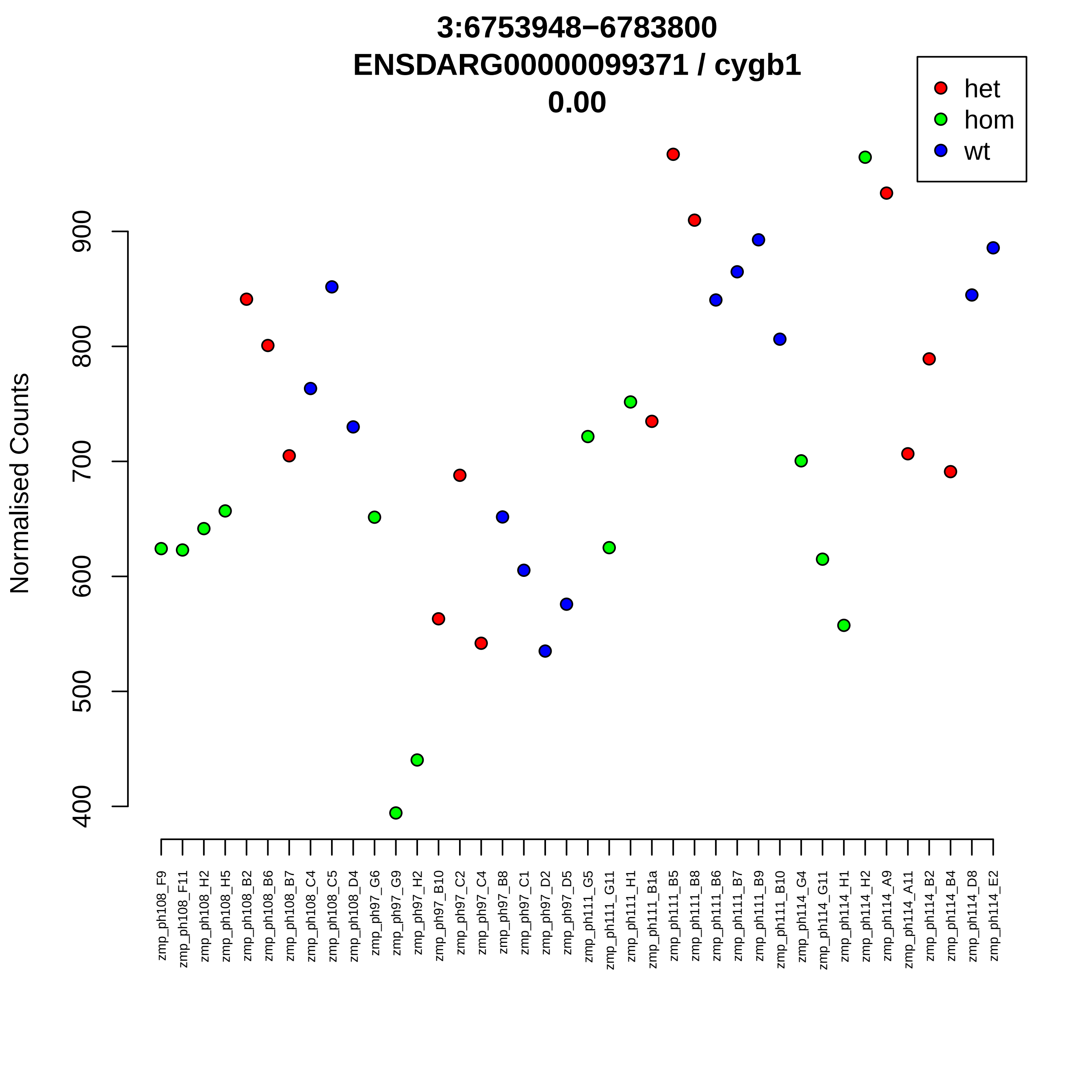

3:6753948−6783800
ENSDARG00000099371 / cygb1
0.00
het
hom
wt
Normalised Counts
400
500
600
700
800
900
zmp_ph108_F9
zmp_ph108_F11
zmp_ph108_H2
zmp_ph108_H5
zmp_ph108_B2
zmp_ph108_B6
zmp_ph108_B7
zmp_ph108_C4
zmp_ph108_C5
zmp_ph108_D4
zmp_ph97_G6
zmp_ph97_G9
zmp_ph97_H2
zmp_ph97_B10
zmp_ph97_C2
zmp_ph97_C4
zmp_ph97_B8
zmp_ph97_C1
zmp_ph97_D2
zmp_ph97_D5
zmp_ph111_G5
zmp_ph111_G11
zmp_ph111_H1
zmp_ph111_B1a
zmp_ph111_B5
zmp_ph111_B8
zmp_ph111_B6
zmp_ph111_B7
zmp_ph111_B9
zmp_ph111_B10
zmp_ph114_G4
zmp_ph114_G11
zmp_ph114_H1
zmp_ph114_H2
zmp_ph114_A9
zmp_ph114_A11
zmp_ph114_B2
zmp_ph114_B4
zmp_ph114_D8
zmp_ph114_E2

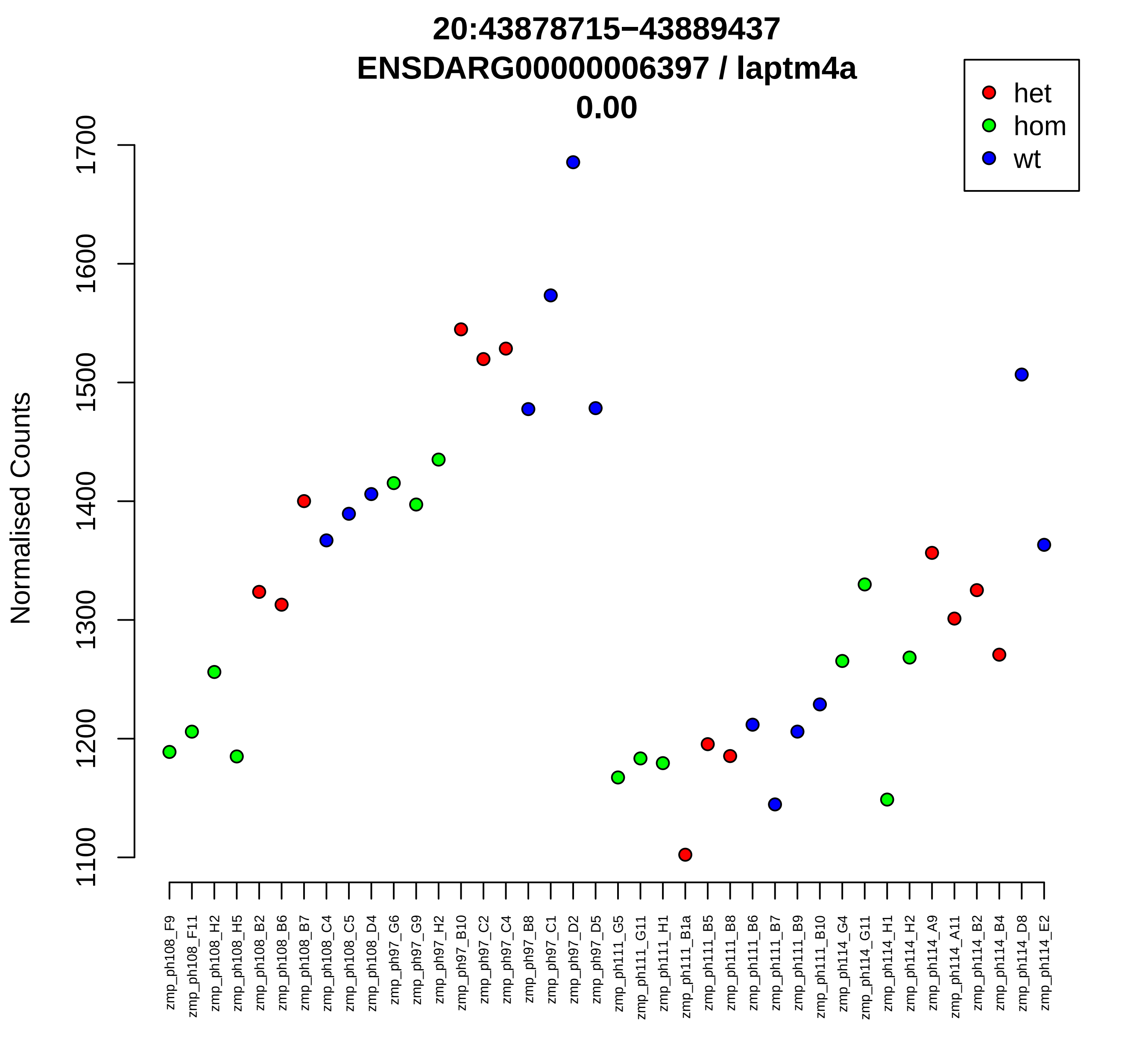

20:43878715−43889437
ENSDARG00000006397 / laptm4a
0.00
het
hom
wt
Normalised Counts
1100
1200
1300
1400
1500
1600
1700
zmp_ph108_F9
zmp_ph108_F11
zmp_ph108_H2
zmp_ph108_H5
zmp_ph108_B2
zmp_ph108_B6
zmp_ph108_B7
zmp_ph108_C4
zmp_ph108_C5
zmp_ph108_D4
zmp_ph97_G6
zmp_ph97_G9
zmp_ph97_H2
zmp_ph97_B10
zmp_ph97_C2
zmp_ph97_C4
zmp_ph97_B8
zmp_ph97_C1
zmp_ph97_D2
zmp_ph97_D5
zmp_ph111_G5
zmp_ph111_G11
zmp_ph111_H1
zmp_ph111_B1a
zmp_ph111_B5
zmp_ph111_B8
zmp_ph111_B6
zmp_ph111_B7
zmp_ph111_B9
zmp_ph111_B10
zmp_ph114_G4
zmp_ph114_G11
zmp_ph114_H1
zmp_ph114_H2
zmp_ph114_A9
zmp_ph114_A11
zmp_ph114_B2
zmp_ph114_B4
zmp_ph114_D8
zmp_ph114_E2

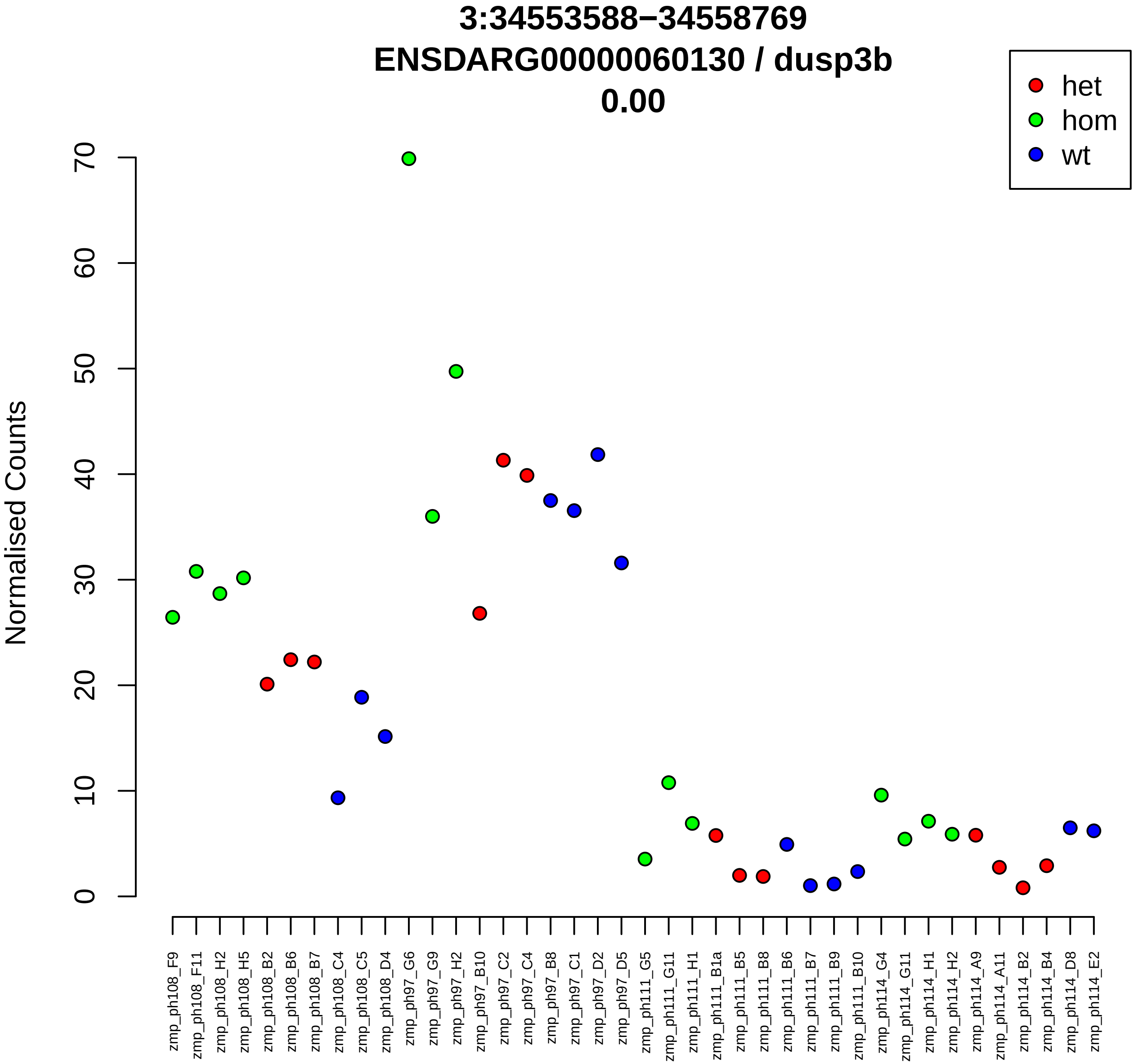
3:34553588−34558769
ENSDARG00000060130 / dusp3b
0.00
het
hom
wt
Normalised Counts
0
10
20
30
40
50
60
70
zmp_ph108_F9
zmp_ph108_F11
zmp_ph108_H2
zmp_ph108_H5
zmp_ph108_B2
zmp_ph108_B6
zmp_ph108_B7
zmp_ph108_C4
zmp_ph108_C5
zmp_ph108_D4
zmp_ph97_G6
zmp_ph97_G9
zmp_ph97_H2
zmp_ph97_B10
zmp_ph97_C2
zmp_ph97_C4
zmp_ph97_B8
zmp_ph97_C1
zmp_ph97_D2
zmp_ph97_D5
zmp_ph111_G5
zmp_ph111_G11
zmp_ph111_H1
zmp_ph111_B1a
zmp_ph111_B5
zmp_ph111_B8
zmp_ph111_B6
zmp_ph111_B7
zmp_ph111_B9
zmp_ph111_B10
zmp_ph114_G4
zmp_ph114_G11
zmp_ph114_H1
zmp_ph114_H2
zmp_ph114_A9
zmp_ph114_A11
zmp_ph114_B2
zmp_ph114_B4
zmp_ph114_D8
zmp_ph114_E2

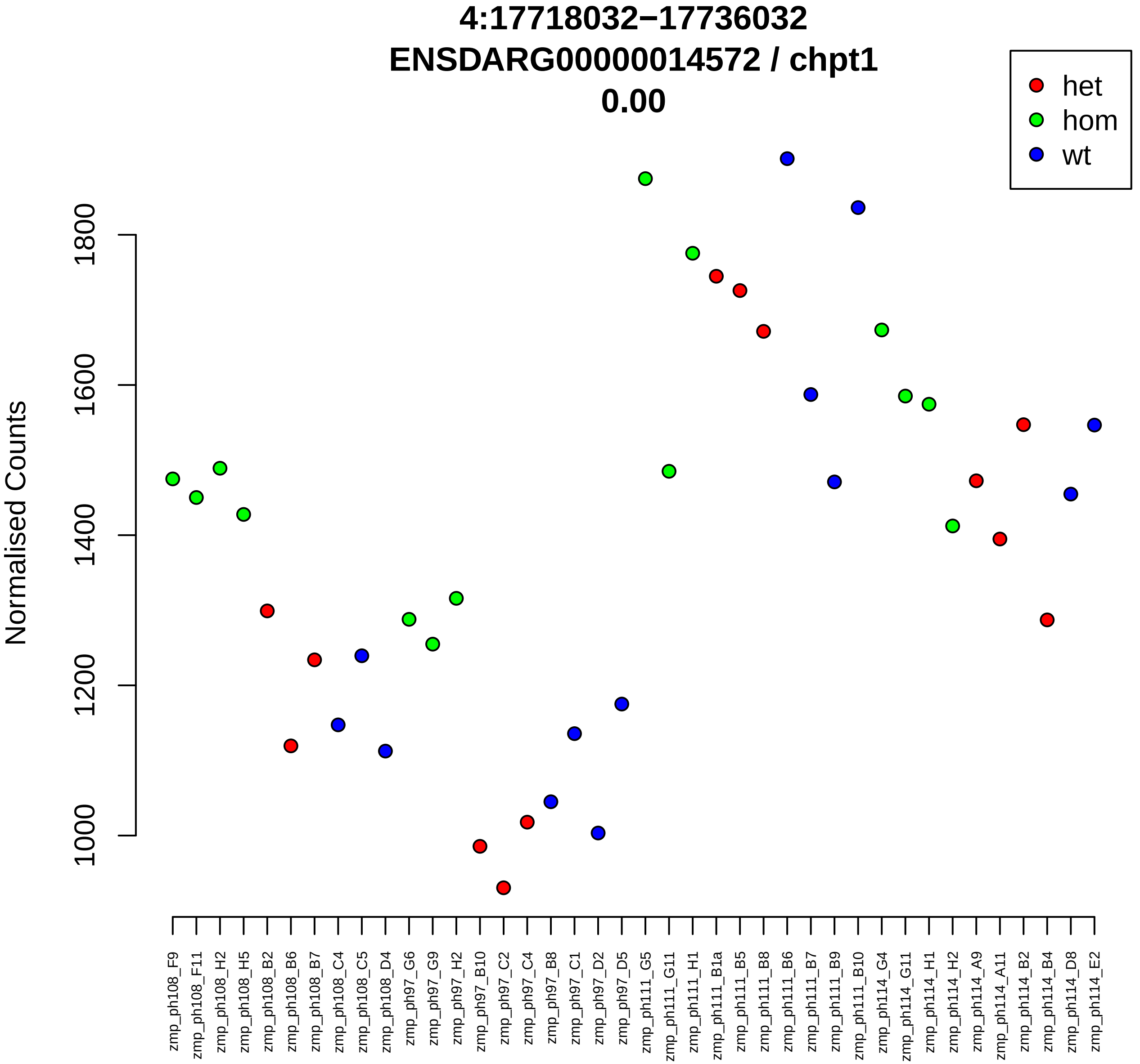

4:17718032−17736032
ENSDARG00000014572 / chpt1
0.00
het
hom
wt
Normalised Counts
1000
1200
1400
1600
1800
zmp_ph108_F9
zmp_ph108_F11
zmp_ph108_H2
zmp_ph108_H5
zmp_ph108_B2
zmp_ph108_B6
zmp_ph108_B7
zmp_ph108_C4
zmp_ph108_C5
zmp_ph108_D4
zmp_ph97_G6
zmp_ph97_G9
zmp_ph97_H2
zmp_ph97_B10
zmp_ph97_C2
zmp_ph97_C4
zmp_ph97_B8
zmp_ph97_C1
zmp_ph97_D2
zmp_ph97_D5
zmp_ph111_G5
zmp_ph111_G11
zmp_ph111_H1
zmp_ph111_B1a
zmp_ph111_B5
zmp_ph111_B8
zmp_ph111_B6
zmp_ph111_B7
zmp_ph111_B9
zmp_ph111_B10
zmp_ph114_G4
zmp_ph114_G11
zmp_ph114_H1
zmp_ph114_H2
zmp_ph114_A9
zmp_ph114_A11
zmp_ph114_B2
zmp_ph114_B4
zmp_ph114_D8
zmp_ph114_E2

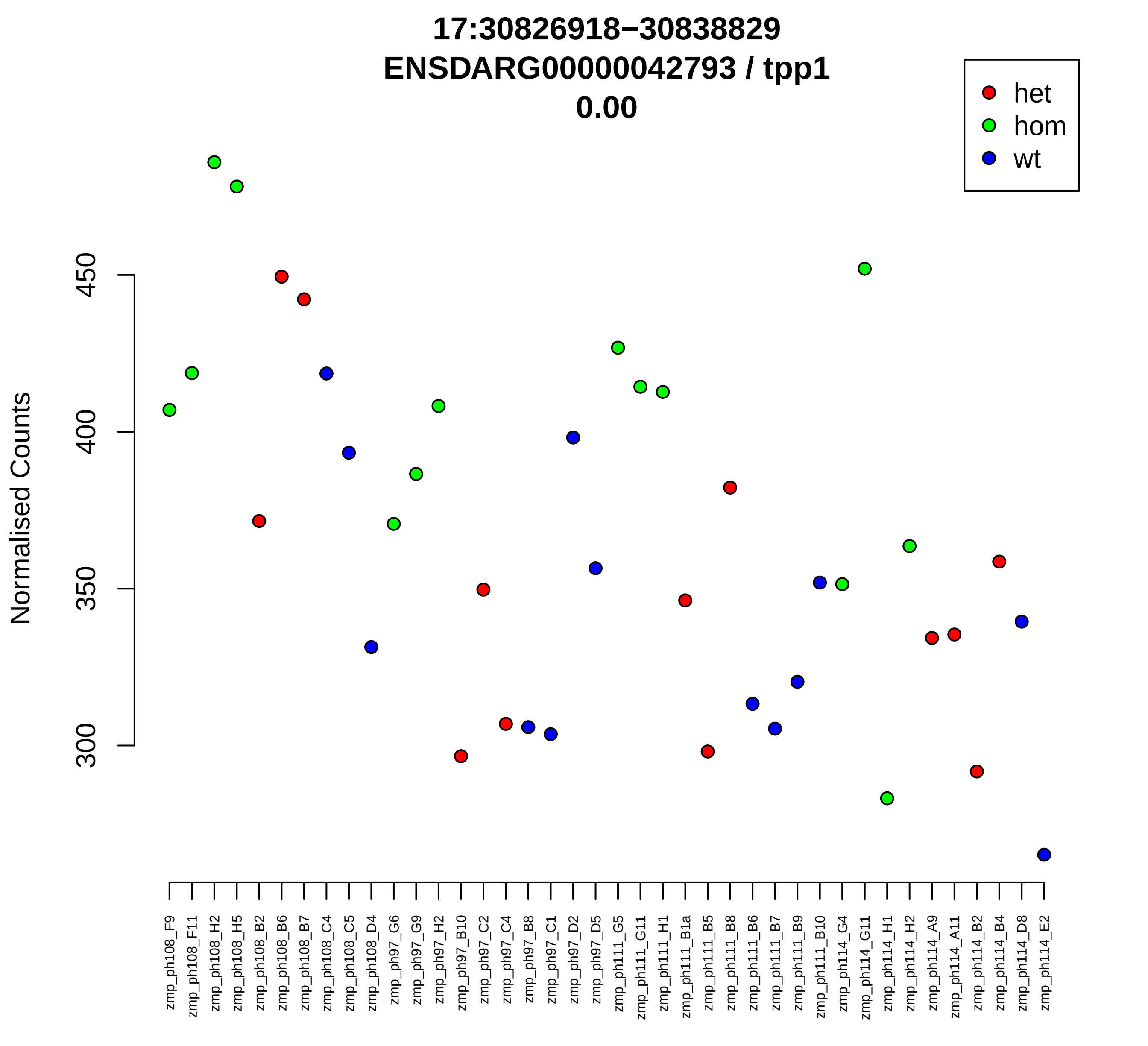

17:30826918−30838829
ENSDARG00000042793 / tpp1
0.00
het
hom
wt
Normalised Counts
300
350
400
450
zmp_ph108_F9
zmp_ph108_F11
zmp_ph108_H2
zmp_ph108_H5
zmp_ph108_B2
zmp_ph108_B6
zmp_ph108_B7
zmp_ph108_C4
zmp_ph108_C5
zmp_ph108_D4
zmp_ph97_G6
zmp_ph97_G9
zmp_ph97_H2
zmp_ph97_B10
zmp_ph97_C2
zmp_ph97_C4
zmp_ph97_B8
zmp_ph97_C1
zmp_ph97_D2
zmp_ph97_D5
zmp_ph111_G5
zmp_ph111_G11
zmp_ph111_H1
zmp_ph111_B1a
zmp_ph111_B5
zmp_ph111_B8
zmp_ph111_B6
zmp_ph111_B7
zmp_ph111_B9
zmp_ph111_B10
zmp_ph114_G4
zmp_ph114_G11
zmp_ph114_H1
zmp_ph114_H2
zmp_ph114_A9
zmp_ph114_A11
zmp_ph114_B2
zmp_ph114_B4
zmp_ph114_D8
zmp_ph114_E2

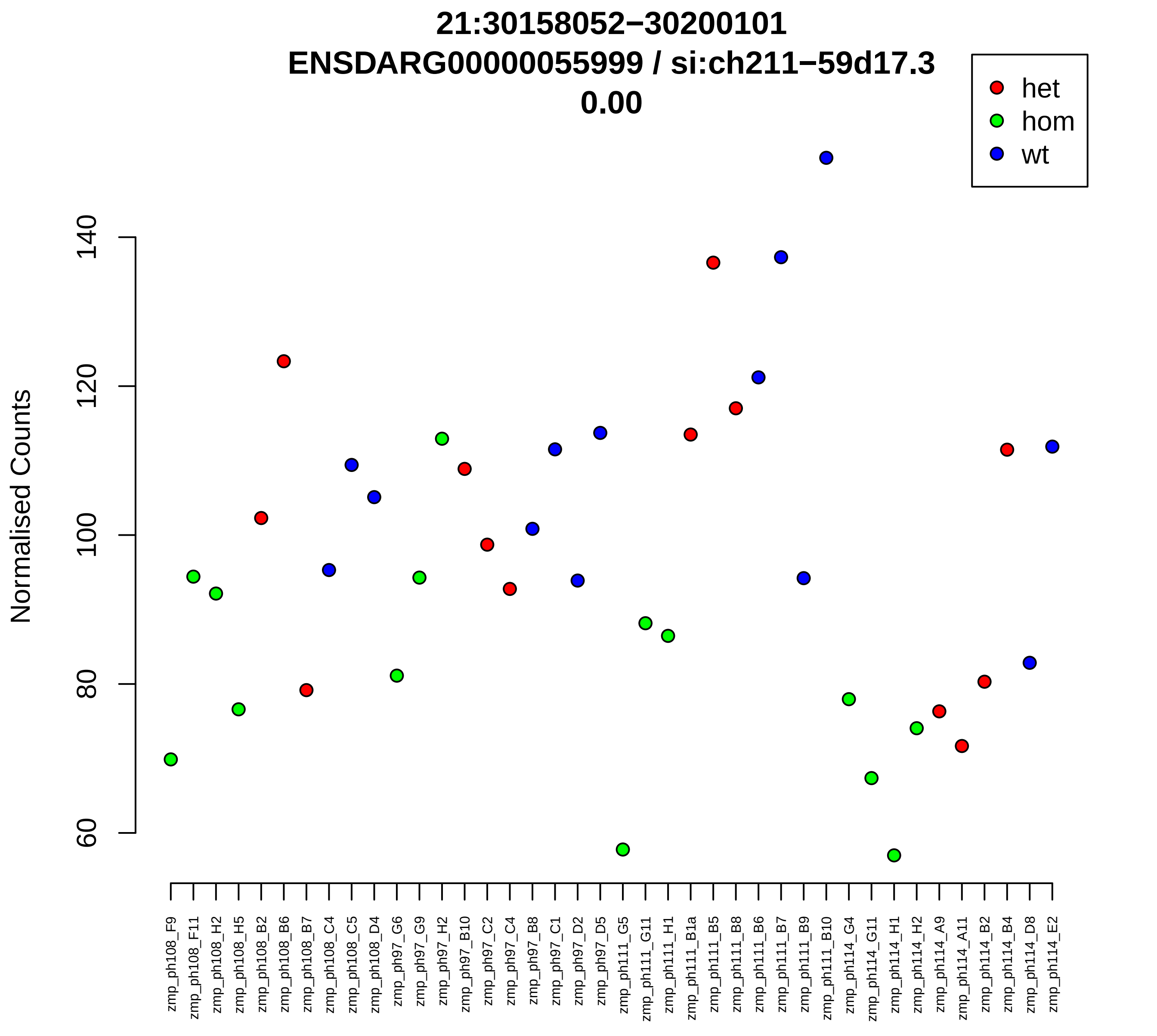
21:30158052−30200101
ENSDARG00000055999 / si:ch211−59d17.3
0.00
het
hom
wt
Normalised Counts
60
80
100
120
140
zmp_ph108_F9
zmp_ph108_F11
zmp_ph108_H2
zmp_ph108_H5
zmp_ph108_B2
zmp_ph108_B6
zmp_ph108_B7
zmp_ph108_C4
zmp_ph108_C5
zmp_ph108_D4
zmp_ph97_G6
zmp_ph97_G9
zmp_ph97_H2
zmp_ph97_B10
zmp_ph97_C2
zmp_ph97_C4
zmp_ph97_B8
zmp_ph97_C1
zmp_ph97_D2
zmp_ph97_D5
zmp_ph111_G5
zmp_ph111_G11
zmp_ph111_H1
zmp_ph111_B1a
zmp_ph111_B5
zmp_ph111_B8
zmp_ph111_B6
zmp_ph111_B7
zmp_ph111_B9
zmp_ph111_B10
zmp_ph114_G4
zmp_ph114_G11
zmp_ph114_H1
zmp_ph114_H2
zmp_ph114_A9
zmp_ph114_A11
zmp_ph114_B2
zmp_ph114_B4
zmp_ph114_D8
zmp_ph114_E2

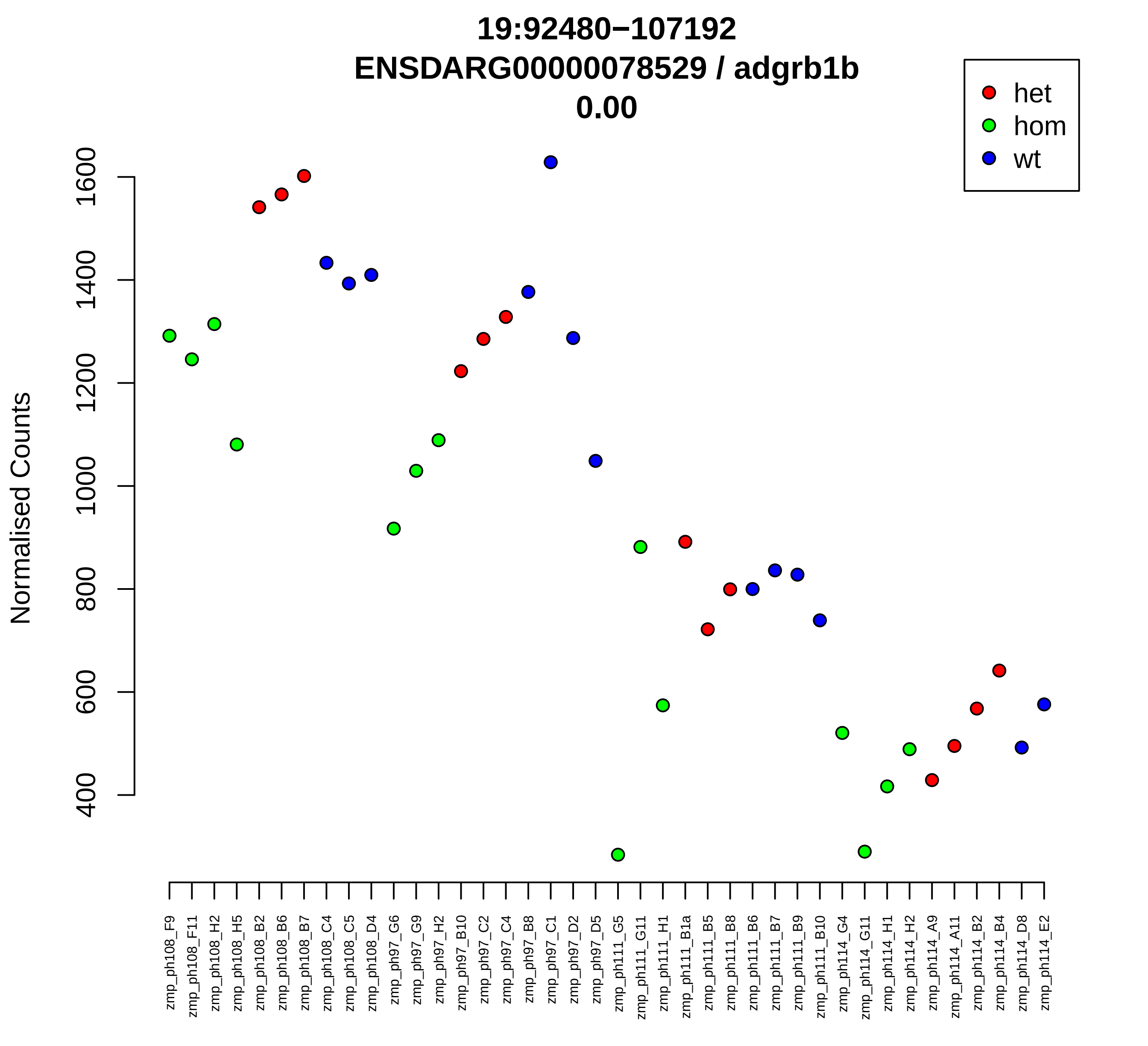

19:92480−107192
ENSDARG00000078529 / adgrb1b
0.00
het
hom
wt
Normalised Counts
400
600
800
1000
1200
1400
1600
zmp_ph108_F9
zmp_ph108_F11
zmp_ph108_H2
zmp_ph108_H5
zmp_ph108_B2
zmp_ph108_B6
zmp_ph108_B7
zmp_ph108_C4
zmp_ph108_C5
zmp_ph108_D4
zmp_ph97_G6
zmp_ph97_G9
zmp_ph97_H2
zmp_ph97_B10
zmp_ph97_C2
zmp_ph97_C4
zmp_ph97_B8
zmp_ph97_C1
zmp_ph97_D2
zmp_ph97_D5
zmp_ph111_G5
zmp_ph111_G11
zmp_ph111_H1
zmp_ph111_B1a
zmp_ph111_B5
zmp_ph111_B8
zmp_ph111_B6
zmp_ph111_B7
zmp_ph111_B9
zmp_ph111_B10
zmp_ph114_G4
zmp_ph114_G11
zmp_ph114_H1
zmp_ph114_H2
zmp_ph114_A9
zmp_ph114_A11
zmp_ph114_B2
zmp_ph114_B4
zmp_ph114_D8
zmp_ph114_E2

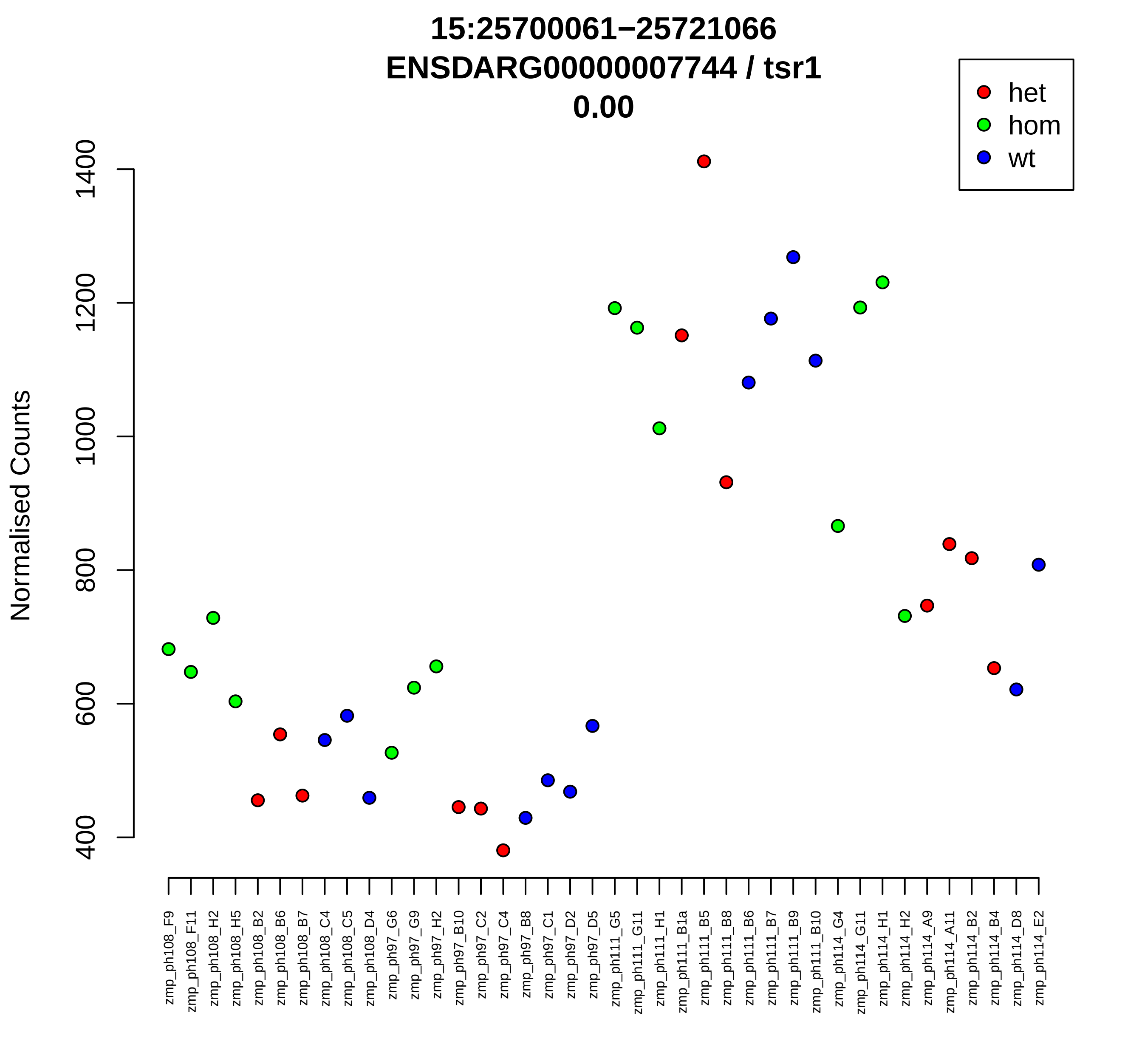

15:25700061−25721066
ENSDARG00000007744 / tsr1
0.00
het
hom
wt
Normalised Counts
400
600
800
1000
1200
1400
zmp_ph108_F9
zmp_ph108_F11
zmp_ph108_H2
zmp_ph108_H5
zmp_ph108_B2
zmp_ph108_B6
zmp_ph108_B7
zmp_ph108_C4
zmp_ph108_C5
zmp_ph108_D4
zmp_ph97_G6
zmp_ph97_G9
zmp_ph97_H2
zmp_ph97_B10
zmp_ph97_C2
zmp_ph97_C4
zmp_ph97_B8
zmp_ph97_C1
zmp_ph97_D2
zmp_ph97_D5
zmp_ph111_G5
zmp_ph111_G11
zmp_ph111_H1
zmp_ph111_B1a
zmp_ph111_B5
zmp_ph111_B8
zmp_ph111_B6
zmp_ph111_B7
zmp_ph111_B9
zmp_ph111_B10
zmp_ph114_G4
zmp_ph114_G11
zmp_ph114_H1
zmp_ph114_H2
zmp_ph114_A9
zmp_ph114_A11
zmp_ph114_B2
zmp_ph114_B4
zmp_ph114_D8
zmp_ph114_E2

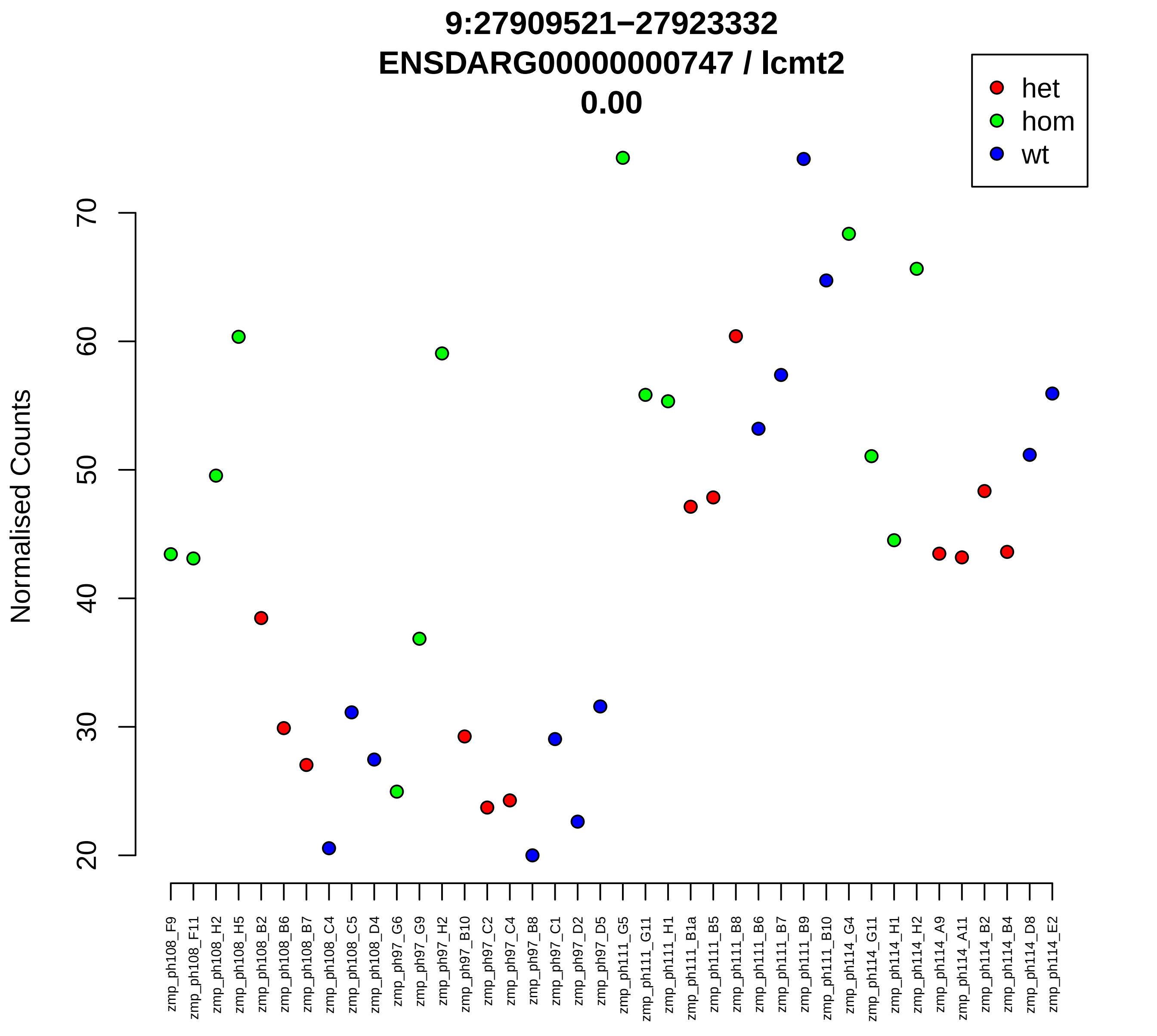

9:27909521−27923332
ENSDARG00000000747 / lcmt2
0.00
het
hom
wt
Normalised Counts
20
30
40
50
60
70
zmp_ph108_F9
zmp_ph108_F11
zmp_ph108_H2
zmp_ph108_H5
zmp_ph108_B2
zmp_ph108_B6
zmp_ph108_B7
zmp_ph108_C4
zmp_ph108_C5
zmp_ph108_D4
zmp_ph97_G6
zmp_ph97_G9
zmp_ph97_H2
zmp_ph97_B10
zmp_ph97_C2
zmp_ph97_C4
zmp_ph97_B8
zmp_ph97_C1
zmp_ph97_D2
zmp_ph97_D5
zmp_ph111_G5
zmp_ph111_G11
zmp_ph111_H1
zmp_ph111_B1a
zmp_ph111_B5
zmp_ph111_B8
zmp_ph111_B6
zmp_ph111_B7
zmp_ph111_B9
zmp_ph111_B10
zmp_ph114_G4
zmp_ph114_G11
zmp_ph114_H1
zmp_ph114_H2
zmp_ph114_A9
zmp_ph114_A11
zmp_ph114_B2
zmp_ph114_B4
zmp_ph114_D8
zmp_ph114_E2

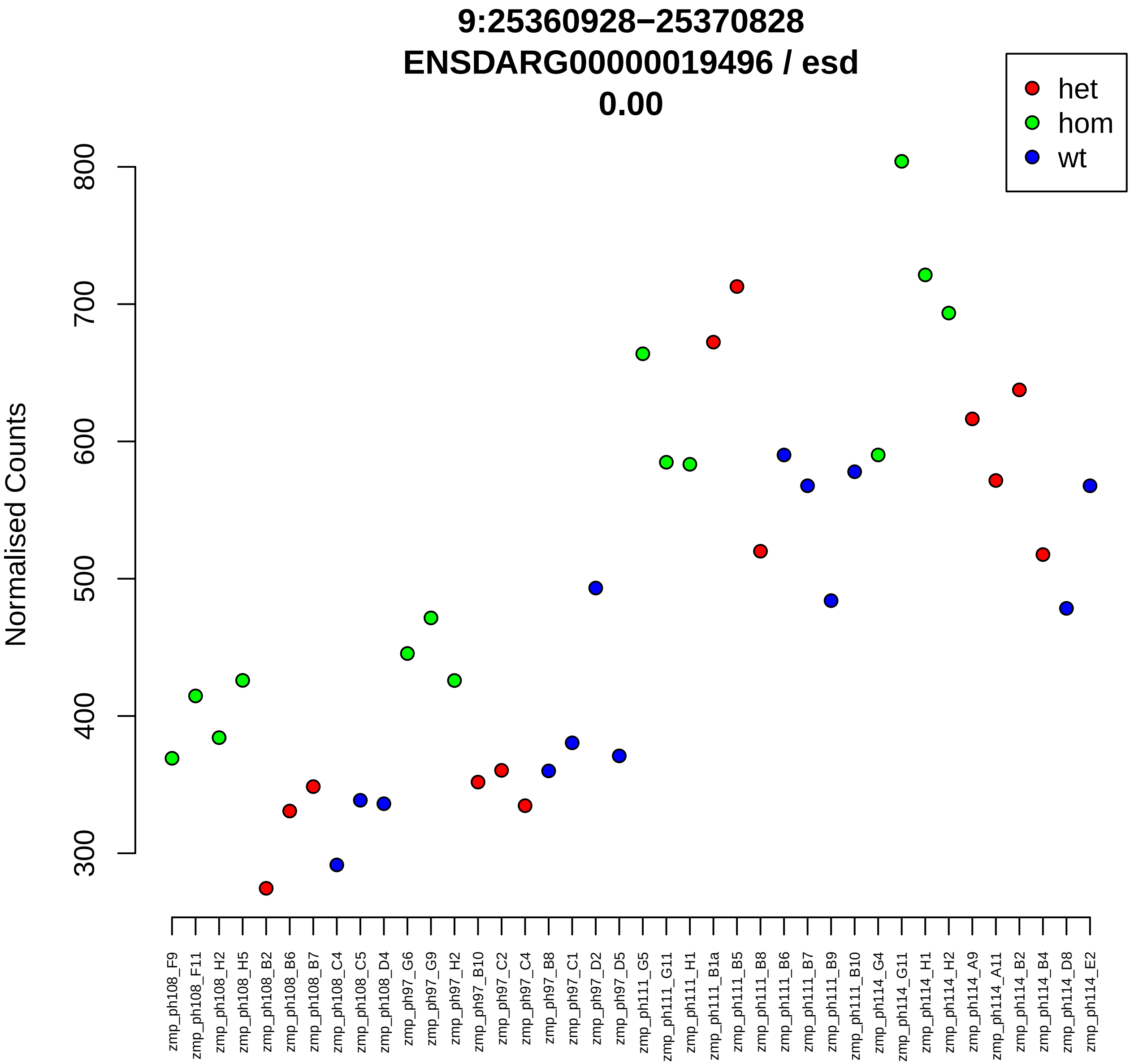
9:25360928−25370828
ENSDARG00000019496 / esd
0.00
het
hom
wt
Normalised Counts
300
400
500
600
700
800
zmp_ph108_F9
zmp_ph108_F11
zmp_ph108_H2
zmp_ph108_H5
zmp_ph108_B2
zmp_ph108_B6
zmp_ph108_B7
zmp_ph108_C4
zmp_ph108_C5
zmp_ph108_D4
zmp_ph97_G6
zmp_ph97_G9
zmp_ph97_H2
zmp_ph97_B10
zmp_ph97_C2
zmp_ph97_C4
zmp_ph97_B8
zmp_ph97_C1
zmp_ph97_D2
zmp_ph97_D5
zmp_ph111_G5
zmp_ph111_G11
zmp_ph111_H1
zmp_ph111_B1a
zmp_ph111_B5
zmp_ph111_B8
zmp_ph111_B6
zmp_ph111_B7
zmp_ph111_B9
zmp_ph111_B10
zmp_ph114_G4
zmp_ph114_G11
zmp_ph114_H1
zmp_ph114_H2
zmp_ph114_A9
zmp_ph114_A11
zmp_ph114_B2
zmp_ph114_B4
zmp_ph114_D8
zmp_ph114_E2

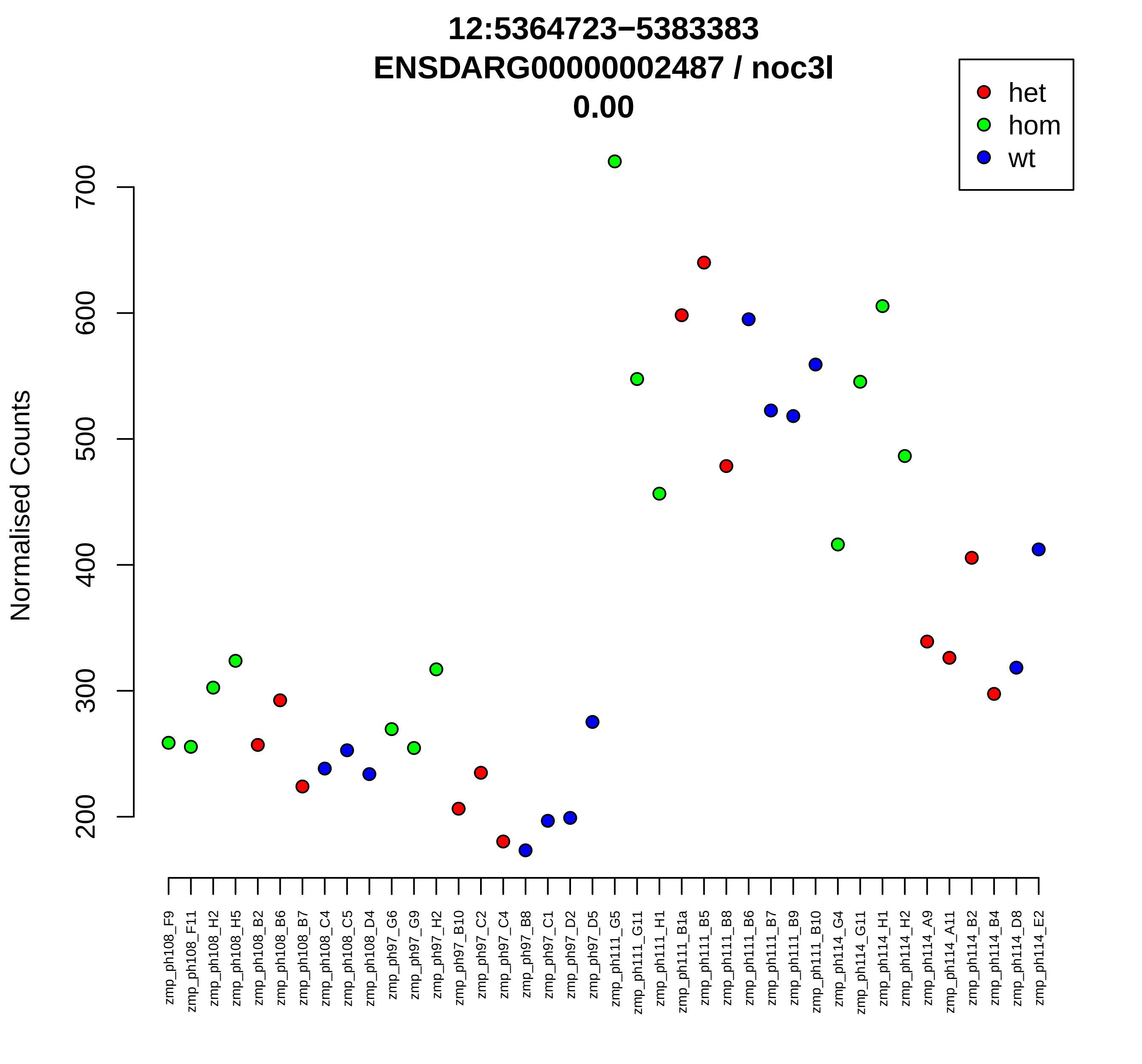

12:5364723−5383383
ENSDARG00000002487 / noc3l
0.00
het
hom
wt
Normalised Counts
200
300
400
500
600
700
zmp_ph108_F9
zmp_ph108_F11
zmp_ph108_H2
zmp_ph108_H5
zmp_ph108_B2
zmp_ph108_B6
zmp_ph108_B7
zmp_ph108_C4
zmp_ph108_C5
zmp_ph108_D4
zmp_ph97_G6
zmp_ph97_G9
zmp_ph97_H2
zmp_ph97_B10
zmp_ph97_C2
zmp_ph97_C4
zmp_ph97_B8
zmp_ph97_C1
zmp_ph97_D2
zmp_ph97_D5
zmp_ph111_G5
zmp_ph111_G11
zmp_ph111_H1
zmp_ph111_B1a
zmp_ph111_B5
zmp_ph111_B8
zmp_ph111_B6
zmp_ph111_B7
zmp_ph111_B9
zmp_ph111_B10
zmp_ph114_G4
zmp_ph114_G11
zmp_ph114_H1
zmp_ph114_H2
zmp_ph114_A9
zmp_ph114_A11
zmp_ph114_B2
zmp_ph114_B4
zmp_ph114_D8
zmp_ph114_E2

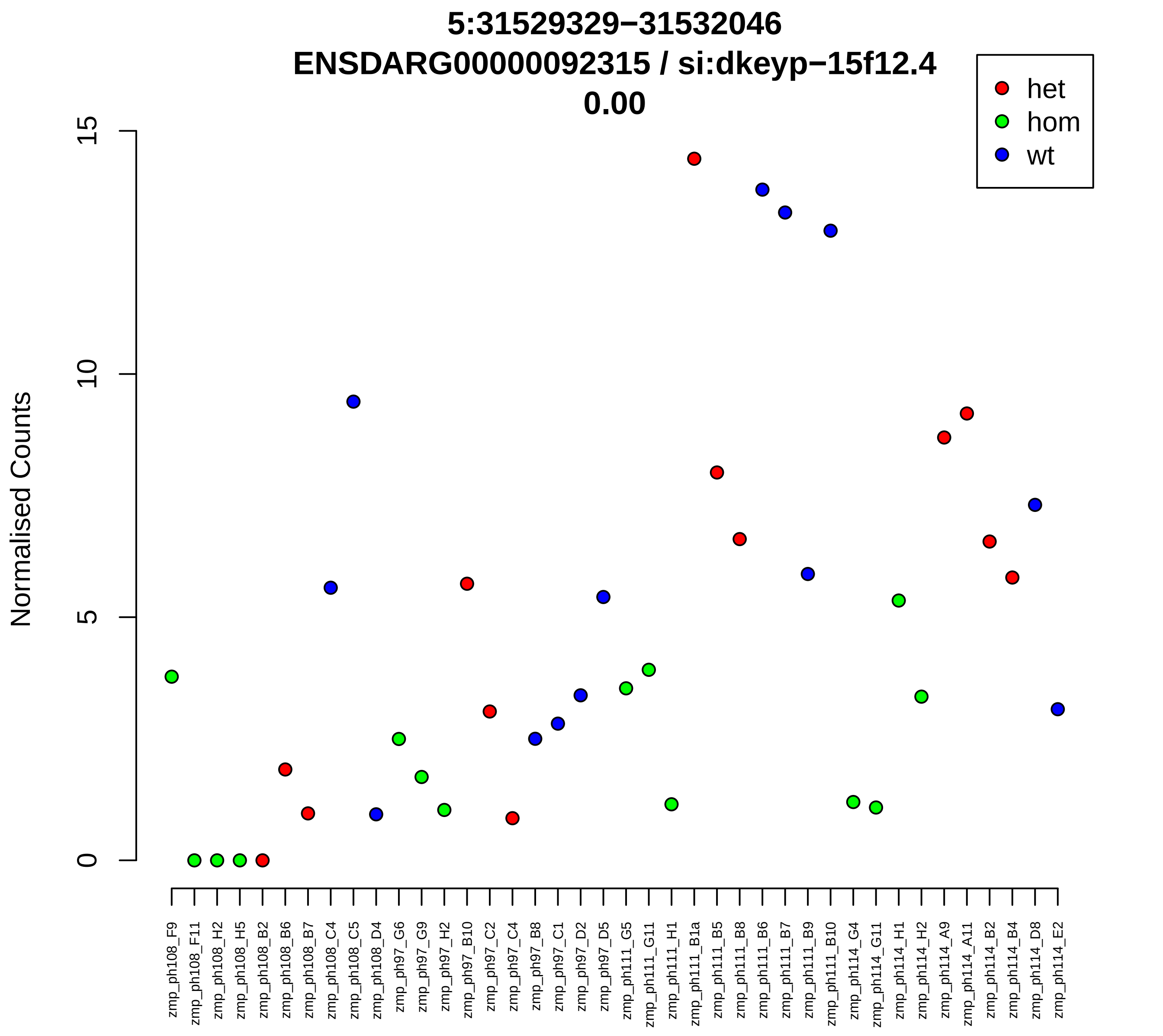
5:31529329−31532046
ENSDARG00000092315 / si:dkeyp−15f12.4
0.00
Normalised Counts
0
5
10
15
het
hom
wt
zmp_ph108_F9
zmp_ph108_F11
zmp_ph108_H2
zmp_ph108_H5
zmp_ph108_B2
zmp_ph108_B6
zmp_ph108_B7
zmp_ph108_C4
zmp_ph108_C5
zmp_ph108_D4
zmp_ph97_G6
zmp_ph97_G9
zmp_ph97_H2
zmp_ph97_B10
zmp_ph97_C2
zmp_ph97_C4
zmp_ph97_B8
zmp_ph97_C1
zmp_ph97_D2
zmp_ph97_D5
zmp_ph111_G5
zmp_ph111_G11
zmp_ph111_H1
zmp_ph111_B1a
zmp_ph111_B5
zmp_ph111_B8
zmp_ph111_B6
zmp_ph111_B7
zmp_ph111_B9
zmp_ph111_B10
zmp_ph114_G4
zmp_ph114_G11
zmp_ph114_H1
zmp_ph114_H2
zmp_ph114_A9
zmp_ph114_A11
zmp_ph114_B2
zmp_ph114_B4
zmp_ph114_D8
zmp_ph114_E2

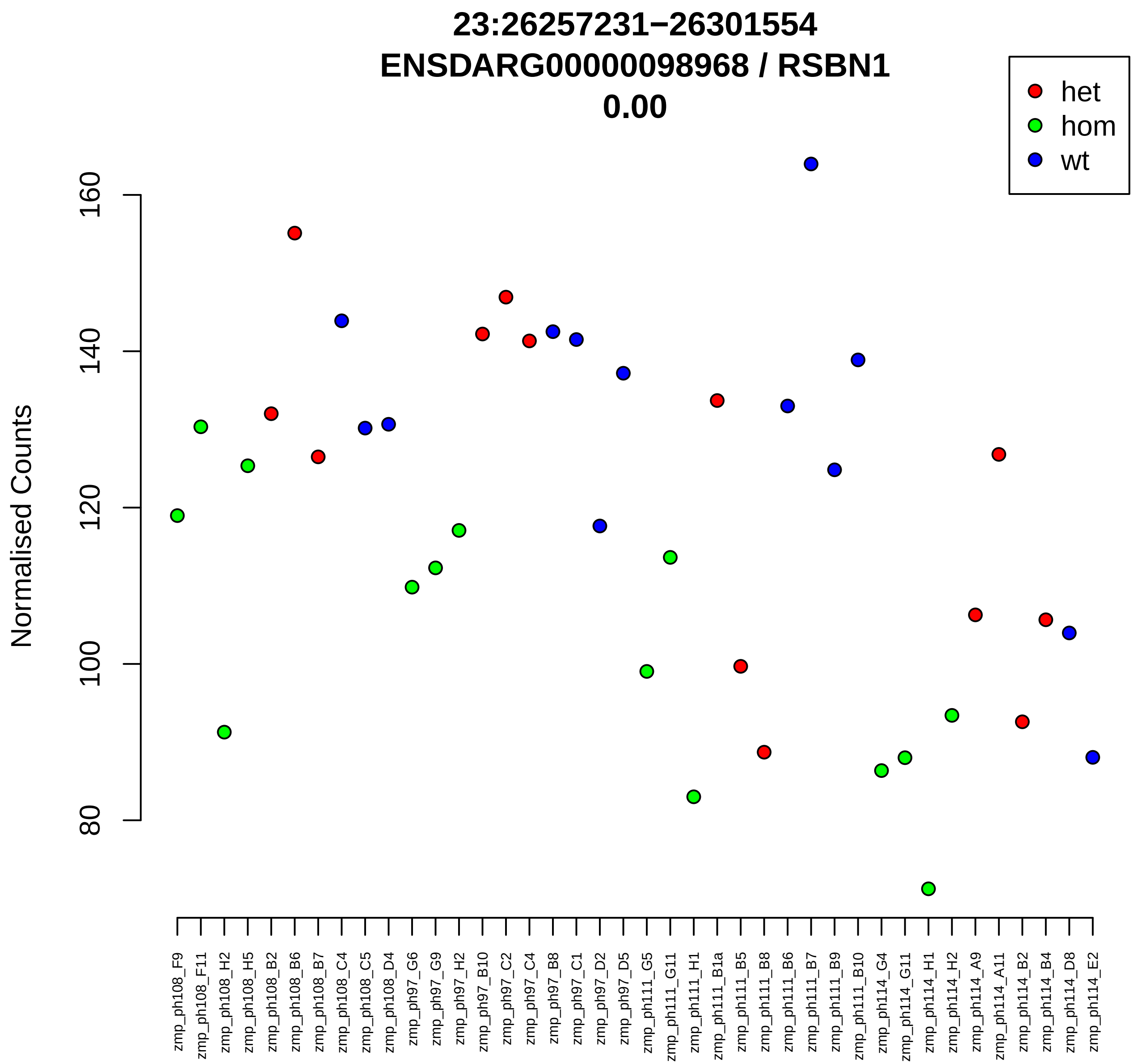

23:26257231−26301554
ENSDARG00000098968 / RSBN1
0.00
het
hom
wt
Normalised Counts
80
100
120
140
160
zmp_ph108_F9
zmp_ph108_F11
zmp_ph108_H2
zmp_ph108_H5
zmp_ph108_B2
zmp_ph108_B6
zmp_ph108_B7
zmp_ph108_C4
zmp_ph108_C5
zmp_ph108_D4
zmp_ph97_G6
zmp_ph97_G9
zmp_ph97_H2
zmp_ph97_B10
zmp_ph97_C2
zmp_ph97_C4
zmp_ph97_B8
zmp_ph97_C1
zmp_ph97_D2
zmp_ph97_D5
zmp_ph111_G5
zmp_ph111_G11
zmp_ph111_H1
zmp_ph111_B1a
zmp_ph111_B5
zmp_ph111_B8
zmp_ph111_B6
zmp_ph111_B7
zmp_ph111_B9
zmp_ph111_B10
zmp_ph114_G4
zmp_ph114_G11
zmp_ph114_H1
zmp_ph114_H2
zmp_ph114_A9
zmp_ph114_A11
zmp_ph114_B2
zmp_ph114_B4
zmp_ph114_D8
zmp_ph114_E2

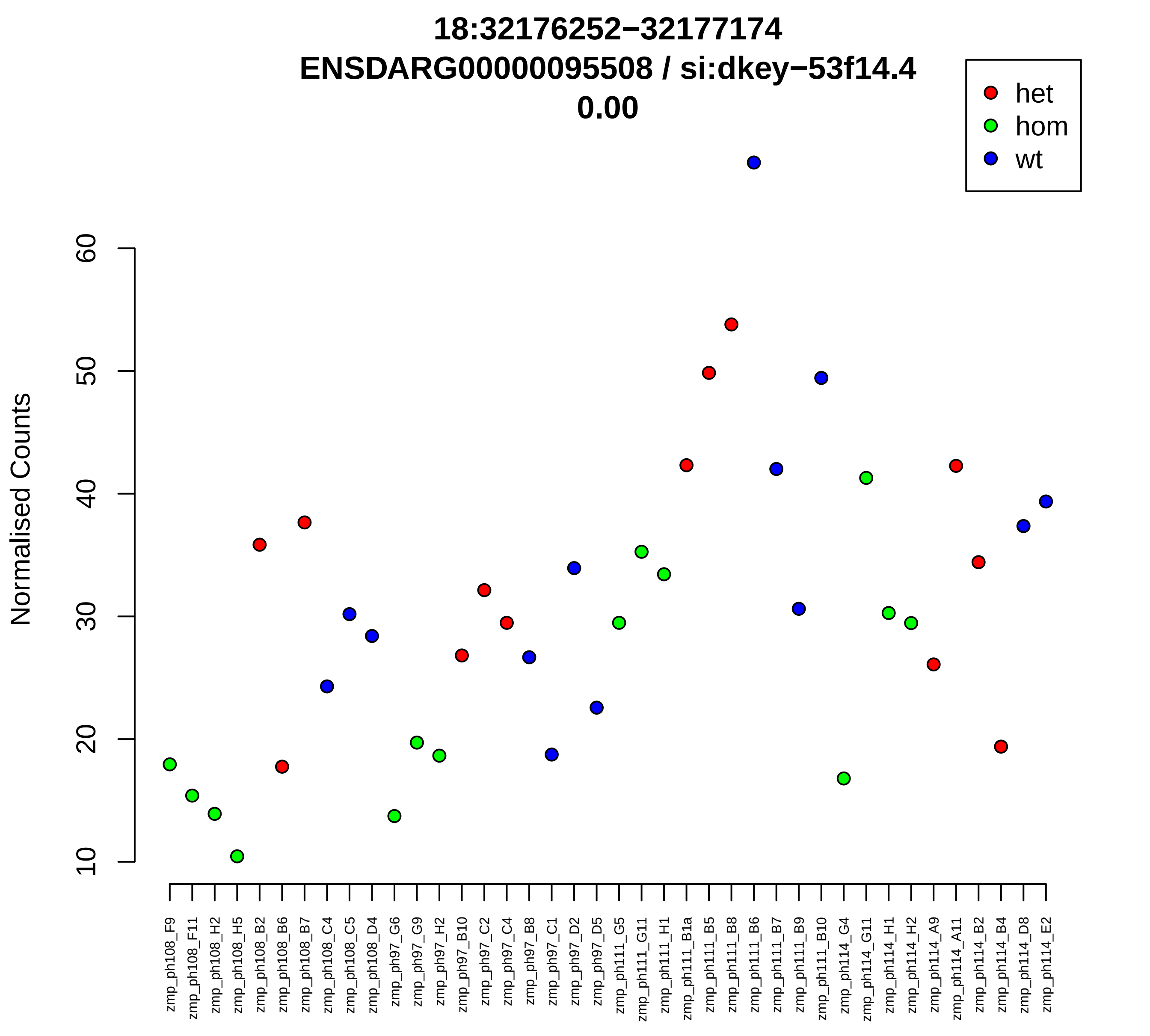
18:32176252−32177174
ENSDARG00000095508 / si:dkey−53f14.4
0.00
het
hom
wt
Normalised Counts
10
20
30
40
50
60
zmp_ph108_F9
zmp_ph108_F11
zmp_ph108_H2
zmp_ph108_H5
zmp_ph108_B2
zmp_ph108_B6
zmp_ph108_B7
zmp_ph108_C4
zmp_ph108_C5
zmp_ph108_D4
zmp_ph97_G6
zmp_ph97_G9
zmp_ph97_H2
zmp_ph97_B10
zmp_ph97_C2
zmp_ph97_C4
zmp_ph97_B8
zmp_ph97_C1
zmp_ph97_D2
zmp_ph97_D5
zmp_ph111_G5
zmp_ph111_G11
zmp_ph111_H1
zmp_ph111_B1a
zmp_ph111_B5
zmp_ph111_B8
zmp_ph111_B6
zmp_ph111_B7
zmp_ph111_B9
zmp_ph111_B10
zmp_ph114_G4
zmp_ph114_G11
zmp_ph114_H1
zmp_ph114_H2
zmp_ph114_A9
zmp_ph114_A11
zmp_ph114_B2
zmp_ph114_B4
zmp_ph114_D8
zmp_ph114_E2

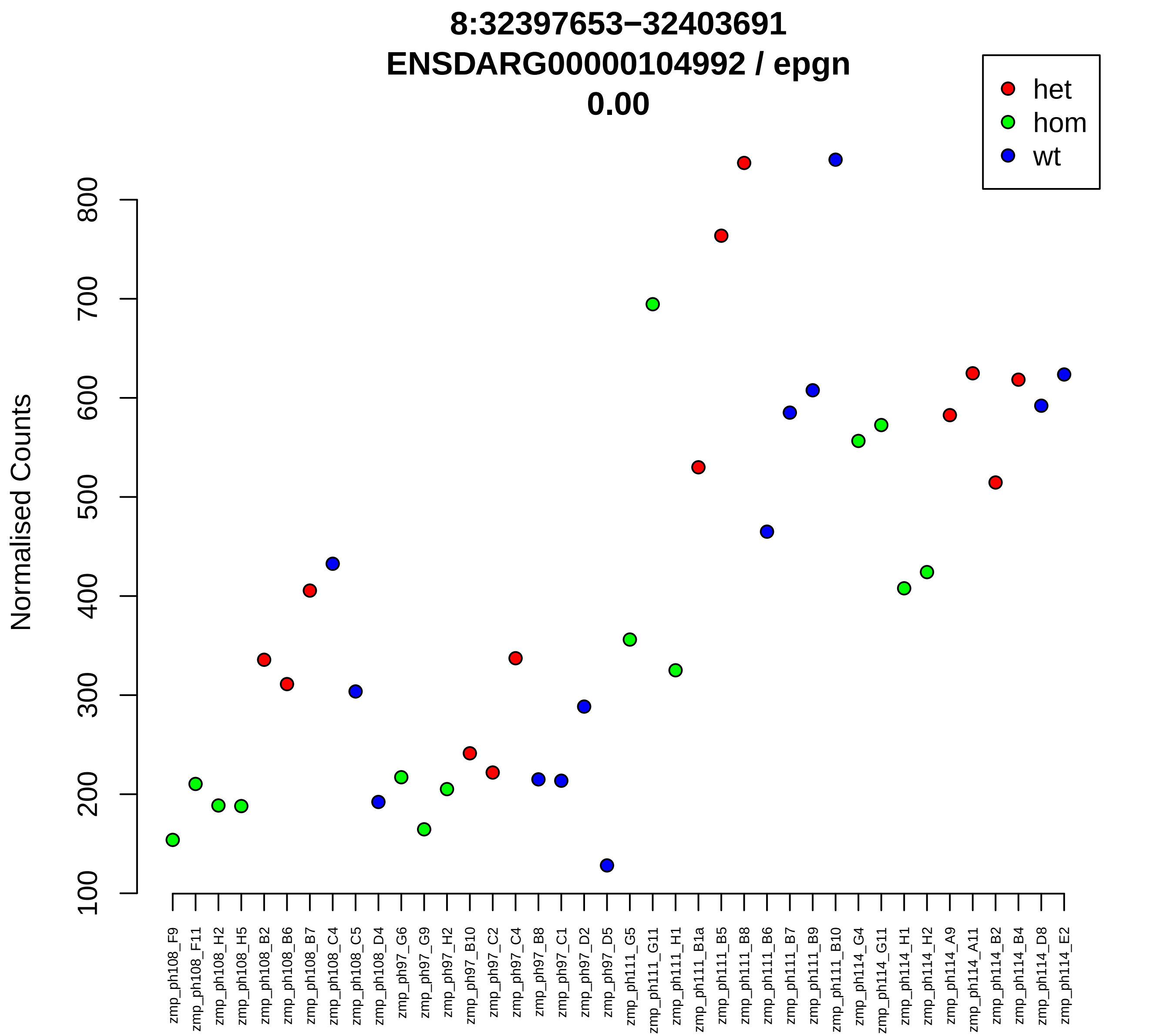
8:32397653−32403691
ENSDARG00000104992 / epgn
0.00
het
hom
wt
Normalised Counts
100
200
300
400
500
600
700
800
zmp_ph108_F9
zmp_ph108_F11
zmp_ph108_H2
zmp_ph108_H5
zmp_ph108_B2
zmp_ph108_B6
zmp_ph108_B7
zmp_ph108_C4
zmp_ph108_C5
zmp_ph108_D4
zmp_ph97_G6
zmp_ph97_G9
zmp_ph97_H2
zmp_ph97_B10
zmp_ph97_C2
zmp_ph97_C4
zmp_ph97_B8
zmp_ph97_C1
zmp_ph97_D2
zmp_ph97_D5
zmp_ph111_G5
zmp_ph111_G11
zmp_ph111_H1
zmp_ph111_B1a
zmp_ph111_B5
zmp_ph111_B8
zmp_ph111_B6
zmp_ph111_B7
zmp_ph111_B9
zmp_ph111_B10
zmp_ph114_G4
zmp_ph114_G11
zmp_ph114_H1
zmp_ph114_H2
zmp_ph114_A9
zmp_ph114_A11
zmp_ph114_B2
zmp_ph114_B4
zmp_ph114_D8
zmp_ph114_E2

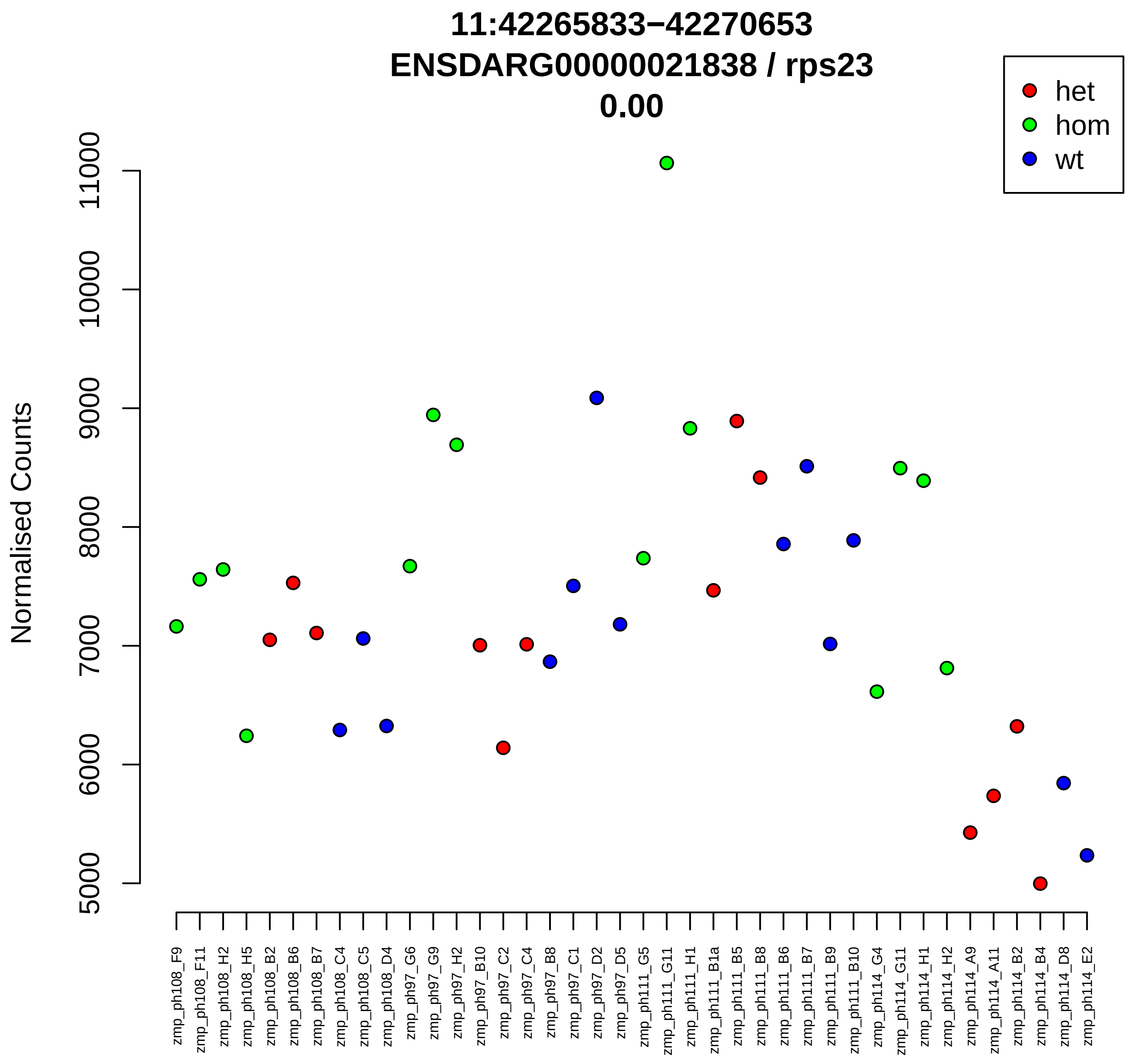

11:42265833−42270653
ENSDARG00000021838 / rps23
0.00
het
hom
wt
Normalised Counts
5000
6000
7000
8000
9000
10000
11000
zmp_ph108_F9
zmp_ph108_F11
zmp_ph108_H2
zmp_ph108_H5
zmp_ph108_B2
zmp_ph108_B6
zmp_ph108_B7
zmp_ph108_C4
zmp_ph108_C5
zmp_ph108_D4
zmp_ph97_G6
zmp_ph97_G9
zmp_ph97_H2
zmp_ph97_B10
zmp_ph97_C2
zmp_ph97_C4
zmp_ph97_B8
zmp_ph97_C1
zmp_ph97_D2
zmp_ph97_D5
zmp_ph111_G5
zmp_ph111_G11
zmp_ph111_H1
zmp_ph111_B1a
zmp_ph111_B5
zmp_ph111_B8
zmp_ph111_B6
zmp_ph111_B7
zmp_ph111_B9
zmp_ph111_B10
zmp_ph114_G4
zmp_ph114_G11
zmp_ph114_H1
zmp_ph114_H2
zmp_ph114_A9
zmp_ph114_A11
zmp_ph114_B2
zmp_ph114_B4
zmp_ph114_D8
zmp_ph114_E2

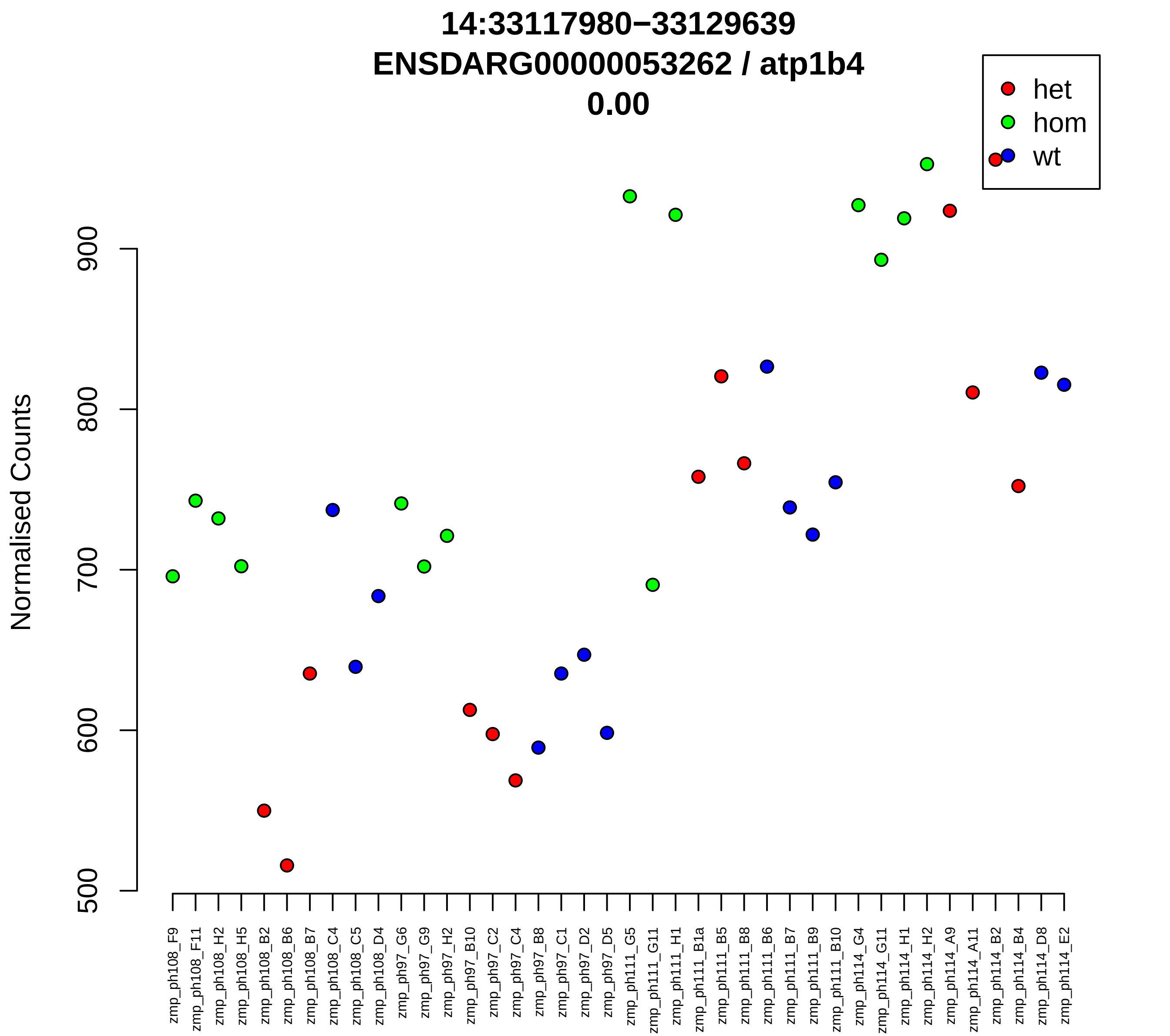

14:33117980−33129639
ENSDARG00000053262 / atp1b4
0.00
het
hom
wt
Normalised Counts
500
600
700
800
900
zmp_ph108_F9
zmp_ph108_F11
zmp_ph108_H2
zmp_ph108_H5
zmp_ph108_B2
zmp_ph108_B6
zmp_ph108_B7
zmp_ph108_C4
zmp_ph108_C5
zmp_ph108_D4
zmp_ph97_G6
zmp_ph97_G9
zmp_ph97_H2
zmp_ph97_B10
zmp_ph97_C2
zmp_ph97_C4
zmp_ph97_B8
zmp_ph97_C1
zmp_ph97_D2
zmp_ph97_D5
zmp_ph111_G5
zmp_ph111_G11
zmp_ph111_H1
zmp_ph111_B1a
zmp_ph111_B5
zmp_ph111_B8
zmp_ph111_B6
zmp_ph111_B7
zmp_ph111_B9
zmp_ph111_B10
zmp_ph114_G4
zmp_ph114_G11
zmp_ph114_H1
zmp_ph114_H2
zmp_ph114_A9
zmp_ph114_A11
zmp_ph114_B2
zmp_ph114_B4
zmp_ph114_D8
zmp_ph114_E2

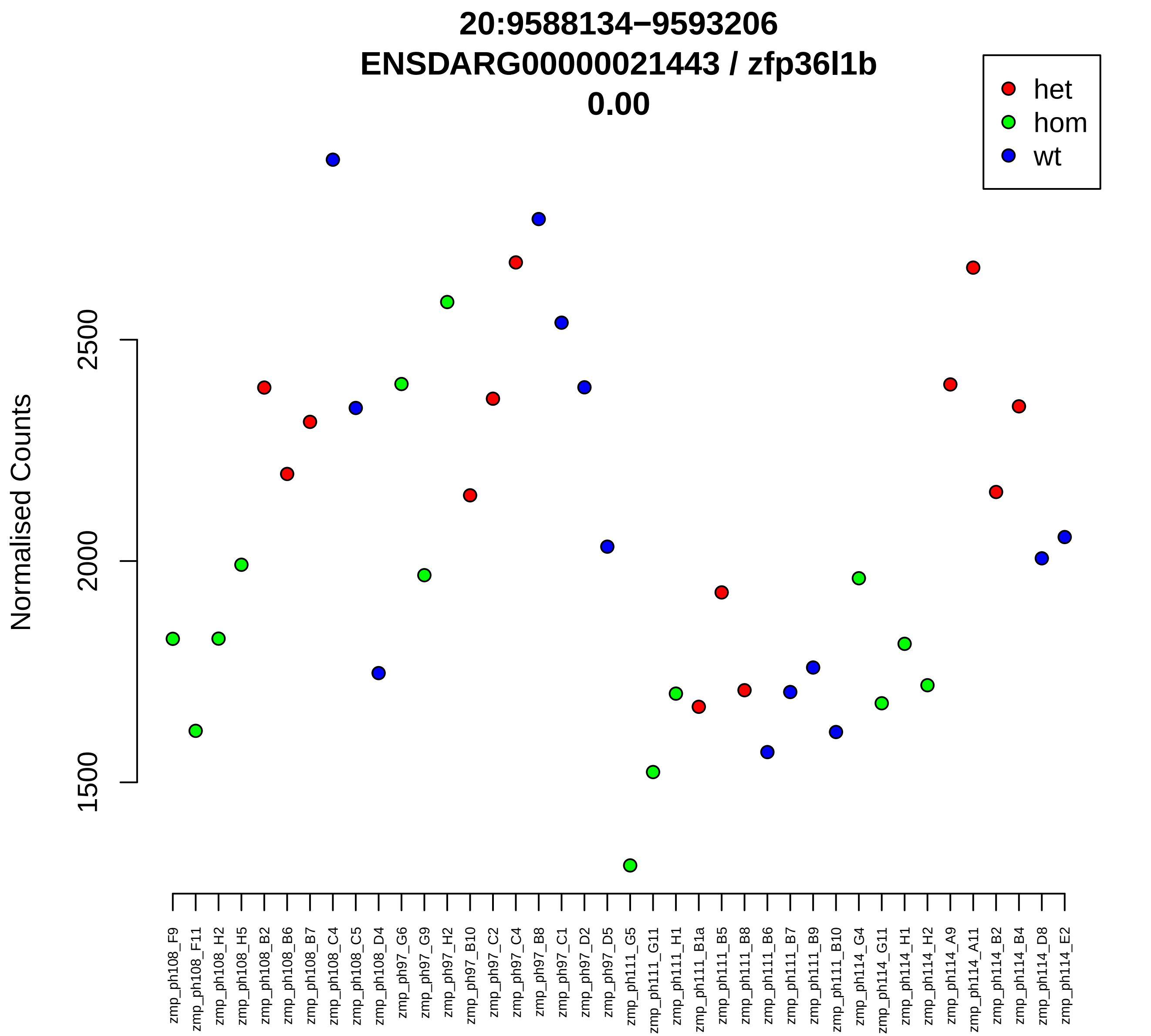
20:9588134−9593206
ENSDARG00000021443 / zfp36l1b
0.00
het
hom
wt
Normalised Counts
1500
2000
2500
zmp_ph108_F9
zmp_ph108_F11
zmp_ph108_H2
zmp_ph108_H5
zmp_ph108_B2
zmp_ph108_B6
zmp_ph108_B7
zmp_ph108_C4
zmp_ph108_C5
zmp_ph108_D4
zmp_ph97_G6
zmp_ph97_G9
zmp_ph97_H2
zmp_ph97_B10
zmp_ph97_C2
zmp_ph97_C4
zmp_ph97_B8
zmp_ph97_C1
zmp_ph97_D2
zmp_ph97_D5
zmp_ph111_G5
zmp_ph111_G11
zmp_ph111_H1
zmp_ph111_B1a
zmp_ph111_B5
zmp_ph111_B8
zmp_ph111_B6
zmp_ph111_B7
zmp_ph111_B9
zmp_ph111_B10
zmp_ph114_G4
zmp_ph114_G11
zmp_ph114_H1
zmp_ph114_H2
zmp_ph114_A9
zmp_ph114_A11
zmp_ph114_B2
zmp_ph114_B4
zmp_ph114_D8
zmp_ph114_E2

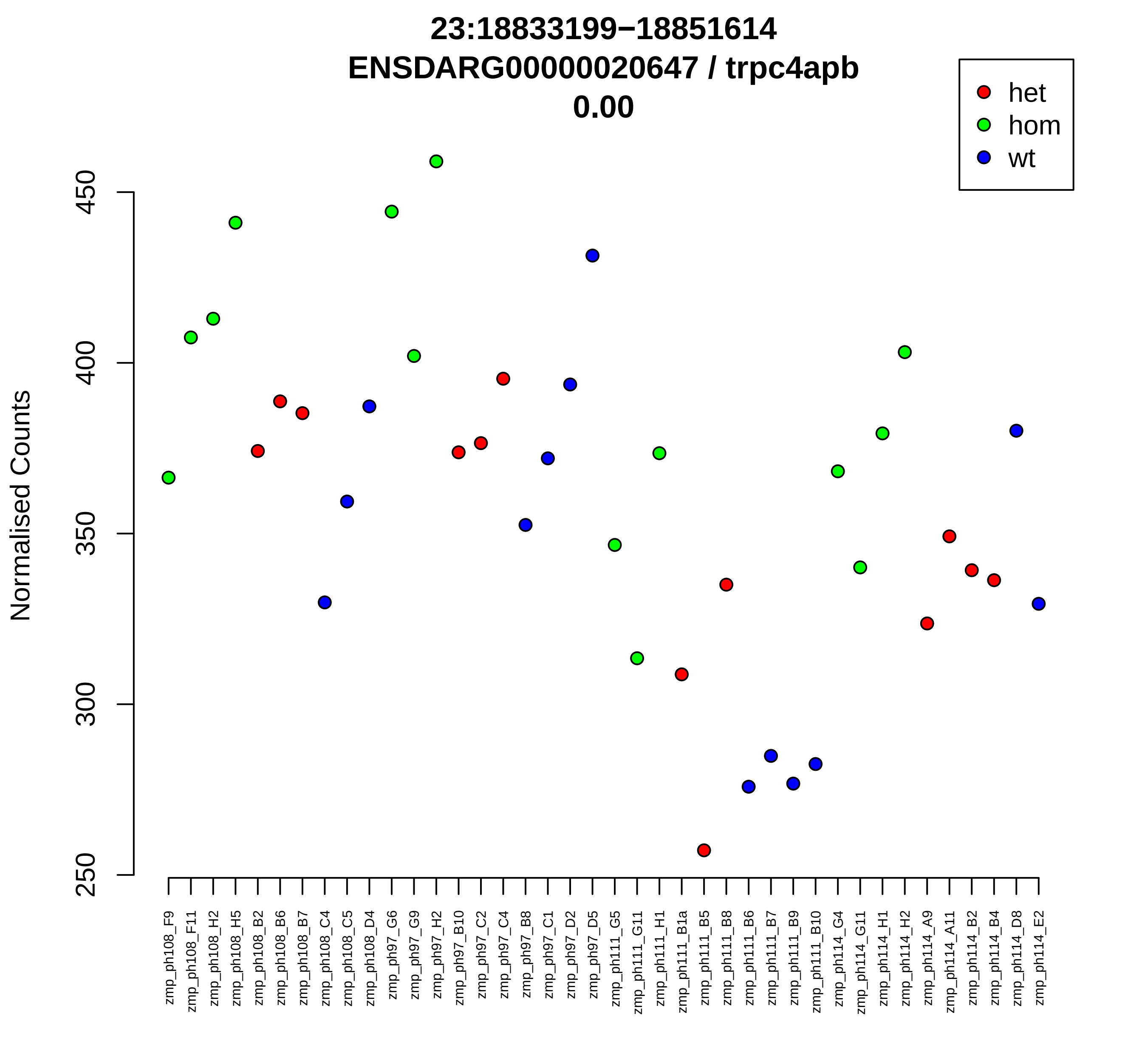
23:18833199−18851614
ENSDARG00000020647 / trpc4apb
0.00
het
hom
wt
Normalised Counts
250
300
350
400
450
zmp_ph108_F9
zmp_ph108_F11
zmp_ph108_H2
zmp_ph108_H5
zmp_ph108_B2
zmp_ph108_B6
zmp_ph108_B7
zmp_ph108_C4
zmp_ph108_C5
zmp_ph108_D4
zmp_ph97_G6
zmp_ph97_G9
zmp_ph97_H2
zmp_ph97_B10
zmp_ph97_C2
zmp_ph97_C4
zmp_ph97_B8
zmp_ph97_C1
zmp_ph97_D2
zmp_ph97_D5
zmp_ph111_G5
zmp_ph111_G11
zmp_ph111_H1
zmp_ph111_B1a
zmp_ph111_B5
zmp_ph111_B8
zmp_ph111_B6
zmp_ph111_B7
zmp_ph111_B9
zmp_ph111_B10
zmp_ph114_G4
zmp_ph114_G11
zmp_ph114_H1
zmp_ph114_H2
zmp_ph114_A9
zmp_ph114_A11
zmp_ph114_B2
zmp_ph114_B4
zmp_ph114_D8
zmp_ph114_E2

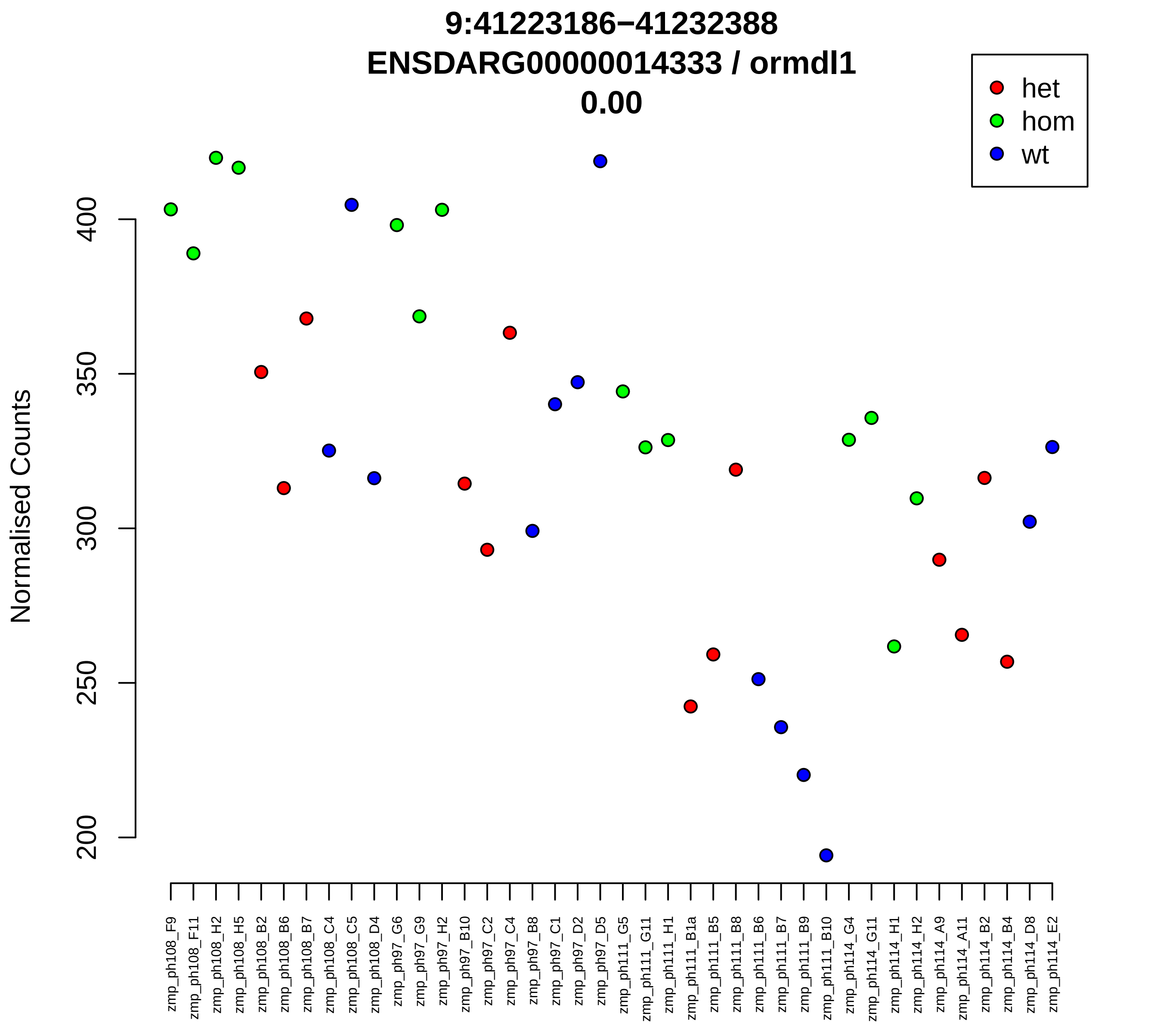

9:41223186−41232388
ENSDARG00000014333 / ormdl1
0.00
het
hom
wt
Normalised Counts
200
250
300
350
400
zmp_ph108_F9
zmp_ph108_F11
zmp_ph108_H2
zmp_ph108_H5
zmp_ph108_B2
zmp_ph108_B6
zmp_ph108_B7
zmp_ph108_C4
zmp_ph108_C5
zmp_ph108_D4
zmp_ph97_G6
zmp_ph97_G9
zmp_ph97_H2
zmp_ph97_B10
zmp_ph97_C2
zmp_ph97_C4
zmp_ph97_B8
zmp_ph97_C1
zmp_ph97_D2
zmp_ph97_D5
zmp_ph111_G5
zmp_ph111_G11
zmp_ph111_H1
zmp_ph111_B1a
zmp_ph111_B5
zmp_ph111_B8
zmp_ph111_B6
zmp_ph111_B7
zmp_ph111_B9
zmp_ph111_B10
zmp_ph114_G4
zmp_ph114_G11
zmp_ph114_H1
zmp_ph114_H2
zmp_ph114_A9
zmp_ph114_A11
zmp_ph114_B2
zmp_ph114_B4
zmp_ph114_D8
zmp_ph114_E2

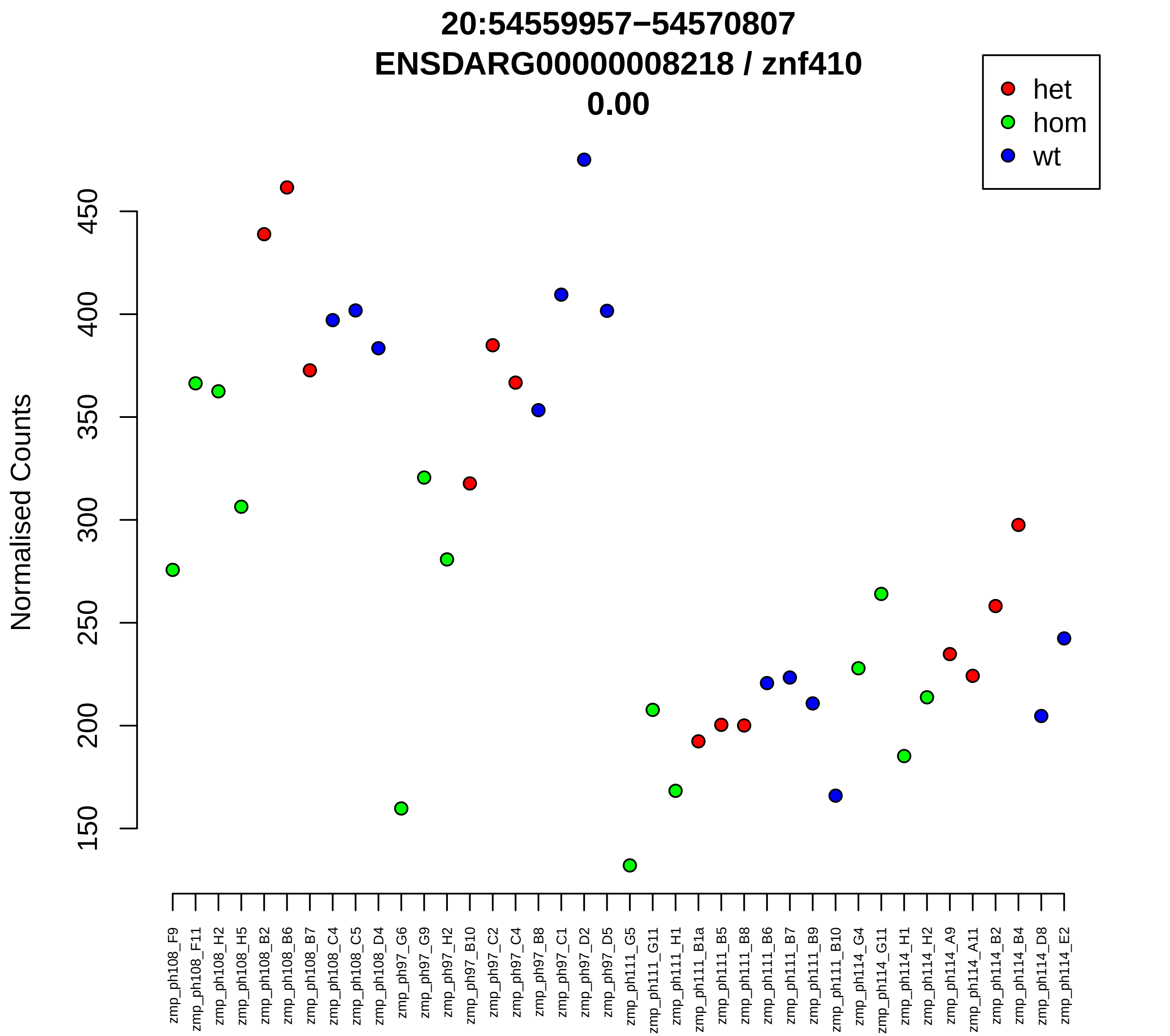

20:54559957−54570807
ENSDARG00000008218 / znf410
0.00
het
hom
wt
Normalised Counts
150
200
250
300
350
400
450
zmp_ph108_F9
zmp_ph108_F11
zmp_ph108_H2
zmp_ph108_H5
zmp_ph108_B2
zmp_ph108_B6
zmp_ph108_B7
zmp_ph108_C4
zmp_ph108_C5
zmp_ph108_D4
zmp_ph97_G6
zmp_ph97_G9
zmp_ph97_H2
zmp_ph97_B10
zmp_ph97_C2
zmp_ph97_C4
zmp_ph97_B8
zmp_ph97_C1
zmp_ph97_D2
zmp_ph97_D5
zmp_ph111_G5
zmp_ph111_G11
zmp_ph111_H1
zmp_ph111_B1a
zmp_ph111_B5
zmp_ph111_B8
zmp_ph111_B6
zmp_ph111_B7
zmp_ph111_B9
zmp_ph111_B10
zmp_ph114_G4
zmp_ph114_G11
zmp_ph114_H1
zmp_ph114_H2
zmp_ph114_A9
zmp_ph114_A11
zmp_ph114_B2
zmp_ph114_B4
zmp_ph114_D8
zmp_ph114_E2

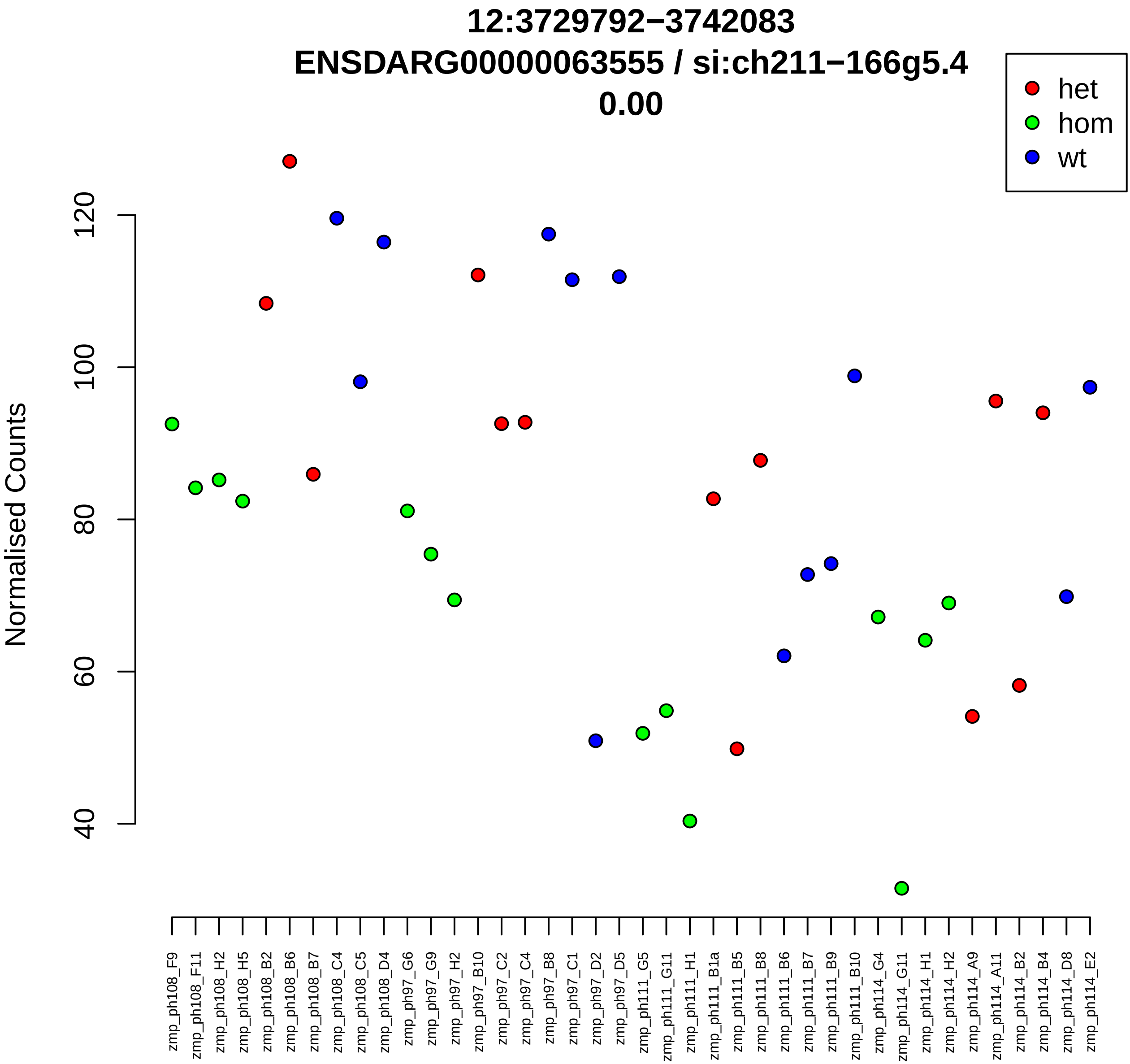
12:3729792−3742083
ENSDARG00000063555 / si:ch211−166g5.4
0.00
het
hom
wt
Normalised Counts
40
60
80
100
120
zmp_ph108_F9
zmp_ph108_F11
zmp_ph108_H2
zmp_ph108_H5
zmp_ph108_B2
zmp_ph108_B6
zmp_ph108_B7
zmp_ph108_C4
zmp_ph108_C5
zmp_ph108_D4
zmp_ph97_G6
zmp_ph97_G9
zmp_ph97_H2
zmp_ph97_B10
zmp_ph97_C2
zmp_ph97_C4
zmp_ph97_B8
zmp_ph97_C1
zmp_ph97_D2
zmp_ph97_D5
zmp_ph111_G5
zmp_ph111_G11
zmp_ph111_H1
zmp_ph111_B1a
zmp_ph111_B5
zmp_ph111_B8
zmp_ph111_B6
zmp_ph111_B7
zmp_ph111_B9
zmp_ph111_B10
zmp_ph114_G4
zmp_ph114_G11
zmp_ph114_H1
zmp_ph114_H2
zmp_ph114_A9
zmp_ph114_A11
zmp_ph114_B2
zmp_ph114_B4
zmp_ph114_D8
zmp_ph114_E2

5:57021762−57045055
ENSDARG00000052411 / si:dkey−71l1.1
0.00

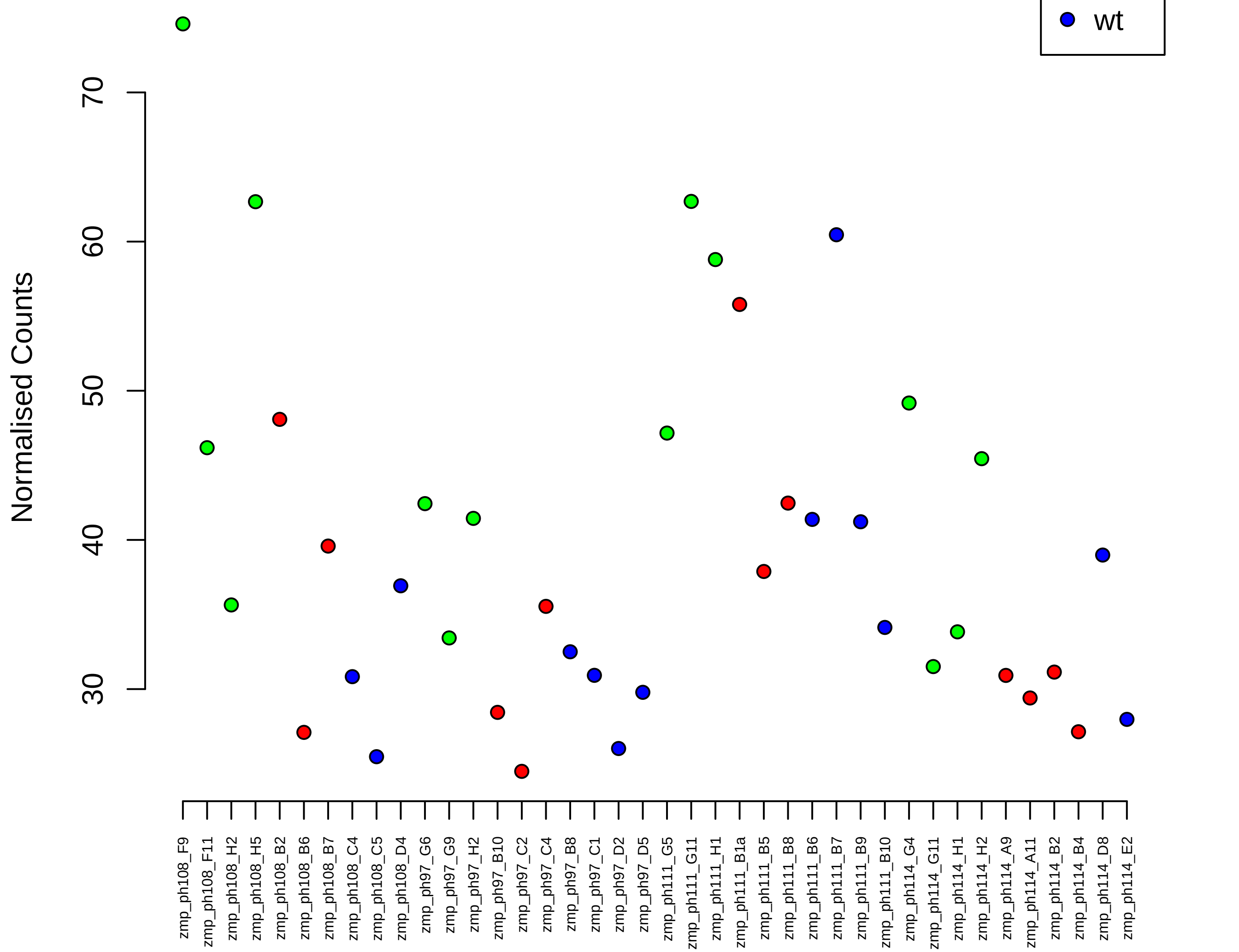

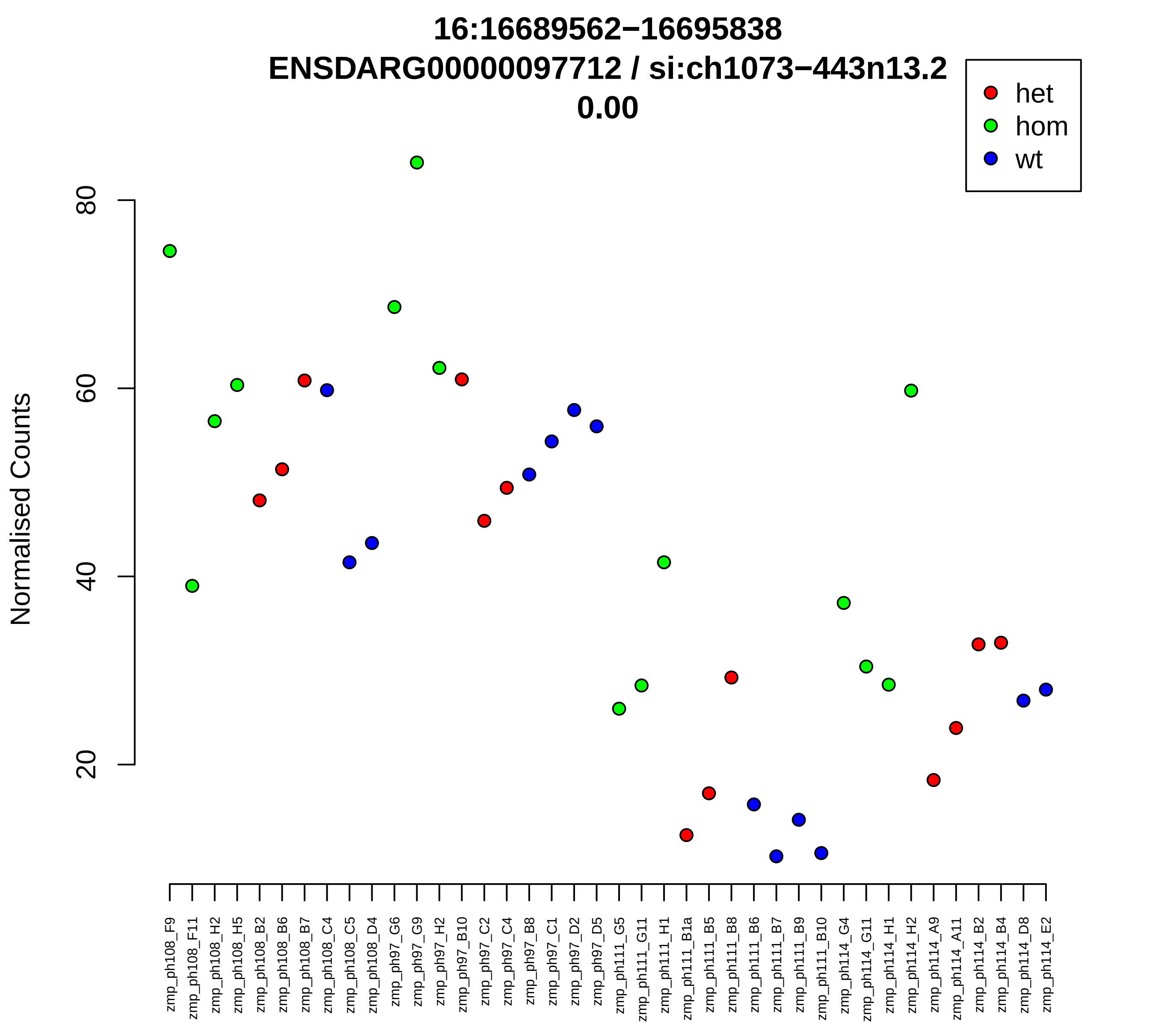

16:16689562−16695838
ENSDARG00000097712 / si:ch1073−443n13.2
0.00
het
hom
wt
Normalised Counts
20
40
60
80
zmp_ph108_F9
zmp_ph108_F11
zmp_ph108_H2
zmp_ph108_H5
zmp_ph108_B2
zmp_ph108_B6
zmp_ph108_B7
zmp_ph108_C4
zmp_ph108_C5
zmp_ph108_D4
zmp_ph97_G6
zmp_ph97_G9
zmp_ph97_H2
zmp_ph97_B10
zmp_ph97_C2
zmp_ph97_C4
zmp_ph97_B8
zmp_ph97_C1
zmp_ph97_D2
zmp_ph97_D5
zmp_ph111_G5
zmp_ph111_G11
zmp_ph111_H1
zmp_ph111_B1a
zmp_ph111_B5
zmp_ph111_B8
zmp_ph111_B6
zmp_ph111_B7
zmp_ph111_B9
zmp_ph111_B10
zmp_ph114_G4
zmp_ph114_G11
zmp_ph114_H1
zmp_ph114_H2
zmp_ph114_A9
zmp_ph114_A11
zmp_ph114_B2
zmp_ph114_B4
zmp_ph114_D8
zmp_ph114_E2

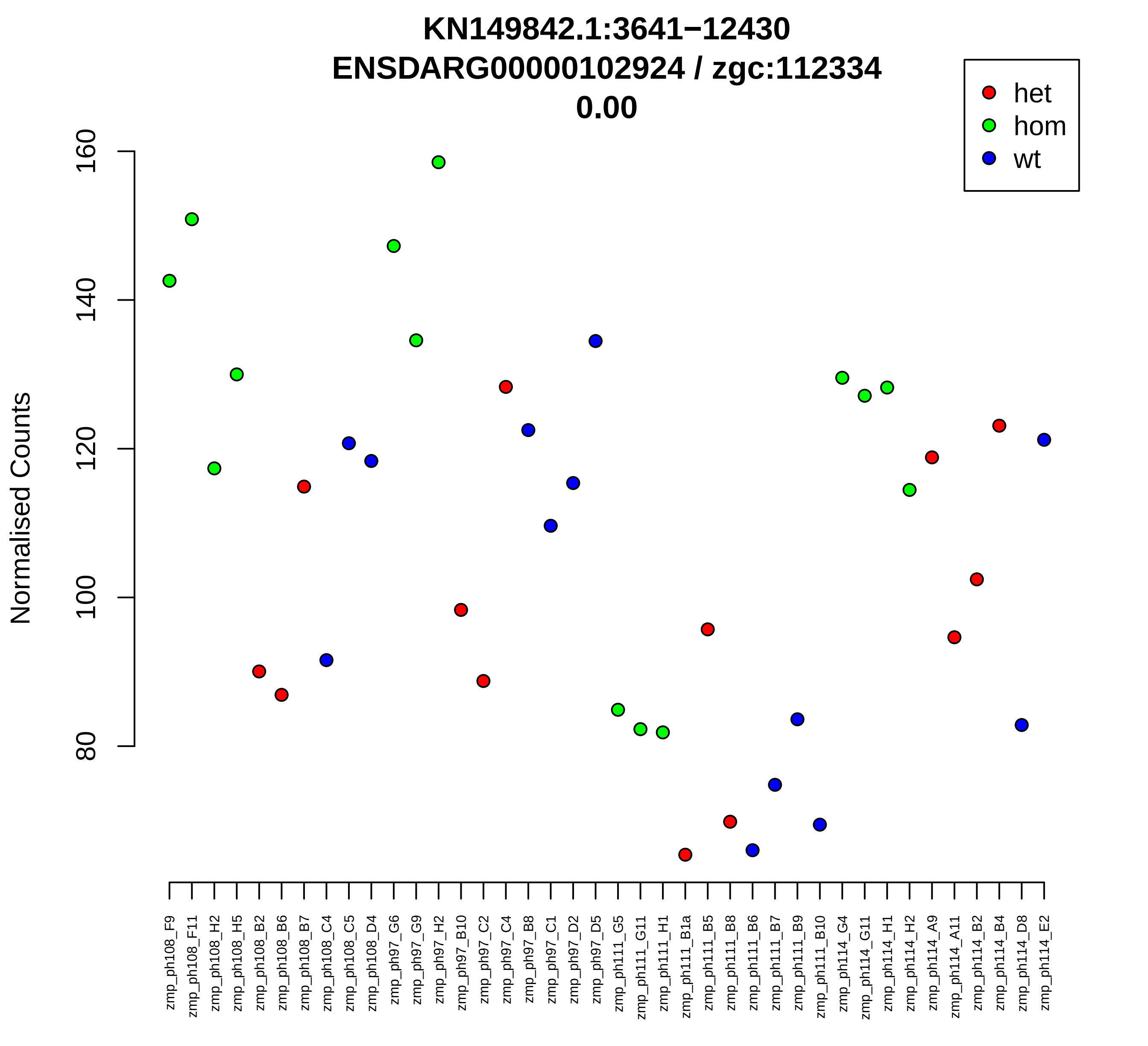
KN149842.1:3641−12430
ENSDARG00000102924 / zgc:112334
0.00
het
hom
wt
Normalised Counts
80
100
120
140
160
zmp_ph108_F9
zmp_ph108_F11
zmp_ph108_H2
zmp_ph108_H5
zmp_ph108_B2
zmp_ph108_B6
zmp_ph108_B7
zmp_ph108_C4
zmp_ph108_C5
zmp_ph108_D4
zmp_ph97_G6
zmp_ph97_G9
zmp_ph97_H2
zmp_ph97_B10
zmp_ph97_C2
zmp_ph97_C4
zmp_ph97_B8
zmp_ph97_C1
zmp_ph97_D2
zmp_ph97_D5
zmp_ph111_G5
zmp_ph111_G11
zmp_ph111_H1
zmp_ph111_B1a
zmp_ph111_B5
zmp_ph111_B8
zmp_ph111_B6
zmp_ph111_B7
zmp_ph111_B9
zmp_ph111_B10
zmp_ph114_G4
zmp_ph114_G11
zmp_ph114_H1
zmp_ph114_H2
zmp_ph114_A9
zmp_ph114_A11
zmp_ph114_B2
zmp_ph114_B4
zmp_ph114_D8
zmp_ph114_E2

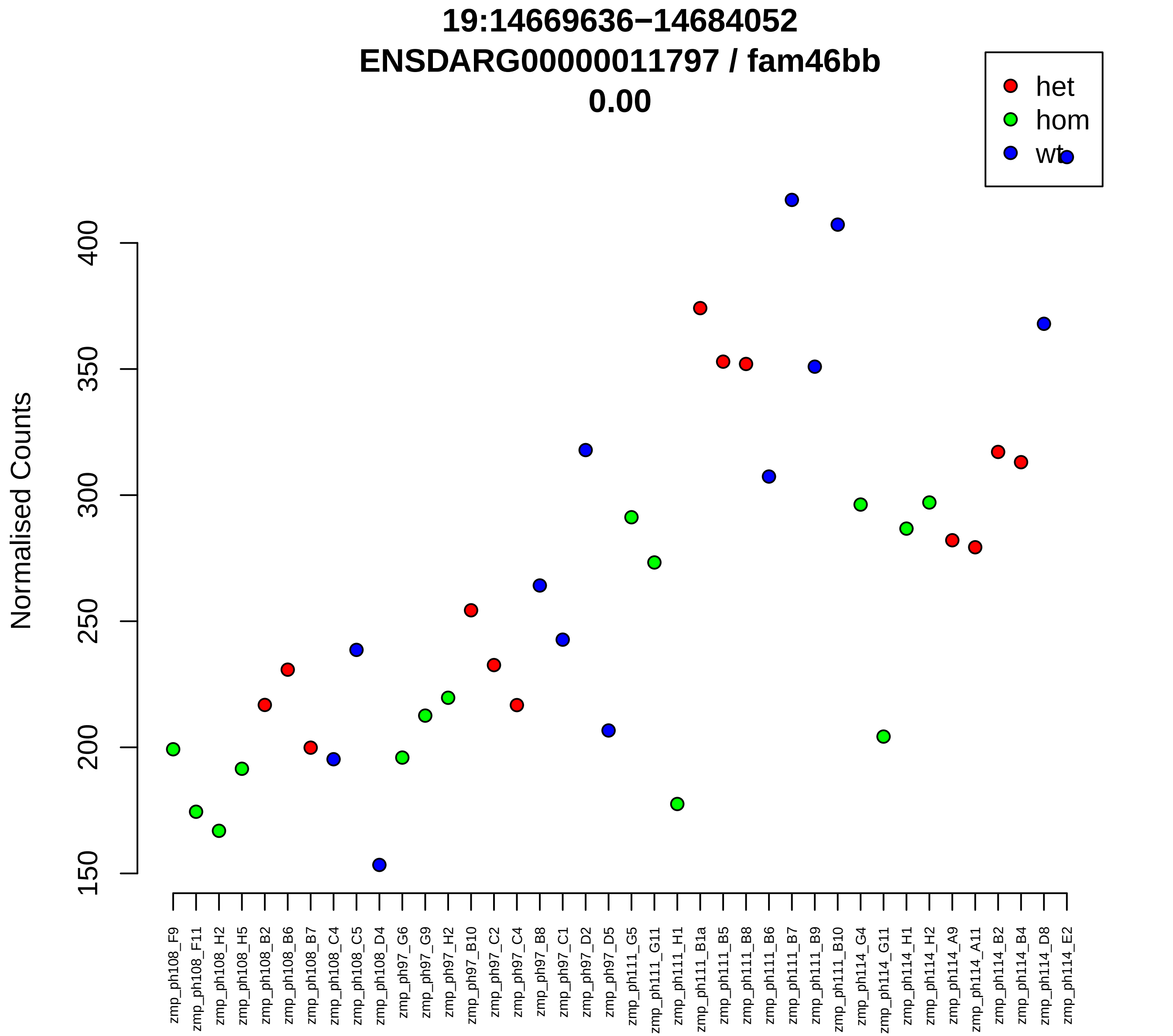
19:14669636−14684052
ENSDARG00000011797 / fam46bb
0.00
het
hom
wt
Normalised Counts
150
200
250
300
350
400
zmp_ph108_F9
zmp_ph108_F11
zmp_ph108_H2
zmp_ph108_H5
zmp_ph108_B2
zmp_ph108_B6
zmp_ph108_B7
zmp_ph108_C4
zmp_ph108_C5
zmp_ph108_D4
zmp_ph97_G6
zmp_ph97_G9
zmp_ph97_H2
zmp_ph97_B10
zmp_ph97_C2
zmp_ph97_C4
zmp_ph97_B8
zmp_ph97_C1
zmp_ph97_D2
zmp_ph97_D5
zmp_ph111_G5
zmp_ph111_G11
zmp_ph111_H1
zmp_ph111_B1a
zmp_ph111_B5
zmp_ph111_B8
zmp_ph111_B6
zmp_ph111_B7
zmp_ph111_B9
zmp_ph111_B10
zmp_ph114_G4
zmp_ph114_G11
zmp_ph114_H1
zmp_ph114_H2
zmp_ph114_A9
zmp_ph114_A11
zmp_ph114_B2
zmp_ph114_B4
zmp_ph114_D8
zmp_ph114_E2

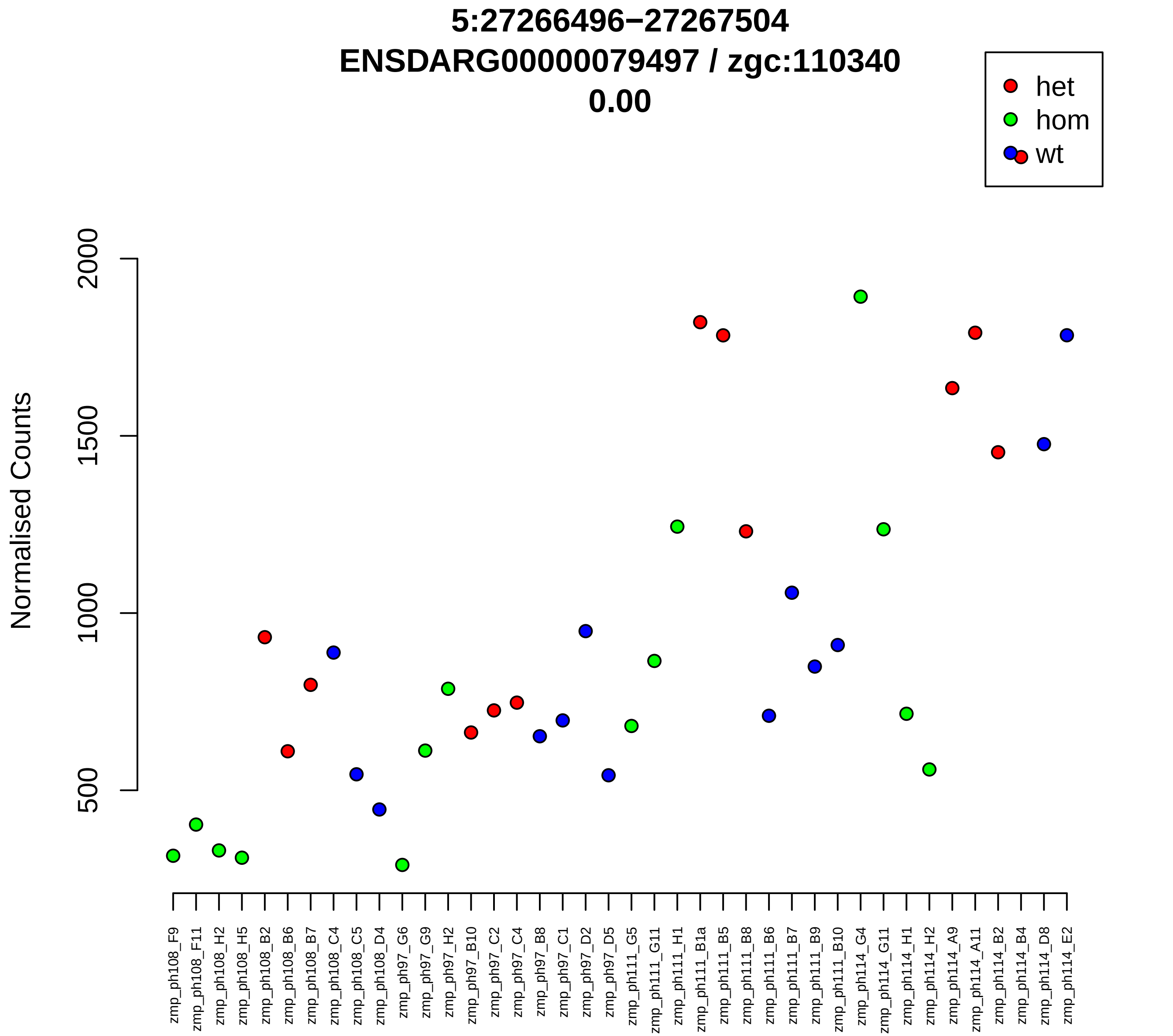
5:27266496−27267504
ENSDARG00000079497 / zgc:110340
0.00
het
hom
wt
Normalised Counts
500
1000
1500
2000
zmp_ph108_F9
zmp_ph108_F11
zmp_ph108_H2
zmp_ph108_H5
zmp_ph108_B2
zmp_ph108_B6
zmp_ph108_B7
zmp_ph108_C4
zmp_ph108_C5
zmp_ph108_D4
zmp_ph97_G6
zmp_ph97_G9
zmp_ph97_H2
zmp_ph97_B10
zmp_ph97_C2
zmp_ph97_C4
zmp_ph97_B8
zmp_ph97_C1
zmp_ph97_D2
zmp_ph97_D5
zmp_ph111_G5
zmp_ph111_G11
zmp_ph111_H1
zmp_ph111_B1a
zmp_ph111_B5
zmp_ph111_B8
zmp_ph111_B6
zmp_ph111_B7
zmp_ph111_B9
zmp_ph111_B10
zmp_ph114_G4
zmp_ph114_G11
zmp_ph114_H1
zmp_ph114_H2
zmp_ph114_A9
zmp_ph114_A11
zmp_ph114_B2
zmp_ph114_B4
zmp_ph114_D8
zmp_ph114_E2

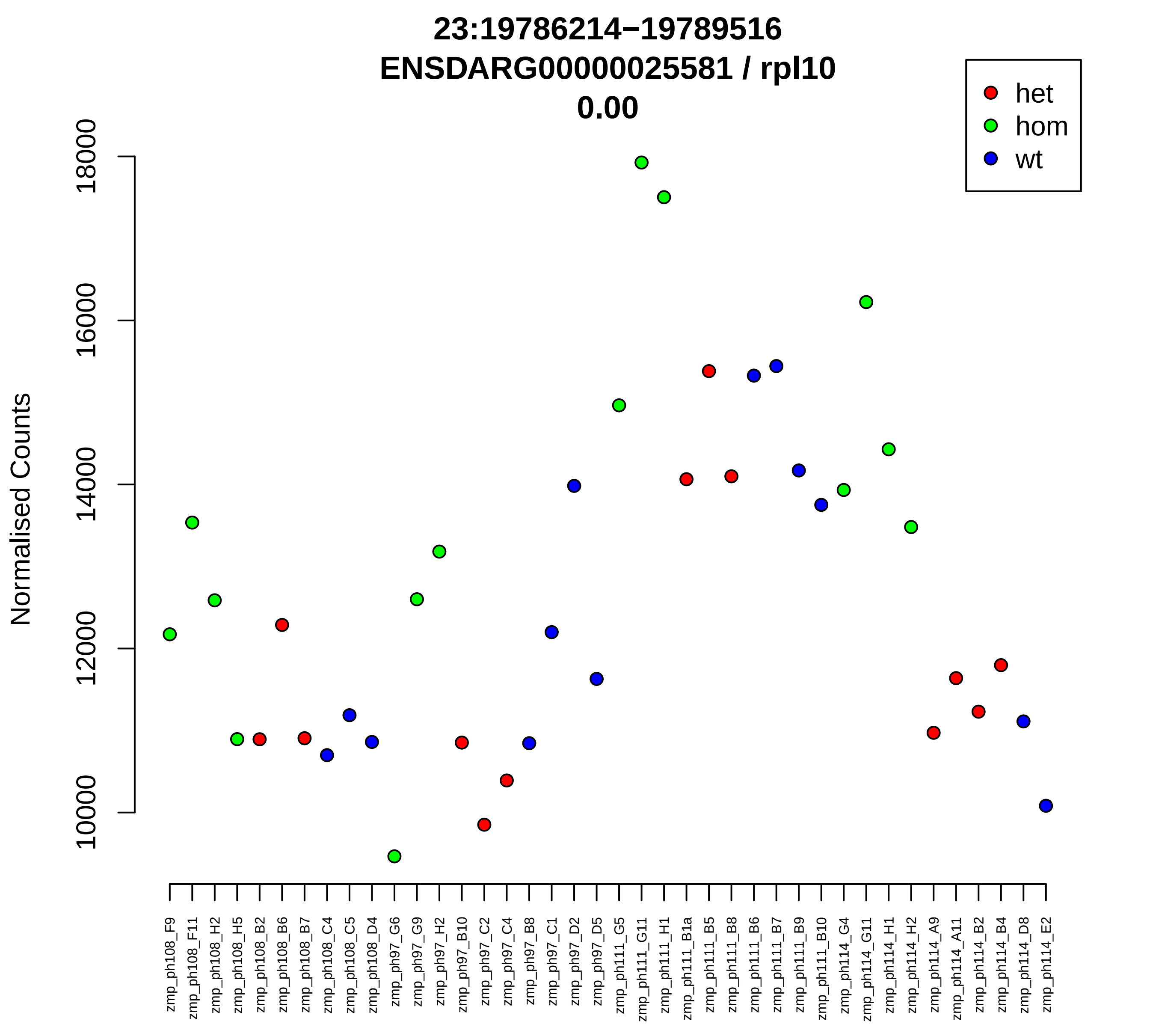

23:19786214−19789516
ENSDARG00000025581 / rpl10
0.00
het
hom
wt
Normalised Counts
10000
12000
14000
16000
18000
zmp_ph108_F9
zmp_ph108_F11
zmp_ph108_H2
zmp_ph108_H5
zmp_ph108_B2
zmp_ph108_B6
zmp_ph108_B7
zmp_ph108_C4
zmp_ph108_C5
zmp_ph108_D4
zmp_ph97_G6
zmp_ph97_G9
zmp_ph97_H2
zmp_ph97_B10
zmp_ph97_C2
zmp_ph97_C4
zmp_ph97_B8
zmp_ph97_C1
zmp_ph97_D2
zmp_ph97_D5
zmp_ph111_G5
zmp_ph111_G11
zmp_ph111_H1
zmp_ph111_B1a
zmp_ph111_B5
zmp_ph111_B8
zmp_ph111_B6
zmp_ph111_B7
zmp_ph111_B9
zmp_ph111_B10
zmp_ph114_G4
zmp_ph114_G11
zmp_ph114_H1
zmp_ph114_H2
zmp_ph114_A9
zmp_ph114_A11
zmp_ph114_B2
zmp_ph114_B4
zmp_ph114_D8
zmp_ph114_E2

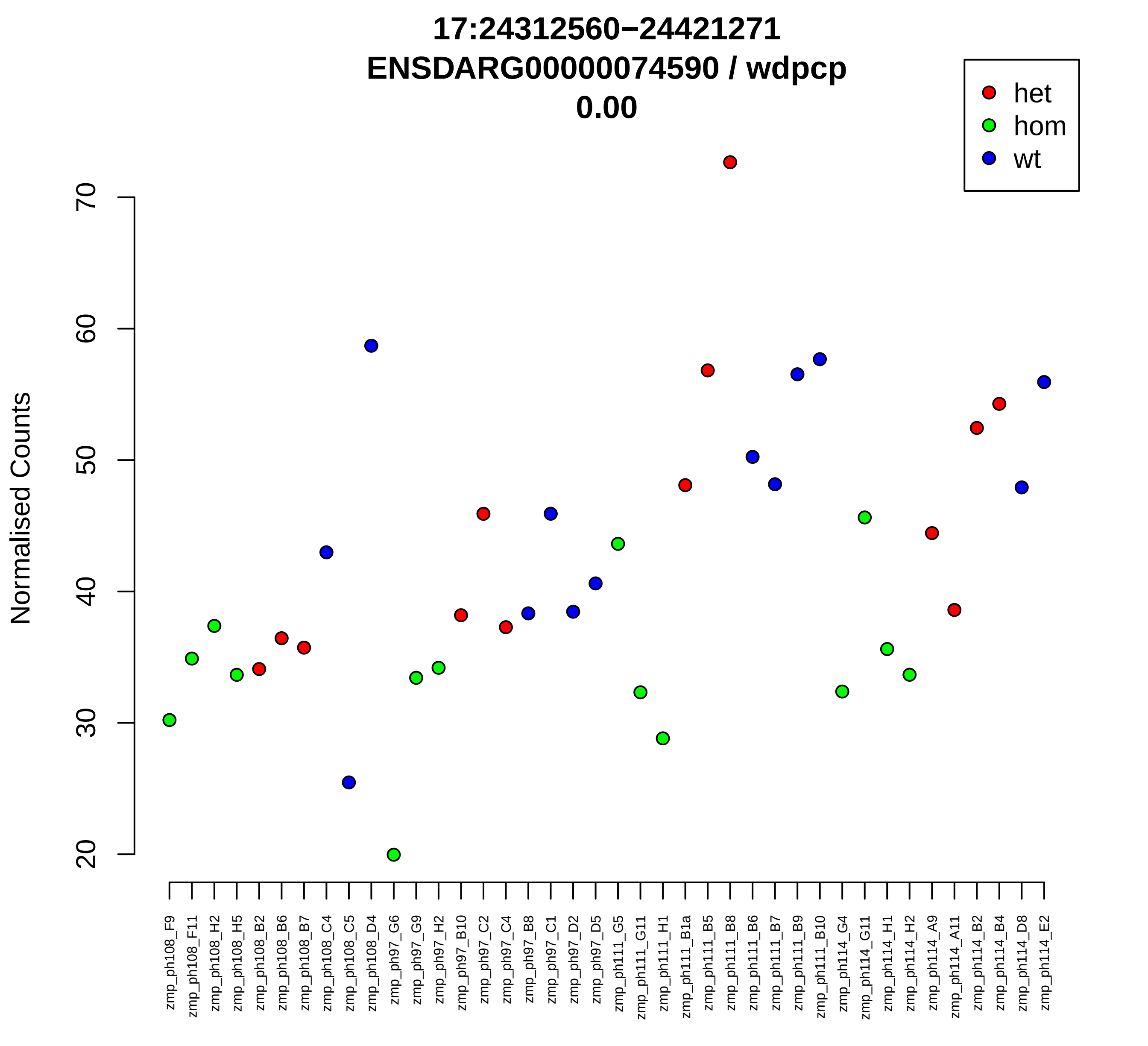

17:24312560−24421271
ENSDARG00000074590 / wdpcp
0.00
het
hom
wt
Normalised Counts
20
30
40
50
60
70
zmp_ph108_F9
zmp_ph108_F11
zmp_ph108_H2
zmp_ph108_H5
zmp_ph108_B2
zmp_ph108_B6
zmp_ph108_B7
zmp_ph108_C4
zmp_ph108_C5
zmp_ph108_D4
zmp_ph97_G6
zmp_ph97_G9
zmp_ph97_H2
zmp_ph97_B10
zmp_ph97_C2
zmp_ph97_C4
zmp_ph97_B8
zmp_ph97_C1
zmp_ph97_D2
zmp_ph97_D5
zmp_ph111_G5
zmp_ph111_G11
zmp_ph111_H1
zmp_ph111_B1a
zmp_ph111_B5
zmp_ph111_B8
zmp_ph111_B6
zmp_ph111_B7
zmp_ph111_B9
zmp_ph111_B10
zmp_ph114_G4
zmp_ph114_G11
zmp_ph114_H1
zmp_ph114_H2
zmp_ph114_A9
zmp_ph114_A11
zmp_ph114_B2
zmp_ph114_B4
zmp_ph114_D8
zmp_ph114_E2

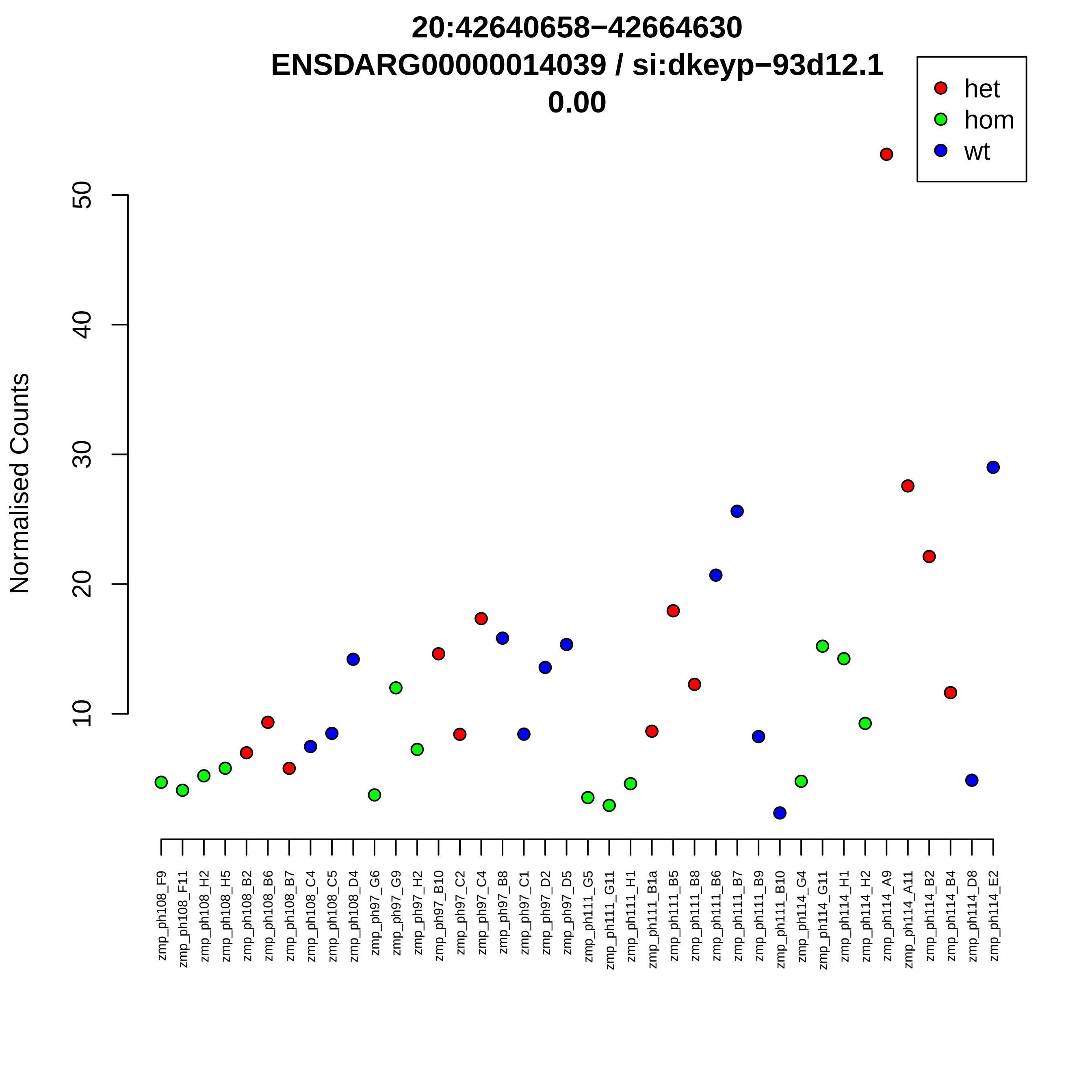

20:42640658−42664630
ENSDARG00000014039 / si:dkeyp−93d12.1
0.00
het
hom
wt
Normalised Counts
10
20
30
40
50
zmp_ph108_F9
zmp_ph108_F11
zmp_ph108_H2
zmp_ph108_H5
zmp_ph108_B2
zmp_ph108_B6
zmp_ph108_B7
zmp_ph108_C4
zmp_ph108_C5
zmp_ph108_D4
zmp_ph97_G6
zmp_ph97_G9
zmp_ph97_H2
zmp_ph97_B10
zmp_ph97_C2
zmp_ph97_C4
zmp_ph97_B8
zmp_ph97_C1
zmp_ph97_D2
zmp_ph97_D5
zmp_ph111_G5
zmp_ph111_G11
zmp_ph111_H1
zmp_ph111_B1a
zmp_ph111_B5
zmp_ph111_B8
zmp_ph111_B6
zmp_ph111_B7
zmp_ph111_B9
zmp_ph111_B10
zmp_ph114_G4
zmp_ph114_G11
zmp_ph114_H1
zmp_ph114_H2
zmp_ph114_A9
zmp_ph114_A11
zmp_ph114_B2
zmp_ph114_B4
zmp_ph114_D8
zmp_ph114_E2

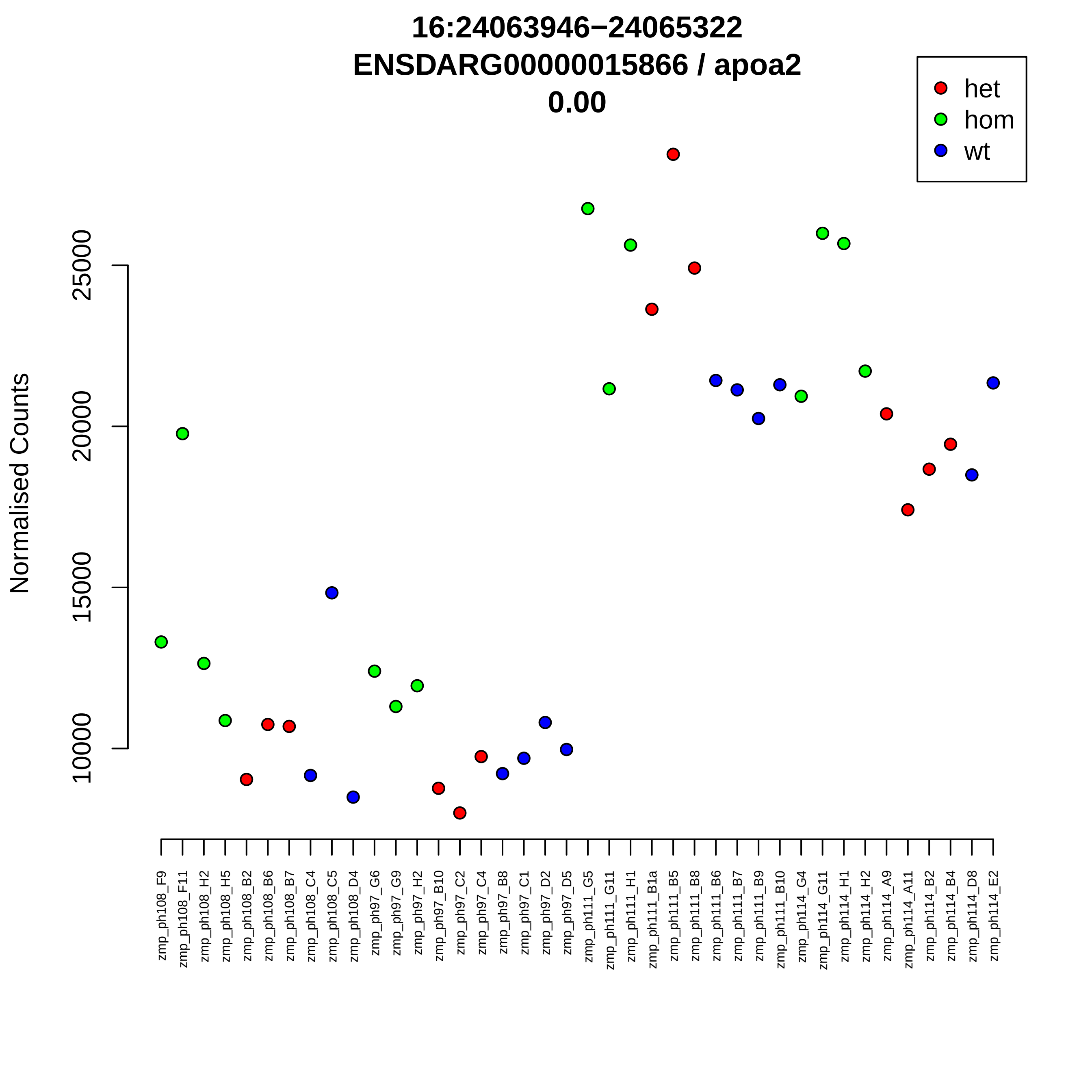
16:24063946−24065322
ENSDARG00000015866 / apoa2
0.00
Normalised Counts
10000
15000
20000
25000
het
hom
wt
zmp_ph108_F9
zmp_ph108_F11
zmp_ph108_H2
zmp_ph108_H5
zmp_ph108_B2
zmp_ph108_B6
zmp_ph108_B7
zmp_ph108_C4
zmp_ph108_C5
zmp_ph108_D4
zmp_ph97_G6
zmp_ph97_G9
zmp_ph97_H2
zmp_ph97_B10
zmp_ph97_C2
zmp_ph97_C4
zmp_ph97_B8
zmp_ph97_C1
zmp_ph97_D2
zmp_ph97_D5
zmp_ph111_G5
zmp_ph111_G11
zmp_ph111_H1
zmp_ph111_B1a
zmp_ph111_B5
zmp_ph111_B8
zmp_ph111_B6
zmp_ph111_B7
zmp_ph111_B9
zmp_ph111_B10
zmp_ph114_G4
zmp_ph114_G11
zmp_ph114_H1
zmp_ph114_H2
zmp_ph114_A9
zmp_ph114_A11
zmp_ph114_B2
zmp_ph114_B4
zmp_ph114_D8
zmp_ph114_E2

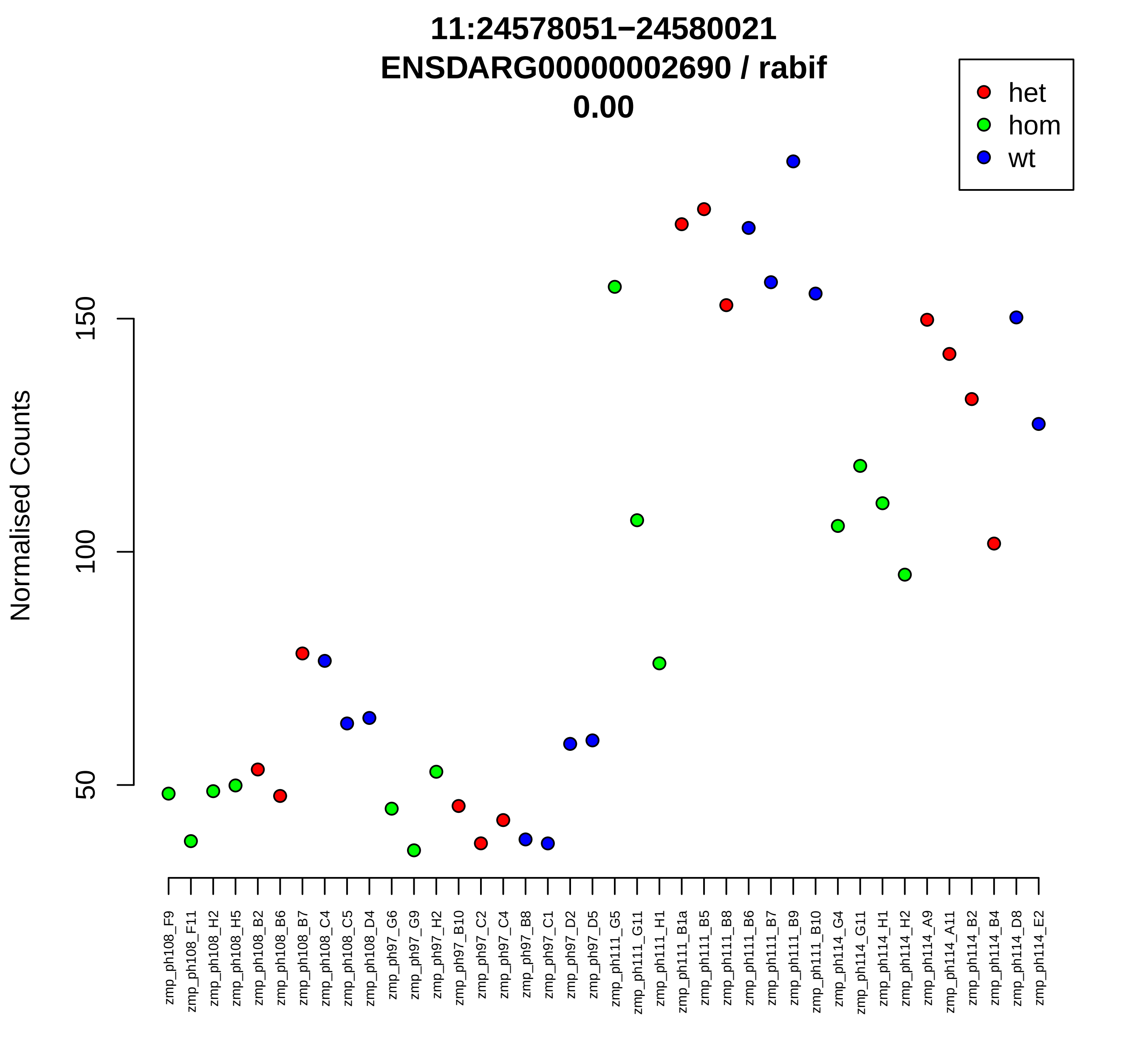

11:24578051−24580021
ENSDARG00000002690 / rabif
0.00
het
hom
wt
Normalised Counts
50
100
150
zmp_ph108_F9
zmp_ph108_F11
zmp_ph108_H2
zmp_ph108_H5
zmp_ph108_B2
zmp_ph108_B6
zmp_ph108_B7
zmp_ph108_C4
zmp_ph108_C5
zmp_ph108_D4
zmp_ph97_G6
zmp_ph97_G9
zmp_ph97_H2
zmp_ph97_B10
zmp_ph97_C2
zmp_ph97_C4
zmp_ph97_B8
zmp_ph97_C1
zmp_ph97_D2
zmp_ph97_D5
zmp_ph111_G5
zmp_ph111_G11
zmp_ph111_H1
zmp_ph111_B1a
zmp_ph111_B5
zmp_ph111_B8
zmp_ph111_B6
zmp_ph111_B7
zmp_ph111_B9
zmp_ph111_B10
zmp_ph114_G4
zmp_ph114_G11
zmp_ph114_H1
zmp_ph114_H2
zmp_ph114_A9
zmp_ph114_A11
zmp_ph114_B2
zmp_ph114_B4
zmp_ph114_D8
zmp_ph114_E2

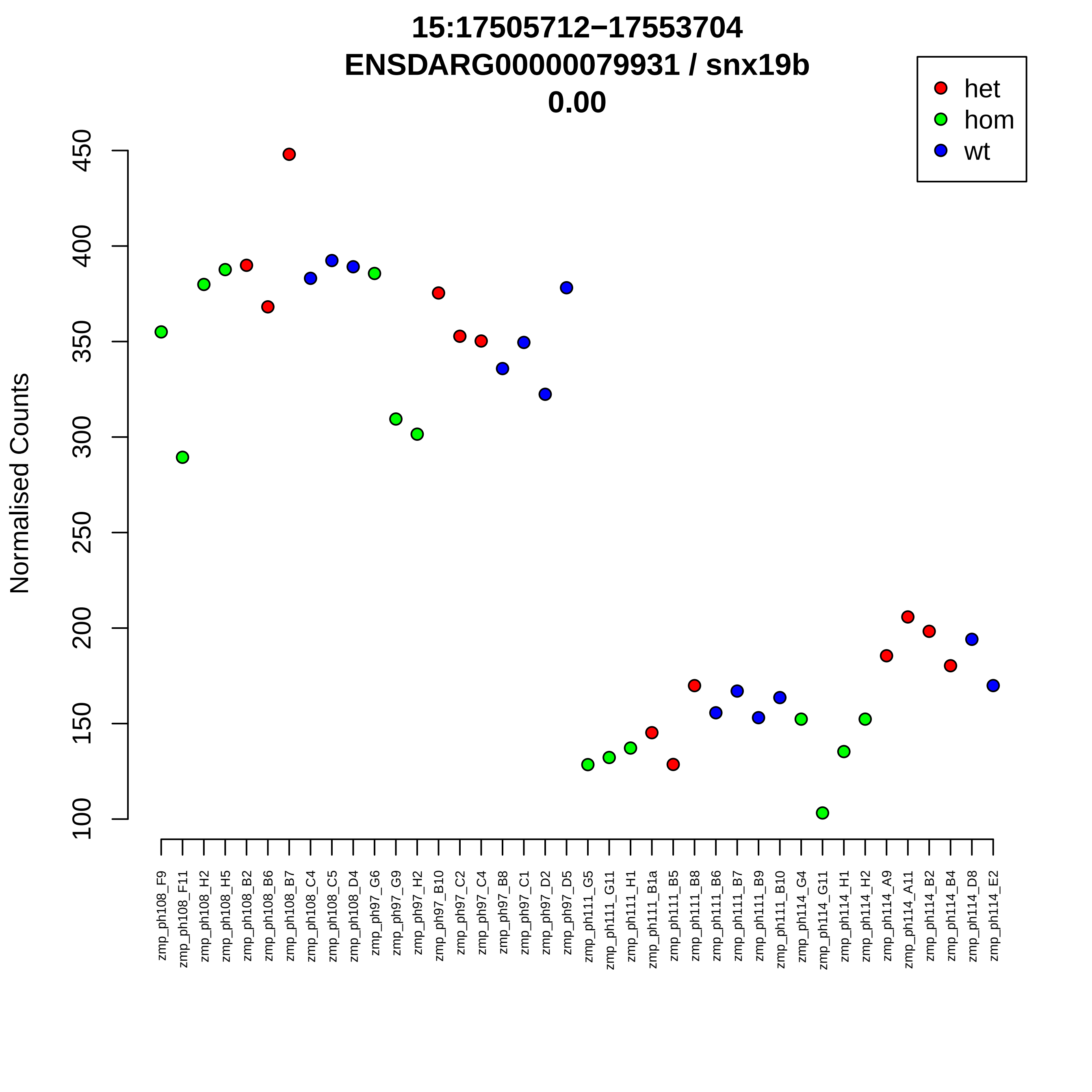

15:17505712−17553704
ENSDARG00000079931 / snx19b
0.00
het
hom
wt
Normalised Counts
100
150
200
250
300
350
400
450
zmp_ph108_F9
zmp_ph108_F11
zmp_ph108_H2
zmp_ph108_H5
zmp_ph108_B2
zmp_ph108_B6
zmp_ph108_B7
zmp_ph108_C4
zmp_ph108_C5
zmp_ph108_D4
zmp_ph97_G6
zmp_ph97_G9
zmp_ph97_H2
zmp_ph97_B10
zmp_ph97_C2
zmp_ph97_C4
zmp_ph97_B8
zmp_ph97_C1
zmp_ph97_D2
zmp_ph97_D5
zmp_ph111_G5
zmp_ph111_G11
zmp_ph111_H1
zmp_ph111_B1a
zmp_ph111_B5
zmp_ph111_B8
zmp_ph111_B6
zmp_ph111_B7
zmp_ph111_B9
zmp_ph111_B10
zmp_ph114_G4
zmp_ph114_G11
zmp_ph114_H1
zmp_ph114_H2
zmp_ph114_A9
zmp_ph114_A11
zmp_ph114_B2
zmp_ph114_B4
zmp_ph114_D8
zmp_ph114_E2

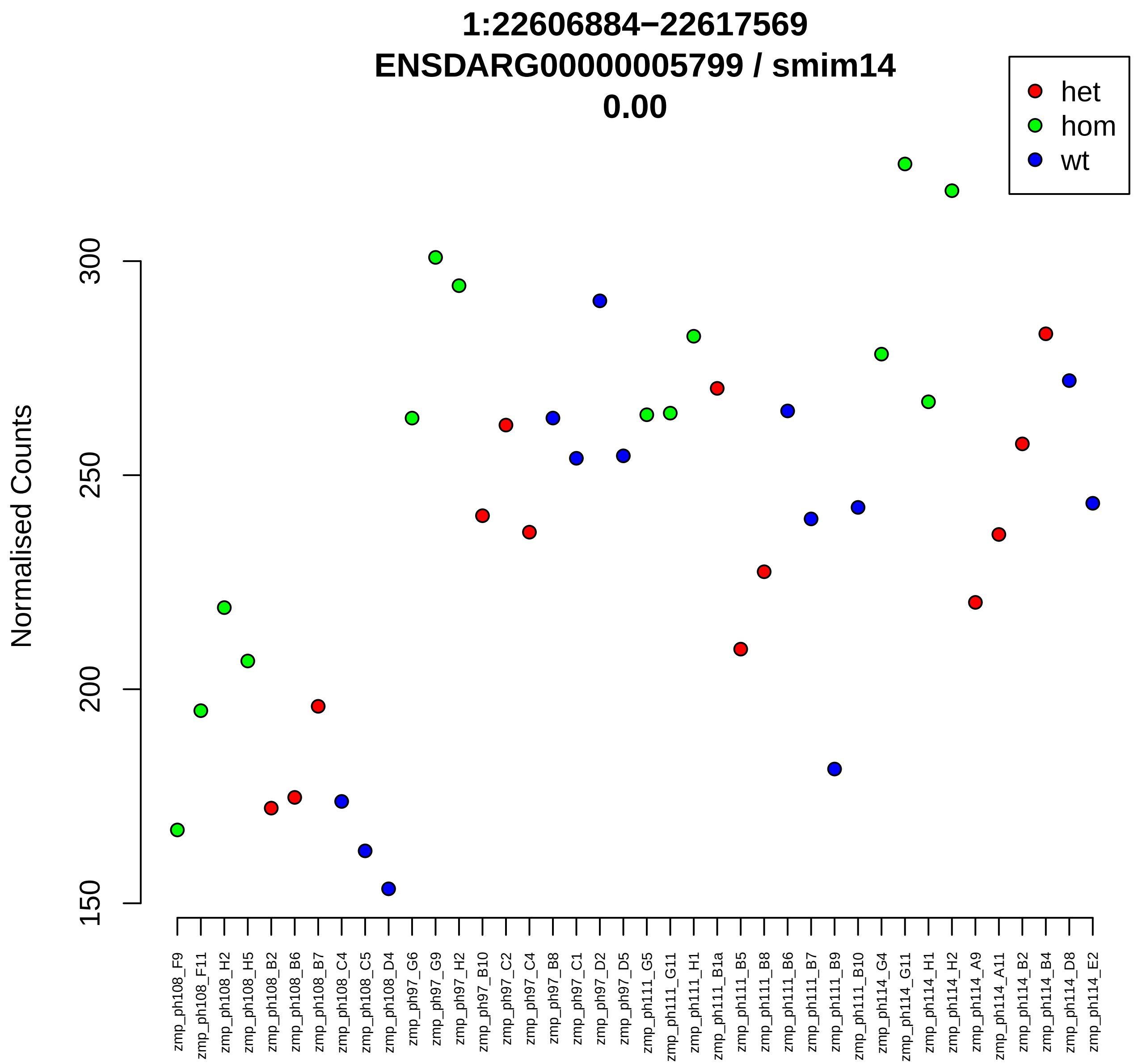
1:22606884−22617569
ENSDARG00000005799 / smim14
0.00
het
hom
wt
Normalised Counts
150
200
250
300
zmp_ph108_F9
zmp_ph108_F11
zmp_ph108_H2
zmp_ph108_H5
zmp_ph108_B2
zmp_ph108_B6
zmp_ph108_B7
zmp_ph108_C4
zmp_ph108_C5
zmp_ph108_D4
zmp_ph97_G6
zmp_ph97_G9
zmp_ph97_H2
zmp_ph97_B10
zmp_ph97_C2
zmp_ph97_C4
zmp_ph97_B8
zmp_ph97_C1
zmp_ph97_D2
zmp_ph97_D5
zmp_ph111_G5
zmp_ph111_G11
zmp_ph111_H1
zmp_ph111_B1a
zmp_ph111_B5
zmp_ph111_B8
zmp_ph111_B6
zmp_ph111_B7
zmp_ph111_B9
zmp_ph111_B10
zmp_ph114_G4
zmp_ph114_G11
zmp_ph114_H1
zmp_ph114_H2
zmp_ph114_A9
zmp_ph114_A11
zmp_ph114_B2
zmp_ph114_B4
zmp_ph114_D8
zmp_ph114_E2

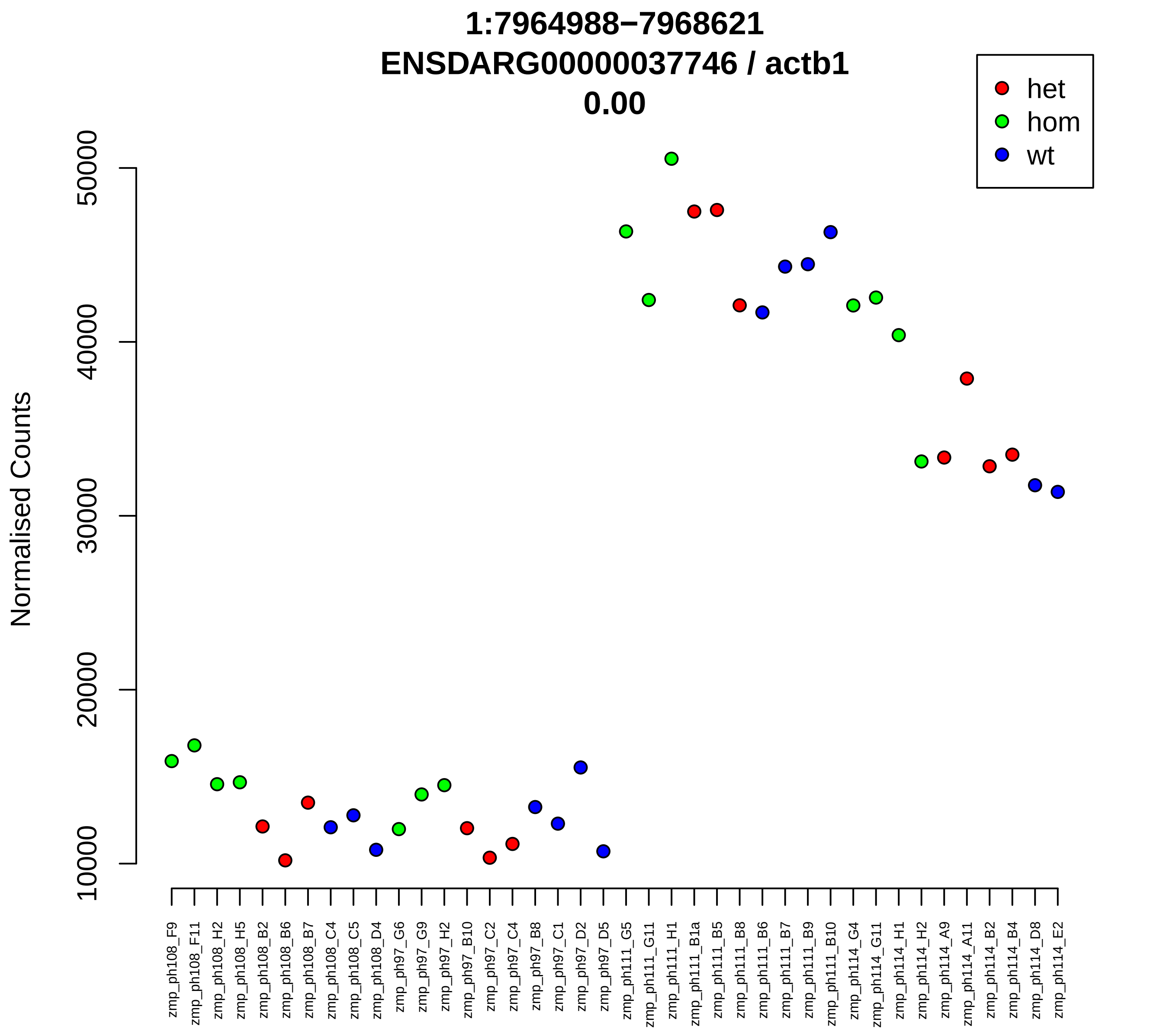

1:7964988−7968621
ENSDARG00000037746 / actb1
0.00
het
hom
wt
Normalised Counts
10000
20000
30000
40000
50000
zmp_ph108_F9
zmp_ph108_F11
zmp_ph108_H2
zmp_ph108_H5
zmp_ph108_B2
zmp_ph108_B6
zmp_ph108_B7
zmp_ph108_C4
zmp_ph108_C5
zmp_ph108_D4
zmp_ph97_G6
zmp_ph97_G9
zmp_ph97_H2
zmp_ph97_B10
zmp_ph97_C2
zmp_ph97_C4
zmp_ph97_B8
zmp_ph97_C1
zmp_ph97_D2
zmp_ph97_D5
zmp_ph111_G5
zmp_ph111_G11
zmp_ph111_H1
zmp_ph111_B1a
zmp_ph111_B5
zmp_ph111_B8
zmp_ph111_B6
zmp_ph111_B7
zmp_ph111_B9
zmp_ph111_B10
zmp_ph114_G4
zmp_ph114_G11
zmp_ph114_H1
zmp_ph114_H2
zmp_ph114_A9
zmp_ph114_A11
zmp_ph114_B2
zmp_ph114_B4
zmp_ph114_D8
zmp_ph114_E2

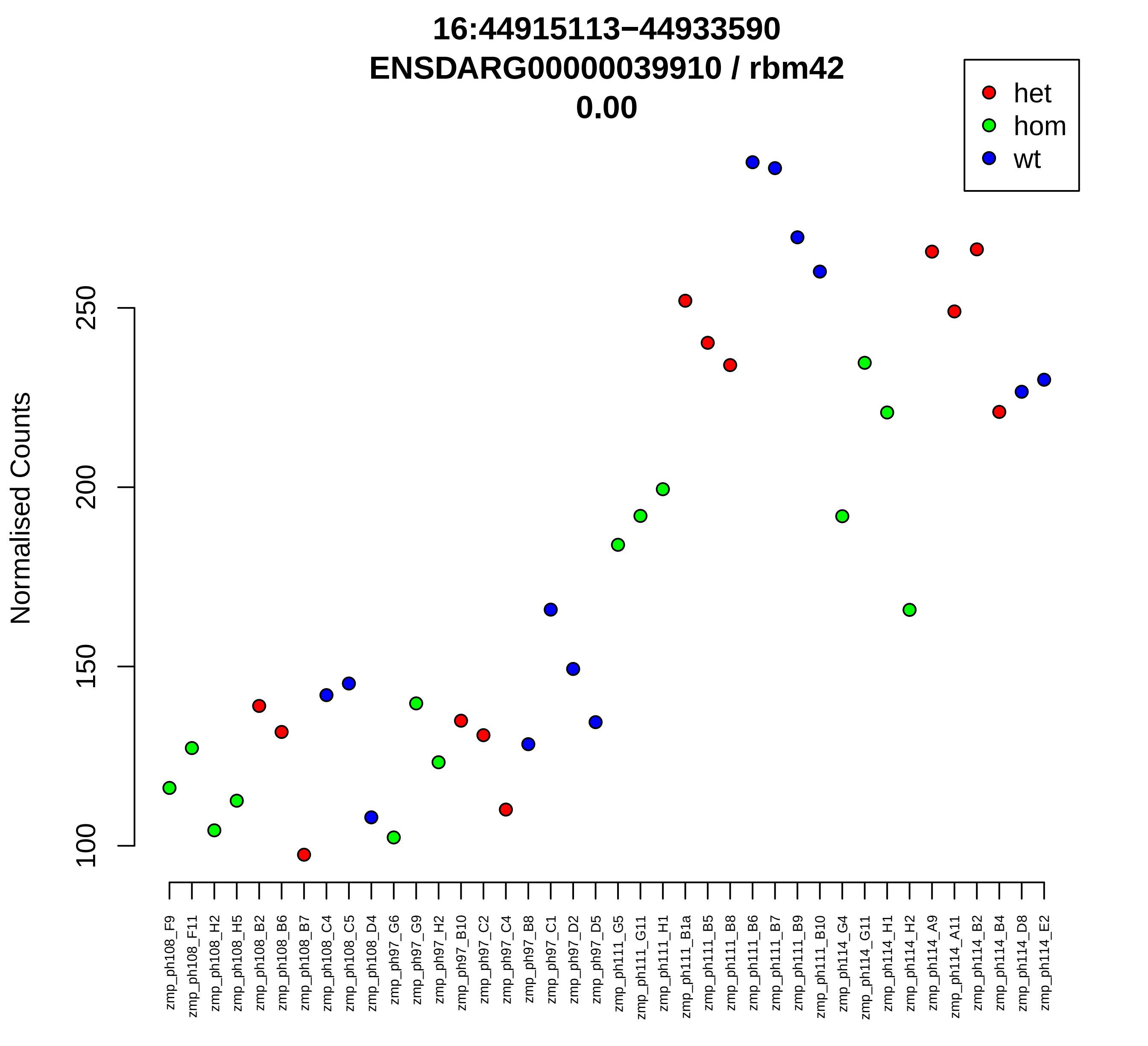
16:44915113−44933590
ENSDARG00000039910 / rbm42
0.00
het
hom
wt
Normalised Counts
100
150
200
250
zmp_ph108_F9
zmp_ph108_F11
zmp_ph108_H2
zmp_ph108_H5
zmp_ph108_B2
zmp_ph108_B6
zmp_ph108_B7
zmp_ph108_C4
zmp_ph108_C5
zmp_ph108_D4
zmp_ph97_G6
zmp_ph97_G9
zmp_ph97_H2
zmp_ph97_B10
zmp_ph97_C2
zmp_ph97_C4
zmp_ph97_B8
zmp_ph97_C1
zmp_ph97_D2
zmp_ph97_D5
zmp_ph111_G5
zmp_ph111_G11
zmp_ph111_H1
zmp_ph111_B1a
zmp_ph111_B5
zmp_ph111_B8
zmp_ph111_B6
zmp_ph111_B7
zmp_ph111_B9
zmp_ph111_B10
zmp_ph114_G4
zmp_ph114_G11
zmp_ph114_H1
zmp_ph114_H2
zmp_ph114_A9
zmp_ph114_A11
zmp_ph114_B2
zmp_ph114_B4
zmp_ph114_D8
zmp_ph114_E2

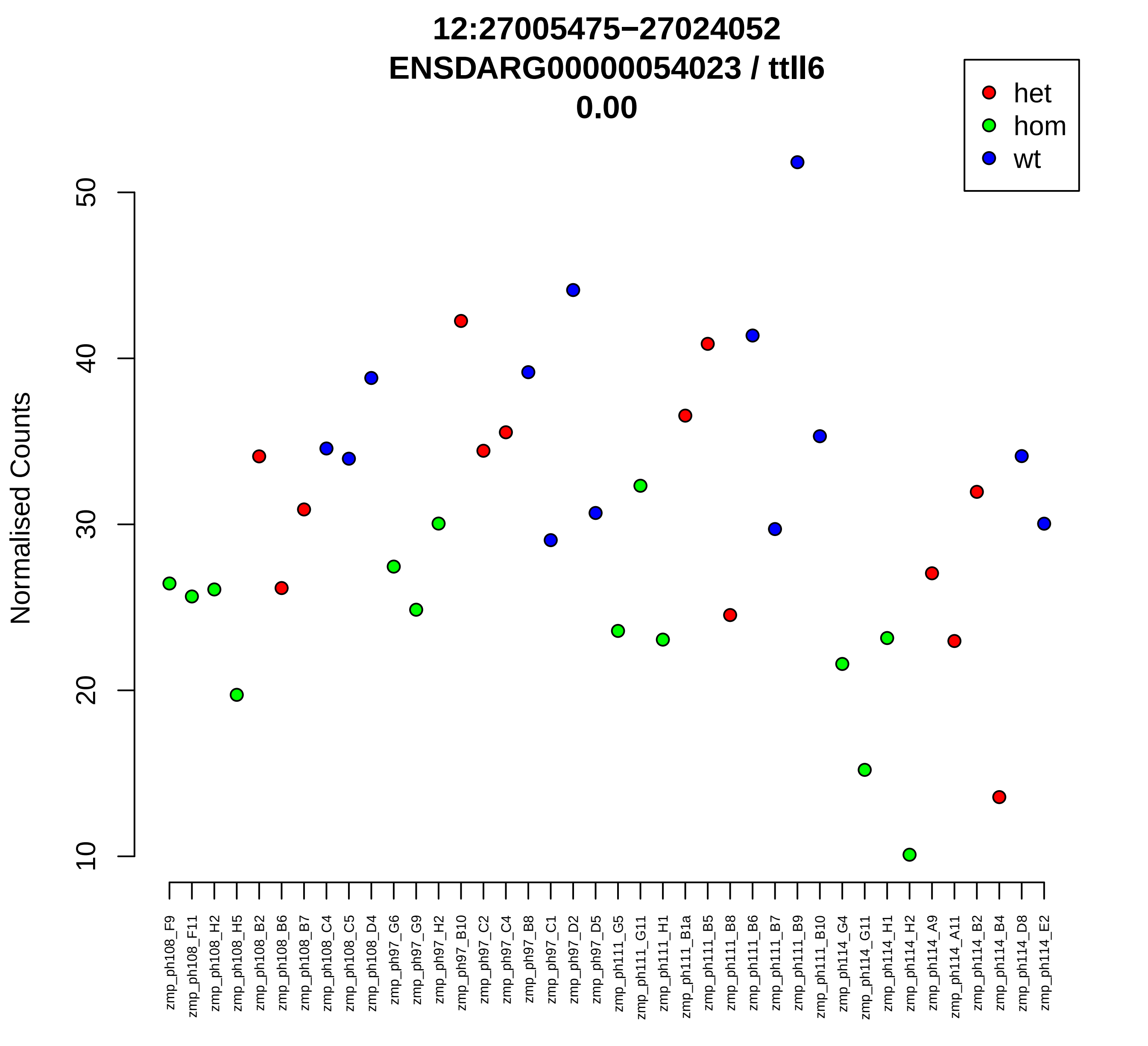
12:27005475−27024052
ENSDARG00000054023 / ttll6
0.00
het
hom
wt
Normalised Counts
10
20
30
40
50
zmp_ph108_F9
zmp_ph108_F11
zmp_ph108_H2
zmp_ph108_H5
zmp_ph108_B2
zmp_ph108_B6
zmp_ph108_B7
zmp_ph108_C4
zmp_ph108_C5
zmp_ph108_D4
zmp_ph97_G6
zmp_ph97_G9
zmp_ph97_H2
zmp_ph97_B10
zmp_ph97_C2
zmp_ph97_C4
zmp_ph97_B8
zmp_ph97_C1
zmp_ph97_D2
zmp_ph97_D5
zmp_ph111_G5
zmp_ph111_G11
zmp_ph111_H1
zmp_ph111_B1a
zmp_ph111_B5
zmp_ph111_B8
zmp_ph111_B6
zmp_ph111_B7
zmp_ph111_B9
zmp_ph111_B10
zmp_ph114_G4
zmp_ph114_G11
zmp_ph114_H1
zmp_ph114_H2
zmp_ph114_A9
zmp_ph114_A11
zmp_ph114_B2
zmp_ph114_B4
zmp_ph114_D8
zmp_ph114_E2

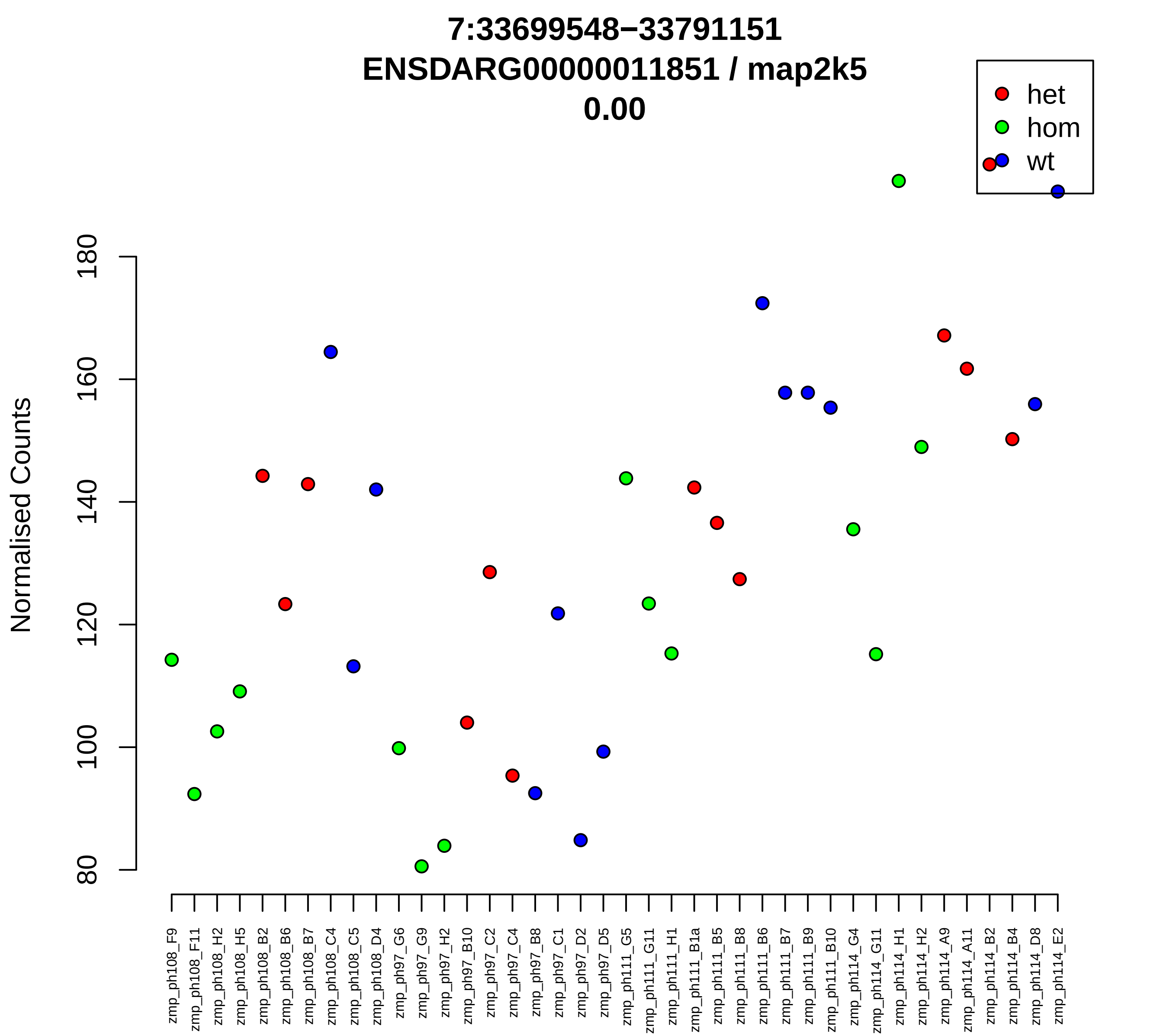

7:33699548−33791151
ENSDARG00000011851 / map2k5
0.00
het
hom
wt
Normalised Counts
80
100
120
140
160
180
zmp_ph108_F9
zmp_ph108_F11
zmp_ph108_H2
zmp_ph108_H5
zmp_ph108_B2
zmp_ph108_B6
zmp_ph108_B7
zmp_ph108_C4
zmp_ph108_C5
zmp_ph108_D4
zmp_ph97_G6
zmp_ph97_G9
zmp_ph97_H2
zmp_ph97_B10
zmp_ph97_C2
zmp_ph97_C4
zmp_ph97_B8
zmp_ph97_C1
zmp_ph97_D2
zmp_ph97_D5
zmp_ph111_G5
zmp_ph111_G11
zmp_ph111_H1
zmp_ph111_B1a
zmp_ph111_B5
zmp_ph111_B8
zmp_ph111_B6
zmp_ph111_B7
zmp_ph111_B9
zmp_ph111_B10
zmp_ph114_G4
zmp_ph114_G11
zmp_ph114_H1
zmp_ph114_H2
zmp_ph114_A9
zmp_ph114_A11
zmp_ph114_B2
zmp_ph114_B4
zmp_ph114_D8
zmp_ph114_E2

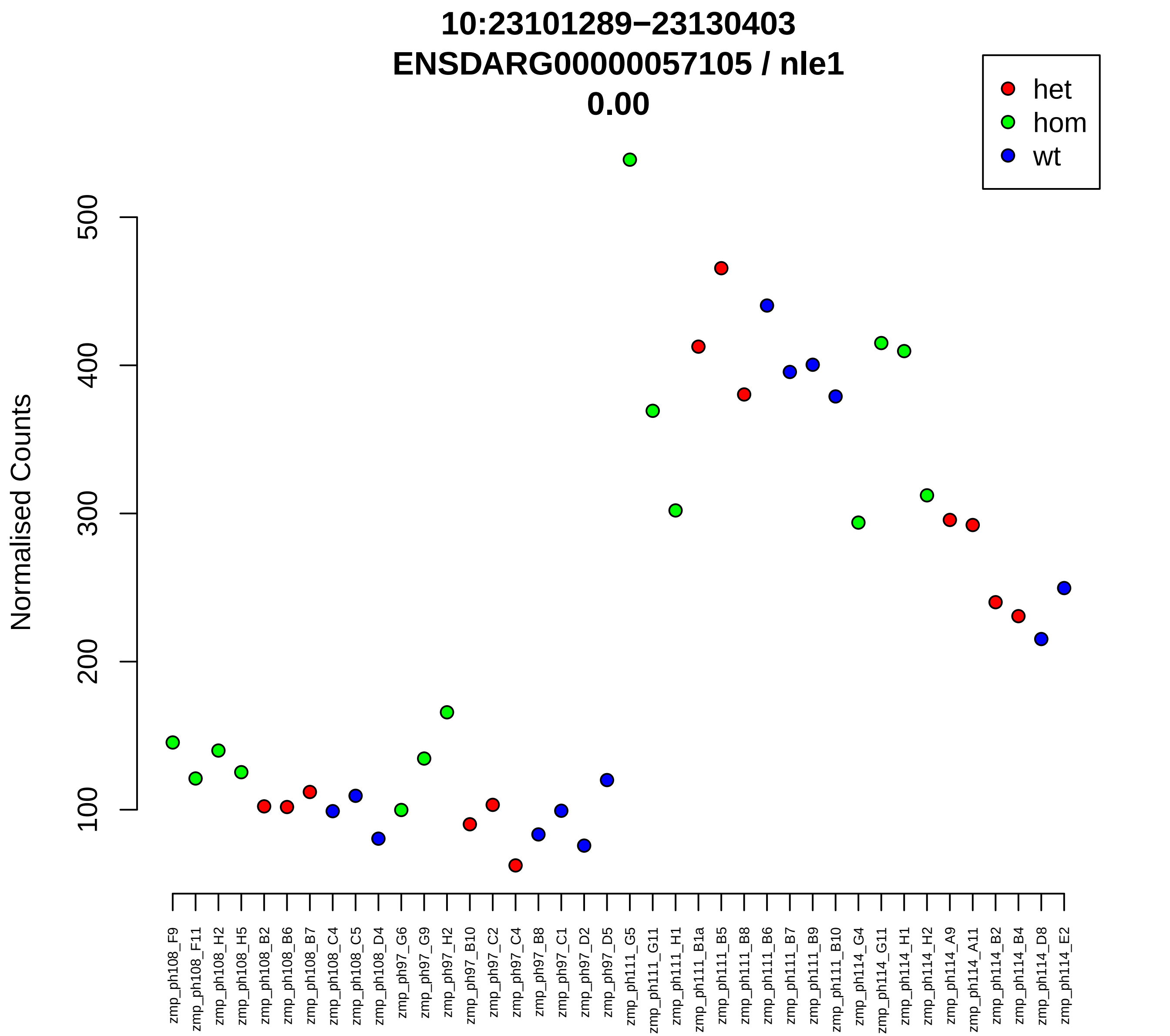
10:23101289−23130403
ENSDARG00000057105 / nle1
0.00
het
hom
wt
Normalised Counts
100
200
300
400
500
zmp_ph108_F9
zmp_ph108_F11
zmp_ph108_H2
zmp_ph108_H5
zmp_ph108_B2
zmp_ph108_B6
zmp_ph108_B7
zmp_ph108_C4
zmp_ph108_C5
zmp_ph108_D4
zmp_ph97_G6
zmp_ph97_G9
zmp_ph97_H2
zmp_ph97_B10
zmp_ph97_C2
zmp_ph97_C4
zmp_ph97_B8
zmp_ph97_C1
zmp_ph97_D2
zmp_ph97_D5
zmp_ph111_G5
zmp_ph111_G11
zmp_ph111_H1
zmp_ph111_B1a
zmp_ph111_B5
zmp_ph111_B8
zmp_ph111_B6
zmp_ph111_B7
zmp_ph111_B9
zmp_ph111_B10
zmp_ph114_G4
zmp_ph114_G11
zmp_ph114_H1
zmp_ph114_H2
zmp_ph114_A9
zmp_ph114_A11
zmp_ph114_B2
zmp_ph114_B4
zmp_ph114_D8
zmp_ph114_E2

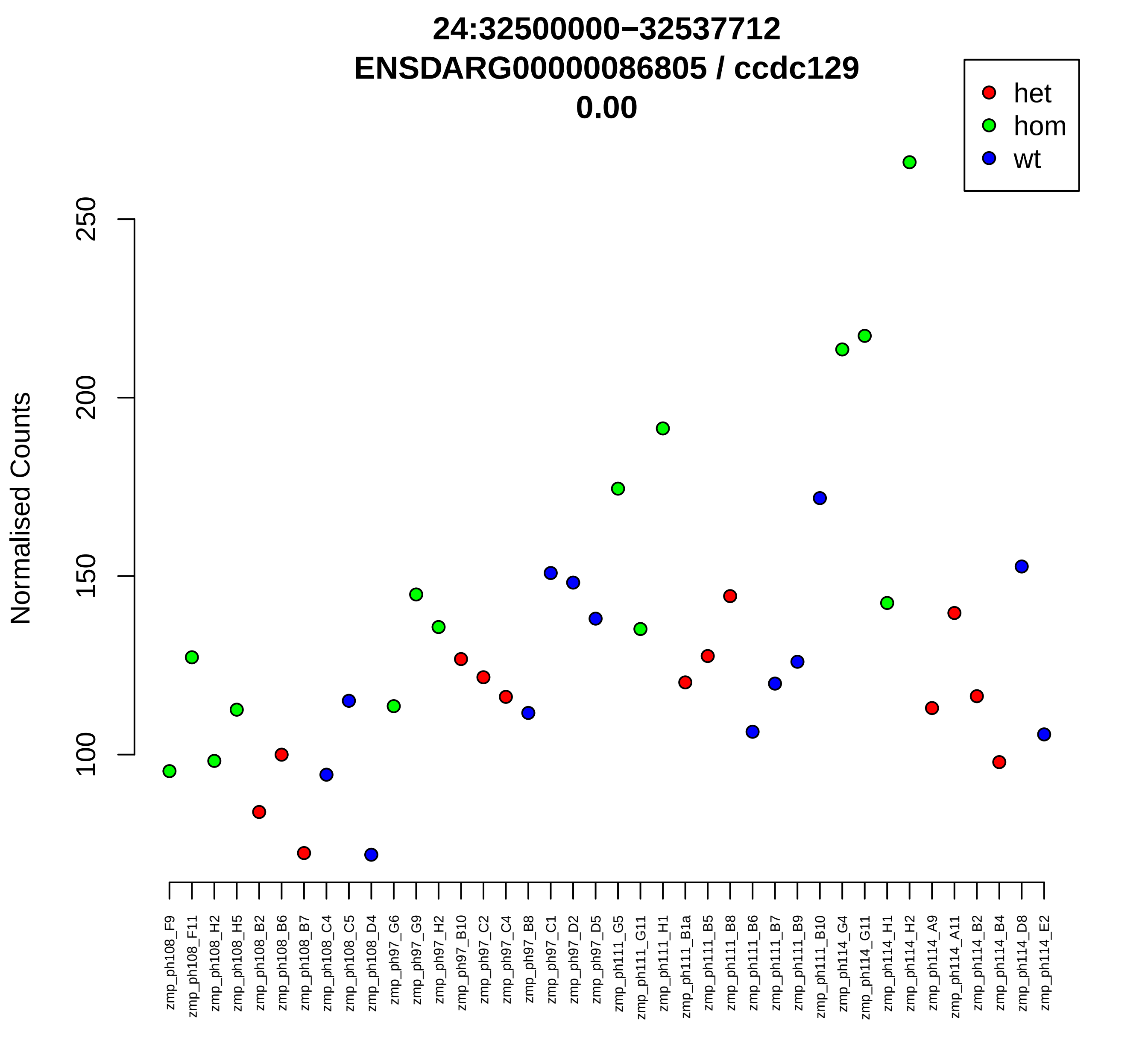
24:32500000−32537712
ENSDARG00000086805 / ccdc129
0.00
het
hom
wt
Normalised Counts
250
200
150
100
zmp_ph108_F9
zmp_ph108_F11
zmp_ph108_H2
zmp_ph108_H5
zmp_ph108_B2
zmp_ph108_B6
zmp_ph108_B7
zmp_ph108_C4
zmp_ph108_C5
zmp_ph108_D4
zmp_ph97_G6
zmp_ph97_G9
zmp_ph97_H2
zmp_ph97_B10
zmp_ph97_C2
zmp_ph97_C4
zmp_ph97_B8
zmp_ph97_C1
zmp_ph97_D2
zmp_ph97_D5
zmp_ph111_G5
zmp_ph111_G11
zmp_ph111_H1
zmp_ph111_B1a
zmp_ph111_B5
zmp_ph111_B8
zmp_ph111_B6
zmp_ph111_B7
zmp_ph111_B9
zmp_ph111_B10
zmp_ph114_G4
zmp_ph114_G11
zmp_ph114_H1
zmp_ph114_H2
zmp_ph114_A9
zmp_ph114_A11
zmp_ph114_B2
zmp_ph114_B4
zmp_ph114_D8
zmp_ph114_E2

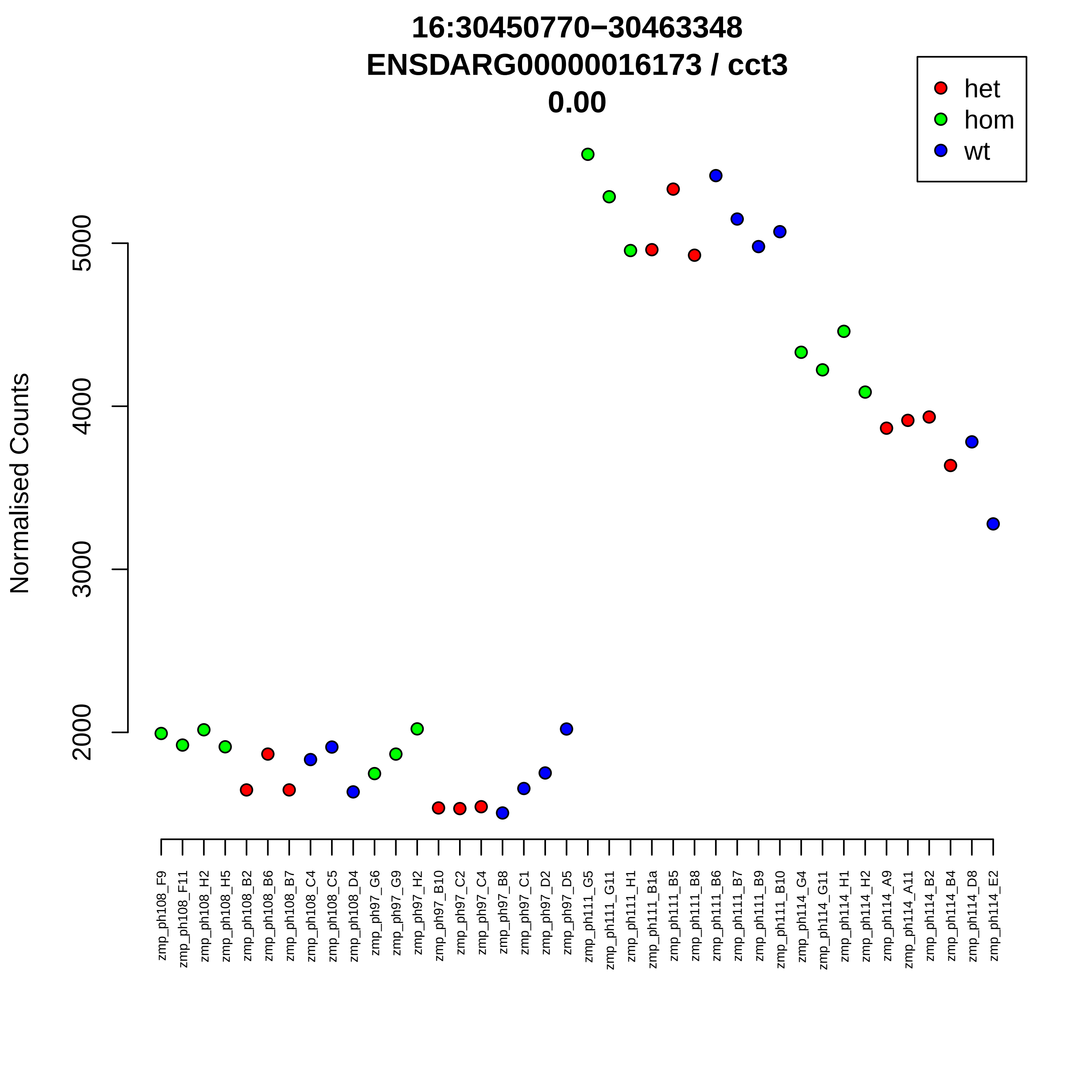
16:30450770−30463348
ENSDARG00000016173 / cct3
0.00
het
hom
wt
Normalised Counts
2000
3000
4000
5000
zmp_ph108_F9
zmp_ph108_F11
zmp_ph108_H2
zmp_ph108_H5
zmp_ph108_B2
zmp_ph108_B6
zmp_ph108_B7
zmp_ph108_C4
zmp_ph108_C5
zmp_ph108_D4
zmp_ph97_G6
zmp_ph97_G9
zmp_ph97_H2
zmp_ph97_B10
zmp_ph97_C2
zmp_ph97_C4
zmp_ph97_B8
zmp_ph97_C1
zmp_ph97_D2
zmp_ph97_D5
zmp_ph111_G5
zmp_ph111_G11
zmp_ph111_H1
zmp_ph111_B1a
zmp_ph111_B5
zmp_ph111_B8
zmp_ph111_B6
zmp_ph111_B7
zmp_ph111_B9
zmp_ph111_B10
zmp_ph114_G4
zmp_ph114_G11
zmp_ph114_H1
zmp_ph114_H2
zmp_ph114_A9
zmp_ph114_A11
zmp_ph114_B2
zmp_ph114_B4
zmp_ph114_D8
zmp_ph114_E2

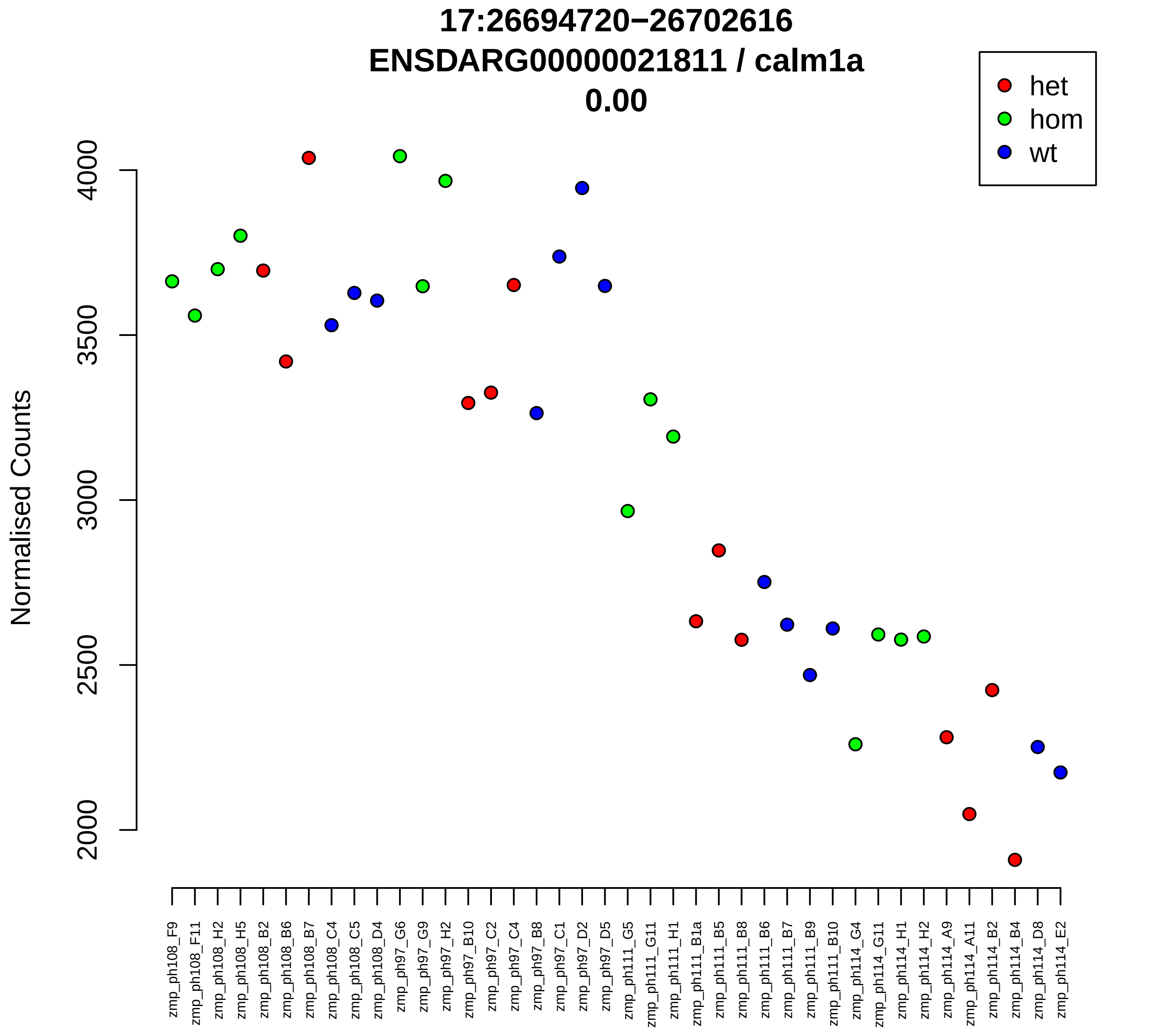

17:26694720−26702616
ENSDARG00000021811 / calm1a
0.00
het
hom
wt
Normalised Counts
2000
2500
3000
3500
4000
zmp_ph108_F9
zmp_ph108_F11
zmp_ph108_H2
zmp_ph108_H5
zmp_ph108_B2
zmp_ph108_B6
zmp_ph108_B7
zmp_ph108_C4
zmp_ph108_C5
zmp_ph108_D4
zmp_ph97_G6
zmp_ph97_G9
zmp_ph97_H2
zmp_ph97_B10
zmp_ph97_C2
zmp_ph97_C4
zmp_ph97_B8
zmp_ph97_C1
zmp_ph97_D2
zmp_ph97_D5
zmp_ph111_G5
zmp_ph111_G11
zmp_ph111_H1
zmp_ph111_B1a
zmp_ph111_B5
zmp_ph111_B8
zmp_ph111_B6
zmp_ph111_B7
zmp_ph111_B9
zmp_ph111_B10
zmp_ph114_G4
zmp_ph114_G11
zmp_ph114_H1
zmp_ph114_H2
zmp_ph114_A9
zmp_ph114_A11
zmp_ph114_B2
zmp_ph114_B4
zmp_ph114_D8
zmp_ph114_E2

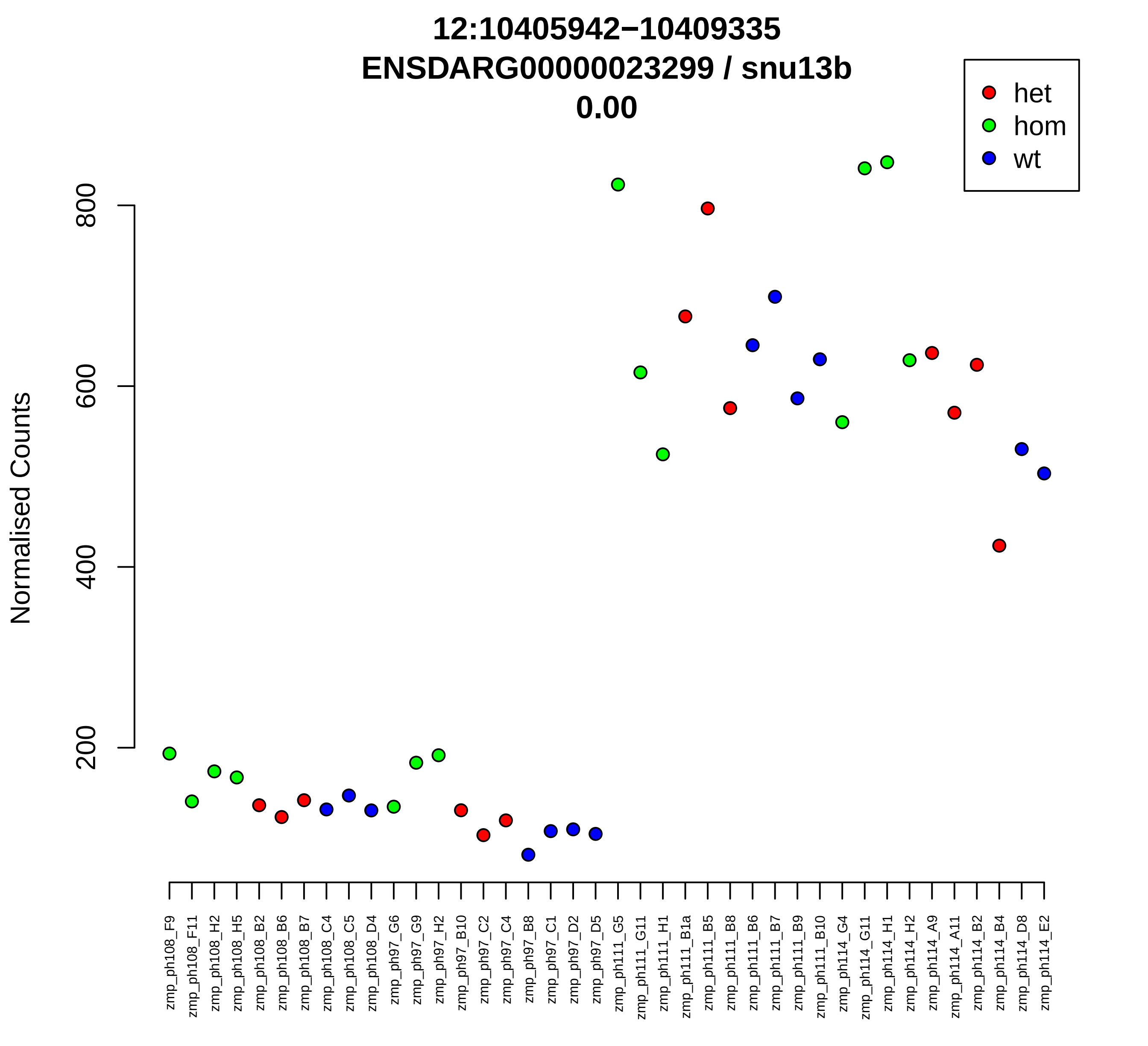

12:10405942−10409335
ENSDARG00000023299 / snu13b
0.00
het
hom
wt
Normalised Counts
200
400
600
800
zmp_ph108_F9
zmp_ph108_F11
zmp_ph108_H2
zmp_ph108_H5
zmp_ph108_B2
zmp_ph108_B6
zmp_ph108_B7
zmp_ph108_C4
zmp_ph108_C5
zmp_ph108_D4
zmp_ph97_G6
zmp_ph97_G9
zmp_ph97_H2
zmp_ph97_B10
zmp_ph97_C2
zmp_ph97_C4
zmp_ph97_B8
zmp_ph97_C1
zmp_ph97_D2
zmp_ph97_D5
zmp_ph111_G5
zmp_ph111_G11
zmp_ph111_H1
zmp_ph111_B1a
zmp_ph111_B5
zmp_ph111_B8
zmp_ph111_B6
zmp_ph111_B7
zmp_ph111_B9
zmp_ph111_B10
zmp_ph114_G4
zmp_ph114_G11
zmp_ph114_H1
zmp_ph114_H2
zmp_ph114_A9
zmp_ph114_A11
zmp_ph114_B2
zmp_ph114_B4
zmp_ph114_D8
zmp_ph114_E2

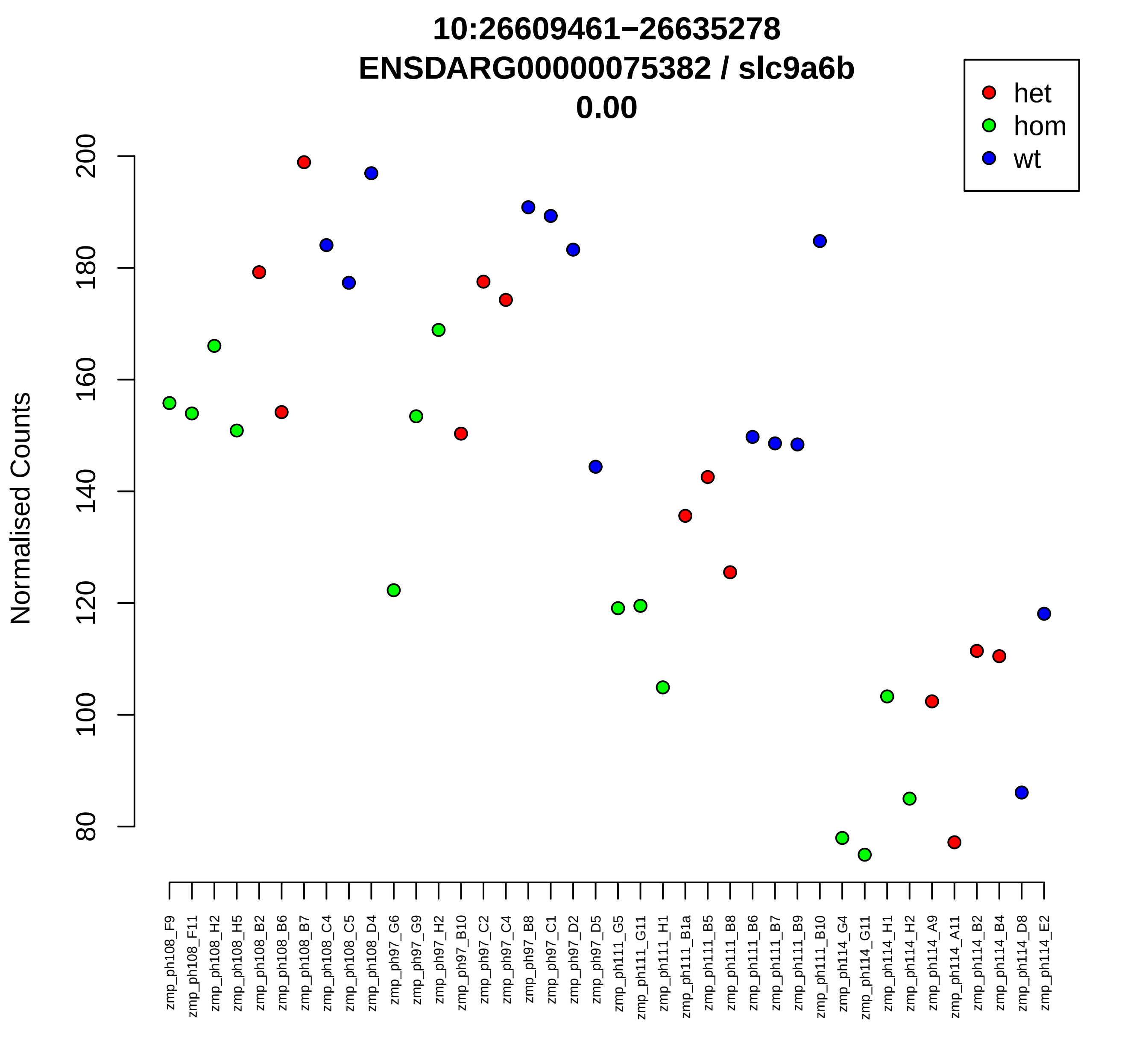
10:26609461−26635278
ENSDARG00000075382 / slc9a6b
0.00
het
hom
wt
Normalised Counts
80
100
120
140
160
180
200
zmp_ph108_F9
zmp_ph108_F11
zmp_ph108_H2
zmp_ph108_H5
zmp_ph108_B2
zmp_ph108_B6
zmp_ph108_B7
zmp_ph108_C4
zmp_ph108_C5
zmp_ph108_D4
zmp_ph97_G6
zmp_ph97_G9
zmp_ph97_H2
zmp_ph97_B10
zmp_ph97_C2
zmp_ph97_C4
zmp_ph97_B8
zmp_ph97_C1
zmp_ph97_D2
zmp_ph97_D5
zmp_ph111_G5
zmp_ph111_G11
zmp_ph111_H1
zmp_ph111_B1a
zmp_ph111_B5
zmp_ph111_B8
zmp_ph111_B6
zmp_ph111_B7
zmp_ph111_B9
zmp_ph111_B10
zmp_ph114_G4
zmp_ph114_G11
zmp_ph114_H1
zmp_ph114_H2
zmp_ph114_A9
zmp_ph114_A11
zmp_ph114_B2
zmp_ph114_B4
zmp_ph114_D8
zmp_ph114_E2

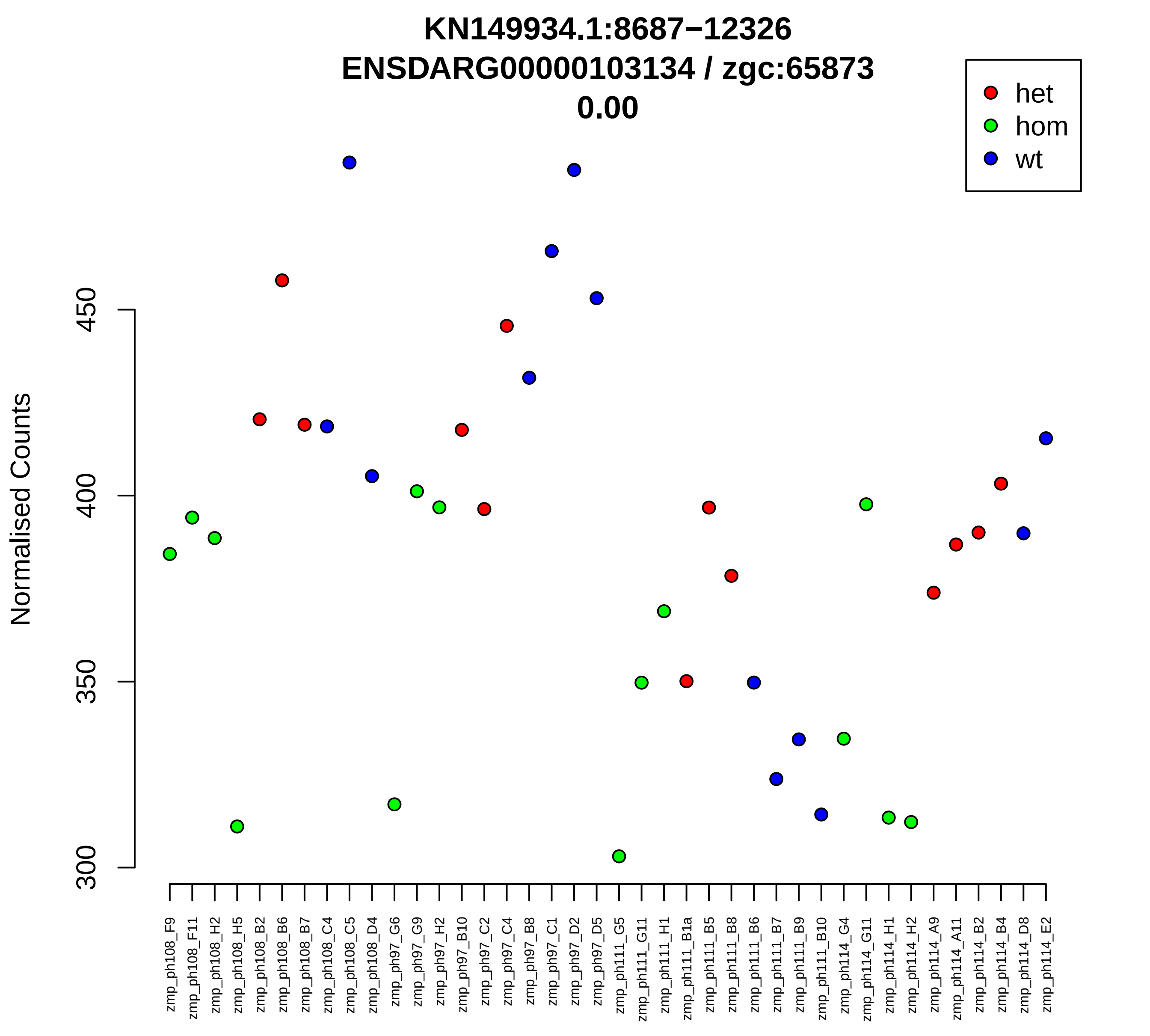

KN149934.1:8687−12326
ENSDARG00000103134 / zgc:65873
0.00
het
hom
wt
Normalised Counts
300
350
400
450
zmp_ph108_F9
zmp_ph108_F11
zmp_ph108_H2
zmp_ph108_H5
zmp_ph108_B2
zmp_ph108_B6
zmp_ph108_B7
zmp_ph108_C4
zmp_ph108_C5
zmp_ph108_D4
zmp_ph97_G6
zmp_ph97_G9
zmp_ph97_H2
zmp_ph97_B10
zmp_ph97_C2
zmp_ph97_C4
zmp_ph97_B8
zmp_ph97_C1
zmp_ph97_D2
zmp_ph97_D5
zmp_ph111_G5
zmp_ph111_G11
zmp_ph111_H1
zmp_ph111_B1a
zmp_ph111_B5
zmp_ph111_B8
zmp_ph111_B6
zmp_ph111_B7
zmp_ph111_B9
zmp_ph111_B10
zmp_ph114_G4
zmp_ph114_G11
zmp_ph114_H1
zmp_ph114_H2
zmp_ph114_A9
zmp_ph114_A11
zmp_ph114_B2
zmp_ph114_B4
zmp_ph114_D8
zmp_ph114_E2

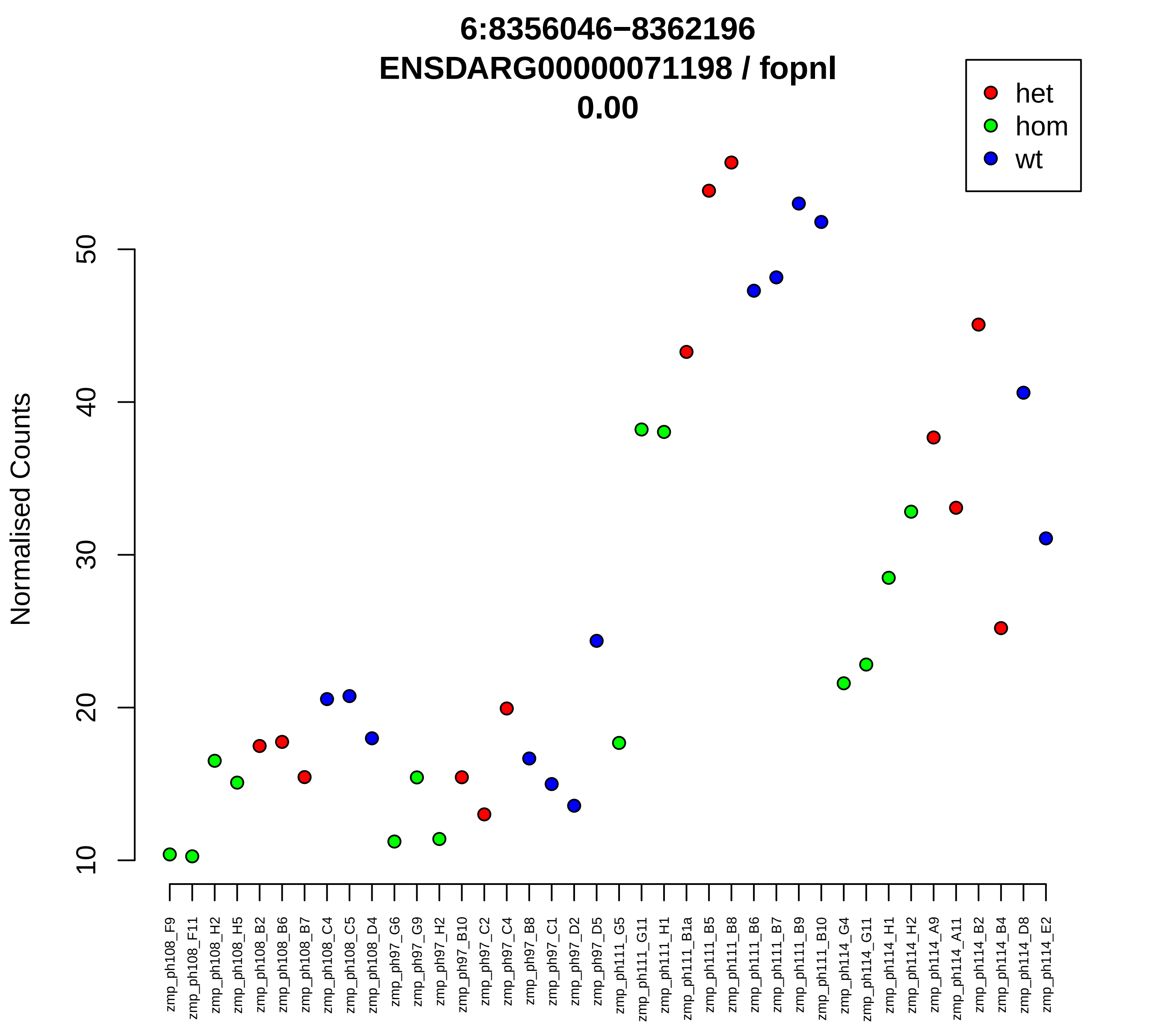

6:8356046−8362196
ENSDARG00000071198 / fopnl
0.00
het
hom
wt
Normalised Counts
10
20
30
40
50
zmp_ph108_F9
zmp_ph108_F11
zmp_ph108_H2
zmp_ph108_H5
zmp_ph108_B2
zmp_ph108_B6
zmp_ph108_B7
zmp_ph108_C4
zmp_ph108_C5
zmp_ph108_D4
zmp_ph97_G6
zmp_ph97_G9
zmp_ph97_H2
zmp_ph97_B10
zmp_ph97_C2
zmp_ph97_C4
zmp_ph97_B8
zmp_ph97_C1
zmp_ph97_D2
zmp_ph97_D5
zmp_ph111_G5
zmp_ph111_G11
zmp_ph111_H1
zmp_ph111_B1a
zmp_ph111_B5
zmp_ph111_B8
zmp_ph111_B6
zmp_ph111_B7
zmp_ph111_B9
zmp_ph111_B10
zmp_ph114_G4
zmp_ph114_G11
zmp_ph114_H1
zmp_ph114_H2
zmp_ph114_A9
zmp_ph114_A11
zmp_ph114_B2
zmp_ph114_B4
zmp_ph114_D8
zmp_ph114_E2

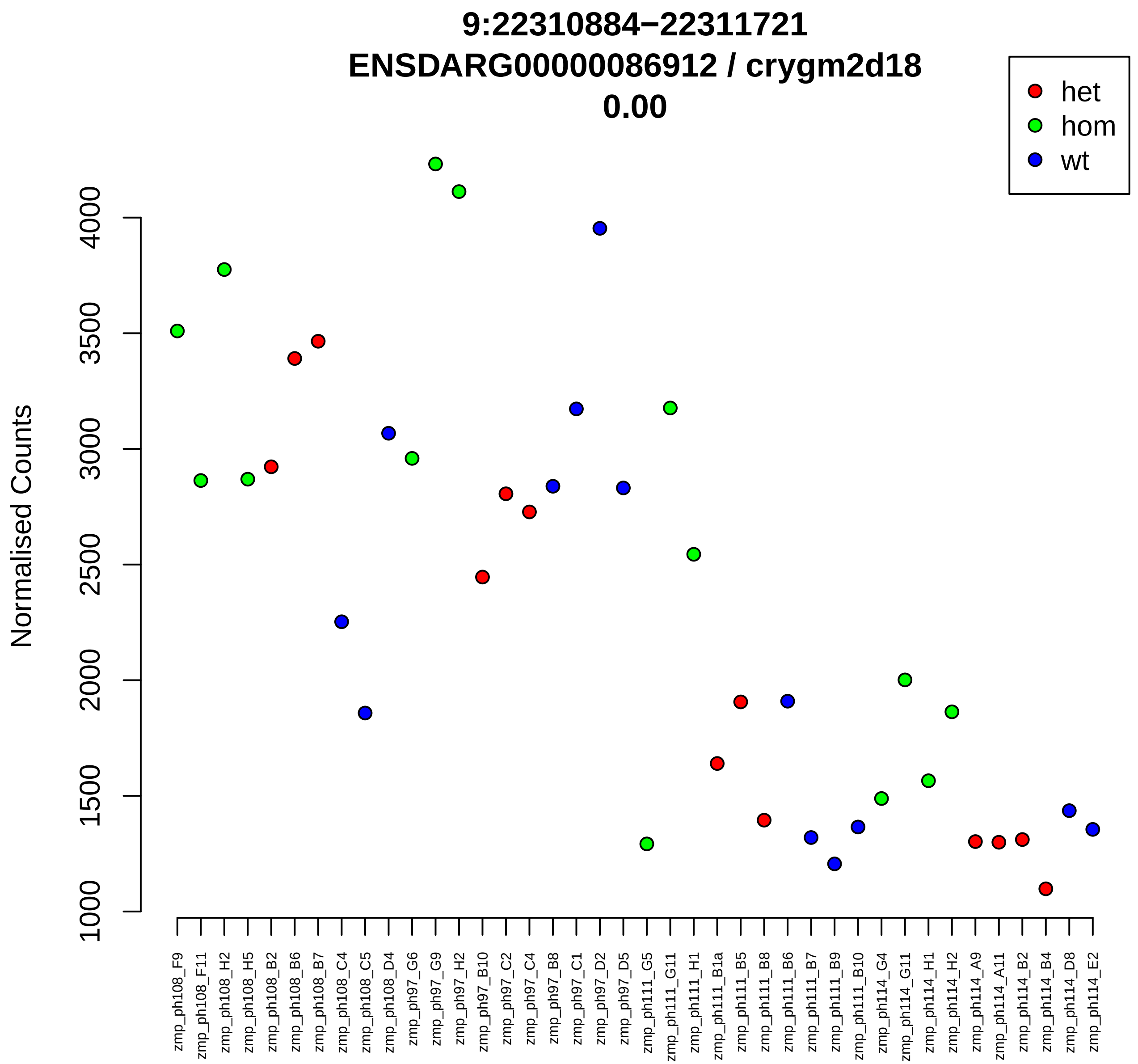
9:22310884−22311721
ENSDARG00000086912 / crygm2d18
0.00
het
hom
wt
Normalised Counts
1000
1500
2000
2500
3000
3500
4000
zmp_ph108_F9
zmp_ph108_F11
zmp_ph108_H2
zmp_ph108_H5
zmp_ph108_B2
zmp_ph108_B6
zmp_ph108_B7
zmp_ph108_C4
zmp_ph108_C5
zmp_ph108_D4
zmp_ph97_G6
zmp_ph97_G9
zmp_ph97_H2
zmp_ph97_B10
zmp_ph97_C2
zmp_ph97_C4
zmp_ph97_B8
zmp_ph97_C1
zmp_ph97_D2
zmp_ph97_D5
zmp_ph111_G5
zmp_ph111_G11
zmp_ph111_H1
zmp_ph111_B1a
zmp_ph111_B5
zmp_ph111_B8
zmp_ph111_B6
zmp_ph111_B7
zmp_ph111_B9
zmp_ph111_B10
zmp_ph114_G4
zmp_ph114_G11
zmp_ph114_H1
zmp_ph114_H2
zmp_ph114_A9
zmp_ph114_A11
zmp_ph114_B2
zmp_ph114_B4
zmp_ph114_D8
zmp_ph114_E2

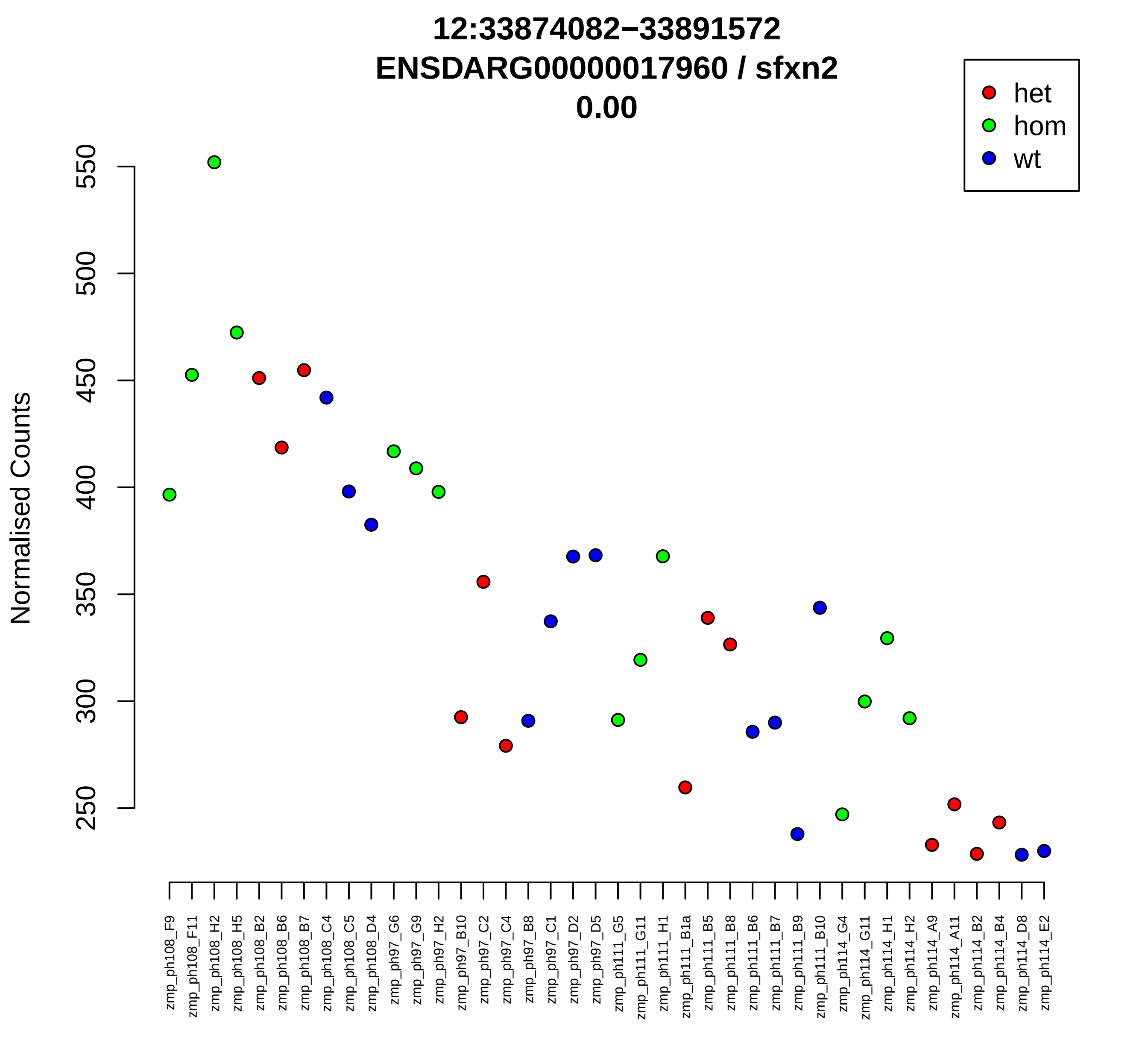
12:33874082−33891572
ENSDARG00000017960 / sfxn2
0.00
het
hom
wt
Normalised Counts
550
500
450
400
350
300
250
zmp_ph108_F9
zmp_ph108_F11
zmp_ph108_H2
zmp_ph108_H5
zmp_ph108_B2
zmp_ph108_B6
zmp_ph108_B7
zmp_ph108_C4
zmp_ph108_C5
zmp_ph108_D4
zmp_ph97_G6
zmp_ph97_G9
zmp_ph97_H2
zmp_ph97_B10
zmp_ph97_C2
zmp_ph97_C4
zmp_ph97_B8
zmp_ph97_C1
zmp_ph97_D2
zmp_ph97_D5
zmp_ph111_G5
zmp_ph111_G11
zmp_ph111_H1
zmp_ph111_B1a
zmp_ph111_B5
zmp_ph111_B8
zmp_ph111_B6
zmp_ph111_B7
zmp_ph111_B9
zmp_ph111_B10
zmp_ph114_G4
zmp_ph114_G11
zmp_ph114_H1
zmp_ph114_H2
zmp_ph114_A9
zmp_ph114_A11
zmp_ph114_B2
zmp_ph114_B4
zmp_ph114_D8
zmp_ph114_E2

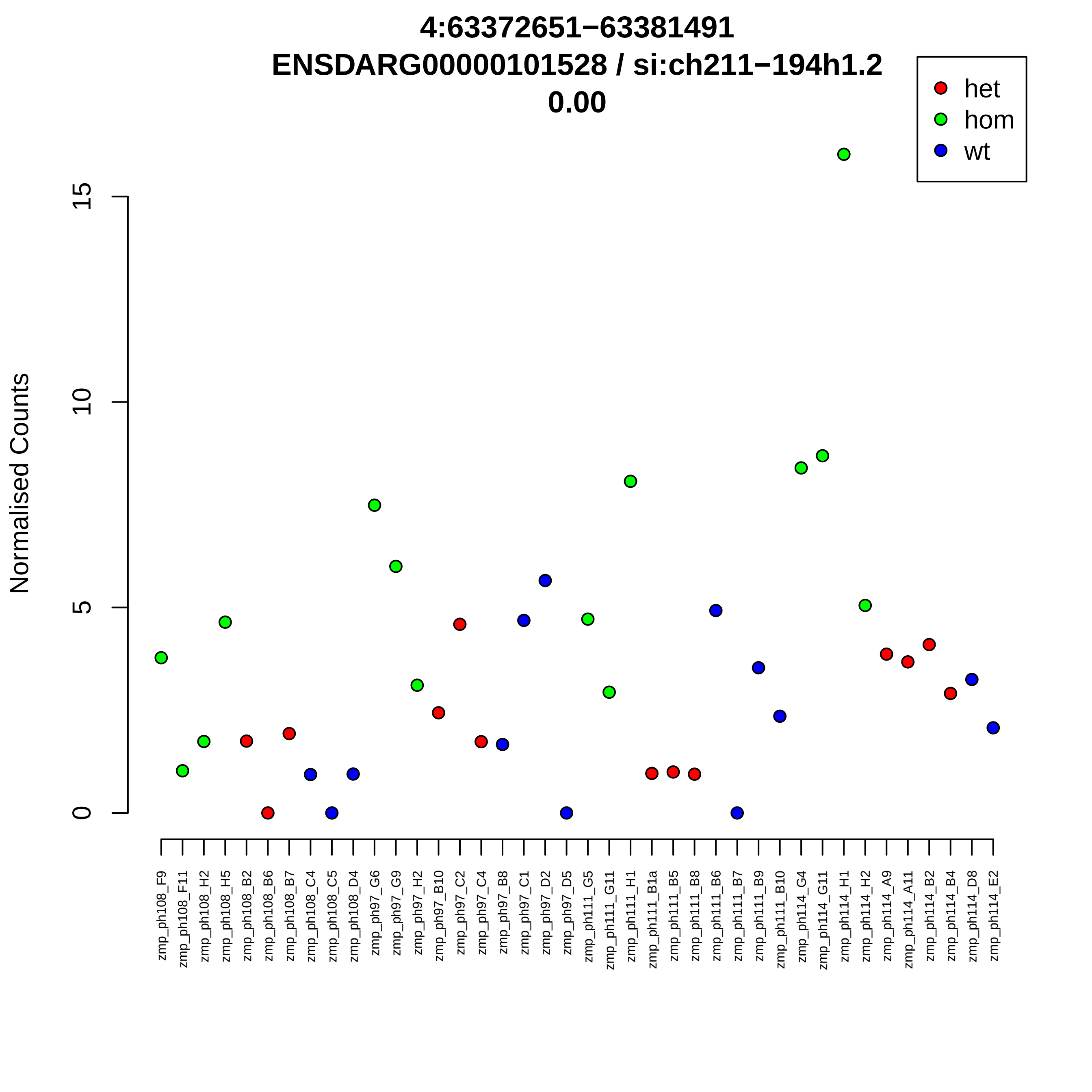
4:63372651−63381491
ENSDARG00000101528 / si:ch211−194h1.2
0.00
het
hom
wt
Normalised Counts
0
5
10
15
zmp_ph108_F9
zmp_ph108_F11
zmp_ph108_H2
zmp_ph108_H5
zmp_ph108_B2
zmp_ph108_B6
zmp_ph108_B7
zmp_ph108_C4
zmp_ph108_C5
zmp_ph108_D4
zmp_ph97_G6
zmp_ph97_G9
zmp_ph97_H2
zmp_ph97_B10
zmp_ph97_C2
zmp_ph97_C4
zmp_ph97_B8
zmp_ph97_C1
zmp_ph97_D2
zmp_ph97_D5
zmp_ph111_G5
zmp_ph111_G11
zmp_ph111_H1
zmp_ph111_B1a
zmp_ph111_B5
zmp_ph111_B8
zmp_ph111_B6
zmp_ph111_B7
zmp_ph111_B9
zmp_ph111_B10
zmp_ph114_G4
zmp_ph114_G11
zmp_ph114_H1
zmp_ph114_H2
zmp_ph114_A9
zmp_ph114_A11
zmp_ph114_B2
zmp_ph114_B4
zmp_ph114_D8
zmp_ph114_E2

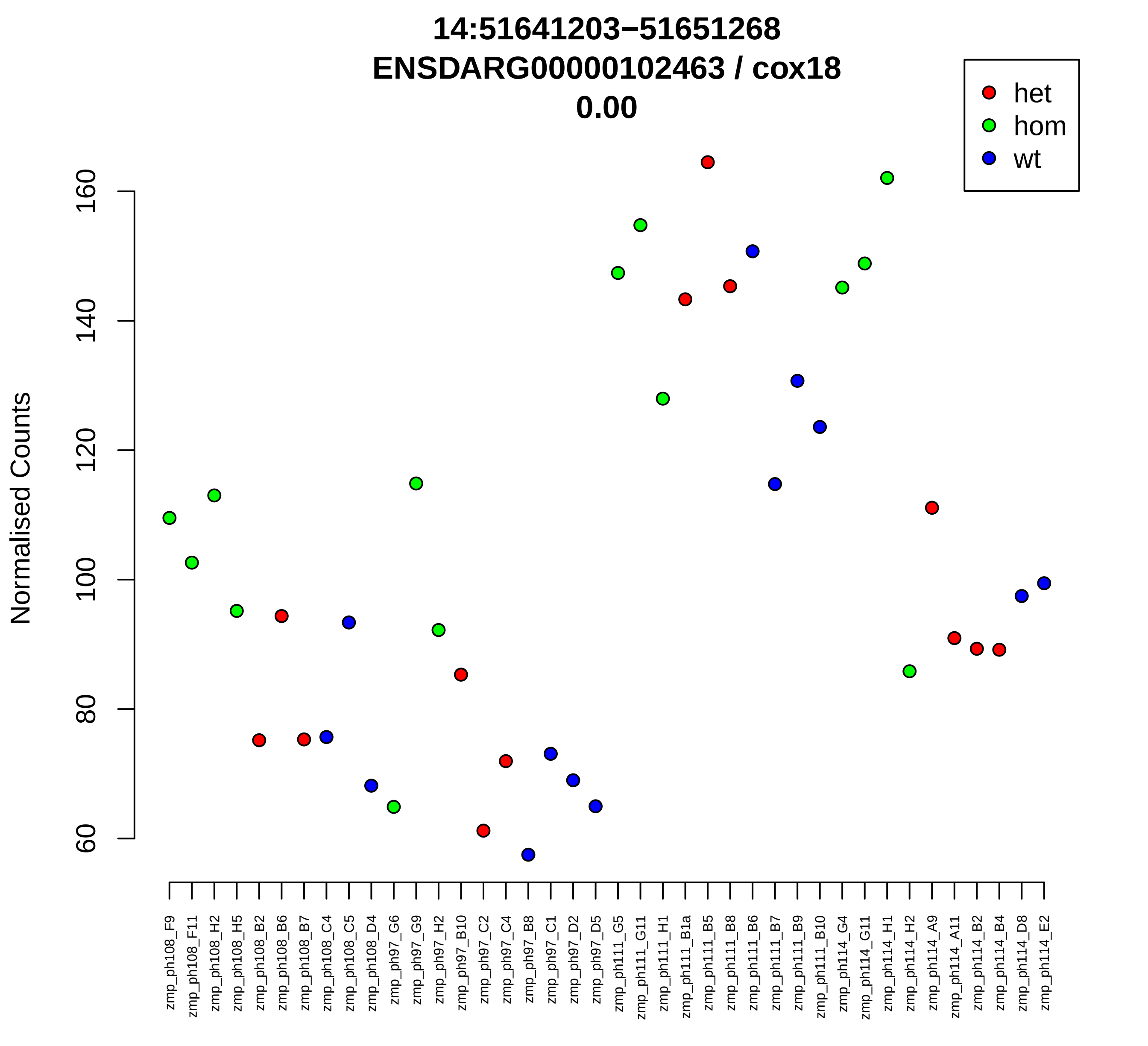
14:51641203−51651268
ENSDARG00000102463 / cox18
0.00
het
hom
wt
Normalised Counts
60
80
100
120
140
160
zmp_ph108_F9
zmp_ph108_F11
zmp_ph108_H2
zmp_ph108_H5
zmp_ph108_B2
zmp_ph108_B6
zmp_ph108_B7
zmp_ph108_C4
zmp_ph108_C5
zmp_ph108_D4
zmp_ph97_G6
zmp_ph97_G9
zmp_ph97_H2
zmp_ph97_B10
zmp_ph97_C2
zmp_ph97_C4
zmp_ph97_B8
zmp_ph97_C1
zmp_ph97_D2
zmp_ph97_D5
zmp_ph111_G5
zmp_ph111_G11
zmp_ph111_H1
zmp_ph111_B1a
zmp_ph111_B5
zmp_ph111_B8
zmp_ph111_B6
zmp_ph111_B7
zmp_ph111_B9
zmp_ph111_B10
zmp_ph114_G4
zmp_ph114_G11
zmp_ph114_H1
zmp_ph114_H2
zmp_ph114_A9
zmp_ph114_A11
zmp_ph114_B2
zmp_ph114_B4
zmp_ph114_D8
zmp_ph114_E2

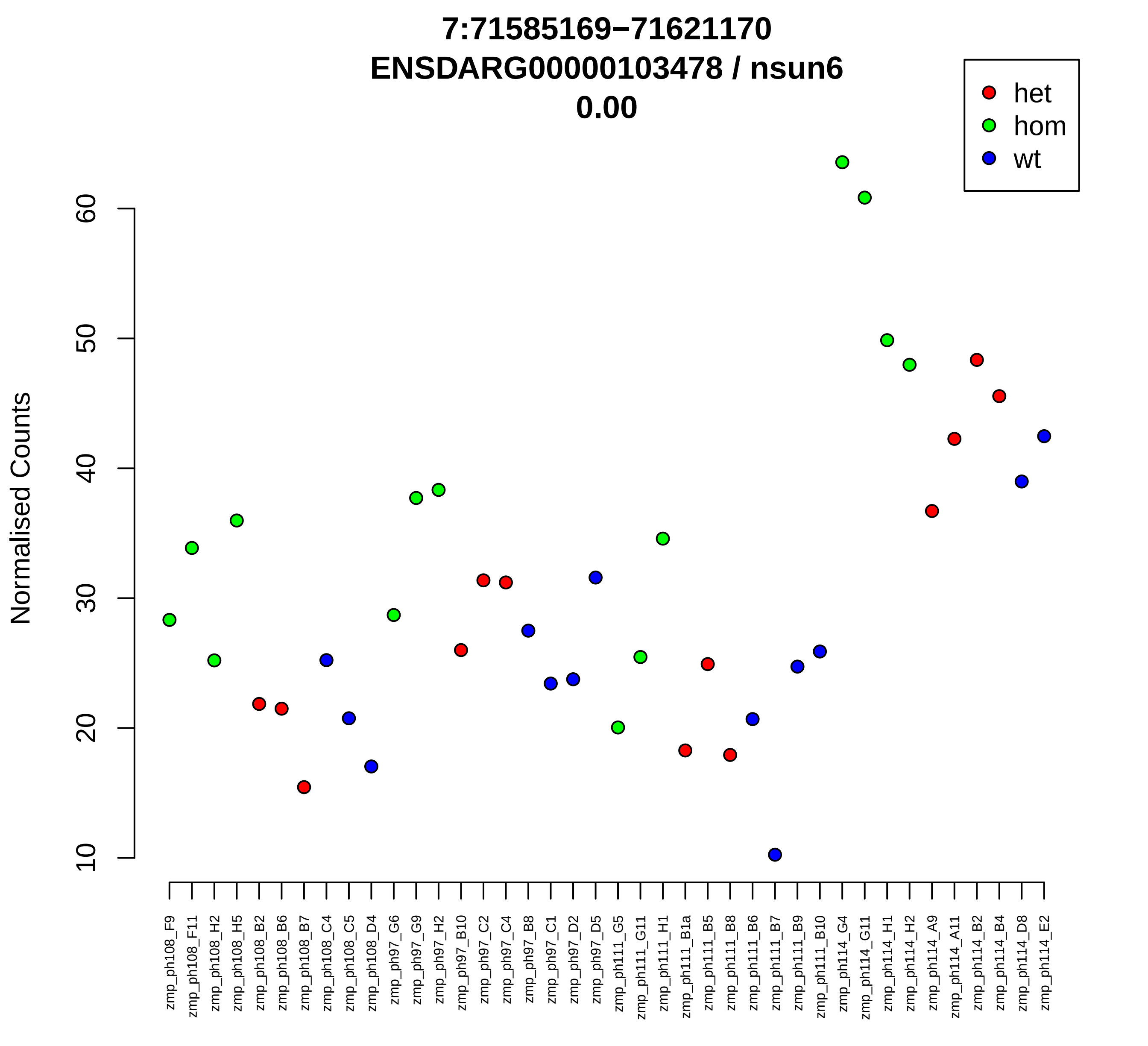
7:71585169−71621170
ENSDARG00000103478 / nsun6
0.00
het
hom
wt
Normalised Counts
10
20
30
40
50
60
zmp_ph108_F9
zmp_ph108_F11
zmp_ph108_H2
zmp_ph108_H5
zmp_ph108_B2
zmp_ph108_B6
zmp_ph108_B7
zmp_ph108_C4
zmp_ph108_C5
zmp_ph108_D4
zmp_ph97_G6
zmp_ph97_G9
zmp_ph97_H2
zmp_ph97_B10
zmp_ph97_C2
zmp_ph97_C4
zmp_ph97_B8
zmp_ph97_C1
zmp_ph97_D2
zmp_ph97_D5
zmp_ph111_G5
zmp_ph111_G11
zmp_ph111_H1
zmp_ph111_B1a
zmp_ph111_B5
zmp_ph111_B8
zmp_ph111_B6
zmp_ph111_B7
zmp_ph111_B9
zmp_ph111_B10
zmp_ph114_G4
zmp_ph114_G11
zmp_ph114_H1
zmp_ph114_H2
zmp_ph114_A9
zmp_ph114_A11
zmp_ph114_B2
zmp_ph114_B4
zmp_ph114_D8
zmp_ph114_E2

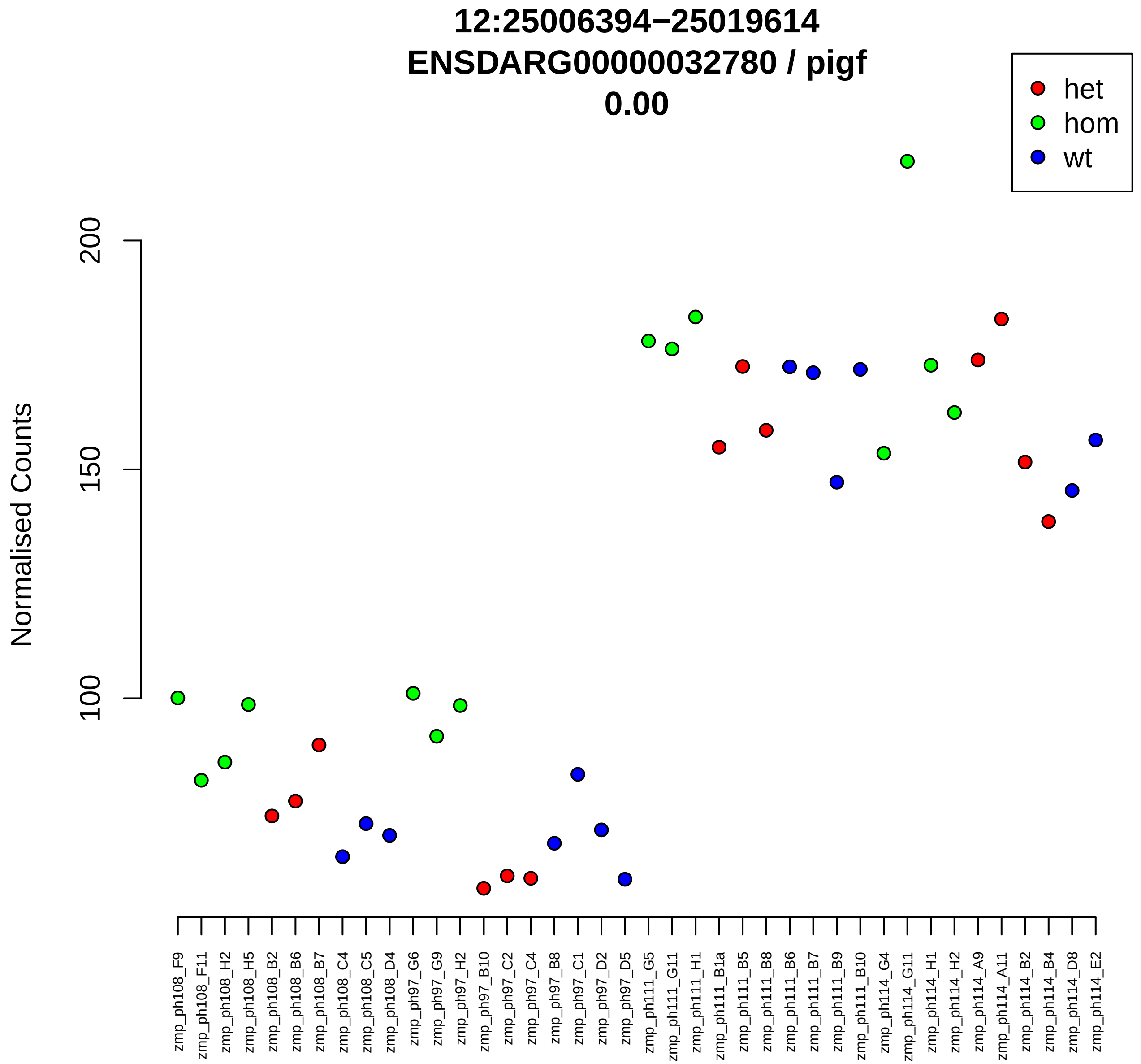

12:25006394−25019614
ENSDARG00000032780 / pigf
0.00
het
hom
wt
Normalised Counts
100
150
200
zmp_ph108_F9
zmp_ph108_F11
zmp_ph108_H2
zmp_ph108_H5
zmp_ph108_B2
zmp_ph108_B6
zmp_ph108_B7
zmp_ph108_C4
zmp_ph108_C5
zmp_ph108_D4
zmp_ph97_G6
zmp_ph97_G9
zmp_ph97_H2
zmp_ph97_B10
zmp_ph97_C2
zmp_ph97_C4
zmp_ph97_B8
zmp_ph97_C1
zmp_ph97_D2
zmp_ph97_D5
zmp_ph111_G5
zmp_ph111_G11
zmp_ph111_H1
zmp_ph111_B1a
zmp_ph111_B5
zmp_ph111_B8
zmp_ph111_B6
zmp_ph111_B7
zmp_ph111_B9
zmp_ph111_B10
zmp_ph114_G4
zmp_ph114_G11
zmp_ph114_H1
zmp_ph114_H2
zmp_ph114_A9
zmp_ph114_A11
zmp_ph114_B2
zmp_ph114_B4
zmp_ph114_D8
zmp_ph114_E2

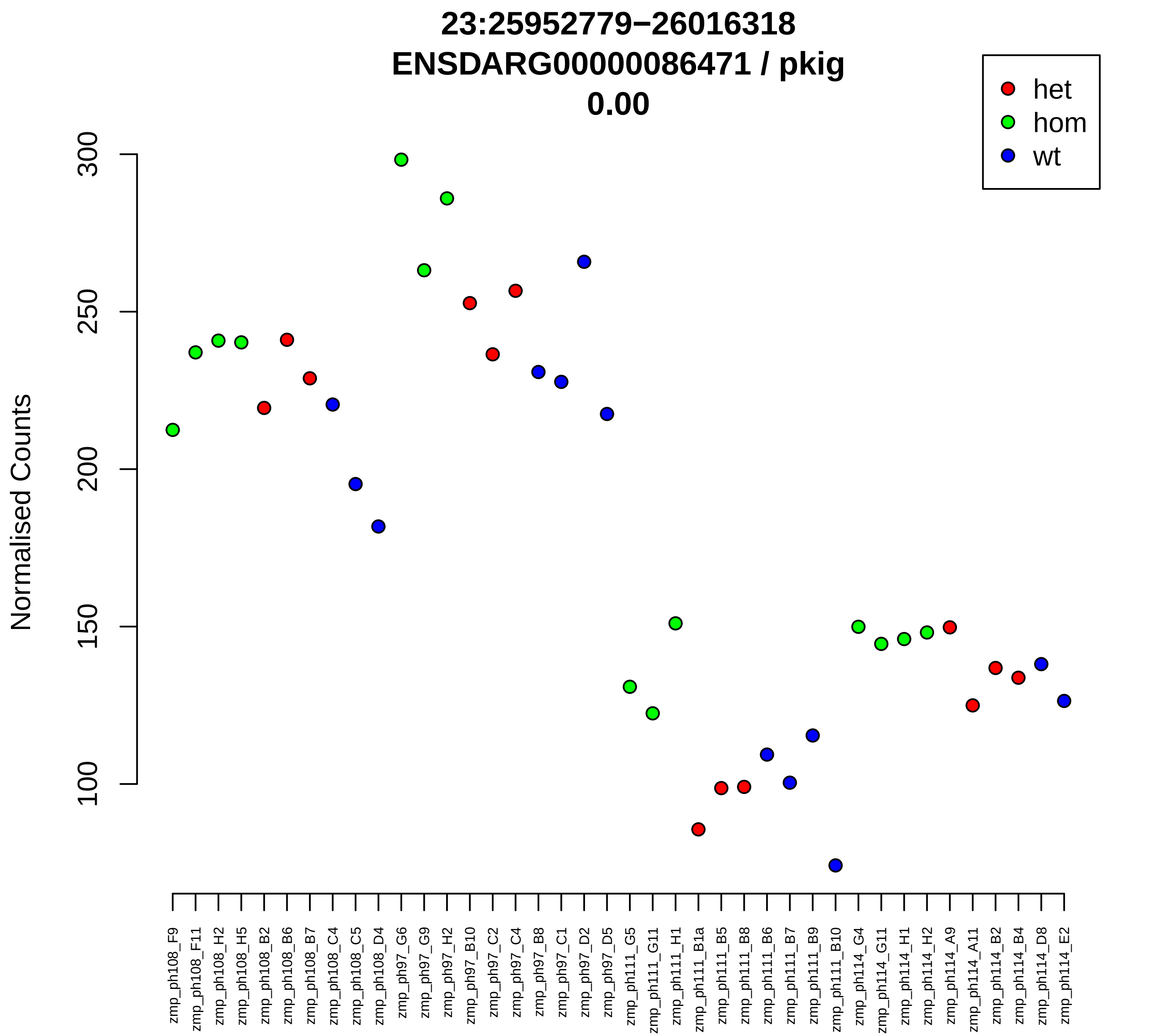

23:25952779−26016318
ENSDARG00000086471 / pkig
0.00
het
hom
wt
Normalised Counts
300
250
200
150
100
zmp_ph108_F9
zmp_ph108_F11
zmp_ph108_H2
zmp_ph108_H5
zmp_ph108_B2
zmp_ph108_B6
zmp_ph108_B7
zmp_ph108_C4
zmp_ph108_C5
zmp_ph108_D4
zmp_ph97_G6
zmp_ph97_G9
zmp_ph97_H2
zmp_ph97_B10
zmp_ph97_C2
zmp_ph97_C4
zmp_ph97_B8
zmp_ph97_C1
zmp_ph97_D2
zmp_ph97_D5
zmp_ph111_G5
zmp_ph111_G11
zmp_ph111_H1
zmp_ph111_B1a
zmp_ph111_B5
zmp_ph111_B8
zmp_ph111_B6
zmp_ph111_B7
zmp_ph111_B9
zmp_ph111_B10
zmp_ph114_G4
zmp_ph114_G11
zmp_ph114_H1
zmp_ph114_H2
zmp_ph114_A9
zmp_ph114_A11
zmp_ph114_B2
zmp_ph114_B4
zmp_ph114_D8
zmp_ph114_E2

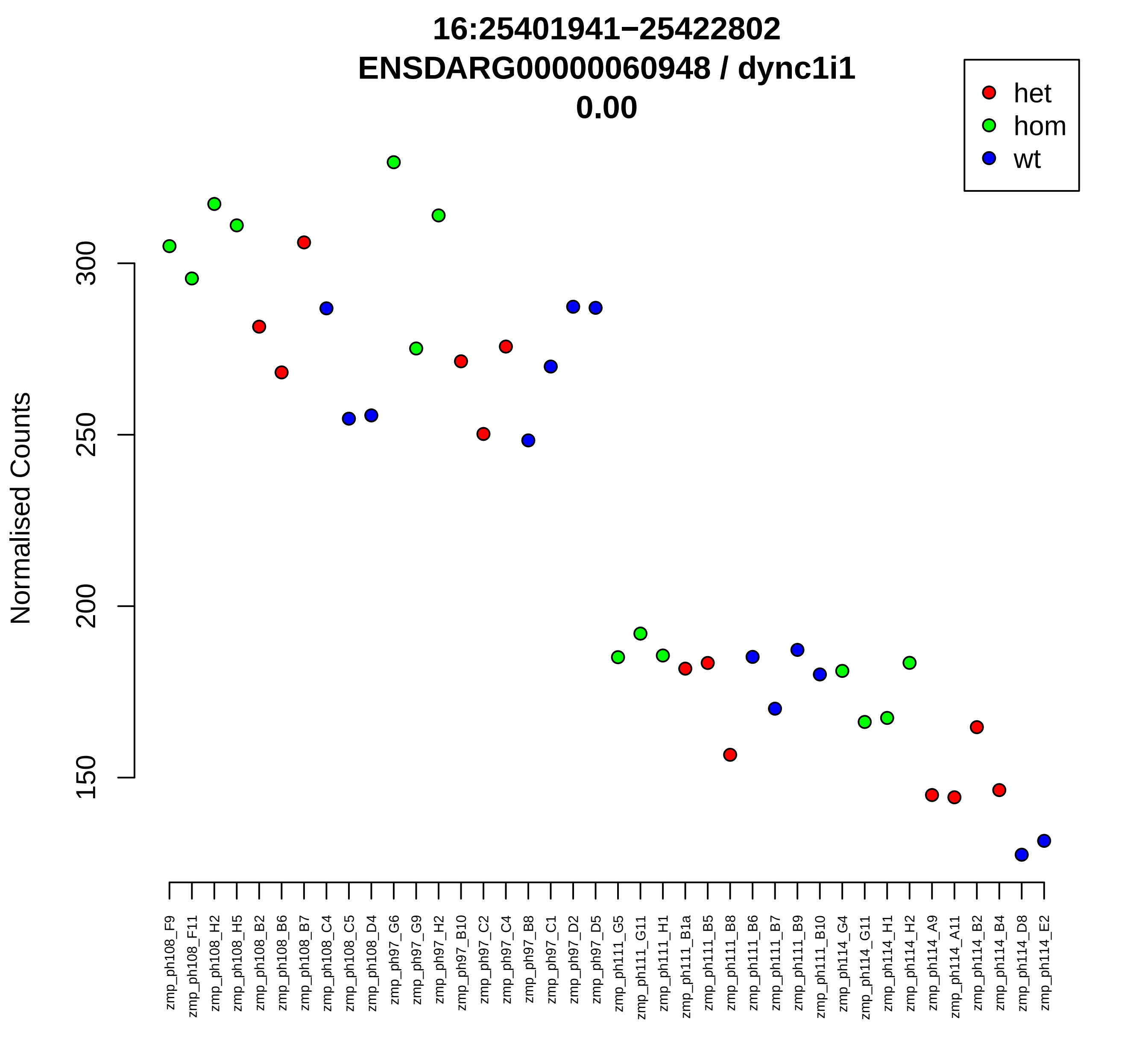

16:25401941−25422802
ENSDARG00000060948 / dync1i1
0.00
het
hom
wt
Normalised Counts
150
200
250
300
zmp_ph108_F9
zmp_ph108_F11
zmp_ph108_H2
zmp_ph108_H5
zmp_ph108_B2
zmp_ph108_B6
zmp_ph108_B7
zmp_ph108_C4
zmp_ph108_C5
zmp_ph108_D4
zmp_ph97_G6
zmp_ph97_G9
zmp_ph97_H2
zmp_ph97_B10
zmp_ph97_C2
zmp_ph97_C4
zmp_ph97_B8
zmp_ph97_C1
zmp_ph97_D2
zmp_ph97_D5
zmp_ph111_G5
zmp_ph111_G11
zmp_ph111_H1
zmp_ph111_B1a
zmp_ph111_B5
zmp_ph111_B8
zmp_ph111_B6
zmp_ph111_B7
zmp_ph111_B9
zmp_ph111_B10
zmp_ph114_G4
zmp_ph114_G11
zmp_ph114_H1
zmp_ph114_H2
zmp_ph114_A9
zmp_ph114_A11
zmp_ph114_B2
zmp_ph114_B4
zmp_ph114_D8
zmp_ph114_E2

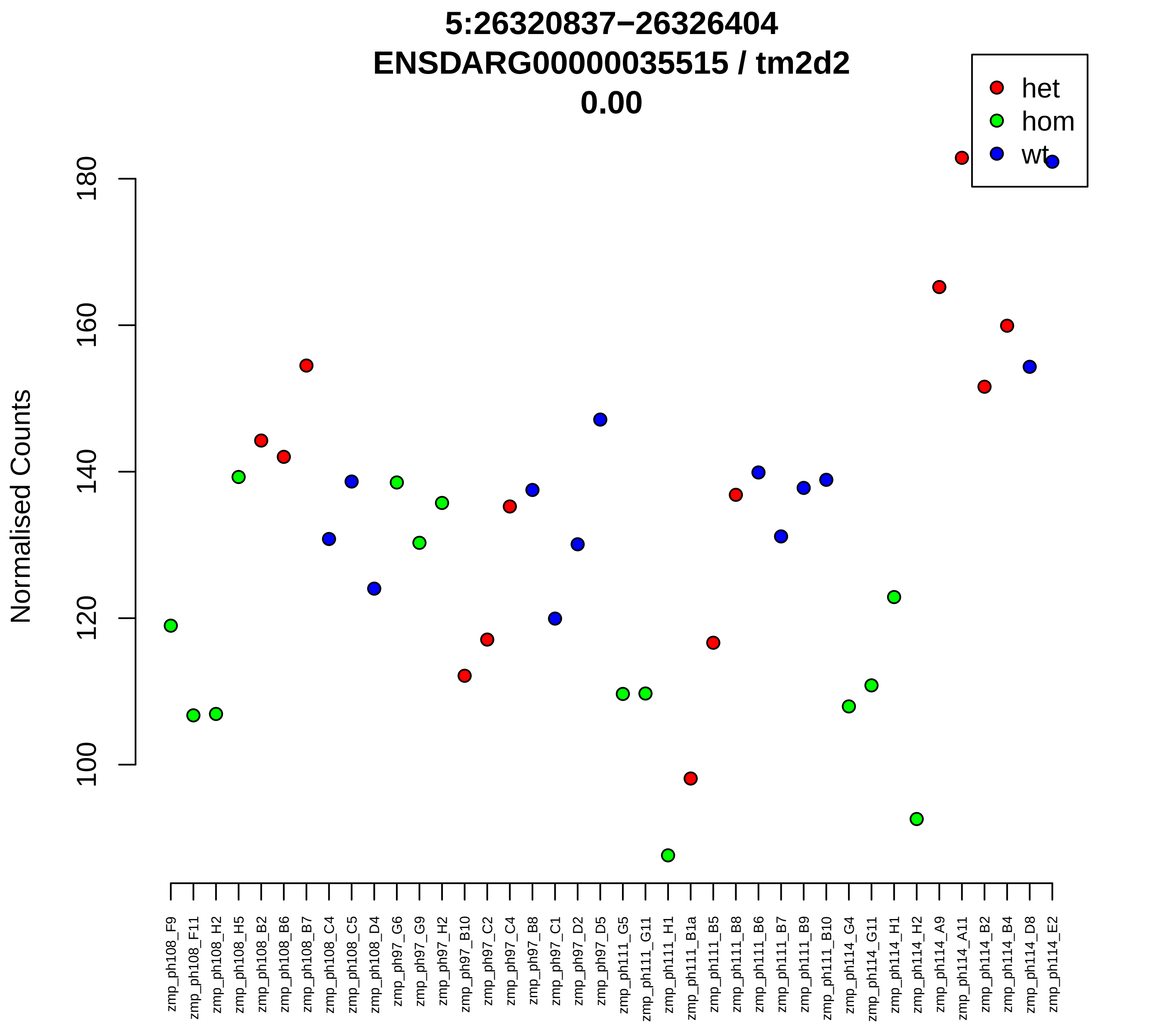
5:26320837−26326404
ENSDARG00000035515 / tm2d2
0.00
het
hom
wt
Normalised Counts
100
120
140
160
180
zmp_ph108_F9
zmp_ph108_F11
zmp_ph108_H2
zmp_ph108_H5
zmp_ph108_B2
zmp_ph108_B6
zmp_ph108_B7
zmp_ph108_C4
zmp_ph108_C5
zmp_ph108_D4
zmp_ph97_G6
zmp_ph97_G9
zmp_ph97_H2
zmp_ph97_B10
zmp_ph97_C2
zmp_ph97_C4
zmp_ph97_B8
zmp_ph97_C1
zmp_ph97_D2
zmp_ph97_D5
zmp_ph111_G5
zmp_ph111_G11
zmp_ph111_H1
zmp_ph111_B1a
zmp_ph111_B5
zmp_ph111_B8
zmp_ph111_B6
zmp_ph111_B7
zmp_ph111_B9
zmp_ph111_B10
zmp_ph114_G4
zmp_ph114_G11
zmp_ph114_H1
zmp_ph114_H2
zmp_ph114_A9
zmp_ph114_A11
zmp_ph114_B2
zmp_ph114_B4
zmp_ph114_D8
zmp_ph114_E2

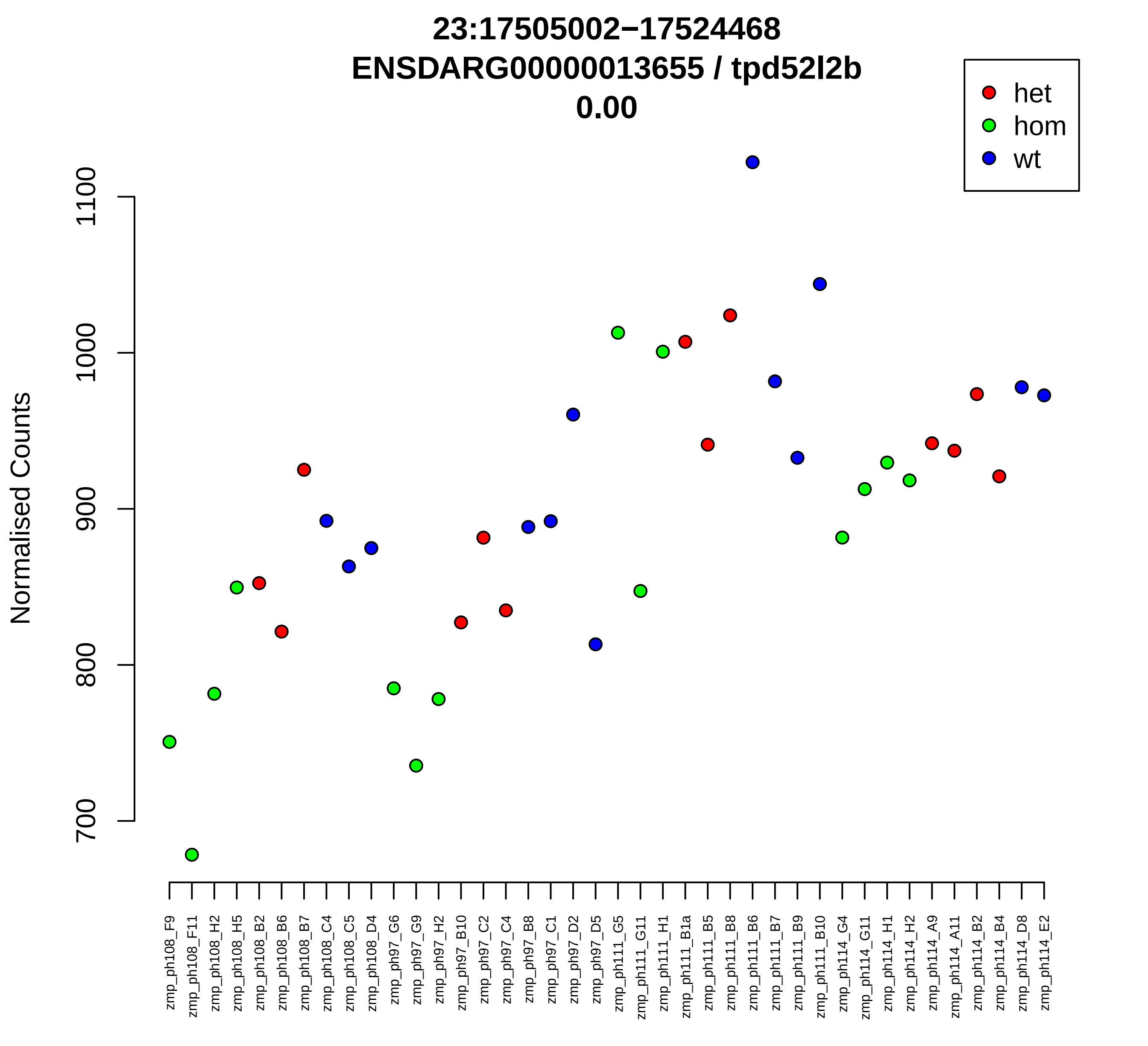
23:17505002−17524468
ENSDARG00000013655 / tpd52l2b
0.00
het
hom
wt
Normalised Counts
700
800
900
1000
1100
zmp_ph108_F9
zmp_ph108_F11
zmp_ph108_H2
zmp_ph108_H5
zmp_ph108_B2
zmp_ph108_B6
zmp_ph108_B7
zmp_ph108_C4
zmp_ph108_C5
zmp_ph108_D4
zmp_ph97_G6
zmp_ph97_G9
zmp_ph97_H2
zmp_ph97_B10
zmp_ph97_C2
zmp_ph97_C4
zmp_ph97_B8
zmp_ph97_C1
zmp_ph97_D2
zmp_ph97_D5
zmp_ph111_G5
zmp_ph111_G11
zmp_ph111_H1
zmp_ph111_B1a
zmp_ph111_B5
zmp_ph111_B8
zmp_ph111_B6
zmp_ph111_B7
zmp_ph111_B9
zmp_ph111_B10
zmp_ph114_G4
zmp_ph114_G11
zmp_ph114_H1
zmp_ph114_H2
zmp_ph114_A9
zmp_ph114_A11
zmp_ph114_B2
zmp_ph114_B4
zmp_ph114_D8
zmp_ph114_E2

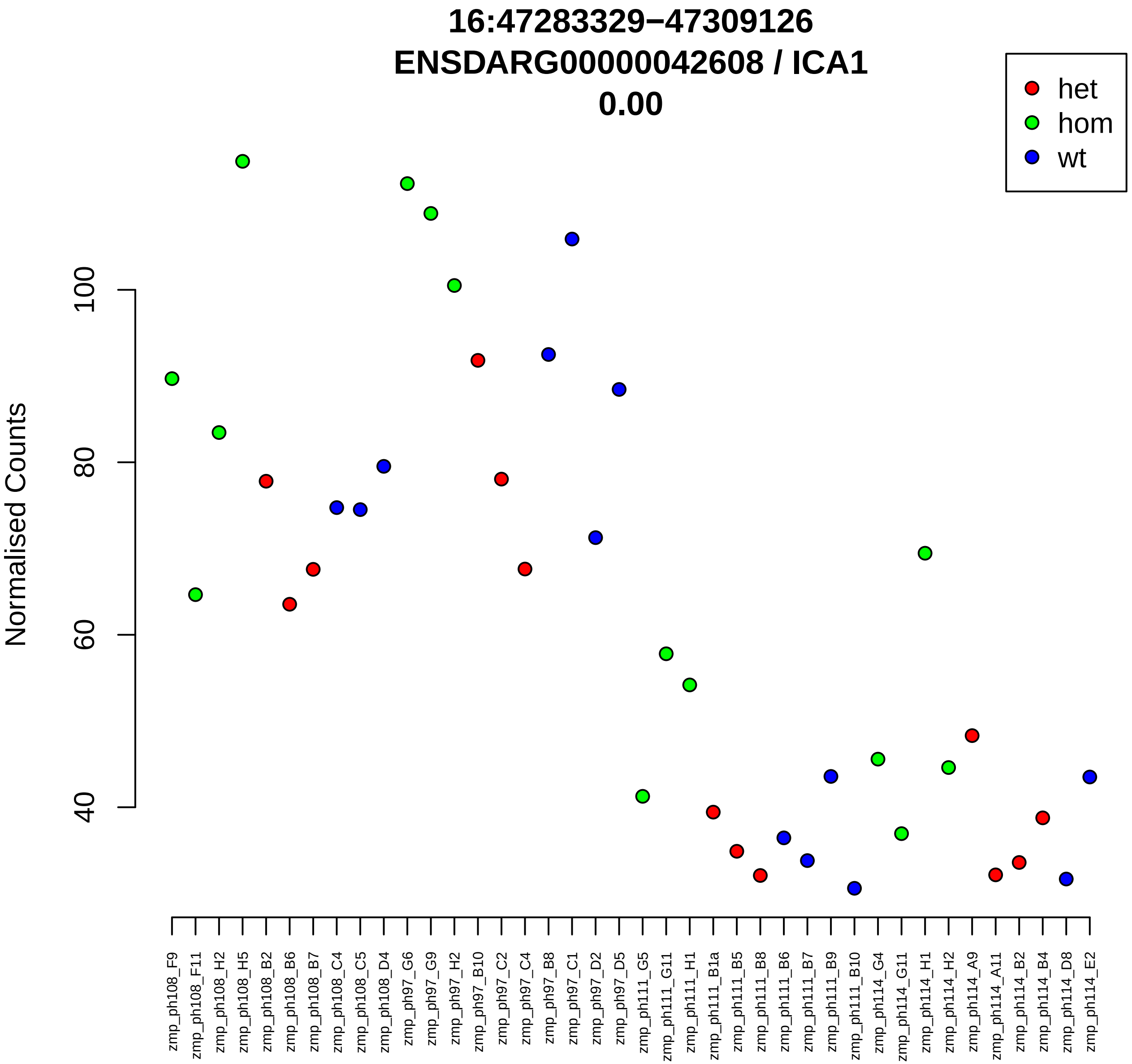
16:47283329−47309126
ENSDARG00000042608 / ICA1
0.00
het
hom
wt
Normalised Counts
40
60
80
100
zmp_ph108_F9
zmp_ph108_F11
zmp_ph108_H2
zmp_ph108_H5
zmp_ph108_B2
zmp_ph108_B6
zmp_ph108_B7
zmp_ph108_C4
zmp_ph108_C5
zmp_ph108_D4
zmp_ph97_G6
zmp_ph97_G9
zmp_ph97_H2
zmp_ph97_B10
zmp_ph97_C2
zmp_ph97_C4
zmp_ph97_B8
zmp_ph97_C1
zmp_ph97_D2
zmp_ph97_D5
zmp_ph111_G5
zmp_ph111_G11
zmp_ph111_H1
zmp_ph111_B1a
zmp_ph111_B5
zmp_ph111_B8
zmp_ph111_B6
zmp_ph111_B7
zmp_ph111_B9
zmp_ph111_B10
zmp_ph114_G4
zmp_ph114_G11
zmp_ph114_H1
zmp_ph114_H2
zmp_ph114_A9
zmp_ph114_A11
zmp_ph114_B2
zmp_ph114_B4
zmp_ph114_D8
zmp_ph114_E2

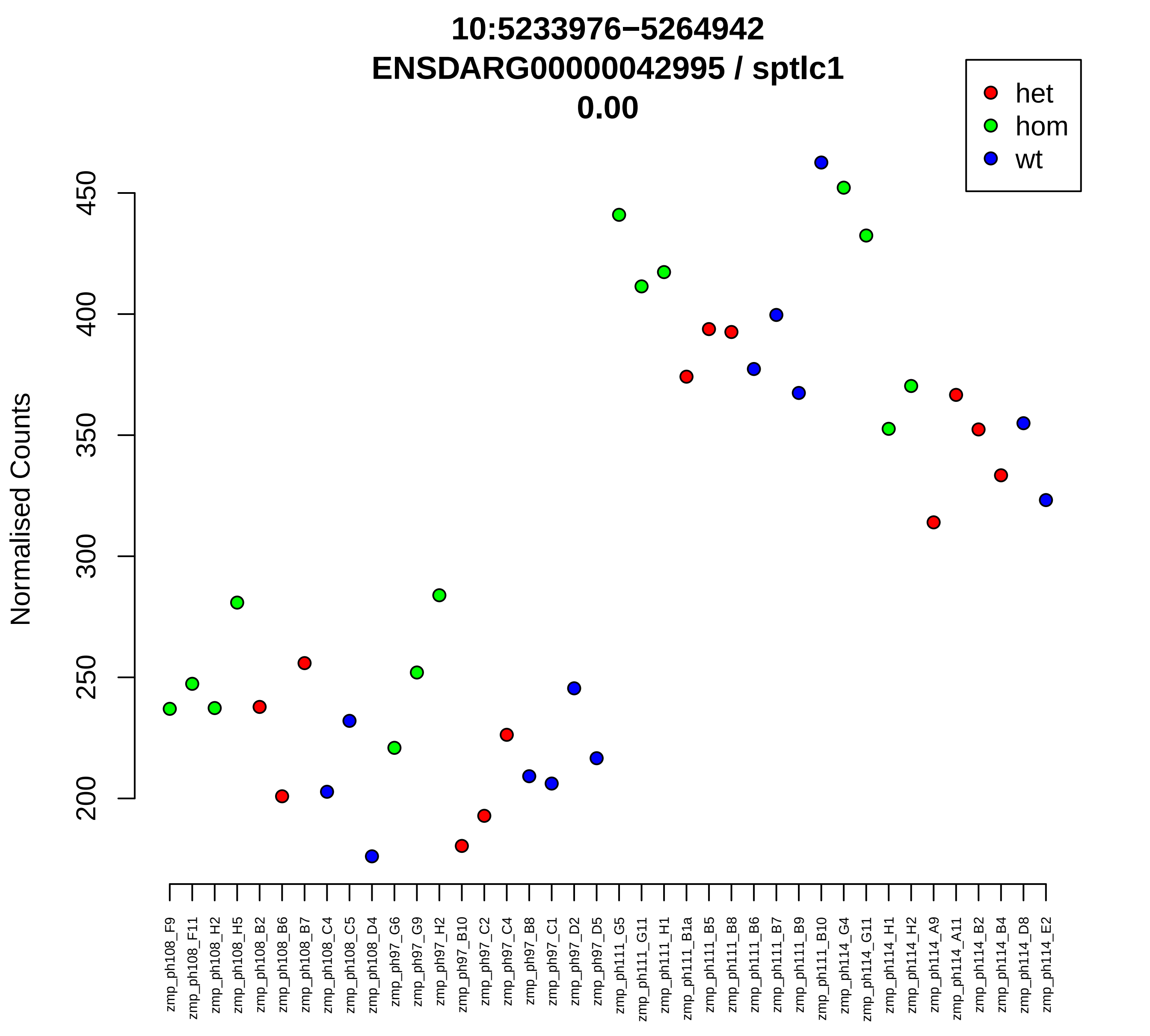

10:5233976−5264942
ENSDARG00000042995 / sptlc1
0.00
het
hom
wt
Normalised Counts
200
250
300
350
400
450
zmp_ph108_F9
zmp_ph108_F11
zmp_ph108_H2
zmp_ph108_H5
zmp_ph108_B2
zmp_ph108_B6
zmp_ph108_B7
zmp_ph108_C4
zmp_ph108_C5
zmp_ph108_D4
zmp_ph97_G6
zmp_ph97_G9
zmp_ph97_H2
zmp_ph97_B10
zmp_ph97_C2
zmp_ph97_C4
zmp_ph97_B8
zmp_ph97_C1
zmp_ph97_D2
zmp_ph97_D5
zmp_ph111_G5
zmp_ph111_G11
zmp_ph111_H1
zmp_ph111_B1a
zmp_ph111_B5
zmp_ph111_B8
zmp_ph111_B6
zmp_ph111_B7
zmp_ph111_B9
zmp_ph111_B10
zmp_ph114_G4
zmp_ph114_G11
zmp_ph114_H1
zmp_ph114_H2
zmp_ph114_A9
zmp_ph114_A11
zmp_ph114_B2
zmp_ph114_B4
zmp_ph114_D8
zmp_ph114_E2

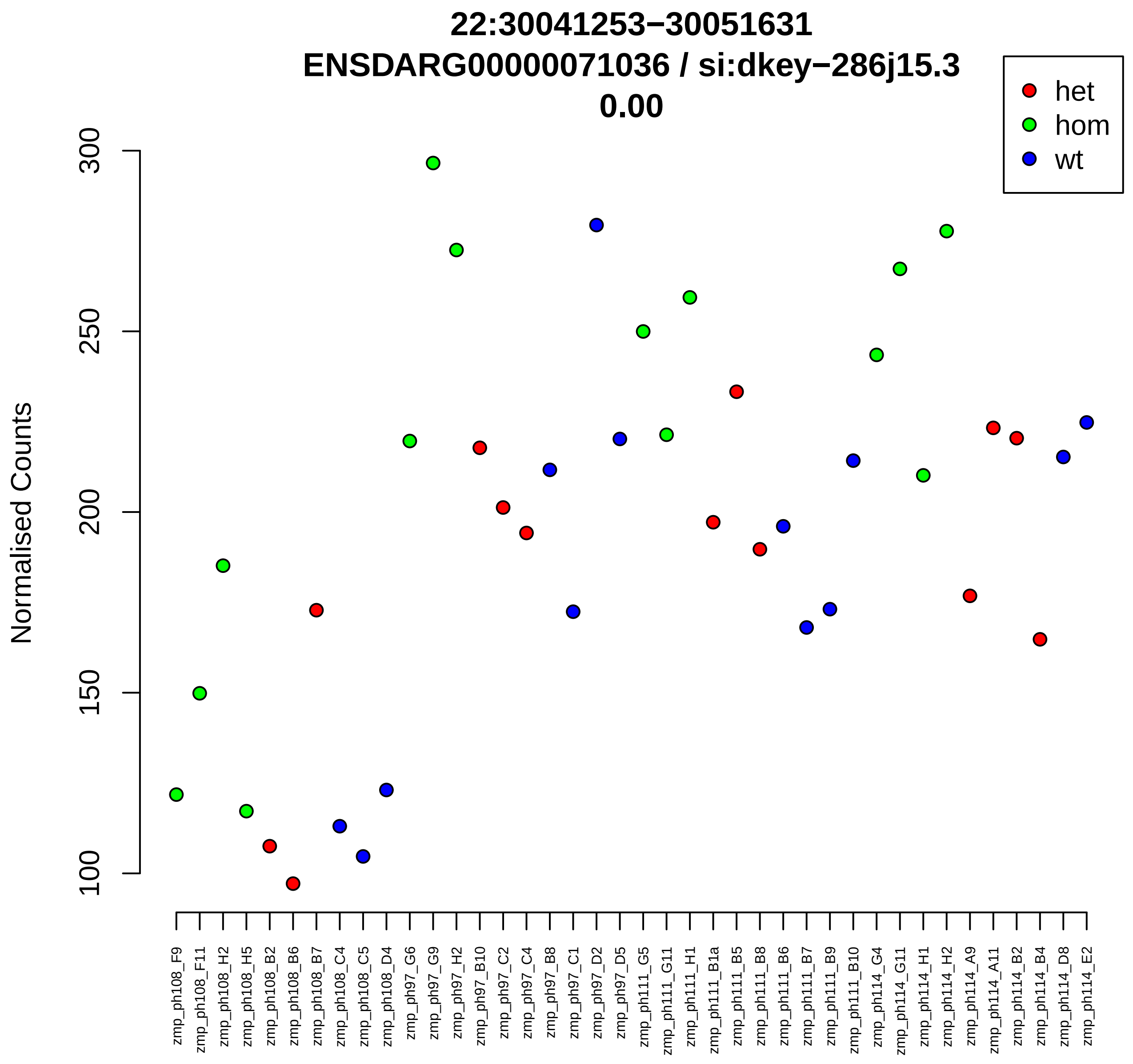

22:30041253−30051631
ENSDARG00000071036 / si:dkey−286j15.3
0.00
het
hom
wt
Normalised Counts
100
150
200
250
300
zmp_ph108_F9
zmp_ph108_F11
zmp_ph108_H2
zmp_ph108_H5
zmp_ph108_B2
zmp_ph108_B6
zmp_ph108_B7
zmp_ph108_C4
zmp_ph108_C5
zmp_ph108_D4
zmp_ph97_G6
zmp_ph97_G9
zmp_ph97_H2
zmp_ph97_B10
zmp_ph97_C2
zmp_ph97_C4
zmp_ph97_B8
zmp_ph97_C1
zmp_ph97_D2
zmp_ph97_D5
zmp_ph111_G5
zmp_ph111_G11
zmp_ph111_H1
zmp_ph111_B1a
zmp_ph111_B5
zmp_ph111_B8
zmp_ph111_B6
zmp_ph111_B7
zmp_ph111_B9
zmp_ph111_B10
zmp_ph114_G4
zmp_ph114_G11
zmp_ph114_H1
zmp_ph114_H2
zmp_ph114_A9
zmp_ph114_A11
zmp_ph114_B2
zmp_ph114_B4
zmp_ph114_D8
zmp_ph114_E2

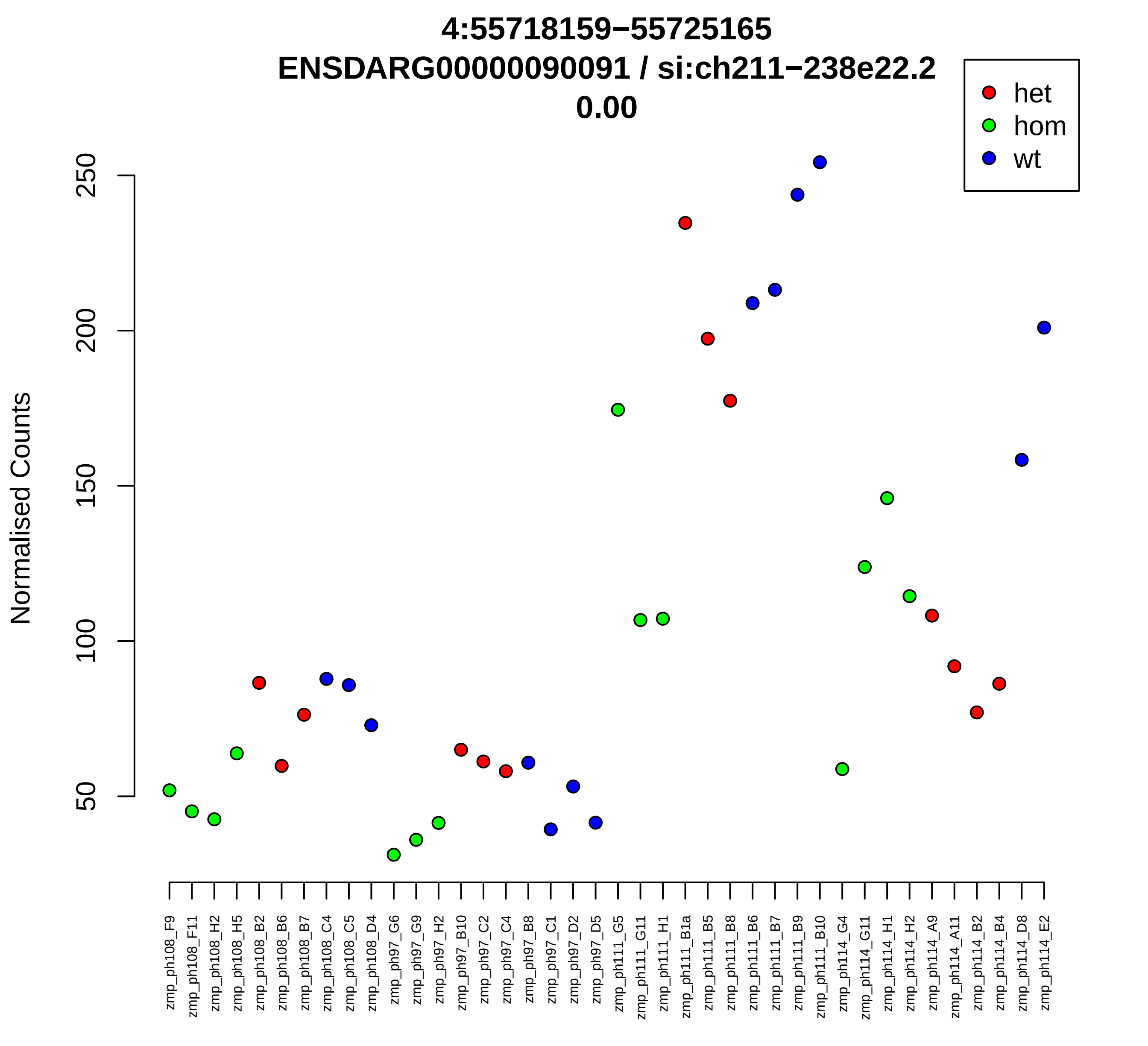

4:55718159−55725165
ENSDARG00000090091 / si:ch211−238e22.2
0.00
het
hom
wt
Normalised Counts
50
100
150
200
250
zmp_ph108_F9
zmp_ph108_F11
zmp_ph108_H2
zmp_ph108_H5
zmp_ph108_B2
zmp_ph108_B6
zmp_ph108_B7
zmp_ph108_C4
zmp_ph108_C5
zmp_ph108_D4
zmp_ph97_G6
zmp_ph97_G9
zmp_ph97_H2
zmp_ph97_B10
zmp_ph97_C2
zmp_ph97_C4
zmp_ph97_B8
zmp_ph97_C1
zmp_ph97_D2
zmp_ph97_D5
zmp_ph111_G5
zmp_ph111_G11
zmp_ph111_H1
zmp_ph111_B1a
zmp_ph111_B5
zmp_ph111_B8
zmp_ph111_B6
zmp_ph111_B7
zmp_ph111_B9
zmp_ph111_B10
zmp_ph114_G4
zmp_ph114_G11
zmp_ph114_H1
zmp_ph114_H2
zmp_ph114_A9
zmp_ph114_A11
zmp_ph114_B2
zmp_ph114_B4
zmp_ph114_D8
zmp_ph114_E2

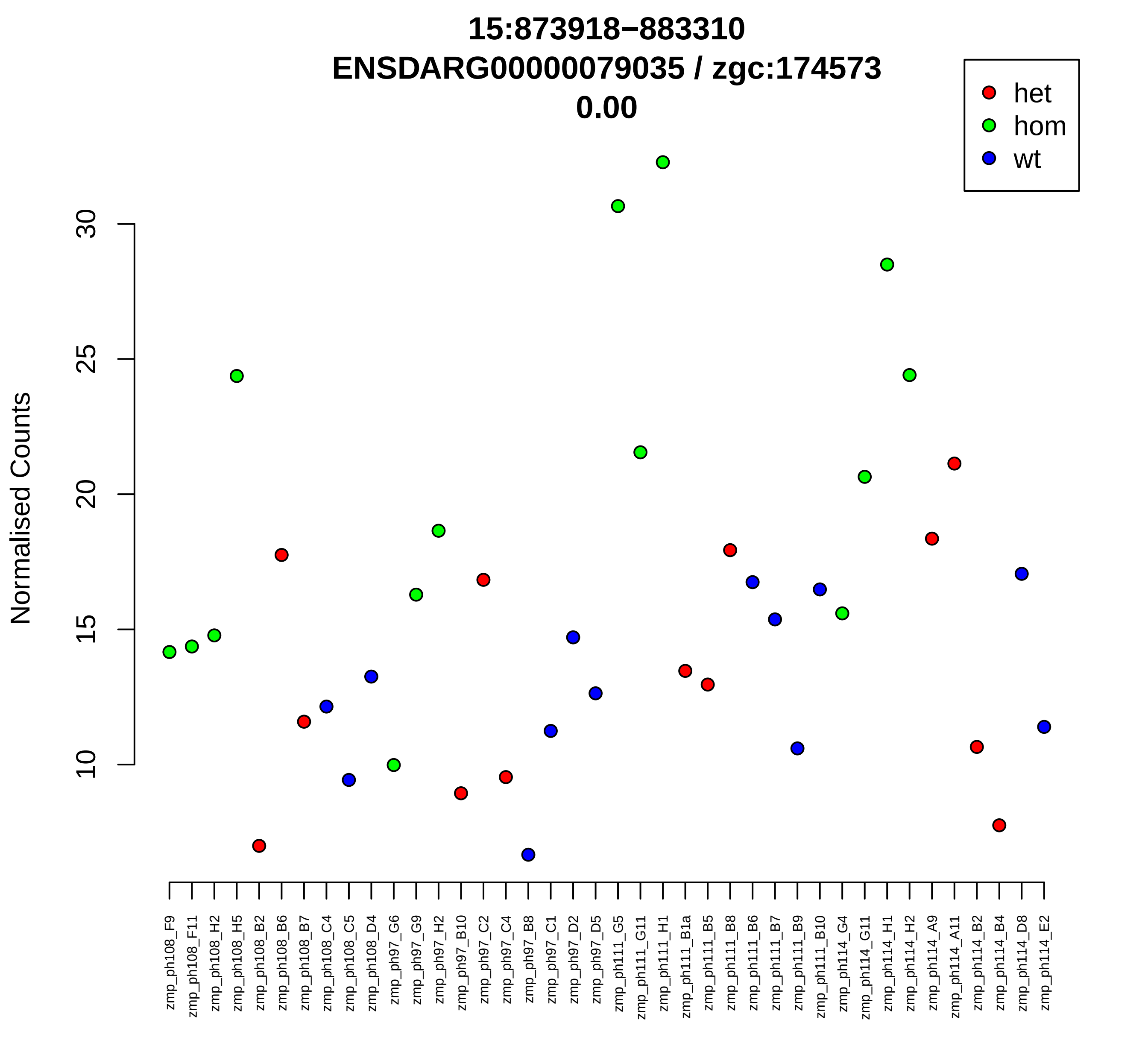

15:873918−883310
ENSDARG00000079035 / zgc:174573
0.00
het
hom
wt
Normalised Counts
10
15
20
25
30
zmp_ph108_F9
zmp_ph108_F11
zmp_ph108_H2
zmp_ph108_H5
zmp_ph108_B2
zmp_ph108_B6
zmp_ph108_B7
zmp_ph108_C4
zmp_ph108_C5
zmp_ph108_D4
zmp_ph97_G6
zmp_ph97_G9
zmp_ph97_H2
zmp_ph97_B10
zmp_ph97_C2
zmp_ph97_C4
zmp_ph97_B8
zmp_ph97_C1
zmp_ph97_D2
zmp_ph97_D5
zmp_ph111_G5
zmp_ph111_G11
zmp_ph111_H1
zmp_ph111_B1a
zmp_ph111_B5
zmp_ph111_B8
zmp_ph111_B6
zmp_ph111_B7
zmp_ph111_B9
zmp_ph111_B10
zmp_ph114_G4
zmp_ph114_G11
zmp_ph114_H1
zmp_ph114_H2
zmp_ph114_A9
zmp_ph114_A11
zmp_ph114_B2
zmp_ph114_B4
zmp_ph114_D8
zmp_ph114_E2

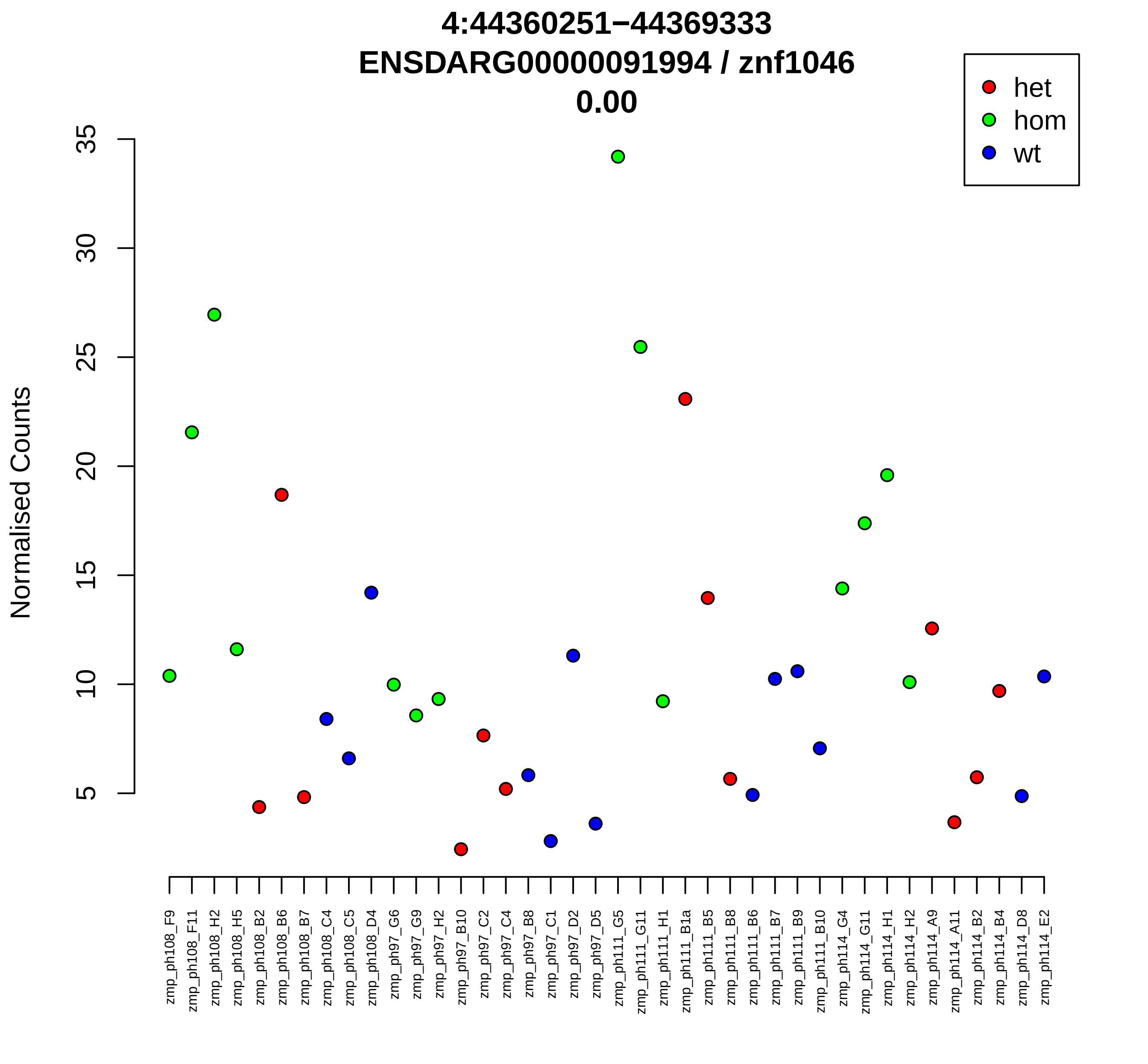

4:44360251−44369333
ENSDARG00000091994 / znf1046
0.00
het
hom
wt
Normalised Counts
5
10
15
20
25
30
35
zmp_ph108_F9
zmp_ph108_F11
zmp_ph108_H2
zmp_ph108_H5
zmp_ph108_B2
zmp_ph108_B6
zmp_ph108_B7
zmp_ph108_C4
zmp_ph108_C5
zmp_ph108_D4
zmp_ph97_G6
zmp_ph97_G9
zmp_ph97_H2
zmp_ph97_B10
zmp_ph97_C2
zmp_ph97_C4
zmp_ph97_B8
zmp_ph97_C1
zmp_ph97_D2
zmp_ph97_D5
zmp_ph111_G5
zmp_ph111_G11
zmp_ph111_H1
zmp_ph111_B1a
zmp_ph111_B5
zmp_ph111_B8
zmp_ph111_B6
zmp_ph111_B7
zmp_ph111_B9
zmp_ph111_B10
zmp_ph114_G4
zmp_ph114_G11
zmp_ph114_H1
zmp_ph114_H2
zmp_ph114_A9
zmp_ph114_A11
zmp_ph114_B2
zmp_ph114_B4
zmp_ph114_D8
zmp_ph114_E2

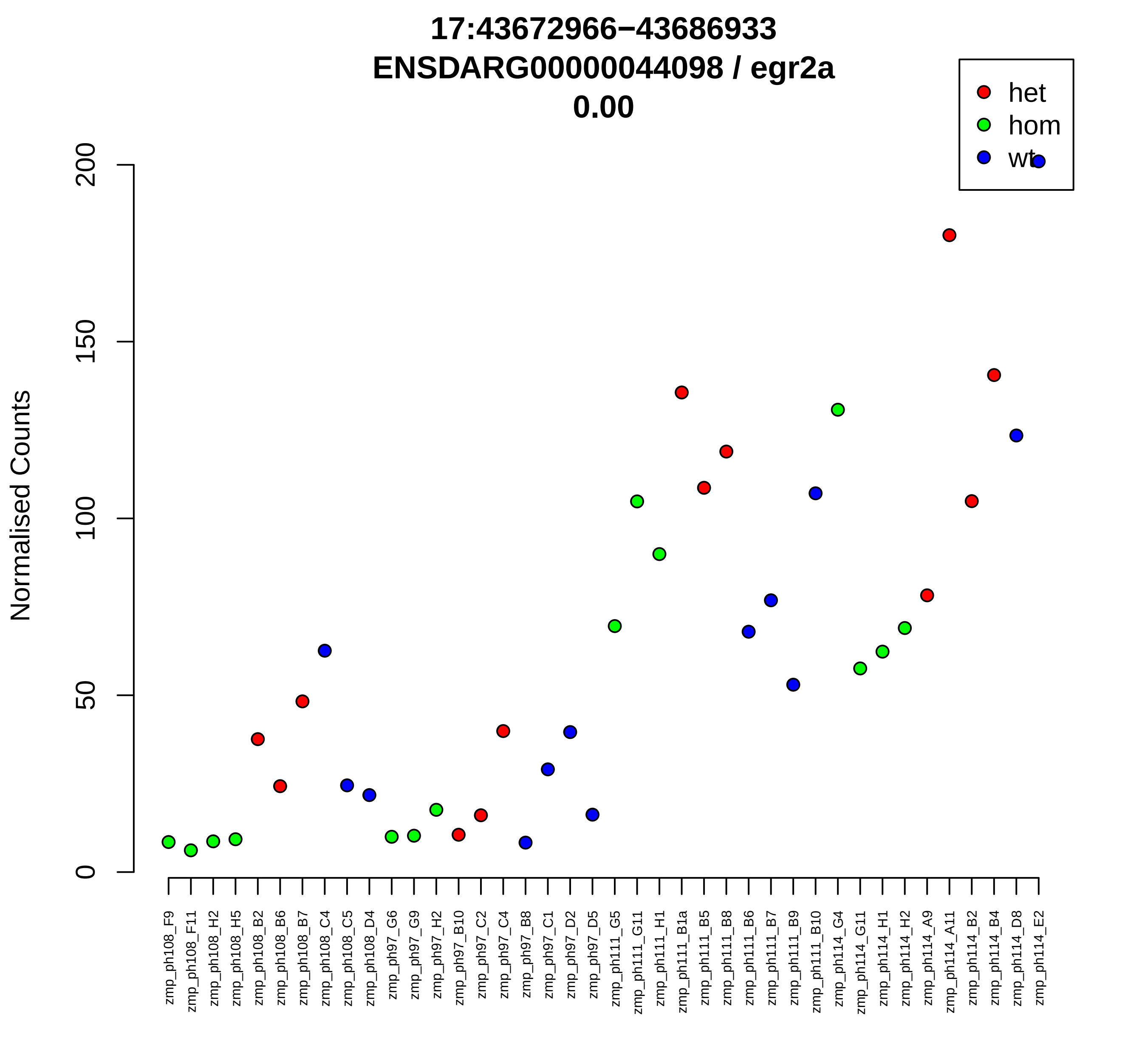

17:43672966−43686933
ENSDARG00000044098 / egr2a
0.00
het
hom
wt
Normalised Counts
0
50
100
150
200
zmp_ph108_F9
zmp_ph108_F11
zmp_ph108_H2
zmp_ph108_H5
zmp_ph108_B2
zmp_ph108_B6
zmp_ph108_B7
zmp_ph108_C4
zmp_ph108_C5
zmp_ph108_D4
zmp_ph97_G6
zmp_ph97_G9
zmp_ph97_H2
zmp_ph97_B10
zmp_ph97_C2
zmp_ph97_C4
zmp_ph97_B8
zmp_ph97_C1
zmp_ph97_D2
zmp_ph97_D5
zmp_ph111_G5
zmp_ph111_G11
zmp_ph111_H1
zmp_ph111_B1a
zmp_ph111_B5
zmp_ph111_B8
zmp_ph111_B6
zmp_ph111_B7
zmp_ph111_B9
zmp_ph111_B10
zmp_ph114_G4
zmp_ph114_G11
zmp_ph114_H1
zmp_ph114_H2
zmp_ph114_A9
zmp_ph114_A11
zmp_ph114_B2
zmp_ph114_B4
zmp_ph114_D8
zmp_ph114_E2

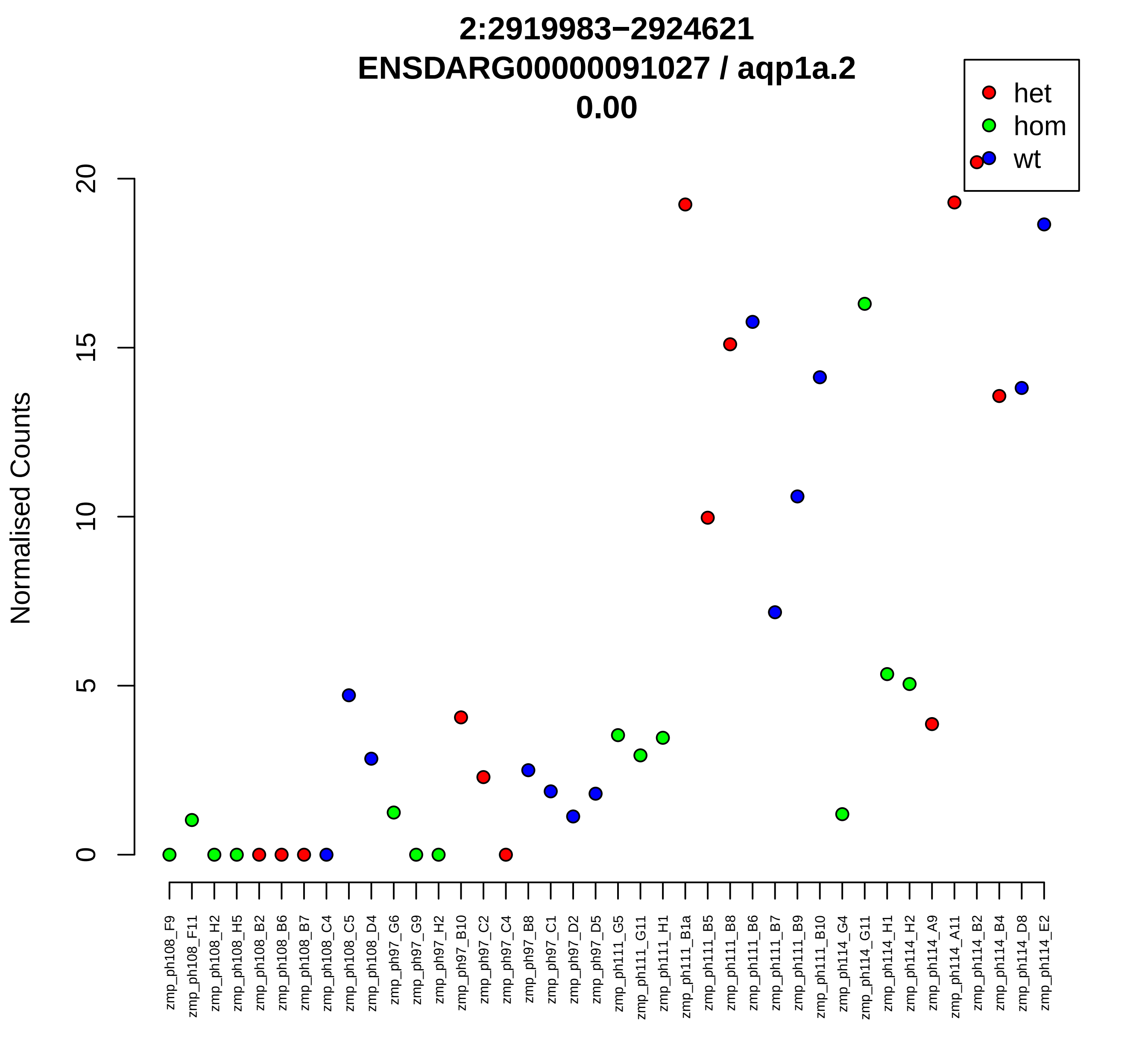

2:2919983−2924621
ENSDARG00000091027 / aqp1a.2
0.00
het
hom
wt
Normalised Counts
0
5
10
15
20
zmp_ph108_F9
zmp_ph108_F11
zmp_ph108_H2
zmp_ph108_H5
zmp_ph108_B2
zmp_ph108_B6
zmp_ph108_B7
zmp_ph108_C4
zmp_ph108_C5
zmp_ph108_D4
zmp_ph97_G6
zmp_ph97_G9
zmp_ph97_H2
zmp_ph97_B10
zmp_ph97_C2
zmp_ph97_C4
zmp_ph97_B8
zmp_ph97_C1
zmp_ph97_D2
zmp_ph97_D5
zmp_ph111_G5
zmp_ph111_G11
zmp_ph111_H1
zmp_ph111_B1a
zmp_ph111_B5
zmp_ph111_B8
zmp_ph111_B6
zmp_ph111_B7
zmp_ph111_B9
zmp_ph111_B10
zmp_ph114_G4
zmp_ph114_G11
zmp_ph114_H1
zmp_ph114_H2
zmp_ph114_A9
zmp_ph114_A11
zmp_ph114_B2
zmp_ph114_B4
zmp_ph114_D8
zmp_ph114_E2

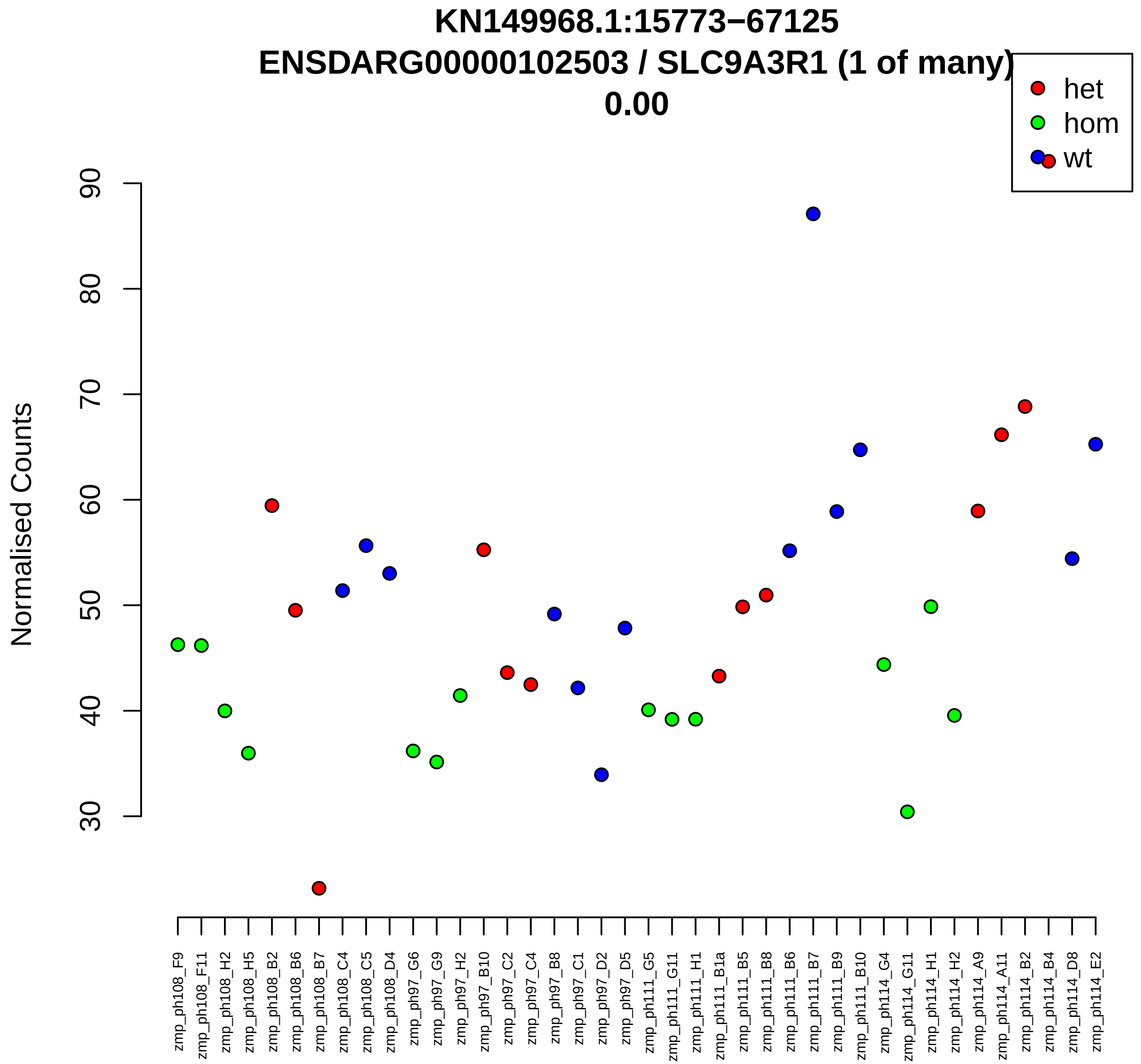
KN149968.1:15773−67125
ENSDARG00000102503 / SLC9A3R1 (1 of many)
0.00
het
hom
wt
Normalised Counts
30
40
50
60
70
80
90
zmp_ph108_F9
zmp_ph108_F11
zmp_ph108_H2
zmp_ph108_H5
zmp_ph108_B2
zmp_ph108_B6
zmp_ph108_B7
zmp_ph108_C4
zmp_ph108_C5
zmp_ph108_D4
zmp_ph97_G6
zmp_ph97_G9
zmp_ph97_H2
zmp_ph97_B10
zmp_ph97_C2
zmp_ph97_C4
zmp_ph97_B8
zmp_ph97_C1
zmp_ph97_D2
zmp_ph97_D5
zmp_ph111_G5
zmp_ph111_G11
zmp_ph111_H1
zmp_ph111_B1a
zmp_ph111_B5
zmp_ph111_B8
zmp_ph111_B6
zmp_ph111_B7
zmp_ph111_B9
zmp_ph111_B10
zmp_ph114_G4
zmp_ph114_G11
zmp_ph114_H1
zmp_ph114_H2
zmp_ph114_A9
zmp_ph114_A11
zmp_ph114_B2
zmp_ph114_B4
zmp_ph114_D8
zmp_ph114_E2

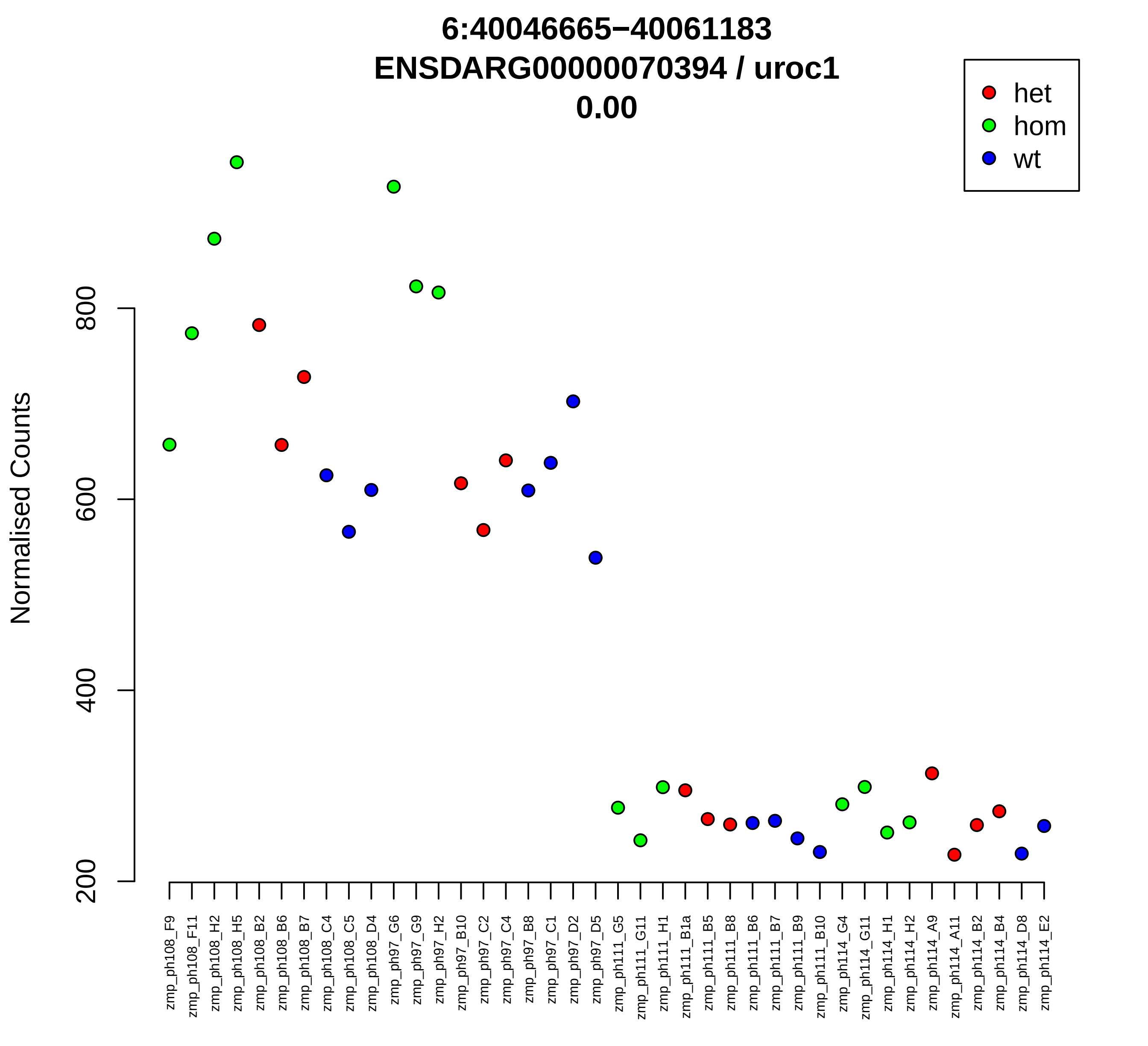

6:40046665−40061183
ENSDARG00000070394 / uroc1
0.00
het
hom
wt
Normalised Counts
200
400
600
800
zmp_ph108_F9
zmp_ph108_F11
zmp_ph108_H2
zmp_ph108_H5
zmp_ph108_B2
zmp_ph108_B6
zmp_ph108_B7
zmp_ph108_C4
zmp_ph108_C5
zmp_ph108_D4
zmp_ph97_G6
zmp_ph97_G9
zmp_ph97_H2
zmp_ph97_B10
zmp_ph97_C2
zmp_ph97_C4
zmp_ph97_B8
zmp_ph97_C1
zmp_ph97_D2
zmp_ph97_D5
zmp_ph111_G5
zmp_ph111_G11
zmp_ph111_H1
zmp_ph111_B1a
zmp_ph111_B5
zmp_ph111_B8
zmp_ph111_B6
zmp_ph111_B7
zmp_ph111_B9
zmp_ph111_B10
zmp_ph114_G4
zmp_ph114_G11
zmp_ph114_H1
zmp_ph114_H2
zmp_ph114_A9
zmp_ph114_A11
zmp_ph114_B2
zmp_ph114_B4
zmp_ph114_D8
zmp_ph114_E2

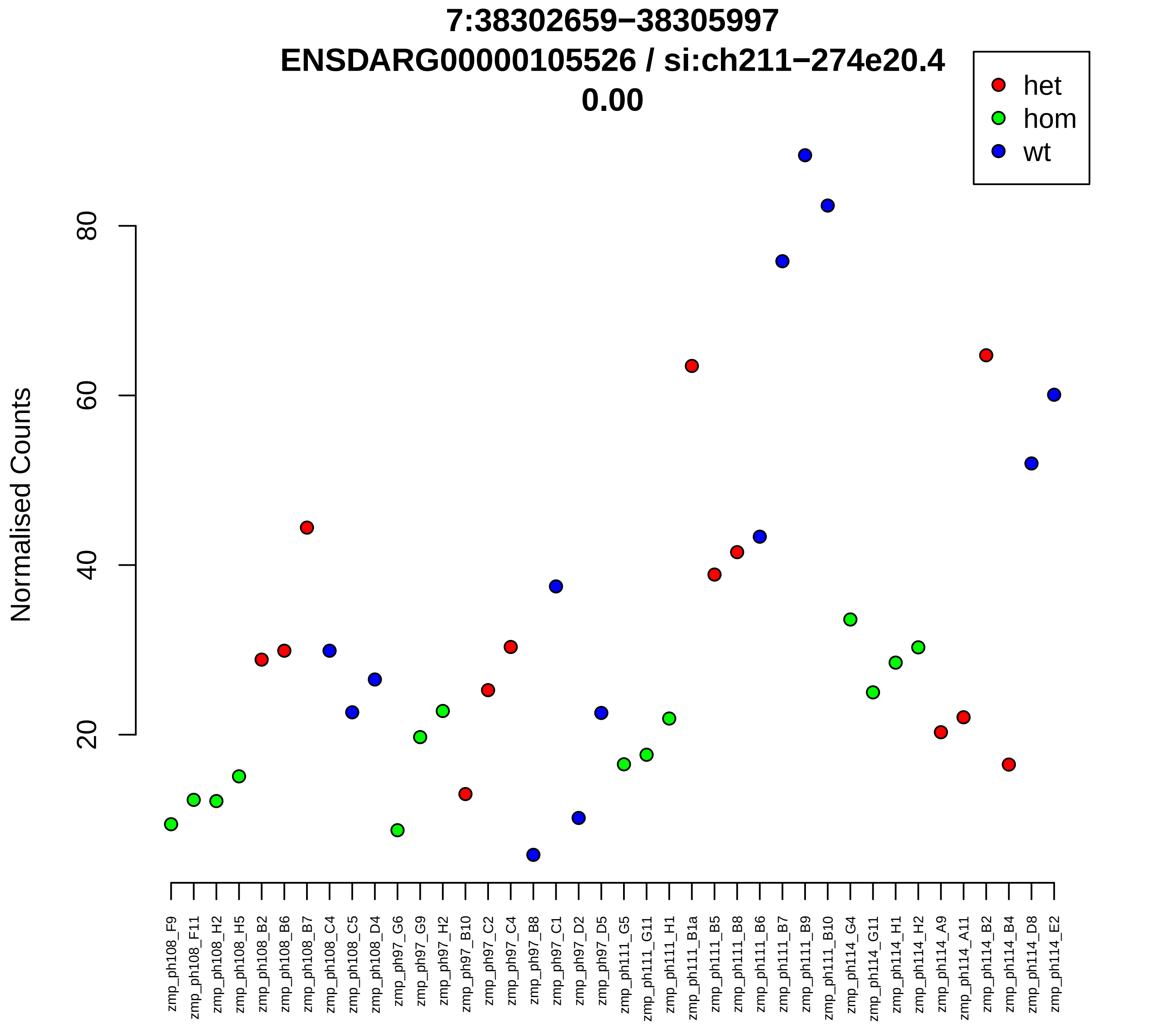
7:38302659−38305997
ENSDARG00000105526 / si:ch211−274e20.4
0.00
het
hom
wt
Normalised Counts
20
40
60
80
zmp_ph108_F9
zmp_ph108_F11
zmp_ph108_H2
zmp_ph108_H5
zmp_ph108_B2
zmp_ph108_B6
zmp_ph108_B7
zmp_ph108_C4
zmp_ph108_C5
zmp_ph108_D4
zmp_ph97_G6
zmp_ph97_G9
zmp_ph97_H2
zmp_ph97_B10
zmp_ph97_C2
zmp_ph97_C4
zmp_ph97_B8
zmp_ph97_C1
zmp_ph97_D2
zmp_ph97_D5
zmp_ph111_G5
zmp_ph111_G11
zmp_ph111_H1
zmp_ph111_B1a
zmp_ph111_B5
zmp_ph111_B8
zmp_ph111_B6
zmp_ph111_B7
zmp_ph111_B9
zmp_ph111_B10
zmp_ph114_G4
zmp_ph114_G11
zmp_ph114_H1
zmp_ph114_H2
zmp_ph114_A9
zmp_ph114_A11
zmp_ph114_B2
zmp_ph114_B4
zmp_ph114_D8
zmp_ph114_E2

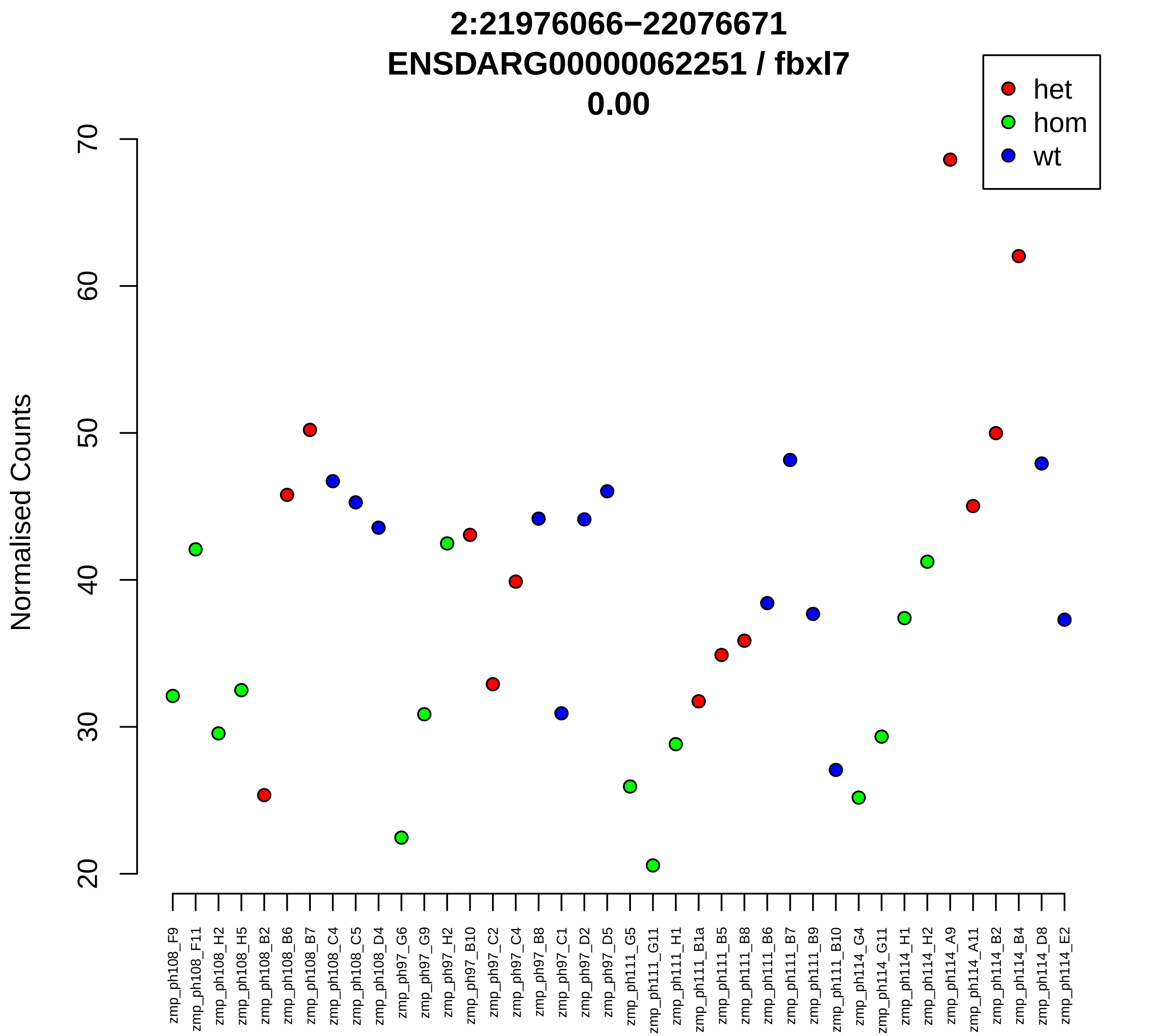

2:21976066−22076671
ENSDARG00000062251 / fbxl7
0.00
het
hom
wt
Normalised Counts
20
30
40
50
60
70
zmp_ph108_F9
zmp_ph108_F11
zmp_ph108_H2
zmp_ph108_H5
zmp_ph108_B2
zmp_ph108_B6
zmp_ph108_B7
zmp_ph108_C4
zmp_ph108_C5
zmp_ph108_D4
zmp_ph97_G6
zmp_ph97_G9
zmp_ph97_H2
zmp_ph97_B10
zmp_ph97_C2
zmp_ph97_C4
zmp_ph97_B8
zmp_ph97_C1
zmp_ph97_D2
zmp_ph97_D5
zmp_ph111_G5
zmp_ph111_G11
zmp_ph111_H1
zmp_ph111_B1a
zmp_ph111_B5
zmp_ph111_B8
zmp_ph111_B6
zmp_ph111_B7
zmp_ph111_B9
zmp_ph111_B10
zmp_ph114_G4
zmp_ph114_G11
zmp_ph114_H1
zmp_ph114_H2
zmp_ph114_A9
zmp_ph114_A11
zmp_ph114_B2
zmp_ph114_B4
zmp_ph114_D8
zmp_ph114_E2

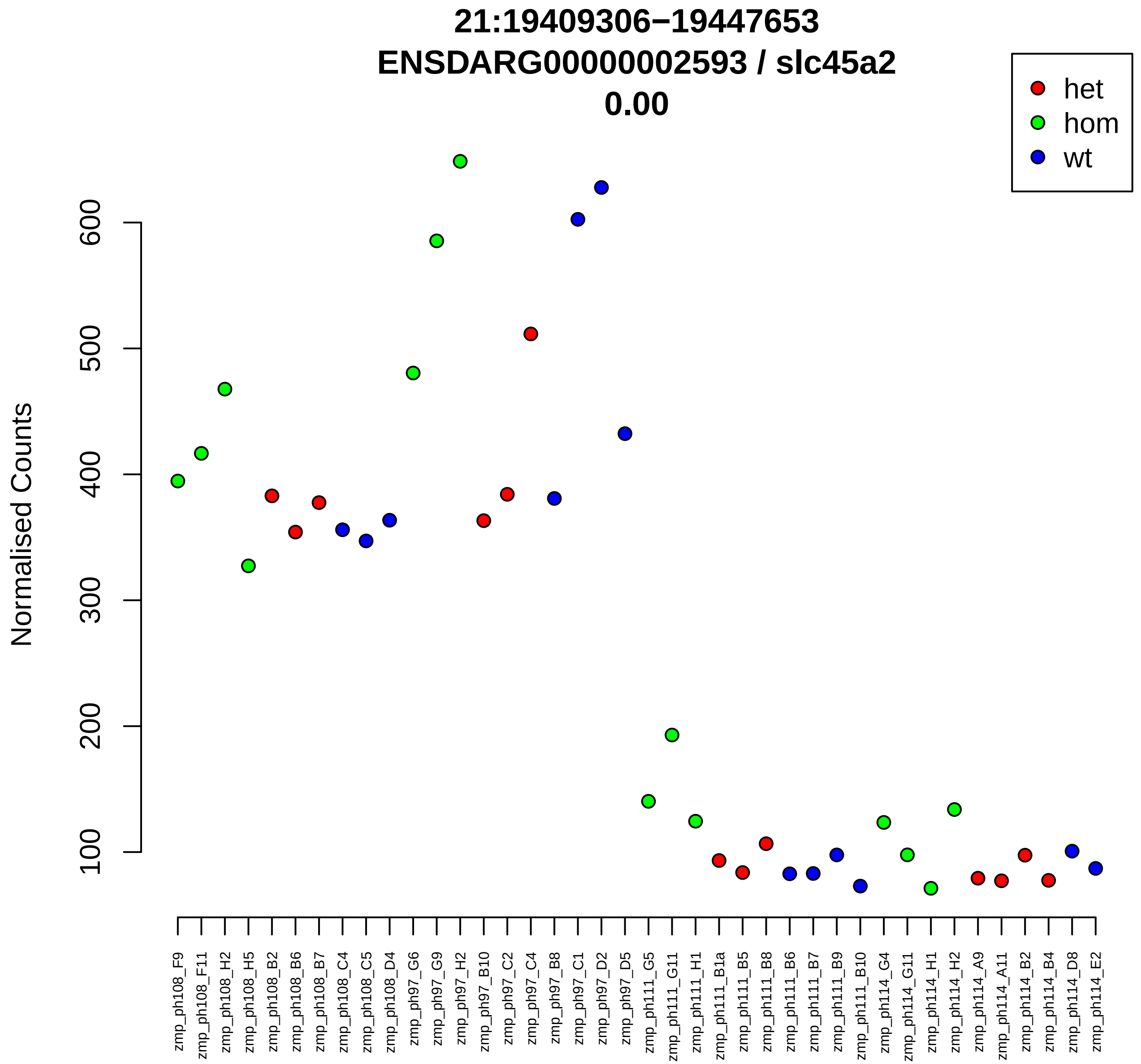

21:19409306−19447653
ENSDARG00000002593 / slc45a2
0.00
het
hom
wt
Normalised Counts
100
200
300
400
500
600
zmp_ph108_F9
zmp_ph108_F11
zmp_ph108_H2
zmp_ph108_H5
zmp_ph108_B2
zmp_ph108_B6
zmp_ph108_B7
zmp_ph108_C4
zmp_ph108_C5
zmp_ph108_D4
zmp_ph97_G6
zmp_ph97_G9
zmp_ph97_H2
zmp_ph97_B10
zmp_ph97_C2
zmp_ph97_C4
zmp_ph97_B8
zmp_ph97_C1
zmp_ph97_D2
zmp_ph97_D5
zmp_ph111_G5
zmp_ph111_G11
zmp_ph111_H1
zmp_ph111_B1a
zmp_ph111_B5
zmp_ph111_B8
zmp_ph111_B6
zmp_ph111_B7
zmp_ph111_B9
zmp_ph111_B10
zmp_ph114_G4
zmp_ph114_G11
zmp_ph114_H1
zmp_ph114_H2
zmp_ph114_A9
zmp_ph114_A11
zmp_ph114_B2
zmp_ph114_B4
zmp_ph114_D8
zmp_ph114_E2

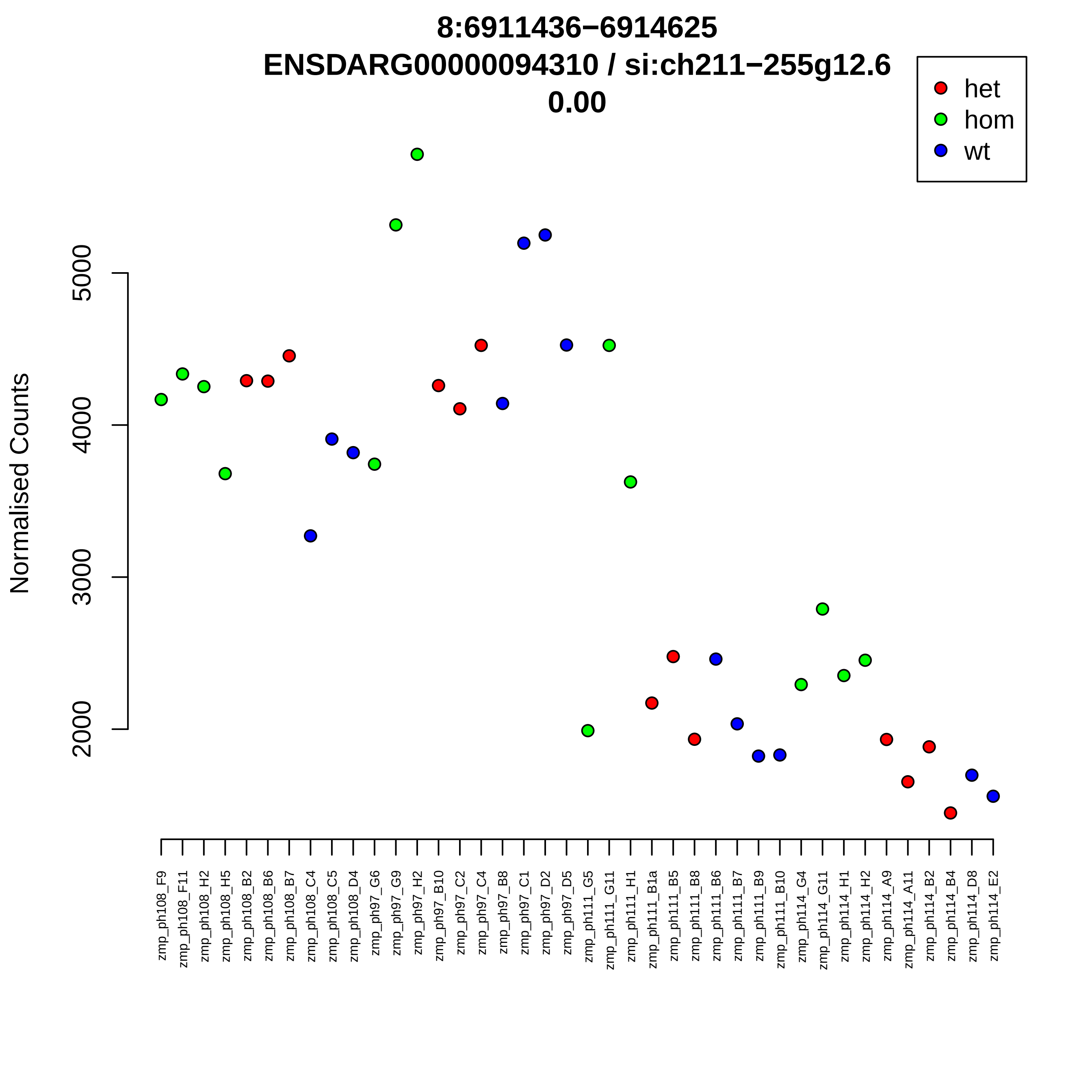

8:6911436−6914625
ENSDARG00000094310 / si:ch211−255g12.6
0.00
het
hom
wt
Normalised Counts
2000
3000
4000
5000
zmp_ph108_F9
zmp_ph108_F11
zmp_ph108_H2
zmp_ph108_H5
zmp_ph108_B2
zmp_ph108_B6
zmp_ph108_B7
zmp_ph108_C4
zmp_ph108_C5
zmp_ph108_D4
zmp_ph97_G6
zmp_ph97_G9
zmp_ph97_H2
zmp_ph97_B10
zmp_ph97_C2
zmp_ph97_C4
zmp_ph97_B8
zmp_ph97_C1
zmp_ph97_D2
zmp_ph97_D5
zmp_ph111_G5
zmp_ph111_G11
zmp_ph111_H1
zmp_ph111_B1a
zmp_ph111_B5
zmp_ph111_B8
zmp_ph111_B6
zmp_ph111_B7
zmp_ph111_B9
zmp_ph111_B10
zmp_ph114_G4
zmp_ph114_G11
zmp_ph114_H1
zmp_ph114_H2
zmp_ph114_A9
zmp_ph114_A11
zmp_ph114_B2
zmp_ph114_B4
zmp_ph114_D8
zmp_ph114_E2

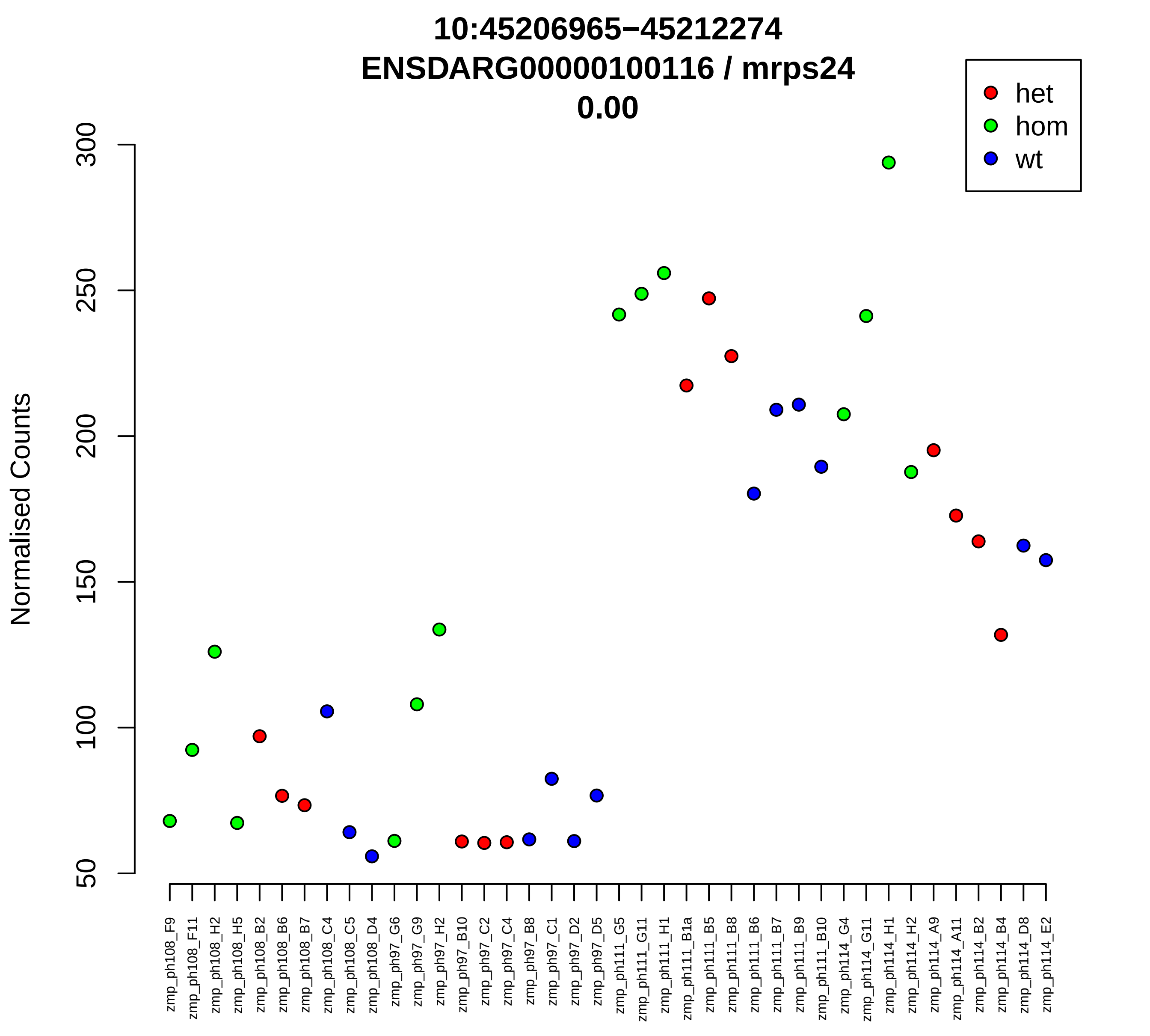

10:45206965−45212274
ENSDARG00000100116 / mrps24
0.00
het
hom
wt
Normalised Counts
300
250
200
150
100
50
zmp_ph108_F9
zmp_ph108_F11
zmp_ph108_H2
zmp_ph108_H5
zmp_ph108_B2
zmp_ph108_B6
zmp_ph108_B7
zmp_ph108_C4
zmp_ph108_C5
zmp_ph108_D4
zmp_ph97_G6
zmp_ph97_G9
zmp_ph97_H2
zmp_ph97_B10
zmp_ph97_C2
zmp_ph97_C4
zmp_ph97_B8
zmp_ph97_C1
zmp_ph97_D2
zmp_ph97_D5
zmp_ph111_G5
zmp_ph111_G11
zmp_ph111_H1
zmp_ph111_B1a
zmp_ph111_B5
zmp_ph111_B8
zmp_ph111_B6
zmp_ph111_B7
zmp_ph111_B9
zmp_ph111_B10
zmp_ph114_G4
zmp_ph114_G11
zmp_ph114_H1
zmp_ph114_H2
zmp_ph114_A9
zmp_ph114_A11
zmp_ph114_B2
zmp_ph114_B4
zmp_ph114_D8
zmp_ph114_E2

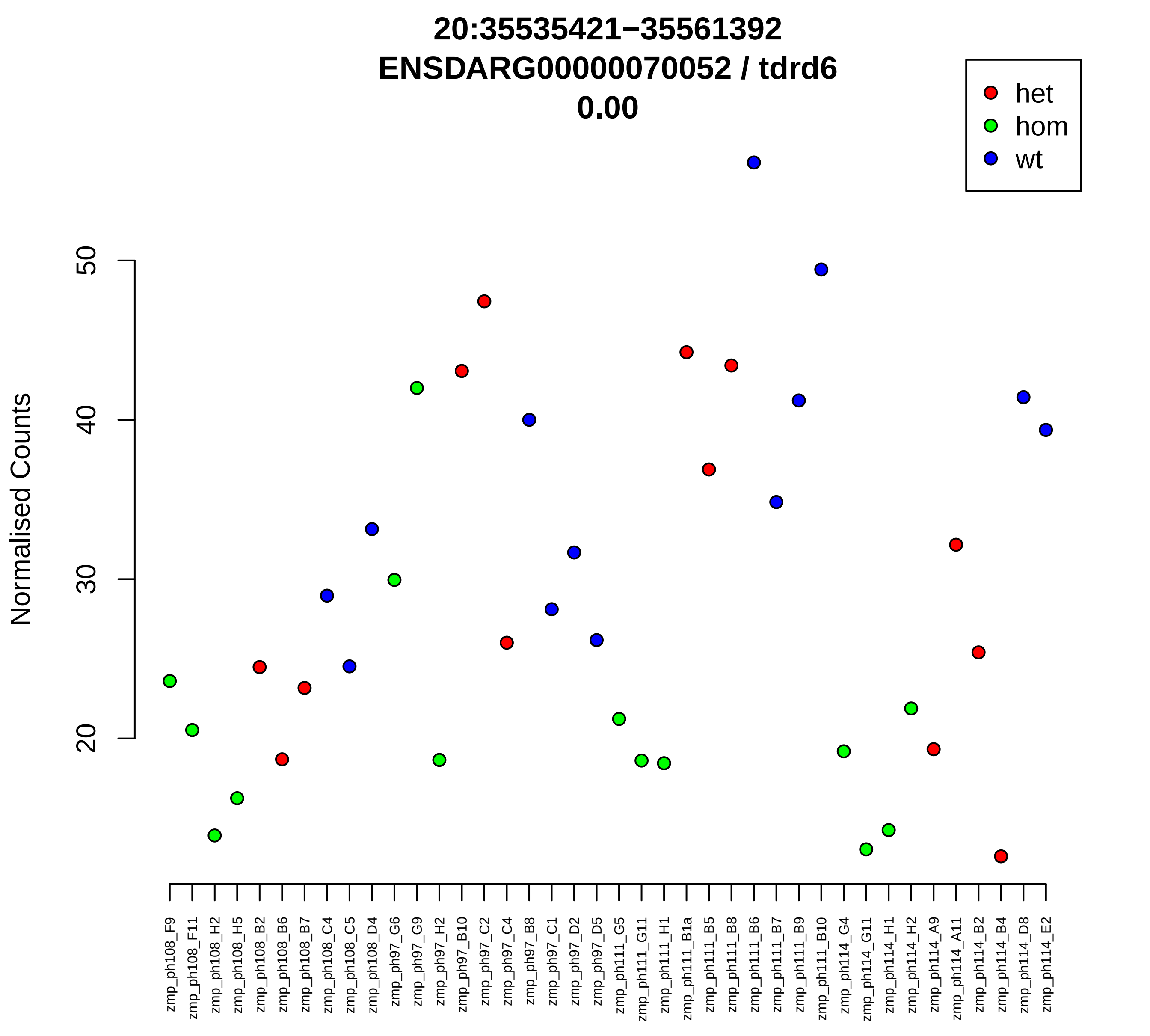

20:35535421−35561392
ENSDARG00000070052 / tdrd6
0.00
het
hom
wt
Normalised Counts
20
30
40
50
zmp_ph108_F9
zmp_ph108_F11
zmp_ph108_H2
zmp_ph108_H5
zmp_ph108_B2
zmp_ph108_B6
zmp_ph108_B7
zmp_ph108_C4
zmp_ph108_C5
zmp_ph108_D4
zmp_ph97_G6
zmp_ph97_G9
zmp_ph97_H2
zmp_ph97_B10
zmp_ph97_C2
zmp_ph97_C4
zmp_ph97_B8
zmp_ph97_C1
zmp_ph97_D2
zmp_ph97_D5
zmp_ph111_G5
zmp_ph111_G11
zmp_ph111_H1
zmp_ph111_B1a
zmp_ph111_B5
zmp_ph111_B8
zmp_ph111_B6
zmp_ph111_B7
zmp_ph111_B9
zmp_ph111_B10
zmp_ph114_G4
zmp_ph114_G11
zmp_ph114_H1
zmp_ph114_H2
zmp_ph114_A9
zmp_ph114_A11
zmp_ph114_B2
zmp_ph114_B4
zmp_ph114_D8
zmp_ph114_E2

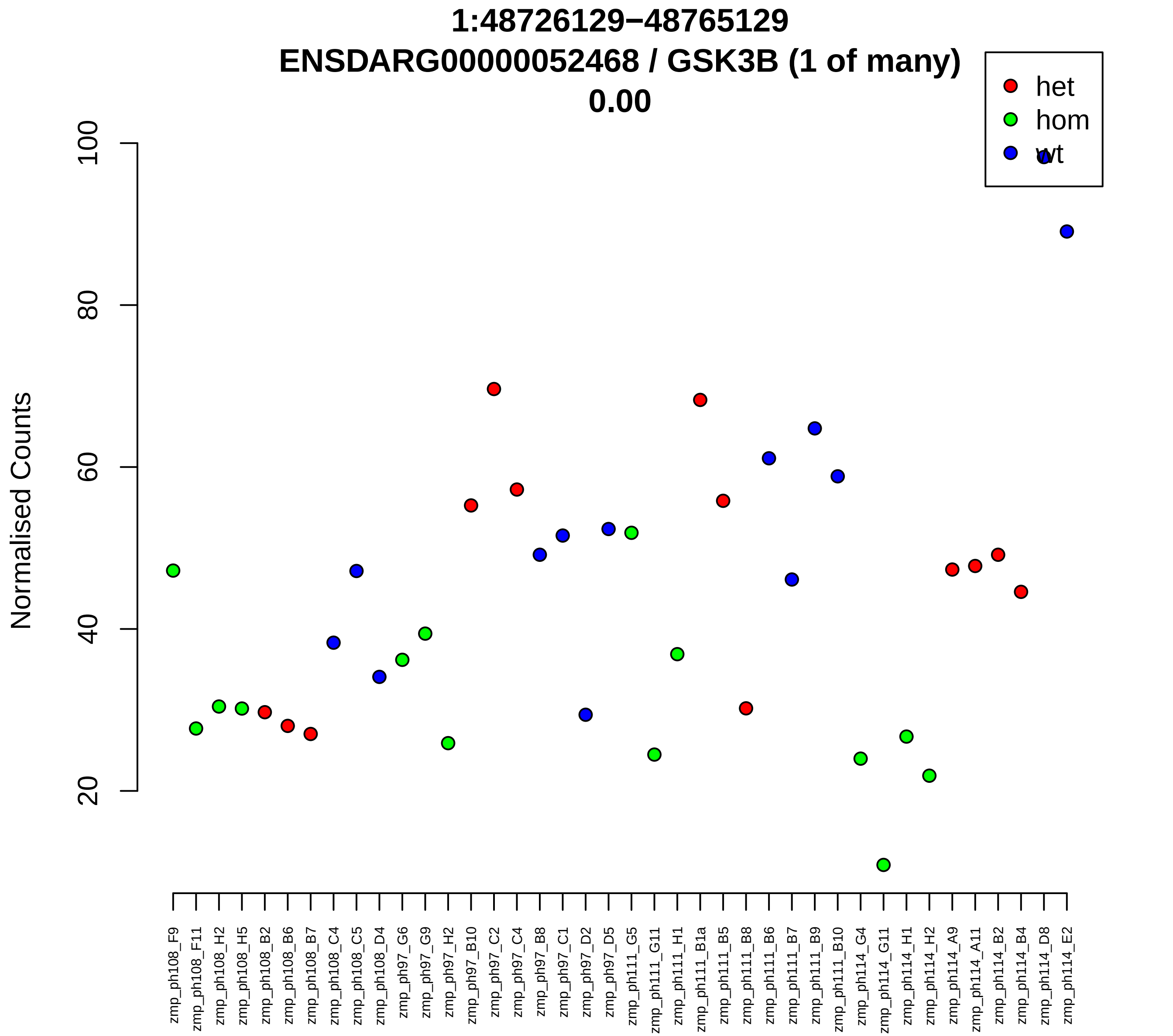
1:48726129−48765129
ENSDARG00000052468 / GSK3B (1 of many)
0.00
het
hom
wt
Normalised Counts
100
80
60
40
20
zmp_ph108_F9
zmp_ph108_F11
zmp_ph108_H2
zmp_ph108_H5
zmp_ph108_B2
zmp_ph108_B6
zmp_ph108_B7
zmp_ph108_C4
zmp_ph108_C5
zmp_ph108_D4
zmp_ph97_G6
zmp_ph97_G9
zmp_ph97_H2
zmp_ph97_B10
zmp_ph97_C2
zmp_ph97_C4
zmp_ph97_B8
zmp_ph97_C1
zmp_ph97_D2
zmp_ph97_D5
zmp_ph111_G5
zmp_ph111_G11
zmp_ph111_H1
zmp_ph111_B1a
zmp_ph111_B5
zmp_ph111_B8
zmp_ph111_B6
zmp_ph111_B7
zmp_ph111_B9
zmp_ph111_B10
zmp_ph114_G4
zmp_ph114_G11
zmp_ph114_H1
zmp_ph114_H2
zmp_ph114_A9
zmp_ph114_A11
zmp_ph114_B2
zmp_ph114_B4
zmp_ph114_D8
zmp_ph114_E2

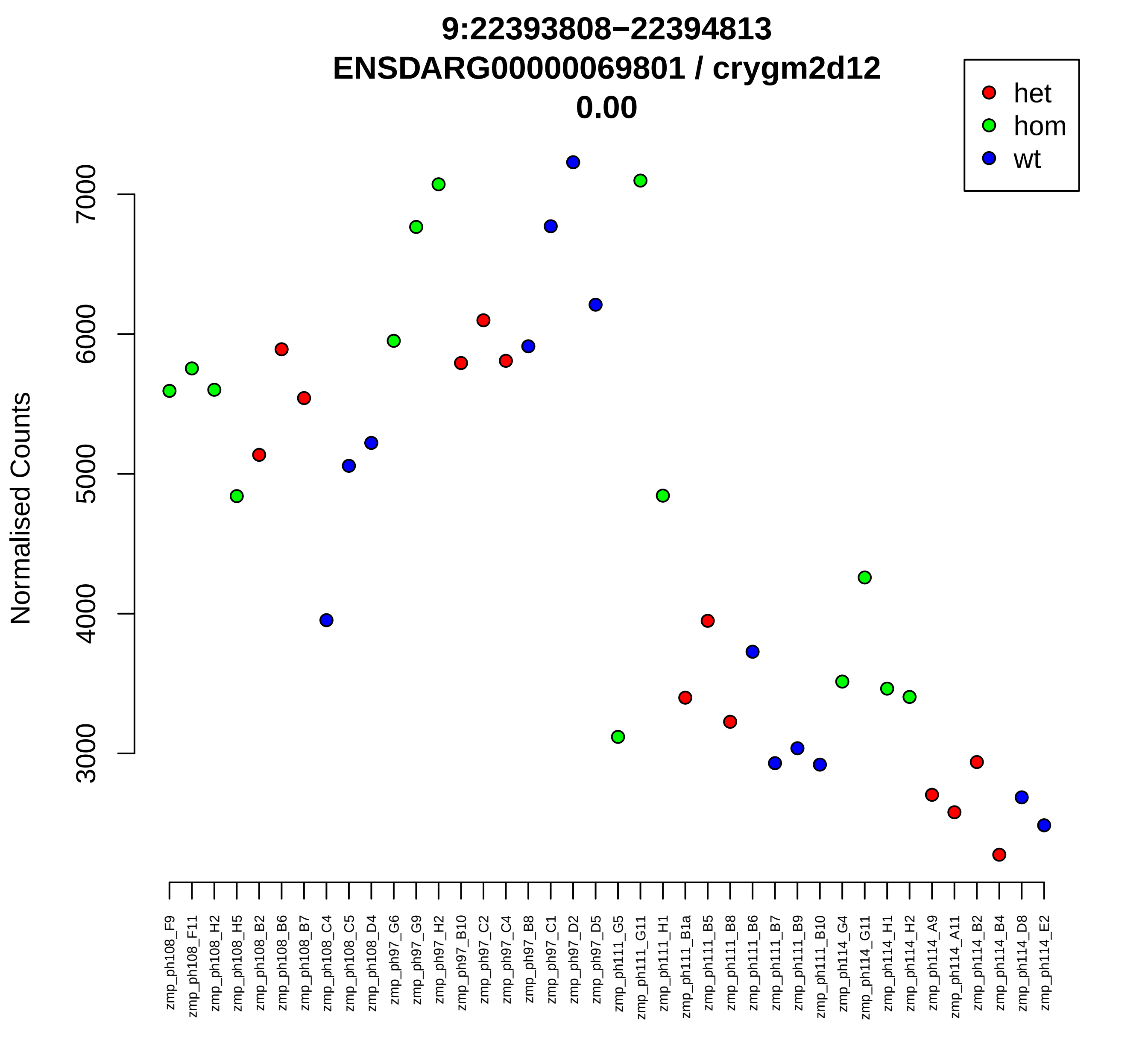

9:22393808−22394813
ENSDARG00000069801 / crygm2d12
0.00
het
hom
wt
Normalised Counts
3000
4000
5000
6000
7000
zmp_ph108_F9
zmp_ph108_F11
zmp_ph108_H2
zmp_ph108_H5
zmp_ph108_B2
zmp_ph108_B6
zmp_ph108_B7
zmp_ph108_C4
zmp_ph108_C5
zmp_ph108_D4
zmp_ph97_G6
zmp_ph97_G9
zmp_ph97_H2
zmp_ph97_B10
zmp_ph97_C2
zmp_ph97_C4
zmp_ph97_B8
zmp_ph97_C1
zmp_ph97_D2
zmp_ph97_D5
zmp_ph111_G5
zmp_ph111_G11
zmp_ph111_H1
zmp_ph111_B1a
zmp_ph111_B5
zmp_ph111_B8
zmp_ph111_B6
zmp_ph111_B7
zmp_ph111_B9
zmp_ph111_B10
zmp_ph114_G4
zmp_ph114_G11
zmp_ph114_H1
zmp_ph114_H2
zmp_ph114_A9
zmp_ph114_A11
zmp_ph114_B2
zmp_ph114_B4
zmp_ph114_D8
zmp_ph114_E2

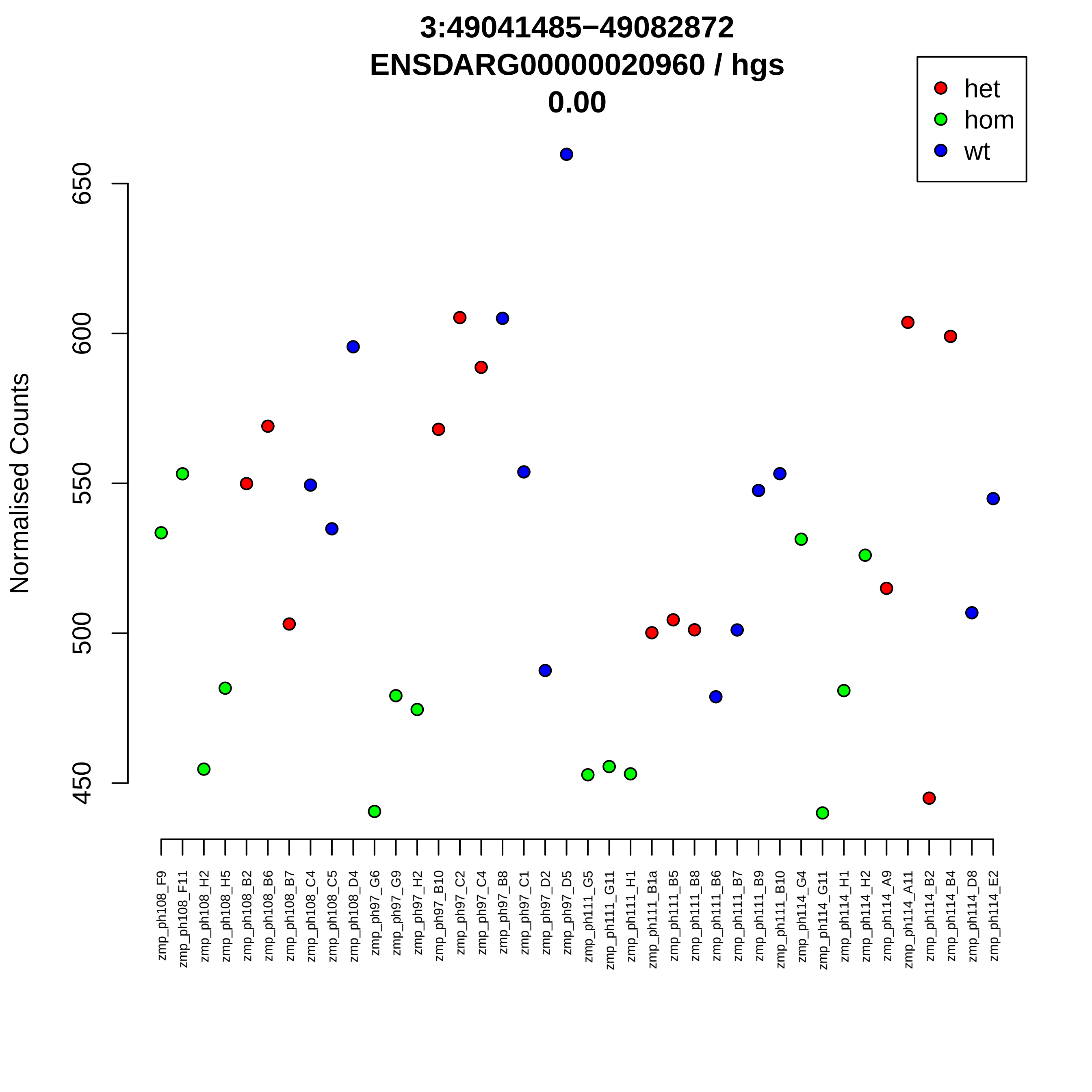
3:49041485−49082872
ENSDARG00000020960 / hgs
0.00
het
hom
wt
Normalised Counts
450
500
550
600
650
zmp_ph108_F9
zmp_ph108_F11
zmp_ph108_H2
zmp_ph108_H5
zmp_ph108_B2
zmp_ph108_B6
zmp_ph108_B7
zmp_ph108_C4
zmp_ph108_C5
zmp_ph108_D4
zmp_ph97_G6
zmp_ph97_G9
zmp_ph97_H2
zmp_ph97_B10
zmp_ph97_C2
zmp_ph97_C4
zmp_ph97_B8
zmp_ph97_C1
zmp_ph97_D2
zmp_ph97_D5
zmp_ph111_G5
zmp_ph111_G11
zmp_ph111_H1
zmp_ph111_B1a
zmp_ph111_B5
zmp_ph111_B8
zmp_ph111_B6
zmp_ph111_B7
zmp_ph111_B9
zmp_ph111_B10
zmp_ph114_G4
zmp_ph114_G11
zmp_ph114_H1
zmp_ph114_H2
zmp_ph114_A9
zmp_ph114_A11
zmp_ph114_B2
zmp_ph114_B4
zmp_ph114_D8
zmp_ph114_E2

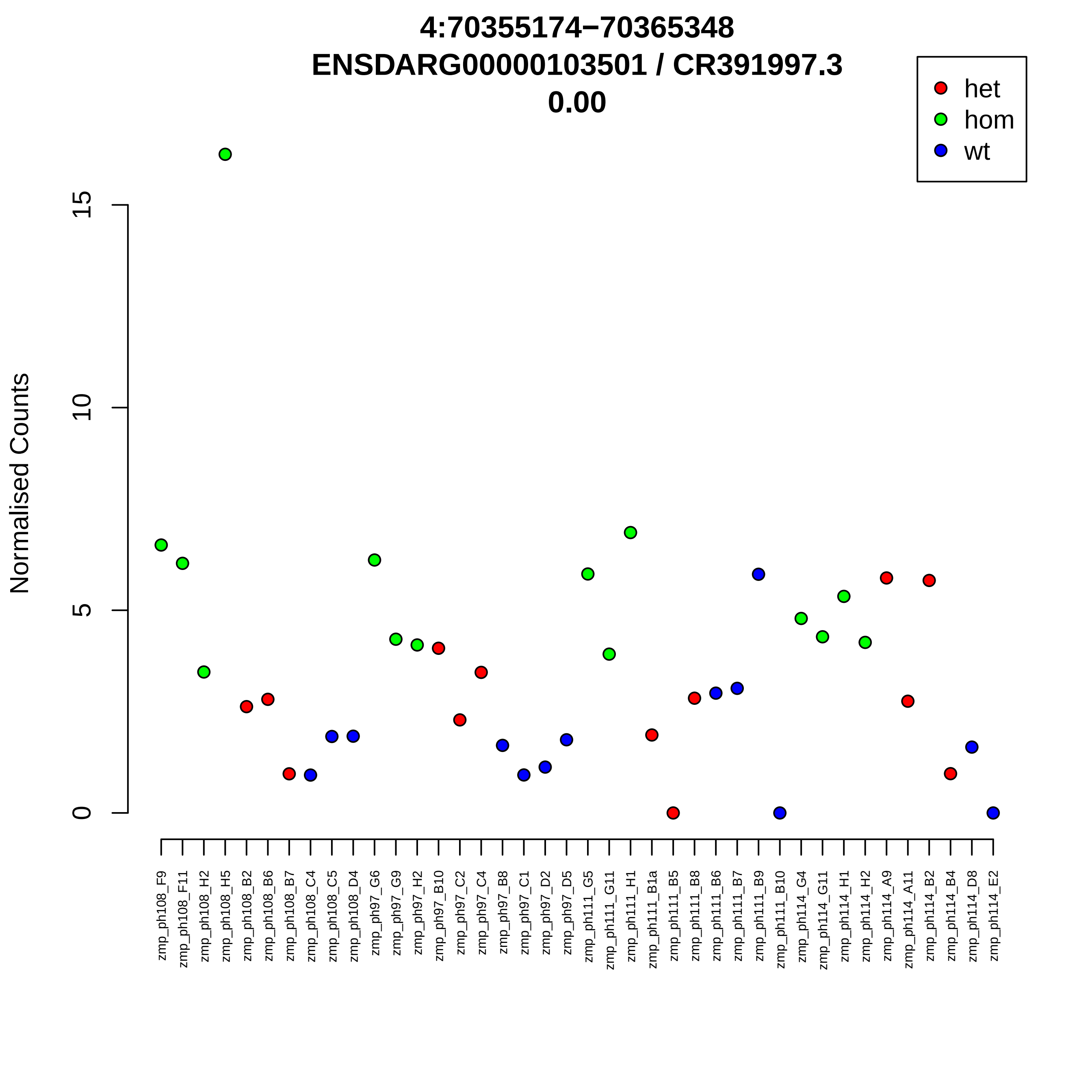

4:70355174−70365348
ENSDARG00000103501 / CR391997.3
0.00
het
hom
wt
Normalised Counts
0
5
10
15
zmp_ph108_F9
zmp_ph108_F11
zmp_ph108_H2
zmp_ph108_H5
zmp_ph108_B2
zmp_ph108_B6
zmp_ph108_B7
zmp_ph108_C4
zmp_ph108_C5
zmp_ph108_D4
zmp_ph97_G6
zmp_ph97_G9
zmp_ph97_H2
zmp_ph97_B10
zmp_ph97_C2
zmp_ph97_C4
zmp_ph97_B8
zmp_ph97_C1
zmp_ph97_D2
zmp_ph97_D5
zmp_ph111_G5
zmp_ph111_G11
zmp_ph111_H1
zmp_ph111_B1a
zmp_ph111_B5
zmp_ph111_B8
zmp_ph111_B6
zmp_ph111_B7
zmp_ph111_B9
zmp_ph111_B10
zmp_ph114_G4
zmp_ph114_G11
zmp_ph114_H1
zmp_ph114_H2
zmp_ph114_A9
zmp_ph114_A11
zmp_ph114_B2
zmp_ph114_B4
zmp_ph114_D8
zmp_ph114_E2

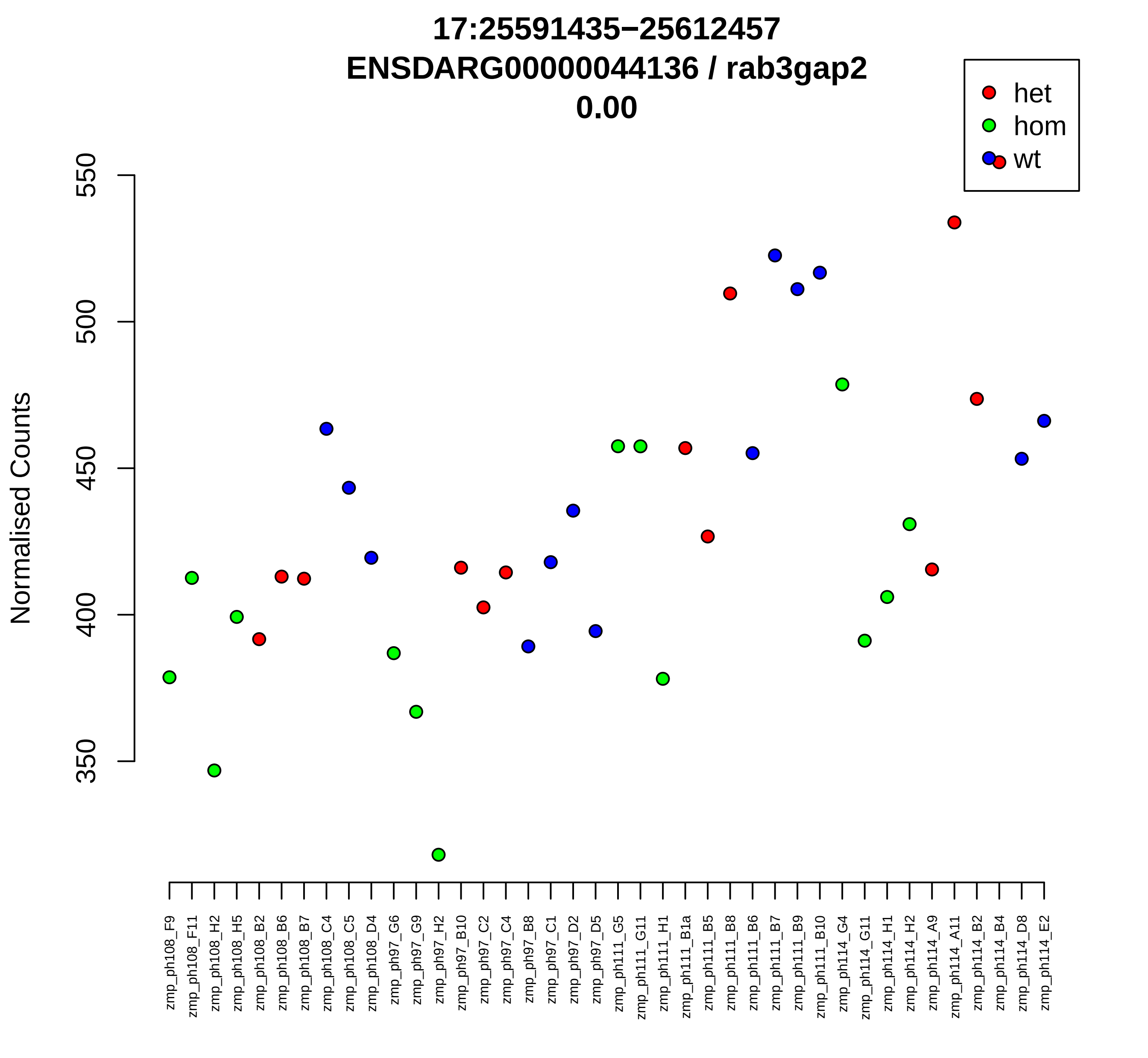
17:25591435−25612457
ENSDARG00000044136 / rab3gap2
0.00
het
hom
wt
Normalised Counts
350
400
450
500
550
zmp_ph108_F9
zmp_ph108_F11
zmp_ph108_H2
zmp_ph108_H5
zmp_ph108_B2
zmp_ph108_B6
zmp_ph108_B7
zmp_ph108_C4
zmp_ph108_C5
zmp_ph108_D4
zmp_ph97_G6
zmp_ph97_G9
zmp_ph97_H2
zmp_ph97_B10
zmp_ph97_C2
zmp_ph97_C4
zmp_ph97_B8
zmp_ph97_C1
zmp_ph97_D2
zmp_ph97_D5
zmp_ph111_G5
zmp_ph111_G11
zmp_ph111_H1
zmp_ph111_B1a
zmp_ph111_B5
zmp_ph111_B8
zmp_ph111_B6
zmp_ph111_B7
zmp_ph111_B9
zmp_ph111_B10
zmp_ph114_G4
zmp_ph114_G11
zmp_ph114_H1
zmp_ph114_H2
zmp_ph114_A9
zmp_ph114_A11
zmp_ph114_B2
zmp_ph114_B4
zmp_ph114_D8
zmp_ph114_E2

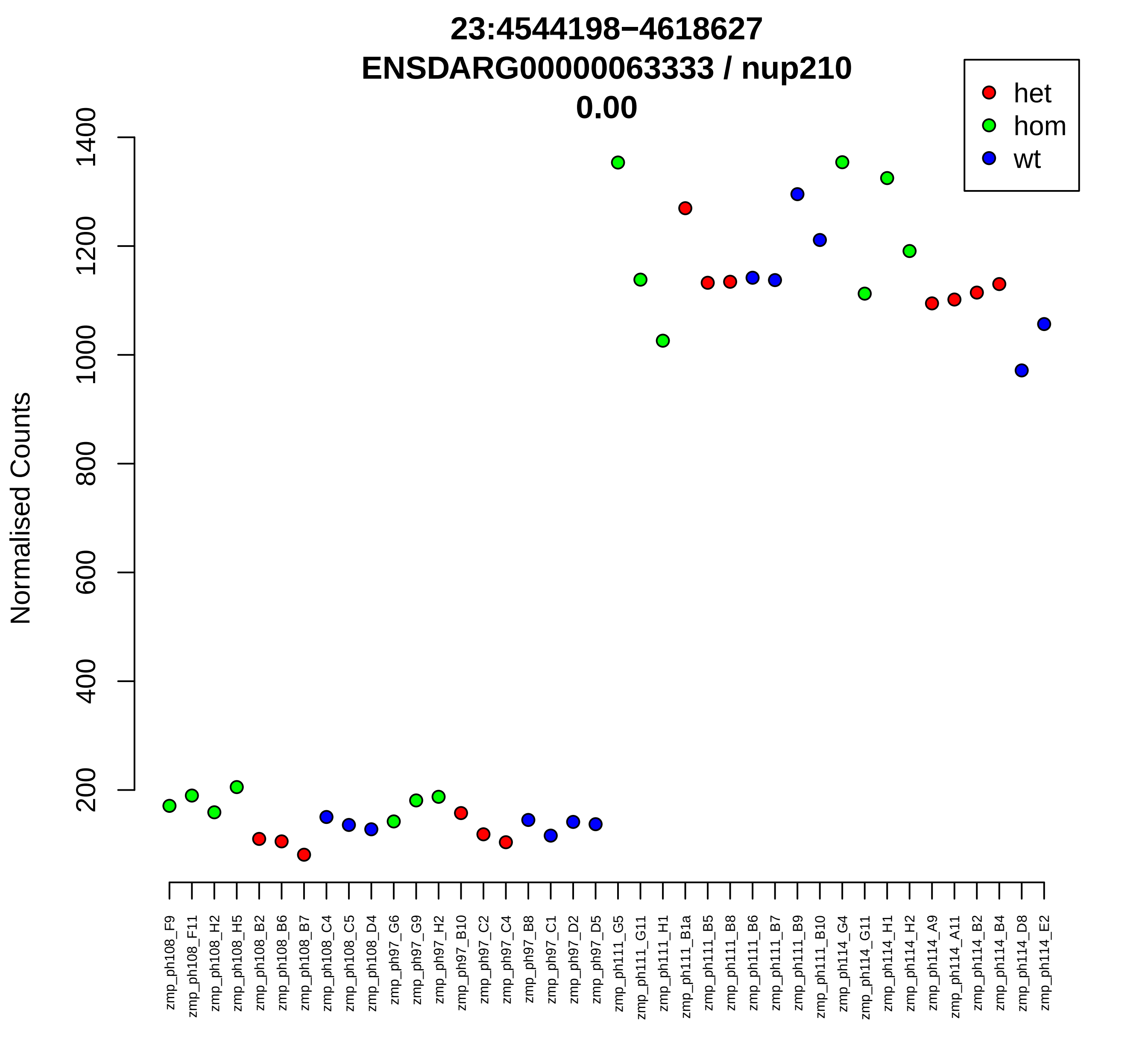

23:4544198−4618627
ENSDARG00000063333 / nup210
0.00
het
hom
wt
Normalised Counts
200
400
600
800
1000
1200
1400
zmp_ph108_F9
zmp_ph108_F11
zmp_ph108_H2
zmp_ph108_H5
zmp_ph108_B2
zmp_ph108_B6
zmp_ph108_B7
zmp_ph108_C4
zmp_ph108_C5
zmp_ph108_D4
zmp_ph97_G6
zmp_ph97_G9
zmp_ph97_H2
zmp_ph97_B10
zmp_ph97_C2
zmp_ph97_C4
zmp_ph97_B8
zmp_ph97_C1
zmp_ph97_D2
zmp_ph97_D5
zmp_ph111_G5
zmp_ph111_G11
zmp_ph111_H1
zmp_ph111_B1a
zmp_ph111_B5
zmp_ph111_B8
zmp_ph111_B6
zmp_ph111_B7
zmp_ph111_B9
zmp_ph111_B10
zmp_ph114_G4
zmp_ph114_G11
zmp_ph114_H1
zmp_ph114_H2
zmp_ph114_A9
zmp_ph114_A11
zmp_ph114_B2
zmp_ph114_B4
zmp_ph114_D8
zmp_ph114_E2

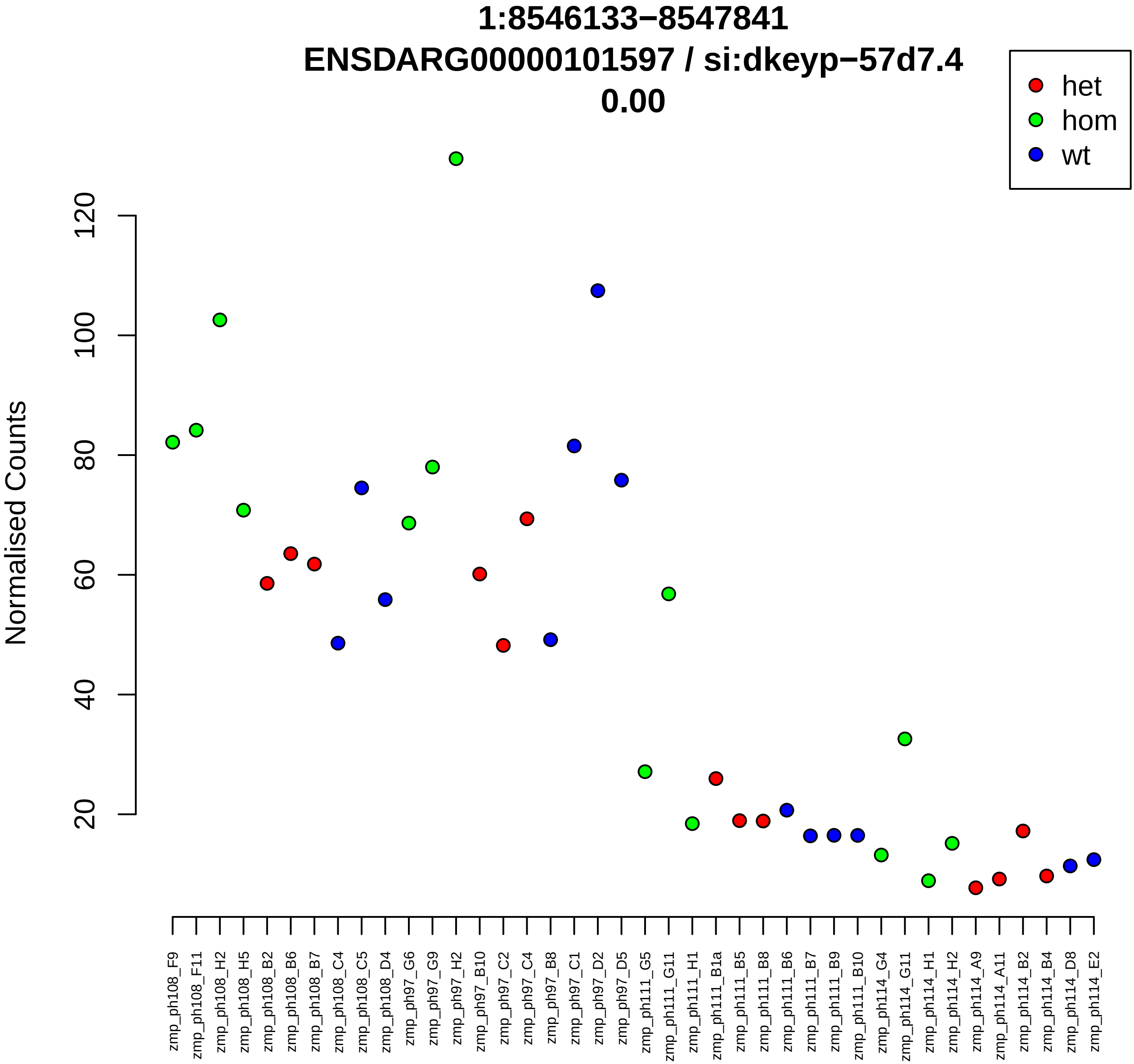

1:8546133−8547841
ENSDARG00000101597 / si:dkeyp−57d7.4
0.00
het
hom
wt
Normalised Counts
20
40
60
80
100
120
zmp_ph108_F9
zmp_ph108_F11
zmp_ph108_H2
zmp_ph108_H5
zmp_ph108_B2
zmp_ph108_B6
zmp_ph108_B7
zmp_ph108_C4
zmp_ph108_C5
zmp_ph108_D4
zmp_ph97_G6
zmp_ph97_G9
zmp_ph97_H2
zmp_ph97_B10
zmp_ph97_C2
zmp_ph97_C4
zmp_ph97_B8
zmp_ph97_C1
zmp_ph97_D2
zmp_ph97_D5
zmp_ph111_G5
zmp_ph111_G11
zmp_ph111_H1
zmp_ph111_B1a
zmp_ph111_B5
zmp_ph111_B8
zmp_ph111_B6
zmp_ph111_B7
zmp_ph111_B9
zmp_ph111_B10
zmp_ph114_G4
zmp_ph114_G11
zmp_ph114_H1
zmp_ph114_H2
zmp_ph114_A9
zmp_ph114_A11
zmp_ph114_B2
zmp_ph114_B4
zmp_ph114_D8
zmp_ph114_E2

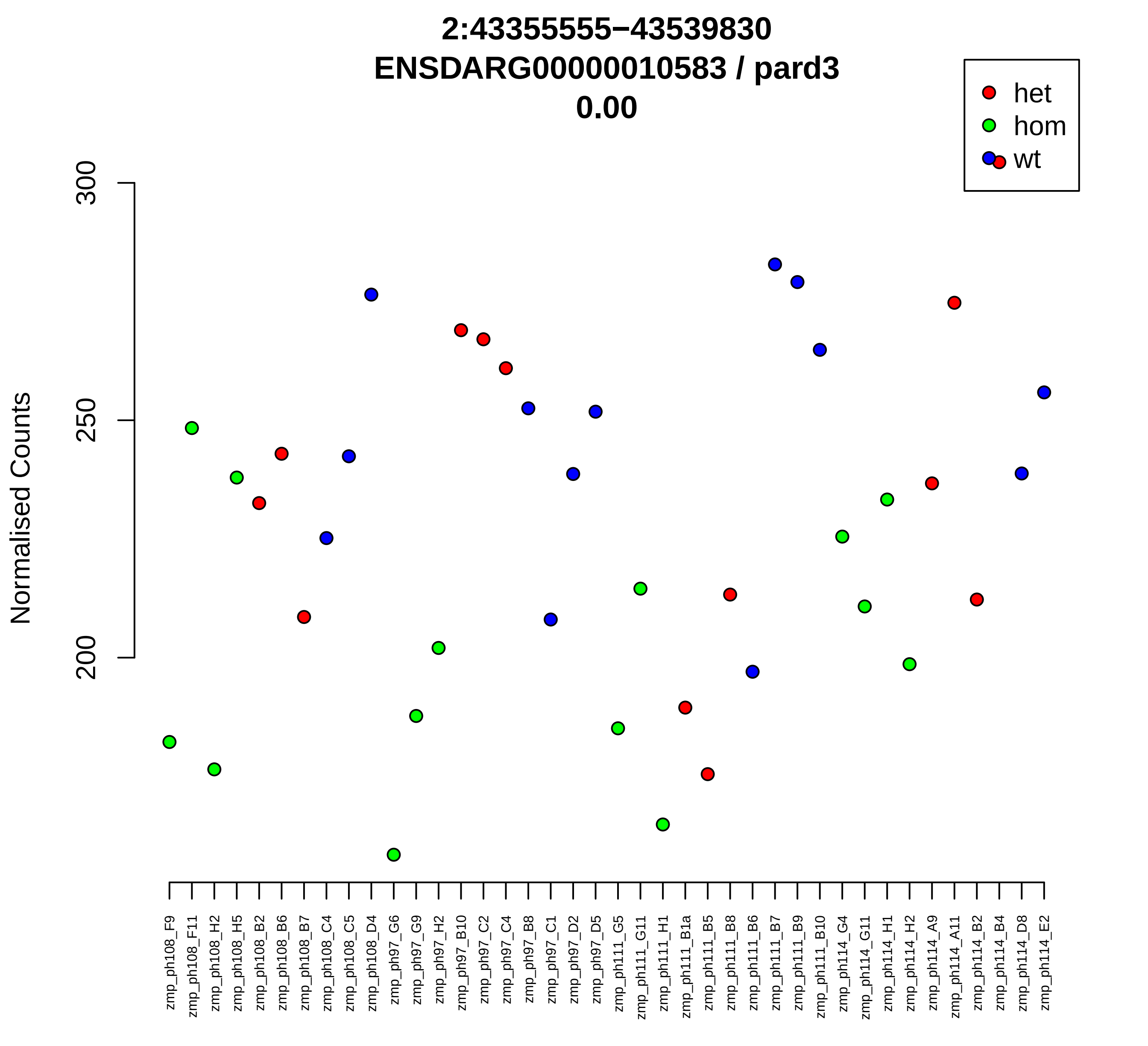

2:43355555−43539830
ENSDARG00000010583 / pard3
0.00
het
hom
wt
Normalised Counts
300
250
200
zmp_ph108_F9
zmp_ph108_F11
zmp_ph108_H2
zmp_ph108_H5
zmp_ph108_B2
zmp_ph108_B6
zmp_ph108_B7
zmp_ph108_C4
zmp_ph108_C5
zmp_ph108_D4
zmp_ph97_G6
zmp_ph97_G9
zmp_ph97_H2
zmp_ph97_B10
zmp_ph97_C2
zmp_ph97_C4
zmp_ph97_B8
zmp_ph97_C1
zmp_ph97_D2
zmp_ph97_D5
zmp_ph111_G5
zmp_ph111_G11
zmp_ph111_H1
zmp_ph111_B1a
zmp_ph111_B5
zmp_ph111_B8
zmp_ph111_B6
zmp_ph111_B7
zmp_ph111_B9
zmp_ph111_B10
zmp_ph114_G4
zmp_ph114_G11
zmp_ph114_H1
zmp_ph114_H2
zmp_ph114_A9
zmp_ph114_A11
zmp_ph114_B2
zmp_ph114_B4
zmp_ph114_D8
zmp_ph114_E2

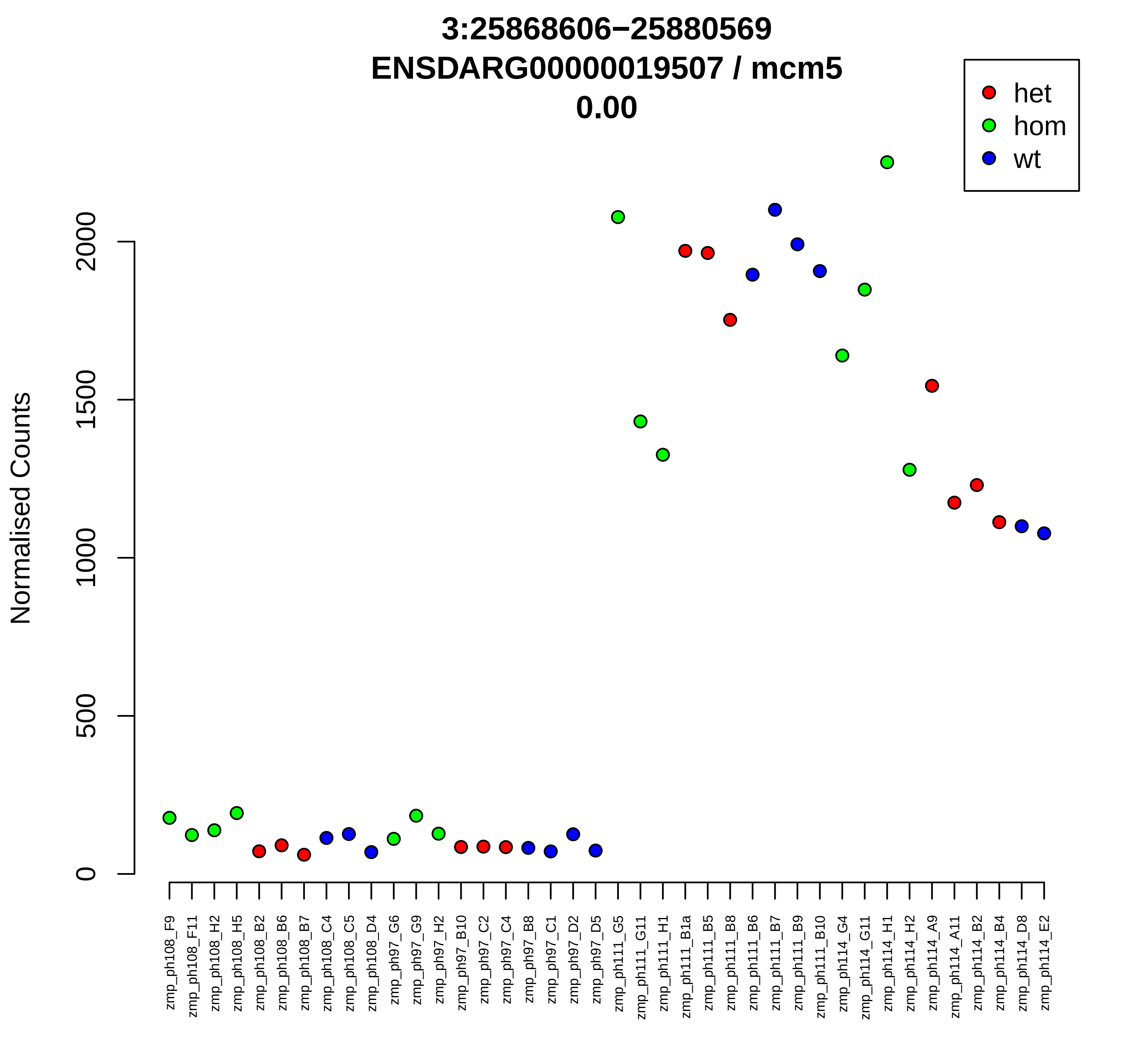

3:25868606−25880569
ENSDARG00000019507 / mcm5
0.00
het
hom
wt
Normalised Counts
0
500
1000
1500
2000
zmp_ph108_F9
zmp_ph108_F11
zmp_ph108_H2
zmp_ph108_H5
zmp_ph108_B2
zmp_ph108_B6
zmp_ph108_B7
zmp_ph108_C4
zmp_ph108_C5
zmp_ph108_D4
zmp_ph97_G6
zmp_ph97_G9
zmp_ph97_H2
zmp_ph97_B10
zmp_ph97_C2
zmp_ph97_C4
zmp_ph97_B8
zmp_ph97_C1
zmp_ph97_D2
zmp_ph97_D5
zmp_ph111_G5
zmp_ph111_G11
zmp_ph111_H1
zmp_ph111_B1a
zmp_ph111_B5
zmp_ph111_B8
zmp_ph111_B6
zmp_ph111_B7
zmp_ph111_B9
zmp_ph111_B10
zmp_ph114_G4
zmp_ph114_G11
zmp_ph114_H1
zmp_ph114_H2
zmp_ph114_A9
zmp_ph114_A11
zmp_ph114_B2
zmp_ph114_B4
zmp_ph114_D8
zmp_ph114_E2

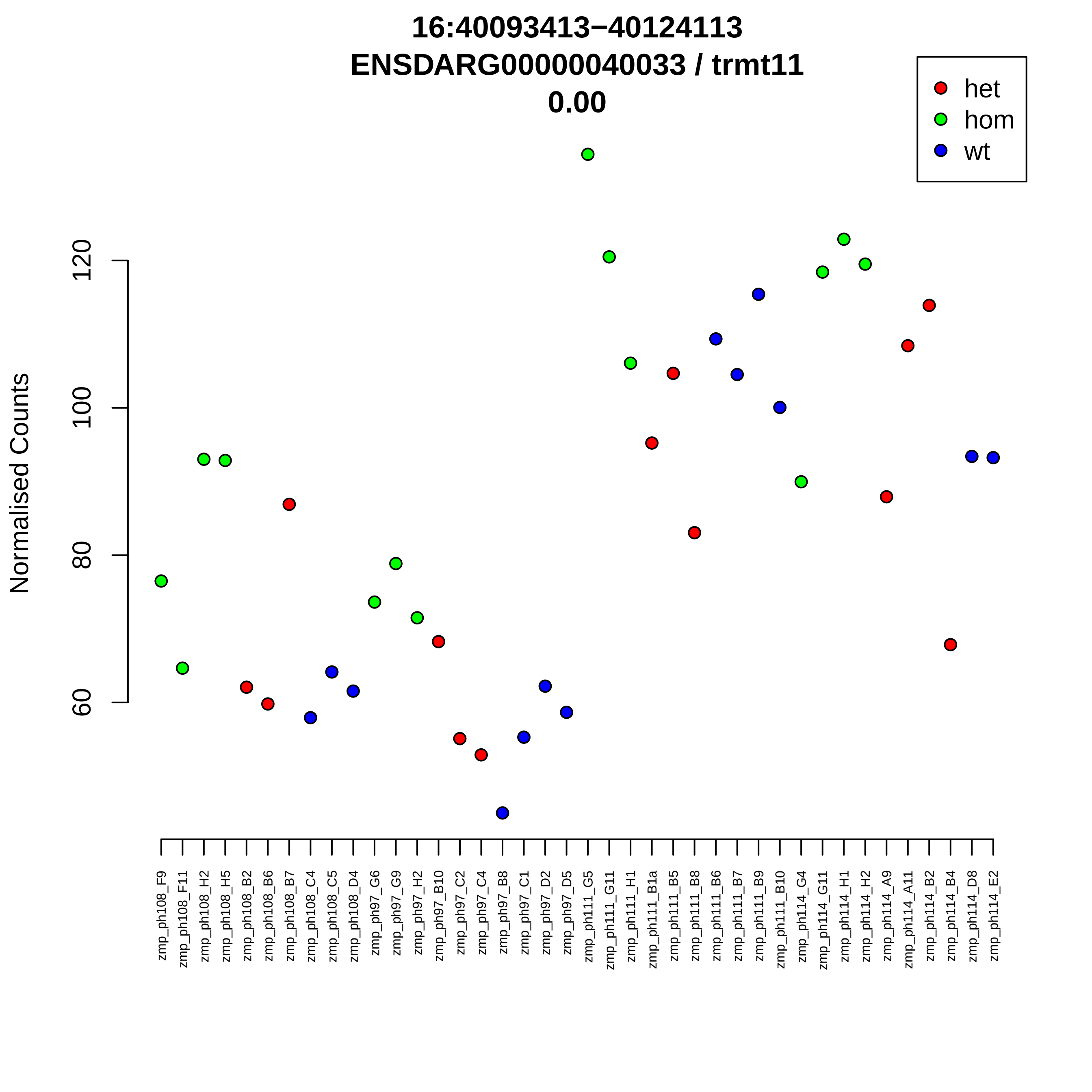

16:40093413−40124113
ENSDARG00000040033 / trmt11
0.00
het
hom
wt
Normalised Counts
60
80
100
120
zmp_ph108_F9
zmp_ph108_F11
zmp_ph108_H2
zmp_ph108_H5
zmp_ph108_B2
zmp_ph108_B6
zmp_ph108_B7
zmp_ph108_C4
zmp_ph108_C5
zmp_ph108_D4
zmp_ph97_G6
zmp_ph97_G9
zmp_ph97_H2
zmp_ph97_B10
zmp_ph97_C2
zmp_ph97_C4
zmp_ph97_B8
zmp_ph97_C1
zmp_ph97_D2
zmp_ph97_D5
zmp_ph111_G5
zmp_ph111_G11
zmp_ph111_H1
zmp_ph111_B1a
zmp_ph111_B5
zmp_ph111_B8
zmp_ph111_B6
zmp_ph111_B7
zmp_ph111_B9
zmp_ph111_B10
zmp_ph114_G4
zmp_ph114_G11
zmp_ph114_H1
zmp_ph114_H2
zmp_ph114_A9
zmp_ph114_A11
zmp_ph114_B2
zmp_ph114_B4
zmp_ph114_D8
zmp_ph114_E2

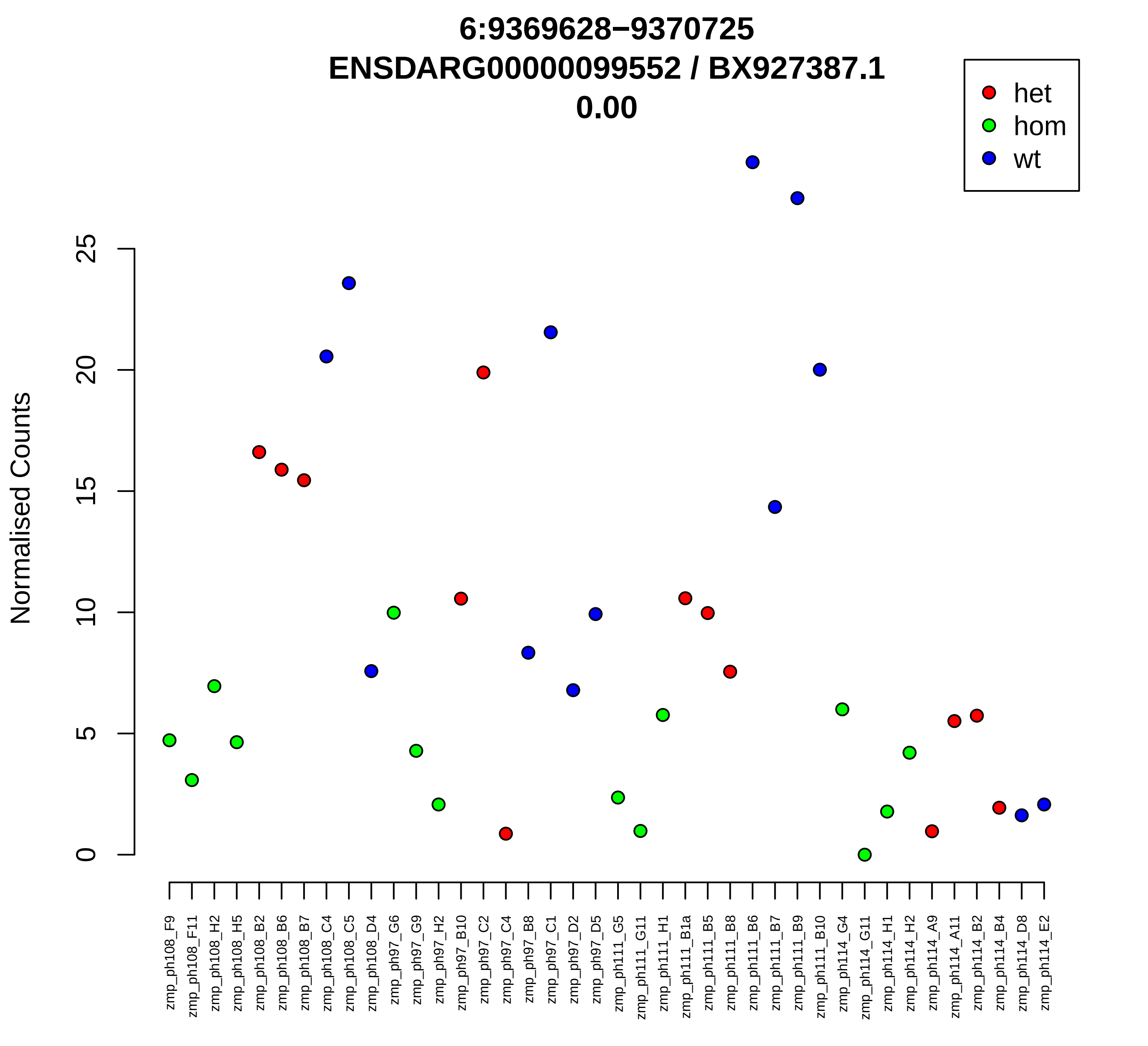

6:9369628−9370725
ENSDARG00000099552 / BX927387.1
0.00
het
hom
wt
Normalised Counts
0
5
10
15
20
25
zmp_ph108_F9
zmp_ph108_F11
zmp_ph108_H2
zmp_ph108_H5
zmp_ph108_B2
zmp_ph108_B6
zmp_ph108_B7
zmp_ph108_C4
zmp_ph108_C5
zmp_ph108_D4
zmp_ph97_G6
zmp_ph97_G9
zmp_ph97_H2
zmp_ph97_B10
zmp_ph97_C2
zmp_ph97_C4
zmp_ph97_B8
zmp_ph97_C1
zmp_ph97_D2
zmp_ph97_D5
zmp_ph111_G5
zmp_ph111_G11
zmp_ph111_H1
zmp_ph111_B1a
zmp_ph111_B5
zmp_ph111_B8
zmp_ph111_B6
zmp_ph111_B7
zmp_ph111_B9
zmp_ph111_B10
zmp_ph114_G4
zmp_ph114_G11
zmp_ph114_H1
zmp_ph114_H2
zmp_ph114_A9
zmp_ph114_A11
zmp_ph114_B2
zmp_ph114_B4
zmp_ph114_D8
zmp_ph114_E2

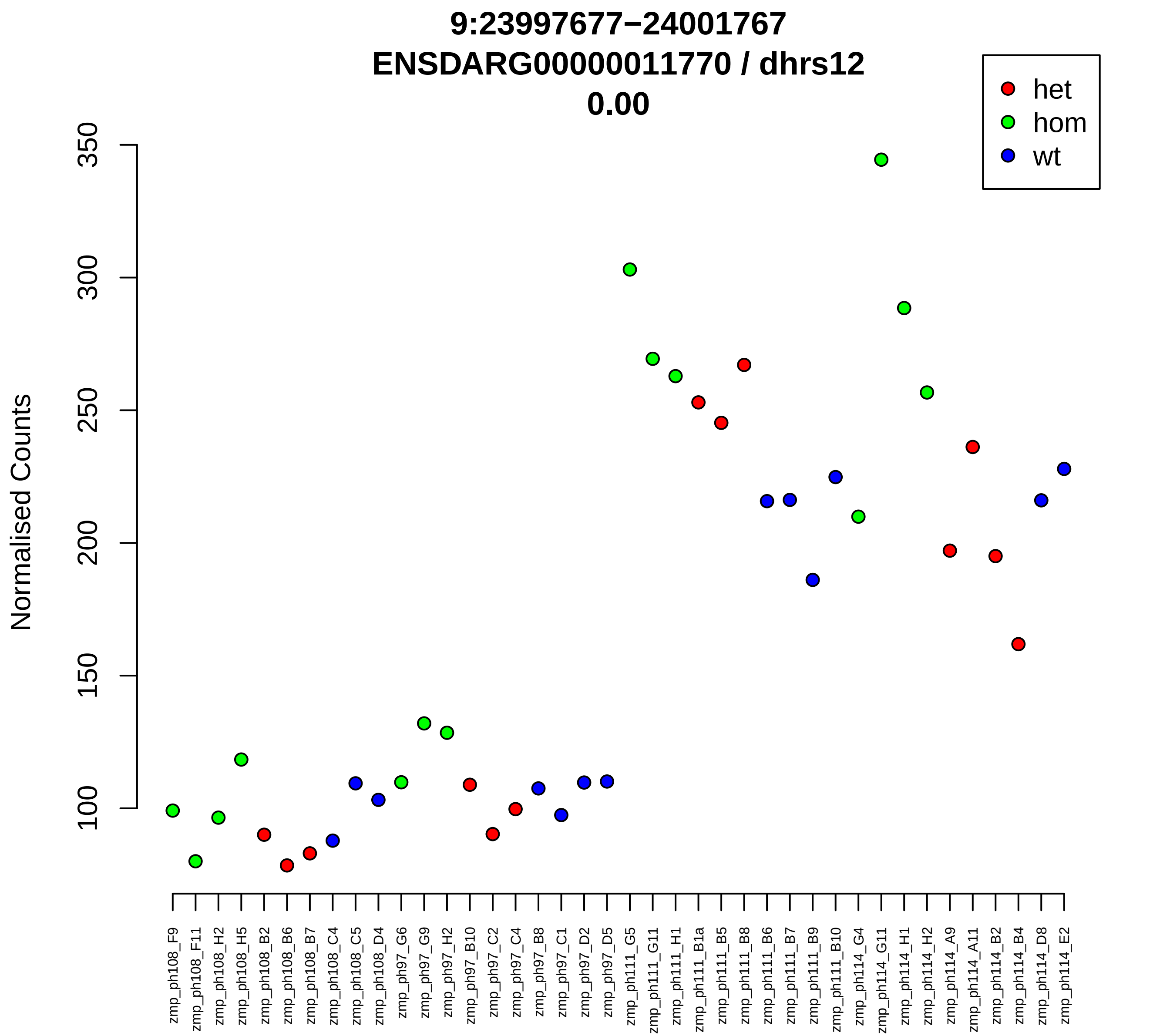
9:23997677−24001767
ENSDARG00000011770 / dhrs12
0.00
het
hom
wt
Normalised Counts
100
150
200
250
300
350
zmp_ph108_F9
zmp_ph108_F11
zmp_ph108_H2
zmp_ph108_H5
zmp_ph108_B2
zmp_ph108_B6
zmp_ph108_B7
zmp_ph108_C4
zmp_ph108_C5
zmp_ph108_D4
zmp_ph97_G6
zmp_ph97_G9
zmp_ph97_H2
zmp_ph97_B10
zmp_ph97_C2
zmp_ph97_C4
zmp_ph97_B8
zmp_ph97_C1
zmp_ph97_D2
zmp_ph97_D5
zmp_ph111_G5
zmp_ph111_G11
zmp_ph111_H1
zmp_ph111_B1a
zmp_ph111_B5
zmp_ph111_B8
zmp_ph111_B6
zmp_ph111_B7
zmp_ph111_B9
zmp_ph111_B10
zmp_ph114_G4
zmp_ph114_G11
zmp_ph114_H1
zmp_ph114_H2
zmp_ph114_A9
zmp_ph114_A11
zmp_ph114_B2
zmp_ph114_B4
zmp_ph114_D8
zmp_ph114_E2

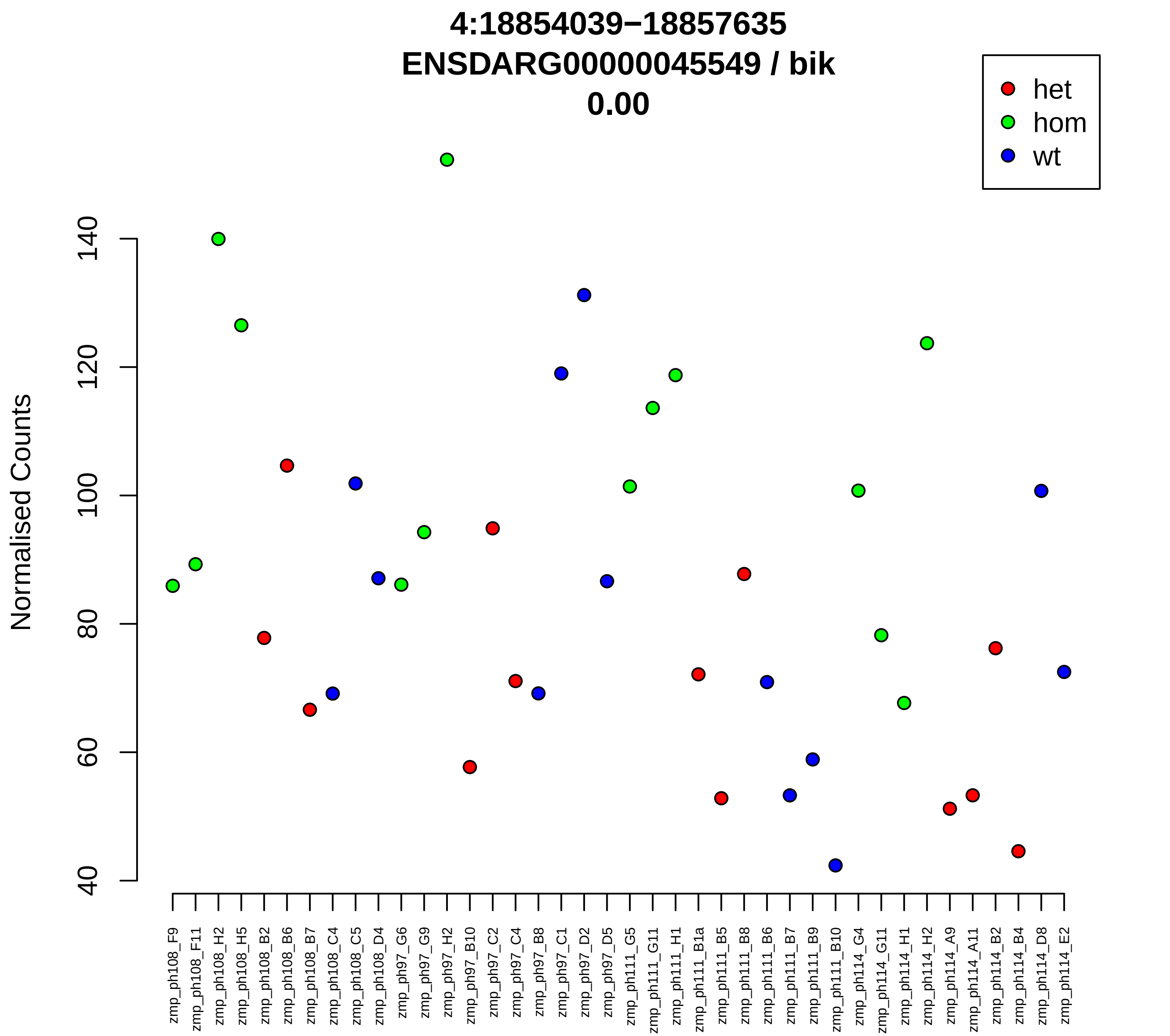

4:18854039−18857635
ENSDARG00000045549 / bik
0.00
het
hom
wt
Normalised Counts
40
60
80
100
120
140
zmp_ph108_F9
zmp_ph108_F11
zmp_ph108_H2
zmp_ph108_H5
zmp_ph108_B2
zmp_ph108_B6
zmp_ph108_B7
zmp_ph108_C4
zmp_ph108_C5
zmp_ph108_D4
zmp_ph97_G6
zmp_ph97_G9
zmp_ph97_H2
zmp_ph97_B10
zmp_ph97_C2
zmp_ph97_C4
zmp_ph97_B8
zmp_ph97_C1
zmp_ph97_D2
zmp_ph97_D5
zmp_ph111_G5
zmp_ph111_G11
zmp_ph111_H1
zmp_ph111_B1a
zmp_ph111_B5
zmp_ph111_B8
zmp_ph111_B6
zmp_ph111_B7
zmp_ph111_B9
zmp_ph111_B10
zmp_ph114_G4
zmp_ph114_G11
zmp_ph114_H1
zmp_ph114_H2
zmp_ph114_A9
zmp_ph114_A11
zmp_ph114_B2
zmp_ph114_B4
zmp_ph114_D8
zmp_ph114_E2

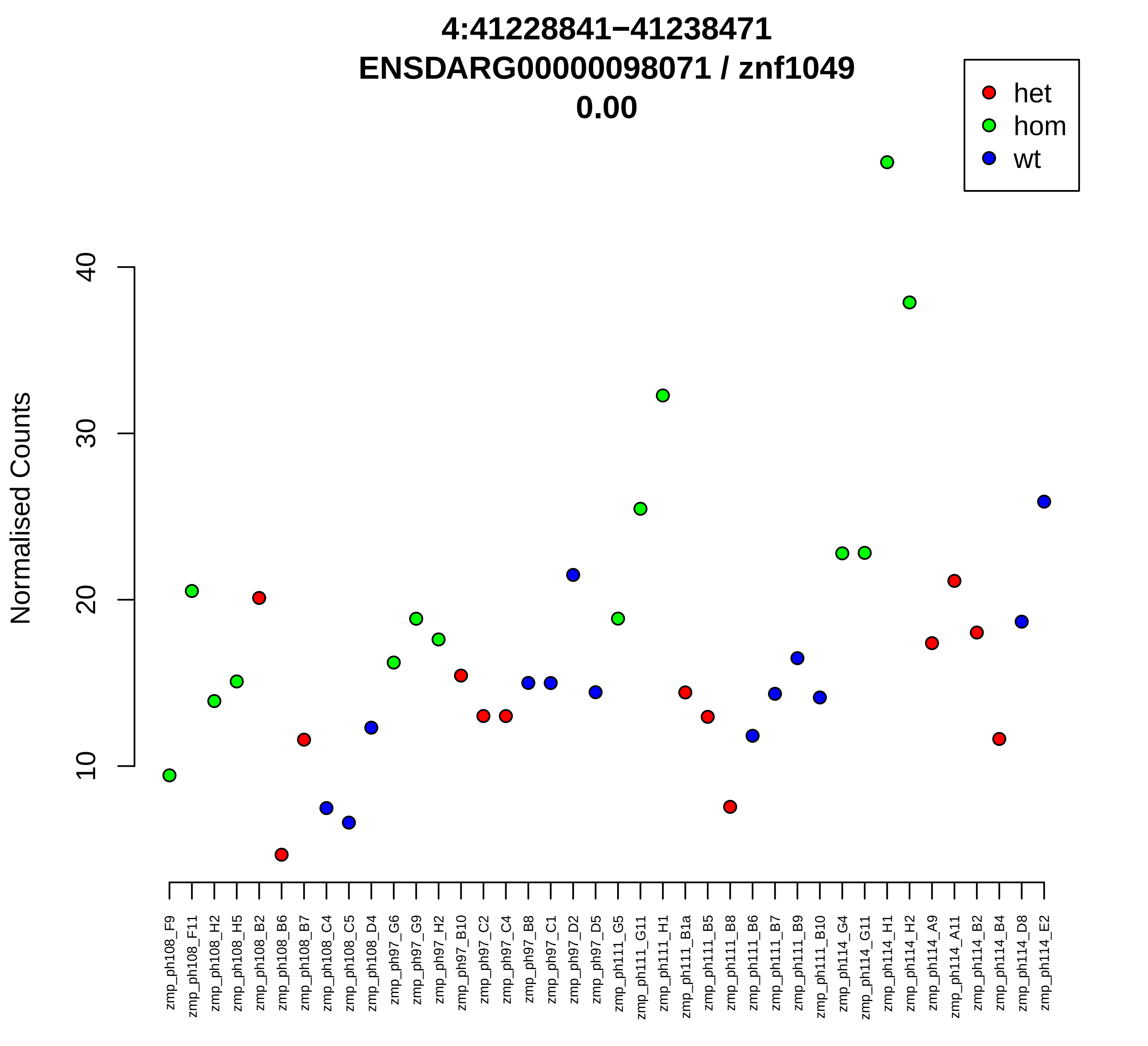

4:41228841−41238471
ENSDARG00000098071 / znf1049
0.00
het
hom
wt
Normalised Counts
10
20
30
40
zmp_ph108_F9
zmp_ph108_F11
zmp_ph108_H2
zmp_ph108_H5
zmp_ph108_B2
zmp_ph108_B6
zmp_ph108_B7
zmp_ph108_C4
zmp_ph108_C5
zmp_ph108_D4
zmp_ph97_G6
zmp_ph97_G9
zmp_ph97_H2
zmp_ph97_B10
zmp_ph97_C2
zmp_ph97_C4
zmp_ph97_B8
zmp_ph97_C1
zmp_ph97_D2
zmp_ph97_D5
zmp_ph111_G5
zmp_ph111_G11
zmp_ph111_H1
zmp_ph111_B1a
zmp_ph111_B5
zmp_ph111_B8
zmp_ph111_B6
zmp_ph111_B7
zmp_ph111_B9
zmp_ph111_B10
zmp_ph114_G4
zmp_ph114_G11
zmp_ph114_H1
zmp_ph114_H2
zmp_ph114_A9
zmp_ph114_A11
zmp_ph114_B2
zmp_ph114_B4
zmp_ph114_D8
zmp_ph114_E2

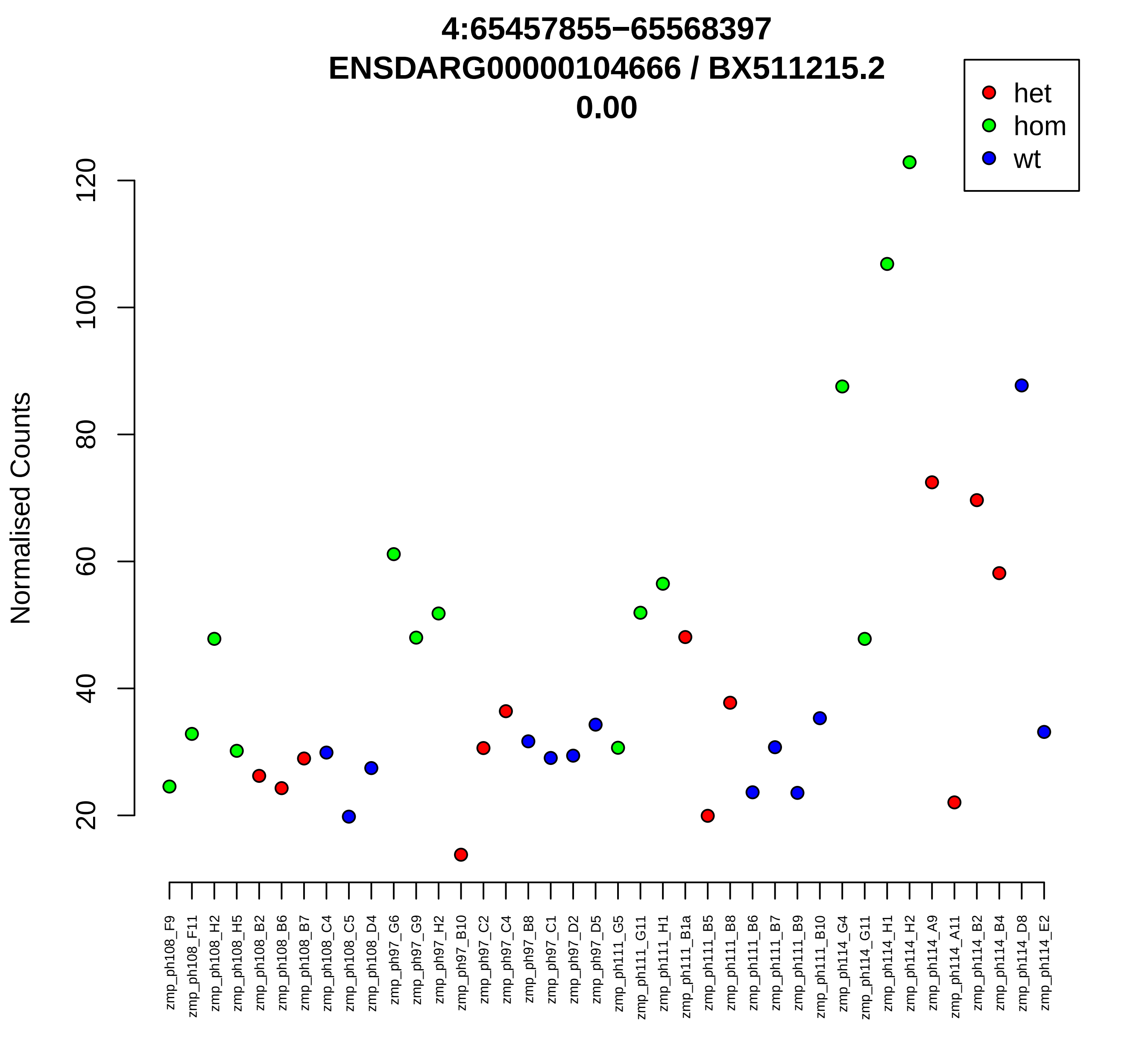
4:65457855−65568397
ENSDARG00000104666 / BX511215.2
0.00
het
hom
wt
Normalised Counts
20
40
60
80
100
120
zmp_ph108_F9
zmp_ph108_F11
zmp_ph108_H2
zmp_ph108_H5
zmp_ph108_B2
zmp_ph108_B6
zmp_ph108_B7
zmp_ph108_C4
zmp_ph108_C5
zmp_ph108_D4
zmp_ph97_G6
zmp_ph97_G9
zmp_ph97_H2
zmp_ph97_B10
zmp_ph97_C2
zmp_ph97_C4
zmp_ph97_B8
zmp_ph97_C1
zmp_ph97_D2
zmp_ph97_D5
zmp_ph111_G5
zmp_ph111_G11
zmp_ph111_H1
zmp_ph111_B1a
zmp_ph111_B5
zmp_ph111_B8
zmp_ph111_B6
zmp_ph111_B7
zmp_ph111_B9
zmp_ph111_B10
zmp_ph114_G4
zmp_ph114_G11
zmp_ph114_H1
zmp_ph114_H2
zmp_ph114_A9
zmp_ph114_A11
zmp_ph114_B2
zmp_ph114_B4
zmp_ph114_D8
zmp_ph114_E2

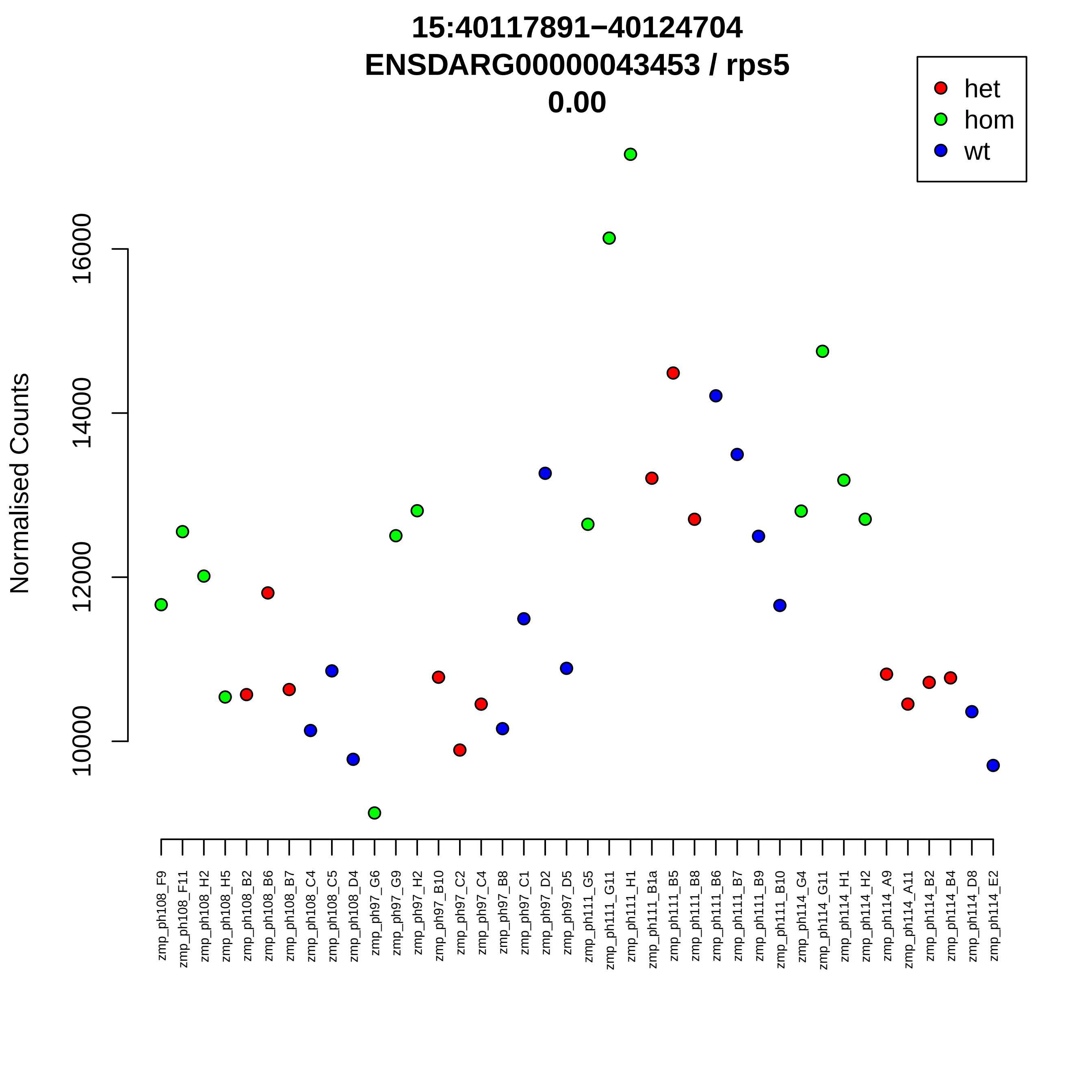
15:40117891−40124704
ENSDARG00000043453 / rps5
0.00
het
hom
wt
Normalised Counts
10000
12000
14000
16000
zmp_ph108_F9
zmp_ph108_F11
zmp_ph108_H2
zmp_ph108_H5
zmp_ph108_B2
zmp_ph108_B6
zmp_ph108_B7
zmp_ph108_C4
zmp_ph108_C5
zmp_ph108_D4
zmp_ph97_G6
zmp_ph97_G9
zmp_ph97_H2
zmp_ph97_B10
zmp_ph97_C2
zmp_ph97_C4
zmp_ph97_B8
zmp_ph97_C1
zmp_ph97_D2
zmp_ph97_D5
zmp_ph111_G5
zmp_ph111_G11
zmp_ph111_H1
zmp_ph111_B1a
zmp_ph111_B5
zmp_ph111_B8
zmp_ph111_B6
zmp_ph111_B7
zmp_ph111_B9
zmp_ph111_B10
zmp_ph114_G4
zmp_ph114_G11
zmp_ph114_H1
zmp_ph114_H2
zmp_ph114_A9
zmp_ph114_A11
zmp_ph114_B2
zmp_ph114_B4
zmp_ph114_D8
zmp_ph114_E2

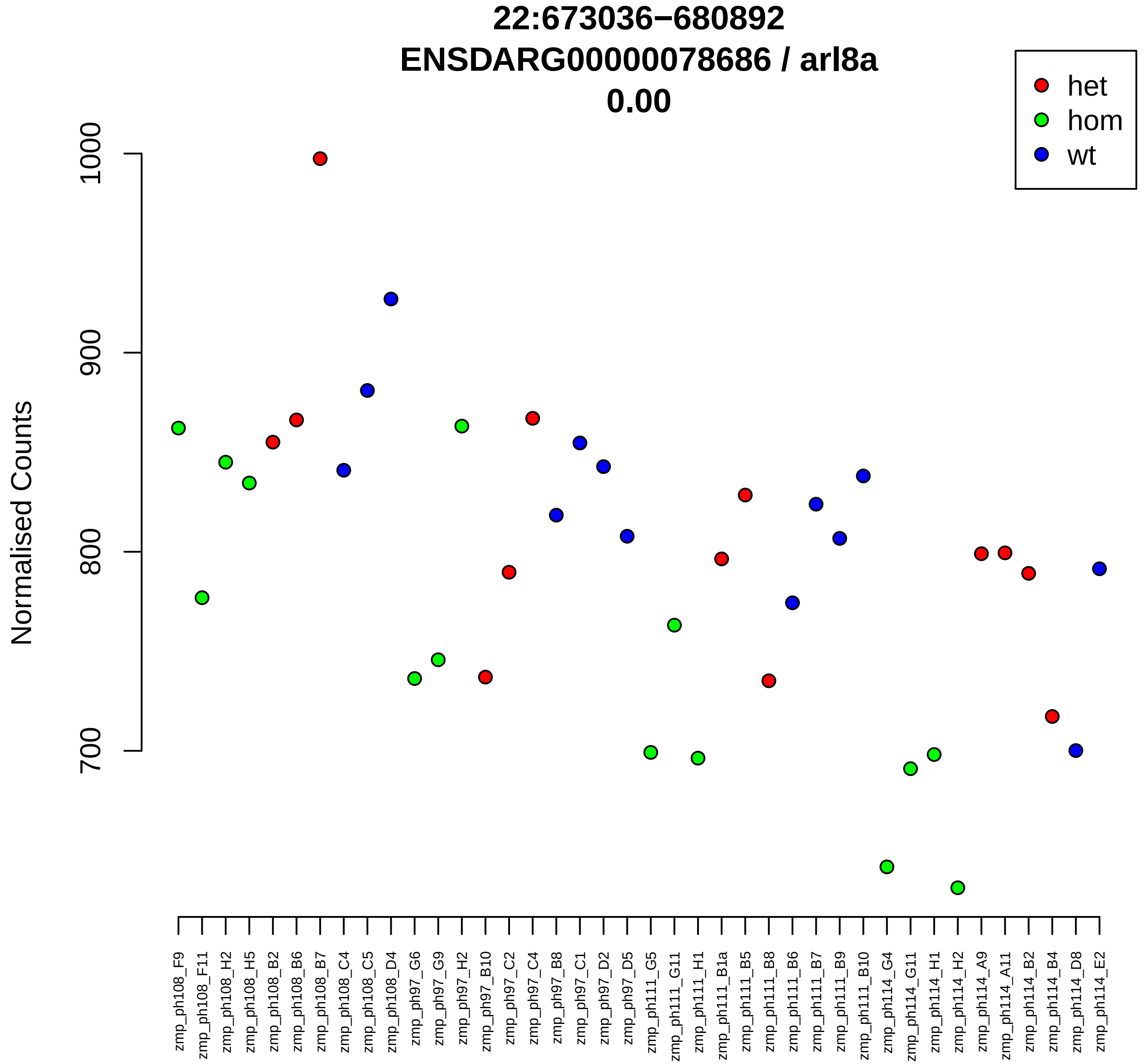
22:673036−680892
ENSDARG00000078686 / arl8a
0.00
het
hom
wt
Normalised Counts
1000
900
800
700
zmp_ph108_F9
zmp_ph108_F11
zmp_ph108_H2
zmp_ph108_H5
zmp_ph108_B2
zmp_ph108_B6
zmp_ph108_B7
zmp_ph108_C4
zmp_ph108_C5
zmp_ph108_D4
zmp_ph97_G6
zmp_ph97_G9
zmp_ph97_H2
zmp_ph97_B10
zmp_ph97_C2
zmp_ph97_C4
zmp_ph97_B8
zmp_ph97_C1
zmp_ph97_D2
zmp_ph97_D5
zmp_ph111_G5
zmp_ph111_G11
zmp_ph111_H1
zmp_ph111_B1a
zmp_ph111_B5
zmp_ph111_B8
zmp_ph111_B6
zmp_ph111_B7
zmp_ph111_B9
zmp_ph111_B10
zmp_ph114_G4
zmp_ph114_G11
zmp_ph114_H1
zmp_ph114_H2
zmp_ph114_A9
zmp_ph114_A11
zmp_ph114_B2
zmp_ph114_B4
zmp_ph114_D8
zmp_ph114_E2

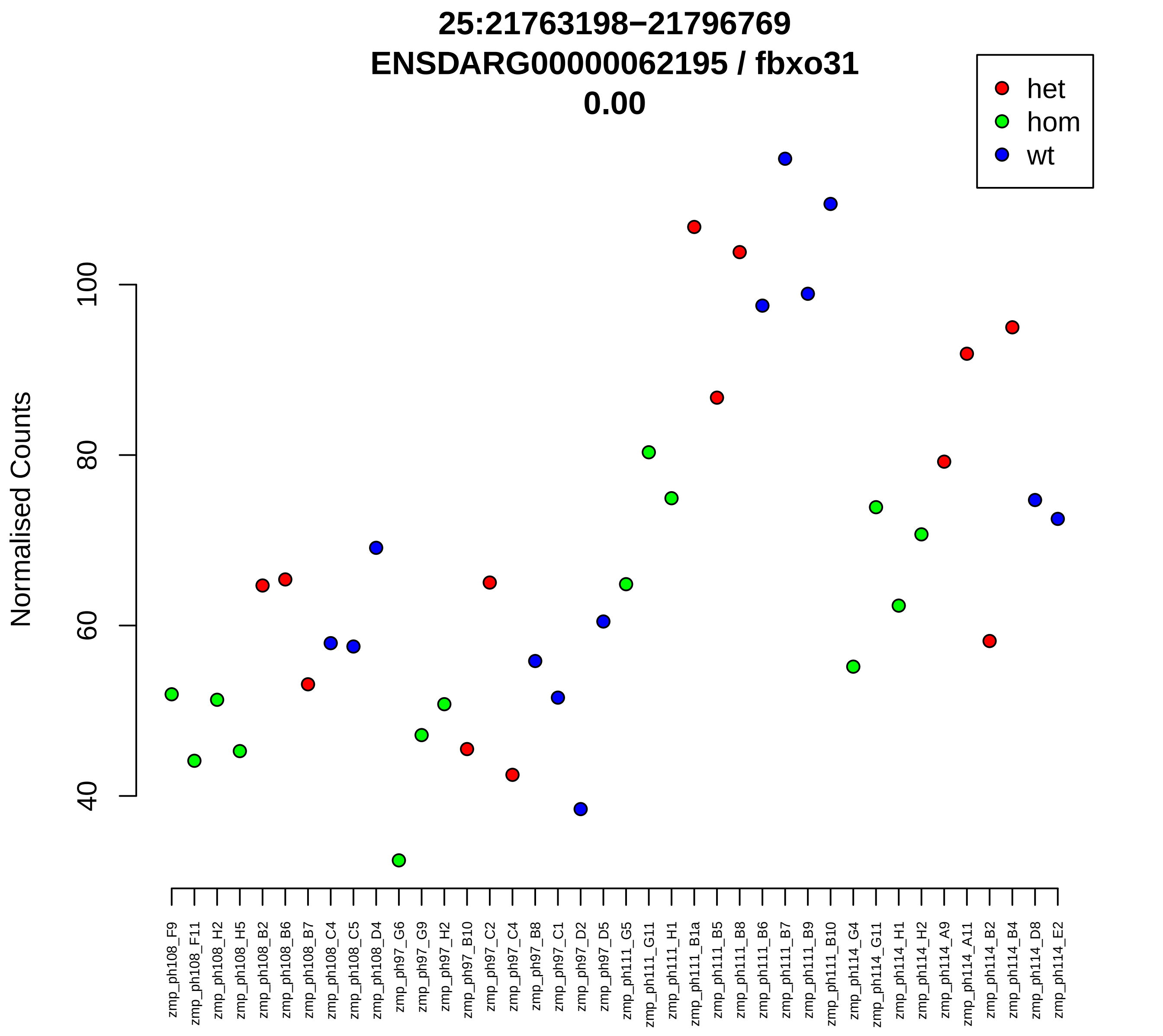

25:21763198−21796769
ENSDARG00000062195 / fbxo31
0.00
het
hom
wt
Normalised Counts
40
60
80
100
zmp_ph108_F9
zmp_ph108_F11
zmp_ph108_H2
zmp_ph108_H5
zmp_ph108_B2
zmp_ph108_B6
zmp_ph108_B7
zmp_ph108_C4
zmp_ph108_C5
zmp_ph108_D4
zmp_ph97_G6
zmp_ph97_G9
zmp_ph97_H2
zmp_ph97_B10
zmp_ph97_C2
zmp_ph97_C4
zmp_ph97_B8
zmp_ph97_C1
zmp_ph97_D2
zmp_ph97_D5
zmp_ph111_G5
zmp_ph111_G11
zmp_ph111_H1
zmp_ph111_B1a
zmp_ph111_B5
zmp_ph111_B8
zmp_ph111_B6
zmp_ph111_B7
zmp_ph111_B9
zmp_ph111_B10
zmp_ph114_G4
zmp_ph114_G11
zmp_ph114_H1
zmp_ph114_H2
zmp_ph114_A9
zmp_ph114_A11
zmp_ph114_B2
zmp_ph114_B4
zmp_ph114_D8
zmp_ph114_E2

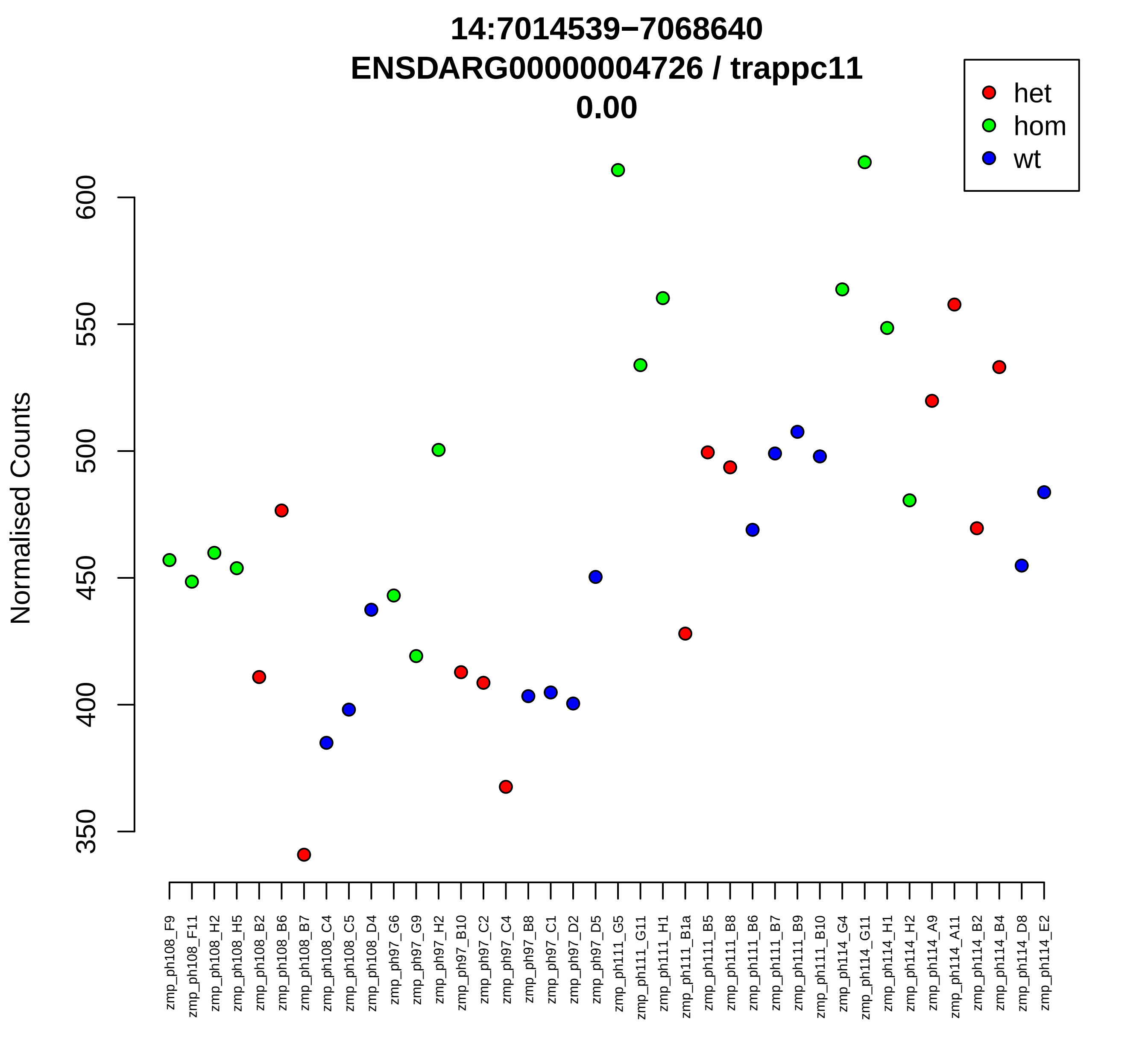

14:7014539−7068640
ENSDARG00000004726 / trappc11
0.00
het
hom
wt
Normalised Counts
350
400
450
500
550
600
zmp_ph108_F9
zmp_ph108_F11
zmp_ph108_H2
zmp_ph108_H5
zmp_ph108_B2
zmp_ph108_B6
zmp_ph108_B7
zmp_ph108_C4
zmp_ph108_C5
zmp_ph108_D4
zmp_ph97_G6
zmp_ph97_G9
zmp_ph97_H2
zmp_ph97_B10
zmp_ph97_C2
zmp_ph97_C4
zmp_ph97_B8
zmp_ph97_C1
zmp_ph97_D2
zmp_ph97_D5
zmp_ph111_G5
zmp_ph111_G11
zmp_ph111_H1
zmp_ph111_B1a
zmp_ph111_B5
zmp_ph111_B8
zmp_ph111_B6
zmp_ph111_B7
zmp_ph111_B9
zmp_ph111_B10
zmp_ph114_G4
zmp_ph114_G11
zmp_ph114_H1
zmp_ph114_H2
zmp_ph114_A9
zmp_ph114_A11
zmp_ph114_B2
zmp_ph114_B4
zmp_ph114_D8
zmp_ph114_E2

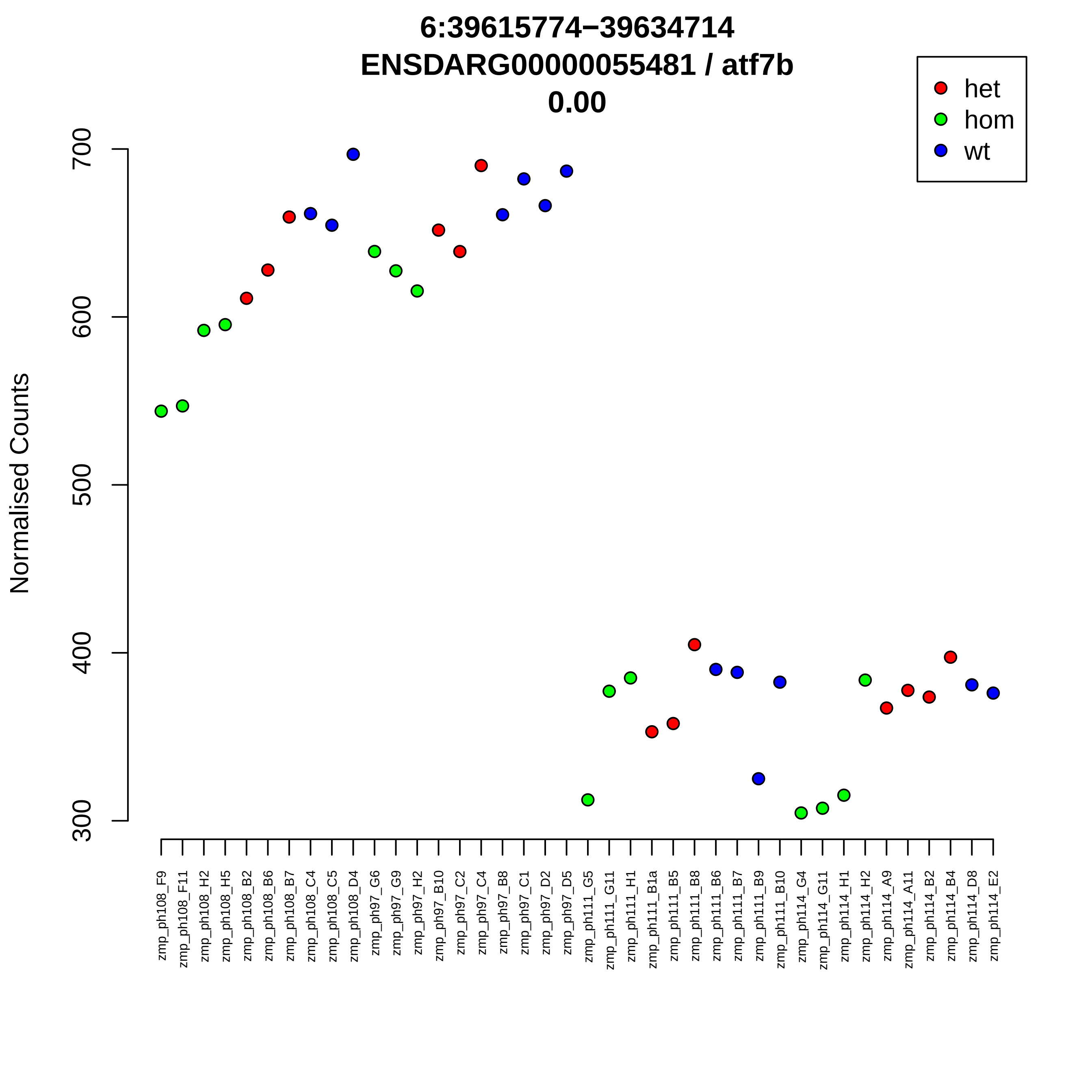

6:39615774−39634714
ENSDARG00000055481 / atf7b
0.00
het
hom
wt
Normalised Counts
300
400
500
600
700
zmp_ph108_F9
zmp_ph108_F11
zmp_ph108_H2
zmp_ph108_H5
zmp_ph108_B2
zmp_ph108_B6
zmp_ph108_B7
zmp_ph108_C4
zmp_ph108_C5
zmp_ph108_D4
zmp_ph97_G6
zmp_ph97_G9
zmp_ph97_H2
zmp_ph97_B10
zmp_ph97_C2
zmp_ph97_C4
zmp_ph97_B8
zmp_ph97_C1
zmp_ph97_D2
zmp_ph97_D5
zmp_ph111_G5
zmp_ph111_G11
zmp_ph111_H1
zmp_ph111_B1a
zmp_ph111_B5
zmp_ph111_B8
zmp_ph111_B6
zmp_ph111_B7
zmp_ph111_B9
zmp_ph111_B10
zmp_ph114_G4
zmp_ph114_G11
zmp_ph114_H1
zmp_ph114_H2
zmp_ph114_A9
zmp_ph114_A11
zmp_ph114_B2
zmp_ph114_B4
zmp_ph114_D8
zmp_ph114_E2

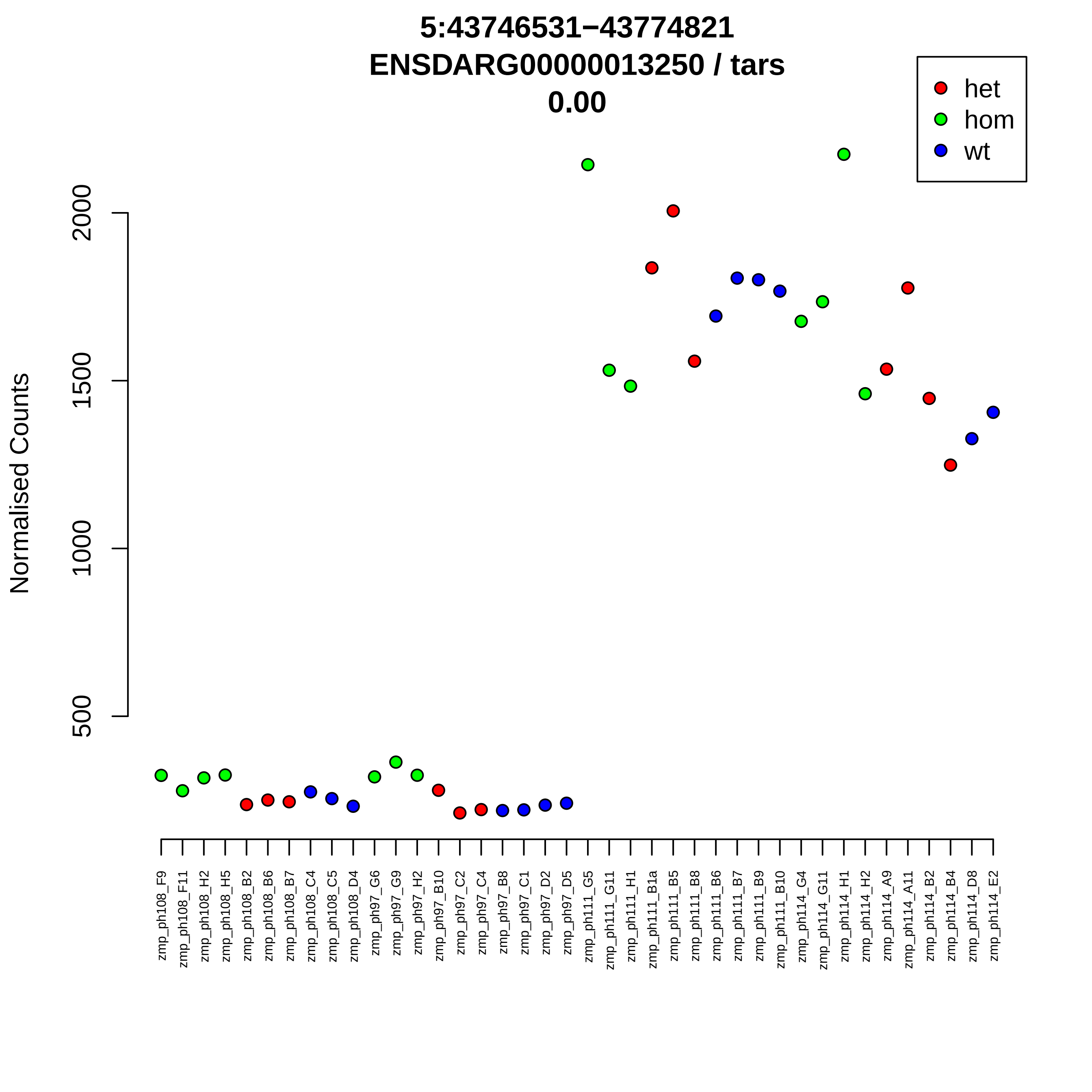
5:43746531−43774821
ENSDARG00000013250 / tars
0.00
het
hom
wt
Normalised Counts
500
1000
1500
2000
zmp_ph108_F9
zmp_ph108_F11
zmp_ph108_H2
zmp_ph108_H5
zmp_ph108_B2
zmp_ph108_B6
zmp_ph108_B7
zmp_ph108_C4
zmp_ph108_C5
zmp_ph108_D4
zmp_ph97_G6
zmp_ph97_G9
zmp_ph97_H2
zmp_ph97_B10
zmp_ph97_C2
zmp_ph97_C4
zmp_ph97_B8
zmp_ph97_C1
zmp_ph97_D2
zmp_ph97_D5
zmp_ph111_G5
zmp_ph111_G11
zmp_ph111_H1
zmp_ph111_B1a
zmp_ph111_B5
zmp_ph111_B8
zmp_ph111_B6
zmp_ph111_B7
zmp_ph111_B9
zmp_ph111_B10
zmp_ph114_G4
zmp_ph114_G11
zmp_ph114_H1
zmp_ph114_H2
zmp_ph114_A9
zmp_ph114_A11
zmp_ph114_B2
zmp_ph114_B4
zmp_ph114_D8
zmp_ph114_E2

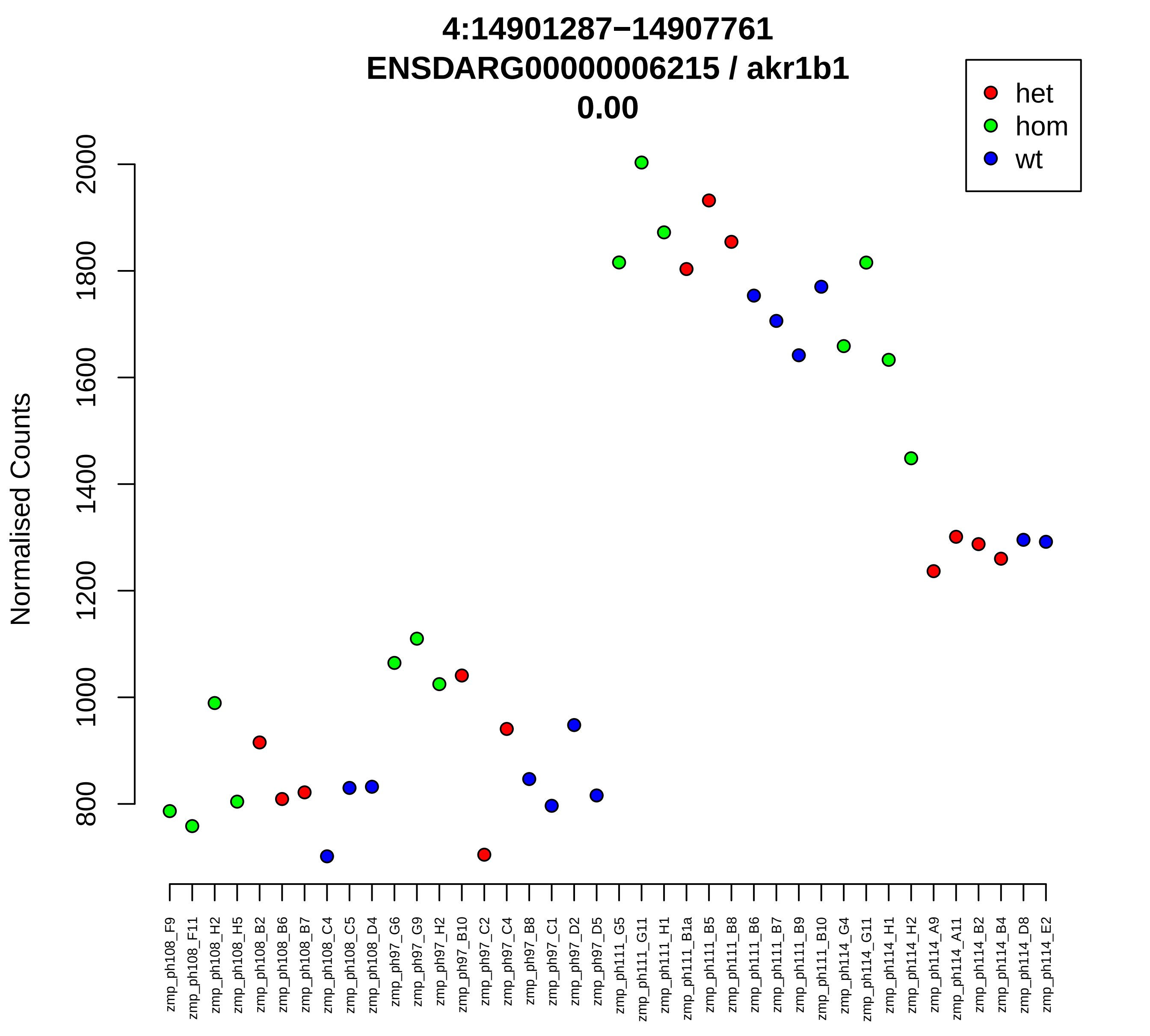

4:14901287−14907761
ENSDARG00000006215 / akr1b1
0.00
het
hom
wt
Normalised Counts
800
1000
1200
1400
1600
1800
2000
zmp_ph108_F9
zmp_ph108_F11
zmp_ph108_H2
zmp_ph108_H5
zmp_ph108_B2
zmp_ph108_B6
zmp_ph108_B7
zmp_ph108_C4
zmp_ph108_C5
zmp_ph108_D4
zmp_ph97_G6
zmp_ph97_G9
zmp_ph97_H2
zmp_ph97_B10
zmp_ph97_C2
zmp_ph97_C4
zmp_ph97_B8
zmp_ph97_C1
zmp_ph97_D2
zmp_ph97_D5
zmp_ph111_G5
zmp_ph111_G11
zmp_ph111_H1
zmp_ph111_B1a
zmp_ph111_B5
zmp_ph111_B8
zmp_ph111_B6
zmp_ph111_B7
zmp_ph111_B9
zmp_ph111_B10
zmp_ph114_G4
zmp_ph114_G11
zmp_ph114_H1
zmp_ph114_H2
zmp_ph114_A9
zmp_ph114_A11
zmp_ph114_B2
zmp_ph114_B4
zmp_ph114_D8
zmp_ph114_E2

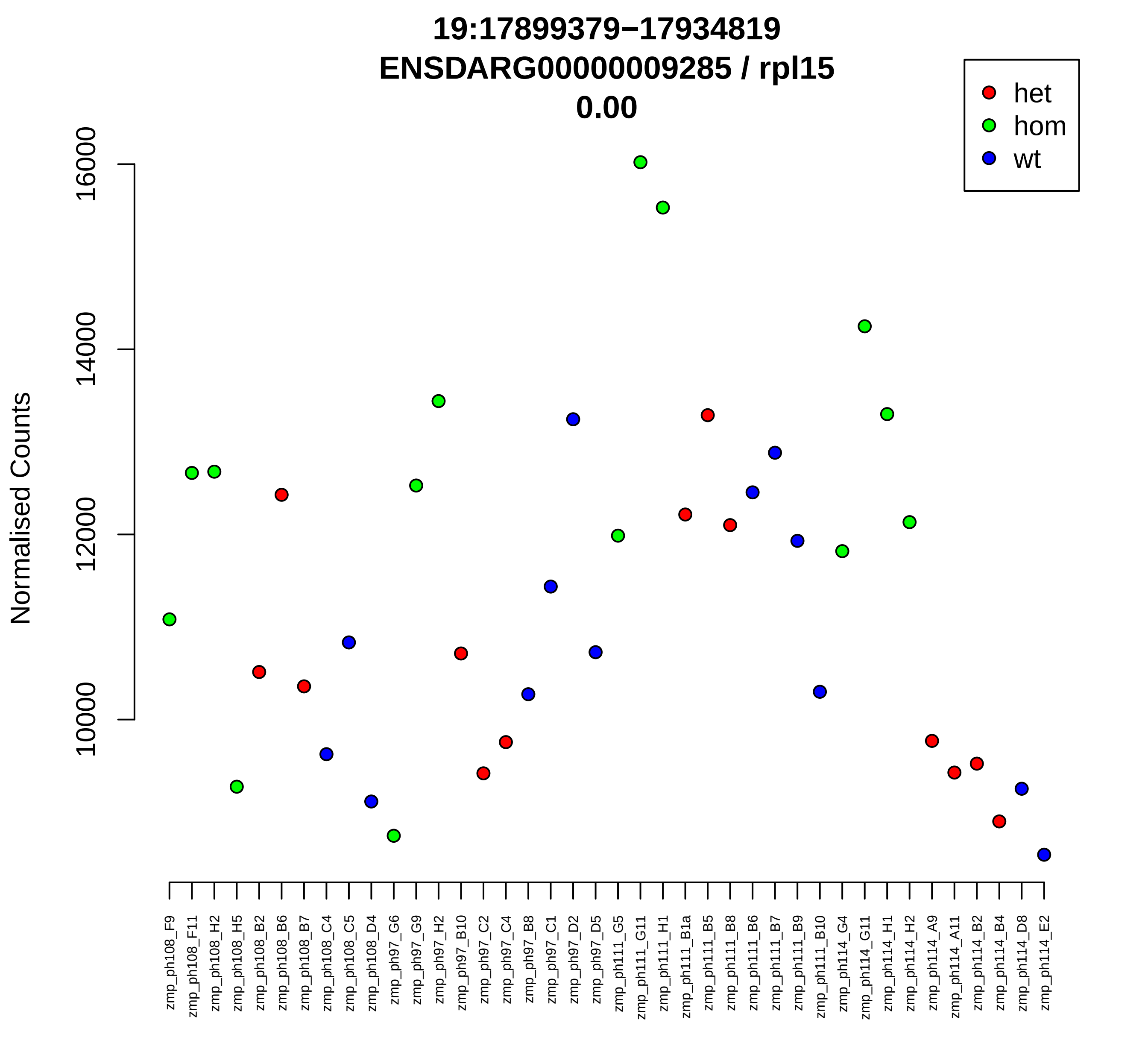

19:17899379−17934819
ENSDARG00000009285 / rpl15
0.00
het
hom
wt
Normalised Counts
10000
12000
14000
16000
zmp_ph108_F9
zmp_ph108_F11
zmp_ph108_H2
zmp_ph108_H5
zmp_ph108_B2
zmp_ph108_B6
zmp_ph108_B7
zmp_ph108_C4
zmp_ph108_C5
zmp_ph108_D4
zmp_ph97_G6
zmp_ph97_G9
zmp_ph97_H2
zmp_ph97_B10
zmp_ph97_C2
zmp_ph97_C4
zmp_ph97_B8
zmp_ph97_C1
zmp_ph97_D2
zmp_ph97_D5
zmp_ph111_G5
zmp_ph111_G11
zmp_ph111_H1
zmp_ph111_B1a
zmp_ph111_B5
zmp_ph111_B8
zmp_ph111_B6
zmp_ph111_B7
zmp_ph111_B9
zmp_ph111_B10
zmp_ph114_G4
zmp_ph114_G11
zmp_ph114_H1
zmp_ph114_H2
zmp_ph114_A9
zmp_ph114_A11
zmp_ph114_B2
zmp_ph114_B4
zmp_ph114_D8
zmp_ph114_E2

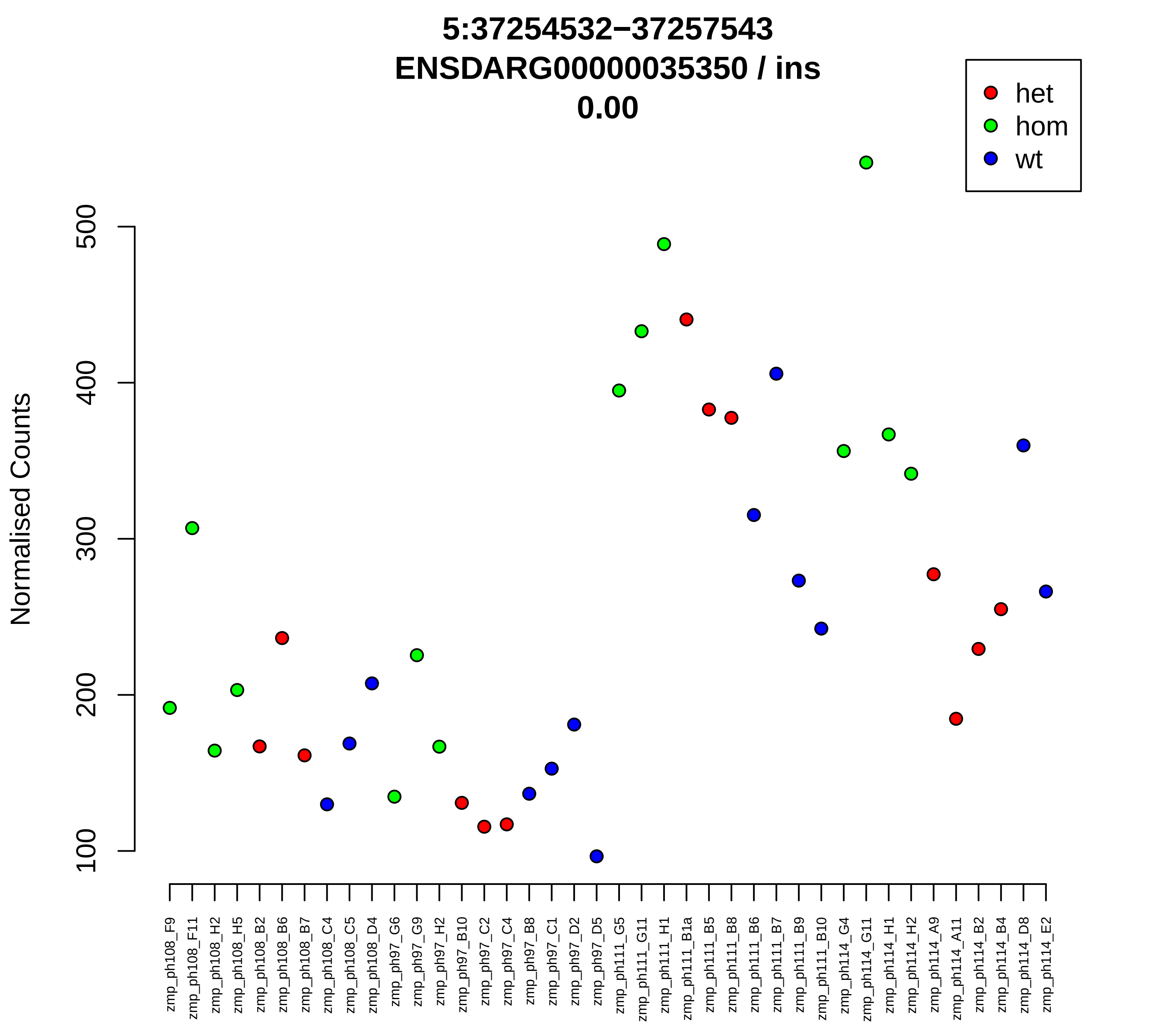
5:37254532−37257543
ENSDARG00000035350 / ins
0.00
het
hom
wt
Normalised Counts
100
200
300
400
500
zmp_ph108_F9
zmp_ph108_F11
zmp_ph108_H2
zmp_ph108_H5
zmp_ph108_B2
zmp_ph108_B6
zmp_ph108_B7
zmp_ph108_C4
zmp_ph108_C5
zmp_ph108_D4
zmp_ph97_G6
zmp_ph97_G9
zmp_ph97_H2
zmp_ph97_B10
zmp_ph97_C2
zmp_ph97_C4
zmp_ph97_B8
zmp_ph97_C1
zmp_ph97_D2
zmp_ph97_D5
zmp_ph111_G5
zmp_ph111_G11
zmp_ph111_H1
zmp_ph111_B1a
zmp_ph111_B5
zmp_ph111_B8
zmp_ph111_B6
zmp_ph111_B7
zmp_ph111_B9
zmp_ph111_B10
zmp_ph114_G4
zmp_ph114_G11
zmp_ph114_H1
zmp_ph114_H2
zmp_ph114_A9
zmp_ph114_A11
zmp_ph114_B2
zmp_ph114_B4
zmp_ph114_D8
zmp_ph114_E2

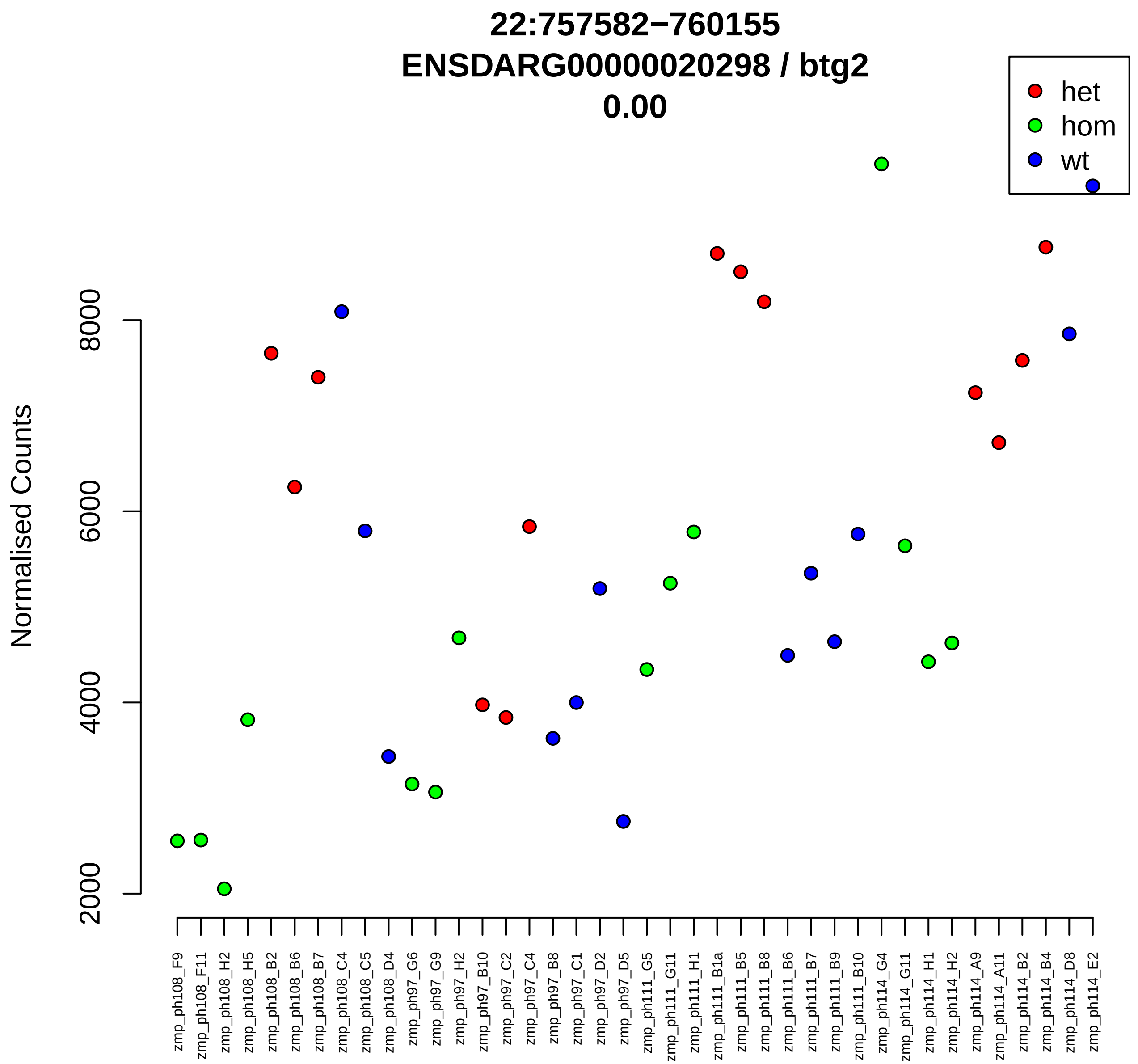

22:757582−760155
ENSDARG00000020298 / btg2
0.00
het
hom
wt
Normalised Counts
2000
4000
6000
8000
zmp_ph108_F9
zmp_ph108_F11
zmp_ph108_H2
zmp_ph108_H5
zmp_ph108_B2
zmp_ph108_B6
zmp_ph108_B7
zmp_ph108_C4
zmp_ph108_C5
zmp_ph108_D4
zmp_ph97_G6
zmp_ph97_G9
zmp_ph97_H2
zmp_ph97_B10
zmp_ph97_C2
zmp_ph97_C4
zmp_ph97_B8
zmp_ph97_C1
zmp_ph97_D2
zmp_ph97_D5
zmp_ph111_G5
zmp_ph111_G11
zmp_ph111_H1
zmp_ph111_B1a
zmp_ph111_B5
zmp_ph111_B8
zmp_ph111_B6
zmp_ph111_B7
zmp_ph111_B9
zmp_ph111_B10
zmp_ph114_G4
zmp_ph114_G11
zmp_ph114_H1
zmp_ph114_H2
zmp_ph114_A9
zmp_ph114_A11
zmp_ph114_B2
zmp_ph114_B4
zmp_ph114_D8
zmp_ph114_E2

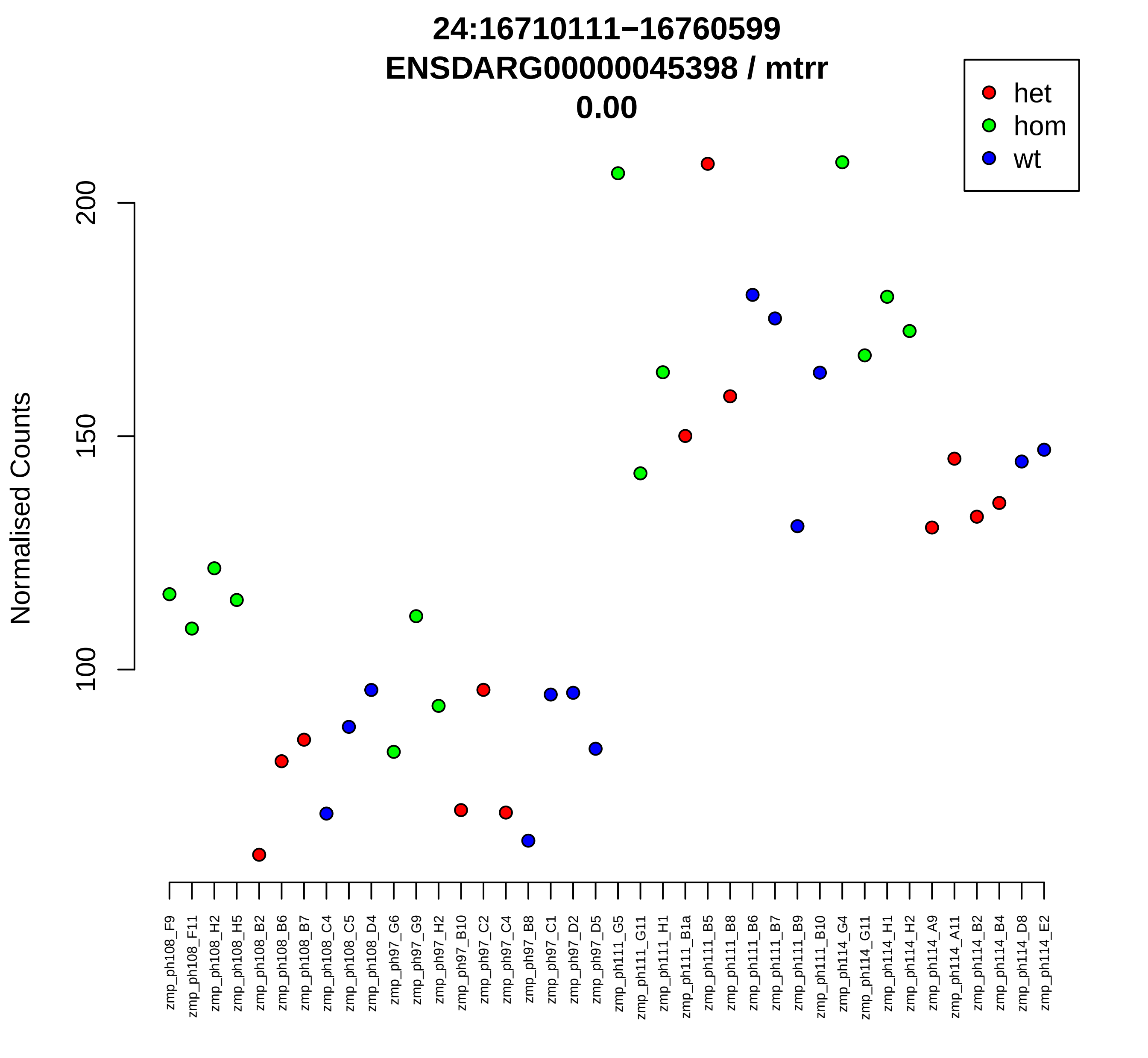
24:16710111−16760599
ENSDARG00000045398 / mtrr
0.00
het
hom
wt
Normalised Counts
100
150
200
zmp_ph108_F9
zmp_ph108_F11
zmp_ph108_H2
zmp_ph108_H5
zmp_ph108_B2
zmp_ph108_B6
zmp_ph108_B7
zmp_ph108_C4
zmp_ph108_C5
zmp_ph108_D4
zmp_ph97_G6
zmp_ph97_G9
zmp_ph97_H2
zmp_ph97_B10
zmp_ph97_C2
zmp_ph97_C4
zmp_ph97_B8
zmp_ph97_C1
zmp_ph97_D2
zmp_ph97_D5
zmp_ph111_G5
zmp_ph111_G11
zmp_ph111_H1
zmp_ph111_B1a
zmp_ph111_B5
zmp_ph111_B8
zmp_ph111_B6
zmp_ph111_B7
zmp_ph111_B9
zmp_ph111_B10
zmp_ph114_G4
zmp_ph114_G11
zmp_ph114_H1
zmp_ph114_H2
zmp_ph114_A9
zmp_ph114_A11
zmp_ph114_B2
zmp_ph114_B4
zmp_ph114_D8
zmp_ph114_E2

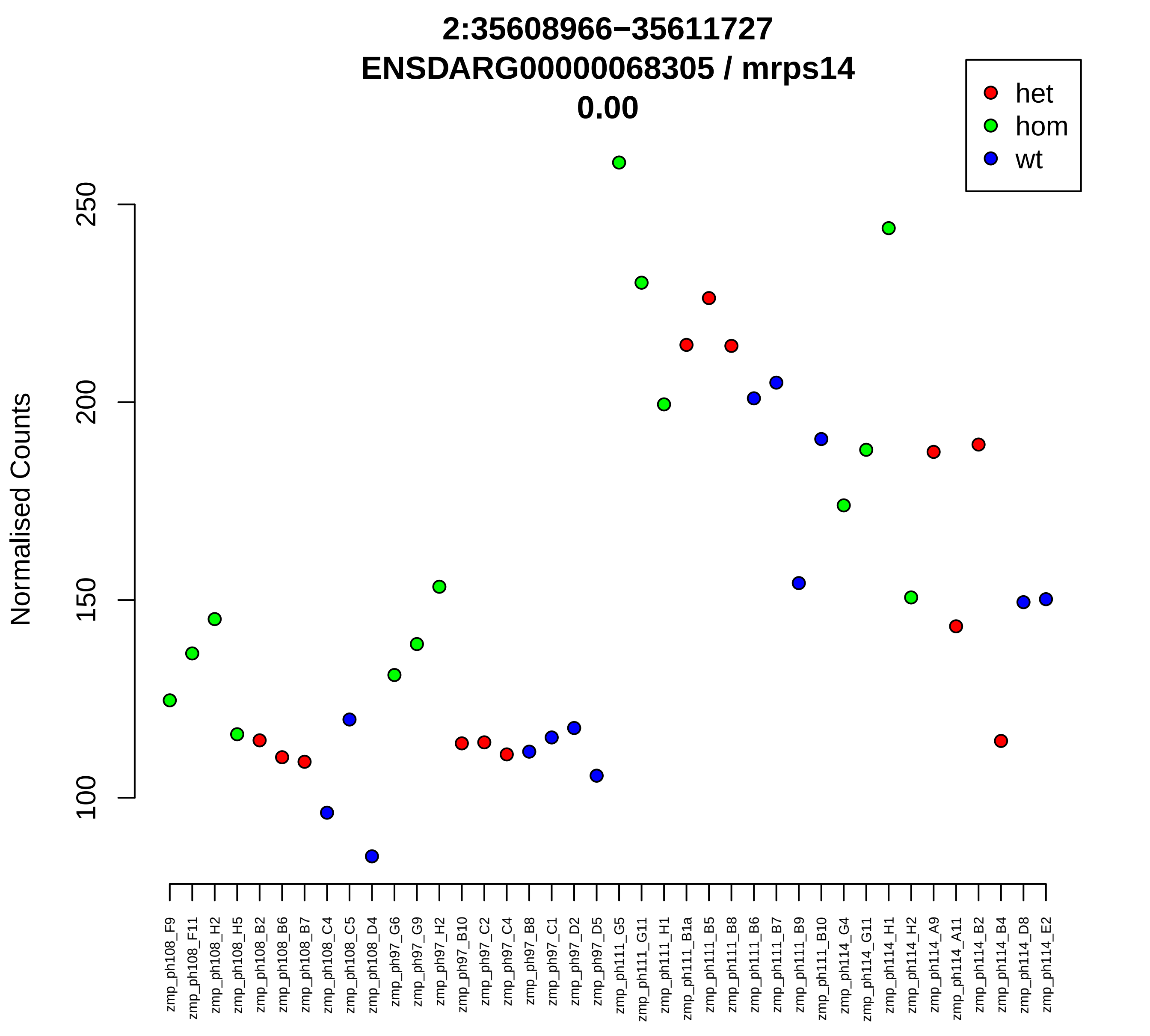

2:35608966−35611727
ENSDARG00000068305 / mrps14
0.00
het
hom
wt
Normalised Counts
100
150
200
250
zmp_ph108_F9
zmp_ph108_F11
zmp_ph108_H2
zmp_ph108_H5
zmp_ph108_B2
zmp_ph108_B6
zmp_ph108_B7
zmp_ph108_C4
zmp_ph108_C5
zmp_ph108_D4
zmp_ph97_G6
zmp_ph97_G9
zmp_ph97_H2
zmp_ph97_B10
zmp_ph97_C2
zmp_ph97_C4
zmp_ph97_B8
zmp_ph97_C1
zmp_ph97_D2
zmp_ph97_D5
zmp_ph111_G5
zmp_ph111_G11
zmp_ph111_H1
zmp_ph111_B1a
zmp_ph111_B5
zmp_ph111_B8
zmp_ph111_B6
zmp_ph111_B7
zmp_ph111_B9
zmp_ph111_B10
zmp_ph114_G4
zmp_ph114_G11
zmp_ph114_H1
zmp_ph114_H2
zmp_ph114_A9
zmp_ph114_A11
zmp_ph114_B2
zmp_ph114_B4
zmp_ph114_D8
zmp_ph114_E2

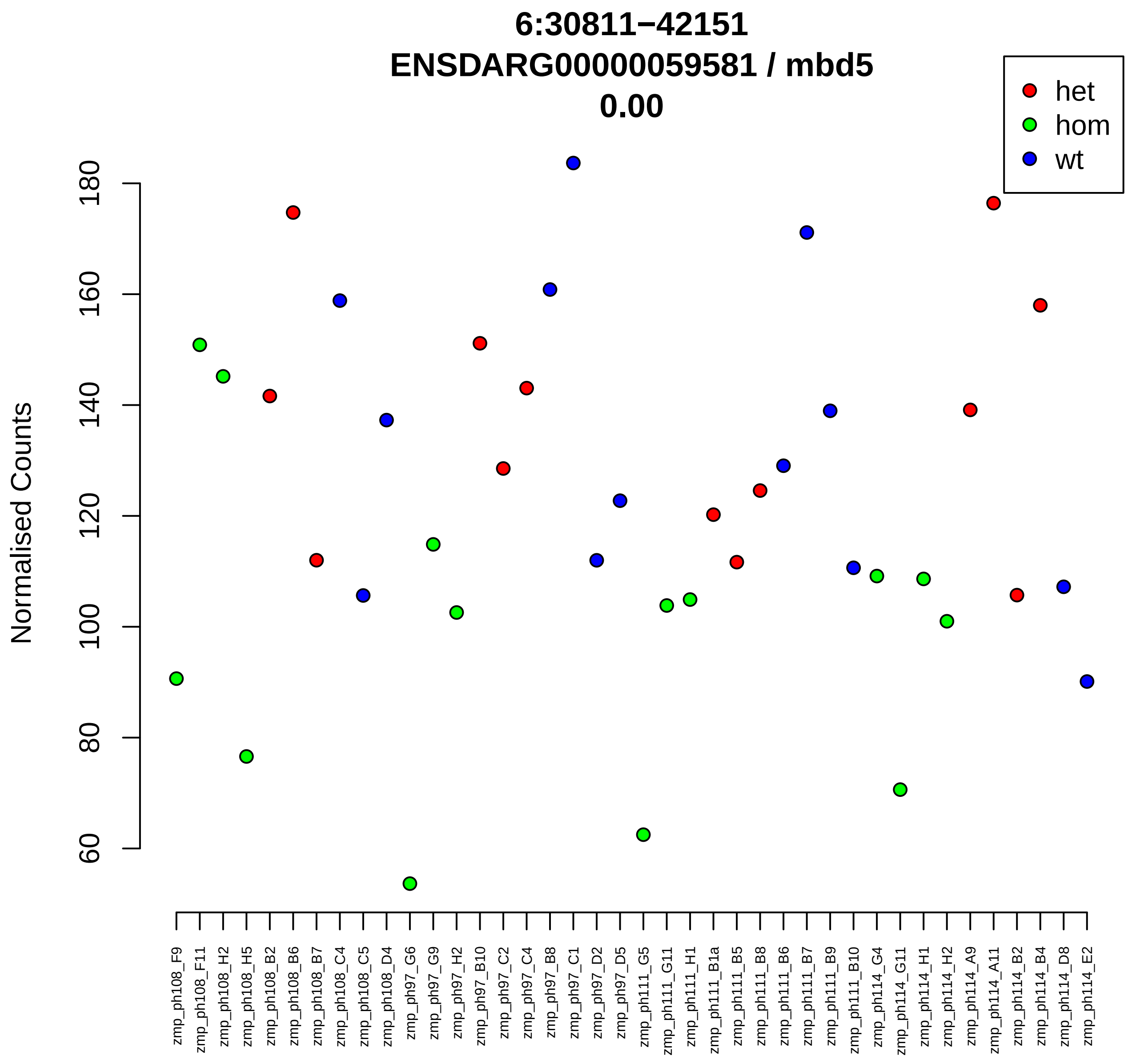
6:30811−42151
ENSDARG00000059581 / mbd5
0.00
Normalised Counts
het
hom
wt
60
80
100
120
140
160
180
zmp_ph108_F9
zmp_ph108_F11
zmp_ph108_H2
zmp_ph108_H5
zmp_ph108_B2
zmp_ph108_B6
zmp_ph108_B7
zmp_ph108_C4
zmp_ph108_C5
zmp_ph108_D4
zmp_ph97_G6
zmp_ph97_G9
zmp_ph97_H2
zmp_ph97_B10
zmp_ph97_C2
zmp_ph97_C4
zmp_ph97_B8
zmp_ph97_C1
zmp_ph97_D2
zmp_ph97_D5
zmp_ph111_G5
zmp_ph111_G11
zmp_ph111_H1
zmp_ph111_B1a
zmp_ph111_B5
zmp_ph111_B8
zmp_ph111_B6
zmp_ph111_B7
zmp_ph111_B9
zmp_ph111_B10
zmp_ph114_G4
zmp_ph114_G11
zmp_ph114_H1
zmp_ph114_H2
zmp_ph114_A9
zmp_ph114_A11
zmp_ph114_B2
zmp_ph114_B4
zmp_ph114_D8
zmp_ph114_E2

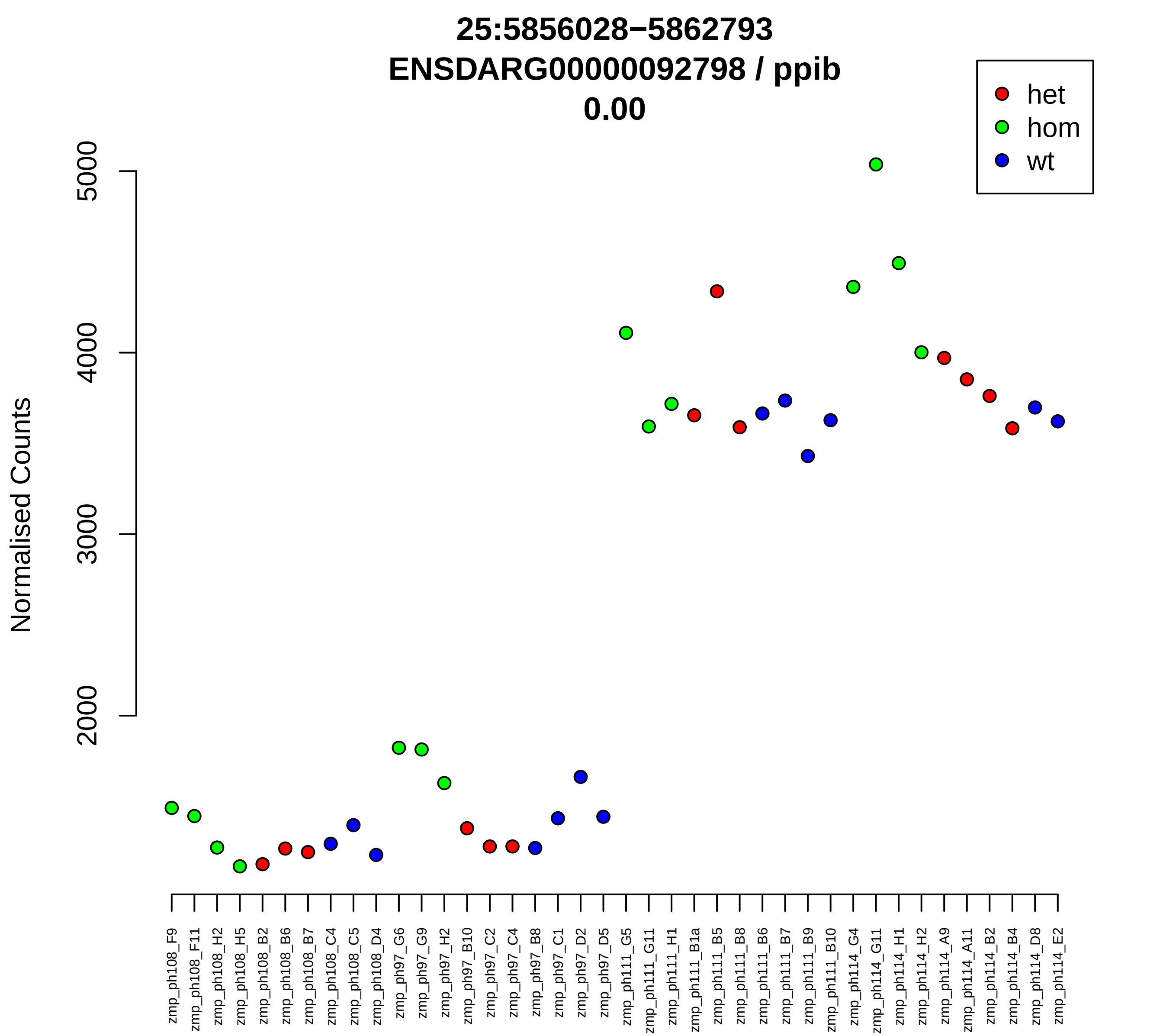
25:5856028−5862793
ENSDARG00000092798 / ppib
0.00
het
hom
wt
Normalised Counts
5000
4000
3000
2000
zmp_ph108_F9
zmp_ph108_F11
zmp_ph108_H2
zmp_ph108_H5
zmp_ph108_B2
zmp_ph108_B6
zmp_ph108_B7
zmp_ph108_C4
zmp_ph108_C5
zmp_ph108_D4
zmp_ph97_G6
zmp_ph97_G9
zmp_ph97_H2
zmp_ph97_B10
zmp_ph97_C2
zmp_ph97_C4
zmp_ph97_B8
zmp_ph97_C1
zmp_ph97_D2
zmp_ph97_D5
zmp_ph111_G5
zmp_ph111_G11
zmp_ph111_H1
zmp_ph111_B1a
zmp_ph111_B5
zmp_ph111_B8
zmp_ph111_B6
zmp_ph111_B7
zmp_ph111_B9
zmp_ph111_B10
zmp_ph114_G4
zmp_ph114_G11
zmp_ph114_H1
zmp_ph114_H2
zmp_ph114_A9
zmp_ph114_A11
zmp_ph114_B2
zmp_ph114_B4
zmp_ph114_D8
zmp_ph114_E2

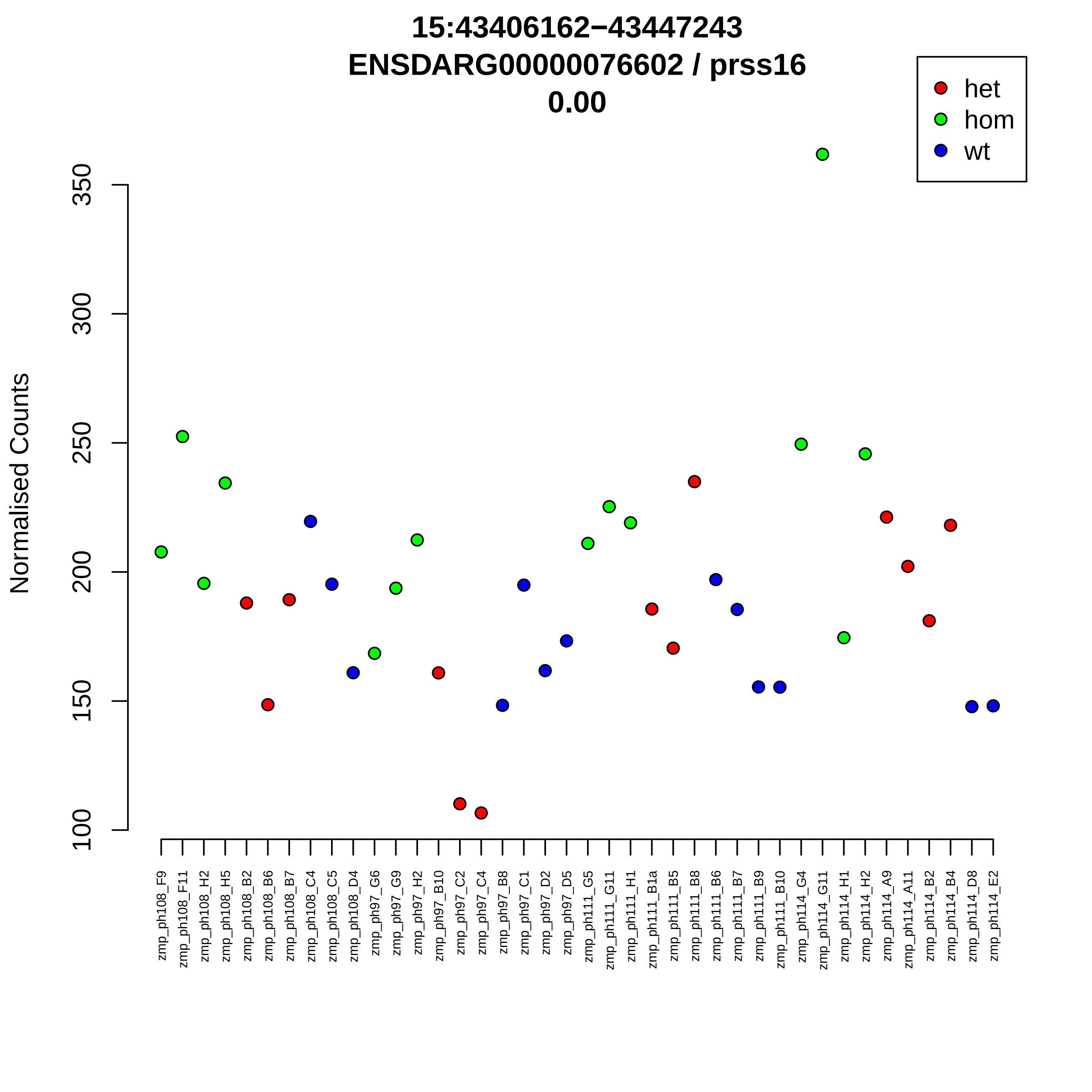

15:43406162−43447243
ENSDARG00000076602 / prss16
0.00
het
hom
wt
Normalised Counts
100
150
200
250
300
350
zmp_ph108_F9
zmp_ph108_F11
zmp_ph108_H2
zmp_ph108_H5
zmp_ph108_B2
zmp_ph108_B6
zmp_ph108_B7
zmp_ph108_C4
zmp_ph108_C5
zmp_ph108_D4
zmp_ph97_G6
zmp_ph97_G9
zmp_ph97_H2
zmp_ph97_B10
zmp_ph97_C2
zmp_ph97_C4
zmp_ph97_B8
zmp_ph97_C1
zmp_ph97_D2
zmp_ph97_D5
zmp_ph111_G5
zmp_ph111_G11
zmp_ph111_H1
zmp_ph111_B1a
zmp_ph111_B5
zmp_ph111_B8
zmp_ph111_B6
zmp_ph111_B7
zmp_ph111_B9
zmp_ph111_B10
zmp_ph114_G4
zmp_ph114_G11
zmp_ph114_H1
zmp_ph114_H2
zmp_ph114_A9
zmp_ph114_A11
zmp_ph114_B2
zmp_ph114_B4
zmp_ph114_D8
zmp_ph114_E2

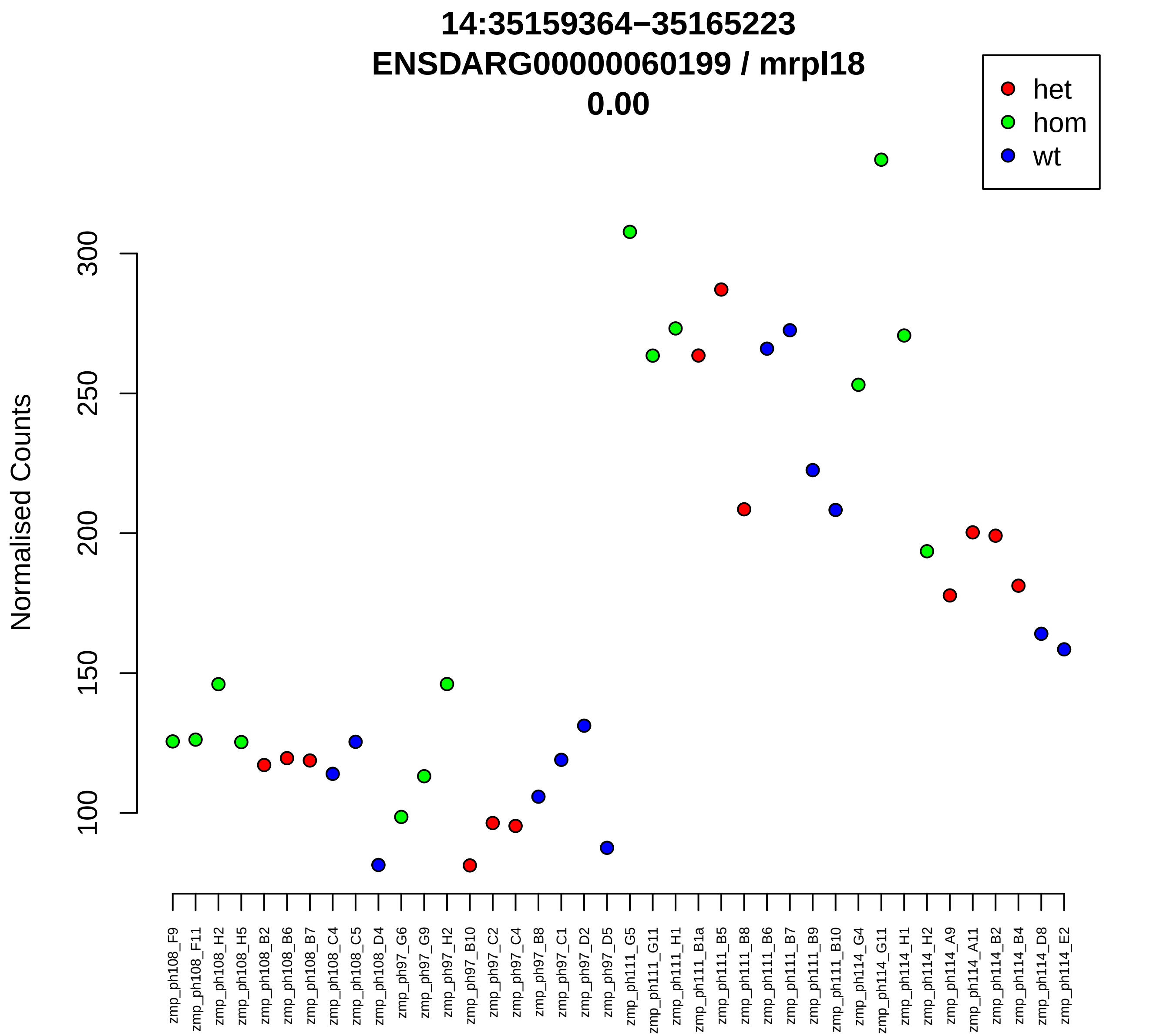

14:35159364−35165223
ENSDARG00000060199 / mrpl18
0.00
het
hom
wt
Normalised Counts
100
150
200
250
300
zmp_ph108_F9
zmp_ph108_F11
zmp_ph108_H2
zmp_ph108_H5
zmp_ph108_B2
zmp_ph108_B6
zmp_ph108_B7
zmp_ph108_C4
zmp_ph108_C5
zmp_ph108_D4
zmp_ph97_G6
zmp_ph97_G9
zmp_ph97_H2
zmp_ph97_B10
zmp_ph97_C2
zmp_ph97_C4
zmp_ph97_B8
zmp_ph97_C1
zmp_ph97_D2
zmp_ph97_D5
zmp_ph111_G5
zmp_ph111_G11
zmp_ph111_H1
zmp_ph111_B1a
zmp_ph111_B5
zmp_ph111_B8
zmp_ph111_B6
zmp_ph111_B7
zmp_ph111_B9
zmp_ph111_B10
zmp_ph114_G4
zmp_ph114_G11
zmp_ph114_H1
zmp_ph114_H2
zmp_ph114_A9
zmp_ph114_A11
zmp_ph114_B2
zmp_ph114_B4
zmp_ph114_D8
zmp_ph114_E2

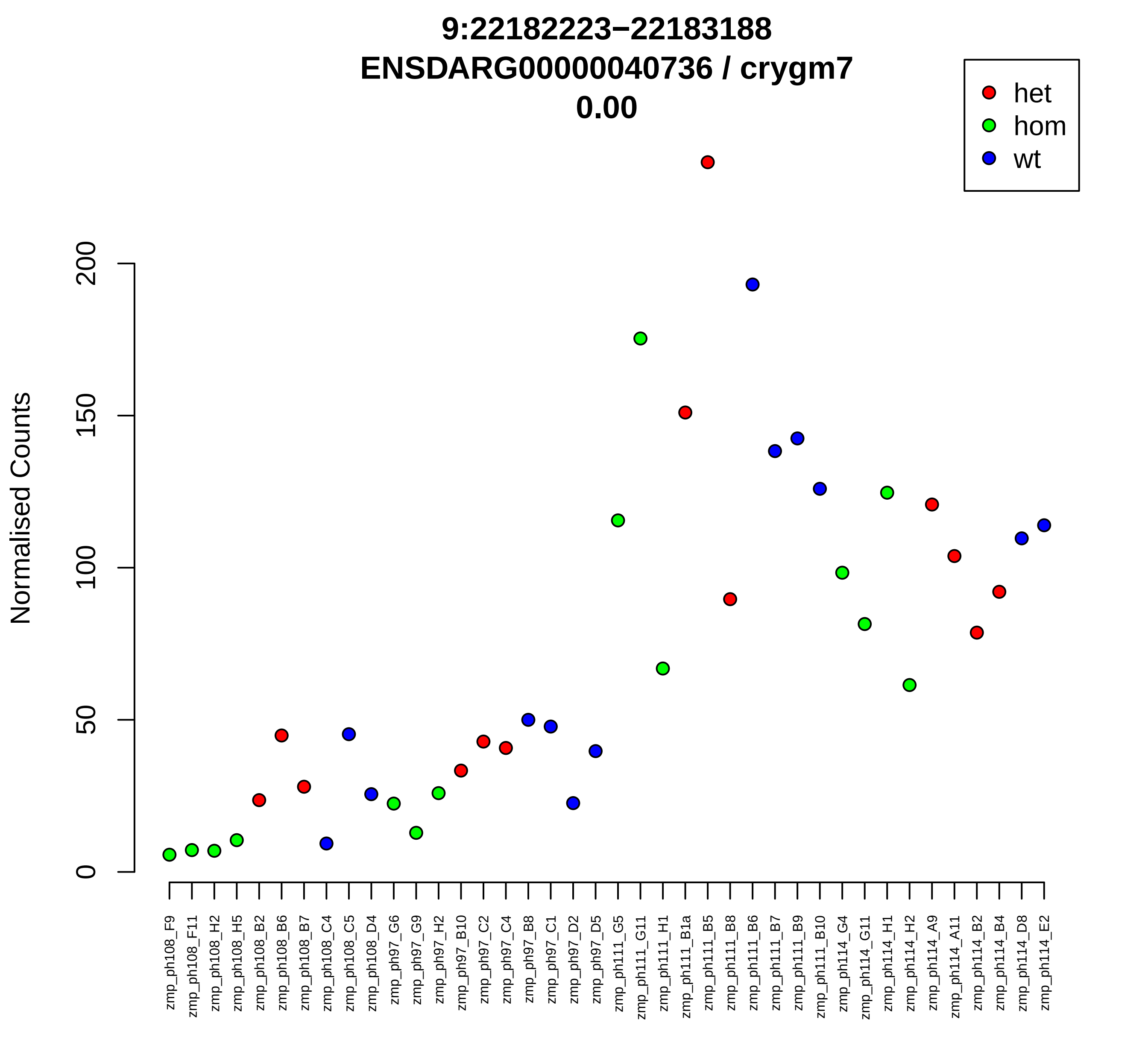

9:22182223−22183188
ENSDARG00000040736 / crygm7
0.00
het
hom
wt
Normalised Counts
0
50
100
150
200
zmp_ph108_F9
zmp_ph108_F11
zmp_ph108_H2
zmp_ph108_H5
zmp_ph108_B2
zmp_ph108_B6
zmp_ph108_B7
zmp_ph108_C4
zmp_ph108_C5
zmp_ph108_D4
zmp_ph97_G6
zmp_ph97_G9
zmp_ph97_H2
zmp_ph97_B10
zmp_ph97_C2
zmp_ph97_C4
zmp_ph97_B8
zmp_ph97_C1
zmp_ph97_D2
zmp_ph97_D5
zmp_ph111_G5
zmp_ph111_G11
zmp_ph111_H1
zmp_ph111_B1a
zmp_ph111_B5
zmp_ph111_B8
zmp_ph111_B6
zmp_ph111_B7
zmp_ph111_B9
zmp_ph111_B10
zmp_ph114_G4
zmp_ph114_G11
zmp_ph114_H1
zmp_ph114_H2
zmp_ph114_A9
zmp_ph114_A11
zmp_ph114_B2
zmp_ph114_B4
zmp_ph114_D8
zmp_ph114_E2

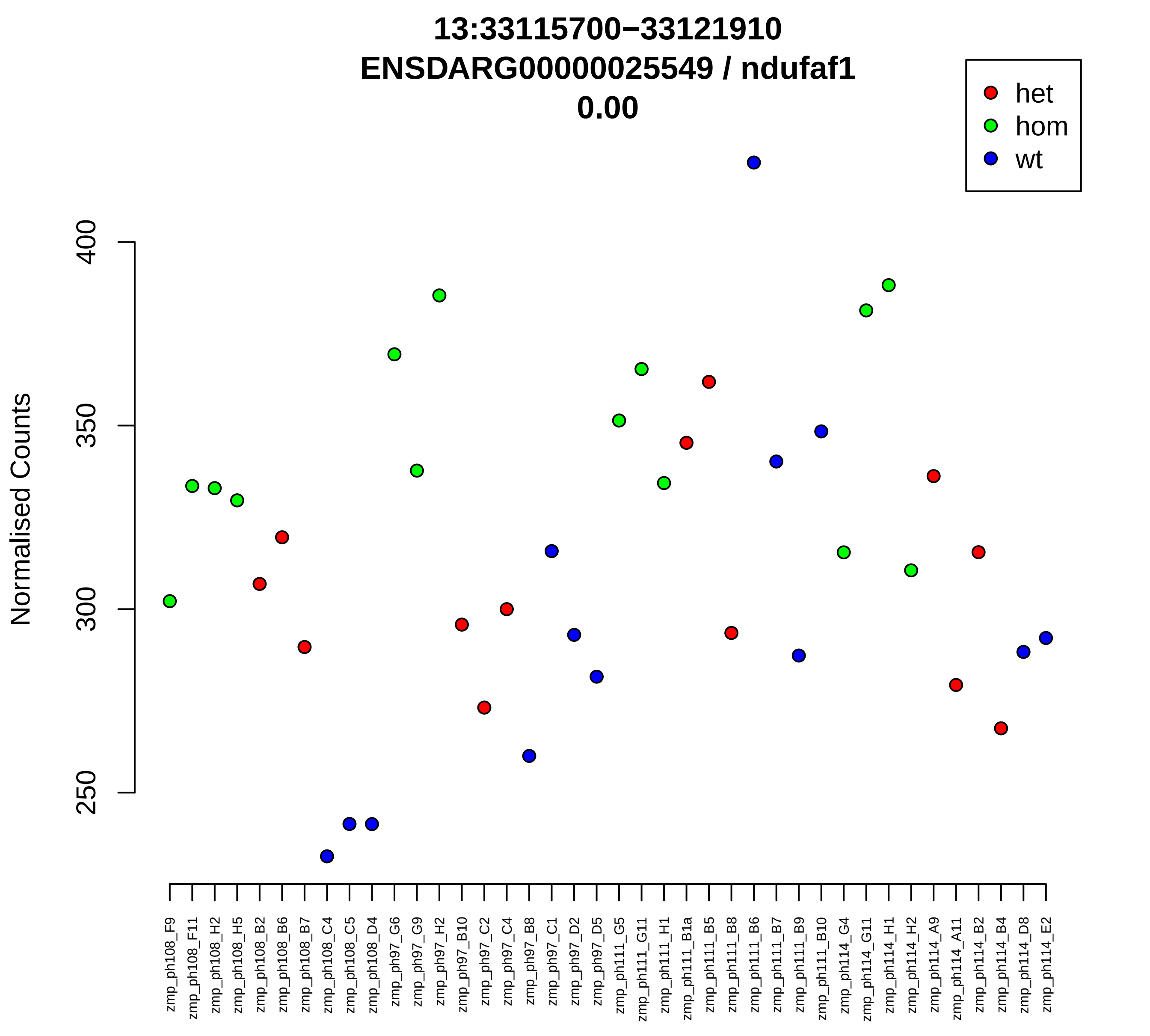

13:33115700−33121910
ENSDARG00000025549 / ndufaf1
0.00
het
hom
wt
Normalised Counts
250
300
350
400
zmp_ph108_F9
zmp_ph108_F11
zmp_ph108_H2
zmp_ph108_H5
zmp_ph108_B2
zmp_ph108_B6
zmp_ph108_B7
zmp_ph108_C4
zmp_ph108_C5
zmp_ph108_D4
zmp_ph97_G6
zmp_ph97_G9
zmp_ph97_H2
zmp_ph97_B10
zmp_ph97_C2
zmp_ph97_C4
zmp_ph97_B8
zmp_ph97_C1
zmp_ph97_D2
zmp_ph97_D5
zmp_ph111_G5
zmp_ph111_G11
zmp_ph111_H1
zmp_ph111_B1a
zmp_ph111_B5
zmp_ph111_B8
zmp_ph111_B6
zmp_ph111_B7
zmp_ph111_B9
zmp_ph111_B10
zmp_ph114_G4
zmp_ph114_G11
zmp_ph114_H1
zmp_ph114_H2
zmp_ph114_A9
zmp_ph114_A11
zmp_ph114_B2
zmp_ph114_B4
zmp_ph114_D8
zmp_ph114_E2

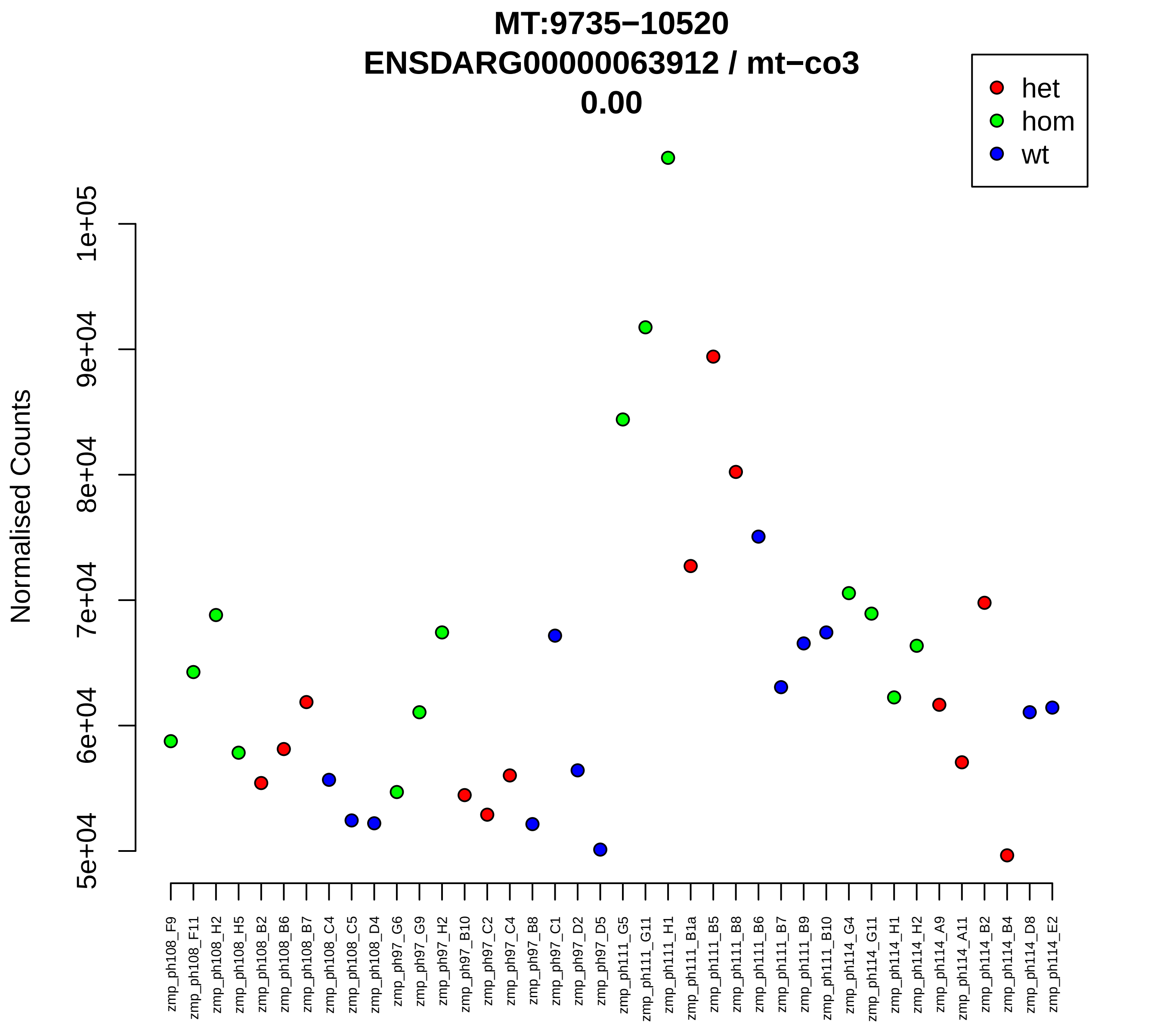
MT:9735−10520
ENSDARG00000063912 / mt−co3
0.00
het
hom
wt
Normalised Counts
1e+05
9e+04
8e+04
7e+04
6e+04
5e+04
zmp_ph108_F9
zmp_ph108_F11
zmp_ph108_H2
zmp_ph108_H5
zmp_ph108_B2
zmp_ph108_B6
zmp_ph108_B7
zmp_ph108_C4
zmp_ph108_C5
zmp_ph108_D4
zmp_ph97_G6
zmp_ph97_G9
zmp_ph97_H2
zmp_ph97_B10
zmp_ph97_C2
zmp_ph97_C4
zmp_ph97_B8
zmp_ph97_C1
zmp_ph97_D2
zmp_ph97_D5
zmp_ph111_G5
zmp_ph111_G11
zmp_ph111_H1
zmp_ph111_B1a
zmp_ph111_B5
zmp_ph111_B8
zmp_ph111_B6
zmp_ph111_B7
zmp_ph111_B9
zmp_ph111_B10
zmp_ph114_G4
zmp_ph114_G11
zmp_ph114_H1
zmp_ph114_H2
zmp_ph114_A9
zmp_ph114_A11
zmp_ph114_B2
zmp_ph114_B4
zmp_ph114_D8
zmp_ph114_E2

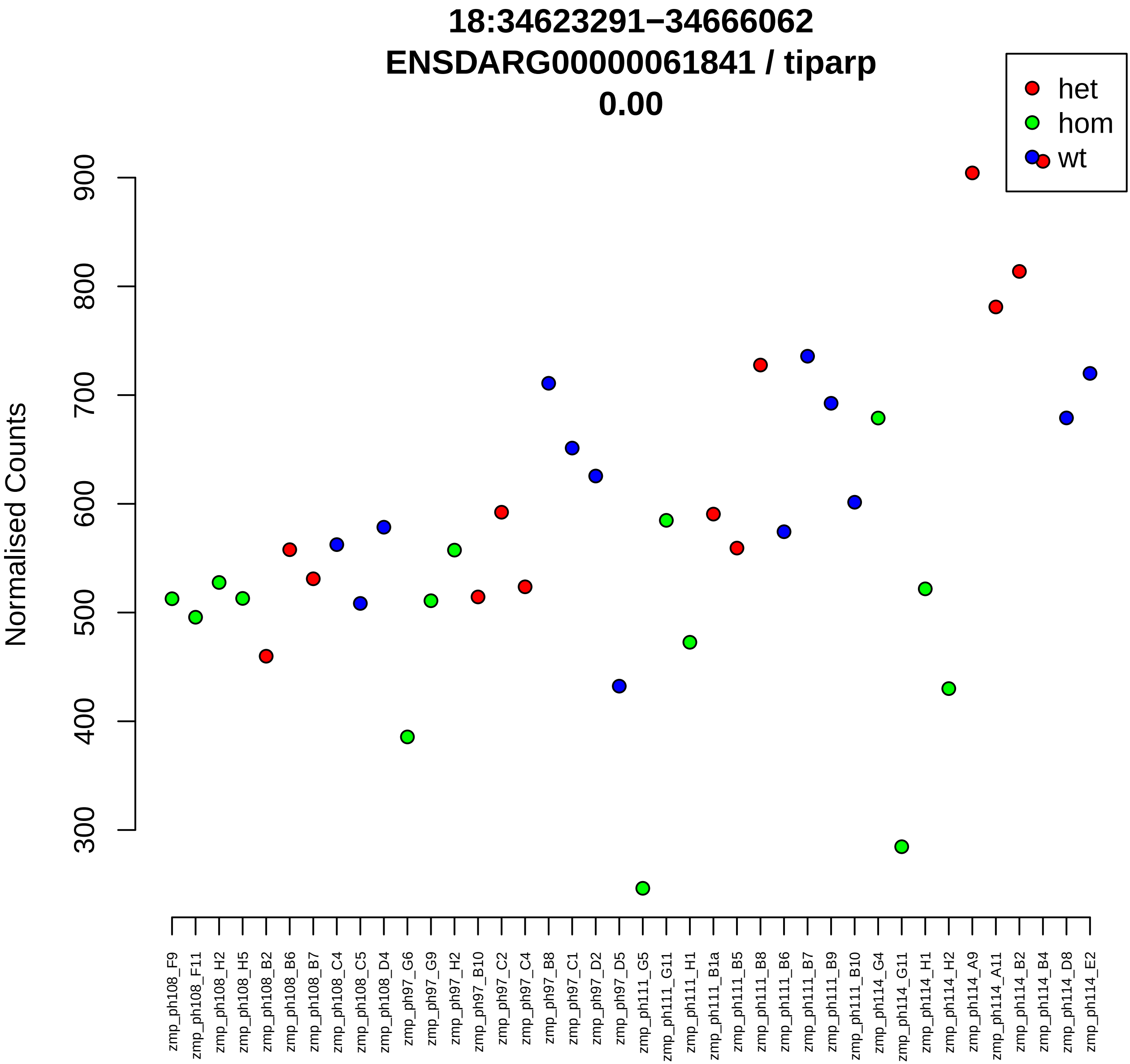

18:34623291−34666062
ENSDARG00000061841 / tiparp
0.00
het
hom
wt
Normalised Counts
300
400
500
600
700
800
900
zmp_ph108_F9
zmp_ph108_F11
zmp_ph108_H2
zmp_ph108_H5
zmp_ph108_B2
zmp_ph108_B6
zmp_ph108_B7
zmp_ph108_C4
zmp_ph108_C5
zmp_ph108_D4
zmp_ph97_G6
zmp_ph97_G9
zmp_ph97_H2
zmp_ph97_B10
zmp_ph97_C2
zmp_ph97_C4
zmp_ph97_B8
zmp_ph97_C1
zmp_ph97_D2
zmp_ph97_D5
zmp_ph111_G5
zmp_ph111_G11
zmp_ph111_H1
zmp_ph111_B1a
zmp_ph111_B5
zmp_ph111_B8
zmp_ph111_B6
zmp_ph111_B7
zmp_ph111_B9
zmp_ph111_B10
zmp_ph114_G4
zmp_ph114_G11
zmp_ph114_H1
zmp_ph114_H2
zmp_ph114_A9
zmp_ph114_A11
zmp_ph114_B2
zmp_ph114_B4
zmp_ph114_D8
zmp_ph114_E2

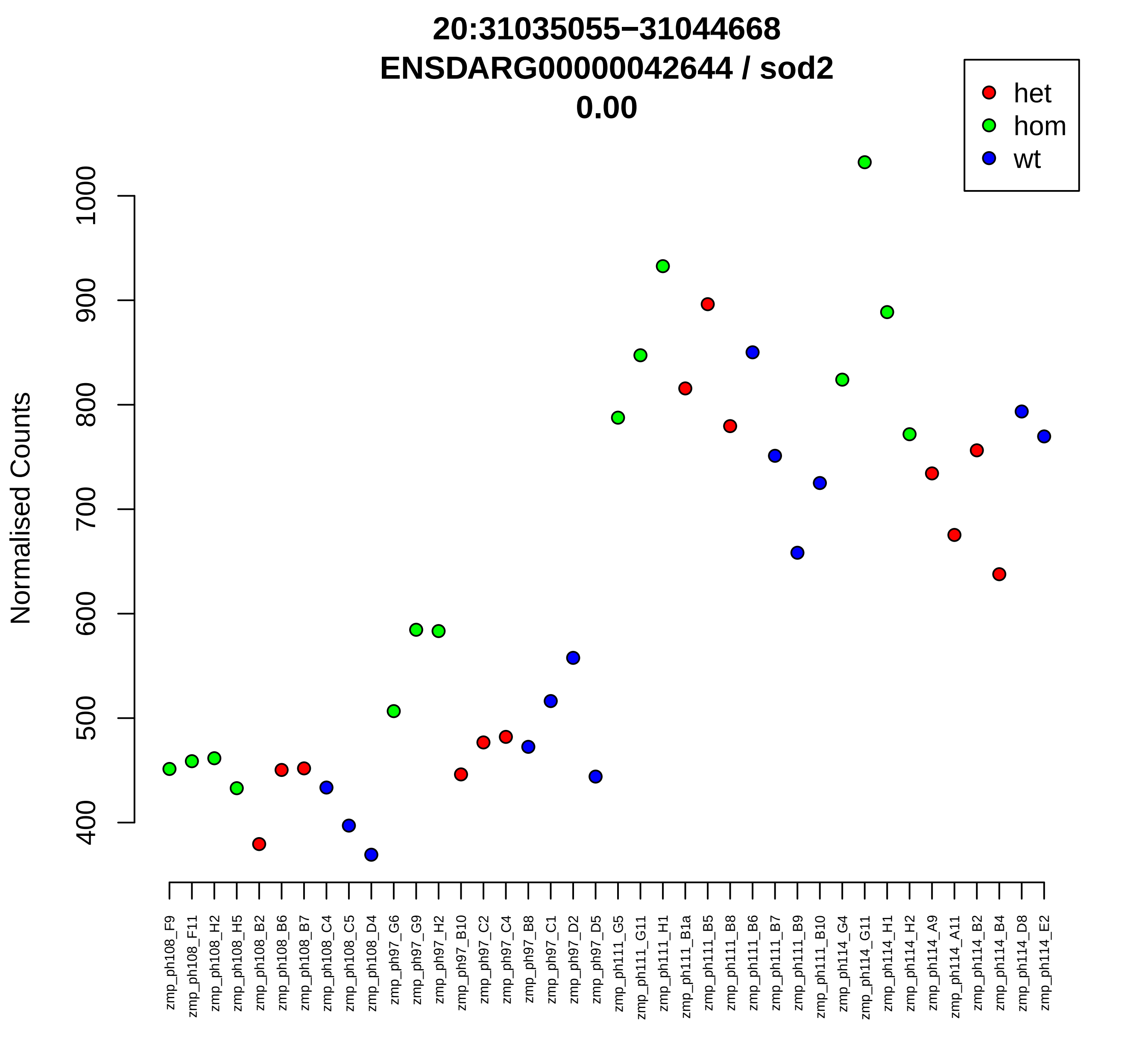

20:31035055−31044668
ENSDARG00000042644 / sod2
0.00
het
hom
wt
Normalised Counts
400
500
600
700
800
900
1000
zmp_ph108_F9
zmp_ph108_F11
zmp_ph108_H2
zmp_ph108_H5
zmp_ph108_B2
zmp_ph108_B6
zmp_ph108_B7
zmp_ph108_C4
zmp_ph108_C5
zmp_ph108_D4
zmp_ph97_G6
zmp_ph97_G9
zmp_ph97_H2
zmp_ph97_B10
zmp_ph97_C2
zmp_ph97_C4
zmp_ph97_B8
zmp_ph97_C1
zmp_ph97_D2
zmp_ph97_D5
zmp_ph111_G5
zmp_ph111_G11
zmp_ph111_H1
zmp_ph111_B1a
zmp_ph111_B5
zmp_ph111_B8
zmp_ph111_B6
zmp_ph111_B7
zmp_ph111_B9
zmp_ph111_B10
zmp_ph114_G4
zmp_ph114_G11
zmp_ph114_H1
zmp_ph114_H2
zmp_ph114_A9
zmp_ph114_A11
zmp_ph114_B2
zmp_ph114_B4
zmp_ph114_D8
zmp_ph114_E2

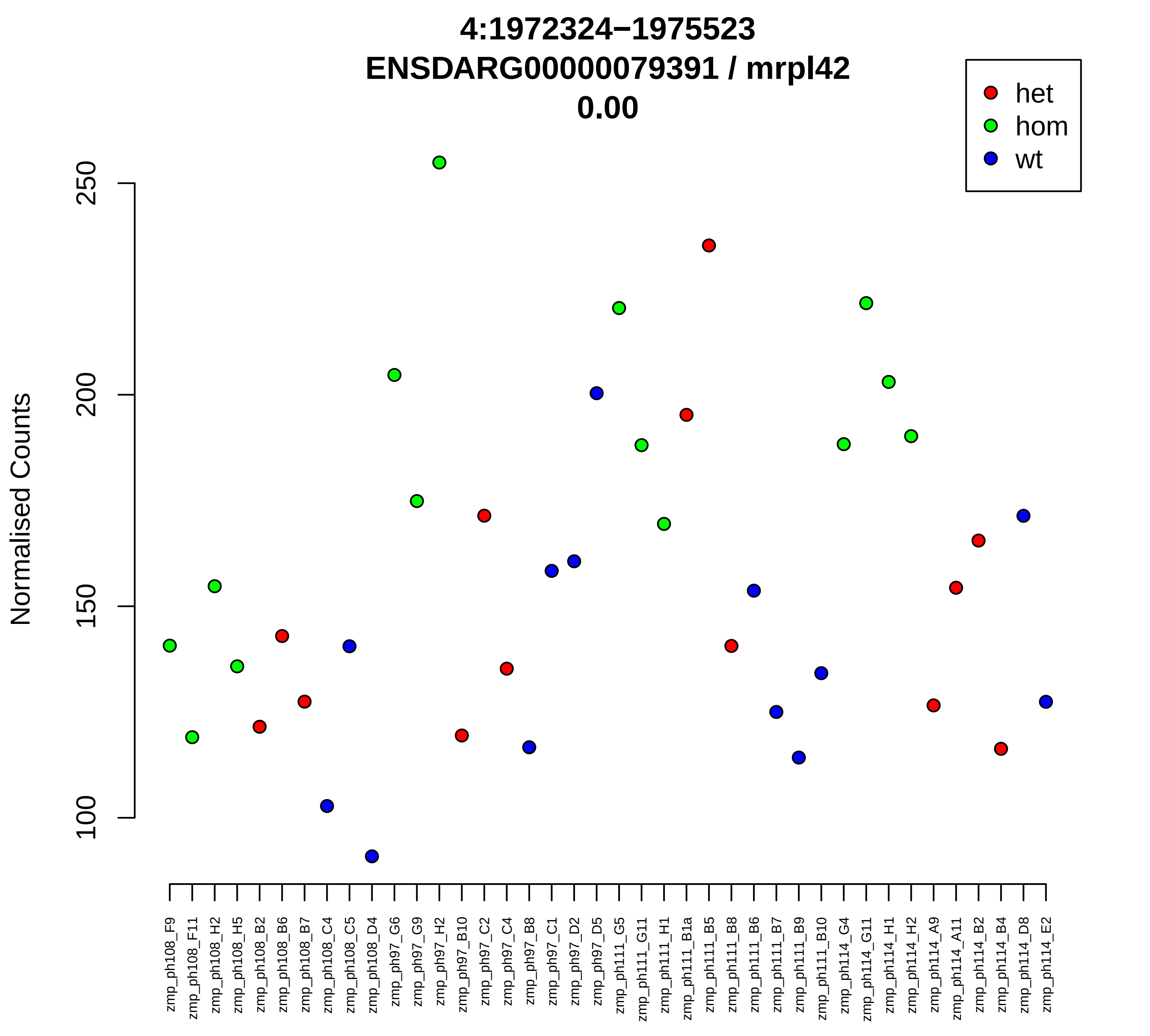
4:1972324−1975523
ENSDARG00000079391 / mrpl42
0.00
het
hom
wt
Normalised Counts
100
150
200
250
zmp_ph108_F9
zmp_ph108_F11
zmp_ph108_H2
zmp_ph108_H5
zmp_ph108_B2
zmp_ph108_B6
zmp_ph108_B7
zmp_ph108_C4
zmp_ph108_C5
zmp_ph108_D4
zmp_ph97_G6
zmp_ph97_G9
zmp_ph97_H2
zmp_ph97_B10
zmp_ph97_C2
zmp_ph97_C4
zmp_ph97_B8
zmp_ph97_C1
zmp_ph97_D2
zmp_ph97_D5
zmp_ph111_G5
zmp_ph111_G11
zmp_ph111_H1
zmp_ph111_B1a
zmp_ph111_B5
zmp_ph111_B8
zmp_ph111_B6
zmp_ph111_B7
zmp_ph111_B9
zmp_ph111_B10
zmp_ph114_G4
zmp_ph114_G11
zmp_ph114_H1
zmp_ph114_H2
zmp_ph114_A9
zmp_ph114_A11
zmp_ph114_B2
zmp_ph114_B4
zmp_ph114_D8
zmp_ph114_E2

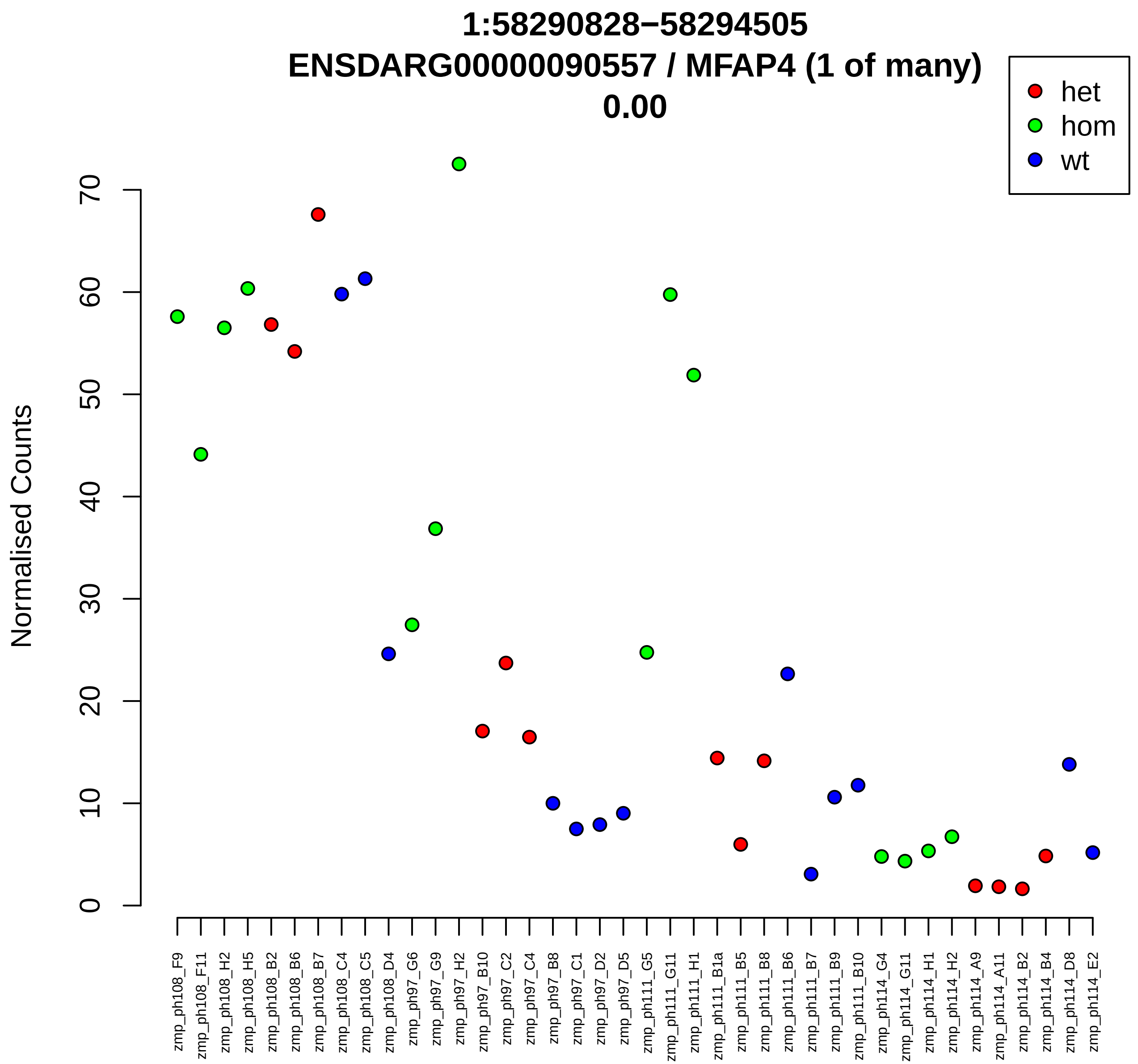
1:58290828−58294505
ENSDARG00000090557 / MFAP4 (1 of many)
0.00
het
hom
wt
Normalised Counts
0
10
20
30
40
50
60
70
zmp_ph108_F9
zmp_ph108_F11
zmp_ph108_H2
zmp_ph108_H5
zmp_ph108_B2
zmp_ph108_B6
zmp_ph108_B7
zmp_ph108_C4
zmp_ph108_C5
zmp_ph108_D4
zmp_ph97_G6
zmp_ph97_G9
zmp_ph97_H2
zmp_ph97_B10
zmp_ph97_C2
zmp_ph97_C4
zmp_ph97_B8
zmp_ph97_C1
zmp_ph97_D2
zmp_ph97_D5
zmp_ph111_G5
zmp_ph111_G11
zmp_ph111_H1
zmp_ph111_B1a
zmp_ph111_B5
zmp_ph111_B8
zmp_ph111_B6
zmp_ph111_B7
zmp_ph111_B9
zmp_ph111_B10
zmp_ph114_G4
zmp_ph114_G11
zmp_ph114_H1
zmp_ph114_H2
zmp_ph114_A9
zmp_ph114_A11
zmp_ph114_B2
zmp_ph114_B4
zmp_ph114_D8
zmp_ph114_E2

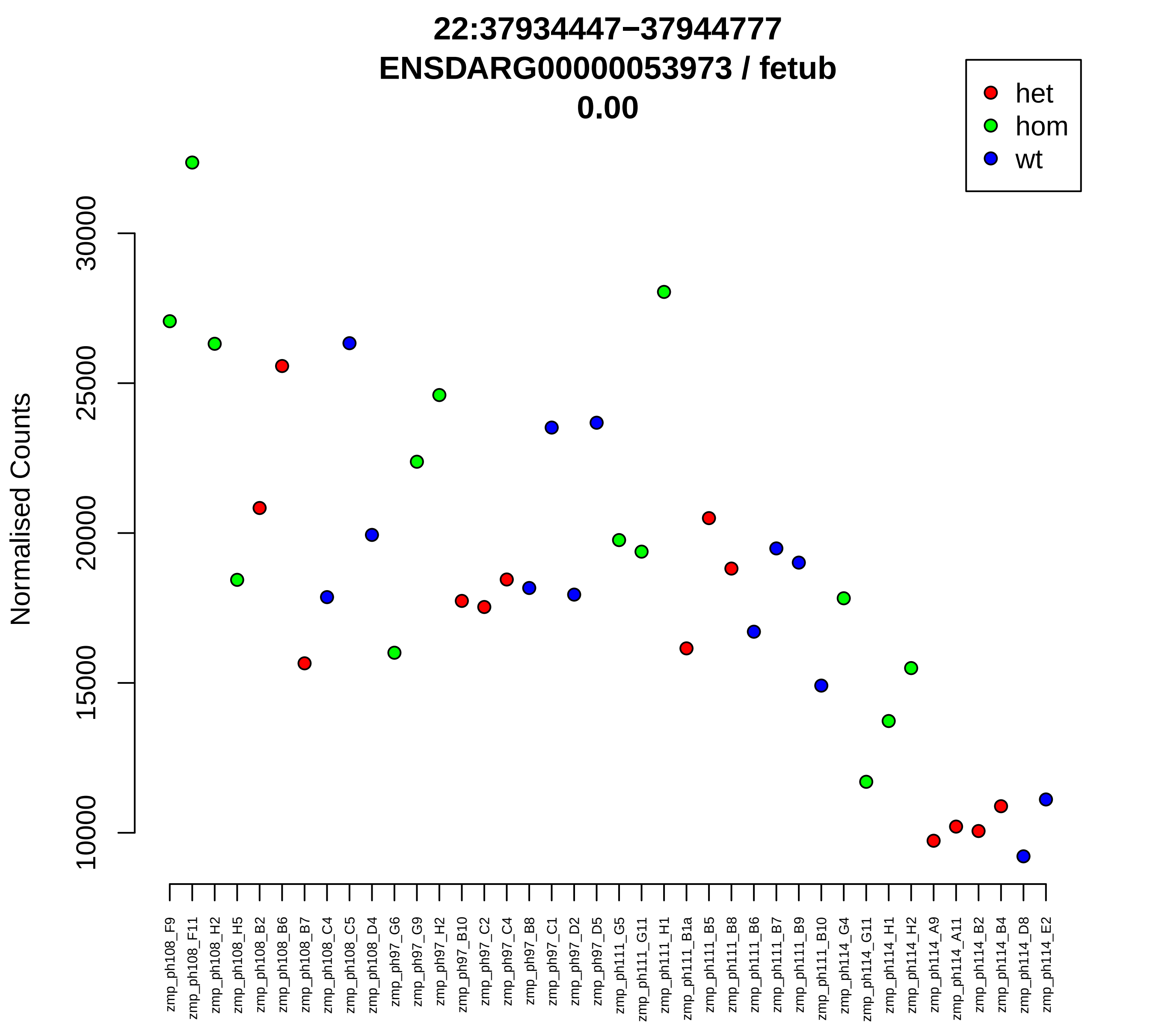

22:37934447−37944777
ENSDARG00000053973 / fetub
0.00
het
hom
wt
Normalised Counts
10000
15000
20000
25000
30000
zmp_ph108_F9
zmp_ph108_F11
zmp_ph108_H2
zmp_ph108_H5
zmp_ph108_B2
zmp_ph108_B6
zmp_ph108_B7
zmp_ph108_C4
zmp_ph108_C5
zmp_ph108_D4
zmp_ph97_G6
zmp_ph97_G9
zmp_ph97_H2
zmp_ph97_B10
zmp_ph97_C2
zmp_ph97_C4
zmp_ph97_B8
zmp_ph97_C1
zmp_ph97_D2
zmp_ph97_D5
zmp_ph111_G5
zmp_ph111_G11
zmp_ph111_H1
zmp_ph111_B1a
zmp_ph111_B5
zmp_ph111_B8
zmp_ph111_B6
zmp_ph111_B7
zmp_ph111_B9
zmp_ph111_B10
zmp_ph114_G4
zmp_ph114_G11
zmp_ph114_H1
zmp_ph114_H2
zmp_ph114_A9
zmp_ph114_A11
zmp_ph114_B2
zmp_ph114_B4
zmp_ph114_D8
zmp_ph114_E2

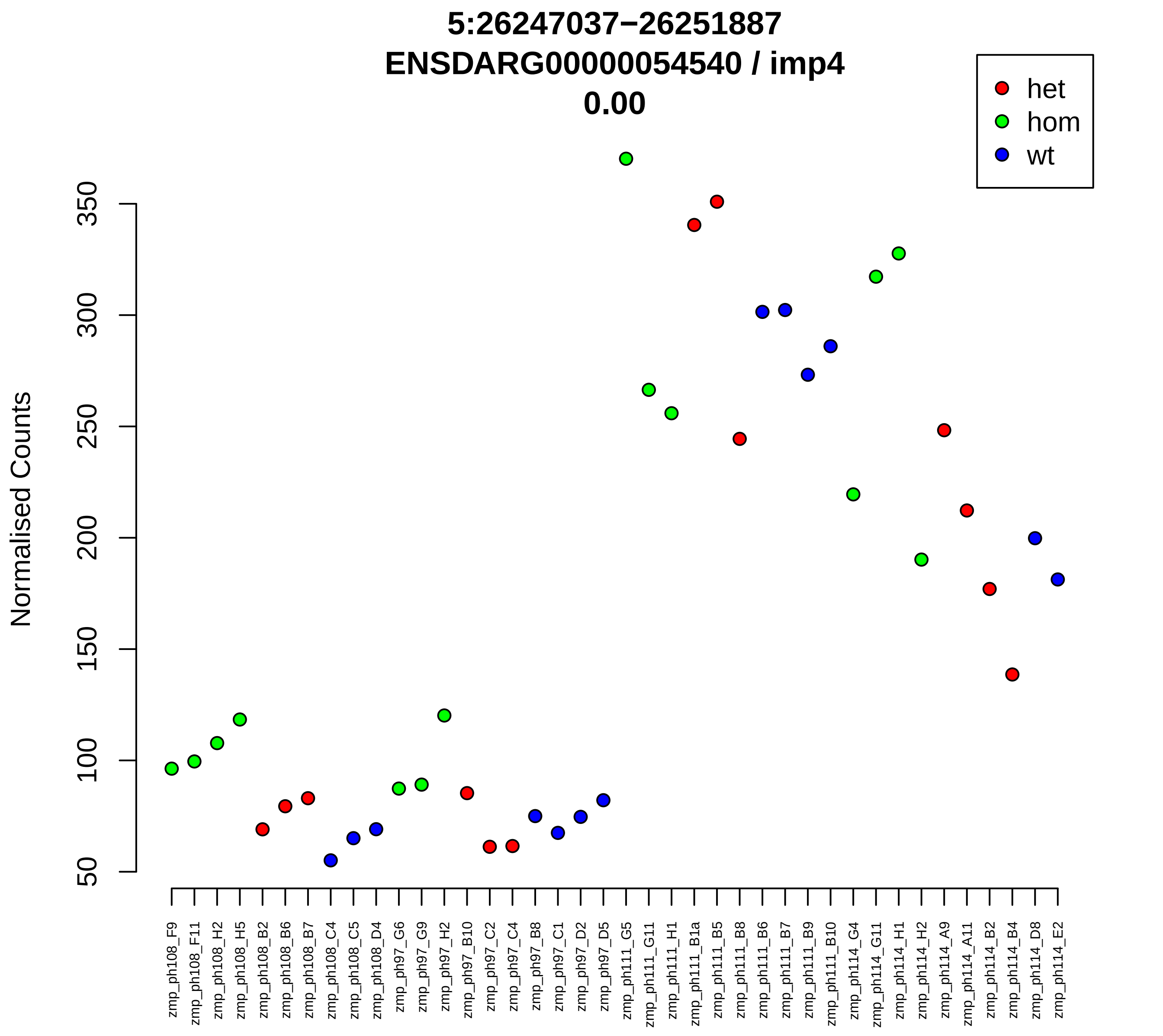

5:26247037−26251887
ENSDARG00000054540 / imp4
0.00
het
hom
wt
Normalised Counts
50
100
150
200
250
300
350
zmp_ph108_F9
zmp_ph108_F11
zmp_ph108_H2
zmp_ph108_H5
zmp_ph108_B2
zmp_ph108_B6
zmp_ph108_B7
zmp_ph108_C4
zmp_ph108_C5
zmp_ph108_D4
zmp_ph97_G6
zmp_ph97_G9
zmp_ph97_H2
zmp_ph97_B10
zmp_ph97_C2
zmp_ph97_C4
zmp_ph97_B8
zmp_ph97_C1
zmp_ph97_D2
zmp_ph97_D5
zmp_ph111_G5
zmp_ph111_G11
zmp_ph111_H1
zmp_ph111_B1a
zmp_ph111_B5
zmp_ph111_B8
zmp_ph111_B6
zmp_ph111_B7
zmp_ph111_B9
zmp_ph111_B10
zmp_ph114_G4
zmp_ph114_G11
zmp_ph114_H1
zmp_ph114_H2
zmp_ph114_A9
zmp_ph114_A11
zmp_ph114_B2
zmp_ph114_B4
zmp_ph114_D8
zmp_ph114_E2

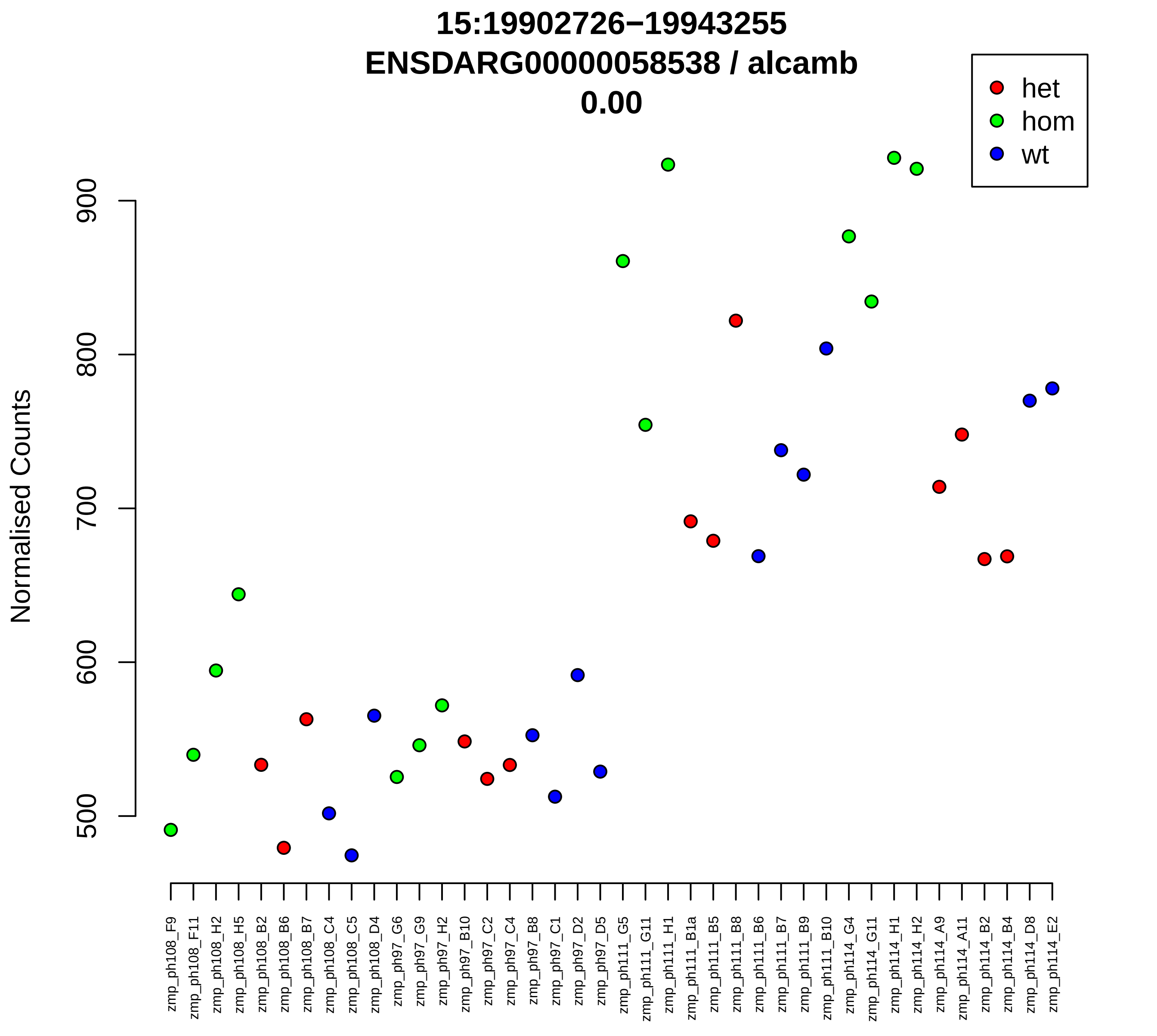

15:19902726−19943255
ENSDARG00000058538 / alcamb
0.00
het
hom
wt
Normalised Counts
500
600
700
800
900
zmp_ph108_F9
zmp_ph108_F11
zmp_ph108_H2
zmp_ph108_H5
zmp_ph108_B2
zmp_ph108_B6
zmp_ph108_B7
zmp_ph108_C4
zmp_ph108_C5
zmp_ph108_D4
zmp_ph97_G6
zmp_ph97_G9
zmp_ph97_H2
zmp_ph97_B10
zmp_ph97_C2
zmp_ph97_C4
zmp_ph97_B8
zmp_ph97_C1
zmp_ph97_D2
zmp_ph97_D5
zmp_ph111_G5
zmp_ph111_G11
zmp_ph111_H1
zmp_ph111_B1a
zmp_ph111_B5
zmp_ph111_B8
zmp_ph111_B6
zmp_ph111_B7
zmp_ph111_B9
zmp_ph111_B10
zmp_ph114_G4
zmp_ph114_G11
zmp_ph114_H1
zmp_ph114_H2
zmp_ph114_A9
zmp_ph114_A11
zmp_ph114_B2
zmp_ph114_B4
zmp_ph114_D8
zmp_ph114_E2

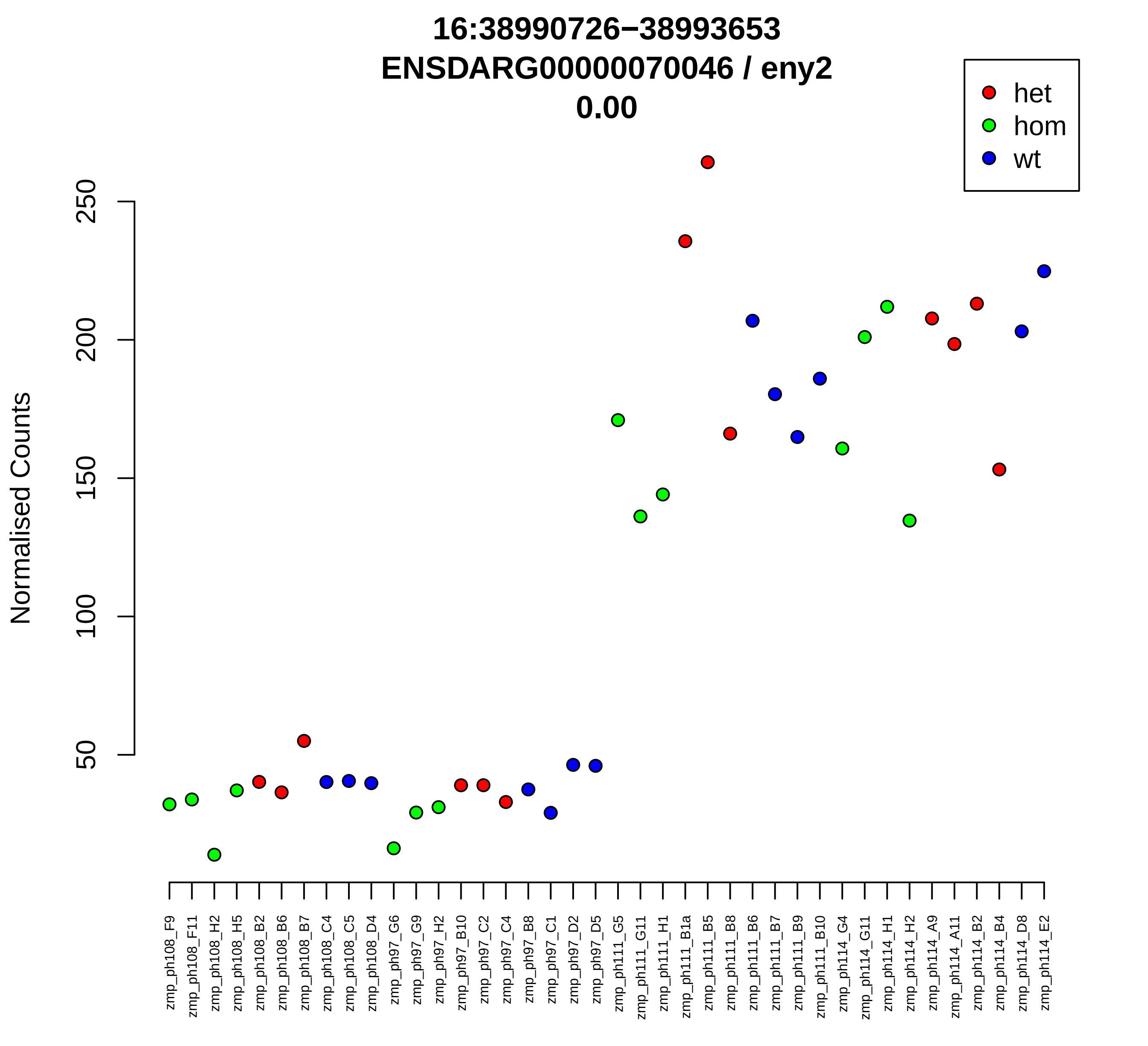
16:38990726−38993653
ENSDARG00000070046 / eny2
0.00
het
hom
wt
Normalised Counts
50
100
150
200
250
zmp_ph108_F9
zmp_ph108_F11
zmp_ph108_H2
zmp_ph108_H5
zmp_ph108_B2
zmp_ph108_B6
zmp_ph108_B7
zmp_ph108_C4
zmp_ph108_C5
zmp_ph108_D4
zmp_ph97_G6
zmp_ph97_G9
zmp_ph97_H2
zmp_ph97_B10
zmp_ph97_C2
zmp_ph97_C4
zmp_ph97_B8
zmp_ph97_C1
zmp_ph97_D2
zmp_ph97_D5
zmp_ph111_G5
zmp_ph111_G11
zmp_ph111_H1
zmp_ph111_B1a
zmp_ph111_B5
zmp_ph111_B8
zmp_ph111_B6
zmp_ph111_B7
zmp_ph111_B9
zmp_ph111_B10
zmp_ph114_G4
zmp_ph114_G11
zmp_ph114_H1
zmp_ph114_H2
zmp_ph114_A9
zmp_ph114_A11
zmp_ph114_B2
zmp_ph114_B4
zmp_ph114_D8
zmp_ph114_E2

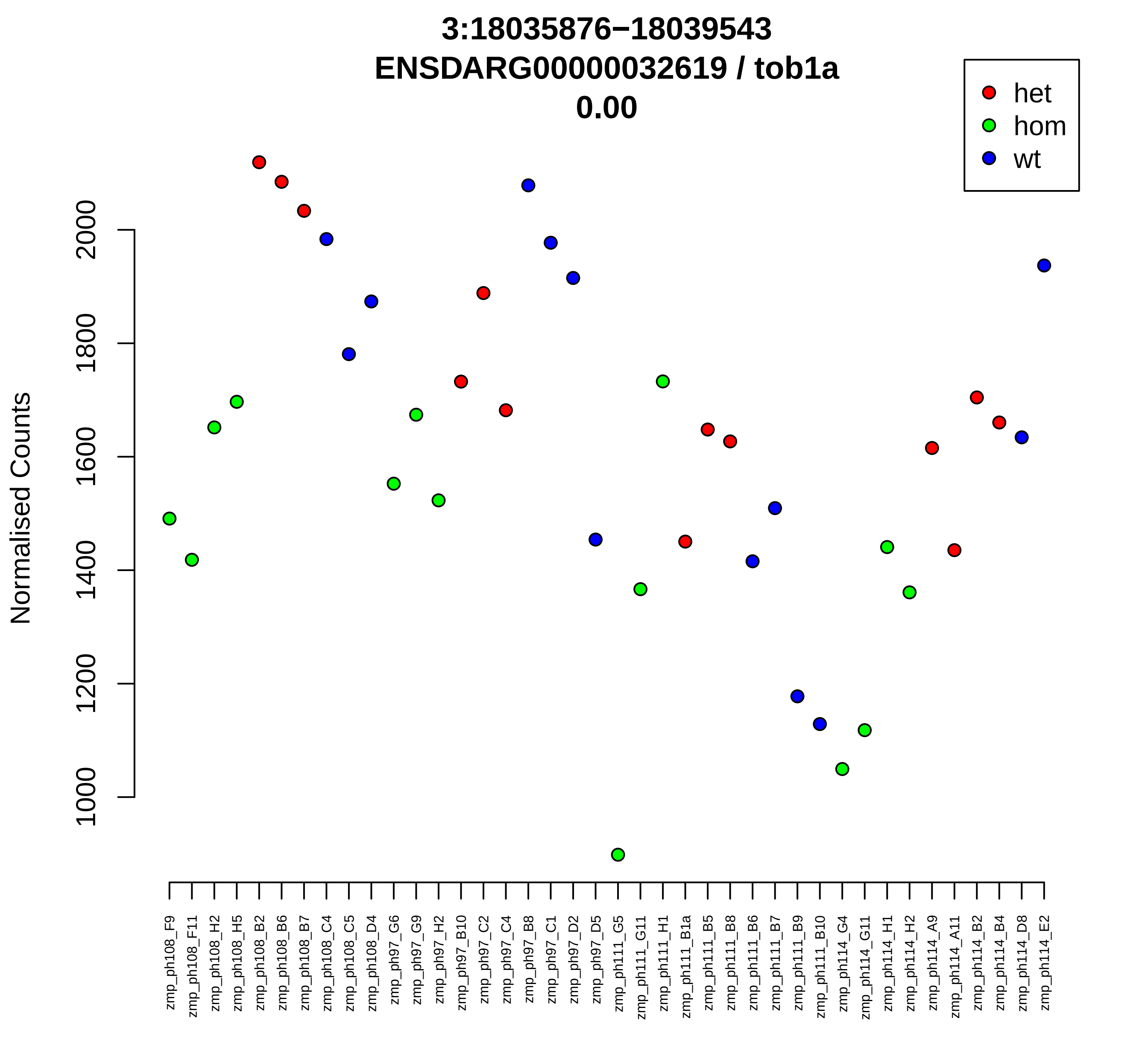

3:18035876−18039543
ENSDARG00000032619 / tob1a
0.00
het
hom
wt
Normalised Counts
1000
1200
1400
1600
1800
2000
zmp_ph108_F9
zmp_ph108_F11
zmp_ph108_H2
zmp_ph108_H5
zmp_ph108_B2
zmp_ph108_B6
zmp_ph108_B7
zmp_ph108_C4
zmp_ph108_C5
zmp_ph108_D4
zmp_ph97_G6
zmp_ph97_G9
zmp_ph97_H2
zmp_ph97_B10
zmp_ph97_C2
zmp_ph97_C4
zmp_ph97_B8
zmp_ph97_C1
zmp_ph97_D2
zmp_ph97_D5
zmp_ph111_G5
zmp_ph111_G11
zmp_ph111_H1
zmp_ph111_B1a
zmp_ph111_B5
zmp_ph111_B8
zmp_ph111_B6
zmp_ph111_B7
zmp_ph111_B9
zmp_ph111_B10
zmp_ph114_G4
zmp_ph114_G11
zmp_ph114_H1
zmp_ph114_H2
zmp_ph114_A9
zmp_ph114_A11
zmp_ph114_B2
zmp_ph114_B4
zmp_ph114_D8
zmp_ph114_E2

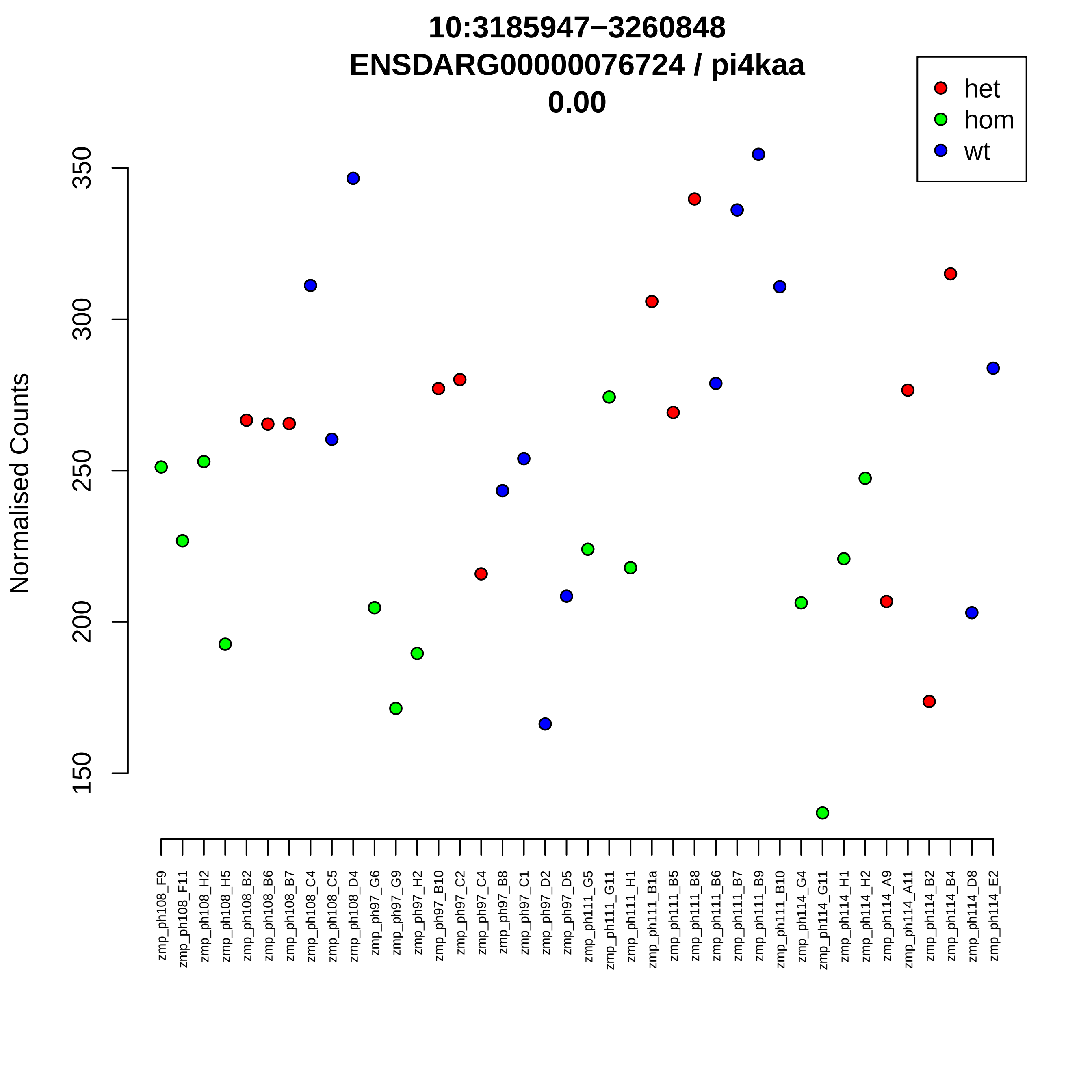
10:3185947−3260848
ENSDARG00000076724 / pi4kaa
0.00
het
hom
wt
Normalised Counts
350
300
250
200
150
zmp_ph108_F9
zmp_ph108_F11
zmp_ph108_H2
zmp_ph108_H5
zmp_ph108_B2
zmp_ph108_B6
zmp_ph108_B7
zmp_ph108_C4
zmp_ph108_C5
zmp_ph108_D4
zmp_ph97_G6
zmp_ph97_G9
zmp_ph97_H2
zmp_ph97_B10
zmp_ph97_C2
zmp_ph97_C4
zmp_ph97_B8
zmp_ph97_C1
zmp_ph97_D2
zmp_ph97_D5
zmp_ph111_G5
zmp_ph111_G11
zmp_ph111_H1
zmp_ph111_B1a
zmp_ph111_B5
zmp_ph111_B8
zmp_ph111_B6
zmp_ph111_B7
zmp_ph111_B9
zmp_ph111_B10
zmp_ph114_G4
zmp_ph114_G11
zmp_ph114_H1
zmp_ph114_H2
zmp_ph114_A9
zmp_ph114_A11
zmp_ph114_B2
zmp_ph114_B4
zmp_ph114_D8
zmp_ph114_E2

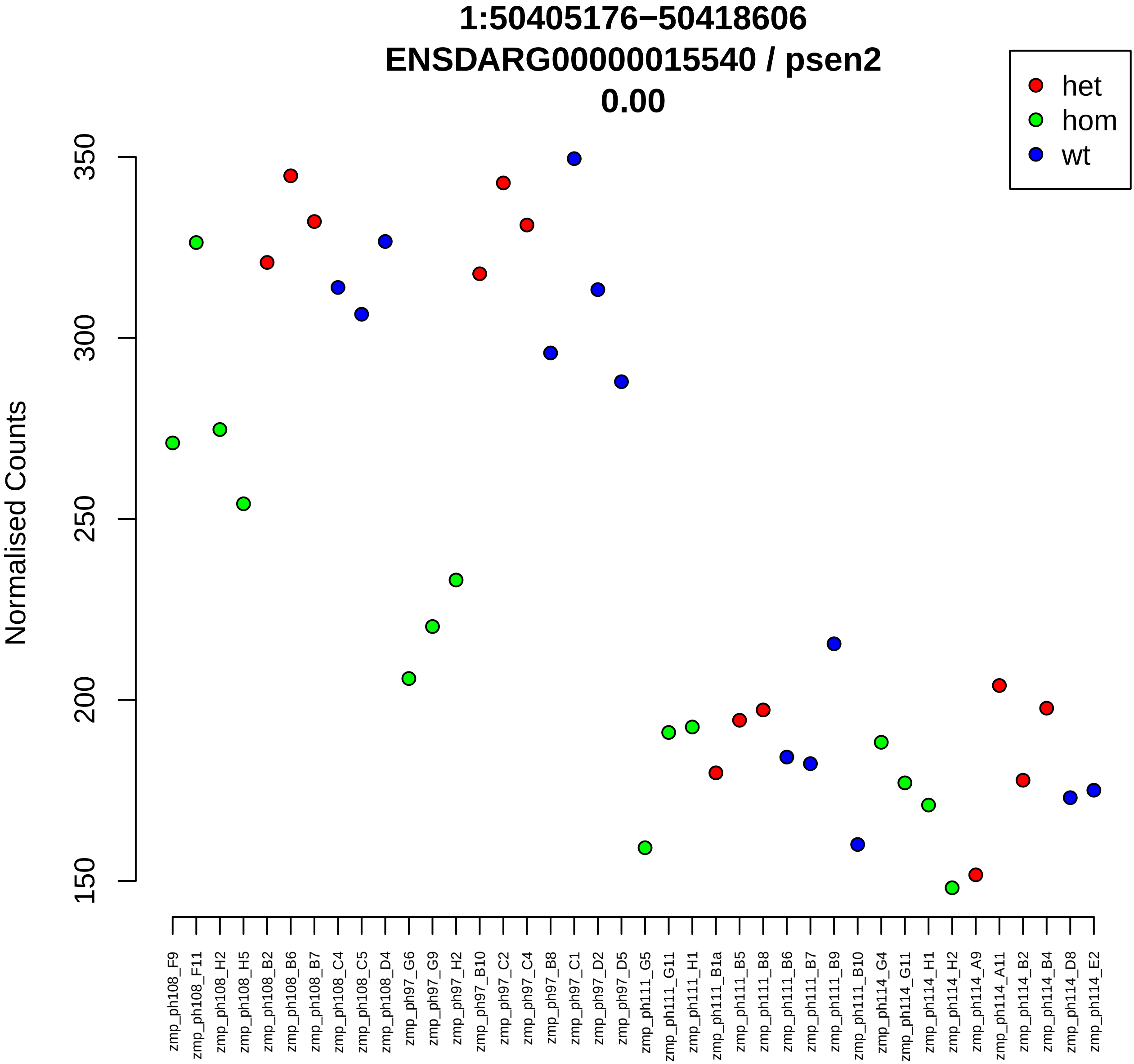

1:50405176−50418606
ENSDARG00000015540 / psen2
0.00
het
hom
wt
Normalised Counts
350
300
250
200
150
zmp_ph108_F9
zmp_ph108_F11
zmp_ph108_H2
zmp_ph108_H5
zmp_ph108_B2
zmp_ph108_B6
zmp_ph108_B7
zmp_ph108_C4
zmp_ph108_C5
zmp_ph108_D4
zmp_ph97_G6
zmp_ph97_G9
zmp_ph97_H2
zmp_ph97_B10
zmp_ph97_C2
zmp_ph97_C4
zmp_ph97_B8
zmp_ph97_C1
zmp_ph97_D2
zmp_ph97_D5
zmp_ph111_G5
zmp_ph111_G11
zmp_ph111_H1
zmp_ph111_B1a
zmp_ph111_B5
zmp_ph111_B8
zmp_ph111_B6
zmp_ph111_B7
zmp_ph111_B9
zmp_ph111_B10
zmp_ph114_G4
zmp_ph114_G11
zmp_ph114_H1
zmp_ph114_H2
zmp_ph114_A9
zmp_ph114_A11
zmp_ph114_B2
zmp_ph114_B4
zmp_ph114_D8
zmp_ph114_E2

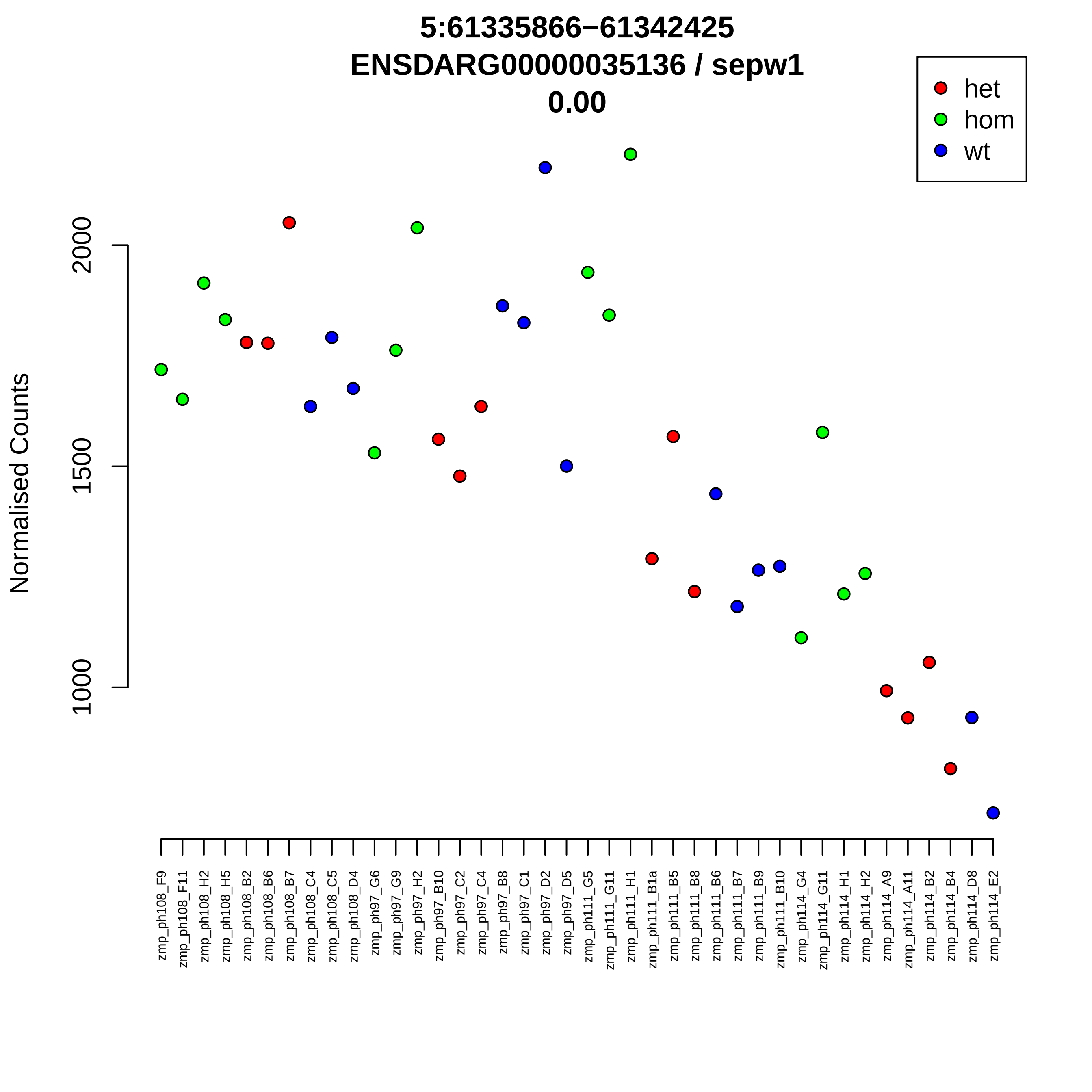

5:61335866−61342425
ENSDARG00000035136 / sepw1
0.00
het
hom
wt
Normalised Counts
2000
1500
1000
zmp_ph108_F9
zmp_ph108_F11
zmp_ph108_H2
zmp_ph108_H5
zmp_ph108_B2
zmp_ph108_B6
zmp_ph108_B7
zmp_ph108_C4
zmp_ph108_C5
zmp_ph108_D4
zmp_ph97_G6
zmp_ph97_G9
zmp_ph97_H2
zmp_ph97_B10
zmp_ph97_C2
zmp_ph97_C4
zmp_ph97_B8
zmp_ph97_C1
zmp_ph97_D2
zmp_ph97_D5
zmp_ph111_G5
zmp_ph111_G11
zmp_ph111_H1
zmp_ph111_B1a
zmp_ph111_B5
zmp_ph111_B8
zmp_ph111_B6
zmp_ph111_B7
zmp_ph111_B9
zmp_ph111_B10
zmp_ph114_G4
zmp_ph114_G11
zmp_ph114_H1
zmp_ph114_H2
zmp_ph114_A9
zmp_ph114_A11
zmp_ph114_B2
zmp_ph114_B4
zmp_ph114_D8
zmp_ph114_E2

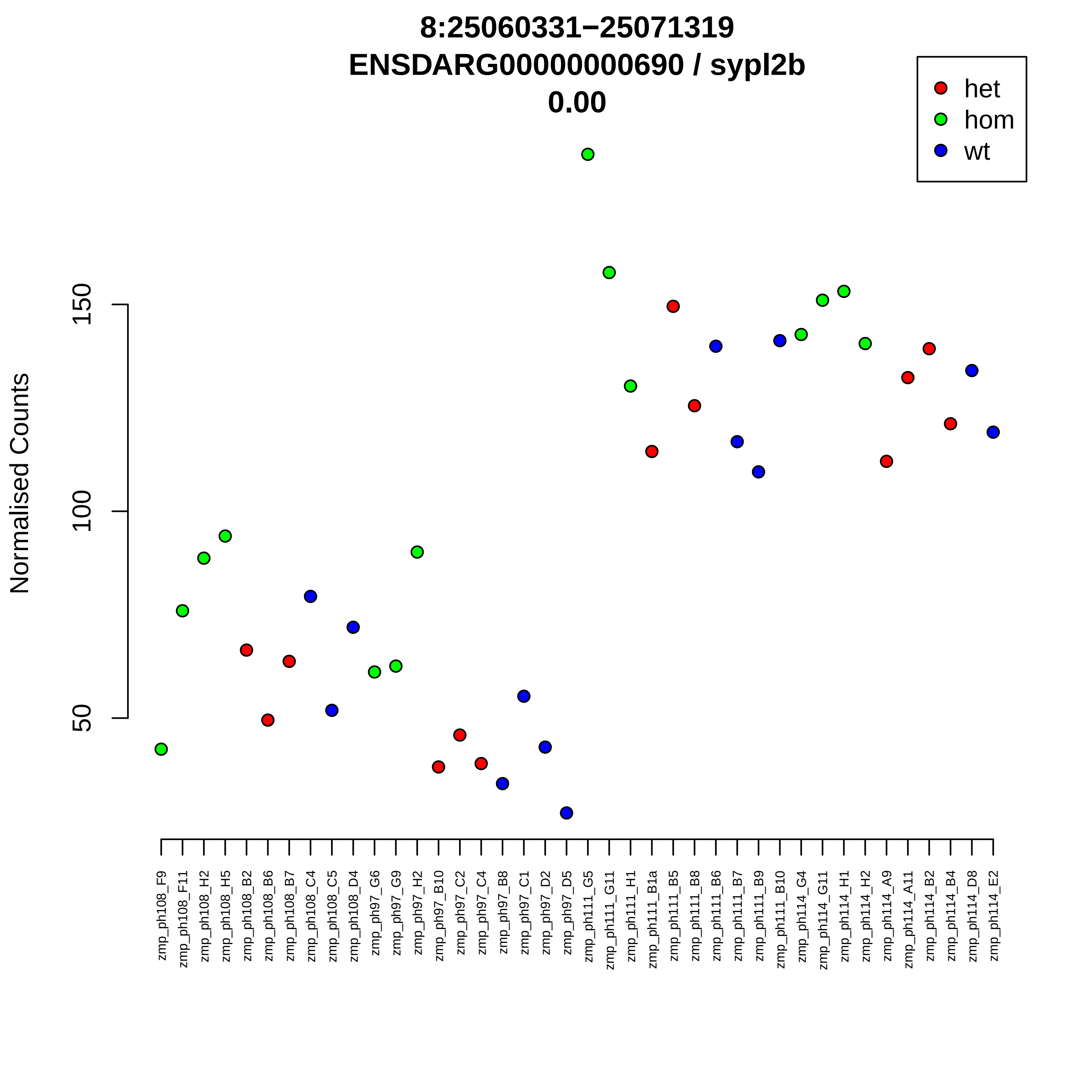

8:25060331−25071319
ENSDARG00000000690 / sypl2b
0.00
het
hom
wt
Normalised Counts
50
100
150
zmp_ph108_F9
zmp_ph108_F11
zmp_ph108_H2
zmp_ph108_H5
zmp_ph108_B2
zmp_ph108_B6
zmp_ph108_B7
zmp_ph108_C4
zmp_ph108_C5
zmp_ph108_D4
zmp_ph97_G6
zmp_ph97_G9
zmp_ph97_H2
zmp_ph97_B10
zmp_ph97_C2
zmp_ph97_C4
zmp_ph97_B8
zmp_ph97_C1
zmp_ph97_D2
zmp_ph97_D5
zmp_ph111_G5
zmp_ph111_G11
zmp_ph111_H1
zmp_ph111_B1a
zmp_ph111_B5
zmp_ph111_B8
zmp_ph111_B6
zmp_ph111_B7
zmp_ph111_B9
zmp_ph111_B10
zmp_ph114_G4
zmp_ph114_G11
zmp_ph114_H1
zmp_ph114_H2
zmp_ph114_A9
zmp_ph114_A11
zmp_ph114_B2
zmp_ph114_B4
zmp_ph114_D8
zmp_ph114_E2

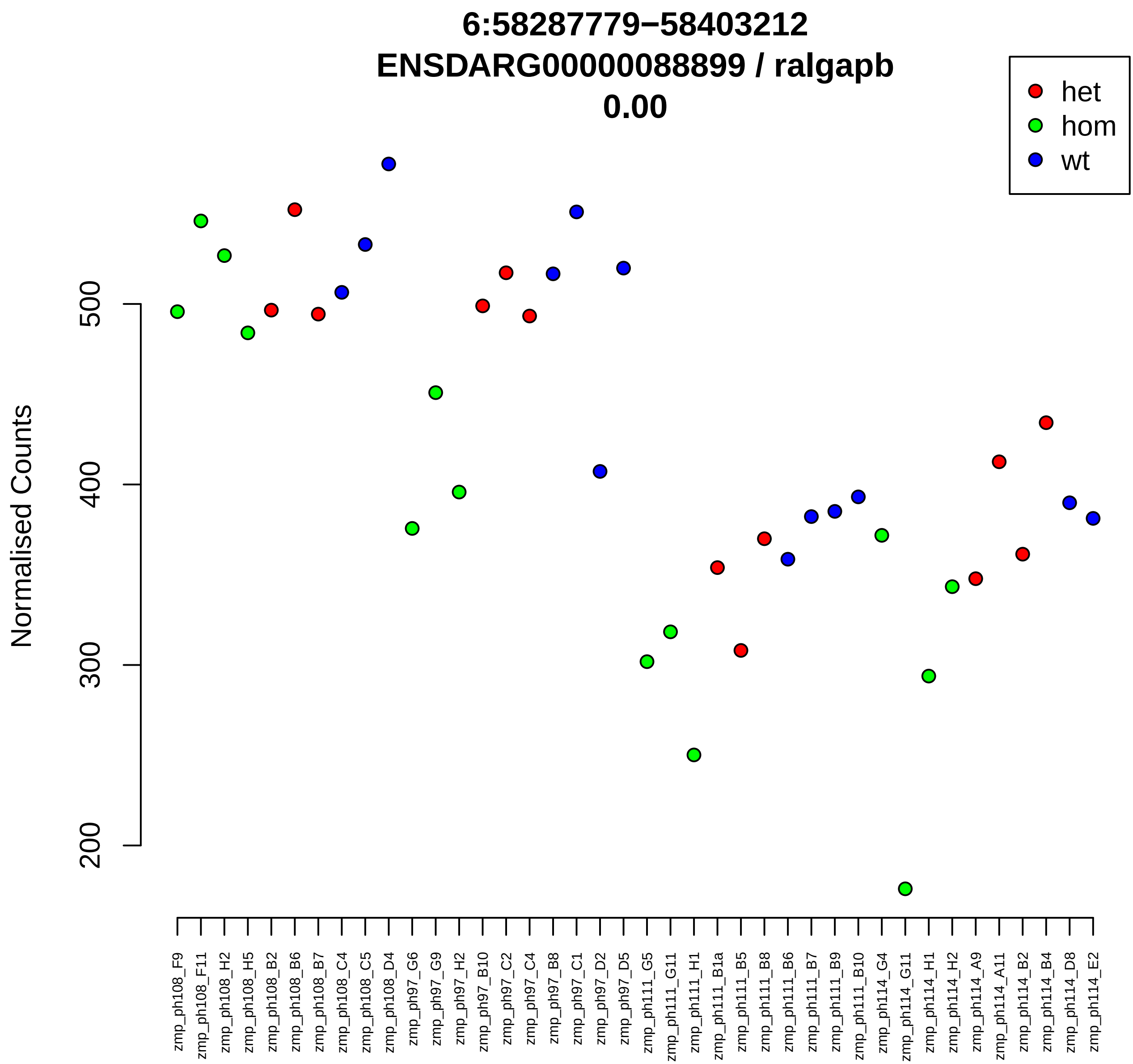
6:58287779−58403212
ENSDARG00000088899 / ralgapb
0.00
het
hom
wt
Normalised Counts
200
300
400
500
zmp_ph108_F9
zmp_ph108_F11
zmp_ph108_H2
zmp_ph108_H5
zmp_ph108_B2
zmp_ph108_B6
zmp_ph108_B7
zmp_ph108_C4
zmp_ph108_C5
zmp_ph108_D4
zmp_ph97_G6
zmp_ph97_G9
zmp_ph97_H2
zmp_ph97_B10
zmp_ph97_C2
zmp_ph97_C4
zmp_ph97_B8
zmp_ph97_C1
zmp_ph97_D2
zmp_ph97_D5
zmp_ph111_G5
zmp_ph111_G11
zmp_ph111_H1
zmp_ph111_B1a
zmp_ph111_B5
zmp_ph111_B8
zmp_ph111_B6
zmp_ph111_B7
zmp_ph111_B9
zmp_ph111_B10
zmp_ph114_G4
zmp_ph114_G11
zmp_ph114_H1
zmp_ph114_H2
zmp_ph114_A9
zmp_ph114_A11
zmp_ph114_B2
zmp_ph114_B4
zmp_ph114_D8
zmp_ph114_E2

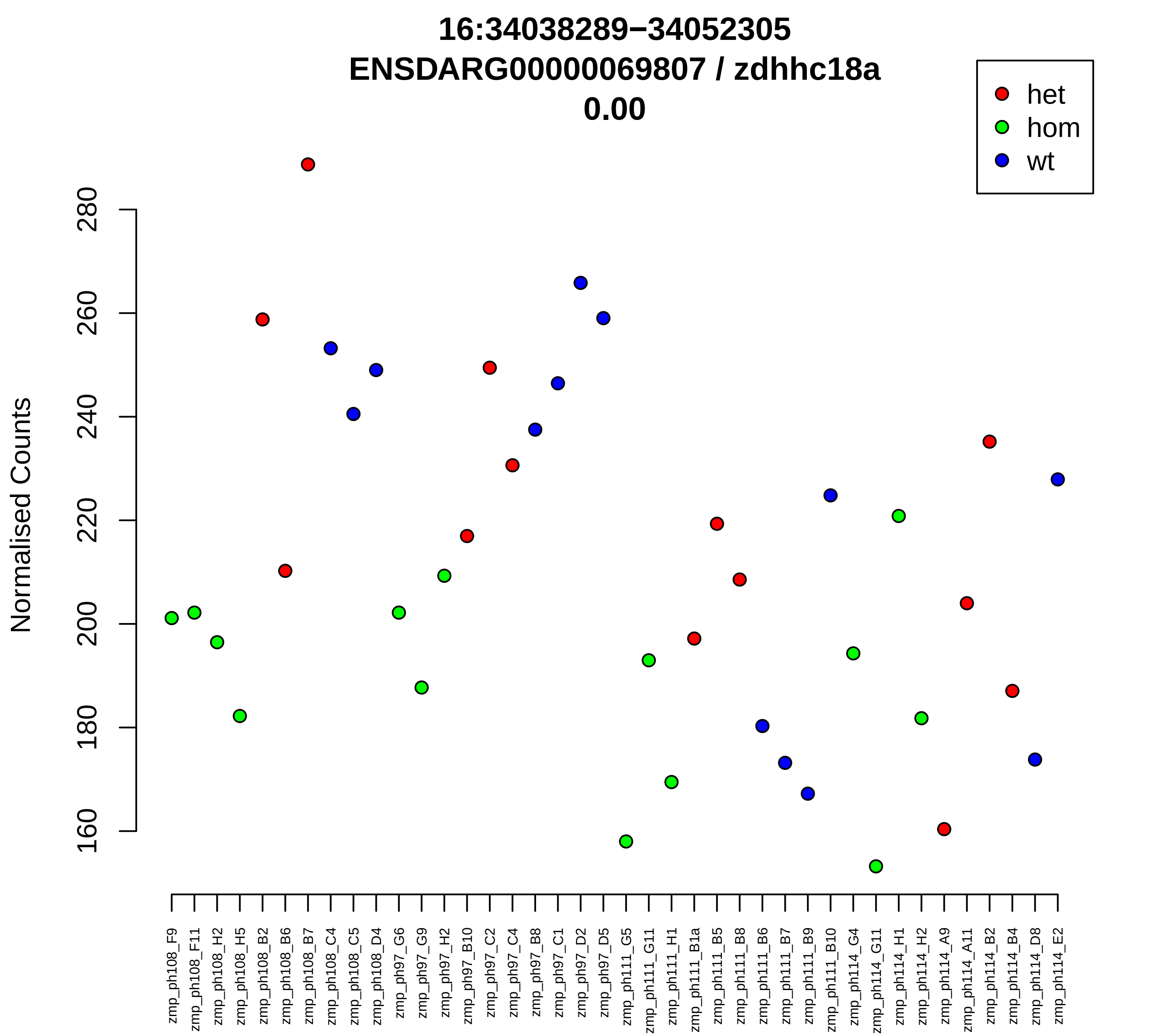

16:34038289−34052305
ENSDARG00000069807 / zdhhc18a
0.00
het
hom
wt
Normalised Counts
160
180
200
220
240
260
280
zmp_ph108_F9
zmp_ph108_F11
zmp_ph108_H2
zmp_ph108_H5
zmp_ph108_B2
zmp_ph108_B6
zmp_ph108_B7
zmp_ph108_C4
zmp_ph108_C5
zmp_ph108_D4
zmp_ph97_G6
zmp_ph97_G9
zmp_ph97_H2
zmp_ph97_B10
zmp_ph97_C2
zmp_ph97_C4
zmp_ph97_B8
zmp_ph97_C1
zmp_ph97_D2
zmp_ph97_D5
zmp_ph111_G5
zmp_ph111_G11
zmp_ph111_H1
zmp_ph111_B1a
zmp_ph111_B5
zmp_ph111_B8
zmp_ph111_B6
zmp_ph111_B7
zmp_ph111_B9
zmp_ph111_B10
zmp_ph114_G4
zmp_ph114_G11
zmp_ph114_H1
zmp_ph114_H2
zmp_ph114_A9
zmp_ph114_A11
zmp_ph114_B2
zmp_ph114_B4
zmp_ph114_D8
zmp_ph114_E2

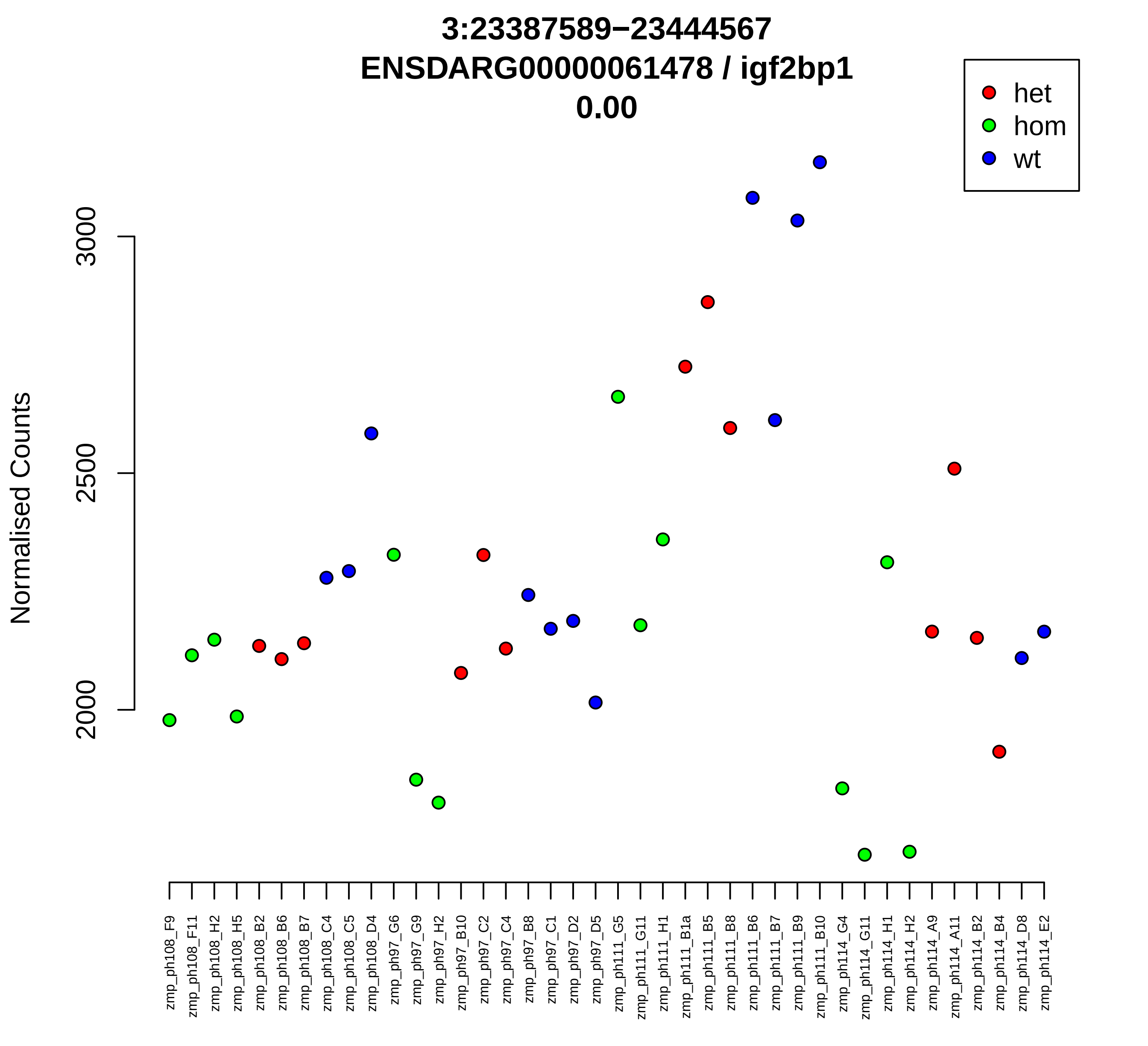

3:23387589−23444567
ENSDARG00000061478 / igf2bp1
0.00
het
hom
wt
Normalised Counts
2000
2500
3000
zmp_ph108_F9
zmp_ph108_F11
zmp_ph108_H2
zmp_ph108_H5
zmp_ph108_B2
zmp_ph108_B6
zmp_ph108_B7
zmp_ph108_C4
zmp_ph108_C5
zmp_ph108_D4
zmp_ph97_G6
zmp_ph97_G9
zmp_ph97_H2
zmp_ph97_B10
zmp_ph97_C2
zmp_ph97_C4
zmp_ph97_B8
zmp_ph97_C1
zmp_ph97_D2
zmp_ph97_D5
zmp_ph111_G5
zmp_ph111_G11
zmp_ph111_H1
zmp_ph111_B1a
zmp_ph111_B5
zmp_ph111_B8
zmp_ph111_B6
zmp_ph111_B7
zmp_ph111_B9
zmp_ph111_B10
zmp_ph114_G4
zmp_ph114_G11
zmp_ph114_H1
zmp_ph114_H2
zmp_ph114_A9
zmp_ph114_A11
zmp_ph114_B2
zmp_ph114_B4
zmp_ph114_D8
zmp_ph114_E2

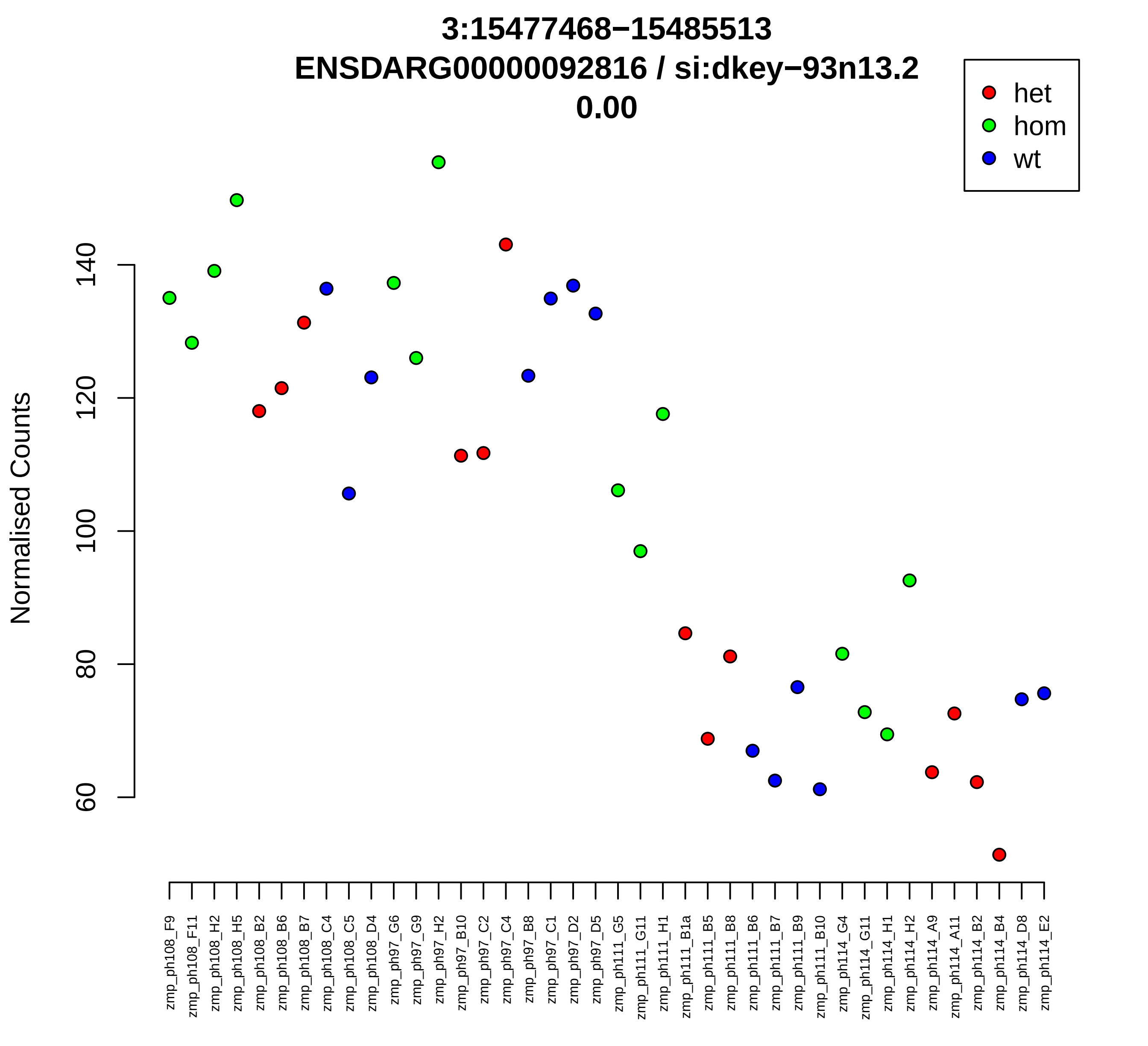

3:15477468−15485513
ENSDARG00000092816 / si:dkey−93n13.2
0.00
het
hom
wt
Normalised Counts
60
80
100
120
140
zmp_ph108_F9
zmp_ph108_F11
zmp_ph108_H2
zmp_ph108_H5
zmp_ph108_B2
zmp_ph108_B6
zmp_ph108_B7
zmp_ph108_C4
zmp_ph108_C5
zmp_ph108_D4
zmp_ph97_G6
zmp_ph97_G9
zmp_ph97_H2
zmp_ph97_B10
zmp_ph97_C2
zmp_ph97_C4
zmp_ph97_B8
zmp_ph97_C1
zmp_ph97_D2
zmp_ph97_D5
zmp_ph111_G5
zmp_ph111_G11
zmp_ph111_H1
zmp_ph111_B1a
zmp_ph111_B5
zmp_ph111_B8
zmp_ph111_B6
zmp_ph111_B7
zmp_ph111_B9
zmp_ph111_B10
zmp_ph114_G4
zmp_ph114_G11
zmp_ph114_H1
zmp_ph114_H2
zmp_ph114_A9
zmp_ph114_A11
zmp_ph114_B2
zmp_ph114_B4
zmp_ph114_D8
zmp_ph114_E2

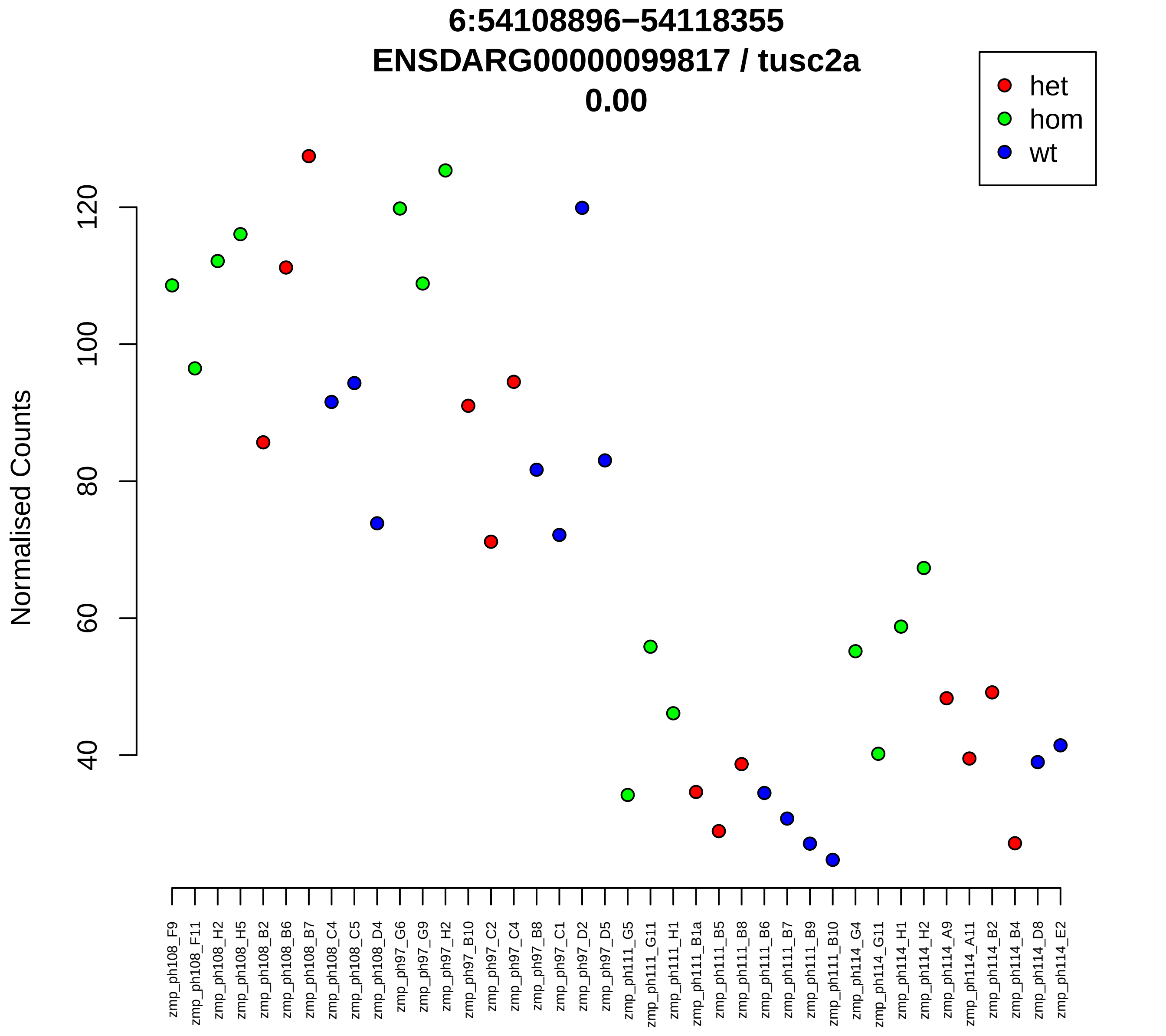
6:54108896−54118355
ENSDARG00000099817 / tusc2a
0.00
het
hom
wt
Normalised Counts
40
60
80
100
120
zmp_ph108_F9
zmp_ph108_F11
zmp_ph108_H2
zmp_ph108_H5
zmp_ph108_B2
zmp_ph108_B6
zmp_ph108_B7
zmp_ph108_C4
zmp_ph108_C5
zmp_ph108_D4
zmp_ph97_G6
zmp_ph97_G9
zmp_ph97_H2
zmp_ph97_B10
zmp_ph97_C2
zmp_ph97_C4
zmp_ph97_B8
zmp_ph97_C1
zmp_ph97_D2
zmp_ph97_D5
zmp_ph111_G5
zmp_ph111_G11
zmp_ph111_H1
zmp_ph111_B1a
zmp_ph111_B5
zmp_ph111_B8
zmp_ph111_B6
zmp_ph111_B7
zmp_ph111_B9
zmp_ph111_B10
zmp_ph114_G4
zmp_ph114_G11
zmp_ph114_H1
zmp_ph114_H2
zmp_ph114_A9
zmp_ph114_A11
zmp_ph114_B2
zmp_ph114_B4
zmp_ph114_D8
zmp_ph114_E2

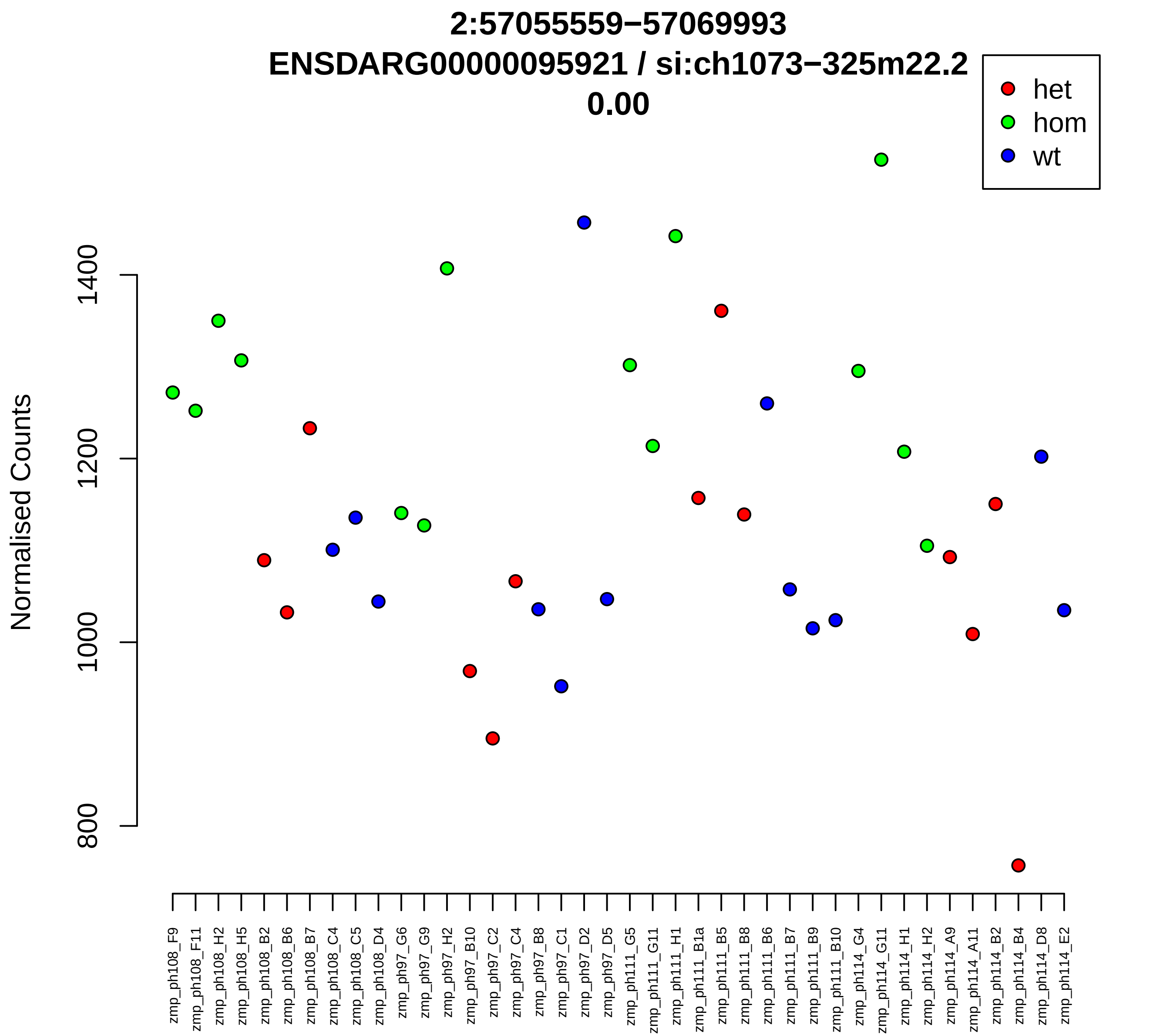

2:57055559−57069993
ENSDARG00000095921 / si:ch1073−325m22.2
0.00
het
hom
wt
Normalised Counts
800
1000
1200
1400
zmp_ph108_F9
zmp_ph108_F11
zmp_ph108_H2
zmp_ph108_H5
zmp_ph108_B2
zmp_ph108_B6
zmp_ph108_B7
zmp_ph108_C4
zmp_ph108_C5
zmp_ph108_D4
zmp_ph97_G6
zmp_ph97_G9
zmp_ph97_H2
zmp_ph97_B10
zmp_ph97_C2
zmp_ph97_C4
zmp_ph97_B8
zmp_ph97_C1
zmp_ph97_D2
zmp_ph97_D5
zmp_ph111_G5
zmp_ph111_G11
zmp_ph111_H1
zmp_ph111_B1a
zmp_ph111_B5
zmp_ph111_B8
zmp_ph111_B6
zmp_ph111_B7
zmp_ph111_B9
zmp_ph111_B10
zmp_ph114_G4
zmp_ph114_G11
zmp_ph114_H1
zmp_ph114_H2
zmp_ph114_A9
zmp_ph114_A11
zmp_ph114_B2
zmp_ph114_B4
zmp_ph114_D8
zmp_ph114_E2

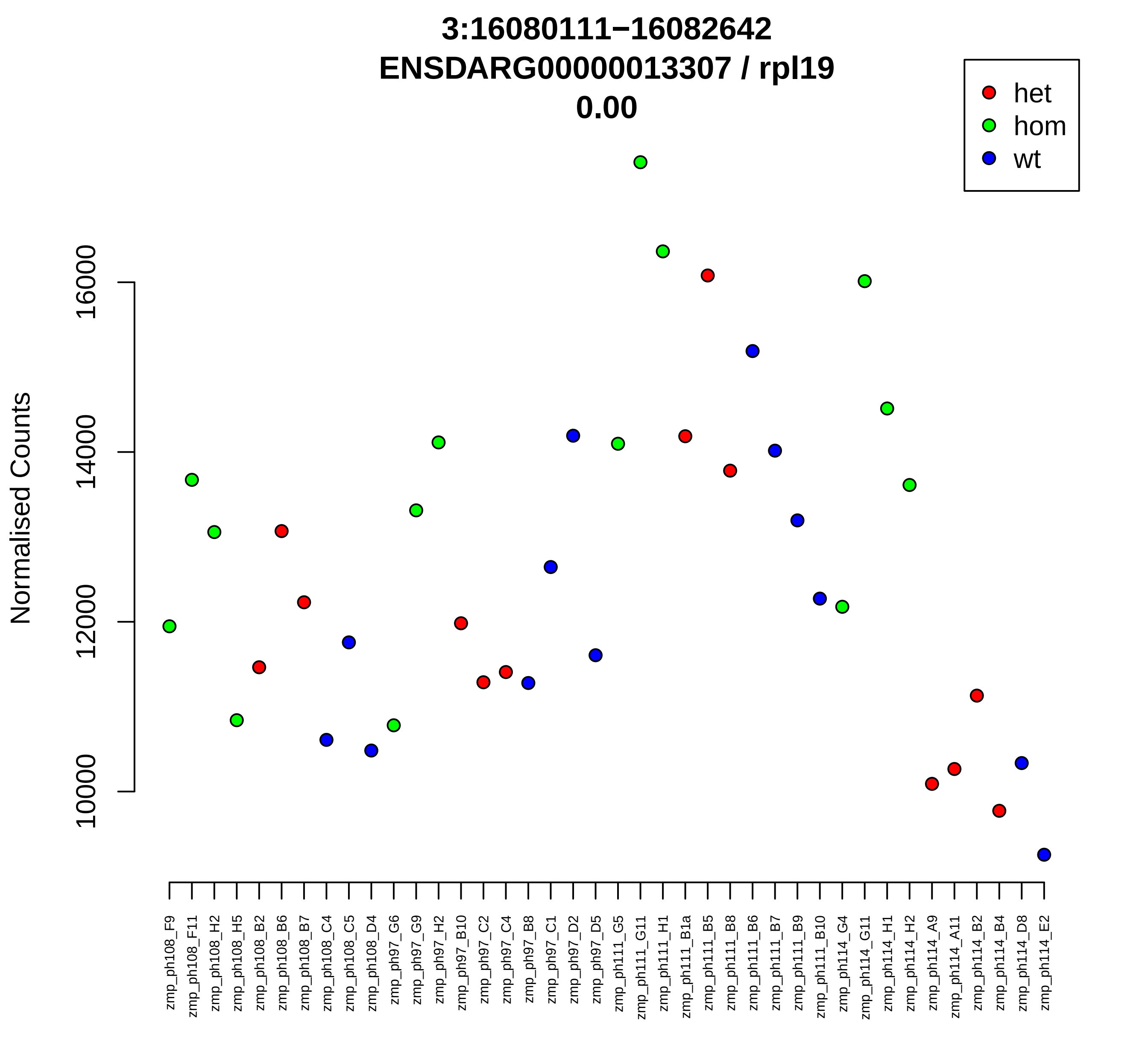

3:16080111−16082642
ENSDARG00000013307 / rpl19
0.00
het
hom
wt
Normalised Counts
10000
12000
14000
16000
zmp_ph108_F9
zmp_ph108_F11
zmp_ph108_H2
zmp_ph108_H5
zmp_ph108_B2
zmp_ph108_B6
zmp_ph108_B7
zmp_ph108_C4
zmp_ph108_C5
zmp_ph108_D4
zmp_ph97_G6
zmp_ph97_G9
zmp_ph97_H2
zmp_ph97_B10
zmp_ph97_C2
zmp_ph97_C4
zmp_ph97_B8
zmp_ph97_C1
zmp_ph97_D2
zmp_ph97_D5
zmp_ph111_G5
zmp_ph111_G11
zmp_ph111_H1
zmp_ph111_B1a
zmp_ph111_B5
zmp_ph111_B8
zmp_ph111_B6
zmp_ph111_B7
zmp_ph111_B9
zmp_ph111_B10
zmp_ph114_G4
zmp_ph114_G11
zmp_ph114_H1
zmp_ph114_H2
zmp_ph114_A9
zmp_ph114_A11
zmp_ph114_B2
zmp_ph114_B4
zmp_ph114_D8
zmp_ph114_E2

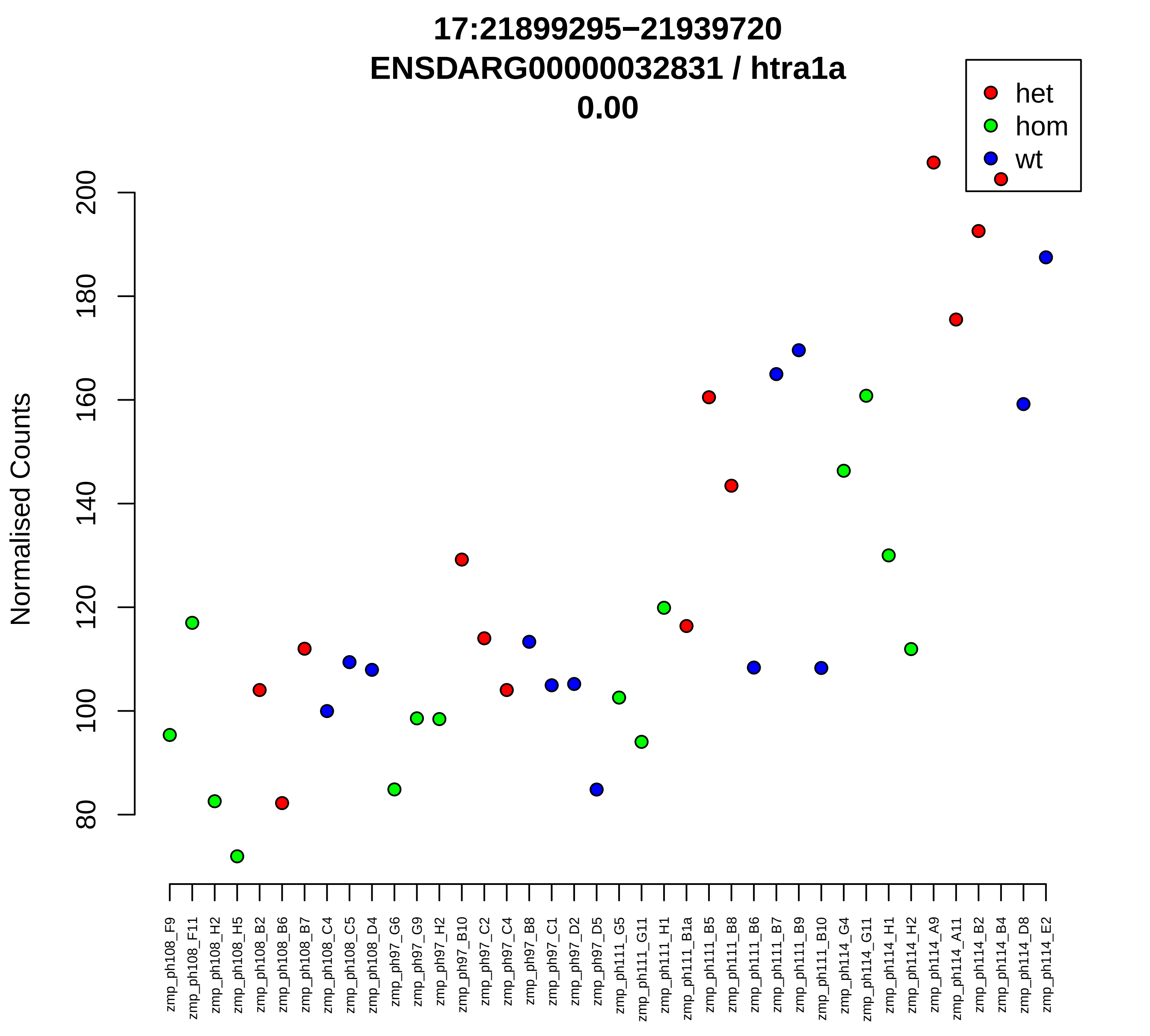
17:21899295−21939720
ENSDARG00000032831 / htra1a
0.00
het
hom
wt
Normalised Counts
80
100
120
140
160
180
200
zmp_ph108_F9
zmp_ph108_F11
zmp_ph108_H2
zmp_ph108_H5
zmp_ph108_B2
zmp_ph108_B6
zmp_ph108_B7
zmp_ph108_C4
zmp_ph108_C5
zmp_ph108_D4
zmp_ph97_G6
zmp_ph97_G9
zmp_ph97_H2
zmp_ph97_B10
zmp_ph97_C2
zmp_ph97_C4
zmp_ph97_B8
zmp_ph97_C1
zmp_ph97_D2
zmp_ph97_D5
zmp_ph111_G5
zmp_ph111_G11
zmp_ph111_H1
zmp_ph111_B1a
zmp_ph111_B5
zmp_ph111_B8
zmp_ph111_B6
zmp_ph111_B7
zmp_ph111_B9
zmp_ph111_B10
zmp_ph114_G4
zmp_ph114_G11
zmp_ph114_H1
zmp_ph114_H2
zmp_ph114_A9
zmp_ph114_A11
zmp_ph114_B2
zmp_ph114_B4
zmp_ph114_D8
zmp_ph114_E2

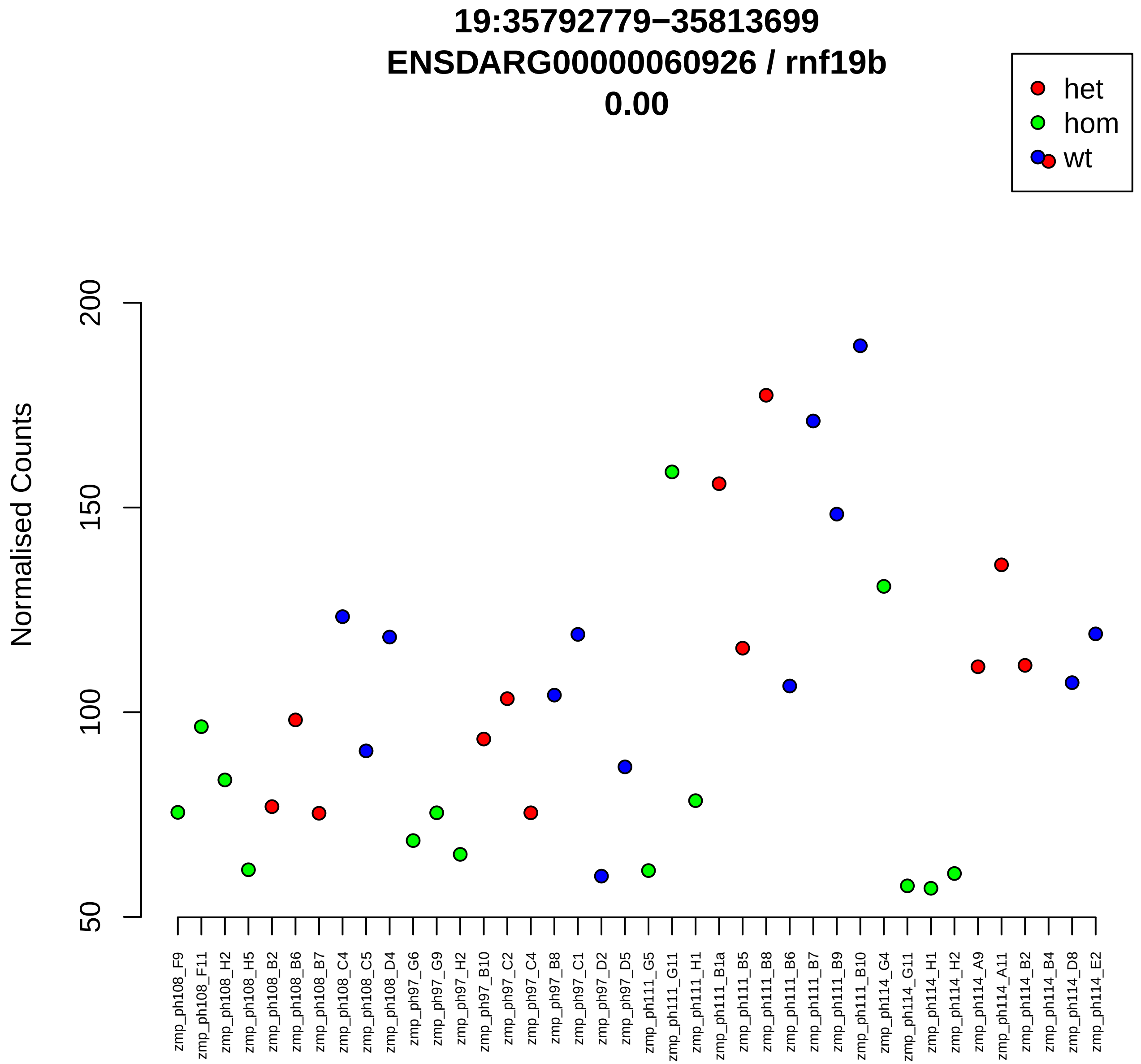

19:35792779−35813699
ENSDARG00000060926 / rnf19b
0.00
het
hom
wt
Normalised Counts
50
100
150
200
zmp_ph108_F9
zmp_ph108_F11
zmp_ph108_H2
zmp_ph108_H5
zmp_ph108_B2
zmp_ph108_B6
zmp_ph108_B7
zmp_ph108_C4
zmp_ph108_C5
zmp_ph108_D4
zmp_ph97_G6
zmp_ph97_G9
zmp_ph97_H2
zmp_ph97_B10
zmp_ph97_C2
zmp_ph97_C4
zmp_ph97_B8
zmp_ph97_C1
zmp_ph97_D2
zmp_ph97_D5
zmp_ph111_G5
zmp_ph111_G11
zmp_ph111_H1
zmp_ph111_B1a
zmp_ph111_B5
zmp_ph111_B8
zmp_ph111_B6
zmp_ph111_B7
zmp_ph111_B9
zmp_ph111_B10
zmp_ph114_G4
zmp_ph114_G11
zmp_ph114_H1
zmp_ph114_H2
zmp_ph114_A9
zmp_ph114_A11
zmp_ph114_B2
zmp_ph114_B4
zmp_ph114_D8
zmp_ph114_E2

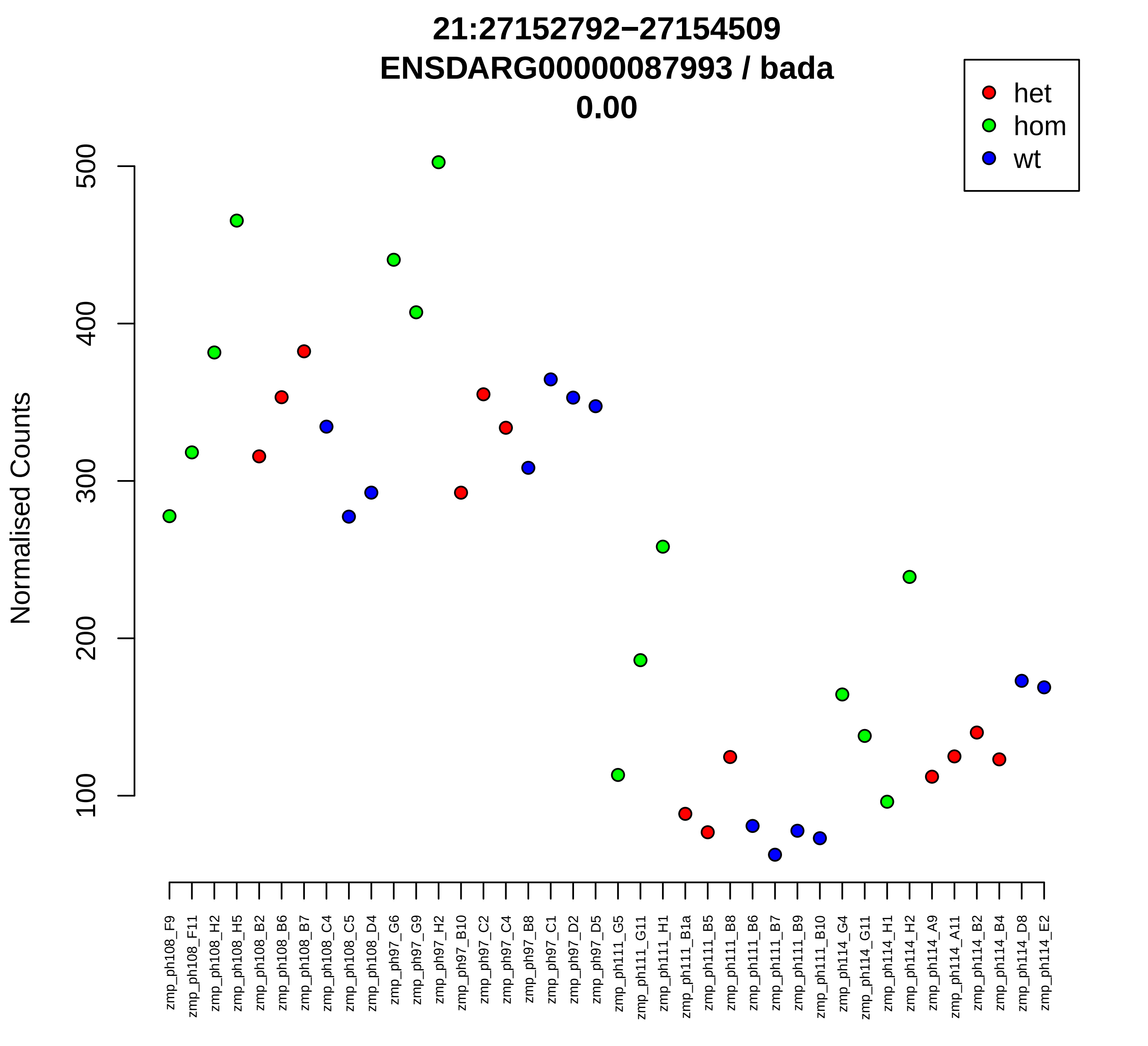

21:27152792−27154509
ENSDARG00000087993 / bada
0.00
het
hom
wt
Normalised Counts
100
200
300
400
500
zmp_ph108_F9
zmp_ph108_F11
zmp_ph108_H2
zmp_ph108_H5
zmp_ph108_B2
zmp_ph108_B6
zmp_ph108_B7
zmp_ph108_C4
zmp_ph108_C5
zmp_ph108_D4
zmp_ph97_G6
zmp_ph97_G9
zmp_ph97_H2
zmp_ph97_B10
zmp_ph97_C2
zmp_ph97_C4
zmp_ph97_B8
zmp_ph97_C1
zmp_ph97_D2
zmp_ph97_D5
zmp_ph111_G5
zmp_ph111_G11
zmp_ph111_H1
zmp_ph111_B1a
zmp_ph111_B5
zmp_ph111_B8
zmp_ph111_B6
zmp_ph111_B7
zmp_ph111_B9
zmp_ph111_B10
zmp_ph114_G4
zmp_ph114_G11
zmp_ph114_H1
zmp_ph114_H2
zmp_ph114_A9
zmp_ph114_A11
zmp_ph114_B2
zmp_ph114_B4
zmp_ph114_D8
zmp_ph114_E2

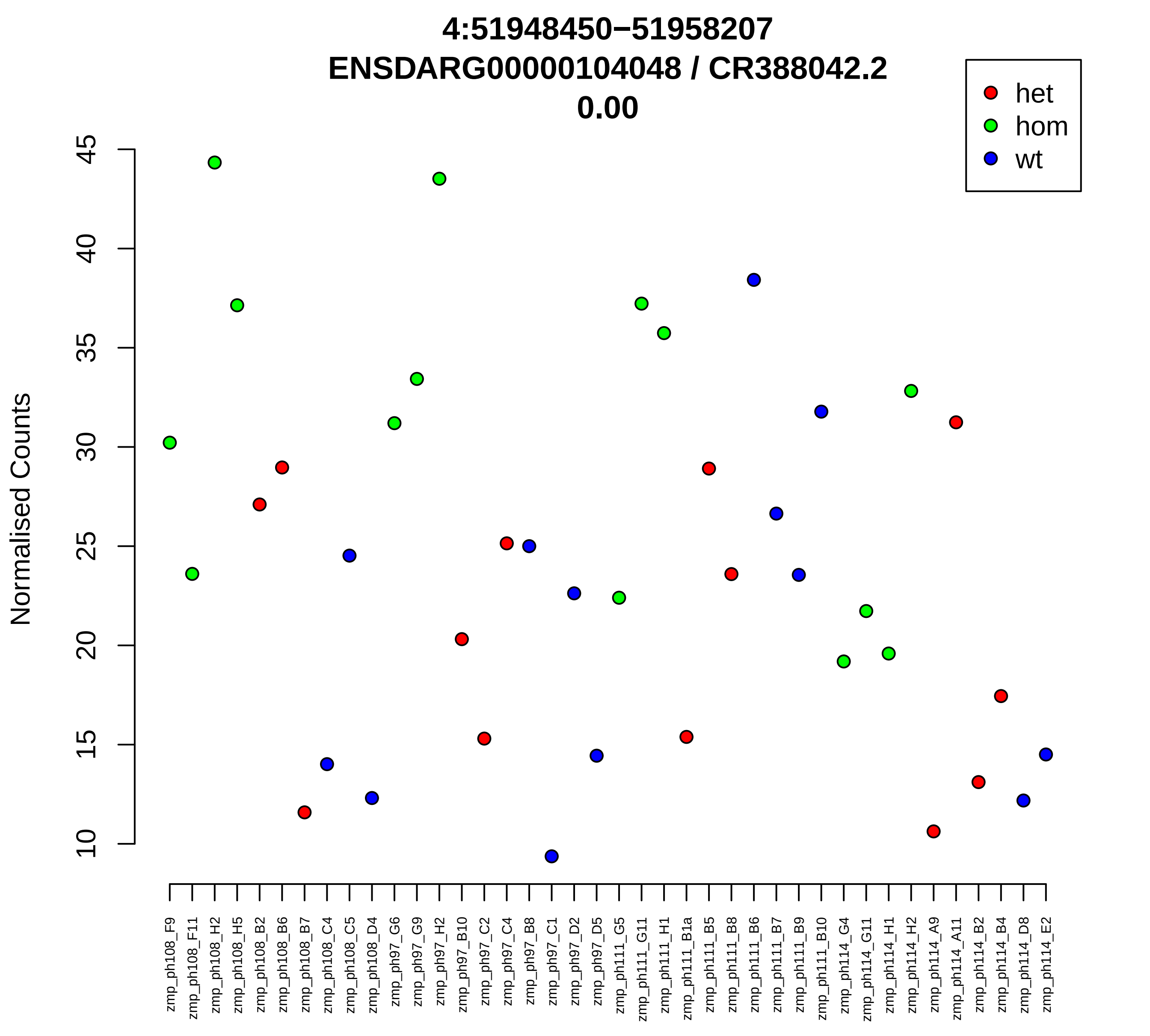

4:51948450−51958207
ENSDARG00000104048 / CR388042.2
0.00
het
hom
wt
Normalised Counts
10
15
20
25
30
35
40
45
zmp_ph108_F9
zmp_ph108_F11
zmp_ph108_H2
zmp_ph108_H5
zmp_ph108_B2
zmp_ph108_B6
zmp_ph108_B7
zmp_ph108_C4
zmp_ph108_C5
zmp_ph108_D4
zmp_ph97_G6
zmp_ph97_G9
zmp_ph97_H2
zmp_ph97_B10
zmp_ph97_C2
zmp_ph97_C4
zmp_ph97_B8
zmp_ph97_C1
zmp_ph97_D2
zmp_ph97_D5
zmp_ph111_G5
zmp_ph111_G11
zmp_ph111_H1
zmp_ph111_B1a
zmp_ph111_B5
zmp_ph111_B8
zmp_ph111_B6
zmp_ph111_B7
zmp_ph111_B9
zmp_ph111_B10
zmp_ph114_G4
zmp_ph114_G11
zmp_ph114_H1
zmp_ph114_H2
zmp_ph114_A9
zmp_ph114_A11
zmp_ph114_B2
zmp_ph114_B4
zmp_ph114_D8
zmp_ph114_E2

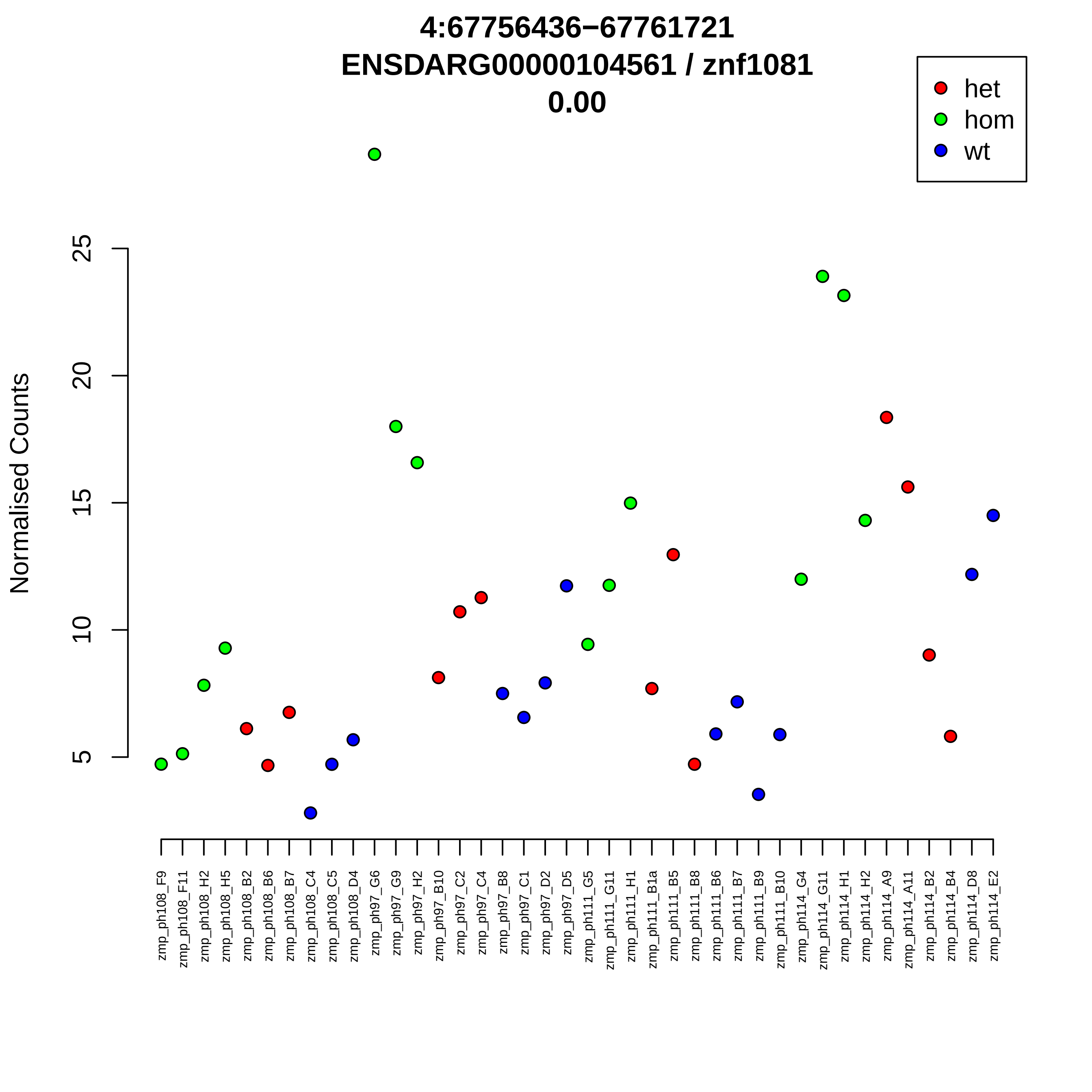

4:67756436−67761721
ENSDARG00000104561 / znf1081
0.00
het
hom
wt
Normalised Counts
5
10
15
20
25
zmp_ph108_F9
zmp_ph108_F11
zmp_ph108_H2
zmp_ph108_H5
zmp_ph108_B2
zmp_ph108_B6
zmp_ph108_B7
zmp_ph108_C4
zmp_ph108_C5
zmp_ph108_D4
zmp_ph97_G6
zmp_ph97_G9
zmp_ph97_H2
zmp_ph97_B10
zmp_ph97_C2
zmp_ph97_C4
zmp_ph97_B8
zmp_ph97_C1
zmp_ph97_D2
zmp_ph97_D5
zmp_ph111_G5
zmp_ph111_G11
zmp_ph111_H1
zmp_ph111_B1a
zmp_ph111_B5
zmp_ph111_B8
zmp_ph111_B6
zmp_ph111_B7
zmp_ph111_B9
zmp_ph111_B10
zmp_ph114_G4
zmp_ph114_G11
zmp_ph114_H1
zmp_ph114_H2
zmp_ph114_A9
zmp_ph114_A11
zmp_ph114_B2
zmp_ph114_B4
zmp_ph114_D8
zmp_ph114_E2

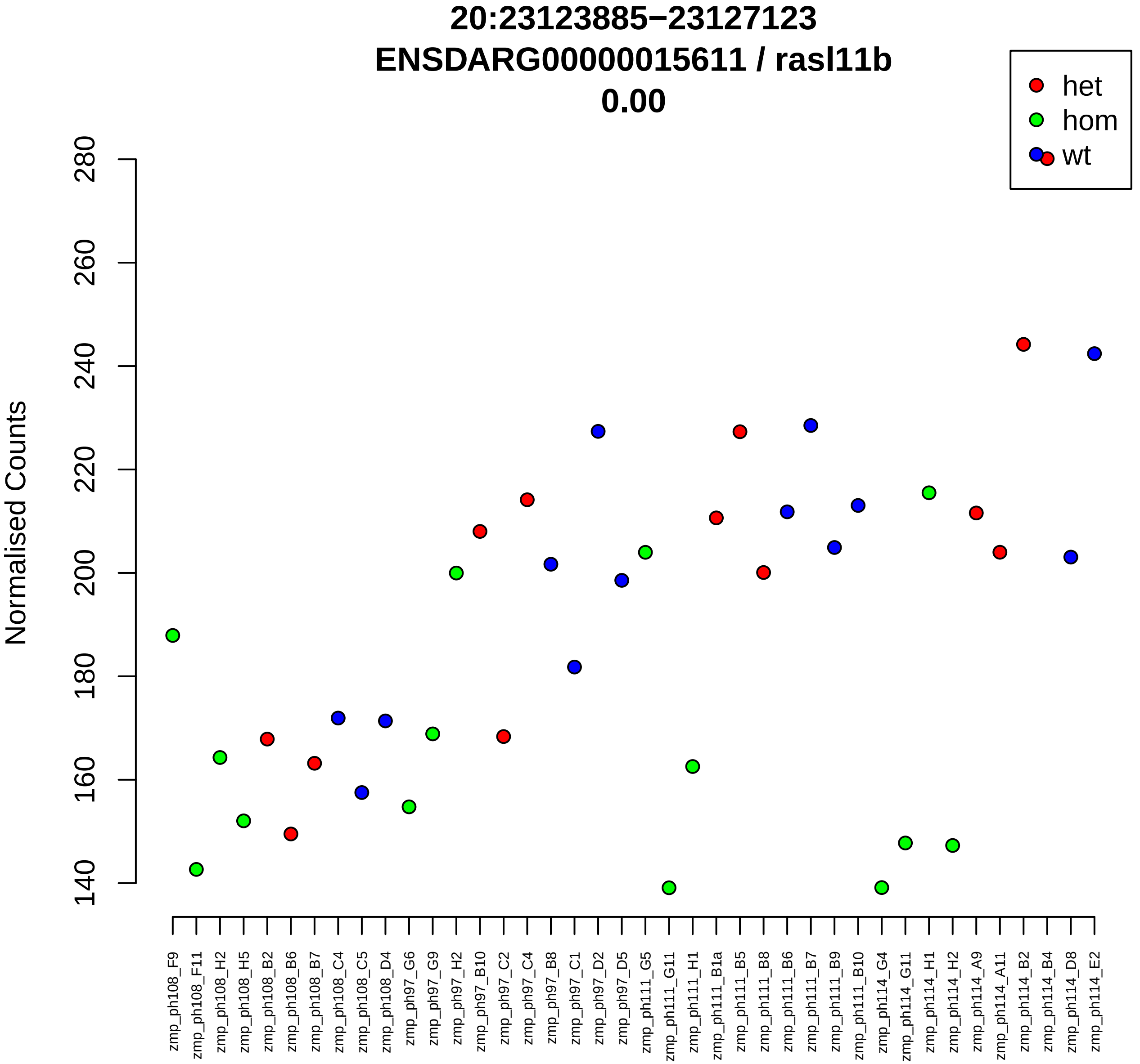

20:23123885−23127123
ENSDARG00000015611 / rasl11b
0.00
het
hom
wt
Normalised Counts
140
160
180
200
220
240
260
280
zmp_ph108_F9
zmp_ph108_F11
zmp_ph108_H2
zmp_ph108_H5
zmp_ph108_B2
zmp_ph108_B6
zmp_ph108_B7
zmp_ph108_C4
zmp_ph108_C5
zmp_ph108_D4
zmp_ph97_G6
zmp_ph97_G9
zmp_ph97_H2
zmp_ph97_B10
zmp_ph97_C2
zmp_ph97_C4
zmp_ph97_B8
zmp_ph97_C1
zmp_ph97_D2
zmp_ph97_D5
zmp_ph111_G5
zmp_ph111_G11
zmp_ph111_H1
zmp_ph111_B1a
zmp_ph111_B5
zmp_ph111_B8
zmp_ph111_B6
zmp_ph111_B7
zmp_ph111_B9
zmp_ph111_B10
zmp_ph114_G4
zmp_ph114_G11
zmp_ph114_H1
zmp_ph114_H2
zmp_ph114_A9
zmp_ph114_A11
zmp_ph114_B2
zmp_ph114_B4
zmp_ph114_D8
zmp_ph114_E2

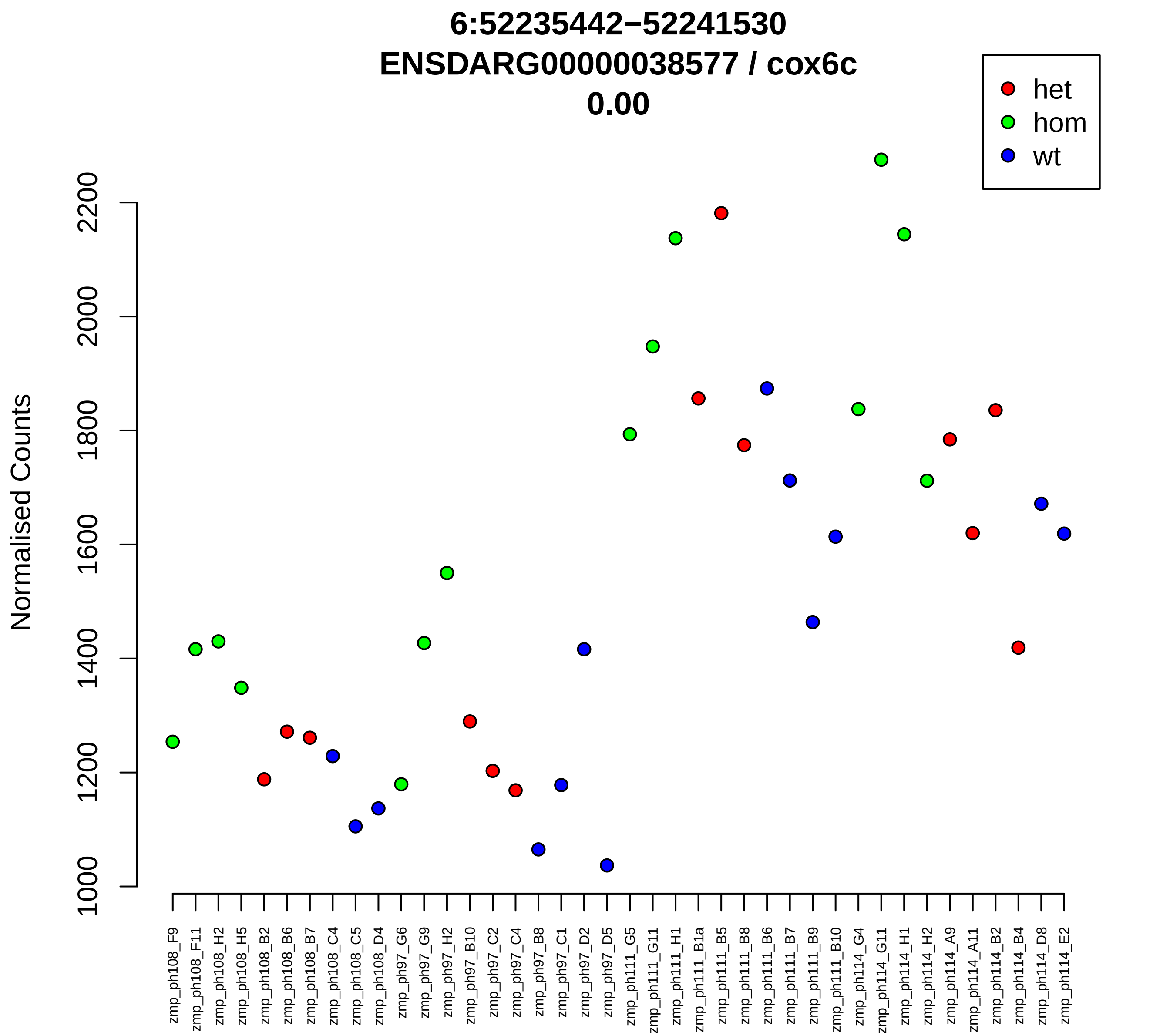

6:52235442−52241530
ENSDARG00000038577 / cox6c
0.00
het
hom
wt
Normalised Counts
1000
1200
1400
1600
1800
2000
2200
zmp_ph108_F9
zmp_ph108_F11
zmp_ph108_H2
zmp_ph108_H5
zmp_ph108_B2
zmp_ph108_B6
zmp_ph108_B7
zmp_ph108_C4
zmp_ph108_C5
zmp_ph108_D4
zmp_ph97_G6
zmp_ph97_G9
zmp_ph97_H2
zmp_ph97_B10
zmp_ph97_C2
zmp_ph97_C4
zmp_ph97_B8
zmp_ph97_C1
zmp_ph97_D2
zmp_ph97_D5
zmp_ph111_G5
zmp_ph111_G11
zmp_ph111_H1
zmp_ph111_B1a
zmp_ph111_B5
zmp_ph111_B8
zmp_ph111_B6
zmp_ph111_B7
zmp_ph111_B9
zmp_ph111_B10
zmp_ph114_G4
zmp_ph114_G11
zmp_ph114_H1
zmp_ph114_H2
zmp_ph114_A9
zmp_ph114_A11
zmp_ph114_B2
zmp_ph114_B4
zmp_ph114_D8
zmp_ph114_E2

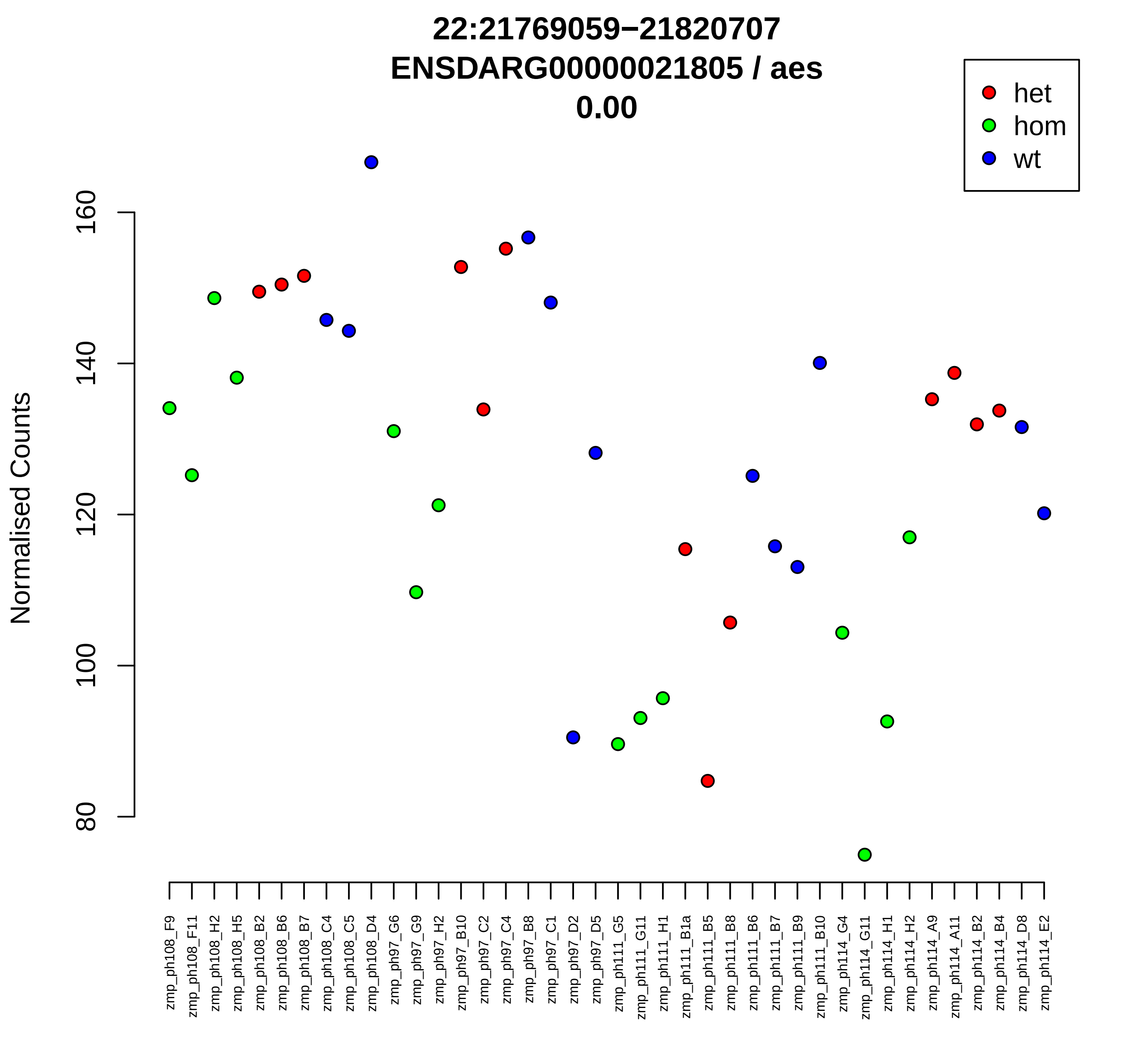

22:21769059−21820707
ENSDARG00000021805 / aes
0.00
het
hom
wt
Normalised Counts
80
100
120
140
160
zmp_ph108_F9
zmp_ph108_F11
zmp_ph108_H2
zmp_ph108_H5
zmp_ph108_B2
zmp_ph108_B6
zmp_ph108_B7
zmp_ph108_C4
zmp_ph108_C5
zmp_ph108_D4
zmp_ph97_G6
zmp_ph97_G9
zmp_ph97_H2
zmp_ph97_B10
zmp_ph97_C2
zmp_ph97_C4
zmp_ph97_B8
zmp_ph97_C1
zmp_ph97_D2
zmp_ph97_D5
zmp_ph111_G5
zmp_ph111_G11
zmp_ph111_H1
zmp_ph111_B1a
zmp_ph111_B5
zmp_ph111_B8
zmp_ph111_B6
zmp_ph111_B7
zmp_ph111_B9
zmp_ph111_B10
zmp_ph114_G4
zmp_ph114_G11
zmp_ph114_H1
zmp_ph114_H2
zmp_ph114_A9
zmp_ph114_A11
zmp_ph114_B2
zmp_ph114_B4
zmp_ph114_D8
zmp_ph114_E2

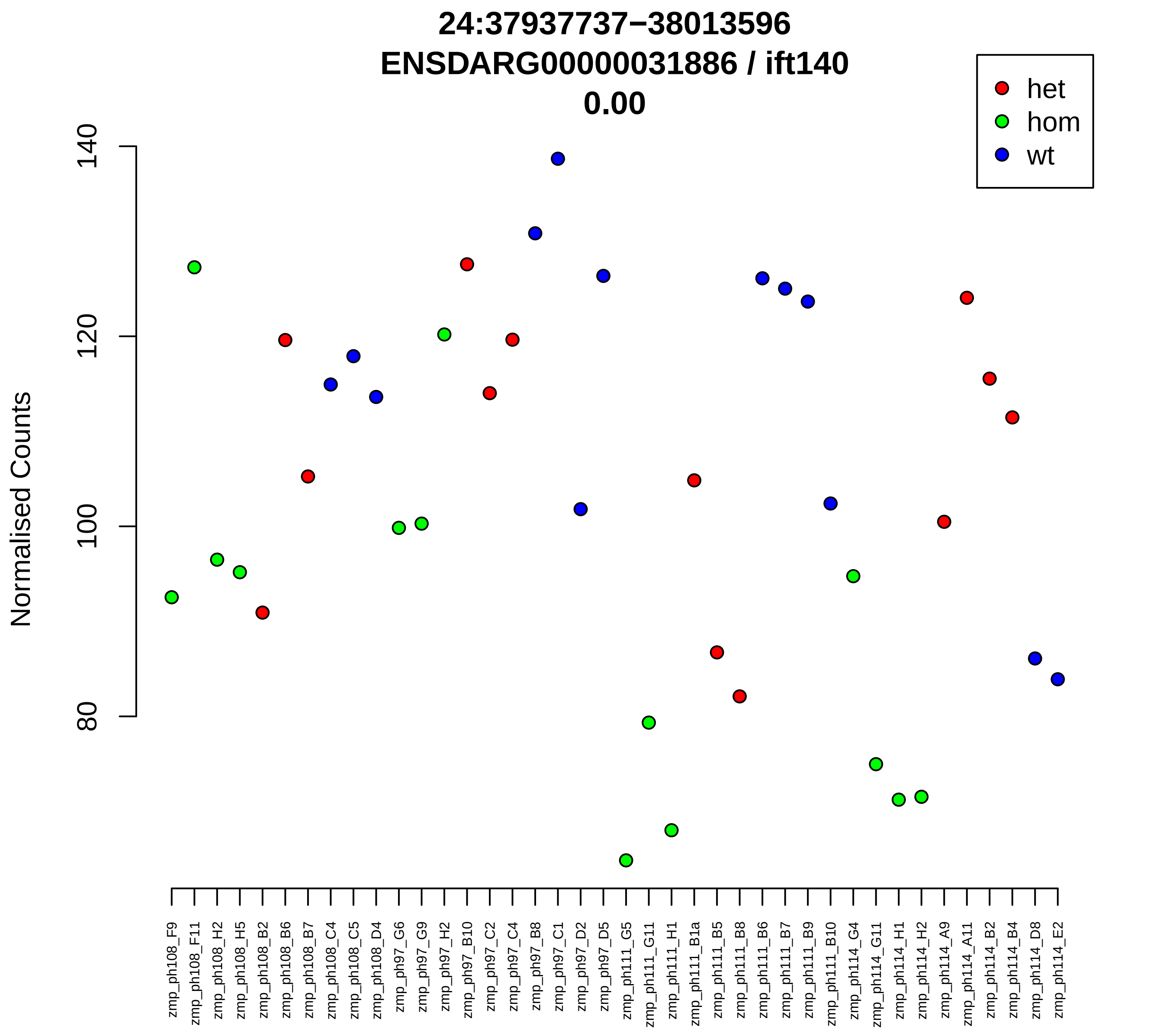
24:37937737−38013596
ENSDARG00000031886 / ift140
0.00
Normalised Counts
140
120
100
80
het
hom
wt
zmp_ph108_F9
zmp_ph108_F11
zmp_ph108_H2
zmp_ph108_H5
zmp_ph108_B2
zmp_ph108_B6
zmp_ph108_B7
zmp_ph108_C4
zmp_ph108_C5
zmp_ph108_D4
zmp_ph97_G6
zmp_ph97_G9
zmp_ph97_H2
zmp_ph97_B10
zmp_ph97_C2
zmp_ph97_C4
zmp_ph97_B8
zmp_ph97_C1
zmp_ph97_D2
zmp_ph97_D5
zmp_ph111_G5
zmp_ph111_G11
zmp_ph111_H1
zmp_ph111_B1a
zmp_ph111_B5
zmp_ph111_B8
zmp_ph111_B6
zmp_ph111_B7
zmp_ph111_B9
zmp_ph111_B10
zmp_ph114_G4
zmp_ph114_G11
zmp_ph114_H1
zmp_ph114_H2
zmp_ph114_A9
zmp_ph114_A11
zmp_ph114_B2
zmp_ph114_B4
zmp_ph114_D8
zmp_ph114_E2

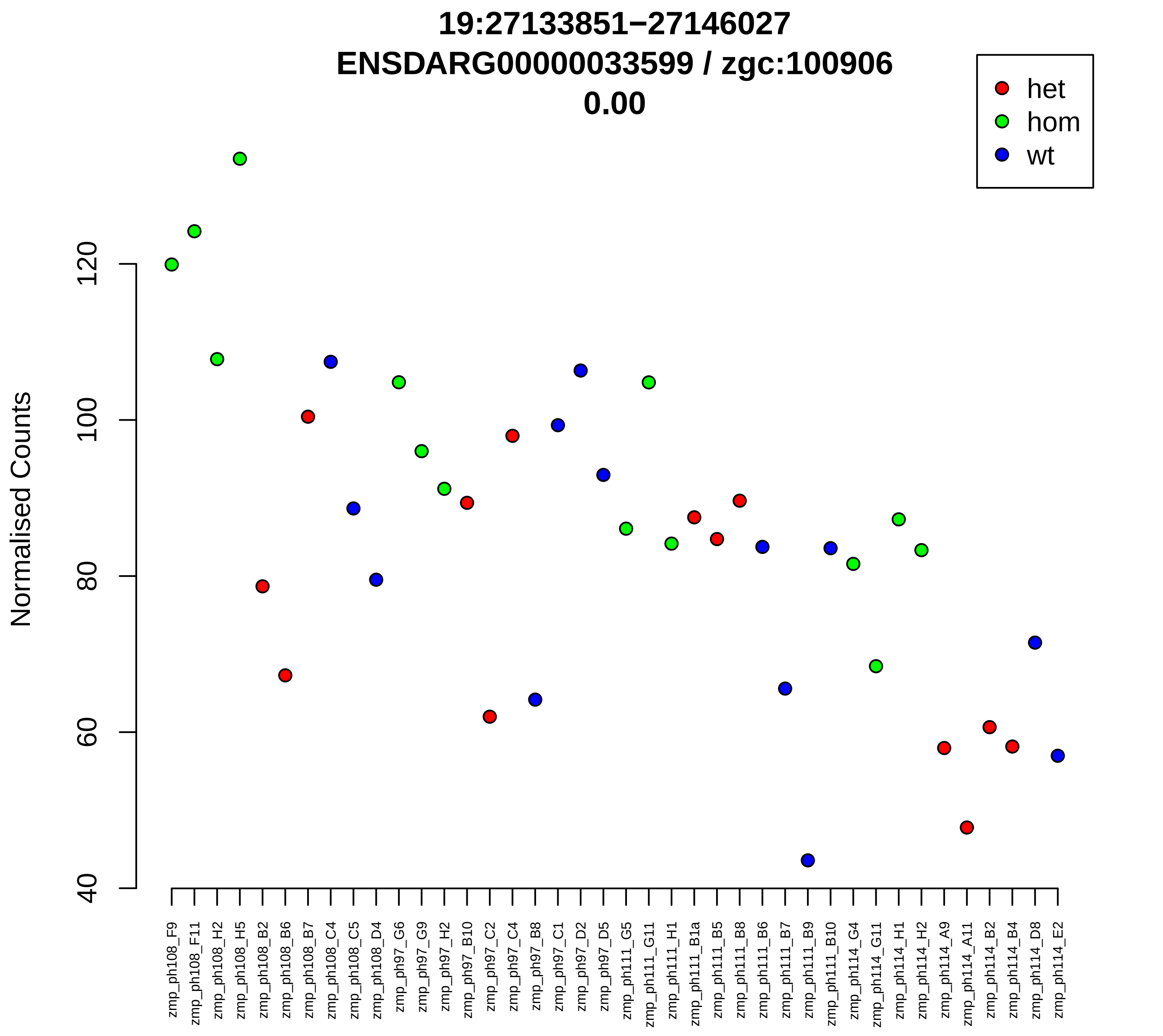
19:27133851−27146027
ENSDARG00000033599 / zgc:100906
0.00
het
hom
wt
Normalised Counts
40
60
80
100
120
zmp_ph108_F9
zmp_ph108_F11
zmp_ph108_H2
zmp_ph108_H5
zmp_ph108_B2
zmp_ph108_B6
zmp_ph108_B7
zmp_ph108_C4
zmp_ph108_C5
zmp_ph108_D4
zmp_ph97_G6
zmp_ph97_G9
zmp_ph97_H2
zmp_ph97_B10
zmp_ph97_C2
zmp_ph97_C4
zmp_ph97_B8
zmp_ph97_C1
zmp_ph97_D2
zmp_ph97_D5
zmp_ph111_G5
zmp_ph111_G11
zmp_ph111_H1
zmp_ph111_B1a
zmp_ph111_B5
zmp_ph111_B8
zmp_ph111_B6
zmp_ph111_B7
zmp_ph111_B9
zmp_ph111_B10
zmp_ph114_G4
zmp_ph114_G11
zmp_ph114_H1
zmp_ph114_H2
zmp_ph114_A9
zmp_ph114_A11
zmp_ph114_B2
zmp_ph114_B4
zmp_ph114_D8
zmp_ph114_E2

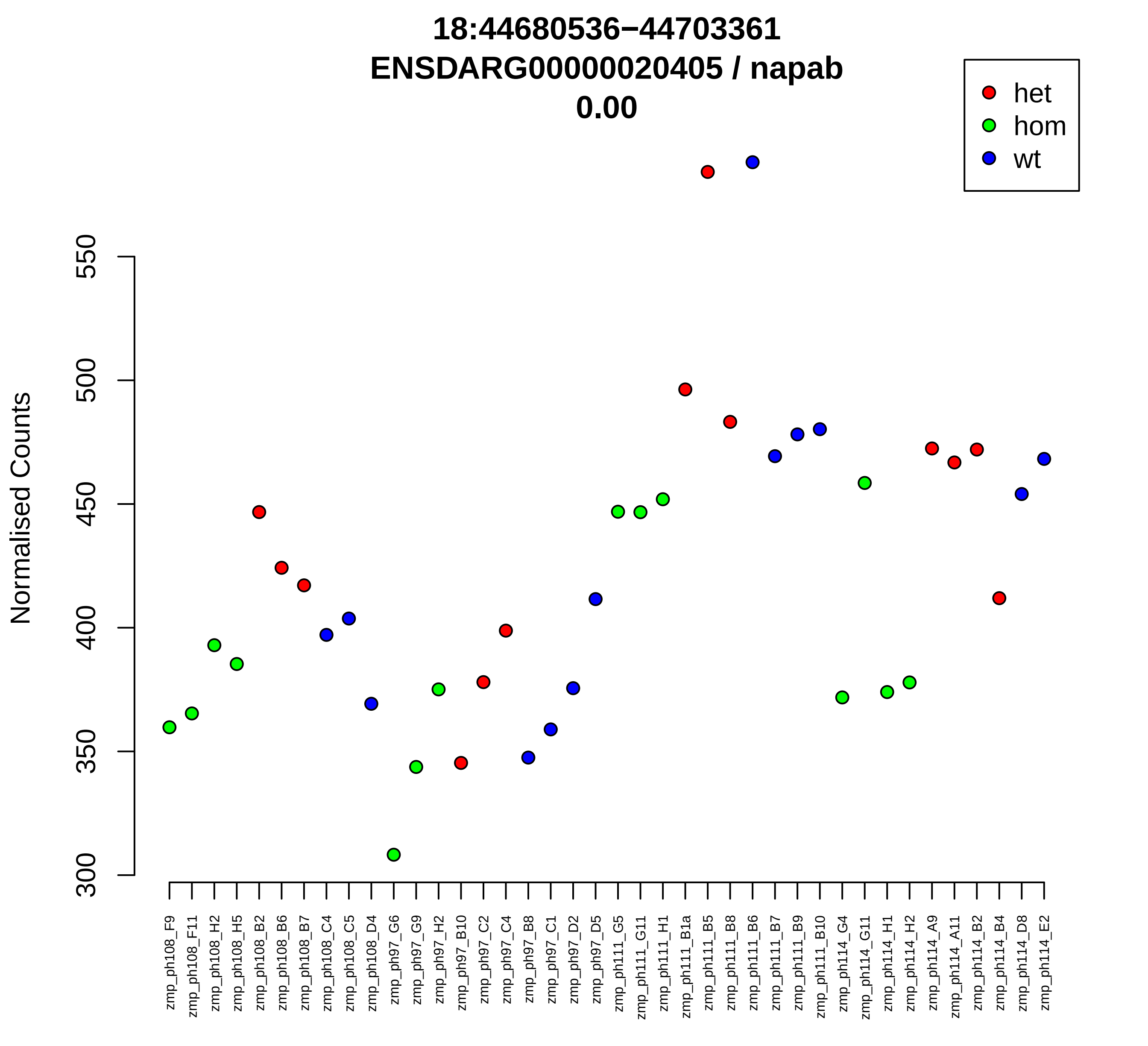

18:44680536−44703361
ENSDARG00000020405 / napab
0.00
het
hom
wt
Normalised Counts
300
350
400
450
500
550
zmp_ph108_F9
zmp_ph108_F11
zmp_ph108_H2
zmp_ph108_H5
zmp_ph108_B2
zmp_ph108_B6
zmp_ph108_B7
zmp_ph108_C4
zmp_ph108_C5
zmp_ph108_D4
zmp_ph97_G6
zmp_ph97_G9
zmp_ph97_H2
zmp_ph97_B10
zmp_ph97_C2
zmp_ph97_C4
zmp_ph97_B8
zmp_ph97_C1
zmp_ph97_D2
zmp_ph97_D5
zmp_ph111_G5
zmp_ph111_G11
zmp_ph111_H1
zmp_ph111_B1a
zmp_ph111_B5
zmp_ph111_B8
zmp_ph111_B6
zmp_ph111_B7
zmp_ph111_B9
zmp_ph111_B10
zmp_ph114_G4
zmp_ph114_G11
zmp_ph114_H1
zmp_ph114_H2
zmp_ph114_A9
zmp_ph114_A11
zmp_ph114_B2
zmp_ph114_B4
zmp_ph114_D8
zmp_ph114_E2

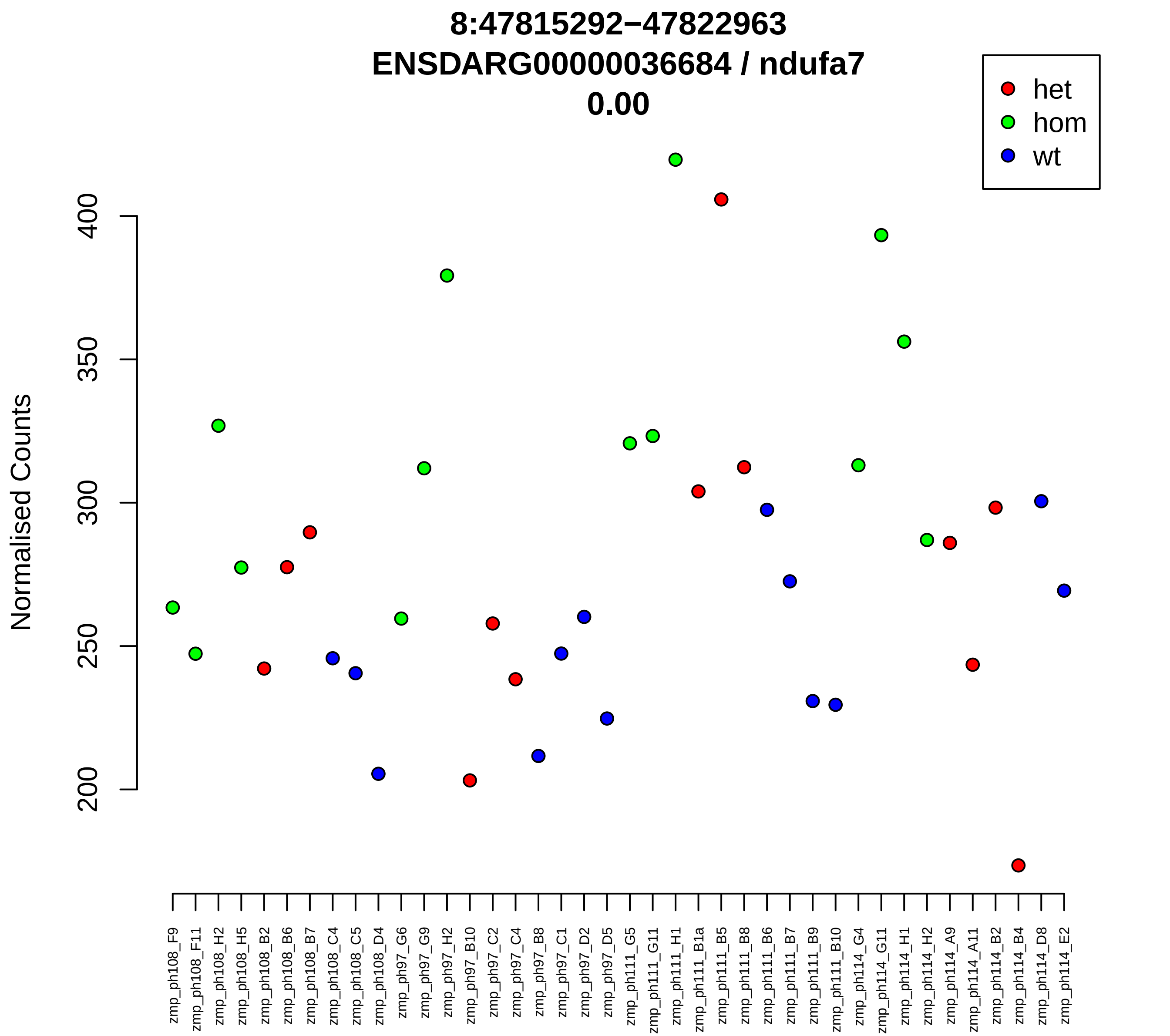

8:47815292−47822963
ENSDARG00000036684 / ndufa7
0.00
het
hom
wt
Normalised Counts
200
250
300
350
400
zmp_ph108_F9
zmp_ph108_F11
zmp_ph108_H2
zmp_ph108_H5
zmp_ph108_B2
zmp_ph108_B6
zmp_ph108_B7
zmp_ph108_C4
zmp_ph108_C5
zmp_ph108_D4
zmp_ph97_G6
zmp_ph97_G9
zmp_ph97_H2
zmp_ph97_B10
zmp_ph97_C2
zmp_ph97_C4
zmp_ph97_B8
zmp_ph97_C1
zmp_ph97_D2
zmp_ph97_D5
zmp_ph111_G5
zmp_ph111_G11
zmp_ph111_H1
zmp_ph111_B1a
zmp_ph111_B5
zmp_ph111_B8
zmp_ph111_B6
zmp_ph111_B7
zmp_ph111_B9
zmp_ph111_B10
zmp_ph114_G4
zmp_ph114_G11
zmp_ph114_H1
zmp_ph114_H2
zmp_ph114_A9
zmp_ph114_A11
zmp_ph114_B2
zmp_ph114_B4
zmp_ph114_D8
zmp_ph114_E2

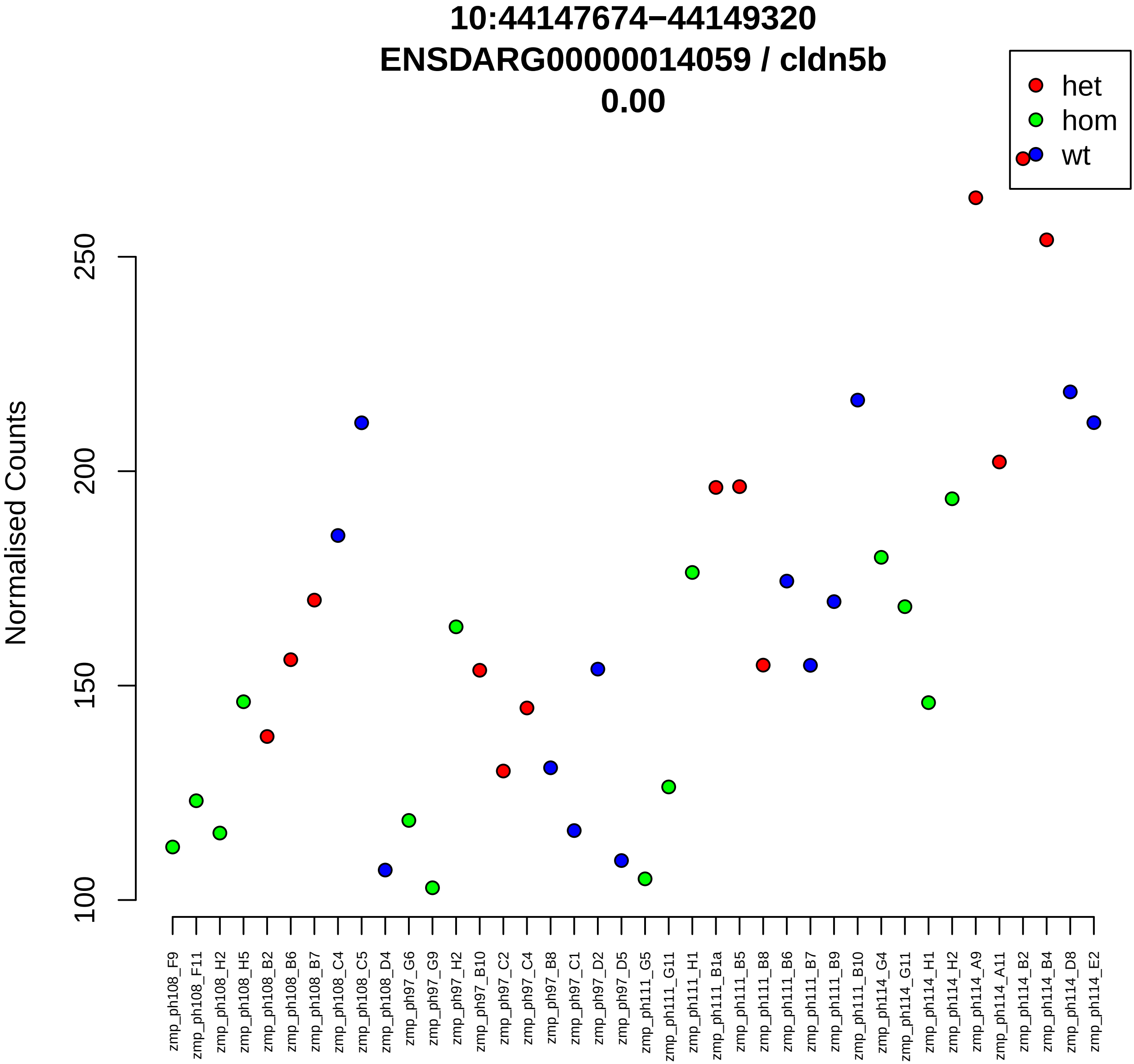

10:44147674−44149320
ENSDARG00000014059 / cldn5b
0.00
het
hom
wt
Normalised Counts
100
150
200
250
zmp_ph108_F9
zmp_ph108_F11
zmp_ph108_H2
zmp_ph108_H5
zmp_ph108_B2
zmp_ph108_B6
zmp_ph108_B7
zmp_ph108_C4
zmp_ph108_C5
zmp_ph108_D4
zmp_ph97_G6
zmp_ph97_G9
zmp_ph97_H2
zmp_ph97_B10
zmp_ph97_C2
zmp_ph97_C4
zmp_ph97_B8
zmp_ph97_C1
zmp_ph97_D2
zmp_ph97_D5
zmp_ph111_G5
zmp_ph111_G11
zmp_ph111_H1
zmp_ph111_B1a
zmp_ph111_B5
zmp_ph111_B8
zmp_ph111_B6
zmp_ph111_B7
zmp_ph111_B9
zmp_ph111_B10
zmp_ph114_G4
zmp_ph114_G11
zmp_ph114_H1
zmp_ph114_H2
zmp_ph114_A9
zmp_ph114_A11
zmp_ph114_B2
zmp_ph114_B4
zmp_ph114_D8
zmp_ph114_E2

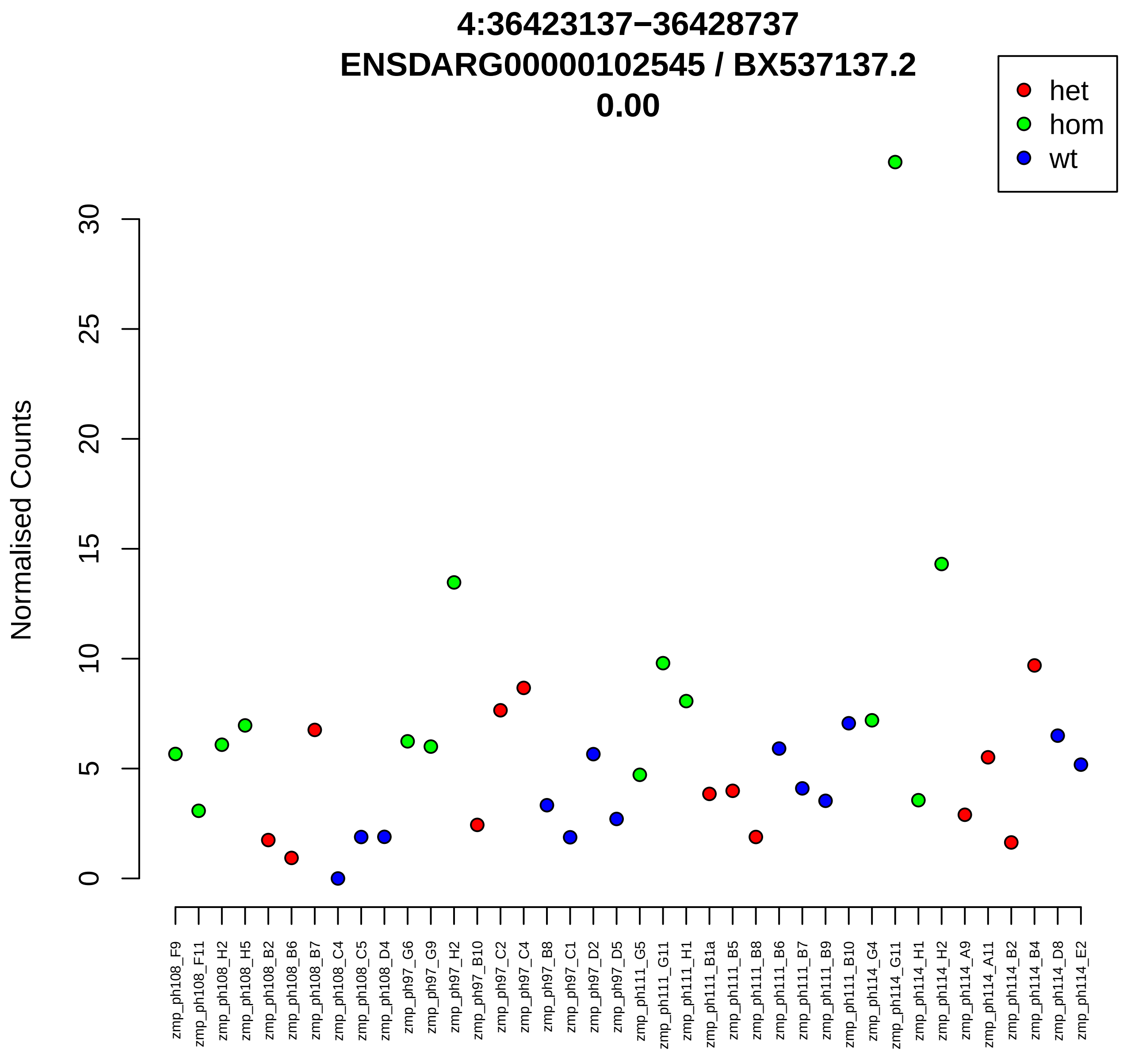
4:36423137−36428737
ENSDARG00000102545 / BX537137.2
0.00
het
hom
wt
Normalised Counts
0
5
10
15
20
25
30
zmp_ph108_F9
zmp_ph108_F11
zmp_ph108_H2
zmp_ph108_H5
zmp_ph108_B2
zmp_ph108_B6
zmp_ph108_B7
zmp_ph108_C4
zmp_ph108_C5
zmp_ph108_D4
zmp_ph97_G6
zmp_ph97_G9
zmp_ph97_H2
zmp_ph97_B10
zmp_ph97_C2
zmp_ph97_C4
zmp_ph97_B8
zmp_ph97_C1
zmp_ph97_D2
zmp_ph97_D5
zmp_ph111_G5
zmp_ph111_G11
zmp_ph111_H1
zmp_ph111_B1a
zmp_ph111_B5
zmp_ph111_B8
zmp_ph111_B6
zmp_ph111_B7
zmp_ph111_B9
zmp_ph111_B10
zmp_ph114_G4
zmp_ph114_G11
zmp_ph114_H1
zmp_ph114_H2
zmp_ph114_A9
zmp_ph114_A11
zmp_ph114_B2
zmp_ph114_B4
zmp_ph114_D8
zmp_ph114_E2

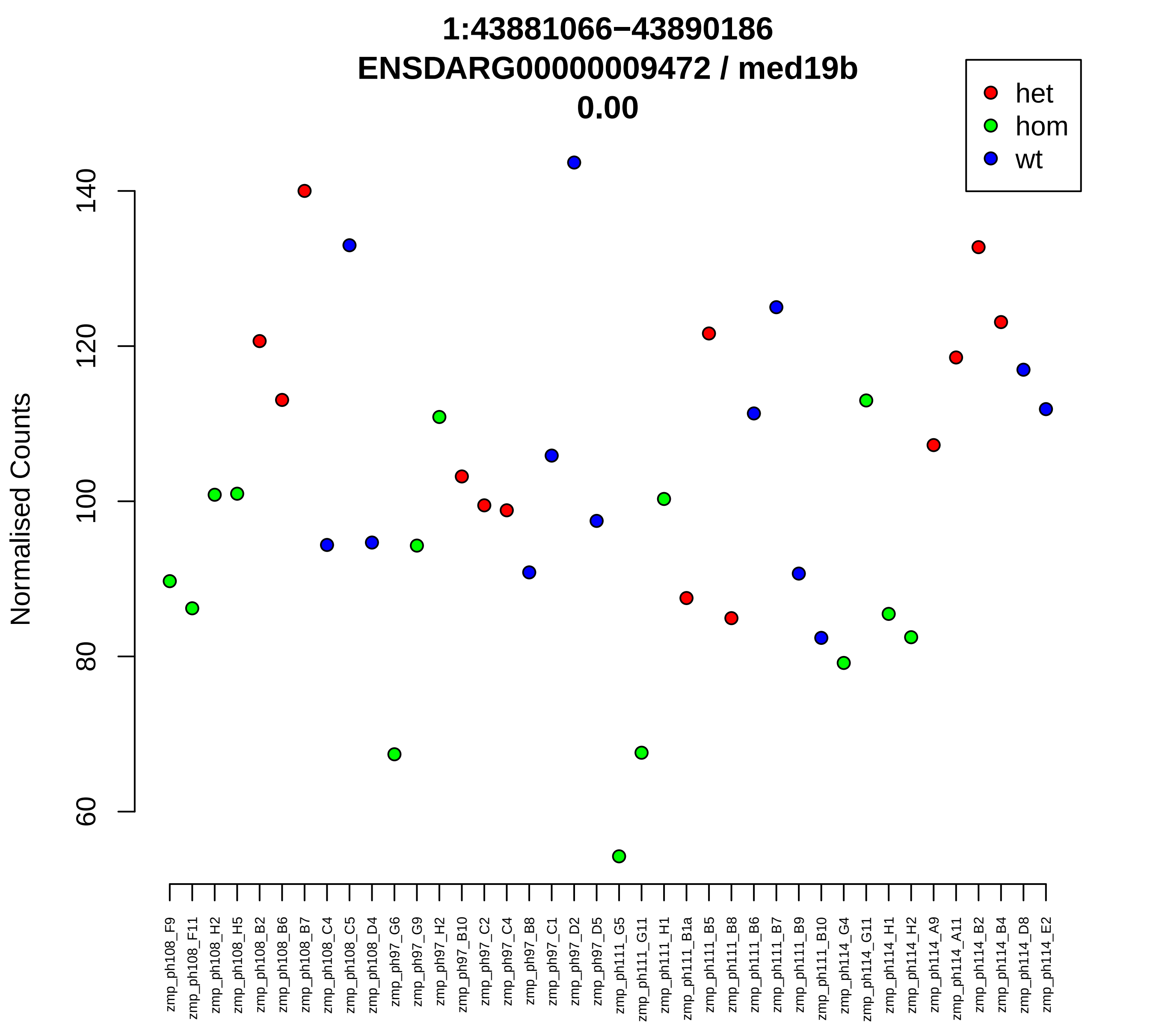
1:43881066−43890186
ENSDARG00000009472 / med19b
0.00
het
hom
wt
Normalised Counts
60
80
100
120
140
zmp_ph108_F9
zmp_ph108_F11
zmp_ph108_H2
zmp_ph108_H5
zmp_ph108_B2
zmp_ph108_B6
zmp_ph108_B7
zmp_ph108_C4
zmp_ph108_C5
zmp_ph108_D4
zmp_ph97_G6
zmp_ph97_G9
zmp_ph97_H2
zmp_ph97_B10
zmp_ph97_C2
zmp_ph97_C4
zmp_ph97_B8
zmp_ph97_C1
zmp_ph97_D2
zmp_ph97_D5
zmp_ph111_G5
zmp_ph111_G11
zmp_ph111_H1
zmp_ph111_B1a
zmp_ph111_B5
zmp_ph111_B8
zmp_ph111_B6
zmp_ph111_B7
zmp_ph111_B9
zmp_ph111_B10
zmp_ph114_G4
zmp_ph114_G11
zmp_ph114_H1
zmp_ph114_H2
zmp_ph114_A9
zmp_ph114_A11
zmp_ph114_B2
zmp_ph114_B4
zmp_ph114_D8
zmp_ph114_E2

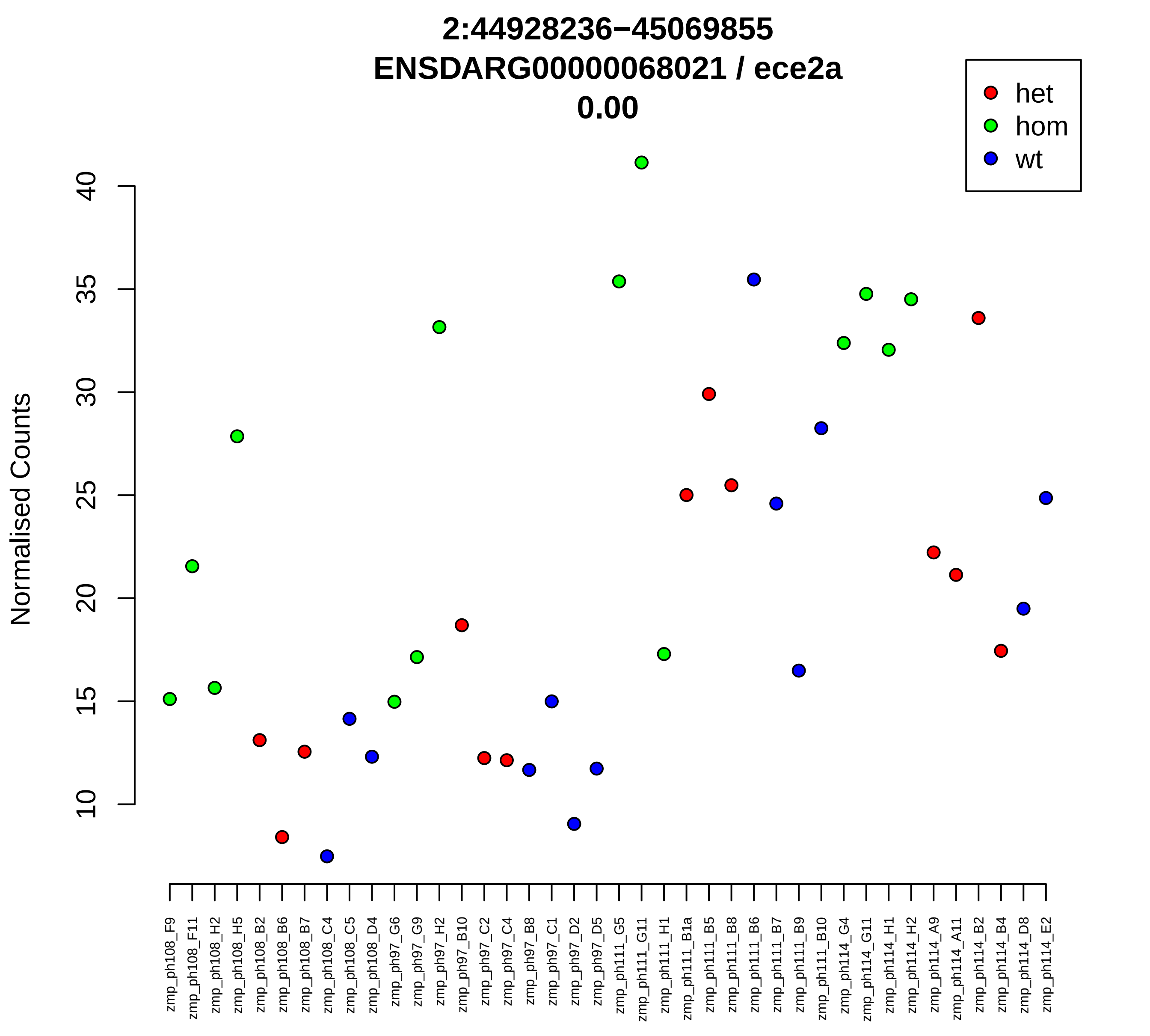

2:44928236−45069855
ENSDARG00000068021 / ece2a
0.00
het
hom
wt
Normalised Counts
10
15
20
25
30
35
40
zmp_ph108_F9
zmp_ph108_F11
zmp_ph108_H2
zmp_ph108_H5
zmp_ph108_B2
zmp_ph108_B6
zmp_ph108_B7
zmp_ph108_C4
zmp_ph108_C5
zmp_ph108_D4
zmp_ph97_G6
zmp_ph97_G9
zmp_ph97_H2
zmp_ph97_B10
zmp_ph97_C2
zmp_ph97_C4
zmp_ph97_B8
zmp_ph97_C1
zmp_ph97_D2
zmp_ph97_D5
zmp_ph111_G5
zmp_ph111_G11
zmp_ph111_H1
zmp_ph111_B1a
zmp_ph111_B5
zmp_ph111_B8
zmp_ph111_B6
zmp_ph111_B7
zmp_ph111_B9
zmp_ph111_B10
zmp_ph114_G4
zmp_ph114_G11
zmp_ph114_H1
zmp_ph114_H2
zmp_ph114_A9
zmp_ph114_A11
zmp_ph114_B2
zmp_ph114_B4
zmp_ph114_D8
zmp_ph114_E2

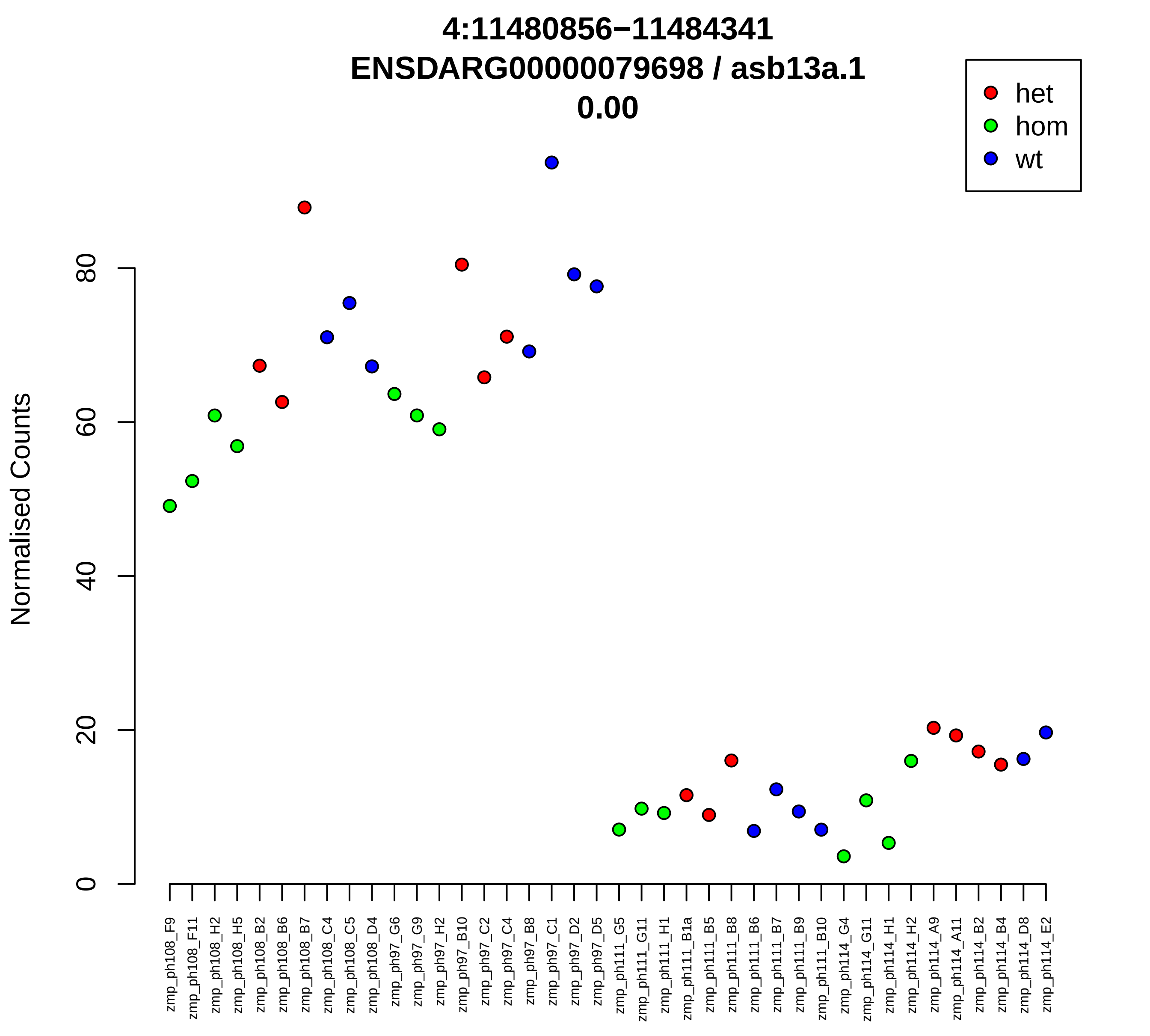

4:11480856−11484341
ENSDARG00000079698 / asb13a.1
0.00
het
hom
wt
Normalised Counts
0
20
40
60
80
zmp_ph108_F9
zmp_ph108_F11
zmp_ph108_H2
zmp_ph108_H5
zmp_ph108_B2
zmp_ph108_B6
zmp_ph108_B7
zmp_ph108_C4
zmp_ph108_C5
zmp_ph108_D4
zmp_ph97_G6
zmp_ph97_G9
zmp_ph97_H2
zmp_ph97_B10
zmp_ph97_C2
zmp_ph97_C4
zmp_ph97_B8
zmp_ph97_C1
zmp_ph97_D2
zmp_ph97_D5
zmp_ph111_G5
zmp_ph111_G11
zmp_ph111_H1
zmp_ph111_B1a
zmp_ph111_B5
zmp_ph111_B8
zmp_ph111_B6
zmp_ph111_B7
zmp_ph111_B9
zmp_ph111_B10
zmp_ph114_G4
zmp_ph114_G11
zmp_ph114_H1
zmp_ph114_H2
zmp_ph114_A9
zmp_ph114_A11
zmp_ph114_B2
zmp_ph114_B4
zmp_ph114_D8
zmp_ph114_E2

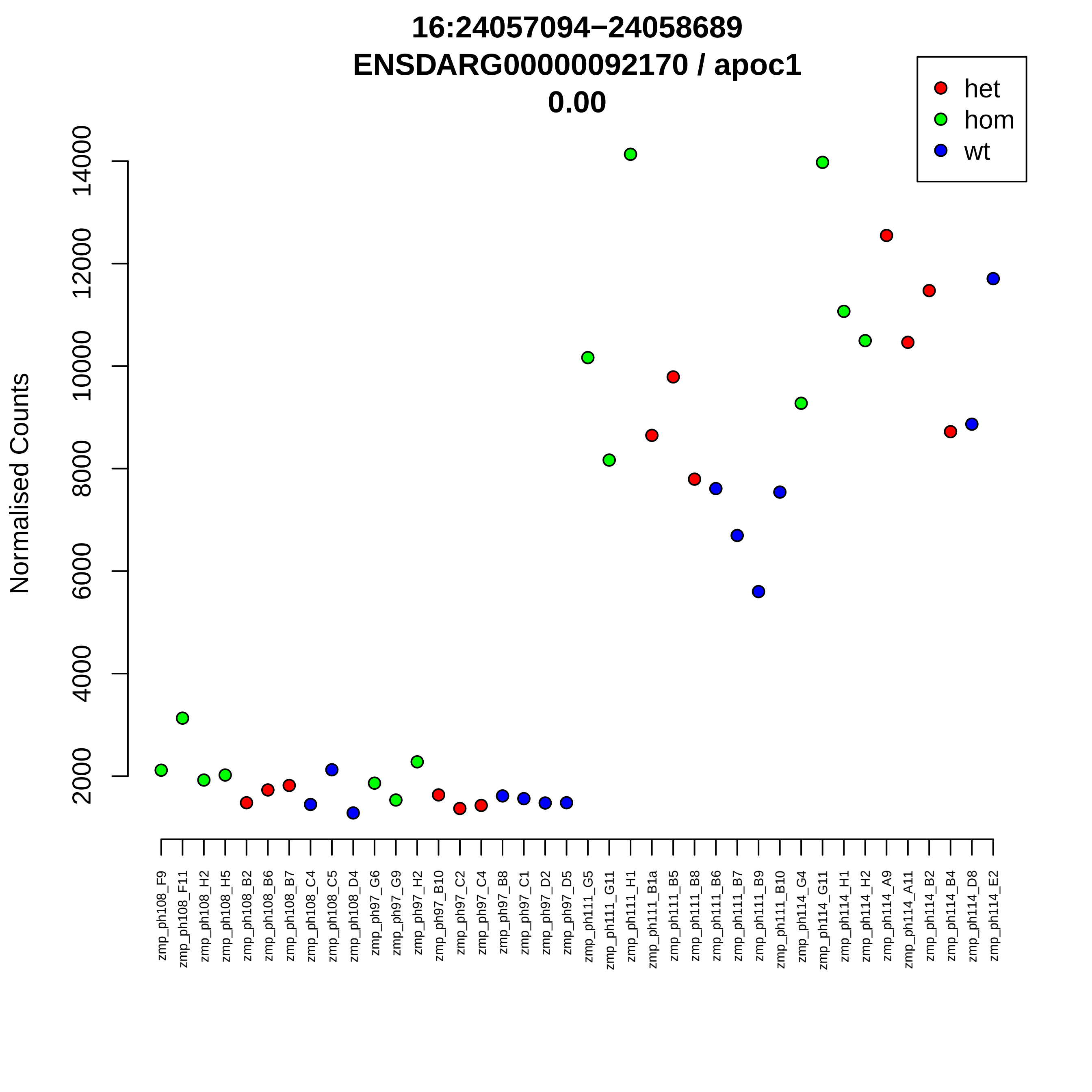

16:24057094−24058689
ENSDARG00000092170 / apoc1
0.00
het
hom
wt
Normalised Counts
2000
4000
6000
8000
10000
12000
14000
zmp_ph108_F9
zmp_ph108_F11
zmp_ph108_H2
zmp_ph108_H5
zmp_ph108_B2
zmp_ph108_B6
zmp_ph108_B7
zmp_ph108_C4
zmp_ph108_C5
zmp_ph108_D4
zmp_ph97_G6
zmp_ph97_G9
zmp_ph97_H2
zmp_ph97_B10
zmp_ph97_C2
zmp_ph97_C4
zmp_ph97_B8
zmp_ph97_C1
zmp_ph97_D2
zmp_ph97_D5
zmp_ph111_G5
zmp_ph111_G11
zmp_ph111_H1
zmp_ph111_B1a
zmp_ph111_B5
zmp_ph111_B8
zmp_ph111_B6
zmp_ph111_B7
zmp_ph111_B9
zmp_ph111_B10
zmp_ph114_G4
zmp_ph114_G11
zmp_ph114_H1
zmp_ph114_H2
zmp_ph114_A9
zmp_ph114_A11
zmp_ph114_B2
zmp_ph114_B4
zmp_ph114_D8
zmp_ph114_E2

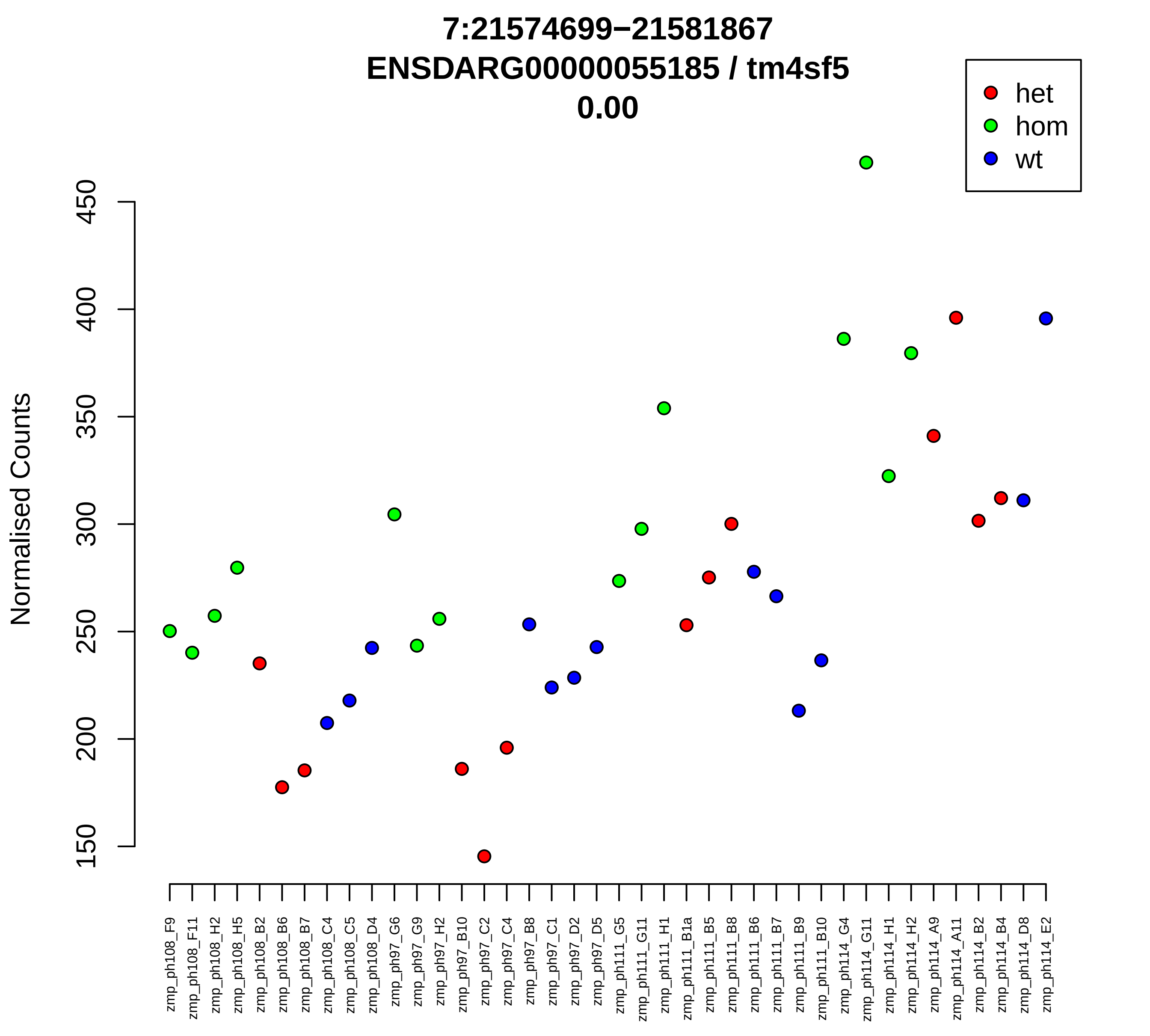

7:21574699−21581867
ENSDARG00000055185 / tm4sf5
0.00
het
hom
wt
Normalised Counts
150
200
250
300
350
400
450
zmp_ph108_F9
zmp_ph108_F11
zmp_ph108_H2
zmp_ph108_H5
zmp_ph108_B2
zmp_ph108_B6
zmp_ph108_B7
zmp_ph108_C4
zmp_ph108_C5
zmp_ph108_D4
zmp_ph97_G6
zmp_ph97_G9
zmp_ph97_H2
zmp_ph97_B10
zmp_ph97_C2
zmp_ph97_C4
zmp_ph97_B8
zmp_ph97_C1
zmp_ph97_D2
zmp_ph97_D5
zmp_ph111_G5
zmp_ph111_G11
zmp_ph111_H1
zmp_ph111_B1a
zmp_ph111_B5
zmp_ph111_B8
zmp_ph111_B6
zmp_ph111_B7
zmp_ph111_B9
zmp_ph111_B10
zmp_ph114_G4
zmp_ph114_G11
zmp_ph114_H1
zmp_ph114_H2
zmp_ph114_A9
zmp_ph114_A11
zmp_ph114_B2
zmp_ph114_B4
zmp_ph114_D8
zmp_ph114_E2

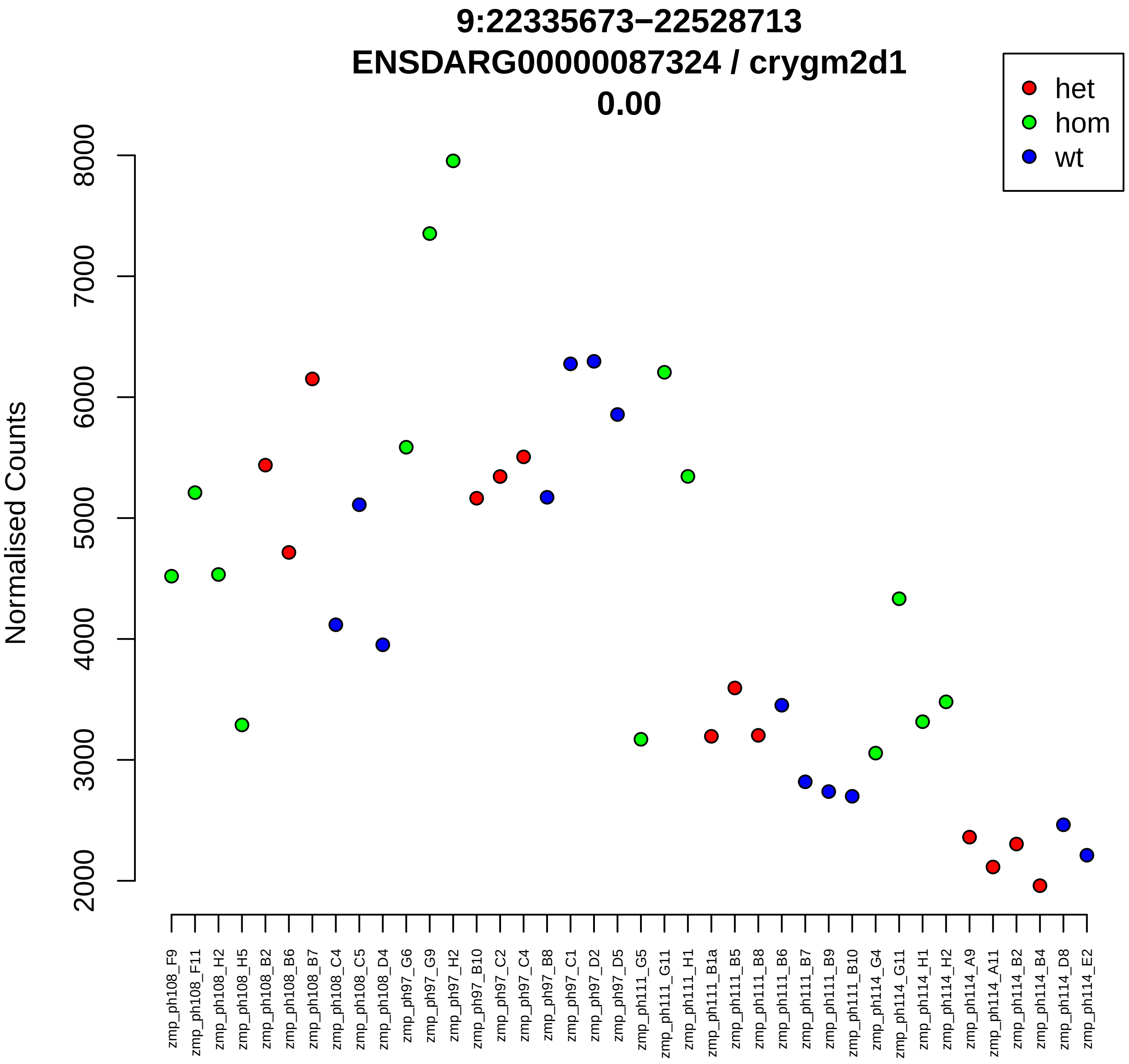
9:22335673−22528713
ENSDARG00000087324 / crygm2d1
0.00
het
hom
wt
Normalised Counts
2000
3000
4000
5000
6000
7000
8000
zmp_ph108_F9
zmp_ph108_F11
zmp_ph108_H2
zmp_ph108_H5
zmp_ph108_B2
zmp_ph108_B6
zmp_ph108_B7
zmp_ph108_C4
zmp_ph108_C5
zmp_ph108_D4
zmp_ph97_G6
zmp_ph97_G9
zmp_ph97_H2
zmp_ph97_B10
zmp_ph97_C2
zmp_ph97_C4
zmp_ph97_B8
zmp_ph97_C1
zmp_ph97_D2
zmp_ph97_D5
zmp_ph111_G5
zmp_ph111_G11
zmp_ph111_H1
zmp_ph111_B1a
zmp_ph111_B5
zmp_ph111_B8
zmp_ph111_B6
zmp_ph111_B7
zmp_ph111_B9
zmp_ph111_B10
zmp_ph114_G4
zmp_ph114_G11
zmp_ph114_H1
zmp_ph114_H2
zmp_ph114_A9
zmp_ph114_A11
zmp_ph114_B2
zmp_ph114_B4
zmp_ph114_D8
zmp_ph114_E2

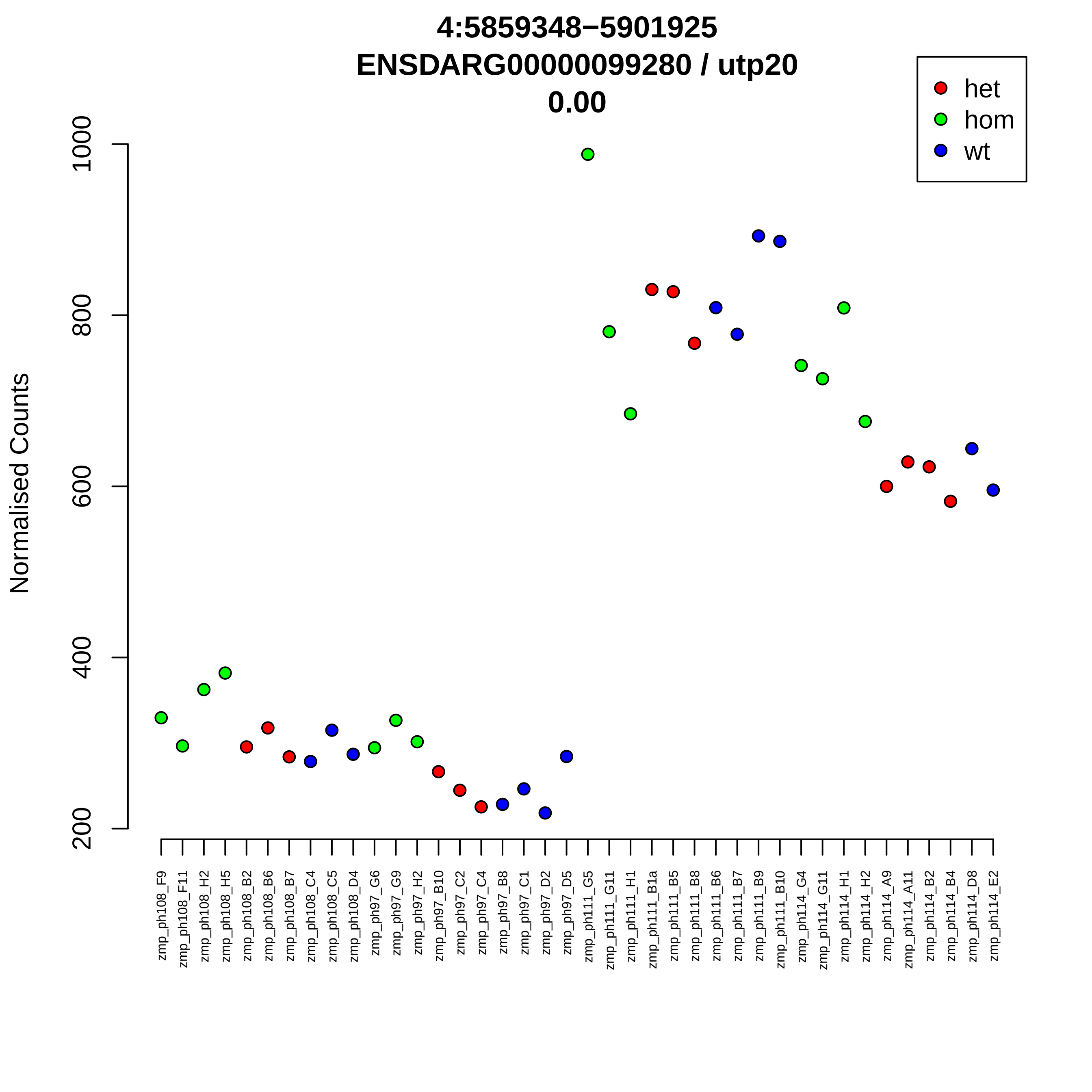
4:5859348−5901925
ENSDARG00000099280 / utp20
0.00
het
hom
wt
Normalised Counts
200
400
600
800
1000
zmp_ph108_F9
zmp_ph108_F11
zmp_ph108_H2
zmp_ph108_H5
zmp_ph108_B2
zmp_ph108_B6
zmp_ph108_B7
zmp_ph108_C4
zmp_ph108_C5
zmp_ph108_D4
zmp_ph97_G6
zmp_ph97_G9
zmp_ph97_H2
zmp_ph97_B10
zmp_ph97_C2
zmp_ph97_C4
zmp_ph97_B8
zmp_ph97_C1
zmp_ph97_D2
zmp_ph97_D5
zmp_ph111_G5
zmp_ph111_G11
zmp_ph111_H1
zmp_ph111_B1a
zmp_ph111_B5
zmp_ph111_B8
zmp_ph111_B6
zmp_ph111_B7
zmp_ph111_B9
zmp_ph111_B10
zmp_ph114_G4
zmp_ph114_G11
zmp_ph114_H1
zmp_ph114_H2
zmp_ph114_A9
zmp_ph114_A11
zmp_ph114_B2
zmp_ph114_B4
zmp_ph114_D8
zmp_ph114_E2

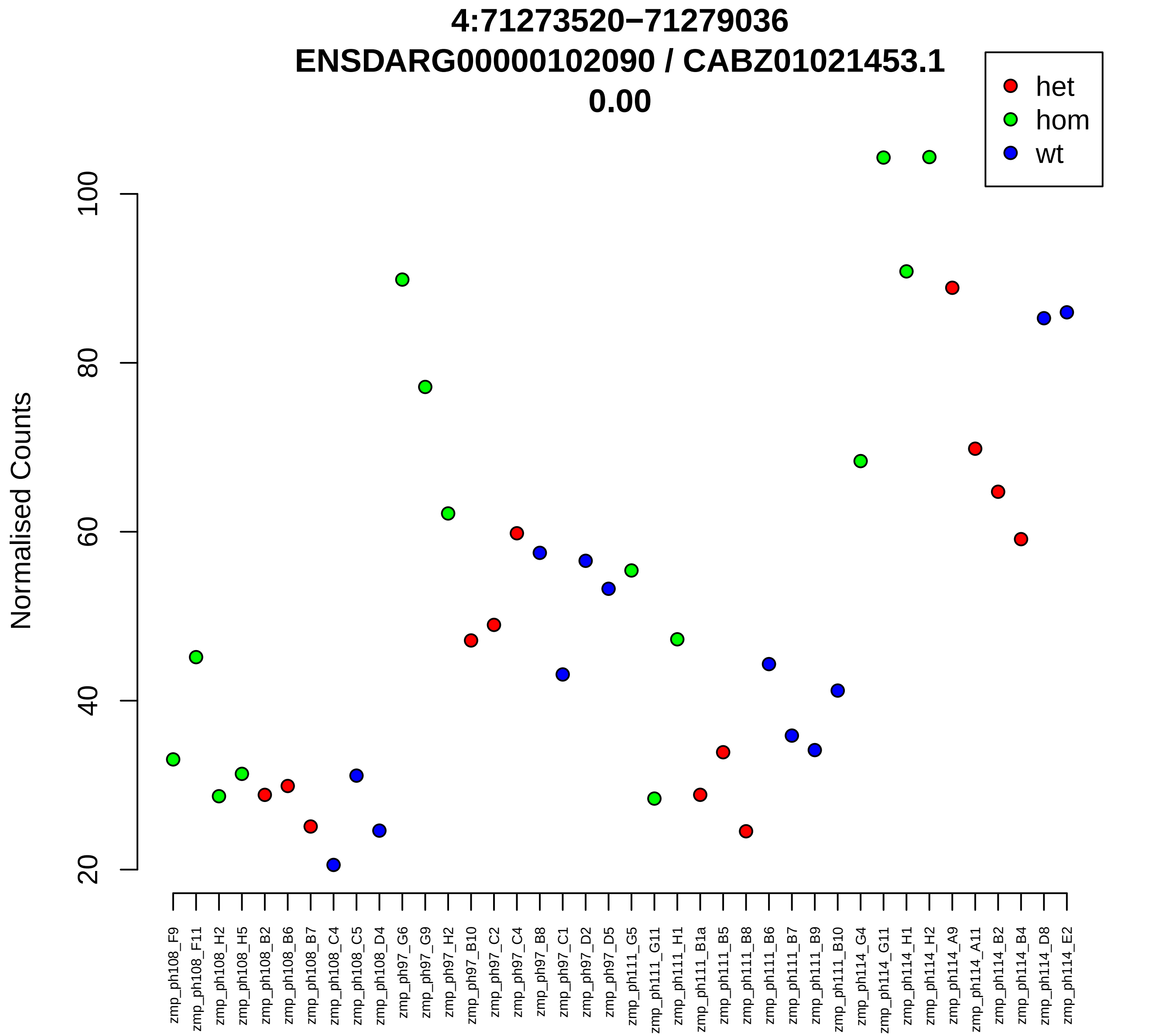

4:71273520−71279036
ENSDARG00000102090 / CABZ01021453.1
0.00
het
hom
wt
Normalised Counts
20
40
60
80
100
zmp_ph108_F9
zmp_ph108_F11
zmp_ph108_H2
zmp_ph108_H5
zmp_ph108_B2
zmp_ph108_B6
zmp_ph108_B7
zmp_ph108_C4
zmp_ph108_C5
zmp_ph108_D4
zmp_ph97_G6
zmp_ph97_G9
zmp_ph97_H2
zmp_ph97_B10
zmp_ph97_C2
zmp_ph97_C4
zmp_ph97_B8
zmp_ph97_C1
zmp_ph97_D2
zmp_ph97_D5
zmp_ph111_G5
zmp_ph111_G11
zmp_ph111_H1
zmp_ph111_B1a
zmp_ph111_B5
zmp_ph111_B8
zmp_ph111_B6
zmp_ph111_B7
zmp_ph111_B9
zmp_ph111_B10
zmp_ph114_G4
zmp_ph114_G11
zmp_ph114_H1
zmp_ph114_H2
zmp_ph114_A9
zmp_ph114_A11
zmp_ph114_B2
zmp_ph114_B4
zmp_ph114_D8
zmp_ph114_E2

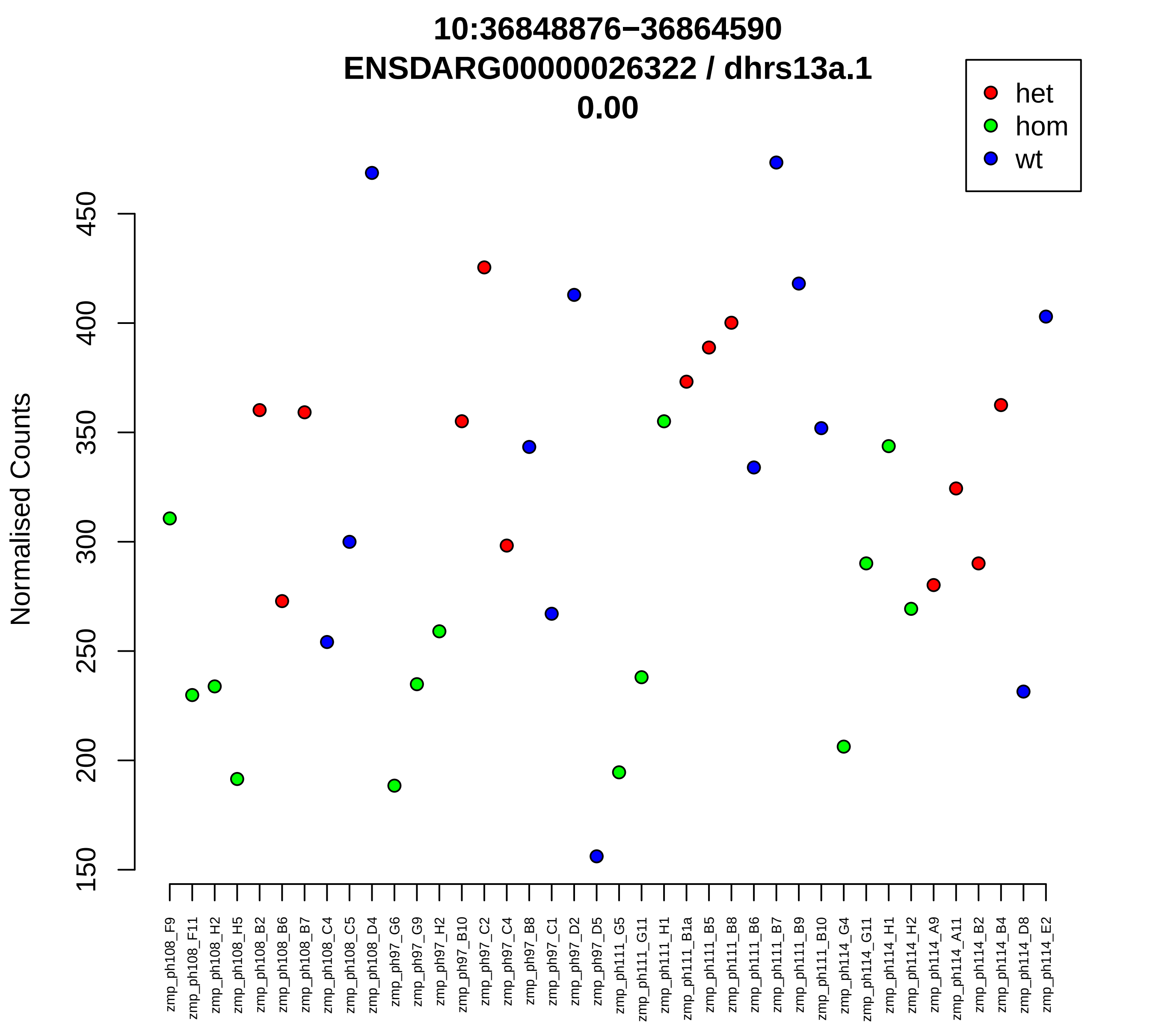

10:36848876−36864590
ENSDARG00000026322 / dhrs13a.1
0.00
het
hom
wt
Normalised Counts
150
200
250
300
350
400
450
zmp_ph108_F9
zmp_ph108_F11
zmp_ph108_H2
zmp_ph108_H5
zmp_ph108_B2
zmp_ph108_B6
zmp_ph108_B7
zmp_ph108_C4
zmp_ph108_C5
zmp_ph108_D4
zmp_ph97_G6
zmp_ph97_G9
zmp_ph97_H2
zmp_ph97_B10
zmp_ph97_C2
zmp_ph97_C4
zmp_ph97_B8
zmp_ph97_C1
zmp_ph97_D2
zmp_ph97_D5
zmp_ph111_G5
zmp_ph111_G11
zmp_ph111_H1
zmp_ph111_B1a
zmp_ph111_B5
zmp_ph111_B8
zmp_ph111_B6
zmp_ph111_B7
zmp_ph111_B9
zmp_ph111_B10
zmp_ph114_G4
zmp_ph114_G11
zmp_ph114_H1
zmp_ph114_H2
zmp_ph114_A9
zmp_ph114_A11
zmp_ph114_B2
zmp_ph114_B4
zmp_ph114_D8
zmp_ph114_E2

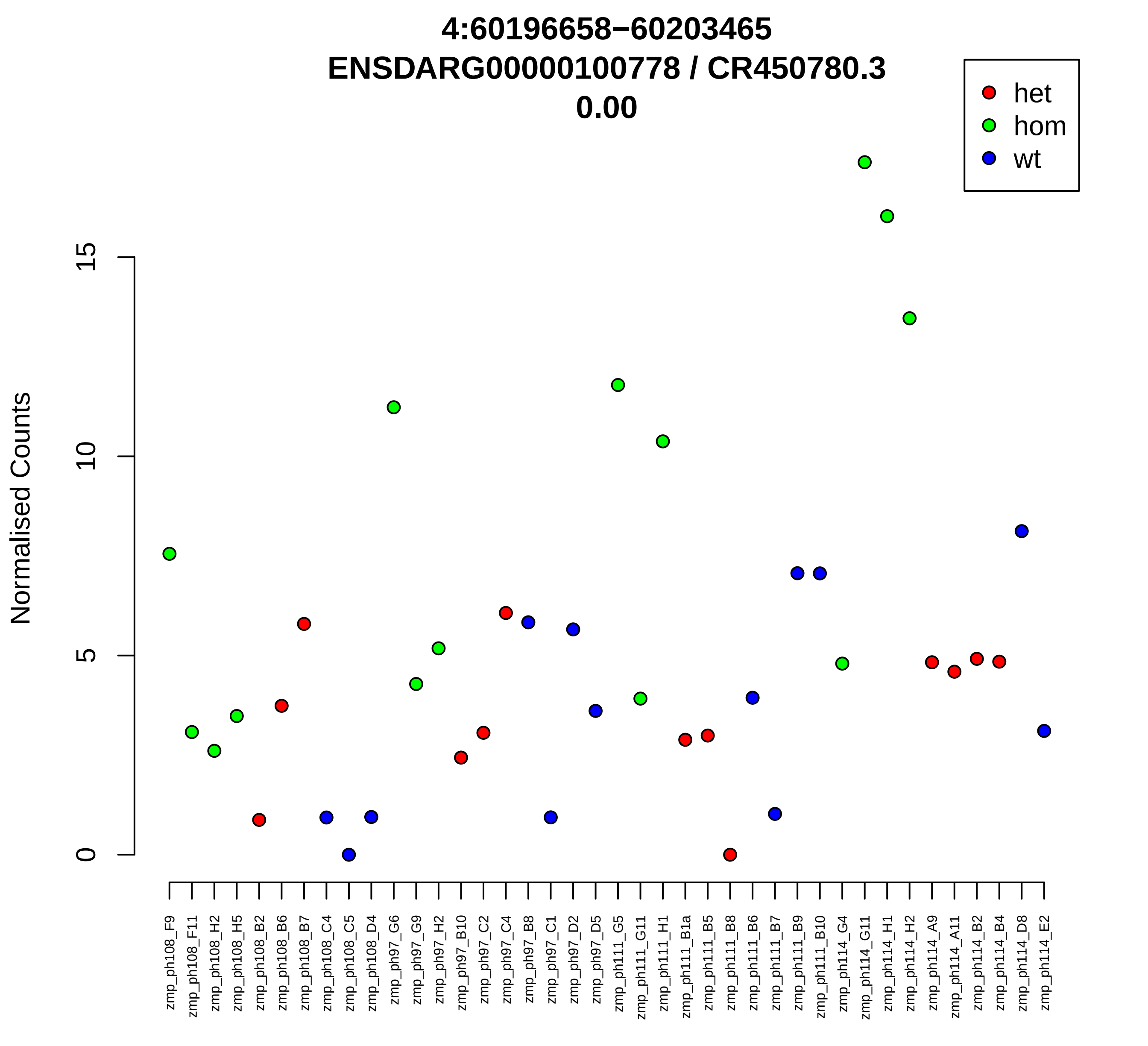

4:60196658−60203465
ENSDARG00000100778 / CR450780.3
0.00
het
hom
wt
Normalised Counts
0
5
10
15
zmp_ph108_F9
zmp_ph108_F11
zmp_ph108_H2
zmp_ph108_H5
zmp_ph108_B2
zmp_ph108_B6
zmp_ph108_B7
zmp_ph108_C4
zmp_ph108_C5
zmp_ph108_D4
zmp_ph97_G6
zmp_ph97_G9
zmp_ph97_H2
zmp_ph97_B10
zmp_ph97_C2
zmp_ph97_C4
zmp_ph97_B8
zmp_ph97_C1
zmp_ph97_D2
zmp_ph97_D5
zmp_ph111_G5
zmp_ph111_G11
zmp_ph111_H1
zmp_ph111_B1a
zmp_ph111_B5
zmp_ph111_B8
zmp_ph111_B6
zmp_ph111_B7
zmp_ph111_B9
zmp_ph111_B10
zmp_ph114_G4
zmp_ph114_G11
zmp_ph114_H1
zmp_ph114_H2
zmp_ph114_A9
zmp_ph114_A11
zmp_ph114_B2
zmp_ph114_B4
zmp_ph114_D8
zmp_ph114_E2

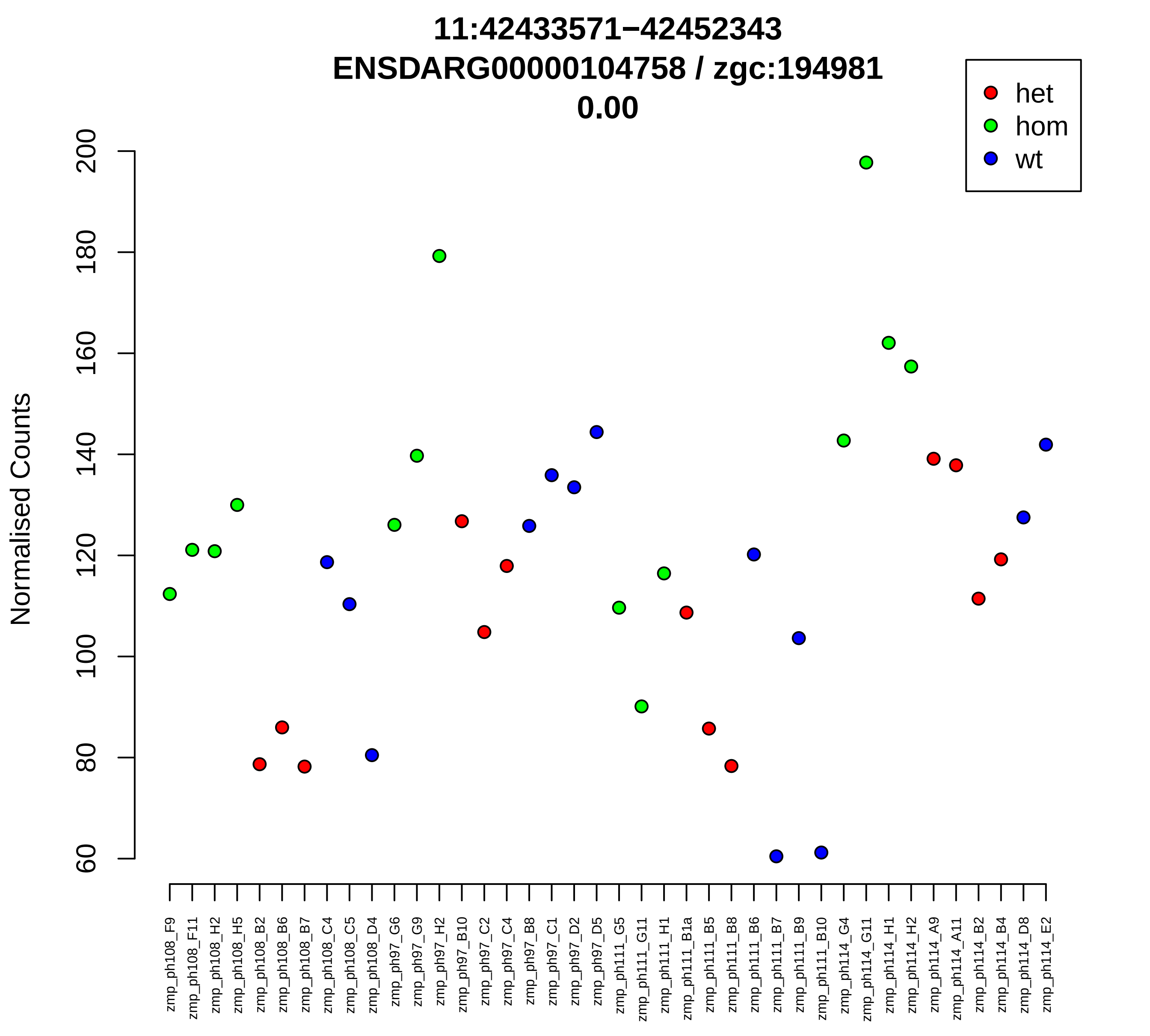

11:42433571−42452343
ENSDARG00000104758 / zgc:194981
0.00
het
hom
wt
Normalised Counts
60
80
100
120
140
160
180
200
zmp_ph108_F9
zmp_ph108_F11
zmp_ph108_H2
zmp_ph108_H5
zmp_ph108_B2
zmp_ph108_B6
zmp_ph108_B7
zmp_ph108_C4
zmp_ph108_C5
zmp_ph108_D4
zmp_ph97_G6
zmp_ph97_G9
zmp_ph97_H2
zmp_ph97_B10
zmp_ph97_C2
zmp_ph97_C4
zmp_ph97_B8
zmp_ph97_C1
zmp_ph97_D2
zmp_ph97_D5
zmp_ph111_G5
zmp_ph111_G11
zmp_ph111_H1
zmp_ph111_B1a
zmp_ph111_B5
zmp_ph111_B8
zmp_ph111_B6
zmp_ph111_B7
zmp_ph111_B9
zmp_ph111_B10
zmp_ph114_G4
zmp_ph114_G11
zmp_ph114_H1
zmp_ph114_H2
zmp_ph114_A9
zmp_ph114_A11
zmp_ph114_B2
zmp_ph114_B4
zmp_ph114_D8
zmp_ph114_E2

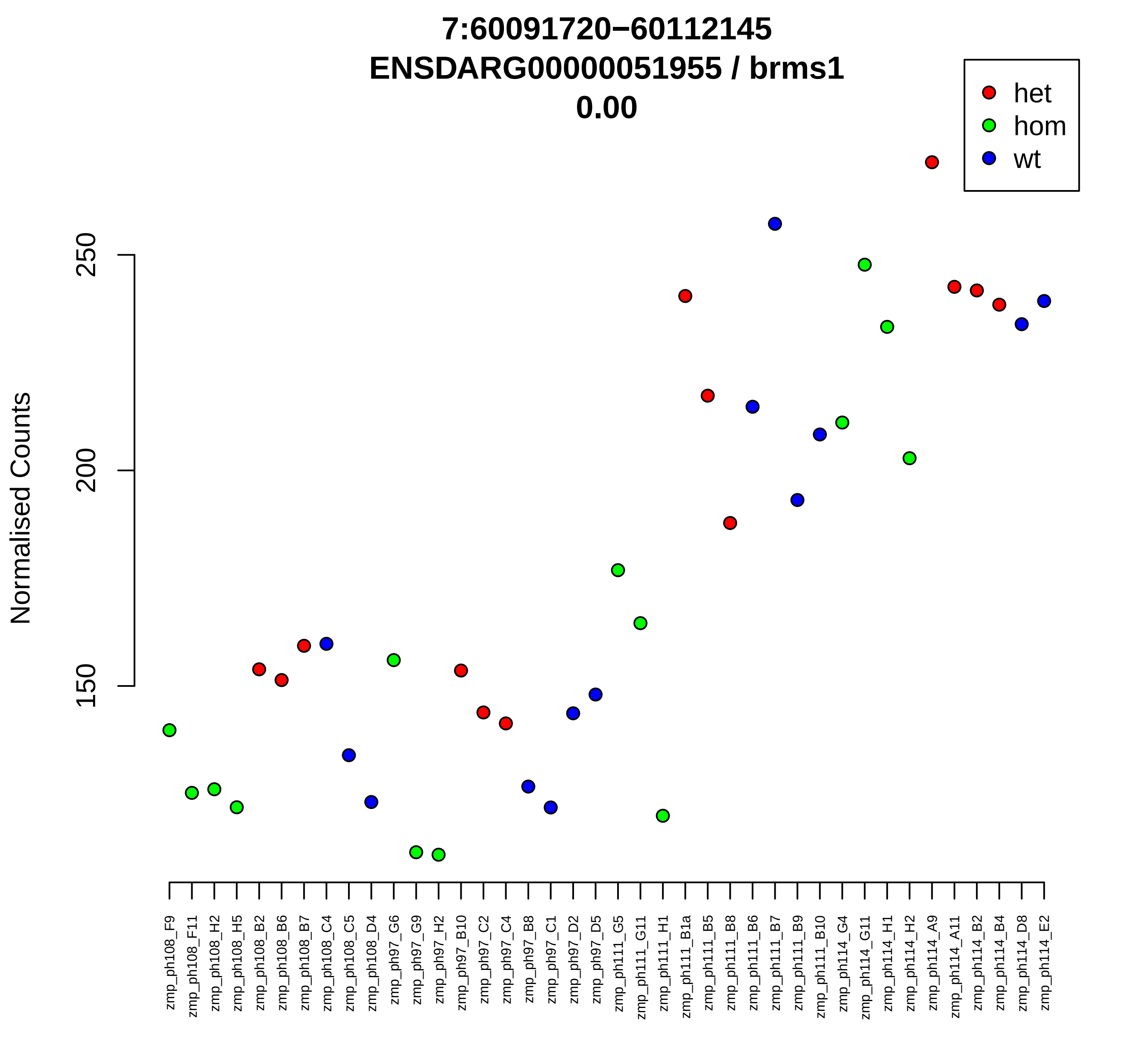
7:60091720−60112145
ENSDARG00000051955 / brms1
0.00
het
hom
wt
Normalised Counts
150
200
250
zmp_ph108_F9
zmp_ph108_F11
zmp_ph108_H2
zmp_ph108_H5
zmp_ph108_B2
zmp_ph108_B6
zmp_ph108_B7
zmp_ph108_C4
zmp_ph108_C5
zmp_ph108_D4
zmp_ph97_G6
zmp_ph97_G9
zmp_ph97_H2
zmp_ph97_B10
zmp_ph97_C2
zmp_ph97_C4
zmp_ph97_B8
zmp_ph97_C1
zmp_ph97_D2
zmp_ph97_D5
zmp_ph111_G5
zmp_ph111_G11
zmp_ph111_H1
zmp_ph111_B1a
zmp_ph111_B5
zmp_ph111_B8
zmp_ph111_B6
zmp_ph111_B7
zmp_ph111_B9
zmp_ph111_B10
zmp_ph114_G4
zmp_ph114_G11
zmp_ph114_H1
zmp_ph114_H2
zmp_ph114_A9
zmp_ph114_A11
zmp_ph114_B2
zmp_ph114_B4
zmp_ph114_D8
zmp_ph114_E2

5:37237092−37239157
ENSDARG00000103498 / epd
0.00

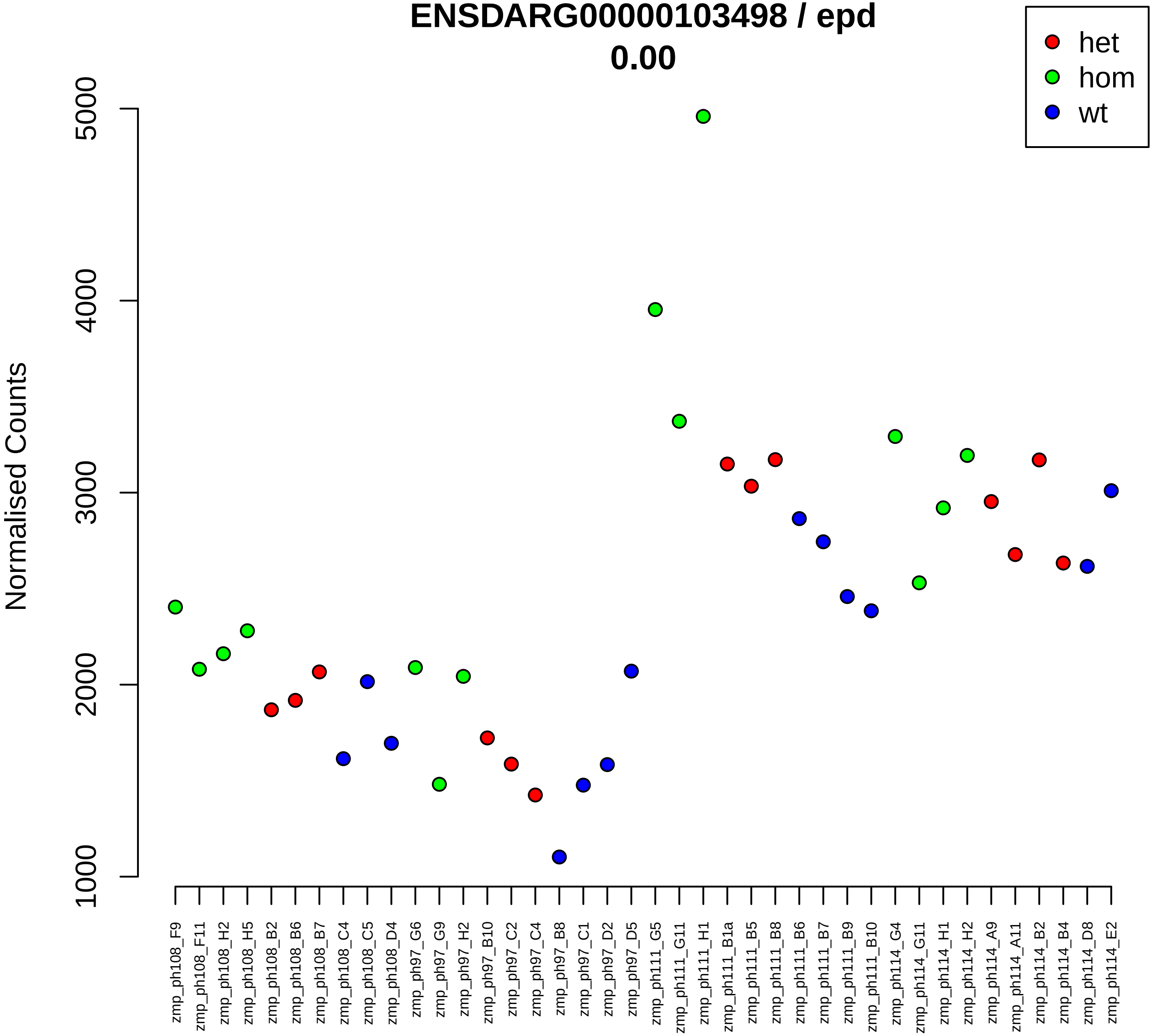

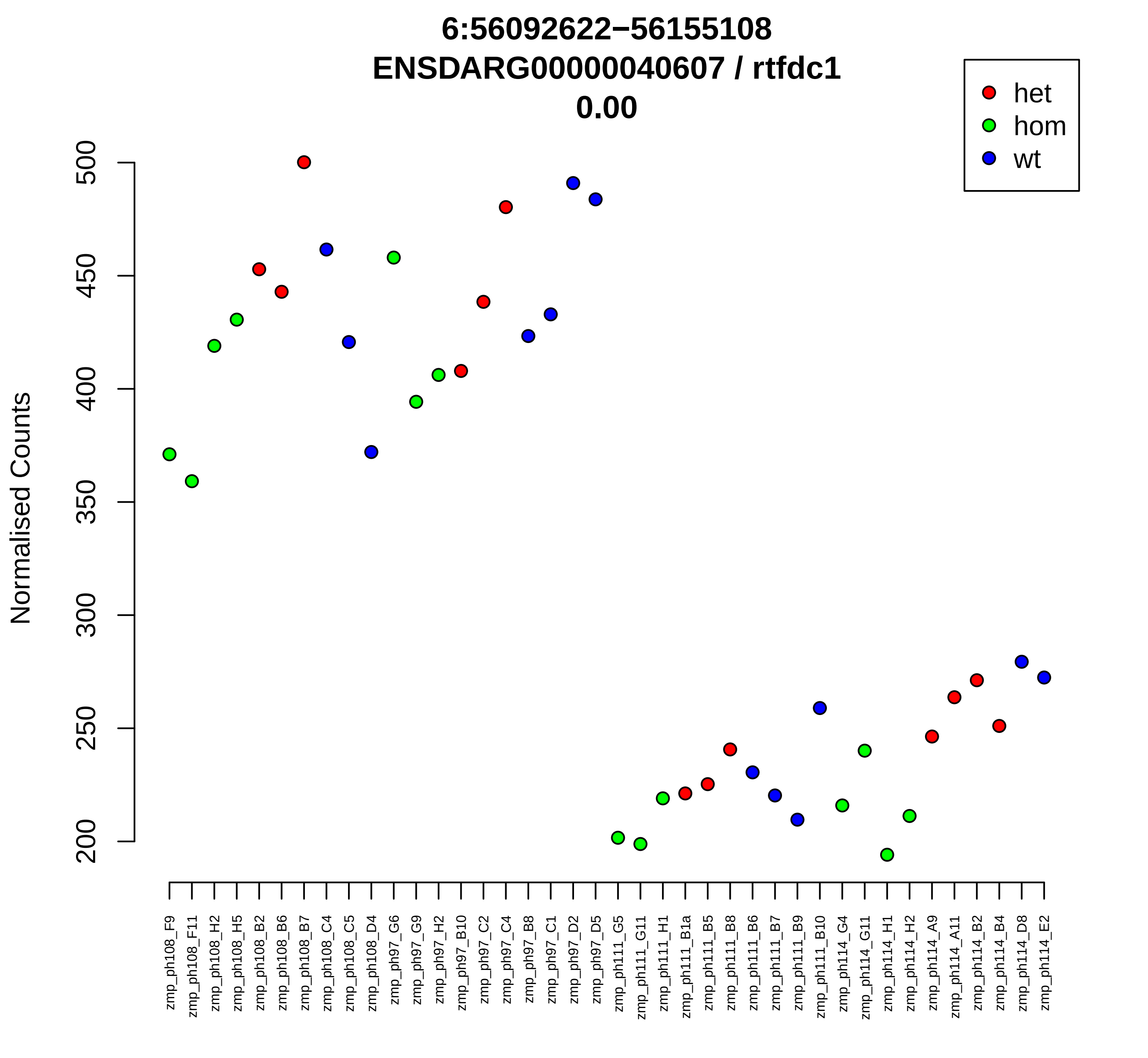
6:56092622−56155108
ENSDARG00000040607 / rtfdc1
0.00
het
hom
wt
Normalised Counts
200
250
300
350
400
450
500
zmp_ph108_F9
zmp_ph108_F11
zmp_ph108_H2
zmp_ph108_H5
zmp_ph108_B2
zmp_ph108_B6
zmp_ph108_B7
zmp_ph108_C4
zmp_ph108_C5
zmp_ph108_D4
zmp_ph97_G6
zmp_ph97_G9
zmp_ph97_H2
zmp_ph97_B10
zmp_ph97_C2
zmp_ph97_C4
zmp_ph97_B8
zmp_ph97_C1
zmp_ph97_D2
zmp_ph97_D5
zmp_ph111_G5
zmp_ph111_G11
zmp_ph111_H1
zmp_ph111_B1a
zmp_ph111_B5
zmp_ph111_B8
zmp_ph111_B6
zmp_ph111_B7
zmp_ph111_B9
zmp_ph111_B10
zmp_ph114_G4
zmp_ph114_G11
zmp_ph114_H1
zmp_ph114_H2
zmp_ph114_A9
zmp_ph114_A11
zmp_ph114_B2
zmp_ph114_B4
zmp_ph114_D8
zmp_ph114_E2

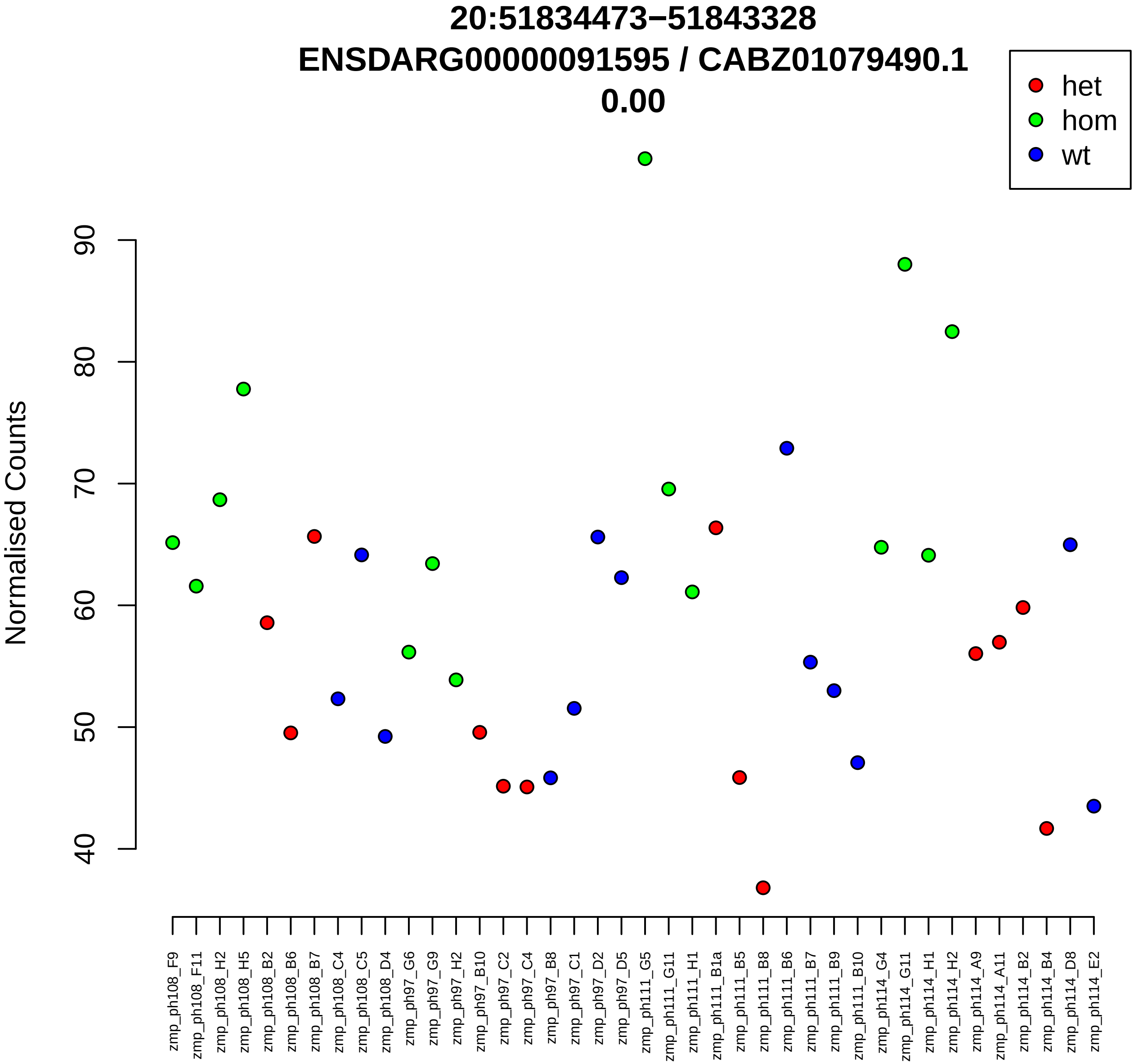
20:51834473−51843328
ENSDARG00000091595 / CABZ01079490.1
0.00
het
hom
wt
Normalised Counts
40
50
60
70
80
90
zmp_ph108_F9
zmp_ph108_F11
zmp_ph108_H2
zmp_ph108_H5
zmp_ph108_B2
zmp_ph108_B6
zmp_ph108_B7
zmp_ph108_C4
zmp_ph108_C5
zmp_ph108_D4
zmp_ph97_G6
zmp_ph97_G9
zmp_ph97_H2
zmp_ph97_B10
zmp_ph97_C2
zmp_ph97_C4
zmp_ph97_B8
zmp_ph97_C1
zmp_ph97_D2
zmp_ph97_D5
zmp_ph111_G5
zmp_ph111_G11
zmp_ph111_H1
zmp_ph111_B1a
zmp_ph111_B5
zmp_ph111_B8
zmp_ph111_B6
zmp_ph111_B7
zmp_ph111_B9
zmp_ph111_B10
zmp_ph114_G4
zmp_ph114_G11
zmp_ph114_H1
zmp_ph114_H2
zmp_ph114_A9
zmp_ph114_A11
zmp_ph114_B2
zmp_ph114_B4
zmp_ph114_D8
zmp_ph114_E2

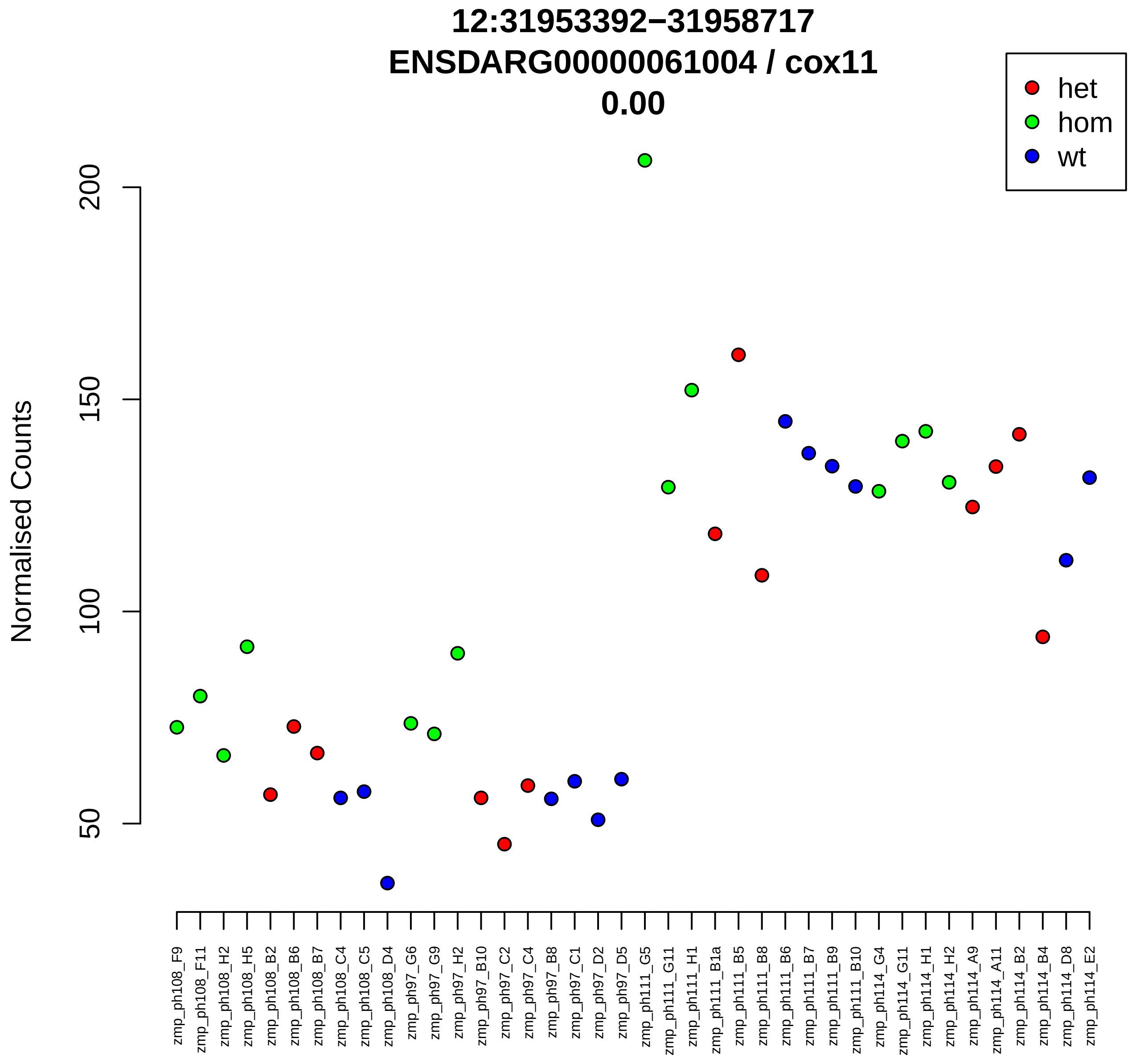
12:31953392−31958717
ENSDARG00000061004 / cox11
0.00
het
hom
wt
Normalised Counts
200
150
100
50
zmp_ph108_F9
zmp_ph108_F11
zmp_ph108_H2
zmp_ph108_H5
zmp_ph108_B2
zmp_ph108_B6
zmp_ph108_B7
zmp_ph108_C4
zmp_ph108_C5
zmp_ph108_D4
zmp_ph97_G6
zmp_ph97_G9
zmp_ph97_H2
zmp_ph97_B10
zmp_ph97_C2
zmp_ph97_C4
zmp_ph97_B8
zmp_ph97_C1
zmp_ph97_D2
zmp_ph97_D5
zmp_ph111_G5
zmp_ph111_G11
zmp_ph111_H1
zmp_ph111_B1a
zmp_ph111_B5
zmp_ph111_B8
zmp_ph111_B6
zmp_ph111_B7
zmp_ph111_B9
zmp_ph111_B10
zmp_ph114_G4
zmp_ph114_G11
zmp_ph114_H1
zmp_ph114_H2
zmp_ph114_A9
zmp_ph114_A11
zmp_ph114_B2
zmp_ph114_B4
zmp_ph114_D8
zmp_ph114_E2

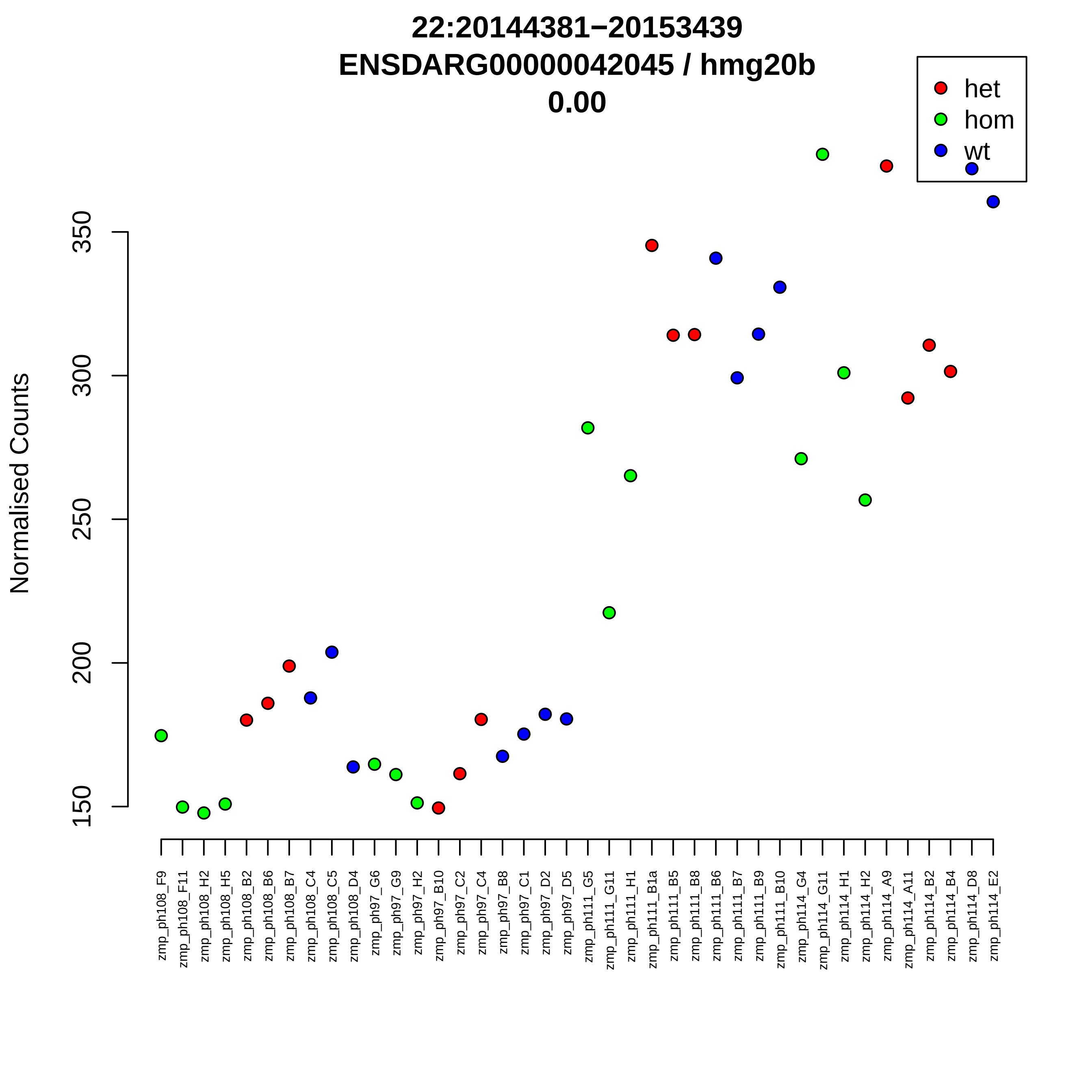

22:20144381−20153439
ENSDARG00000042045 / hmg20b
0.00
het
hom
wt
Normalised Counts
150
200
250
300
350
zmp_ph108_F9
zmp_ph108_F11
zmp_ph108_H2
zmp_ph108_H5
zmp_ph108_B2
zmp_ph108_B6
zmp_ph108_B7
zmp_ph108_C4
zmp_ph108_C5
zmp_ph108_D4
zmp_ph97_G6
zmp_ph97_G9
zmp_ph97_H2
zmp_ph97_B10
zmp_ph97_C2
zmp_ph97_C4
zmp_ph97_B8
zmp_ph97_C1
zmp_ph97_D2
zmp_ph97_D5
zmp_ph111_G5
zmp_ph111_G11
zmp_ph111_H1
zmp_ph111_B1a
zmp_ph111_B5
zmp_ph111_B8
zmp_ph111_B6
zmp_ph111_B7
zmp_ph111_B9
zmp_ph111_B10
zmp_ph114_G4
zmp_ph114_G11
zmp_ph114_H1
zmp_ph114_H2
zmp_ph114_A9
zmp_ph114_A11
zmp_ph114_B2
zmp_ph114_B4
zmp_ph114_D8
zmp_ph114_E2

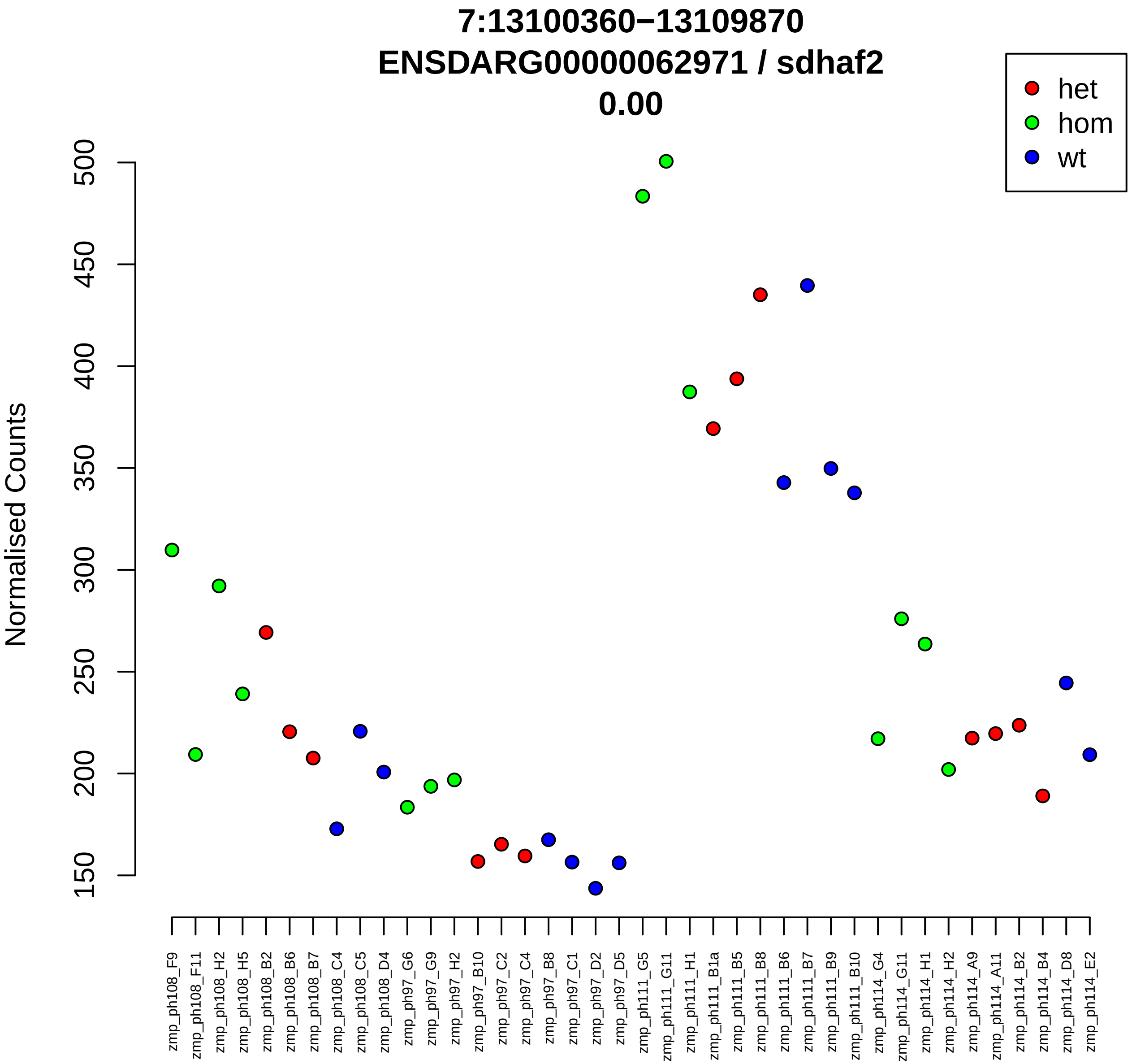

7:13100360−13109870
ENSDARG00000062971 / sdhaf2
0.00
het
hom
wt
Normalised Counts
150
200
250
300
350
400
450
500
zmp_ph108_F9
zmp_ph108_F11
zmp_ph108_H2
zmp_ph108_H5
zmp_ph108_B2
zmp_ph108_B6
zmp_ph108_B7
zmp_ph108_C4
zmp_ph108_C5
zmp_ph108_D4
zmp_ph97_G6
zmp_ph97_G9
zmp_ph97_H2
zmp_ph97_B10
zmp_ph97_C2
zmp_ph97_C4
zmp_ph97_B8
zmp_ph97_C1
zmp_ph97_D2
zmp_ph97_D5
zmp_ph111_G5
zmp_ph111_G11
zmp_ph111_H1
zmp_ph111_B1a
zmp_ph111_B5
zmp_ph111_B8
zmp_ph111_B6
zmp_ph111_B7
zmp_ph111_B9
zmp_ph111_B10
zmp_ph114_G4
zmp_ph114_G11
zmp_ph114_H1
zmp_ph114_H2
zmp_ph114_A9
zmp_ph114_A11
zmp_ph114_B2
zmp_ph114_B4
zmp_ph114_D8
zmp_ph114_E2

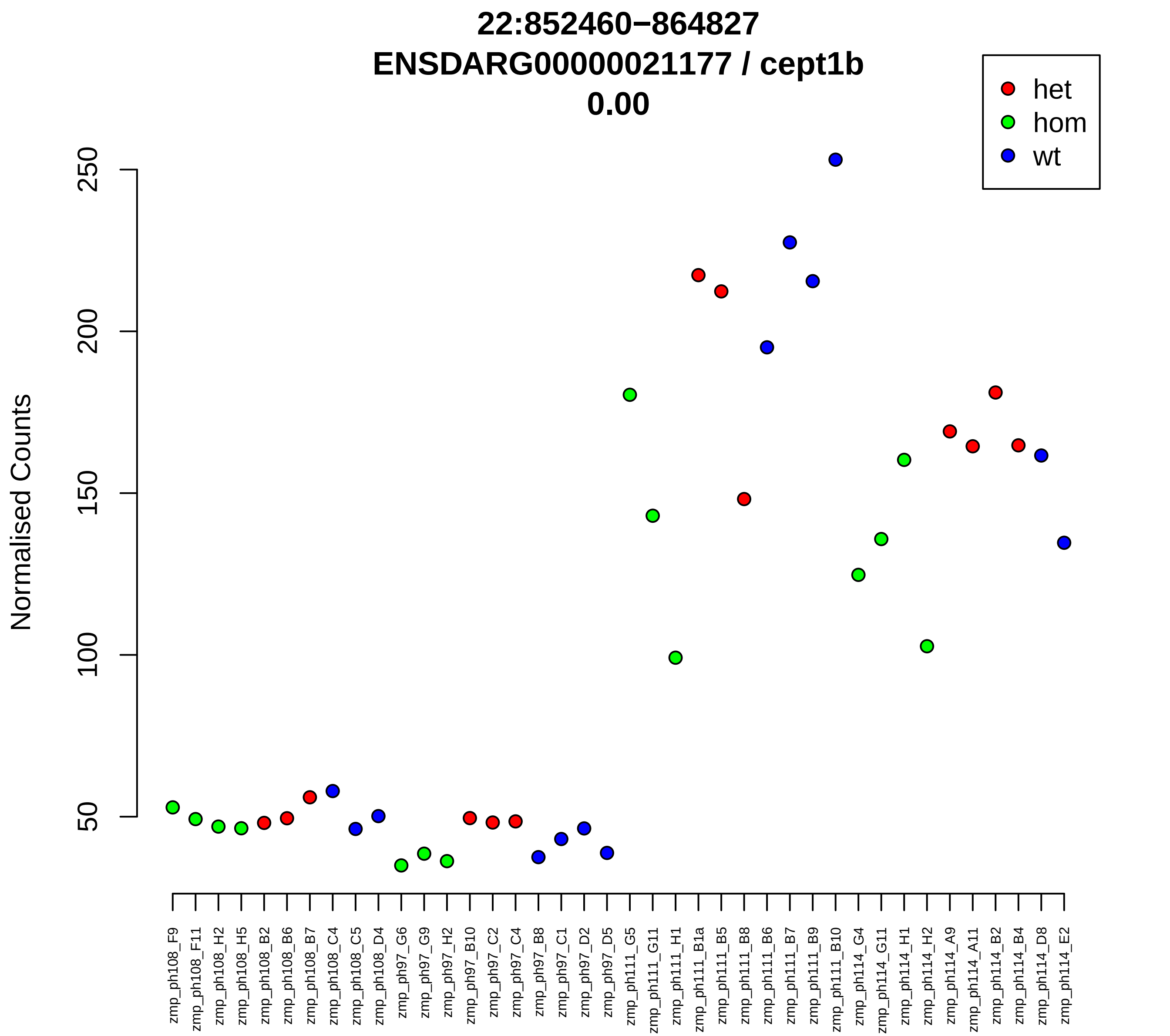

22:852460−864827
ENSDARG00000021177 / cept1b
0.00
het
hom
wt
Normalised Counts
50
100
150
200
250
zmp_ph108_F9
zmp_ph108_F11
zmp_ph108_H2
zmp_ph108_H5
zmp_ph108_B2
zmp_ph108_B6
zmp_ph108_B7
zmp_ph108_C4
zmp_ph108_C5
zmp_ph108_D4
zmp_ph97_G6
zmp_ph97_G9
zmp_ph97_H2
zmp_ph97_B10
zmp_ph97_C2
zmp_ph97_C4
zmp_ph97_B8
zmp_ph97_C1
zmp_ph97_D2
zmp_ph97_D5
zmp_ph111_G5
zmp_ph111_G11
zmp_ph111_H1
zmp_ph111_B1a
zmp_ph111_B5
zmp_ph111_B8
zmp_ph111_B6
zmp_ph111_B7
zmp_ph111_B9
zmp_ph111_B10
zmp_ph114_G4
zmp_ph114_G11
zmp_ph114_H1
zmp_ph114_H2
zmp_ph114_A9
zmp_ph114_A11
zmp_ph114_B2
zmp_ph114_B4
zmp_ph114_D8
zmp_ph114_E2

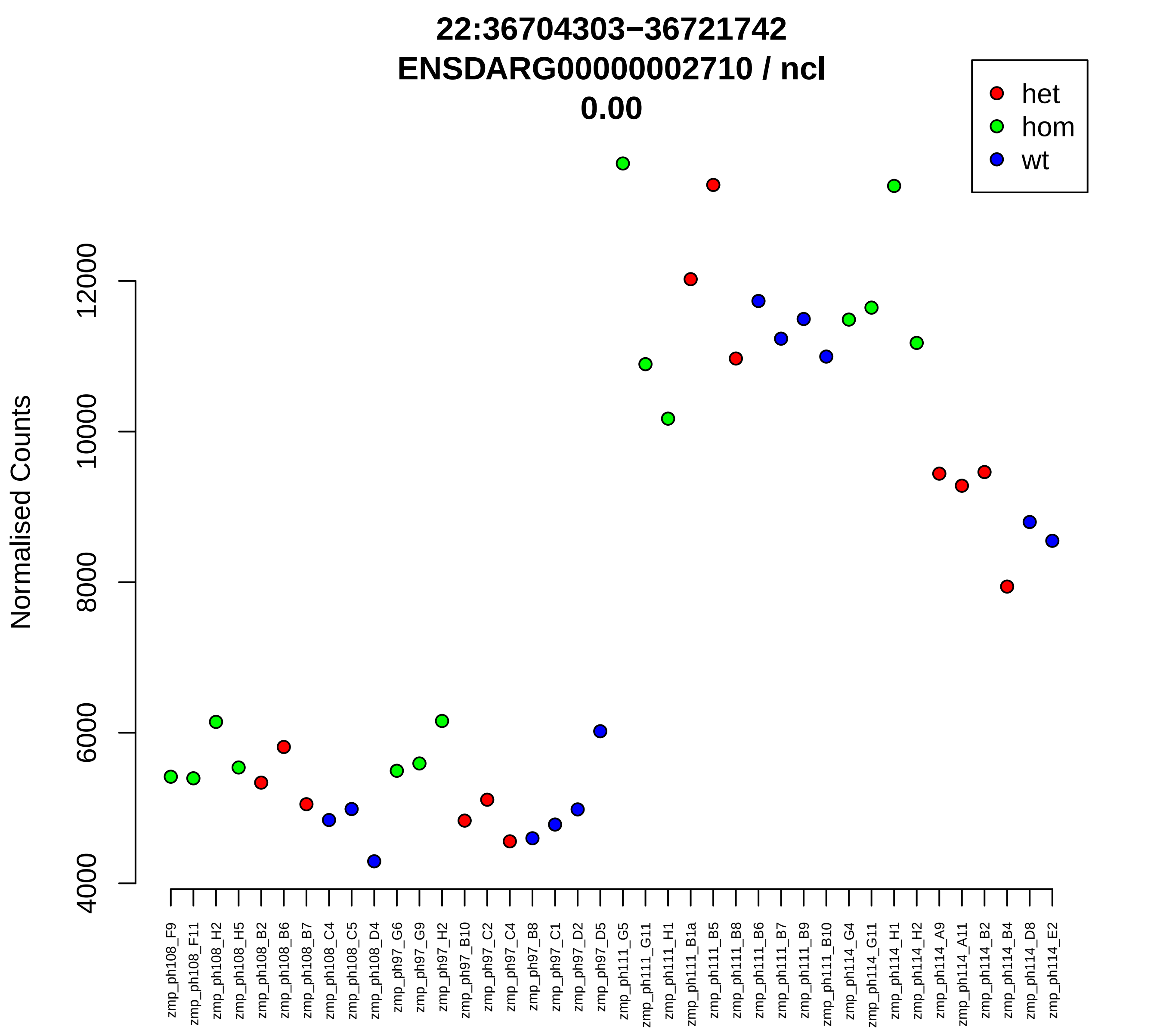

22:36704303−36721742
ENSDARG00000002710 / ncl
0.00
het
hom
wt
Normalised Counts
4000
6000
8000
10000
12000
zmp_ph108_F9
zmp_ph108_F11
zmp_ph108_H2
zmp_ph108_H5
zmp_ph108_B2
zmp_ph108_B6
zmp_ph108_B7
zmp_ph108_C4
zmp_ph108_C5
zmp_ph108_D4
zmp_ph97_G6
zmp_ph97_G9
zmp_ph97_H2
zmp_ph97_B10
zmp_ph97_C2
zmp_ph97_C4
zmp_ph97_B8
zmp_ph97_C1
zmp_ph97_D2
zmp_ph97_D5
zmp_ph111_G5
zmp_ph111_G11
zmp_ph111_H1
zmp_ph111_B1a
zmp_ph111_B5
zmp_ph111_B8
zmp_ph111_B6
zmp_ph111_B7
zmp_ph111_B9
zmp_ph111_B10
zmp_ph114_G4
zmp_ph114_G11
zmp_ph114_H1
zmp_ph114_H2
zmp_ph114_A9
zmp_ph114_A11
zmp_ph114_B2
zmp_ph114_B4
zmp_ph114_D8
zmp_ph114_E2

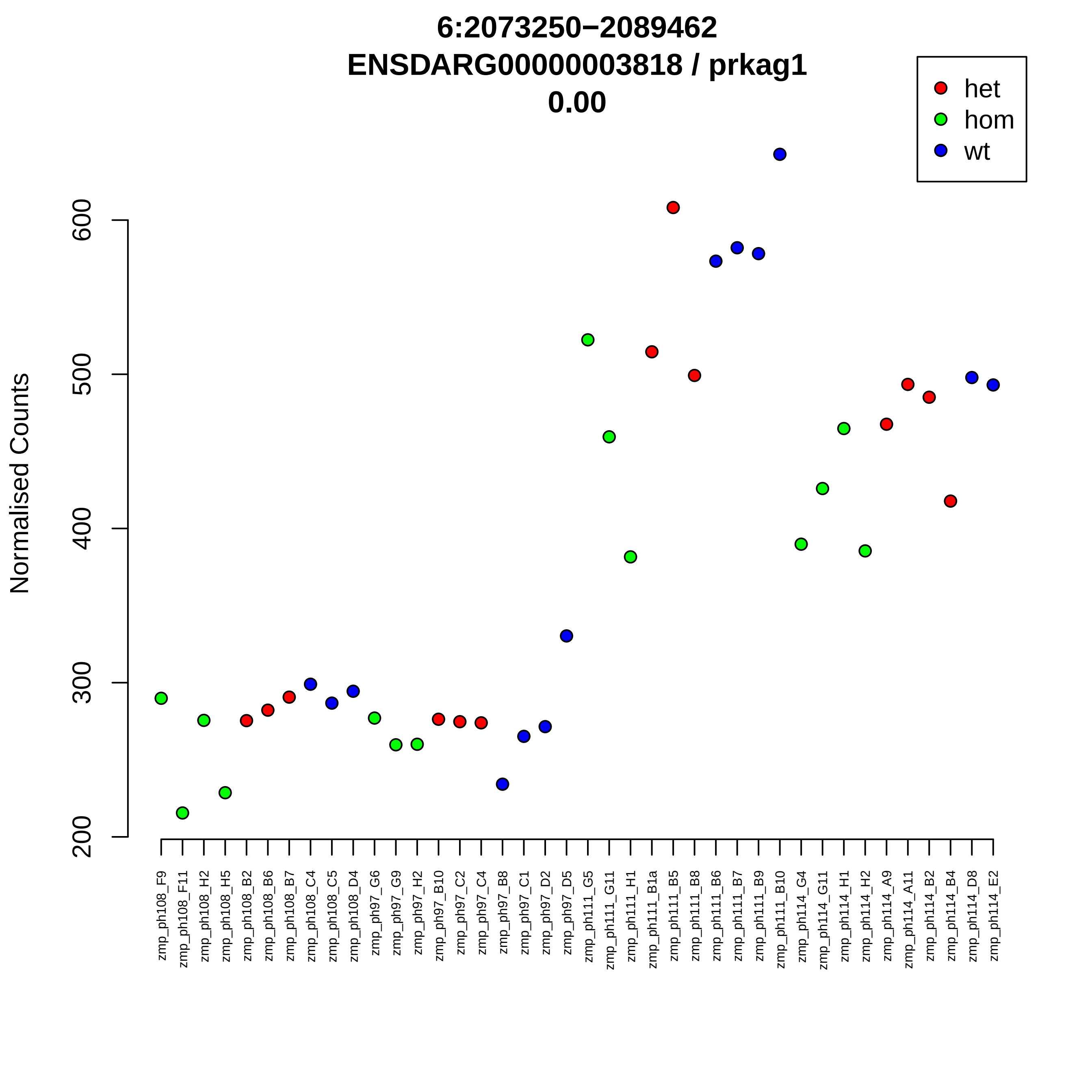
6:2073250−2089462
ENSDARG00000003818 / prkag1
0.00
het
hom
wt
Normalised Counts
200
300
400
500
600
zmp_ph108_F9
zmp_ph108_F11
zmp_ph108_H2
zmp_ph108_H5
zmp_ph108_B2
zmp_ph108_B6
zmp_ph108_B7
zmp_ph108_C4
zmp_ph108_C5
zmp_ph108_D4
zmp_ph97_G6
zmp_ph97_G9
zmp_ph97_H2
zmp_ph97_B10
zmp_ph97_C2
zmp_ph97_C4
zmp_ph97_B8
zmp_ph97_C1
zmp_ph97_D2
zmp_ph97_D5
zmp_ph111_G5
zmp_ph111_G11
zmp_ph111_H1
zmp_ph111_B1a
zmp_ph111_B5
zmp_ph111_B8
zmp_ph111_B6
zmp_ph111_B7
zmp_ph111_B9
zmp_ph111_B10
zmp_ph114_G4
zmp_ph114_G11
zmp_ph114_H1
zmp_ph114_H2
zmp_ph114_A9
zmp_ph114_A11
zmp_ph114_B2
zmp_ph114_B4
zmp_ph114_D8
zmp_ph114_E2

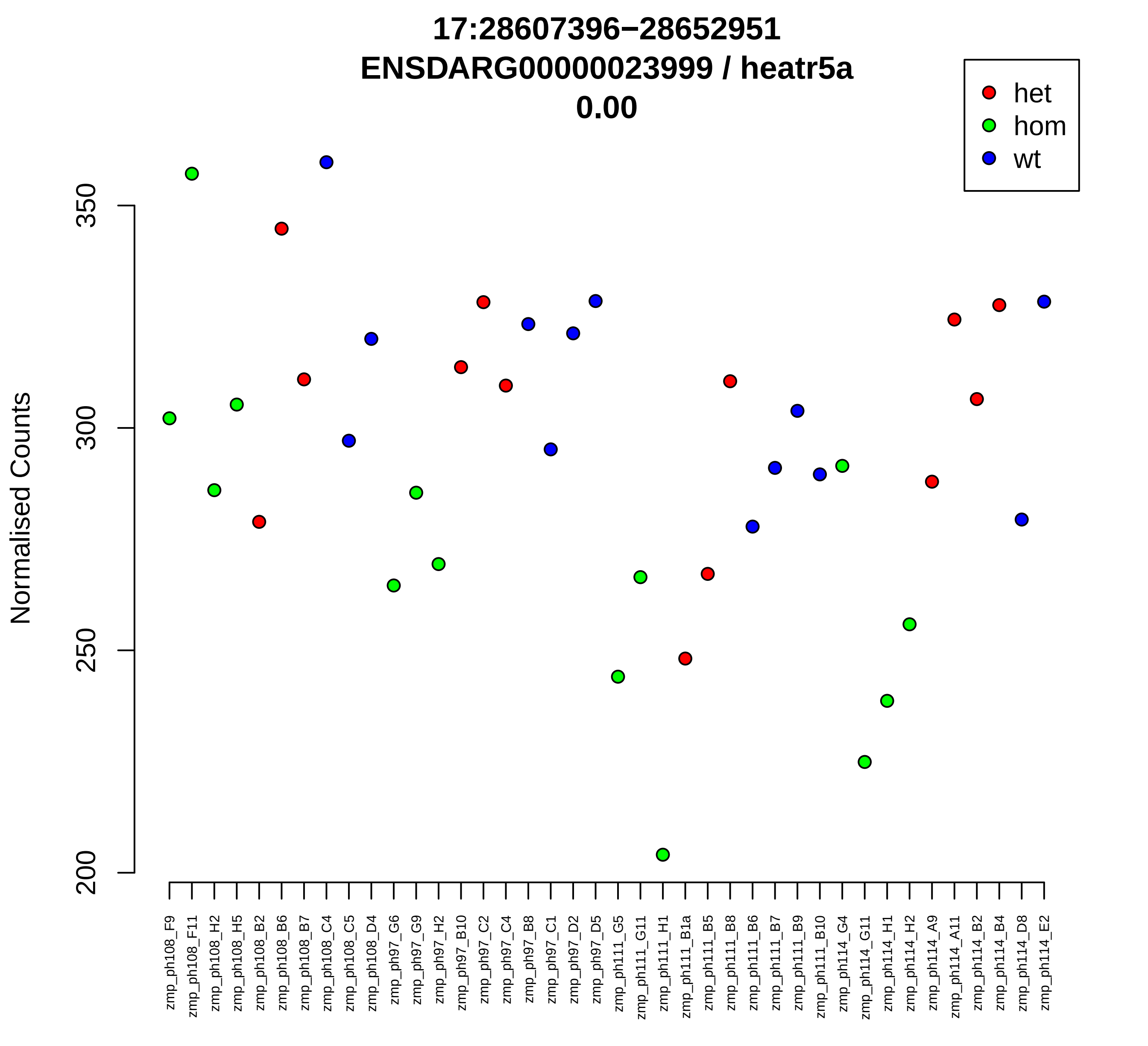

17:28607396−28652951
ENSDARG00000023999 / heatr5a
0.00
het
hom
wt
Normalised Counts
200
250
300
350
zmp_ph108_F9
zmp_ph108_F11
zmp_ph108_H2
zmp_ph108_H5
zmp_ph108_B2
zmp_ph108_B6
zmp_ph108_B7
zmp_ph108_C4
zmp_ph108_C5
zmp_ph108_D4
zmp_ph97_G6
zmp_ph97_G9
zmp_ph97_H2
zmp_ph97_B10
zmp_ph97_C2
zmp_ph97_C4
zmp_ph97_B8
zmp_ph97_C1
zmp_ph97_D2
zmp_ph97_D5
zmp_ph111_G5
zmp_ph111_G11
zmp_ph111_H1
zmp_ph111_B1a
zmp_ph111_B5
zmp_ph111_B8
zmp_ph111_B6
zmp_ph111_B7
zmp_ph111_B9
zmp_ph111_B10
zmp_ph114_G4
zmp_ph114_G11
zmp_ph114_H1
zmp_ph114_H2
zmp_ph114_A9
zmp_ph114_A11
zmp_ph114_B2
zmp_ph114_B4
zmp_ph114_D8
zmp_ph114_E2

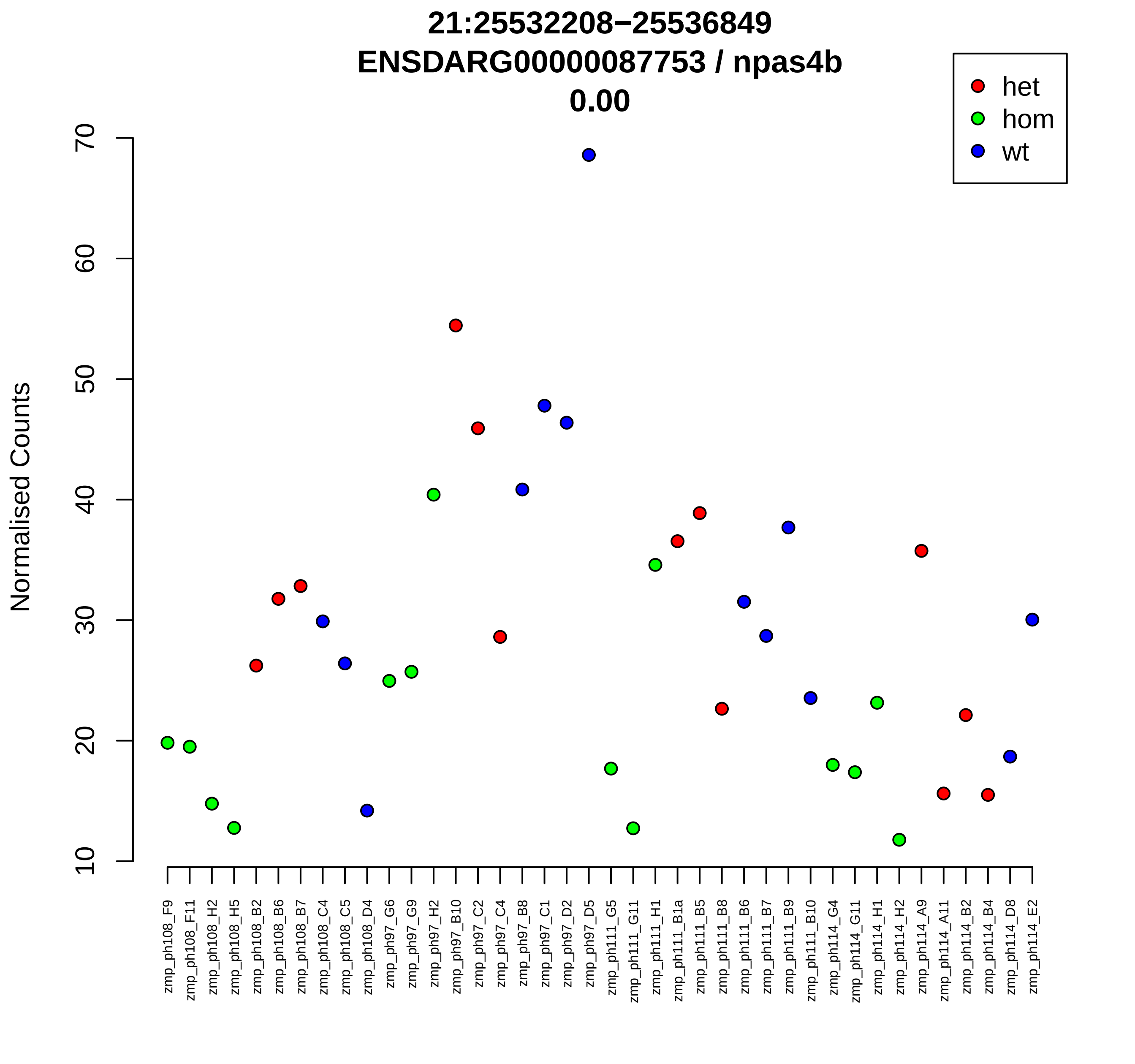
21:25532208−25536849
ENSDARG00000087753 / npas4b
0.00
het
hom
wt
Normalised Counts
10
20
30
40
50
60
70
zmp_ph108_F9
zmp_ph108_F11
zmp_ph108_H2
zmp_ph108_H5
zmp_ph108_B2
zmp_ph108_B6
zmp_ph108_B7
zmp_ph108_C4
zmp_ph108_C5
zmp_ph108_D4
zmp_ph97_G6
zmp_ph97_G9
zmp_ph97_H2
zmp_ph97_B10
zmp_ph97_C2
zmp_ph97_C4
zmp_ph97_B8
zmp_ph97_C1
zmp_ph97_D2
zmp_ph97_D5
zmp_ph111_G5
zmp_ph111_G11
zmp_ph111_H1
zmp_ph111_B1a
zmp_ph111_B5
zmp_ph111_B8
zmp_ph111_B6
zmp_ph111_B7
zmp_ph111_B9
zmp_ph111_B10
zmp_ph114_G4
zmp_ph114_G11
zmp_ph114_H1
zmp_ph114_H2
zmp_ph114_A9
zmp_ph114_A11
zmp_ph114_B2
zmp_ph114_B4
zmp_ph114_D8
zmp_ph114_E2

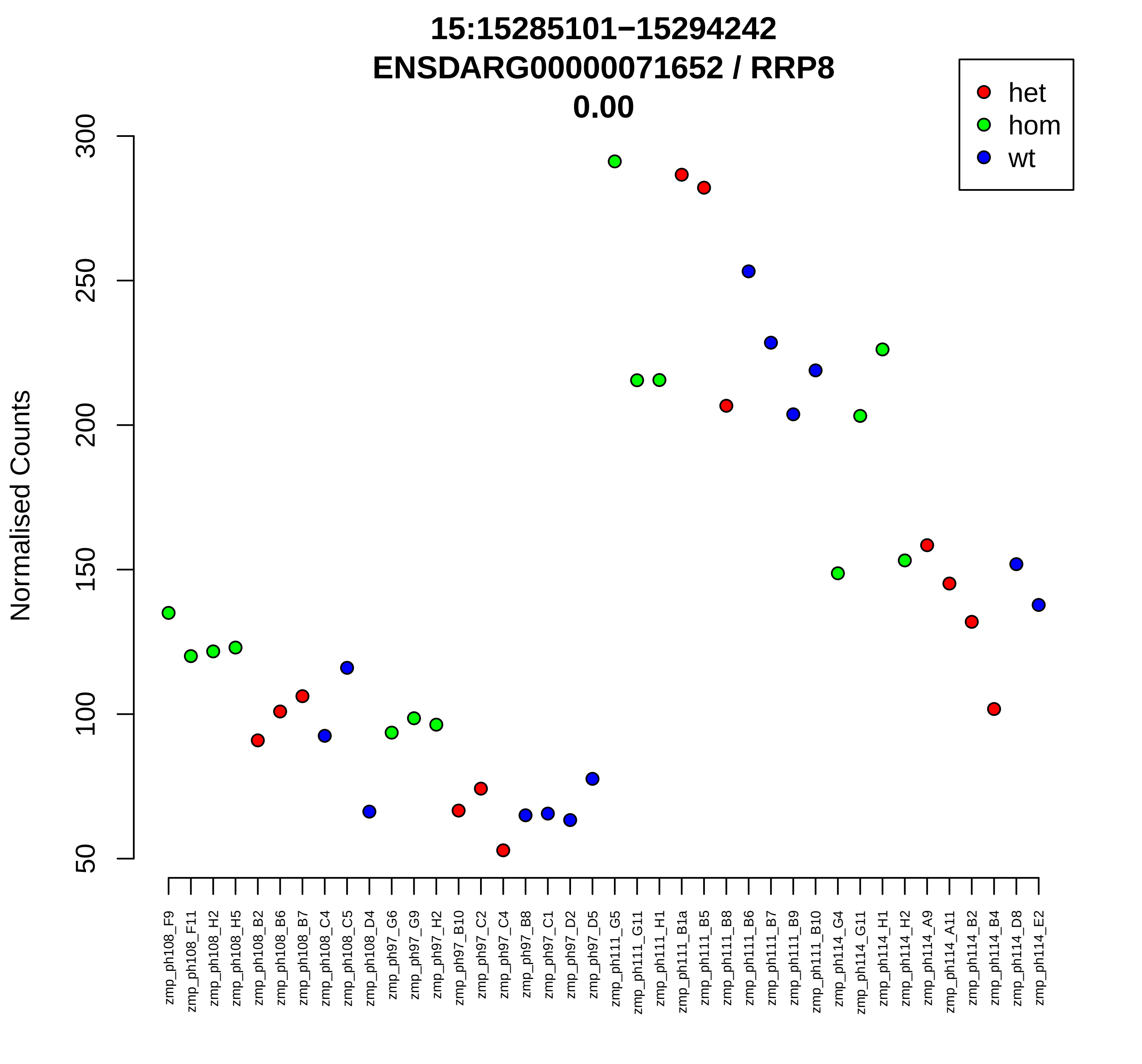

15:15285101−15294242
ENSDARG00000071652 / RRP8
0.00
het
hom
wt
Normalised Counts
50
100
150
200
250
300
zmp_ph108_F9
zmp_ph108_F11
zmp_ph108_H2
zmp_ph108_H5
zmp_ph108_B2
zmp_ph108_B6
zmp_ph108_B7
zmp_ph108_C4
zmp_ph108_C5
zmp_ph108_D4
zmp_ph97_G6
zmp_ph97_G9
zmp_ph97_H2
zmp_ph97_B10
zmp_ph97_C2
zmp_ph97_C4
zmp_ph97_B8
zmp_ph97_C1
zmp_ph97_D2
zmp_ph97_D5
zmp_ph111_G5
zmp_ph111_G11
zmp_ph111_H1
zmp_ph111_B1a
zmp_ph111_B5
zmp_ph111_B8
zmp_ph111_B6
zmp_ph111_B7
zmp_ph111_B9
zmp_ph111_B10
zmp_ph114_G4
zmp_ph114_G11
zmp_ph114_H1
zmp_ph114_H2
zmp_ph114_A9
zmp_ph114_A11
zmp_ph114_B2
zmp_ph114_B4
zmp_ph114_D8
zmp_ph114_E2

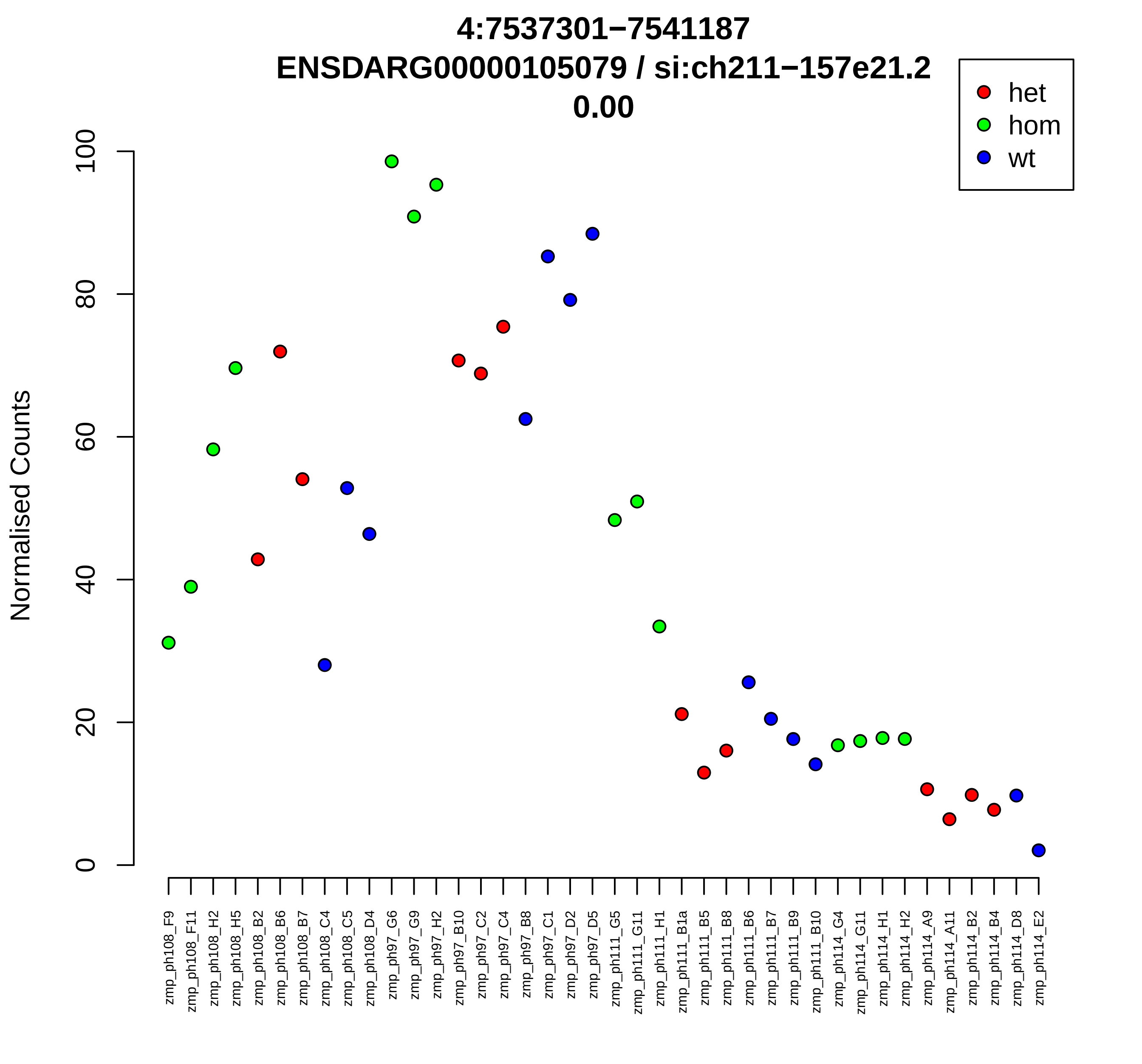
4:7537301−7541187
ENSDARG00000105079 / si:ch211−157e21.2
0.00
het
hom
wt
Normalised Counts
0
20
40
60
80
100
zmp_ph108_F9
zmp_ph108_F11
zmp_ph108_H2
zmp_ph108_H5
zmp_ph108_B2
zmp_ph108_B6
zmp_ph108_B7
zmp_ph108_C4
zmp_ph108_C5
zmp_ph108_D4
zmp_ph97_G6
zmp_ph97_G9
zmp_ph97_H2
zmp_ph97_B10
zmp_ph97_C2
zmp_ph97_C4
zmp_ph97_B8
zmp_ph97_C1
zmp_ph97_D2
zmp_ph97_D5
zmp_ph111_G5
zmp_ph111_G11
zmp_ph111_H1
zmp_ph111_B1a
zmp_ph111_B5
zmp_ph111_B8
zmp_ph111_B6
zmp_ph111_B7
zmp_ph111_B9
zmp_ph111_B10
zmp_ph114_G4
zmp_ph114_G11
zmp_ph114_H1
zmp_ph114_H2
zmp_ph114_A9
zmp_ph114_A11
zmp_ph114_B2
zmp_ph114_B4
zmp_ph114_D8
zmp_ph114_E2

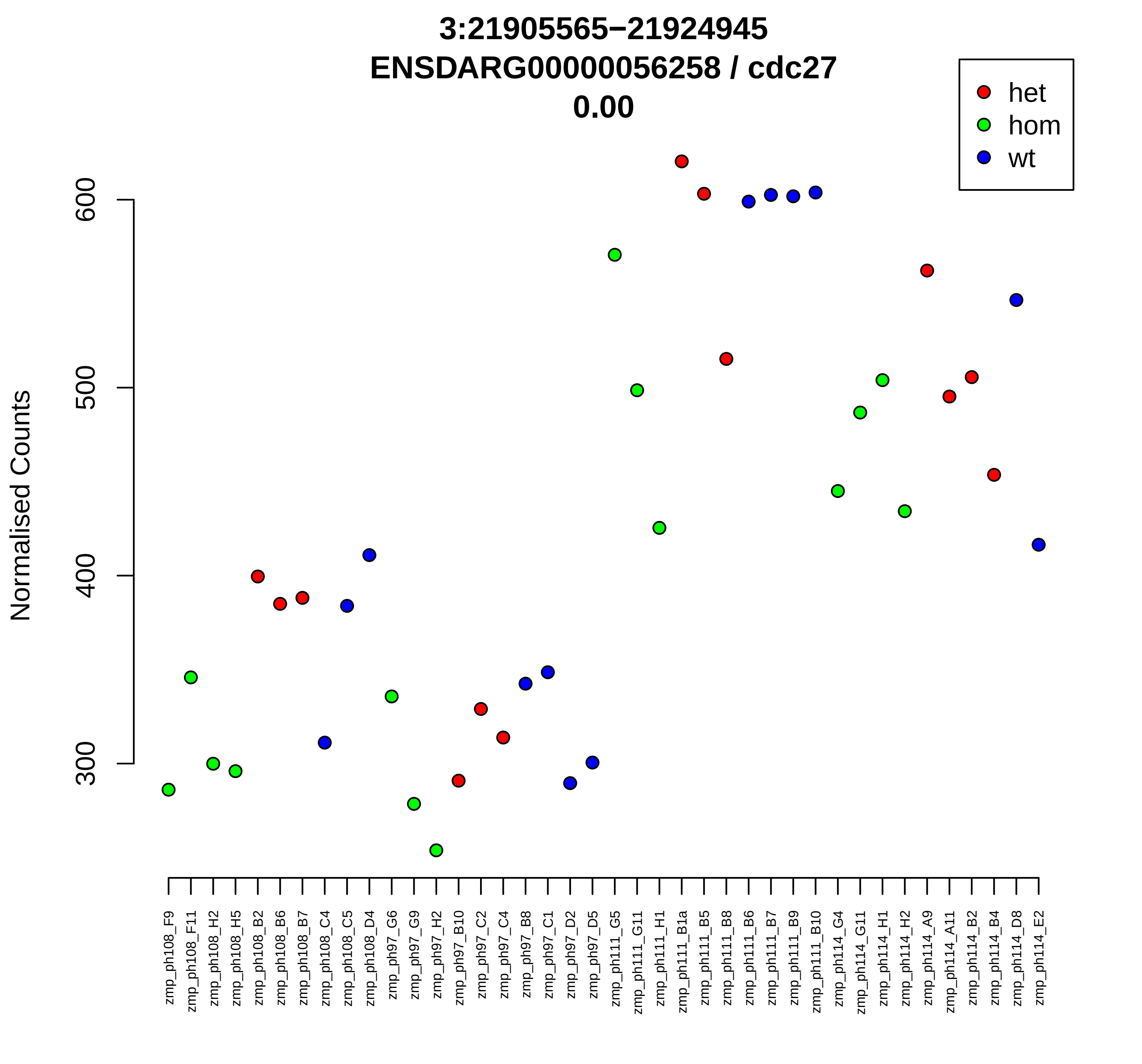
3:21905565−21924945
ENSDARG00000056258 / cdc27
0.00
het
hom
wt
Normalised Counts
300
400
500
600
zmp_ph108_F9
zmp_ph108_F11
zmp_ph108_H2
zmp_ph108_H5
zmp_ph108_B2
zmp_ph108_B6
zmp_ph108_B7
zmp_ph108_C4
zmp_ph108_C5
zmp_ph108_D4
zmp_ph97_G6
zmp_ph97_G9
zmp_ph97_H2
zmp_ph97_B10
zmp_ph97_C2
zmp_ph97_C4
zmp_ph97_B8
zmp_ph97_C1
zmp_ph97_D2
zmp_ph97_D5
zmp_ph111_G5
zmp_ph111_G11
zmp_ph111_H1
zmp_ph111_B1a
zmp_ph111_B5
zmp_ph111_B8
zmp_ph111_B6
zmp_ph111_B7
zmp_ph111_B9
zmp_ph111_B10
zmp_ph114_G4
zmp_ph114_G11
zmp_ph114_H1
zmp_ph114_H2
zmp_ph114_A9
zmp_ph114_A11
zmp_ph114_B2
zmp_ph114_B4
zmp_ph114_D8
zmp_ph114_E2

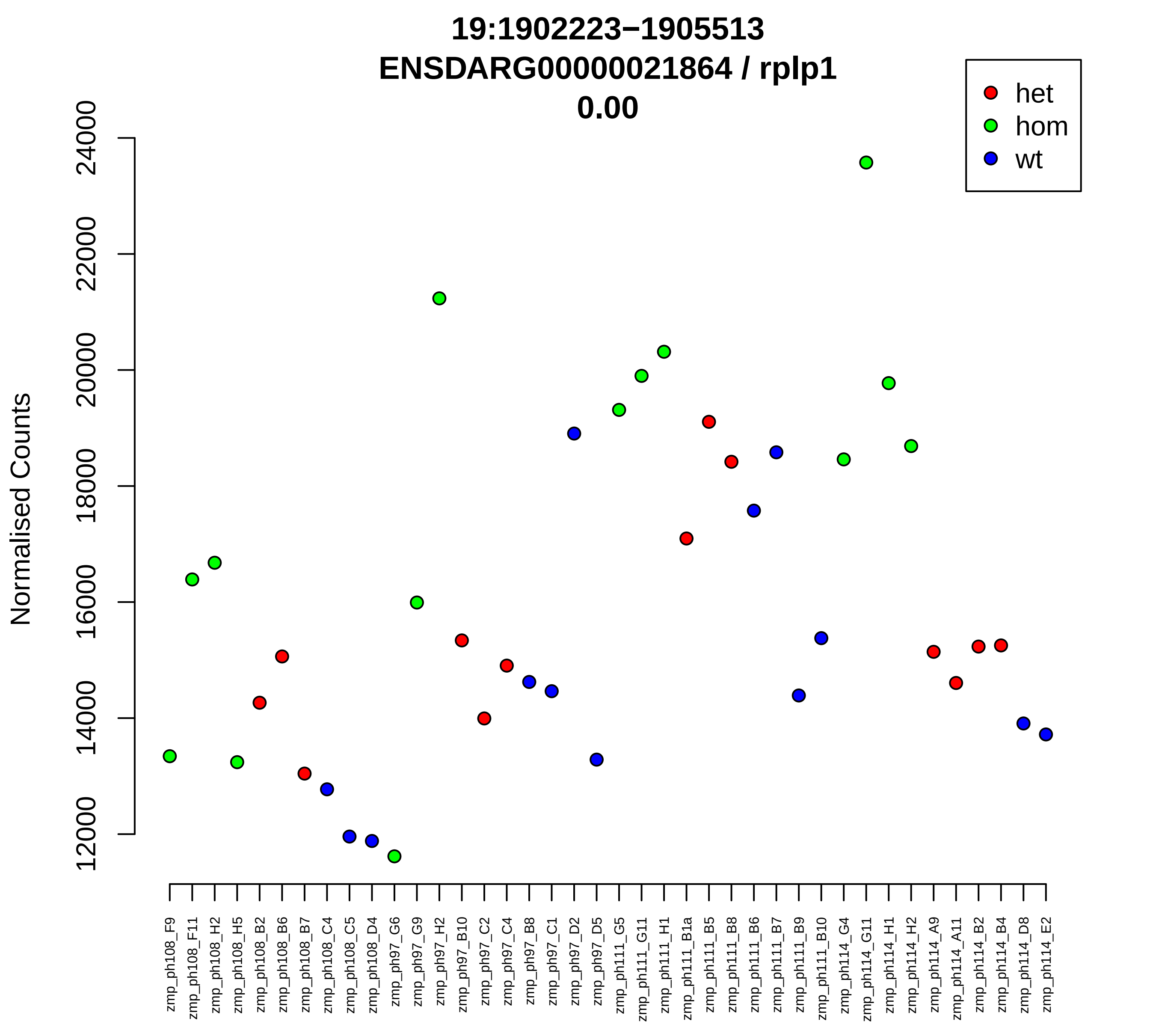

19:1902223−1905513
ENSDARG00000021864 / rplp1
0.00
het
hom
wt
Normalised Counts
12000
14000
16000
18000
20000
22000
24000
zmp_ph108_F9
zmp_ph108_F11
zmp_ph108_H2
zmp_ph108_H5
zmp_ph108_B2
zmp_ph108_B6
zmp_ph108_B7
zmp_ph108_C4
zmp_ph108_C5
zmp_ph108_D4
zmp_ph97_G6
zmp_ph97_G9
zmp_ph97_H2
zmp_ph97_B10
zmp_ph97_C2
zmp_ph97_C4
zmp_ph97_B8
zmp_ph97_C1
zmp_ph97_D2
zmp_ph97_D5
zmp_ph111_G5
zmp_ph111_G11
zmp_ph111_H1
zmp_ph111_B1a
zmp_ph111_B5
zmp_ph111_B8
zmp_ph111_B6
zmp_ph111_B7
zmp_ph111_B9
zmp_ph111_B10
zmp_ph114_G4
zmp_ph114_G11
zmp_ph114_H1
zmp_ph114_H2
zmp_ph114_A9
zmp_ph114_A11
zmp_ph114_B2
zmp_ph114_B4
zmp_ph114_D8
zmp_ph114_E2

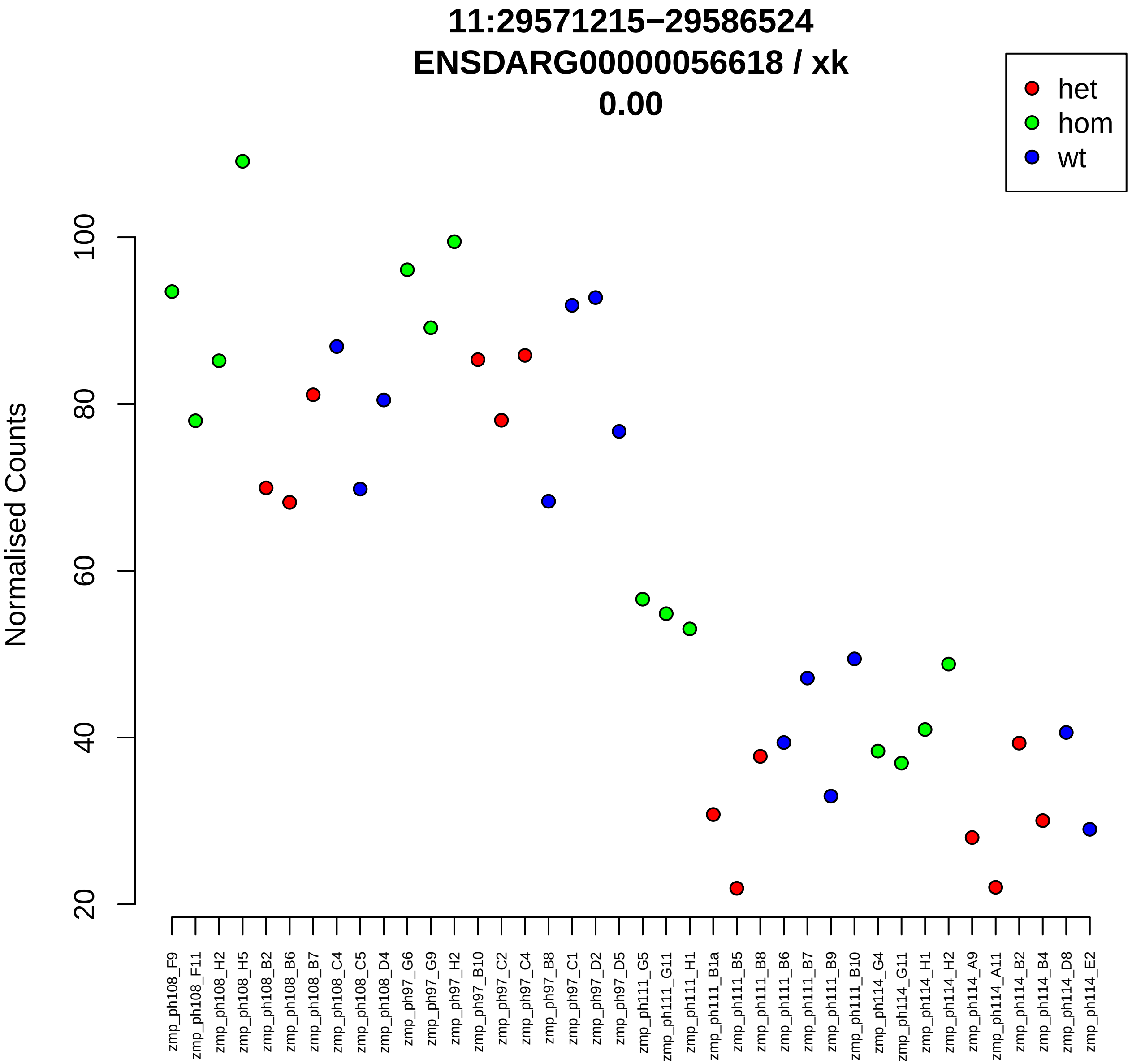
11:29571215−29586524
ENSDARG00000056618 / xk
0.00
het
hom
wt
Normalised Counts
20
40
60
80
100
zmp_ph108_F9
zmp_ph108_F11
zmp_ph108_H2
zmp_ph108_H5
zmp_ph108_B2
zmp_ph108_B6
zmp_ph108_B7
zmp_ph108_C4
zmp_ph108_C5
zmp_ph108_D4
zmp_ph97_G6
zmp_ph97_G9
zmp_ph97_H2
zmp_ph97_B10
zmp_ph97_C2
zmp_ph97_C4
zmp_ph97_B8
zmp_ph97_C1
zmp_ph97_D2
zmp_ph97_D5
zmp_ph111_G5
zmp_ph111_G11
zmp_ph111_H1
zmp_ph111_B1a
zmp_ph111_B5
zmp_ph111_B8
zmp_ph111_B6
zmp_ph111_B7
zmp_ph111_B9
zmp_ph111_B10
zmp_ph114_G4
zmp_ph114_G11
zmp_ph114_H1
zmp_ph114_H2
zmp_ph114_A9
zmp_ph114_A11
zmp_ph114_B2
zmp_ph114_B4
zmp_ph114_D8
zmp_ph114_E2

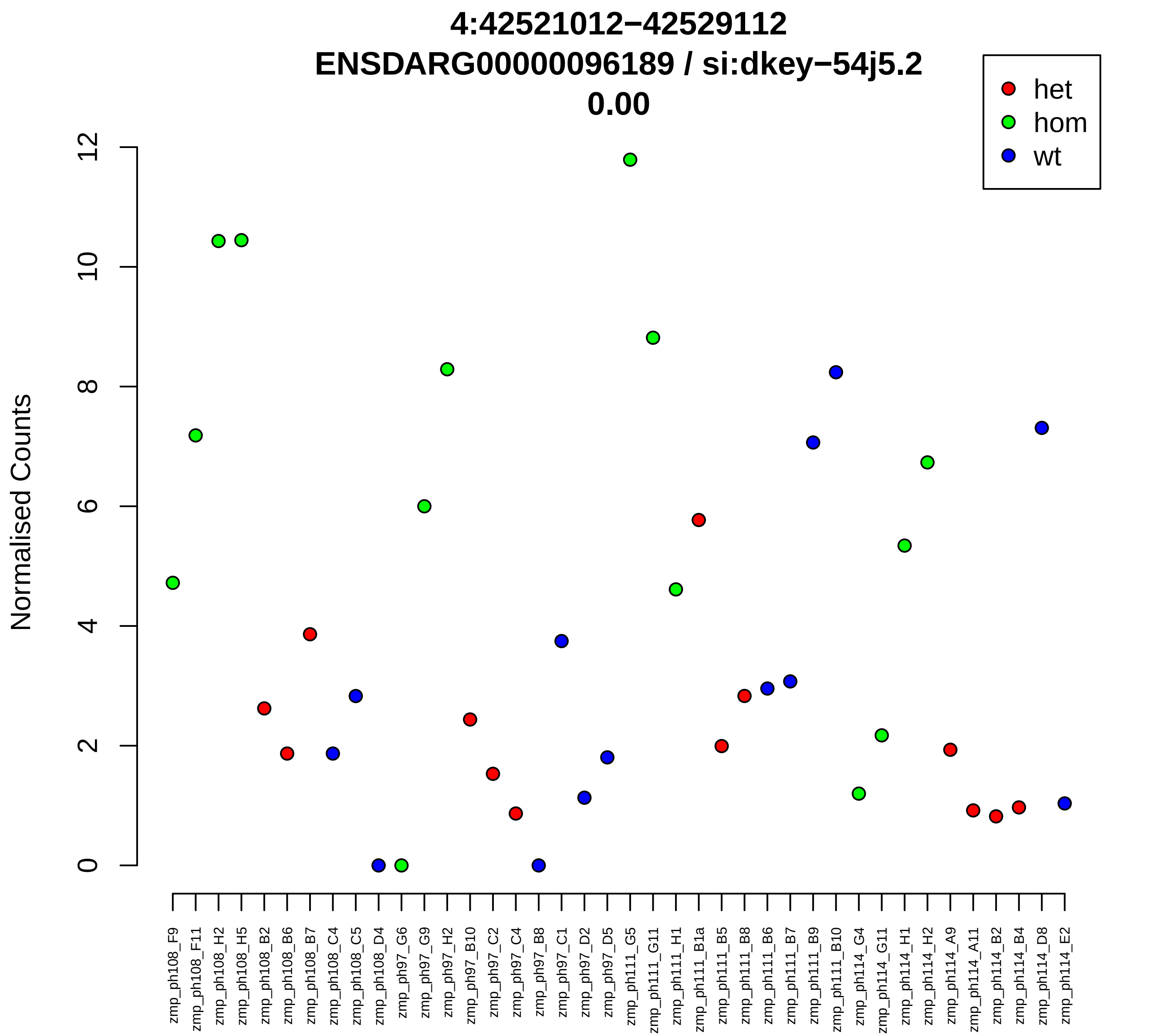
4:42521012−42529112
ENSDARG00000096189 / si:dkey−54j5.2
0.00
het
hom
wt
Normalised Counts
0
2
4
6
8
10
12
zmp_ph108_F9
zmp_ph108_F11
zmp_ph108_H2
zmp_ph108_H5
zmp_ph108_B2
zmp_ph108_B6
zmp_ph108_B7
zmp_ph108_C4
zmp_ph108_C5
zmp_ph108_D4
zmp_ph97_G6
zmp_ph97_G9
zmp_ph97_H2
zmp_ph97_B10
zmp_ph97_C2
zmp_ph97_C4
zmp_ph97_B8
zmp_ph97_C1
zmp_ph97_D2
zmp_ph97_D5
zmp_ph111_G5
zmp_ph111_G11
zmp_ph111_H1
zmp_ph111_B1a
zmp_ph111_B5
zmp_ph111_B8
zmp_ph111_B6
zmp_ph111_B7
zmp_ph111_B9
zmp_ph111_B10
zmp_ph114_G4
zmp_ph114_G11
zmp_ph114_H1
zmp_ph114_H2
zmp_ph114_A9
zmp_ph114_A11
zmp_ph114_B2
zmp_ph114_B4
zmp_ph114_D8
zmp_ph114_E2

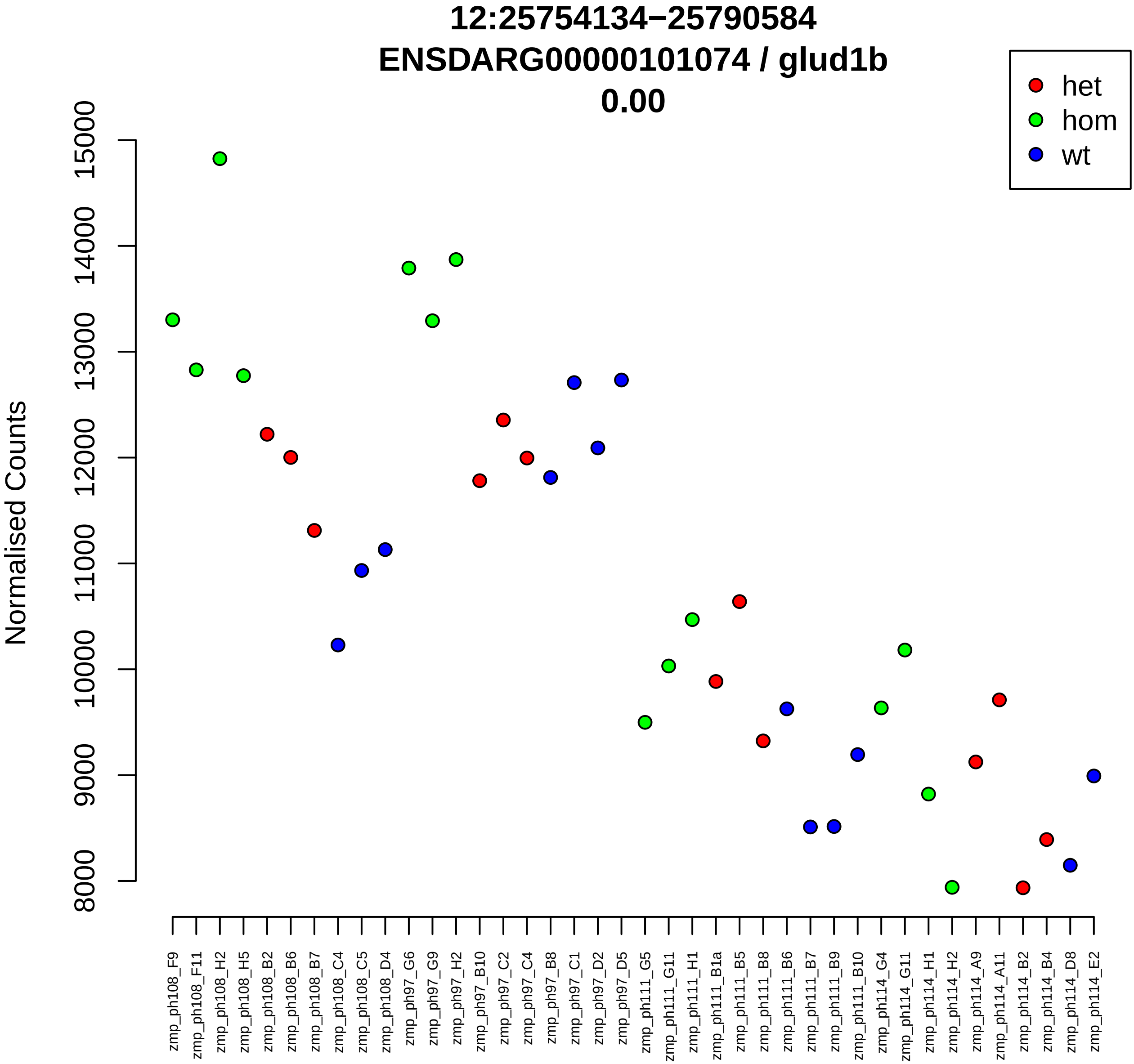

12:25754134−25790584
ENSDARG00000101074 / glud1b
0.00
het
hom
wt
Normalised Counts
8000
9000
10000
11000
12000
13000
14000
15000
zmp_ph108_F9
zmp_ph108_F11
zmp_ph108_H2
zmp_ph108_H5
zmp_ph108_B2
zmp_ph108_B6
zmp_ph108_B7
zmp_ph108_C4
zmp_ph108_C5
zmp_ph108_D4
zmp_ph97_G6
zmp_ph97_G9
zmp_ph97_H2
zmp_ph97_B10
zmp_ph97_C2
zmp_ph97_C4
zmp_ph97_B8
zmp_ph97_C1
zmp_ph97_D2
zmp_ph97_D5
zmp_ph111_G5
zmp_ph111_G11
zmp_ph111_H1
zmp_ph111_B1a
zmp_ph111_B5
zmp_ph111_B8
zmp_ph111_B6
zmp_ph111_B7
zmp_ph111_B9
zmp_ph111_B10
zmp_ph114_G4
zmp_ph114_G11
zmp_ph114_H1
zmp_ph114_H2
zmp_ph114_A9
zmp_ph114_A11
zmp_ph114_B2
zmp_ph114_B4
zmp_ph114_D8
zmp_ph114_E2

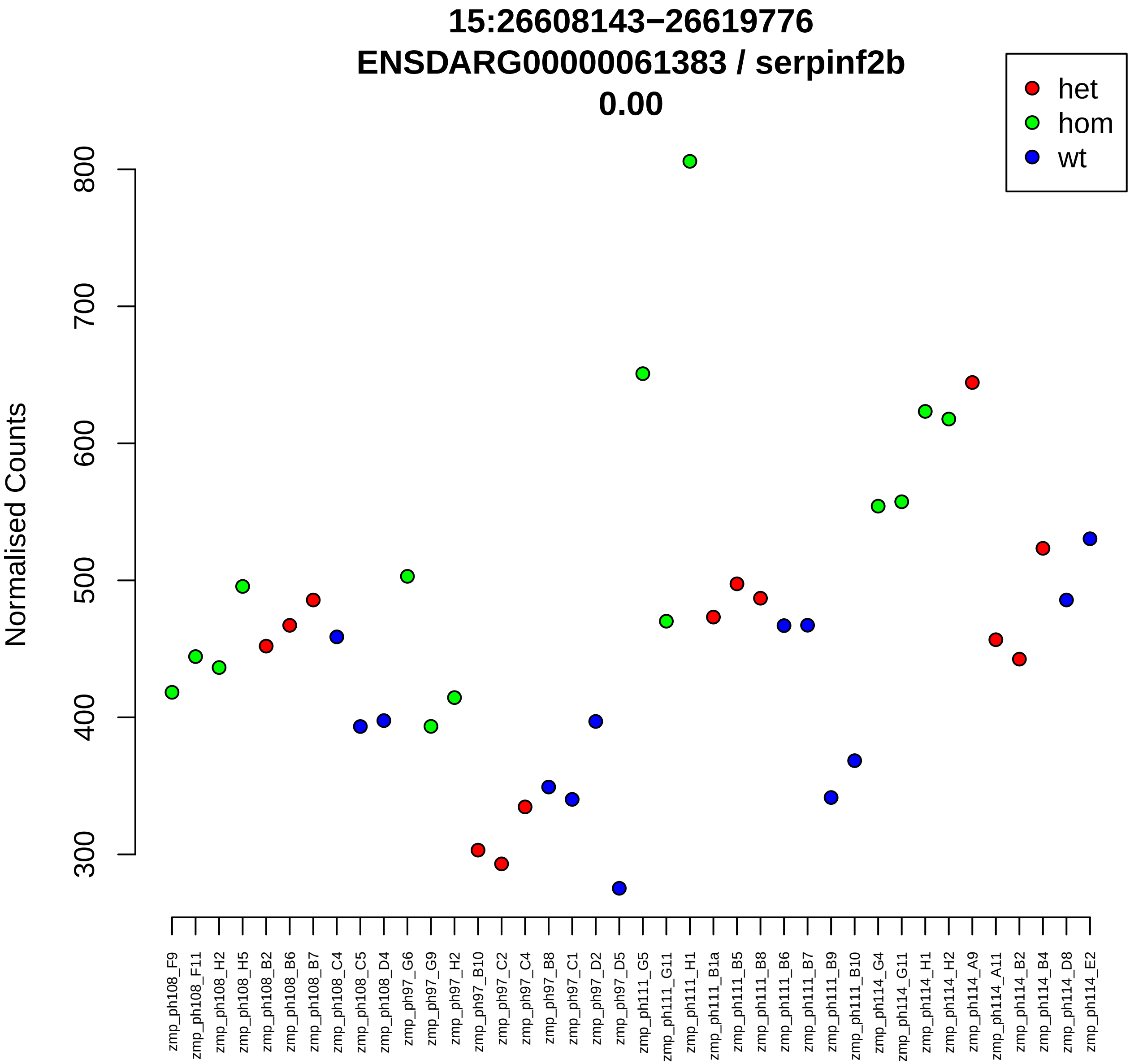
15:26608143−26619776
ENSDARG00000061383 / serpinf2b
0.00
het
hom
wt
Normalised Counts
300
400
500
600
700
800
zmp_ph108_F9
zmp_ph108_F11
zmp_ph108_H2
zmp_ph108_H5
zmp_ph108_B2
zmp_ph108_B6
zmp_ph108_B7
zmp_ph108_C4
zmp_ph108_C5
zmp_ph108_D4
zmp_ph97_G6
zmp_ph97_G9
zmp_ph97_H2
zmp_ph97_B10
zmp_ph97_C2
zmp_ph97_C4
zmp_ph97_B8
zmp_ph97_C1
zmp_ph97_D2
zmp_ph97_D5
zmp_ph111_G5
zmp_ph111_G11
zmp_ph111_H1
zmp_ph111_B1a
zmp_ph111_B5
zmp_ph111_B8
zmp_ph111_B6
zmp_ph111_B7
zmp_ph111_B9
zmp_ph111_B10
zmp_ph114_G4
zmp_ph114_G11
zmp_ph114_H1
zmp_ph114_H2
zmp_ph114_A9
zmp_ph114_A11
zmp_ph114_B2
zmp_ph114_B4
zmp_ph114_D8
zmp_ph114_E2

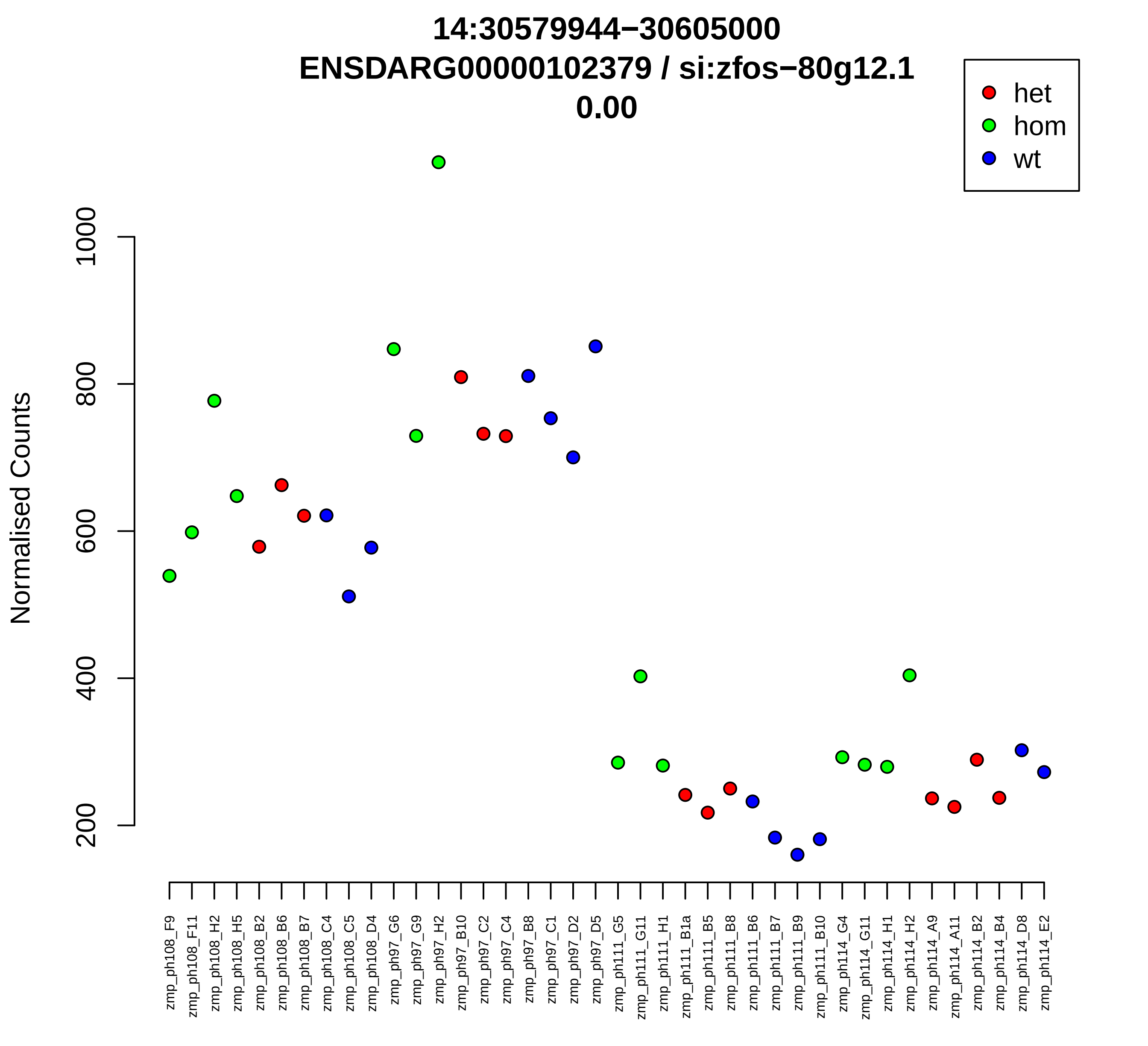
14:30579944−30605000
ENSDARG00000102379 / si:zfos−80g12.1
0.00
het
hom
wt
Normalised Counts
1000
800
600
400
200
zmp_ph108_F9
zmp_ph108_F11
zmp_ph108_H2
zmp_ph108_H5
zmp_ph108_B2
zmp_ph108_B6
zmp_ph108_B7
zmp_ph108_C4
zmp_ph108_C5
zmp_ph108_D4
zmp_ph97_G6
zmp_ph97_G9
zmp_ph97_H2
zmp_ph97_B10
zmp_ph97_C2
zmp_ph97_C4
zmp_ph97_B8
zmp_ph97_C1
zmp_ph97_D2
zmp_ph97_D5
zmp_ph111_G5
zmp_ph111_G11
zmp_ph111_H1
zmp_ph111_B1a
zmp_ph111_B5
zmp_ph111_B8
zmp_ph111_B6
zmp_ph111_B7
zmp_ph111_B9
zmp_ph111_B10
zmp_ph114_G4
zmp_ph114_G11
zmp_ph114_H1
zmp_ph114_H2
zmp_ph114_A9
zmp_ph114_A11
zmp_ph114_B2
zmp_ph114_B4
zmp_ph114_D8
zmp_ph114_E2

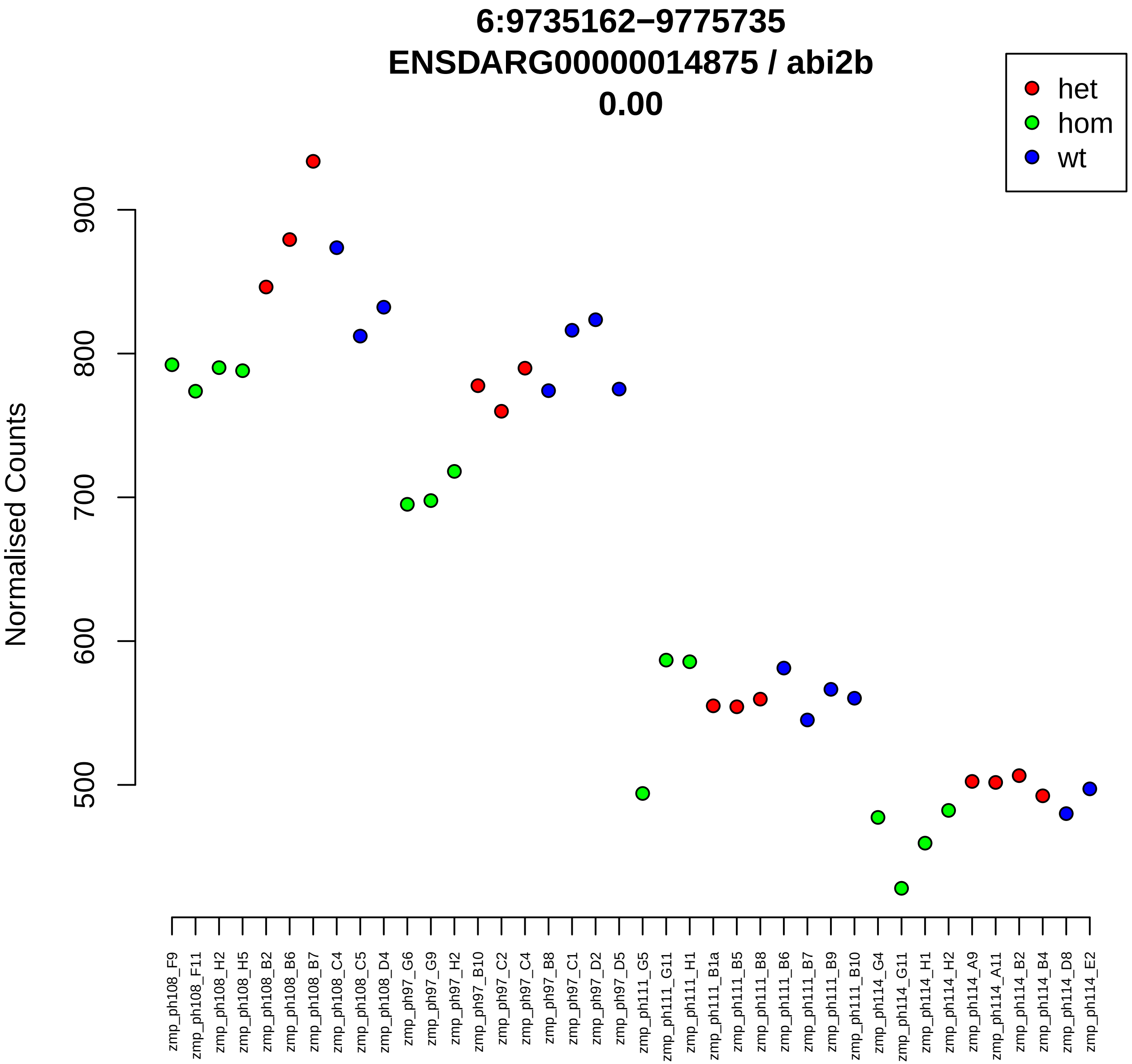

6:9735162−9775735
ENSDARG00000014875 / abi2b
0.00
het
hom
wt
Normalised Counts
500
600
700
800
900
zmp_ph108_F9
zmp_ph108_F11
zmp_ph108_H2
zmp_ph108_H5
zmp_ph108_B2
zmp_ph108_B6
zmp_ph108_B7
zmp_ph108_C4
zmp_ph108_C5
zmp_ph108_D4
zmp_ph97_G6
zmp_ph97_G9
zmp_ph97_H2
zmp_ph97_B10
zmp_ph97_C2
zmp_ph97_C4
zmp_ph97_B8
zmp_ph97_C1
zmp_ph97_D2
zmp_ph97_D5
zmp_ph111_G5
zmp_ph111_G11
zmp_ph111_H1
zmp_ph111_B1a
zmp_ph111_B5
zmp_ph111_B8
zmp_ph111_B6
zmp_ph111_B7
zmp_ph111_B9
zmp_ph111_B10
zmp_ph114_G4
zmp_ph114_G11
zmp_ph114_H1
zmp_ph114_H2
zmp_ph114_A9
zmp_ph114_A11
zmp_ph114_B2
zmp_ph114_B4
zmp_ph114_D8
zmp_ph114_E2

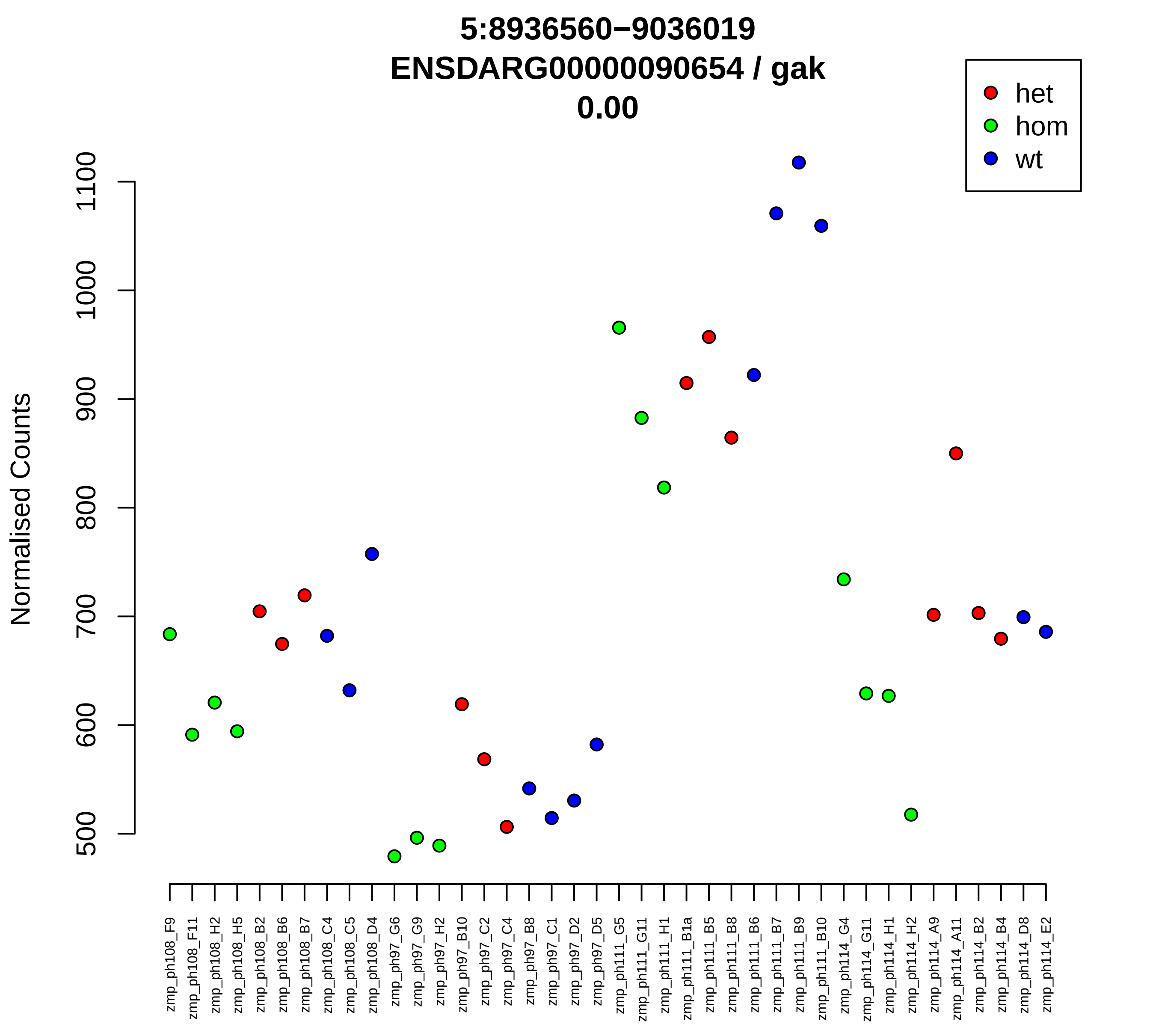

5:8936560−9036019
ENSDARG00000090654 / gak
0.00
het
hom
wt
Normalised Counts
500
600
700
800
900
1000
1100
zmp_ph108_F9
zmp_ph108_F11
zmp_ph108_H2
zmp_ph108_H5
zmp_ph108_B2
zmp_ph108_B6
zmp_ph108_B7
zmp_ph108_C4
zmp_ph108_C5
zmp_ph108_D4
zmp_ph97_G6
zmp_ph97_G9
zmp_ph97_H2
zmp_ph97_B10
zmp_ph97_C2
zmp_ph97_C4
zmp_ph97_B8
zmp_ph97_C1
zmp_ph97_D2
zmp_ph97_D5
zmp_ph111_G5
zmp_ph111_G11
zmp_ph111_H1
zmp_ph111_B1a
zmp_ph111_B5
zmp_ph111_B8
zmp_ph111_B6
zmp_ph111_B7
zmp_ph111_B9
zmp_ph111_B10
zmp_ph114_G4
zmp_ph114_G11
zmp_ph114_H1
zmp_ph114_H2
zmp_ph114_A9
zmp_ph114_A11
zmp_ph114_B2
zmp_ph114_B4
zmp_ph114_D8
zmp_ph114_E2

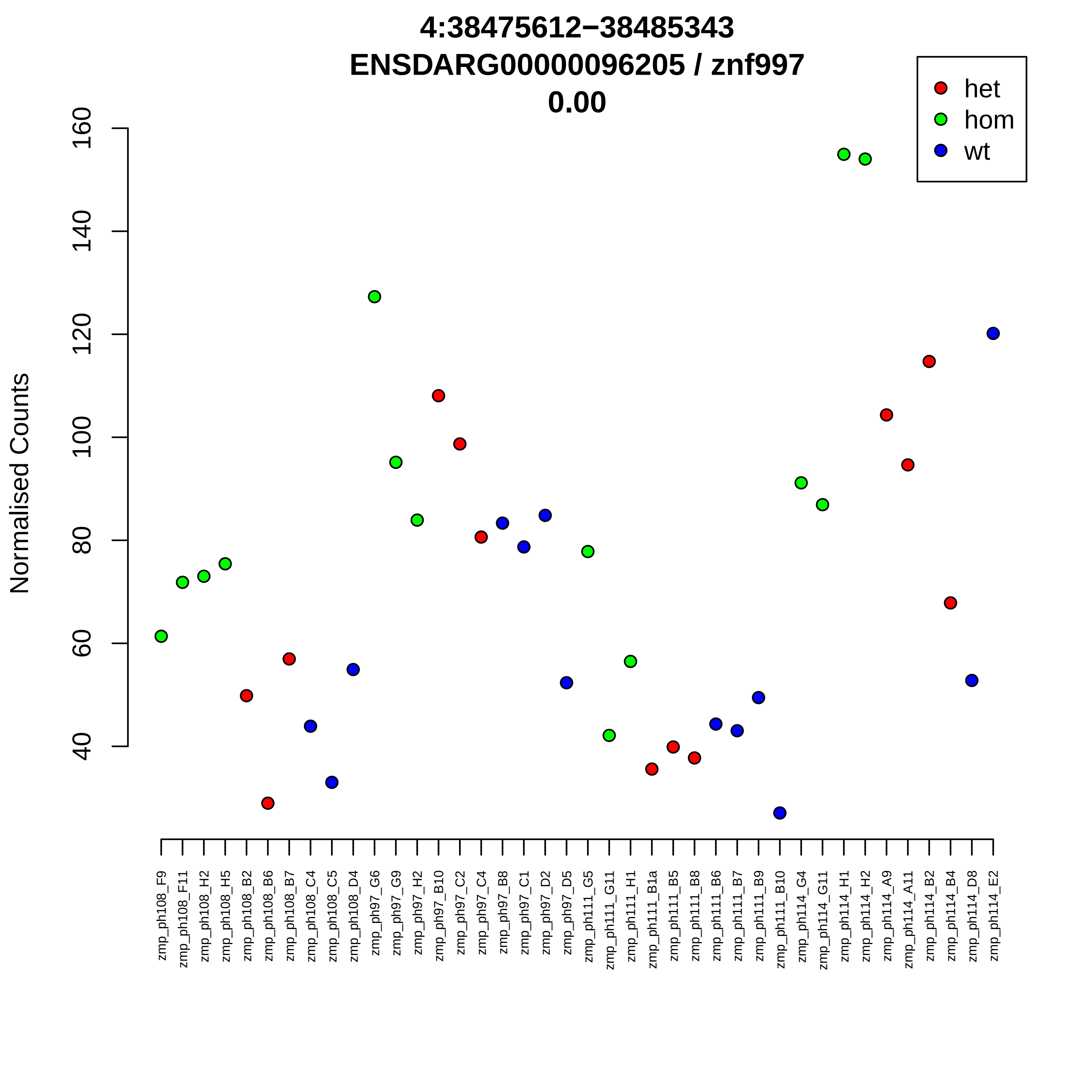
4:38475612−38485343
ENSDARG00000096205 / znf997
0.00
het
hom
wt
Normalised Counts
160
140
120
100
80
60
40
zmp_ph108_F9
zmp_ph108_F11
zmp_ph108_H2
zmp_ph108_H5
zmp_ph108_B2
zmp_ph108_B6
zmp_ph108_B7
zmp_ph108_C4
zmp_ph108_C5
zmp_ph108_D4
zmp_ph97_G6
zmp_ph97_G9
zmp_ph97_H2
zmp_ph97_B10
zmp_ph97_C2
zmp_ph97_C4
zmp_ph97_B8
zmp_ph97_C1
zmp_ph97_D2
zmp_ph97_D5
zmp_ph111_G5
zmp_ph111_G11
zmp_ph111_H1
zmp_ph111_B1a
zmp_ph111_B5
zmp_ph111_B8
zmp_ph111_B6
zmp_ph111_B7
zmp_ph111_B9
zmp_ph111_B10
zmp_ph114_G4
zmp_ph114_G11
zmp_ph114_H1
zmp_ph114_H2
zmp_ph114_A9
zmp_ph114_A11
zmp_ph114_B2
zmp_ph114_B4
zmp_ph114_D8
zmp_ph114_E2

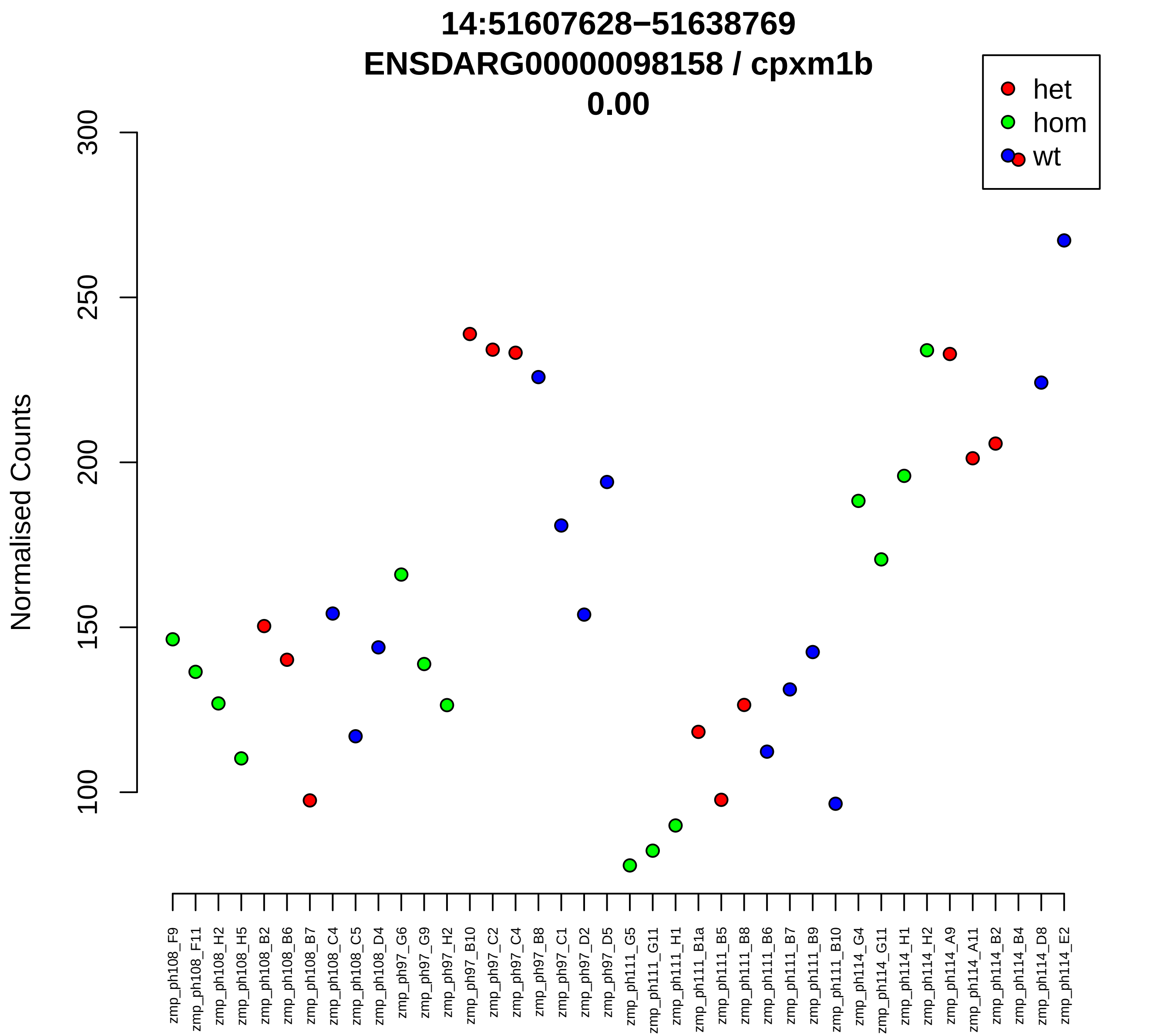

14:51607628−51638769
ENSDARG00000098158 / cpxm1b
0.00
het
hom
wt
Normalised Counts
100
150
200
250
300
zmp_ph108_F9
zmp_ph108_F11
zmp_ph108_H2
zmp_ph108_H5
zmp_ph108_B2
zmp_ph108_B6
zmp_ph108_B7
zmp_ph108_C4
zmp_ph108_C5
zmp_ph108_D4
zmp_ph97_G6
zmp_ph97_G9
zmp_ph97_H2
zmp_ph97_B10
zmp_ph97_C2
zmp_ph97_C4
zmp_ph97_B8
zmp_ph97_C1
zmp_ph97_D2
zmp_ph97_D5
zmp_ph111_G5
zmp_ph111_G11
zmp_ph111_H1
zmp_ph111_B1a
zmp_ph111_B5
zmp_ph111_B8
zmp_ph111_B6
zmp_ph111_B7
zmp_ph111_B9
zmp_ph111_B10
zmp_ph114_G4
zmp_ph114_G11
zmp_ph114_H1
zmp_ph114_H2
zmp_ph114_A9
zmp_ph114_A11
zmp_ph114_B2
zmp_ph114_B4
zmp_ph114_D8
zmp_ph114_E2

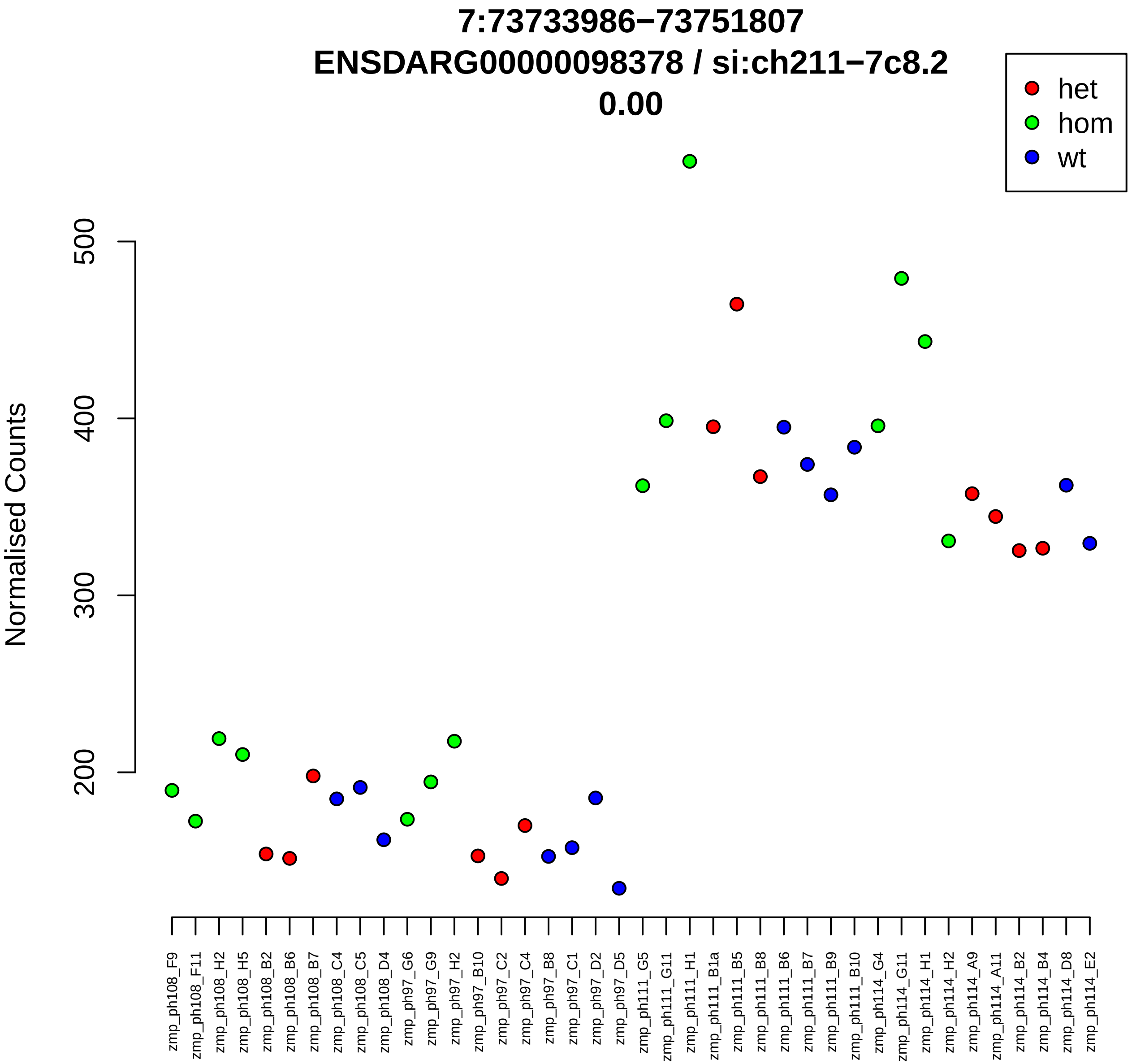
7:73733986−73751807
ENSDARG00000098378 / si:ch211−7c8.2
0.00
het
hom
wt
Normalised Counts
200
300
400
500
zmp_ph108_F9
zmp_ph108_F11
zmp_ph108_H2
zmp_ph108_H5
zmp_ph108_B2
zmp_ph108_B6
zmp_ph108_B7
zmp_ph108_C4
zmp_ph108_C5
zmp_ph108_D4
zmp_ph97_G6
zmp_ph97_G9
zmp_ph97_H2
zmp_ph97_B10
zmp_ph97_C2
zmp_ph97_C4
zmp_ph97_B8
zmp_ph97_C1
zmp_ph97_D2
zmp_ph97_D5
zmp_ph111_G5
zmp_ph111_G11
zmp_ph111_H1
zmp_ph111_B1a
zmp_ph111_B5
zmp_ph111_B8
zmp_ph111_B6
zmp_ph111_B7
zmp_ph111_B9
zmp_ph111_B10
zmp_ph114_G4
zmp_ph114_G11
zmp_ph114_H1
zmp_ph114_H2
zmp_ph114_A9
zmp_ph114_A11
zmp_ph114_B2
zmp_ph114_B4
zmp_ph114_D8
zmp_ph114_E2

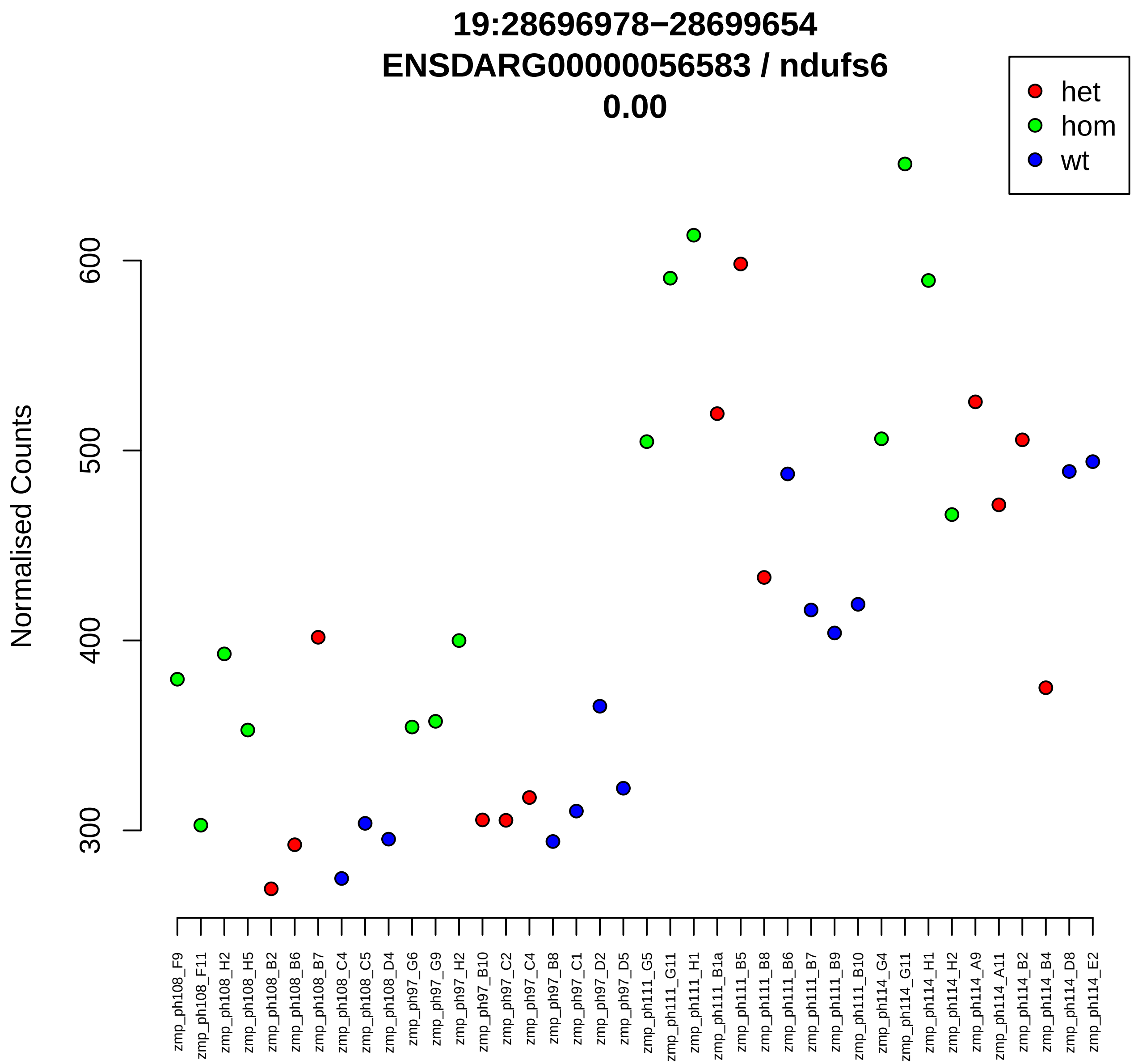

19:28696978−28699654
ENSDARG00000056583 / ndufs6
0.00
het
hom
wt
Normalised Counts
300
400
500
600
zmp_ph108_F9
zmp_ph108_F11
zmp_ph108_H2
zmp_ph108_H5
zmp_ph108_B2
zmp_ph108_B6
zmp_ph108_B7
zmp_ph108_C4
zmp_ph108_C5
zmp_ph108_D4
zmp_ph97_G6
zmp_ph97_G9
zmp_ph97_H2
zmp_ph97_B10
zmp_ph97_C2
zmp_ph97_C4
zmp_ph97_B8
zmp_ph97_C1
zmp_ph97_D2
zmp_ph97_D5
zmp_ph111_G5
zmp_ph111_G11
zmp_ph111_H1
zmp_ph111_B1a
zmp_ph111_B5
zmp_ph111_B8
zmp_ph111_B6
zmp_ph111_B7
zmp_ph111_B9
zmp_ph111_B10
zmp_ph114_G4
zmp_ph114_G11
zmp_ph114_H1
zmp_ph114_H2
zmp_ph114_A9
zmp_ph114_A11
zmp_ph114_B2
zmp_ph114_B4
zmp_ph114_D8
zmp_ph114_E2

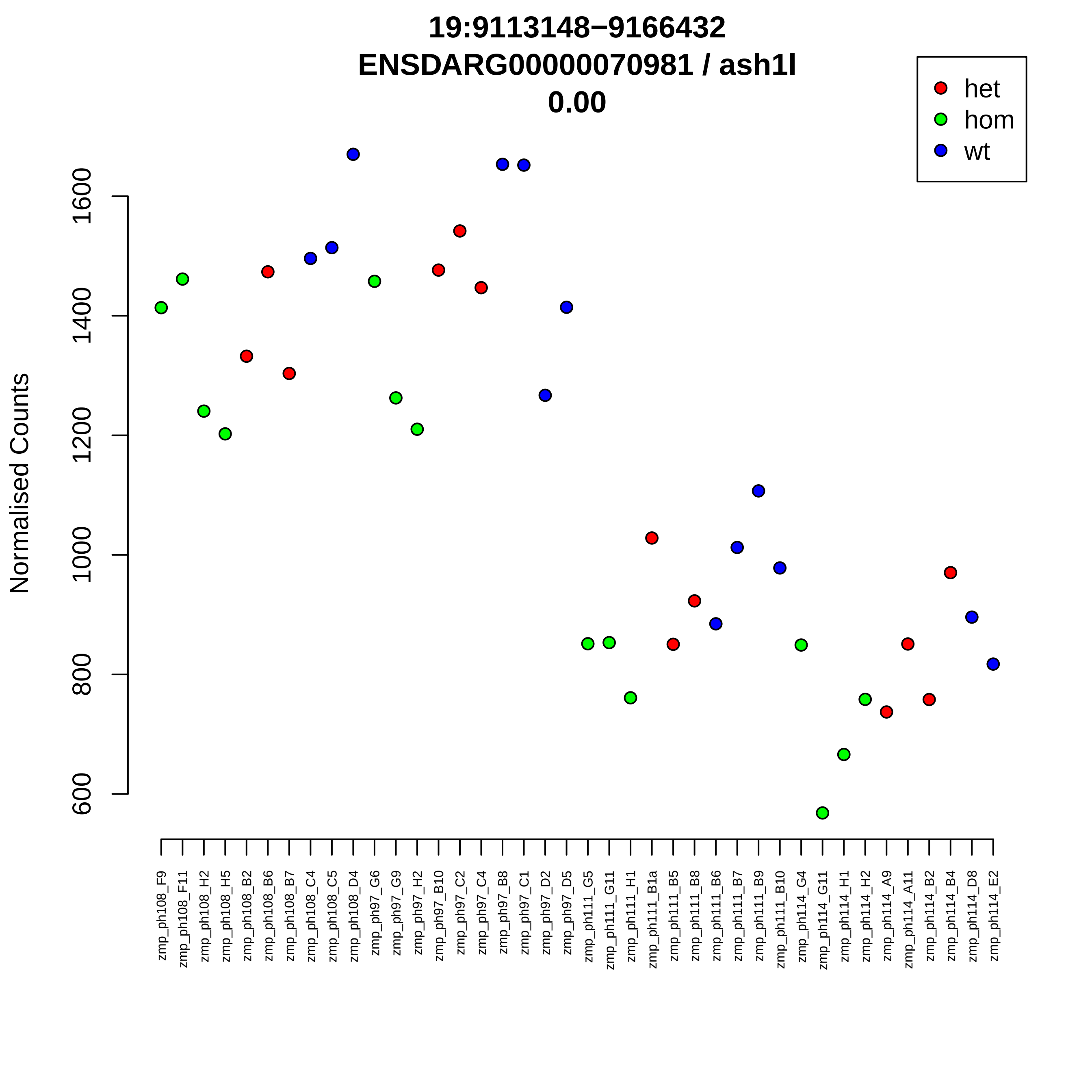
19:9113148−9166432
ENSDARG00000070981 / ash1l
0.00
het
hom
wt
Normalised Counts
600
800
1000
1200
1400
1600
zmp_ph108_F9
zmp_ph108_F11
zmp_ph108_H2
zmp_ph108_H5
zmp_ph108_B2
zmp_ph108_B6
zmp_ph108_B7
zmp_ph108_C4
zmp_ph108_C5
zmp_ph108_D4
zmp_ph97_G6
zmp_ph97_G9
zmp_ph97_H2
zmp_ph97_B10
zmp_ph97_C2
zmp_ph97_C4
zmp_ph97_B8
zmp_ph97_C1
zmp_ph97_D2
zmp_ph97_D5
zmp_ph111_G5
zmp_ph111_G11
zmp_ph111_H1
zmp_ph111_B1a
zmp_ph111_B5
zmp_ph111_B8
zmp_ph111_B6
zmp_ph111_B7
zmp_ph111_B9
zmp_ph111_B10
zmp_ph114_G4
zmp_ph114_G11
zmp_ph114_H1
zmp_ph114_H2
zmp_ph114_A9
zmp_ph114_A11
zmp_ph114_B2
zmp_ph114_B4
zmp_ph114_D8
zmp_ph114_E2

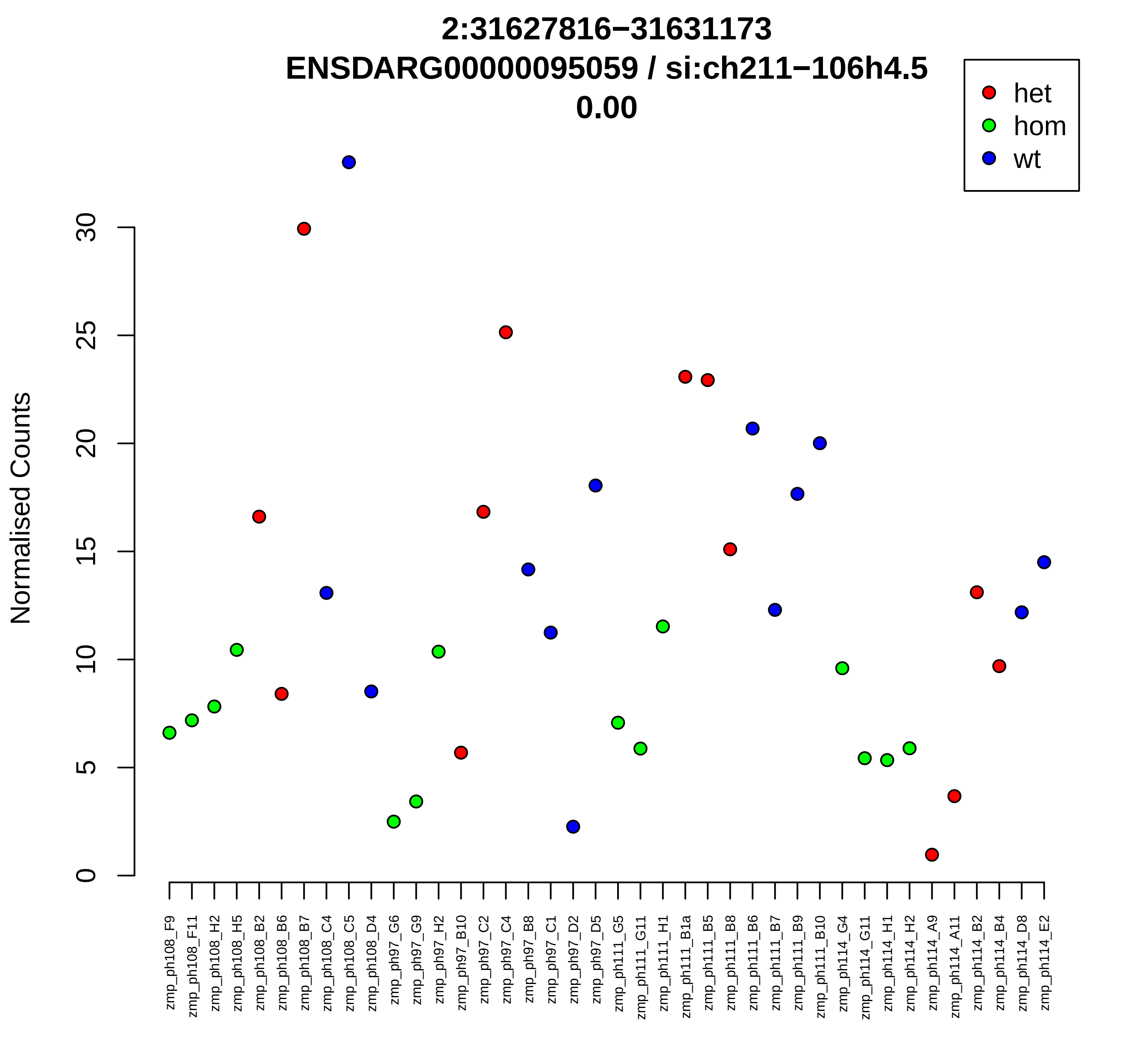
2:31627816−31631173
ENSDARG00000095059 / si:ch211−106h4.5
0.00
het
hom
wt
Normalised Counts
0
5
10
15
20
25
30
zmp_ph108_F9
zmp_ph108_F11
zmp_ph108_H2
zmp_ph108_H5
zmp_ph108_B2
zmp_ph108_B6
zmp_ph108_B7
zmp_ph108_C4
zmp_ph108_C5
zmp_ph108_D4
zmp_ph97_G6
zmp_ph97_G9
zmp_ph97_H2
zmp_ph97_B10
zmp_ph97_C2
zmp_ph97_C4
zmp_ph97_B8
zmp_ph97_C1
zmp_ph97_D2
zmp_ph97_D5
zmp_ph111_G5
zmp_ph111_G11
zmp_ph111_H1
zmp_ph111_B1a
zmp_ph111_B5
zmp_ph111_B8
zmp_ph111_B6
zmp_ph111_B7
zmp_ph111_B9
zmp_ph111_B10
zmp_ph114_G4
zmp_ph114_G11
zmp_ph114_H1
zmp_ph114_H2
zmp_ph114_A9
zmp_ph114_A11
zmp_ph114_B2
zmp_ph114_B4
zmp_ph114_D8
zmp_ph114_E2

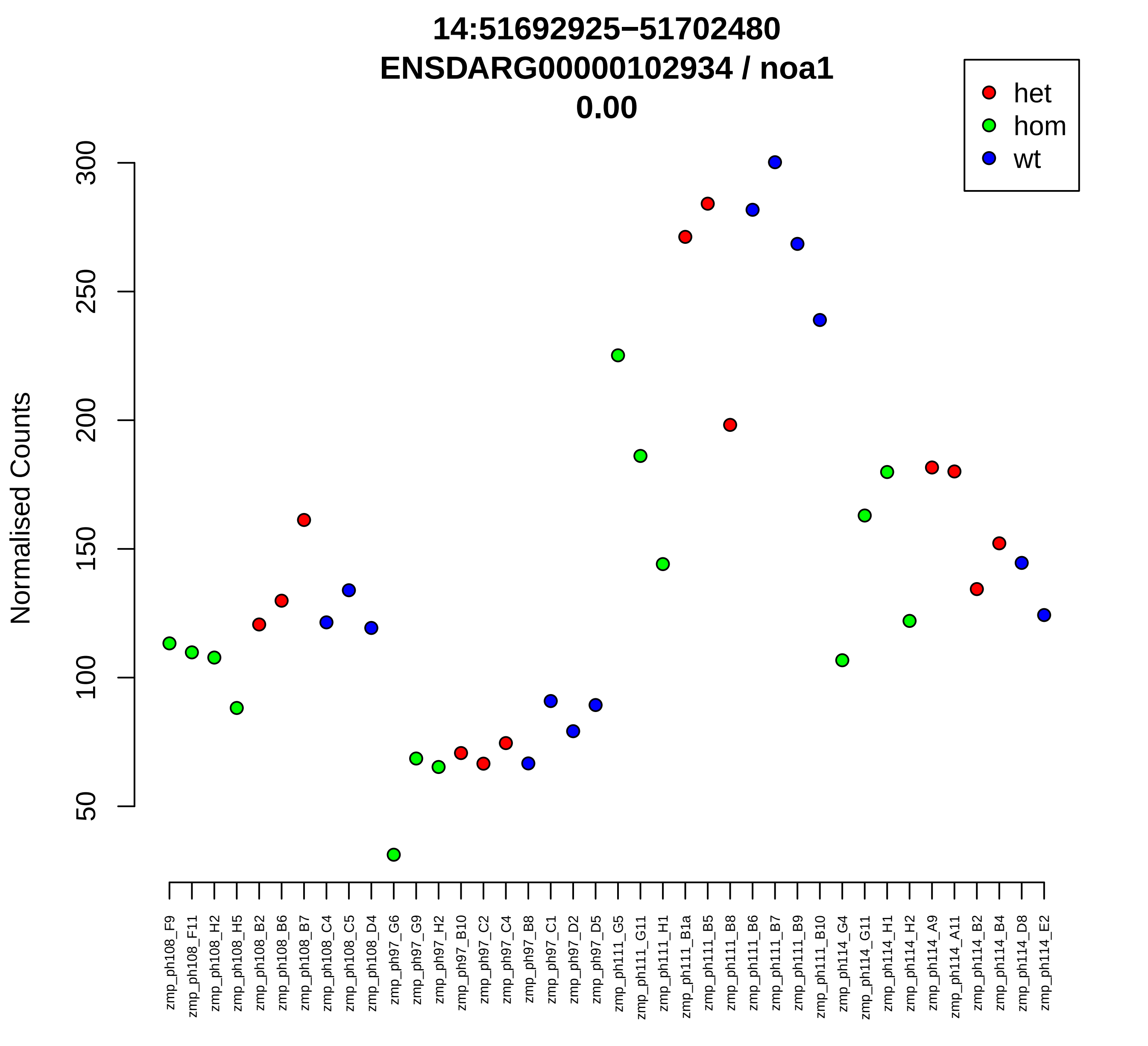
14:51692925−51702480
ENSDARG00000102934 / noa1
0.00
het
hom
wt
Normalised Counts
50
100
150
200
250
300
zmp_ph108_F9
zmp_ph108_F11
zmp_ph108_H2
zmp_ph108_H5
zmp_ph108_B2
zmp_ph108_B6
zmp_ph108_B7
zmp_ph108_C4
zmp_ph108_C5
zmp_ph108_D4
zmp_ph97_G6
zmp_ph97_G9
zmp_ph97_H2
zmp_ph97_B10
zmp_ph97_C2
zmp_ph97_C4
zmp_ph97_B8
zmp_ph97_C1
zmp_ph97_D2
zmp_ph97_D5
zmp_ph111_G5
zmp_ph111_G11
zmp_ph111_H1
zmp_ph111_B1a
zmp_ph111_B5
zmp_ph111_B8
zmp_ph111_B6
zmp_ph111_B7
zmp_ph111_B9
zmp_ph111_B10
zmp_ph114_G4
zmp_ph114_G11
zmp_ph114_H1
zmp_ph114_H2
zmp_ph114_A9
zmp_ph114_A11
zmp_ph114_B2
zmp_ph114_B4
zmp_ph114_D8
zmp_ph114_E2

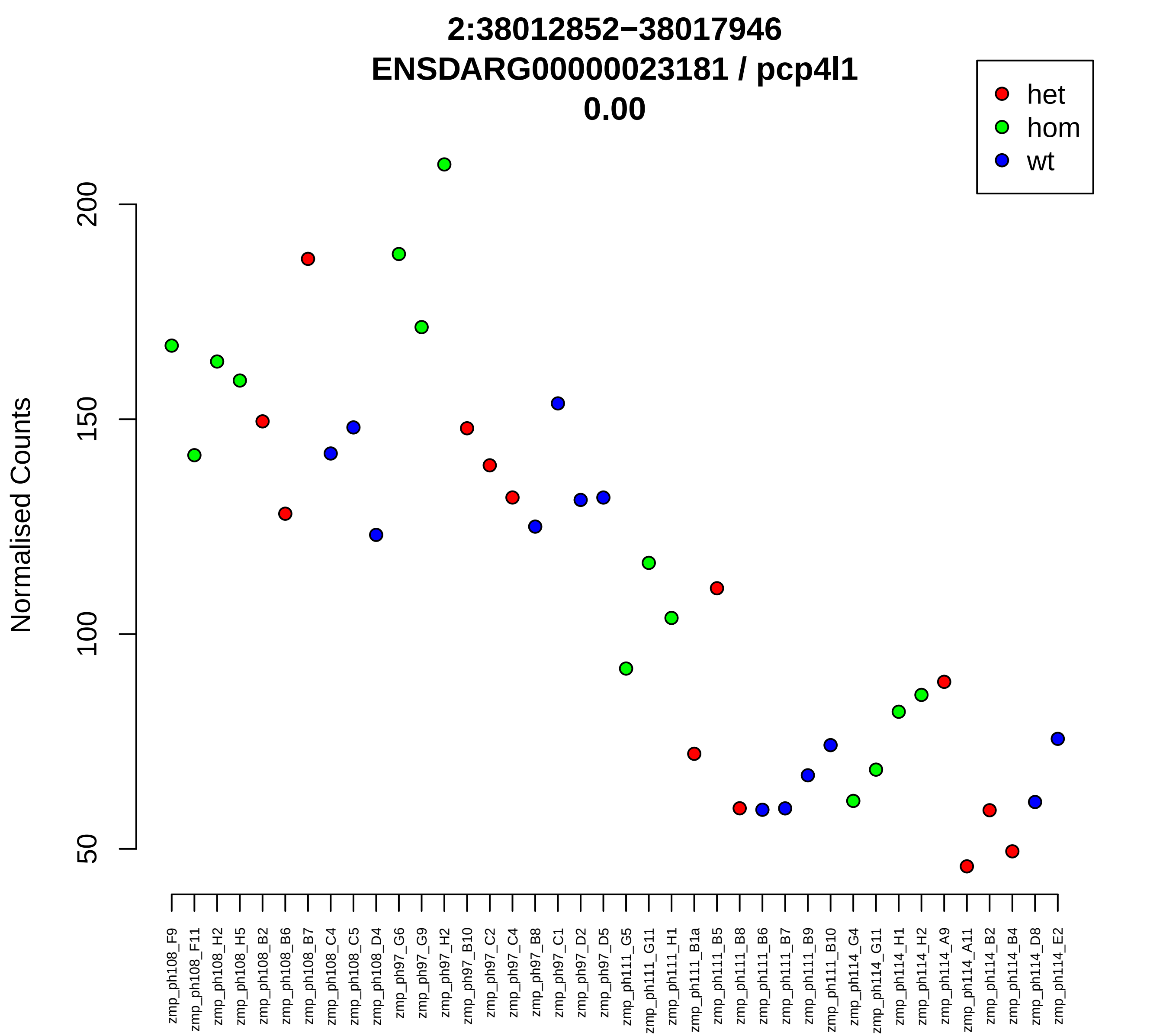
2:38012852−38017946
ENSDARG00000023181 / pcp4l1
0.00
het
hom
wt
Normalised Counts
200
150
100
50
zmp_ph108_F9
zmp_ph108_F11
zmp_ph108_H2
zmp_ph108_H5
zmp_ph108_B2
zmp_ph108_B6
zmp_ph108_B7
zmp_ph108_C4
zmp_ph108_C5
zmp_ph108_D4
zmp_ph97_G6
zmp_ph97_G9
zmp_ph97_H2
zmp_ph97_B10
zmp_ph97_C2
zmp_ph97_C4
zmp_ph97_B8
zmp_ph97_C1
zmp_ph97_D2
zmp_ph97_D5
zmp_ph111_G5
zmp_ph111_G11
zmp_ph111_H1
zmp_ph111_B1a
zmp_ph111_B5
zmp_ph111_B8
zmp_ph111_B6
zmp_ph111_B7
zmp_ph111_B9
zmp_ph111_B10
zmp_ph114_G4
zmp_ph114_G11
zmp_ph114_H1
zmp_ph114_H2
zmp_ph114_A9
zmp_ph114_A11
zmp_ph114_B2
zmp_ph114_B4
zmp_ph114_D8
zmp_ph114_E2

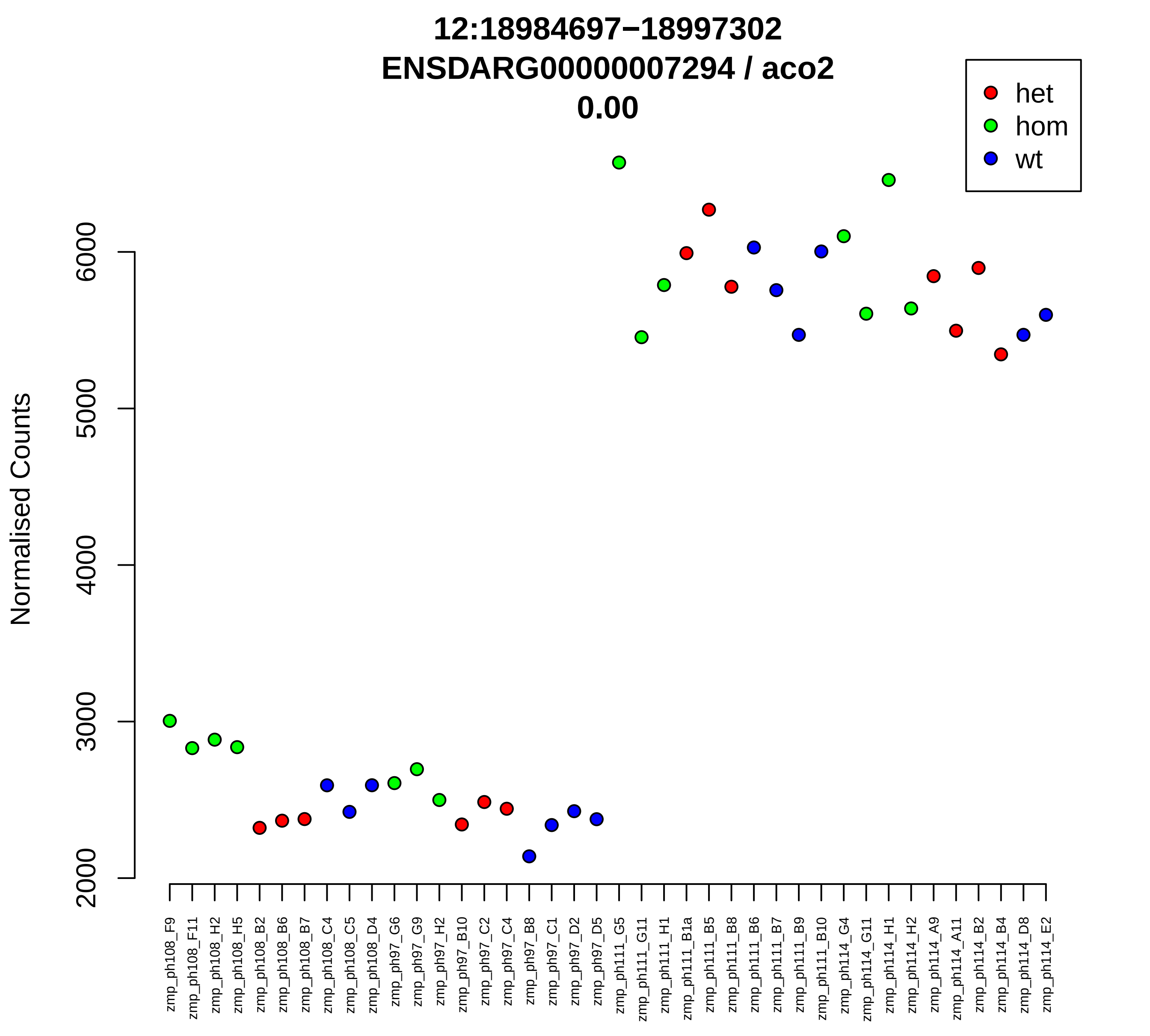

12:18984697−18997302
ENSDARG00000007294 / aco2
0.00
het
hom
wt
Normalised Counts
2000
3000
4000
5000
6000
zmp_ph108_F9
zmp_ph108_F11
zmp_ph108_H2
zmp_ph108_H5
zmp_ph108_B2
zmp_ph108_B6
zmp_ph108_B7
zmp_ph108_C4
zmp_ph108_C5
zmp_ph108_D4
zmp_ph97_G6
zmp_ph97_G9
zmp_ph97_H2
zmp_ph97_B10
zmp_ph97_C2
zmp_ph97_C4
zmp_ph97_B8
zmp_ph97_C1
zmp_ph97_D2
zmp_ph97_D5
zmp_ph111_G5
zmp_ph111_G11
zmp_ph111_H1
zmp_ph111_B1a
zmp_ph111_B5
zmp_ph111_B8
zmp_ph111_B6
zmp_ph111_B7
zmp_ph111_B9
zmp_ph111_B10
zmp_ph114_G4
zmp_ph114_G11
zmp_ph114_H1
zmp_ph114_H2
zmp_ph114_A9
zmp_ph114_A11
zmp_ph114_B2
zmp_ph114_B4
zmp_ph114_D8
zmp_ph114_E2

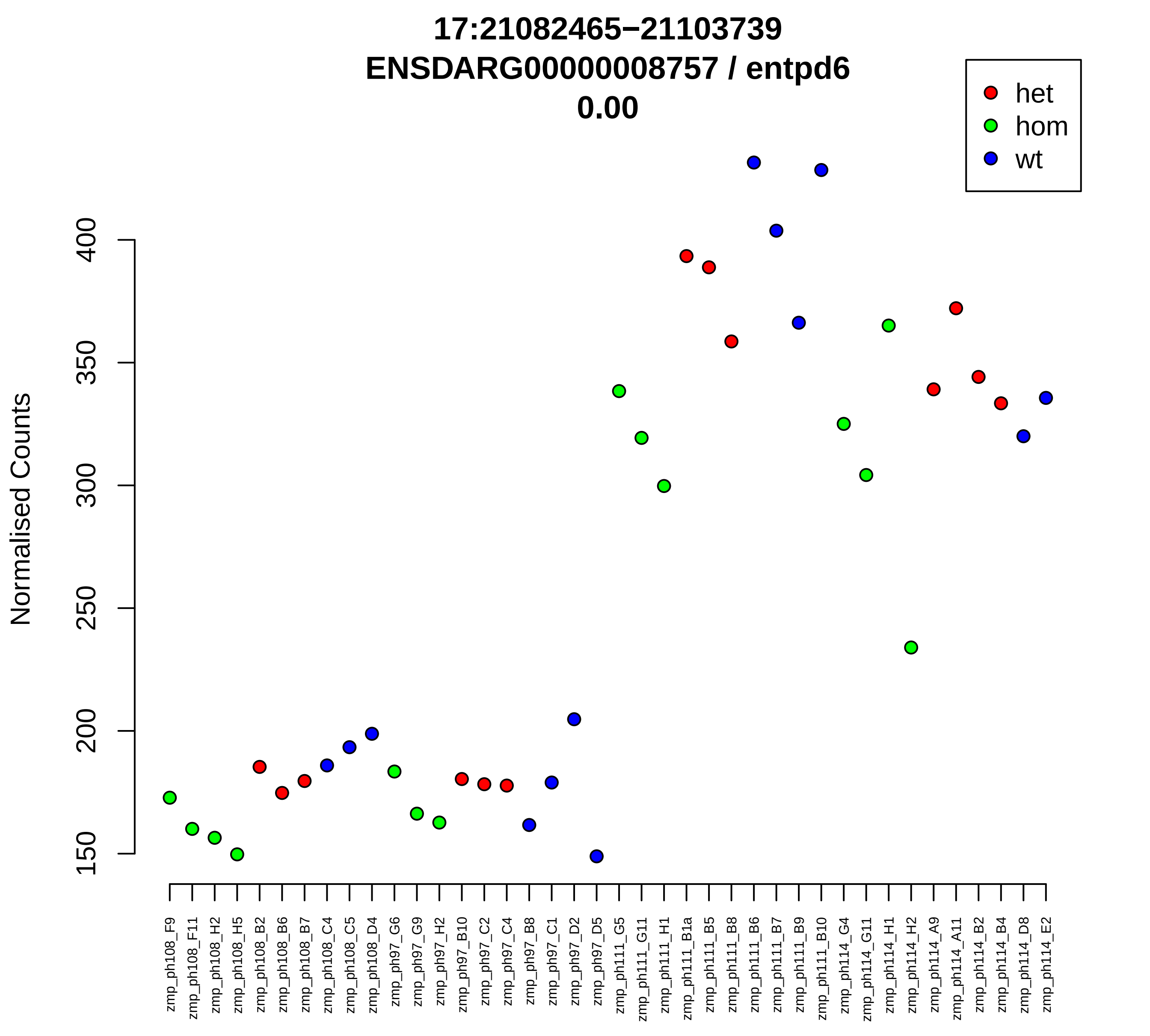

17:21082465−21103739
ENSDARG00000008757 / entpd6
0.00
het
hom
wt
Normalised Counts
150
200
250
300
350
400
zmp_ph108_F9
zmp_ph108_F11
zmp_ph108_H2
zmp_ph108_H5
zmp_ph108_B2
zmp_ph108_B6
zmp_ph108_B7
zmp_ph108_C4
zmp_ph108_C5
zmp_ph108_D4
zmp_ph97_G6
zmp_ph97_G9
zmp_ph97_H2
zmp_ph97_B10
zmp_ph97_C2
zmp_ph97_C4
zmp_ph97_B8
zmp_ph97_C1
zmp_ph97_D2
zmp_ph97_D5
zmp_ph111_G5
zmp_ph111_G11
zmp_ph111_H1
zmp_ph111_B1a
zmp_ph111_B5
zmp_ph111_B8
zmp_ph111_B6
zmp_ph111_B7
zmp_ph111_B9
zmp_ph111_B10
zmp_ph114_G4
zmp_ph114_G11
zmp_ph114_H1
zmp_ph114_H2
zmp_ph114_A9
zmp_ph114_A11
zmp_ph114_B2
zmp_ph114_B4
zmp_ph114_D8
zmp_ph114_E2

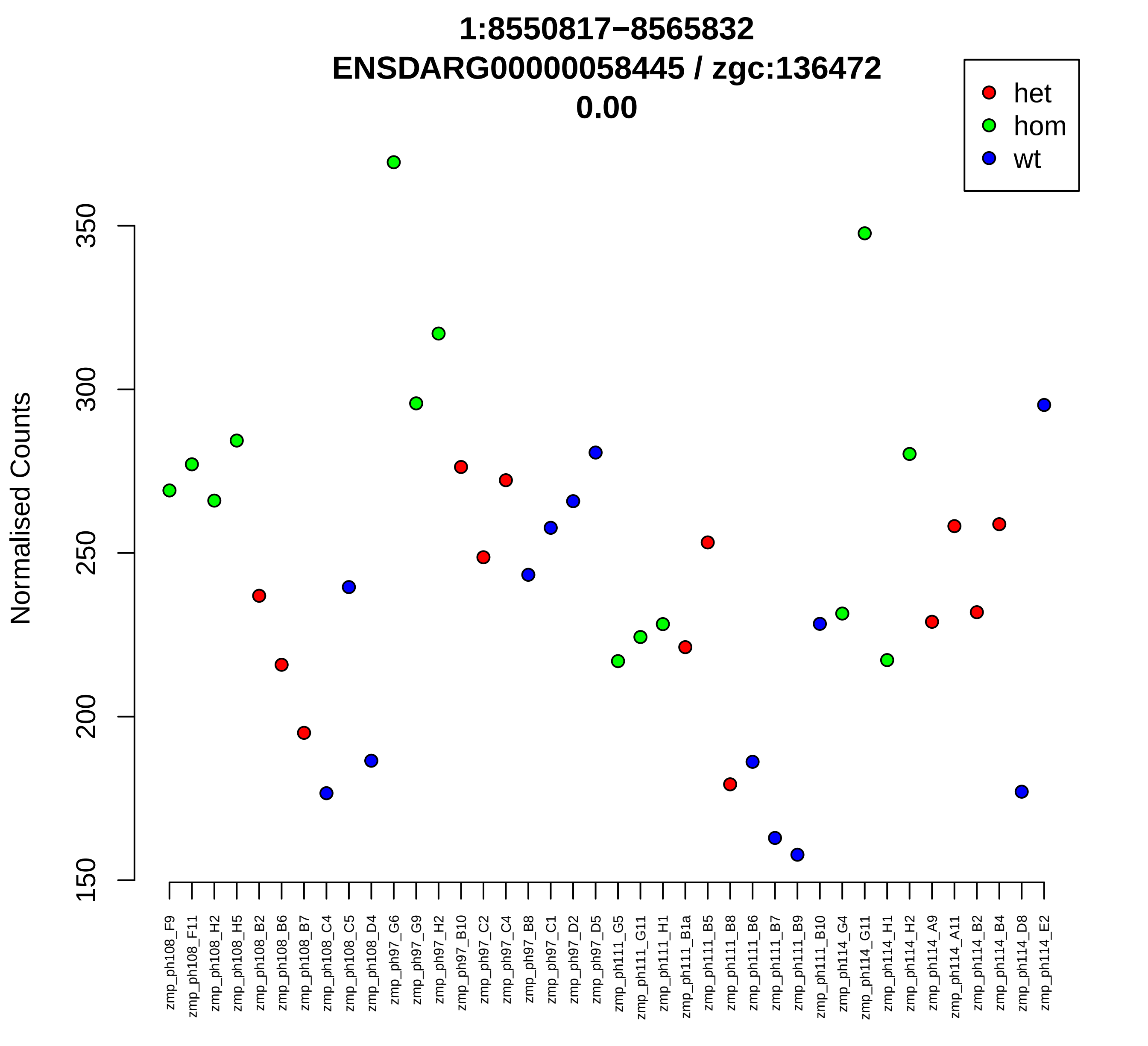

1:8550817−8565832
ENSDARG00000058445 / zgc:136472
0.00
het
hom
wt
Normalised Counts
150
200
250
300
350
zmp_ph108_F9
zmp_ph108_F11
zmp_ph108_H2
zmp_ph108_H5
zmp_ph108_B2
zmp_ph108_B6
zmp_ph108_B7
zmp_ph108_C4
zmp_ph108_C5
zmp_ph108_D4
zmp_ph97_G6
zmp_ph97_G9
zmp_ph97_H2
zmp_ph97_B10
zmp_ph97_C2
zmp_ph97_C4
zmp_ph97_B8
zmp_ph97_C1
zmp_ph97_D2
zmp_ph97_D5
zmp_ph111_G5
zmp_ph111_G11
zmp_ph111_H1
zmp_ph111_B1a
zmp_ph111_B5
zmp_ph111_B8
zmp_ph111_B6
zmp_ph111_B7
zmp_ph111_B9
zmp_ph111_B10
zmp_ph114_G4
zmp_ph114_G11
zmp_ph114_H1
zmp_ph114_H2
zmp_ph114_A9
zmp_ph114_A11
zmp_ph114_B2
zmp_ph114_B4
zmp_ph114_D8
zmp_ph114_E2

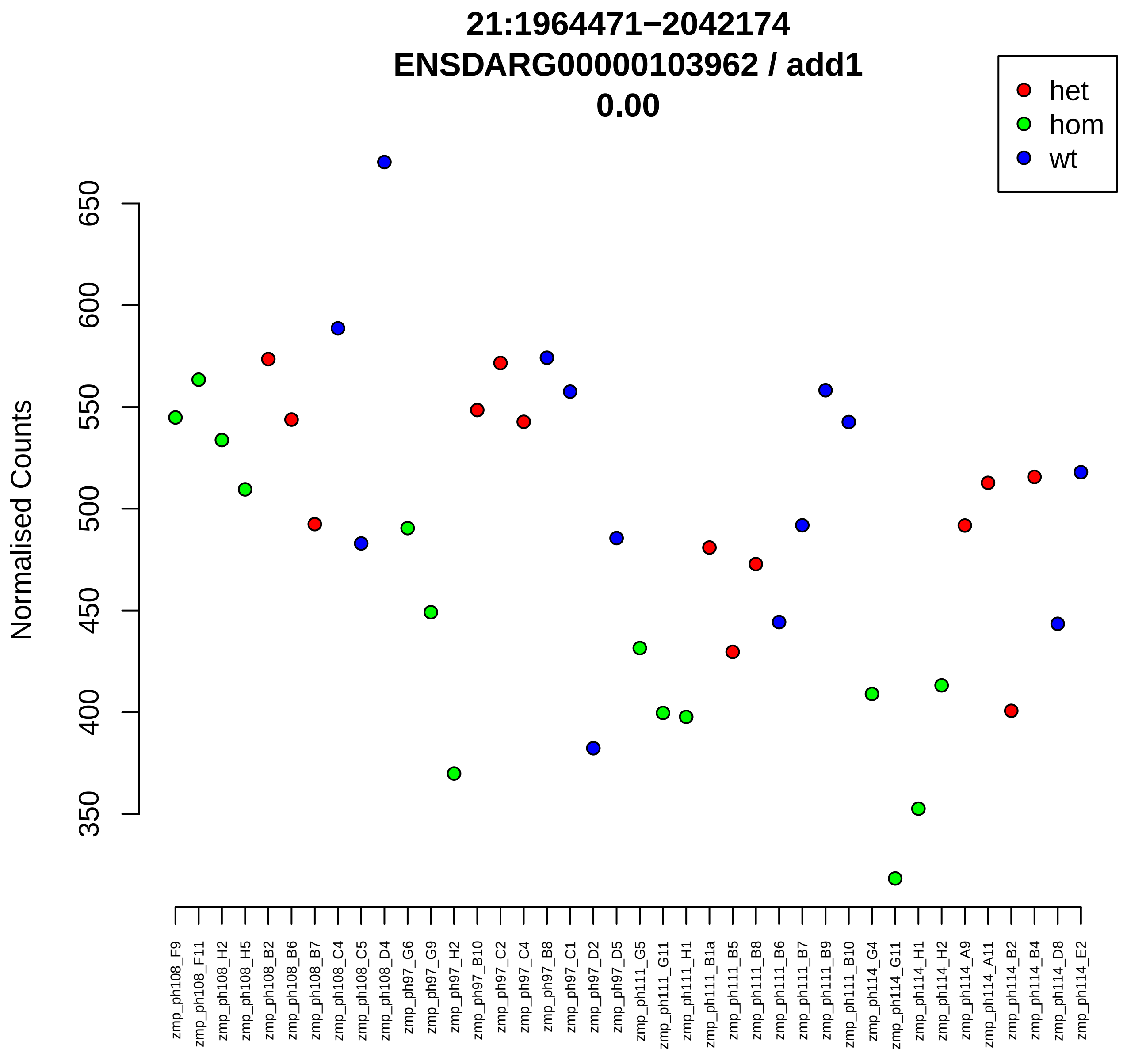

21:1964471−2042174
ENSDARG00000103962 / add1
0.00
het
hom
wt
Normalised Counts
350
400
450
500
550
600
650
zmp_ph108_F9
zmp_ph108_F11
zmp_ph108_H2
zmp_ph108_H5
zmp_ph108_B2
zmp_ph108_B6
zmp_ph108_B7
zmp_ph108_C4
zmp_ph108_C5
zmp_ph108_D4
zmp_ph97_G6
zmp_ph97_G9
zmp_ph97_H2
zmp_ph97_B10
zmp_ph97_C2
zmp_ph97_C4
zmp_ph97_B8
zmp_ph97_C1
zmp_ph97_D2
zmp_ph97_D5
zmp_ph111_G5
zmp_ph111_G11
zmp_ph111_H1
zmp_ph111_B1a
zmp_ph111_B5
zmp_ph111_B8
zmp_ph111_B6
zmp_ph111_B7
zmp_ph111_B9
zmp_ph111_B10
zmp_ph114_G4
zmp_ph114_G11
zmp_ph114_H1
zmp_ph114_H2
zmp_ph114_A9
zmp_ph114_A11
zmp_ph114_B2
zmp_ph114_B4
zmp_ph114_D8
zmp_ph114_E2

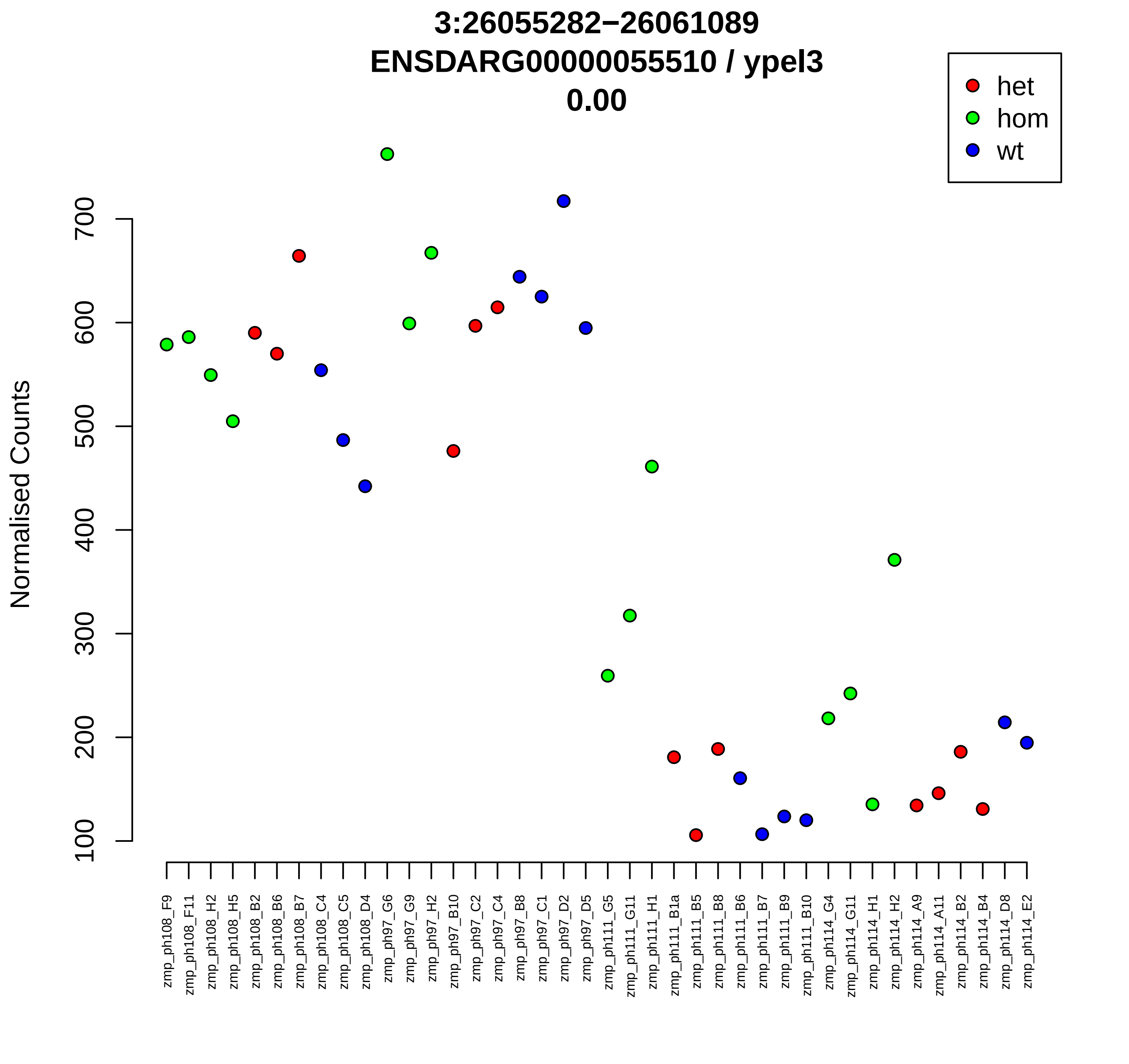
3:26055282−26061089
ENSDARG00000055510 / ypel3
0.00
het
hom
wt
Normalised Counts
100
200
300
400
500
600
700
zmp_ph108_F9
zmp_ph108_F11
zmp_ph108_H2
zmp_ph108_H5
zmp_ph108_B2
zmp_ph108_B6
zmp_ph108_B7
zmp_ph108_C4
zmp_ph108_C5
zmp_ph108_D4
zmp_ph97_G6
zmp_ph97_G9
zmp_ph97_H2
zmp_ph97_B10
zmp_ph97_C2
zmp_ph97_C4
zmp_ph97_B8
zmp_ph97_C1
zmp_ph97_D2
zmp_ph97_D5
zmp_ph111_G5
zmp_ph111_G11
zmp_ph111_H1
zmp_ph111_B1a
zmp_ph111_B5
zmp_ph111_B8
zmp_ph111_B6
zmp_ph111_B7
zmp_ph111_B9
zmp_ph111_B10
zmp_ph114_G4
zmp_ph114_G11
zmp_ph114_H1
zmp_ph114_H2
zmp_ph114_A9
zmp_ph114_A11
zmp_ph114_B2
zmp_ph114_B4
zmp_ph114_D8
zmp_ph114_E2

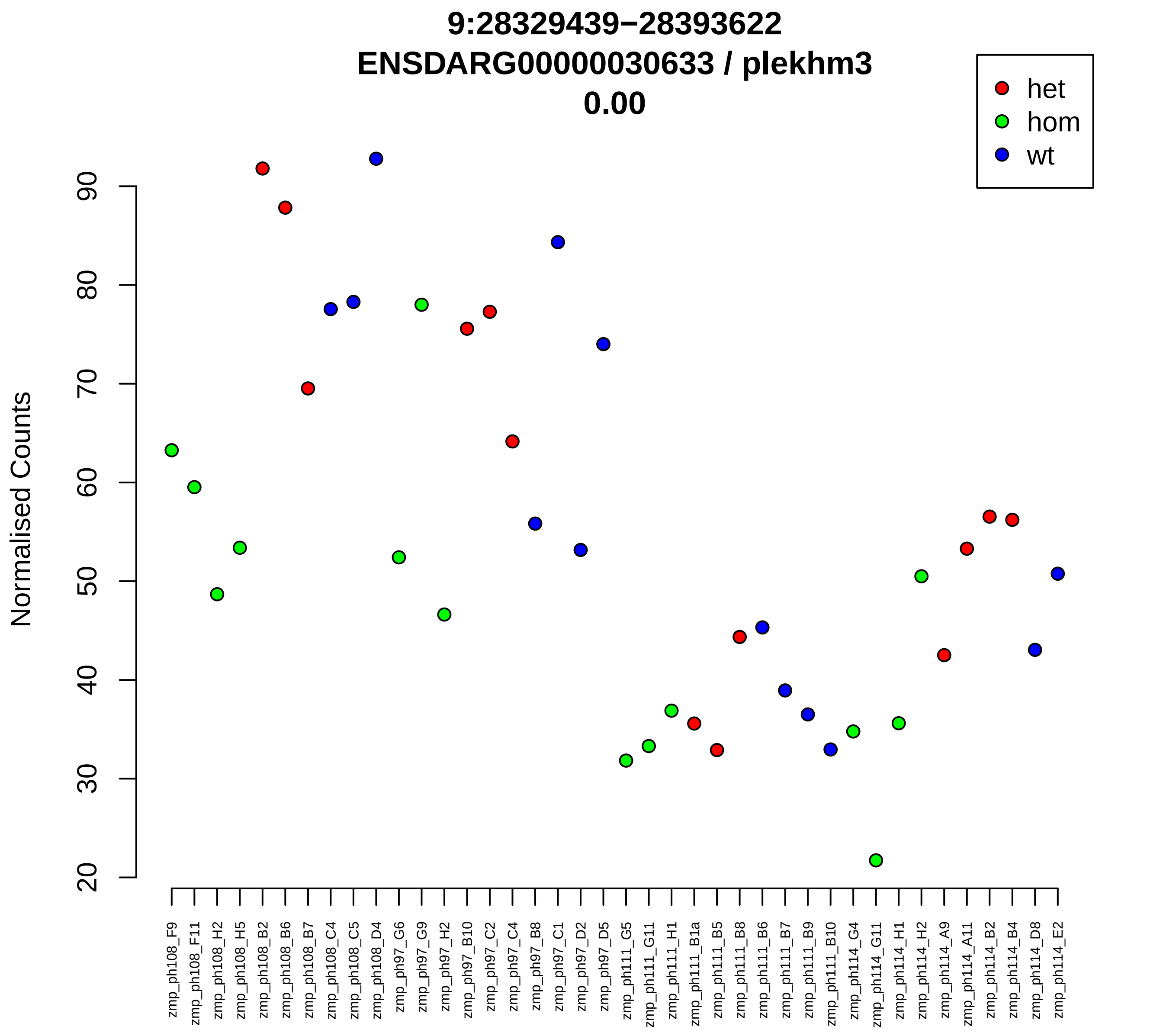

9:28329439−28393622
ENSDARG00000030633 / plekhm3
0.00
het
hom
wt
Normalised Counts
20
30
40
50
60
70
80
90
zmp_ph108_F9
zmp_ph108_F11
zmp_ph108_H2
zmp_ph108_H5
zmp_ph108_B2
zmp_ph108_B6
zmp_ph108_B7
zmp_ph108_C4
zmp_ph108_C5
zmp_ph108_D4
zmp_ph97_G6
zmp_ph97_G9
zmp_ph97_H2
zmp_ph97_B10
zmp_ph97_C2
zmp_ph97_C4
zmp_ph97_B8
zmp_ph97_C1
zmp_ph97_D2
zmp_ph97_D5
zmp_ph111_G5
zmp_ph111_G11
zmp_ph111_H1
zmp_ph111_B1a
zmp_ph111_B5
zmp_ph111_B8
zmp_ph111_B6
zmp_ph111_B7
zmp_ph111_B9
zmp_ph111_B10
zmp_ph114_G4
zmp_ph114_G11
zmp_ph114_H1
zmp_ph114_H2
zmp_ph114_A9
zmp_ph114_A11
zmp_ph114_B2
zmp_ph114_B4
zmp_ph114_D8
zmp_ph114_E2

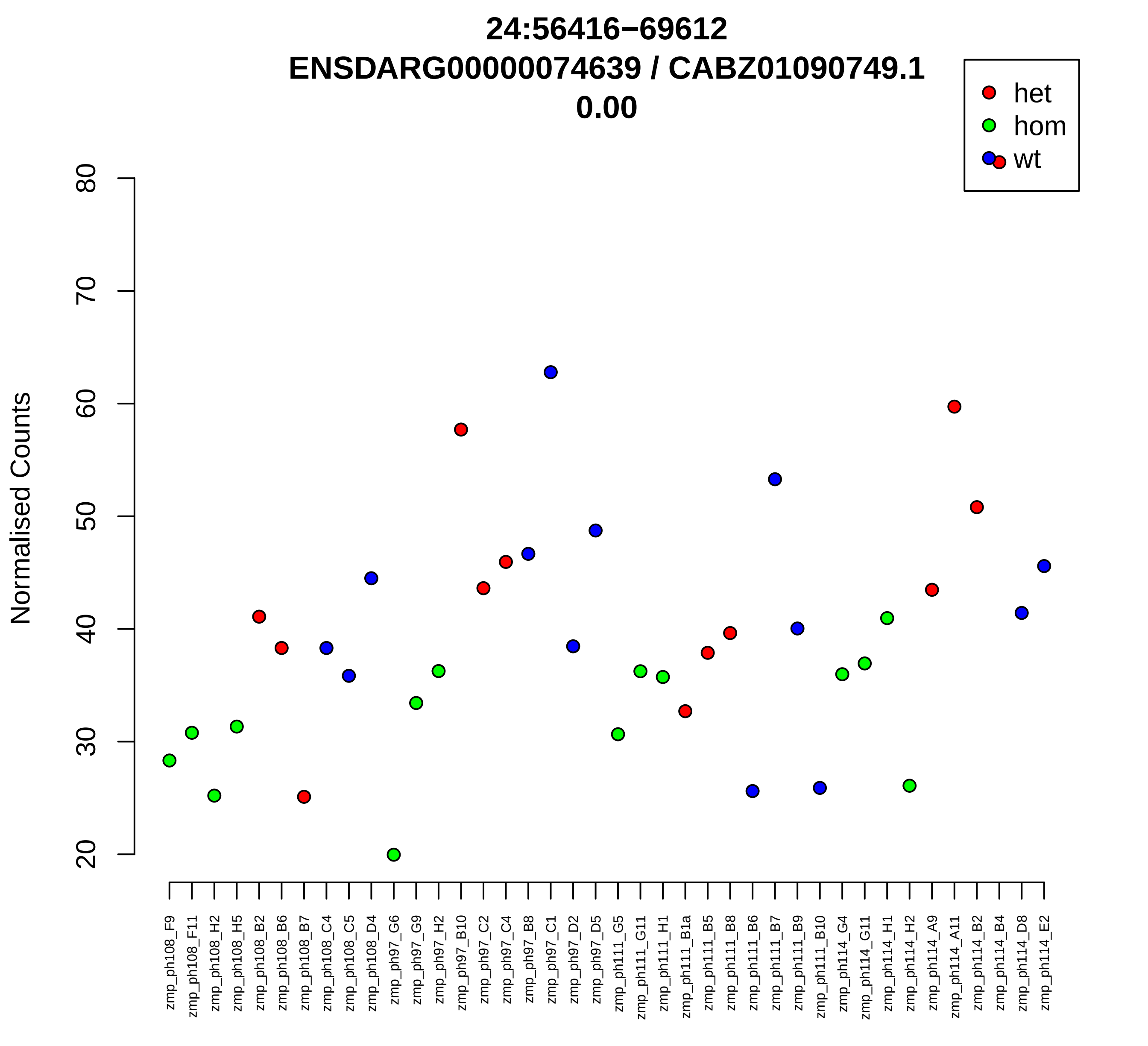
24:56416−69612
ENSDARG00000074639 / CABZ01090749.1
0.00
het
hom
wt
Normalised Counts
20
30
40
50
60
70
80
zmp_ph108_F9
zmp_ph108_F11
zmp_ph108_H2
zmp_ph108_H5
zmp_ph108_B2
zmp_ph108_B6
zmp_ph108_B7
zmp_ph108_C4
zmp_ph108_C5
zmp_ph108_D4
zmp_ph97_G6
zmp_ph97_G9
zmp_ph97_H2
zmp_ph97_B10
zmp_ph97_C2
zmp_ph97_C4
zmp_ph97_B8
zmp_ph97_C1
zmp_ph97_D2
zmp_ph97_D5
zmp_ph111_G5
zmp_ph111_G11
zmp_ph111_H1
zmp_ph111_B1a
zmp_ph111_B5
zmp_ph111_B8
zmp_ph111_B6
zmp_ph111_B7
zmp_ph111_B9
zmp_ph111_B10
zmp_ph114_G4
zmp_ph114_G11
zmp_ph114_H1
zmp_ph114_H2
zmp_ph114_A9
zmp_ph114_A11
zmp_ph114_B2
zmp_ph114_B4
zmp_ph114_D8
zmp_ph114_E2

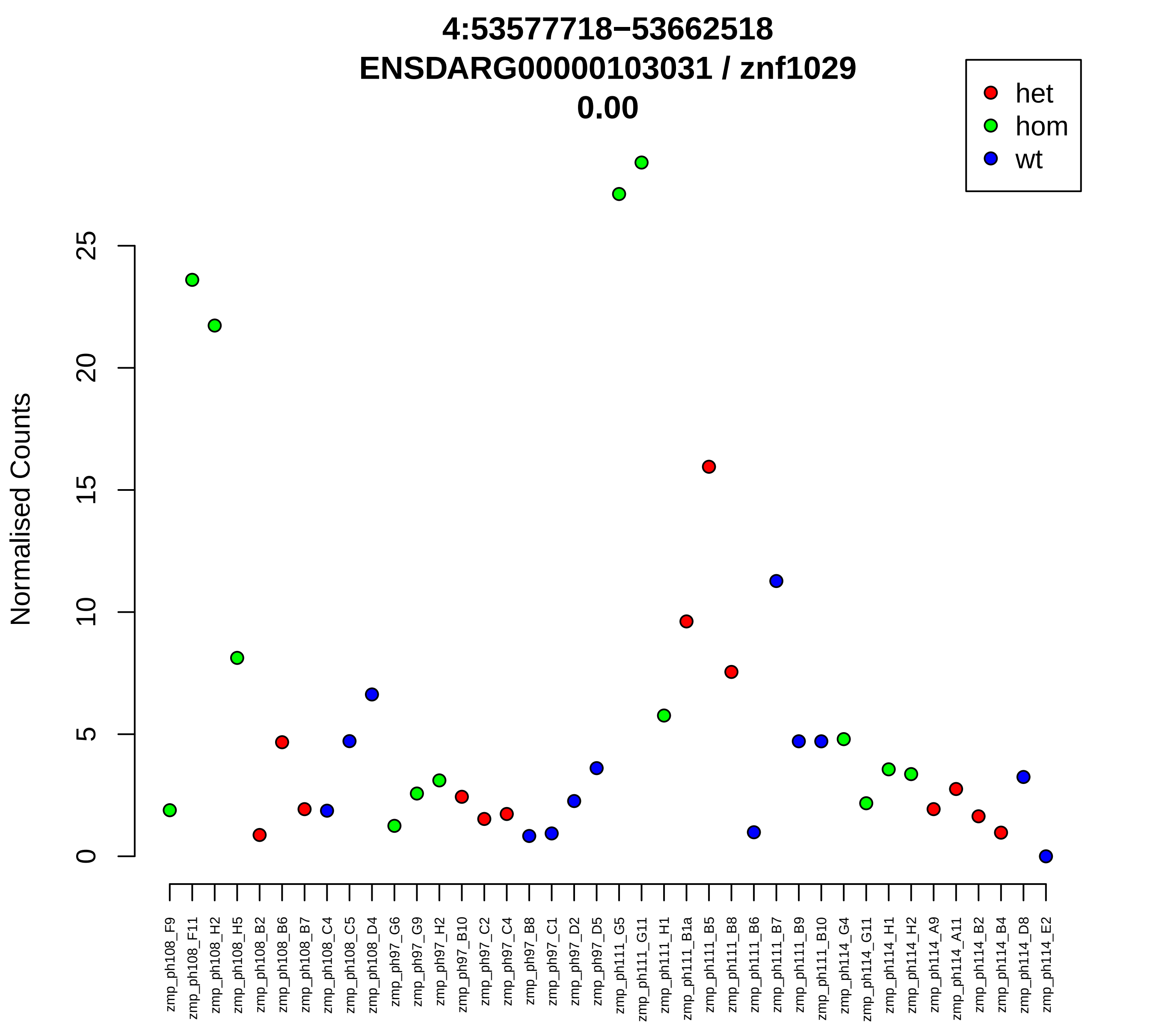
4:53577718−53662518
ENSDARG00000103031 / znf1029
0.00
het
hom
wt
Normalised Counts
0
5
10
15
20
25
zmp_ph108_F9
zmp_ph108_F11
zmp_ph108_H2
zmp_ph108_H5
zmp_ph108_B2
zmp_ph108_B6
zmp_ph108_B7
zmp_ph108_C4
zmp_ph108_C5
zmp_ph108_D4
zmp_ph97_G6
zmp_ph97_G9
zmp_ph97_H2
zmp_ph97_B10
zmp_ph97_C2
zmp_ph97_C4
zmp_ph97_B8
zmp_ph97_C1
zmp_ph97_D2
zmp_ph97_D5
zmp_ph111_G5
zmp_ph111_G11
zmp_ph111_H1
zmp_ph111_B1a
zmp_ph111_B5
zmp_ph111_B8
zmp_ph111_B6
zmp_ph111_B7
zmp_ph111_B9
zmp_ph111_B10
zmp_ph114_G4
zmp_ph114_G11
zmp_ph114_H1
zmp_ph114_H2
zmp_ph114_A9
zmp_ph114_A11
zmp_ph114_B2
zmp_ph114_B4
zmp_ph114_D8
zmp_ph114_E2

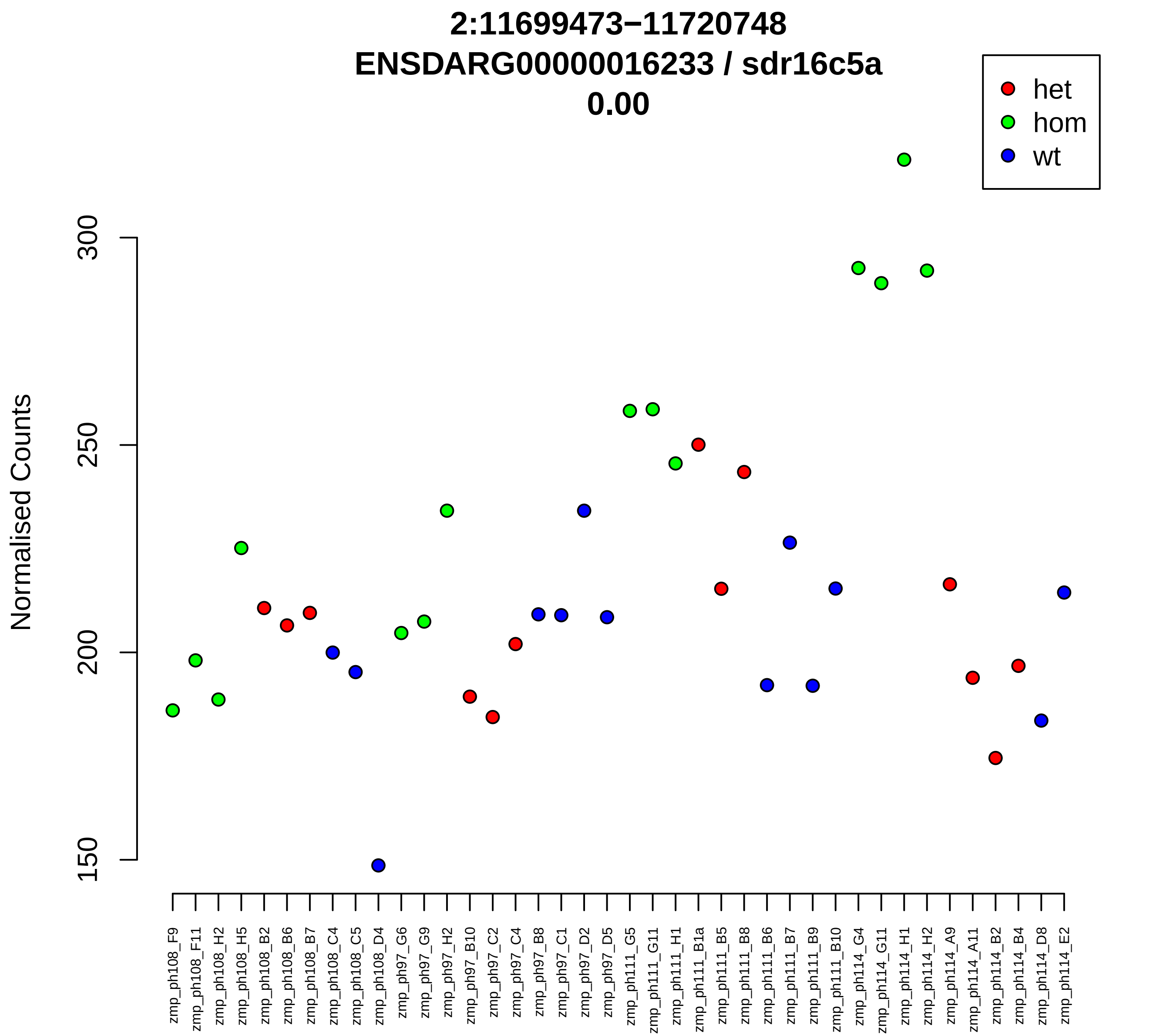

2:11699473−11720748
ENSDARG00000016233 / sdr16c5a
0.00
het
hom
wt
Normalised Counts
150
200
250
300
zmp_ph108_F9
zmp_ph108_F11
zmp_ph108_H2
zmp_ph108_H5
zmp_ph108_B2
zmp_ph108_B6
zmp_ph108_B7
zmp_ph108_C4
zmp_ph108_C5
zmp_ph108_D4
zmp_ph97_G6
zmp_ph97_G9
zmp_ph97_H2
zmp_ph97_B10
zmp_ph97_C2
zmp_ph97_C4
zmp_ph97_B8
zmp_ph97_C1
zmp_ph97_D2
zmp_ph97_D5
zmp_ph111_G5
zmp_ph111_G11
zmp_ph111_H1
zmp_ph111_B1a
zmp_ph111_B5
zmp_ph111_B8
zmp_ph111_B6
zmp_ph111_B7
zmp_ph111_B9
zmp_ph111_B10
zmp_ph114_G4
zmp_ph114_G11
zmp_ph114_H1
zmp_ph114_H2
zmp_ph114_A9
zmp_ph114_A11
zmp_ph114_B2
zmp_ph114_B4
zmp_ph114_D8
zmp_ph114_E2

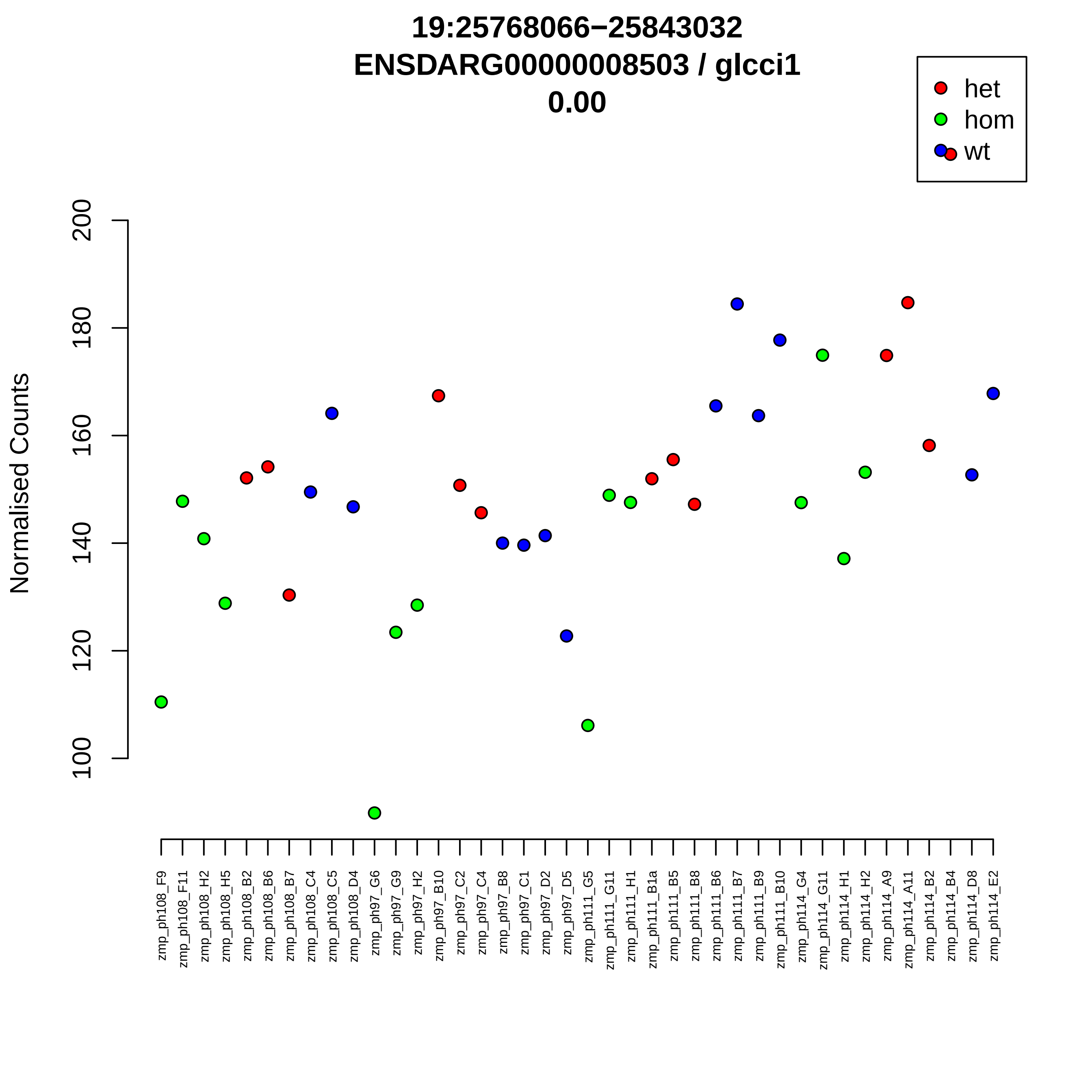

19:25768066−25843032
ENSDARG00000008503 / glcci1
0.00
het
hom
wt
Normalised Counts
100
120
140
160
180
200
zmp_ph108_F9
zmp_ph108_F11
zmp_ph108_H2
zmp_ph108_H5
zmp_ph108_B2
zmp_ph108_B6
zmp_ph108_B7
zmp_ph108_C4
zmp_ph108_C5
zmp_ph108_D4
zmp_ph97_G6
zmp_ph97_G9
zmp_ph97_H2
zmp_ph97_B10
zmp_ph97_C2
zmp_ph97_C4
zmp_ph97_B8
zmp_ph97_C1
zmp_ph97_D2
zmp_ph97_D5
zmp_ph111_G5
zmp_ph111_G11
zmp_ph111_H1
zmp_ph111_B1a
zmp_ph111_B5
zmp_ph111_B8
zmp_ph111_B6
zmp_ph111_B7
zmp_ph111_B9
zmp_ph111_B10
zmp_ph114_G4
zmp_ph114_G11
zmp_ph114_H1
zmp_ph114_H2
zmp_ph114_A9
zmp_ph114_A11
zmp_ph114_B2
zmp_ph114_B4
zmp_ph114_D8
zmp_ph114_E2

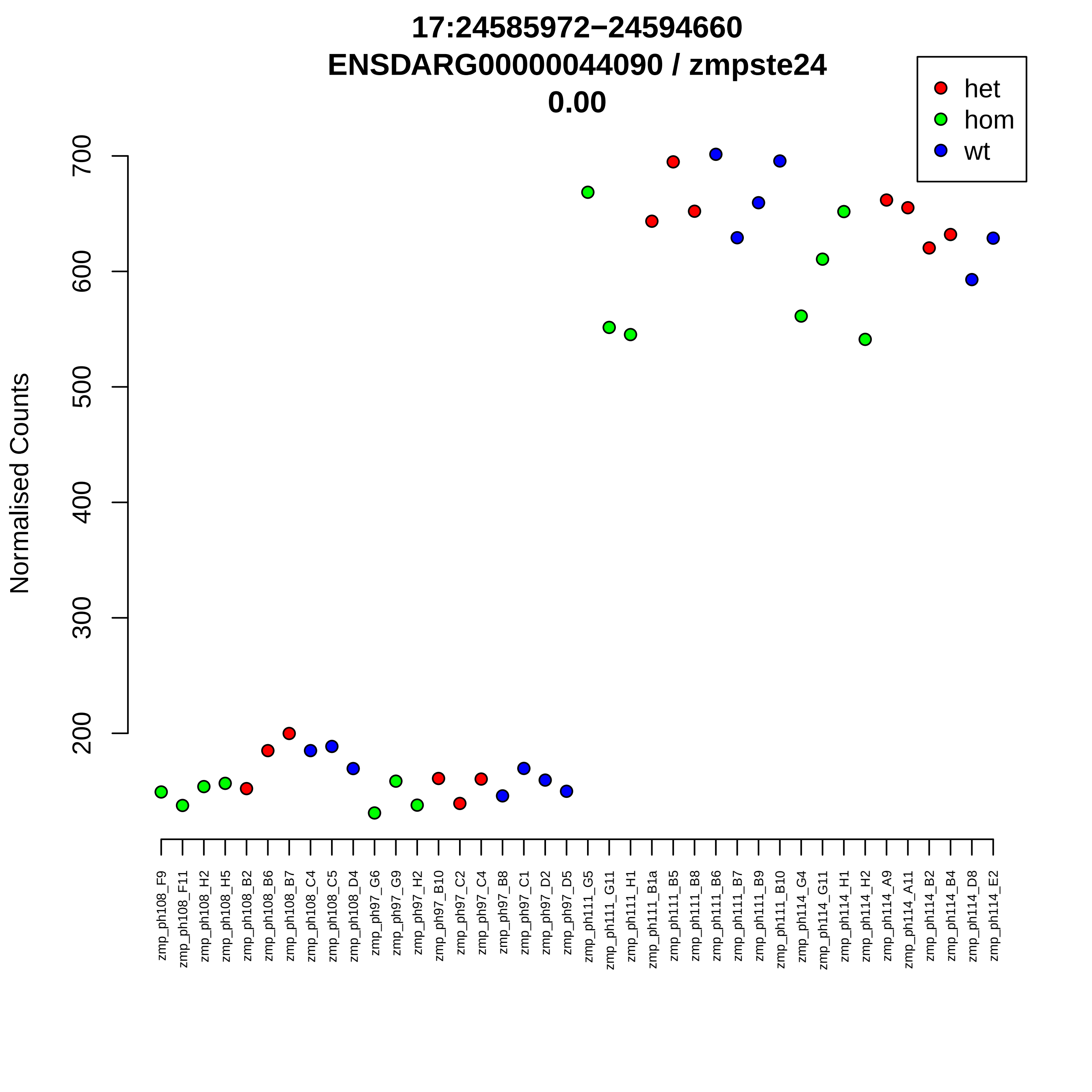
17:24585972−24594660
ENSDARG00000044090 / zmpste24
0.00
het
hom
wt
Normalised Counts
200
300
400
500
600
700
zmp_ph108_F9
zmp_ph108_F11
zmp_ph108_H2
zmp_ph108_H5
zmp_ph108_B2
zmp_ph108_B6
zmp_ph108_B7
zmp_ph108_C4
zmp_ph108_C5
zmp_ph108_D4
zmp_ph97_G6
zmp_ph97_G9
zmp_ph97_H2
zmp_ph97_B10
zmp_ph97_C2
zmp_ph97_C4
zmp_ph97_B8
zmp_ph97_C1
zmp_ph97_D2
zmp_ph97_D5
zmp_ph111_G5
zmp_ph111_G11
zmp_ph111_H1
zmp_ph111_B1a
zmp_ph111_B5
zmp_ph111_B8
zmp_ph111_B6
zmp_ph111_B7
zmp_ph111_B9
zmp_ph111_B10
zmp_ph114_G4
zmp_ph114_G11
zmp_ph114_H1
zmp_ph114_H2
zmp_ph114_A9
zmp_ph114_A11
zmp_ph114_B2
zmp_ph114_B4
zmp_ph114_D8
zmp_ph114_E2

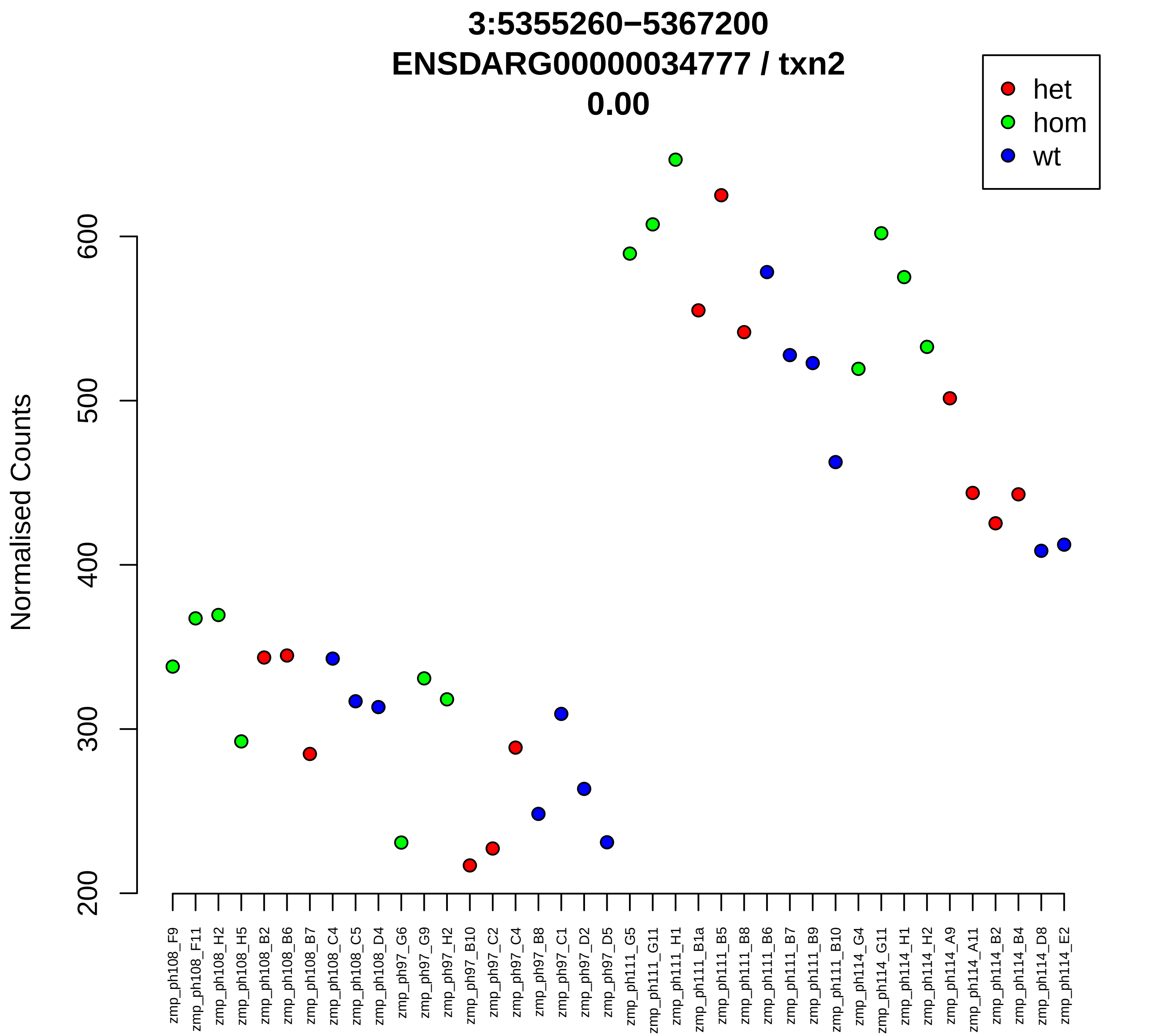
3:5355260−5367200
ENSDARG00000034777 / txn2
0.00
het
hom
wt
Normalised Counts
200
300
400
500
600
zmp_ph108_F9
zmp_ph108_F11
zmp_ph108_H2
zmp_ph108_H5
zmp_ph108_B2
zmp_ph108_B6
zmp_ph108_B7
zmp_ph108_C4
zmp_ph108_C5
zmp_ph108_D4
zmp_ph97_G6
zmp_ph97_G9
zmp_ph97_H2
zmp_ph97_B10
zmp_ph97_C2
zmp_ph97_C4
zmp_ph97_B8
zmp_ph97_C1
zmp_ph97_D2
zmp_ph97_D5
zmp_ph111_G5
zmp_ph111_G11
zmp_ph111_H1
zmp_ph111_B1a
zmp_ph111_B5
zmp_ph111_B8
zmp_ph111_B6
zmp_ph111_B7
zmp_ph111_B9
zmp_ph111_B10
zmp_ph114_G4
zmp_ph114_G11
zmp_ph114_H1
zmp_ph114_H2
zmp_ph114_A9
zmp_ph114_A11
zmp_ph114_B2
zmp_ph114_B4
zmp_ph114_D8
zmp_ph114_E2

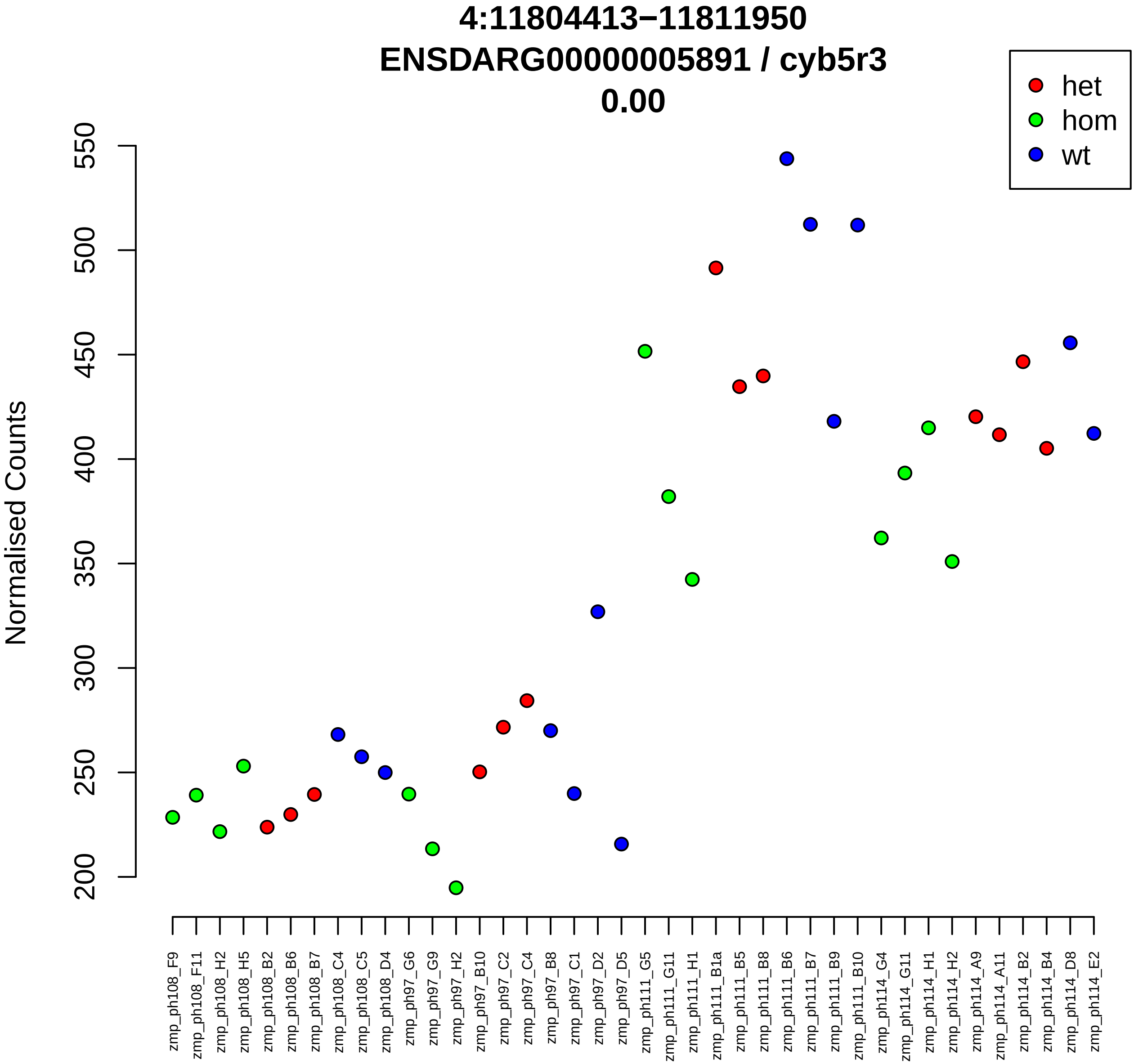

4:11804413−11811950
ENSDARG00000005891 / cyb5r3
0.00
het
hom
wt
Normalised Counts
200
250
300
350
400
450
500
550
zmp_ph108_F9
zmp_ph108_F11
zmp_ph108_H2
zmp_ph108_H5
zmp_ph108_B2
zmp_ph108_B6
zmp_ph108_B7
zmp_ph108_C4
zmp_ph108_C5
zmp_ph108_D4
zmp_ph97_G6
zmp_ph97_G9
zmp_ph97_H2
zmp_ph97_B10
zmp_ph97_C2
zmp_ph97_C4
zmp_ph97_B8
zmp_ph97_C1
zmp_ph97_D2
zmp_ph97_D5
zmp_ph111_G5
zmp_ph111_G11
zmp_ph111_H1
zmp_ph111_B1a
zmp_ph111_B5
zmp_ph111_B8
zmp_ph111_B6
zmp_ph111_B7
zmp_ph111_B9
zmp_ph111_B10
zmp_ph114_G4
zmp_ph114_G11
zmp_ph114_H1
zmp_ph114_H2
zmp_ph114_A9
zmp_ph114_A11
zmp_ph114_B2
zmp_ph114_B4
zmp_ph114_D8
zmp_ph114_E2

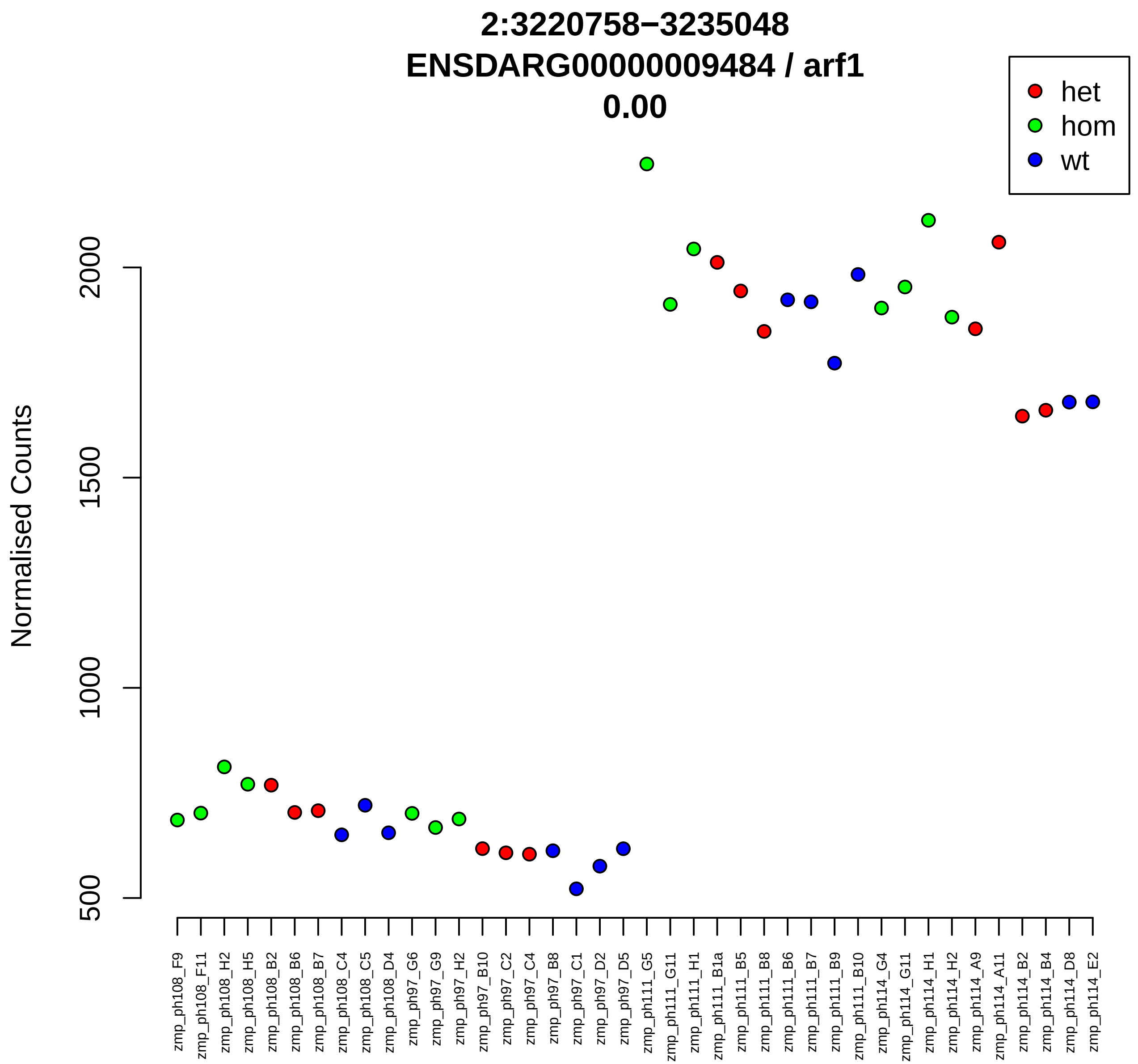
2:3220758−3235048
ENSDARG00000009484 / arf1
0.00
het
hom
wt
Normalised Counts
500
1000
1500
2000
zmp_ph108_F9
zmp_ph108_F11
zmp_ph108_H2
zmp_ph108_H5
zmp_ph108_B2
zmp_ph108_B6
zmp_ph108_B7
zmp_ph108_C4
zmp_ph108_C5
zmp_ph108_D4
zmp_ph97_G6
zmp_ph97_G9
zmp_ph97_H2
zmp_ph97_B10
zmp_ph97_C2
zmp_ph97_C4
zmp_ph97_B8
zmp_ph97_C1
zmp_ph97_D2
zmp_ph97_D5
zmp_ph111_G5
zmp_ph111_G11
zmp_ph111_H1
zmp_ph111_B1a
zmp_ph111_B5
zmp_ph111_B8
zmp_ph111_B6
zmp_ph111_B7
zmp_ph111_B9
zmp_ph111_B10
zmp_ph114_G4
zmp_ph114_G11
zmp_ph114_H1
zmp_ph114_H2
zmp_ph114_A9
zmp_ph114_A11
zmp_ph114_B2
zmp_ph114_B4
zmp_ph114_D8
zmp_ph114_E2

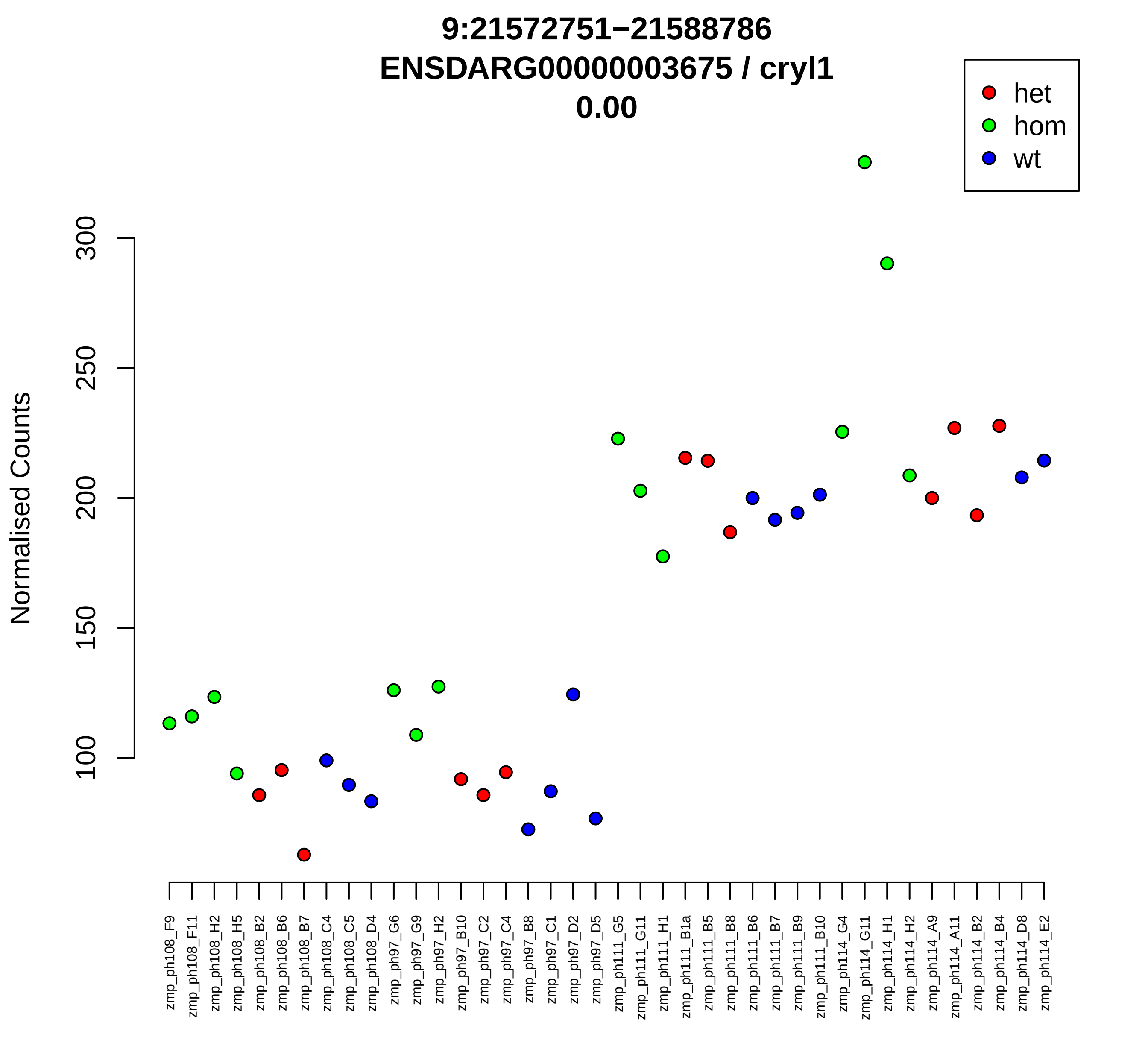
9:21572751−21588786
ENSDARG00000003675 / cryl1
0.00
het
hom
wt
Normalised Counts
100
150
200
250
300
zmp_ph108_F9
zmp_ph108_F11
zmp_ph108_H2
zmp_ph108_H5
zmp_ph108_B2
zmp_ph108_B6
zmp_ph108_B7
zmp_ph108_C4
zmp_ph108_C5
zmp_ph108_D4
zmp_ph97_G6
zmp_ph97_G9
zmp_ph97_H2
zmp_ph97_B10
zmp_ph97_C2
zmp_ph97_C4
zmp_ph97_B8
zmp_ph97_C1
zmp_ph97_D2
zmp_ph97_D5
zmp_ph111_G5
zmp_ph111_G11
zmp_ph111_H1
zmp_ph111_B1a
zmp_ph111_B5
zmp_ph111_B8
zmp_ph111_B6
zmp_ph111_B7
zmp_ph111_B9
zmp_ph111_B10
zmp_ph114_G4
zmp_ph114_G11
zmp_ph114_H1
zmp_ph114_H2
zmp_ph114_A9
zmp_ph114_A11
zmp_ph114_B2
zmp_ph114_B4
zmp_ph114_D8
zmp_ph114_E2

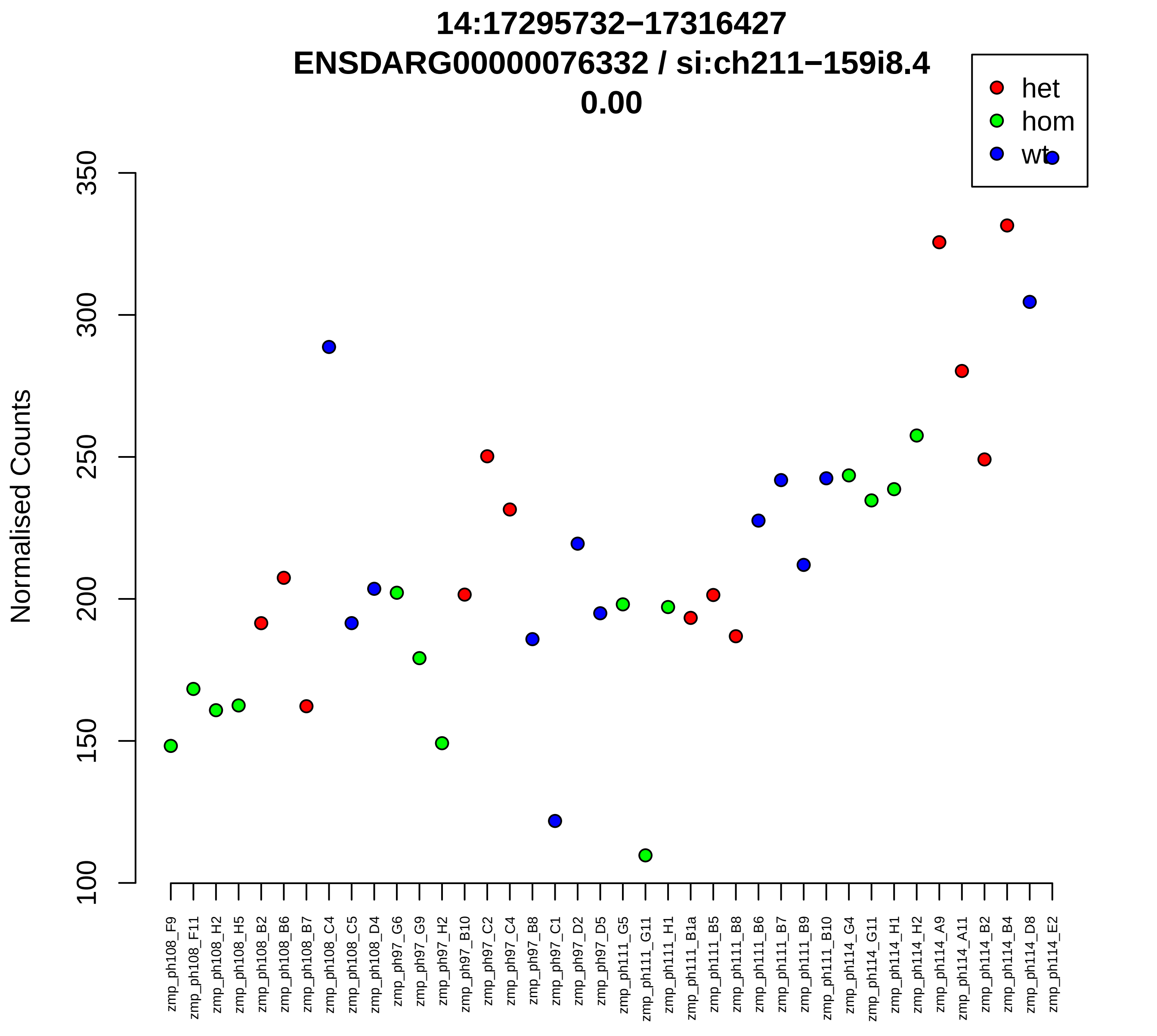
14:17295732−17316427
ENSDARG00000076332 / si:ch211−159i8.4
0.00
het
hom
wt
Normalised Counts
100
150
200
250
300
350
zmp_ph108_F9
zmp_ph108_F11
zmp_ph108_H2
zmp_ph108_H5
zmp_ph108_B2
zmp_ph108_B6
zmp_ph108_B7
zmp_ph108_C4
zmp_ph108_C5
zmp_ph108_D4
zmp_ph97_G6
zmp_ph97_G9
zmp_ph97_H2
zmp_ph97_B10
zmp_ph97_C2
zmp_ph97_C4
zmp_ph97_B8
zmp_ph97_C1
zmp_ph97_D2
zmp_ph97_D5
zmp_ph111_G5
zmp_ph111_G11
zmp_ph111_H1
zmp_ph111_B1a
zmp_ph111_B5
zmp_ph111_B8
zmp_ph111_B6
zmp_ph111_B7
zmp_ph111_B9
zmp_ph111_B10
zmp_ph114_G4
zmp_ph114_G11
zmp_ph114_H1
zmp_ph114_H2
zmp_ph114_A9
zmp_ph114_A11
zmp_ph114_B2
zmp_ph114_B4
zmp_ph114_D8
zmp_ph114_E2

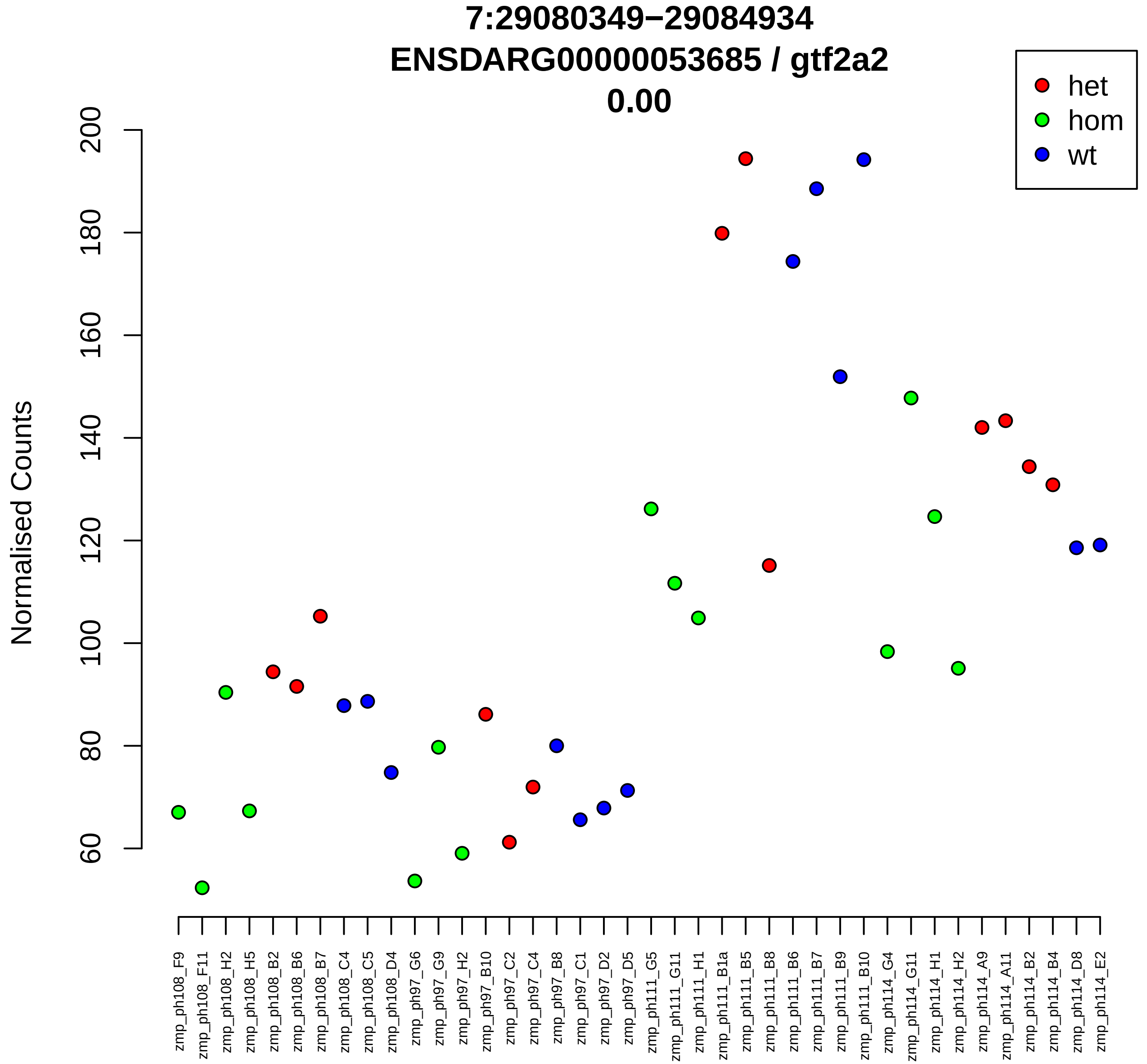

7:29080349−29084934
ENSDARG00000053685 / gtf2a2
0.00
het
hom
wt
Normalised Counts
60
80
100
120
140
160
180
200
zmp_ph108_F9
zmp_ph108_F11
zmp_ph108_H2
zmp_ph108_H5
zmp_ph108_B2
zmp_ph108_B6
zmp_ph108_B7
zmp_ph108_C4
zmp_ph108_C5
zmp_ph108_D4
zmp_ph97_G6
zmp_ph97_G9
zmp_ph97_H2
zmp_ph97_B10
zmp_ph97_C2
zmp_ph97_C4
zmp_ph97_B8
zmp_ph97_C1
zmp_ph97_D2
zmp_ph97_D5
zmp_ph111_G5
zmp_ph111_G11
zmp_ph111_H1
zmp_ph111_B1a
zmp_ph111_B5
zmp_ph111_B8
zmp_ph111_B6
zmp_ph111_B7
zmp_ph111_B9
zmp_ph111_B10
zmp_ph114_G4
zmp_ph114_G11
zmp_ph114_H1
zmp_ph114_H2
zmp_ph114_A9
zmp_ph114_A11
zmp_ph114_B2
zmp_ph114_B4
zmp_ph114_D8
zmp_ph114_E2

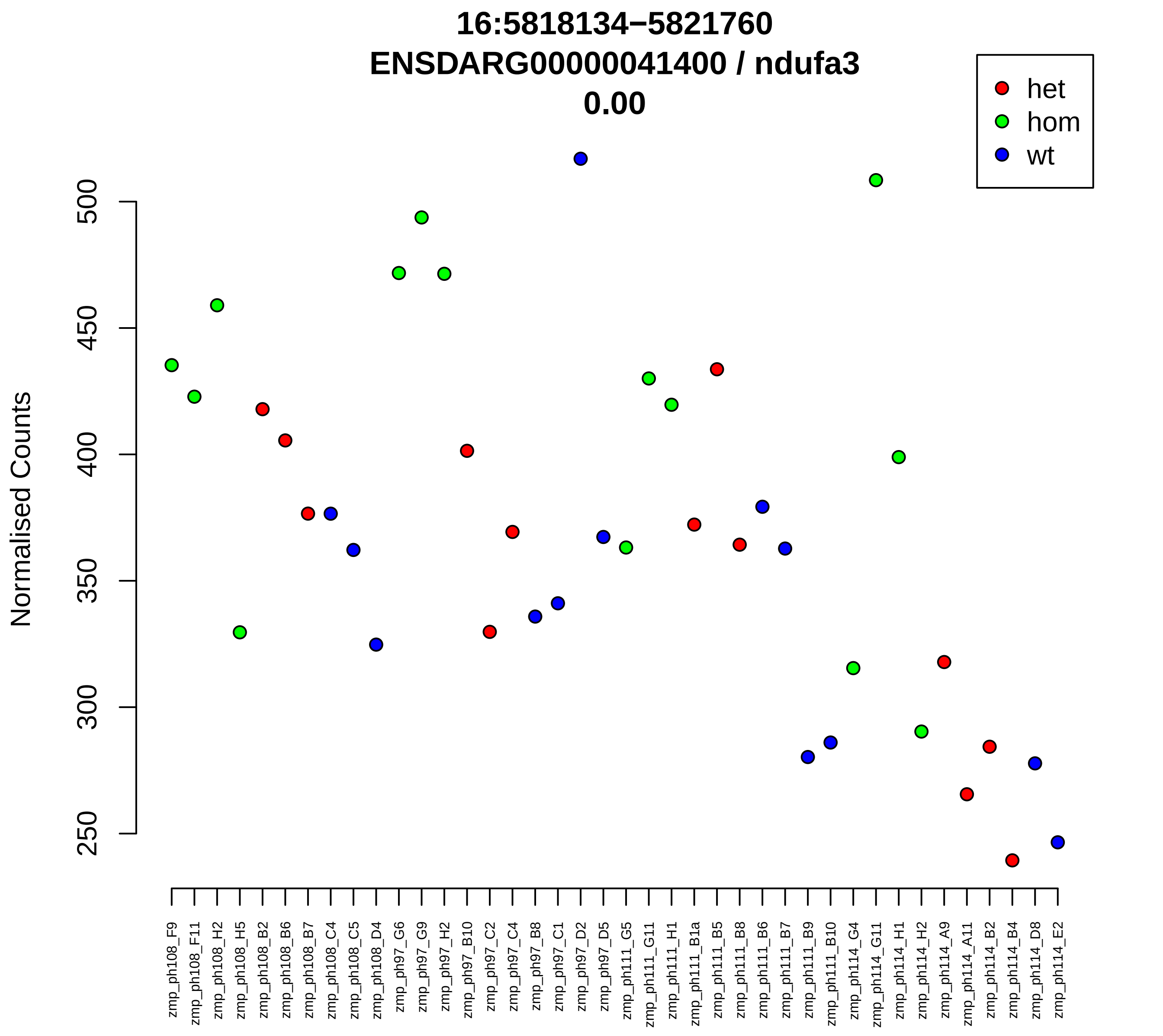
16:5818134−5821760
ENSDARG00000041400 / ndufa3
0.00
het
hom
wt
Normalised Counts
250
300
350
400
450
500
zmp_ph108_F9
zmp_ph108_F11
zmp_ph108_H2
zmp_ph108_H5
zmp_ph108_B2
zmp_ph108_B6
zmp_ph108_B7
zmp_ph108_C4
zmp_ph108_C5
zmp_ph108_D4
zmp_ph97_G6
zmp_ph97_G9
zmp_ph97_H2
zmp_ph97_B10
zmp_ph97_C2
zmp_ph97_C4
zmp_ph97_B8
zmp_ph97_C1
zmp_ph97_D2
zmp_ph97_D5
zmp_ph111_G5
zmp_ph111_G11
zmp_ph111_H1
zmp_ph111_B1a
zmp_ph111_B5
zmp_ph111_B8
zmp_ph111_B6
zmp_ph111_B7
zmp_ph111_B9
zmp_ph111_B10
zmp_ph114_G4
zmp_ph114_G11
zmp_ph114_H1
zmp_ph114_H2
zmp_ph114_A9
zmp_ph114_A11
zmp_ph114_B2
zmp_ph114_B4
zmp_ph114_D8
zmp_ph114_E2

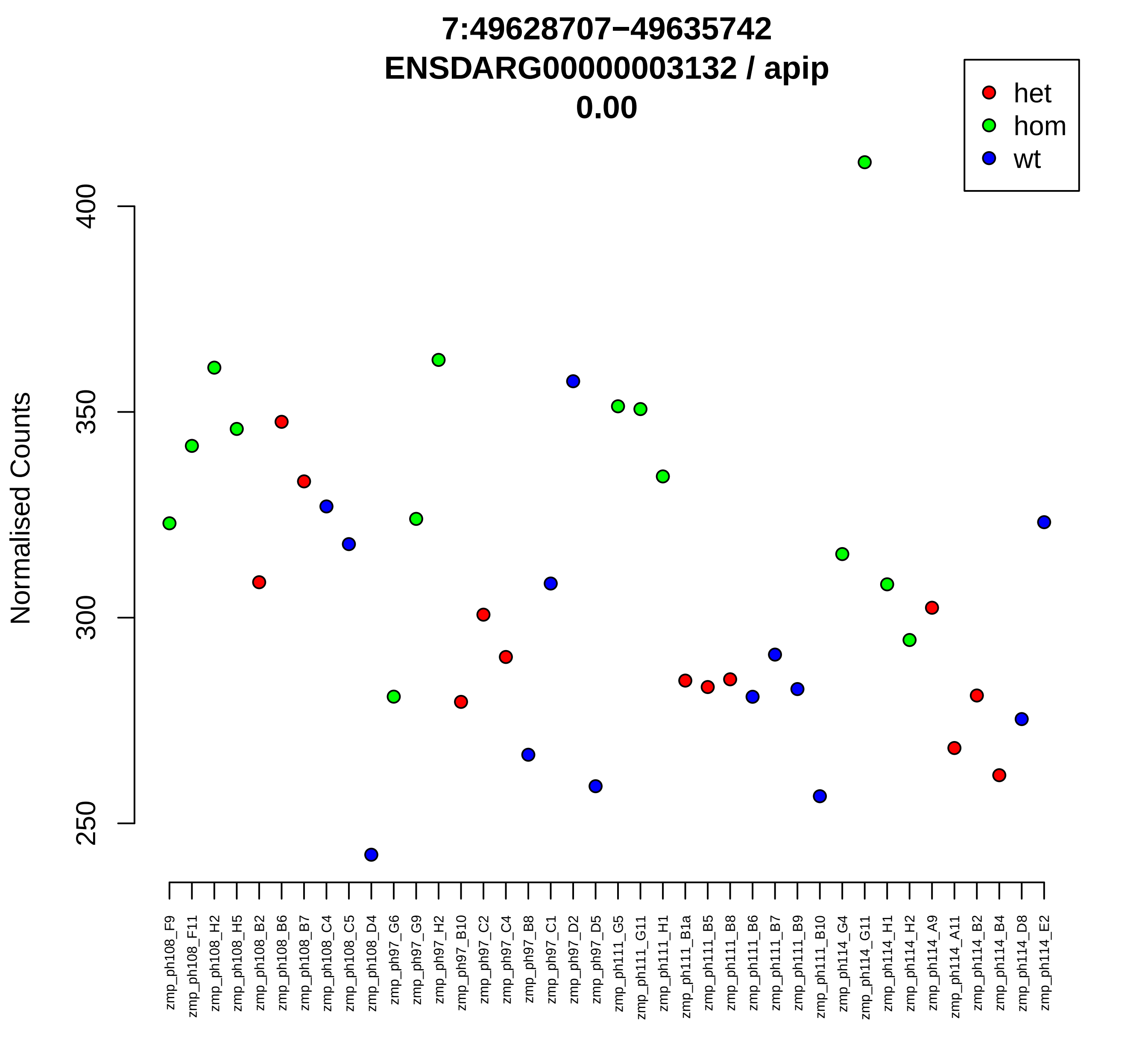

7:49628707−49635742
ENSDARG00000003132 / apip
0.00
het
hom
wt
Normalised Counts
250
300
350
400
zmp_ph108_F9
zmp_ph108_F11
zmp_ph108_H2
zmp_ph108_H5
zmp_ph108_B2
zmp_ph108_B6
zmp_ph108_B7
zmp_ph108_C4
zmp_ph108_C5
zmp_ph108_D4
zmp_ph97_G6
zmp_ph97_G9
zmp_ph97_H2
zmp_ph97_B10
zmp_ph97_C2
zmp_ph97_C4
zmp_ph97_B8
zmp_ph97_C1
zmp_ph97_D2
zmp_ph97_D5
zmp_ph111_G5
zmp_ph111_G11
zmp_ph111_H1
zmp_ph111_B1a
zmp_ph111_B5
zmp_ph111_B8
zmp_ph111_B6
zmp_ph111_B7
zmp_ph111_B9
zmp_ph111_B10
zmp_ph114_G4
zmp_ph114_G11
zmp_ph114_H1
zmp_ph114_H2
zmp_ph114_A9
zmp_ph114_A11
zmp_ph114_B2
zmp_ph114_B4
zmp_ph114_D8
zmp_ph114_E2

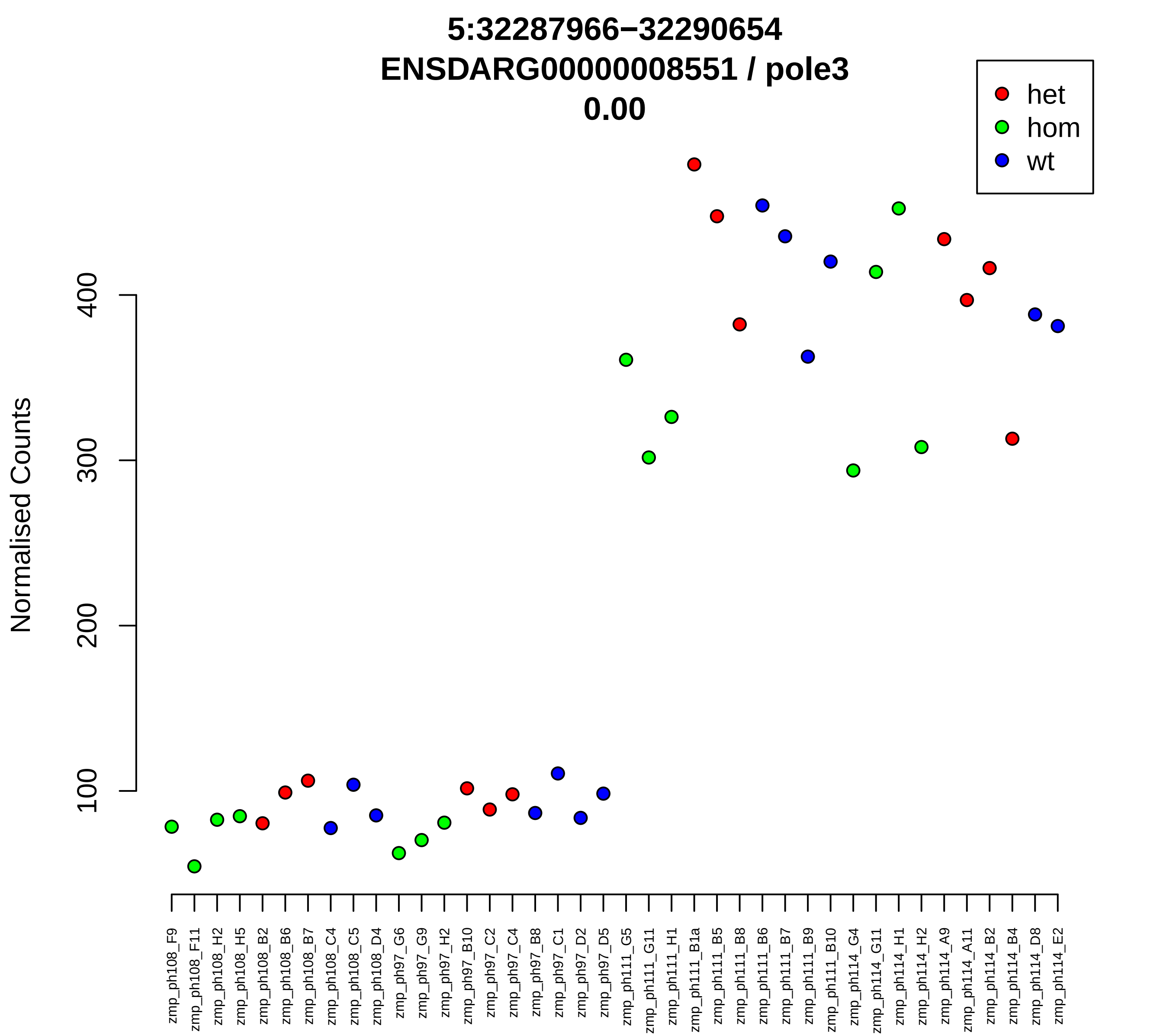

5:32287966−32290654
ENSDARG00000008551 / pole3
0.00
het
hom
wt
Normalised Counts
100
200
300
400
zmp_ph108_F9
zmp_ph108_F11
zmp_ph108_H2
zmp_ph108_H5
zmp_ph108_B2
zmp_ph108_B6
zmp_ph108_B7
zmp_ph108_C4
zmp_ph108_C5
zmp_ph108_D4
zmp_ph97_G6
zmp_ph97_G9
zmp_ph97_H2
zmp_ph97_B10
zmp_ph97_C2
zmp_ph97_C4
zmp_ph97_B8
zmp_ph97_C1
zmp_ph97_D2
zmp_ph97_D5
zmp_ph111_G5
zmp_ph111_G11
zmp_ph111_H1
zmp_ph111_B1a
zmp_ph111_B5
zmp_ph111_B8
zmp_ph111_B6
zmp_ph111_B7
zmp_ph111_B9
zmp_ph111_B10
zmp_ph114_G4
zmp_ph114_G11
zmp_ph114_H1
zmp_ph114_H2
zmp_ph114_A9
zmp_ph114_A11
zmp_ph114_B2
zmp_ph114_B4
zmp_ph114_D8
zmp_ph114_E2

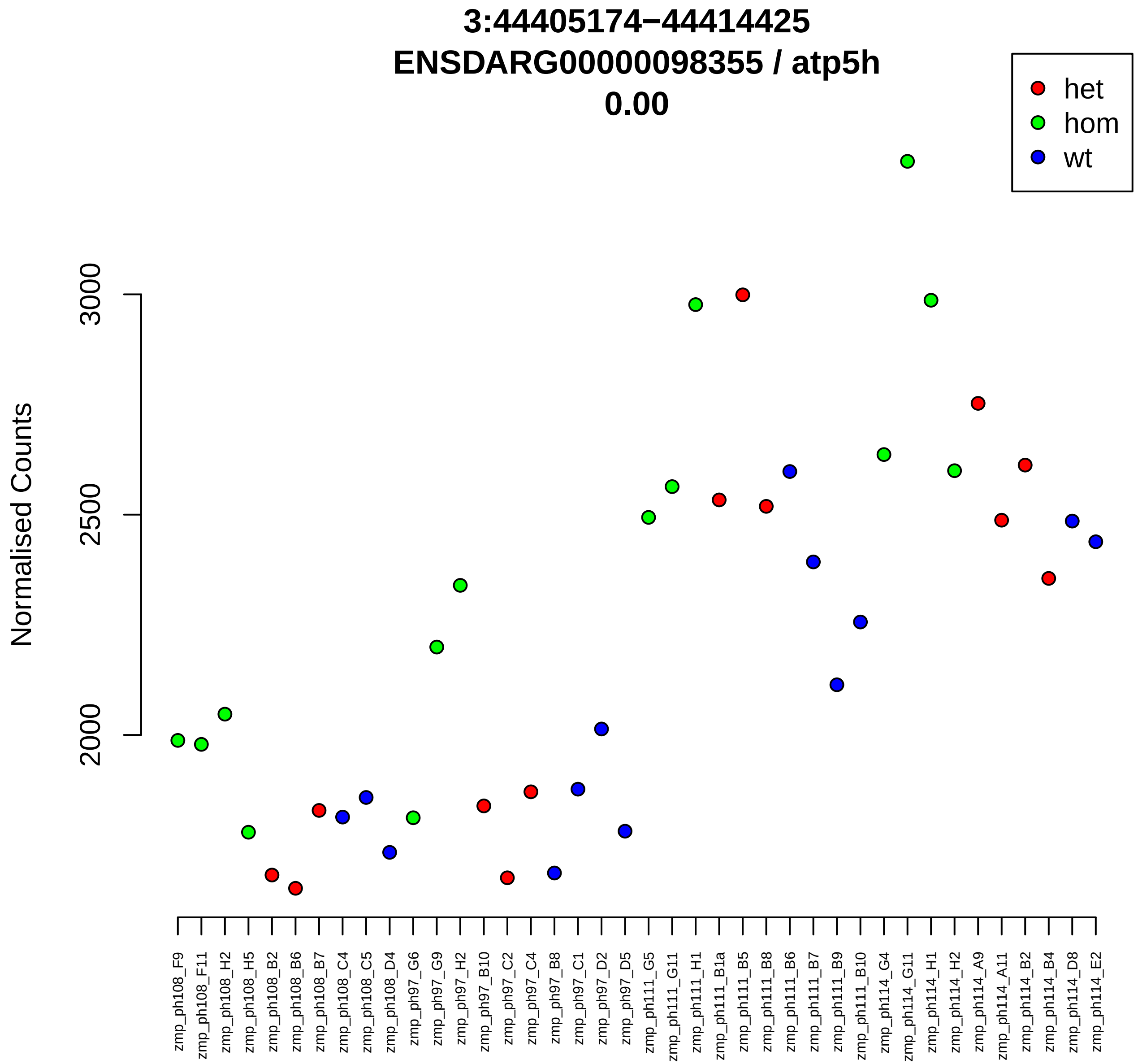

3:44405174−44414425
ENSDARG00000098355 / atp5h
0.00
het
hom
wt
Normalised Counts
3000
2500
2000
zmp_ph108_F9
zmp_ph108_F11
zmp_ph108_H2
zmp_ph108_H5
zmp_ph108_B2
zmp_ph108_B6
zmp_ph108_B7
zmp_ph108_C4
zmp_ph108_C5
zmp_ph108_D4
zmp_ph97_G6
zmp_ph97_G9
zmp_ph97_H2
zmp_ph97_B10
zmp_ph97_C2
zmp_ph97_C4
zmp_ph97_B8
zmp_ph97_C1
zmp_ph97_D2
zmp_ph97_D5
zmp_ph111_G5
zmp_ph111_G11
zmp_ph111_H1
zmp_ph111_B1a
zmp_ph111_B5
zmp_ph111_B8
zmp_ph111_B6
zmp_ph111_B7
zmp_ph111_B9
zmp_ph111_B10
zmp_ph114_G4
zmp_ph114_G11
zmp_ph114_H1
zmp_ph114_H2
zmp_ph114_A9
zmp_ph114_A11
zmp_ph114_B2
zmp_ph114_B4
zmp_ph114_D8
zmp_ph114_E2

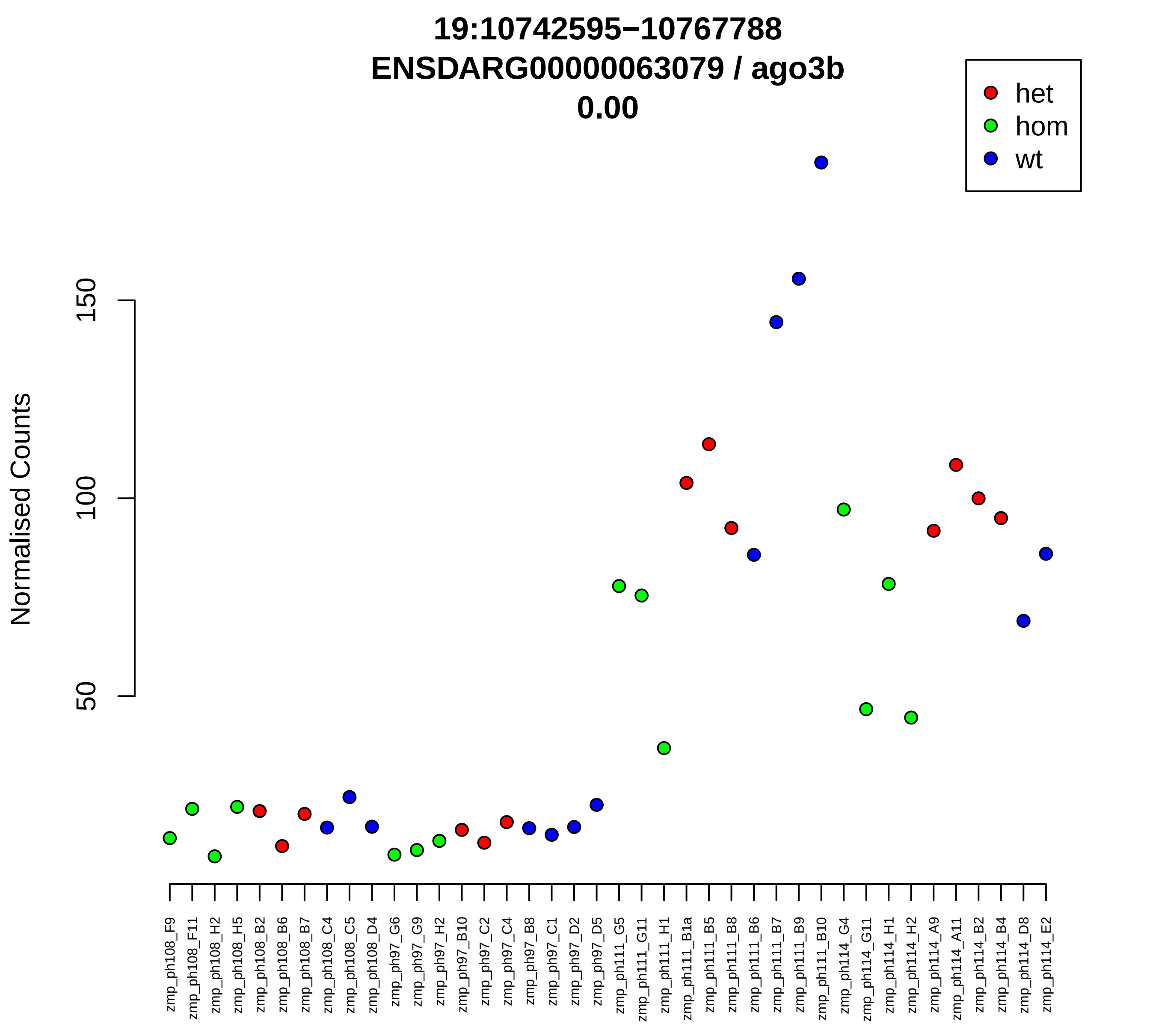
19:10742595−10767788
ENSDARG00000063079 / ago3b
0.00
het
hom
wt
Normalised Counts
50
100
150
zmp_ph108_F9
zmp_ph108_F11
zmp_ph108_H2
zmp_ph108_H5
zmp_ph108_B2
zmp_ph108_B6
zmp_ph108_B7
zmp_ph108_C4
zmp_ph108_C5
zmp_ph108_D4
zmp_ph97_G6
zmp_ph97_G9
zmp_ph97_H2
zmp_ph97_B10
zmp_ph97_C2
zmp_ph97_C4
zmp_ph97_B8
zmp_ph97_C1
zmp_ph97_D2
zmp_ph97_D5
zmp_ph111_G5
zmp_ph111_G11
zmp_ph111_H1
zmp_ph111_B1a
zmp_ph111_B5
zmp_ph111_B8
zmp_ph111_B6
zmp_ph111_B7
zmp_ph111_B9
zmp_ph111_B10
zmp_ph114_G4
zmp_ph114_G11
zmp_ph114_H1
zmp_ph114_H2
zmp_ph114_A9
zmp_ph114_A11
zmp_ph114_B2
zmp_ph114_B4
zmp_ph114_D8
zmp_ph114_E2

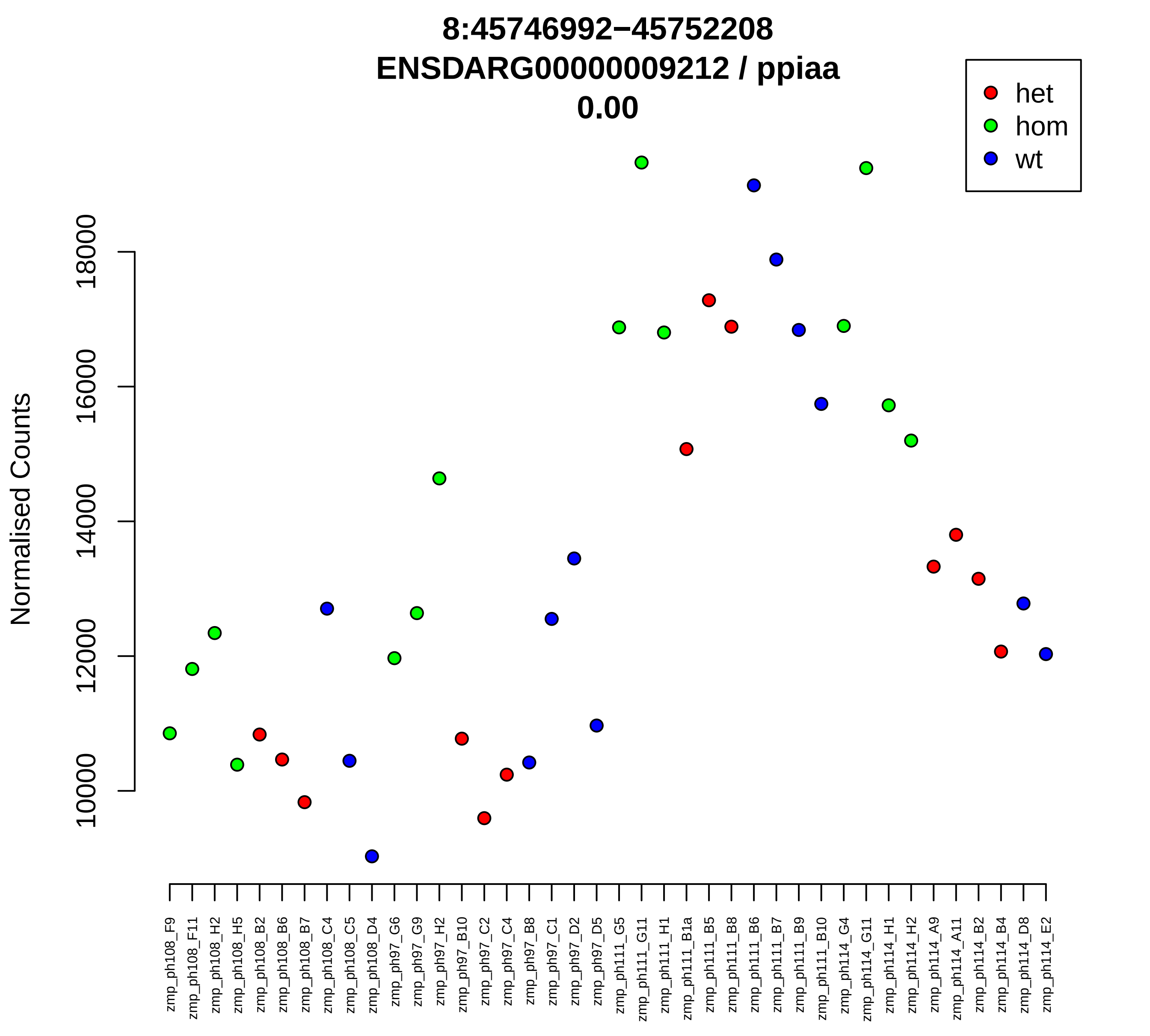

8:45746992−45752208
ENSDARG00000009212 / ppiaa
0.00
het
hom
wt
Normalised Counts
10000
12000
14000
16000
18000
zmp_ph108_F9
zmp_ph108_F11
zmp_ph108_H2
zmp_ph108_H5
zmp_ph108_B2
zmp_ph108_B6
zmp_ph108_B7
zmp_ph108_C4
zmp_ph108_C5
zmp_ph108_D4
zmp_ph97_G6
zmp_ph97_G9
zmp_ph97_H2
zmp_ph97_B10
zmp_ph97_C2
zmp_ph97_C4
zmp_ph97_B8
zmp_ph97_C1
zmp_ph97_D2
zmp_ph97_D5
zmp_ph111_G5
zmp_ph111_G11
zmp_ph111_H1
zmp_ph111_B1a
zmp_ph111_B5
zmp_ph111_B8
zmp_ph111_B6
zmp_ph111_B7
zmp_ph111_B9
zmp_ph111_B10
zmp_ph114_G4
zmp_ph114_G11
zmp_ph114_H1
zmp_ph114_H2
zmp_ph114_A9
zmp_ph114_A11
zmp_ph114_B2
zmp_ph114_B4
zmp_ph114_D8
zmp_ph114_E2

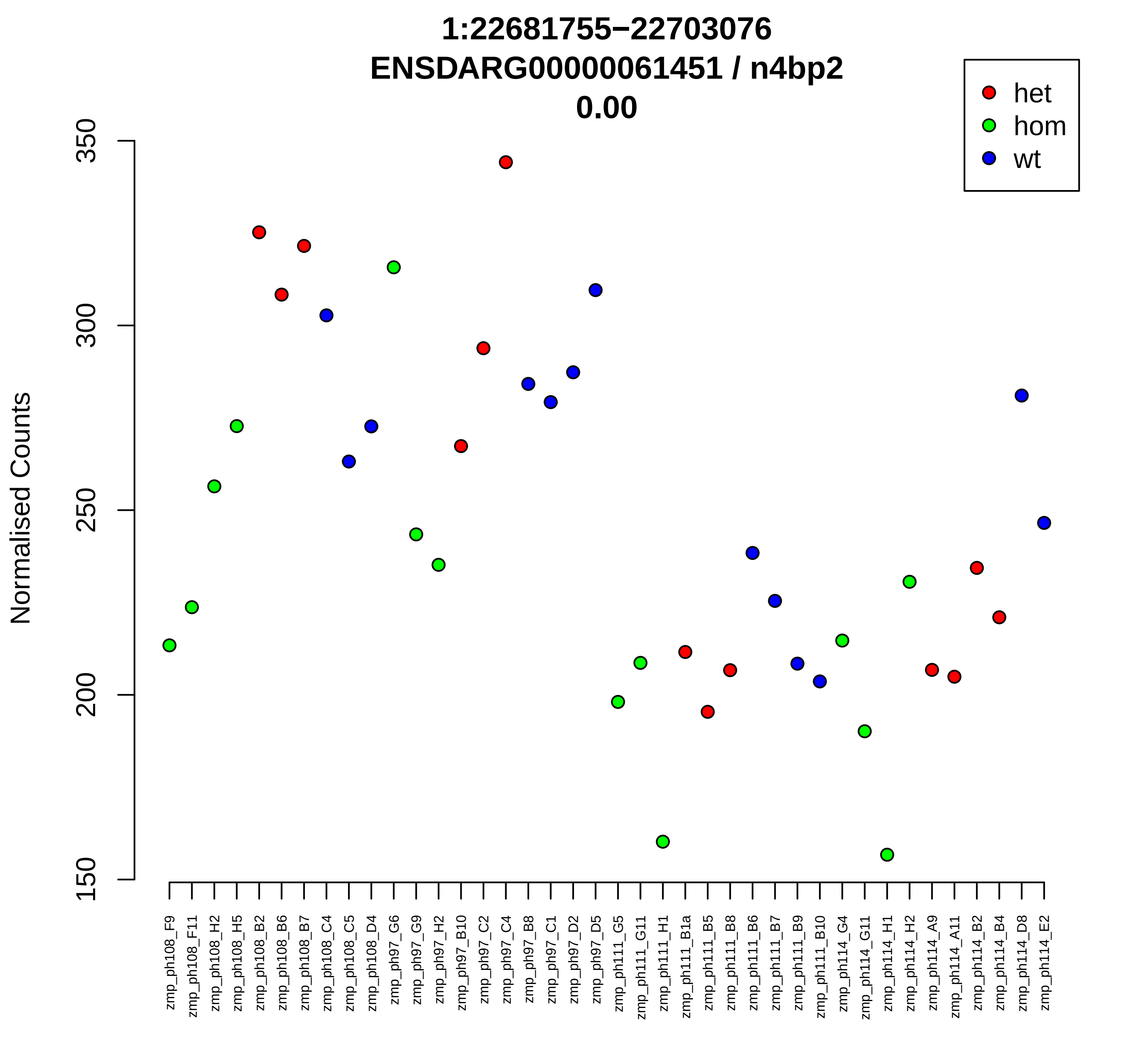
1:22681755−22703076
ENSDARG00000061451 / n4bp2
0.00
het
hom
wt
Normalised Counts
150
200
250
300
350
zmp_ph108_F9
zmp_ph108_F11
zmp_ph108_H2
zmp_ph108_H5
zmp_ph108_B2
zmp_ph108_B6
zmp_ph108_B7
zmp_ph108_C4
zmp_ph108_C5
zmp_ph108_D4
zmp_ph97_G6
zmp_ph97_G9
zmp_ph97_H2
zmp_ph97_B10
zmp_ph97_C2
zmp_ph97_C4
zmp_ph97_B8
zmp_ph97_C1
zmp_ph97_D2
zmp_ph97_D5
zmp_ph111_G5
zmp_ph111_G11
zmp_ph111_H1
zmp_ph111_B1a
zmp_ph111_B5
zmp_ph111_B8
zmp_ph111_B6
zmp_ph111_B7
zmp_ph111_B9
zmp_ph111_B10
zmp_ph114_G4
zmp_ph114_G11
zmp_ph114_H1
zmp_ph114_H2
zmp_ph114_A9
zmp_ph114_A11
zmp_ph114_B2
zmp_ph114_B4
zmp_ph114_D8
zmp_ph114_E2

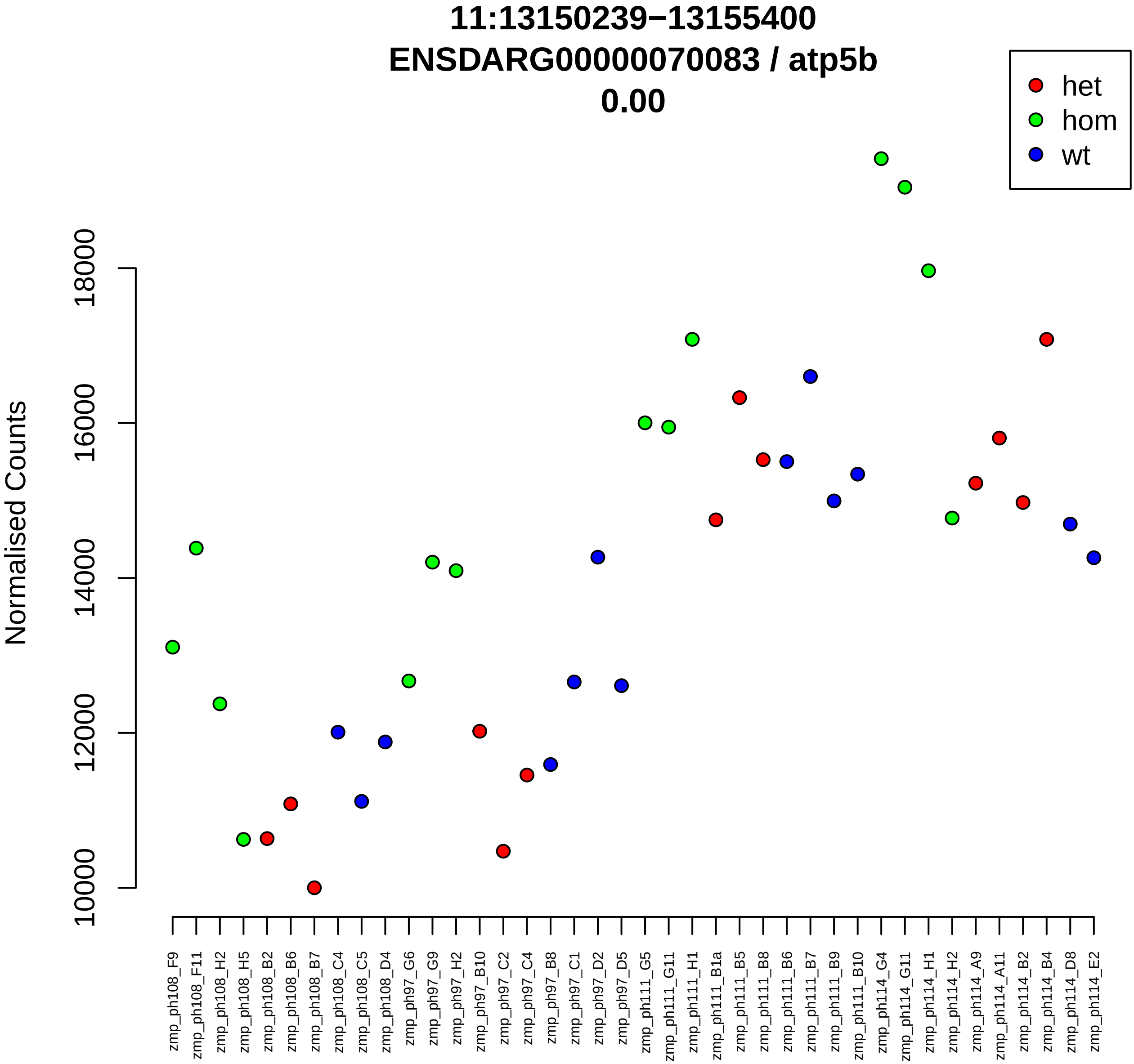

11:13150239−13155400
ENSDARG00000070083 / atp5b
0.00
het
hom
wt
Normalised Counts
10000
12000
14000
16000
18000
zmp_ph108_F9
zmp_ph108_F11
zmp_ph108_H2
zmp_ph108_H5
zmp_ph108_B2
zmp_ph108_B6
zmp_ph108_B7
zmp_ph108_C4
zmp_ph108_C5
zmp_ph108_D4
zmp_ph97_G6
zmp_ph97_G9
zmp_ph97_H2
zmp_ph97_B10
zmp_ph97_C2
zmp_ph97_C4
zmp_ph97_B8
zmp_ph97_C1
zmp_ph97_D2
zmp_ph97_D5
zmp_ph111_G5
zmp_ph111_G11
zmp_ph111_H1
zmp_ph111_B1a
zmp_ph111_B5
zmp_ph111_B8
zmp_ph111_B6
zmp_ph111_B7
zmp_ph111_B9
zmp_ph111_B10
zmp_ph114_G4
zmp_ph114_G11
zmp_ph114_H1
zmp_ph114_H2
zmp_ph114_A9
zmp_ph114_A11
zmp_ph114_B2
zmp_ph114_B4
zmp_ph114_D8
zmp_ph114_E2

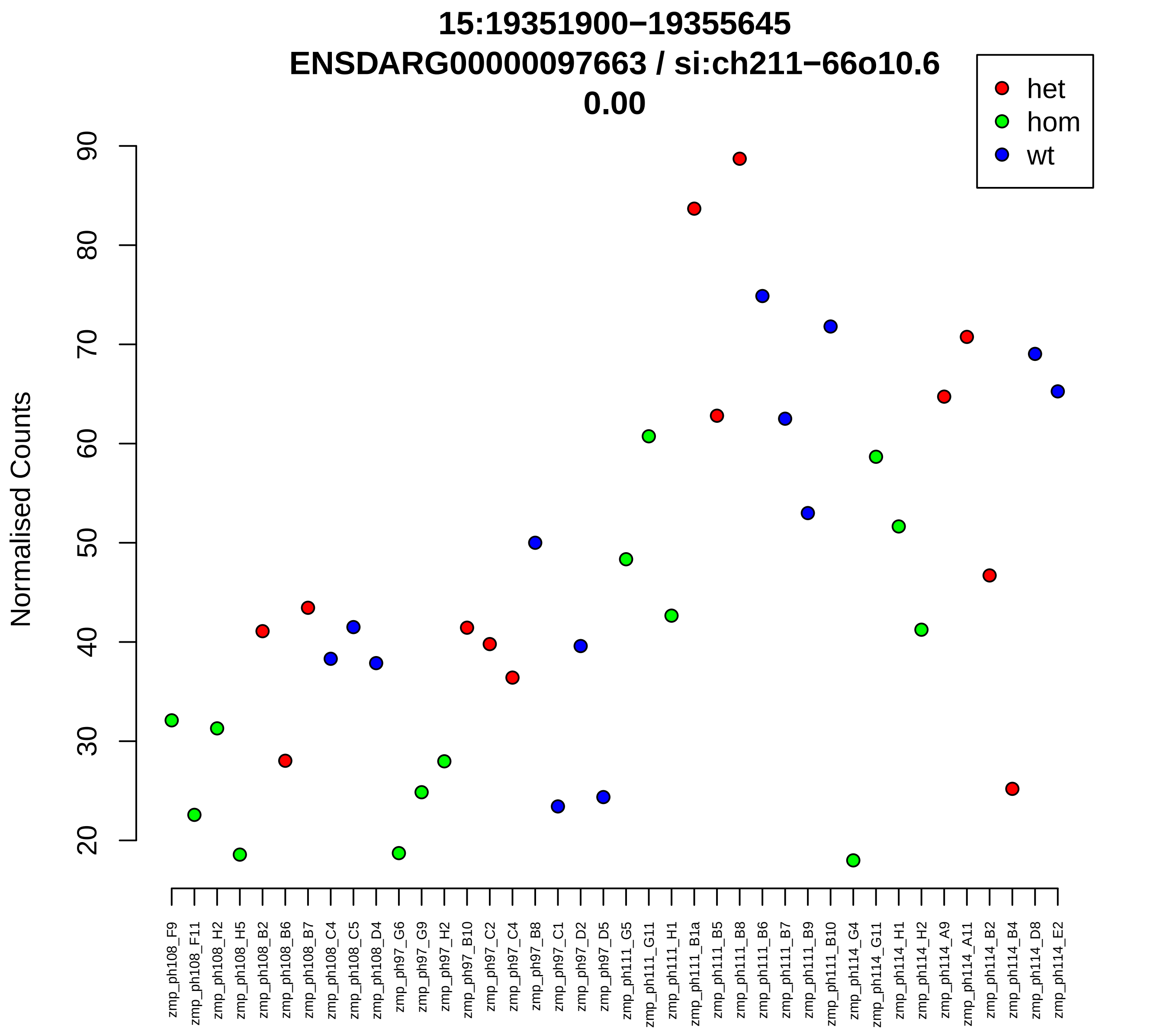
15:19351900−19355645
ENSDARG00000097663 / si:ch211−66o10.6
0.00
het
hom
wt
Normalised Counts
20
30
40
50
60
70
80
90
zmp_ph108_F9
zmp_ph108_F11
zmp_ph108_H2
zmp_ph108_H5
zmp_ph108_B2
zmp_ph108_B6
zmp_ph108_B7
zmp_ph108_C4
zmp_ph108_C5
zmp_ph108_D4
zmp_ph97_G6
zmp_ph97_G9
zmp_ph97_H2
zmp_ph97_B10
zmp_ph97_C2
zmp_ph97_C4
zmp_ph97_B8
zmp_ph97_C1
zmp_ph97_D2
zmp_ph97_D5
zmp_ph111_G5
zmp_ph111_G11
zmp_ph111_H1
zmp_ph111_B1a
zmp_ph111_B5
zmp_ph111_B8
zmp_ph111_B6
zmp_ph111_B7
zmp_ph111_B9
zmp_ph111_B10
zmp_ph114_G4
zmp_ph114_G11
zmp_ph114_H1
zmp_ph114_H2
zmp_ph114_A9
zmp_ph114_A11
zmp_ph114_B2
zmp_ph114_B4
zmp_ph114_D8
zmp_ph114_E2

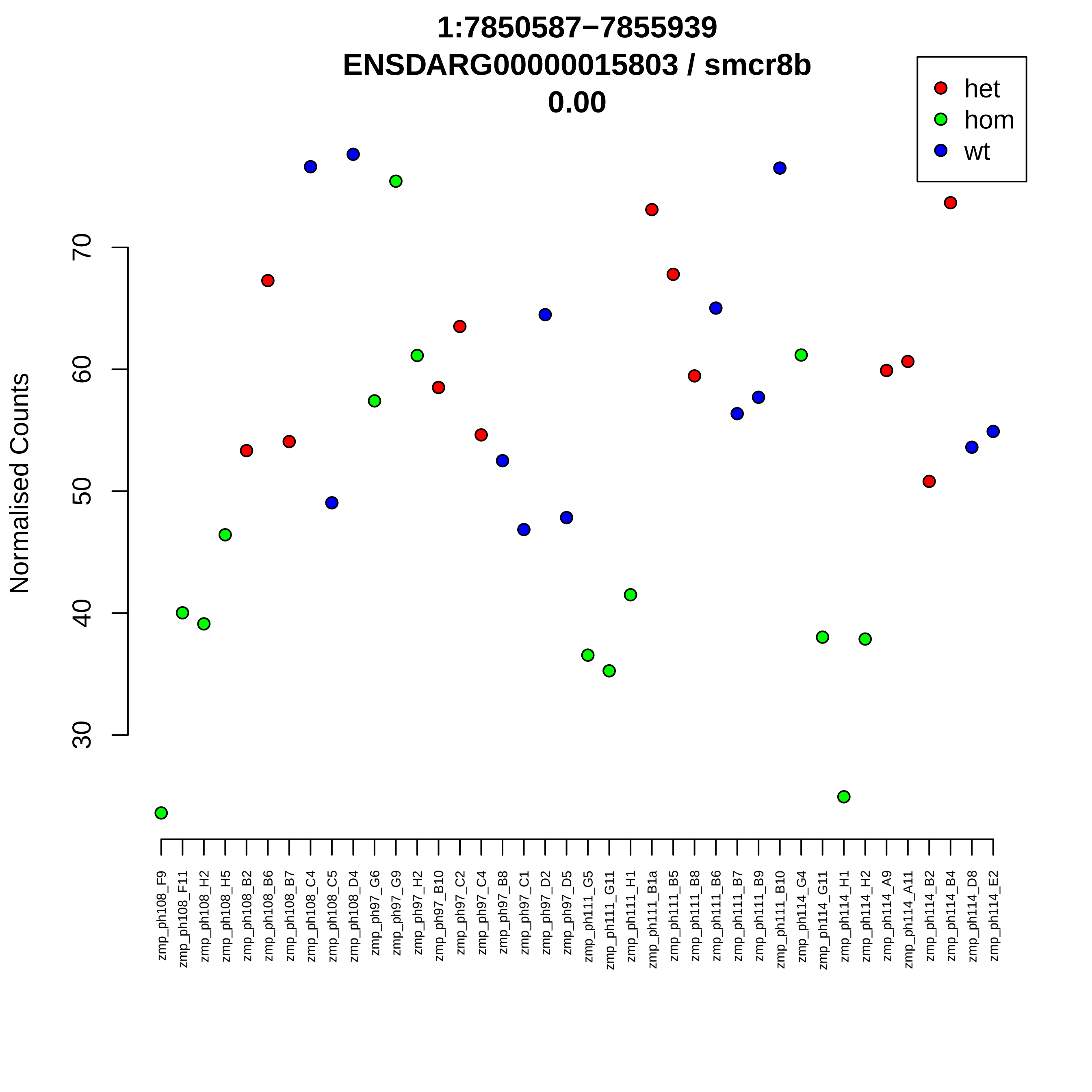

1:7850587−7855939
ENSDARG00000015803 / smcr8b
0.00
het
hom
wt
Normalised Counts
30
40
50
60
70
zmp_ph108_F9
zmp_ph108_F11
zmp_ph108_H2
zmp_ph108_H5
zmp_ph108_B2
zmp_ph108_B6
zmp_ph108_B7
zmp_ph108_C4
zmp_ph108_C5
zmp_ph108_D4
zmp_ph97_G6
zmp_ph97_G9
zmp_ph97_H2
zmp_ph97_B10
zmp_ph97_C2
zmp_ph97_C4
zmp_ph97_B8
zmp_ph97_C1
zmp_ph97_D2
zmp_ph97_D5
zmp_ph111_G5
zmp_ph111_G11
zmp_ph111_H1
zmp_ph111_B1a
zmp_ph111_B5
zmp_ph111_B8
zmp_ph111_B6
zmp_ph111_B7
zmp_ph111_B9
zmp_ph111_B10
zmp_ph114_G4
zmp_ph114_G11
zmp_ph114_H1
zmp_ph114_H2
zmp_ph114_A9
zmp_ph114_A11
zmp_ph114_B2
zmp_ph114_B4
zmp_ph114_D8
zmp_ph114_E2

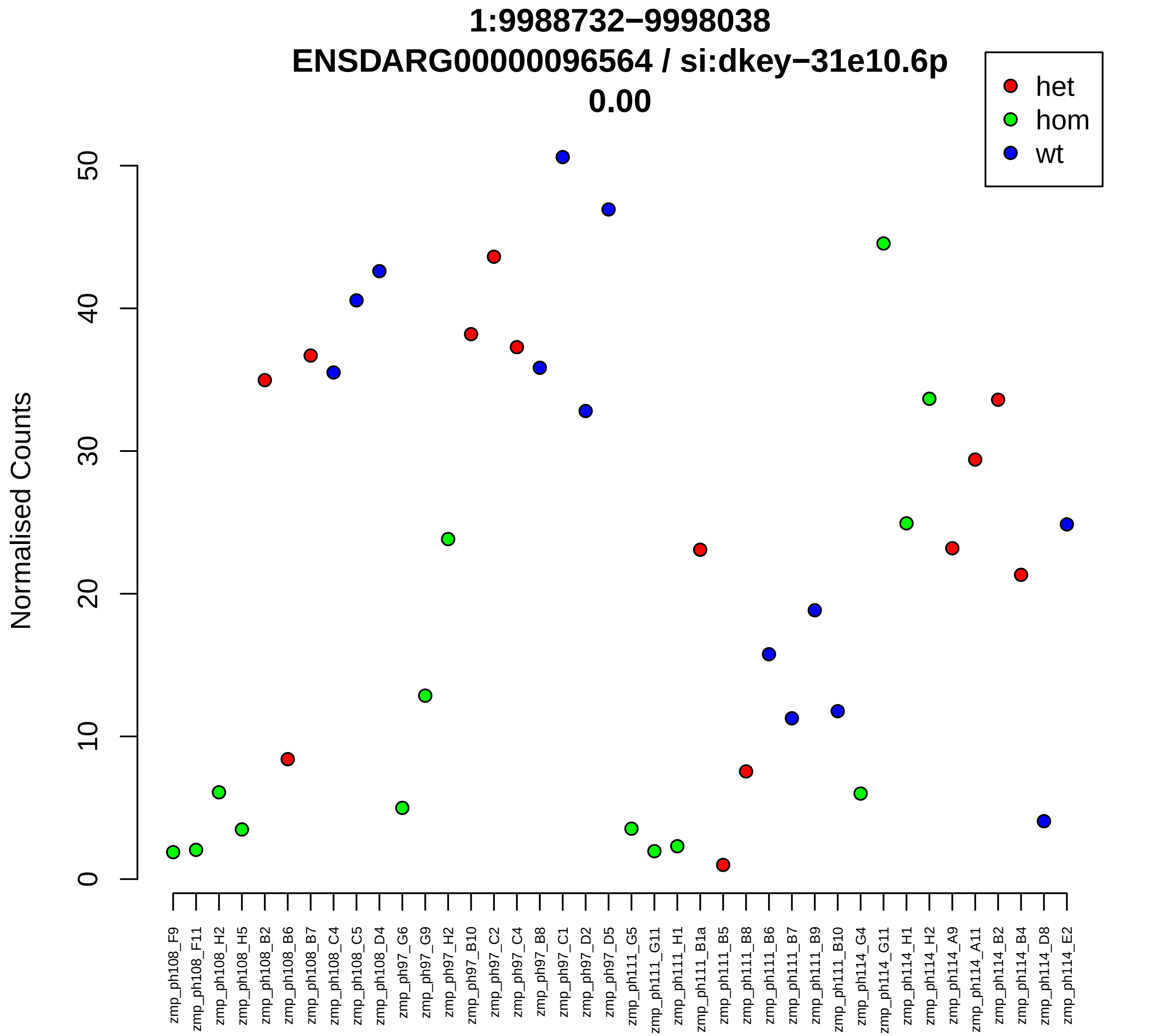

1:9988732−9998038
ENSDARG00000096564 / si:dkey−31e10.6p
0.00
het
hom
wt
Normalised Counts
0
10
20
30
40
50
zmp_ph108_F9
zmp_ph108_F11
zmp_ph108_H2
zmp_ph108_H5
zmp_ph108_B2
zmp_ph108_B6
zmp_ph108_B7
zmp_ph108_C4
zmp_ph108_C5
zmp_ph108_D4
zmp_ph97_G6
zmp_ph97_G9
zmp_ph97_H2
zmp_ph97_B10
zmp_ph97_C2
zmp_ph97_C4
zmp_ph97_B8
zmp_ph97_C1
zmp_ph97_D2
zmp_ph97_D5
zmp_ph111_G5
zmp_ph111_G11
zmp_ph111_H1
zmp_ph111_B1a
zmp_ph111_B5
zmp_ph111_B8
zmp_ph111_B6
zmp_ph111_B7
zmp_ph111_B9
zmp_ph111_B10
zmp_ph114_G4
zmp_ph114_G11
zmp_ph114_H1
zmp_ph114_H2
zmp_ph114_A9
zmp_ph114_A11
zmp_ph114_B2
zmp_ph114_B4
zmp_ph114_D8
zmp_ph114_E2

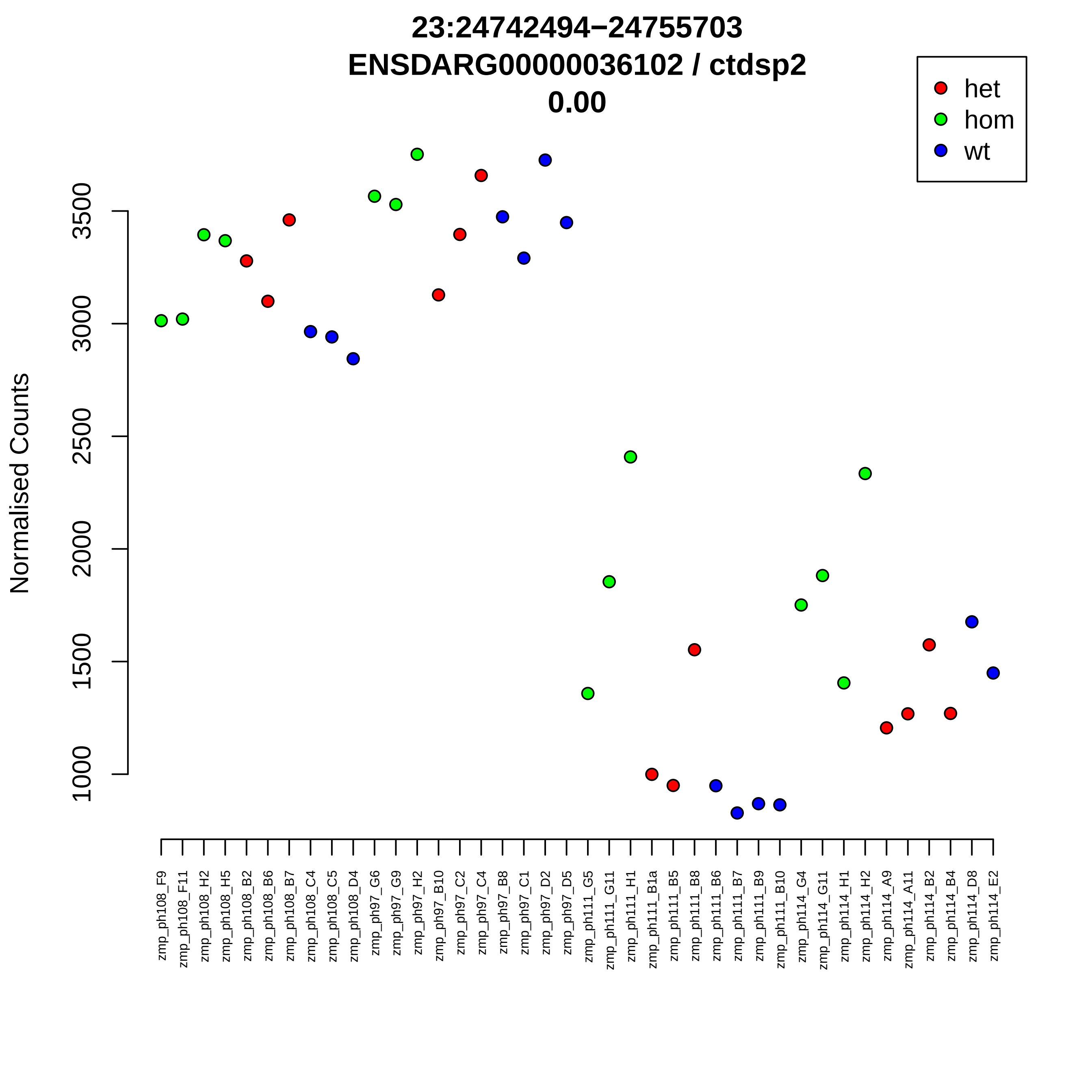
23:24742494−24755703
ENSDARG00000036102 / ctdsp2
0.00
het
hom
wt
Normalised Counts
1000
1500
2000
2500
3000
3500
zmp_ph108_F9
zmp_ph108_F11
zmp_ph108_H2
zmp_ph108_H5
zmp_ph108_B2
zmp_ph108_B6
zmp_ph108_B7
zmp_ph108_C4
zmp_ph108_C5
zmp_ph108_D4
zmp_ph97_G6
zmp_ph97_G9
zmp_ph97_H2
zmp_ph97_B10
zmp_ph97_C2
zmp_ph97_C4
zmp_ph97_B8
zmp_ph97_C1
zmp_ph97_D2
zmp_ph97_D5
zmp_ph111_G5
zmp_ph111_G11
zmp_ph111_H1
zmp_ph111_B1a
zmp_ph111_B5
zmp_ph111_B8
zmp_ph111_B6
zmp_ph111_B7
zmp_ph111_B9
zmp_ph111_B10
zmp_ph114_G4
zmp_ph114_G11
zmp_ph114_H1
zmp_ph114_H2
zmp_ph114_A9
zmp_ph114_A11
zmp_ph114_B2
zmp_ph114_B4
zmp_ph114_D8
zmp_ph114_E2

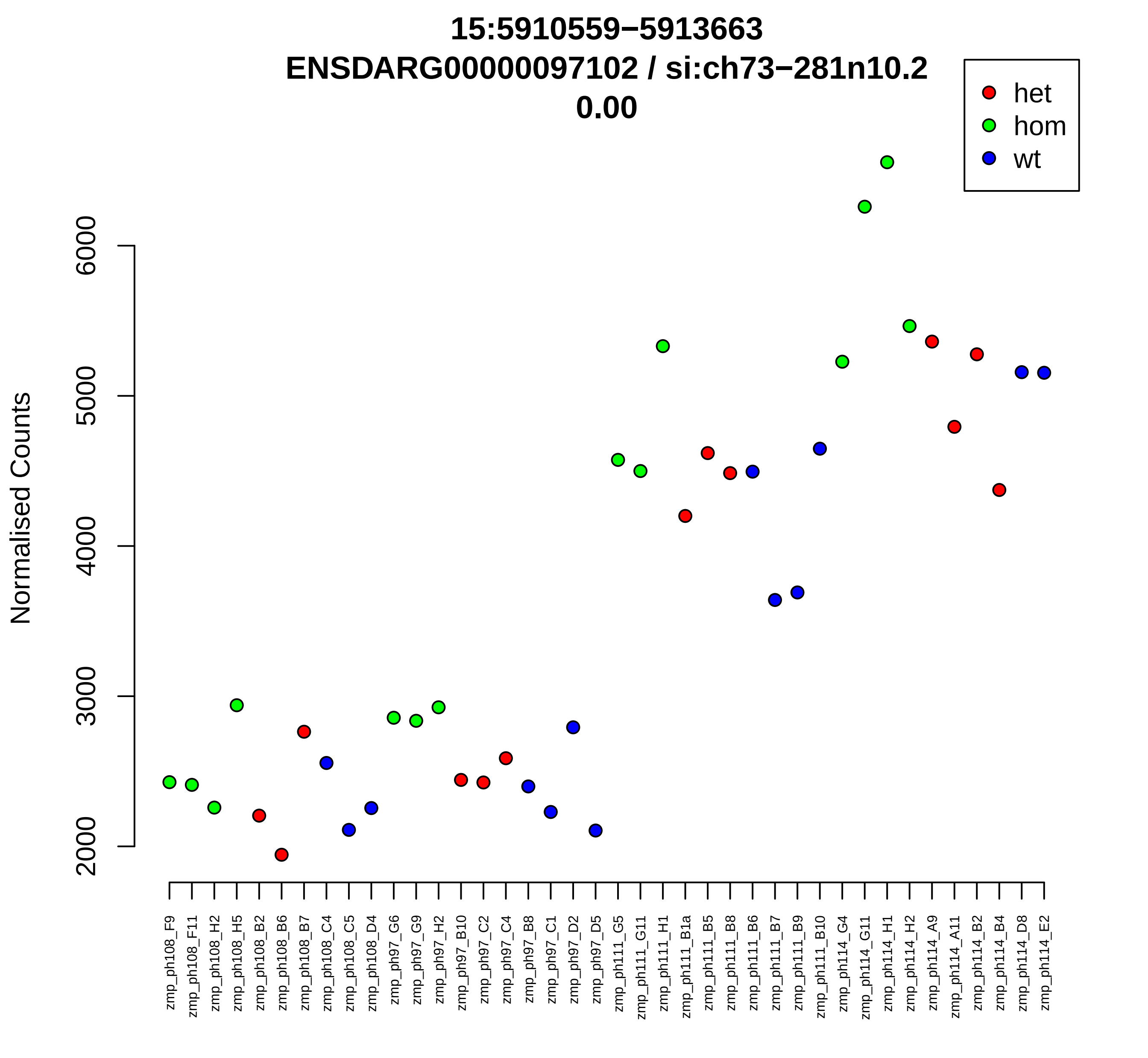

15:5910559−5913663
ENSDARG00000097102 / si:ch73−281n10.2
0.00
het
hom
wt
Normalised Counts
2000
3000
4000
5000
6000
zmp_ph108_F9
zmp_ph108_F11
zmp_ph108_H2
zmp_ph108_H5
zmp_ph108_B2
zmp_ph108_B6
zmp_ph108_B7
zmp_ph108_C4
zmp_ph108_C5
zmp_ph108_D4
zmp_ph97_G6
zmp_ph97_G9
zmp_ph97_H2
zmp_ph97_B10
zmp_ph97_C2
zmp_ph97_C4
zmp_ph97_B8
zmp_ph97_C1
zmp_ph97_D2
zmp_ph97_D5
zmp_ph111_G5
zmp_ph111_G11
zmp_ph111_H1
zmp_ph111_B1a
zmp_ph111_B5
zmp_ph111_B8
zmp_ph111_B6
zmp_ph111_B7
zmp_ph111_B9
zmp_ph111_B10
zmp_ph114_G4
zmp_ph114_G11
zmp_ph114_H1
zmp_ph114_H2
zmp_ph114_A9
zmp_ph114_A11
zmp_ph114_B2
zmp_ph114_B4
zmp_ph114_D8
zmp_ph114_E2

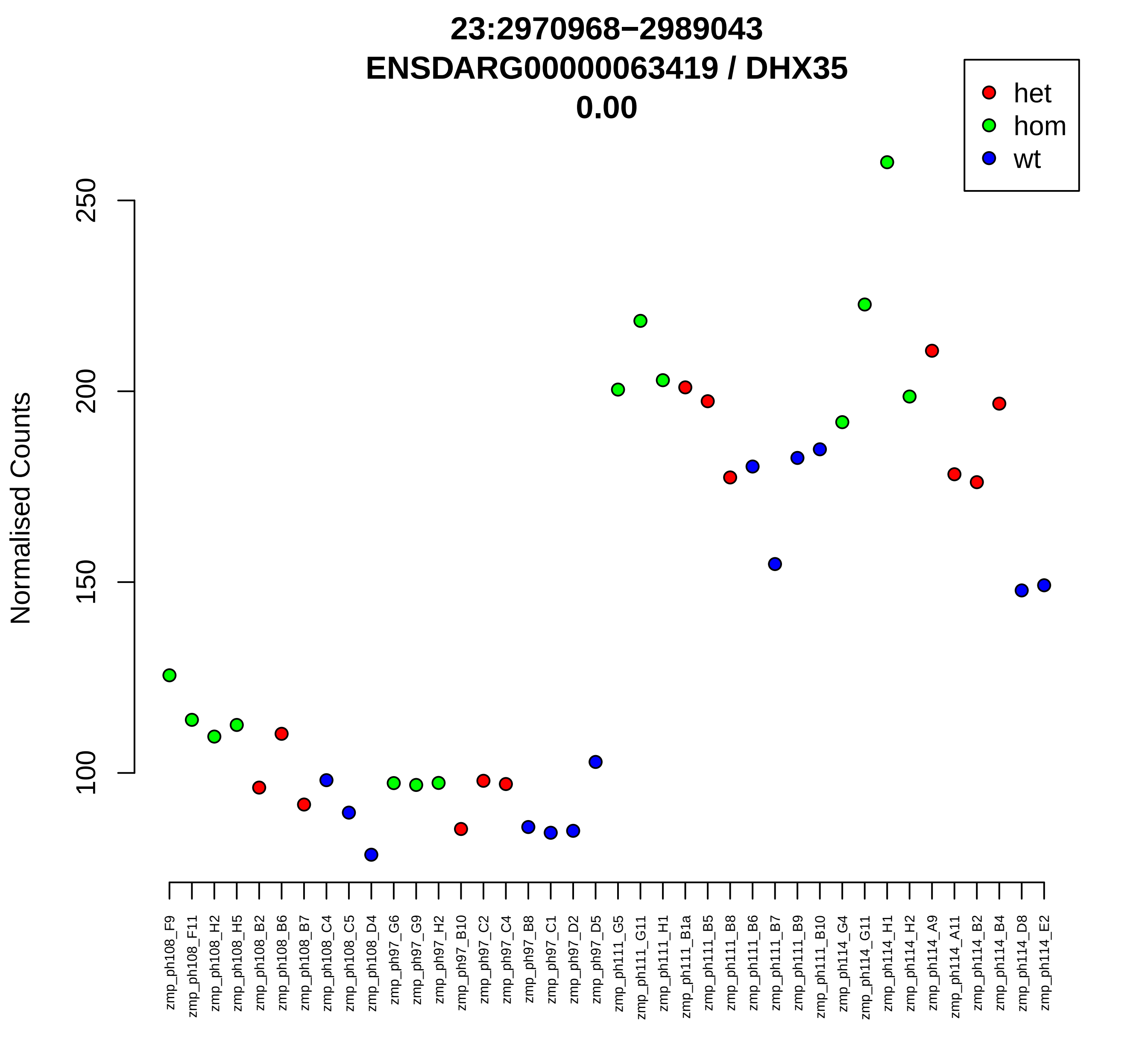

23:2970968−2989043
ENSDARG00000063419 / DHX35
0.00
het
hom
wt
Normalised Counts
100
150
200
250
zmp_ph108_F9
zmp_ph108_F11
zmp_ph108_H2
zmp_ph108_H5
zmp_ph108_B2
zmp_ph108_B6
zmp_ph108_B7
zmp_ph108_C4
zmp_ph108_C5
zmp_ph108_D4
zmp_ph97_G6
zmp_ph97_G9
zmp_ph97_H2
zmp_ph97_B10
zmp_ph97_C2
zmp_ph97_C4
zmp_ph97_B8
zmp_ph97_C1
zmp_ph97_D2
zmp_ph97_D5
zmp_ph111_G5
zmp_ph111_G11
zmp_ph111_H1
zmp_ph111_B1a
zmp_ph111_B5
zmp_ph111_B8
zmp_ph111_B6
zmp_ph111_B7
zmp_ph111_B9
zmp_ph111_B10
zmp_ph114_G4
zmp_ph114_G11
zmp_ph114_H1
zmp_ph114_H2
zmp_ph114_A9
zmp_ph114_A11
zmp_ph114_B2
zmp_ph114_B4
zmp_ph114_D8
zmp_ph114_E2

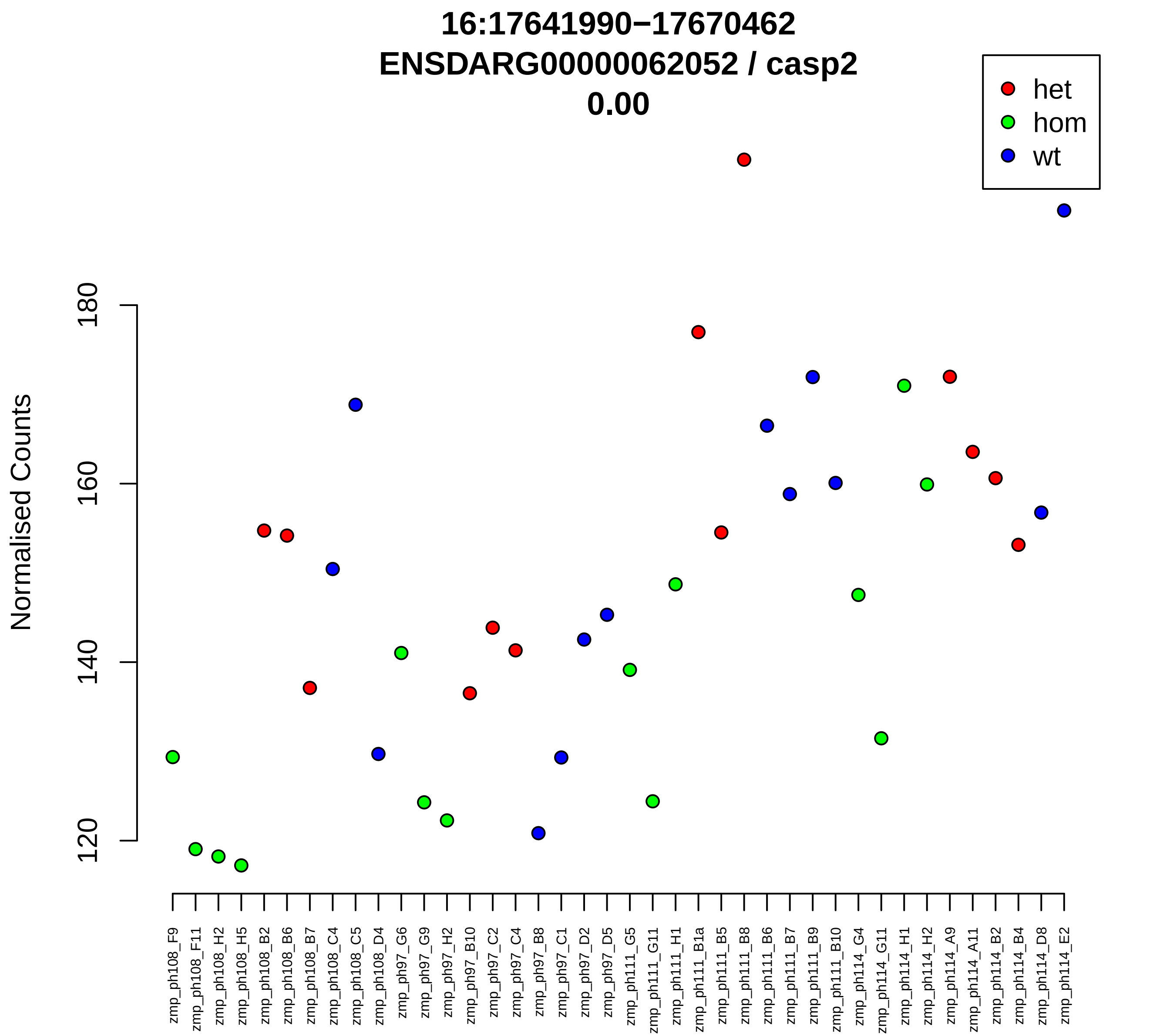

16:17641990−17670462
ENSDARG00000062052 / casp2
0.00
het
hom
wt
Normalised Counts
120
140
160
180
zmp_ph108_F9
zmp_ph108_F11
zmp_ph108_H2
zmp_ph108_H5
zmp_ph108_B2
zmp_ph108_B6
zmp_ph108_B7
zmp_ph108_C4
zmp_ph108_C5
zmp_ph108_D4
zmp_ph97_G6
zmp_ph97_G9
zmp_ph97_H2
zmp_ph97_B10
zmp_ph97_C2
zmp_ph97_C4
zmp_ph97_B8
zmp_ph97_C1
zmp_ph97_D2
zmp_ph97_D5
zmp_ph111_G5
zmp_ph111_G11
zmp_ph111_H1
zmp_ph111_B1a
zmp_ph111_B5
zmp_ph111_B8
zmp_ph111_B6
zmp_ph111_B7
zmp_ph111_B9
zmp_ph111_B10
zmp_ph114_G4
zmp_ph114_G11
zmp_ph114_H1
zmp_ph114_H2
zmp_ph114_A9
zmp_ph114_A11
zmp_ph114_B2
zmp_ph114_B4
zmp_ph114_D8
zmp_ph114_E2

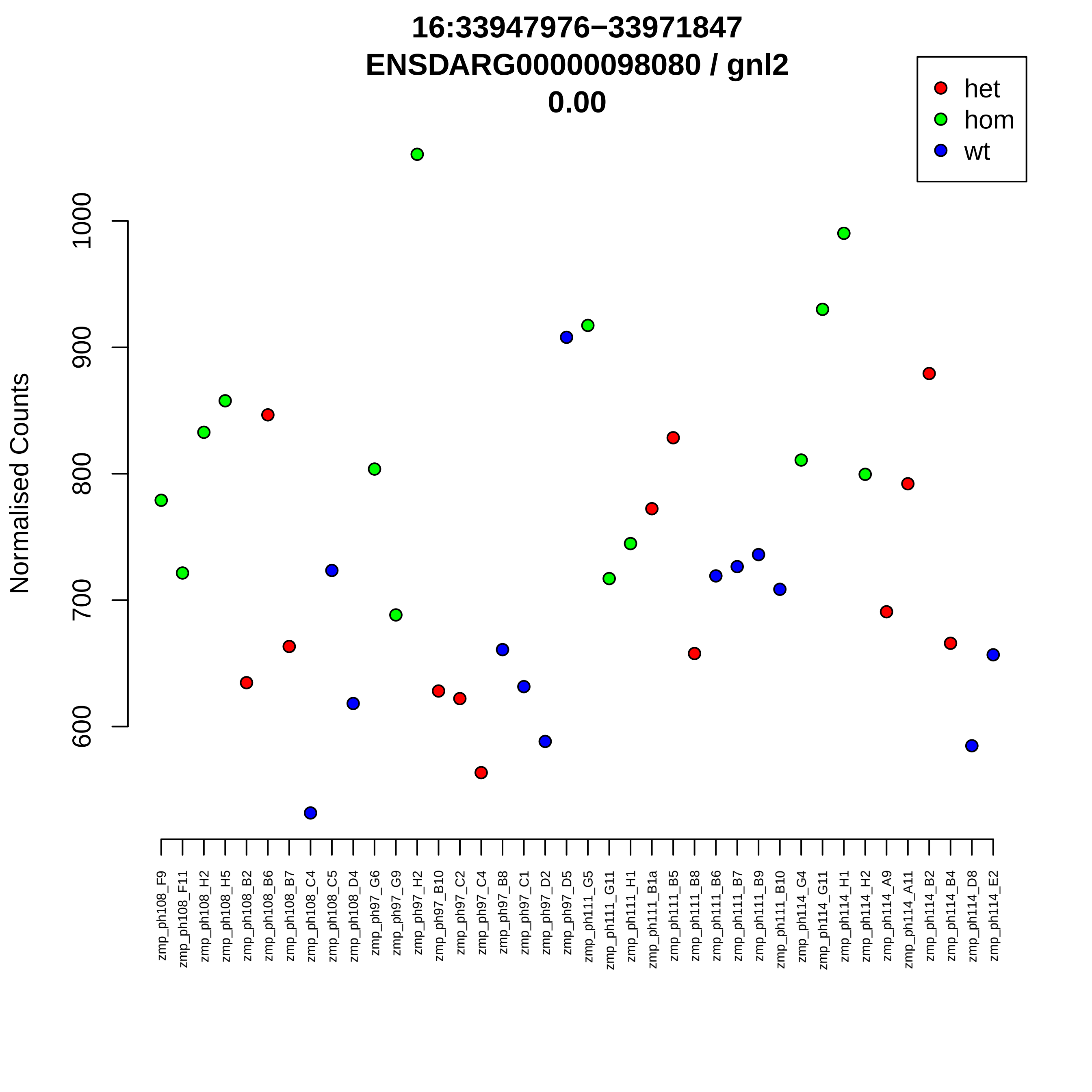

16:33947976−33971847
ENSDARG00000098080 / gnl2
0.00
het
hom
wt
Normalised Counts
600
700
800
900
1000
zmp_ph108_F9
zmp_ph108_F11
zmp_ph108_H2
zmp_ph108_H5
zmp_ph108_B2
zmp_ph108_B6
zmp_ph108_B7
zmp_ph108_C4
zmp_ph108_C5
zmp_ph108_D4
zmp_ph97_G6
zmp_ph97_G9
zmp_ph97_H2
zmp_ph97_B10
zmp_ph97_C2
zmp_ph97_C4
zmp_ph97_B8
zmp_ph97_C1
zmp_ph97_D2
zmp_ph97_D5
zmp_ph111_G5
zmp_ph111_G11
zmp_ph111_H1
zmp_ph111_B1a
zmp_ph111_B5
zmp_ph111_B8
zmp_ph111_B6
zmp_ph111_B7
zmp_ph111_B9
zmp_ph111_B10
zmp_ph114_G4
zmp_ph114_G11
zmp_ph114_H1
zmp_ph114_H2
zmp_ph114_A9
zmp_ph114_A11
zmp_ph114_B2
zmp_ph114_B4
zmp_ph114_D8
zmp_ph114_E2

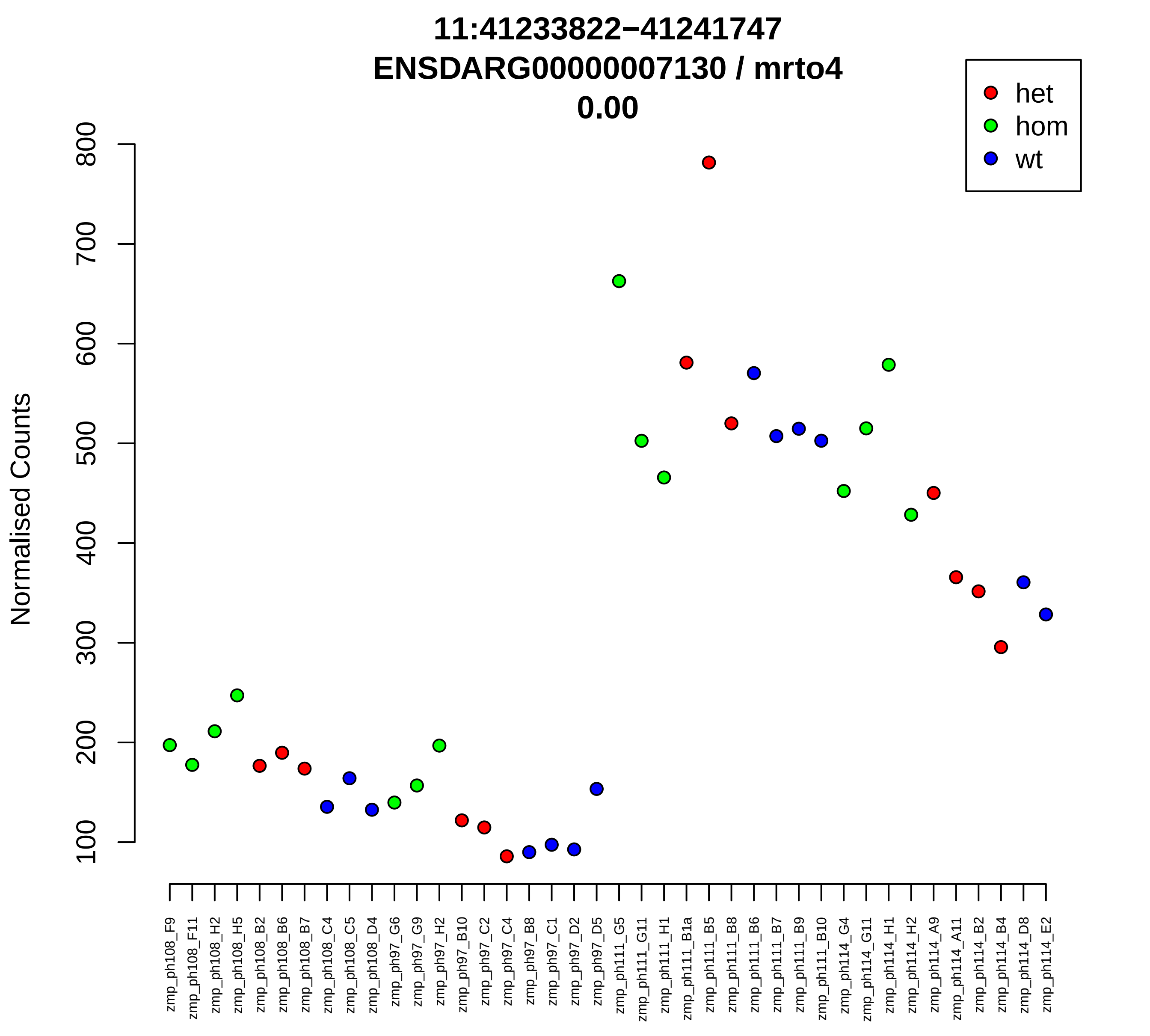
11:41233822−41241747
ENSDARG00000007130 / mrto4
0.00
het
hom
wt
Normalised Counts
100
200
300
400
500
600
700
800
zmp_ph108_F9
zmp_ph108_F11
zmp_ph108_H2
zmp_ph108_H5
zmp_ph108_B2
zmp_ph108_B6
zmp_ph108_B7
zmp_ph108_C4
zmp_ph108_C5
zmp_ph108_D4
zmp_ph97_G6
zmp_ph97_G9
zmp_ph97_H2
zmp_ph97_B10
zmp_ph97_C2
zmp_ph97_C4
zmp_ph97_B8
zmp_ph97_C1
zmp_ph97_D2
zmp_ph97_D5
zmp_ph111_G5
zmp_ph111_G11
zmp_ph111_H1
zmp_ph111_B1a
zmp_ph111_B5
zmp_ph111_B8
zmp_ph111_B6
zmp_ph111_B7
zmp_ph111_B9
zmp_ph111_B10
zmp_ph114_G4
zmp_ph114_G11
zmp_ph114_H1
zmp_ph114_H2
zmp_ph114_A9
zmp_ph114_A11
zmp_ph114_B2
zmp_ph114_B4
zmp_ph114_D8
zmp_ph114_E2

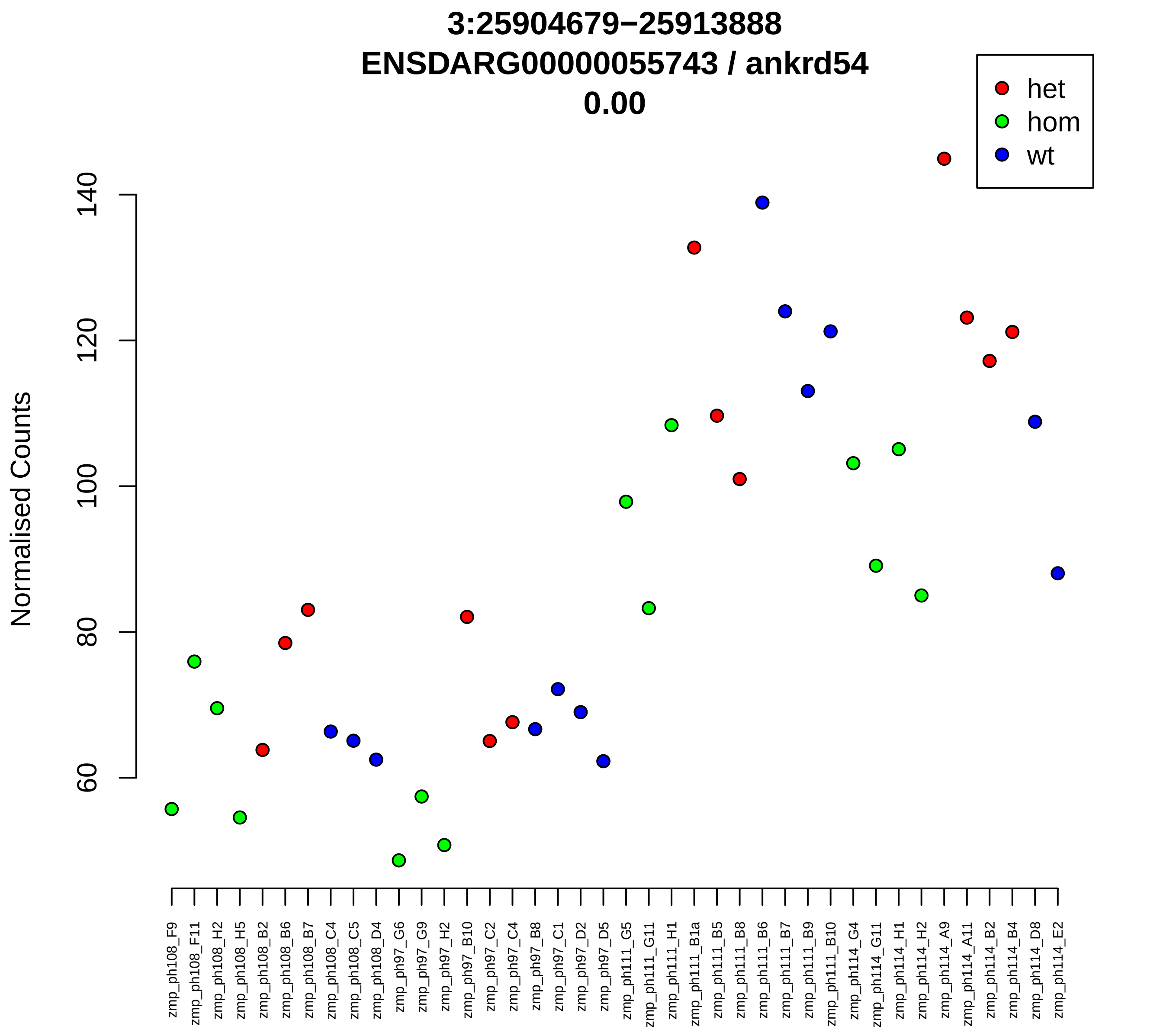
3:25904679−25913888
ENSDARG00000055743 / ankrd54
0.00
het
hom
wt
Normalised Counts
60
80
100
120
140
zmp_ph108_F9
zmp_ph108_F11
zmp_ph108_H2
zmp_ph108_H5
zmp_ph108_B2
zmp_ph108_B6
zmp_ph108_B7
zmp_ph108_C4
zmp_ph108_C5
zmp_ph108_D4
zmp_ph97_G6
zmp_ph97_G9
zmp_ph97_H2
zmp_ph97_B10
zmp_ph97_C2
zmp_ph97_C4
zmp_ph97_B8
zmp_ph97_C1
zmp_ph97_D2
zmp_ph97_D5
zmp_ph111_G5
zmp_ph111_G11
zmp_ph111_H1
zmp_ph111_B1a
zmp_ph111_B5
zmp_ph111_B8
zmp_ph111_B6
zmp_ph111_B7
zmp_ph111_B9
zmp_ph111_B10
zmp_ph114_G4
zmp_ph114_G11
zmp_ph114_H1
zmp_ph114_H2
zmp_ph114_A9
zmp_ph114_A11
zmp_ph114_B2
zmp_ph114_B4
zmp_ph114_D8
zmp_ph114_E2

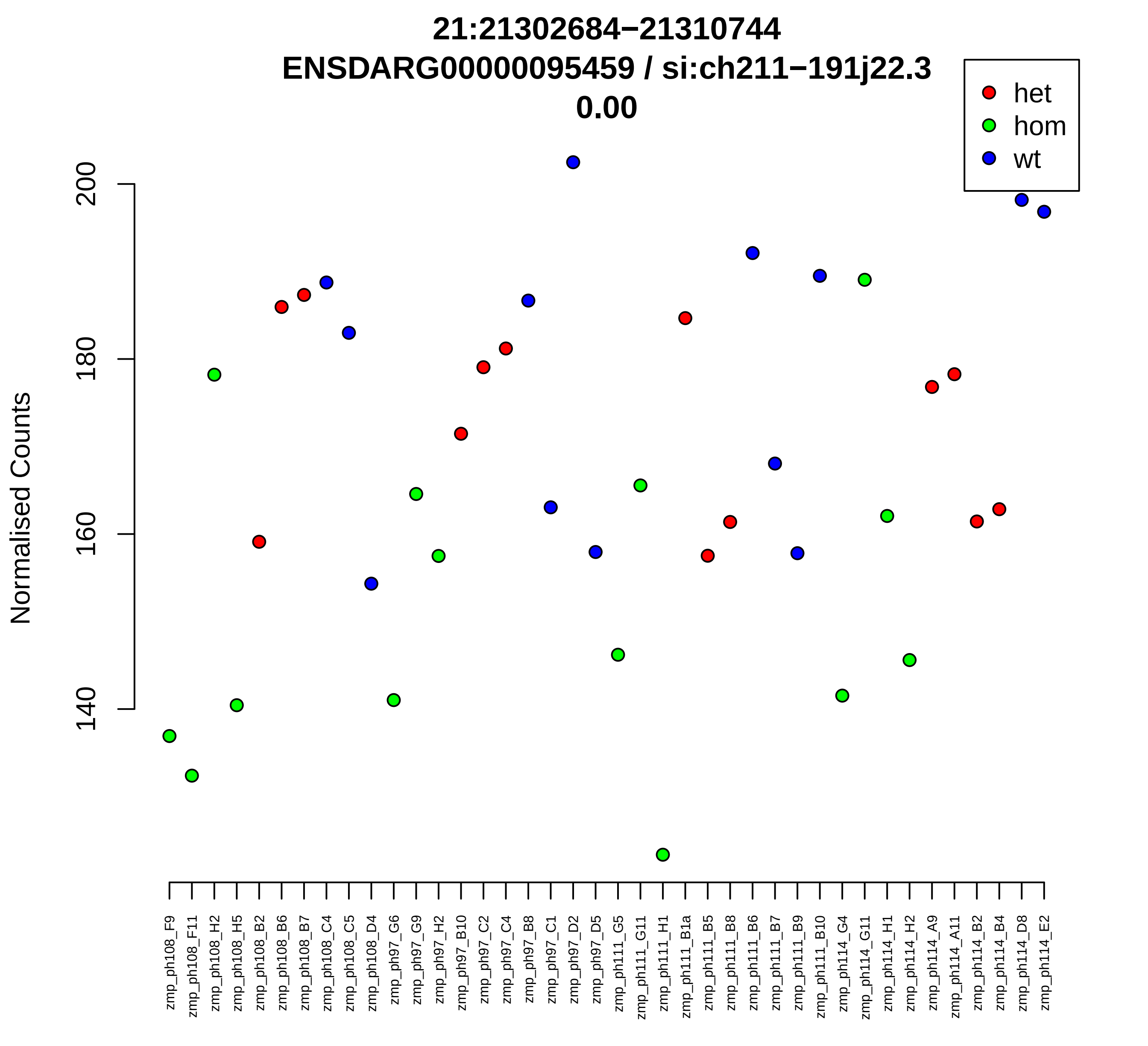

21:21302684−21310744
ENSDARG00000095459 / si:ch211−191j22.3
0.00
het
hom
wt
Normalised Counts
200
180
160
140
zmp_ph108_F9
zmp_ph108_F11
zmp_ph108_H2
zmp_ph108_H5
zmp_ph108_B2
zmp_ph108_B6
zmp_ph108_B7
zmp_ph108_C4
zmp_ph108_C5
zmp_ph108_D4
zmp_ph97_G6
zmp_ph97_G9
zmp_ph97_H2
zmp_ph97_B10
zmp_ph97_C2
zmp_ph97_C4
zmp_ph97_B8
zmp_ph97_C1
zmp_ph97_D2
zmp_ph97_D5
zmp_ph111_G5
zmp_ph111_G11
zmp_ph111_H1
zmp_ph111_B1a
zmp_ph111_B5
zmp_ph111_B8
zmp_ph111_B6
zmp_ph111_B7
zmp_ph111_B9
zmp_ph111_B10
zmp_ph114_G4
zmp_ph114_G11
zmp_ph114_H1
zmp_ph114_H2
zmp_ph114_A9
zmp_ph114_A11
zmp_ph114_B2
zmp_ph114_B4
zmp_ph114_D8
zmp_ph114_E2

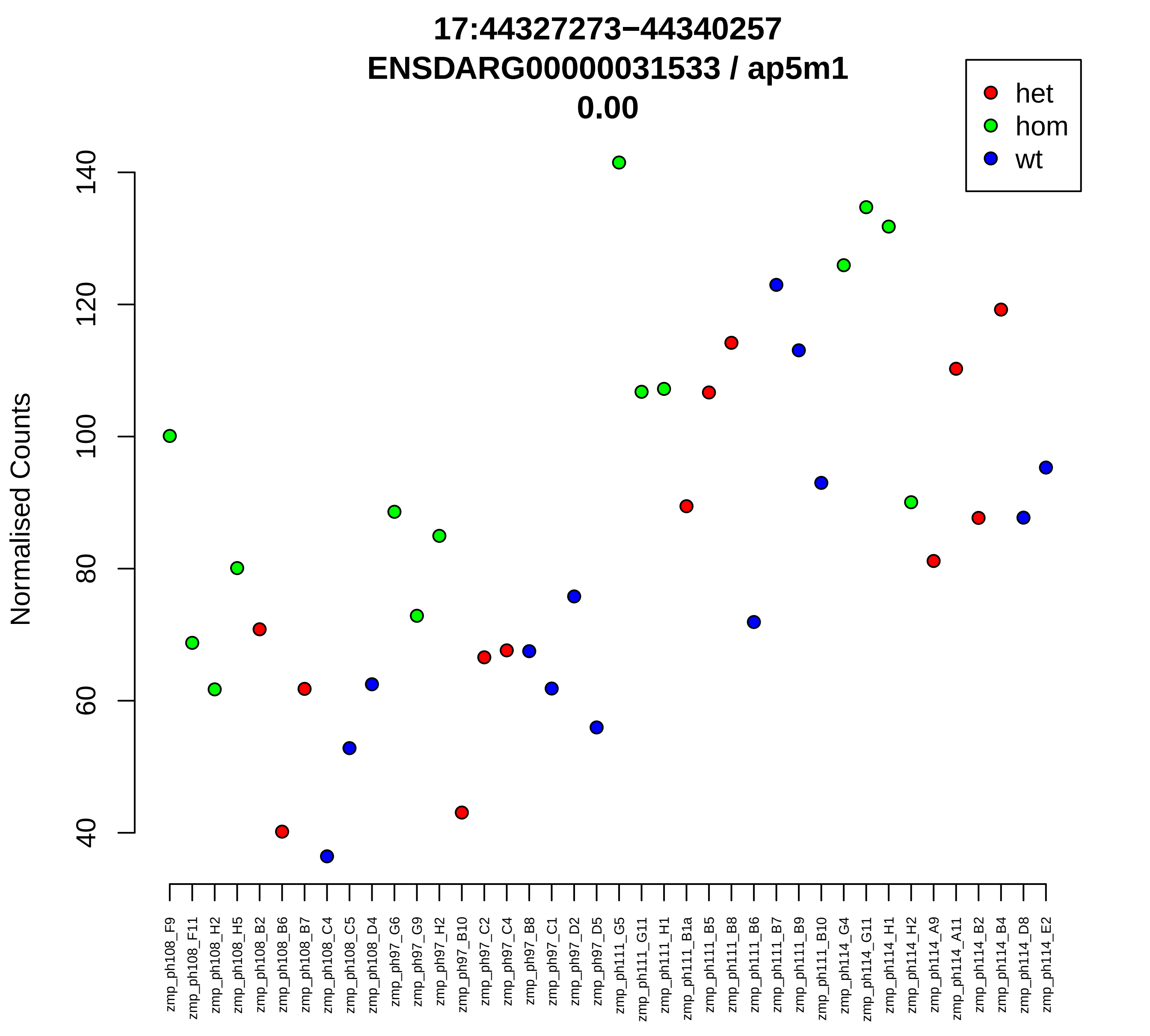

17:44327273−44340257
ENSDARG00000031533 / ap5m1
0.00
het
hom
wt
Normalised Counts
40
60
80
100
120
140
zmp_ph108_F9
zmp_ph108_F11
zmp_ph108_H2
zmp_ph108_H5
zmp_ph108_B2
zmp_ph108_B6
zmp_ph108_B7
zmp_ph108_C4
zmp_ph108_C5
zmp_ph108_D4
zmp_ph97_G6
zmp_ph97_G9
zmp_ph97_H2
zmp_ph97_B10
zmp_ph97_C2
zmp_ph97_C4
zmp_ph97_B8
zmp_ph97_C1
zmp_ph97_D2
zmp_ph97_D5
zmp_ph111_G5
zmp_ph111_G11
zmp_ph111_H1
zmp_ph111_B1a
zmp_ph111_B5
zmp_ph111_B8
zmp_ph111_B6
zmp_ph111_B7
zmp_ph111_B9
zmp_ph111_B10
zmp_ph114_G4
zmp_ph114_G11
zmp_ph114_H1
zmp_ph114_H2
zmp_ph114_A9
zmp_ph114_A11
zmp_ph114_B2
zmp_ph114_B4
zmp_ph114_D8
zmp_ph114_E2

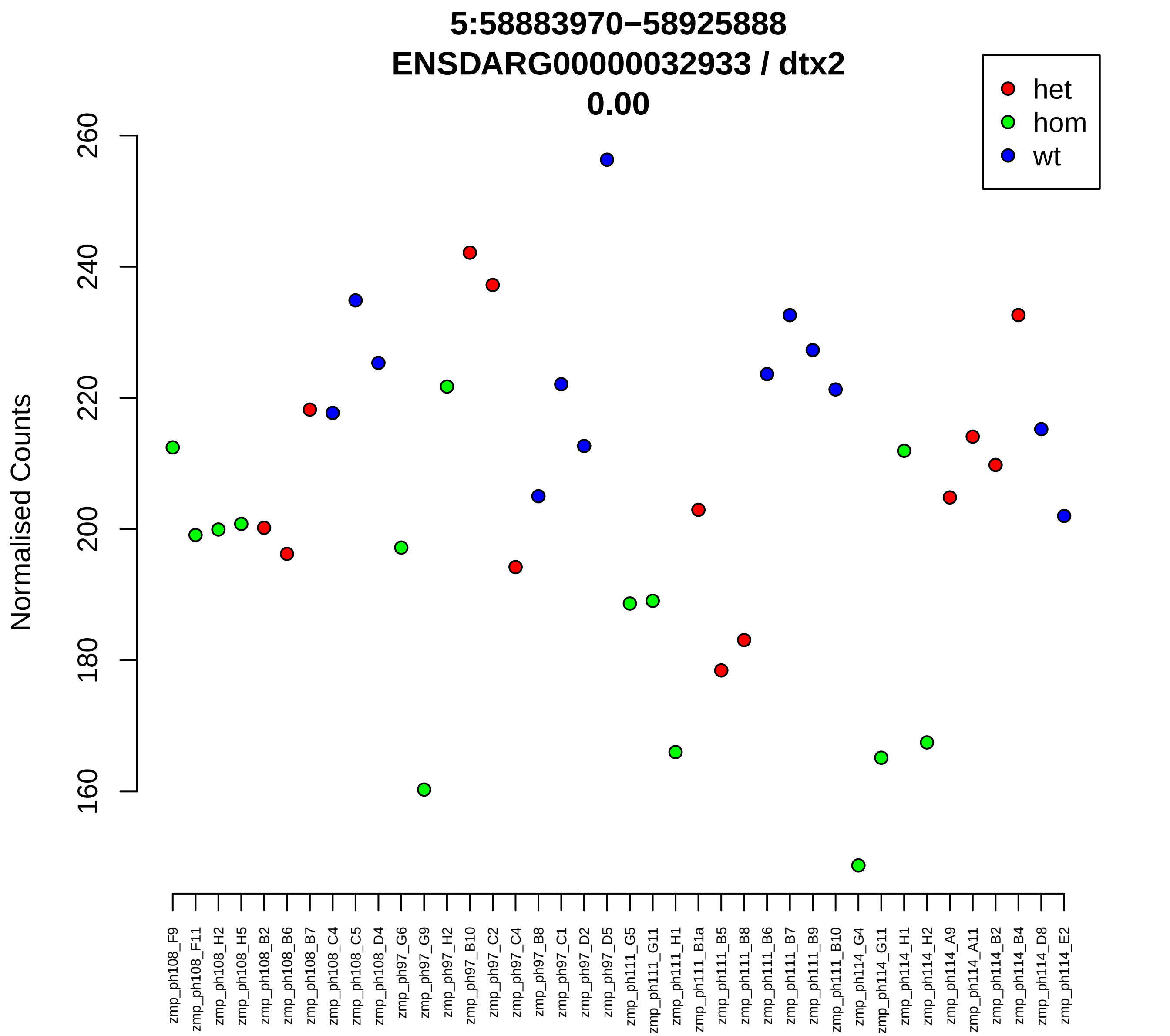

5:58883970−58925888
ENSDARG00000032933 / dtx2
0.00
het
hom
wt
Normalised Counts
160
180
200
220
240
260
zmp_ph108_F9
zmp_ph108_F11
zmp_ph108_H2
zmp_ph108_H5
zmp_ph108_B2
zmp_ph108_B6
zmp_ph108_B7
zmp_ph108_C4
zmp_ph108_C5
zmp_ph108_D4
zmp_ph97_G6
zmp_ph97_G9
zmp_ph97_H2
zmp_ph97_B10
zmp_ph97_C2
zmp_ph97_C4
zmp_ph97_B8
zmp_ph97_C1
zmp_ph97_D2
zmp_ph97_D5
zmp_ph111_G5
zmp_ph111_G11
zmp_ph111_H1
zmp_ph111_B1a
zmp_ph111_B5
zmp_ph111_B8
zmp_ph111_B6
zmp_ph111_B7
zmp_ph111_B9
zmp_ph111_B10
zmp_ph114_G4
zmp_ph114_G11
zmp_ph114_H1
zmp_ph114_H2
zmp_ph114_A9
zmp_ph114_A11
zmp_ph114_B2
zmp_ph114_B4
zmp_ph114_D8
zmp_ph114_E2

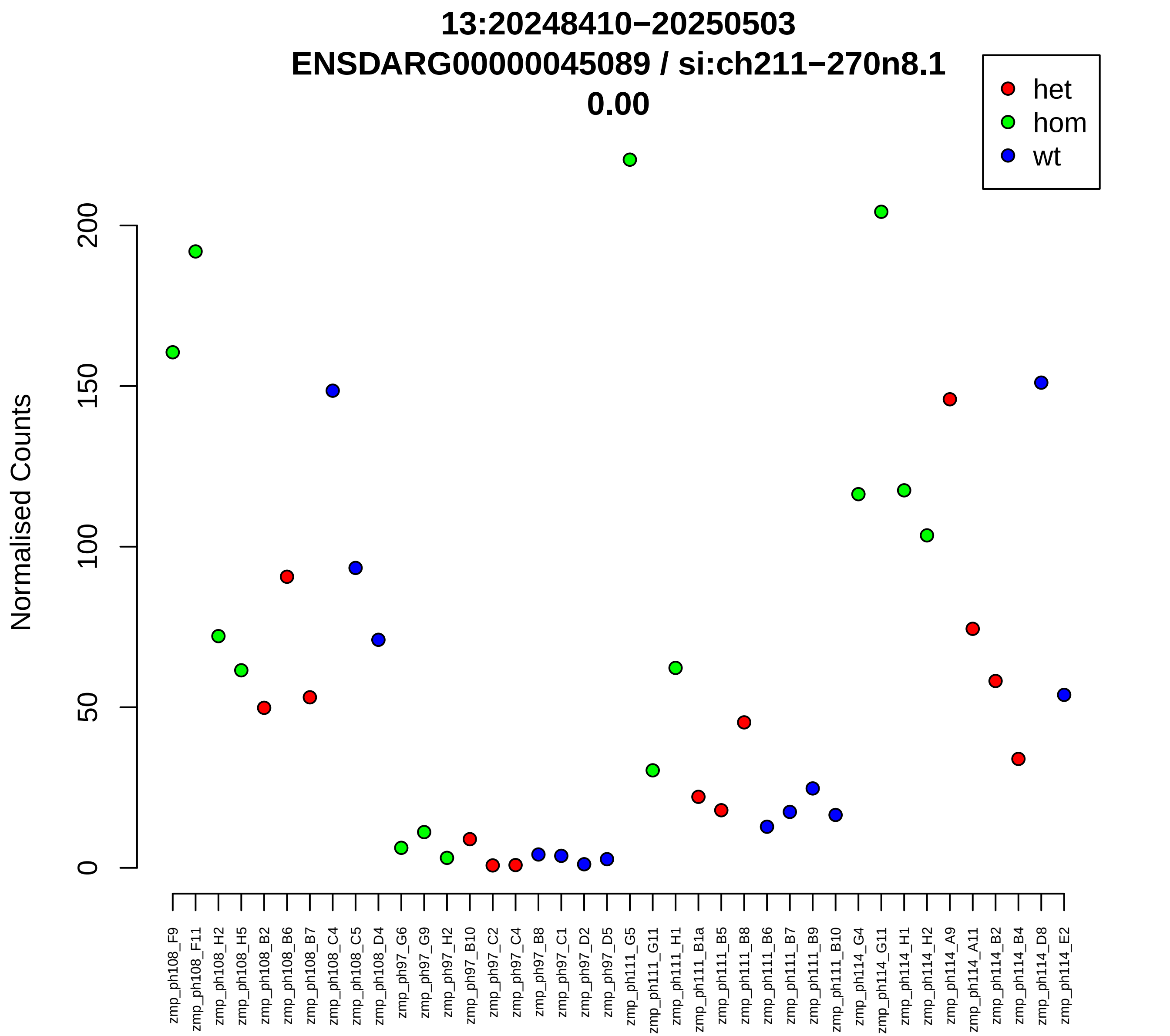

13:20248410−20250503
ENSDARG00000045089 / si:ch211−270n8.1
0.00
het
hom
wt
Normalised Counts
0
50
100
150
200
zmp_ph108_F9
zmp_ph108_F11
zmp_ph108_H2
zmp_ph108_H5
zmp_ph108_B2
zmp_ph108_B6
zmp_ph108_B7
zmp_ph108_C4
zmp_ph108_C5
zmp_ph108_D4
zmp_ph97_G6
zmp_ph97_G9
zmp_ph97_H2
zmp_ph97_B10
zmp_ph97_C2
zmp_ph97_C4
zmp_ph97_B8
zmp_ph97_C1
zmp_ph97_D2
zmp_ph97_D5
zmp_ph111_G5
zmp_ph111_G11
zmp_ph111_H1
zmp_ph111_B1a
zmp_ph111_B5
zmp_ph111_B8
zmp_ph111_B6
zmp_ph111_B7
zmp_ph111_B9
zmp_ph111_B10
zmp_ph114_G4
zmp_ph114_G11
zmp_ph114_H1
zmp_ph114_H2
zmp_ph114_A9
zmp_ph114_A11
zmp_ph114_B2
zmp_ph114_B4
zmp_ph114_D8
zmp_ph114_E2

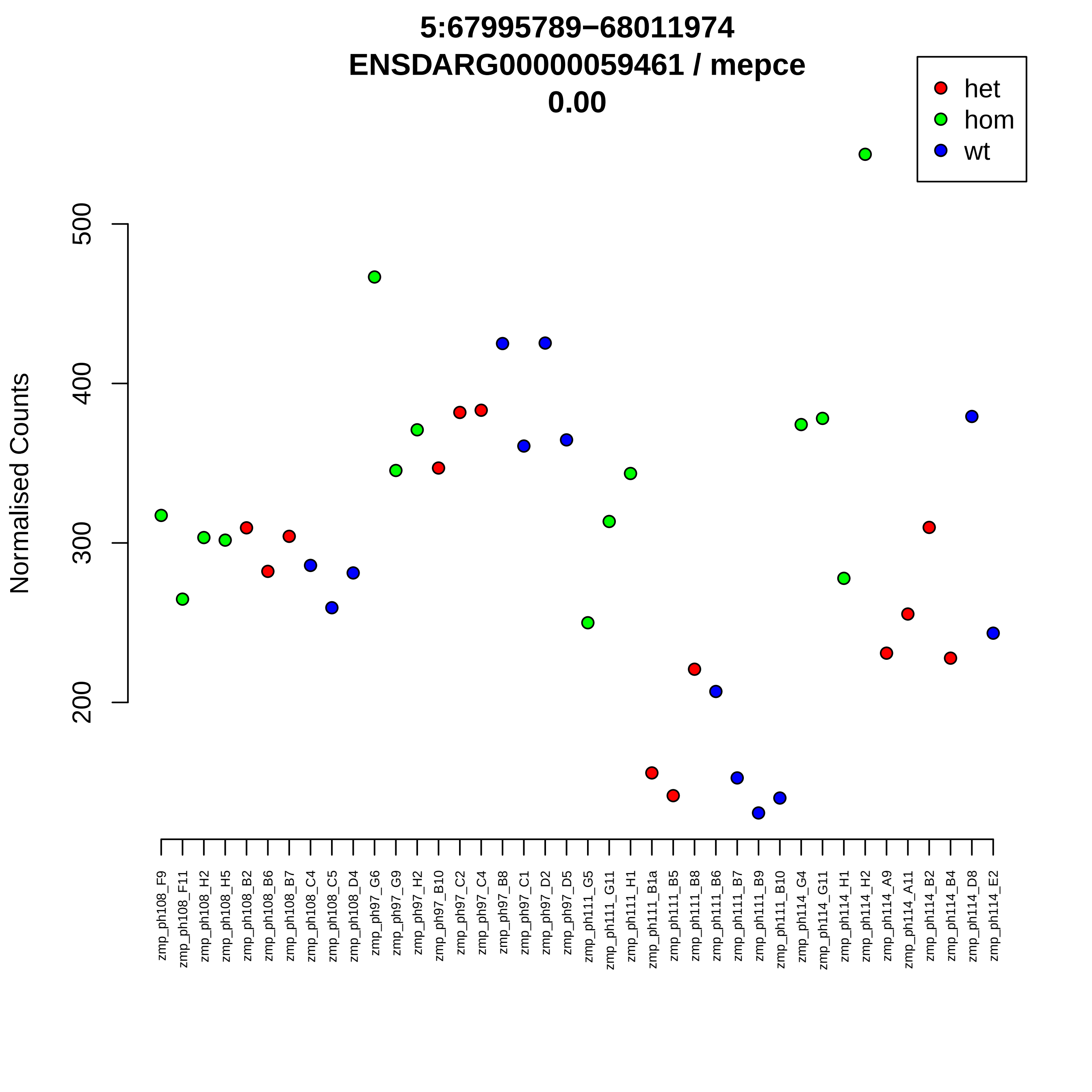

5:67995789−68011974
ENSDARG00000059461 / mepce
0.00
het
hom
wt
Normalised Counts
200
300
400
500
zmp_ph108_F9
zmp_ph108_F11
zmp_ph108_H2
zmp_ph108_H5
zmp_ph108_B2
zmp_ph108_B6
zmp_ph108_B7
zmp_ph108_C4
zmp_ph108_C5
zmp_ph108_D4
zmp_ph97_G6
zmp_ph97_G9
zmp_ph97_H2
zmp_ph97_B10
zmp_ph97_C2
zmp_ph97_C4
zmp_ph97_B8
zmp_ph97_C1
zmp_ph97_D2
zmp_ph97_D5
zmp_ph111_G5
zmp_ph111_G11
zmp_ph111_H1
zmp_ph111_B1a
zmp_ph111_B5
zmp_ph111_B8
zmp_ph111_B6
zmp_ph111_B7
zmp_ph111_B9
zmp_ph111_B10
zmp_ph114_G4
zmp_ph114_G11
zmp_ph114_H1
zmp_ph114_H2
zmp_ph114_A9
zmp_ph114_A11
zmp_ph114_B2
zmp_ph114_B4
zmp_ph114_D8
zmp_ph114_E2

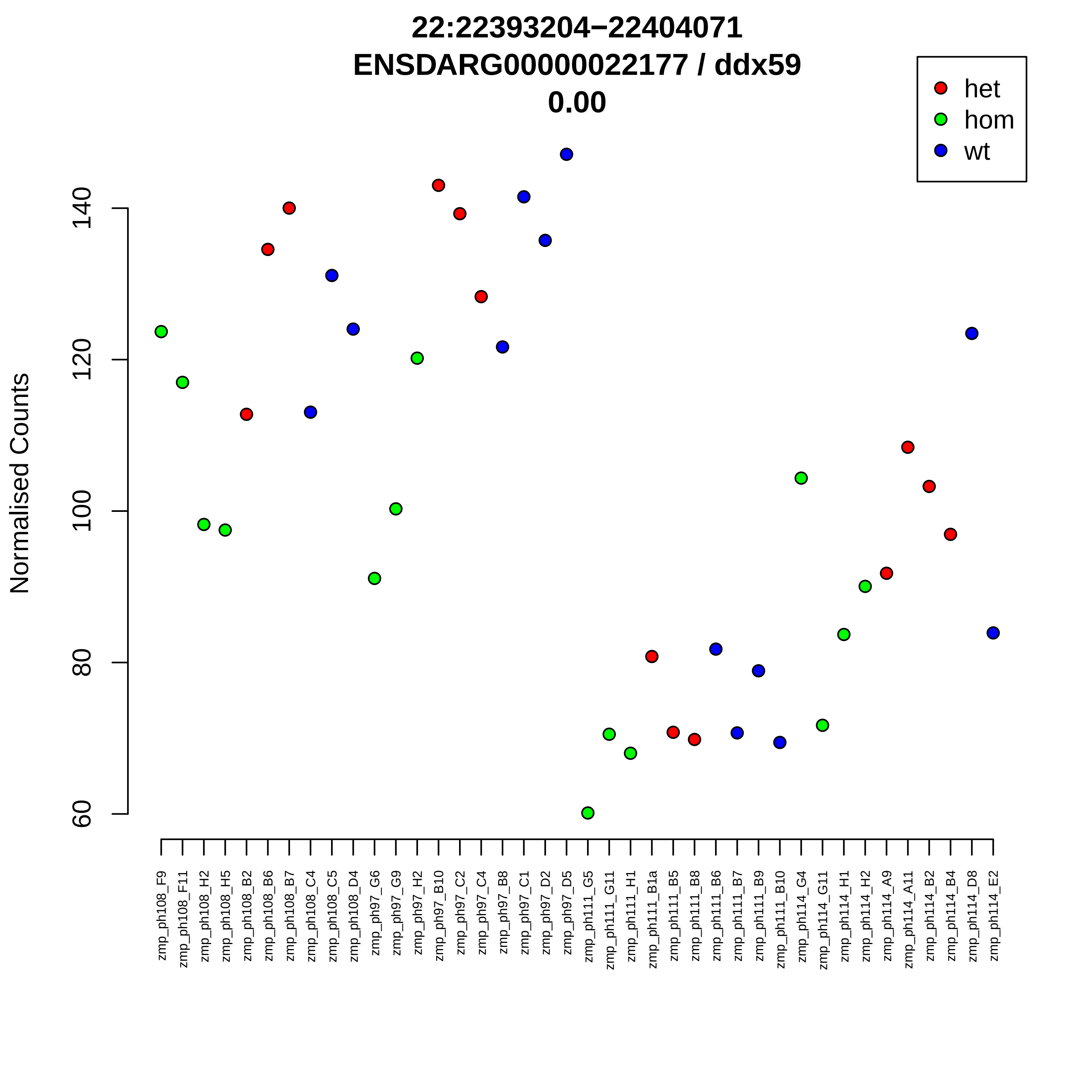
22:22393204−22404071
ENSDARG00000022177 / ddx59
0.00
het
hom
wt
Normalised Counts
60
80
100
120
140
zmp_ph108_F9
zmp_ph108_F11
zmp_ph108_H2
zmp_ph108_H5
zmp_ph108_B2
zmp_ph108_B6
zmp_ph108_B7
zmp_ph108_C4
zmp_ph108_C5
zmp_ph108_D4
zmp_ph97_G6
zmp_ph97_G9
zmp_ph97_H2
zmp_ph97_B10
zmp_ph97_C2
zmp_ph97_C4
zmp_ph97_B8
zmp_ph97_C1
zmp_ph97_D2
zmp_ph97_D5
zmp_ph111_G5
zmp_ph111_G11
zmp_ph111_H1
zmp_ph111_B1a
zmp_ph111_B5
zmp_ph111_B8
zmp_ph111_B6
zmp_ph111_B7
zmp_ph111_B9
zmp_ph111_B10
zmp_ph114_G4
zmp_ph114_G11
zmp_ph114_H1
zmp_ph114_H2
zmp_ph114_A9
zmp_ph114_A11
zmp_ph114_B2
zmp_ph114_B4
zmp_ph114_D8
zmp_ph114_E2

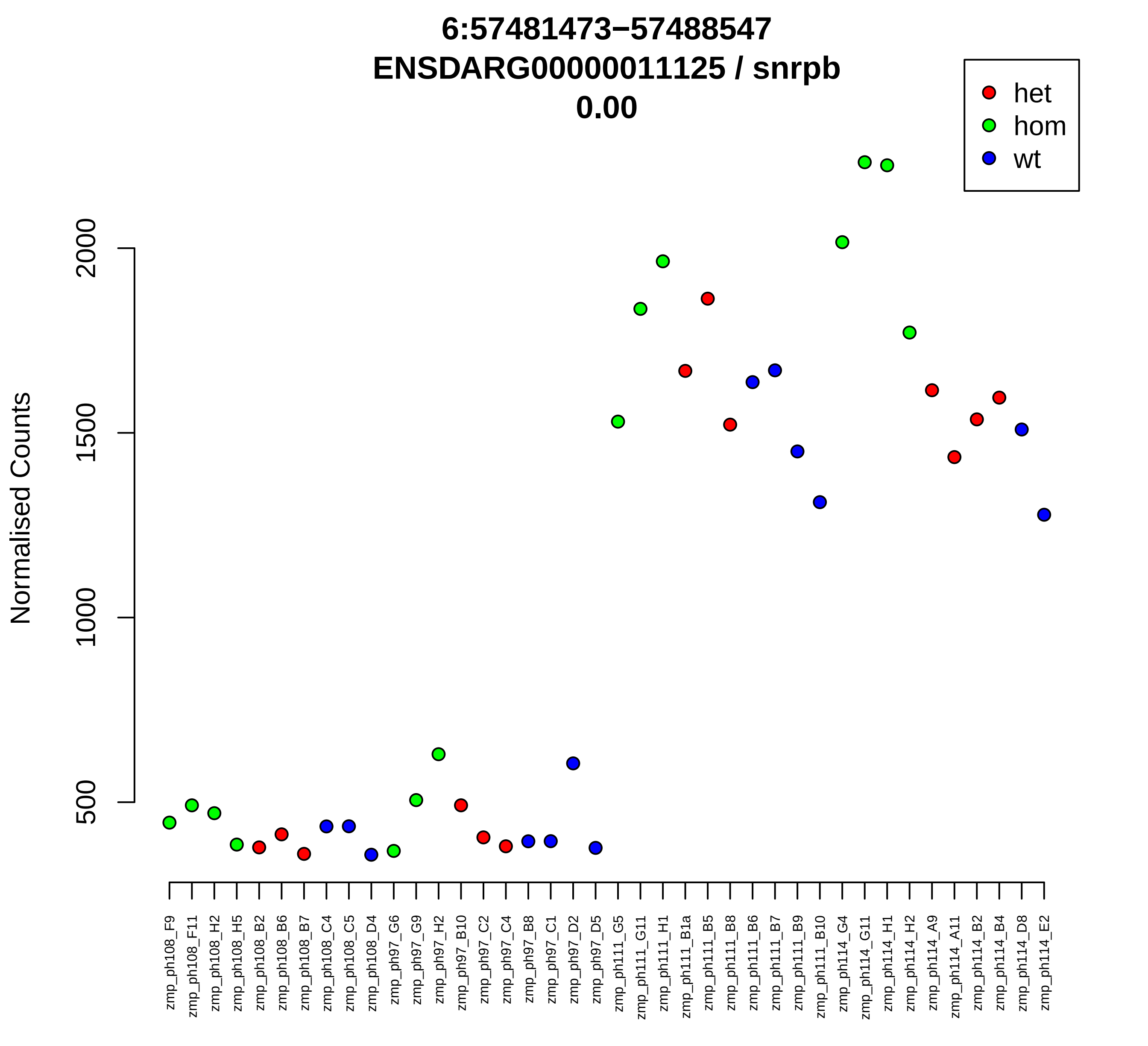
6:57481473−57488547
ENSDARG00000011125 / snrpb
0.00
het
hom
wt
Normalised Counts
500
1000
1500
2000
zmp_ph108_F9
zmp_ph108_F11
zmp_ph108_H2
zmp_ph108_H5
zmp_ph108_B2
zmp_ph108_B6
zmp_ph108_B7
zmp_ph108_C4
zmp_ph108_C5
zmp_ph108_D4
zmp_ph97_G6
zmp_ph97_G9
zmp_ph97_H2
zmp_ph97_B10
zmp_ph97_C2
zmp_ph97_C4
zmp_ph97_B8
zmp_ph97_C1
zmp_ph97_D2
zmp_ph97_D5
zmp_ph111_G5
zmp_ph111_G11
zmp_ph111_H1
zmp_ph111_B1a
zmp_ph111_B5
zmp_ph111_B8
zmp_ph111_B6
zmp_ph111_B7
zmp_ph111_B9
zmp_ph111_B10
zmp_ph114_G4
zmp_ph114_G11
zmp_ph114_H1
zmp_ph114_H2
zmp_ph114_A9
zmp_ph114_A11
zmp_ph114_B2
zmp_ph114_B4
zmp_ph114_D8
zmp_ph114_E2

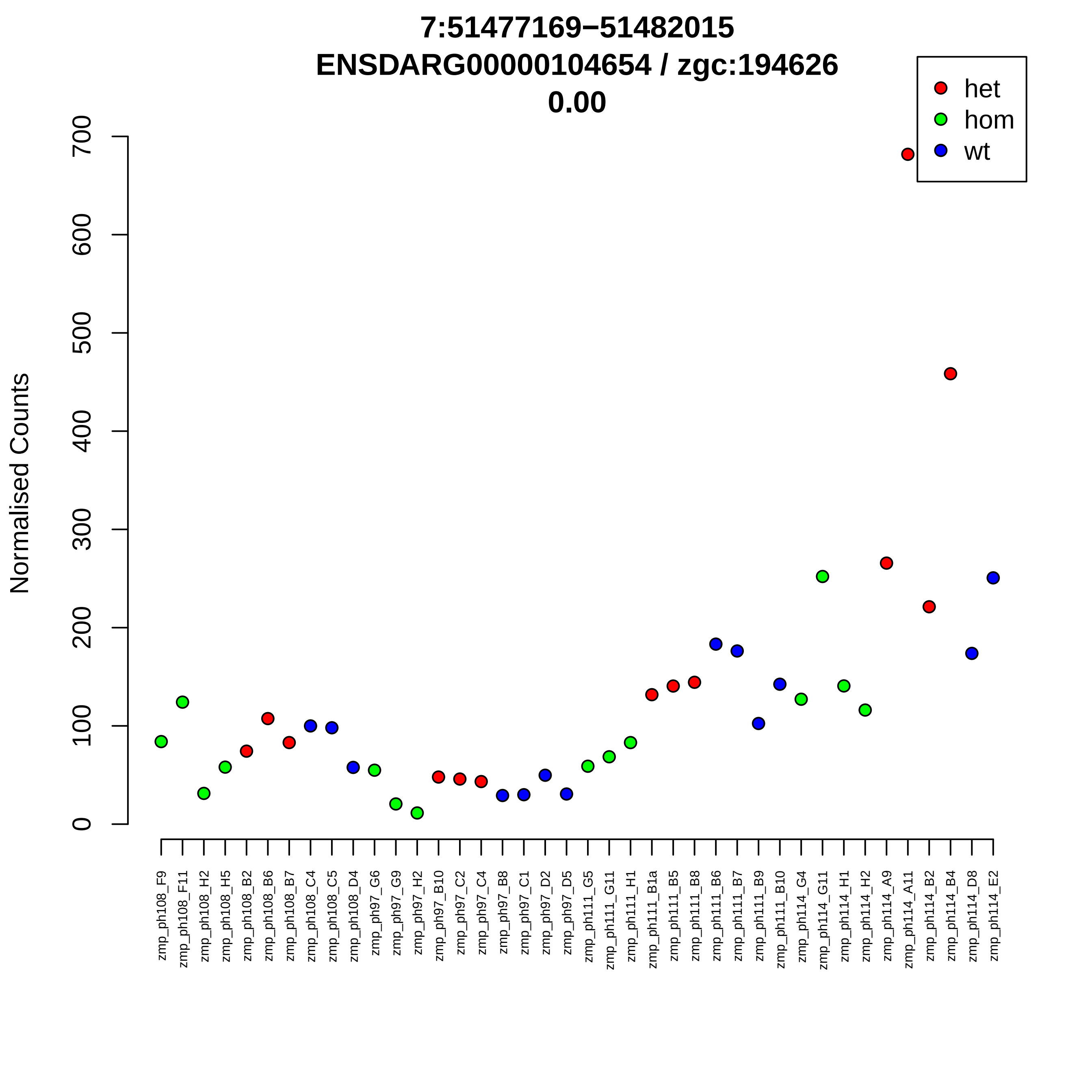
7:51477169−51482015
ENSDARG00000104654 / zgc:194626
0.00
het
hom
wt
Normalised Counts
0
100
200
300
400
500
600
700
zmp_ph108_F9
zmp_ph108_F11
zmp_ph108_H2
zmp_ph108_H5
zmp_ph108_B2
zmp_ph108_B6
zmp_ph108_B7
zmp_ph108_C4
zmp_ph108_C5
zmp_ph108_D4
zmp_ph97_G6
zmp_ph97_G9
zmp_ph97_H2
zmp_ph97_B10
zmp_ph97_C2
zmp_ph97_C4
zmp_ph97_B8
zmp_ph97_C1
zmp_ph97_D2
zmp_ph97_D5
zmp_ph111_G5
zmp_ph111_G11
zmp_ph111_H1
zmp_ph111_B1a
zmp_ph111_B5
zmp_ph111_B8
zmp_ph111_B6
zmp_ph111_B7
zmp_ph111_B9
zmp_ph111_B10
zmp_ph114_G4
zmp_ph114_G11
zmp_ph114_H1
zmp_ph114_H2
zmp_ph114_A9
zmp_ph114_A11
zmp_ph114_B2
zmp_ph114_B4
zmp_ph114_D8
zmp_ph114_E2

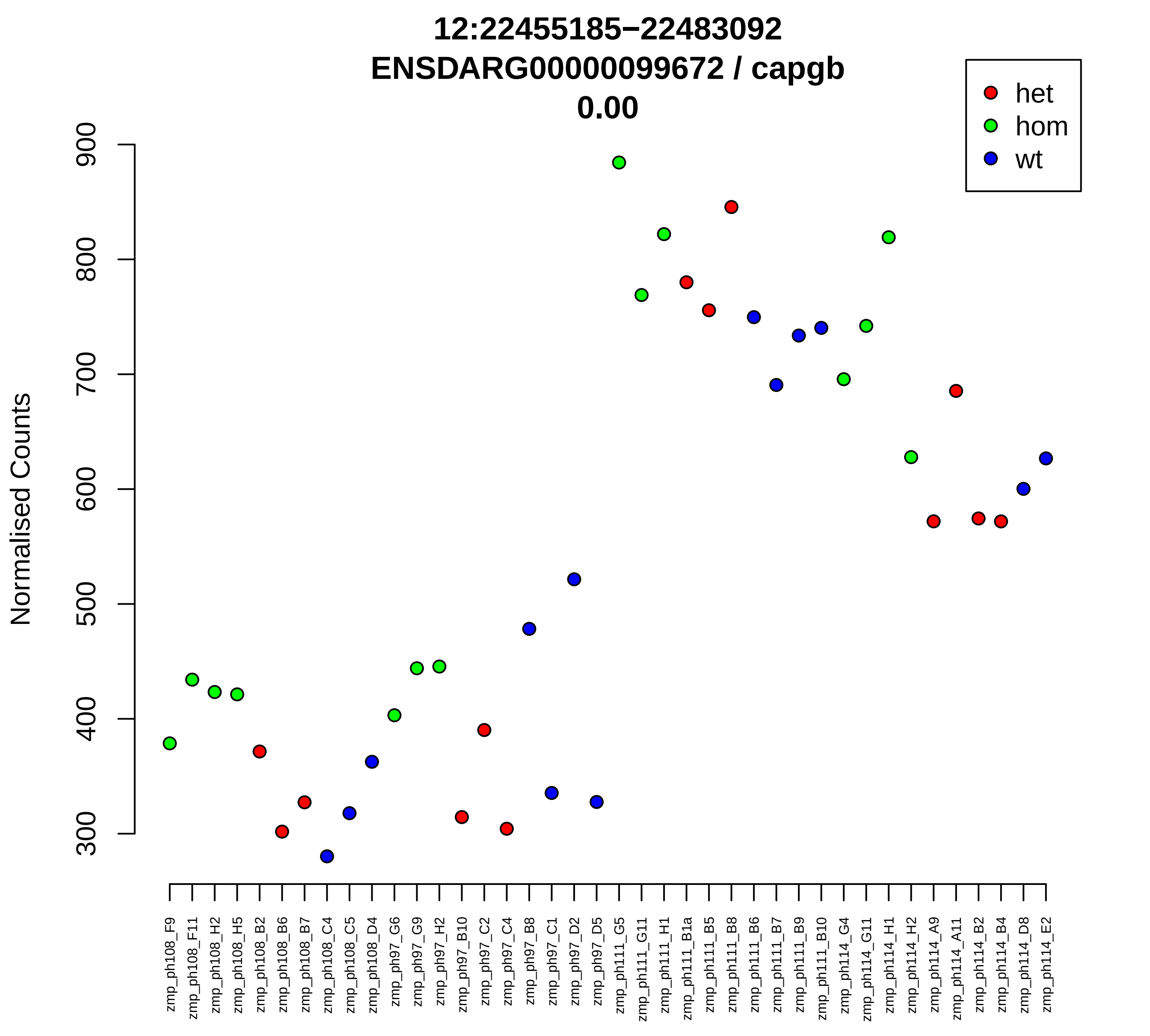

12:22455185−22483092
ENSDARG00000099672 / capgb
0.00
Normalised Counts
300
400
500
600
700
800
900
het
hom
wt
zmp_ph108_F9
zmp_ph108_F11
zmp_ph108_H2
zmp_ph108_H5
zmp_ph108_B2
zmp_ph108_B6
zmp_ph108_B7
zmp_ph108_C4
zmp_ph108_C5
zmp_ph108_D4
zmp_ph97_G6
zmp_ph97_G9
zmp_ph97_H2
zmp_ph97_B10
zmp_ph97_C2
zmp_ph97_C4
zmp_ph97_B8
zmp_ph97_C1
zmp_ph97_D2
zmp_ph97_D5
zmp_ph111_G5
zmp_ph111_G11
zmp_ph111_H1
zmp_ph111_B1a
zmp_ph111_B5
zmp_ph111_B8
zmp_ph111_B6
zmp_ph111_B7
zmp_ph111_B9
zmp_ph111_B10
zmp_ph114_G4
zmp_ph114_G11
zmp_ph114_H1
zmp_ph114_H2
zmp_ph114_A9
zmp_ph114_A11
zmp_ph114_B2
zmp_ph114_B4
zmp_ph114_D8
zmp_ph114_E2

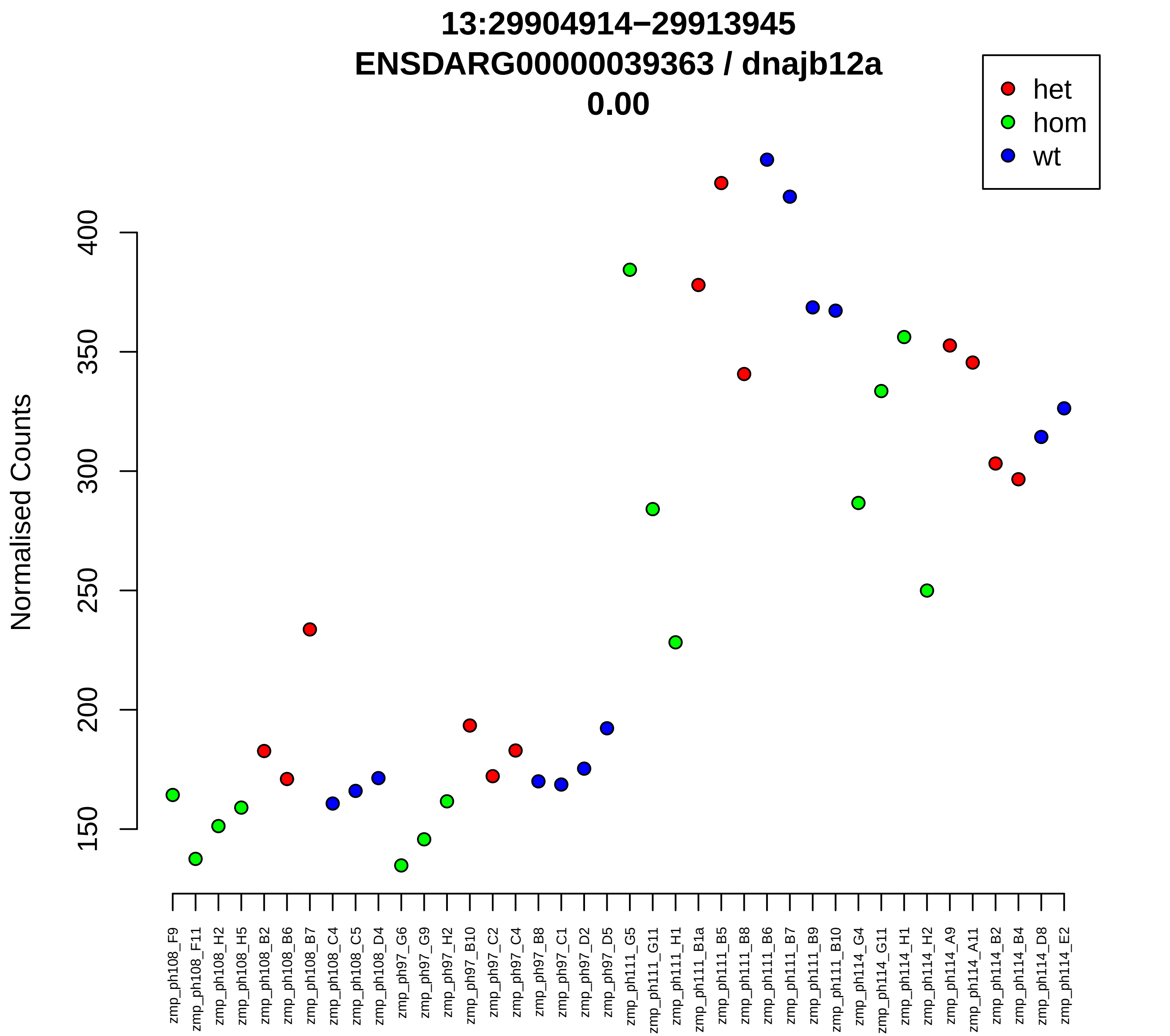

13:29904914−29913945
ENSDARG00000039363 / dnajb12a
0.00
het
hom
wt
Normalised Counts
150
200
250
300
350
400
zmp_ph108_F9
zmp_ph108_F11
zmp_ph108_H2
zmp_ph108_H5
zmp_ph108_B2
zmp_ph108_B6
zmp_ph108_B7
zmp_ph108_C4
zmp_ph108_C5
zmp_ph108_D4
zmp_ph97_G6
zmp_ph97_G9
zmp_ph97_H2
zmp_ph97_B10
zmp_ph97_C2
zmp_ph97_C4
zmp_ph97_B8
zmp_ph97_C1
zmp_ph97_D2
zmp_ph97_D5
zmp_ph111_G5
zmp_ph111_G11
zmp_ph111_H1
zmp_ph111_B1a
zmp_ph111_B5
zmp_ph111_B8
zmp_ph111_B6
zmp_ph111_B7
zmp_ph111_B9
zmp_ph111_B10
zmp_ph114_G4
zmp_ph114_G11
zmp_ph114_H1
zmp_ph114_H2
zmp_ph114_A9
zmp_ph114_A11
zmp_ph114_B2
zmp_ph114_B4
zmp_ph114_D8
zmp_ph114_E2

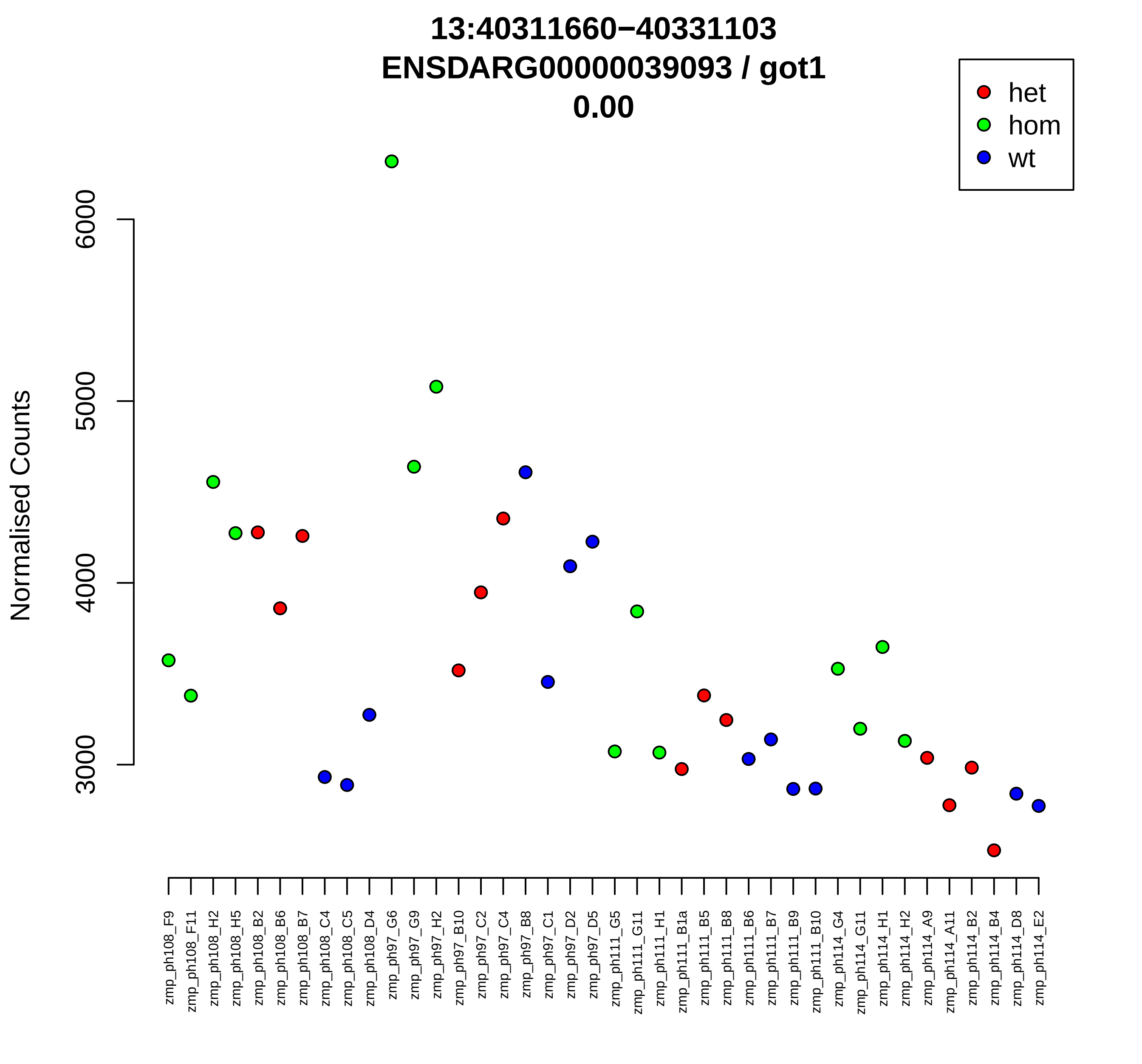

13:40311660−40331103
ENSDARG00000039093 / got1
0.00
het
hom
wt
Normalised Counts
6000
5000
4000
3000
zmp_ph108_F9
zmp_ph108_F11
zmp_ph108_H2
zmp_ph108_H5
zmp_ph108_B2
zmp_ph108_B6
zmp_ph108_B7
zmp_ph108_C4
zmp_ph108_C5
zmp_ph108_D4
zmp_ph97_G6
zmp_ph97_G9
zmp_ph97_H2
zmp_ph97_B10
zmp_ph97_C2
zmp_ph97_C4
zmp_ph97_B8
zmp_ph97_C1
zmp_ph97_D2
zmp_ph97_D5
zmp_ph111_G5
zmp_ph111_G11
zmp_ph111_H1
zmp_ph111_B1a
zmp_ph111_B5
zmp_ph111_B8
zmp_ph111_B6
zmp_ph111_B7
zmp_ph111_B9
zmp_ph111_B10
zmp_ph114_G4
zmp_ph114_G11
zmp_ph114_H1
zmp_ph114_H2
zmp_ph114_A9
zmp_ph114_A11
zmp_ph114_B2
zmp_ph114_B4
zmp_ph114_D8
zmp_ph114_E2

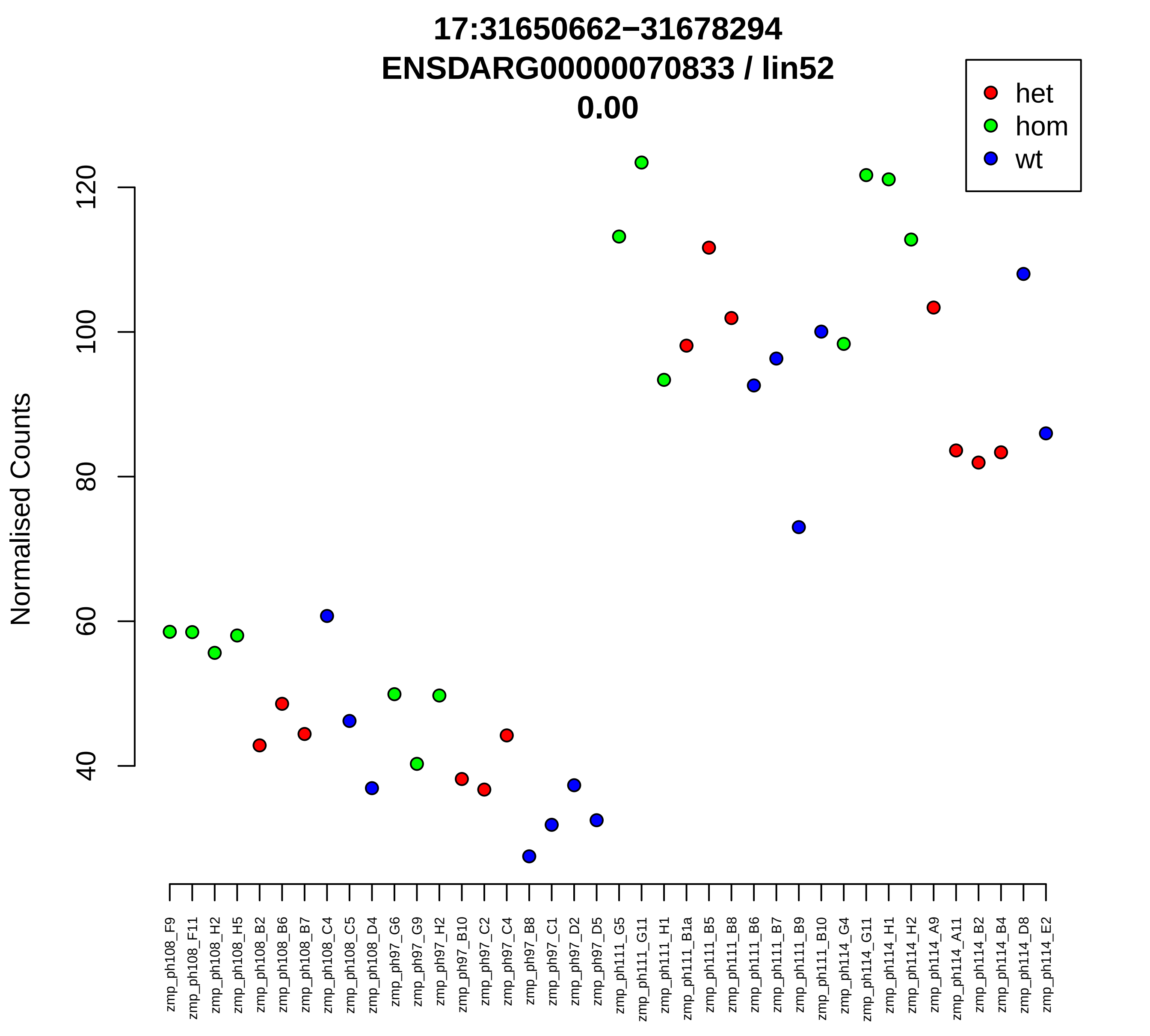
17:31650662−31678294
ENSDARG00000070833 / lin52
0.00
het
hom
wt
Normalised Counts
40
60
80
100
120
zmp_ph108_F9
zmp_ph108_F11
zmp_ph108_H2
zmp_ph108_H5
zmp_ph108_B2
zmp_ph108_B6
zmp_ph108_B7
zmp_ph108_C4
zmp_ph108_C5
zmp_ph108_D4
zmp_ph97_G6
zmp_ph97_G9
zmp_ph97_H2
zmp_ph97_B10
zmp_ph97_C2
zmp_ph97_C4
zmp_ph97_B8
zmp_ph97_C1
zmp_ph97_D2
zmp_ph97_D5
zmp_ph111_G5
zmp_ph111_G11
zmp_ph111_H1
zmp_ph111_B1a
zmp_ph111_B5
zmp_ph111_B8
zmp_ph111_B6
zmp_ph111_B7
zmp_ph111_B9
zmp_ph111_B10
zmp_ph114_G4
zmp_ph114_G11
zmp_ph114_H1
zmp_ph114_H2
zmp_ph114_A9
zmp_ph114_A11
zmp_ph114_B2
zmp_ph114_B4
zmp_ph114_D8
zmp_ph114_E2

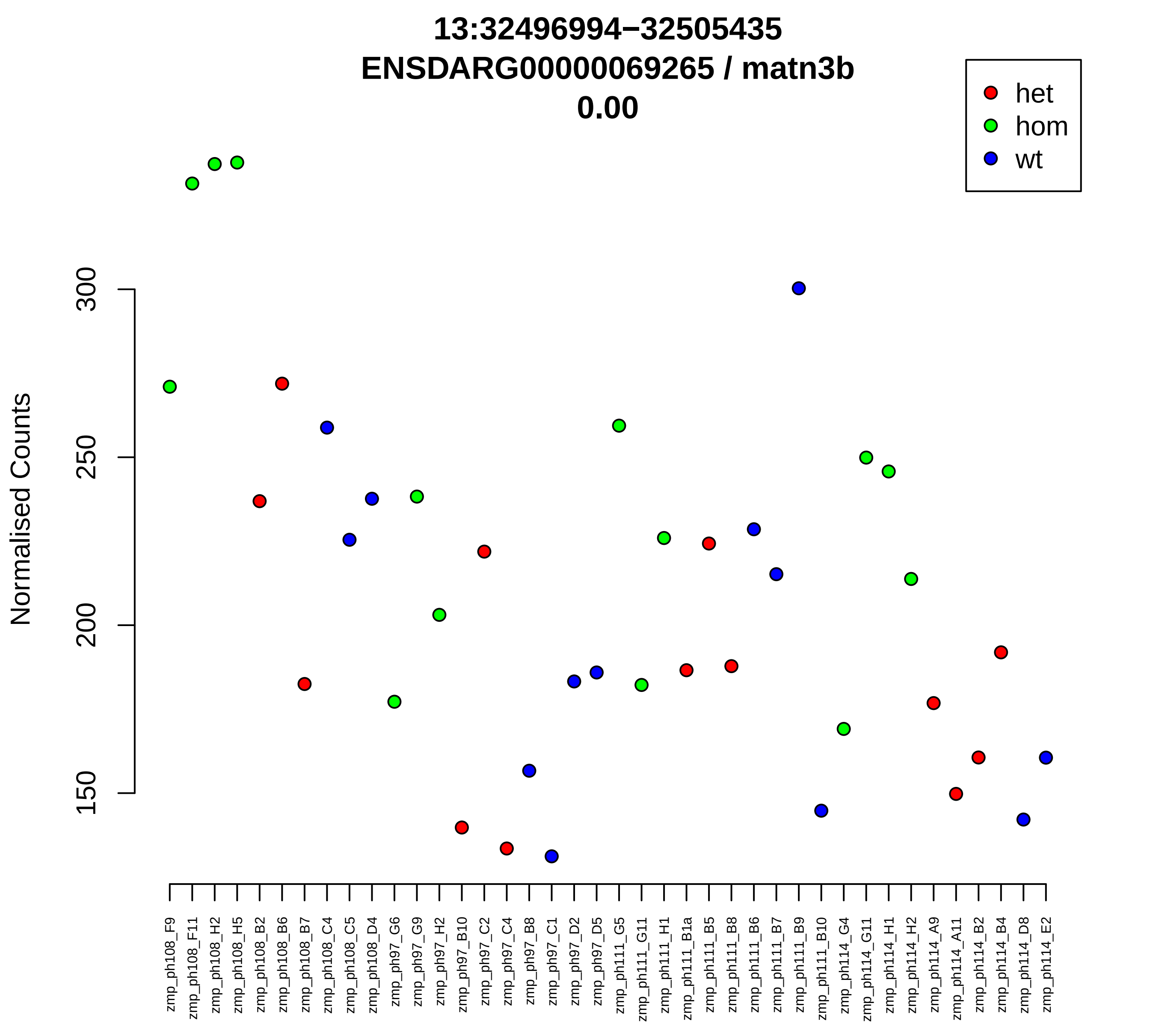
13:32496994−32505435
ENSDARG00000069265 / matn3b
0.00
het
hom
wt
Normalised Counts
150
200
250
300
zmp_ph108_F9
zmp_ph108_F11
zmp_ph108_H2
zmp_ph108_H5
zmp_ph108_B2
zmp_ph108_B6
zmp_ph108_B7
zmp_ph108_C4
zmp_ph108_C5
zmp_ph108_D4
zmp_ph97_G6
zmp_ph97_G9
zmp_ph97_H2
zmp_ph97_B10
zmp_ph97_C2
zmp_ph97_C4
zmp_ph97_B8
zmp_ph97_C1
zmp_ph97_D2
zmp_ph97_D5
zmp_ph111_G5
zmp_ph111_G11
zmp_ph111_H1
zmp_ph111_B1a
zmp_ph111_B5
zmp_ph111_B8
zmp_ph111_B6
zmp_ph111_B7
zmp_ph111_B9
zmp_ph111_B10
zmp_ph114_G4
zmp_ph114_G11
zmp_ph114_H1
zmp_ph114_H2
zmp_ph114_A9
zmp_ph114_A11
zmp_ph114_B2
zmp_ph114_B4
zmp_ph114_D8
zmp_ph114_E2

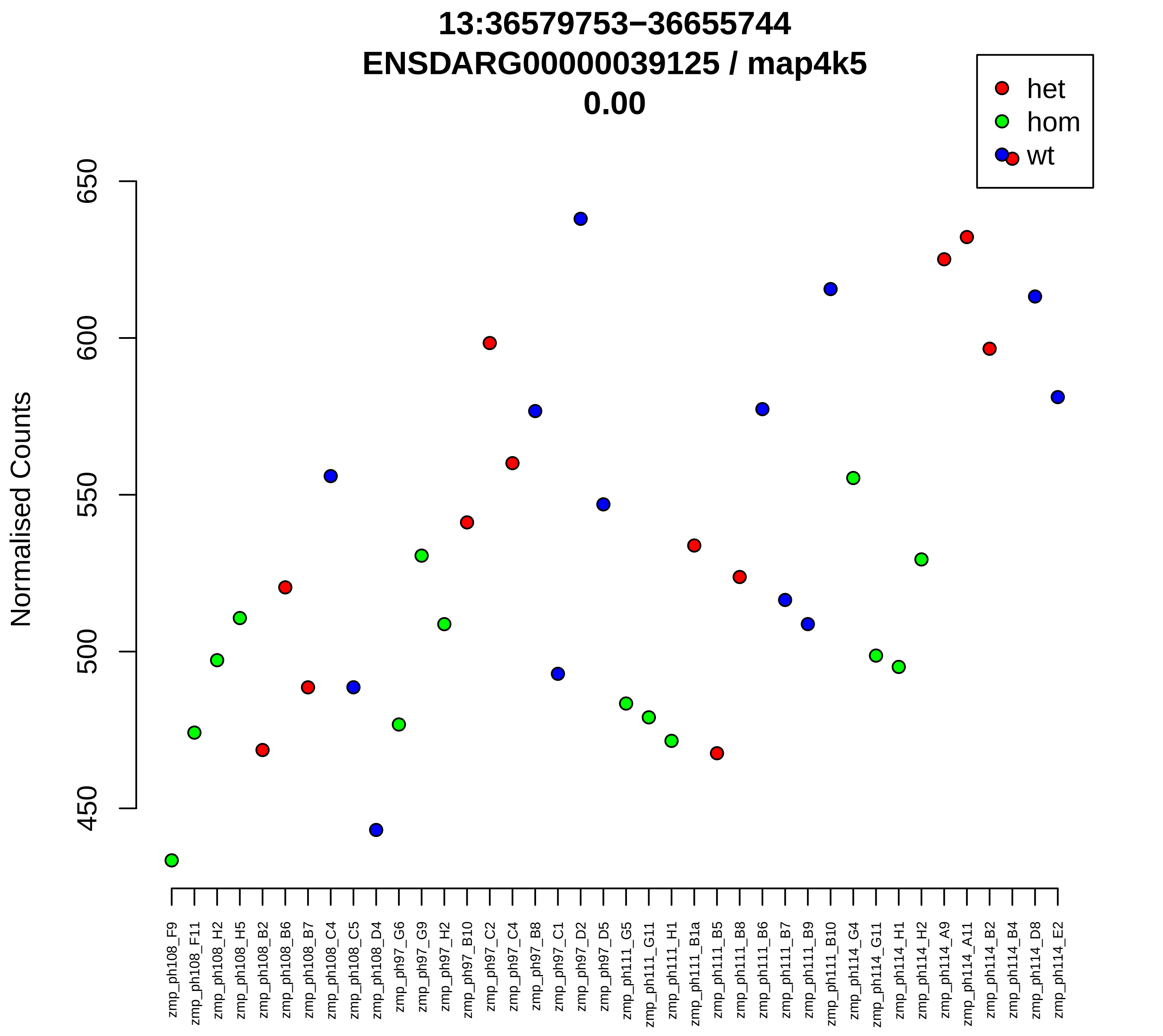

13:36579753−36655744
ENSDARG00000039125 / map4k5
0.00
het
hom
wt
Normalised Counts
450
500
550
600
650
zmp_ph108_F9
zmp_ph108_F11
zmp_ph108_H2
zmp_ph108_H5
zmp_ph108_B2
zmp_ph108_B6
zmp_ph108_B7
zmp_ph108_C4
zmp_ph108_C5
zmp_ph108_D4
zmp_ph97_G6
zmp_ph97_G9
zmp_ph97_H2
zmp_ph97_B10
zmp_ph97_C2
zmp_ph97_C4
zmp_ph97_B8
zmp_ph97_C1
zmp_ph97_D2
zmp_ph97_D5
zmp_ph111_G5
zmp_ph111_G11
zmp_ph111_H1
zmp_ph111_B1a
zmp_ph111_B5
zmp_ph111_B8
zmp_ph111_B6
zmp_ph111_B7
zmp_ph111_B9
zmp_ph111_B10
zmp_ph114_G4
zmp_ph114_G11
zmp_ph114_H1
zmp_ph114_H2
zmp_ph114_A9
zmp_ph114_A11
zmp_ph114_B2
zmp_ph114_B4
zmp_ph114_D8
zmp_ph114_E2

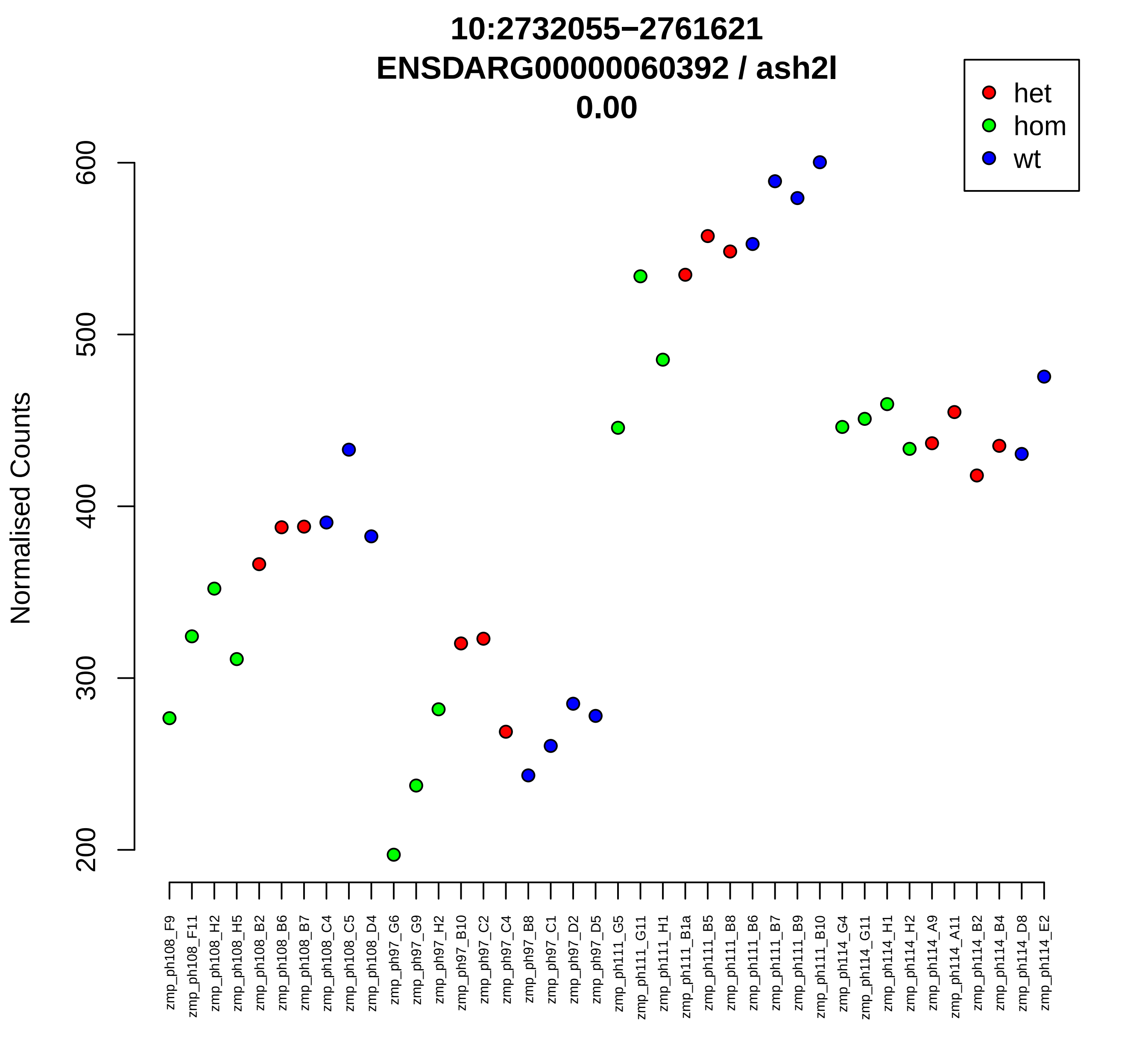

10:2732055−2761621
ENSDARG00000060392 / ash2l
0.00
het
hom
wt
Normalised Counts
200
300
400
500
600
zmp_ph108_F9
zmp_ph108_F11
zmp_ph108_H2
zmp_ph108_H5
zmp_ph108_B2
zmp_ph108_B6
zmp_ph108_B7
zmp_ph108_C4
zmp_ph108_C5
zmp_ph108_D4
zmp_ph97_G6
zmp_ph97_G9
zmp_ph97_H2
zmp_ph97_B10
zmp_ph97_C2
zmp_ph97_C4
zmp_ph97_B8
zmp_ph97_C1
zmp_ph97_D2
zmp_ph97_D5
zmp_ph111_G5
zmp_ph111_G11
zmp_ph111_H1
zmp_ph111_B1a
zmp_ph111_B5
zmp_ph111_B8
zmp_ph111_B6
zmp_ph111_B7
zmp_ph111_B9
zmp_ph111_B10
zmp_ph114_G4
zmp_ph114_G11
zmp_ph114_H1
zmp_ph114_H2
zmp_ph114_A9
zmp_ph114_A11
zmp_ph114_B2
zmp_ph114_B4
zmp_ph114_D8
zmp_ph114_E2

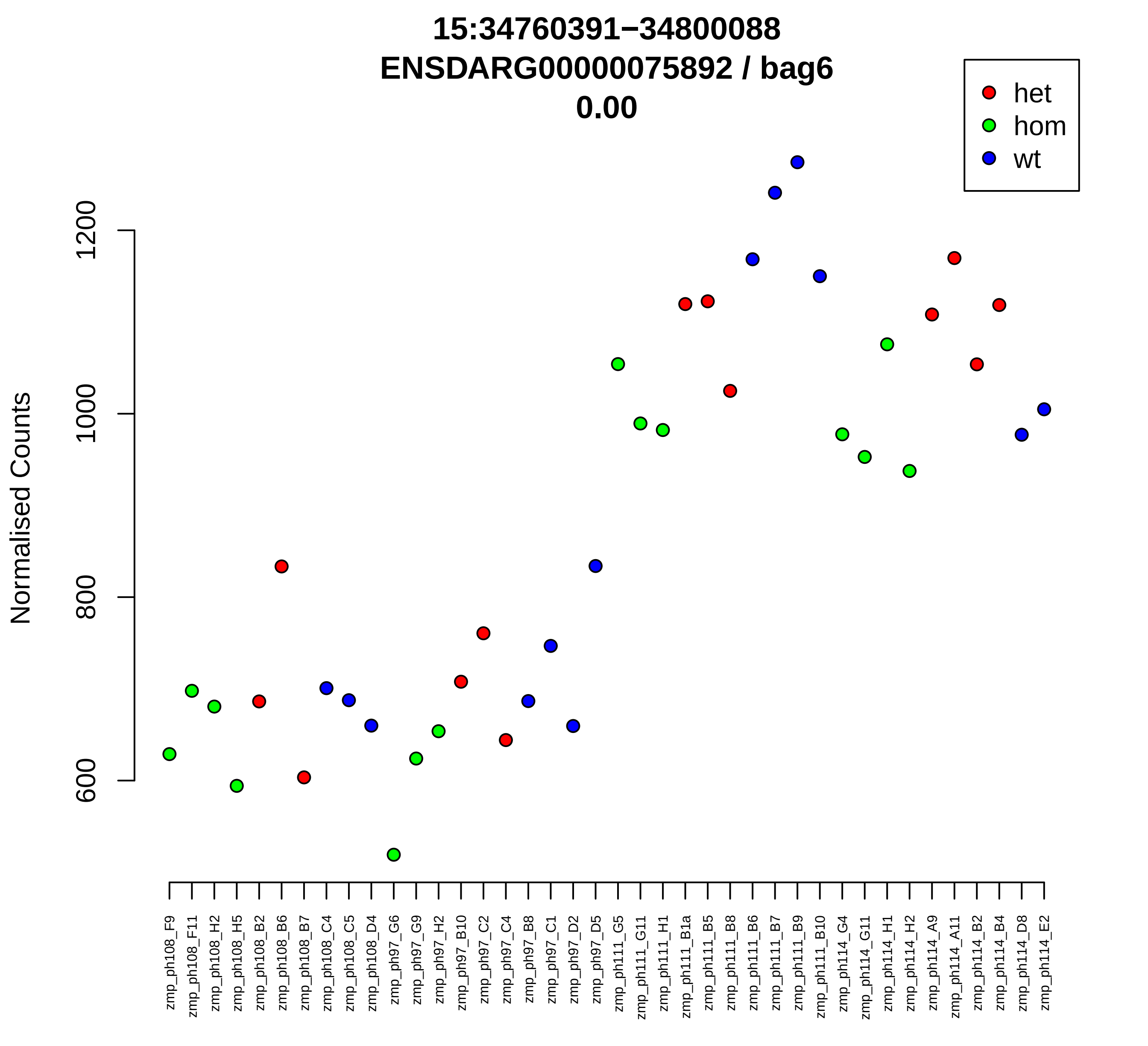
15:34760391−34800088
ENSDARG00000075892 / bag6
0.00
het
hom
wt
Normalised Counts
600
800
1000
1200
zmp_ph108_F9
zmp_ph108_F11
zmp_ph108_H2
zmp_ph108_H5
zmp_ph108_B2
zmp_ph108_B6
zmp_ph108_B7
zmp_ph108_C4
zmp_ph108_C5
zmp_ph108_D4
zmp_ph97_G6
zmp_ph97_G9
zmp_ph97_H2
zmp_ph97_B10
zmp_ph97_C2
zmp_ph97_C4
zmp_ph97_B8
zmp_ph97_C1
zmp_ph97_D2
zmp_ph97_D5
zmp_ph111_G5
zmp_ph111_G11
zmp_ph111_H1
zmp_ph111_B1a
zmp_ph111_B5
zmp_ph111_B8
zmp_ph111_B6
zmp_ph111_B7
zmp_ph111_B9
zmp_ph111_B10
zmp_ph114_G4
zmp_ph114_G11
zmp_ph114_H1
zmp_ph114_H2
zmp_ph114_A9
zmp_ph114_A11
zmp_ph114_B2
zmp_ph114_B4
zmp_ph114_D8
zmp_ph114_E2

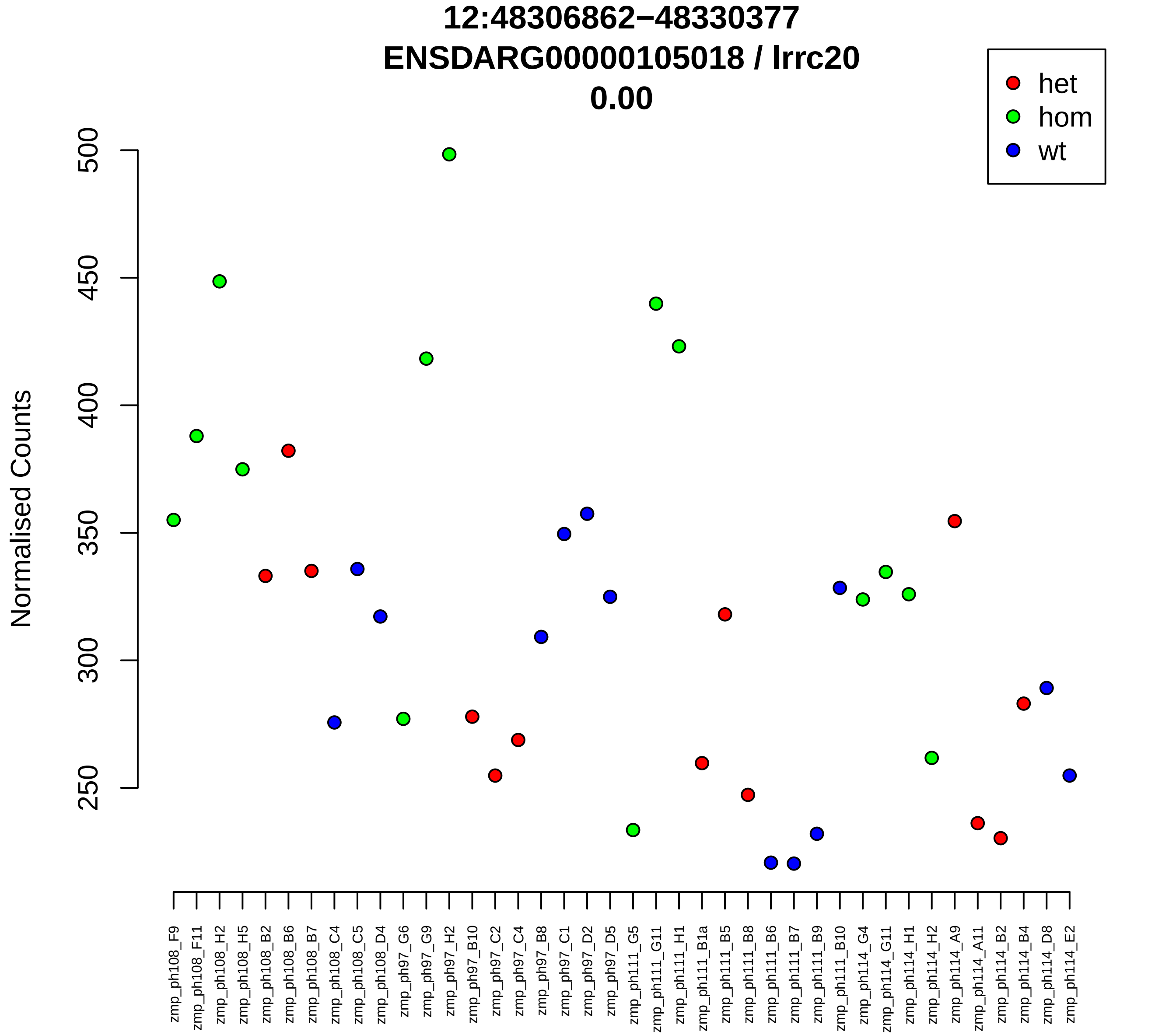

12:48306862−48330377
ENSDARG00000105018 / lrrc20
0.00
Normalised Counts
250
300
350
400
450
500
het
hom
wt
zmp_ph108_F9
zmp_ph108_F11
zmp_ph108_H2
zmp_ph108_H5
zmp_ph108_B2
zmp_ph108_B6
zmp_ph108_B7
zmp_ph108_C4
zmp_ph108_C5
zmp_ph108_D4
zmp_ph97_G6
zmp_ph97_G9
zmp_ph97_H2
zmp_ph97_B10
zmp_ph97_C2
zmp_ph97_C4
zmp_ph97_B8
zmp_ph97_C1
zmp_ph97_D2
zmp_ph97_D5
zmp_ph111_G5
zmp_ph111_G11
zmp_ph111_H1
zmp_ph111_B1a
zmp_ph111_B5
zmp_ph111_B8
zmp_ph111_B6
zmp_ph111_B7
zmp_ph111_B9
zmp_ph111_B10
zmp_ph114_G4
zmp_ph114_G11
zmp_ph114_H1
zmp_ph114_H2
zmp_ph114_A9
zmp_ph114_A11
zmp_ph114_B2
zmp_ph114_B4
zmp_ph114_D8
zmp_ph114_E2

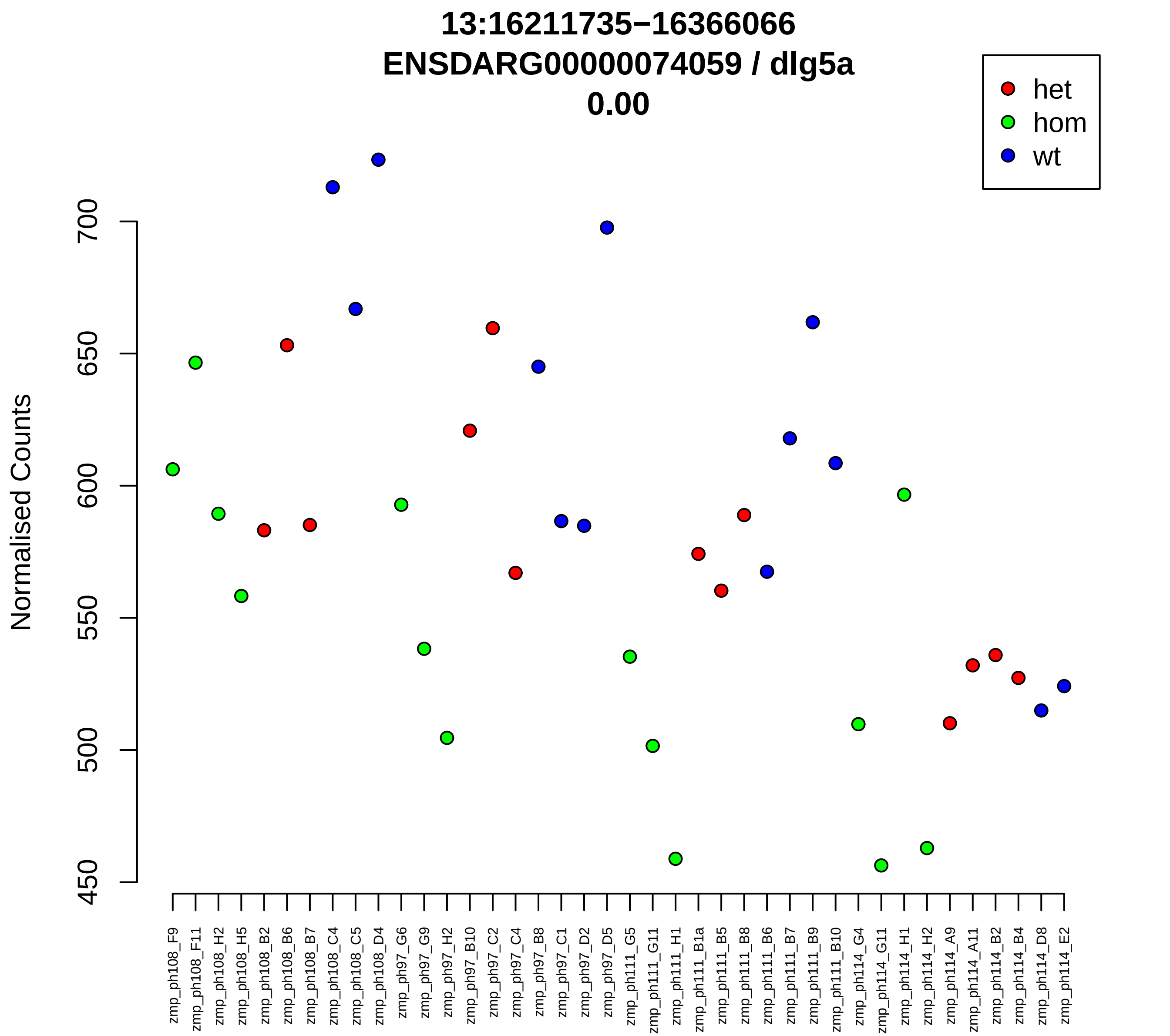

13:16211735−16366066
ENSDARG00000074059 / dlg5a
0.00
het
hom
wt
Normalised Counts
450
500
550
600
650
700
zmp_ph108_F9
zmp_ph108_F11
zmp_ph108_H2
zmp_ph108_H5
zmp_ph108_B2
zmp_ph108_B6
zmp_ph108_B7
zmp_ph108_C4
zmp_ph108_C5
zmp_ph108_D4
zmp_ph97_G6
zmp_ph97_G9
zmp_ph97_H2
zmp_ph97_B10
zmp_ph97_C2
zmp_ph97_C4
zmp_ph97_B8
zmp_ph97_C1
zmp_ph97_D2
zmp_ph97_D5
zmp_ph111_G5
zmp_ph111_G11
zmp_ph111_H1
zmp_ph111_B1a
zmp_ph111_B5
zmp_ph111_B8
zmp_ph111_B6
zmp_ph111_B7
zmp_ph111_B9
zmp_ph111_B10
zmp_ph114_G4
zmp_ph114_G11
zmp_ph114_H1
zmp_ph114_H2
zmp_ph114_A9
zmp_ph114_A11
zmp_ph114_B2
zmp_ph114_B4
zmp_ph114_D8
zmp_ph114_E2

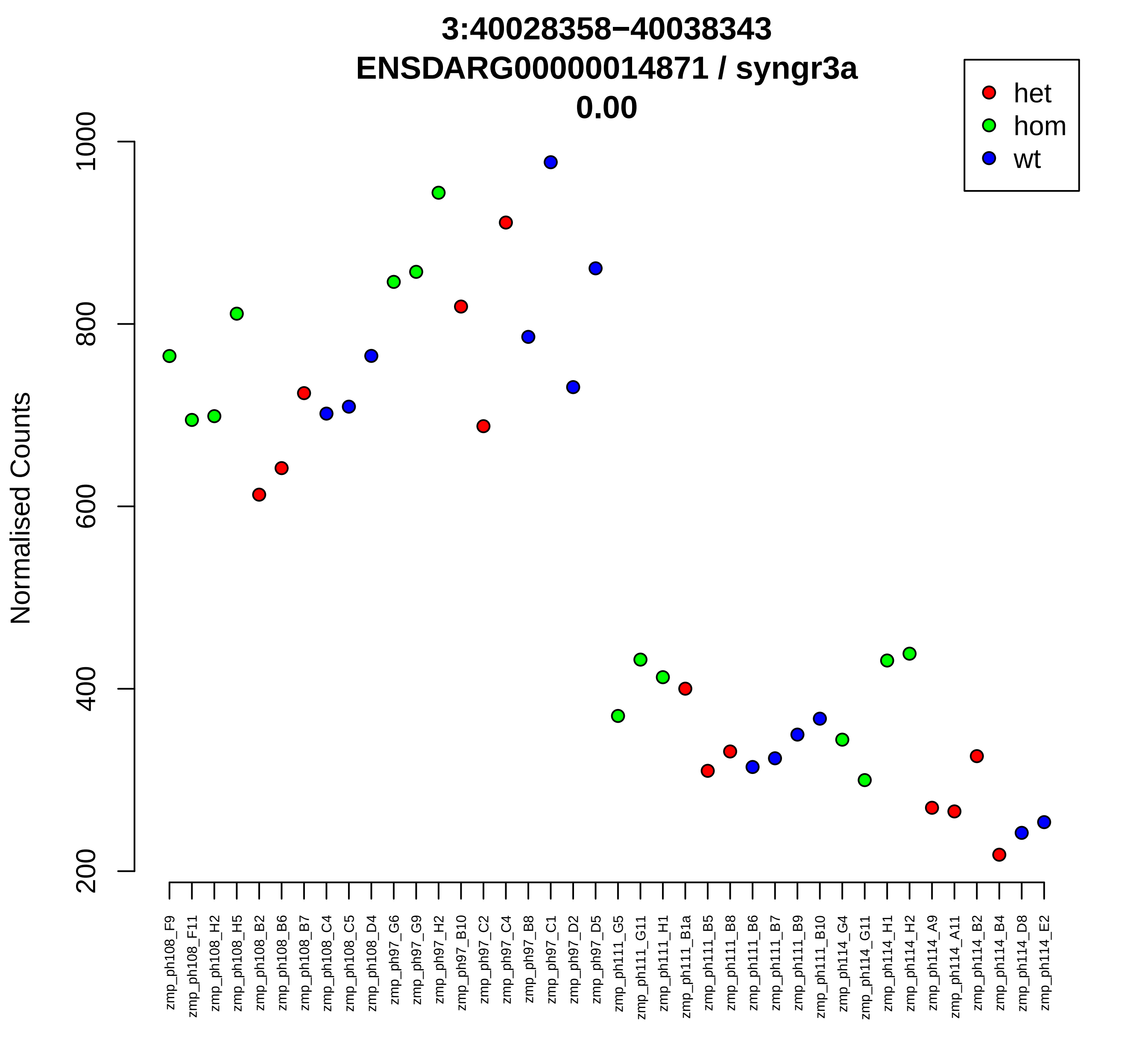

3:40028358−40038343
ENSDARG00000014871 / syngr3a
0.00
het
hom
wt
Normalised Counts
200
400
600
800
1000
zmp_ph108_F9
zmp_ph108_F11
zmp_ph108_H2
zmp_ph108_H5
zmp_ph108_B2
zmp_ph108_B6
zmp_ph108_B7
zmp_ph108_C4
zmp_ph108_C5
zmp_ph108_D4
zmp_ph97_G6
zmp_ph97_G9
zmp_ph97_H2
zmp_ph97_B10
zmp_ph97_C2
zmp_ph97_C4
zmp_ph97_B8
zmp_ph97_C1
zmp_ph97_D2
zmp_ph97_D5
zmp_ph111_G5
zmp_ph111_G11
zmp_ph111_H1
zmp_ph111_B1a
zmp_ph111_B5
zmp_ph111_B8
zmp_ph111_B6
zmp_ph111_B7
zmp_ph111_B9
zmp_ph111_B10
zmp_ph114_G4
zmp_ph114_G11
zmp_ph114_H1
zmp_ph114_H2
zmp_ph114_A9
zmp_ph114_A11
zmp_ph114_B2
zmp_ph114_B4
zmp_ph114_D8
zmp_ph114_E2

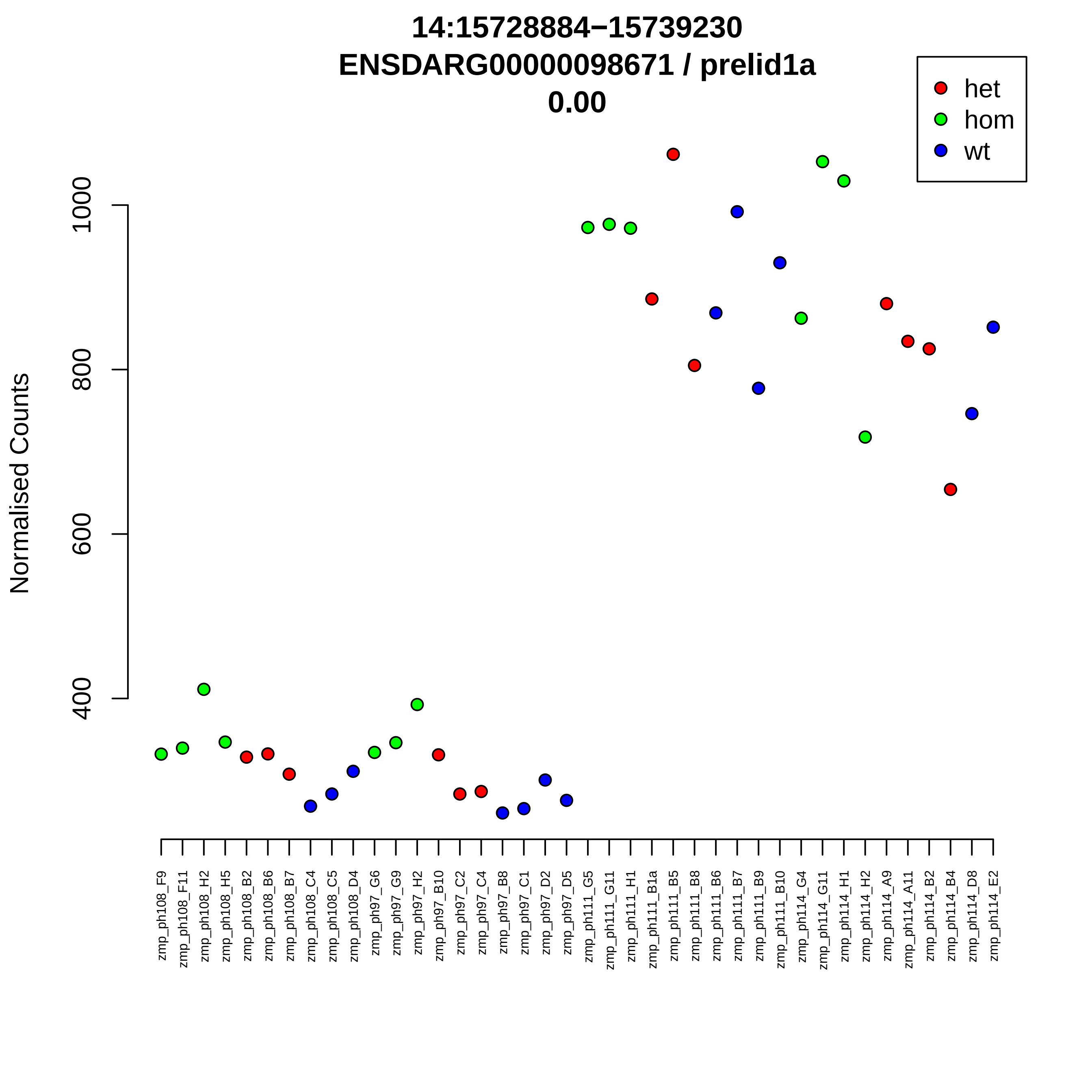

14:15728884−15739230
ENSDARG00000098671 / prelid1a
0.00
het
hom
wt
Normalised Counts
400
600
800
1000
zmp_ph108_F9
zmp_ph108_F11
zmp_ph108_H2
zmp_ph108_H5
zmp_ph108_B2
zmp_ph108_B6
zmp_ph108_B7
zmp_ph108_C4
zmp_ph108_C5
zmp_ph108_D4
zmp_ph97_G6
zmp_ph97_G9
zmp_ph97_H2
zmp_ph97_B10
zmp_ph97_C2
zmp_ph97_C4
zmp_ph97_B8
zmp_ph97_C1
zmp_ph97_D2
zmp_ph97_D5
zmp_ph111_G5
zmp_ph111_G11
zmp_ph111_H1
zmp_ph111_B1a
zmp_ph111_B5
zmp_ph111_B8
zmp_ph111_B6
zmp_ph111_B7
zmp_ph111_B9
zmp_ph111_B10
zmp_ph114_G4
zmp_ph114_G11
zmp_ph114_H1
zmp_ph114_H2
zmp_ph114_A9
zmp_ph114_A11
zmp_ph114_B2
zmp_ph114_B4
zmp_ph114_D8
zmp_ph114_E2

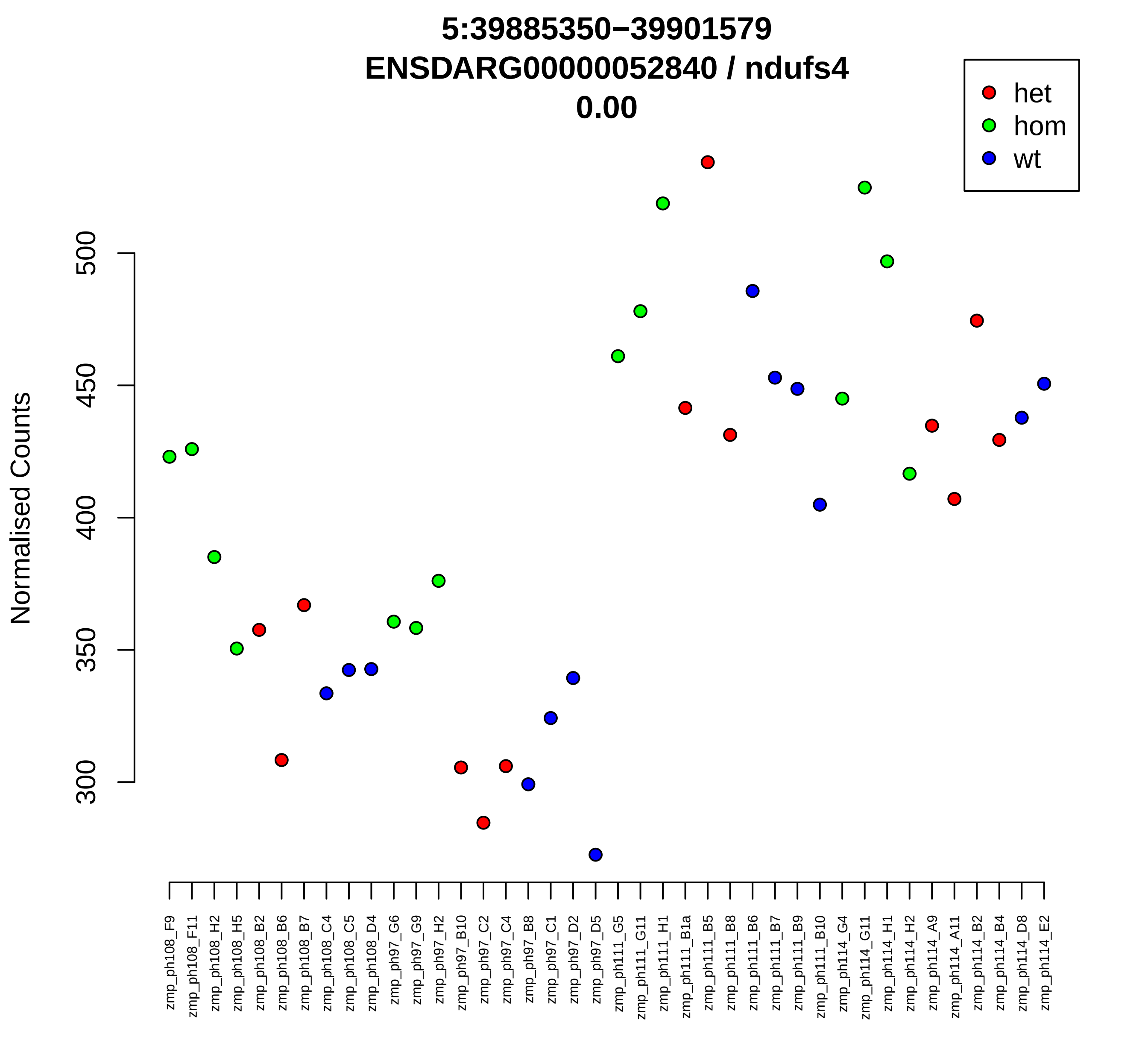
5:39885350−39901579
ENSDARG00000052840 / ndufs4
0.00
het
hom
wt
Normalised Counts
300
350
400
450
500
zmp_ph108_F9
zmp_ph108_F11
zmp_ph108_H2
zmp_ph108_H5
zmp_ph108_B2
zmp_ph108_B6
zmp_ph108_B7
zmp_ph108_C4
zmp_ph108_C5
zmp_ph108_D4
zmp_ph97_G6
zmp_ph97_G9
zmp_ph97_H2
zmp_ph97_B10
zmp_ph97_C2
zmp_ph97_C4
zmp_ph97_B8
zmp_ph97_C1
zmp_ph97_D2
zmp_ph97_D5
zmp_ph111_G5
zmp_ph111_G11
zmp_ph111_H1
zmp_ph111_B1a
zmp_ph111_B5
zmp_ph111_B8
zmp_ph111_B6
zmp_ph111_B7
zmp_ph111_B9
zmp_ph111_B10
zmp_ph114_G4
zmp_ph114_G11
zmp_ph114_H1
zmp_ph114_H2
zmp_ph114_A9
zmp_ph114_A11
zmp_ph114_B2
zmp_ph114_B4
zmp_ph114_D8
zmp_ph114_E2

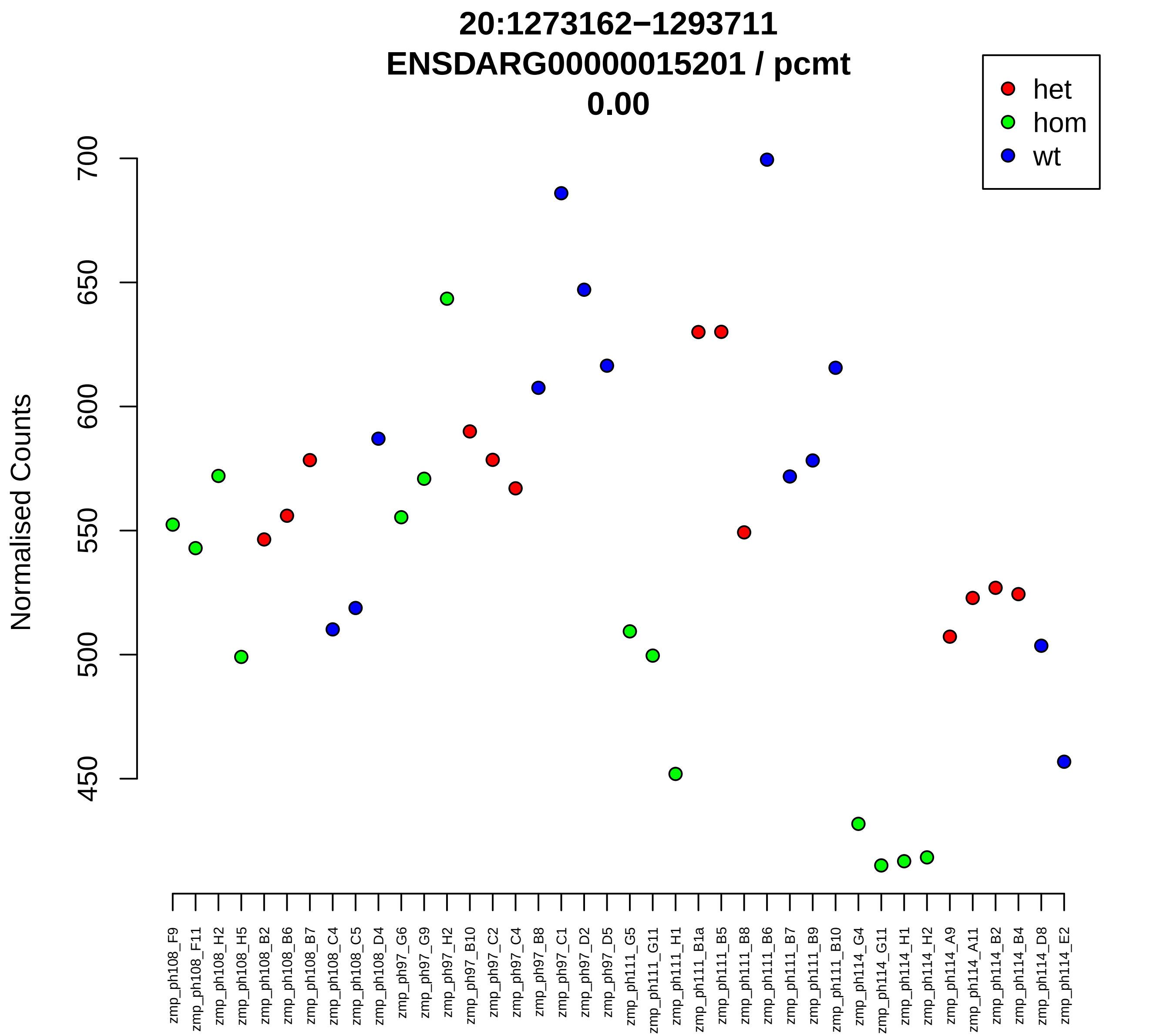

20:1273162−1293711
ENSDARG00000015201 / pcmt
0.00
het
hom
wt
Normalised Counts
700
650
600
550
500
450
zmp_ph108_F9
zmp_ph108_F11
zmp_ph108_H2
zmp_ph108_H5
zmp_ph108_B2
zmp_ph108_B6
zmp_ph108_B7
zmp_ph108_C4
zmp_ph108_C5
zmp_ph108_D4
zmp_ph97_G6
zmp_ph97_G9
zmp_ph97_H2
zmp_ph97_B10
zmp_ph97_C2
zmp_ph97_C4
zmp_ph97_B8
zmp_ph97_C1
zmp_ph97_D2
zmp_ph97_D5
zmp_ph111_G5
zmp_ph111_G11
zmp_ph111_H1
zmp_ph111_B1a
zmp_ph111_B5
zmp_ph111_B8
zmp_ph111_B6
zmp_ph111_B7
zmp_ph111_B9
zmp_ph111_B10
zmp_ph114_G4
zmp_ph114_G11
zmp_ph114_H1
zmp_ph114_H2
zmp_ph114_A9
zmp_ph114_A11
zmp_ph114_B2
zmp_ph114_B4
zmp_ph114_D8
zmp_ph114_E2

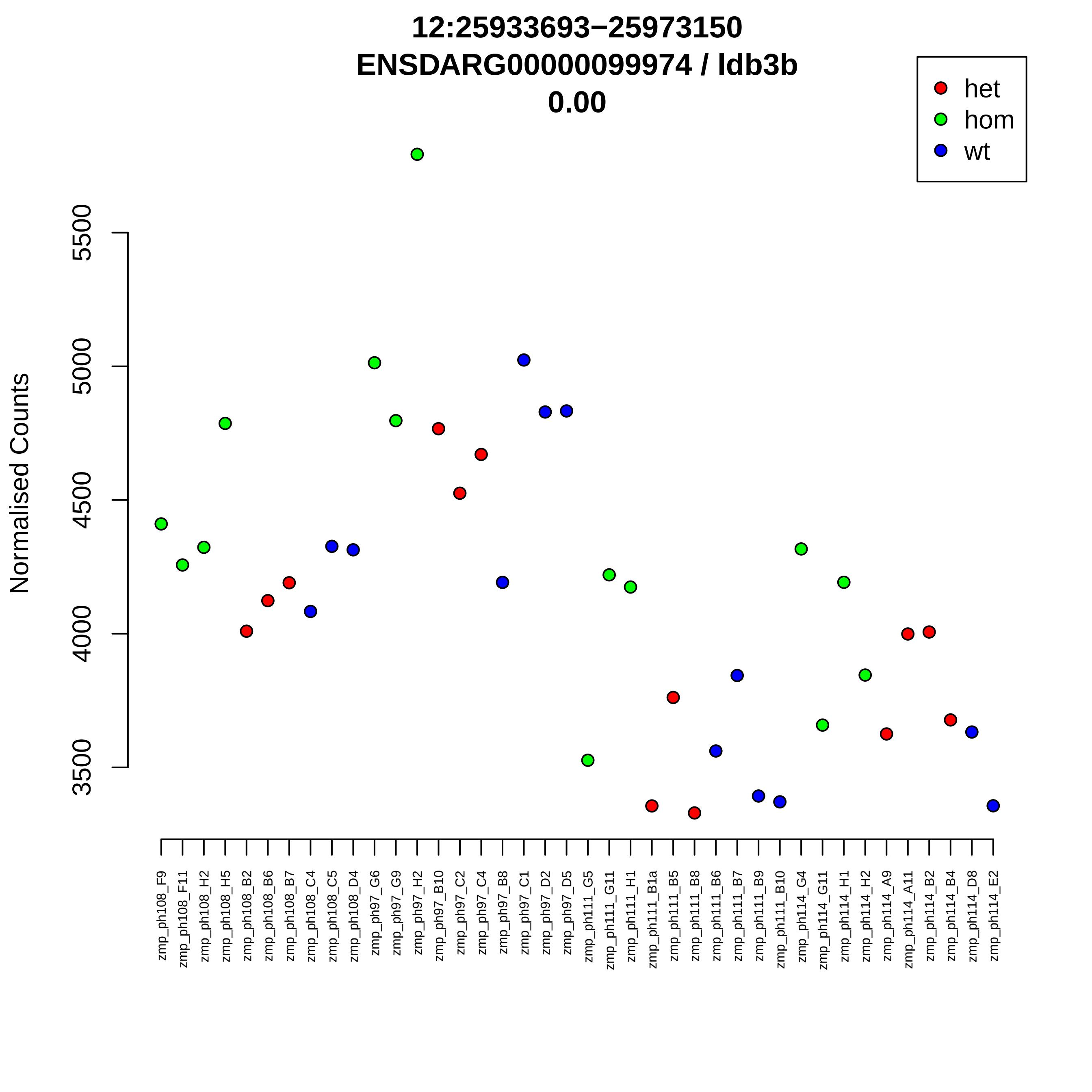

12:25933693−25973150
ENSDARG00000099974 / ldb3b
0.00
het
hom
wt
Normalised Counts
3500
4000
4500
5000
5500
zmp_ph108_F9
zmp_ph108_F11
zmp_ph108_H2
zmp_ph108_H5
zmp_ph108_B2
zmp_ph108_B6
zmp_ph108_B7
zmp_ph108_C4
zmp_ph108_C5
zmp_ph108_D4
zmp_ph97_G6
zmp_ph97_G9
zmp_ph97_H2
zmp_ph97_B10
zmp_ph97_C2
zmp_ph97_C4
zmp_ph97_B8
zmp_ph97_C1
zmp_ph97_D2
zmp_ph97_D5
zmp_ph111_G5
zmp_ph111_G11
zmp_ph111_H1
zmp_ph111_B1a
zmp_ph111_B5
zmp_ph111_B8
zmp_ph111_B6
zmp_ph111_B7
zmp_ph111_B9
zmp_ph111_B10
zmp_ph114_G4
zmp_ph114_G11
zmp_ph114_H1
zmp_ph114_H2
zmp_ph114_A9
zmp_ph114_A11
zmp_ph114_B2
zmp_ph114_B4
zmp_ph114_D8
zmp_ph114_E2

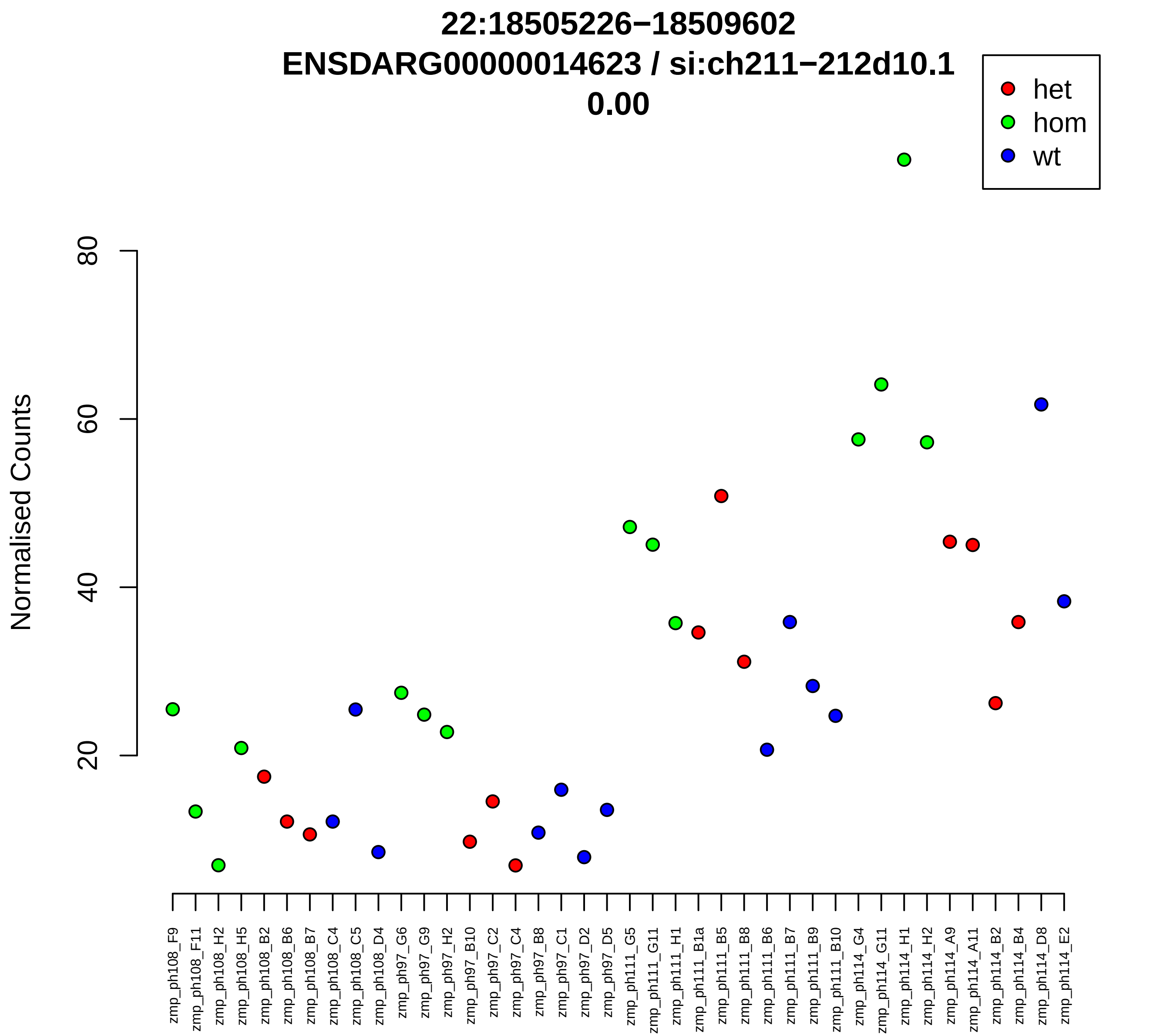

22:18505226−18509602
ENSDARG00000014623 / si:ch211−212d10.1
0.00
het
hom
wt
Normalised Counts
20
40
60
80
zmp_ph108_F9
zmp_ph108_F11
zmp_ph108_H2
zmp_ph108_H5
zmp_ph108_B2
zmp_ph108_B6
zmp_ph108_B7
zmp_ph108_C4
zmp_ph108_C5
zmp_ph108_D4
zmp_ph97_G6
zmp_ph97_G9
zmp_ph97_H2
zmp_ph97_B10
zmp_ph97_C2
zmp_ph97_C4
zmp_ph97_B8
zmp_ph97_C1
zmp_ph97_D2
zmp_ph97_D5
zmp_ph111_G5
zmp_ph111_G11
zmp_ph111_H1
zmp_ph111_B1a
zmp_ph111_B5
zmp_ph111_B8
zmp_ph111_B6
zmp_ph111_B7
zmp_ph111_B9
zmp_ph111_B10
zmp_ph114_G4
zmp_ph114_G11
zmp_ph114_H1
zmp_ph114_H2
zmp_ph114_A9
zmp_ph114_A11
zmp_ph114_B2
zmp_ph114_B4
zmp_ph114_D8
zmp_ph114_E2

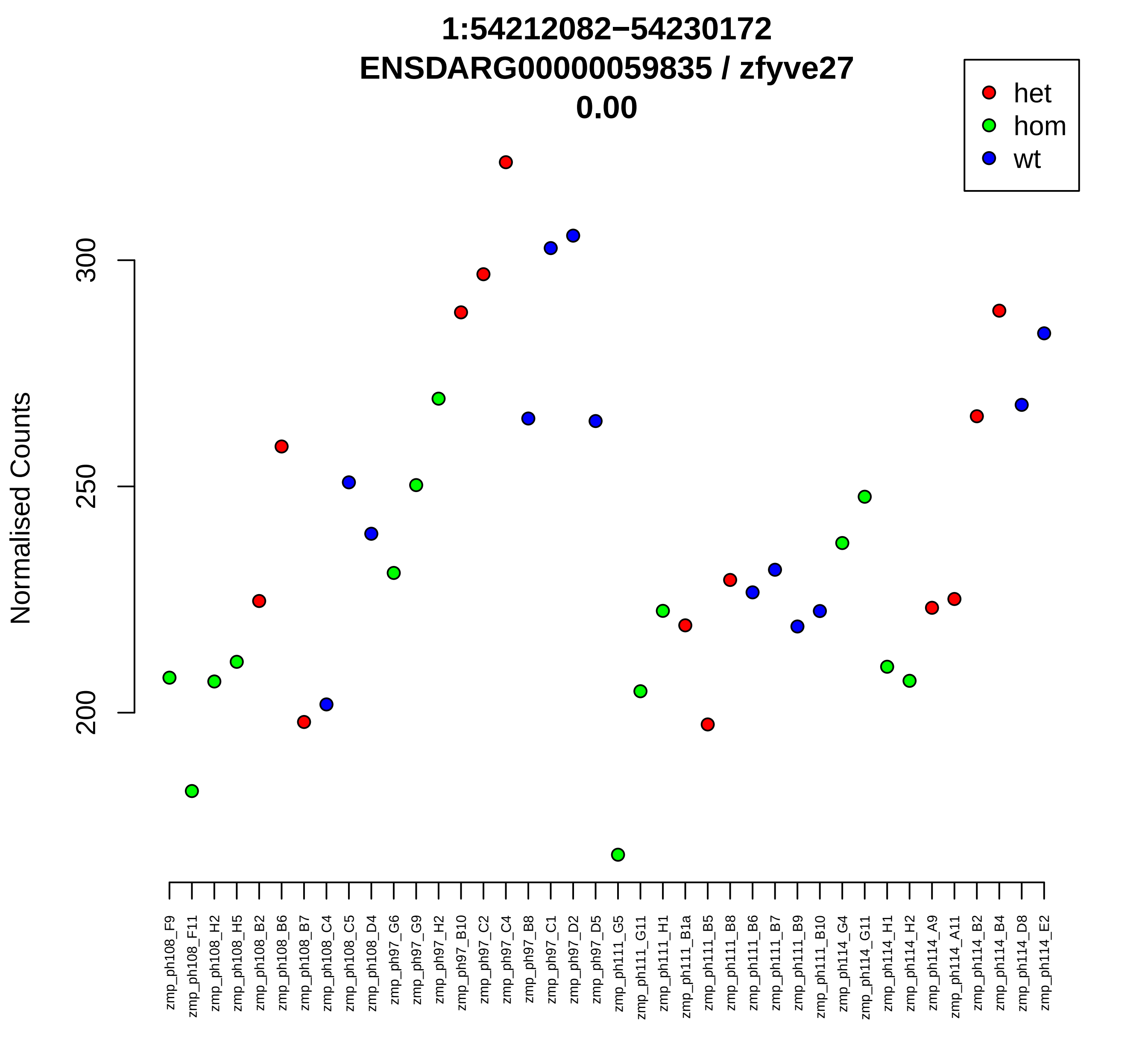
1:54212082−54230172
ENSDARG00000059835 / zfyve27
0.00
het
hom
wt
Normalised Counts
200
250
300
zmp_ph108_F9
zmp_ph108_F11
zmp_ph108_H2
zmp_ph108_H5
zmp_ph108_B2
zmp_ph108_B6
zmp_ph108_B7
zmp_ph108_C4
zmp_ph108_C5
zmp_ph108_D4
zmp_ph97_G6
zmp_ph97_G9
zmp_ph97_H2
zmp_ph97_B10
zmp_ph97_C2
zmp_ph97_C4
zmp_ph97_B8
zmp_ph97_C1
zmp_ph97_D2
zmp_ph97_D5
zmp_ph111_G5
zmp_ph111_G11
zmp_ph111_H1
zmp_ph111_B1a
zmp_ph111_B5
zmp_ph111_B8
zmp_ph111_B6
zmp_ph111_B7
zmp_ph111_B9
zmp_ph111_B10
zmp_ph114_G4
zmp_ph114_G11
zmp_ph114_H1
zmp_ph114_H2
zmp_ph114_A9
zmp_ph114_A11
zmp_ph114_B2
zmp_ph114_B4
zmp_ph114_D8
zmp_ph114_E2

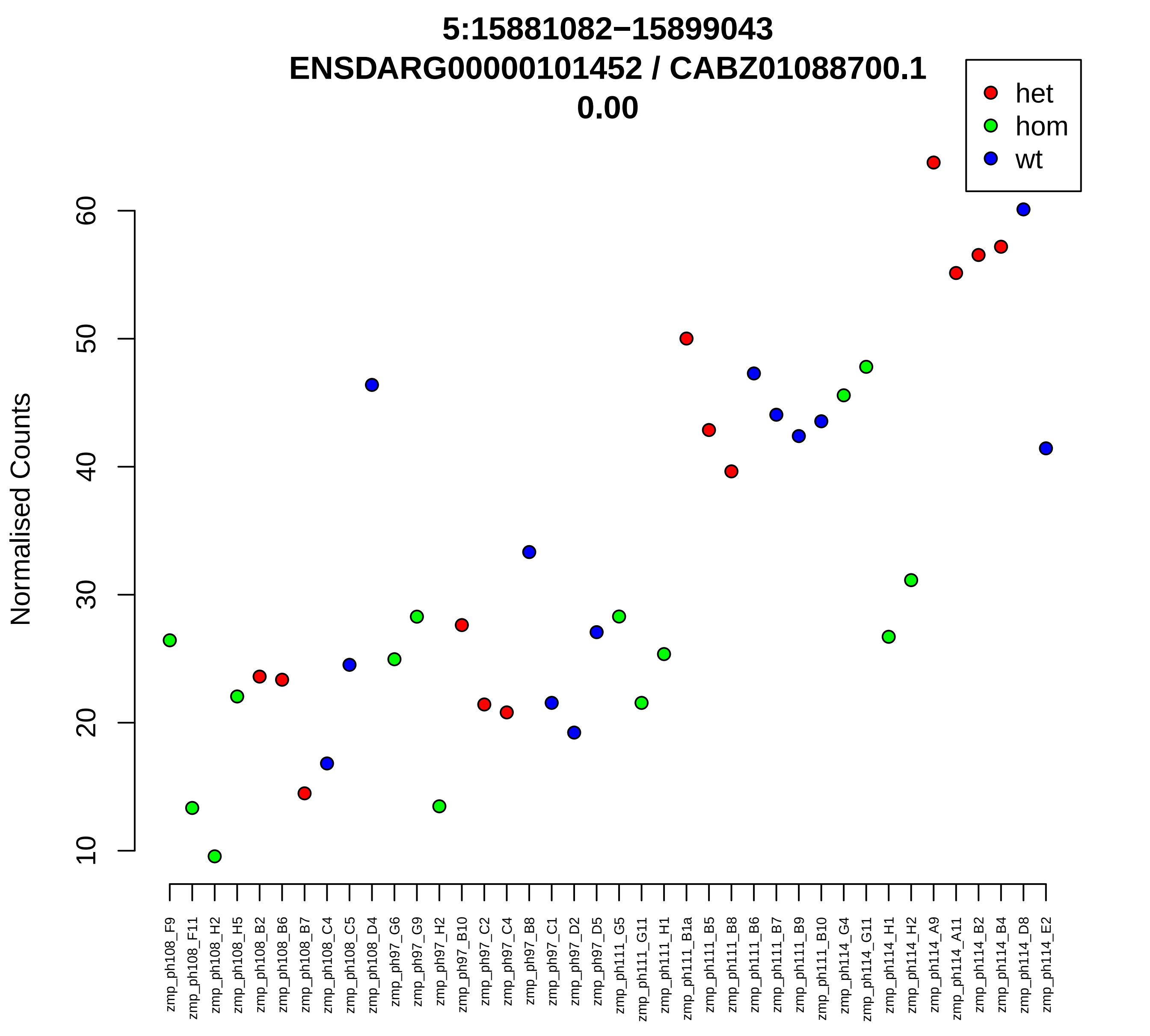

5:15881082−15899043
ENSDARG00000101452 / CABZ01088700.1
0.00
het
hom
wt
Normalised Counts
10
20
30
40
50
60
zmp_ph108_F9
zmp_ph108_F11
zmp_ph108_H2
zmp_ph108_H5
zmp_ph108_B2
zmp_ph108_B6
zmp_ph108_B7
zmp_ph108_C4
zmp_ph108_C5
zmp_ph108_D4
zmp_ph97_G6
zmp_ph97_G9
zmp_ph97_H2
zmp_ph97_B10
zmp_ph97_C2
zmp_ph97_C4
zmp_ph97_B8
zmp_ph97_C1
zmp_ph97_D2
zmp_ph97_D5
zmp_ph111_G5
zmp_ph111_G11
zmp_ph111_H1
zmp_ph111_B1a
zmp_ph111_B5
zmp_ph111_B8
zmp_ph111_B6
zmp_ph111_B7
zmp_ph111_B9
zmp_ph111_B10
zmp_ph114_G4
zmp_ph114_G11
zmp_ph114_H1
zmp_ph114_H2
zmp_ph114_A9
zmp_ph114_A11
zmp_ph114_B2
zmp_ph114_B4
zmp_ph114_D8
zmp_ph114_E2

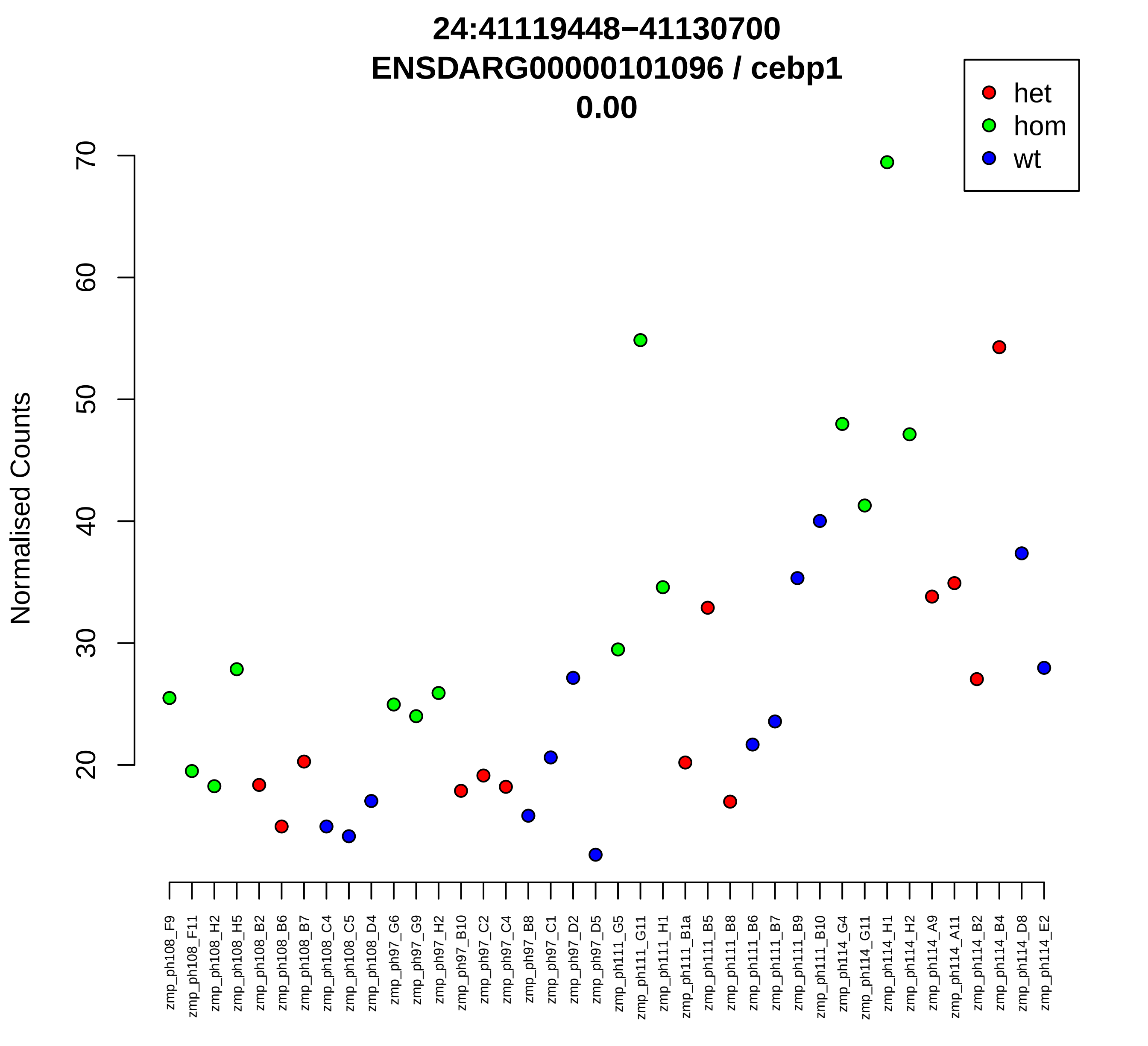

24:41119448−41130700
ENSDARG00000101096 / cebp1
0.00
het
hom
wt
Normalised Counts
20
30
40
50
60
70
zmp_ph108_F9
zmp_ph108_F11
zmp_ph108_H2
zmp_ph108_H5
zmp_ph108_B2
zmp_ph108_B6
zmp_ph108_B7
zmp_ph108_C4
zmp_ph108_C5
zmp_ph108_D4
zmp_ph97_G6
zmp_ph97_G9
zmp_ph97_H2
zmp_ph97_B10
zmp_ph97_C2
zmp_ph97_C4
zmp_ph97_B8
zmp_ph97_C1
zmp_ph97_D2
zmp_ph97_D5
zmp_ph111_G5
zmp_ph111_G11
zmp_ph111_H1
zmp_ph111_B1a
zmp_ph111_B5
zmp_ph111_B8
zmp_ph111_B6
zmp_ph111_B7
zmp_ph111_B9
zmp_ph111_B10
zmp_ph114_G4
zmp_ph114_G11
zmp_ph114_H1
zmp_ph114_H2
zmp_ph114_A9
zmp_ph114_A11
zmp_ph114_B2
zmp_ph114_B4
zmp_ph114_D8
zmp_ph114_E2

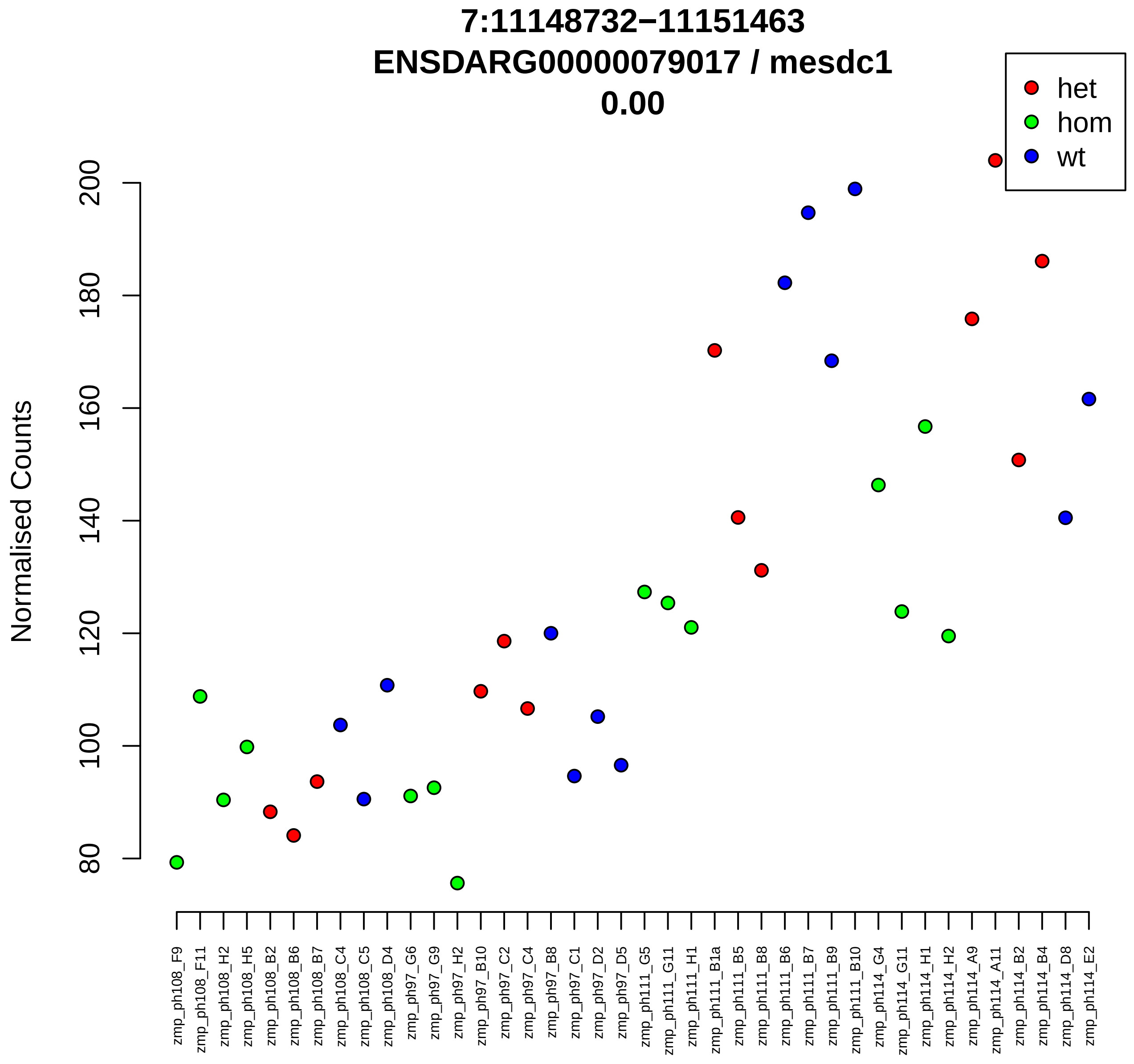
7:11148732−11151463
ENSDARG00000079017 / mesdc1
0.00
het
hom
wt
Normalised Counts
80
100
120
140
160
180
200
zmp_ph108_F9
zmp_ph108_F11
zmp_ph108_H2
zmp_ph108_H5
zmp_ph108_B2
zmp_ph108_B6
zmp_ph108_B7
zmp_ph108_C4
zmp_ph108_C5
zmp_ph108_D4
zmp_ph97_G6
zmp_ph97_G9
zmp_ph97_H2
zmp_ph97_B10
zmp_ph97_C2
zmp_ph97_C4
zmp_ph97_B8
zmp_ph97_C1
zmp_ph97_D2
zmp_ph97_D5
zmp_ph111_G5
zmp_ph111_G11
zmp_ph111_H1
zmp_ph111_B1a
zmp_ph111_B5
zmp_ph111_B8
zmp_ph111_B6
zmp_ph111_B7
zmp_ph111_B9
zmp_ph111_B10
zmp_ph114_G4
zmp_ph114_G11
zmp_ph114_H1
zmp_ph114_H2
zmp_ph114_A9
zmp_ph114_A11
zmp_ph114_B2
zmp_ph114_B4
zmp_ph114_D8
zmp_ph114_E2

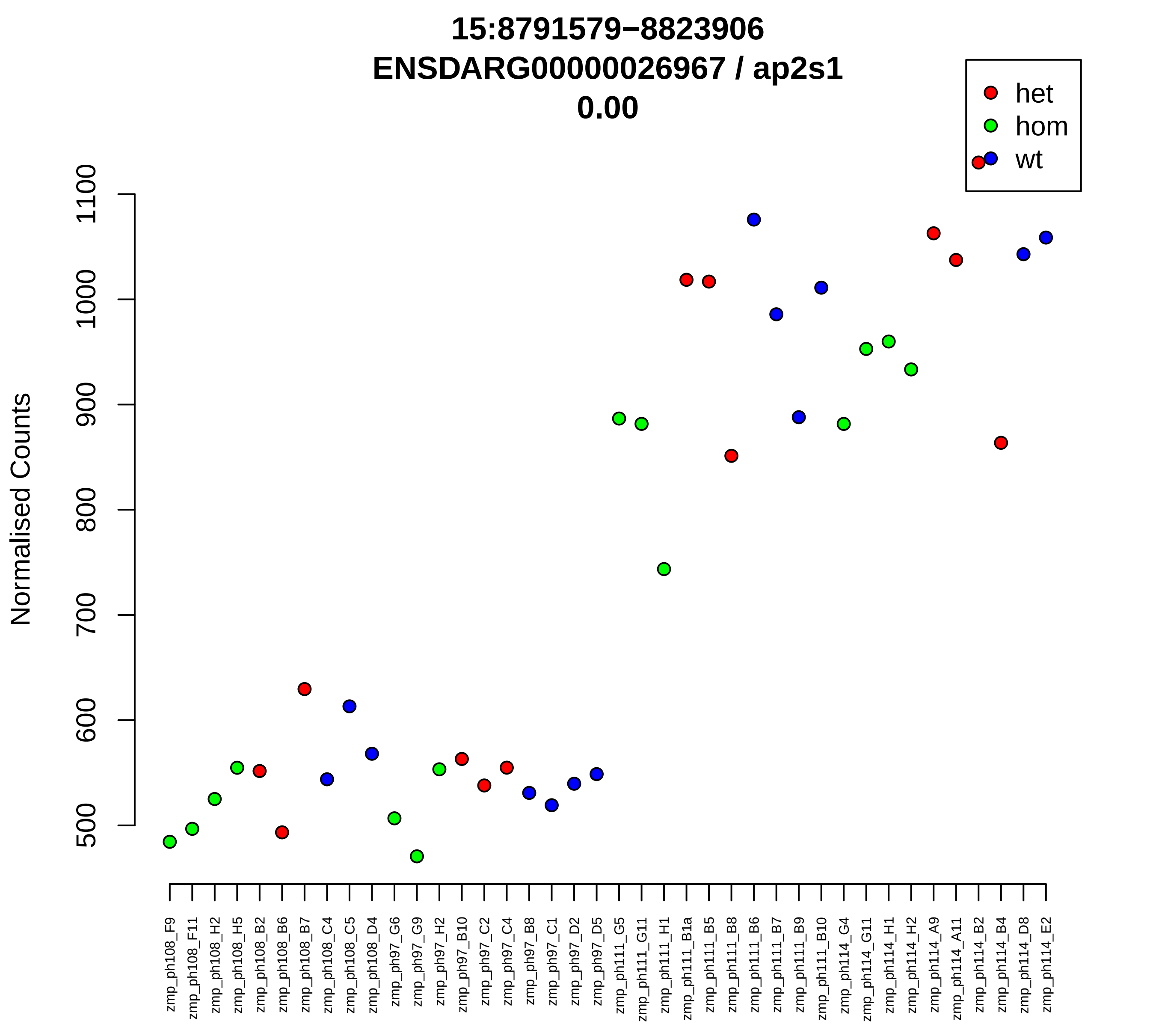
15:8791579−8823906
ENSDARG00000026967 / ap2s1
0.00
het
hom
wt
Normalised Counts
500
600
700
800
900
1000
1100
zmp_ph108_F9
zmp_ph108_F11
zmp_ph108_H2
zmp_ph108_H5
zmp_ph108_B2
zmp_ph108_B6
zmp_ph108_B7
zmp_ph108_C4
zmp_ph108_C5
zmp_ph108_D4
zmp_ph97_G6
zmp_ph97_G9
zmp_ph97_H2
zmp_ph97_B10
zmp_ph97_C2
zmp_ph97_C4
zmp_ph97_B8
zmp_ph97_C1
zmp_ph97_D2
zmp_ph97_D5
zmp_ph111_G5
zmp_ph111_G11
zmp_ph111_H1
zmp_ph111_B1a
zmp_ph111_B5
zmp_ph111_B8
zmp_ph111_B6
zmp_ph111_B7
zmp_ph111_B9
zmp_ph111_B10
zmp_ph114_G4
zmp_ph114_G11
zmp_ph114_H1
zmp_ph114_H2
zmp_ph114_A9
zmp_ph114_A11
zmp_ph114_B2
zmp_ph114_B4
zmp_ph114_D8
zmp_ph114_E2

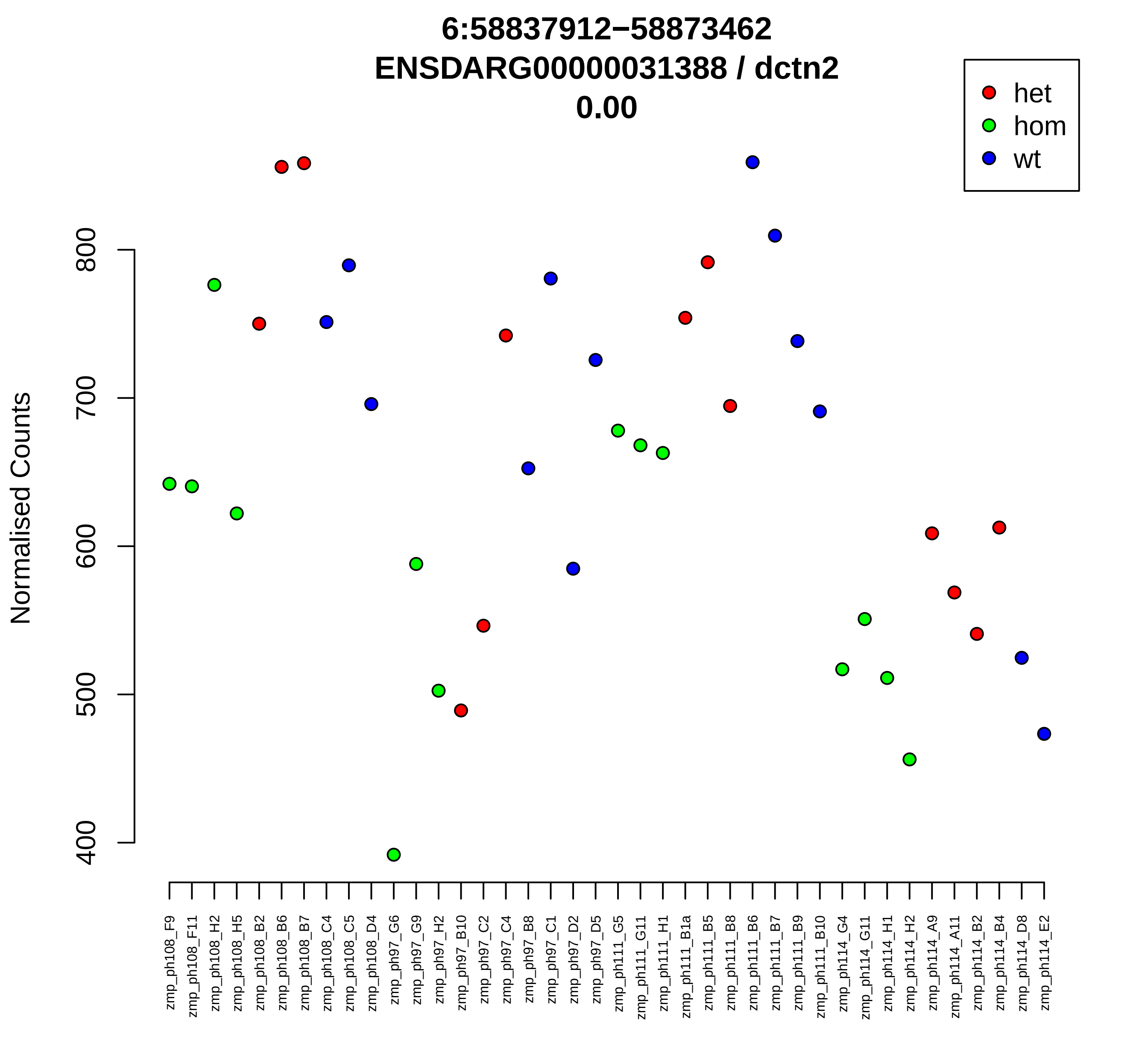

6:58837912−58873462
ENSDARG00000031388 / dctn2
0.00
het
hom
wt
Normalised Counts
400
500
600
700
800
zmp_ph108_F9
zmp_ph108_F11
zmp_ph108_H2
zmp_ph108_H5
zmp_ph108_B2
zmp_ph108_B6
zmp_ph108_B7
zmp_ph108_C4
zmp_ph108_C5
zmp_ph108_D4
zmp_ph97_G6
zmp_ph97_G9
zmp_ph97_H2
zmp_ph97_B10
zmp_ph97_C2
zmp_ph97_C4
zmp_ph97_B8
zmp_ph97_C1
zmp_ph97_D2
zmp_ph97_D5
zmp_ph111_G5
zmp_ph111_G11
zmp_ph111_H1
zmp_ph111_B1a
zmp_ph111_B5
zmp_ph111_B8
zmp_ph111_B6
zmp_ph111_B7
zmp_ph111_B9
zmp_ph111_B10
zmp_ph114_G4
zmp_ph114_G11
zmp_ph114_H1
zmp_ph114_H2
zmp_ph114_A9
zmp_ph114_A11
zmp_ph114_B2
zmp_ph114_B4
zmp_ph114_D8
zmp_ph114_E2

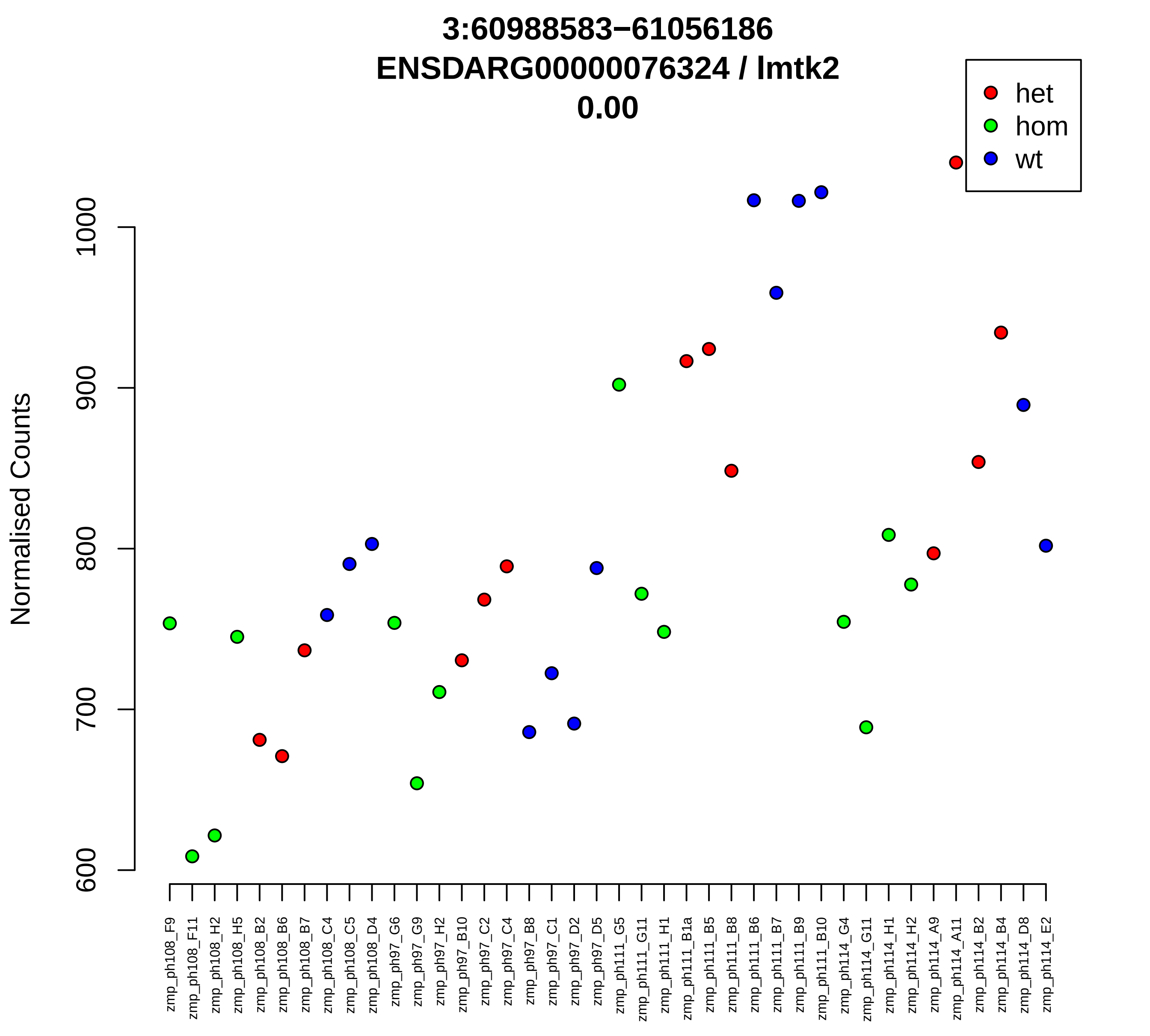

3:60988583−61056186
ENSDARG00000076324 / lmtk2
0.00
het
hom
wt
Normalised Counts
600
700
800
900
1000
zmp_ph108_F9
zmp_ph108_F11
zmp_ph108_H2
zmp_ph108_H5
zmp_ph108_B2
zmp_ph108_B6
zmp_ph108_B7
zmp_ph108_C4
zmp_ph108_C5
zmp_ph108_D4
zmp_ph97_G6
zmp_ph97_G9
zmp_ph97_H2
zmp_ph97_B10
zmp_ph97_C2
zmp_ph97_C4
zmp_ph97_B8
zmp_ph97_C1
zmp_ph97_D2
zmp_ph97_D5
zmp_ph111_G5
zmp_ph111_G11
zmp_ph111_H1
zmp_ph111_B1a
zmp_ph111_B5
zmp_ph111_B8
zmp_ph111_B6
zmp_ph111_B7
zmp_ph111_B9
zmp_ph111_B10
zmp_ph114_G4
zmp_ph114_G11
zmp_ph114_H1
zmp_ph114_H2
zmp_ph114_A9
zmp_ph114_A11
zmp_ph114_B2
zmp_ph114_B4
zmp_ph114_D8
zmp_ph114_E2

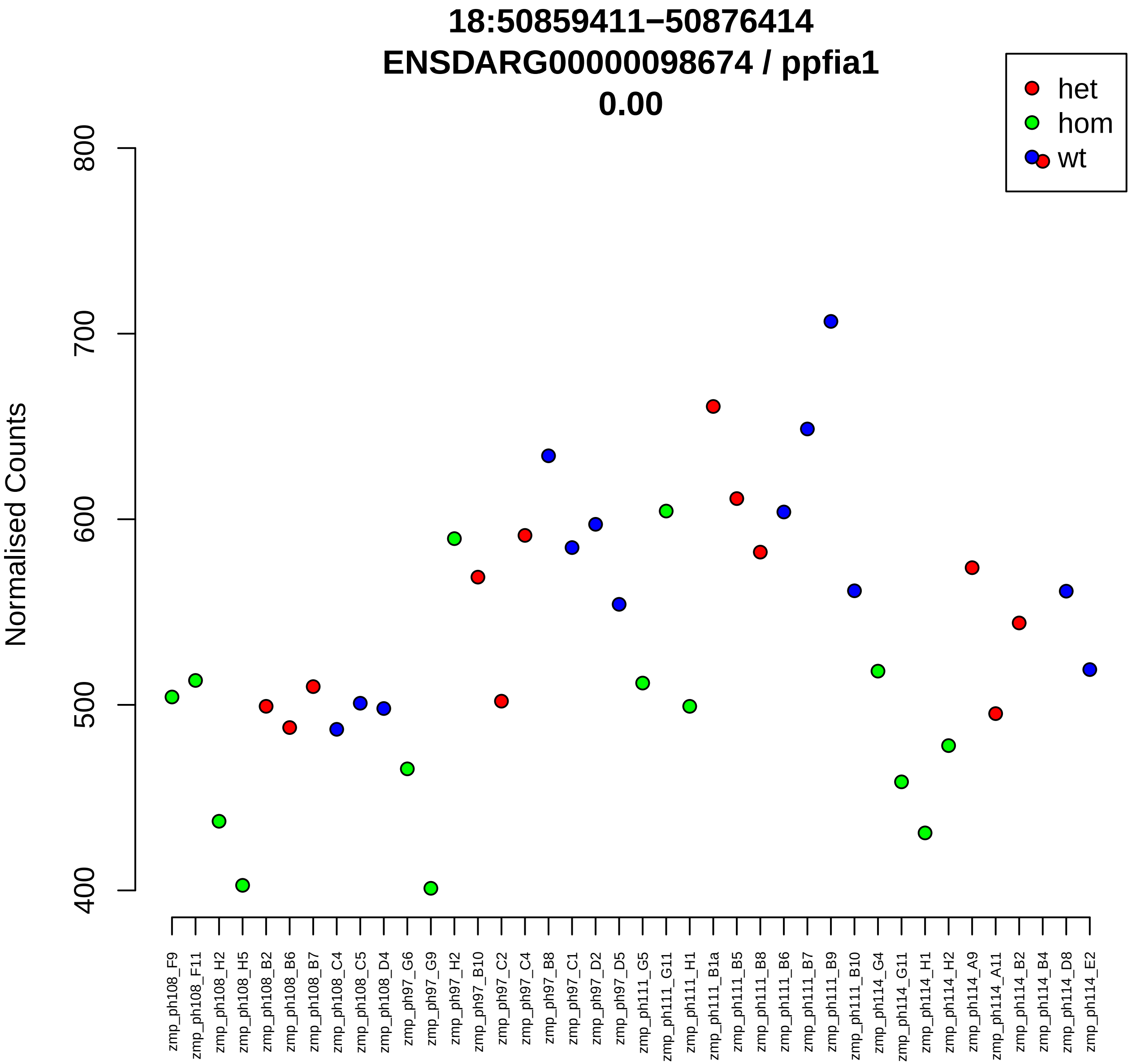
18:50859411−50876414
ENSDARG00000098674 / ppfia1
0.00
het
hom
wt
Normalised Counts
400
500
600
700
800
zmp_ph108_F9
zmp_ph108_F11
zmp_ph108_H2
zmp_ph108_H5
zmp_ph108_B2
zmp_ph108_B6
zmp_ph108_B7
zmp_ph108_C4
zmp_ph108_C5
zmp_ph108_D4
zmp_ph97_G6
zmp_ph97_G9
zmp_ph97_H2
zmp_ph97_B10
zmp_ph97_C2
zmp_ph97_C4
zmp_ph97_B8
zmp_ph97_C1
zmp_ph97_D2
zmp_ph97_D5
zmp_ph111_G5
zmp_ph111_G11
zmp_ph111_H1
zmp_ph111_B1a
zmp_ph111_B5
zmp_ph111_B8
zmp_ph111_B6
zmp_ph111_B7
zmp_ph111_B9
zmp_ph111_B10
zmp_ph114_G4
zmp_ph114_G11
zmp_ph114_H1
zmp_ph114_H2
zmp_ph114_A9
zmp_ph114_A11
zmp_ph114_B2
zmp_ph114_B4
zmp_ph114_D8
zmp_ph114_E2

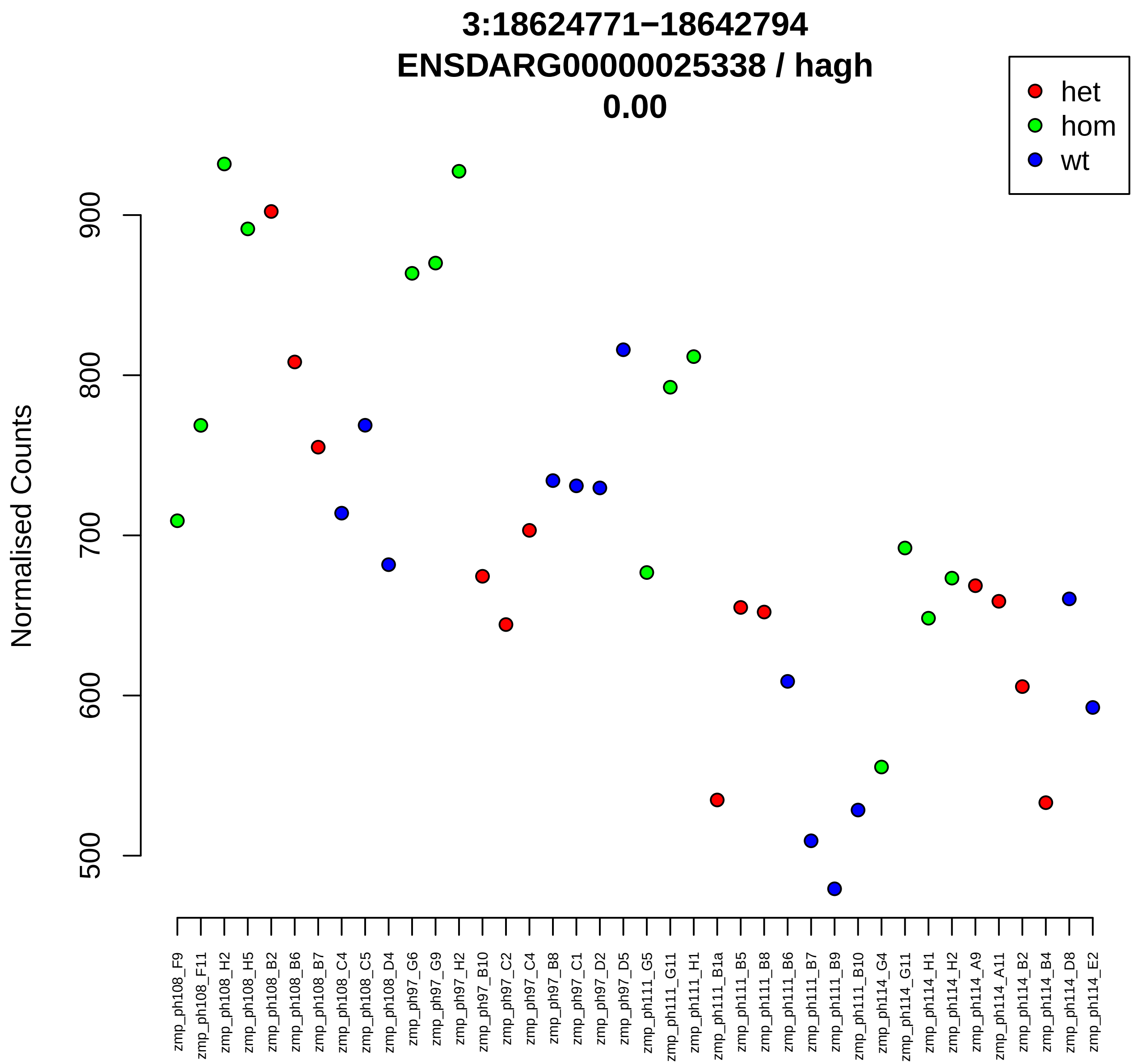

3:18624771−18642794
ENSDARG00000025338 / hagh
0.00
het
hom
wt
Normalised Counts
500
600
700
800
900
zmp_ph108_F9
zmp_ph108_F11
zmp_ph108_H2
zmp_ph108_H5
zmp_ph108_B2
zmp_ph108_B6
zmp_ph108_B7
zmp_ph108_C4
zmp_ph108_C5
zmp_ph108_D4
zmp_ph97_G6
zmp_ph97_G9
zmp_ph97_H2
zmp_ph97_B10
zmp_ph97_C2
zmp_ph97_C4
zmp_ph97_B8
zmp_ph97_C1
zmp_ph97_D2
zmp_ph97_D5
zmp_ph111_G5
zmp_ph111_G11
zmp_ph111_H1
zmp_ph111_B1a
zmp_ph111_B5
zmp_ph111_B8
zmp_ph111_B6
zmp_ph111_B7
zmp_ph111_B9
zmp_ph111_B10
zmp_ph114_G4
zmp_ph114_G11
zmp_ph114_H1
zmp_ph114_H2
zmp_ph114_A9
zmp_ph114_A11
zmp_ph114_B2
zmp_ph114_B4
zmp_ph114_D8
zmp_ph114_E2

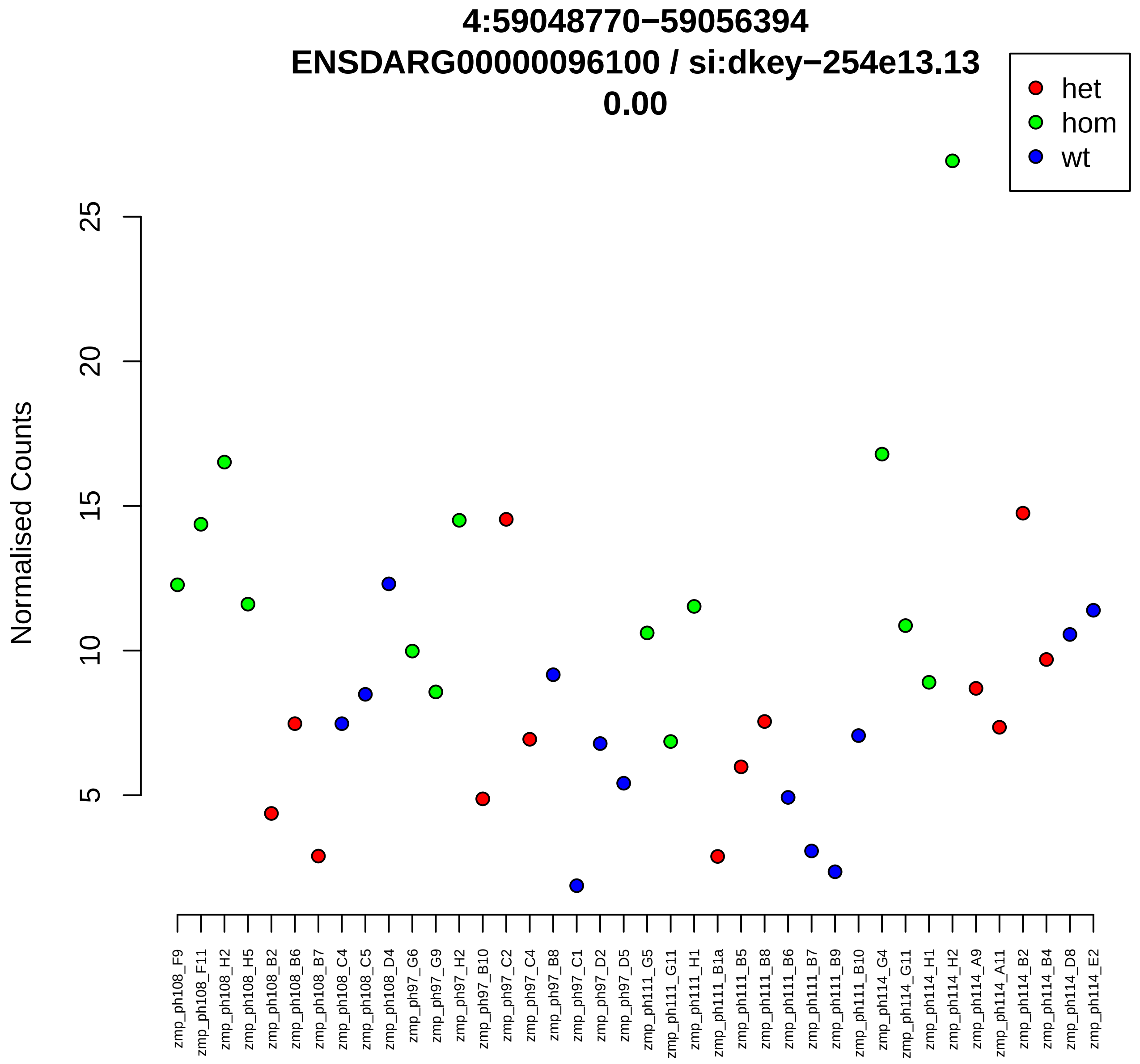
4:59048770−59056394
ENSDARG00000096100 / si:dkey−254e13.13
0.00
het
hom
wt
Normalised Counts
5
10
15
20
25
zmp_ph108_F9
zmp_ph108_F11
zmp_ph108_H2
zmp_ph108_H5
zmp_ph108_B2
zmp_ph108_B6
zmp_ph108_B7
zmp_ph108_C4
zmp_ph108_C5
zmp_ph108_D4
zmp_ph97_G6
zmp_ph97_G9
zmp_ph97_H2
zmp_ph97_B10
zmp_ph97_C2
zmp_ph97_C4
zmp_ph97_B8
zmp_ph97_C1
zmp_ph97_D2
zmp_ph97_D5
zmp_ph111_G5
zmp_ph111_G11
zmp_ph111_H1
zmp_ph111_B1a
zmp_ph111_B5
zmp_ph111_B8
zmp_ph111_B6
zmp_ph111_B7
zmp_ph111_B9
zmp_ph111_B10
zmp_ph114_G4
zmp_ph114_G11
zmp_ph114_H1
zmp_ph114_H2
zmp_ph114_A9
zmp_ph114_A11
zmp_ph114_B2
zmp_ph114_B4
zmp_ph114_D8
zmp_ph114_E2

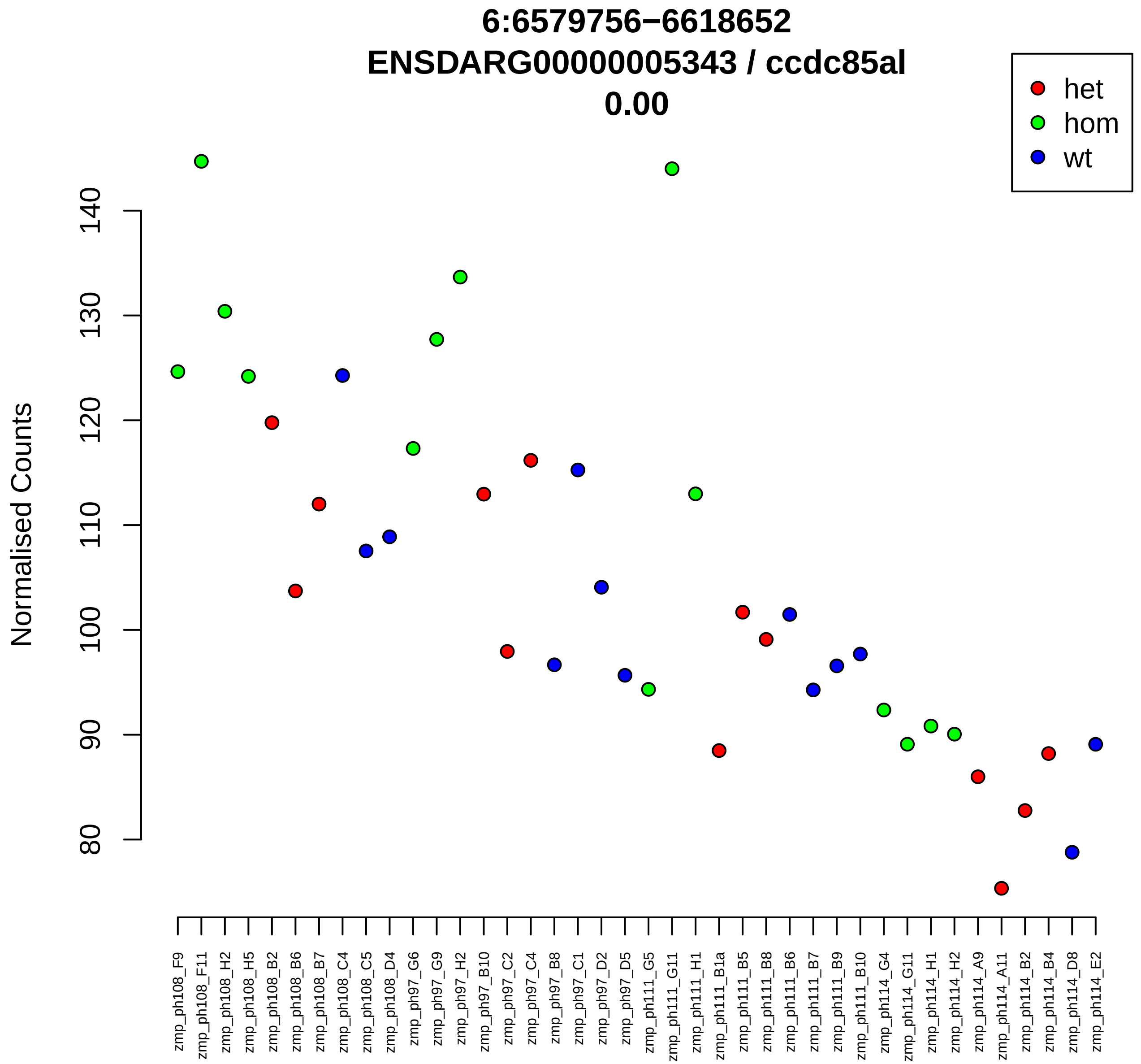

6:6579756−6618652
ENSDARG00000005343 / ccdc85al
0.00
het
hom
wt
Normalised Counts
80
90
100
110
120
130
140
zmp_ph108_F9
zmp_ph108_F11
zmp_ph108_H2
zmp_ph108_H5
zmp_ph108_B2
zmp_ph108_B6
zmp_ph108_B7
zmp_ph108_C4
zmp_ph108_C5
zmp_ph108_D4
zmp_ph97_G6
zmp_ph97_G9
zmp_ph97_H2
zmp_ph97_B10
zmp_ph97_C2
zmp_ph97_C4
zmp_ph97_B8
zmp_ph97_C1
zmp_ph97_D2
zmp_ph97_D5
zmp_ph111_G5
zmp_ph111_G11
zmp_ph111_H1
zmp_ph111_B1a
zmp_ph111_B5
zmp_ph111_B8
zmp_ph111_B6
zmp_ph111_B7
zmp_ph111_B9
zmp_ph111_B10
zmp_ph114_G4
zmp_ph114_G11
zmp_ph114_H1
zmp_ph114_H2
zmp_ph114_A9
zmp_ph114_A11
zmp_ph114_B2
zmp_ph114_B4
zmp_ph114_D8
zmp_ph114_E2

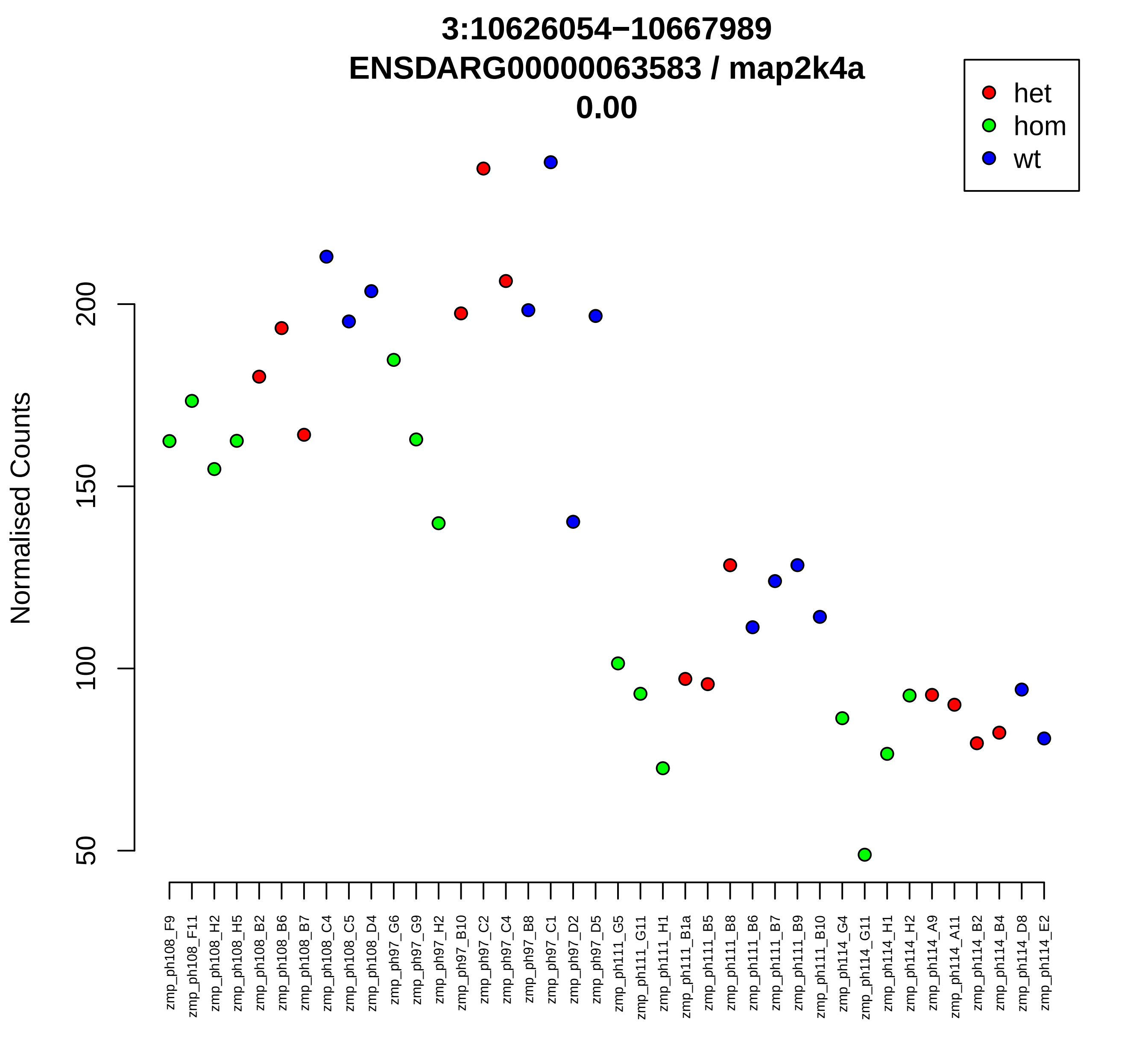

3:10626054−10667989
ENSDARG00000063583 / map2k4a
0.00
het
hom
wt
Normalised Counts
50
100
150
200
zmp_ph108_F9
zmp_ph108_F11
zmp_ph108_H2
zmp_ph108_H5
zmp_ph108_B2
zmp_ph108_B6
zmp_ph108_B7
zmp_ph108_C4
zmp_ph108_C5
zmp_ph108_D4
zmp_ph97_G6
zmp_ph97_G9
zmp_ph97_H2
zmp_ph97_B10
zmp_ph97_C2
zmp_ph97_C4
zmp_ph97_B8
zmp_ph97_C1
zmp_ph97_D2
zmp_ph97_D5
zmp_ph111_G5
zmp_ph111_G11
zmp_ph111_H1
zmp_ph111_B1a
zmp_ph111_B5
zmp_ph111_B8
zmp_ph111_B6
zmp_ph111_B7
zmp_ph111_B9
zmp_ph111_B10
zmp_ph114_G4
zmp_ph114_G11
zmp_ph114_H1
zmp_ph114_H2
zmp_ph114_A9
zmp_ph114_A11
zmp_ph114_B2
zmp_ph114_B4
zmp_ph114_D8
zmp_ph114_E2

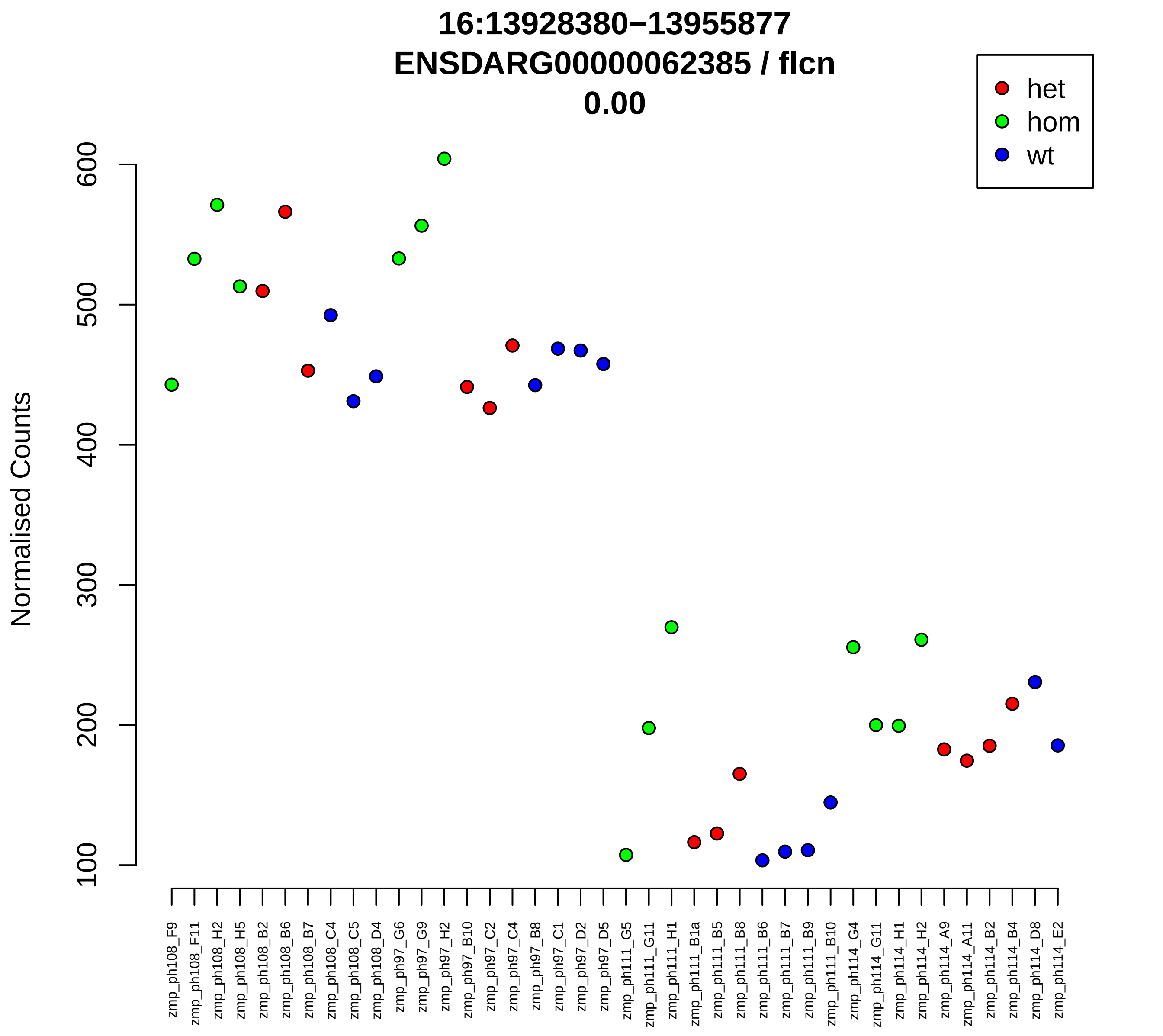
16:13928380−13955877
ENSDARG00000062385 / flcn
0.00
het
hom
wt
Normalised Counts
100
200
300
400
500
600
zmp_ph108_F9
zmp_ph108_F11
zmp_ph108_H2
zmp_ph108_H5
zmp_ph108_B2
zmp_ph108_B6
zmp_ph108_B7
zmp_ph108_C4
zmp_ph108_C5
zmp_ph108_D4
zmp_ph97_G6
zmp_ph97_G9
zmp_ph97_H2
zmp_ph97_B10
zmp_ph97_C2
zmp_ph97_C4
zmp_ph97_B8
zmp_ph97_C1
zmp_ph97_D2
zmp_ph97_D5
zmp_ph111_G5
zmp_ph111_G11
zmp_ph111_H1
zmp_ph111_B1a
zmp_ph111_B5
zmp_ph111_B8
zmp_ph111_B6
zmp_ph111_B7
zmp_ph111_B9
zmp_ph111_B10
zmp_ph114_G4
zmp_ph114_G11
zmp_ph114_H1
zmp_ph114_H2
zmp_ph114_A9
zmp_ph114_A11
zmp_ph114_B2
zmp_ph114_B4
zmp_ph114_D8
zmp_ph114_E2

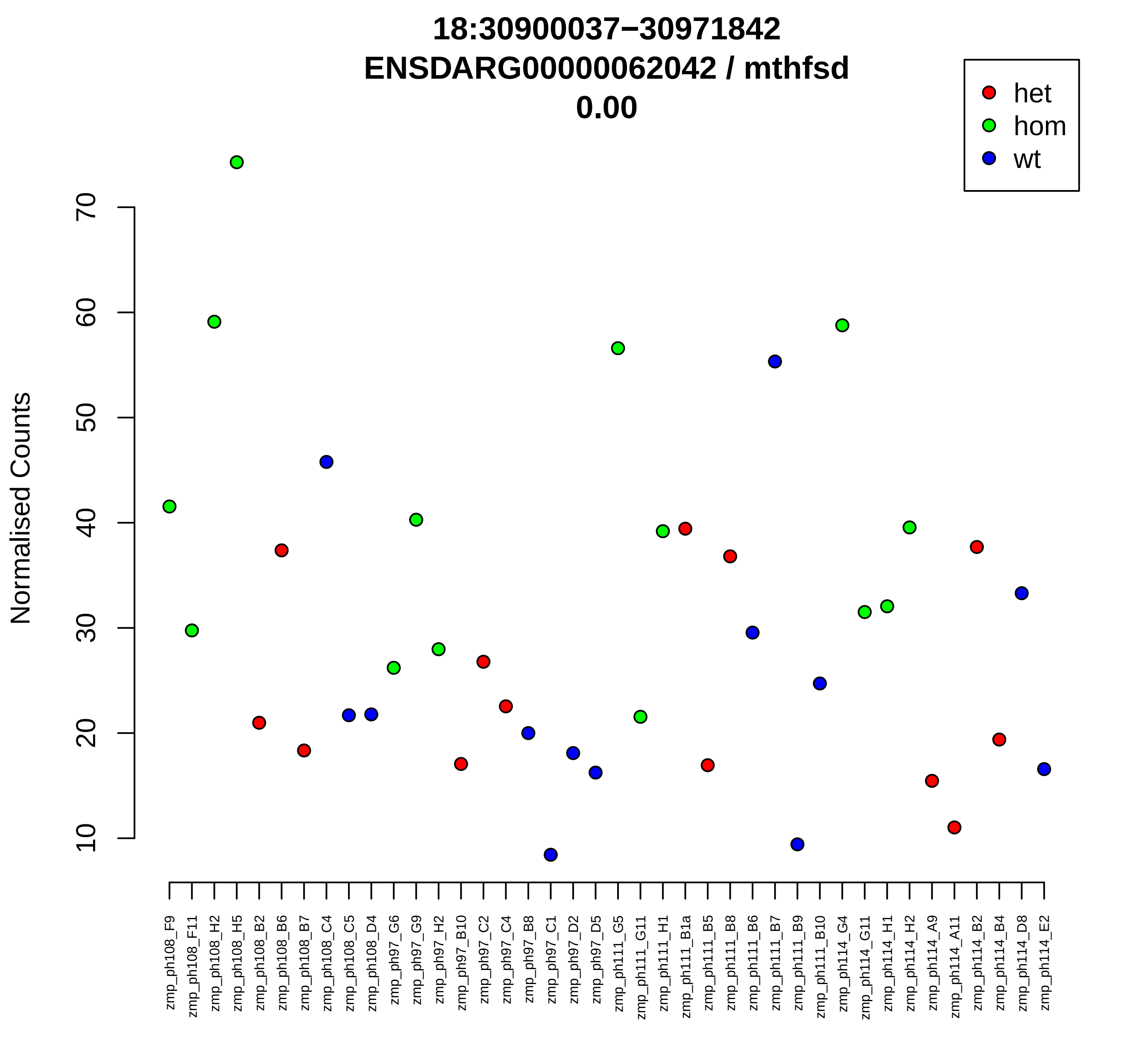

18:30900037−30971842
ENSDARG00000062042 / mthfsd
0.00
het
hom
wt
Normalised Counts
10
20
30
40
50
60
70
zmp_ph108_F9
zmp_ph108_F11
zmp_ph108_H2
zmp_ph108_H5
zmp_ph108_B2
zmp_ph108_B6
zmp_ph108_B7
zmp_ph108_C4
zmp_ph108_C5
zmp_ph108_D4
zmp_ph97_G6
zmp_ph97_G9
zmp_ph97_H2
zmp_ph97_B10
zmp_ph97_C2
zmp_ph97_C4
zmp_ph97_B8
zmp_ph97_C1
zmp_ph97_D2
zmp_ph97_D5
zmp_ph111_G5
zmp_ph111_G11
zmp_ph111_H1
zmp_ph111_B1a
zmp_ph111_B5
zmp_ph111_B8
zmp_ph111_B6
zmp_ph111_B7
zmp_ph111_B9
zmp_ph111_B10
zmp_ph114_G4
zmp_ph114_G11
zmp_ph114_H1
zmp_ph114_H2
zmp_ph114_A9
zmp_ph114_A11
zmp_ph114_B2
zmp_ph114_B4
zmp_ph114_D8
zmp_ph114_E2

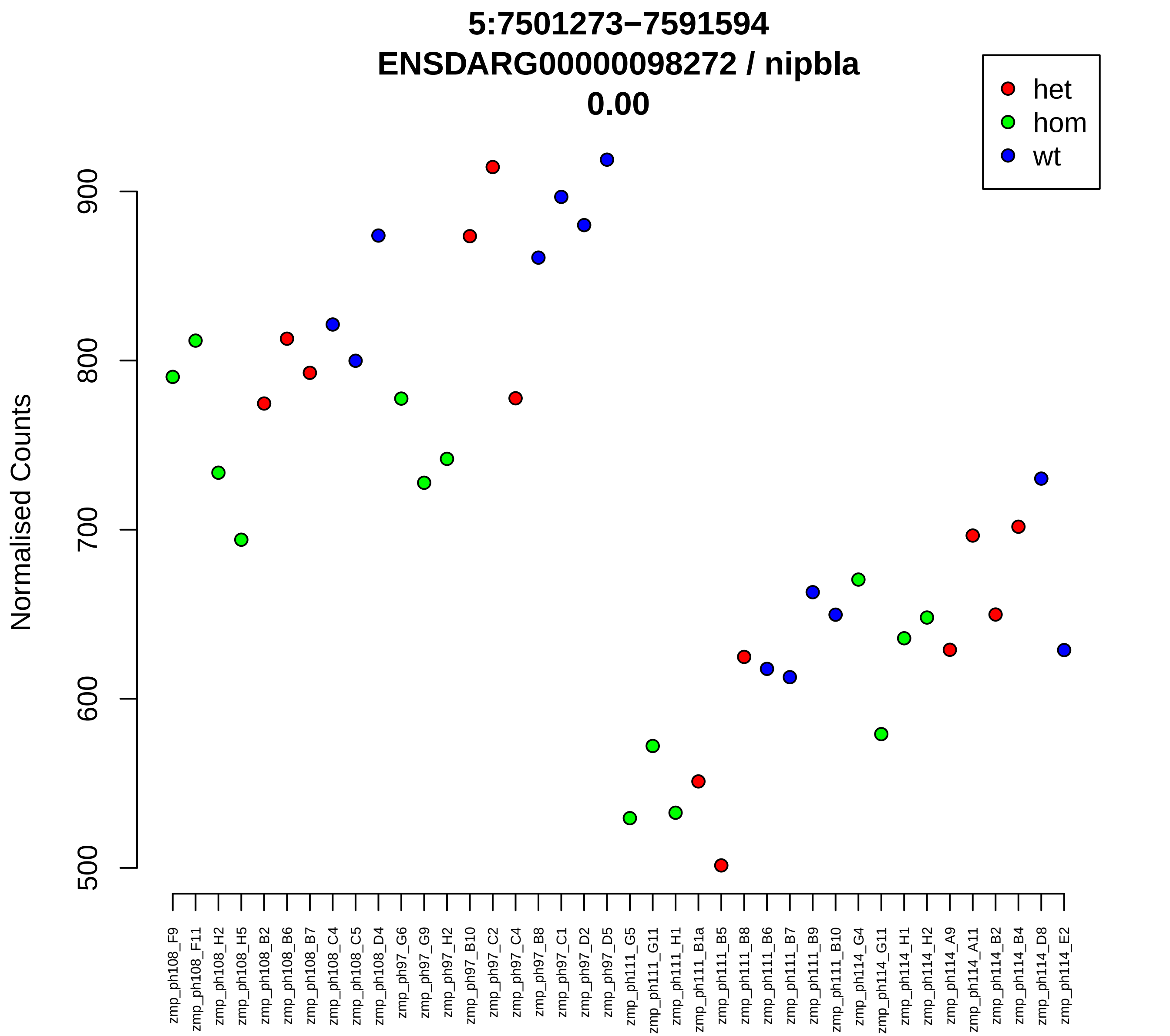

5:7501273−7591594
ENSDARG00000098272 / nipbla
0.00
het
hom
wt
Normalised Counts
500
600
700
800
900
zmp_ph108_F9
zmp_ph108_F11
zmp_ph108_H2
zmp_ph108_H5
zmp_ph108_B2
zmp_ph108_B6
zmp_ph108_B7
zmp_ph108_C4
zmp_ph108_C5
zmp_ph108_D4
zmp_ph97_G6
zmp_ph97_G9
zmp_ph97_H2
zmp_ph97_B10
zmp_ph97_C2
zmp_ph97_C4
zmp_ph97_B8
zmp_ph97_C1
zmp_ph97_D2
zmp_ph97_D5
zmp_ph111_G5
zmp_ph111_G11
zmp_ph111_H1
zmp_ph111_B1a
zmp_ph111_B5
zmp_ph111_B8
zmp_ph111_B6
zmp_ph111_B7
zmp_ph111_B9
zmp_ph111_B10
zmp_ph114_G4
zmp_ph114_G11
zmp_ph114_H1
zmp_ph114_H2
zmp_ph114_A9
zmp_ph114_A11
zmp_ph114_B2
zmp_ph114_B4
zmp_ph114_D8
zmp_ph114_E2

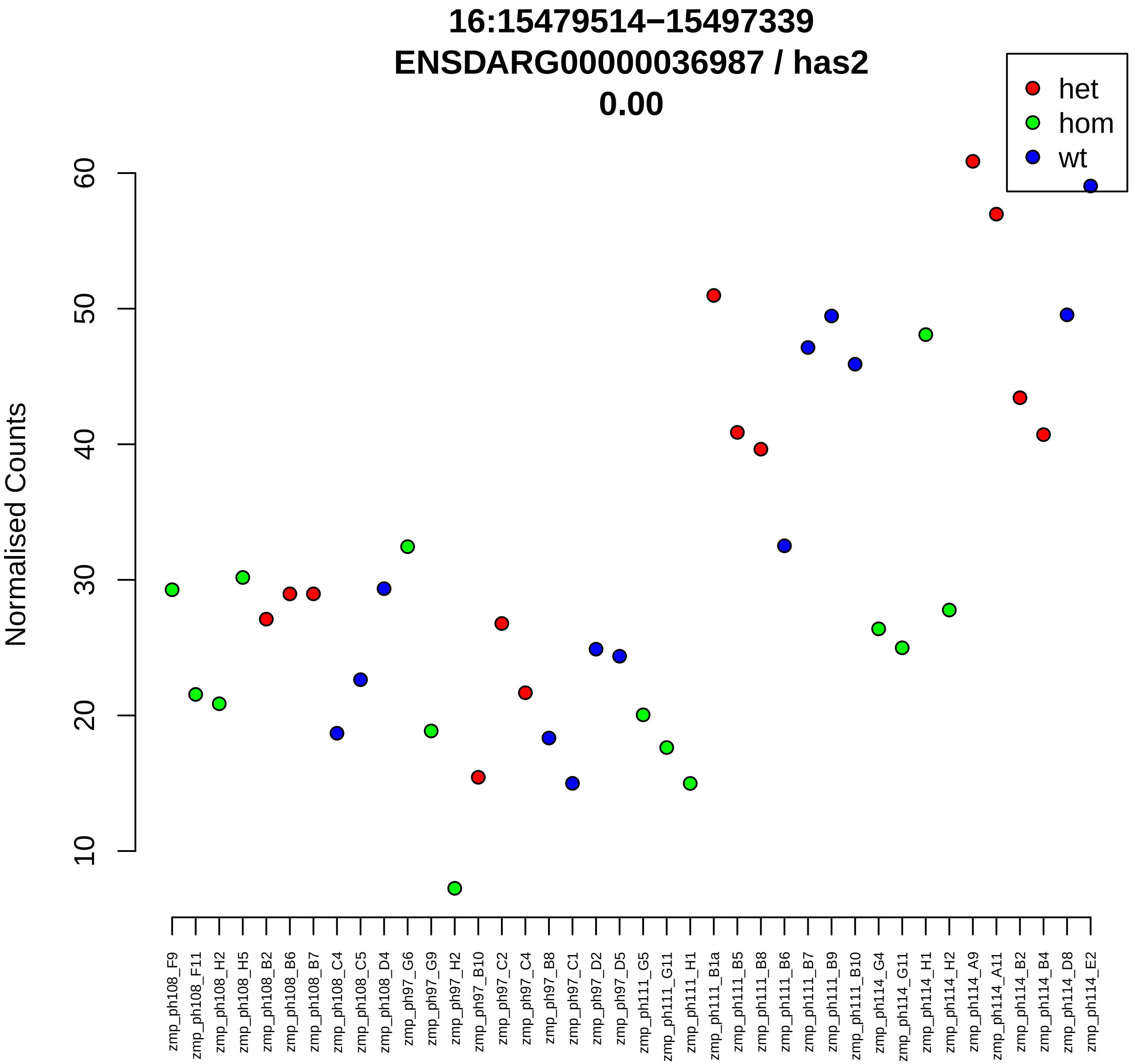

16:15479514−15497339
ENSDARG00000036987 / has2
0.00
het
hom
wt
Normalised Counts
10
20
30
40
50
60
zmp_ph108_F9
zmp_ph108_F11
zmp_ph108_H2
zmp_ph108_H5
zmp_ph108_B2
zmp_ph108_B6
zmp_ph108_B7
zmp_ph108_C4
zmp_ph108_C5
zmp_ph108_D4
zmp_ph97_G6
zmp_ph97_G9
zmp_ph97_H2
zmp_ph97_B10
zmp_ph97_C2
zmp_ph97_C4
zmp_ph97_B8
zmp_ph97_C1
zmp_ph97_D2
zmp_ph97_D5
zmp_ph111_G5
zmp_ph111_G11
zmp_ph111_H1
zmp_ph111_B1a
zmp_ph111_B5
zmp_ph111_B8
zmp_ph111_B6
zmp_ph111_B7
zmp_ph111_B9
zmp_ph111_B10
zmp_ph114_G4
zmp_ph114_G11
zmp_ph114_H1
zmp_ph114_H2
zmp_ph114_A9
zmp_ph114_A11
zmp_ph114_B2
zmp_ph114_B4
zmp_ph114_D8
zmp_ph114_E2

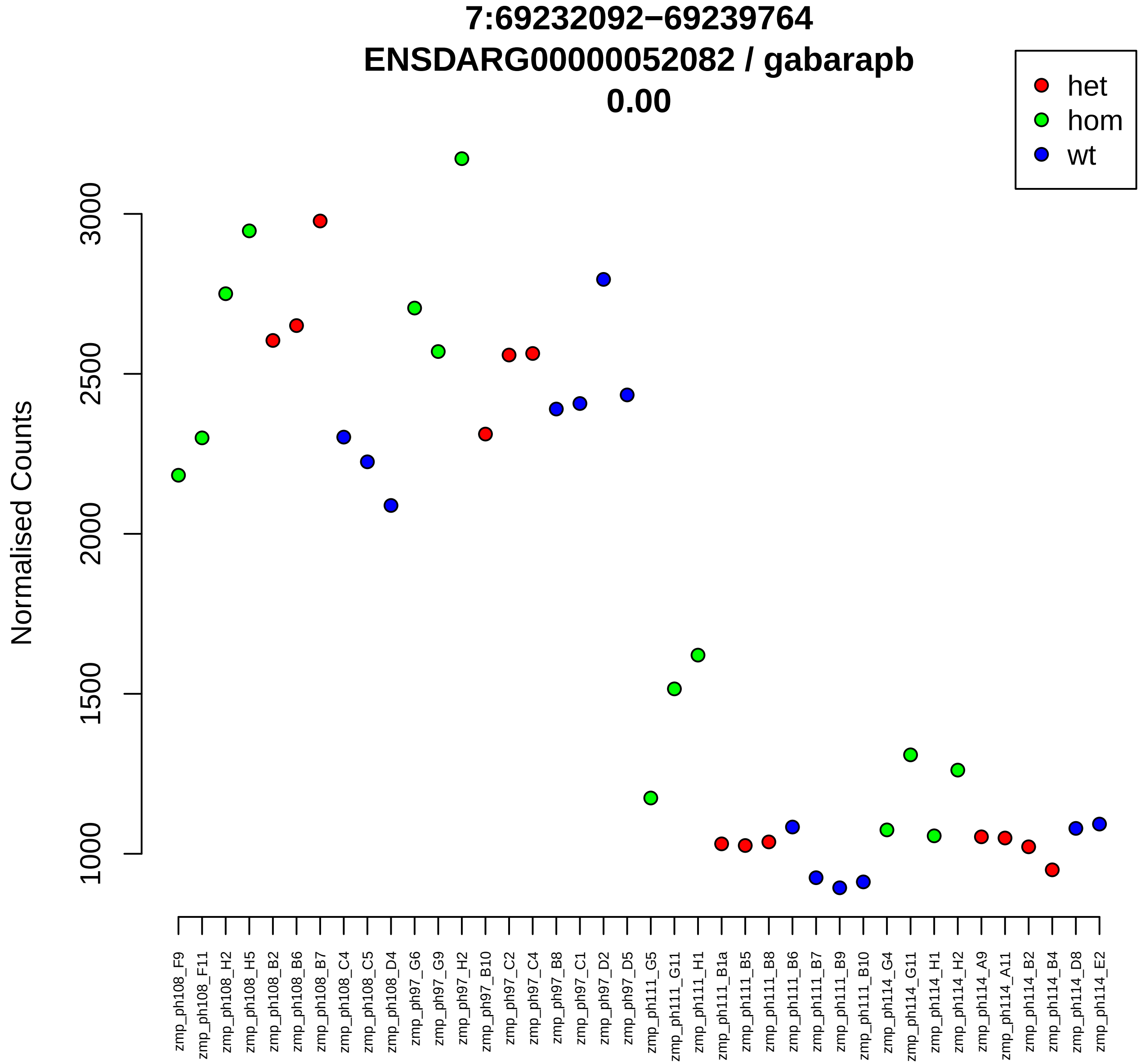

7:69232092−69239764
ENSDARG00000052082 / gabarapb
0.00
het
hom
wt
Normalised Counts
1000
1500
2000
2500
3000
zmp_ph108_F9
zmp_ph108_F11
zmp_ph108_H2
zmp_ph108_H5
zmp_ph108_B2
zmp_ph108_B6
zmp_ph108_B7
zmp_ph108_C4
zmp_ph108_C5
zmp_ph108_D4
zmp_ph97_G6
zmp_ph97_G9
zmp_ph97_H2
zmp_ph97_B10
zmp_ph97_C2
zmp_ph97_C4
zmp_ph97_B8
zmp_ph97_C1
zmp_ph97_D2
zmp_ph97_D5
zmp_ph111_G5
zmp_ph111_G11
zmp_ph111_H1
zmp_ph111_B1a
zmp_ph111_B5
zmp_ph111_B8
zmp_ph111_B6
zmp_ph111_B7
zmp_ph111_B9
zmp_ph111_B10
zmp_ph114_G4
zmp_ph114_G11
zmp_ph114_H1
zmp_ph114_H2
zmp_ph114_A9
zmp_ph114_A11
zmp_ph114_B2
zmp_ph114_B4
zmp_ph114_D8
zmp_ph114_E2

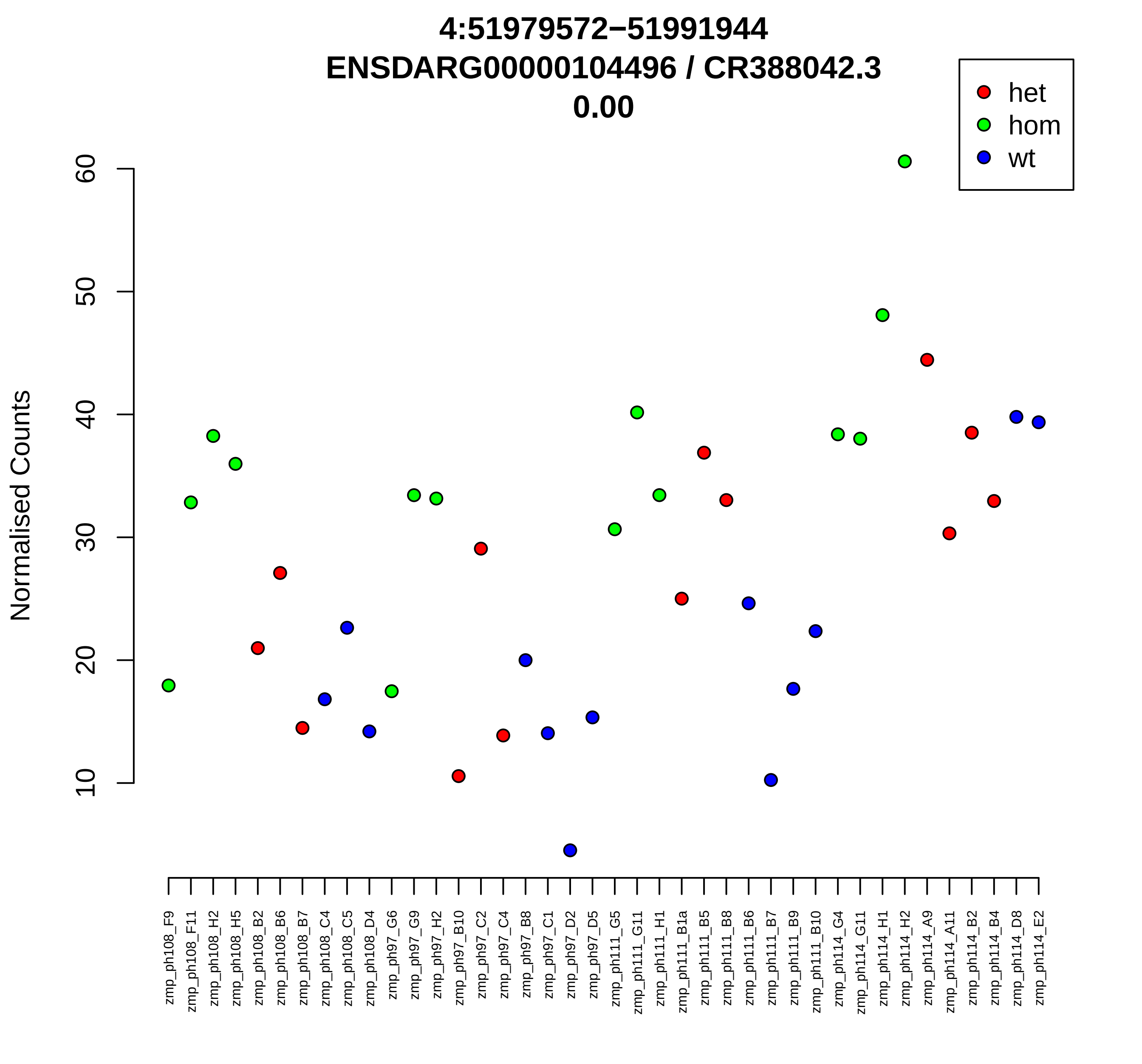
4:51979572−51991944
ENSDARG00000104496 / CR388042.3
0.00
het
hom
wt
Normalised Counts
10
20
30
40
50
60
zmp_ph108_F9
zmp_ph108_F11
zmp_ph108_H2
zmp_ph108_H5
zmp_ph108_B2
zmp_ph108_B6
zmp_ph108_B7
zmp_ph108_C4
zmp_ph108_C5
zmp_ph108_D4
zmp_ph97_G6
zmp_ph97_G9
zmp_ph97_H2
zmp_ph97_B10
zmp_ph97_C2
zmp_ph97_C4
zmp_ph97_B8
zmp_ph97_C1
zmp_ph97_D2
zmp_ph97_D5
zmp_ph111_G5
zmp_ph111_G11
zmp_ph111_H1
zmp_ph111_B1a
zmp_ph111_B5
zmp_ph111_B8
zmp_ph111_B6
zmp_ph111_B7
zmp_ph111_B9
zmp_ph111_B10
zmp_ph114_G4
zmp_ph114_G11
zmp_ph114_H1
zmp_ph114_H2
zmp_ph114_A9
zmp_ph114_A11
zmp_ph114_B2
zmp_ph114_B4
zmp_ph114_D8
zmp_ph114_E2

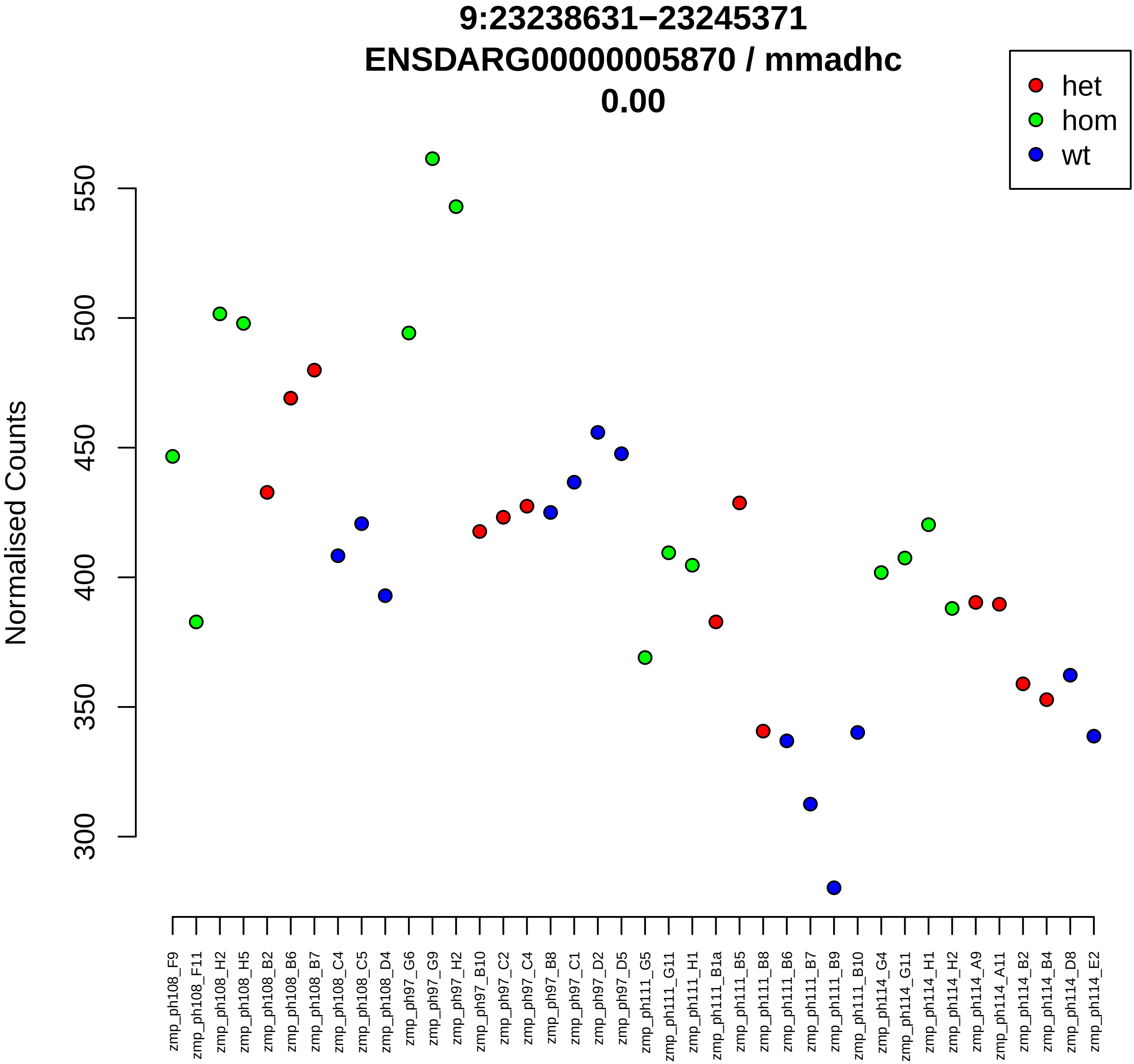

9:23238631−23245371
ENSDARG00000005870 / mmadhc
0.00
het
hom
wt
Normalised Counts
300
350
400
450
500
550
zmp_ph108_F9
zmp_ph108_F11
zmp_ph108_H2
zmp_ph108_H5
zmp_ph108_B2
zmp_ph108_B6
zmp_ph108_B7
zmp_ph108_C4
zmp_ph108_C5
zmp_ph108_D4
zmp_ph97_G6
zmp_ph97_G9
zmp_ph97_H2
zmp_ph97_B10
zmp_ph97_C2
zmp_ph97_C4
zmp_ph97_B8
zmp_ph97_C1
zmp_ph97_D2
zmp_ph97_D5
zmp_ph111_G5
zmp_ph111_G11
zmp_ph111_H1
zmp_ph111_B1a
zmp_ph111_B5
zmp_ph111_B8
zmp_ph111_B6
zmp_ph111_B7
zmp_ph111_B9
zmp_ph111_B10
zmp_ph114_G4
zmp_ph114_G11
zmp_ph114_H1
zmp_ph114_H2
zmp_ph114_A9
zmp_ph114_A11
zmp_ph114_B2
zmp_ph114_B4
zmp_ph114_D8
zmp_ph114_E2

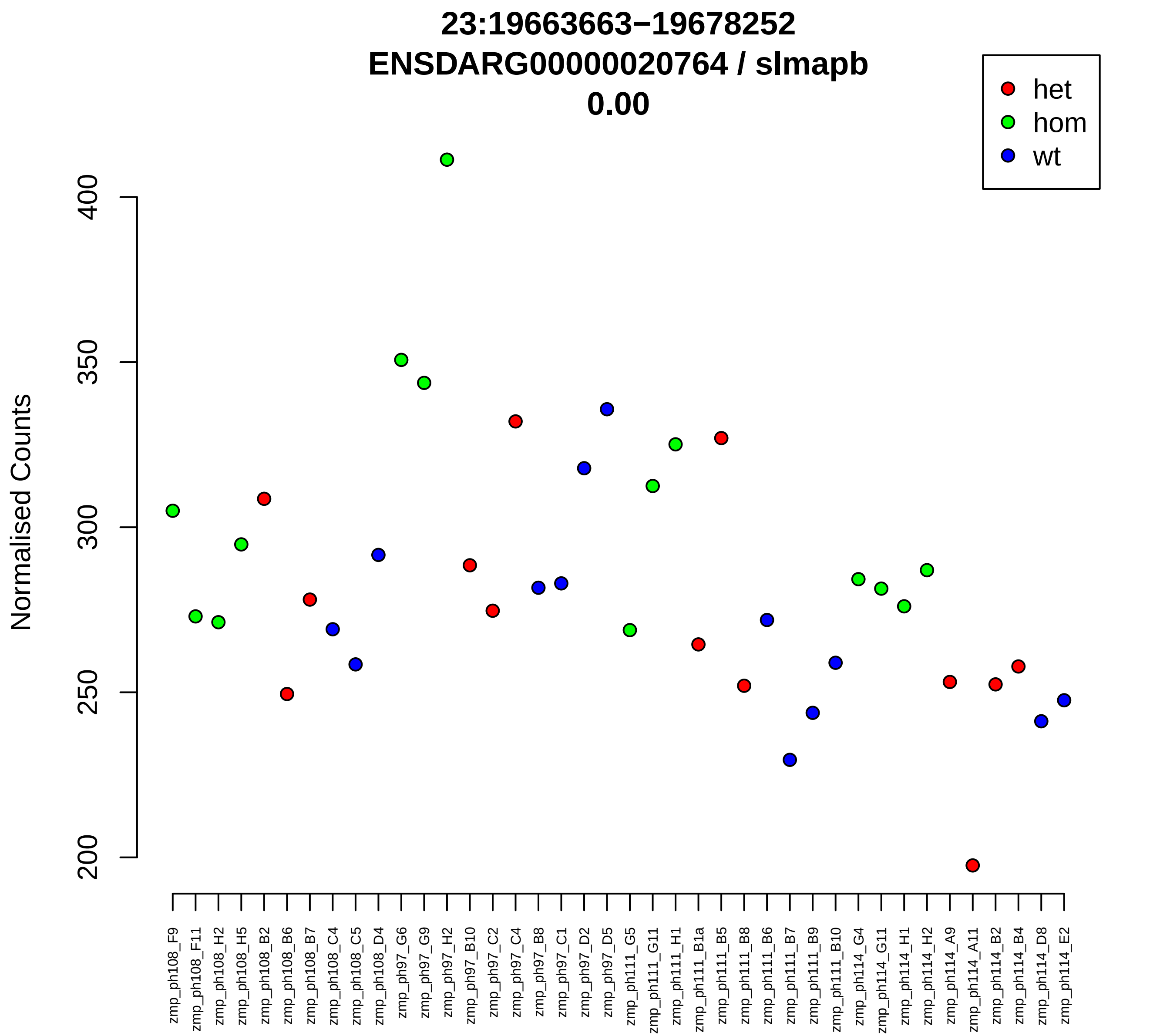

23:19663663−19678252
ENSDARG00000020764 / slmapb
0.00
het
hom
wt
Normalised Counts
200
250
300
350
400
zmp_ph108_F9
zmp_ph108_F11
zmp_ph108_H2
zmp_ph108_H5
zmp_ph108_B2
zmp_ph108_B6
zmp_ph108_B7
zmp_ph108_C4
zmp_ph108_C5
zmp_ph108_D4
zmp_ph97_G6
zmp_ph97_G9
zmp_ph97_H2
zmp_ph97_B10
zmp_ph97_C2
zmp_ph97_C4
zmp_ph97_B8
zmp_ph97_C1
zmp_ph97_D2
zmp_ph97_D5
zmp_ph111_G5
zmp_ph111_G11
zmp_ph111_H1
zmp_ph111_B1a
zmp_ph111_B5
zmp_ph111_B8
zmp_ph111_B6
zmp_ph111_B7
zmp_ph111_B9
zmp_ph111_B10
zmp_ph114_G4
zmp_ph114_G11
zmp_ph114_H1
zmp_ph114_H2
zmp_ph114_A9
zmp_ph114_A11
zmp_ph114_B2
zmp_ph114_B4
zmp_ph114_D8
zmp_ph114_E2

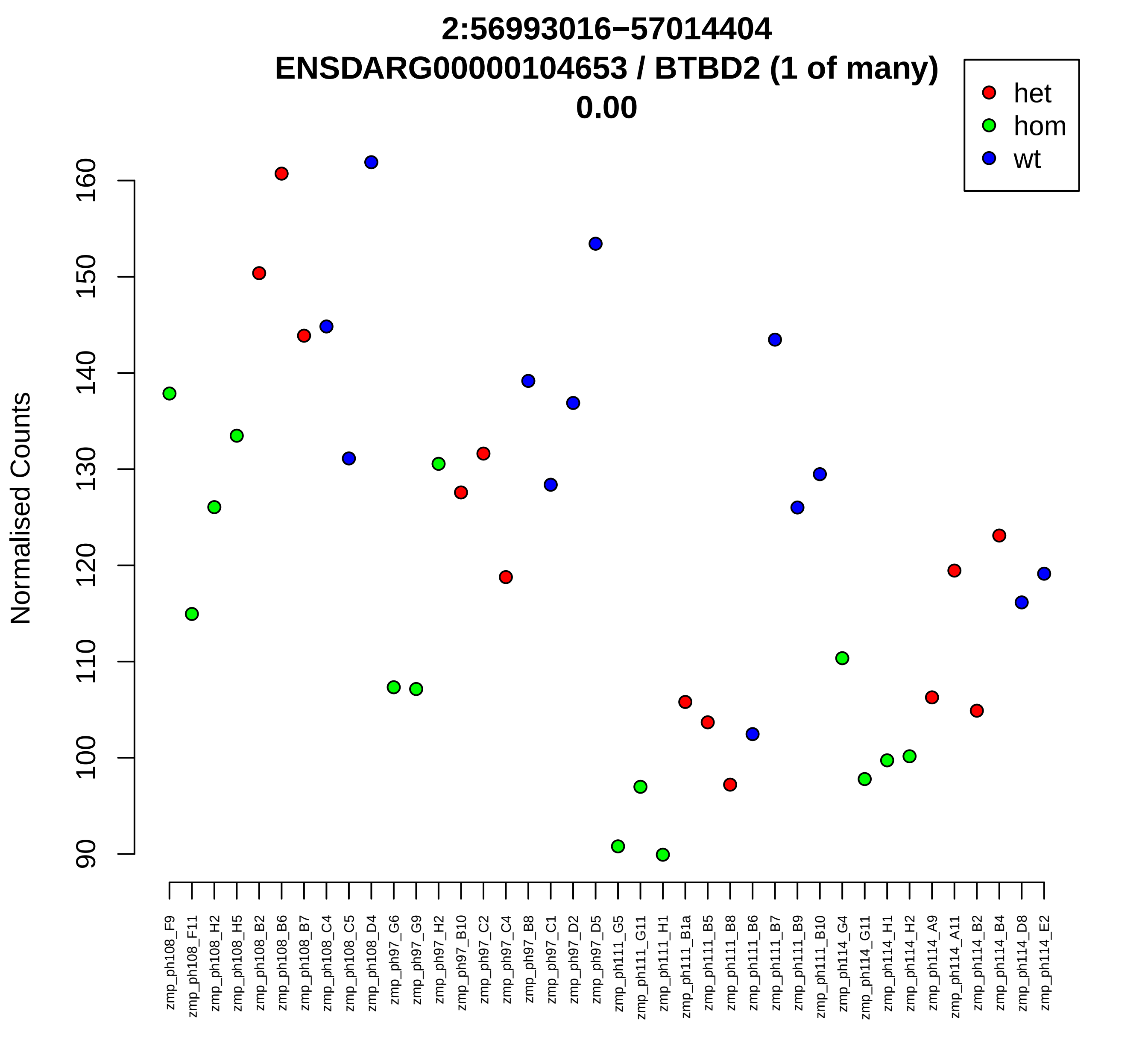

2:56993016−57014404
ENSDARG00000104653 / BTBD2 (1 of many)
0.00
het
hom
wt
Normalised Counts
90
100
110
120
130
140
150
160
zmp_ph108_F9
zmp_ph108_F11
zmp_ph108_H2
zmp_ph108_H5
zmp_ph108_B2
zmp_ph108_B6
zmp_ph108_B7
zmp_ph108_C4
zmp_ph108_C5
zmp_ph108_D4
zmp_ph97_G6
zmp_ph97_G9
zmp_ph97_H2
zmp_ph97_B10
zmp_ph97_C2
zmp_ph97_C4
zmp_ph97_B8
zmp_ph97_C1
zmp_ph97_D2
zmp_ph97_D5
zmp_ph111_G5
zmp_ph111_G11
zmp_ph111_H1
zmp_ph111_B1a
zmp_ph111_B5
zmp_ph111_B8
zmp_ph111_B6
zmp_ph111_B7
zmp_ph111_B9
zmp_ph111_B10
zmp_ph114_G4
zmp_ph114_G11
zmp_ph114_H1
zmp_ph114_H2
zmp_ph114_A9
zmp_ph114_A11
zmp_ph114_B2
zmp_ph114_B4
zmp_ph114_D8
zmp_ph114_E2

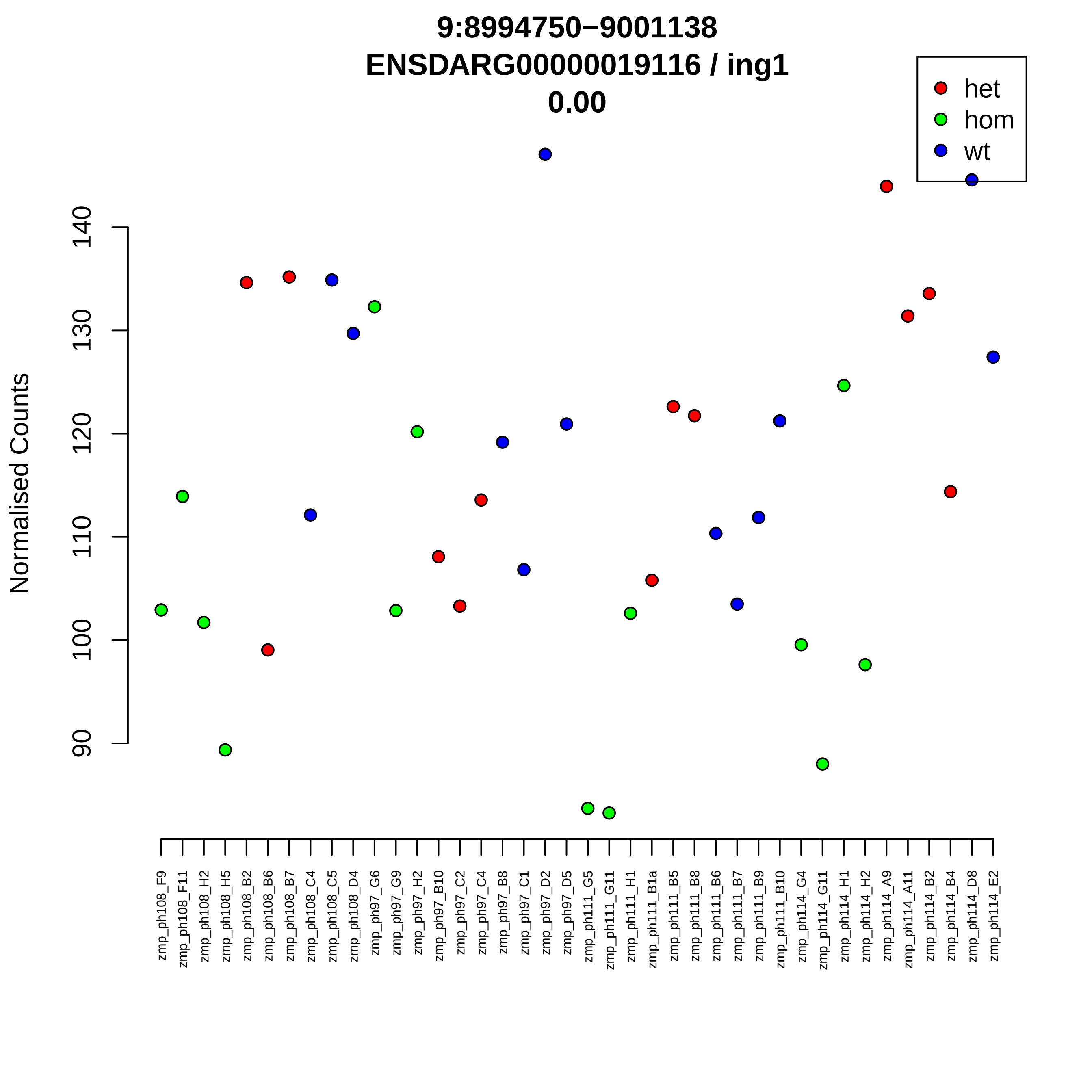
9:8994750−9001138
ENSDARG00000019116 / ing1
0.00
het
hom
wt
Normalised Counts
90
100
110
120
130
140
zmp_ph108_F9
zmp_ph108_F11
zmp_ph108_H2
zmp_ph108_H5
zmp_ph108_B2
zmp_ph108_B6
zmp_ph108_B7
zmp_ph108_C4
zmp_ph108_C5
zmp_ph108_D4
zmp_ph97_G6
zmp_ph97_G9
zmp_ph97_H2
zmp_ph97_B10
zmp_ph97_C2
zmp_ph97_C4
zmp_ph97_B8
zmp_ph97_C1
zmp_ph97_D2
zmp_ph97_D5
zmp_ph111_G5
zmp_ph111_G11
zmp_ph111_H1
zmp_ph111_B1a
zmp_ph111_B5
zmp_ph111_B8
zmp_ph111_B6
zmp_ph111_B7
zmp_ph111_B9
zmp_ph111_B10
zmp_ph114_G4
zmp_ph114_G11
zmp_ph114_H1
zmp_ph114_H2
zmp_ph114_A9
zmp_ph114_A11
zmp_ph114_B2
zmp_ph114_B4
zmp_ph114_D8
zmp_ph114_E2

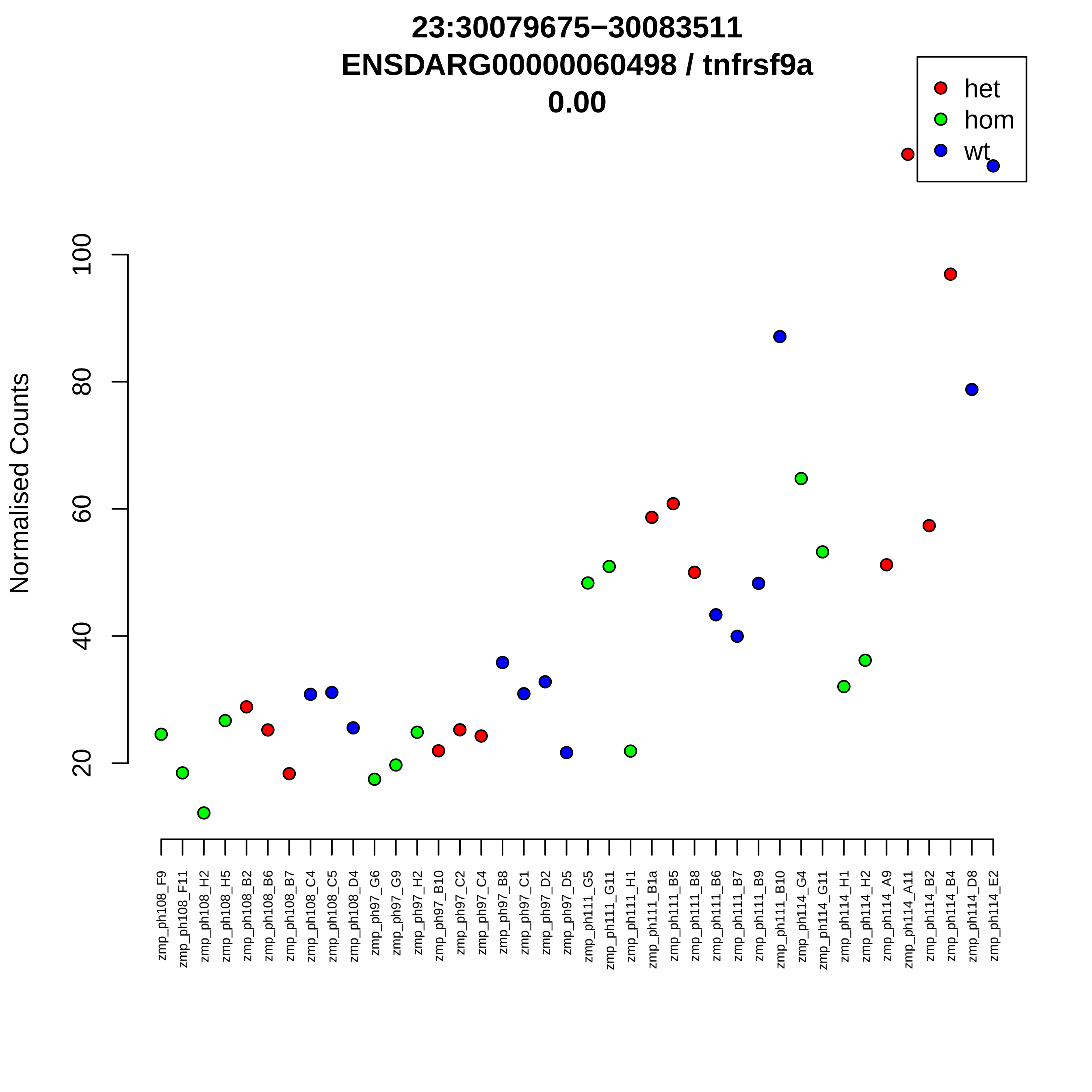
23:30079675−30083511
ENSDARG00000060498 / tnfrsf9a
0.00
het
hom
wt
Normalised Counts
20
40
60
80
100
zmp_ph108_F9
zmp_ph108_F11
zmp_ph108_H2
zmp_ph108_H5
zmp_ph108_B2
zmp_ph108_B6
zmp_ph108_B7
zmp_ph108_C4
zmp_ph108_C5
zmp_ph108_D4
zmp_ph97_G6
zmp_ph97_G9
zmp_ph97_H2
zmp_ph97_B10
zmp_ph97_C2
zmp_ph97_C4
zmp_ph97_B8
zmp_ph97_C1
zmp_ph97_D2
zmp_ph97_D5
zmp_ph111_G5
zmp_ph111_G11
zmp_ph111_H1
zmp_ph111_B1a
zmp_ph111_B5
zmp_ph111_B8
zmp_ph111_B6
zmp_ph111_B7
zmp_ph111_B9
zmp_ph111_B10
zmp_ph114_G4
zmp_ph114_G11
zmp_ph114_H1
zmp_ph114_H2
zmp_ph114_A9
zmp_ph114_A11
zmp_ph114_B2
zmp_ph114_B4
zmp_ph114_D8
zmp_ph114_E2

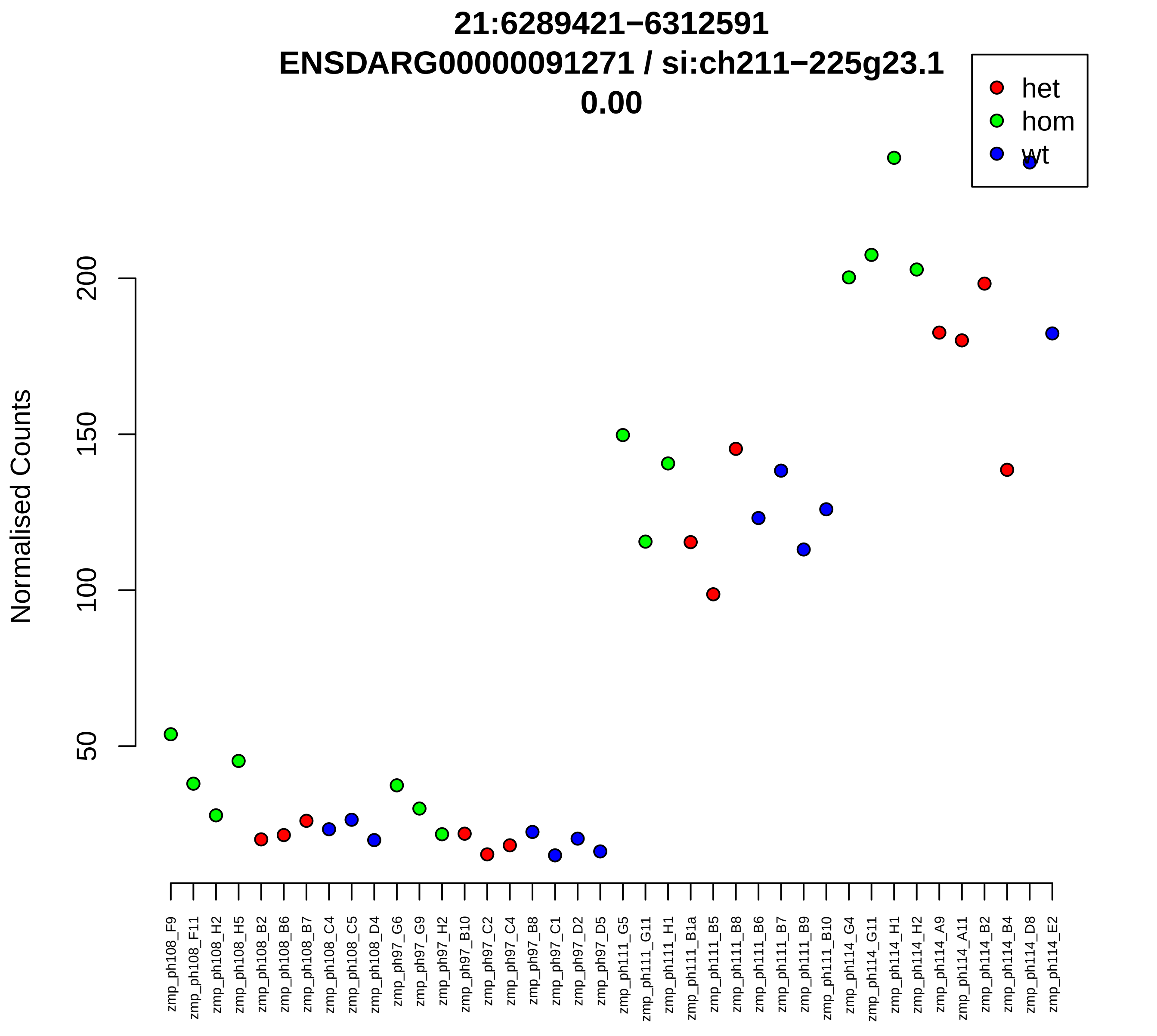

21:6289421−6312591
ENSDARG00000091271 / si:ch211−225g23.1
0.00
het
hom
wt
Normalised Counts
50
100
150
200
zmp_ph108_F9
zmp_ph108_F11
zmp_ph108_H2
zmp_ph108_H5
zmp_ph108_B2
zmp_ph108_B6
zmp_ph108_B7
zmp_ph108_C4
zmp_ph108_C5
zmp_ph108_D4
zmp_ph97_G6
zmp_ph97_G9
zmp_ph97_H2
zmp_ph97_B10
zmp_ph97_C2
zmp_ph97_C4
zmp_ph97_B8
zmp_ph97_C1
zmp_ph97_D2
zmp_ph97_D5
zmp_ph111_G5
zmp_ph111_G11
zmp_ph111_H1
zmp_ph111_B1a
zmp_ph111_B5
zmp_ph111_B8
zmp_ph111_B6
zmp_ph111_B7
zmp_ph111_B9
zmp_ph111_B10
zmp_ph114_G4
zmp_ph114_G11
zmp_ph114_H1
zmp_ph114_H2
zmp_ph114_A9
zmp_ph114_A11
zmp_ph114_B2
zmp_ph114_B4
zmp_ph114_D8
zmp_ph114_E2

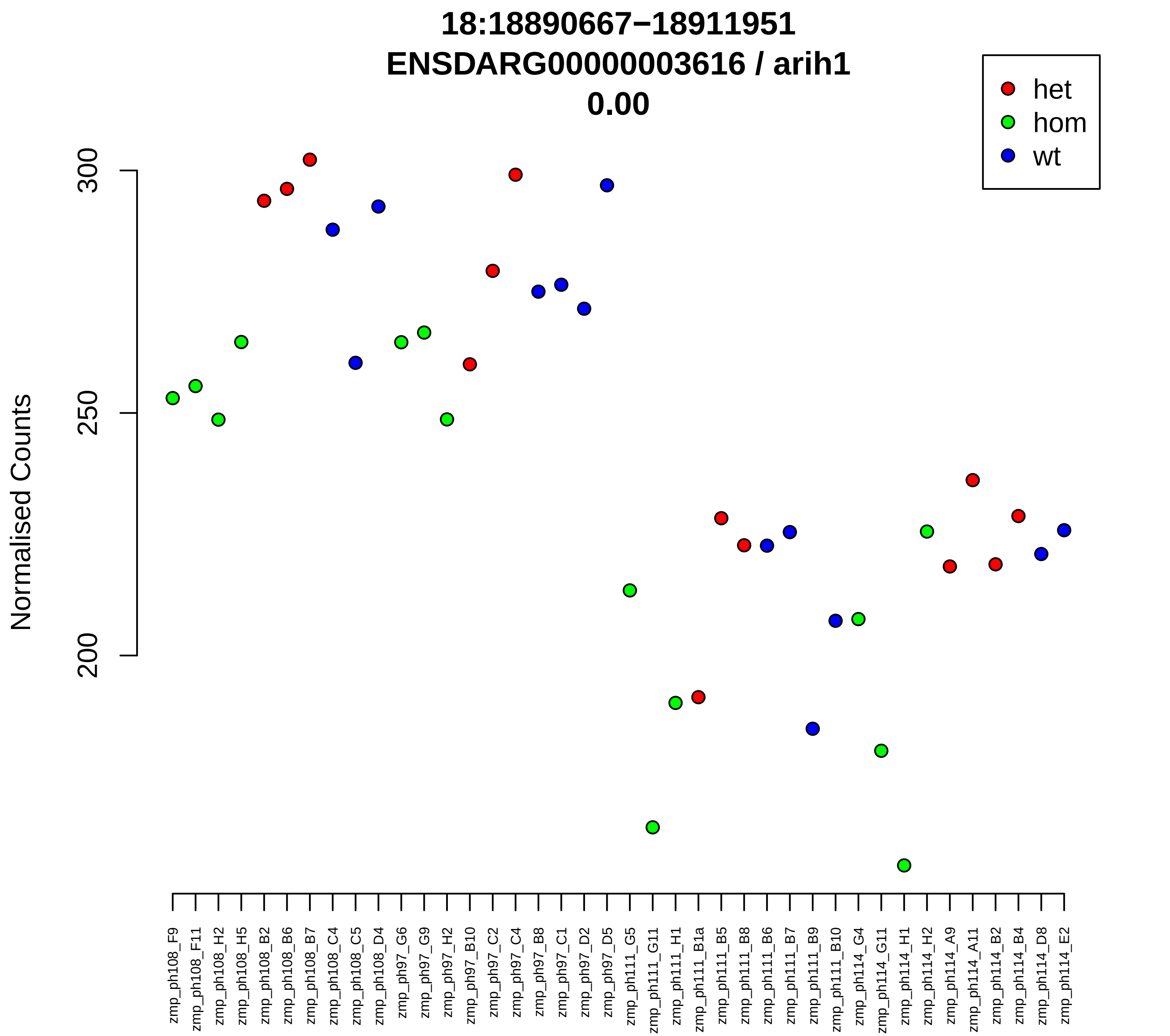
18:18890667−18911951
ENSDARG00000003616 / arih1
0.00
het
hom
wt
Normalised Counts
300
250
200
zmp_ph108_F9
zmp_ph108_F11
zmp_ph108_H2
zmp_ph108_H5
zmp_ph108_B2
zmp_ph108_B6
zmp_ph108_B7
zmp_ph108_C4
zmp_ph108_C5
zmp_ph108_D4
zmp_ph97_G6
zmp_ph97_G9
zmp_ph97_H2
zmp_ph97_B10
zmp_ph97_C2
zmp_ph97_C4
zmp_ph97_B8
zmp_ph97_C1
zmp_ph97_D2
zmp_ph97_D5
zmp_ph111_G5
zmp_ph111_G11
zmp_ph111_H1
zmp_ph111_B1a
zmp_ph111_B5
zmp_ph111_B8
zmp_ph111_B6
zmp_ph111_B7
zmp_ph111_B9
zmp_ph111_B10
zmp_ph114_G4
zmp_ph114_G11
zmp_ph114_H1
zmp_ph114_H2
zmp_ph114_A9
zmp_ph114_A11
zmp_ph114_B2
zmp_ph114_B4
zmp_ph114_D8
zmp_ph114_E2

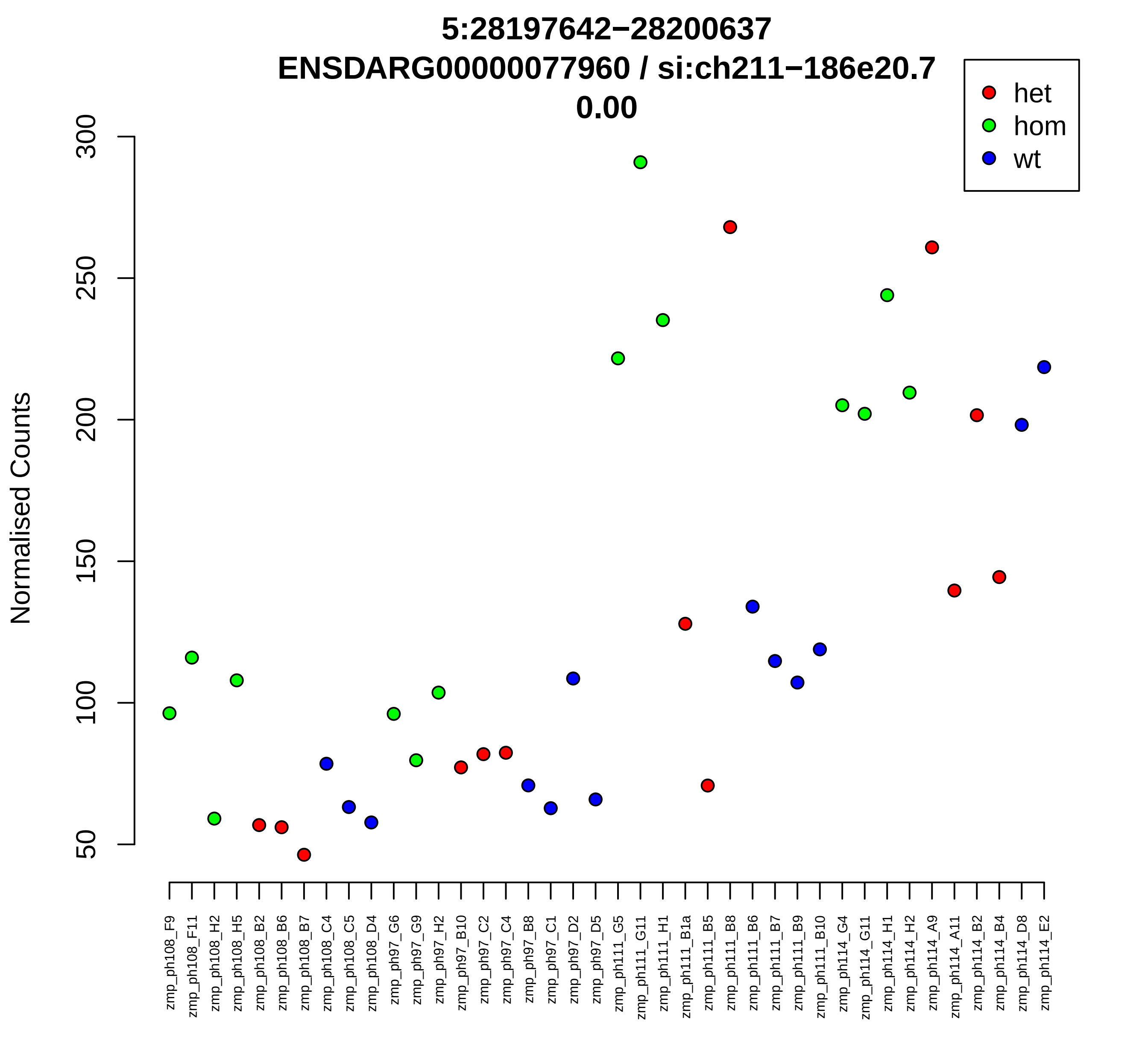

5:28197642−28200637
ENSDARG00000077960 / si:ch211−186e20.7
0.00
het
hom
wt
Normalised Counts
50
100
150
200
250
300
zmp_ph108_F9
zmp_ph108_F11
zmp_ph108_H2
zmp_ph108_H5
zmp_ph108_B2
zmp_ph108_B6
zmp_ph108_B7
zmp_ph108_C4
zmp_ph108_C5
zmp_ph108_D4
zmp_ph97_G6
zmp_ph97_G9
zmp_ph97_H2
zmp_ph97_B10
zmp_ph97_C2
zmp_ph97_C4
zmp_ph97_B8
zmp_ph97_C1
zmp_ph97_D2
zmp_ph97_D5
zmp_ph111_G5
zmp_ph111_G11
zmp_ph111_H1
zmp_ph111_B1a
zmp_ph111_B5
zmp_ph111_B8
zmp_ph111_B6
zmp_ph111_B7
zmp_ph111_B9
zmp_ph111_B10
zmp_ph114_G4
zmp_ph114_G11
zmp_ph114_H1
zmp_ph114_H2
zmp_ph114_A9
zmp_ph114_A11
zmp_ph114_B2
zmp_ph114_B4
zmp_ph114_D8
zmp_ph114_E2

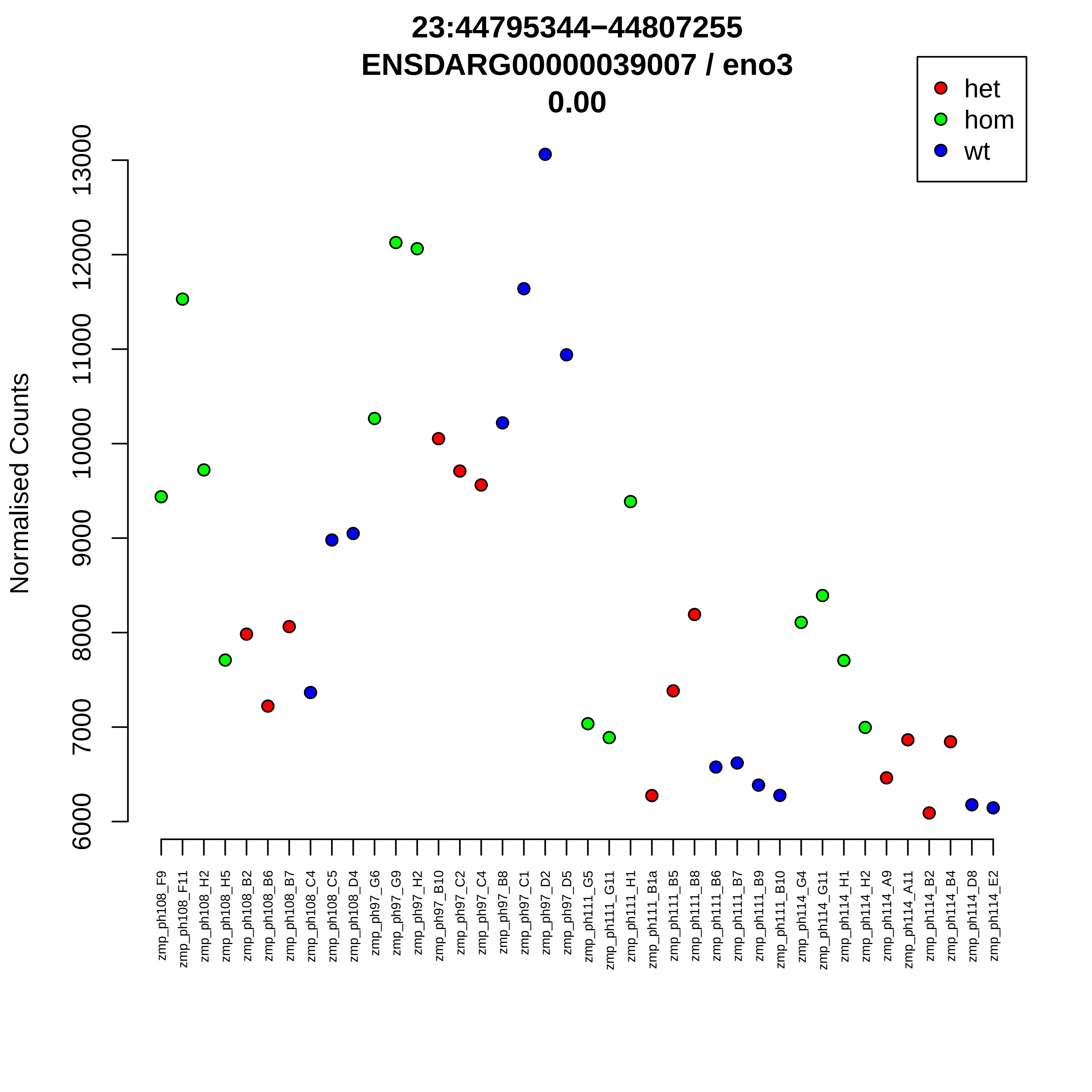

23:44795344−44807255
ENSDARG00000039007 / eno3
0.00
het
hom
wt
Normalised Counts
6000
7000
8000
9000
10000
11000
12000
13000
zmp_ph108_F9
zmp_ph108_F11
zmp_ph108_H2
zmp_ph108_H5
zmp_ph108_B2
zmp_ph108_B6
zmp_ph108_B7
zmp_ph108_C4
zmp_ph108_C5
zmp_ph108_D4
zmp_ph97_G6
zmp_ph97_G9
zmp_ph97_H2
zmp_ph97_B10
zmp_ph97_C2
zmp_ph97_C4
zmp_ph97_B8
zmp_ph97_C1
zmp_ph97_D2
zmp_ph97_D5
zmp_ph111_G5
zmp_ph111_G11
zmp_ph111_H1
zmp_ph111_B1a
zmp_ph111_B5
zmp_ph111_B8
zmp_ph111_B6
zmp_ph111_B7
zmp_ph111_B9
zmp_ph111_B10
zmp_ph114_G4
zmp_ph114_G11
zmp_ph114_H1
zmp_ph114_H2
zmp_ph114_A9
zmp_ph114_A11
zmp_ph114_B2
zmp_ph114_B4
zmp_ph114_D8
zmp_ph114_E2

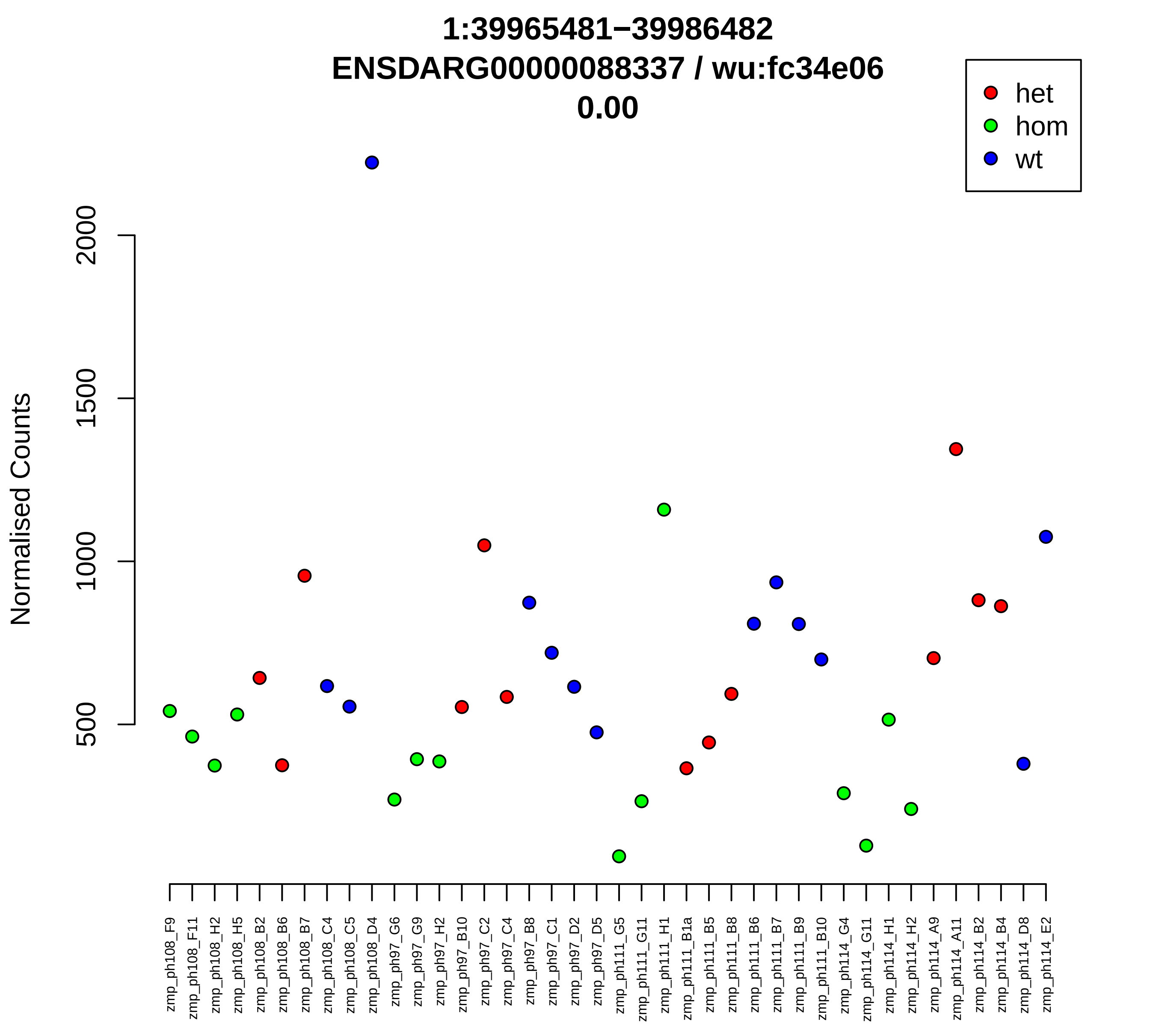

1:39965481−39986482
ENSDARG00000088337 / wu:fc34e06
0.00
het
hom
wt
Normalised Counts
500
1000
1500
2000
zmp_ph108_F9
zmp_ph108_F11
zmp_ph108_H2
zmp_ph108_H5
zmp_ph108_B2
zmp_ph108_B6
zmp_ph108_B7
zmp_ph108_C4
zmp_ph108_C5
zmp_ph108_D4
zmp_ph97_G6
zmp_ph97_G9
zmp_ph97_H2
zmp_ph97_B10
zmp_ph97_C2
zmp_ph97_C4
zmp_ph97_B8
zmp_ph97_C1
zmp_ph97_D2
zmp_ph97_D5
zmp_ph111_G5
zmp_ph111_G11
zmp_ph111_H1
zmp_ph111_B1a
zmp_ph111_B5
zmp_ph111_B8
zmp_ph111_B6
zmp_ph111_B7
zmp_ph111_B9
zmp_ph111_B10
zmp_ph114_G4
zmp_ph114_G11
zmp_ph114_H1
zmp_ph114_H2
zmp_ph114_A9
zmp_ph114_A11
zmp_ph114_B2
zmp_ph114_B4
zmp_ph114_D8
zmp_ph114_E2

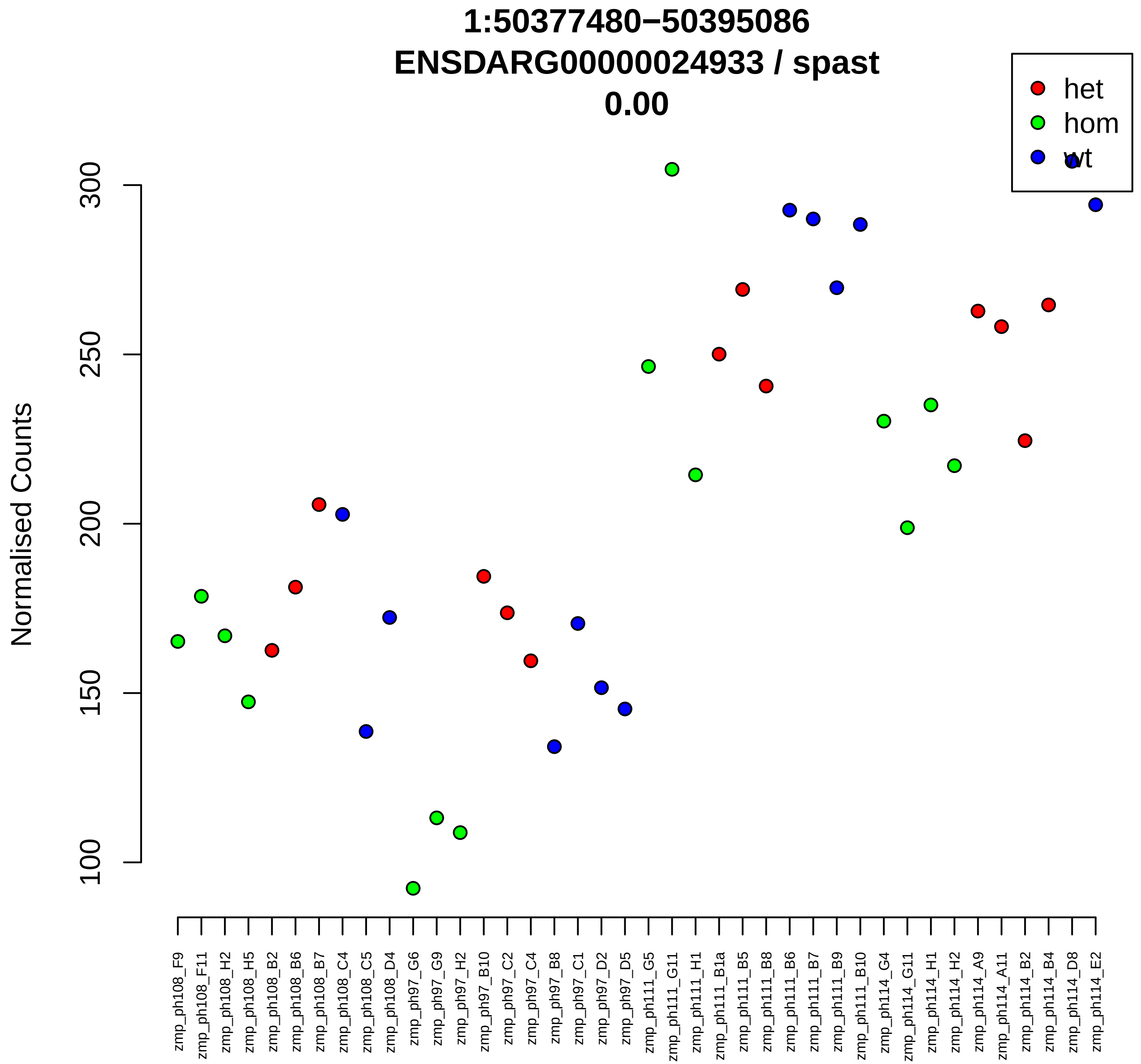
1:50377480−50395086
ENSDARG00000024933 / spast
0.00
het
hom
wt
Normalised Counts
100
150
200
250
300
zmp_ph108_F9
zmp_ph108_F11
zmp_ph108_H2
zmp_ph108_H5
zmp_ph108_B2
zmp_ph108_B6
zmp_ph108_B7
zmp_ph108_C4
zmp_ph108_C5
zmp_ph108_D4
zmp_ph97_G6
zmp_ph97_G9
zmp_ph97_H2
zmp_ph97_B10
zmp_ph97_C2
zmp_ph97_C4
zmp_ph97_B8
zmp_ph97_C1
zmp_ph97_D2
zmp_ph97_D5
zmp_ph111_G5
zmp_ph111_G11
zmp_ph111_H1
zmp_ph111_B1a
zmp_ph111_B5
zmp_ph111_B8
zmp_ph111_B6
zmp_ph111_B7
zmp_ph111_B9
zmp_ph111_B10
zmp_ph114_G4
zmp_ph114_G11
zmp_ph114_H1
zmp_ph114_H2
zmp_ph114_A9
zmp_ph114_A11
zmp_ph114_B2
zmp_ph114_B4
zmp_ph114_D8
zmp_ph114_E2

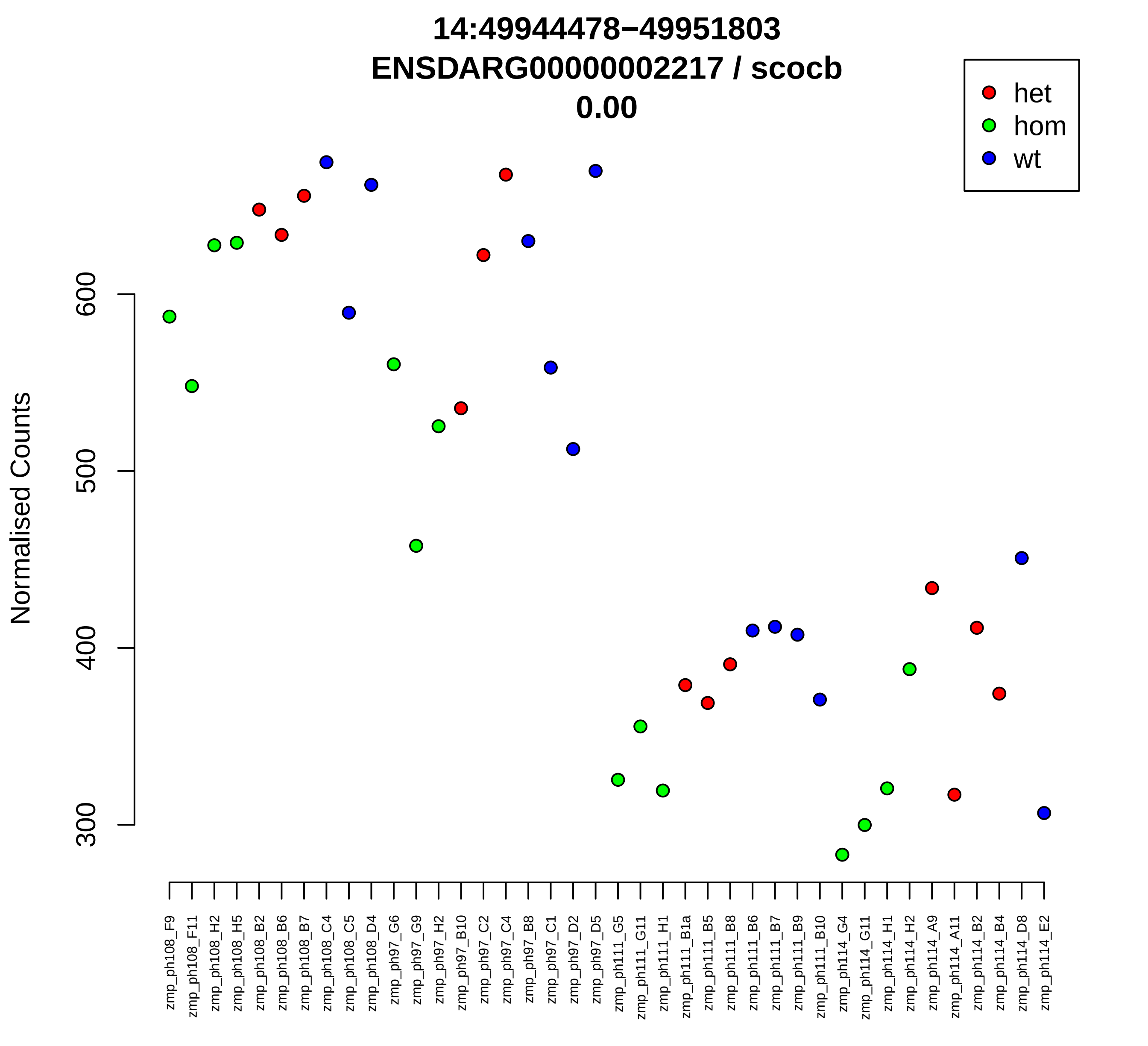
14:49944478−49951803
ENSDARG00000002217 / scocb
0.00
het
hom
wt
Normalised Counts
300
400
500
600
zmp_ph108_F9
zmp_ph108_F11
zmp_ph108_H2
zmp_ph108_H5
zmp_ph108_B2
zmp_ph108_B6
zmp_ph108_B7
zmp_ph108_C4
zmp_ph108_C5
zmp_ph108_D4
zmp_ph97_G6
zmp_ph97_G9
zmp_ph97_H2
zmp_ph97_B10
zmp_ph97_C2
zmp_ph97_C4
zmp_ph97_B8
zmp_ph97_C1
zmp_ph97_D2
zmp_ph97_D5
zmp_ph111_G5
zmp_ph111_G11
zmp_ph111_H1
zmp_ph111_B1a
zmp_ph111_B5
zmp_ph111_B8
zmp_ph111_B6
zmp_ph111_B7
zmp_ph111_B9
zmp_ph111_B10
zmp_ph114_G4
zmp_ph114_G11
zmp_ph114_H1
zmp_ph114_H2
zmp_ph114_A9
zmp_ph114_A11
zmp_ph114_B2
zmp_ph114_B4
zmp_ph114_D8
zmp_ph114_E2

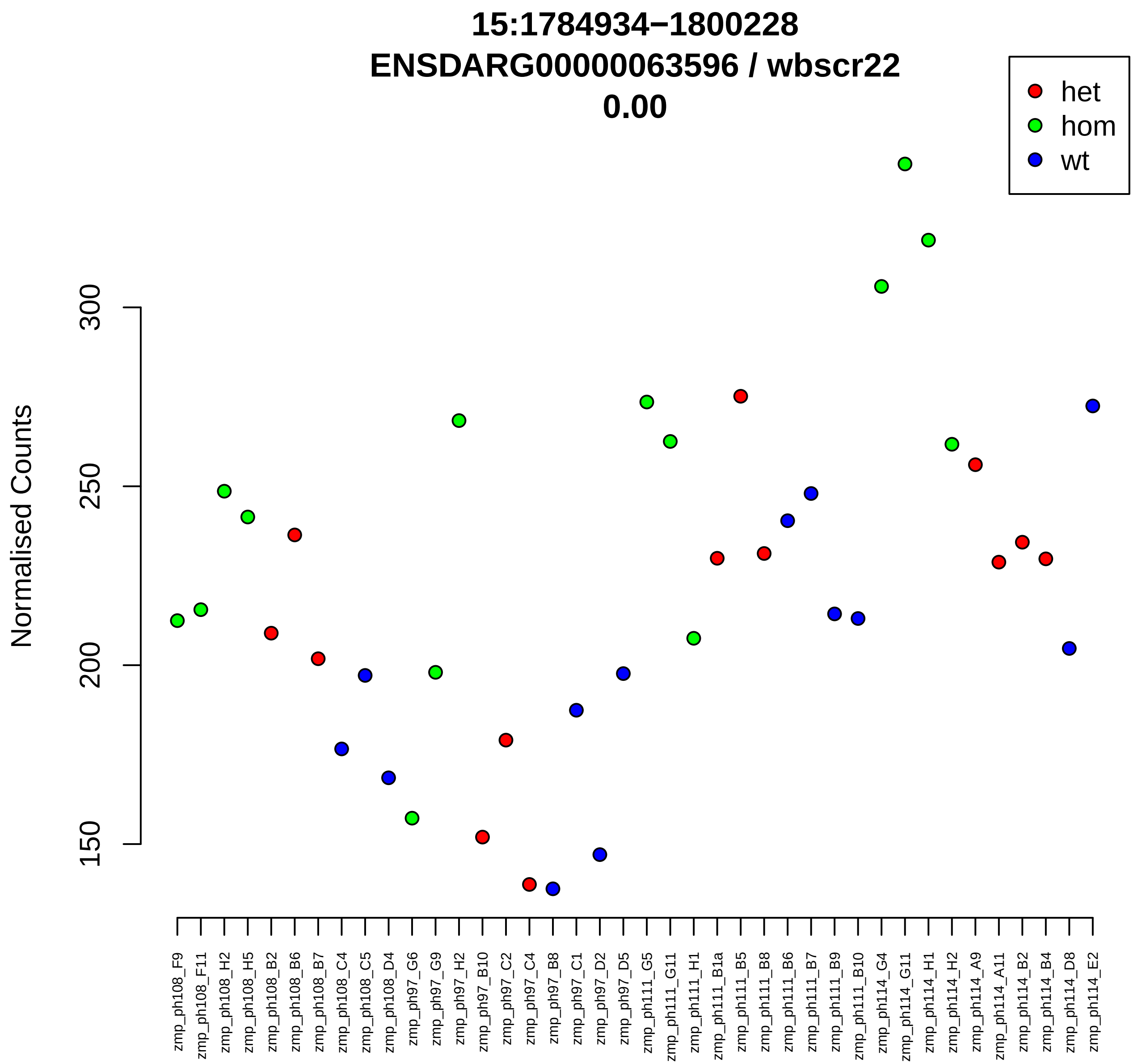
15:1784934−1800228
ENSDARG00000063596 / wbscr22
0.00
het
hom
wt
Normalised Counts
150
200
250
300
zmp_ph108_F9
zmp_ph108_F11
zmp_ph108_H2
zmp_ph108_H5
zmp_ph108_B2
zmp_ph108_B6
zmp_ph108_B7
zmp_ph108_C4
zmp_ph108_C5
zmp_ph108_D4
zmp_ph97_G6
zmp_ph97_G9
zmp_ph97_H2
zmp_ph97_B10
zmp_ph97_C2
zmp_ph97_C4
zmp_ph97_B8
zmp_ph97_C1
zmp_ph97_D2
zmp_ph97_D5
zmp_ph111_G5
zmp_ph111_G11
zmp_ph111_H1
zmp_ph111_B1a
zmp_ph111_B5
zmp_ph111_B8
zmp_ph111_B6
zmp_ph111_B7
zmp_ph111_B9
zmp_ph111_B10
zmp_ph114_G4
zmp_ph114_G11
zmp_ph114_H1
zmp_ph114_H2
zmp_ph114_A9
zmp_ph114_A11
zmp_ph114_B2
zmp_ph114_B4
zmp_ph114_D8
zmp_ph114_E2

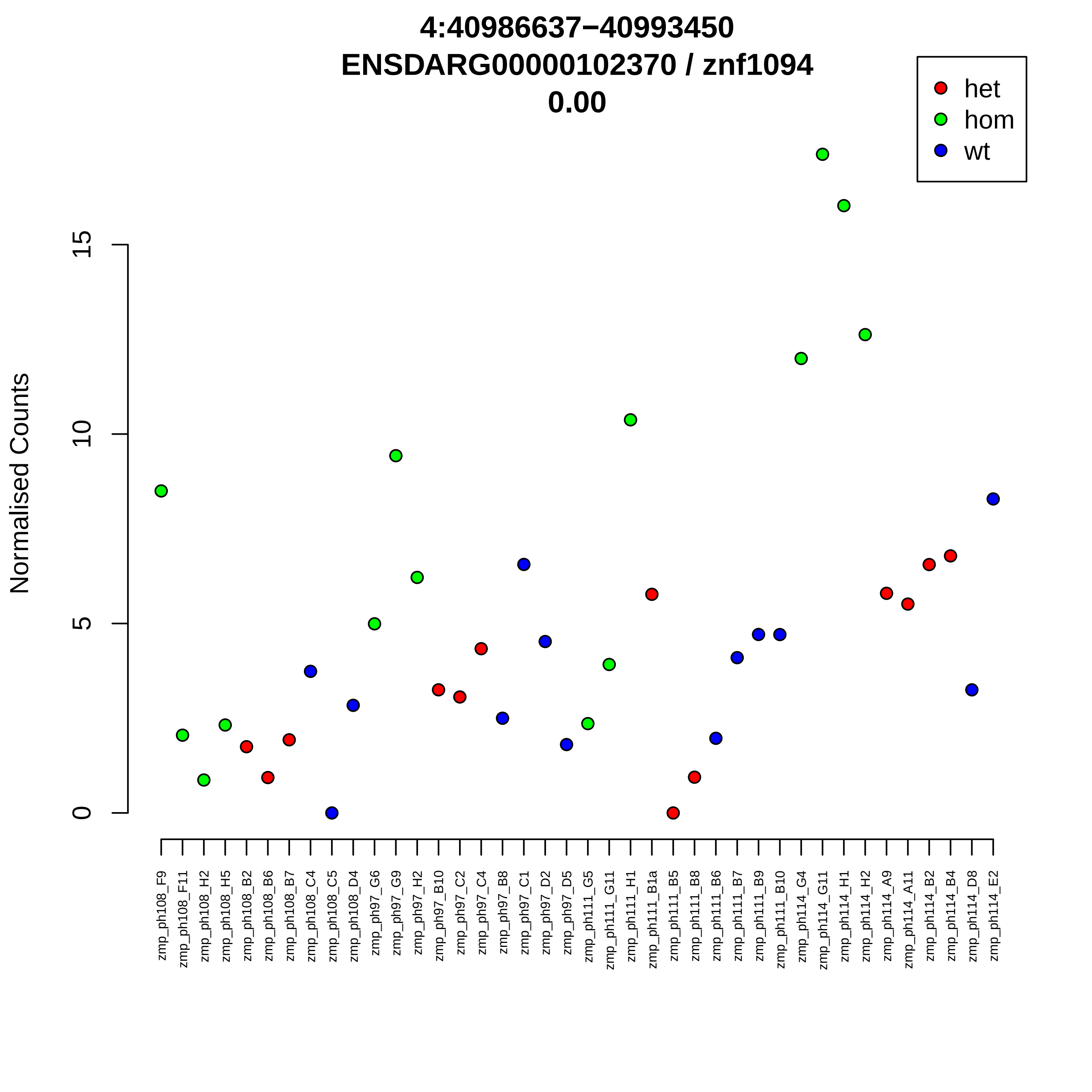

4:40986637−40993450
ENSDARG00000102370 / znf1094
0.00
het
hom
wt
Normalised Counts
0
5
10
15
zmp_ph108_F9
zmp_ph108_F11
zmp_ph108_H2
zmp_ph108_H5
zmp_ph108_B2
zmp_ph108_B6
zmp_ph108_B7
zmp_ph108_C4
zmp_ph108_C5
zmp_ph108_D4
zmp_ph97_G6
zmp_ph97_G9
zmp_ph97_H2
zmp_ph97_B10
zmp_ph97_C2
zmp_ph97_C4
zmp_ph97_B8
zmp_ph97_C1
zmp_ph97_D2
zmp_ph97_D5
zmp_ph111_G5
zmp_ph111_G11
zmp_ph111_H1
zmp_ph111_B1a
zmp_ph111_B5
zmp_ph111_B8
zmp_ph111_B6
zmp_ph111_B7
zmp_ph111_B9
zmp_ph111_B10
zmp_ph114_G4
zmp_ph114_G11
zmp_ph114_H1
zmp_ph114_H2
zmp_ph114_A9
zmp_ph114_A11
zmp_ph114_B2
zmp_ph114_B4
zmp_ph114_D8
zmp_ph114_E2

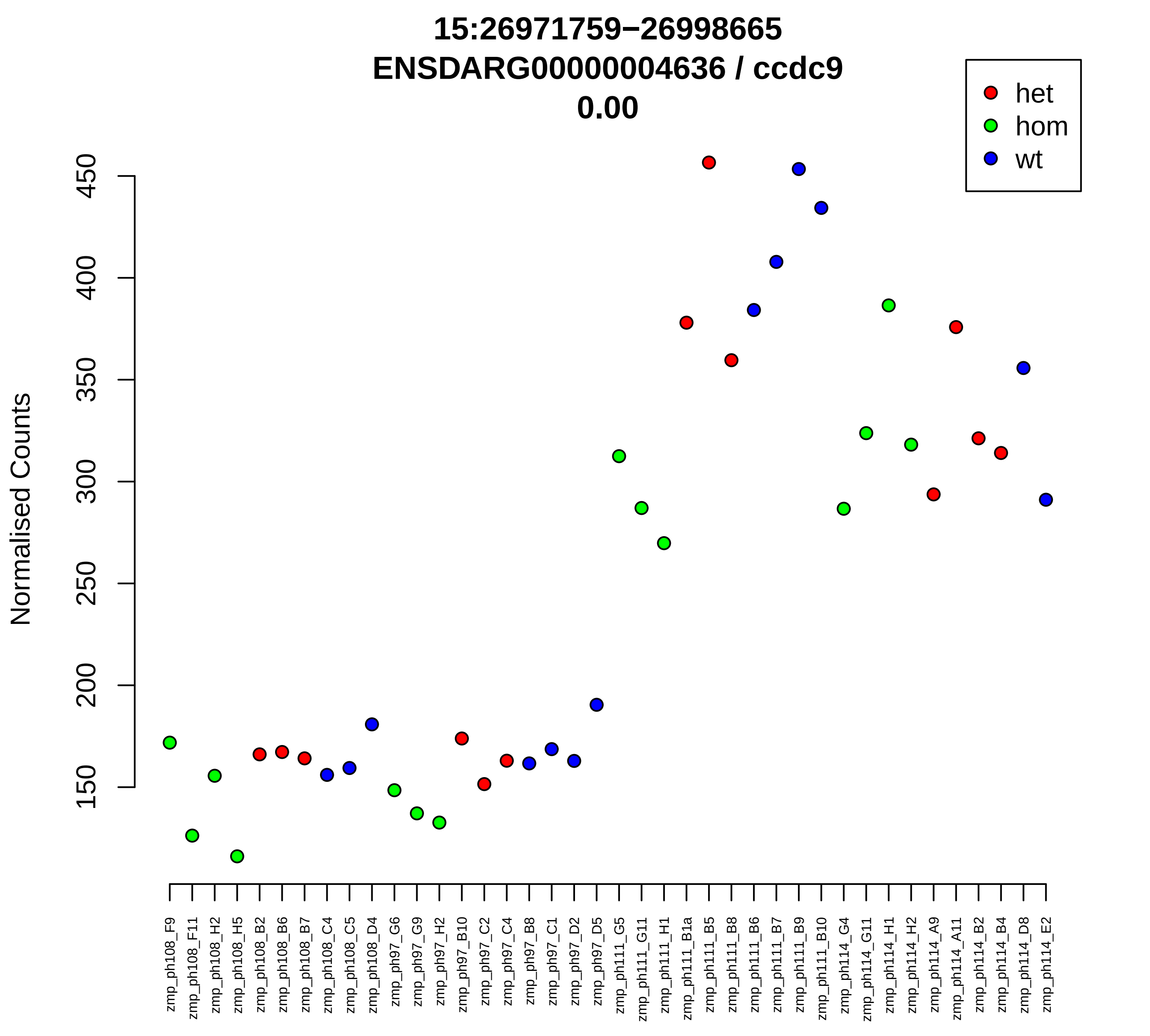

15:26971759−26998665
ENSDARG00000004636 / ccdc9
0.00
het
hom
wt
Normalised Counts
150
200
250
300
350
400
450
zmp_ph108_F9
zmp_ph108_F11
zmp_ph108_H2
zmp_ph108_H5
zmp_ph108_B2
zmp_ph108_B6
zmp_ph108_B7
zmp_ph108_C4
zmp_ph108_C5
zmp_ph108_D4
zmp_ph97_G6
zmp_ph97_G9
zmp_ph97_H2
zmp_ph97_B10
zmp_ph97_C2
zmp_ph97_C4
zmp_ph97_B8
zmp_ph97_C1
zmp_ph97_D2
zmp_ph97_D5
zmp_ph111_G5
zmp_ph111_G11
zmp_ph111_H1
zmp_ph111_B1a
zmp_ph111_B5
zmp_ph111_B8
zmp_ph111_B6
zmp_ph111_B7
zmp_ph111_B9
zmp_ph111_B10
zmp_ph114_G4
zmp_ph114_G11
zmp_ph114_H1
zmp_ph114_H2
zmp_ph114_A9
zmp_ph114_A11
zmp_ph114_B2
zmp_ph114_B4
zmp_ph114_D8
zmp_ph114_E2

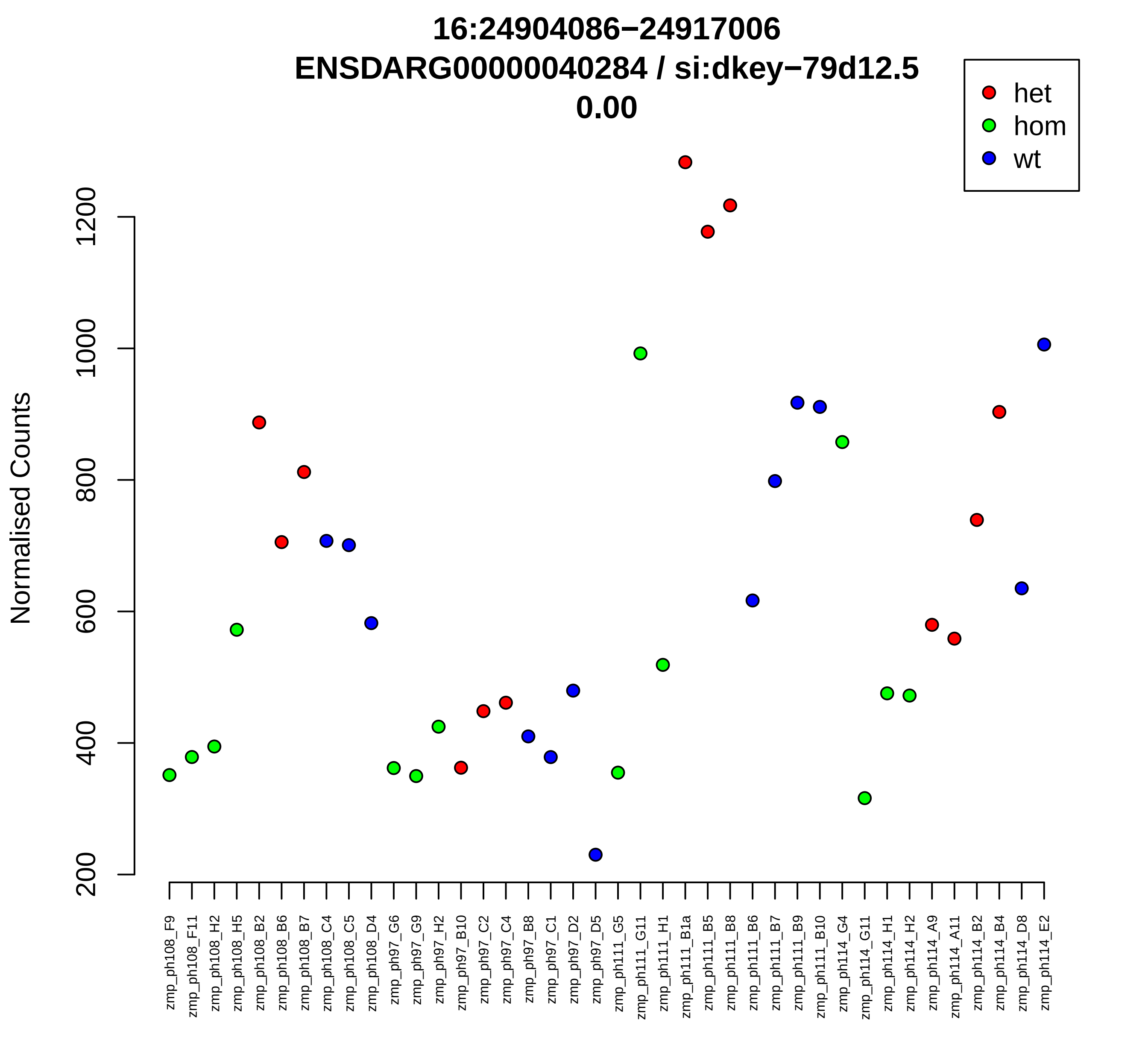
16:24904086−24917006
ENSDARG00000040284 / si:dkey−79d12.5
0.00
het
hom
wt
Normalised Counts
200
400
600
800
1000
1200
zmp_ph108_F9
zmp_ph108_F11
zmp_ph108_H2
zmp_ph108_H5
zmp_ph108_B2
zmp_ph108_B6
zmp_ph108_B7
zmp_ph108_C4
zmp_ph108_C5
zmp_ph108_D4
zmp_ph97_G6
zmp_ph97_G9
zmp_ph97_H2
zmp_ph97_B10
zmp_ph97_C2
zmp_ph97_C4
zmp_ph97_B8
zmp_ph97_C1
zmp_ph97_D2
zmp_ph97_D5
zmp_ph111_G5
zmp_ph111_G11
zmp_ph111_H1
zmp_ph111_B1a
zmp_ph111_B5
zmp_ph111_B8
zmp_ph111_B6
zmp_ph111_B7
zmp_ph111_B9
zmp_ph111_B10
zmp_ph114_G4
zmp_ph114_G11
zmp_ph114_H1
zmp_ph114_H2
zmp_ph114_A9
zmp_ph114_A11
zmp_ph114_B2
zmp_ph114_B4
zmp_ph114_D8
zmp_ph114_E2

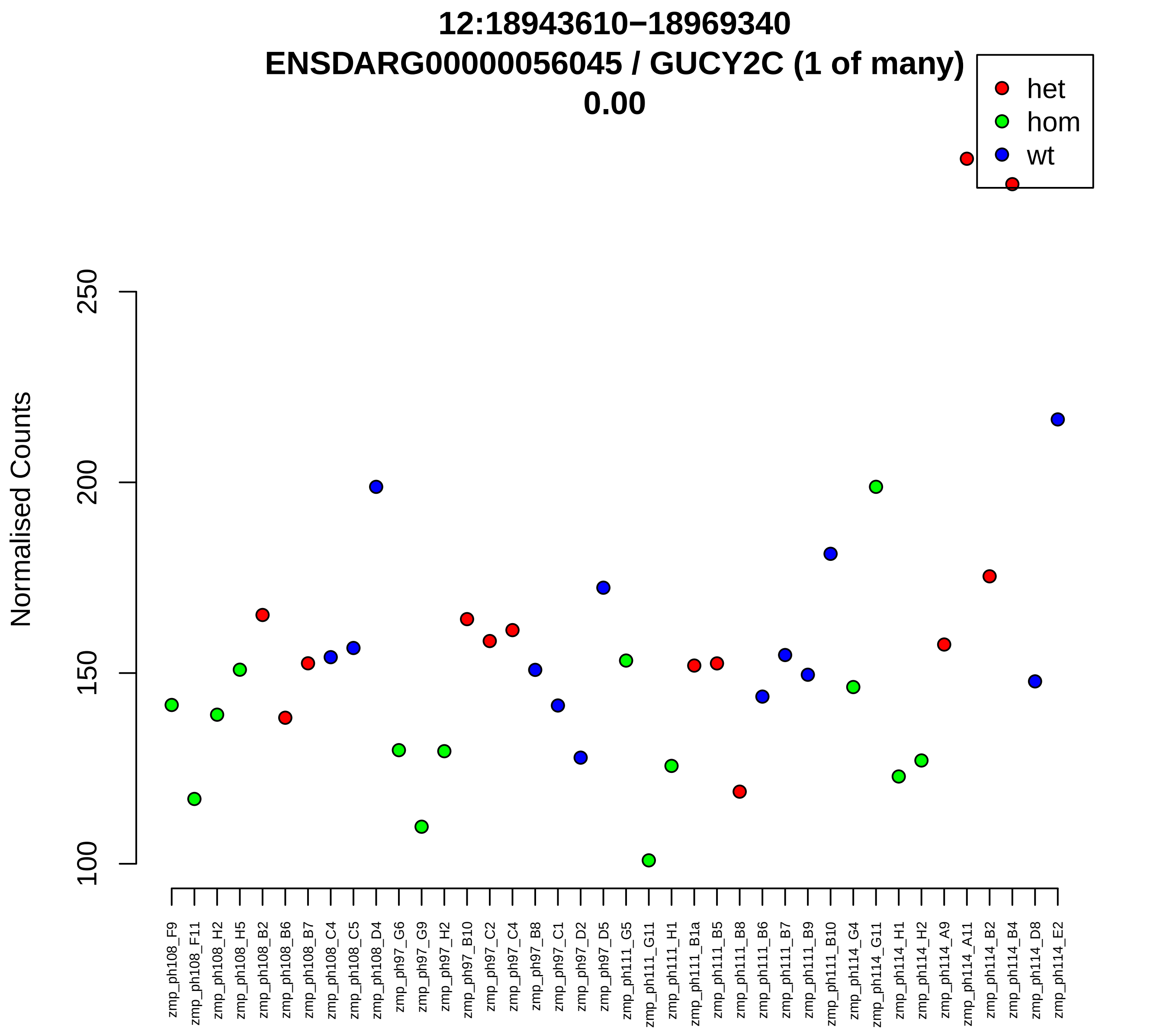
12:18943610−18969340
ENSDARG00000056045 / GUCY2C (1 of many)
0.00
het
hom
wt
Normalised Counts
100
150
200
250
zmp_ph108_F9
zmp_ph108_F11
zmp_ph108_H2
zmp_ph108_H5
zmp_ph108_B2
zmp_ph108_B6
zmp_ph108_B7
zmp_ph108_C4
zmp_ph108_C5
zmp_ph108_D4
zmp_ph97_G6
zmp_ph97_G9
zmp_ph97_H2
zmp_ph97_B10
zmp_ph97_C2
zmp_ph97_C4
zmp_ph97_B8
zmp_ph97_C1
zmp_ph97_D2
zmp_ph97_D5
zmp_ph111_G5
zmp_ph111_G11
zmp_ph111_H1
zmp_ph111_B1a
zmp_ph111_B5
zmp_ph111_B8
zmp_ph111_B6
zmp_ph111_B7
zmp_ph111_B9
zmp_ph111_B10
zmp_ph114_G4
zmp_ph114_G11
zmp_ph114_H1
zmp_ph114_H2
zmp_ph114_A9
zmp_ph114_A11
zmp_ph114_B2
zmp_ph114_B4
zmp_ph114_D8
zmp_ph114_E2

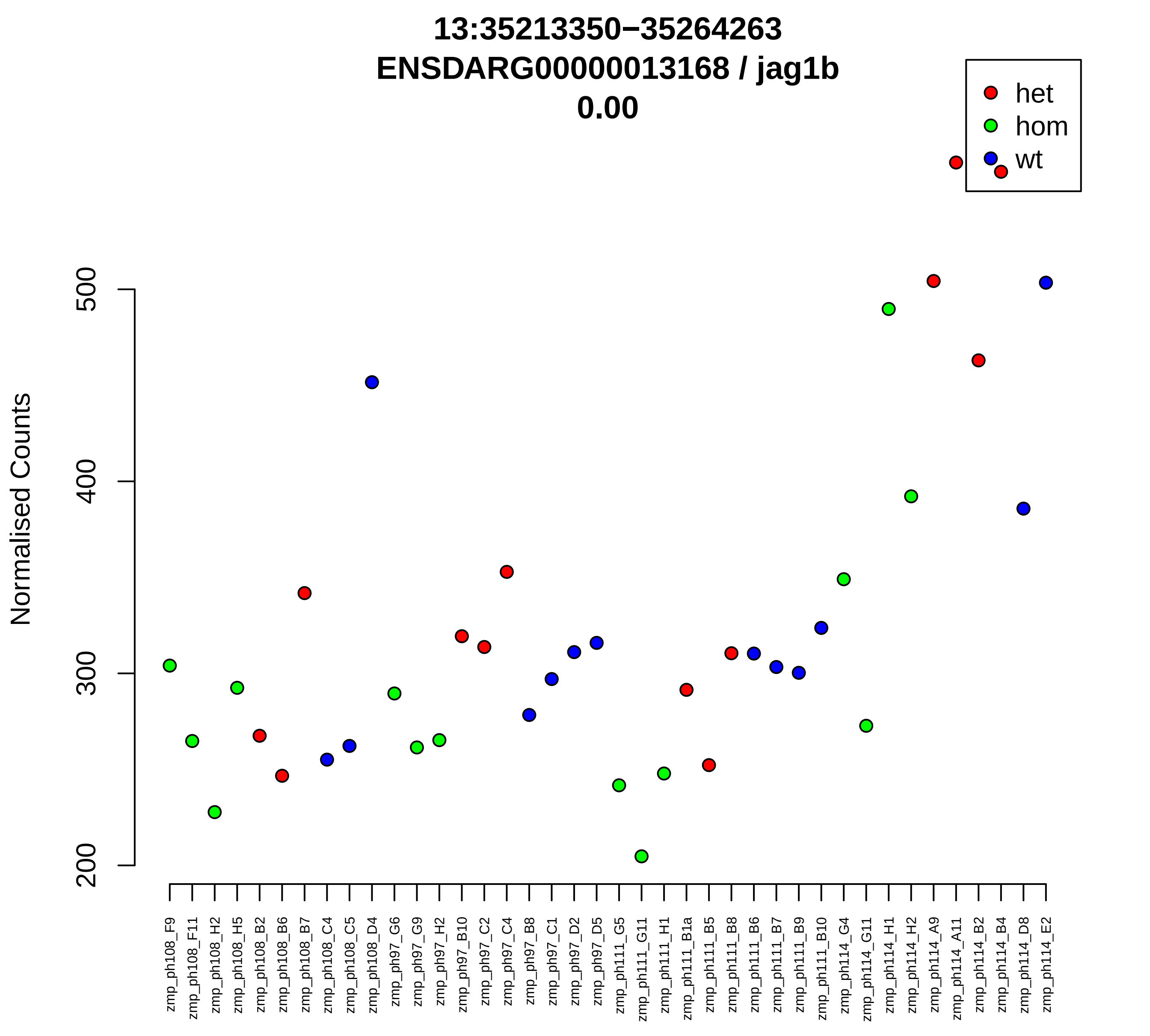
13:35213350−35264263
ENSDARG00000013168 / jag1b
0.00
het
hom
wt
Normalised Counts
200
300
400
500
zmp_ph108_F9
zmp_ph108_F11
zmp_ph108_H2
zmp_ph108_H5
zmp_ph108_B2
zmp_ph108_B6
zmp_ph108_B7
zmp_ph108_C4
zmp_ph108_C5
zmp_ph108_D4
zmp_ph97_G6
zmp_ph97_G9
zmp_ph97_H2
zmp_ph97_B10
zmp_ph97_C2
zmp_ph97_C4
zmp_ph97_B8
zmp_ph97_C1
zmp_ph97_D2
zmp_ph97_D5
zmp_ph111_G5
zmp_ph111_G11
zmp_ph111_H1
zmp_ph111_B1a
zmp_ph111_B5
zmp_ph111_B8
zmp_ph111_B6
zmp_ph111_B7
zmp_ph111_B9
zmp_ph111_B10
zmp_ph114_G4
zmp_ph114_G11
zmp_ph114_H1
zmp_ph114_H2
zmp_ph114_A9
zmp_ph114_A11
zmp_ph114_B2
zmp_ph114_B4
zmp_ph114_D8
zmp_ph114_E2

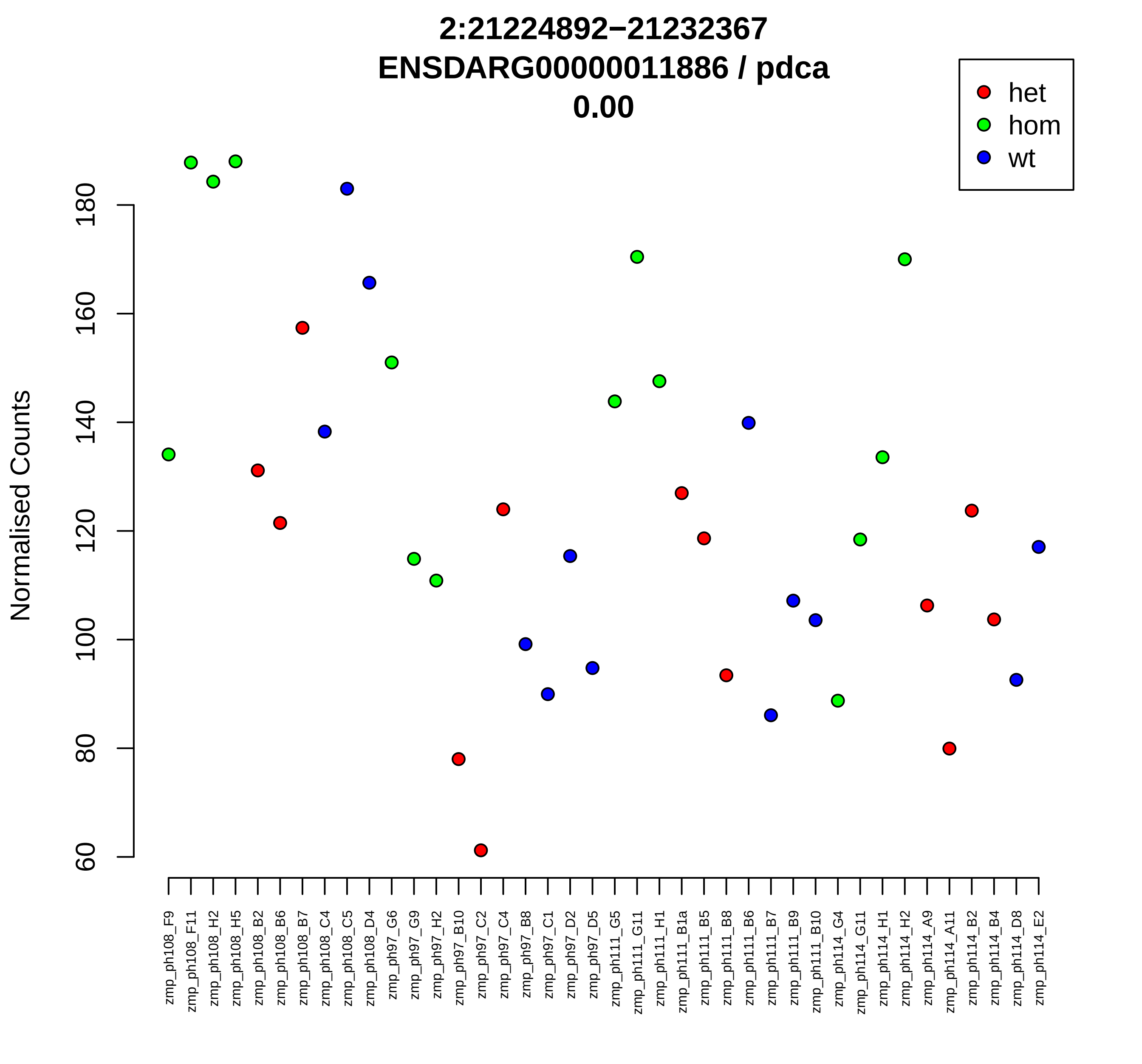

2:21224892−21232367
ENSDARG00000011886 / pdca
0.00
het
hom
wt
Normalised Counts
60
80
100
120
140
160
180
zmp_ph108_F9
zmp_ph108_F11
zmp_ph108_H2
zmp_ph108_H5
zmp_ph108_B2
zmp_ph108_B6
zmp_ph108_B7
zmp_ph108_C4
zmp_ph108_C5
zmp_ph108_D4
zmp_ph97_G6
zmp_ph97_G9
zmp_ph97_H2
zmp_ph97_B10
zmp_ph97_C2
zmp_ph97_C4
zmp_ph97_B8
zmp_ph97_C1
zmp_ph97_D2
zmp_ph97_D5
zmp_ph111_G5
zmp_ph111_G11
zmp_ph111_H1
zmp_ph111_B1a
zmp_ph111_B5
zmp_ph111_B8
zmp_ph111_B6
zmp_ph111_B7
zmp_ph111_B9
zmp_ph111_B10
zmp_ph114_G4
zmp_ph114_G11
zmp_ph114_H1
zmp_ph114_H2
zmp_ph114_A9
zmp_ph114_A11
zmp_ph114_B2
zmp_ph114_B4
zmp_ph114_D8
zmp_ph114_E2

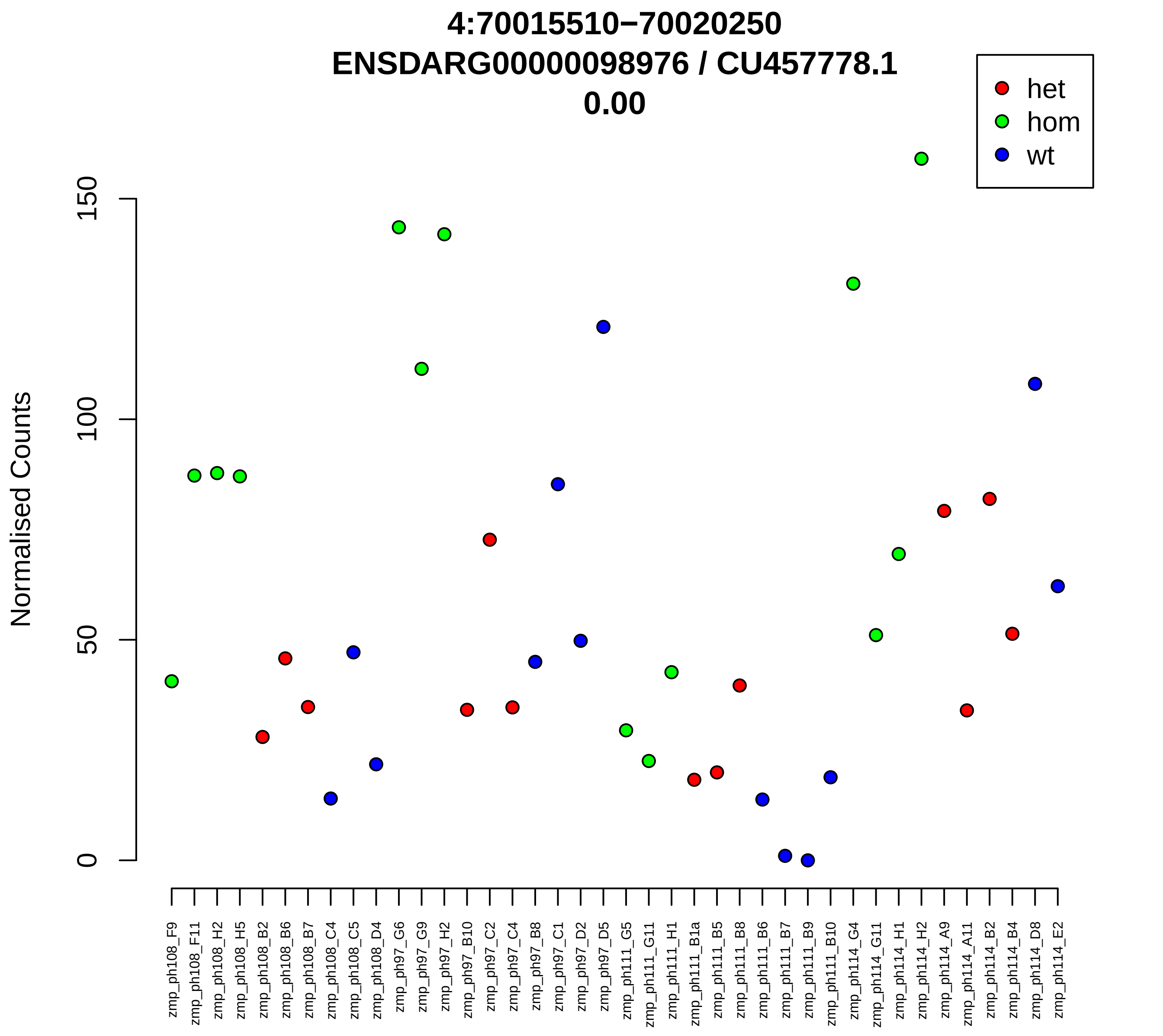
4:70015510−70020250
ENSDARG00000098976 / CU457778.1
0.00
het
hom
wt
Normalised Counts
0
50
100
150
zmp_ph108_F9
zmp_ph108_F11
zmp_ph108_H2
zmp_ph108_H5
zmp_ph108_B2
zmp_ph108_B6
zmp_ph108_B7
zmp_ph108_C4
zmp_ph108_C5
zmp_ph108_D4
zmp_ph97_G6
zmp_ph97_G9
zmp_ph97_H2
zmp_ph97_B10
zmp_ph97_C2
zmp_ph97_C4
zmp_ph97_B8
zmp_ph97_C1
zmp_ph97_D2
zmp_ph97_D5
zmp_ph111_G5
zmp_ph111_G11
zmp_ph111_H1
zmp_ph111_B1a
zmp_ph111_B5
zmp_ph111_B8
zmp_ph111_B6
zmp_ph111_B7
zmp_ph111_B9
zmp_ph111_B10
zmp_ph114_G4
zmp_ph114_G11
zmp_ph114_H1
zmp_ph114_H2
zmp_ph114_A9
zmp_ph114_A11
zmp_ph114_B2
zmp_ph114_B4
zmp_ph114_D8
zmp_ph114_E2

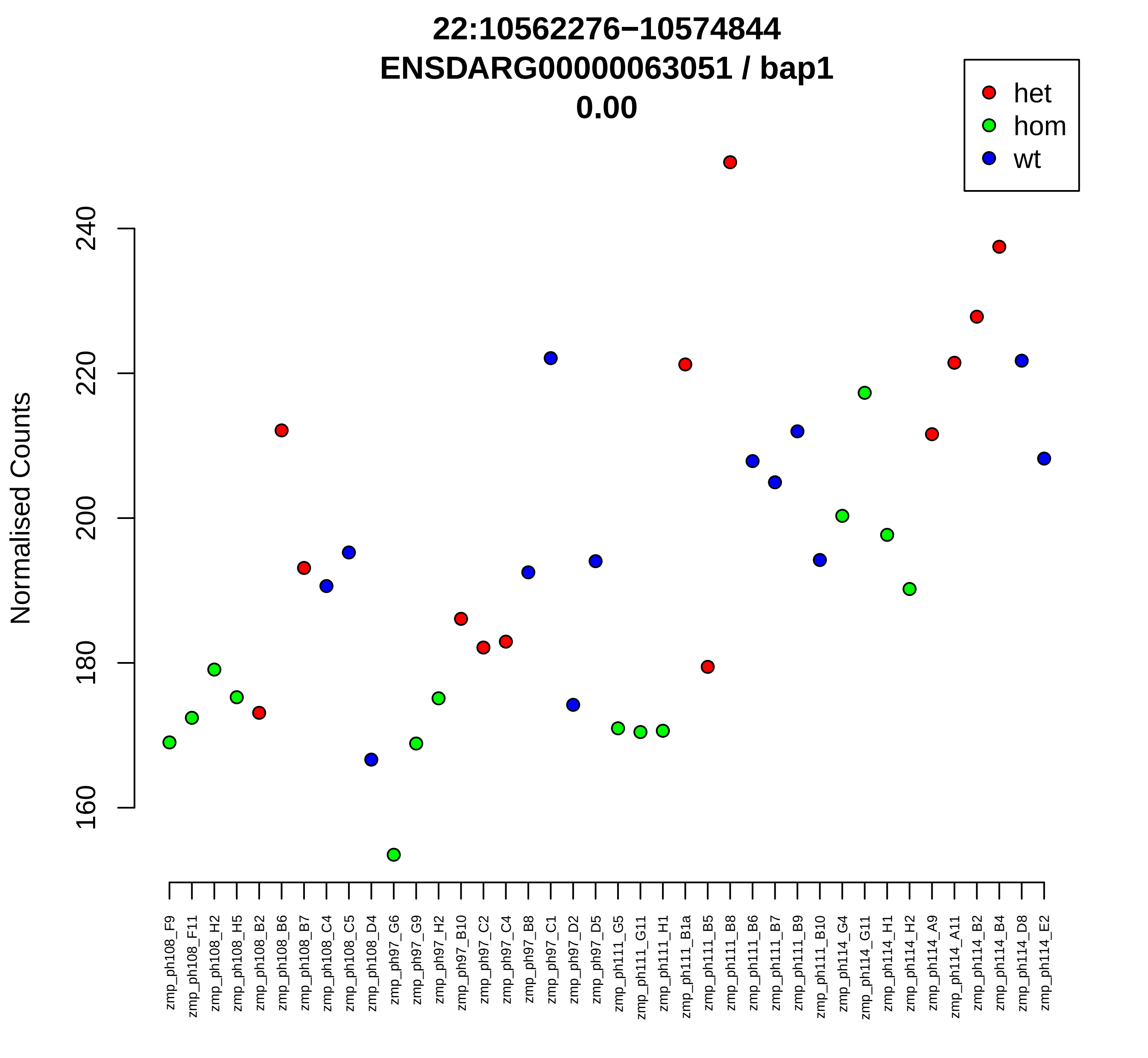
22:10562276−10574844
ENSDARG00000063051 / bap1
0.00
het
hom
wt
Normalised Counts
160
180
200
220
240
zmp_ph108_F9
zmp_ph108_F11
zmp_ph108_H2
zmp_ph108_H5
zmp_ph108_B2
zmp_ph108_B6
zmp_ph108_B7
zmp_ph108_C4
zmp_ph108_C5
zmp_ph108_D4
zmp_ph97_G6
zmp_ph97_G9
zmp_ph97_H2
zmp_ph97_B10
zmp_ph97_C2
zmp_ph97_C4
zmp_ph97_B8
zmp_ph97_C1
zmp_ph97_D2
zmp_ph97_D5
zmp_ph111_G5
zmp_ph111_G11
zmp_ph111_H1
zmp_ph111_B1a
zmp_ph111_B5
zmp_ph111_B8
zmp_ph111_B6
zmp_ph111_B7
zmp_ph111_B9
zmp_ph111_B10
zmp_ph114_G4
zmp_ph114_G11
zmp_ph114_H1
zmp_ph114_H2
zmp_ph114_A9
zmp_ph114_A11
zmp_ph114_B2
zmp_ph114_B4
zmp_ph114_D8
zmp_ph114_E2

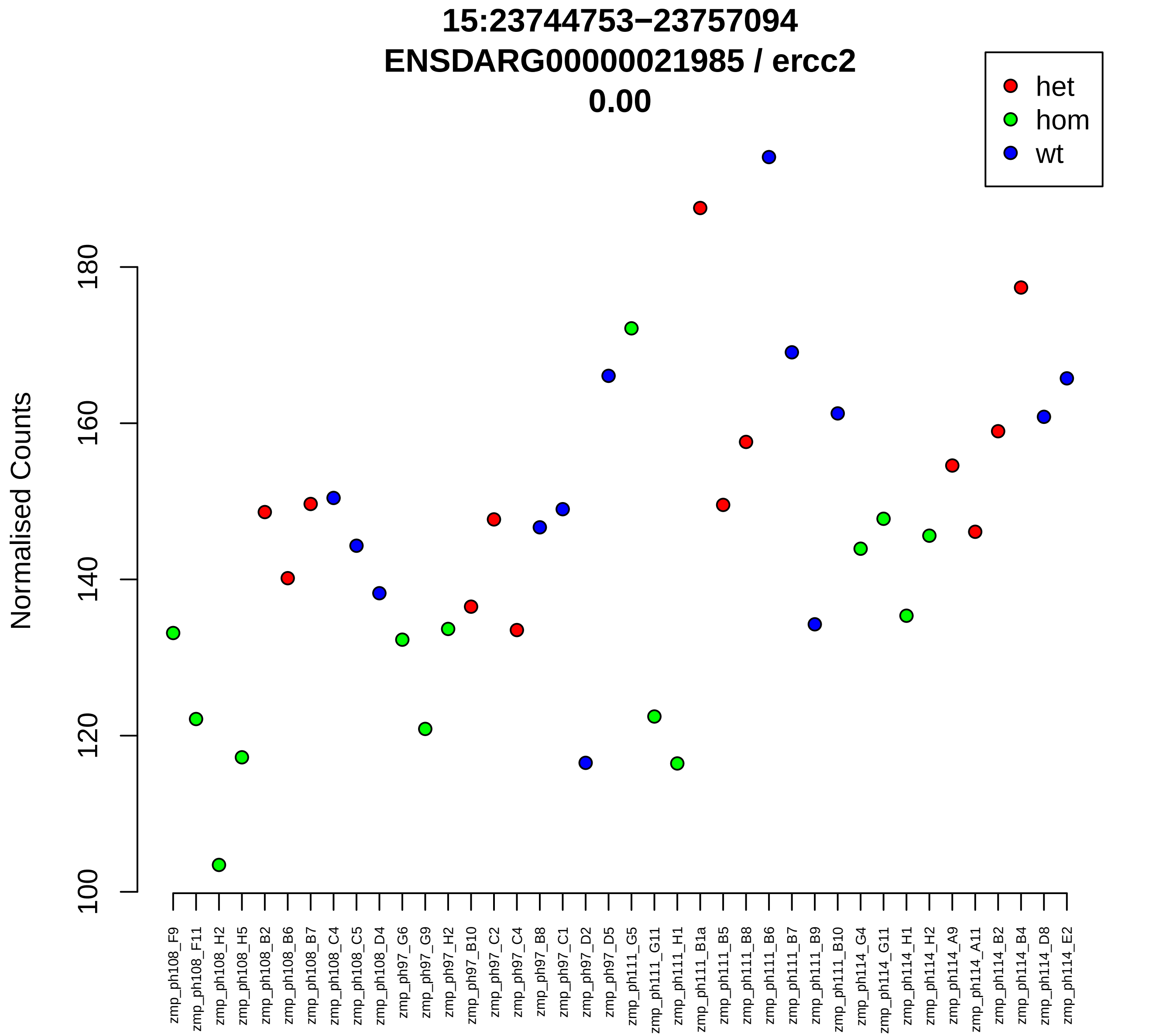

15:23744753−23757094
ENSDARG00000021985 / ercc2
0.00
het
hom
wt
Normalised Counts
100
120
140
160
180
zmp_ph108_F9
zmp_ph108_F11
zmp_ph108_H2
zmp_ph108_H5
zmp_ph108_B2
zmp_ph108_B6
zmp_ph108_B7
zmp_ph108_C4
zmp_ph108_C5
zmp_ph108_D4
zmp_ph97_G6
zmp_ph97_G9
zmp_ph97_H2
zmp_ph97_B10
zmp_ph97_C2
zmp_ph97_C4
zmp_ph97_B8
zmp_ph97_C1
zmp_ph97_D2
zmp_ph97_D5
zmp_ph111_G5
zmp_ph111_G11
zmp_ph111_H1
zmp_ph111_B1a
zmp_ph111_B5
zmp_ph111_B8
zmp_ph111_B6
zmp_ph111_B7
zmp_ph111_B9
zmp_ph111_B10
zmp_ph114_G4
zmp_ph114_G11
zmp_ph114_H1
zmp_ph114_H2
zmp_ph114_A9
zmp_ph114_A11
zmp_ph114_B2
zmp_ph114_B4
zmp_ph114_D8
zmp_ph114_E2

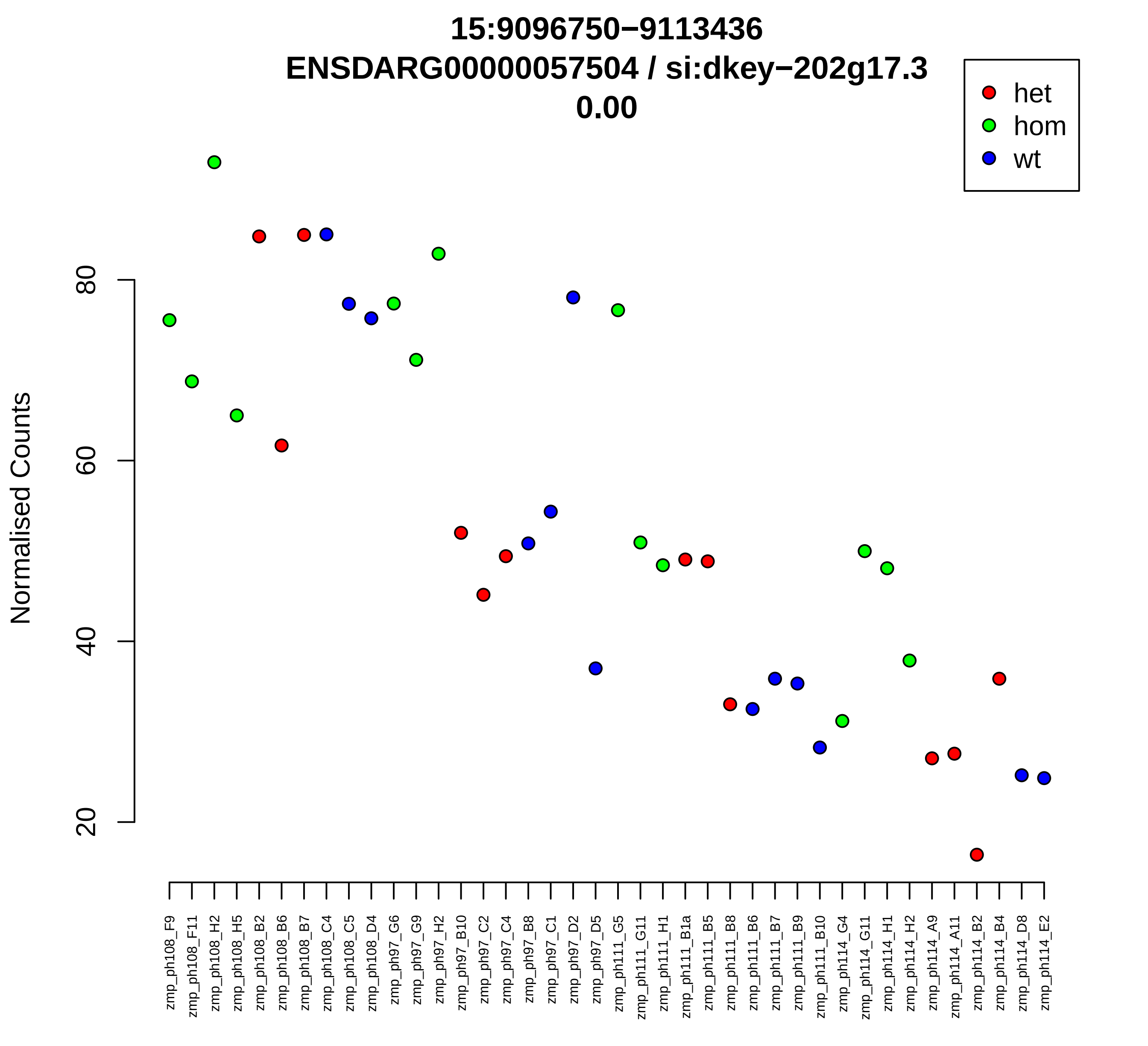
15:9096750−9113436
ENSDARG00000057504 / si:dkey−202g17.3
0.00
het
hom
wt
Normalised Counts
20
40
60
80
zmp_ph108_F9
zmp_ph108_F11
zmp_ph108_H2
zmp_ph108_H5
zmp_ph108_B2
zmp_ph108_B6
zmp_ph108_B7
zmp_ph108_C4
zmp_ph108_C5
zmp_ph108_D4
zmp_ph97_G6
zmp_ph97_G9
zmp_ph97_H2
zmp_ph97_B10
zmp_ph97_C2
zmp_ph97_C4
zmp_ph97_B8
zmp_ph97_C1
zmp_ph97_D2
zmp_ph97_D5
zmp_ph111_G5
zmp_ph111_G11
zmp_ph111_H1
zmp_ph111_B1a
zmp_ph111_B5
zmp_ph111_B8
zmp_ph111_B6
zmp_ph111_B7
zmp_ph111_B9
zmp_ph111_B10
zmp_ph114_G4
zmp_ph114_G11
zmp_ph114_H1
zmp_ph114_H2
zmp_ph114_A9
zmp_ph114_A11
zmp_ph114_B2
zmp_ph114_B4
zmp_ph114_D8
zmp_ph114_E2

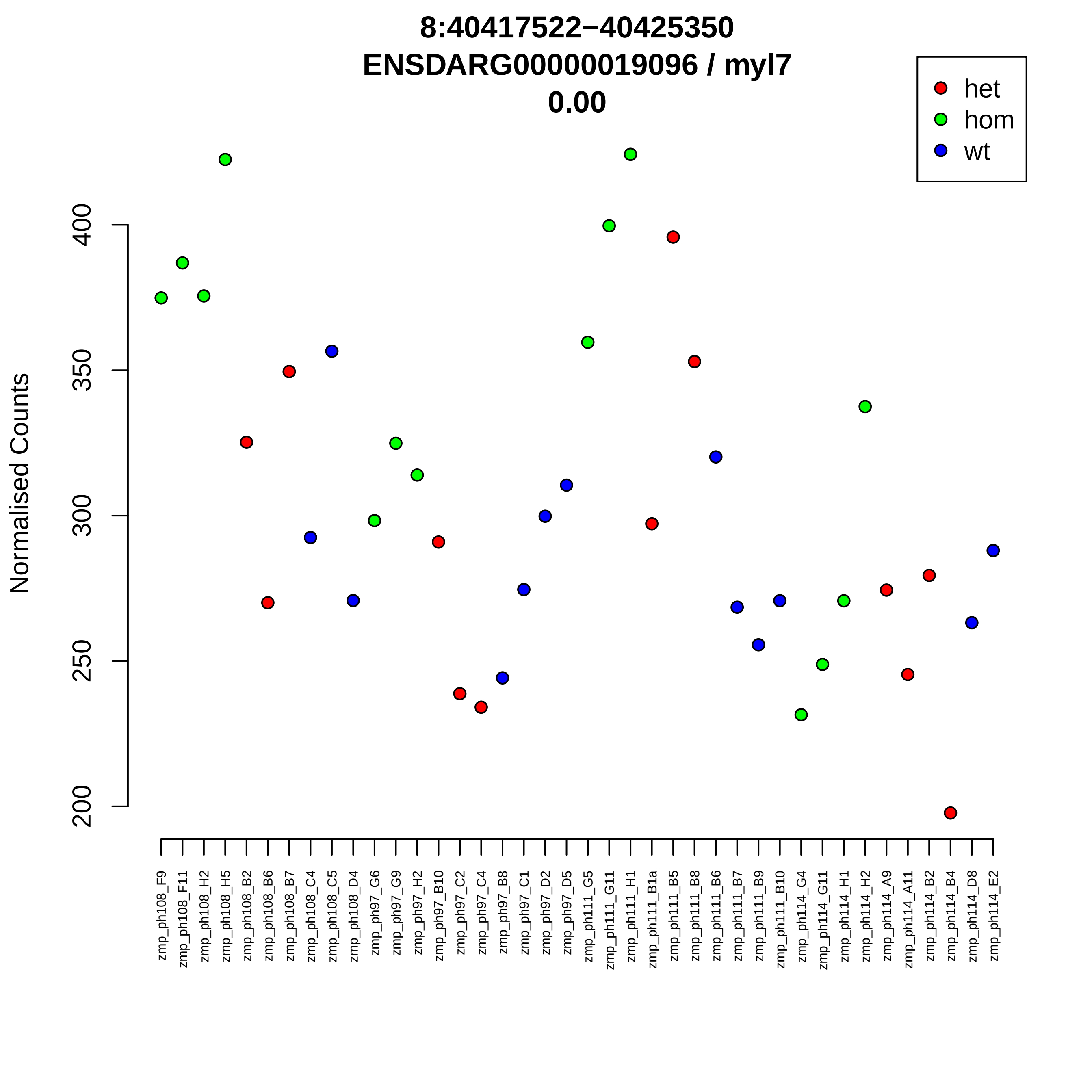
8:40417522−40425350
ENSDARG00000019096 / myl7
0.00
het
hom
wt
Normalised Counts
200
250
300
350
400
zmp_ph108_F9
zmp_ph108_F11
zmp_ph108_H2
zmp_ph108_H5
zmp_ph108_B2
zmp_ph108_B6
zmp_ph108_B7
zmp_ph108_C4
zmp_ph108_C5
zmp_ph108_D4
zmp_ph97_G6
zmp_ph97_G9
zmp_ph97_H2
zmp_ph97_B10
zmp_ph97_C2
zmp_ph97_C4
zmp_ph97_B8
zmp_ph97_C1
zmp_ph97_D2
zmp_ph97_D5
zmp_ph111_G5
zmp_ph111_G11
zmp_ph111_H1
zmp_ph111_B1a
zmp_ph111_B5
zmp_ph111_B8
zmp_ph111_B6
zmp_ph111_B7
zmp_ph111_B9
zmp_ph111_B10
zmp_ph114_G4
zmp_ph114_G11
zmp_ph114_H1
zmp_ph114_H2
zmp_ph114_A9
zmp_ph114_A11
zmp_ph114_B2
zmp_ph114_B4
zmp_ph114_D8
zmp_ph114_E2

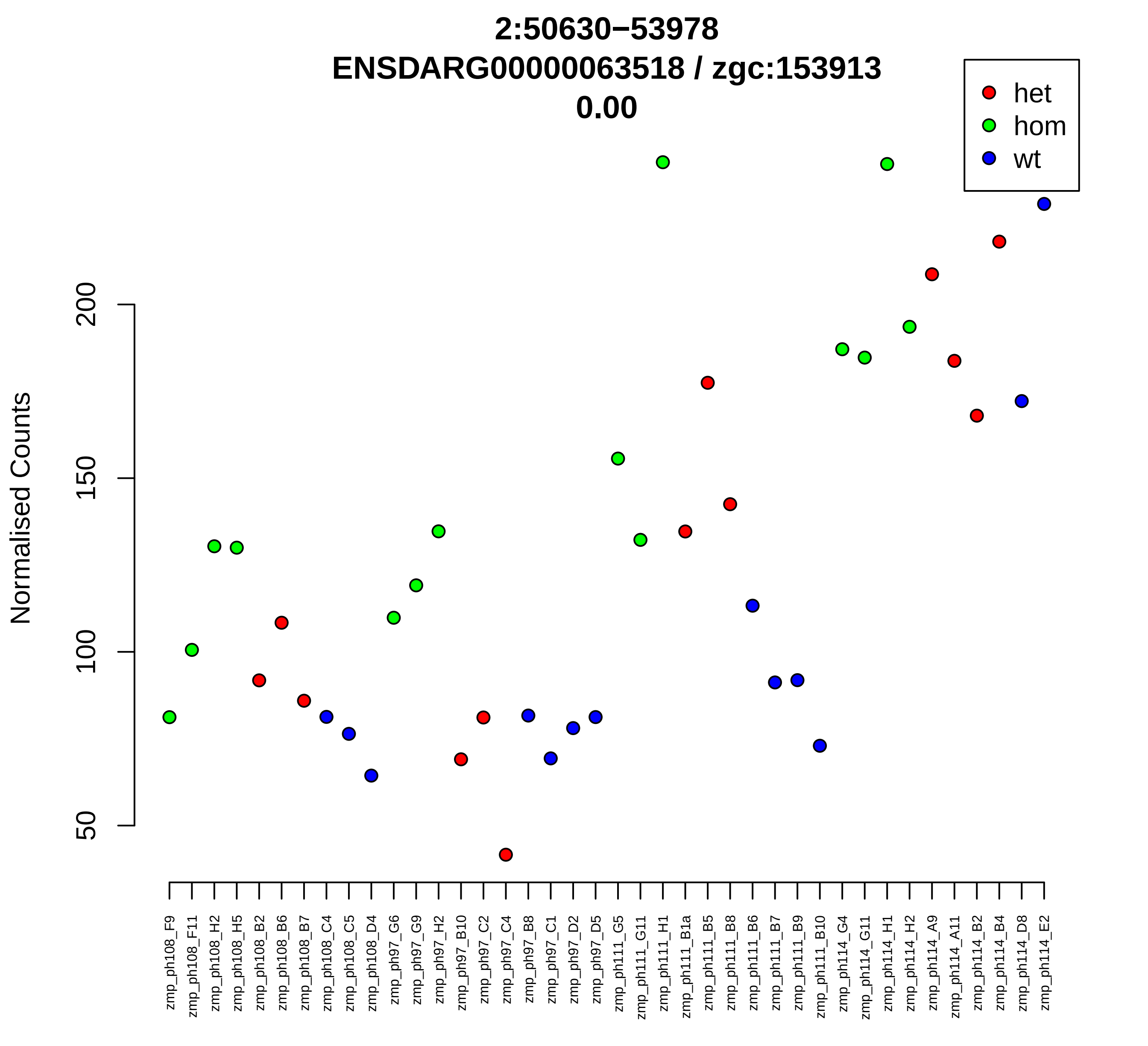
2:50630−53978
ENSDARG00000063518 / zgc:153913
0.00
het
hom
wt
Normalised Counts
50
100
150
200
zmp_ph108_F9
zmp_ph108_F11
zmp_ph108_H2
zmp_ph108_H5
zmp_ph108_B2
zmp_ph108_B6
zmp_ph108_B7
zmp_ph108_C4
zmp_ph108_C5
zmp_ph108_D4
zmp_ph97_G6
zmp_ph97_G9
zmp_ph97_H2
zmp_ph97_B10
zmp_ph97_C2
zmp_ph97_C4
zmp_ph97_B8
zmp_ph97_C1
zmp_ph97_D2
zmp_ph97_D5
zmp_ph111_G5
zmp_ph111_G11
zmp_ph111_H1
zmp_ph111_B1a
zmp_ph111_B5
zmp_ph111_B8
zmp_ph111_B6
zmp_ph111_B7
zmp_ph111_B9
zmp_ph111_B10
zmp_ph114_G4
zmp_ph114_G11
zmp_ph114_H1
zmp_ph114_H2
zmp_ph114_A9
zmp_ph114_A11
zmp_ph114_B2
zmp_ph114_B4
zmp_ph114_D8
zmp_ph114_E2

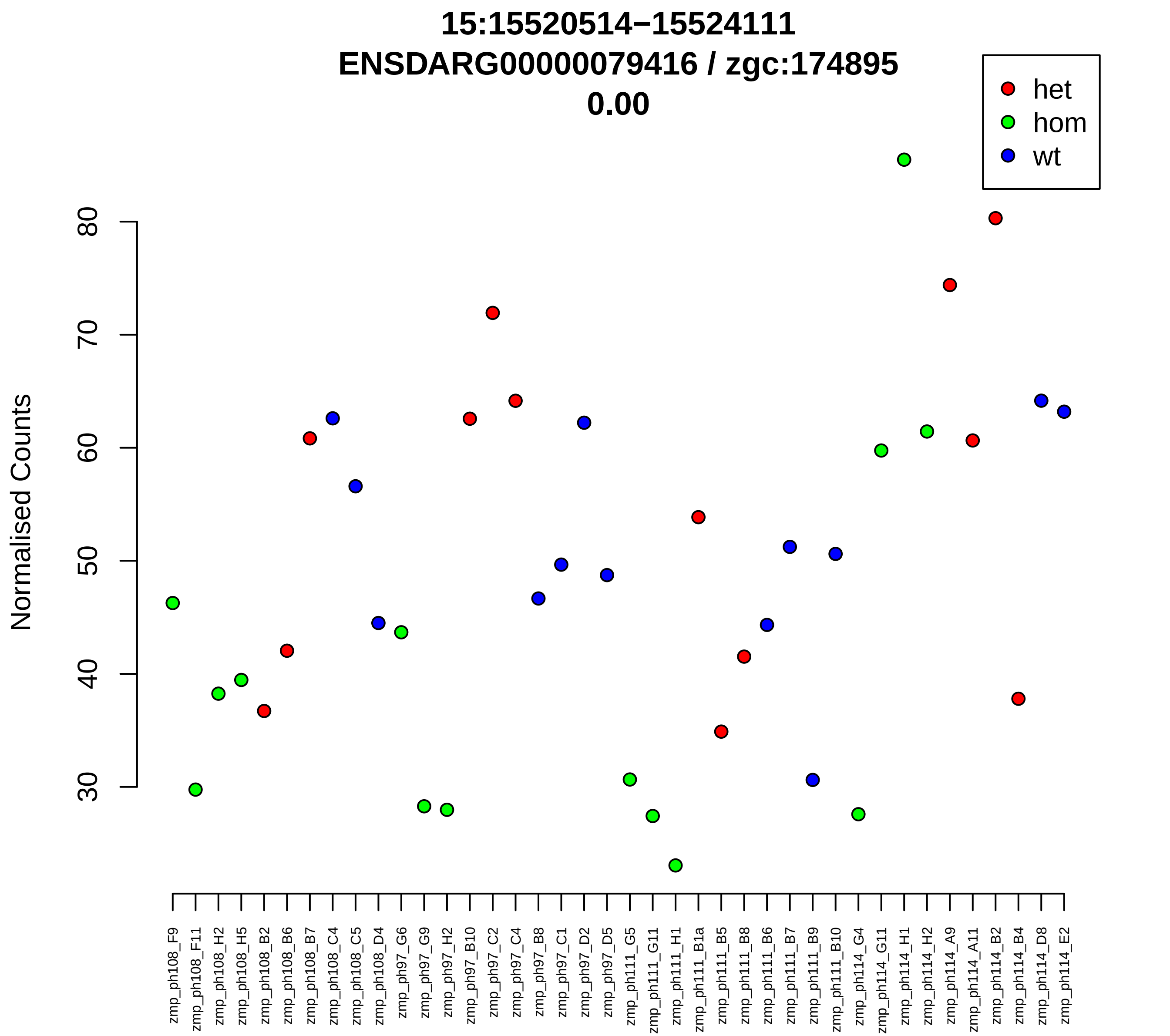

15:15520514−15524111
ENSDARG00000079416 / zgc:174895
0.00
het
hom
wt
Normalised Counts
30
40
50
60
70
80
zmp_ph108_F9
zmp_ph108_F11
zmp_ph108_H2
zmp_ph108_H5
zmp_ph108_B2
zmp_ph108_B6
zmp_ph108_B7
zmp_ph108_C4
zmp_ph108_C5
zmp_ph108_D4
zmp_ph97_G6
zmp_ph97_G9
zmp_ph97_H2
zmp_ph97_B10
zmp_ph97_C2
zmp_ph97_C4
zmp_ph97_B8
zmp_ph97_C1
zmp_ph97_D2
zmp_ph97_D5
zmp_ph111_G5
zmp_ph111_G11
zmp_ph111_H1
zmp_ph111_B1a
zmp_ph111_B5
zmp_ph111_B8
zmp_ph111_B6
zmp_ph111_B7
zmp_ph111_B9
zmp_ph111_B10
zmp_ph114_G4
zmp_ph114_G11
zmp_ph114_H1
zmp_ph114_H2
zmp_ph114_A9
zmp_ph114_A11
zmp_ph114_B2
zmp_ph114_B4
zmp_ph114_D8
zmp_ph114_E2

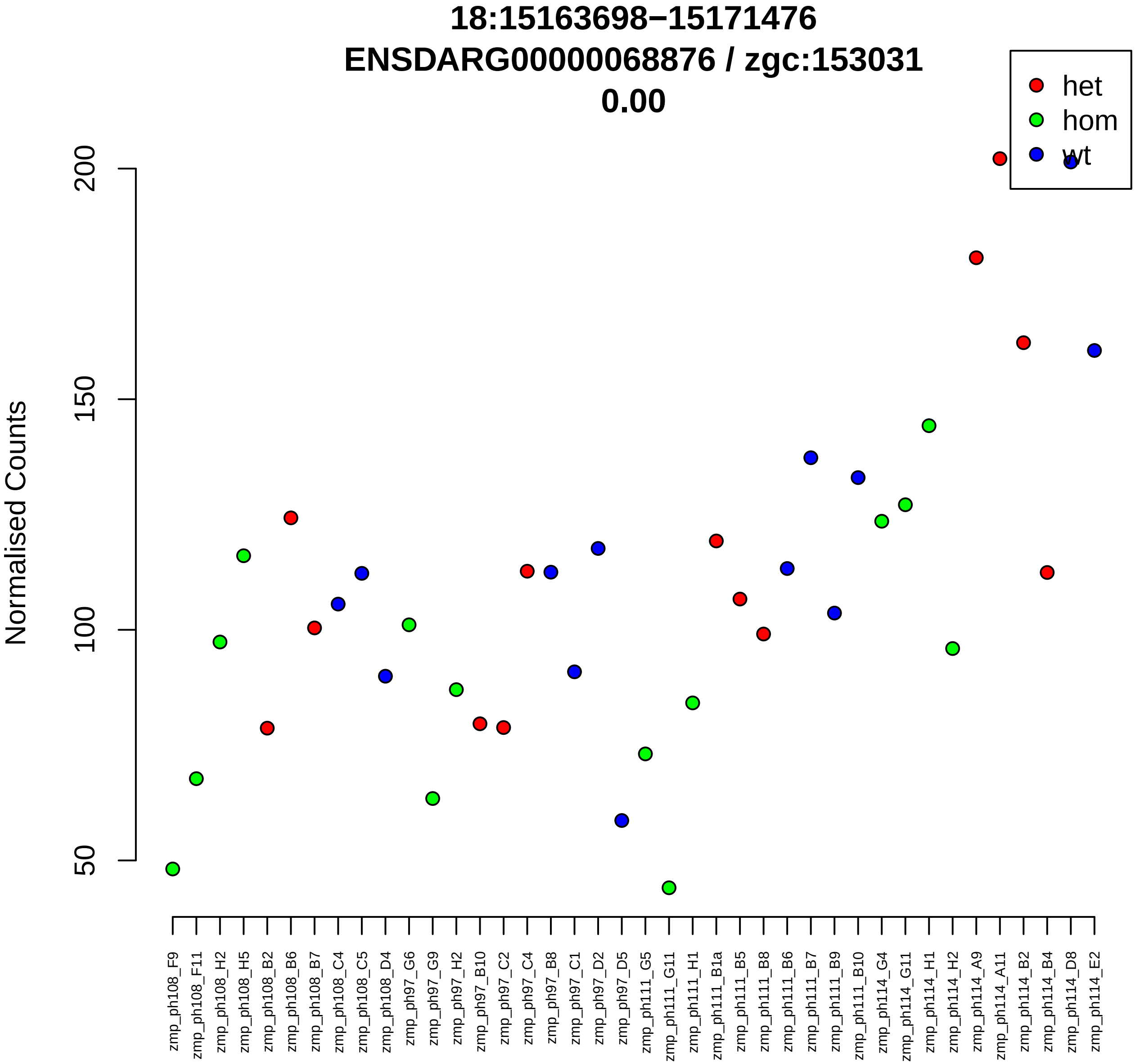
18:15163698−15171476
ENSDARG00000068876 / zgc:153031
0.00
het
hom
wt
Normalised Counts
50
100
150
200
zmp_ph108_F9
zmp_ph108_F11
zmp_ph108_H2
zmp_ph108_H5
zmp_ph108_B2
zmp_ph108_B6
zmp_ph108_B7
zmp_ph108_C4
zmp_ph108_C5
zmp_ph108_D4
zmp_ph97_G6
zmp_ph97_G9
zmp_ph97_H2
zmp_ph97_B10
zmp_ph97_C2
zmp_ph97_C4
zmp_ph97_B8
zmp_ph97_C1
zmp_ph97_D2
zmp_ph97_D5
zmp_ph111_G5
zmp_ph111_G11
zmp_ph111_H1
zmp_ph111_B1a
zmp_ph111_B5
zmp_ph111_B8
zmp_ph111_B6
zmp_ph111_B7
zmp_ph111_B9
zmp_ph111_B10
zmp_ph114_G4
zmp_ph114_G11
zmp_ph114_H1
zmp_ph114_H2
zmp_ph114_A9
zmp_ph114_A11
zmp_ph114_B2
zmp_ph114_B4
zmp_ph114_D8
zmp_ph114_E2

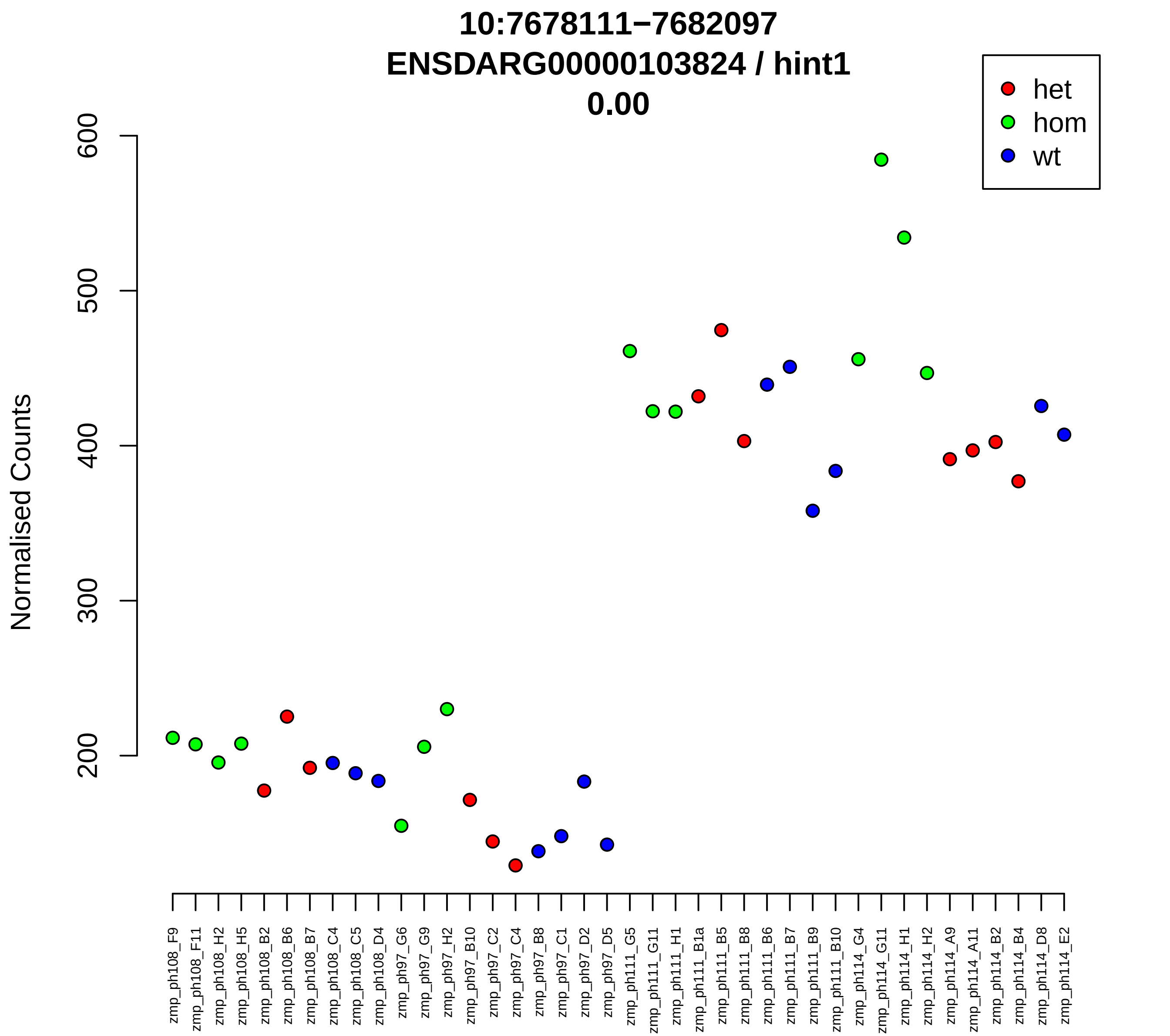

10:7678111−7682097
ENSDARG00000103824 / hint1
0.00
het
hom
wt
Normalised Counts
200
300
400
500
600
zmp_ph108_F9
zmp_ph108_F11
zmp_ph108_H2
zmp_ph108_H5
zmp_ph108_B2
zmp_ph108_B6
zmp_ph108_B7
zmp_ph108_C4
zmp_ph108_C5
zmp_ph108_D4
zmp_ph97_G6
zmp_ph97_G9
zmp_ph97_H2
zmp_ph97_B10
zmp_ph97_C2
zmp_ph97_C4
zmp_ph97_B8
zmp_ph97_C1
zmp_ph97_D2
zmp_ph97_D5
zmp_ph111_G5
zmp_ph111_G11
zmp_ph111_H1
zmp_ph111_B1a
zmp_ph111_B5
zmp_ph111_B8
zmp_ph111_B6
zmp_ph111_B7
zmp_ph111_B9
zmp_ph111_B10
zmp_ph114_G4
zmp_ph114_G11
zmp_ph114_H1
zmp_ph114_H2
zmp_ph114_A9
zmp_ph114_A11
zmp_ph114_B2
zmp_ph114_B4
zmp_ph114_D8
zmp_ph114_E2

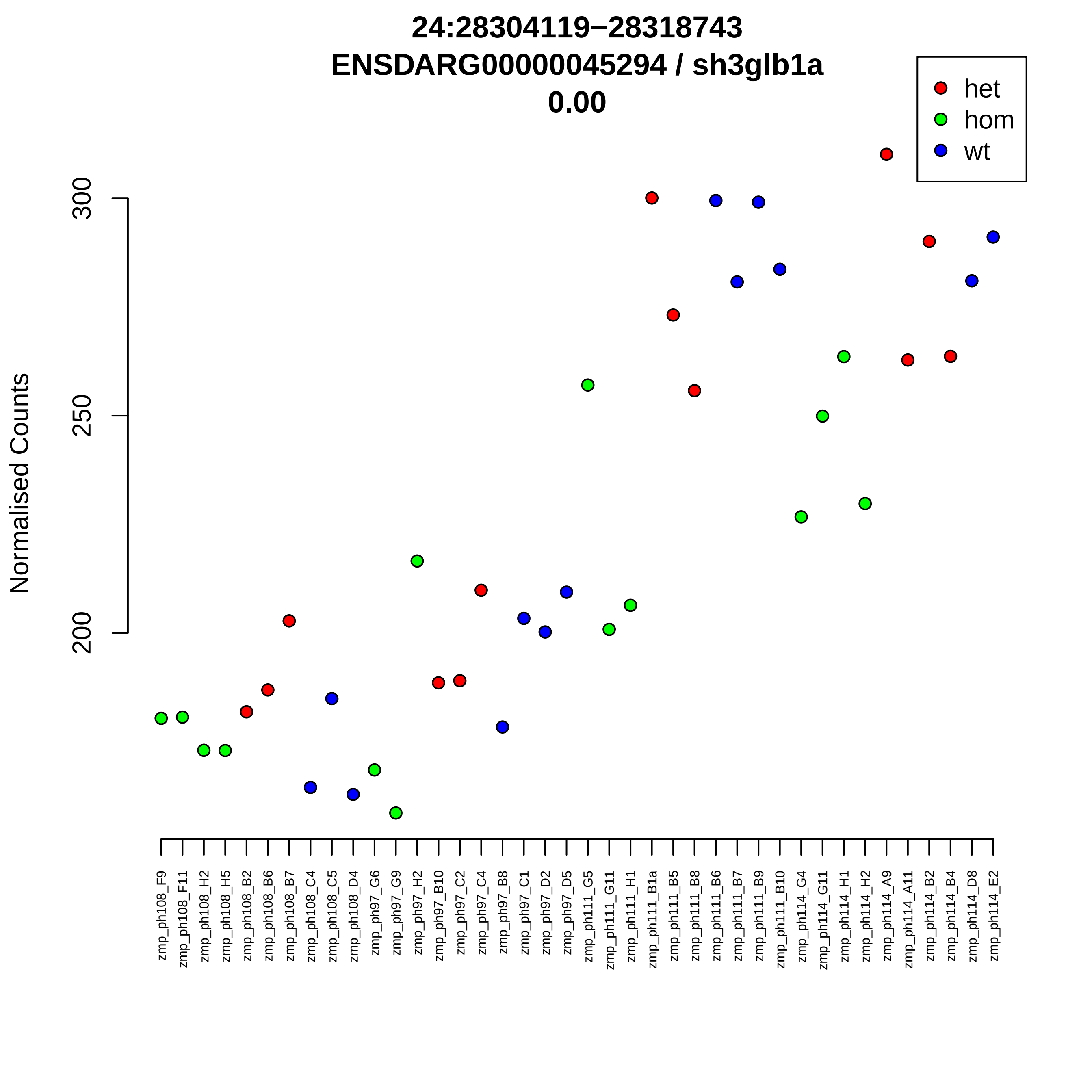

24:28304119−28318743
ENSDARG00000045294 / sh3glb1a
0.00
het
hom
wt
Normalised Counts
200
250
300
zmp_ph108_F9
zmp_ph108_F11
zmp_ph108_H2
zmp_ph108_H5
zmp_ph108_B2
zmp_ph108_B6
zmp_ph108_B7
zmp_ph108_C4
zmp_ph108_C5
zmp_ph108_D4
zmp_ph97_G6
zmp_ph97_G9
zmp_ph97_H2
zmp_ph97_B10
zmp_ph97_C2
zmp_ph97_C4
zmp_ph97_B8
zmp_ph97_C1
zmp_ph97_D2
zmp_ph97_D5
zmp_ph111_G5
zmp_ph111_G11
zmp_ph111_H1
zmp_ph111_B1a
zmp_ph111_B5
zmp_ph111_B8
zmp_ph111_B6
zmp_ph111_B7
zmp_ph111_B9
zmp_ph111_B10
zmp_ph114_G4
zmp_ph114_G11
zmp_ph114_H1
zmp_ph114_H2
zmp_ph114_A9
zmp_ph114_A11
zmp_ph114_B2
zmp_ph114_B4
zmp_ph114_D8
zmp_ph114_E2

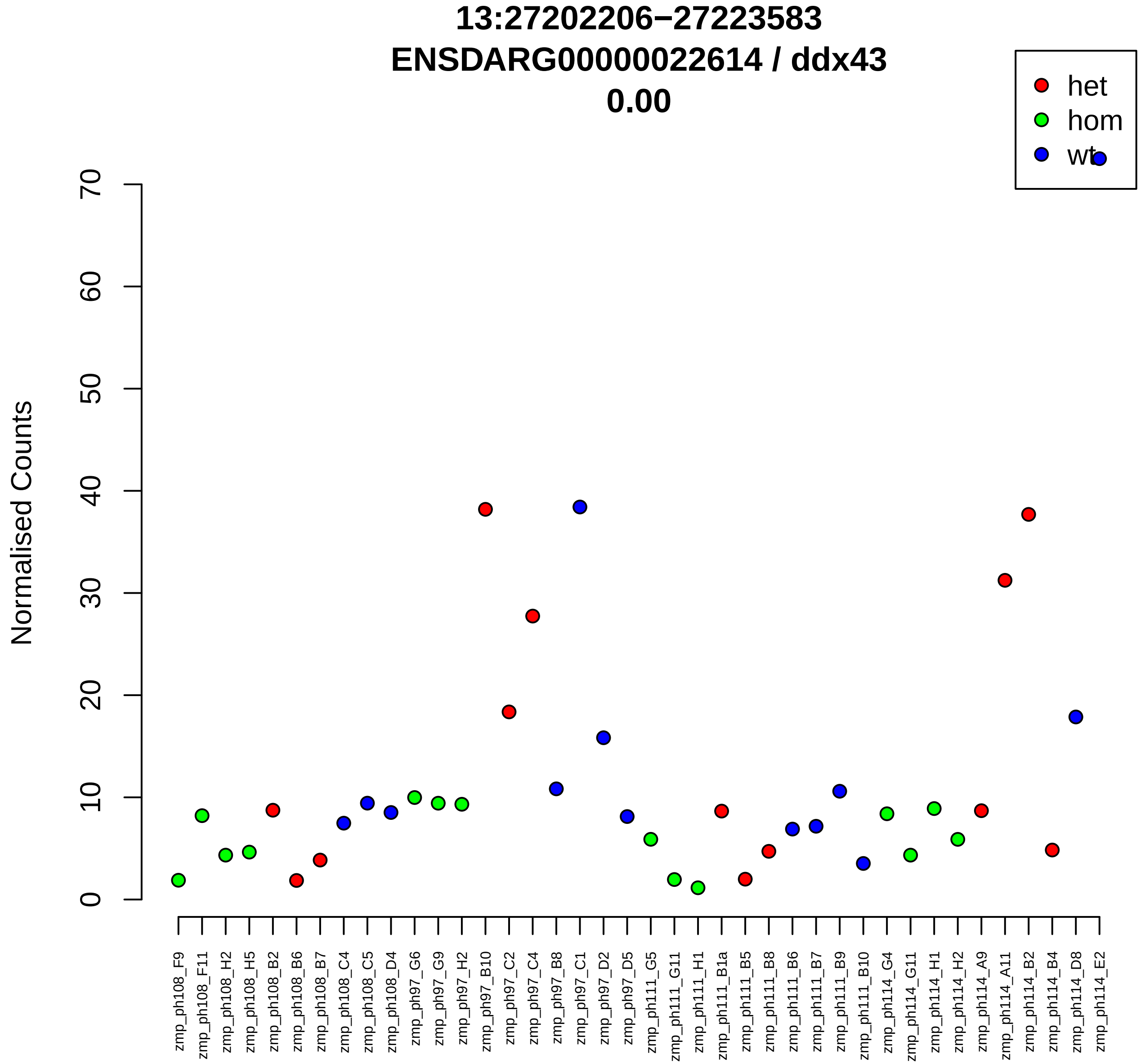

13:27202206−27223583
ENSDARG00000022614 / ddx43
0.00
het
hom
wt
Normalised Counts
0
10
20
30
40
50
60
70
zmp_ph108_F9
zmp_ph108_F11
zmp_ph108_H2
zmp_ph108_H5
zmp_ph108_B2
zmp_ph108_B6
zmp_ph108_B7
zmp_ph108_C4
zmp_ph108_C5
zmp_ph108_D4
zmp_ph97_G6
zmp_ph97_G9
zmp_ph97_H2
zmp_ph97_B10
zmp_ph97_C2
zmp_ph97_C4
zmp_ph97_B8
zmp_ph97_C1
zmp_ph97_D2
zmp_ph97_D5
zmp_ph111_G5
zmp_ph111_G11
zmp_ph111_H1
zmp_ph111_B1a
zmp_ph111_B5
zmp_ph111_B8
zmp_ph111_B6
zmp_ph111_B7
zmp_ph111_B9
zmp_ph111_B10
zmp_ph114_G4
zmp_ph114_G11
zmp_ph114_H1
zmp_ph114_H2
zmp_ph114_A9
zmp_ph114_A11
zmp_ph114_B2
zmp_ph114_B4
zmp_ph114_D8
zmp_ph114_E2

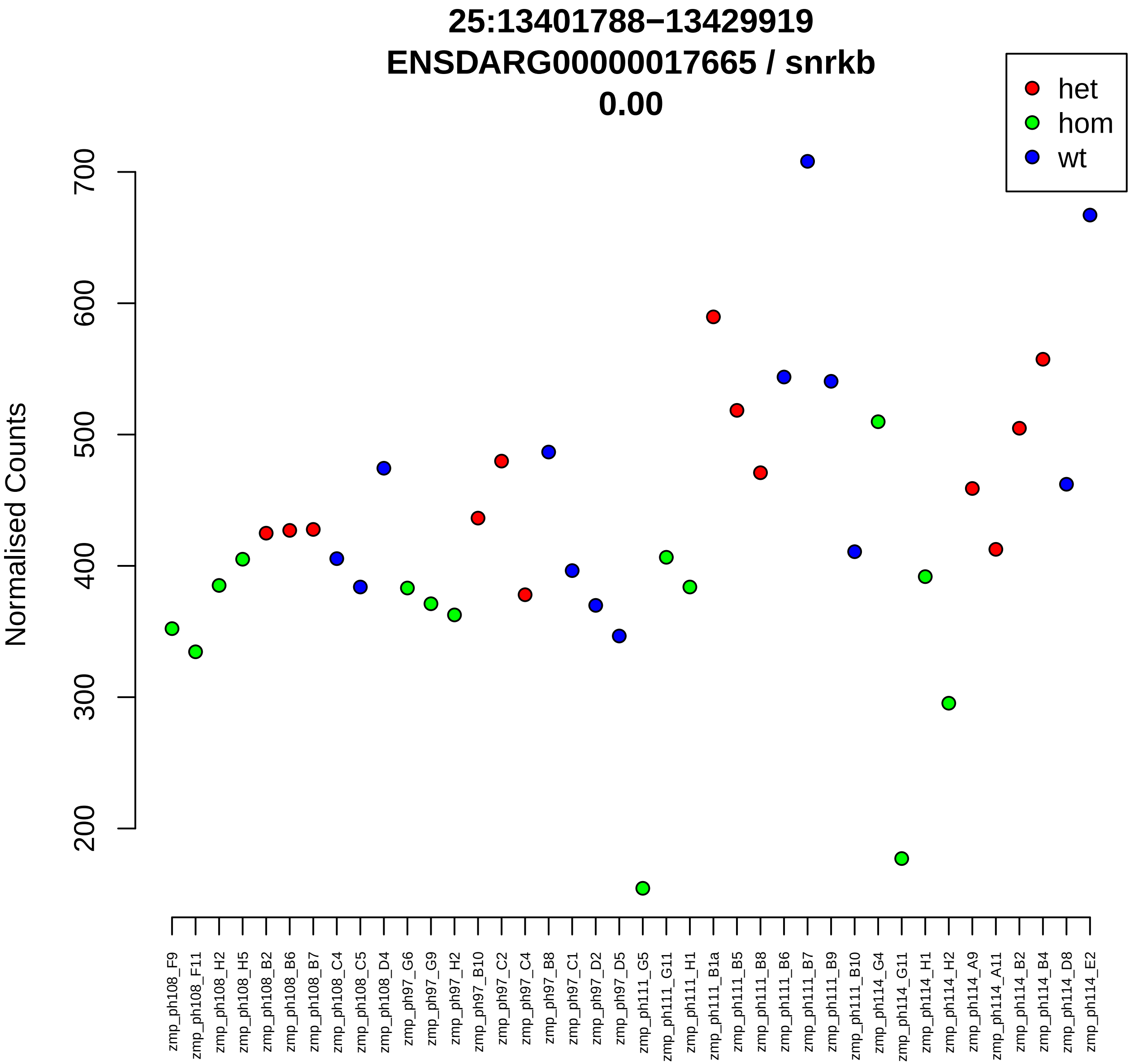

25:13401788−13429919
ENSDARG00000017665 / snrkb
0.00
het
hom
wt
Normalised Counts
200
300
400
500
600
700
zmp_ph108_F9
zmp_ph108_F11
zmp_ph108_H2
zmp_ph108_H5
zmp_ph108_B2
zmp_ph108_B6
zmp_ph108_B7
zmp_ph108_C4
zmp_ph108_C5
zmp_ph108_D4
zmp_ph97_G6
zmp_ph97_G9
zmp_ph97_H2
zmp_ph97_B10
zmp_ph97_C2
zmp_ph97_C4
zmp_ph97_B8
zmp_ph97_C1
zmp_ph97_D2
zmp_ph97_D5
zmp_ph111_G5
zmp_ph111_G11
zmp_ph111_H1
zmp_ph111_B1a
zmp_ph111_B5
zmp_ph111_B8
zmp_ph111_B6
zmp_ph111_B7
zmp_ph111_B9
zmp_ph111_B10
zmp_ph114_G4
zmp_ph114_G11
zmp_ph114_H1
zmp_ph114_H2
zmp_ph114_A9
zmp_ph114_A11
zmp_ph114_B2
zmp_ph114_B4
zmp_ph114_D8
zmp_ph114_E2

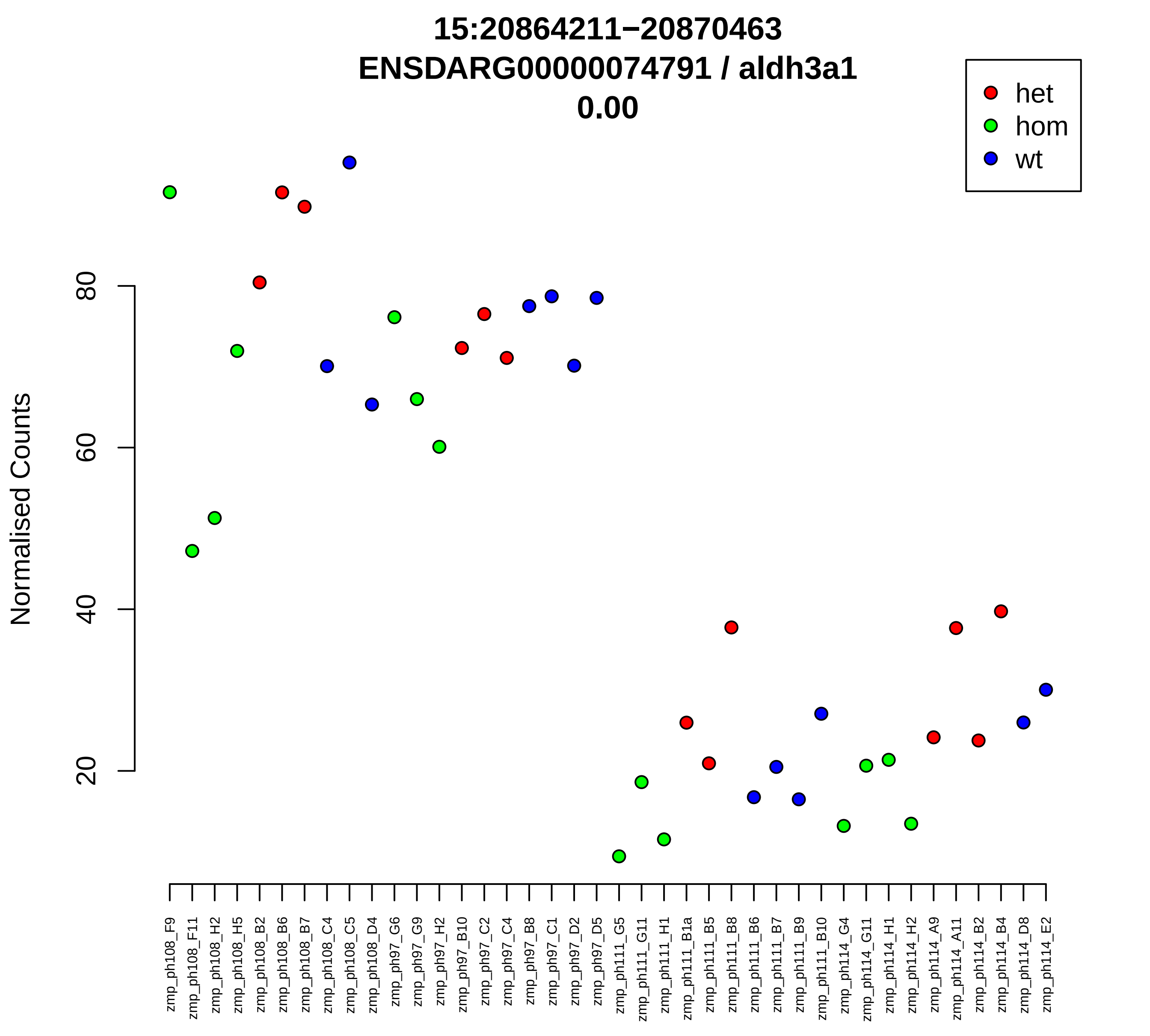
15:20864211−20870463
ENSDARG00000074791 / aldh3a1
0.00
het
hom
wt
Normalised Counts
20
40
60
80
zmp_ph108_F9
zmp_ph108_F11
zmp_ph108_H2
zmp_ph108_H5
zmp_ph108_B2
zmp_ph108_B6
zmp_ph108_B7
zmp_ph108_C4
zmp_ph108_C5
zmp_ph108_D4
zmp_ph97_G6
zmp_ph97_G9
zmp_ph97_H2
zmp_ph97_B10
zmp_ph97_C2
zmp_ph97_C4
zmp_ph97_B8
zmp_ph97_C1
zmp_ph97_D2
zmp_ph97_D5
zmp_ph111_G5
zmp_ph111_G11
zmp_ph111_H1
zmp_ph111_B1a
zmp_ph111_B5
zmp_ph111_B8
zmp_ph111_B6
zmp_ph111_B7
zmp_ph111_B9
zmp_ph111_B10
zmp_ph114_G4
zmp_ph114_G11
zmp_ph114_H1
zmp_ph114_H2
zmp_ph114_A9
zmp_ph114_A11
zmp_ph114_B2
zmp_ph114_B4
zmp_ph114_D8
zmp_ph114_E2

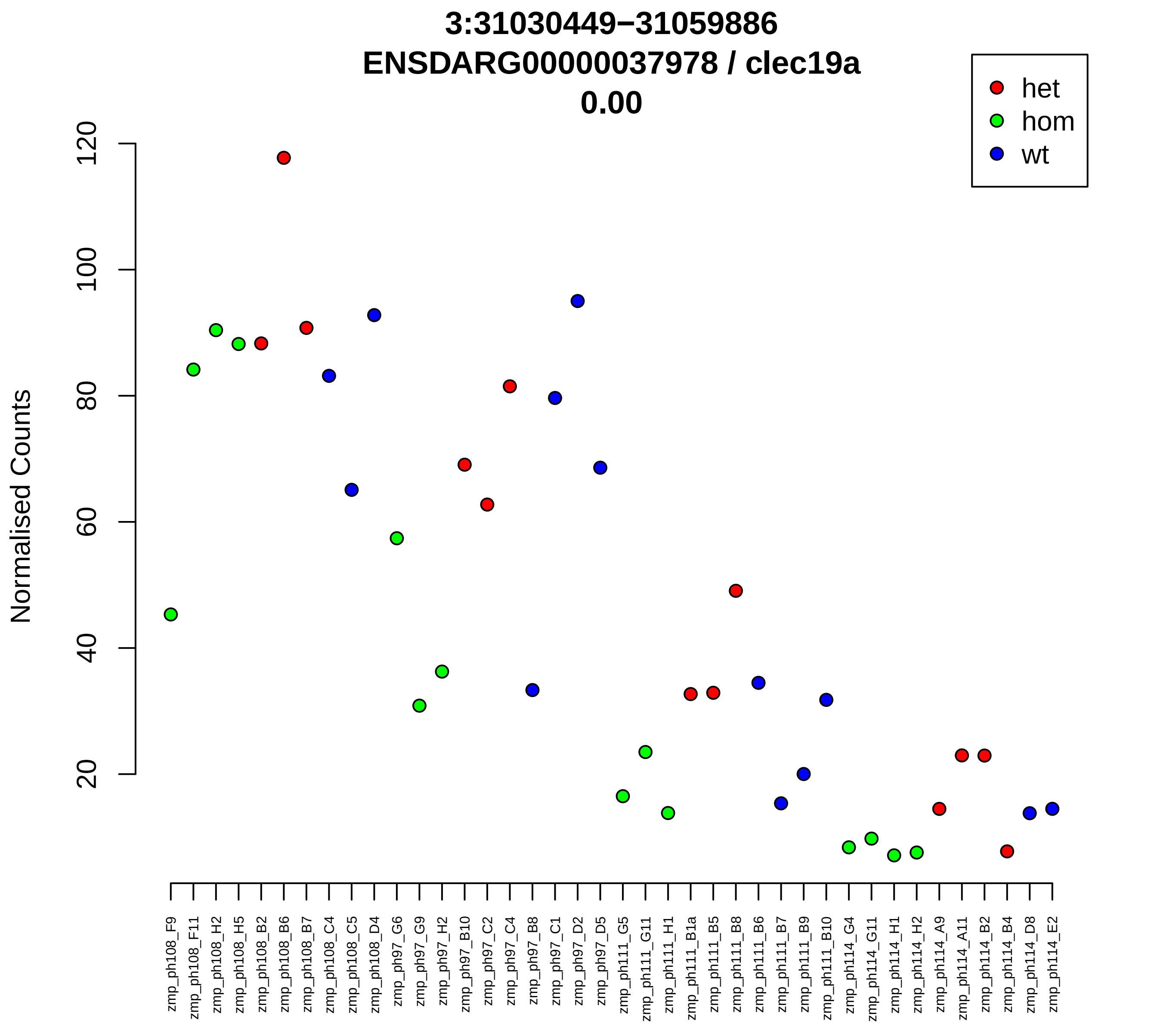

3:31030449−31059886
ENSDARG00000037978 / clec19a
0.00
het
hom
wt
Normalised Counts
20
40
60
80
100
120
zmp_ph108_F9
zmp_ph108_F11
zmp_ph108_H2
zmp_ph108_H5
zmp_ph108_B2
zmp_ph108_B6
zmp_ph108_B7
zmp_ph108_C4
zmp_ph108_C5
zmp_ph108_D4
zmp_ph97_G6
zmp_ph97_G9
zmp_ph97_H2
zmp_ph97_B10
zmp_ph97_C2
zmp_ph97_C4
zmp_ph97_B8
zmp_ph97_C1
zmp_ph97_D2
zmp_ph97_D5
zmp_ph111_G5
zmp_ph111_G11
zmp_ph111_H1
zmp_ph111_B1a
zmp_ph111_B5
zmp_ph111_B8
zmp_ph111_B6
zmp_ph111_B7
zmp_ph111_B9
zmp_ph111_B10
zmp_ph114_G4
zmp_ph114_G11
zmp_ph114_H1
zmp_ph114_H2
zmp_ph114_A9
zmp_ph114_A11
zmp_ph114_B2
zmp_ph114_B4
zmp_ph114_D8
zmp_ph114_E2

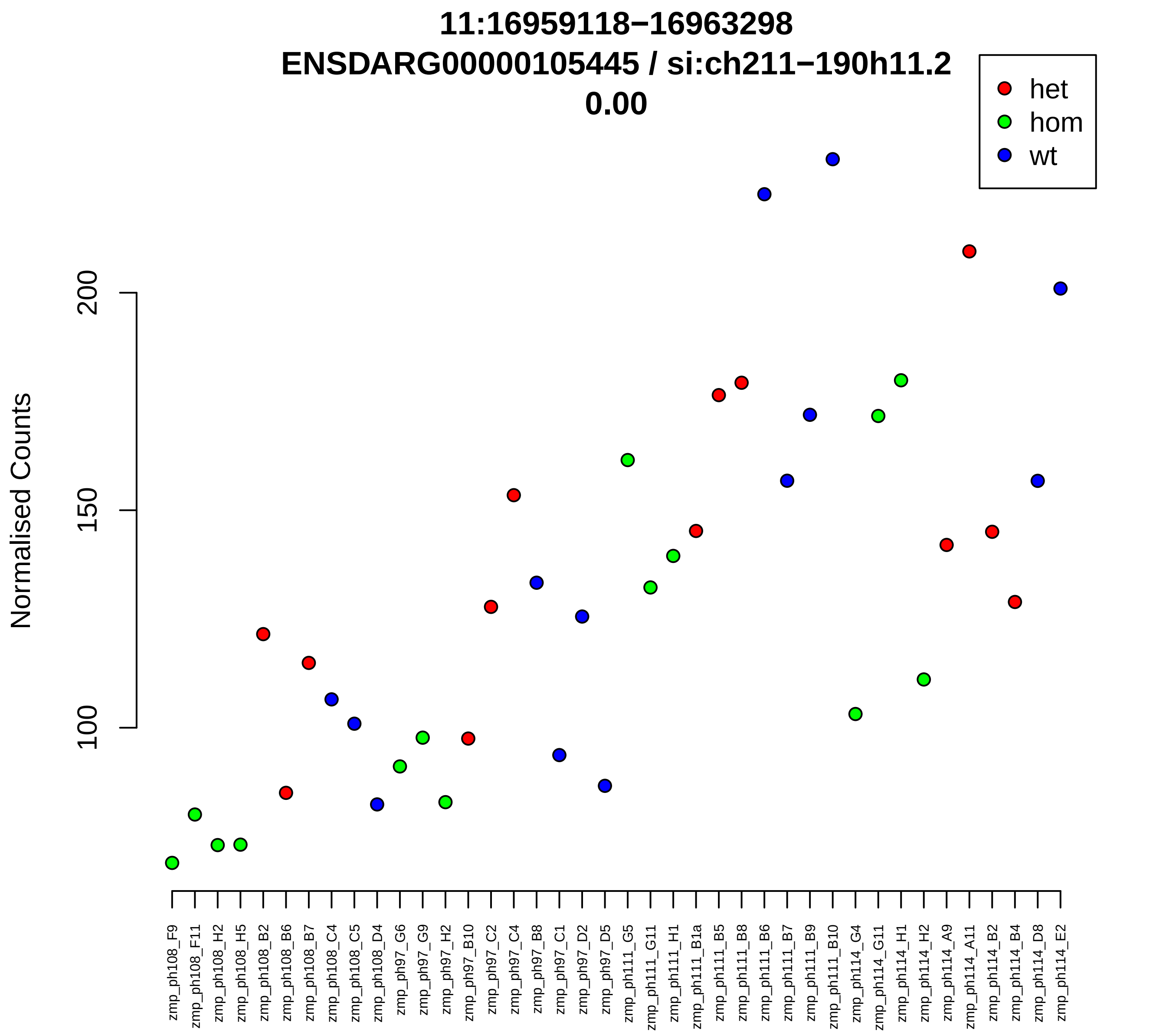
11:16959118−16963298
ENSDARG00000105445 / si:ch211−190h11.2
0.00
Normalised Counts
100
150
200
het
hom
wt
zmp_ph108_F9
zmp_ph108_F11
zmp_ph108_H2
zmp_ph108_H5
zmp_ph108_B2
zmp_ph108_B6
zmp_ph108_B7
zmp_ph108_C4
zmp_ph108_C5
zmp_ph108_D4
zmp_ph97_G6
zmp_ph97_G9
zmp_ph97_H2
zmp_ph97_B10
zmp_ph97_C2
zmp_ph97_C4
zmp_ph97_B8
zmp_ph97_C1
zmp_ph97_D2
zmp_ph97_D5
zmp_ph111_G5
zmp_ph111_G11
zmp_ph111_H1
zmp_ph111_B1a
zmp_ph111_B5
zmp_ph111_B8
zmp_ph111_B6
zmp_ph111_B7
zmp_ph111_B9
zmp_ph111_B10
zmp_ph114_G4
zmp_ph114_G11
zmp_ph114_H1
zmp_ph114_H2
zmp_ph114_A9
zmp_ph114_A11
zmp_ph114_B2
zmp_ph114_B4
zmp_ph114_D8
zmp_ph114_E2

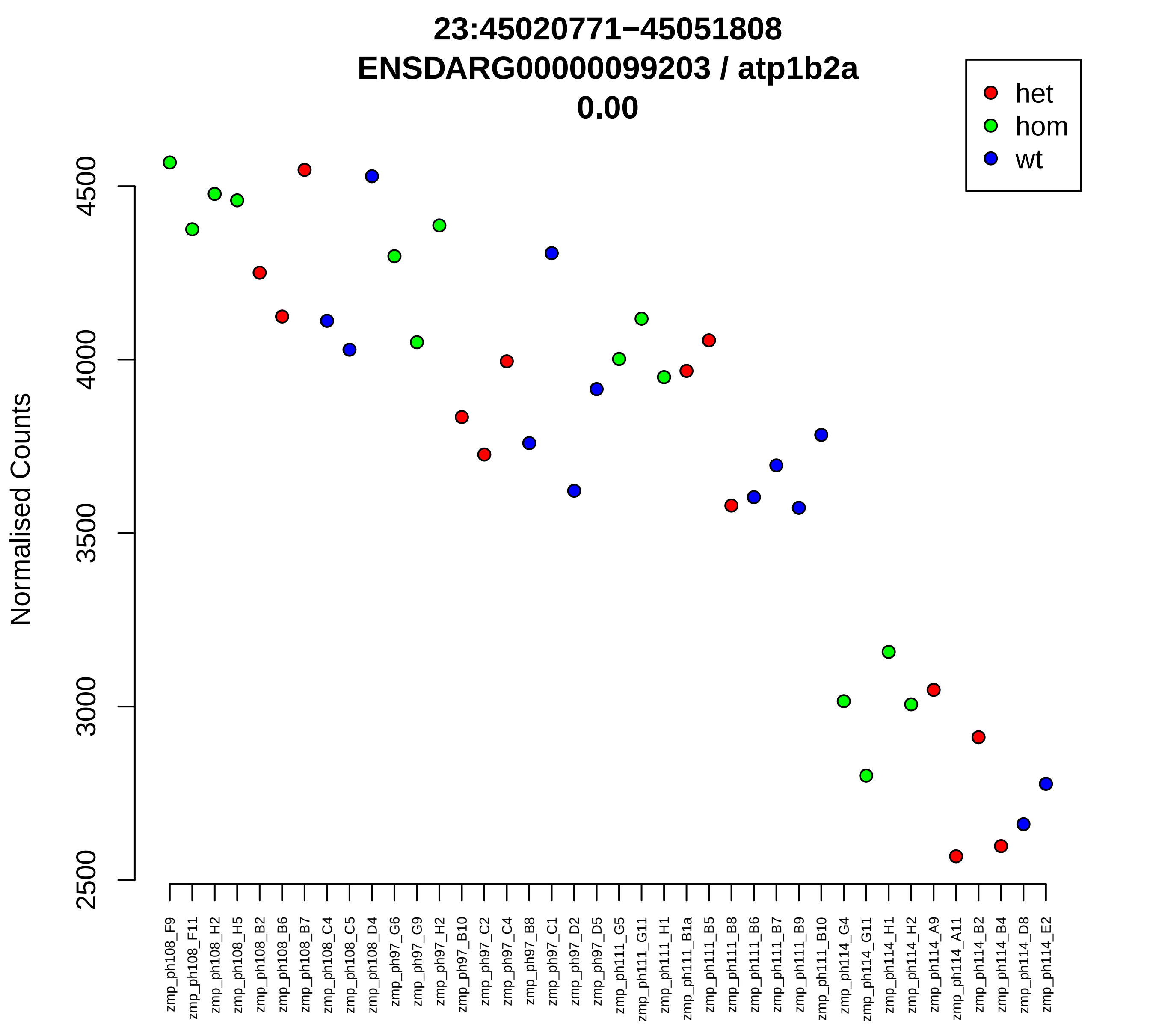
23:45020771−45051808
ENSDARG00000099203 / atp1b2a
0.00
het
hom
wt
Normalised Counts
2500
3000
3500
4000
4500
zmp_ph108_F9
zmp_ph108_F11
zmp_ph108_H2
zmp_ph108_H5
zmp_ph108_B2
zmp_ph108_B6
zmp_ph108_B7
zmp_ph108_C4
zmp_ph108_C5
zmp_ph108_D4
zmp_ph97_G6
zmp_ph97_G9
zmp_ph97_H2
zmp_ph97_B10
zmp_ph97_C2
zmp_ph97_C4
zmp_ph97_B8
zmp_ph97_C1
zmp_ph97_D2
zmp_ph97_D5
zmp_ph111_G5
zmp_ph111_G11
zmp_ph111_H1
zmp_ph111_B1a
zmp_ph111_B5
zmp_ph111_B8
zmp_ph111_B6
zmp_ph111_B7
zmp_ph111_B9
zmp_ph111_B10
zmp_ph114_G4
zmp_ph114_G11
zmp_ph114_H1
zmp_ph114_H2
zmp_ph114_A9
zmp_ph114_A11
zmp_ph114_B2
zmp_ph114_B4
zmp_ph114_D8
zmp_ph114_E2

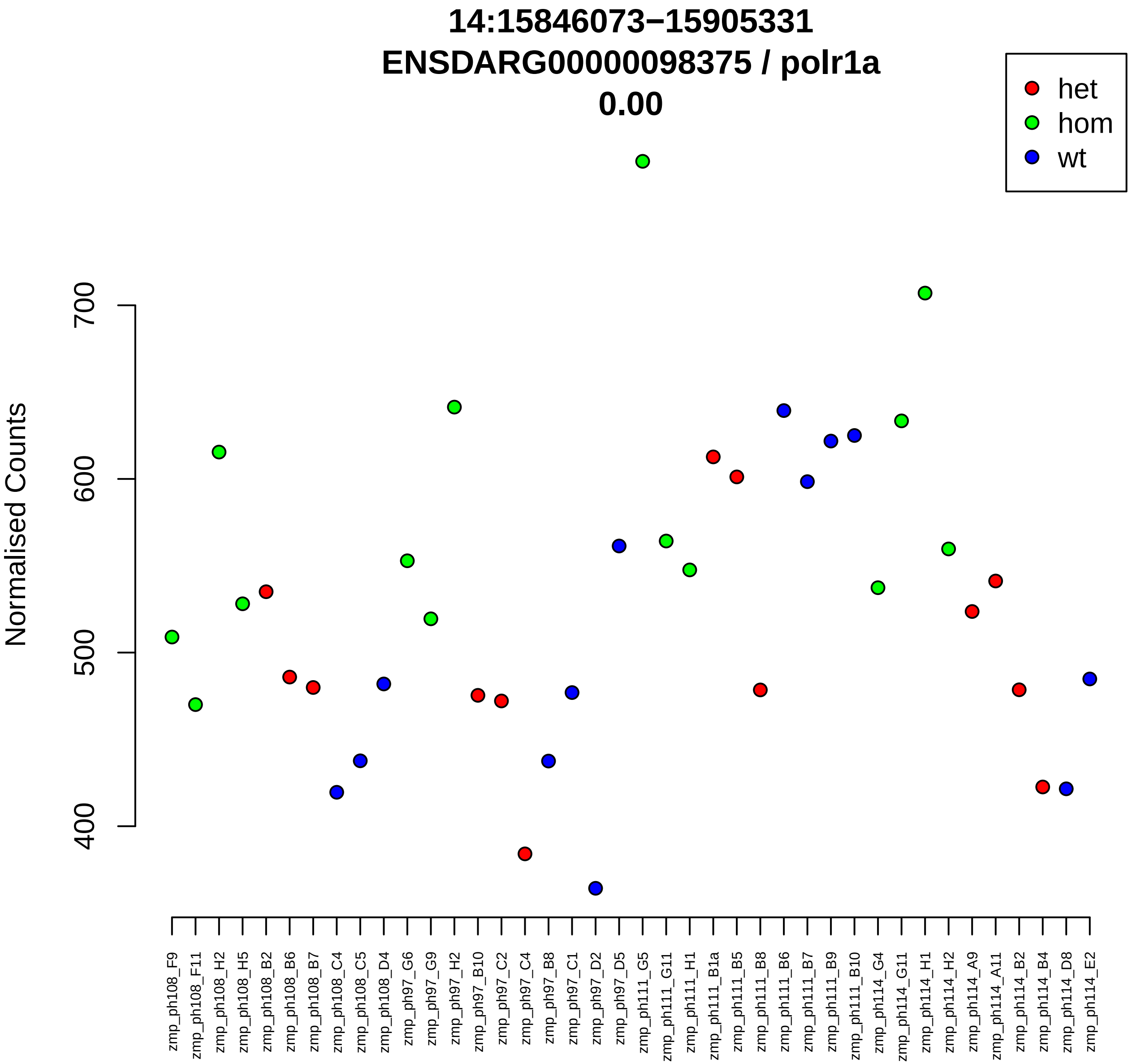

14:15846073−15905331
ENSDARG00000098375 / polr1a
0.00
het
hom
wt
Normalised Counts
400
500
600
700
zmp_ph108_F9
zmp_ph108_F11
zmp_ph108_H2
zmp_ph108_H5
zmp_ph108_B2
zmp_ph108_B6
zmp_ph108_B7
zmp_ph108_C4
zmp_ph108_C5
zmp_ph108_D4
zmp_ph97_G6
zmp_ph97_G9
zmp_ph97_H2
zmp_ph97_B10
zmp_ph97_C2
zmp_ph97_C4
zmp_ph97_B8
zmp_ph97_C1
zmp_ph97_D2
zmp_ph97_D5
zmp_ph111_G5
zmp_ph111_G11
zmp_ph111_H1
zmp_ph111_B1a
zmp_ph111_B5
zmp_ph111_B8
zmp_ph111_B6
zmp_ph111_B7
zmp_ph111_B9
zmp_ph111_B10
zmp_ph114_G4
zmp_ph114_G11
zmp_ph114_H1
zmp_ph114_H2
zmp_ph114_A9
zmp_ph114_A11
zmp_ph114_B2
zmp_ph114_B4
zmp_ph114_D8
zmp_ph114_E2

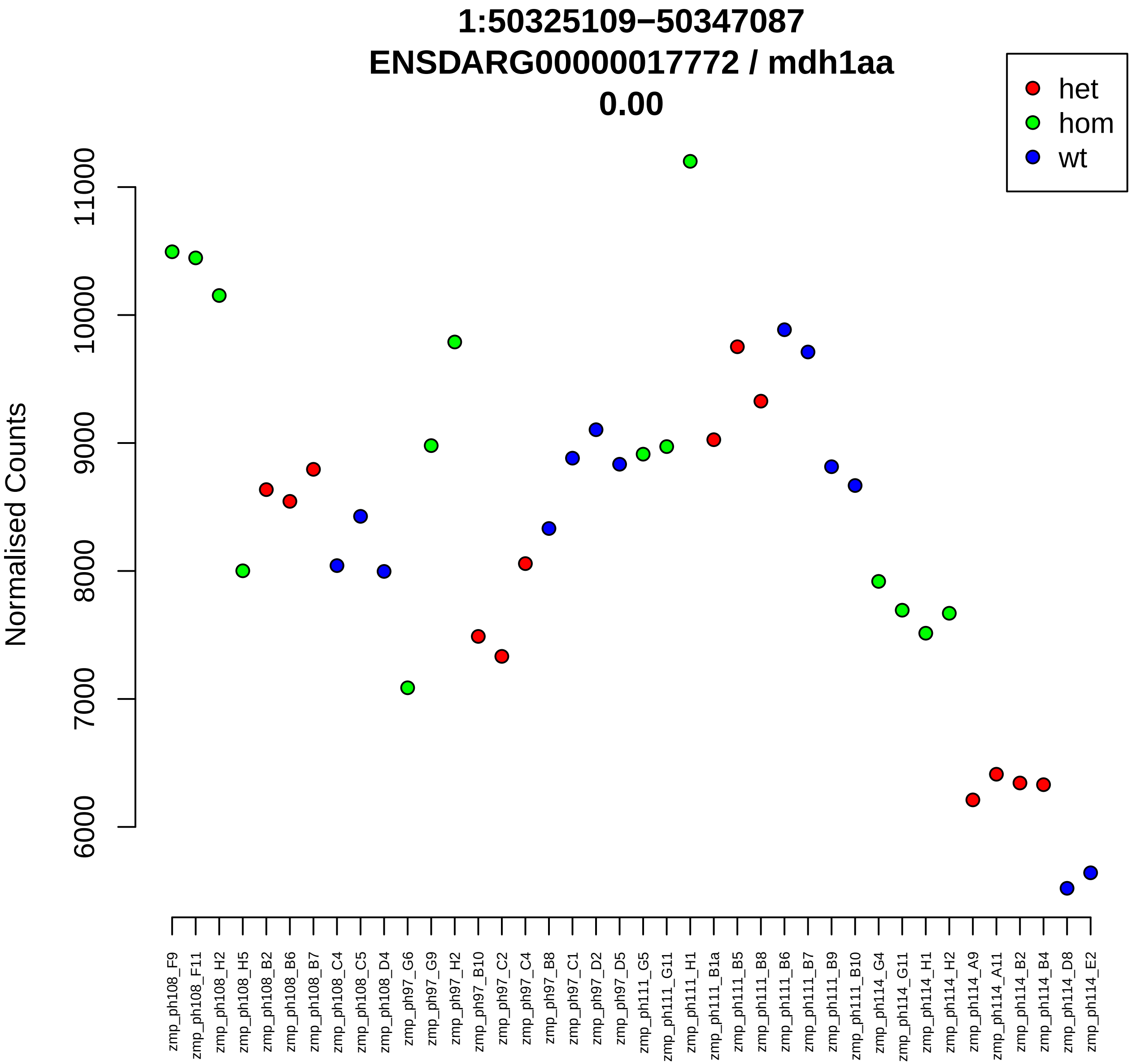

1:50325109−50347087
ENSDARG00000017772 / mdh1aa
0.00
het
hom
wt
Normalised Counts
6000
7000
8000
9000
10000
11000
zmp_ph108_F9
zmp_ph108_F11
zmp_ph108_H2
zmp_ph108_H5
zmp_ph108_B2
zmp_ph108_B6
zmp_ph108_B7
zmp_ph108_C4
zmp_ph108_C5
zmp_ph108_D4
zmp_ph97_G6
zmp_ph97_G9
zmp_ph97_H2
zmp_ph97_B10
zmp_ph97_C2
zmp_ph97_C4
zmp_ph97_B8
zmp_ph97_C1
zmp_ph97_D2
zmp_ph97_D5
zmp_ph111_G5
zmp_ph111_G11
zmp_ph111_H1
zmp_ph111_B1a
zmp_ph111_B5
zmp_ph111_B8
zmp_ph111_B6
zmp_ph111_B7
zmp_ph111_B9
zmp_ph111_B10
zmp_ph114_G4
zmp_ph114_G11
zmp_ph114_H1
zmp_ph114_H2
zmp_ph114_A9
zmp_ph114_A11
zmp_ph114_B2
zmp_ph114_B4
zmp_ph114_D8
zmp_ph114_E2

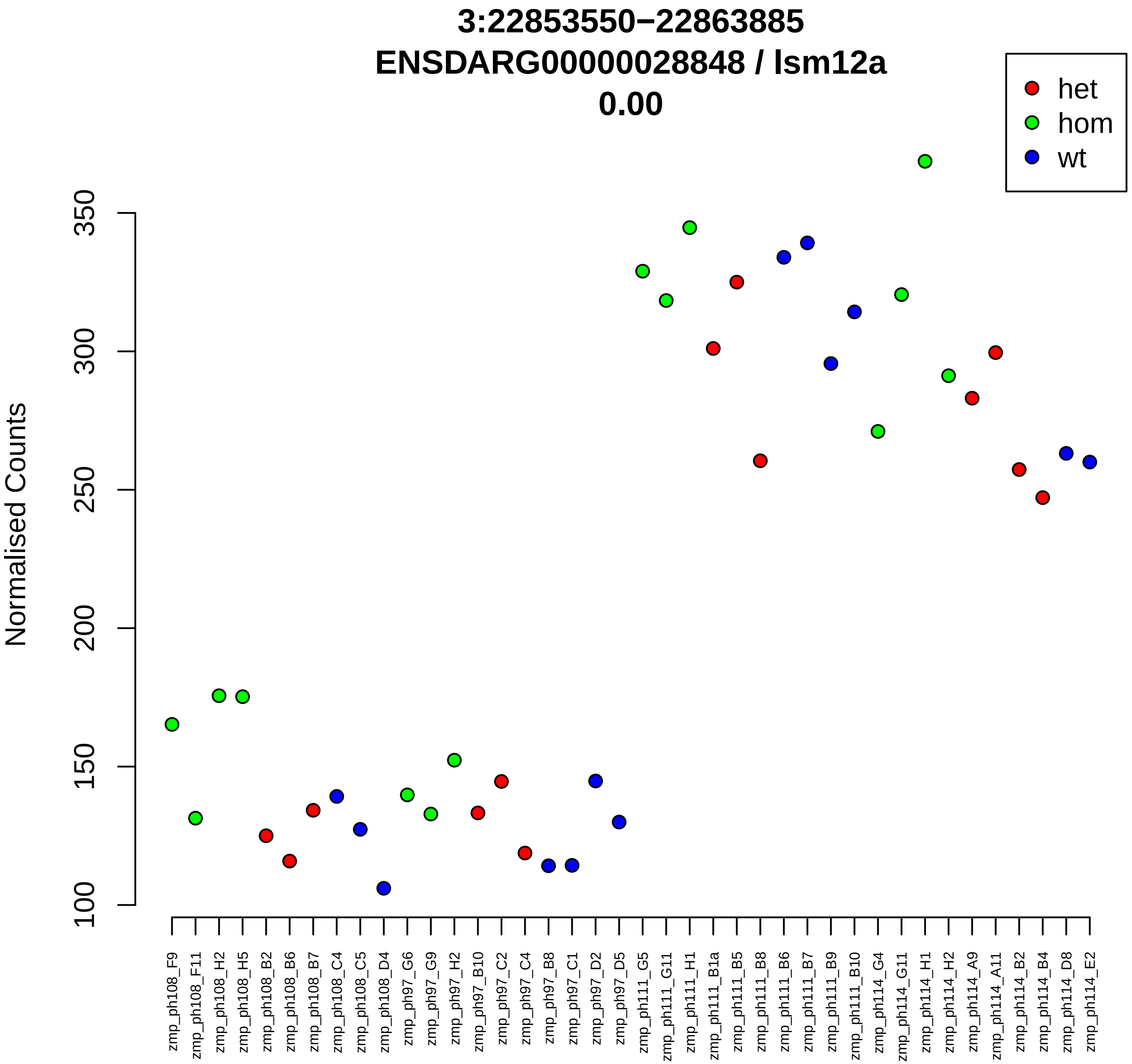

3:22853550−22863885
ENSDARG00000028848 / lsm12a
0.00
het
hom
wt
Normalised Counts
100
150
200
250
300
350
zmp_ph108_F9
zmp_ph108_F11
zmp_ph108_H2
zmp_ph108_H5
zmp_ph108_B2
zmp_ph108_B6
zmp_ph108_B7
zmp_ph108_C4
zmp_ph108_C5
zmp_ph108_D4
zmp_ph97_G6
zmp_ph97_G9
zmp_ph97_H2
zmp_ph97_B10
zmp_ph97_C2
zmp_ph97_C4
zmp_ph97_B8
zmp_ph97_C1
zmp_ph97_D2
zmp_ph97_D5
zmp_ph111_G5
zmp_ph111_G11
zmp_ph111_H1
zmp_ph111_B1a
zmp_ph111_B5
zmp_ph111_B8
zmp_ph111_B6
zmp_ph111_B7
zmp_ph111_B9
zmp_ph111_B10
zmp_ph114_G4
zmp_ph114_G11
zmp_ph114_H1
zmp_ph114_H2
zmp_ph114_A9
zmp_ph114_A11
zmp_ph114_B2
zmp_ph114_B4
zmp_ph114_D8
zmp_ph114_E2

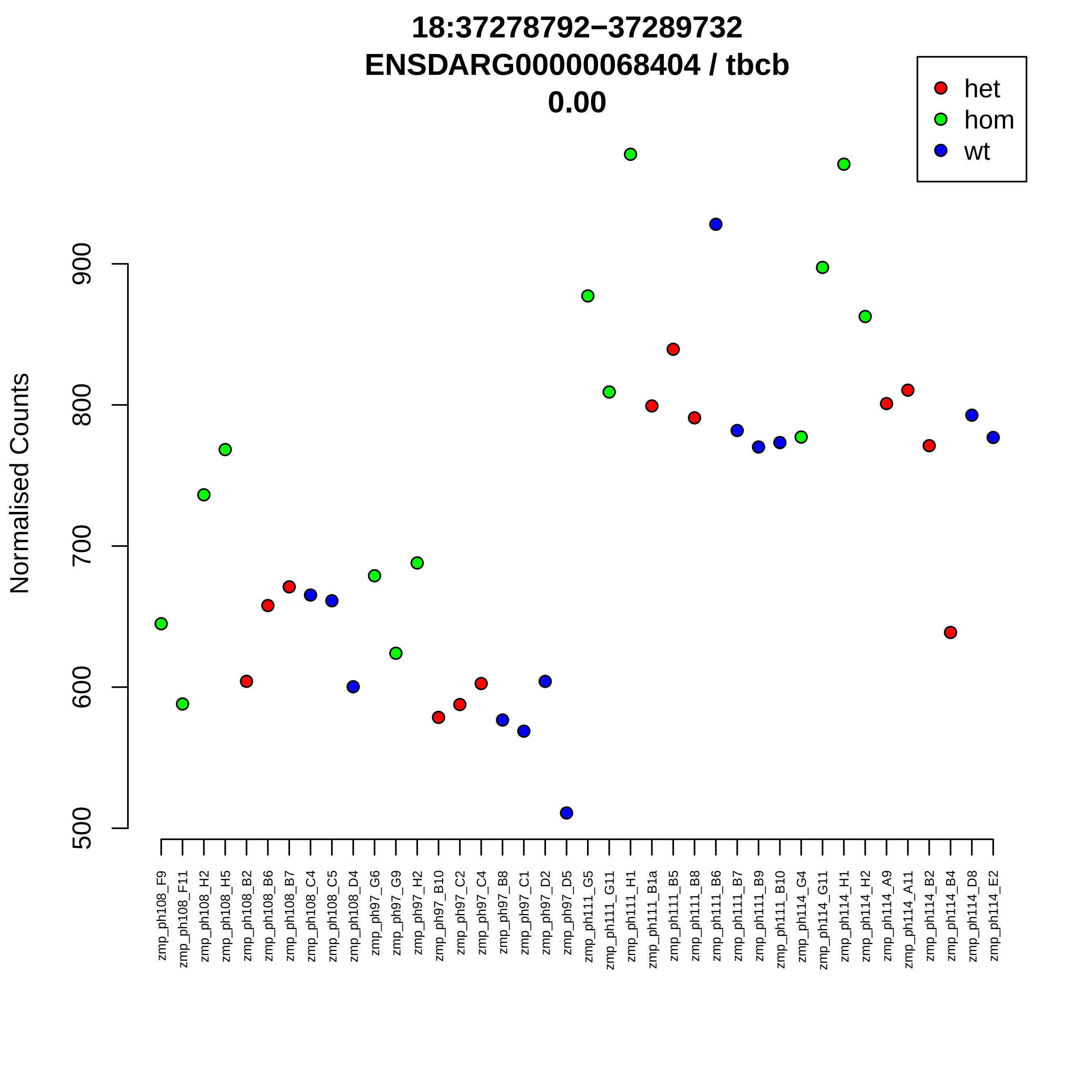
18:37278792−37289732
ENSDARG00000068404 / tbcb
0.00
het
hom
wt
Normalised Counts
500
600
700
800
900
zmp_ph108_F9
zmp_ph108_F11
zmp_ph108_H2
zmp_ph108_H5
zmp_ph108_B2
zmp_ph108_B6
zmp_ph108_B7
zmp_ph108_C4
zmp_ph108_C5
zmp_ph108_D4
zmp_ph97_G6
zmp_ph97_G9
zmp_ph97_H2
zmp_ph97_B10
zmp_ph97_C2
zmp_ph97_C4
zmp_ph97_B8
zmp_ph97_C1
zmp_ph97_D2
zmp_ph97_D5
zmp_ph111_G5
zmp_ph111_G11
zmp_ph111_H1
zmp_ph111_B1a
zmp_ph111_B5
zmp_ph111_B8
zmp_ph111_B6
zmp_ph111_B7
zmp_ph111_B9
zmp_ph111_B10
zmp_ph114_G4
zmp_ph114_G11
zmp_ph114_H1
zmp_ph114_H2
zmp_ph114_A9
zmp_ph114_A11
zmp_ph114_B2
zmp_ph114_B4
zmp_ph114_D8
zmp_ph114_E2

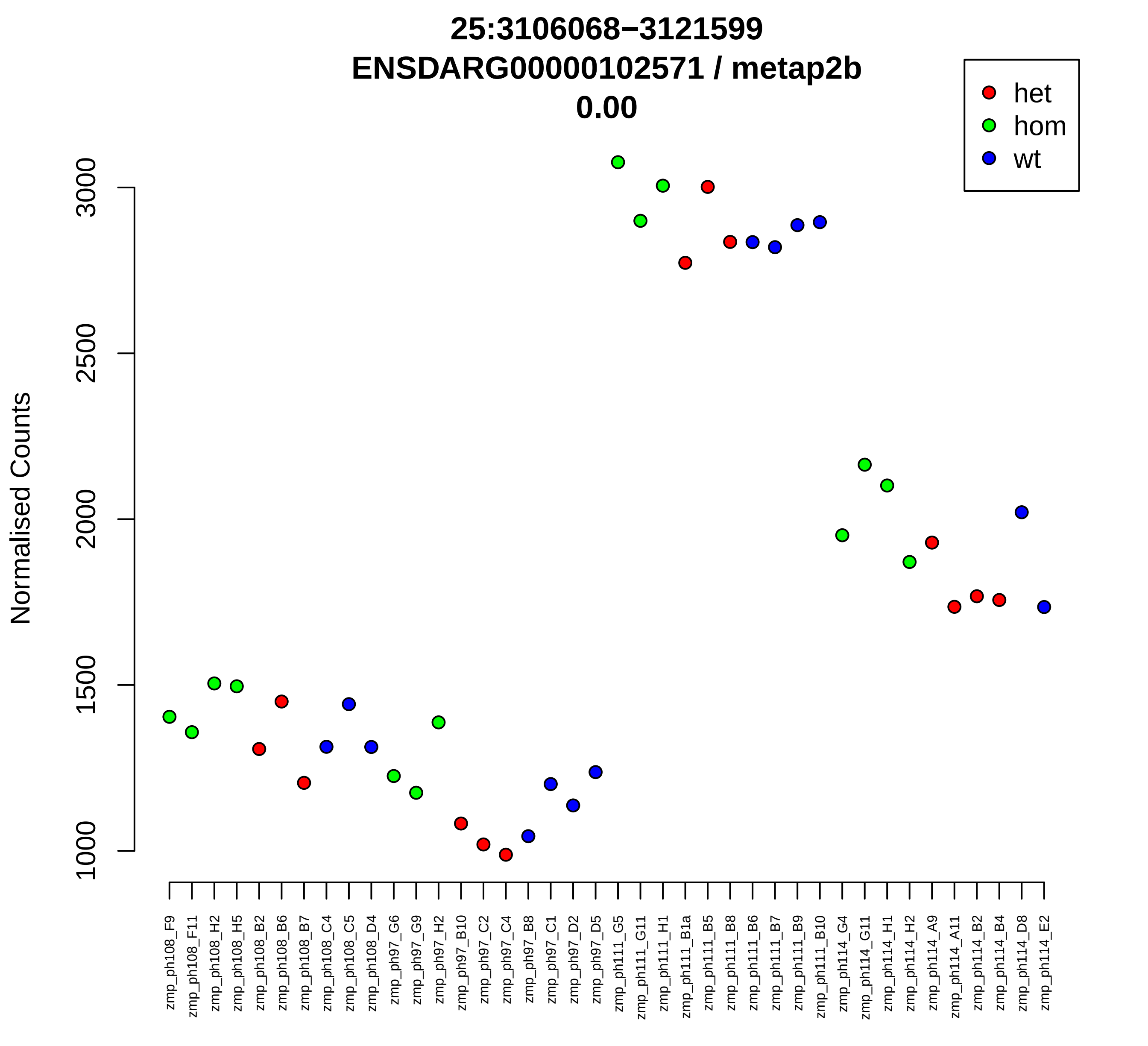
25:3106068−3121599
ENSDARG00000102571 / metap2b
0.00
het
hom
wt
Normalised Counts
1000
1500
2000
2500
3000
zmp_ph108_F9
zmp_ph108_F11
zmp_ph108_H2
zmp_ph108_H5
zmp_ph108_B2
zmp_ph108_B6
zmp_ph108_B7
zmp_ph108_C4
zmp_ph108_C5
zmp_ph108_D4
zmp_ph97_G6
zmp_ph97_G9
zmp_ph97_H2
zmp_ph97_B10
zmp_ph97_C2
zmp_ph97_C4
zmp_ph97_B8
zmp_ph97_C1
zmp_ph97_D2
zmp_ph97_D5
zmp_ph111_G5
zmp_ph111_G11
zmp_ph111_H1
zmp_ph111_B1a
zmp_ph111_B5
zmp_ph111_B8
zmp_ph111_B6
zmp_ph111_B7
zmp_ph111_B9
zmp_ph111_B10
zmp_ph114_G4
zmp_ph114_G11
zmp_ph114_H1
zmp_ph114_H2
zmp_ph114_A9
zmp_ph114_A11
zmp_ph114_B2
zmp_ph114_B4
zmp_ph114_D8
zmp_ph114_E2

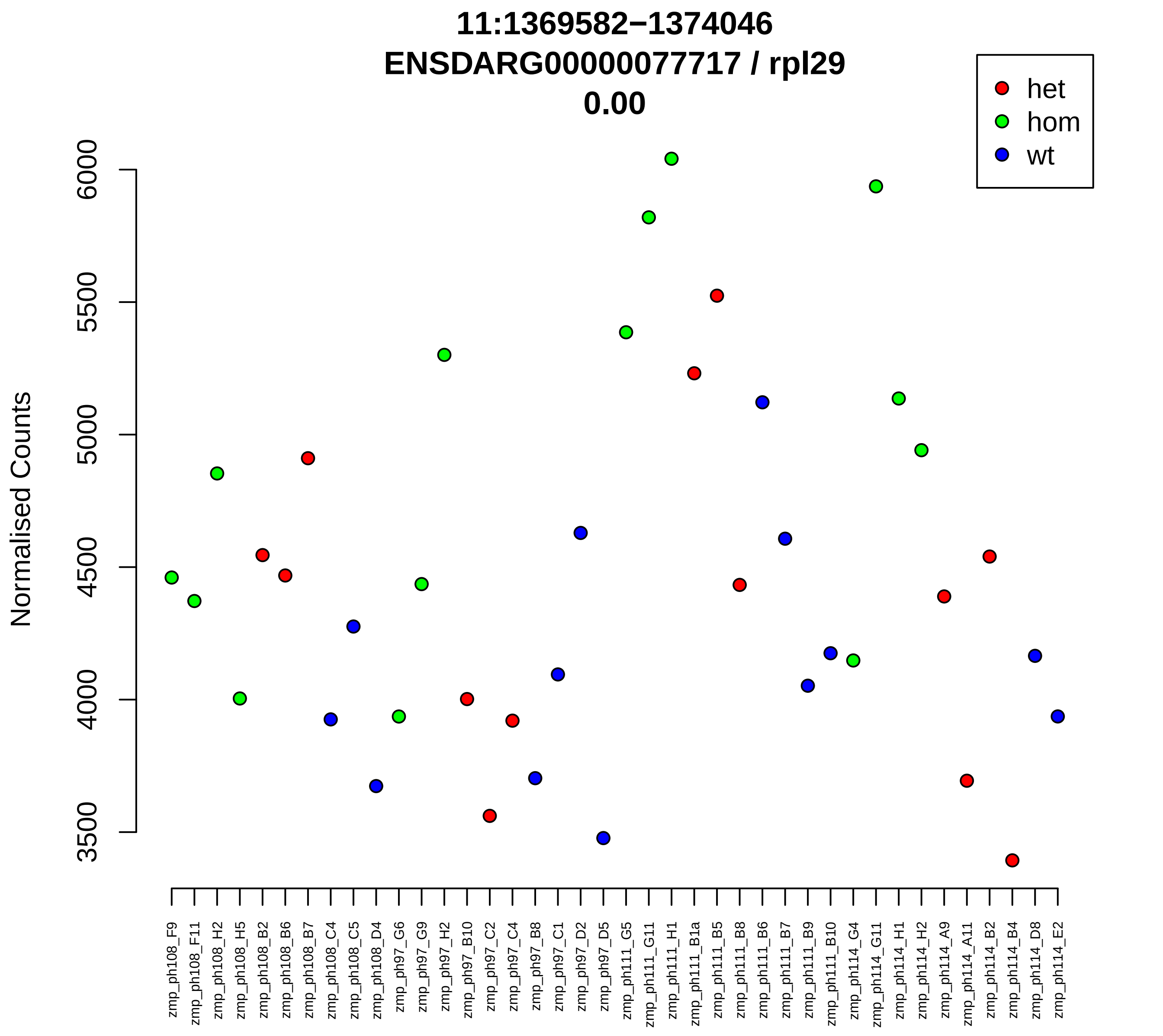
11:1369582−1374046
ENSDARG00000077717 / rpl29
0.00
het
hom
wt
Normalised Counts
3500
4000
4500
5000
5500
6000
zmp_ph108_F9
zmp_ph108_F11
zmp_ph108_H2
zmp_ph108_H5
zmp_ph108_B2
zmp_ph108_B6
zmp_ph108_B7
zmp_ph108_C4
zmp_ph108_C5
zmp_ph108_D4
zmp_ph97_G6
zmp_ph97_G9
zmp_ph97_H2
zmp_ph97_B10
zmp_ph97_C2
zmp_ph97_C4
zmp_ph97_B8
zmp_ph97_C1
zmp_ph97_D2
zmp_ph97_D5
zmp_ph111_G5
zmp_ph111_G11
zmp_ph111_H1
zmp_ph111_B1a
zmp_ph111_B5
zmp_ph111_B8
zmp_ph111_B6
zmp_ph111_B7
zmp_ph111_B9
zmp_ph111_B10
zmp_ph114_G4
zmp_ph114_G11
zmp_ph114_H1
zmp_ph114_H2
zmp_ph114_A9
zmp_ph114_A11
zmp_ph114_B2
zmp_ph114_B4
zmp_ph114_D8
zmp_ph114_E2

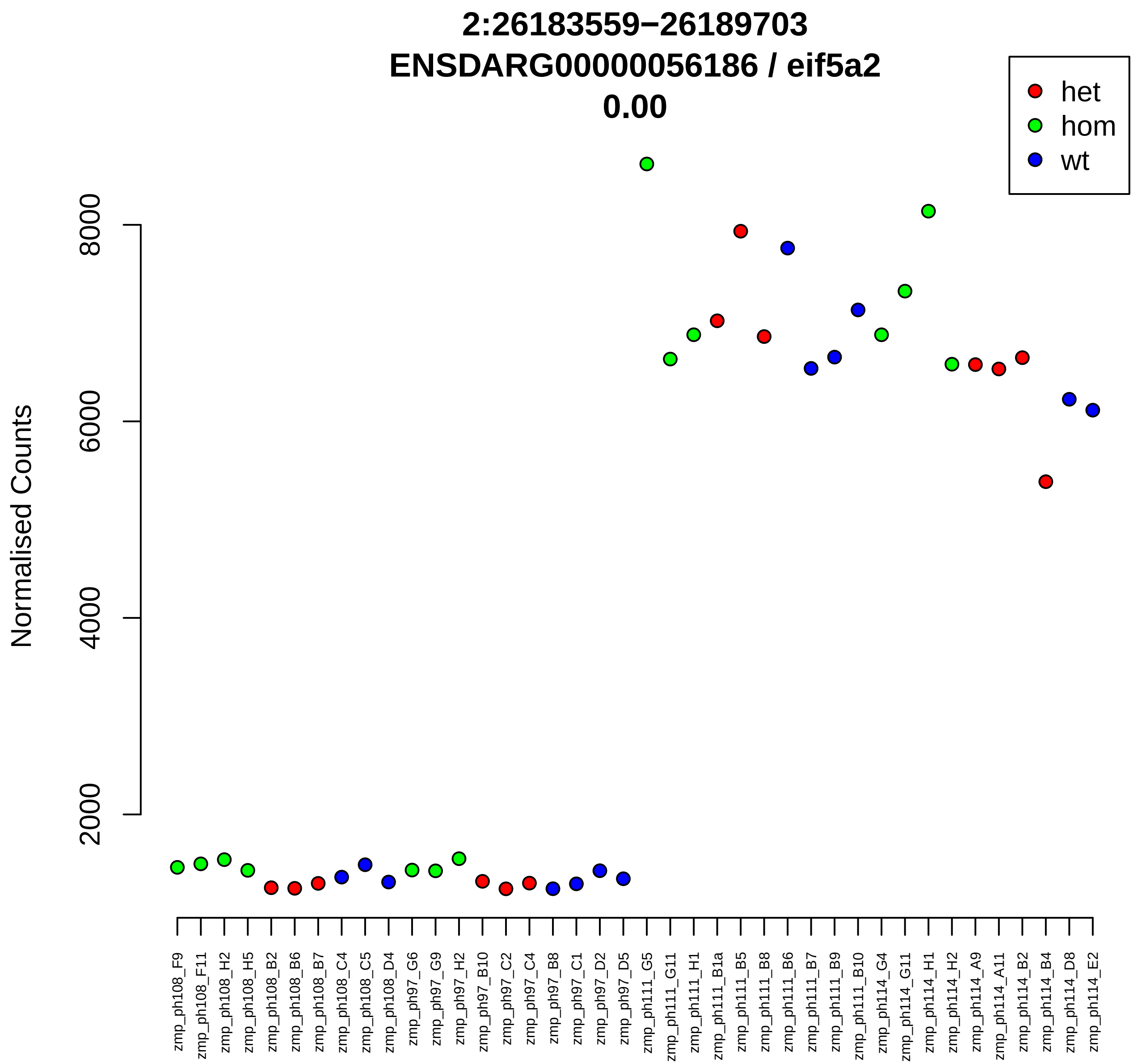
2:26183559−26189703
ENSDARG00000056186 / eif5a2
0.00
het
hom
wt
Normalised Counts
2000
4000
6000
8000
zmp_ph108_F9
zmp_ph108_F11
zmp_ph108_H2
zmp_ph108_H5
zmp_ph108_B2
zmp_ph108_B6
zmp_ph108_B7
zmp_ph108_C4
zmp_ph108_C5
zmp_ph108_D4
zmp_ph97_G6
zmp_ph97_G9
zmp_ph97_H2
zmp_ph97_B10
zmp_ph97_C2
zmp_ph97_C4
zmp_ph97_B8
zmp_ph97_C1
zmp_ph97_D2
zmp_ph97_D5
zmp_ph111_G5
zmp_ph111_G11
zmp_ph111_H1
zmp_ph111_B1a
zmp_ph111_B5
zmp_ph111_B8
zmp_ph111_B6
zmp_ph111_B7
zmp_ph111_B9
zmp_ph111_B10
zmp_ph114_G4
zmp_ph114_G11
zmp_ph114_H1
zmp_ph114_H2
zmp_ph114_A9
zmp_ph114_A11
zmp_ph114_B2
zmp_ph114_B4
zmp_ph114_D8
zmp_ph114_E2

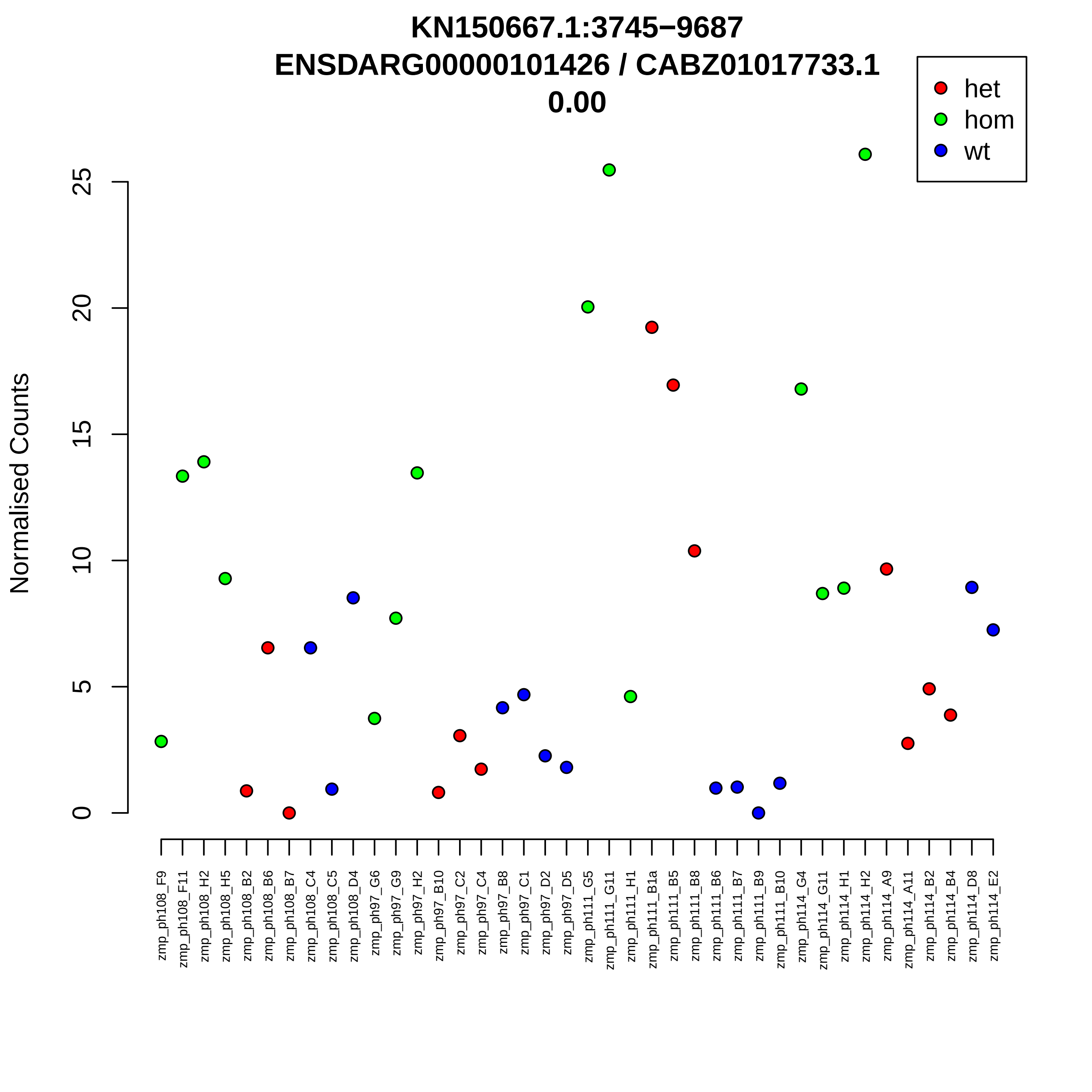

KN150667.1:3745−9687
ENSDARG00000101426 / CABZ01017733.1
0.00
het
hom
wt
Normalised Counts
0
5
10
15
20
25
zmp_ph108_F9
zmp_ph108_F11
zmp_ph108_H2
zmp_ph108_H5
zmp_ph108_B2
zmp_ph108_B6
zmp_ph108_B7
zmp_ph108_C4
zmp_ph108_C5
zmp_ph108_D4
zmp_ph97_G6
zmp_ph97_G9
zmp_ph97_H2
zmp_ph97_B10
zmp_ph97_C2
zmp_ph97_C4
zmp_ph97_B8
zmp_ph97_C1
zmp_ph97_D2
zmp_ph97_D5
zmp_ph111_G5
zmp_ph111_G11
zmp_ph111_H1
zmp_ph111_B1a
zmp_ph111_B5
zmp_ph111_B8
zmp_ph111_B6
zmp_ph111_B7
zmp_ph111_B9
zmp_ph111_B10
zmp_ph114_G4
zmp_ph114_G11
zmp_ph114_H1
zmp_ph114_H2
zmp_ph114_A9
zmp_ph114_A11
zmp_ph114_B2
zmp_ph114_B4
zmp_ph114_D8
zmp_ph114_E2

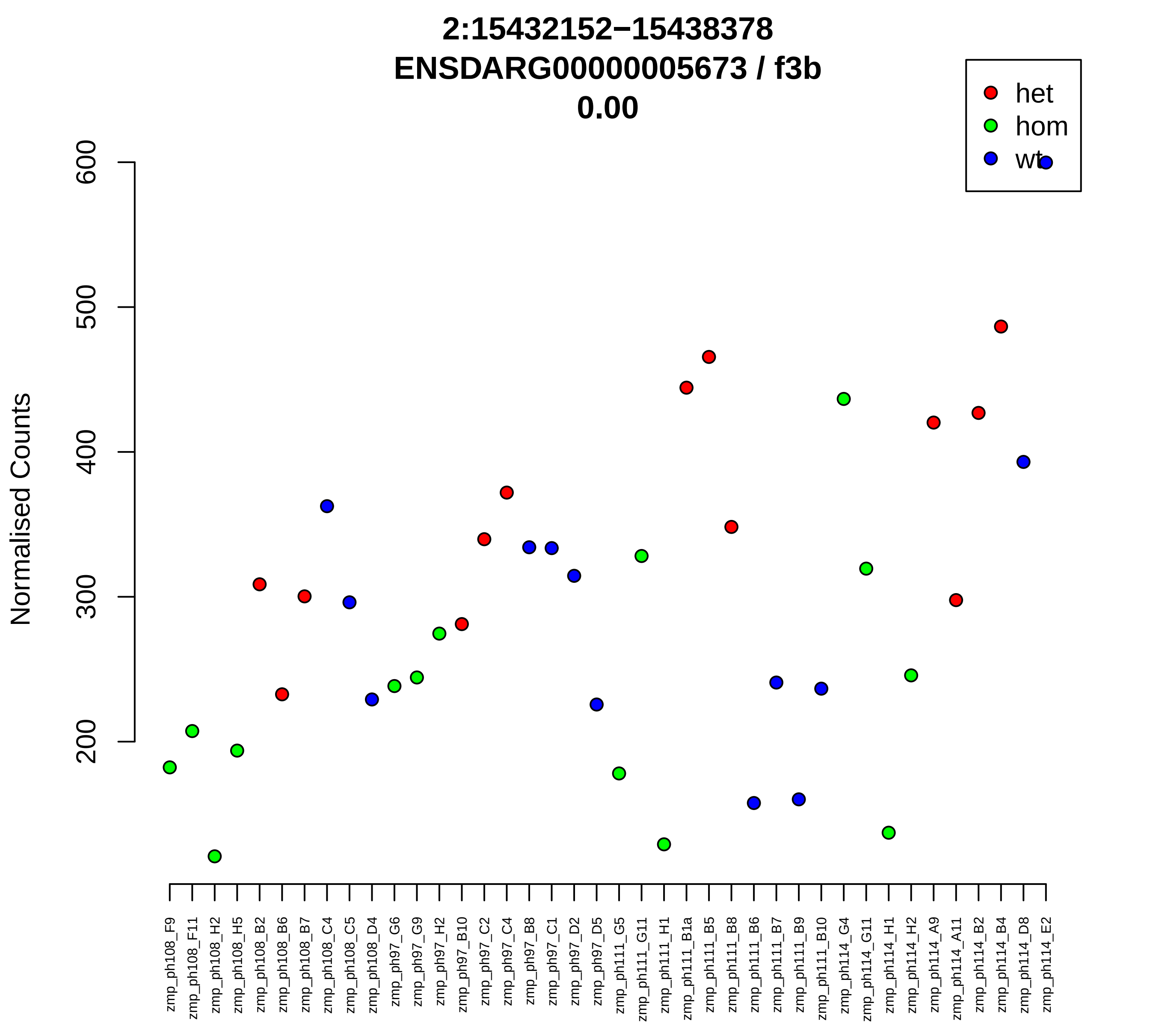
2:15432152−15438378
ENSDARG00000005673 / f3b
0.00
het
hom
wt
Normalised Counts
200
300
400
500
600
zmp_ph108_F9
zmp_ph108_F11
zmp_ph108_H2
zmp_ph108_H5
zmp_ph108_B2
zmp_ph108_B6
zmp_ph108_B7
zmp_ph108_C4
zmp_ph108_C5
zmp_ph108_D4
zmp_ph97_G6
zmp_ph97_G9
zmp_ph97_H2
zmp_ph97_B10
zmp_ph97_C2
zmp_ph97_C4
zmp_ph97_B8
zmp_ph97_C1
zmp_ph97_D2
zmp_ph97_D5
zmp_ph111_G5
zmp_ph111_G11
zmp_ph111_H1
zmp_ph111_B1a
zmp_ph111_B5
zmp_ph111_B8
zmp_ph111_B6
zmp_ph111_B7
zmp_ph111_B9
zmp_ph111_B10
zmp_ph114_G4
zmp_ph114_G11
zmp_ph114_H1
zmp_ph114_H2
zmp_ph114_A9
zmp_ph114_A11
zmp_ph114_B2
zmp_ph114_B4
zmp_ph114_D8
zmp_ph114_E2

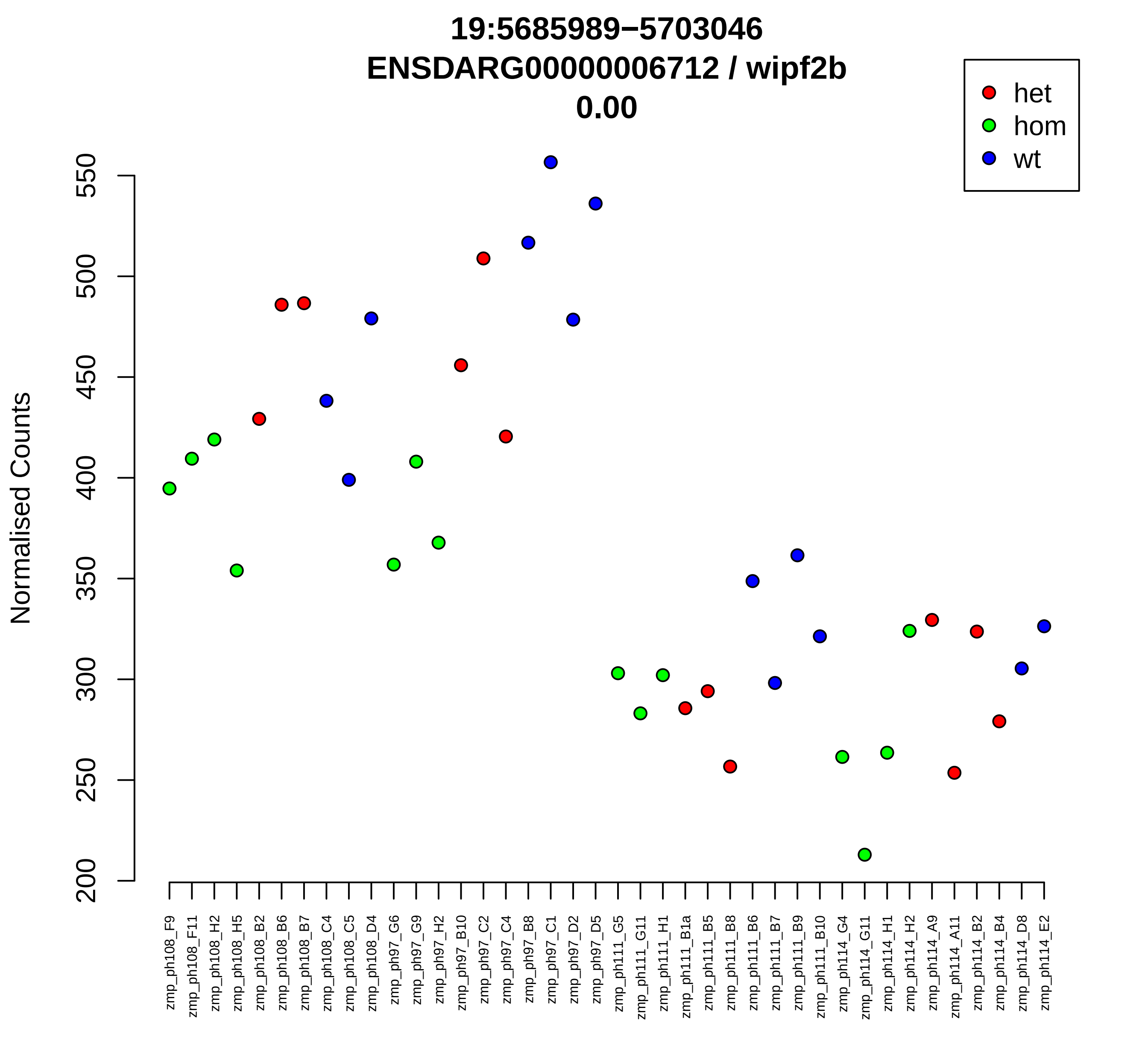
19:5685989−5703046
ENSDARG00000006712 / wipf2b
0.00
het
hom
wt
Normalised Counts
200
250
300
350
400
450
500
550
zmp_ph108_F9
zmp_ph108_F11
zmp_ph108_H2
zmp_ph108_H5
zmp_ph108_B2
zmp_ph108_B6
zmp_ph108_B7
zmp_ph108_C4
zmp_ph108_C5
zmp_ph108_D4
zmp_ph97_G6
zmp_ph97_G9
zmp_ph97_H2
zmp_ph97_B10
zmp_ph97_C2
zmp_ph97_C4
zmp_ph97_B8
zmp_ph97_C1
zmp_ph97_D2
zmp_ph97_D5
zmp_ph111_G5
zmp_ph111_G11
zmp_ph111_H1
zmp_ph111_B1a
zmp_ph111_B5
zmp_ph111_B8
zmp_ph111_B6
zmp_ph111_B7
zmp_ph111_B9
zmp_ph111_B10
zmp_ph114_G4
zmp_ph114_G11
zmp_ph114_H1
zmp_ph114_H2
zmp_ph114_A9
zmp_ph114_A11
zmp_ph114_B2
zmp_ph114_B4
zmp_ph114_D8
zmp_ph114_E2

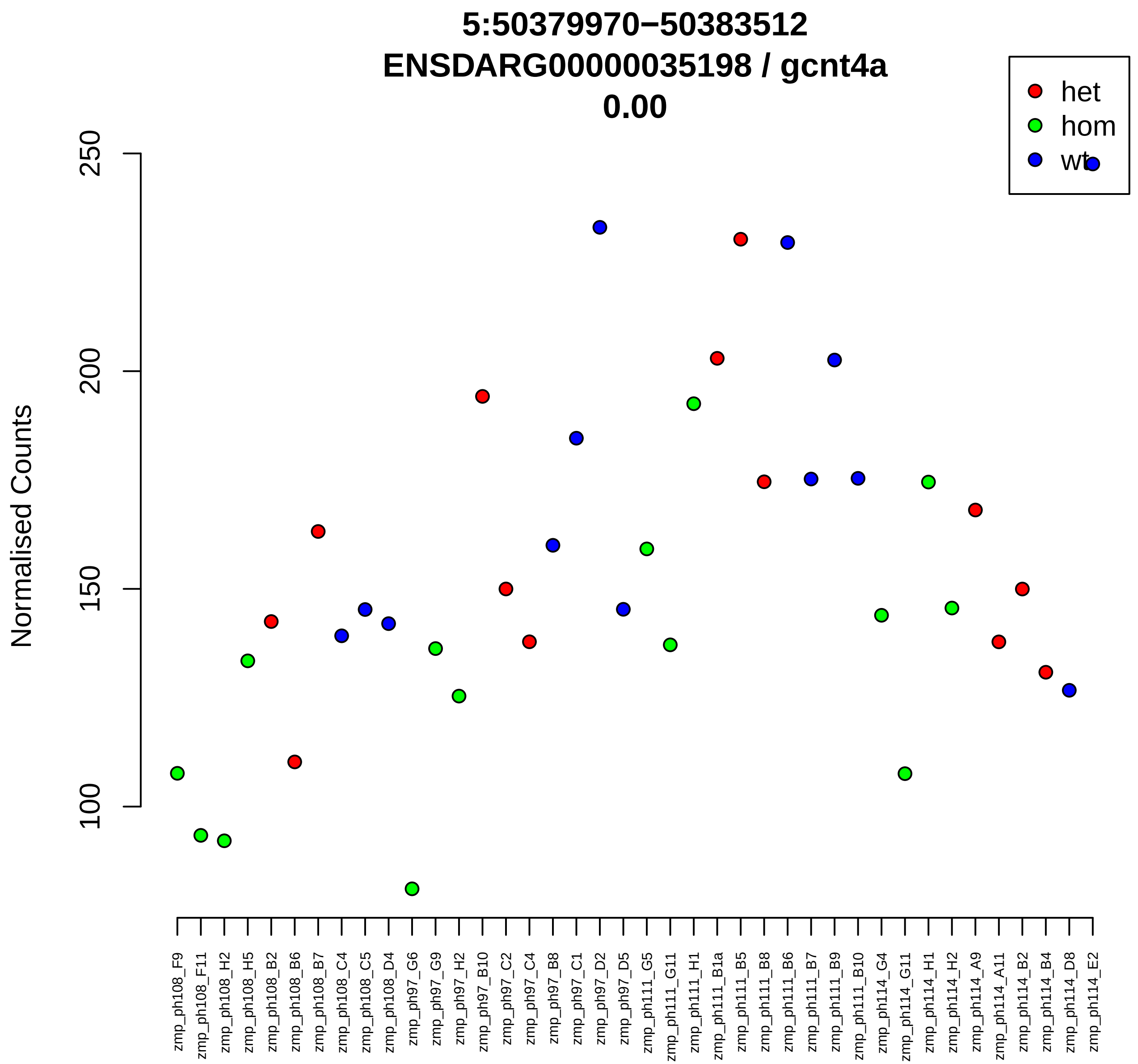
5:50379970−50383512
ENSDARG00000035198 / gcnt4a
0.00
het
hom
wt
Normalised Counts
250
200
150
100
zmp_ph108_F9
zmp_ph108_F11
zmp_ph108_H2
zmp_ph108_H5
zmp_ph108_B2
zmp_ph108_B6
zmp_ph108_B7
zmp_ph108_C4
zmp_ph108_C5
zmp_ph108_D4
zmp_ph97_G6
zmp_ph97_G9
zmp_ph97_H2
zmp_ph97_B10
zmp_ph97_C2
zmp_ph97_C4
zmp_ph97_B8
zmp_ph97_C1
zmp_ph97_D2
zmp_ph97_D5
zmp_ph111_G5
zmp_ph111_G11
zmp_ph111_H1
zmp_ph111_B1a
zmp_ph111_B5
zmp_ph111_B8
zmp_ph111_B6
zmp_ph111_B7
zmp_ph111_B9
zmp_ph111_B10
zmp_ph114_G4
zmp_ph114_G11
zmp_ph114_H1
zmp_ph114_H2
zmp_ph114_A9
zmp_ph114_A11
zmp_ph114_B2
zmp_ph114_B4
zmp_ph114_D8
zmp_ph114_E2

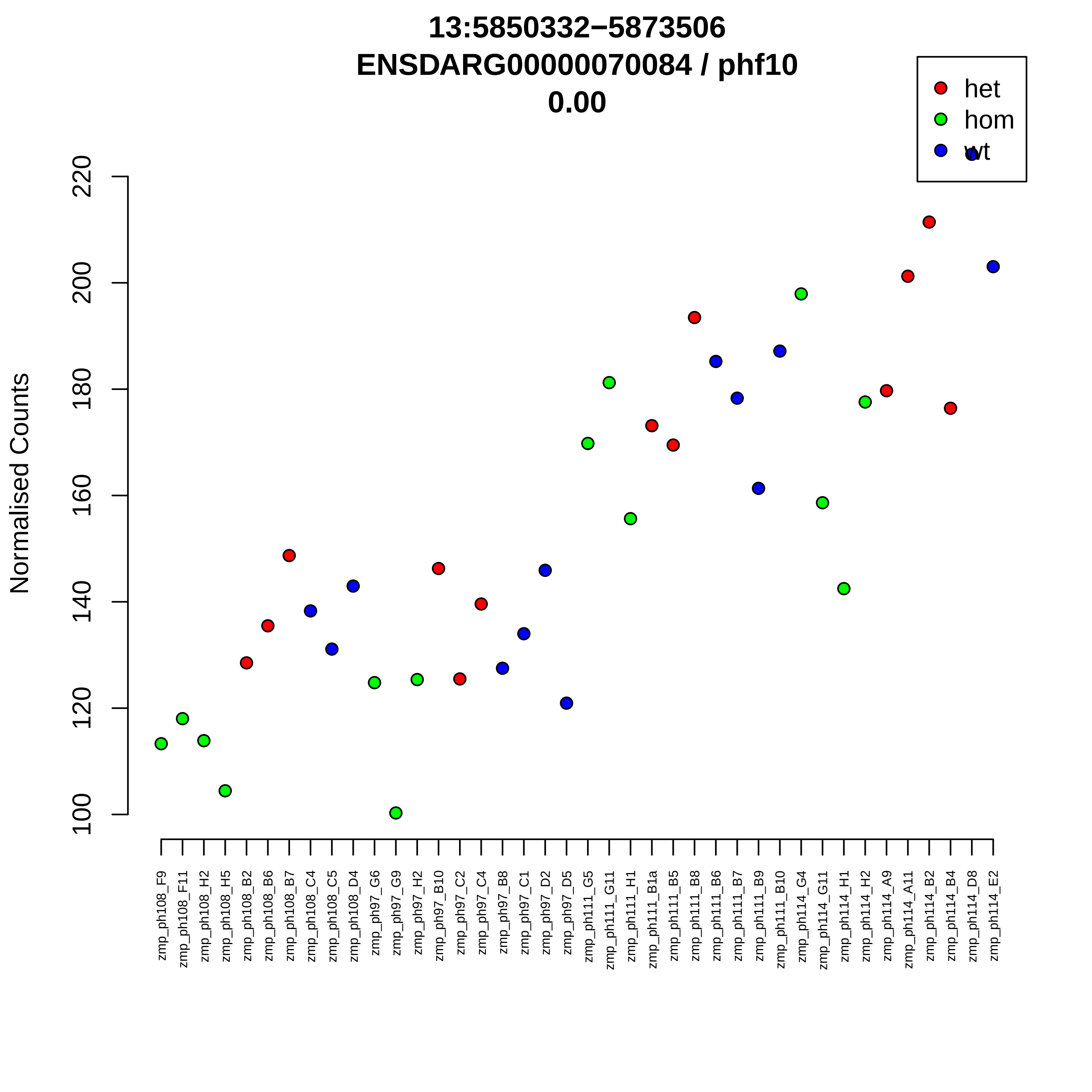

13:5850332−5873506
ENSDARG00000070084 / phf10
0.00
het
hom
wt
Normalised Counts
220
200
180
160
140
120
100
zmp_ph108_F9
zmp_ph108_F11
zmp_ph108_H2
zmp_ph108_H5
zmp_ph108_B2
zmp_ph108_B6
zmp_ph108_B7
zmp_ph108_C4
zmp_ph108_C5
zmp_ph108_D4
zmp_ph97_G6
zmp_ph97_G9
zmp_ph97_H2
zmp_ph97_B10
zmp_ph97_C2
zmp_ph97_C4
zmp_ph97_B8
zmp_ph97_C1
zmp_ph97_D2
zmp_ph97_D5
zmp_ph111_G5
zmp_ph111_G11
zmp_ph111_H1
zmp_ph111_B1a
zmp_ph111_B5
zmp_ph111_B8
zmp_ph111_B6
zmp_ph111_B7
zmp_ph111_B9
zmp_ph111_B10
zmp_ph114_G4
zmp_ph114_G11
zmp_ph114_H1
zmp_ph114_H2
zmp_ph114_A9
zmp_ph114_A11
zmp_ph114_B2
zmp_ph114_B4
zmp_ph114_D8
zmp_ph114_E2

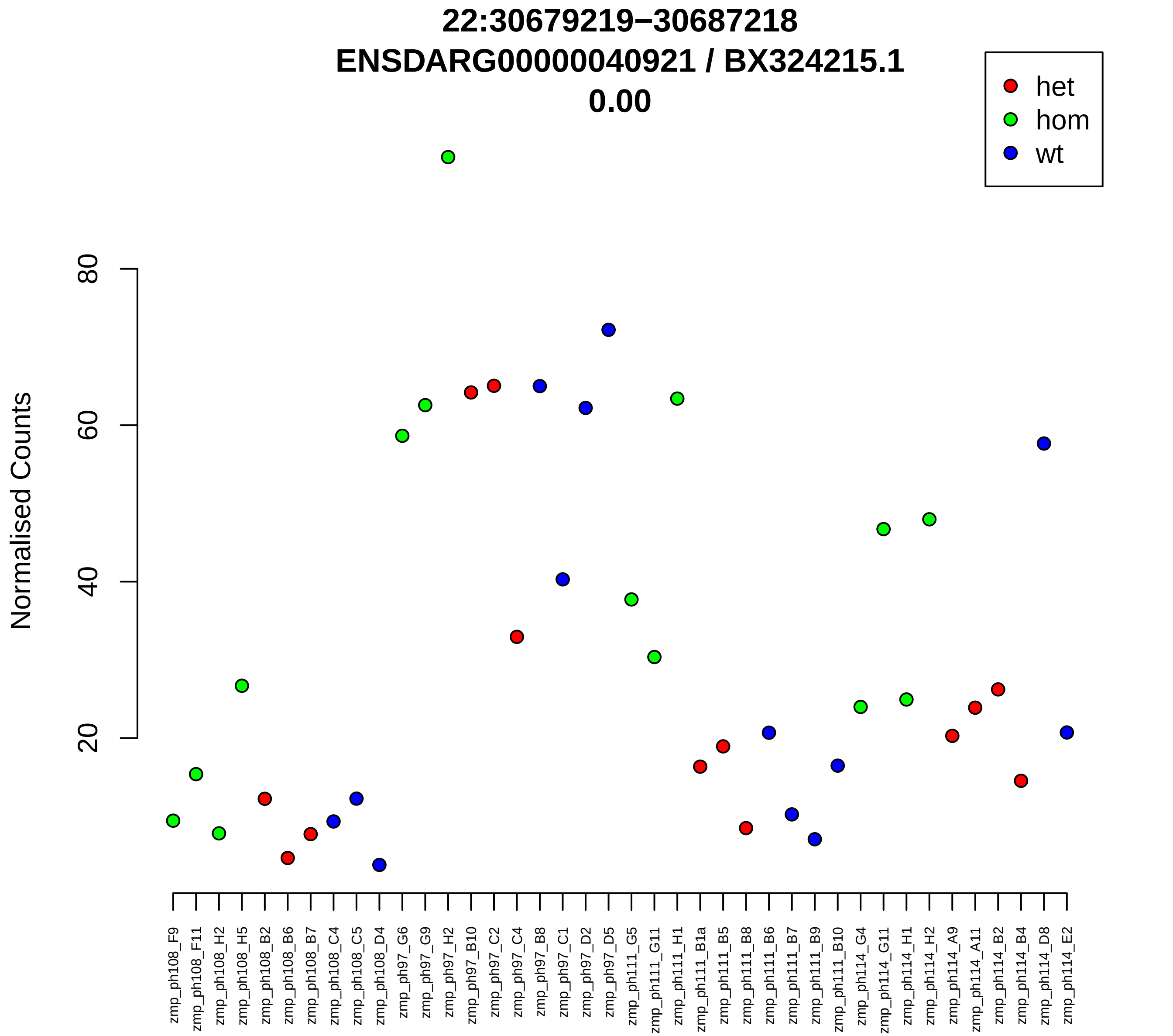
22:30679219−30687218
ENSDARG00000040921 / BX324215.1
0.00
het
hom
wt
Normalised Counts
20
40
60
80
zmp_ph108_F9
zmp_ph108_F11
zmp_ph108_H2
zmp_ph108_H5
zmp_ph108_B2
zmp_ph108_B6
zmp_ph108_B7
zmp_ph108_C4
zmp_ph108_C5
zmp_ph108_D4
zmp_ph97_G6
zmp_ph97_G9
zmp_ph97_H2
zmp_ph97_B10
zmp_ph97_C2
zmp_ph97_C4
zmp_ph97_B8
zmp_ph97_C1
zmp_ph97_D2
zmp_ph97_D5
zmp_ph111_G5
zmp_ph111_G11
zmp_ph111_H1
zmp_ph111_B1a
zmp_ph111_B5
zmp_ph111_B8
zmp_ph111_B6
zmp_ph111_B7
zmp_ph111_B9
zmp_ph111_B10
zmp_ph114_G4
zmp_ph114_G11
zmp_ph114_H1
zmp_ph114_H2
zmp_ph114_A9
zmp_ph114_A11
zmp_ph114_B2
zmp_ph114_B4
zmp_ph114_D8
zmp_ph114_E2

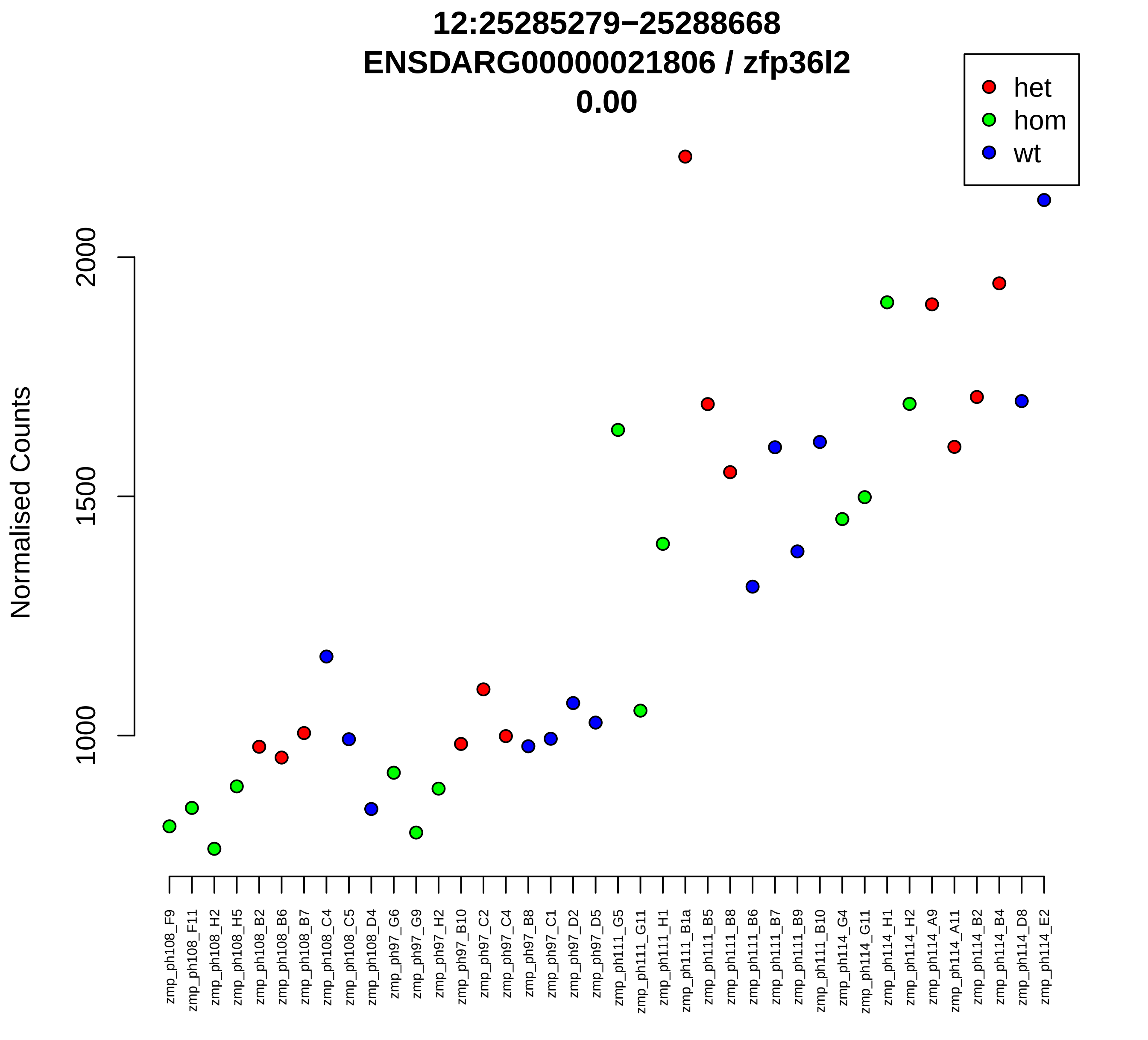
12:25285279−25288668
ENSDARG00000021806 / zfp36l2
0.00
het
hom
wt
Normalised Counts
1000
1500
2000
zmp_ph108_F9
zmp_ph108_F11
zmp_ph108_H2
zmp_ph108_H5
zmp_ph108_B2
zmp_ph108_B6
zmp_ph108_B7
zmp_ph108_C4
zmp_ph108_C5
zmp_ph108_D4
zmp_ph97_G6
zmp_ph97_G9
zmp_ph97_H2
zmp_ph97_B10
zmp_ph97_C2
zmp_ph97_C4
zmp_ph97_B8
zmp_ph97_C1
zmp_ph97_D2
zmp_ph97_D5
zmp_ph111_G5
zmp_ph111_G11
zmp_ph111_H1
zmp_ph111_B1a
zmp_ph111_B5
zmp_ph111_B8
zmp_ph111_B6
zmp_ph111_B7
zmp_ph111_B9
zmp_ph111_B10
zmp_ph114_G4
zmp_ph114_G11
zmp_ph114_H1
zmp_ph114_H2
zmp_ph114_A9
zmp_ph114_A11
zmp_ph114_B2
zmp_ph114_B4
zmp_ph114_D8
zmp_ph114_E2

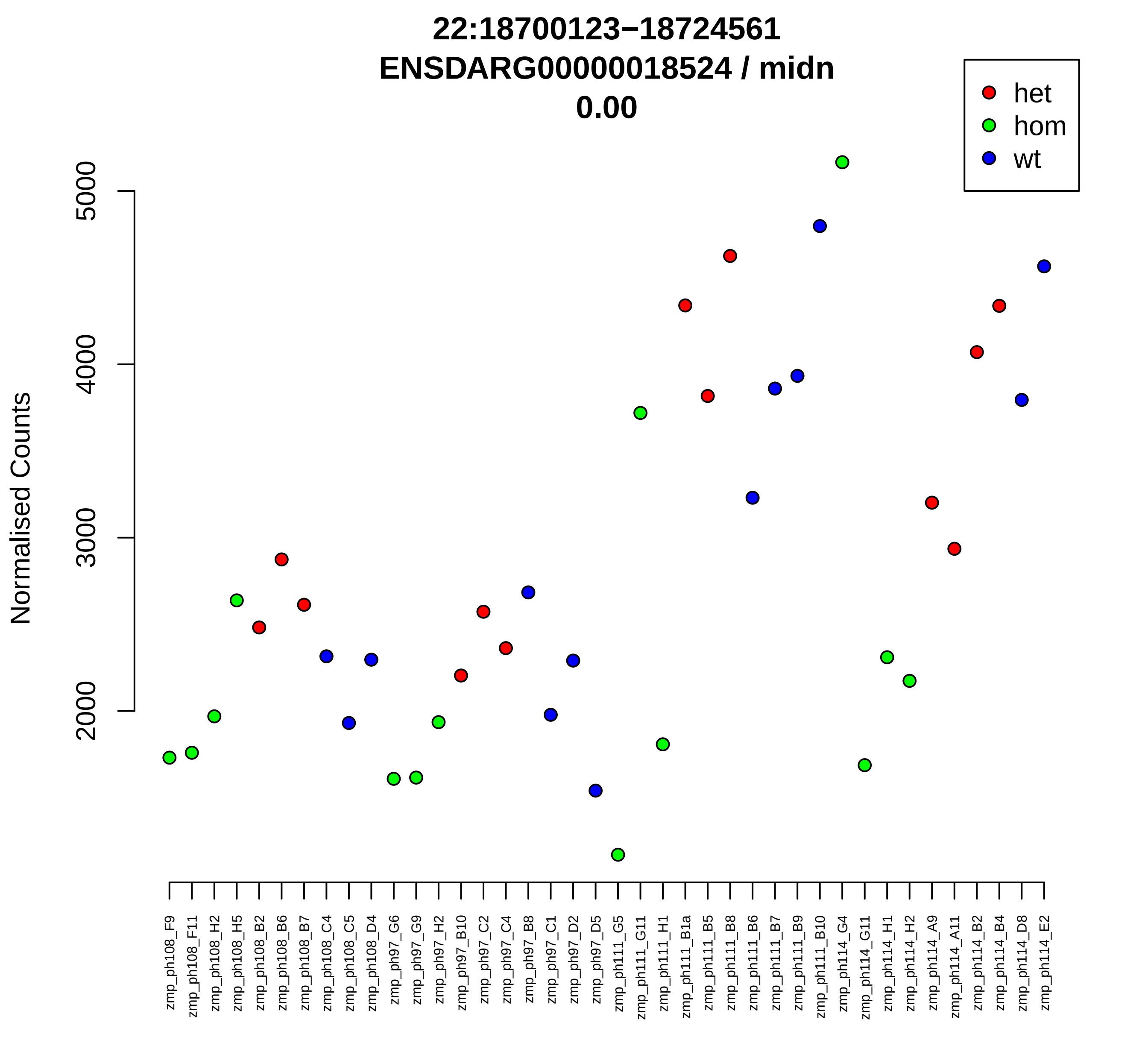
22:18700123−18724561
ENSDARG00000018524 / midn
0.00
het
hom
wt
Normalised Counts
2000
3000
4000
5000
zmp_ph108_F9
zmp_ph108_F11
zmp_ph108_H2
zmp_ph108_H5
zmp_ph108_B2
zmp_ph108_B6
zmp_ph108_B7
zmp_ph108_C4
zmp_ph108_C5
zmp_ph108_D4
zmp_ph97_G6
zmp_ph97_G9
zmp_ph97_H2
zmp_ph97_B10
zmp_ph97_C2
zmp_ph97_C4
zmp_ph97_B8
zmp_ph97_C1
zmp_ph97_D2
zmp_ph97_D5
zmp_ph111_G5
zmp_ph111_G11
zmp_ph111_H1
zmp_ph111_B1a
zmp_ph111_B5
zmp_ph111_B8
zmp_ph111_B6
zmp_ph111_B7
zmp_ph111_B9
zmp_ph111_B10
zmp_ph114_G4
zmp_ph114_G11
zmp_ph114_H1
zmp_ph114_H2
zmp_ph114_A9
zmp_ph114_A11
zmp_ph114_B2
zmp_ph114_B4
zmp_ph114_D8
zmp_ph114_E2

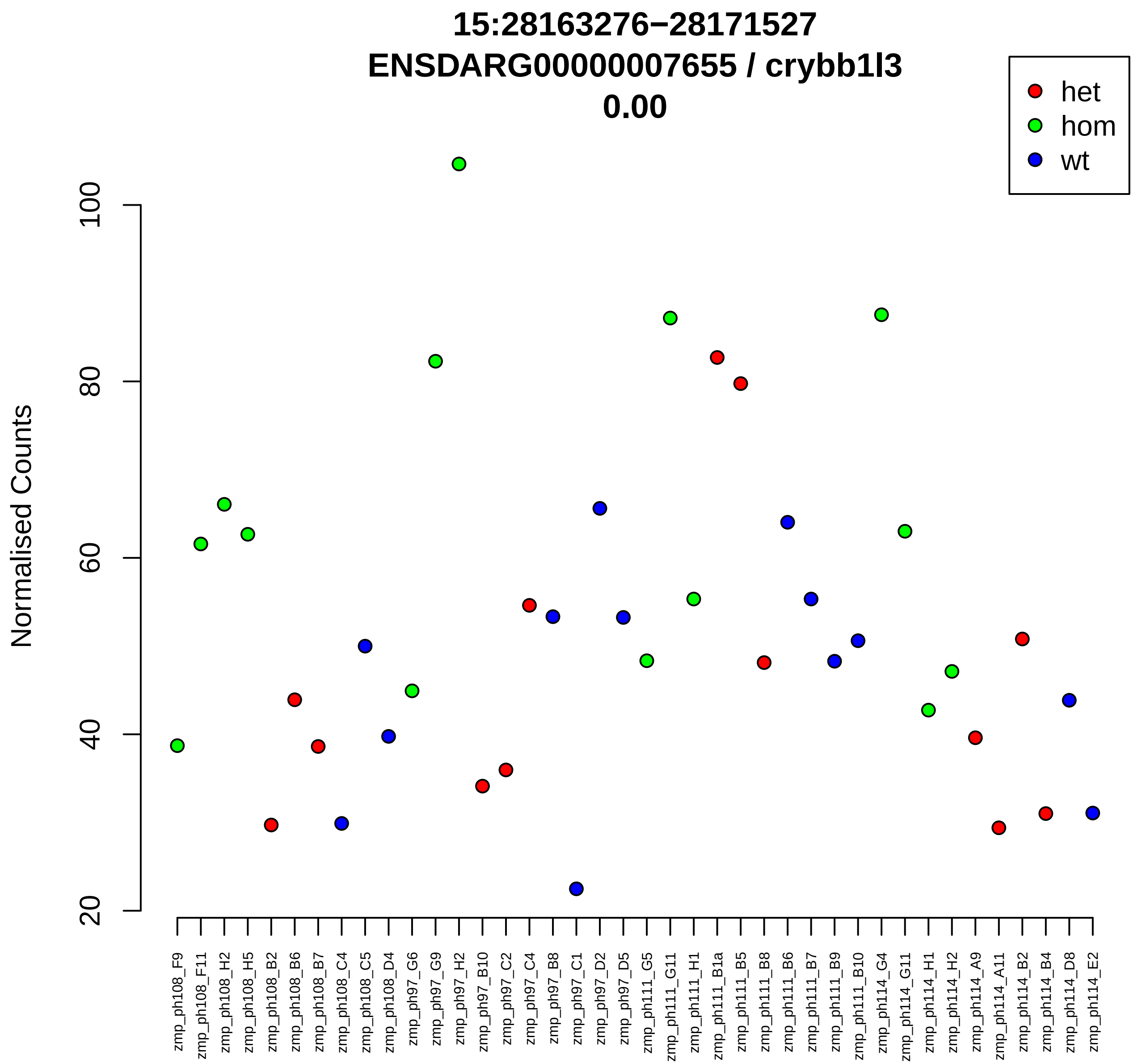

15:28163276−28171527
ENSDARG00000007655 / crybb1l3
0.00
het
hom
wt
Normalised Counts
20
40
60
80
100
zmp_ph108_F9
zmp_ph108_F11
zmp_ph108_H2
zmp_ph108_H5
zmp_ph108_B2
zmp_ph108_B6
zmp_ph108_B7
zmp_ph108_C4
zmp_ph108_C5
zmp_ph108_D4
zmp_ph97_G6
zmp_ph97_G9
zmp_ph97_H2
zmp_ph97_B10
zmp_ph97_C2
zmp_ph97_C4
zmp_ph97_B8
zmp_ph97_C1
zmp_ph97_D2
zmp_ph97_D5
zmp_ph111_G5
zmp_ph111_G11
zmp_ph111_H1
zmp_ph111_B1a
zmp_ph111_B5
zmp_ph111_B8
zmp_ph111_B6
zmp_ph111_B7
zmp_ph111_B9
zmp_ph111_B10
zmp_ph114_G4
zmp_ph114_G11
zmp_ph114_H1
zmp_ph114_H2
zmp_ph114_A9
zmp_ph114_A11
zmp_ph114_B2
zmp_ph114_B4
zmp_ph114_D8
zmp_ph114_E2

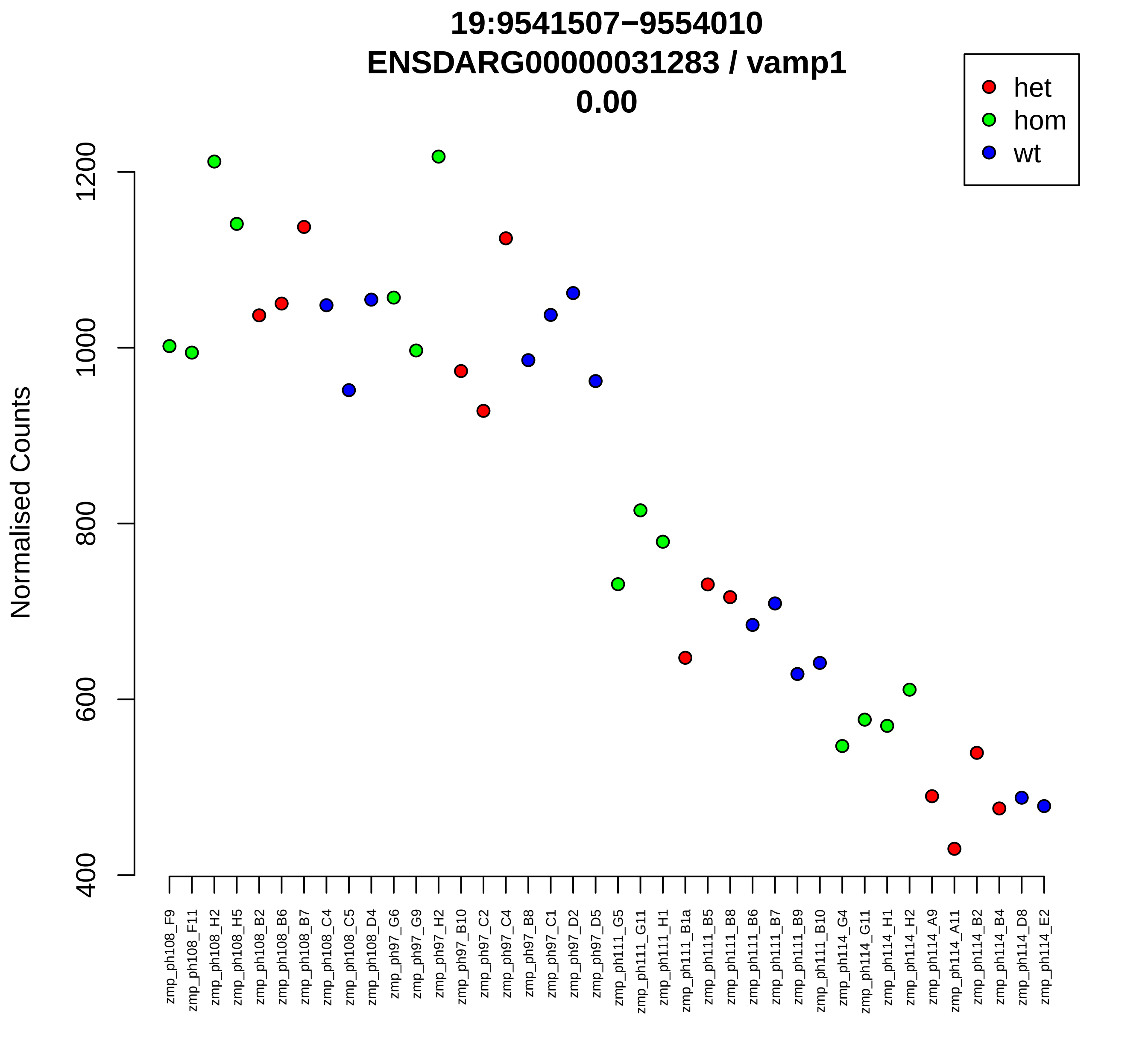
19:9541507−9554010
ENSDARG00000031283 / vamp1
0.00
het
hom
wt
Normalised Counts
400
600
800
1000
1200
zmp_ph108_F9
zmp_ph108_F11
zmp_ph108_H2
zmp_ph108_H5
zmp_ph108_B2
zmp_ph108_B6
zmp_ph108_B7
zmp_ph108_C4
zmp_ph108_C5
zmp_ph108_D4
zmp_ph97_G6
zmp_ph97_G9
zmp_ph97_H2
zmp_ph97_B10
zmp_ph97_C2
zmp_ph97_C4
zmp_ph97_B8
zmp_ph97_C1
zmp_ph97_D2
zmp_ph97_D5
zmp_ph111_G5
zmp_ph111_G11
zmp_ph111_H1
zmp_ph111_B1a
zmp_ph111_B5
zmp_ph111_B8
zmp_ph111_B6
zmp_ph111_B7
zmp_ph111_B9
zmp_ph111_B10
zmp_ph114_G4
zmp_ph114_G11
zmp_ph114_H1
zmp_ph114_H2
zmp_ph114_A9
zmp_ph114_A11
zmp_ph114_B2
zmp_ph114_B4
zmp_ph114_D8
zmp_ph114_E2

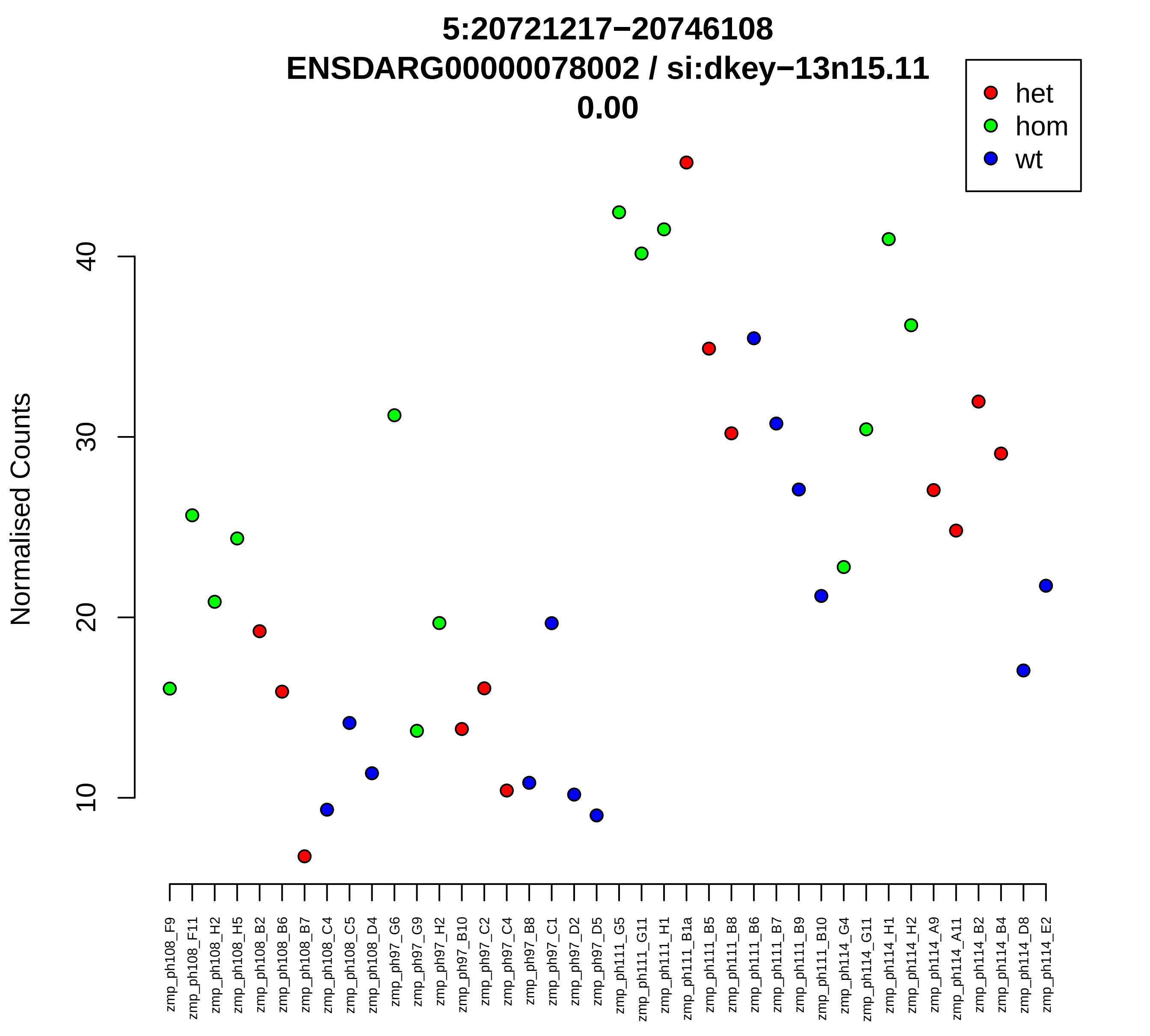

5:20721217−20746108
ENSDARG00000078002 / si:dkey−13n15.11
0.00
het
hom
wt
Normalised Counts
10
20
30
40
zmp_ph108_F9
zmp_ph108_F11
zmp_ph108_H2
zmp_ph108_H5
zmp_ph108_B2
zmp_ph108_B6
zmp_ph108_B7
zmp_ph108_C4
zmp_ph108_C5
zmp_ph108_D4
zmp_ph97_G6
zmp_ph97_G9
zmp_ph97_H2
zmp_ph97_B10
zmp_ph97_C2
zmp_ph97_C4
zmp_ph97_B8
zmp_ph97_C1
zmp_ph97_D2
zmp_ph97_D5
zmp_ph111_G5
zmp_ph111_G11
zmp_ph111_H1
zmp_ph111_B1a
zmp_ph111_B5
zmp_ph111_B8
zmp_ph111_B6
zmp_ph111_B7
zmp_ph111_B9
zmp_ph111_B10
zmp_ph114_G4
zmp_ph114_G11
zmp_ph114_H1
zmp_ph114_H2
zmp_ph114_A9
zmp_ph114_A11
zmp_ph114_B2
zmp_ph114_B4
zmp_ph114_D8
zmp_ph114_E2

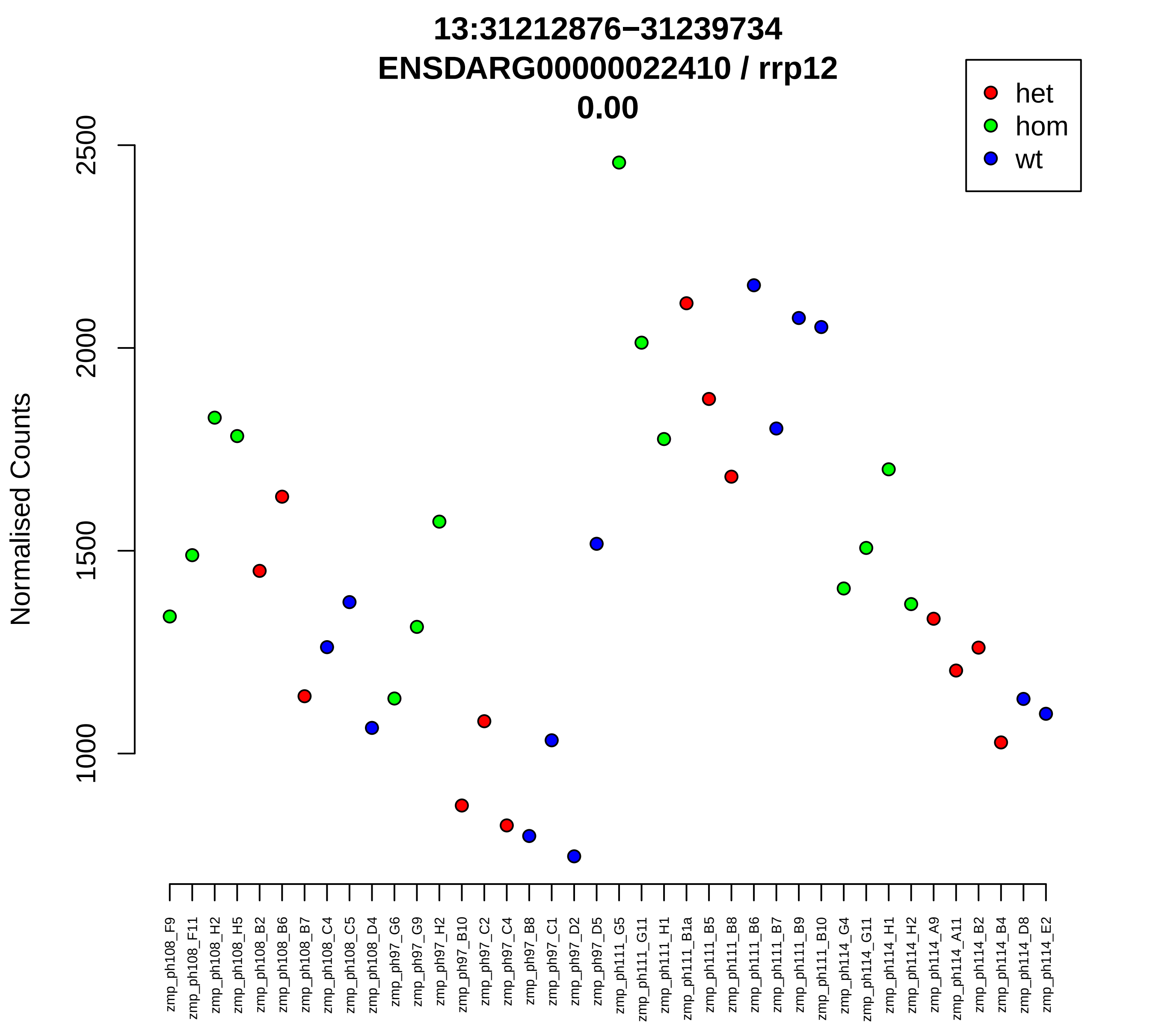

13:31212876−31239734
ENSDARG00000022410 / rrp12
0.00
het
hom
wt
Normalised Counts
1000
1500
2000
2500
zmp_ph108_F9
zmp_ph108_F11
zmp_ph108_H2
zmp_ph108_H5
zmp_ph108_B2
zmp_ph108_B6
zmp_ph108_B7
zmp_ph108_C4
zmp_ph108_C5
zmp_ph108_D4
zmp_ph97_G6
zmp_ph97_G9
zmp_ph97_H2
zmp_ph97_B10
zmp_ph97_C2
zmp_ph97_C4
zmp_ph97_B8
zmp_ph97_C1
zmp_ph97_D2
zmp_ph97_D5
zmp_ph111_G5
zmp_ph111_G11
zmp_ph111_H1
zmp_ph111_B1a
zmp_ph111_B5
zmp_ph111_B8
zmp_ph111_B6
zmp_ph111_B7
zmp_ph111_B9
zmp_ph111_B10
zmp_ph114_G4
zmp_ph114_G11
zmp_ph114_H1
zmp_ph114_H2
zmp_ph114_A9
zmp_ph114_A11
zmp_ph114_B2
zmp_ph114_B4
zmp_ph114_D8
zmp_ph114_E2

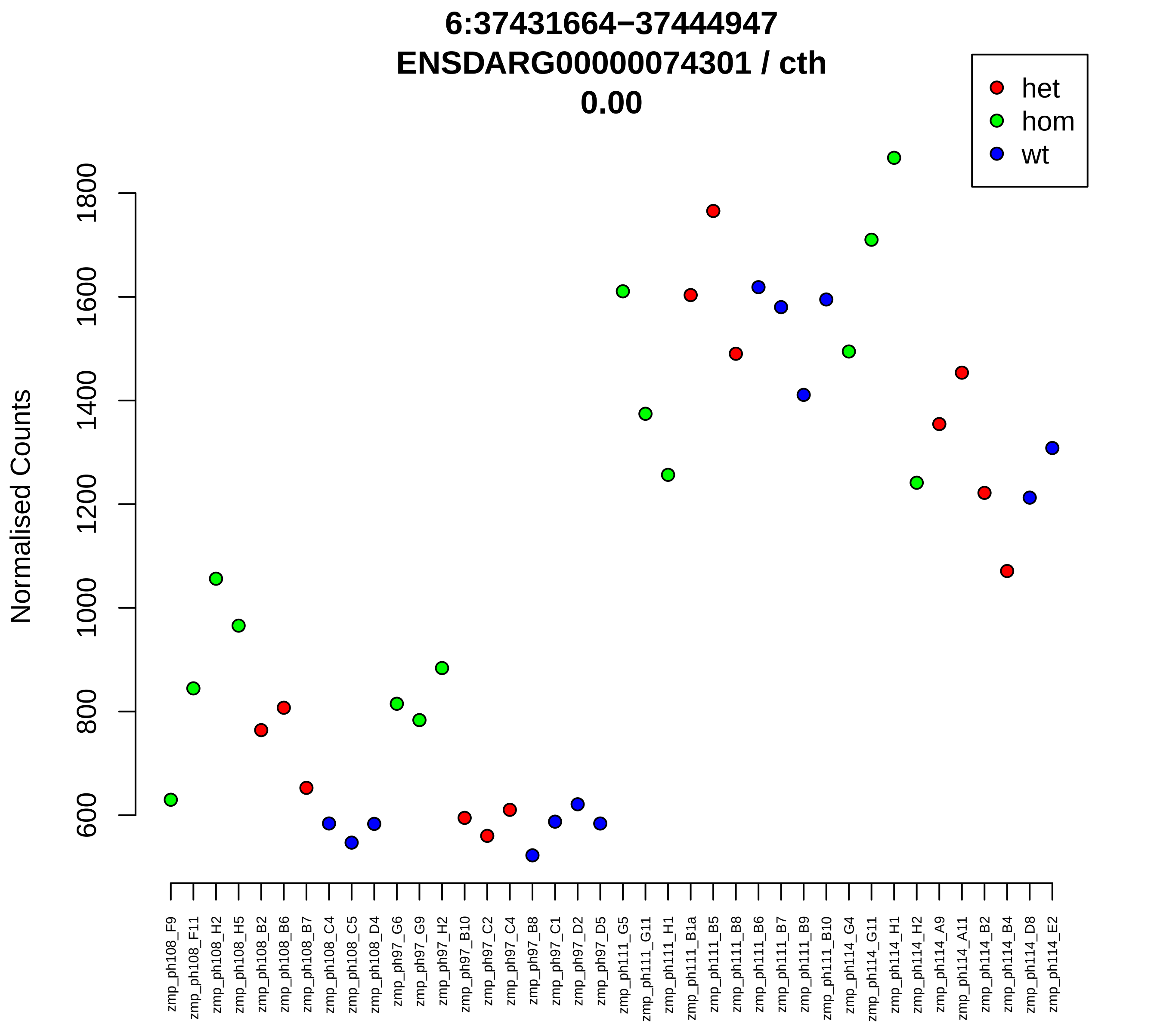

6:37431664−37444947
ENSDARG00000074301 / cth
0.00
het
hom
wt
Normalised Counts
600
800
1000
1200
1400
1600
1800
zmp_ph108_F9
zmp_ph108_F11
zmp_ph108_H2
zmp_ph108_H5
zmp_ph108_B2
zmp_ph108_B6
zmp_ph108_B7
zmp_ph108_C4
zmp_ph108_C5
zmp_ph108_D4
zmp_ph97_G6
zmp_ph97_G9
zmp_ph97_H2
zmp_ph97_B10
zmp_ph97_C2
zmp_ph97_C4
zmp_ph97_B8
zmp_ph97_C1
zmp_ph97_D2
zmp_ph97_D5
zmp_ph111_G5
zmp_ph111_G11
zmp_ph111_H1
zmp_ph111_B1a
zmp_ph111_B5
zmp_ph111_B8
zmp_ph111_B6
zmp_ph111_B7
zmp_ph111_B9
zmp_ph111_B10
zmp_ph114_G4
zmp_ph114_G11
zmp_ph114_H1
zmp_ph114_H2
zmp_ph114_A9
zmp_ph114_A11
zmp_ph114_B2
zmp_ph114_B4
zmp_ph114_D8
zmp_ph114_E2

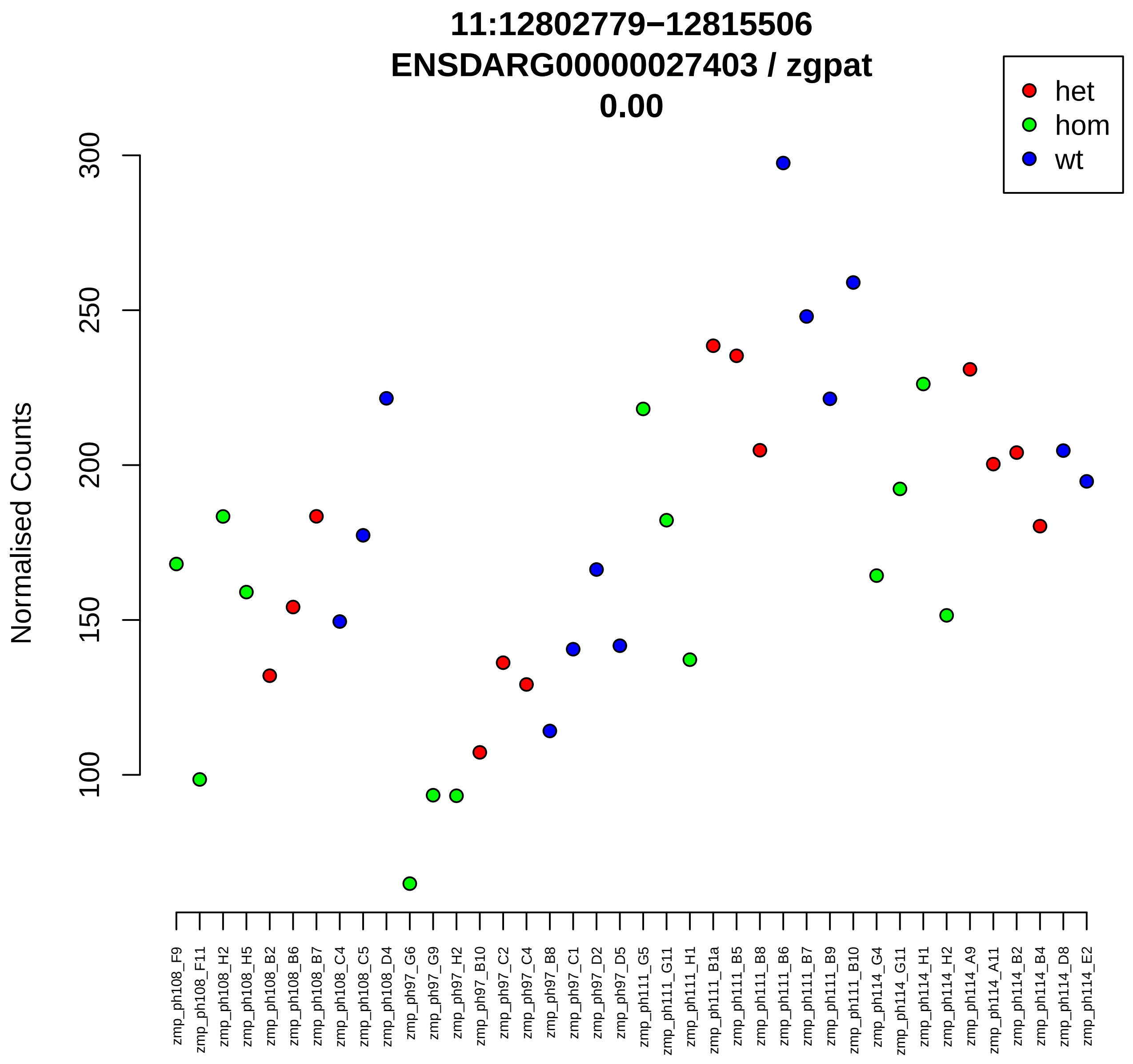

11:12802779−12815506
ENSDARG00000027403 / zgpat
0.00
het
hom
wt
Normalised Counts
100
150
200
250
300
zmp_ph108_F9
zmp_ph108_F11
zmp_ph108_H2
zmp_ph108_H5
zmp_ph108_B2
zmp_ph108_B6
zmp_ph108_B7
zmp_ph108_C4
zmp_ph108_C5
zmp_ph108_D4
zmp_ph97_G6
zmp_ph97_G9
zmp_ph97_H2
zmp_ph97_B10
zmp_ph97_C2
zmp_ph97_C4
zmp_ph97_B8
zmp_ph97_C1
zmp_ph97_D2
zmp_ph97_D5
zmp_ph111_G5
zmp_ph111_G11
zmp_ph111_H1
zmp_ph111_B1a
zmp_ph111_B5
zmp_ph111_B8
zmp_ph111_B6
zmp_ph111_B7
zmp_ph111_B9
zmp_ph111_B10
zmp_ph114_G4
zmp_ph114_G11
zmp_ph114_H1
zmp_ph114_H2
zmp_ph114_A9
zmp_ph114_A11
zmp_ph114_B2
zmp_ph114_B4
zmp_ph114_D8
zmp_ph114_E2

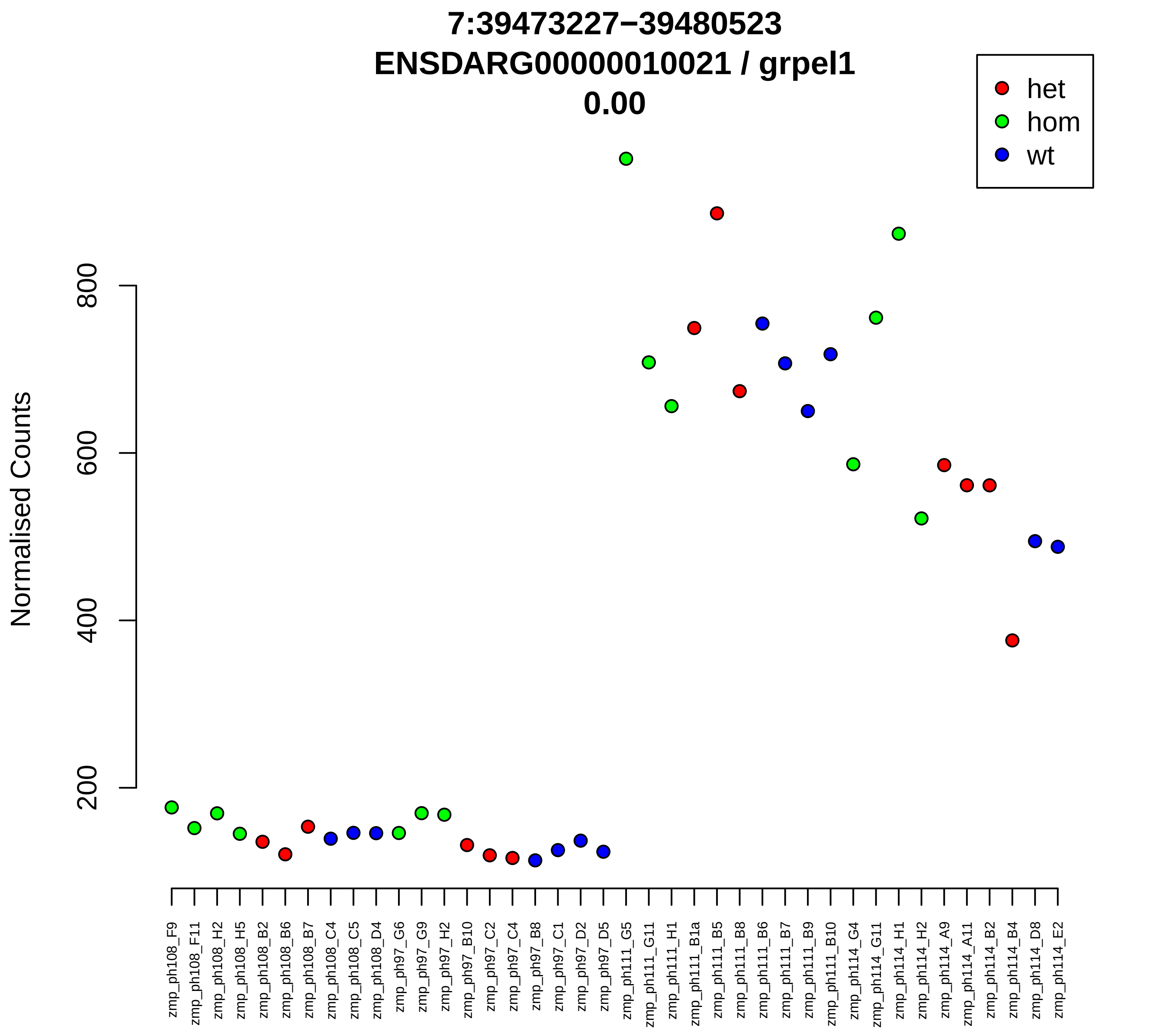

7:39473227−39480523
ENSDARG00000010021 / grpel1
0.00
het
hom
wt
Normalised Counts
200
400
600
800
zmp_ph108_F9
zmp_ph108_F11
zmp_ph108_H2
zmp_ph108_H5
zmp_ph108_B2
zmp_ph108_B6
zmp_ph108_B7
zmp_ph108_C4
zmp_ph108_C5
zmp_ph108_D4
zmp_ph97_G6
zmp_ph97_G9
zmp_ph97_H2
zmp_ph97_B10
zmp_ph97_C2
zmp_ph97_C4
zmp_ph97_B8
zmp_ph97_C1
zmp_ph97_D2
zmp_ph97_D5
zmp_ph111_G5
zmp_ph111_G11
zmp_ph111_H1
zmp_ph111_B1a
zmp_ph111_B5
zmp_ph111_B8
zmp_ph111_B6
zmp_ph111_B7
zmp_ph111_B9
zmp_ph111_B10
zmp_ph114_G4
zmp_ph114_G11
zmp_ph114_H1
zmp_ph114_H2
zmp_ph114_A9
zmp_ph114_A11
zmp_ph114_B2
zmp_ph114_B4
zmp_ph114_D8
zmp_ph114_E2

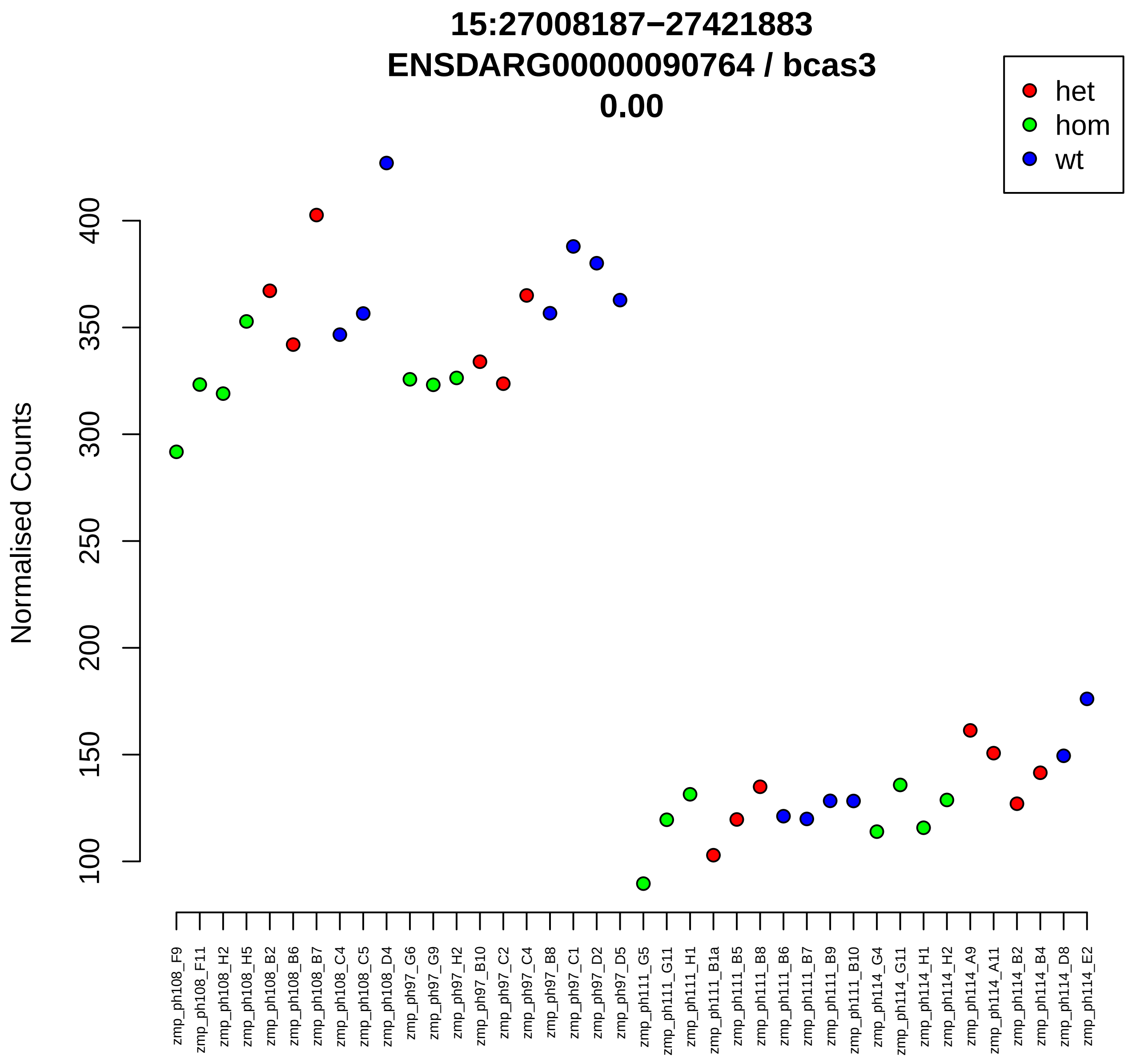

15:27008187−27421883
ENSDARG00000090764 / bcas3
0.00
het
hom
wt
Normalised Counts
100
150
200
250
300
350
400
zmp_ph108_F9
zmp_ph108_F11
zmp_ph108_H2
zmp_ph108_H5
zmp_ph108_B2
zmp_ph108_B6
zmp_ph108_B7
zmp_ph108_C4
zmp_ph108_C5
zmp_ph108_D4
zmp_ph97_G6
zmp_ph97_G9
zmp_ph97_H2
zmp_ph97_B10
zmp_ph97_C2
zmp_ph97_C4
zmp_ph97_B8
zmp_ph97_C1
zmp_ph97_D2
zmp_ph97_D5
zmp_ph111_G5
zmp_ph111_G11
zmp_ph111_H1
zmp_ph111_B1a
zmp_ph111_B5
zmp_ph111_B8
zmp_ph111_B6
zmp_ph111_B7
zmp_ph111_B9
zmp_ph111_B10
zmp_ph114_G4
zmp_ph114_G11
zmp_ph114_H1
zmp_ph114_H2
zmp_ph114_A9
zmp_ph114_A11
zmp_ph114_B2
zmp_ph114_B4
zmp_ph114_D8
zmp_ph114_E2

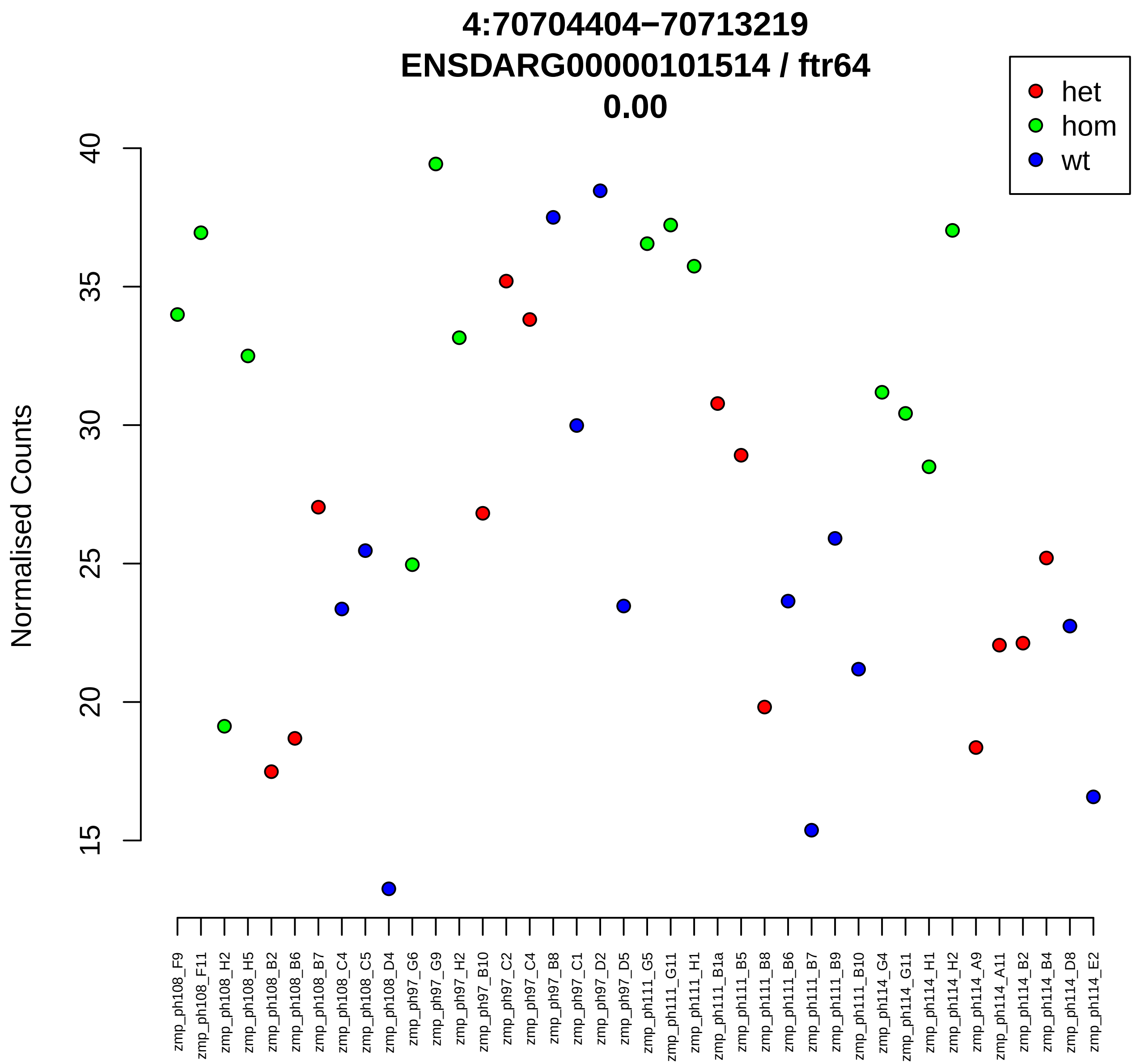
4:70704404−70713219
ENSDARG00000101514 / ftr64
0.00
het
hom
wt
Normalised Counts
15
20
25
30
35
40
zmp_ph108_F9
zmp_ph108_F11
zmp_ph108_H2
zmp_ph108_H5
zmp_ph108_B2
zmp_ph108_B6
zmp_ph108_B7
zmp_ph108_C4
zmp_ph108_C5
zmp_ph108_D4
zmp_ph97_G6
zmp_ph97_G9
zmp_ph97_H2
zmp_ph97_B10
zmp_ph97_C2
zmp_ph97_C4
zmp_ph97_B8
zmp_ph97_C1
zmp_ph97_D2
zmp_ph97_D5
zmp_ph111_G5
zmp_ph111_G11
zmp_ph111_H1
zmp_ph111_B1a
zmp_ph111_B5
zmp_ph111_B8
zmp_ph111_B6
zmp_ph111_B7
zmp_ph111_B9
zmp_ph111_B10
zmp_ph114_G4
zmp_ph114_G11
zmp_ph114_H1
zmp_ph114_H2
zmp_ph114_A9
zmp_ph114_A11
zmp_ph114_B2
zmp_ph114_B4
zmp_ph114_D8
zmp_ph114_E2

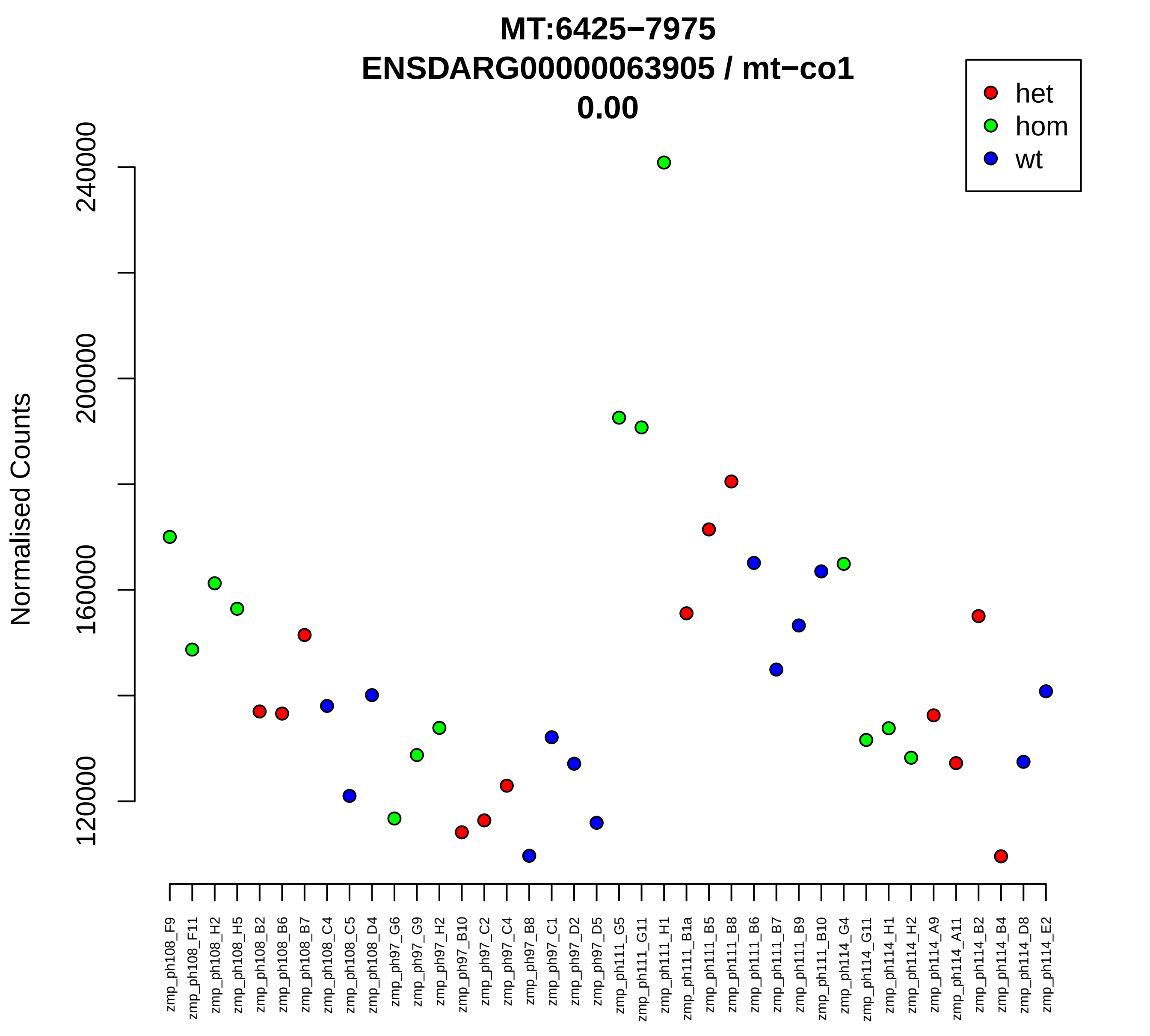
MT:6425−7975
ENSDARG00000063905 / mt−co1
0.00
het
hom
wt
Normalised Counts
120000
160000
200000
240000
zmp_ph108_F9
zmp_ph108_F11
zmp_ph108_H2
zmp_ph108_H5
zmp_ph108_B2
zmp_ph108_B6
zmp_ph108_B7
zmp_ph108_C4
zmp_ph108_C5
zmp_ph108_D4
zmp_ph97_G6
zmp_ph97_G9
zmp_ph97_H2
zmp_ph97_B10
zmp_ph97_C2
zmp_ph97_C4
zmp_ph97_B8
zmp_ph97_C1
zmp_ph97_D2
zmp_ph97_D5
zmp_ph111_G5
zmp_ph111_G11
zmp_ph111_H1
zmp_ph111_B1a
zmp_ph111_B5
zmp_ph111_B8
zmp_ph111_B6
zmp_ph111_B7
zmp_ph111_B9
zmp_ph111_B10
zmp_ph114_G4
zmp_ph114_G11
zmp_ph114_H1
zmp_ph114_H2
zmp_ph114_A9
zmp_ph114_A11
zmp_ph114_B2
zmp_ph114_B4
zmp_ph114_D8
zmp_ph114_E2

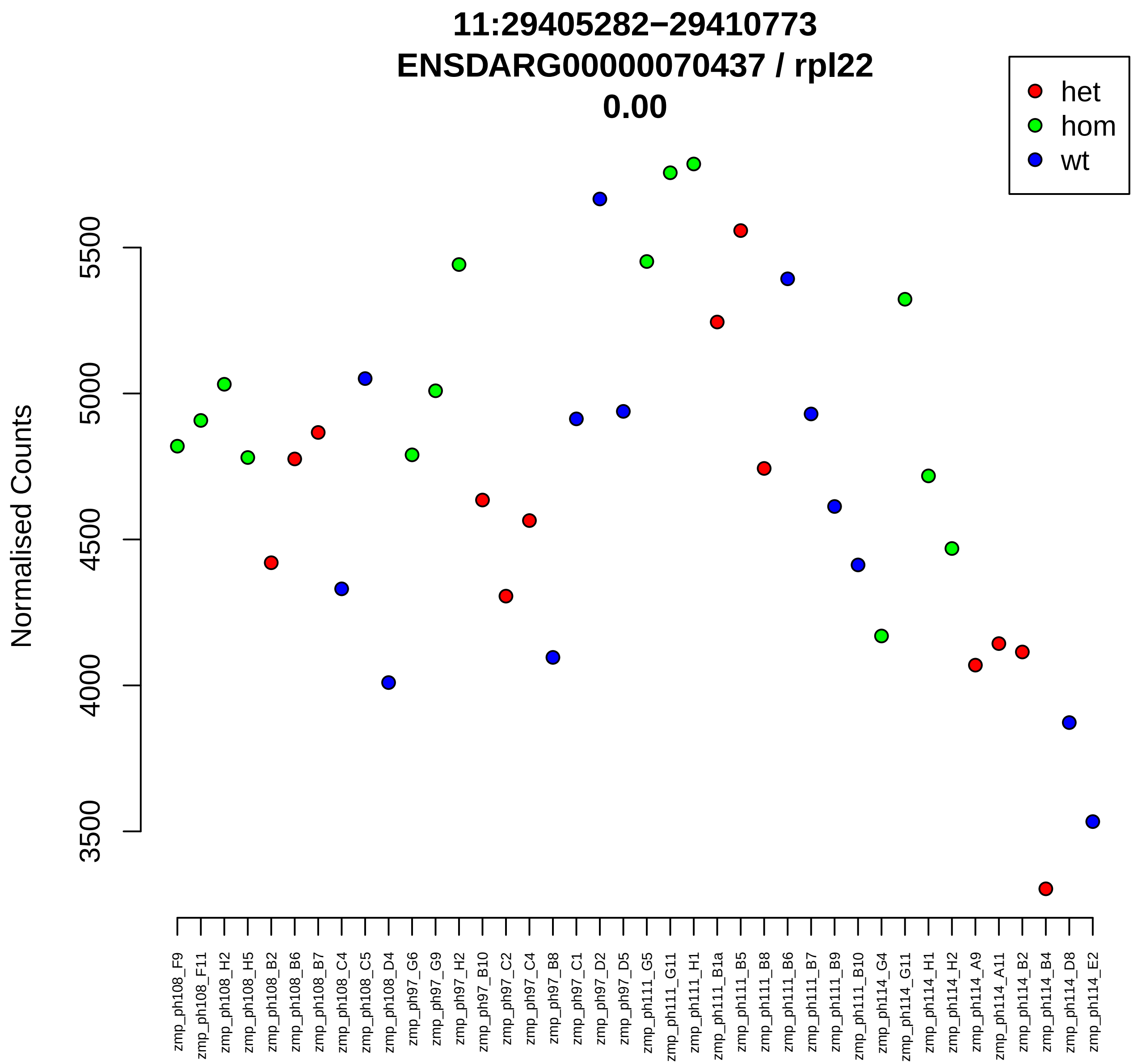
11:29405282−29410773
ENSDARG00000070437 / rpl22
0.00
het
hom
wt
Normalised Counts
3500
4000
4500
5000
5500
zmp_ph108_F9
zmp_ph108_F11
zmp_ph108_H2
zmp_ph108_H5
zmp_ph108_B2
zmp_ph108_B6
zmp_ph108_B7
zmp_ph108_C4
zmp_ph108_C5
zmp_ph108_D4
zmp_ph97_G6
zmp_ph97_G9
zmp_ph97_H2
zmp_ph97_B10
zmp_ph97_C2
zmp_ph97_C4
zmp_ph97_B8
zmp_ph97_C1
zmp_ph97_D2
zmp_ph97_D5
zmp_ph111_G5
zmp_ph111_G11
zmp_ph111_H1
zmp_ph111_B1a
zmp_ph111_B5
zmp_ph111_B8
zmp_ph111_B6
zmp_ph111_B7
zmp_ph111_B9
zmp_ph111_B10
zmp_ph114_G4
zmp_ph114_G11
zmp_ph114_H1
zmp_ph114_H2
zmp_ph114_A9
zmp_ph114_A11
zmp_ph114_B2
zmp_ph114_B4
zmp_ph114_D8
zmp_ph114_E2

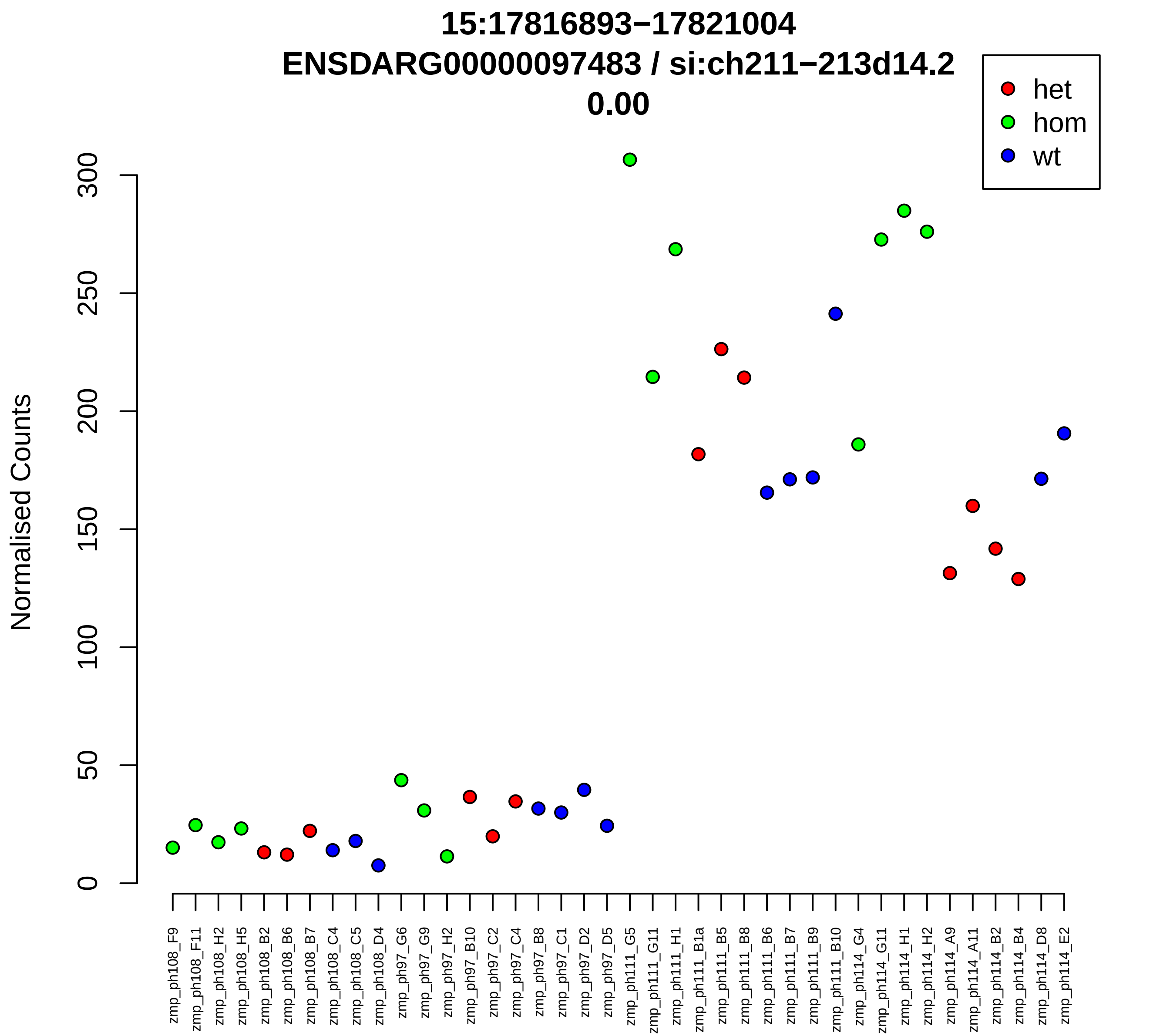

15:17816893−17821004
ENSDARG00000097483 / si:ch211−213d14.2
0.00
het
hom
wt
Normalised Counts
0
50
100
150
200
250
300
zmp_ph108_F9
zmp_ph108_F11
zmp_ph108_H2
zmp_ph108_H5
zmp_ph108_B2
zmp_ph108_B6
zmp_ph108_B7
zmp_ph108_C4
zmp_ph108_C5
zmp_ph108_D4
zmp_ph97_G6
zmp_ph97_G9
zmp_ph97_H2
zmp_ph97_B10
zmp_ph97_C2
zmp_ph97_C4
zmp_ph97_B8
zmp_ph97_C1
zmp_ph97_D2
zmp_ph97_D5
zmp_ph111_G5
zmp_ph111_G11
zmp_ph111_H1
zmp_ph111_B1a
zmp_ph111_B5
zmp_ph111_B8
zmp_ph111_B6
zmp_ph111_B7
zmp_ph111_B9
zmp_ph111_B10
zmp_ph114_G4
zmp_ph114_G11
zmp_ph114_H1
zmp_ph114_H2
zmp_ph114_A9
zmp_ph114_A11
zmp_ph114_B2
zmp_ph114_B4
zmp_ph114_D8
zmp_ph114_E2

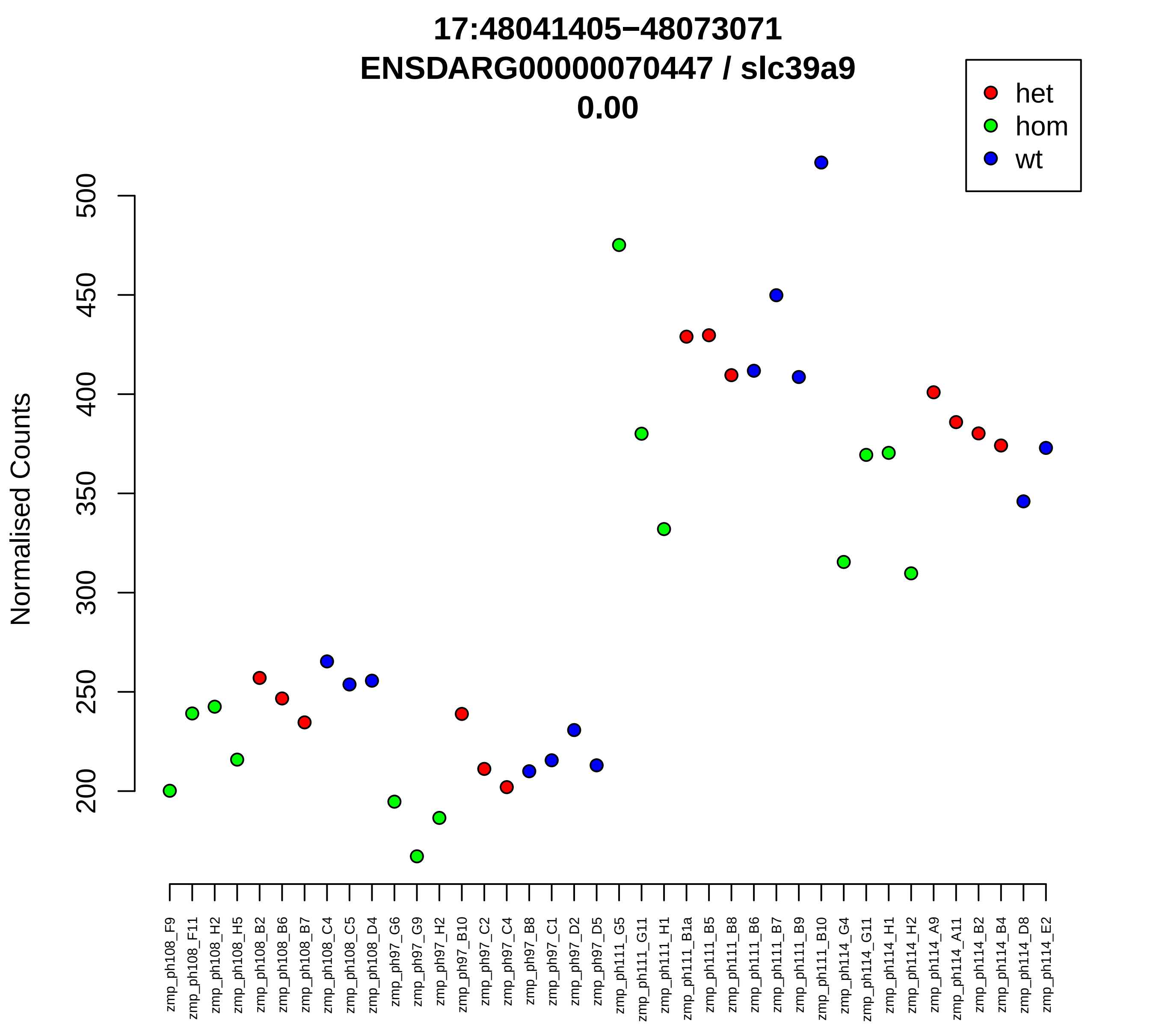

17:48041405−48073071
ENSDARG00000070447 / slc39a9
0.00
het
hom
wt
Normalised Counts
200
250
300
350
400
450
500
zmp_ph108_F9
zmp_ph108_F11
zmp_ph108_H2
zmp_ph108_H5
zmp_ph108_B2
zmp_ph108_B6
zmp_ph108_B7
zmp_ph108_C4
zmp_ph108_C5
zmp_ph108_D4
zmp_ph97_G6
zmp_ph97_G9
zmp_ph97_H2
zmp_ph97_B10
zmp_ph97_C2
zmp_ph97_C4
zmp_ph97_B8
zmp_ph97_C1
zmp_ph97_D2
zmp_ph97_D5
zmp_ph111_G5
zmp_ph111_G11
zmp_ph111_H1
zmp_ph111_B1a
zmp_ph111_B5
zmp_ph111_B8
zmp_ph111_B6
zmp_ph111_B7
zmp_ph111_B9
zmp_ph111_B10
zmp_ph114_G4
zmp_ph114_G11
zmp_ph114_H1
zmp_ph114_H2
zmp_ph114_A9
zmp_ph114_A11
zmp_ph114_B2
zmp_ph114_B4
zmp_ph114_D8
zmp_ph114_E2

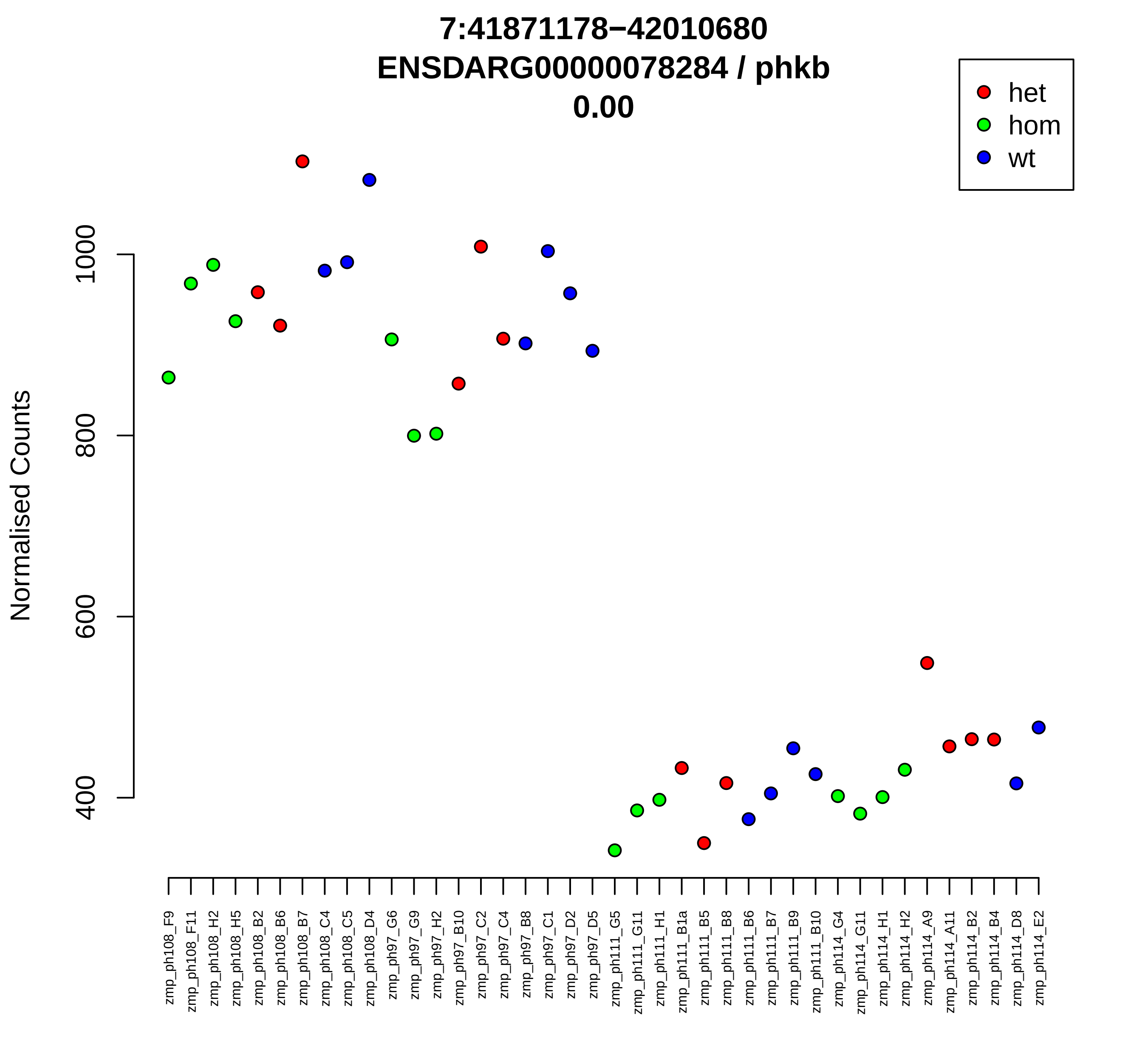

7:41871178−42010680
ENSDARG00000078284 / phkb
0.00
het
hom
wt
Normalised Counts
400
600
800
1000
zmp_ph108_F9
zmp_ph108_F11
zmp_ph108_H2
zmp_ph108_H5
zmp_ph108_B2
zmp_ph108_B6
zmp_ph108_B7
zmp_ph108_C4
zmp_ph108_C5
zmp_ph108_D4
zmp_ph97_G6
zmp_ph97_G9
zmp_ph97_H2
zmp_ph97_B10
zmp_ph97_C2
zmp_ph97_C4
zmp_ph97_B8
zmp_ph97_C1
zmp_ph97_D2
zmp_ph97_D5
zmp_ph111_G5
zmp_ph111_G11
zmp_ph111_H1
zmp_ph111_B1a
zmp_ph111_B5
zmp_ph111_B8
zmp_ph111_B6
zmp_ph111_B7
zmp_ph111_B9
zmp_ph111_B10
zmp_ph114_G4
zmp_ph114_G11
zmp_ph114_H1
zmp_ph114_H2
zmp_ph114_A9
zmp_ph114_A11
zmp_ph114_B2
zmp_ph114_B4
zmp_ph114_D8
zmp_ph114_E2

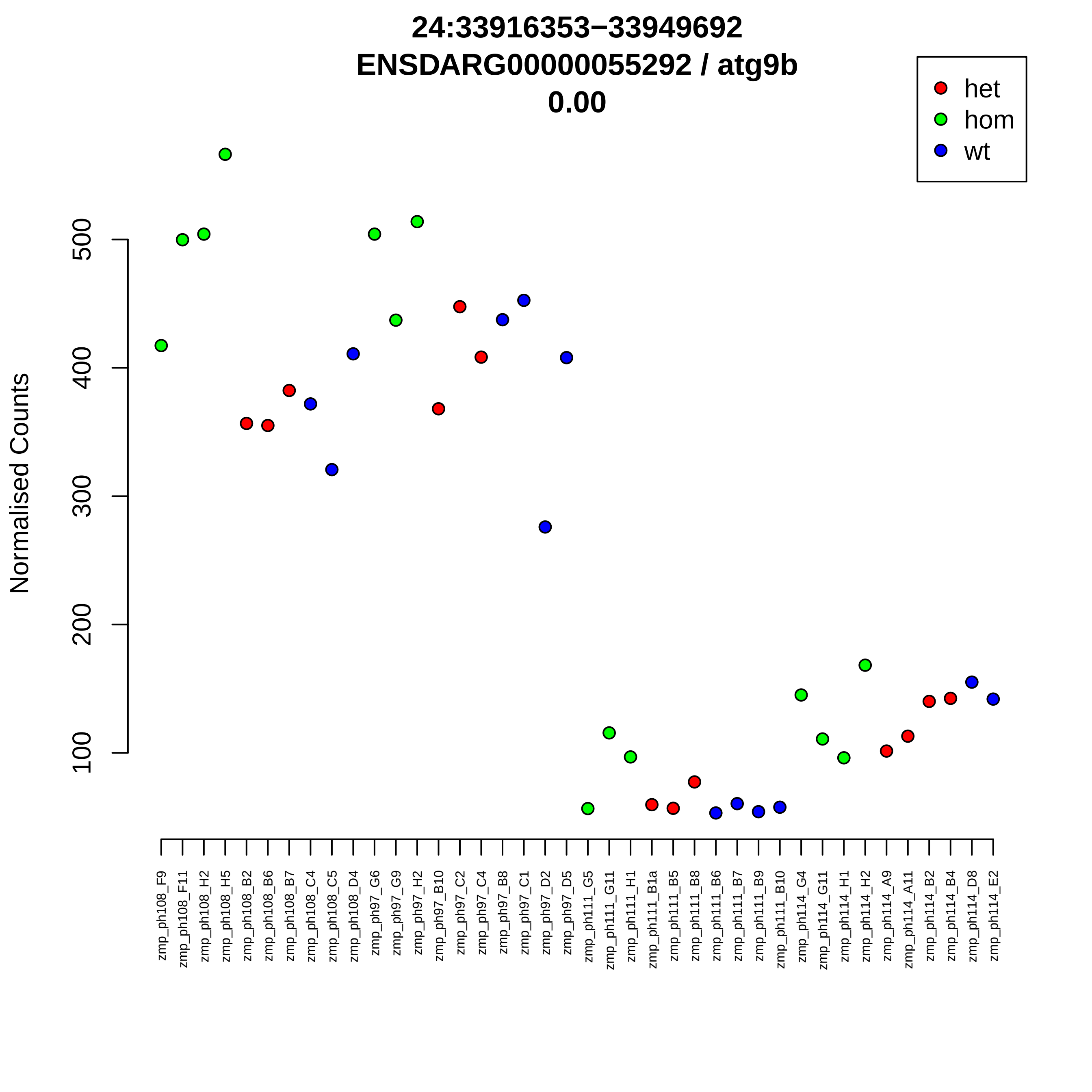

24:33916353−33949692
ENSDARG00000055292 / atg9b
0.00
het
hom
wt
Normalised Counts
100
200
300
400
500
zmp_ph108_F9
zmp_ph108_F11
zmp_ph108_H2
zmp_ph108_H5
zmp_ph108_B2
zmp_ph108_B6
zmp_ph108_B7
zmp_ph108_C4
zmp_ph108_C5
zmp_ph108_D4
zmp_ph97_G6
zmp_ph97_G9
zmp_ph97_H2
zmp_ph97_B10
zmp_ph97_C2
zmp_ph97_C4
zmp_ph97_B8
zmp_ph97_C1
zmp_ph97_D2
zmp_ph97_D5
zmp_ph111_G5
zmp_ph111_G11
zmp_ph111_H1
zmp_ph111_B1a
zmp_ph111_B5
zmp_ph111_B8
zmp_ph111_B6
zmp_ph111_B7
zmp_ph111_B9
zmp_ph111_B10
zmp_ph114_G4
zmp_ph114_G11
zmp_ph114_H1
zmp_ph114_H2
zmp_ph114_A9
zmp_ph114_A11
zmp_ph114_B2
zmp_ph114_B4
zmp_ph114_D8
zmp_ph114_E2

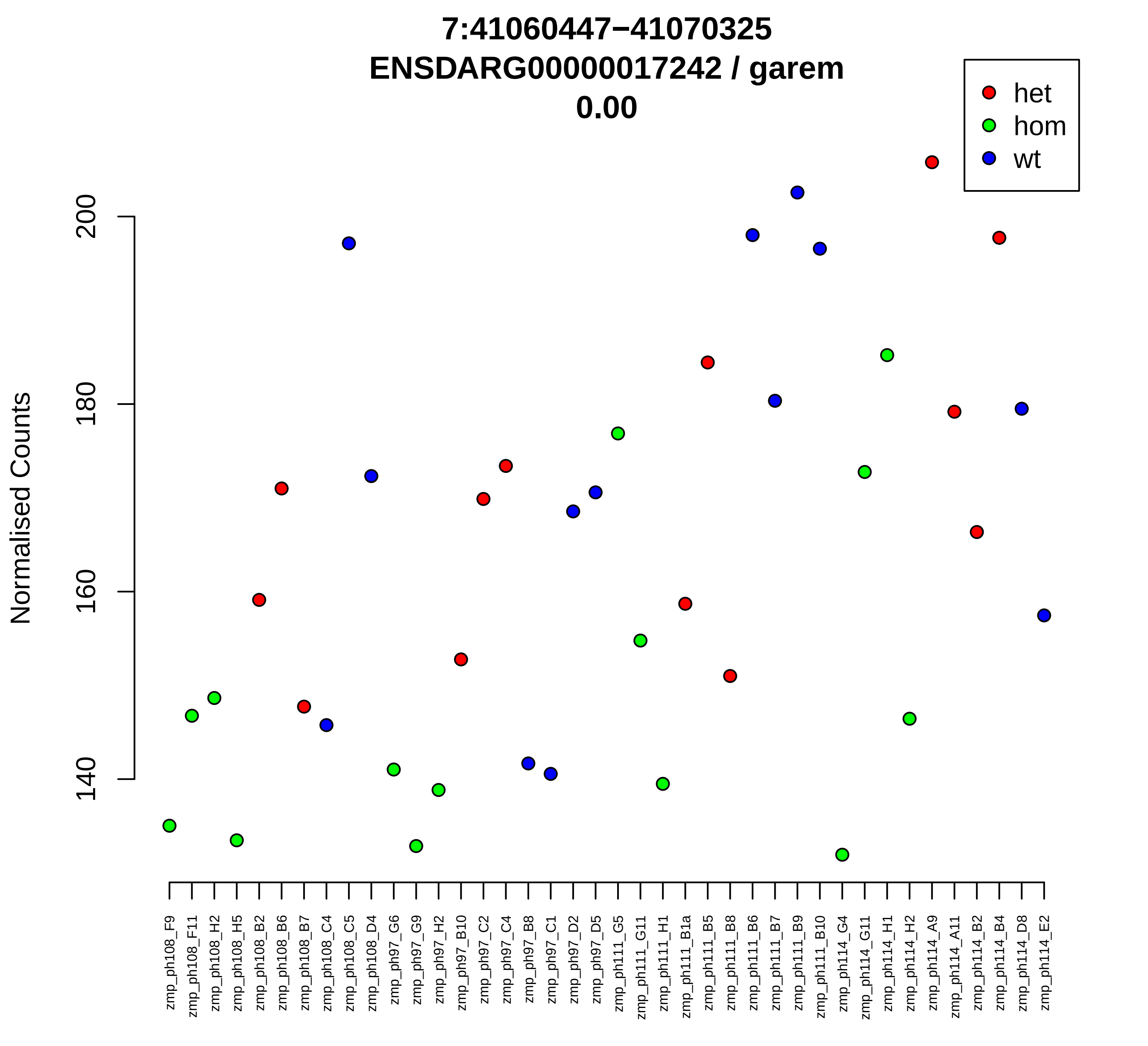

7:41060447−41070325
ENSDARG00000017242 / garem
0.00
het
hom
wt
Normalised Counts
140
160
180
200
zmp_ph108_F9
zmp_ph108_F11
zmp_ph108_H2
zmp_ph108_H5
zmp_ph108_B2
zmp_ph108_B6
zmp_ph108_B7
zmp_ph108_C4
zmp_ph108_C5
zmp_ph108_D4
zmp_ph97_G6
zmp_ph97_G9
zmp_ph97_H2
zmp_ph97_B10
zmp_ph97_C2
zmp_ph97_C4
zmp_ph97_B8
zmp_ph97_C1
zmp_ph97_D2
zmp_ph97_D5
zmp_ph111_G5
zmp_ph111_G11
zmp_ph111_H1
zmp_ph111_B1a
zmp_ph111_B5
zmp_ph111_B8
zmp_ph111_B6
zmp_ph111_B7
zmp_ph111_B9
zmp_ph111_B10
zmp_ph114_G4
zmp_ph114_G11
zmp_ph114_H1
zmp_ph114_H2
zmp_ph114_A9
zmp_ph114_A11
zmp_ph114_B2
zmp_ph114_B4
zmp_ph114_D8
zmp_ph114_E2

3:61116567−61121651
ENSDARG00000097842 / znf1134
0.00

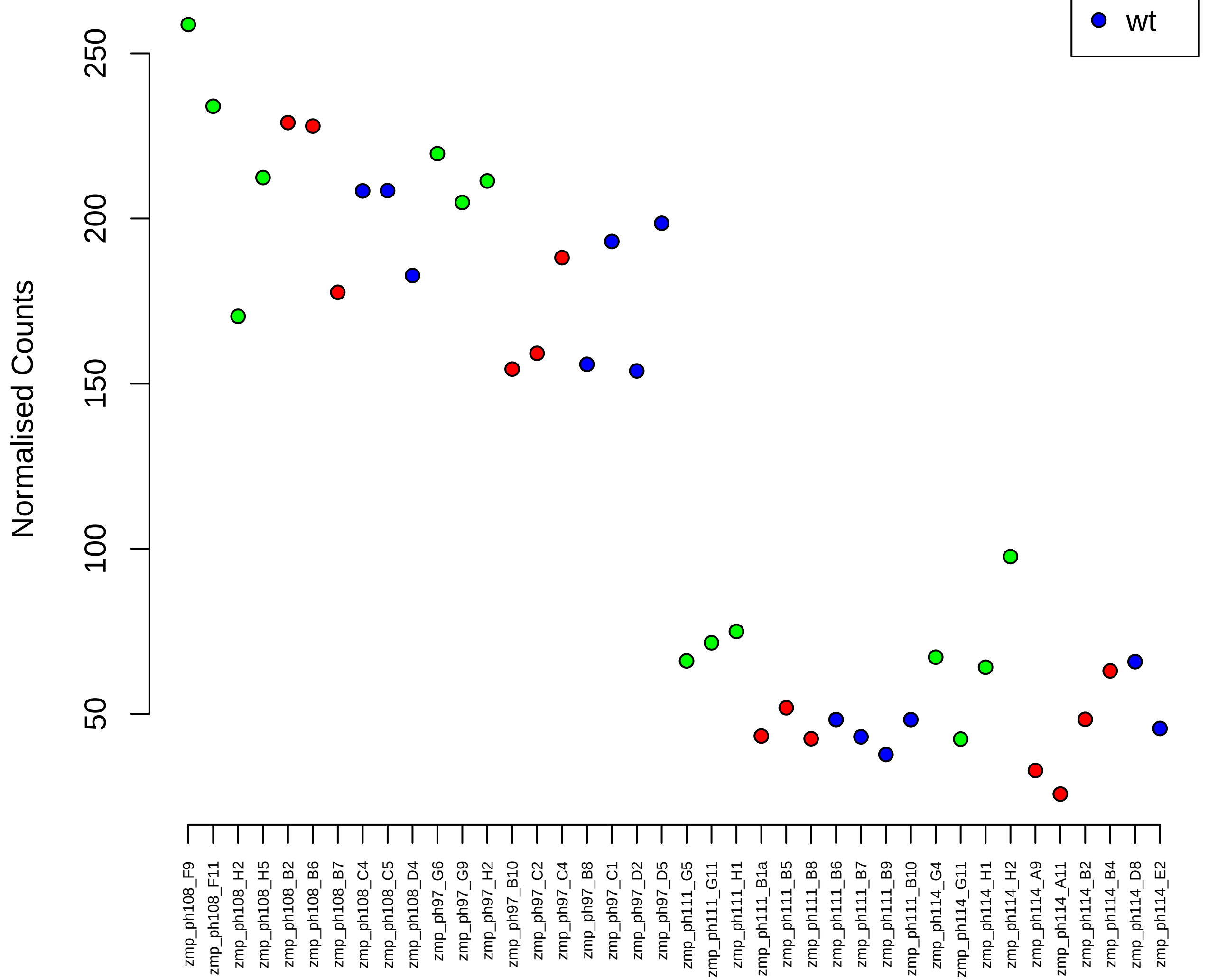

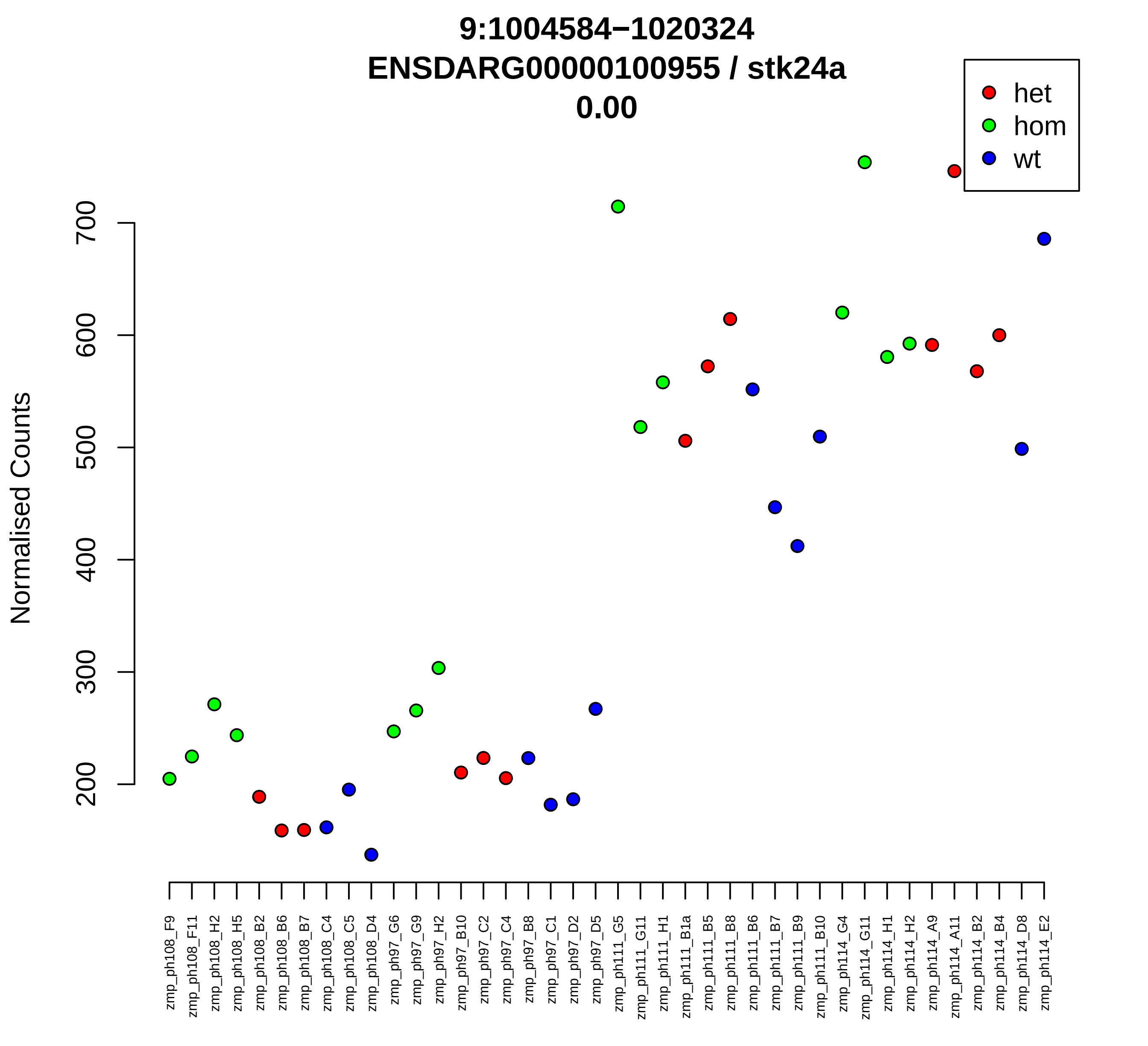

9:1004584−1020324
ENSDARG00000100955 / stk24a
0.00
het
hom
wt
Normalised Counts
200
300
400
500
600
700
zmp_ph108_F9
zmp_ph108_F11
zmp_ph108_H2
zmp_ph108_H5
zmp_ph108_B2
zmp_ph108_B6
zmp_ph108_B7
zmp_ph108_C4
zmp_ph108_C5
zmp_ph108_D4
zmp_ph97_G6
zmp_ph97_G9
zmp_ph97_H2
zmp_ph97_B10
zmp_ph97_C2
zmp_ph97_C4
zmp_ph97_B8
zmp_ph97_C1
zmp_ph97_D2
zmp_ph97_D5
zmp_ph111_G5
zmp_ph111_G11
zmp_ph111_H1
zmp_ph111_B1a
zmp_ph111_B5
zmp_ph111_B8
zmp_ph111_B6
zmp_ph111_B7
zmp_ph111_B9
zmp_ph111_B10
zmp_ph114_G4
zmp_ph114_G11
zmp_ph114_H1
zmp_ph114_H2
zmp_ph114_A9
zmp_ph114_A11
zmp_ph114_B2
zmp_ph114_B4
zmp_ph114_D8
zmp_ph114_E2

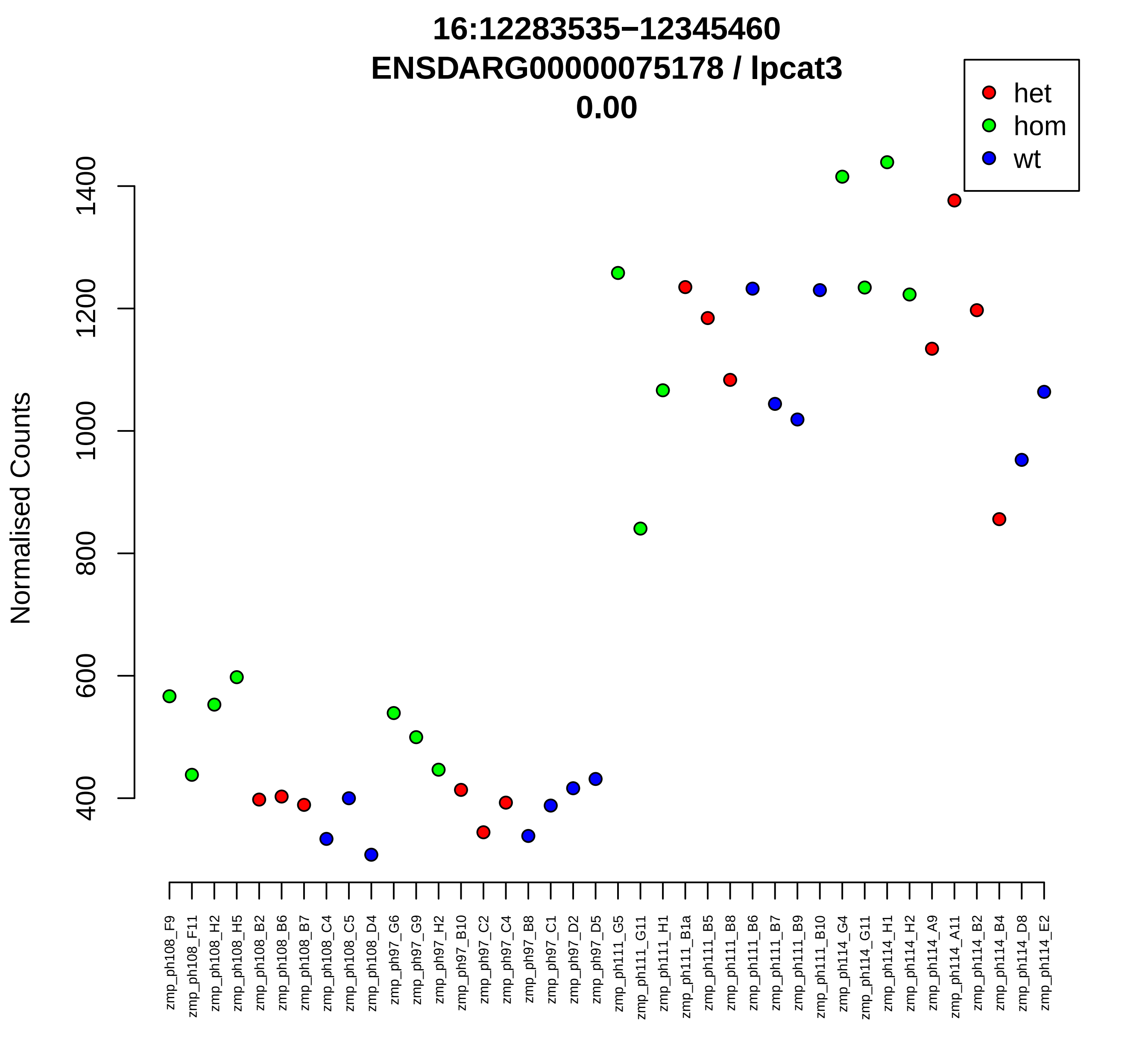

16:12283535−12345460
ENSDARG00000075178 / lpcat3
0.00
het
hom
wt
Normalised Counts
400
600
800
1000
1200
1400
zmp_ph108_F9
zmp_ph108_F11
zmp_ph108_H2
zmp_ph108_H5
zmp_ph108_B2
zmp_ph108_B6
zmp_ph108_B7
zmp_ph108_C4
zmp_ph108_C5
zmp_ph108_D4
zmp_ph97_G6
zmp_ph97_G9
zmp_ph97_H2
zmp_ph97_B10
zmp_ph97_C2
zmp_ph97_C4
zmp_ph97_B8
zmp_ph97_C1
zmp_ph97_D2
zmp_ph97_D5
zmp_ph111_G5
zmp_ph111_G11
zmp_ph111_H1
zmp_ph111_B1a
zmp_ph111_B5
zmp_ph111_B8
zmp_ph111_B6
zmp_ph111_B7
zmp_ph111_B9
zmp_ph111_B10
zmp_ph114_G4
zmp_ph114_G11
zmp_ph114_H1
zmp_ph114_H2
zmp_ph114_A9
zmp_ph114_A11
zmp_ph114_B2
zmp_ph114_B4
zmp_ph114_D8
zmp_ph114_E2

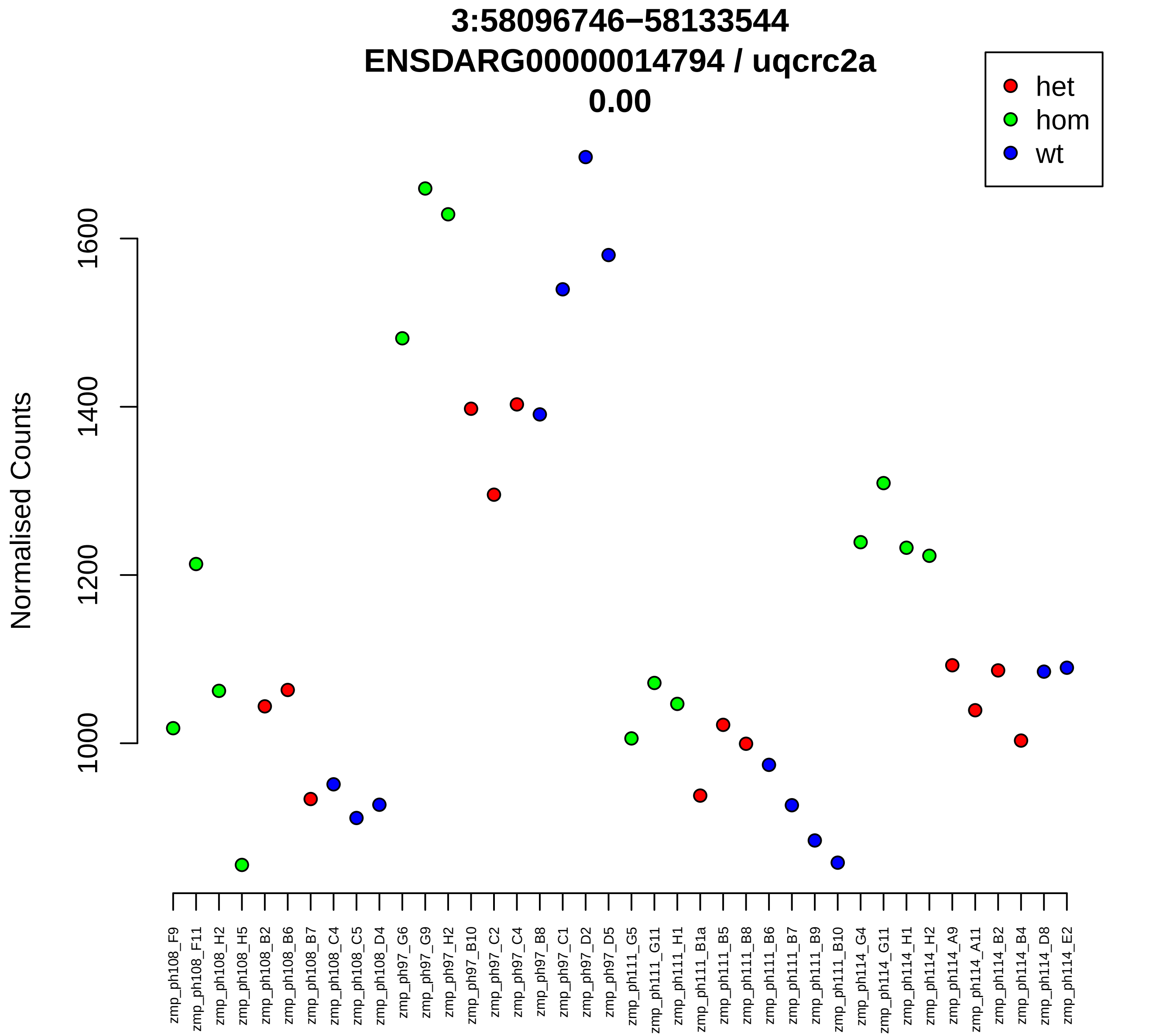

3:58096746−58133544
ENSDARG00000014794 / uqcrc2a
0.00
het
hom
wt
Normalised Counts
1000
1200
1400
1600
zmp_ph108_F9
zmp_ph108_F11
zmp_ph108_H2
zmp_ph108_H5
zmp_ph108_B2
zmp_ph108_B6
zmp_ph108_B7
zmp_ph108_C4
zmp_ph108_C5
zmp_ph108_D4
zmp_ph97_G6
zmp_ph97_G9
zmp_ph97_H2
zmp_ph97_B10
zmp_ph97_C2
zmp_ph97_C4
zmp_ph97_B8
zmp_ph97_C1
zmp_ph97_D2
zmp_ph97_D5
zmp_ph111_G5
zmp_ph111_G11
zmp_ph111_H1
zmp_ph111_B1a
zmp_ph111_B5
zmp_ph111_B8
zmp_ph111_B6
zmp_ph111_B7
zmp_ph111_B9
zmp_ph111_B10
zmp_ph114_G4
zmp_ph114_G11
zmp_ph114_H1
zmp_ph114_H2
zmp_ph114_A9
zmp_ph114_A11
zmp_ph114_B2
zmp_ph114_B4
zmp_ph114_D8
zmp_ph114_E2

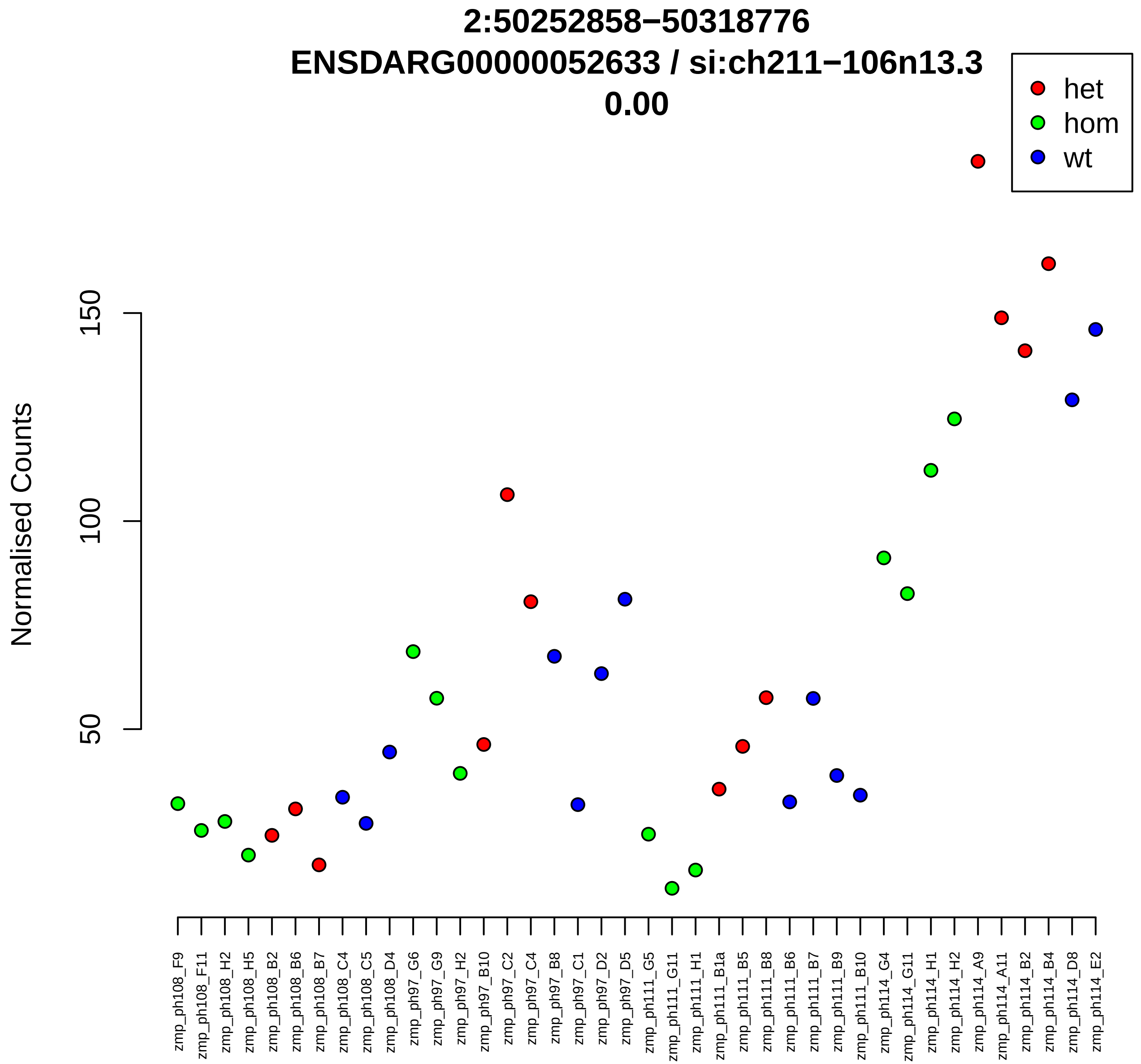
2:50252858−50318776
ENSDARG00000052633 / si:ch211−106n13.3
0.00
het
hom
wt
Normalised Counts
50
100
150
zmp_ph108_F9
zmp_ph108_F11
zmp_ph108_H2
zmp_ph108_H5
zmp_ph108_B2
zmp_ph108_B6
zmp_ph108_B7
zmp_ph108_C4
zmp_ph108_C5
zmp_ph108_D4
zmp_ph97_G6
zmp_ph97_G9
zmp_ph97_H2
zmp_ph97_B10
zmp_ph97_C2
zmp_ph97_C4
zmp_ph97_B8
zmp_ph97_C1
zmp_ph97_D2
zmp_ph97_D5
zmp_ph111_G5
zmp_ph111_G11
zmp_ph111_H1
zmp_ph111_B1a
zmp_ph111_B5
zmp_ph111_B8
zmp_ph111_B6
zmp_ph111_B7
zmp_ph111_B9
zmp_ph111_B10
zmp_ph114_G4
zmp_ph114_G11
zmp_ph114_H1
zmp_ph114_H2
zmp_ph114_A9
zmp_ph114_A11
zmp_ph114_B2
zmp_ph114_B4
zmp_ph114_D8
zmp_ph114_E2

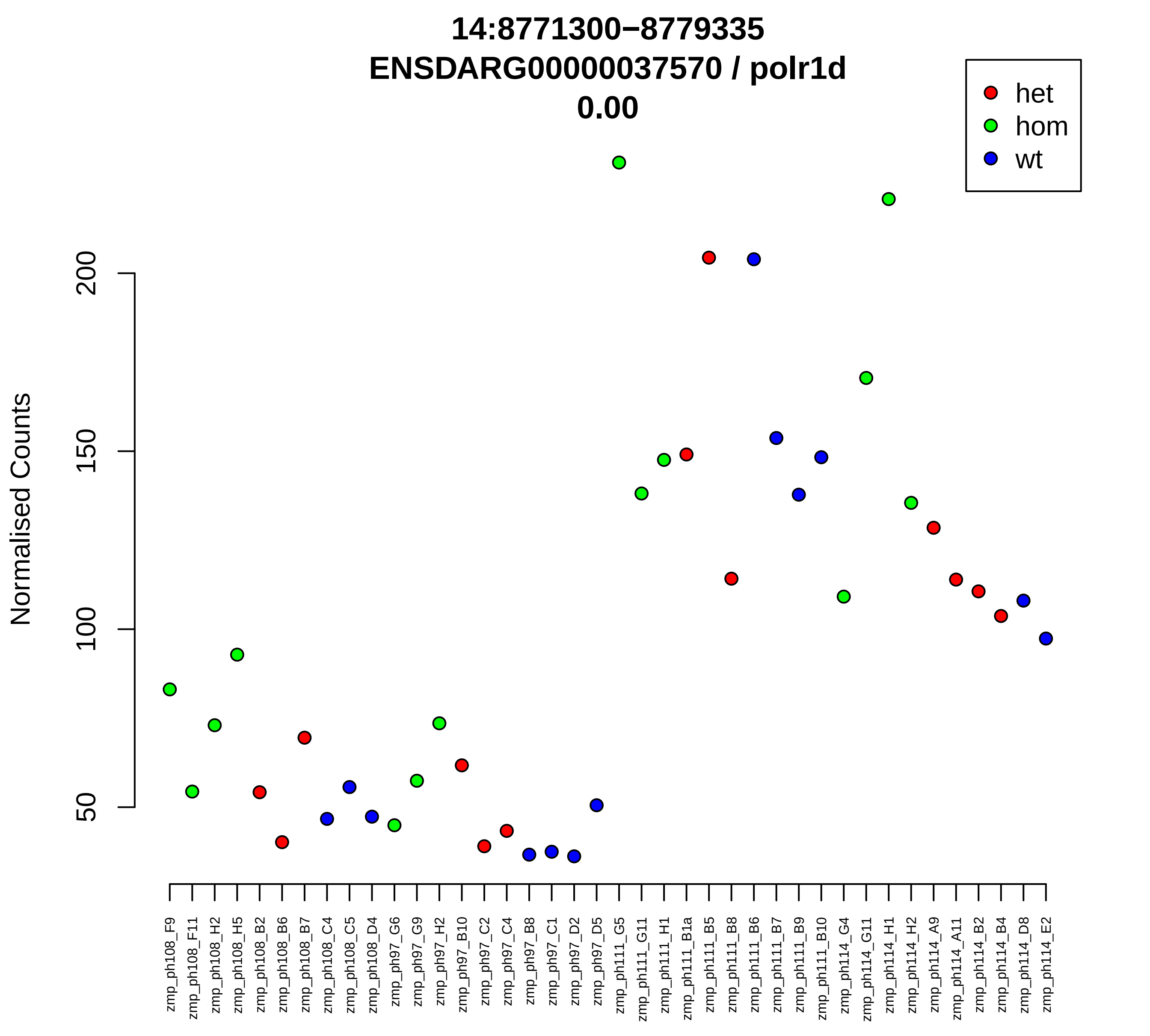

14:8771300−8779335
ENSDARG00000037570 / polr1d
0.00
het
hom
wt
Normalised Counts
50
100
150
200
zmp_ph108_F9
zmp_ph108_F11
zmp_ph108_H2
zmp_ph108_H5
zmp_ph108_B2
zmp_ph108_B6
zmp_ph108_B7
zmp_ph108_C4
zmp_ph108_C5
zmp_ph108_D4
zmp_ph97_G6
zmp_ph97_G9
zmp_ph97_H2
zmp_ph97_B10
zmp_ph97_C2
zmp_ph97_C4
zmp_ph97_B8
zmp_ph97_C1
zmp_ph97_D2
zmp_ph97_D5
zmp_ph111_G5
zmp_ph111_G11
zmp_ph111_H1
zmp_ph111_B1a
zmp_ph111_B5
zmp_ph111_B8
zmp_ph111_B6
zmp_ph111_B7
zmp_ph111_B9
zmp_ph111_B10
zmp_ph114_G4
zmp_ph114_G11
zmp_ph114_H1
zmp_ph114_H2
zmp_ph114_A9
zmp_ph114_A11
zmp_ph114_B2
zmp_ph114_B4
zmp_ph114_D8
zmp_ph114_E2

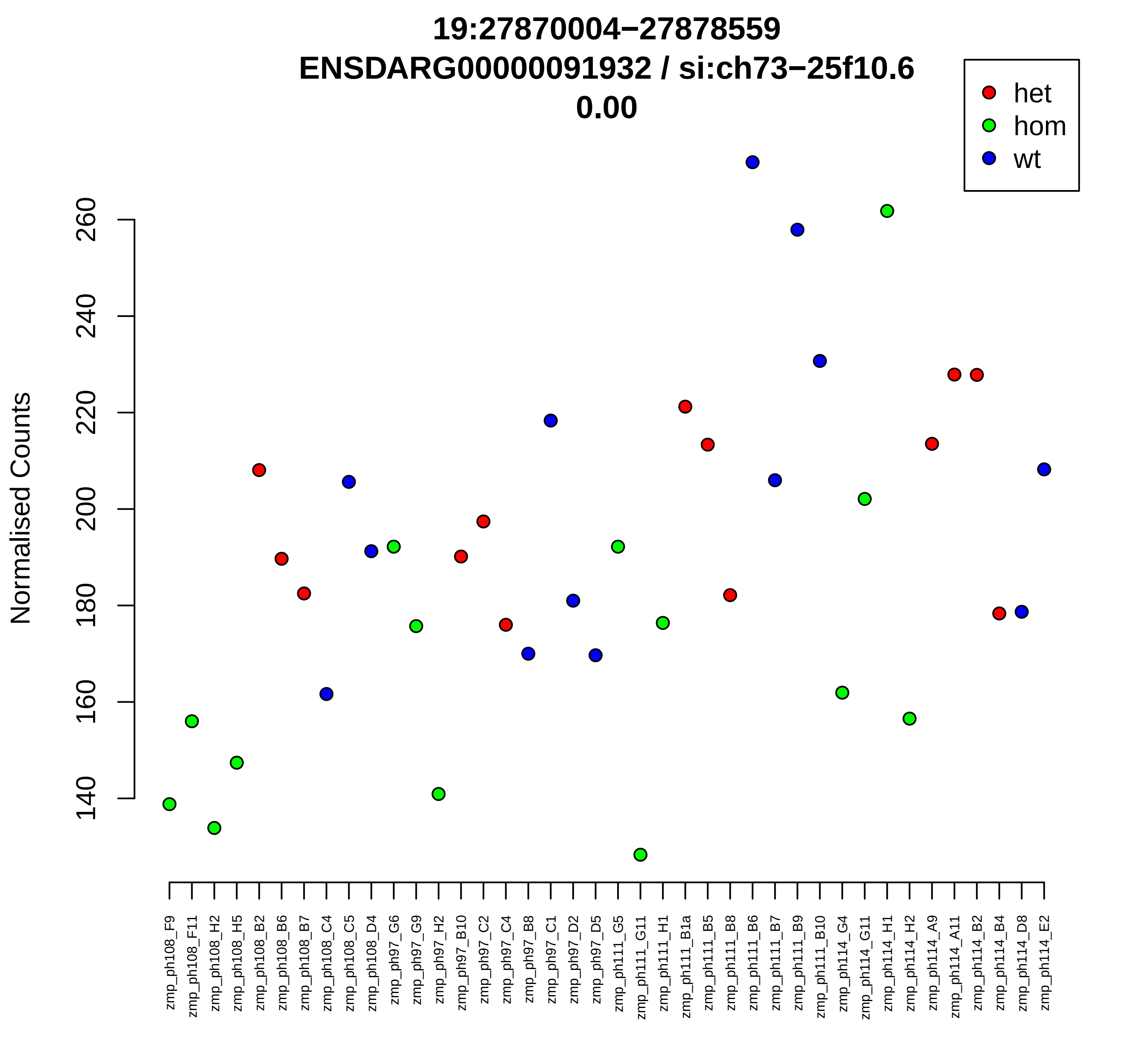

19:27870004−27878559
ENSDARG00000091932 / si:ch73−25f10.6
0.00
het
hom
wt
Normalised Counts
140
160
180
200
220
240
260
zmp_ph108_F9
zmp_ph108_F11
zmp_ph108_H2
zmp_ph108_H5
zmp_ph108_B2
zmp_ph108_B6
zmp_ph108_B7
zmp_ph108_C4
zmp_ph108_C5
zmp_ph108_D4
zmp_ph97_G6
zmp_ph97_G9
zmp_ph97_H2
zmp_ph97_B10
zmp_ph97_C2
zmp_ph97_C4
zmp_ph97_B8
zmp_ph97_C1
zmp_ph97_D2
zmp_ph97_D5
zmp_ph111_G5
zmp_ph111_G11
zmp_ph111_H1
zmp_ph111_B1a
zmp_ph111_B5
zmp_ph111_B8
zmp_ph111_B6
zmp_ph111_B7
zmp_ph111_B9
zmp_ph111_B10
zmp_ph114_G4
zmp_ph114_G11
zmp_ph114_H1
zmp_ph114_H2
zmp_ph114_A9
zmp_ph114_A11
zmp_ph114_B2
zmp_ph114_B4
zmp_ph114_D8
zmp_ph114_E2

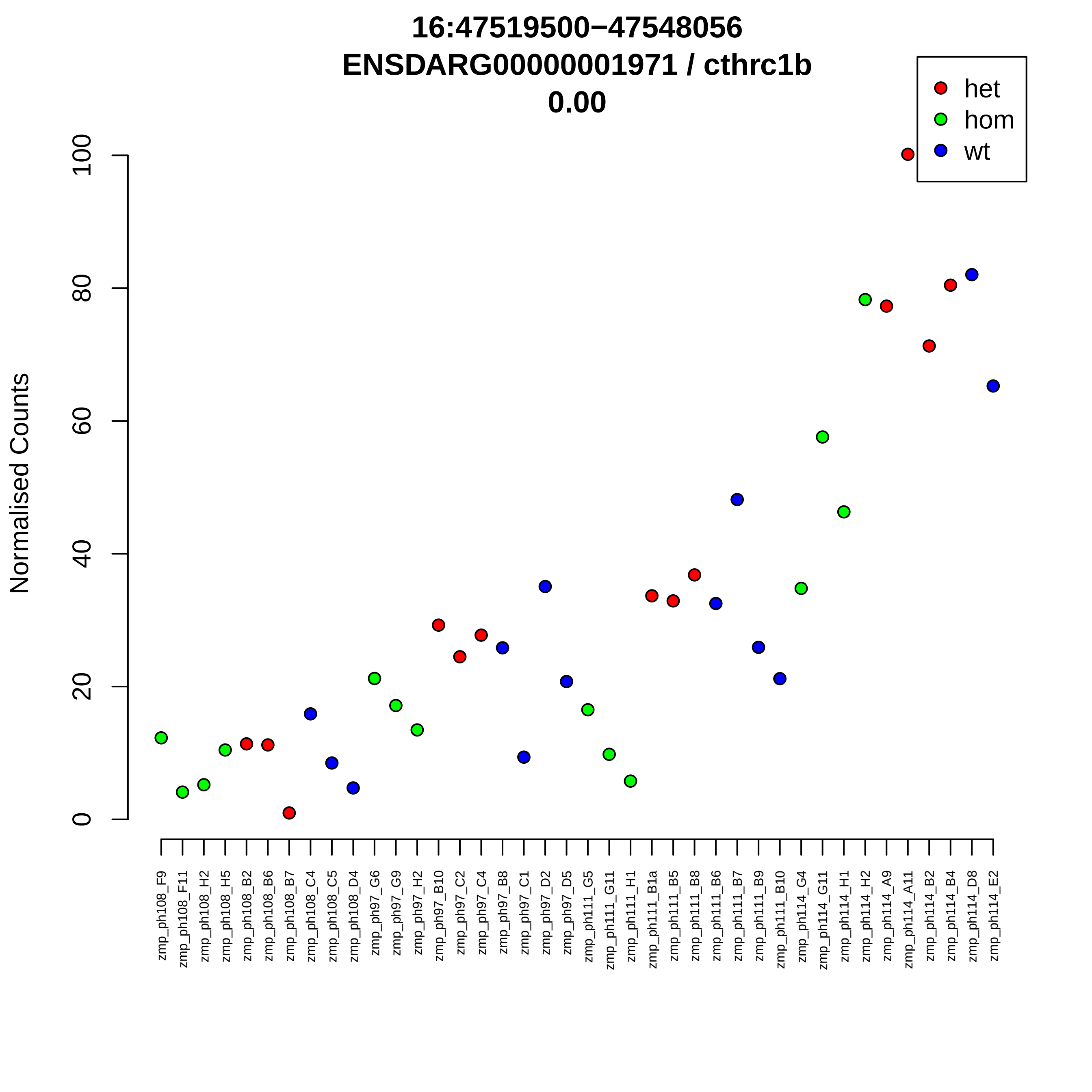

16:47519500−47548056
ENSDARG00000001971 / cthrc1b
0.00
het
hom
wt
Normalised Counts
0
20
40
60
80
100
zmp_ph108_F9
zmp_ph108_F11
zmp_ph108_H2
zmp_ph108_H5
zmp_ph108_B2
zmp_ph108_B6
zmp_ph108_B7
zmp_ph108_C4
zmp_ph108_C5
zmp_ph108_D4
zmp_ph97_G6
zmp_ph97_G9
zmp_ph97_H2
zmp_ph97_B10
zmp_ph97_C2
zmp_ph97_C4
zmp_ph97_B8
zmp_ph97_C1
zmp_ph97_D2
zmp_ph97_D5
zmp_ph111_G5
zmp_ph111_G11
zmp_ph111_H1
zmp_ph111_B1a
zmp_ph111_B5
zmp_ph111_B8
zmp_ph111_B6
zmp_ph111_B7
zmp_ph111_B9
zmp_ph111_B10
zmp_ph114_G4
zmp_ph114_G11
zmp_ph114_H1
zmp_ph114_H2
zmp_ph114_A9
zmp_ph114_A11
zmp_ph114_B2
zmp_ph114_B4
zmp_ph114_D8
zmp_ph114_E2

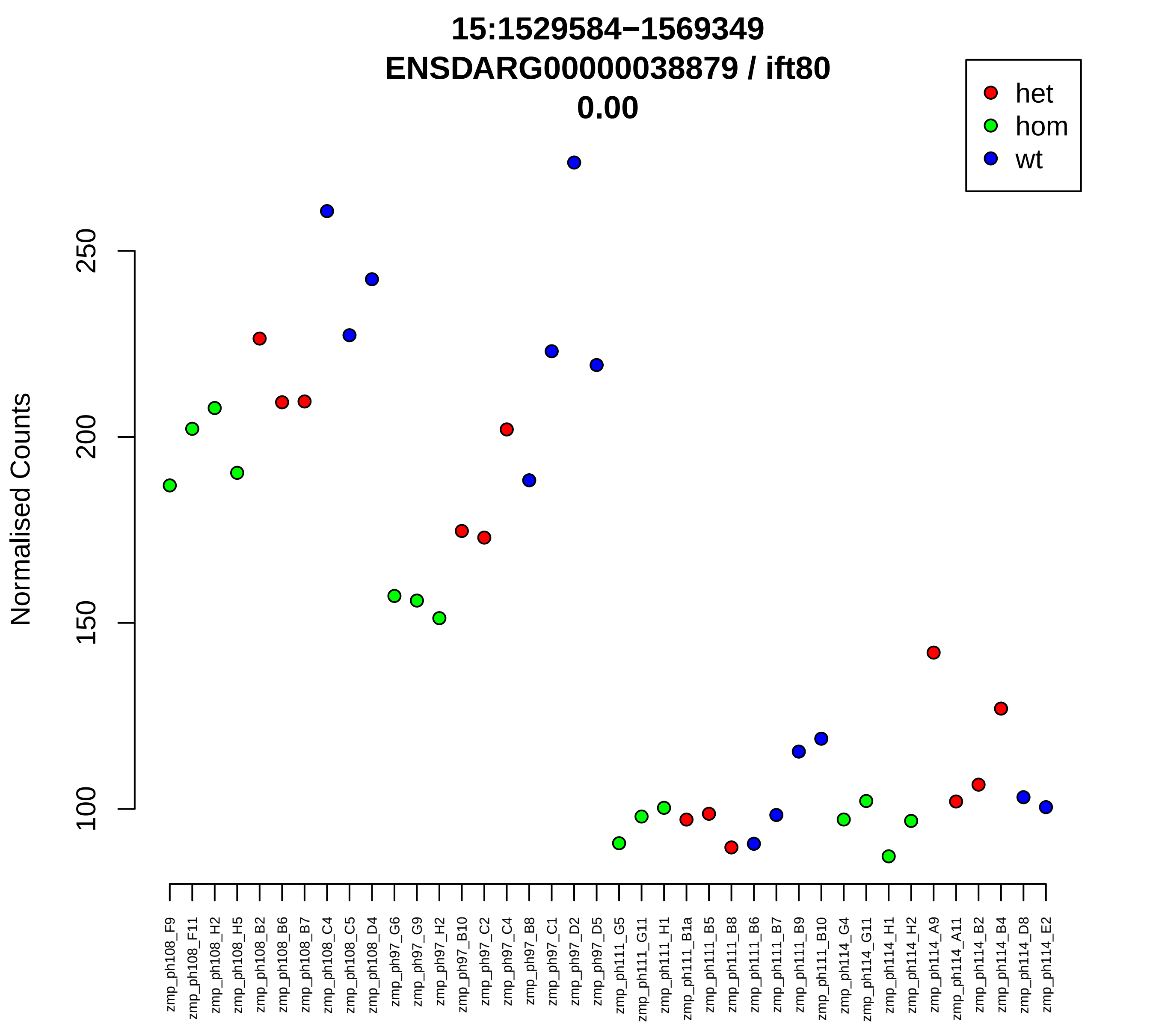
15:1529584−1569349
ENSDARG00000038879 / ift80
0.00
het
hom
wt
Normalised Counts
100
150
200
250
zmp_ph108_F9
zmp_ph108_F11
zmp_ph108_H2
zmp_ph108_H5
zmp_ph108_B2
zmp_ph108_B6
zmp_ph108_B7
zmp_ph108_C4
zmp_ph108_C5
zmp_ph108_D4
zmp_ph97_G6
zmp_ph97_G9
zmp_ph97_H2
zmp_ph97_B10
zmp_ph97_C2
zmp_ph97_C4
zmp_ph97_B8
zmp_ph97_C1
zmp_ph97_D2
zmp_ph97_D5
zmp_ph111_G5
zmp_ph111_G11
zmp_ph111_H1
zmp_ph111_B1a
zmp_ph111_B5
zmp_ph111_B8
zmp_ph111_B6
zmp_ph111_B7
zmp_ph111_B9
zmp_ph111_B10
zmp_ph114_G4
zmp_ph114_G11
zmp_ph114_H1
zmp_ph114_H2
zmp_ph114_A9
zmp_ph114_A11
zmp_ph114_B2
zmp_ph114_B4
zmp_ph114_D8
zmp_ph114_E2

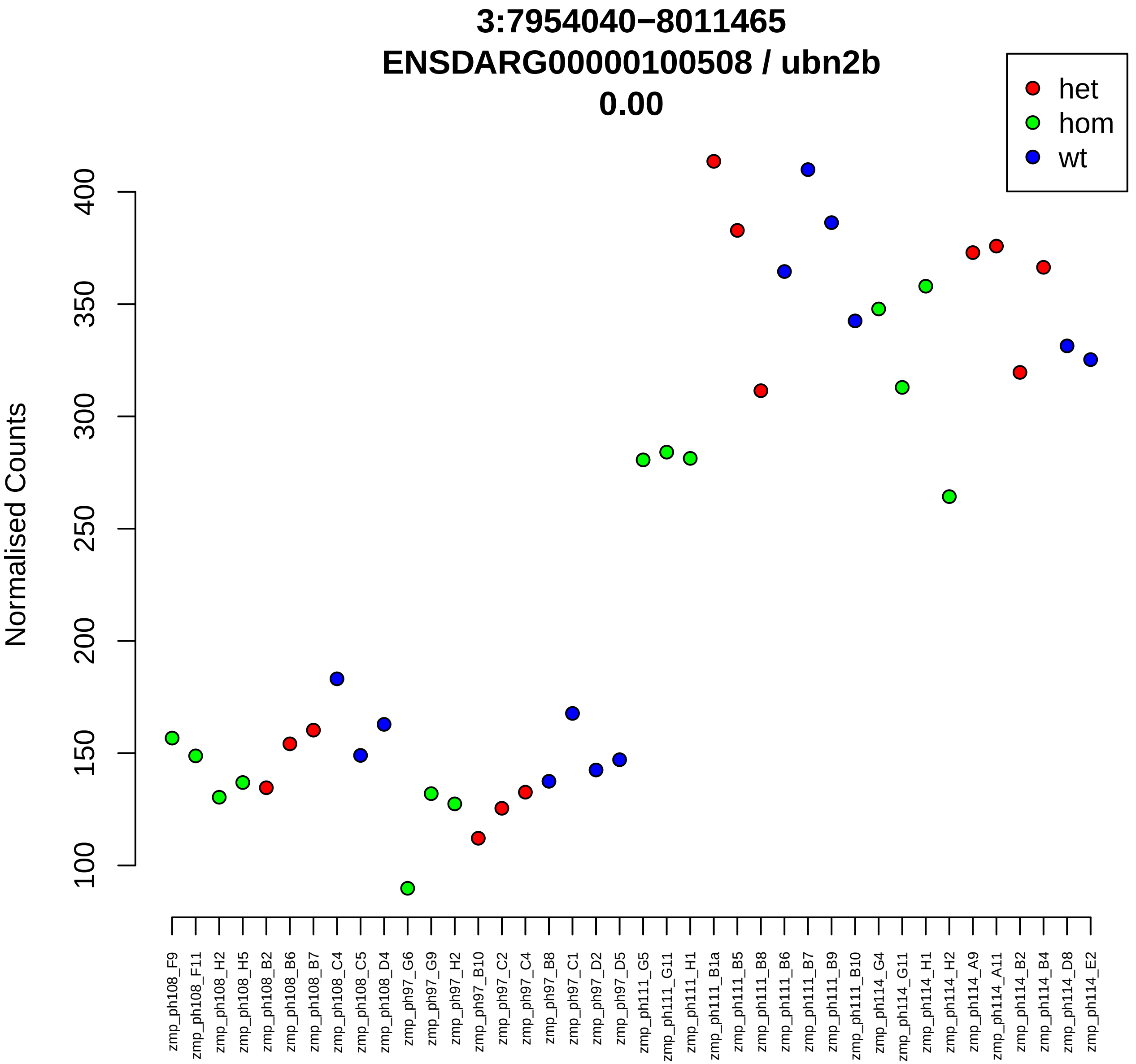
3:7954040−8011465
ENSDARG00000100508 / ubn2b
0.00
het
hom
wt
Normalised Counts
100
150
200
250
300
350
400
zmp_ph108_F9
zmp_ph108_F11
zmp_ph108_H2
zmp_ph108_H5
zmp_ph108_B2
zmp_ph108_B6
zmp_ph108_B7
zmp_ph108_C4
zmp_ph108_C5
zmp_ph108_D4
zmp_ph97_G6
zmp_ph97_G9
zmp_ph97_H2
zmp_ph97_B10
zmp_ph97_C2
zmp_ph97_C4
zmp_ph97_B8
zmp_ph97_C1
zmp_ph97_D2
zmp_ph97_D5
zmp_ph111_G5
zmp_ph111_G11
zmp_ph111_H1
zmp_ph111_B1a
zmp_ph111_B5
zmp_ph111_B8
zmp_ph111_B6
zmp_ph111_B7
zmp_ph111_B9
zmp_ph111_B10
zmp_ph114_G4
zmp_ph114_G11
zmp_ph114_H1
zmp_ph114_H2
zmp_ph114_A9
zmp_ph114_A11
zmp_ph114_B2
zmp_ph114_B4
zmp_ph114_D8
zmp_ph114_E2

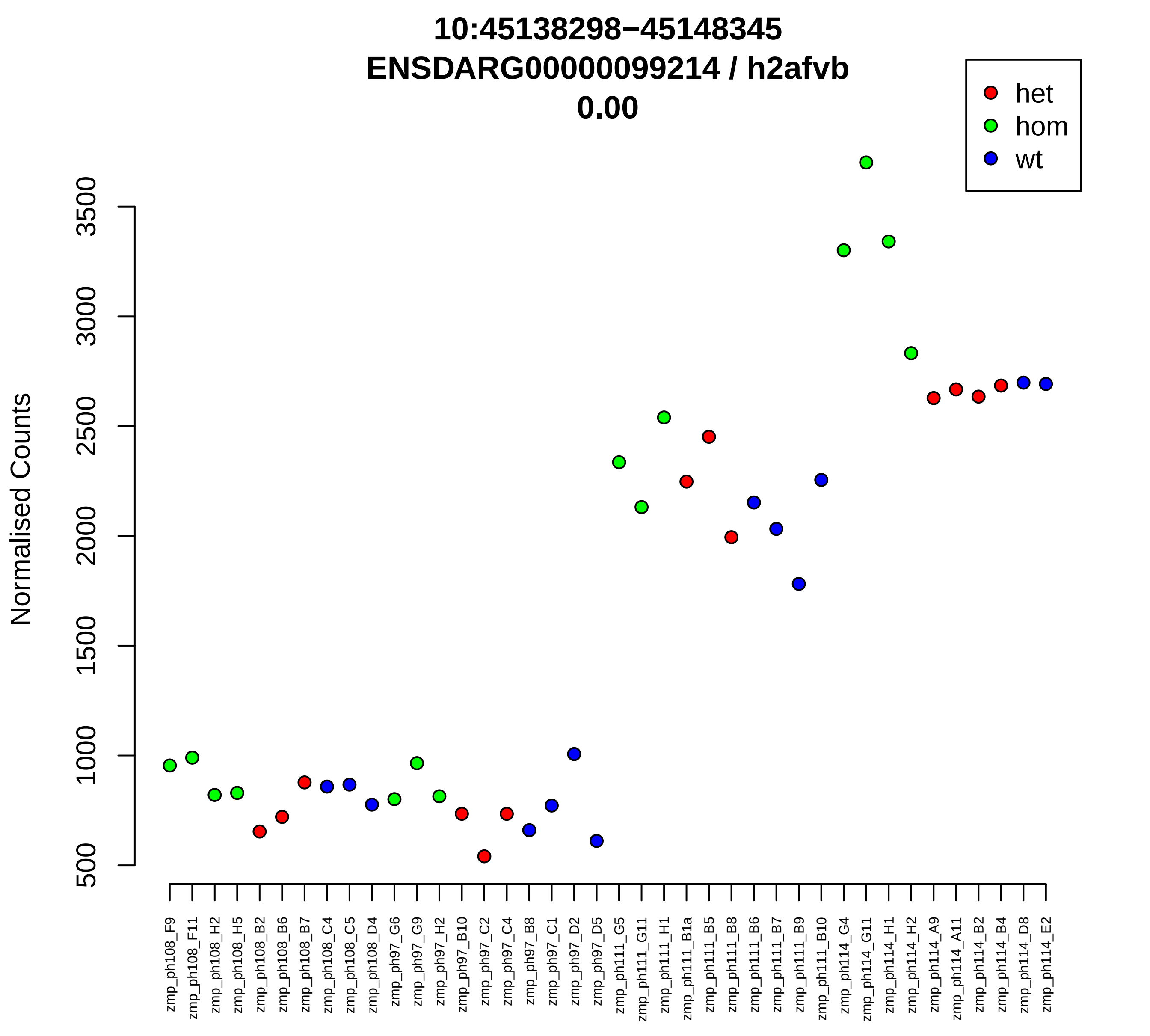
10:45138298−45148345
ENSDARG00000099214 / h2afvb
0.00
het
hom
wt
Normalised Counts
500
1000
1500
2000
2500
3000
3500
zmp_ph108_F9
zmp_ph108_F11
zmp_ph108_H2
zmp_ph108_H5
zmp_ph108_B2
zmp_ph108_B6
zmp_ph108_B7
zmp_ph108_C4
zmp_ph108_C5
zmp_ph108_D4
zmp_ph97_G6
zmp_ph97_G9
zmp_ph97_H2
zmp_ph97_B10
zmp_ph97_C2
zmp_ph97_C4
zmp_ph97_B8
zmp_ph97_C1
zmp_ph97_D2
zmp_ph97_D5
zmp_ph111_G5
zmp_ph111_G11
zmp_ph111_H1
zmp_ph111_B1a
zmp_ph111_B5
zmp_ph111_B8
zmp_ph111_B6
zmp_ph111_B7
zmp_ph111_B9
zmp_ph111_B10
zmp_ph114_G4
zmp_ph114_G11
zmp_ph114_H1
zmp_ph114_H2
zmp_ph114_A9
zmp_ph114_A11
zmp_ph114_B2
zmp_ph114_B4
zmp_ph114_D8
zmp_ph114_E2

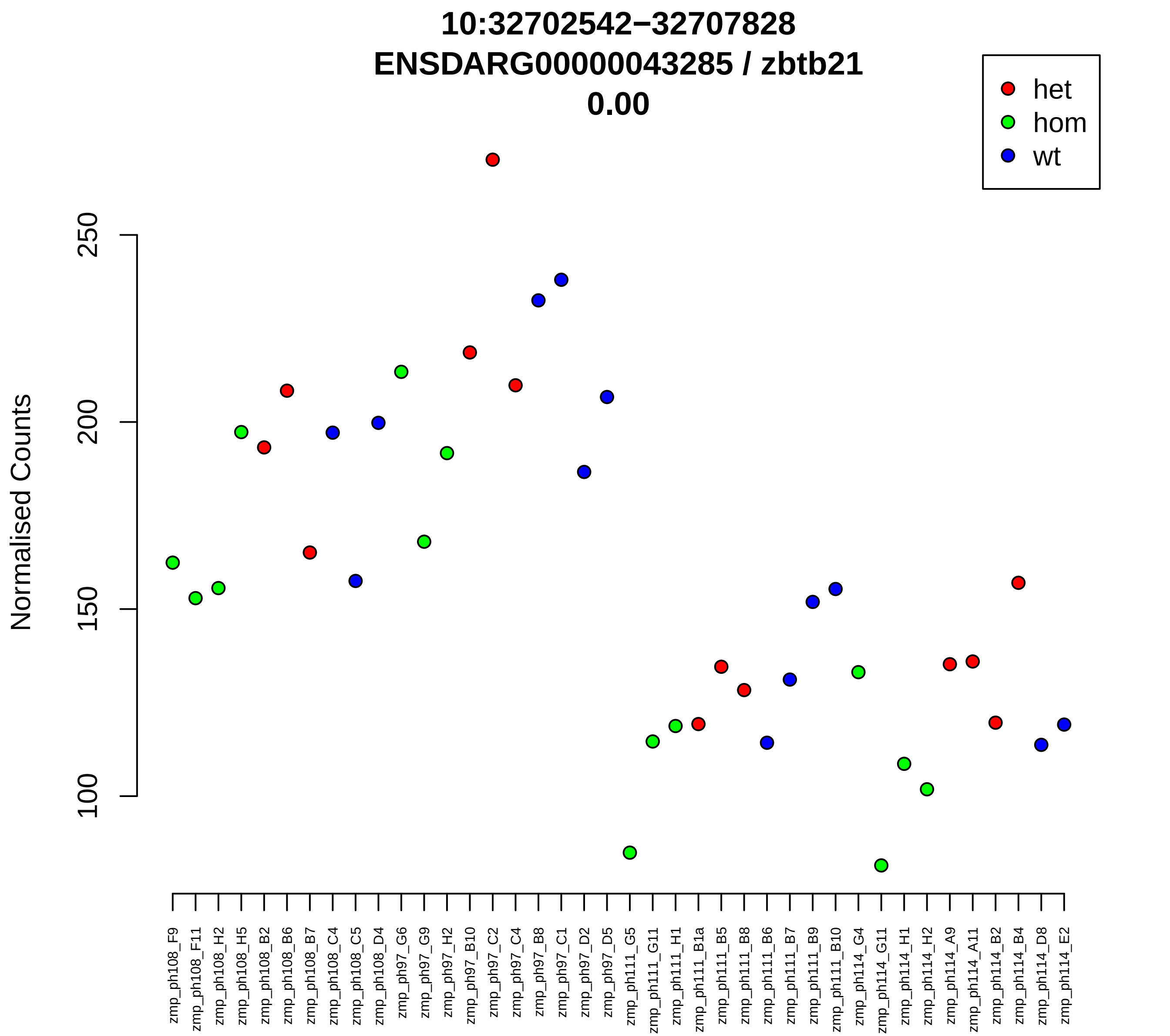

10:32702542−32707828
ENSDARG00000043285 / zbtb21
0.00
het
hom
wt
Normalised Counts
100
150
200
250
zmp_ph108_F9
zmp_ph108_F11
zmp_ph108_H2
zmp_ph108_H5
zmp_ph108_B2
zmp_ph108_B6
zmp_ph108_B7
zmp_ph108_C4
zmp_ph108_C5
zmp_ph108_D4
zmp_ph97_G6
zmp_ph97_G9
zmp_ph97_H2
zmp_ph97_B10
zmp_ph97_C2
zmp_ph97_C4
zmp_ph97_B8
zmp_ph97_C1
zmp_ph97_D2
zmp_ph97_D5
zmp_ph111_G5
zmp_ph111_G11
zmp_ph111_H1
zmp_ph111_B1a
zmp_ph111_B5
zmp_ph111_B8
zmp_ph111_B6
zmp_ph111_B7
zmp_ph111_B9
zmp_ph111_B10
zmp_ph114_G4
zmp_ph114_G11
zmp_ph114_H1
zmp_ph114_H2
zmp_ph114_A9
zmp_ph114_A11
zmp_ph114_B2
zmp_ph114_B4
zmp_ph114_D8
zmp_ph114_E2

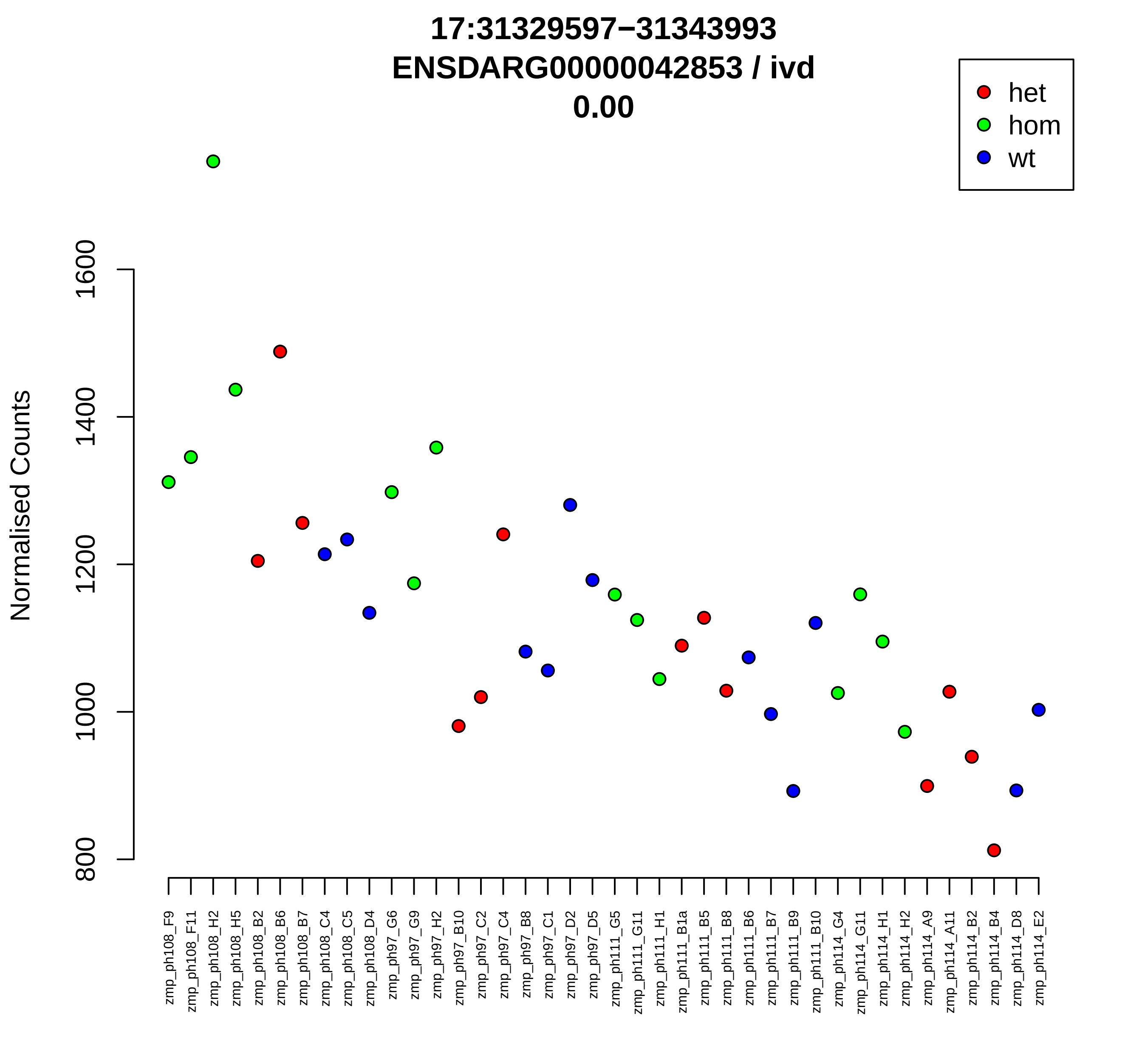

17:31329597−31343993
ENSDARG00000042853 / ivd
0.00
het
hom
wt
Normalised Counts
800
1000
1200
1400
1600
zmp_ph108_F9
zmp_ph108_F11
zmp_ph108_H2
zmp_ph108_H5
zmp_ph108_B2
zmp_ph108_B6
zmp_ph108_B7
zmp_ph108_C4
zmp_ph108_C5
zmp_ph108_D4
zmp_ph97_G6
zmp_ph97_G9
zmp_ph97_H2
zmp_ph97_B10
zmp_ph97_C2
zmp_ph97_C4
zmp_ph97_B8
zmp_ph97_C1
zmp_ph97_D2
zmp_ph97_D5
zmp_ph111_G5
zmp_ph111_G11
zmp_ph111_H1
zmp_ph111_B1a
zmp_ph111_B5
zmp_ph111_B8
zmp_ph111_B6
zmp_ph111_B7
zmp_ph111_B9
zmp_ph111_B10
zmp_ph114_G4
zmp_ph114_G11
zmp_ph114_H1
zmp_ph114_H2
zmp_ph114_A9
zmp_ph114_A11
zmp_ph114_B2
zmp_ph114_B4
zmp_ph114_D8
zmp_ph114_E2

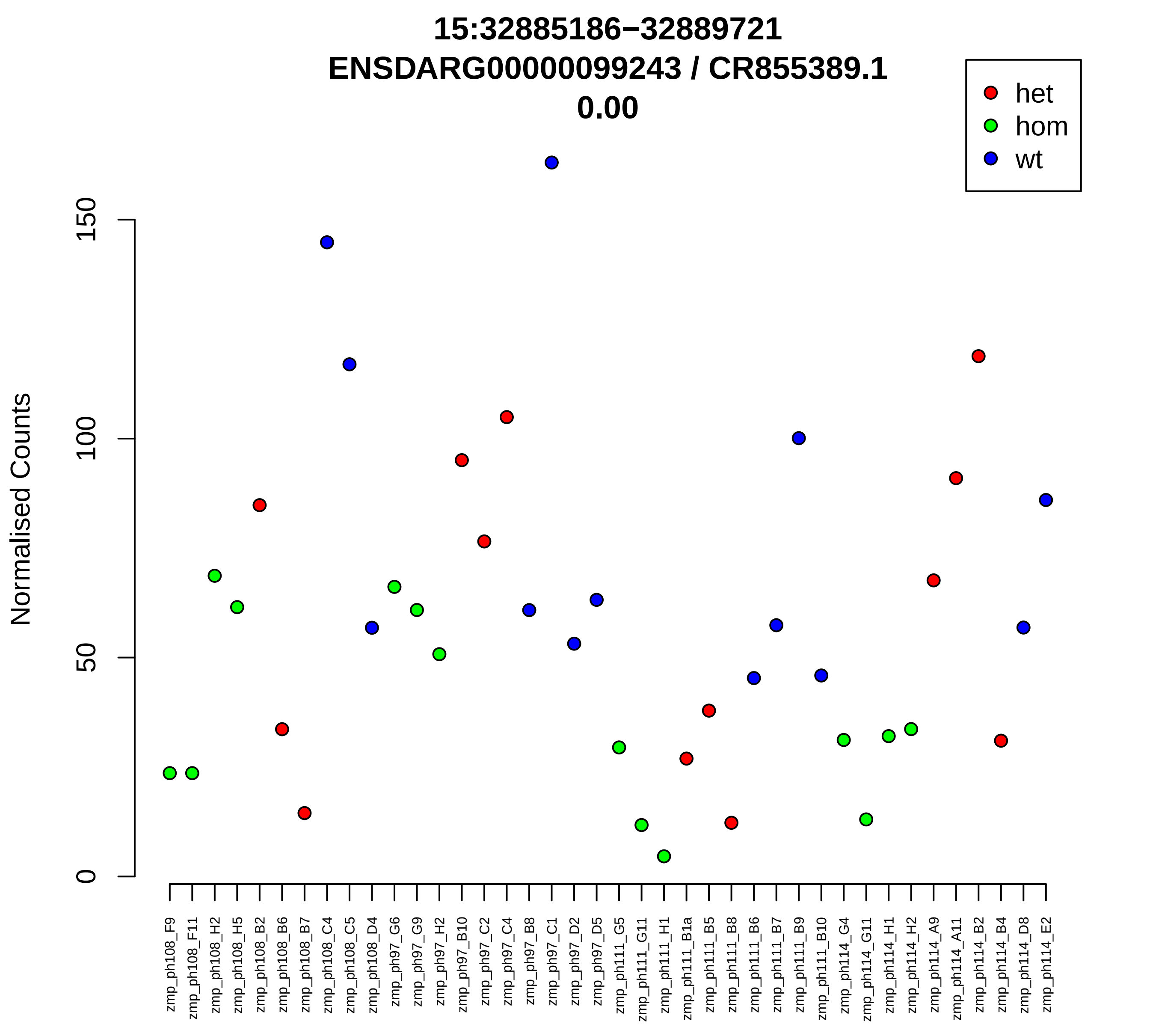
15:32885186−32889721
ENSDARG00000099243 / CR855389.1
0.00
het
hom
wt
Normalised Counts
0
50
100
150
zmp_ph108_F9
zmp_ph108_F11
zmp_ph108_H2
zmp_ph108_H5
zmp_ph108_B2
zmp_ph108_B6
zmp_ph108_B7
zmp_ph108_C4
zmp_ph108_C5
zmp_ph108_D4
zmp_ph97_G6
zmp_ph97_G9
zmp_ph97_H2
zmp_ph97_B10
zmp_ph97_C2
zmp_ph97_C4
zmp_ph97_B8
zmp_ph97_C1
zmp_ph97_D2
zmp_ph97_D5
zmp_ph111_G5
zmp_ph111_G11
zmp_ph111_H1
zmp_ph111_B1a
zmp_ph111_B5
zmp_ph111_B8
zmp_ph111_B6
zmp_ph111_B7
zmp_ph111_B9
zmp_ph111_B10
zmp_ph114_G4
zmp_ph114_G11
zmp_ph114_H1
zmp_ph114_H2
zmp_ph114_A9
zmp_ph114_A11
zmp_ph114_B2
zmp_ph114_B4
zmp_ph114_D8
zmp_ph114_E2

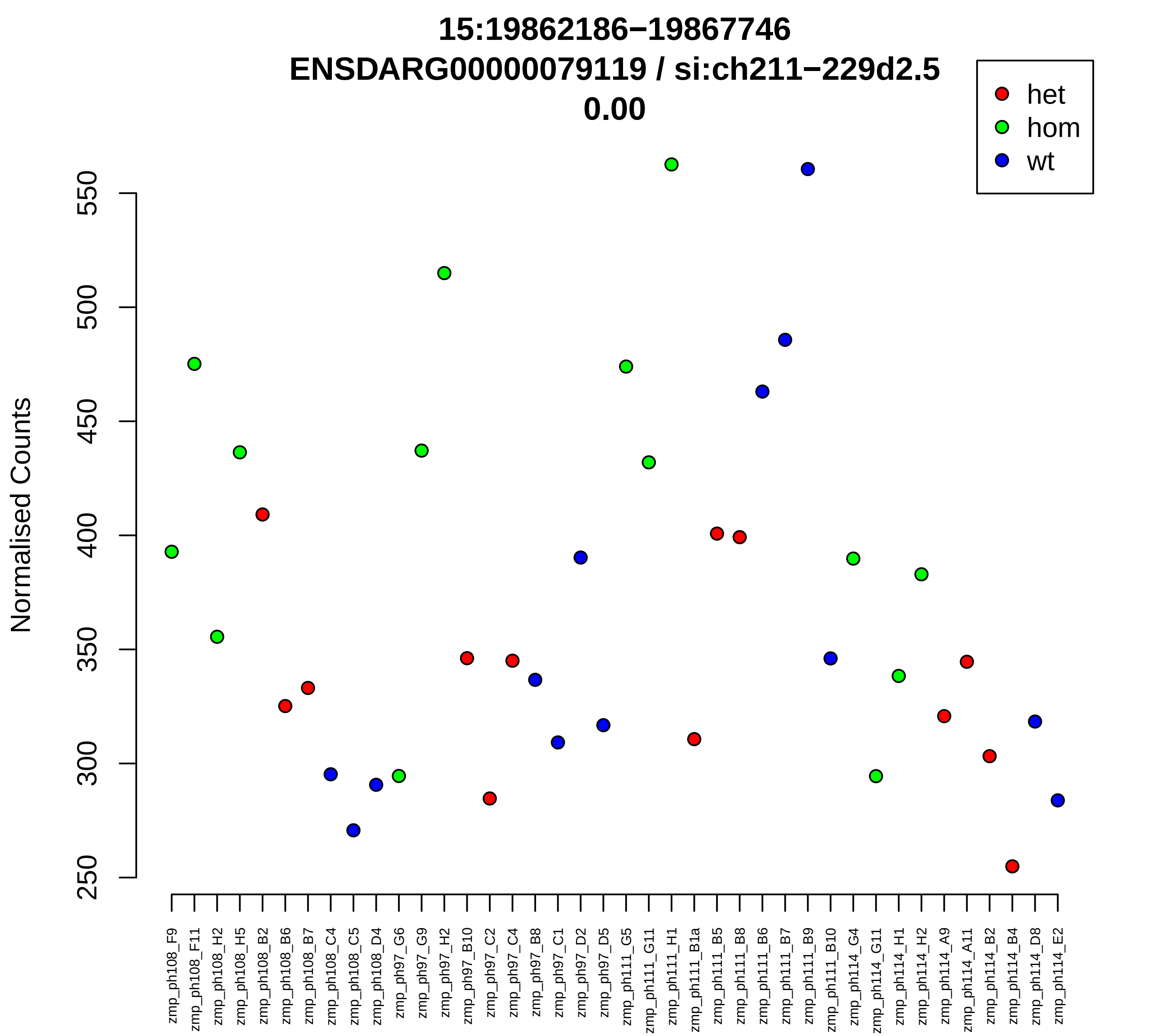

15:19862186−19867746
ENSDARG00000079119 / si:ch211−229d2.5
0.00
het
hom
wt
Normalised Counts
250
300
350
400
450
500
550
zmp_ph108_F9
zmp_ph108_F11
zmp_ph108_H2
zmp_ph108_H5
zmp_ph108_B2
zmp_ph108_B6
zmp_ph108_B7
zmp_ph108_C4
zmp_ph108_C5
zmp_ph108_D4
zmp_ph97_G6
zmp_ph97_G9
zmp_ph97_H2
zmp_ph97_B10
zmp_ph97_C2
zmp_ph97_C4
zmp_ph97_B8
zmp_ph97_C1
zmp_ph97_D2
zmp_ph97_D5
zmp_ph111_G5
zmp_ph111_G11
zmp_ph111_H1
zmp_ph111_B1a
zmp_ph111_B5
zmp_ph111_B8
zmp_ph111_B6
zmp_ph111_B7
zmp_ph111_B9
zmp_ph111_B10
zmp_ph114_G4
zmp_ph114_G11
zmp_ph114_H1
zmp_ph114_H2
zmp_ph114_A9
zmp_ph114_A11
zmp_ph114_B2
zmp_ph114_B4
zmp_ph114_D8
zmp_ph114_E2

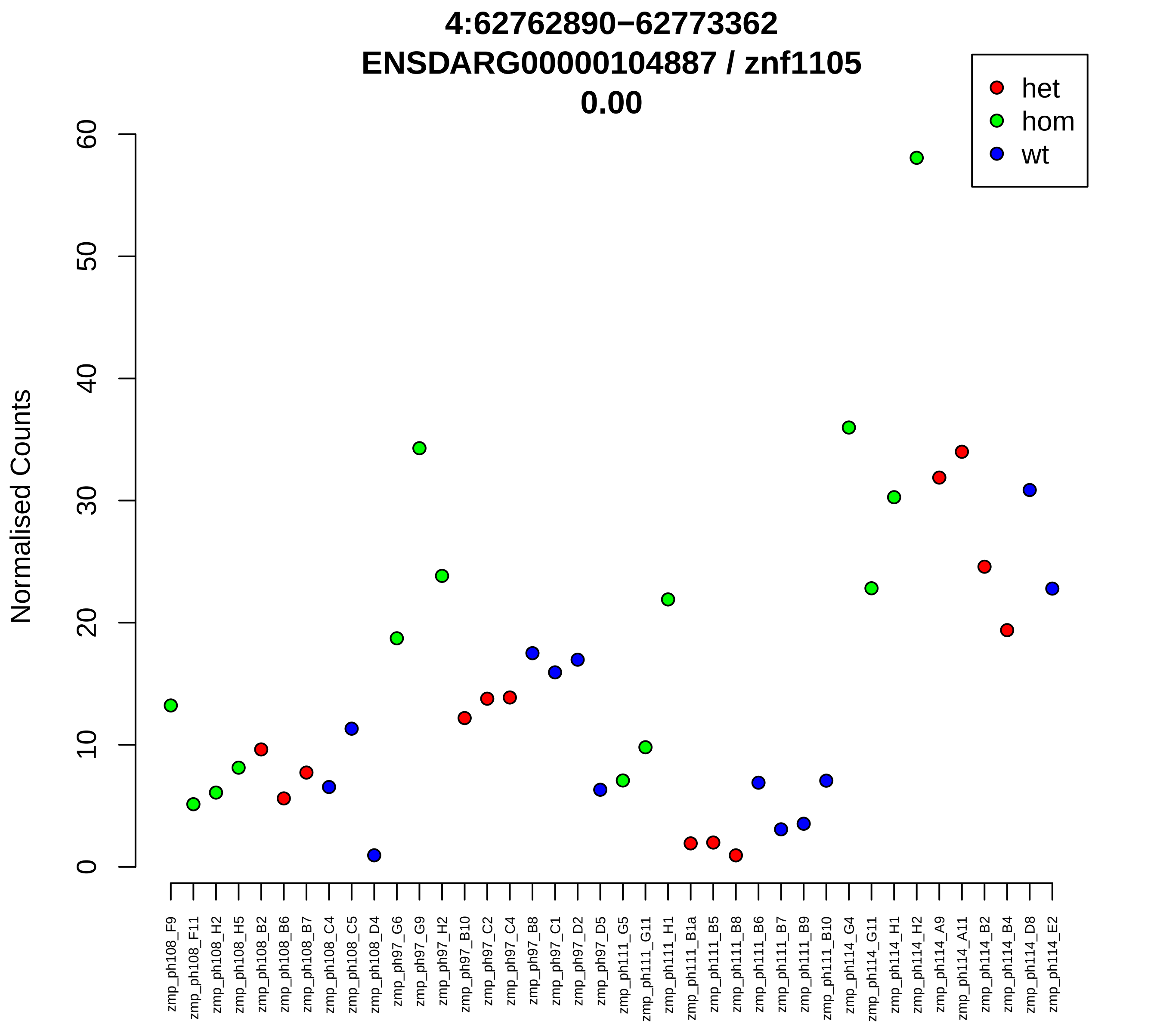
4:62762890−62773362
ENSDARG00000104887 / znf1105
0.00
het
hom
wt
Normalised Counts
0
10
20
30
40
50
60
zmp_ph108_F9
zmp_ph108_F11
zmp_ph108_H2
zmp_ph108_H5
zmp_ph108_B2
zmp_ph108_B6
zmp_ph108_B7
zmp_ph108_C4
zmp_ph108_C5
zmp_ph108_D4
zmp_ph97_G6
zmp_ph97_G9
zmp_ph97_H2
zmp_ph97_B10
zmp_ph97_C2
zmp_ph97_C4
zmp_ph97_B8
zmp_ph97_C1
zmp_ph97_D2
zmp_ph97_D5
zmp_ph111_G5
zmp_ph111_G11
zmp_ph111_H1
zmp_ph111_B1a
zmp_ph111_B5
zmp_ph111_B8
zmp_ph111_B6
zmp_ph111_B7
zmp_ph111_B9
zmp_ph111_B10
zmp_ph114_G4
zmp_ph114_G11
zmp_ph114_H1
zmp_ph114_H2
zmp_ph114_A9
zmp_ph114_A11
zmp_ph114_B2
zmp_ph114_B4
zmp_ph114_D8
zmp_ph114_E2

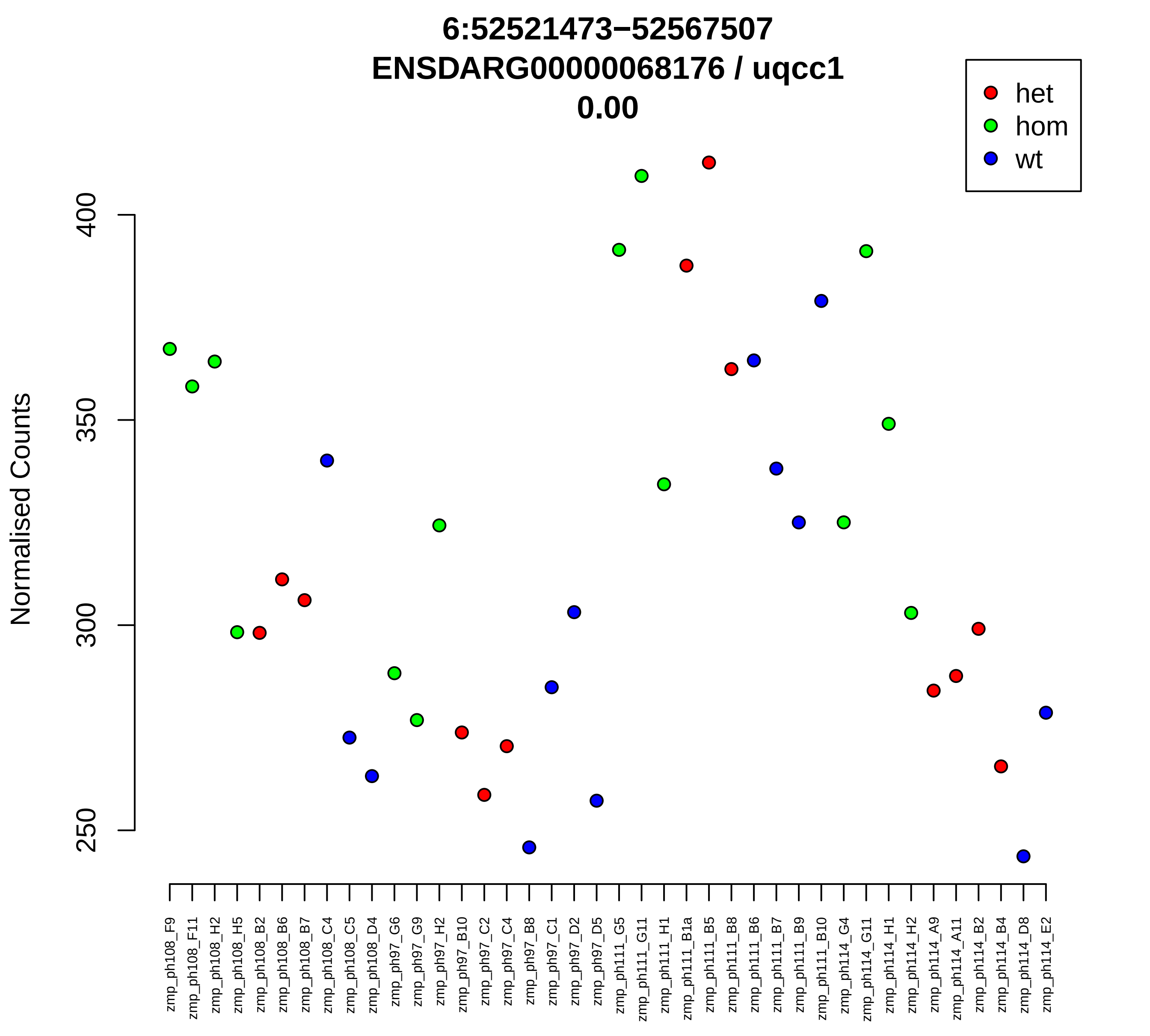
6:52521473−52567507
ENSDARG00000068176 / uqcc1
0.00
het
hom
wt
Normalised Counts
400
350
300
250
zmp_ph108_F9
zmp_ph108_F11
zmp_ph108_H2
zmp_ph108_H5
zmp_ph108_B2
zmp_ph108_B6
zmp_ph108_B7
zmp_ph108_C4
zmp_ph108_C5
zmp_ph108_D4
zmp_ph97_G6
zmp_ph97_G9
zmp_ph97_H2
zmp_ph97_B10
zmp_ph97_C2
zmp_ph97_C4
zmp_ph97_B8
zmp_ph97_C1
zmp_ph97_D2
zmp_ph97_D5
zmp_ph111_G5
zmp_ph111_G11
zmp_ph111_H1
zmp_ph111_B1a
zmp_ph111_B5
zmp_ph111_B8
zmp_ph111_B6
zmp_ph111_B7
zmp_ph111_B9
zmp_ph111_B10
zmp_ph114_G4
zmp_ph114_G11
zmp_ph114_H1
zmp_ph114_H2
zmp_ph114_A9
zmp_ph114_A11
zmp_ph114_B2
zmp_ph114_B4
zmp_ph114_D8
zmp_ph114_E2

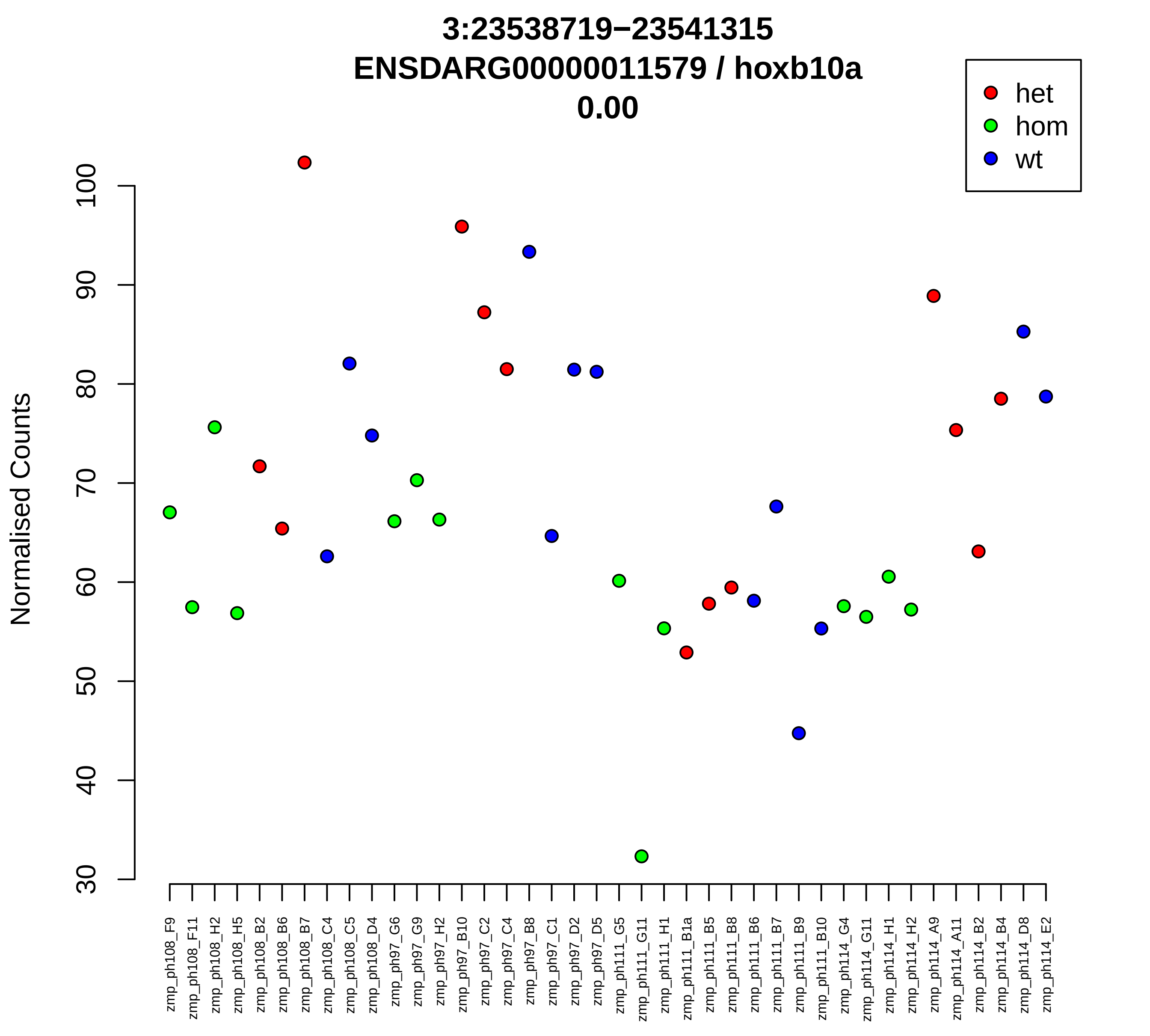

3:23538719−23541315
ENSDARG00000011579 / hoxb10a
0.00
het
hom
wt
Normalised Counts
30
40
50
60
70
80
90
100
zmp_ph108_F9
zmp_ph108_F11
zmp_ph108_H2
zmp_ph108_H5
zmp_ph108_B2
zmp_ph108_B6
zmp_ph108_B7
zmp_ph108_C4
zmp_ph108_C5
zmp_ph108_D4
zmp_ph97_G6
zmp_ph97_G9
zmp_ph97_H2
zmp_ph97_B10
zmp_ph97_C2
zmp_ph97_C4
zmp_ph97_B8
zmp_ph97_C1
zmp_ph97_D2
zmp_ph97_D5
zmp_ph111_G5
zmp_ph111_G11
zmp_ph111_H1
zmp_ph111_B1a
zmp_ph111_B5
zmp_ph111_B8
zmp_ph111_B6
zmp_ph111_B7
zmp_ph111_B9
zmp_ph111_B10
zmp_ph114_G4
zmp_ph114_G11
zmp_ph114_H1
zmp_ph114_H2
zmp_ph114_A9
zmp_ph114_A11
zmp_ph114_B2
zmp_ph114_B4
zmp_ph114_D8
zmp_ph114_E2

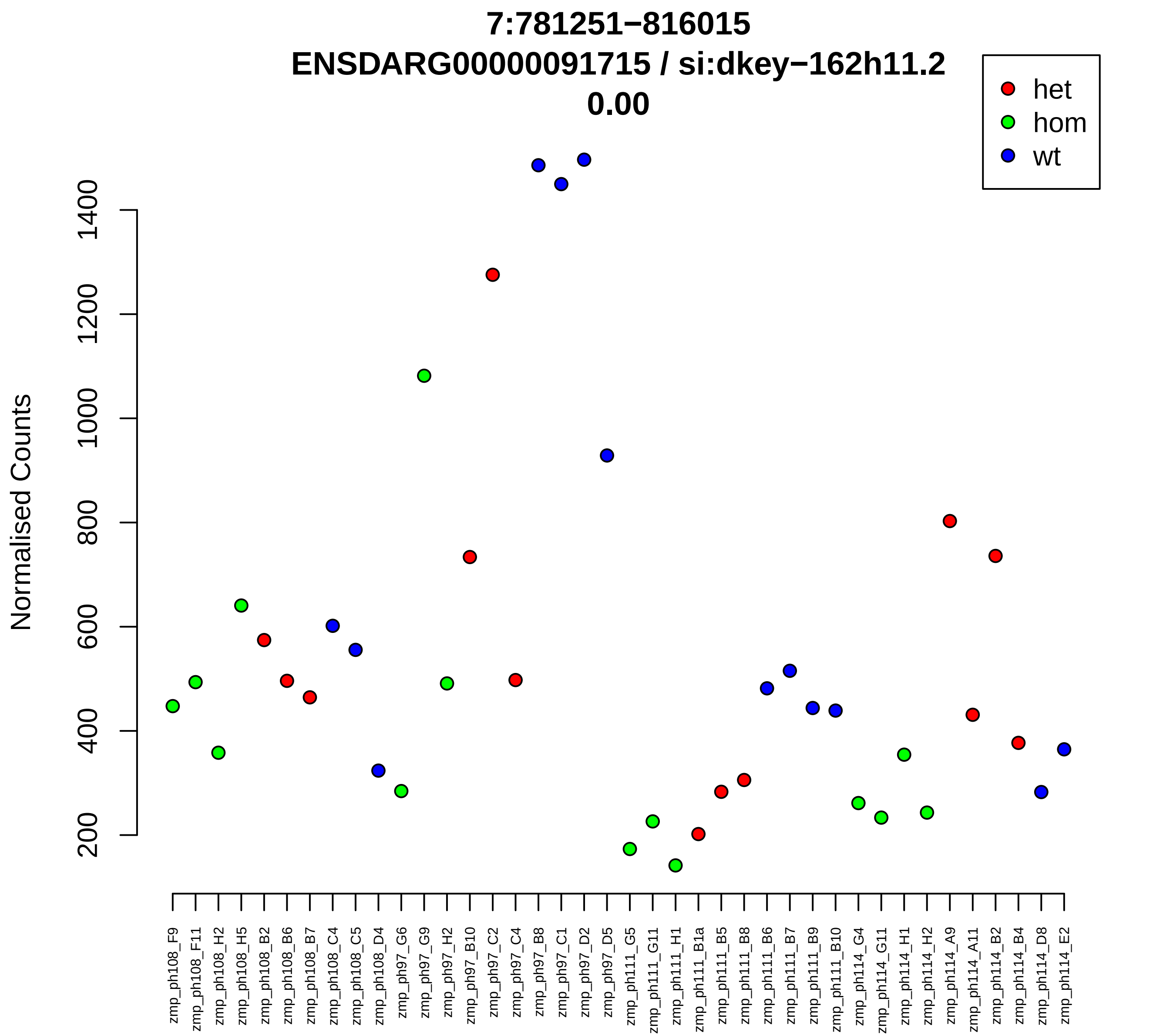

7:781251−816015
ENSDARG00000091715 / si:dkey−162h11.2
0.00
het
hom
wt
Normalised Counts
200
400
600
800
1000
1200
1400
zmp_ph108_F9
zmp_ph108_F11
zmp_ph108_H2
zmp_ph108_H5
zmp_ph108_B2
zmp_ph108_B6
zmp_ph108_B7
zmp_ph108_C4
zmp_ph108_C5
zmp_ph108_D4
zmp_ph97_G6
zmp_ph97_G9
zmp_ph97_H2
zmp_ph97_B10
zmp_ph97_C2
zmp_ph97_C4
zmp_ph97_B8
zmp_ph97_C1
zmp_ph97_D2
zmp_ph97_D5
zmp_ph111_G5
zmp_ph111_G11
zmp_ph111_H1
zmp_ph111_B1a
zmp_ph111_B5
zmp_ph111_B8
zmp_ph111_B6
zmp_ph111_B7
zmp_ph111_B9
zmp_ph111_B10
zmp_ph114_G4
zmp_ph114_G11
zmp_ph114_H1
zmp_ph114_H2
zmp_ph114_A9
zmp_ph114_A11
zmp_ph114_B2
zmp_ph114_B4
zmp_ph114_D8
zmp_ph114_E2

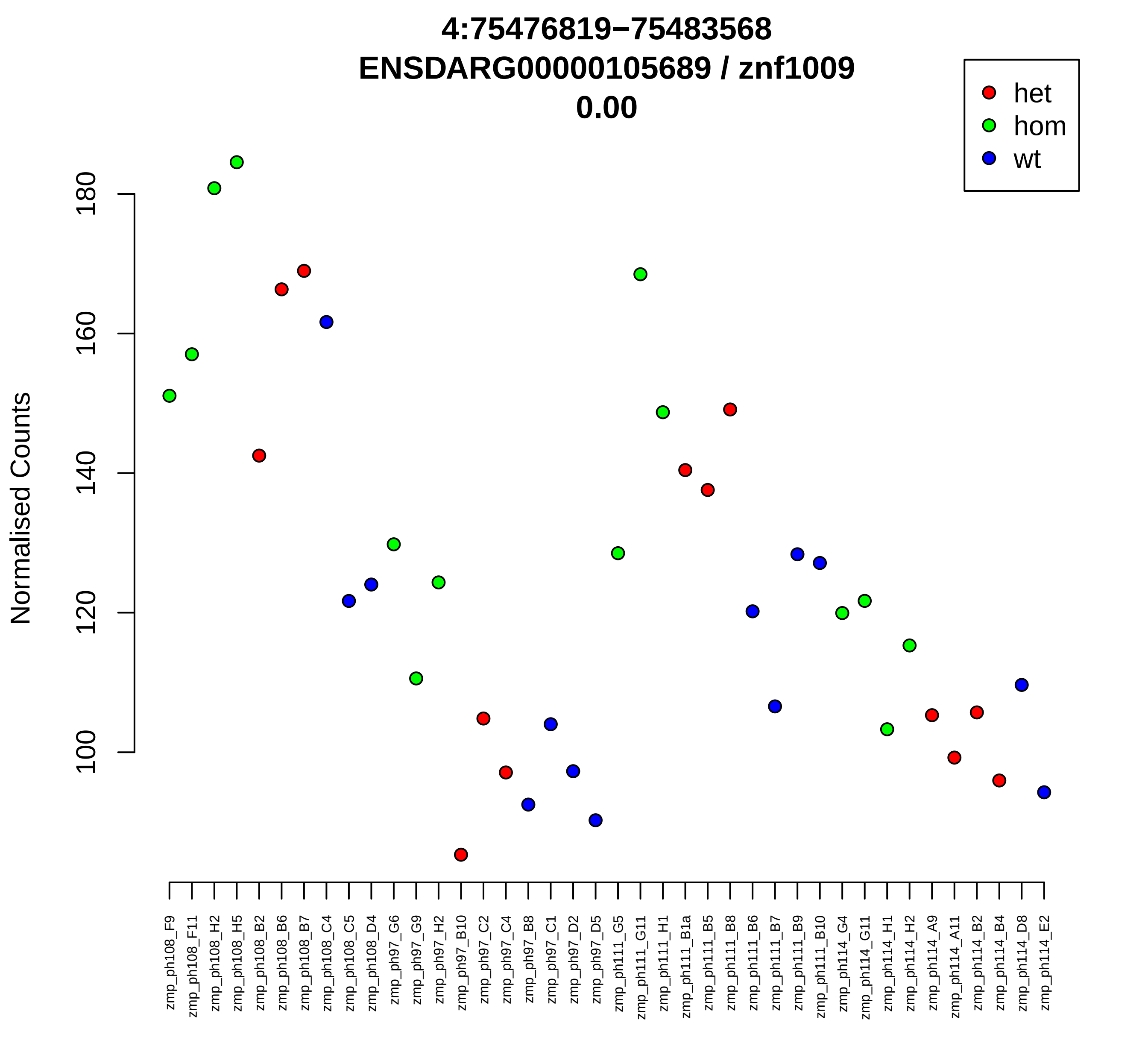
4:75476819−75483568
ENSDARG00000105689 / znf1009
0.00
het
hom
wt
Normalised Counts
100
120
140
160
180
zmp_ph108_F9
zmp_ph108_F11
zmp_ph108_H2
zmp_ph108_H5
zmp_ph108_B2
zmp_ph108_B6
zmp_ph108_B7
zmp_ph108_C4
zmp_ph108_C5
zmp_ph108_D4
zmp_ph97_G6
zmp_ph97_G9
zmp_ph97_H2
zmp_ph97_B10
zmp_ph97_C2
zmp_ph97_C4
zmp_ph97_B8
zmp_ph97_C1
zmp_ph97_D2
zmp_ph97_D5
zmp_ph111_G5
zmp_ph111_G11
zmp_ph111_H1
zmp_ph111_B1a
zmp_ph111_B5
zmp_ph111_B8
zmp_ph111_B6
zmp_ph111_B7
zmp_ph111_B9
zmp_ph111_B10
zmp_ph114_G4
zmp_ph114_G11
zmp_ph114_H1
zmp_ph114_H2
zmp_ph114_A9
zmp_ph114_A11
zmp_ph114_B2
zmp_ph114_B4
zmp_ph114_D8
zmp_ph114_E2

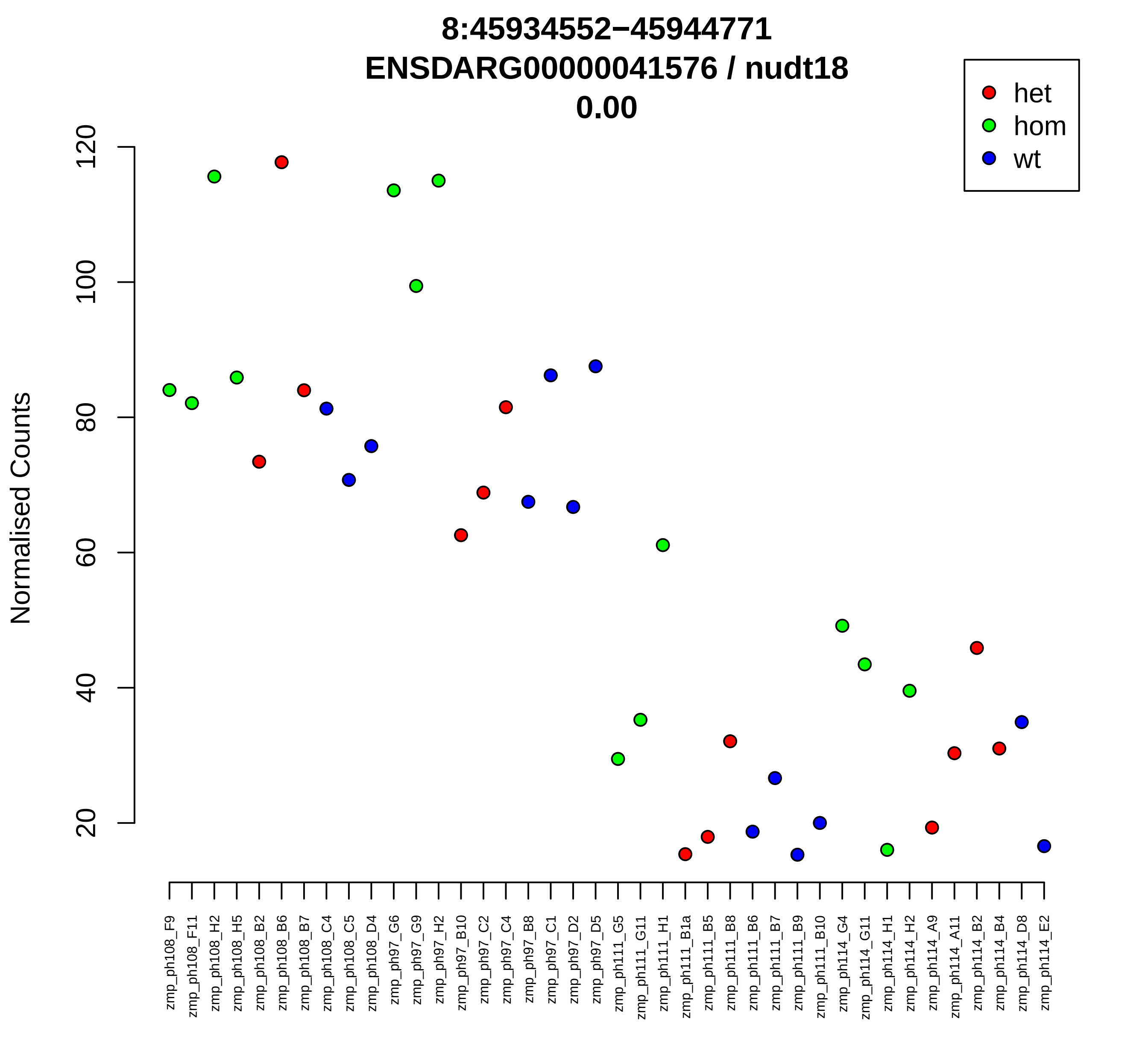
8:45934552−45944771
ENSDARG00000041576 / nudt18
0.00
het
hom
wt
Normalised Counts
20
40
60
80
100
120
zmp_ph108_F9
zmp_ph108_F11
zmp_ph108_H2
zmp_ph108_H5
zmp_ph108_B2
zmp_ph108_B6
zmp_ph108_B7
zmp_ph108_C4
zmp_ph108_C5
zmp_ph108_D4
zmp_ph97_G6
zmp_ph97_G9
zmp_ph97_H2
zmp_ph97_B10
zmp_ph97_C2
zmp_ph97_C4
zmp_ph97_B8
zmp_ph97_C1
zmp_ph97_D2
zmp_ph97_D5
zmp_ph111_G5
zmp_ph111_G11
zmp_ph111_H1
zmp_ph111_B1a
zmp_ph111_B5
zmp_ph111_B8
zmp_ph111_B6
zmp_ph111_B7
zmp_ph111_B9
zmp_ph111_B10
zmp_ph114_G4
zmp_ph114_G11
zmp_ph114_H1
zmp_ph114_H2
zmp_ph114_A9
zmp_ph114_A11
zmp_ph114_B2
zmp_ph114_B4
zmp_ph114_D8
zmp_ph114_E2

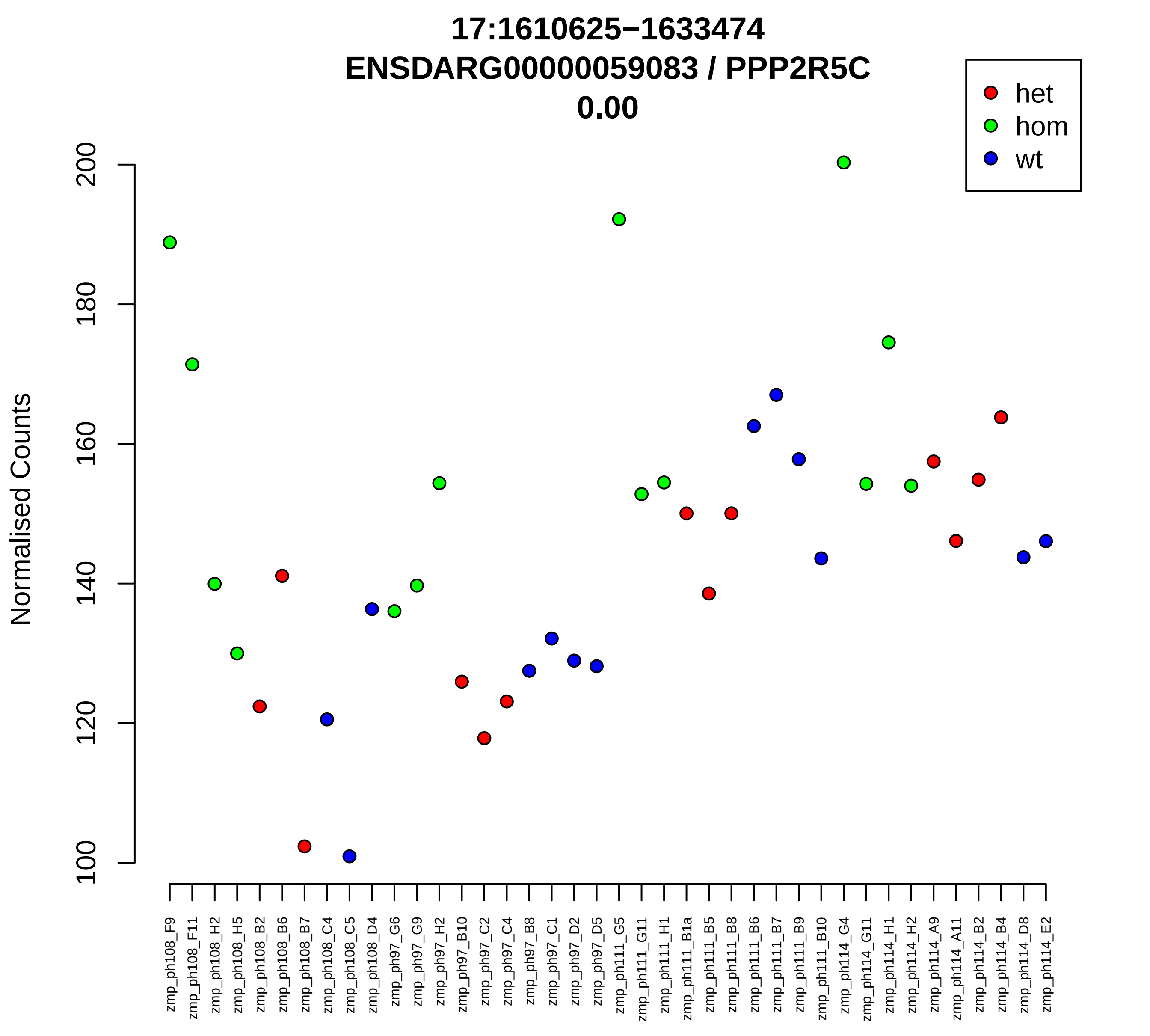

17:1610625−1633474
ENSDARG00000059083 / PPP2R5C
0.00
het
hom
wt
Normalised Counts
100
120
140
160
180
200
zmp_ph108_F9
zmp_ph108_F11
zmp_ph108_H2
zmp_ph108_H5
zmp_ph108_B2
zmp_ph108_B6
zmp_ph108_B7
zmp_ph108_C4
zmp_ph108_C5
zmp_ph108_D4
zmp_ph97_G6
zmp_ph97_G9
zmp_ph97_H2
zmp_ph97_B10
zmp_ph97_C2
zmp_ph97_C4
zmp_ph97_B8
zmp_ph97_C1
zmp_ph97_D2
zmp_ph97_D5
zmp_ph111_G5
zmp_ph111_G11
zmp_ph111_H1
zmp_ph111_B1a
zmp_ph111_B5
zmp_ph111_B8
zmp_ph111_B6
zmp_ph111_B7
zmp_ph111_B9
zmp_ph111_B10
zmp_ph114_G4
zmp_ph114_G11
zmp_ph114_H1
zmp_ph114_H2
zmp_ph114_A9
zmp_ph114_A11
zmp_ph114_B2
zmp_ph114_B4
zmp_ph114_D8
zmp_ph114_E2

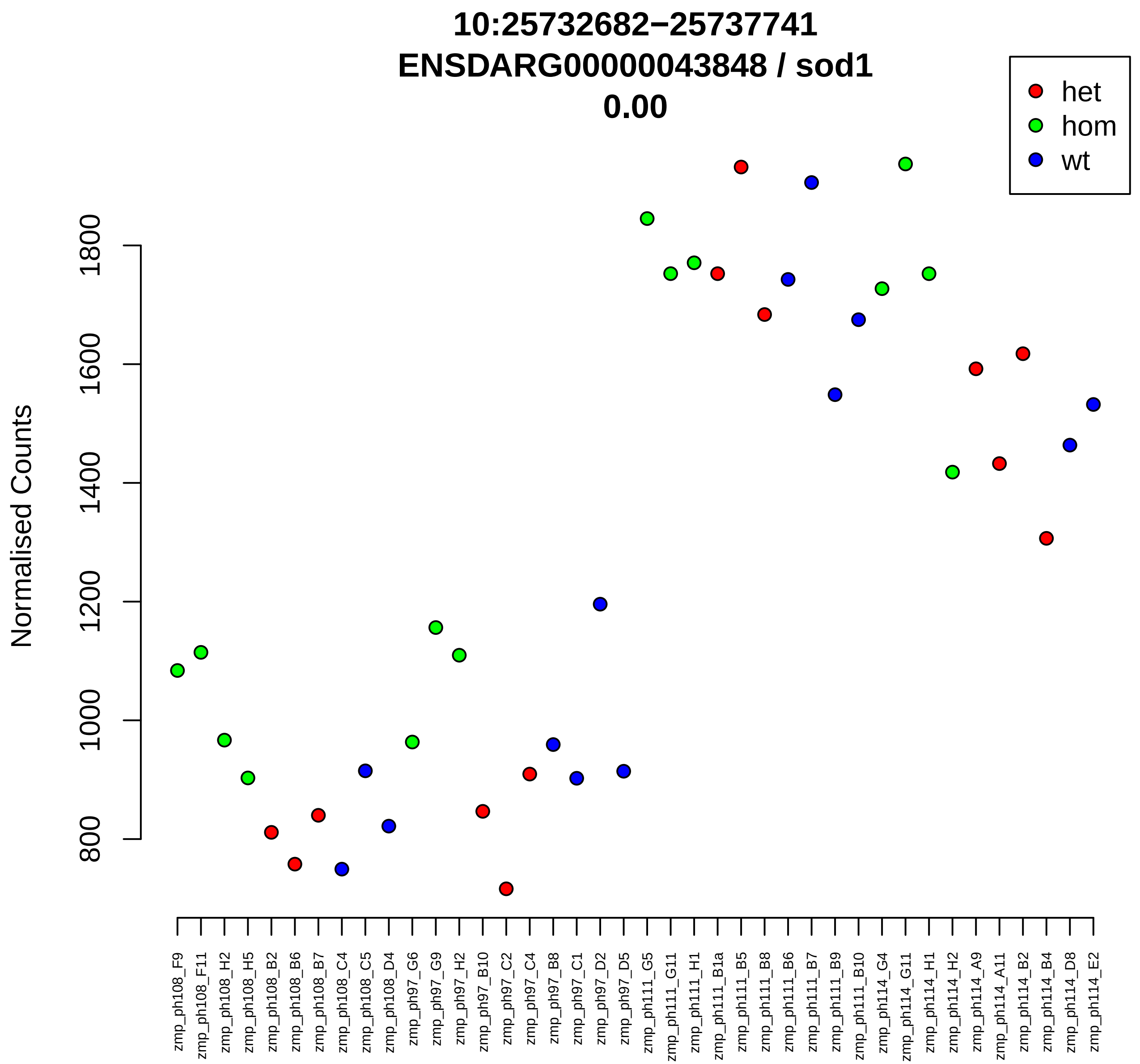

10:25732682−25737741
ENSDARG00000043848 / sod1
0.00
het
hom
wt
Normalised Counts
800
1000
1200
1400
1600
1800
zmp_ph108_F9
zmp_ph108_F11
zmp_ph108_H2
zmp_ph108_H5
zmp_ph108_B2
zmp_ph108_B6
zmp_ph108_B7
zmp_ph108_C4
zmp_ph108_C5
zmp_ph108_D4
zmp_ph97_G6
zmp_ph97_G9
zmp_ph97_H2
zmp_ph97_B10
zmp_ph97_C2
zmp_ph97_C4
zmp_ph97_B8
zmp_ph97_C1
zmp_ph97_D2
zmp_ph97_D5
zmp_ph111_G5
zmp_ph111_G11
zmp_ph111_H1
zmp_ph111_B1a
zmp_ph111_B5
zmp_ph111_B8
zmp_ph111_B6
zmp_ph111_B7
zmp_ph111_B9
zmp_ph111_B10
zmp_ph114_G4
zmp_ph114_G11
zmp_ph114_H1
zmp_ph114_H2
zmp_ph114_A9
zmp_ph114_A11
zmp_ph114_B2
zmp_ph114_B4
zmp_ph114_D8
zmp_ph114_E2

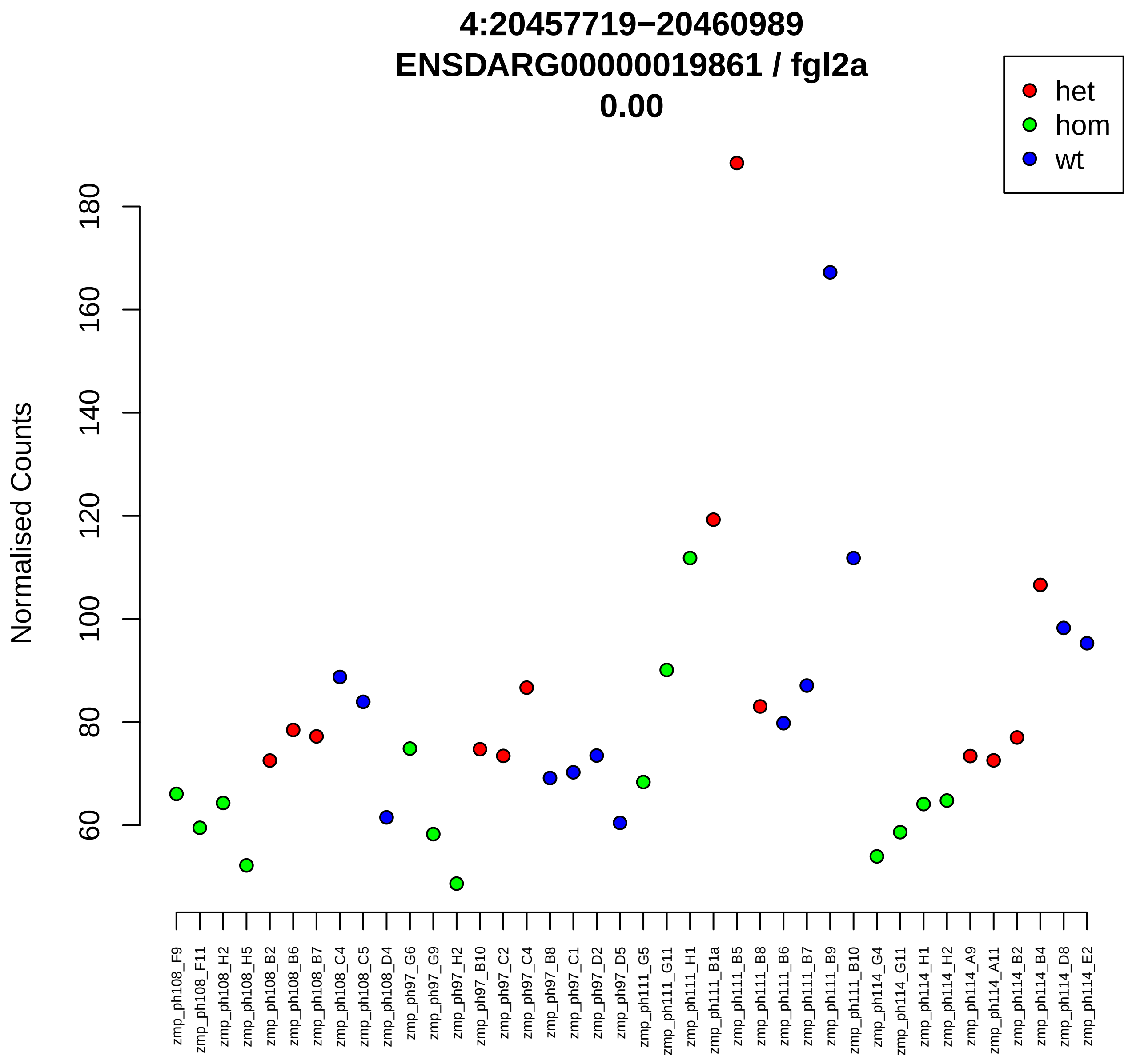

4:20457719−20460989
ENSDARG00000019861 / fgl2a
0.00
het
hom
wt
Normalised Counts
60
80
100
120
140
160
180
zmp_ph108_F9
zmp_ph108_F11
zmp_ph108_H2
zmp_ph108_H5
zmp_ph108_B2
zmp_ph108_B6
zmp_ph108_B7
zmp_ph108_C4
zmp_ph108_C5
zmp_ph108_D4
zmp_ph97_G6
zmp_ph97_G9
zmp_ph97_H2
zmp_ph97_B10
zmp_ph97_C2
zmp_ph97_C4
zmp_ph97_B8
zmp_ph97_C1
zmp_ph97_D2
zmp_ph97_D5
zmp_ph111_G5
zmp_ph111_G11
zmp_ph111_H1
zmp_ph111_B1a
zmp_ph111_B5
zmp_ph111_B8
zmp_ph111_B6
zmp_ph111_B7
zmp_ph111_B9
zmp_ph111_B10
zmp_ph114_G4
zmp_ph114_G11
zmp_ph114_H1
zmp_ph114_H2
zmp_ph114_A9
zmp_ph114_A11
zmp_ph114_B2
zmp_ph114_B4
zmp_ph114_D8
zmp_ph114_E2

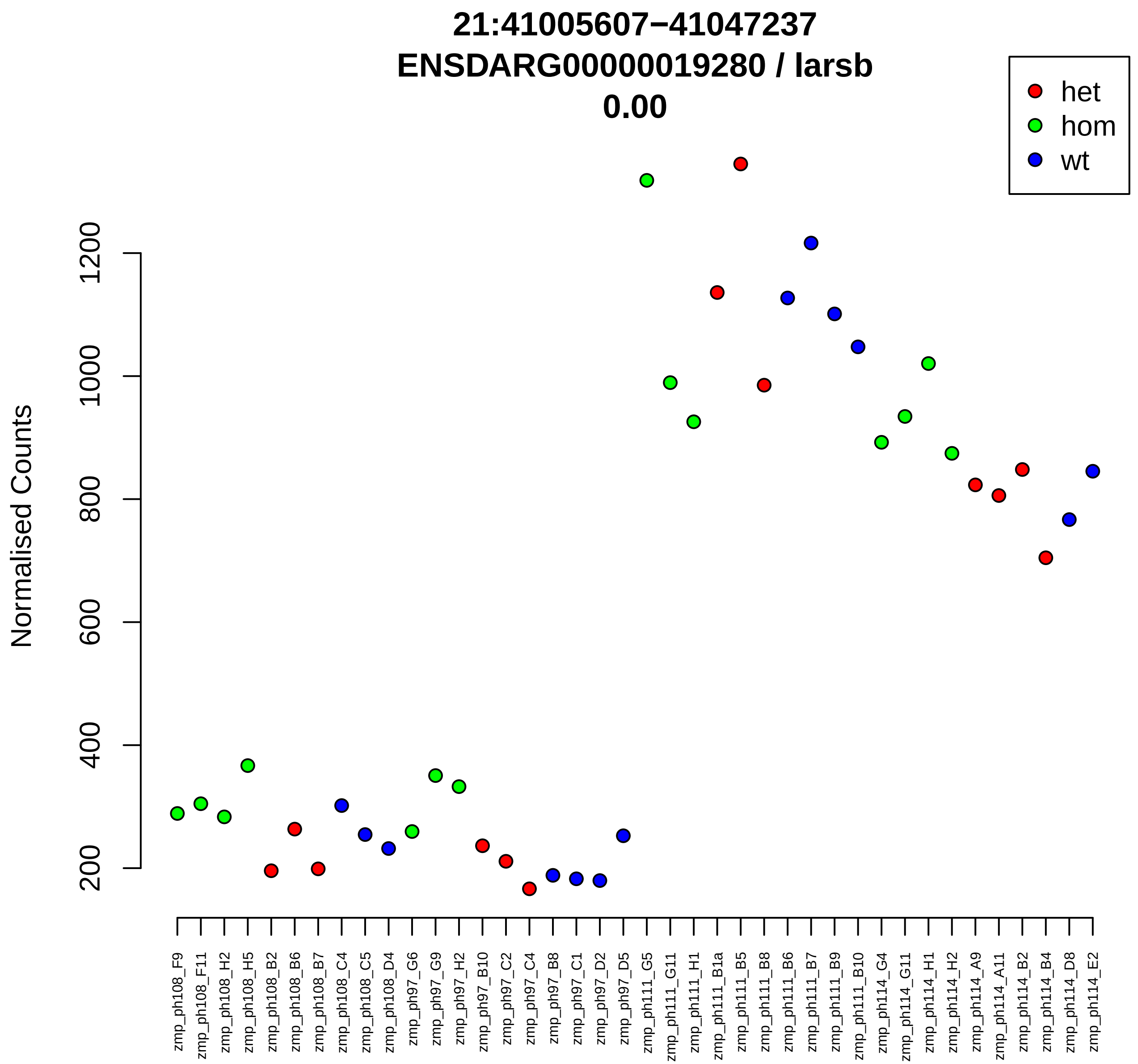
21:41005607−41047237
ENSDARG00000019280 / larsb
0.00
het
hom
wt
Normalised Counts
200
400
600
800
1000
1200
zmp_ph108_F9
zmp_ph108_F11
zmp_ph108_H2
zmp_ph108_H5
zmp_ph108_B2
zmp_ph108_B6
zmp_ph108_B7
zmp_ph108_C4
zmp_ph108_C5
zmp_ph108_D4
zmp_ph97_G6
zmp_ph97_G9
zmp_ph97_H2
zmp_ph97_B10
zmp_ph97_C2
zmp_ph97_C4
zmp_ph97_B8
zmp_ph97_C1
zmp_ph97_D2
zmp_ph97_D5
zmp_ph111_G5
zmp_ph111_G11
zmp_ph111_H1
zmp_ph111_B1a
zmp_ph111_B5
zmp_ph111_B8
zmp_ph111_B6
zmp_ph111_B7
zmp_ph111_B9
zmp_ph111_B10
zmp_ph114_G4
zmp_ph114_G11
zmp_ph114_H1
zmp_ph114_H2
zmp_ph114_A9
zmp_ph114_A11
zmp_ph114_B2
zmp_ph114_B4
zmp_ph114_D8
zmp_ph114_E2

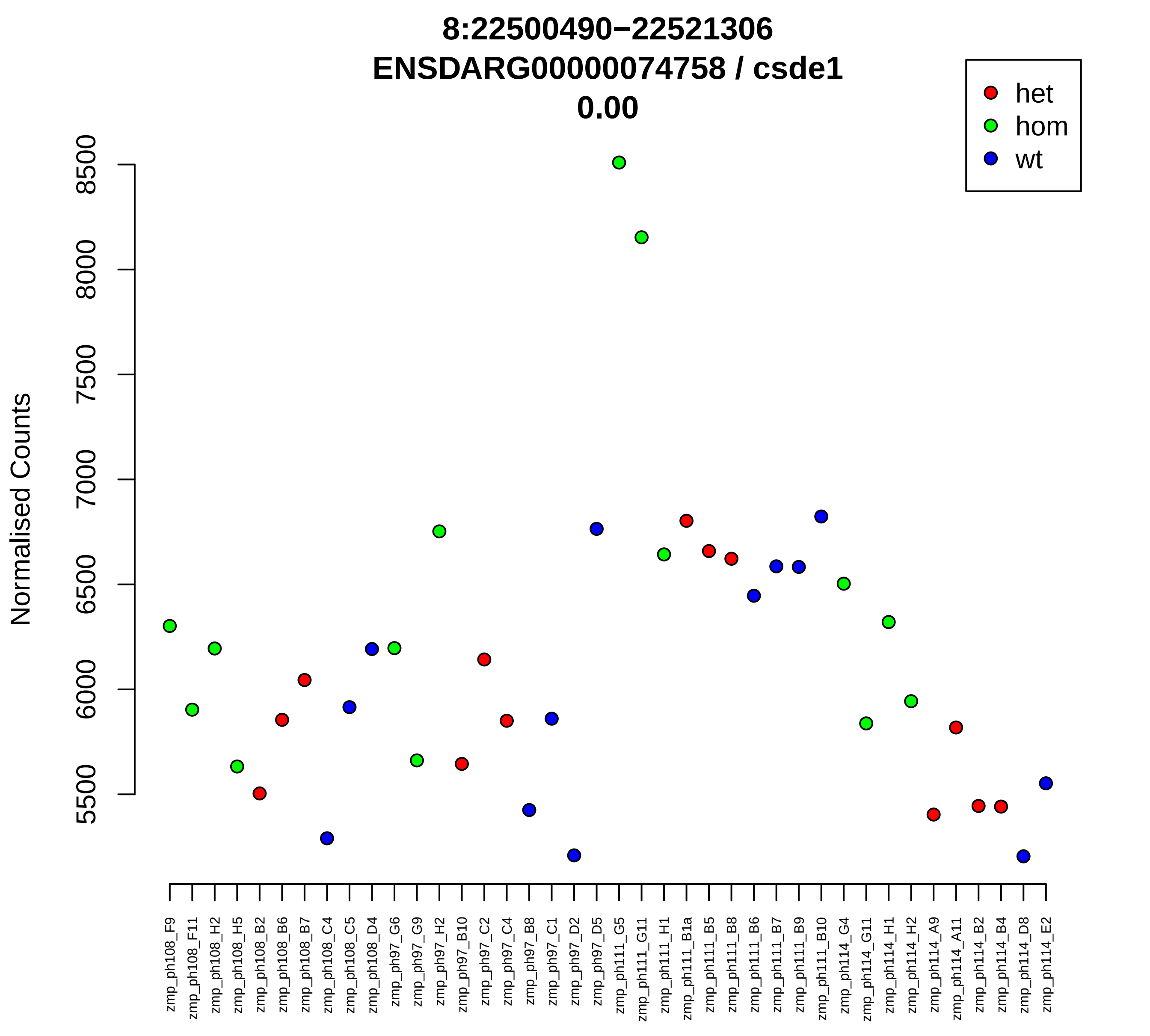

8:22500490−22521306
ENSDARG00000074758 / csde1
0.00
het
hom
wt
Normalised Counts
5500
6000
6500
7000
7500
8000
8500
zmp_ph108_F9
zmp_ph108_F11
zmp_ph108_H2
zmp_ph108_H5
zmp_ph108_B2
zmp_ph108_B6
zmp_ph108_B7
zmp_ph108_C4
zmp_ph108_C5
zmp_ph108_D4
zmp_ph97_G6
zmp_ph97_G9
zmp_ph97_H2
zmp_ph97_B10
zmp_ph97_C2
zmp_ph97_C4
zmp_ph97_B8
zmp_ph97_C1
zmp_ph97_D2
zmp_ph97_D5
zmp_ph111_G5
zmp_ph111_G11
zmp_ph111_H1
zmp_ph111_B1a
zmp_ph111_B5
zmp_ph111_B8
zmp_ph111_B6
zmp_ph111_B7
zmp_ph111_B9
zmp_ph111_B10
zmp_ph114_G4
zmp_ph114_G11
zmp_ph114_H1
zmp_ph114_H2
zmp_ph114_A9
zmp_ph114_A11
zmp_ph114_B2
zmp_ph114_B4
zmp_ph114_D8
zmp_ph114_E2

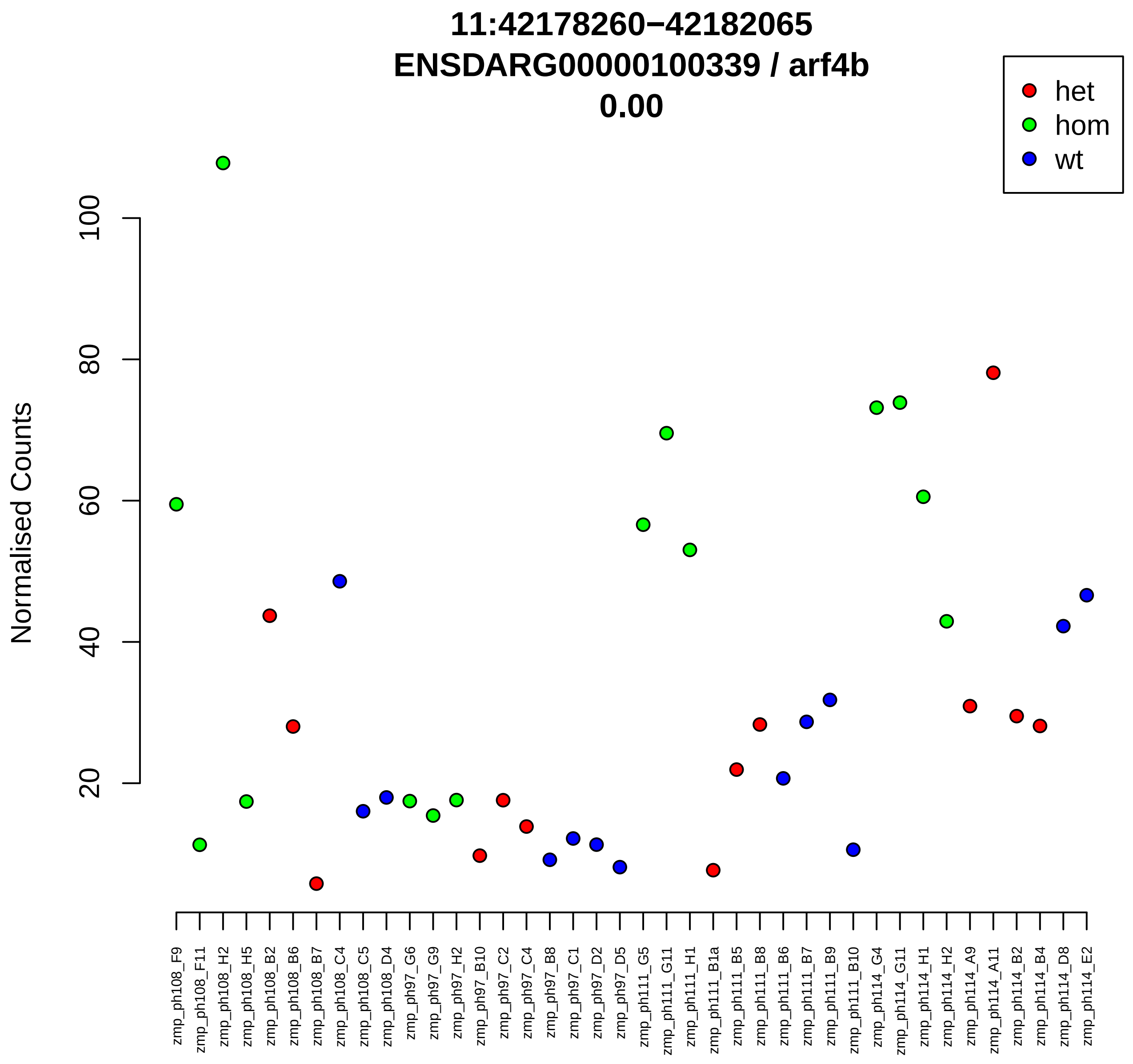

11:42178260−42182065
ENSDARG00000100339 / arf4b
0.00
het
hom
wt
Normalised Counts
20
40
60
80
100
zmp_ph108_F9
zmp_ph108_F11
zmp_ph108_H2
zmp_ph108_H5
zmp_ph108_B2
zmp_ph108_B6
zmp_ph108_B7
zmp_ph108_C4
zmp_ph108_C5
zmp_ph108_D4
zmp_ph97_G6
zmp_ph97_G9
zmp_ph97_H2
zmp_ph97_B10
zmp_ph97_C2
zmp_ph97_C4
zmp_ph97_B8
zmp_ph97_C1
zmp_ph97_D2
zmp_ph97_D5
zmp_ph111_G5
zmp_ph111_G11
zmp_ph111_H1
zmp_ph111_B1a
zmp_ph111_B5
zmp_ph111_B8
zmp_ph111_B6
zmp_ph111_B7
zmp_ph111_B9
zmp_ph111_B10
zmp_ph114_G4
zmp_ph114_G11
zmp_ph114_H1
zmp_ph114_H2
zmp_ph114_A9
zmp_ph114_A11
zmp_ph114_B2
zmp_ph114_B4
zmp_ph114_D8
zmp_ph114_E2

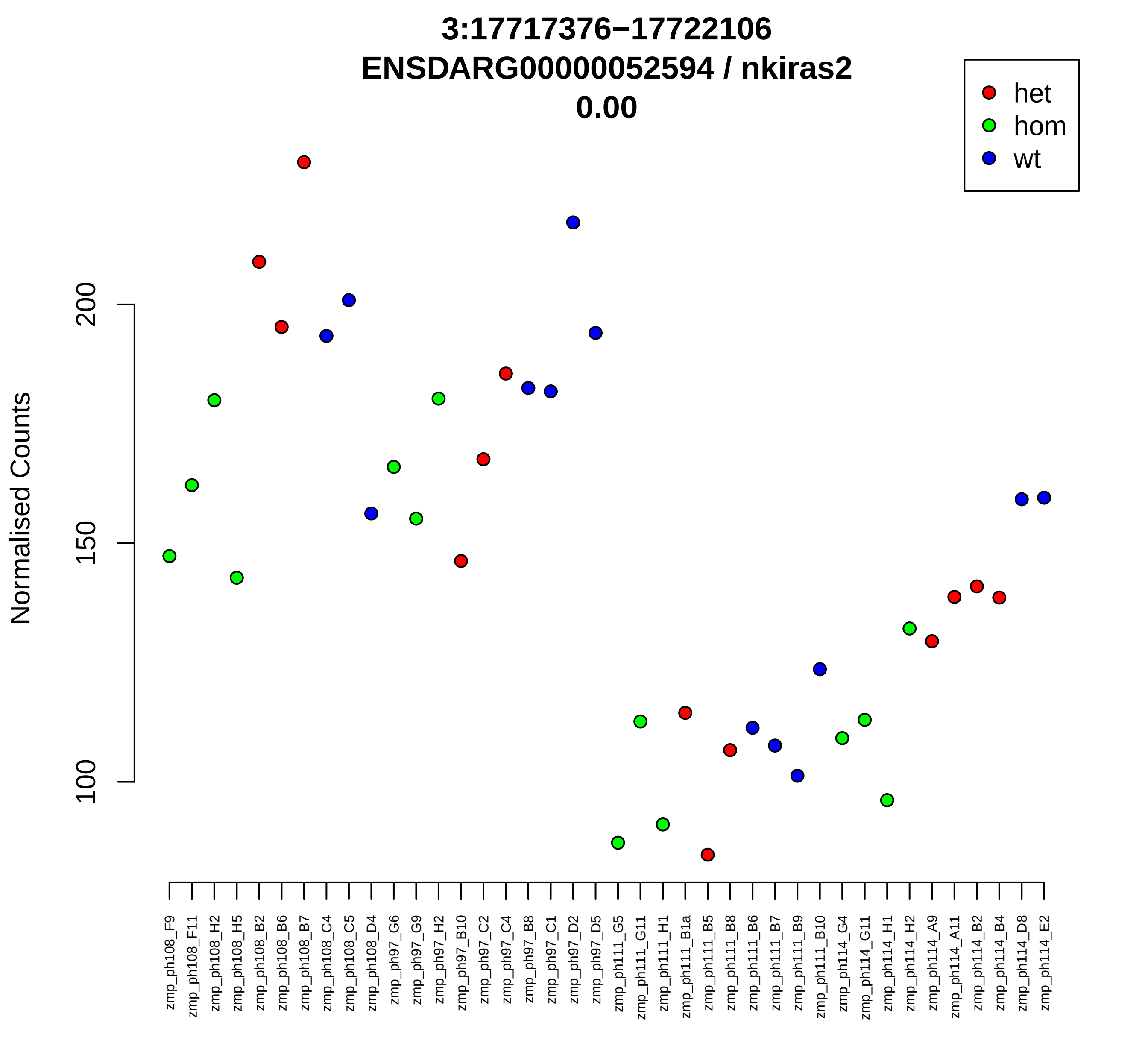

3:17717376−17722106
ENSDARG00000052594 / nkiras2
0.00
het
hom
wt
Normalised Counts
100
150
200
zmp_ph108_F9
zmp_ph108_F11
zmp_ph108_H2
zmp_ph108_H5
zmp_ph108_B2
zmp_ph108_B6
zmp_ph108_B7
zmp_ph108_C4
zmp_ph108_C5
zmp_ph108_D4
zmp_ph97_G6
zmp_ph97_G9
zmp_ph97_H2
zmp_ph97_B10
zmp_ph97_C2
zmp_ph97_C4
zmp_ph97_B8
zmp_ph97_C1
zmp_ph97_D2
zmp_ph97_D5
zmp_ph111_G5
zmp_ph111_G11
zmp_ph111_H1
zmp_ph111_B1a
zmp_ph111_B5
zmp_ph111_B8
zmp_ph111_B6
zmp_ph111_B7
zmp_ph111_B9
zmp_ph111_B10
zmp_ph114_G4
zmp_ph114_G11
zmp_ph114_H1
zmp_ph114_H2
zmp_ph114_A9
zmp_ph114_A11
zmp_ph114_B2
zmp_ph114_B4
zmp_ph114_D8
zmp_ph114_E2

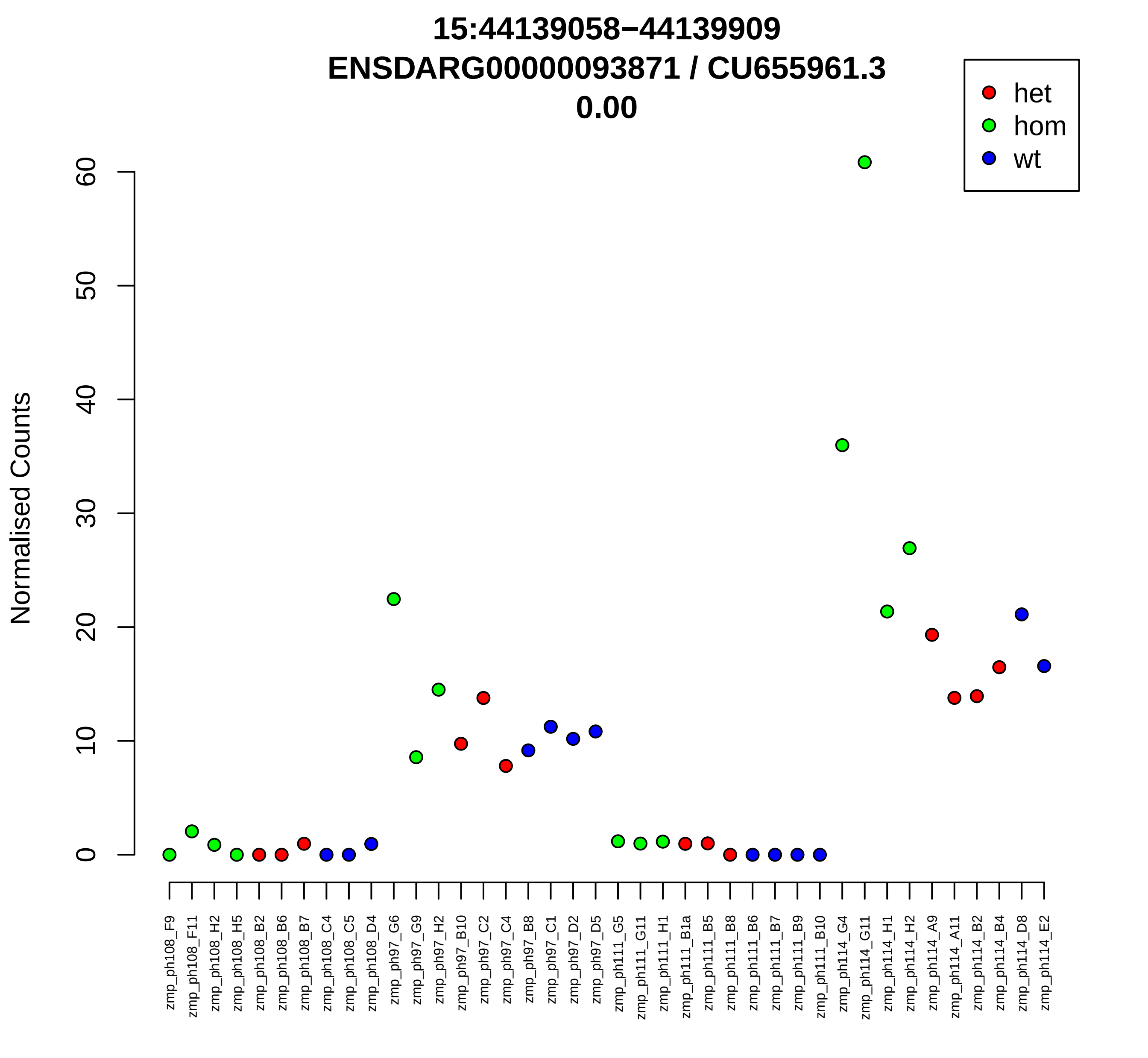
15:44139058−44139909
ENSDARG00000093871 / CU655961.3
0.00
het
hom
wt
Normalised Counts
0
10
20
30
40
50
60
zmp_ph108_F9
zmp_ph108_F11
zmp_ph108_H2
zmp_ph108_H5
zmp_ph108_B2
zmp_ph108_B6
zmp_ph108_B7
zmp_ph108_C4
zmp_ph108_C5
zmp_ph108_D4
zmp_ph97_G6
zmp_ph97_G9
zmp_ph97_H2
zmp_ph97_B10
zmp_ph97_C2
zmp_ph97_C4
zmp_ph97_B8
zmp_ph97_C1
zmp_ph97_D2
zmp_ph97_D5
zmp_ph111_G5
zmp_ph111_G11
zmp_ph111_H1
zmp_ph111_B1a
zmp_ph111_B5
zmp_ph111_B8
zmp_ph111_B6
zmp_ph111_B7
zmp_ph111_B9
zmp_ph111_B10
zmp_ph114_G4
zmp_ph114_G11
zmp_ph114_H1
zmp_ph114_H2
zmp_ph114_A9
zmp_ph114_A11
zmp_ph114_B2
zmp_ph114_B4
zmp_ph114_D8
zmp_ph114_E2

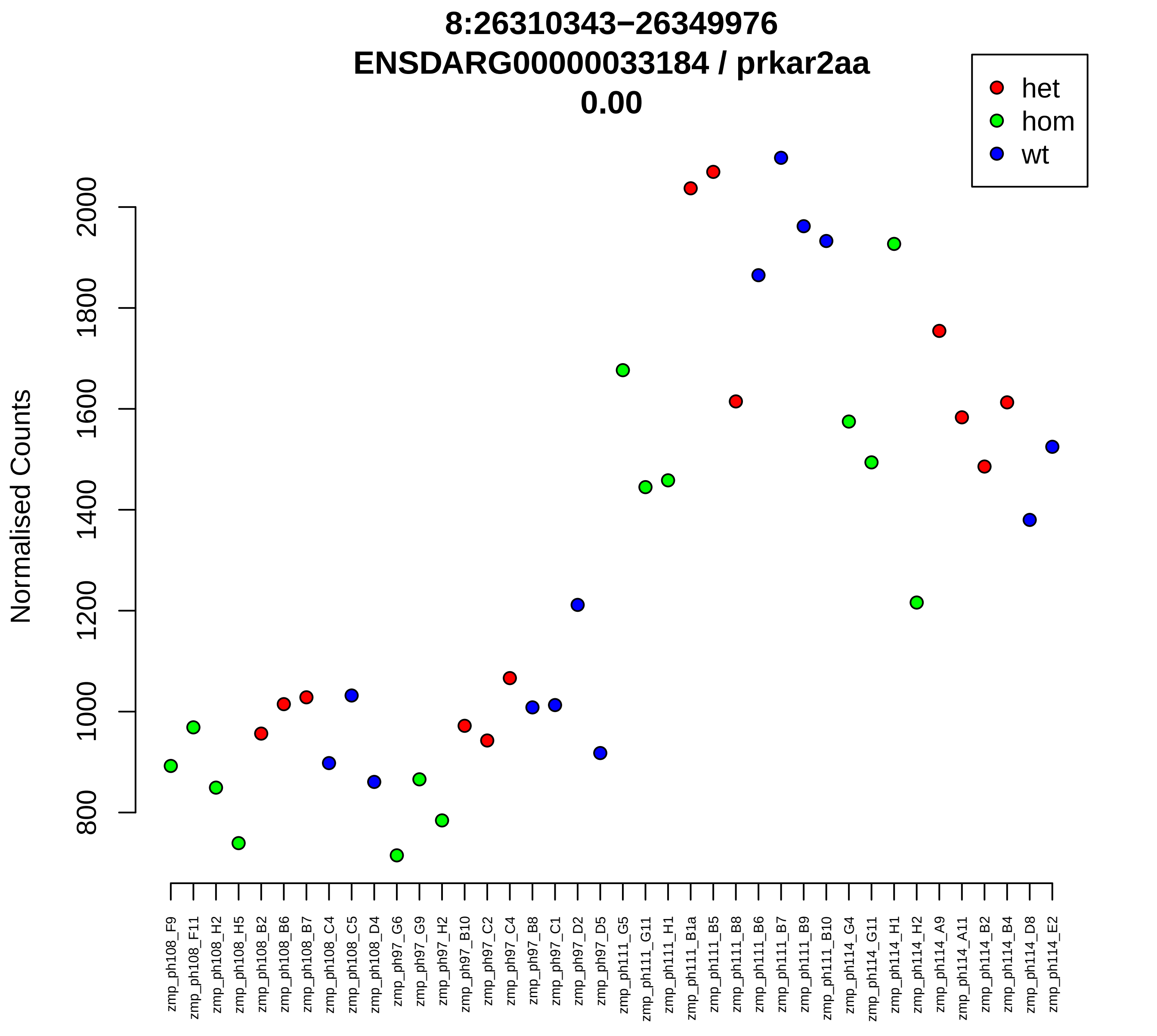

8:26310343−26349976
ENSDARG00000033184 / prkar2aa
0.00
het
hom
wt
Normalised Counts
800
1000
1200
1400
1600
1800
2000
zmp_ph108_F9
zmp_ph108_F11
zmp_ph108_H2
zmp_ph108_H5
zmp_ph108_B2
zmp_ph108_B6
zmp_ph108_B7
zmp_ph108_C4
zmp_ph108_C5
zmp_ph108_D4
zmp_ph97_G6
zmp_ph97_G9
zmp_ph97_H2
zmp_ph97_B10
zmp_ph97_C2
zmp_ph97_C4
zmp_ph97_B8
zmp_ph97_C1
zmp_ph97_D2
zmp_ph97_D5
zmp_ph111_G5
zmp_ph111_G11
zmp_ph111_H1
zmp_ph111_B1a
zmp_ph111_B5
zmp_ph111_B8
zmp_ph111_B6
zmp_ph111_B7
zmp_ph111_B9
zmp_ph111_B10
zmp_ph114_G4
zmp_ph114_G11
zmp_ph114_H1
zmp_ph114_H2
zmp_ph114_A9
zmp_ph114_A11
zmp_ph114_B2
zmp_ph114_B4
zmp_ph114_D8
zmp_ph114_E2

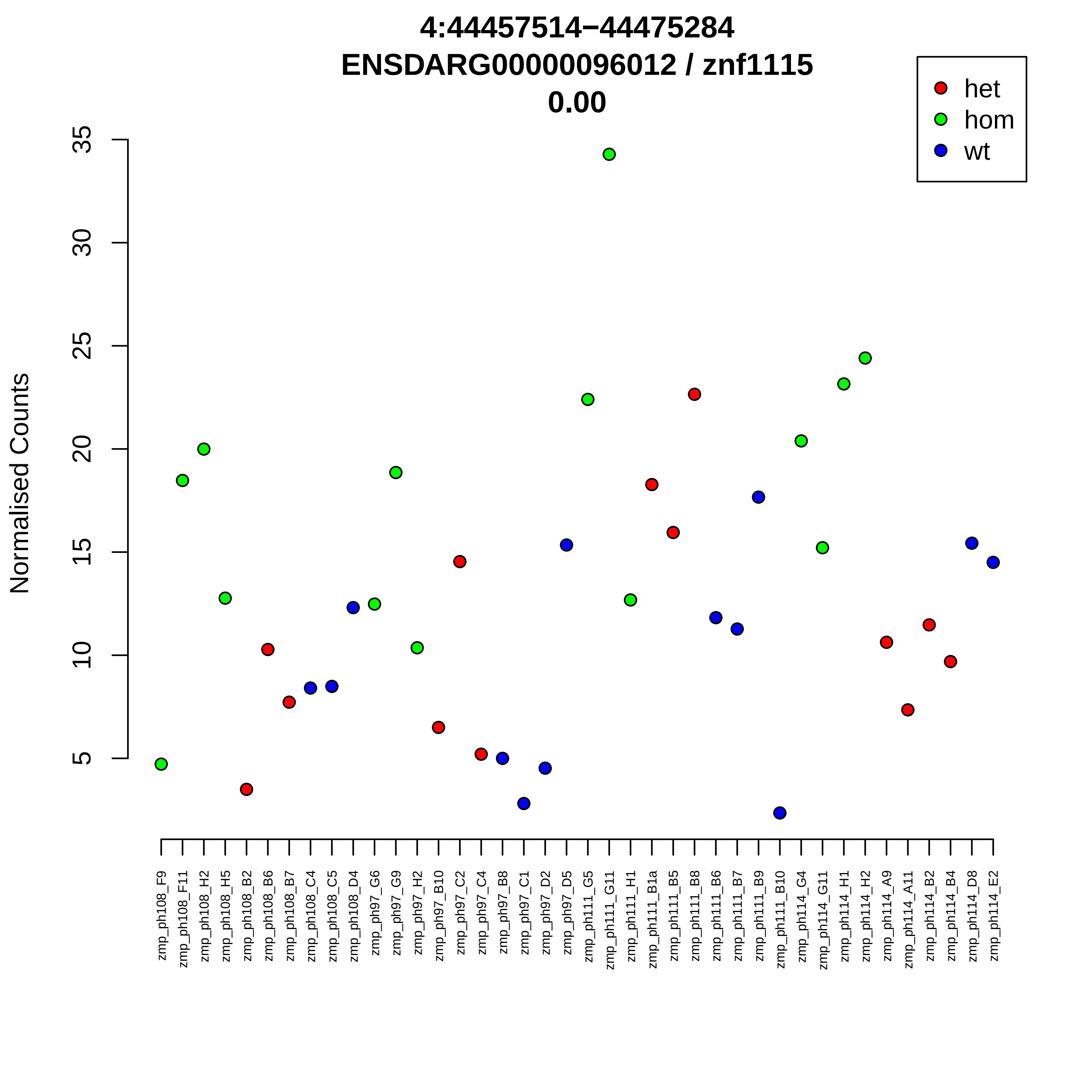

4:44457514−44475284
ENSDARG00000096012 / znf1115
0.00
het
hom
wt
Normalised Counts
5
10
15
20
25
30
35
zmp_ph108_F9
zmp_ph108_F11
zmp_ph108_H2
zmp_ph108_H5
zmp_ph108_B2
zmp_ph108_B6
zmp_ph108_B7
zmp_ph108_C4
zmp_ph108_C5
zmp_ph108_D4
zmp_ph97_G6
zmp_ph97_G9
zmp_ph97_H2
zmp_ph97_B10
zmp_ph97_C2
zmp_ph97_C4
zmp_ph97_B8
zmp_ph97_C1
zmp_ph97_D2
zmp_ph97_D5
zmp_ph111_G5
zmp_ph111_G11
zmp_ph111_H1
zmp_ph111_B1a
zmp_ph111_B5
zmp_ph111_B8
zmp_ph111_B6
zmp_ph111_B7
zmp_ph111_B9
zmp_ph111_B10
zmp_ph114_G4
zmp_ph114_G11
zmp_ph114_H1
zmp_ph114_H2
zmp_ph114_A9
zmp_ph114_A11
zmp_ph114_B2
zmp_ph114_B4
zmp_ph114_D8
zmp_ph114_E2

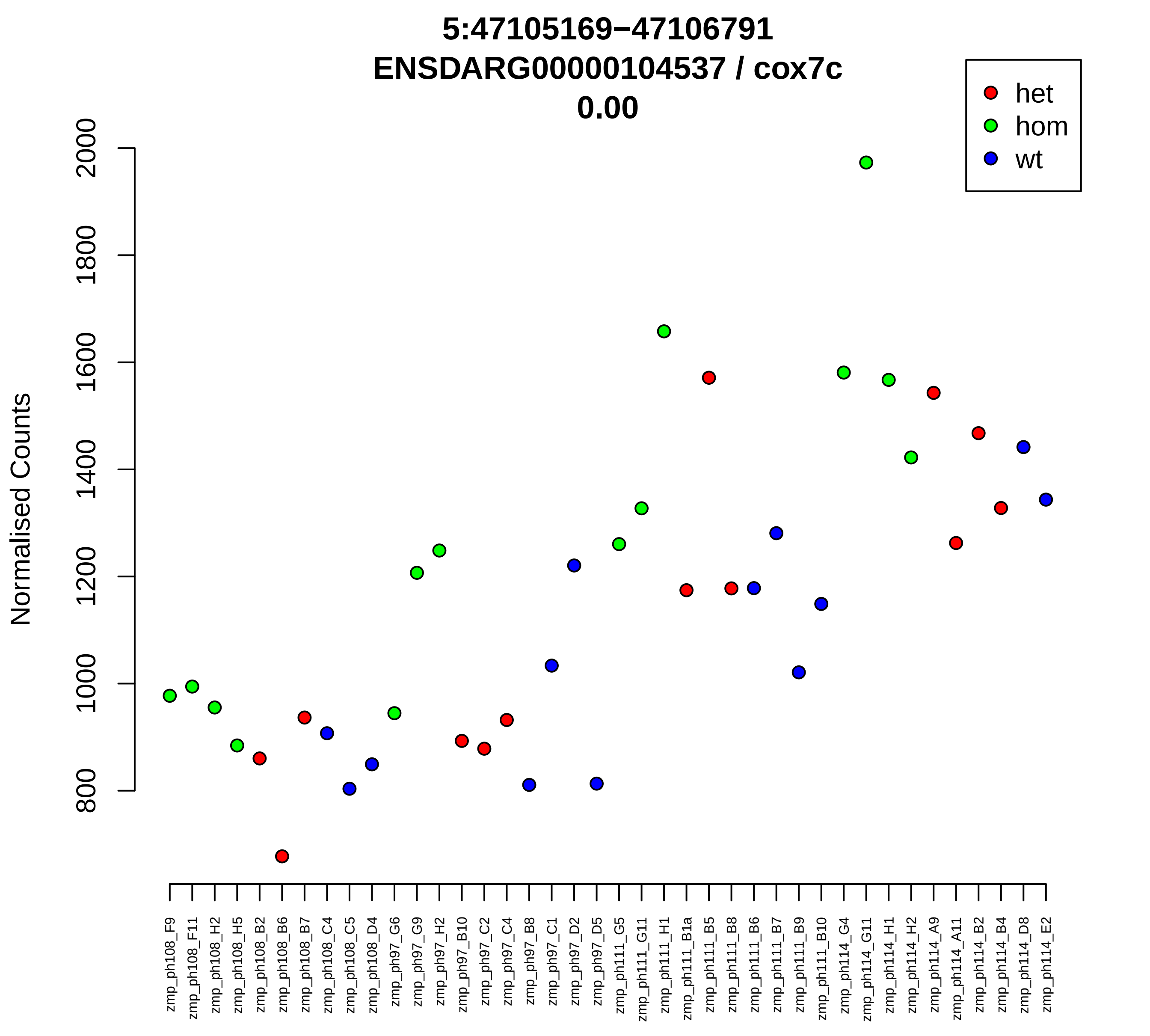

5:47105169−47106791
ENSDARG00000104537 / cox7c
0.00
het
hom
wt
Normalised Counts
2000
1800
1600
1400
1200
1000
800
zmp_ph108_F9
zmp_ph108_F11
zmp_ph108_H2
zmp_ph108_H5
zmp_ph108_B2
zmp_ph108_B6
zmp_ph108_B7
zmp_ph108_C4
zmp_ph108_C5
zmp_ph108_D4
zmp_ph97_G6
zmp_ph97_G9
zmp_ph97_H2
zmp_ph97_B10
zmp_ph97_C2
zmp_ph97_C4
zmp_ph97_B8
zmp_ph97_C1
zmp_ph97_D2
zmp_ph97_D5
zmp_ph111_G5
zmp_ph111_G11
zmp_ph111_H1
zmp_ph111_B1a
zmp_ph111_B5
zmp_ph111_B8
zmp_ph111_B6
zmp_ph111_B7
zmp_ph111_B9
zmp_ph111_B10
zmp_ph114_G4
zmp_ph114_G11
zmp_ph114_H1
zmp_ph114_H2
zmp_ph114_A9
zmp_ph114_A11
zmp_ph114_B2
zmp_ph114_B4
zmp_ph114_D8
zmp_ph114_E2

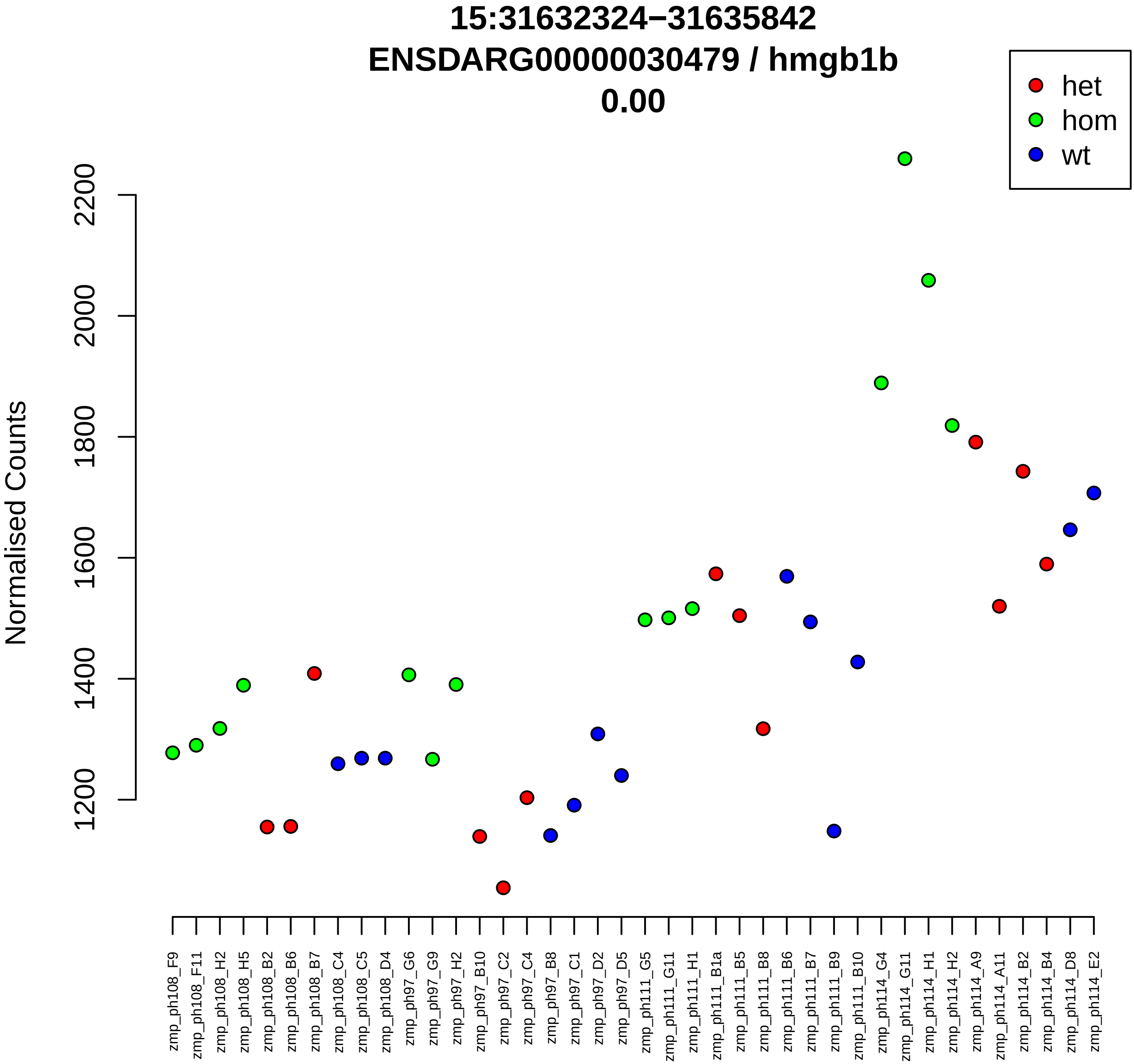

15:31632324−31635842
ENSDARG00000030479 / hmgb1b
0.00
het
hom
wt
Normalised Counts
1200
1400
1600
1800
2000
2200
zmp_ph108_F9
zmp_ph108_F11
zmp_ph108_H2
zmp_ph108_H5
zmp_ph108_B2
zmp_ph108_B6
zmp_ph108_B7
zmp_ph108_C4
zmp_ph108_C5
zmp_ph108_D4
zmp_ph97_G6
zmp_ph97_G9
zmp_ph97_H2
zmp_ph97_B10
zmp_ph97_C2
zmp_ph97_C4
zmp_ph97_B8
zmp_ph97_C1
zmp_ph97_D2
zmp_ph97_D5
zmp_ph111_G5
zmp_ph111_G11
zmp_ph111_H1
zmp_ph111_B1a
zmp_ph111_B5
zmp_ph111_B8
zmp_ph111_B6
zmp_ph111_B7
zmp_ph111_B9
zmp_ph111_B10
zmp_ph114_G4
zmp_ph114_G11
zmp_ph114_H1
zmp_ph114_H2
zmp_ph114_A9
zmp_ph114_A11
zmp_ph114_B2
zmp_ph114_B4
zmp_ph114_D8
zmp_ph114_E2

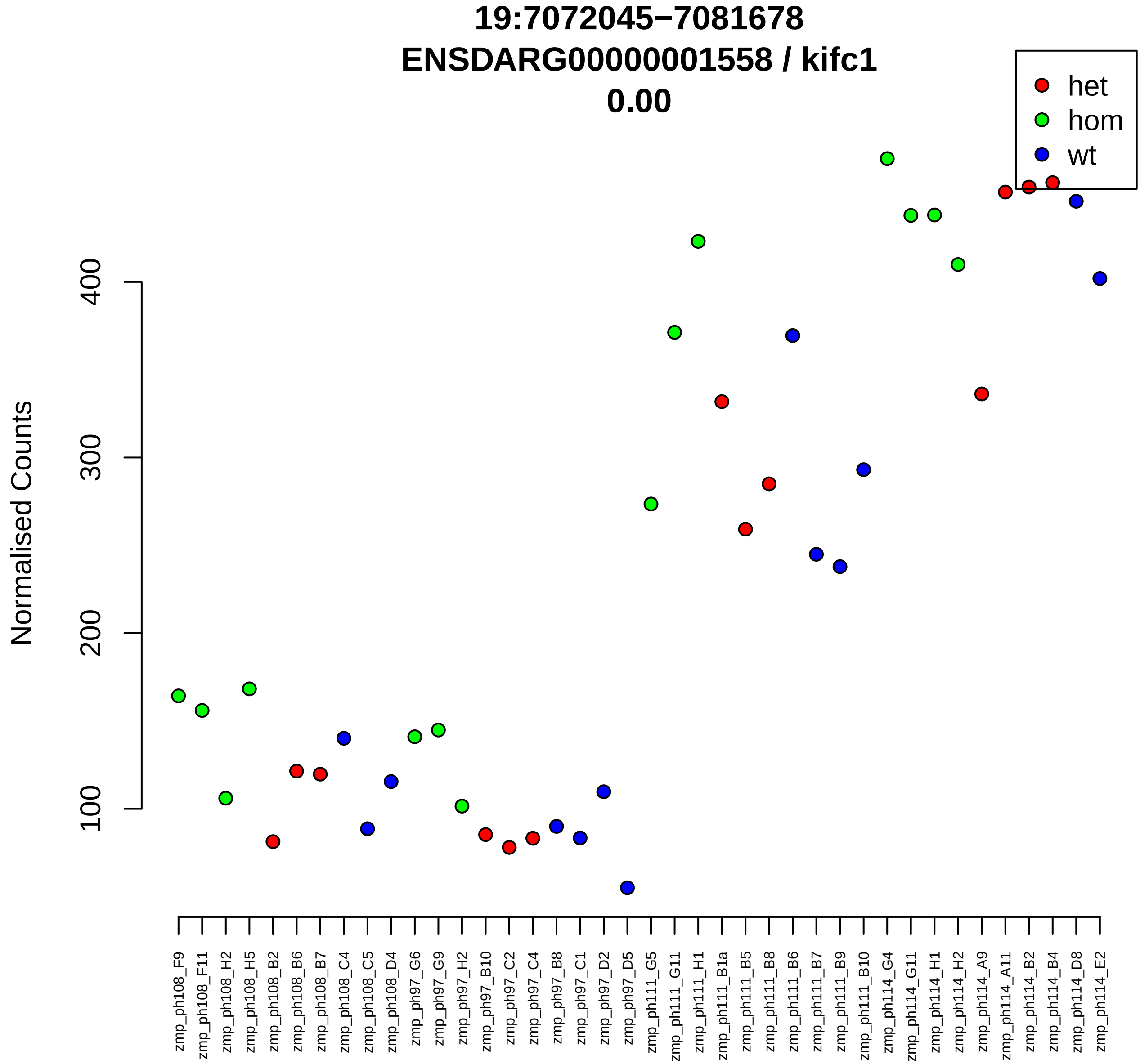

19:7072045−7081678
ENSDARG00000001558 / kifc1
0.00
het
hom
wt
Normalised Counts
100
200
300
400
zmp_ph108_F9
zmp_ph108_F11
zmp_ph108_H2
zmp_ph108_H5
zmp_ph108_B2
zmp_ph108_B6
zmp_ph108_B7
zmp_ph108_C4
zmp_ph108_C5
zmp_ph108_D4
zmp_ph97_G6
zmp_ph97_G9
zmp_ph97_H2
zmp_ph97_B10
zmp_ph97_C2
zmp_ph97_C4
zmp_ph97_B8
zmp_ph97_C1
zmp_ph97_D2
zmp_ph97_D5
zmp_ph111_G5
zmp_ph111_G11
zmp_ph111_H1
zmp_ph111_B1a
zmp_ph111_B5
zmp_ph111_B8
zmp_ph111_B6
zmp_ph111_B7
zmp_ph111_B9
zmp_ph111_B10
zmp_ph114_G4
zmp_ph114_G11
zmp_ph114_H1
zmp_ph114_H2
zmp_ph114_A9
zmp_ph114_A11
zmp_ph114_B2
zmp_ph114_B4
zmp_ph114_D8
zmp_ph114_E2

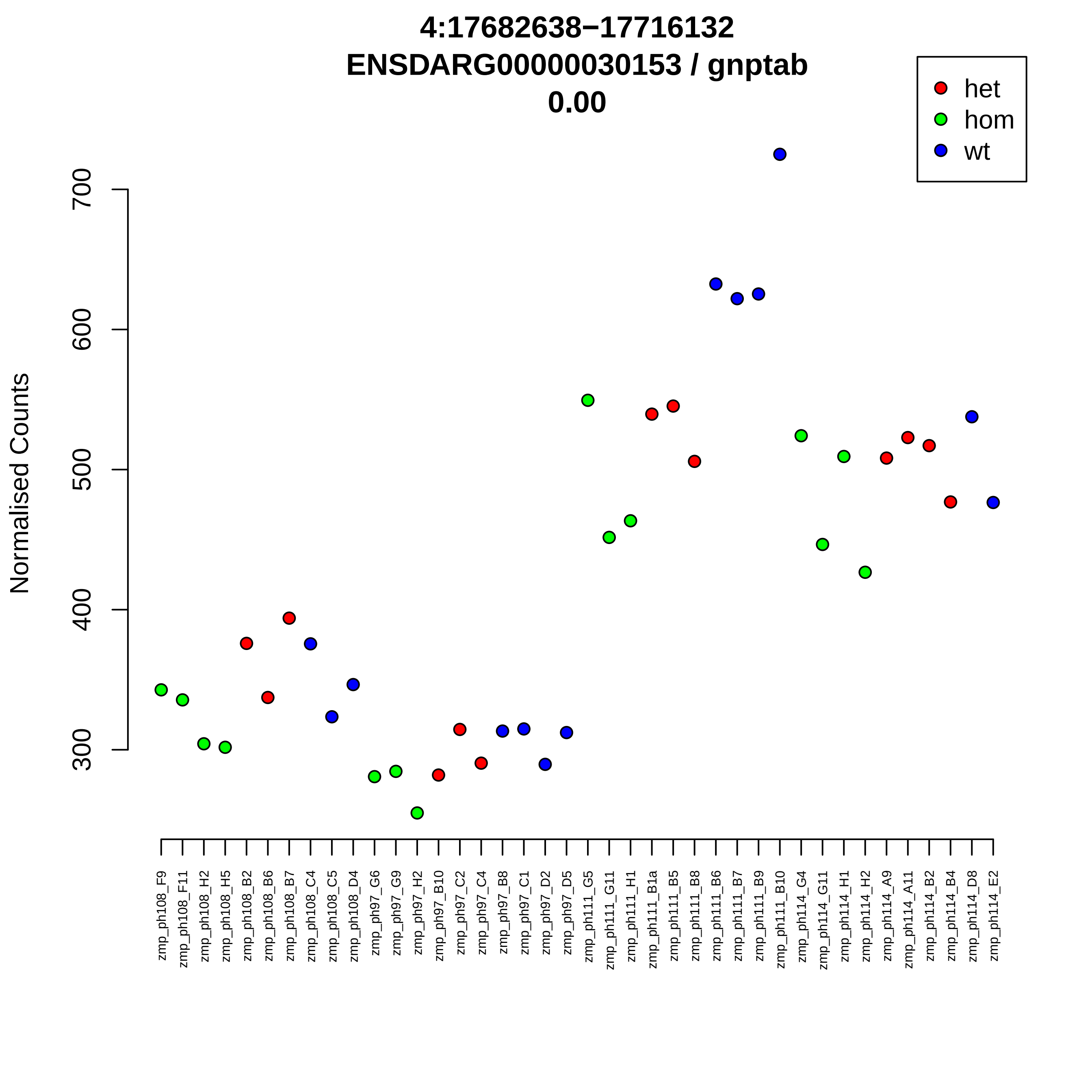

4:17682638−17716132
ENSDARG00000030153 / gnptab
0.00
het
hom
wt
Normalised Counts
300
400
500
600
700
zmp_ph108_F9
zmp_ph108_F11
zmp_ph108_H2
zmp_ph108_H5
zmp_ph108_B2
zmp_ph108_B6
zmp_ph108_B7
zmp_ph108_C4
zmp_ph108_C5
zmp_ph108_D4
zmp_ph97_G6
zmp_ph97_G9
zmp_ph97_H2
zmp_ph97_B10
zmp_ph97_C2
zmp_ph97_C4
zmp_ph97_B8
zmp_ph97_C1
zmp_ph97_D2
zmp_ph97_D5
zmp_ph111_G5
zmp_ph111_G11
zmp_ph111_H1
zmp_ph111_B1a
zmp_ph111_B5
zmp_ph111_B8
zmp_ph111_B6
zmp_ph111_B7
zmp_ph111_B9
zmp_ph111_B10
zmp_ph114_G4
zmp_ph114_G11
zmp_ph114_H1
zmp_ph114_H2
zmp_ph114_A9
zmp_ph114_A11
zmp_ph114_B2
zmp_ph114_B4
zmp_ph114_D8
zmp_ph114_E2

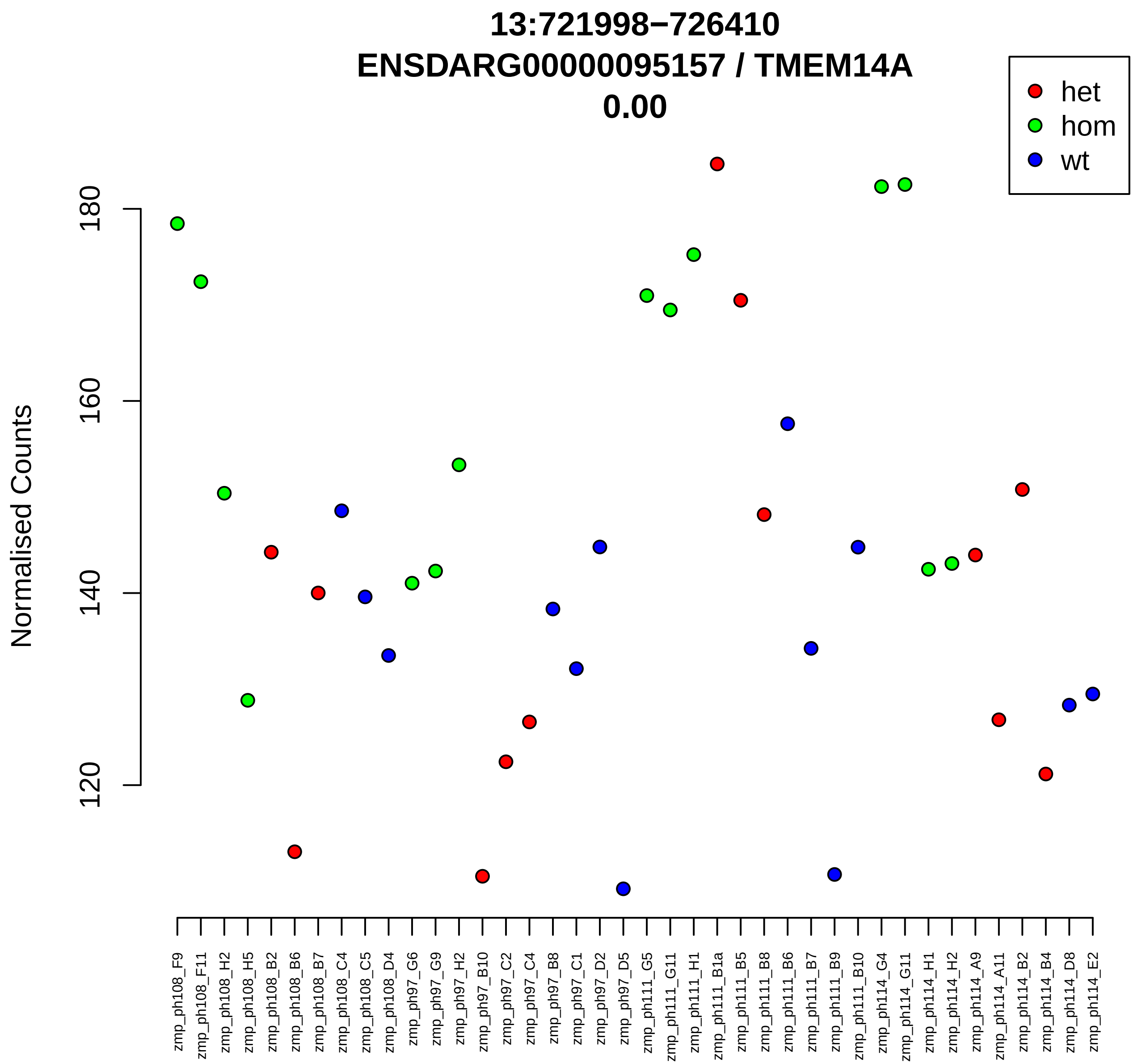
13:721998−726410
ENSDARG00000095157 / TMEM14A
0.00
het
hom
wt
Normalised Counts
120
140
160
180
zmp_ph108_F9
zmp_ph108_F11
zmp_ph108_H2
zmp_ph108_H5
zmp_ph108_B2
zmp_ph108_B6
zmp_ph108_B7
zmp_ph108_C4
zmp_ph108_C5
zmp_ph108_D4
zmp_ph97_G6
zmp_ph97_G9
zmp_ph97_H2
zmp_ph97_B10
zmp_ph97_C2
zmp_ph97_C4
zmp_ph97_B8
zmp_ph97_C1
zmp_ph97_D2
zmp_ph97_D5
zmp_ph111_G5
zmp_ph111_G11
zmp_ph111_H1
zmp_ph111_B1a
zmp_ph111_B5
zmp_ph111_B8
zmp_ph111_B6
zmp_ph111_B7
zmp_ph111_B9
zmp_ph111_B10
zmp_ph114_G4
zmp_ph114_G11
zmp_ph114_H1
zmp_ph114_H2
zmp_ph114_A9
zmp_ph114_A11
zmp_ph114_B2
zmp_ph114_B4
zmp_ph114_D8
zmp_ph114_E2

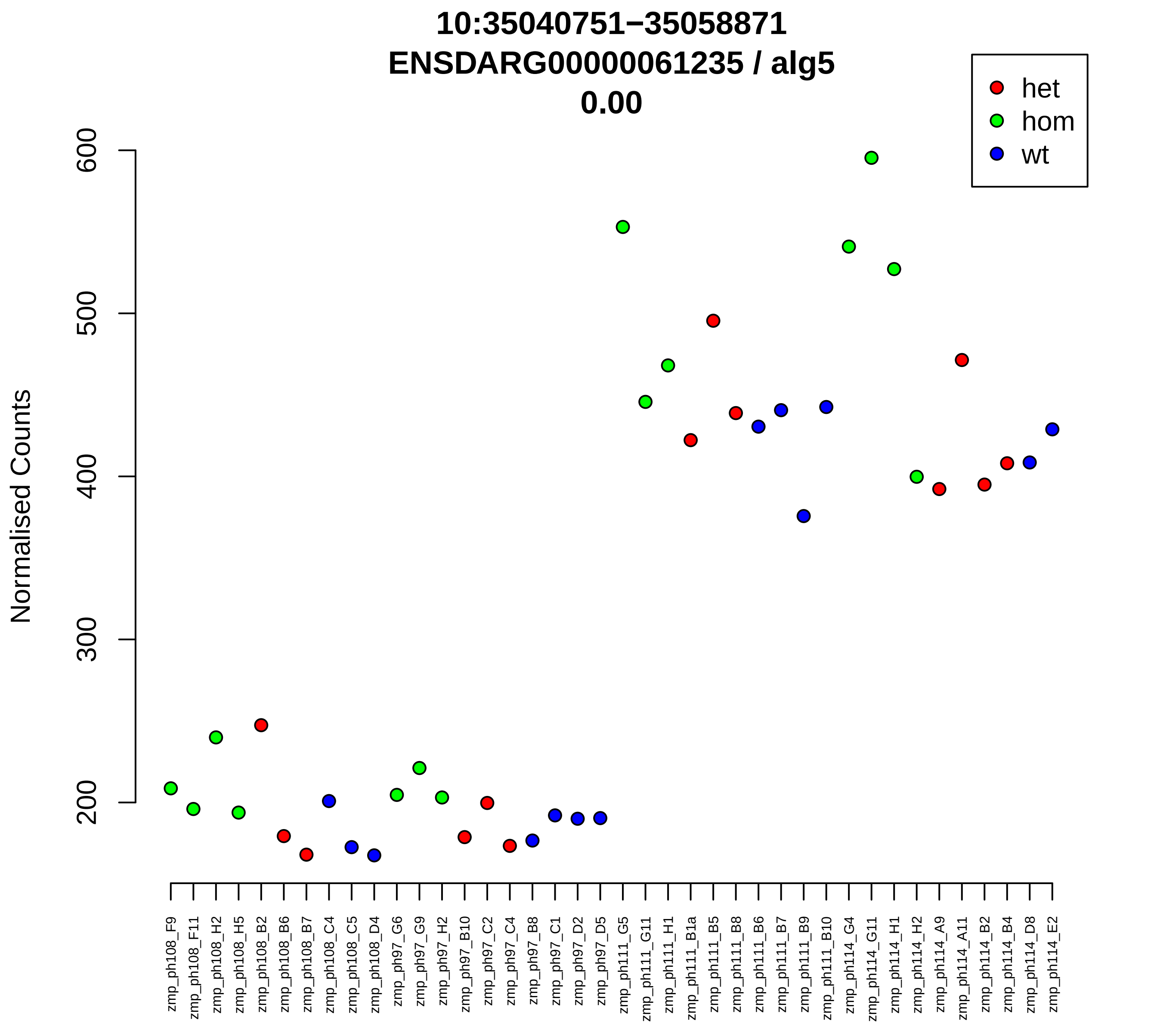

10:35040751−35058871
ENSDARG00000061235 / alg5
0.00
het
hom
wt
Normalised Counts
200
300
400
500
600
zmp_ph108_F9
zmp_ph108_F11
zmp_ph108_H2
zmp_ph108_H5
zmp_ph108_B2
zmp_ph108_B6
zmp_ph108_B7
zmp_ph108_C4
zmp_ph108_C5
zmp_ph108_D4
zmp_ph97_G6
zmp_ph97_G9
zmp_ph97_H2
zmp_ph97_B10
zmp_ph97_C2
zmp_ph97_C4
zmp_ph97_B8
zmp_ph97_C1
zmp_ph97_D2
zmp_ph97_D5
zmp_ph111_G5
zmp_ph111_G11
zmp_ph111_H1
zmp_ph111_B1a
zmp_ph111_B5
zmp_ph111_B8
zmp_ph111_B6
zmp_ph111_B7
zmp_ph111_B9
zmp_ph111_B10
zmp_ph114_G4
zmp_ph114_G11
zmp_ph114_H1
zmp_ph114_H2
zmp_ph114_A9
zmp_ph114_A11
zmp_ph114_B2
zmp_ph114_B4
zmp_ph114_D8
zmp_ph114_E2

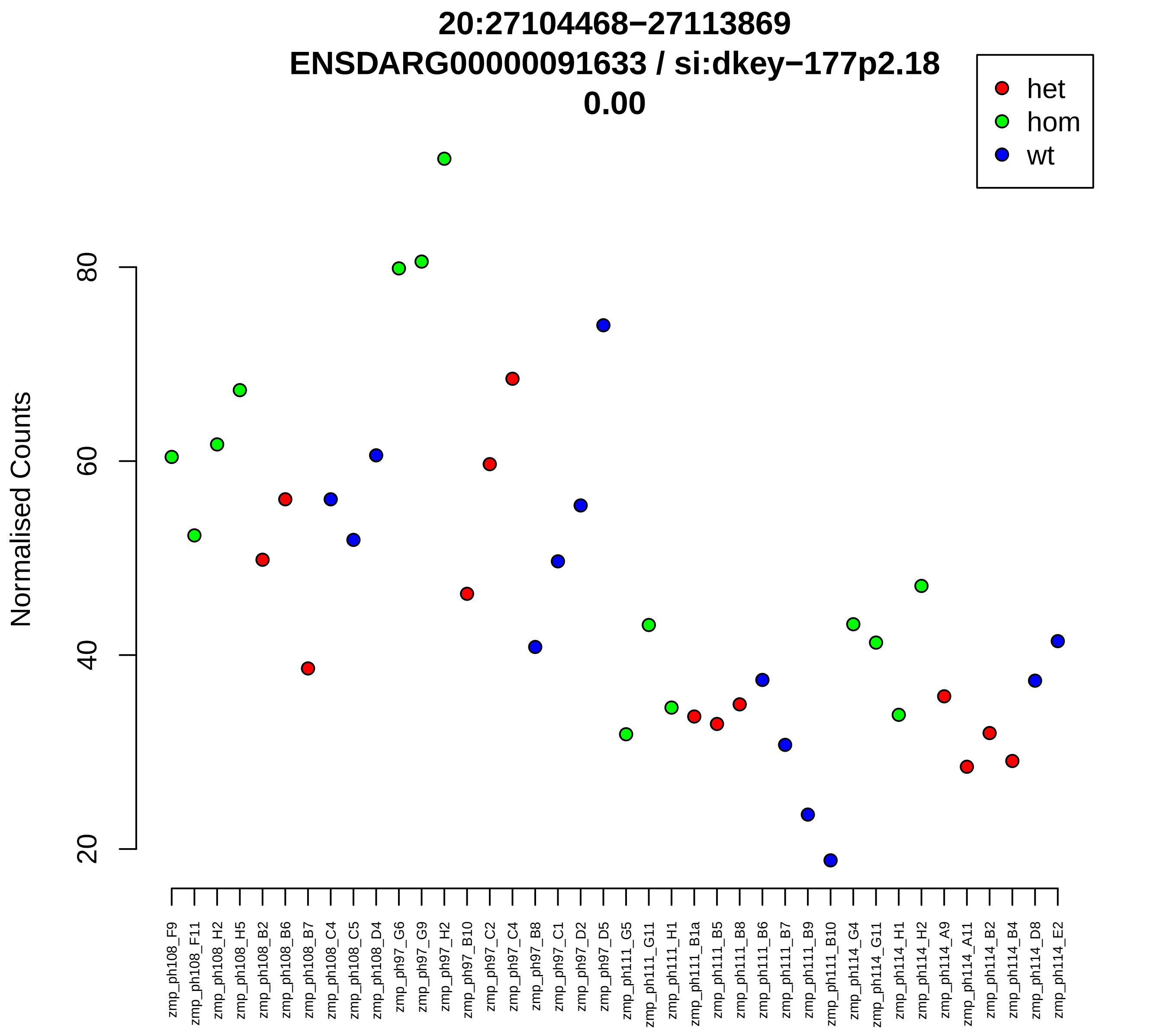
20:27104468−27113869
ENSDARG00000091633 / si:dkey−177p2.18
0.00
het
hom
wt
Normalised Counts
20
40
60
80
zmp_ph108_F9
zmp_ph108_F11
zmp_ph108_H2
zmp_ph108_H5
zmp_ph108_B2
zmp_ph108_B6
zmp_ph108_B7
zmp_ph108_C4
zmp_ph108_C5
zmp_ph108_D4
zmp_ph97_G6
zmp_ph97_G9
zmp_ph97_H2
zmp_ph97_B10
zmp_ph97_C2
zmp_ph97_C4
zmp_ph97_B8
zmp_ph97_C1
zmp_ph97_D2
zmp_ph97_D5
zmp_ph111_G5
zmp_ph111_G11
zmp_ph111_H1
zmp_ph111_B1a
zmp_ph111_B5
zmp_ph111_B8
zmp_ph111_B6
zmp_ph111_B7
zmp_ph111_B9
zmp_ph111_B10
zmp_ph114_G4
zmp_ph114_G11
zmp_ph114_H1
zmp_ph114_H2
zmp_ph114_A9
zmp_ph114_A11
zmp_ph114_B2
zmp_ph114_B4
zmp_ph114_D8
zmp_ph114_E2

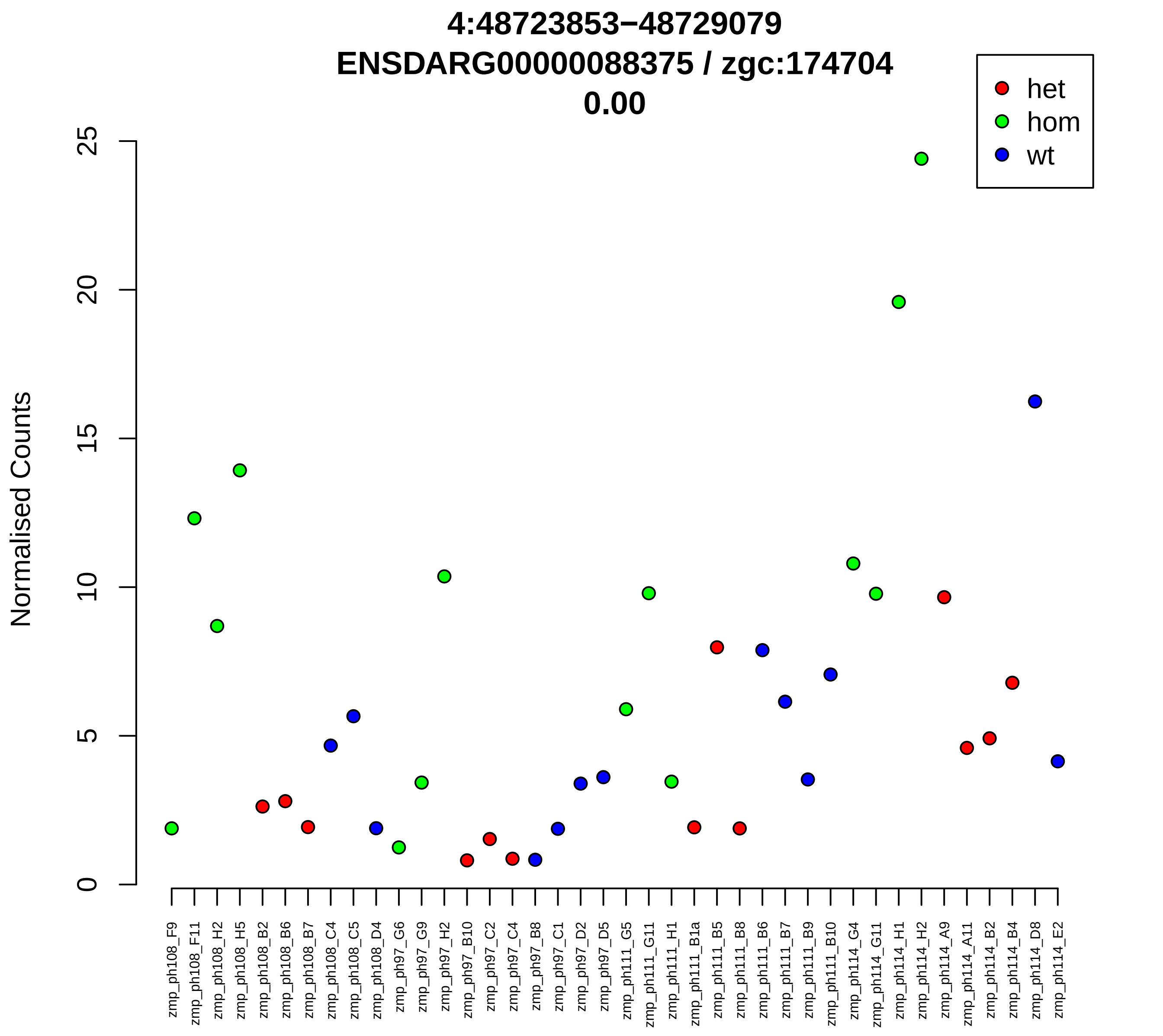
4:48723853−48729079
ENSDARG00000088375 / zgc:174704
0.00
het
hom
wt
Normalised Counts
0
5
10
15
20
25
zmp_ph108_F9
zmp_ph108_F11
zmp_ph108_H2
zmp_ph108_H5
zmp_ph108_B2
zmp_ph108_B6
zmp_ph108_B7
zmp_ph108_C4
zmp_ph108_C5
zmp_ph108_D4
zmp_ph97_G6
zmp_ph97_G9
zmp_ph97_H2
zmp_ph97_B10
zmp_ph97_C2
zmp_ph97_C4
zmp_ph97_B8
zmp_ph97_C1
zmp_ph97_D2
zmp_ph97_D5
zmp_ph111_G5
zmp_ph111_G11
zmp_ph111_H1
zmp_ph111_B1a
zmp_ph111_B5
zmp_ph111_B8
zmp_ph111_B6
zmp_ph111_B7
zmp_ph111_B9
zmp_ph111_B10
zmp_ph114_G4
zmp_ph114_G11
zmp_ph114_H1
zmp_ph114_H2
zmp_ph114_A9
zmp_ph114_A11
zmp_ph114_B2
zmp_ph114_B4
zmp_ph114_D8
zmp_ph114_E2

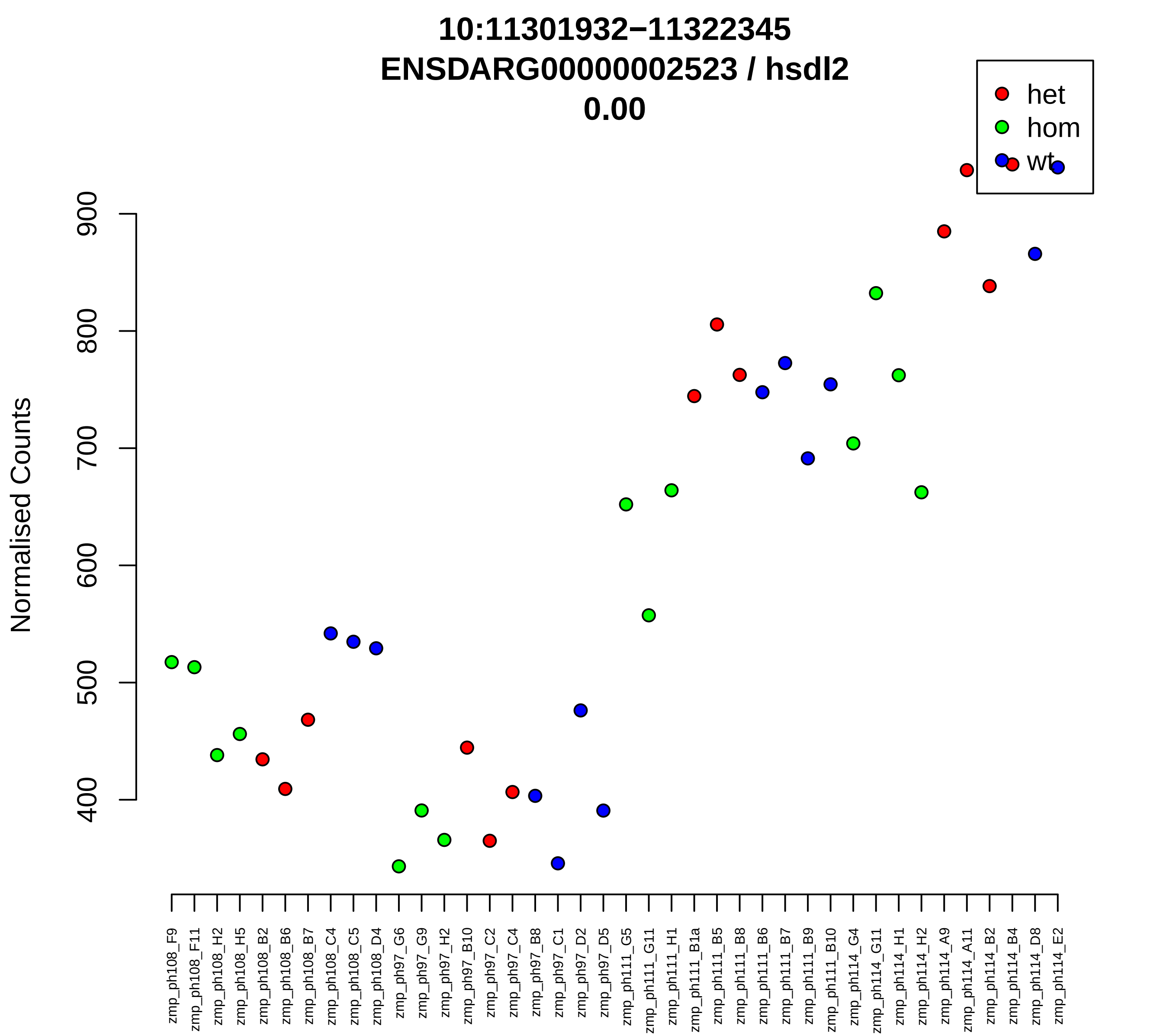
10:11301932−11322345
ENSDARG00000002523 / hsdl2
0.00
het
hom
wt
Normalised Counts
400
500
600
700
800
900
zmp_ph108_F9
zmp_ph108_F11
zmp_ph108_H2
zmp_ph108_H5
zmp_ph108_B2
zmp_ph108_B6
zmp_ph108_B7
zmp_ph108_C4
zmp_ph108_C5
zmp_ph108_D4
zmp_ph97_G6
zmp_ph97_G9
zmp_ph97_H2
zmp_ph97_B10
zmp_ph97_C2
zmp_ph97_C4
zmp_ph97_B8
zmp_ph97_C1
zmp_ph97_D2
zmp_ph97_D5
zmp_ph111_G5
zmp_ph111_G11
zmp_ph111_H1
zmp_ph111_B1a
zmp_ph111_B5
zmp_ph111_B8
zmp_ph111_B6
zmp_ph111_B7
zmp_ph111_B9
zmp_ph111_B10
zmp_ph114_G4
zmp_ph114_G11
zmp_ph114_H1
zmp_ph114_H2
zmp_ph114_A9
zmp_ph114_A11
zmp_ph114_B2
zmp_ph114_B4
zmp_ph114_D8
zmp_ph114_E2

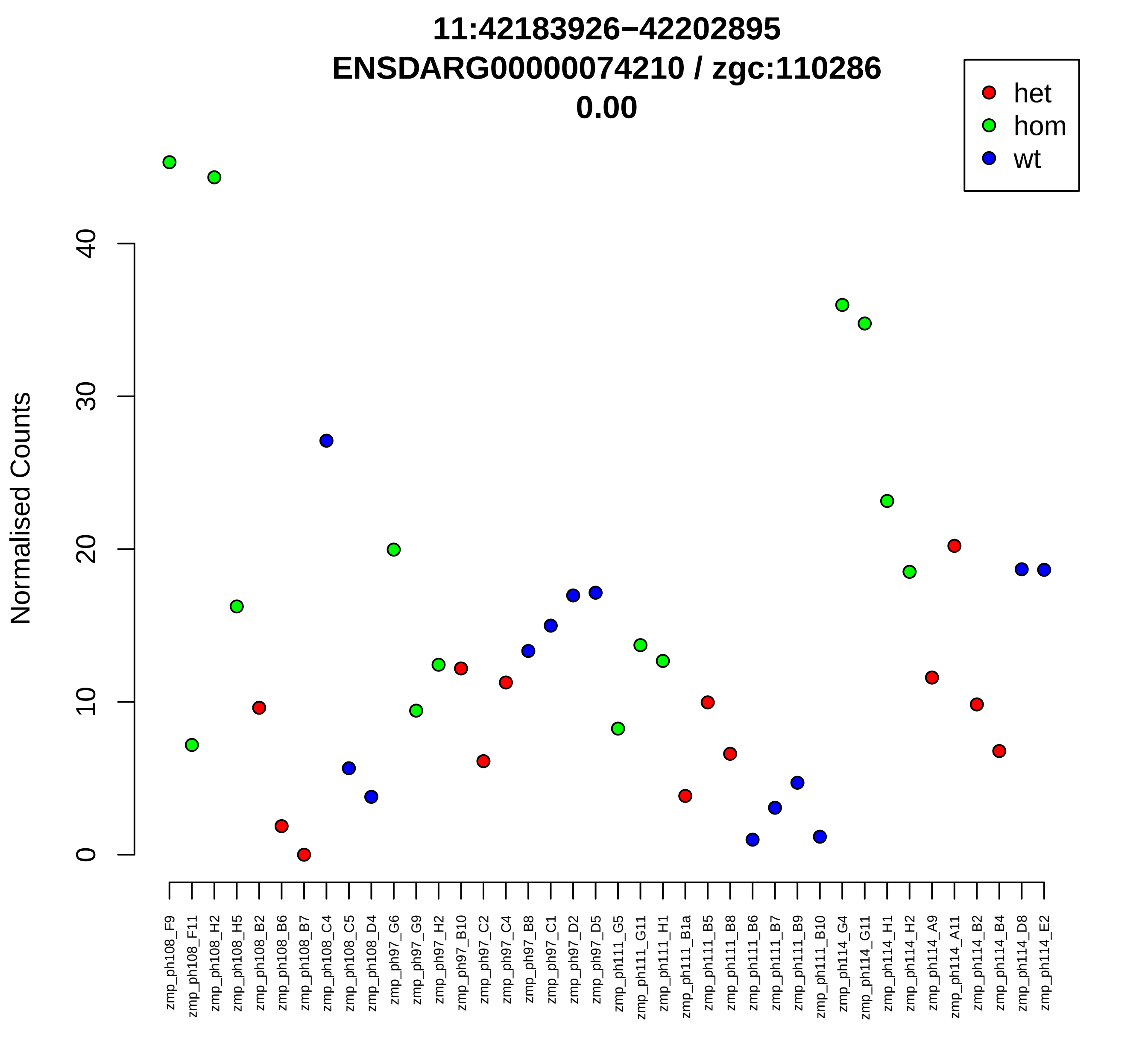

11:42183926−42202895
ENSDARG00000074210 / zgc:110286
0.00
het
hom
wt
Normalised Counts
0
10
20
30
40
zmp_ph108_F9
zmp_ph108_F11
zmp_ph108_H2
zmp_ph108_H5
zmp_ph108_B2
zmp_ph108_B6
zmp_ph108_B7
zmp_ph108_C4
zmp_ph108_C5
zmp_ph108_D4
zmp_ph97_G6
zmp_ph97_G9
zmp_ph97_H2
zmp_ph97_B10
zmp_ph97_C2
zmp_ph97_C4
zmp_ph97_B8
zmp_ph97_C1
zmp_ph97_D2
zmp_ph97_D5
zmp_ph111_G5
zmp_ph111_G11
zmp_ph111_H1
zmp_ph111_B1a
zmp_ph111_B5
zmp_ph111_B8
zmp_ph111_B6
zmp_ph111_B7
zmp_ph111_B9
zmp_ph111_B10
zmp_ph114_G4
zmp_ph114_G11
zmp_ph114_H1
zmp_ph114_H2
zmp_ph114_A9
zmp_ph114_A11
zmp_ph114_B2
zmp_ph114_B4
zmp_ph114_D8
zmp_ph114_E2

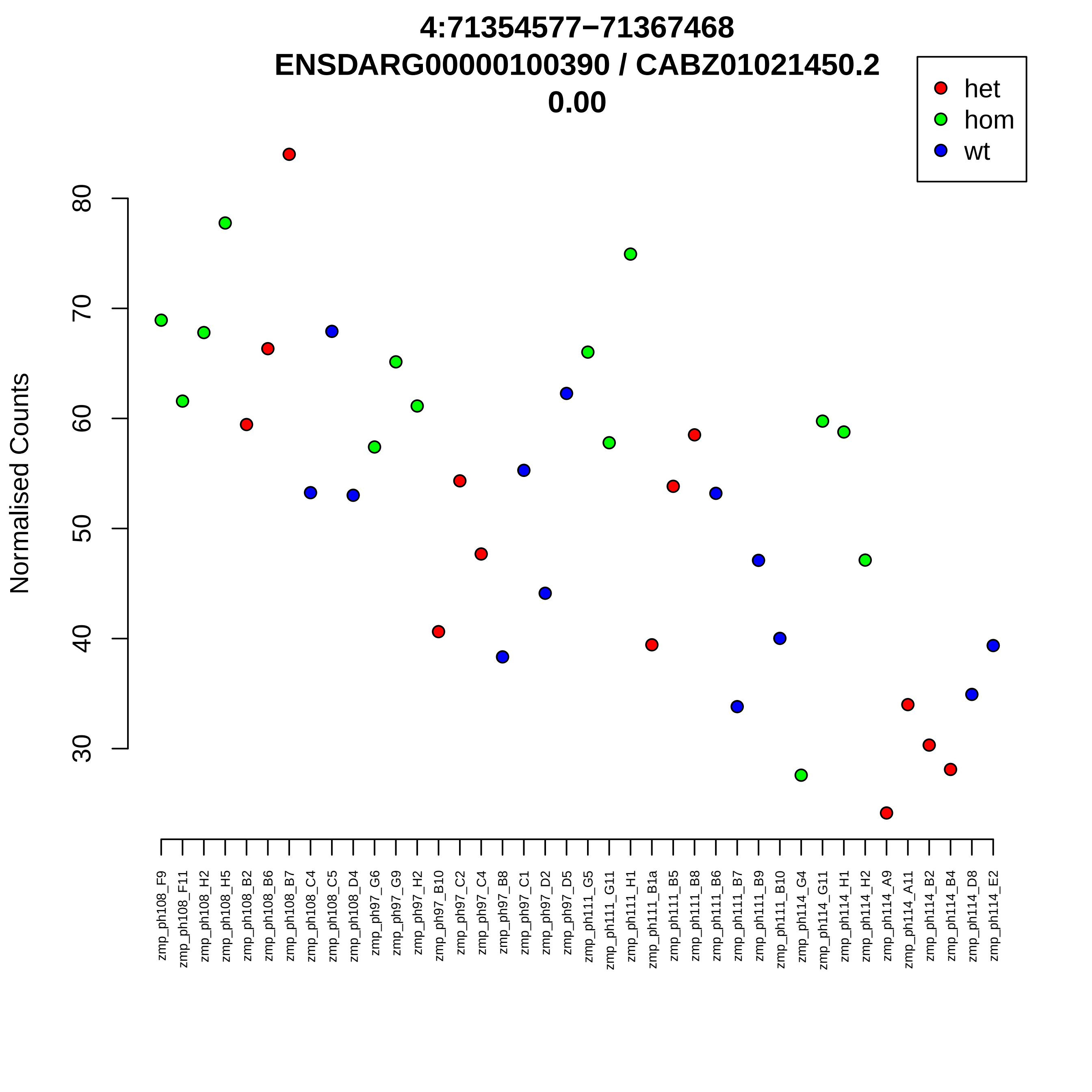
4:71354577−71367468
ENSDARG00000100390 / CABZ01021450.2
0.00
het
hom
wt
Normalised Counts
30
40
50
60
70
80
zmp_ph108_F9
zmp_ph108_F11
zmp_ph108_H2
zmp_ph108_H5
zmp_ph108_B2
zmp_ph108_B6
zmp_ph108_B7
zmp_ph108_C4
zmp_ph108_C5
zmp_ph108_D4
zmp_ph97_G6
zmp_ph97_G9
zmp_ph97_H2
zmp_ph97_B10
zmp_ph97_C2
zmp_ph97_C4
zmp_ph97_B8
zmp_ph97_C1
zmp_ph97_D2
zmp_ph97_D5
zmp_ph111_G5
zmp_ph111_G11
zmp_ph111_H1
zmp_ph111_B1a
zmp_ph111_B5
zmp_ph111_B8
zmp_ph111_B6
zmp_ph111_B7
zmp_ph111_B9
zmp_ph111_B10
zmp_ph114_G4
zmp_ph114_G11
zmp_ph114_H1
zmp_ph114_H2
zmp_ph114_A9
zmp_ph114_A11
zmp_ph114_B2
zmp_ph114_B4
zmp_ph114_D8
zmp_ph114_E2

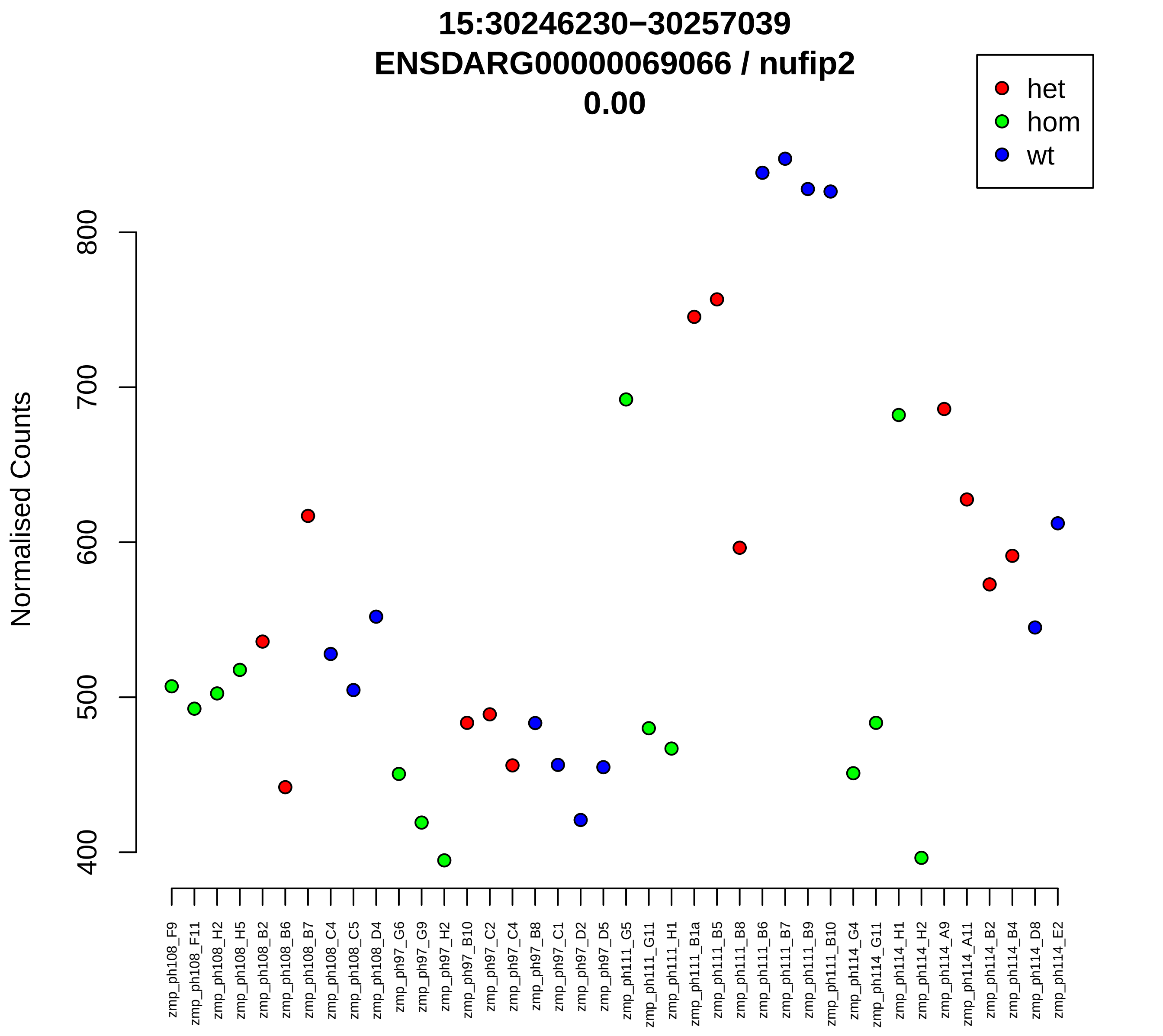
15:30246230−30257039
ENSDARG00000069066 / nufip2
0.00
het
hom
wt
Normalised Counts
400
500
600
700
800
zmp_ph108_F9
zmp_ph108_F11
zmp_ph108_H2
zmp_ph108_H5
zmp_ph108_B2
zmp_ph108_B6
zmp_ph108_B7
zmp_ph108_C4
zmp_ph108_C5
zmp_ph108_D4
zmp_ph97_G6
zmp_ph97_G9
zmp_ph97_H2
zmp_ph97_B10
zmp_ph97_C2
zmp_ph97_C4
zmp_ph97_B8
zmp_ph97_C1
zmp_ph97_D2
zmp_ph97_D5
zmp_ph111_G5
zmp_ph111_G11
zmp_ph111_H1
zmp_ph111_B1a
zmp_ph111_B5
zmp_ph111_B8
zmp_ph111_B6
zmp_ph111_B7
zmp_ph111_B9
zmp_ph111_B10
zmp_ph114_G4
zmp_ph114_G11
zmp_ph114_H1
zmp_ph114_H2
zmp_ph114_A9
zmp_ph114_A11
zmp_ph114_B2
zmp_ph114_B4
zmp_ph114_D8
zmp_ph114_E2

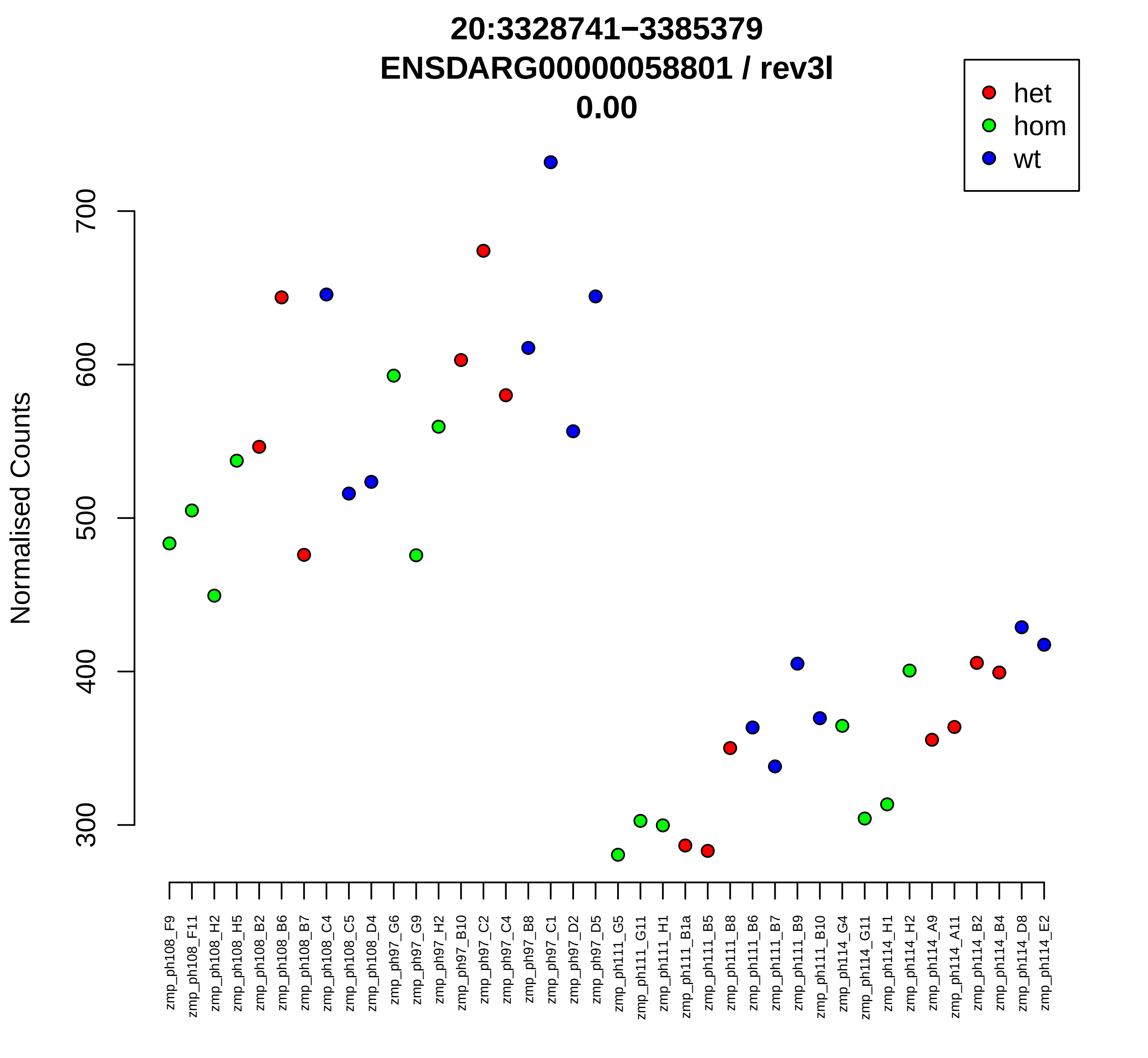

20:3328741−3385379
ENSDARG00000058801 / rev3l
0.00
het
hom
wt
Normalised Counts
300
400
500
600
700
zmp_ph108_F9
zmp_ph108_F11
zmp_ph108_H2
zmp_ph108_H5
zmp_ph108_B2
zmp_ph108_B6
zmp_ph108_B7
zmp_ph108_C4
zmp_ph108_C5
zmp_ph108_D4
zmp_ph97_G6
zmp_ph97_G9
zmp_ph97_H2
zmp_ph97_B10
zmp_ph97_C2
zmp_ph97_C4
zmp_ph97_B8
zmp_ph97_C1
zmp_ph97_D2
zmp_ph97_D5
zmp_ph111_G5
zmp_ph111_G11
zmp_ph111_H1
zmp_ph111_B1a
zmp_ph111_B5
zmp_ph111_B8
zmp_ph111_B6
zmp_ph111_B7
zmp_ph111_B9
zmp_ph111_B10
zmp_ph114_G4
zmp_ph114_G11
zmp_ph114_H1
zmp_ph114_H2
zmp_ph114_A9
zmp_ph114_A11
zmp_ph114_B2
zmp_ph114_B4
zmp_ph114_D8
zmp_ph114_E2

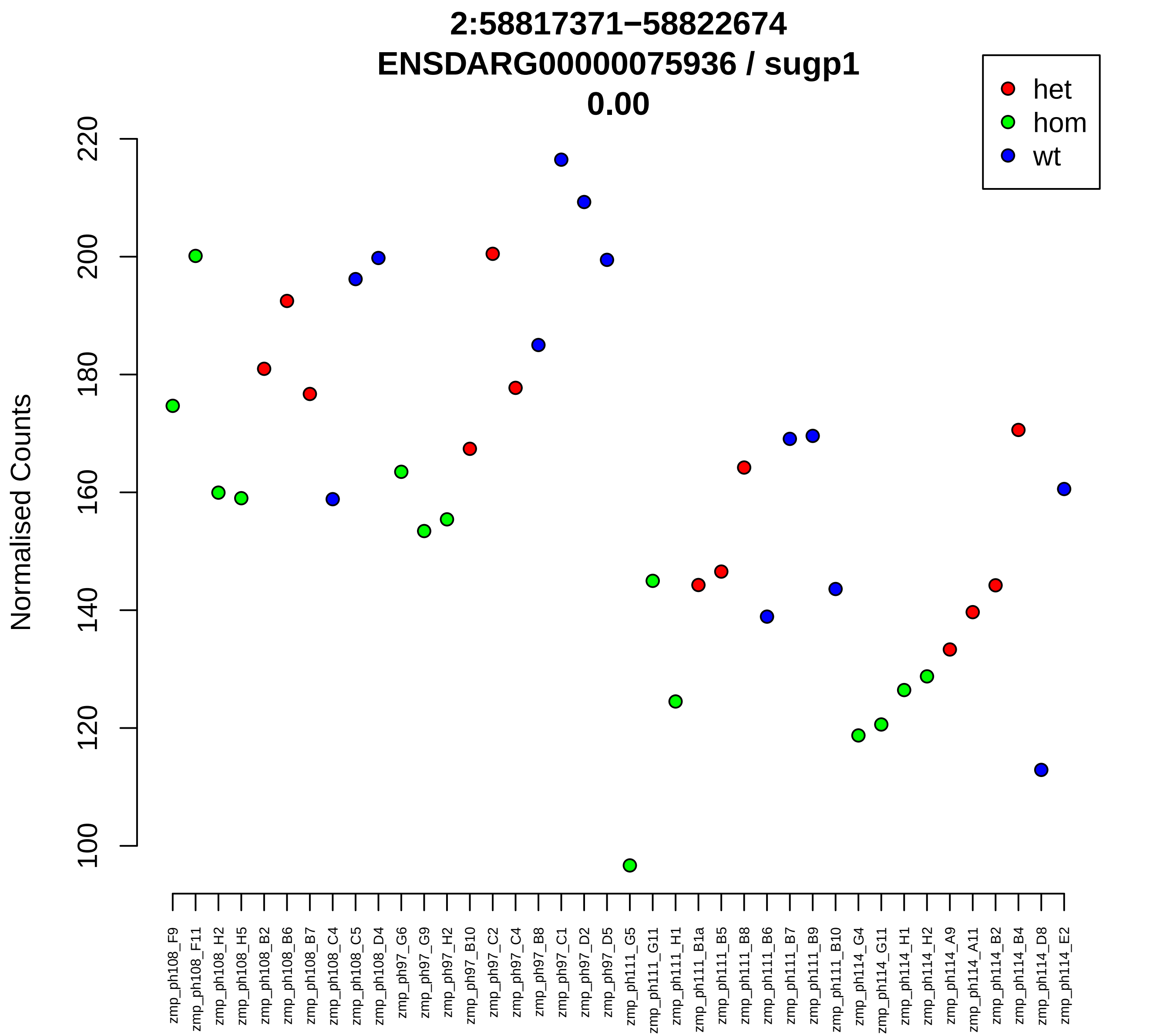

2:58817371−58822674
ENSDARG00000075936 / sugp1
0.00
het
hom
wt
Normalised Counts
100
120
140
160
180
200
220
zmp_ph108_F9
zmp_ph108_F11
zmp_ph108_H2
zmp_ph108_H5
zmp_ph108_B2
zmp_ph108_B6
zmp_ph108_B7
zmp_ph108_C4
zmp_ph108_C5
zmp_ph108_D4
zmp_ph97_G6
zmp_ph97_G9
zmp_ph97_H2
zmp_ph97_B10
zmp_ph97_C2
zmp_ph97_C4
zmp_ph97_B8
zmp_ph97_C1
zmp_ph97_D2
zmp_ph97_D5
zmp_ph111_G5
zmp_ph111_G11
zmp_ph111_H1
zmp_ph111_B1a
zmp_ph111_B5
zmp_ph111_B8
zmp_ph111_B6
zmp_ph111_B7
zmp_ph111_B9
zmp_ph111_B10
zmp_ph114_G4
zmp_ph114_G11
zmp_ph114_H1
zmp_ph114_H2
zmp_ph114_A9
zmp_ph114_A11
zmp_ph114_B2
zmp_ph114_B4
zmp_ph114_D8
zmp_ph114_E2

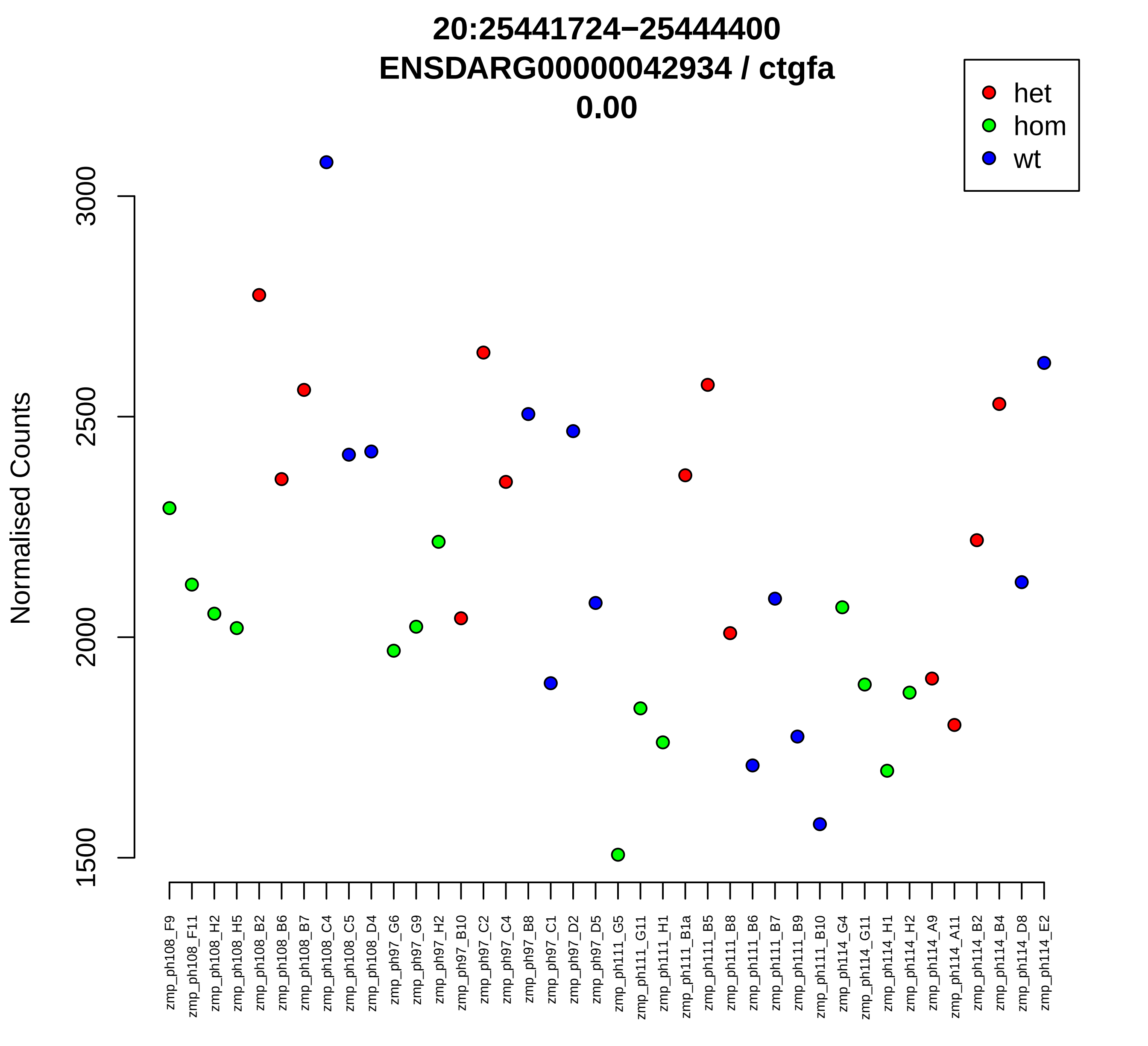

20:25441724−25444400
ENSDARG00000042934 / ctgfa
0.00
het
hom
wt
Normalised Counts
3000
2500
2000
1500
zmp_ph108_F9
zmp_ph108_F11
zmp_ph108_H2
zmp_ph108_H5
zmp_ph108_B2
zmp_ph108_B6
zmp_ph108_B7
zmp_ph108_C4
zmp_ph108_C5
zmp_ph108_D4
zmp_ph97_G6
zmp_ph97_G9
zmp_ph97_H2
zmp_ph97_B10
zmp_ph97_C2
zmp_ph97_C4
zmp_ph97_B8
zmp_ph97_C1
zmp_ph97_D2
zmp_ph97_D5
zmp_ph111_G5
zmp_ph111_G11
zmp_ph111_H1
zmp_ph111_B1a
zmp_ph111_B5
zmp_ph111_B8
zmp_ph111_B6
zmp_ph111_B7
zmp_ph111_B9
zmp_ph111_B10
zmp_ph114_G4
zmp_ph114_G11
zmp_ph114_H1
zmp_ph114_H2
zmp_ph114_A9
zmp_ph114_A11
zmp_ph114_B2
zmp_ph114_B4
zmp_ph114_D8
zmp_ph114_E2

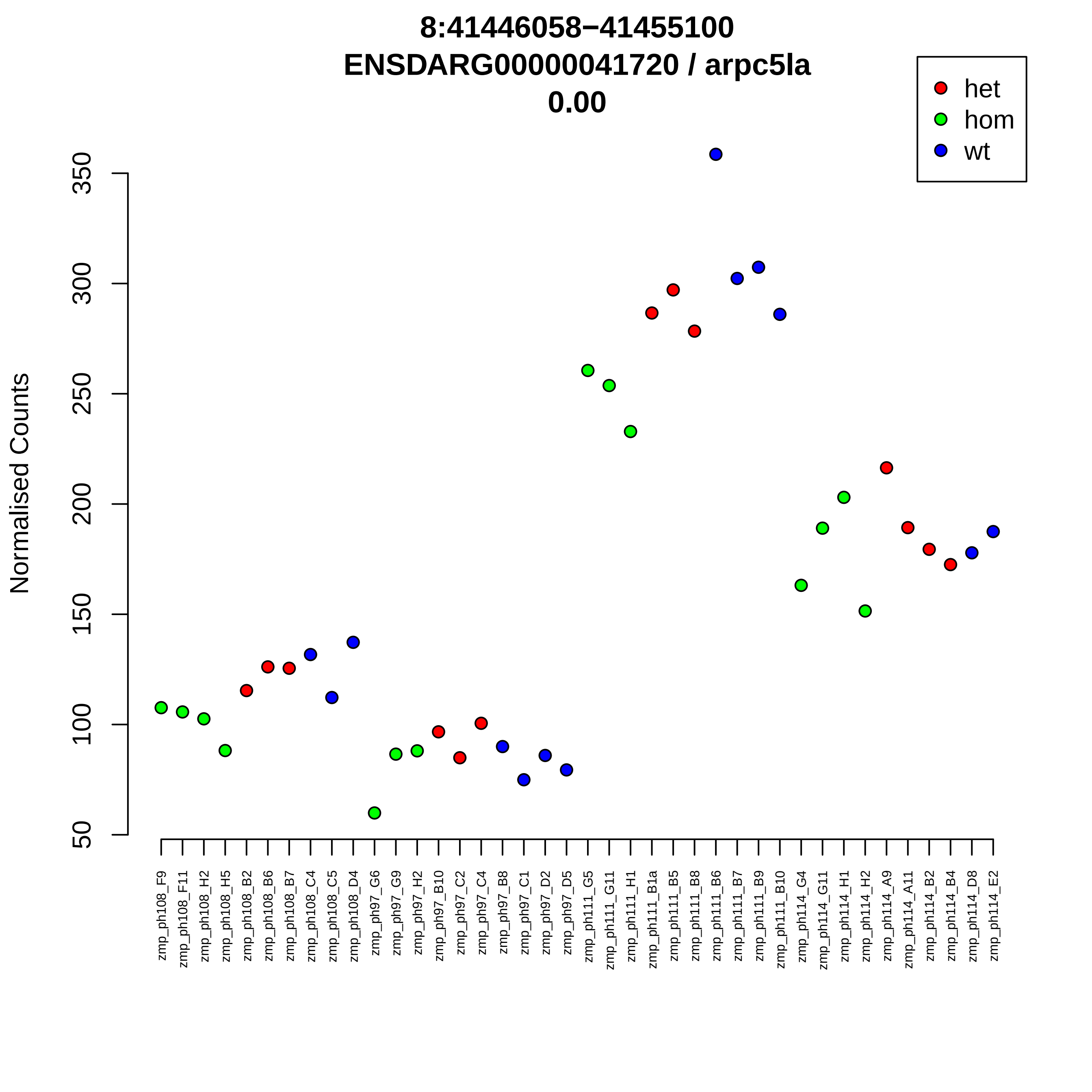
8:41446058−41455100
ENSDARG00000041720 / arpc5la
0.00
het
hom
wt
Normalised Counts
50
100
150
200
250
300
350
zmp_ph108_F9
zmp_ph108_F11
zmp_ph108_H2
zmp_ph108_H5
zmp_ph108_B2
zmp_ph108_B6
zmp_ph108_B7
zmp_ph108_C4
zmp_ph108_C5
zmp_ph108_D4
zmp_ph97_G6
zmp_ph97_G9
zmp_ph97_H2
zmp_ph97_B10
zmp_ph97_C2
zmp_ph97_C4
zmp_ph97_B8
zmp_ph97_C1
zmp_ph97_D2
zmp_ph97_D5
zmp_ph111_G5
zmp_ph111_G11
zmp_ph111_H1
zmp_ph111_B1a
zmp_ph111_B5
zmp_ph111_B8
zmp_ph111_B6
zmp_ph111_B7
zmp_ph111_B9
zmp_ph111_B10
zmp_ph114_G4
zmp_ph114_G11
zmp_ph114_H1
zmp_ph114_H2
zmp_ph114_A9
zmp_ph114_A11
zmp_ph114_B2
zmp_ph114_B4
zmp_ph114_D8
zmp_ph114_E2

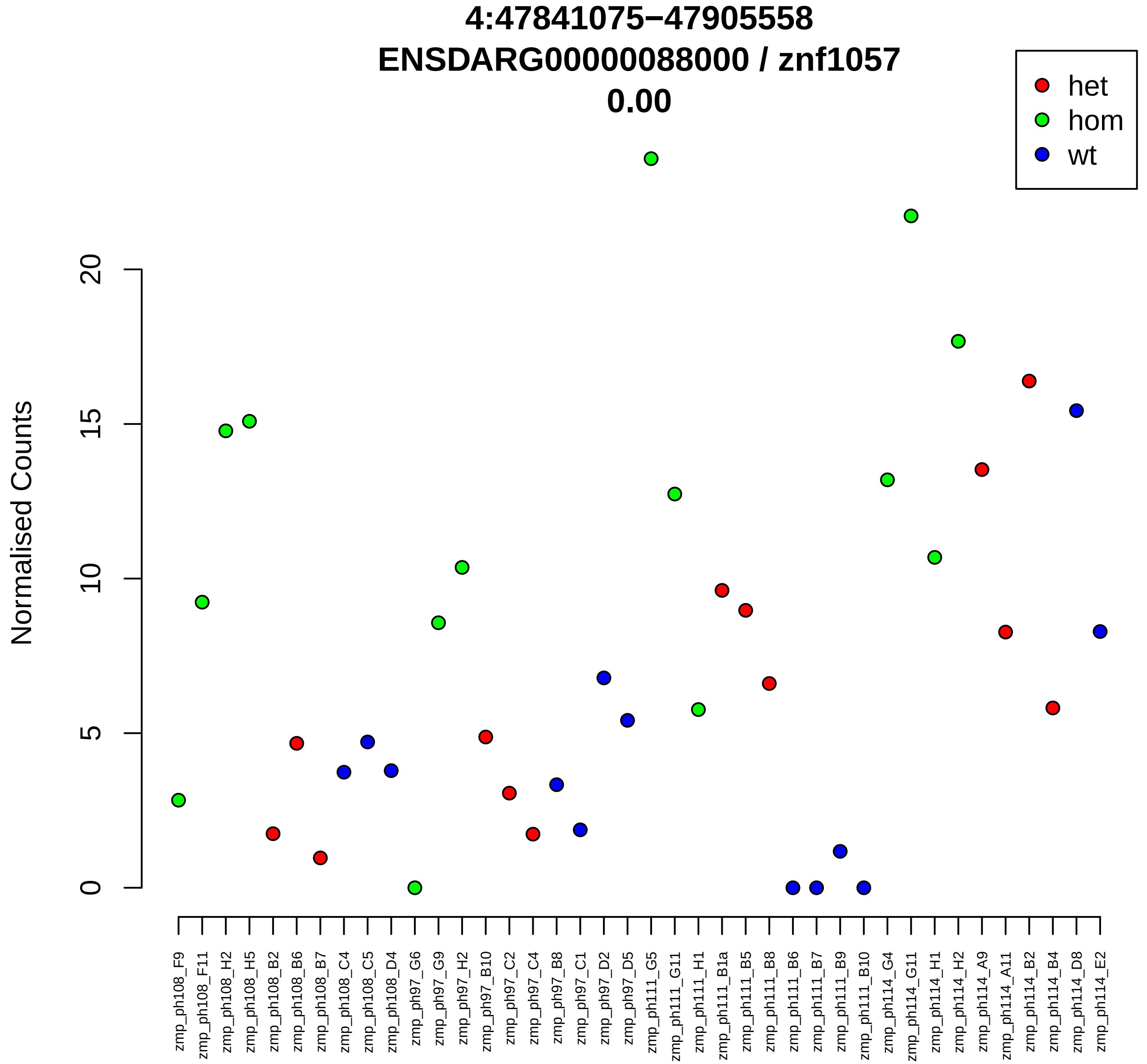

4:47841075−47905558
ENSDARG00000088000 / znf1057
0.00
het
hom
wt
Normalised Counts
0
5
10
15
20
zmp_ph108_F9
zmp_ph108_F11
zmp_ph108_H2
zmp_ph108_H5
zmp_ph108_B2
zmp_ph108_B6
zmp_ph108_B7
zmp_ph108_C4
zmp_ph108_C5
zmp_ph108_D4
zmp_ph97_G6
zmp_ph97_G9
zmp_ph97_H2
zmp_ph97_B10
zmp_ph97_C2
zmp_ph97_C4
zmp_ph97_B8
zmp_ph97_C1
zmp_ph97_D2
zmp_ph97_D5
zmp_ph111_G5
zmp_ph111_G11
zmp_ph111_H1
zmp_ph111_B1a
zmp_ph111_B5
zmp_ph111_B8
zmp_ph111_B6
zmp_ph111_B7
zmp_ph111_B9
zmp_ph111_B10
zmp_ph114_G4
zmp_ph114_G11
zmp_ph114_H1
zmp_ph114_H2
zmp_ph114_A9
zmp_ph114_A11
zmp_ph114_B2
zmp_ph114_B4
zmp_ph114_D8
zmp_ph114_E2

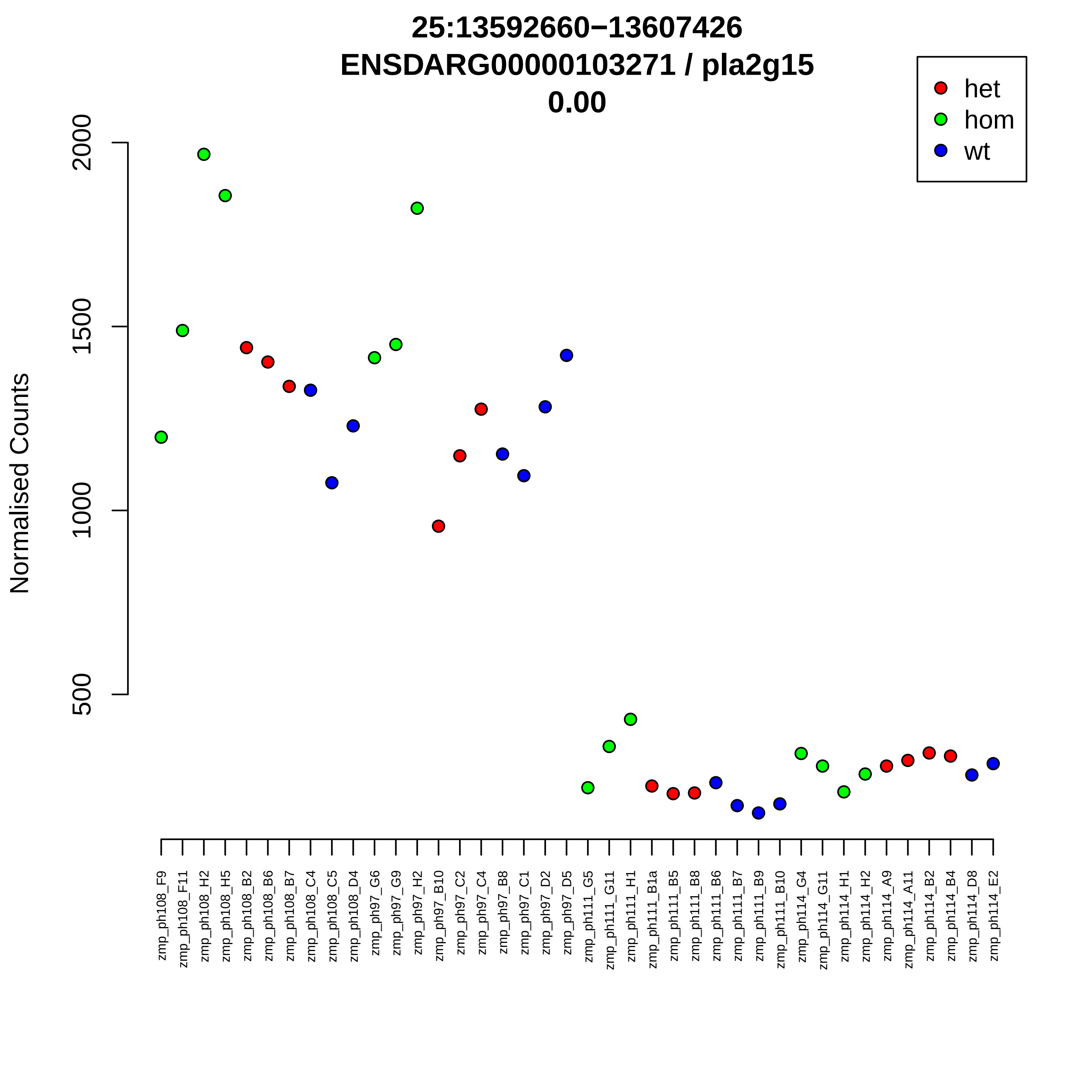

25:13592660−13607426
ENSDARG00000103271 / pla2g15
0.00
het
hom
wt
Normalised Counts
2000
1500
1000
500
zmp_ph108_F9
zmp_ph108_F11
zmp_ph108_H2
zmp_ph108_H5
zmp_ph108_B2
zmp_ph108_B6
zmp_ph108_B7
zmp_ph108_C4
zmp_ph108_C5
zmp_ph108_D4
zmp_ph97_G6
zmp_ph97_G9
zmp_ph97_H2
zmp_ph97_B10
zmp_ph97_C2
zmp_ph97_C4
zmp_ph97_B8
zmp_ph97_C1
zmp_ph97_D2
zmp_ph97_D5
zmp_ph111_G5
zmp_ph111_G11
zmp_ph111_H1
zmp_ph111_B1a
zmp_ph111_B5
zmp_ph111_B8
zmp_ph111_B6
zmp_ph111_B7
zmp_ph111_B9
zmp_ph111_B10
zmp_ph114_G4
zmp_ph114_G11
zmp_ph114_H1
zmp_ph114_H2
zmp_ph114_A9
zmp_ph114_A11
zmp_ph114_B2
zmp_ph114_B4
zmp_ph114_D8
zmp_ph114_E2

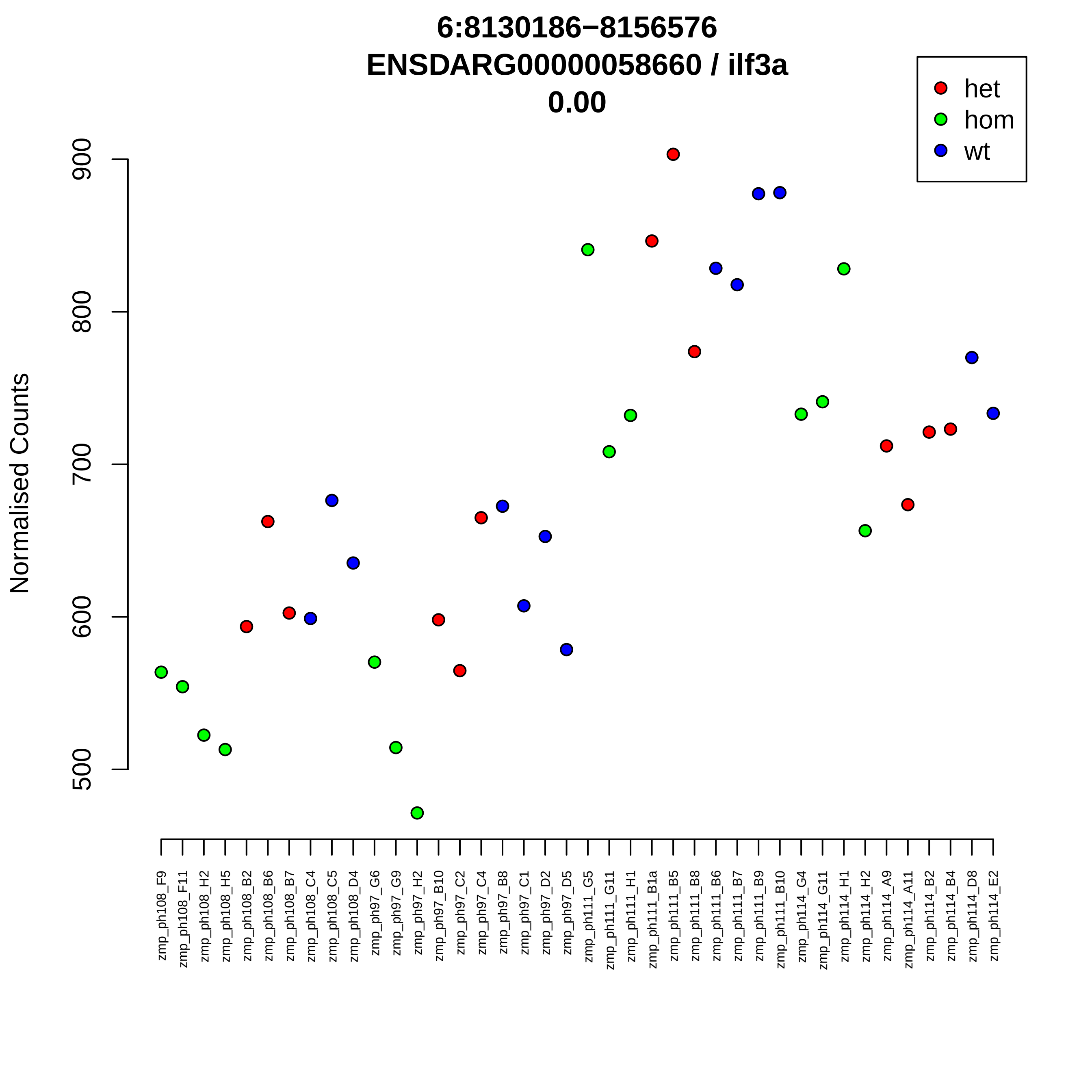
6:8130186−8156576
ENSDARG00000058660 / ilf3a
0.00
het
hom
wt
Normalised Counts
500
600
700
800
900
zmp_ph108_F9
zmp_ph108_F11
zmp_ph108_H2
zmp_ph108_H5
zmp_ph108_B2
zmp_ph108_B6
zmp_ph108_B7
zmp_ph108_C4
zmp_ph108_C5
zmp_ph108_D4
zmp_ph97_G6
zmp_ph97_G9
zmp_ph97_H2
zmp_ph97_B10
zmp_ph97_C2
zmp_ph97_C4
zmp_ph97_B8
zmp_ph97_C1
zmp_ph97_D2
zmp_ph97_D5
zmp_ph111_G5
zmp_ph111_G11
zmp_ph111_H1
zmp_ph111_B1a
zmp_ph111_B5
zmp_ph111_B8
zmp_ph111_B6
zmp_ph111_B7
zmp_ph111_B9
zmp_ph111_B10
zmp_ph114_G4
zmp_ph114_G11
zmp_ph114_H1
zmp_ph114_H2
zmp_ph114_A9
zmp_ph114_A11
zmp_ph114_B2
zmp_ph114_B4
zmp_ph114_D8
zmp_ph114_E2

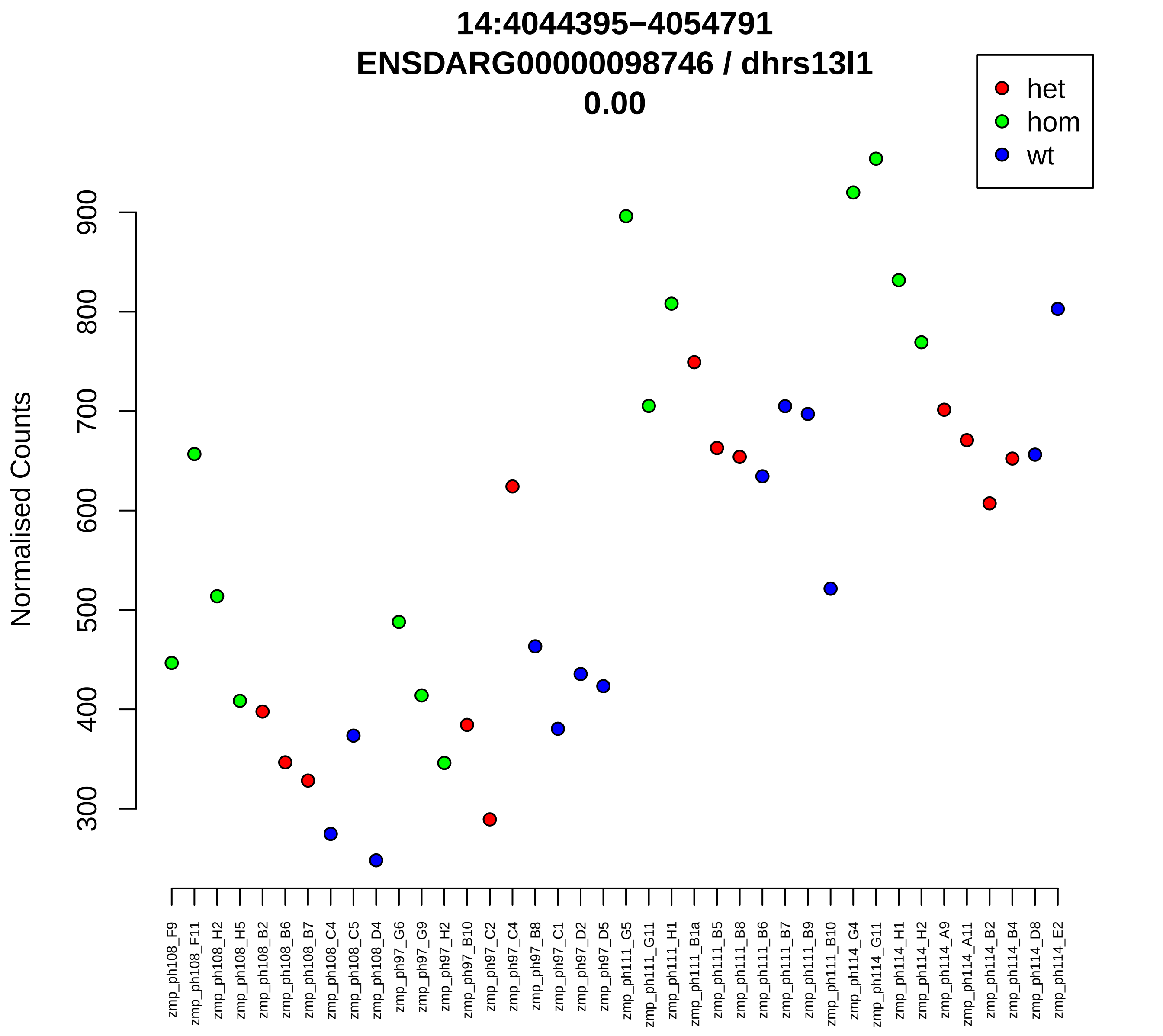
14:4044395−4054791
ENSDARG00000098746 / dhrs13l1
0.00
het
hom
wt
Normalised Counts
300
400
500
600
700
800
900
zmp_ph108_F9
zmp_ph108_F11
zmp_ph108_H2
zmp_ph108_H5
zmp_ph108_B2
zmp_ph108_B6
zmp_ph108_B7
zmp_ph108_C4
zmp_ph108_C5
zmp_ph108_D4
zmp_ph97_G6
zmp_ph97_G9
zmp_ph97_H2
zmp_ph97_B10
zmp_ph97_C2
zmp_ph97_C4
zmp_ph97_B8
zmp_ph97_C1
zmp_ph97_D2
zmp_ph97_D5
zmp_ph111_G5
zmp_ph111_G11
zmp_ph111_H1
zmp_ph111_B1a
zmp_ph111_B5
zmp_ph111_B8
zmp_ph111_B6
zmp_ph111_B7
zmp_ph111_B9
zmp_ph111_B10
zmp_ph114_G4
zmp_ph114_G11
zmp_ph114_H1
zmp_ph114_H2
zmp_ph114_A9
zmp_ph114_A11
zmp_ph114_B2
zmp_ph114_B4
zmp_ph114_D8
zmp_ph114_E2

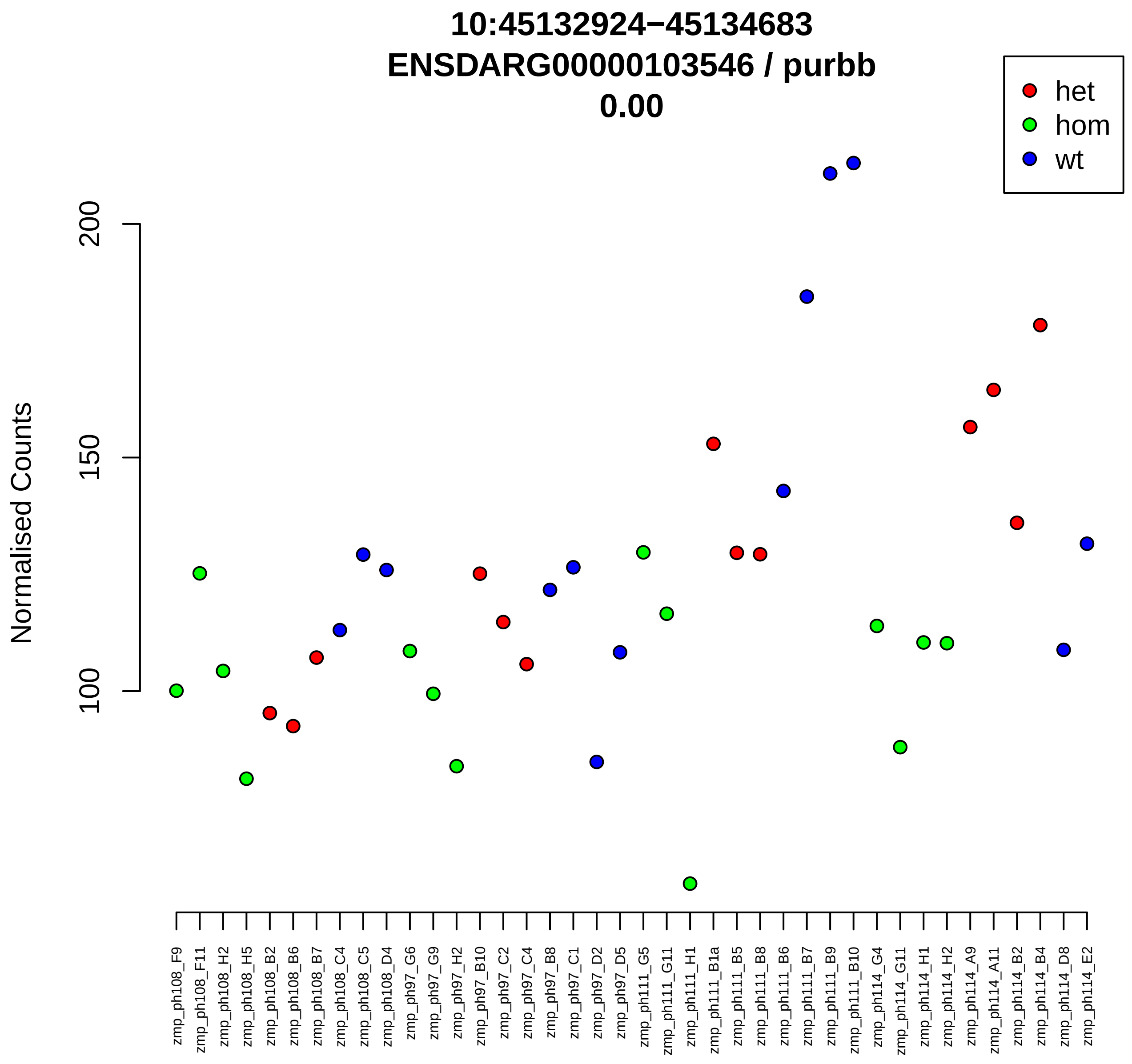

10:45132924−45134683
ENSDARG00000103546 / purbb
0.00
het
hom
wt
Normalised Counts
100
150
200
zmp_ph108_F9
zmp_ph108_F11
zmp_ph108_H2
zmp_ph108_H5
zmp_ph108_B2
zmp_ph108_B6
zmp_ph108_B7
zmp_ph108_C4
zmp_ph108_C5
zmp_ph108_D4
zmp_ph97_G6
zmp_ph97_G9
zmp_ph97_H2
zmp_ph97_B10
zmp_ph97_C2
zmp_ph97_C4
zmp_ph97_B8
zmp_ph97_C1
zmp_ph97_D2
zmp_ph97_D5
zmp_ph111_G5
zmp_ph111_G11
zmp_ph111_H1
zmp_ph111_B1a
zmp_ph111_B5
zmp_ph111_B8
zmp_ph111_B6
zmp_ph111_B7
zmp_ph111_B9
zmp_ph111_B10
zmp_ph114_G4
zmp_ph114_G11
zmp_ph114_H1
zmp_ph114_H2
zmp_ph114_A9
zmp_ph114_A11
zmp_ph114_B2
zmp_ph114_B4
zmp_ph114_D8
zmp_ph114_E2

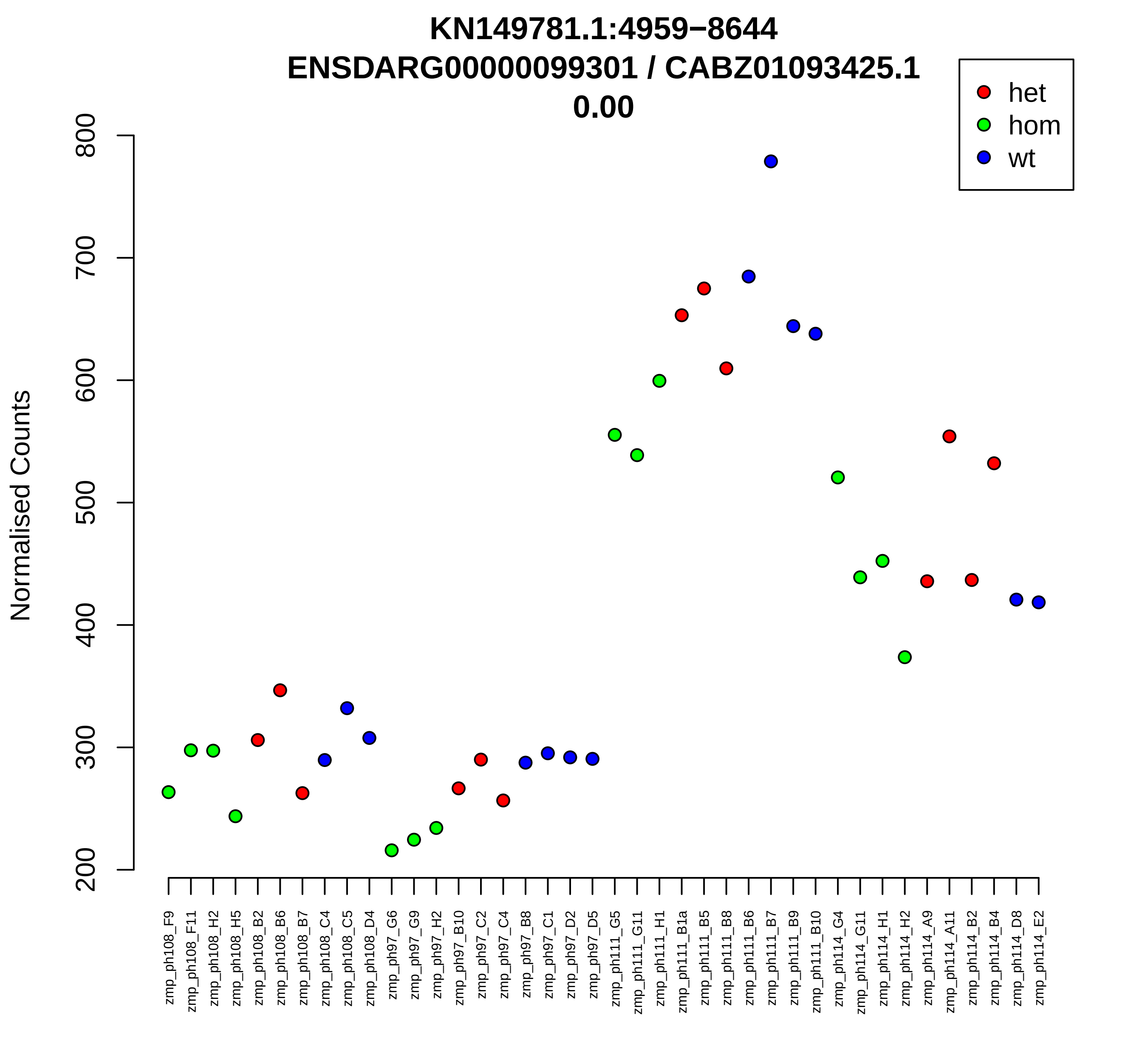

KN149781.1:4959−8644
ENSDARG00000099301 / CABZ01093425.1
0.00
het
hom
wt
Normalised Counts
200
300
400
500
600
700
800
zmp_ph108_F9
zmp_ph108_F11
zmp_ph108_H2
zmp_ph108_H5
zmp_ph108_B2
zmp_ph108_B6
zmp_ph108_B7
zmp_ph108_C4
zmp_ph108_C5
zmp_ph108_D4
zmp_ph97_G6
zmp_ph97_G9
zmp_ph97_H2
zmp_ph97_B10
zmp_ph97_C2
zmp_ph97_C4
zmp_ph97_B8
zmp_ph97_C1
zmp_ph97_D2
zmp_ph97_D5
zmp_ph111_G5
zmp_ph111_G11
zmp_ph111_H1
zmp_ph111_B1a
zmp_ph111_B5
zmp_ph111_B8
zmp_ph111_B6
zmp_ph111_B7
zmp_ph111_B9
zmp_ph111_B10
zmp_ph114_G4
zmp_ph114_G11
zmp_ph114_H1
zmp_ph114_H2
zmp_ph114_A9
zmp_ph114_A11
zmp_ph114_B2
zmp_ph114_B4
zmp_ph114_D8
zmp_ph114_E2

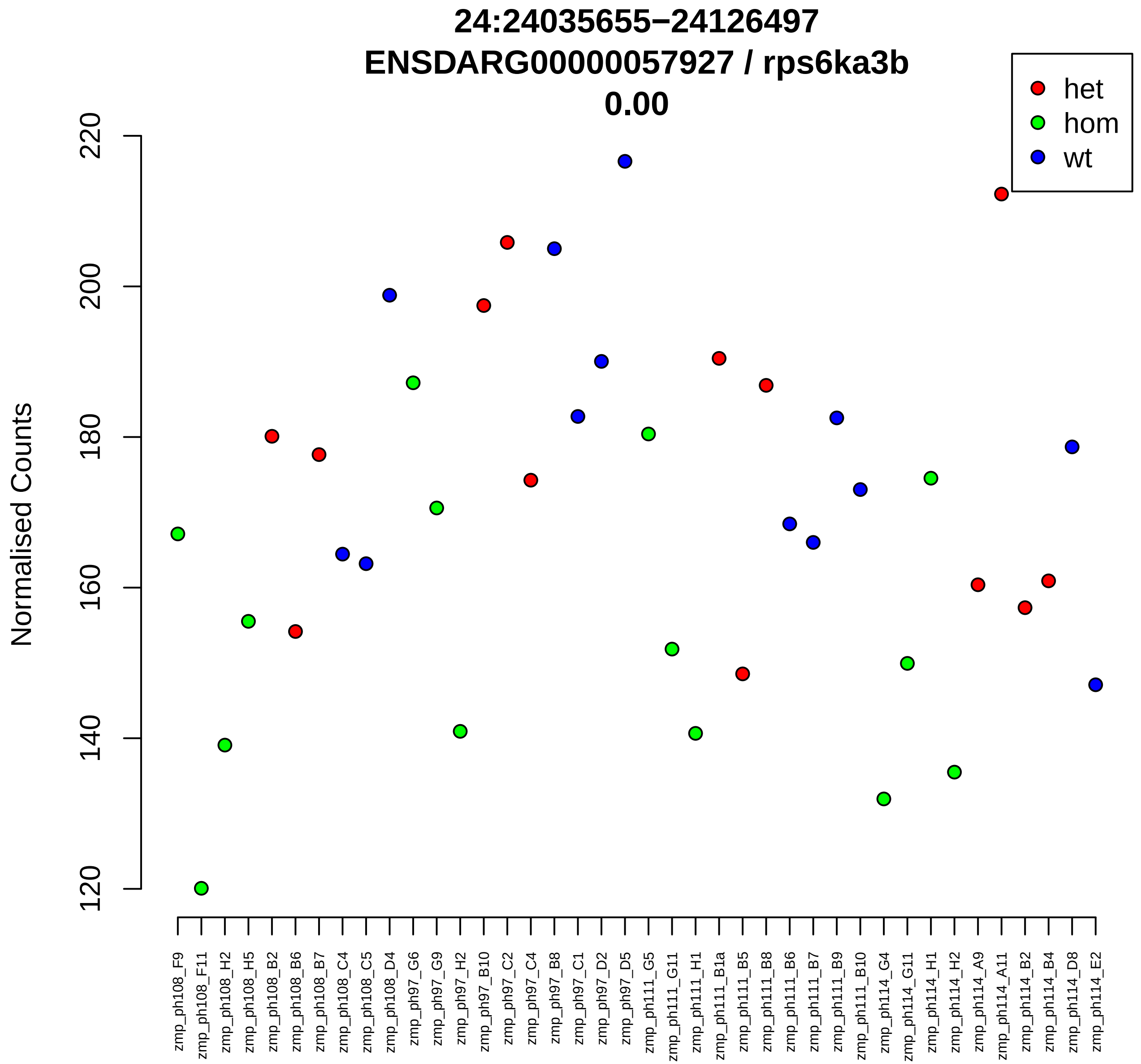

24:24035655−24126497
ENSDARG00000057927 / rps6ka3b
0.00
het
hom
wt
Normalised Counts
120
140
160
180
200
220
zmp_ph108_F9
zmp_ph108_F11
zmp_ph108_H2
zmp_ph108_H5
zmp_ph108_B2
zmp_ph108_B6
zmp_ph108_B7
zmp_ph108_C4
zmp_ph108_C5
zmp_ph108_D4
zmp_ph97_G6
zmp_ph97_G9
zmp_ph97_H2
zmp_ph97_B10
zmp_ph97_C2
zmp_ph97_C4
zmp_ph97_B8
zmp_ph97_C1
zmp_ph97_D2
zmp_ph97_D5
zmp_ph111_G5
zmp_ph111_G11
zmp_ph111_H1
zmp_ph111_B1a
zmp_ph111_B5
zmp_ph111_B8
zmp_ph111_B6
zmp_ph111_B7
zmp_ph111_B9
zmp_ph111_B10
zmp_ph114_G4
zmp_ph114_G11
zmp_ph114_H1
zmp_ph114_H2
zmp_ph114_A9
zmp_ph114_A11
zmp_ph114_B2
zmp_ph114_B4
zmp_ph114_D8
zmp_ph114_E2

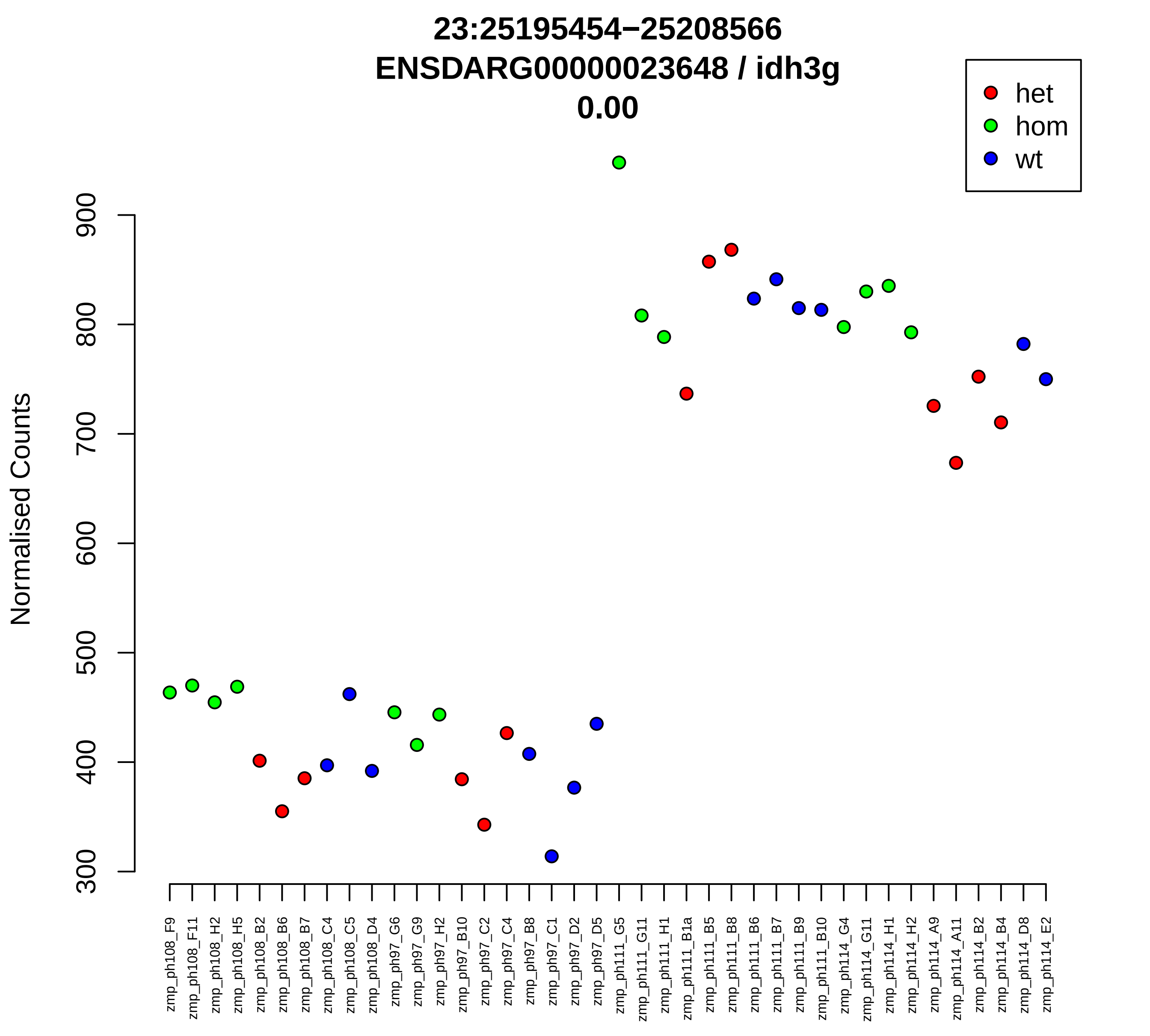
23:25195454−25208566
ENSDARG00000023648 / idh3g
0.00
het
hom
wt
Normalised Counts
300
400
500
600
700
800
900
zmp_ph108_F9
zmp_ph108_F11
zmp_ph108_H2
zmp_ph108_H5
zmp_ph108_B2
zmp_ph108_B6
zmp_ph108_B7
zmp_ph108_C4
zmp_ph108_C5
zmp_ph108_D4
zmp_ph97_G6
zmp_ph97_G9
zmp_ph97_H2
zmp_ph97_B10
zmp_ph97_C2
zmp_ph97_C4
zmp_ph97_B8
zmp_ph97_C1
zmp_ph97_D2
zmp_ph97_D5
zmp_ph111_G5
zmp_ph111_G11
zmp_ph111_H1
zmp_ph111_B1a
zmp_ph111_B5
zmp_ph111_B8
zmp_ph111_B6
zmp_ph111_B7
zmp_ph111_B9
zmp_ph111_B10
zmp_ph114_G4
zmp_ph114_G11
zmp_ph114_H1
zmp_ph114_H2
zmp_ph114_A9
zmp_ph114_A11
zmp_ph114_B2
zmp_ph114_B4
zmp_ph114_D8
zmp_ph114_E2

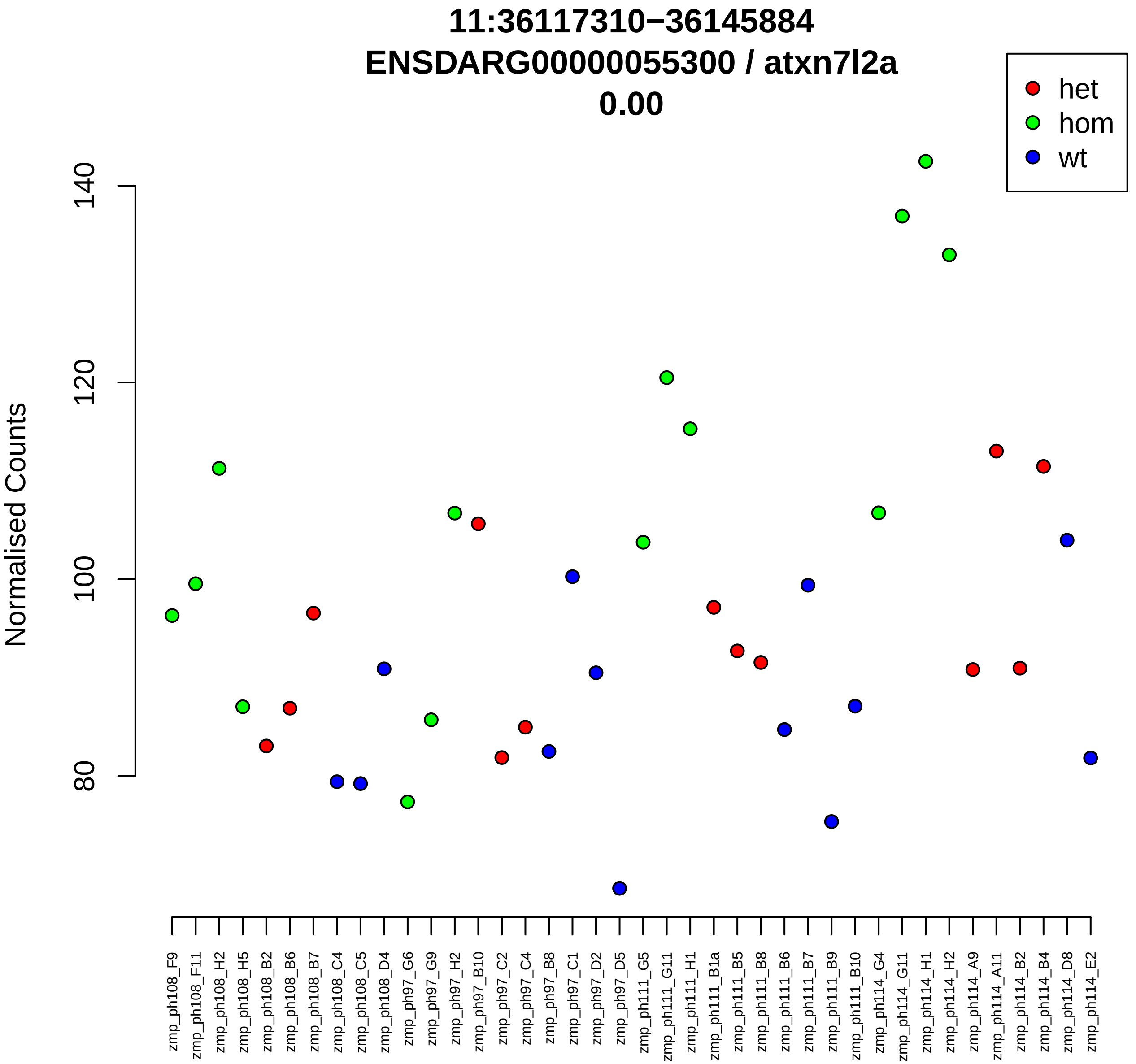

11:36117310−36145884
ENSDARG00000055300 / atxn7l2a
0.00
het
hom
wt
Normalised Counts
80
100
120
140
zmp_ph108_F9
zmp_ph108_F11
zmp_ph108_H2
zmp_ph108_H5
zmp_ph108_B2
zmp_ph108_B6
zmp_ph108_B7
zmp_ph108_C4
zmp_ph108_C5
zmp_ph108_D4
zmp_ph97_G6
zmp_ph97_G9
zmp_ph97_H2
zmp_ph97_B10
zmp_ph97_C2
zmp_ph97_C4
zmp_ph97_B8
zmp_ph97_C1
zmp_ph97_D2
zmp_ph97_D5
zmp_ph111_G5
zmp_ph111_G11
zmp_ph111_H1
zmp_ph111_B1a
zmp_ph111_B5
zmp_ph111_B8
zmp_ph111_B6
zmp_ph111_B7
zmp_ph111_B9
zmp_ph111_B10
zmp_ph114_G4
zmp_ph114_G11
zmp_ph114_H1
zmp_ph114_H2
zmp_ph114_A9
zmp_ph114_A11
zmp_ph114_B2
zmp_ph114_B4
zmp_ph114_D8
zmp_ph114_E2

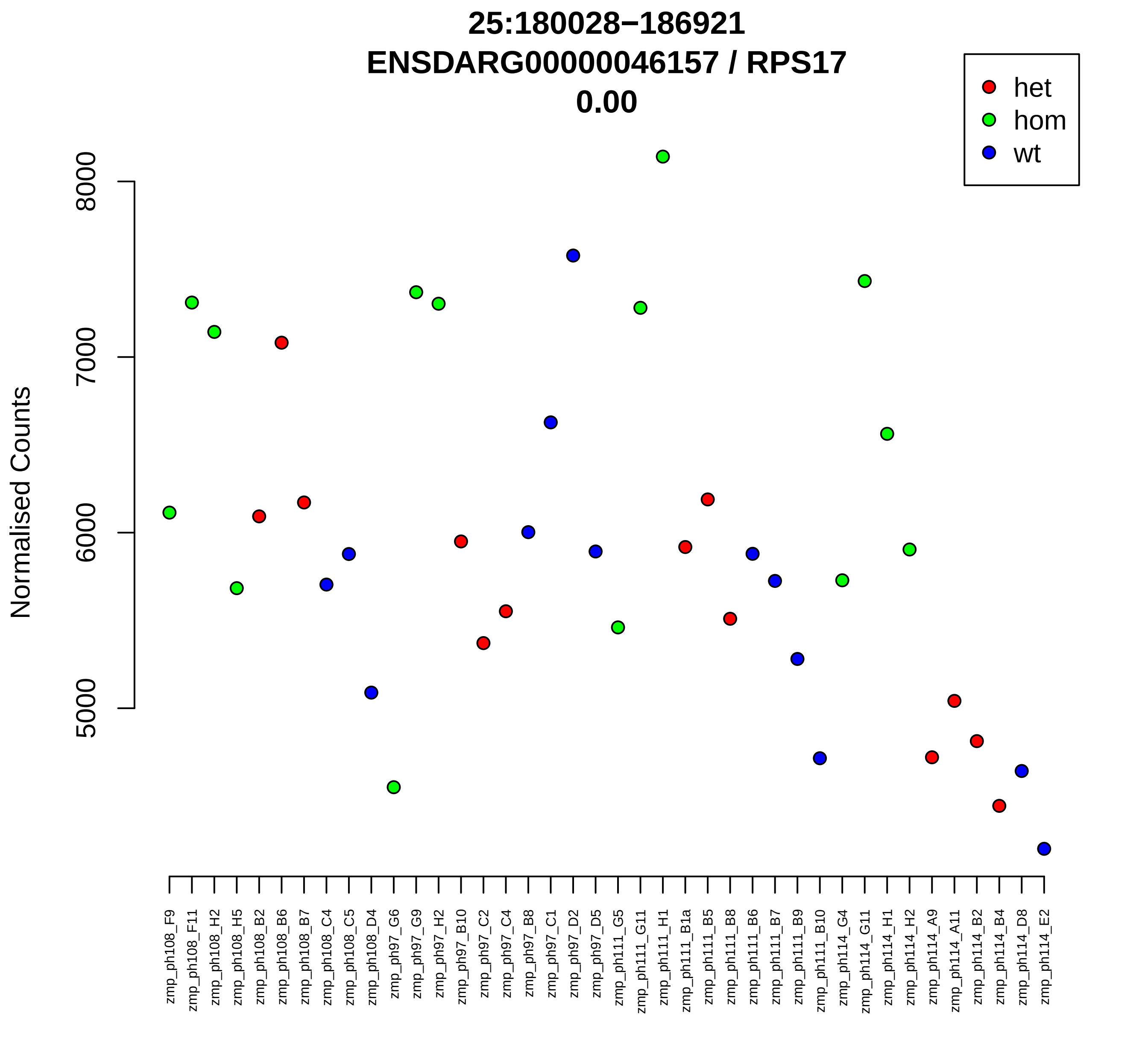

25:180028−186921
ENSDARG00000046157 / RPS17
0.00
het
hom
wt
Normalised Counts
5000
6000
7000
8000
zmp_ph108_F9
zmp_ph108_F11
zmp_ph108_H2
zmp_ph108_H5
zmp_ph108_B2
zmp_ph108_B6
zmp_ph108_B7
zmp_ph108_C4
zmp_ph108_C5
zmp_ph108_D4
zmp_ph97_G6
zmp_ph97_G9
zmp_ph97_H2
zmp_ph97_B10
zmp_ph97_C2
zmp_ph97_C4
zmp_ph97_B8
zmp_ph97_C1
zmp_ph97_D2
zmp_ph97_D5
zmp_ph111_G5
zmp_ph111_G11
zmp_ph111_H1
zmp_ph111_B1a
zmp_ph111_B5
zmp_ph111_B8
zmp_ph111_B6
zmp_ph111_B7
zmp_ph111_B9
zmp_ph111_B10
zmp_ph114_G4
zmp_ph114_G11
zmp_ph114_H1
zmp_ph114_H2
zmp_ph114_A9
zmp_ph114_A11
zmp_ph114_B2
zmp_ph114_B4
zmp_ph114_D8
zmp_ph114_E2

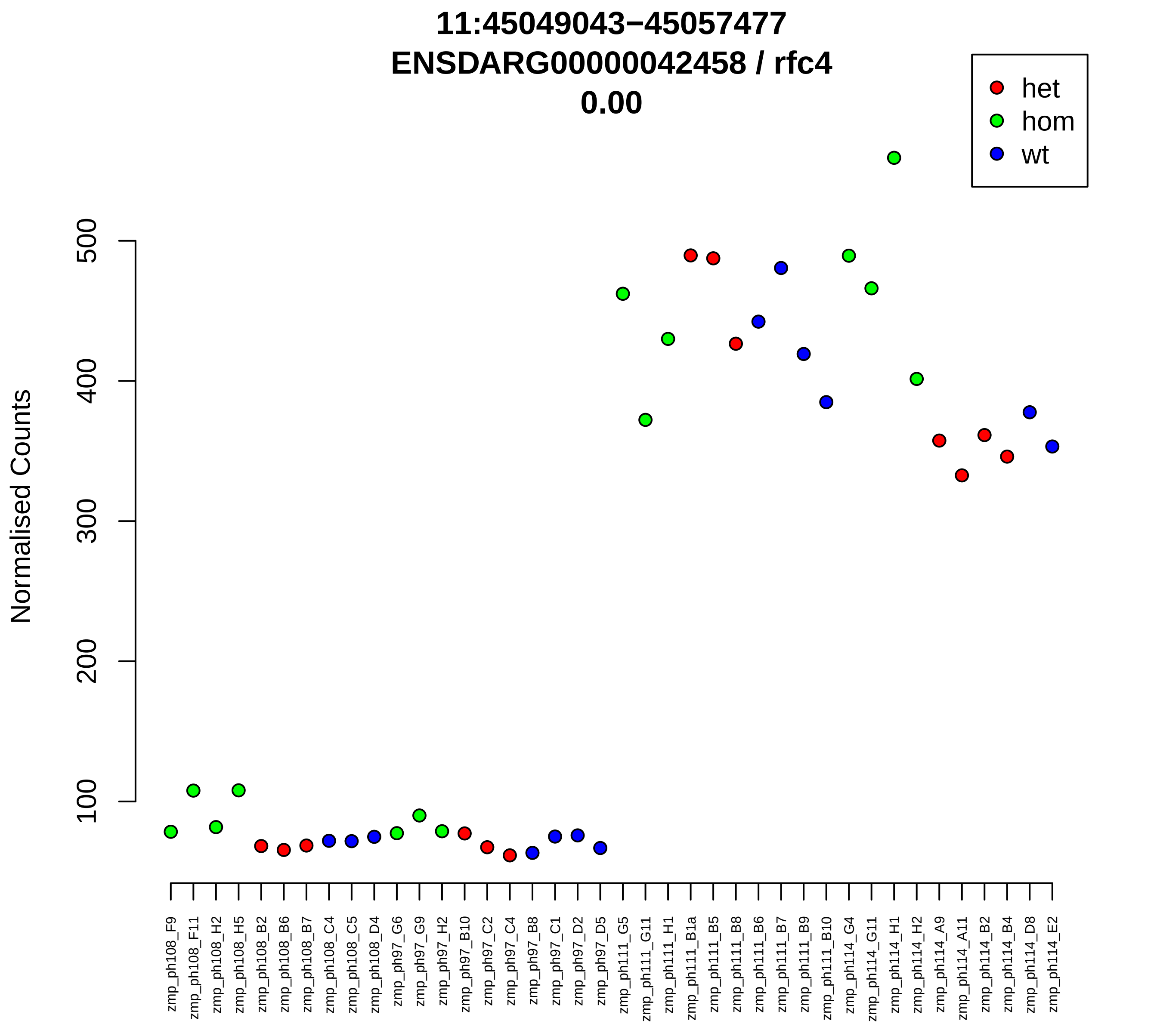

11:45049043−45057477
ENSDARG00000042458 / rfc4
0.00
het
hom
wt
Normalised Counts
100
200
300
400
500
zmp_ph108_F9
zmp_ph108_F11
zmp_ph108_H2
zmp_ph108_H5
zmp_ph108_B2
zmp_ph108_B6
zmp_ph108_B7
zmp_ph108_C4
zmp_ph108_C5
zmp_ph108_D4
zmp_ph97_G6
zmp_ph97_G9
zmp_ph97_H2
zmp_ph97_B10
zmp_ph97_C2
zmp_ph97_C4
zmp_ph97_B8
zmp_ph97_C1
zmp_ph97_D2
zmp_ph97_D5
zmp_ph111_G5
zmp_ph111_G11
zmp_ph111_H1
zmp_ph111_B1a
zmp_ph111_B5
zmp_ph111_B8
zmp_ph111_B6
zmp_ph111_B7
zmp_ph111_B9
zmp_ph111_B10
zmp_ph114_G4
zmp_ph114_G11
zmp_ph114_H1
zmp_ph114_H2
zmp_ph114_A9
zmp_ph114_A11
zmp_ph114_B2
zmp_ph114_B4
zmp_ph114_D8
zmp_ph114_E2

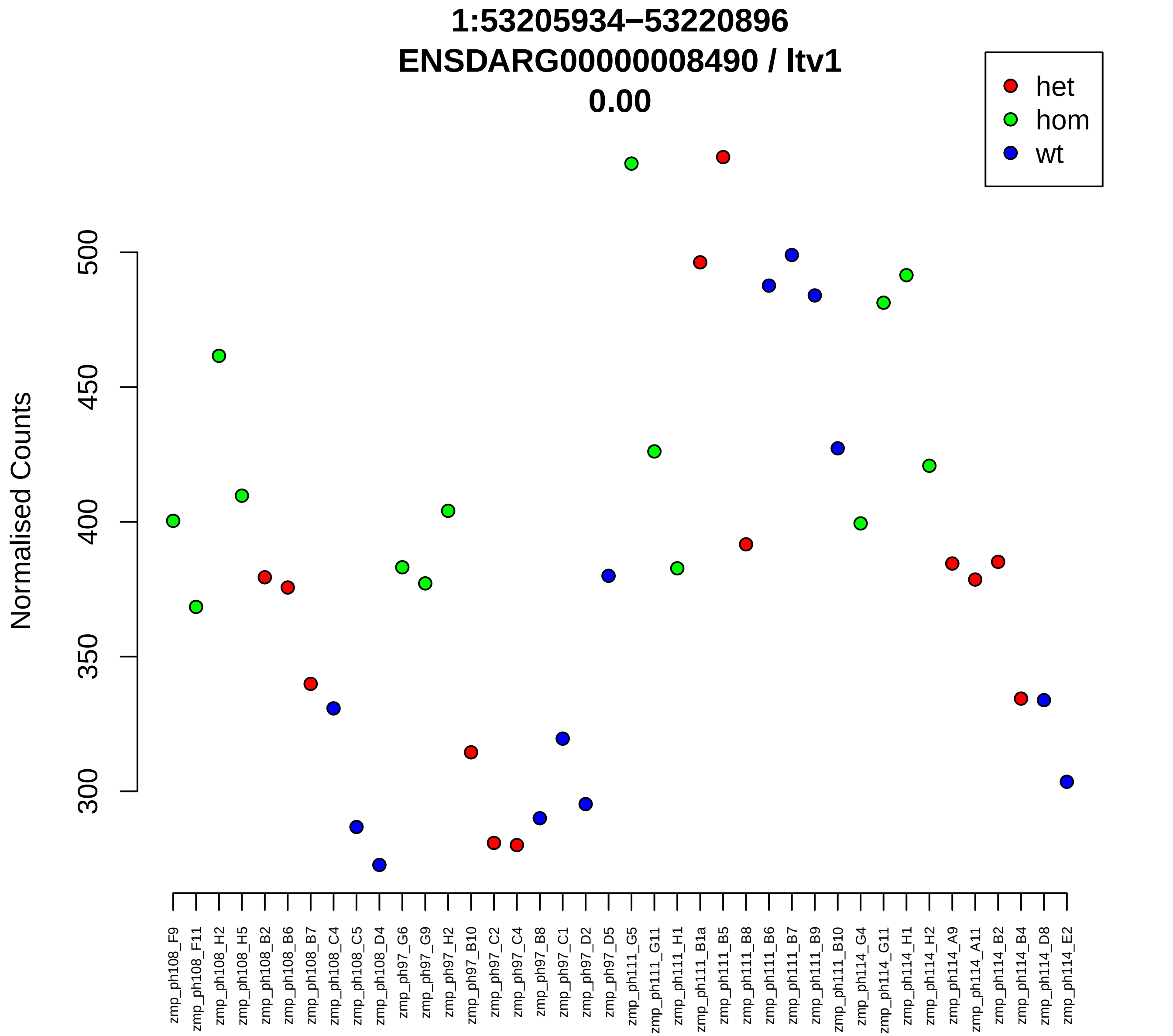
1:53205934−53220896
ENSDARG00000008490 / ltv1
0.00
het
hom
wt
Normalised Counts
300
350
400
450
500
zmp_ph108_F9
zmp_ph108_F11
zmp_ph108_H2
zmp_ph108_H5
zmp_ph108_B2
zmp_ph108_B6
zmp_ph108_B7
zmp_ph108_C4
zmp_ph108_C5
zmp_ph108_D4
zmp_ph97_G6
zmp_ph97_G9
zmp_ph97_H2
zmp_ph97_B10
zmp_ph97_C2
zmp_ph97_C4
zmp_ph97_B8
zmp_ph97_C1
zmp_ph97_D2
zmp_ph97_D5
zmp_ph111_G5
zmp_ph111_G11
zmp_ph111_H1
zmp_ph111_B1a
zmp_ph111_B5
zmp_ph111_B8
zmp_ph111_B6
zmp_ph111_B7
zmp_ph111_B9
zmp_ph111_B10
zmp_ph114_G4
zmp_ph114_G11
zmp_ph114_H1
zmp_ph114_H2
zmp_ph114_A9
zmp_ph114_A11
zmp_ph114_B2
zmp_ph114_B4
zmp_ph114_D8
zmp_ph114_E2

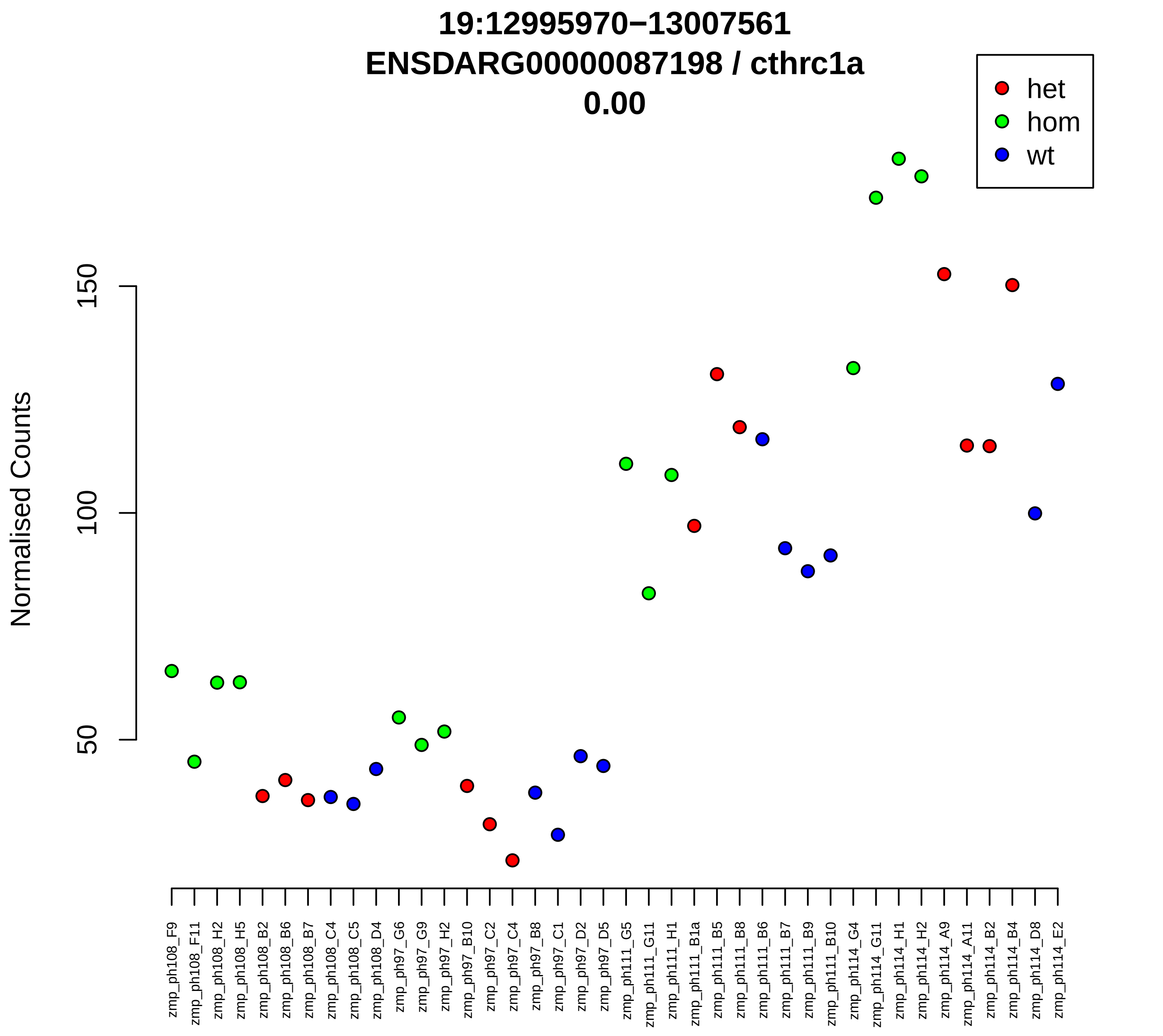
19:12995970−13007561
ENSDARG00000087198 / cthrc1a
0.00
het
hom
wt
Normalised Counts
50
100
150
zmp_ph108_F9
zmp_ph108_F11
zmp_ph108_H2
zmp_ph108_H5
zmp_ph108_B2
zmp_ph108_B6
zmp_ph108_B7
zmp_ph108_C4
zmp_ph108_C5
zmp_ph108_D4
zmp_ph97_G6
zmp_ph97_G9
zmp_ph97_H2
zmp_ph97_B10
zmp_ph97_C2
zmp_ph97_C4
zmp_ph97_B8
zmp_ph97_C1
zmp_ph97_D2
zmp_ph97_D5
zmp_ph111_G5
zmp_ph111_G11
zmp_ph111_H1
zmp_ph111_B1a
zmp_ph111_B5
zmp_ph111_B8
zmp_ph111_B6
zmp_ph111_B7
zmp_ph111_B9
zmp_ph111_B10
zmp_ph114_G4
zmp_ph114_G11
zmp_ph114_H1
zmp_ph114_H2
zmp_ph114_A9
zmp_ph114_A11
zmp_ph114_B2
zmp_ph114_B4
zmp_ph114_D8
zmp_ph114_E2

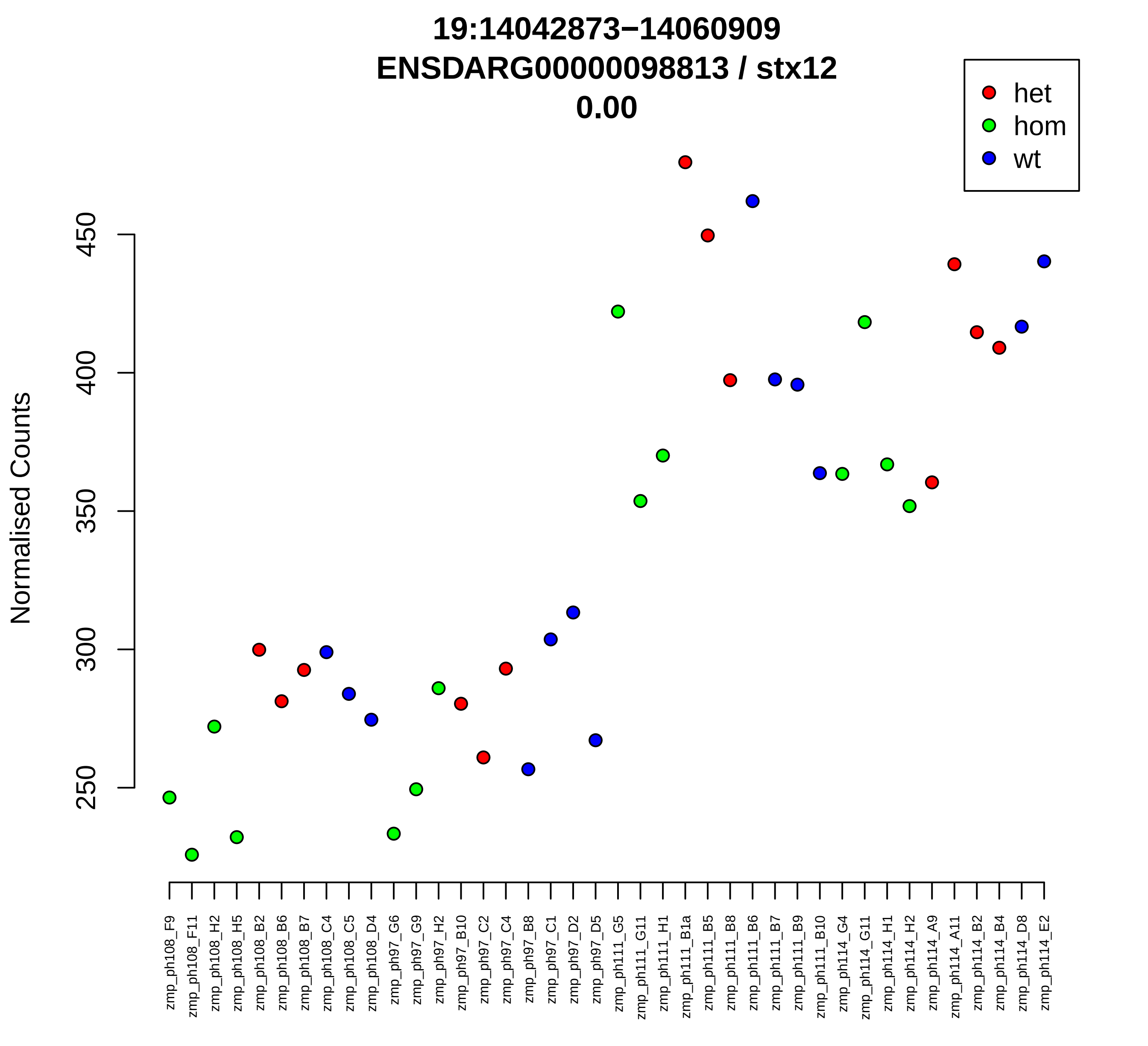
19:14042873−14060909
ENSDARG00000098813 / stx12
0.00
het
hom
wt
Normalised Counts
250
300
350
400
450
zmp_ph108_F9
zmp_ph108_F11
zmp_ph108_H2
zmp_ph108_H5
zmp_ph108_B2
zmp_ph108_B6
zmp_ph108_B7
zmp_ph108_C4
zmp_ph108_C5
zmp_ph108_D4
zmp_ph97_G6
zmp_ph97_G9
zmp_ph97_H2
zmp_ph97_B10
zmp_ph97_C2
zmp_ph97_C4
zmp_ph97_B8
zmp_ph97_C1
zmp_ph97_D2
zmp_ph97_D5
zmp_ph111_G5
zmp_ph111_G11
zmp_ph111_H1
zmp_ph111_B1a
zmp_ph111_B5
zmp_ph111_B8
zmp_ph111_B6
zmp_ph111_B7
zmp_ph111_B9
zmp_ph111_B10
zmp_ph114_G4
zmp_ph114_G11
zmp_ph114_H1
zmp_ph114_H2
zmp_ph114_A9
zmp_ph114_A11
zmp_ph114_B2
zmp_ph114_B4
zmp_ph114_D8
zmp_ph114_E2

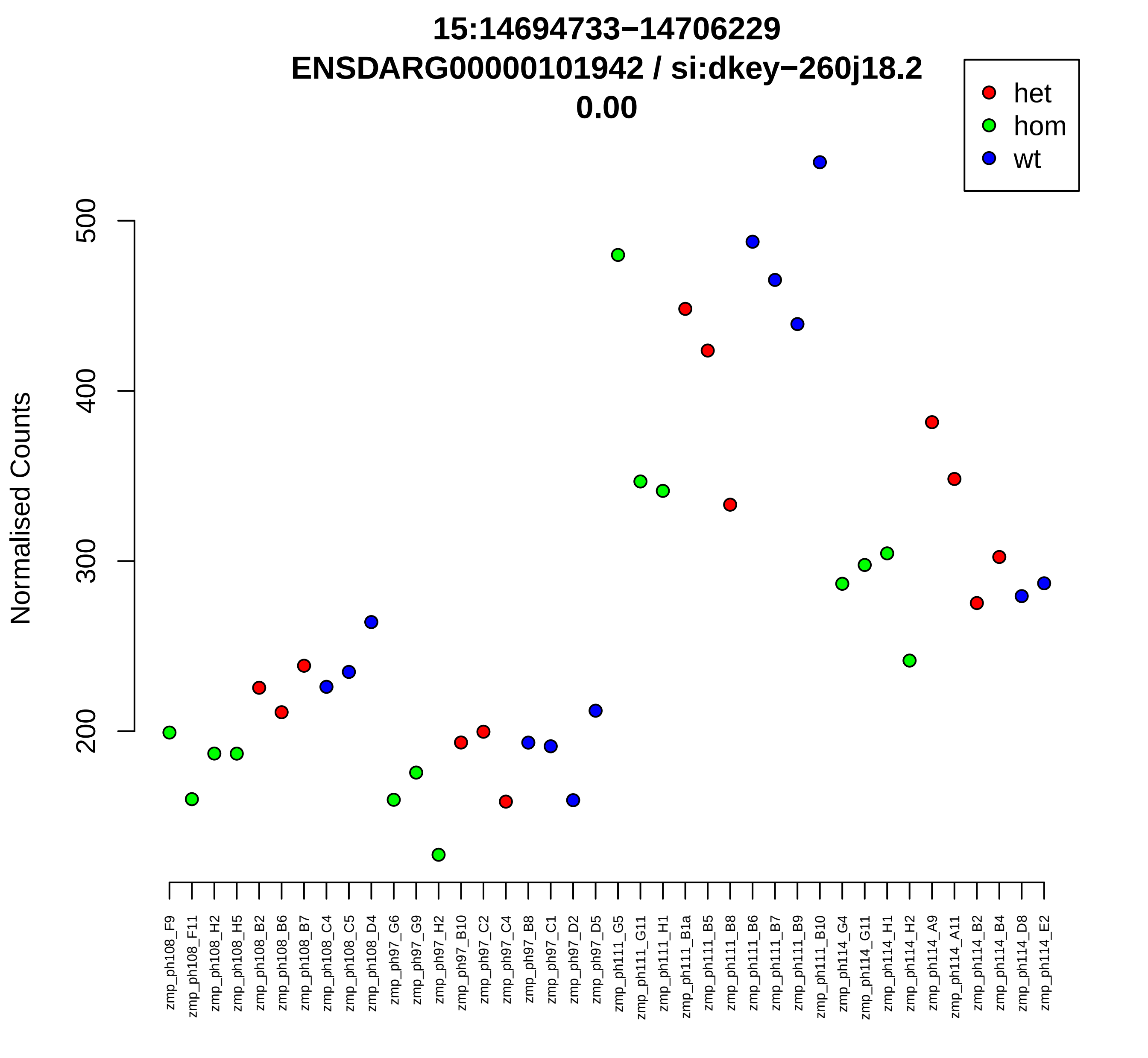

15:14694733−14706229
ENSDARG00000101942 / si:dkey−260j18.2
0.00
het
hom
wt
Normalised Counts
500
400
300
200
zmp_ph108_F9
zmp_ph108_F11
zmp_ph108_H2
zmp_ph108_H5
zmp_ph108_B2
zmp_ph108_B6
zmp_ph108_B7
zmp_ph108_C4
zmp_ph108_C5
zmp_ph108_D4
zmp_ph97_G6
zmp_ph97_G9
zmp_ph97_H2
zmp_ph97_B10
zmp_ph97_C2
zmp_ph97_C4
zmp_ph97_B8
zmp_ph97_C1
zmp_ph97_D2
zmp_ph97_D5
zmp_ph111_G5
zmp_ph111_G11
zmp_ph111_H1
zmp_ph111_B1a
zmp_ph111_B5
zmp_ph111_B8
zmp_ph111_B6
zmp_ph111_B7
zmp_ph111_B9
zmp_ph111_B10
zmp_ph114_G4
zmp_ph114_G11
zmp_ph114_H1
zmp_ph114_H2
zmp_ph114_A9
zmp_ph114_A11
zmp_ph114_B2
zmp_ph114_B4
zmp_ph114_D8
zmp_ph114_E2

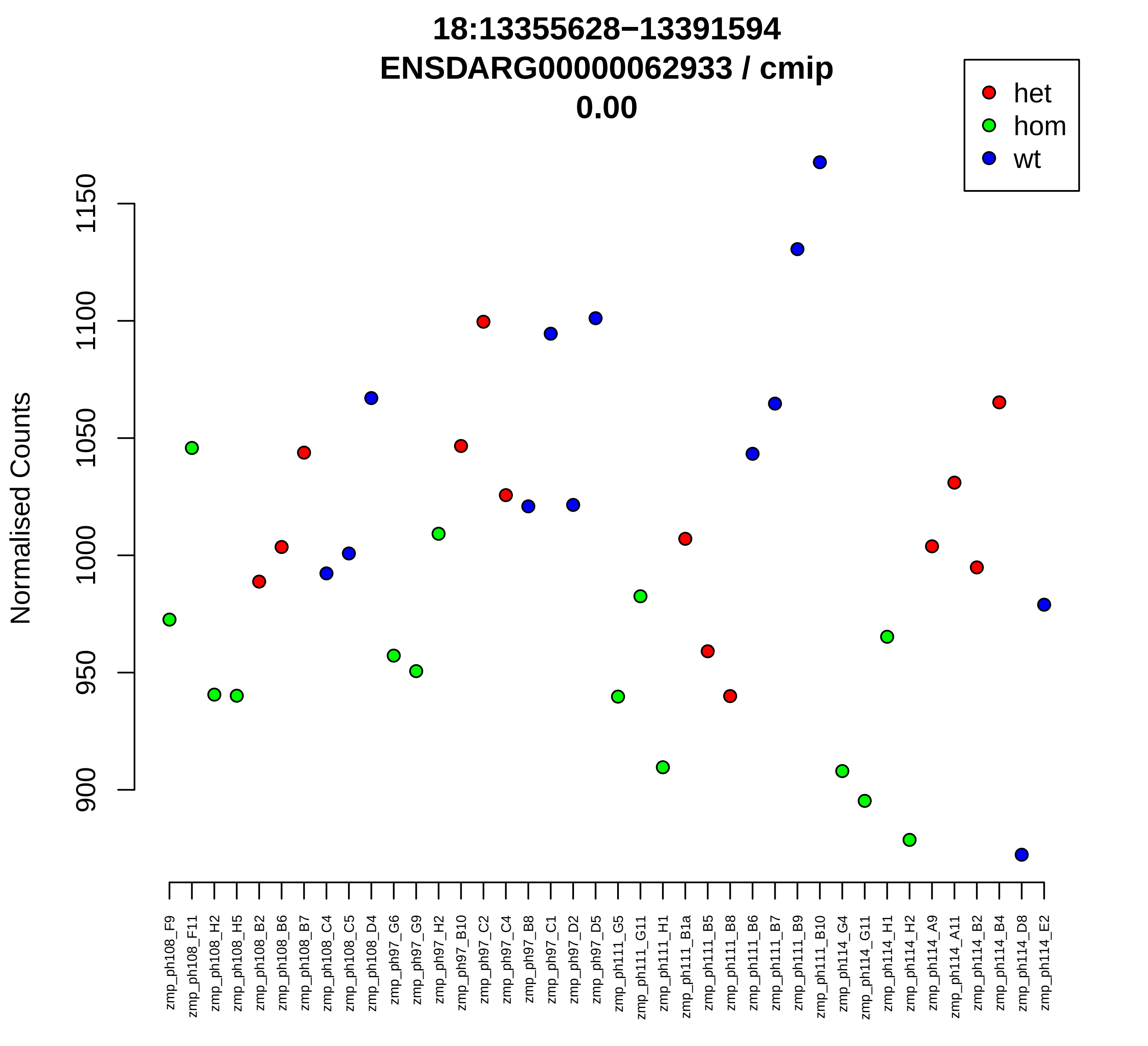

18:13355628−13391594
ENSDARG00000062933 / cmip
0.00
het
hom
wt
Normalised Counts
900
950
1000
1050
1100
1150
zmp_ph108_F9
zmp_ph108_F11
zmp_ph108_H2
zmp_ph108_H5
zmp_ph108_B2
zmp_ph108_B6
zmp_ph108_B7
zmp_ph108_C4
zmp_ph108_C5
zmp_ph108_D4
zmp_ph97_G6
zmp_ph97_G9
zmp_ph97_H2
zmp_ph97_B10
zmp_ph97_C2
zmp_ph97_C4
zmp_ph97_B8
zmp_ph97_C1
zmp_ph97_D2
zmp_ph97_D5
zmp_ph111_G5
zmp_ph111_G11
zmp_ph111_H1
zmp_ph111_B1a
zmp_ph111_B5
zmp_ph111_B8
zmp_ph111_B6
zmp_ph111_B7
zmp_ph111_B9
zmp_ph111_B10
zmp_ph114_G4
zmp_ph114_G11
zmp_ph114_H1
zmp_ph114_H2
zmp_ph114_A9
zmp_ph114_A11
zmp_ph114_B2
zmp_ph114_B4
zmp_ph114_D8
zmp_ph114_E2

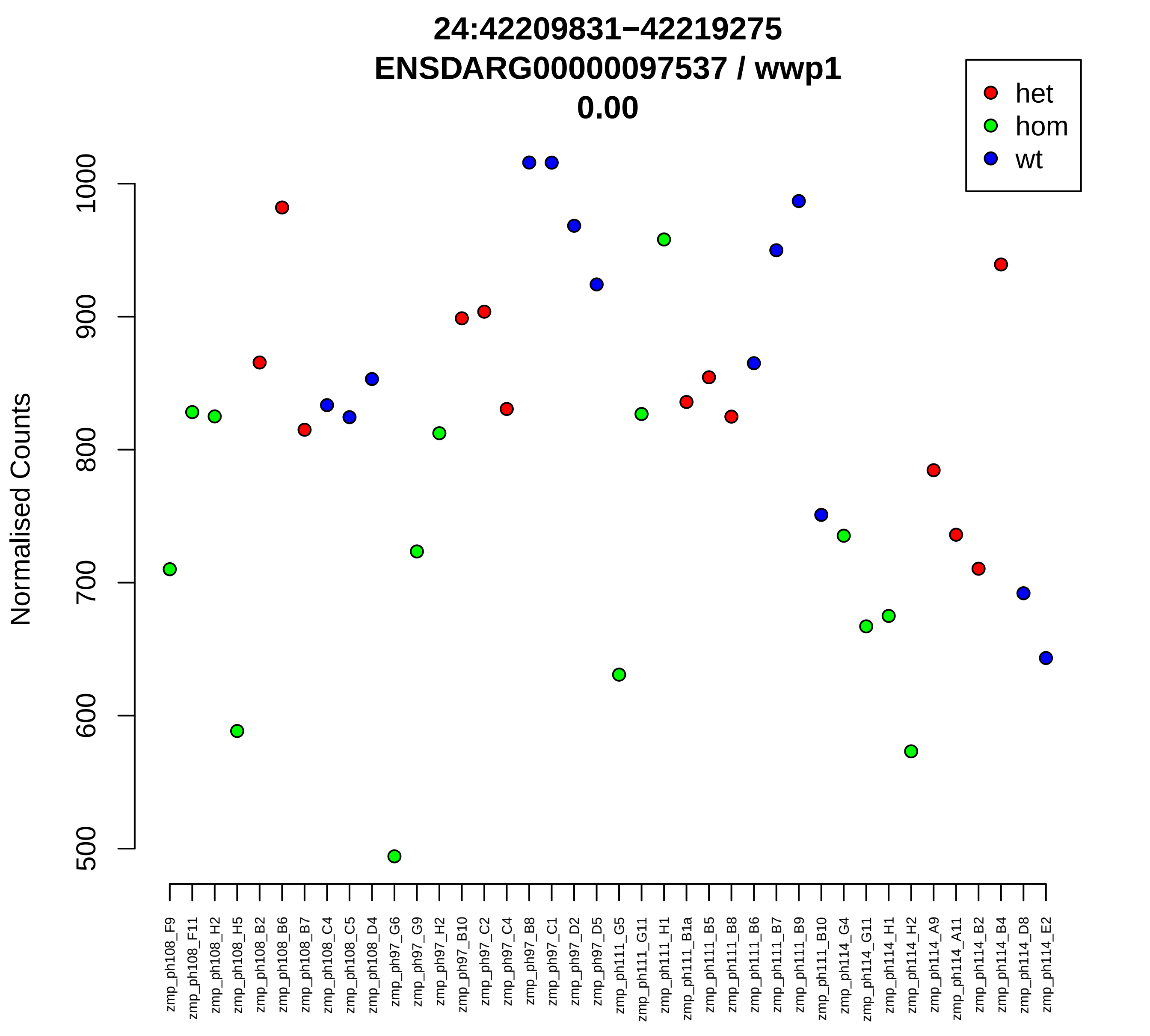
24:42209831−42219275
ENSDARG00000097537 / wwp1
0.00
Normalised Counts
500
600
700
800
900
1000
het
hom
wt
zmp_ph108_F9
zmp_ph108_F11
zmp_ph108_H2
zmp_ph108_H5
zmp_ph108_B2
zmp_ph108_B6
zmp_ph108_B7
zmp_ph108_C4
zmp_ph108_C5
zmp_ph108_D4
zmp_ph97_G6
zmp_ph97_G9
zmp_ph97_H2
zmp_ph97_B10
zmp_ph97_C2
zmp_ph97_C4
zmp_ph97_B8
zmp_ph97_C1
zmp_ph97_D2
zmp_ph97_D5
zmp_ph111_G5
zmp_ph111_G11
zmp_ph111_H1
zmp_ph111_B1a
zmp_ph111_B5
zmp_ph111_B8
zmp_ph111_B6
zmp_ph111_B7
zmp_ph111_B9
zmp_ph111_B10
zmp_ph114_G4
zmp_ph114_G11
zmp_ph114_H1
zmp_ph114_H2
zmp_ph114_A9
zmp_ph114_A11
zmp_ph114_B2
zmp_ph114_B4
zmp_ph114_D8
zmp_ph114_E2

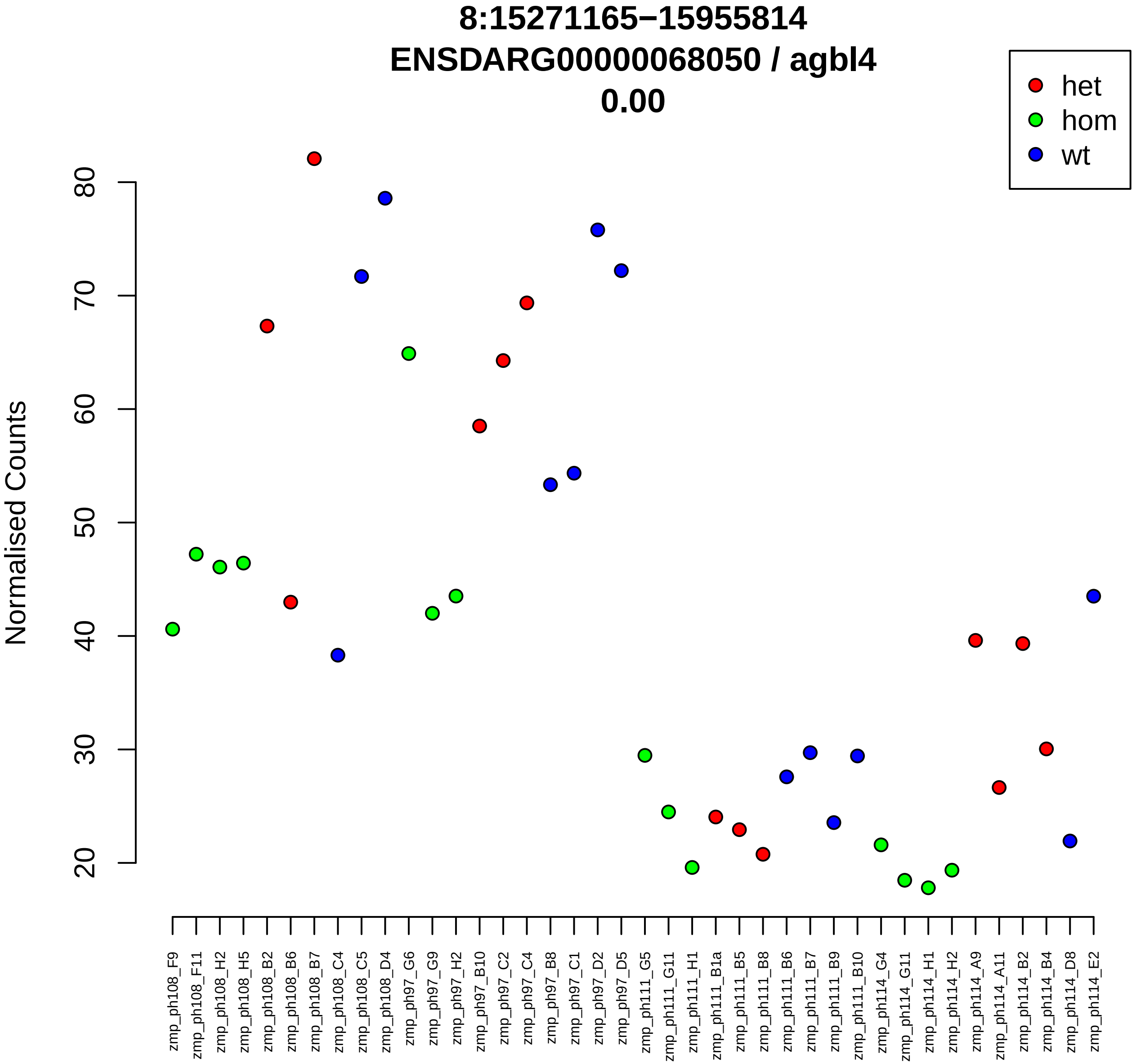

8:15271165−15955814
ENSDARG00000068050 / agbl4
0.00
het
hom
wt
Normalised Counts
20
30
40
50
60
70
80
zmp_ph108_F9
zmp_ph108_F11
zmp_ph108_H2
zmp_ph108_H5
zmp_ph108_B2
zmp_ph108_B6
zmp_ph108_B7
zmp_ph108_C4
zmp_ph108_C5
zmp_ph108_D4
zmp_ph97_G6
zmp_ph97_G9
zmp_ph97_H2
zmp_ph97_B10
zmp_ph97_C2
zmp_ph97_C4
zmp_ph97_B8
zmp_ph97_C1
zmp_ph97_D2
zmp_ph97_D5
zmp_ph111_G5
zmp_ph111_G11
zmp_ph111_H1
zmp_ph111_B1a
zmp_ph111_B5
zmp_ph111_B8
zmp_ph111_B6
zmp_ph111_B7
zmp_ph111_B9
zmp_ph111_B10
zmp_ph114_G4
zmp_ph114_G11
zmp_ph114_H1
zmp_ph114_H2
zmp_ph114_A9
zmp_ph114_A11
zmp_ph114_B2
zmp_ph114_B4
zmp_ph114_D8
zmp_ph114_E2

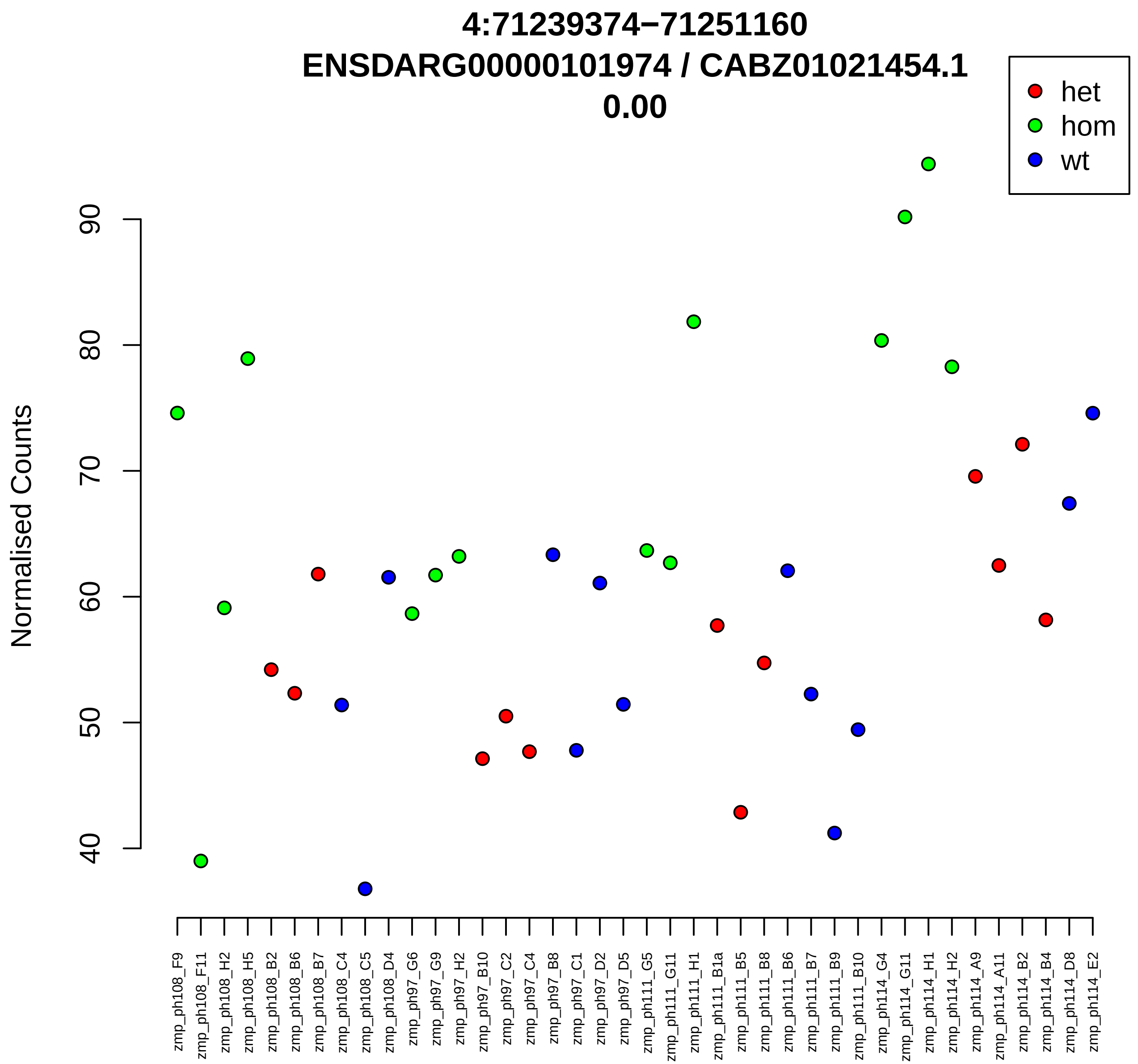
4:71239374−71251160
ENSDARG00000101974 / CABZ01021454.1
0.00
het
hom
wt
Normalised Counts
40
50
60
70
80
90
zmp_ph108_F9
zmp_ph108_F11
zmp_ph108_H2
zmp_ph108_H5
zmp_ph108_B2
zmp_ph108_B6
zmp_ph108_B7
zmp_ph108_C4
zmp_ph108_C5
zmp_ph108_D4
zmp_ph97_G6
zmp_ph97_G9
zmp_ph97_H2
zmp_ph97_B10
zmp_ph97_C2
zmp_ph97_C4
zmp_ph97_B8
zmp_ph97_C1
zmp_ph97_D2
zmp_ph97_D5
zmp_ph111_G5
zmp_ph111_G11
zmp_ph111_H1
zmp_ph111_B1a
zmp_ph111_B5
zmp_ph111_B8
zmp_ph111_B6
zmp_ph111_B7
zmp_ph111_B9
zmp_ph111_B10
zmp_ph114_G4
zmp_ph114_G11
zmp_ph114_H1
zmp_ph114_H2
zmp_ph114_A9
zmp_ph114_A11
zmp_ph114_B2
zmp_ph114_B4
zmp_ph114_D8
zmp_ph114_E2

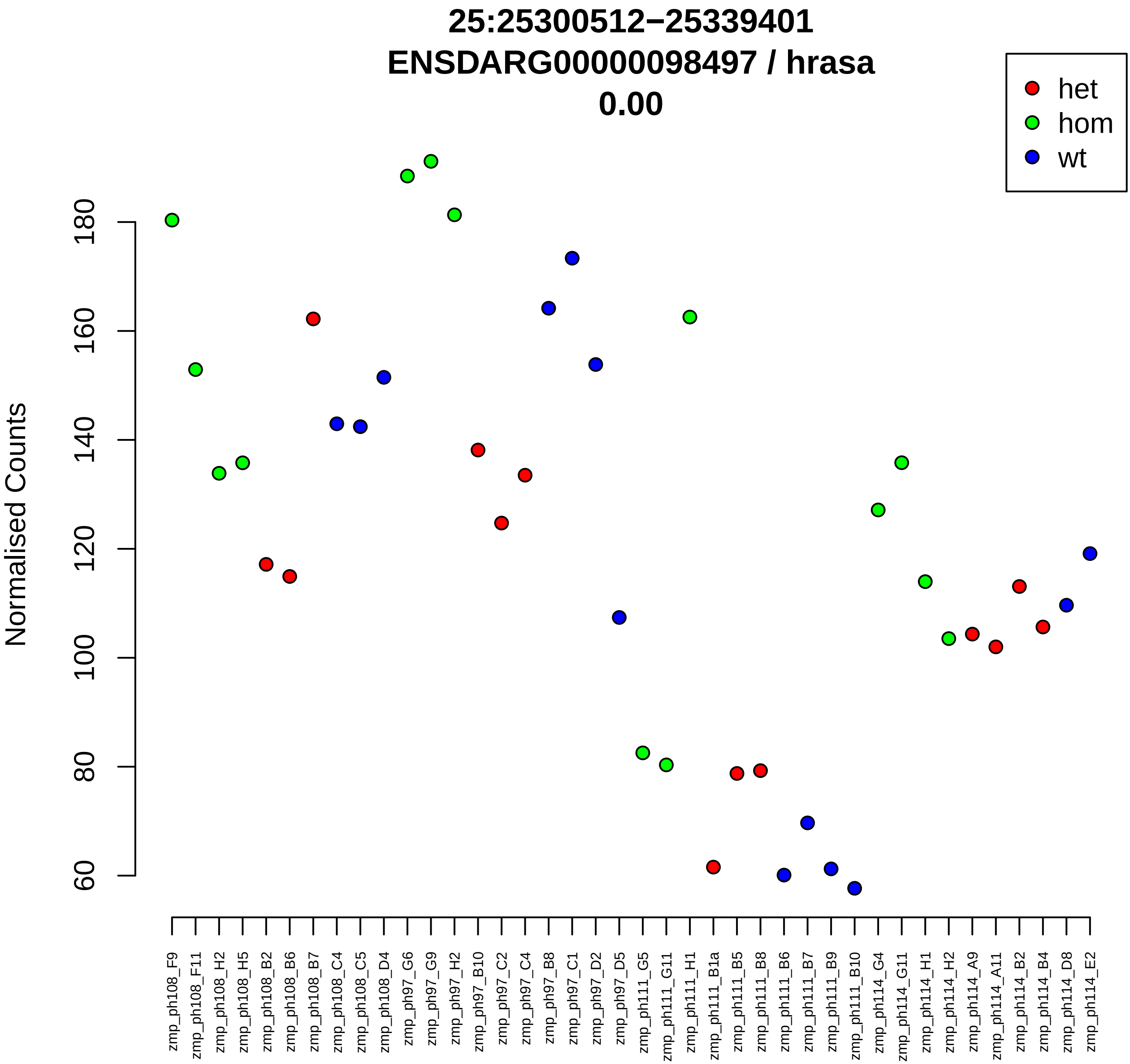
25:25300512−25339401
ENSDARG00000098497 / hrasa
0.00
het
hom
wt
Normalised Counts
60
80
100
120
140
160
180
zmp_ph108_F9
zmp_ph108_F11
zmp_ph108_H2
zmp_ph108_H5
zmp_ph108_B2
zmp_ph108_B6
zmp_ph108_B7
zmp_ph108_C4
zmp_ph108_C5
zmp_ph108_D4
zmp_ph97_G6
zmp_ph97_G9
zmp_ph97_H2
zmp_ph97_B10
zmp_ph97_C2
zmp_ph97_C4
zmp_ph97_B8
zmp_ph97_C1
zmp_ph97_D2
zmp_ph97_D5
zmp_ph111_G5
zmp_ph111_G11
zmp_ph111_H1
zmp_ph111_B1a
zmp_ph111_B5
zmp_ph111_B8
zmp_ph111_B6
zmp_ph111_B7
zmp_ph111_B9
zmp_ph111_B10
zmp_ph114_G4
zmp_ph114_G11
zmp_ph114_H1
zmp_ph114_H2
zmp_ph114_A9
zmp_ph114_A11
zmp_ph114_B2
zmp_ph114_B4
zmp_ph114_D8
zmp_ph114_E2

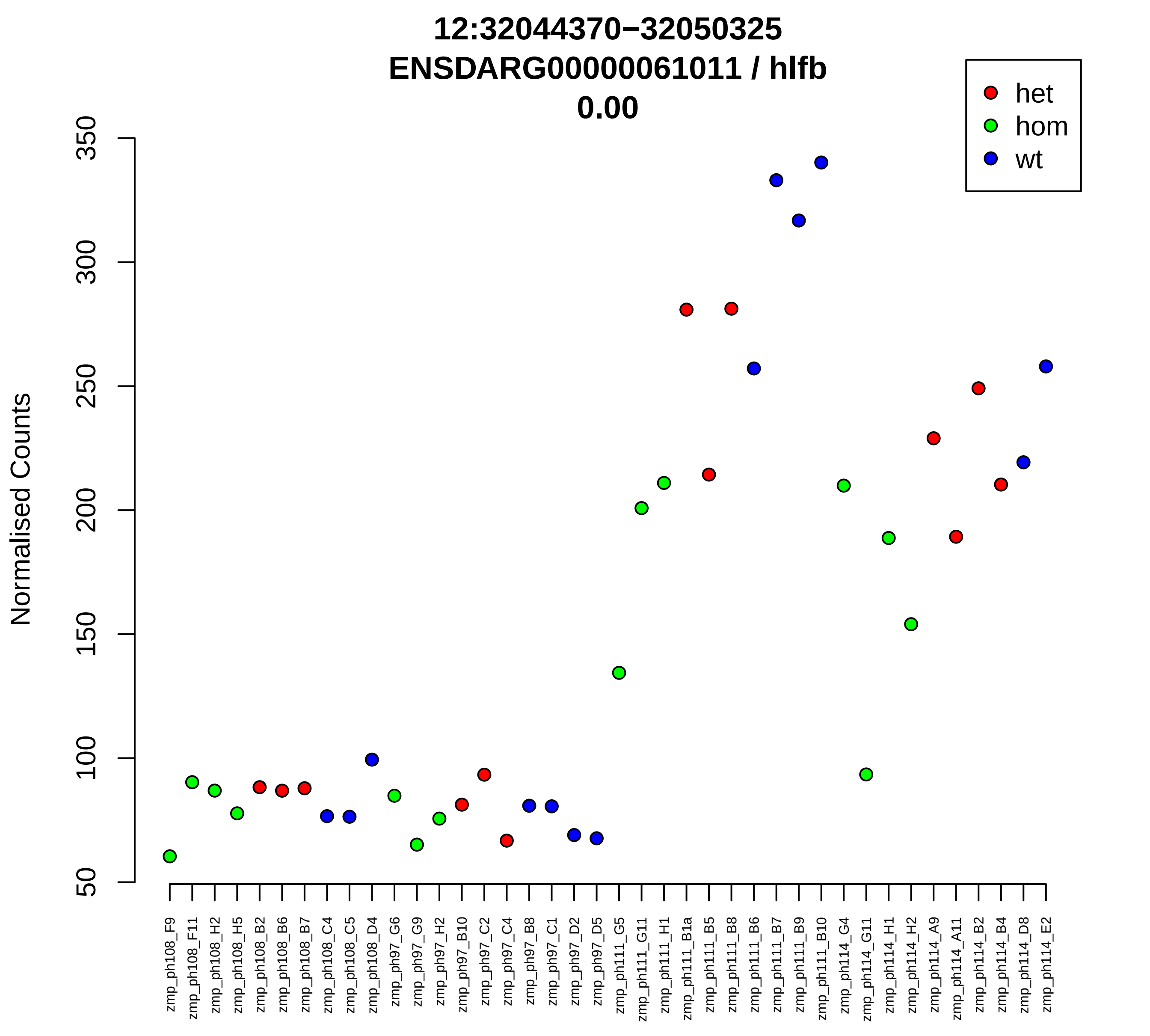
12:32044370−32050325
ENSDARG00000061011 / hlfb
0.00
het
hom
wt
Normalised Counts
50
100
150
200
250
300
350
zmp_ph108_F9
zmp_ph108_F11
zmp_ph108_H2
zmp_ph108_H5
zmp_ph108_B2
zmp_ph108_B6
zmp_ph108_B7
zmp_ph108_C4
zmp_ph108_C5
zmp_ph108_D4
zmp_ph97_G6
zmp_ph97_G9
zmp_ph97_H2
zmp_ph97_B10
zmp_ph97_C2
zmp_ph97_C4
zmp_ph97_B8
zmp_ph97_C1
zmp_ph97_D2
zmp_ph97_D5
zmp_ph111_G5
zmp_ph111_G11
zmp_ph111_H1
zmp_ph111_B1a
zmp_ph111_B5
zmp_ph111_B8
zmp_ph111_B6
zmp_ph111_B7
zmp_ph111_B9
zmp_ph111_B10
zmp_ph114_G4
zmp_ph114_G11
zmp_ph114_H1
zmp_ph114_H2
zmp_ph114_A9
zmp_ph114_A11
zmp_ph114_B2
zmp_ph114_B4
zmp_ph114_D8
zmp_ph114_E2

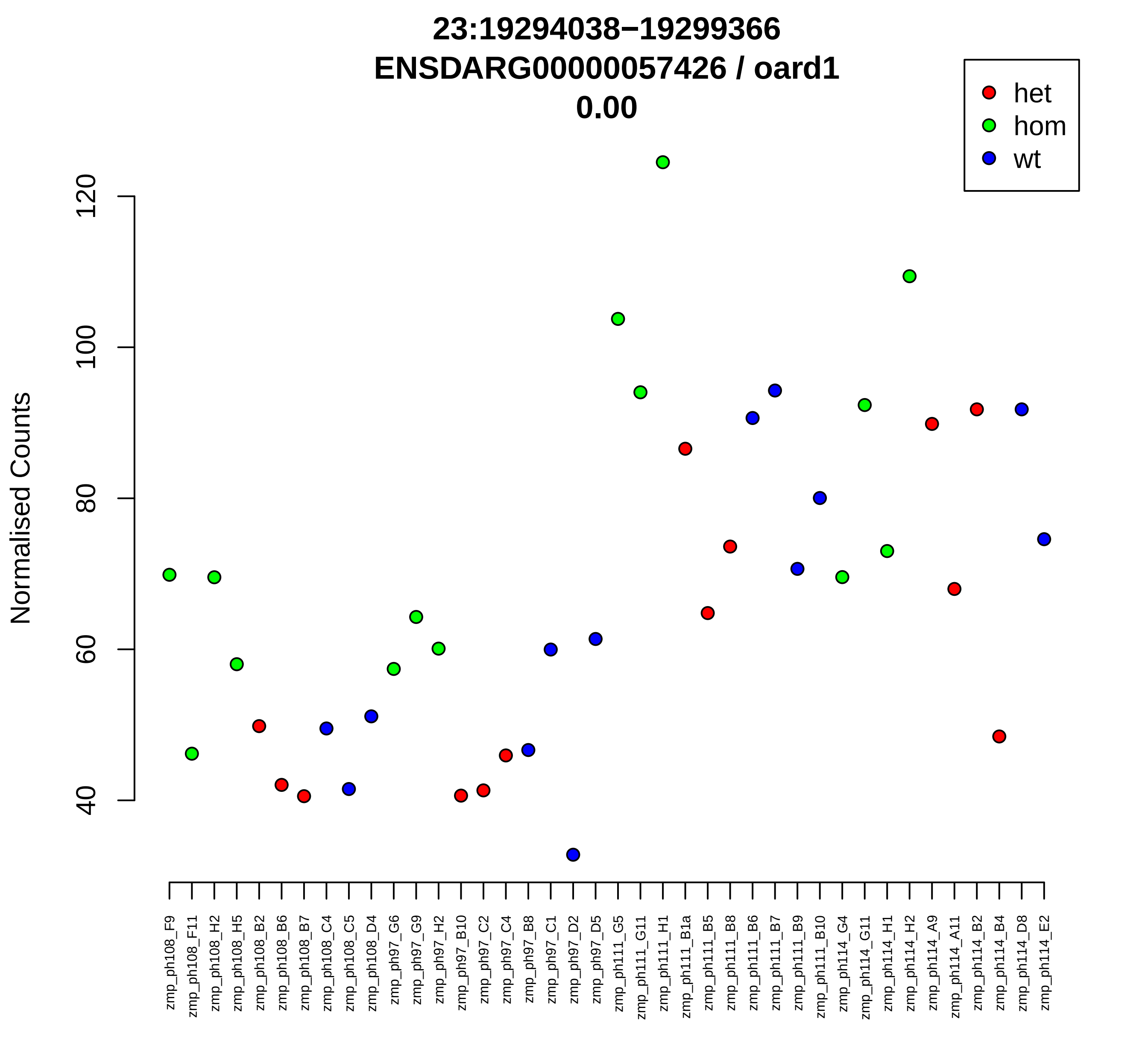

23:19294038−19299366
ENSDARG00000057426 / oard1
0.00
het
hom
wt
Normalised Counts
40
60
80
100
120
zmp_ph108_F9
zmp_ph108_F11
zmp_ph108_H2
zmp_ph108_H5
zmp_ph108_B2
zmp_ph108_B6
zmp_ph108_B7
zmp_ph108_C4
zmp_ph108_C5
zmp_ph108_D4
zmp_ph97_G6
zmp_ph97_G9
zmp_ph97_H2
zmp_ph97_B10
zmp_ph97_C2
zmp_ph97_C4
zmp_ph97_B8
zmp_ph97_C1
zmp_ph97_D2
zmp_ph97_D5
zmp_ph111_G5
zmp_ph111_G11
zmp_ph111_H1
zmp_ph111_B1a
zmp_ph111_B5
zmp_ph111_B8
zmp_ph111_B6
zmp_ph111_B7
zmp_ph111_B9
zmp_ph111_B10
zmp_ph114_G4
zmp_ph114_G11
zmp_ph114_H1
zmp_ph114_H2
zmp_ph114_A9
zmp_ph114_A11
zmp_ph114_B2
zmp_ph114_B4
zmp_ph114_D8
zmp_ph114_E2

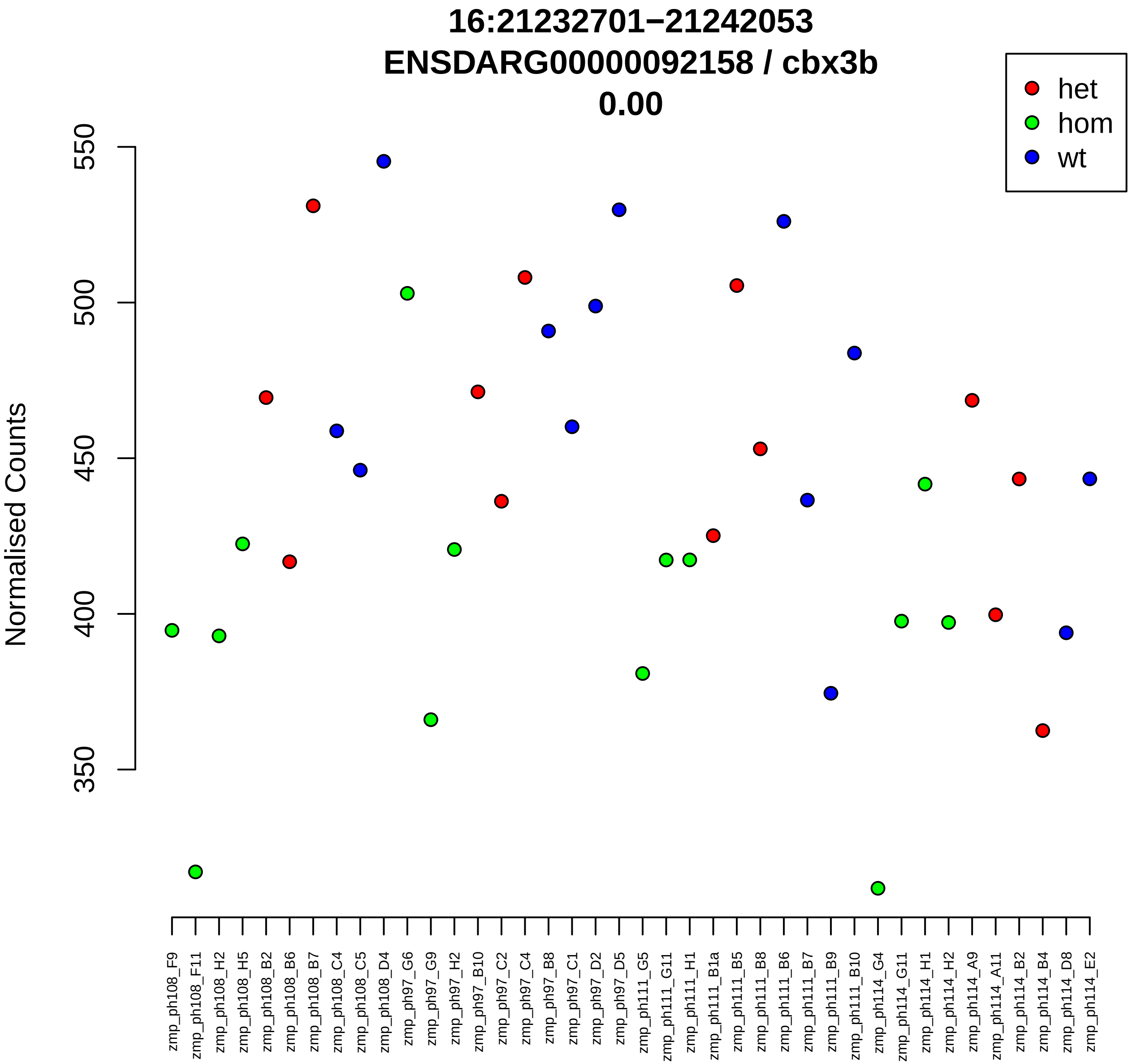

16:21232701−21242053
ENSDARG00000092158 / cbx3b
0.00
het
hom
wt
Normalised Counts
350
400
450
500
550
zmp_ph108_F9
zmp_ph108_F11
zmp_ph108_H2
zmp_ph108_H5
zmp_ph108_B2
zmp_ph108_B6
zmp_ph108_B7
zmp_ph108_C4
zmp_ph108_C5
zmp_ph108_D4
zmp_ph97_G6
zmp_ph97_G9
zmp_ph97_H2
zmp_ph97_B10
zmp_ph97_C2
zmp_ph97_C4
zmp_ph97_B8
zmp_ph97_C1
zmp_ph97_D2
zmp_ph97_D5
zmp_ph111_G5
zmp_ph111_G11
zmp_ph111_H1
zmp_ph111_B1a
zmp_ph111_B5
zmp_ph111_B8
zmp_ph111_B6
zmp_ph111_B7
zmp_ph111_B9
zmp_ph111_B10
zmp_ph114_G4
zmp_ph114_G11
zmp_ph114_H1
zmp_ph114_H2
zmp_ph114_A9
zmp_ph114_A11
zmp_ph114_B2
zmp_ph114_B4
zmp_ph114_D8
zmp_ph114_E2

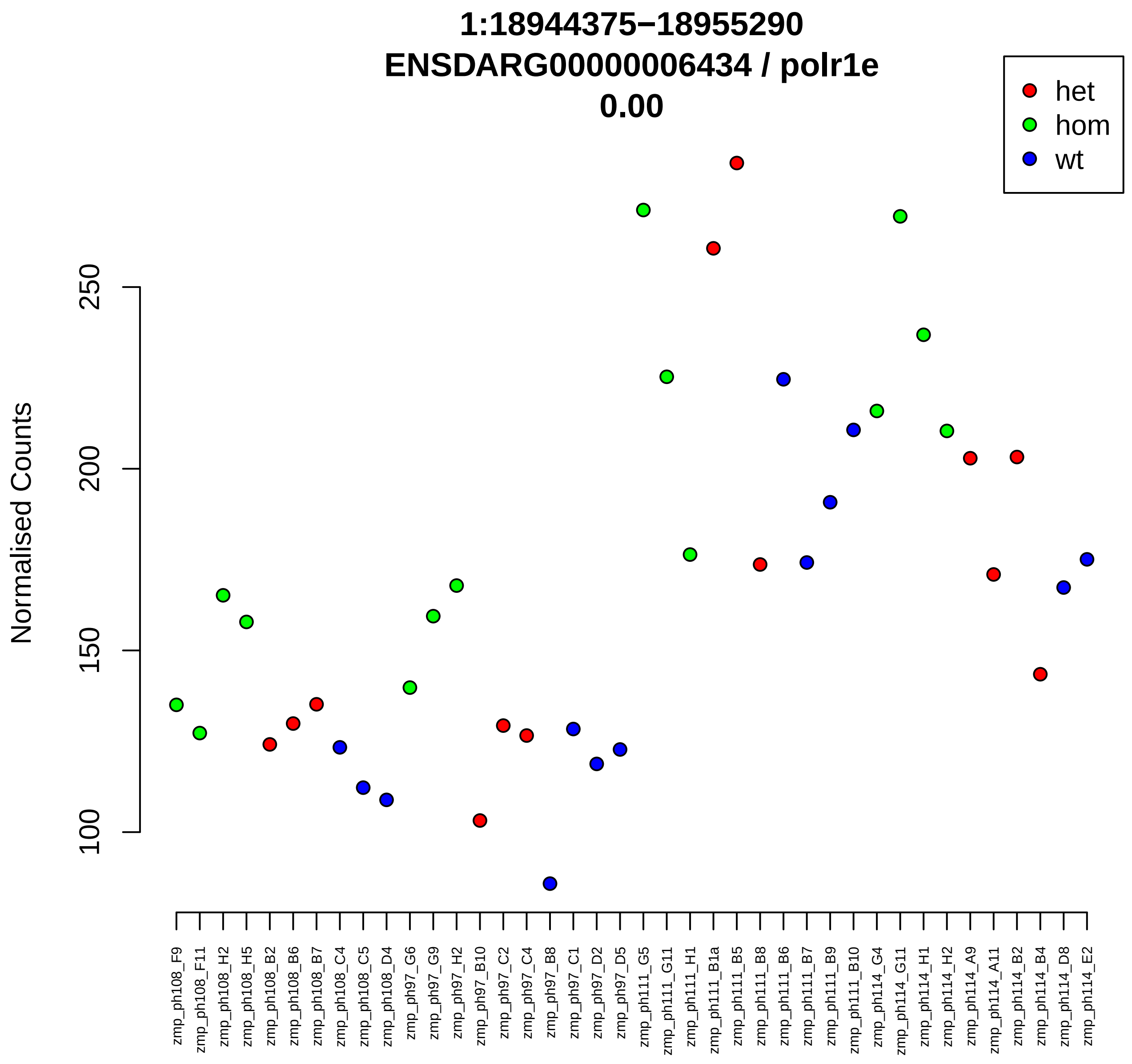
1:18944375−18955290
ENSDARG00000006434 / polr1e
0.00
het
hom
wt
Normalised Counts
100
150
200
250
zmp_ph108_F9
zmp_ph108_F11
zmp_ph108_H2
zmp_ph108_H5
zmp_ph108_B2
zmp_ph108_B6
zmp_ph108_B7
zmp_ph108_C4
zmp_ph108_C5
zmp_ph108_D4
zmp_ph97_G6
zmp_ph97_G9
zmp_ph97_H2
zmp_ph97_B10
zmp_ph97_C2
zmp_ph97_C4
zmp_ph97_B8
zmp_ph97_C1
zmp_ph97_D2
zmp_ph97_D5
zmp_ph111_G5
zmp_ph111_G11
zmp_ph111_H1
zmp_ph111_B1a
zmp_ph111_B5
zmp_ph111_B8
zmp_ph111_B6
zmp_ph111_B7
zmp_ph111_B9
zmp_ph111_B10
zmp_ph114_G4
zmp_ph114_G11
zmp_ph114_H1
zmp_ph114_H2
zmp_ph114_A9
zmp_ph114_A11
zmp_ph114_B2
zmp_ph114_B4
zmp_ph114_D8
zmp_ph114_E2

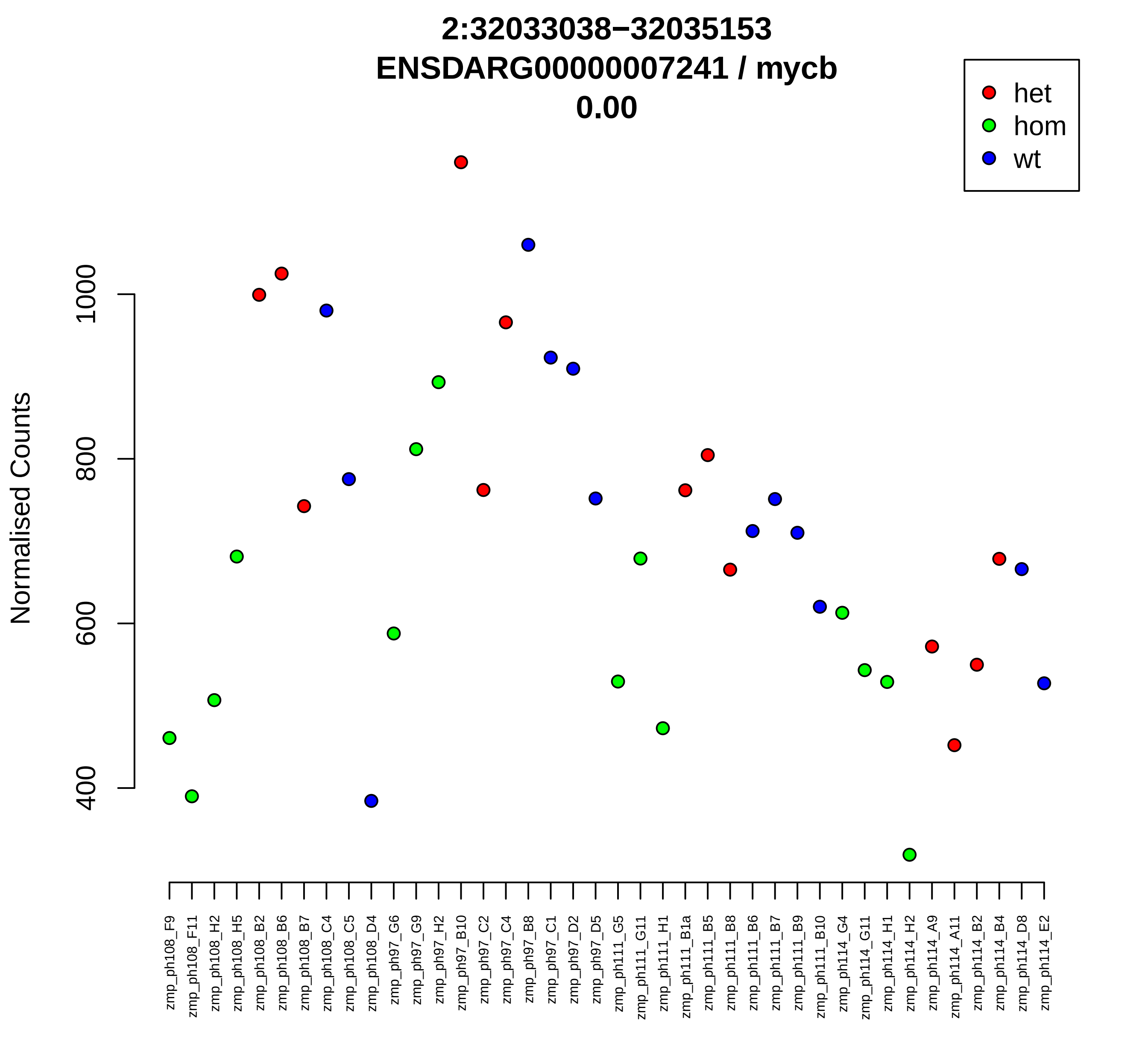
2:32033038−32035153
ENSDARG00000007241 / mycb
0.00
het
hom
wt
Normalised Counts
400
600
800
1000
zmp_ph108_F9
zmp_ph108_F11
zmp_ph108_H2
zmp_ph108_H5
zmp_ph108_B2
zmp_ph108_B6
zmp_ph108_B7
zmp_ph108_C4
zmp_ph108_C5
zmp_ph108_D4
zmp_ph97_G6
zmp_ph97_G9
zmp_ph97_H2
zmp_ph97_B10
zmp_ph97_C2
zmp_ph97_C4
zmp_ph97_B8
zmp_ph97_C1
zmp_ph97_D2
zmp_ph97_D5
zmp_ph111_G5
zmp_ph111_G11
zmp_ph111_H1
zmp_ph111_B1a
zmp_ph111_B5
zmp_ph111_B8
zmp_ph111_B6
zmp_ph111_B7
zmp_ph111_B9
zmp_ph111_B10
zmp_ph114_G4
zmp_ph114_G11
zmp_ph114_H1
zmp_ph114_H2
zmp_ph114_A9
zmp_ph114_A11
zmp_ph114_B2
zmp_ph114_B4
zmp_ph114_D8
zmp_ph114_E2

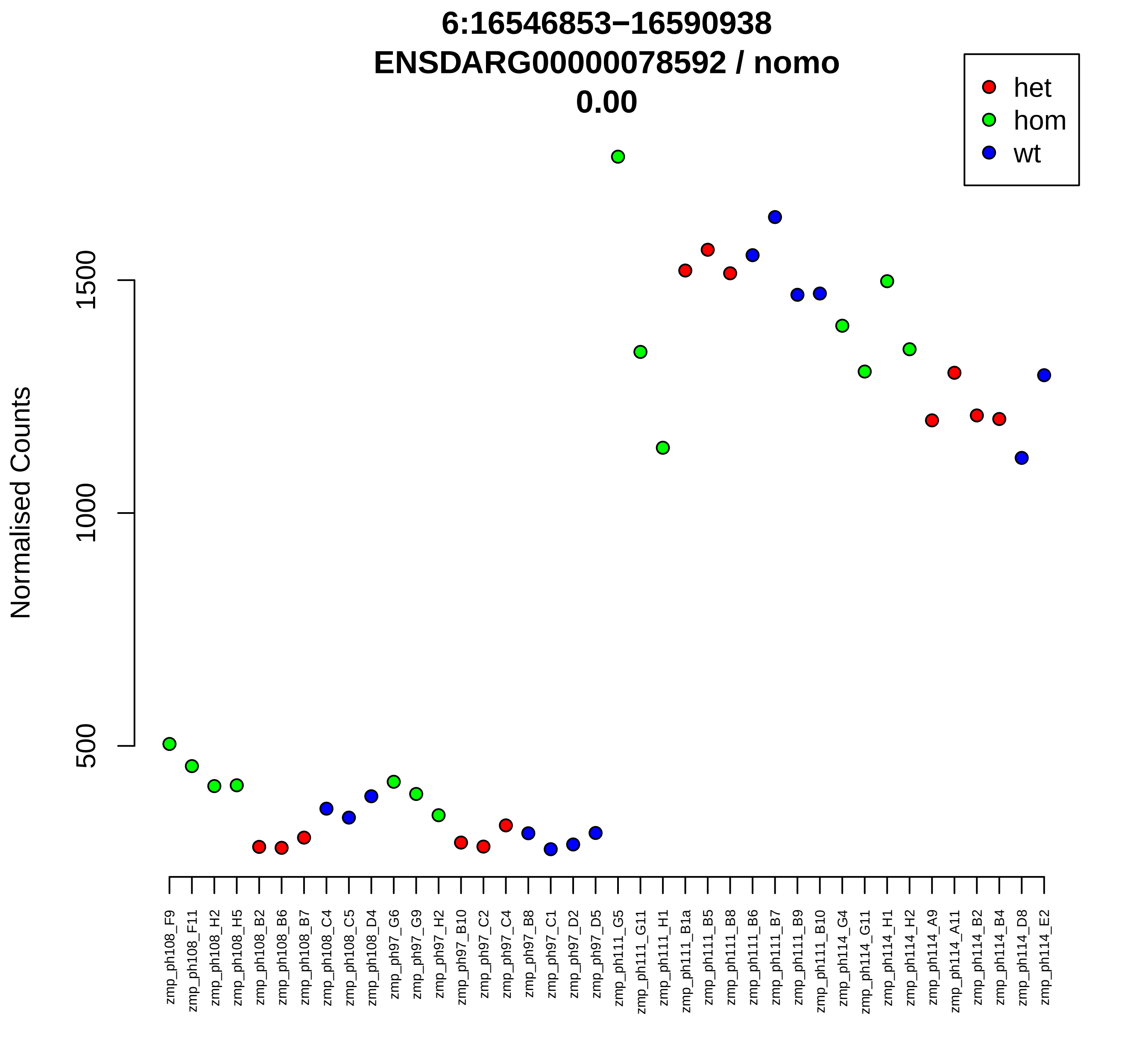

6:16546853−16590938
ENSDARG00000078592 / nomo
0.00
het
hom
wt
Normalised Counts
500
1000
1500
zmp_ph108_F9
zmp_ph108_F11
zmp_ph108_H2
zmp_ph108_H5
zmp_ph108_B2
zmp_ph108_B6
zmp_ph108_B7
zmp_ph108_C4
zmp_ph108_C5
zmp_ph108_D4
zmp_ph97_G6
zmp_ph97_G9
zmp_ph97_H2
zmp_ph97_B10
zmp_ph97_C2
zmp_ph97_C4
zmp_ph97_B8
zmp_ph97_C1
zmp_ph97_D2
zmp_ph97_D5
zmp_ph111_G5
zmp_ph111_G11
zmp_ph111_H1
zmp_ph111_B1a
zmp_ph111_B5
zmp_ph111_B8
zmp_ph111_B6
zmp_ph111_B7
zmp_ph111_B9
zmp_ph111_B10
zmp_ph114_G4
zmp_ph114_G11
zmp_ph114_H1
zmp_ph114_H2
zmp_ph114_A9
zmp_ph114_A11
zmp_ph114_B2
zmp_ph114_B4
zmp_ph114_D8
zmp_ph114_E2

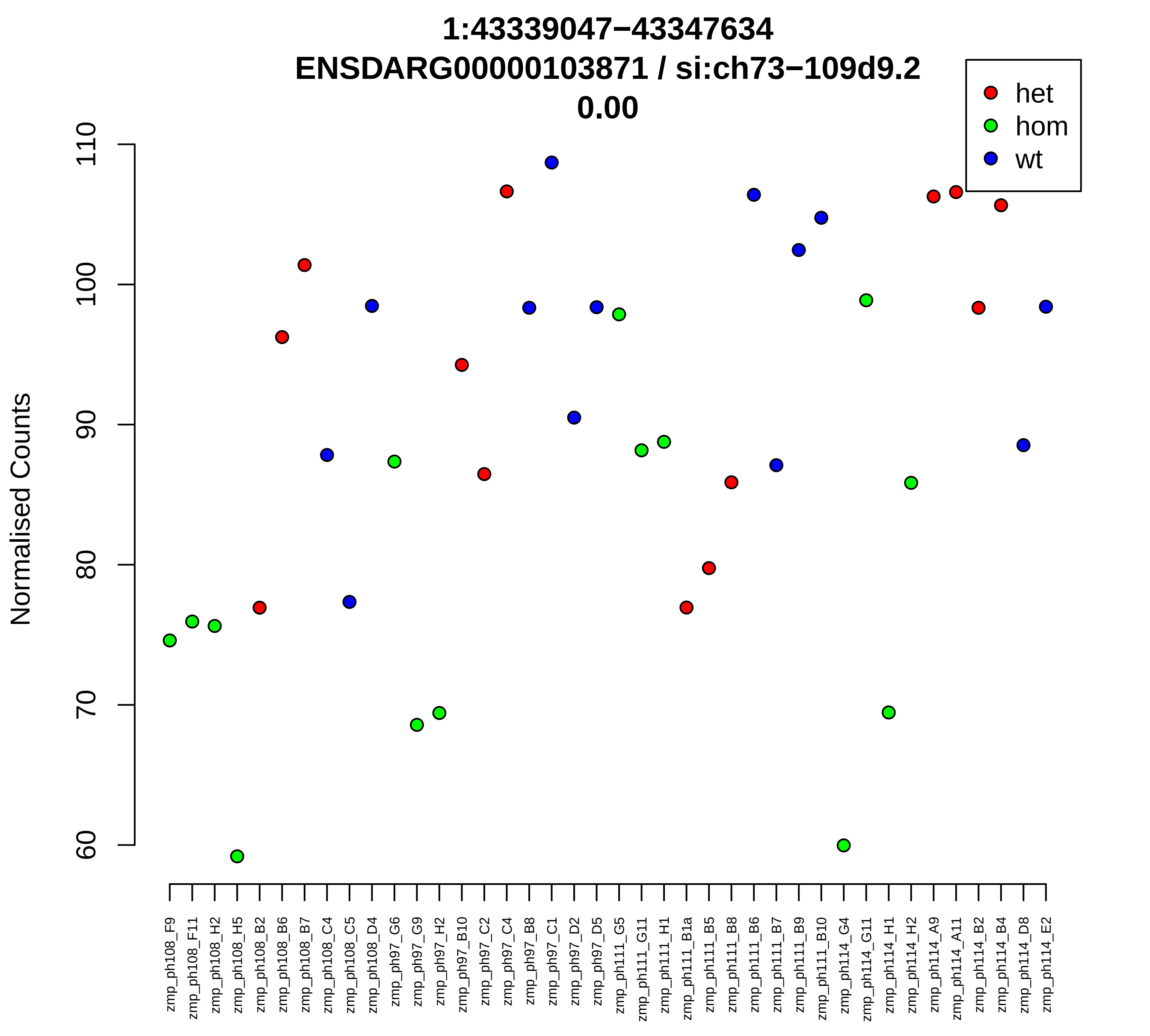

1:43339047−43347634
ENSDARG00000103871 / si:ch73−109d9.2
0.00
het
hom
wt
Normalised Counts
60
70
80
90
100
110
zmp_ph108_F9
zmp_ph108_F11
zmp_ph108_H2
zmp_ph108_H5
zmp_ph108_B2
zmp_ph108_B6
zmp_ph108_B7
zmp_ph108_C4
zmp_ph108_C5
zmp_ph108_D4
zmp_ph97_G6
zmp_ph97_G9
zmp_ph97_H2
zmp_ph97_B10
zmp_ph97_C2
zmp_ph97_C4
zmp_ph97_B8
zmp_ph97_C1
zmp_ph97_D2
zmp_ph97_D5
zmp_ph111_G5
zmp_ph111_G11
zmp_ph111_H1
zmp_ph111_B1a
zmp_ph111_B5
zmp_ph111_B8
zmp_ph111_B6
zmp_ph111_B7
zmp_ph111_B9
zmp_ph111_B10
zmp_ph114_G4
zmp_ph114_G11
zmp_ph114_H1
zmp_ph114_H2
zmp_ph114_A9
zmp_ph114_A11
zmp_ph114_B2
zmp_ph114_B4
zmp_ph114_D8
zmp_ph114_E2

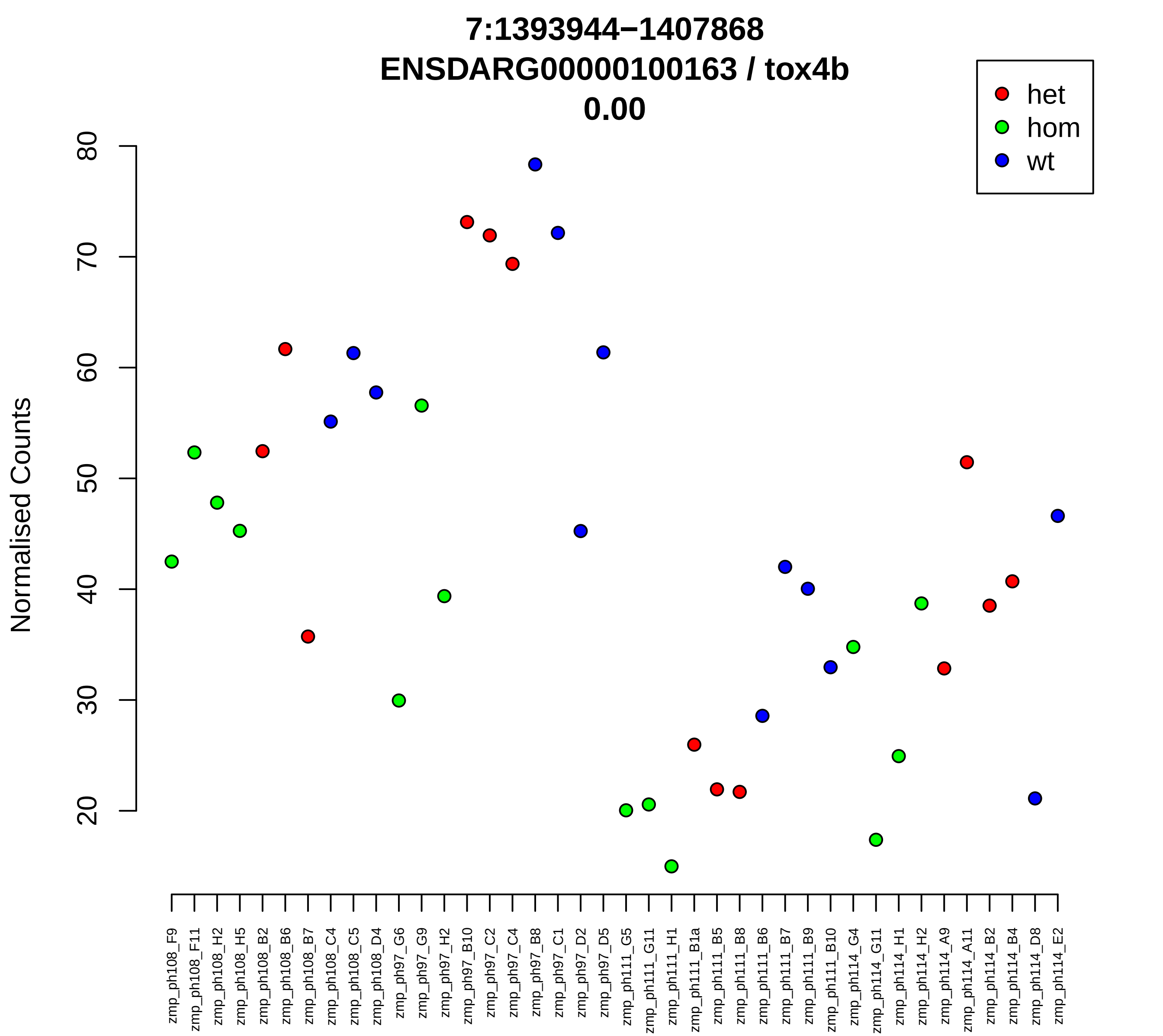

7:1393944−1407868
ENSDARG00000100163 / tox4b
0.00
het
hom
wt
Normalised Counts
20
30
40
50
60
70
80
zmp_ph108_F9
zmp_ph108_F11
zmp_ph108_H2
zmp_ph108_H5
zmp_ph108_B2
zmp_ph108_B6
zmp_ph108_B7
zmp_ph108_C4
zmp_ph108_C5
zmp_ph108_D4
zmp_ph97_G6
zmp_ph97_G9
zmp_ph97_H2
zmp_ph97_B10
zmp_ph97_C2
zmp_ph97_C4
zmp_ph97_B8
zmp_ph97_C1
zmp_ph97_D2
zmp_ph97_D5
zmp_ph111_G5
zmp_ph111_G11
zmp_ph111_H1
zmp_ph111_B1a
zmp_ph111_B5
zmp_ph111_B8
zmp_ph111_B6
zmp_ph111_B7
zmp_ph111_B9
zmp_ph111_B10
zmp_ph114_G4
zmp_ph114_G11
zmp_ph114_H1
zmp_ph114_H2
zmp_ph114_A9
zmp_ph114_A11
zmp_ph114_B2
zmp_ph114_B4
zmp_ph114_D8
zmp_ph114_E2

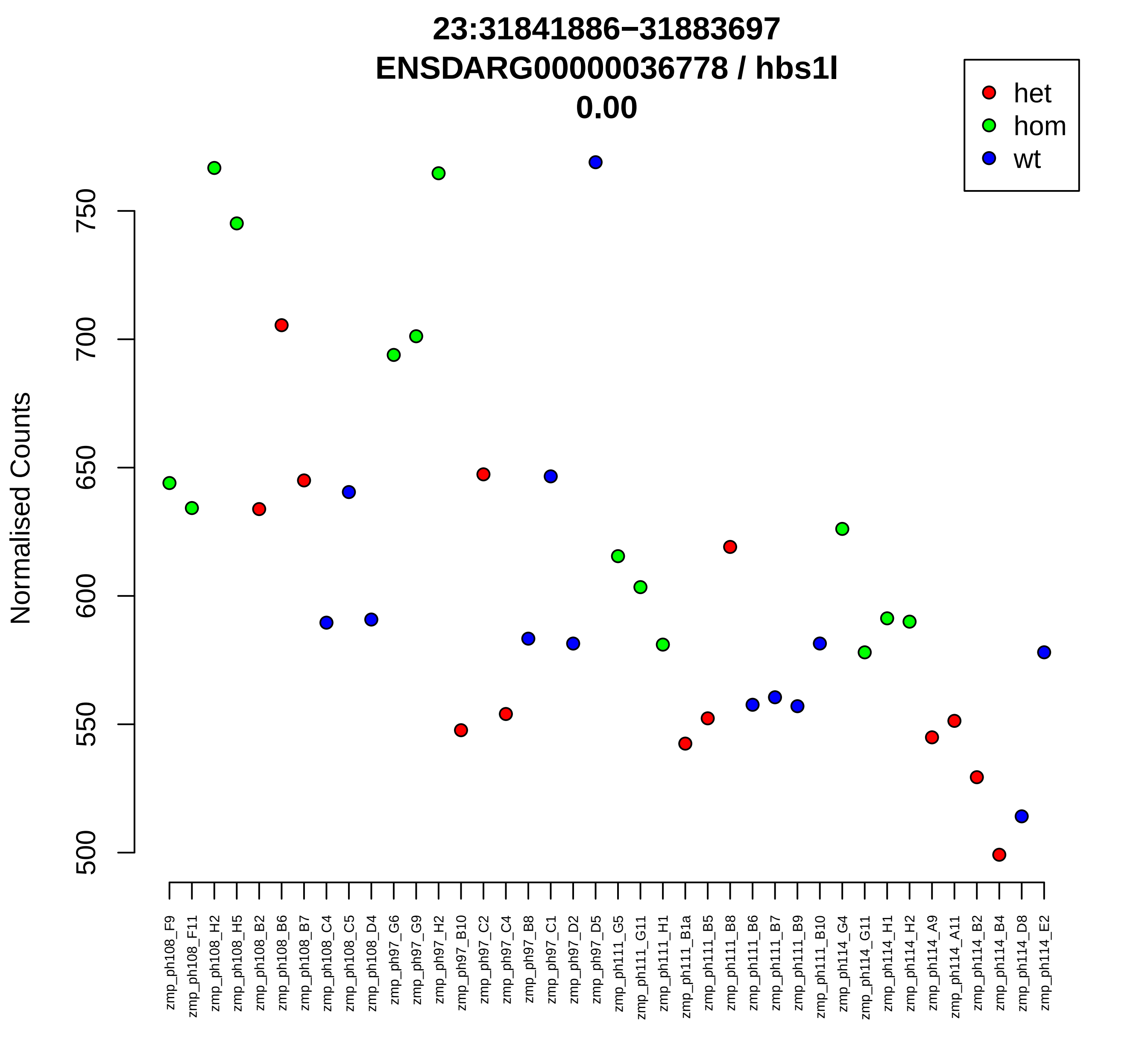

23:31841886−31883697
ENSDARG00000036778 / hbs1l
0.00
het
hom
wt
Normalised Counts
500
550
600
650
700
750
zmp_ph108_F9
zmp_ph108_F11
zmp_ph108_H2
zmp_ph108_H5
zmp_ph108_B2
zmp_ph108_B6
zmp_ph108_B7
zmp_ph108_C4
zmp_ph108_C5
zmp_ph108_D4
zmp_ph97_G6
zmp_ph97_G9
zmp_ph97_H2
zmp_ph97_B10
zmp_ph97_C2
zmp_ph97_C4
zmp_ph97_B8
zmp_ph97_C1
zmp_ph97_D2
zmp_ph97_D5
zmp_ph111_G5
zmp_ph111_G11
zmp_ph111_H1
zmp_ph111_B1a
zmp_ph111_B5
zmp_ph111_B8
zmp_ph111_B6
zmp_ph111_B7
zmp_ph111_B9
zmp_ph111_B10
zmp_ph114_G4
zmp_ph114_G11
zmp_ph114_H1
zmp_ph114_H2
zmp_ph114_A9
zmp_ph114_A11
zmp_ph114_B2
zmp_ph114_B4
zmp_ph114_D8
zmp_ph114_E2

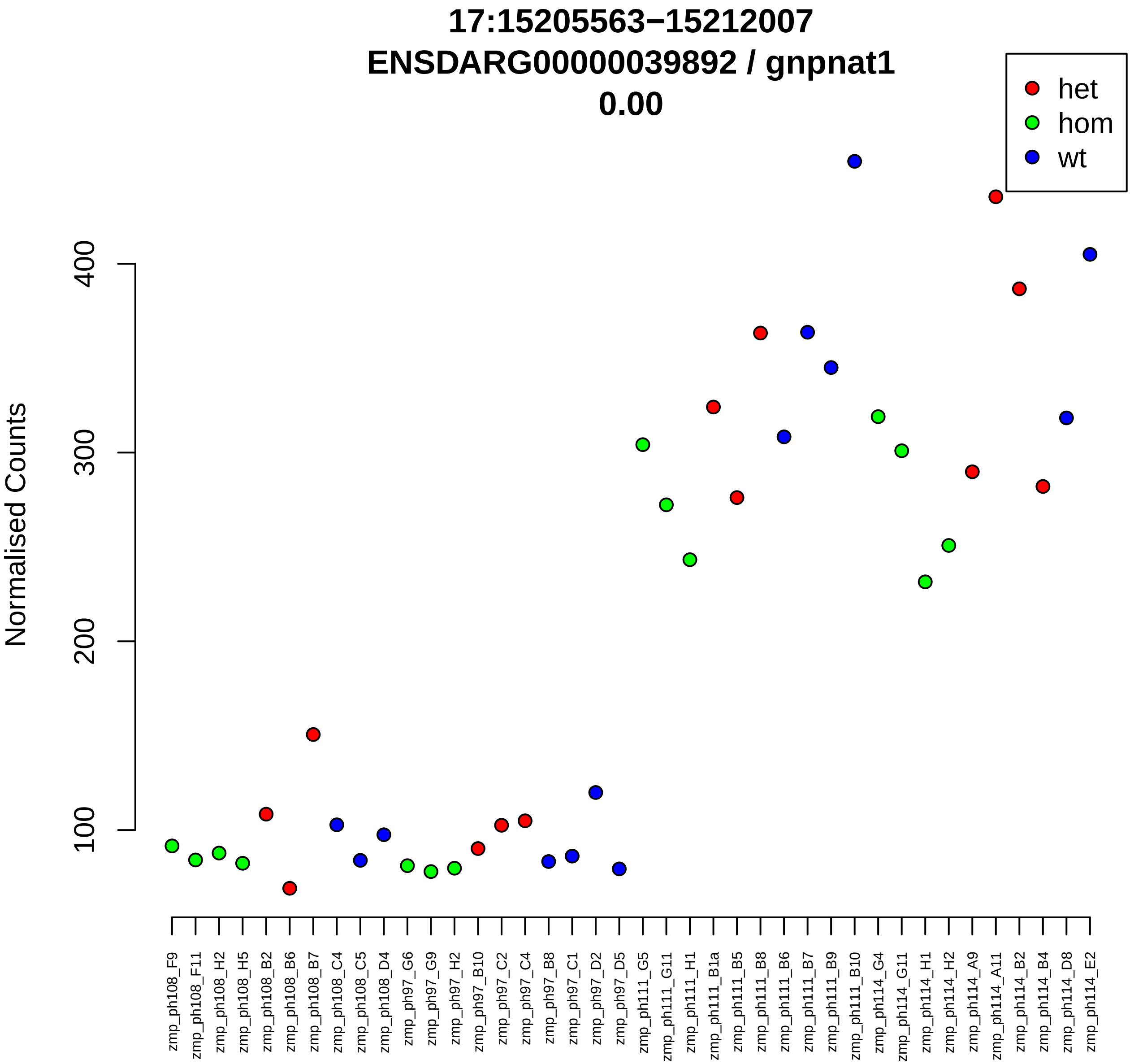

17:15205563−15212007
ENSDARG00000039892 / gnpnat1
0.00
het
hom
wt
Normalised Counts
100
200
300
400
zmp_ph108_F9
zmp_ph108_F11
zmp_ph108_H2
zmp_ph108_H5
zmp_ph108_B2
zmp_ph108_B6
zmp_ph108_B7
zmp_ph108_C4
zmp_ph108_C5
zmp_ph108_D4
zmp_ph97_G6
zmp_ph97_G9
zmp_ph97_H2
zmp_ph97_B10
zmp_ph97_C2
zmp_ph97_C4
zmp_ph97_B8
zmp_ph97_C1
zmp_ph97_D2
zmp_ph97_D5
zmp_ph111_G5
zmp_ph111_G11
zmp_ph111_H1
zmp_ph111_B1a
zmp_ph111_B5
zmp_ph111_B8
zmp_ph111_B6
zmp_ph111_B7
zmp_ph111_B9
zmp_ph111_B10
zmp_ph114_G4
zmp_ph114_G11
zmp_ph114_H1
zmp_ph114_H2
zmp_ph114_A9
zmp_ph114_A11
zmp_ph114_B2
zmp_ph114_B4
zmp_ph114_D8
zmp_ph114_E2

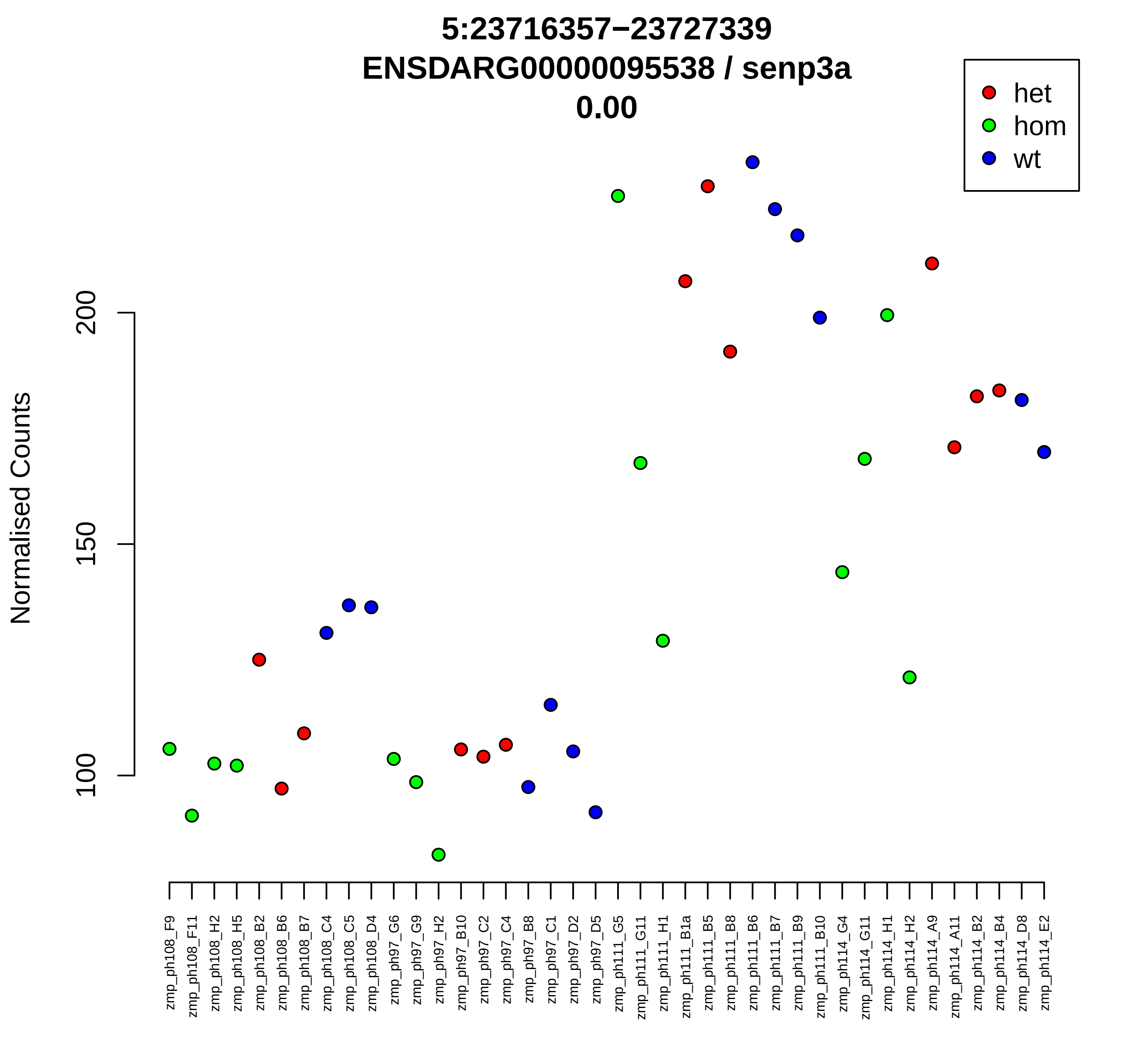
5:23716357−23727339
ENSDARG00000095538 / senp3a
0.00
het
hom
wt
Normalised Counts
100
150
200
zmp_ph108_F9
zmp_ph108_F11
zmp_ph108_H2
zmp_ph108_H5
zmp_ph108_B2
zmp_ph108_B6
zmp_ph108_B7
zmp_ph108_C4
zmp_ph108_C5
zmp_ph108_D4
zmp_ph97_G6
zmp_ph97_G9
zmp_ph97_H2
zmp_ph97_B10
zmp_ph97_C2
zmp_ph97_C4
zmp_ph97_B8
zmp_ph97_C1
zmp_ph97_D2
zmp_ph97_D5
zmp_ph111_G5
zmp_ph111_G11
zmp_ph111_H1
zmp_ph111_B1a
zmp_ph111_B5
zmp_ph111_B8
zmp_ph111_B6
zmp_ph111_B7
zmp_ph111_B9
zmp_ph111_B10
zmp_ph114_G4
zmp_ph114_G11
zmp_ph114_H1
zmp_ph114_H2
zmp_ph114_A9
zmp_ph114_A11
zmp_ph114_B2
zmp_ph114_B4
zmp_ph114_D8
zmp_ph114_E2

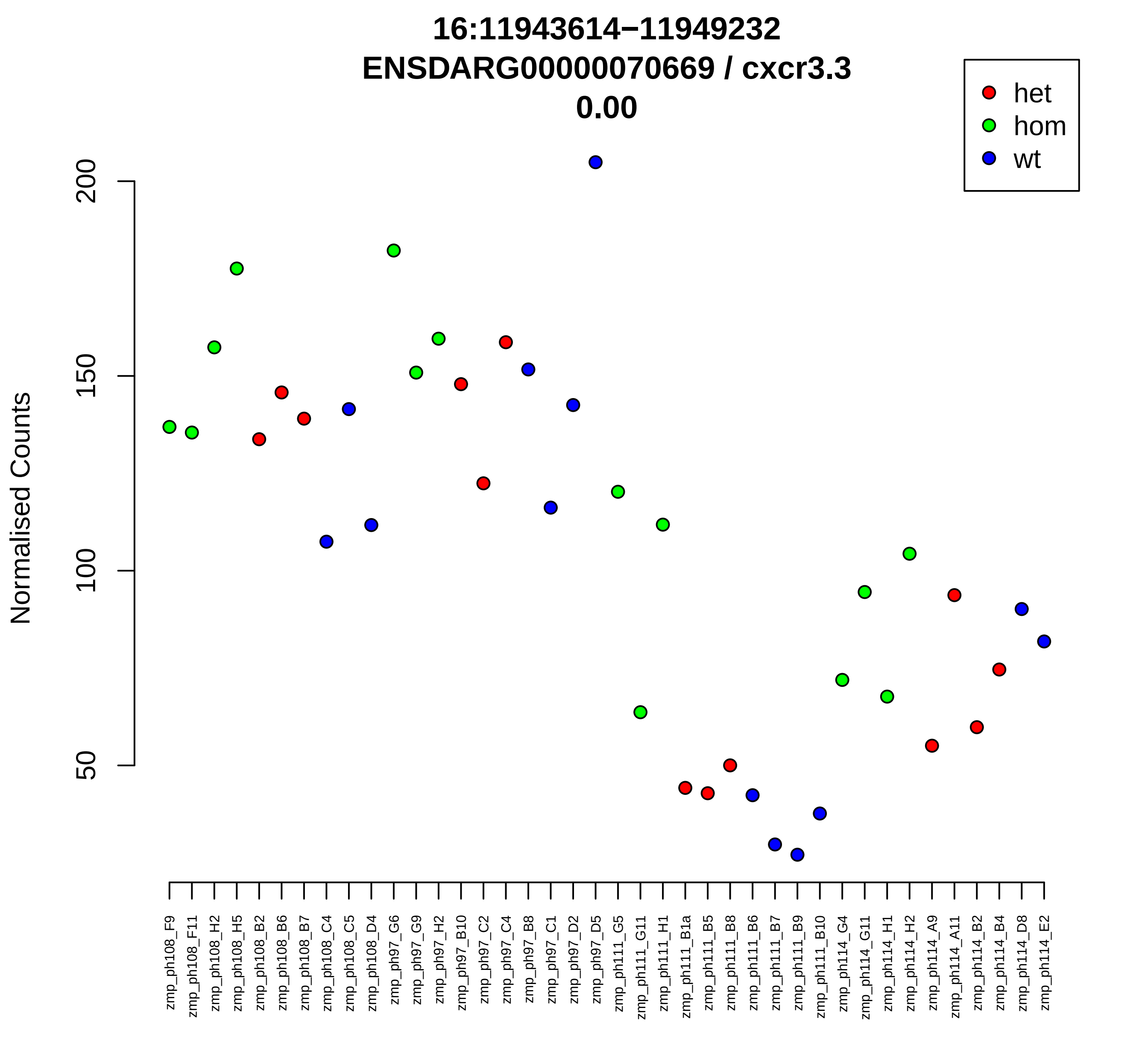

16:11943614−11949232
ENSDARG00000070669 / cxcr3.3
0.00
het
hom
wt
Normalised Counts
200
150
100
50
zmp_ph108_F9
zmp_ph108_F11
zmp_ph108_H2
zmp_ph108_H5
zmp_ph108_B2
zmp_ph108_B6
zmp_ph108_B7
zmp_ph108_C4
zmp_ph108_C5
zmp_ph108_D4
zmp_ph97_G6
zmp_ph97_G9
zmp_ph97_H2
zmp_ph97_B10
zmp_ph97_C2
zmp_ph97_C4
zmp_ph97_B8
zmp_ph97_C1
zmp_ph97_D2
zmp_ph97_D5
zmp_ph111_G5
zmp_ph111_G11
zmp_ph111_H1
zmp_ph111_B1a
zmp_ph111_B5
zmp_ph111_B8
zmp_ph111_B6
zmp_ph111_B7
zmp_ph111_B9
zmp_ph111_B10
zmp_ph114_G4
zmp_ph114_G11
zmp_ph114_H1
zmp_ph114_H2
zmp_ph114_A9
zmp_ph114_A11
zmp_ph114_B2
zmp_ph114_B4
zmp_ph114_D8
zmp_ph114_E2

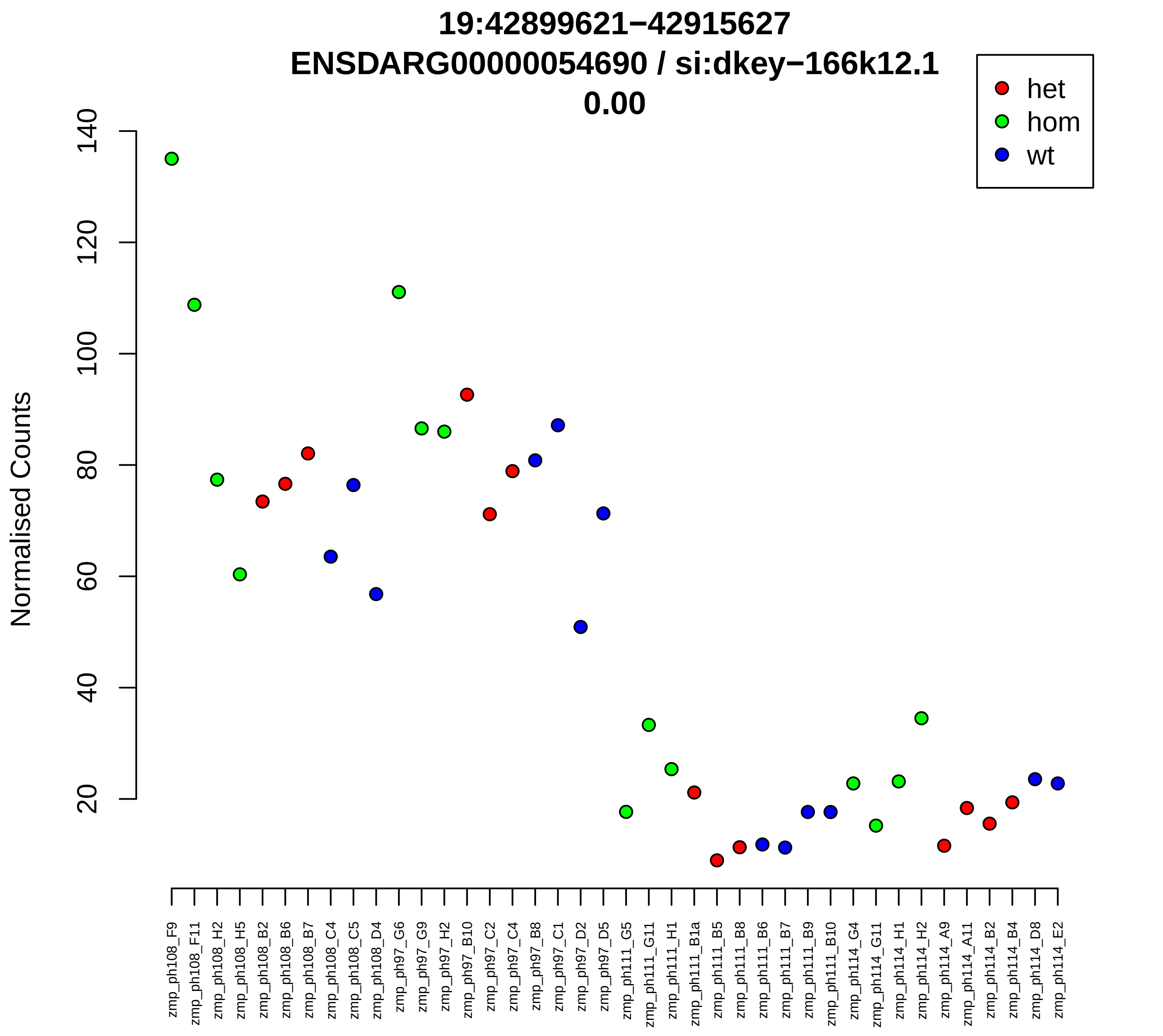

19:42899621−42915627
ENSDARG00000054690 / si:dkey−166k12.1
0.00
het
hom
wt
Normalised Counts
20
40
60
80
100
120
140
zmp_ph108_F9
zmp_ph108_F11
zmp_ph108_H2
zmp_ph108_H5
zmp_ph108_B2
zmp_ph108_B6
zmp_ph108_B7
zmp_ph108_C4
zmp_ph108_C5
zmp_ph108_D4
zmp_ph97_G6
zmp_ph97_G9
zmp_ph97_H2
zmp_ph97_B10
zmp_ph97_C2
zmp_ph97_C4
zmp_ph97_B8
zmp_ph97_C1
zmp_ph97_D2
zmp_ph97_D5
zmp_ph111_G5
zmp_ph111_G11
zmp_ph111_H1
zmp_ph111_B1a
zmp_ph111_B5
zmp_ph111_B8
zmp_ph111_B6
zmp_ph111_B7
zmp_ph111_B9
zmp_ph111_B10
zmp_ph114_G4
zmp_ph114_G11
zmp_ph114_H1
zmp_ph114_H2
zmp_ph114_A9
zmp_ph114_A11
zmp_ph114_B2
zmp_ph114_B4
zmp_ph114_D8
zmp_ph114_E2

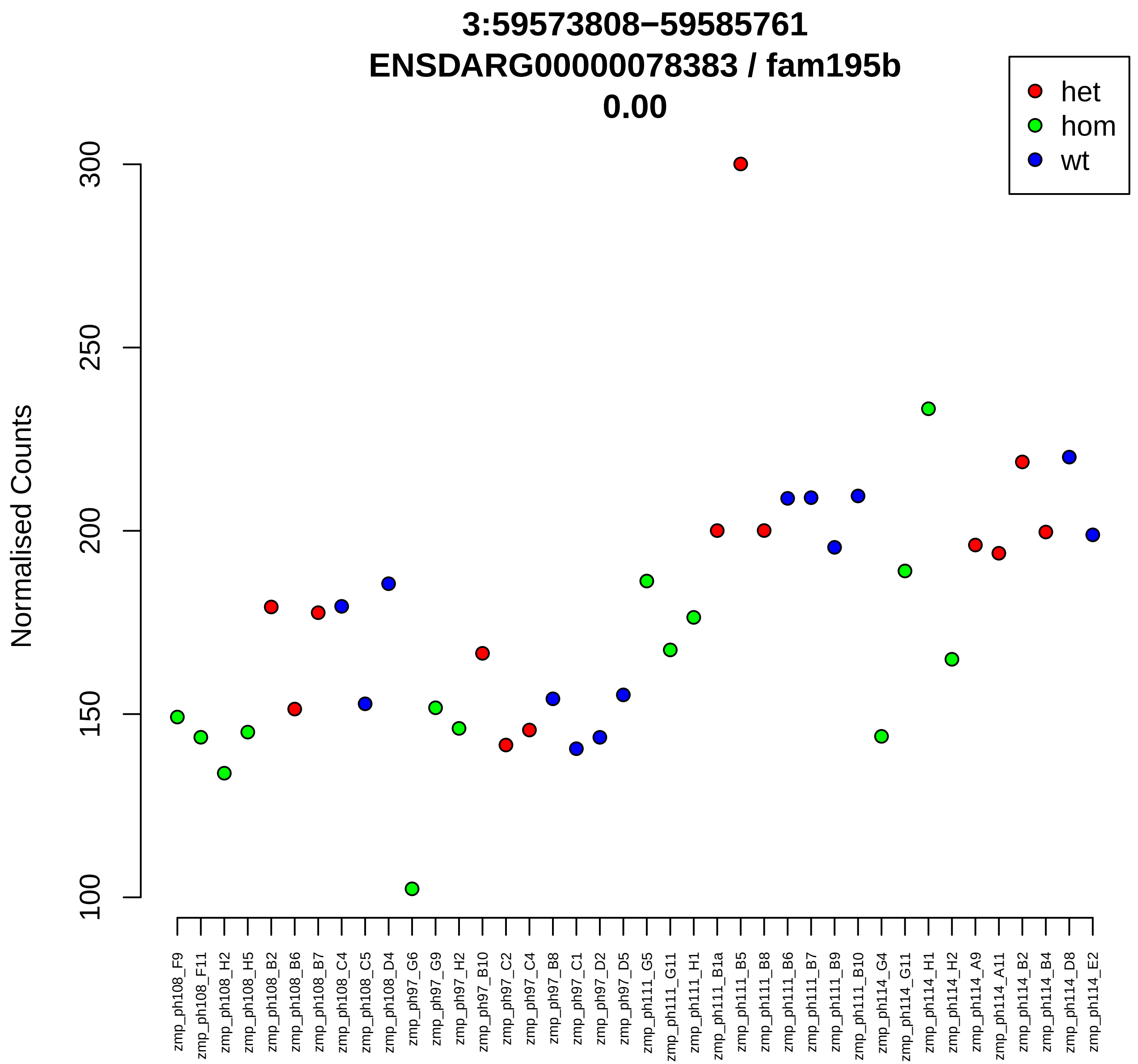
3:59573808−59585761
ENSDARG00000078383 / fam195b
0.00
het
hom
wt
Normalised Counts
100
150
200
250
300
zmp_ph108_F9
zmp_ph108_F11
zmp_ph108_H2
zmp_ph108_H5
zmp_ph108_B2
zmp_ph108_B6
zmp_ph108_B7
zmp_ph108_C4
zmp_ph108_C5
zmp_ph108_D4
zmp_ph97_G6
zmp_ph97_G9
zmp_ph97_H2
zmp_ph97_B10
zmp_ph97_C2
zmp_ph97_C4
zmp_ph97_B8
zmp_ph97_C1
zmp_ph97_D2
zmp_ph97_D5
zmp_ph111_G5
zmp_ph111_G11
zmp_ph111_H1
zmp_ph111_B1a
zmp_ph111_B5
zmp_ph111_B8
zmp_ph111_B6
zmp_ph111_B7
zmp_ph111_B9
zmp_ph111_B10
zmp_ph114_G4
zmp_ph114_G11
zmp_ph114_H1
zmp_ph114_H2
zmp_ph114_A9
zmp_ph114_A11
zmp_ph114_B2
zmp_ph114_B4
zmp_ph114_D8
zmp_ph114_E2

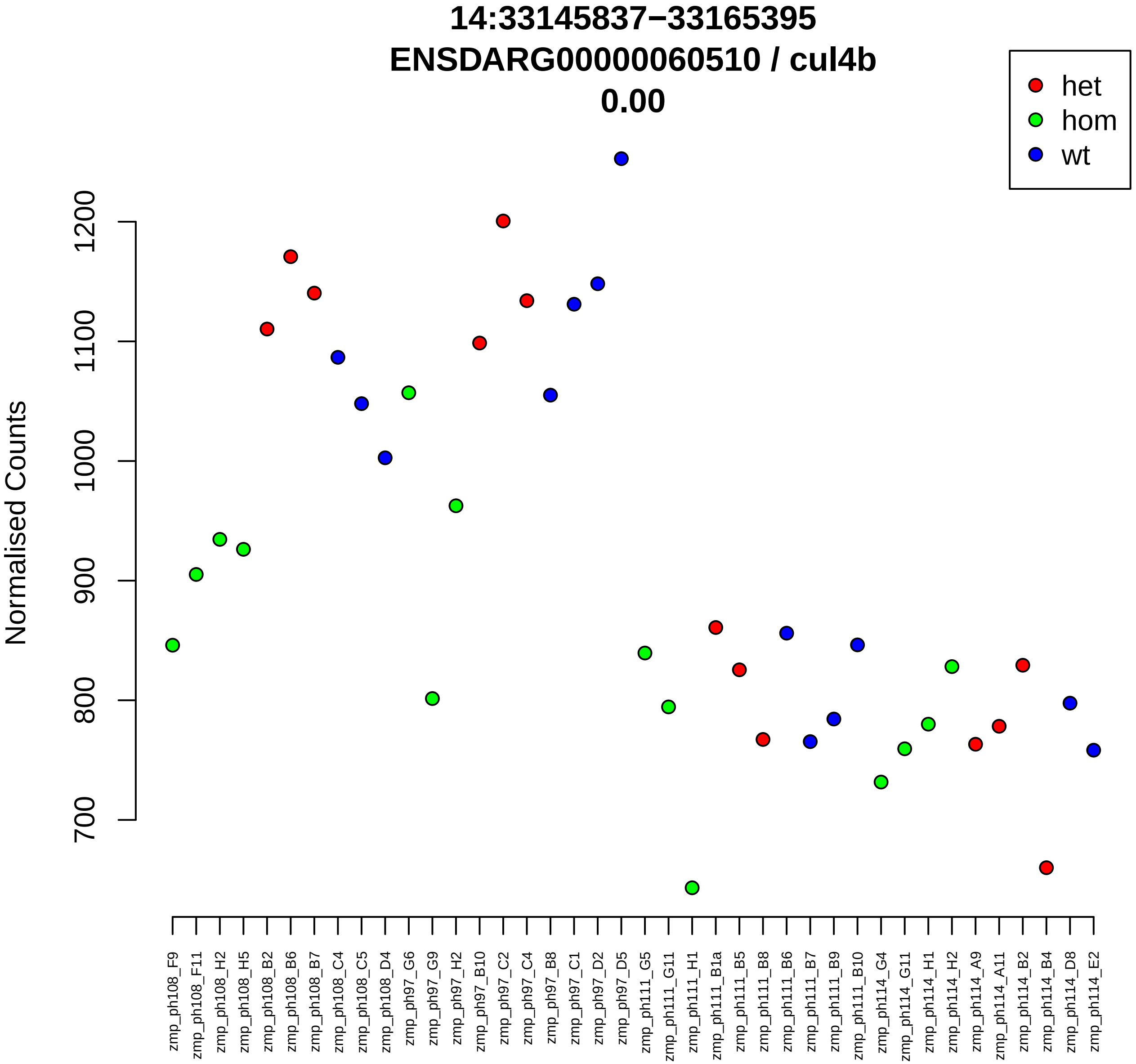

14:33145837−33165395
ENSDARG00000060510 / cul4b
0.00
het
hom
wt
Normalised Counts
700
800
900
1000
1100
1200
zmp_ph108_F9
zmp_ph108_F11
zmp_ph108_H2
zmp_ph108_H5
zmp_ph108_B2
zmp_ph108_B6
zmp_ph108_B7
zmp_ph108_C4
zmp_ph108_C5
zmp_ph108_D4
zmp_ph97_G6
zmp_ph97_G9
zmp_ph97_H2
zmp_ph97_B10
zmp_ph97_C2
zmp_ph97_C4
zmp_ph97_B8
zmp_ph97_C1
zmp_ph97_D2
zmp_ph97_D5
zmp_ph111_G5
zmp_ph111_G11
zmp_ph111_H1
zmp_ph111_B1a
zmp_ph111_B5
zmp_ph111_B8
zmp_ph111_B6
zmp_ph111_B7
zmp_ph111_B9
zmp_ph111_B10
zmp_ph114_G4
zmp_ph114_G11
zmp_ph114_H1
zmp_ph114_H2
zmp_ph114_A9
zmp_ph114_A11
zmp_ph114_B2
zmp_ph114_B4
zmp_ph114_D8
zmp_ph114_E2

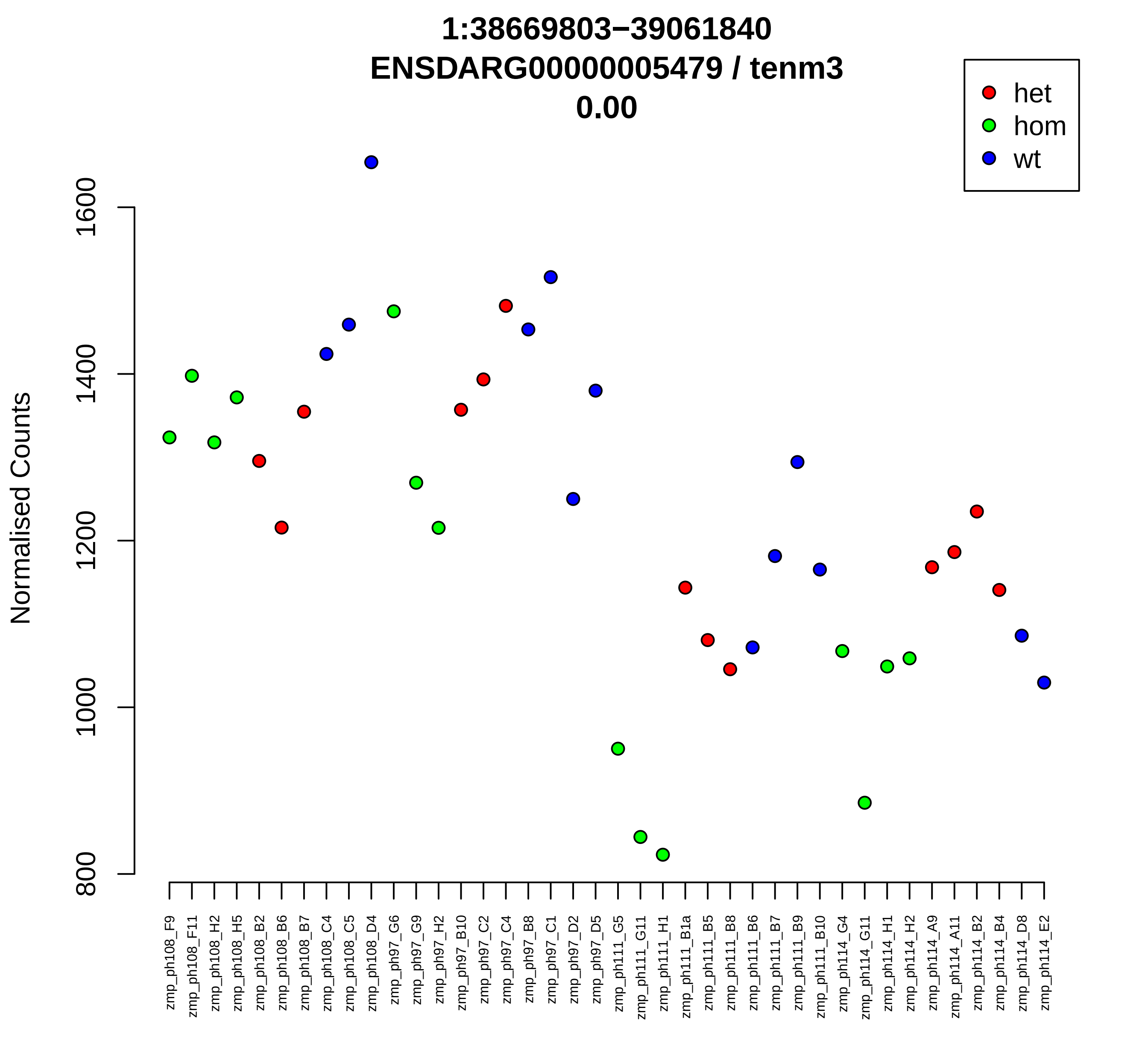
1:38669803−39061840
ENSDARG00000005479 / tenm3
0.00
het
hom
wt
Normalised Counts
800
1000
1200
1400
1600
zmp_ph108_F9
zmp_ph108_F11
zmp_ph108_H2
zmp_ph108_H5
zmp_ph108_B2
zmp_ph108_B6
zmp_ph108_B7
zmp_ph108_C4
zmp_ph108_C5
zmp_ph108_D4
zmp_ph97_G6
zmp_ph97_G9
zmp_ph97_H2
zmp_ph97_B10
zmp_ph97_C2
zmp_ph97_C4
zmp_ph97_B8
zmp_ph97_C1
zmp_ph97_D2
zmp_ph97_D5
zmp_ph111_G5
zmp_ph111_G11
zmp_ph111_H1
zmp_ph111_B1a
zmp_ph111_B5
zmp_ph111_B8
zmp_ph111_B6
zmp_ph111_B7
zmp_ph111_B9
zmp_ph111_B10
zmp_ph114_G4
zmp_ph114_G11
zmp_ph114_H1
zmp_ph114_H2
zmp_ph114_A9
zmp_ph114_A11
zmp_ph114_B2
zmp_ph114_B4
zmp_ph114_D8
zmp_ph114_E2

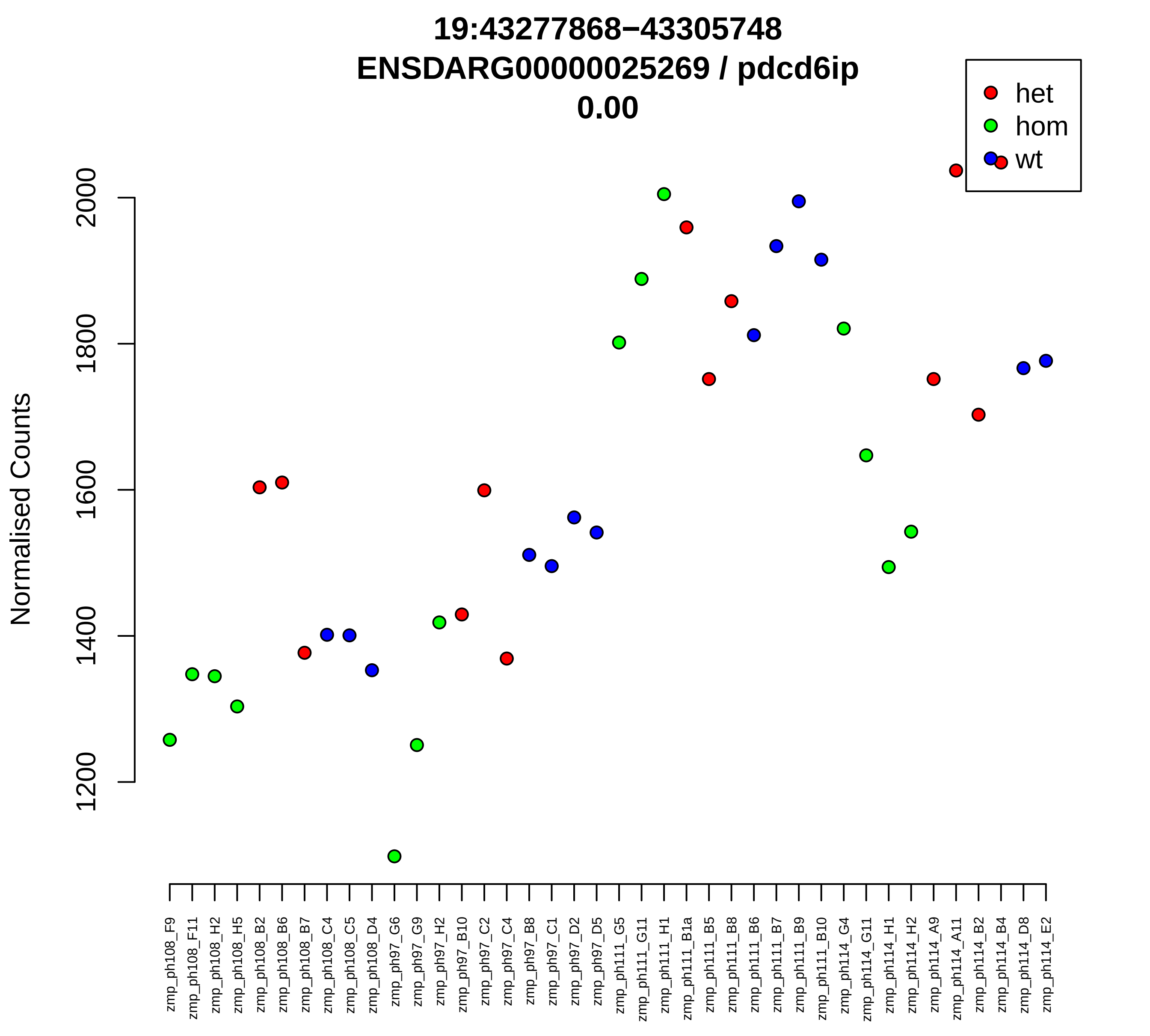
19:43277868−43305748
ENSDARG00000025269 / pdcd6ip
0.00
het
hom
wt
Normalised Counts
1200
1400
1600
1800
2000
zmp_ph108_F9
zmp_ph108_F11
zmp_ph108_H2
zmp_ph108_H5
zmp_ph108_B2
zmp_ph108_B6
zmp_ph108_B7
zmp_ph108_C4
zmp_ph108_C5
zmp_ph108_D4
zmp_ph97_G6
zmp_ph97_G9
zmp_ph97_H2
zmp_ph97_B10
zmp_ph97_C2
zmp_ph97_C4
zmp_ph97_B8
zmp_ph97_C1
zmp_ph97_D2
zmp_ph97_D5
zmp_ph111_G5
zmp_ph111_G11
zmp_ph111_H1
zmp_ph111_B1a
zmp_ph111_B5
zmp_ph111_B8
zmp_ph111_B6
zmp_ph111_B7
zmp_ph111_B9
zmp_ph111_B10
zmp_ph114_G4
zmp_ph114_G11
zmp_ph114_H1
zmp_ph114_H2
zmp_ph114_A9
zmp_ph114_A11
zmp_ph114_B2
zmp_ph114_B4
zmp_ph114_D8
zmp_ph114_E2

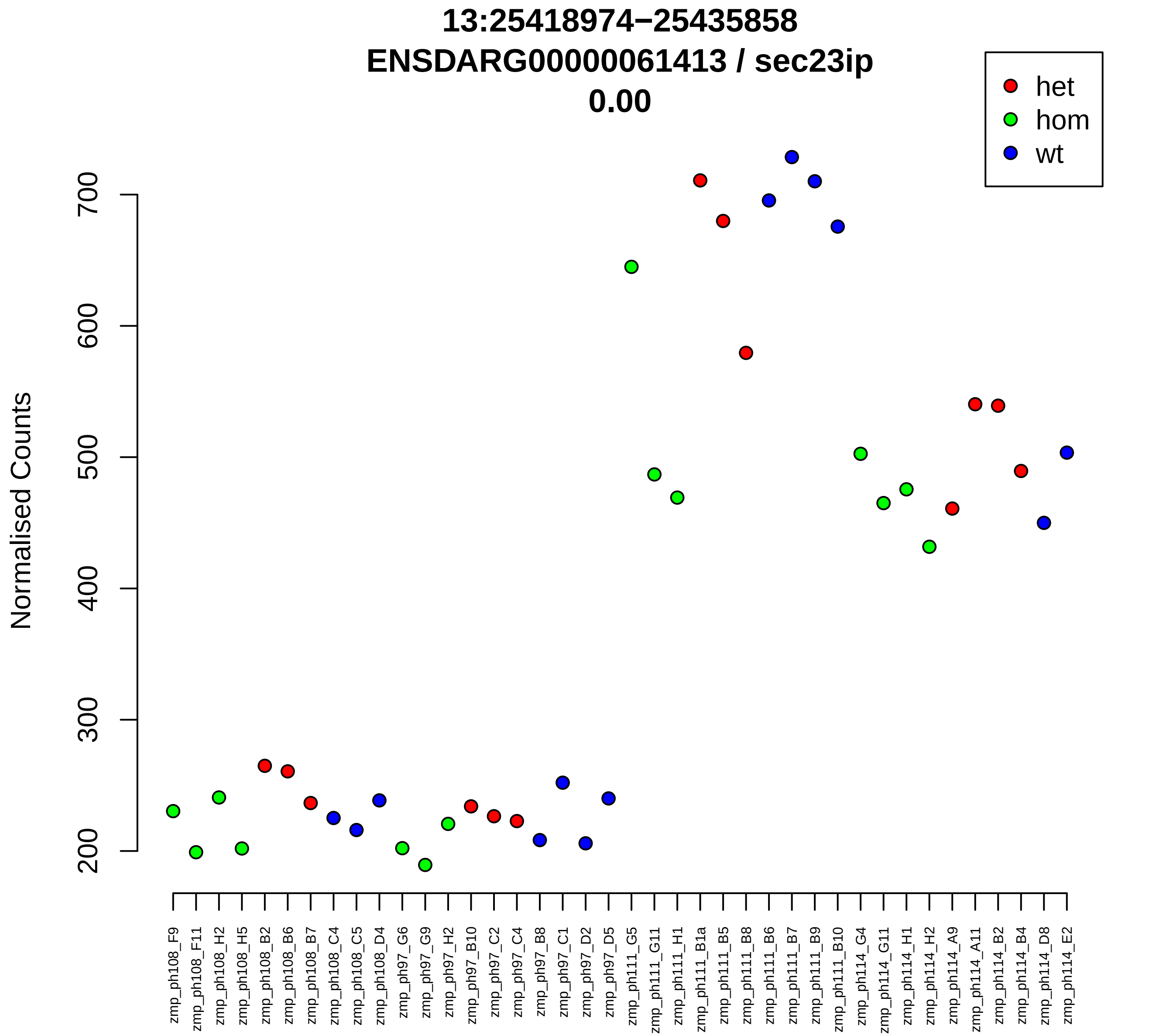
13:25418974−25435858
ENSDARG00000061413 / sec23ip
0.00
het
hom
wt
Normalised Counts
200
300
400
500
600
700
zmp_ph108_F9
zmp_ph108_F11
zmp_ph108_H2
zmp_ph108_H5
zmp_ph108_B2
zmp_ph108_B6
zmp_ph108_B7
zmp_ph108_C4
zmp_ph108_C5
zmp_ph108_D4
zmp_ph97_G6
zmp_ph97_G9
zmp_ph97_H2
zmp_ph97_B10
zmp_ph97_C2
zmp_ph97_C4
zmp_ph97_B8
zmp_ph97_C1
zmp_ph97_D2
zmp_ph97_D5
zmp_ph111_G5
zmp_ph111_G11
zmp_ph111_H1
zmp_ph111_B1a
zmp_ph111_B5
zmp_ph111_B8
zmp_ph111_B6
zmp_ph111_B7
zmp_ph111_B9
zmp_ph111_B10
zmp_ph114_G4
zmp_ph114_G11
zmp_ph114_H1
zmp_ph114_H2
zmp_ph114_A9
zmp_ph114_A11
zmp_ph114_B2
zmp_ph114_B4
zmp_ph114_D8
zmp_ph114_E2

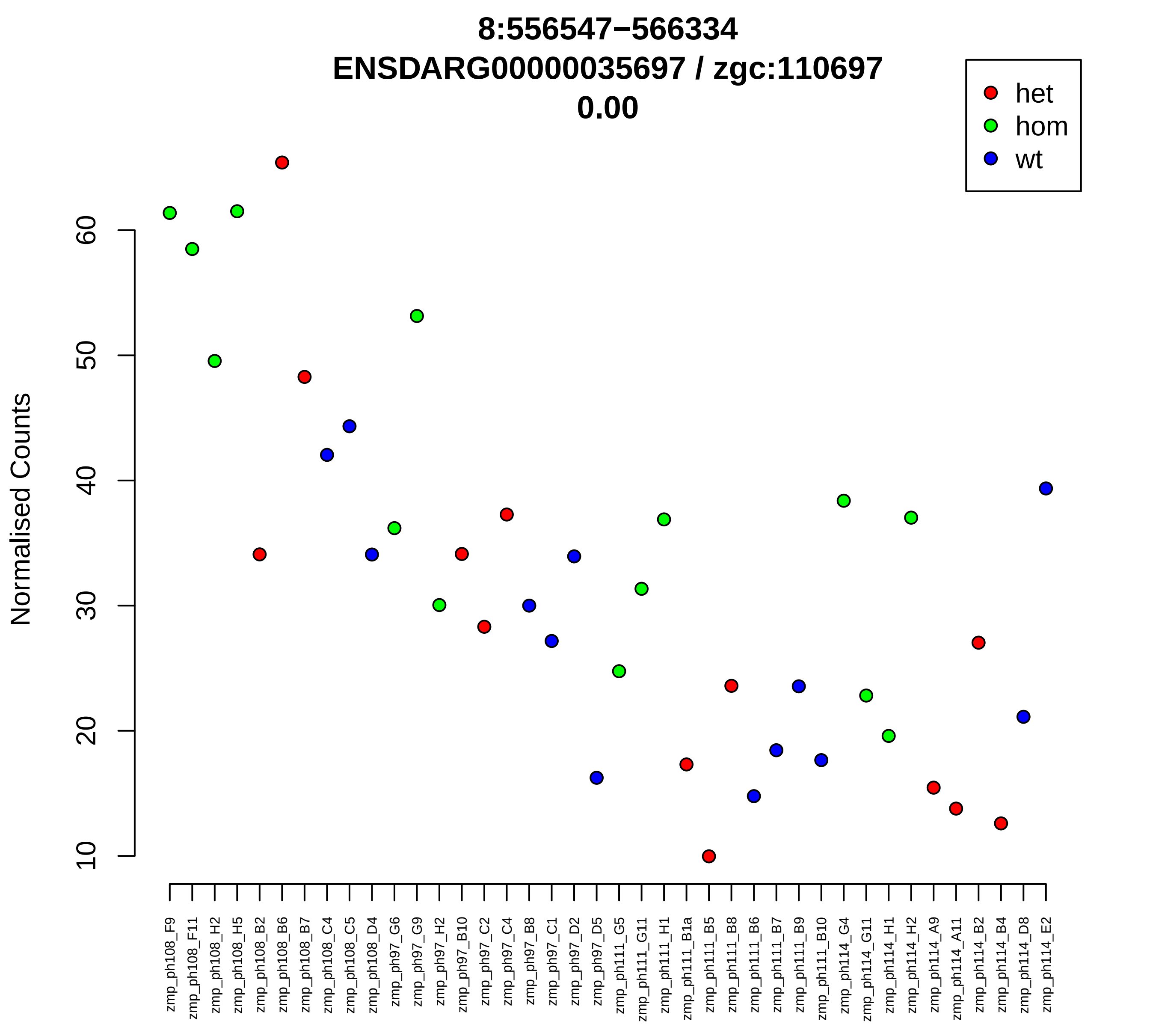
8:556547−566334
ENSDARG00000035697 / zgc:110697
0.00
het
hom
wt
Normalised Counts
10
20
30
40
50
60
zmp_ph108_F9
zmp_ph108_F11
zmp_ph108_H2
zmp_ph108_H5
zmp_ph108_B2
zmp_ph108_B6
zmp_ph108_B7
zmp_ph108_C4
zmp_ph108_C5
zmp_ph108_D4
zmp_ph97_G6
zmp_ph97_G9
zmp_ph97_H2
zmp_ph97_B10
zmp_ph97_C2
zmp_ph97_C4
zmp_ph97_B8
zmp_ph97_C1
zmp_ph97_D2
zmp_ph97_D5
zmp_ph111_G5
zmp_ph111_G11
zmp_ph111_H1
zmp_ph111_B1a
zmp_ph111_B5
zmp_ph111_B8
zmp_ph111_B6
zmp_ph111_B7
zmp_ph111_B9
zmp_ph111_B10
zmp_ph114_G4
zmp_ph114_G11
zmp_ph114_H1
zmp_ph114_H2
zmp_ph114_A9
zmp_ph114_A11
zmp_ph114_B2
zmp_ph114_B4
zmp_ph114_D8
zmp_ph114_E2

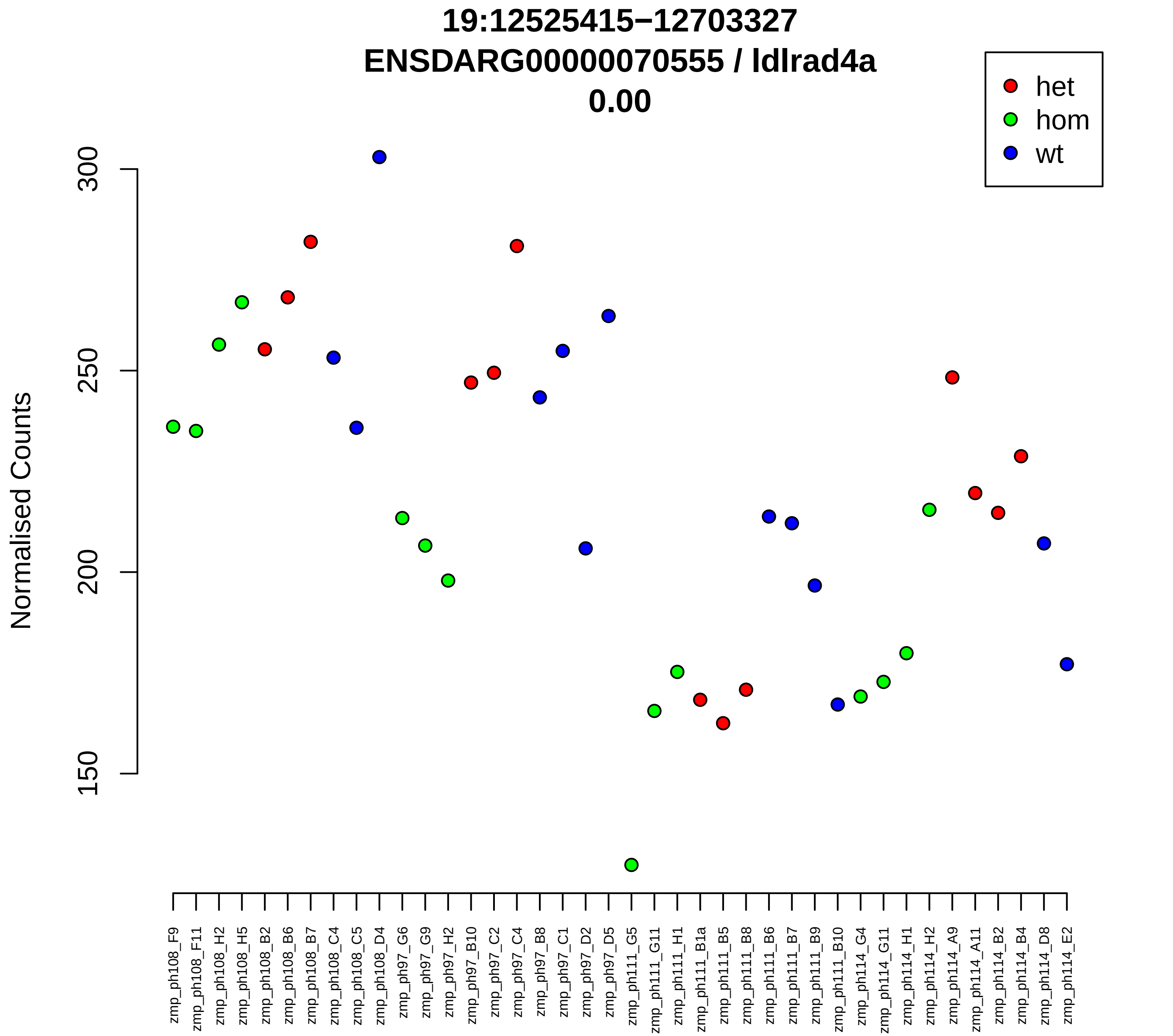
19:12525415−12703327
ENSDARG00000070555 / ldlrad4a
0.00
het
hom
wt
Normalised Counts
300
250
200
150
zmp_ph108_F9
zmp_ph108_F11
zmp_ph108_H2
zmp_ph108_H5
zmp_ph108_B2
zmp_ph108_B6
zmp_ph108_B7
zmp_ph108_C4
zmp_ph108_C5
zmp_ph108_D4
zmp_ph97_G6
zmp_ph97_G9
zmp_ph97_H2
zmp_ph97_B10
zmp_ph97_C2
zmp_ph97_C4
zmp_ph97_B8
zmp_ph97_C1
zmp_ph97_D2
zmp_ph97_D5
zmp_ph111_G5
zmp_ph111_G11
zmp_ph111_H1
zmp_ph111_B1a
zmp_ph111_B5
zmp_ph111_B8
zmp_ph111_B6
zmp_ph111_B7
zmp_ph111_B9
zmp_ph111_B10
zmp_ph114_G4
zmp_ph114_G11
zmp_ph114_H1
zmp_ph114_H2
zmp_ph114_A9
zmp_ph114_A11
zmp_ph114_B2
zmp_ph114_B4
zmp_ph114_D8
zmp_ph114_E2

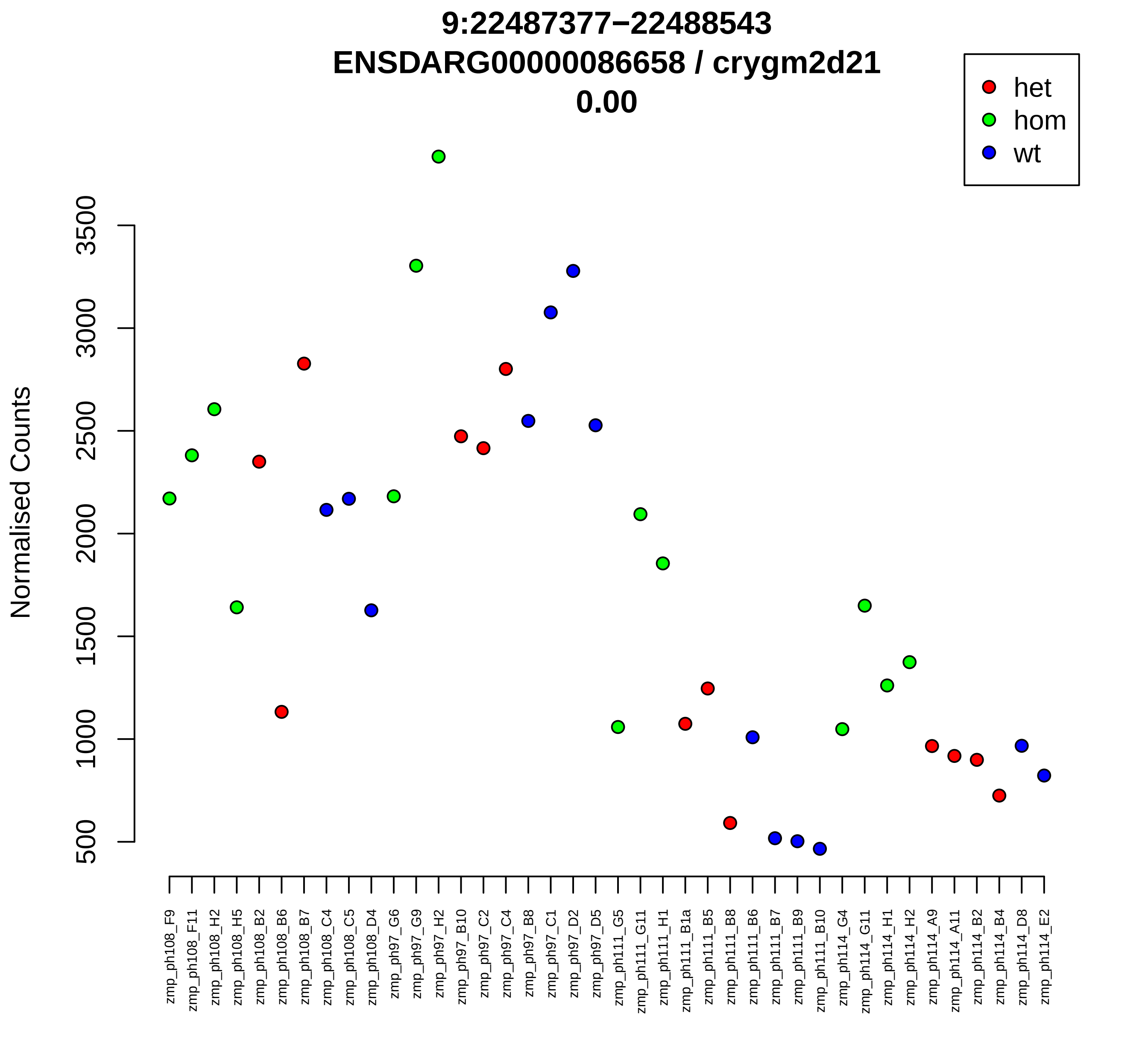

9:22487377−22488543
ENSDARG00000086658 / crygm2d21
0.00
het
hom
wt
Normalised Counts
500
1000
1500
2000
2500
3000
3500
zmp_ph108_F9
zmp_ph108_F11
zmp_ph108_H2
zmp_ph108_H5
zmp_ph108_B2
zmp_ph108_B6
zmp_ph108_B7
zmp_ph108_C4
zmp_ph108_C5
zmp_ph108_D4
zmp_ph97_G6
zmp_ph97_G9
zmp_ph97_H2
zmp_ph97_B10
zmp_ph97_C2
zmp_ph97_C4
zmp_ph97_B8
zmp_ph97_C1
zmp_ph97_D2
zmp_ph97_D5
zmp_ph111_G5
zmp_ph111_G11
zmp_ph111_H1
zmp_ph111_B1a
zmp_ph111_B5
zmp_ph111_B8
zmp_ph111_B6
zmp_ph111_B7
zmp_ph111_B9
zmp_ph111_B10
zmp_ph114_G4
zmp_ph114_G11
zmp_ph114_H1
zmp_ph114_H2
zmp_ph114_A9
zmp_ph114_A11
zmp_ph114_B2
zmp_ph114_B4
zmp_ph114_D8
zmp_ph114_E2

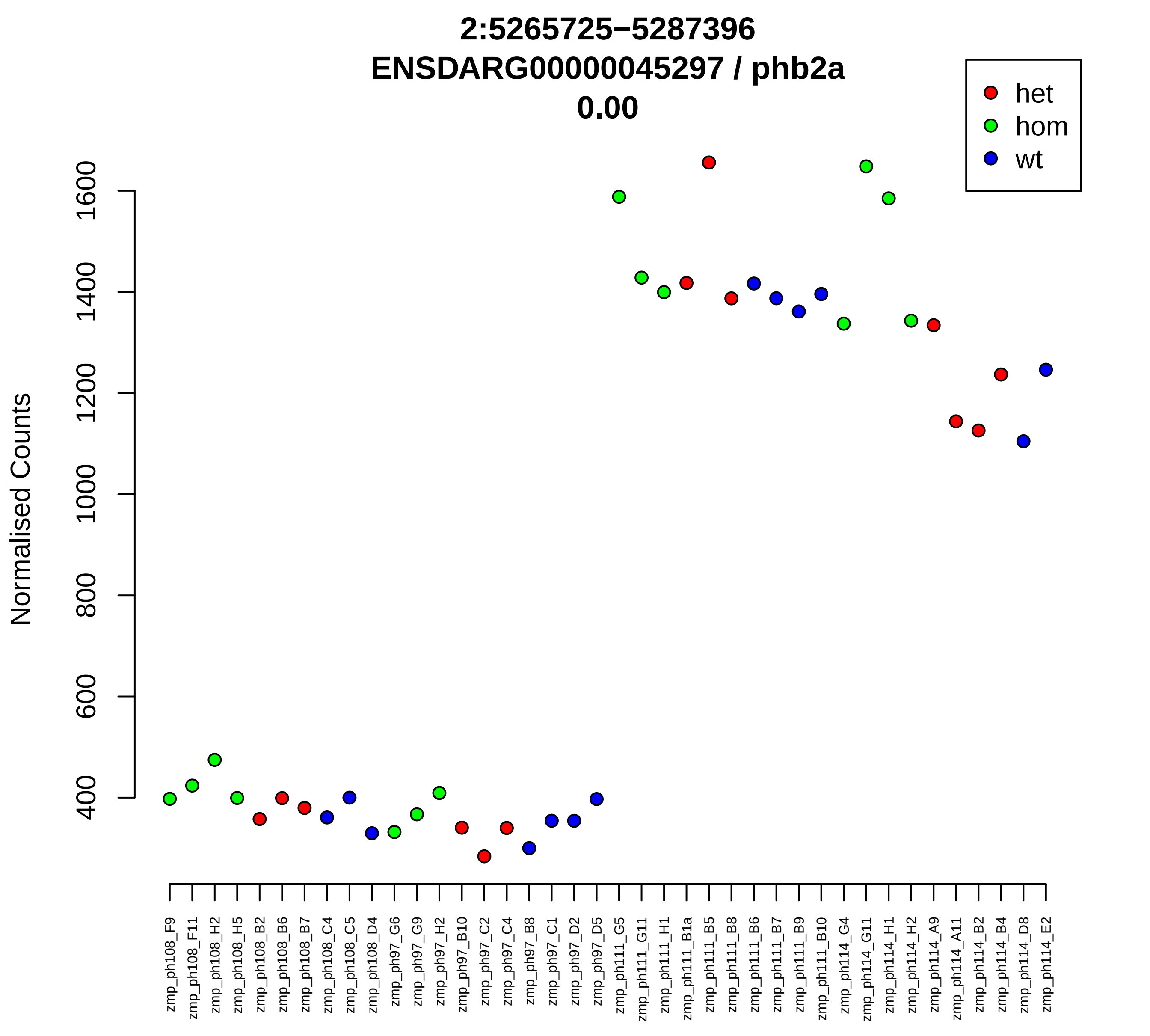

2:5265725−5287396
ENSDARG00000045297 / phb2a
0.00
het
hom
wt
Normalised Counts
400
600
800
1000
1200
1400
1600
zmp_ph108_F9
zmp_ph108_F11
zmp_ph108_H2
zmp_ph108_H5
zmp_ph108_B2
zmp_ph108_B6
zmp_ph108_B7
zmp_ph108_C4
zmp_ph108_C5
zmp_ph108_D4
zmp_ph97_G6
zmp_ph97_G9
zmp_ph97_H2
zmp_ph97_B10
zmp_ph97_C2
zmp_ph97_C4
zmp_ph97_B8
zmp_ph97_C1
zmp_ph97_D2
zmp_ph97_D5
zmp_ph111_G5
zmp_ph111_G11
zmp_ph111_H1
zmp_ph111_B1a
zmp_ph111_B5
zmp_ph111_B8
zmp_ph111_B6
zmp_ph111_B7
zmp_ph111_B9
zmp_ph111_B10
zmp_ph114_G4
zmp_ph114_G11
zmp_ph114_H1
zmp_ph114_H2
zmp_ph114_A9
zmp_ph114_A11
zmp_ph114_B2
zmp_ph114_B4
zmp_ph114_D8
zmp_ph114_E2

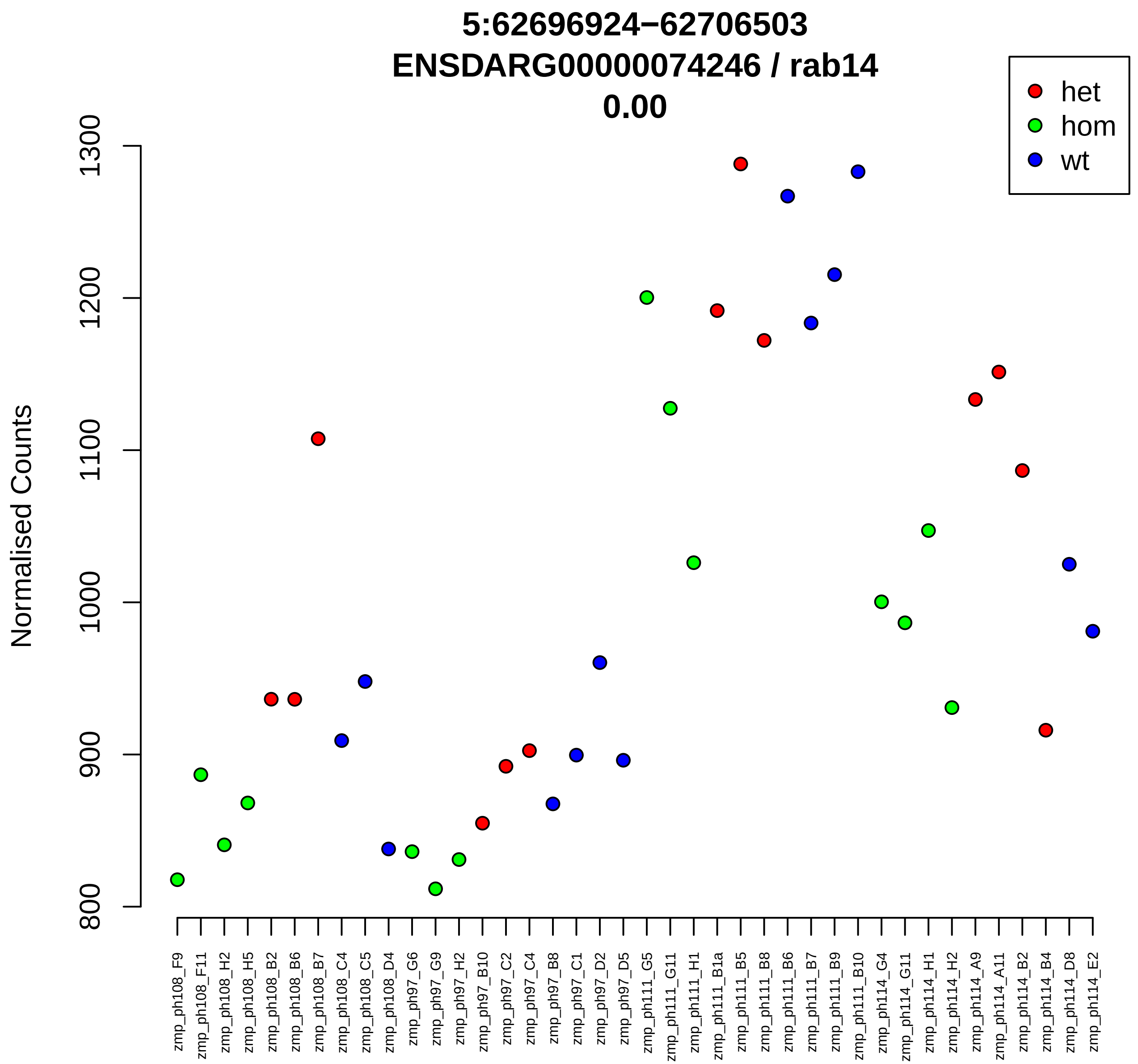

5:62696924−62706503
ENSDARG00000074246 / rab14
0.00
het
hom
wt
Normalised Counts
800
900
1000
1100
1200
1300
zmp_ph108_F9
zmp_ph108_F11
zmp_ph108_H2
zmp_ph108_H5
zmp_ph108_B2
zmp_ph108_B6
zmp_ph108_B7
zmp_ph108_C4
zmp_ph108_C5
zmp_ph108_D4
zmp_ph97_G6
zmp_ph97_G9
zmp_ph97_H2
zmp_ph97_B10
zmp_ph97_C2
zmp_ph97_C4
zmp_ph97_B8
zmp_ph97_C1
zmp_ph97_D2
zmp_ph97_D5
zmp_ph111_G5
zmp_ph111_G11
zmp_ph111_H1
zmp_ph111_B1a
zmp_ph111_B5
zmp_ph111_B8
zmp_ph111_B6
zmp_ph111_B7
zmp_ph111_B9
zmp_ph111_B10
zmp_ph114_G4
zmp_ph114_G11
zmp_ph114_H1
zmp_ph114_H2
zmp_ph114_A9
zmp_ph114_A11
zmp_ph114_B2
zmp_ph114_B4
zmp_ph114_D8
zmp_ph114_E2

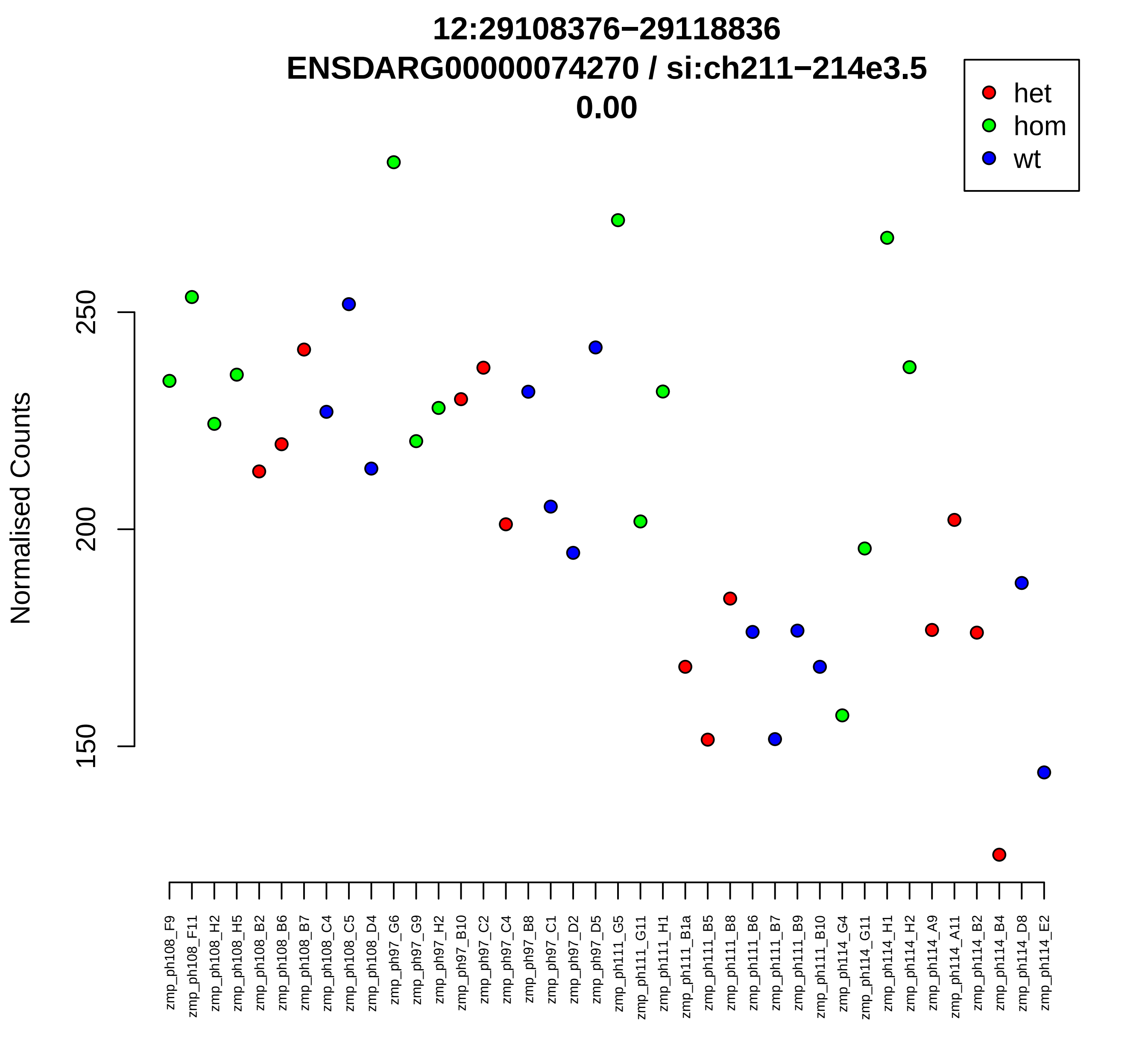

12:29108376−29118836
ENSDARG00000074270 / si:ch211−214e3.5
0.00
het
hom
wt
Normalised Counts
150
200
250
zmp_ph108_F9
zmp_ph108_F11
zmp_ph108_H2
zmp_ph108_H5
zmp_ph108_B2
zmp_ph108_B6
zmp_ph108_B7
zmp_ph108_C4
zmp_ph108_C5
zmp_ph108_D4
zmp_ph97_G6
zmp_ph97_G9
zmp_ph97_H2
zmp_ph97_B10
zmp_ph97_C2
zmp_ph97_C4
zmp_ph97_B8
zmp_ph97_C1
zmp_ph97_D2
zmp_ph97_D5
zmp_ph111_G5
zmp_ph111_G11
zmp_ph111_H1
zmp_ph111_B1a
zmp_ph111_B5
zmp_ph111_B8
zmp_ph111_B6
zmp_ph111_B7
zmp_ph111_B9
zmp_ph111_B10
zmp_ph114_G4
zmp_ph114_G11
zmp_ph114_H1
zmp_ph114_H2
zmp_ph114_A9
zmp_ph114_A11
zmp_ph114_B2
zmp_ph114_B4
zmp_ph114_D8
zmp_ph114_E2

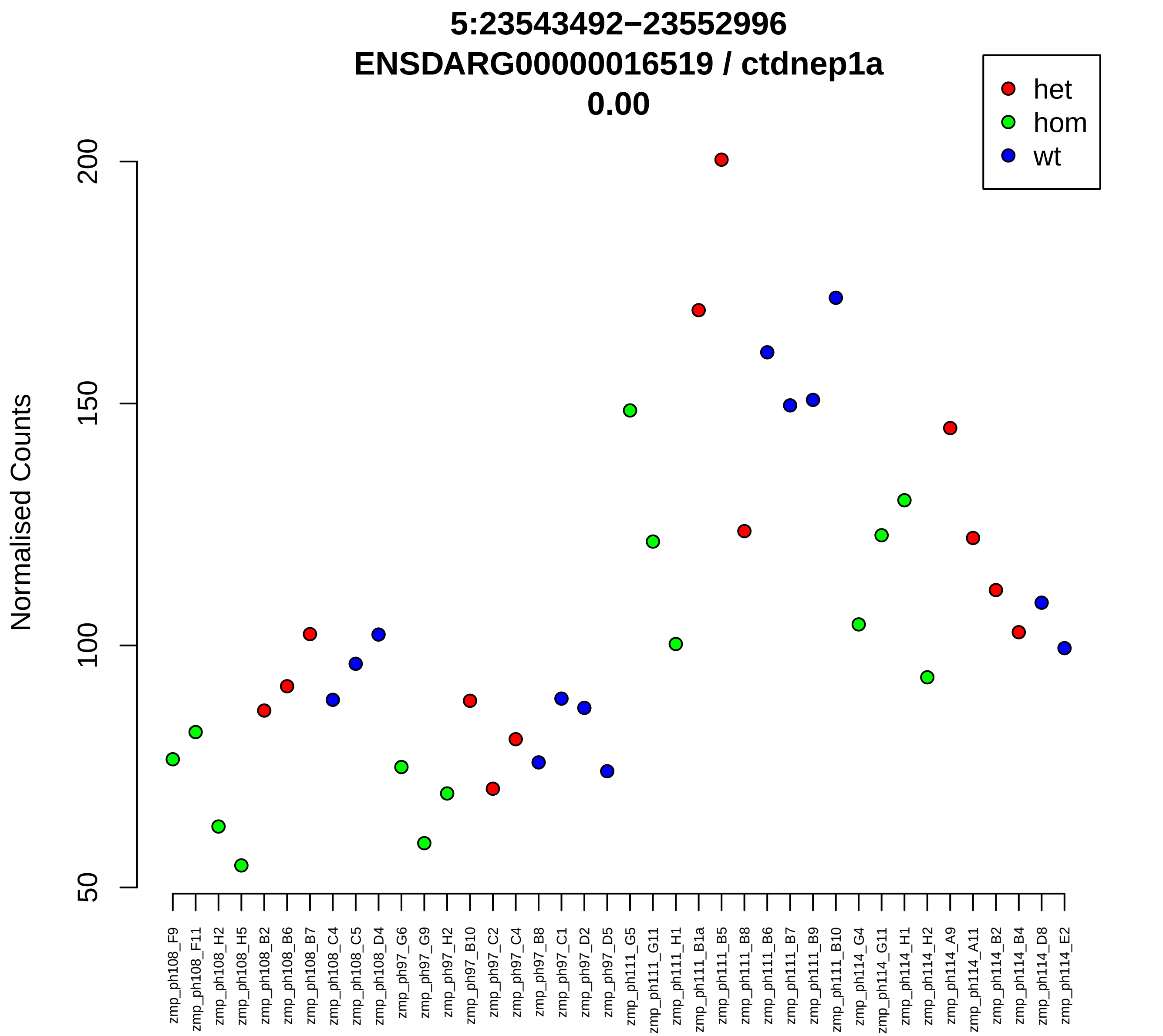
5:23543492−23552996
ENSDARG00000016519 / ctdnep1a
0.00
het
hom
wt
Normalised Counts
50
100
150
200
zmp_ph108_F9
zmp_ph108_F11
zmp_ph108_H2
zmp_ph108_H5
zmp_ph108_B2
zmp_ph108_B6
zmp_ph108_B7
zmp_ph108_C4
zmp_ph108_C5
zmp_ph108_D4
zmp_ph97_G6
zmp_ph97_G9
zmp_ph97_H2
zmp_ph97_B10
zmp_ph97_C2
zmp_ph97_C4
zmp_ph97_B8
zmp_ph97_C1
zmp_ph97_D2
zmp_ph97_D5
zmp_ph111_G5
zmp_ph111_G11
zmp_ph111_H1
zmp_ph111_B1a
zmp_ph111_B5
zmp_ph111_B8
zmp_ph111_B6
zmp_ph111_B7
zmp_ph111_B9
zmp_ph111_B10
zmp_ph114_G4
zmp_ph114_G11
zmp_ph114_H1
zmp_ph114_H2
zmp_ph114_A9
zmp_ph114_A11
zmp_ph114_B2
zmp_ph114_B4
zmp_ph114_D8
zmp_ph114_E2

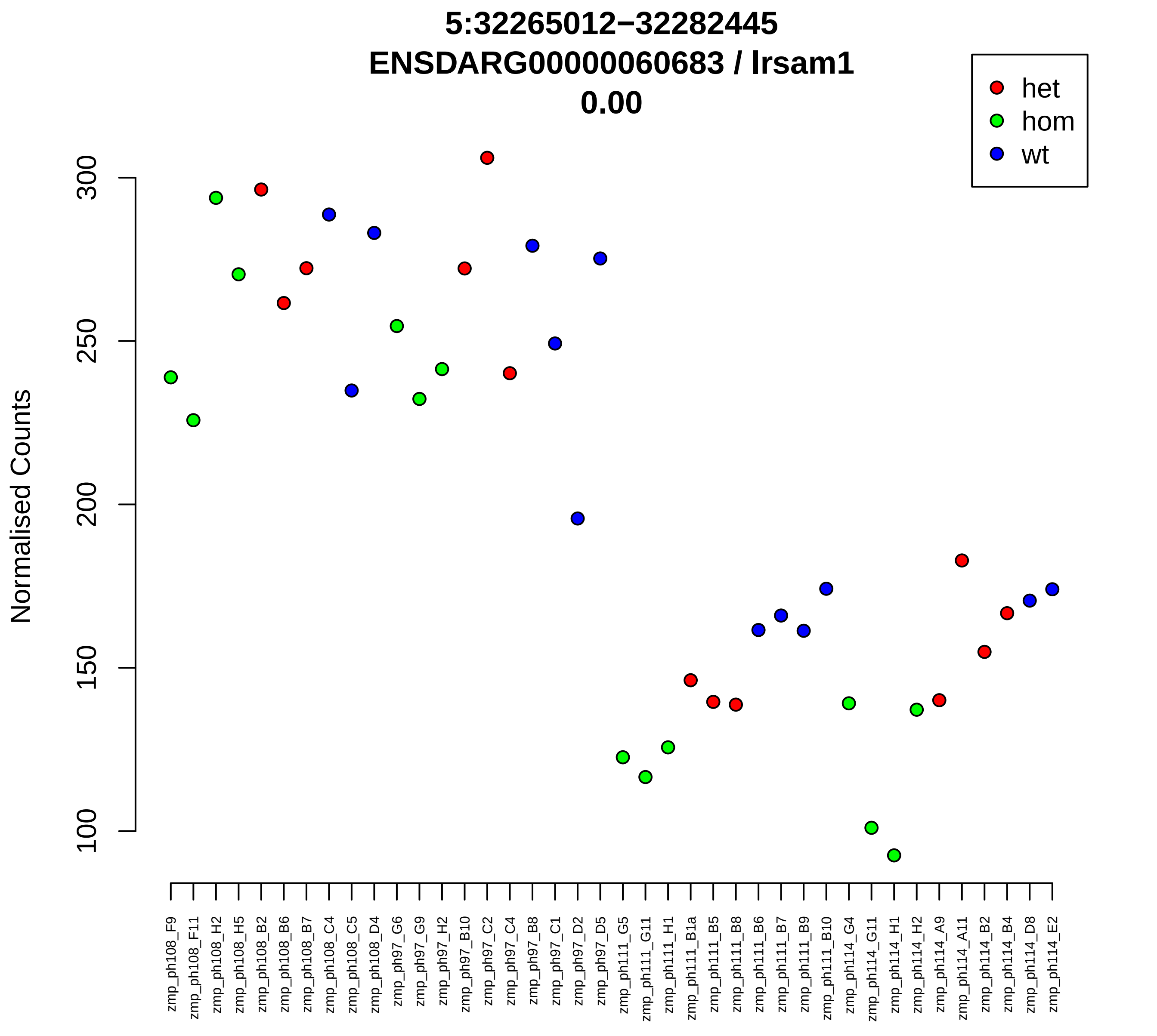

5:32265012−32282445
ENSDARG00000060683 / lrsam1
0.00
het
hom
wt
Normalised Counts
100
150
200
250
300
zmp_ph108_F9
zmp_ph108_F11
zmp_ph108_H2
zmp_ph108_H5
zmp_ph108_B2
zmp_ph108_B6
zmp_ph108_B7
zmp_ph108_C4
zmp_ph108_C5
zmp_ph108_D4
zmp_ph97_G6
zmp_ph97_G9
zmp_ph97_H2
zmp_ph97_B10
zmp_ph97_C2
zmp_ph97_C4
zmp_ph97_B8
zmp_ph97_C1
zmp_ph97_D2
zmp_ph97_D5
zmp_ph111_G5
zmp_ph111_G11
zmp_ph111_H1
zmp_ph111_B1a
zmp_ph111_B5
zmp_ph111_B8
zmp_ph111_B6
zmp_ph111_B7
zmp_ph111_B9
zmp_ph111_B10
zmp_ph114_G4
zmp_ph114_G11
zmp_ph114_H1
zmp_ph114_H2
zmp_ph114_A9
zmp_ph114_A11
zmp_ph114_B2
zmp_ph114_B4
zmp_ph114_D8
zmp_ph114_E2

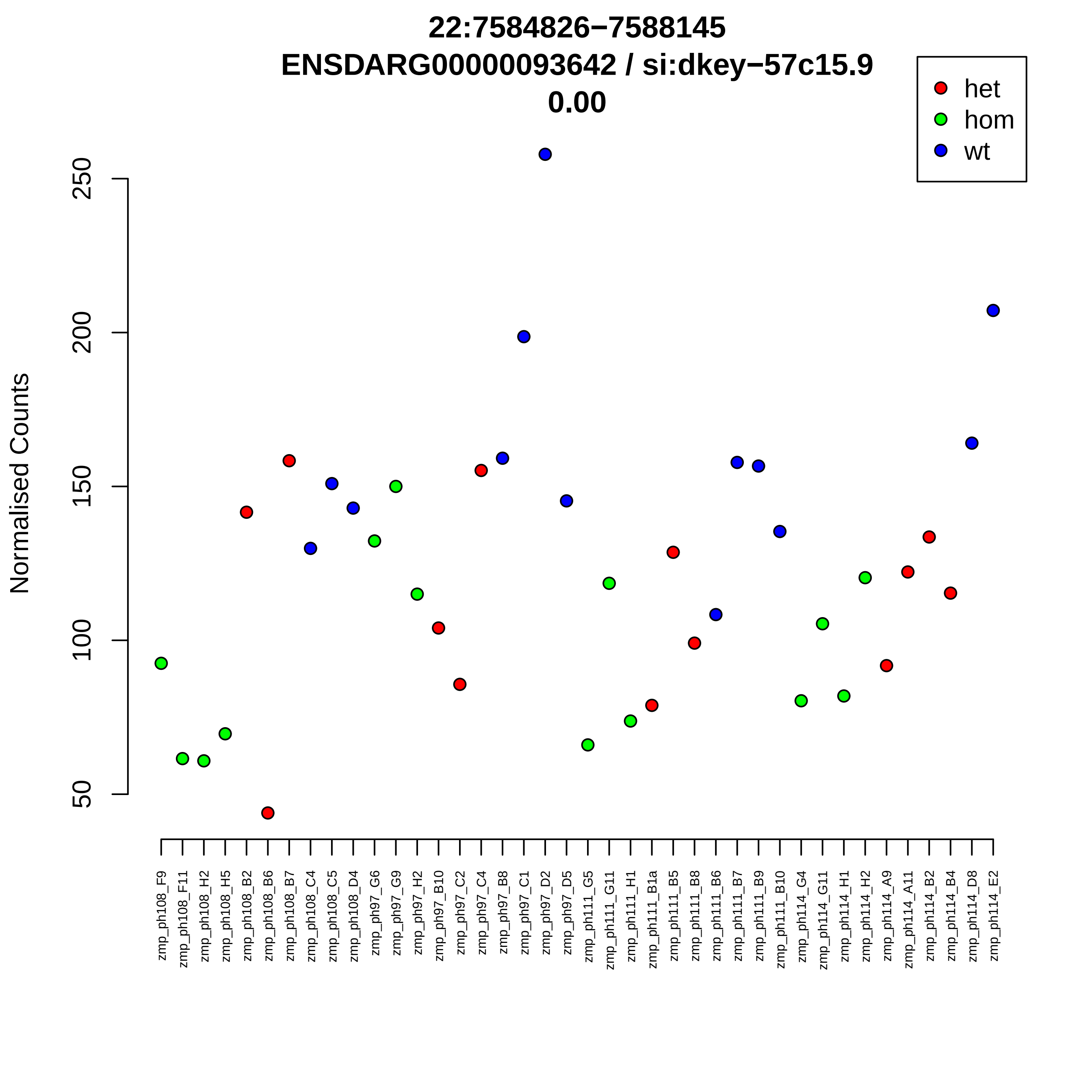

22:7584826−7588145
ENSDARG00000093642 / si:dkey−57c15.9
0.00
het
hom
wt
Normalised Counts
50
100
150
200
250
zmp_ph108_F9
zmp_ph108_F11
zmp_ph108_H2
zmp_ph108_H5
zmp_ph108_B2
zmp_ph108_B6
zmp_ph108_B7
zmp_ph108_C4
zmp_ph108_C5
zmp_ph108_D4
zmp_ph97_G6
zmp_ph97_G9
zmp_ph97_H2
zmp_ph97_B10
zmp_ph97_C2
zmp_ph97_C4
zmp_ph97_B8
zmp_ph97_C1
zmp_ph97_D2
zmp_ph97_D5
zmp_ph111_G5
zmp_ph111_G11
zmp_ph111_H1
zmp_ph111_B1a
zmp_ph111_B5
zmp_ph111_B8
zmp_ph111_B6
zmp_ph111_B7
zmp_ph111_B9
zmp_ph111_B10
zmp_ph114_G4
zmp_ph114_G11
zmp_ph114_H1
zmp_ph114_H2
zmp_ph114_A9
zmp_ph114_A11
zmp_ph114_B2
zmp_ph114_B4
zmp_ph114_D8
zmp_ph114_E2

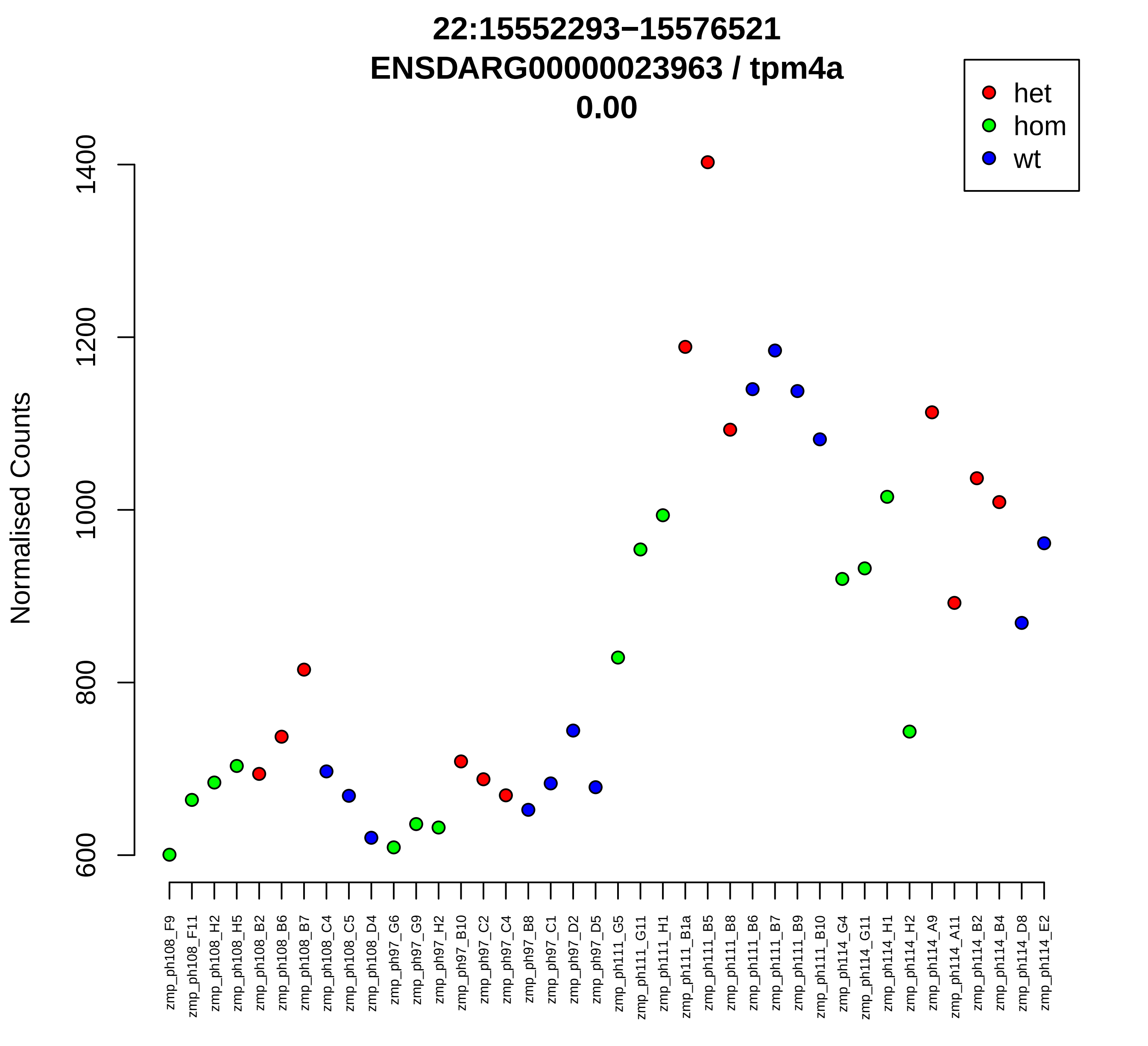
22:15552293−15576521
ENSDARG00000023963 / tpm4a
0.00
het
hom
wt
Normalised Counts
600
800
1000
1200
1400
zmp_ph108_F9
zmp_ph108_F11
zmp_ph108_H2
zmp_ph108_H5
zmp_ph108_B2
zmp_ph108_B6
zmp_ph108_B7
zmp_ph108_C4
zmp_ph108_C5
zmp_ph108_D4
zmp_ph97_G6
zmp_ph97_G9
zmp_ph97_H2
zmp_ph97_B10
zmp_ph97_C2
zmp_ph97_C4
zmp_ph97_B8
zmp_ph97_C1
zmp_ph97_D2
zmp_ph97_D5
zmp_ph111_G5
zmp_ph111_G11
zmp_ph111_H1
zmp_ph111_B1a
zmp_ph111_B5
zmp_ph111_B8
zmp_ph111_B6
zmp_ph111_B7
zmp_ph111_B9
zmp_ph111_B10
zmp_ph114_G4
zmp_ph114_G11
zmp_ph114_H1
zmp_ph114_H2
zmp_ph114_A9
zmp_ph114_A11
zmp_ph114_B2
zmp_ph114_B4
zmp_ph114_D8
zmp_ph114_E2

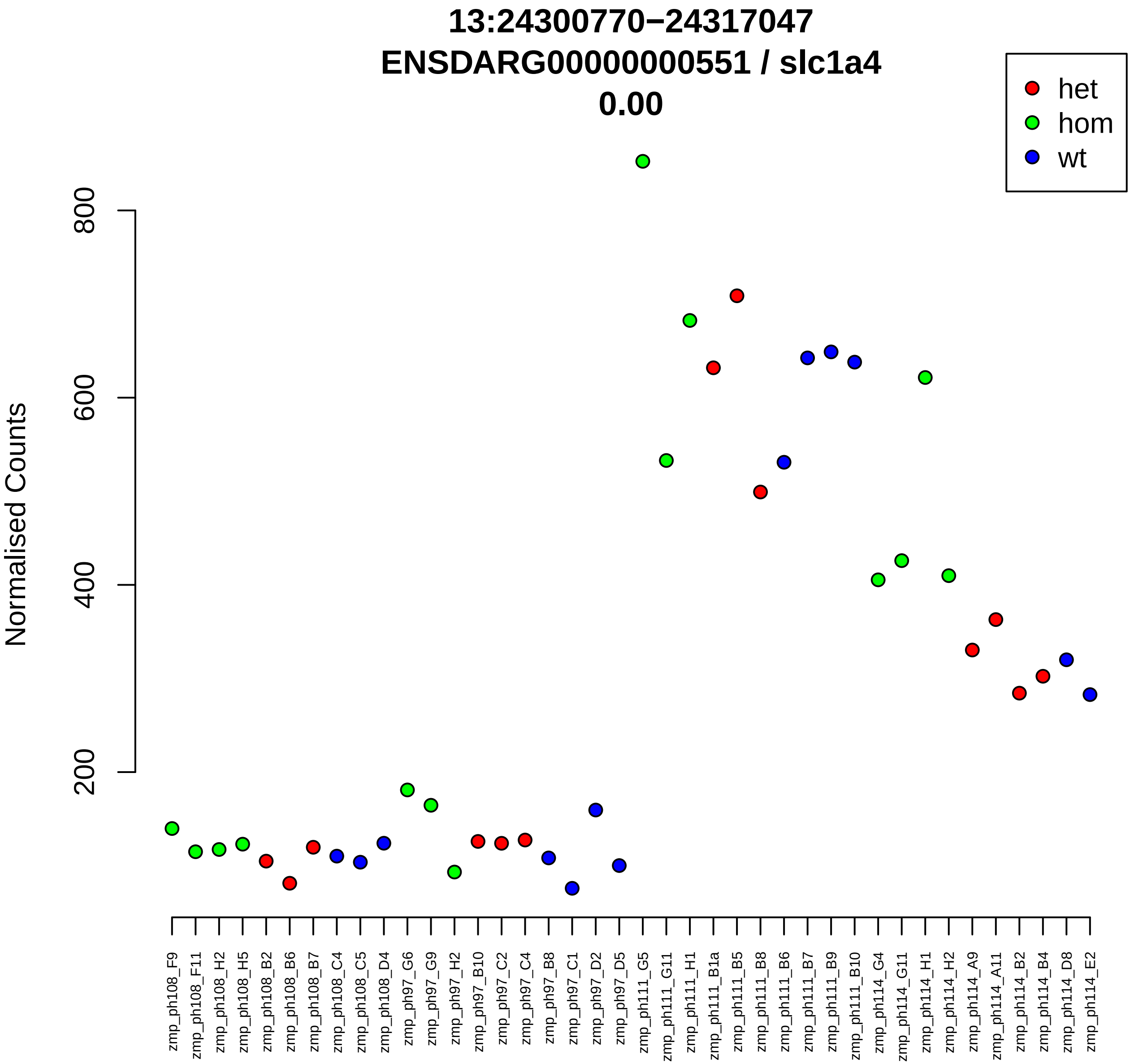

13:24300770−24317047
ENSDARG00000000551 / slc1a4
0.00
het
hom
wt
Normalised Counts
200
400
600
800
zmp_ph108_F9
zmp_ph108_F11
zmp_ph108_H2
zmp_ph108_H5
zmp_ph108_B2
zmp_ph108_B6
zmp_ph108_B7
zmp_ph108_C4
zmp_ph108_C5
zmp_ph108_D4
zmp_ph97_G6
zmp_ph97_G9
zmp_ph97_H2
zmp_ph97_B10
zmp_ph97_C2
zmp_ph97_C4
zmp_ph97_B8
zmp_ph97_C1
zmp_ph97_D2
zmp_ph97_D5
zmp_ph111_G5
zmp_ph111_G11
zmp_ph111_H1
zmp_ph111_B1a
zmp_ph111_B5
zmp_ph111_B8
zmp_ph111_B6
zmp_ph111_B7
zmp_ph111_B9
zmp_ph111_B10
zmp_ph114_G4
zmp_ph114_G11
zmp_ph114_H1
zmp_ph114_H2
zmp_ph114_A9
zmp_ph114_A11
zmp_ph114_B2
zmp_ph114_B4
zmp_ph114_D8
zmp_ph114_E2

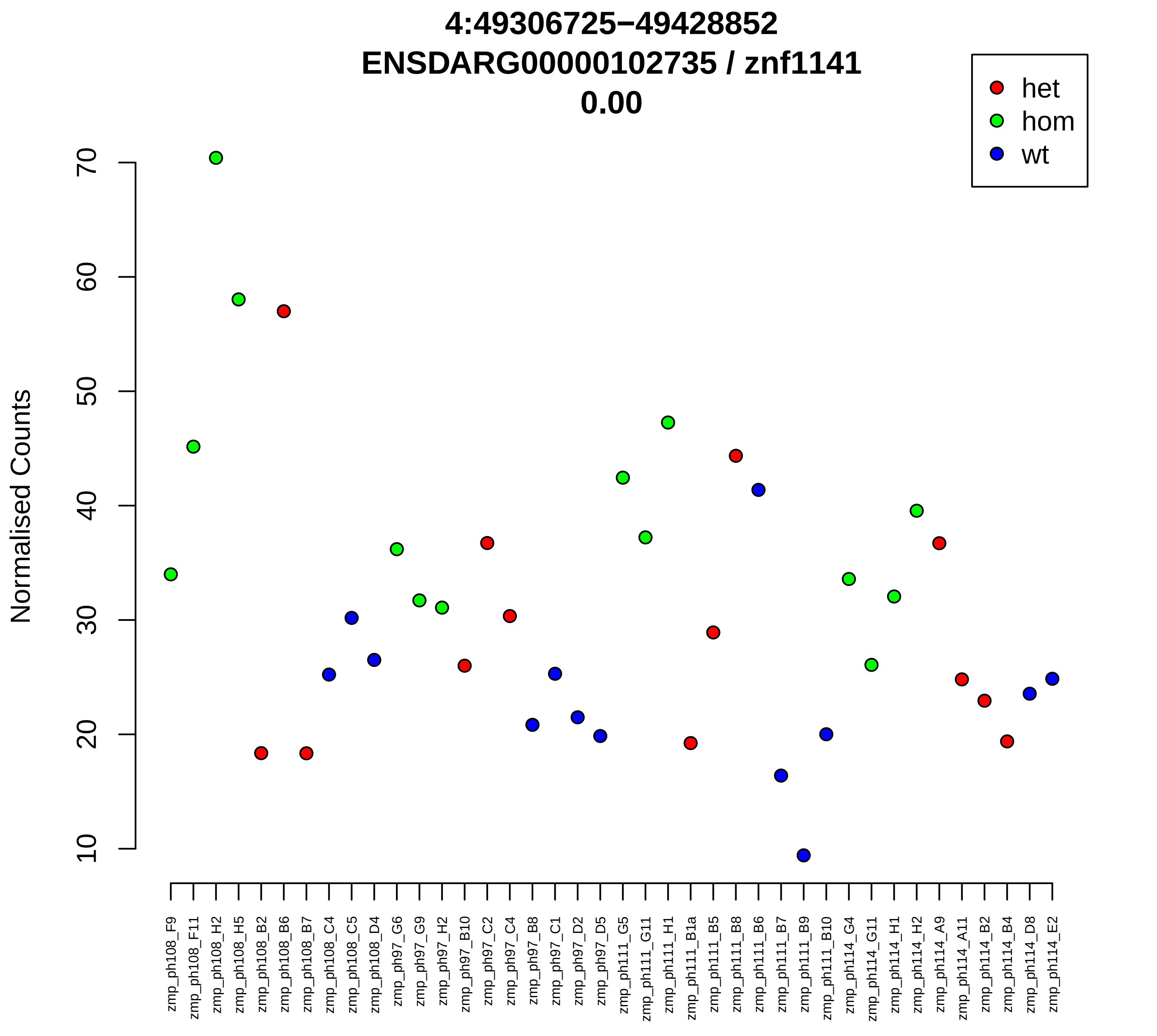

4:49306725−49428852
ENSDARG00000102735 / znf1141
0.00
het
hom
wt
Normalised Counts
10
20
30
40
50
60
70
zmp_ph108_F9
zmp_ph108_F11
zmp_ph108_H2
zmp_ph108_H5
zmp_ph108_B2
zmp_ph108_B6
zmp_ph108_B7
zmp_ph108_C4
zmp_ph108_C5
zmp_ph108_D4
zmp_ph97_G6
zmp_ph97_G9
zmp_ph97_H2
zmp_ph97_B10
zmp_ph97_C2
zmp_ph97_C4
zmp_ph97_B8
zmp_ph97_C1
zmp_ph97_D2
zmp_ph97_D5
zmp_ph111_G5
zmp_ph111_G11
zmp_ph111_H1
zmp_ph111_B1a
zmp_ph111_B5
zmp_ph111_B8
zmp_ph111_B6
zmp_ph111_B7
zmp_ph111_B9
zmp_ph111_B10
zmp_ph114_G4
zmp_ph114_G11
zmp_ph114_H1
zmp_ph114_H2
zmp_ph114_A9
zmp_ph114_A11
zmp_ph114_B2
zmp_ph114_B4
zmp_ph114_D8
zmp_ph114_E2

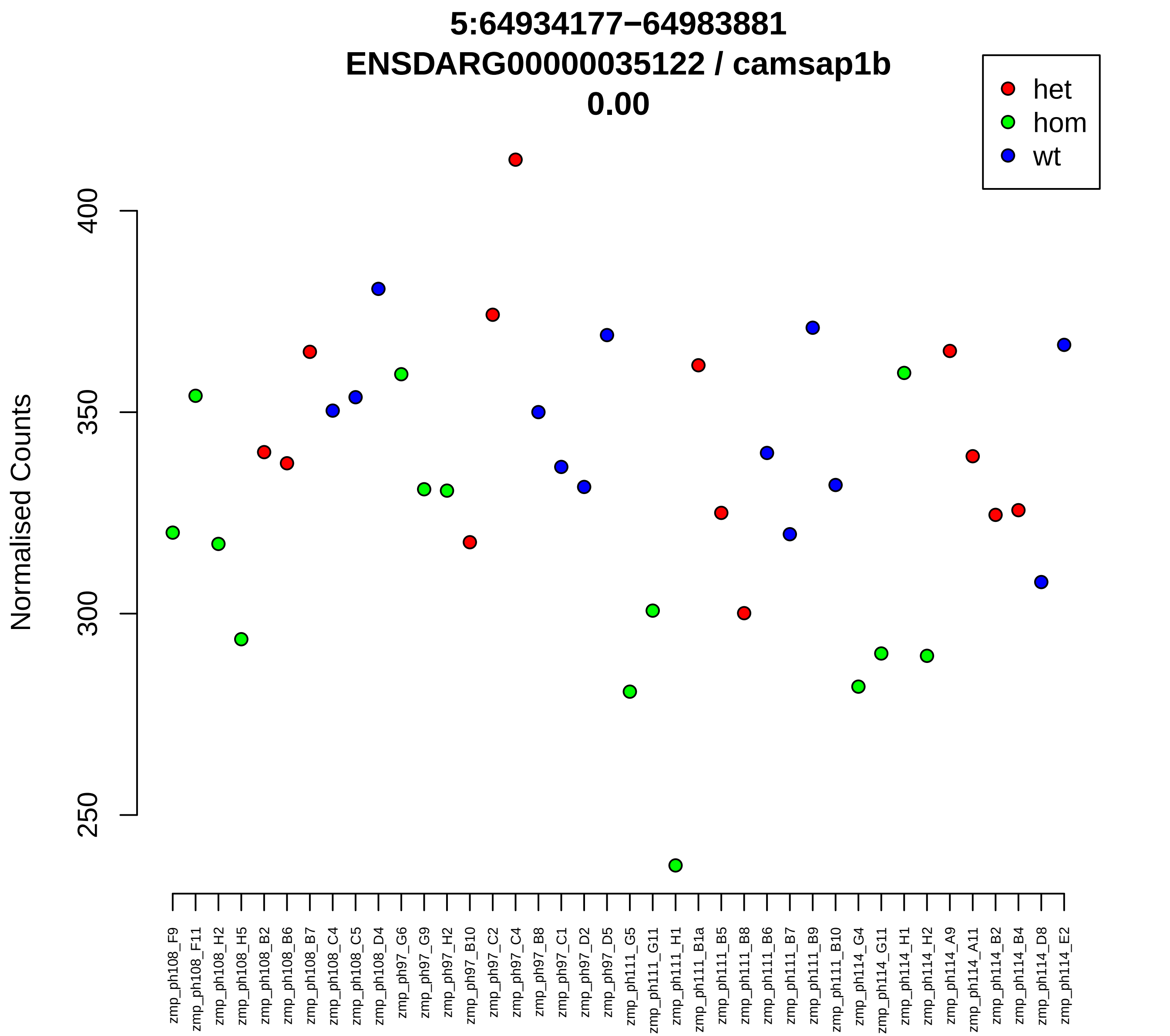

5:64934177−64983881
ENSDARG00000035122 / camsap1b
0.00
het
hom
wt
Normalised Counts
400
350
300
250
zmp_ph108_F9
zmp_ph108_F11
zmp_ph108_H2
zmp_ph108_H5
zmp_ph108_B2
zmp_ph108_B6
zmp_ph108_B7
zmp_ph108_C4
zmp_ph108_C5
zmp_ph108_D4
zmp_ph97_G6
zmp_ph97_G9
zmp_ph97_H2
zmp_ph97_B10
zmp_ph97_C2
zmp_ph97_C4
zmp_ph97_B8
zmp_ph97_C1
zmp_ph97_D2
zmp_ph97_D5
zmp_ph111_G5
zmp_ph111_G11
zmp_ph111_H1
zmp_ph111_B1a
zmp_ph111_B5
zmp_ph111_B8
zmp_ph111_B6
zmp_ph111_B7
zmp_ph111_B9
zmp_ph111_B10
zmp_ph114_G4
zmp_ph114_G11
zmp_ph114_H1
zmp_ph114_H2
zmp_ph114_A9
zmp_ph114_A11
zmp_ph114_B2
zmp_ph114_B4
zmp_ph114_D8
zmp_ph114_E2

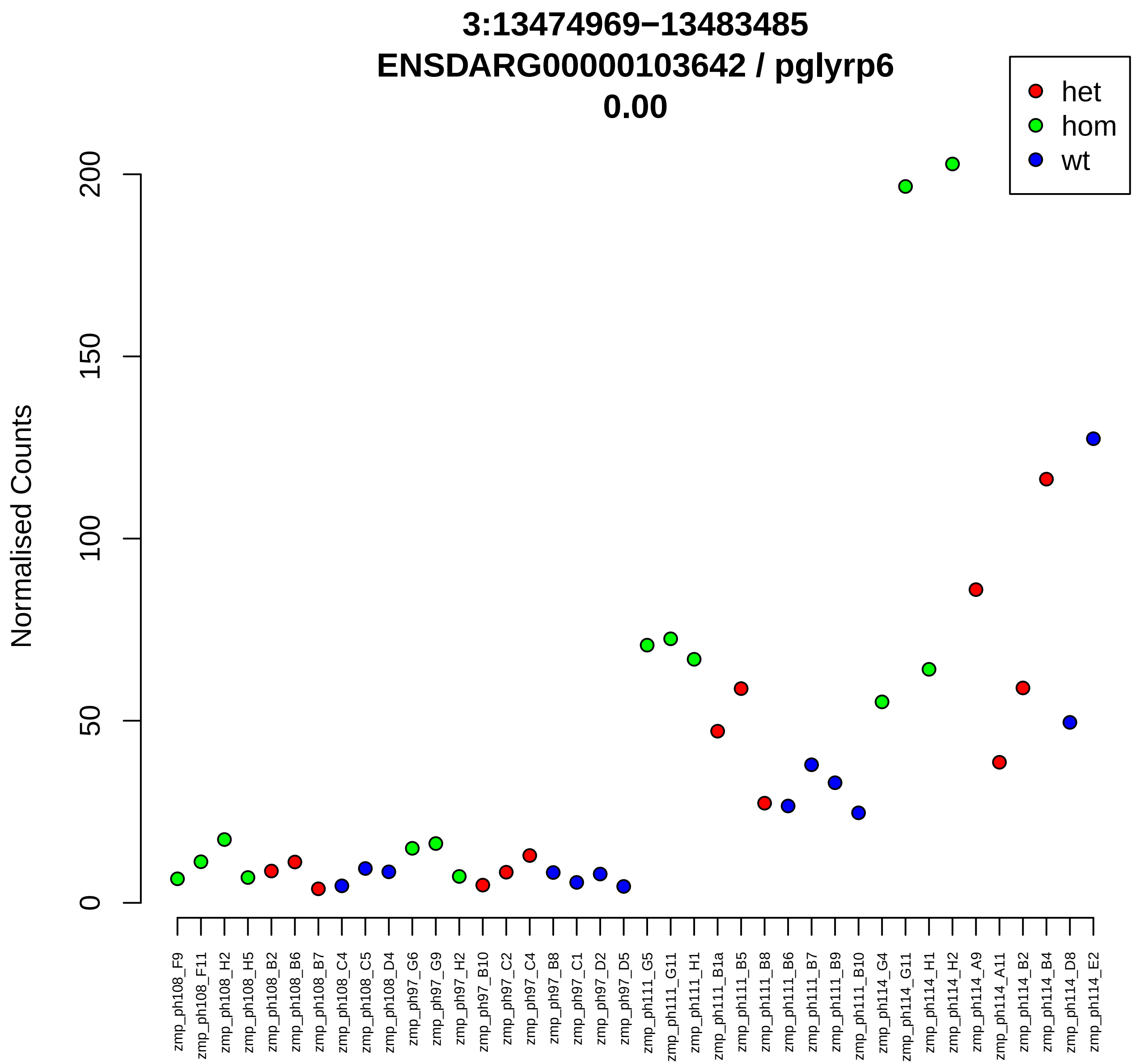
3:13474969−13483485
ENSDARG00000103642 / pglyrp6
0.00
het
hom
wt
Normalised Counts
0
50
100
150
200
zmp_ph108_F9
zmp_ph108_F11
zmp_ph108_H2
zmp_ph108_H5
zmp_ph108_B2
zmp_ph108_B6
zmp_ph108_B7
zmp_ph108_C4
zmp_ph108_C5
zmp_ph108_D4
zmp_ph97_G6
zmp_ph97_G9
zmp_ph97_H2
zmp_ph97_B10
zmp_ph97_C2
zmp_ph97_C4
zmp_ph97_B8
zmp_ph97_C1
zmp_ph97_D2
zmp_ph97_D5
zmp_ph111_G5
zmp_ph111_G11
zmp_ph111_H1
zmp_ph111_B1a
zmp_ph111_B5
zmp_ph111_B8
zmp_ph111_B6
zmp_ph111_B7
zmp_ph111_B9
zmp_ph111_B10
zmp_ph114_G4
zmp_ph114_G11
zmp_ph114_H1
zmp_ph114_H2
zmp_ph114_A9
zmp_ph114_A11
zmp_ph114_B2
zmp_ph114_B4
zmp_ph114_D8
zmp_ph114_E2

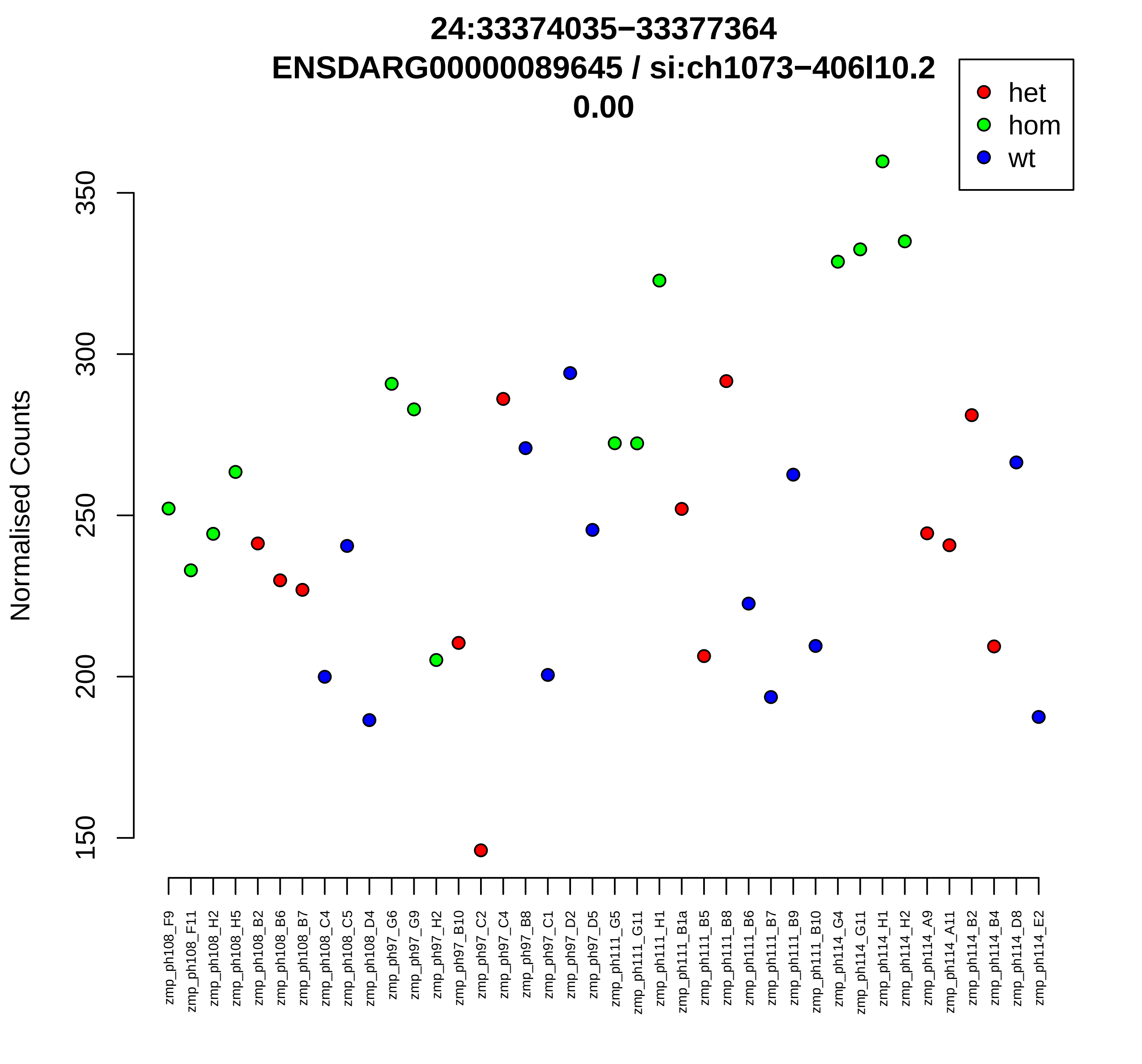

24:33374035−33377364
ENSDARG00000089645 / si:ch1073−406l10.2
0.00
het
hom
wt
Normalised Counts
150
200
250
300
350
zmp_ph108_F9
zmp_ph108_F11
zmp_ph108_H2
zmp_ph108_H5
zmp_ph108_B2
zmp_ph108_B6
zmp_ph108_B7
zmp_ph108_C4
zmp_ph108_C5
zmp_ph108_D4
zmp_ph97_G6
zmp_ph97_G9
zmp_ph97_H2
zmp_ph97_B10
zmp_ph97_C2
zmp_ph97_C4
zmp_ph97_B8
zmp_ph97_C1
zmp_ph97_D2
zmp_ph97_D5
zmp_ph111_G5
zmp_ph111_G11
zmp_ph111_H1
zmp_ph111_B1a
zmp_ph111_B5
zmp_ph111_B8
zmp_ph111_B6
zmp_ph111_B7
zmp_ph111_B9
zmp_ph111_B10
zmp_ph114_G4
zmp_ph114_G11
zmp_ph114_H1
zmp_ph114_H2
zmp_ph114_A9
zmp_ph114_A11
zmp_ph114_B2
zmp_ph114_B4
zmp_ph114_D8
zmp_ph114_E2

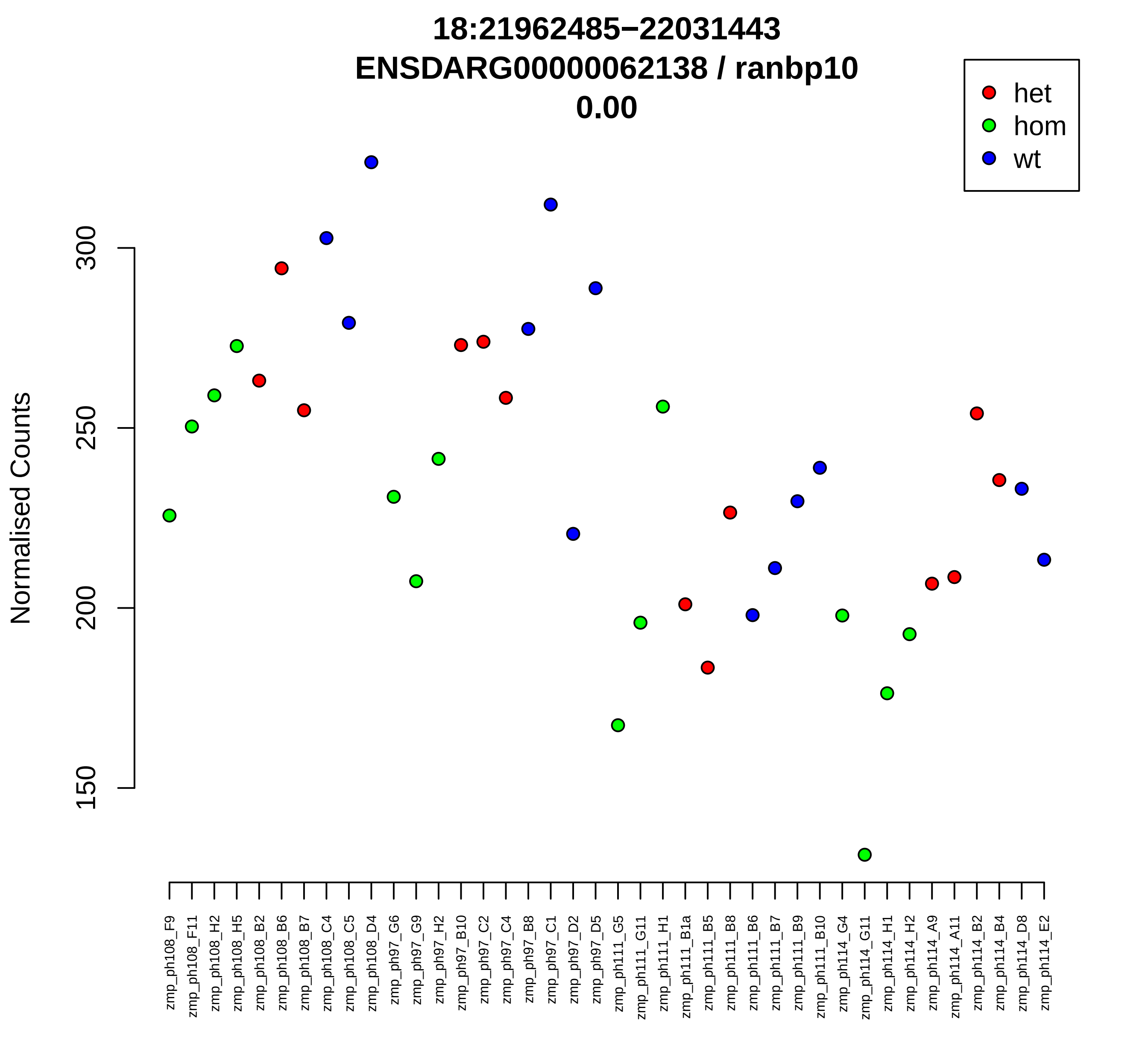

18:21962485−22031443
ENSDARG00000062138 / ranbp10
0.00
het
hom
wt
Normalised Counts
150
200
250
300
zmp_ph108_F9
zmp_ph108_F11
zmp_ph108_H2
zmp_ph108_H5
zmp_ph108_B2
zmp_ph108_B6
zmp_ph108_B7
zmp_ph108_C4
zmp_ph108_C5
zmp_ph108_D4
zmp_ph97_G6
zmp_ph97_G9
zmp_ph97_H2
zmp_ph97_B10
zmp_ph97_C2
zmp_ph97_C4
zmp_ph97_B8
zmp_ph97_C1
zmp_ph97_D2
zmp_ph97_D5
zmp_ph111_G5
zmp_ph111_G11
zmp_ph111_H1
zmp_ph111_B1a
zmp_ph111_B5
zmp_ph111_B8
zmp_ph111_B6
zmp_ph111_B7
zmp_ph111_B9
zmp_ph111_B10
zmp_ph114_G4
zmp_ph114_G11
zmp_ph114_H1
zmp_ph114_H2
zmp_ph114_A9
zmp_ph114_A11
zmp_ph114_B2
zmp_ph114_B4
zmp_ph114_D8
zmp_ph114_E2

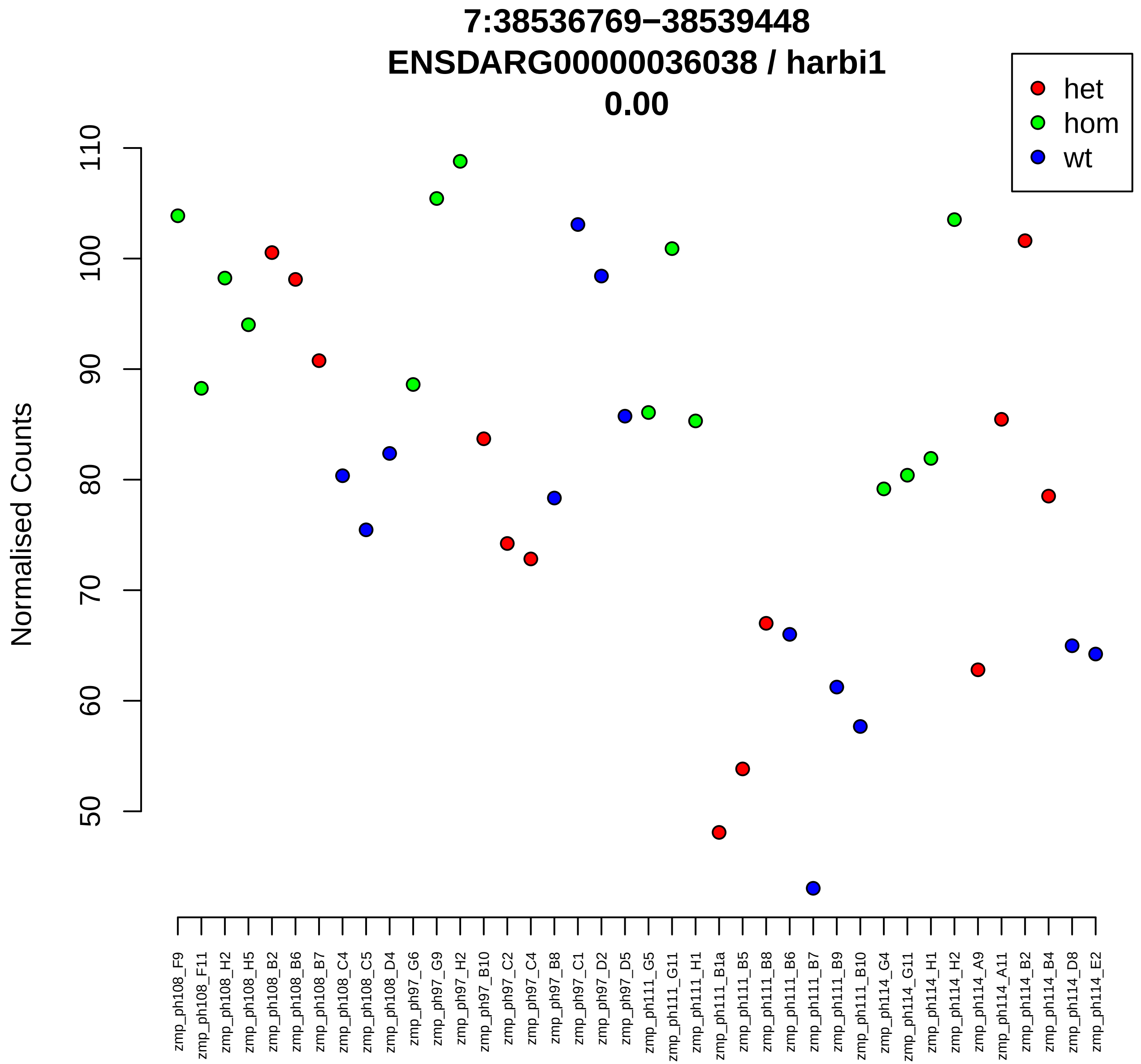
7:38536769−38539448
ENSDARG00000036038 / harbi1
0.00
Normalised Counts
50
60
70
80
90
100
110
het
hom
wt
zmp_ph108_F9
zmp_ph108_F11
zmp_ph108_H2
zmp_ph108_H5
zmp_ph108_B2
zmp_ph108_B6
zmp_ph108_B7
zmp_ph108_C4
zmp_ph108_C5
zmp_ph108_D4
zmp_ph97_G6
zmp_ph97_G9
zmp_ph97_H2
zmp_ph97_B10
zmp_ph97_C2
zmp_ph97_C4
zmp_ph97_B8
zmp_ph97_C1
zmp_ph97_D2
zmp_ph97_D5
zmp_ph111_G5
zmp_ph111_G11
zmp_ph111_H1
zmp_ph111_B1a
zmp_ph111_B5
zmp_ph111_B8
zmp_ph111_B6
zmp_ph111_B7
zmp_ph111_B9
zmp_ph111_B10
zmp_ph114_G4
zmp_ph114_G11
zmp_ph114_H1
zmp_ph114_H2
zmp_ph114_A9
zmp_ph114_A11
zmp_ph114_B2
zmp_ph114_B4
zmp_ph114_D8
zmp_ph114_E2

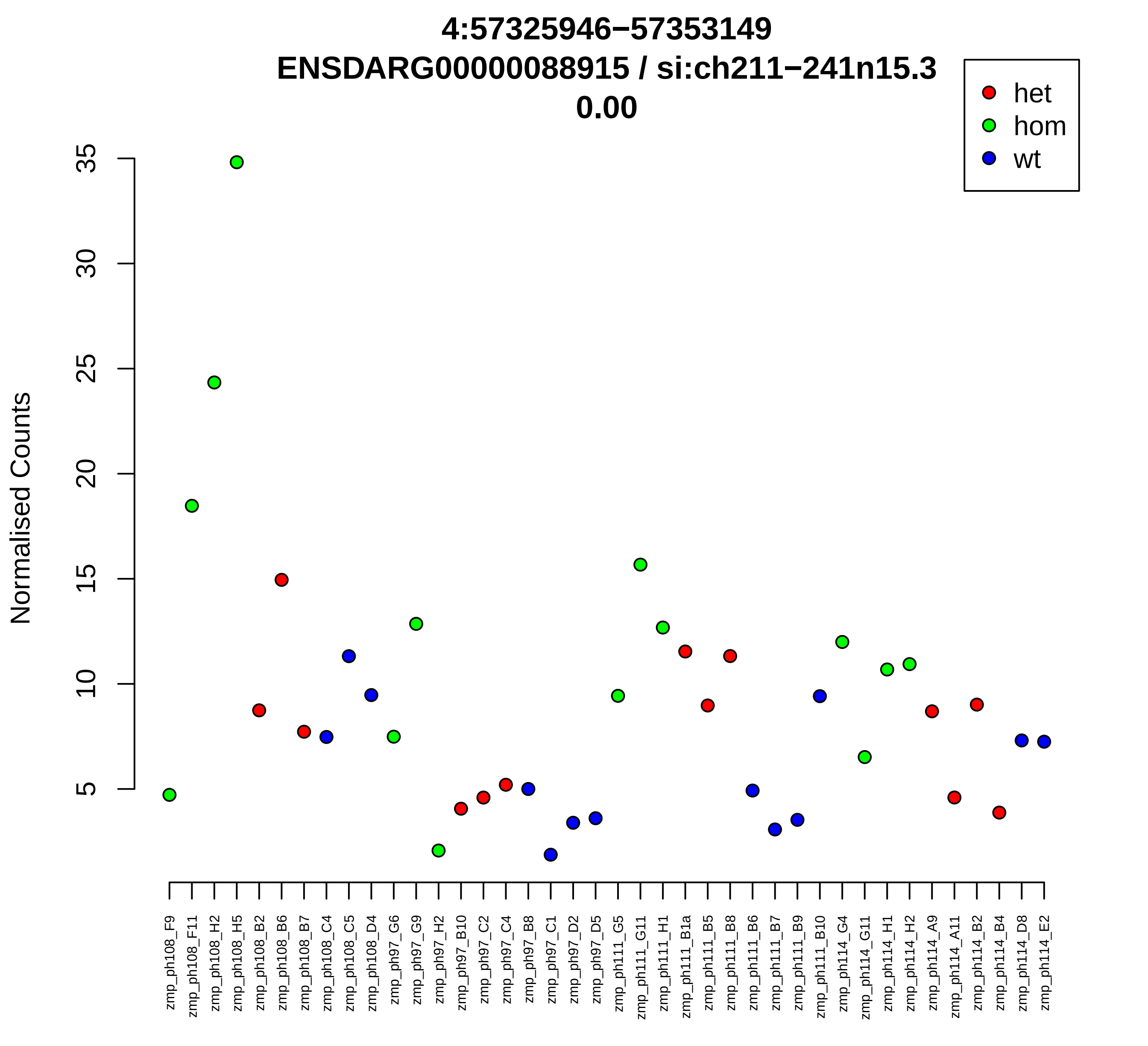

4:57325946−57353149
ENSDARG00000088915 / si:ch211−241n15.3
0.00
het
hom
wt
Normalised Counts
5
10
15
20
25
30
35
zmp_ph108_F9
zmp_ph108_F11
zmp_ph108_H2
zmp_ph108_H5
zmp_ph108_B2
zmp_ph108_B6
zmp_ph108_B7
zmp_ph108_C4
zmp_ph108_C5
zmp_ph108_D4
zmp_ph97_G6
zmp_ph97_G9
zmp_ph97_H2
zmp_ph97_B10
zmp_ph97_C2
zmp_ph97_C4
zmp_ph97_B8
zmp_ph97_C1
zmp_ph97_D2
zmp_ph97_D5
zmp_ph111_G5
zmp_ph111_G11
zmp_ph111_H1
zmp_ph111_B1a
zmp_ph111_B5
zmp_ph111_B8
zmp_ph111_B6
zmp_ph111_B7
zmp_ph111_B9
zmp_ph111_B10
zmp_ph114_G4
zmp_ph114_G11
zmp_ph114_H1
zmp_ph114_H2
zmp_ph114_A9
zmp_ph114_A11
zmp_ph114_B2
zmp_ph114_B4
zmp_ph114_D8
zmp_ph114_E2

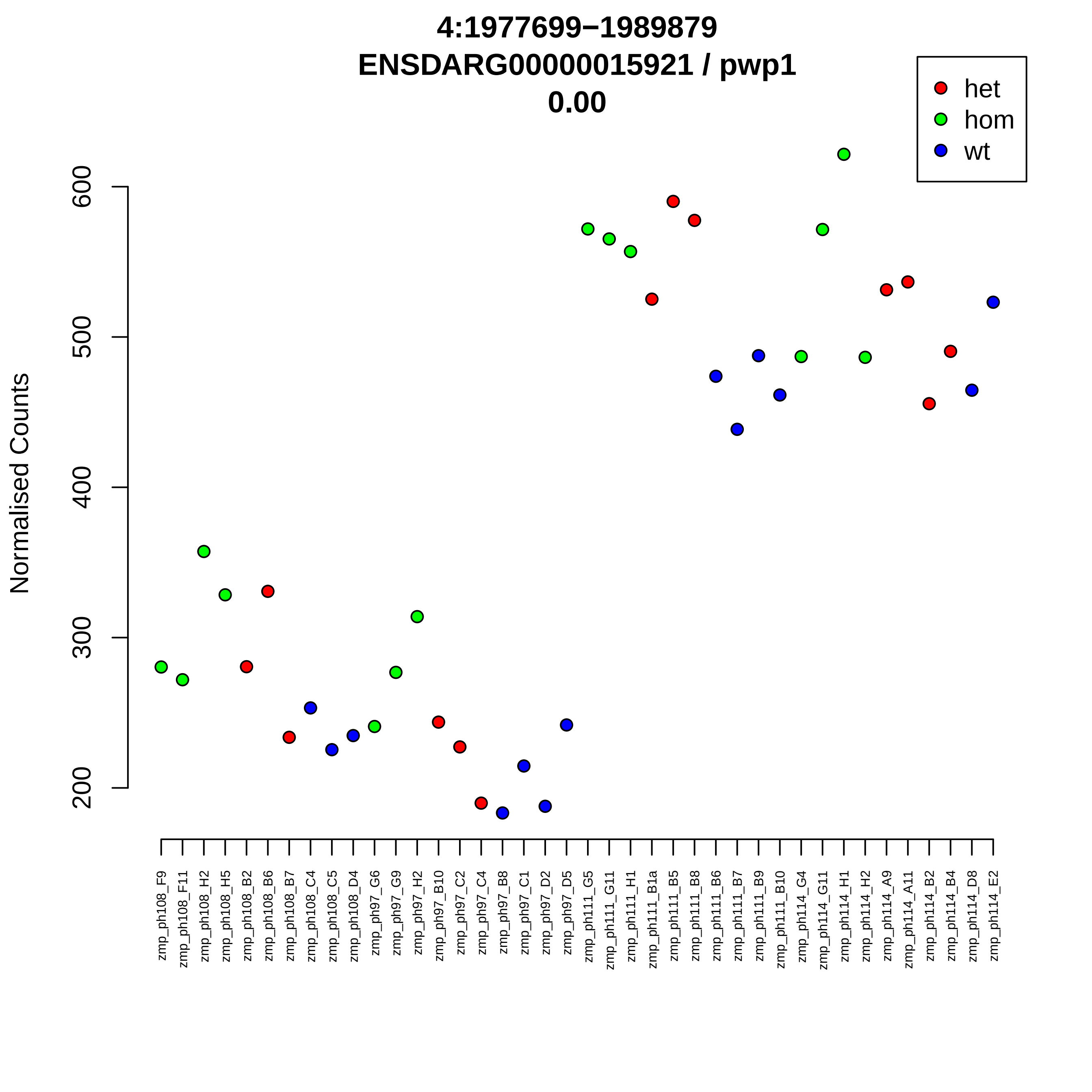
4:1977699−1989879
ENSDARG00000015921 / pwp1
0.00
het
hom
wt
Normalised Counts
200
300
400
500
600
zmp_ph108_F9
zmp_ph108_F11
zmp_ph108_H2
zmp_ph108_H5
zmp_ph108_B2
zmp_ph108_B6
zmp_ph108_B7
zmp_ph108_C4
zmp_ph108_C5
zmp_ph108_D4
zmp_ph97_G6
zmp_ph97_G9
zmp_ph97_H2
zmp_ph97_B10
zmp_ph97_C2
zmp_ph97_C4
zmp_ph97_B8
zmp_ph97_C1
zmp_ph97_D2
zmp_ph97_D5
zmp_ph111_G5
zmp_ph111_G11
zmp_ph111_H1
zmp_ph111_B1a
zmp_ph111_B5
zmp_ph111_B8
zmp_ph111_B6
zmp_ph111_B7
zmp_ph111_B9
zmp_ph111_B10
zmp_ph114_G4
zmp_ph114_G11
zmp_ph114_H1
zmp_ph114_H2
zmp_ph114_A9
zmp_ph114_A11
zmp_ph114_B2
zmp_ph114_B4
zmp_ph114_D8
zmp_ph114_E2

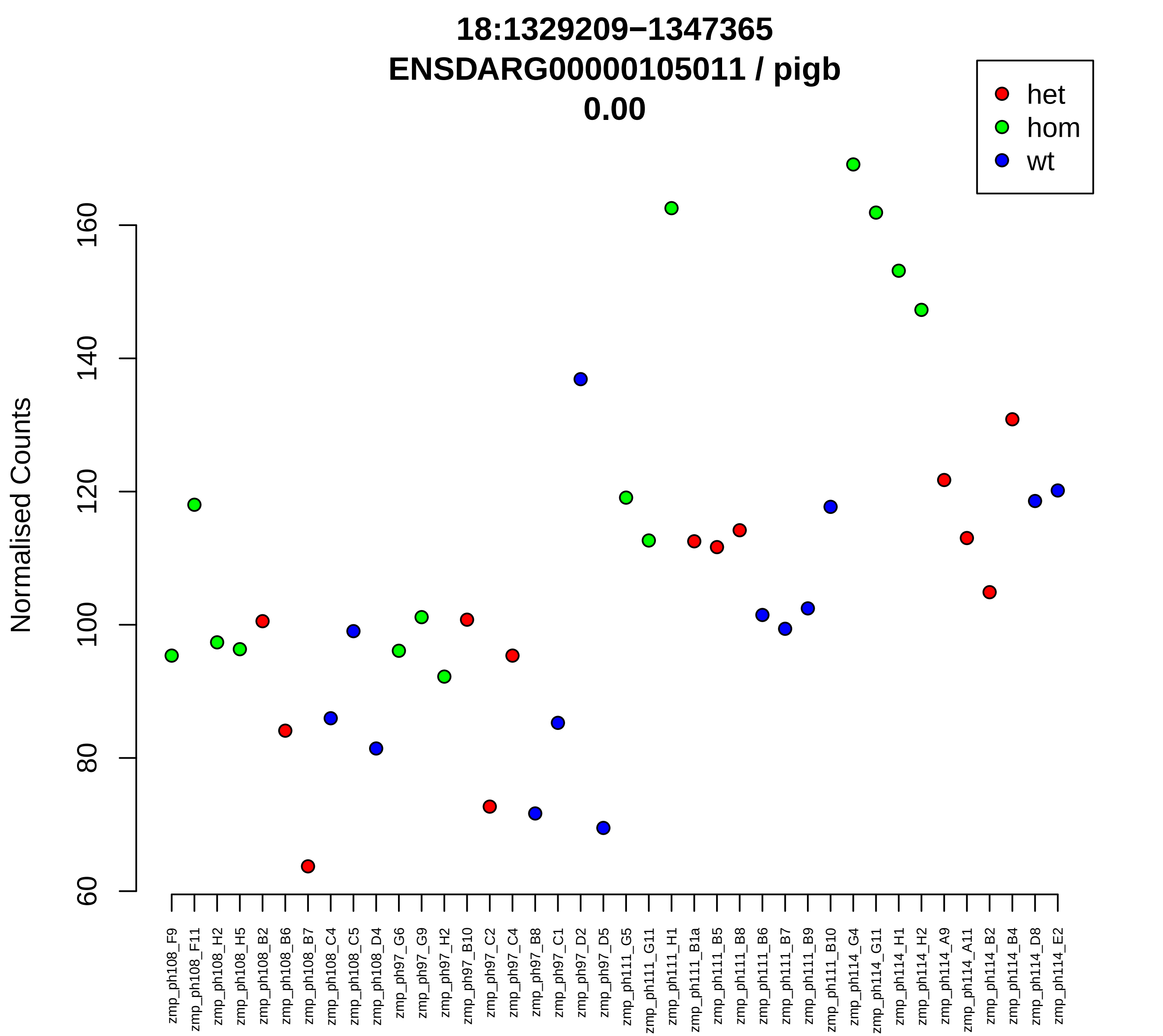

18:1329209−1347365
ENSDARG00000105011 / pigb
0.00
het
hom
wt
Normalised Counts
60
80
100
120
140
160
zmp_ph108_F9
zmp_ph108_F11
zmp_ph108_H2
zmp_ph108_H5
zmp_ph108_B2
zmp_ph108_B6
zmp_ph108_B7
zmp_ph108_C4
zmp_ph108_C5
zmp_ph108_D4
zmp_ph97_G6
zmp_ph97_G9
zmp_ph97_H2
zmp_ph97_B10
zmp_ph97_C2
zmp_ph97_C4
zmp_ph97_B8
zmp_ph97_C1
zmp_ph97_D2
zmp_ph97_D5
zmp_ph111_G5
zmp_ph111_G11
zmp_ph111_H1
zmp_ph111_B1a
zmp_ph111_B5
zmp_ph111_B8
zmp_ph111_B6
zmp_ph111_B7
zmp_ph111_B9
zmp_ph111_B10
zmp_ph114_G4
zmp_ph114_G11
zmp_ph114_H1
zmp_ph114_H2
zmp_ph114_A9
zmp_ph114_A11
zmp_ph114_B2
zmp_ph114_B4
zmp_ph114_D8
zmp_ph114_E2

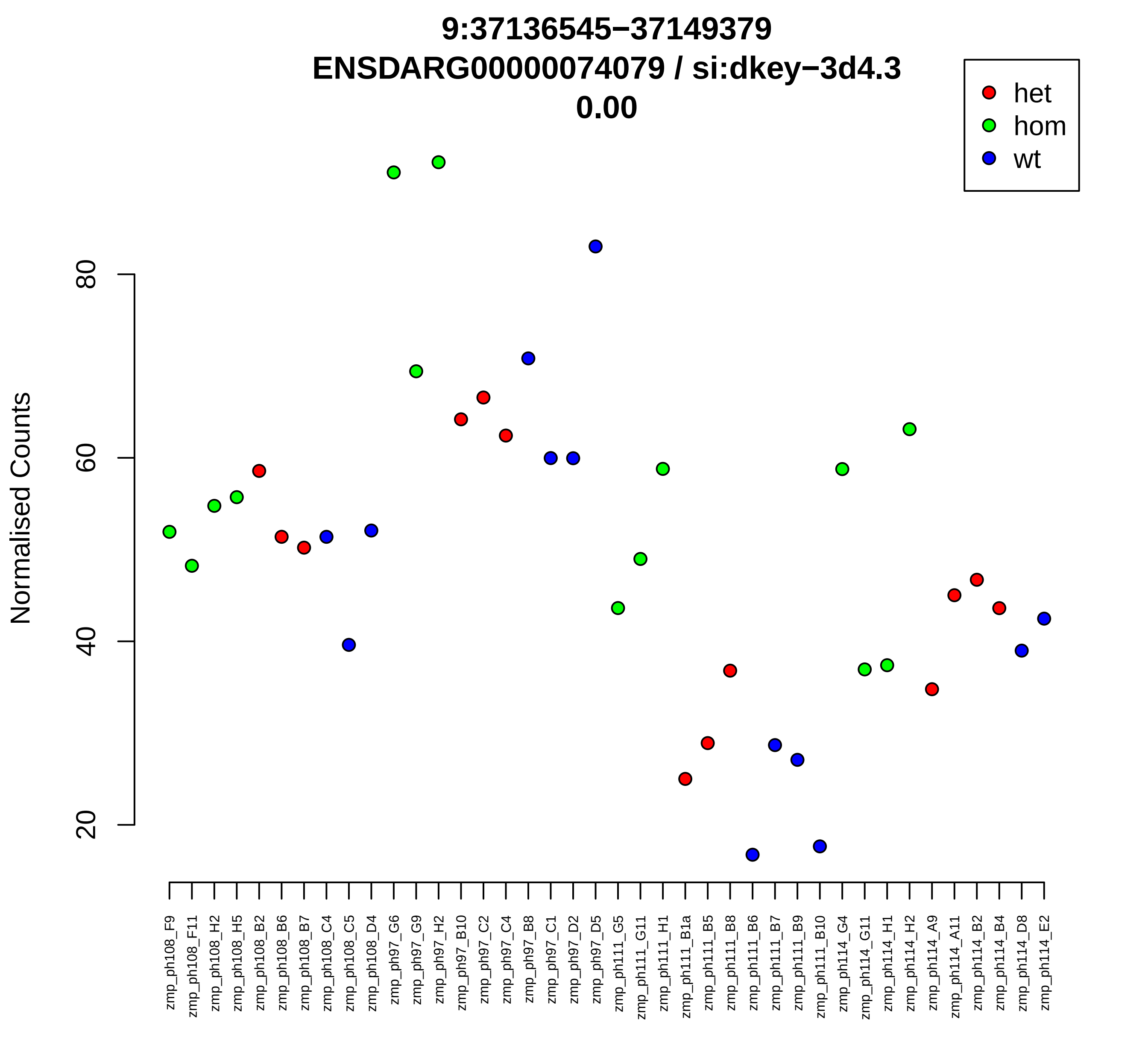

9:37136545−37149379
ENSDARG00000074079 / si:dkey−3d4.3
0.00
het
hom
wt
Normalised Counts
20
40
60
80
zmp_ph108_F9
zmp_ph108_F11
zmp_ph108_H2
zmp_ph108_H5
zmp_ph108_B2
zmp_ph108_B6
zmp_ph108_B7
zmp_ph108_C4
zmp_ph108_C5
zmp_ph108_D4
zmp_ph97_G6
zmp_ph97_G9
zmp_ph97_H2
zmp_ph97_B10
zmp_ph97_C2
zmp_ph97_C4
zmp_ph97_B8
zmp_ph97_C1
zmp_ph97_D2
zmp_ph97_D5
zmp_ph111_G5
zmp_ph111_G11
zmp_ph111_H1
zmp_ph111_B1a
zmp_ph111_B5
zmp_ph111_B8
zmp_ph111_B6
zmp_ph111_B7
zmp_ph111_B9
zmp_ph111_B10
zmp_ph114_G4
zmp_ph114_G11
zmp_ph114_H1
zmp_ph114_H2
zmp_ph114_A9
zmp_ph114_A11
zmp_ph114_B2
zmp_ph114_B4
zmp_ph114_D8
zmp_ph114_E2

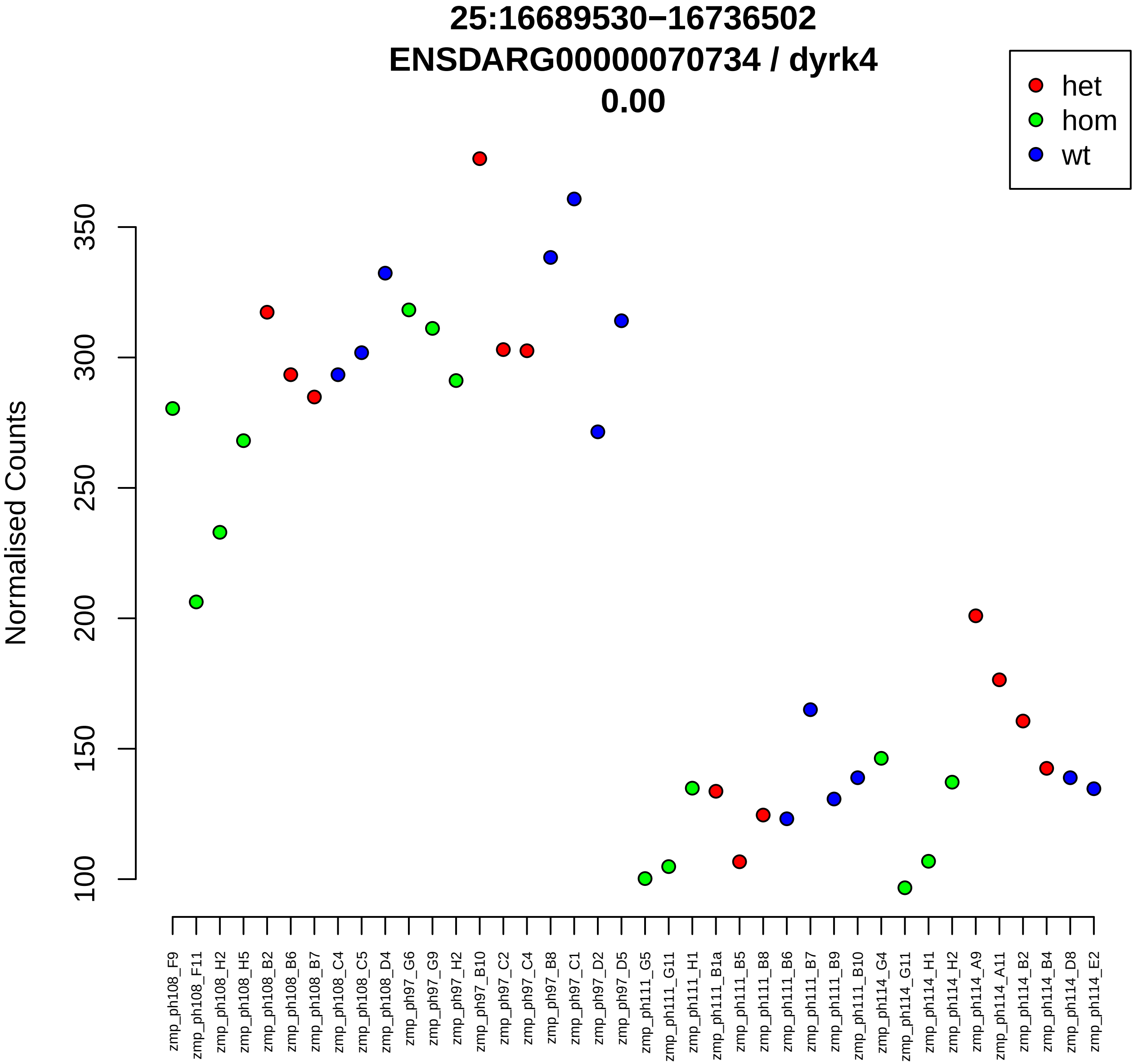

25:16689530−16736502
ENSDARG00000070734 / dyrk4
0.00
het
hom
wt
Normalised Counts
100
150
200
250
300
350
zmp_ph108_F9
zmp_ph108_F11
zmp_ph108_H2
zmp_ph108_H5
zmp_ph108_B2
zmp_ph108_B6
zmp_ph108_B7
zmp_ph108_C4
zmp_ph108_C5
zmp_ph108_D4
zmp_ph97_G6
zmp_ph97_G9
zmp_ph97_H2
zmp_ph97_B10
zmp_ph97_C2
zmp_ph97_C4
zmp_ph97_B8
zmp_ph97_C1
zmp_ph97_D2
zmp_ph97_D5
zmp_ph111_G5
zmp_ph111_G11
zmp_ph111_H1
zmp_ph111_B1a
zmp_ph111_B5
zmp_ph111_B8
zmp_ph111_B6
zmp_ph111_B7
zmp_ph111_B9
zmp_ph111_B10
zmp_ph114_G4
zmp_ph114_G11
zmp_ph114_H1
zmp_ph114_H2
zmp_ph114_A9
zmp_ph114_A11
zmp_ph114_B2
zmp_ph114_B4
zmp_ph114_D8
zmp_ph114_E2

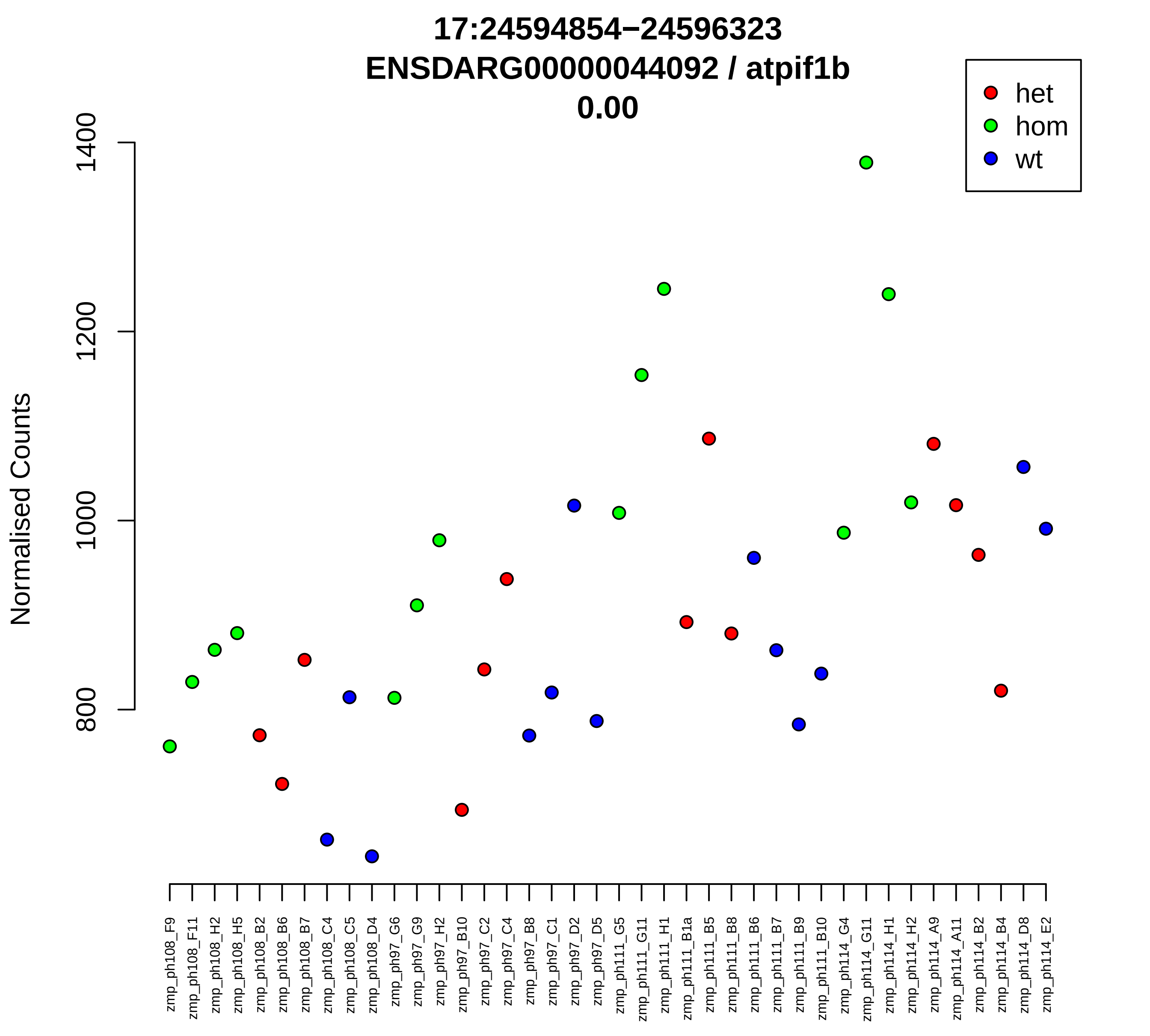

17:24594854−24596323
ENSDARG00000044092 / atpif1b
0.00
Normalised Counts
800
1000
1200
1400
het
hom
wt
zmp_ph108_F9
zmp_ph108_F11
zmp_ph108_H2
zmp_ph108_H5
zmp_ph108_B2
zmp_ph108_B6
zmp_ph108_B7
zmp_ph108_C4
zmp_ph108_C5
zmp_ph108_D4
zmp_ph97_G6
zmp_ph97_G9
zmp_ph97_H2
zmp_ph97_B10
zmp_ph97_C2
zmp_ph97_C4
zmp_ph97_B8
zmp_ph97_C1
zmp_ph97_D2
zmp_ph97_D5
zmp_ph111_G5
zmp_ph111_G11
zmp_ph111_H1
zmp_ph111_B1a
zmp_ph111_B5
zmp_ph111_B8
zmp_ph111_B6
zmp_ph111_B7
zmp_ph111_B9
zmp_ph111_B10
zmp_ph114_G4
zmp_ph114_G11
zmp_ph114_H1
zmp_ph114_H2
zmp_ph114_A9
zmp_ph114_A11
zmp_ph114_B2
zmp_ph114_B4
zmp_ph114_D8
zmp_ph114_E2

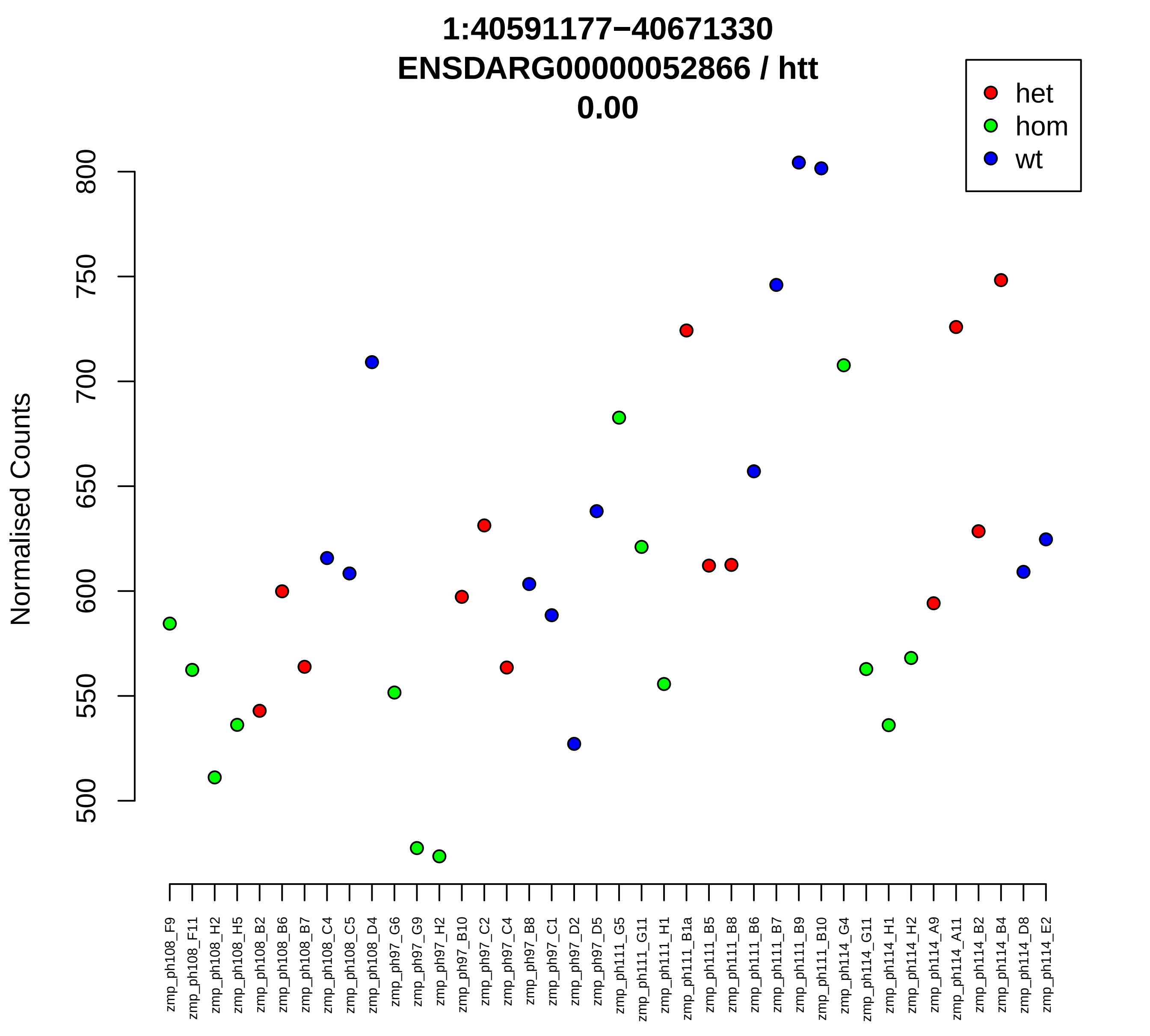

1:40591177−40671330
ENSDARG00000052866 / htt
0.00
het
hom
wt
Normalised Counts
800
750
700
650
600
550
500
zmp_ph108_F9
zmp_ph108_F11
zmp_ph108_H2
zmp_ph108_H5
zmp_ph108_B2
zmp_ph108_B6
zmp_ph108_B7
zmp_ph108_C4
zmp_ph108_C5
zmp_ph108_D4
zmp_ph97_G6
zmp_ph97_G9
zmp_ph97_H2
zmp_ph97_B10
zmp_ph97_C2
zmp_ph97_C4
zmp_ph97_B8
zmp_ph97_C1
zmp_ph97_D2
zmp_ph97_D5
zmp_ph111_G5
zmp_ph111_G11
zmp_ph111_H1
zmp_ph111_B1a
zmp_ph111_B5
zmp_ph111_B8
zmp_ph111_B6
zmp_ph111_B7
zmp_ph111_B9
zmp_ph111_B10
zmp_ph114_G4
zmp_ph114_G11
zmp_ph114_H1
zmp_ph114_H2
zmp_ph114_A9
zmp_ph114_A11
zmp_ph114_B2
zmp_ph114_B4
zmp_ph114_D8
zmp_ph114_E2

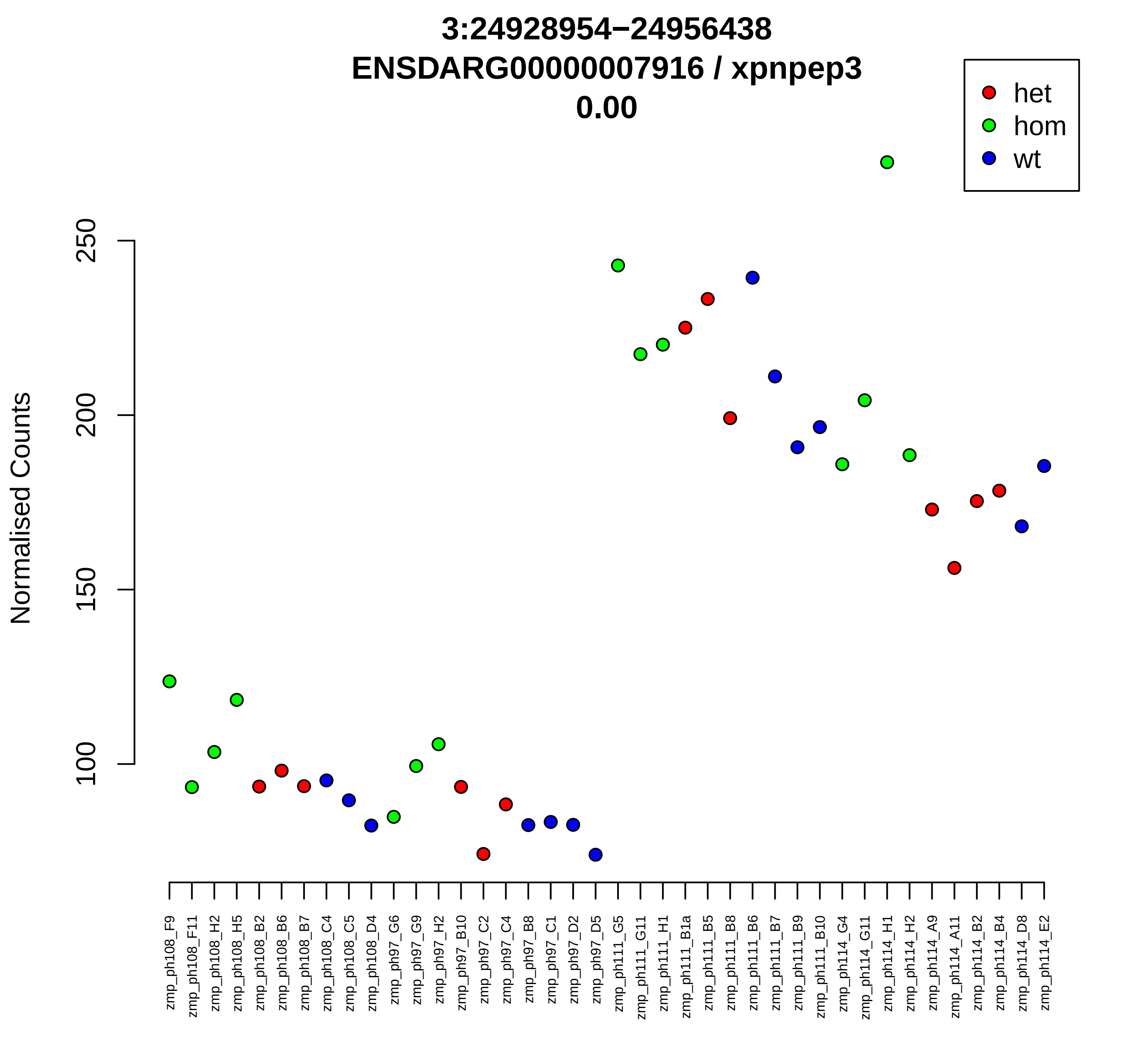

3:24928954−24956438
ENSDARG00000007916 / xpnpep3
0.00
het
hom
wt
Normalised Counts
100
150
200
250
zmp_ph108_F9
zmp_ph108_F11
zmp_ph108_H2
zmp_ph108_H5
zmp_ph108_B2
zmp_ph108_B6
zmp_ph108_B7
zmp_ph108_C4
zmp_ph108_C5
zmp_ph108_D4
zmp_ph97_G6
zmp_ph97_G9
zmp_ph97_H2
zmp_ph97_B10
zmp_ph97_C2
zmp_ph97_C4
zmp_ph97_B8
zmp_ph97_C1
zmp_ph97_D2
zmp_ph97_D5
zmp_ph111_G5
zmp_ph111_G11
zmp_ph111_H1
zmp_ph111_B1a
zmp_ph111_B5
zmp_ph111_B8
zmp_ph111_B6
zmp_ph111_B7
zmp_ph111_B9
zmp_ph111_B10
zmp_ph114_G4
zmp_ph114_G11
zmp_ph114_H1
zmp_ph114_H2
zmp_ph114_A9
zmp_ph114_A11
zmp_ph114_B2
zmp_ph114_B4
zmp_ph114_D8
zmp_ph114_E2

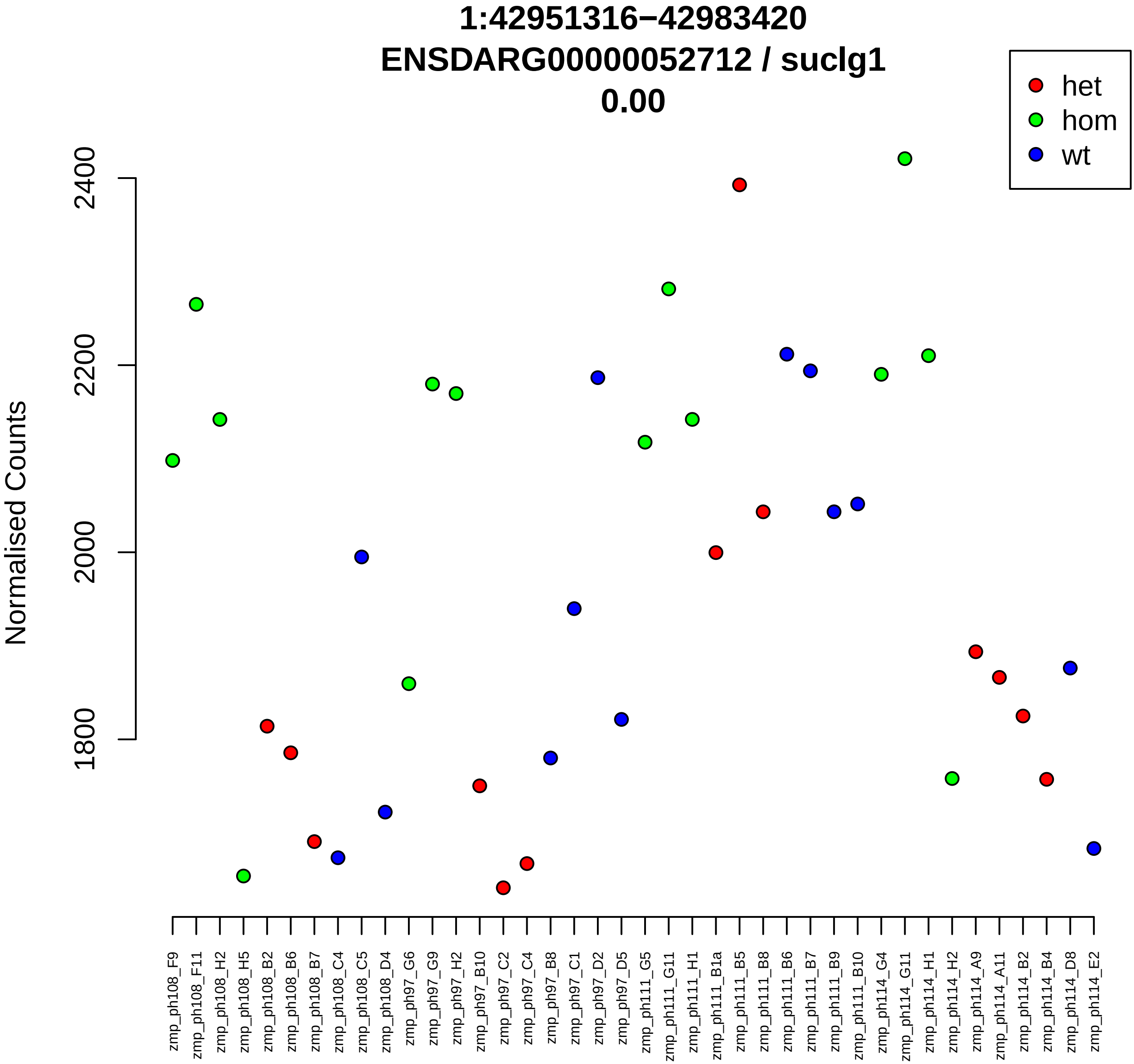
1:42951316−42983420
ENSDARG00000052712 / suclg1
0.00
het
hom
wt
Normalised Counts
2400
2200
2000
1800
zmp_ph108_F9
zmp_ph108_F11
zmp_ph108_H2
zmp_ph108_H5
zmp_ph108_B2
zmp_ph108_B6
zmp_ph108_B7
zmp_ph108_C4
zmp_ph108_C5
zmp_ph108_D4
zmp_ph97_G6
zmp_ph97_G9
zmp_ph97_H2
zmp_ph97_B10
zmp_ph97_C2
zmp_ph97_C4
zmp_ph97_B8
zmp_ph97_C1
zmp_ph97_D2
zmp_ph97_D5
zmp_ph111_G5
zmp_ph111_G11
zmp_ph111_H1
zmp_ph111_B1a
zmp_ph111_B5
zmp_ph111_B8
zmp_ph111_B6
zmp_ph111_B7
zmp_ph111_B9
zmp_ph111_B10
zmp_ph114_G4
zmp_ph114_G11
zmp_ph114_H1
zmp_ph114_H2
zmp_ph114_A9
zmp_ph114_A11
zmp_ph114_B2
zmp_ph114_B4
zmp_ph114_D8
zmp_ph114_E2

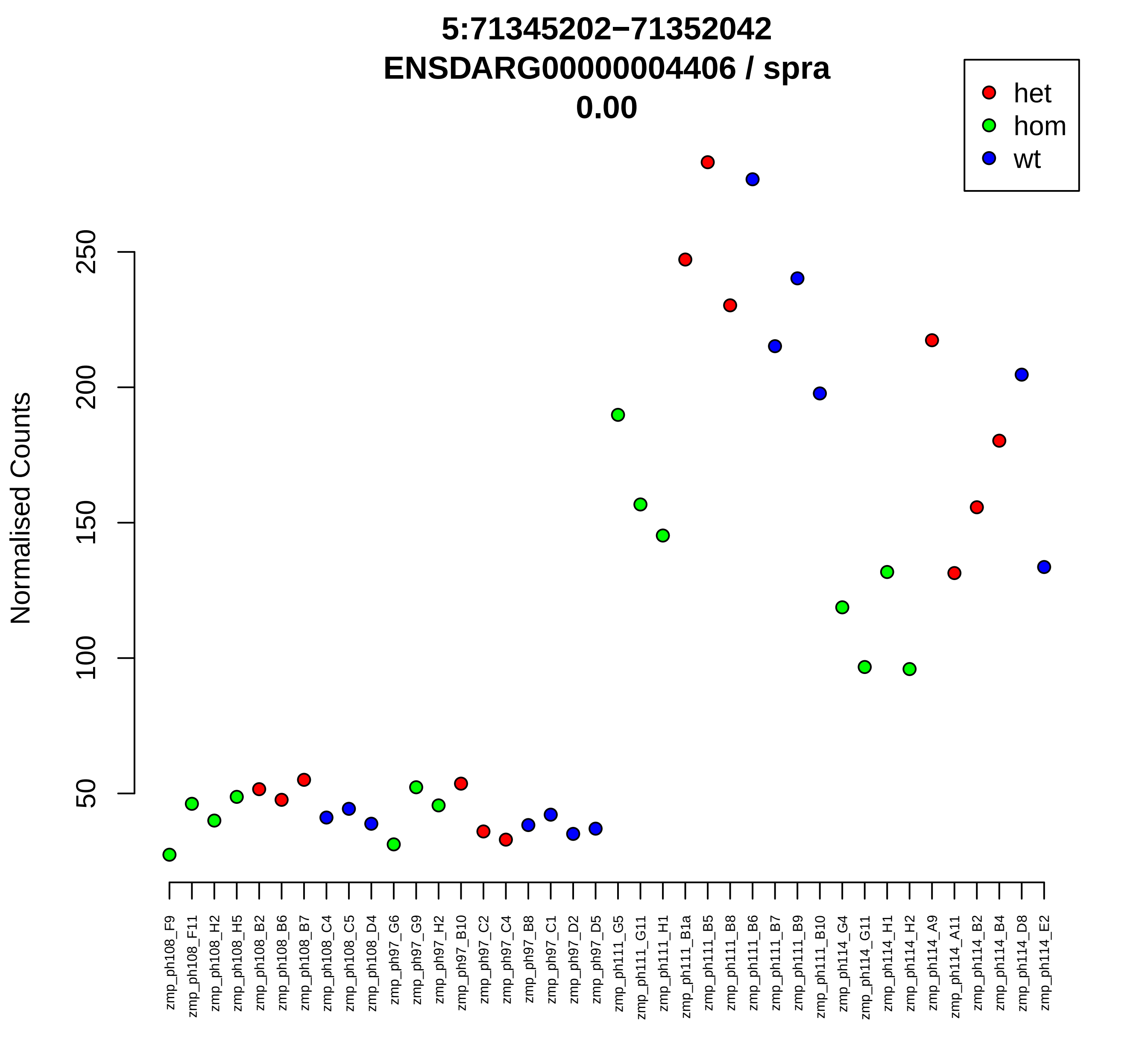

5:71345202−71352042
ENSDARG00000004406 / spra
0.00
het
hom
wt
Normalised Counts
250
200
150
100
50
zmp_ph108_F9
zmp_ph108_F11
zmp_ph108_H2
zmp_ph108_H5
zmp_ph108_B2
zmp_ph108_B6
zmp_ph108_B7
zmp_ph108_C4
zmp_ph108_C5
zmp_ph108_D4
zmp_ph97_G6
zmp_ph97_G9
zmp_ph97_H2
zmp_ph97_B10
zmp_ph97_C2
zmp_ph97_C4
zmp_ph97_B8
zmp_ph97_C1
zmp_ph97_D2
zmp_ph97_D5
zmp_ph111_G5
zmp_ph111_G11
zmp_ph111_H1
zmp_ph111_B1a
zmp_ph111_B5
zmp_ph111_B8
zmp_ph111_B6
zmp_ph111_B7
zmp_ph111_B9
zmp_ph111_B10
zmp_ph114_G4
zmp_ph114_G11
zmp_ph114_H1
zmp_ph114_H2
zmp_ph114_A9
zmp_ph114_A11
zmp_ph114_B2
zmp_ph114_B4
zmp_ph114_D8
zmp_ph114_E2

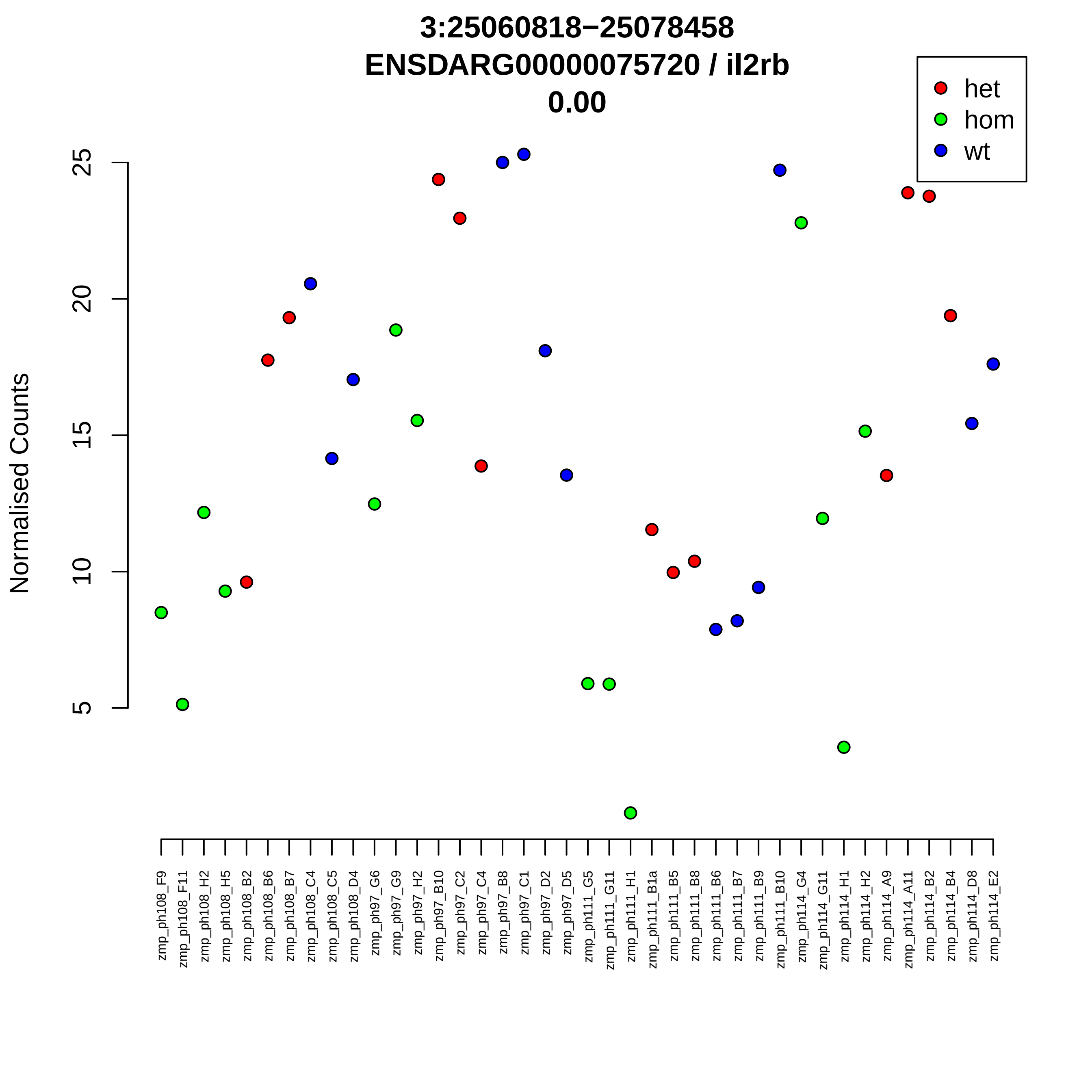
3:25060818−25078458
ENSDARG00000075720 / il2rb
0.00
het
hom
wt
Normalised Counts
5
10
15
20
25
zmp_ph108_F9
zmp_ph108_F11
zmp_ph108_H2
zmp_ph108_H5
zmp_ph108_B2
zmp_ph108_B6
zmp_ph108_B7
zmp_ph108_C4
zmp_ph108_C5
zmp_ph108_D4
zmp_ph97_G6
zmp_ph97_G9
zmp_ph97_H2
zmp_ph97_B10
zmp_ph97_C2
zmp_ph97_C4
zmp_ph97_B8
zmp_ph97_C1
zmp_ph97_D2
zmp_ph97_D5
zmp_ph111_G5
zmp_ph111_G11
zmp_ph111_H1
zmp_ph111_B1a
zmp_ph111_B5
zmp_ph111_B8
zmp_ph111_B6
zmp_ph111_B7
zmp_ph111_B9
zmp_ph111_B10
zmp_ph114_G4
zmp_ph114_G11
zmp_ph114_H1
zmp_ph114_H2
zmp_ph114_A9
zmp_ph114_A11
zmp_ph114_B2
zmp_ph114_B4
zmp_ph114_D8
zmp_ph114_E2

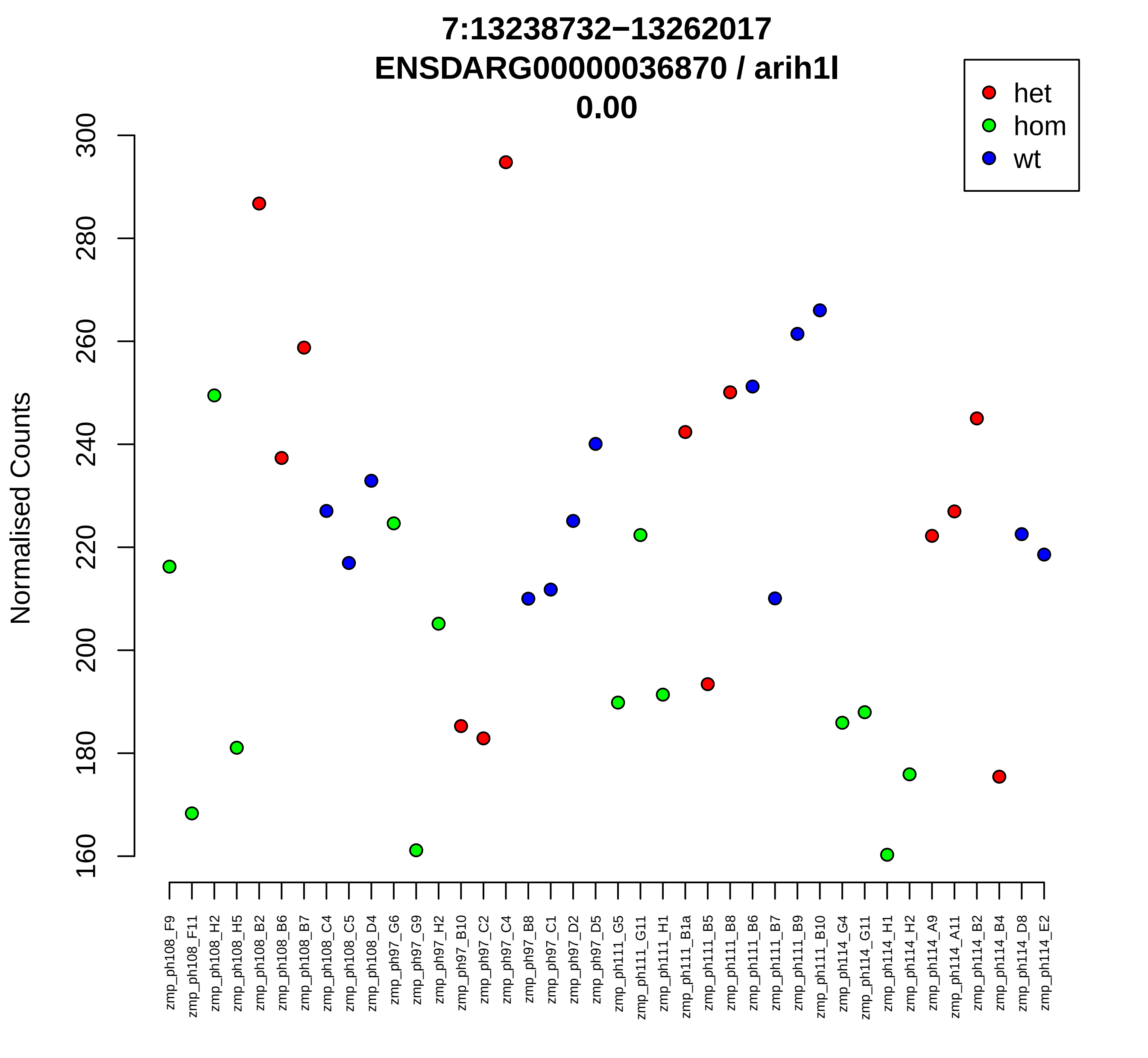

7:13238732−13262017
ENSDARG00000036870 / arih1l
0.00
het
hom
wt
Normalised Counts
160
180
200
220
240
260
280
300
zmp_ph108_F9
zmp_ph108_F11
zmp_ph108_H2
zmp_ph108_H5
zmp_ph108_B2
zmp_ph108_B6
zmp_ph108_B7
zmp_ph108_C4
zmp_ph108_C5
zmp_ph108_D4
zmp_ph97_G6
zmp_ph97_G9
zmp_ph97_H2
zmp_ph97_B10
zmp_ph97_C2
zmp_ph97_C4
zmp_ph97_B8
zmp_ph97_C1
zmp_ph97_D2
zmp_ph97_D5
zmp_ph111_G5
zmp_ph111_G11
zmp_ph111_H1
zmp_ph111_B1a
zmp_ph111_B5
zmp_ph111_B8
zmp_ph111_B6
zmp_ph111_B7
zmp_ph111_B9
zmp_ph111_B10
zmp_ph114_G4
zmp_ph114_G11
zmp_ph114_H1
zmp_ph114_H2
zmp_ph114_A9
zmp_ph114_A11
zmp_ph114_B2
zmp_ph114_B4
zmp_ph114_D8
zmp_ph114_E2

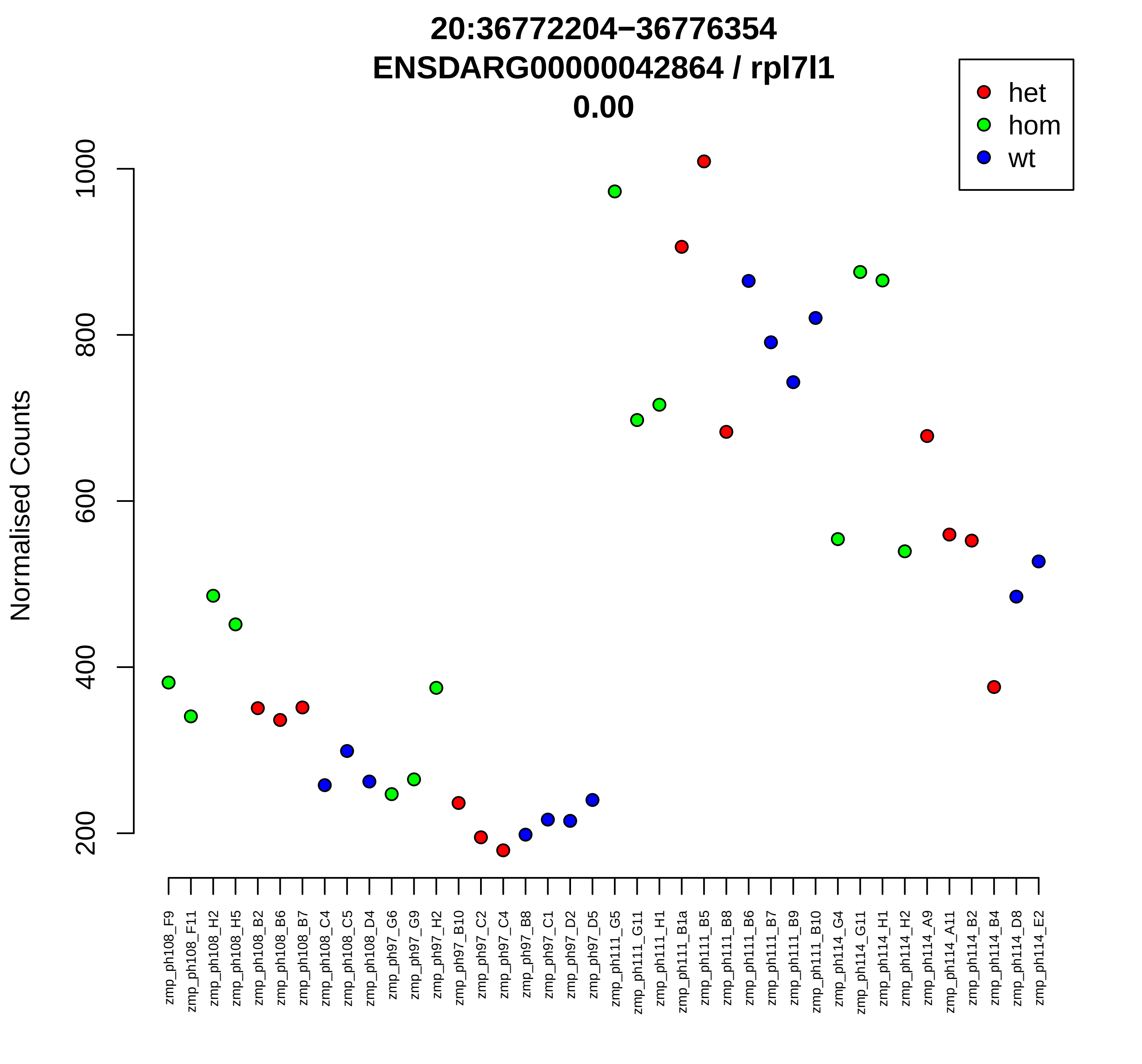
20:36772204−36776354
ENSDARG00000042864 / rpl7l1
0.00
het
hom
wt
Normalised Counts
1000
800
600
400
200
zmp_ph108_F9
zmp_ph108_F11
zmp_ph108_H2
zmp_ph108_H5
zmp_ph108_B2
zmp_ph108_B6
zmp_ph108_B7
zmp_ph108_C4
zmp_ph108_C5
zmp_ph108_D4
zmp_ph97_G6
zmp_ph97_G9
zmp_ph97_H2
zmp_ph97_B10
zmp_ph97_C2
zmp_ph97_C4
zmp_ph97_B8
zmp_ph97_C1
zmp_ph97_D2
zmp_ph97_D5
zmp_ph111_G5
zmp_ph111_G11
zmp_ph111_H1
zmp_ph111_B1a
zmp_ph111_B5
zmp_ph111_B8
zmp_ph111_B6
zmp_ph111_B7
zmp_ph111_B9
zmp_ph111_B10
zmp_ph114_G4
zmp_ph114_G11
zmp_ph114_H1
zmp_ph114_H2
zmp_ph114_A9
zmp_ph114_A11
zmp_ph114_B2
zmp_ph114_B4
zmp_ph114_D8
zmp_ph114_E2

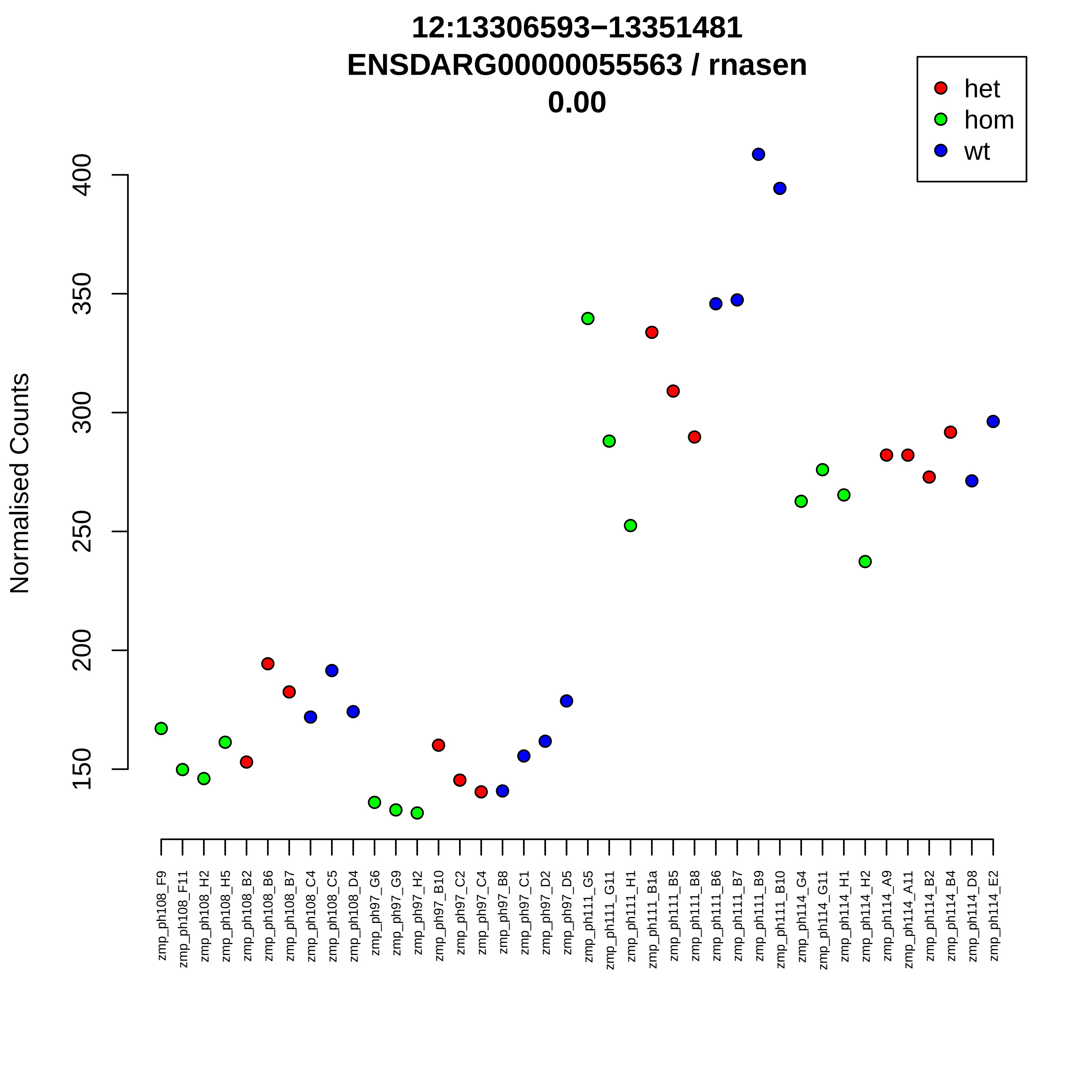
12:13306593−13351481
ENSDARG00000055563 / rnasen
0.00
het
hom
wt
Normalised Counts
150
200
250
300
350
400
zmp_ph108_F9
zmp_ph108_F11
zmp_ph108_H2
zmp_ph108_H5
zmp_ph108_B2
zmp_ph108_B6
zmp_ph108_B7
zmp_ph108_C4
zmp_ph108_C5
zmp_ph108_D4
zmp_ph97_G6
zmp_ph97_G9
zmp_ph97_H2
zmp_ph97_B10
zmp_ph97_C2
zmp_ph97_C4
zmp_ph97_B8
zmp_ph97_C1
zmp_ph97_D2
zmp_ph97_D5
zmp_ph111_G5
zmp_ph111_G11
zmp_ph111_H1
zmp_ph111_B1a
zmp_ph111_B5
zmp_ph111_B8
zmp_ph111_B6
zmp_ph111_B7
zmp_ph111_B9
zmp_ph111_B10
zmp_ph114_G4
zmp_ph114_G11
zmp_ph114_H1
zmp_ph114_H2
zmp_ph114_A9
zmp_ph114_A11
zmp_ph114_B2
zmp_ph114_B4
zmp_ph114_D8
zmp_ph114_E2

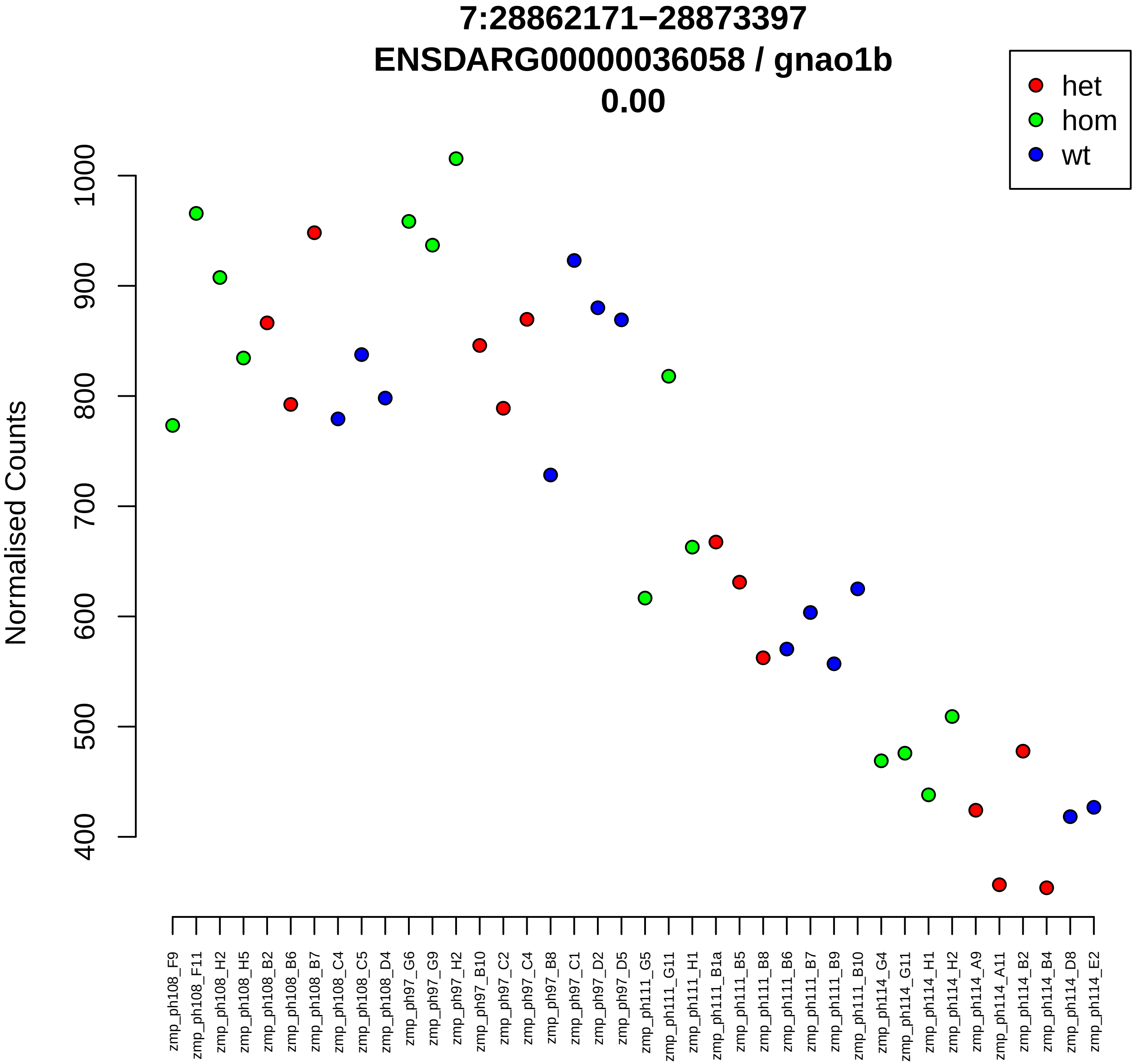

7:28862171−28873397
ENSDARG00000036058 / gnao1b
0.00
het
hom
wt
Normalised Counts
400
500
600
700
800
900
1000
zmp_ph108_F9
zmp_ph108_F11
zmp_ph108_H2
zmp_ph108_H5
zmp_ph108_B2
zmp_ph108_B6
zmp_ph108_B7
zmp_ph108_C4
zmp_ph108_C5
zmp_ph108_D4
zmp_ph97_G6
zmp_ph97_G9
zmp_ph97_H2
zmp_ph97_B10
zmp_ph97_C2
zmp_ph97_C4
zmp_ph97_B8
zmp_ph97_C1
zmp_ph97_D2
zmp_ph97_D5
zmp_ph111_G5
zmp_ph111_G11
zmp_ph111_H1
zmp_ph111_B1a
zmp_ph111_B5
zmp_ph111_B8
zmp_ph111_B6
zmp_ph111_B7
zmp_ph111_B9
zmp_ph111_B10
zmp_ph114_G4
zmp_ph114_G11
zmp_ph114_H1
zmp_ph114_H2
zmp_ph114_A9
zmp_ph114_A11
zmp_ph114_B2
zmp_ph114_B4
zmp_ph114_D8
zmp_ph114_E2

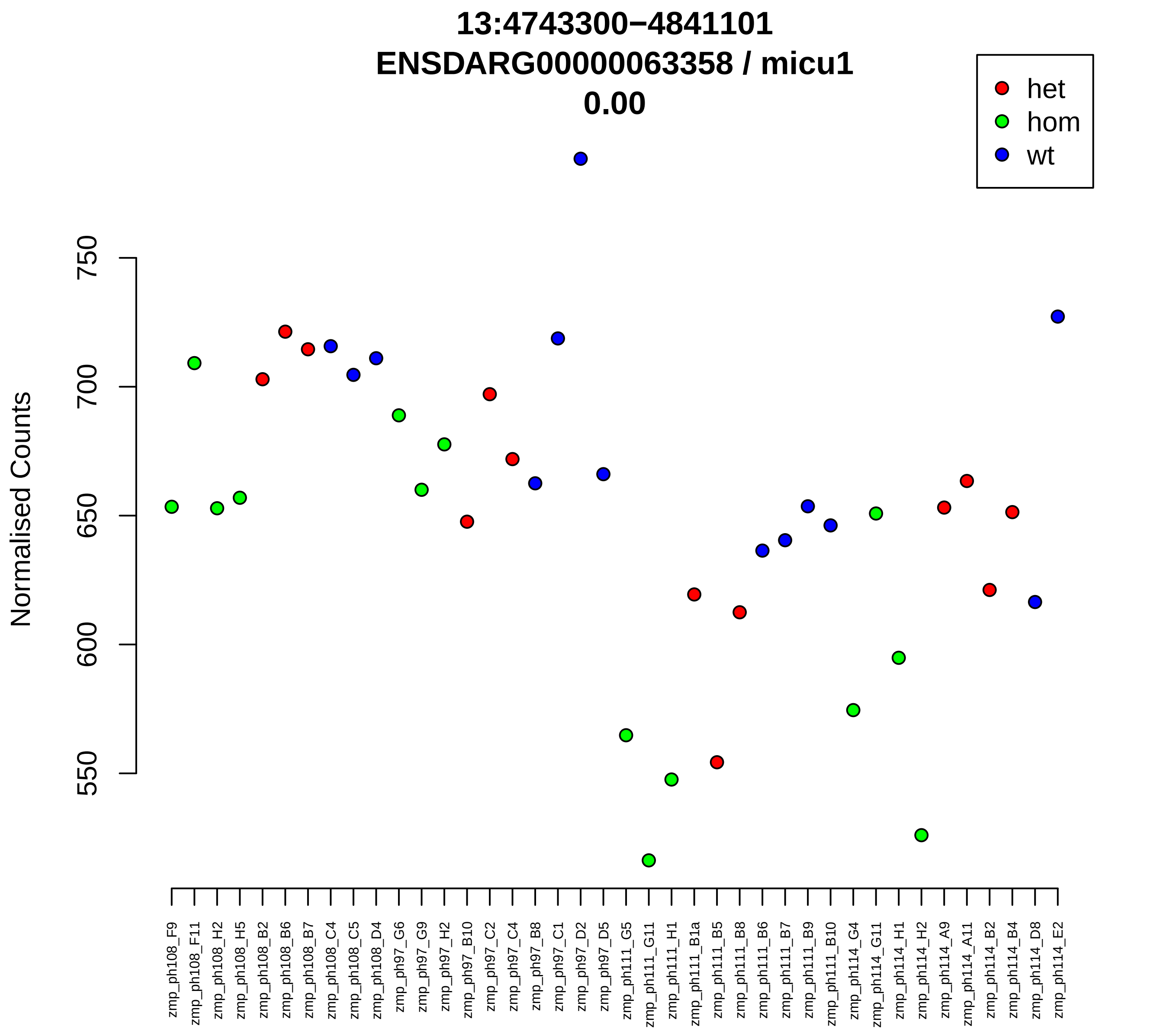
13:4743300−4841101
ENSDARG00000063358 / micu1
0.00
het
hom
wt
Normalised Counts
550
600
650
700
750
zmp_ph108_F9
zmp_ph108_F11
zmp_ph108_H2
zmp_ph108_H5
zmp_ph108_B2
zmp_ph108_B6
zmp_ph108_B7
zmp_ph108_C4
zmp_ph108_C5
zmp_ph108_D4
zmp_ph97_G6
zmp_ph97_G9
zmp_ph97_H2
zmp_ph97_B10
zmp_ph97_C2
zmp_ph97_C4
zmp_ph97_B8
zmp_ph97_C1
zmp_ph97_D2
zmp_ph97_D5
zmp_ph111_G5
zmp_ph111_G11
zmp_ph111_H1
zmp_ph111_B1a
zmp_ph111_B5
zmp_ph111_B8
zmp_ph111_B6
zmp_ph111_B7
zmp_ph111_B9
zmp_ph111_B10
zmp_ph114_G4
zmp_ph114_G11
zmp_ph114_H1
zmp_ph114_H2
zmp_ph114_A9
zmp_ph114_A11
zmp_ph114_B2
zmp_ph114_B4
zmp_ph114_D8
zmp_ph114_E2

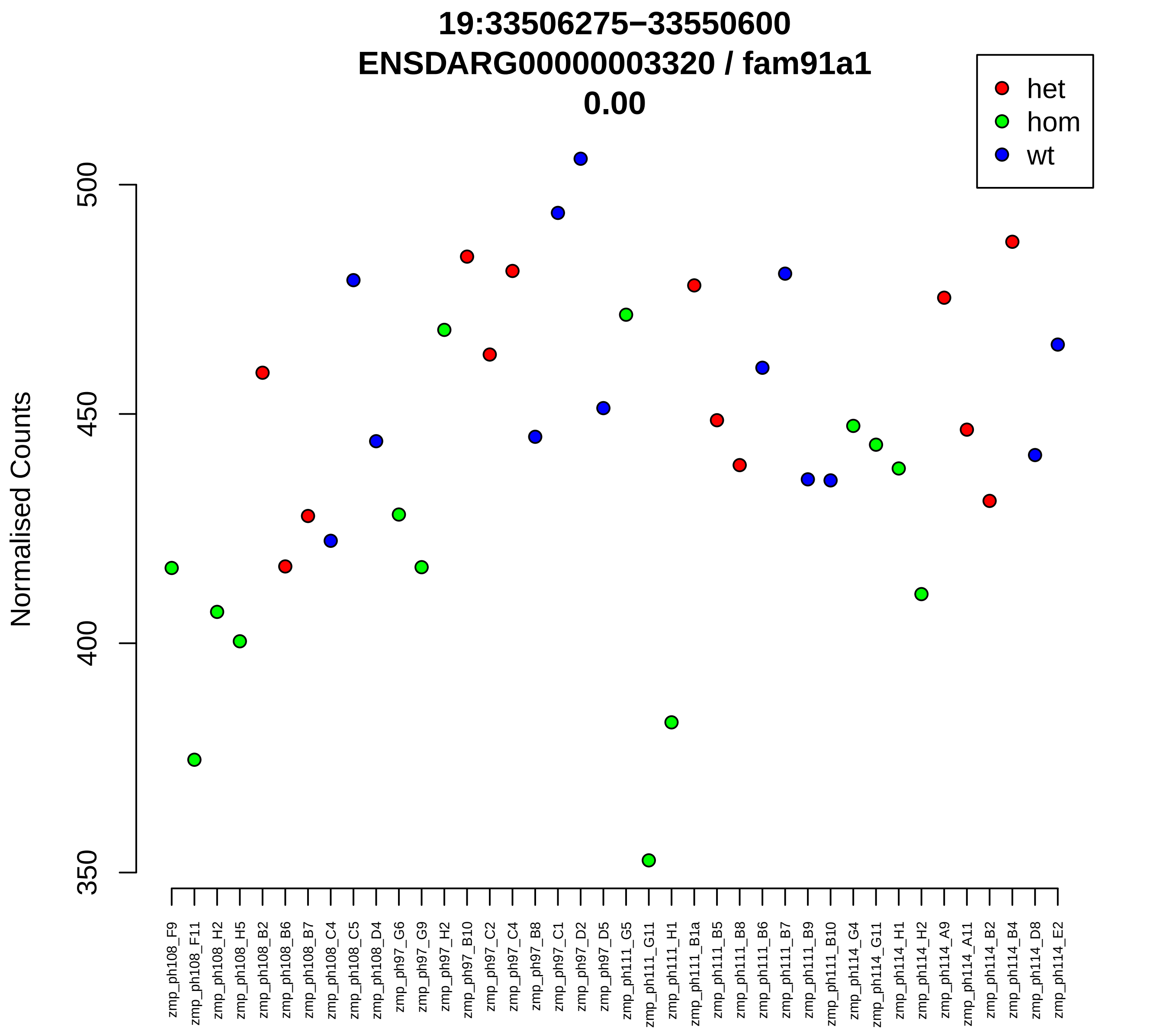

19:33506275−33550600
ENSDARG00000003320 / fam91a1
0.00
het
hom
wt
Normalised Counts
350
400
450
500
zmp_ph108_F9
zmp_ph108_F11
zmp_ph108_H2
zmp_ph108_H5
zmp_ph108_B2
zmp_ph108_B6
zmp_ph108_B7
zmp_ph108_C4
zmp_ph108_C5
zmp_ph108_D4
zmp_ph97_G6
zmp_ph97_G9
zmp_ph97_H2
zmp_ph97_B10
zmp_ph97_C2
zmp_ph97_C4
zmp_ph97_B8
zmp_ph97_C1
zmp_ph97_D2
zmp_ph97_D5
zmp_ph111_G5
zmp_ph111_G11
zmp_ph111_H1
zmp_ph111_B1a
zmp_ph111_B5
zmp_ph111_B8
zmp_ph111_B6
zmp_ph111_B7
zmp_ph111_B9
zmp_ph111_B10
zmp_ph114_G4
zmp_ph114_G11
zmp_ph114_H1
zmp_ph114_H2
zmp_ph114_A9
zmp_ph114_A11
zmp_ph114_B2
zmp_ph114_B4
zmp_ph114_D8
zmp_ph114_E2

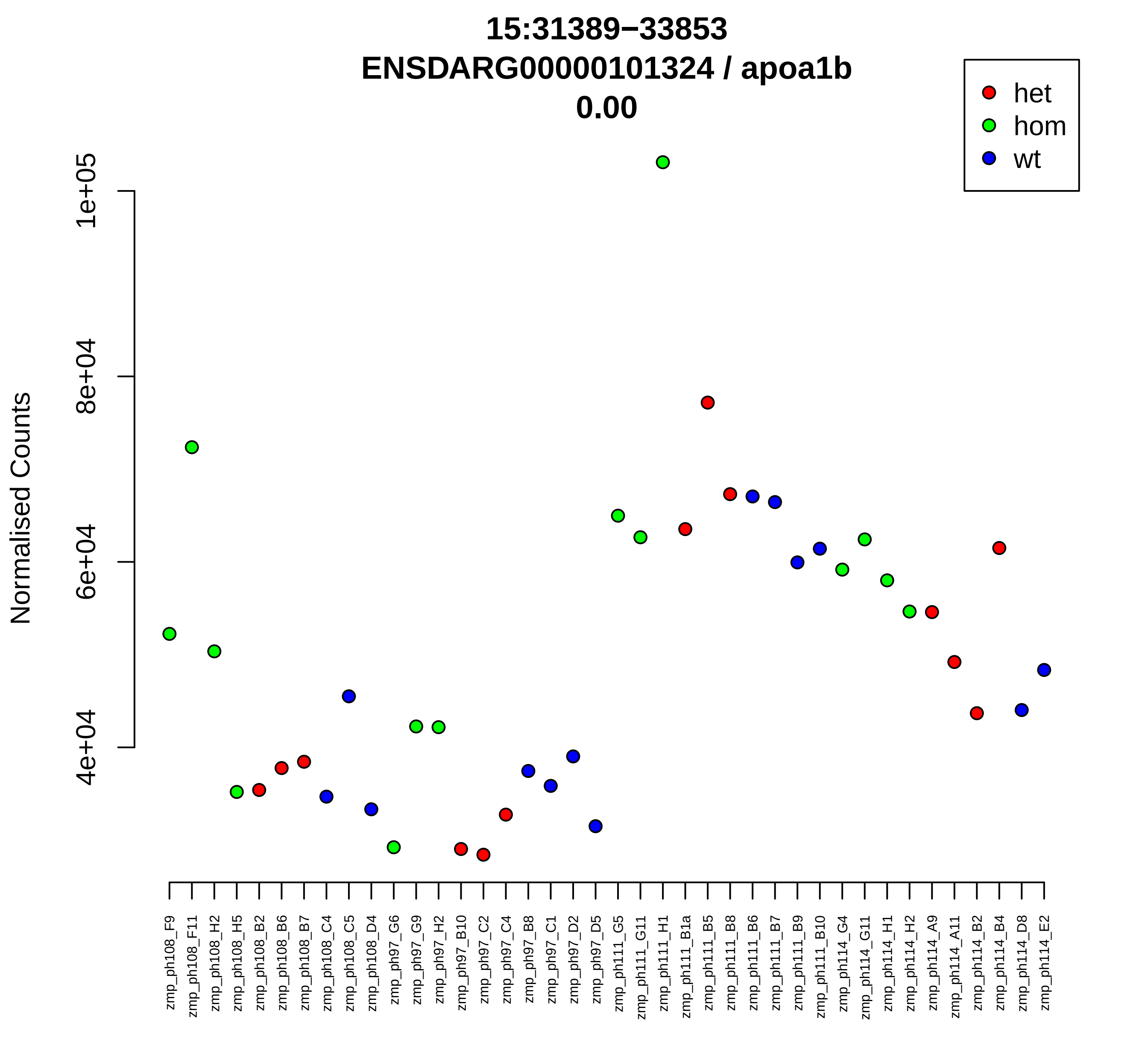
15:31389−33853
ENSDARG00000101324 / apoa1b
0.00
het
hom
wt
Normalised Counts
1e+05
8e+04
6e+04
4e+04
zmp_ph108_F9
zmp_ph108_F11
zmp_ph108_H2
zmp_ph108_H5
zmp_ph108_B2
zmp_ph108_B6
zmp_ph108_B7
zmp_ph108_C4
zmp_ph108_C5
zmp_ph108_D4
zmp_ph97_G6
zmp_ph97_G9
zmp_ph97_H2
zmp_ph97_B10
zmp_ph97_C2
zmp_ph97_C4
zmp_ph97_B8
zmp_ph97_C1
zmp_ph97_D2
zmp_ph97_D5
zmp_ph111_G5
zmp_ph111_G11
zmp_ph111_H1
zmp_ph111_B1a
zmp_ph111_B5
zmp_ph111_B8
zmp_ph111_B6
zmp_ph111_B7
zmp_ph111_B9
zmp_ph111_B10
zmp_ph114_G4
zmp_ph114_G11
zmp_ph114_H1
zmp_ph114_H2
zmp_ph114_A9
zmp_ph114_A11
zmp_ph114_B2
zmp_ph114_B4
zmp_ph114_D8
zmp_ph114_E2

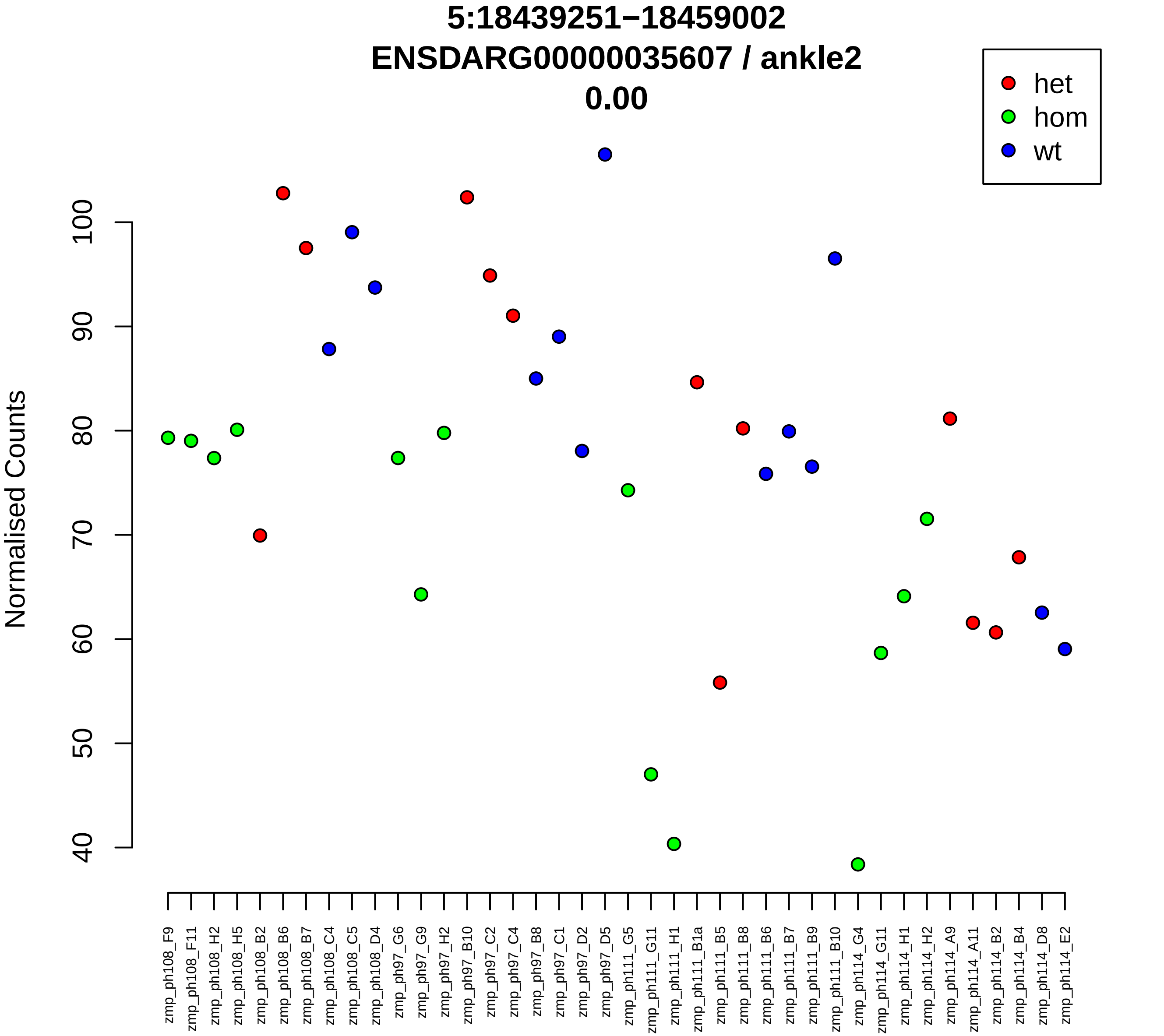

5:18439251−18459002
ENSDARG00000035607 / ankle2
0.00
het
hom
wt
Normalised Counts
40
50
60
70
80
90
100
zmp_ph108_F9
zmp_ph108_F11
zmp_ph108_H2
zmp_ph108_H5
zmp_ph108_B2
zmp_ph108_B6
zmp_ph108_B7
zmp_ph108_C4
zmp_ph108_C5
zmp_ph108_D4
zmp_ph97_G6
zmp_ph97_G9
zmp_ph97_H2
zmp_ph97_B10
zmp_ph97_C2
zmp_ph97_C4
zmp_ph97_B8
zmp_ph97_C1
zmp_ph97_D2
zmp_ph97_D5
zmp_ph111_G5
zmp_ph111_G11
zmp_ph111_H1
zmp_ph111_B1a
zmp_ph111_B5
zmp_ph111_B8
zmp_ph111_B6
zmp_ph111_B7
zmp_ph111_B9
zmp_ph111_B10
zmp_ph114_G4
zmp_ph114_G11
zmp_ph114_H1
zmp_ph114_H2
zmp_ph114_A9
zmp_ph114_A11
zmp_ph114_B2
zmp_ph114_B4
zmp_ph114_D8
zmp_ph114_E2

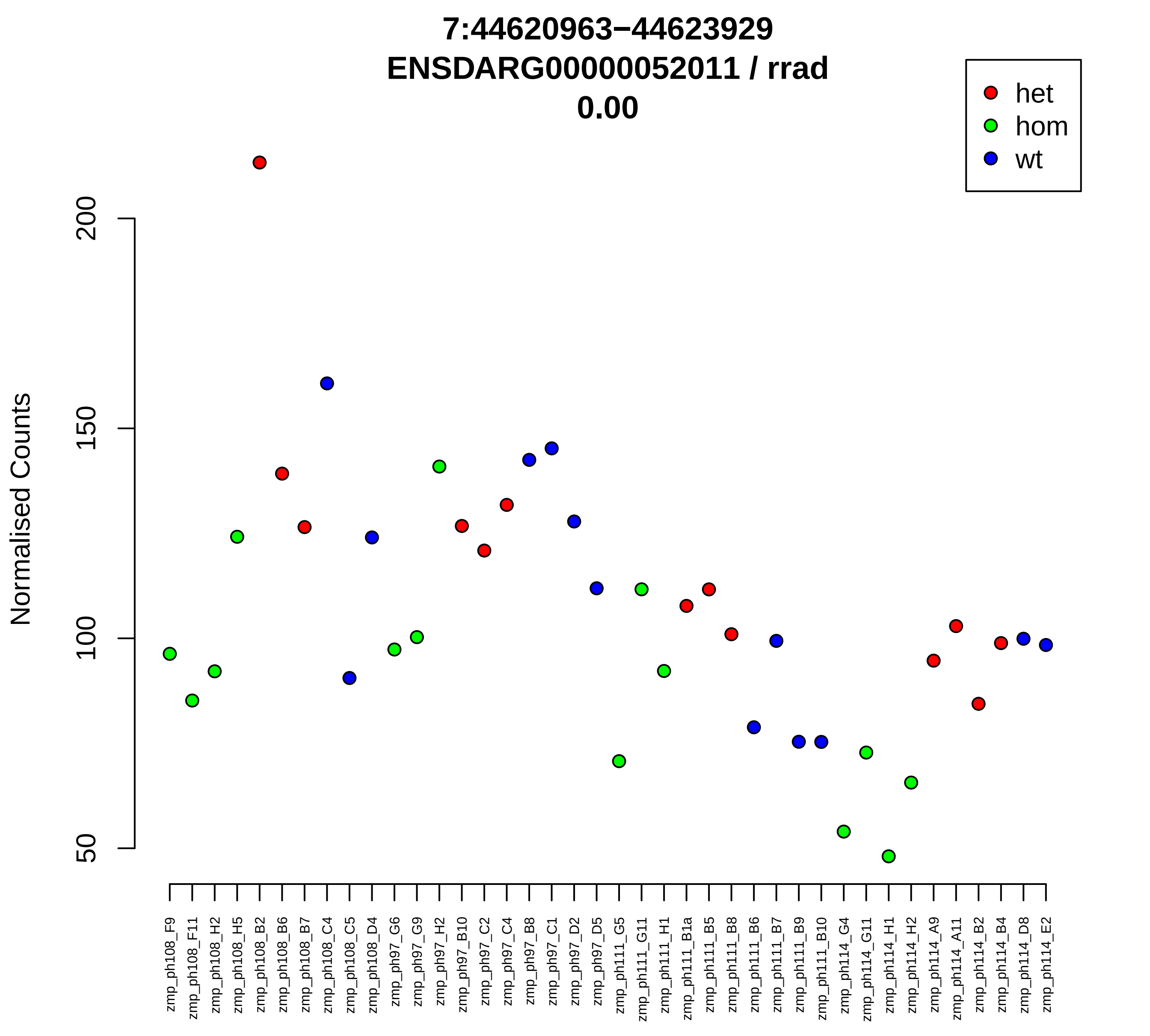

7:44620963−44623929
ENSDARG00000052011 / rrad
0.00
het
hom
wt
Normalised Counts
50
100
150
200
zmp_ph108_F9
zmp_ph108_F11
zmp_ph108_H2
zmp_ph108_H5
zmp_ph108_B2
zmp_ph108_B6
zmp_ph108_B7
zmp_ph108_C4
zmp_ph108_C5
zmp_ph108_D4
zmp_ph97_G6
zmp_ph97_G9
zmp_ph97_H2
zmp_ph97_B10
zmp_ph97_C2
zmp_ph97_C4
zmp_ph97_B8
zmp_ph97_C1
zmp_ph97_D2
zmp_ph97_D5
zmp_ph111_G5
zmp_ph111_G11
zmp_ph111_H1
zmp_ph111_B1a
zmp_ph111_B5
zmp_ph111_B8
zmp_ph111_B6
zmp_ph111_B7
zmp_ph111_B9
zmp_ph111_B10
zmp_ph114_G4
zmp_ph114_G11
zmp_ph114_H1
zmp_ph114_H2
zmp_ph114_A9
zmp_ph114_A11
zmp_ph114_B2
zmp_ph114_B4
zmp_ph114_D8
zmp_ph114_E2

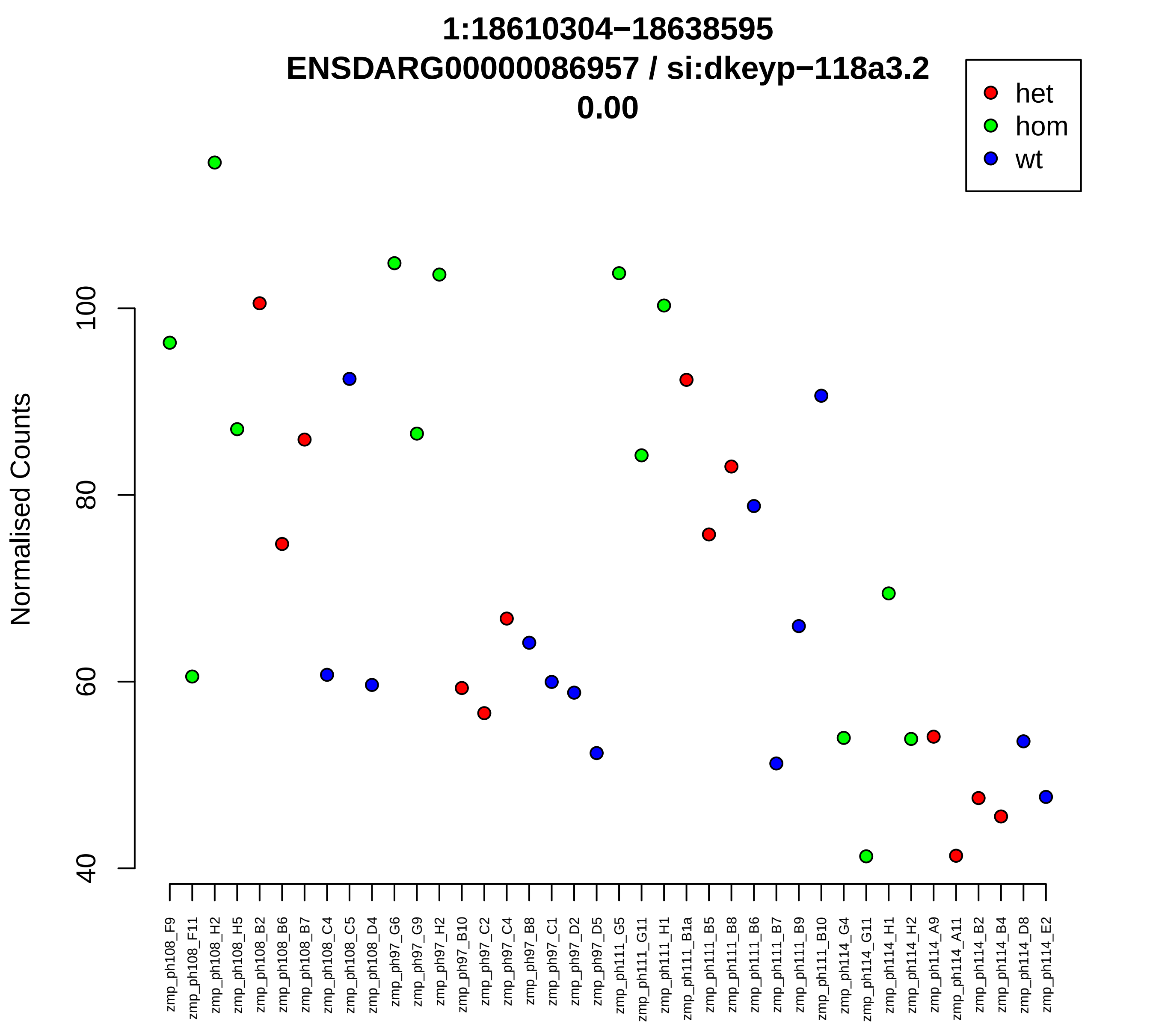
1:18610304−18638595
ENSDARG00000086957 / si:dkeyp−118a3.2
0.00
het
hom
wt
Normalised Counts
40
60
80
100
zmp_ph108_F9
zmp_ph108_F11
zmp_ph108_H2
zmp_ph108_H5
zmp_ph108_B2
zmp_ph108_B6
zmp_ph108_B7
zmp_ph108_C4
zmp_ph108_C5
zmp_ph108_D4
zmp_ph97_G6
zmp_ph97_G9
zmp_ph97_H2
zmp_ph97_B10
zmp_ph97_C2
zmp_ph97_C4
zmp_ph97_B8
zmp_ph97_C1
zmp_ph97_D2
zmp_ph97_D5
zmp_ph111_G5
zmp_ph111_G11
zmp_ph111_H1
zmp_ph111_B1a
zmp_ph111_B5
zmp_ph111_B8
zmp_ph111_B6
zmp_ph111_B7
zmp_ph111_B9
zmp_ph111_B10
zmp_ph114_G4
zmp_ph114_G11
zmp_ph114_H1
zmp_ph114_H2
zmp_ph114_A9
zmp_ph114_A11
zmp_ph114_B2
zmp_ph114_B4
zmp_ph114_D8
zmp_ph114_E2

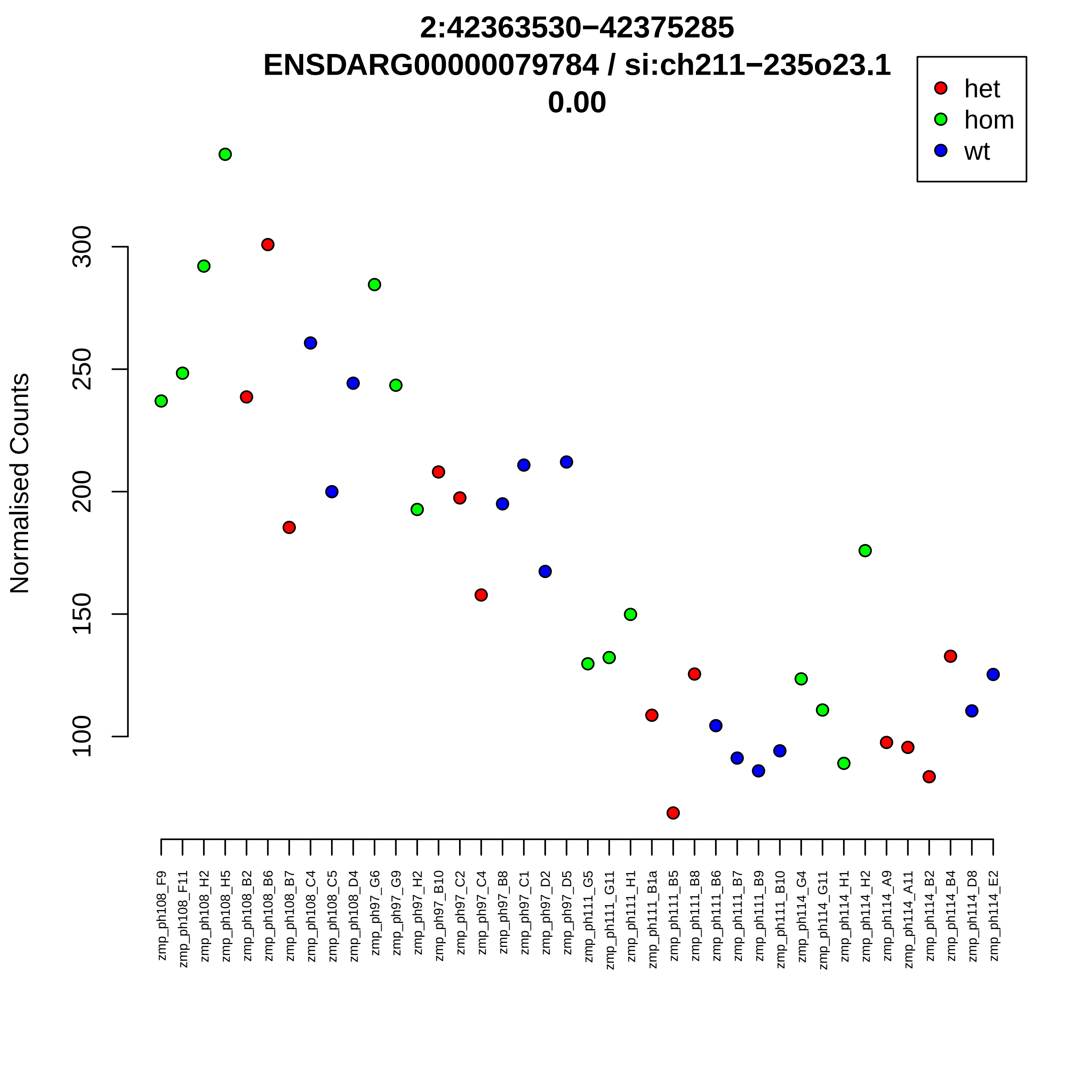

2:42363530−42375285
ENSDARG00000079784 / si:ch211−235o23.1
0.00
het
hom
wt
Normalised Counts
100
150
200
250
300
zmp_ph108_F9
zmp_ph108_F11
zmp_ph108_H2
zmp_ph108_H5
zmp_ph108_B2
zmp_ph108_B6
zmp_ph108_B7
zmp_ph108_C4
zmp_ph108_C5
zmp_ph108_D4
zmp_ph97_G6
zmp_ph97_G9
zmp_ph97_H2
zmp_ph97_B10
zmp_ph97_C2
zmp_ph97_C4
zmp_ph97_B8
zmp_ph97_C1
zmp_ph97_D2
zmp_ph97_D5
zmp_ph111_G5
zmp_ph111_G11
zmp_ph111_H1
zmp_ph111_B1a
zmp_ph111_B5
zmp_ph111_B8
zmp_ph111_B6
zmp_ph111_B7
zmp_ph111_B9
zmp_ph111_B10
zmp_ph114_G4
zmp_ph114_G11
zmp_ph114_H1
zmp_ph114_H2
zmp_ph114_A9
zmp_ph114_A11
zmp_ph114_B2
zmp_ph114_B4
zmp_ph114_D8
zmp_ph114_E2

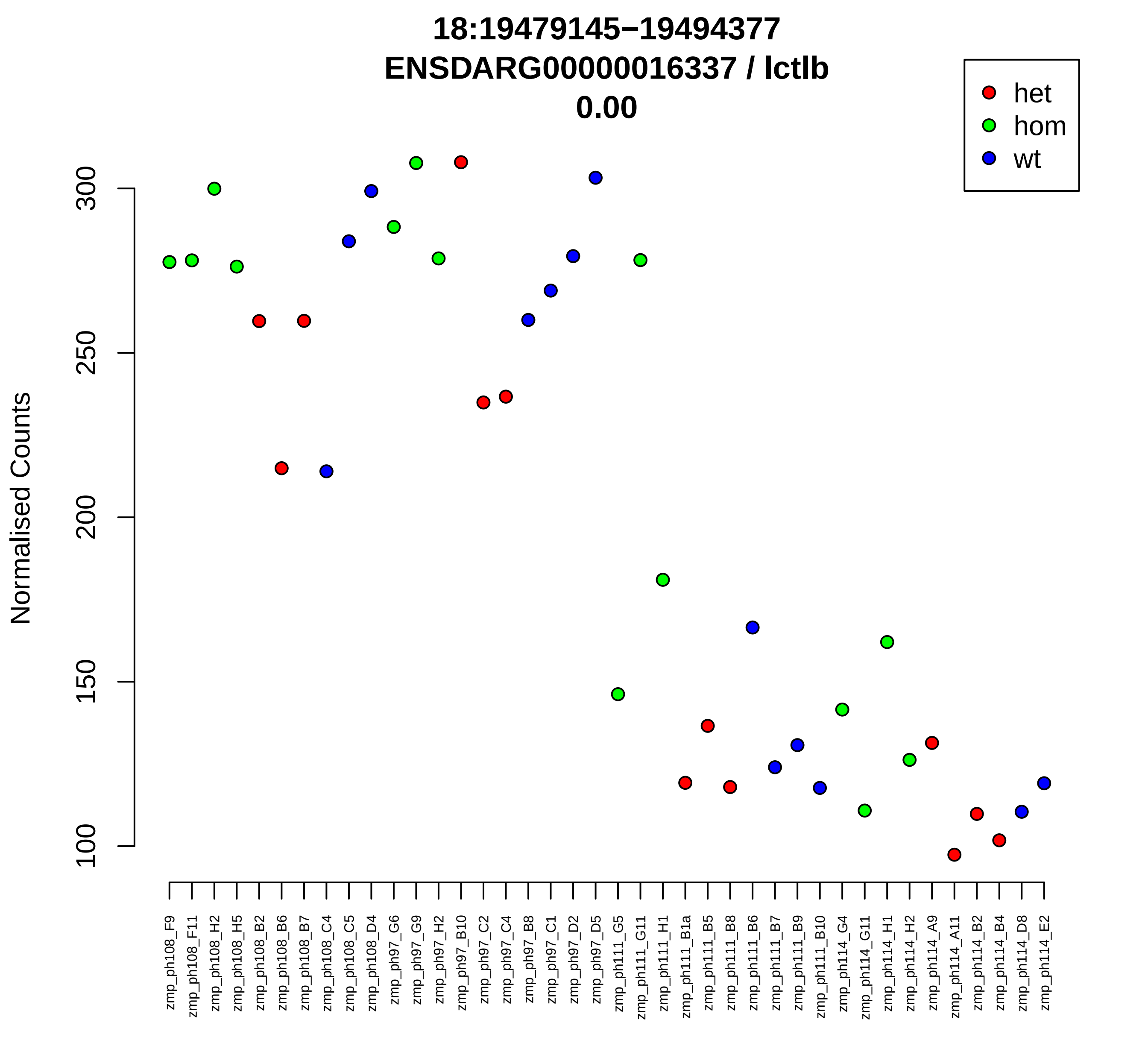
18:19479145−19494377
ENSDARG00000016337 / lctlb
0.00
het
hom
wt
Normalised Counts
100
150
200
250
300
zmp_ph108_F9
zmp_ph108_F11
zmp_ph108_H2
zmp_ph108_H5
zmp_ph108_B2
zmp_ph108_B6
zmp_ph108_B7
zmp_ph108_C4
zmp_ph108_C5
zmp_ph108_D4
zmp_ph97_G6
zmp_ph97_G9
zmp_ph97_H2
zmp_ph97_B10
zmp_ph97_C2
zmp_ph97_C4
zmp_ph97_B8
zmp_ph97_C1
zmp_ph97_D2
zmp_ph97_D5
zmp_ph111_G5
zmp_ph111_G11
zmp_ph111_H1
zmp_ph111_B1a
zmp_ph111_B5
zmp_ph111_B8
zmp_ph111_B6
zmp_ph111_B7
zmp_ph111_B9
zmp_ph111_B10
zmp_ph114_G4
zmp_ph114_G11
zmp_ph114_H1
zmp_ph114_H2
zmp_ph114_A9
zmp_ph114_A11
zmp_ph114_B2
zmp_ph114_B4
zmp_ph114_D8
zmp_ph114_E2

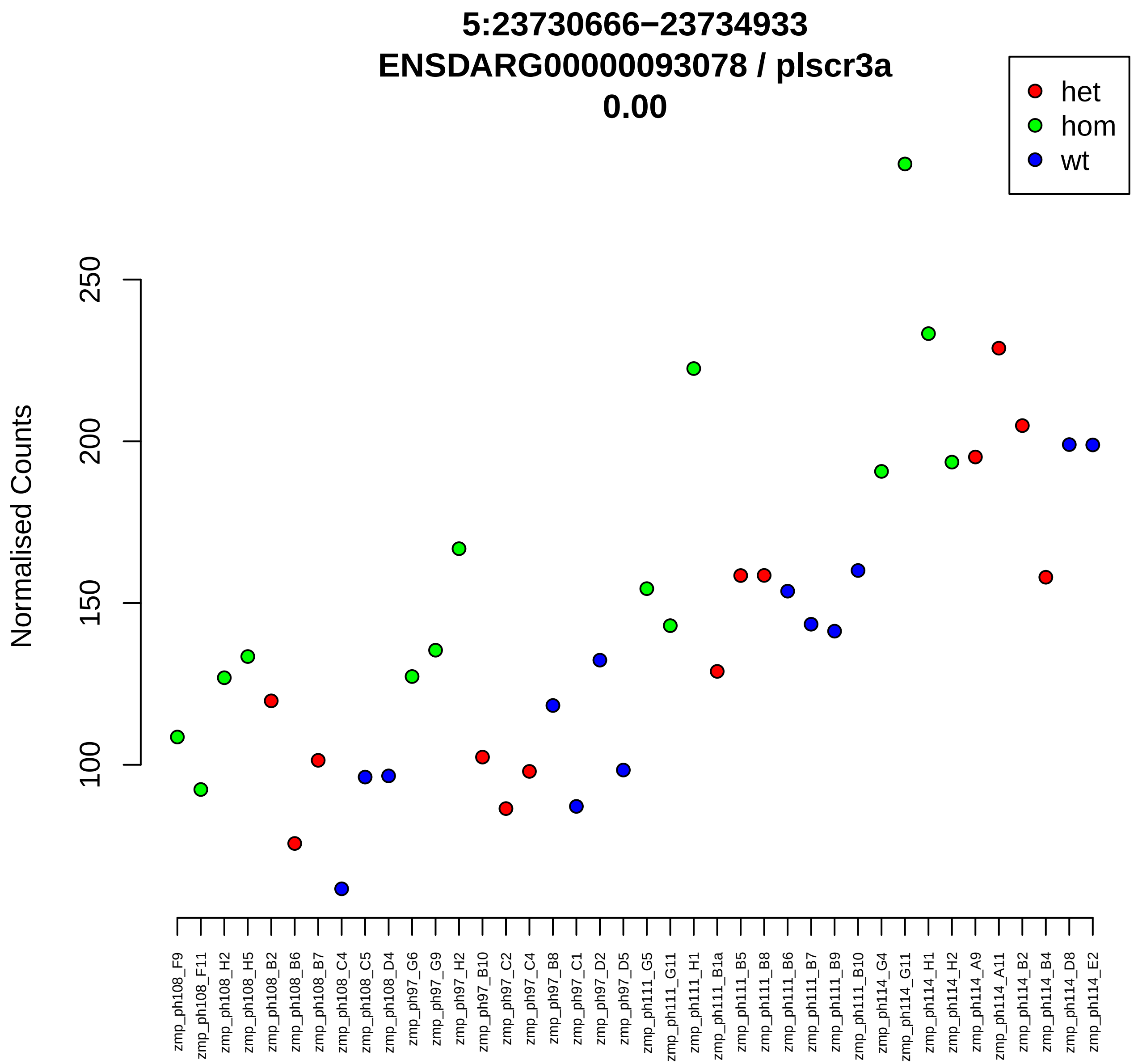

5:23730666−23734933
ENSDARG00000093078 / plscr3a
0.00
het
hom
wt
Normalised Counts
100
150
200
250
zmp_ph108_F9
zmp_ph108_F11
zmp_ph108_H2
zmp_ph108_H5
zmp_ph108_B2
zmp_ph108_B6
zmp_ph108_B7
zmp_ph108_C4
zmp_ph108_C5
zmp_ph108_D4
zmp_ph97_G6
zmp_ph97_G9
zmp_ph97_H2
zmp_ph97_B10
zmp_ph97_C2
zmp_ph97_C4
zmp_ph97_B8
zmp_ph97_C1
zmp_ph97_D2
zmp_ph97_D5
zmp_ph111_G5
zmp_ph111_G11
zmp_ph111_H1
zmp_ph111_B1a
zmp_ph111_B5
zmp_ph111_B8
zmp_ph111_B6
zmp_ph111_B7
zmp_ph111_B9
zmp_ph111_B10
zmp_ph114_G4
zmp_ph114_G11
zmp_ph114_H1
zmp_ph114_H2
zmp_ph114_A9
zmp_ph114_A11
zmp_ph114_B2
zmp_ph114_B4
zmp_ph114_D8
zmp_ph114_E2

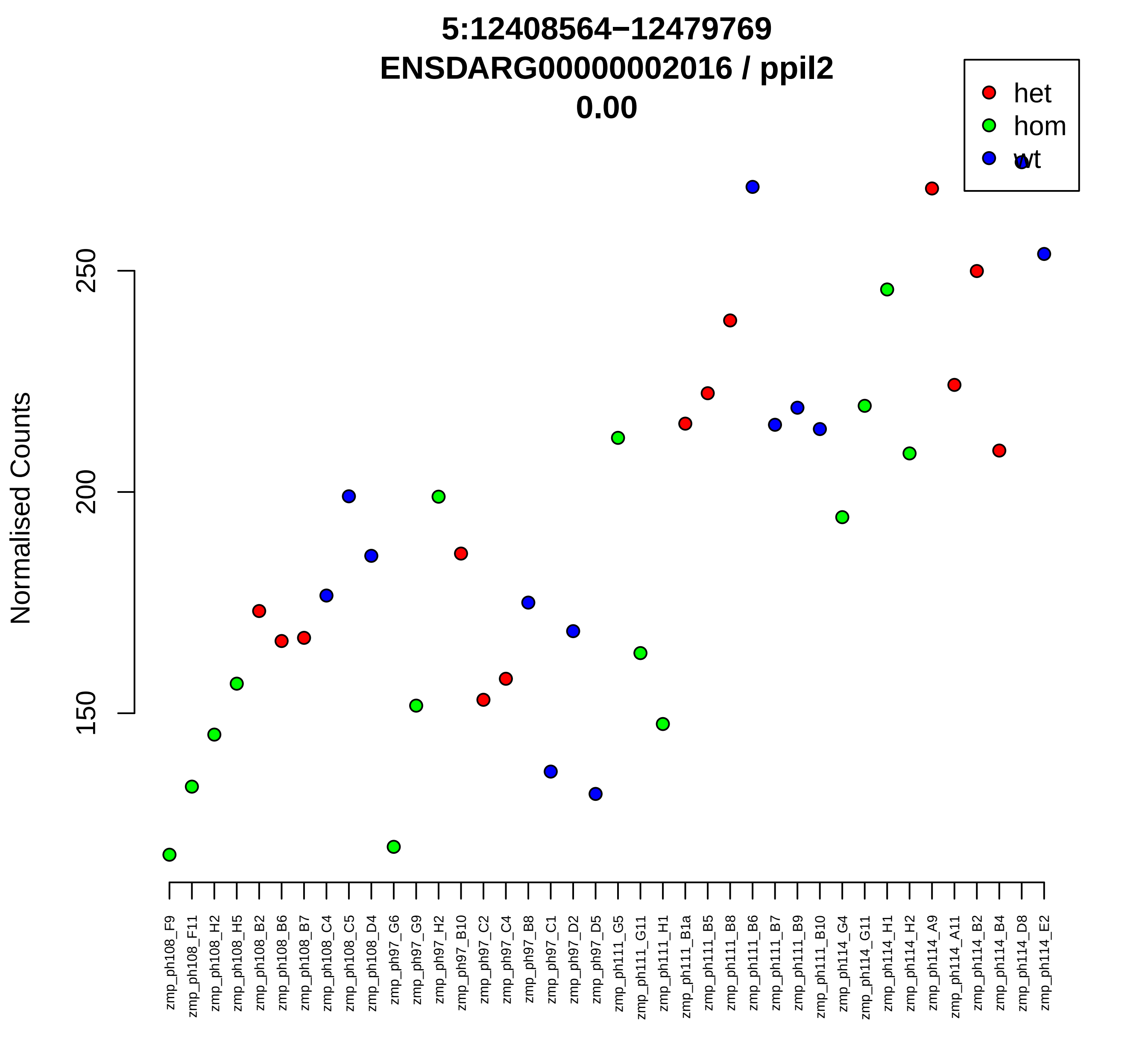

5:12408564−12479769
ENSDARG00000002016 / ppil2
0.00
het
hom
wt
Normalised Counts
150
200
250
zmp_ph108_F9
zmp_ph108_F11
zmp_ph108_H2
zmp_ph108_H5
zmp_ph108_B2
zmp_ph108_B6
zmp_ph108_B7
zmp_ph108_C4
zmp_ph108_C5
zmp_ph108_D4
zmp_ph97_G6
zmp_ph97_G9
zmp_ph97_H2
zmp_ph97_B10
zmp_ph97_C2
zmp_ph97_C4
zmp_ph97_B8
zmp_ph97_C1
zmp_ph97_D2
zmp_ph97_D5
zmp_ph111_G5
zmp_ph111_G11
zmp_ph111_H1
zmp_ph111_B1a
zmp_ph111_B5
zmp_ph111_B8
zmp_ph111_B6
zmp_ph111_B7
zmp_ph111_B9
zmp_ph111_B10
zmp_ph114_G4
zmp_ph114_G11
zmp_ph114_H1
zmp_ph114_H2
zmp_ph114_A9
zmp_ph114_A11
zmp_ph114_B2
zmp_ph114_B4
zmp_ph114_D8
zmp_ph114_E2

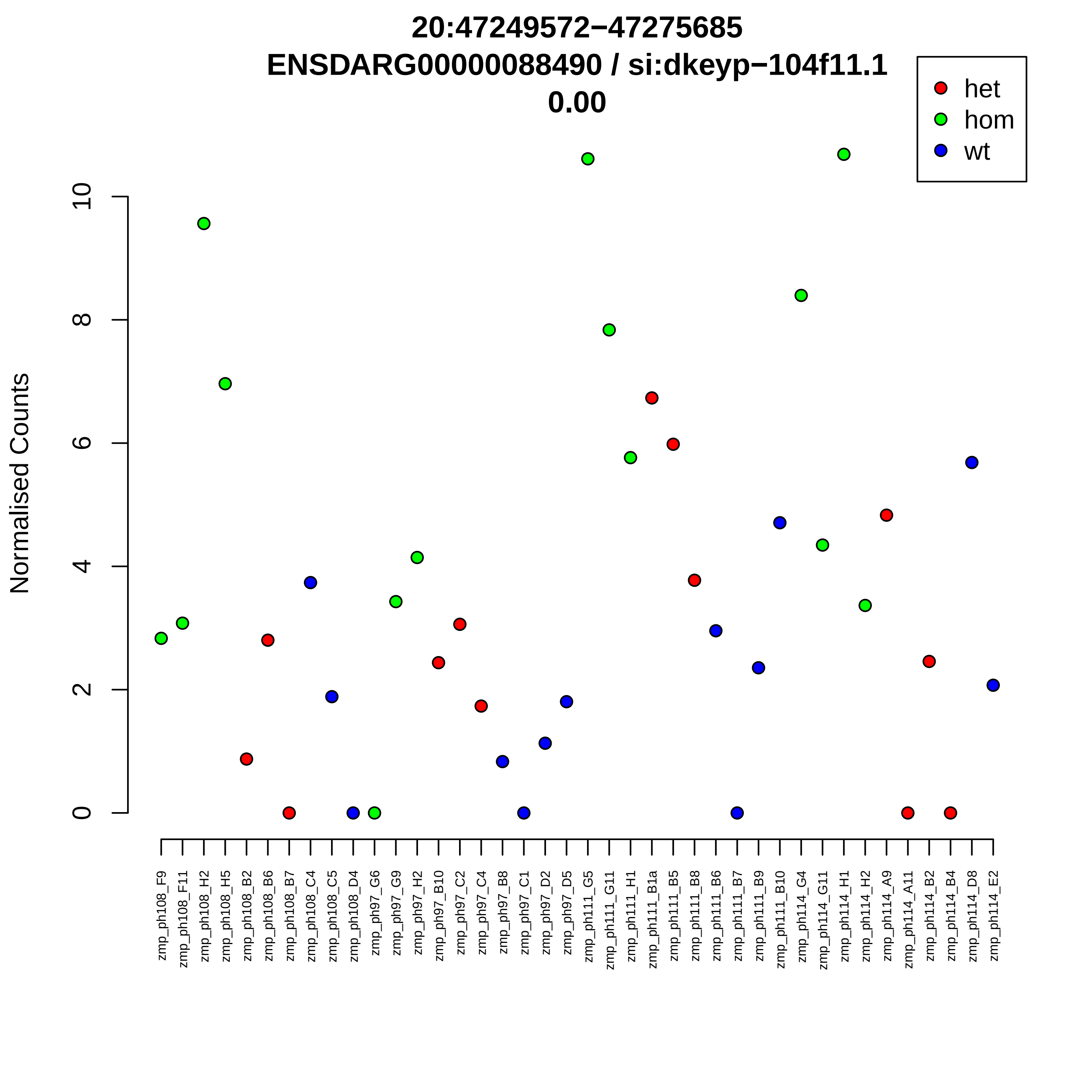

20:47249572−47275685
ENSDARG00000088490 / si:dkeyp−104f11.1
0.00
het
hom
wt
Normalised Counts
0
2
4
6
8
10
zmp_ph108_F9
zmp_ph108_F11
zmp_ph108_H2
zmp_ph108_H5
zmp_ph108_B2
zmp_ph108_B6
zmp_ph108_B7
zmp_ph108_C4
zmp_ph108_C5
zmp_ph108_D4
zmp_ph97_G6
zmp_ph97_G9
zmp_ph97_H2
zmp_ph97_B10
zmp_ph97_C2
zmp_ph97_C4
zmp_ph97_B8
zmp_ph97_C1
zmp_ph97_D2
zmp_ph97_D5
zmp_ph111_G5
zmp_ph111_G11
zmp_ph111_H1
zmp_ph111_B1a
zmp_ph111_B5
zmp_ph111_B8
zmp_ph111_B6
zmp_ph111_B7
zmp_ph111_B9
zmp_ph111_B10
zmp_ph114_G4
zmp_ph114_G11
zmp_ph114_H1
zmp_ph114_H2
zmp_ph114_A9
zmp_ph114_A11
zmp_ph114_B2
zmp_ph114_B4
zmp_ph114_D8
zmp_ph114_E2

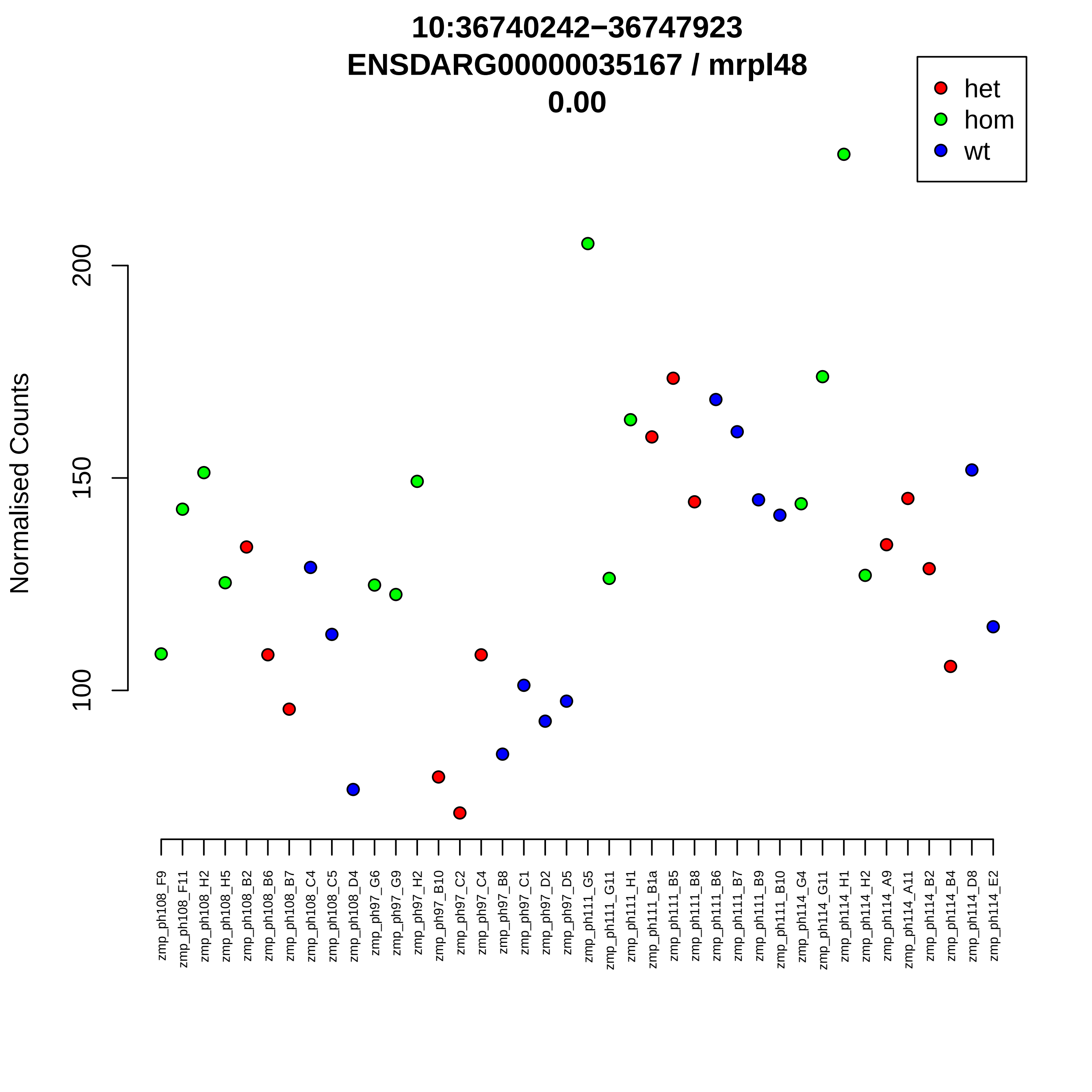
10:36740242−36747923
ENSDARG00000035167 / mrpl48
0.00
het
hom
wt
Normalised Counts
100
150
200
zmp_ph108_F9
zmp_ph108_F11
zmp_ph108_H2
zmp_ph108_H5
zmp_ph108_B2
zmp_ph108_B6
zmp_ph108_B7
zmp_ph108_C4
zmp_ph108_C5
zmp_ph108_D4
zmp_ph97_G6
zmp_ph97_G9
zmp_ph97_H2
zmp_ph97_B10
zmp_ph97_C2
zmp_ph97_C4
zmp_ph97_B8
zmp_ph97_C1
zmp_ph97_D2
zmp_ph97_D5
zmp_ph111_G5
zmp_ph111_G11
zmp_ph111_H1
zmp_ph111_B1a
zmp_ph111_B5
zmp_ph111_B8
zmp_ph111_B6
zmp_ph111_B7
zmp_ph111_B9
zmp_ph111_B10
zmp_ph114_G4
zmp_ph114_G11
zmp_ph114_H1
zmp_ph114_H2
zmp_ph114_A9
zmp_ph114_A11
zmp_ph114_B2
zmp_ph114_B4
zmp_ph114_D8
zmp_ph114_E2

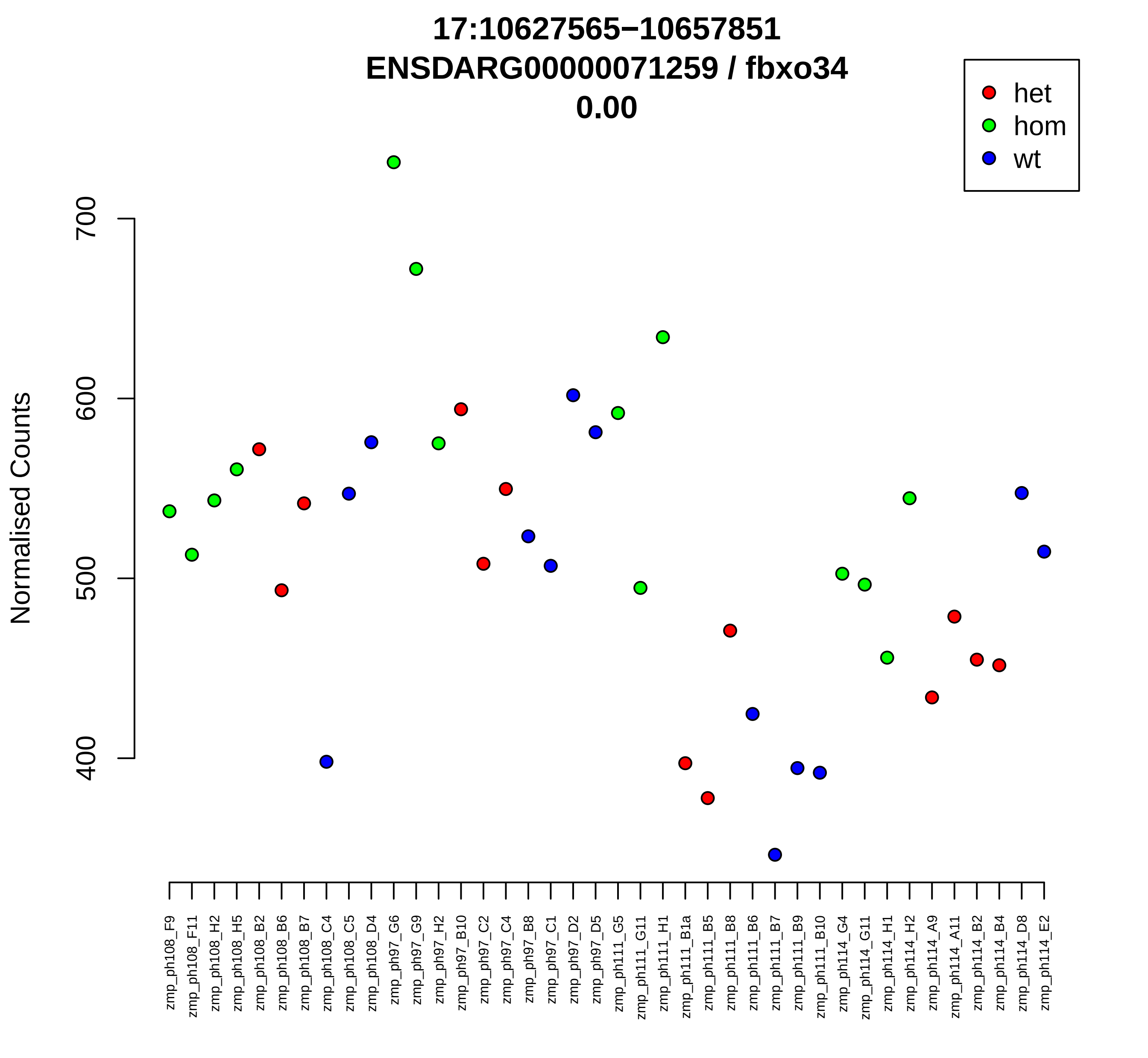

17:10627565−10657851
ENSDARG00000071259 / fbxo34
0.00
het
hom
wt
Normalised Counts
700
600
500
400
zmp_ph108_F9
zmp_ph108_F11
zmp_ph108_H2
zmp_ph108_H5
zmp_ph108_B2
zmp_ph108_B6
zmp_ph108_B7
zmp_ph108_C4
zmp_ph108_C5
zmp_ph108_D4
zmp_ph97_G6
zmp_ph97_G9
zmp_ph97_H2
zmp_ph97_B10
zmp_ph97_C2
zmp_ph97_C4
zmp_ph97_B8
zmp_ph97_C1
zmp_ph97_D2
zmp_ph97_D5
zmp_ph111_G5
zmp_ph111_G11
zmp_ph111_H1
zmp_ph111_B1a
zmp_ph111_B5
zmp_ph111_B8
zmp_ph111_B6
zmp_ph111_B7
zmp_ph111_B9
zmp_ph111_B10
zmp_ph114_G4
zmp_ph114_G11
zmp_ph114_H1
zmp_ph114_H2
zmp_ph114_A9
zmp_ph114_A11
zmp_ph114_B2
zmp_ph114_B4
zmp_ph114_D8
zmp_ph114_E2

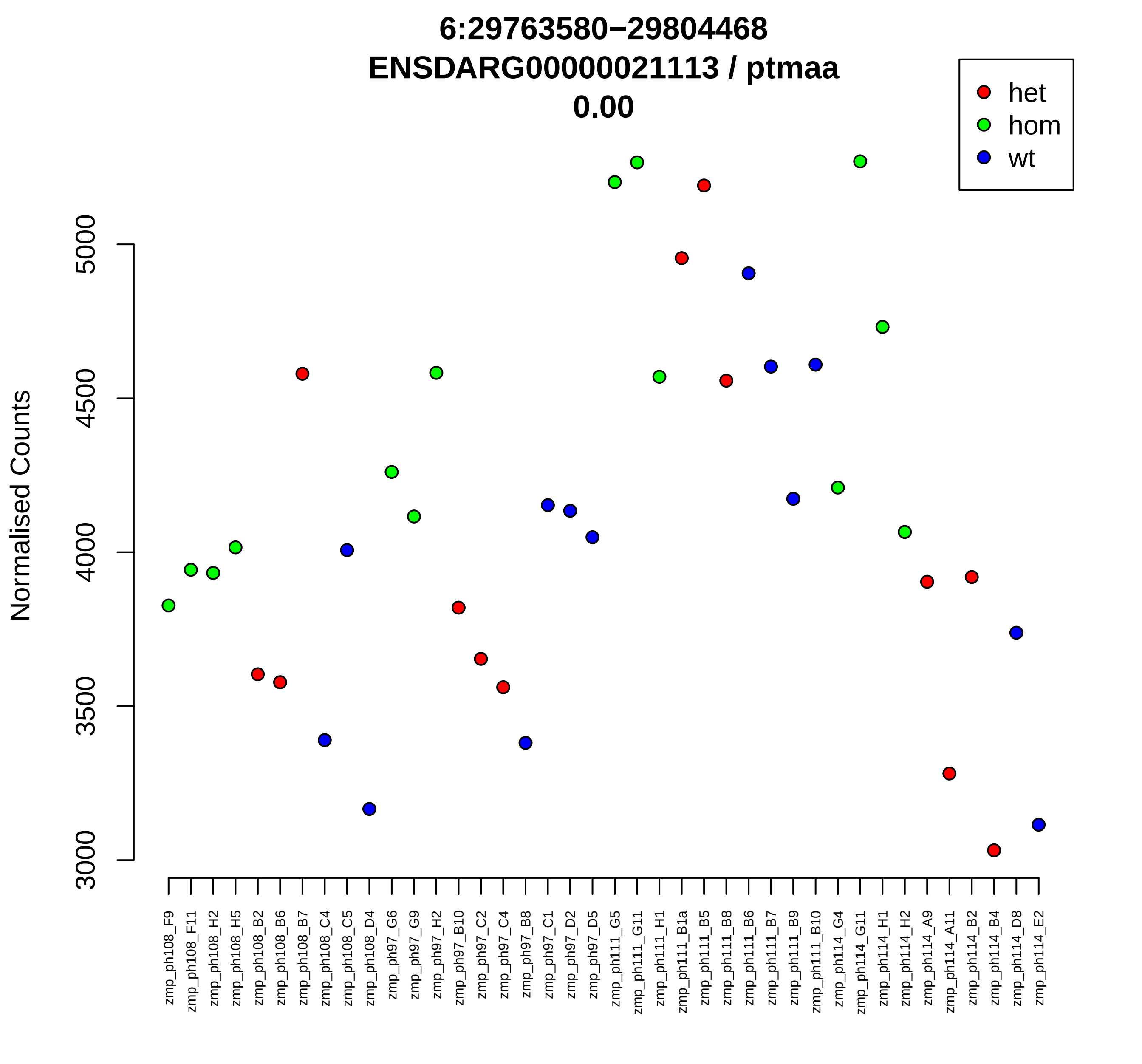
6:29763580−29804468
ENSDARG00000021113 / ptmaa
0.00
het
hom
wt
Normalised Counts
3000
3500
4000
4500
5000
zmp_ph108_F9
zmp_ph108_F11
zmp_ph108_H2
zmp_ph108_H5
zmp_ph108_B2
zmp_ph108_B6
zmp_ph108_B7
zmp_ph108_C4
zmp_ph108_C5
zmp_ph108_D4
zmp_ph97_G6
zmp_ph97_G9
zmp_ph97_H2
zmp_ph97_B10
zmp_ph97_C2
zmp_ph97_C4
zmp_ph97_B8
zmp_ph97_C1
zmp_ph97_D2
zmp_ph97_D5
zmp_ph111_G5
zmp_ph111_G11
zmp_ph111_H1
zmp_ph111_B1a
zmp_ph111_B5
zmp_ph111_B8
zmp_ph111_B6
zmp_ph111_B7
zmp_ph111_B9
zmp_ph111_B10
zmp_ph114_G4
zmp_ph114_G11
zmp_ph114_H1
zmp_ph114_H2
zmp_ph114_A9
zmp_ph114_A11
zmp_ph114_B2
zmp_ph114_B4
zmp_ph114_D8
zmp_ph114_E2

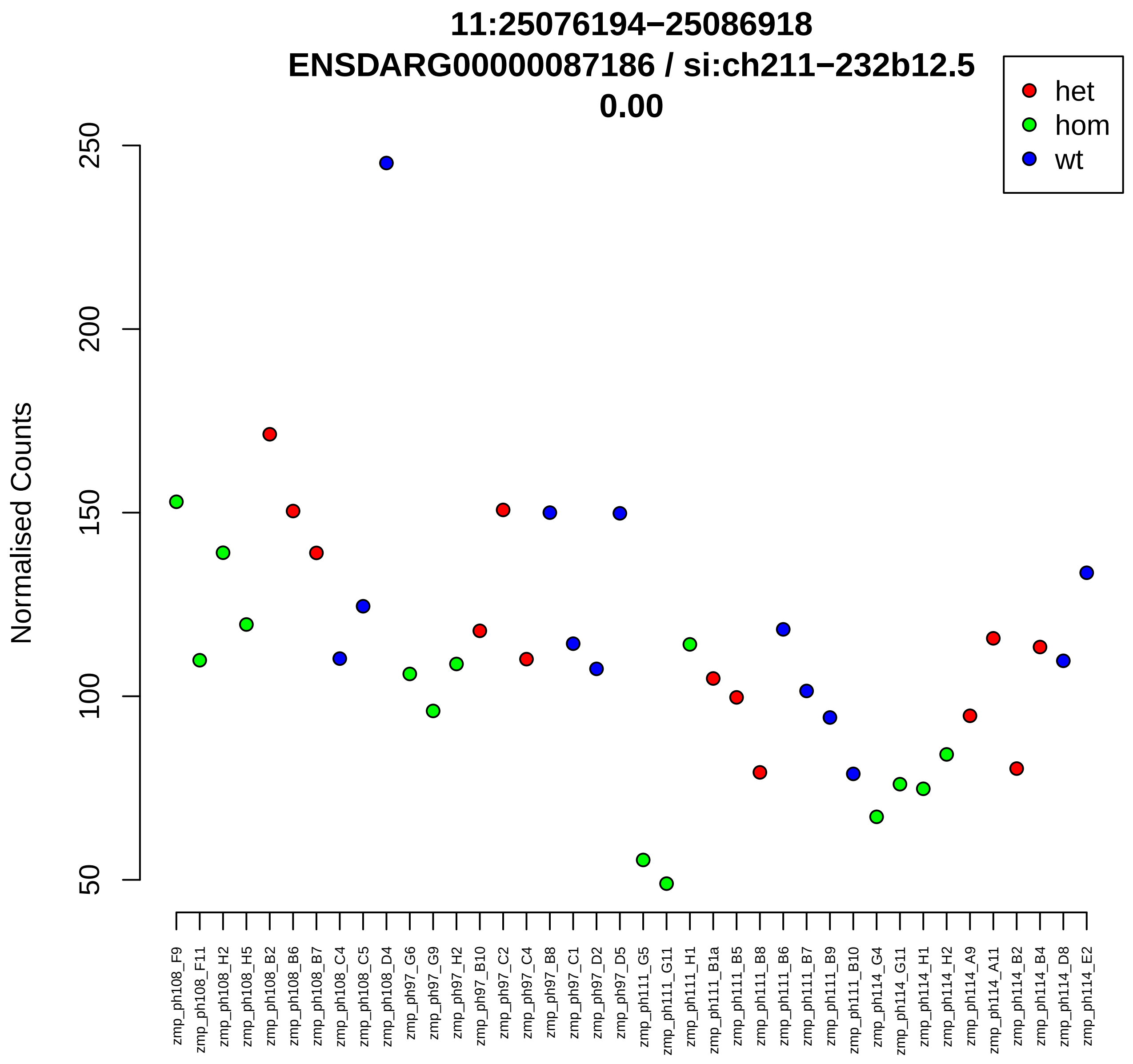
11:25076194−25086918
ENSDARG00000087186 / si:ch211−232b12.5
0.00
het
hom
wt
Normalised Counts
50
100
150
200
250
zmp_ph108_F9
zmp_ph108_F11
zmp_ph108_H2
zmp_ph108_H5
zmp_ph108_B2
zmp_ph108_B6
zmp_ph108_B7
zmp_ph108_C4
zmp_ph108_C5
zmp_ph108_D4
zmp_ph97_G6
zmp_ph97_G9
zmp_ph97_H2
zmp_ph97_B10
zmp_ph97_C2
zmp_ph97_C4
zmp_ph97_B8
zmp_ph97_C1
zmp_ph97_D2
zmp_ph97_D5
zmp_ph111_G5
zmp_ph111_G11
zmp_ph111_H1
zmp_ph111_B1a
zmp_ph111_B5
zmp_ph111_B8
zmp_ph111_B6
zmp_ph111_B7
zmp_ph111_B9
zmp_ph111_B10
zmp_ph114_G4
zmp_ph114_G11
zmp_ph114_H1
zmp_ph114_H2
zmp_ph114_A9
zmp_ph114_A11
zmp_ph114_B2
zmp_ph114_B4
zmp_ph114_D8
zmp_ph114_E2

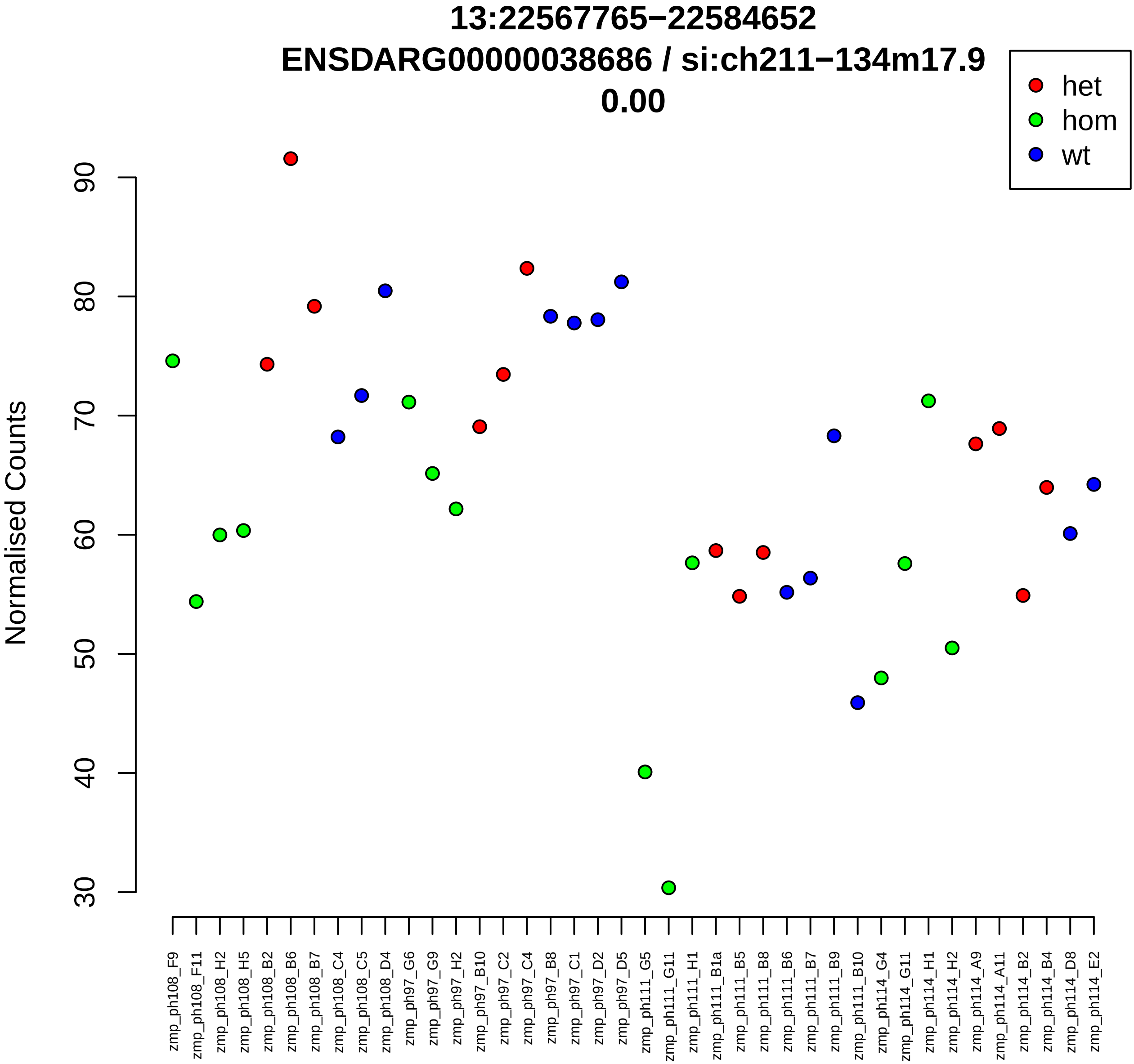
13:22567765−22584652
ENSDARG00000038686 / si:ch211−134m17.9
0.00
het
hom
wt
Normalised Counts
30
40
50
60
70
80
90
zmp_ph108_F9
zmp_ph108_F11
zmp_ph108_H2
zmp_ph108_H5
zmp_ph108_B2
zmp_ph108_B6
zmp_ph108_B7
zmp_ph108_C4
zmp_ph108_C5
zmp_ph108_D4
zmp_ph97_G6
zmp_ph97_G9
zmp_ph97_H2
zmp_ph97_B10
zmp_ph97_C2
zmp_ph97_C4
zmp_ph97_B8
zmp_ph97_C1
zmp_ph97_D2
zmp_ph97_D5
zmp_ph111_G5
zmp_ph111_G11
zmp_ph111_H1
zmp_ph111_B1a
zmp_ph111_B5
zmp_ph111_B8
zmp_ph111_B6
zmp_ph111_B7
zmp_ph111_B9
zmp_ph111_B10
zmp_ph114_G4
zmp_ph114_G11
zmp_ph114_H1
zmp_ph114_H2
zmp_ph114_A9
zmp_ph114_A11
zmp_ph114_B2
zmp_ph114_B4
zmp_ph114_D8
zmp_ph114_E2

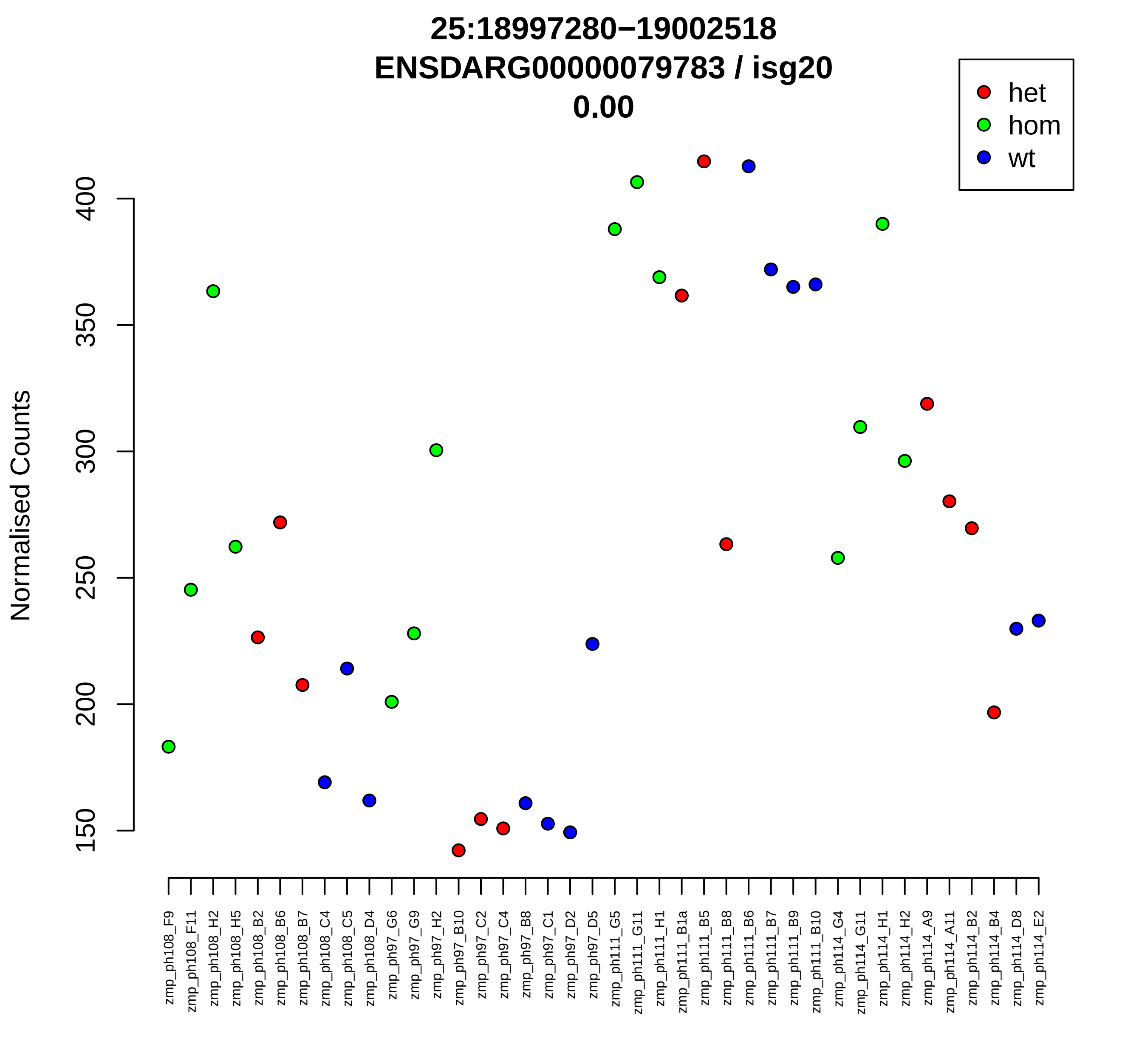
25:18997280−19002518
ENSDARG00000079783 / isg20
0.00
het
hom
wt
Normalised Counts
150
200
250
300
350
400
zmp_ph108_F9
zmp_ph108_F11
zmp_ph108_H2
zmp_ph108_H5
zmp_ph108_B2
zmp_ph108_B6
zmp_ph108_B7
zmp_ph108_C4
zmp_ph108_C5
zmp_ph108_D4
zmp_ph97_G6
zmp_ph97_G9
zmp_ph97_H2
zmp_ph97_B10
zmp_ph97_C2
zmp_ph97_C4
zmp_ph97_B8
zmp_ph97_C1
zmp_ph97_D2
zmp_ph97_D5
zmp_ph111_G5
zmp_ph111_G11
zmp_ph111_H1
zmp_ph111_B1a
zmp_ph111_B5
zmp_ph111_B8
zmp_ph111_B6
zmp_ph111_B7
zmp_ph111_B9
zmp_ph111_B10
zmp_ph114_G4
zmp_ph114_G11
zmp_ph114_H1
zmp_ph114_H2
zmp_ph114_A9
zmp_ph114_A11
zmp_ph114_B2
zmp_ph114_B4
zmp_ph114_D8
zmp_ph114_E2

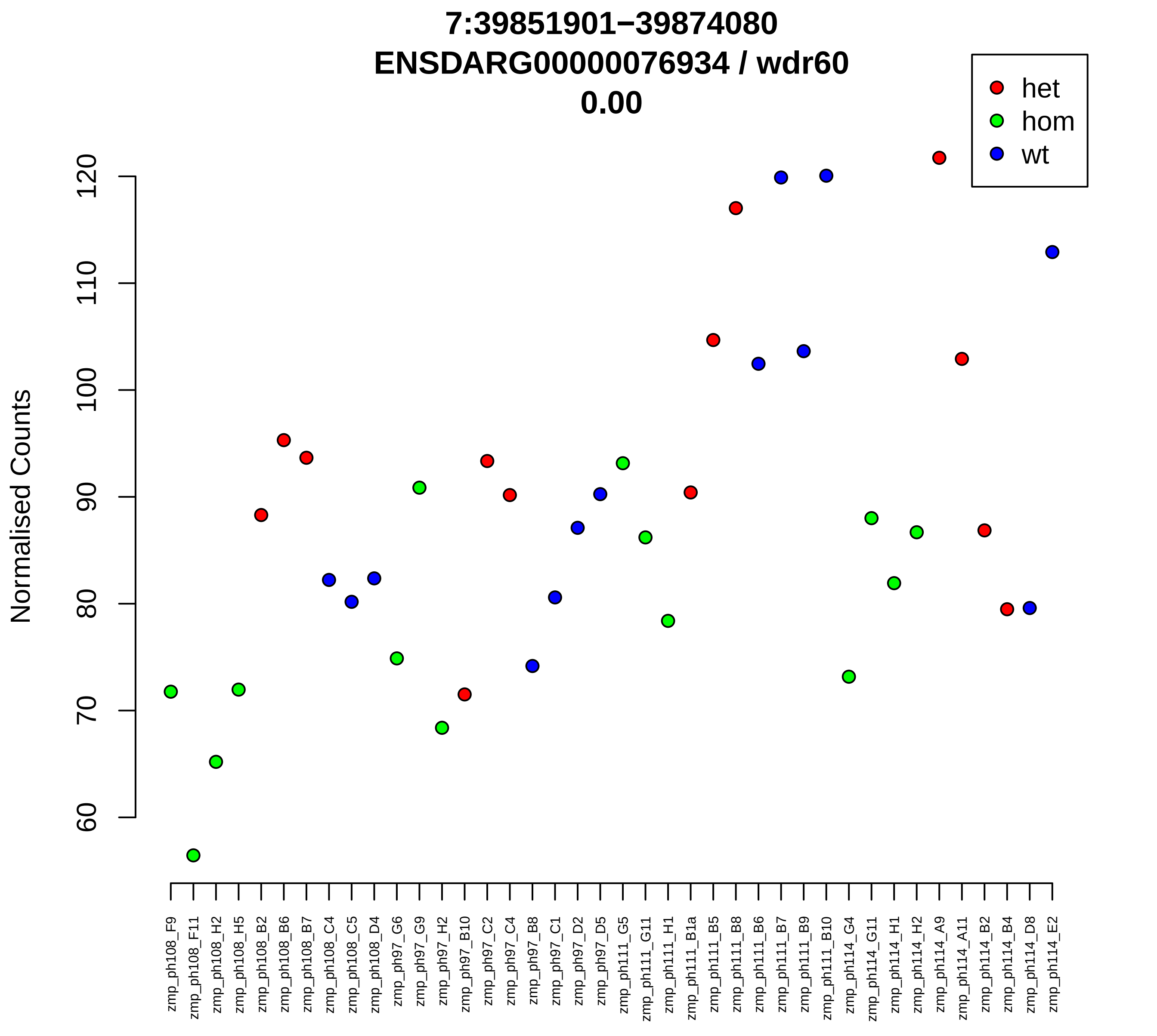
7:39851901−39874080
ENSDARG00000076934 / wdr60
0.00
het
hom
wt
Normalised Counts
60
70
80
90
100
110
120
zmp_ph108_F9
zmp_ph108_F11
zmp_ph108_H2
zmp_ph108_H5
zmp_ph108_B2
zmp_ph108_B6
zmp_ph108_B7
zmp_ph108_C4
zmp_ph108_C5
zmp_ph108_D4
zmp_ph97_G6
zmp_ph97_G9
zmp_ph97_H2
zmp_ph97_B10
zmp_ph97_C2
zmp_ph97_C4
zmp_ph97_B8
zmp_ph97_C1
zmp_ph97_D2
zmp_ph97_D5
zmp_ph111_G5
zmp_ph111_G11
zmp_ph111_H1
zmp_ph111_B1a
zmp_ph111_B5
zmp_ph111_B8
zmp_ph111_B6
zmp_ph111_B7
zmp_ph111_B9
zmp_ph111_B10
zmp_ph114_G4
zmp_ph114_G11
zmp_ph114_H1
zmp_ph114_H2
zmp_ph114_A9
zmp_ph114_A11
zmp_ph114_B2
zmp_ph114_B4
zmp_ph114_D8
zmp_ph114_E2

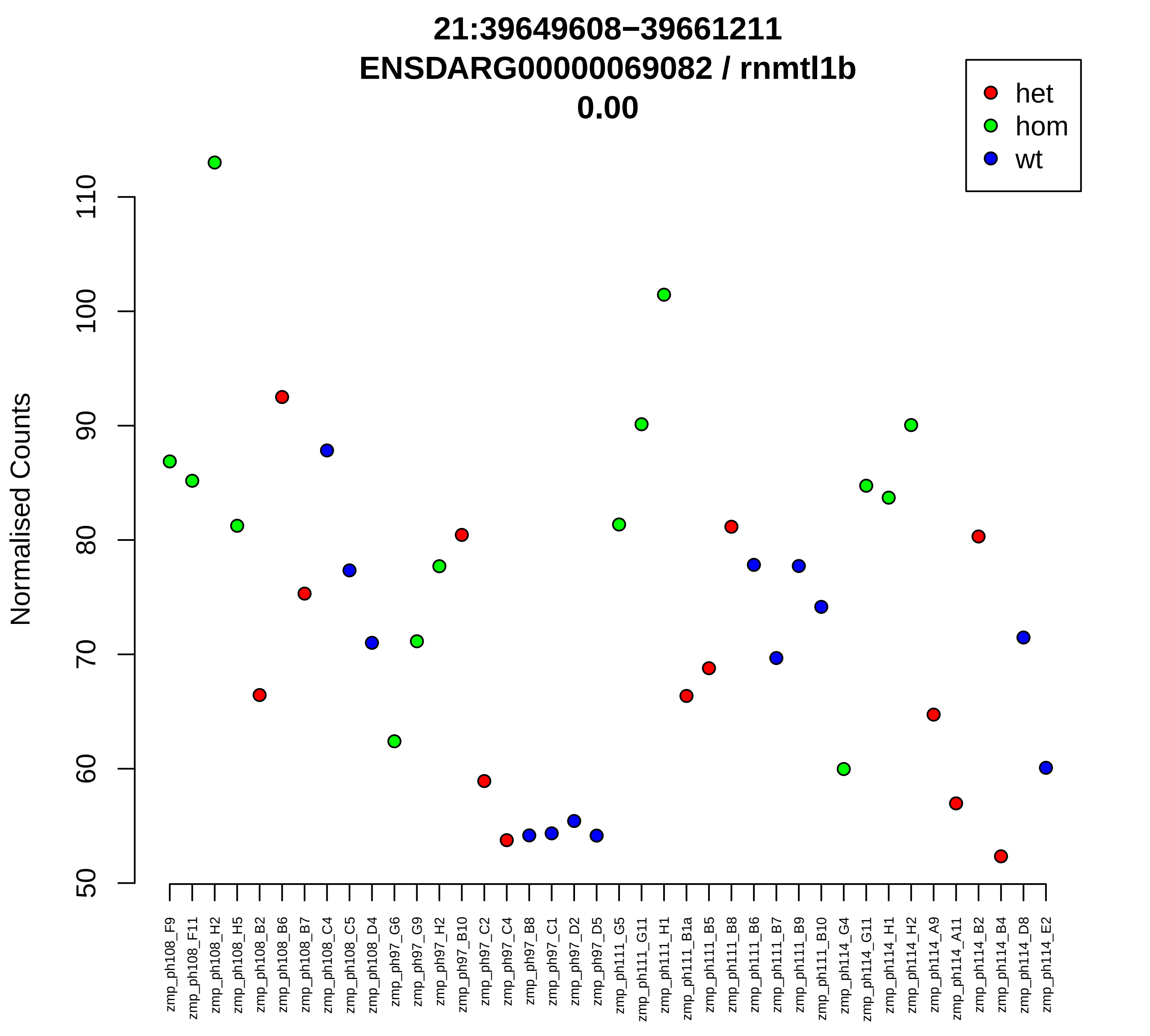

21:39649608−39661211
ENSDARG00000069082 / rnmtl1b
0.00
het
hom
wt
Normalised Counts
50
60
70
80
90
100
110
zmp_ph108_F9
zmp_ph108_F11
zmp_ph108_H2
zmp_ph108_H5
zmp_ph108_B2
zmp_ph108_B6
zmp_ph108_B7
zmp_ph108_C4
zmp_ph108_C5
zmp_ph108_D4
zmp_ph97_G6
zmp_ph97_G9
zmp_ph97_H2
zmp_ph97_B10
zmp_ph97_C2
zmp_ph97_C4
zmp_ph97_B8
zmp_ph97_C1
zmp_ph97_D2
zmp_ph97_D5
zmp_ph111_G5
zmp_ph111_G11
zmp_ph111_H1
zmp_ph111_B1a
zmp_ph111_B5
zmp_ph111_B8
zmp_ph111_B6
zmp_ph111_B7
zmp_ph111_B9
zmp_ph111_B10
zmp_ph114_G4
zmp_ph114_G11
zmp_ph114_H1
zmp_ph114_H2
zmp_ph114_A9
zmp_ph114_A11
zmp_ph114_B2
zmp_ph114_B4
zmp_ph114_D8
zmp_ph114_E2

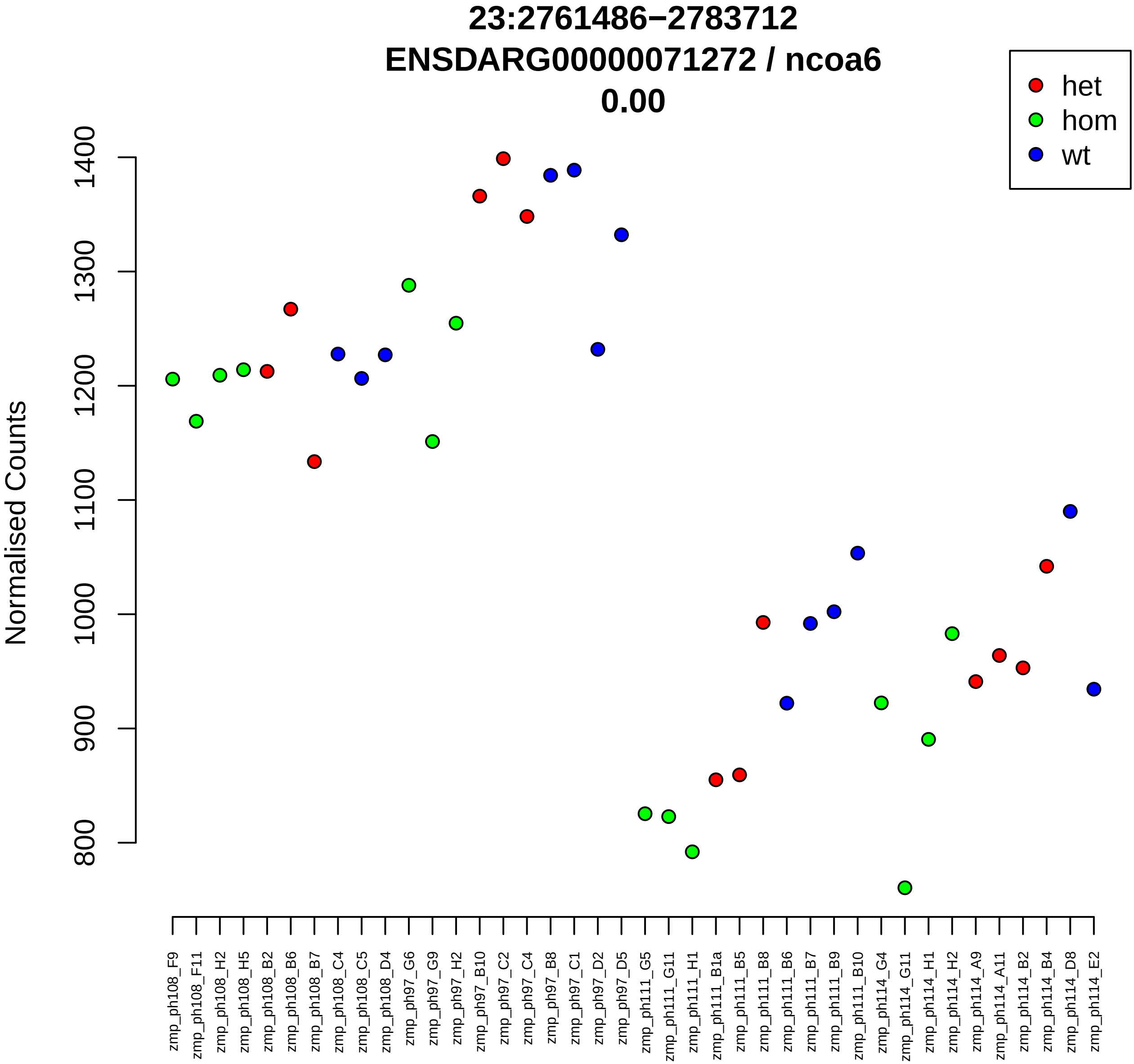

23:2761486−2783712
ENSDARG00000071272 / ncoa6
0.00
Normalised Counts
800
900
1000
1100
1200
1300
1400
het
hom
wt
zmp_ph108_F9
zmp_ph108_F11
zmp_ph108_H2
zmp_ph108_H5
zmp_ph108_B2
zmp_ph108_B6
zmp_ph108_B7
zmp_ph108_C4
zmp_ph108_C5
zmp_ph108_D4
zmp_ph97_G6
zmp_ph97_G9
zmp_ph97_H2
zmp_ph97_B10
zmp_ph97_C2
zmp_ph97_C4
zmp_ph97_B8
zmp_ph97_C1
zmp_ph97_D2
zmp_ph97_D5
zmp_ph111_G5
zmp_ph111_G11
zmp_ph111_H1
zmp_ph111_B1a
zmp_ph111_B5
zmp_ph111_B8
zmp_ph111_B6
zmp_ph111_B7
zmp_ph111_B9
zmp_ph111_B10
zmp_ph114_G4
zmp_ph114_G11
zmp_ph114_H1
zmp_ph114_H2
zmp_ph114_A9
zmp_ph114_A11
zmp_ph114_B2
zmp_ph114_B4
zmp_ph114_D8
zmp_ph114_E2

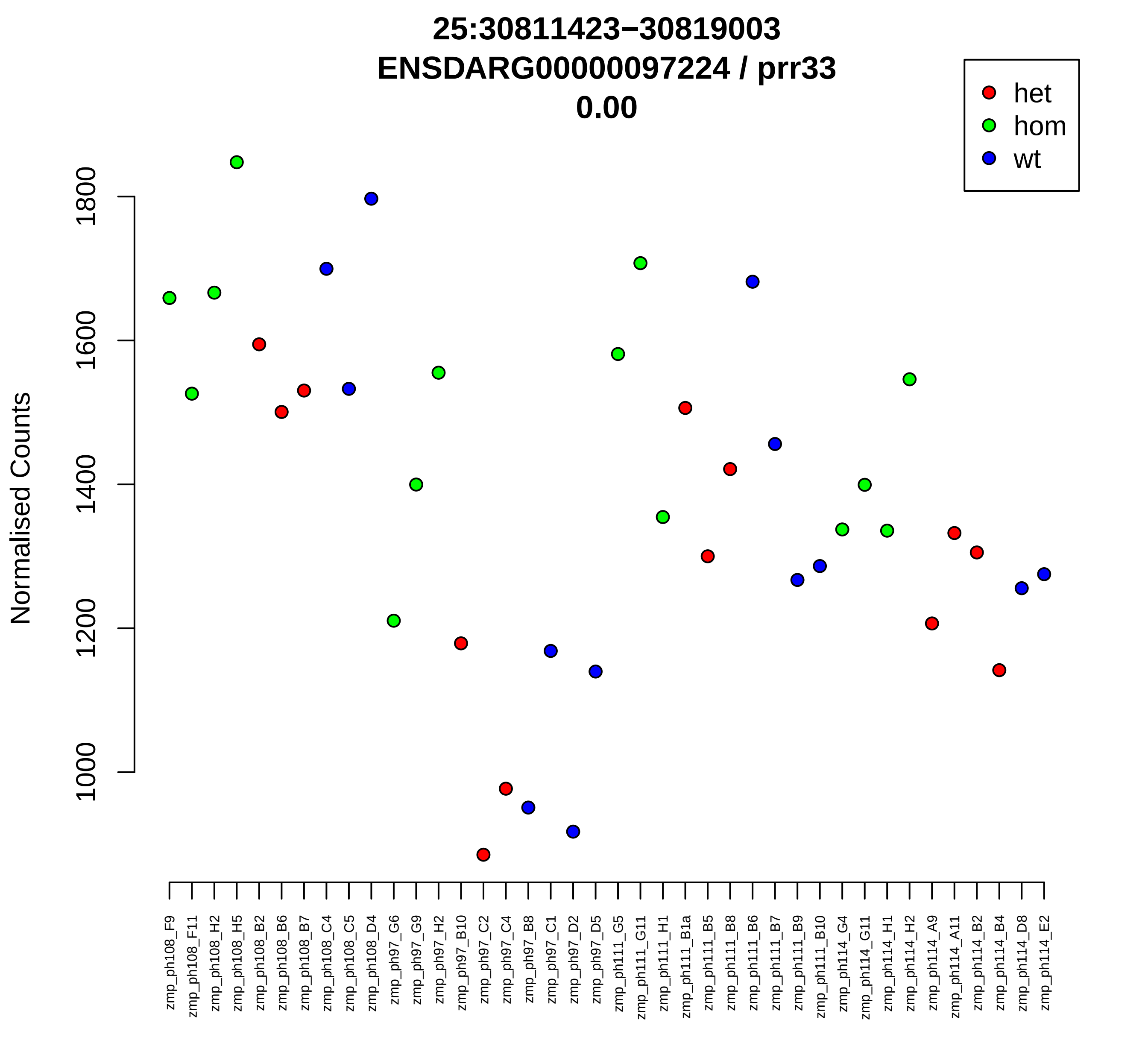
25:30811423−30819003
ENSDARG00000097224 / prr33
0.00
het
hom
wt
Normalised Counts
1000
1200
1400
1600
1800
zmp_ph108_F9
zmp_ph108_F11
zmp_ph108_H2
zmp_ph108_H5
zmp_ph108_B2
zmp_ph108_B6
zmp_ph108_B7
zmp_ph108_C4
zmp_ph108_C5
zmp_ph108_D4
zmp_ph97_G6
zmp_ph97_G9
zmp_ph97_H2
zmp_ph97_B10
zmp_ph97_C2
zmp_ph97_C4
zmp_ph97_B8
zmp_ph97_C1
zmp_ph97_D2
zmp_ph97_D5
zmp_ph111_G5
zmp_ph111_G11
zmp_ph111_H1
zmp_ph111_B1a
zmp_ph111_B5
zmp_ph111_B8
zmp_ph111_B6
zmp_ph111_B7
zmp_ph111_B9
zmp_ph111_B10
zmp_ph114_G4
zmp_ph114_G11
zmp_ph114_H1
zmp_ph114_H2
zmp_ph114_A9
zmp_ph114_A11
zmp_ph114_B2
zmp_ph114_B4
zmp_ph114_D8
zmp_ph114_E2

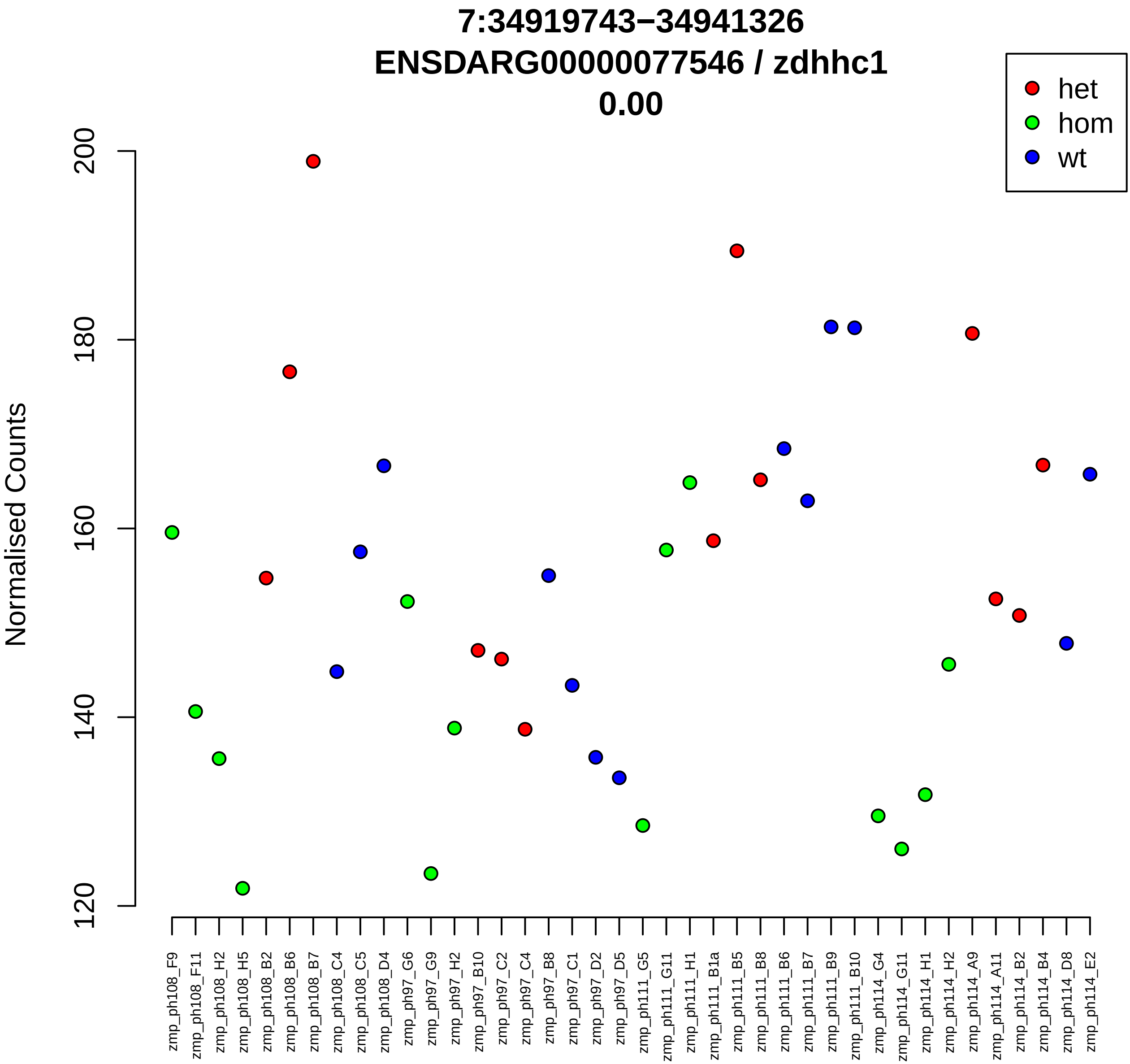

7:34919743−34941326
ENSDARG00000077546 / zdhhc1
0.00
het
hom
wt
Normalised Counts
120
140
160
180
200
zmp_ph108_F9
zmp_ph108_F11
zmp_ph108_H2
zmp_ph108_H5
zmp_ph108_B2
zmp_ph108_B6
zmp_ph108_B7
zmp_ph108_C4
zmp_ph108_C5
zmp_ph108_D4
zmp_ph97_G6
zmp_ph97_G9
zmp_ph97_H2
zmp_ph97_B10
zmp_ph97_C2
zmp_ph97_C4
zmp_ph97_B8
zmp_ph97_C1
zmp_ph97_D2
zmp_ph97_D5
zmp_ph111_G5
zmp_ph111_G11
zmp_ph111_H1
zmp_ph111_B1a
zmp_ph111_B5
zmp_ph111_B8
zmp_ph111_B6
zmp_ph111_B7
zmp_ph111_B9
zmp_ph111_B10
zmp_ph114_G4
zmp_ph114_G11
zmp_ph114_H1
zmp_ph114_H2
zmp_ph114_A9
zmp_ph114_A11
zmp_ph114_B2
zmp_ph114_B4
zmp_ph114_D8
zmp_ph114_E2

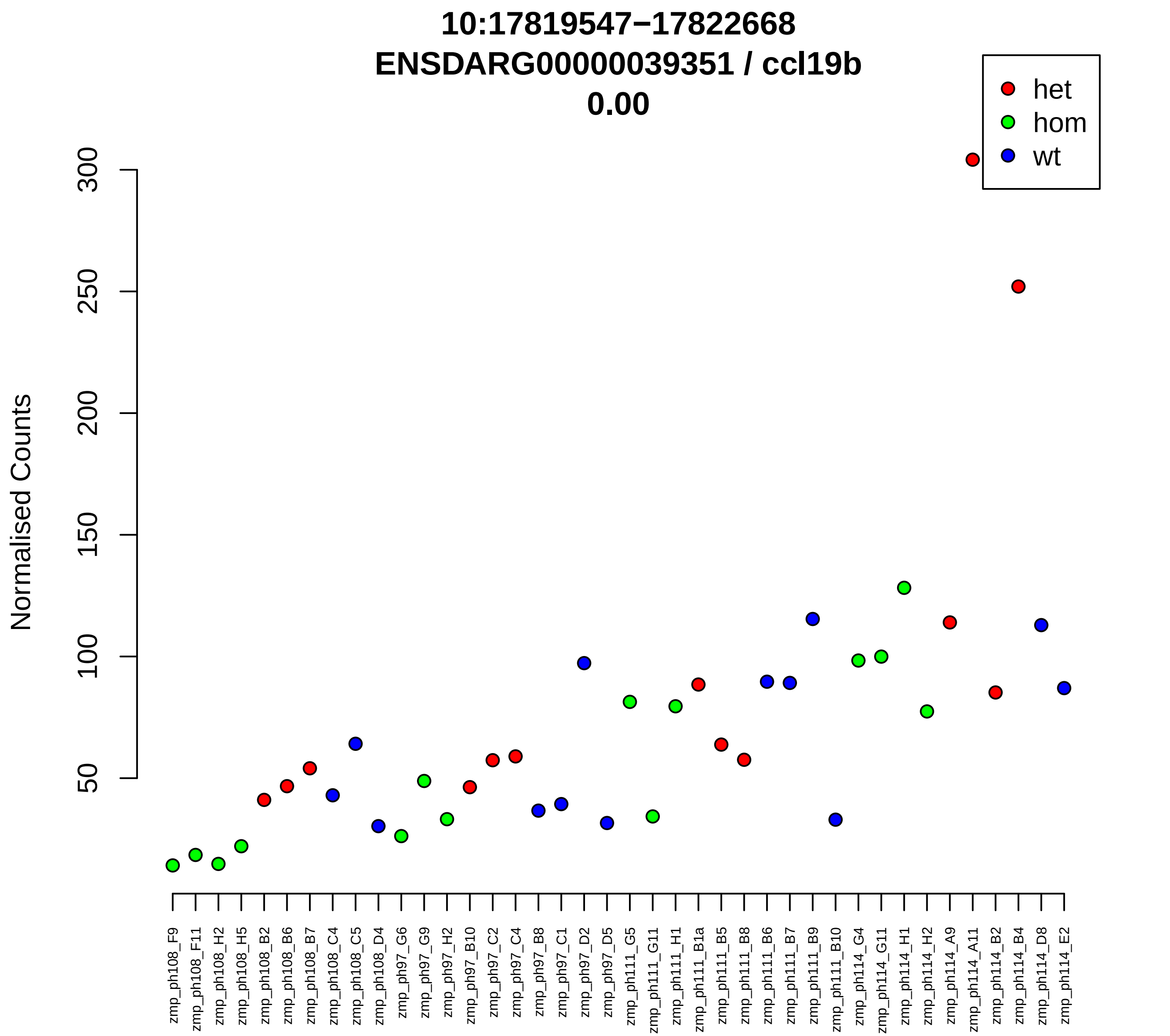
10:17819547−17822668
ENSDARG00000039351 / ccl19b
0.00
het
hom
wt
Normalised Counts
50
100
150
200
250
300
zmp_ph108_F9
zmp_ph108_F11
zmp_ph108_H2
zmp_ph108_H5
zmp_ph108_B2
zmp_ph108_B6
zmp_ph108_B7
zmp_ph108_C4
zmp_ph108_C5
zmp_ph108_D4
zmp_ph97_G6
zmp_ph97_G9
zmp_ph97_H2
zmp_ph97_B10
zmp_ph97_C2
zmp_ph97_C4
zmp_ph97_B8
zmp_ph97_C1
zmp_ph97_D2
zmp_ph97_D5
zmp_ph111_G5
zmp_ph111_G11
zmp_ph111_H1
zmp_ph111_B1a
zmp_ph111_B5
zmp_ph111_B8
zmp_ph111_B6
zmp_ph111_B7
zmp_ph111_B9
zmp_ph111_B10
zmp_ph114_G4
zmp_ph114_G11
zmp_ph114_H1
zmp_ph114_H2
zmp_ph114_A9
zmp_ph114_A11
zmp_ph114_B2
zmp_ph114_B4
zmp_ph114_D8
zmp_ph114_E2

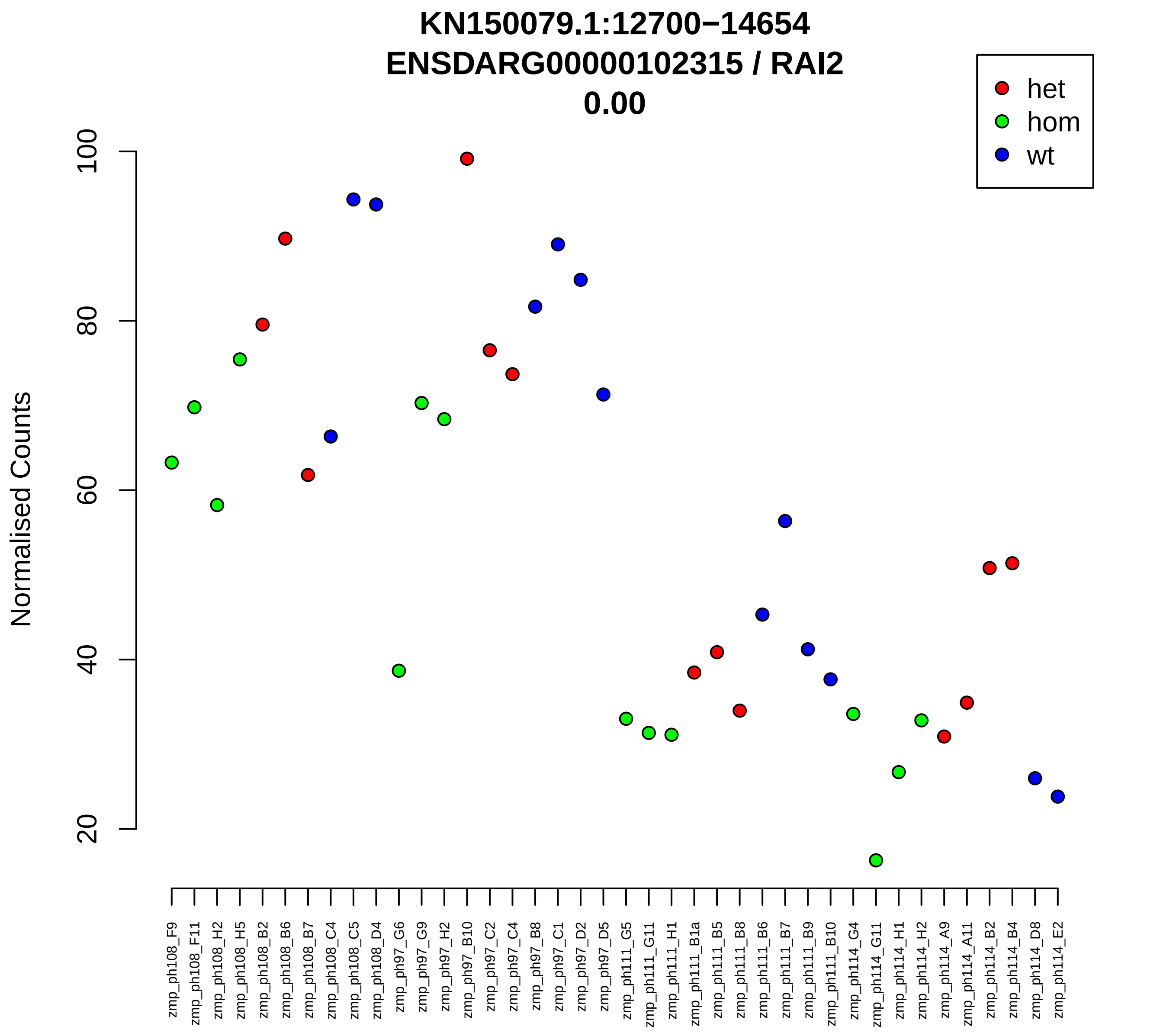
KN150079.1:12700−14654
ENSDARG00000102315 / RAI2
0.00
het
hom
wt
Normalised Counts
100
80
60
40
20
zmp_ph108_F9
zmp_ph108_F11
zmp_ph108_H2
zmp_ph108_H5
zmp_ph108_B2
zmp_ph108_B6
zmp_ph108_B7
zmp_ph108_C4
zmp_ph108_C5
zmp_ph108_D4
zmp_ph97_G6
zmp_ph97_G9
zmp_ph97_H2
zmp_ph97_B10
zmp_ph97_C2
zmp_ph97_C4
zmp_ph97_B8
zmp_ph97_C1
zmp_ph97_D2
zmp_ph97_D5
zmp_ph111_G5
zmp_ph111_G11
zmp_ph111_H1
zmp_ph111_B1a
zmp_ph111_B5
zmp_ph111_B8
zmp_ph111_B6
zmp_ph111_B7
zmp_ph111_B9
zmp_ph111_B10
zmp_ph114_G4
zmp_ph114_G11
zmp_ph114_H1
zmp_ph114_H2
zmp_ph114_A9
zmp_ph114_A11
zmp_ph114_B2
zmp_ph114_B4
zmp_ph114_D8
zmp_ph114_E2

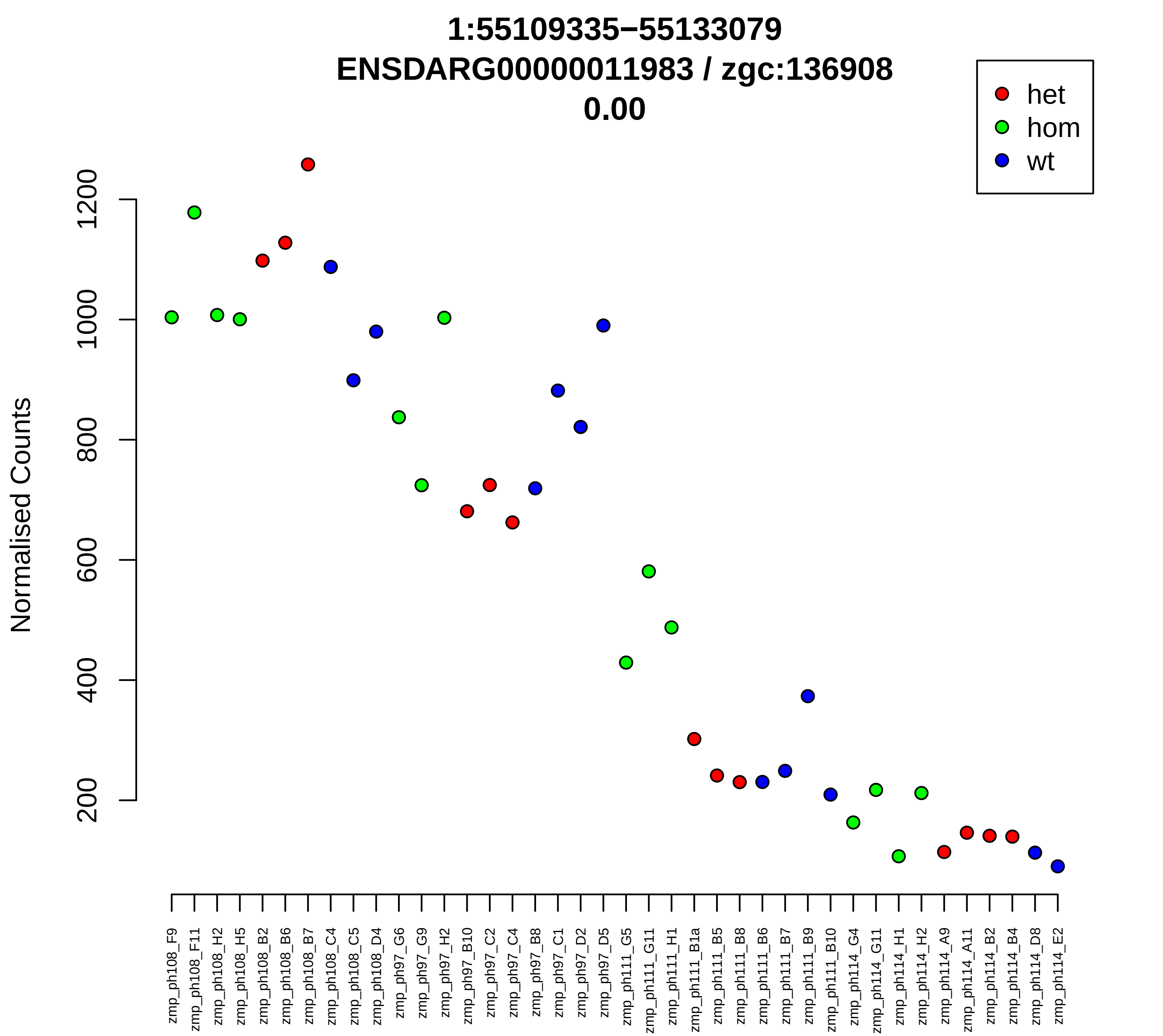

1:55109335−55133079
ENSDARG00000011983 / zgc:136908
0.00
het
hom
wt
Normalised Counts
200
400
600
800
1000
1200
zmp_ph108_F9
zmp_ph108_F11
zmp_ph108_H2
zmp_ph108_H5
zmp_ph108_B2
zmp_ph108_B6
zmp_ph108_B7
zmp_ph108_C4
zmp_ph108_C5
zmp_ph108_D4
zmp_ph97_G6
zmp_ph97_G9
zmp_ph97_H2
zmp_ph97_B10
zmp_ph97_C2
zmp_ph97_C4
zmp_ph97_B8
zmp_ph97_C1
zmp_ph97_D2
zmp_ph97_D5
zmp_ph111_G5
zmp_ph111_G11
zmp_ph111_H1
zmp_ph111_B1a
zmp_ph111_B5
zmp_ph111_B8
zmp_ph111_B6
zmp_ph111_B7
zmp_ph111_B9
zmp_ph111_B10
zmp_ph114_G4
zmp_ph114_G11
zmp_ph114_H1
zmp_ph114_H2
zmp_ph114_A9
zmp_ph114_A11
zmp_ph114_B2
zmp_ph114_B4
zmp_ph114_D8
zmp_ph114_E2

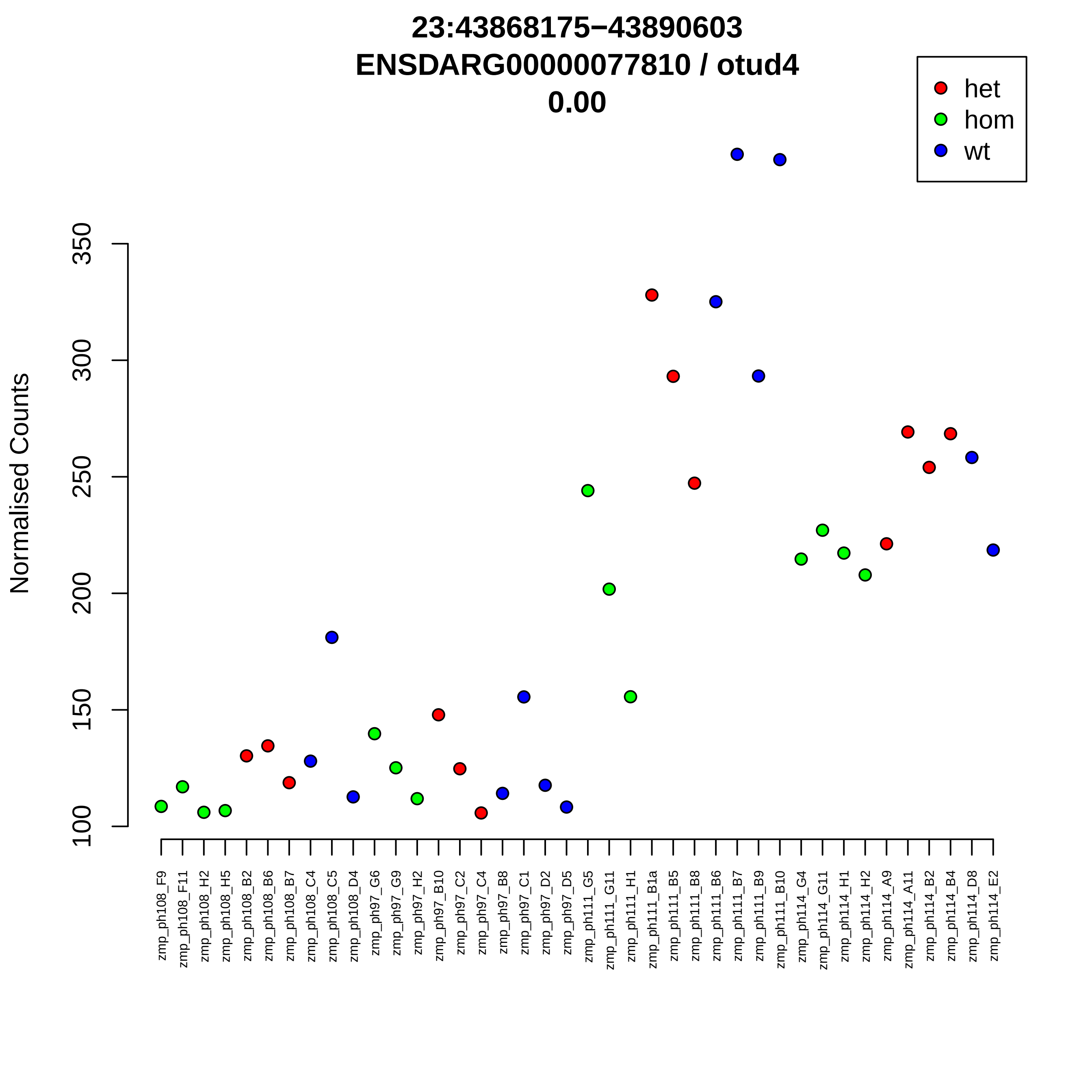
23:43868175−43890603
ENSDARG00000077810 / otud4
0.00
het
hom
wt
Normalised Counts
100
150
200
250
300
350
zmp_ph108_F9
zmp_ph108_F11
zmp_ph108_H2
zmp_ph108_H5
zmp_ph108_B2
zmp_ph108_B6
zmp_ph108_B7
zmp_ph108_C4
zmp_ph108_C5
zmp_ph108_D4
zmp_ph97_G6
zmp_ph97_G9
zmp_ph97_H2
zmp_ph97_B10
zmp_ph97_C2
zmp_ph97_C4
zmp_ph97_B8
zmp_ph97_C1
zmp_ph97_D2
zmp_ph97_D5
zmp_ph111_G5
zmp_ph111_G11
zmp_ph111_H1
zmp_ph111_B1a
zmp_ph111_B5
zmp_ph111_B8
zmp_ph111_B6
zmp_ph111_B7
zmp_ph111_B9
zmp_ph111_B10
zmp_ph114_G4
zmp_ph114_G11
zmp_ph114_H1
zmp_ph114_H2
zmp_ph114_A9
zmp_ph114_A11
zmp_ph114_B2
zmp_ph114_B4
zmp_ph114_D8
zmp_ph114_E2

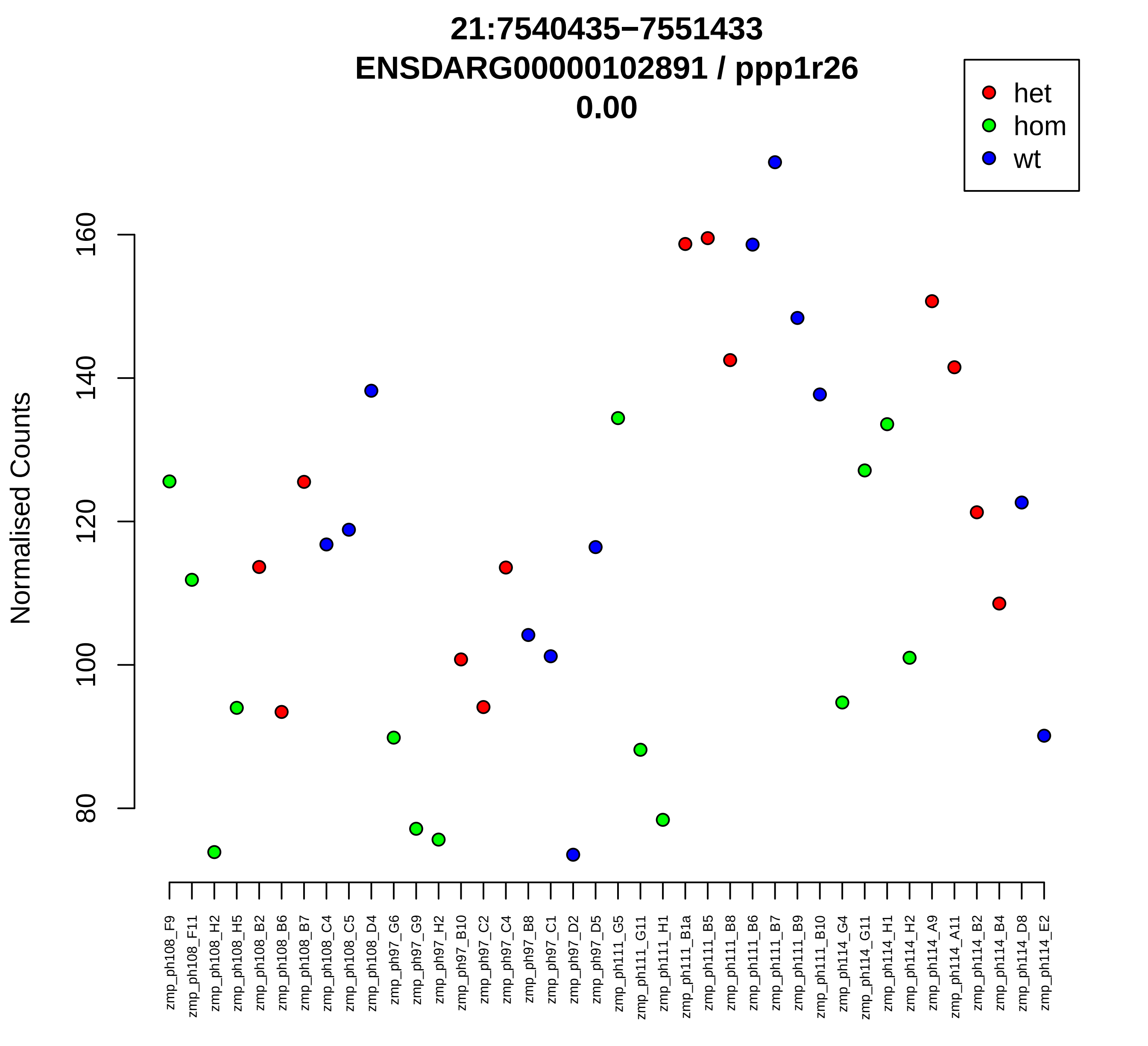
21:7540435−7551433
ENSDARG00000102891 / ppp1r26
0.00
het
hom
wt
Normalised Counts
80
100
120
140
160
zmp_ph108_F9
zmp_ph108_F11
zmp_ph108_H2
zmp_ph108_H5
zmp_ph108_B2
zmp_ph108_B6
zmp_ph108_B7
zmp_ph108_C4
zmp_ph108_C5
zmp_ph108_D4
zmp_ph97_G6
zmp_ph97_G9
zmp_ph97_H2
zmp_ph97_B10
zmp_ph97_C2
zmp_ph97_C4
zmp_ph97_B8
zmp_ph97_C1
zmp_ph97_D2
zmp_ph97_D5
zmp_ph111_G5
zmp_ph111_G11
zmp_ph111_H1
zmp_ph111_B1a
zmp_ph111_B5
zmp_ph111_B8
zmp_ph111_B6
zmp_ph111_B7
zmp_ph111_B9
zmp_ph111_B10
zmp_ph114_G4
zmp_ph114_G11
zmp_ph114_H1
zmp_ph114_H2
zmp_ph114_A9
zmp_ph114_A11
zmp_ph114_B2
zmp_ph114_B4
zmp_ph114_D8
zmp_ph114_E2

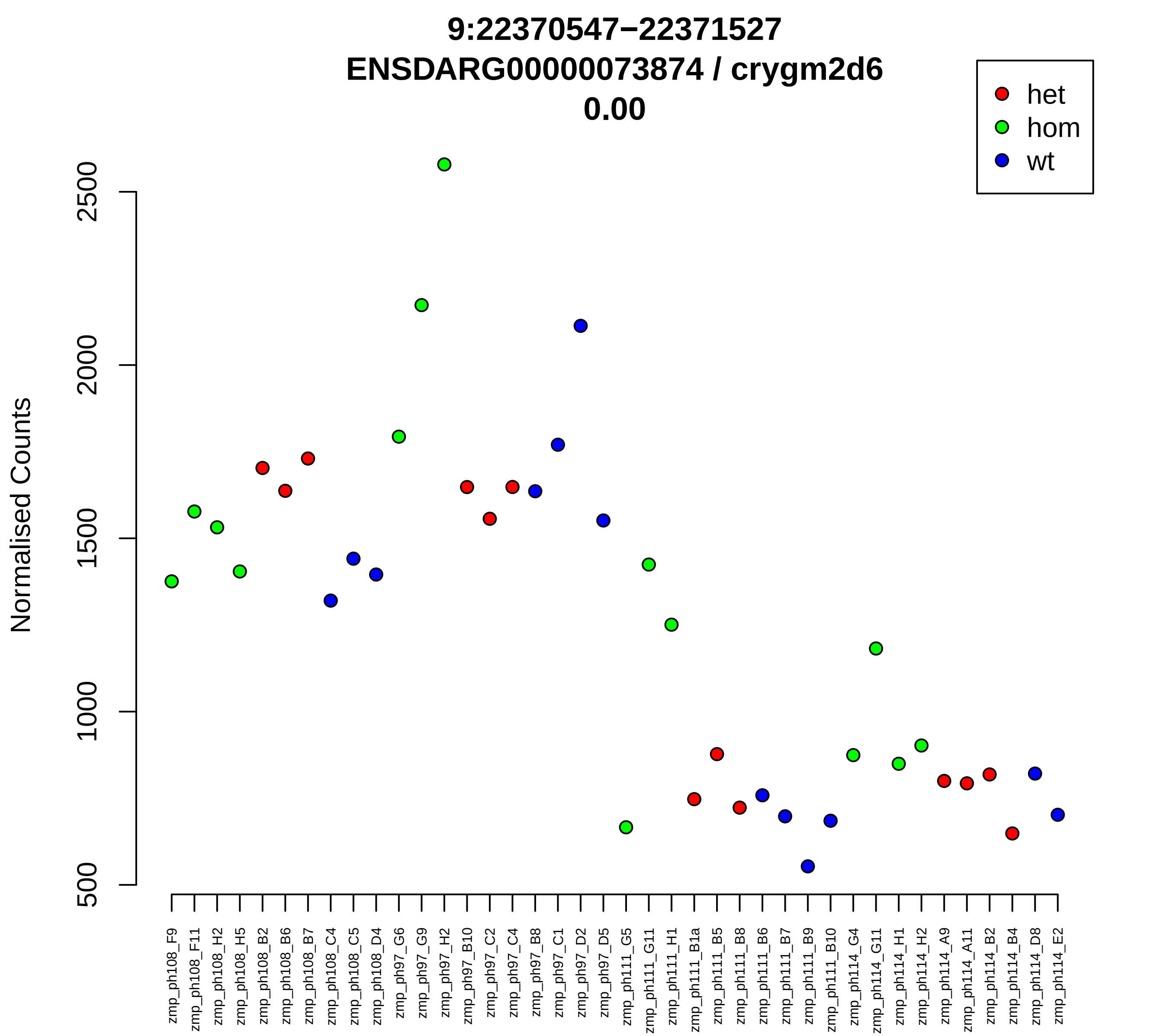

9:22370547−22371527
ENSDARG00000073874 / crygm2d6
0.00
het
hom
wt
Normalised Counts
500
1000
1500
2000
2500
zmp_ph108_F9
zmp_ph108_F11
zmp_ph108_H2
zmp_ph108_H5
zmp_ph108_B2
zmp_ph108_B6
zmp_ph108_B7
zmp_ph108_C4
zmp_ph108_C5
zmp_ph108_D4
zmp_ph97_G6
zmp_ph97_G9
zmp_ph97_H2
zmp_ph97_B10
zmp_ph97_C2
zmp_ph97_C4
zmp_ph97_B8
zmp_ph97_C1
zmp_ph97_D2
zmp_ph97_D5
zmp_ph111_G5
zmp_ph111_G11
zmp_ph111_H1
zmp_ph111_B1a
zmp_ph111_B5
zmp_ph111_B8
zmp_ph111_B6
zmp_ph111_B7
zmp_ph111_B9
zmp_ph111_B10
zmp_ph114_G4
zmp_ph114_G11
zmp_ph114_H1
zmp_ph114_H2
zmp_ph114_A9
zmp_ph114_A11
zmp_ph114_B2
zmp_ph114_B4
zmp_ph114_D8
zmp_ph114_E2

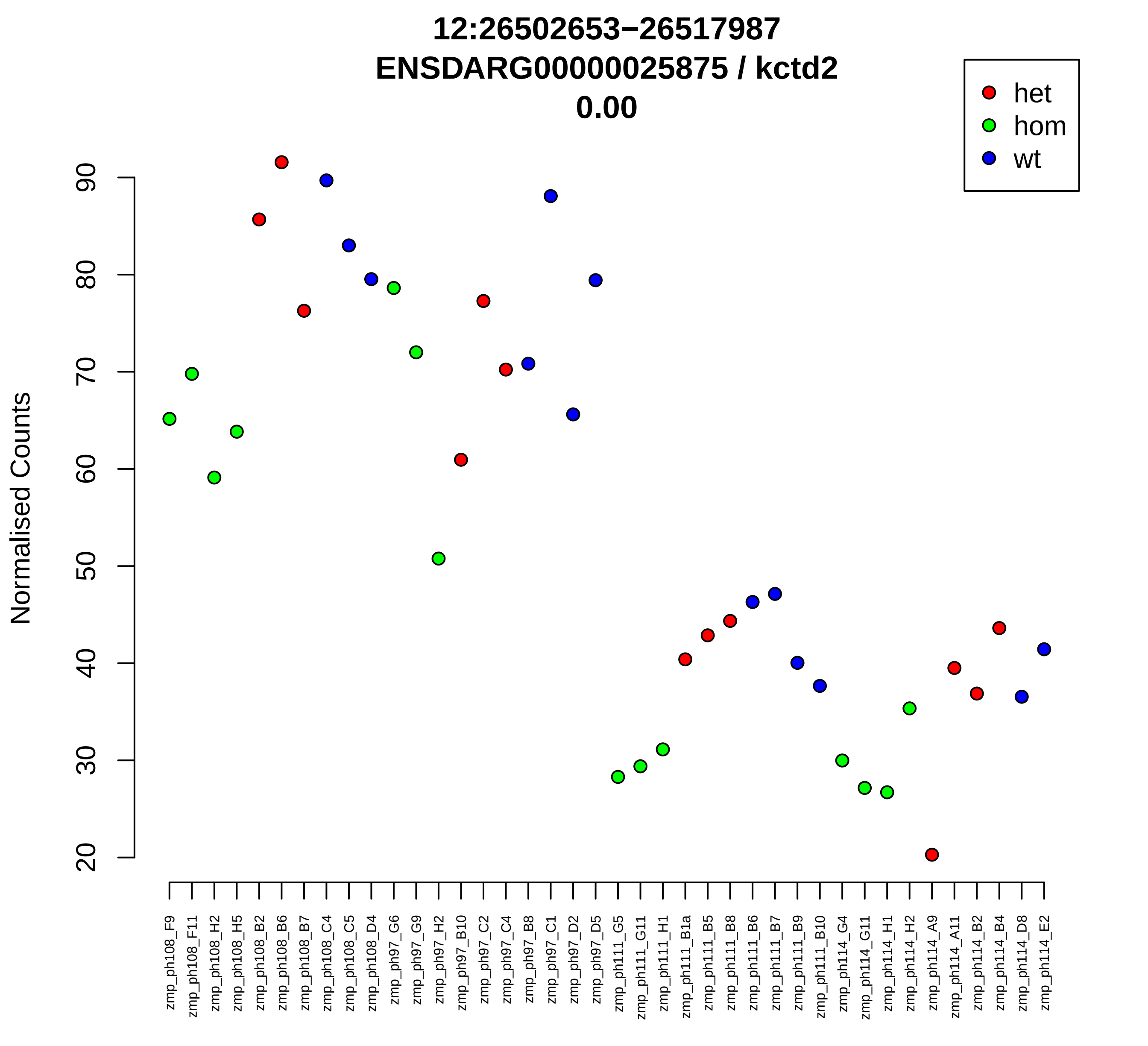

12:26502653−26517987
ENSDARG00000025875 / kctd2
0.00
het
hom
wt
Normalised Counts
20
30
40
50
60
70
80
90
zmp_ph108_F9
zmp_ph108_F11
zmp_ph108_H2
zmp_ph108_H5
zmp_ph108_B2
zmp_ph108_B6
zmp_ph108_B7
zmp_ph108_C4
zmp_ph108_C5
zmp_ph108_D4
zmp_ph97_G6
zmp_ph97_G9
zmp_ph97_H2
zmp_ph97_B10
zmp_ph97_C2
zmp_ph97_C4
zmp_ph97_B8
zmp_ph97_C1
zmp_ph97_D2
zmp_ph97_D5
zmp_ph111_G5
zmp_ph111_G11
zmp_ph111_H1
zmp_ph111_B1a
zmp_ph111_B5
zmp_ph111_B8
zmp_ph111_B6
zmp_ph111_B7
zmp_ph111_B9
zmp_ph111_B10
zmp_ph114_G4
zmp_ph114_G11
zmp_ph114_H1
zmp_ph114_H2
zmp_ph114_A9
zmp_ph114_A11
zmp_ph114_B2
zmp_ph114_B4
zmp_ph114_D8
zmp_ph114_E2

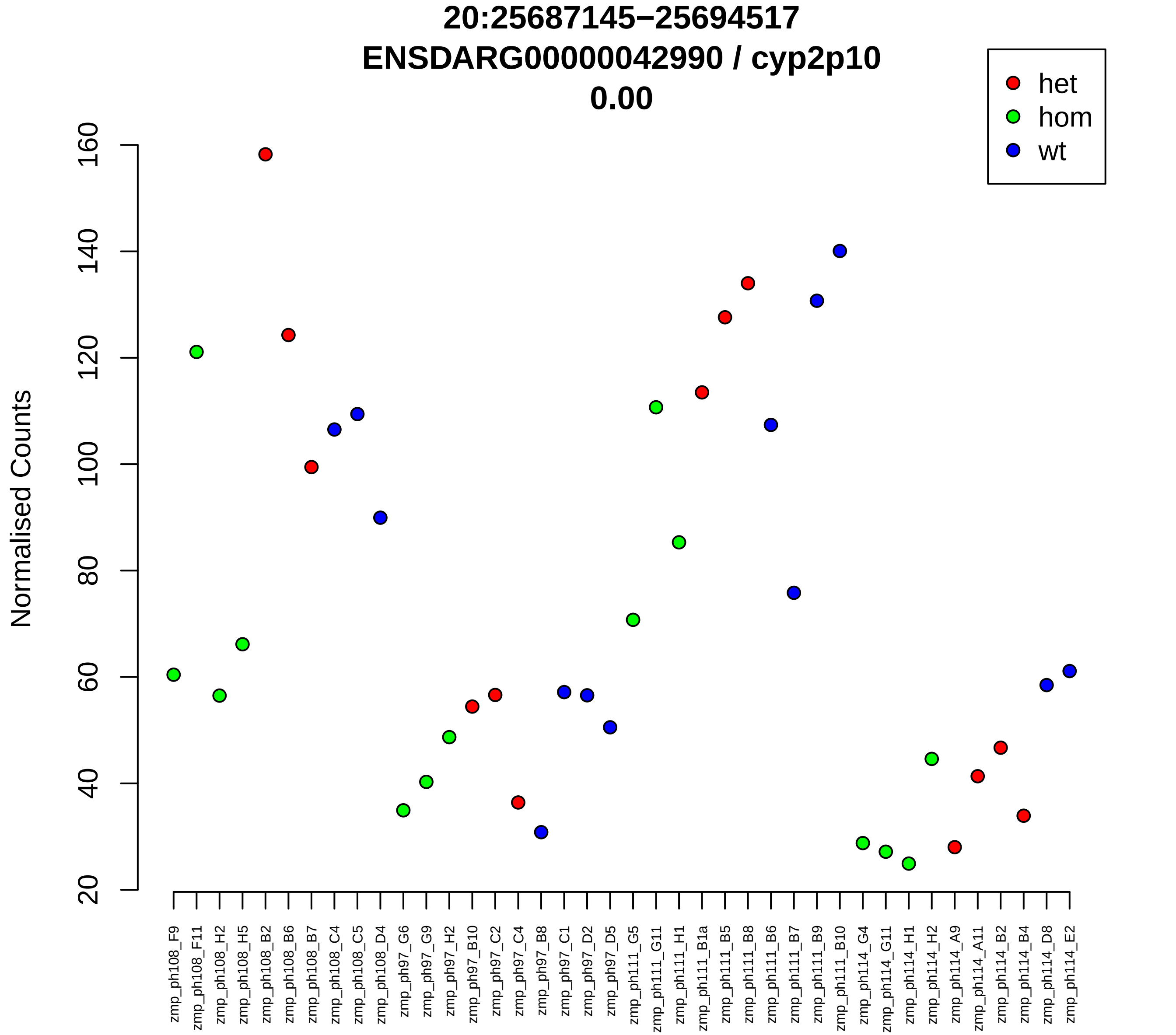

20:25687145−25694517
ENSDARG00000042990 / cyp2p10
0.00
het
hom
wt
Normalised Counts
20
40
60
80
100
120
140
160
zmp_ph108_F9
zmp_ph108_F11
zmp_ph108_H2
zmp_ph108_H5
zmp_ph108_B2
zmp_ph108_B6
zmp_ph108_B7
zmp_ph108_C4
zmp_ph108_C5
zmp_ph108_D4
zmp_ph97_G6
zmp_ph97_G9
zmp_ph97_H2
zmp_ph97_B10
zmp_ph97_C2
zmp_ph97_C4
zmp_ph97_B8
zmp_ph97_C1
zmp_ph97_D2
zmp_ph97_D5
zmp_ph111_G5
zmp_ph111_G11
zmp_ph111_H1
zmp_ph111_B1a
zmp_ph111_B5
zmp_ph111_B8
zmp_ph111_B6
zmp_ph111_B7
zmp_ph111_B9
zmp_ph111_B10
zmp_ph114_G4
zmp_ph114_G11
zmp_ph114_H1
zmp_ph114_H2
zmp_ph114_A9
zmp_ph114_A11
zmp_ph114_B2
zmp_ph114_B4
zmp_ph114_D8
zmp_ph114_E2

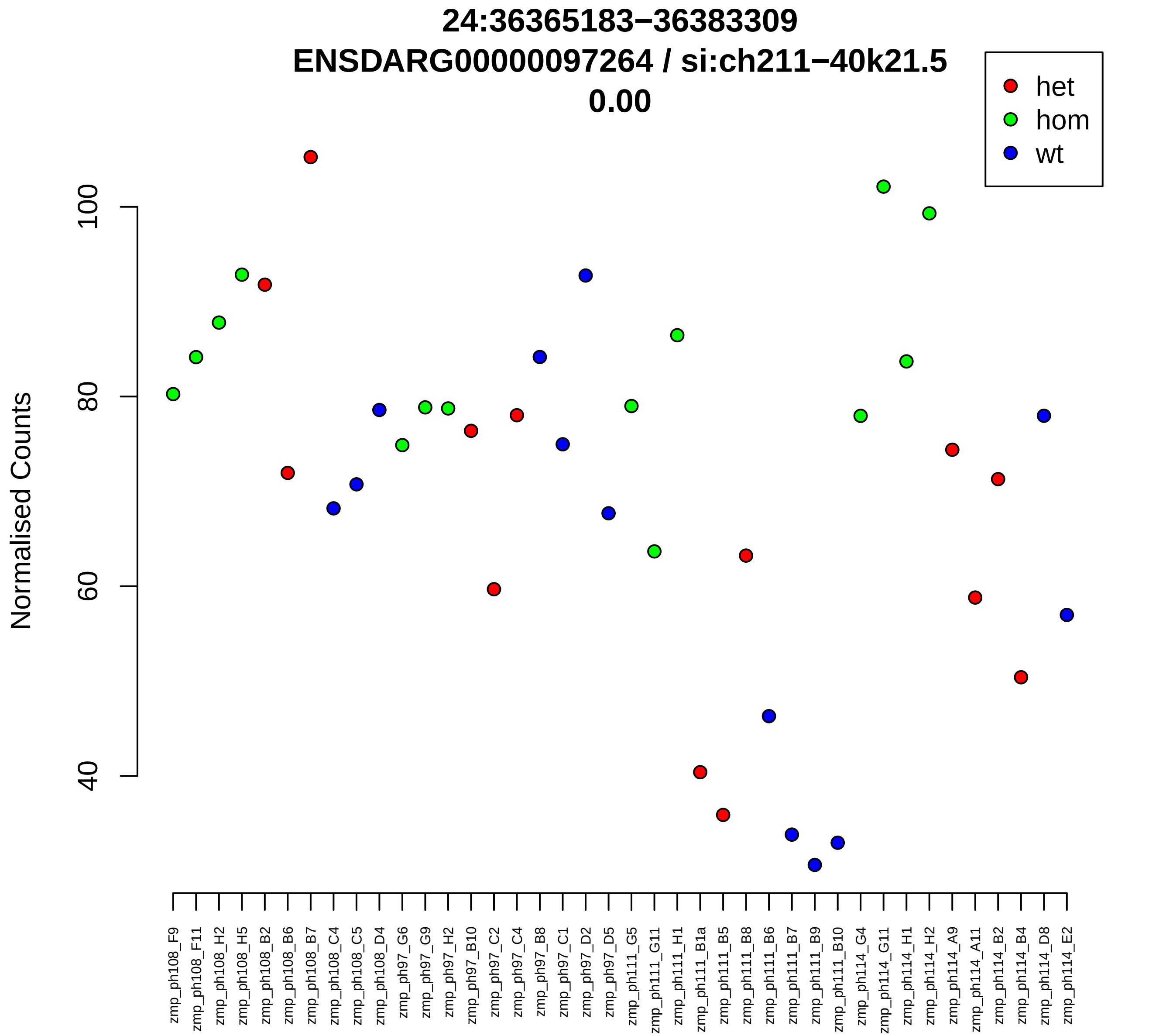
24:36365183−36383309
ENSDARG00000097264 / si:ch211−40k21.5
0.00
het
hom
wt
Normalised Counts
40
60
80
100
zmp_ph108_F9
zmp_ph108_F11
zmp_ph108_H2
zmp_ph108_H5
zmp_ph108_B2
zmp_ph108_B6
zmp_ph108_B7
zmp_ph108_C4
zmp_ph108_C5
zmp_ph108_D4
zmp_ph97_G6
zmp_ph97_G9
zmp_ph97_H2
zmp_ph97_B10
zmp_ph97_C2
zmp_ph97_C4
zmp_ph97_B8
zmp_ph97_C1
zmp_ph97_D2
zmp_ph97_D5
zmp_ph111_G5
zmp_ph111_G11
zmp_ph111_H1
zmp_ph111_B1a
zmp_ph111_B5
zmp_ph111_B8
zmp_ph111_B6
zmp_ph111_B7
zmp_ph111_B9
zmp_ph111_B10
zmp_ph114_G4
zmp_ph114_G11
zmp_ph114_H1
zmp_ph114_H2
zmp_ph114_A9
zmp_ph114_A11
zmp_ph114_B2
zmp_ph114_B4
zmp_ph114_D8
zmp_ph114_E2

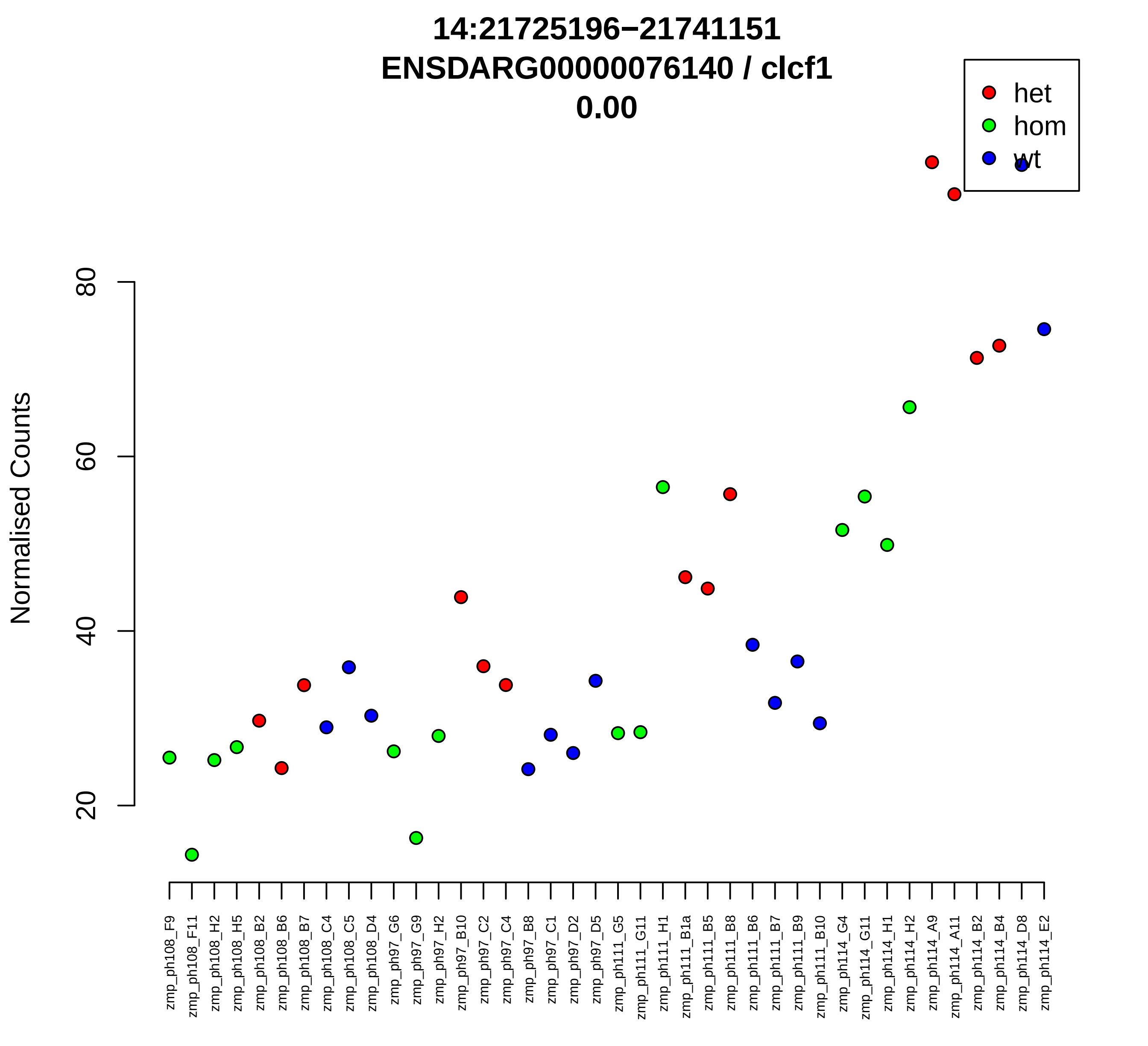

14:21725196−21741151
ENSDARG00000076140 / clcf1
0.00
het
hom
wt
Normalised Counts
20
40
60
80
zmp_ph108_F9
zmp_ph108_F11
zmp_ph108_H2
zmp_ph108_H5
zmp_ph108_B2
zmp_ph108_B6
zmp_ph108_B7
zmp_ph108_C4
zmp_ph108_C5
zmp_ph108_D4
zmp_ph97_G6
zmp_ph97_G9
zmp_ph97_H2
zmp_ph97_B10
zmp_ph97_C2
zmp_ph97_C4
zmp_ph97_B8
zmp_ph97_C1
zmp_ph97_D2
zmp_ph97_D5
zmp_ph111_G5
zmp_ph111_G11
zmp_ph111_H1
zmp_ph111_B1a
zmp_ph111_B5
zmp_ph111_B8
zmp_ph111_B6
zmp_ph111_B7
zmp_ph111_B9
zmp_ph111_B10
zmp_ph114_G4
zmp_ph114_G11
zmp_ph114_H1
zmp_ph114_H2
zmp_ph114_A9
zmp_ph114_A11
zmp_ph114_B2
zmp_ph114_B4
zmp_ph114_D8
zmp_ph114_E2

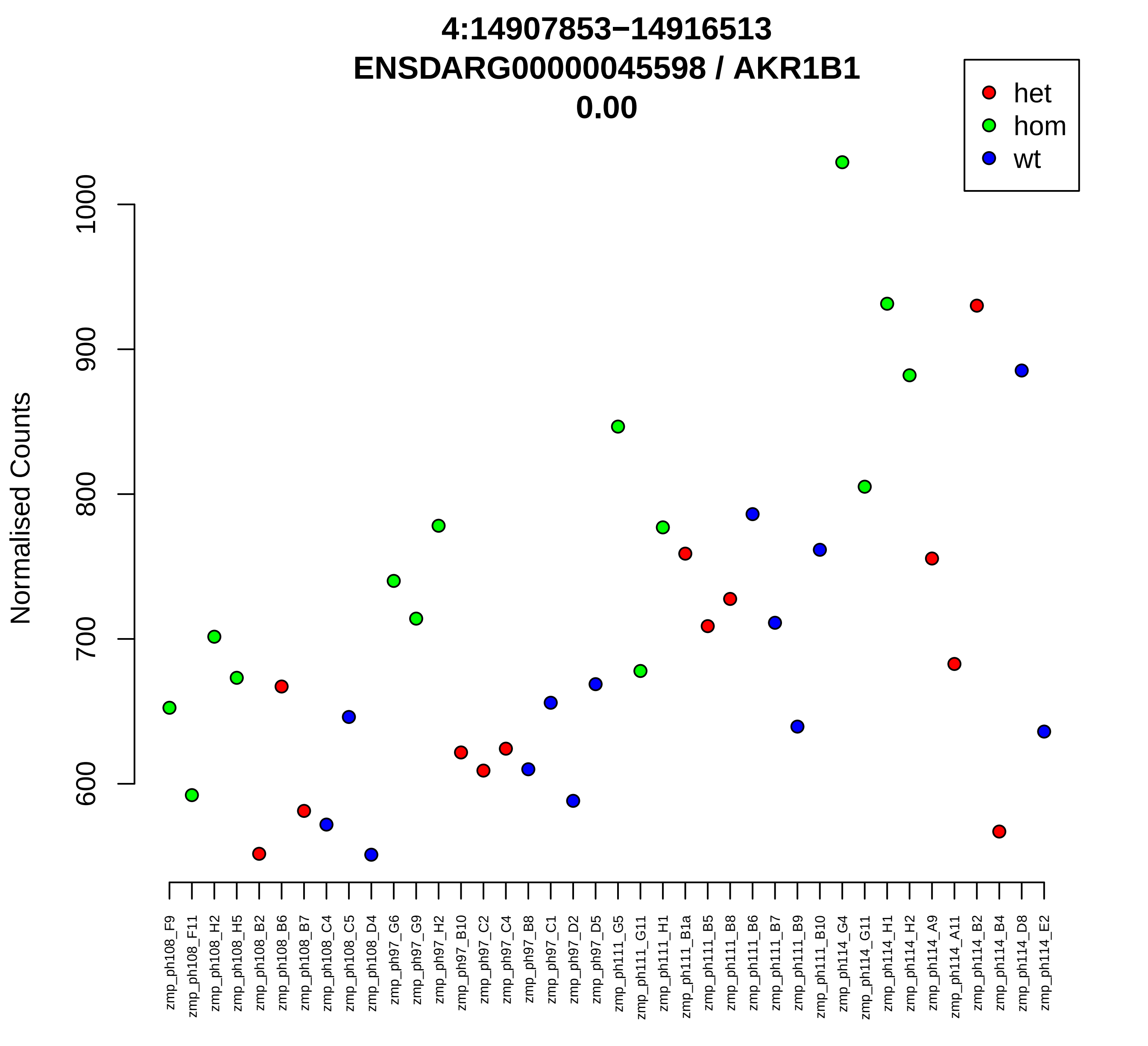
4:14907853−14916513
ENSDARG00000045598 / AKR1B1
0.00
het
hom
wt
Normalised Counts
600
700
800
900
1000
zmp_ph108_F9
zmp_ph108_F11
zmp_ph108_H2
zmp_ph108_H5
zmp_ph108_B2
zmp_ph108_B6
zmp_ph108_B7
zmp_ph108_C4
zmp_ph108_C5
zmp_ph108_D4
zmp_ph97_G6
zmp_ph97_G9
zmp_ph97_H2
zmp_ph97_B10
zmp_ph97_C2
zmp_ph97_C4
zmp_ph97_B8
zmp_ph97_C1
zmp_ph97_D2
zmp_ph97_D5
zmp_ph111_G5
zmp_ph111_G11
zmp_ph111_H1
zmp_ph111_B1a
zmp_ph111_B5
zmp_ph111_B8
zmp_ph111_B6
zmp_ph111_B7
zmp_ph111_B9
zmp_ph111_B10
zmp_ph114_G4
zmp_ph114_G11
zmp_ph114_H1
zmp_ph114_H2
zmp_ph114_A9
zmp_ph114_A11
zmp_ph114_B2
zmp_ph114_B4
zmp_ph114_D8
zmp_ph114_E2

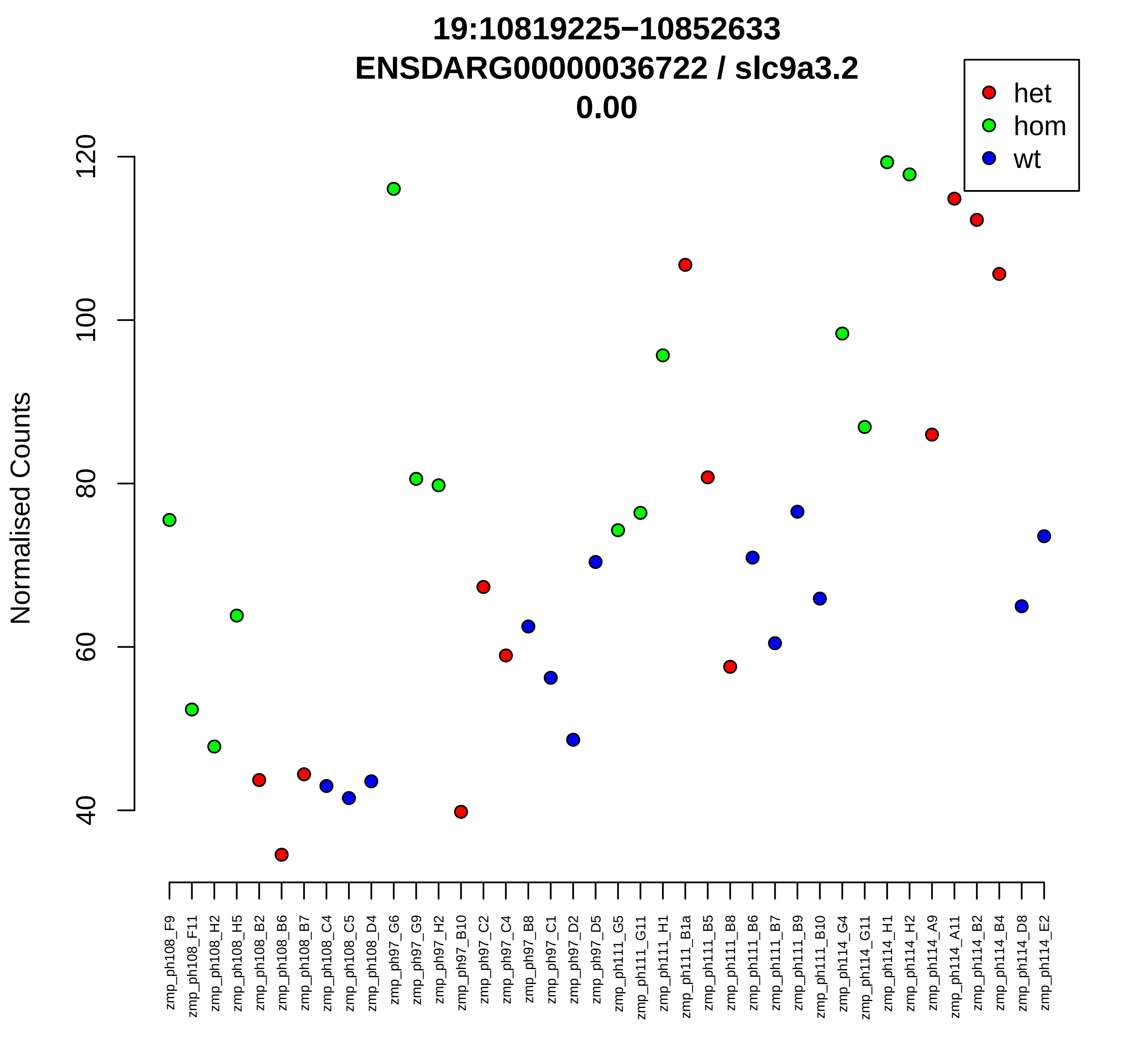

19:10819225−10852633
ENSDARG00000036722 / slc9a3.2
0.00
het
hom
wt
Normalised Counts
40
60
80
100
120
zmp_ph108_F9
zmp_ph108_F11
zmp_ph108_H2
zmp_ph108_H5
zmp_ph108_B2
zmp_ph108_B6
zmp_ph108_B7
zmp_ph108_C4
zmp_ph108_C5
zmp_ph108_D4
zmp_ph97_G6
zmp_ph97_G9
zmp_ph97_H2
zmp_ph97_B10
zmp_ph97_C2
zmp_ph97_C4
zmp_ph97_B8
zmp_ph97_C1
zmp_ph97_D2
zmp_ph97_D5
zmp_ph111_G5
zmp_ph111_G11
zmp_ph111_H1
zmp_ph111_B1a
zmp_ph111_B5
zmp_ph111_B8
zmp_ph111_B6
zmp_ph111_B7
zmp_ph111_B9
zmp_ph111_B10
zmp_ph114_G4
zmp_ph114_G11
zmp_ph114_H1
zmp_ph114_H2
zmp_ph114_A9
zmp_ph114_A11
zmp_ph114_B2
zmp_ph114_B4
zmp_ph114_D8
zmp_ph114_E2

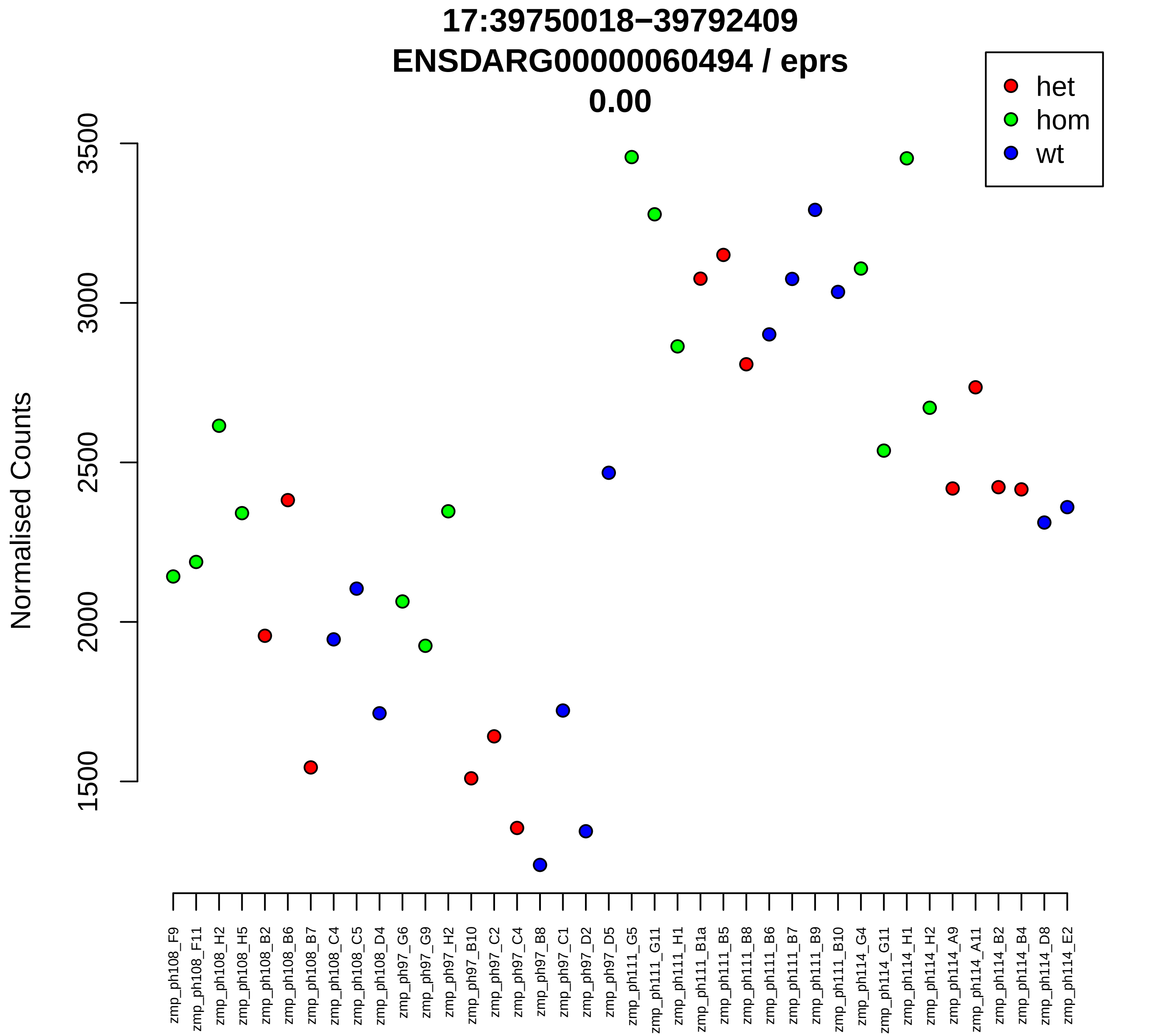
17:39750018−39792409
ENSDARG00000060494 / eprs
0.00
het
hom
wt
Normalised Counts
1500
2000
2500
3000
3500
zmp_ph108_F9
zmp_ph108_F11
zmp_ph108_H2
zmp_ph108_H5
zmp_ph108_B2
zmp_ph108_B6
zmp_ph108_B7
zmp_ph108_C4
zmp_ph108_C5
zmp_ph108_D4
zmp_ph97_G6
zmp_ph97_G9
zmp_ph97_H2
zmp_ph97_B10
zmp_ph97_C2
zmp_ph97_C4
zmp_ph97_B8
zmp_ph97_C1
zmp_ph97_D2
zmp_ph97_D5
zmp_ph111_G5
zmp_ph111_G11
zmp_ph111_H1
zmp_ph111_B1a
zmp_ph111_B5
zmp_ph111_B8
zmp_ph111_B6
zmp_ph111_B7
zmp_ph111_B9
zmp_ph111_B10
zmp_ph114_G4
zmp_ph114_G11
zmp_ph114_H1
zmp_ph114_H2
zmp_ph114_A9
zmp_ph114_A11
zmp_ph114_B2
zmp_ph114_B4
zmp_ph114_D8
zmp_ph114_E2

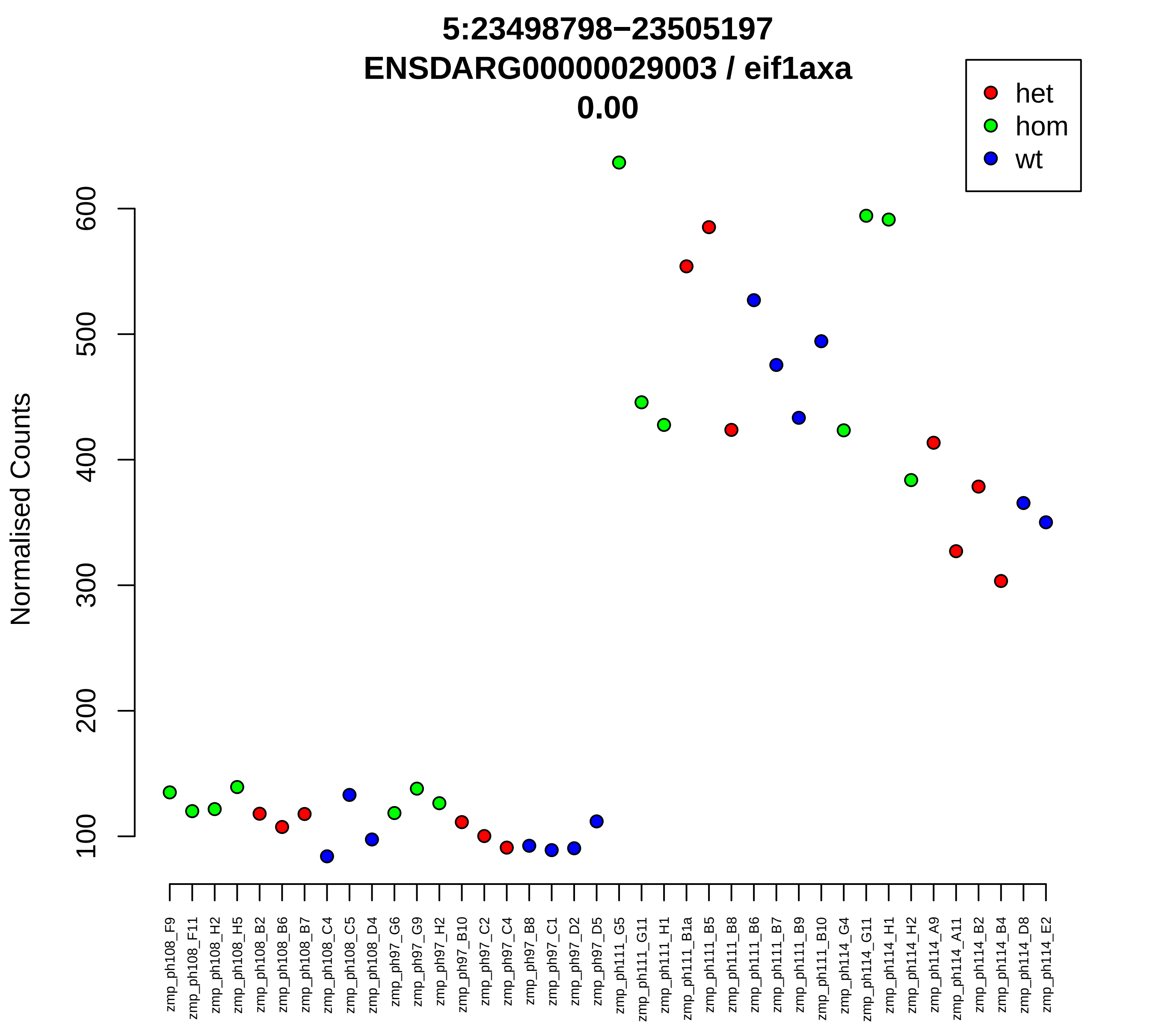
5:23498798−23505197
ENSDARG00000029003 / eif1axa
0.00
het
hom
wt
Normalised Counts
100
200
300
400
500
600
zmp_ph108_F9
zmp_ph108_F11
zmp_ph108_H2
zmp_ph108_H5
zmp_ph108_B2
zmp_ph108_B6
zmp_ph108_B7
zmp_ph108_C4
zmp_ph108_C5
zmp_ph108_D4
zmp_ph97_G6
zmp_ph97_G9
zmp_ph97_H2
zmp_ph97_B10
zmp_ph97_C2
zmp_ph97_C4
zmp_ph97_B8
zmp_ph97_C1
zmp_ph97_D2
zmp_ph97_D5
zmp_ph111_G5
zmp_ph111_G11
zmp_ph111_H1
zmp_ph111_B1a
zmp_ph111_B5
zmp_ph111_B8
zmp_ph111_B6
zmp_ph111_B7
zmp_ph111_B9
zmp_ph111_B10
zmp_ph114_G4
zmp_ph114_G11
zmp_ph114_H1
zmp_ph114_H2
zmp_ph114_A9
zmp_ph114_A11
zmp_ph114_B2
zmp_ph114_B4
zmp_ph114_D8
zmp_ph114_E2

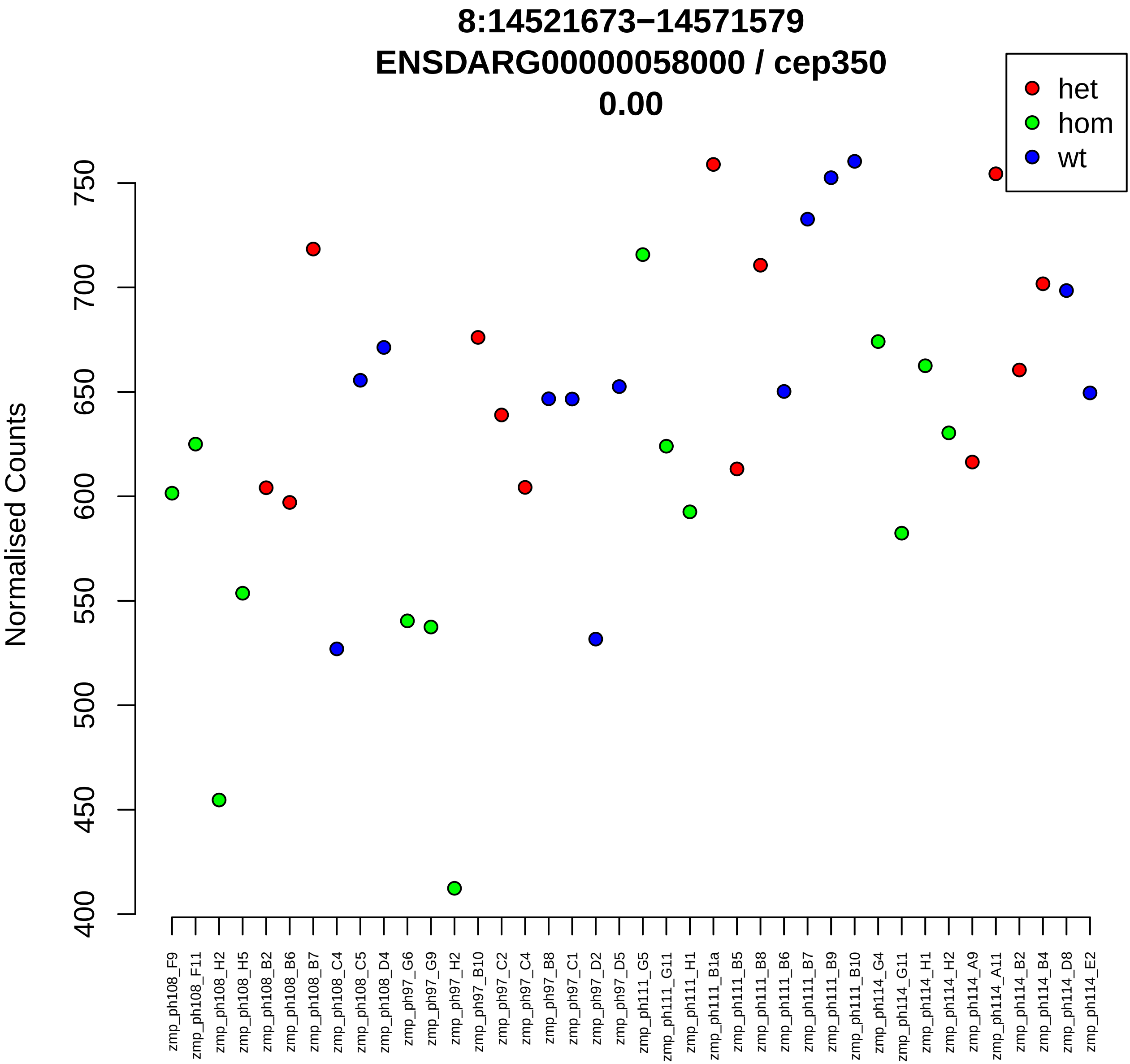
8:14521673−14571579
ENSDARG00000058000 / cep350
0.00
het
hom
wt
Normalised Counts
400
450
500
550
600
650
700
750
zmp_ph108_F9
zmp_ph108_F11
zmp_ph108_H2
zmp_ph108_H5
zmp_ph108_B2
zmp_ph108_B6
zmp_ph108_B7
zmp_ph108_C4
zmp_ph108_C5
zmp_ph108_D4
zmp_ph97_G6
zmp_ph97_G9
zmp_ph97_H2
zmp_ph97_B10
zmp_ph97_C2
zmp_ph97_C4
zmp_ph97_B8
zmp_ph97_C1
zmp_ph97_D2
zmp_ph97_D5
zmp_ph111_G5
zmp_ph111_G11
zmp_ph111_H1
zmp_ph111_B1a
zmp_ph111_B5
zmp_ph111_B8
zmp_ph111_B6
zmp_ph111_B7
zmp_ph111_B9
zmp_ph111_B10
zmp_ph114_G4
zmp_ph114_G11
zmp_ph114_H1
zmp_ph114_H2
zmp_ph114_A9
zmp_ph114_A11
zmp_ph114_B2
zmp_ph114_B4
zmp_ph114_D8
zmp_ph114_E2

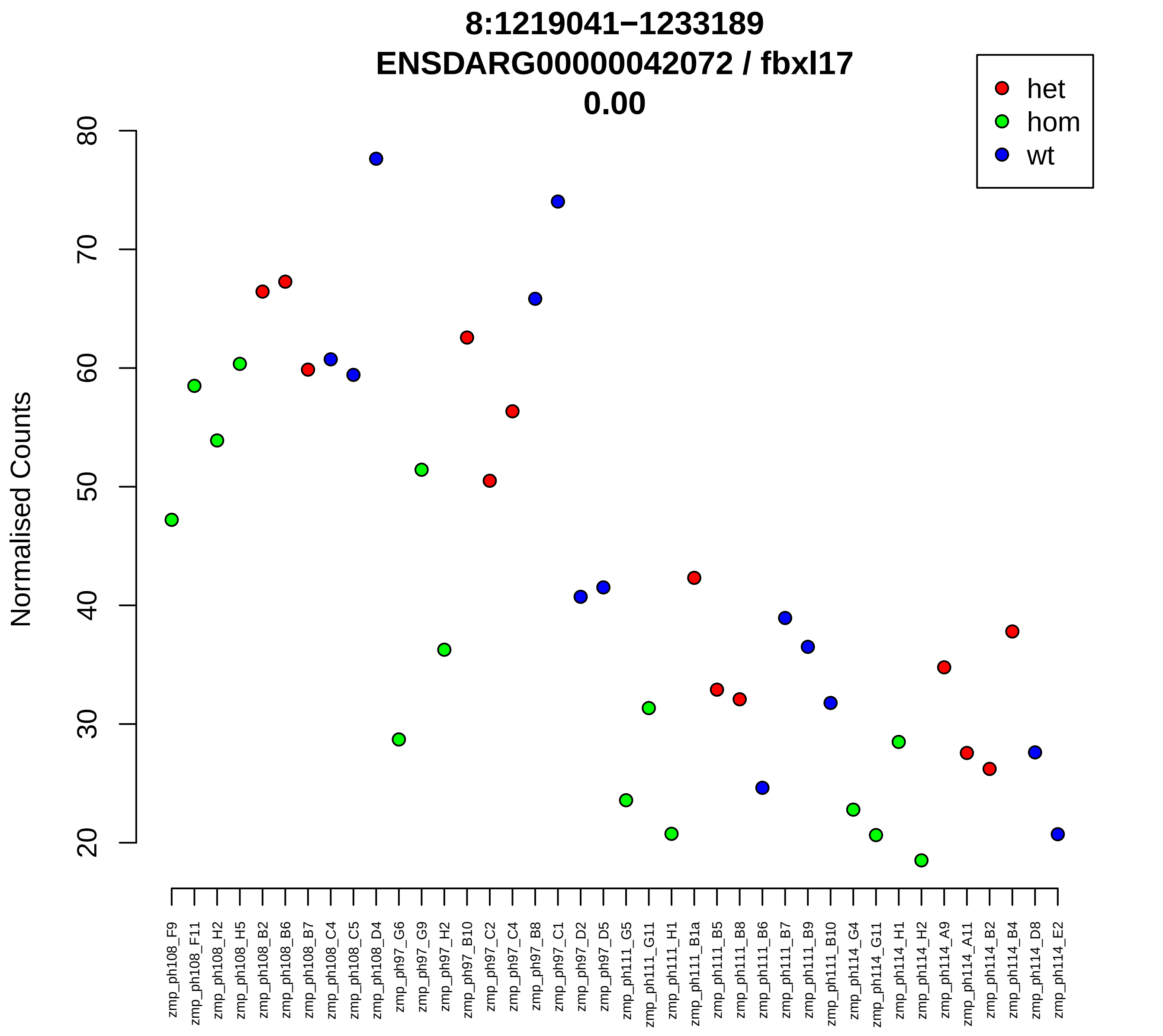

8:1219041−1233189
ENSDARG00000042072 / fbxl17
0.00
het
hom
wt
Normalised Counts
20
30
40
50
60
70
80
zmp_ph108_F9
zmp_ph108_F11
zmp_ph108_H2
zmp_ph108_H5
zmp_ph108_B2
zmp_ph108_B6
zmp_ph108_B7
zmp_ph108_C4
zmp_ph108_C5
zmp_ph108_D4
zmp_ph97_G6
zmp_ph97_G9
zmp_ph97_H2
zmp_ph97_B10
zmp_ph97_C2
zmp_ph97_C4
zmp_ph97_B8
zmp_ph97_C1
zmp_ph97_D2
zmp_ph97_D5
zmp_ph111_G5
zmp_ph111_G11
zmp_ph111_H1
zmp_ph111_B1a
zmp_ph111_B5
zmp_ph111_B8
zmp_ph111_B6
zmp_ph111_B7
zmp_ph111_B9
zmp_ph111_B10
zmp_ph114_G4
zmp_ph114_G11
zmp_ph114_H1
zmp_ph114_H2
zmp_ph114_A9
zmp_ph114_A11
zmp_ph114_B2
zmp_ph114_B4
zmp_ph114_D8
zmp_ph114_E2

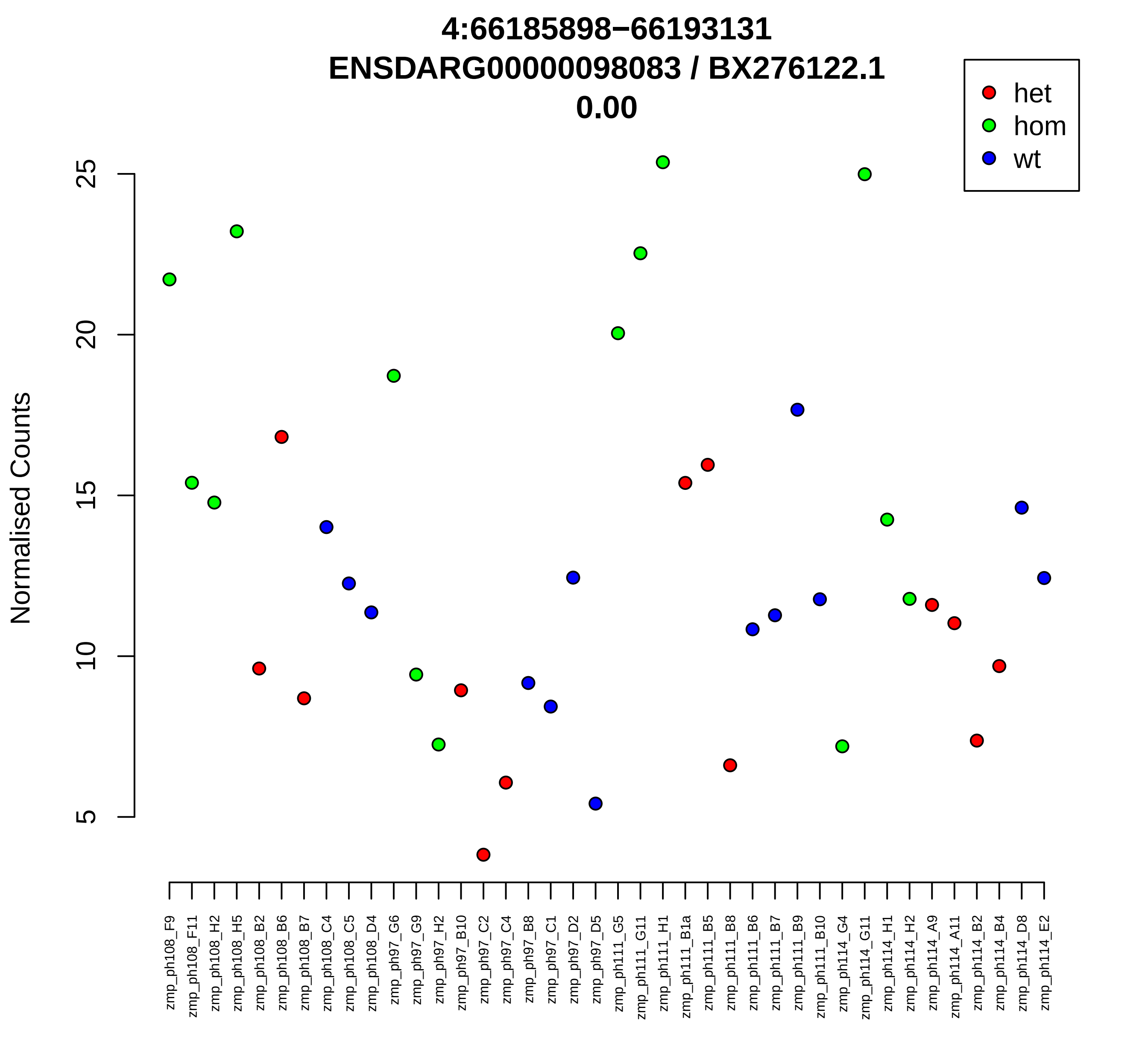
4:66185898−66193131
ENSDARG00000098083 / BX276122.1
0.00
het
hom
wt
Normalised Counts
5
10
15
20
25
zmp_ph108_F9
zmp_ph108_F11
zmp_ph108_H2
zmp_ph108_H5
zmp_ph108_B2
zmp_ph108_B6
zmp_ph108_B7
zmp_ph108_C4
zmp_ph108_C5
zmp_ph108_D4
zmp_ph97_G6
zmp_ph97_G9
zmp_ph97_H2
zmp_ph97_B10
zmp_ph97_C2
zmp_ph97_C4
zmp_ph97_B8
zmp_ph97_C1
zmp_ph97_D2
zmp_ph97_D5
zmp_ph111_G5
zmp_ph111_G11
zmp_ph111_H1
zmp_ph111_B1a
zmp_ph111_B5
zmp_ph111_B8
zmp_ph111_B6
zmp_ph111_B7
zmp_ph111_B9
zmp_ph111_B10
zmp_ph114_G4
zmp_ph114_G11
zmp_ph114_H1
zmp_ph114_H2
zmp_ph114_A9
zmp_ph114_A11
zmp_ph114_B2
zmp_ph114_B4
zmp_ph114_D8
zmp_ph114_E2

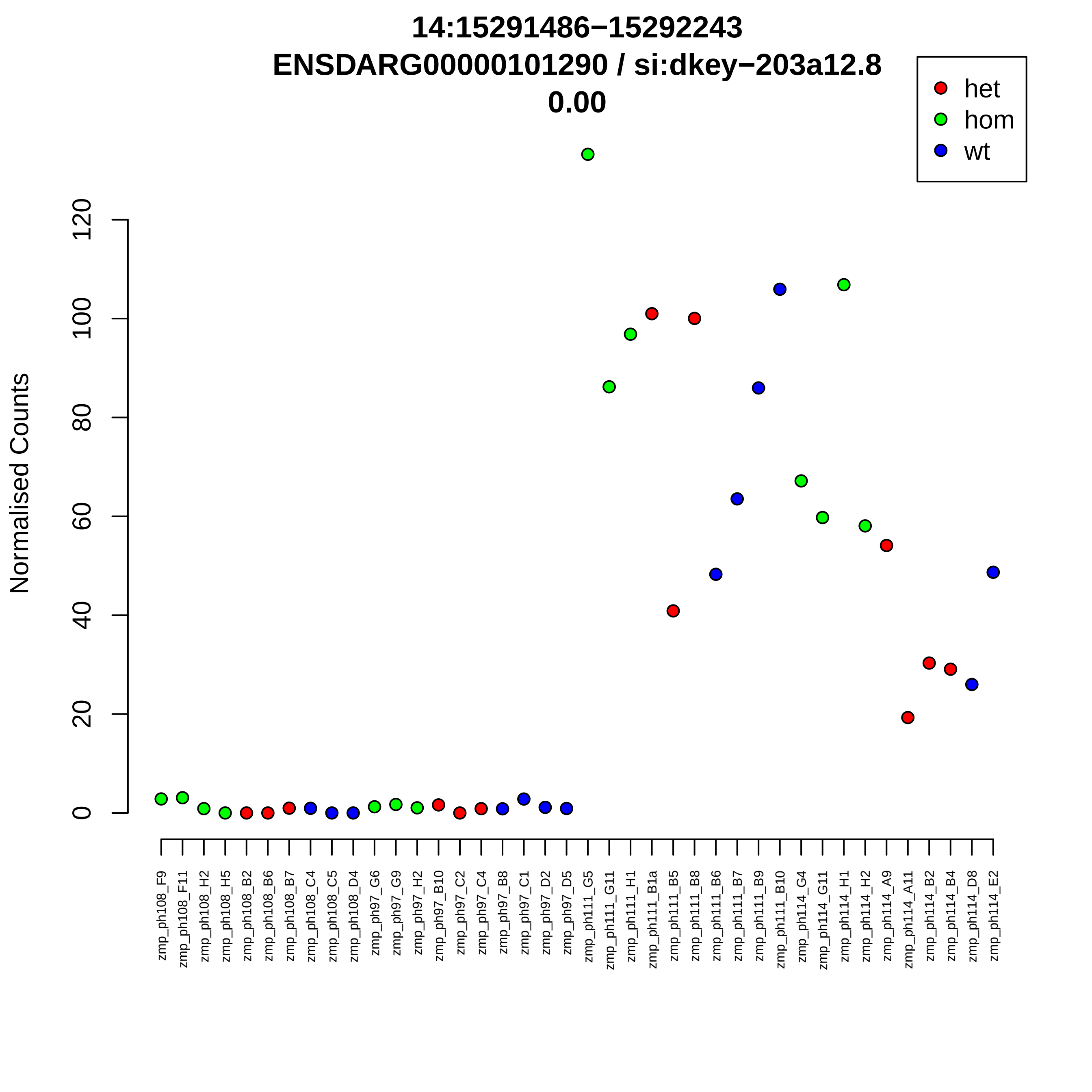

14:15291486−15292243
ENSDARG00000101290 / si:dkey−203a12.8
0.00
het
hom
wt
Normalised Counts
0
20
40
60
80
100
120
zmp_ph108_F9
zmp_ph108_F11
zmp_ph108_H2
zmp_ph108_H5
zmp_ph108_B2
zmp_ph108_B6
zmp_ph108_B7
zmp_ph108_C4
zmp_ph108_C5
zmp_ph108_D4
zmp_ph97_G6
zmp_ph97_G9
zmp_ph97_H2
zmp_ph97_B10
zmp_ph97_C2
zmp_ph97_C4
zmp_ph97_B8
zmp_ph97_C1
zmp_ph97_D2
zmp_ph97_D5
zmp_ph111_G5
zmp_ph111_G11
zmp_ph111_H1
zmp_ph111_B1a
zmp_ph111_B5
zmp_ph111_B8
zmp_ph111_B6
zmp_ph111_B7
zmp_ph111_B9
zmp_ph111_B10
zmp_ph114_G4
zmp_ph114_G11
zmp_ph114_H1
zmp_ph114_H2
zmp_ph114_A9
zmp_ph114_A11
zmp_ph114_B2
zmp_ph114_B4
zmp_ph114_D8
zmp_ph114_E2

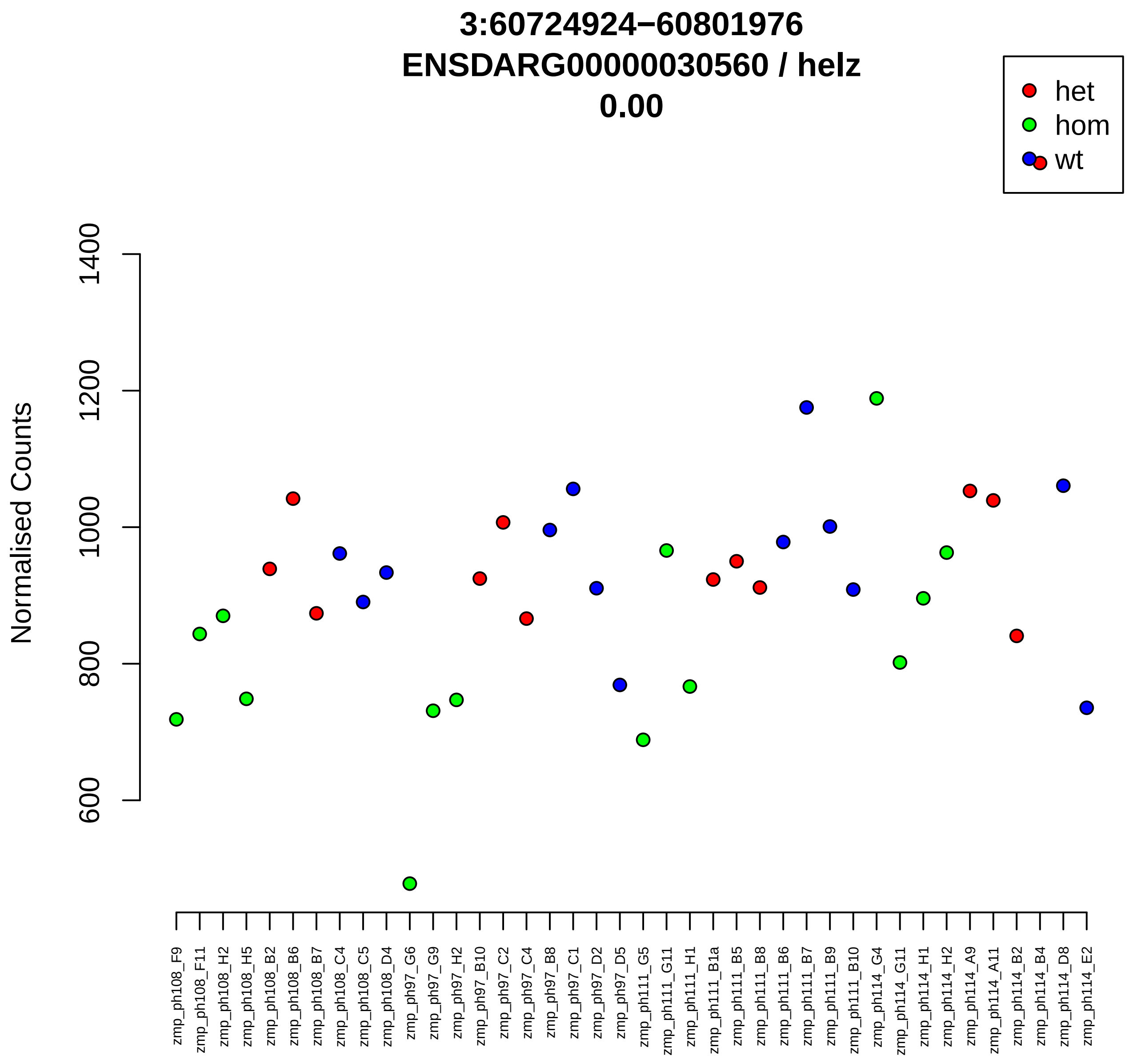
3:60724924−60801976
ENSDARG00000030560 / helz
0.00
het
hom
wt
Normalised Counts
600
800
1000
1200
1400
zmp_ph108_F9
zmp_ph108_F11
zmp_ph108_H2
zmp_ph108_H5
zmp_ph108_B2
zmp_ph108_B6
zmp_ph108_B7
zmp_ph108_C4
zmp_ph108_C5
zmp_ph108_D4
zmp_ph97_G6
zmp_ph97_G9
zmp_ph97_H2
zmp_ph97_B10
zmp_ph97_C2
zmp_ph97_C4
zmp_ph97_B8
zmp_ph97_C1
zmp_ph97_D2
zmp_ph97_D5
zmp_ph111_G5
zmp_ph111_G11
zmp_ph111_H1
zmp_ph111_B1a
zmp_ph111_B5
zmp_ph111_B8
zmp_ph111_B6
zmp_ph111_B7
zmp_ph111_B9
zmp_ph111_B10
zmp_ph114_G4
zmp_ph114_G11
zmp_ph114_H1
zmp_ph114_H2
zmp_ph114_A9
zmp_ph114_A11
zmp_ph114_B2
zmp_ph114_B4
zmp_ph114_D8
zmp_ph114_E2

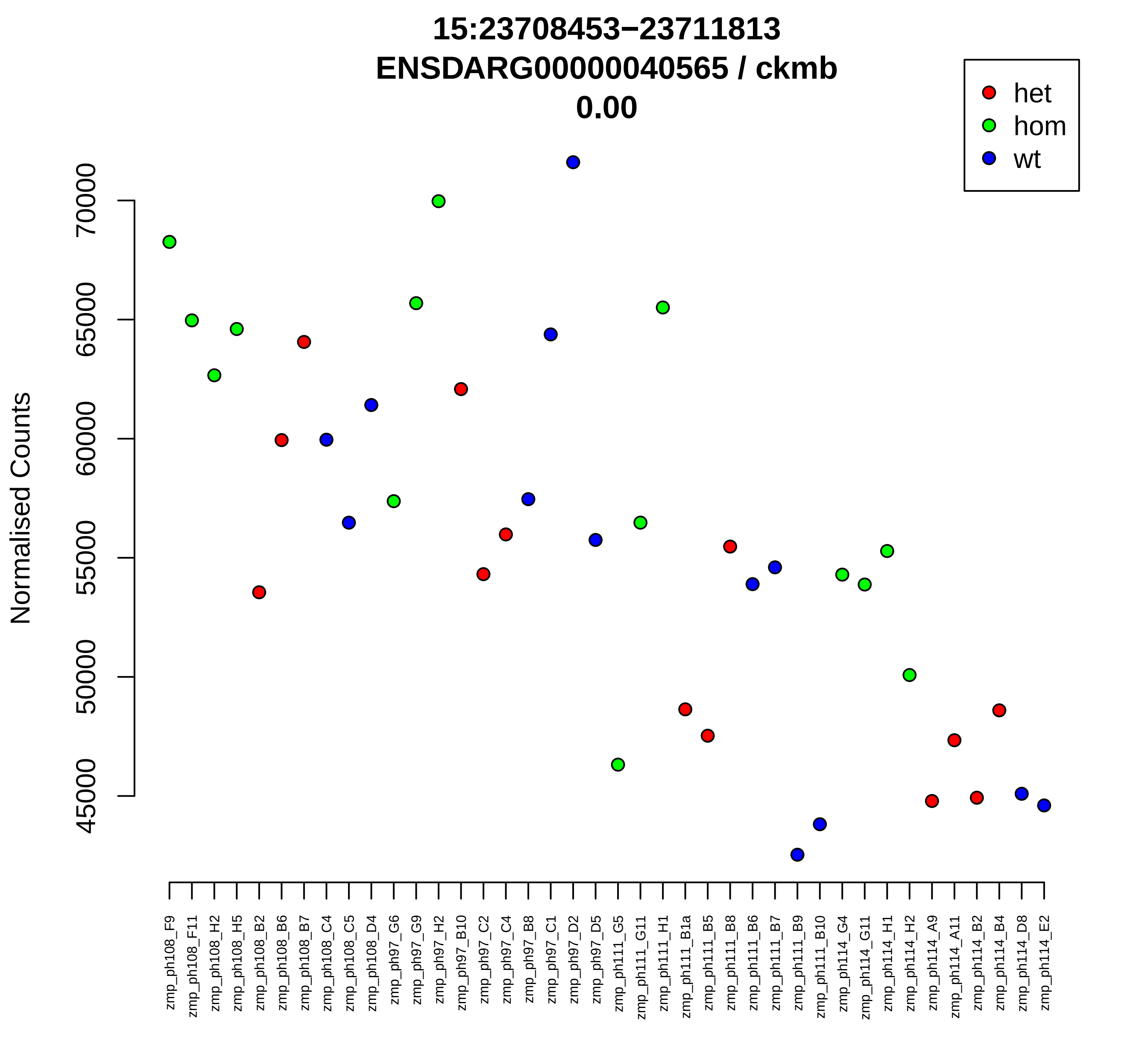

15:23708453−23711813
ENSDARG00000040565 / ckmb
0.00
het
hom
wt
Normalised Counts
45000
50000
55000
60000
65000
70000
zmp_ph108_F9
zmp_ph108_F11
zmp_ph108_H2
zmp_ph108_H5
zmp_ph108_B2
zmp_ph108_B6
zmp_ph108_B7
zmp_ph108_C4
zmp_ph108_C5
zmp_ph108_D4
zmp_ph97_G6
zmp_ph97_G9
zmp_ph97_H2
zmp_ph97_B10
zmp_ph97_C2
zmp_ph97_C4
zmp_ph97_B8
zmp_ph97_C1
zmp_ph97_D2
zmp_ph97_D5
zmp_ph111_G5
zmp_ph111_G11
zmp_ph111_H1
zmp_ph111_B1a
zmp_ph111_B5
zmp_ph111_B8
zmp_ph111_B6
zmp_ph111_B7
zmp_ph111_B9
zmp_ph111_B10
zmp_ph114_G4
zmp_ph114_G11
zmp_ph114_H1
zmp_ph114_H2
zmp_ph114_A9
zmp_ph114_A11
zmp_ph114_B2
zmp_ph114_B4
zmp_ph114_D8
zmp_ph114_E2

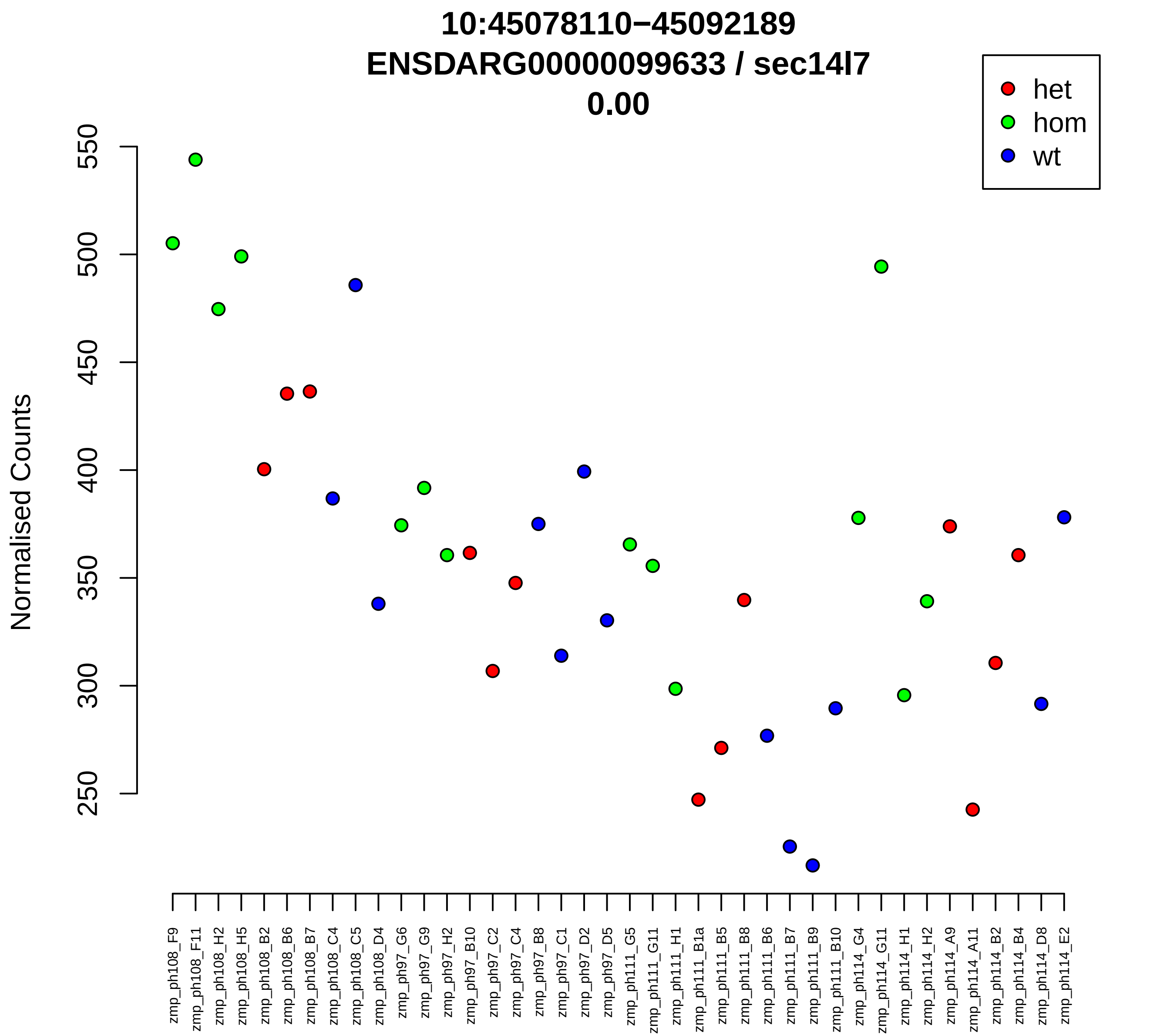
10:45078110−45092189
ENSDARG00000099633 / sec14l7
0.00
het
hom
wt
Normalised Counts
250
300
350
400
450
500
550
zmp_ph108_F9
zmp_ph108_F11
zmp_ph108_H2
zmp_ph108_H5
zmp_ph108_B2
zmp_ph108_B6
zmp_ph108_B7
zmp_ph108_C4
zmp_ph108_C5
zmp_ph108_D4
zmp_ph97_G6
zmp_ph97_G9
zmp_ph97_H2
zmp_ph97_B10
zmp_ph97_C2
zmp_ph97_C4
zmp_ph97_B8
zmp_ph97_C1
zmp_ph97_D2
zmp_ph97_D5
zmp_ph111_G5
zmp_ph111_G11
zmp_ph111_H1
zmp_ph111_B1a
zmp_ph111_B5
zmp_ph111_B8
zmp_ph111_B6
zmp_ph111_B7
zmp_ph111_B9
zmp_ph111_B10
zmp_ph114_G4
zmp_ph114_G11
zmp_ph114_H1
zmp_ph114_H2
zmp_ph114_A9
zmp_ph114_A11
zmp_ph114_B2
zmp_ph114_B4
zmp_ph114_D8
zmp_ph114_E2

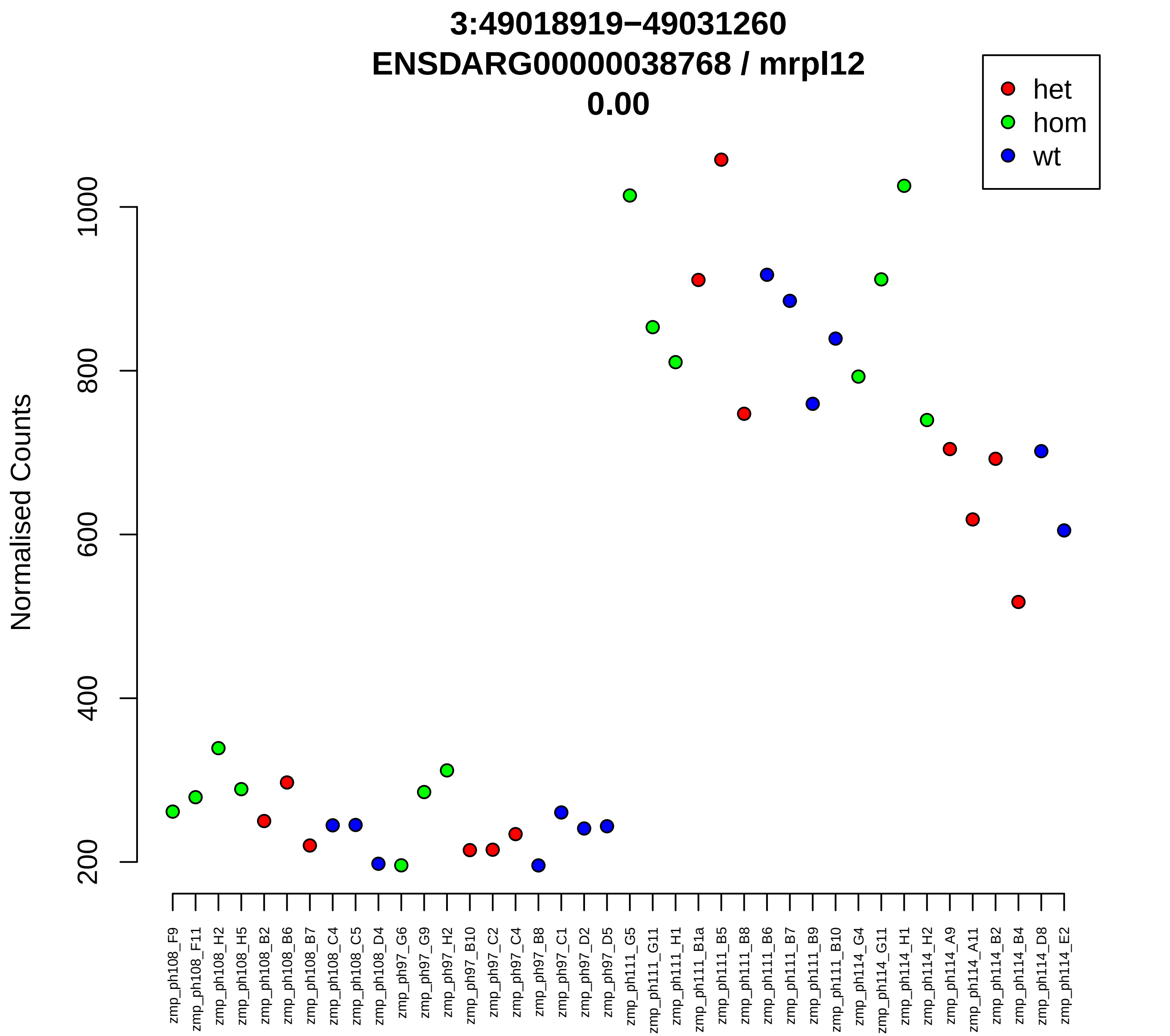

3:49018919−49031260
ENSDARG00000038768 / mrpl12
0.00
het
hom
wt
Normalised Counts
200
400
600
800
1000
zmp_ph108_F9
zmp_ph108_F11
zmp_ph108_H2
zmp_ph108_H5
zmp_ph108_B2
zmp_ph108_B6
zmp_ph108_B7
zmp_ph108_C4
zmp_ph108_C5
zmp_ph108_D4
zmp_ph97_G6
zmp_ph97_G9
zmp_ph97_H2
zmp_ph97_B10
zmp_ph97_C2
zmp_ph97_C4
zmp_ph97_B8
zmp_ph97_C1
zmp_ph97_D2
zmp_ph97_D5
zmp_ph111_G5
zmp_ph111_G11
zmp_ph111_H1
zmp_ph111_B1a
zmp_ph111_B5
zmp_ph111_B8
zmp_ph111_B6
zmp_ph111_B7
zmp_ph111_B9
zmp_ph111_B10
zmp_ph114_G4
zmp_ph114_G11
zmp_ph114_H1
zmp_ph114_H2
zmp_ph114_A9
zmp_ph114_A11
zmp_ph114_B2
zmp_ph114_B4
zmp_ph114_D8
zmp_ph114_E2

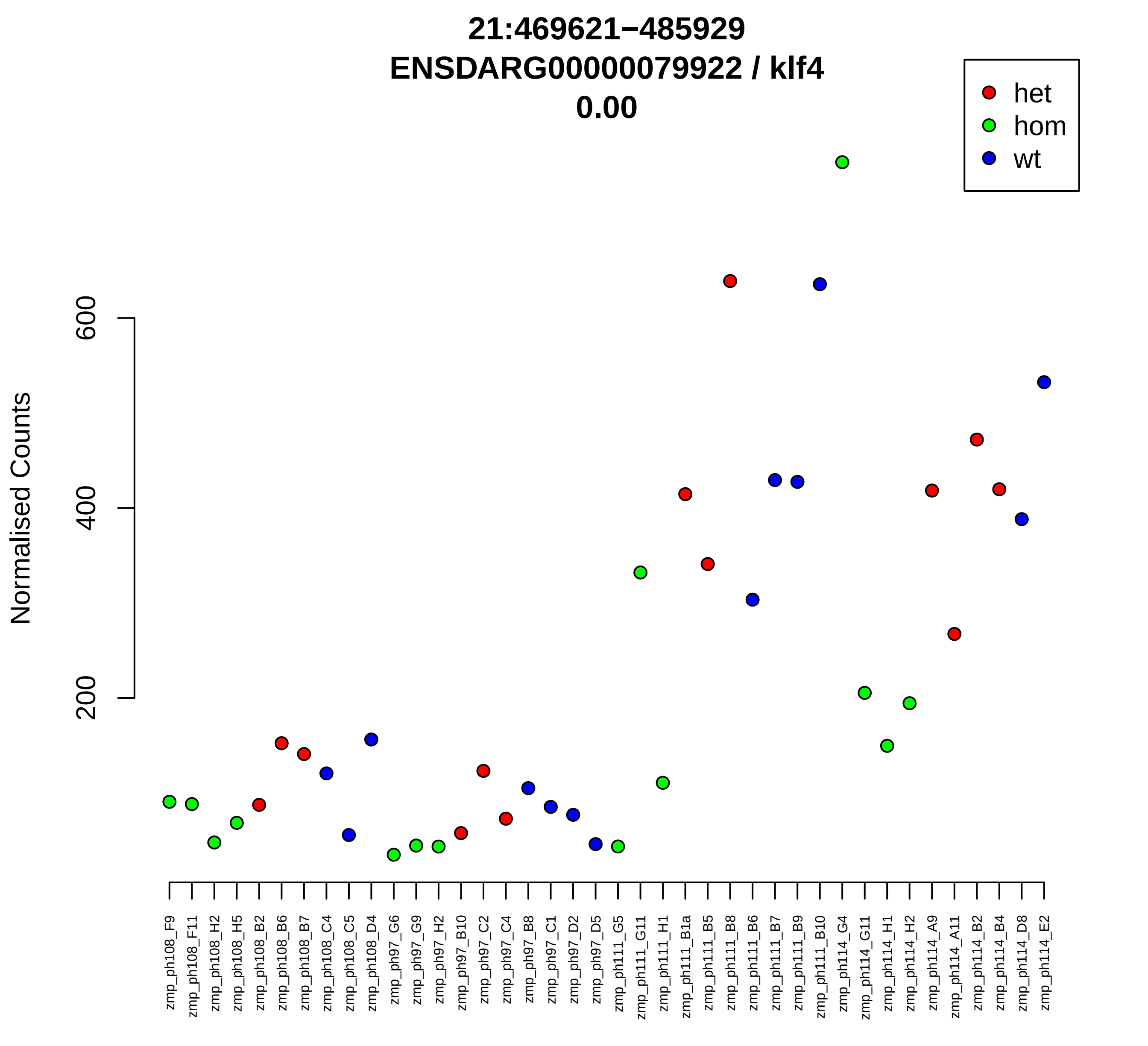
21:469621−485929
ENSDARG00000079922 / klf4
0.00
het
hom
wt
Normalised Counts
200
400
600
zmp_ph108_F9
zmp_ph108_F11
zmp_ph108_H2
zmp_ph108_H5
zmp_ph108_B2
zmp_ph108_B6
zmp_ph108_B7
zmp_ph108_C4
zmp_ph108_C5
zmp_ph108_D4
zmp_ph97_G6
zmp_ph97_G9
zmp_ph97_H2
zmp_ph97_B10
zmp_ph97_C2
zmp_ph97_C4
zmp_ph97_B8
zmp_ph97_C1
zmp_ph97_D2
zmp_ph97_D5
zmp_ph111_G5
zmp_ph111_G11
zmp_ph111_H1
zmp_ph111_B1a
zmp_ph111_B5
zmp_ph111_B8
zmp_ph111_B6
zmp_ph111_B7
zmp_ph111_B9
zmp_ph111_B10
zmp_ph114_G4
zmp_ph114_G11
zmp_ph114_H1
zmp_ph114_H2
zmp_ph114_A9
zmp_ph114_A11
zmp_ph114_B2
zmp_ph114_B4
zmp_ph114_D8
zmp_ph114_E2

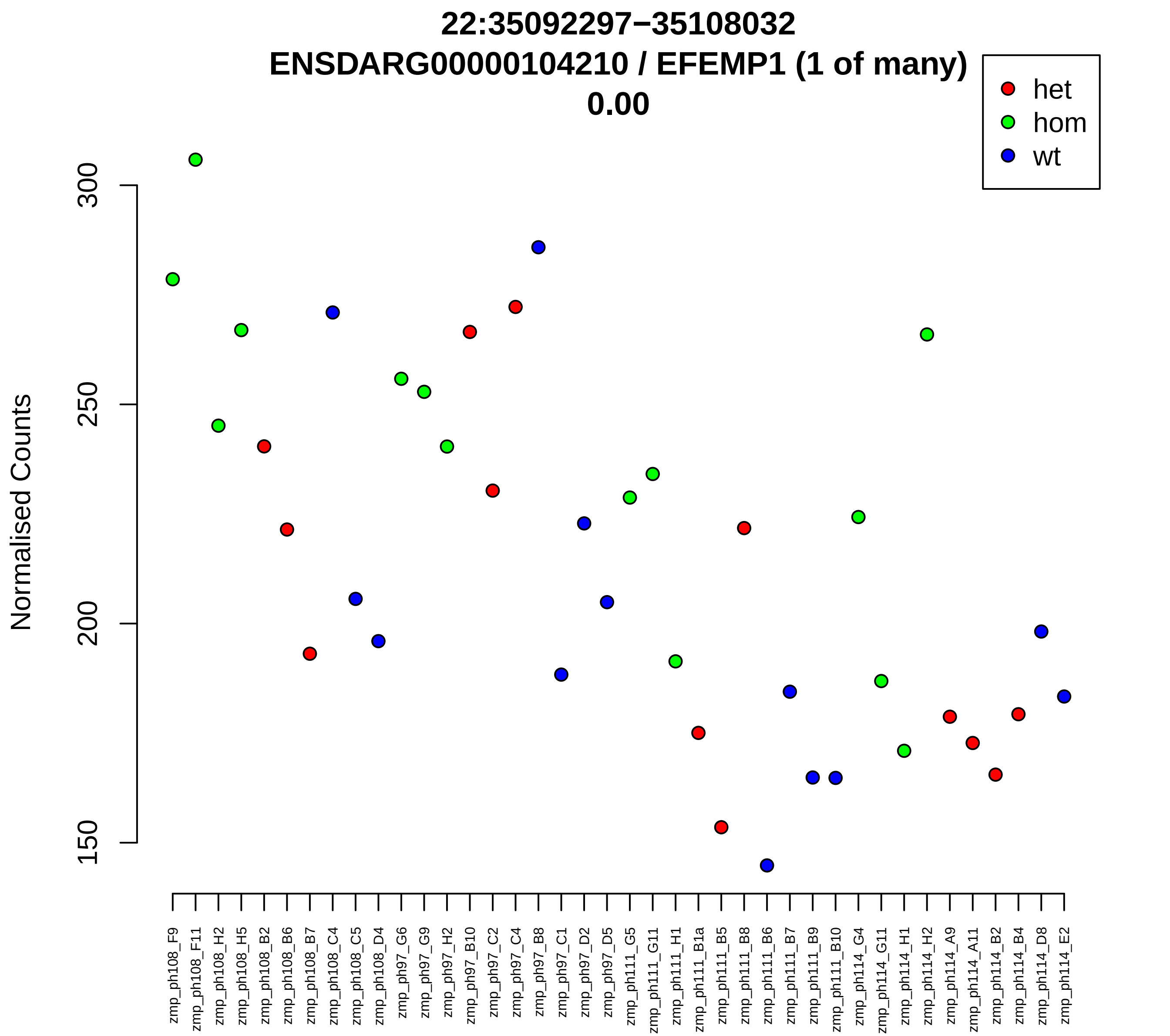
22:35092297−35108032
ENSDARG00000104210 / EFEMP1 (1 of many)
0.00
het
hom
wt
Normalised Counts
150
200
250
300
zmp_ph108_F9
zmp_ph108_F11
zmp_ph108_H2
zmp_ph108_H5
zmp_ph108_B2
zmp_ph108_B6
zmp_ph108_B7
zmp_ph108_C4
zmp_ph108_C5
zmp_ph108_D4
zmp_ph97_G6
zmp_ph97_G9
zmp_ph97_H2
zmp_ph97_B10
zmp_ph97_C2
zmp_ph97_C4
zmp_ph97_B8
zmp_ph97_C1
zmp_ph97_D2
zmp_ph97_D5
zmp_ph111_G5
zmp_ph111_G11
zmp_ph111_H1
zmp_ph111_B1a
zmp_ph111_B5
zmp_ph111_B8
zmp_ph111_B6
zmp_ph111_B7
zmp_ph111_B9
zmp_ph111_B10
zmp_ph114_G4
zmp_ph114_G11
zmp_ph114_H1
zmp_ph114_H2
zmp_ph114_A9
zmp_ph114_A11
zmp_ph114_B2
zmp_ph114_B4
zmp_ph114_D8
zmp_ph114_E2

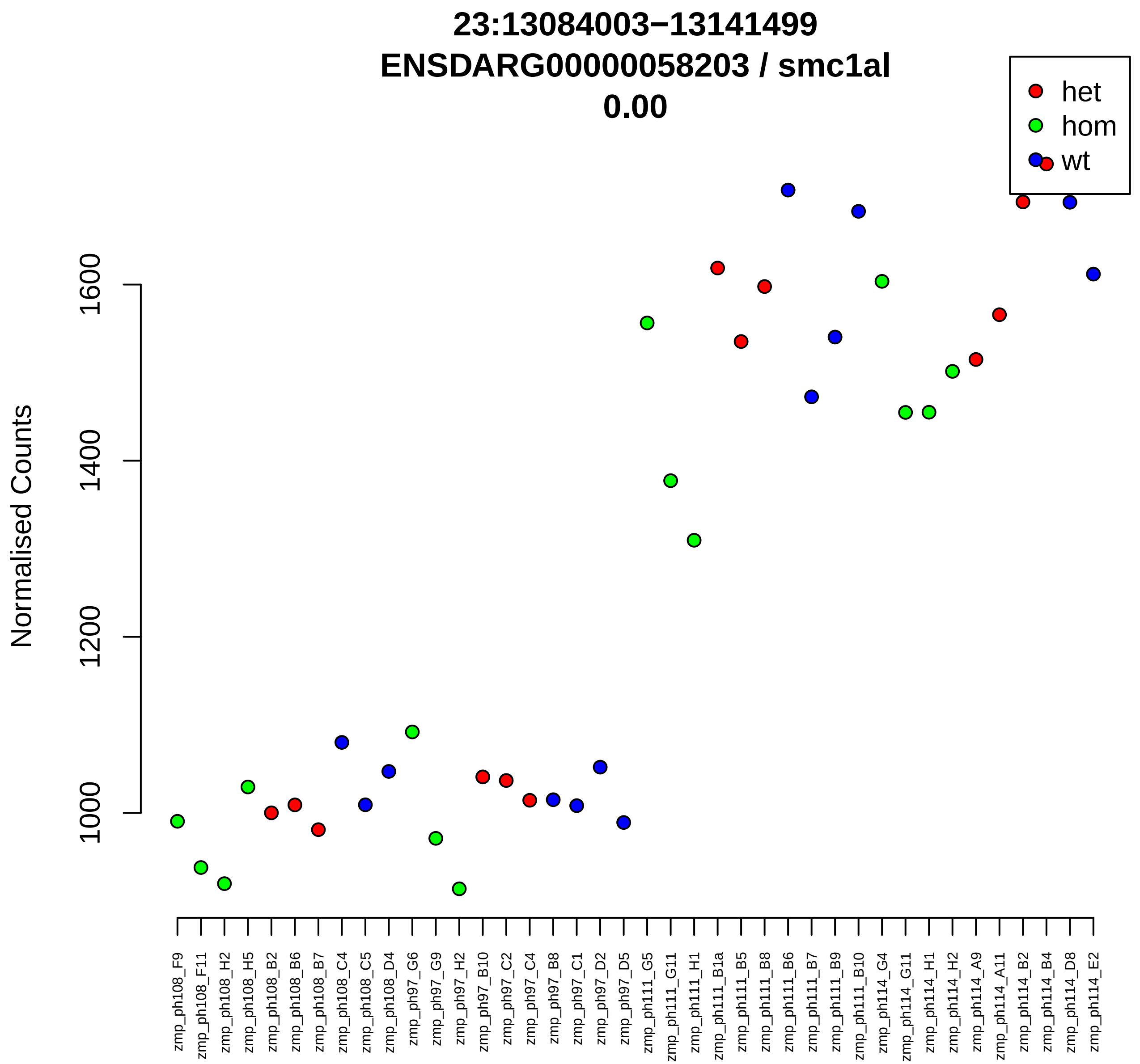

23:13084003−13141499
ENSDARG00000058203 / smc1al
0.00
het
hom
wt
Normalised Counts
1000
1200
1400
1600
zmp_ph108_F9
zmp_ph108_F11
zmp_ph108_H2
zmp_ph108_H5
zmp_ph108_B2
zmp_ph108_B6
zmp_ph108_B7
zmp_ph108_C4
zmp_ph108_C5
zmp_ph108_D4
zmp_ph97_G6
zmp_ph97_G9
zmp_ph97_H2
zmp_ph97_B10
zmp_ph97_C2
zmp_ph97_C4
zmp_ph97_B8
zmp_ph97_C1
zmp_ph97_D2
zmp_ph97_D5
zmp_ph111_G5
zmp_ph111_G11
zmp_ph111_H1
zmp_ph111_B1a
zmp_ph111_B5
zmp_ph111_B8
zmp_ph111_B6
zmp_ph111_B7
zmp_ph111_B9
zmp_ph111_B10
zmp_ph114_G4
zmp_ph114_G11
zmp_ph114_H1
zmp_ph114_H2
zmp_ph114_A9
zmp_ph114_A11
zmp_ph114_B2
zmp_ph114_B4
zmp_ph114_D8
zmp_ph114_E2

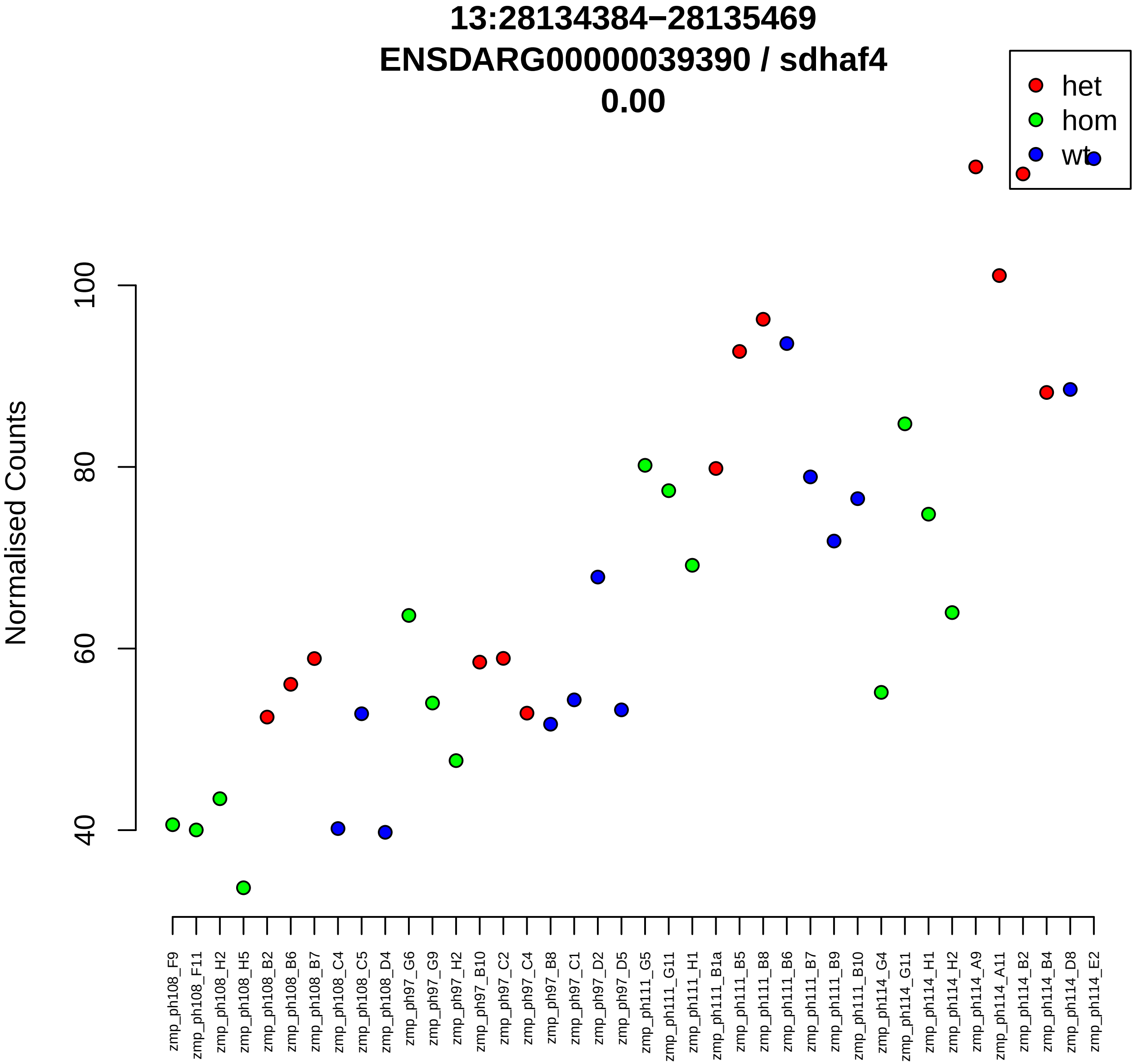
13:28134384−28135469
ENSDARG00000039390 / sdhaf4
0.00
het
hom
wt
Normalised Counts
40
60
80
100
zmp_ph108_F9
zmp_ph108_F11
zmp_ph108_H2
zmp_ph108_H5
zmp_ph108_B2
zmp_ph108_B6
zmp_ph108_B7
zmp_ph108_C4
zmp_ph108_C5
zmp_ph108_D4
zmp_ph97_G6
zmp_ph97_G9
zmp_ph97_H2
zmp_ph97_B10
zmp_ph97_C2
zmp_ph97_C4
zmp_ph97_B8
zmp_ph97_C1
zmp_ph97_D2
zmp_ph97_D5
zmp_ph111_G5
zmp_ph111_G11
zmp_ph111_H1
zmp_ph111_B1a
zmp_ph111_B5
zmp_ph111_B8
zmp_ph111_B6
zmp_ph111_B7
zmp_ph111_B9
zmp_ph111_B10
zmp_ph114_G4
zmp_ph114_G11
zmp_ph114_H1
zmp_ph114_H2
zmp_ph114_A9
zmp_ph114_A11
zmp_ph114_B2
zmp_ph114_B4
zmp_ph114_D8
zmp_ph114_E2

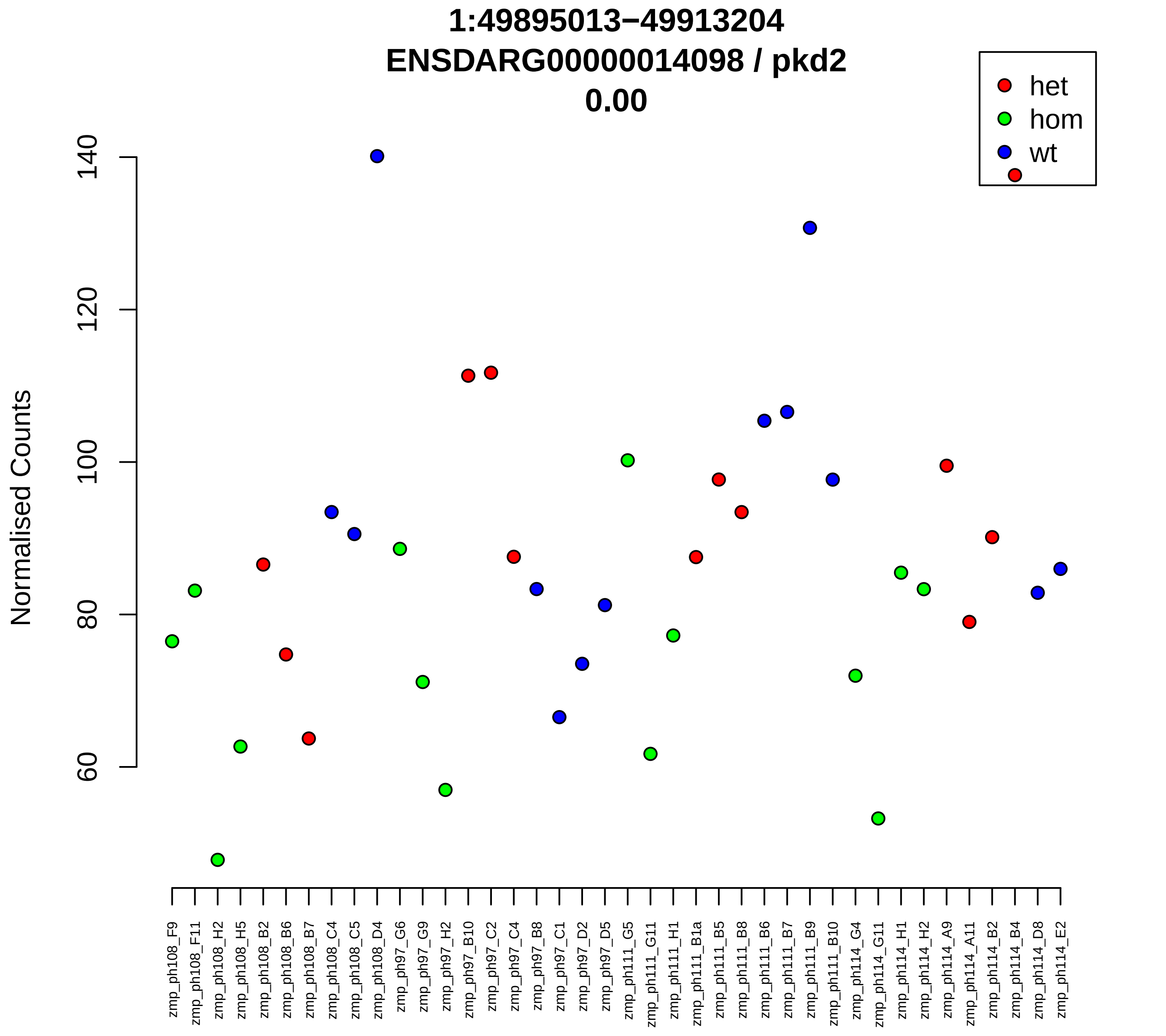
1:49895013−49913204
ENSDARG00000014098 / pkd2
0.00
het
hom
wt
Normalised Counts
60
80
100
120
140
zmp_ph108_F9
zmp_ph108_F11
zmp_ph108_H2
zmp_ph108_H5
zmp_ph108_B2
zmp_ph108_B6
zmp_ph108_B7
zmp_ph108_C4
zmp_ph108_C5
zmp_ph108_D4
zmp_ph97_G6
zmp_ph97_G9
zmp_ph97_H2
zmp_ph97_B10
zmp_ph97_C2
zmp_ph97_C4
zmp_ph97_B8
zmp_ph97_C1
zmp_ph97_D2
zmp_ph97_D5
zmp_ph111_G5
zmp_ph111_G11
zmp_ph111_H1
zmp_ph111_B1a
zmp_ph111_B5
zmp_ph111_B8
zmp_ph111_B6
zmp_ph111_B7
zmp_ph111_B9
zmp_ph111_B10
zmp_ph114_G4
zmp_ph114_G11
zmp_ph114_H1
zmp_ph114_H2
zmp_ph114_A9
zmp_ph114_A11
zmp_ph114_B2
zmp_ph114_B4
zmp_ph114_D8
zmp_ph114_E2

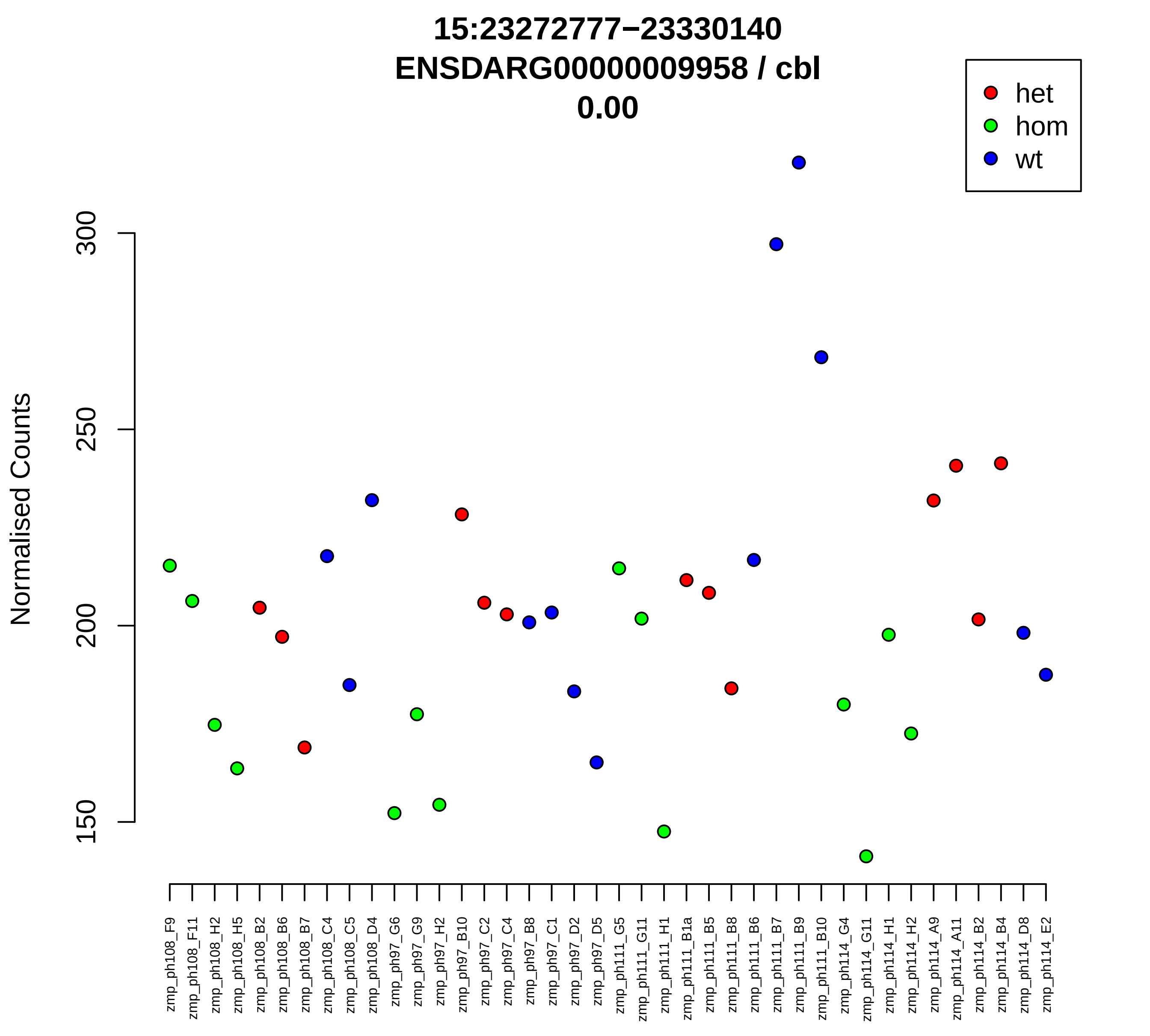

15:23272777−23330140
ENSDARG00000009958 / cbl
0.00
het
hom
wt
Normalised Counts
300
250
200
150
zmp_ph108_F9
zmp_ph108_F11
zmp_ph108_H2
zmp_ph108_H5
zmp_ph108_B2
zmp_ph108_B6
zmp_ph108_B7
zmp_ph108_C4
zmp_ph108_C5
zmp_ph108_D4
zmp_ph97_G6
zmp_ph97_G9
zmp_ph97_H2
zmp_ph97_B10
zmp_ph97_C2
zmp_ph97_C4
zmp_ph97_B8
zmp_ph97_C1
zmp_ph97_D2
zmp_ph97_D5
zmp_ph111_G5
zmp_ph111_G11
zmp_ph111_H1
zmp_ph111_B1a
zmp_ph111_B5
zmp_ph111_B8
zmp_ph111_B6
zmp_ph111_B7
zmp_ph111_B9
zmp_ph111_B10
zmp_ph114_G4
zmp_ph114_G11
zmp_ph114_H1
zmp_ph114_H2
zmp_ph114_A9
zmp_ph114_A11
zmp_ph114_B2
zmp_ph114_B4
zmp_ph114_D8
zmp_ph114_E2

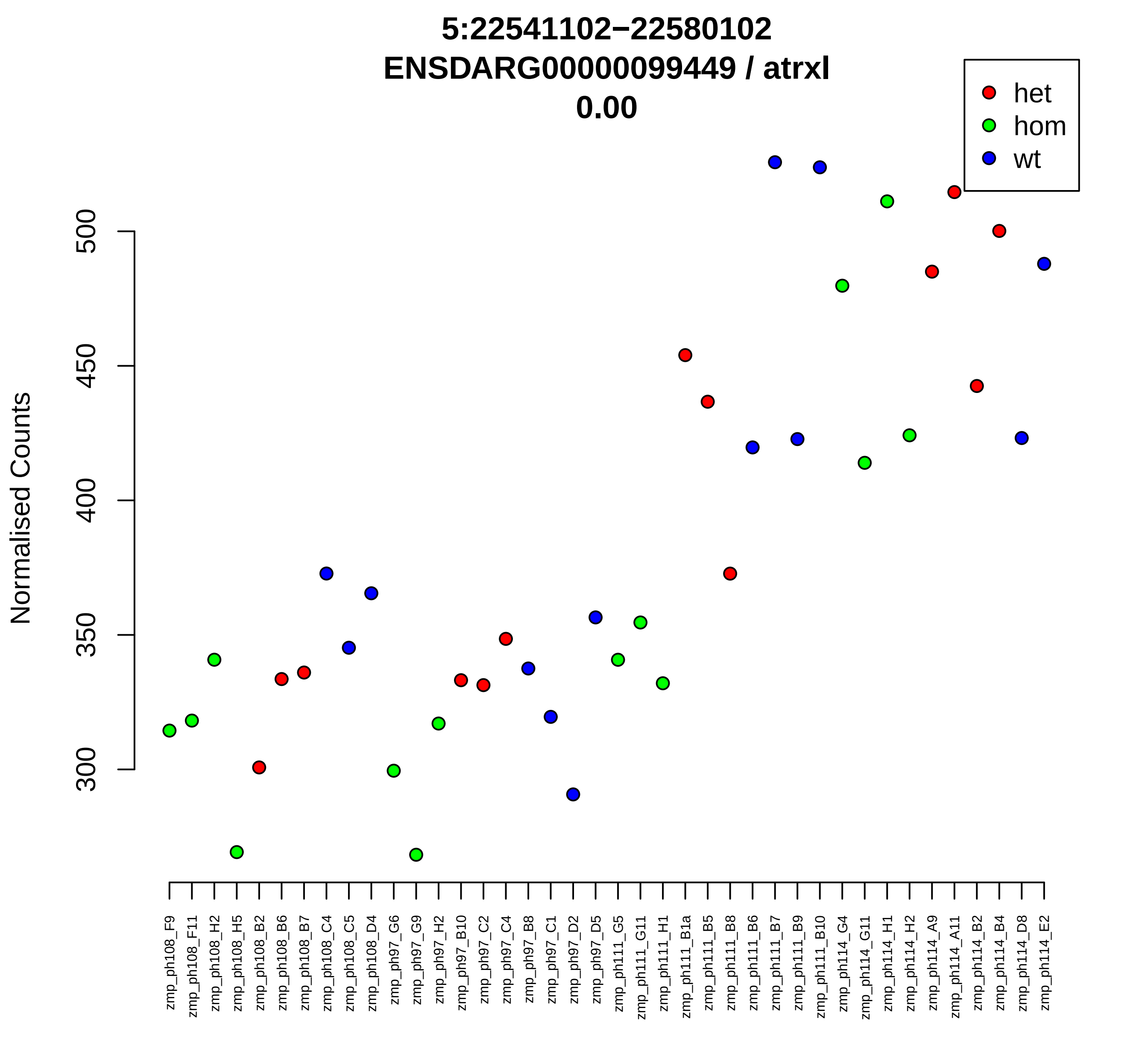
5:22541102−22580102
ENSDARG00000099449 / atrxl
0.00
het
hom
wt
Normalised Counts
500
450
400
350
300
zmp_ph108_F9
zmp_ph108_F11
zmp_ph108_H2
zmp_ph108_H5
zmp_ph108_B2
zmp_ph108_B6
zmp_ph108_B7
zmp_ph108_C4
zmp_ph108_C5
zmp_ph108_D4
zmp_ph97_G6
zmp_ph97_G9
zmp_ph97_H2
zmp_ph97_B10
zmp_ph97_C2
zmp_ph97_C4
zmp_ph97_B8
zmp_ph97_C1
zmp_ph97_D2
zmp_ph97_D5
zmp_ph111_G5
zmp_ph111_G11
zmp_ph111_H1
zmp_ph111_B1a
zmp_ph111_B5
zmp_ph111_B8
zmp_ph111_B6
zmp_ph111_B7
zmp_ph111_B9
zmp_ph111_B10
zmp_ph114_G4
zmp_ph114_G11
zmp_ph114_H1
zmp_ph114_H2
zmp_ph114_A9
zmp_ph114_A11
zmp_ph114_B2
zmp_ph114_B4
zmp_ph114_D8
zmp_ph114_E2

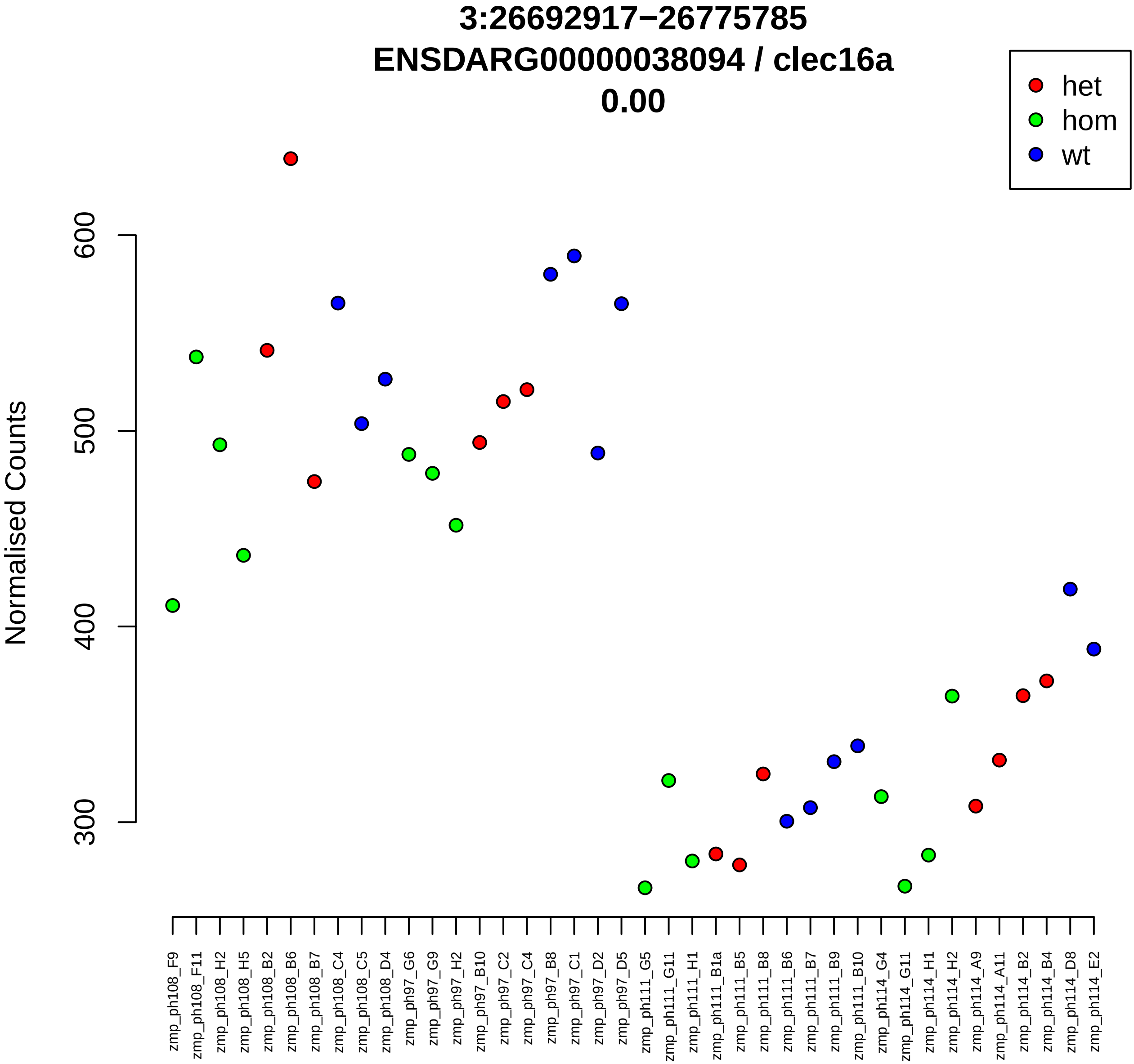
3:26692917−26775785
ENSDARG00000038094 / clec16a
0.00
het
hom
wt
Normalised Counts
300
400
500
600
zmp_ph108_F9
zmp_ph108_F11
zmp_ph108_H2
zmp_ph108_H5
zmp_ph108_B2
zmp_ph108_B6
zmp_ph108_B7
zmp_ph108_C4
zmp_ph108_C5
zmp_ph108_D4
zmp_ph97_G6
zmp_ph97_G9
zmp_ph97_H2
zmp_ph97_B10
zmp_ph97_C2
zmp_ph97_C4
zmp_ph97_B8
zmp_ph97_C1
zmp_ph97_D2
zmp_ph97_D5
zmp_ph111_G5
zmp_ph111_G11
zmp_ph111_H1
zmp_ph111_B1a
zmp_ph111_B5
zmp_ph111_B8
zmp_ph111_B6
zmp_ph111_B7
zmp_ph111_B9
zmp_ph111_B10
zmp_ph114_G4
zmp_ph114_G11
zmp_ph114_H1
zmp_ph114_H2
zmp_ph114_A9
zmp_ph114_A11
zmp_ph114_B2
zmp_ph114_B4
zmp_ph114_D8
zmp_ph114_E2

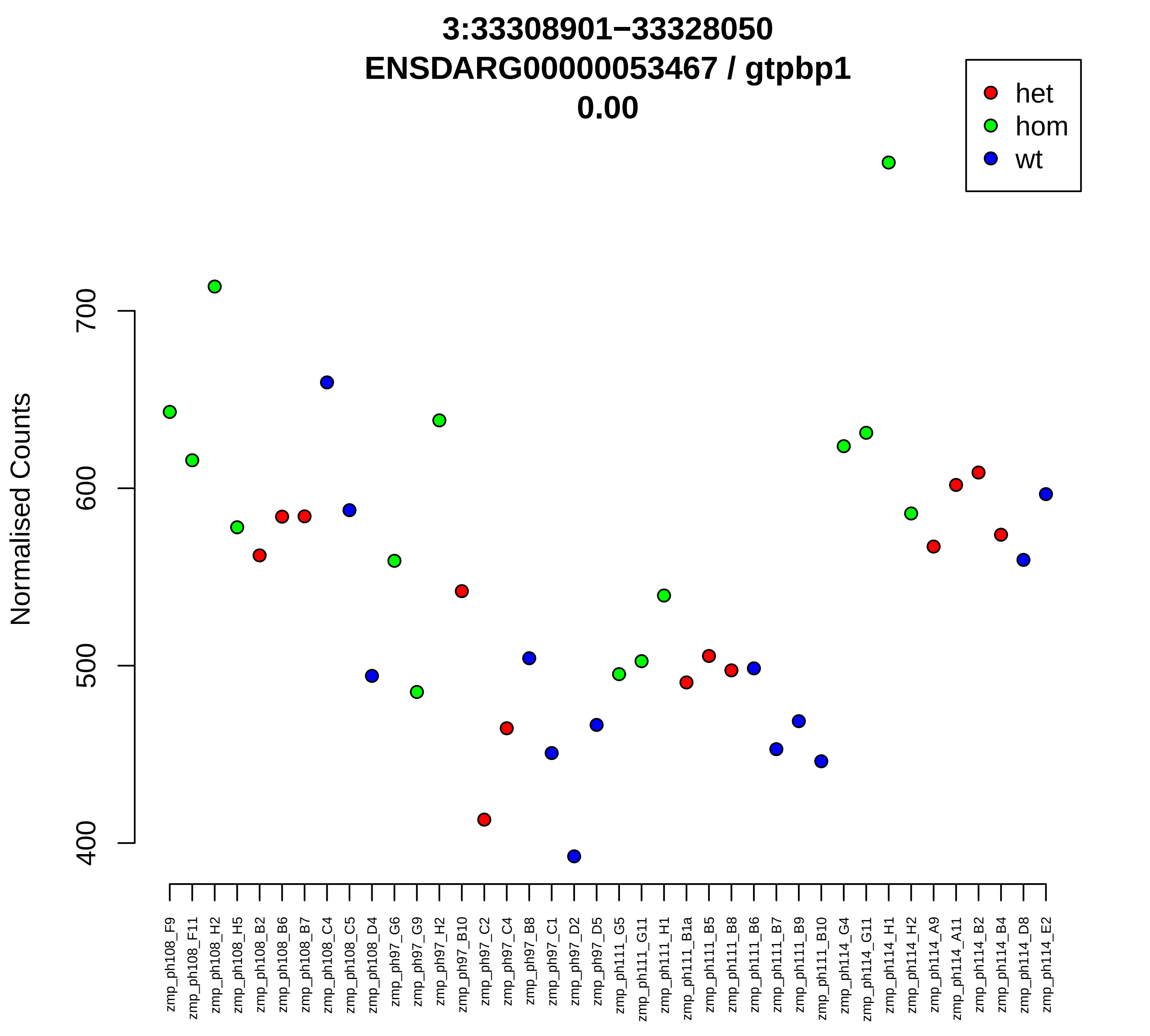

3:33308901−33328050
ENSDARG00000053467 / gtpbp1
0.00
het
hom
wt
Normalised Counts
400
500
600
700
zmp_ph108_F9
zmp_ph108_F11
zmp_ph108_H2
zmp_ph108_H5
zmp_ph108_B2
zmp_ph108_B6
zmp_ph108_B7
zmp_ph108_C4
zmp_ph108_C5
zmp_ph108_D4
zmp_ph97_G6
zmp_ph97_G9
zmp_ph97_H2
zmp_ph97_B10
zmp_ph97_C2
zmp_ph97_C4
zmp_ph97_B8
zmp_ph97_C1
zmp_ph97_D2
zmp_ph97_D5
zmp_ph111_G5
zmp_ph111_G11
zmp_ph111_H1
zmp_ph111_B1a
zmp_ph111_B5
zmp_ph111_B8
zmp_ph111_B6
zmp_ph111_B7
zmp_ph111_B9
zmp_ph111_B10
zmp_ph114_G4
zmp_ph114_G11
zmp_ph114_H1
zmp_ph114_H2
zmp_ph114_A9
zmp_ph114_A11
zmp_ph114_B2
zmp_ph114_B4
zmp_ph114_D8
zmp_ph114_E2

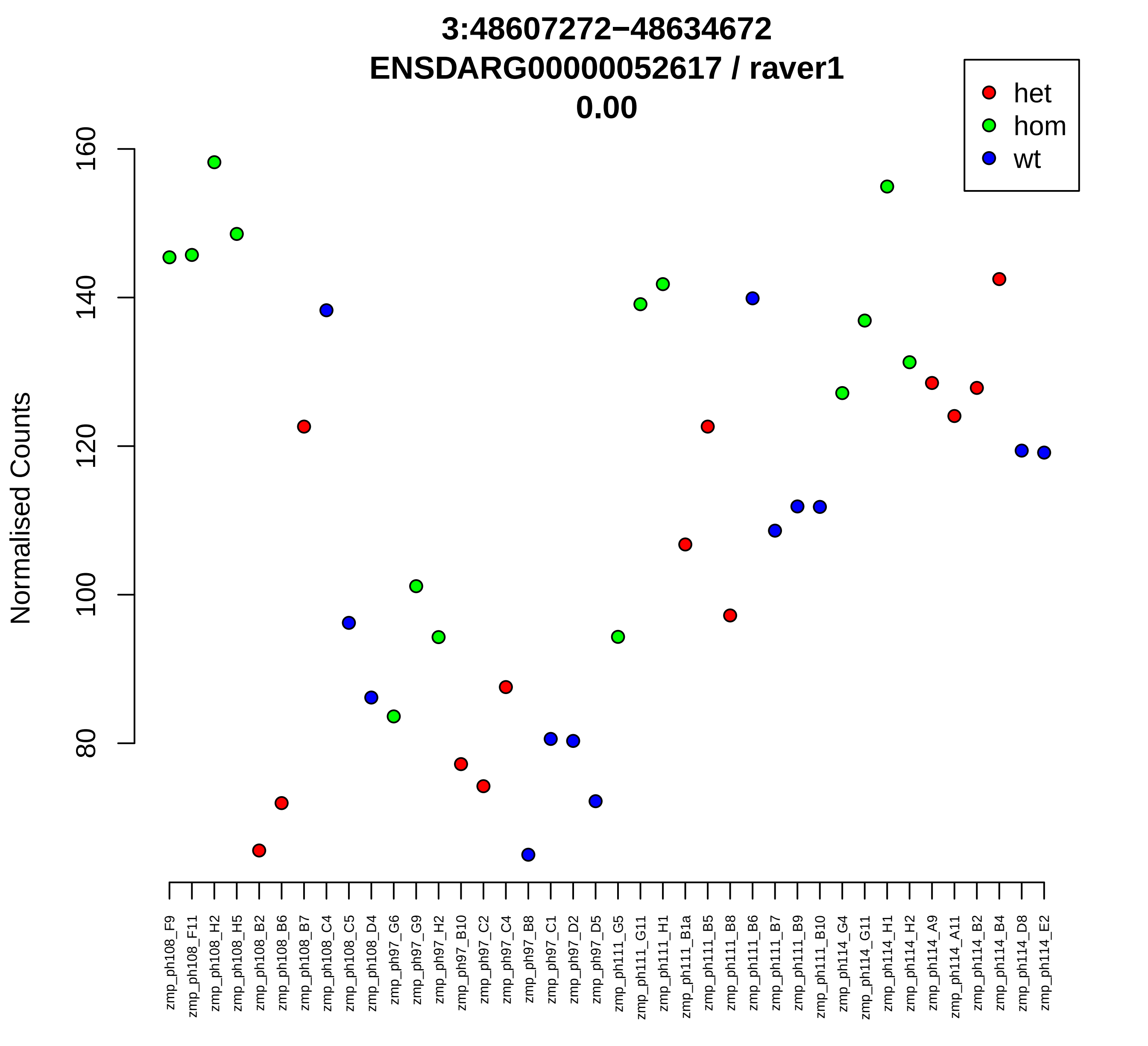

3:48607272−48634672
ENSDARG00000052617 / raver1
0.00
het
hom
wt
Normalised Counts
160
140
120
100
80
zmp_ph108_F9
zmp_ph108_F11
zmp_ph108_H2
zmp_ph108_H5
zmp_ph108_B2
zmp_ph108_B6
zmp_ph108_B7
zmp_ph108_C4
zmp_ph108_C5
zmp_ph108_D4
zmp_ph97_G6
zmp_ph97_G9
zmp_ph97_H2
zmp_ph97_B10
zmp_ph97_C2
zmp_ph97_C4
zmp_ph97_B8
zmp_ph97_C1
zmp_ph97_D2
zmp_ph97_D5
zmp_ph111_G5
zmp_ph111_G11
zmp_ph111_H1
zmp_ph111_B1a
zmp_ph111_B5
zmp_ph111_B8
zmp_ph111_B6
zmp_ph111_B7
zmp_ph111_B9
zmp_ph111_B10
zmp_ph114_G4
zmp_ph114_G11
zmp_ph114_H1
zmp_ph114_H2
zmp_ph114_A9
zmp_ph114_A11
zmp_ph114_B2
zmp_ph114_B4
zmp_ph114_D8
zmp_ph114_E2

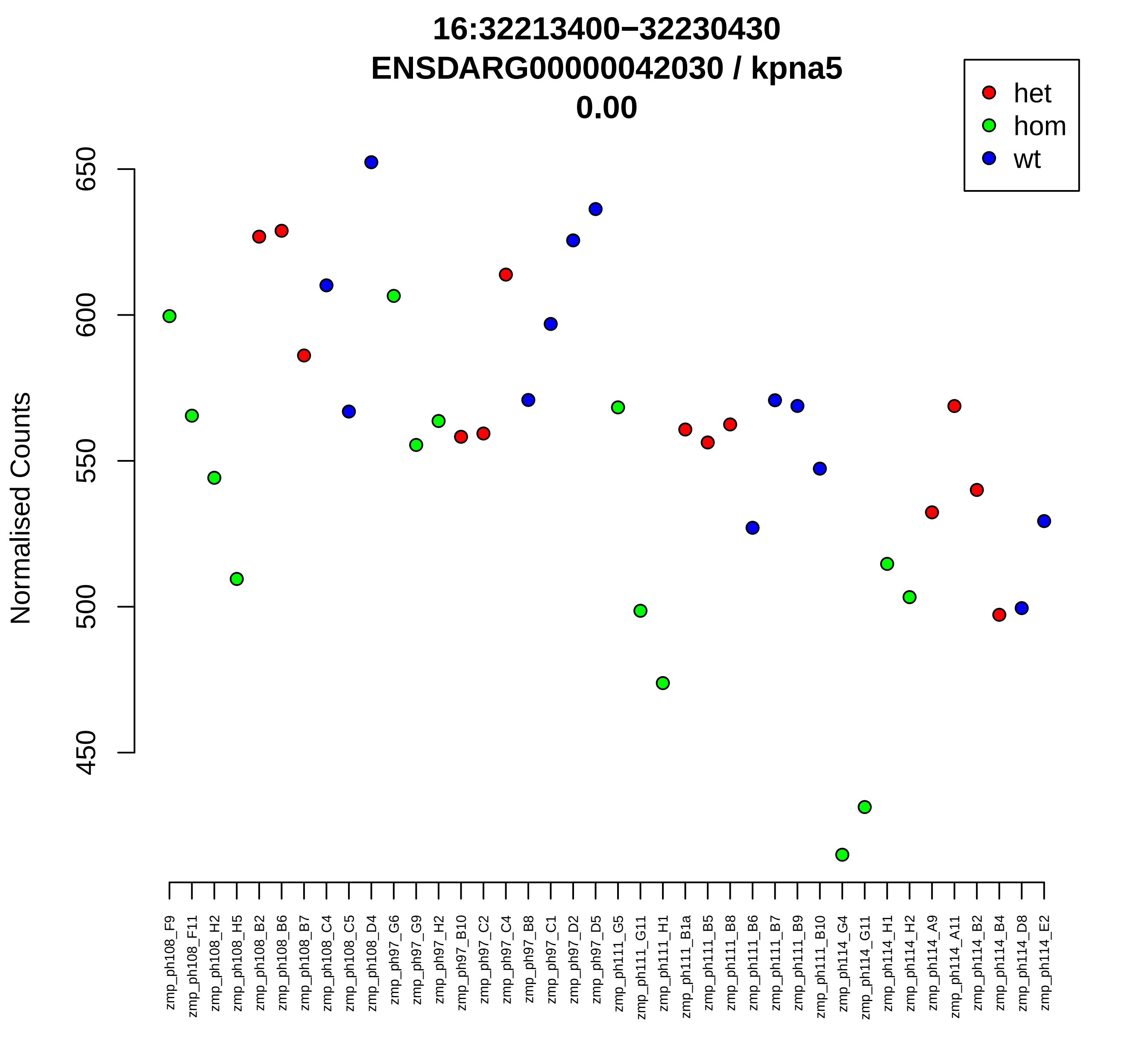

16:32213400−32230430
ENSDARG00000042030 / kpna5
0.00
het
hom
wt
Normalised Counts
650
600
550
500
450
zmp_ph108_F9
zmp_ph108_F11
zmp_ph108_H2
zmp_ph108_H5
zmp_ph108_B2
zmp_ph108_B6
zmp_ph108_B7
zmp_ph108_C4
zmp_ph108_C5
zmp_ph108_D4
zmp_ph97_G6
zmp_ph97_G9
zmp_ph97_H2
zmp_ph97_B10
zmp_ph97_C2
zmp_ph97_C4
zmp_ph97_B8
zmp_ph97_C1
zmp_ph97_D2
zmp_ph97_D5
zmp_ph111_G5
zmp_ph111_G11
zmp_ph111_H1
zmp_ph111_B1a
zmp_ph111_B5
zmp_ph111_B8
zmp_ph111_B6
zmp_ph111_B7
zmp_ph111_B9
zmp_ph111_B10
zmp_ph114_G4
zmp_ph114_G11
zmp_ph114_H1
zmp_ph114_H2
zmp_ph114_A9
zmp_ph114_A11
zmp_ph114_B2
zmp_ph114_B4
zmp_ph114_D8
zmp_ph114_E2

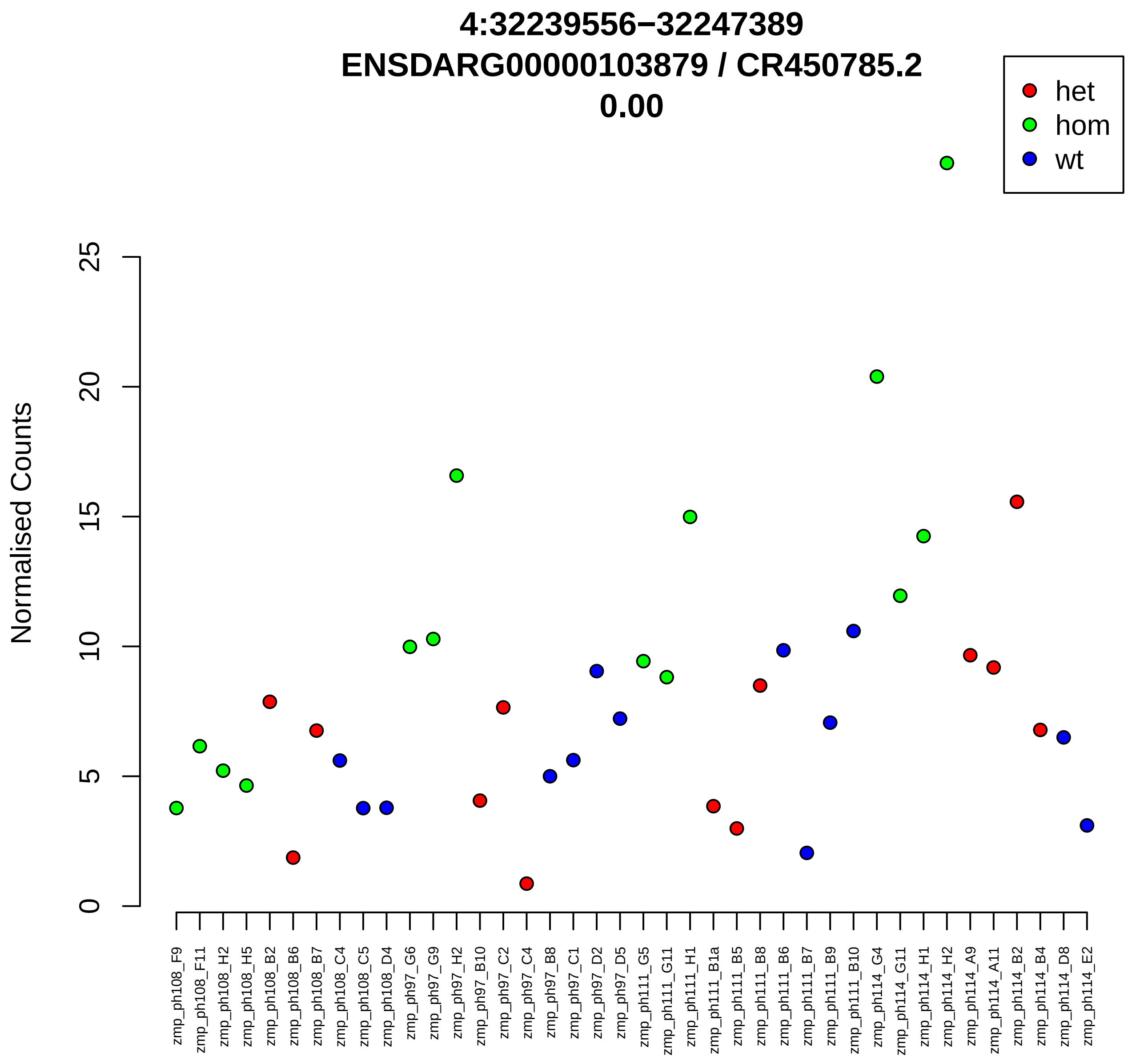
4:32239556−32247389
ENSDARG00000103879 / CR450785.2
0.00
het
hom
wt
Normalised Counts
0
5
10
15
20
25
zmp_ph108_F9
zmp_ph108_F11
zmp_ph108_H2
zmp_ph108_H5
zmp_ph108_B2
zmp_ph108_B6
zmp_ph108_B7
zmp_ph108_C4
zmp_ph108_C5
zmp_ph108_D4
zmp_ph97_G6
zmp_ph97_G9
zmp_ph97_H2
zmp_ph97_B10
zmp_ph97_C2
zmp_ph97_C4
zmp_ph97_B8
zmp_ph97_C1
zmp_ph97_D2
zmp_ph97_D5
zmp_ph111_G5
zmp_ph111_G11
zmp_ph111_H1
zmp_ph111_B1a
zmp_ph111_B5
zmp_ph111_B8
zmp_ph111_B6
zmp_ph111_B7
zmp_ph111_B9
zmp_ph111_B10
zmp_ph114_G4
zmp_ph114_G11
zmp_ph114_H1
zmp_ph114_H2
zmp_ph114_A9
zmp_ph114_A11
zmp_ph114_B2
zmp_ph114_B4
zmp_ph114_D8
zmp_ph114_E2

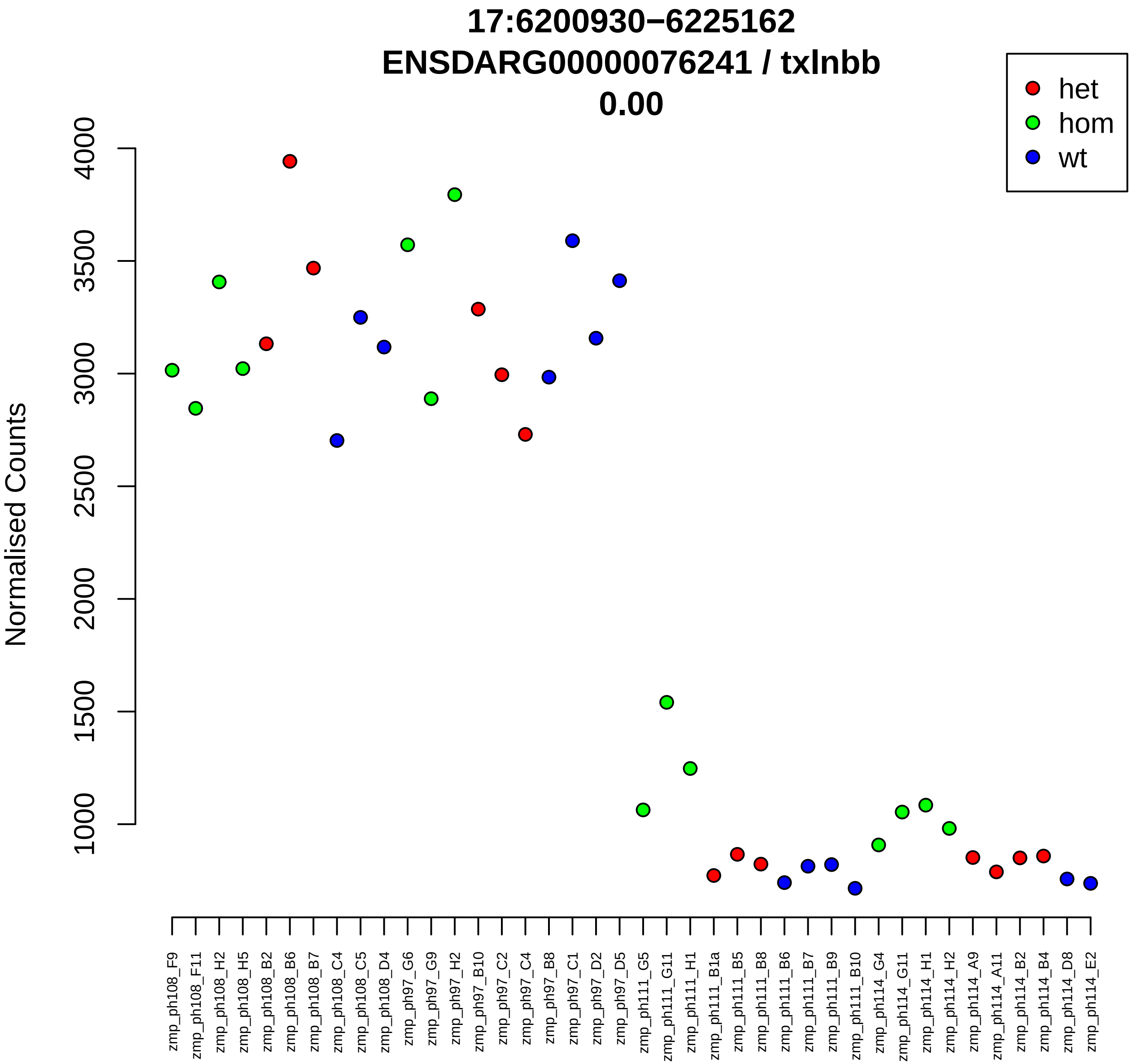

17:6200930−6225162
ENSDARG00000076241 / txlnbb
0.00
het
hom
wt
Normalised Counts
1000
1500
2000
2500
3000
3500
4000
zmp_ph108_F9
zmp_ph108_F11
zmp_ph108_H2
zmp_ph108_H5
zmp_ph108_B2
zmp_ph108_B6
zmp_ph108_B7
zmp_ph108_C4
zmp_ph108_C5
zmp_ph108_D4
zmp_ph97_G6
zmp_ph97_G9
zmp_ph97_H2
zmp_ph97_B10
zmp_ph97_C2
zmp_ph97_C4
zmp_ph97_B8
zmp_ph97_C1
zmp_ph97_D2
zmp_ph97_D5
zmp_ph111_G5
zmp_ph111_G11
zmp_ph111_H1
zmp_ph111_B1a
zmp_ph111_B5
zmp_ph111_B8
zmp_ph111_B6
zmp_ph111_B7
zmp_ph111_B9
zmp_ph111_B10
zmp_ph114_G4
zmp_ph114_G11
zmp_ph114_H1
zmp_ph114_H2
zmp_ph114_A9
zmp_ph114_A11
zmp_ph114_B2
zmp_ph114_B4
zmp_ph114_D8
zmp_ph114_E2

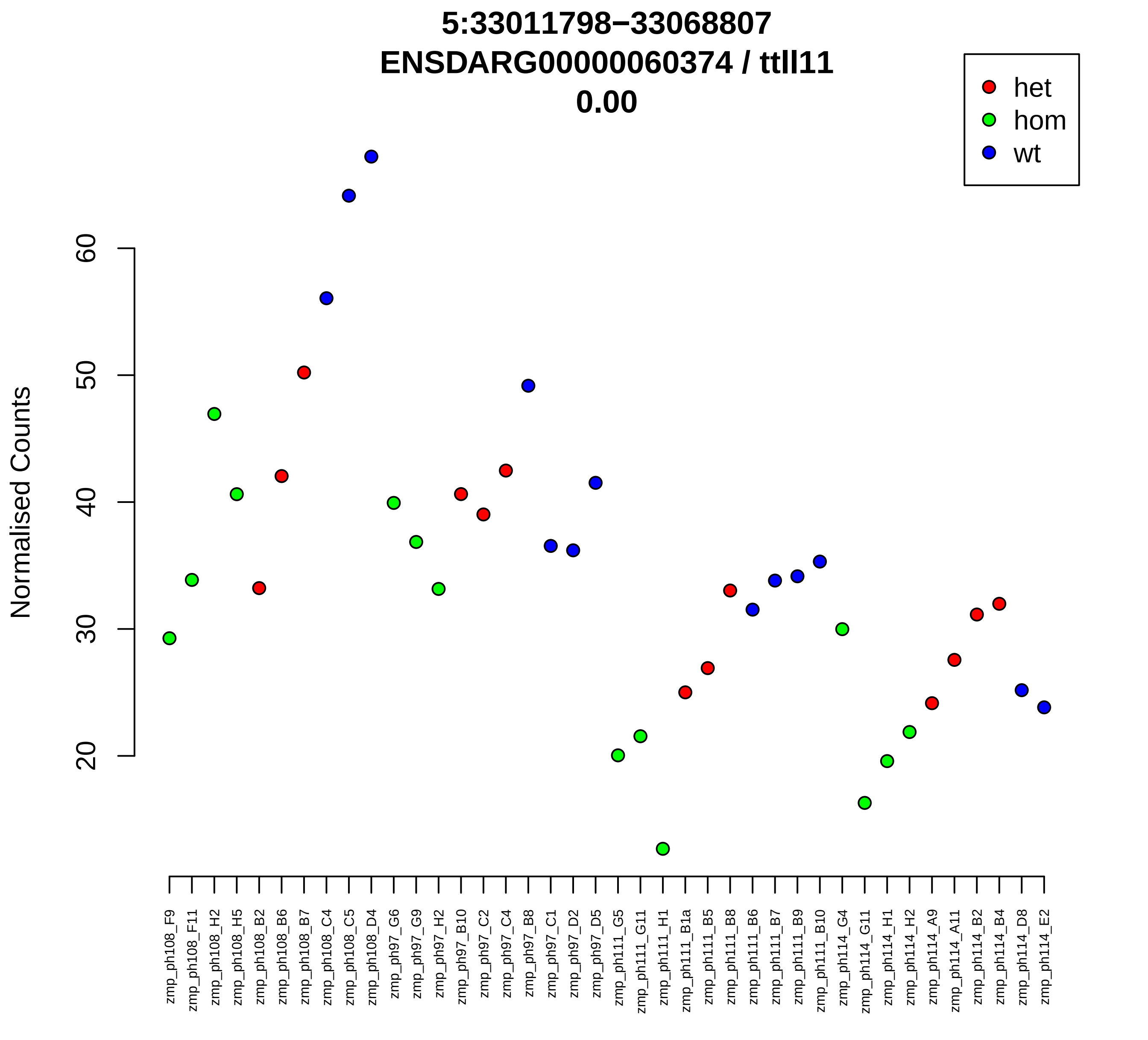
5:33011798−33068807
ENSDARG00000060374 / ttll11
0.00
het
hom
wt
Normalised Counts
20
30
40
50
60
zmp_ph108_F9
zmp_ph108_F11
zmp_ph108_H2
zmp_ph108_H5
zmp_ph108_B2
zmp_ph108_B6
zmp_ph108_B7
zmp_ph108_C4
zmp_ph108_C5
zmp_ph108_D4
zmp_ph97_G6
zmp_ph97_G9
zmp_ph97_H2
zmp_ph97_B10
zmp_ph97_C2
zmp_ph97_C4
zmp_ph97_B8
zmp_ph97_C1
zmp_ph97_D2
zmp_ph97_D5
zmp_ph111_G5
zmp_ph111_G11
zmp_ph111_H1
zmp_ph111_B1a
zmp_ph111_B5
zmp_ph111_B8
zmp_ph111_B6
zmp_ph111_B7
zmp_ph111_B9
zmp_ph111_B10
zmp_ph114_G4
zmp_ph114_G11
zmp_ph114_H1
zmp_ph114_H2
zmp_ph114_A9
zmp_ph114_A11
zmp_ph114_B2
zmp_ph114_B4
zmp_ph114_D8
zmp_ph114_E2

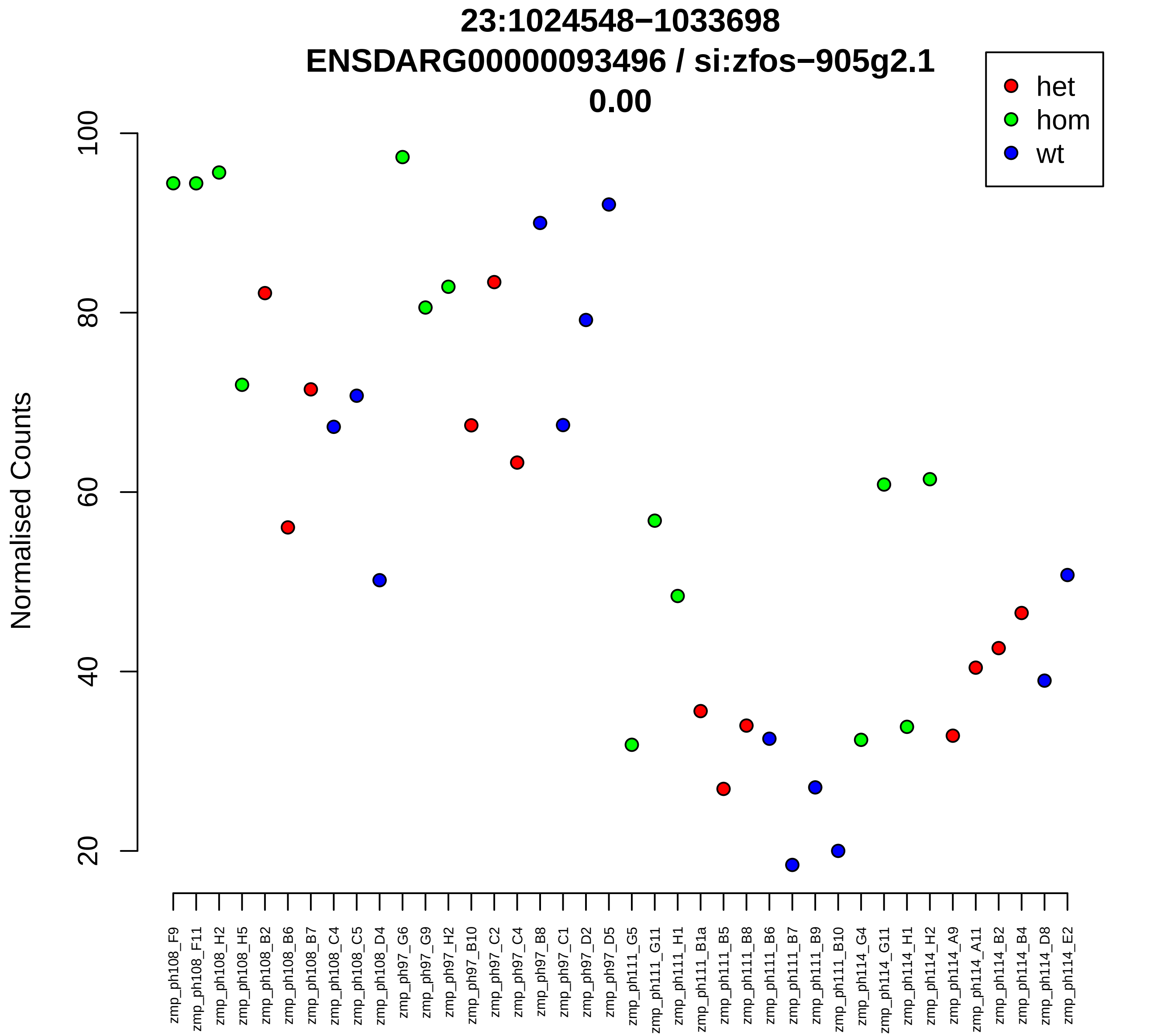

23:1024548−1033698
ENSDARG00000093496 / si:zfos−905g2.1
0.00
het
hom
wt
Normalised Counts
20
40
60
80
100
zmp_ph108_F9
zmp_ph108_F11
zmp_ph108_H2
zmp_ph108_H5
zmp_ph108_B2
zmp_ph108_B6
zmp_ph108_B7
zmp_ph108_C4
zmp_ph108_C5
zmp_ph108_D4
zmp_ph97_G6
zmp_ph97_G9
zmp_ph97_H2
zmp_ph97_B10
zmp_ph97_C2
zmp_ph97_C4
zmp_ph97_B8
zmp_ph97_C1
zmp_ph97_D2
zmp_ph97_D5
zmp_ph111_G5
zmp_ph111_G11
zmp_ph111_H1
zmp_ph111_B1a
zmp_ph111_B5
zmp_ph111_B8
zmp_ph111_B6
zmp_ph111_B7
zmp_ph111_B9
zmp_ph111_B10
zmp_ph114_G4
zmp_ph114_G11
zmp_ph114_H1
zmp_ph114_H2
zmp_ph114_A9
zmp_ph114_A11
zmp_ph114_B2
zmp_ph114_B4
zmp_ph114_D8
zmp_ph114_E2

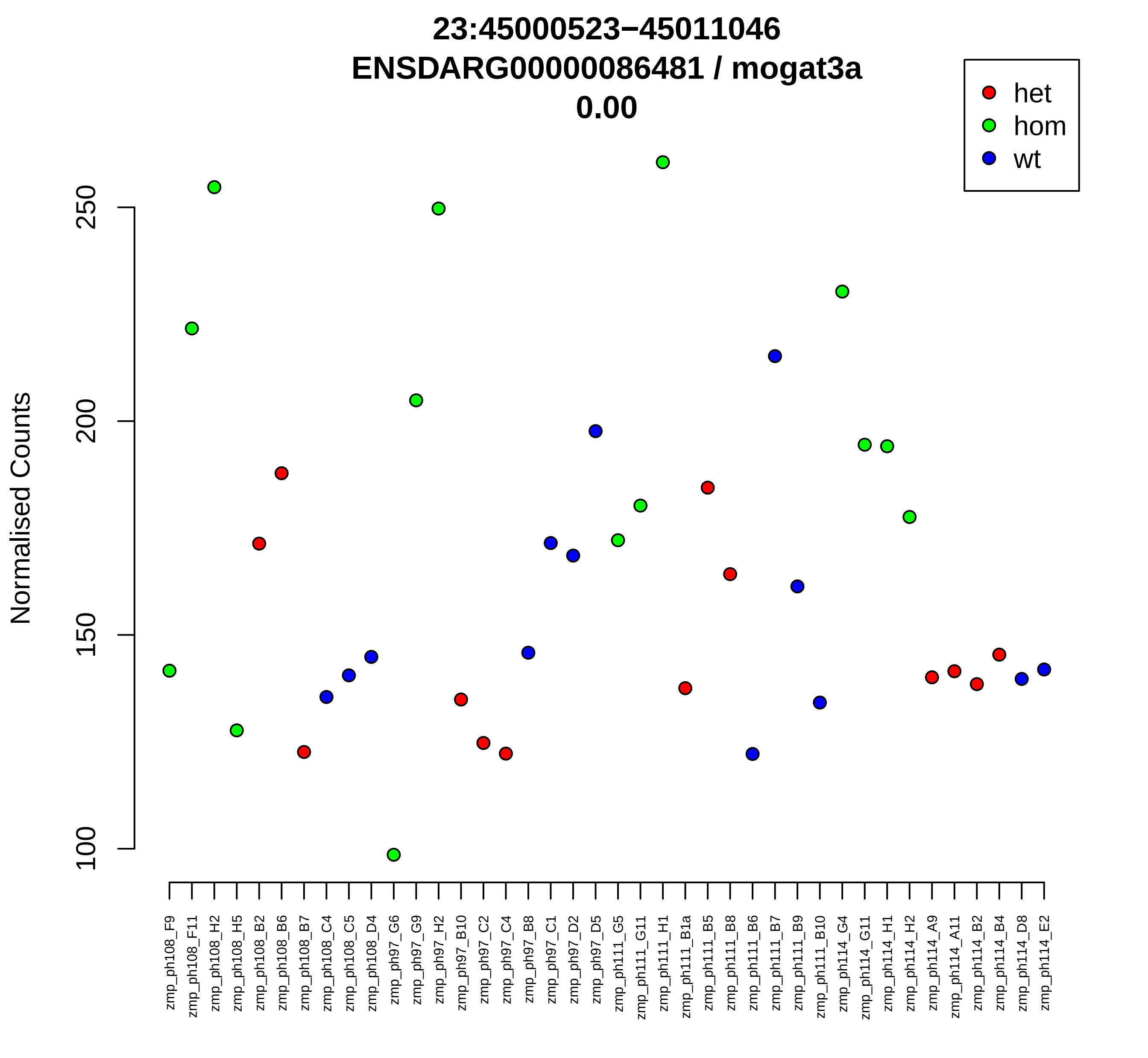

23:45000523−45011046
ENSDARG00000086481 / mogat3a
0.00
het
hom
wt
Normalised Counts
100
150
200
250
zmp_ph108_F9
zmp_ph108_F11
zmp_ph108_H2
zmp_ph108_H5
zmp_ph108_B2
zmp_ph108_B6
zmp_ph108_B7
zmp_ph108_C4
zmp_ph108_C5
zmp_ph108_D4
zmp_ph97_G6
zmp_ph97_G9
zmp_ph97_H2
zmp_ph97_B10
zmp_ph97_C2
zmp_ph97_C4
zmp_ph97_B8
zmp_ph97_C1
zmp_ph97_D2
zmp_ph97_D5
zmp_ph111_G5
zmp_ph111_G11
zmp_ph111_H1
zmp_ph111_B1a
zmp_ph111_B5
zmp_ph111_B8
zmp_ph111_B6
zmp_ph111_B7
zmp_ph111_B9
zmp_ph111_B10
zmp_ph114_G4
zmp_ph114_G11
zmp_ph114_H1
zmp_ph114_H2
zmp_ph114_A9
zmp_ph114_A11
zmp_ph114_B2
zmp_ph114_B4
zmp_ph114_D8
zmp_ph114_E2

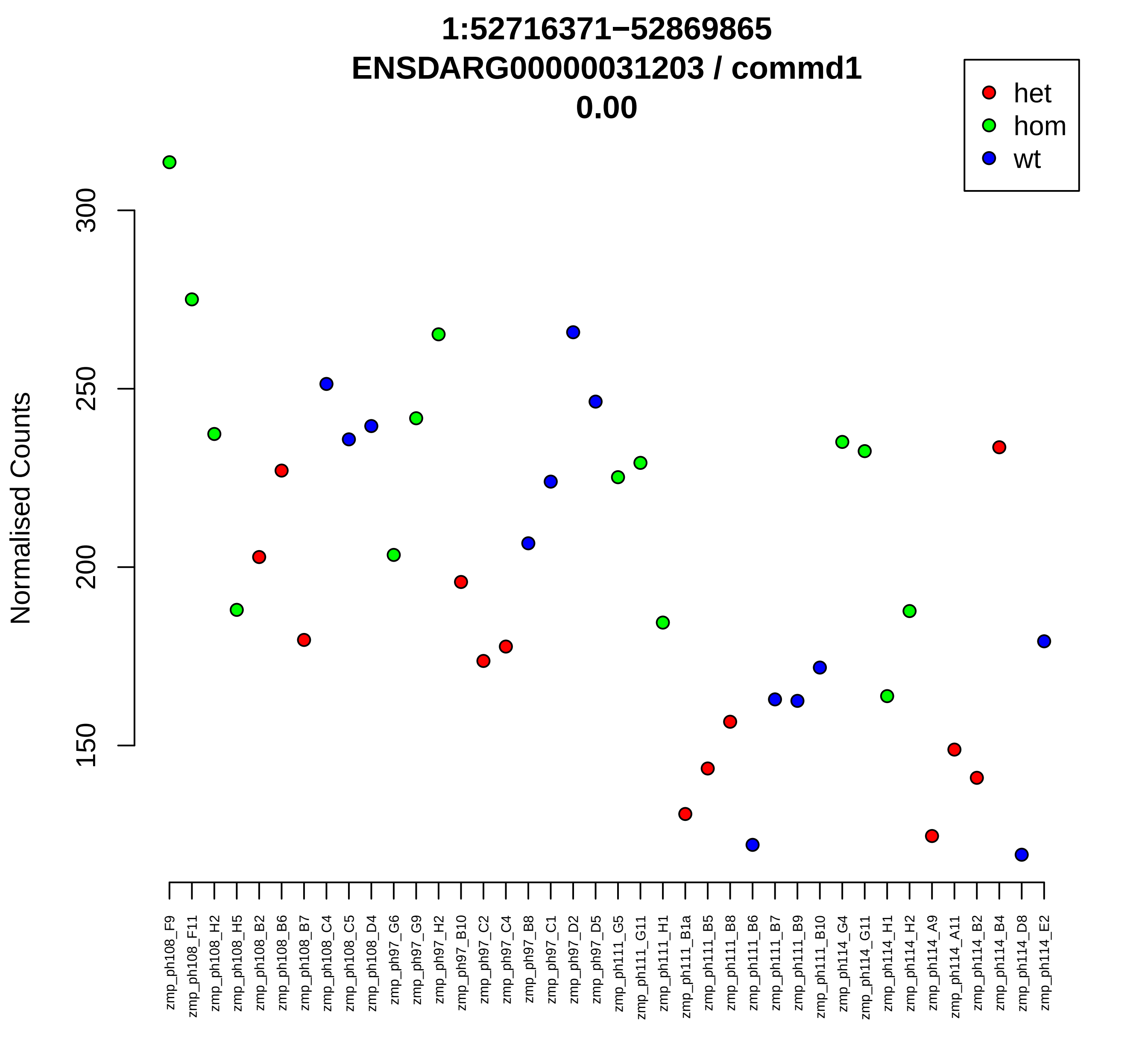

1:52716371−52869865
ENSDARG00000031203 / commd1
0.00
het
hom
wt
Normalised Counts
300
250
200
150
zmp_ph108_F9
zmp_ph108_F11
zmp_ph108_H2
zmp_ph108_H5
zmp_ph108_B2
zmp_ph108_B6
zmp_ph108_B7
zmp_ph108_C4
zmp_ph108_C5
zmp_ph108_D4
zmp_ph97_G6
zmp_ph97_G9
zmp_ph97_H2
zmp_ph97_B10
zmp_ph97_C2
zmp_ph97_C4
zmp_ph97_B8
zmp_ph97_C1
zmp_ph97_D2
zmp_ph97_D5
zmp_ph111_G5
zmp_ph111_G11
zmp_ph111_H1
zmp_ph111_B1a
zmp_ph111_B5
zmp_ph111_B8
zmp_ph111_B6
zmp_ph111_B7
zmp_ph111_B9
zmp_ph111_B10
zmp_ph114_G4
zmp_ph114_G11
zmp_ph114_H1
zmp_ph114_H2
zmp_ph114_A9
zmp_ph114_A11
zmp_ph114_B2
zmp_ph114_B4
zmp_ph114_D8
zmp_ph114_E2

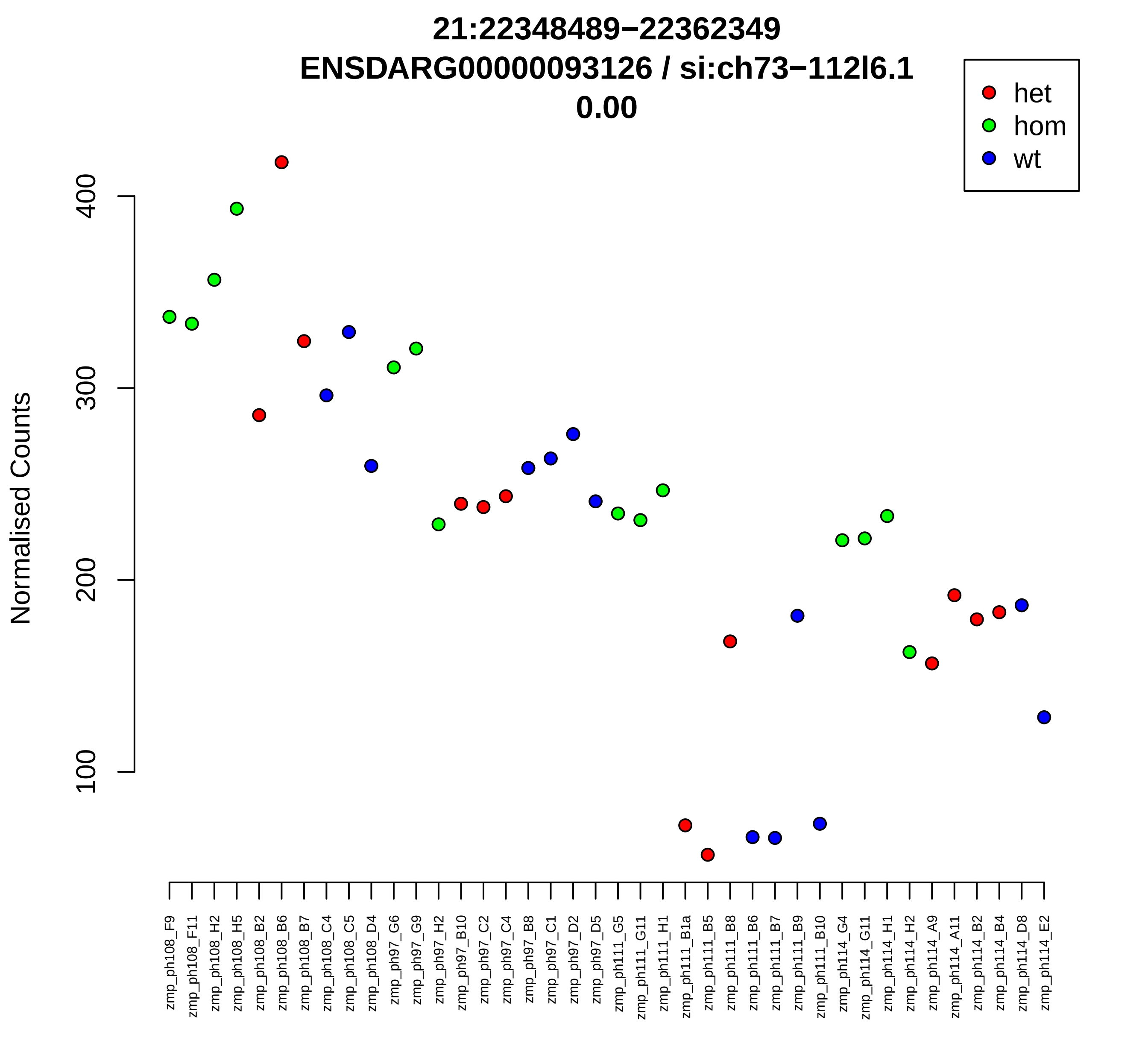
21:22348489−22362349
ENSDARG00000093126 / si:ch73−112l6.1
0.00
het
hom
wt
Normalised Counts
100
200
300
400
zmp_ph108_F9
zmp_ph108_F11
zmp_ph108_H2
zmp_ph108_H5
zmp_ph108_B2
zmp_ph108_B6
zmp_ph108_B7
zmp_ph108_C4
zmp_ph108_C5
zmp_ph108_D4
zmp_ph97_G6
zmp_ph97_G9
zmp_ph97_H2
zmp_ph97_B10
zmp_ph97_C2
zmp_ph97_C4
zmp_ph97_B8
zmp_ph97_C1
zmp_ph97_D2
zmp_ph97_D5
zmp_ph111_G5
zmp_ph111_G11
zmp_ph111_H1
zmp_ph111_B1a
zmp_ph111_B5
zmp_ph111_B8
zmp_ph111_B6
zmp_ph111_B7
zmp_ph111_B9
zmp_ph111_B10
zmp_ph114_G4
zmp_ph114_G11
zmp_ph114_H1
zmp_ph114_H2
zmp_ph114_A9
zmp_ph114_A11
zmp_ph114_B2
zmp_ph114_B4
zmp_ph114_D8
zmp_ph114_E2

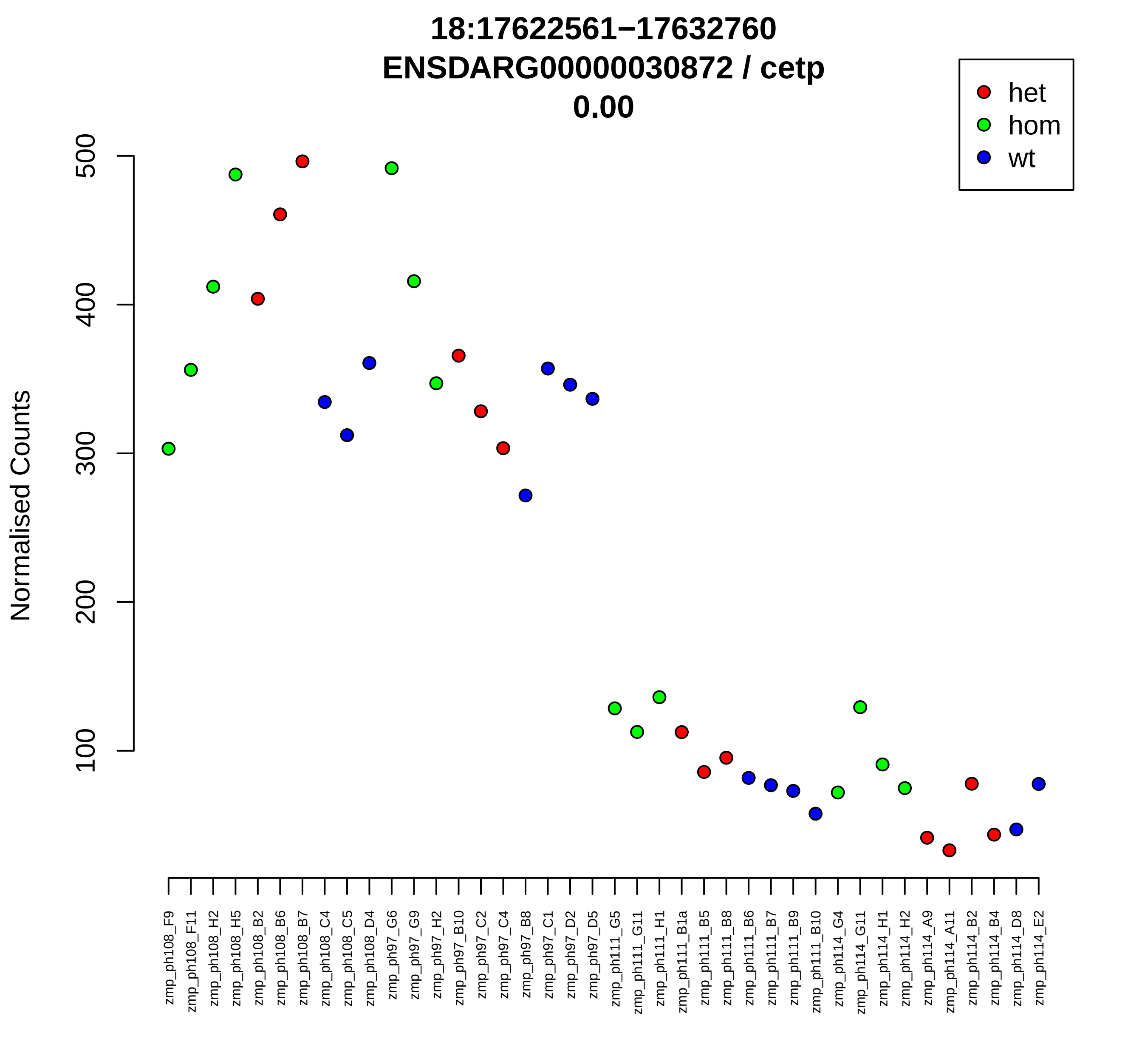

18:17622561−17632760
ENSDARG00000030872 / cetp
0.00
het
hom
wt
Normalised Counts
100
200
300
400
500
zmp_ph108_F9
zmp_ph108_F11
zmp_ph108_H2
zmp_ph108_H5
zmp_ph108_B2
zmp_ph108_B6
zmp_ph108_B7
zmp_ph108_C4
zmp_ph108_C5
zmp_ph108_D4
zmp_ph97_G6
zmp_ph97_G9
zmp_ph97_H2
zmp_ph97_B10
zmp_ph97_C2
zmp_ph97_C4
zmp_ph97_B8
zmp_ph97_C1
zmp_ph97_D2
zmp_ph97_D5
zmp_ph111_G5
zmp_ph111_G11
zmp_ph111_H1
zmp_ph111_B1a
zmp_ph111_B5
zmp_ph111_B8
zmp_ph111_B6
zmp_ph111_B7
zmp_ph111_B9
zmp_ph111_B10
zmp_ph114_G4
zmp_ph114_G11
zmp_ph114_H1
zmp_ph114_H2
zmp_ph114_A9
zmp_ph114_A11
zmp_ph114_B2
zmp_ph114_B4
zmp_ph114_D8
zmp_ph114_E2

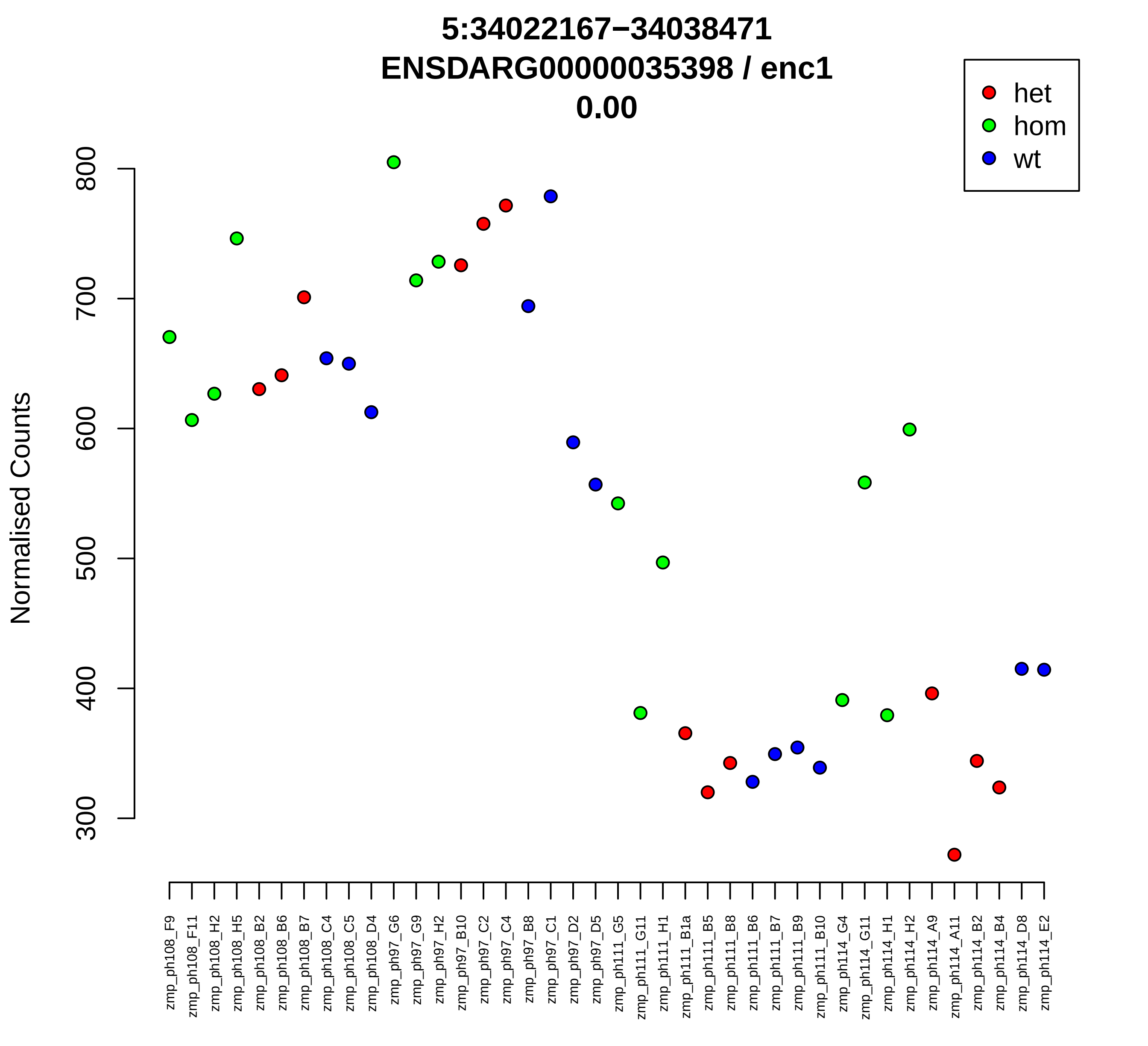

5:34022167−34038471
ENSDARG00000035398 / enc1
0.00
het
hom
wt
Normalised Counts
800
700
600
500
400
300
zmp_ph108_F9
zmp_ph108_F11
zmp_ph108_H2
zmp_ph108_H5
zmp_ph108_B2
zmp_ph108_B6
zmp_ph108_B7
zmp_ph108_C4
zmp_ph108_C5
zmp_ph108_D4
zmp_ph97_G6
zmp_ph97_G9
zmp_ph97_H2
zmp_ph97_B10
zmp_ph97_C2
zmp_ph97_C4
zmp_ph97_B8
zmp_ph97_C1
zmp_ph97_D2
zmp_ph97_D5
zmp_ph111_G5
zmp_ph111_G11
zmp_ph111_H1
zmp_ph111_B1a
zmp_ph111_B5
zmp_ph111_B8
zmp_ph111_B6
zmp_ph111_B7
zmp_ph111_B9
zmp_ph111_B10
zmp_ph114_G4
zmp_ph114_G11
zmp_ph114_H1
zmp_ph114_H2
zmp_ph114_A9
zmp_ph114_A11
zmp_ph114_B2
zmp_ph114_B4
zmp_ph114_D8
zmp_ph114_E2

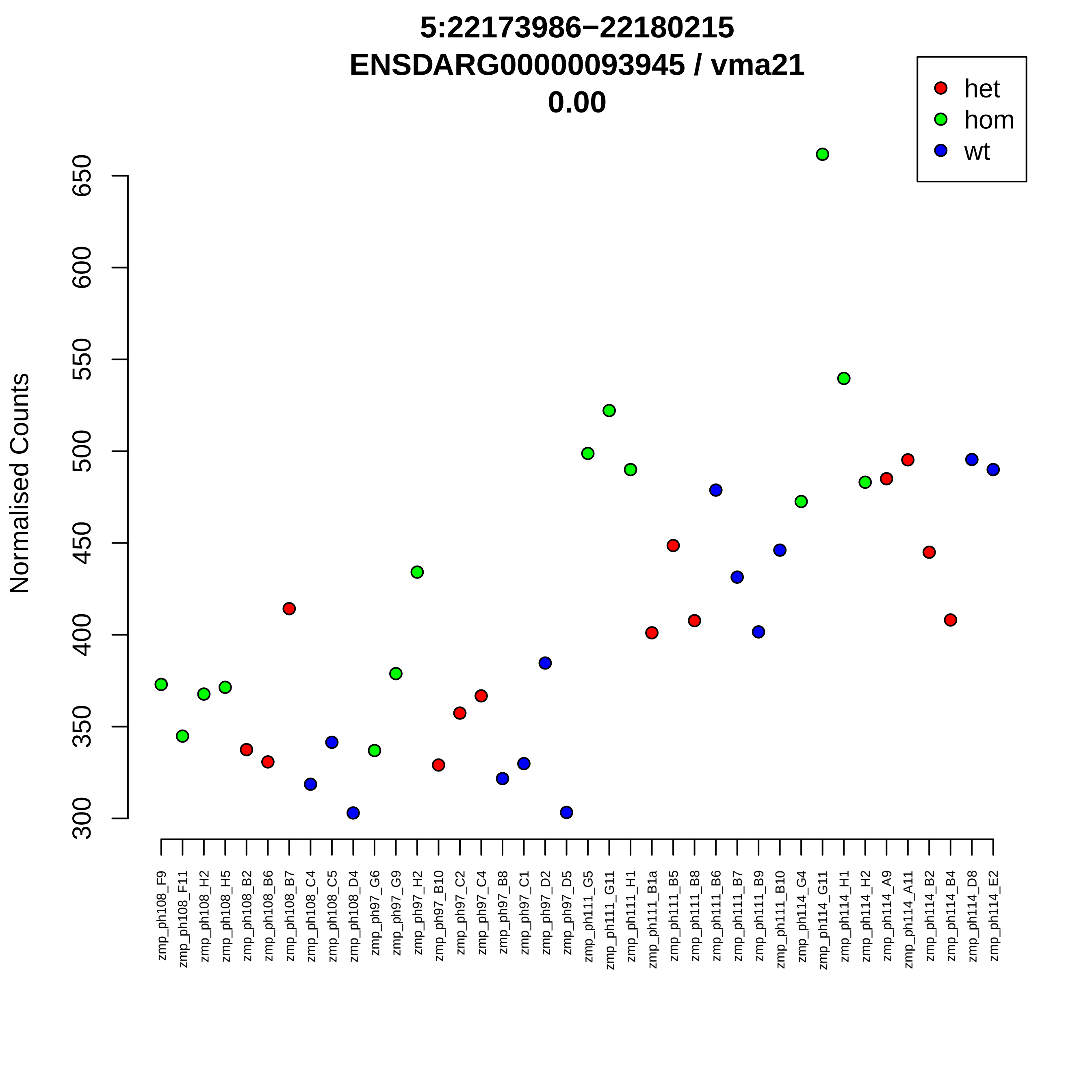

5:22173986−22180215
ENSDARG00000093945 / vma21
0.00
het
hom
wt
Normalised Counts
300
350
400
450
500
550
600
650
zmp_ph108_F9
zmp_ph108_F11
zmp_ph108_H2
zmp_ph108_H5
zmp_ph108_B2
zmp_ph108_B6
zmp_ph108_B7
zmp_ph108_C4
zmp_ph108_C5
zmp_ph108_D4
zmp_ph97_G6
zmp_ph97_G9
zmp_ph97_H2
zmp_ph97_B10
zmp_ph97_C2
zmp_ph97_C4
zmp_ph97_B8
zmp_ph97_C1
zmp_ph97_D2
zmp_ph97_D5
zmp_ph111_G5
zmp_ph111_G11
zmp_ph111_H1
zmp_ph111_B1a
zmp_ph111_B5
zmp_ph111_B8
zmp_ph111_B6
zmp_ph111_B7
zmp_ph111_B9
zmp_ph111_B10
zmp_ph114_G4
zmp_ph114_G11
zmp_ph114_H1
zmp_ph114_H2
zmp_ph114_A9
zmp_ph114_A11
zmp_ph114_B2
zmp_ph114_B4
zmp_ph114_D8
zmp_ph114_E2

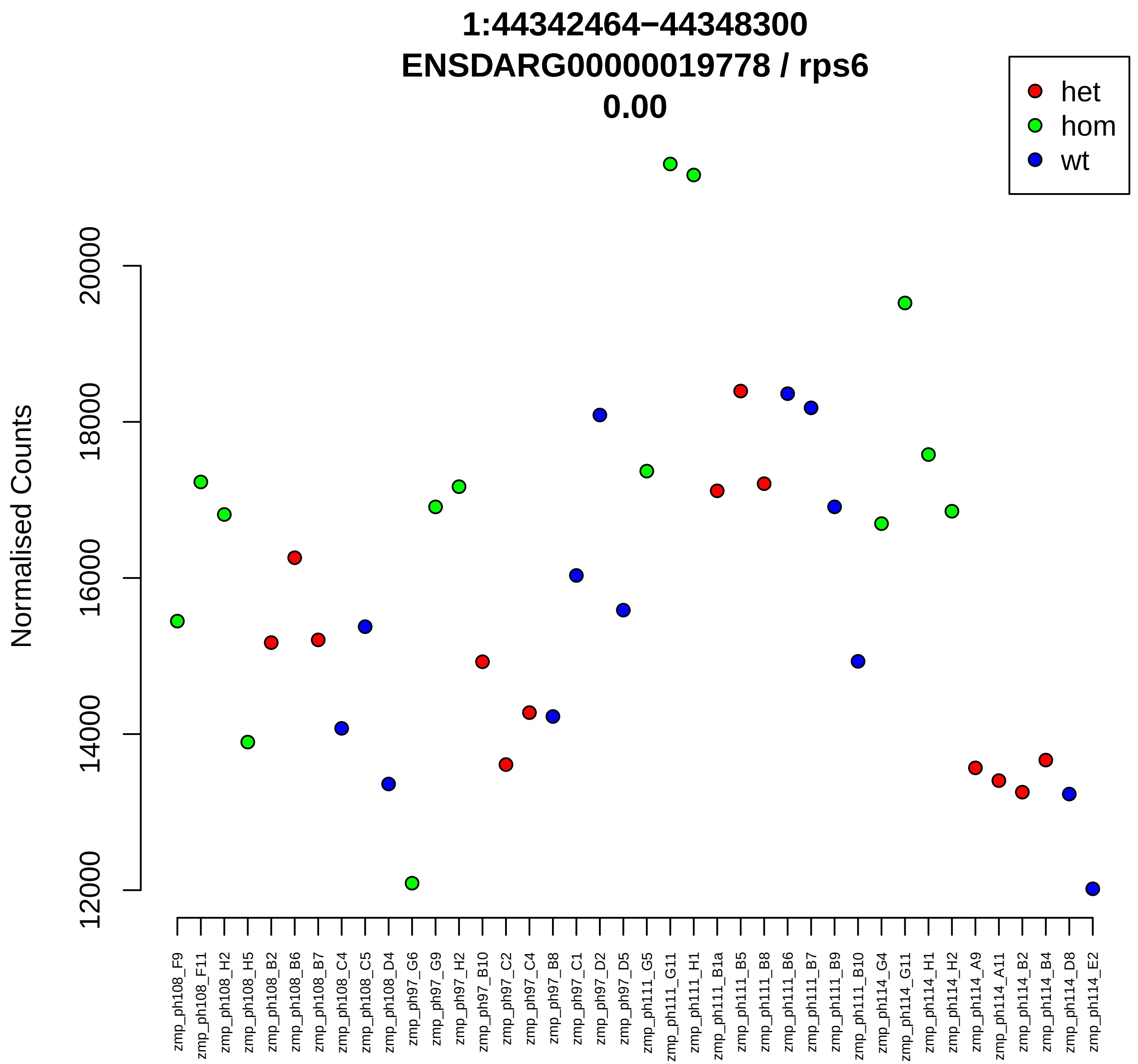
1:44342464−44348300
ENSDARG00000019778 / rps6
0.00
het
hom
wt
Normalised Counts
12000
14000
16000
18000
20000
zmp_ph108_F9
zmp_ph108_F11
zmp_ph108_H2
zmp_ph108_H5
zmp_ph108_B2
zmp_ph108_B6
zmp_ph108_B7
zmp_ph108_C4
zmp_ph108_C5
zmp_ph108_D4
zmp_ph97_G6
zmp_ph97_G9
zmp_ph97_H2
zmp_ph97_B10
zmp_ph97_C2
zmp_ph97_C4
zmp_ph97_B8
zmp_ph97_C1
zmp_ph97_D2
zmp_ph97_D5
zmp_ph111_G5
zmp_ph111_G11
zmp_ph111_H1
zmp_ph111_B1a
zmp_ph111_B5
zmp_ph111_B8
zmp_ph111_B6
zmp_ph111_B7
zmp_ph111_B9
zmp_ph111_B10
zmp_ph114_G4
zmp_ph114_G11
zmp_ph114_H1
zmp_ph114_H2
zmp_ph114_A9
zmp_ph114_A11
zmp_ph114_B2
zmp_ph114_B4
zmp_ph114_D8
zmp_ph114_E2

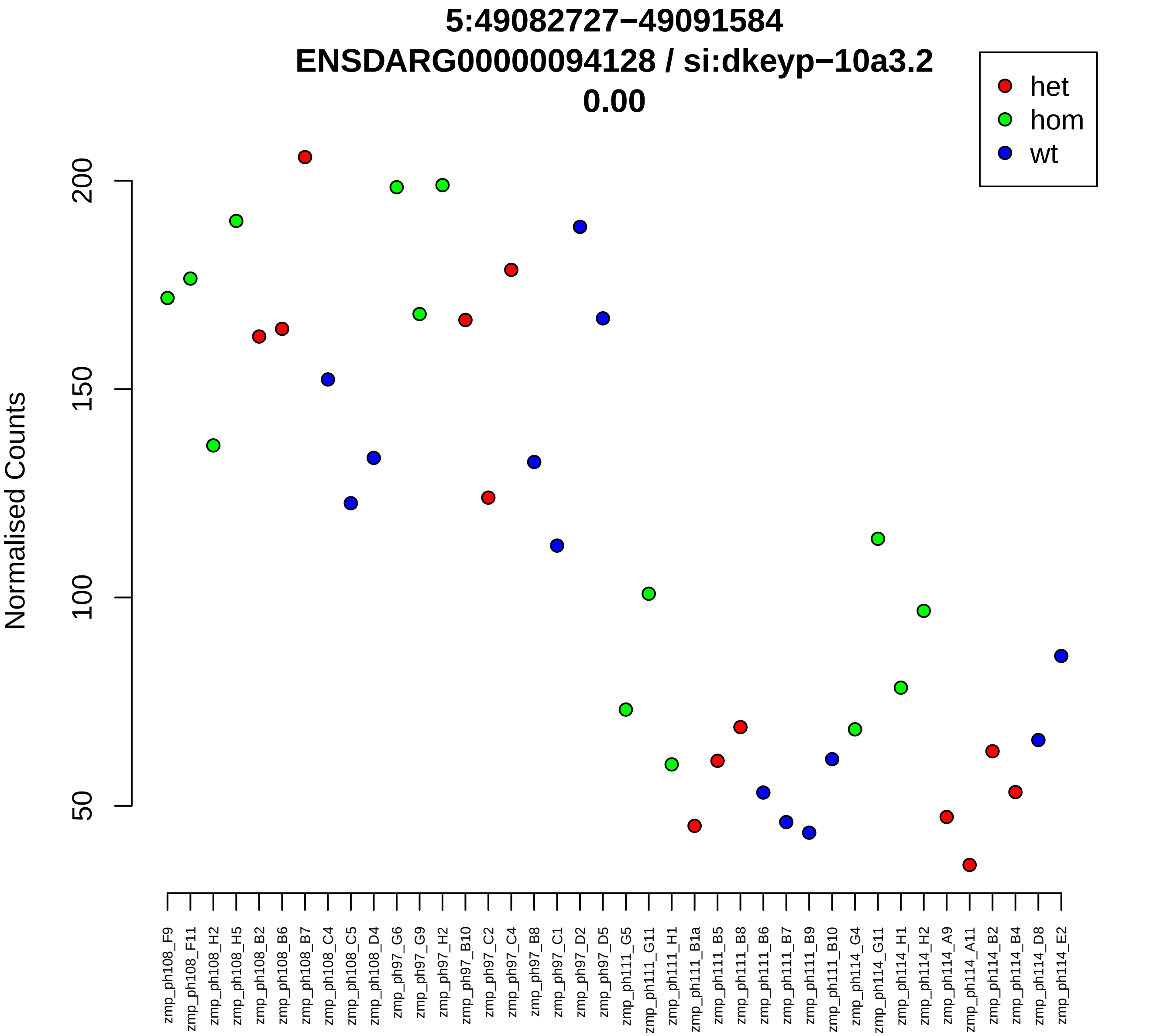

5:49082727−49091584
ENSDARG00000094128 / si:dkeyp−10a3.2
0.00
het
hom
wt
Normalised Counts
50
100
150
200
zmp_ph108_F9
zmp_ph108_F11
zmp_ph108_H2
zmp_ph108_H5
zmp_ph108_B2
zmp_ph108_B6
zmp_ph108_B7
zmp_ph108_C4
zmp_ph108_C5
zmp_ph108_D4
zmp_ph97_G6
zmp_ph97_G9
zmp_ph97_H2
zmp_ph97_B10
zmp_ph97_C2
zmp_ph97_C4
zmp_ph97_B8
zmp_ph97_C1
zmp_ph97_D2
zmp_ph97_D5
zmp_ph111_G5
zmp_ph111_G11
zmp_ph111_H1
zmp_ph111_B1a
zmp_ph111_B5
zmp_ph111_B8
zmp_ph111_B6
zmp_ph111_B7
zmp_ph111_B9
zmp_ph111_B10
zmp_ph114_G4
zmp_ph114_G11
zmp_ph114_H1
zmp_ph114_H2
zmp_ph114_A9
zmp_ph114_A11
zmp_ph114_B2
zmp_ph114_B4
zmp_ph114_D8
zmp_ph114_E2

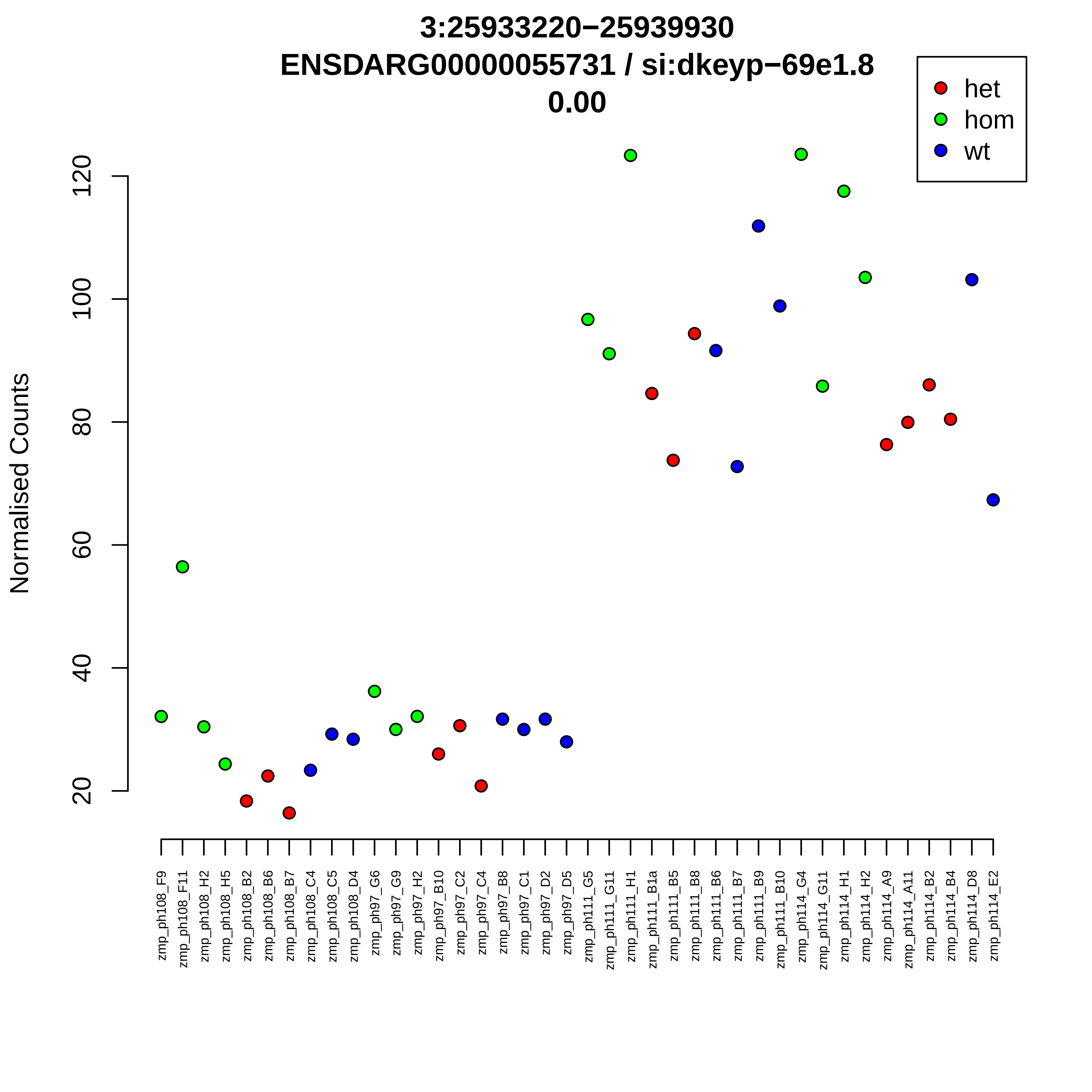
3:25933220−25939930
ENSDARG00000055731 / si:dkeyp−69e1.8
0.00
het
hom
wt
Normalised Counts
20
40
60
80
100
120
zmp_ph108_F9
zmp_ph108_F11
zmp_ph108_H2
zmp_ph108_H5
zmp_ph108_B2
zmp_ph108_B6
zmp_ph108_B7
zmp_ph108_C4
zmp_ph108_C5
zmp_ph108_D4
zmp_ph97_G6
zmp_ph97_G9
zmp_ph97_H2
zmp_ph97_B10
zmp_ph97_C2
zmp_ph97_C4
zmp_ph97_B8
zmp_ph97_C1
zmp_ph97_D2
zmp_ph97_D5
zmp_ph111_G5
zmp_ph111_G11
zmp_ph111_H1
zmp_ph111_B1a
zmp_ph111_B5
zmp_ph111_B8
zmp_ph111_B6
zmp_ph111_B7
zmp_ph111_B9
zmp_ph111_B10
zmp_ph114_G4
zmp_ph114_G11
zmp_ph114_H1
zmp_ph114_H2
zmp_ph114_A9
zmp_ph114_A11
zmp_ph114_B2
zmp_ph114_B4
zmp_ph114_D8
zmp_ph114_E2

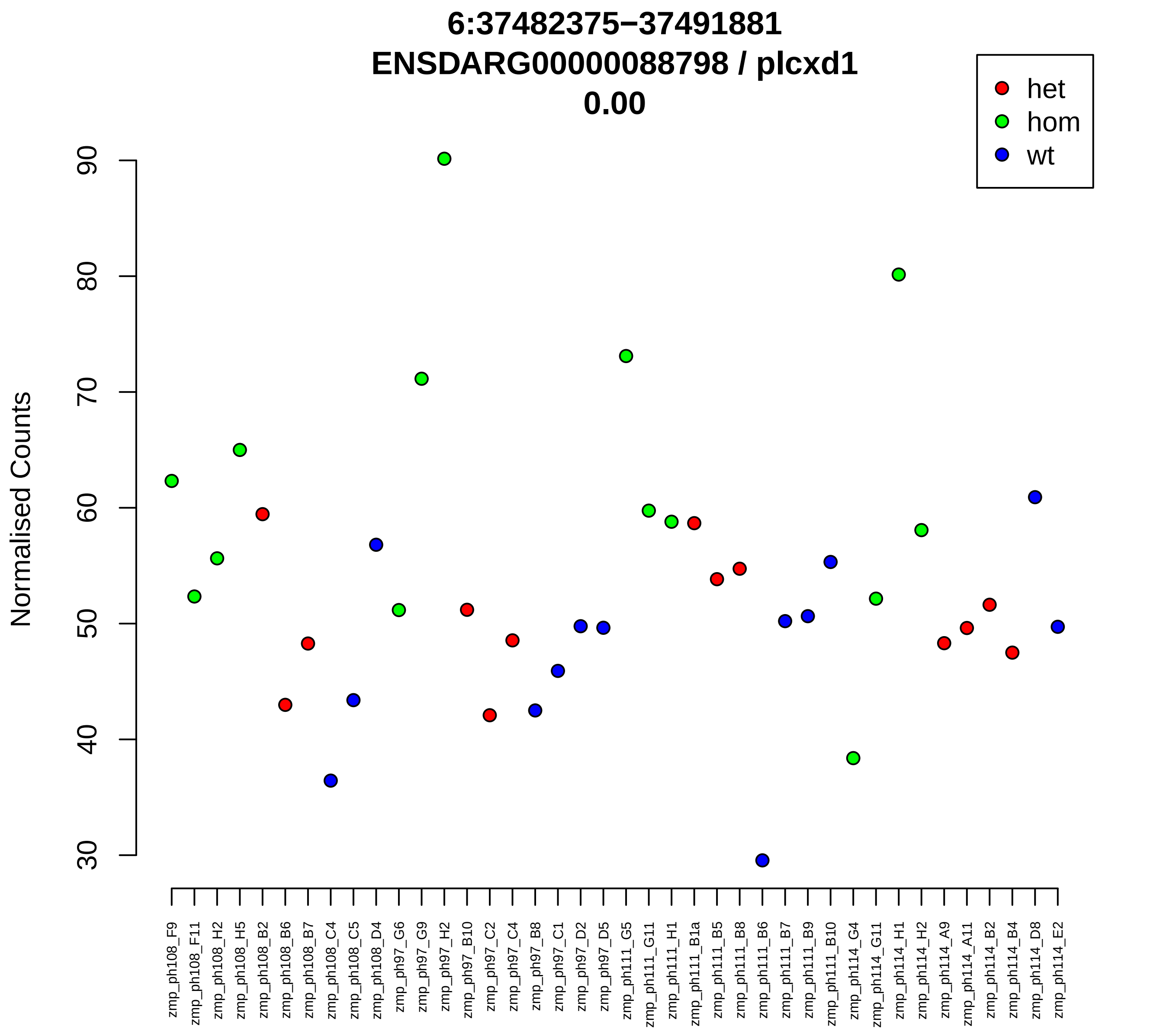

6:37482375−37491881
ENSDARG00000088798 / plcxd1
0.00
het
hom
wt
Normalised Counts
30
40
50
60
70
80
90
zmp_ph108_F9
zmp_ph108_F11
zmp_ph108_H2
zmp_ph108_H5
zmp_ph108_B2
zmp_ph108_B6
zmp_ph108_B7
zmp_ph108_C4
zmp_ph108_C5
zmp_ph108_D4
zmp_ph97_G6
zmp_ph97_G9
zmp_ph97_H2
zmp_ph97_B10
zmp_ph97_C2
zmp_ph97_C4
zmp_ph97_B8
zmp_ph97_C1
zmp_ph97_D2
zmp_ph97_D5
zmp_ph111_G5
zmp_ph111_G11
zmp_ph111_H1
zmp_ph111_B1a
zmp_ph111_B5
zmp_ph111_B8
zmp_ph111_B6
zmp_ph111_B7
zmp_ph111_B9
zmp_ph111_B10
zmp_ph114_G4
zmp_ph114_G11
zmp_ph114_H1
zmp_ph114_H2
zmp_ph114_A9
zmp_ph114_A11
zmp_ph114_B2
zmp_ph114_B4
zmp_ph114_D8
zmp_ph114_E2

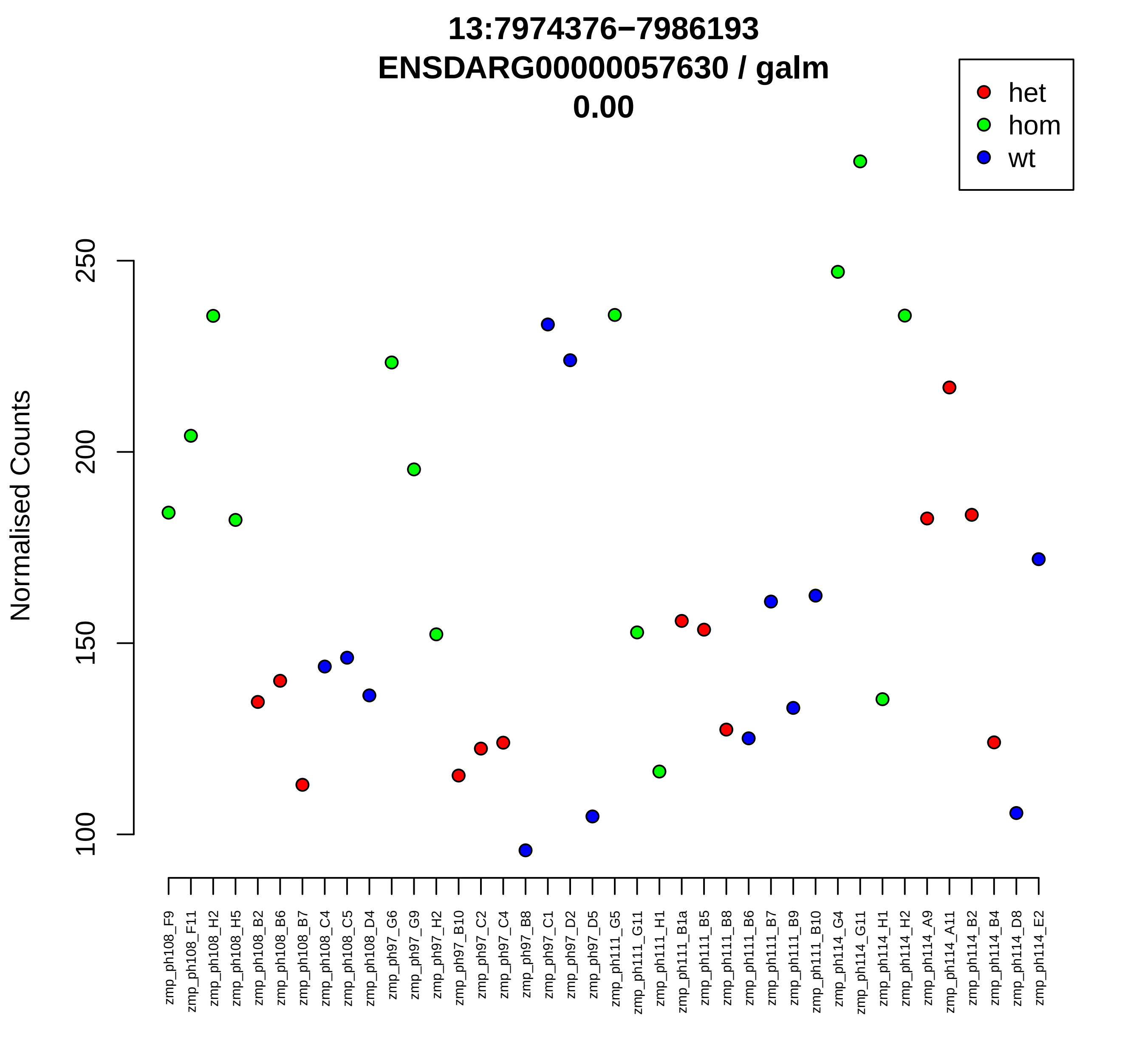
13:7974376−7986193
ENSDARG00000057630 / galm
0.00
het
hom
wt
Normalised Counts
250
200
150
100
zmp_ph108_F9
zmp_ph108_F11
zmp_ph108_H2
zmp_ph108_H5
zmp_ph108_B2
zmp_ph108_B6
zmp_ph108_B7
zmp_ph108_C4
zmp_ph108_C5
zmp_ph108_D4
zmp_ph97_G6
zmp_ph97_G9
zmp_ph97_H2
zmp_ph97_B10
zmp_ph97_C2
zmp_ph97_C4
zmp_ph97_B8
zmp_ph97_C1
zmp_ph97_D2
zmp_ph97_D5
zmp_ph111_G5
zmp_ph111_G11
zmp_ph111_H1
zmp_ph111_B1a
zmp_ph111_B5
zmp_ph111_B8
zmp_ph111_B6
zmp_ph111_B7
zmp_ph111_B9
zmp_ph111_B10
zmp_ph114_G4
zmp_ph114_G11
zmp_ph114_H1
zmp_ph114_H2
zmp_ph114_A9
zmp_ph114_A11
zmp_ph114_B2
zmp_ph114_B4
zmp_ph114_D8
zmp_ph114_E2

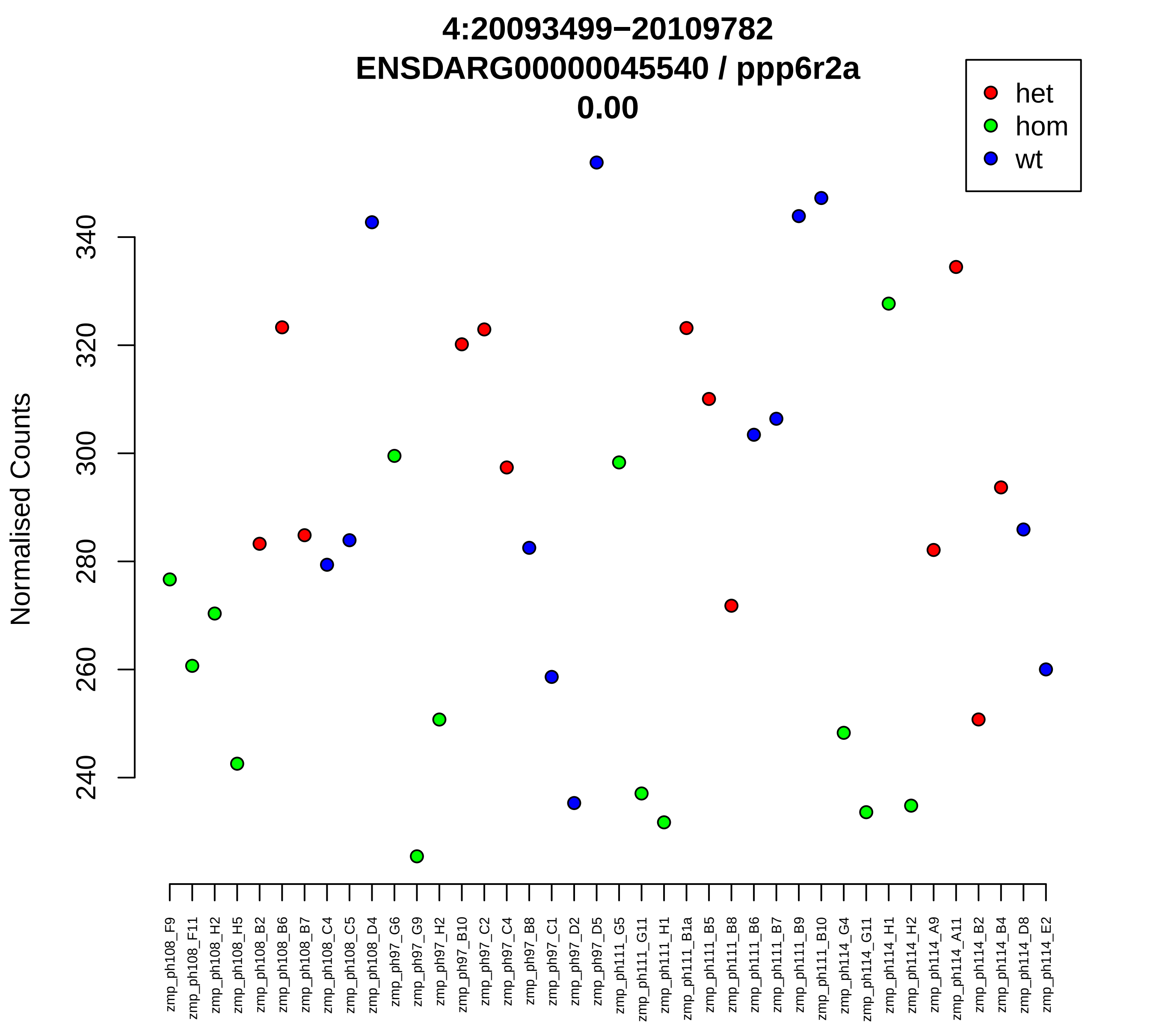
4:20093499−20109782
ENSDARG00000045540 / ppp6r2a
0.00
het
hom
wt
Normalised Counts
240
260
280
300
320
340
zmp_ph108_F9
zmp_ph108_F11
zmp_ph108_H2
zmp_ph108_H5
zmp_ph108_B2
zmp_ph108_B6
zmp_ph108_B7
zmp_ph108_C4
zmp_ph108_C5
zmp_ph108_D4
zmp_ph97_G6
zmp_ph97_G9
zmp_ph97_H2
zmp_ph97_B10
zmp_ph97_C2
zmp_ph97_C4
zmp_ph97_B8
zmp_ph97_C1
zmp_ph97_D2
zmp_ph97_D5
zmp_ph111_G5
zmp_ph111_G11
zmp_ph111_H1
zmp_ph111_B1a
zmp_ph111_B5
zmp_ph111_B8
zmp_ph111_B6
zmp_ph111_B7
zmp_ph111_B9
zmp_ph111_B10
zmp_ph114_G4
zmp_ph114_G11
zmp_ph114_H1
zmp_ph114_H2
zmp_ph114_A9
zmp_ph114_A11
zmp_ph114_B2
zmp_ph114_B4
zmp_ph114_D8
zmp_ph114_E2

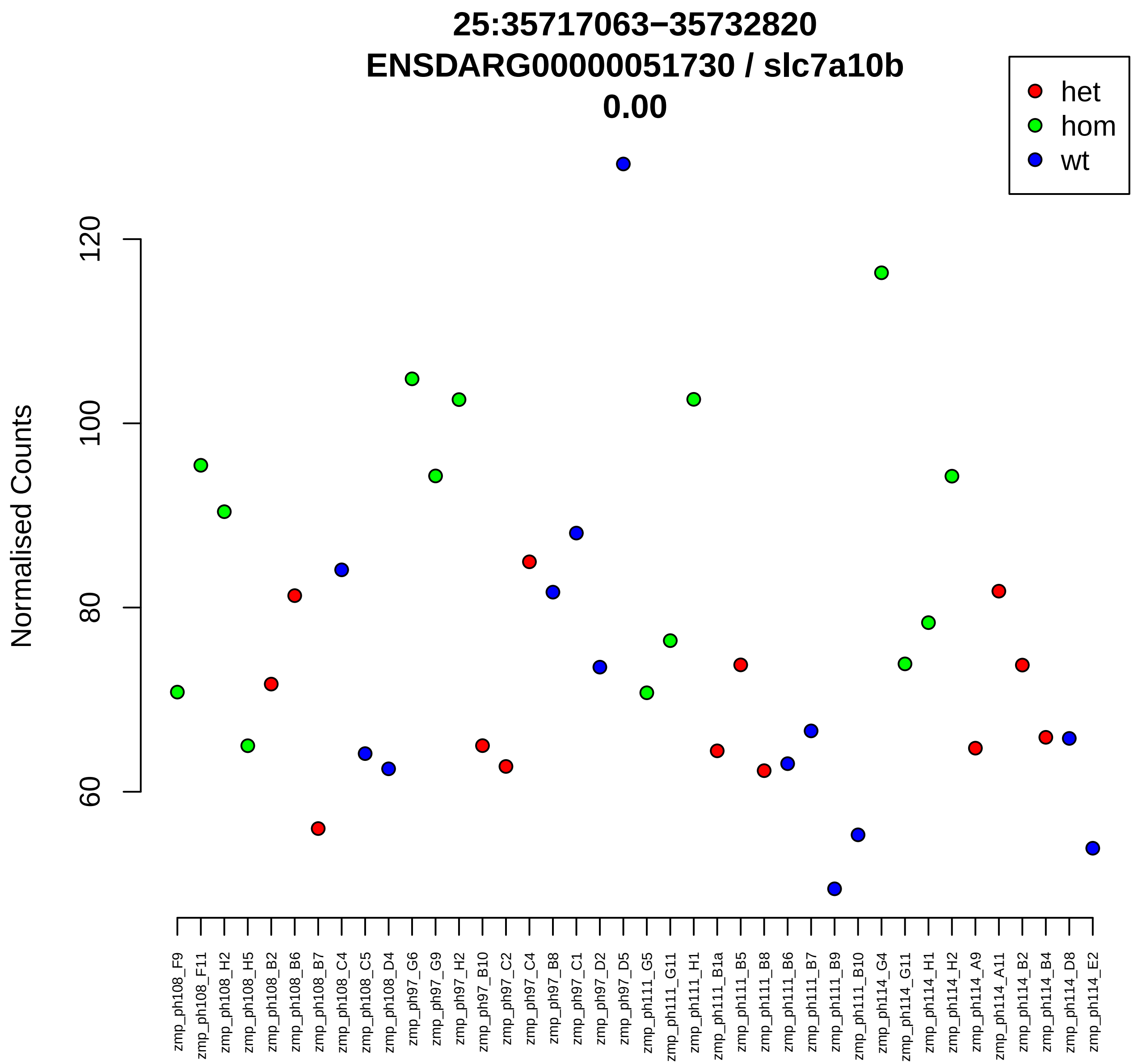
25:35717063−35732820
ENSDARG00000051730 / slc7a10b
0.00
het
hom
wt
Normalised Counts
60
80
100
120
zmp_ph108_F9
zmp_ph108_F11
zmp_ph108_H2
zmp_ph108_H5
zmp_ph108_B2
zmp_ph108_B6
zmp_ph108_B7
zmp_ph108_C4
zmp_ph108_C5
zmp_ph108_D4
zmp_ph97_G6
zmp_ph97_G9
zmp_ph97_H2
zmp_ph97_B10
zmp_ph97_C2
zmp_ph97_C4
zmp_ph97_B8
zmp_ph97_C1
zmp_ph97_D2
zmp_ph97_D5
zmp_ph111_G5
zmp_ph111_G11
zmp_ph111_H1
zmp_ph111_B1a
zmp_ph111_B5
zmp_ph111_B8
zmp_ph111_B6
zmp_ph111_B7
zmp_ph111_B9
zmp_ph111_B10
zmp_ph114_G4
zmp_ph114_G11
zmp_ph114_H1
zmp_ph114_H2
zmp_ph114_A9
zmp_ph114_A11
zmp_ph114_B2
zmp_ph114_B4
zmp_ph114_D8
zmp_ph114_E2

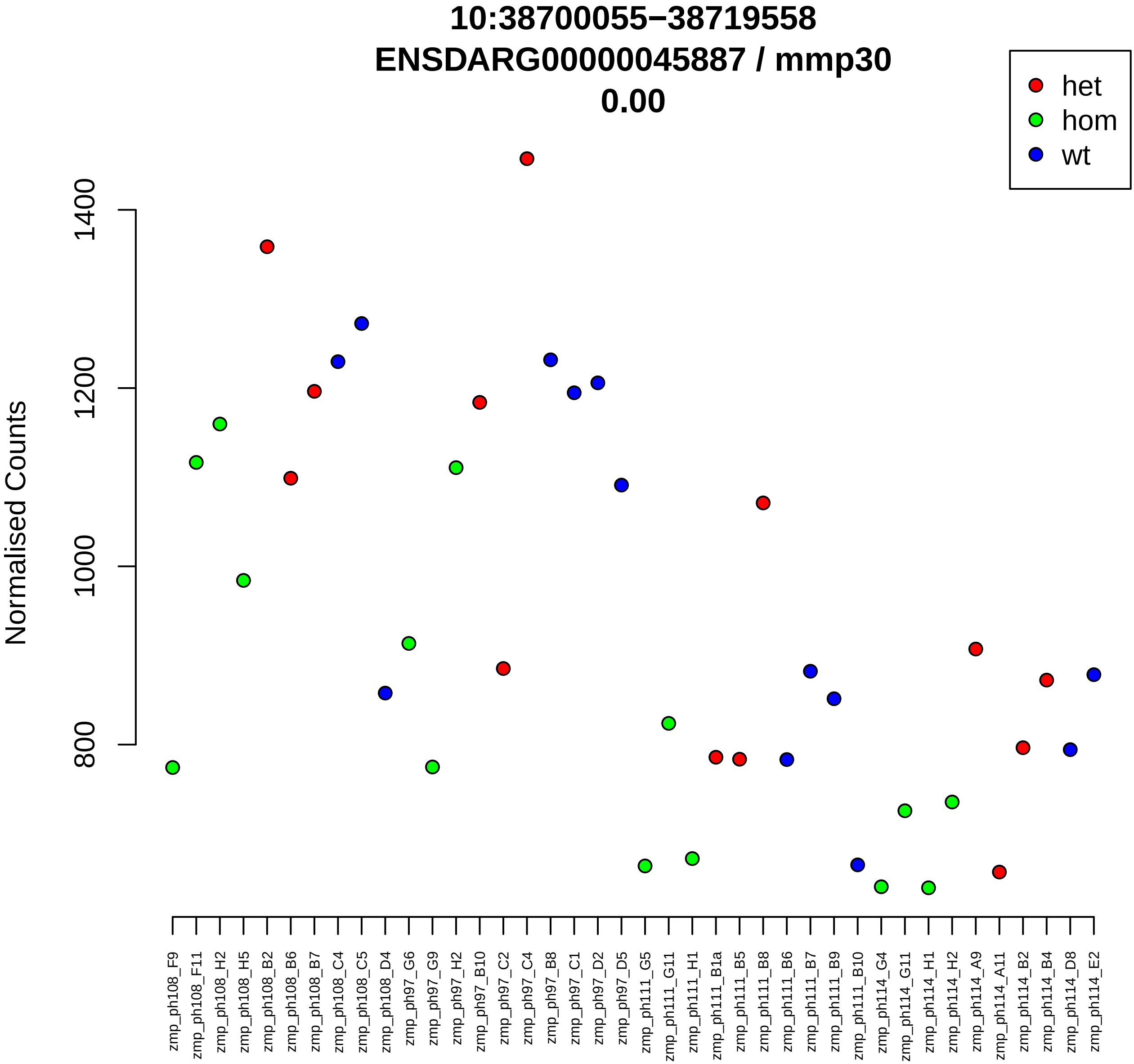
10:38700055−38719558
ENSDARG00000045887 / mmp30
0.00
het
hom
wt
Normalised Counts
1400
1200
1000
800
zmp_ph108_F9
zmp_ph108_F11
zmp_ph108_H2
zmp_ph108_H5
zmp_ph108_B2
zmp_ph108_B6
zmp_ph108_B7
zmp_ph108_C4
zmp_ph108_C5
zmp_ph108_D4
zmp_ph97_G6
zmp_ph97_G9
zmp_ph97_H2
zmp_ph97_B10
zmp_ph97_C2
zmp_ph97_C4
zmp_ph97_B8
zmp_ph97_C1
zmp_ph97_D2
zmp_ph97_D5
zmp_ph111_G5
zmp_ph111_G11
zmp_ph111_H1
zmp_ph111_B1a
zmp_ph111_B5
zmp_ph111_B8
zmp_ph111_B6
zmp_ph111_B7
zmp_ph111_B9
zmp_ph111_B10
zmp_ph114_G4
zmp_ph114_G11
zmp_ph114_H1
zmp_ph114_H2
zmp_ph114_A9
zmp_ph114_A11
zmp_ph114_B2
zmp_ph114_B4
zmp_ph114_D8
zmp_ph114_E2

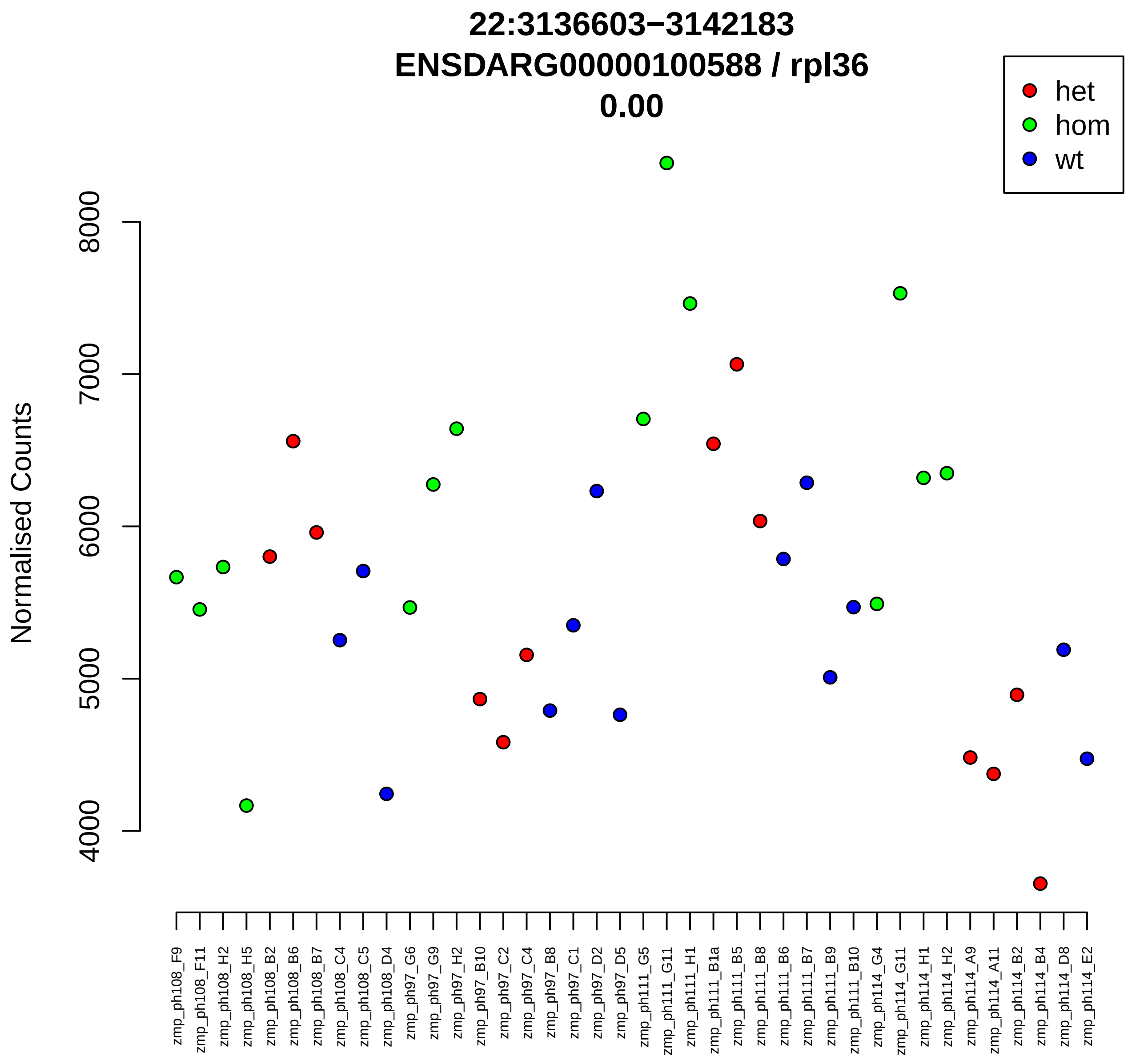
22:3136603−3142183
ENSDARG00000100588 / rpl36
0.00
het
hom
wt
Normalised Counts
4000
5000
6000
7000
8000
zmp_ph108_F9
zmp_ph108_F11
zmp_ph108_H2
zmp_ph108_H5
zmp_ph108_B2
zmp_ph108_B6
zmp_ph108_B7
zmp_ph108_C4
zmp_ph108_C5
zmp_ph108_D4
zmp_ph97_G6
zmp_ph97_G9
zmp_ph97_H2
zmp_ph97_B10
zmp_ph97_C2
zmp_ph97_C4
zmp_ph97_B8
zmp_ph97_C1
zmp_ph97_D2
zmp_ph97_D5
zmp_ph111_G5
zmp_ph111_G11
zmp_ph111_H1
zmp_ph111_B1a
zmp_ph111_B5
zmp_ph111_B8
zmp_ph111_B6
zmp_ph111_B7
zmp_ph111_B9
zmp_ph111_B10
zmp_ph114_G4
zmp_ph114_G11
zmp_ph114_H1
zmp_ph114_H2
zmp_ph114_A9
zmp_ph114_A11
zmp_ph114_B2
zmp_ph114_B4
zmp_ph114_D8
zmp_ph114_E2

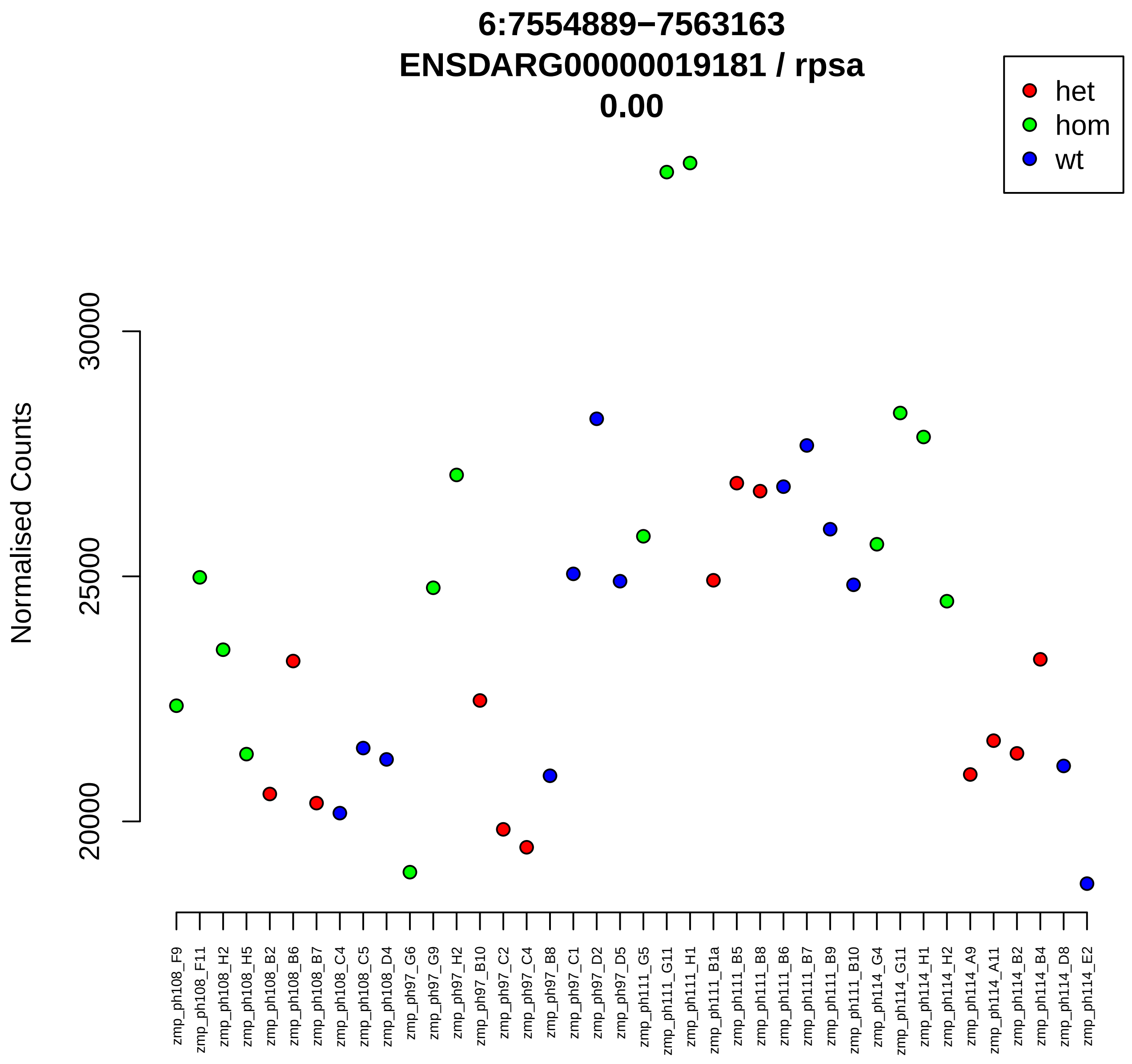

6:7554889−7563163
ENSDARG00000019181 / rpsa
0.00
het
hom
wt
Normalised Counts
20000
25000
30000
zmp_ph108_F9
zmp_ph108_F11
zmp_ph108_H2
zmp_ph108_H5
zmp_ph108_B2
zmp_ph108_B6
zmp_ph108_B7
zmp_ph108_C4
zmp_ph108_C5
zmp_ph108_D4
zmp_ph97_G6
zmp_ph97_G9
zmp_ph97_H2
zmp_ph97_B10
zmp_ph97_C2
zmp_ph97_C4
zmp_ph97_B8
zmp_ph97_C1
zmp_ph97_D2
zmp_ph97_D5
zmp_ph111_G5
zmp_ph111_G11
zmp_ph111_H1
zmp_ph111_B1a
zmp_ph111_B5
zmp_ph111_B8
zmp_ph111_B6
zmp_ph111_B7
zmp_ph111_B9
zmp_ph111_B10
zmp_ph114_G4
zmp_ph114_G11
zmp_ph114_H1
zmp_ph114_H2
zmp_ph114_A9
zmp_ph114_A11
zmp_ph114_B2
zmp_ph114_B4
zmp_ph114_D8
zmp_ph114_E2

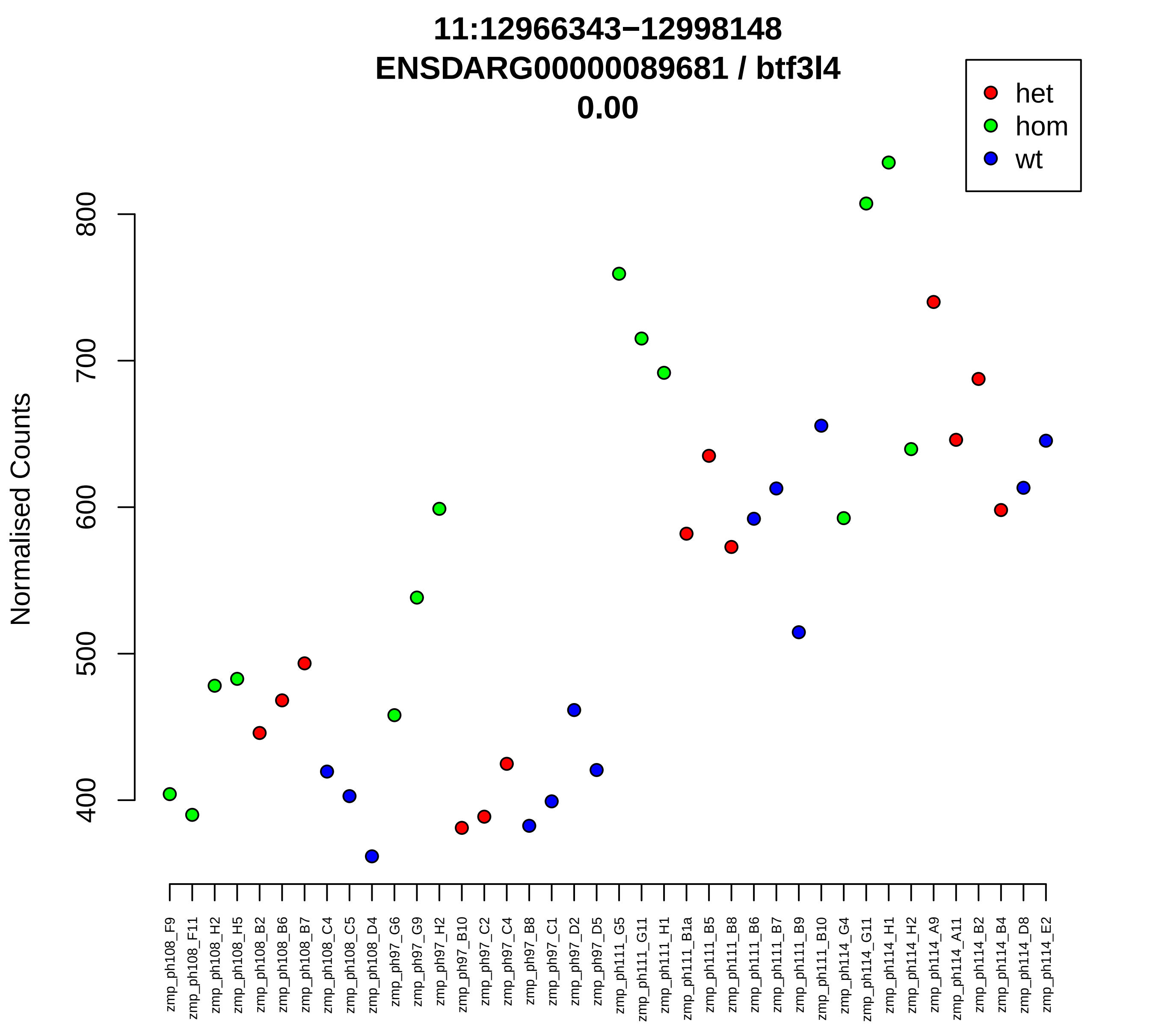
11:12966343−12998148
ENSDARG00000089681 / btf3l4
0.00
het
hom
wt
Normalised Counts
400
500
600
700
800
zmp_ph108_F9
zmp_ph108_F11
zmp_ph108_H2
zmp_ph108_H5
zmp_ph108_B2
zmp_ph108_B6
zmp_ph108_B7
zmp_ph108_C4
zmp_ph108_C5
zmp_ph108_D4
zmp_ph97_G6
zmp_ph97_G9
zmp_ph97_H2
zmp_ph97_B10
zmp_ph97_C2
zmp_ph97_C4
zmp_ph97_B8
zmp_ph97_C1
zmp_ph97_D2
zmp_ph97_D5
zmp_ph111_G5
zmp_ph111_G11
zmp_ph111_H1
zmp_ph111_B1a
zmp_ph111_B5
zmp_ph111_B8
zmp_ph111_B6
zmp_ph111_B7
zmp_ph111_B9
zmp_ph111_B10
zmp_ph114_G4
zmp_ph114_G11
zmp_ph114_H1
zmp_ph114_H2
zmp_ph114_A9
zmp_ph114_A11
zmp_ph114_B2
zmp_ph114_B4
zmp_ph114_D8
zmp_ph114_E2

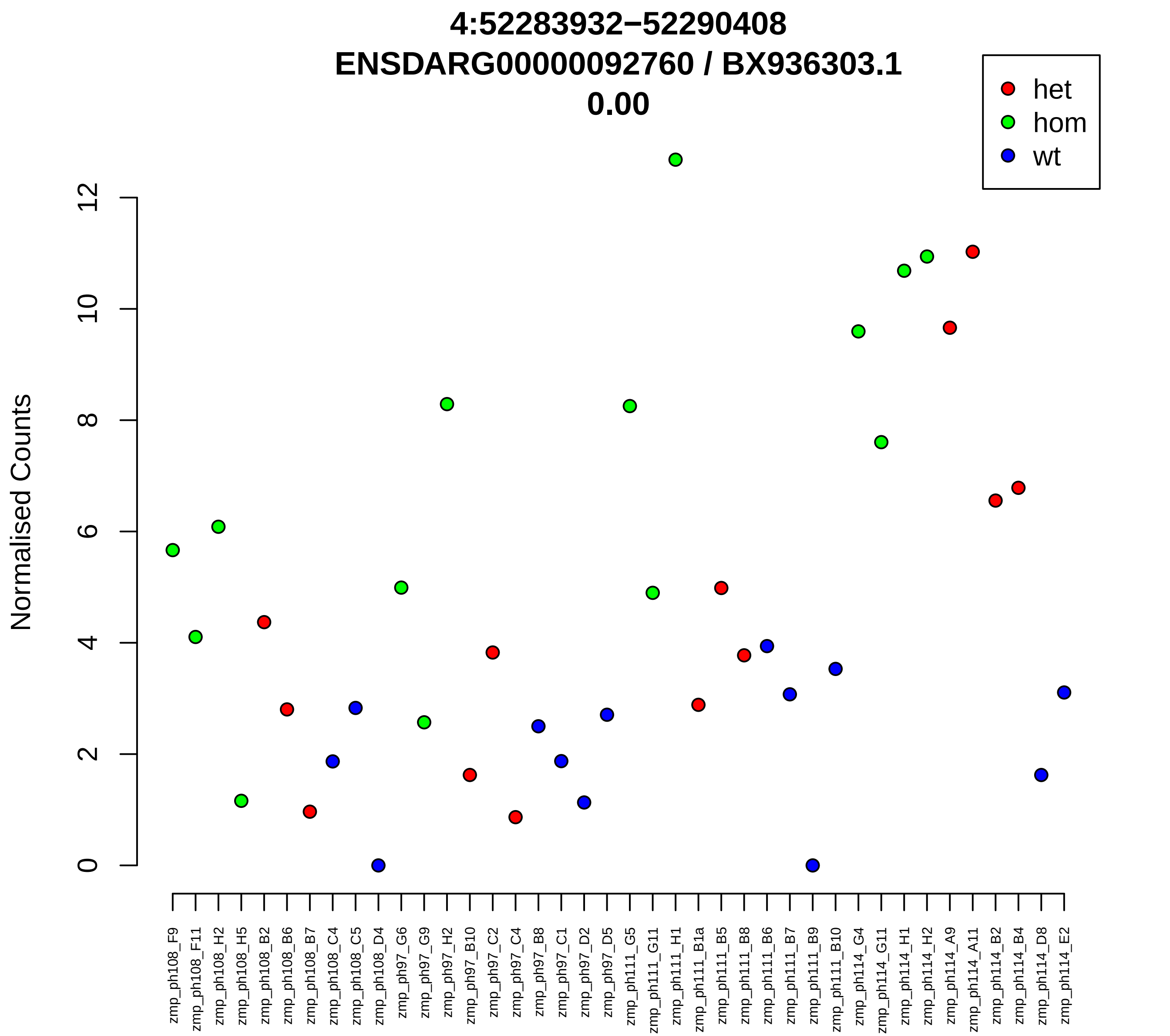

4:52283932−52290408
ENSDARG00000092760 / BX936303.1
0.00
het
hom
wt
Normalised Counts
0
2
4
6
8
10
12
zmp_ph108_F9
zmp_ph108_F11
zmp_ph108_H2
zmp_ph108_H5
zmp_ph108_B2
zmp_ph108_B6
zmp_ph108_B7
zmp_ph108_C4
zmp_ph108_C5
zmp_ph108_D4
zmp_ph97_G6
zmp_ph97_G9
zmp_ph97_H2
zmp_ph97_B10
zmp_ph97_C2
zmp_ph97_C4
zmp_ph97_B8
zmp_ph97_C1
zmp_ph97_D2
zmp_ph97_D5
zmp_ph111_G5
zmp_ph111_G11
zmp_ph111_H1
zmp_ph111_B1a
zmp_ph111_B5
zmp_ph111_B8
zmp_ph111_B6
zmp_ph111_B7
zmp_ph111_B9
zmp_ph111_B10
zmp_ph114_G4
zmp_ph114_G11
zmp_ph114_H1
zmp_ph114_H2
zmp_ph114_A9
zmp_ph114_A11
zmp_ph114_B2
zmp_ph114_B4
zmp_ph114_D8
zmp_ph114_E2

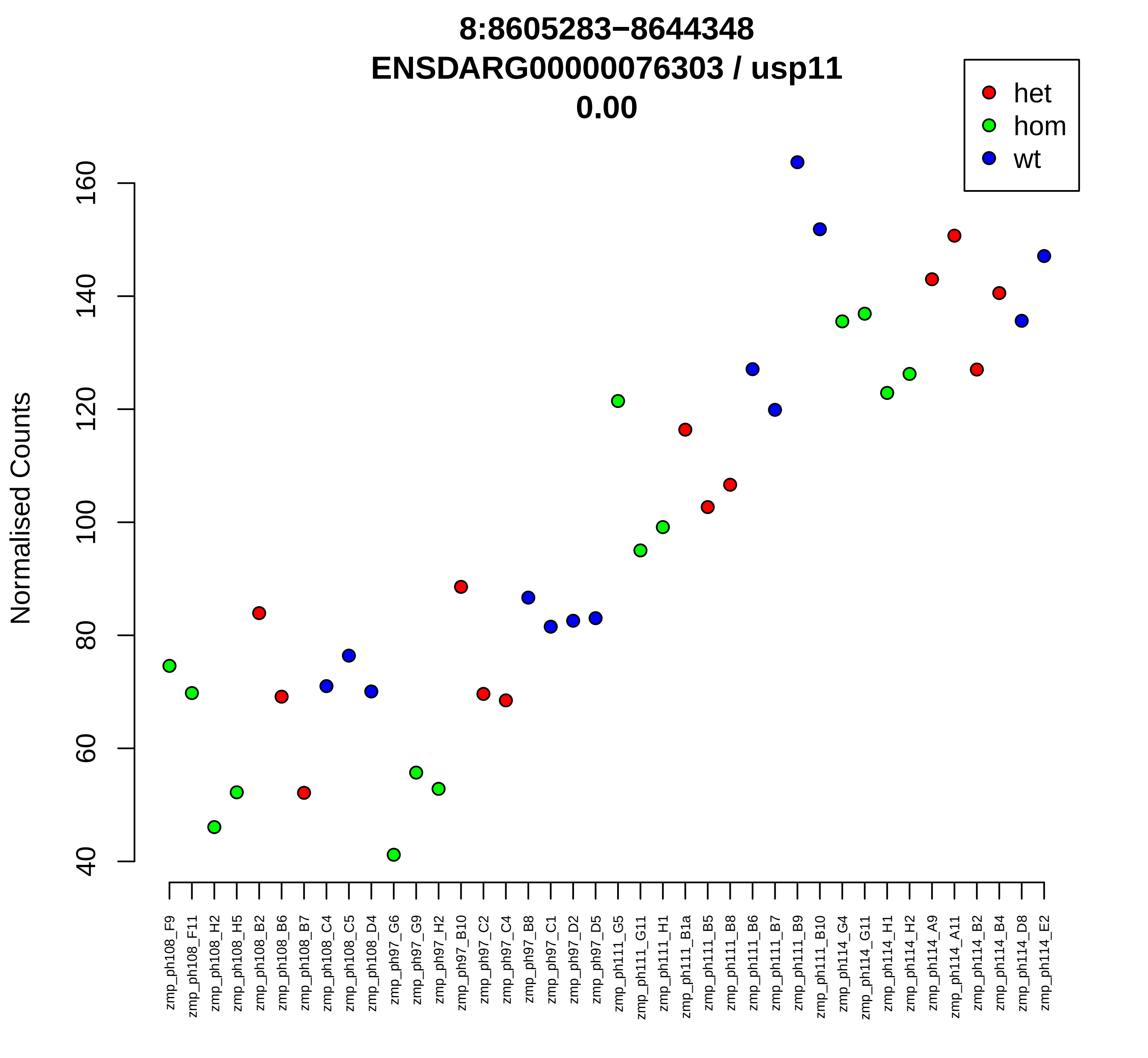

8:8605283−8644348
ENSDARG00000076303 / usp11
0.00
het
hom
wt
Normalised Counts
40
60
80
100
120
140
160
zmp_ph108_F9
zmp_ph108_F11
zmp_ph108_H2
zmp_ph108_H5
zmp_ph108_B2
zmp_ph108_B6
zmp_ph108_B7
zmp_ph108_C4
zmp_ph108_C5
zmp_ph108_D4
zmp_ph97_G6
zmp_ph97_G9
zmp_ph97_H2
zmp_ph97_B10
zmp_ph97_C2
zmp_ph97_C4
zmp_ph97_B8
zmp_ph97_C1
zmp_ph97_D2
zmp_ph97_D5
zmp_ph111_G5
zmp_ph111_G11
zmp_ph111_H1
zmp_ph111_B1a
zmp_ph111_B5
zmp_ph111_B8
zmp_ph111_B6
zmp_ph111_B7
zmp_ph111_B9
zmp_ph111_B10
zmp_ph114_G4
zmp_ph114_G11
zmp_ph114_H1
zmp_ph114_H2
zmp_ph114_A9
zmp_ph114_A11
zmp_ph114_B2
zmp_ph114_B4
zmp_ph114_D8
zmp_ph114_E2

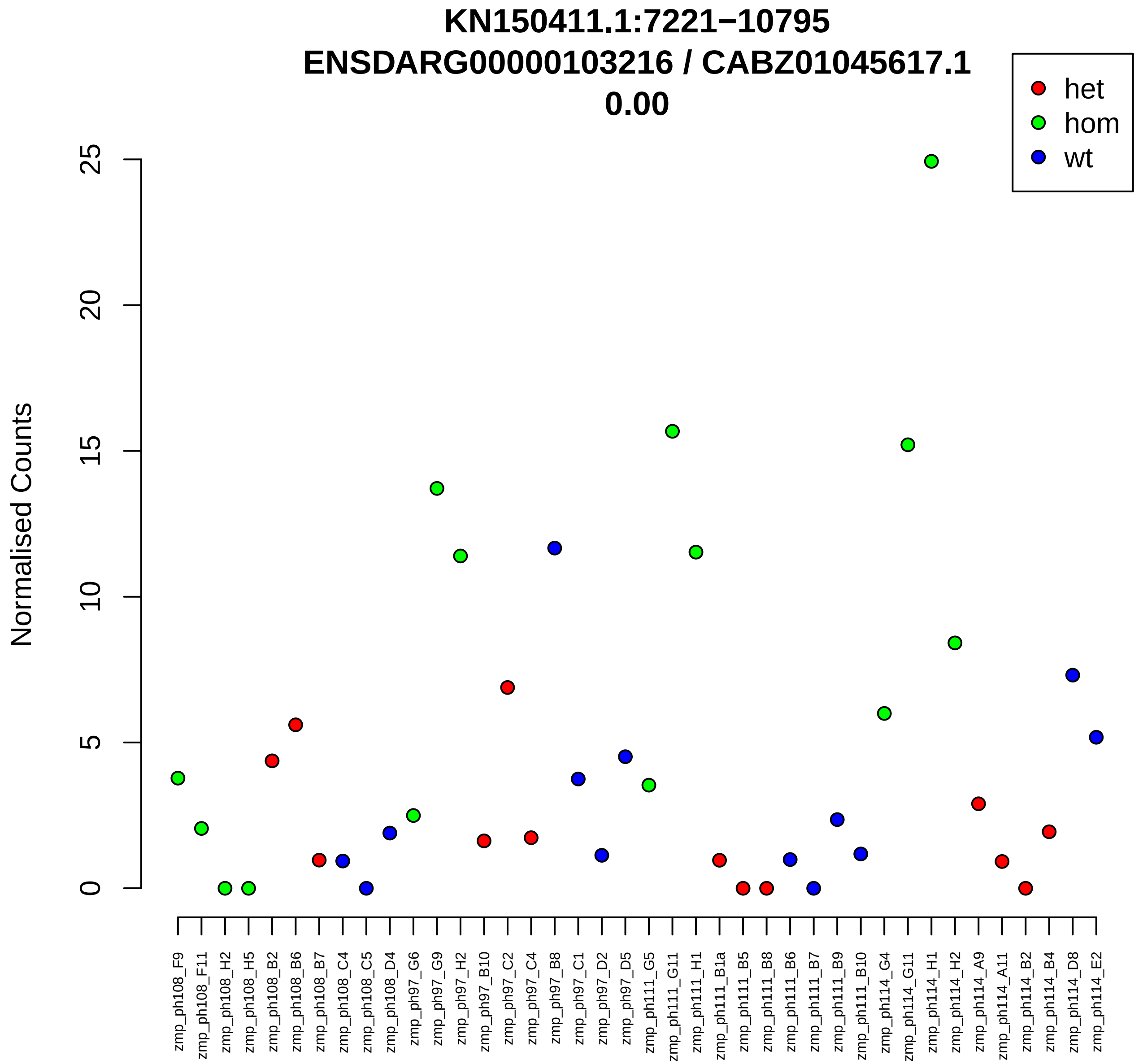

KN150411.1:7221−10795
ENSDARG00000103216 / CABZ01045617.1
0.00
Normalised Counts
het
hom
wt
0
5
10
15
20
25
zmp_ph108_F9
zmp_ph108_F11
zmp_ph108_H2
zmp_ph108_H5
zmp_ph108_B2
zmp_ph108_B6
zmp_ph108_B7
zmp_ph108_C4
zmp_ph108_C5
zmp_ph108_D4
zmp_ph97_G6
zmp_ph97_G9
zmp_ph97_H2
zmp_ph97_B10
zmp_ph97_C2
zmp_ph97_C4
zmp_ph97_B8
zmp_ph97_C1
zmp_ph97_D2
zmp_ph97_D5
zmp_ph111_G5
zmp_ph111_G11
zmp_ph111_H1
zmp_ph111_B1a
zmp_ph111_B5
zmp_ph111_B8
zmp_ph111_B6
zmp_ph111_B7
zmp_ph111_B9
zmp_ph111_B10
zmp_ph114_G4
zmp_ph114_G11
zmp_ph114_H1
zmp_ph114_H2
zmp_ph114_A9
zmp_ph114_A11
zmp_ph114_B2
zmp_ph114_B4
zmp_ph114_D8
zmp_ph114_E2

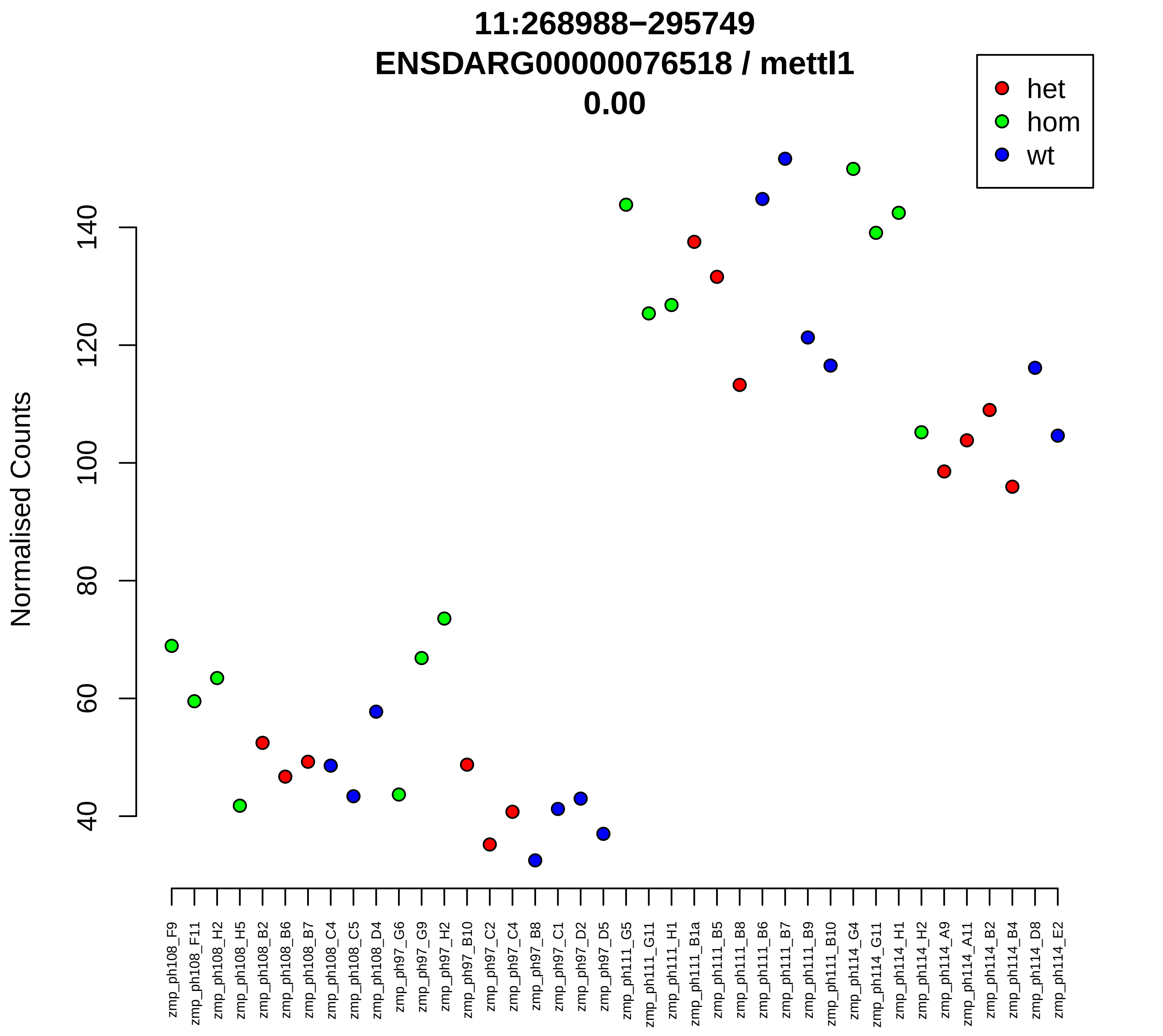

11:268988−295749
ENSDARG00000076518 / mettl1
0.00
het
hom
wt
Normalised Counts
40
60
80
100
120
140
zmp_ph108_F9
zmp_ph108_F11
zmp_ph108_H2
zmp_ph108_H5
zmp_ph108_B2
zmp_ph108_B6
zmp_ph108_B7
zmp_ph108_C4
zmp_ph108_C5
zmp_ph108_D4
zmp_ph97_G6
zmp_ph97_G9
zmp_ph97_H2
zmp_ph97_B10
zmp_ph97_C2
zmp_ph97_C4
zmp_ph97_B8
zmp_ph97_C1
zmp_ph97_D2
zmp_ph97_D5
zmp_ph111_G5
zmp_ph111_G11
zmp_ph111_H1
zmp_ph111_B1a
zmp_ph111_B5
zmp_ph111_B8
zmp_ph111_B6
zmp_ph111_B7
zmp_ph111_B9
zmp_ph111_B10
zmp_ph114_G4
zmp_ph114_G11
zmp_ph114_H1
zmp_ph114_H2
zmp_ph114_A9
zmp_ph114_A11
zmp_ph114_B2
zmp_ph114_B4
zmp_ph114_D8
zmp_ph114_E2

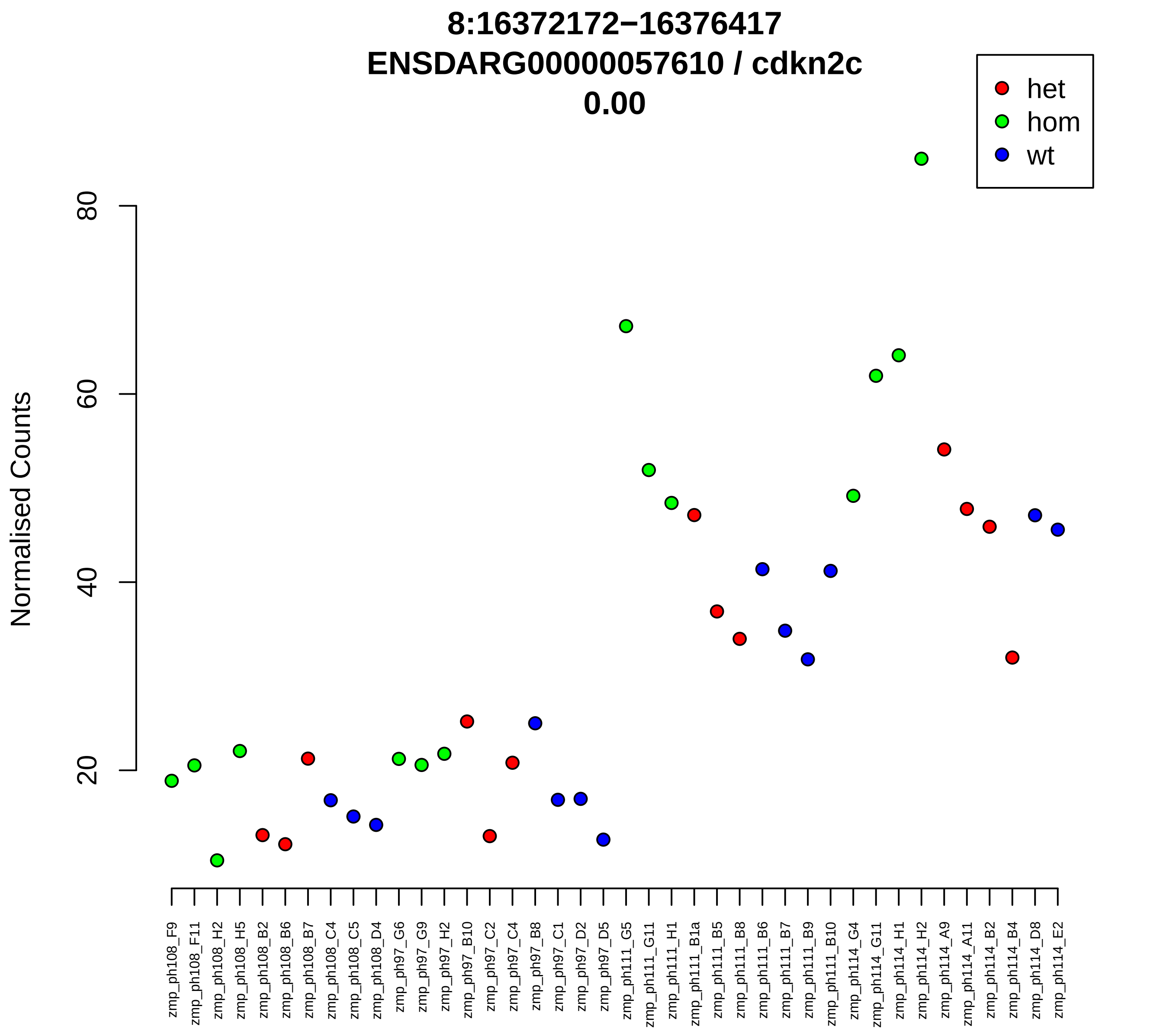
8:16372172−16376417
ENSDARG00000057610 / cdkn2c
0.00
het
hom
wt
Normalised Counts
20
40
60
80
zmp_ph108_F9
zmp_ph108_F11
zmp_ph108_H2
zmp_ph108_H5
zmp_ph108_B2
zmp_ph108_B6
zmp_ph108_B7
zmp_ph108_C4
zmp_ph108_C5
zmp_ph108_D4
zmp_ph97_G6
zmp_ph97_G9
zmp_ph97_H2
zmp_ph97_B10
zmp_ph97_C2
zmp_ph97_C4
zmp_ph97_B8
zmp_ph97_C1
zmp_ph97_D2
zmp_ph97_D5
zmp_ph111_G5
zmp_ph111_G11
zmp_ph111_H1
zmp_ph111_B1a
zmp_ph111_B5
zmp_ph111_B8
zmp_ph111_B6
zmp_ph111_B7
zmp_ph111_B9
zmp_ph111_B10
zmp_ph114_G4
zmp_ph114_G11
zmp_ph114_H1
zmp_ph114_H2
zmp_ph114_A9
zmp_ph114_A11
zmp_ph114_B2
zmp_ph114_B4
zmp_ph114_D8
zmp_ph114_E2

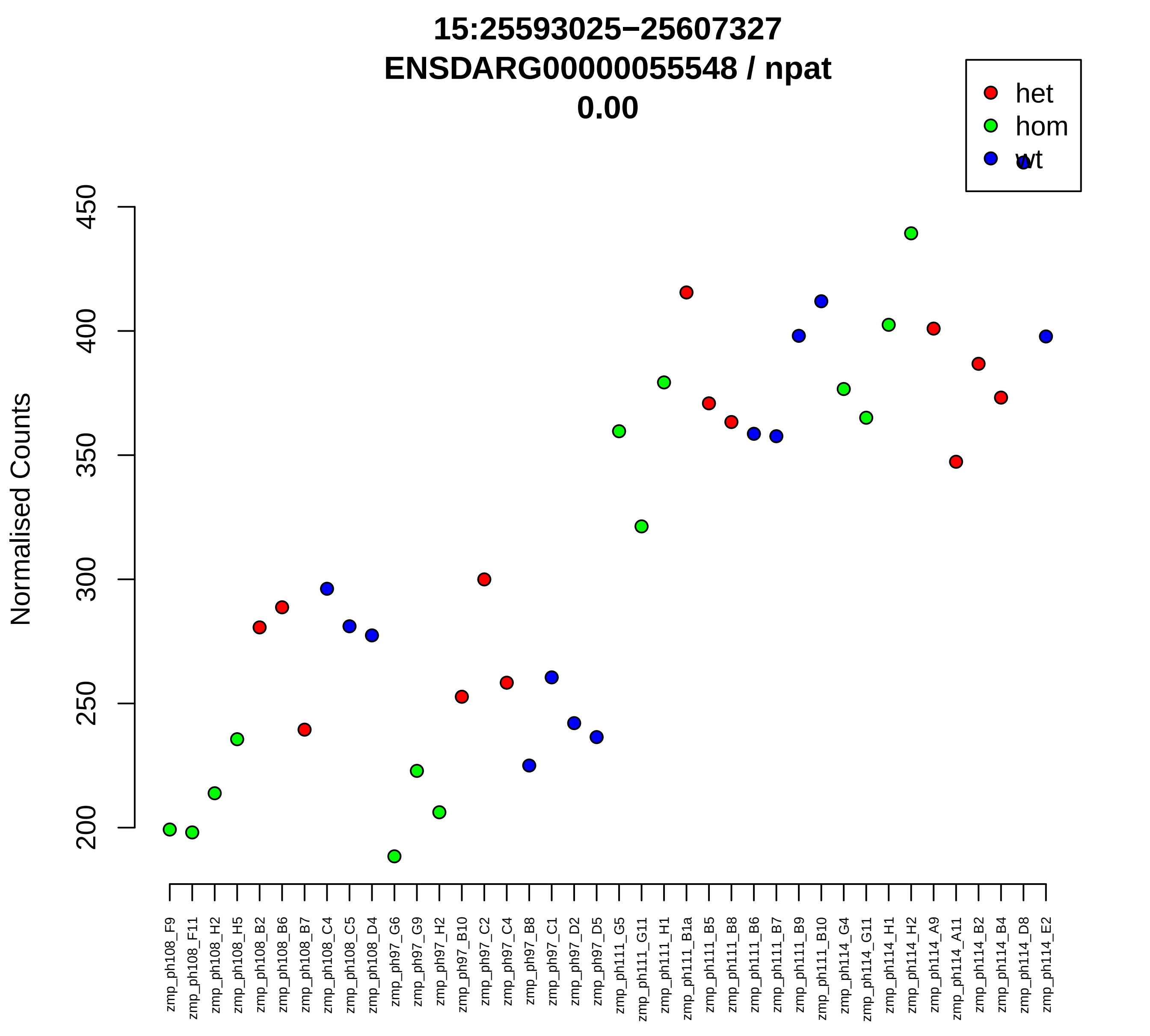

15:25593025−25607327
ENSDARG00000055548 / npat
0.00
het
hom
wt
Normalised Counts
200
250
300
350
400
450
zmp_ph108_F9
zmp_ph108_F11
zmp_ph108_H2
zmp_ph108_H5
zmp_ph108_B2
zmp_ph108_B6
zmp_ph108_B7
zmp_ph108_C4
zmp_ph108_C5
zmp_ph108_D4
zmp_ph97_G6
zmp_ph97_G9
zmp_ph97_H2
zmp_ph97_B10
zmp_ph97_C2
zmp_ph97_C4
zmp_ph97_B8
zmp_ph97_C1
zmp_ph97_D2
zmp_ph97_D5
zmp_ph111_G5
zmp_ph111_G11
zmp_ph111_H1
zmp_ph111_B1a
zmp_ph111_B5
zmp_ph111_B8
zmp_ph111_B6
zmp_ph111_B7
zmp_ph111_B9
zmp_ph111_B10
zmp_ph114_G4
zmp_ph114_G11
zmp_ph114_H1
zmp_ph114_H2
zmp_ph114_A9
zmp_ph114_A11
zmp_ph114_B2
zmp_ph114_B4
zmp_ph114_D8
zmp_ph114_E2

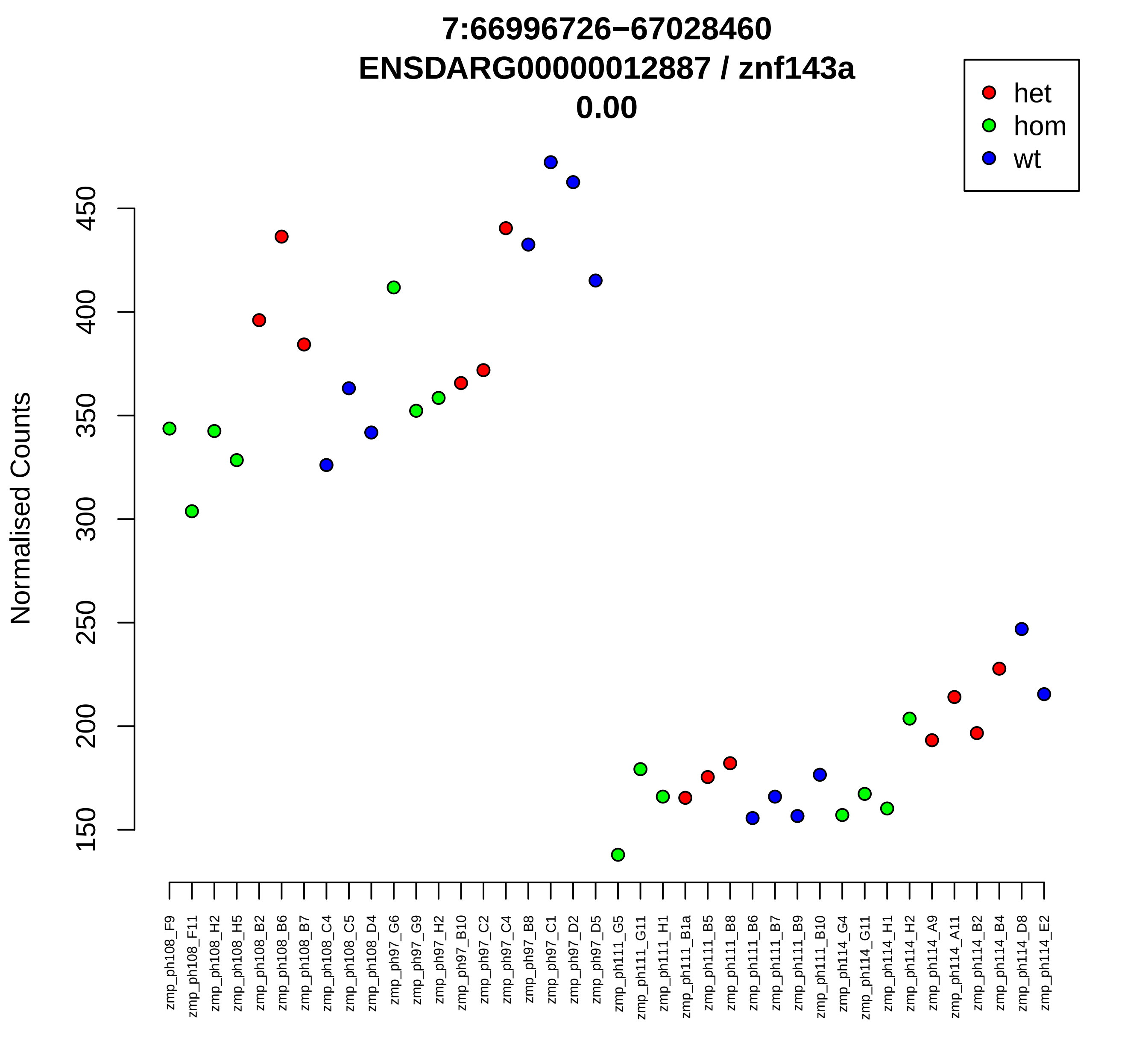
7:66996726−67028460
ENSDARG00000012887 / znf143a
0.00
het
hom
wt
Normalised Counts
150
200
250
300
350
400
450
zmp_ph108_F9
zmp_ph108_F11
zmp_ph108_H2
zmp_ph108_H5
zmp_ph108_B2
zmp_ph108_B6
zmp_ph108_B7
zmp_ph108_C4
zmp_ph108_C5
zmp_ph108_D4
zmp_ph97_G6
zmp_ph97_G9
zmp_ph97_H2
zmp_ph97_B10
zmp_ph97_C2
zmp_ph97_C4
zmp_ph97_B8
zmp_ph97_C1
zmp_ph97_D2
zmp_ph97_D5
zmp_ph111_G5
zmp_ph111_G11
zmp_ph111_H1
zmp_ph111_B1a
zmp_ph111_B5
zmp_ph111_B8
zmp_ph111_B6
zmp_ph111_B7
zmp_ph111_B9
zmp_ph111_B10
zmp_ph114_G4
zmp_ph114_G11
zmp_ph114_H1
zmp_ph114_H2
zmp_ph114_A9
zmp_ph114_A11
zmp_ph114_B2
zmp_ph114_B4
zmp_ph114_D8
zmp_ph114_E2

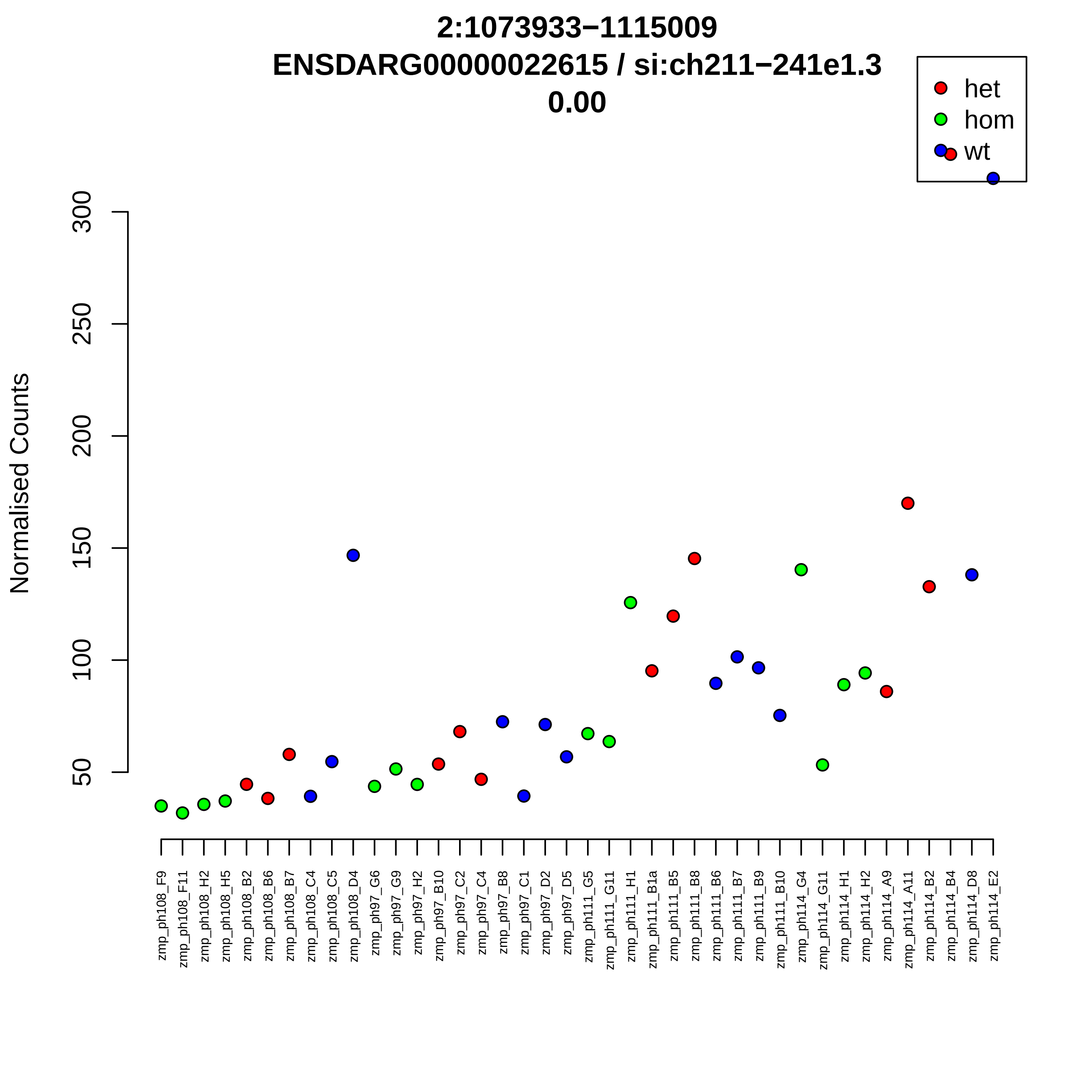

2:1073933−1115009
ENSDARG00000022615 / si:ch211−241e1.3
0.00
het
hom
wt
Normalised Counts
50
100
150
200
250
300
zmp_ph108_F9
zmp_ph108_F11
zmp_ph108_H2
zmp_ph108_H5
zmp_ph108_B2
zmp_ph108_B6
zmp_ph108_B7
zmp_ph108_C4
zmp_ph108_C5
zmp_ph108_D4
zmp_ph97_G6
zmp_ph97_G9
zmp_ph97_H2
zmp_ph97_B10
zmp_ph97_C2
zmp_ph97_C4
zmp_ph97_B8
zmp_ph97_C1
zmp_ph97_D2
zmp_ph97_D5
zmp_ph111_G5
zmp_ph111_G11
zmp_ph111_H1
zmp_ph111_B1a
zmp_ph111_B5
zmp_ph111_B8
zmp_ph111_B6
zmp_ph111_B7
zmp_ph111_B9
zmp_ph111_B10
zmp_ph114_G4
zmp_ph114_G11
zmp_ph114_H1
zmp_ph114_H2
zmp_ph114_A9
zmp_ph114_A11
zmp_ph114_B2
zmp_ph114_B4
zmp_ph114_D8
zmp_ph114_E2

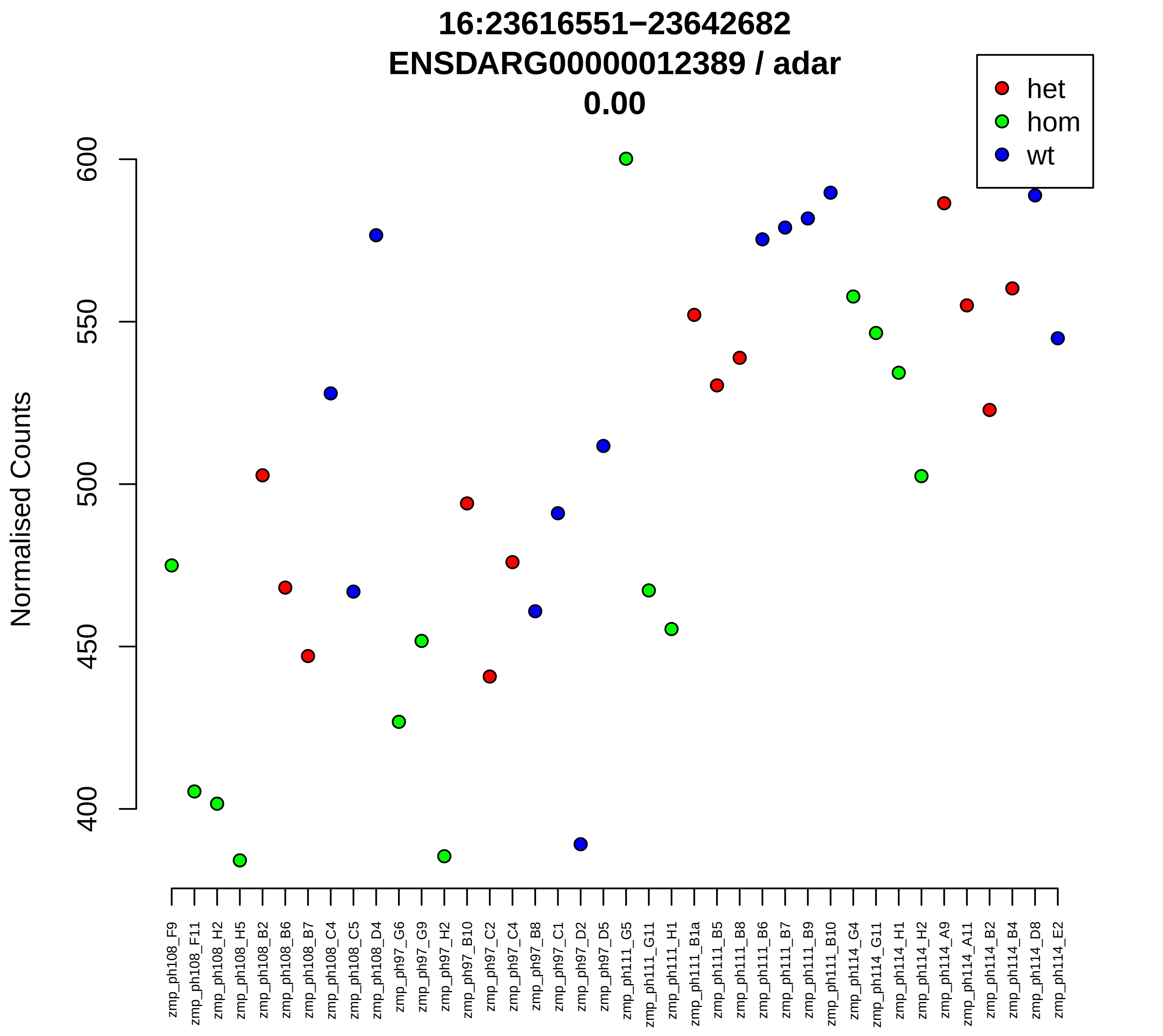
16:23616551−23642682
ENSDARG00000012389 / adar
0.00
het
hom
wt
Normalised Counts
400
450
500
550
600
zmp_ph108_F9
zmp_ph108_F11
zmp_ph108_H2
zmp_ph108_H5
zmp_ph108_B2
zmp_ph108_B6
zmp_ph108_B7
zmp_ph108_C4
zmp_ph108_C5
zmp_ph108_D4
zmp_ph97_G6
zmp_ph97_G9
zmp_ph97_H2
zmp_ph97_B10
zmp_ph97_C2
zmp_ph97_C4
zmp_ph97_B8
zmp_ph97_C1
zmp_ph97_D2
zmp_ph97_D5
zmp_ph111_G5
zmp_ph111_G11
zmp_ph111_H1
zmp_ph111_B1a
zmp_ph111_B5
zmp_ph111_B8
zmp_ph111_B6
zmp_ph111_B7
zmp_ph111_B9
zmp_ph111_B10
zmp_ph114_G4
zmp_ph114_G11
zmp_ph114_H1
zmp_ph114_H2
zmp_ph114_A9
zmp_ph114_A11
zmp_ph114_B2
zmp_ph114_B4
zmp_ph114_D8
zmp_ph114_E2

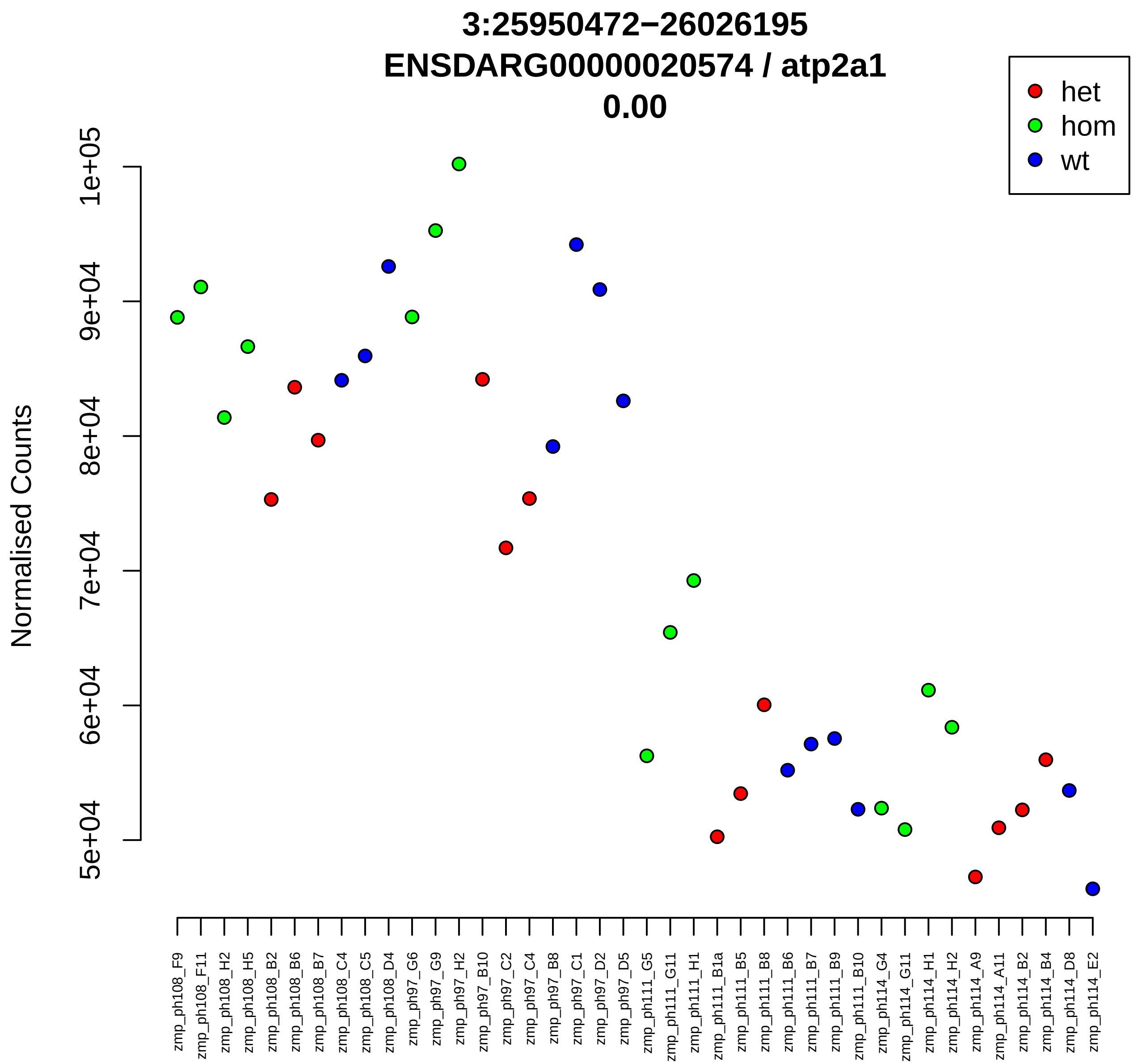
3:25950472−26026195
ENSDARG00000020574 / atp2a1
0.00
het
hom
wt
Normalised Counts
1e+05
9e+04
8e+04
7e+04
6e+04
5e+04
zmp_ph108_F9
zmp_ph108_F11
zmp_ph108_H2
zmp_ph108_H5
zmp_ph108_B2
zmp_ph108_B6
zmp_ph108_B7
zmp_ph108_C4
zmp_ph108_C5
zmp_ph108_D4
zmp_ph97_G6
zmp_ph97_G9
zmp_ph97_H2
zmp_ph97_B10
zmp_ph97_C2
zmp_ph97_C4
zmp_ph97_B8
zmp_ph97_C1
zmp_ph97_D2
zmp_ph97_D5
zmp_ph111_G5
zmp_ph111_G11
zmp_ph111_H1
zmp_ph111_B1a
zmp_ph111_B5
zmp_ph111_B8
zmp_ph111_B6
zmp_ph111_B7
zmp_ph111_B9
zmp_ph111_B10
zmp_ph114_G4
zmp_ph114_G11
zmp_ph114_H1
zmp_ph114_H2
zmp_ph114_A9
zmp_ph114_A11
zmp_ph114_B2
zmp_ph114_B4
zmp_ph114_D8
zmp_ph114_E2

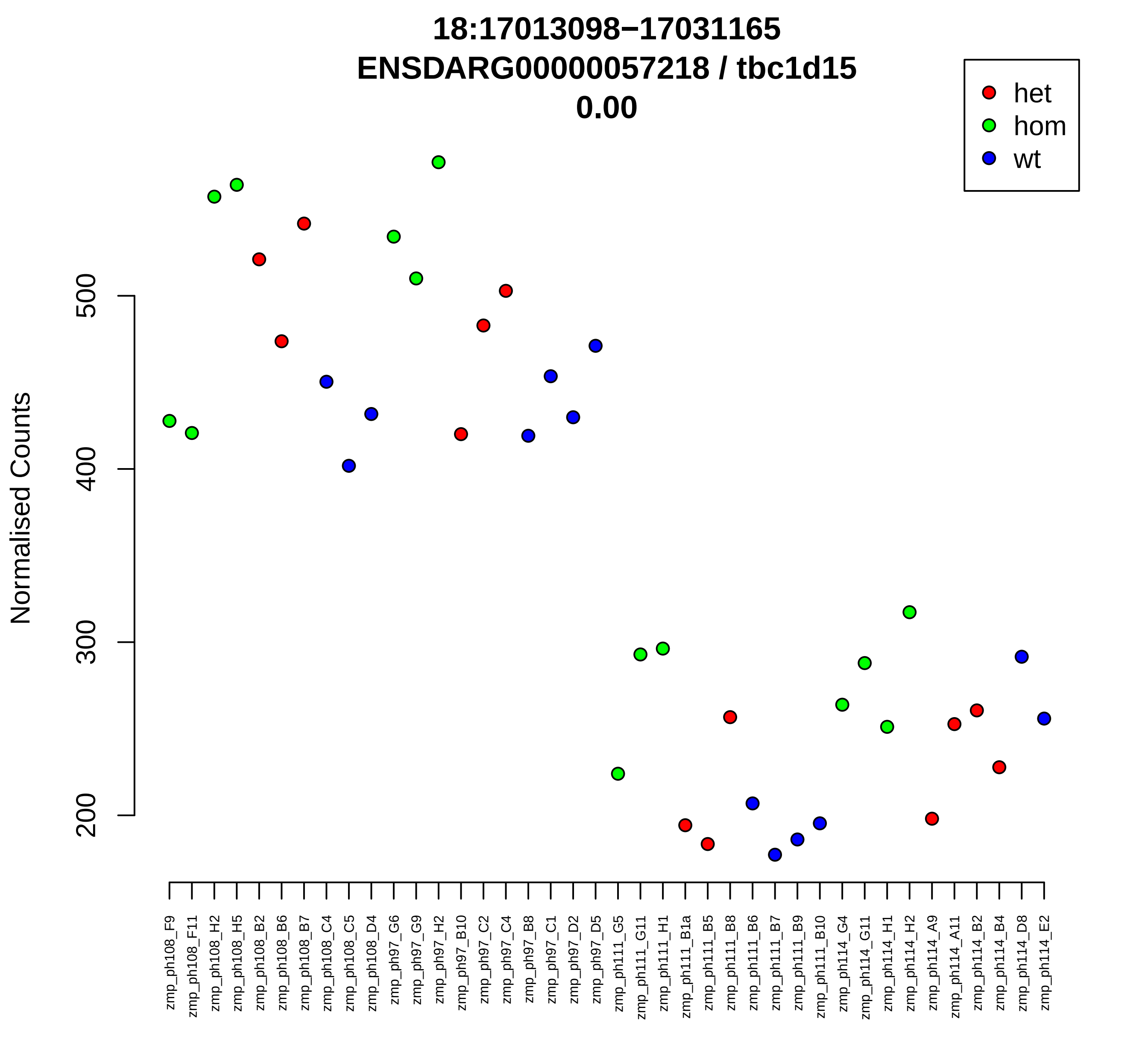
18:17013098−17031165
ENSDARG00000057218 / tbc1d15
0.00
het
hom
wt
Normalised Counts
200
300
400
500
zmp_ph108_F9
zmp_ph108_F11
zmp_ph108_H2
zmp_ph108_H5
zmp_ph108_B2
zmp_ph108_B6
zmp_ph108_B7
zmp_ph108_C4
zmp_ph108_C5
zmp_ph108_D4
zmp_ph97_G6
zmp_ph97_G9
zmp_ph97_H2
zmp_ph97_B10
zmp_ph97_C2
zmp_ph97_C4
zmp_ph97_B8
zmp_ph97_C1
zmp_ph97_D2
zmp_ph97_D5
zmp_ph111_G5
zmp_ph111_G11
zmp_ph111_H1
zmp_ph111_B1a
zmp_ph111_B5
zmp_ph111_B8
zmp_ph111_B6
zmp_ph111_B7
zmp_ph111_B9
zmp_ph111_B10
zmp_ph114_G4
zmp_ph114_G11
zmp_ph114_H1
zmp_ph114_H2
zmp_ph114_A9
zmp_ph114_A11
zmp_ph114_B2
zmp_ph114_B4
zmp_ph114_D8
zmp_ph114_E2

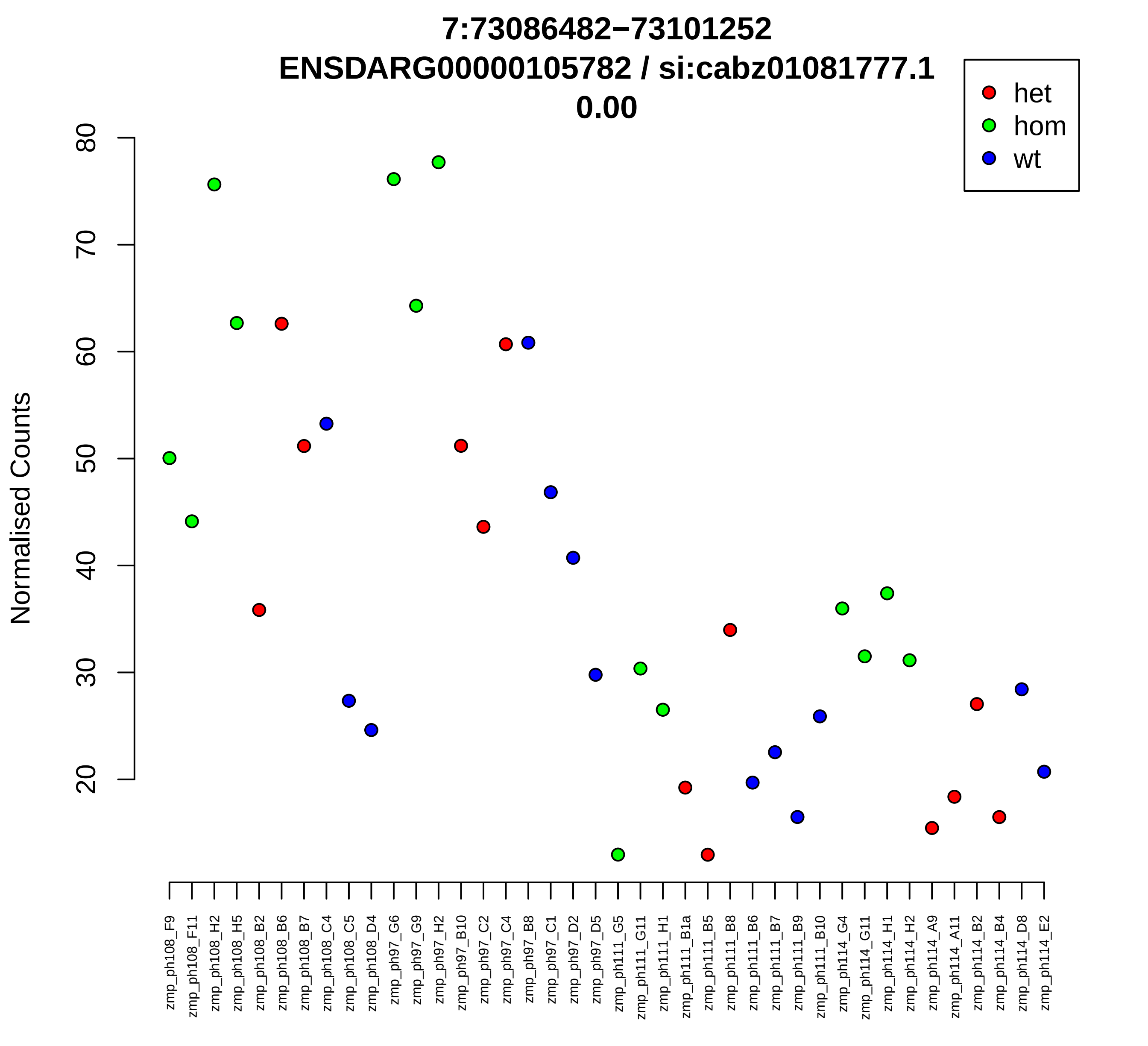

7:73086482−73101252
ENSDARG00000105782 / si:cabz01081777.1
0.00
het
hom
wt
Normalised Counts
20
30
40
50
60
70
80
zmp_ph108_F9
zmp_ph108_F11
zmp_ph108_H2
zmp_ph108_H5
zmp_ph108_B2
zmp_ph108_B6
zmp_ph108_B7
zmp_ph108_C4
zmp_ph108_C5
zmp_ph108_D4
zmp_ph97_G6
zmp_ph97_G9
zmp_ph97_H2
zmp_ph97_B10
zmp_ph97_C2
zmp_ph97_C4
zmp_ph97_B8
zmp_ph97_C1
zmp_ph97_D2
zmp_ph97_D5
zmp_ph111_G5
zmp_ph111_G11
zmp_ph111_H1
zmp_ph111_B1a
zmp_ph111_B5
zmp_ph111_B8
zmp_ph111_B6
zmp_ph111_B7
zmp_ph111_B9
zmp_ph111_B10
zmp_ph114_G4
zmp_ph114_G11
zmp_ph114_H1
zmp_ph114_H2
zmp_ph114_A9
zmp_ph114_A11
zmp_ph114_B2
zmp_ph114_B4
zmp_ph114_D8
zmp_ph114_E2

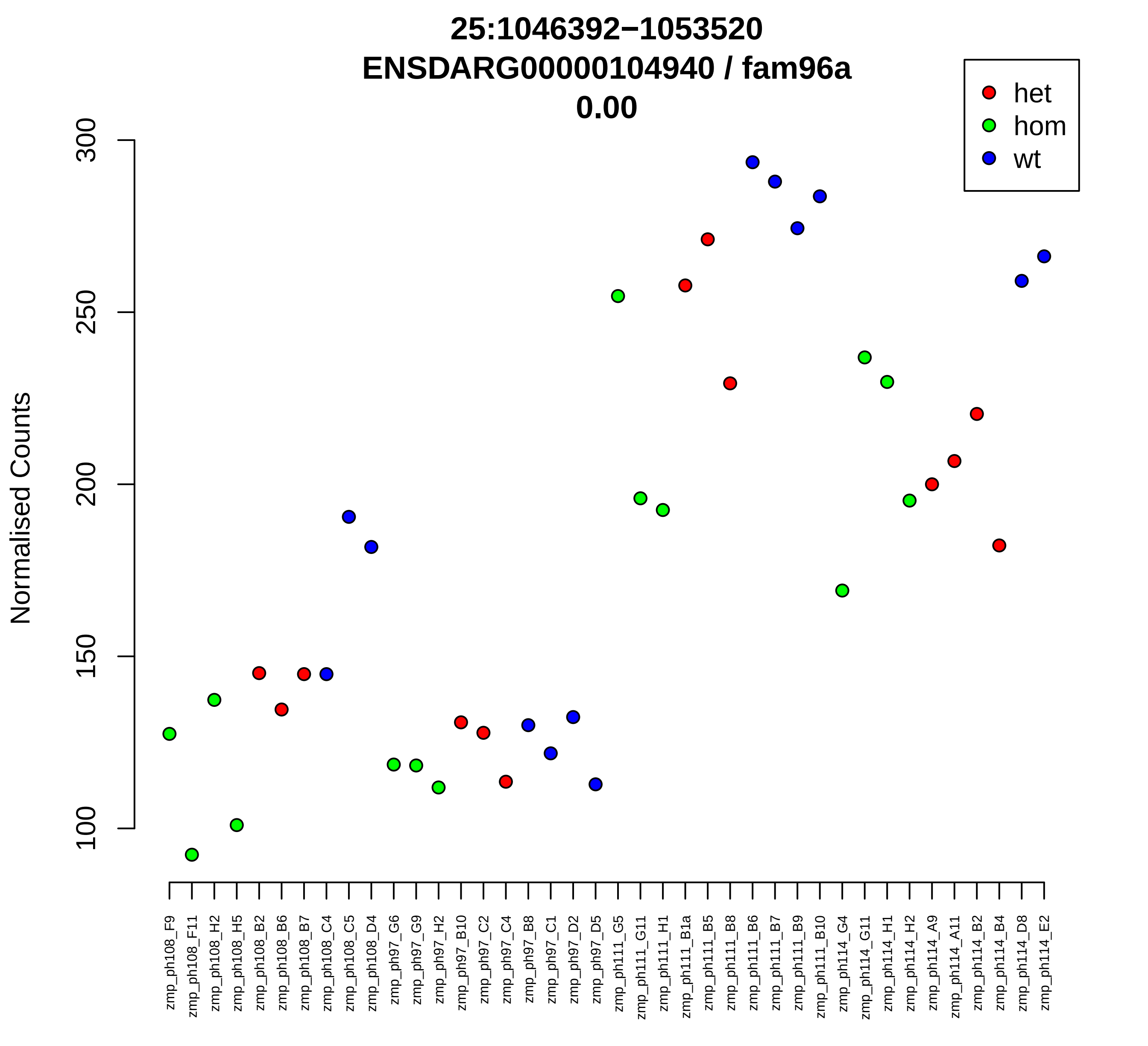
25:1046392−1053520
ENSDARG00000104940 / fam96a
0.00
het
hom
wt
Normalised Counts
100
150
200
250
300
zmp_ph108_F9
zmp_ph108_F11
zmp_ph108_H2
zmp_ph108_H5
zmp_ph108_B2
zmp_ph108_B6
zmp_ph108_B7
zmp_ph108_C4
zmp_ph108_C5
zmp_ph108_D4
zmp_ph97_G6
zmp_ph97_G9
zmp_ph97_H2
zmp_ph97_B10
zmp_ph97_C2
zmp_ph97_C4
zmp_ph97_B8
zmp_ph97_C1
zmp_ph97_D2
zmp_ph97_D5
zmp_ph111_G5
zmp_ph111_G11
zmp_ph111_H1
zmp_ph111_B1a
zmp_ph111_B5
zmp_ph111_B8
zmp_ph111_B6
zmp_ph111_B7
zmp_ph111_B9
zmp_ph111_B10
zmp_ph114_G4
zmp_ph114_G11
zmp_ph114_H1
zmp_ph114_H2
zmp_ph114_A9
zmp_ph114_A11
zmp_ph114_B2
zmp_ph114_B4
zmp_ph114_D8
zmp_ph114_E2

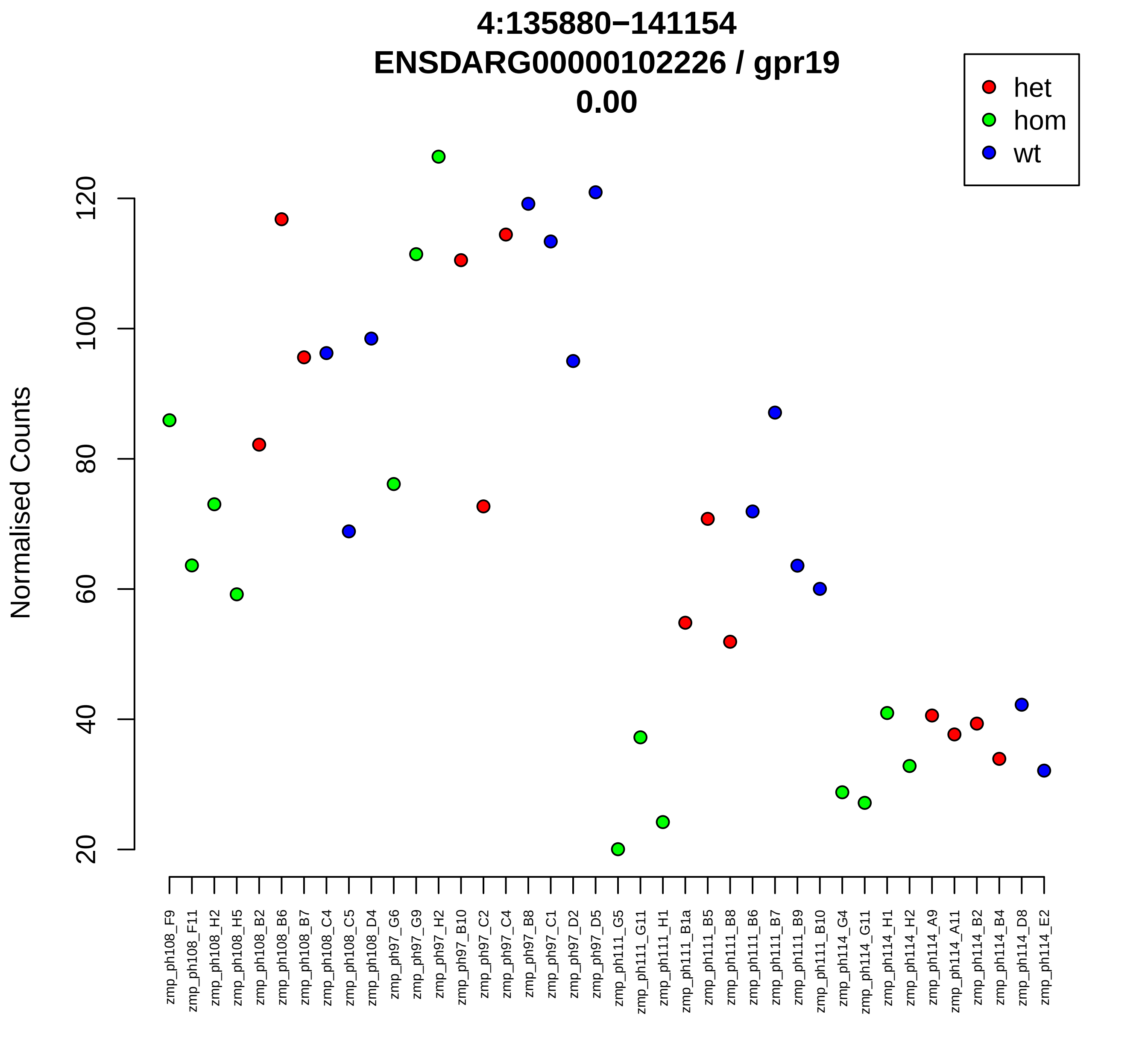

4:135880−141154
ENSDARG00000102226 / gpr19
0.00
het
hom
wt
Normalised Counts
20
40
60
80
100
120
zmp_ph108_F9
zmp_ph108_F11
zmp_ph108_H2
zmp_ph108_H5
zmp_ph108_B2
zmp_ph108_B6
zmp_ph108_B7
zmp_ph108_C4
zmp_ph108_C5
zmp_ph108_D4
zmp_ph97_G6
zmp_ph97_G9
zmp_ph97_H2
zmp_ph97_B10
zmp_ph97_C2
zmp_ph97_C4
zmp_ph97_B8
zmp_ph97_C1
zmp_ph97_D2
zmp_ph97_D5
zmp_ph111_G5
zmp_ph111_G11
zmp_ph111_H1
zmp_ph111_B1a
zmp_ph111_B5
zmp_ph111_B8
zmp_ph111_B6
zmp_ph111_B7
zmp_ph111_B9
zmp_ph111_B10
zmp_ph114_G4
zmp_ph114_G11
zmp_ph114_H1
zmp_ph114_H2
zmp_ph114_A9
zmp_ph114_A11
zmp_ph114_B2
zmp_ph114_B4
zmp_ph114_D8
zmp_ph114_E2

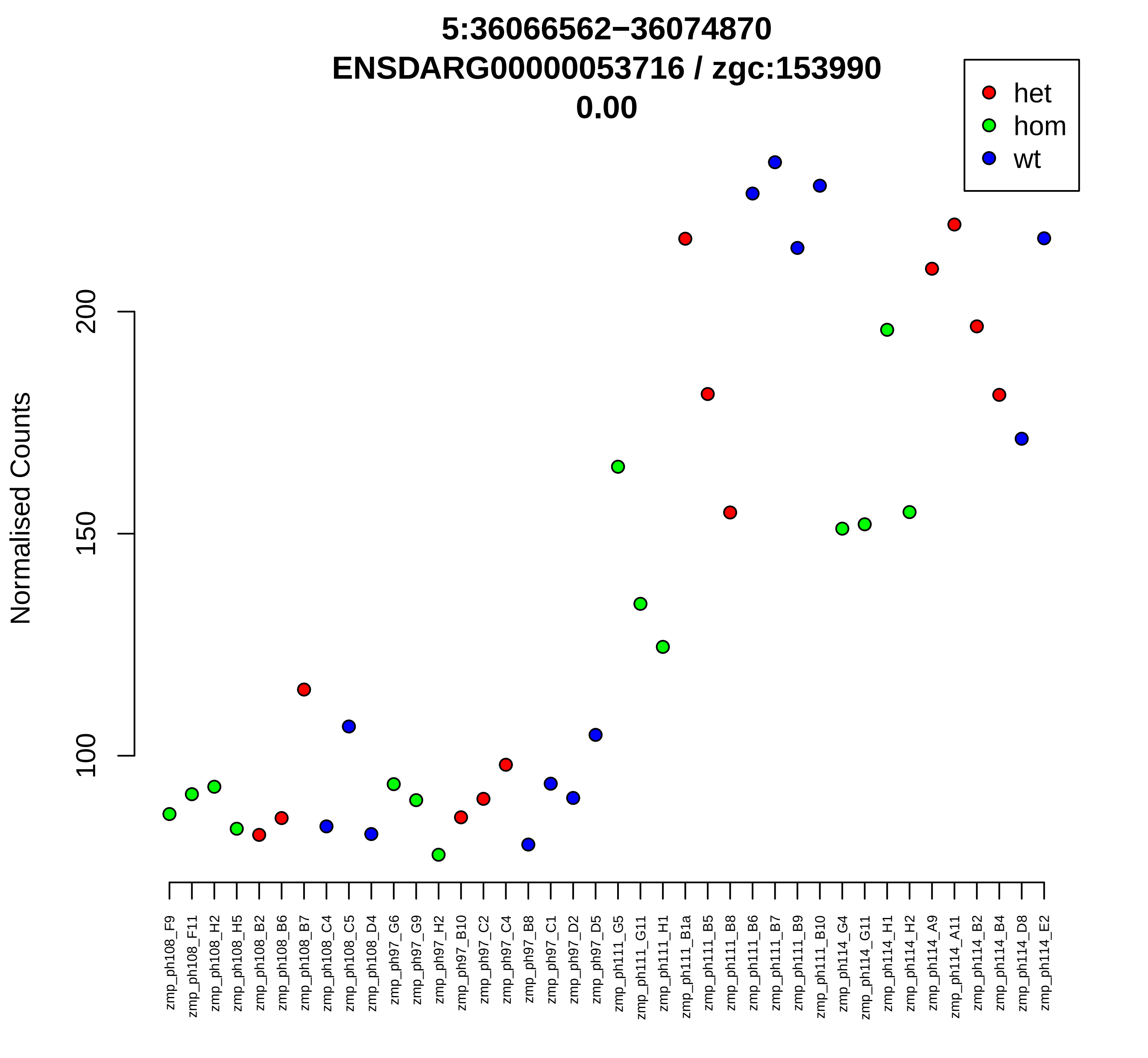

5:36066562−36074870
ENSDARG00000053716 / zgc:153990
0.00
het
hom
wt
Normalised Counts
100
150
200
zmp_ph108_F9
zmp_ph108_F11
zmp_ph108_H2
zmp_ph108_H5
zmp_ph108_B2
zmp_ph108_B6
zmp_ph108_B7
zmp_ph108_C4
zmp_ph108_C5
zmp_ph108_D4
zmp_ph97_G6
zmp_ph97_G9
zmp_ph97_H2
zmp_ph97_B10
zmp_ph97_C2
zmp_ph97_C4
zmp_ph97_B8
zmp_ph97_C1
zmp_ph97_D2
zmp_ph97_D5
zmp_ph111_G5
zmp_ph111_G11
zmp_ph111_H1
zmp_ph111_B1a
zmp_ph111_B5
zmp_ph111_B8
zmp_ph111_B6
zmp_ph111_B7
zmp_ph111_B9
zmp_ph111_B10
zmp_ph114_G4
zmp_ph114_G11
zmp_ph114_H1
zmp_ph114_H2
zmp_ph114_A9
zmp_ph114_A11
zmp_ph114_B2
zmp_ph114_B4
zmp_ph114_D8
zmp_ph114_E2

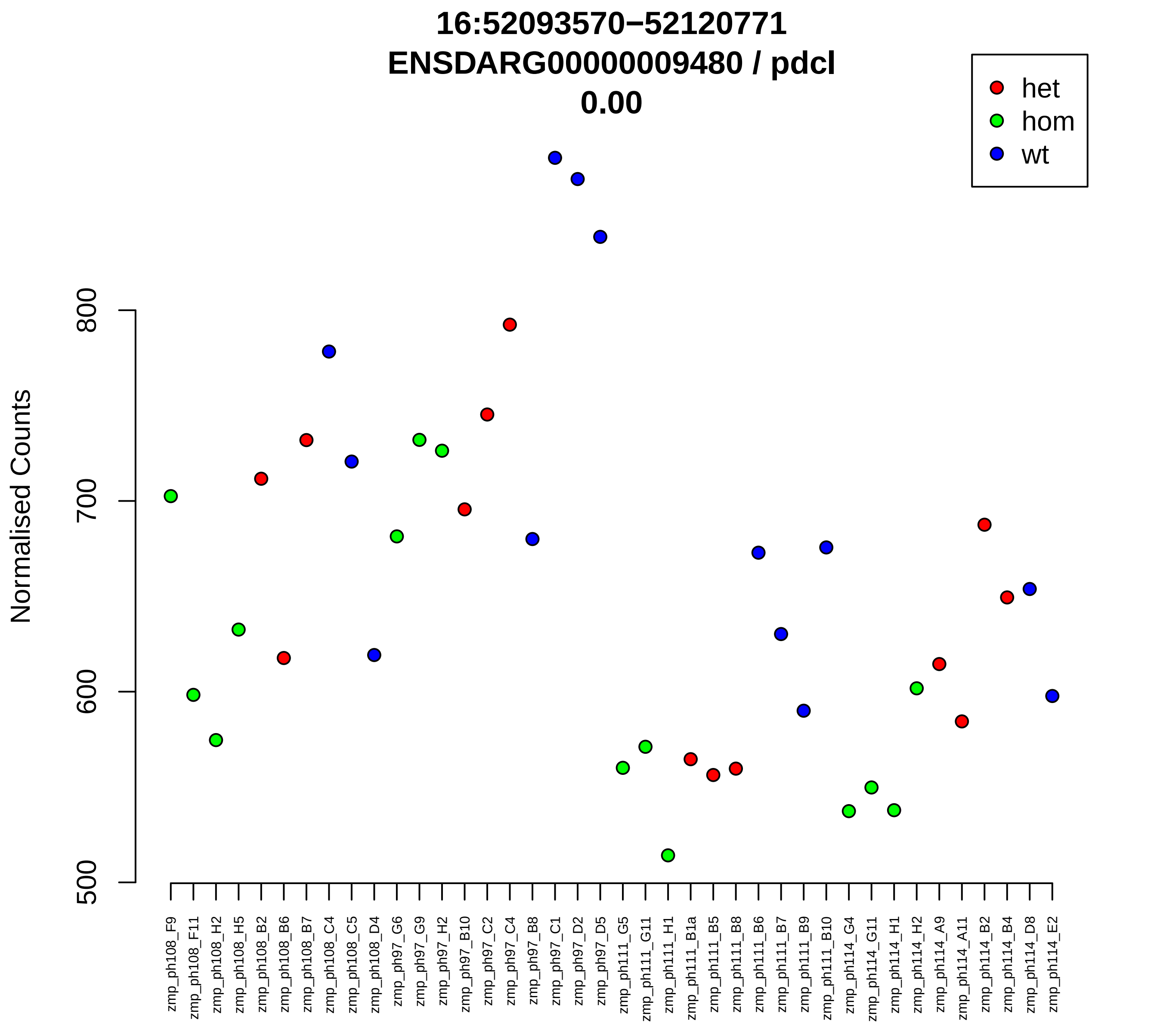
16:52093570−52120771
ENSDARG00000009480 / pdcl
0.00
het
hom
wt
Normalised Counts
500
600
700
800
zmp_ph108_F9
zmp_ph108_F11
zmp_ph108_H2
zmp_ph108_H5
zmp_ph108_B2
zmp_ph108_B6
zmp_ph108_B7
zmp_ph108_C4
zmp_ph108_C5
zmp_ph108_D4
zmp_ph97_G6
zmp_ph97_G9
zmp_ph97_H2
zmp_ph97_B10
zmp_ph97_C2
zmp_ph97_C4
zmp_ph97_B8
zmp_ph97_C1
zmp_ph97_D2
zmp_ph97_D5
zmp_ph111_G5
zmp_ph111_G11
zmp_ph111_H1
zmp_ph111_B1a
zmp_ph111_B5
zmp_ph111_B8
zmp_ph111_B6
zmp_ph111_B7
zmp_ph111_B9
zmp_ph111_B10
zmp_ph114_G4
zmp_ph114_G11
zmp_ph114_H1
zmp_ph114_H2
zmp_ph114_A9
zmp_ph114_A11
zmp_ph114_B2
zmp_ph114_B4
zmp_ph114_D8
zmp_ph114_E2

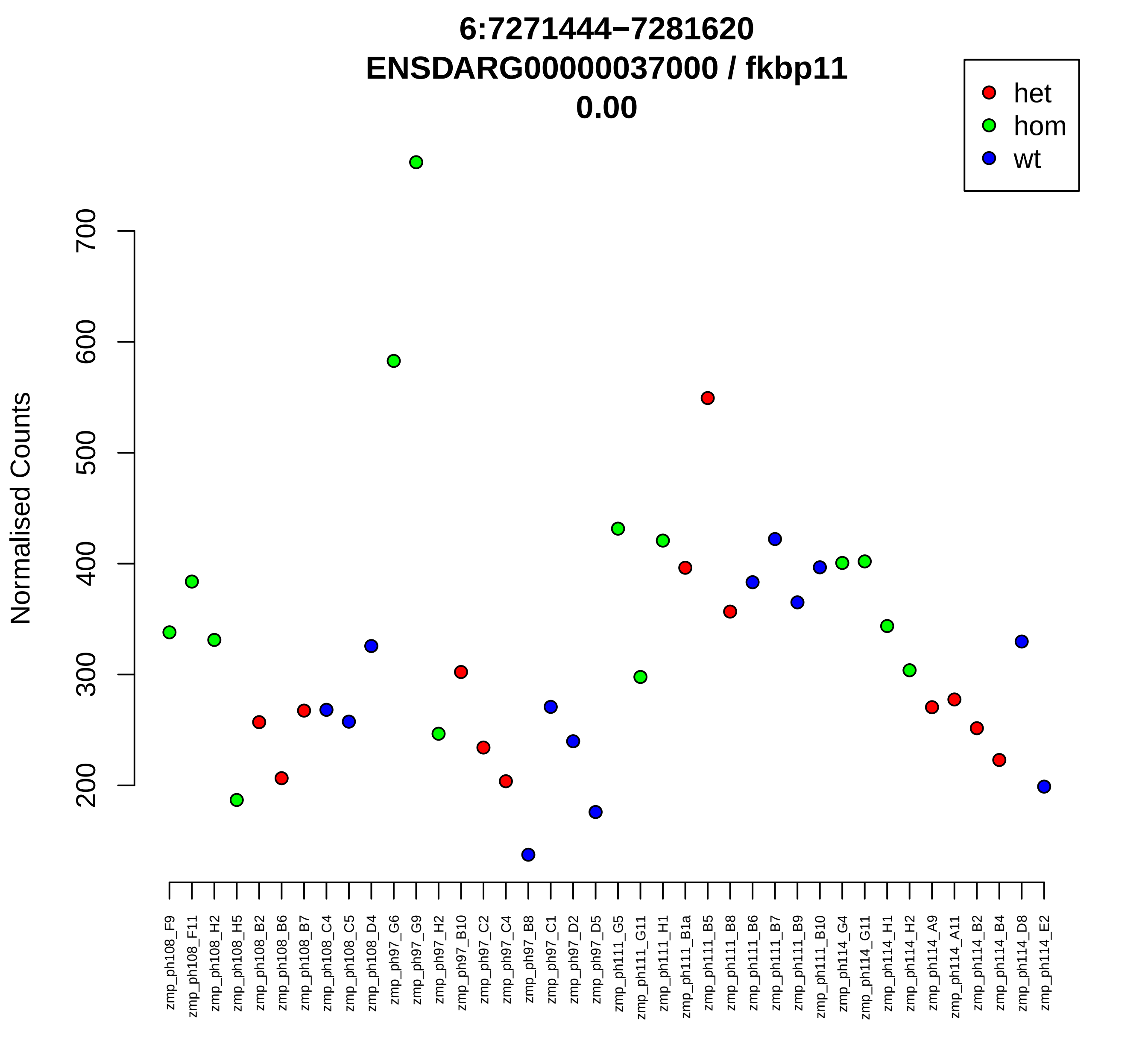

6:7271444−7281620
ENSDARG00000037000 / fkbp11
0.00
het
hom
wt
Normalised Counts
200
300
400
500
600
700
zmp_ph108_F9
zmp_ph108_F11
zmp_ph108_H2
zmp_ph108_H5
zmp_ph108_B2
zmp_ph108_B6
zmp_ph108_B7
zmp_ph108_C4
zmp_ph108_C5
zmp_ph108_D4
zmp_ph97_G6
zmp_ph97_G9
zmp_ph97_H2
zmp_ph97_B10
zmp_ph97_C2
zmp_ph97_C4
zmp_ph97_B8
zmp_ph97_C1
zmp_ph97_D2
zmp_ph97_D5
zmp_ph111_G5
zmp_ph111_G11
zmp_ph111_H1
zmp_ph111_B1a
zmp_ph111_B5
zmp_ph111_B8
zmp_ph111_B6
zmp_ph111_B7
zmp_ph111_B9
zmp_ph111_B10
zmp_ph114_G4
zmp_ph114_G11
zmp_ph114_H1
zmp_ph114_H2
zmp_ph114_A9
zmp_ph114_A11
zmp_ph114_B2
zmp_ph114_B4
zmp_ph114_D8
zmp_ph114_E2

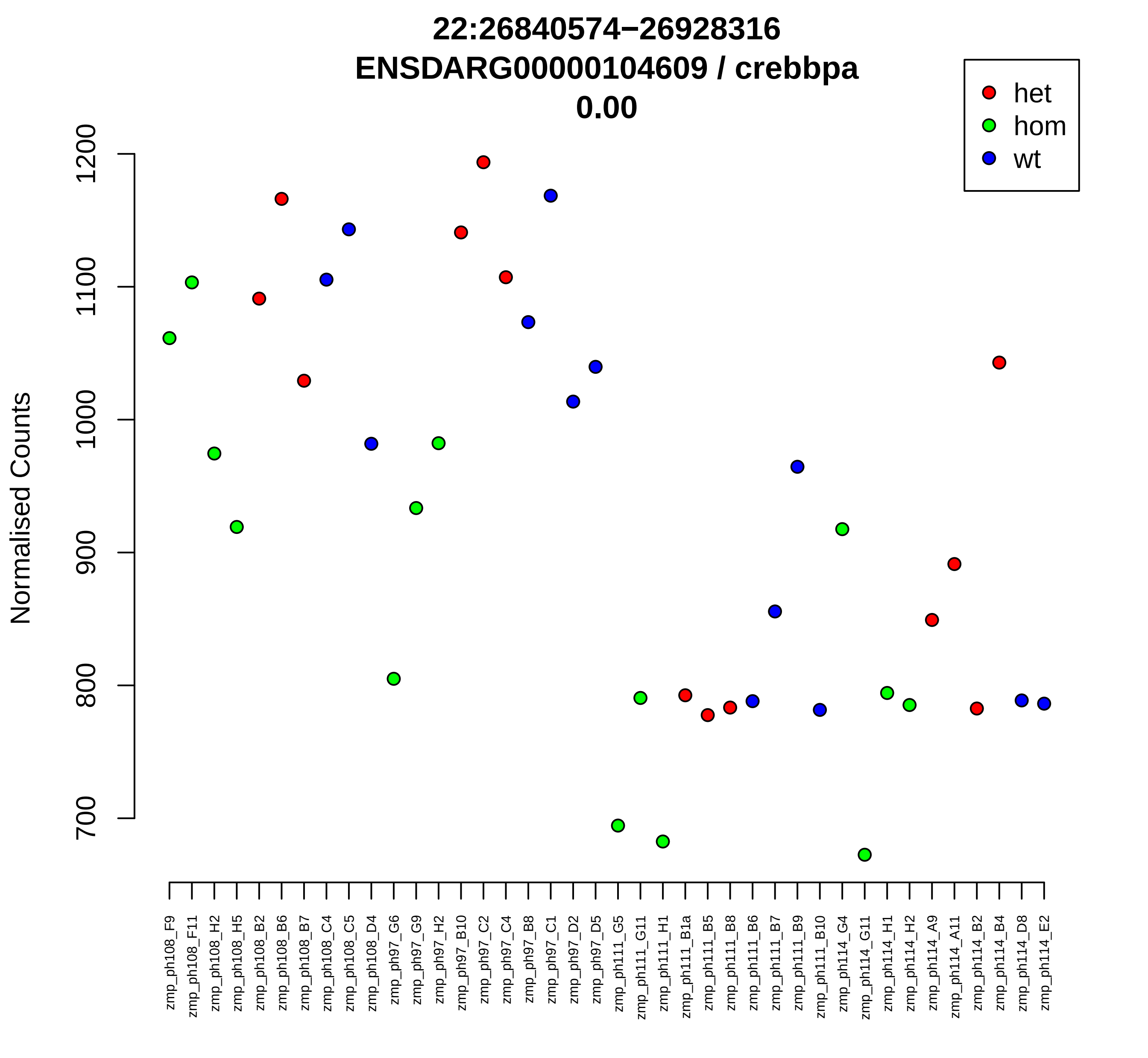

22:26840574−26928316
ENSDARG00000104609 / crebbpa
0.00
Normalised Counts
700
800
900
1000
1100
1200
het
hom
wt
zmp_ph108_F9
zmp_ph108_F11
zmp_ph108_H2
zmp_ph108_H5
zmp_ph108_B2
zmp_ph108_B6
zmp_ph108_B7
zmp_ph108_C4
zmp_ph108_C5
zmp_ph108_D4
zmp_ph97_G6
zmp_ph97_G9
zmp_ph97_H2
zmp_ph97_B10
zmp_ph97_C2
zmp_ph97_C4
zmp_ph97_B8
zmp_ph97_C1
zmp_ph97_D2
zmp_ph97_D5
zmp_ph111_G5
zmp_ph111_G11
zmp_ph111_H1
zmp_ph111_B1a
zmp_ph111_B5
zmp_ph111_B8
zmp_ph111_B6
zmp_ph111_B7
zmp_ph111_B9
zmp_ph111_B10
zmp_ph114_G4
zmp_ph114_G11
zmp_ph114_H1
zmp_ph114_H2
zmp_ph114_A9
zmp_ph114_A11
zmp_ph114_B2
zmp_ph114_B4
zmp_ph114_D8
zmp_ph114_E2

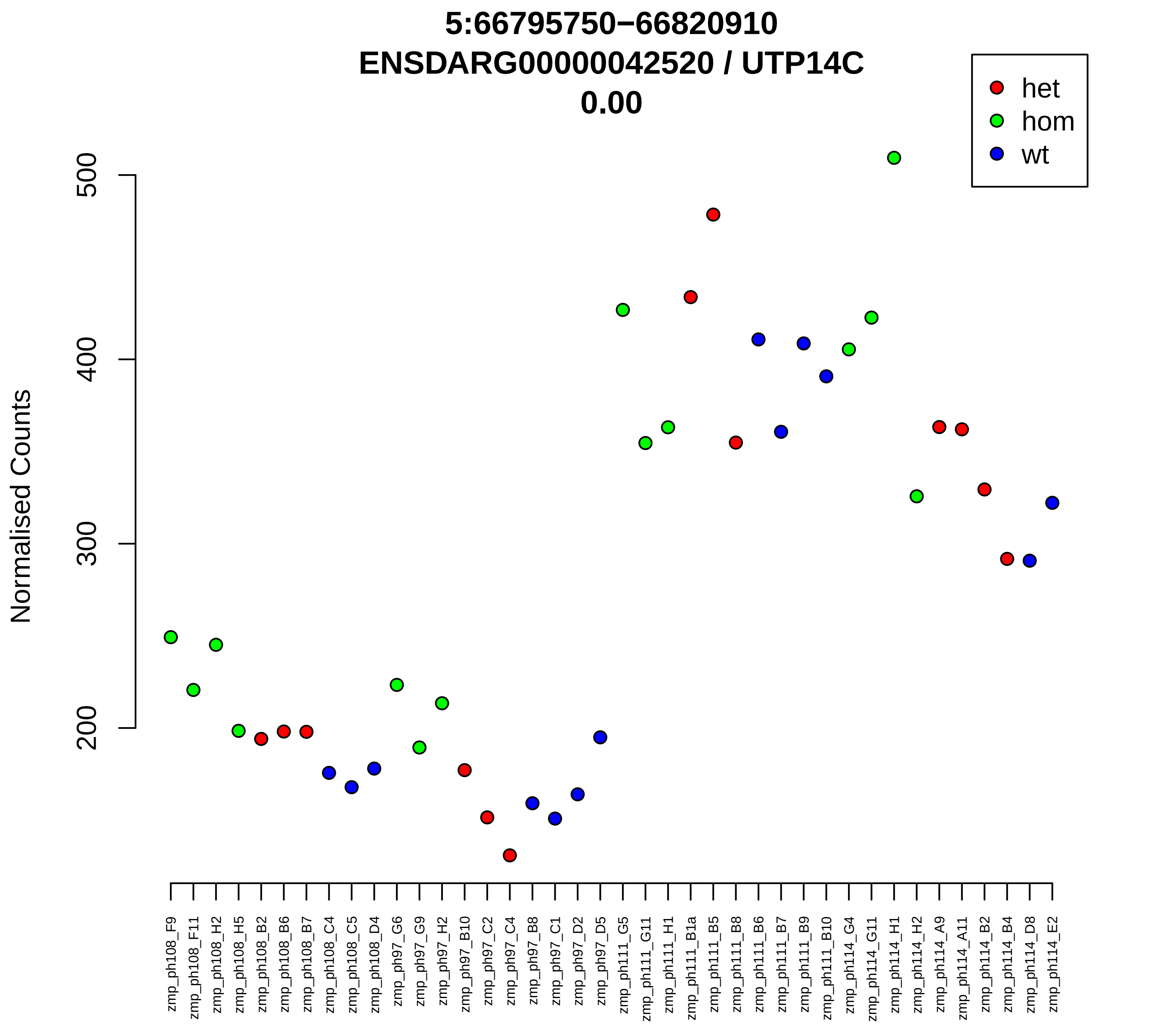

5:66795750−66820910
ENSDARG00000042520 / UTP14C
0.00
het
hom
wt
Normalised Counts
500
400
300
200
zmp_ph108_F9
zmp_ph108_F11
zmp_ph108_H2
zmp_ph108_H5
zmp_ph108_B2
zmp_ph108_B6
zmp_ph108_B7
zmp_ph108_C4
zmp_ph108_C5
zmp_ph108_D4
zmp_ph97_G6
zmp_ph97_G9
zmp_ph97_H2
zmp_ph97_B10
zmp_ph97_C2
zmp_ph97_C4
zmp_ph97_B8
zmp_ph97_C1
zmp_ph97_D2
zmp_ph97_D5
zmp_ph111_G5
zmp_ph111_G11
zmp_ph111_H1
zmp_ph111_B1a
zmp_ph111_B5
zmp_ph111_B8
zmp_ph111_B6
zmp_ph111_B7
zmp_ph111_B9
zmp_ph111_B10
zmp_ph114_G4
zmp_ph114_G11
zmp_ph114_H1
zmp_ph114_H2
zmp_ph114_A9
zmp_ph114_A11
zmp_ph114_B2
zmp_ph114_B4
zmp_ph114_D8
zmp_ph114_E2

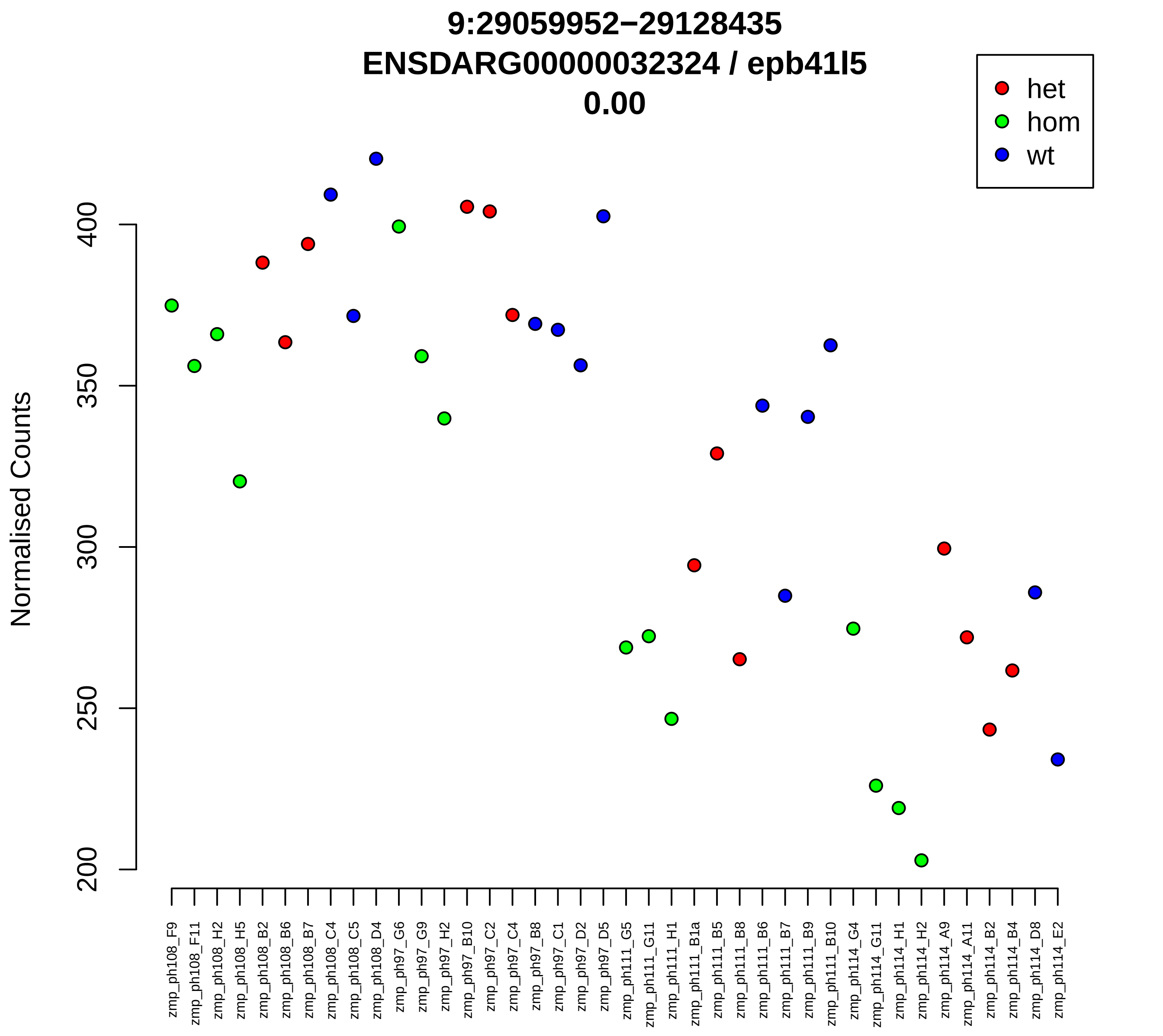

9:29059952−29128435
ENSDARG00000032324 / epb41l5
0.00
het
hom
wt
Normalised Counts
200
250
300
350
400
zmp_ph108_F9
zmp_ph108_F11
zmp_ph108_H2
zmp_ph108_H5
zmp_ph108_B2
zmp_ph108_B6
zmp_ph108_B7
zmp_ph108_C4
zmp_ph108_C5
zmp_ph108_D4
zmp_ph97_G6
zmp_ph97_G9
zmp_ph97_H2
zmp_ph97_B10
zmp_ph97_C2
zmp_ph97_C4
zmp_ph97_B8
zmp_ph97_C1
zmp_ph97_D2
zmp_ph97_D5
zmp_ph111_G5
zmp_ph111_G11
zmp_ph111_H1
zmp_ph111_B1a
zmp_ph111_B5
zmp_ph111_B8
zmp_ph111_B6
zmp_ph111_B7
zmp_ph111_B9
zmp_ph111_B10
zmp_ph114_G4
zmp_ph114_G11
zmp_ph114_H1
zmp_ph114_H2
zmp_ph114_A9
zmp_ph114_A11
zmp_ph114_B2
zmp_ph114_B4
zmp_ph114_D8
zmp_ph114_E2

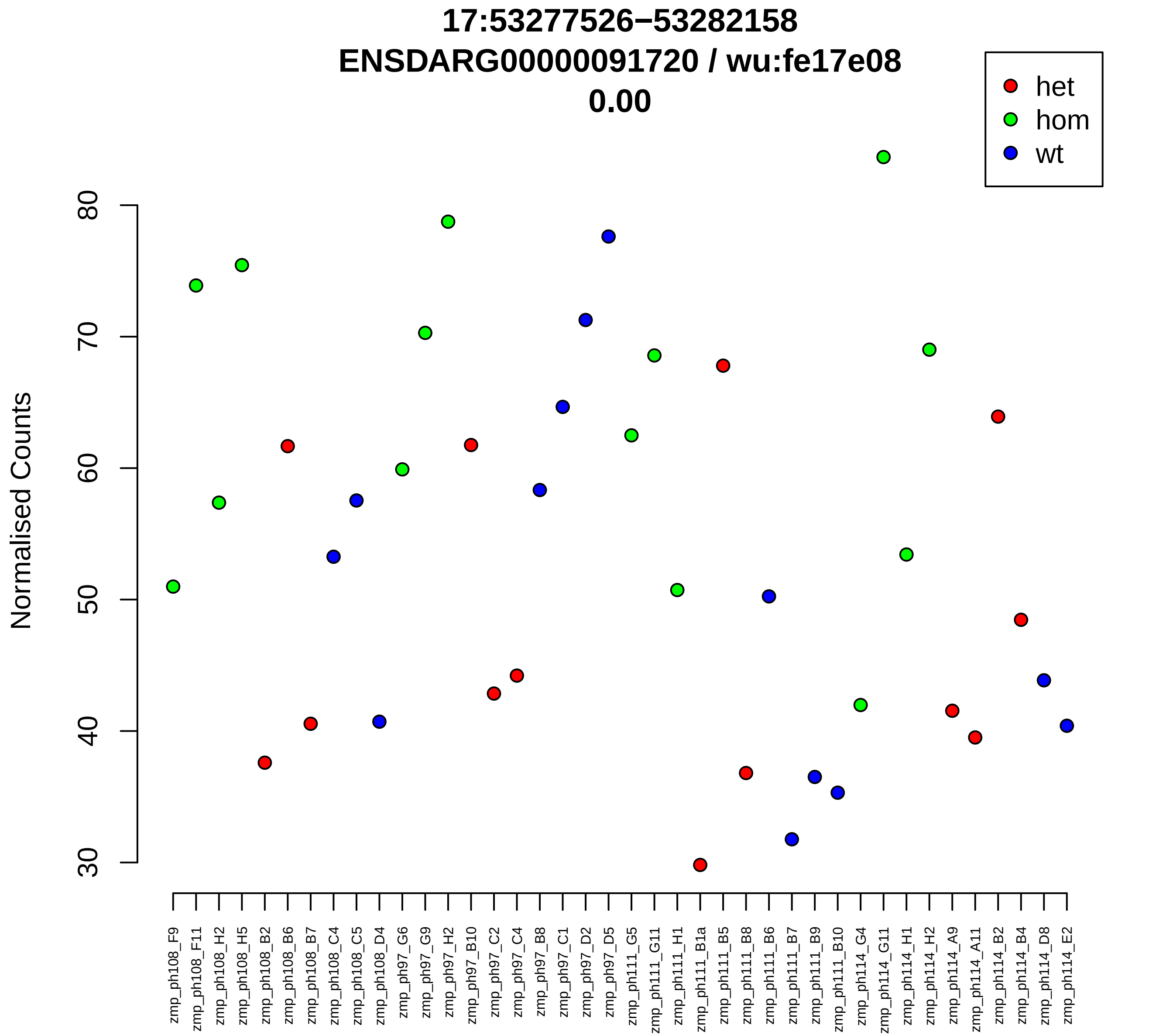

17:53277526−53282158
ENSDARG00000091720 / wu:fe17e08
0.00
het
hom
wt
Normalised Counts
30
40
50
60
70
80
zmp_ph108_F9
zmp_ph108_F11
zmp_ph108_H2
zmp_ph108_H5
zmp_ph108_B2
zmp_ph108_B6
zmp_ph108_B7
zmp_ph108_C4
zmp_ph108_C5
zmp_ph108_D4
zmp_ph97_G6
zmp_ph97_G9
zmp_ph97_H2
zmp_ph97_B10
zmp_ph97_C2
zmp_ph97_C4
zmp_ph97_B8
zmp_ph97_C1
zmp_ph97_D2
zmp_ph97_D5
zmp_ph111_G5
zmp_ph111_G11
zmp_ph111_H1
zmp_ph111_B1a
zmp_ph111_B5
zmp_ph111_B8
zmp_ph111_B6
zmp_ph111_B7
zmp_ph111_B9
zmp_ph111_B10
zmp_ph114_G4
zmp_ph114_G11
zmp_ph114_H1
zmp_ph114_H2
zmp_ph114_A9
zmp_ph114_A11
zmp_ph114_B2
zmp_ph114_B4
zmp_ph114_D8
zmp_ph114_E2

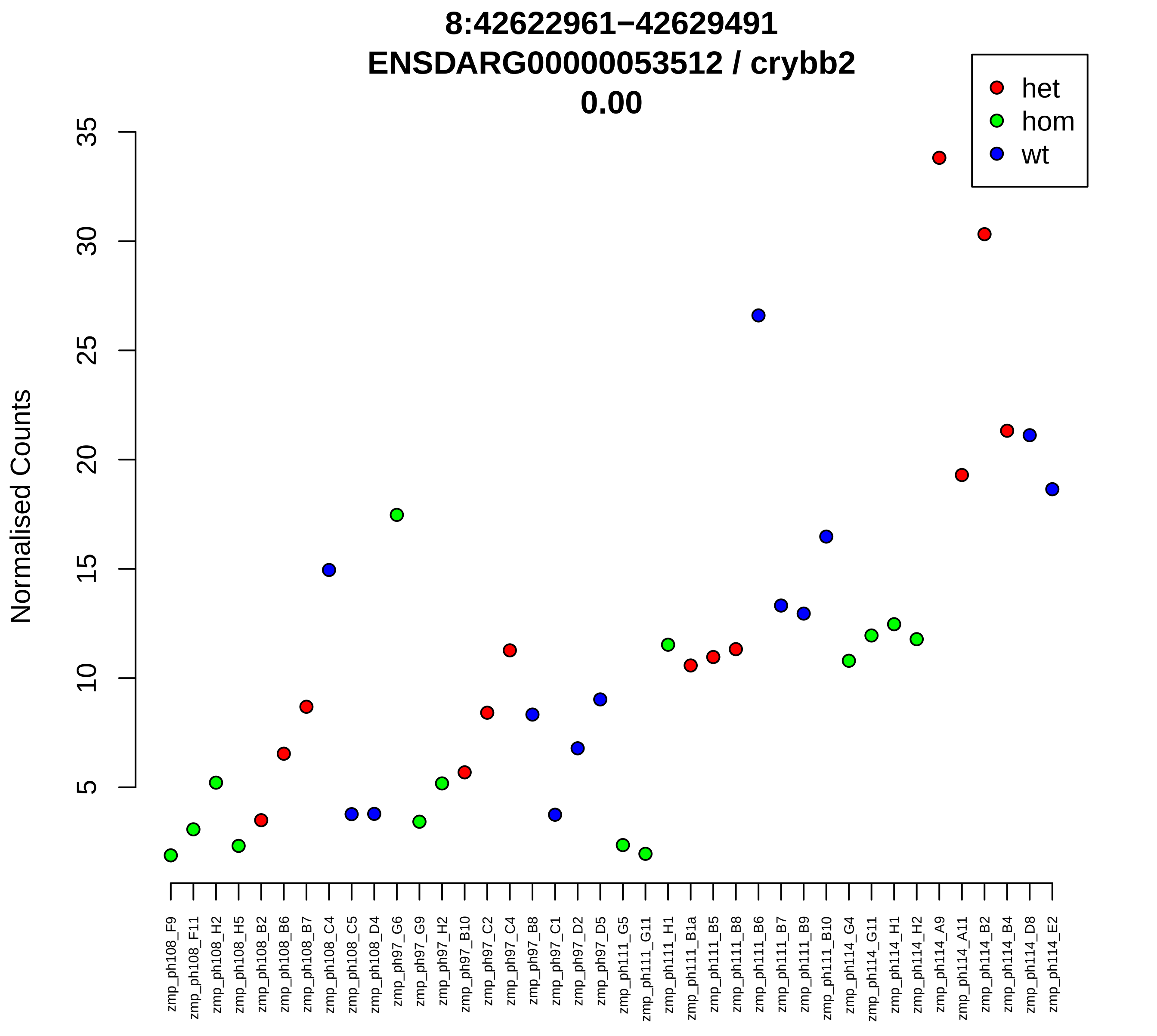

8:42622961−42629491
ENSDARG00000053512 / crybb2
0.00
het
hom
wt
Normalised Counts
5
10
15
20
25
30
35
zmp_ph108_F9
zmp_ph108_F11
zmp_ph108_H2
zmp_ph108_H5
zmp_ph108_B2
zmp_ph108_B6
zmp_ph108_B7
zmp_ph108_C4
zmp_ph108_C5
zmp_ph108_D4
zmp_ph97_G6
zmp_ph97_G9
zmp_ph97_H2
zmp_ph97_B10
zmp_ph97_C2
zmp_ph97_C4
zmp_ph97_B8
zmp_ph97_C1
zmp_ph97_D2
zmp_ph97_D5
zmp_ph111_G5
zmp_ph111_G11
zmp_ph111_H1
zmp_ph111_B1a
zmp_ph111_B5
zmp_ph111_B8
zmp_ph111_B6
zmp_ph111_B7
zmp_ph111_B9
zmp_ph111_B10
zmp_ph114_G4
zmp_ph114_G11
zmp_ph114_H1
zmp_ph114_H2
zmp_ph114_A9
zmp_ph114_A11
zmp_ph114_B2
zmp_ph114_B4
zmp_ph114_D8
zmp_ph114_E2

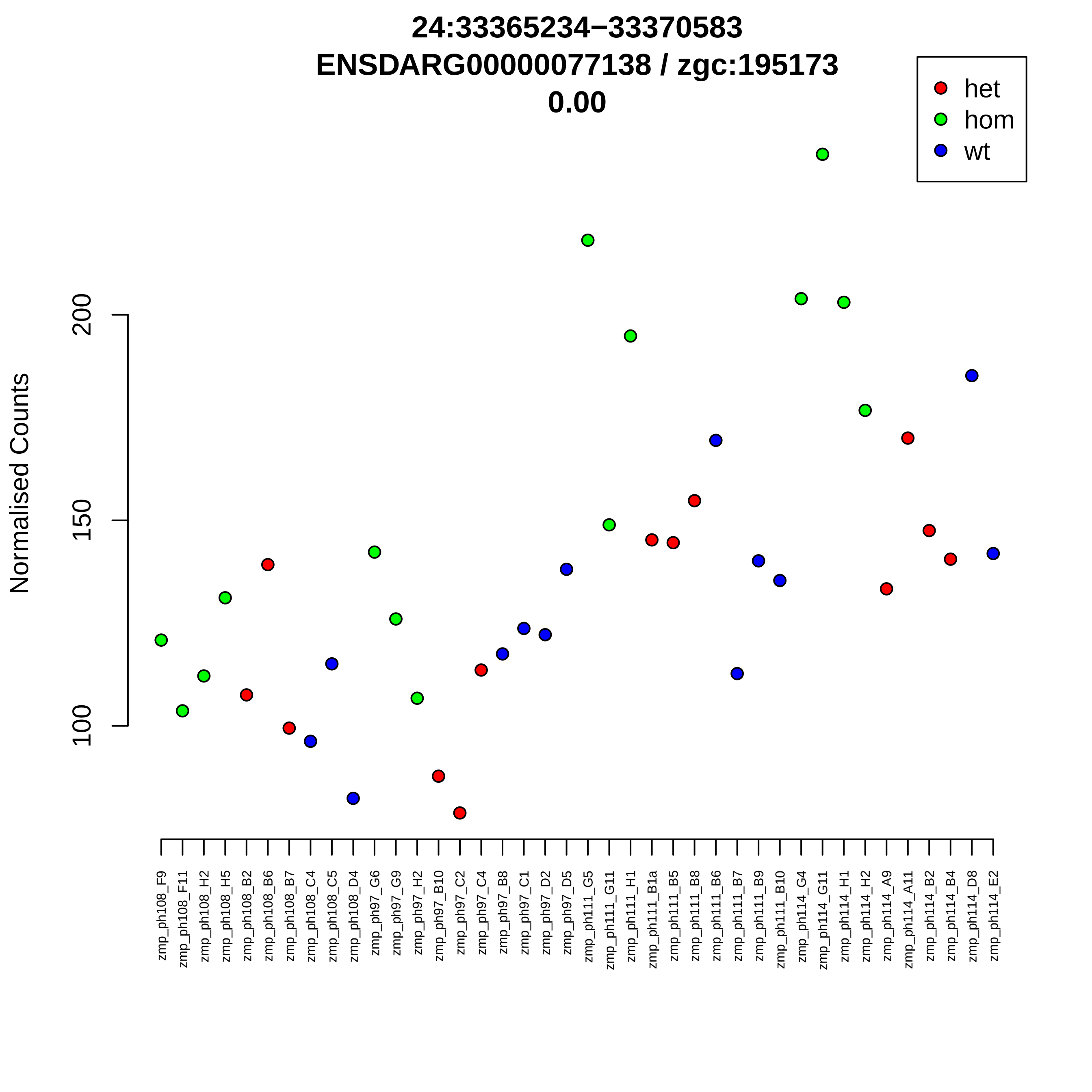
24:33365234−33370583
ENSDARG00000077138 / zgc:195173
0.00
het
hom
wt
Normalised Counts
100
150
200
zmp_ph108_F9
zmp_ph108_F11
zmp_ph108_H2
zmp_ph108_H5
zmp_ph108_B2
zmp_ph108_B6
zmp_ph108_B7
zmp_ph108_C4
zmp_ph108_C5
zmp_ph108_D4
zmp_ph97_G6
zmp_ph97_G9
zmp_ph97_H2
zmp_ph97_B10
zmp_ph97_C2
zmp_ph97_C4
zmp_ph97_B8
zmp_ph97_C1
zmp_ph97_D2
zmp_ph97_D5
zmp_ph111_G5
zmp_ph111_G11
zmp_ph111_H1
zmp_ph111_B1a
zmp_ph111_B5
zmp_ph111_B8
zmp_ph111_B6
zmp_ph111_B7
zmp_ph111_B9
zmp_ph111_B10
zmp_ph114_G4
zmp_ph114_G11
zmp_ph114_H1
zmp_ph114_H2
zmp_ph114_A9
zmp_ph114_A11
zmp_ph114_B2
zmp_ph114_B4
zmp_ph114_D8
zmp_ph114_E2

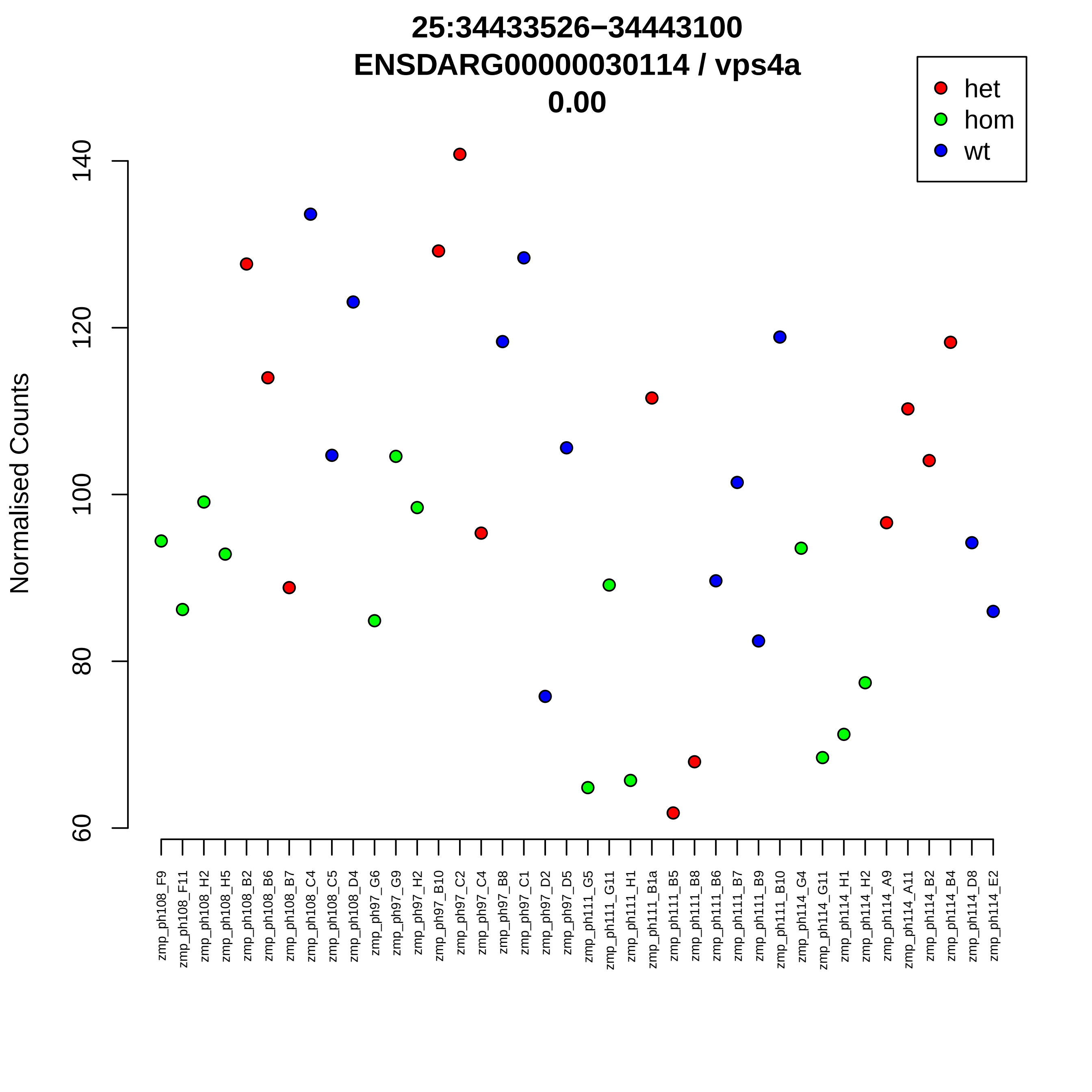

25:34433526−34443100
ENSDARG00000030114 / vps4a
0.00
het
hom
wt
Normalised Counts
60
80
100
120
140
zmp_ph108_F9
zmp_ph108_F11
zmp_ph108_H2
zmp_ph108_H5
zmp_ph108_B2
zmp_ph108_B6
zmp_ph108_B7
zmp_ph108_C4
zmp_ph108_C5
zmp_ph108_D4
zmp_ph97_G6
zmp_ph97_G9
zmp_ph97_H2
zmp_ph97_B10
zmp_ph97_C2
zmp_ph97_C4
zmp_ph97_B8
zmp_ph97_C1
zmp_ph97_D2
zmp_ph97_D5
zmp_ph111_G5
zmp_ph111_G11
zmp_ph111_H1
zmp_ph111_B1a
zmp_ph111_B5
zmp_ph111_B8
zmp_ph111_B6
zmp_ph111_B7
zmp_ph111_B9
zmp_ph111_B10
zmp_ph114_G4
zmp_ph114_G11
zmp_ph114_H1
zmp_ph114_H2
zmp_ph114_A9
zmp_ph114_A11
zmp_ph114_B2
zmp_ph114_B4
zmp_ph114_D8
zmp_ph114_E2

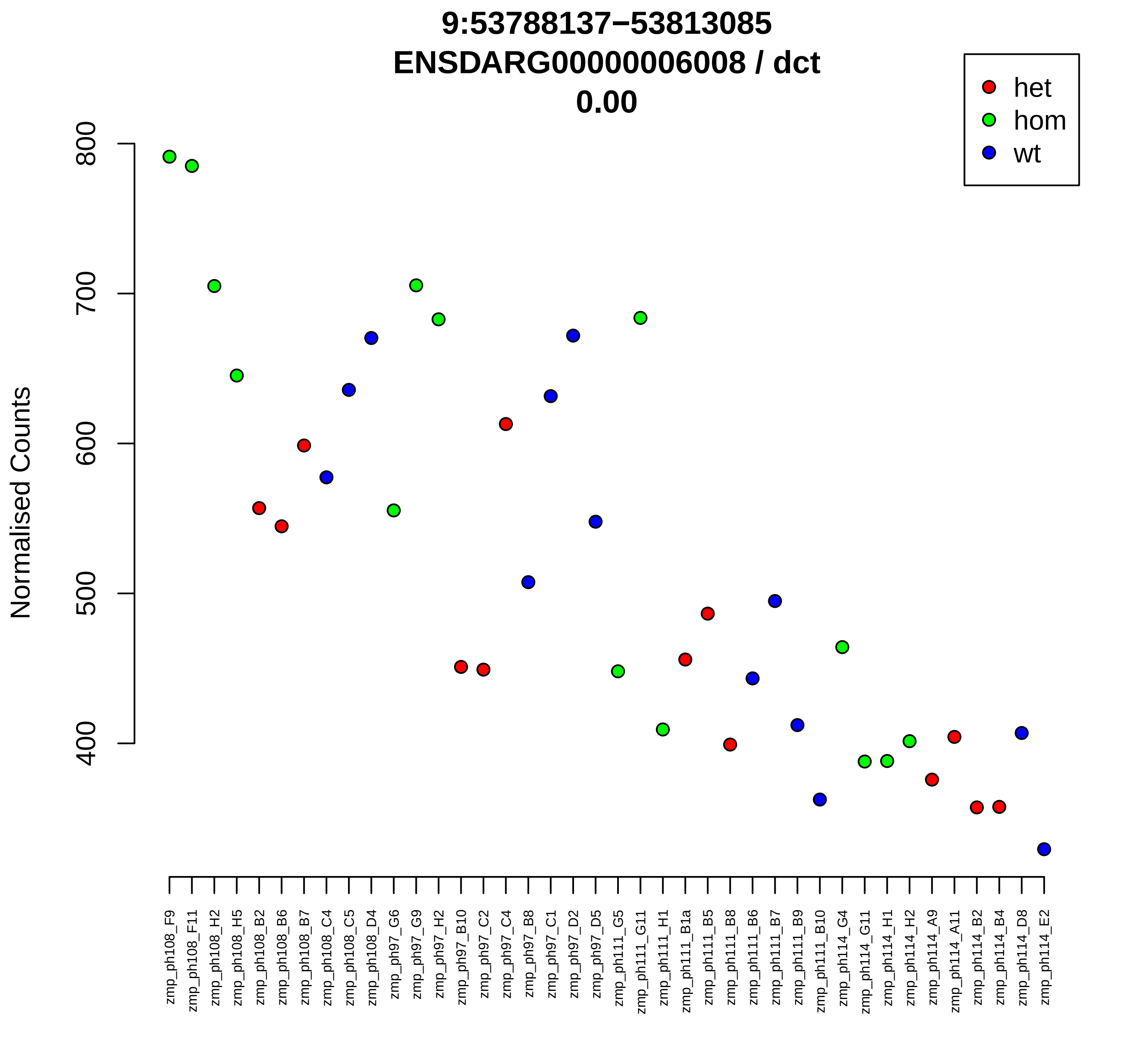
9:53788137−53813085
ENSDARG00000006008 / dct
0.00
het
hom
wt
Normalised Counts
800
700
600
500
400
zmp_ph108_F9
zmp_ph108_F11
zmp_ph108_H2
zmp_ph108_H5
zmp_ph108_B2
zmp_ph108_B6
zmp_ph108_B7
zmp_ph108_C4
zmp_ph108_C5
zmp_ph108_D4
zmp_ph97_G6
zmp_ph97_G9
zmp_ph97_H2
zmp_ph97_B10
zmp_ph97_C2
zmp_ph97_C4
zmp_ph97_B8
zmp_ph97_C1
zmp_ph97_D2
zmp_ph97_D5
zmp_ph111_G5
zmp_ph111_G11
zmp_ph111_H1
zmp_ph111_B1a
zmp_ph111_B5
zmp_ph111_B8
zmp_ph111_B6
zmp_ph111_B7
zmp_ph111_B9
zmp_ph111_B10
zmp_ph114_G4
zmp_ph114_G11
zmp_ph114_H1
zmp_ph114_H2
zmp_ph114_A9
zmp_ph114_A11
zmp_ph114_B2
zmp_ph114_B4
zmp_ph114_D8
zmp_ph114_E2

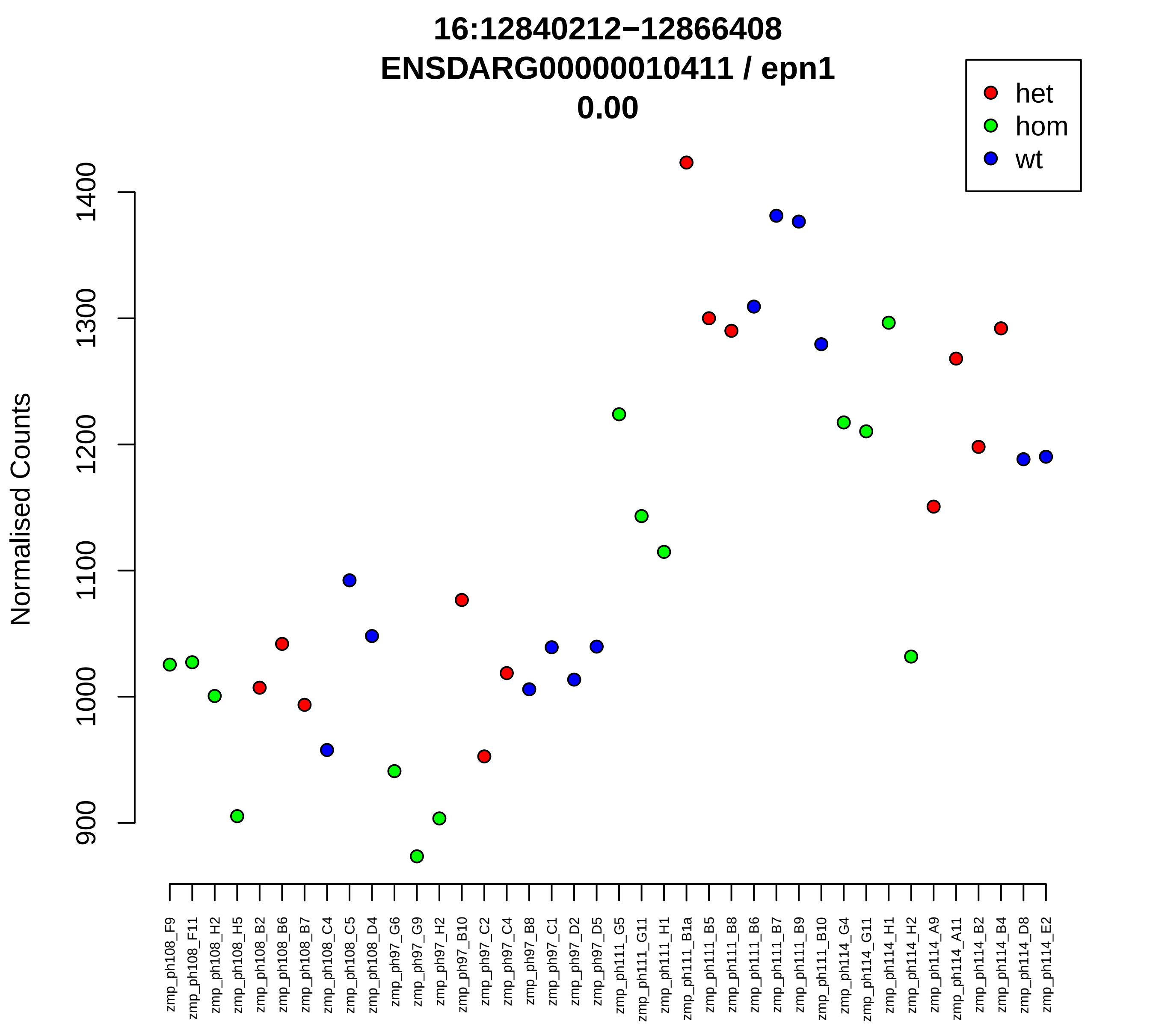
16:12840212−12866408
ENSDARG00000010411 / epn1
0.00
het
hom
wt
Normalised Counts
900
1000
1100
1200
1300
1400
zmp_ph108_F9
zmp_ph108_F11
zmp_ph108_H2
zmp_ph108_H5
zmp_ph108_B2
zmp_ph108_B6
zmp_ph108_B7
zmp_ph108_C4
zmp_ph108_C5
zmp_ph108_D4
zmp_ph97_G6
zmp_ph97_G9
zmp_ph97_H2
zmp_ph97_B10
zmp_ph97_C2
zmp_ph97_C4
zmp_ph97_B8
zmp_ph97_C1
zmp_ph97_D2
zmp_ph97_D5
zmp_ph111_G5
zmp_ph111_G11
zmp_ph111_H1
zmp_ph111_B1a
zmp_ph111_B5
zmp_ph111_B8
zmp_ph111_B6
zmp_ph111_B7
zmp_ph111_B9
zmp_ph111_B10
zmp_ph114_G4
zmp_ph114_G11
zmp_ph114_H1
zmp_ph114_H2
zmp_ph114_A9
zmp_ph114_A11
zmp_ph114_B2
zmp_ph114_B4
zmp_ph114_D8
zmp_ph114_E2

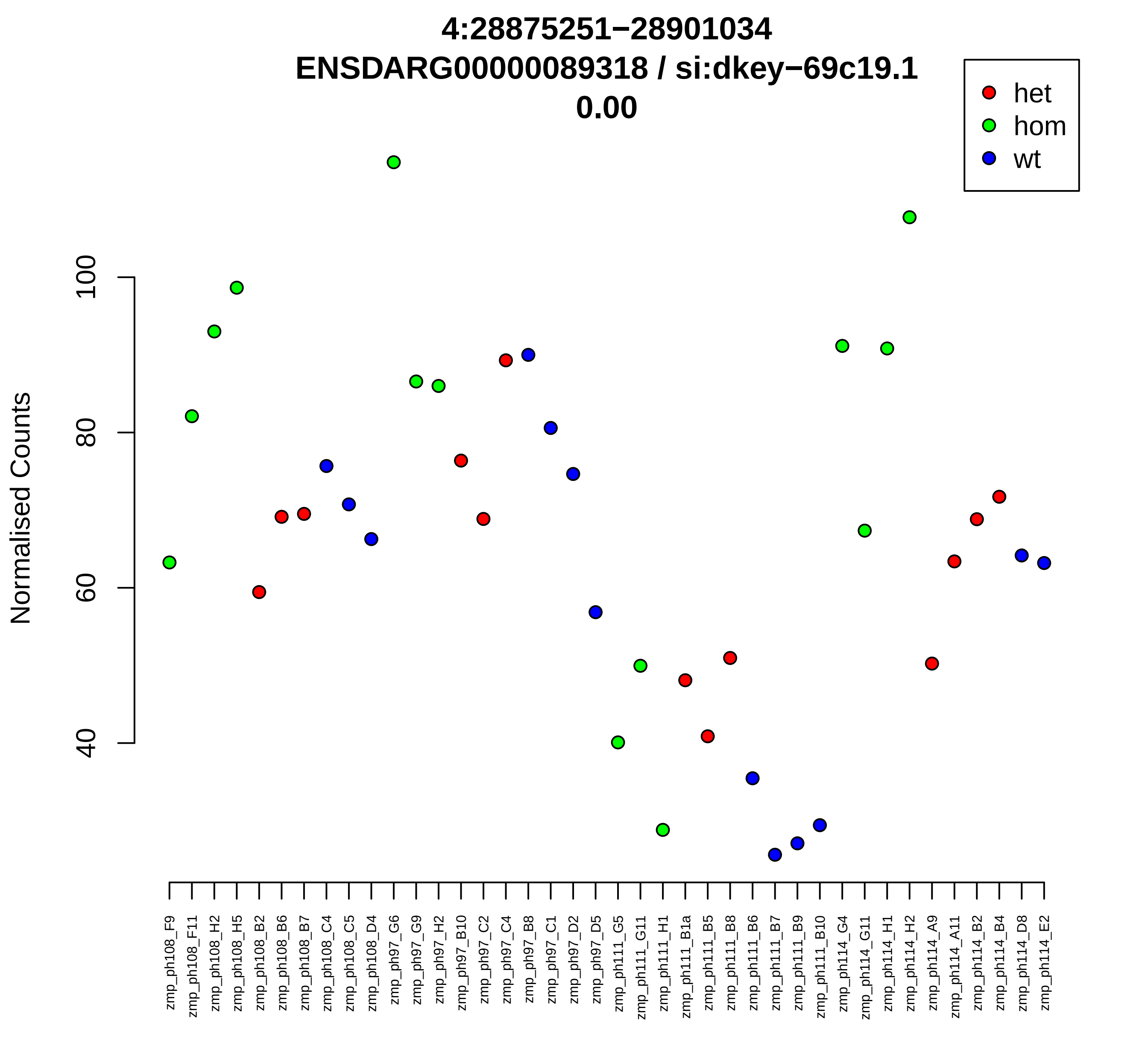
4:28875251−28901034
ENSDARG00000089318 / si:dkey−69c19.1
0.00
het
hom
wt
Normalised Counts
40
60
80
100
zmp_ph108_F9
zmp_ph108_F11
zmp_ph108_H2
zmp_ph108_H5
zmp_ph108_B2
zmp_ph108_B6
zmp_ph108_B7
zmp_ph108_C4
zmp_ph108_C5
zmp_ph108_D4
zmp_ph97_G6
zmp_ph97_G9
zmp_ph97_H2
zmp_ph97_B10
zmp_ph97_C2
zmp_ph97_C4
zmp_ph97_B8
zmp_ph97_C1
zmp_ph97_D2
zmp_ph97_D5
zmp_ph111_G5
zmp_ph111_G11
zmp_ph111_H1
zmp_ph111_B1a
zmp_ph111_B5
zmp_ph111_B8
zmp_ph111_B6
zmp_ph111_B7
zmp_ph111_B9
zmp_ph111_B10
zmp_ph114_G4
zmp_ph114_G11
zmp_ph114_H1
zmp_ph114_H2
zmp_ph114_A9
zmp_ph114_A11
zmp_ph114_B2
zmp_ph114_B4
zmp_ph114_D8
zmp_ph114_E2

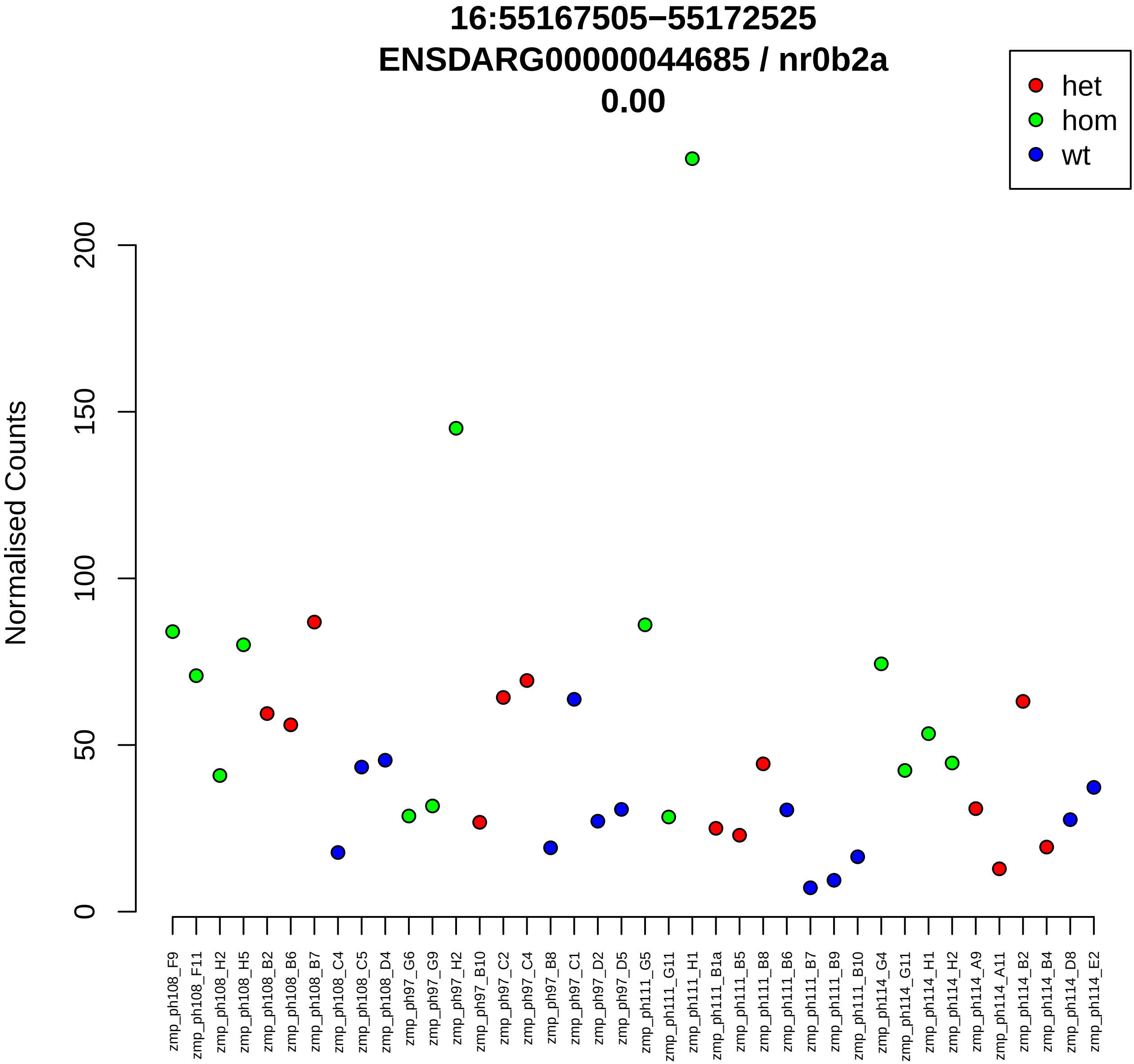
16:55167505−55172525
ENSDARG00000044685 / nr0b2a
0.00
het
hom
wt
Normalised Counts
0
50
100
150
200
zmp_ph108_F9
zmp_ph108_F11
zmp_ph108_H2
zmp_ph108_H5
zmp_ph108_B2
zmp_ph108_B6
zmp_ph108_B7
zmp_ph108_C4
zmp_ph108_C5
zmp_ph108_D4
zmp_ph97_G6
zmp_ph97_G9
zmp_ph97_H2
zmp_ph97_B10
zmp_ph97_C2
zmp_ph97_C4
zmp_ph97_B8
zmp_ph97_C1
zmp_ph97_D2
zmp_ph97_D5
zmp_ph111_G5
zmp_ph111_G11
zmp_ph111_H1
zmp_ph111_B1a
zmp_ph111_B5
zmp_ph111_B8
zmp_ph111_B6
zmp_ph111_B7
zmp_ph111_B9
zmp_ph111_B10
zmp_ph114_G4
zmp_ph114_G11
zmp_ph114_H1
zmp_ph114_H2
zmp_ph114_A9
zmp_ph114_A11
zmp_ph114_B2
zmp_ph114_B4
zmp_ph114_D8
zmp_ph114_E2

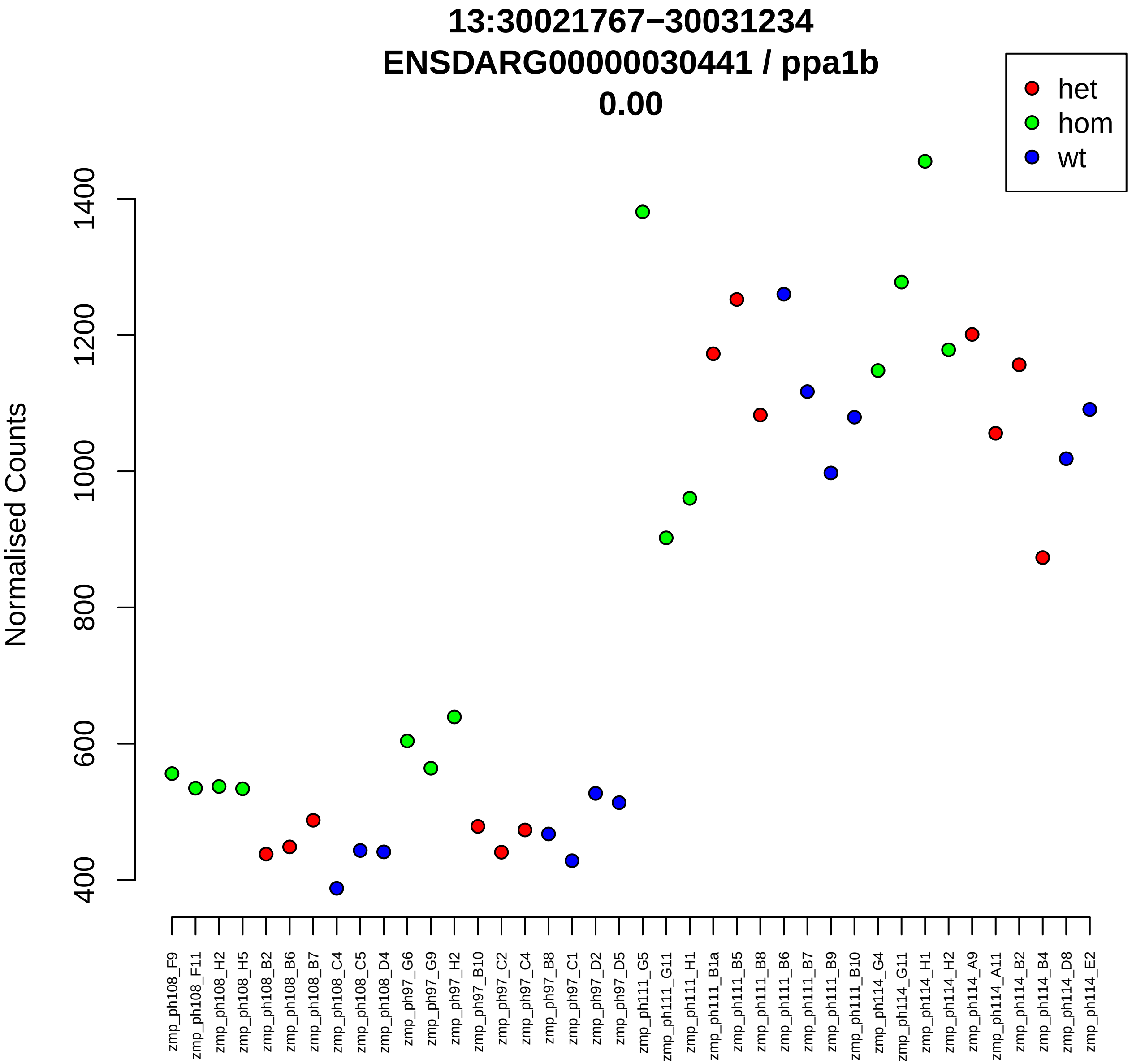

13:30021767−30031234
ENSDARG00000030441 / ppa1b
0.00
het
hom
wt
Normalised Counts
400
600
800
1000
1200
1400
zmp_ph108_F9
zmp_ph108_F11
zmp_ph108_H2
zmp_ph108_H5
zmp_ph108_B2
zmp_ph108_B6
zmp_ph108_B7
zmp_ph108_C4
zmp_ph108_C5
zmp_ph108_D4
zmp_ph97_G6
zmp_ph97_G9
zmp_ph97_H2
zmp_ph97_B10
zmp_ph97_C2
zmp_ph97_C4
zmp_ph97_B8
zmp_ph97_C1
zmp_ph97_D2
zmp_ph97_D5
zmp_ph111_G5
zmp_ph111_G11
zmp_ph111_H1
zmp_ph111_B1a
zmp_ph111_B5
zmp_ph111_B8
zmp_ph111_B6
zmp_ph111_B7
zmp_ph111_B9
zmp_ph111_B10
zmp_ph114_G4
zmp_ph114_G11
zmp_ph114_H1
zmp_ph114_H2
zmp_ph114_A9
zmp_ph114_A11
zmp_ph114_B2
zmp_ph114_B4
zmp_ph114_D8
zmp_ph114_E2

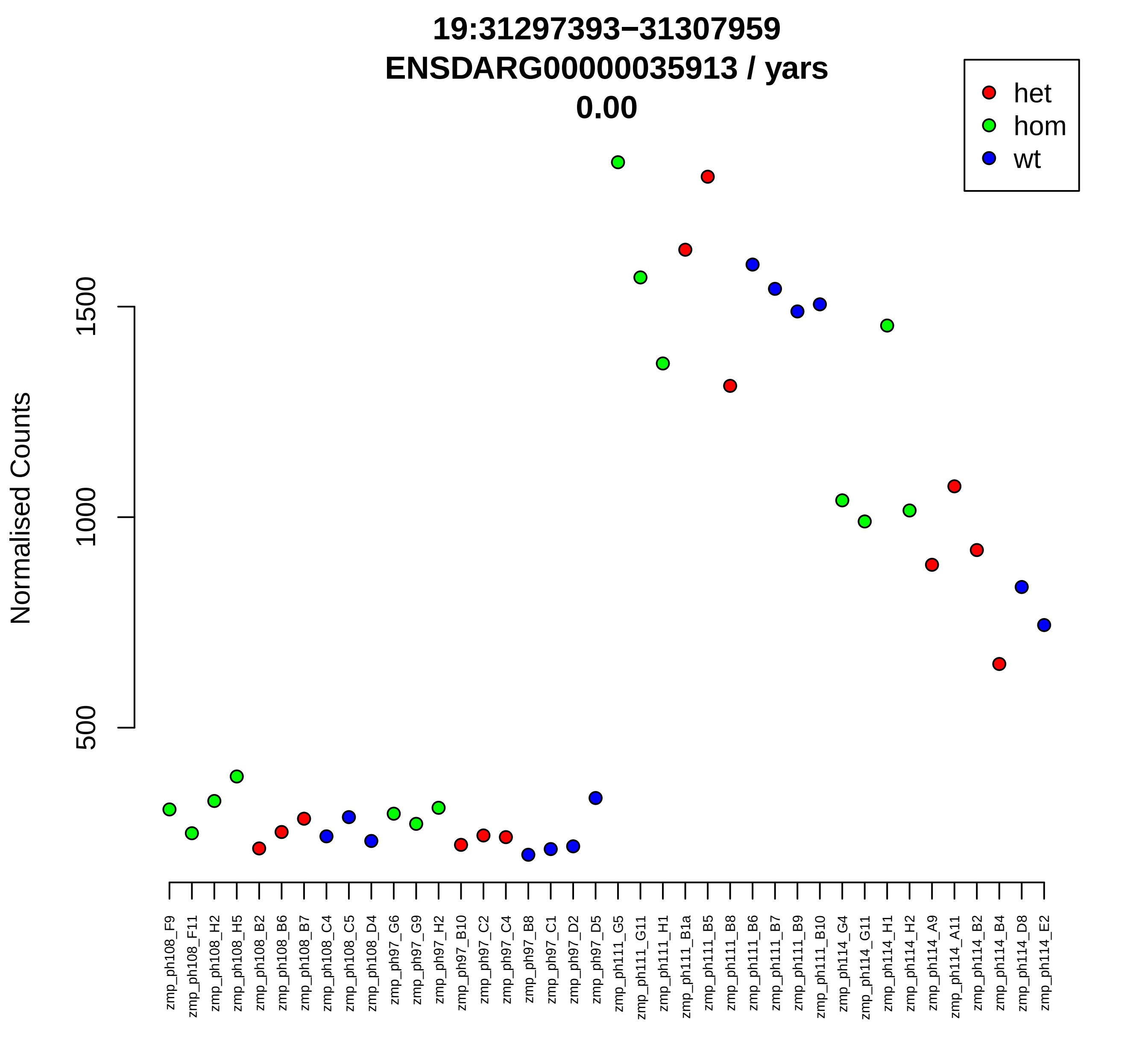

19:31297393−31307959
ENSDARG00000035913 / yars
0.00
het
hom
wt
Normalised Counts
500
1000
1500
zmp_ph108_F9
zmp_ph108_F11
zmp_ph108_H2
zmp_ph108_H5
zmp_ph108_B2
zmp_ph108_B6
zmp_ph108_B7
zmp_ph108_C4
zmp_ph108_C5
zmp_ph108_D4
zmp_ph97_G6
zmp_ph97_G9
zmp_ph97_H2
zmp_ph97_B10
zmp_ph97_C2
zmp_ph97_C4
zmp_ph97_B8
zmp_ph97_C1
zmp_ph97_D2
zmp_ph97_D5
zmp_ph111_G5
zmp_ph111_G11
zmp_ph111_H1
zmp_ph111_B1a
zmp_ph111_B5
zmp_ph111_B8
zmp_ph111_B6
zmp_ph111_B7
zmp_ph111_B9
zmp_ph111_B10
zmp_ph114_G4
zmp_ph114_G11
zmp_ph114_H1
zmp_ph114_H2
zmp_ph114_A9
zmp_ph114_A11
zmp_ph114_B2
zmp_ph114_B4
zmp_ph114_D8
zmp_ph114_E2

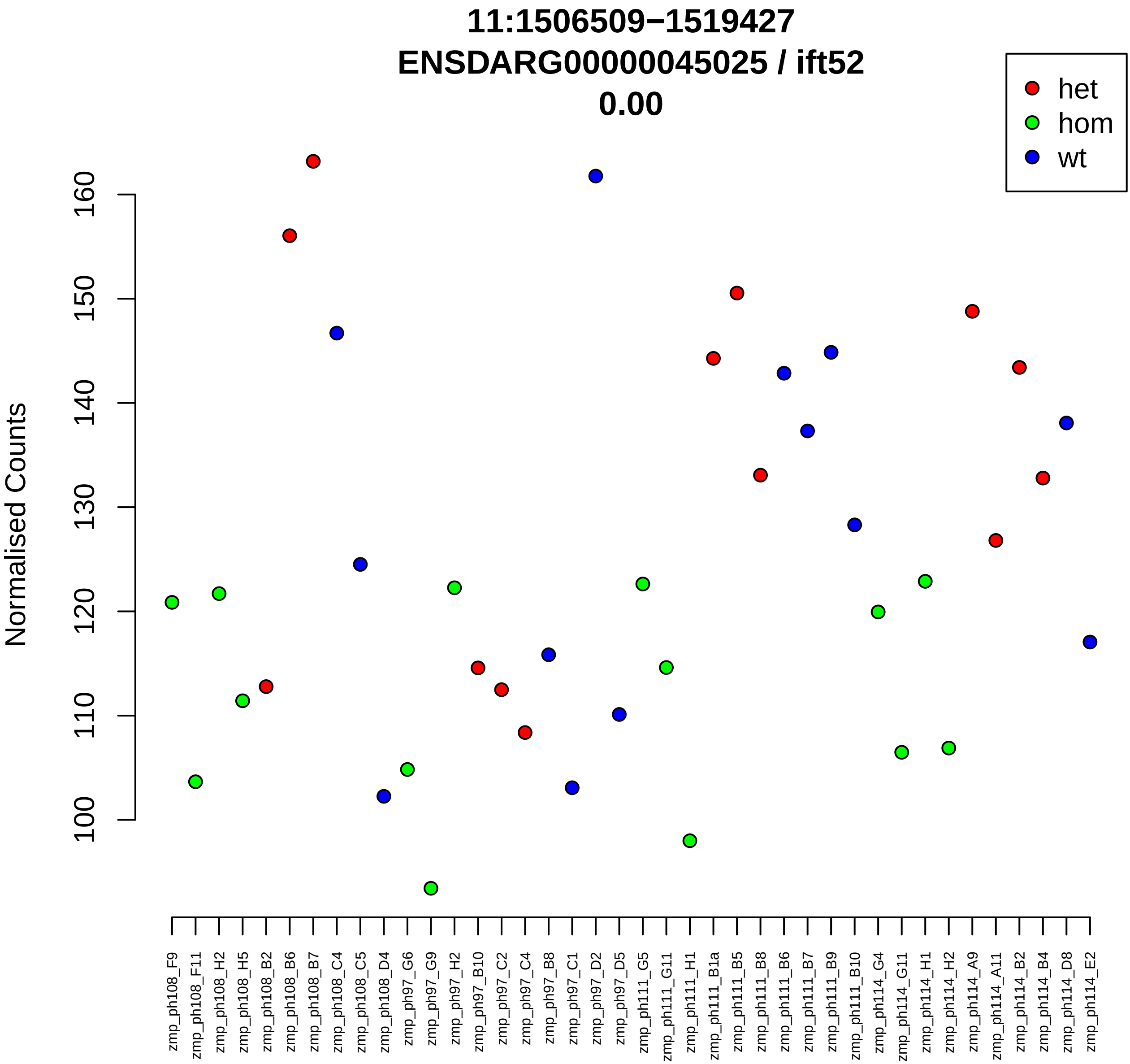
11:1506509−1519427
ENSDARG00000045025 / ift52
0.00
het
hom
wt
Normalised Counts
100
110
120
130
140
150
160
zmp_ph108_F9
zmp_ph108_F11
zmp_ph108_H2
zmp_ph108_H5
zmp_ph108_B2
zmp_ph108_B6
zmp_ph108_B7
zmp_ph108_C4
zmp_ph108_C5
zmp_ph108_D4
zmp_ph97_G6
zmp_ph97_G9
zmp_ph97_H2
zmp_ph97_B10
zmp_ph97_C2
zmp_ph97_C4
zmp_ph97_B8
zmp_ph97_C1
zmp_ph97_D2
zmp_ph97_D5
zmp_ph111_G5
zmp_ph111_G11
zmp_ph111_H1
zmp_ph111_B1a
zmp_ph111_B5
zmp_ph111_B8
zmp_ph111_B6
zmp_ph111_B7
zmp_ph111_B9
zmp_ph111_B10
zmp_ph114_G4
zmp_ph114_G11
zmp_ph114_H1
zmp_ph114_H2
zmp_ph114_A9
zmp_ph114_A11
zmp_ph114_B2
zmp_ph114_B4
zmp_ph114_D8
zmp_ph114_E2

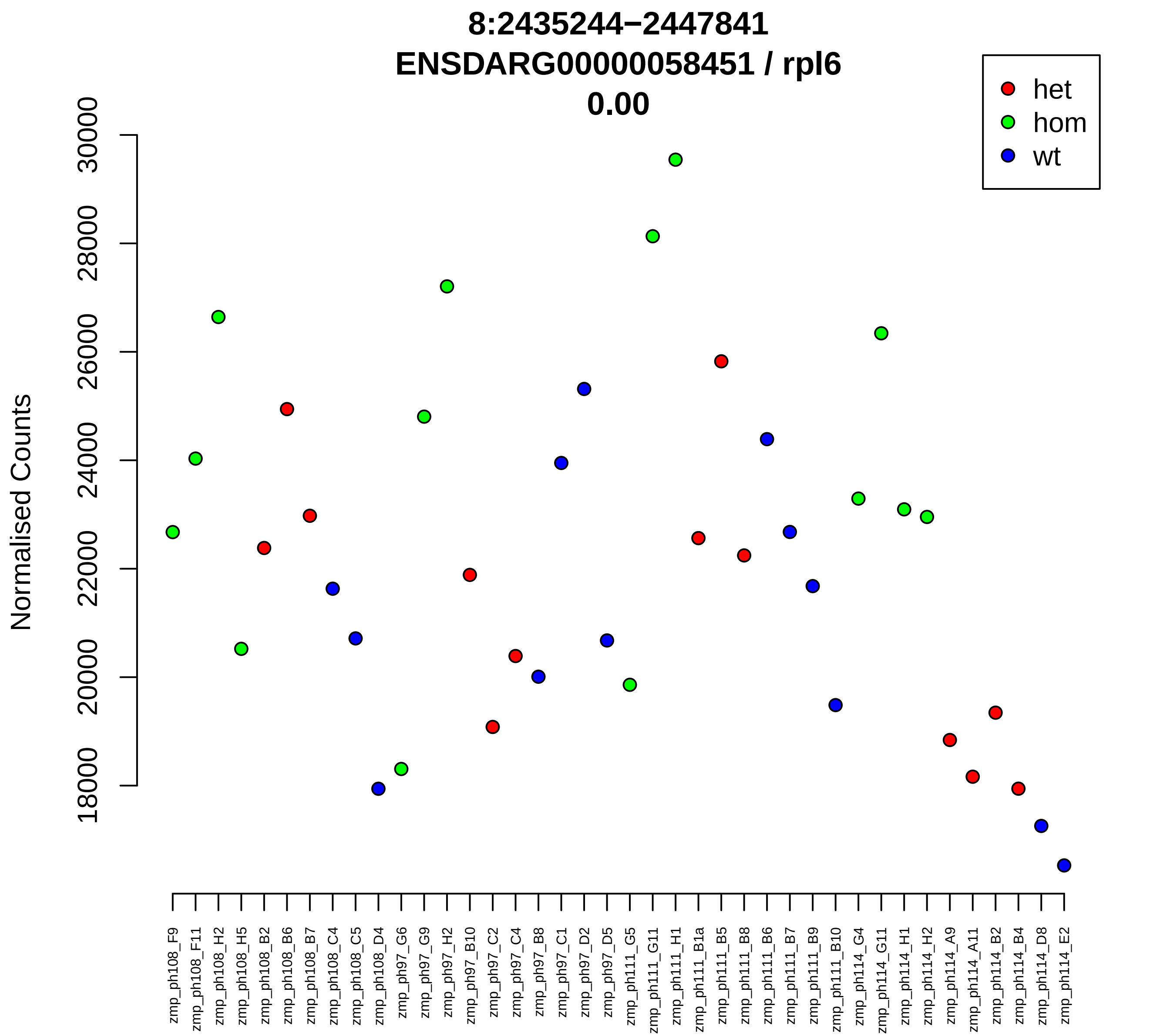

8:2435244−2447841
ENSDARG00000058451 / rpl6
0.00
Normalised Counts
18000
20000
22000
24000
26000
28000
30000
het
hom
wt
zmp_ph108_F9
zmp_ph108_F11
zmp_ph108_H2
zmp_ph108_H5
zmp_ph108_B2
zmp_ph108_B6
zmp_ph108_B7
zmp_ph108_C4
zmp_ph108_C5
zmp_ph108_D4
zmp_ph97_G6
zmp_ph97_G9
zmp_ph97_H2
zmp_ph97_B10
zmp_ph97_C2
zmp_ph97_C4
zmp_ph97_B8
zmp_ph97_C1
zmp_ph97_D2
zmp_ph97_D5
zmp_ph111_G5
zmp_ph111_G11
zmp_ph111_H1
zmp_ph111_B1a
zmp_ph111_B5
zmp_ph111_B8
zmp_ph111_B6
zmp_ph111_B7
zmp_ph111_B9
zmp_ph111_B10
zmp_ph114_G4
zmp_ph114_G11
zmp_ph114_H1
zmp_ph114_H2
zmp_ph114_A9
zmp_ph114_A11
zmp_ph114_B2
zmp_ph114_B4
zmp_ph114_D8
zmp_ph114_E2

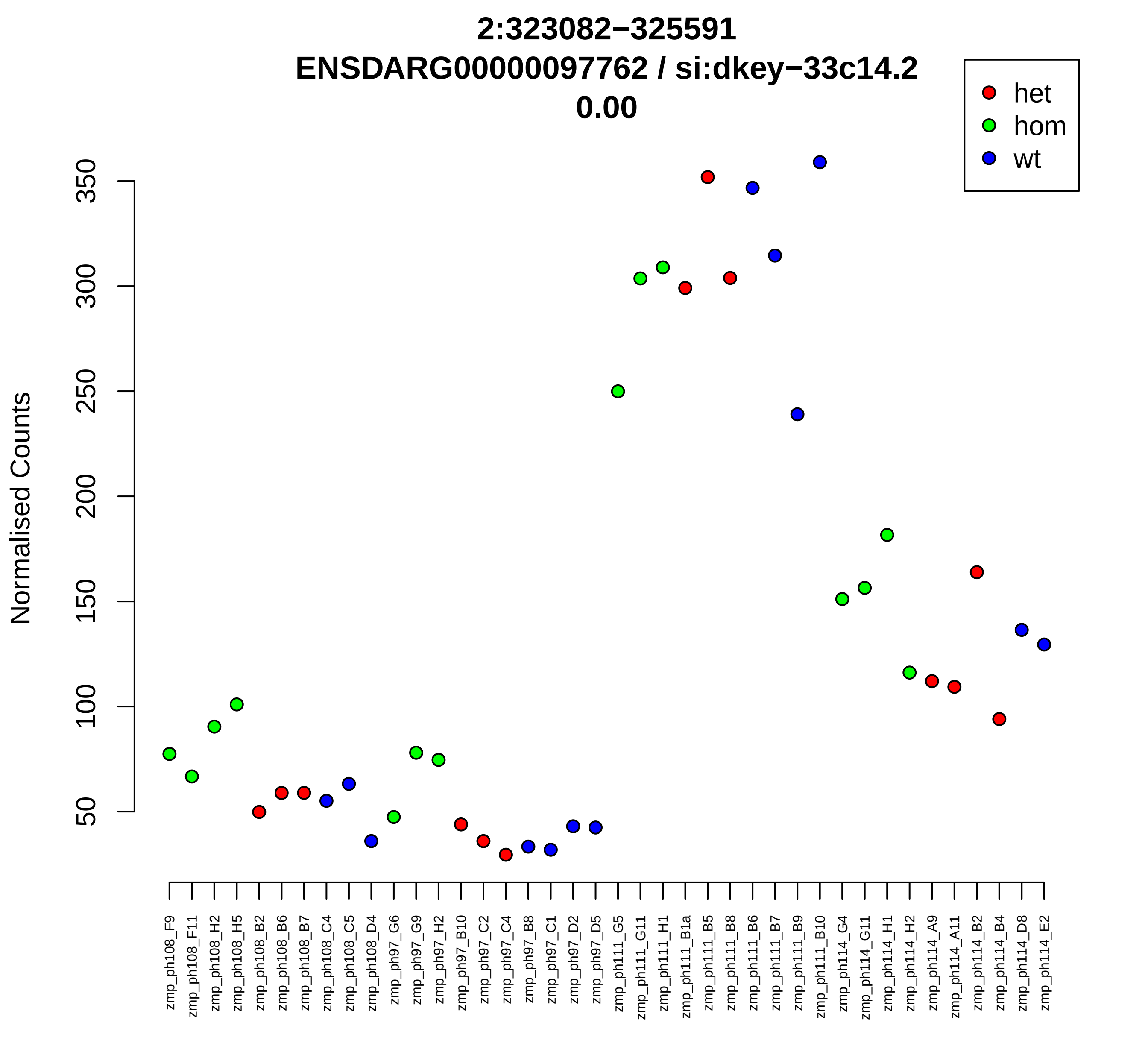

2:323082−325591
ENSDARG00000097762 / si:dkey−33c14.2
0.00
het
hom
wt
Normalised Counts
50
100
150
200
250
300
350
zmp_ph108_F9
zmp_ph108_F11
zmp_ph108_H2
zmp_ph108_H5
zmp_ph108_B2
zmp_ph108_B6
zmp_ph108_B7
zmp_ph108_C4
zmp_ph108_C5
zmp_ph108_D4
zmp_ph97_G6
zmp_ph97_G9
zmp_ph97_H2
zmp_ph97_B10
zmp_ph97_C2
zmp_ph97_C4
zmp_ph97_B8
zmp_ph97_C1
zmp_ph97_D2
zmp_ph97_D5
zmp_ph111_G5
zmp_ph111_G11
zmp_ph111_H1
zmp_ph111_B1a
zmp_ph111_B5
zmp_ph111_B8
zmp_ph111_B6
zmp_ph111_B7
zmp_ph111_B9
zmp_ph111_B10
zmp_ph114_G4
zmp_ph114_G11
zmp_ph114_H1
zmp_ph114_H2
zmp_ph114_A9
zmp_ph114_A11
zmp_ph114_B2
zmp_ph114_B4
zmp_ph114_D8
zmp_ph114_E2

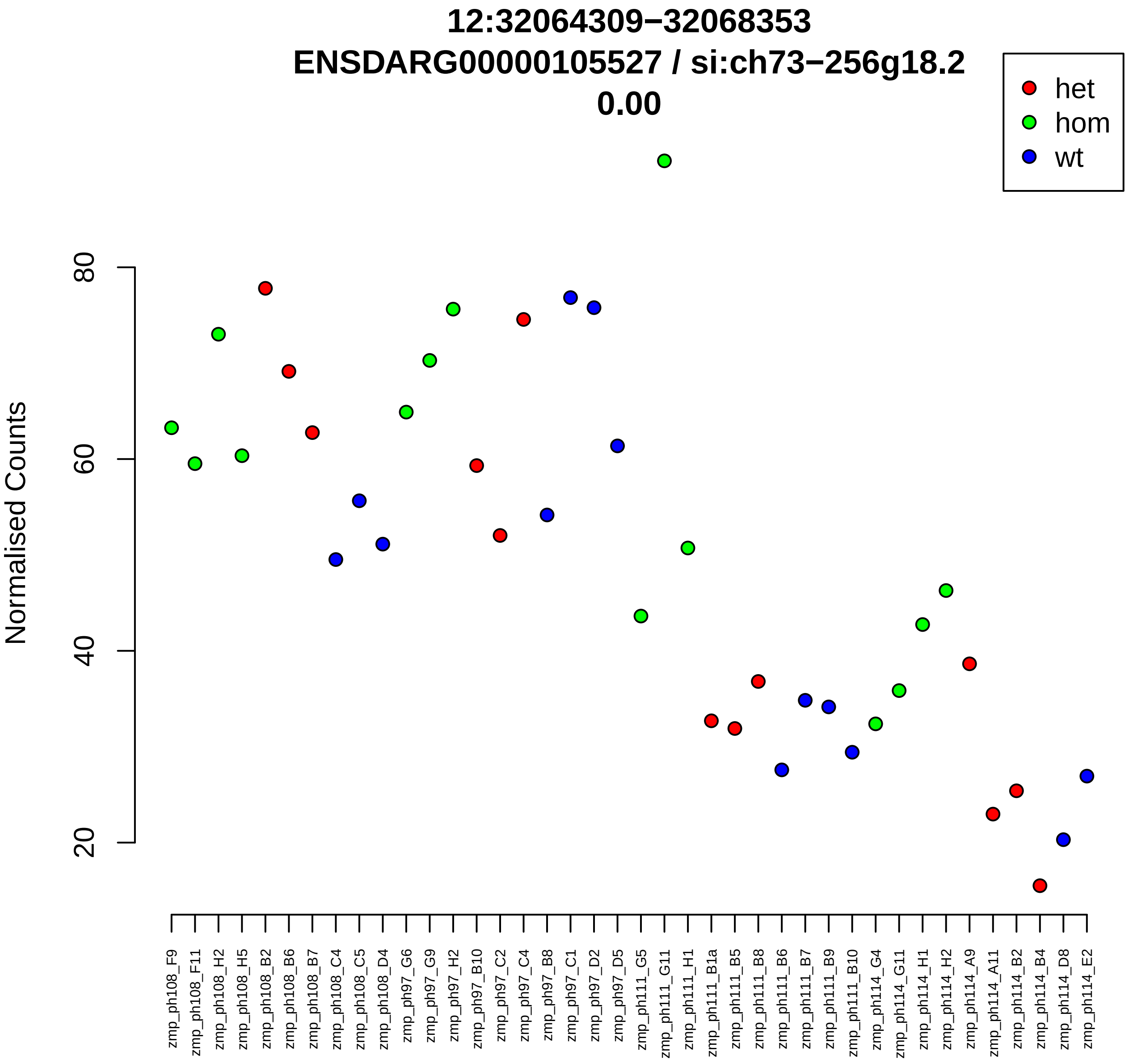

12:32064309−32068353
ENSDARG00000105527 / si:ch73−256g18.2
0.00
het
hom
wt
Normalised Counts
20
40
60
80
zmp_ph108_F9
zmp_ph108_F11
zmp_ph108_H2
zmp_ph108_H5
zmp_ph108_B2
zmp_ph108_B6
zmp_ph108_B7
zmp_ph108_C4
zmp_ph108_C5
zmp_ph108_D4
zmp_ph97_G6
zmp_ph97_G9
zmp_ph97_H2
zmp_ph97_B10
zmp_ph97_C2
zmp_ph97_C4
zmp_ph97_B8
zmp_ph97_C1
zmp_ph97_D2
zmp_ph97_D5
zmp_ph111_G5
zmp_ph111_G11
zmp_ph111_H1
zmp_ph111_B1a
zmp_ph111_B5
zmp_ph111_B8
zmp_ph111_B6
zmp_ph111_B7
zmp_ph111_B9
zmp_ph111_B10
zmp_ph114_G4
zmp_ph114_G11
zmp_ph114_H1
zmp_ph114_H2
zmp_ph114_A9
zmp_ph114_A11
zmp_ph114_B2
zmp_ph114_B4
zmp_ph114_D8
zmp_ph114_E2

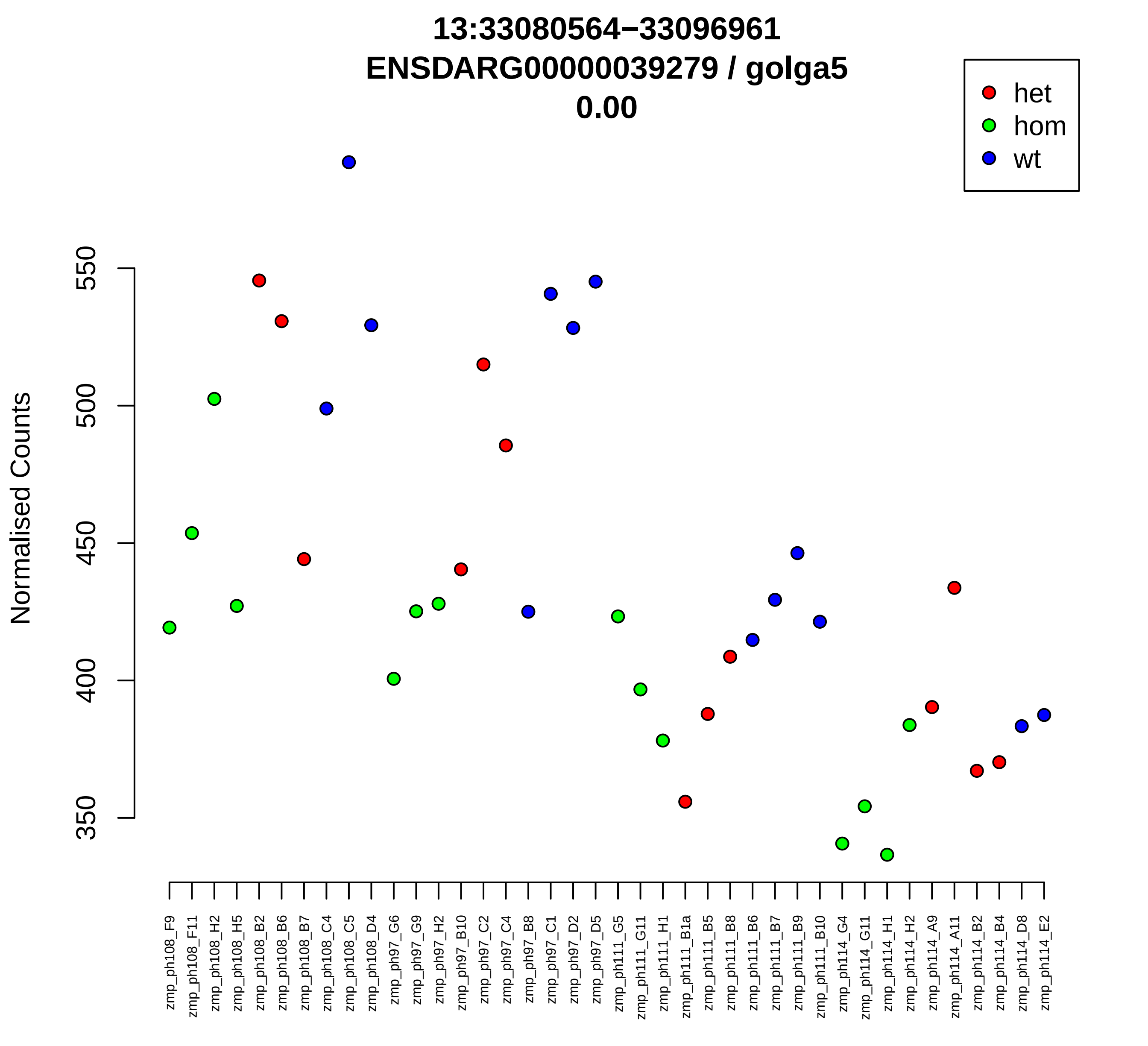

13:33080564−33096961
ENSDARG00000039279 / golga5
0.00
het
hom
wt
Normalised Counts
550
500
450
400
350
zmp_ph108_F9
zmp_ph108_F11
zmp_ph108_H2
zmp_ph108_H5
zmp_ph108_B2
zmp_ph108_B6
zmp_ph108_B7
zmp_ph108_C4
zmp_ph108_C5
zmp_ph108_D4
zmp_ph97_G6
zmp_ph97_G9
zmp_ph97_H2
zmp_ph97_B10
zmp_ph97_C2
zmp_ph97_C4
zmp_ph97_B8
zmp_ph97_C1
zmp_ph97_D2
zmp_ph97_D5
zmp_ph111_G5
zmp_ph111_G11
zmp_ph111_H1
zmp_ph111_B1a
zmp_ph111_B5
zmp_ph111_B8
zmp_ph111_B6
zmp_ph111_B7
zmp_ph111_B9
zmp_ph111_B10
zmp_ph114_G4
zmp_ph114_G11
zmp_ph114_H1
zmp_ph114_H2
zmp_ph114_A9
zmp_ph114_A11
zmp_ph114_B2
zmp_ph114_B4
zmp_ph114_D8
zmp_ph114_E2

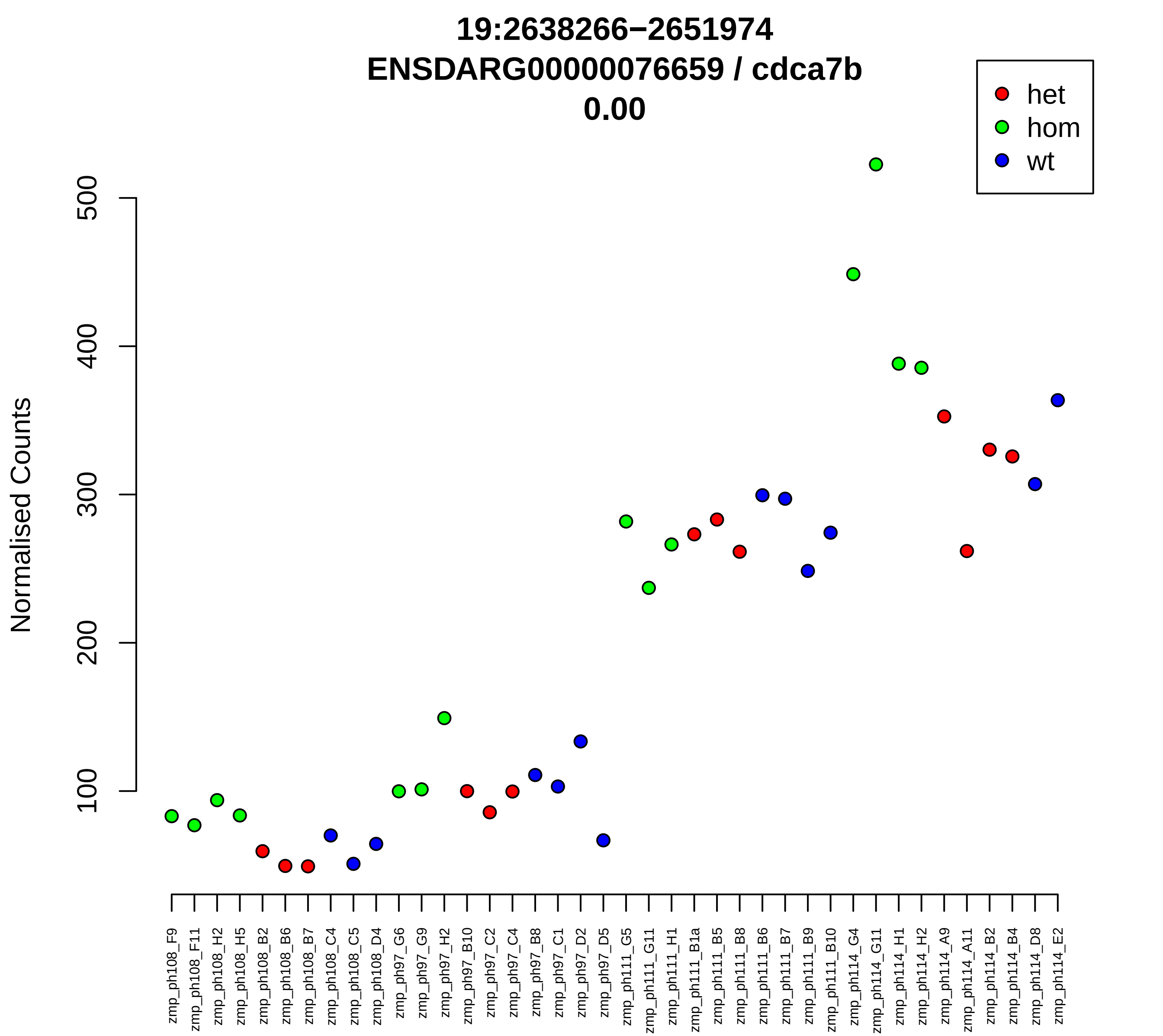
19:2638266−2651974
ENSDARG00000076659 / cdca7b
0.00
Normalised Counts
100
200
300
400
500
het
hom
wt
zmp_ph108_F9
zmp_ph108_F11
zmp_ph108_H2
zmp_ph108_H5
zmp_ph108_B2
zmp_ph108_B6
zmp_ph108_B7
zmp_ph108_C4
zmp_ph108_C5
zmp_ph108_D4
zmp_ph97_G6
zmp_ph97_G9
zmp_ph97_H2
zmp_ph97_B10
zmp_ph97_C2
zmp_ph97_C4
zmp_ph97_B8
zmp_ph97_C1
zmp_ph97_D2
zmp_ph97_D5
zmp_ph111_G5
zmp_ph111_G11
zmp_ph111_H1
zmp_ph111_B1a
zmp_ph111_B5
zmp_ph111_B8
zmp_ph111_B6
zmp_ph111_B7
zmp_ph111_B9
zmp_ph111_B10
zmp_ph114_G4
zmp_ph114_G11
zmp_ph114_H1
zmp_ph114_H2
zmp_ph114_A9
zmp_ph114_A11
zmp_ph114_B2
zmp_ph114_B4
zmp_ph114_D8
zmp_ph114_E2

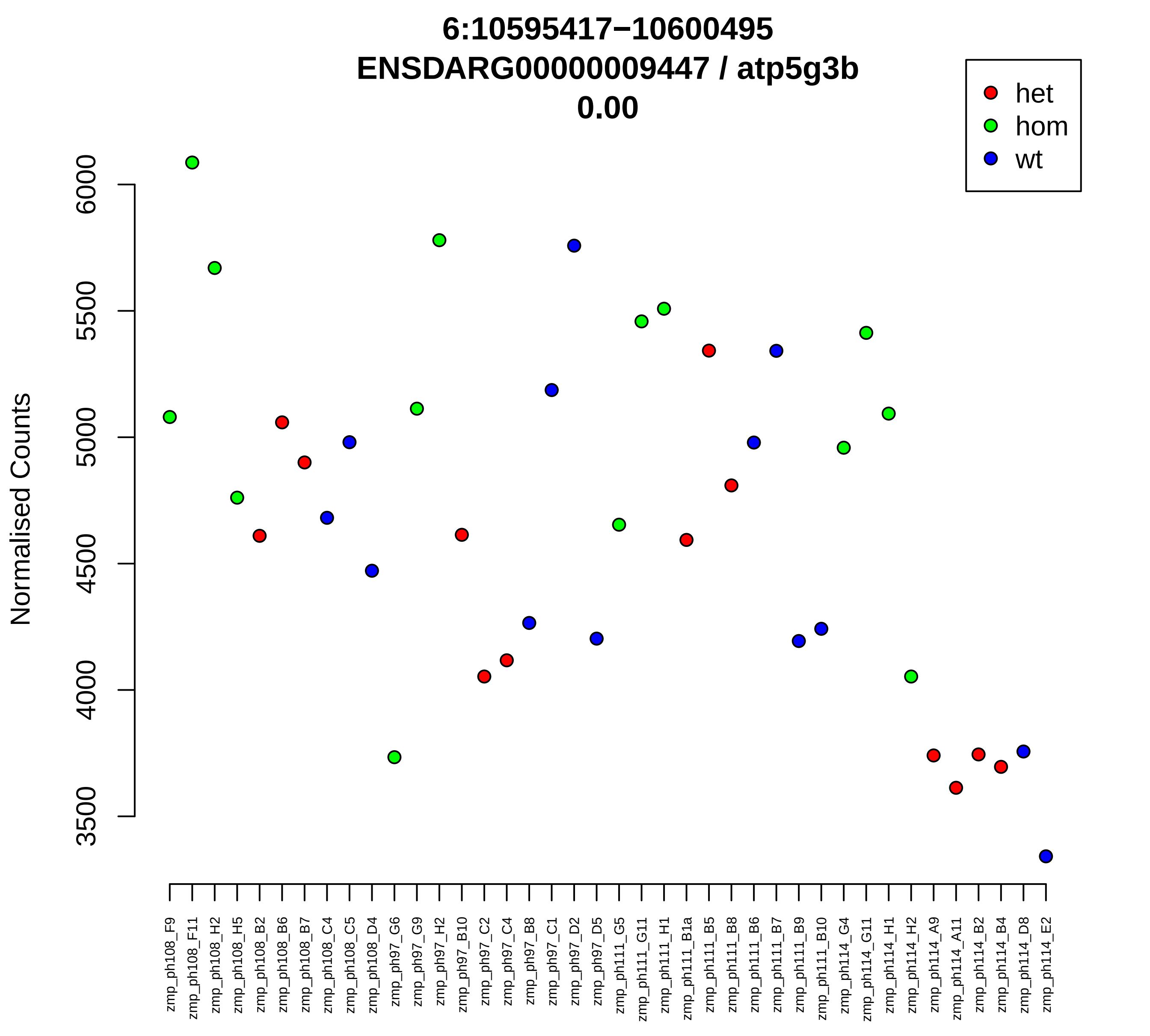

6:10595417−10600495
ENSDARG00000009447 / atp5g3b
0.00
het
hom
wt
Normalised Counts
3500
4000
4500
5000
5500
6000
zmp_ph108_F9
zmp_ph108_F11
zmp_ph108_H2
zmp_ph108_H5
zmp_ph108_B2
zmp_ph108_B6
zmp_ph108_B7
zmp_ph108_C4
zmp_ph108_C5
zmp_ph108_D4
zmp_ph97_G6
zmp_ph97_G9
zmp_ph97_H2
zmp_ph97_B10
zmp_ph97_C2
zmp_ph97_C4
zmp_ph97_B8
zmp_ph97_C1
zmp_ph97_D2
zmp_ph97_D5
zmp_ph111_G5
zmp_ph111_G11
zmp_ph111_H1
zmp_ph111_B1a
zmp_ph111_B5
zmp_ph111_B8
zmp_ph111_B6
zmp_ph111_B7
zmp_ph111_B9
zmp_ph111_B10
zmp_ph114_G4
zmp_ph114_G11
zmp_ph114_H1
zmp_ph114_H2
zmp_ph114_A9
zmp_ph114_A11
zmp_ph114_B2
zmp_ph114_B4
zmp_ph114_D8
zmp_ph114_E2

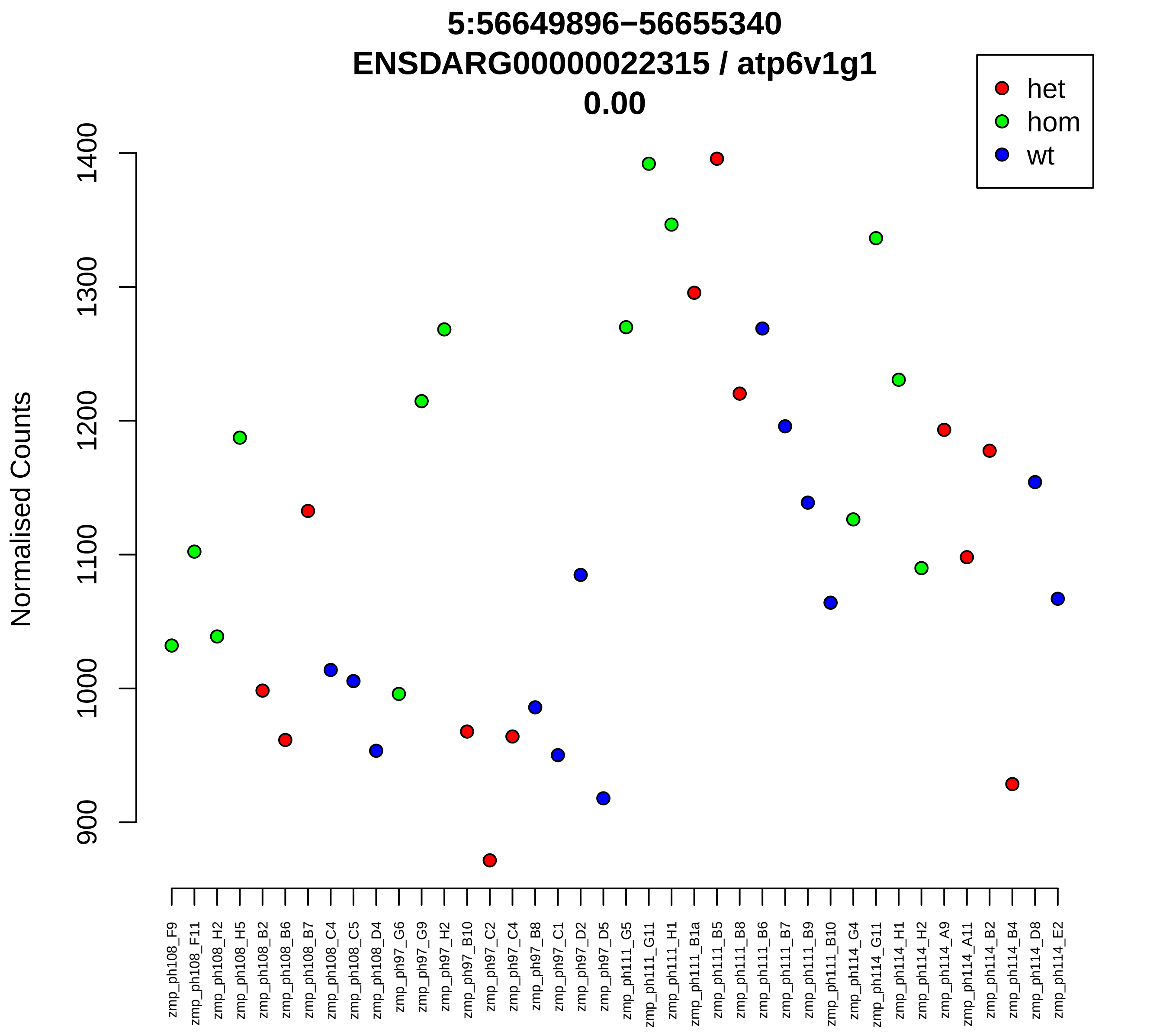
5:56649896−56655340
ENSDARG00000022315 / atp6v1g1
0.00
het
hom
wt
Normalised Counts
900
1000
1100
1200
1300
1400
zmp_ph108_F9
zmp_ph108_F11
zmp_ph108_H2
zmp_ph108_H5
zmp_ph108_B2
zmp_ph108_B6
zmp_ph108_B7
zmp_ph108_C4
zmp_ph108_C5
zmp_ph108_D4
zmp_ph97_G6
zmp_ph97_G9
zmp_ph97_H2
zmp_ph97_B10
zmp_ph97_C2
zmp_ph97_C4
zmp_ph97_B8
zmp_ph97_C1
zmp_ph97_D2
zmp_ph97_D5
zmp_ph111_G5
zmp_ph111_G11
zmp_ph111_H1
zmp_ph111_B1a
zmp_ph111_B5
zmp_ph111_B8
zmp_ph111_B6
zmp_ph111_B7
zmp_ph111_B9
zmp_ph111_B10
zmp_ph114_G4
zmp_ph114_G11
zmp_ph114_H1
zmp_ph114_H2
zmp_ph114_A9
zmp_ph114_A11
zmp_ph114_B2
zmp_ph114_B4
zmp_ph114_D8
zmp_ph114_E2

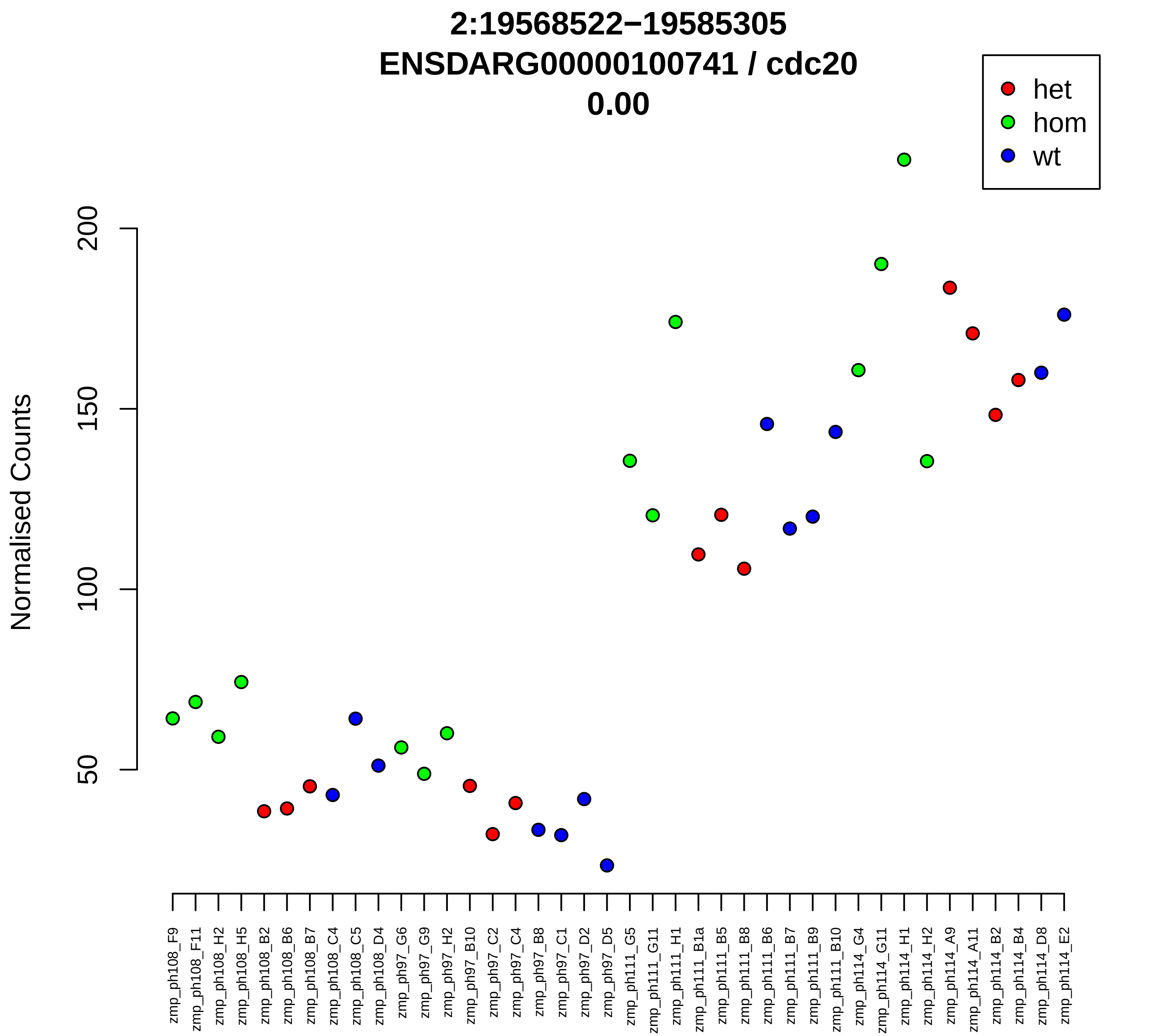
2:19568522−19585305
ENSDARG00000100741 / cdc20
0.00
het
hom
wt
Normalised Counts
50
100
150
200
zmp_ph108_F9
zmp_ph108_F11
zmp_ph108_H2
zmp_ph108_H5
zmp_ph108_B2
zmp_ph108_B6
zmp_ph108_B7
zmp_ph108_C4
zmp_ph108_C5
zmp_ph108_D4
zmp_ph97_G6
zmp_ph97_G9
zmp_ph97_H2
zmp_ph97_B10
zmp_ph97_C2
zmp_ph97_C4
zmp_ph97_B8
zmp_ph97_C1
zmp_ph97_D2
zmp_ph97_D5
zmp_ph111_G5
zmp_ph111_G11
zmp_ph111_H1
zmp_ph111_B1a
zmp_ph111_B5
zmp_ph111_B8
zmp_ph111_B6
zmp_ph111_B7
zmp_ph111_B9
zmp_ph111_B10
zmp_ph114_G4
zmp_ph114_G11
zmp_ph114_H1
zmp_ph114_H2
zmp_ph114_A9
zmp_ph114_A11
zmp_ph114_B2
zmp_ph114_B4
zmp_ph114_D8
zmp_ph114_E2

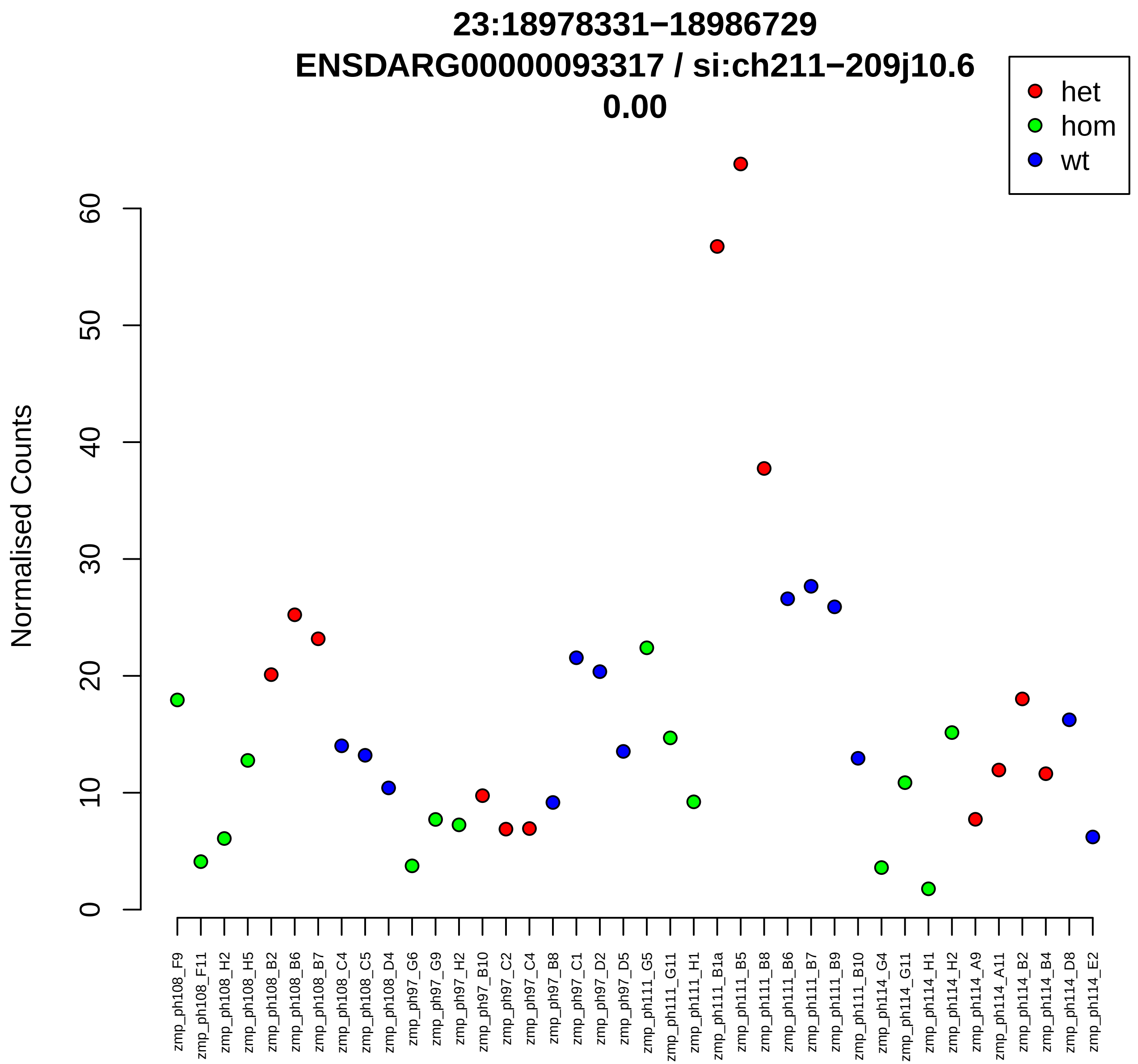

23:18978331−18986729
ENSDARG00000093317 / si:ch211−209j10.6
0.00
het
hom
wt
Normalised Counts
0
10
20
30
40
50
60
zmp_ph108_F9
zmp_ph108_F11
zmp_ph108_H2
zmp_ph108_H5
zmp_ph108_B2
zmp_ph108_B6
zmp_ph108_B7
zmp_ph108_C4
zmp_ph108_C5
zmp_ph108_D4
zmp_ph97_G6
zmp_ph97_G9
zmp_ph97_H2
zmp_ph97_B10
zmp_ph97_C2
zmp_ph97_C4
zmp_ph97_B8
zmp_ph97_C1
zmp_ph97_D2
zmp_ph97_D5
zmp_ph111_G5
zmp_ph111_G11
zmp_ph111_H1
zmp_ph111_B1a
zmp_ph111_B5
zmp_ph111_B8
zmp_ph111_B6
zmp_ph111_B7
zmp_ph111_B9
zmp_ph111_B10
zmp_ph114_G4
zmp_ph114_G11
zmp_ph114_H1
zmp_ph114_H2
zmp_ph114_A9
zmp_ph114_A11
zmp_ph114_B2
zmp_ph114_B4
zmp_ph114_D8
zmp_ph114_E2

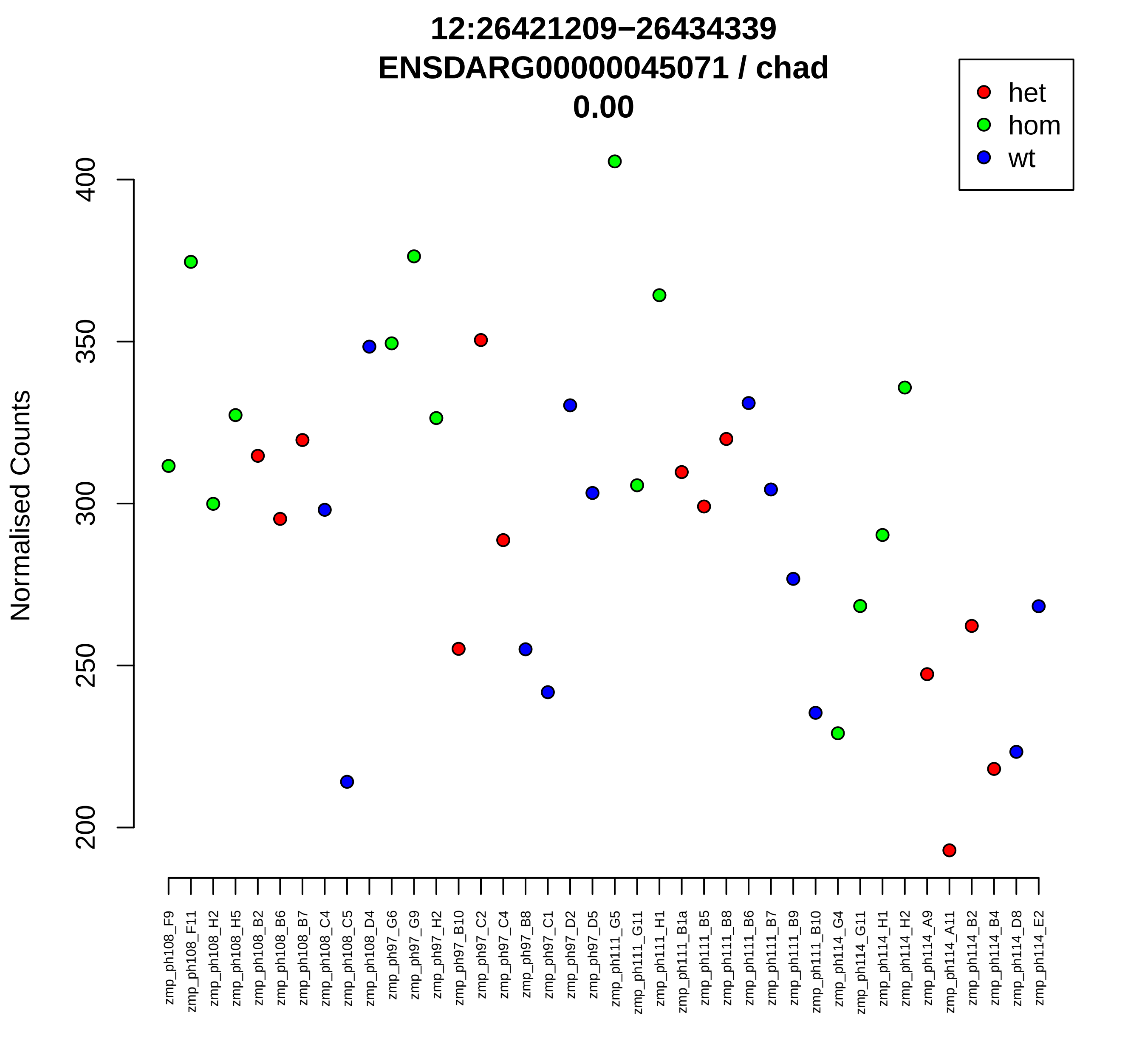

12:26421209−26434339
ENSDARG00000045071 / chad
0.00
het
hom
wt
Normalised Counts
200
250
300
350
400
zmp_ph108_F9
zmp_ph108_F11
zmp_ph108_H2
zmp_ph108_H5
zmp_ph108_B2
zmp_ph108_B6
zmp_ph108_B7
zmp_ph108_C4
zmp_ph108_C5
zmp_ph108_D4
zmp_ph97_G6
zmp_ph97_G9
zmp_ph97_H2
zmp_ph97_B10
zmp_ph97_C2
zmp_ph97_C4
zmp_ph97_B8
zmp_ph97_C1
zmp_ph97_D2
zmp_ph97_D5
zmp_ph111_G5
zmp_ph111_G11
zmp_ph111_H1
zmp_ph111_B1a
zmp_ph111_B5
zmp_ph111_B8
zmp_ph111_B6
zmp_ph111_B7
zmp_ph111_B9
zmp_ph111_B10
zmp_ph114_G4
zmp_ph114_G11
zmp_ph114_H1
zmp_ph114_H2
zmp_ph114_A9
zmp_ph114_A11
zmp_ph114_B2
zmp_ph114_B4
zmp_ph114_D8
zmp_ph114_E2

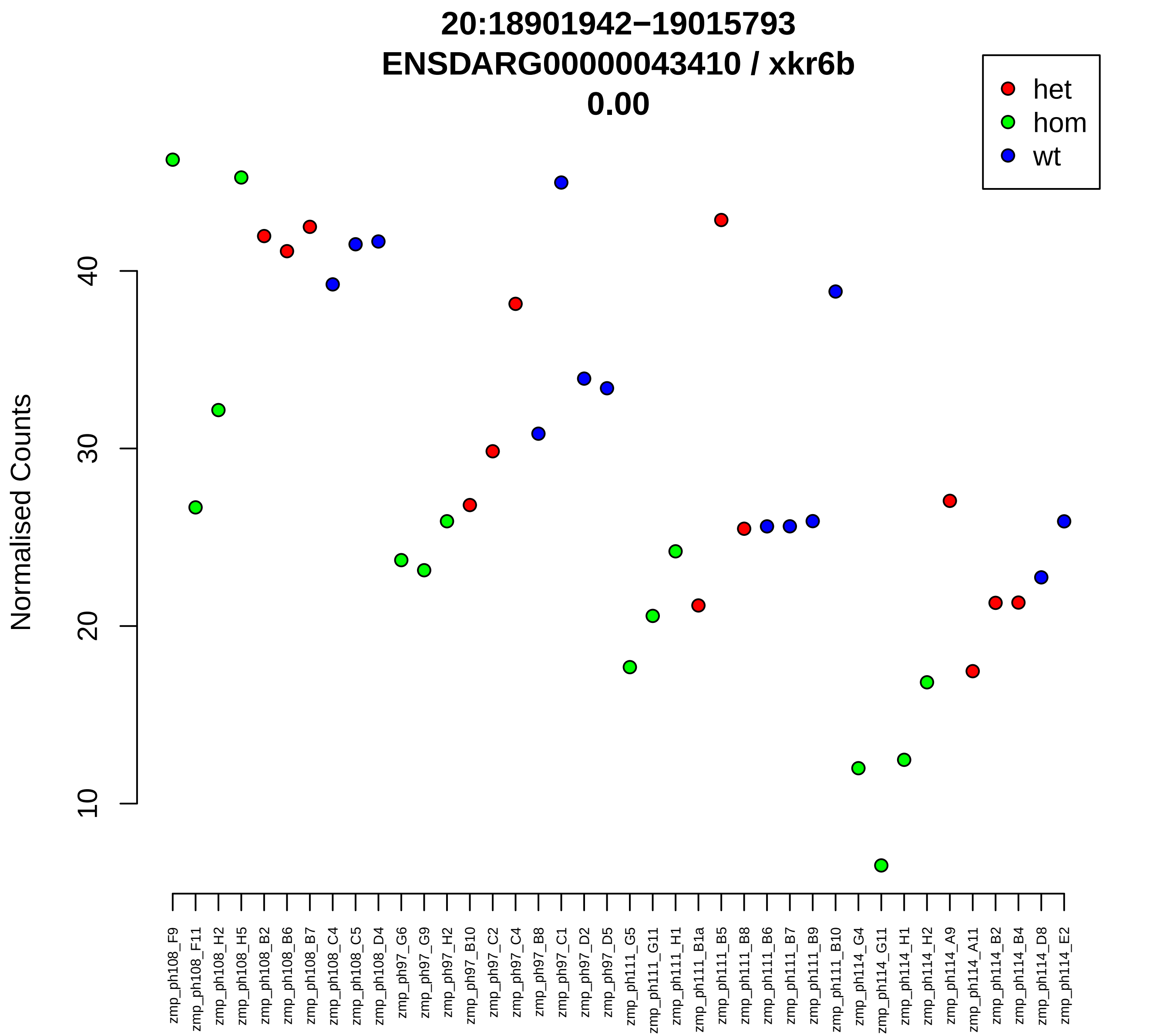

20:18901942−19015793
ENSDARG00000043410 / xkr6b
0.00
het
hom
wt
Normalised Counts
10
20
30
40
zmp_ph108_F9
zmp_ph108_F11
zmp_ph108_H2
zmp_ph108_H5
zmp_ph108_B2
zmp_ph108_B6
zmp_ph108_B7
zmp_ph108_C4
zmp_ph108_C5
zmp_ph108_D4
zmp_ph97_G6
zmp_ph97_G9
zmp_ph97_H2
zmp_ph97_B10
zmp_ph97_C2
zmp_ph97_C4
zmp_ph97_B8
zmp_ph97_C1
zmp_ph97_D2
zmp_ph97_D5
zmp_ph111_G5
zmp_ph111_G11
zmp_ph111_H1
zmp_ph111_B1a
zmp_ph111_B5
zmp_ph111_B8
zmp_ph111_B6
zmp_ph111_B7
zmp_ph111_B9
zmp_ph111_B10
zmp_ph114_G4
zmp_ph114_G11
zmp_ph114_H1
zmp_ph114_H2
zmp_ph114_A9
zmp_ph114_A11
zmp_ph114_B2
zmp_ph114_B4
zmp_ph114_D8
zmp_ph114_E2

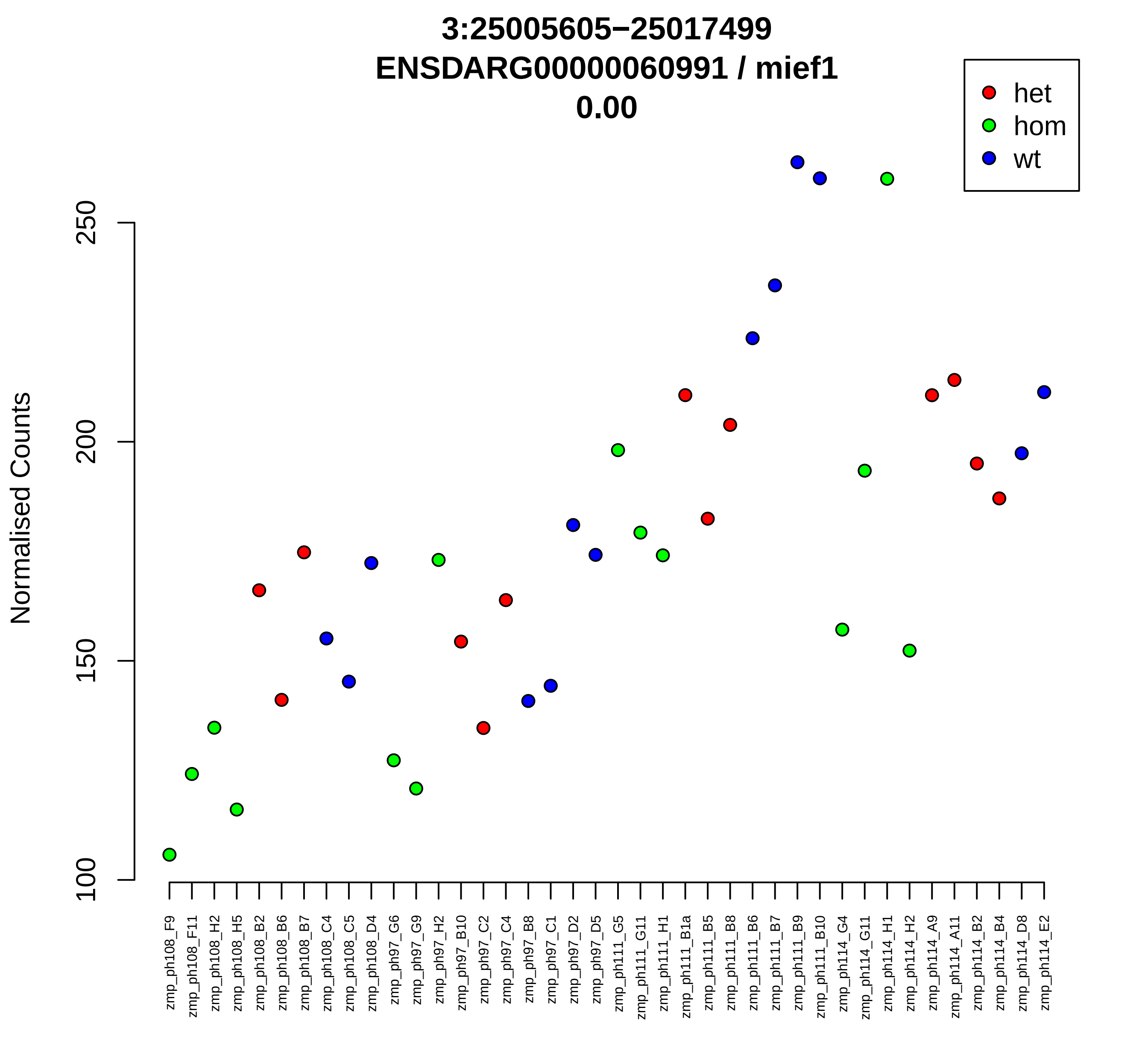

3:25005605−25017499
ENSDARG00000060991 / mief1
0.00
het
hom
wt
Normalised Counts
100
150
200
250
zmp_ph108_F9
zmp_ph108_F11
zmp_ph108_H2
zmp_ph108_H5
zmp_ph108_B2
zmp_ph108_B6
zmp_ph108_B7
zmp_ph108_C4
zmp_ph108_C5
zmp_ph108_D4
zmp_ph97_G6
zmp_ph97_G9
zmp_ph97_H2
zmp_ph97_B10
zmp_ph97_C2
zmp_ph97_C4
zmp_ph97_B8
zmp_ph97_C1
zmp_ph97_D2
zmp_ph97_D5
zmp_ph111_G5
zmp_ph111_G11
zmp_ph111_H1
zmp_ph111_B1a
zmp_ph111_B5
zmp_ph111_B8
zmp_ph111_B6
zmp_ph111_B7
zmp_ph111_B9
zmp_ph111_B10
zmp_ph114_G4
zmp_ph114_G11
zmp_ph114_H1
zmp_ph114_H2
zmp_ph114_A9
zmp_ph114_A11
zmp_ph114_B2
zmp_ph114_B4
zmp_ph114_D8
zmp_ph114_E2

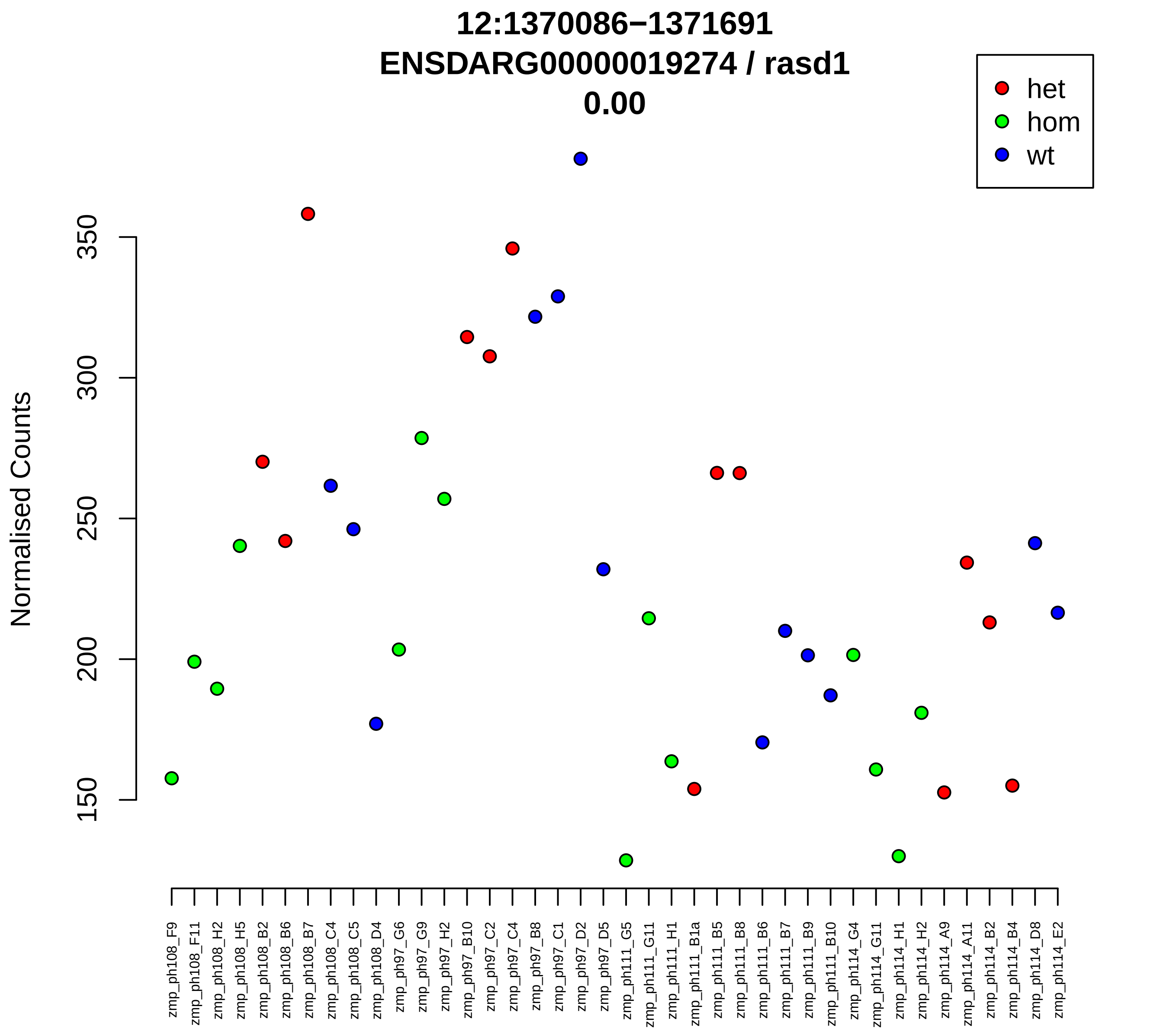
12:1370086−1371691
ENSDARG00000019274 / rasd1
0.00
het
hom
wt
Normalised Counts
150
200
250
300
350
zmp_ph108_F9
zmp_ph108_F11
zmp_ph108_H2
zmp_ph108_H5
zmp_ph108_B2
zmp_ph108_B6
zmp_ph108_B7
zmp_ph108_C4
zmp_ph108_C5
zmp_ph108_D4
zmp_ph97_G6
zmp_ph97_G9
zmp_ph97_H2
zmp_ph97_B10
zmp_ph97_C2
zmp_ph97_C4
zmp_ph97_B8
zmp_ph97_C1
zmp_ph97_D2
zmp_ph97_D5
zmp_ph111_G5
zmp_ph111_G11
zmp_ph111_H1
zmp_ph111_B1a
zmp_ph111_B5
zmp_ph111_B8
zmp_ph111_B6
zmp_ph111_B7
zmp_ph111_B9
zmp_ph111_B10
zmp_ph114_G4
zmp_ph114_G11
zmp_ph114_H1
zmp_ph114_H2
zmp_ph114_A9
zmp_ph114_A11
zmp_ph114_B2
zmp_ph114_B4
zmp_ph114_D8
zmp_ph114_E2

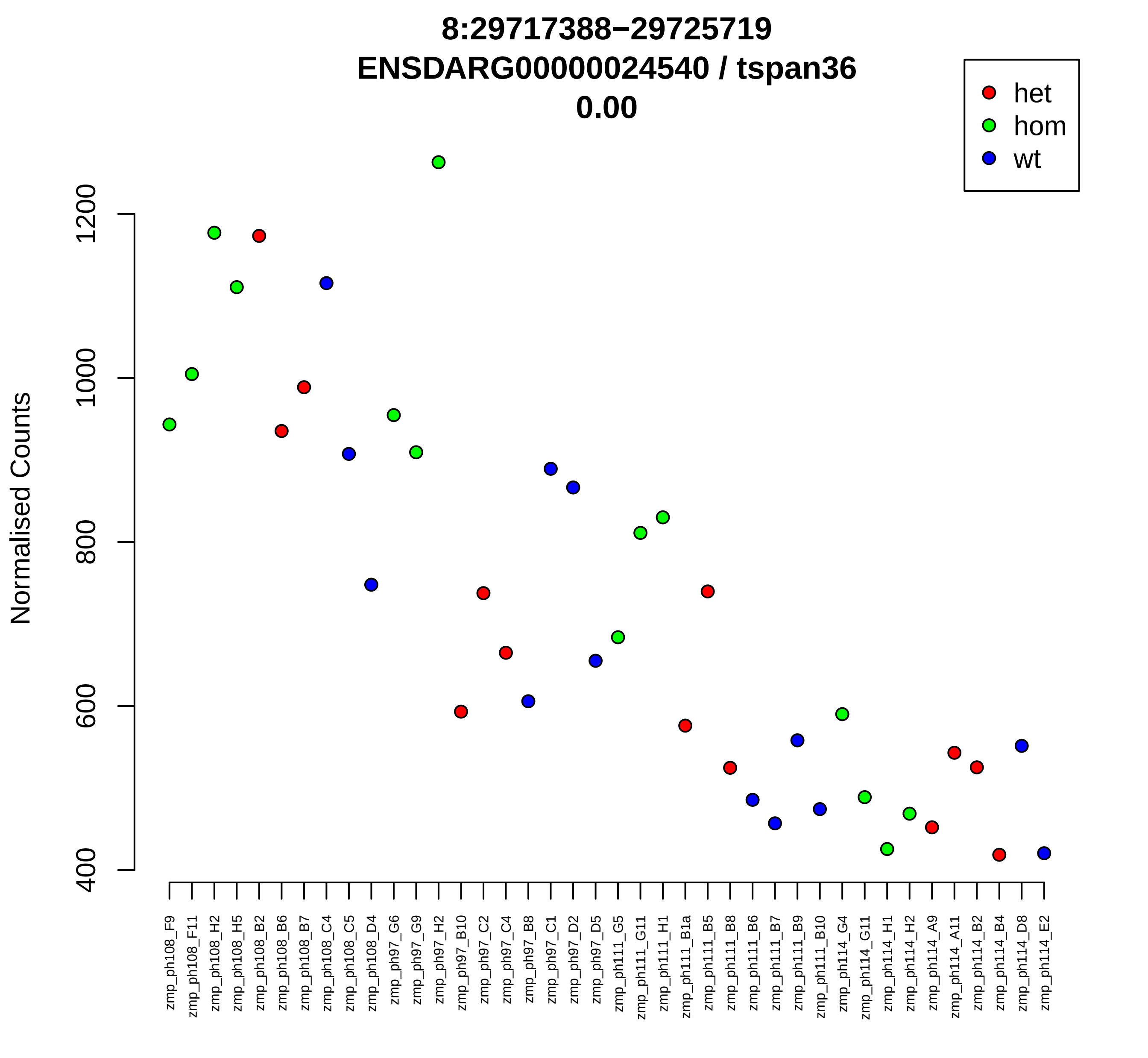
8:29717388−29725719
ENSDARG00000024540 / tspan36
0.00
het
hom
wt
Normalised Counts
400
600
800
1000
1200
zmp_ph108_F9
zmp_ph108_F11
zmp_ph108_H2
zmp_ph108_H5
zmp_ph108_B2
zmp_ph108_B6
zmp_ph108_B7
zmp_ph108_C4
zmp_ph108_C5
zmp_ph108_D4
zmp_ph97_G6
zmp_ph97_G9
zmp_ph97_H2
zmp_ph97_B10
zmp_ph97_C2
zmp_ph97_C4
zmp_ph97_B8
zmp_ph97_C1
zmp_ph97_D2
zmp_ph97_D5
zmp_ph111_G5
zmp_ph111_G11
zmp_ph111_H1
zmp_ph111_B1a
zmp_ph111_B5
zmp_ph111_B8
zmp_ph111_B6
zmp_ph111_B7
zmp_ph111_B9
zmp_ph111_B10
zmp_ph114_G4
zmp_ph114_G11
zmp_ph114_H1
zmp_ph114_H2
zmp_ph114_A9
zmp_ph114_A11
zmp_ph114_B2
zmp_ph114_B4
zmp_ph114_D8
zmp_ph114_E2

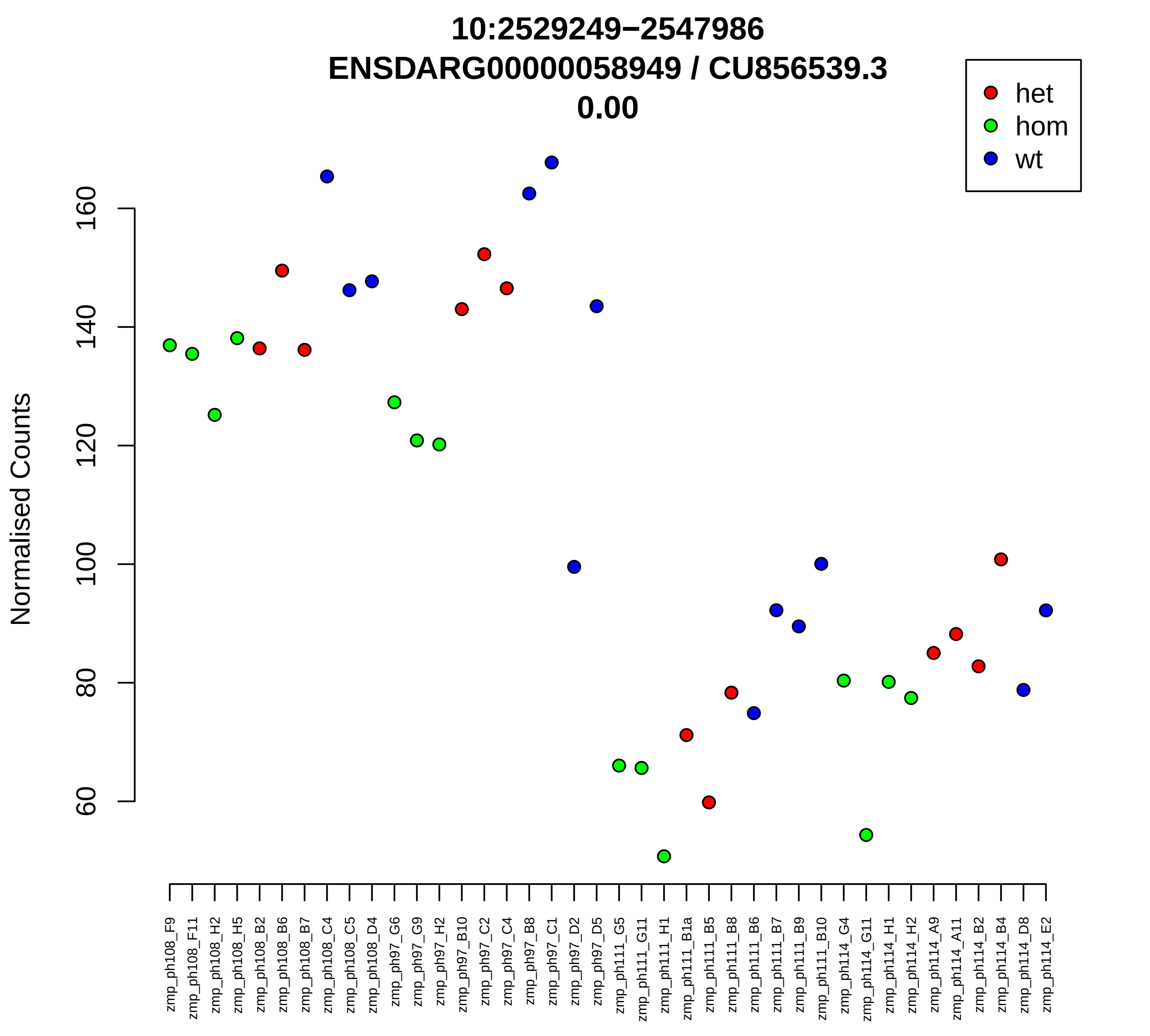

10:2529249−2547986
ENSDARG00000058949 / CU856539.3
0.00
het
hom
wt
Normalised Counts
60
80
100
120
140
160
zmp_ph108_F9
zmp_ph108_F11
zmp_ph108_H2
zmp_ph108_H5
zmp_ph108_B2
zmp_ph108_B6
zmp_ph108_B7
zmp_ph108_C4
zmp_ph108_C5
zmp_ph108_D4
zmp_ph97_G6
zmp_ph97_G9
zmp_ph97_H2
zmp_ph97_B10
zmp_ph97_C2
zmp_ph97_C4
zmp_ph97_B8
zmp_ph97_C1
zmp_ph97_D2
zmp_ph97_D5
zmp_ph111_G5
zmp_ph111_G11
zmp_ph111_H1
zmp_ph111_B1a
zmp_ph111_B5
zmp_ph111_B8
zmp_ph111_B6
zmp_ph111_B7
zmp_ph111_B9
zmp_ph111_B10
zmp_ph114_G4
zmp_ph114_G11
zmp_ph114_H1
zmp_ph114_H2
zmp_ph114_A9
zmp_ph114_A11
zmp_ph114_B2
zmp_ph114_B4
zmp_ph114_D8
zmp_ph114_E2

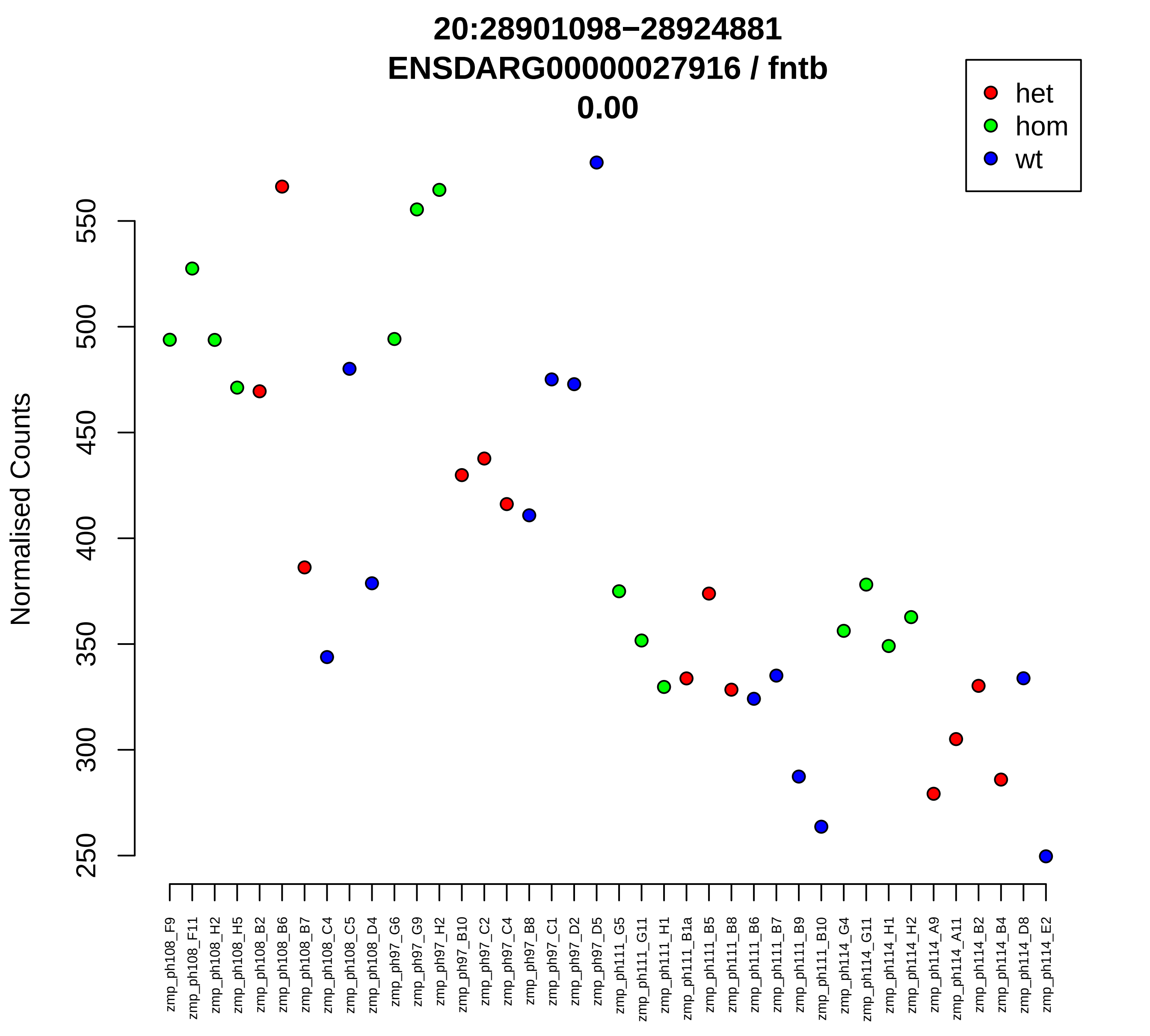

20:28901098−28924881
ENSDARG00000027916 / fntb
0.00
het
hom
wt
Normalised Counts
250
300
350
400
450
500
550
zmp_ph108_F9
zmp_ph108_F11
zmp_ph108_H2
zmp_ph108_H5
zmp_ph108_B2
zmp_ph108_B6
zmp_ph108_B7
zmp_ph108_C4
zmp_ph108_C5
zmp_ph108_D4
zmp_ph97_G6
zmp_ph97_G9
zmp_ph97_H2
zmp_ph97_B10
zmp_ph97_C2
zmp_ph97_C4
zmp_ph97_B8
zmp_ph97_C1
zmp_ph97_D2
zmp_ph97_D5
zmp_ph111_G5
zmp_ph111_G11
zmp_ph111_H1
zmp_ph111_B1a
zmp_ph111_B5
zmp_ph111_B8
zmp_ph111_B6
zmp_ph111_B7
zmp_ph111_B9
zmp_ph111_B10
zmp_ph114_G4
zmp_ph114_G11
zmp_ph114_H1
zmp_ph114_H2
zmp_ph114_A9
zmp_ph114_A11
zmp_ph114_B2
zmp_ph114_B4
zmp_ph114_D8
zmp_ph114_E2

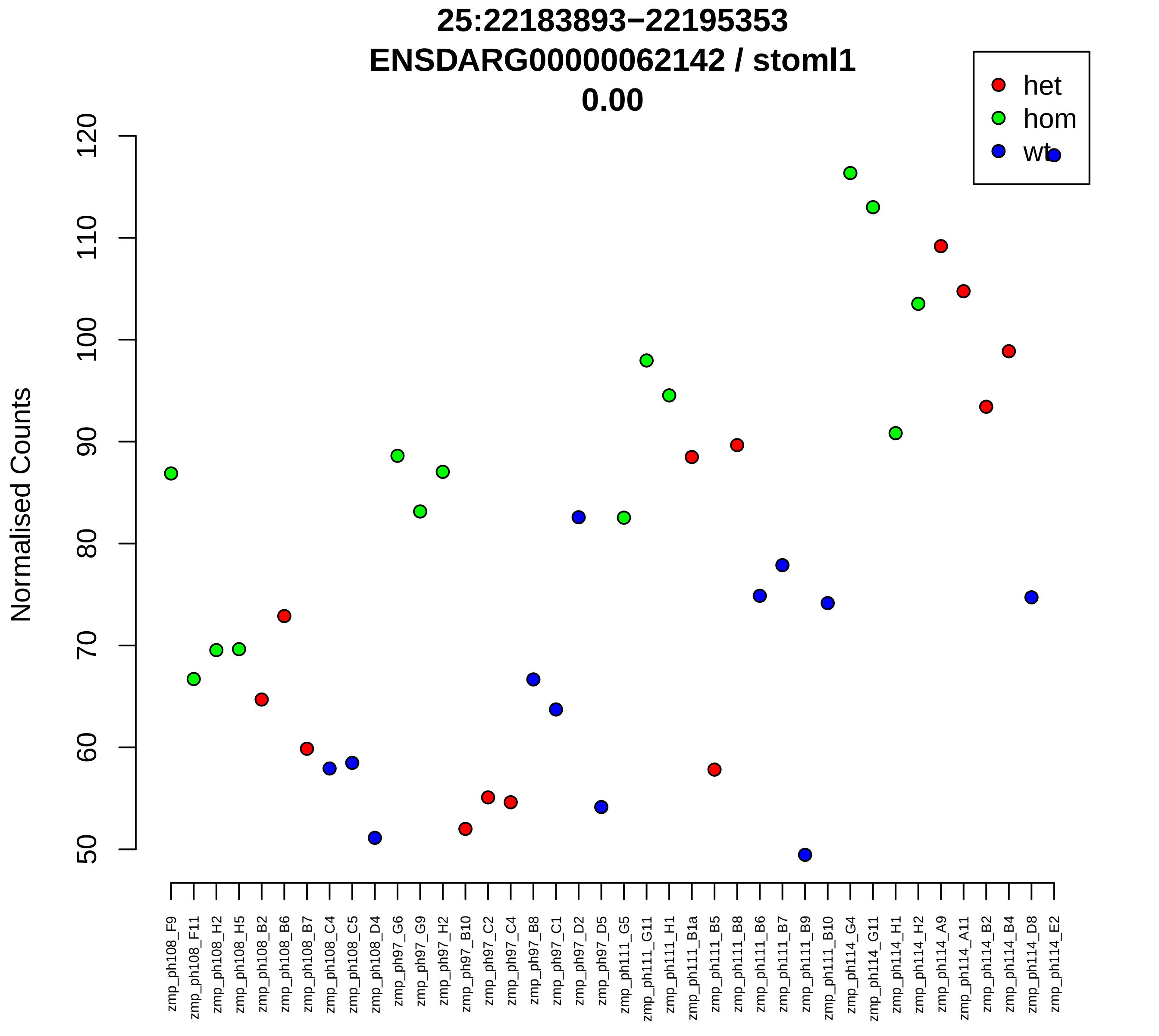

25:22183893−22195353
ENSDARG00000062142 / stoml1
0.00
het
hom
wt
Normalised Counts
50
60
70
80
90
100
110
120
zmp_ph108_F9
zmp_ph108_F11
zmp_ph108_H2
zmp_ph108_H5
zmp_ph108_B2
zmp_ph108_B6
zmp_ph108_B7
zmp_ph108_C4
zmp_ph108_C5
zmp_ph108_D4
zmp_ph97_G6
zmp_ph97_G9
zmp_ph97_H2
zmp_ph97_B10
zmp_ph97_C2
zmp_ph97_C4
zmp_ph97_B8
zmp_ph97_C1
zmp_ph97_D2
zmp_ph97_D5
zmp_ph111_G5
zmp_ph111_G11
zmp_ph111_H1
zmp_ph111_B1a
zmp_ph111_B5
zmp_ph111_B8
zmp_ph111_B6
zmp_ph111_B7
zmp_ph111_B9
zmp_ph111_B10
zmp_ph114_G4
zmp_ph114_G11
zmp_ph114_H1
zmp_ph114_H2
zmp_ph114_A9
zmp_ph114_A11
zmp_ph114_B2
zmp_ph114_B4
zmp_ph114_D8
zmp_ph114_E2

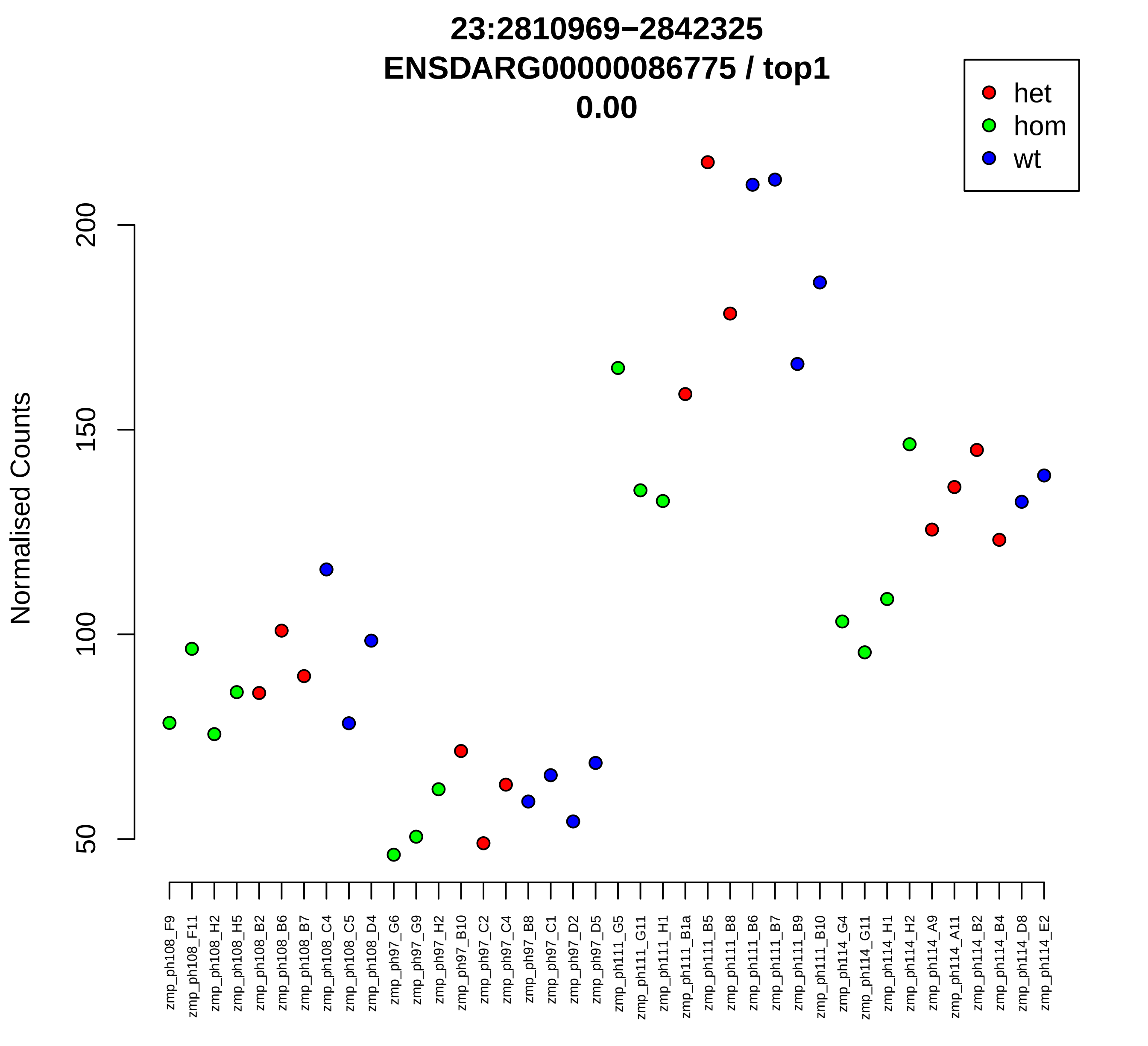

23:2810969−2842325
ENSDARG00000086775 / top1
0.00
het
hom
wt
Normalised Counts
50
100
150
200
zmp_ph108_F9
zmp_ph108_F11
zmp_ph108_H2
zmp_ph108_H5
zmp_ph108_B2
zmp_ph108_B6
zmp_ph108_B7
zmp_ph108_C4
zmp_ph108_C5
zmp_ph108_D4
zmp_ph97_G6
zmp_ph97_G9
zmp_ph97_H2
zmp_ph97_B10
zmp_ph97_C2
zmp_ph97_C4
zmp_ph97_B8
zmp_ph97_C1
zmp_ph97_D2
zmp_ph97_D5
zmp_ph111_G5
zmp_ph111_G11
zmp_ph111_H1
zmp_ph111_B1a
zmp_ph111_B5
zmp_ph111_B8
zmp_ph111_B6
zmp_ph111_B7
zmp_ph111_B9
zmp_ph111_B10
zmp_ph114_G4
zmp_ph114_G11
zmp_ph114_H1
zmp_ph114_H2
zmp_ph114_A9
zmp_ph114_A11
zmp_ph114_B2
zmp_ph114_B4
zmp_ph114_D8
zmp_ph114_E2

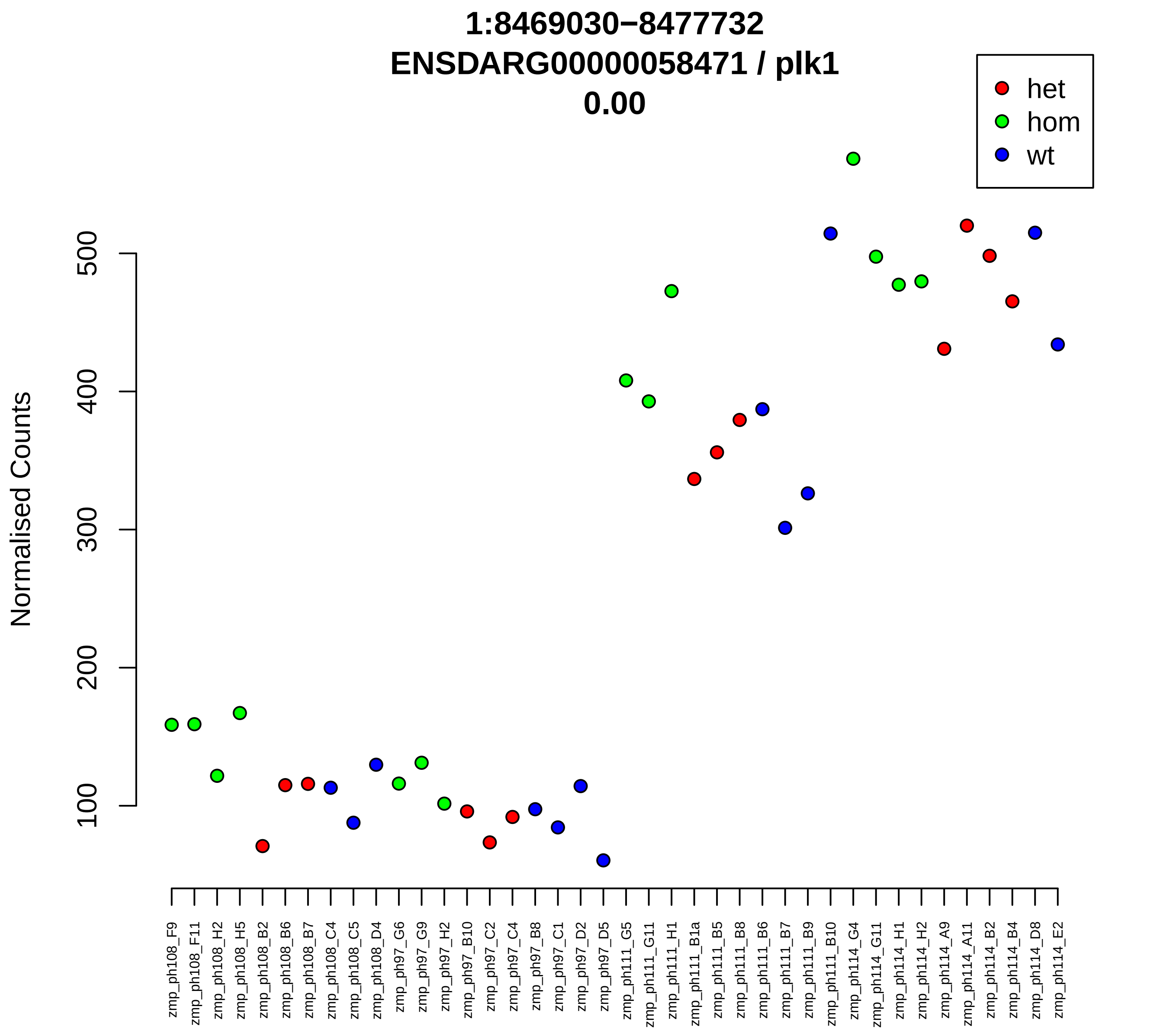

1:8469030−8477732
ENSDARG00000058471 / plk1
0.00
het
hom
wt
Normalised Counts
100
200
300
400
500
zmp_ph108_F9
zmp_ph108_F11
zmp_ph108_H2
zmp_ph108_H5
zmp_ph108_B2
zmp_ph108_B6
zmp_ph108_B7
zmp_ph108_C4
zmp_ph108_C5
zmp_ph108_D4
zmp_ph97_G6
zmp_ph97_G9
zmp_ph97_H2
zmp_ph97_B10
zmp_ph97_C2
zmp_ph97_C4
zmp_ph97_B8
zmp_ph97_C1
zmp_ph97_D2
zmp_ph97_D5
zmp_ph111_G5
zmp_ph111_G11
zmp_ph111_H1
zmp_ph111_B1a
zmp_ph111_B5
zmp_ph111_B8
zmp_ph111_B6
zmp_ph111_B7
zmp_ph111_B9
zmp_ph111_B10
zmp_ph114_G4
zmp_ph114_G11
zmp_ph114_H1
zmp_ph114_H2
zmp_ph114_A9
zmp_ph114_A11
zmp_ph114_B2
zmp_ph114_B4
zmp_ph114_D8
zmp_ph114_E2

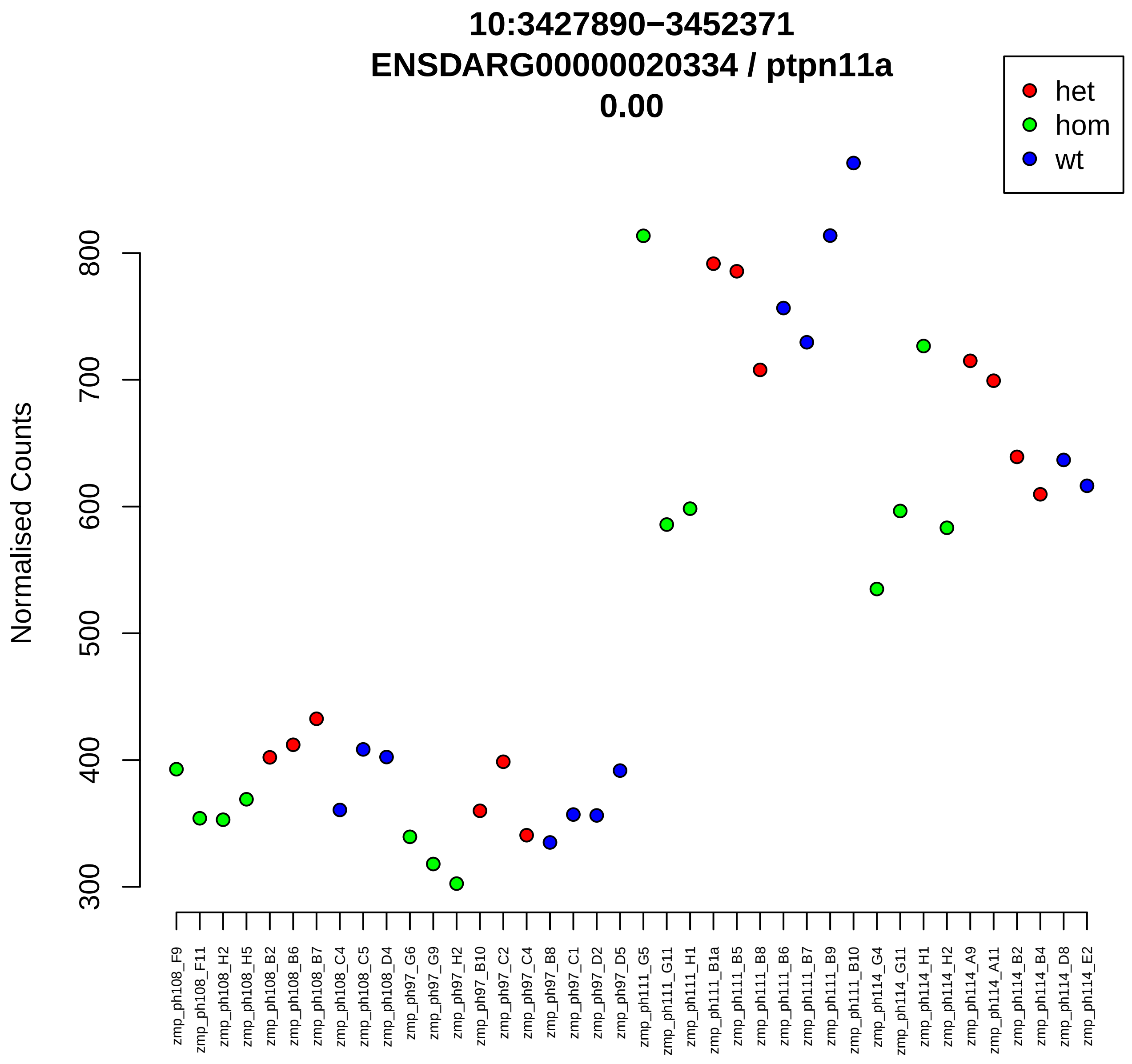
10:3427890−3452371
ENSDARG00000020334 / ptpn11a
0.00
het
hom
wt
Normalised Counts
300
400
500
600
700
800
zmp_ph108_F9
zmp_ph108_F11
zmp_ph108_H2
zmp_ph108_H5
zmp_ph108_B2
zmp_ph108_B6
zmp_ph108_B7
zmp_ph108_C4
zmp_ph108_C5
zmp_ph108_D4
zmp_ph97_G6
zmp_ph97_G9
zmp_ph97_H2
zmp_ph97_B10
zmp_ph97_C2
zmp_ph97_C4
zmp_ph97_B8
zmp_ph97_C1
zmp_ph97_D2
zmp_ph97_D5
zmp_ph111_G5
zmp_ph111_G11
zmp_ph111_H1
zmp_ph111_B1a
zmp_ph111_B5
zmp_ph111_B8
zmp_ph111_B6
zmp_ph111_B7
zmp_ph111_B9
zmp_ph111_B10
zmp_ph114_G4
zmp_ph114_G11
zmp_ph114_H1
zmp_ph114_H2
zmp_ph114_A9
zmp_ph114_A11
zmp_ph114_B2
zmp_ph114_B4
zmp_ph114_D8
zmp_ph114_E2

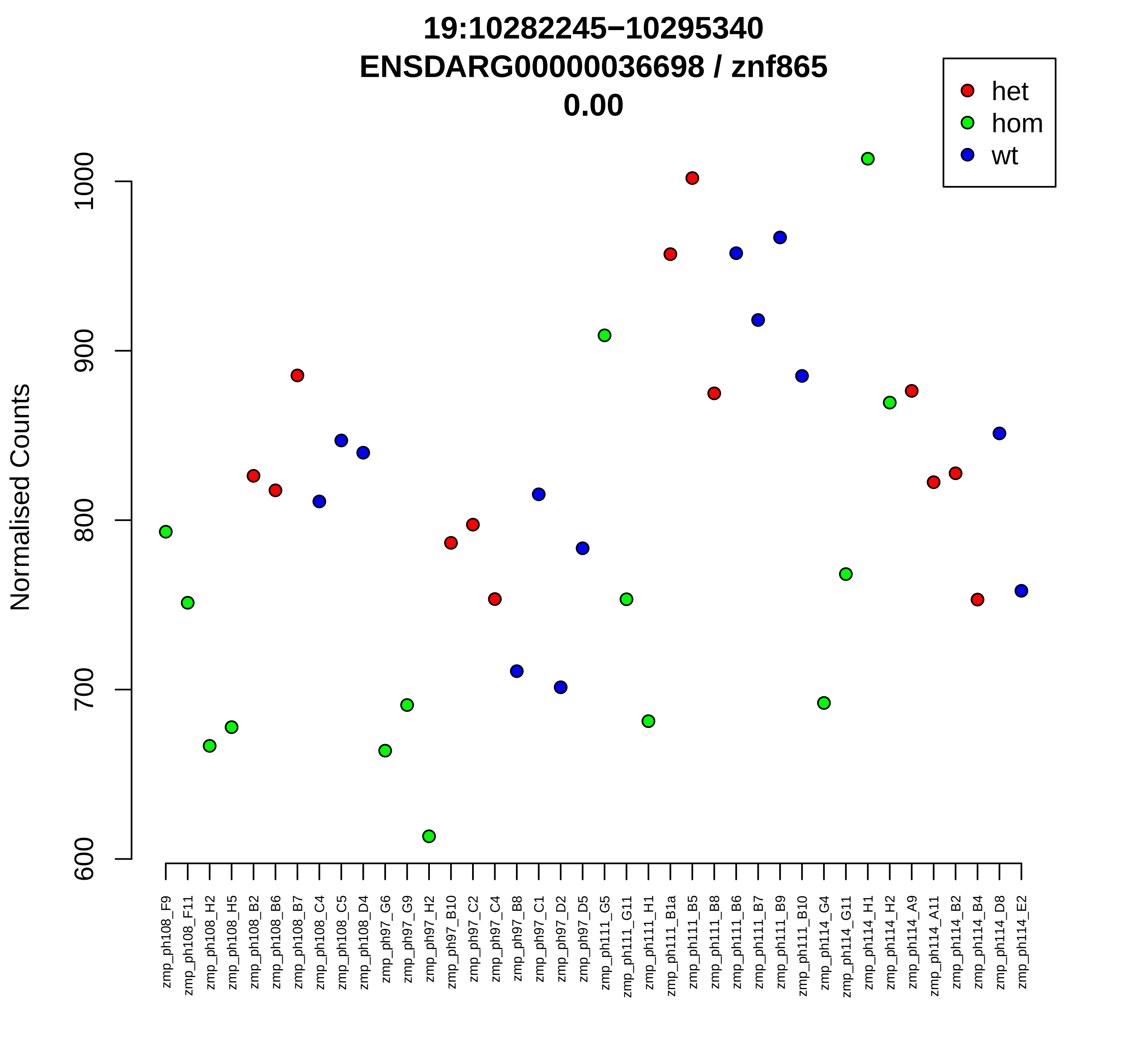

19:10282245−10295340
ENSDARG00000036698 / znf865
0.00
het
hom
wt
Normalised Counts
600
700
800
900
1000
zmp_ph108_F9
zmp_ph108_F11
zmp_ph108_H2
zmp_ph108_H5
zmp_ph108_B2
zmp_ph108_B6
zmp_ph108_B7
zmp_ph108_C4
zmp_ph108_C5
zmp_ph108_D4
zmp_ph97_G6
zmp_ph97_G9
zmp_ph97_H2
zmp_ph97_B10
zmp_ph97_C2
zmp_ph97_C4
zmp_ph97_B8
zmp_ph97_C1
zmp_ph97_D2
zmp_ph97_D5
zmp_ph111_G5
zmp_ph111_G11
zmp_ph111_H1
zmp_ph111_B1a
zmp_ph111_B5
zmp_ph111_B8
zmp_ph111_B6
zmp_ph111_B7
zmp_ph111_B9
zmp_ph111_B10
zmp_ph114_G4
zmp_ph114_G11
zmp_ph114_H1
zmp_ph114_H2
zmp_ph114_A9
zmp_ph114_A11
zmp_ph114_B2
zmp_ph114_B4
zmp_ph114_D8
zmp_ph114_E2

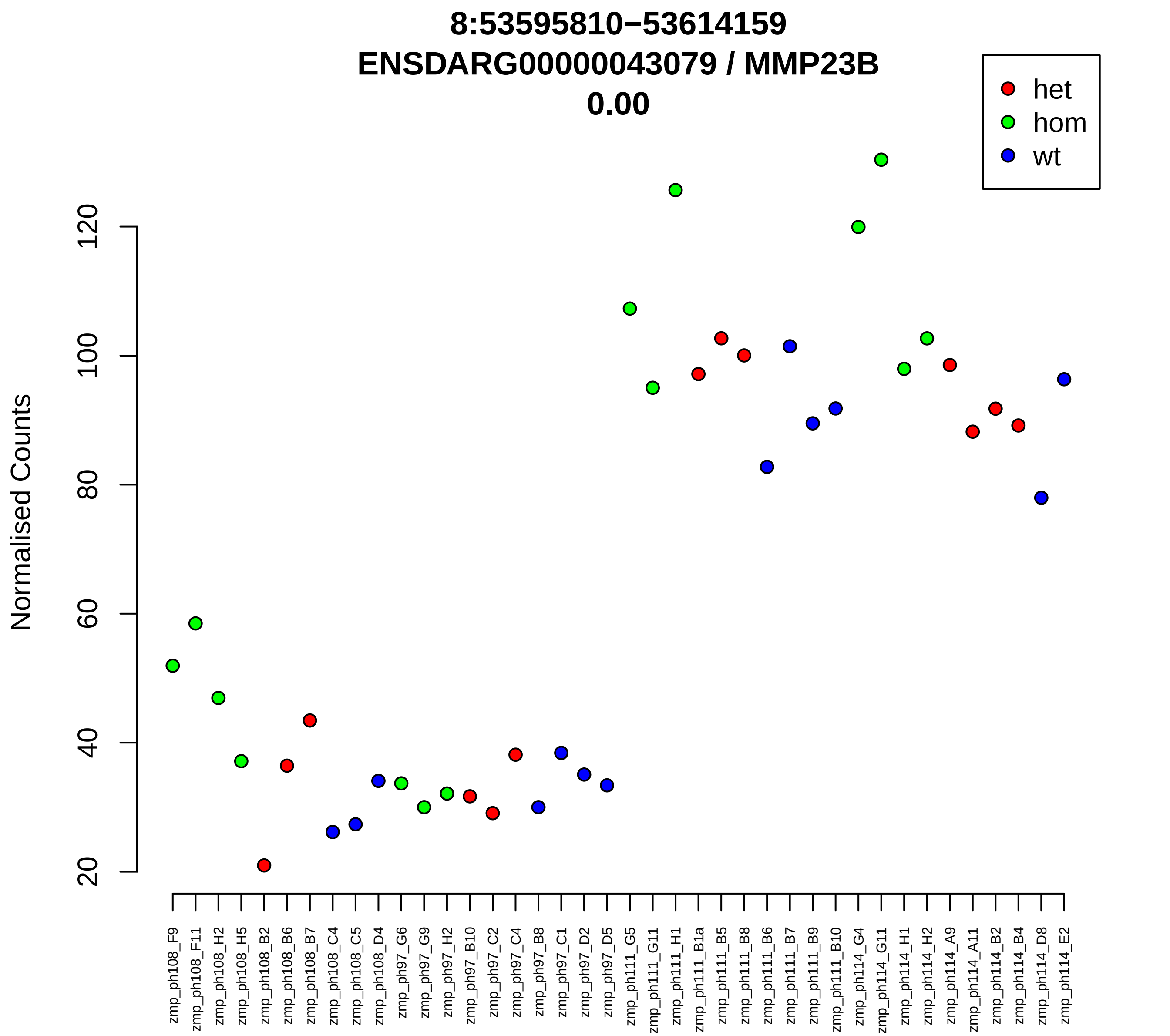

8:53595810−53614159
ENSDARG00000043079 / MMP23B
0.00
het
hom
wt
Normalised Counts
20
40
60
80
100
120
zmp_ph108_F9
zmp_ph108_F11
zmp_ph108_H2
zmp_ph108_H5
zmp_ph108_B2
zmp_ph108_B6
zmp_ph108_B7
zmp_ph108_C4
zmp_ph108_C5
zmp_ph108_D4
zmp_ph97_G6
zmp_ph97_G9
zmp_ph97_H2
zmp_ph97_B10
zmp_ph97_C2
zmp_ph97_C4
zmp_ph97_B8
zmp_ph97_C1
zmp_ph97_D2
zmp_ph97_D5
zmp_ph111_G5
zmp_ph111_G11
zmp_ph111_H1
zmp_ph111_B1a
zmp_ph111_B5
zmp_ph111_B8
zmp_ph111_B6
zmp_ph111_B7
zmp_ph111_B9
zmp_ph111_B10
zmp_ph114_G4
zmp_ph114_G11
zmp_ph114_H1
zmp_ph114_H2
zmp_ph114_A9
zmp_ph114_A11
zmp_ph114_B2
zmp_ph114_B4
zmp_ph114_D8
zmp_ph114_E2

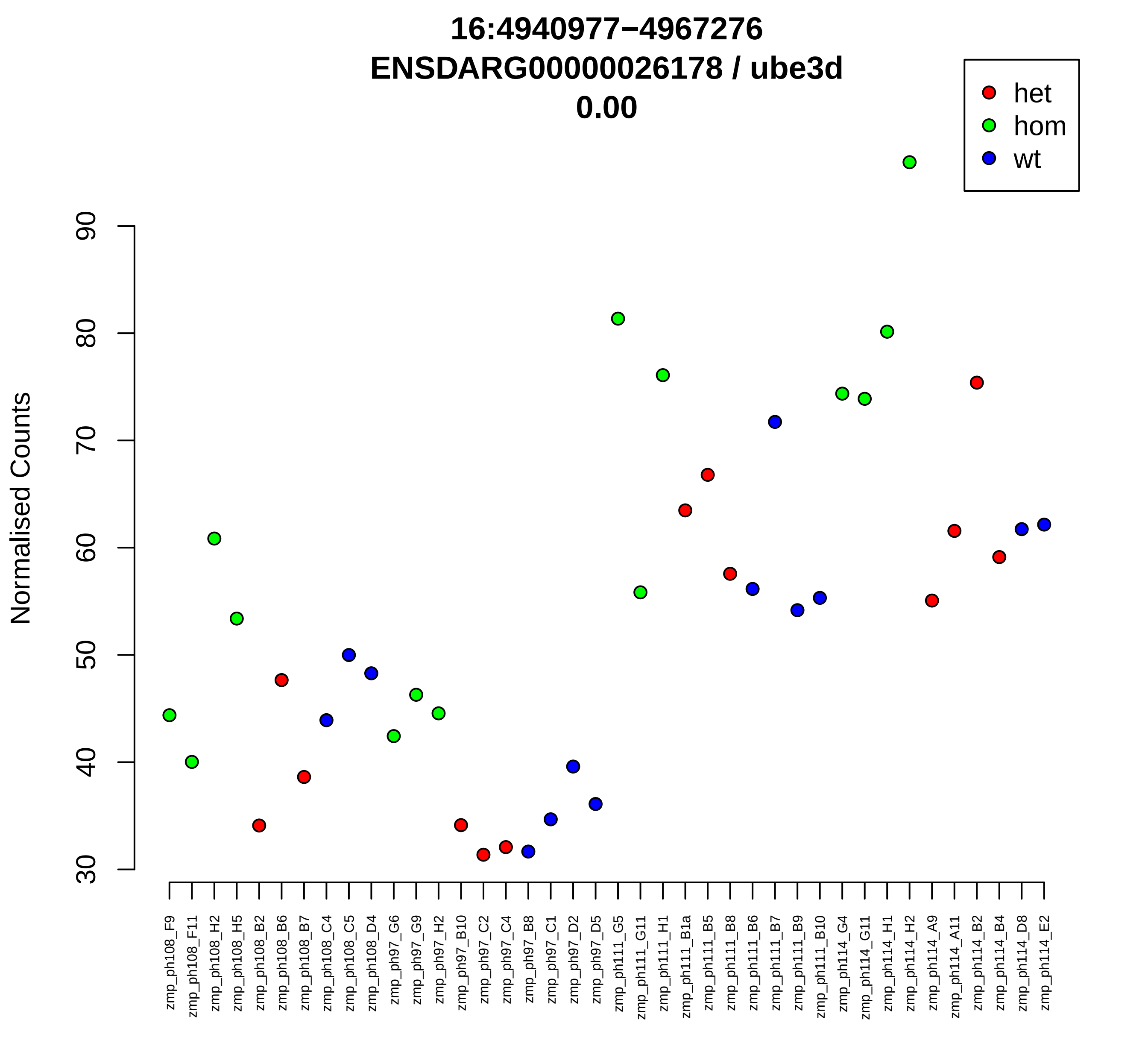
16:4940977−4967276
ENSDARG00000026178 / ube3d
0.00
het
hom
wt
Normalised Counts
30
40
50
60
70
80
90
zmp_ph108_F9
zmp_ph108_F11
zmp_ph108_H2
zmp_ph108_H5
zmp_ph108_B2
zmp_ph108_B6
zmp_ph108_B7
zmp_ph108_C4
zmp_ph108_C5
zmp_ph108_D4
zmp_ph97_G6
zmp_ph97_G9
zmp_ph97_H2
zmp_ph97_B10
zmp_ph97_C2
zmp_ph97_C4
zmp_ph97_B8
zmp_ph97_C1
zmp_ph97_D2
zmp_ph97_D5
zmp_ph111_G5
zmp_ph111_G11
zmp_ph111_H1
zmp_ph111_B1a
zmp_ph111_B5
zmp_ph111_B8
zmp_ph111_B6
zmp_ph111_B7
zmp_ph111_B9
zmp_ph111_B10
zmp_ph114_G4
zmp_ph114_G11
zmp_ph114_H1
zmp_ph114_H2
zmp_ph114_A9
zmp_ph114_A11
zmp_ph114_B2
zmp_ph114_B4
zmp_ph114_D8
zmp_ph114_E2

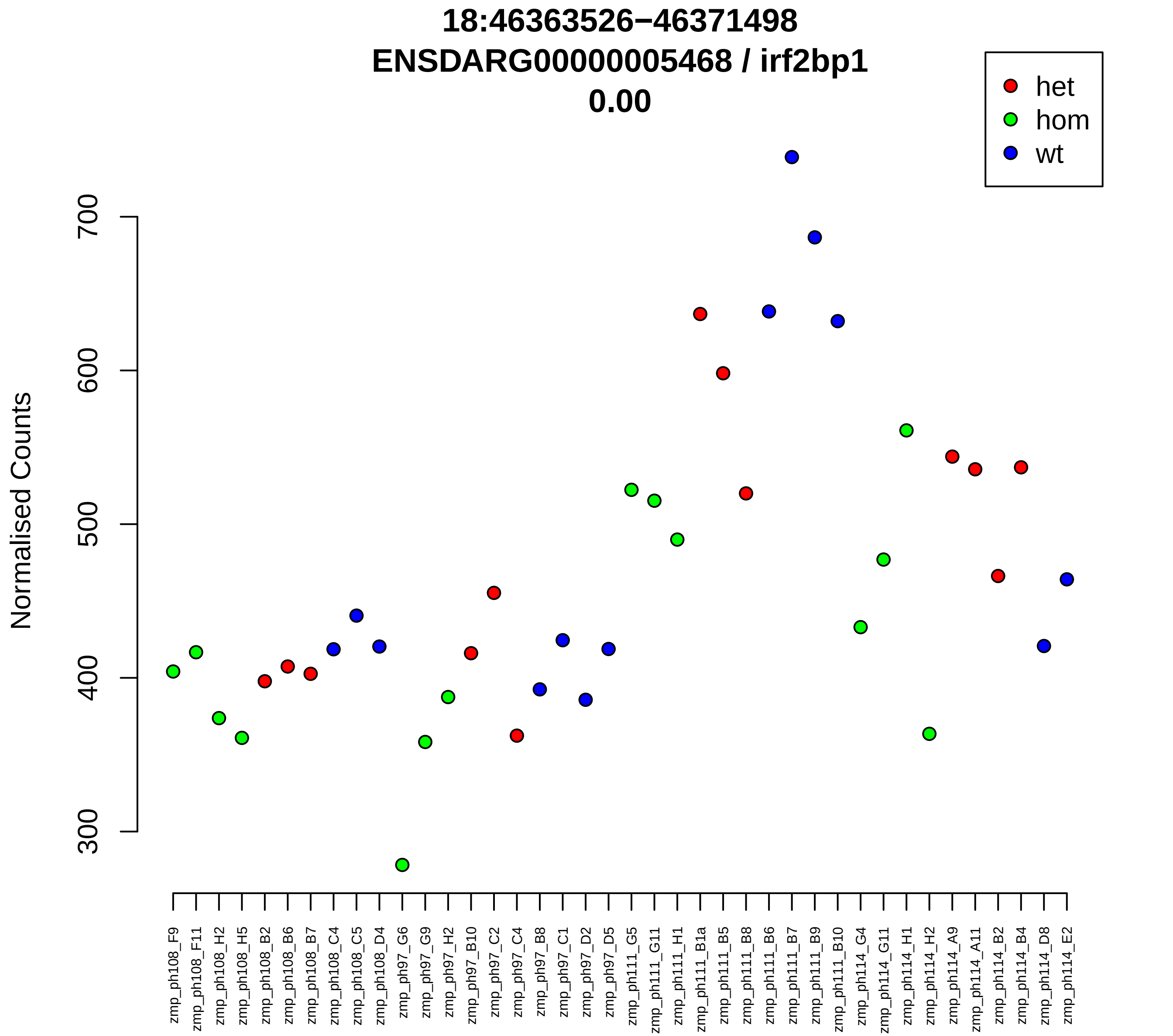

18:46363526−46371498
ENSDARG00000005468 / irf2bp1
0.00
het
hom
wt
Normalised Counts
300
400
500
600
700
zmp_ph108_F9
zmp_ph108_F11
zmp_ph108_H2
zmp_ph108_H5
zmp_ph108_B2
zmp_ph108_B6
zmp_ph108_B7
zmp_ph108_C4
zmp_ph108_C5
zmp_ph108_D4
zmp_ph97_G6
zmp_ph97_G9
zmp_ph97_H2
zmp_ph97_B10
zmp_ph97_C2
zmp_ph97_C4
zmp_ph97_B8
zmp_ph97_C1
zmp_ph97_D2
zmp_ph97_D5
zmp_ph111_G5
zmp_ph111_G11
zmp_ph111_H1
zmp_ph111_B1a
zmp_ph111_B5
zmp_ph111_B8
zmp_ph111_B6
zmp_ph111_B7
zmp_ph111_B9
zmp_ph111_B10
zmp_ph114_G4
zmp_ph114_G11
zmp_ph114_H1
zmp_ph114_H2
zmp_ph114_A9
zmp_ph114_A11
zmp_ph114_B2
zmp_ph114_B4
zmp_ph114_D8
zmp_ph114_E2

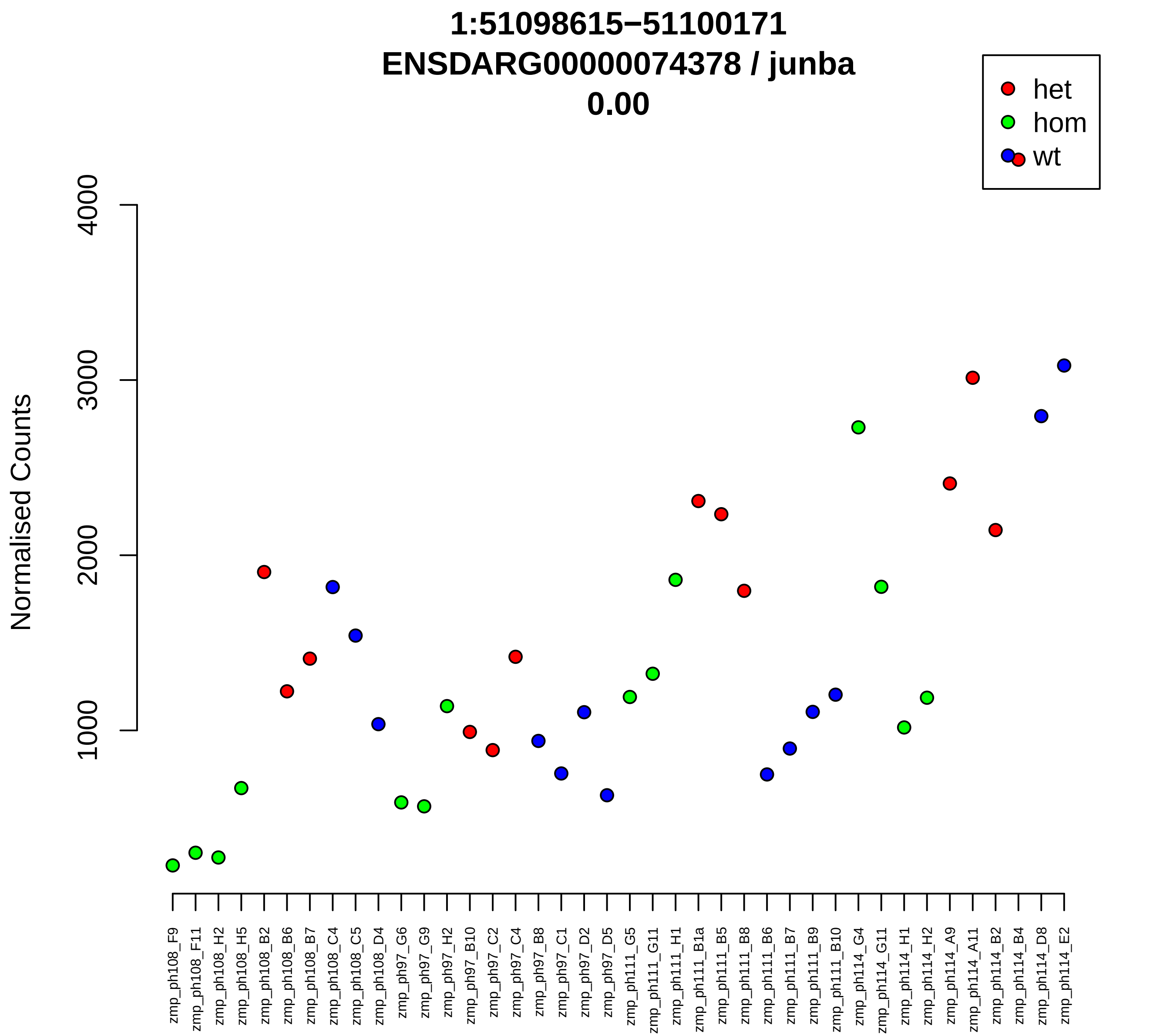

1:51098615−51100171
ENSDARG00000074378 / junba
0.00
het
hom
wt
Normalised Counts
1000
2000
3000
4000
zmp_ph108_F9
zmp_ph108_F11
zmp_ph108_H2
zmp_ph108_H5
zmp_ph108_B2
zmp_ph108_B6
zmp_ph108_B7
zmp_ph108_C4
zmp_ph108_C5
zmp_ph108_D4
zmp_ph97_G6
zmp_ph97_G9
zmp_ph97_H2
zmp_ph97_B10
zmp_ph97_C2
zmp_ph97_C4
zmp_ph97_B8
zmp_ph97_C1
zmp_ph97_D2
zmp_ph97_D5
zmp_ph111_G5
zmp_ph111_G11
zmp_ph111_H1
zmp_ph111_B1a
zmp_ph111_B5
zmp_ph111_B8
zmp_ph111_B6
zmp_ph111_B7
zmp_ph111_B9
zmp_ph111_B10
zmp_ph114_G4
zmp_ph114_G11
zmp_ph114_H1
zmp_ph114_H2
zmp_ph114_A9
zmp_ph114_A11
zmp_ph114_B2
zmp_ph114_B4
zmp_ph114_D8
zmp_ph114_E2

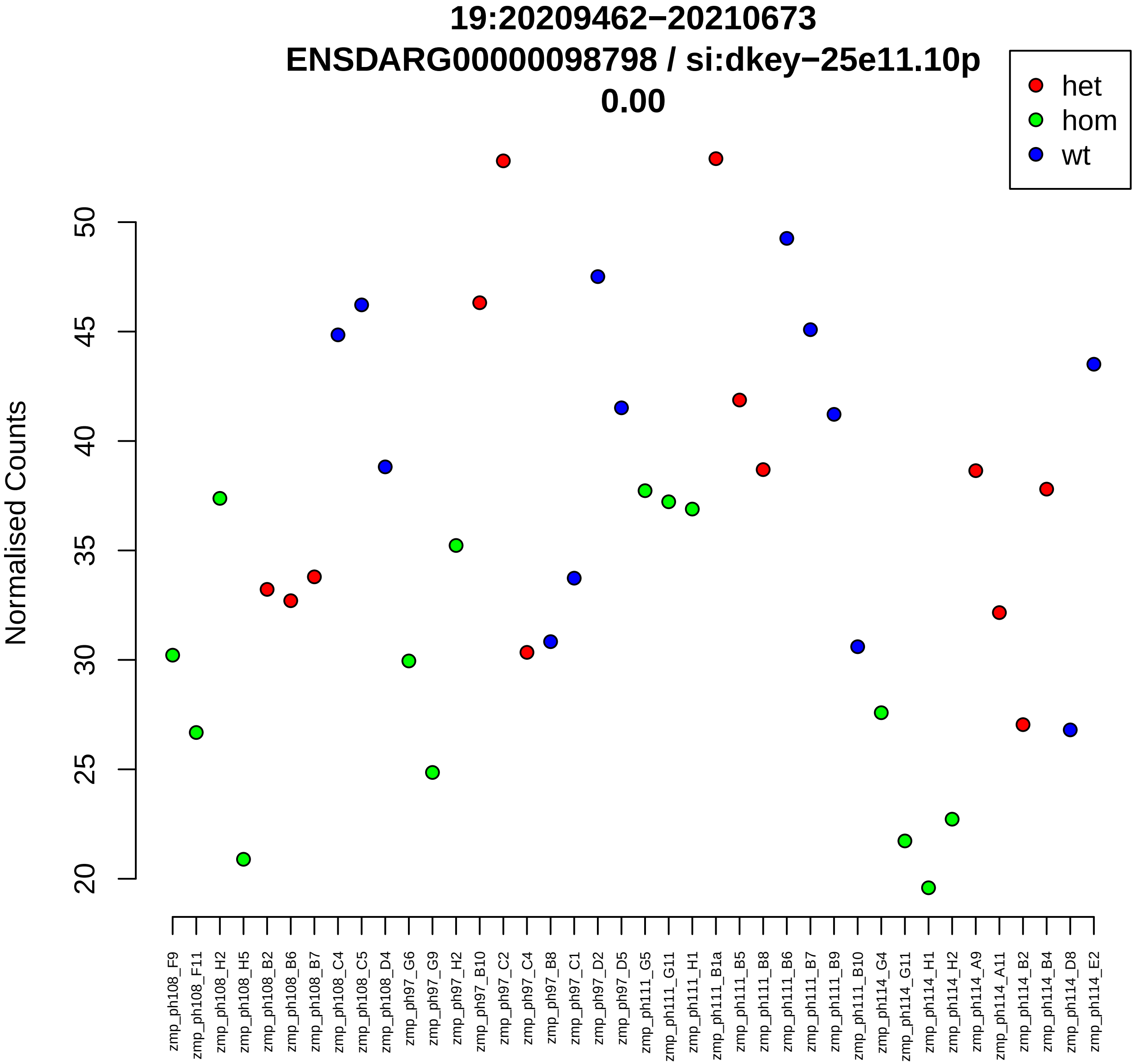
19:20209462−20210673
ENSDARG00000098798 / si:dkey−25e11.10p
0.00
het
hom
wt
Normalised Counts
20
25
30
35
40
45
50
zmp_ph108_F9
zmp_ph108_F11
zmp_ph108_H2
zmp_ph108_H5
zmp_ph108_B2
zmp_ph108_B6
zmp_ph108_B7
zmp_ph108_C4
zmp_ph108_C5
zmp_ph108_D4
zmp_ph97_G6
zmp_ph97_G9
zmp_ph97_H2
zmp_ph97_B10
zmp_ph97_C2
zmp_ph97_C4
zmp_ph97_B8
zmp_ph97_C1
zmp_ph97_D2
zmp_ph97_D5
zmp_ph111_G5
zmp_ph111_G11
zmp_ph111_H1
zmp_ph111_B1a
zmp_ph111_B5
zmp_ph111_B8
zmp_ph111_B6
zmp_ph111_B7
zmp_ph111_B9
zmp_ph111_B10
zmp_ph114_G4
zmp_ph114_G11
zmp_ph114_H1
zmp_ph114_H2
zmp_ph114_A9
zmp_ph114_A11
zmp_ph114_B2
zmp_ph114_B4
zmp_ph114_D8
zmp_ph114_E2

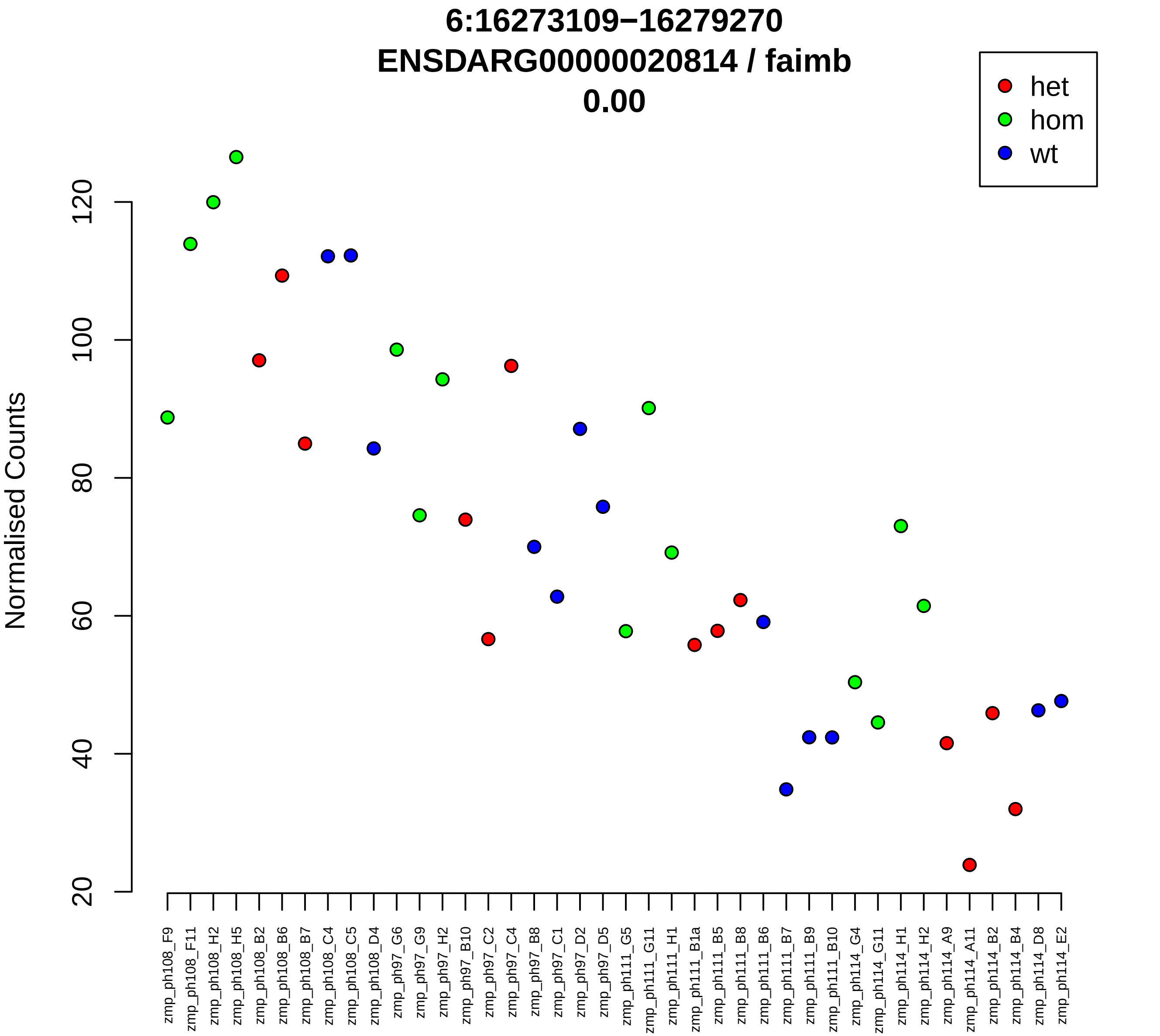
6:16273109−16279270
ENSDARG00000020814 / faimb
0.00
het
hom
wt
Normalised Counts
20
40
60
80
100
120
zmp_ph108_F9
zmp_ph108_F11
zmp_ph108_H2
zmp_ph108_H5
zmp_ph108_B2
zmp_ph108_B6
zmp_ph108_B7
zmp_ph108_C4
zmp_ph108_C5
zmp_ph108_D4
zmp_ph97_G6
zmp_ph97_G9
zmp_ph97_H2
zmp_ph97_B10
zmp_ph97_C2
zmp_ph97_C4
zmp_ph97_B8
zmp_ph97_C1
zmp_ph97_D2
zmp_ph97_D5
zmp_ph111_G5
zmp_ph111_G11
zmp_ph111_H1
zmp_ph111_B1a
zmp_ph111_B5
zmp_ph111_B8
zmp_ph111_B6
zmp_ph111_B7
zmp_ph111_B9
zmp_ph111_B10
zmp_ph114_G4
zmp_ph114_G11
zmp_ph114_H1
zmp_ph114_H2
zmp_ph114_A9
zmp_ph114_A11
zmp_ph114_B2
zmp_ph114_B4
zmp_ph114_D8
zmp_ph114_E2

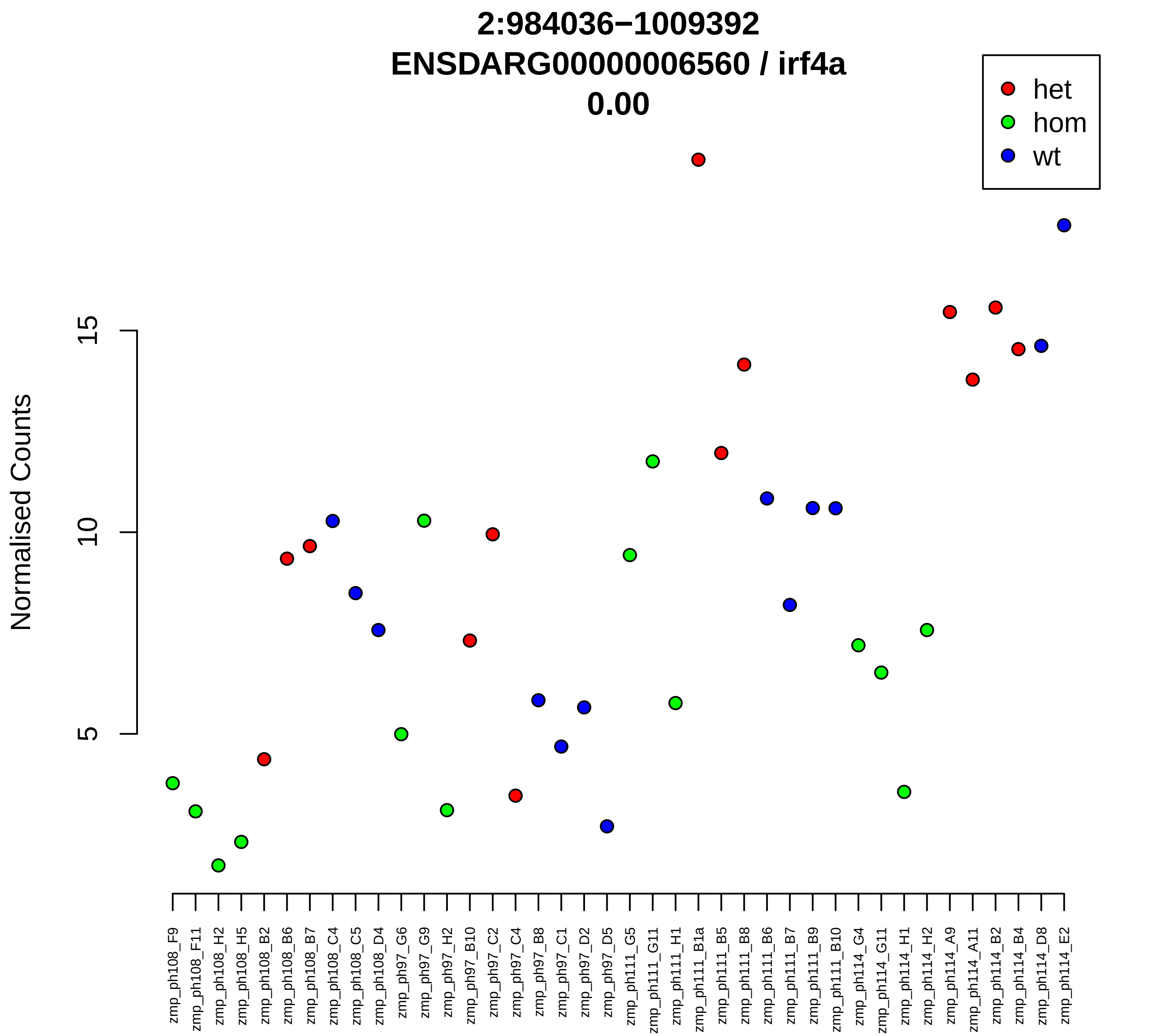
2:984036−1009392
ENSDARG00000006560 / irf4a
0.00
het
hom
wt
Normalised Counts
5
10
15
zmp_ph108_F9
zmp_ph108_F11
zmp_ph108_H2
zmp_ph108_H5
zmp_ph108_B2
zmp_ph108_B6
zmp_ph108_B7
zmp_ph108_C4
zmp_ph108_C5
zmp_ph108_D4
zmp_ph97_G6
zmp_ph97_G9
zmp_ph97_H2
zmp_ph97_B10
zmp_ph97_C2
zmp_ph97_C4
zmp_ph97_B8
zmp_ph97_C1
zmp_ph97_D2
zmp_ph97_D5
zmp_ph111_G5
zmp_ph111_G11
zmp_ph111_H1
zmp_ph111_B1a
zmp_ph111_B5
zmp_ph111_B8
zmp_ph111_B6
zmp_ph111_B7
zmp_ph111_B9
zmp_ph111_B10
zmp_ph114_G4
zmp_ph114_G11
zmp_ph114_H1
zmp_ph114_H2
zmp_ph114_A9
zmp_ph114_A11
zmp_ph114_B2
zmp_ph114_B4
zmp_ph114_D8
zmp_ph114_E2

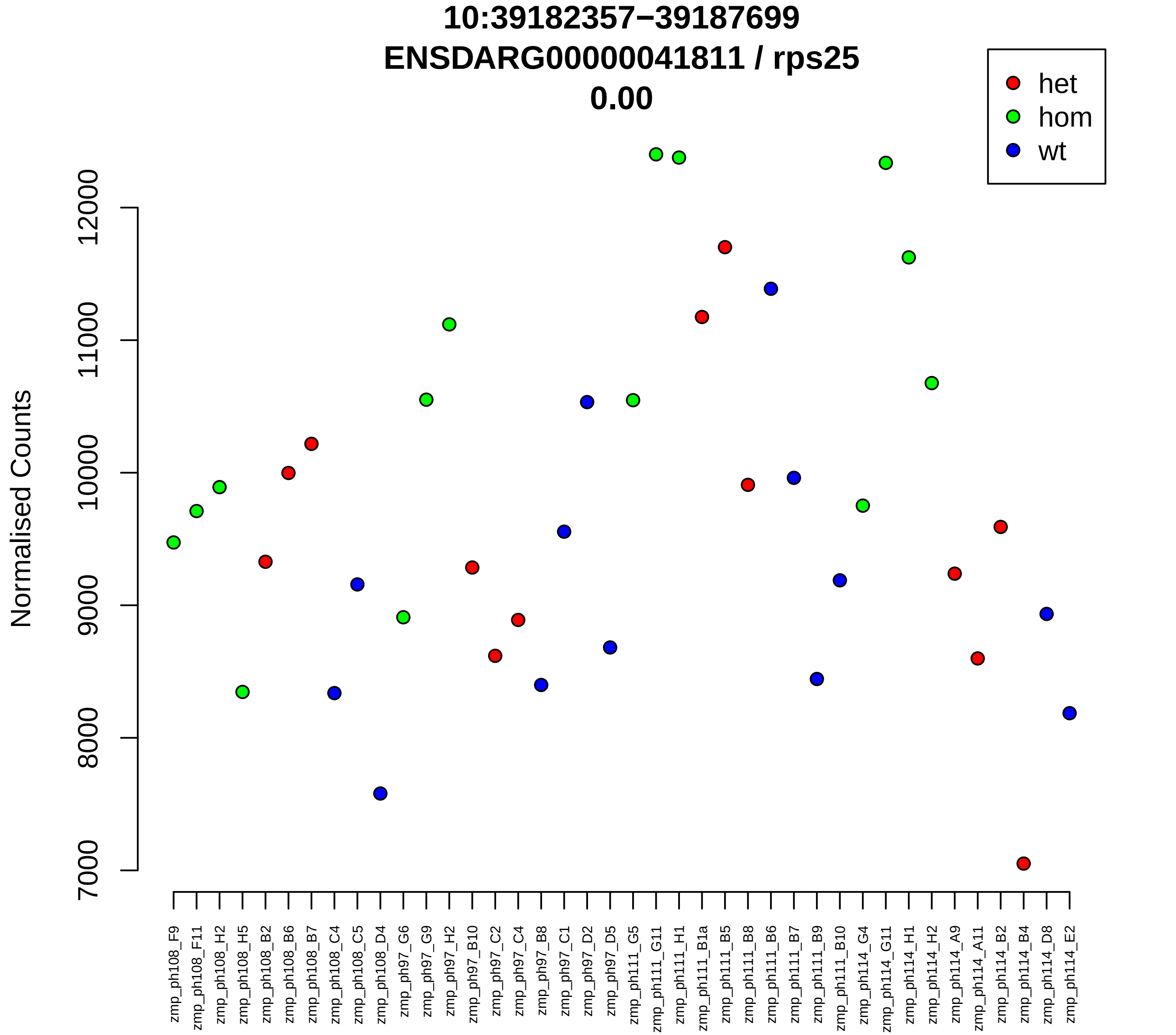

10:39182357−39187699
ENSDARG00000041811 / rps25
0.00
het
hom
wt
Normalised Counts
7000
8000
9000
10000
11000
12000
zmp_ph108_F9
zmp_ph108_F11
zmp_ph108_H2
zmp_ph108_H5
zmp_ph108_B2
zmp_ph108_B6
zmp_ph108_B7
zmp_ph108_C4
zmp_ph108_C5
zmp_ph108_D4
zmp_ph97_G6
zmp_ph97_G9
zmp_ph97_H2
zmp_ph97_B10
zmp_ph97_C2
zmp_ph97_C4
zmp_ph97_B8
zmp_ph97_C1
zmp_ph97_D2
zmp_ph97_D5
zmp_ph111_G5
zmp_ph111_G11
zmp_ph111_H1
zmp_ph111_B1a
zmp_ph111_B5
zmp_ph111_B8
zmp_ph111_B6
zmp_ph111_B7
zmp_ph111_B9
zmp_ph111_B10
zmp_ph114_G4
zmp_ph114_G11
zmp_ph114_H1
zmp_ph114_H2
zmp_ph114_A9
zmp_ph114_A11
zmp_ph114_B2
zmp_ph114_B4
zmp_ph114_D8
zmp_ph114_E2

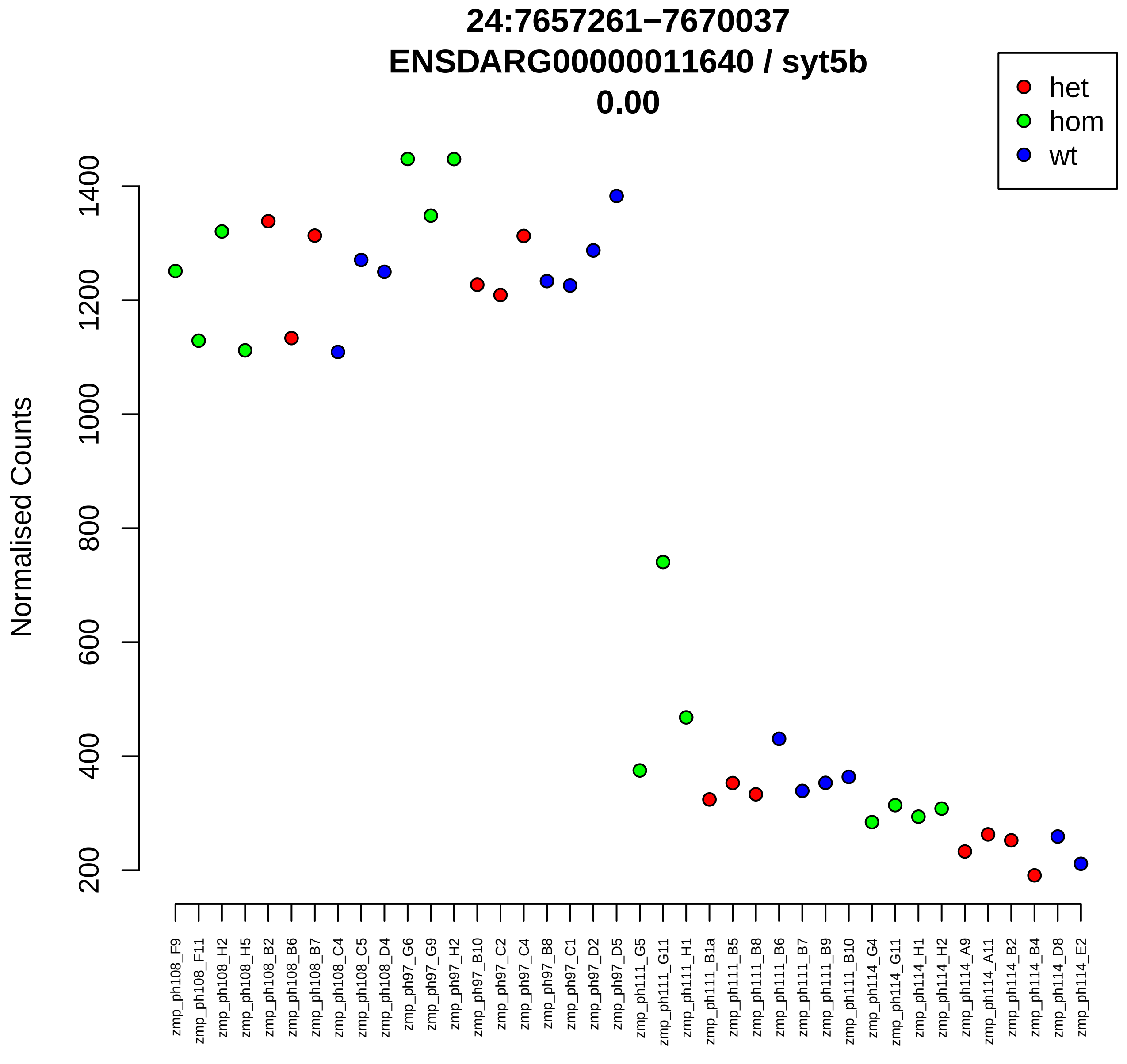

24:7657261−7670037
ENSDARG00000011640 / syt5b
0.00
het
hom
wt
Normalised Counts
200
400
600
800
1000
1200
1400
zmp_ph108_F9
zmp_ph108_F11
zmp_ph108_H2
zmp_ph108_H5
zmp_ph108_B2
zmp_ph108_B6
zmp_ph108_B7
zmp_ph108_C4
zmp_ph108_C5
zmp_ph108_D4
zmp_ph97_G6
zmp_ph97_G9
zmp_ph97_H2
zmp_ph97_B10
zmp_ph97_C2
zmp_ph97_C4
zmp_ph97_B8
zmp_ph97_C1
zmp_ph97_D2
zmp_ph97_D5
zmp_ph111_G5
zmp_ph111_G11
zmp_ph111_H1
zmp_ph111_B1a
zmp_ph111_B5
zmp_ph111_B8
zmp_ph111_B6
zmp_ph111_B7
zmp_ph111_B9
zmp_ph111_B10
zmp_ph114_G4
zmp_ph114_G11
zmp_ph114_H1
zmp_ph114_H2
zmp_ph114_A9
zmp_ph114_A11
zmp_ph114_B2
zmp_ph114_B4
zmp_ph114_D8
zmp_ph114_E2

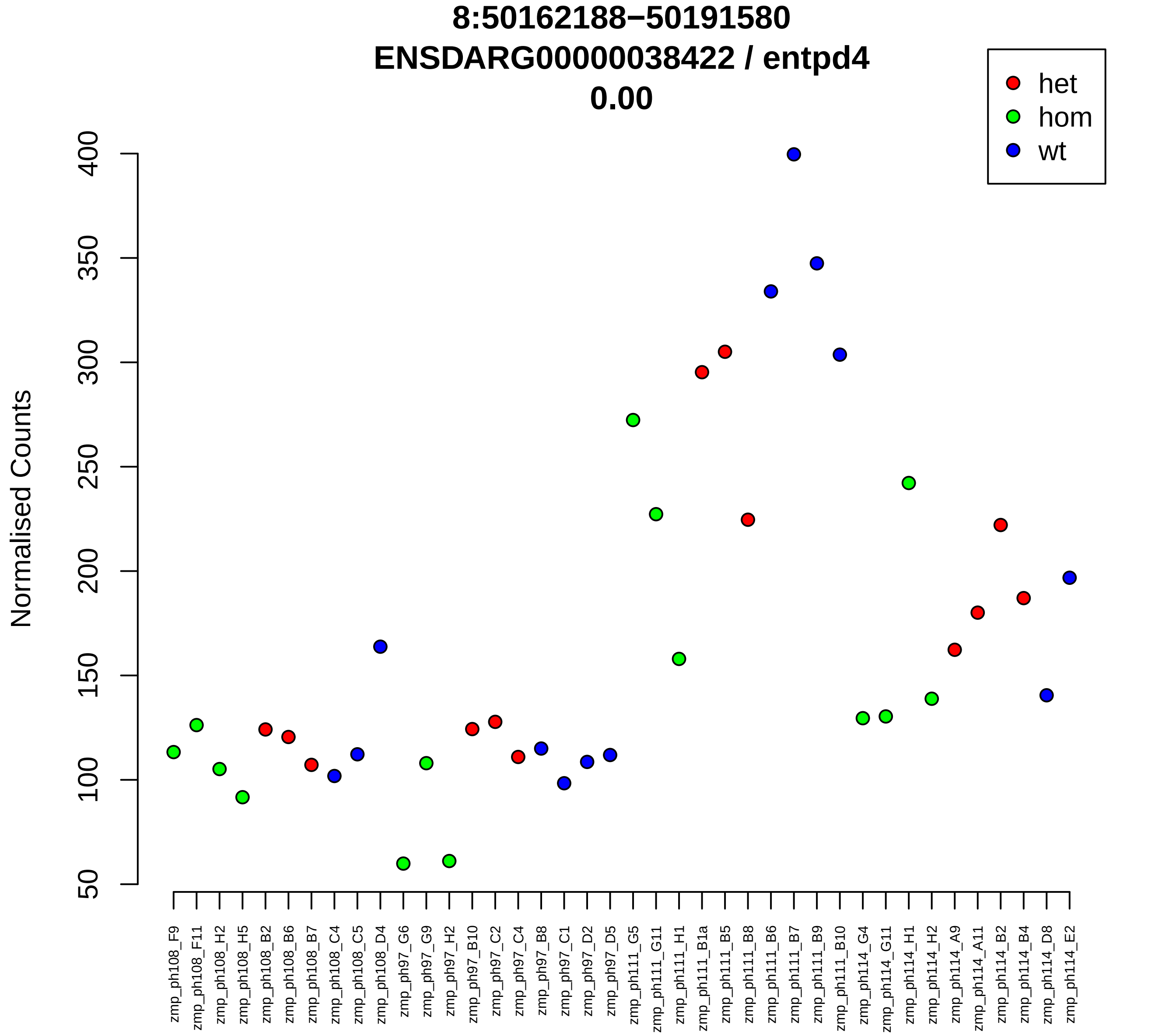
8:50162188−50191580
ENSDARG00000038422 / entpd4
0.00
het
hom
wt
Normalised Counts
50
100
150
200
250
300
350
400
zmp_ph108_F9
zmp_ph108_F11
zmp_ph108_H2
zmp_ph108_H5
zmp_ph108_B2
zmp_ph108_B6
zmp_ph108_B7
zmp_ph108_C4
zmp_ph108_C5
zmp_ph108_D4
zmp_ph97_G6
zmp_ph97_G9
zmp_ph97_H2
zmp_ph97_B10
zmp_ph97_C2
zmp_ph97_C4
zmp_ph97_B8
zmp_ph97_C1
zmp_ph97_D2
zmp_ph97_D5
zmp_ph111_G5
zmp_ph111_G11
zmp_ph111_H1
zmp_ph111_B1a
zmp_ph111_B5
zmp_ph111_B8
zmp_ph111_B6
zmp_ph111_B7
zmp_ph111_B9
zmp_ph111_B10
zmp_ph114_G4
zmp_ph114_G11
zmp_ph114_H1
zmp_ph114_H2
zmp_ph114_A9
zmp_ph114_A11
zmp_ph114_B2
zmp_ph114_B4
zmp_ph114_D8
zmp_ph114_E2

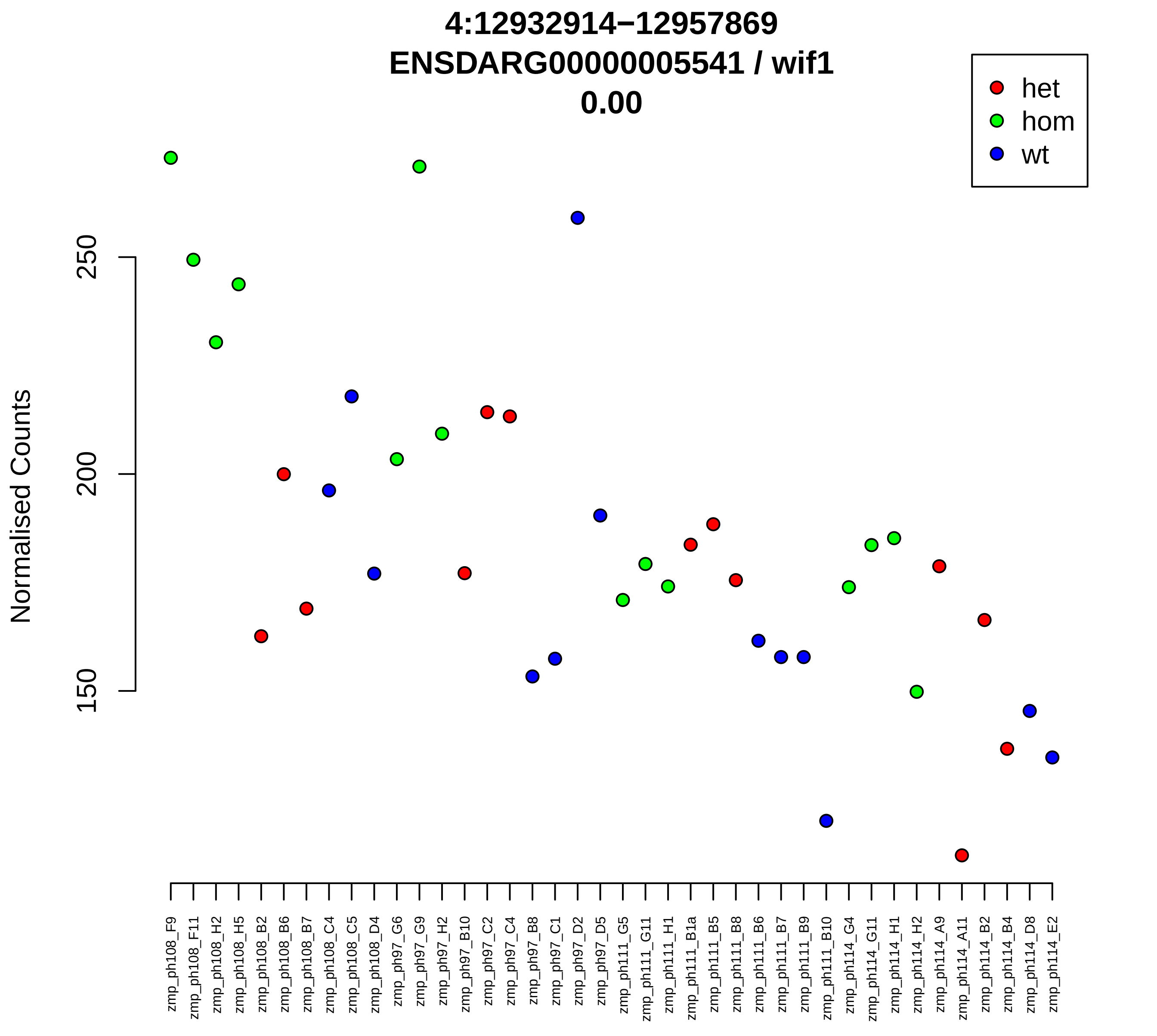

4:12932914−12957869
ENSDARG00000005541 / wif1
0.00
het
hom
wt
Normalised Counts
150
200
250
zmp_ph108_F9
zmp_ph108_F11
zmp_ph108_H2
zmp_ph108_H5
zmp_ph108_B2
zmp_ph108_B6
zmp_ph108_B7
zmp_ph108_C4
zmp_ph108_C5
zmp_ph108_D4
zmp_ph97_G6
zmp_ph97_G9
zmp_ph97_H2
zmp_ph97_B10
zmp_ph97_C2
zmp_ph97_C4
zmp_ph97_B8
zmp_ph97_C1
zmp_ph97_D2
zmp_ph97_D5
zmp_ph111_G5
zmp_ph111_G11
zmp_ph111_H1
zmp_ph111_B1a
zmp_ph111_B5
zmp_ph111_B8
zmp_ph111_B6
zmp_ph111_B7
zmp_ph111_B9
zmp_ph111_B10
zmp_ph114_G4
zmp_ph114_G11
zmp_ph114_H1
zmp_ph114_H2
zmp_ph114_A9
zmp_ph114_A11
zmp_ph114_B2
zmp_ph114_B4
zmp_ph114_D8
zmp_ph114_E2

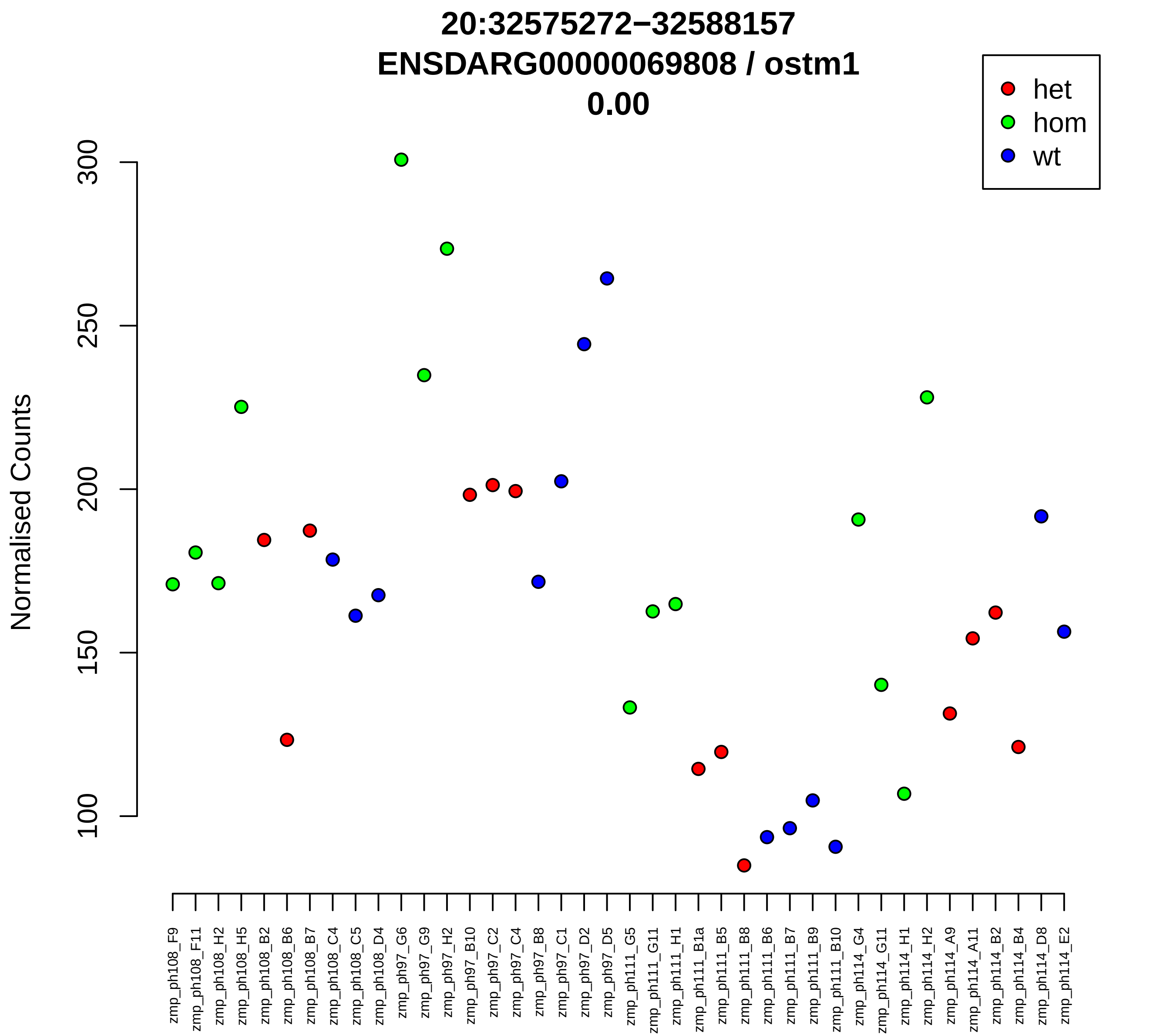
20:32575272−32588157
ENSDARG00000069808 / ostm1
0.00
het
hom
wt
Normalised Counts
100
150
200
250
300
zmp_ph108_F9
zmp_ph108_F11
zmp_ph108_H2
zmp_ph108_H5
zmp_ph108_B2
zmp_ph108_B6
zmp_ph108_B7
zmp_ph108_C4
zmp_ph108_C5
zmp_ph108_D4
zmp_ph97_G6
zmp_ph97_G9
zmp_ph97_H2
zmp_ph97_B10
zmp_ph97_C2
zmp_ph97_C4
zmp_ph97_B8
zmp_ph97_C1
zmp_ph97_D2
zmp_ph97_D5
zmp_ph111_G5
zmp_ph111_G11
zmp_ph111_H1
zmp_ph111_B1a
zmp_ph111_B5
zmp_ph111_B8
zmp_ph111_B6
zmp_ph111_B7
zmp_ph111_B9
zmp_ph111_B10
zmp_ph114_G4
zmp_ph114_G11
zmp_ph114_H1
zmp_ph114_H2
zmp_ph114_A9
zmp_ph114_A11
zmp_ph114_B2
zmp_ph114_B4
zmp_ph114_D8
zmp_ph114_E2

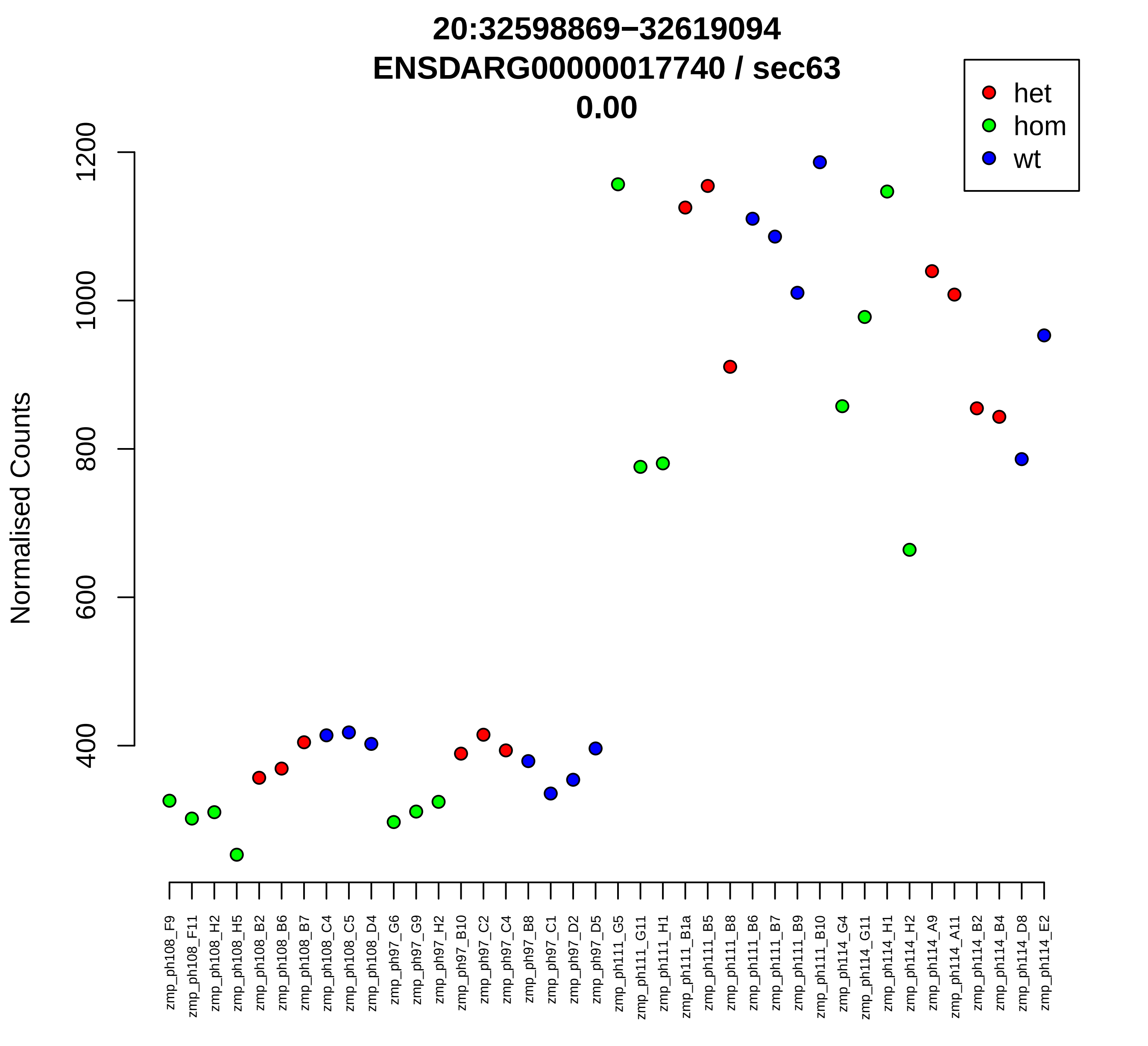

20:32598869−32619094
ENSDARG00000017740 / sec63
0.00
Normalised Counts
400
600
800
1000
1200
het
hom
wt
zmp_ph108_F9
zmp_ph108_F11
zmp_ph108_H2
zmp_ph108_H5
zmp_ph108_B2
zmp_ph108_B6
zmp_ph108_B7
zmp_ph108_C4
zmp_ph108_C5
zmp_ph108_D4
zmp_ph97_G6
zmp_ph97_G9
zmp_ph97_H2
zmp_ph97_B10
zmp_ph97_C2
zmp_ph97_C4
zmp_ph97_B8
zmp_ph97_C1
zmp_ph97_D2
zmp_ph97_D5
zmp_ph111_G5
zmp_ph111_G11
zmp_ph111_H1
zmp_ph111_B1a
zmp_ph111_B5
zmp_ph111_B8
zmp_ph111_B6
zmp_ph111_B7
zmp_ph111_B9
zmp_ph111_B10
zmp_ph114_G4
zmp_ph114_G11
zmp_ph114_H1
zmp_ph114_H2
zmp_ph114_A9
zmp_ph114_A11
zmp_ph114_B2
zmp_ph114_B4
zmp_ph114_D8
zmp_ph114_E2

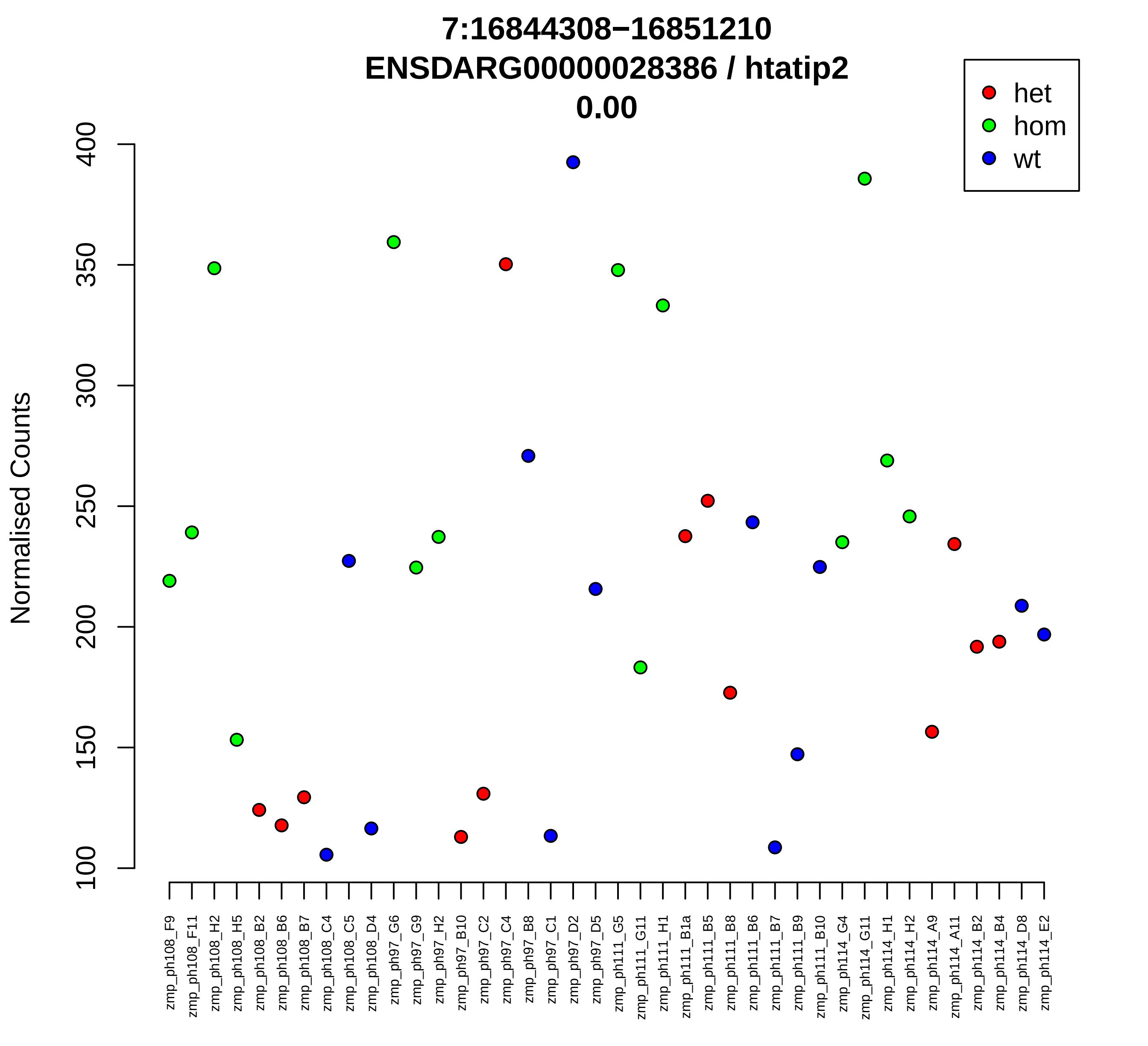

7:16844308−16851210
ENSDARG00000028386 / htatip2
0.00
het
hom
wt
Normalised Counts
100
150
200
250
300
350
400
zmp_ph108_F9
zmp_ph108_F11
zmp_ph108_H2
zmp_ph108_H5
zmp_ph108_B2
zmp_ph108_B6
zmp_ph108_B7
zmp_ph108_C4
zmp_ph108_C5
zmp_ph108_D4
zmp_ph97_G6
zmp_ph97_G9
zmp_ph97_H2
zmp_ph97_B10
zmp_ph97_C2
zmp_ph97_C4
zmp_ph97_B8
zmp_ph97_C1
zmp_ph97_D2
zmp_ph97_D5
zmp_ph111_G5
zmp_ph111_G11
zmp_ph111_H1
zmp_ph111_B1a
zmp_ph111_B5
zmp_ph111_B8
zmp_ph111_B6
zmp_ph111_B7
zmp_ph111_B9
zmp_ph111_B10
zmp_ph114_G4
zmp_ph114_G11
zmp_ph114_H1
zmp_ph114_H2
zmp_ph114_A9
zmp_ph114_A11
zmp_ph114_B2
zmp_ph114_B4
zmp_ph114_D8
zmp_ph114_E2

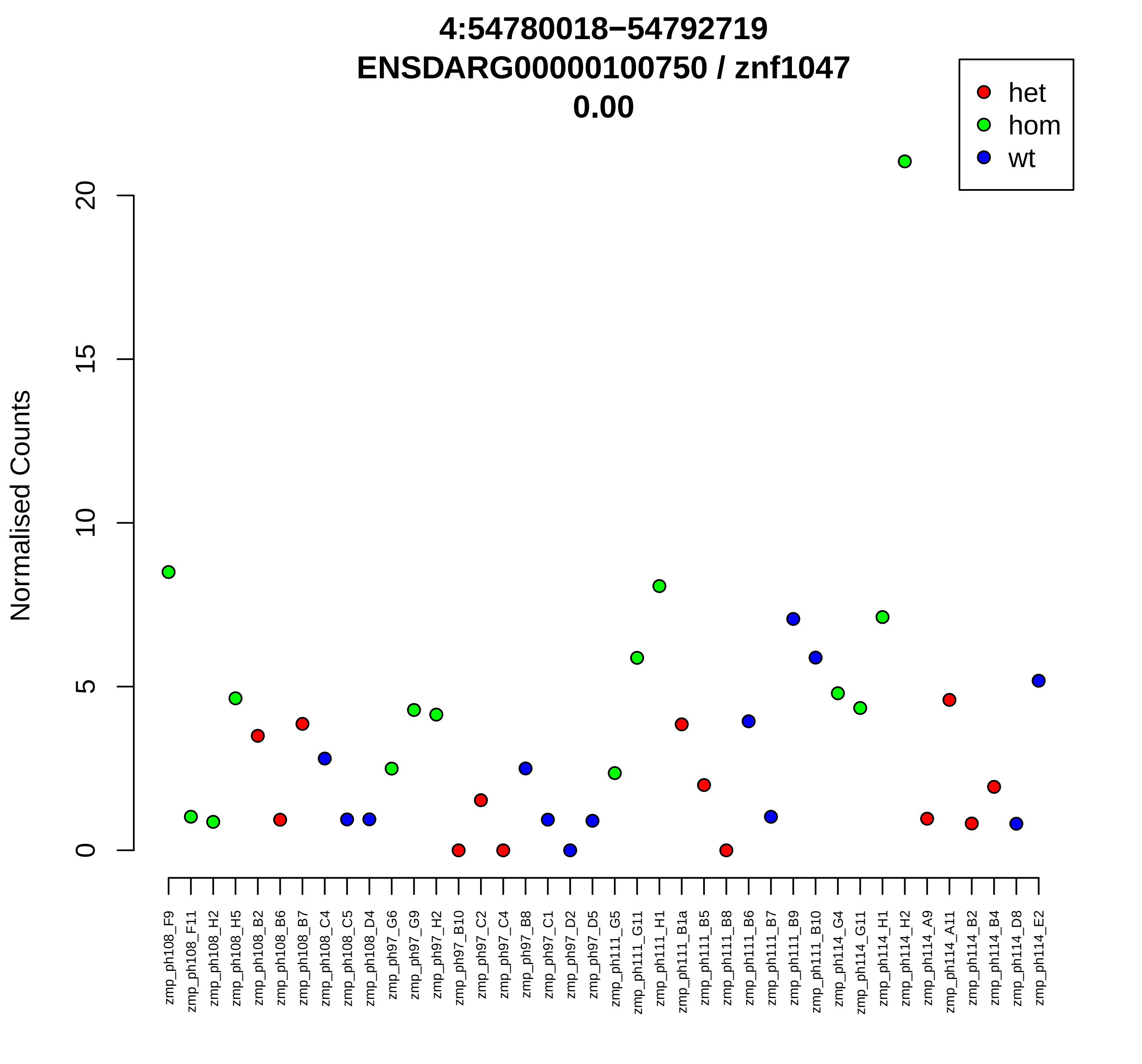

4:54780018−54792719
ENSDARG00000100750 / znf1047
0.00
het
hom
wt
Normalised Counts
0
5
10
15
20
zmp_ph108_F9
zmp_ph108_F11
zmp_ph108_H2
zmp_ph108_H5
zmp_ph108_B2
zmp_ph108_B6
zmp_ph108_B7
zmp_ph108_C4
zmp_ph108_C5
zmp_ph108_D4
zmp_ph97_G6
zmp_ph97_G9
zmp_ph97_H2
zmp_ph97_B10
zmp_ph97_C2
zmp_ph97_C4
zmp_ph97_B8
zmp_ph97_C1
zmp_ph97_D2
zmp_ph97_D5
zmp_ph111_G5
zmp_ph111_G11
zmp_ph111_H1
zmp_ph111_B1a
zmp_ph111_B5
zmp_ph111_B8
zmp_ph111_B6
zmp_ph111_B7
zmp_ph111_B9
zmp_ph111_B10
zmp_ph114_G4
zmp_ph114_G11
zmp_ph114_H1
zmp_ph114_H2
zmp_ph114_A9
zmp_ph114_A11
zmp_ph114_B2
zmp_ph114_B4
zmp_ph114_D8
zmp_ph114_E2

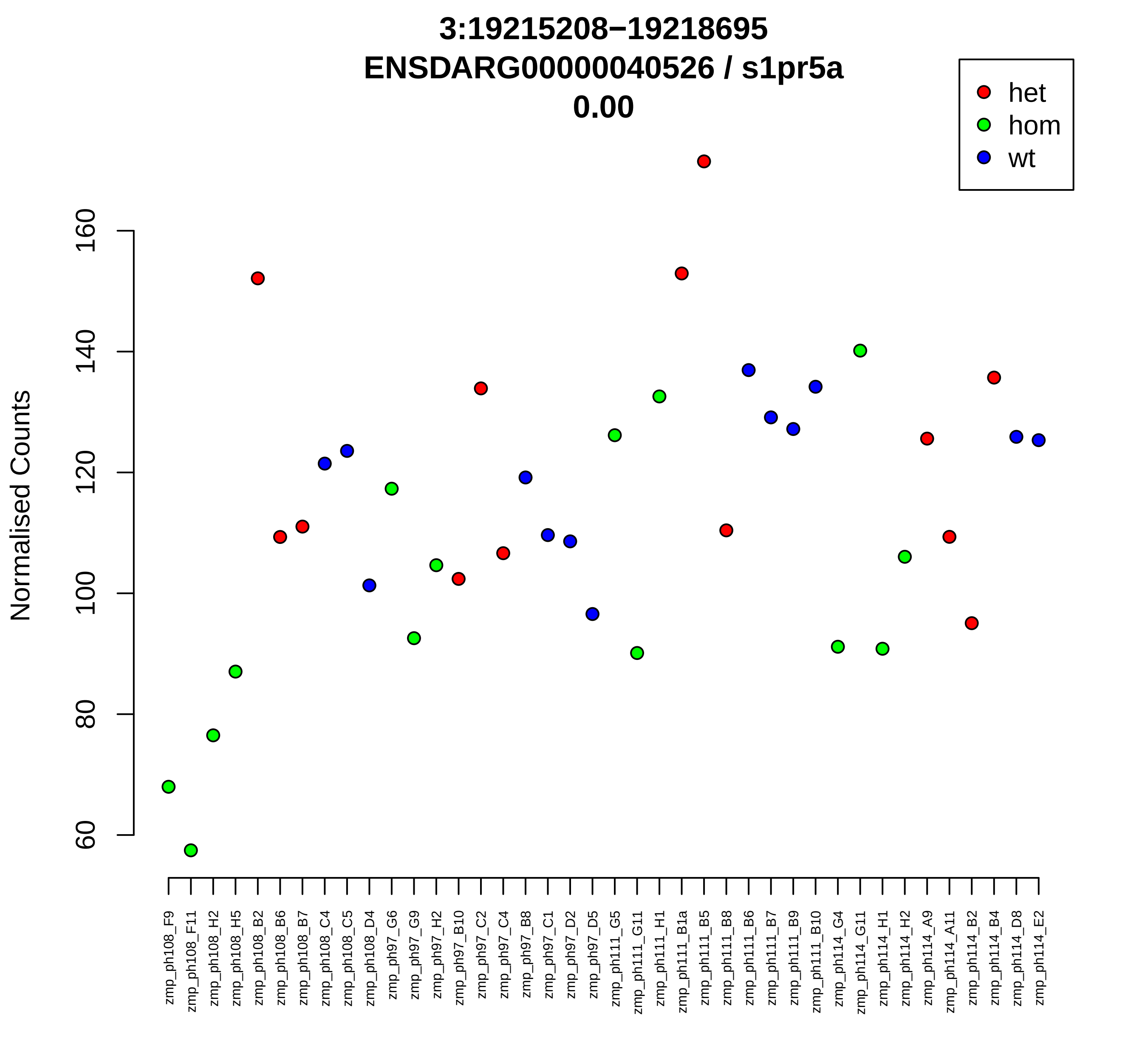
3:19215208−19218695
ENSDARG00000040526 / s1pr5a
0.00
het
hom
wt
Normalised Counts
60
80
100
120
140
160
zmp_ph108_F9
zmp_ph108_F11
zmp_ph108_H2
zmp_ph108_H5
zmp_ph108_B2
zmp_ph108_B6
zmp_ph108_B7
zmp_ph108_C4
zmp_ph108_C5
zmp_ph108_D4
zmp_ph97_G6
zmp_ph97_G9
zmp_ph97_H2
zmp_ph97_B10
zmp_ph97_C2
zmp_ph97_C4
zmp_ph97_B8
zmp_ph97_C1
zmp_ph97_D2
zmp_ph97_D5
zmp_ph111_G5
zmp_ph111_G11
zmp_ph111_H1
zmp_ph111_B1a
zmp_ph111_B5
zmp_ph111_B8
zmp_ph111_B6
zmp_ph111_B7
zmp_ph111_B9
zmp_ph111_B10
zmp_ph114_G4
zmp_ph114_G11
zmp_ph114_H1
zmp_ph114_H2
zmp_ph114_A9
zmp_ph114_A11
zmp_ph114_B2
zmp_ph114_B4
zmp_ph114_D8
zmp_ph114_E2

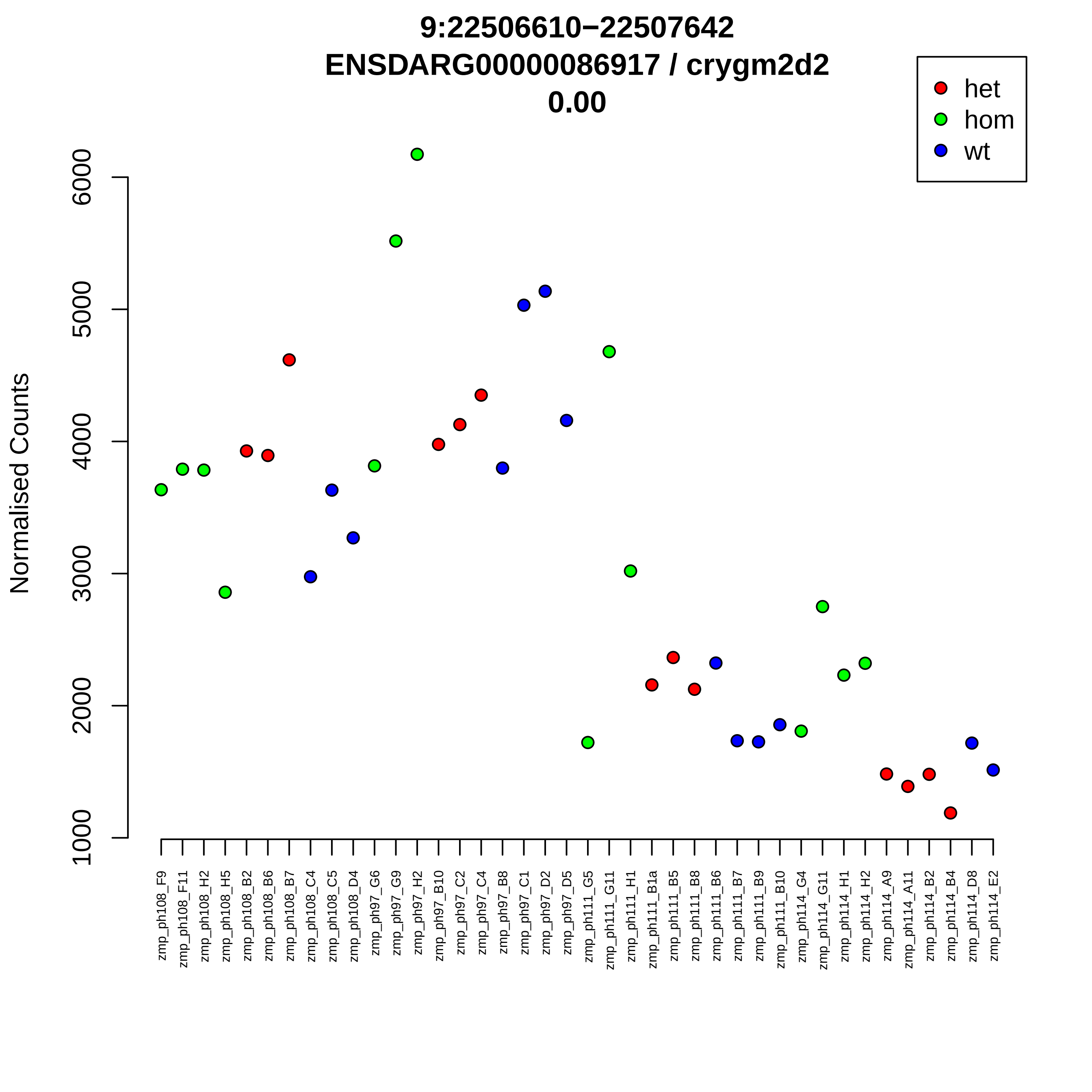
9:22506610−22507642
ENSDARG00000086917 / crygm2d2
0.00
het
hom
wt
Normalised Counts
1000
2000
3000
4000
5000
6000
zmp_ph108_F9
zmp_ph108_F11
zmp_ph108_H2
zmp_ph108_H5
zmp_ph108_B2
zmp_ph108_B6
zmp_ph108_B7
zmp_ph108_C4
zmp_ph108_C5
zmp_ph108_D4
zmp_ph97_G6
zmp_ph97_G9
zmp_ph97_H2
zmp_ph97_B10
zmp_ph97_C2
zmp_ph97_C4
zmp_ph97_B8
zmp_ph97_C1
zmp_ph97_D2
zmp_ph97_D5
zmp_ph111_G5
zmp_ph111_G11
zmp_ph111_H1
zmp_ph111_B1a
zmp_ph111_B5
zmp_ph111_B8
zmp_ph111_B6
zmp_ph111_B7
zmp_ph111_B9
zmp_ph111_B10
zmp_ph114_G4
zmp_ph114_G11
zmp_ph114_H1
zmp_ph114_H2
zmp_ph114_A9
zmp_ph114_A11
zmp_ph114_B2
zmp_ph114_B4
zmp_ph114_D8
zmp_ph114_E2

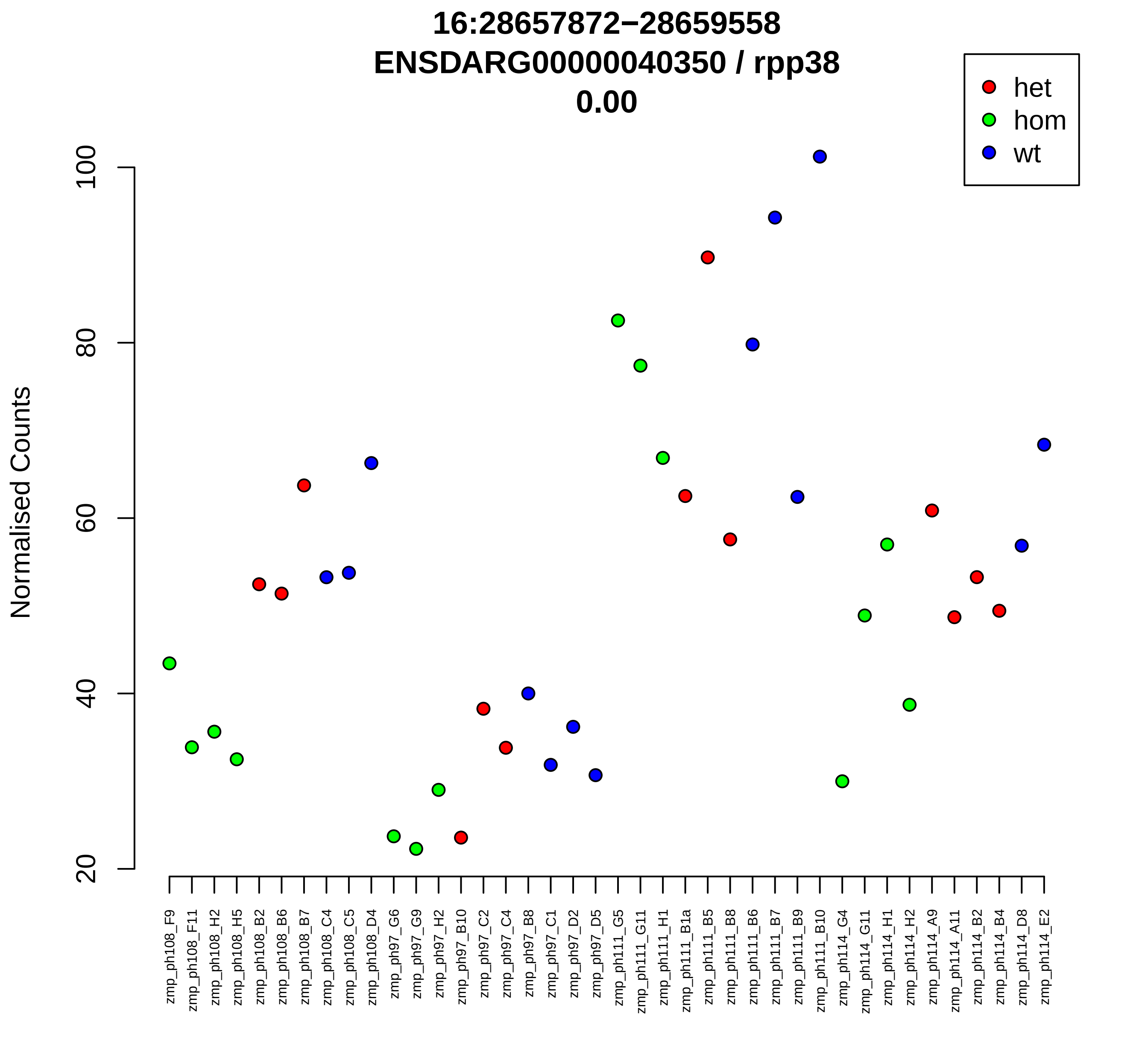

16:28657872−28659558
ENSDARG00000040350 / rpp38
0.00
het
hom
wt
Normalised Counts
20
40
60
80
100
zmp_ph108_F9
zmp_ph108_F11
zmp_ph108_H2
zmp_ph108_H5
zmp_ph108_B2
zmp_ph108_B6
zmp_ph108_B7
zmp_ph108_C4
zmp_ph108_C5
zmp_ph108_D4
zmp_ph97_G6
zmp_ph97_G9
zmp_ph97_H2
zmp_ph97_B10
zmp_ph97_C2
zmp_ph97_C4
zmp_ph97_B8
zmp_ph97_C1
zmp_ph97_D2
zmp_ph97_D5
zmp_ph111_G5
zmp_ph111_G11
zmp_ph111_H1
zmp_ph111_B1a
zmp_ph111_B5
zmp_ph111_B8
zmp_ph111_B6
zmp_ph111_B7
zmp_ph111_B9
zmp_ph111_B10
zmp_ph114_G4
zmp_ph114_G11
zmp_ph114_H1
zmp_ph114_H2
zmp_ph114_A9
zmp_ph114_A11
zmp_ph114_B2
zmp_ph114_B4
zmp_ph114_D8
zmp_ph114_E2

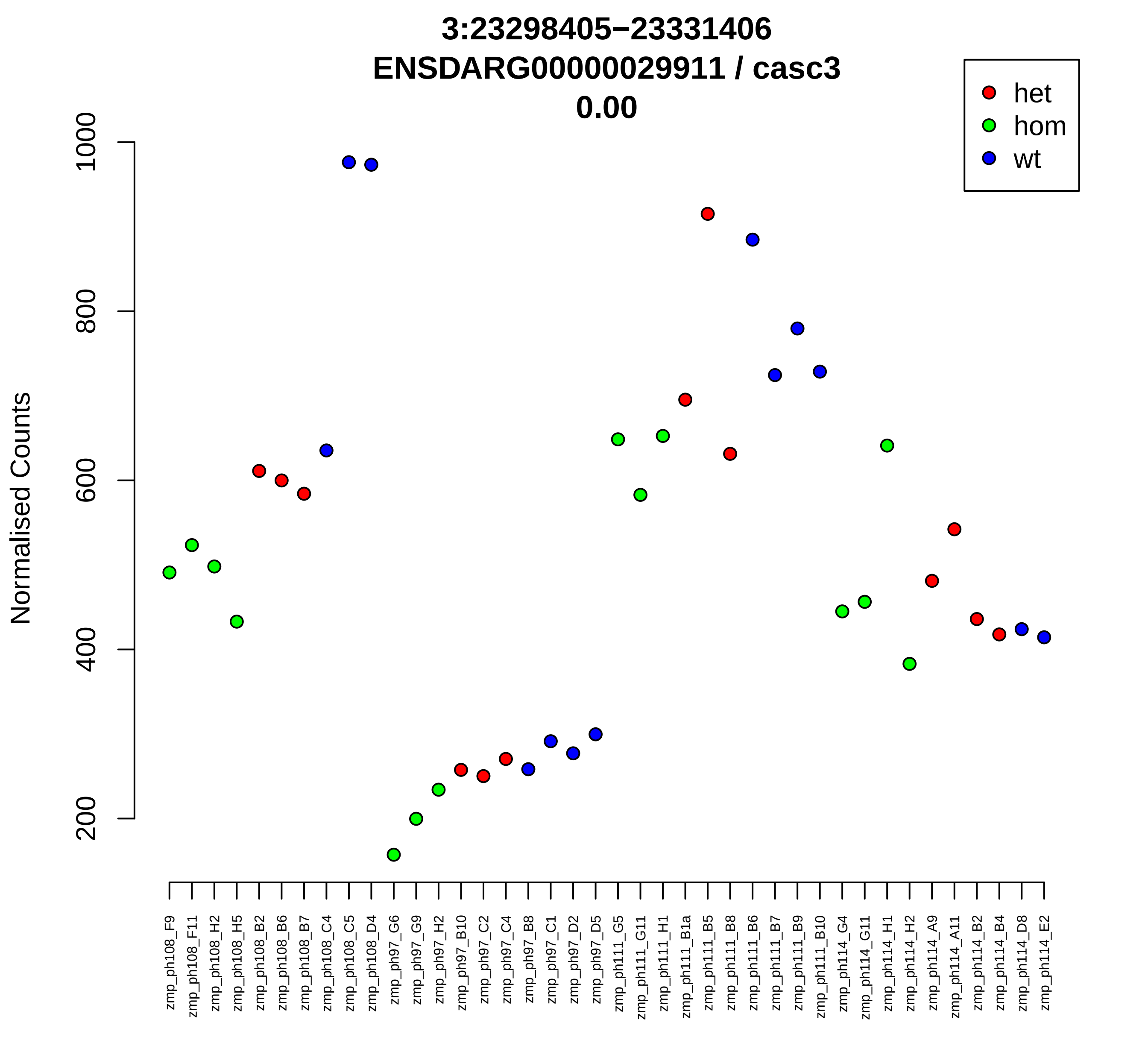
3:23298405−23331406
ENSDARG00000029911 / casc3
0.00
het
hom
wt
Normalised Counts
200
400
600
800
1000
zmp_ph108_F9
zmp_ph108_F11
zmp_ph108_H2
zmp_ph108_H5
zmp_ph108_B2
zmp_ph108_B6
zmp_ph108_B7
zmp_ph108_C4
zmp_ph108_C5
zmp_ph108_D4
zmp_ph97_G6
zmp_ph97_G9
zmp_ph97_H2
zmp_ph97_B10
zmp_ph97_C2
zmp_ph97_C4
zmp_ph97_B8
zmp_ph97_C1
zmp_ph97_D2
zmp_ph97_D5
zmp_ph111_G5
zmp_ph111_G11
zmp_ph111_H1
zmp_ph111_B1a
zmp_ph111_B5
zmp_ph111_B8
zmp_ph111_B6
zmp_ph111_B7
zmp_ph111_B9
zmp_ph111_B10
zmp_ph114_G4
zmp_ph114_G11
zmp_ph114_H1
zmp_ph114_H2
zmp_ph114_A9
zmp_ph114_A11
zmp_ph114_B2
zmp_ph114_B4
zmp_ph114_D8
zmp_ph114_E2

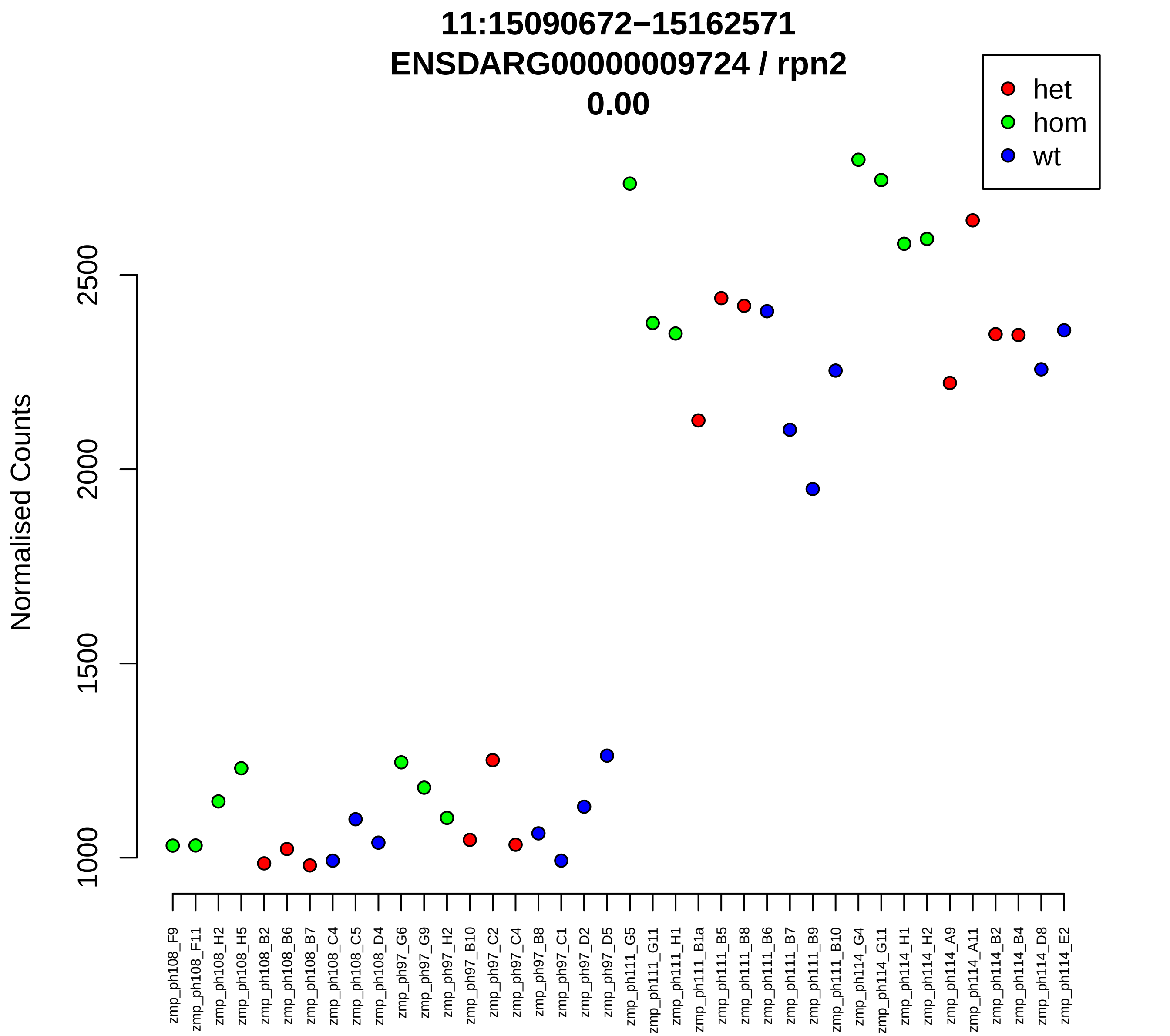

11:15090672−15162571
ENSDARG00000009724 / rpn2
0.00
het
hom
wt
Normalised Counts
1000
1500
2000
2500
zmp_ph108_F9
zmp_ph108_F11
zmp_ph108_H2
zmp_ph108_H5
zmp_ph108_B2
zmp_ph108_B6
zmp_ph108_B7
zmp_ph108_C4
zmp_ph108_C5
zmp_ph108_D4
zmp_ph97_G6
zmp_ph97_G9
zmp_ph97_H2
zmp_ph97_B10
zmp_ph97_C2
zmp_ph97_C4
zmp_ph97_B8
zmp_ph97_C1
zmp_ph97_D2
zmp_ph97_D5
zmp_ph111_G5
zmp_ph111_G11
zmp_ph111_H1
zmp_ph111_B1a
zmp_ph111_B5
zmp_ph111_B8
zmp_ph111_B6
zmp_ph111_B7
zmp_ph111_B9
zmp_ph111_B10
zmp_ph114_G4
zmp_ph114_G11
zmp_ph114_H1
zmp_ph114_H2
zmp_ph114_A9
zmp_ph114_A11
zmp_ph114_B2
zmp_ph114_B4
zmp_ph114_D8
zmp_ph114_E2

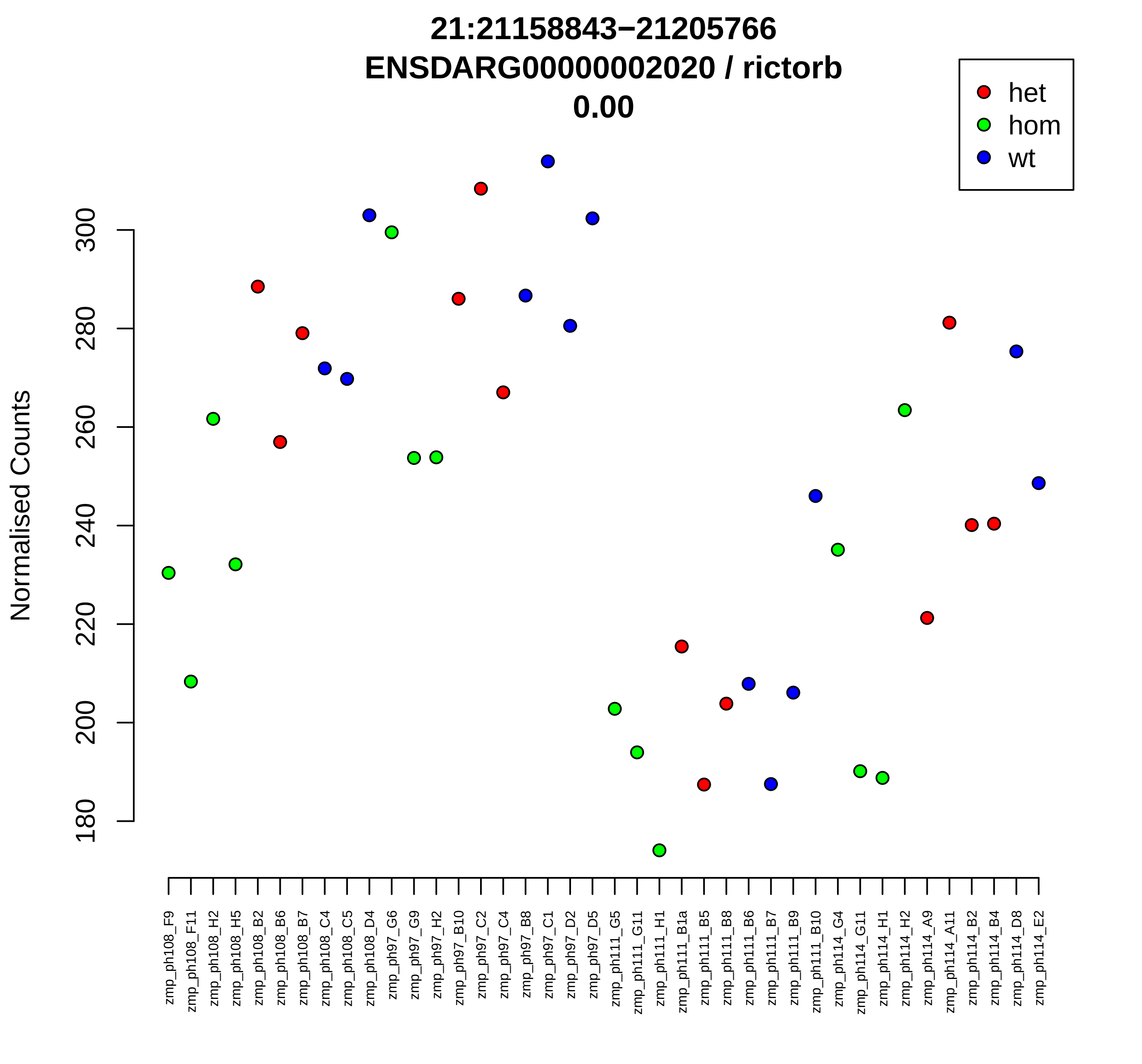

21:21158843−21205766
ENSDARG00000002020 / rictorb
0.00
het
hom
wt
Normalised Counts
180
200
220
240
260
280
300
zmp_ph108_F9
zmp_ph108_F11
zmp_ph108_H2
zmp_ph108_H5
zmp_ph108_B2
zmp_ph108_B6
zmp_ph108_B7
zmp_ph108_C4
zmp_ph108_C5
zmp_ph108_D4
zmp_ph97_G6
zmp_ph97_G9
zmp_ph97_H2
zmp_ph97_B10
zmp_ph97_C2
zmp_ph97_C4
zmp_ph97_B8
zmp_ph97_C1
zmp_ph97_D2
zmp_ph97_D5
zmp_ph111_G5
zmp_ph111_G11
zmp_ph111_H1
zmp_ph111_B1a
zmp_ph111_B5
zmp_ph111_B8
zmp_ph111_B6
zmp_ph111_B7
zmp_ph111_B9
zmp_ph111_B10
zmp_ph114_G4
zmp_ph114_G11
zmp_ph114_H1
zmp_ph114_H2
zmp_ph114_A9
zmp_ph114_A11
zmp_ph114_B2
zmp_ph114_B4
zmp_ph114_D8
zmp_ph114_E2

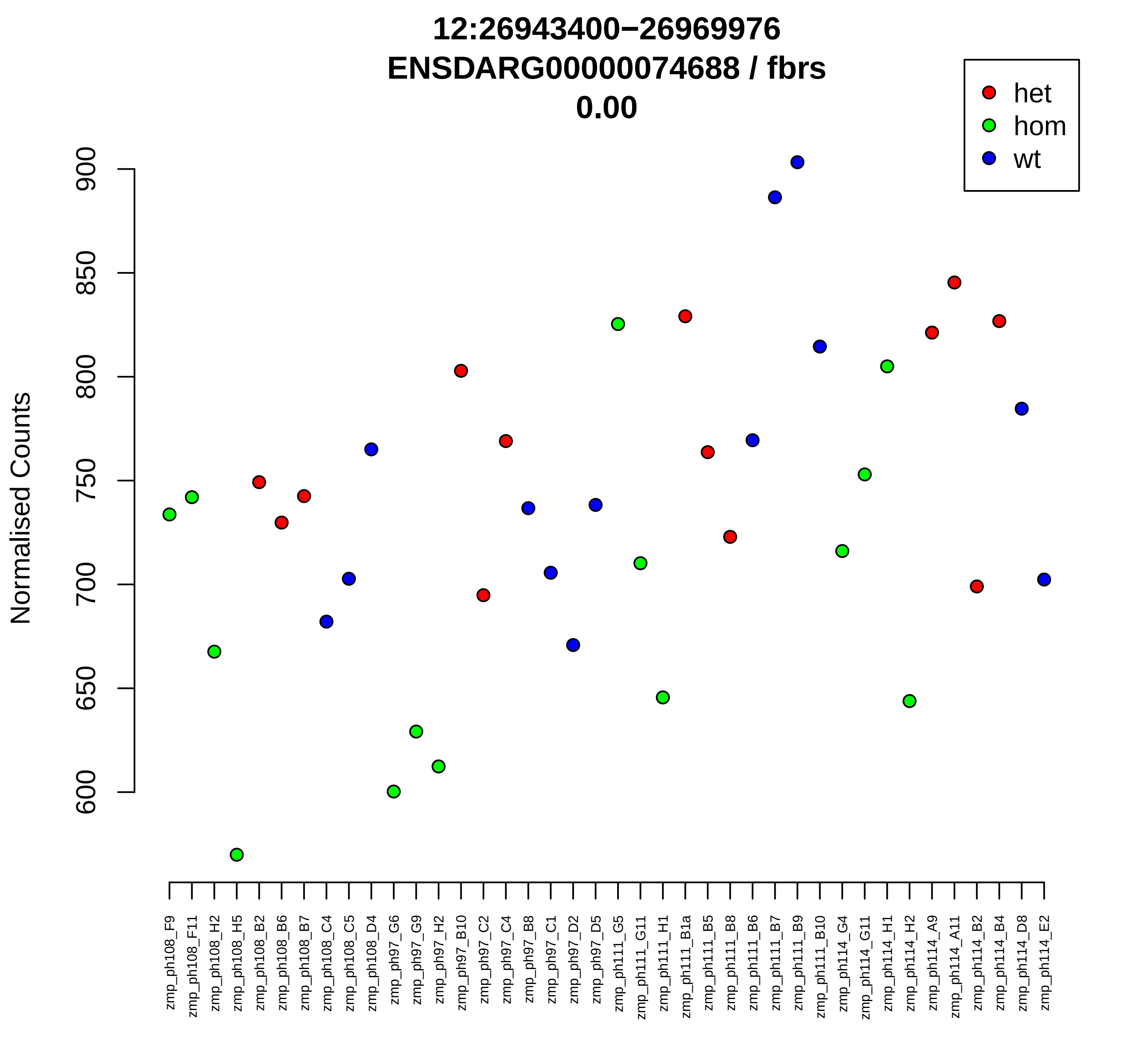
12:26943400−26969976
ENSDARG00000074688 / fbrs
0.00
het
hom
wt
Normalised Counts
600
650
700
750
800
850
900
zmp_ph108_F9
zmp_ph108_F11
zmp_ph108_H2
zmp_ph108_H5
zmp_ph108_B2
zmp_ph108_B6
zmp_ph108_B7
zmp_ph108_C4
zmp_ph108_C5
zmp_ph108_D4
zmp_ph97_G6
zmp_ph97_G9
zmp_ph97_H2
zmp_ph97_B10
zmp_ph97_C2
zmp_ph97_C4
zmp_ph97_B8
zmp_ph97_C1
zmp_ph97_D2
zmp_ph97_D5
zmp_ph111_G5
zmp_ph111_G11
zmp_ph111_H1
zmp_ph111_B1a
zmp_ph111_B5
zmp_ph111_B8
zmp_ph111_B6
zmp_ph111_B7
zmp_ph111_B9
zmp_ph111_B10
zmp_ph114_G4
zmp_ph114_G11
zmp_ph114_H1
zmp_ph114_H2
zmp_ph114_A9
zmp_ph114_A11
zmp_ph114_B2
zmp_ph114_B4
zmp_ph114_D8
zmp_ph114_E2

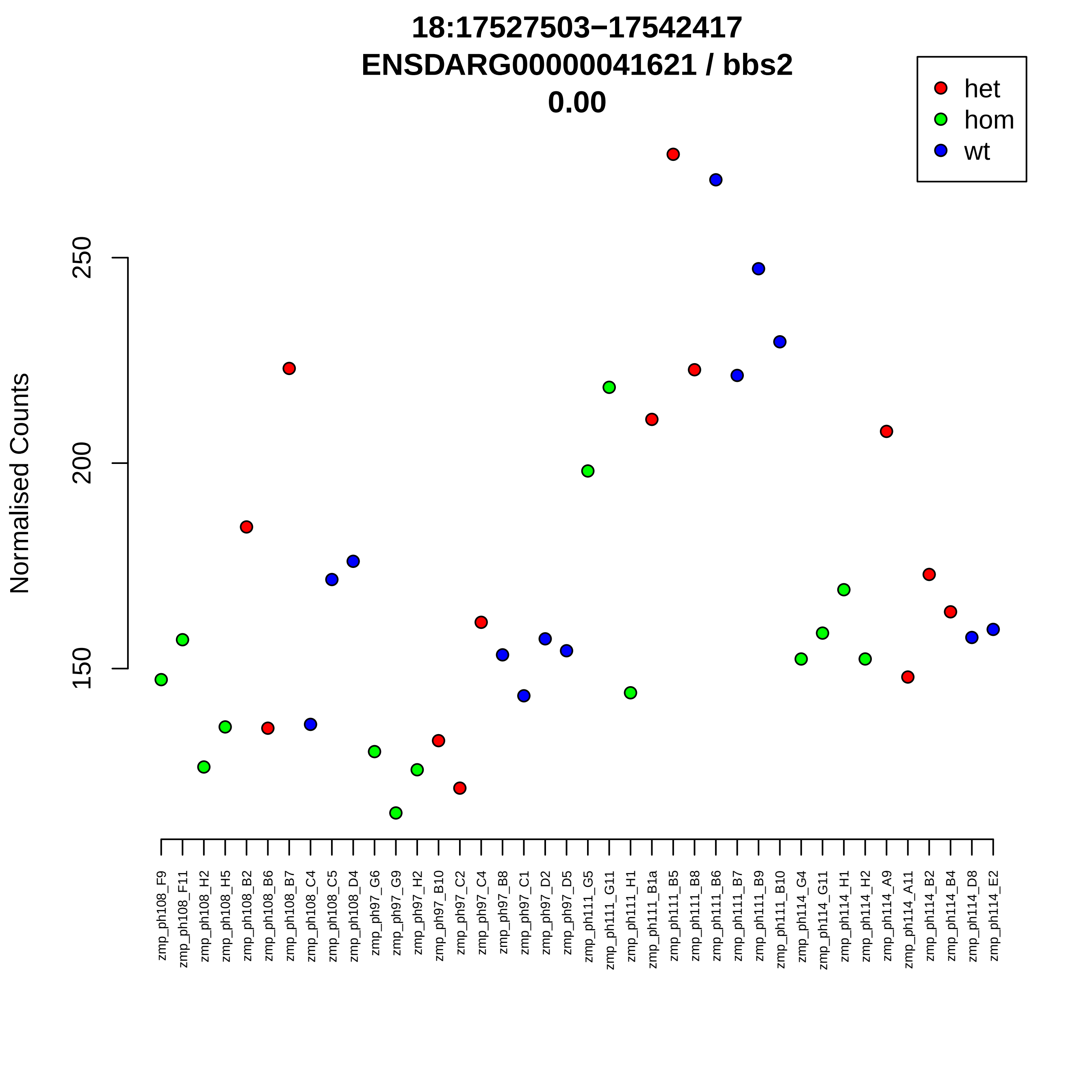

18:17527503−17542417
ENSDARG00000041621 / bbs2
0.00
het
hom
wt
Normalised Counts
150
200
250
zmp_ph108_F9
zmp_ph108_F11
zmp_ph108_H2
zmp_ph108_H5
zmp_ph108_B2
zmp_ph108_B6
zmp_ph108_B7
zmp_ph108_C4
zmp_ph108_C5
zmp_ph108_D4
zmp_ph97_G6
zmp_ph97_G9
zmp_ph97_H2
zmp_ph97_B10
zmp_ph97_C2
zmp_ph97_C4
zmp_ph97_B8
zmp_ph97_C1
zmp_ph97_D2
zmp_ph97_D5
zmp_ph111_G5
zmp_ph111_G11
zmp_ph111_H1
zmp_ph111_B1a
zmp_ph111_B5
zmp_ph111_B8
zmp_ph111_B6
zmp_ph111_B7
zmp_ph111_B9
zmp_ph111_B10
zmp_ph114_G4
zmp_ph114_G11
zmp_ph114_H1
zmp_ph114_H2
zmp_ph114_A9
zmp_ph114_A11
zmp_ph114_B2
zmp_ph114_B4
zmp_ph114_D8
zmp_ph114_E2

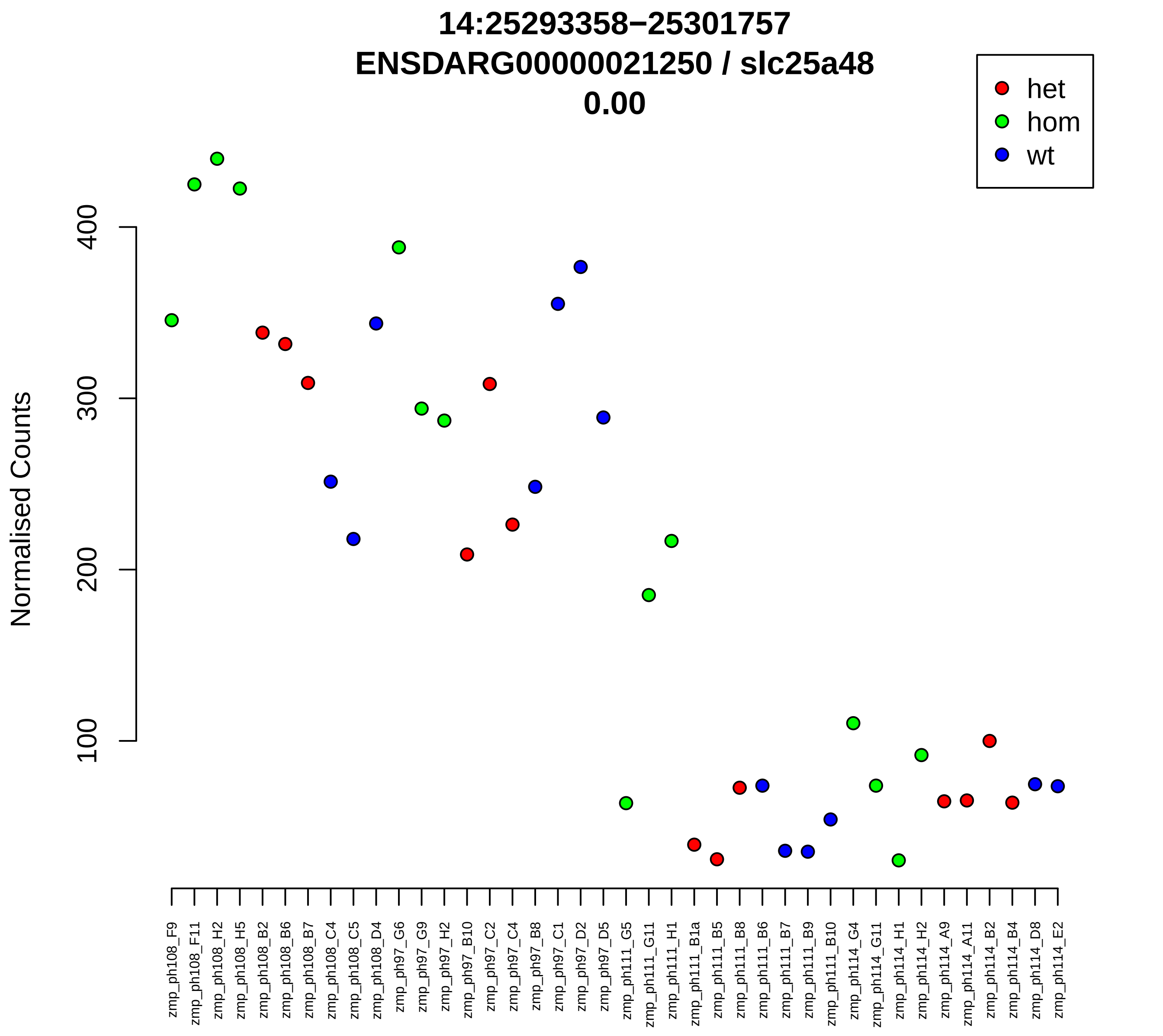

14:25293358−25301757
ENSDARG00000021250 / slc25a48
0.00
het
hom
wt
Normalised Counts
100
200
300
400
zmp_ph108_F9
zmp_ph108_F11
zmp_ph108_H2
zmp_ph108_H5
zmp_ph108_B2
zmp_ph108_B6
zmp_ph108_B7
zmp_ph108_C4
zmp_ph108_C5
zmp_ph108_D4
zmp_ph97_G6
zmp_ph97_G9
zmp_ph97_H2
zmp_ph97_B10
zmp_ph97_C2
zmp_ph97_C4
zmp_ph97_B8
zmp_ph97_C1
zmp_ph97_D2
zmp_ph97_D5
zmp_ph111_G5
zmp_ph111_G11
zmp_ph111_H1
zmp_ph111_B1a
zmp_ph111_B5
zmp_ph111_B8
zmp_ph111_B6
zmp_ph111_B7
zmp_ph111_B9
zmp_ph111_B10
zmp_ph114_G4
zmp_ph114_G11
zmp_ph114_H1
zmp_ph114_H2
zmp_ph114_A9
zmp_ph114_A11
zmp_ph114_B2
zmp_ph114_B4
zmp_ph114_D8
zmp_ph114_E2

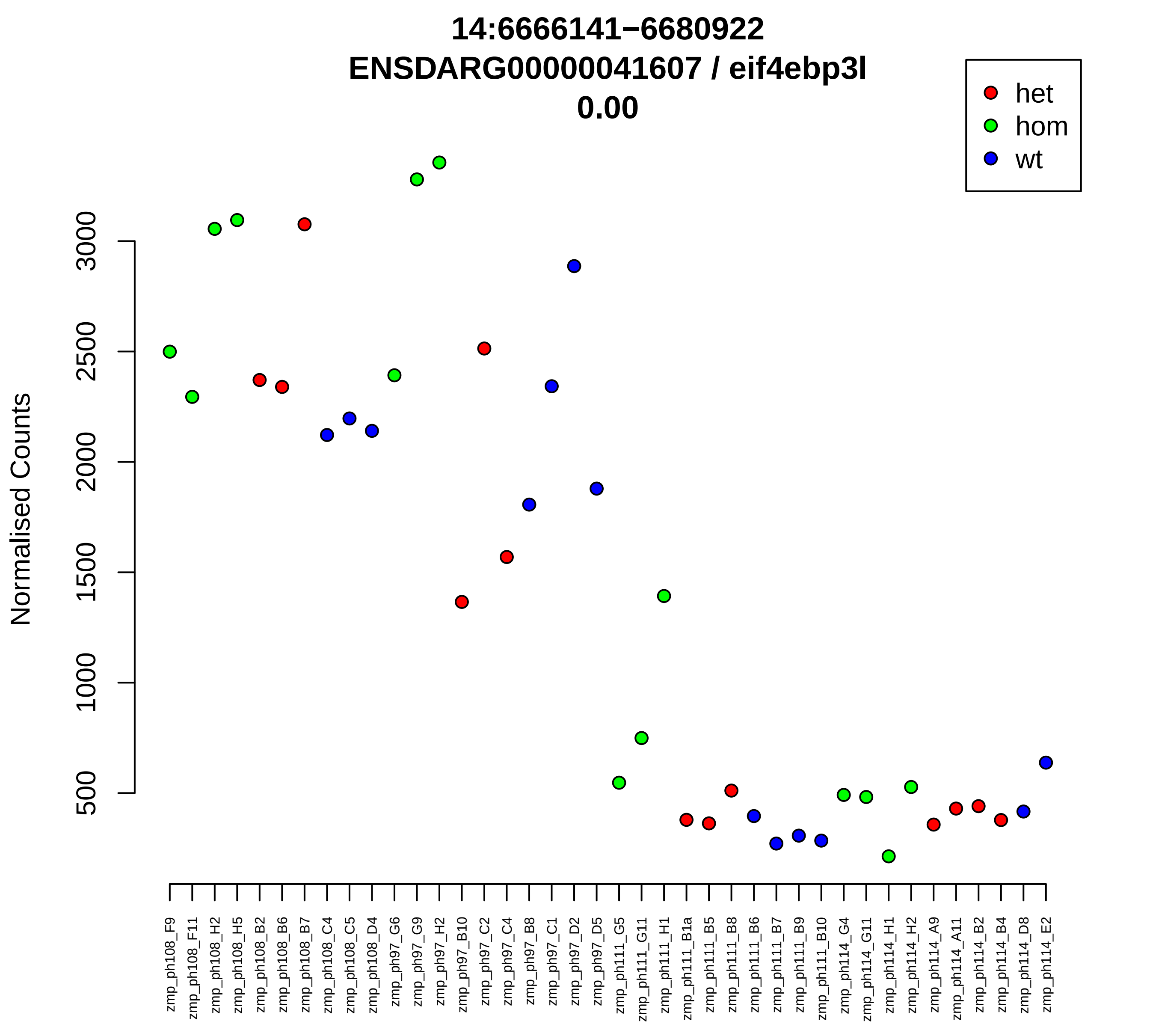

14:6666141−6680922
ENSDARG00000041607 / eif4ebp3l
0.00
het
hom
wt
Normalised Counts
500
1000
1500
2000
2500
3000
zmp_ph108_F9
zmp_ph108_F11
zmp_ph108_H2
zmp_ph108_H5
zmp_ph108_B2
zmp_ph108_B6
zmp_ph108_B7
zmp_ph108_C4
zmp_ph108_C5
zmp_ph108_D4
zmp_ph97_G6
zmp_ph97_G9
zmp_ph97_H2
zmp_ph97_B10
zmp_ph97_C2
zmp_ph97_C4
zmp_ph97_B8
zmp_ph97_C1
zmp_ph97_D2
zmp_ph97_D5
zmp_ph111_G5
zmp_ph111_G11
zmp_ph111_H1
zmp_ph111_B1a
zmp_ph111_B5
zmp_ph111_B8
zmp_ph111_B6
zmp_ph111_B7
zmp_ph111_B9
zmp_ph111_B10
zmp_ph114_G4
zmp_ph114_G11
zmp_ph114_H1
zmp_ph114_H2
zmp_ph114_A9
zmp_ph114_A11
zmp_ph114_B2
zmp_ph114_B4
zmp_ph114_D8
zmp_ph114_E2

22:27067102−27083041
ENSDARG00000055100 / cxcl12b
0.00

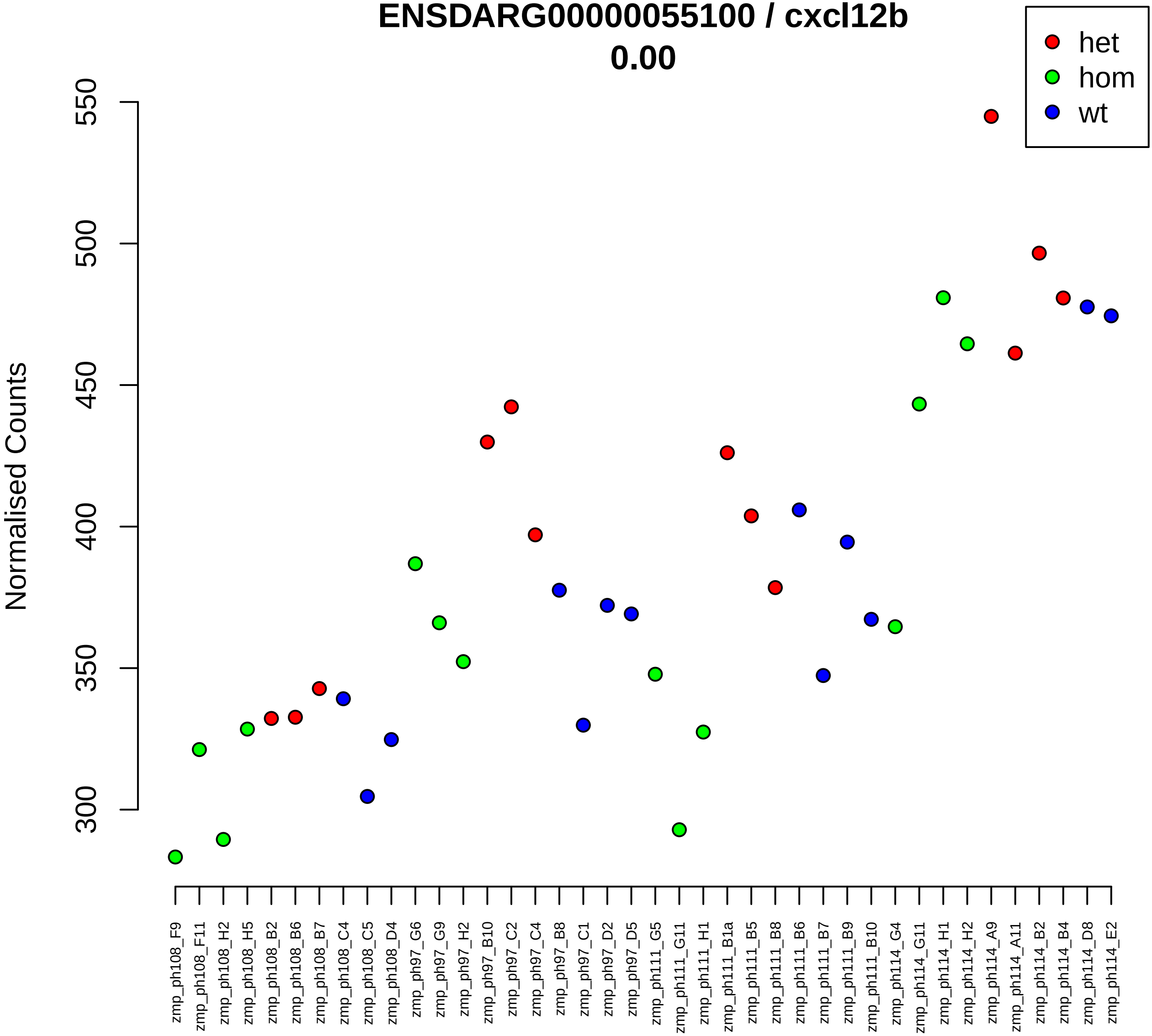

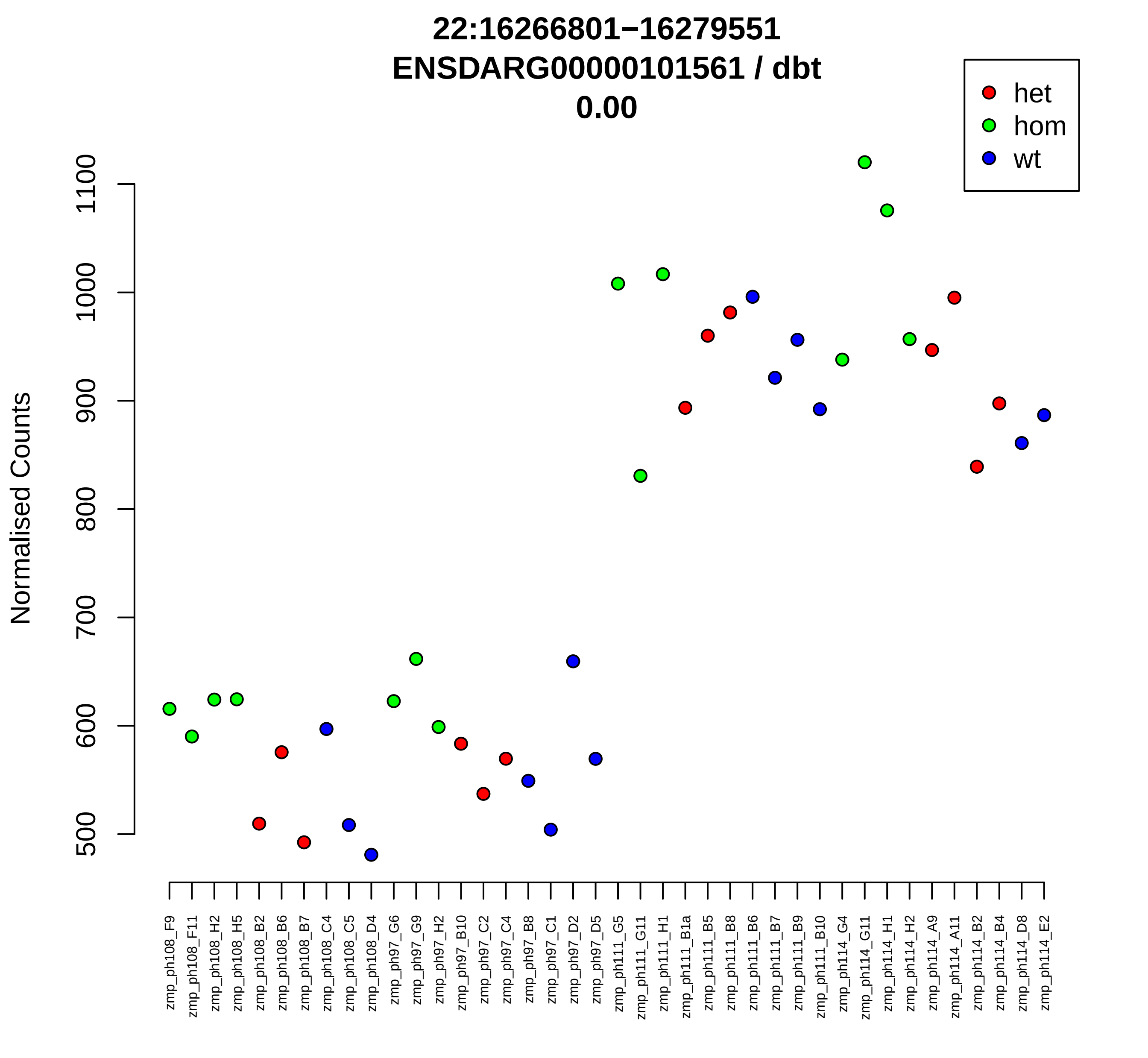

22:16266801−16279551
ENSDARG00000101561 / dbt
0.00
het
hom
wt
Normalised Counts
500
600
700
800
900
1000
1100
zmp_ph108_F9
zmp_ph108_F11
zmp_ph108_H2
zmp_ph108_H5
zmp_ph108_B2
zmp_ph108_B6
zmp_ph108_B7
zmp_ph108_C4
zmp_ph108_C5
zmp_ph108_D4
zmp_ph97_G6
zmp_ph97_G9
zmp_ph97_H2
zmp_ph97_B10
zmp_ph97_C2
zmp_ph97_C4
zmp_ph97_B8
zmp_ph97_C1
zmp_ph97_D2
zmp_ph97_D5
zmp_ph111_G5
zmp_ph111_G11
zmp_ph111_H1
zmp_ph111_B1a
zmp_ph111_B5
zmp_ph111_B8
zmp_ph111_B6
zmp_ph111_B7
zmp_ph111_B9
zmp_ph111_B10
zmp_ph114_G4
zmp_ph114_G11
zmp_ph114_H1
zmp_ph114_H2
zmp_ph114_A9
zmp_ph114_A11
zmp_ph114_B2
zmp_ph114_B4
zmp_ph114_D8
zmp_ph114_E2

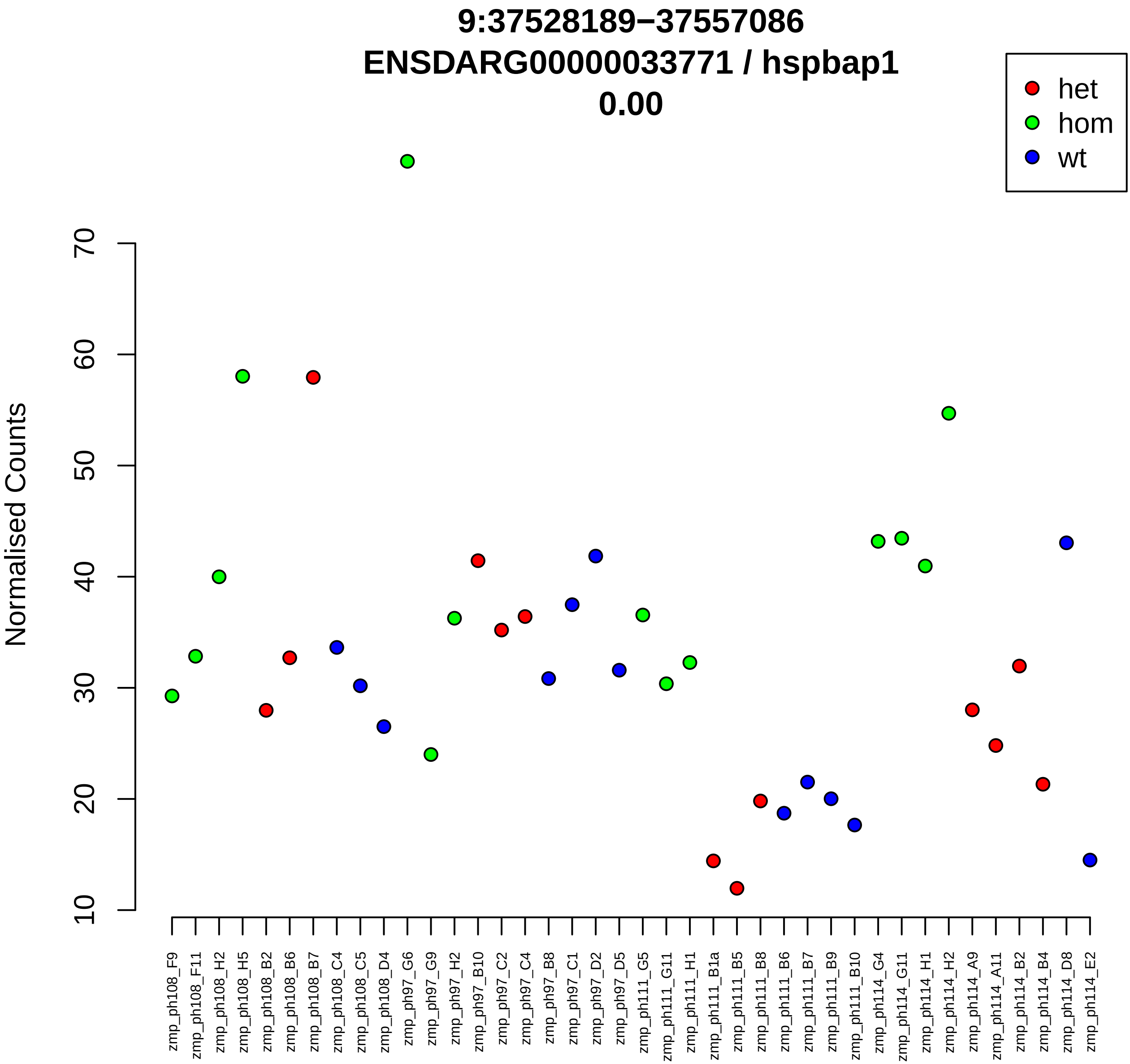

9:37528189−37557086
ENSDARG00000033771 / hspbap1
0.00
het
hom
wt
Normalised Counts
10
20
30
40
50
60
70
zmp_ph108_F9
zmp_ph108_F11
zmp_ph108_H2
zmp_ph108_H5
zmp_ph108_B2
zmp_ph108_B6
zmp_ph108_B7
zmp_ph108_C4
zmp_ph108_C5
zmp_ph108_D4
zmp_ph97_G6
zmp_ph97_G9
zmp_ph97_H2
zmp_ph97_B10
zmp_ph97_C2
zmp_ph97_C4
zmp_ph97_B8
zmp_ph97_C1
zmp_ph97_D2
zmp_ph97_D5
zmp_ph111_G5
zmp_ph111_G11
zmp_ph111_H1
zmp_ph111_B1a
zmp_ph111_B5
zmp_ph111_B8
zmp_ph111_B6
zmp_ph111_B7
zmp_ph111_B9
zmp_ph111_B10
zmp_ph114_G4
zmp_ph114_G11
zmp_ph114_H1
zmp_ph114_H2
zmp_ph114_A9
zmp_ph114_A11
zmp_ph114_B2
zmp_ph114_B4
zmp_ph114_D8
zmp_ph114_E2

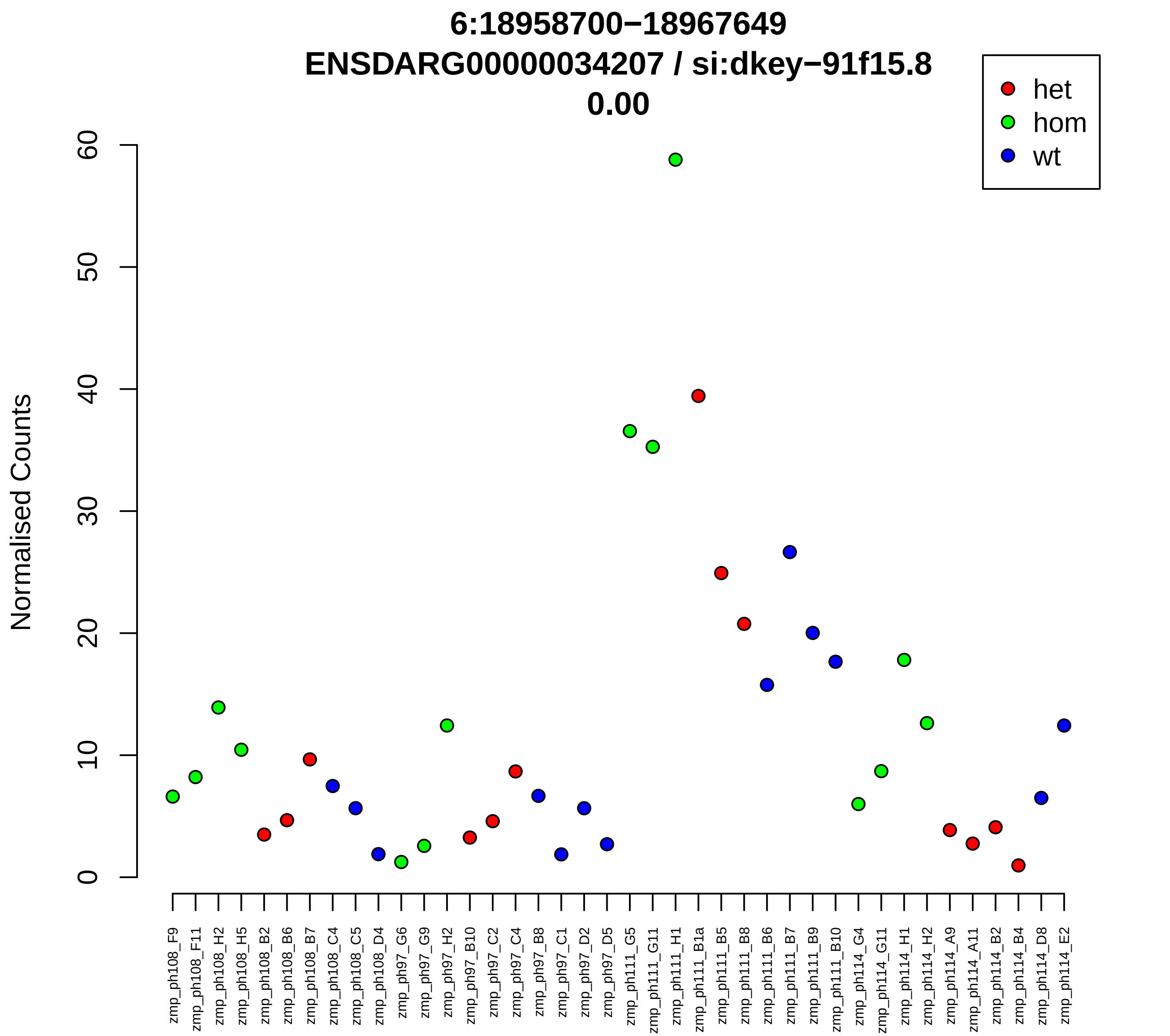
6:18958700−18967649
ENSDARG00000034207 / si:dkey−91f15.8
0.00
het
hom
wt
Normalised Counts
0
10
20
30
40
50
60
zmp_ph108_F9
zmp_ph108_F11
zmp_ph108_H2
zmp_ph108_H5
zmp_ph108_B2
zmp_ph108_B6
zmp_ph108_B7
zmp_ph108_C4
zmp_ph108_C5
zmp_ph108_D4
zmp_ph97_G6
zmp_ph97_G9
zmp_ph97_H2
zmp_ph97_B10
zmp_ph97_C2
zmp_ph97_C4
zmp_ph97_B8
zmp_ph97_C1
zmp_ph97_D2
zmp_ph97_D5
zmp_ph111_G5
zmp_ph111_G11
zmp_ph111_H1
zmp_ph111_B1a
zmp_ph111_B5
zmp_ph111_B8
zmp_ph111_B6
zmp_ph111_B7
zmp_ph111_B9
zmp_ph111_B10
zmp_ph114_G4
zmp_ph114_G11
zmp_ph114_H1
zmp_ph114_H2
zmp_ph114_A9
zmp_ph114_A11
zmp_ph114_B2
zmp_ph114_B4
zmp_ph114_D8
zmp_ph114_E2

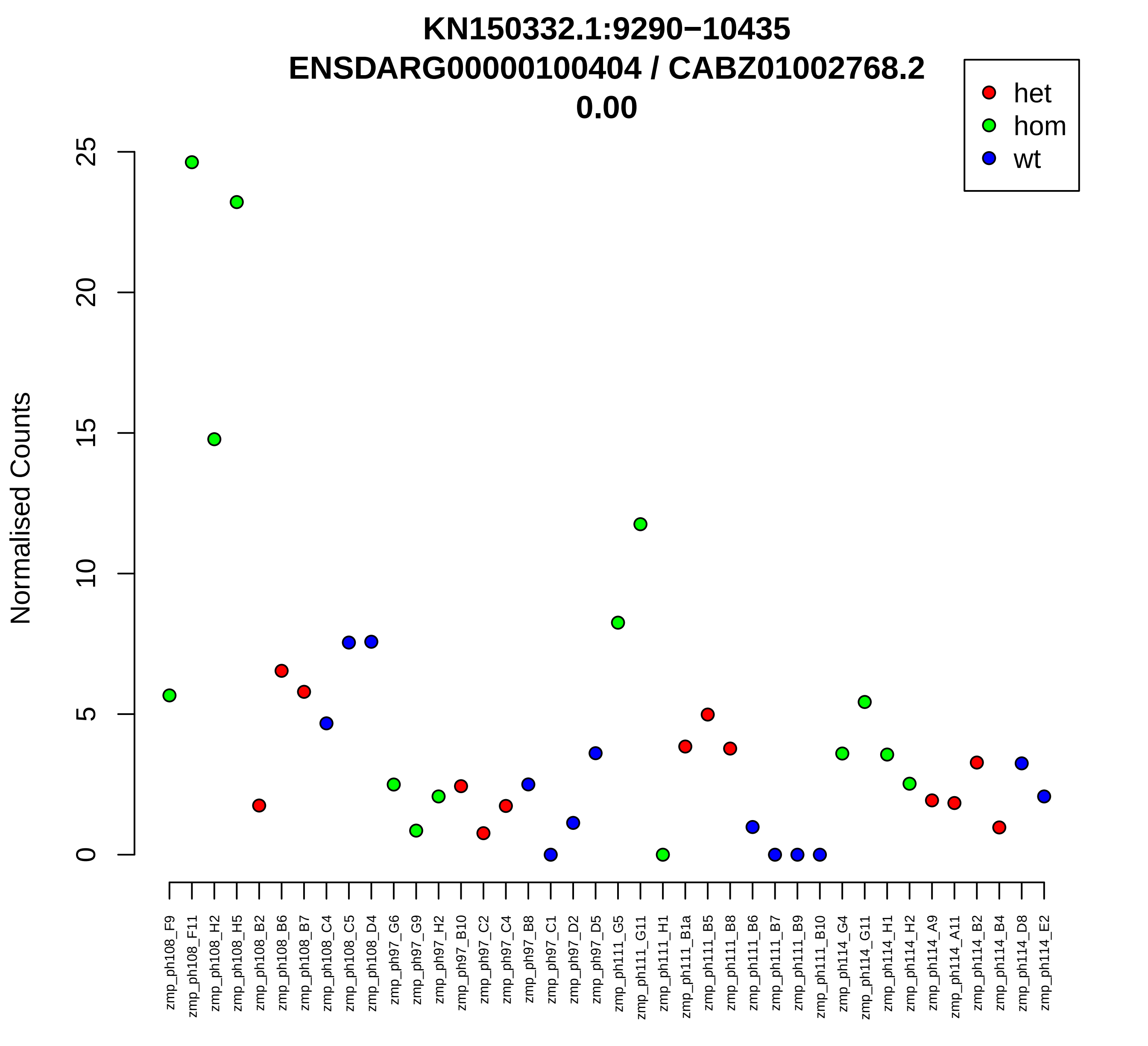
KN150332.1:9290−10435
ENSDARG00000100404 / CABZ01002768.2
0.00
het
hom
wt
Normalised Counts
0
5
10
15
20
25
zmp_ph108_F9
zmp_ph108_F11
zmp_ph108_H2
zmp_ph108_H5
zmp_ph108_B2
zmp_ph108_B6
zmp_ph108_B7
zmp_ph108_C4
zmp_ph108_C5
zmp_ph108_D4
zmp_ph97_G6
zmp_ph97_G9
zmp_ph97_H2
zmp_ph97_B10
zmp_ph97_C2
zmp_ph97_C4
zmp_ph97_B8
zmp_ph97_C1
zmp_ph97_D2
zmp_ph97_D5
zmp_ph111_G5
zmp_ph111_G11
zmp_ph111_H1
zmp_ph111_B1a
zmp_ph111_B5
zmp_ph111_B8
zmp_ph111_B6
zmp_ph111_B7
zmp_ph111_B9
zmp_ph111_B10
zmp_ph114_G4
zmp_ph114_G11
zmp_ph114_H1
zmp_ph114_H2
zmp_ph114_A9
zmp_ph114_A11
zmp_ph114_B2
zmp_ph114_B4
zmp_ph114_D8
zmp_ph114_E2

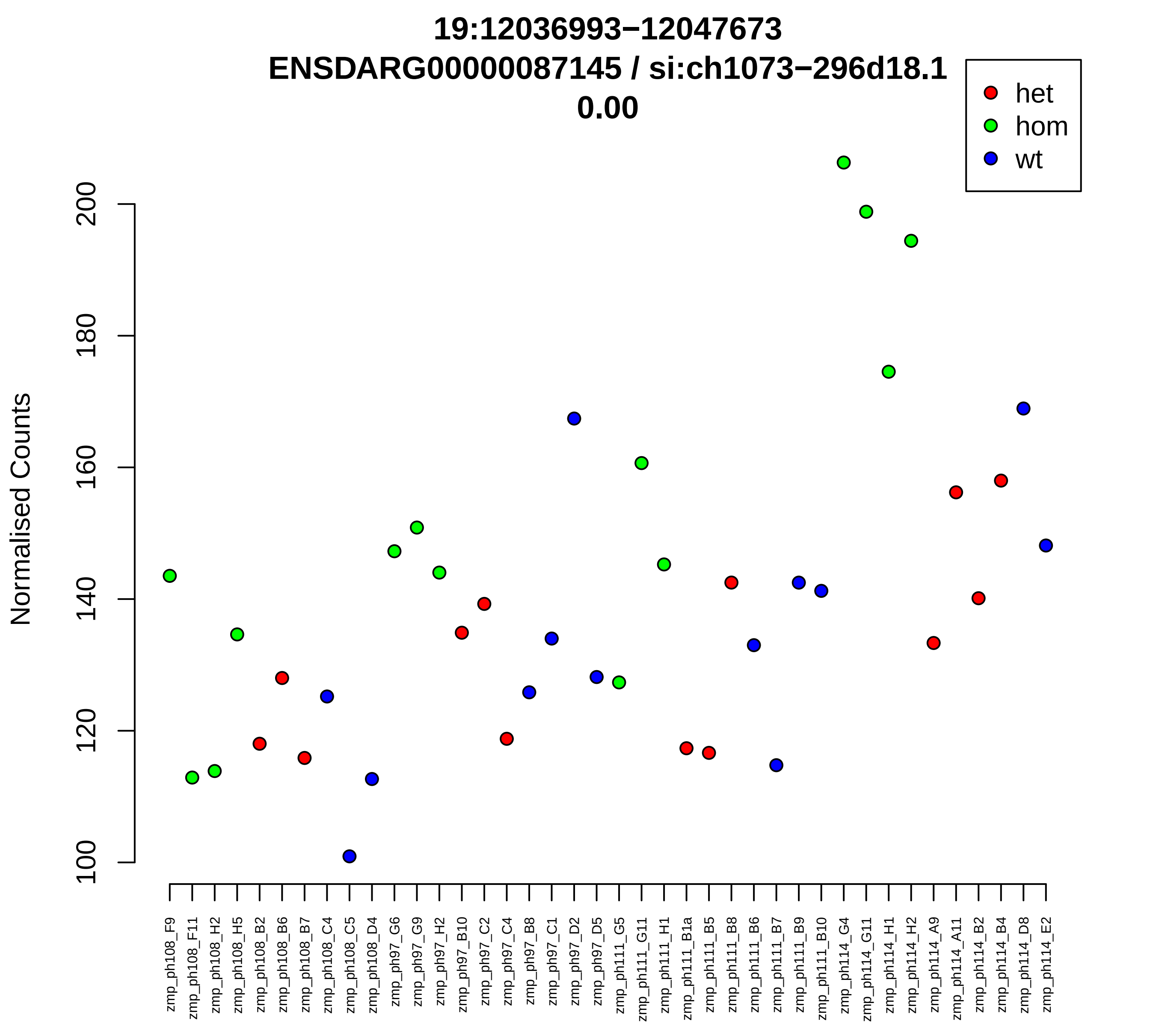
19:12036993−12047673
ENSDARG00000087145 / si:ch1073−296d18.1
0.00
het
hom
wt
Normalised Counts
100
120
140
160
180
200
zmp_ph108_F9
zmp_ph108_F11
zmp_ph108_H2
zmp_ph108_H5
zmp_ph108_B2
zmp_ph108_B6
zmp_ph108_B7
zmp_ph108_C4
zmp_ph108_C5
zmp_ph108_D4
zmp_ph97_G6
zmp_ph97_G9
zmp_ph97_H2
zmp_ph97_B10
zmp_ph97_C2
zmp_ph97_C4
zmp_ph97_B8
zmp_ph97_C1
zmp_ph97_D2
zmp_ph97_D5
zmp_ph111_G5
zmp_ph111_G11
zmp_ph111_H1
zmp_ph111_B1a
zmp_ph111_B5
zmp_ph111_B8
zmp_ph111_B6
zmp_ph111_B7
zmp_ph111_B9
zmp_ph111_B10
zmp_ph114_G4
zmp_ph114_G11
zmp_ph114_H1
zmp_ph114_H2
zmp_ph114_A9
zmp_ph114_A11
zmp_ph114_B2
zmp_ph114_B4
zmp_ph114_D8
zmp_ph114_E2

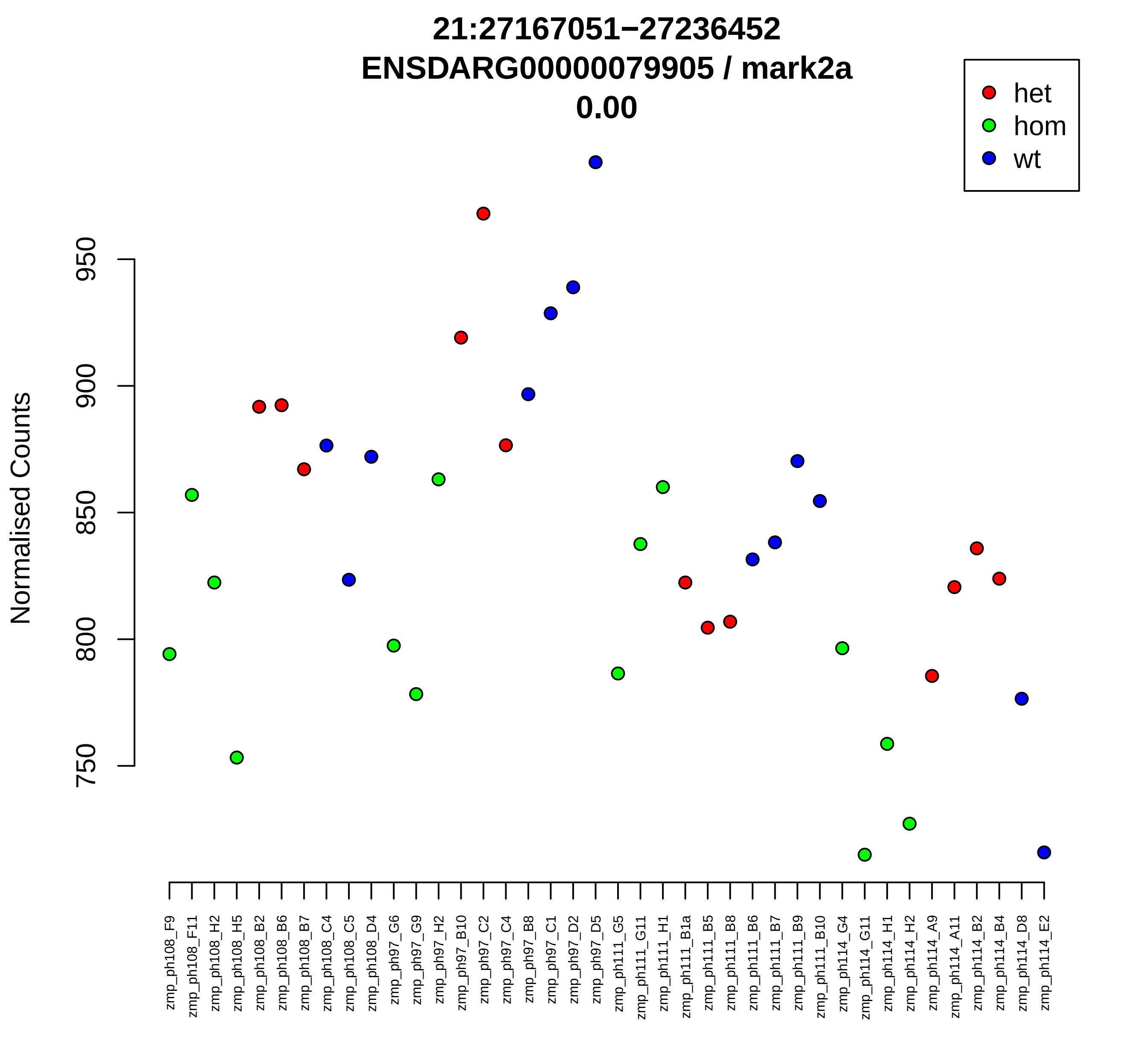

21:27167051−27236452
ENSDARG00000079905 / mark2a
0.00
het
hom
wt
Normalised Counts
750
800
850
900
950
zmp_ph108_F9
zmp_ph108_F11
zmp_ph108_H2
zmp_ph108_H5
zmp_ph108_B2
zmp_ph108_B6
zmp_ph108_B7
zmp_ph108_C4
zmp_ph108_C5
zmp_ph108_D4
zmp_ph97_G6
zmp_ph97_G9
zmp_ph97_H2
zmp_ph97_B10
zmp_ph97_C2
zmp_ph97_C4
zmp_ph97_B8
zmp_ph97_C1
zmp_ph97_D2
zmp_ph97_D5
zmp_ph111_G5
zmp_ph111_G11
zmp_ph111_H1
zmp_ph111_B1a
zmp_ph111_B5
zmp_ph111_B8
zmp_ph111_B6
zmp_ph111_B7
zmp_ph111_B9
zmp_ph111_B10
zmp_ph114_G4
zmp_ph114_G11
zmp_ph114_H1
zmp_ph114_H2
zmp_ph114_A9
zmp_ph114_A11
zmp_ph114_B2
zmp_ph114_B4
zmp_ph114_D8
zmp_ph114_E2

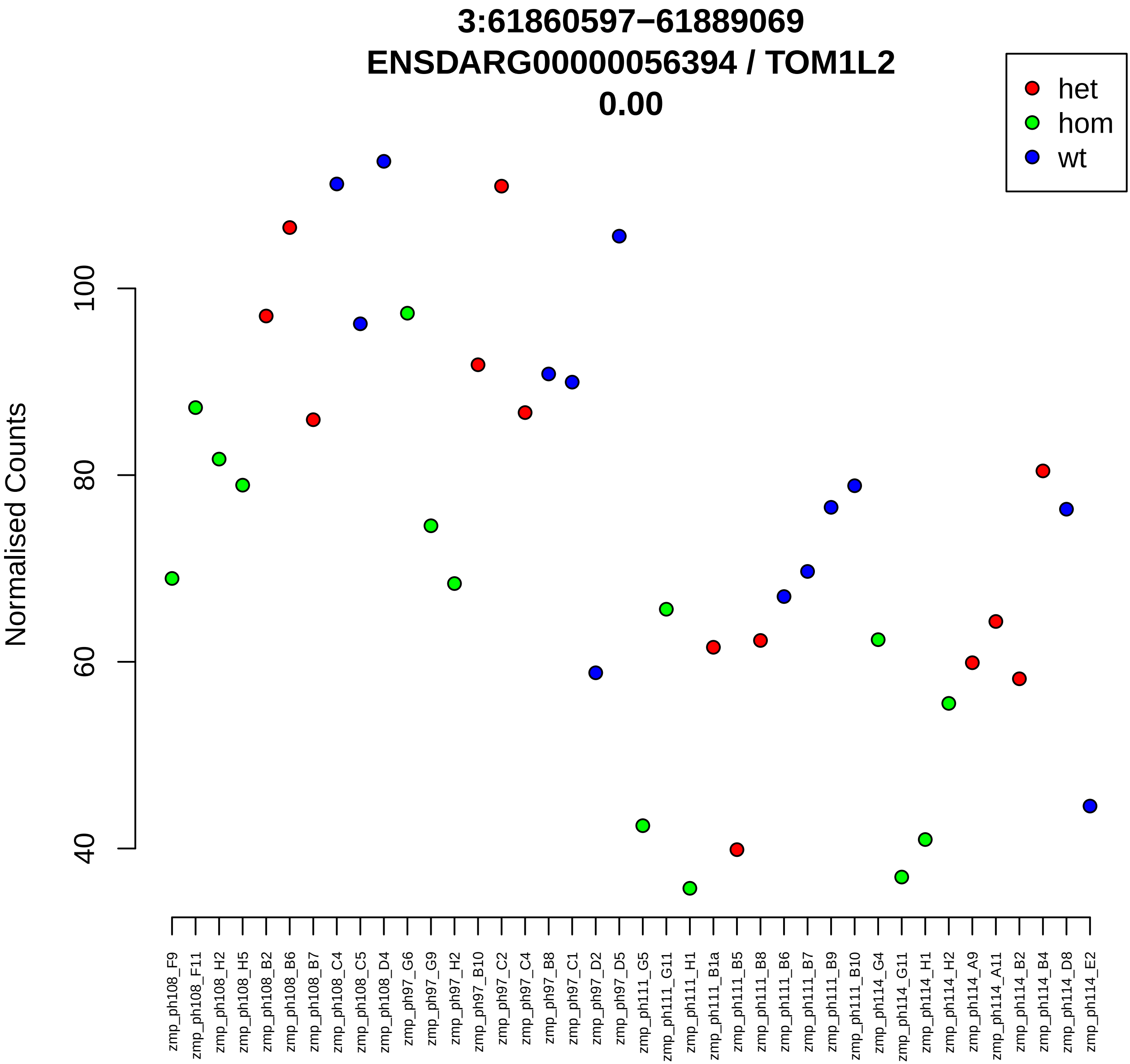

3:61860597−61889069
ENSDARG00000056394 / TOM1L2
0.00
het
hom
wt
Normalised Counts
40
60
80
100
zmp_ph108_F9
zmp_ph108_F11
zmp_ph108_H2
zmp_ph108_H5
zmp_ph108_B2
zmp_ph108_B6
zmp_ph108_B7
zmp_ph108_C4
zmp_ph108_C5
zmp_ph108_D4
zmp_ph97_G6
zmp_ph97_G9
zmp_ph97_H2
zmp_ph97_B10
zmp_ph97_C2
zmp_ph97_C4
zmp_ph97_B8
zmp_ph97_C1
zmp_ph97_D2
zmp_ph97_D5
zmp_ph111_G5
zmp_ph111_G11
zmp_ph111_H1
zmp_ph111_B1a
zmp_ph111_B5
zmp_ph111_B8
zmp_ph111_B6
zmp_ph111_B7
zmp_ph111_B9
zmp_ph111_B10
zmp_ph114_G4
zmp_ph114_G11
zmp_ph114_H1
zmp_ph114_H2
zmp_ph114_A9
zmp_ph114_A11
zmp_ph114_B2
zmp_ph114_B4
zmp_ph114_D8
zmp_ph114_E2

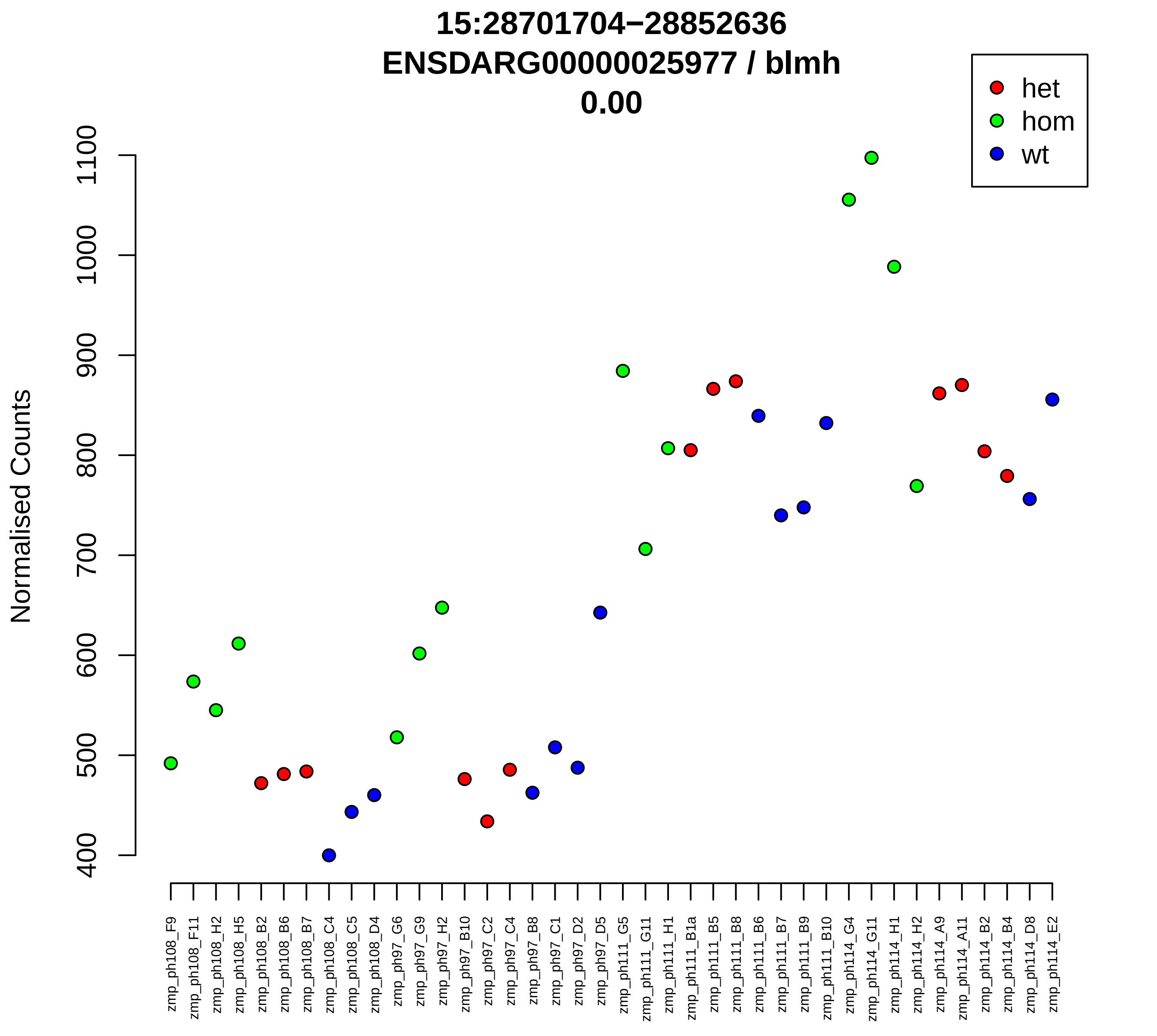

15:28701704−28852636
ENSDARG00000025977 / blmh
0.00
het
hom
wt
Normalised Counts
400
500
600
700
800
900
1000
1100
zmp_ph108_F9
zmp_ph108_F11
zmp_ph108_H2
zmp_ph108_H5
zmp_ph108_B2
zmp_ph108_B6
zmp_ph108_B7
zmp_ph108_C4
zmp_ph108_C5
zmp_ph108_D4
zmp_ph97_G6
zmp_ph97_G9
zmp_ph97_H2
zmp_ph97_B10
zmp_ph97_C2
zmp_ph97_C4
zmp_ph97_B8
zmp_ph97_C1
zmp_ph97_D2
zmp_ph97_D5
zmp_ph111_G5
zmp_ph111_G11
zmp_ph111_H1
zmp_ph111_B1a
zmp_ph111_B5
zmp_ph111_B8
zmp_ph111_B6
zmp_ph111_B7
zmp_ph111_B9
zmp_ph111_B10
zmp_ph114_G4
zmp_ph114_G11
zmp_ph114_H1
zmp_ph114_H2
zmp_ph114_A9
zmp_ph114_A11
zmp_ph114_B2
zmp_ph114_B4
zmp_ph114_D8
zmp_ph114_E2

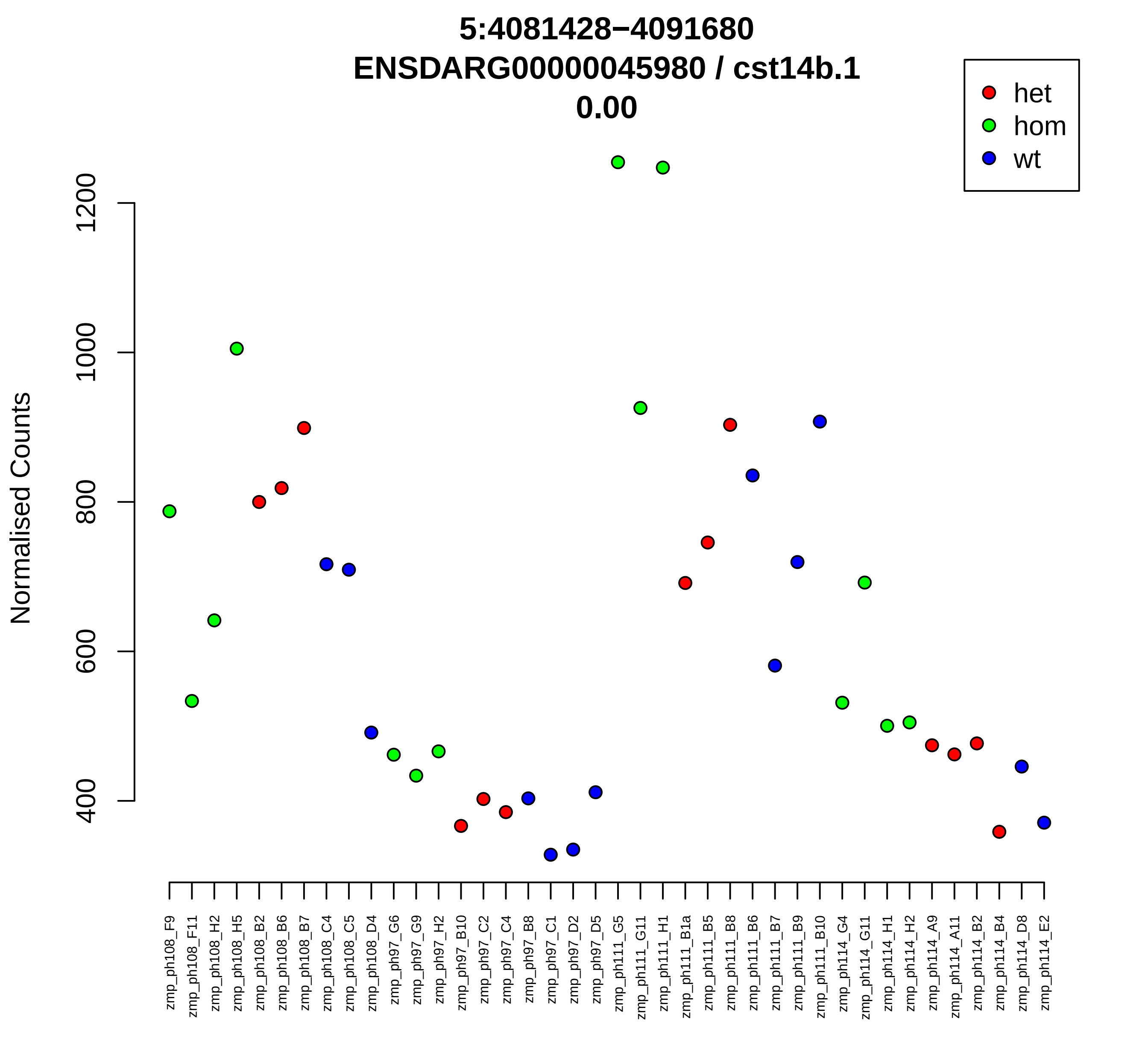
5:4081428−4091680
ENSDARG00000045980 / cst14b.1
0.00
het
hom
wt
Normalised Counts
400
600
800
1000
1200
zmp_ph108_F9
zmp_ph108_F11
zmp_ph108_H2
zmp_ph108_H5
zmp_ph108_B2
zmp_ph108_B6
zmp_ph108_B7
zmp_ph108_C4
zmp_ph108_C5
zmp_ph108_D4
zmp_ph97_G6
zmp_ph97_G9
zmp_ph97_H2
zmp_ph97_B10
zmp_ph97_C2
zmp_ph97_C4
zmp_ph97_B8
zmp_ph97_C1
zmp_ph97_D2
zmp_ph97_D5
zmp_ph111_G5
zmp_ph111_G11
zmp_ph111_H1
zmp_ph111_B1a
zmp_ph111_B5
zmp_ph111_B8
zmp_ph111_B6
zmp_ph111_B7
zmp_ph111_B9
zmp_ph111_B10
zmp_ph114_G4
zmp_ph114_G11
zmp_ph114_H1
zmp_ph114_H2
zmp_ph114_A9
zmp_ph114_A11
zmp_ph114_B2
zmp_ph114_B4
zmp_ph114_D8
zmp_ph114_E2

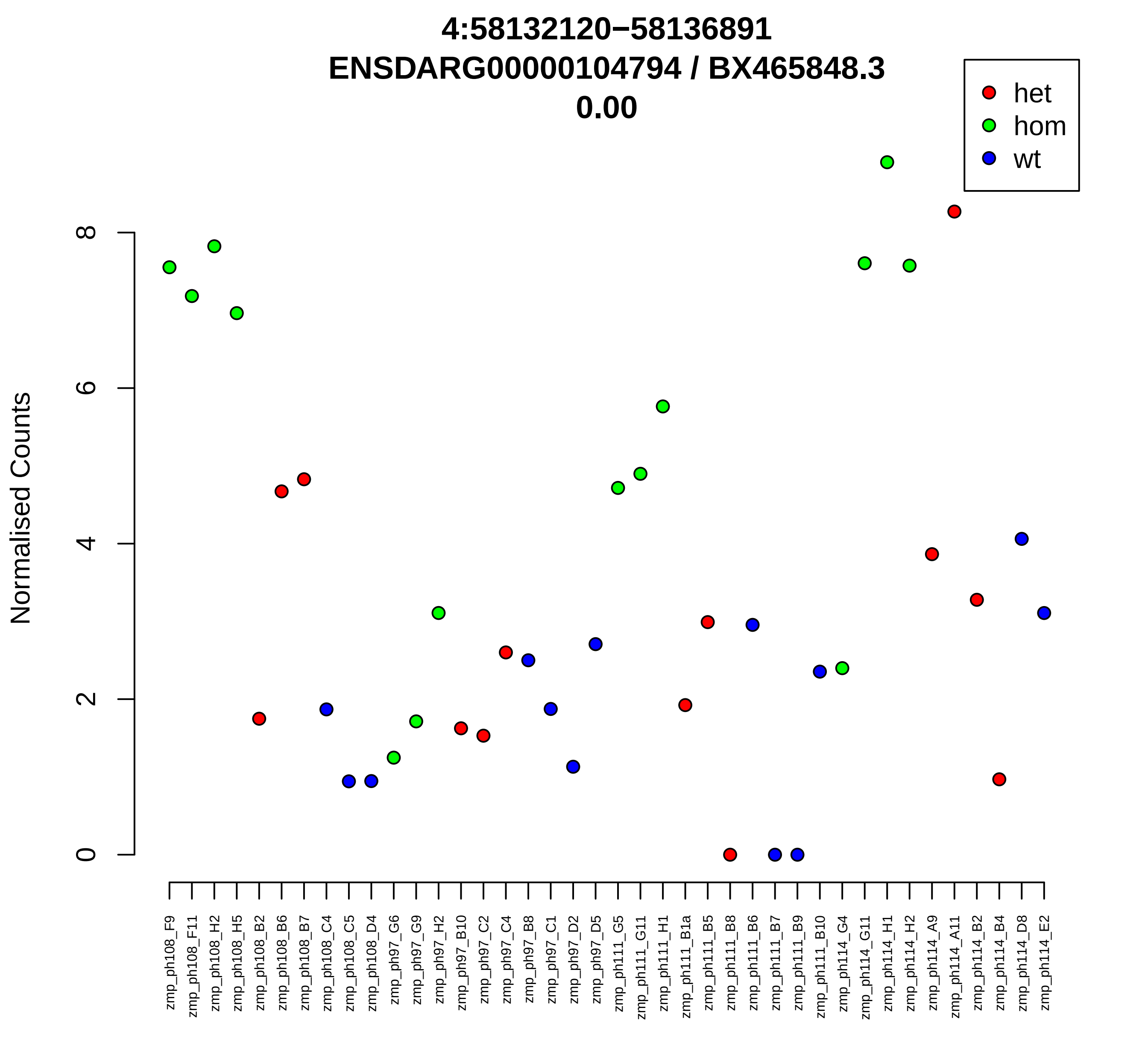
4:58132120−58136891
ENSDARG00000104794 / BX465848.3
0.00
het
hom
wt
Normalised Counts
0
2
4
6
8
zmp_ph108_F9
zmp_ph108_F11
zmp_ph108_H2
zmp_ph108_H5
zmp_ph108_B2
zmp_ph108_B6
zmp_ph108_B7
zmp_ph108_C4
zmp_ph108_C5
zmp_ph108_D4
zmp_ph97_G6
zmp_ph97_G9
zmp_ph97_H2
zmp_ph97_B10
zmp_ph97_C2
zmp_ph97_C4
zmp_ph97_B8
zmp_ph97_C1
zmp_ph97_D2
zmp_ph97_D5
zmp_ph111_G5
zmp_ph111_G11
zmp_ph111_H1
zmp_ph111_B1a
zmp_ph111_B5
zmp_ph111_B8
zmp_ph111_B6
zmp_ph111_B7
zmp_ph111_B9
zmp_ph111_B10
zmp_ph114_G4
zmp_ph114_G11
zmp_ph114_H1
zmp_ph114_H2
zmp_ph114_A9
zmp_ph114_A11
zmp_ph114_B2
zmp_ph114_B4
zmp_ph114_D8
zmp_ph114_E2

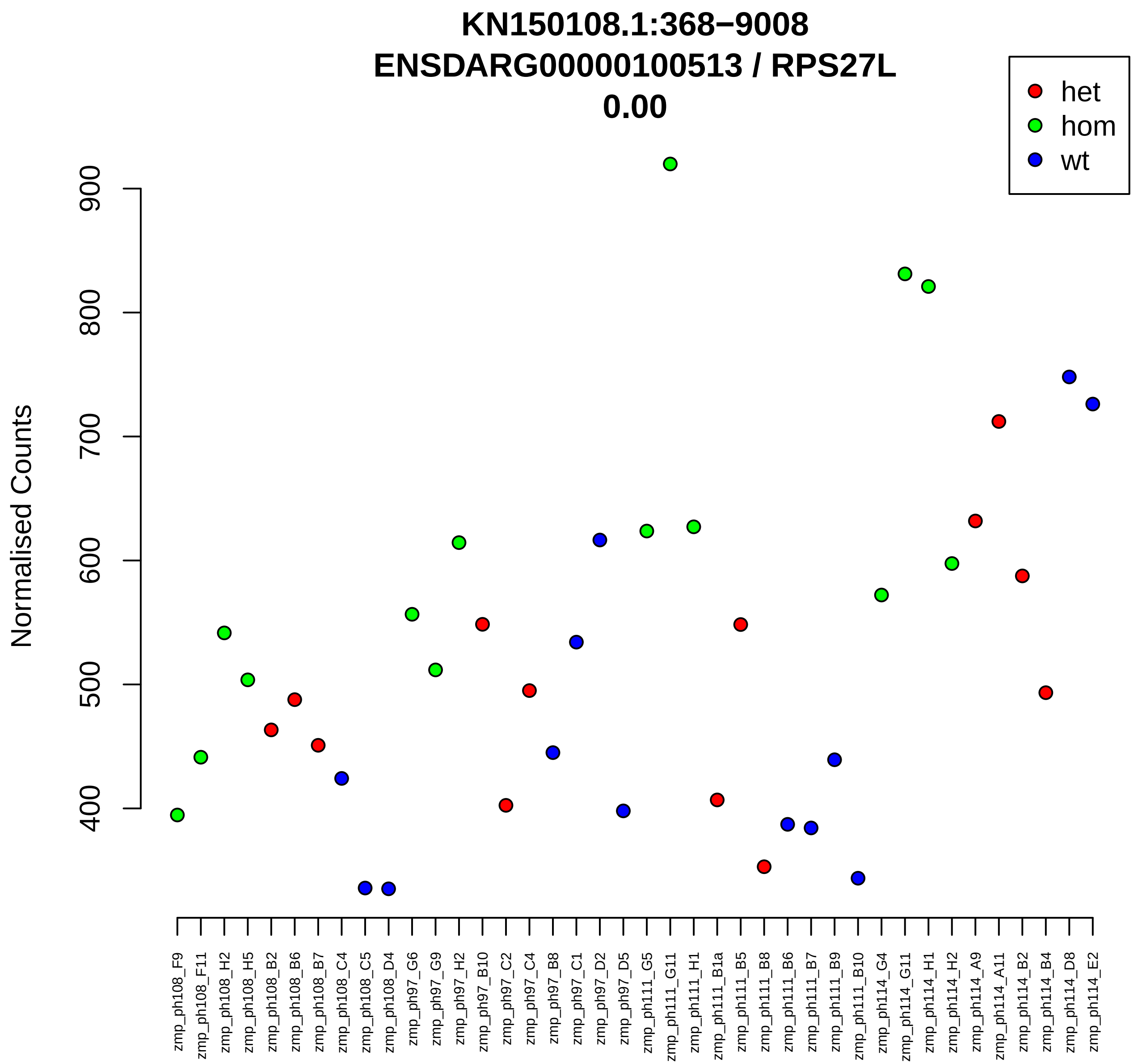

KN150108.1:368−9008
ENSDARG00000100513 / RPS27L
0.00
het
hom
wt
Normalised Counts
900
800
700
600
500
400
zmp_ph108_F9
zmp_ph108_F11
zmp_ph108_H2
zmp_ph108_H5
zmp_ph108_B2
zmp_ph108_B6
zmp_ph108_B7
zmp_ph108_C4
zmp_ph108_C5
zmp_ph108_D4
zmp_ph97_G6
zmp_ph97_G9
zmp_ph97_H2
zmp_ph97_B10
zmp_ph97_C2
zmp_ph97_C4
zmp_ph97_B8
zmp_ph97_C1
zmp_ph97_D2
zmp_ph97_D5
zmp_ph111_G5
zmp_ph111_G11
zmp_ph111_H1
zmp_ph111_B1a
zmp_ph111_B5
zmp_ph111_B8
zmp_ph111_B6
zmp_ph111_B7
zmp_ph111_B9
zmp_ph111_B10
zmp_ph114_G4
zmp_ph114_G11
zmp_ph114_H1
zmp_ph114_H2
zmp_ph114_A9
zmp_ph114_A11
zmp_ph114_B2
zmp_ph114_B4
zmp_ph114_D8
zmp_ph114_E2

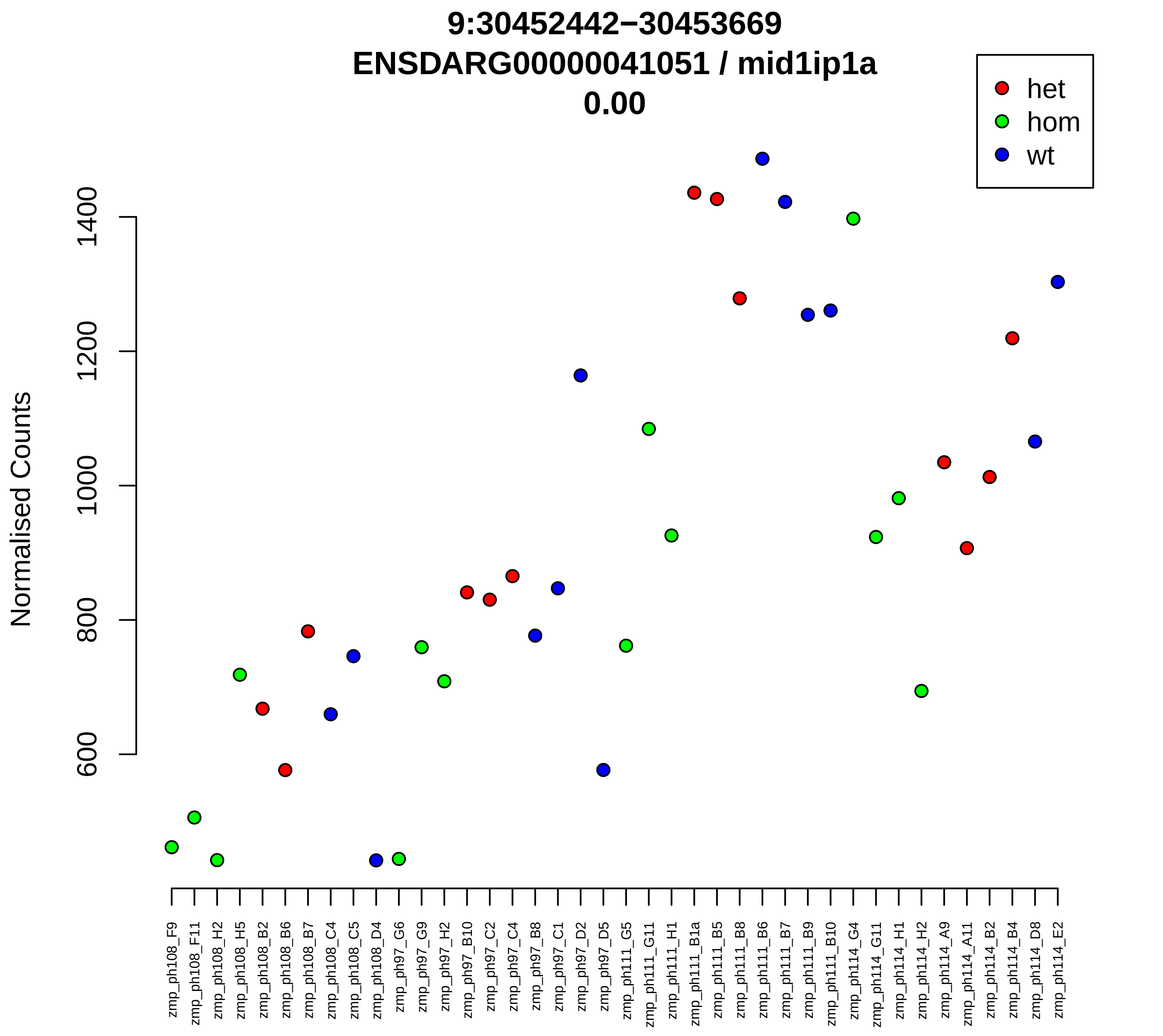
9:30452442−30453669
ENSDARG00000041051 / mid1ip1a
0.00
het
hom
wt
Normalised Counts
600
800
1000
1200
1400
zmp_ph108_F9
zmp_ph108_F11
zmp_ph108_H2
zmp_ph108_H5
zmp_ph108_B2
zmp_ph108_B6
zmp_ph108_B7
zmp_ph108_C4
zmp_ph108_C5
zmp_ph108_D4
zmp_ph97_G6
zmp_ph97_G9
zmp_ph97_H2
zmp_ph97_B10
zmp_ph97_C2
zmp_ph97_C4
zmp_ph97_B8
zmp_ph97_C1
zmp_ph97_D2
zmp_ph97_D5
zmp_ph111_G5
zmp_ph111_G11
zmp_ph111_H1
zmp_ph111_B1a
zmp_ph111_B5
zmp_ph111_B8
zmp_ph111_B6
zmp_ph111_B7
zmp_ph111_B9
zmp_ph111_B10
zmp_ph114_G4
zmp_ph114_G11
zmp_ph114_H1
zmp_ph114_H2
zmp_ph114_A9
zmp_ph114_A11
zmp_ph114_B2
zmp_ph114_B4
zmp_ph114_D8
zmp_ph114_E2

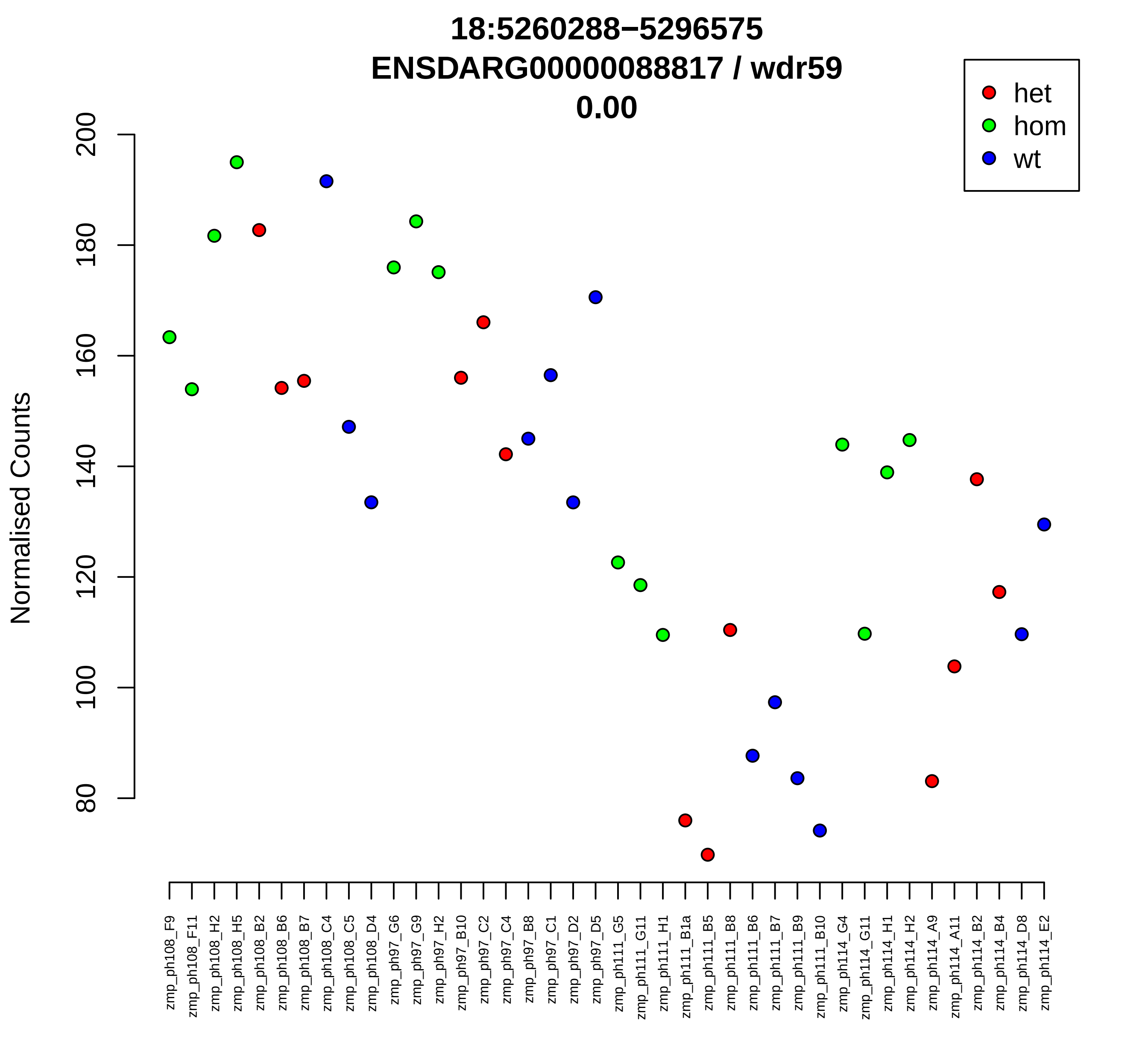

18:5260288−5296575
ENSDARG00000088817 / wdr59
0.00
het
hom
wt
Normalised Counts
80
100
120
140
160
180
200
zmp_ph108_F9
zmp_ph108_F11
zmp_ph108_H2
zmp_ph108_H5
zmp_ph108_B2
zmp_ph108_B6
zmp_ph108_B7
zmp_ph108_C4
zmp_ph108_C5
zmp_ph108_D4
zmp_ph97_G6
zmp_ph97_G9
zmp_ph97_H2
zmp_ph97_B10
zmp_ph97_C2
zmp_ph97_C4
zmp_ph97_B8
zmp_ph97_C1
zmp_ph97_D2
zmp_ph97_D5
zmp_ph111_G5
zmp_ph111_G11
zmp_ph111_H1
zmp_ph111_B1a
zmp_ph111_B5
zmp_ph111_B8
zmp_ph111_B6
zmp_ph111_B7
zmp_ph111_B9
zmp_ph111_B10
zmp_ph114_G4
zmp_ph114_G11
zmp_ph114_H1
zmp_ph114_H2
zmp_ph114_A9
zmp_ph114_A11
zmp_ph114_B2
zmp_ph114_B4
zmp_ph114_D8
zmp_ph114_E2

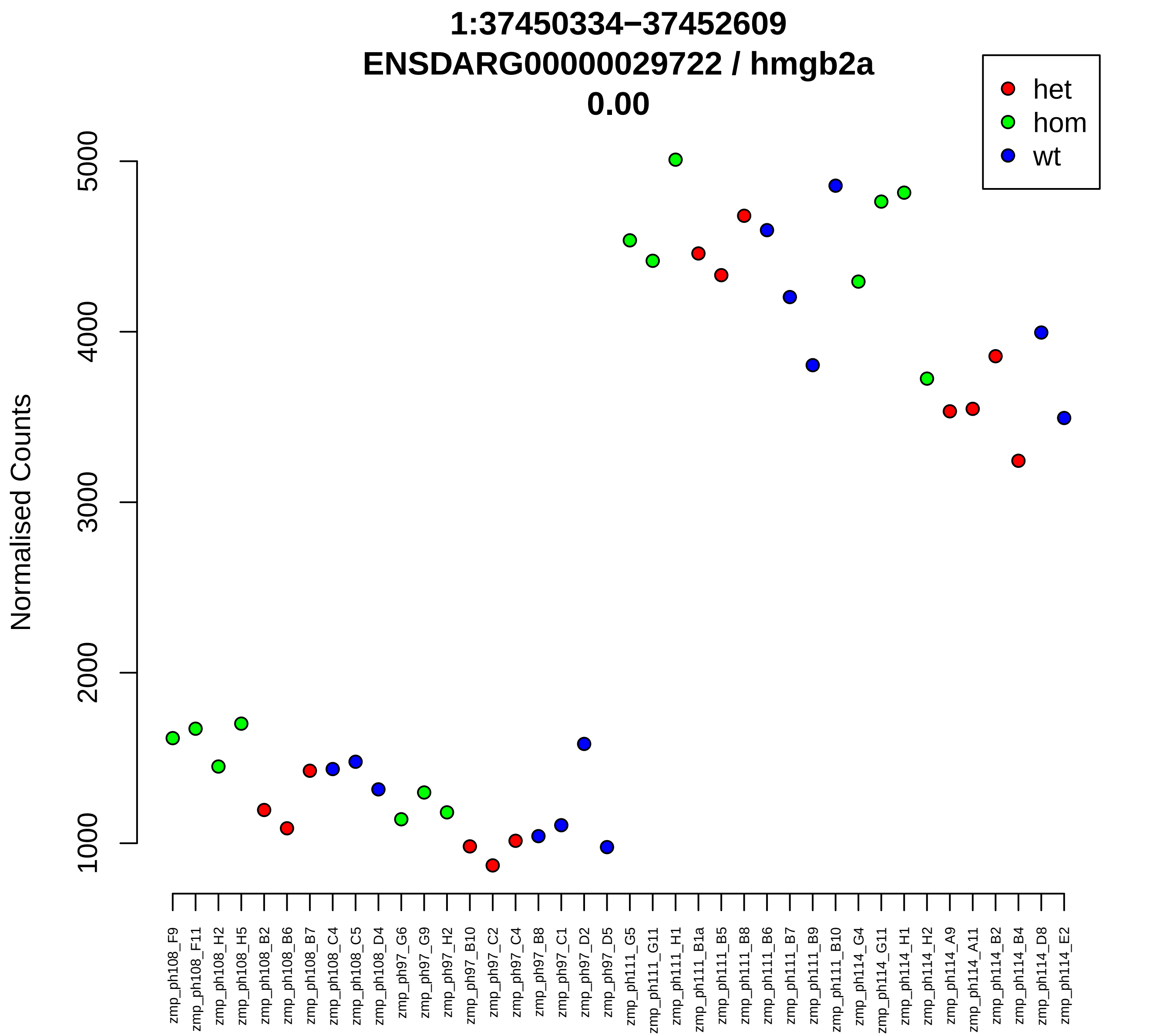

1:37450334−37452609
ENSDARG00000029722 / hmgb2a
0.00
het
hom
wt
Normalised Counts
1000
2000
3000
4000
5000
zmp_ph108_F9
zmp_ph108_F11
zmp_ph108_H2
zmp_ph108_H5
zmp_ph108_B2
zmp_ph108_B6
zmp_ph108_B7
zmp_ph108_C4
zmp_ph108_C5
zmp_ph108_D4
zmp_ph97_G6
zmp_ph97_G9
zmp_ph97_H2
zmp_ph97_B10
zmp_ph97_C2
zmp_ph97_C4
zmp_ph97_B8
zmp_ph97_C1
zmp_ph97_D2
zmp_ph97_D5
zmp_ph111_G5
zmp_ph111_G11
zmp_ph111_H1
zmp_ph111_B1a
zmp_ph111_B5
zmp_ph111_B8
zmp_ph111_B6
zmp_ph111_B7
zmp_ph111_B9
zmp_ph111_B10
zmp_ph114_G4
zmp_ph114_G11
zmp_ph114_H1
zmp_ph114_H2
zmp_ph114_A9
zmp_ph114_A11
zmp_ph114_B2
zmp_ph114_B4
zmp_ph114_D8
zmp_ph114_E2

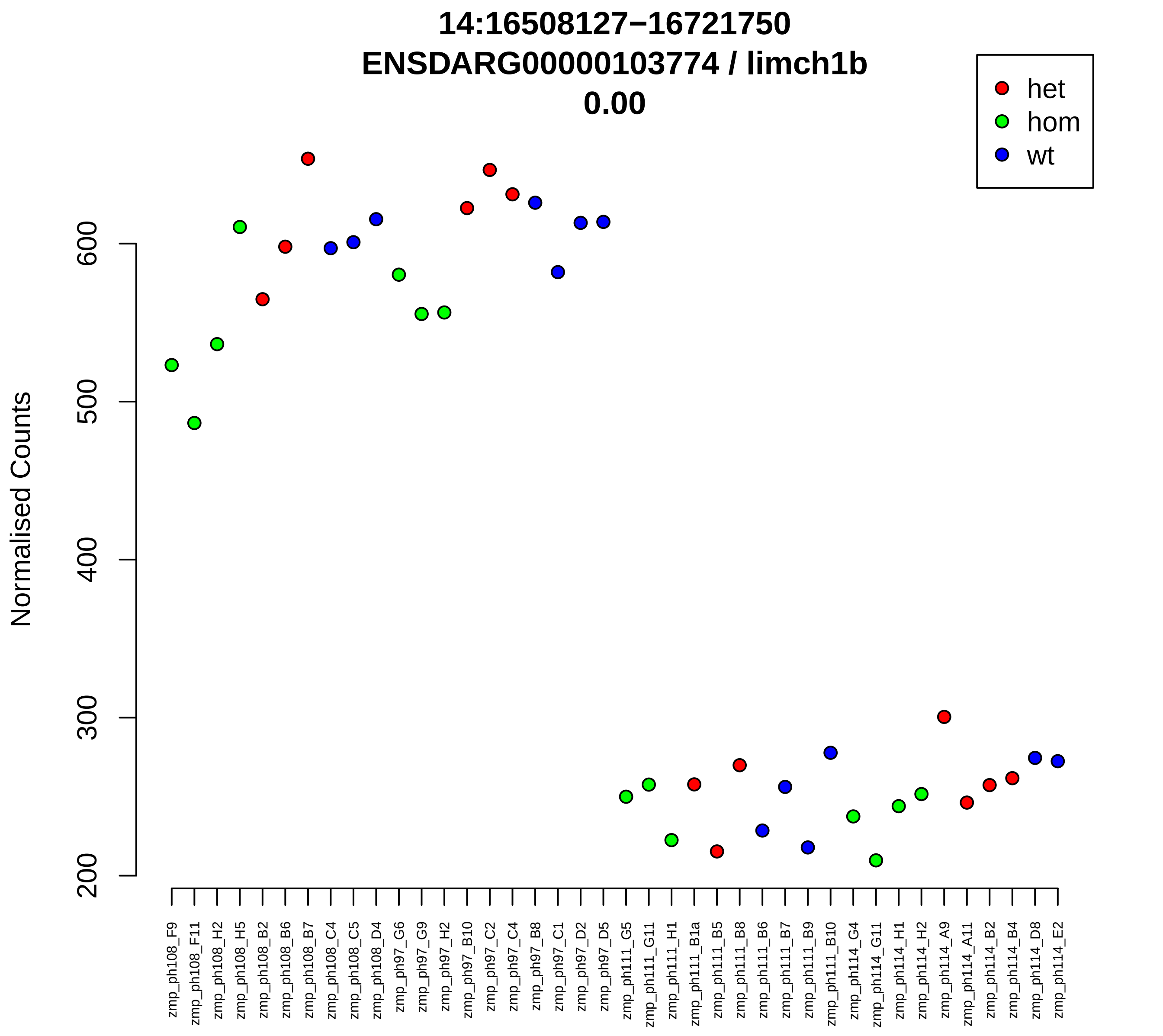
14:16508127−16721750
ENSDARG00000103774 / limch1b
0.00
het
hom
wt
Normalised Counts
200
300
400
500
600
zmp_ph108_F9
zmp_ph108_F11
zmp_ph108_H2
zmp_ph108_H5
zmp_ph108_B2
zmp_ph108_B6
zmp_ph108_B7
zmp_ph108_C4
zmp_ph108_C5
zmp_ph108_D4
zmp_ph97_G6
zmp_ph97_G9
zmp_ph97_H2
zmp_ph97_B10
zmp_ph97_C2
zmp_ph97_C4
zmp_ph97_B8
zmp_ph97_C1
zmp_ph97_D2
zmp_ph97_D5
zmp_ph111_G5
zmp_ph111_G11
zmp_ph111_H1
zmp_ph111_B1a
zmp_ph111_B5
zmp_ph111_B8
zmp_ph111_B6
zmp_ph111_B7
zmp_ph111_B9
zmp_ph111_B10
zmp_ph114_G4
zmp_ph114_G11
zmp_ph114_H1
zmp_ph114_H2
zmp_ph114_A9
zmp_ph114_A11
zmp_ph114_B2
zmp_ph114_B4
zmp_ph114_D8
zmp_ph114_E2

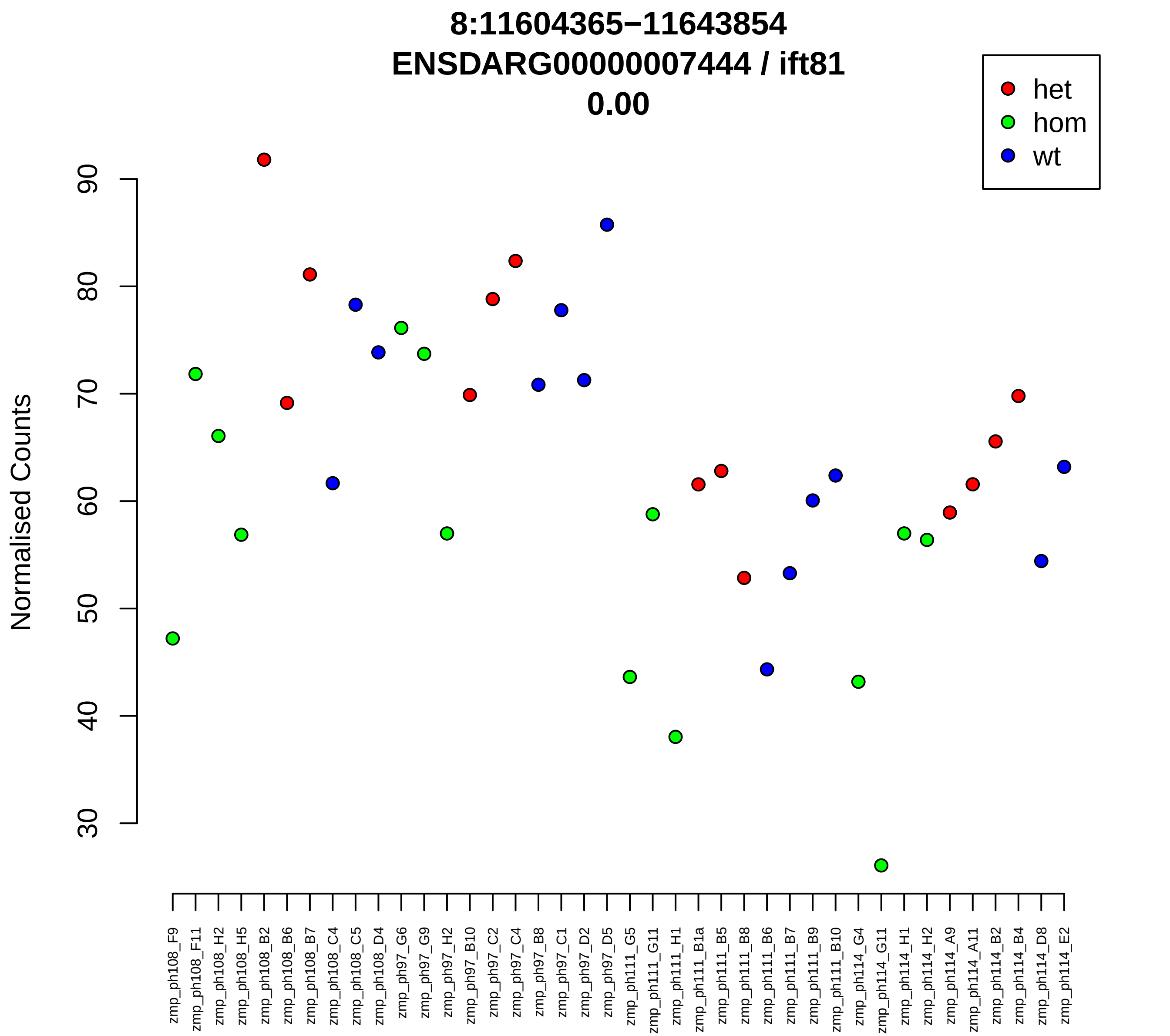

8:11604365−11643854
ENSDARG00000007444 / ift81
0.00
het
hom
wt
Normalised Counts
30
40
50
60
70
80
90
zmp_ph108_F9
zmp_ph108_F11
zmp_ph108_H2
zmp_ph108_H5
zmp_ph108_B2
zmp_ph108_B6
zmp_ph108_B7
zmp_ph108_C4
zmp_ph108_C5
zmp_ph108_D4
zmp_ph97_G6
zmp_ph97_G9
zmp_ph97_H2
zmp_ph97_B10
zmp_ph97_C2
zmp_ph97_C4
zmp_ph97_B8
zmp_ph97_C1
zmp_ph97_D2
zmp_ph97_D5
zmp_ph111_G5
zmp_ph111_G11
zmp_ph111_H1
zmp_ph111_B1a
zmp_ph111_B5
zmp_ph111_B8
zmp_ph111_B6
zmp_ph111_B7
zmp_ph111_B9
zmp_ph111_B10
zmp_ph114_G4
zmp_ph114_G11
zmp_ph114_H1
zmp_ph114_H2
zmp_ph114_A9
zmp_ph114_A11
zmp_ph114_B2
zmp_ph114_B4
zmp_ph114_D8
zmp_ph114_E2

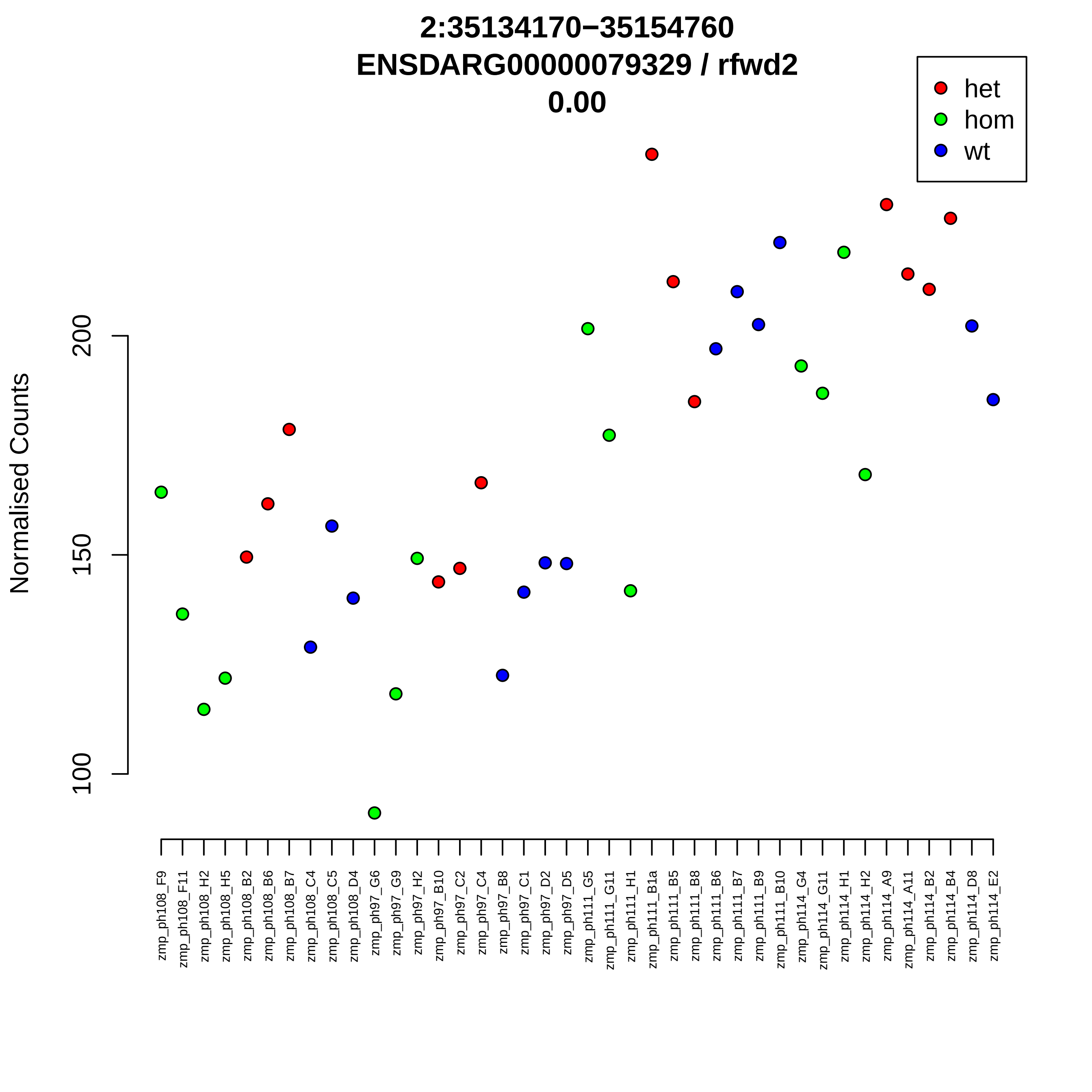
2:35134170−35154760
ENSDARG00000079329 / rfwd2
0.00
het
hom
wt
Normalised Counts
100
150
200
zmp_ph108_F9
zmp_ph108_F11
zmp_ph108_H2
zmp_ph108_H5
zmp_ph108_B2
zmp_ph108_B6
zmp_ph108_B7
zmp_ph108_C4
zmp_ph108_C5
zmp_ph108_D4
zmp_ph97_G6
zmp_ph97_G9
zmp_ph97_H2
zmp_ph97_B10
zmp_ph97_C2
zmp_ph97_C4
zmp_ph97_B8
zmp_ph97_C1
zmp_ph97_D2
zmp_ph97_D5
zmp_ph111_G5
zmp_ph111_G11
zmp_ph111_H1
zmp_ph111_B1a
zmp_ph111_B5
zmp_ph111_B8
zmp_ph111_B6
zmp_ph111_B7
zmp_ph111_B9
zmp_ph111_B10
zmp_ph114_G4
zmp_ph114_G11
zmp_ph114_H1
zmp_ph114_H2
zmp_ph114_A9
zmp_ph114_A11
zmp_ph114_B2
zmp_ph114_B4
zmp_ph114_D8
zmp_ph114_E2

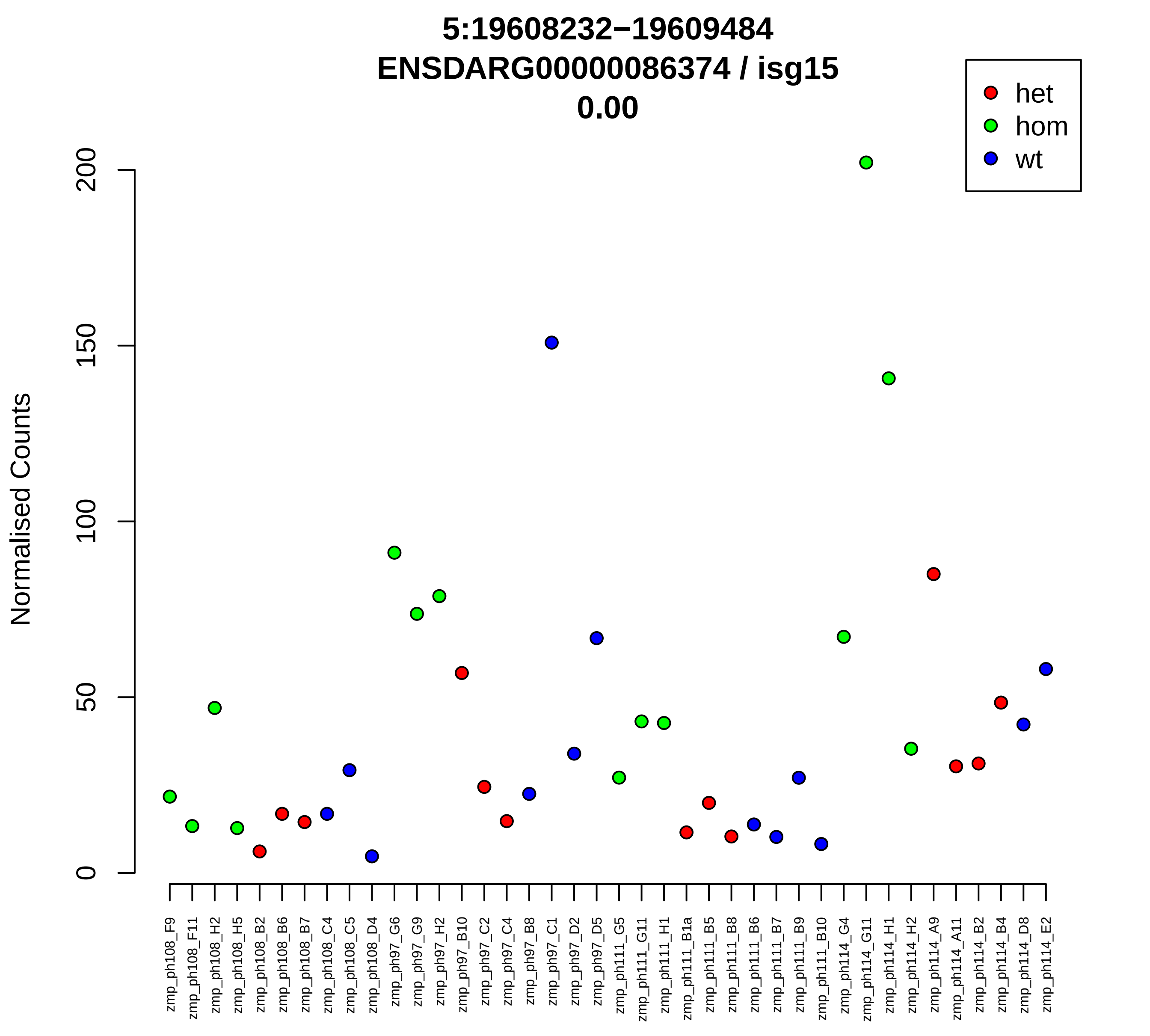

5:19608232−19609484
ENSDARG00000086374 / isg15
0.00
het
hom
wt
Normalised Counts
0
50
100
150
200
zmp_ph108_F9
zmp_ph108_F11
zmp_ph108_H2
zmp_ph108_H5
zmp_ph108_B2
zmp_ph108_B6
zmp_ph108_B7
zmp_ph108_C4
zmp_ph108_C5
zmp_ph108_D4
zmp_ph97_G6
zmp_ph97_G9
zmp_ph97_H2
zmp_ph97_B10
zmp_ph97_C2
zmp_ph97_C4
zmp_ph97_B8
zmp_ph97_C1
zmp_ph97_D2
zmp_ph97_D5
zmp_ph111_G5
zmp_ph111_G11
zmp_ph111_H1
zmp_ph111_B1a
zmp_ph111_B5
zmp_ph111_B8
zmp_ph111_B6
zmp_ph111_B7
zmp_ph111_B9
zmp_ph111_B10
zmp_ph114_G4
zmp_ph114_G11
zmp_ph114_H1
zmp_ph114_H2
zmp_ph114_A9
zmp_ph114_A11
zmp_ph114_B2
zmp_ph114_B4
zmp_ph114_D8
zmp_ph114_E2

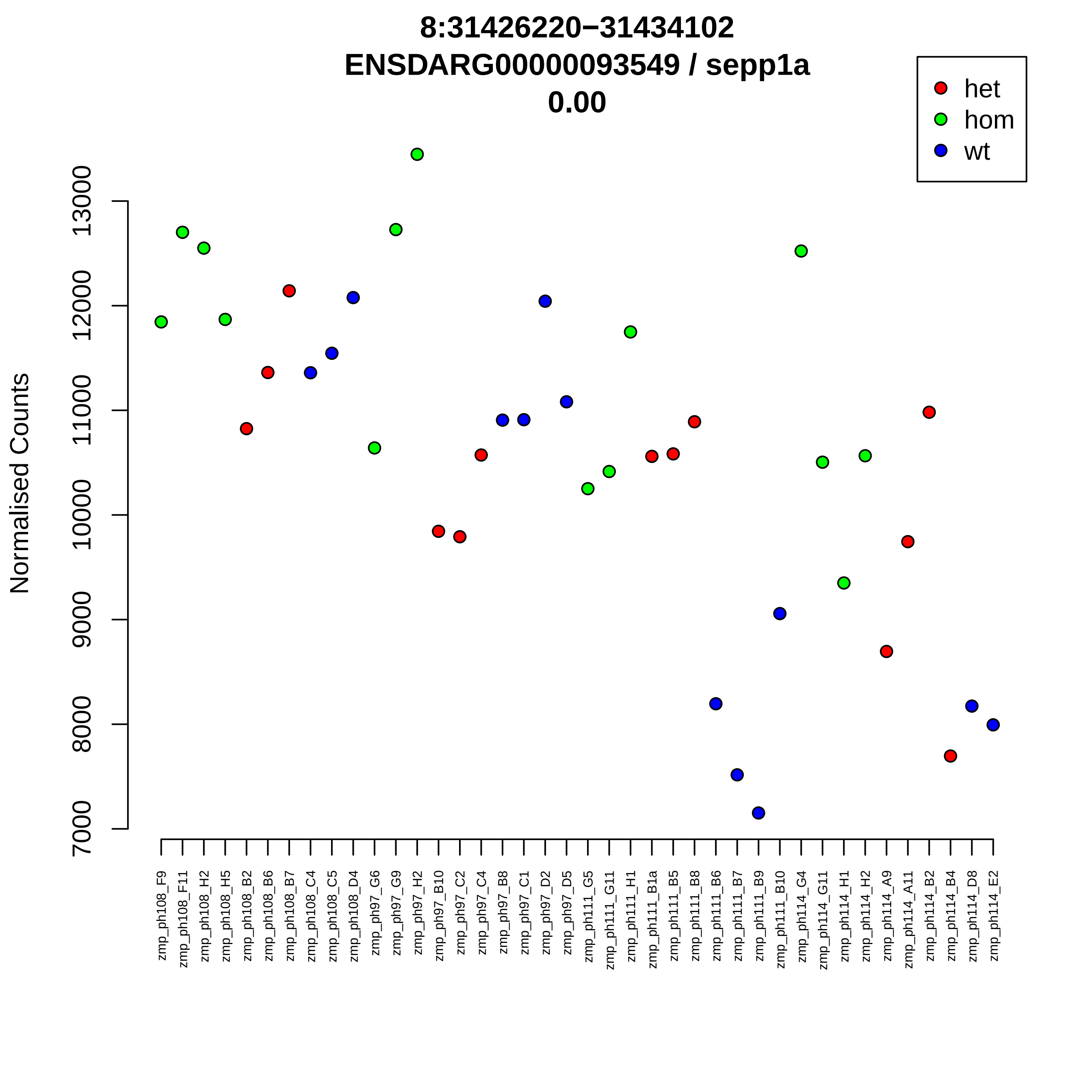

8:31426220−31434102
ENSDARG00000093549 / sepp1a
0.00
het
hom
wt
Normalised Counts
7000
8000
9000
10000
11000
12000
13000
zmp_ph108_F9
zmp_ph108_F11
zmp_ph108_H2
zmp_ph108_H5
zmp_ph108_B2
zmp_ph108_B6
zmp_ph108_B7
zmp_ph108_C4
zmp_ph108_C5
zmp_ph108_D4
zmp_ph97_G6
zmp_ph97_G9
zmp_ph97_H2
zmp_ph97_B10
zmp_ph97_C2
zmp_ph97_C4
zmp_ph97_B8
zmp_ph97_C1
zmp_ph97_D2
zmp_ph97_D5
zmp_ph111_G5
zmp_ph111_G11
zmp_ph111_H1
zmp_ph111_B1a
zmp_ph111_B5
zmp_ph111_B8
zmp_ph111_B6
zmp_ph111_B7
zmp_ph111_B9
zmp_ph111_B10
zmp_ph114_G4
zmp_ph114_G11
zmp_ph114_H1
zmp_ph114_H2
zmp_ph114_A9
zmp_ph114_A11
zmp_ph114_B2
zmp_ph114_B4
zmp_ph114_D8
zmp_ph114_E2

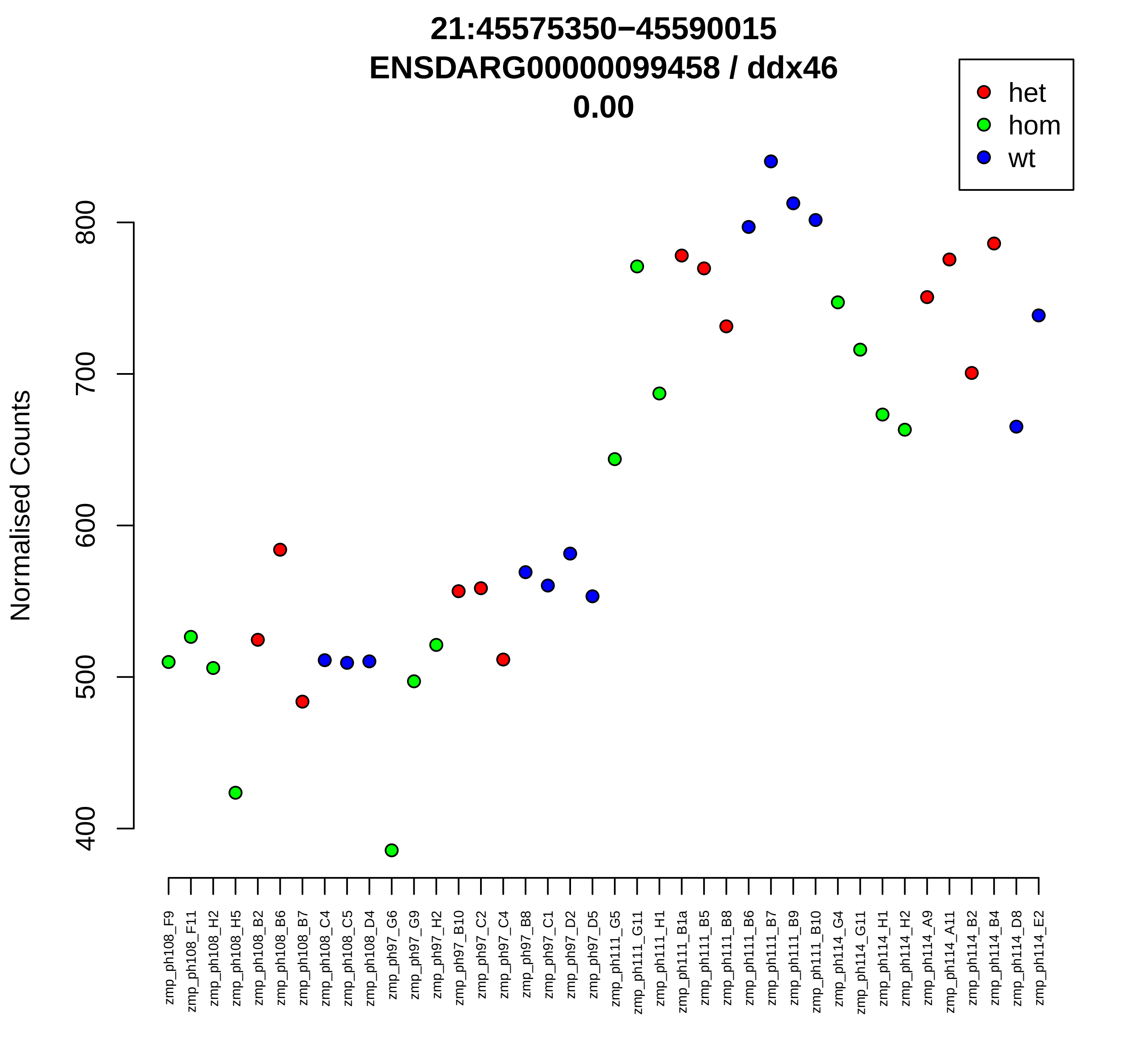

21:45575350−45590015
ENSDARG00000099458 / ddx46
0.00
het
hom
wt
Normalised Counts
400
500
600
700
800
zmp_ph108_F9
zmp_ph108_F11
zmp_ph108_H2
zmp_ph108_H5
zmp_ph108_B2
zmp_ph108_B6
zmp_ph108_B7
zmp_ph108_C4
zmp_ph108_C5
zmp_ph108_D4
zmp_ph97_G6
zmp_ph97_G9
zmp_ph97_H2
zmp_ph97_B10
zmp_ph97_C2
zmp_ph97_C4
zmp_ph97_B8
zmp_ph97_C1
zmp_ph97_D2
zmp_ph97_D5
zmp_ph111_G5
zmp_ph111_G11
zmp_ph111_H1
zmp_ph111_B1a
zmp_ph111_B5
zmp_ph111_B8
zmp_ph111_B6
zmp_ph111_B7
zmp_ph111_B9
zmp_ph111_B10
zmp_ph114_G4
zmp_ph114_G11
zmp_ph114_H1
zmp_ph114_H2
zmp_ph114_A9
zmp_ph114_A11
zmp_ph114_B2
zmp_ph114_B4
zmp_ph114_D8
zmp_ph114_E2

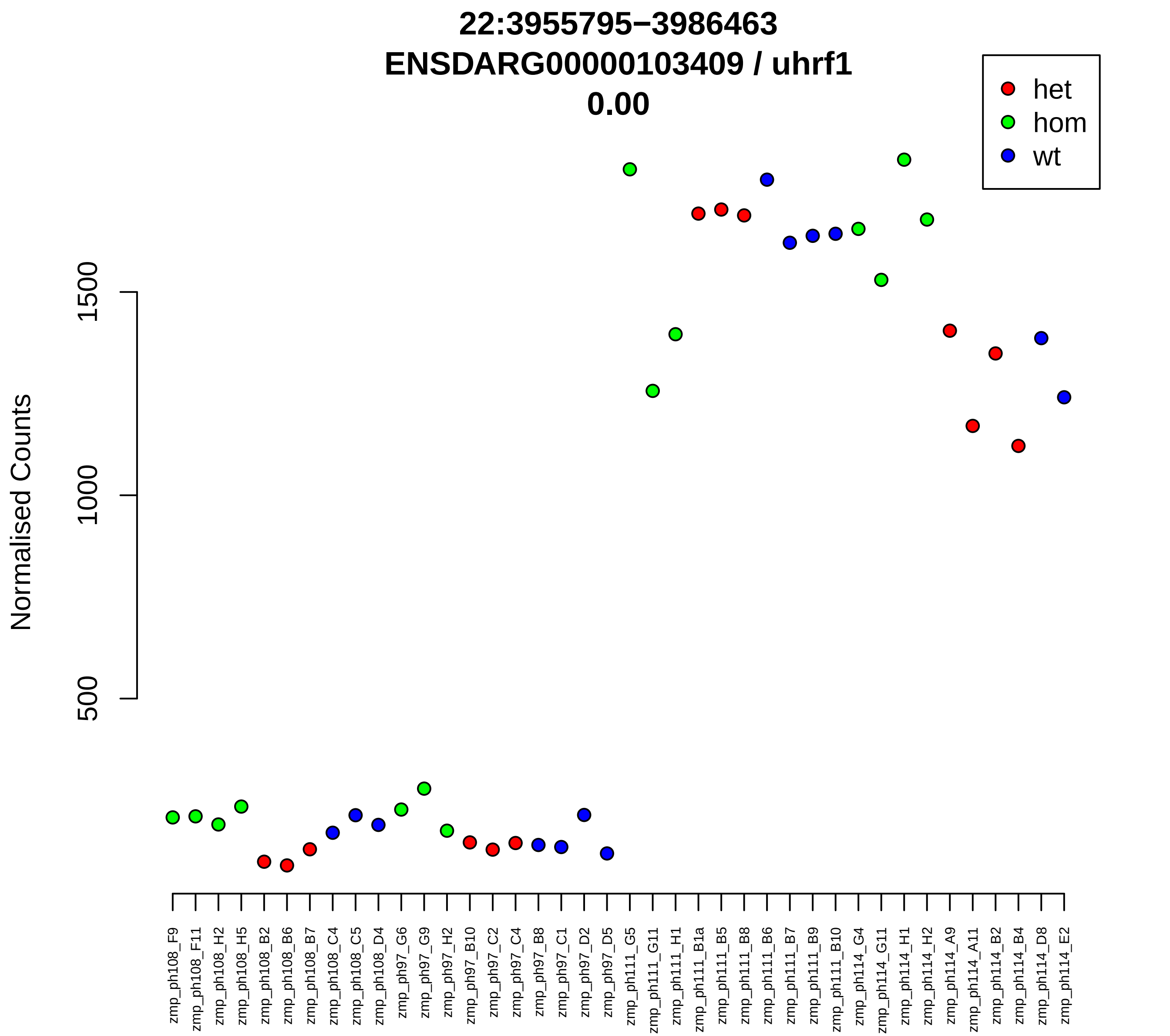
22:3955795−3986463
ENSDARG00000103409 / uhrf1
0.00
het
hom
wt
Normalised Counts
500
1000
1500
zmp_ph108_F9
zmp_ph108_F11
zmp_ph108_H2
zmp_ph108_H5
zmp_ph108_B2
zmp_ph108_B6
zmp_ph108_B7
zmp_ph108_C4
zmp_ph108_C5
zmp_ph108_D4
zmp_ph97_G6
zmp_ph97_G9
zmp_ph97_H2
zmp_ph97_B10
zmp_ph97_C2
zmp_ph97_C4
zmp_ph97_B8
zmp_ph97_C1
zmp_ph97_D2
zmp_ph97_D5
zmp_ph111_G5
zmp_ph111_G11
zmp_ph111_H1
zmp_ph111_B1a
zmp_ph111_B5
zmp_ph111_B8
zmp_ph111_B6
zmp_ph111_B7
zmp_ph111_B9
zmp_ph111_B10
zmp_ph114_G4
zmp_ph114_G11
zmp_ph114_H1
zmp_ph114_H2
zmp_ph114_A9
zmp_ph114_A11
zmp_ph114_B2
zmp_ph114_B4
zmp_ph114_D8
zmp_ph114_E2

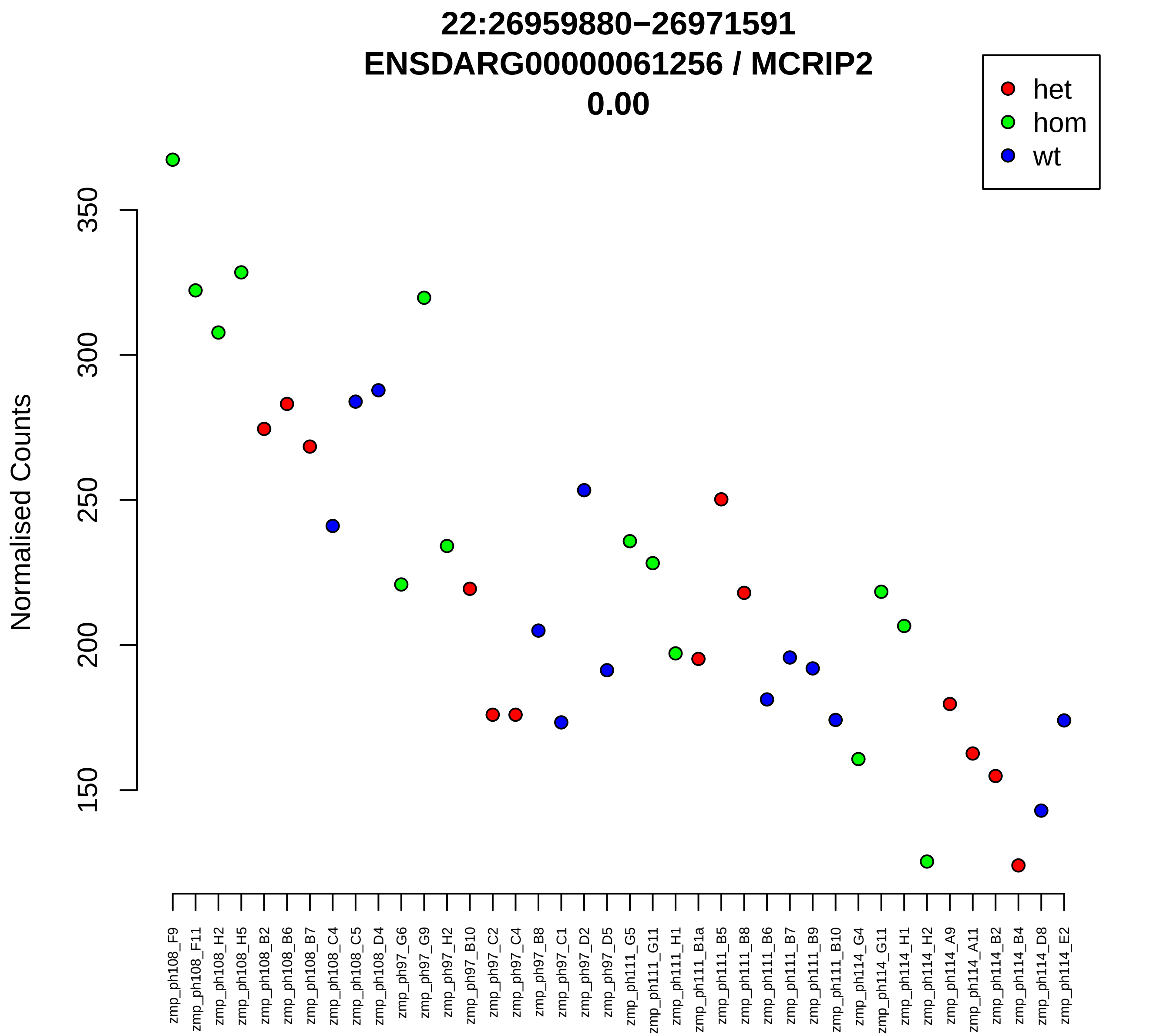
22:26959880−26971591
ENSDARG00000061256 / MCRIP2
0.00
het
hom
wt
Normalised Counts
150
200
250
300
350
zmp_ph108_F9
zmp_ph108_F11
zmp_ph108_H2
zmp_ph108_H5
zmp_ph108_B2
zmp_ph108_B6
zmp_ph108_B7
zmp_ph108_C4
zmp_ph108_C5
zmp_ph108_D4
zmp_ph97_G6
zmp_ph97_G9
zmp_ph97_H2
zmp_ph97_B10
zmp_ph97_C2
zmp_ph97_C4
zmp_ph97_B8
zmp_ph97_C1
zmp_ph97_D2
zmp_ph97_D5
zmp_ph111_G5
zmp_ph111_G11
zmp_ph111_H1
zmp_ph111_B1a
zmp_ph111_B5
zmp_ph111_B8
zmp_ph111_B6
zmp_ph111_B7
zmp_ph111_B9
zmp_ph111_B10
zmp_ph114_G4
zmp_ph114_G11
zmp_ph114_H1
zmp_ph114_H2
zmp_ph114_A9
zmp_ph114_A11
zmp_ph114_B2
zmp_ph114_B4
zmp_ph114_D8
zmp_ph114_E2

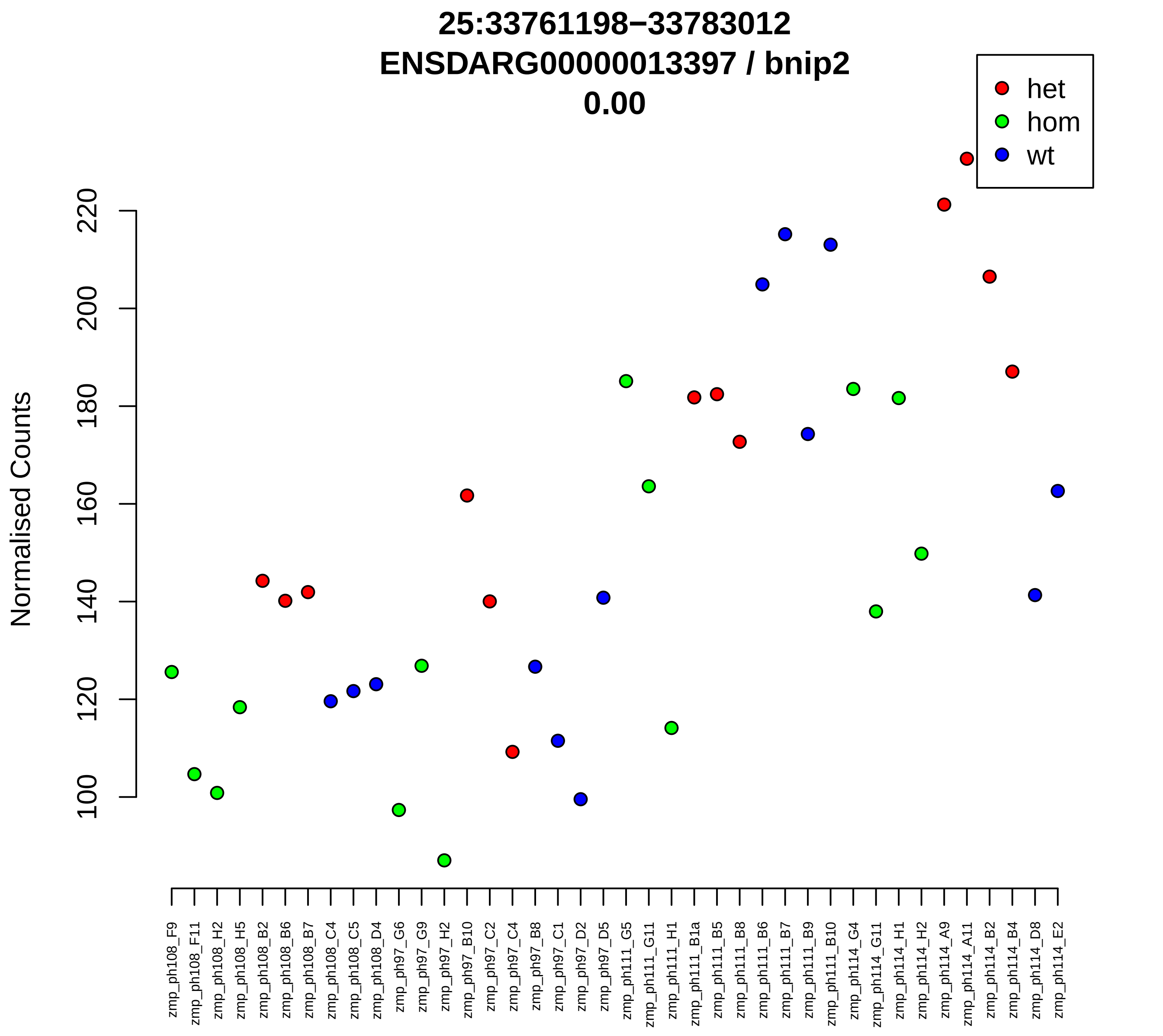

25:33761198−33783012
ENSDARG00000013397 / bnip2
0.00
het
hom
wt
Normalised Counts
100
120
140
160
180
200
220
zmp_ph108_F9
zmp_ph108_F11
zmp_ph108_H2
zmp_ph108_H5
zmp_ph108_B2
zmp_ph108_B6
zmp_ph108_B7
zmp_ph108_C4
zmp_ph108_C5
zmp_ph108_D4
zmp_ph97_G6
zmp_ph97_G9
zmp_ph97_H2
zmp_ph97_B10
zmp_ph97_C2
zmp_ph97_C4
zmp_ph97_B8
zmp_ph97_C1
zmp_ph97_D2
zmp_ph97_D5
zmp_ph111_G5
zmp_ph111_G11
zmp_ph111_H1
zmp_ph111_B1a
zmp_ph111_B5
zmp_ph111_B8
zmp_ph111_B6
zmp_ph111_B7
zmp_ph111_B9
zmp_ph111_B10
zmp_ph114_G4
zmp_ph114_G11
zmp_ph114_H1
zmp_ph114_H2
zmp_ph114_A9
zmp_ph114_A11
zmp_ph114_B2
zmp_ph114_B4
zmp_ph114_D8
zmp_ph114_E2

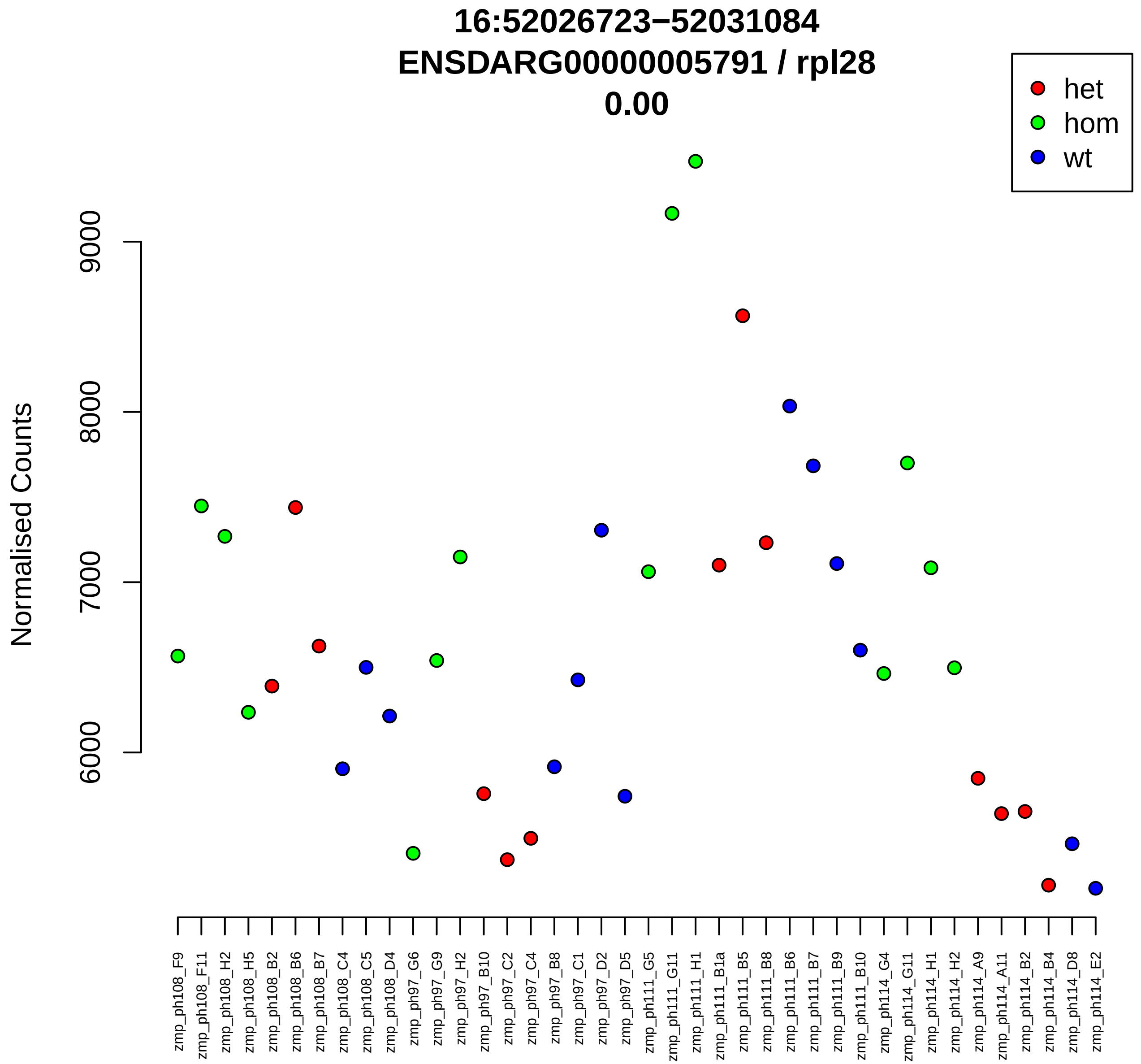
16:52026723−52031084
ENSDARG00000005791 / rpl28
0.00
het
hom
wt
Normalised Counts
6000
7000
8000
9000
zmp_ph108_F9
zmp_ph108_F11
zmp_ph108_H2
zmp_ph108_H5
zmp_ph108_B2
zmp_ph108_B6
zmp_ph108_B7
zmp_ph108_C4
zmp_ph108_C5
zmp_ph108_D4
zmp_ph97_G6
zmp_ph97_G9
zmp_ph97_H2
zmp_ph97_B10
zmp_ph97_C2
zmp_ph97_C4
zmp_ph97_B8
zmp_ph97_C1
zmp_ph97_D2
zmp_ph97_D5
zmp_ph111_G5
zmp_ph111_G11
zmp_ph111_H1
zmp_ph111_B1a
zmp_ph111_B5
zmp_ph111_B8
zmp_ph111_B6
zmp_ph111_B7
zmp_ph111_B9
zmp_ph111_B10
zmp_ph114_G4
zmp_ph114_G11
zmp_ph114_H1
zmp_ph114_H2
zmp_ph114_A9
zmp_ph114_A11
zmp_ph114_B2
zmp_ph114_B4
zmp_ph114_D8
zmp_ph114_E2

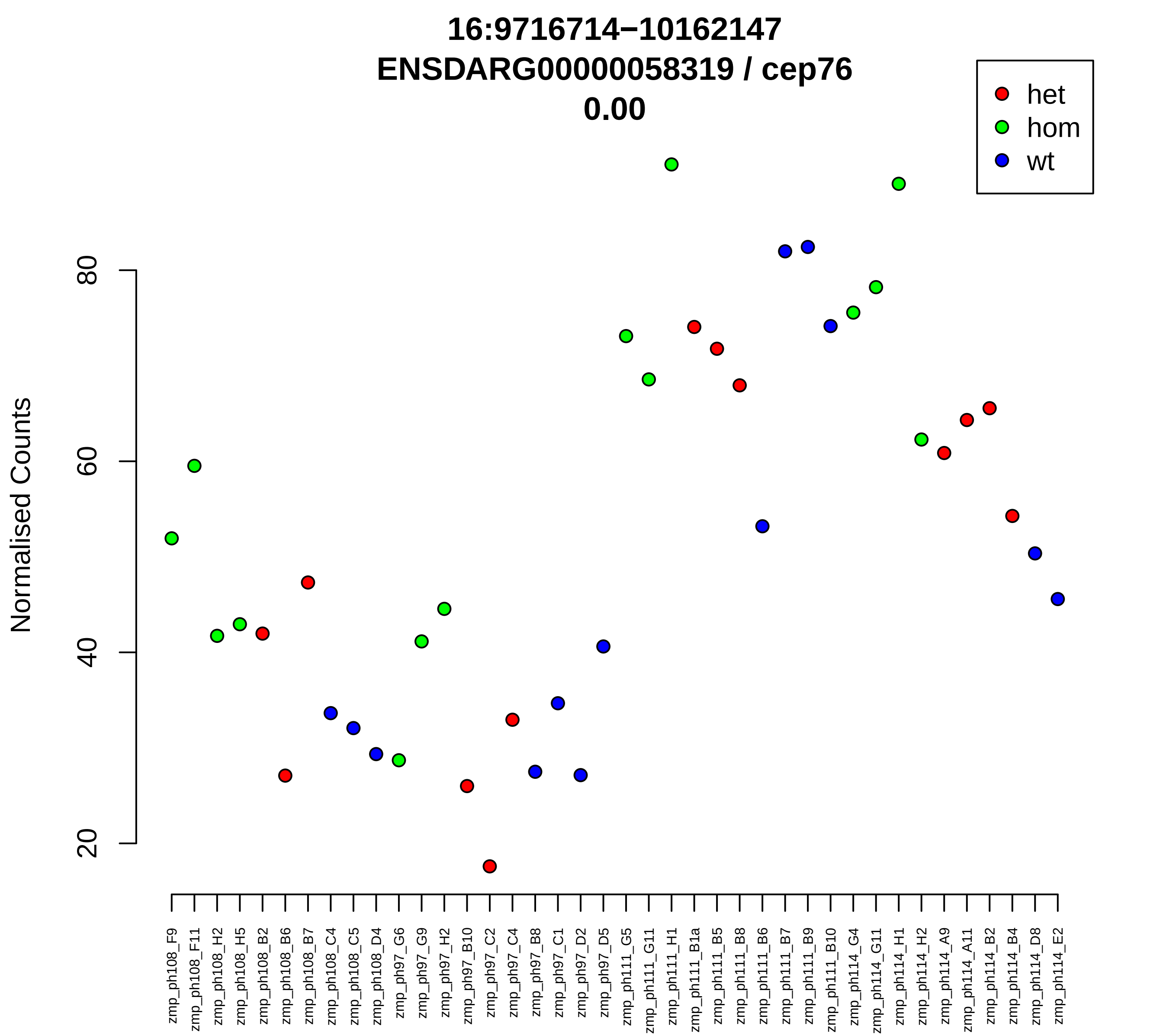
16:9716714−10162147
ENSDARG00000058319 / cep76
0.00
het
hom
wt
Normalised Counts
20
40
60
80
zmp_ph108_F9
zmp_ph108_F11
zmp_ph108_H2
zmp_ph108_H5
zmp_ph108_B2
zmp_ph108_B6
zmp_ph108_B7
zmp_ph108_C4
zmp_ph108_C5
zmp_ph108_D4
zmp_ph97_G6
zmp_ph97_G9
zmp_ph97_H2
zmp_ph97_B10
zmp_ph97_C2
zmp_ph97_C4
zmp_ph97_B8
zmp_ph97_C1
zmp_ph97_D2
zmp_ph97_D5
zmp_ph111_G5
zmp_ph111_G11
zmp_ph111_H1
zmp_ph111_B1a
zmp_ph111_B5
zmp_ph111_B8
zmp_ph111_B6
zmp_ph111_B7
zmp_ph111_B9
zmp_ph111_B10
zmp_ph114_G4
zmp_ph114_G11
zmp_ph114_H1
zmp_ph114_H2
zmp_ph114_A9
zmp_ph114_A11
zmp_ph114_B2
zmp_ph114_B4
zmp_ph114_D8
zmp_ph114_E2

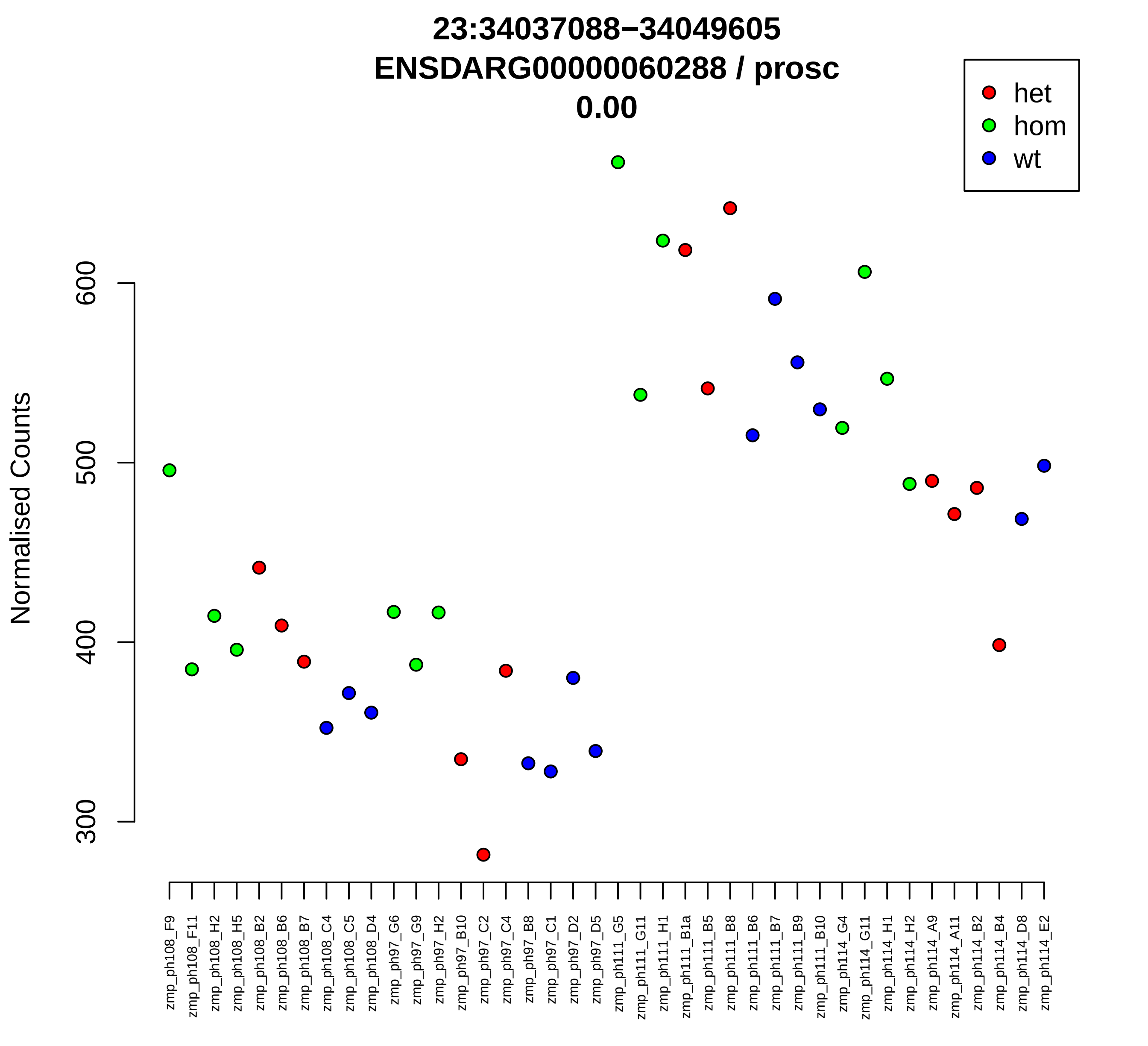
23:34037088−34049605
ENSDARG00000060288 / prosc
0.00
het
hom
wt
Normalised Counts
300
400
500
600
zmp_ph108_F9
zmp_ph108_F11
zmp_ph108_H2
zmp_ph108_H5
zmp_ph108_B2
zmp_ph108_B6
zmp_ph108_B7
zmp_ph108_C4
zmp_ph108_C5
zmp_ph108_D4
zmp_ph97_G6
zmp_ph97_G9
zmp_ph97_H2
zmp_ph97_B10
zmp_ph97_C2
zmp_ph97_C4
zmp_ph97_B8
zmp_ph97_C1
zmp_ph97_D2
zmp_ph97_D5
zmp_ph111_G5
zmp_ph111_G11
zmp_ph111_H1
zmp_ph111_B1a
zmp_ph111_B5
zmp_ph111_B8
zmp_ph111_B6
zmp_ph111_B7
zmp_ph111_B9
zmp_ph111_B10
zmp_ph114_G4
zmp_ph114_G11
zmp_ph114_H1
zmp_ph114_H2
zmp_ph114_A9
zmp_ph114_A11
zmp_ph114_B2
zmp_ph114_B4
zmp_ph114_D8
zmp_ph114_E2

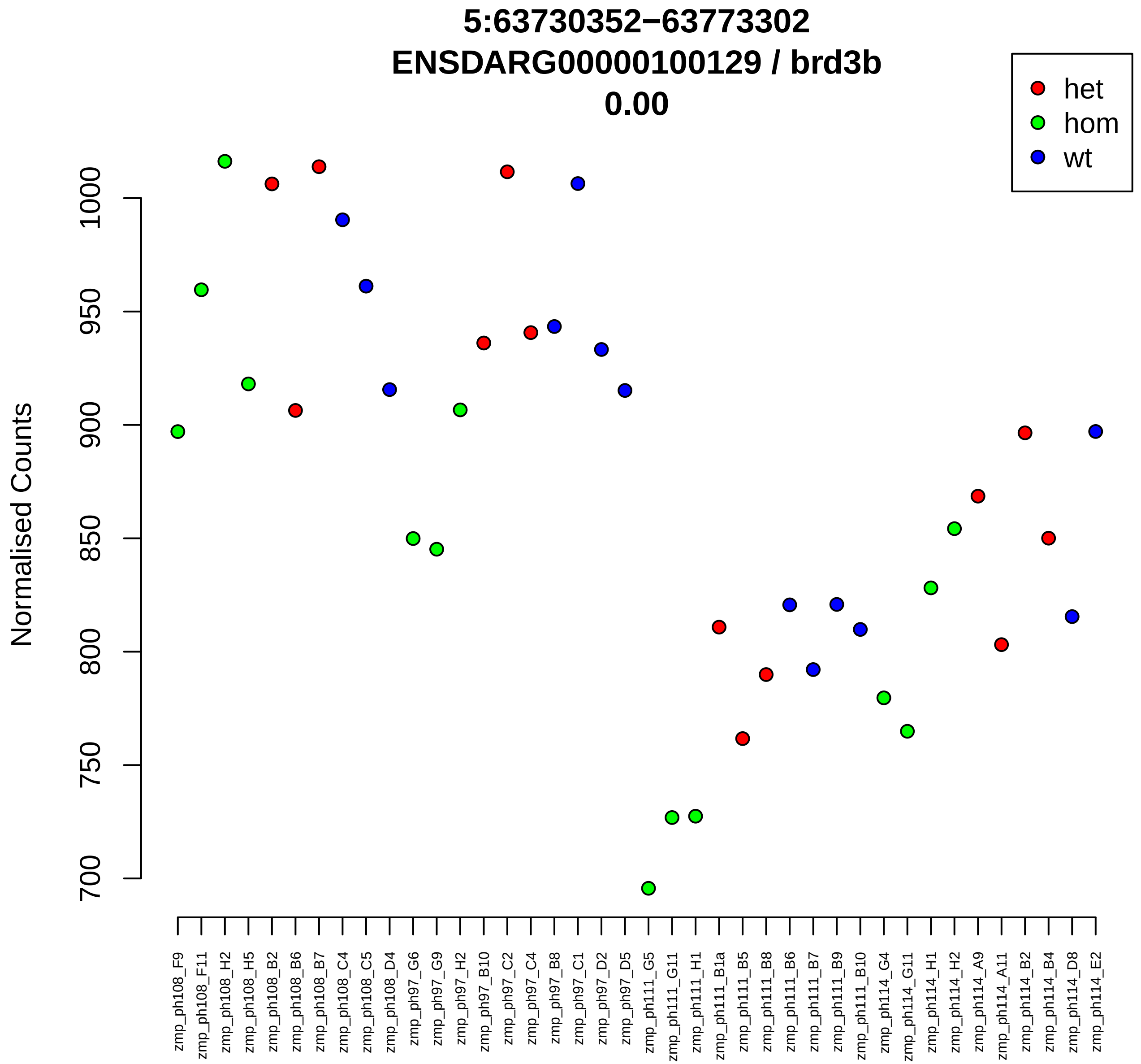
5:63730352−63773302
ENSDARG00000100129 / brd3b
0.00
het
hom
wt
Normalised Counts
700
750
800
850
900
950
1000
zmp_ph108_F9
zmp_ph108_F11
zmp_ph108_H2
zmp_ph108_H5
zmp_ph108_B2
zmp_ph108_B6
zmp_ph108_B7
zmp_ph108_C4
zmp_ph108_C5
zmp_ph108_D4
zmp_ph97_G6
zmp_ph97_G9
zmp_ph97_H2
zmp_ph97_B10
zmp_ph97_C2
zmp_ph97_C4
zmp_ph97_B8
zmp_ph97_C1
zmp_ph97_D2
zmp_ph97_D5
zmp_ph111_G5
zmp_ph111_G11
zmp_ph111_H1
zmp_ph111_B1a
zmp_ph111_B5
zmp_ph111_B8
zmp_ph111_B6
zmp_ph111_B7
zmp_ph111_B9
zmp_ph111_B10
zmp_ph114_G4
zmp_ph114_G11
zmp_ph114_H1
zmp_ph114_H2
zmp_ph114_A9
zmp_ph114_A11
zmp_ph114_B2
zmp_ph114_B4
zmp_ph114_D8
zmp_ph114_E2

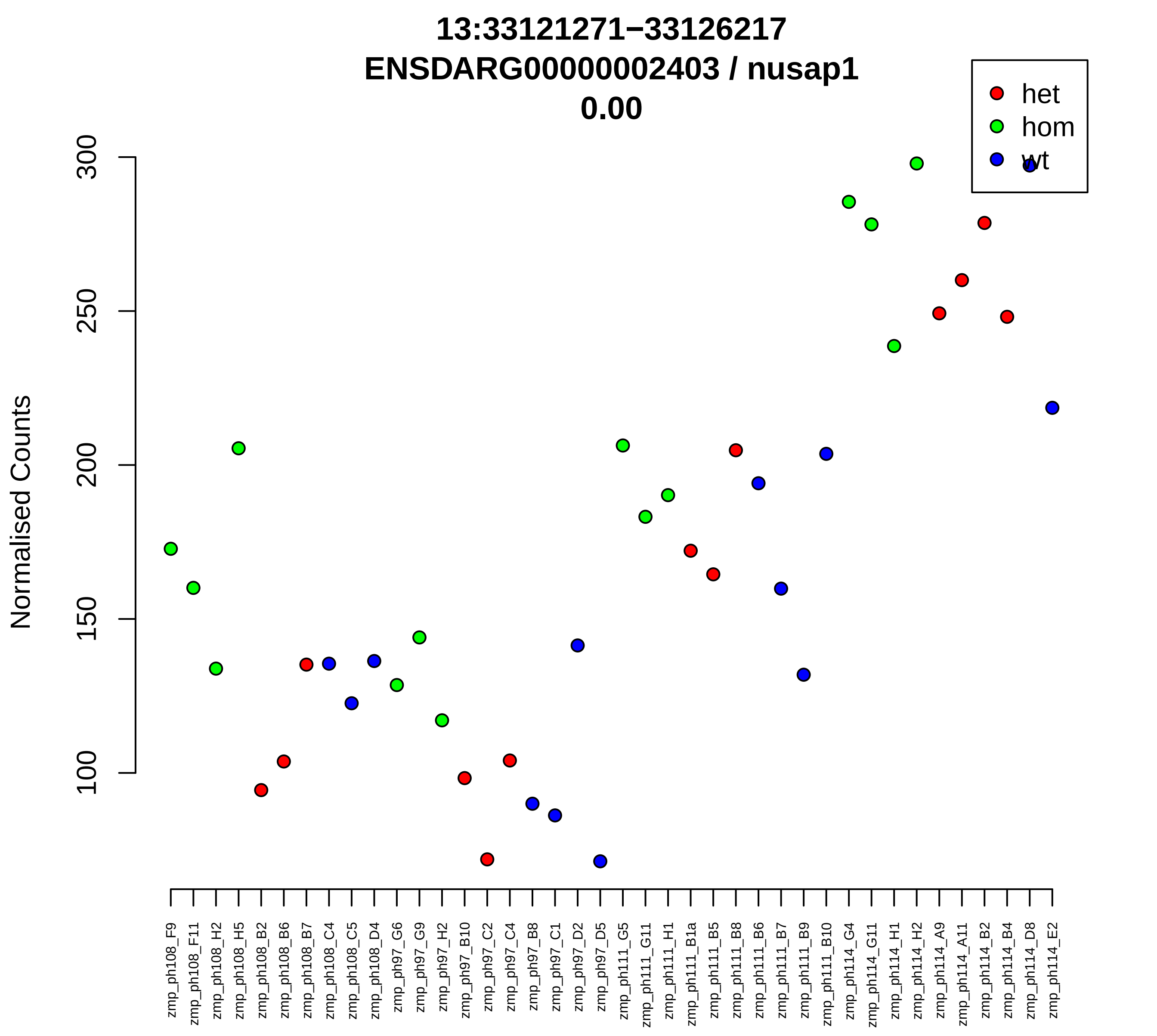
13:33121271−33126217
ENSDARG00000002403 / nusap1
0.00
het
hom
wt
Normalised Counts
100
150
200
250
300
zmp_ph108_F9
zmp_ph108_F11
zmp_ph108_H2
zmp_ph108_H5
zmp_ph108_B2
zmp_ph108_B6
zmp_ph108_B7
zmp_ph108_C4
zmp_ph108_C5
zmp_ph108_D4
zmp_ph97_G6
zmp_ph97_G9
zmp_ph97_H2
zmp_ph97_B10
zmp_ph97_C2
zmp_ph97_C4
zmp_ph97_B8
zmp_ph97_C1
zmp_ph97_D2
zmp_ph97_D5
zmp_ph111_G5
zmp_ph111_G11
zmp_ph111_H1
zmp_ph111_B1a
zmp_ph111_B5
zmp_ph111_B8
zmp_ph111_B6
zmp_ph111_B7
zmp_ph111_B9
zmp_ph111_B10
zmp_ph114_G4
zmp_ph114_G11
zmp_ph114_H1
zmp_ph114_H2
zmp_ph114_A9
zmp_ph114_A11
zmp_ph114_B2
zmp_ph114_B4
zmp_ph114_D8
zmp_ph114_E2

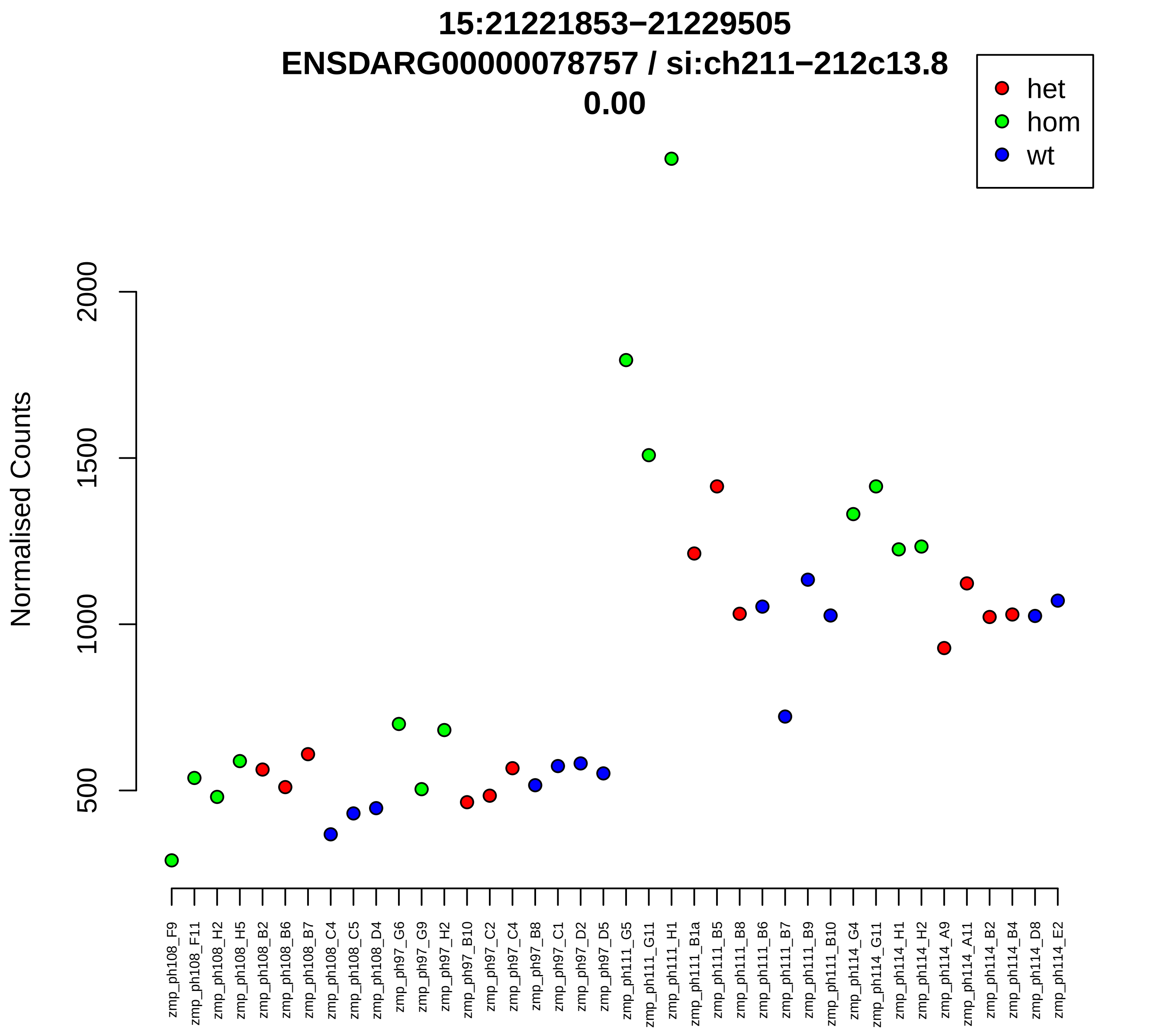
15:21221853−21229505
ENSDARG00000078757 / si:ch211−212c13.8
0.00
het
hom
wt
Normalised Counts
500
1000
1500
2000
zmp_ph108_F9
zmp_ph108_F11
zmp_ph108_H2
zmp_ph108_H5
zmp_ph108_B2
zmp_ph108_B6
zmp_ph108_B7
zmp_ph108_C4
zmp_ph108_C5
zmp_ph108_D4
zmp_ph97_G6
zmp_ph97_G9
zmp_ph97_H2
zmp_ph97_B10
zmp_ph97_C2
zmp_ph97_C4
zmp_ph97_B8
zmp_ph97_C1
zmp_ph97_D2
zmp_ph97_D5
zmp_ph111_G5
zmp_ph111_G11
zmp_ph111_H1
zmp_ph111_B1a
zmp_ph111_B5
zmp_ph111_B8
zmp_ph111_B6
zmp_ph111_B7
zmp_ph111_B9
zmp_ph111_B10
zmp_ph114_G4
zmp_ph114_G11
zmp_ph114_H1
zmp_ph114_H2
zmp_ph114_A9
zmp_ph114_A11
zmp_ph114_B2
zmp_ph114_B4
zmp_ph114_D8
zmp_ph114_E2

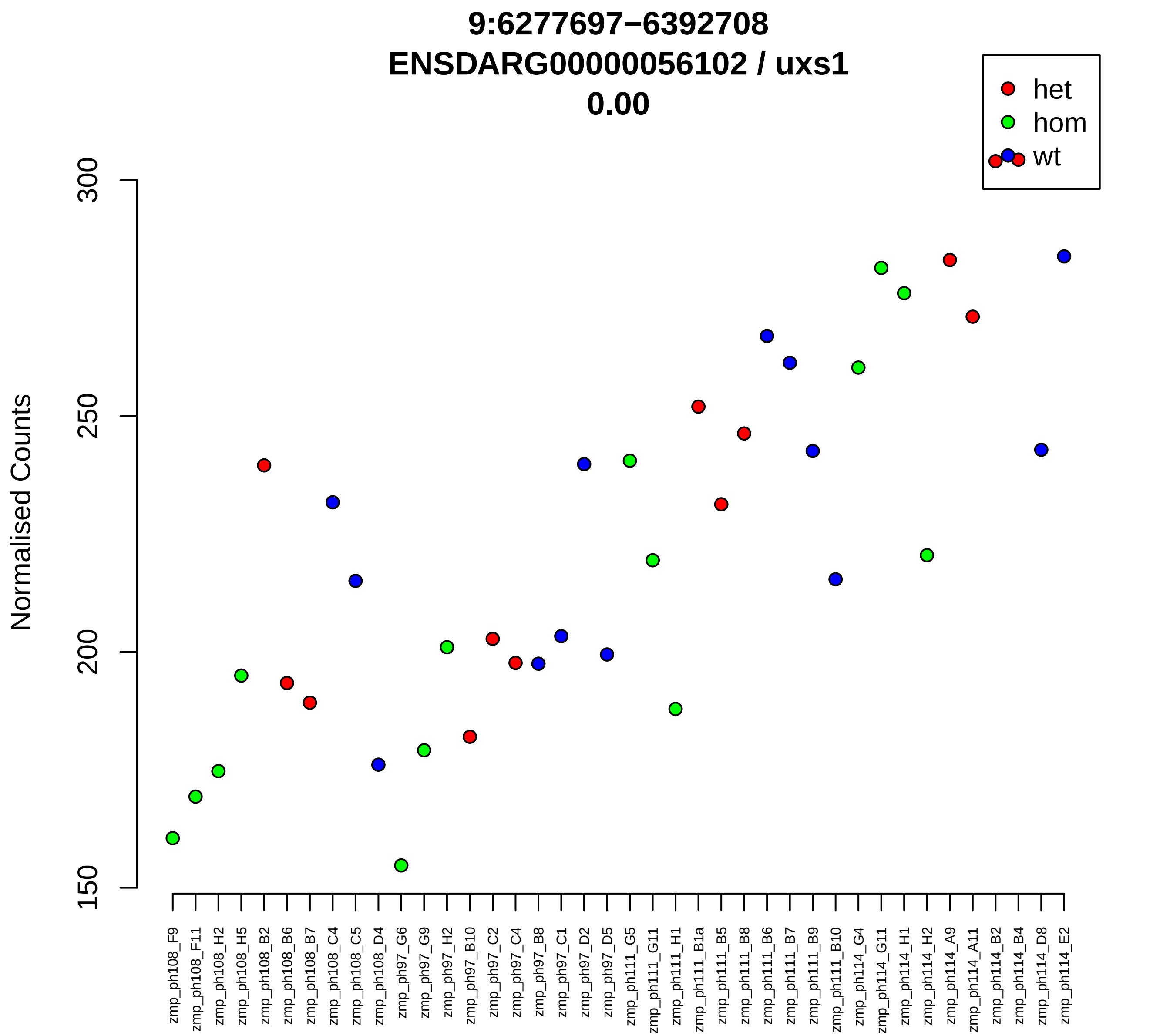

9:6277697−6392708
ENSDARG00000056102 / uxs1
0.00
het
hom
wt
Normalised Counts
150
200
250
300
zmp_ph108_F9
zmp_ph108_F11
zmp_ph108_H2
zmp_ph108_H5
zmp_ph108_B2
zmp_ph108_B6
zmp_ph108_B7
zmp_ph108_C4
zmp_ph108_C5
zmp_ph108_D4
zmp_ph97_G6
zmp_ph97_G9
zmp_ph97_H2
zmp_ph97_B10
zmp_ph97_C2
zmp_ph97_C4
zmp_ph97_B8
zmp_ph97_C1
zmp_ph97_D2
zmp_ph97_D5
zmp_ph111_G5
zmp_ph111_G11
zmp_ph111_H1
zmp_ph111_B1a
zmp_ph111_B5
zmp_ph111_B8
zmp_ph111_B6
zmp_ph111_B7
zmp_ph111_B9
zmp_ph111_B10
zmp_ph114_G4
zmp_ph114_G11
zmp_ph114_H1
zmp_ph114_H2
zmp_ph114_A9
zmp_ph114_A11
zmp_ph114_B2
zmp_ph114_B4
zmp_ph114_D8
zmp_ph114_E2

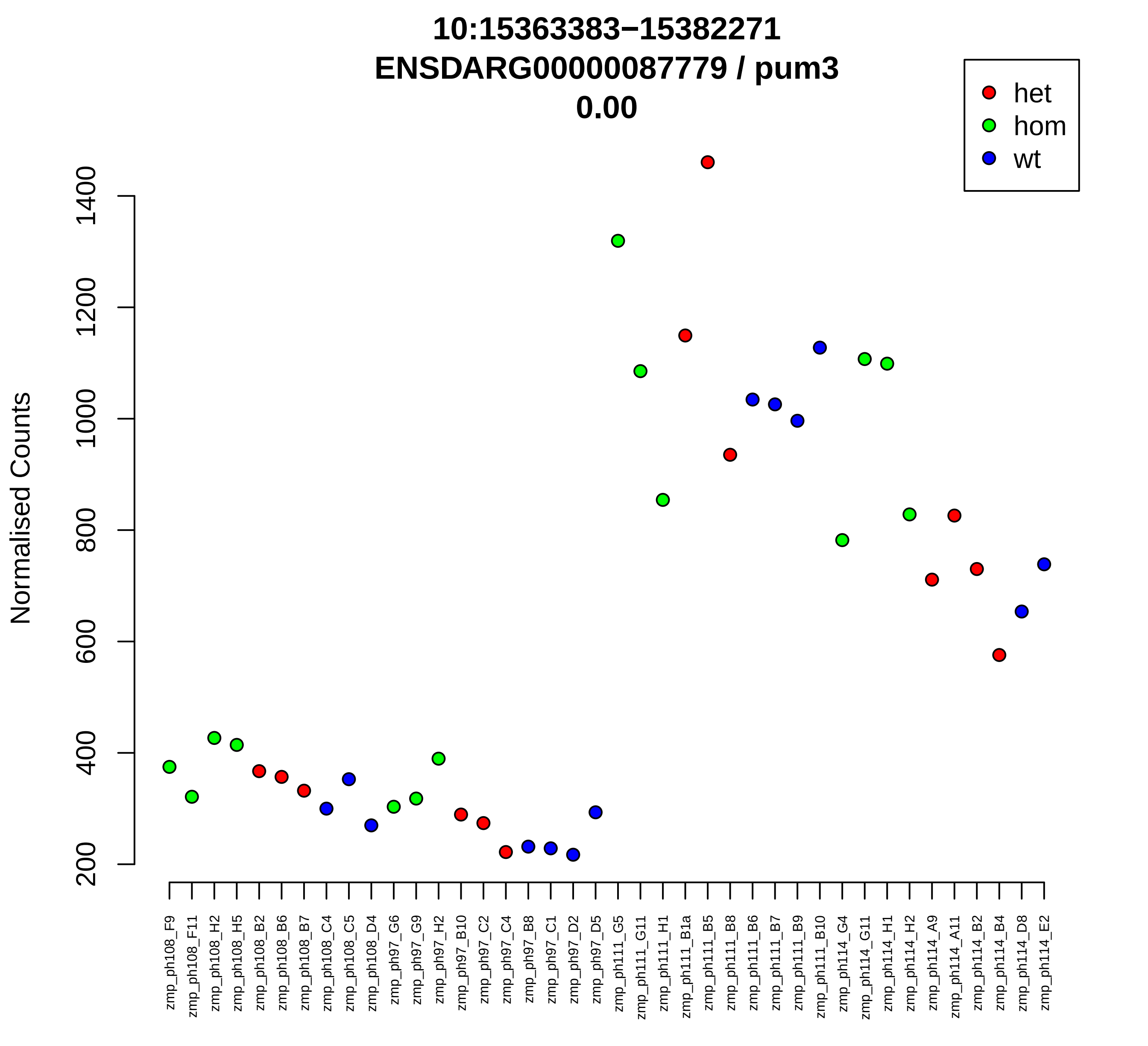

10:15363383−15382271
ENSDARG00000087779 / pum3
0.00
het
hom
wt
Normalised Counts
200
400
600
800
1000
1200
1400
zmp_ph108_F9
zmp_ph108_F11
zmp_ph108_H2
zmp_ph108_H5
zmp_ph108_B2
zmp_ph108_B6
zmp_ph108_B7
zmp_ph108_C4
zmp_ph108_C5
zmp_ph108_D4
zmp_ph97_G6
zmp_ph97_G9
zmp_ph97_H2
zmp_ph97_B10
zmp_ph97_C2
zmp_ph97_C4
zmp_ph97_B8
zmp_ph97_C1
zmp_ph97_D2
zmp_ph97_D5
zmp_ph111_G5
zmp_ph111_G11
zmp_ph111_H1
zmp_ph111_B1a
zmp_ph111_B5
zmp_ph111_B8
zmp_ph111_B6
zmp_ph111_B7
zmp_ph111_B9
zmp_ph111_B10
zmp_ph114_G4
zmp_ph114_G11
zmp_ph114_H1
zmp_ph114_H2
zmp_ph114_A9
zmp_ph114_A11
zmp_ph114_B2
zmp_ph114_B4
zmp_ph114_D8
zmp_ph114_E2

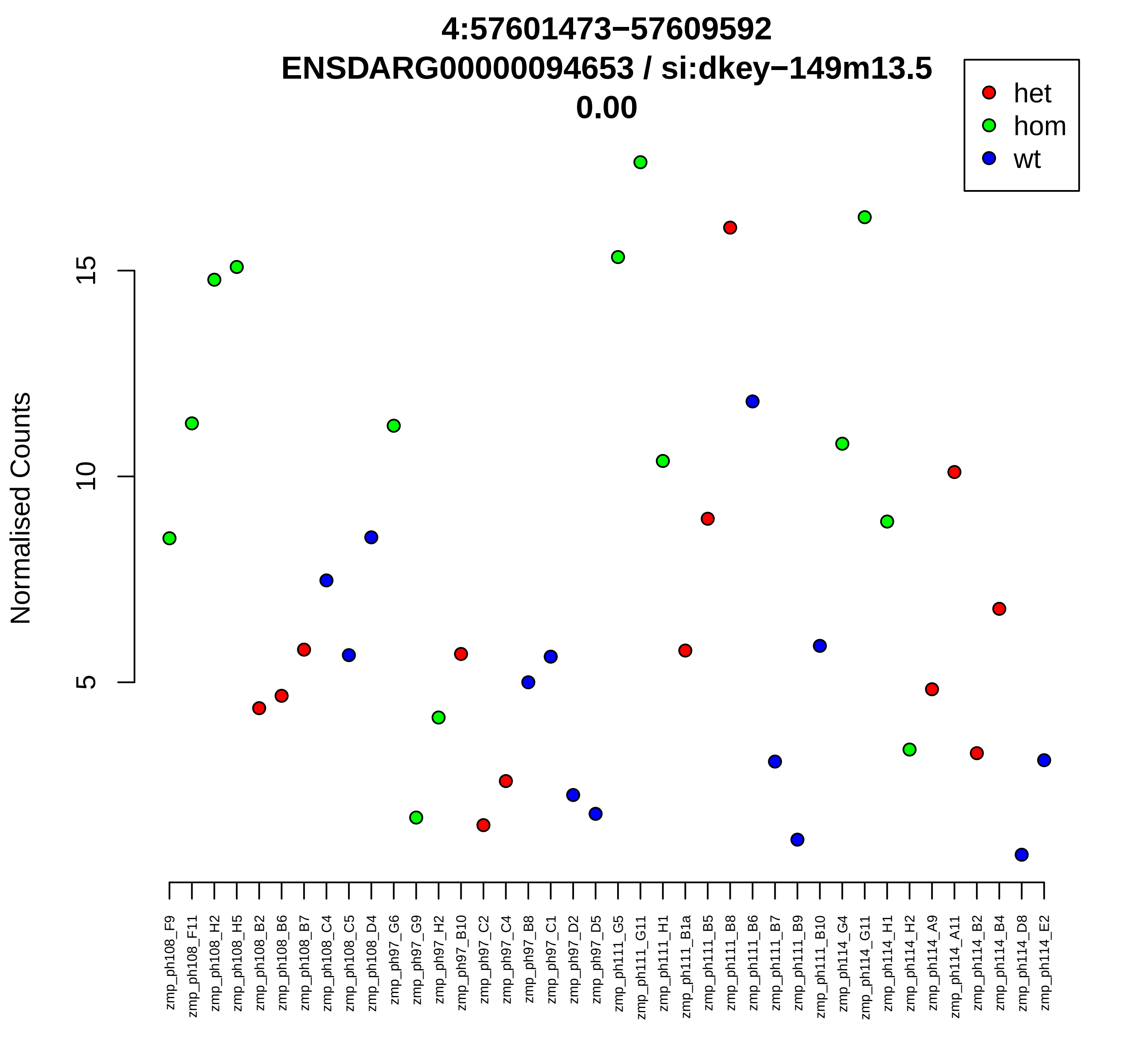

4:57601473−57609592
ENSDARG00000094653 / si:dkey−149m13.5
0.00
het
hom
wt
Normalised Counts
5
10
15
zmp_ph108_F9
zmp_ph108_F11
zmp_ph108_H2
zmp_ph108_H5
zmp_ph108_B2
zmp_ph108_B6
zmp_ph108_B7
zmp_ph108_C4
zmp_ph108_C5
zmp_ph108_D4
zmp_ph97_G6
zmp_ph97_G9
zmp_ph97_H2
zmp_ph97_B10
zmp_ph97_C2
zmp_ph97_C4
zmp_ph97_B8
zmp_ph97_C1
zmp_ph97_D2
zmp_ph97_D5
zmp_ph111_G5
zmp_ph111_G11
zmp_ph111_H1
zmp_ph111_B1a
zmp_ph111_B5
zmp_ph111_B8
zmp_ph111_B6
zmp_ph111_B7
zmp_ph111_B9
zmp_ph111_B10
zmp_ph114_G4
zmp_ph114_G11
zmp_ph114_H1
zmp_ph114_H2
zmp_ph114_A9
zmp_ph114_A11
zmp_ph114_B2
zmp_ph114_B4
zmp_ph114_D8
zmp_ph114_E2

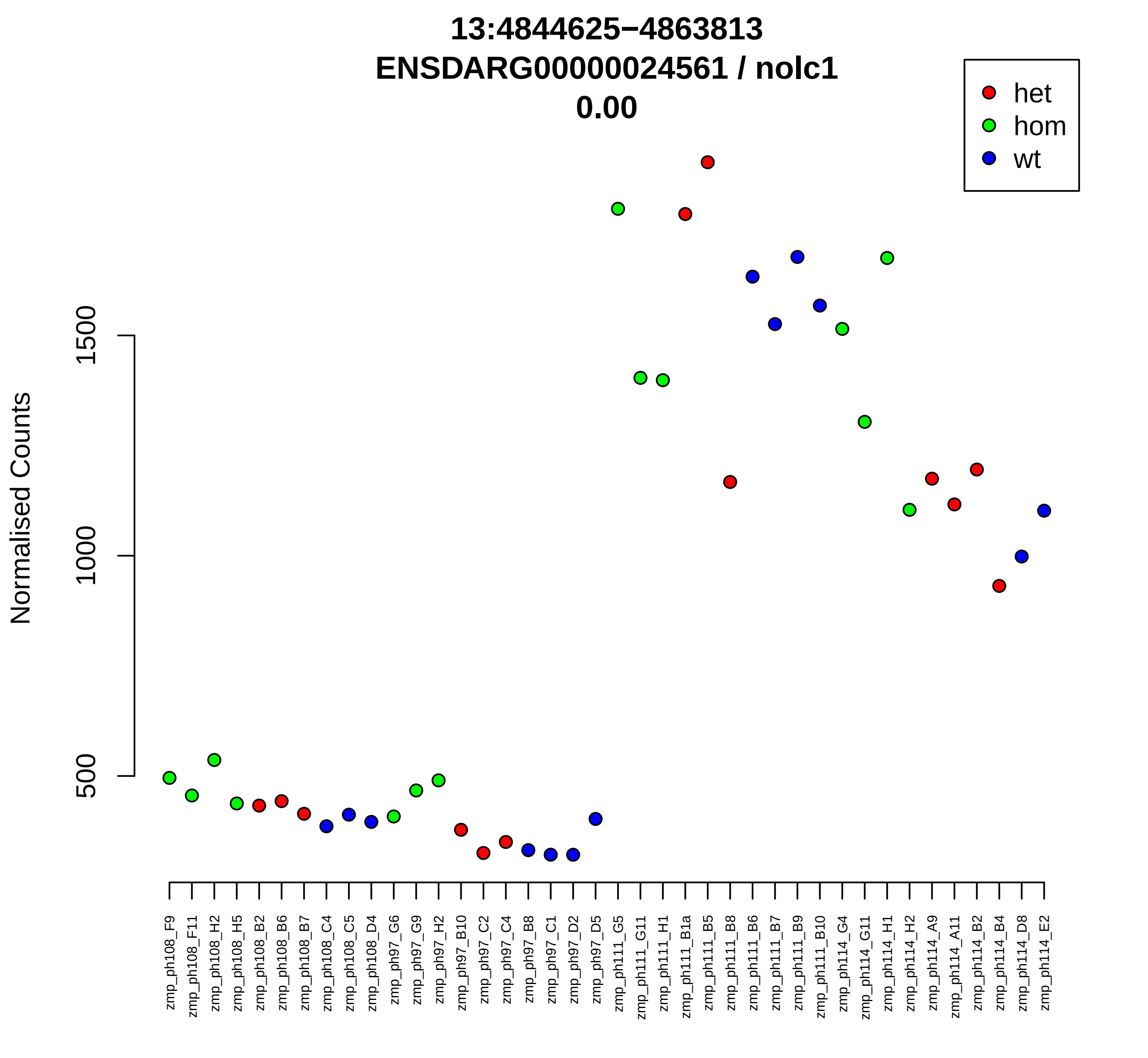

13:4844625−4863813
ENSDARG00000024561 / nolc1
0.00
het
hom
wt
Normalised Counts
500
1000
1500
zmp_ph108_F9
zmp_ph108_F11
zmp_ph108_H2
zmp_ph108_H5
zmp_ph108_B2
zmp_ph108_B6
zmp_ph108_B7
zmp_ph108_C4
zmp_ph108_C5
zmp_ph108_D4
zmp_ph97_G6
zmp_ph97_G9
zmp_ph97_H2
zmp_ph97_B10
zmp_ph97_C2
zmp_ph97_C4
zmp_ph97_B8
zmp_ph97_C1
zmp_ph97_D2
zmp_ph97_D5
zmp_ph111_G5
zmp_ph111_G11
zmp_ph111_H1
zmp_ph111_B1a
zmp_ph111_B5
zmp_ph111_B8
zmp_ph111_B6
zmp_ph111_B7
zmp_ph111_B9
zmp_ph111_B10
zmp_ph114_G4
zmp_ph114_G11
zmp_ph114_H1
zmp_ph114_H2
zmp_ph114_A9
zmp_ph114_A11
zmp_ph114_B2
zmp_ph114_B4
zmp_ph114_D8
zmp_ph114_E2

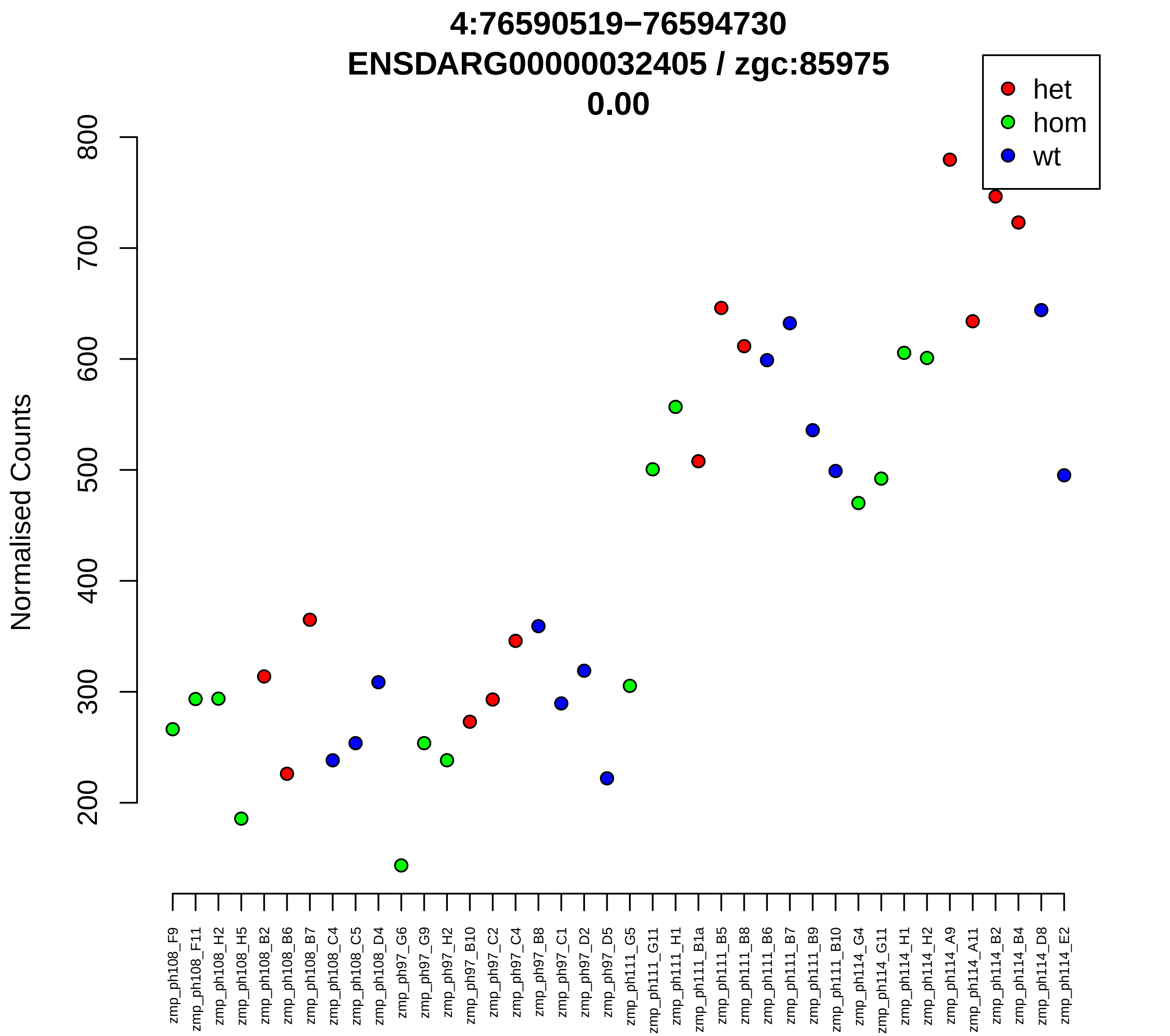

4:76590519−76594730
ENSDARG00000032405 / zgc:85975
0.00
het
hom
wt
Normalised Counts
200
300
400
500
600
700
800
zmp_ph108_F9
zmp_ph108_F11
zmp_ph108_H2
zmp_ph108_H5
zmp_ph108_B2
zmp_ph108_B6
zmp_ph108_B7
zmp_ph108_C4
zmp_ph108_C5
zmp_ph108_D4
zmp_ph97_G6
zmp_ph97_G9
zmp_ph97_H2
zmp_ph97_B10
zmp_ph97_C2
zmp_ph97_C4
zmp_ph97_B8
zmp_ph97_C1
zmp_ph97_D2
zmp_ph97_D5
zmp_ph111_G5
zmp_ph111_G11
zmp_ph111_H1
zmp_ph111_B1a
zmp_ph111_B5
zmp_ph111_B8
zmp_ph111_B6
zmp_ph111_B7
zmp_ph111_B9
zmp_ph111_B10
zmp_ph114_G4
zmp_ph114_G11
zmp_ph114_H1
zmp_ph114_H2
zmp_ph114_A9
zmp_ph114_A11
zmp_ph114_B2
zmp_ph114_B4
zmp_ph114_D8
zmp_ph114_E2

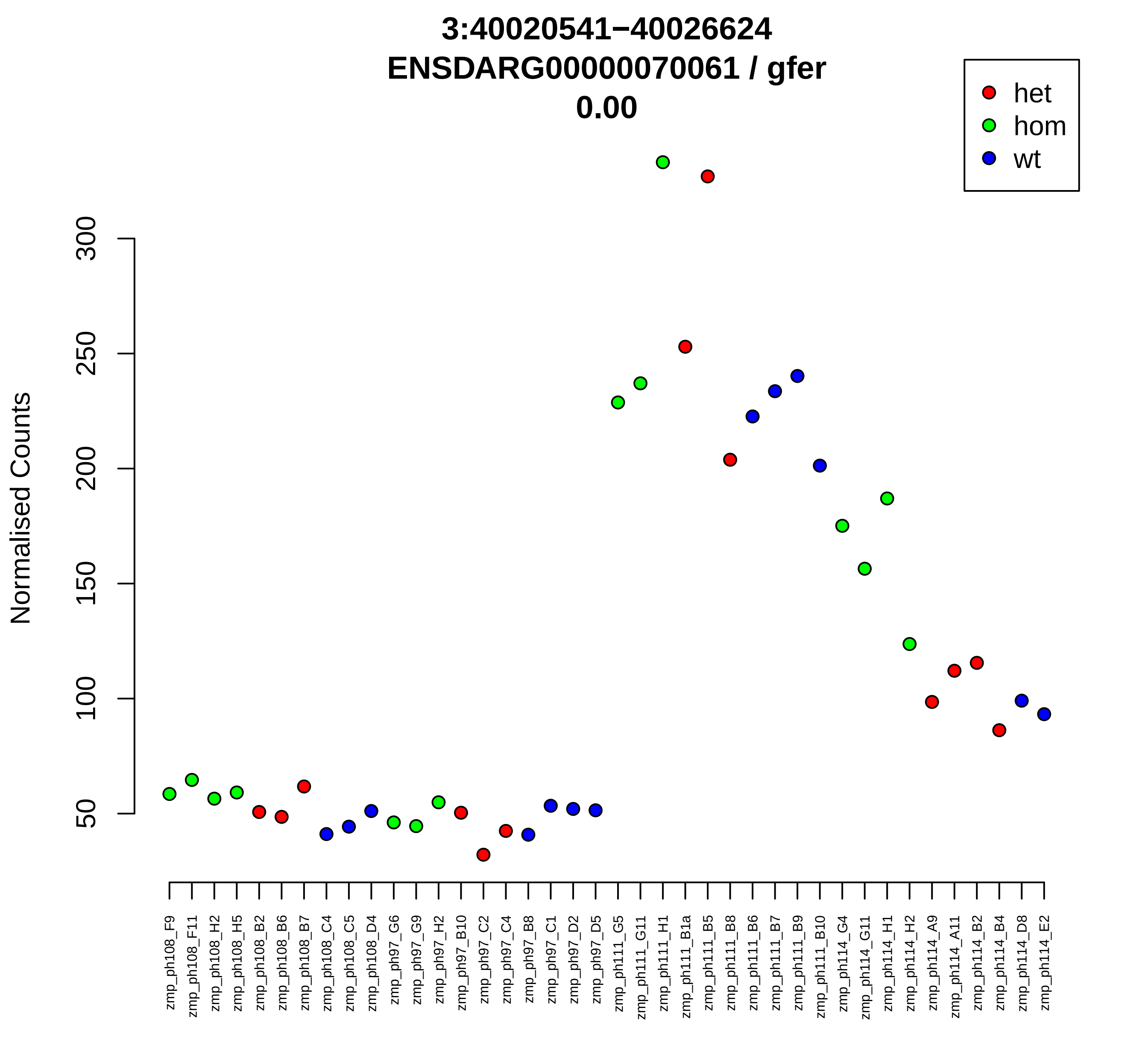

3:40020541−40026624
ENSDARG00000070061 / gfer
0.00
het
hom
wt
Normalised Counts
300
250
200
150
100
50
zmp_ph108_F9
zmp_ph108_F11
zmp_ph108_H2
zmp_ph108_H5
zmp_ph108_B2
zmp_ph108_B6
zmp_ph108_B7
zmp_ph108_C4
zmp_ph108_C5
zmp_ph108_D4
zmp_ph97_G6
zmp_ph97_G9
zmp_ph97_H2
zmp_ph97_B10
zmp_ph97_C2
zmp_ph97_C4
zmp_ph97_B8
zmp_ph97_C1
zmp_ph97_D2
zmp_ph97_D5
zmp_ph111_G5
zmp_ph111_G11
zmp_ph111_H1
zmp_ph111_B1a
zmp_ph111_B5
zmp_ph111_B8
zmp_ph111_B6
zmp_ph111_B7
zmp_ph111_B9
zmp_ph111_B10
zmp_ph114_G4
zmp_ph114_G11
zmp_ph114_H1
zmp_ph114_H2
zmp_ph114_A9
zmp_ph114_A11
zmp_ph114_B2
zmp_ph114_B4
zmp_ph114_D8
zmp_ph114_E2

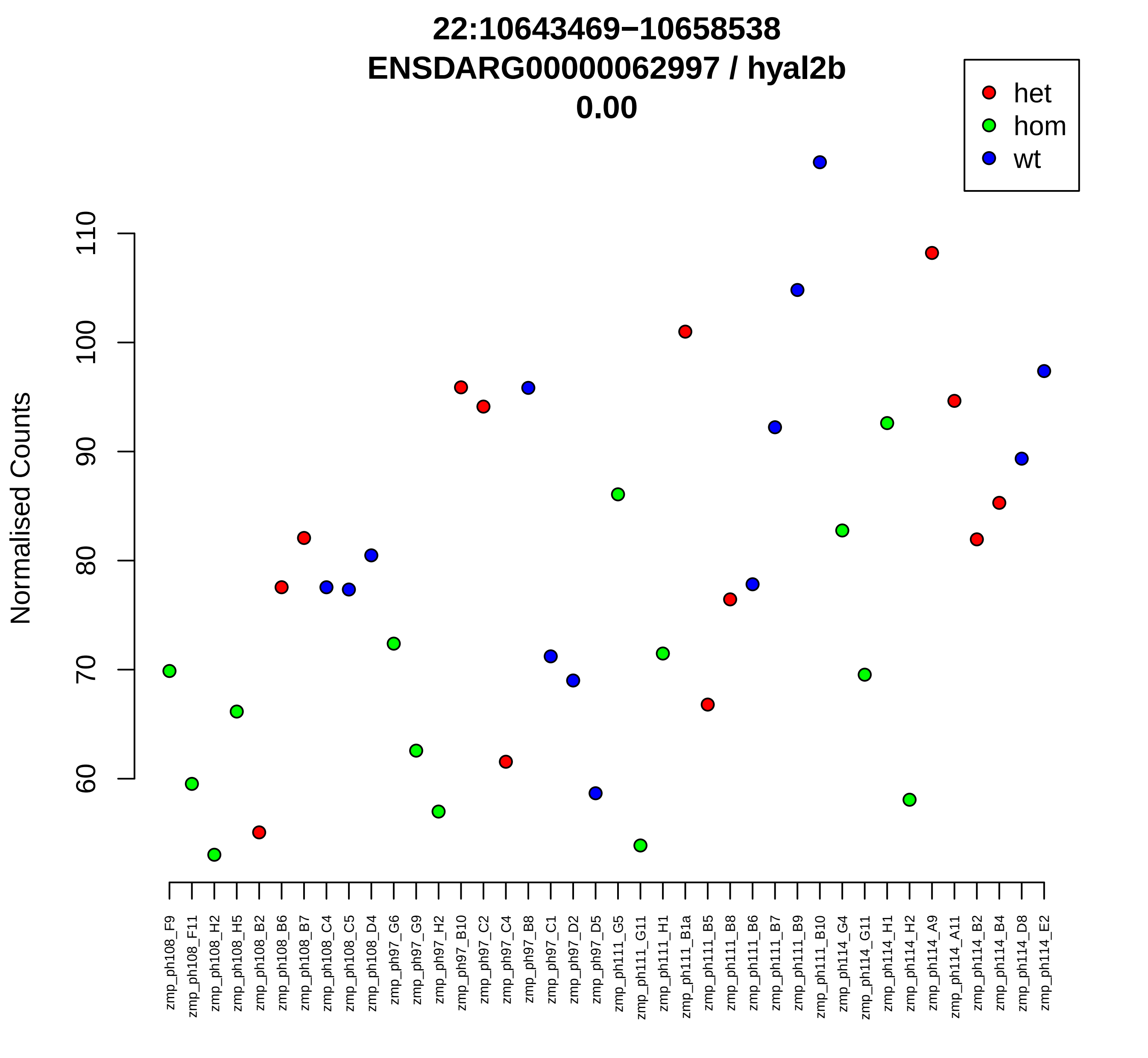
22:10643469−10658538
ENSDARG00000062997 / hyal2b
0.00
het
hom
wt
Normalised Counts
60
70
80
90
100
110
zmp_ph108_F9
zmp_ph108_F11
zmp_ph108_H2
zmp_ph108_H5
zmp_ph108_B2
zmp_ph108_B6
zmp_ph108_B7
zmp_ph108_C4
zmp_ph108_C5
zmp_ph108_D4
zmp_ph97_G6
zmp_ph97_G9
zmp_ph97_H2
zmp_ph97_B10
zmp_ph97_C2
zmp_ph97_C4
zmp_ph97_B8
zmp_ph97_C1
zmp_ph97_D2
zmp_ph97_D5
zmp_ph111_G5
zmp_ph111_G11
zmp_ph111_H1
zmp_ph111_B1a
zmp_ph111_B5
zmp_ph111_B8
zmp_ph111_B6
zmp_ph111_B7
zmp_ph111_B9
zmp_ph111_B10
zmp_ph114_G4
zmp_ph114_G11
zmp_ph114_H1
zmp_ph114_H2
zmp_ph114_A9
zmp_ph114_A11
zmp_ph114_B2
zmp_ph114_B4
zmp_ph114_D8
zmp_ph114_E2

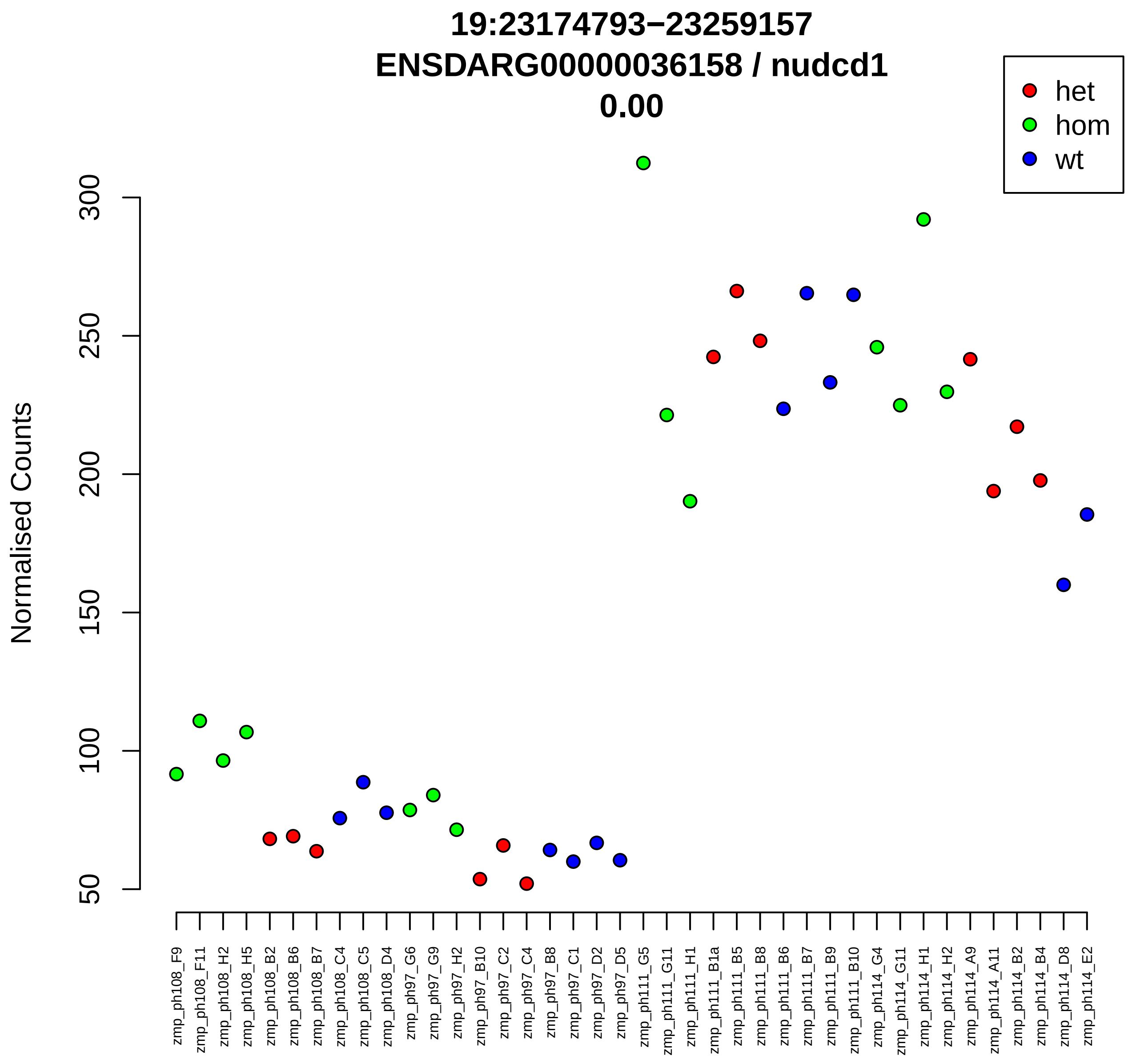
19:23174793−23259157
ENSDARG00000036158 / nudcd1
0.00
het
hom
wt
Normalised Counts
50
100
150
200
250
300
zmp_ph108_F9
zmp_ph108_F11
zmp_ph108_H2
zmp_ph108_H5
zmp_ph108_B2
zmp_ph108_B6
zmp_ph108_B7
zmp_ph108_C4
zmp_ph108_C5
zmp_ph108_D4
zmp_ph97_G6
zmp_ph97_G9
zmp_ph97_H2
zmp_ph97_B10
zmp_ph97_C2
zmp_ph97_C4
zmp_ph97_B8
zmp_ph97_C1
zmp_ph97_D2
zmp_ph97_D5
zmp_ph111_G5
zmp_ph111_G11
zmp_ph111_H1
zmp_ph111_B1a
zmp_ph111_B5
zmp_ph111_B8
zmp_ph111_B6
zmp_ph111_B7
zmp_ph111_B9
zmp_ph111_B10
zmp_ph114_G4
zmp_ph114_G11
zmp_ph114_H1
zmp_ph114_H2
zmp_ph114_A9
zmp_ph114_A11
zmp_ph114_B2
zmp_ph114_B4
zmp_ph114_D8
zmp_ph114_E2

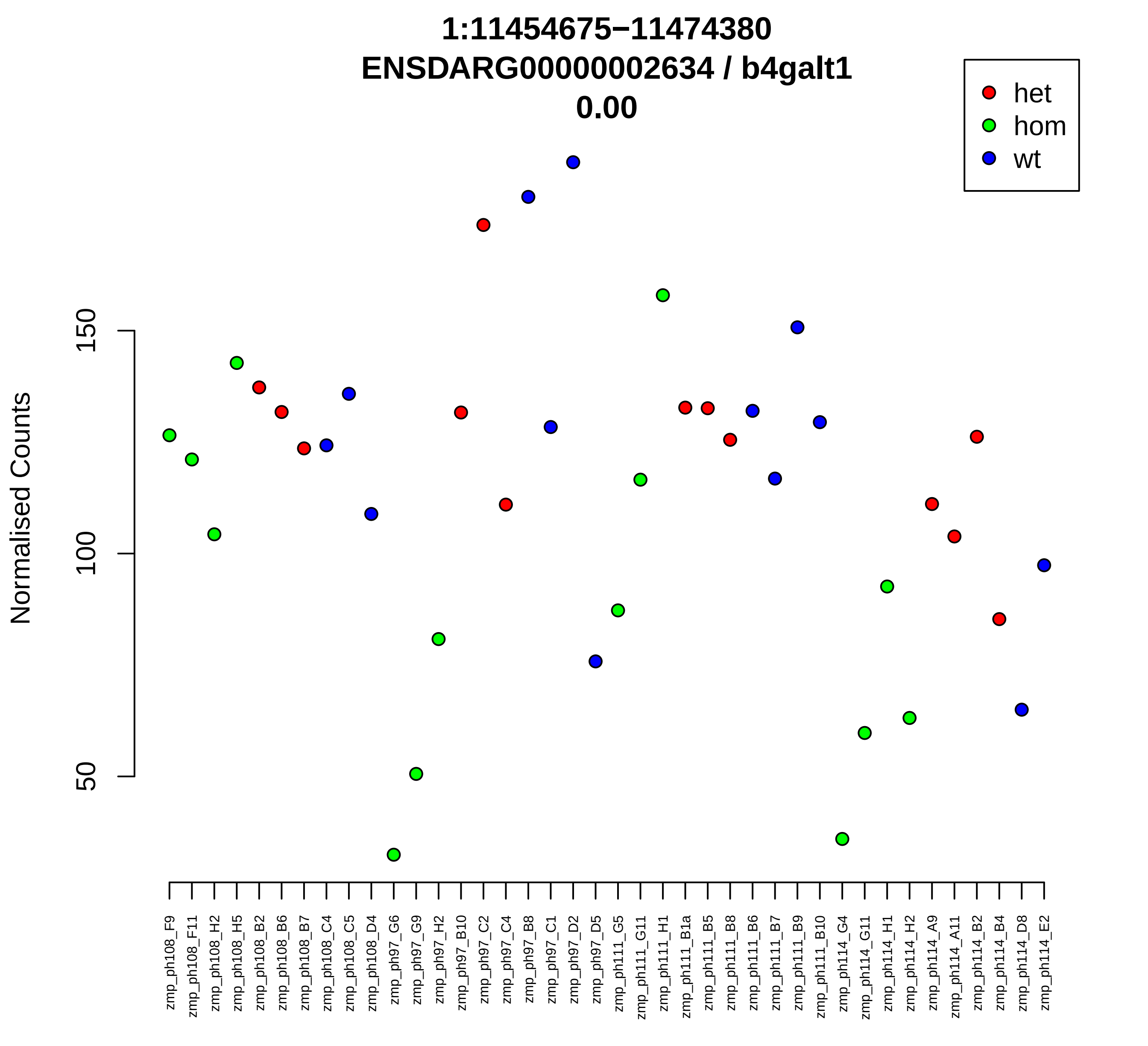
1:11454675−11474380
ENSDARG00000002634 / b4galt1
0.00
het
hom
wt
Normalised Counts
50
100
150
zmp_ph108_F9
zmp_ph108_F11
zmp_ph108_H2
zmp_ph108_H5
zmp_ph108_B2
zmp_ph108_B6
zmp_ph108_B7
zmp_ph108_C4
zmp_ph108_C5
zmp_ph108_D4
zmp_ph97_G6
zmp_ph97_G9
zmp_ph97_H2
zmp_ph97_B10
zmp_ph97_C2
zmp_ph97_C4
zmp_ph97_B8
zmp_ph97_C1
zmp_ph97_D2
zmp_ph97_D5
zmp_ph111_G5
zmp_ph111_G11
zmp_ph111_H1
zmp_ph111_B1a
zmp_ph111_B5
zmp_ph111_B8
zmp_ph111_B6
zmp_ph111_B7
zmp_ph111_B9
zmp_ph111_B10
zmp_ph114_G4
zmp_ph114_G11
zmp_ph114_H1
zmp_ph114_H2
zmp_ph114_A9
zmp_ph114_A11
zmp_ph114_B2
zmp_ph114_B4
zmp_ph114_D8
zmp_ph114_E2

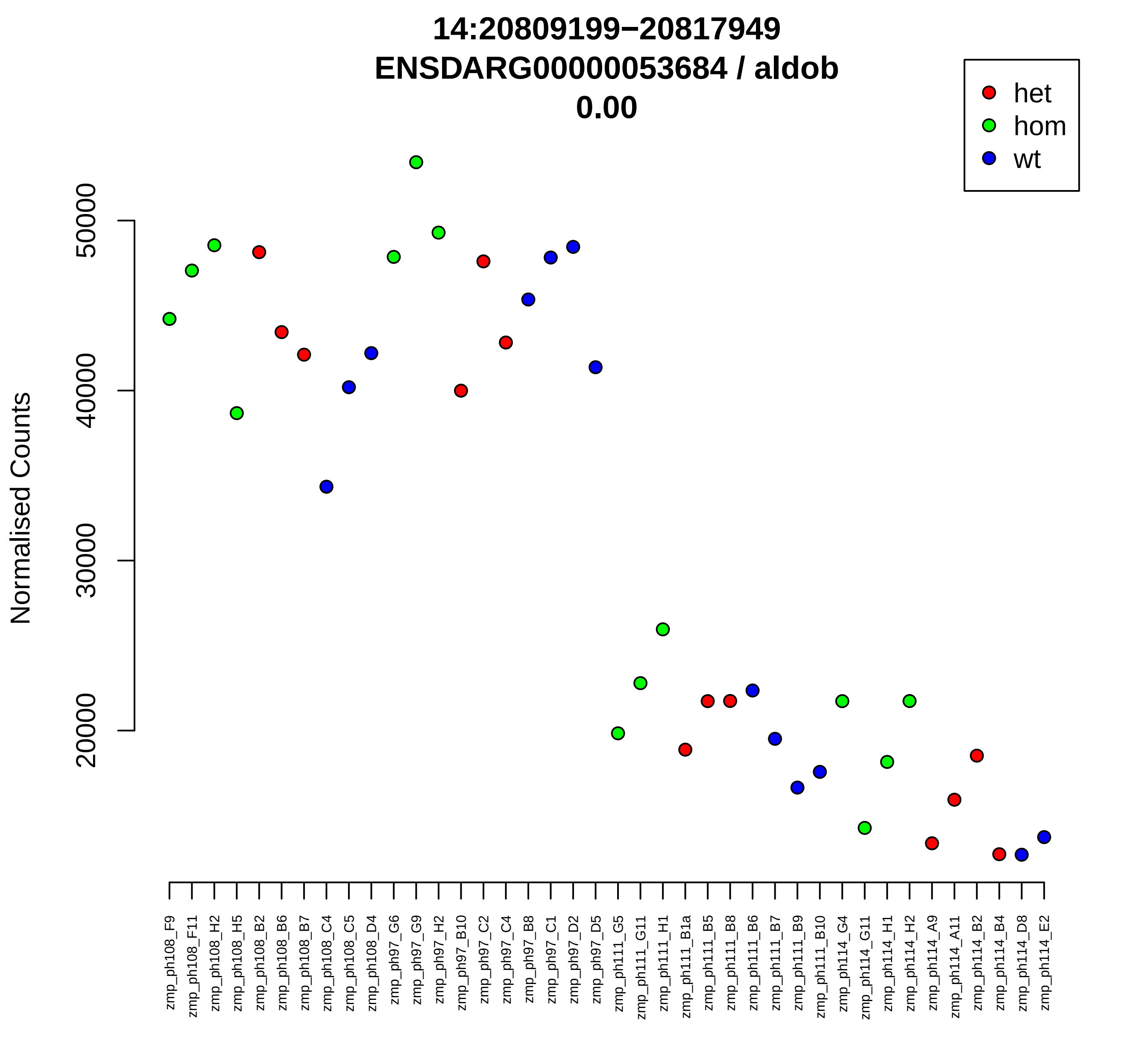

14:20809199−20817949
ENSDARG00000053684 / aldob
0.00
het
hom
wt
Normalised Counts
20000
30000
40000
50000
zmp_ph108_F9
zmp_ph108_F11
zmp_ph108_H2
zmp_ph108_H5
zmp_ph108_B2
zmp_ph108_B6
zmp_ph108_B7
zmp_ph108_C4
zmp_ph108_C5
zmp_ph108_D4
zmp_ph97_G6
zmp_ph97_G9
zmp_ph97_H2
zmp_ph97_B10
zmp_ph97_C2
zmp_ph97_C4
zmp_ph97_B8
zmp_ph97_C1
zmp_ph97_D2
zmp_ph97_D5
zmp_ph111_G5
zmp_ph111_G11
zmp_ph111_H1
zmp_ph111_B1a
zmp_ph111_B5
zmp_ph111_B8
zmp_ph111_B6
zmp_ph111_B7
zmp_ph111_B9
zmp_ph111_B10
zmp_ph114_G4
zmp_ph114_G11
zmp_ph114_H1
zmp_ph114_H2
zmp_ph114_A9
zmp_ph114_A11
zmp_ph114_B2
zmp_ph114_B4
zmp_ph114_D8
zmp_ph114_E2

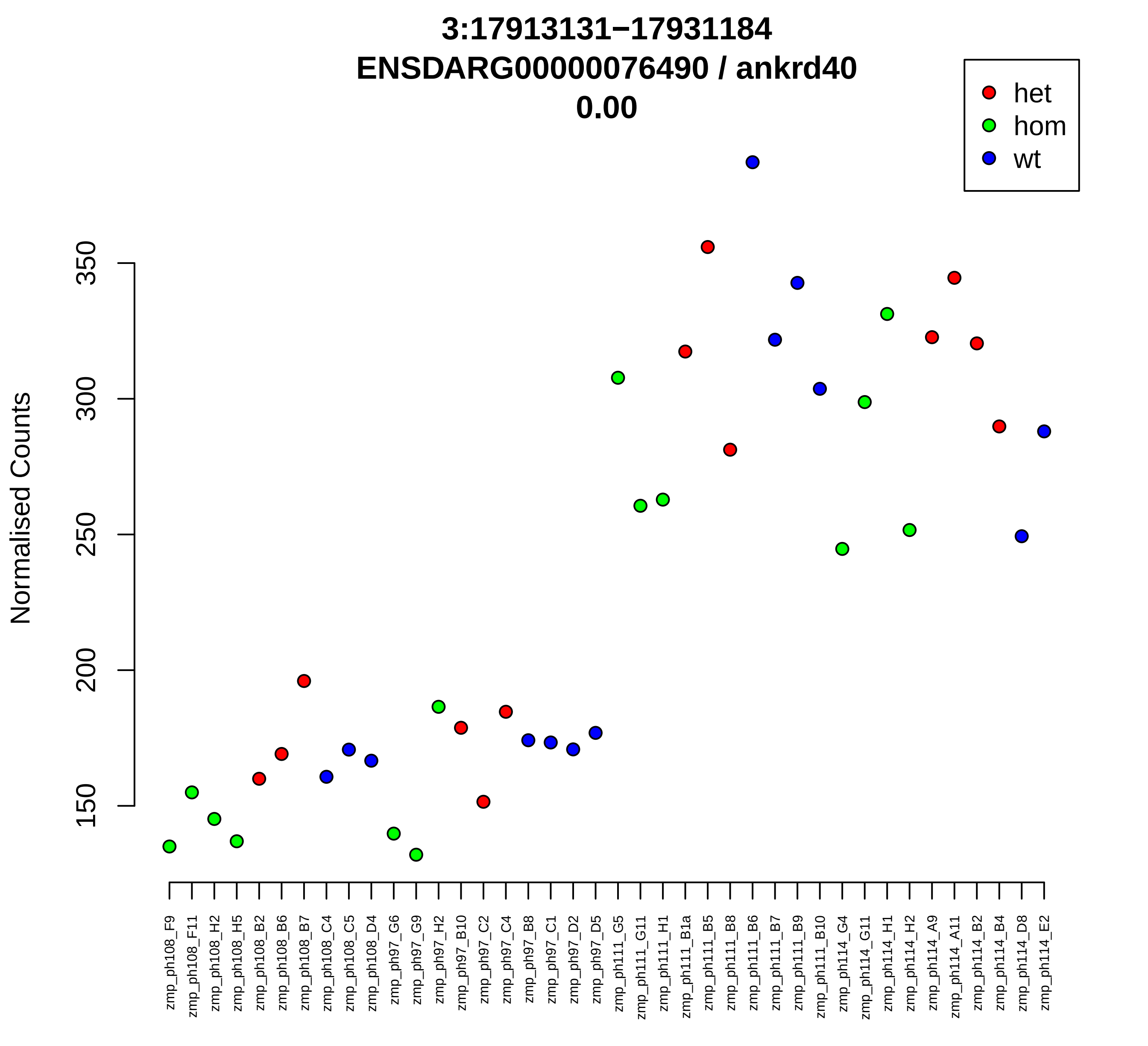
3:17913131−17931184
ENSDARG00000076490 / ankrd40
0.00
het
hom
wt
Normalised Counts
150
200
250
300
350
zmp_ph108_F9
zmp_ph108_F11
zmp_ph108_H2
zmp_ph108_H5
zmp_ph108_B2
zmp_ph108_B6
zmp_ph108_B7
zmp_ph108_C4
zmp_ph108_C5
zmp_ph108_D4
zmp_ph97_G6
zmp_ph97_G9
zmp_ph97_H2
zmp_ph97_B10
zmp_ph97_C2
zmp_ph97_C4
zmp_ph97_B8
zmp_ph97_C1
zmp_ph97_D2
zmp_ph97_D5
zmp_ph111_G5
zmp_ph111_G11
zmp_ph111_H1
zmp_ph111_B1a
zmp_ph111_B5
zmp_ph111_B8
zmp_ph111_B6
zmp_ph111_B7
zmp_ph111_B9
zmp_ph111_B10
zmp_ph114_G4
zmp_ph114_G11
zmp_ph114_H1
zmp_ph114_H2
zmp_ph114_A9
zmp_ph114_A11
zmp_ph114_B2
zmp_ph114_B4
zmp_ph114_D8
zmp_ph114_E2

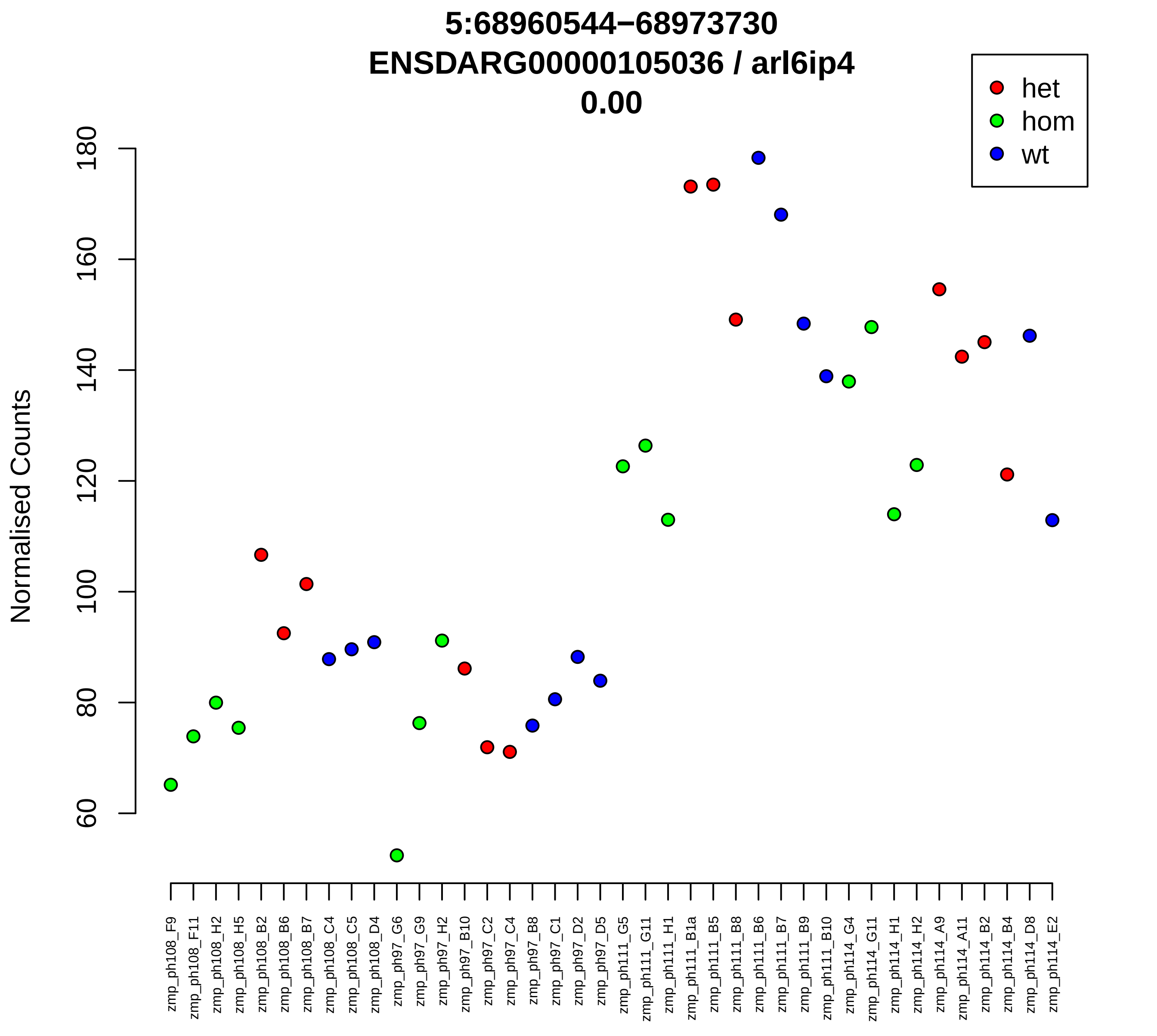

5:68960544−68973730
ENSDARG00000105036 / arl6ip4
0.00
het
hom
wt
Normalised Counts
60
80
100
120
140
160
180
zmp_ph108_F9
zmp_ph108_F11
zmp_ph108_H2
zmp_ph108_H5
zmp_ph108_B2
zmp_ph108_B6
zmp_ph108_B7
zmp_ph108_C4
zmp_ph108_C5
zmp_ph108_D4
zmp_ph97_G6
zmp_ph97_G9
zmp_ph97_H2
zmp_ph97_B10
zmp_ph97_C2
zmp_ph97_C4
zmp_ph97_B8
zmp_ph97_C1
zmp_ph97_D2
zmp_ph97_D5
zmp_ph111_G5
zmp_ph111_G11
zmp_ph111_H1
zmp_ph111_B1a
zmp_ph111_B5
zmp_ph111_B8
zmp_ph111_B6
zmp_ph111_B7
zmp_ph111_B9
zmp_ph111_B10
zmp_ph114_G4
zmp_ph114_G11
zmp_ph114_H1
zmp_ph114_H2
zmp_ph114_A9
zmp_ph114_A11
zmp_ph114_B2
zmp_ph114_B4
zmp_ph114_D8
zmp_ph114_E2

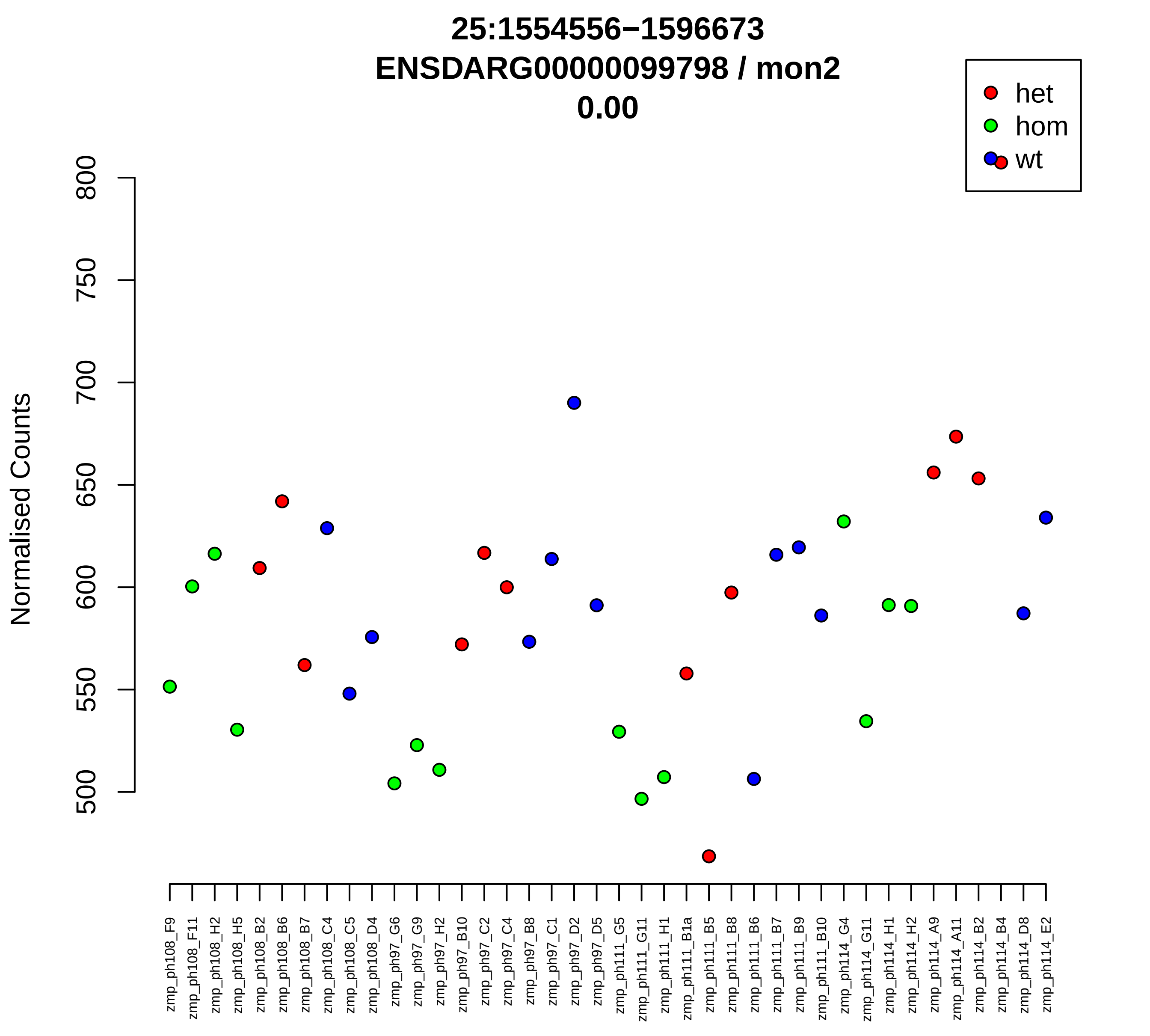

25:1554556−1596673
ENSDARG00000099798 / mon2
0.00
het
hom
wt
Normalised Counts
500
550
600
650
700
750
800
zmp_ph108_F9
zmp_ph108_F11
zmp_ph108_H2
zmp_ph108_H5
zmp_ph108_B2
zmp_ph108_B6
zmp_ph108_B7
zmp_ph108_C4
zmp_ph108_C5
zmp_ph108_D4
zmp_ph97_G6
zmp_ph97_G9
zmp_ph97_H2
zmp_ph97_B10
zmp_ph97_C2
zmp_ph97_C4
zmp_ph97_B8
zmp_ph97_C1
zmp_ph97_D2
zmp_ph97_D5
zmp_ph111_G5
zmp_ph111_G11
zmp_ph111_H1
zmp_ph111_B1a
zmp_ph111_B5
zmp_ph111_B8
zmp_ph111_B6
zmp_ph111_B7
zmp_ph111_B9
zmp_ph111_B10
zmp_ph114_G4
zmp_ph114_G11
zmp_ph114_H1
zmp_ph114_H2
zmp_ph114_A9
zmp_ph114_A11
zmp_ph114_B2
zmp_ph114_B4
zmp_ph114_D8
zmp_ph114_E2

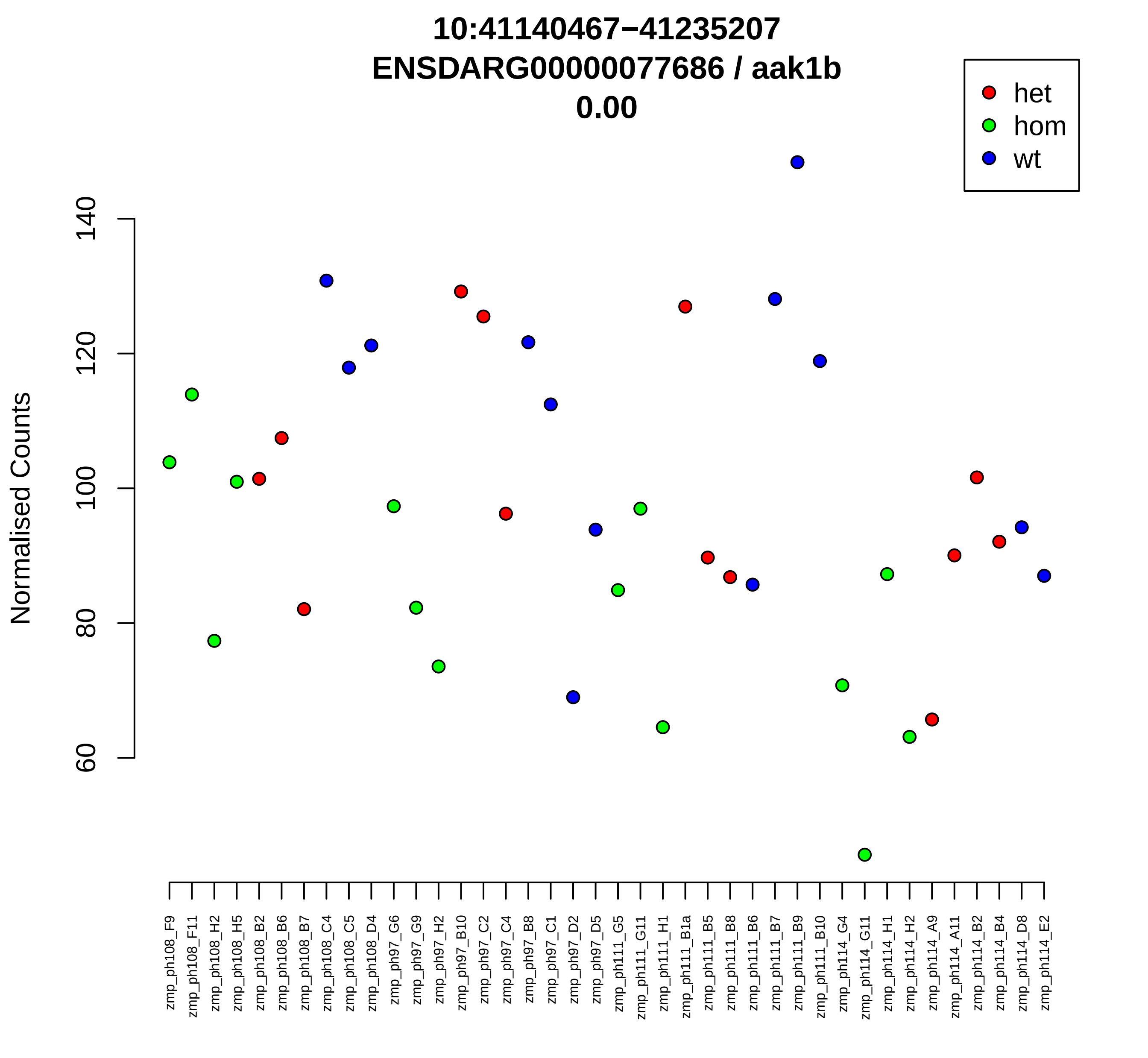

10:41140467−41235207
ENSDARG00000077686 / aak1b
0.00
het
hom
wt
Normalised Counts
140
120
100
80
60
zmp_ph108_F9
zmp_ph108_F11
zmp_ph108_H2
zmp_ph108_H5
zmp_ph108_B2
zmp_ph108_B6
zmp_ph108_B7
zmp_ph108_C4
zmp_ph108_C5
zmp_ph108_D4
zmp_ph97_G6
zmp_ph97_G9
zmp_ph97_H2
zmp_ph97_B10
zmp_ph97_C2
zmp_ph97_C4
zmp_ph97_B8
zmp_ph97_C1
zmp_ph97_D2
zmp_ph97_D5
zmp_ph111_G5
zmp_ph111_G11
zmp_ph111_H1
zmp_ph111_B1a
zmp_ph111_B5
zmp_ph111_B8
zmp_ph111_B6
zmp_ph111_B7
zmp_ph111_B9
zmp_ph111_B10
zmp_ph114_G4
zmp_ph114_G11
zmp_ph114_H1
zmp_ph114_H2
zmp_ph114_A9
zmp_ph114_A11
zmp_ph114_B2
zmp_ph114_B4
zmp_ph114_D8
zmp_ph114_E2

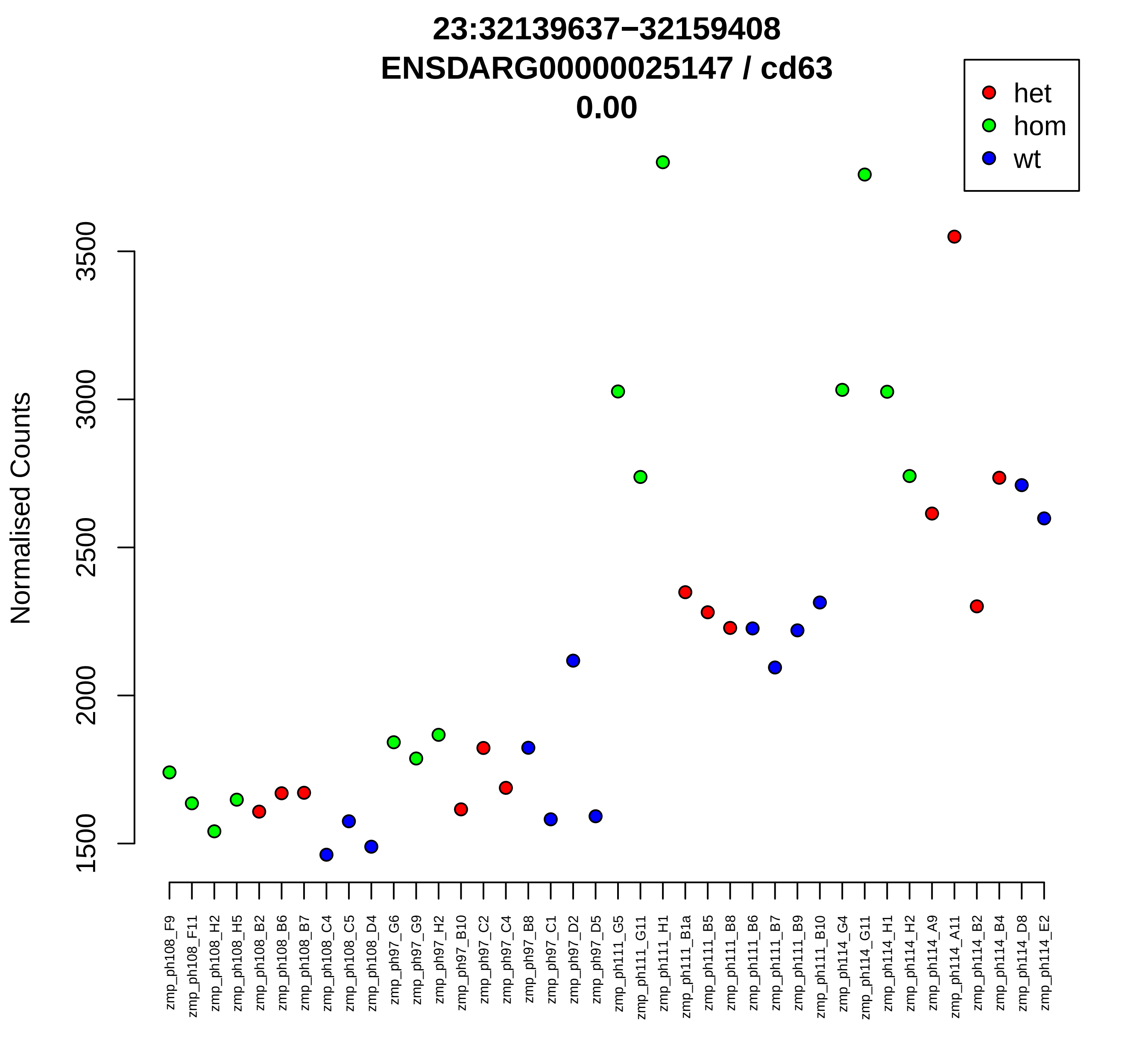
23:32139637−32159408
ENSDARG00000025147 / cd63
0.00
het
hom
wt
Normalised Counts
1500
2000
2500
3000
3500
zmp_ph108_F9
zmp_ph108_F11
zmp_ph108_H2
zmp_ph108_H5
zmp_ph108_B2
zmp_ph108_B6
zmp_ph108_B7
zmp_ph108_C4
zmp_ph108_C5
zmp_ph108_D4
zmp_ph97_G6
zmp_ph97_G9
zmp_ph97_H2
zmp_ph97_B10
zmp_ph97_C2
zmp_ph97_C4
zmp_ph97_B8
zmp_ph97_C1
zmp_ph97_D2
zmp_ph97_D5
zmp_ph111_G5
zmp_ph111_G11
zmp_ph111_H1
zmp_ph111_B1a
zmp_ph111_B5
zmp_ph111_B8
zmp_ph111_B6
zmp_ph111_B7
zmp_ph111_B9
zmp_ph111_B10
zmp_ph114_G4
zmp_ph114_G11
zmp_ph114_H1
zmp_ph114_H2
zmp_ph114_A9
zmp_ph114_A11
zmp_ph114_B2
zmp_ph114_B4
zmp_ph114_D8
zmp_ph114_E2

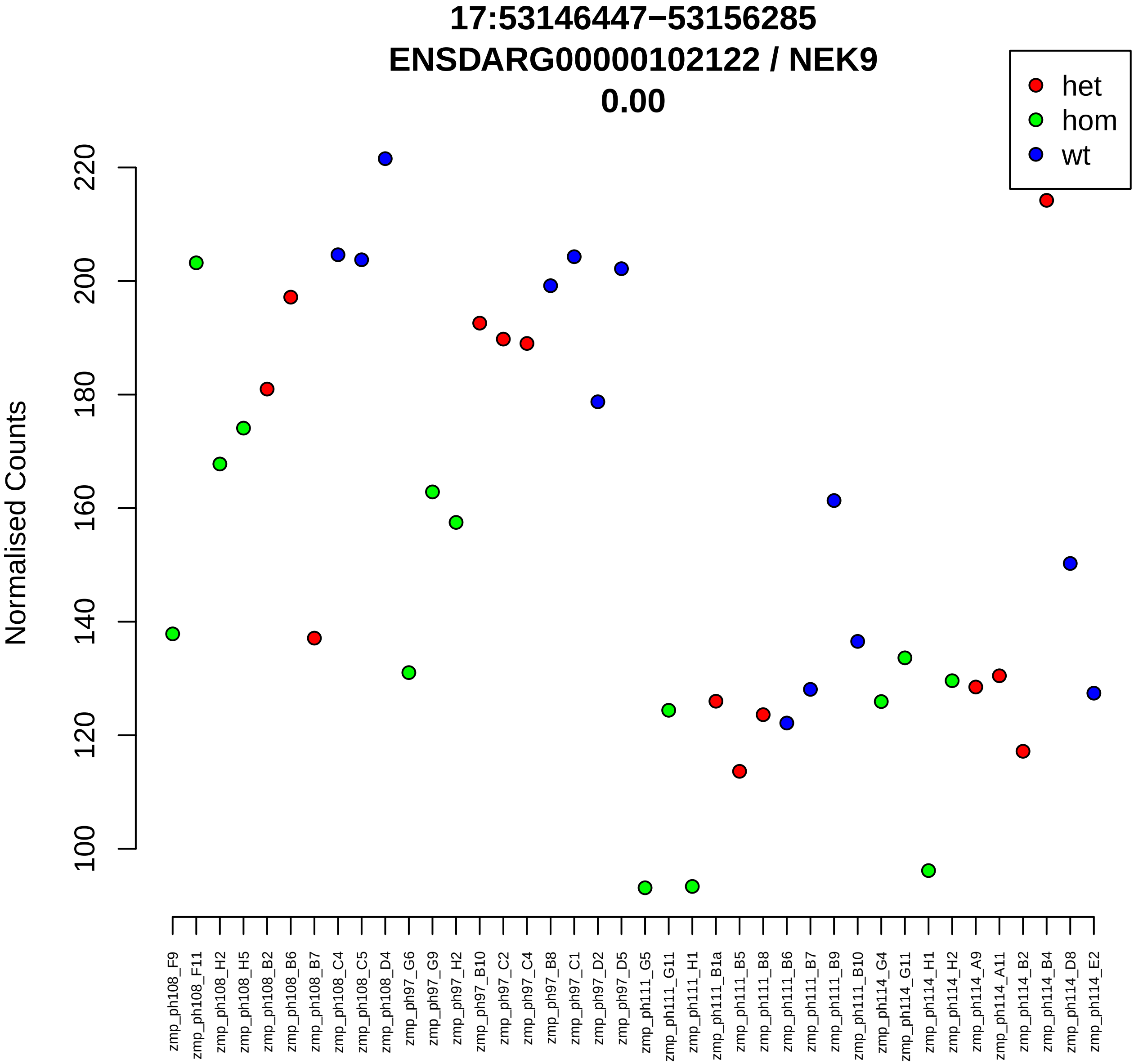

17:53146447−53156285
ENSDARG00000102122 / NEK9
0.00
het
hom
wt
Normalised Counts
100
120
140
160
180
200
220
zmp_ph108_F9
zmp_ph108_F11
zmp_ph108_H2
zmp_ph108_H5
zmp_ph108_B2
zmp_ph108_B6
zmp_ph108_B7
zmp_ph108_C4
zmp_ph108_C5
zmp_ph108_D4
zmp_ph97_G6
zmp_ph97_G9
zmp_ph97_H2
zmp_ph97_B10
zmp_ph97_C2
zmp_ph97_C4
zmp_ph97_B8
zmp_ph97_C1
zmp_ph97_D2
zmp_ph97_D5
zmp_ph111_G5
zmp_ph111_G11
zmp_ph111_H1
zmp_ph111_B1a
zmp_ph111_B5
zmp_ph111_B8
zmp_ph111_B6
zmp_ph111_B7
zmp_ph111_B9
zmp_ph111_B10
zmp_ph114_G4
zmp_ph114_G11
zmp_ph114_H1
zmp_ph114_H2
zmp_ph114_A9
zmp_ph114_A11
zmp_ph114_B2
zmp_ph114_B4
zmp_ph114_D8
zmp_ph114_E2

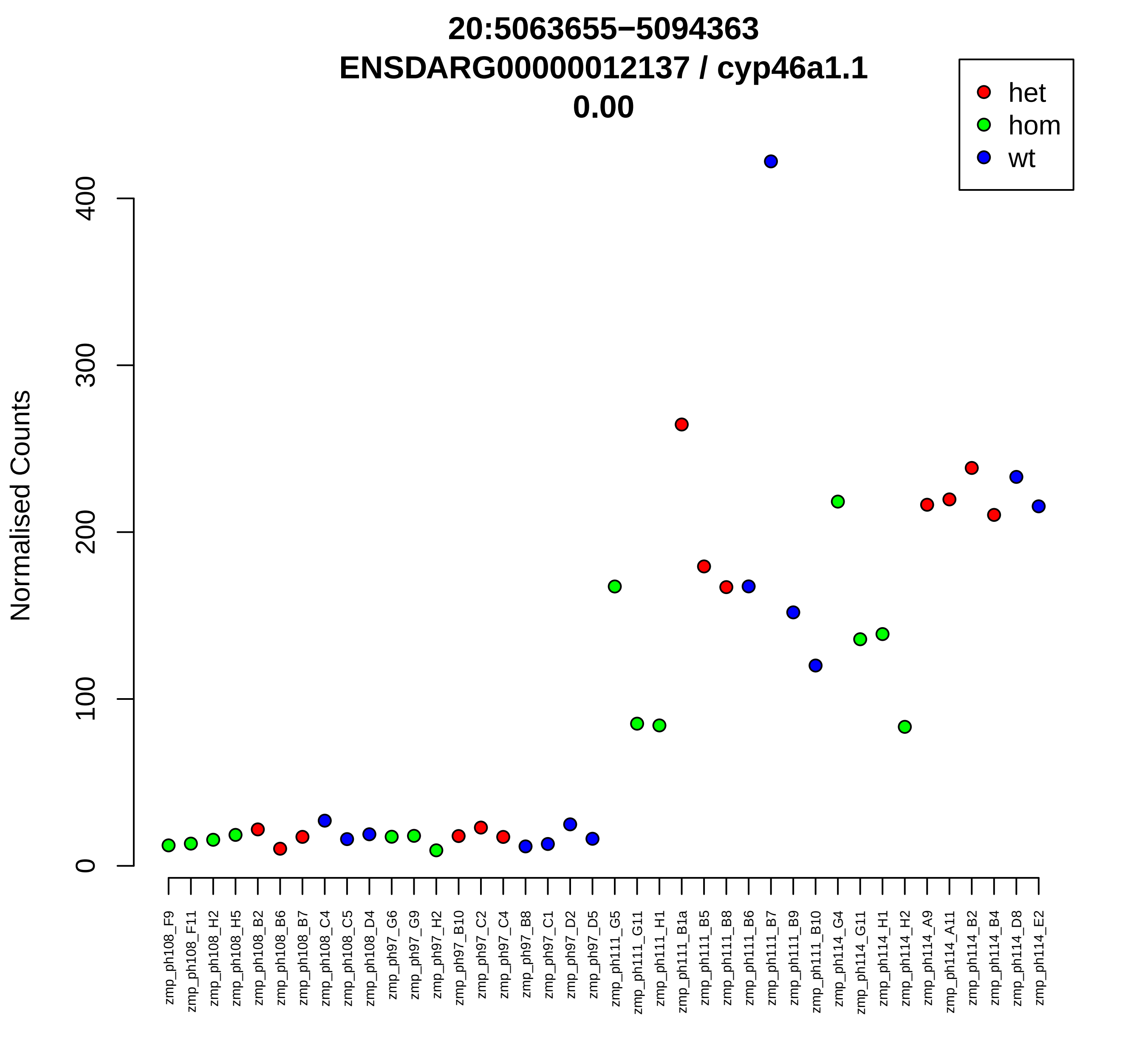

20:5063655−5094363
ENSDARG00000012137 / cyp46a1.1
0.00
het
hom
wt
Normalised Counts
0
100
200
300
400
zmp_ph108_F9
zmp_ph108_F11
zmp_ph108_H2
zmp_ph108_H5
zmp_ph108_B2
zmp_ph108_B6
zmp_ph108_B7
zmp_ph108_C4
zmp_ph108_C5
zmp_ph108_D4
zmp_ph97_G6
zmp_ph97_G9
zmp_ph97_H2
zmp_ph97_B10
zmp_ph97_C2
zmp_ph97_C4
zmp_ph97_B8
zmp_ph97_C1
zmp_ph97_D2
zmp_ph97_D5
zmp_ph111_G5
zmp_ph111_G11
zmp_ph111_H1
zmp_ph111_B1a
zmp_ph111_B5
zmp_ph111_B8
zmp_ph111_B6
zmp_ph111_B7
zmp_ph111_B9
zmp_ph111_B10
zmp_ph114_G4
zmp_ph114_G11
zmp_ph114_H1
zmp_ph114_H2
zmp_ph114_A9
zmp_ph114_A11
zmp_ph114_B2
zmp_ph114_B4
zmp_ph114_D8
zmp_ph114_E2

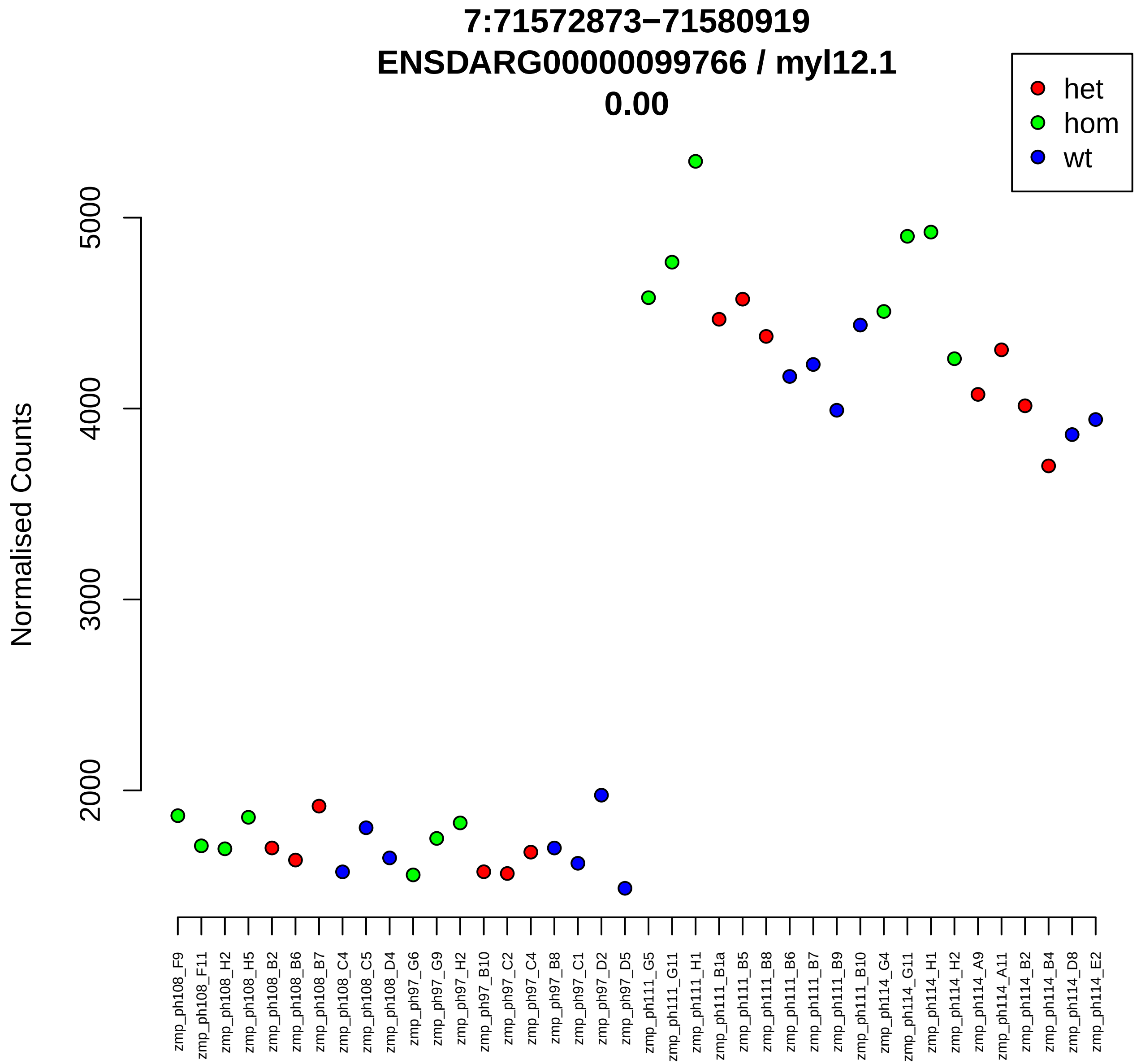

7:71572873−71580919
ENSDARG00000099766 / myl12.1
0.00
het
hom
wt
Normalised Counts
2000
3000
4000
5000
zmp_ph108_F9
zmp_ph108_F11
zmp_ph108_H2
zmp_ph108_H5
zmp_ph108_B2
zmp_ph108_B6
zmp_ph108_B7
zmp_ph108_C4
zmp_ph108_C5
zmp_ph108_D4
zmp_ph97_G6
zmp_ph97_G9
zmp_ph97_H2
zmp_ph97_B10
zmp_ph97_C2
zmp_ph97_C4
zmp_ph97_B8
zmp_ph97_C1
zmp_ph97_D2
zmp_ph97_D5
zmp_ph111_G5
zmp_ph111_G11
zmp_ph111_H1
zmp_ph111_B1a
zmp_ph111_B5
zmp_ph111_B8
zmp_ph111_B6
zmp_ph111_B7
zmp_ph111_B9
zmp_ph111_B10
zmp_ph114_G4
zmp_ph114_G11
zmp_ph114_H1
zmp_ph114_H2
zmp_ph114_A9
zmp_ph114_A11
zmp_ph114_B2
zmp_ph114_B4
zmp_ph114_D8
zmp_ph114_E2

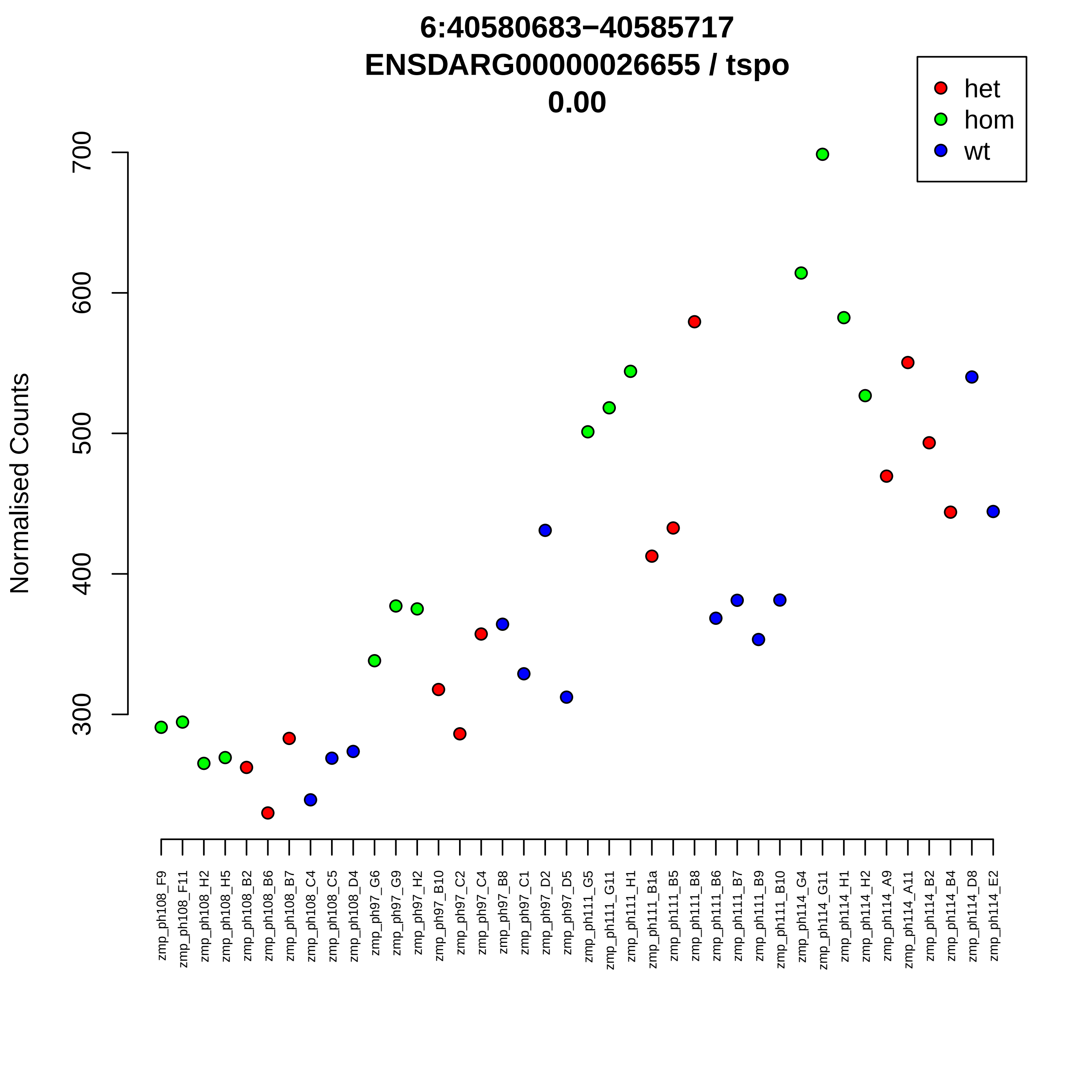

6:40580683−40585717
ENSDARG00000026655 / tspo
0.00
het
hom
wt
Normalised Counts
300
400
500
600
700
zmp_ph108_F9
zmp_ph108_F11
zmp_ph108_H2
zmp_ph108_H5
zmp_ph108_B2
zmp_ph108_B6
zmp_ph108_B7
zmp_ph108_C4
zmp_ph108_C5
zmp_ph108_D4
zmp_ph97_G6
zmp_ph97_G9
zmp_ph97_H2
zmp_ph97_B10
zmp_ph97_C2
zmp_ph97_C4
zmp_ph97_B8
zmp_ph97_C1
zmp_ph97_D2
zmp_ph97_D5
zmp_ph111_G5
zmp_ph111_G11
zmp_ph111_H1
zmp_ph111_B1a
zmp_ph111_B5
zmp_ph111_B8
zmp_ph111_B6
zmp_ph111_B7
zmp_ph111_B9
zmp_ph111_B10
zmp_ph114_G4
zmp_ph114_G11
zmp_ph114_H1
zmp_ph114_H2
zmp_ph114_A9
zmp_ph114_A11
zmp_ph114_B2
zmp_ph114_B4
zmp_ph114_D8
zmp_ph114_E2

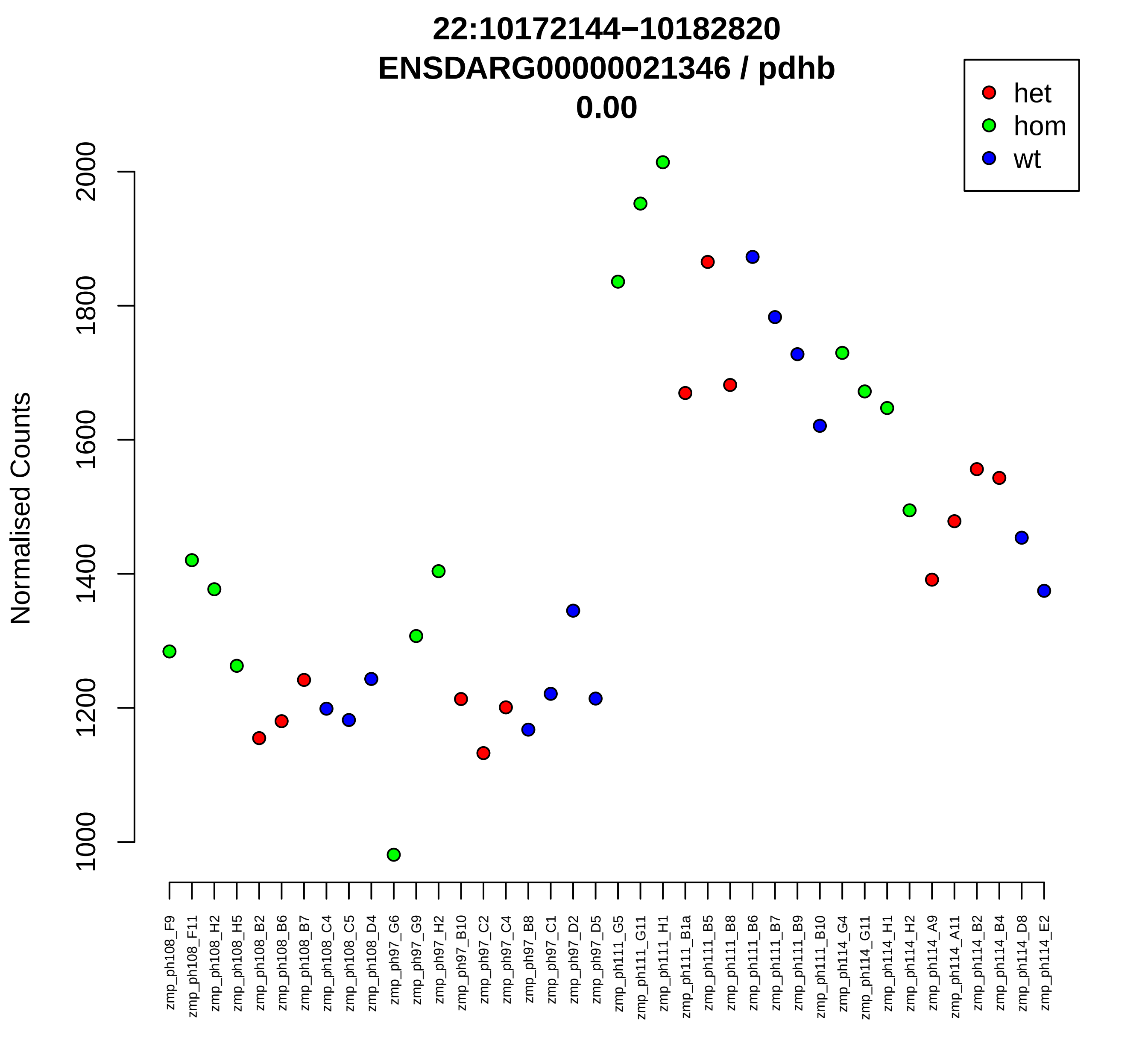

22:10172144−10182820
ENSDARG00000021346 / pdhb
0.00
het
hom
wt
Normalised Counts
2000
1800
1600
1400
1200
1000
zmp_ph108_F9
zmp_ph108_F11
zmp_ph108_H2
zmp_ph108_H5
zmp_ph108_B2
zmp_ph108_B6
zmp_ph108_B7
zmp_ph108_C4
zmp_ph108_C5
zmp_ph108_D4
zmp_ph97_G6
zmp_ph97_G9
zmp_ph97_H2
zmp_ph97_B10
zmp_ph97_C2
zmp_ph97_C4
zmp_ph97_B8
zmp_ph97_C1
zmp_ph97_D2
zmp_ph97_D5
zmp_ph111_G5
zmp_ph111_G11
zmp_ph111_H1
zmp_ph111_B1a
zmp_ph111_B5
zmp_ph111_B8
zmp_ph111_B6
zmp_ph111_B7
zmp_ph111_B9
zmp_ph111_B10
zmp_ph114_G4
zmp_ph114_G11
zmp_ph114_H1
zmp_ph114_H2
zmp_ph114_A9
zmp_ph114_A11
zmp_ph114_B2
zmp_ph114_B4
zmp_ph114_D8
zmp_ph114_E2

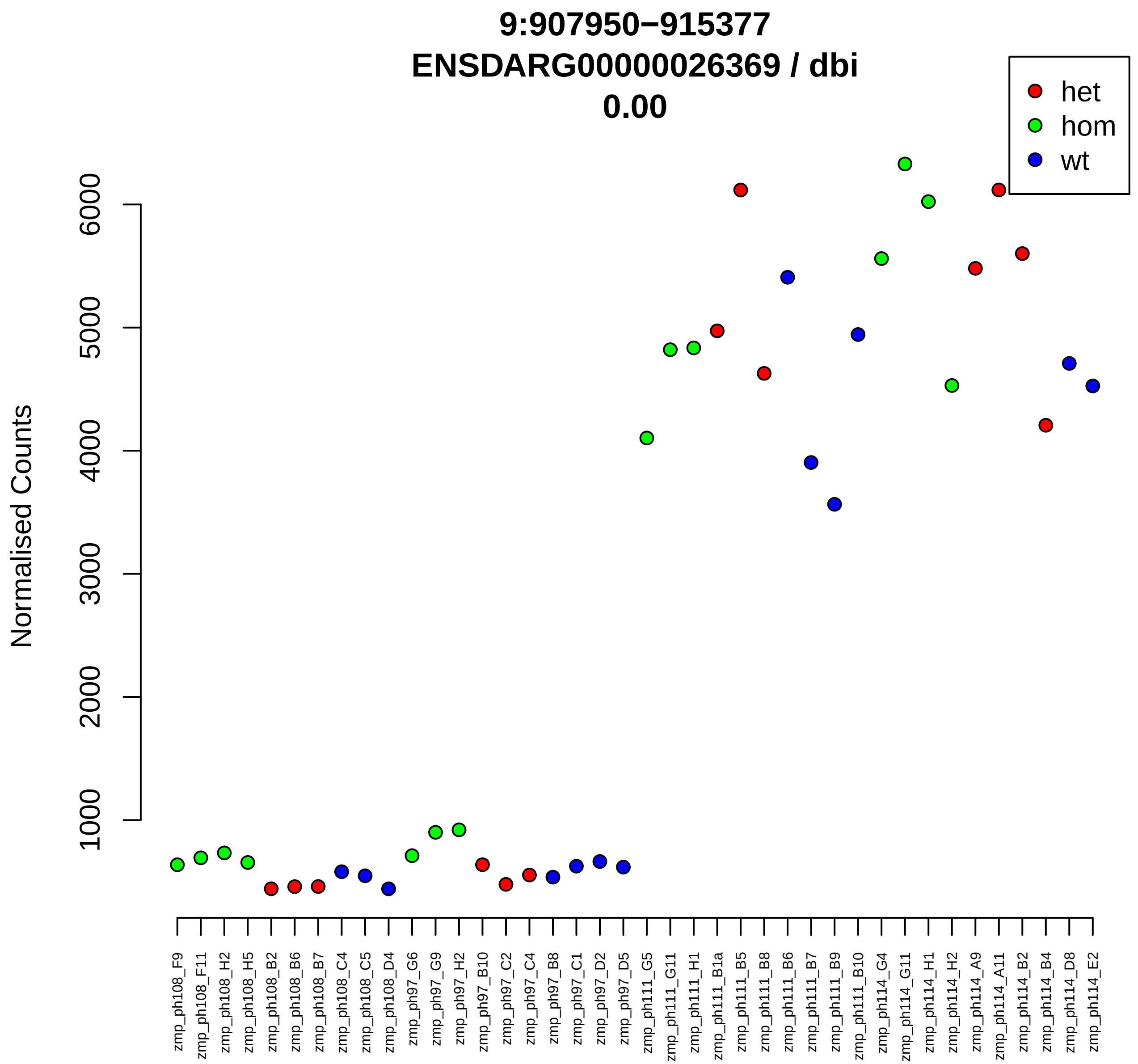

9:907950−915377
ENSDARG00000026369 / dbi
0.00
het
hom
wt
Normalised Counts
1000
2000
3000
4000
5000
6000
zmp_ph108_F9
zmp_ph108_F11
zmp_ph108_H2
zmp_ph108_H5
zmp_ph108_B2
zmp_ph108_B6
zmp_ph108_B7
zmp_ph108_C4
zmp_ph108_C5
zmp_ph108_D4
zmp_ph97_G6
zmp_ph97_G9
zmp_ph97_H2
zmp_ph97_B10
zmp_ph97_C2
zmp_ph97_C4
zmp_ph97_B8
zmp_ph97_C1
zmp_ph97_D2
zmp_ph97_D5
zmp_ph111_G5
zmp_ph111_G11
zmp_ph111_H1
zmp_ph111_B1a
zmp_ph111_B5
zmp_ph111_B8
zmp_ph111_B6
zmp_ph111_B7
zmp_ph111_B9
zmp_ph111_B10
zmp_ph114_G4
zmp_ph114_G11
zmp_ph114_H1
zmp_ph114_H2
zmp_ph114_A9
zmp_ph114_A11
zmp_ph114_B2
zmp_ph114_B4
zmp_ph114_D8
zmp_ph114_E2

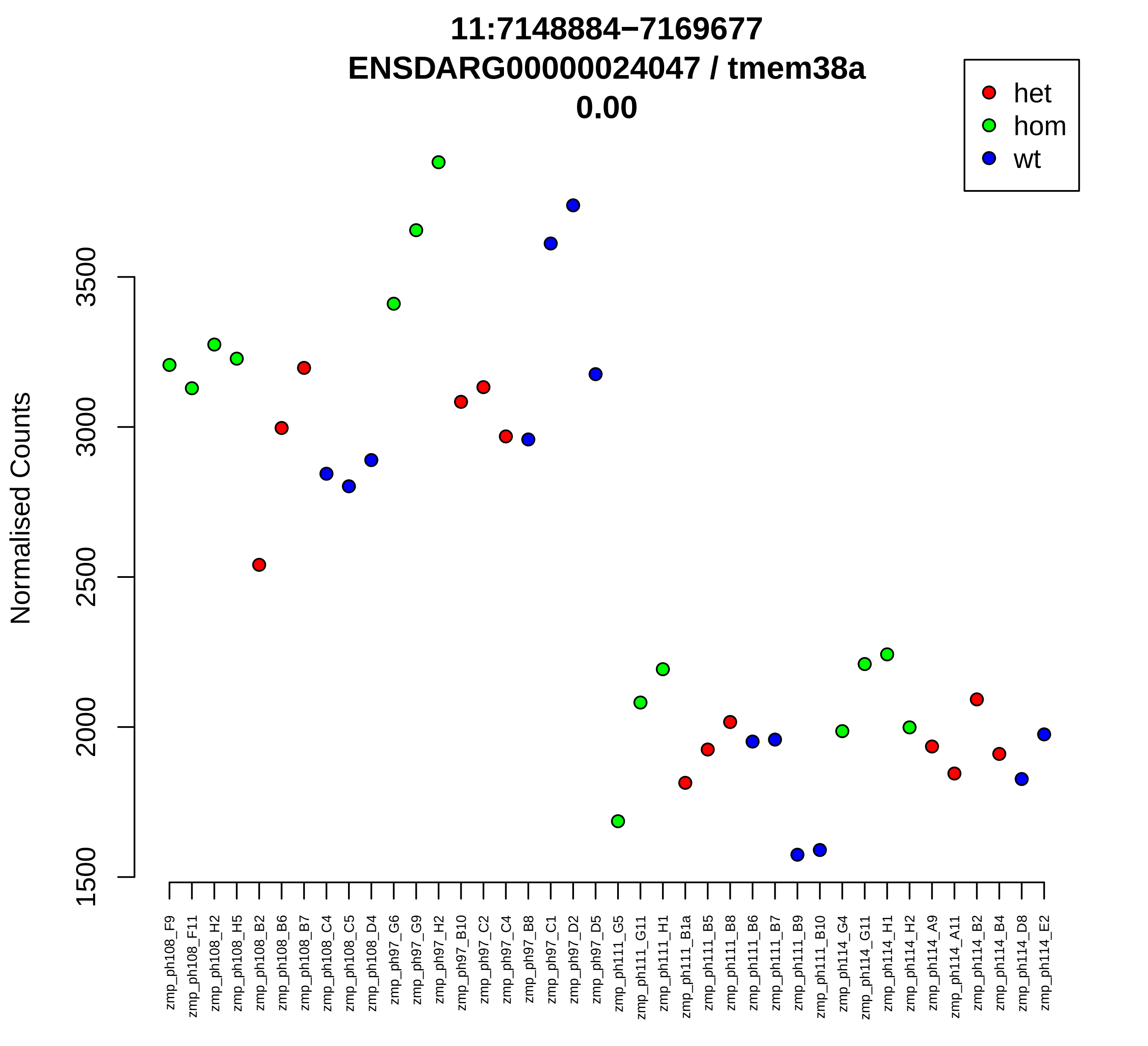

11:7148884−7169677
ENSDARG00000024047 / tmem38a
0.00
het
hom
wt
Normalised Counts
1500
2000
2500
3000
3500
zmp_ph108_F9
zmp_ph108_F11
zmp_ph108_H2
zmp_ph108_H5
zmp_ph108_B2
zmp_ph108_B6
zmp_ph108_B7
zmp_ph108_C4
zmp_ph108_C5
zmp_ph108_D4
zmp_ph97_G6
zmp_ph97_G9
zmp_ph97_H2
zmp_ph97_B10
zmp_ph97_C2
zmp_ph97_C4
zmp_ph97_B8
zmp_ph97_C1
zmp_ph97_D2
zmp_ph97_D5
zmp_ph111_G5
zmp_ph111_G11
zmp_ph111_H1
zmp_ph111_B1a
zmp_ph111_B5
zmp_ph111_B8
zmp_ph111_B6
zmp_ph111_B7
zmp_ph111_B9
zmp_ph111_B10
zmp_ph114_G4
zmp_ph114_G11
zmp_ph114_H1
zmp_ph114_H2
zmp_ph114_A9
zmp_ph114_A11
zmp_ph114_B2
zmp_ph114_B4
zmp_ph114_D8
zmp_ph114_E2

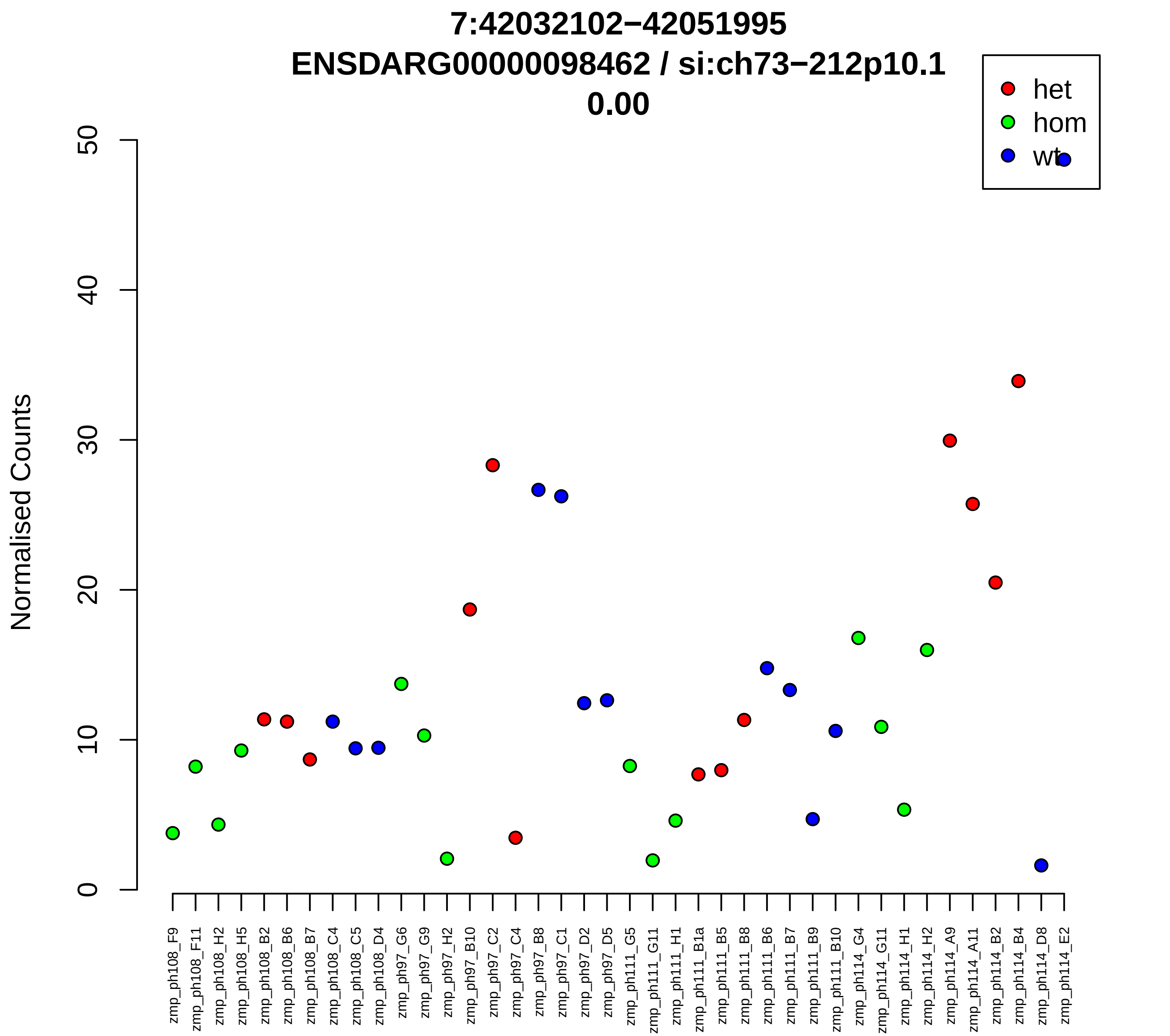

7:42032102−42051995
ENSDARG00000098462 / si:ch73−212p10.1
0.00
het
hom
wt
Normalised Counts
0
10
20
30
40
50
zmp_ph108_F9
zmp_ph108_F11
zmp_ph108_H2
zmp_ph108_H5
zmp_ph108_B2
zmp_ph108_B6
zmp_ph108_B7
zmp_ph108_C4
zmp_ph108_C5
zmp_ph108_D4
zmp_ph97_G6
zmp_ph97_G9
zmp_ph97_H2
zmp_ph97_B10
zmp_ph97_C2
zmp_ph97_C4
zmp_ph97_B8
zmp_ph97_C1
zmp_ph97_D2
zmp_ph97_D5
zmp_ph111_G5
zmp_ph111_G11
zmp_ph111_H1
zmp_ph111_B1a
zmp_ph111_B5
zmp_ph111_B8
zmp_ph111_B6
zmp_ph111_B7
zmp_ph111_B9
zmp_ph111_B10
zmp_ph114_G4
zmp_ph114_G11
zmp_ph114_H1
zmp_ph114_H2
zmp_ph114_A9
zmp_ph114_A11
zmp_ph114_B2
zmp_ph114_B4
zmp_ph114_D8
zmp_ph114_E2

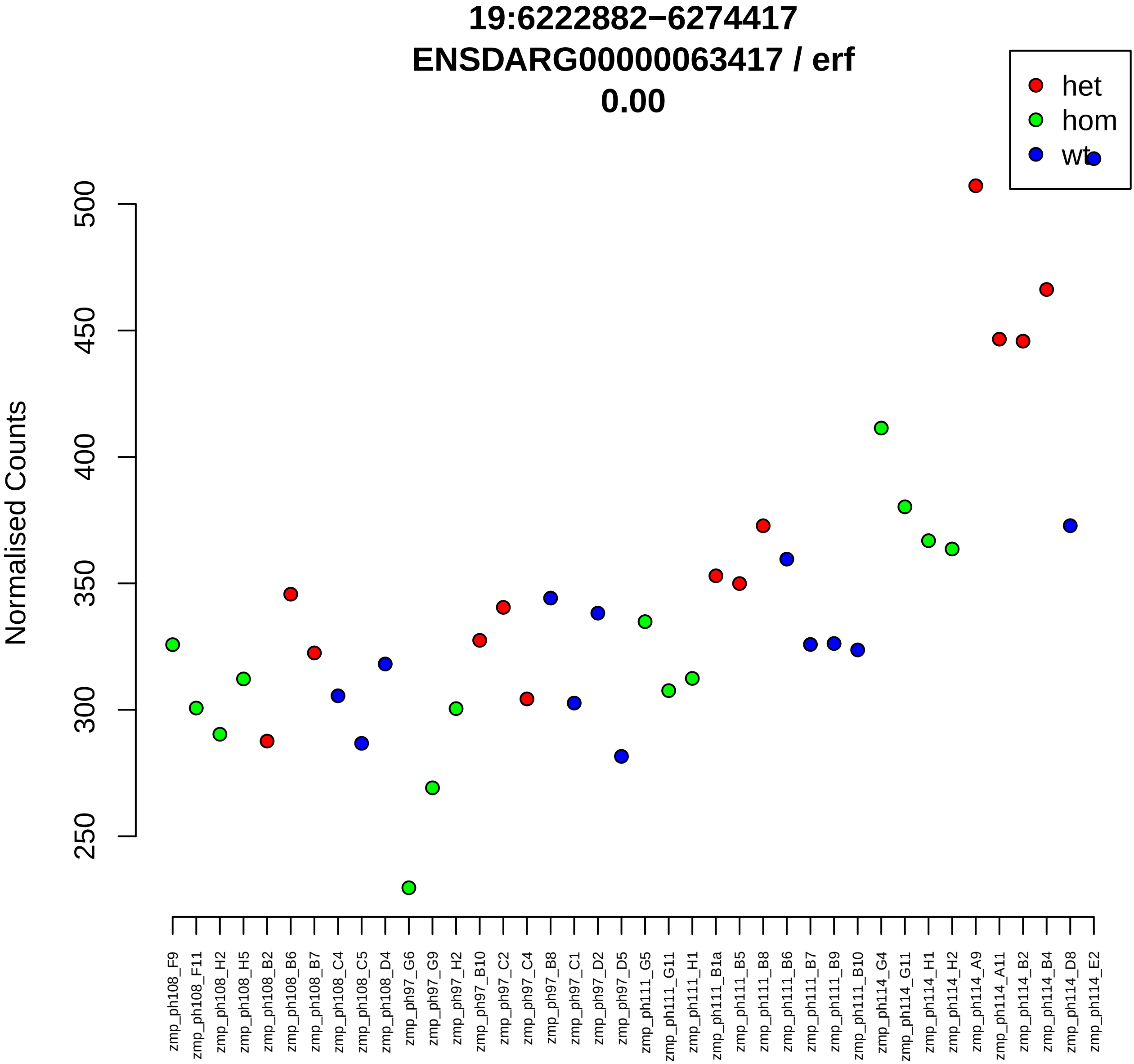

19:6222882−6274417
ENSDARG00000063417 / erf
0.00
het
hom
wt
Normalised Counts
250
300
350
400
450
500
zmp_ph108_F9
zmp_ph108_F11
zmp_ph108_H2
zmp_ph108_H5
zmp_ph108_B2
zmp_ph108_B6
zmp_ph108_B7
zmp_ph108_C4
zmp_ph108_C5
zmp_ph108_D4
zmp_ph97_G6
zmp_ph97_G9
zmp_ph97_H2
zmp_ph97_B10
zmp_ph97_C2
zmp_ph97_C4
zmp_ph97_B8
zmp_ph97_C1
zmp_ph97_D2
zmp_ph97_D5
zmp_ph111_G5
zmp_ph111_G11
zmp_ph111_H1
zmp_ph111_B1a
zmp_ph111_B5
zmp_ph111_B8
zmp_ph111_B6
zmp_ph111_B7
zmp_ph111_B9
zmp_ph111_B10
zmp_ph114_G4
zmp_ph114_G11
zmp_ph114_H1
zmp_ph114_H2
zmp_ph114_A9
zmp_ph114_A11
zmp_ph114_B2
zmp_ph114_B4
zmp_ph114_D8
zmp_ph114_E2

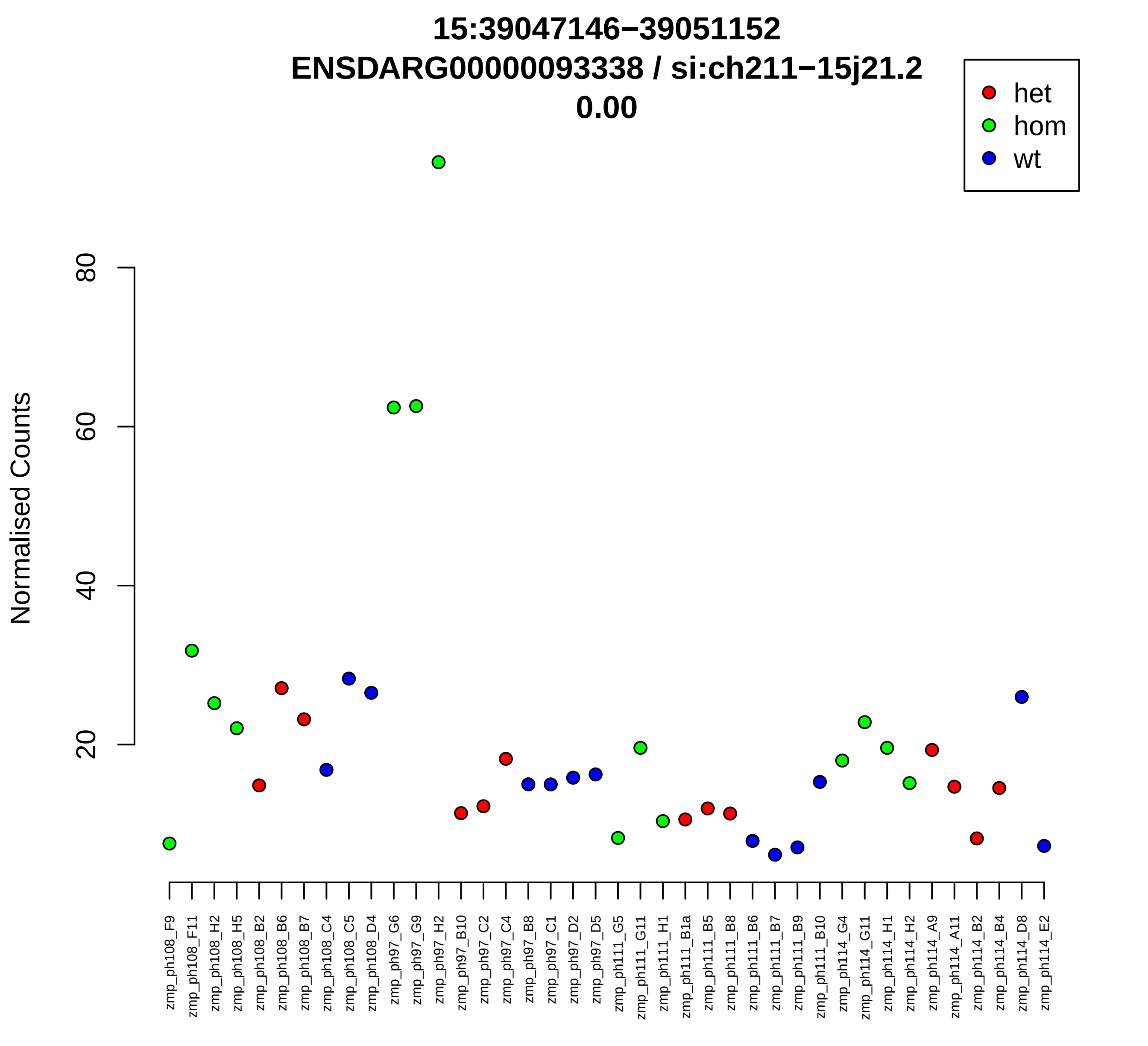

15:39047146−39051152
ENSDARG00000093338 / si:ch211−15j21.2
0.00
het
hom
wt
Normalised Counts
20
40
60
80
zmp_ph108_F9
zmp_ph108_F11
zmp_ph108_H2
zmp_ph108_H5
zmp_ph108_B2
zmp_ph108_B6
zmp_ph108_B7
zmp_ph108_C4
zmp_ph108_C5
zmp_ph108_D4
zmp_ph97_G6
zmp_ph97_G9
zmp_ph97_H2
zmp_ph97_B10
zmp_ph97_C2
zmp_ph97_C4
zmp_ph97_B8
zmp_ph97_C1
zmp_ph97_D2
zmp_ph97_D5
zmp_ph111_G5
zmp_ph111_G11
zmp_ph111_H1
zmp_ph111_B1a
zmp_ph111_B5
zmp_ph111_B8
zmp_ph111_B6
zmp_ph111_B7
zmp_ph111_B9
zmp_ph111_B10
zmp_ph114_G4
zmp_ph114_G11
zmp_ph114_H1
zmp_ph114_H2
zmp_ph114_A9
zmp_ph114_A11
zmp_ph114_B2
zmp_ph114_B4
zmp_ph114_D8
zmp_ph114_E2

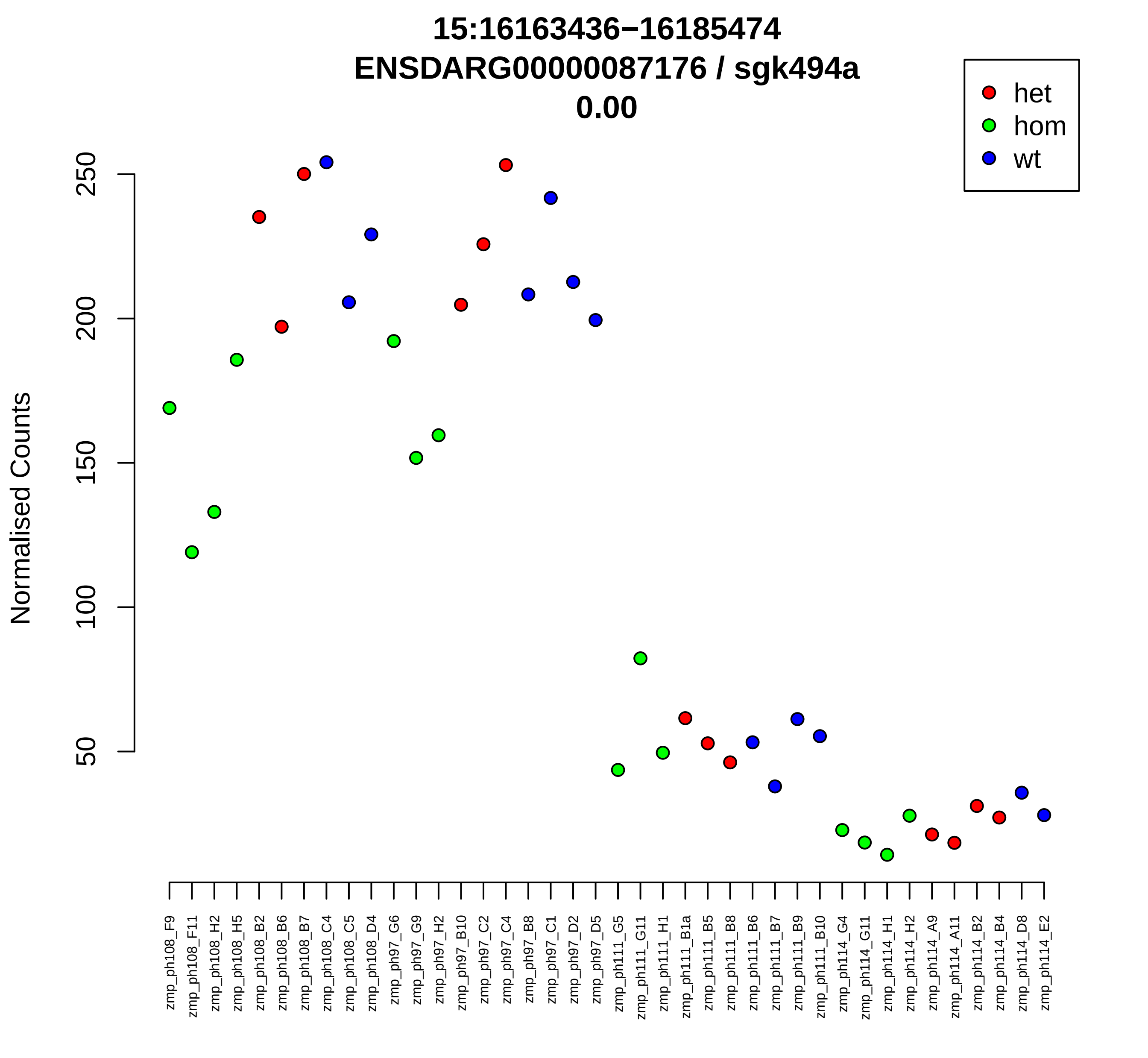
15:16163436−16185474
ENSDARG00000087176 / sgk494a
0.00
het
hom
wt
Normalised Counts
50
100
150
200
250
zmp_ph108_F9
zmp_ph108_F11
zmp_ph108_H2
zmp_ph108_H5
zmp_ph108_B2
zmp_ph108_B6
zmp_ph108_B7
zmp_ph108_C4
zmp_ph108_C5
zmp_ph108_D4
zmp_ph97_G6
zmp_ph97_G9
zmp_ph97_H2
zmp_ph97_B10
zmp_ph97_C2
zmp_ph97_C4
zmp_ph97_B8
zmp_ph97_C1
zmp_ph97_D2
zmp_ph97_D5
zmp_ph111_G5
zmp_ph111_G11
zmp_ph111_H1
zmp_ph111_B1a
zmp_ph111_B5
zmp_ph111_B8
zmp_ph111_B6
zmp_ph111_B7
zmp_ph111_B9
zmp_ph111_B10
zmp_ph114_G4
zmp_ph114_G11
zmp_ph114_H1
zmp_ph114_H2
zmp_ph114_A9
zmp_ph114_A11
zmp_ph114_B2
zmp_ph114_B4
zmp_ph114_D8
zmp_ph114_E2

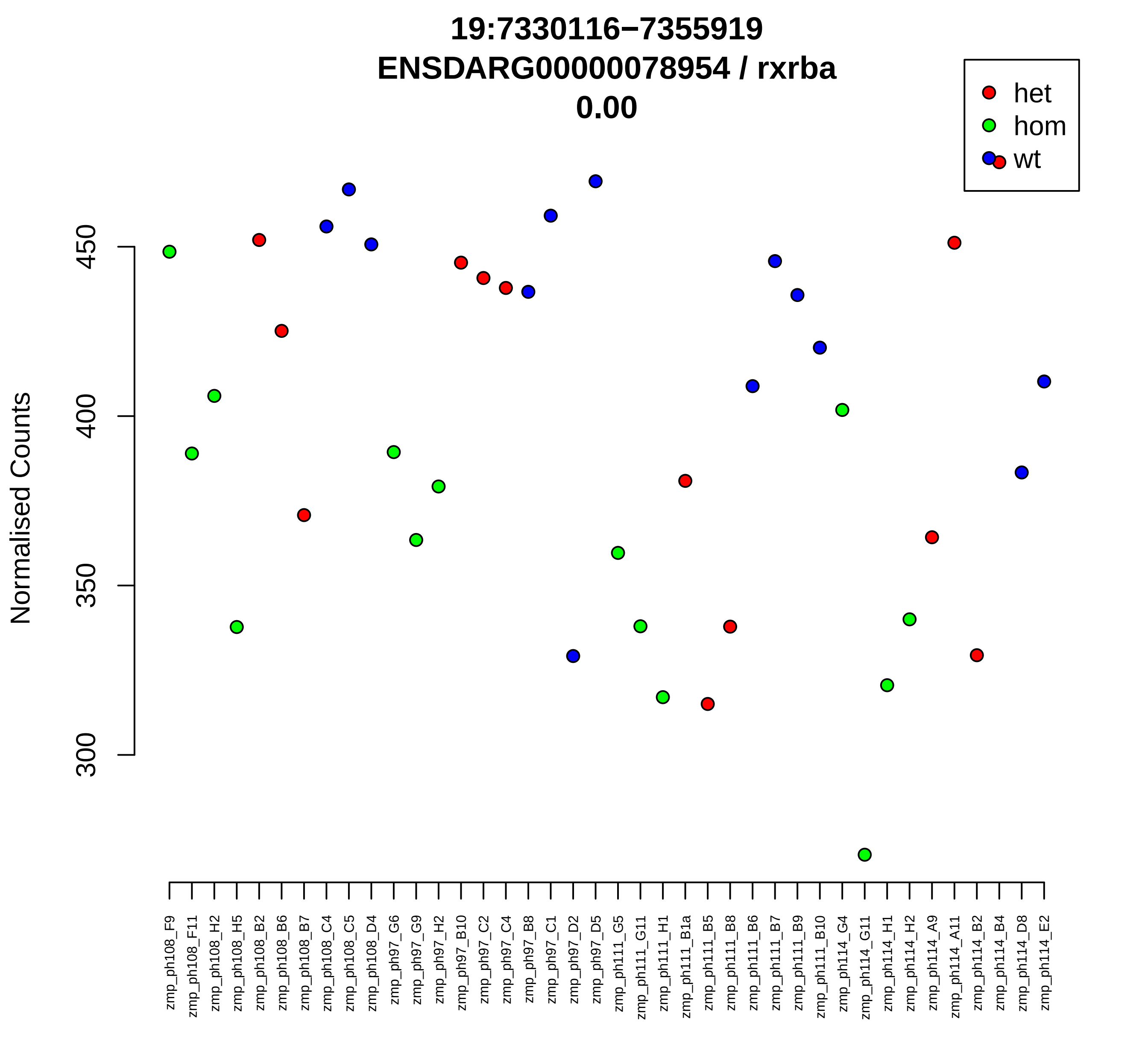

19:7330116−7355919
ENSDARG00000078954 / rxrba
0.00
het
hom
wt
Normalised Counts
300
350
400
450
zmp_ph108_F9
zmp_ph108_F11
zmp_ph108_H2
zmp_ph108_H5
zmp_ph108_B2
zmp_ph108_B6
zmp_ph108_B7
zmp_ph108_C4
zmp_ph108_C5
zmp_ph108_D4
zmp_ph97_G6
zmp_ph97_G9
zmp_ph97_H2
zmp_ph97_B10
zmp_ph97_C2
zmp_ph97_C4
zmp_ph97_B8
zmp_ph97_C1
zmp_ph97_D2
zmp_ph97_D5
zmp_ph111_G5
zmp_ph111_G11
zmp_ph111_H1
zmp_ph111_B1a
zmp_ph111_B5
zmp_ph111_B8
zmp_ph111_B6
zmp_ph111_B7
zmp_ph111_B9
zmp_ph111_B10
zmp_ph114_G4
zmp_ph114_G11
zmp_ph114_H1
zmp_ph114_H2
zmp_ph114_A9
zmp_ph114_A11
zmp_ph114_B2
zmp_ph114_B4
zmp_ph114_D8
zmp_ph114_E2

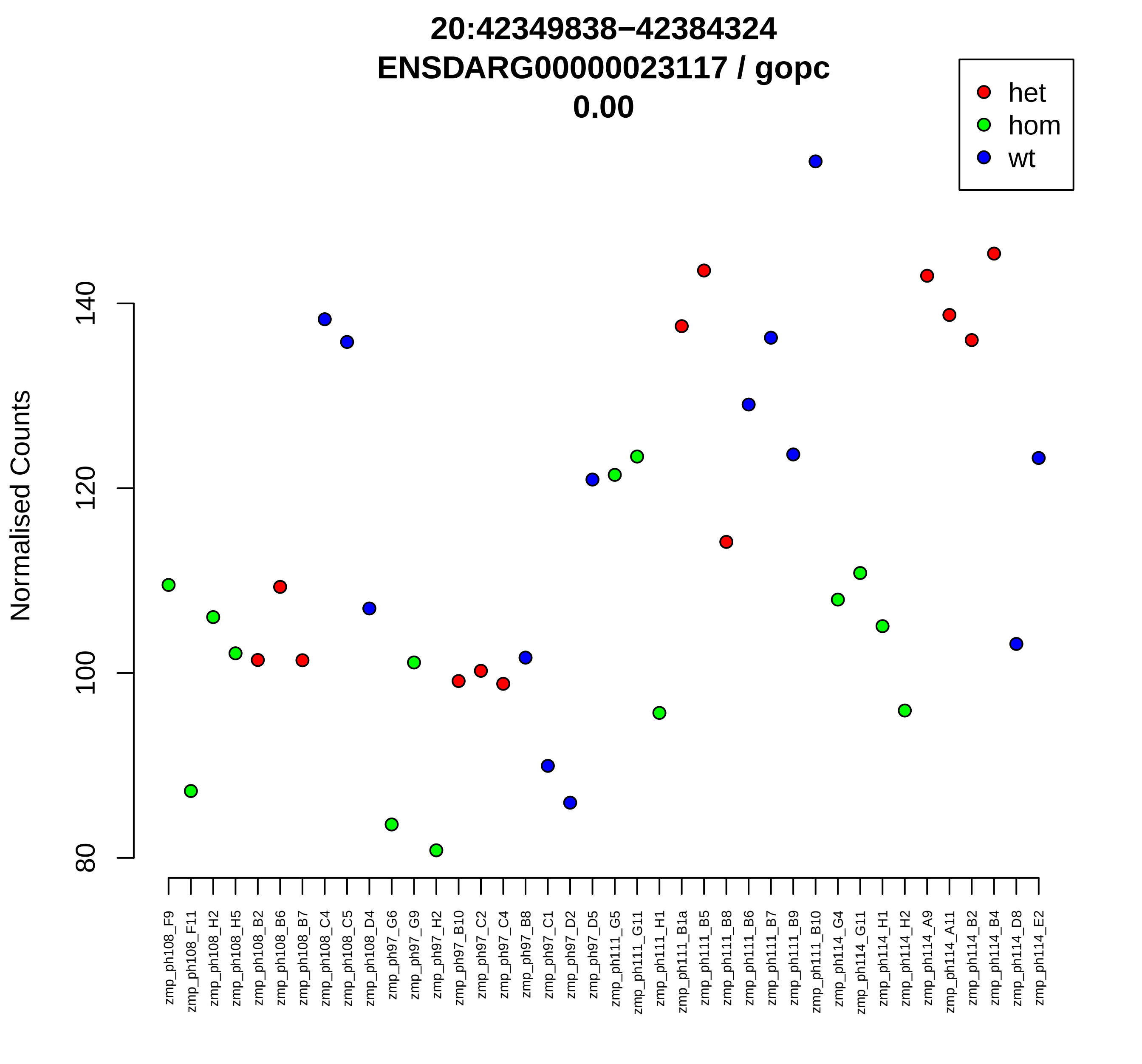

20:42349838−42384324
ENSDARG00000023117 / gopc
0.00
het
hom
wt
Normalised Counts
80
100
120
140
zmp_ph108_F9
zmp_ph108_F11
zmp_ph108_H2
zmp_ph108_H5
zmp_ph108_B2
zmp_ph108_B6
zmp_ph108_B7
zmp_ph108_C4
zmp_ph108_C5
zmp_ph108_D4
zmp_ph97_G6
zmp_ph97_G9
zmp_ph97_H2
zmp_ph97_B10
zmp_ph97_C2
zmp_ph97_C4
zmp_ph97_B8
zmp_ph97_C1
zmp_ph97_D2
zmp_ph97_D5
zmp_ph111_G5
zmp_ph111_G11
zmp_ph111_H1
zmp_ph111_B1a
zmp_ph111_B5
zmp_ph111_B8
zmp_ph111_B6
zmp_ph111_B7
zmp_ph111_B9
zmp_ph111_B10
zmp_ph114_G4
zmp_ph114_G11
zmp_ph114_H1
zmp_ph114_H2
zmp_ph114_A9
zmp_ph114_A11
zmp_ph114_B2
zmp_ph114_B4
zmp_ph114_D8
zmp_ph114_E2

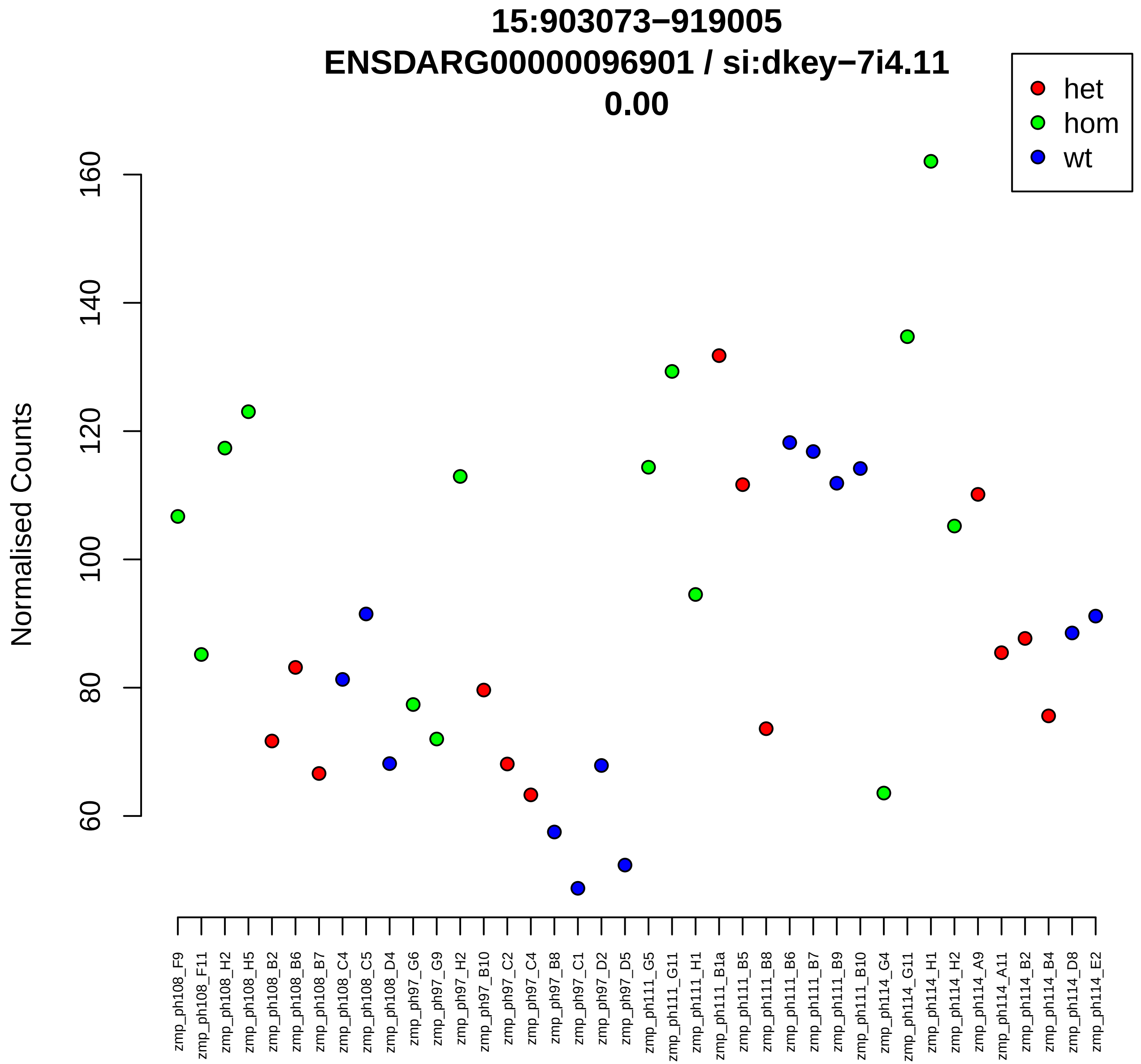
15:903073−919005
ENSDARG00000096901 / si:dkey−7i4.11
0.00
het
hom
wt
Normalised Counts
60
80
100
120
140
160
zmp_ph108_F9
zmp_ph108_F11
zmp_ph108_H2
zmp_ph108_H5
zmp_ph108_B2
zmp_ph108_B6
zmp_ph108_B7
zmp_ph108_C4
zmp_ph108_C5
zmp_ph108_D4
zmp_ph97_G6
zmp_ph97_G9
zmp_ph97_H2
zmp_ph97_B10
zmp_ph97_C2
zmp_ph97_C4
zmp_ph97_B8
zmp_ph97_C1
zmp_ph97_D2
zmp_ph97_D5
zmp_ph111_G5
zmp_ph111_G11
zmp_ph111_H1
zmp_ph111_B1a
zmp_ph111_B5
zmp_ph111_B8
zmp_ph111_B6
zmp_ph111_B7
zmp_ph111_B9
zmp_ph111_B10
zmp_ph114_G4
zmp_ph114_G11
zmp_ph114_H1
zmp_ph114_H2
zmp_ph114_A9
zmp_ph114_A11
zmp_ph114_B2
zmp_ph114_B4
zmp_ph114_D8
zmp_ph114_E2

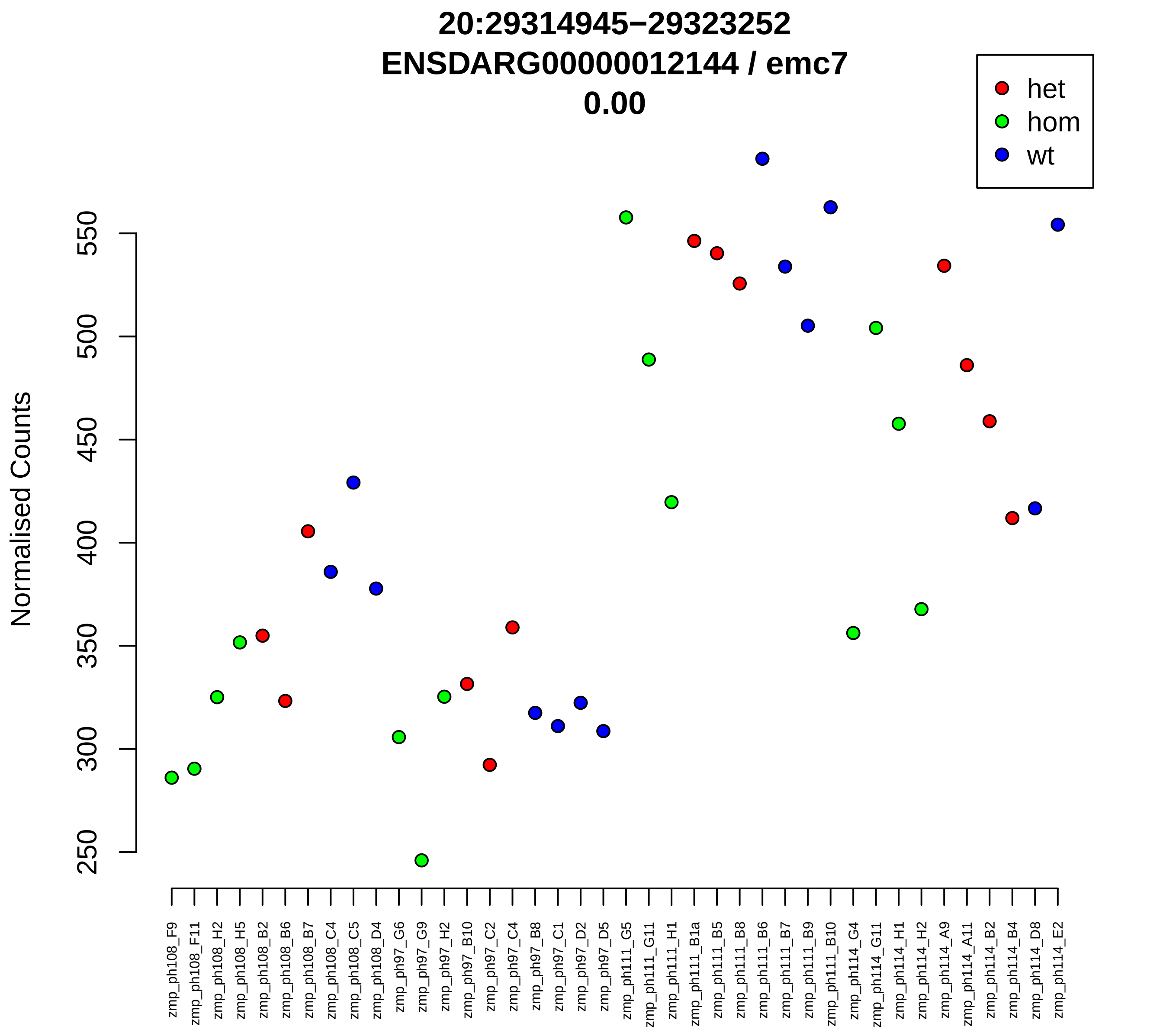

20:29314945−29323252
ENSDARG00000012144 / emc7
0.00
het
hom
wt
Normalised Counts
250
300
350
400
450
500
550
zmp_ph108_F9
zmp_ph108_F11
zmp_ph108_H2
zmp_ph108_H5
zmp_ph108_B2
zmp_ph108_B6
zmp_ph108_B7
zmp_ph108_C4
zmp_ph108_C5
zmp_ph108_D4
zmp_ph97_G6
zmp_ph97_G9
zmp_ph97_H2
zmp_ph97_B10
zmp_ph97_C2
zmp_ph97_C4
zmp_ph97_B8
zmp_ph97_C1
zmp_ph97_D2
zmp_ph97_D5
zmp_ph111_G5
zmp_ph111_G11
zmp_ph111_H1
zmp_ph111_B1a
zmp_ph111_B5
zmp_ph111_B8
zmp_ph111_B6
zmp_ph111_B7
zmp_ph111_B9
zmp_ph111_B10
zmp_ph114_G4
zmp_ph114_G11
zmp_ph114_H1
zmp_ph114_H2
zmp_ph114_A9
zmp_ph114_A11
zmp_ph114_B2
zmp_ph114_B4
zmp_ph114_D8
zmp_ph114_E2

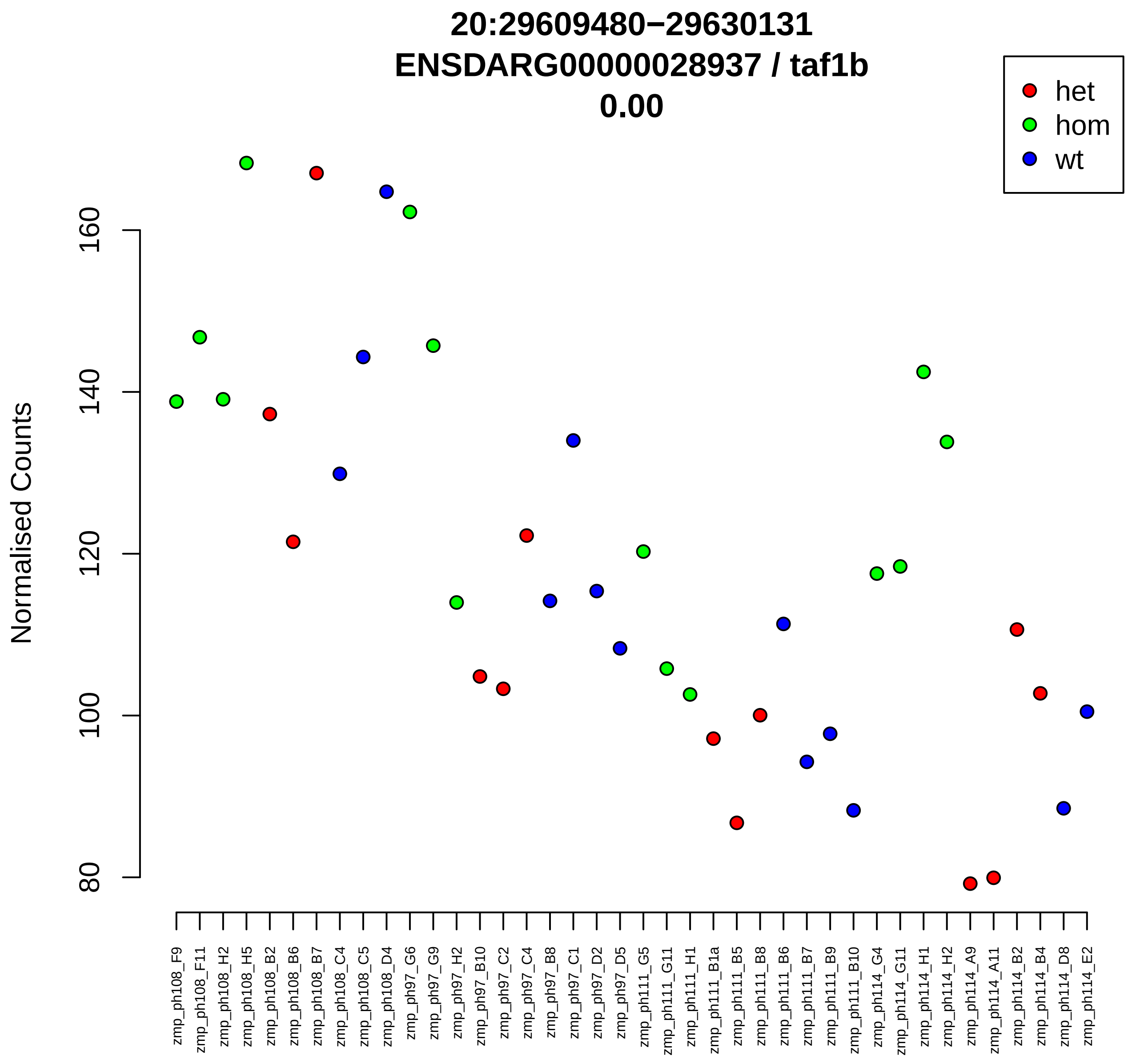
20:29609480−29630131
ENSDARG00000028937 / taf1b
0.00
het
hom
wt
Normalised Counts
80
100
120
140
160
zmp_ph108_F9
zmp_ph108_F11
zmp_ph108_H2
zmp_ph108_H5
zmp_ph108_B2
zmp_ph108_B6
zmp_ph108_B7
zmp_ph108_C4
zmp_ph108_C5
zmp_ph108_D4
zmp_ph97_G6
zmp_ph97_G9
zmp_ph97_H2
zmp_ph97_B10
zmp_ph97_C2
zmp_ph97_C4
zmp_ph97_B8
zmp_ph97_C1
zmp_ph97_D2
zmp_ph97_D5
zmp_ph111_G5
zmp_ph111_G11
zmp_ph111_H1
zmp_ph111_B1a
zmp_ph111_B5
zmp_ph111_B8
zmp_ph111_B6
zmp_ph111_B7
zmp_ph111_B9
zmp_ph111_B10
zmp_ph114_G4
zmp_ph114_G11
zmp_ph114_H1
zmp_ph114_H2
zmp_ph114_A9
zmp_ph114_A11
zmp_ph114_B2
zmp_ph114_B4
zmp_ph114_D8
zmp_ph114_E2

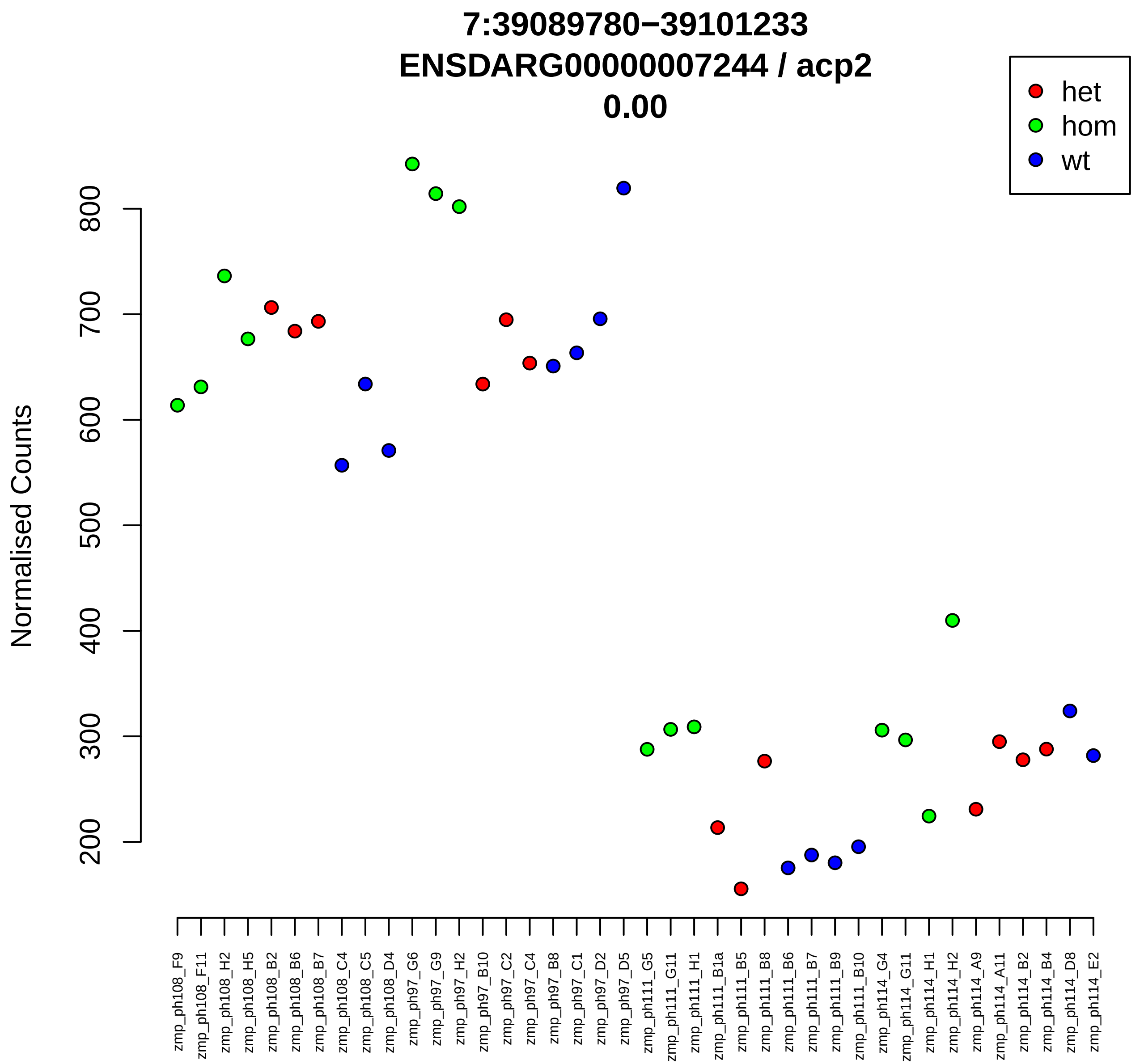

7:39089780−39101233
ENSDARG00000007244 / acp2
0.00
het
hom
wt
Normalised Counts
200
300
400
500
600
700
800
zmp_ph108_F9
zmp_ph108_F11
zmp_ph108_H2
zmp_ph108_H5
zmp_ph108_B2
zmp_ph108_B6
zmp_ph108_B7
zmp_ph108_C4
zmp_ph108_C5
zmp_ph108_D4
zmp_ph97_G6
zmp_ph97_G9
zmp_ph97_H2
zmp_ph97_B10
zmp_ph97_C2
zmp_ph97_C4
zmp_ph97_B8
zmp_ph97_C1
zmp_ph97_D2
zmp_ph97_D5
zmp_ph111_G5
zmp_ph111_G11
zmp_ph111_H1
zmp_ph111_B1a
zmp_ph111_B5
zmp_ph111_B8
zmp_ph111_B6
zmp_ph111_B7
zmp_ph111_B9
zmp_ph111_B10
zmp_ph114_G4
zmp_ph114_G11
zmp_ph114_H1
zmp_ph114_H2
zmp_ph114_A9
zmp_ph114_A11
zmp_ph114_B2
zmp_ph114_B4
zmp_ph114_D8
zmp_ph114_E2

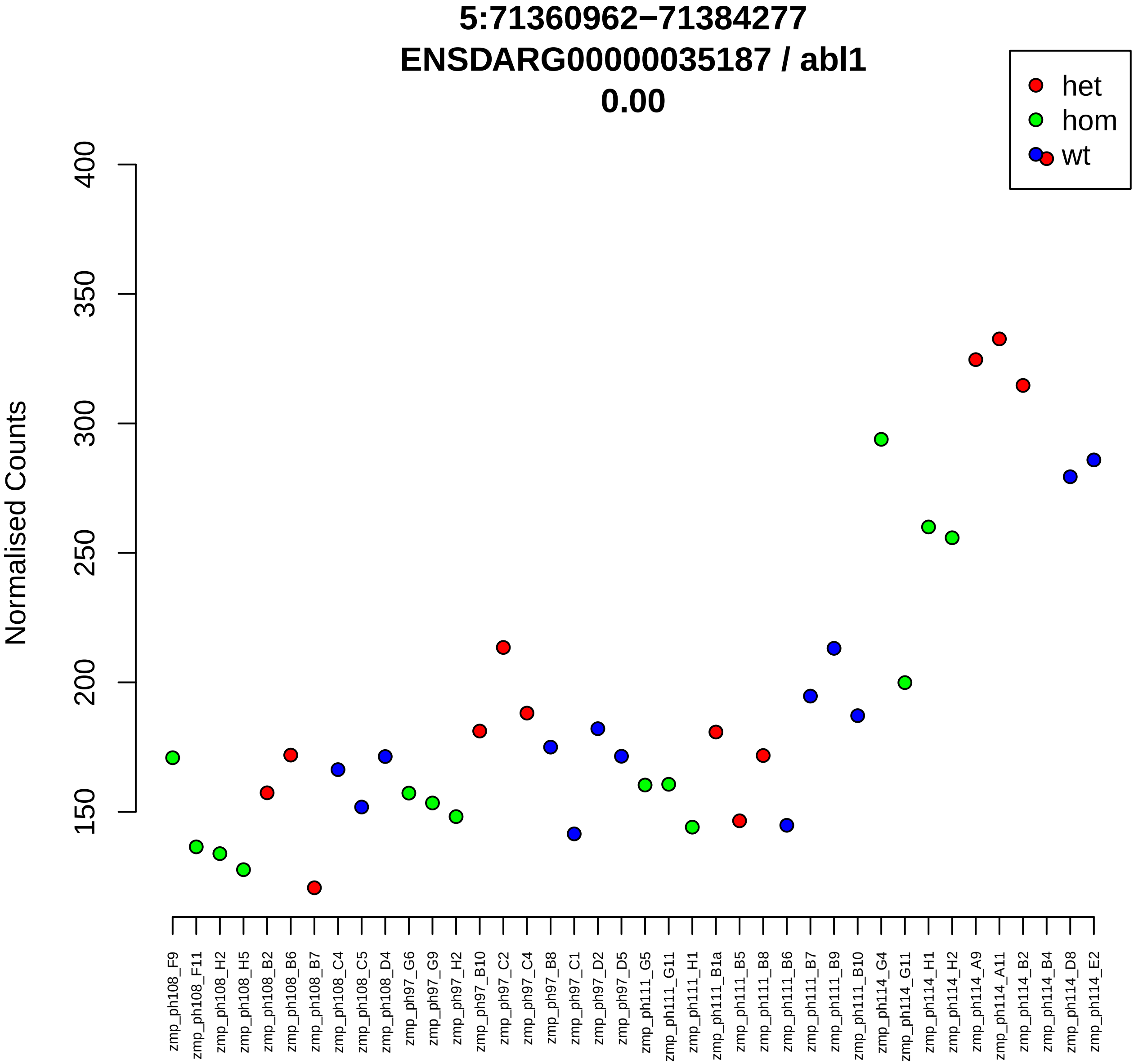

5:71360962−71384277
ENSDARG00000035187 / abl1
0.00
het
hom
wt
Normalised Counts
150
200
250
300
350
400
zmp_ph108_F9
zmp_ph108_F11
zmp_ph108_H2
zmp_ph108_H5
zmp_ph108_B2
zmp_ph108_B6
zmp_ph108_B7
zmp_ph108_C4
zmp_ph108_C5
zmp_ph108_D4
zmp_ph97_G6
zmp_ph97_G9
zmp_ph97_H2
zmp_ph97_B10
zmp_ph97_C2
zmp_ph97_C4
zmp_ph97_B8
zmp_ph97_C1
zmp_ph97_D2
zmp_ph97_D5
zmp_ph111_G5
zmp_ph111_G11
zmp_ph111_H1
zmp_ph111_B1a
zmp_ph111_B5
zmp_ph111_B8
zmp_ph111_B6
zmp_ph111_B7
zmp_ph111_B9
zmp_ph111_B10
zmp_ph114_G4
zmp_ph114_G11
zmp_ph114_H1
zmp_ph114_H2
zmp_ph114_A9
zmp_ph114_A11
zmp_ph114_B2
zmp_ph114_B4
zmp_ph114_D8
zmp_ph114_E2

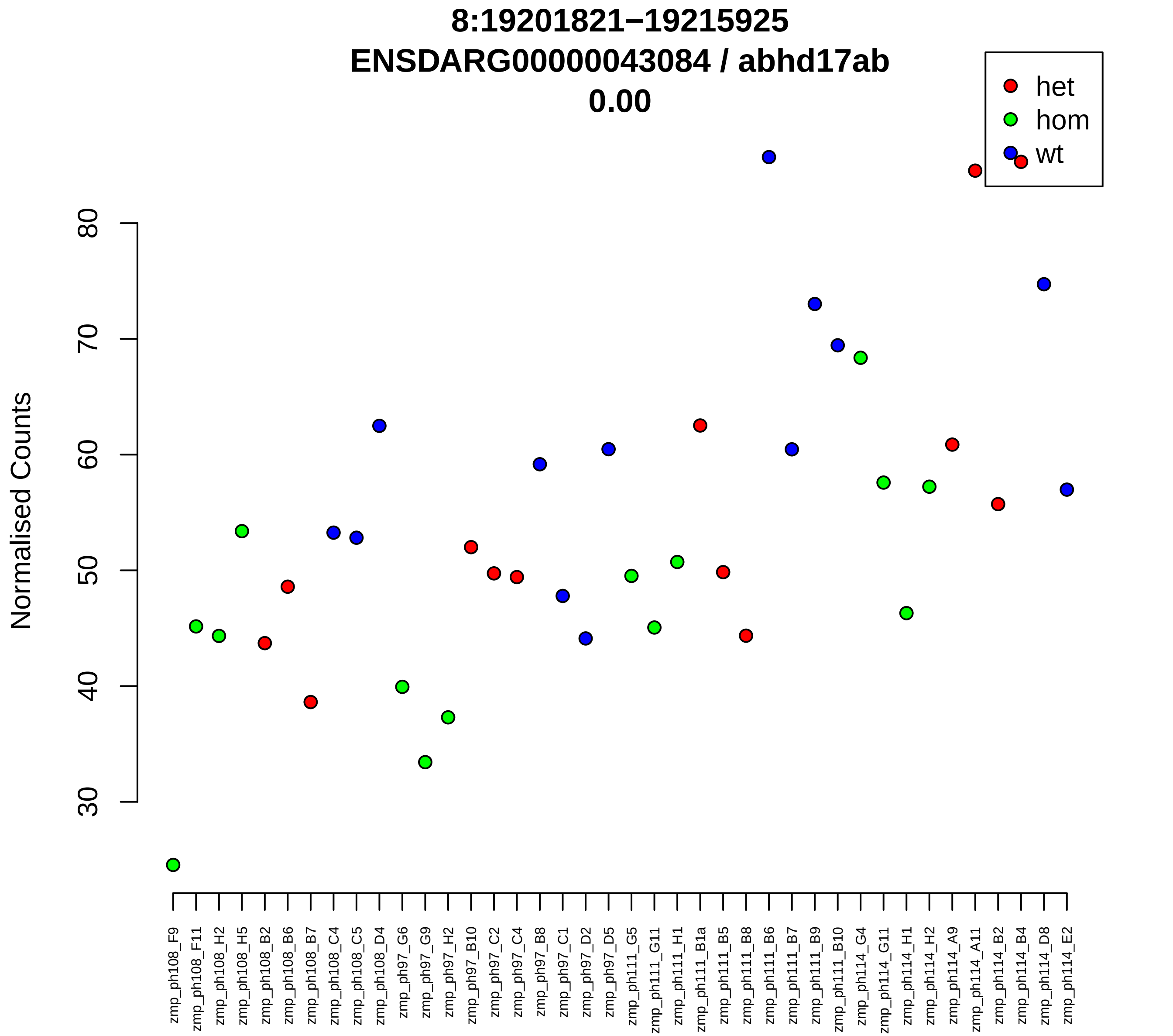
8:19201821−19215925
ENSDARG00000043084 / abhd17ab
0.00
het
hom
wt
Normalised Counts
30
40
50
60
70
80
zmp_ph108_F9
zmp_ph108_F11
zmp_ph108_H2
zmp_ph108_H5
zmp_ph108_B2
zmp_ph108_B6
zmp_ph108_B7
zmp_ph108_C4
zmp_ph108_C5
zmp_ph108_D4
zmp_ph97_G6
zmp_ph97_G9
zmp_ph97_H2
zmp_ph97_B10
zmp_ph97_C2
zmp_ph97_C4
zmp_ph97_B8
zmp_ph97_C1
zmp_ph97_D2
zmp_ph97_D5
zmp_ph111_G5
zmp_ph111_G11
zmp_ph111_H1
zmp_ph111_B1a
zmp_ph111_B5
zmp_ph111_B8
zmp_ph111_B6
zmp_ph111_B7
zmp_ph111_B9
zmp_ph111_B10
zmp_ph114_G4
zmp_ph114_G11
zmp_ph114_H1
zmp_ph114_H2
zmp_ph114_A9
zmp_ph114_A11
zmp_ph114_B2
zmp_ph114_B4
zmp_ph114_D8
zmp_ph114_E2

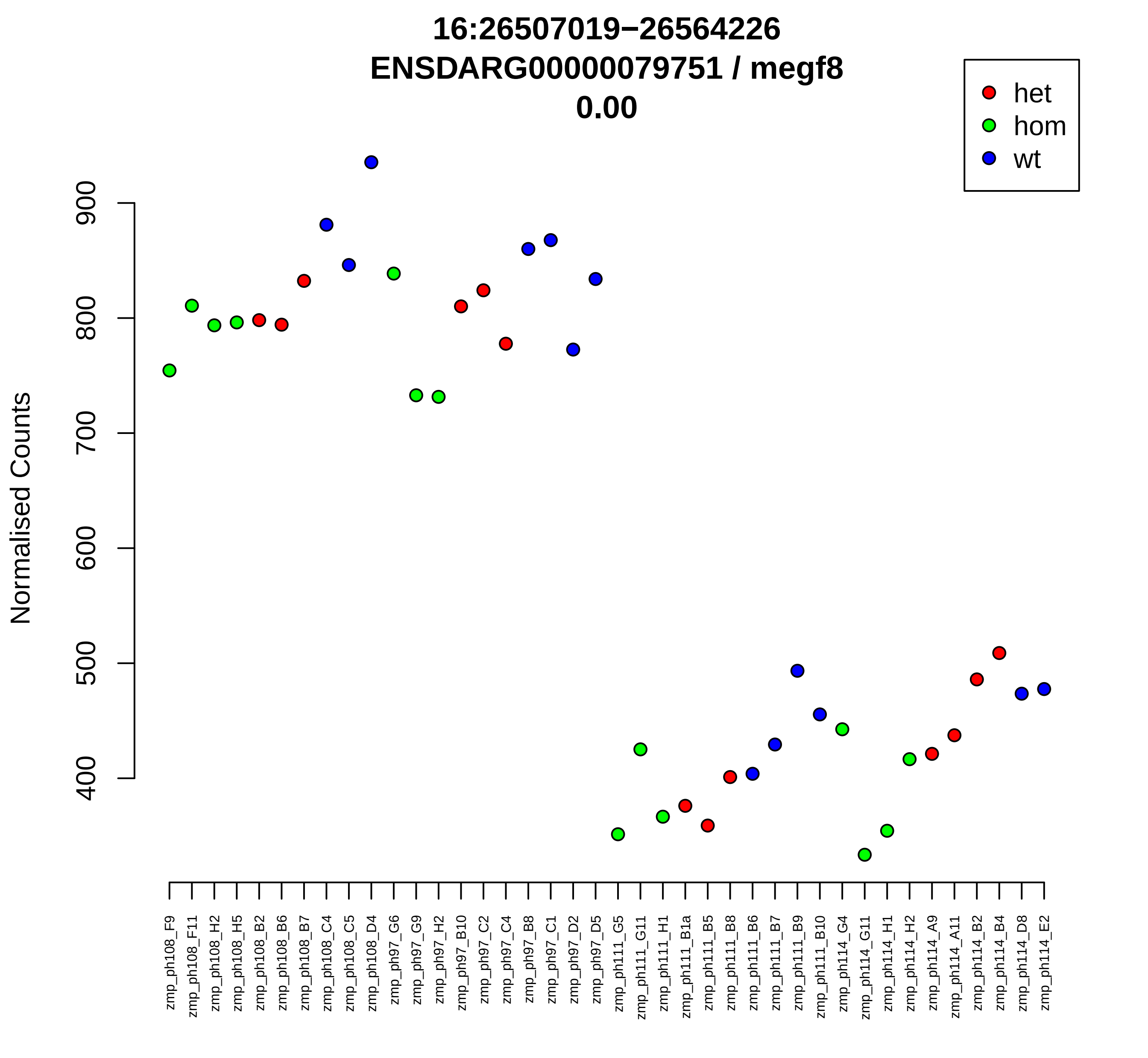

16:26507019−26564226
ENSDARG00000079751 / megf8
0.00
het
hom
wt
Normalised Counts
400
500
600
700
800
900
zmp_ph108_F9
zmp_ph108_F11
zmp_ph108_H2
zmp_ph108_H5
zmp_ph108_B2
zmp_ph108_B6
zmp_ph108_B7
zmp_ph108_C4
zmp_ph108_C5
zmp_ph108_D4
zmp_ph97_G6
zmp_ph97_G9
zmp_ph97_H2
zmp_ph97_B10
zmp_ph97_C2
zmp_ph97_C4
zmp_ph97_B8
zmp_ph97_C1
zmp_ph97_D2
zmp_ph97_D5
zmp_ph111_G5
zmp_ph111_G11
zmp_ph111_H1
zmp_ph111_B1a
zmp_ph111_B5
zmp_ph111_B8
zmp_ph111_B6
zmp_ph111_B7
zmp_ph111_B9
zmp_ph111_B10
zmp_ph114_G4
zmp_ph114_G11
zmp_ph114_H1
zmp_ph114_H2
zmp_ph114_A9
zmp_ph114_A11
zmp_ph114_B2
zmp_ph114_B4
zmp_ph114_D8
zmp_ph114_E2

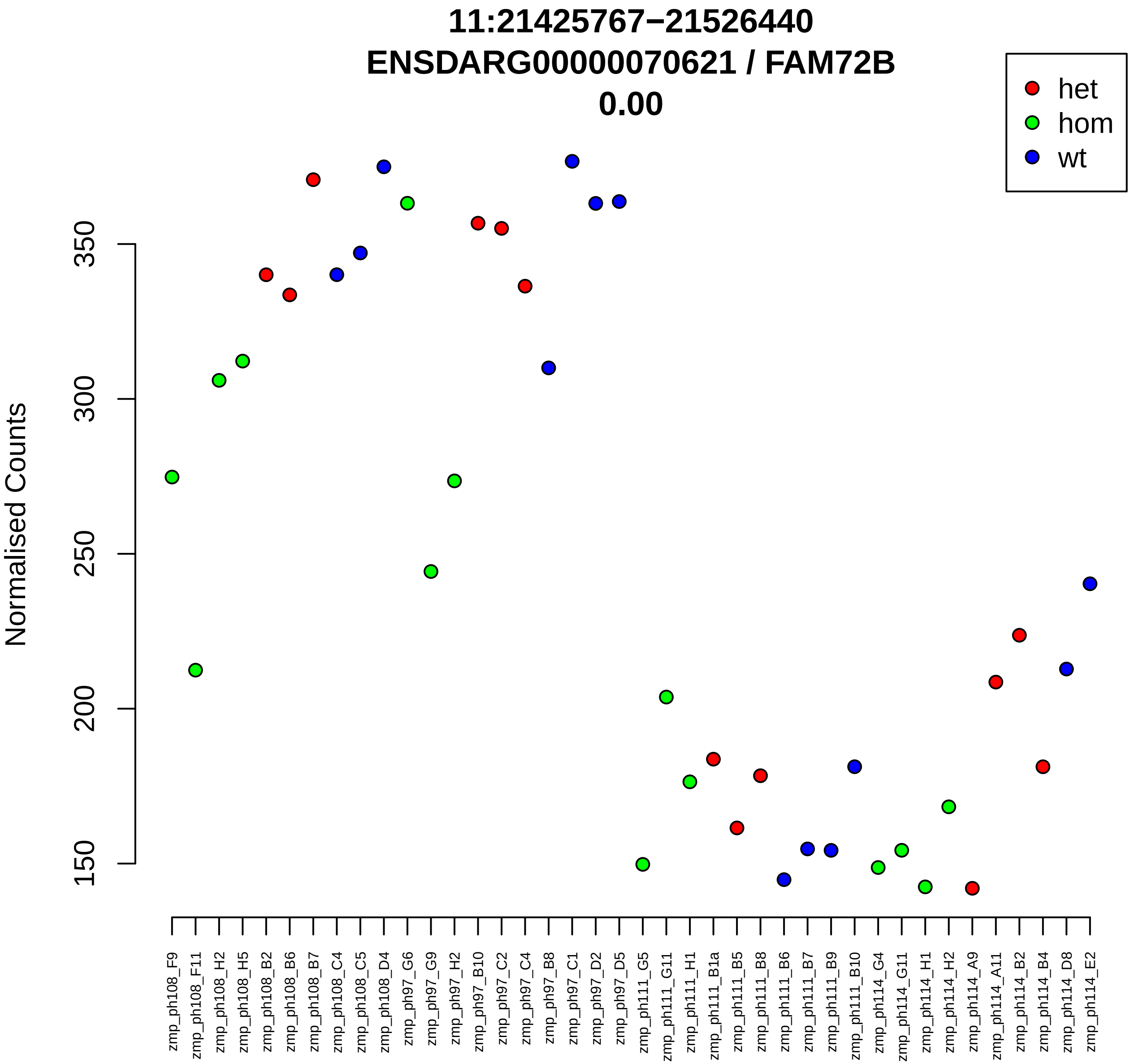
11:21425767−21526440
ENSDARG00000070621 / FAM72B
0.00
het
hom
wt
Normalised Counts
150
200
250
300
350
zmp_ph108_F9
zmp_ph108_F11
zmp_ph108_H2
zmp_ph108_H5
zmp_ph108_B2
zmp_ph108_B6
zmp_ph108_B7
zmp_ph108_C4
zmp_ph108_C5
zmp_ph108_D4
zmp_ph97_G6
zmp_ph97_G9
zmp_ph97_H2
zmp_ph97_B10
zmp_ph97_C2
zmp_ph97_C4
zmp_ph97_B8
zmp_ph97_C1
zmp_ph97_D2
zmp_ph97_D5
zmp_ph111_G5
zmp_ph111_G11
zmp_ph111_H1
zmp_ph111_B1a
zmp_ph111_B5
zmp_ph111_B8
zmp_ph111_B6
zmp_ph111_B7
zmp_ph111_B9
zmp_ph111_B10
zmp_ph114_G4
zmp_ph114_G11
zmp_ph114_H1
zmp_ph114_H2
zmp_ph114_A9
zmp_ph114_A11
zmp_ph114_B2
zmp_ph114_B4
zmp_ph114_D8
zmp_ph114_E2

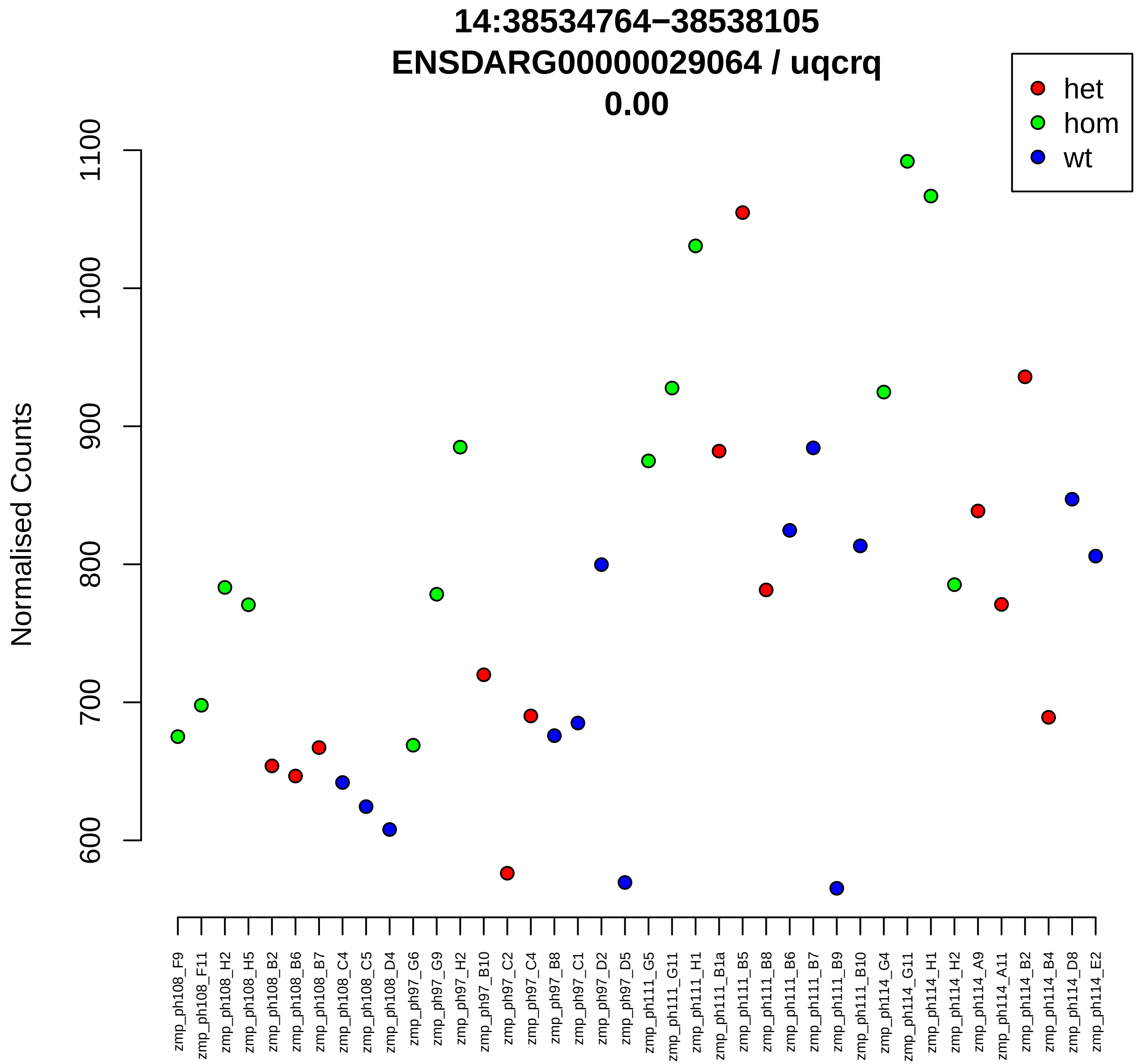
14:38534764−38538105
ENSDARG00000029064 / uqcrq
0.00
Normalised Counts
600
700
800
900
1000
1100
het
hom
wt
zmp_ph108_F9
zmp_ph108_F11
zmp_ph108_H2
zmp_ph108_H5
zmp_ph108_B2
zmp_ph108_B6
zmp_ph108_B7
zmp_ph108_C4
zmp_ph108_C5
zmp_ph108_D4
zmp_ph97_G6
zmp_ph97_G9
zmp_ph97_H2
zmp_ph97_B10
zmp_ph97_C2
zmp_ph97_C4
zmp_ph97_B8
zmp_ph97_C1
zmp_ph97_D2
zmp_ph97_D5
zmp_ph111_G5
zmp_ph111_G11
zmp_ph111_H1
zmp_ph111_B1a
zmp_ph111_B5
zmp_ph111_B8
zmp_ph111_B6
zmp_ph111_B7
zmp_ph111_B9
zmp_ph111_B10
zmp_ph114_G4
zmp_ph114_G11
zmp_ph114_H1
zmp_ph114_H2
zmp_ph114_A9
zmp_ph114_A11
zmp_ph114_B2
zmp_ph114_B4
zmp_ph114_D8
zmp_ph114_E2

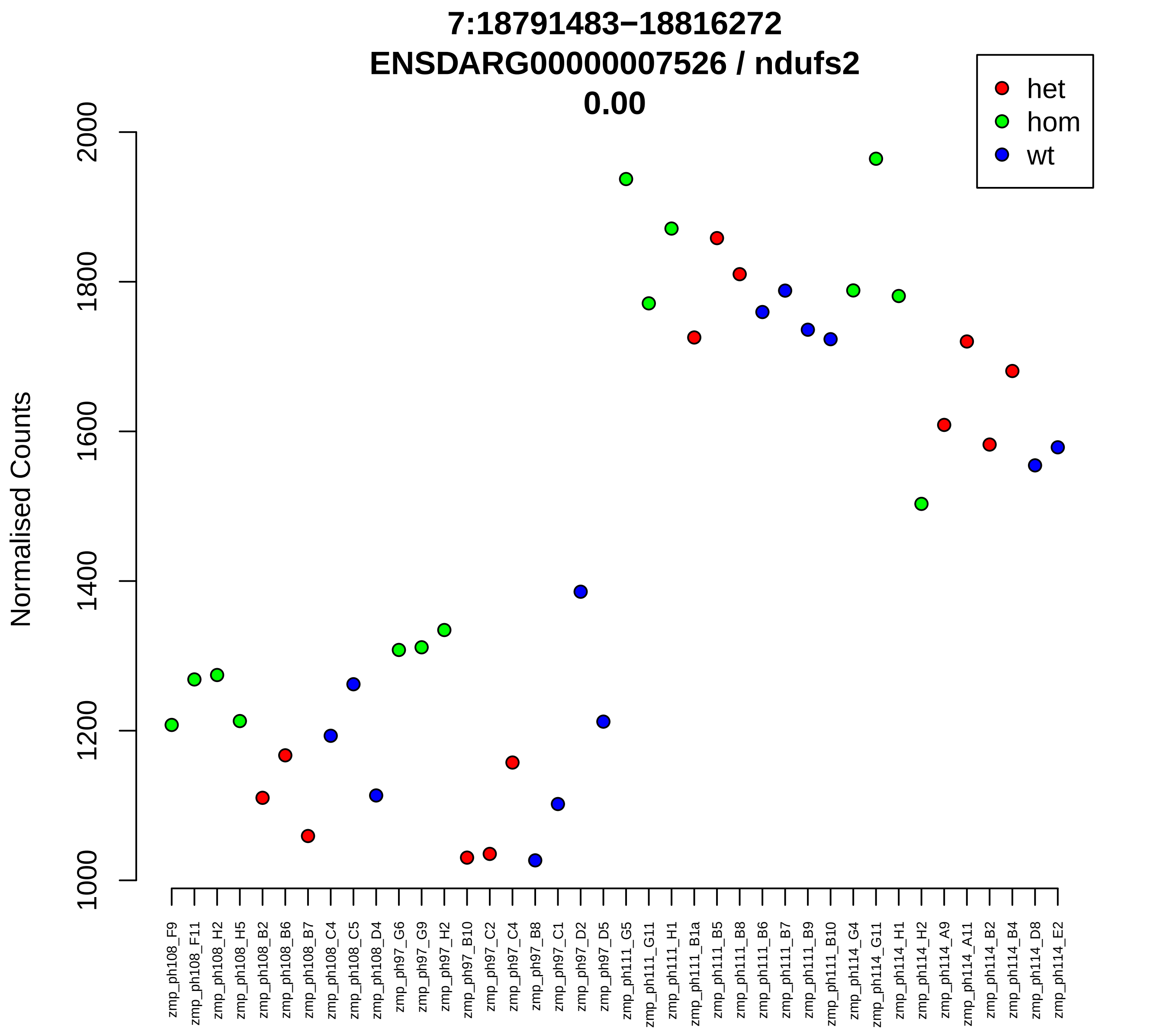

7:18791483−18816272
ENSDARG00000007526 / ndufs2
0.00
het
hom
wt
Normalised Counts
2000
1800
1600
1400
1200
1000
zmp_ph108_F9
zmp_ph108_F11
zmp_ph108_H2
zmp_ph108_H5
zmp_ph108_B2
zmp_ph108_B6
zmp_ph108_B7
zmp_ph108_C4
zmp_ph108_C5
zmp_ph108_D4
zmp_ph97_G6
zmp_ph97_G9
zmp_ph97_H2
zmp_ph97_B10
zmp_ph97_C2
zmp_ph97_C4
zmp_ph97_B8
zmp_ph97_C1
zmp_ph97_D2
zmp_ph97_D5
zmp_ph111_G5
zmp_ph111_G11
zmp_ph111_H1
zmp_ph111_B1a
zmp_ph111_B5
zmp_ph111_B8
zmp_ph111_B6
zmp_ph111_B7
zmp_ph111_B9
zmp_ph111_B10
zmp_ph114_G4
zmp_ph114_G11
zmp_ph114_H1
zmp_ph114_H2
zmp_ph114_A9
zmp_ph114_A11
zmp_ph114_B2
zmp_ph114_B4
zmp_ph114_D8
zmp_ph114_E2

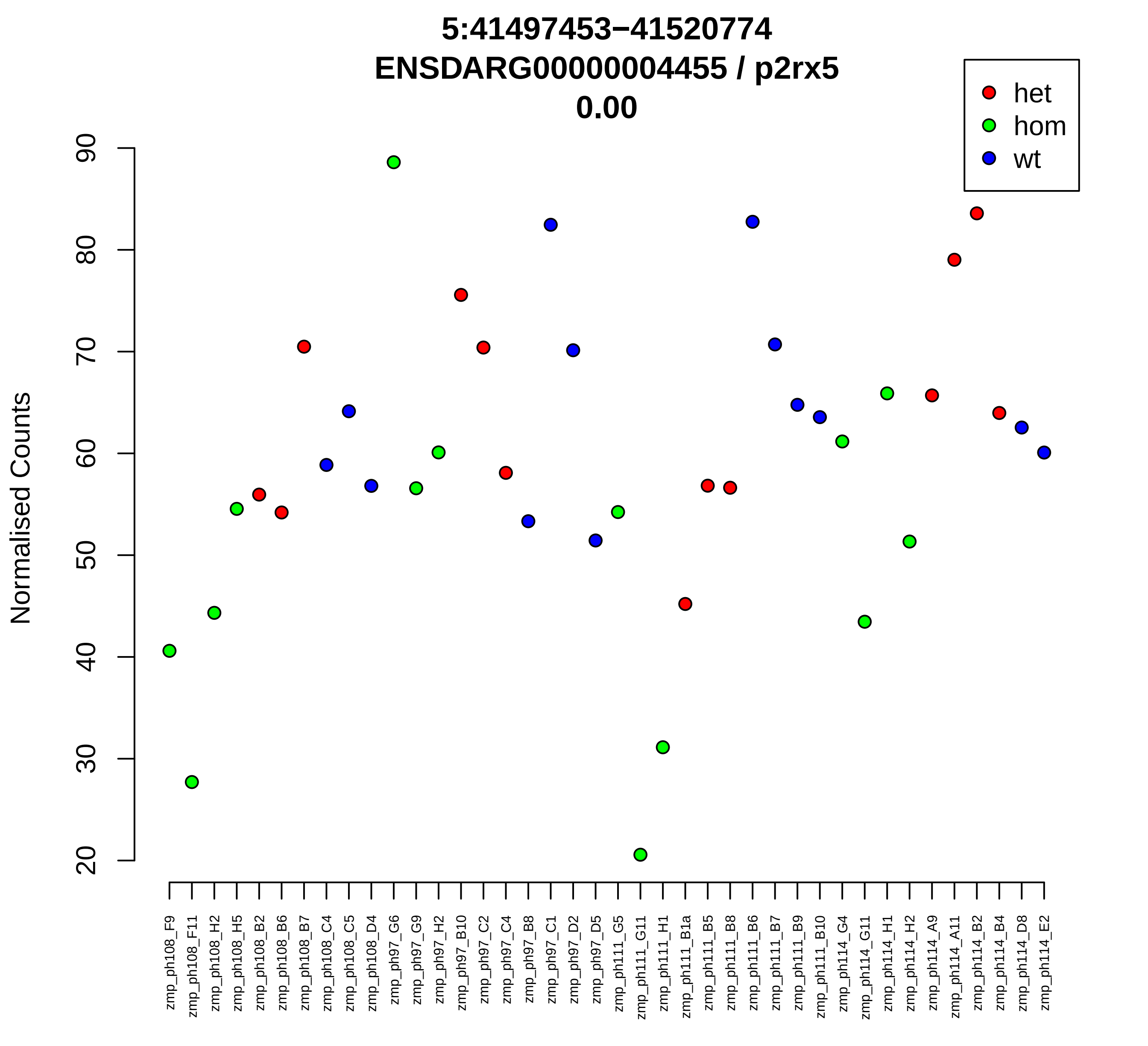

5:41497453−41520774
ENSDARG00000004455 / p2rx5
0.00
het
hom
wt
Normalised Counts
20
30
40
50
60
70
80
90
zmp_ph108_F9
zmp_ph108_F11
zmp_ph108_H2
zmp_ph108_H5
zmp_ph108_B2
zmp_ph108_B6
zmp_ph108_B7
zmp_ph108_C4
zmp_ph108_C5
zmp_ph108_D4
zmp_ph97_G6
zmp_ph97_G9
zmp_ph97_H2
zmp_ph97_B10
zmp_ph97_C2
zmp_ph97_C4
zmp_ph97_B8
zmp_ph97_C1
zmp_ph97_D2
zmp_ph97_D5
zmp_ph111_G5
zmp_ph111_G11
zmp_ph111_H1
zmp_ph111_B1a
zmp_ph111_B5
zmp_ph111_B8
zmp_ph111_B6
zmp_ph111_B7
zmp_ph111_B9
zmp_ph111_B10
zmp_ph114_G4
zmp_ph114_G11
zmp_ph114_H1
zmp_ph114_H2
zmp_ph114_A9
zmp_ph114_A11
zmp_ph114_B2
zmp_ph114_B4
zmp_ph114_D8
zmp_ph114_E2

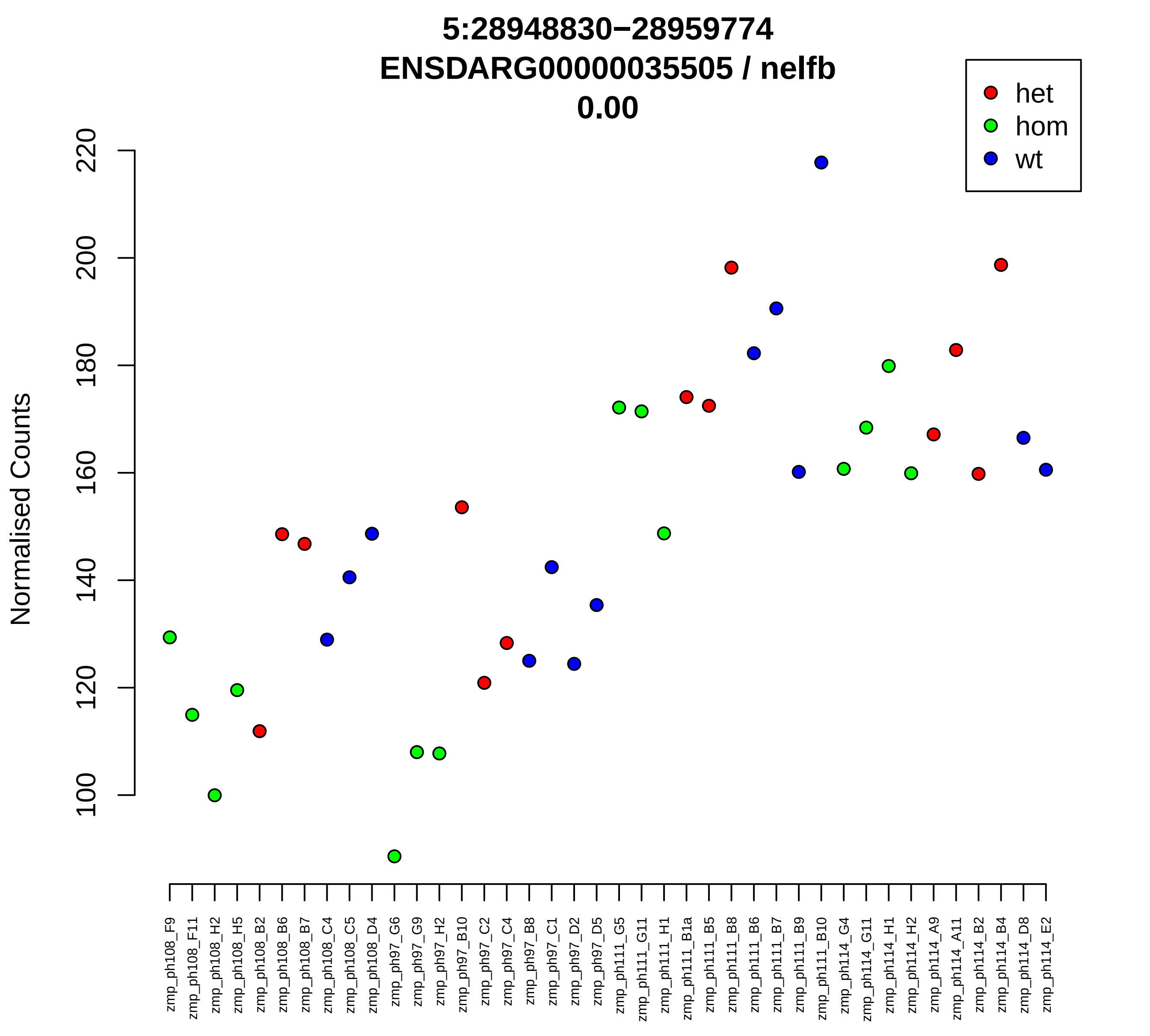

5:28948830−28959774
ENSDARG00000035505 / nelfb
0.00
het
hom
wt
Normalised Counts
100
120
140
160
180
200
220
zmp_ph108_F9
zmp_ph108_F11
zmp_ph108_H2
zmp_ph108_H5
zmp_ph108_B2
zmp_ph108_B6
zmp_ph108_B7
zmp_ph108_C4
zmp_ph108_C5
zmp_ph108_D4
zmp_ph97_G6
zmp_ph97_G9
zmp_ph97_H2
zmp_ph97_B10
zmp_ph97_C2
zmp_ph97_C4
zmp_ph97_B8
zmp_ph97_C1
zmp_ph97_D2
zmp_ph97_D5
zmp_ph111_G5
zmp_ph111_G11
zmp_ph111_H1
zmp_ph111_B1a
zmp_ph111_B5
zmp_ph111_B8
zmp_ph111_B6
zmp_ph111_B7
zmp_ph111_B9
zmp_ph111_B10
zmp_ph114_G4
zmp_ph114_G11
zmp_ph114_H1
zmp_ph114_H2
zmp_ph114_A9
zmp_ph114_A11
zmp_ph114_B2
zmp_ph114_B4
zmp_ph114_D8
zmp_ph114_E2

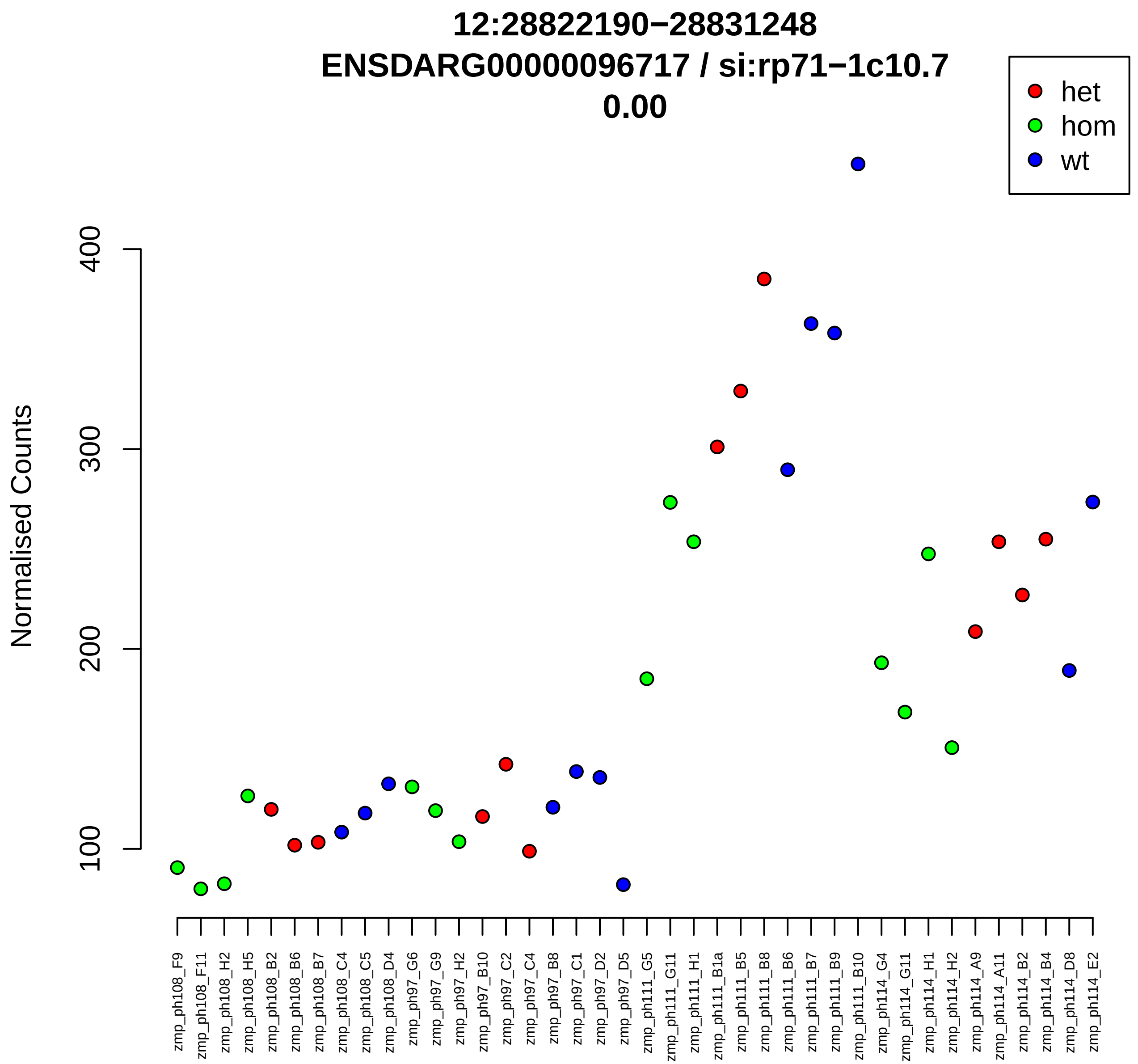

12:28822190−28831248
ENSDARG00000096717 / si:rp71−1c10.7
0.00
het
hom
wt
Normalised Counts
100
200
300
400
zmp_ph108_F9
zmp_ph108_F11
zmp_ph108_H2
zmp_ph108_H5
zmp_ph108_B2
zmp_ph108_B6
zmp_ph108_B7
zmp_ph108_C4
zmp_ph108_C5
zmp_ph108_D4
zmp_ph97_G6
zmp_ph97_G9
zmp_ph97_H2
zmp_ph97_B10
zmp_ph97_C2
zmp_ph97_C4
zmp_ph97_B8
zmp_ph97_C1
zmp_ph97_D2
zmp_ph97_D5
zmp_ph111_G5
zmp_ph111_G11
zmp_ph111_H1
zmp_ph111_B1a
zmp_ph111_B5
zmp_ph111_B8
zmp_ph111_B6
zmp_ph111_B7
zmp_ph111_B9
zmp_ph111_B10
zmp_ph114_G4
zmp_ph114_G11
zmp_ph114_H1
zmp_ph114_H2
zmp_ph114_A9
zmp_ph114_A11
zmp_ph114_B2
zmp_ph114_B4
zmp_ph114_D8
zmp_ph114_E2

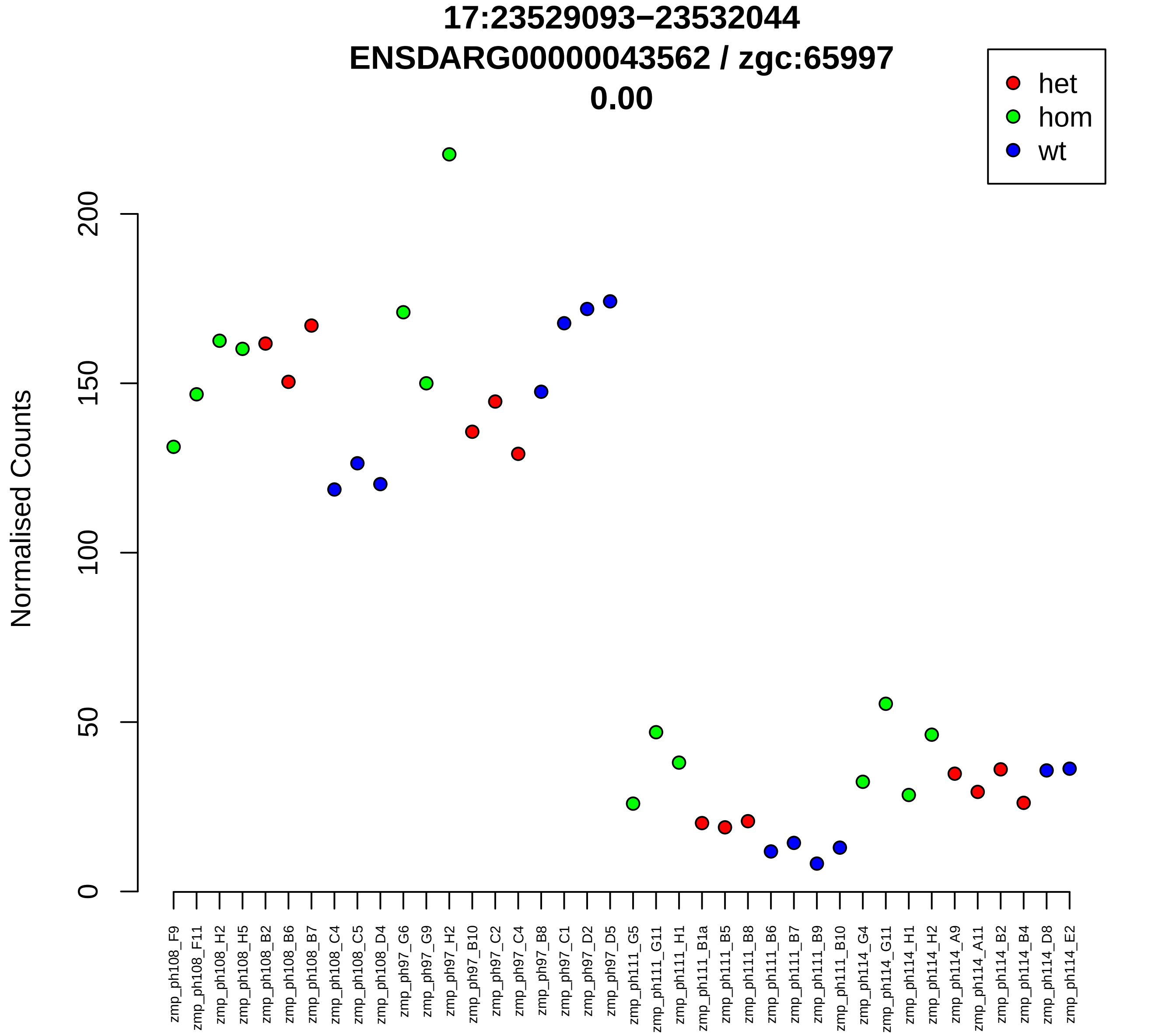
17:23529093−23532044
ENSDARG00000043562 / zgc:65997
0.00
het
hom
wt
Normalised Counts
0
50
100
150
200
zmp_ph108_F9
zmp_ph108_F11
zmp_ph108_H2
zmp_ph108_H5
zmp_ph108_B2
zmp_ph108_B6
zmp_ph108_B7
zmp_ph108_C4
zmp_ph108_C5
zmp_ph108_D4
zmp_ph97_G6
zmp_ph97_G9
zmp_ph97_H2
zmp_ph97_B10
zmp_ph97_C2
zmp_ph97_C4
zmp_ph97_B8
zmp_ph97_C1
zmp_ph97_D2
zmp_ph97_D5
zmp_ph111_G5
zmp_ph111_G11
zmp_ph111_H1
zmp_ph111_B1a
zmp_ph111_B5
zmp_ph111_B8
zmp_ph111_B6
zmp_ph111_B7
zmp_ph111_B9
zmp_ph111_B10
zmp_ph114_G4
zmp_ph114_G11
zmp_ph114_H1
zmp_ph114_H2
zmp_ph114_A9
zmp_ph114_A11
zmp_ph114_B2
zmp_ph114_B4
zmp_ph114_D8
zmp_ph114_E2

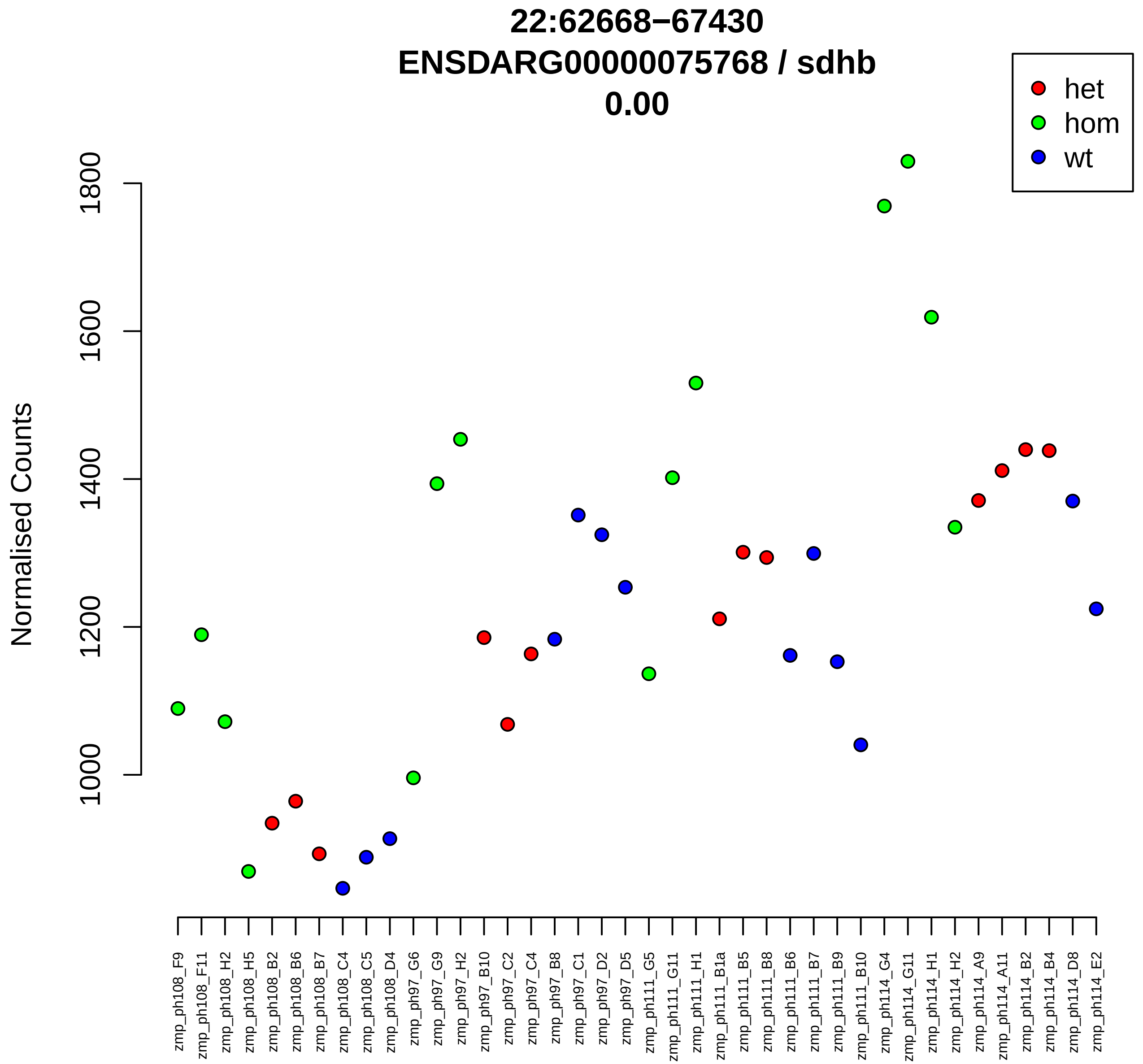

22:62668−67430
ENSDARG00000075768 / sdhb
0.00
het
hom
wt
Normalised Counts
1000
1200
1400
1600
1800
zmp_ph108_F9
zmp_ph108_F11
zmp_ph108_H2
zmp_ph108_H5
zmp_ph108_B2
zmp_ph108_B6
zmp_ph108_B7
zmp_ph108_C4
zmp_ph108_C5
zmp_ph108_D4
zmp_ph97_G6
zmp_ph97_G9
zmp_ph97_H2
zmp_ph97_B10
zmp_ph97_C2
zmp_ph97_C4
zmp_ph97_B8
zmp_ph97_C1
zmp_ph97_D2
zmp_ph97_D5
zmp_ph111_G5
zmp_ph111_G11
zmp_ph111_H1
zmp_ph111_B1a
zmp_ph111_B5
zmp_ph111_B8
zmp_ph111_B6
zmp_ph111_B7
zmp_ph111_B9
zmp_ph111_B10
zmp_ph114_G4
zmp_ph114_G11
zmp_ph114_H1
zmp_ph114_H2
zmp_ph114_A9
zmp_ph114_A11
zmp_ph114_B2
zmp_ph114_B4
zmp_ph114_D8
zmp_ph114_E2

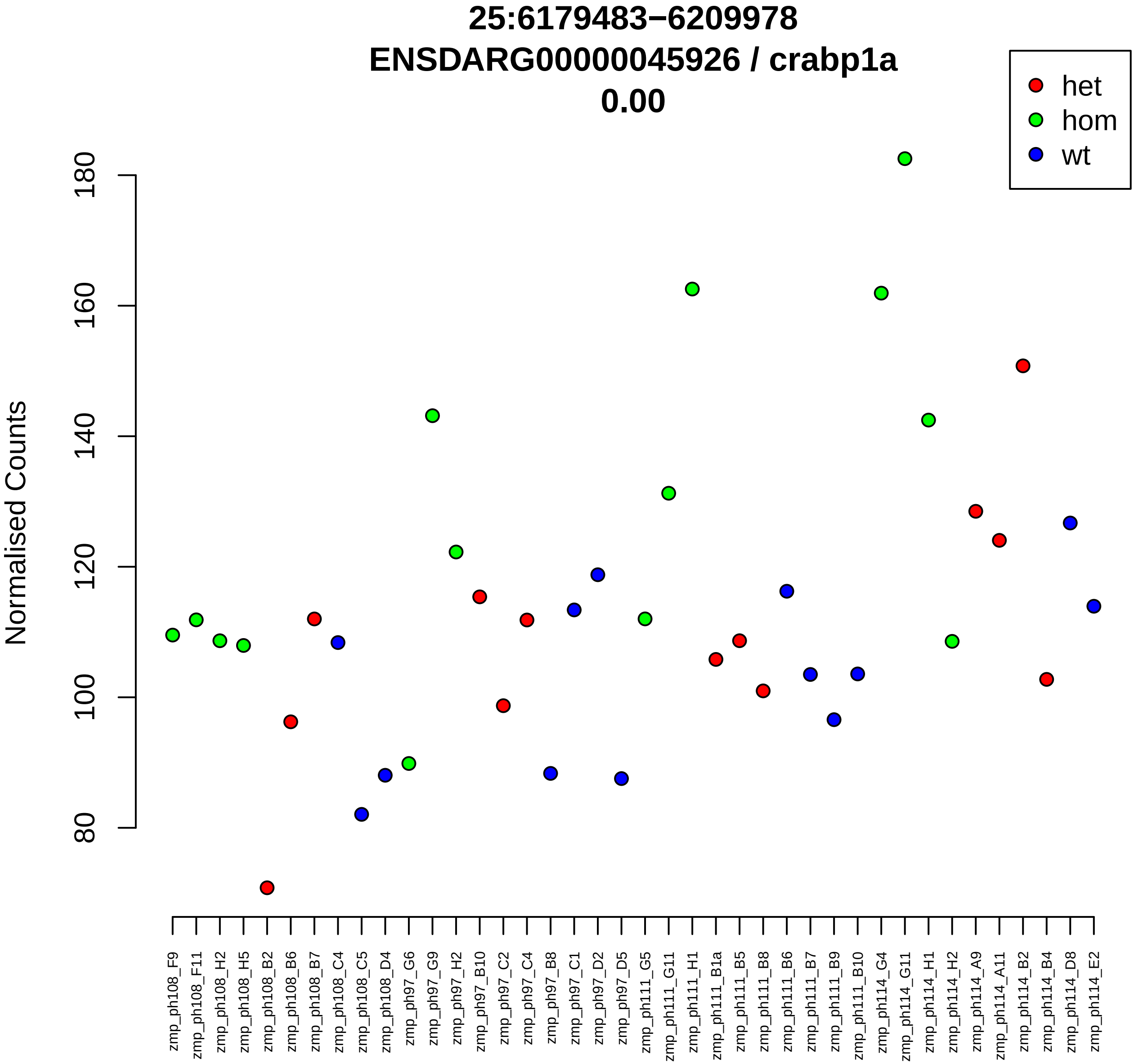
25:6179483−6209978
ENSDARG00000045926 / crabp1a
0.00
het
hom
wt
Normalised Counts
180
160
140
120
100
80
zmp_ph108_F9
zmp_ph108_F11
zmp_ph108_H2
zmp_ph108_H5
zmp_ph108_B2
zmp_ph108_B6
zmp_ph108_B7
zmp_ph108_C4
zmp_ph108_C5
zmp_ph108_D4
zmp_ph97_G6
zmp_ph97_G9
zmp_ph97_H2
zmp_ph97_B10
zmp_ph97_C2
zmp_ph97_C4
zmp_ph97_B8
zmp_ph97_C1
zmp_ph97_D2
zmp_ph97_D5
zmp_ph111_G5
zmp_ph111_G11
zmp_ph111_H1
zmp_ph111_B1a
zmp_ph111_B5
zmp_ph111_B8
zmp_ph111_B6
zmp_ph111_B7
zmp_ph111_B9
zmp_ph111_B10
zmp_ph114_G4
zmp_ph114_G11
zmp_ph114_H1
zmp_ph114_H2
zmp_ph114_A9
zmp_ph114_A11
zmp_ph114_B2
zmp_ph114_B4
zmp_ph114_D8
zmp_ph114_E2

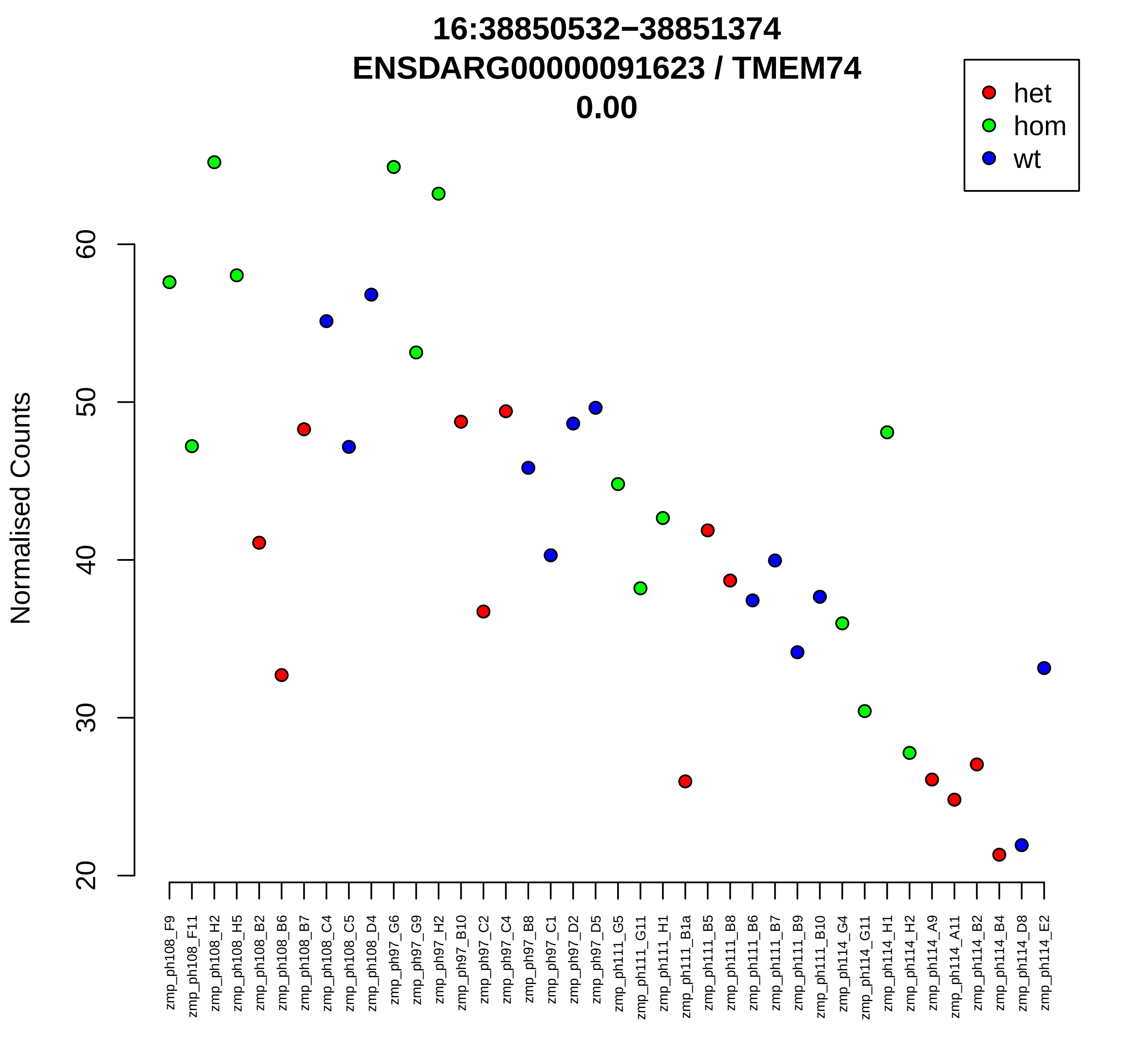

16:38850532−38851374
ENSDARG00000091623 / TMEM74
0.00
het
hom
wt
Normalised Counts
20
30
40
50
60
zmp_ph108_F9
zmp_ph108_F11
zmp_ph108_H2
zmp_ph108_H5
zmp_ph108_B2
zmp_ph108_B6
zmp_ph108_B7
zmp_ph108_C4
zmp_ph108_C5
zmp_ph108_D4
zmp_ph97_G6
zmp_ph97_G9
zmp_ph97_H2
zmp_ph97_B10
zmp_ph97_C2
zmp_ph97_C4
zmp_ph97_B8
zmp_ph97_C1
zmp_ph97_D2
zmp_ph97_D5
zmp_ph111_G5
zmp_ph111_G11
zmp_ph111_H1
zmp_ph111_B1a
zmp_ph111_B5
zmp_ph111_B8
zmp_ph111_B6
zmp_ph111_B7
zmp_ph111_B9
zmp_ph111_B10
zmp_ph114_G4
zmp_ph114_G11
zmp_ph114_H1
zmp_ph114_H2
zmp_ph114_A9
zmp_ph114_A11
zmp_ph114_B2
zmp_ph114_B4
zmp_ph114_D8
zmp_ph114_E2

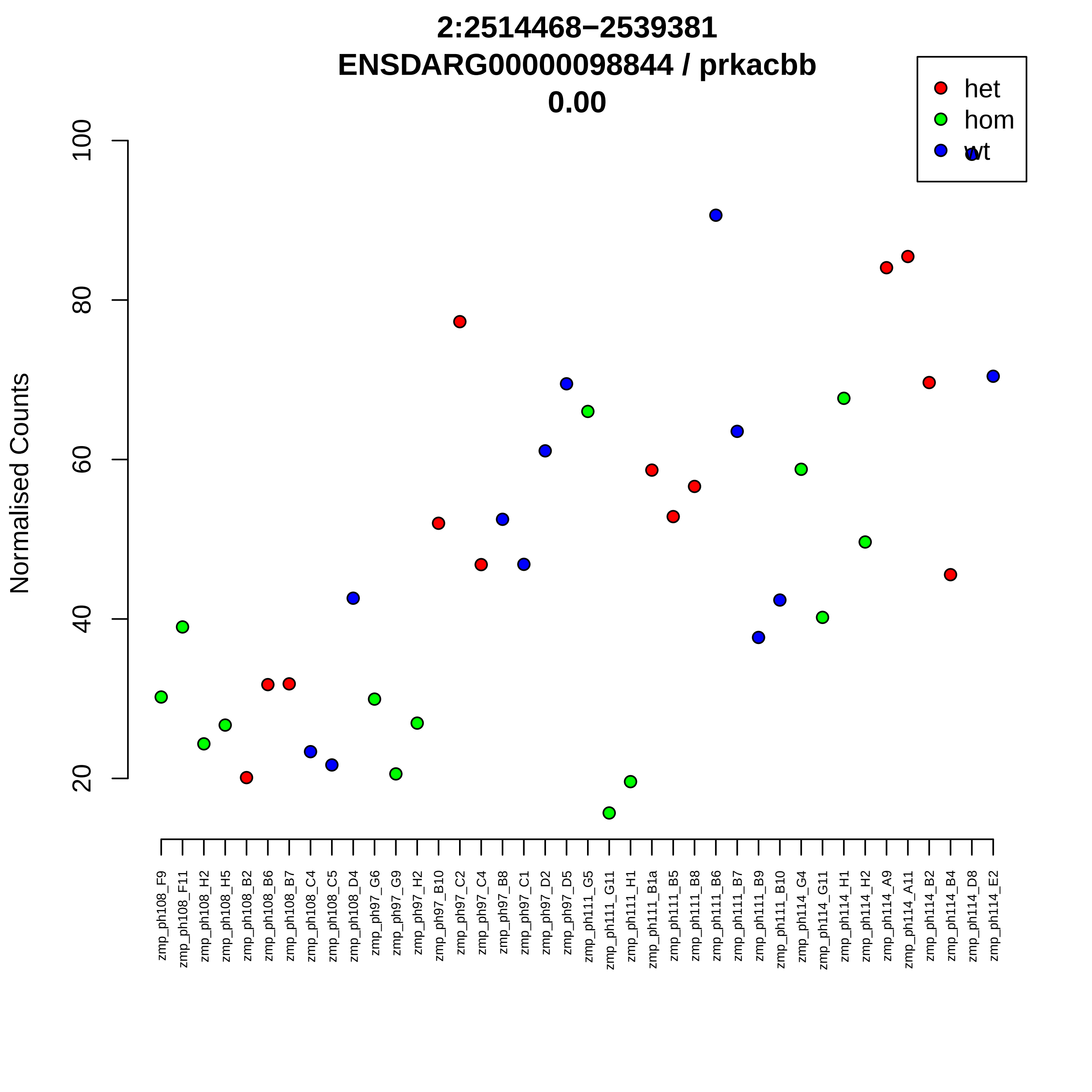

2:2514468−2539381
ENSDARG00000098844 / prkacbb
0.00
het
hom
wt
Normalised Counts
20
40
60
80
100
zmp_ph108_F9
zmp_ph108_F11
zmp_ph108_H2
zmp_ph108_H5
zmp_ph108_B2
zmp_ph108_B6
zmp_ph108_B7
zmp_ph108_C4
zmp_ph108_C5
zmp_ph108_D4
zmp_ph97_G6
zmp_ph97_G9
zmp_ph97_H2
zmp_ph97_B10
zmp_ph97_C2
zmp_ph97_C4
zmp_ph97_B8
zmp_ph97_C1
zmp_ph97_D2
zmp_ph97_D5
zmp_ph111_G5
zmp_ph111_G11
zmp_ph111_H1
zmp_ph111_B1a
zmp_ph111_B5
zmp_ph111_B8
zmp_ph111_B6
zmp_ph111_B7
zmp_ph111_B9
zmp_ph111_B10
zmp_ph114_G4
zmp_ph114_G11
zmp_ph114_H1
zmp_ph114_H2
zmp_ph114_A9
zmp_ph114_A11
zmp_ph114_B2
zmp_ph114_B4
zmp_ph114_D8
zmp_ph114_E2

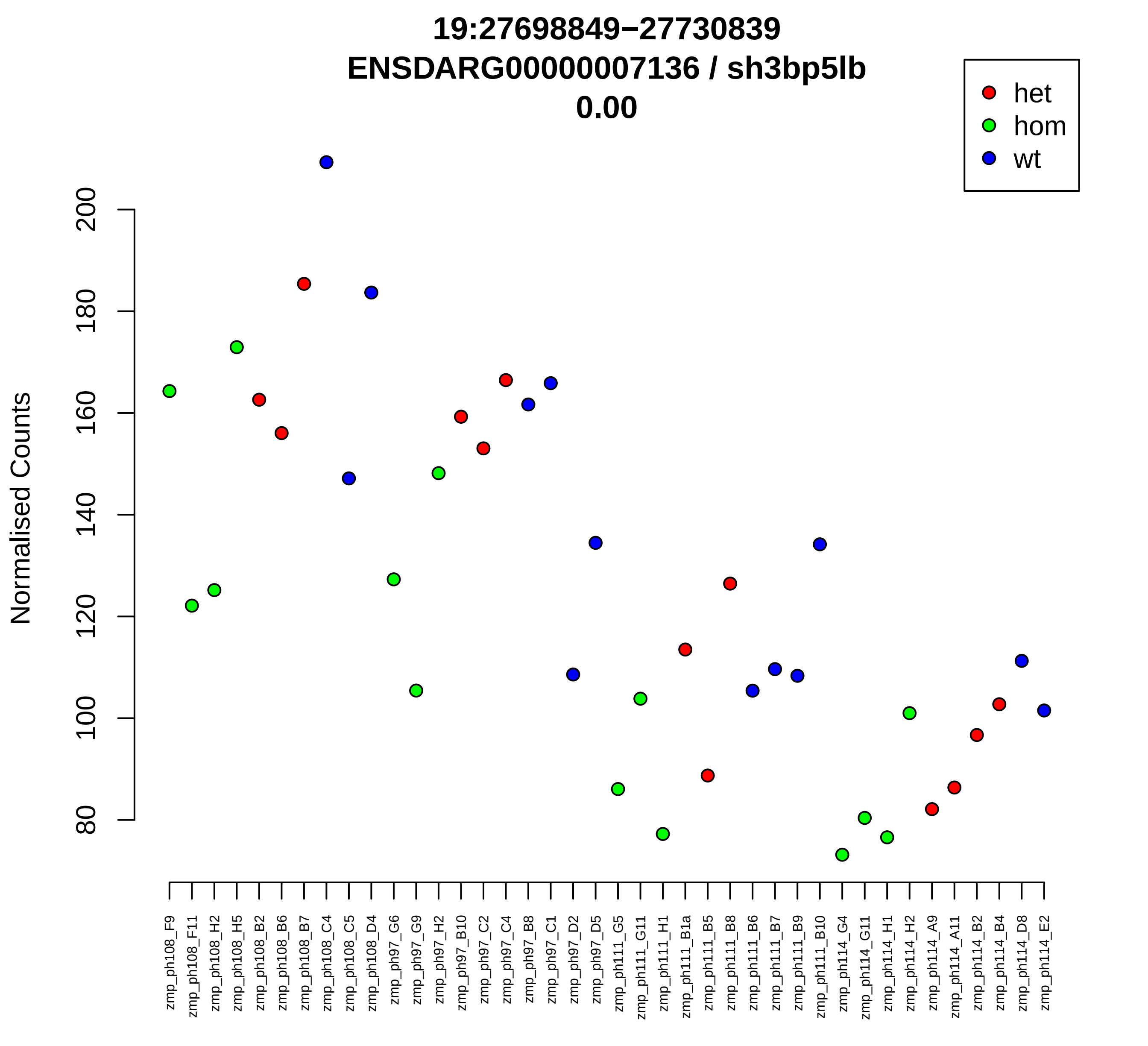
19:27698849−27730839
ENSDARG00000007136 / sh3bp5lb
0.00
het
hom
wt
Normalised Counts
80
100
120
140
160
180
200
zmp_ph108_F9
zmp_ph108_F11
zmp_ph108_H2
zmp_ph108_H5
zmp_ph108_B2
zmp_ph108_B6
zmp_ph108_B7
zmp_ph108_C4
zmp_ph108_C5
zmp_ph108_D4
zmp_ph97_G6
zmp_ph97_G9
zmp_ph97_H2
zmp_ph97_B10
zmp_ph97_C2
zmp_ph97_C4
zmp_ph97_B8
zmp_ph97_C1
zmp_ph97_D2
zmp_ph97_D5
zmp_ph111_G5
zmp_ph111_G11
zmp_ph111_H1
zmp_ph111_B1a
zmp_ph111_B5
zmp_ph111_B8
zmp_ph111_B6
zmp_ph111_B7
zmp_ph111_B9
zmp_ph111_B10
zmp_ph114_G4
zmp_ph114_G11
zmp_ph114_H1
zmp_ph114_H2
zmp_ph114_A9
zmp_ph114_A11
zmp_ph114_B2
zmp_ph114_B4
zmp_ph114_D8
zmp_ph114_E2

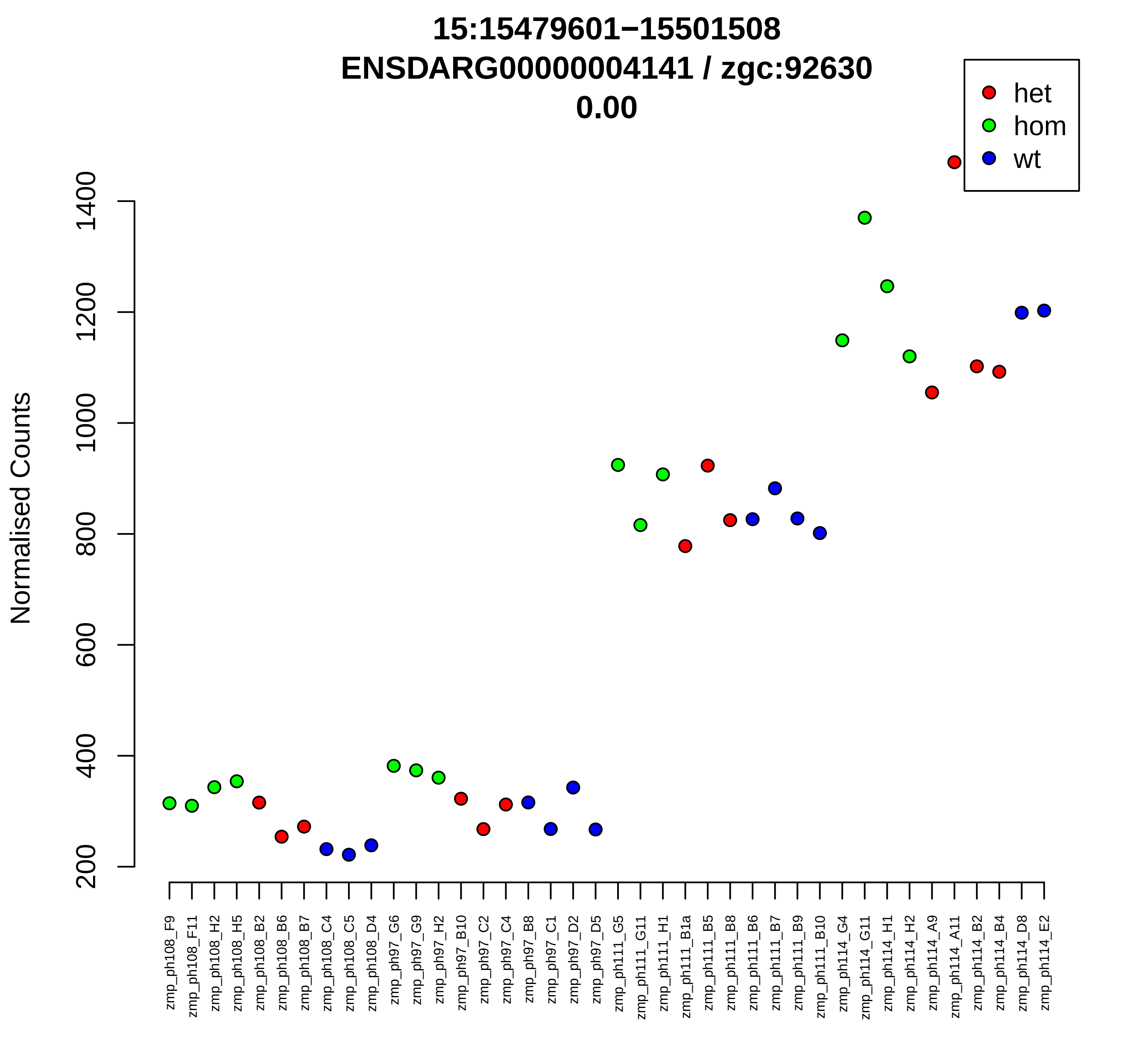
15:15479601−15501508
ENSDARG00000004141 / zgc:92630
0.00
het
hom
wt
Normalised Counts
200
400
600
800
1000
1200
1400
zmp_ph108_F9
zmp_ph108_F11
zmp_ph108_H2
zmp_ph108_H5
zmp_ph108_B2
zmp_ph108_B6
zmp_ph108_B7
zmp_ph108_C4
zmp_ph108_C5
zmp_ph108_D4
zmp_ph97_G6
zmp_ph97_G9
zmp_ph97_H2
zmp_ph97_B10
zmp_ph97_C2
zmp_ph97_C4
zmp_ph97_B8
zmp_ph97_C1
zmp_ph97_D2
zmp_ph97_D5
zmp_ph111_G5
zmp_ph111_G11
zmp_ph111_H1
zmp_ph111_B1a
zmp_ph111_B5
zmp_ph111_B8
zmp_ph111_B6
zmp_ph111_B7
zmp_ph111_B9
zmp_ph111_B10
zmp_ph114_G4
zmp_ph114_G11
zmp_ph114_H1
zmp_ph114_H2
zmp_ph114_A9
zmp_ph114_A11
zmp_ph114_B2
zmp_ph114_B4
zmp_ph114_D8
zmp_ph114_E2

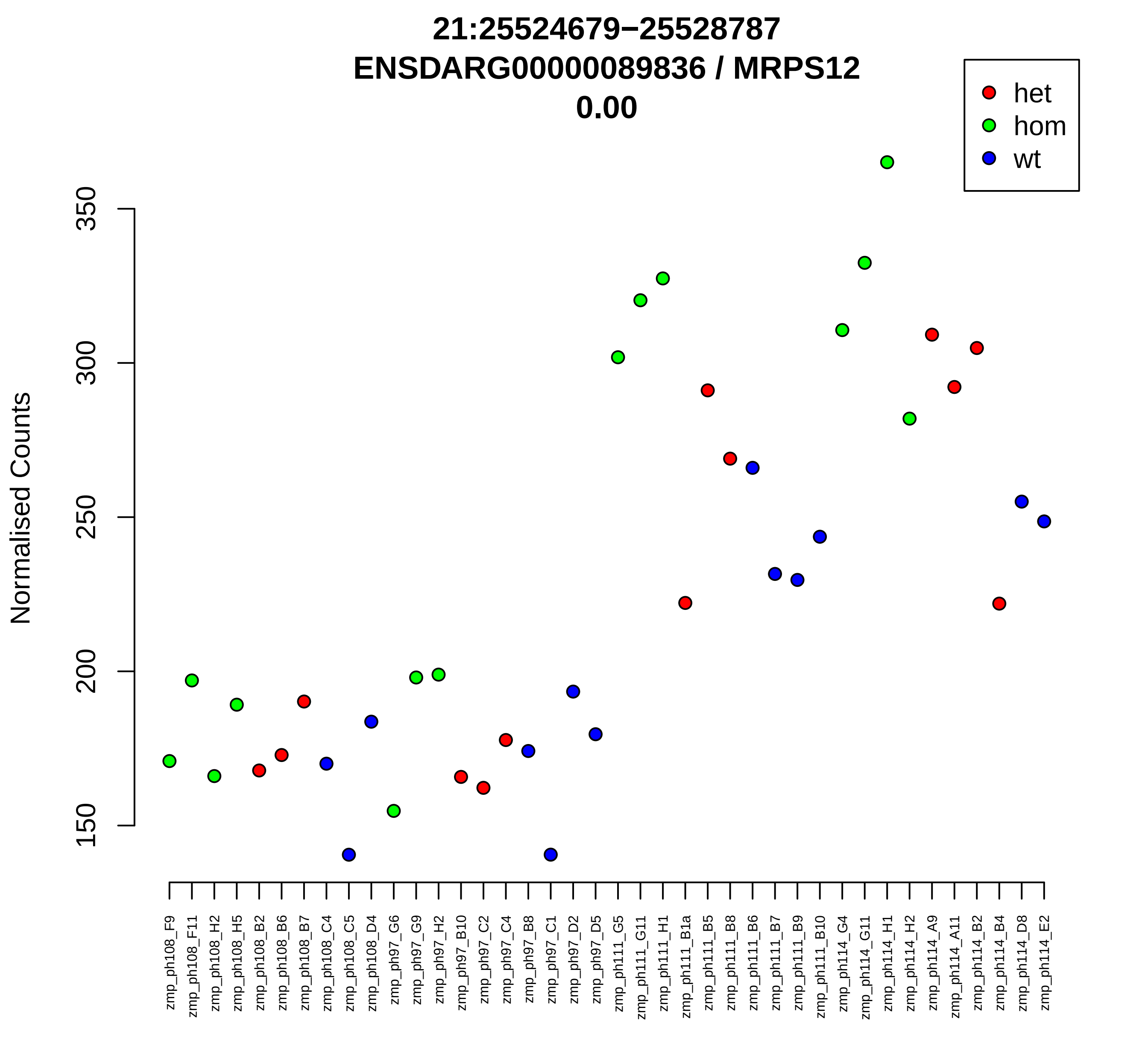

21:25524679−25528787
ENSDARG00000089836 / MRPS12
0.00
het
hom
wt
Normalised Counts
150
200
250
300
350
zmp_ph108_F9
zmp_ph108_F11
zmp_ph108_H2
zmp_ph108_H5
zmp_ph108_B2
zmp_ph108_B6
zmp_ph108_B7
zmp_ph108_C4
zmp_ph108_C5
zmp_ph108_D4
zmp_ph97_G6
zmp_ph97_G9
zmp_ph97_H2
zmp_ph97_B10
zmp_ph97_C2
zmp_ph97_C4
zmp_ph97_B8
zmp_ph97_C1
zmp_ph97_D2
zmp_ph97_D5
zmp_ph111_G5
zmp_ph111_G11
zmp_ph111_H1
zmp_ph111_B1a
zmp_ph111_B5
zmp_ph111_B8
zmp_ph111_B6
zmp_ph111_B7
zmp_ph111_B9
zmp_ph111_B10
zmp_ph114_G4
zmp_ph114_G11
zmp_ph114_H1
zmp_ph114_H2
zmp_ph114_A9
zmp_ph114_A11
zmp_ph114_B2
zmp_ph114_B4
zmp_ph114_D8
zmp_ph114_E2

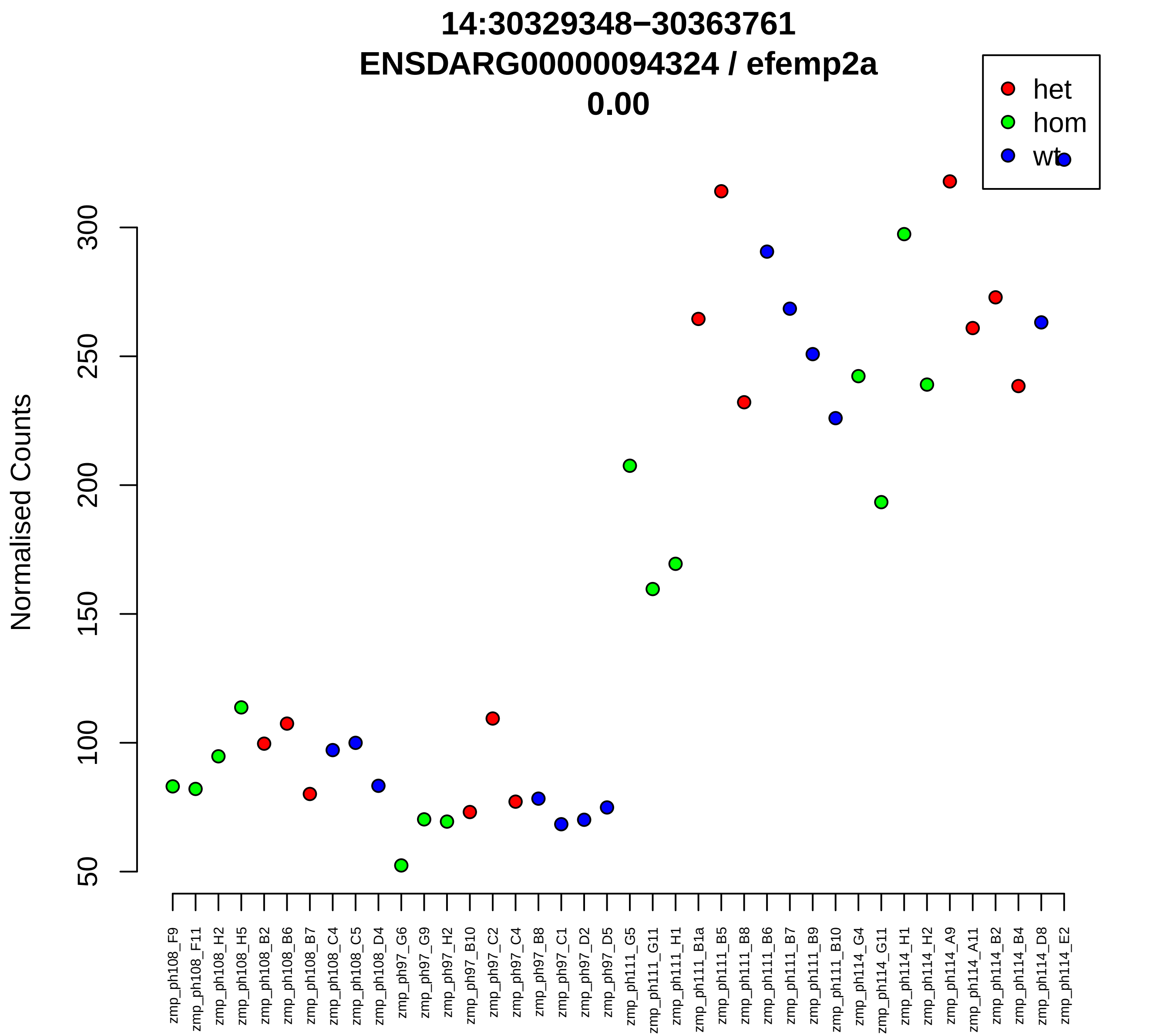
14:30329348−30363761
ENSDARG00000094324 / efemp2a
0.00
het
hom
wt
Normalised Counts
50
100
150
200
250
300
zmp_ph108_F9
zmp_ph108_F11
zmp_ph108_H2
zmp_ph108_H5
zmp_ph108_B2
zmp_ph108_B6
zmp_ph108_B7
zmp_ph108_C4
zmp_ph108_C5
zmp_ph108_D4
zmp_ph97_G6
zmp_ph97_G9
zmp_ph97_H2
zmp_ph97_B10
zmp_ph97_C2
zmp_ph97_C4
zmp_ph97_B8
zmp_ph97_C1
zmp_ph97_D2
zmp_ph97_D5
zmp_ph111_G5
zmp_ph111_G11
zmp_ph111_H1
zmp_ph111_B1a
zmp_ph111_B5
zmp_ph111_B8
zmp_ph111_B6
zmp_ph111_B7
zmp_ph111_B9
zmp_ph111_B10
zmp_ph114_G4
zmp_ph114_G11
zmp_ph114_H1
zmp_ph114_H2
zmp_ph114_A9
zmp_ph114_A11
zmp_ph114_B2
zmp_ph114_B4
zmp_ph114_D8
zmp_ph114_E2

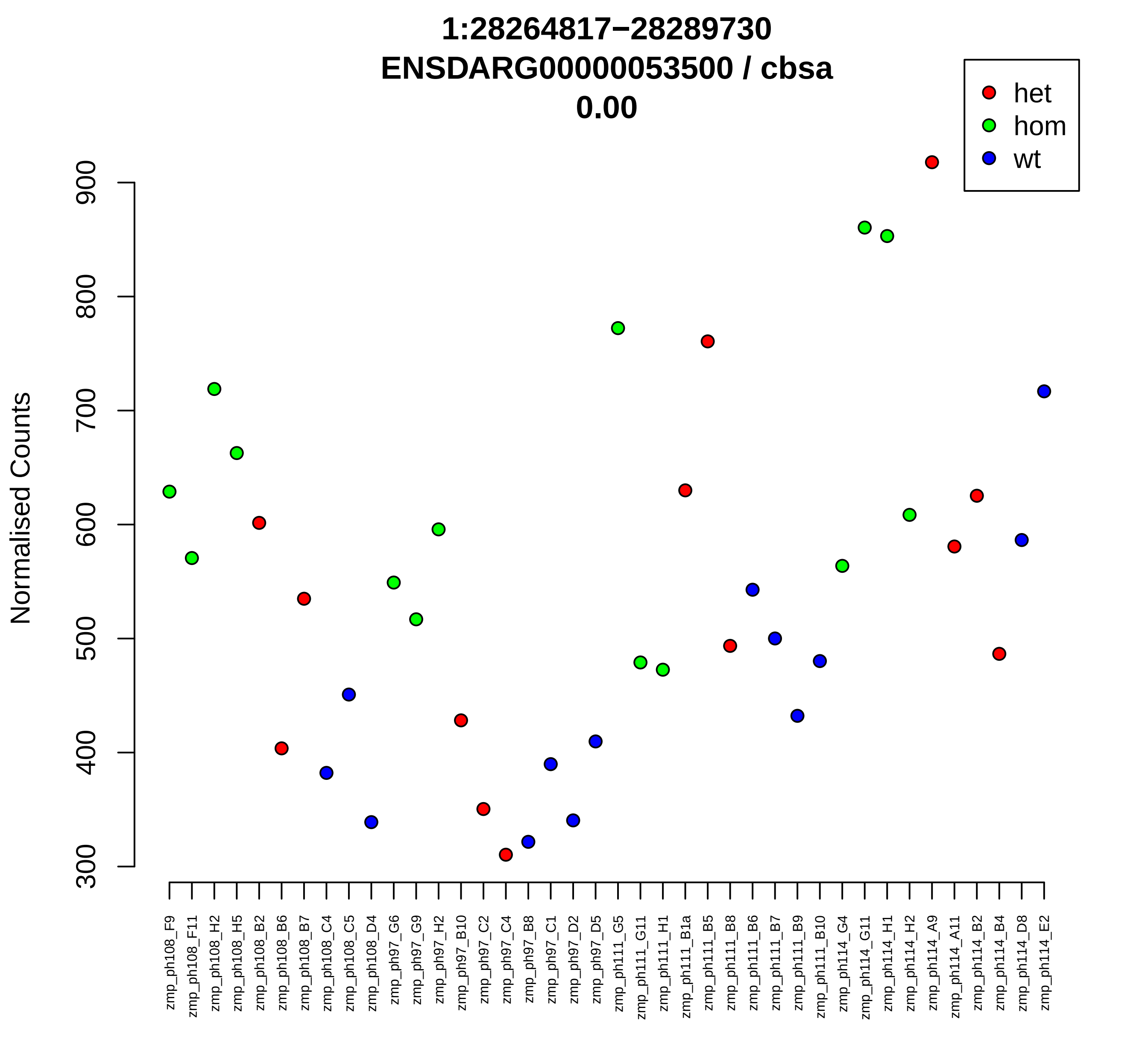
1:28264817−28289730
ENSDARG00000053500 / cbsa
0.00
het
hom
wt
Normalised Counts
300
400
500
600
700
800
900
zmp_ph108_F9
zmp_ph108_F11
zmp_ph108_H2
zmp_ph108_H5
zmp_ph108_B2
zmp_ph108_B6
zmp_ph108_B7
zmp_ph108_C4
zmp_ph108_C5
zmp_ph108_D4
zmp_ph97_G6
zmp_ph97_G9
zmp_ph97_H2
zmp_ph97_B10
zmp_ph97_C2
zmp_ph97_C4
zmp_ph97_B8
zmp_ph97_C1
zmp_ph97_D2
zmp_ph97_D5
zmp_ph111_G5
zmp_ph111_G11
zmp_ph111_H1
zmp_ph111_B1a
zmp_ph111_B5
zmp_ph111_B8
zmp_ph111_B6
zmp_ph111_B7
zmp_ph111_B9
zmp_ph111_B10
zmp_ph114_G4
zmp_ph114_G11
zmp_ph114_H1
zmp_ph114_H2
zmp_ph114_A9
zmp_ph114_A11
zmp_ph114_B2
zmp_ph114_B4
zmp_ph114_D8
zmp_ph114_E2

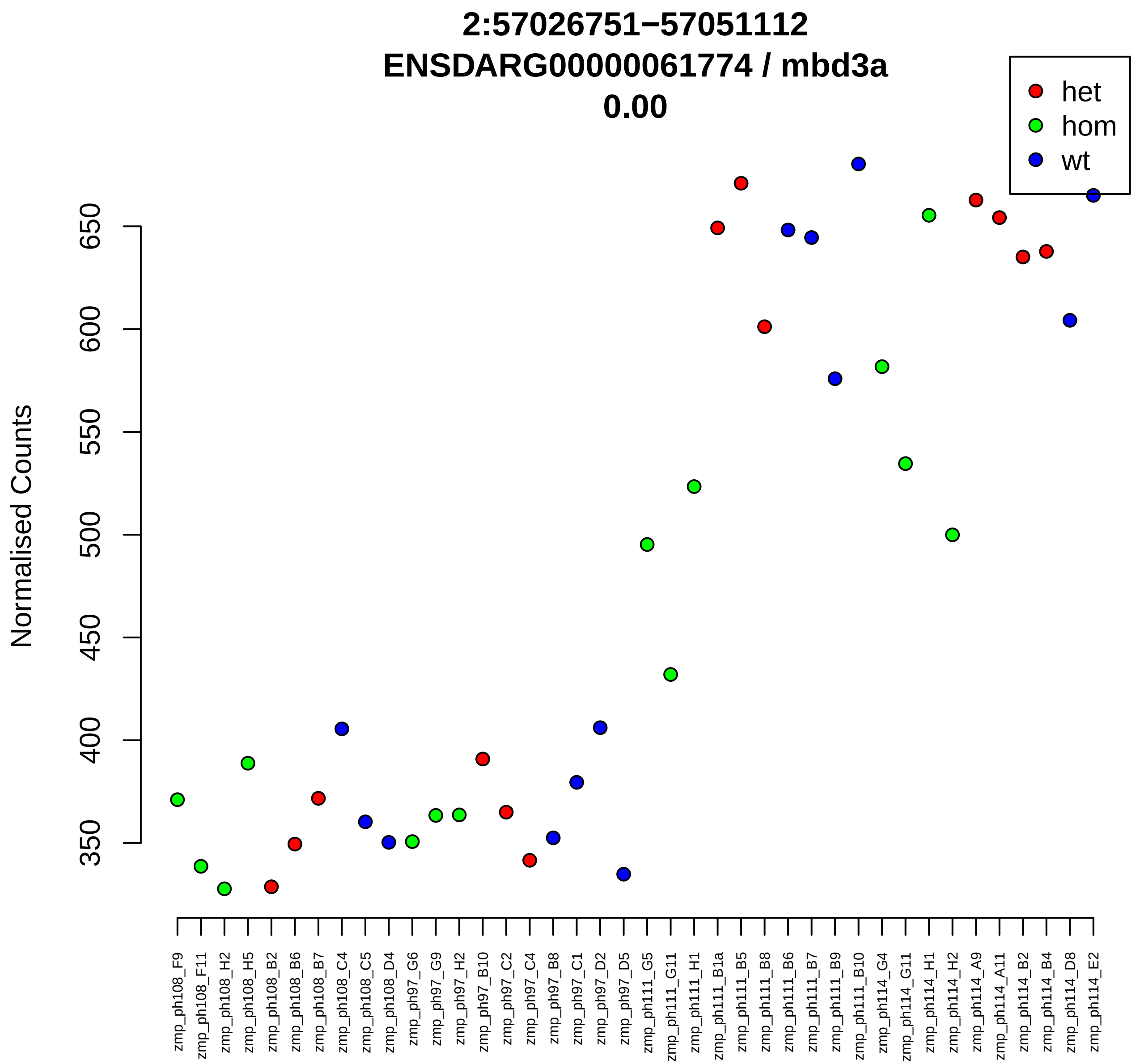
2:57026751−57051112
ENSDARG00000061774 / mbd3a
0.00
het
hom
wt
Normalised Counts
350
400
450
500
550
600
650
zmp_ph108_F9
zmp_ph108_F11
zmp_ph108_H2
zmp_ph108_H5
zmp_ph108_B2
zmp_ph108_B6
zmp_ph108_B7
zmp_ph108_C4
zmp_ph108_C5
zmp_ph108_D4
zmp_ph97_G6
zmp_ph97_G9
zmp_ph97_H2
zmp_ph97_B10
zmp_ph97_C2
zmp_ph97_C4
zmp_ph97_B8
zmp_ph97_C1
zmp_ph97_D2
zmp_ph97_D5
zmp_ph111_G5
zmp_ph111_G11
zmp_ph111_H1
zmp_ph111_B1a
zmp_ph111_B5
zmp_ph111_B8
zmp_ph111_B6
zmp_ph111_B7
zmp_ph111_B9
zmp_ph111_B10
zmp_ph114_G4
zmp_ph114_G11
zmp_ph114_H1
zmp_ph114_H2
zmp_ph114_A9
zmp_ph114_A11
zmp_ph114_B2
zmp_ph114_B4
zmp_ph114_D8
zmp_ph114_E2

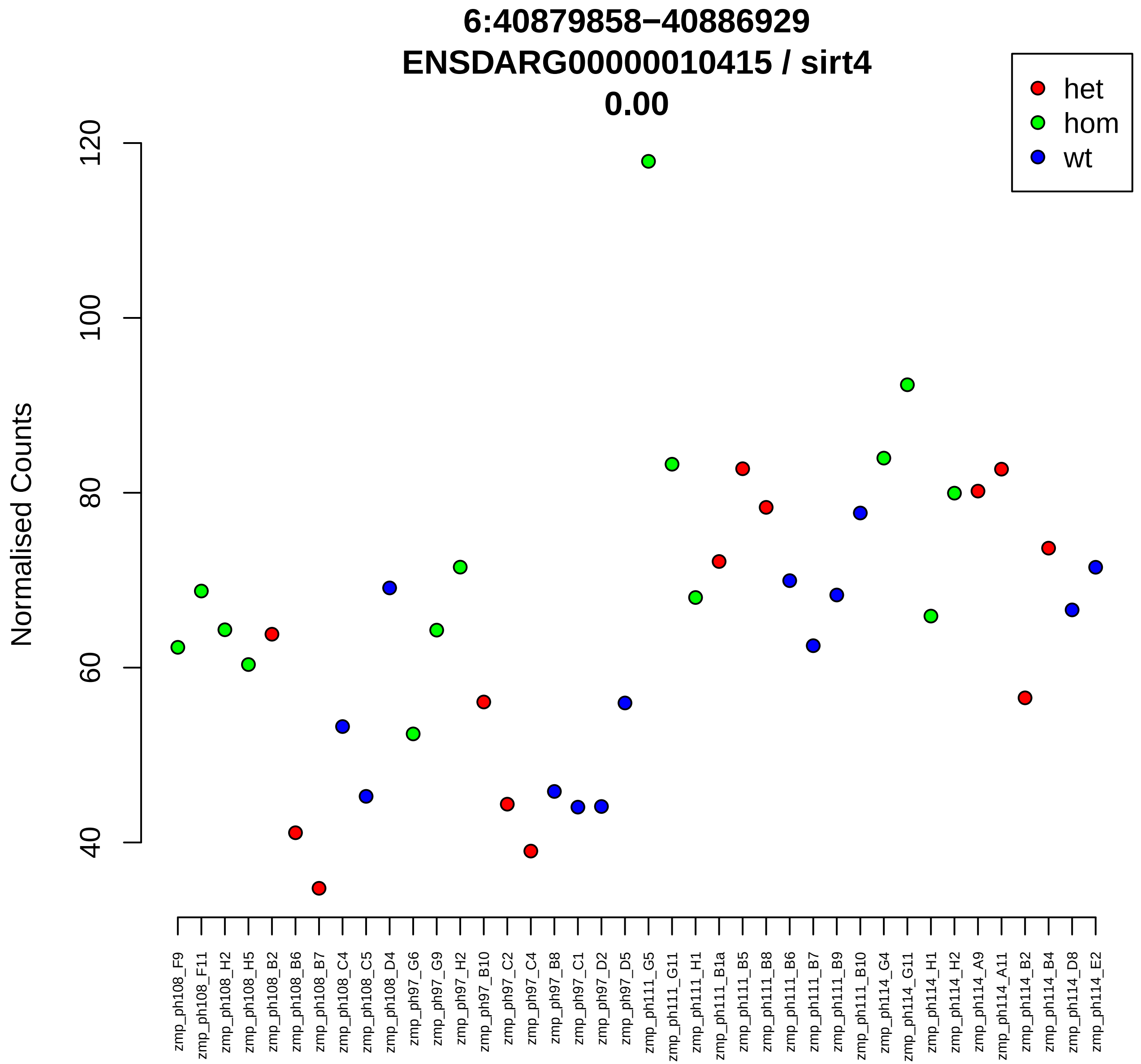
6:40879858−40886929
ENSDARG00000010415 / sirt4
0.00
het
hom
wt
Normalised Counts
40
60
80
100
120
zmp_ph108_F9
zmp_ph108_F11
zmp_ph108_H2
zmp_ph108_H5
zmp_ph108_B2
zmp_ph108_B6
zmp_ph108_B7
zmp_ph108_C4
zmp_ph108_C5
zmp_ph108_D4
zmp_ph97_G6
zmp_ph97_G9
zmp_ph97_H2
zmp_ph97_B10
zmp_ph97_C2
zmp_ph97_C4
zmp_ph97_B8
zmp_ph97_C1
zmp_ph97_D2
zmp_ph97_D5
zmp_ph111_G5
zmp_ph111_G11
zmp_ph111_H1
zmp_ph111_B1a
zmp_ph111_B5
zmp_ph111_B8
zmp_ph111_B6
zmp_ph111_B7
zmp_ph111_B9
zmp_ph111_B10
zmp_ph114_G4
zmp_ph114_G11
zmp_ph114_H1
zmp_ph114_H2
zmp_ph114_A9
zmp_ph114_A11
zmp_ph114_B2
zmp_ph114_B4
zmp_ph114_D8
zmp_ph114_E2

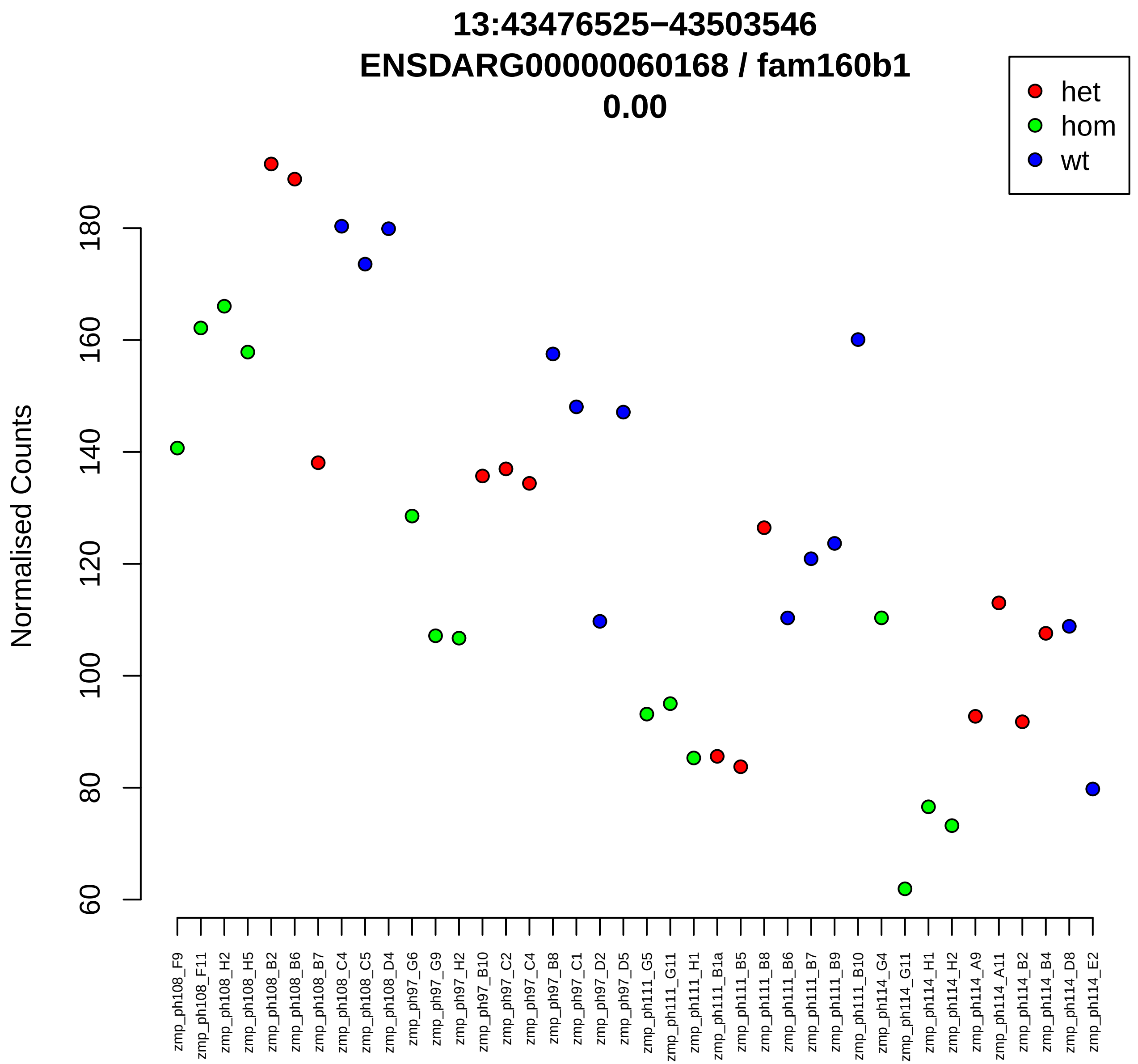

13:43476525−43503546
ENSDARG00000060168 / fam160b1
0.00
het
hom
wt
Normalised Counts
60
80
100
120
140
160
180
zmp_ph108_F9
zmp_ph108_F11
zmp_ph108_H2
zmp_ph108_H5
zmp_ph108_B2
zmp_ph108_B6
zmp_ph108_B7
zmp_ph108_C4
zmp_ph108_C5
zmp_ph108_D4
zmp_ph97_G6
zmp_ph97_G9
zmp_ph97_H2
zmp_ph97_B10
zmp_ph97_C2
zmp_ph97_C4
zmp_ph97_B8
zmp_ph97_C1
zmp_ph97_D2
zmp_ph97_D5
zmp_ph111_G5
zmp_ph111_G11
zmp_ph111_H1
zmp_ph111_B1a
zmp_ph111_B5
zmp_ph111_B8
zmp_ph111_B6
zmp_ph111_B7
zmp_ph111_B9
zmp_ph111_B10
zmp_ph114_G4
zmp_ph114_G11
zmp_ph114_H1
zmp_ph114_H2
zmp_ph114_A9
zmp_ph114_A11
zmp_ph114_B2
zmp_ph114_B4
zmp_ph114_D8
zmp_ph114_E2

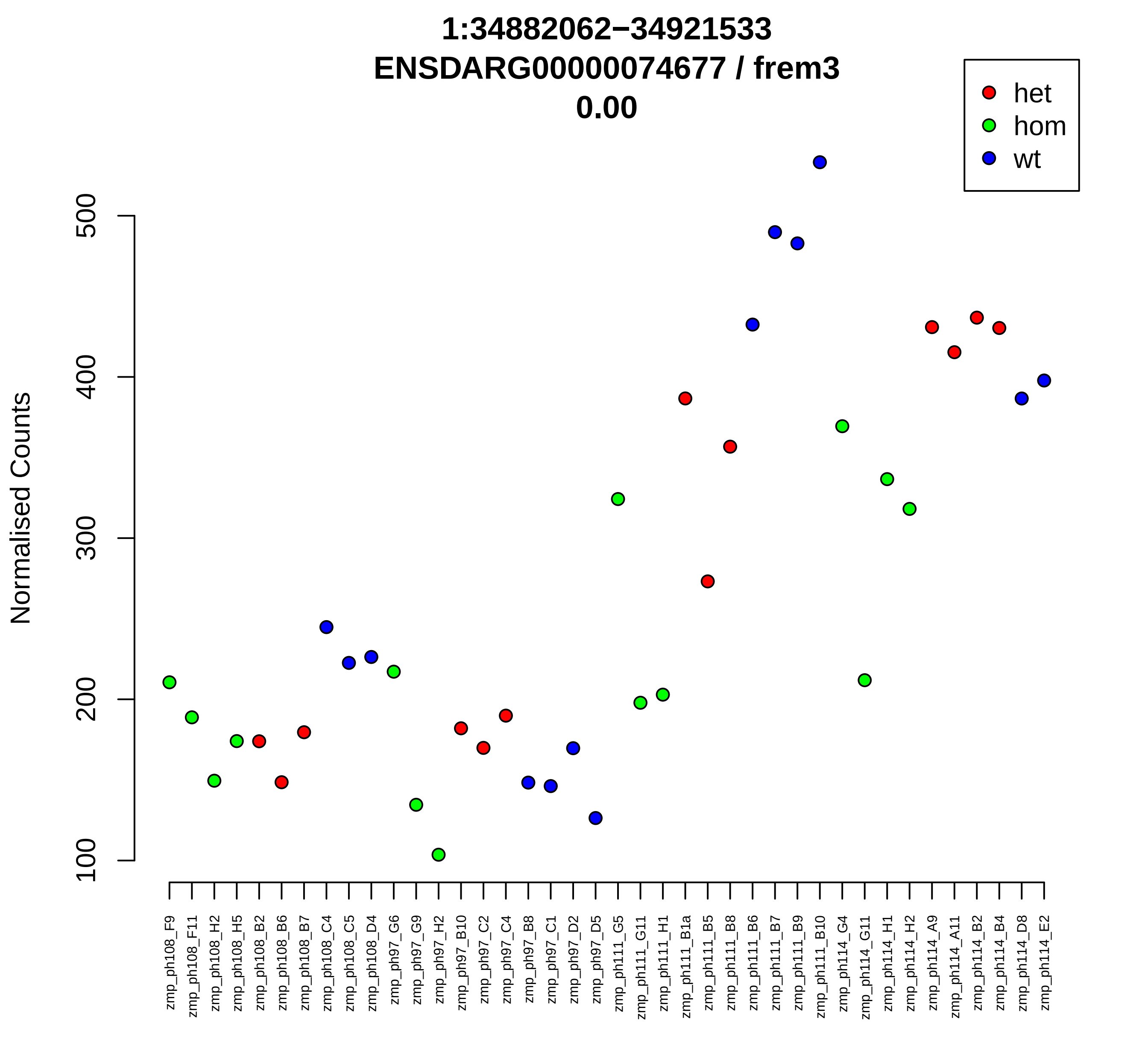
1:34882062−34921533
ENSDARG00000074677 / frem3
0.00
het
hom
wt
Normalised Counts
100
200
300
400
500
zmp_ph108_F9
zmp_ph108_F11
zmp_ph108_H2
zmp_ph108_H5
zmp_ph108_B2
zmp_ph108_B6
zmp_ph108_B7
zmp_ph108_C4
zmp_ph108_C5
zmp_ph108_D4
zmp_ph97_G6
zmp_ph97_G9
zmp_ph97_H2
zmp_ph97_B10
zmp_ph97_C2
zmp_ph97_C4
zmp_ph97_B8
zmp_ph97_C1
zmp_ph97_D2
zmp_ph97_D5
zmp_ph111_G5
zmp_ph111_G11
zmp_ph111_H1
zmp_ph111_B1a
zmp_ph111_B5
zmp_ph111_B8
zmp_ph111_B6
zmp_ph111_B7
zmp_ph111_B9
zmp_ph111_B10
zmp_ph114_G4
zmp_ph114_G11
zmp_ph114_H1
zmp_ph114_H2
zmp_ph114_A9
zmp_ph114_A11
zmp_ph114_B2
zmp_ph114_B4
zmp_ph114_D8
zmp_ph114_E2

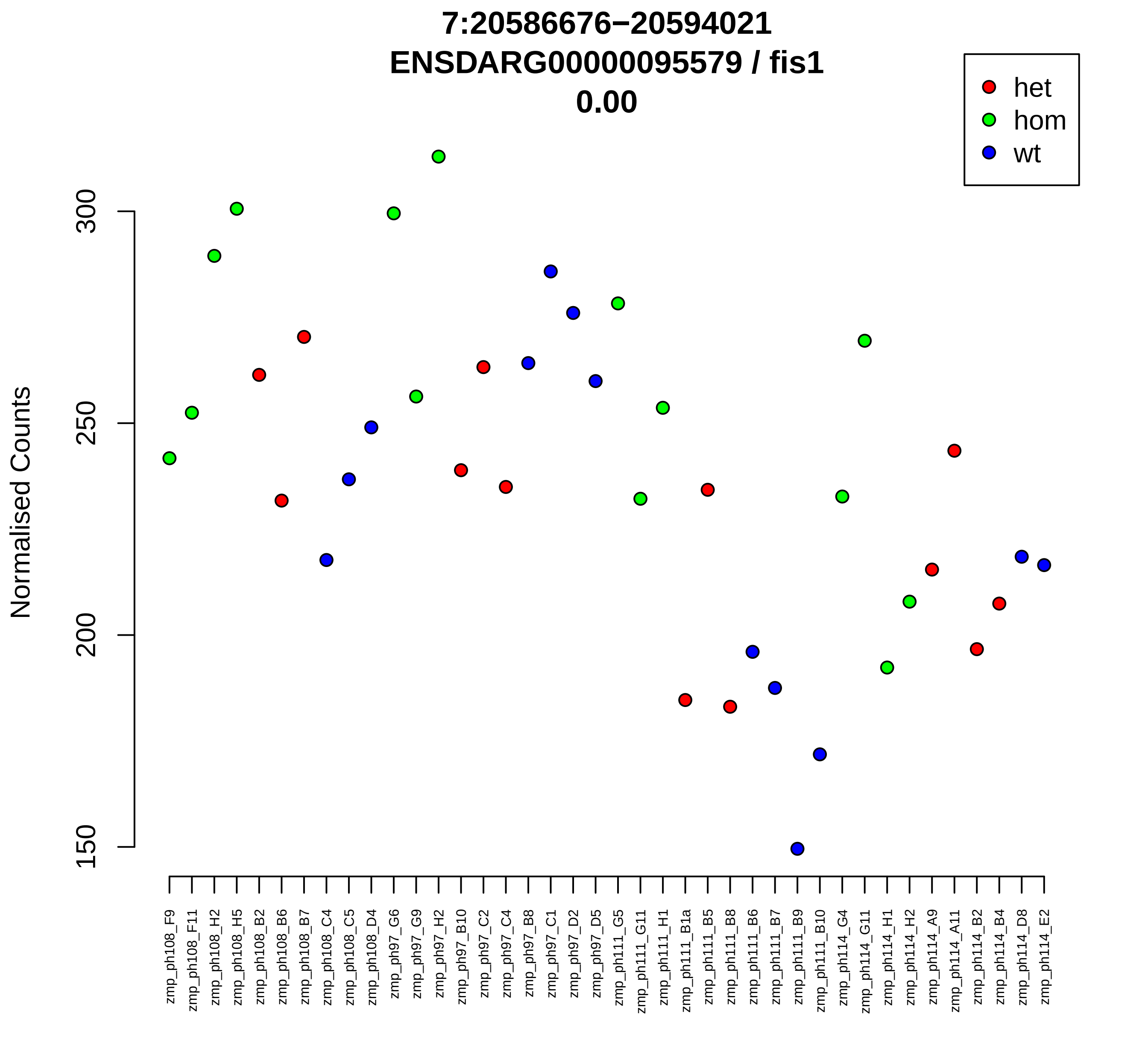
7:20586676−20594021
ENSDARG00000095579 / fis1
0.00
het
hom
wt
Normalised Counts
150
200
250
300
zmp_ph108_F9
zmp_ph108_F11
zmp_ph108_H2
zmp_ph108_H5
zmp_ph108_B2
zmp_ph108_B6
zmp_ph108_B7
zmp_ph108_C4
zmp_ph108_C5
zmp_ph108_D4
zmp_ph97_G6
zmp_ph97_G9
zmp_ph97_H2
zmp_ph97_B10
zmp_ph97_C2
zmp_ph97_C4
zmp_ph97_B8
zmp_ph97_C1
zmp_ph97_D2
zmp_ph97_D5
zmp_ph111_G5
zmp_ph111_G11
zmp_ph111_H1
zmp_ph111_B1a
zmp_ph111_B5
zmp_ph111_B8
zmp_ph111_B6
zmp_ph111_B7
zmp_ph111_B9
zmp_ph111_B10
zmp_ph114_G4
zmp_ph114_G11
zmp_ph114_H1
zmp_ph114_H2
zmp_ph114_A9
zmp_ph114_A11
zmp_ph114_B2
zmp_ph114_B4
zmp_ph114_D8
zmp_ph114_E2

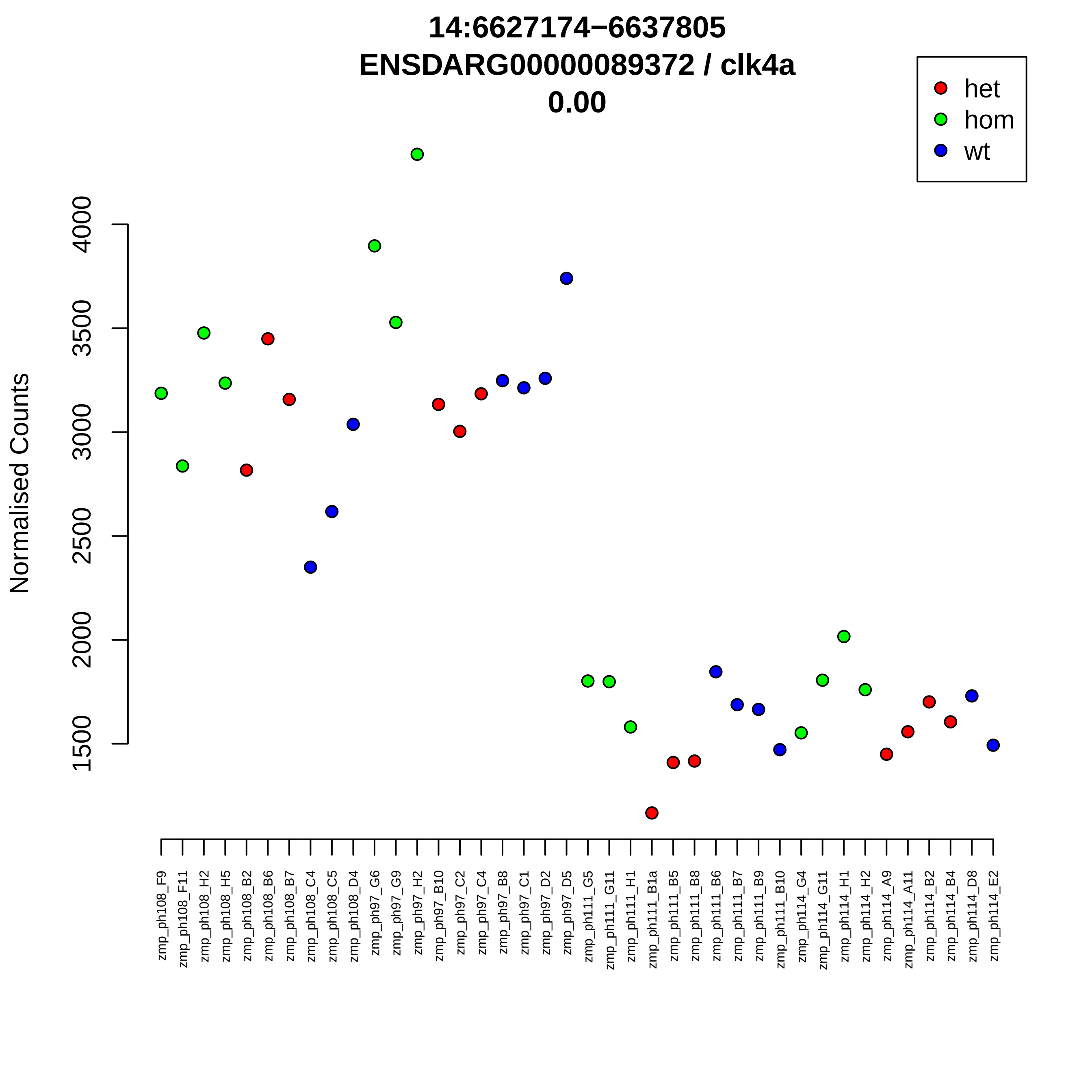

14:6627174−6637805
ENSDARG00000089372 / clk4a
0.00
het
hom
wt
Normalised Counts
1500
2000
2500
3000
3500
4000
zmp_ph108_F9
zmp_ph108_F11
zmp_ph108_H2
zmp_ph108_H5
zmp_ph108_B2
zmp_ph108_B6
zmp_ph108_B7
zmp_ph108_C4
zmp_ph108_C5
zmp_ph108_D4
zmp_ph97_G6
zmp_ph97_G9
zmp_ph97_H2
zmp_ph97_B10
zmp_ph97_C2
zmp_ph97_C4
zmp_ph97_B8
zmp_ph97_C1
zmp_ph97_D2
zmp_ph97_D5
zmp_ph111_G5
zmp_ph111_G11
zmp_ph111_H1
zmp_ph111_B1a
zmp_ph111_B5
zmp_ph111_B8
zmp_ph111_B6
zmp_ph111_B7
zmp_ph111_B9
zmp_ph111_B10
zmp_ph114_G4
zmp_ph114_G11
zmp_ph114_H1
zmp_ph114_H2
zmp_ph114_A9
zmp_ph114_A11
zmp_ph114_B2
zmp_ph114_B4
zmp_ph114_D8
zmp_ph114_E2

8:30777879−30782034
ENSDARG00000042428 / gstt1a
0.00

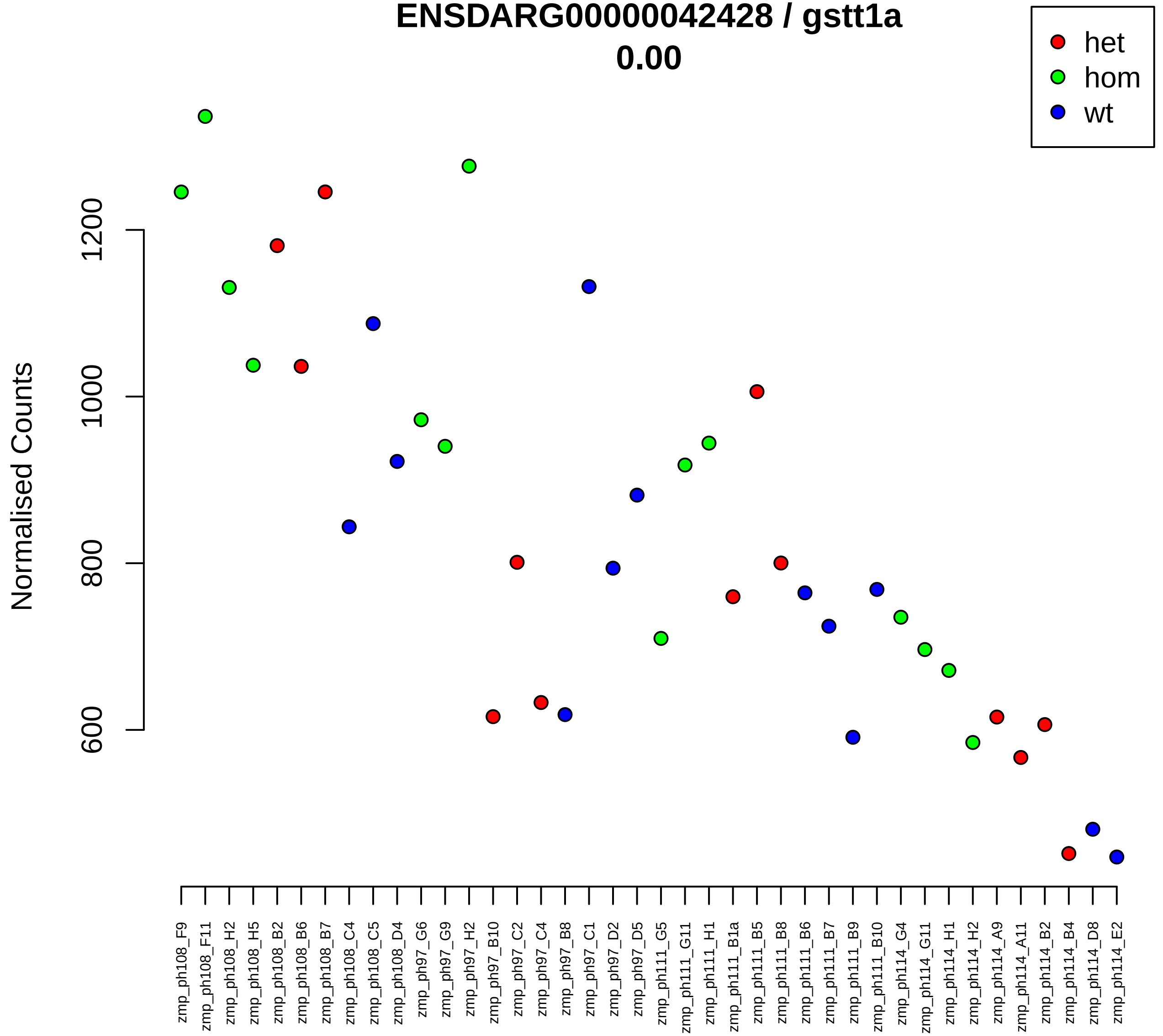

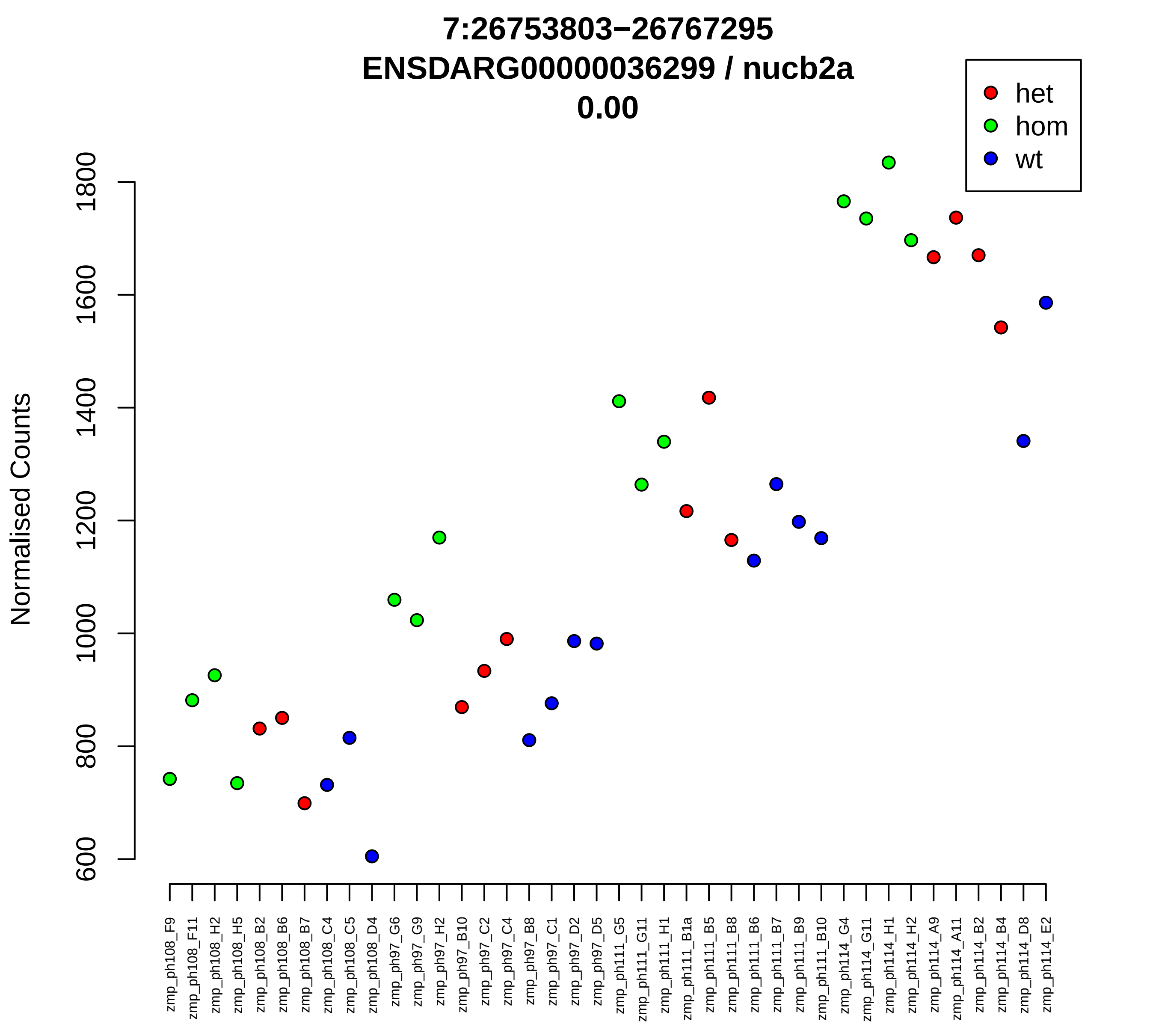

7:26753803−26767295
ENSDARG00000036299 / nucb2a
0.00
het
hom
wt
Normalised Counts
600
800
1000
1200
1400
1600
1800
zmp_ph108_F9
zmp_ph108_F11
zmp_ph108_H2
zmp_ph108_H5
zmp_ph108_B2
zmp_ph108_B6
zmp_ph108_B7
zmp_ph108_C4
zmp_ph108_C5
zmp_ph108_D4
zmp_ph97_G6
zmp_ph97_G9
zmp_ph97_H2
zmp_ph97_B10
zmp_ph97_C2
zmp_ph97_C4
zmp_ph97_B8
zmp_ph97_C1
zmp_ph97_D2
zmp_ph97_D5
zmp_ph111_G5
zmp_ph111_G11
zmp_ph111_H1
zmp_ph111_B1a
zmp_ph111_B5
zmp_ph111_B8
zmp_ph111_B6
zmp_ph111_B7
zmp_ph111_B9
zmp_ph111_B10
zmp_ph114_G4
zmp_ph114_G11
zmp_ph114_H1
zmp_ph114_H2
zmp_ph114_A9
zmp_ph114_A11
zmp_ph114_B2
zmp_ph114_B4
zmp_ph114_D8
zmp_ph114_E2

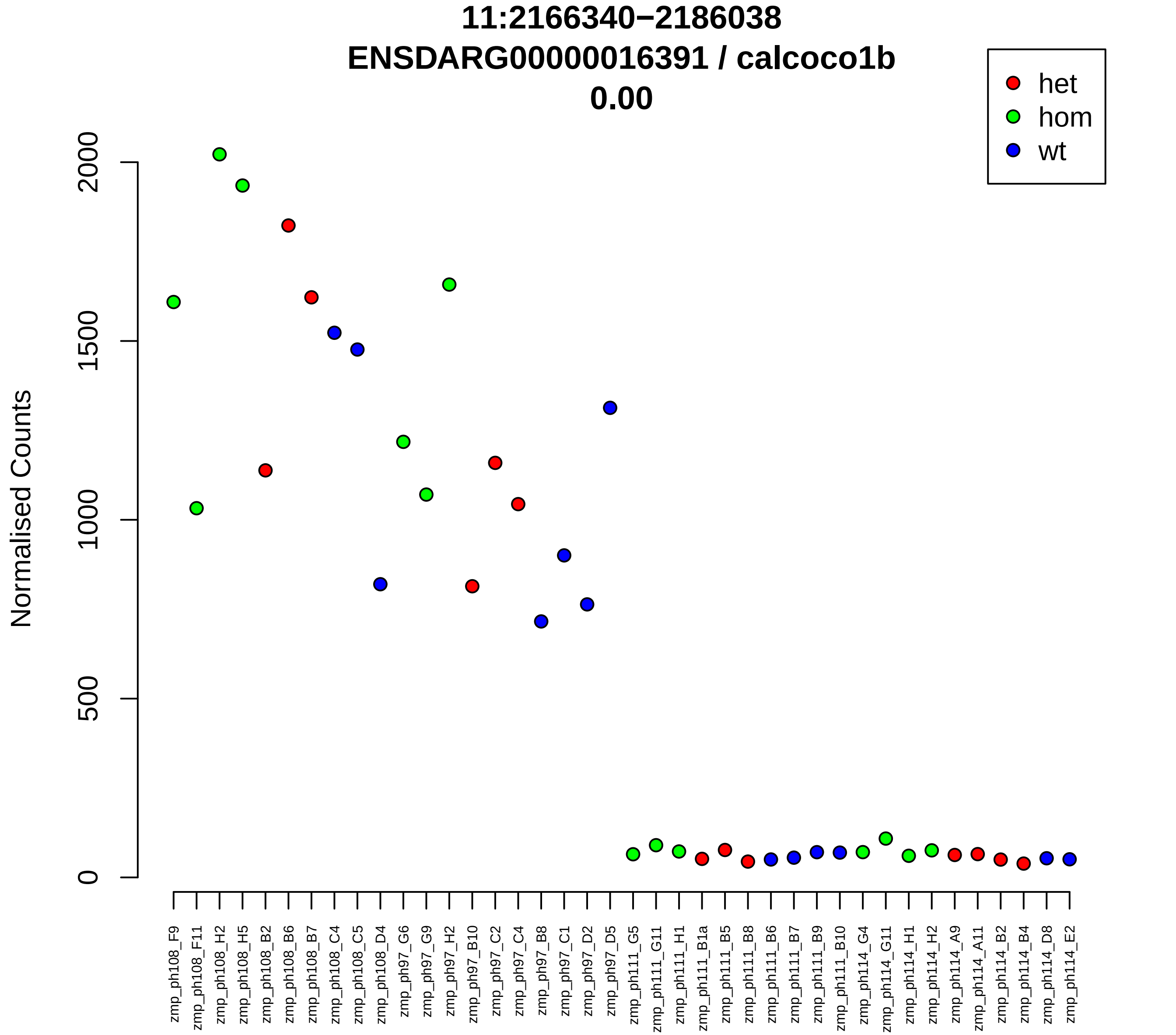
11:2166340−2186038
ENSDARG00000016391 / calcoco1b
0.00
het
hom
wt
Normalised Counts
0
500
1000
1500
2000
zmp_ph108_F9
zmp_ph108_F11
zmp_ph108_H2
zmp_ph108_H5
zmp_ph108_B2
zmp_ph108_B6
zmp_ph108_B7
zmp_ph108_C4
zmp_ph108_C5
zmp_ph108_D4
zmp_ph97_G6
zmp_ph97_G9
zmp_ph97_H2
zmp_ph97_B10
zmp_ph97_C2
zmp_ph97_C4
zmp_ph97_B8
zmp_ph97_C1
zmp_ph97_D2
zmp_ph97_D5
zmp_ph111_G5
zmp_ph111_G11
zmp_ph111_H1
zmp_ph111_B1a
zmp_ph111_B5
zmp_ph111_B8
zmp_ph111_B6
zmp_ph111_B7
zmp_ph111_B9
zmp_ph111_B10
zmp_ph114_G4
zmp_ph114_G11
zmp_ph114_H1
zmp_ph114_H2
zmp_ph114_A9
zmp_ph114_A11
zmp_ph114_B2
zmp_ph114_B4
zmp_ph114_D8
zmp_ph114_E2

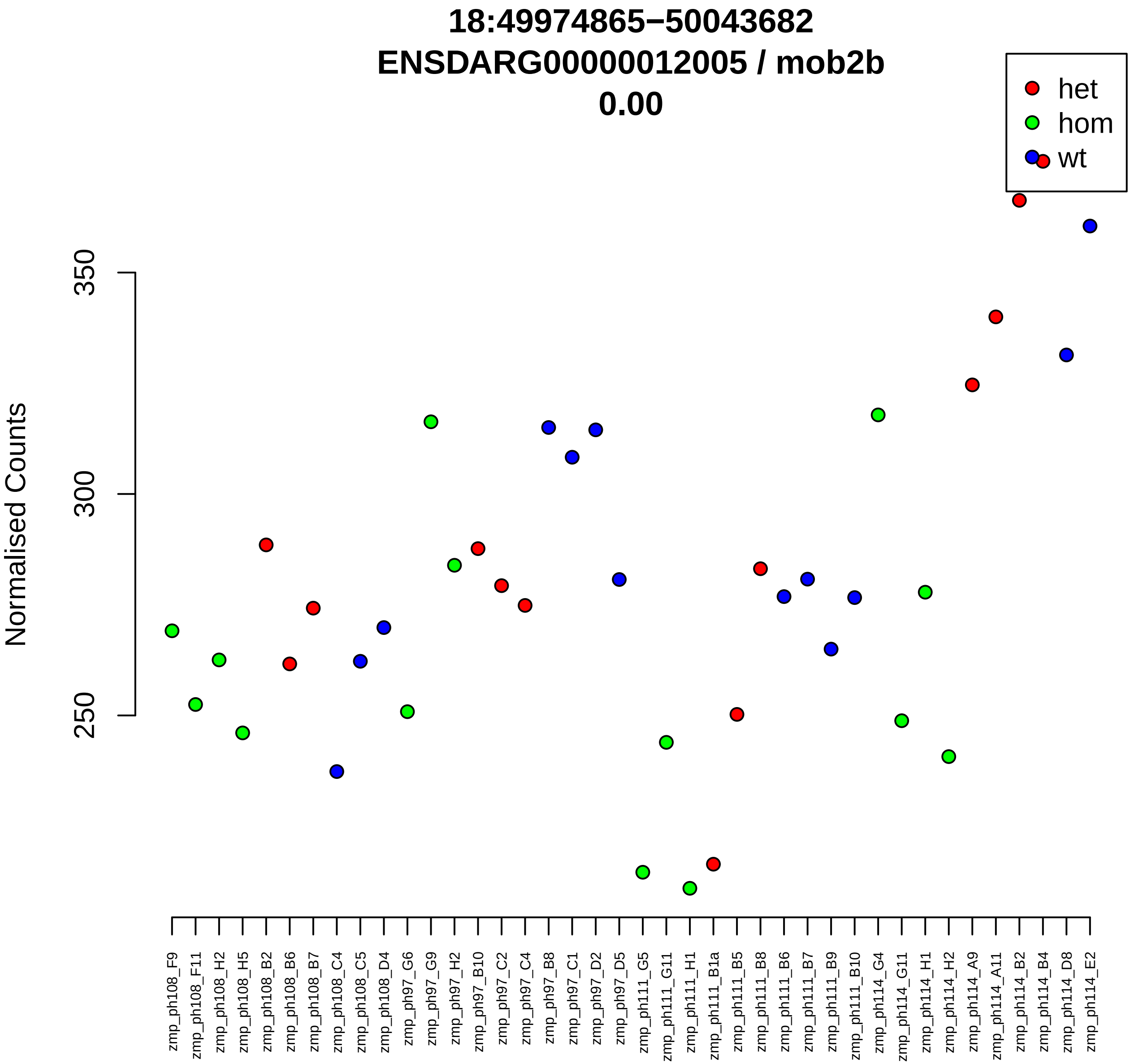

18:49974865−50043682
ENSDARG00000012005 / mob2b
0.00
het
hom
wt
Normalised Counts
250
300
350
zmp_ph108_F9
zmp_ph108_F11
zmp_ph108_H2
zmp_ph108_H5
zmp_ph108_B2
zmp_ph108_B6
zmp_ph108_B7
zmp_ph108_C4
zmp_ph108_C5
zmp_ph108_D4
zmp_ph97_G6
zmp_ph97_G9
zmp_ph97_H2
zmp_ph97_B10
zmp_ph97_C2
zmp_ph97_C4
zmp_ph97_B8
zmp_ph97_C1
zmp_ph97_D2
zmp_ph97_D5
zmp_ph111_G5
zmp_ph111_G11
zmp_ph111_H1
zmp_ph111_B1a
zmp_ph111_B5
zmp_ph111_B8
zmp_ph111_B6
zmp_ph111_B7
zmp_ph111_B9
zmp_ph111_B10
zmp_ph114_G4
zmp_ph114_G11
zmp_ph114_H1
zmp_ph114_H2
zmp_ph114_A9
zmp_ph114_A11
zmp_ph114_B2
zmp_ph114_B4
zmp_ph114_D8
zmp_ph114_E2

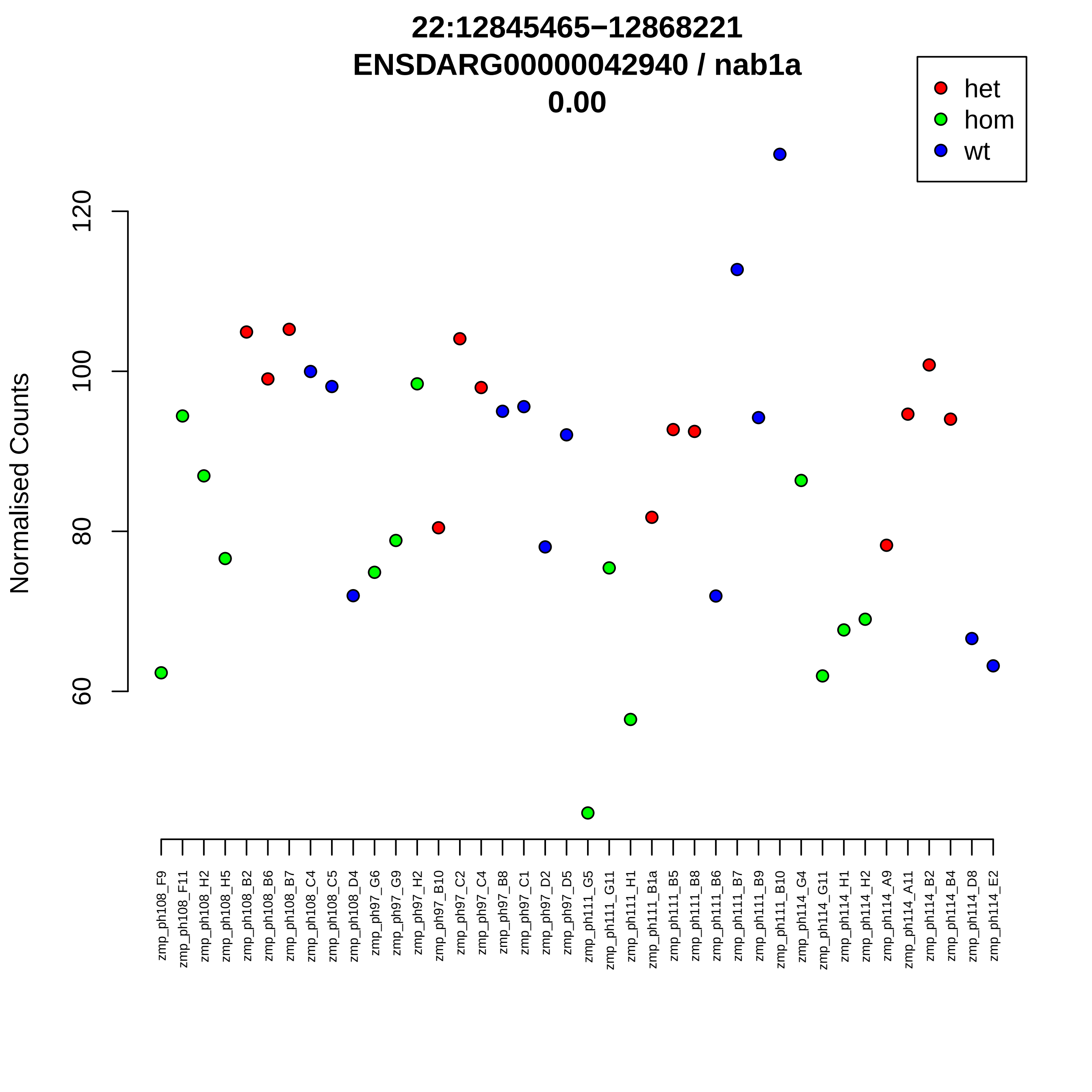

22:12845465−12868221
ENSDARG00000042940 / nab1a
0.00
het
hom
wt
Normalised Counts
60
80
100
120
zmp_ph108_F9
zmp_ph108_F11
zmp_ph108_H2
zmp_ph108_H5
zmp_ph108_B2
zmp_ph108_B6
zmp_ph108_B7
zmp_ph108_C4
zmp_ph108_C5
zmp_ph108_D4
zmp_ph97_G6
zmp_ph97_G9
zmp_ph97_H2
zmp_ph97_B10
zmp_ph97_C2
zmp_ph97_C4
zmp_ph97_B8
zmp_ph97_C1
zmp_ph97_D2
zmp_ph97_D5
zmp_ph111_G5
zmp_ph111_G11
zmp_ph111_H1
zmp_ph111_B1a
zmp_ph111_B5
zmp_ph111_B8
zmp_ph111_B6
zmp_ph111_B7
zmp_ph111_B9
zmp_ph111_B10
zmp_ph114_G4
zmp_ph114_G11
zmp_ph114_H1
zmp_ph114_H2
zmp_ph114_A9
zmp_ph114_A11
zmp_ph114_B2
zmp_ph114_B4
zmp_ph114_D8
zmp_ph114_E2

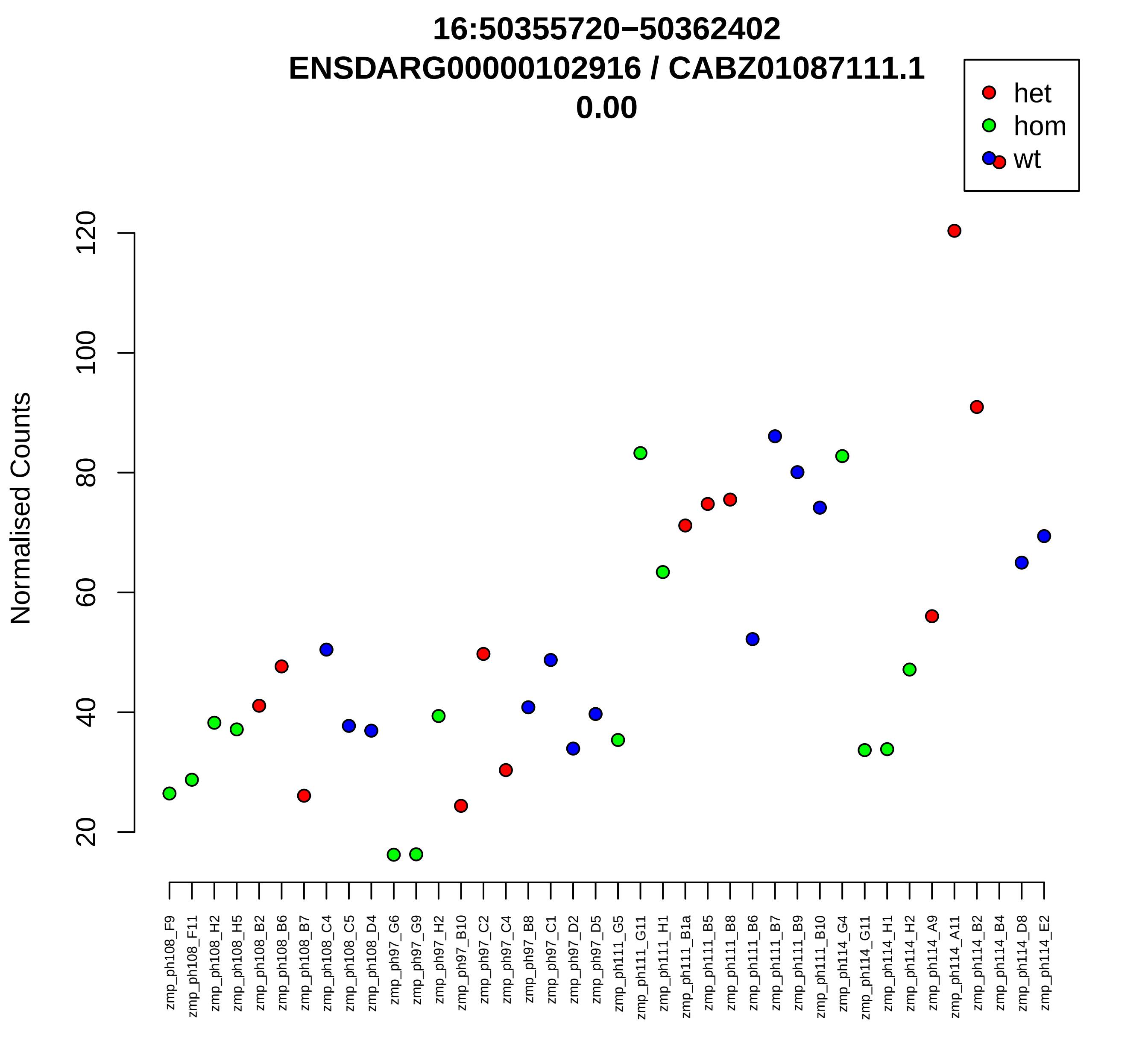

16:50355720−50362402
ENSDARG00000102916 / CABZ01087111.1
0.00
het
hom
wt
Normalised Counts
20
40
60
80
100
120
zmp_ph108_F9
zmp_ph108_F11
zmp_ph108_H2
zmp_ph108_H5
zmp_ph108_B2
zmp_ph108_B6
zmp_ph108_B7
zmp_ph108_C4
zmp_ph108_C5
zmp_ph108_D4
zmp_ph97_G6
zmp_ph97_G9
zmp_ph97_H2
zmp_ph97_B10
zmp_ph97_C2
zmp_ph97_C4
zmp_ph97_B8
zmp_ph97_C1
zmp_ph97_D2
zmp_ph97_D5
zmp_ph111_G5
zmp_ph111_G11
zmp_ph111_H1
zmp_ph111_B1a
zmp_ph111_B5
zmp_ph111_B8
zmp_ph111_B6
zmp_ph111_B7
zmp_ph111_B9
zmp_ph111_B10
zmp_ph114_G4
zmp_ph114_G11
zmp_ph114_H1
zmp_ph114_H2
zmp_ph114_A9
zmp_ph114_A11
zmp_ph114_B2
zmp_ph114_B4
zmp_ph114_D8
zmp_ph114_E2

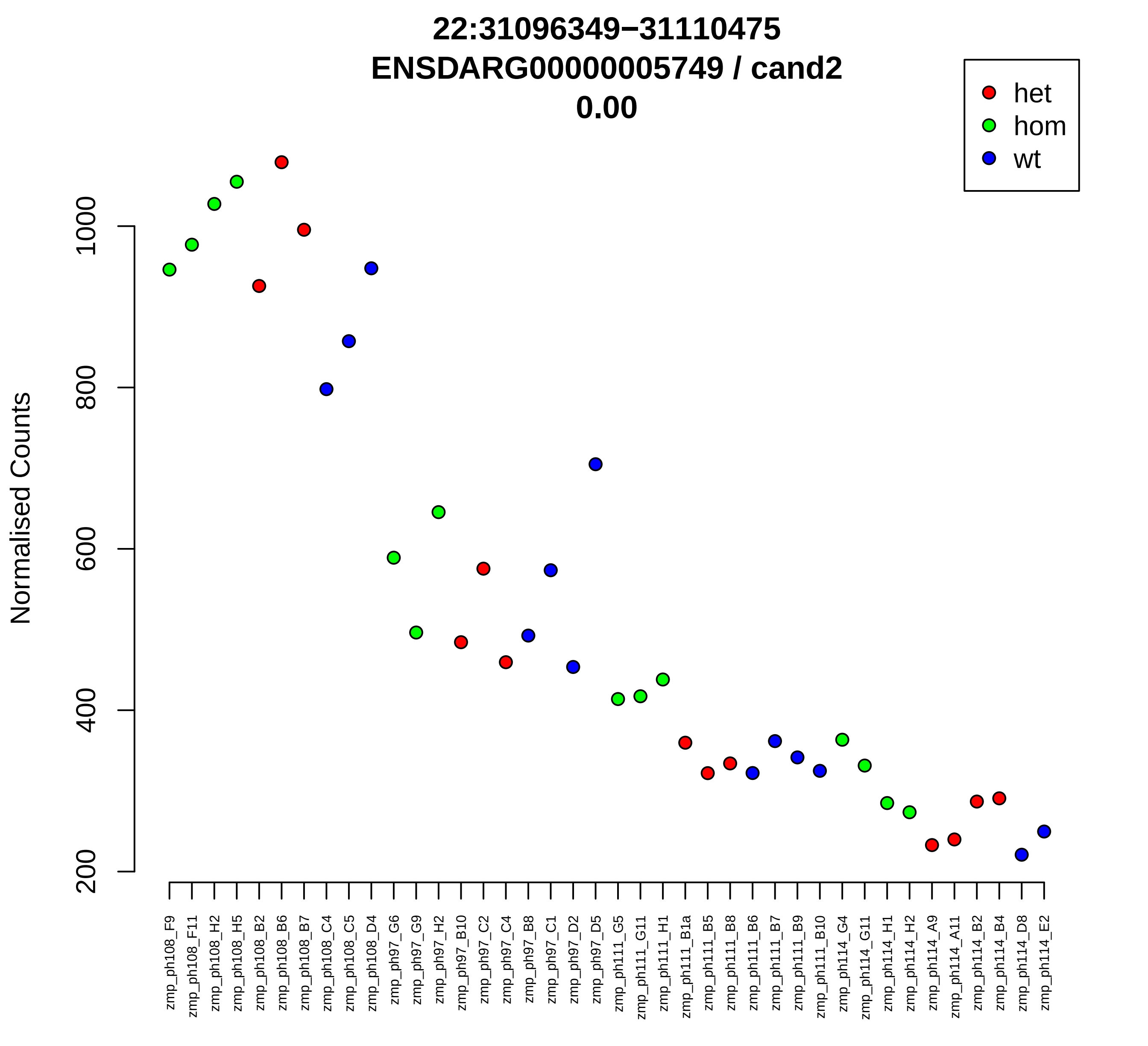
22:31096349−31110475
ENSDARG00000005749 / cand2
0.00
het
hom
wt
Normalised Counts
200
400
600
800
1000
zmp_ph108_F9
zmp_ph108_F11
zmp_ph108_H2
zmp_ph108_H5
zmp_ph108_B2
zmp_ph108_B6
zmp_ph108_B7
zmp_ph108_C4
zmp_ph108_C5
zmp_ph108_D4
zmp_ph97_G6
zmp_ph97_G9
zmp_ph97_H2
zmp_ph97_B10
zmp_ph97_C2
zmp_ph97_C4
zmp_ph97_B8
zmp_ph97_C1
zmp_ph97_D2
zmp_ph97_D5
zmp_ph111_G5
zmp_ph111_G11
zmp_ph111_H1
zmp_ph111_B1a
zmp_ph111_B5
zmp_ph111_B8
zmp_ph111_B6
zmp_ph111_B7
zmp_ph111_B9
zmp_ph111_B10
zmp_ph114_G4
zmp_ph114_G11
zmp_ph114_H1
zmp_ph114_H2
zmp_ph114_A9
zmp_ph114_A11
zmp_ph114_B2
zmp_ph114_B4
zmp_ph114_D8
zmp_ph114_E2

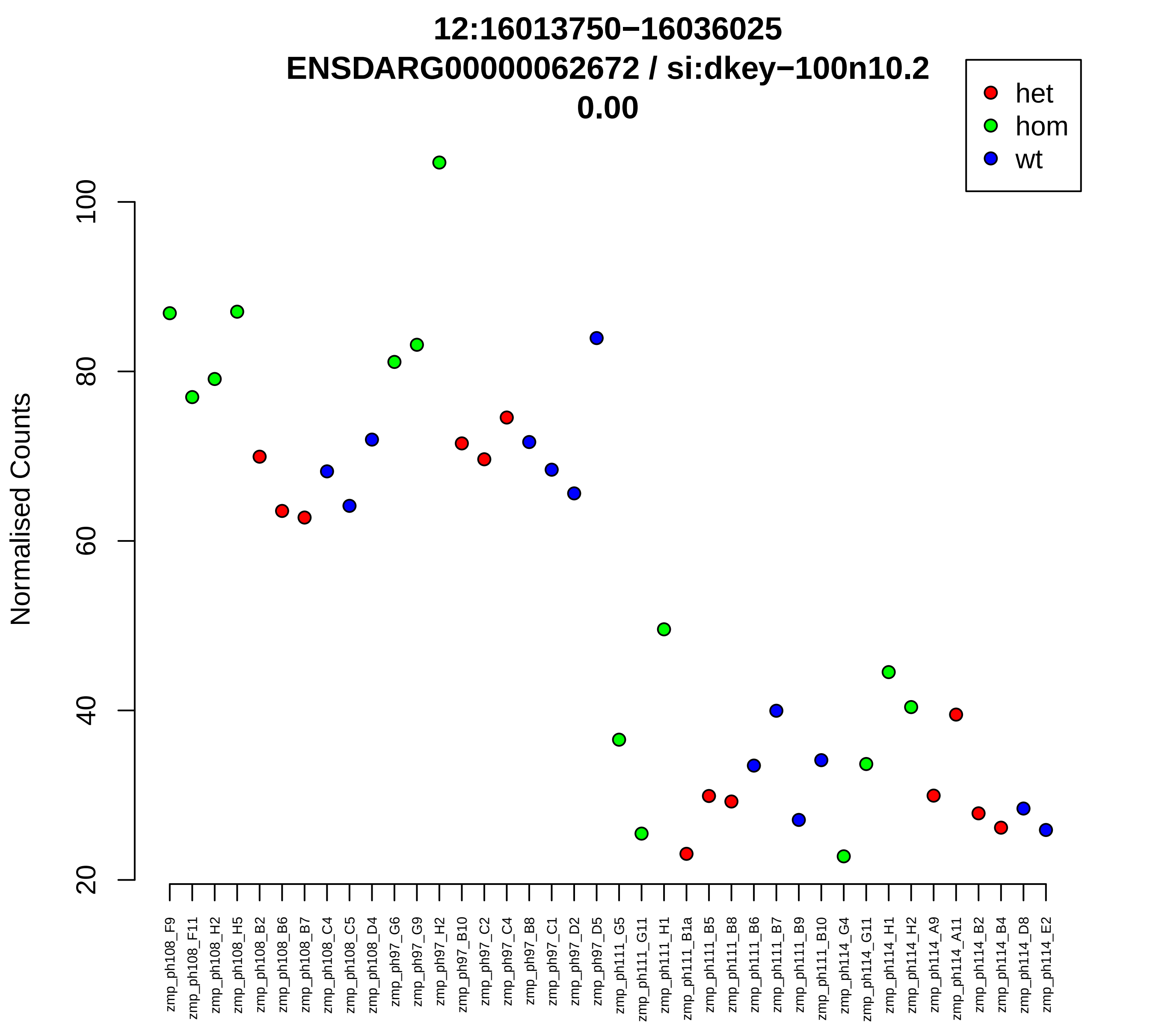
12:16013750−16036025
ENSDARG00000062672 / si:dkey−100n10.2
0.00
het
hom
wt
Normalised Counts
20
40
60
80
100
zmp_ph108_F9
zmp_ph108_F11
zmp_ph108_H2
zmp_ph108_H5
zmp_ph108_B2
zmp_ph108_B6
zmp_ph108_B7
zmp_ph108_C4
zmp_ph108_C5
zmp_ph108_D4
zmp_ph97_G6
zmp_ph97_G9
zmp_ph97_H2
zmp_ph97_B10
zmp_ph97_C2
zmp_ph97_C4
zmp_ph97_B8
zmp_ph97_C1
zmp_ph97_D2
zmp_ph97_D5
zmp_ph111_G5
zmp_ph111_G11
zmp_ph111_H1
zmp_ph111_B1a
zmp_ph111_B5
zmp_ph111_B8
zmp_ph111_B6
zmp_ph111_B7
zmp_ph111_B9
zmp_ph111_B10
zmp_ph114_G4
zmp_ph114_G11
zmp_ph114_H1
zmp_ph114_H2
zmp_ph114_A9
zmp_ph114_A11
zmp_ph114_B2
zmp_ph114_B4
zmp_ph114_D8
zmp_ph114_E2

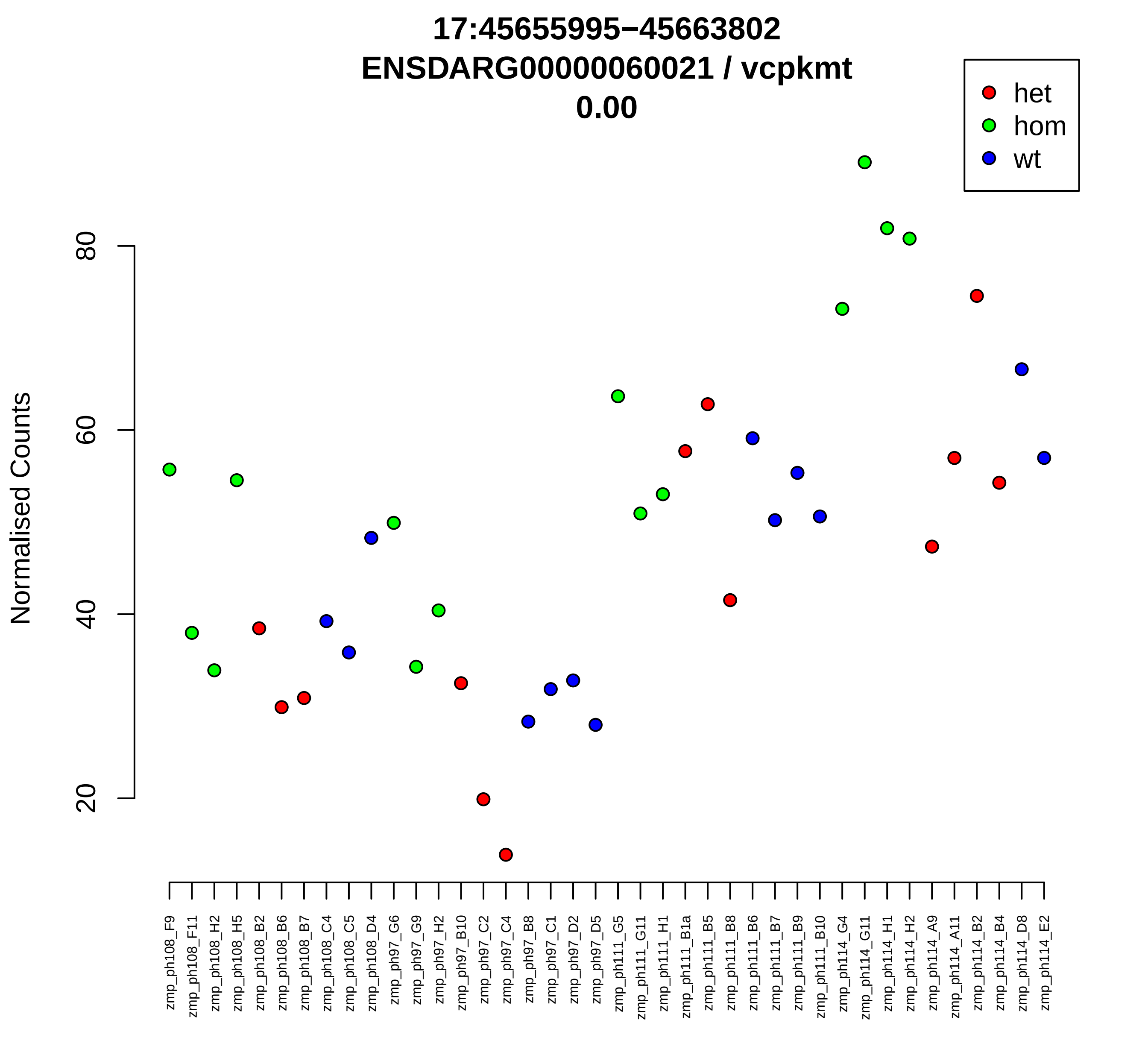

17:45655995−45663802
ENSDARG00000060021 / vcpkmt
0.00
het
hom
wt
Normalised Counts
20
40
60
80
zmp_ph108_F9
zmp_ph108_F11
zmp_ph108_H2
zmp_ph108_H5
zmp_ph108_B2
zmp_ph108_B6
zmp_ph108_B7
zmp_ph108_C4
zmp_ph108_C5
zmp_ph108_D4
zmp_ph97_G6
zmp_ph97_G9
zmp_ph97_H2
zmp_ph97_B10
zmp_ph97_C2
zmp_ph97_C4
zmp_ph97_B8
zmp_ph97_C1
zmp_ph97_D2
zmp_ph97_D5
zmp_ph111_G5
zmp_ph111_G11
zmp_ph111_H1
zmp_ph111_B1a
zmp_ph111_B5
zmp_ph111_B8
zmp_ph111_B6
zmp_ph111_B7
zmp_ph111_B9
zmp_ph111_B10
zmp_ph114_G4
zmp_ph114_G11
zmp_ph114_H1
zmp_ph114_H2
zmp_ph114_A9
zmp_ph114_A11
zmp_ph114_B2
zmp_ph114_B4
zmp_ph114_D8
zmp_ph114_E2

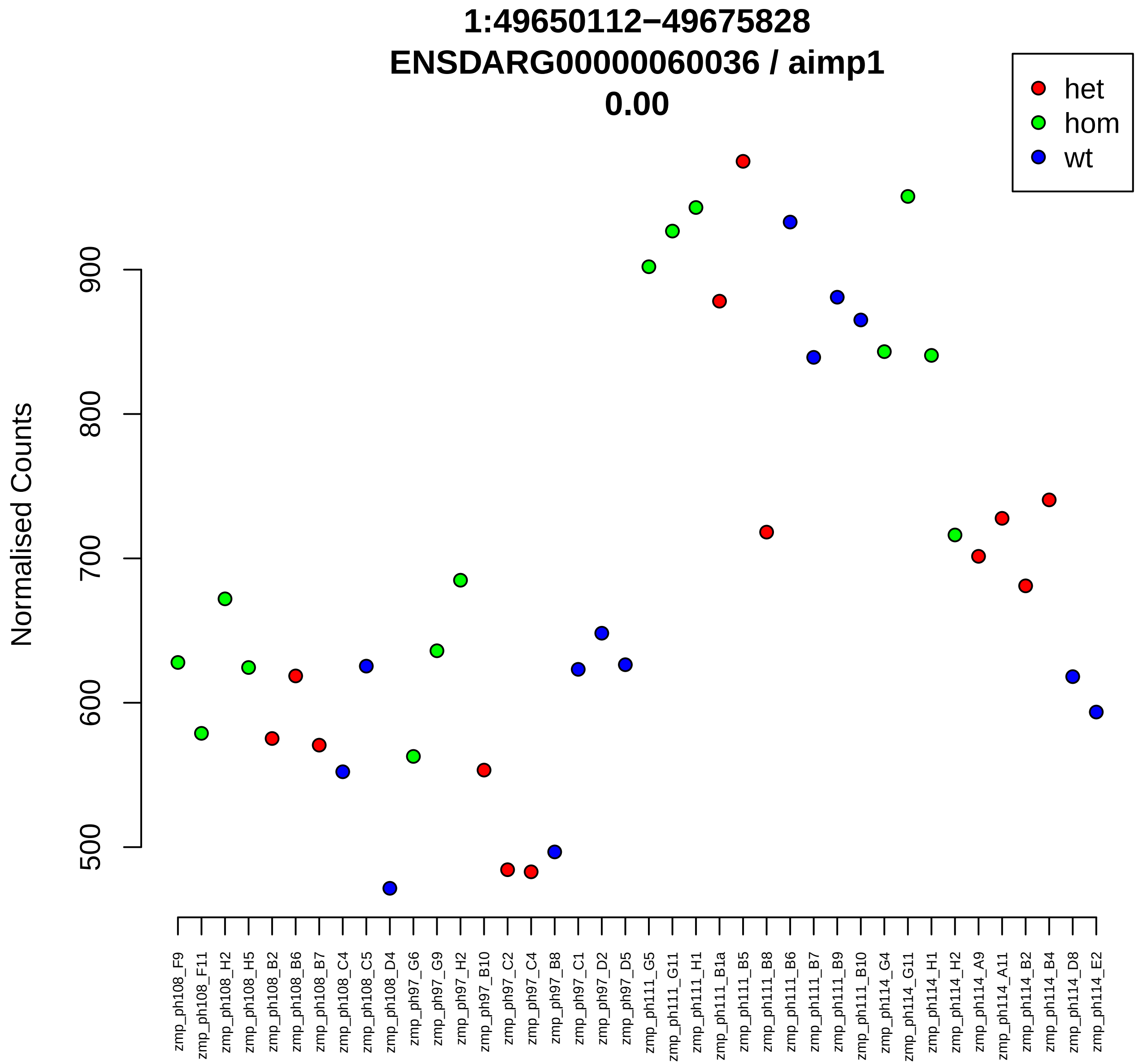
1:49650112−49675828
ENSDARG00000060036 / aimp1
0.00
het
hom
wt
Normalised Counts
500
600
700
800
900
zmp_ph108_F9
zmp_ph108_F11
zmp_ph108_H2
zmp_ph108_H5
zmp_ph108_B2
zmp_ph108_B6
zmp_ph108_B7
zmp_ph108_C4
zmp_ph108_C5
zmp_ph108_D4
zmp_ph97_G6
zmp_ph97_G9
zmp_ph97_H2
zmp_ph97_B10
zmp_ph97_C2
zmp_ph97_C4
zmp_ph97_B8
zmp_ph97_C1
zmp_ph97_D2
zmp_ph97_D5
zmp_ph111_G5
zmp_ph111_G11
zmp_ph111_H1
zmp_ph111_B1a
zmp_ph111_B5
zmp_ph111_B8
zmp_ph111_B6
zmp_ph111_B7
zmp_ph111_B9
zmp_ph111_B10
zmp_ph114_G4
zmp_ph114_G11
zmp_ph114_H1
zmp_ph114_H2
zmp_ph114_A9
zmp_ph114_A11
zmp_ph114_B2
zmp_ph114_B4
zmp_ph114_D8
zmp_ph114_E2

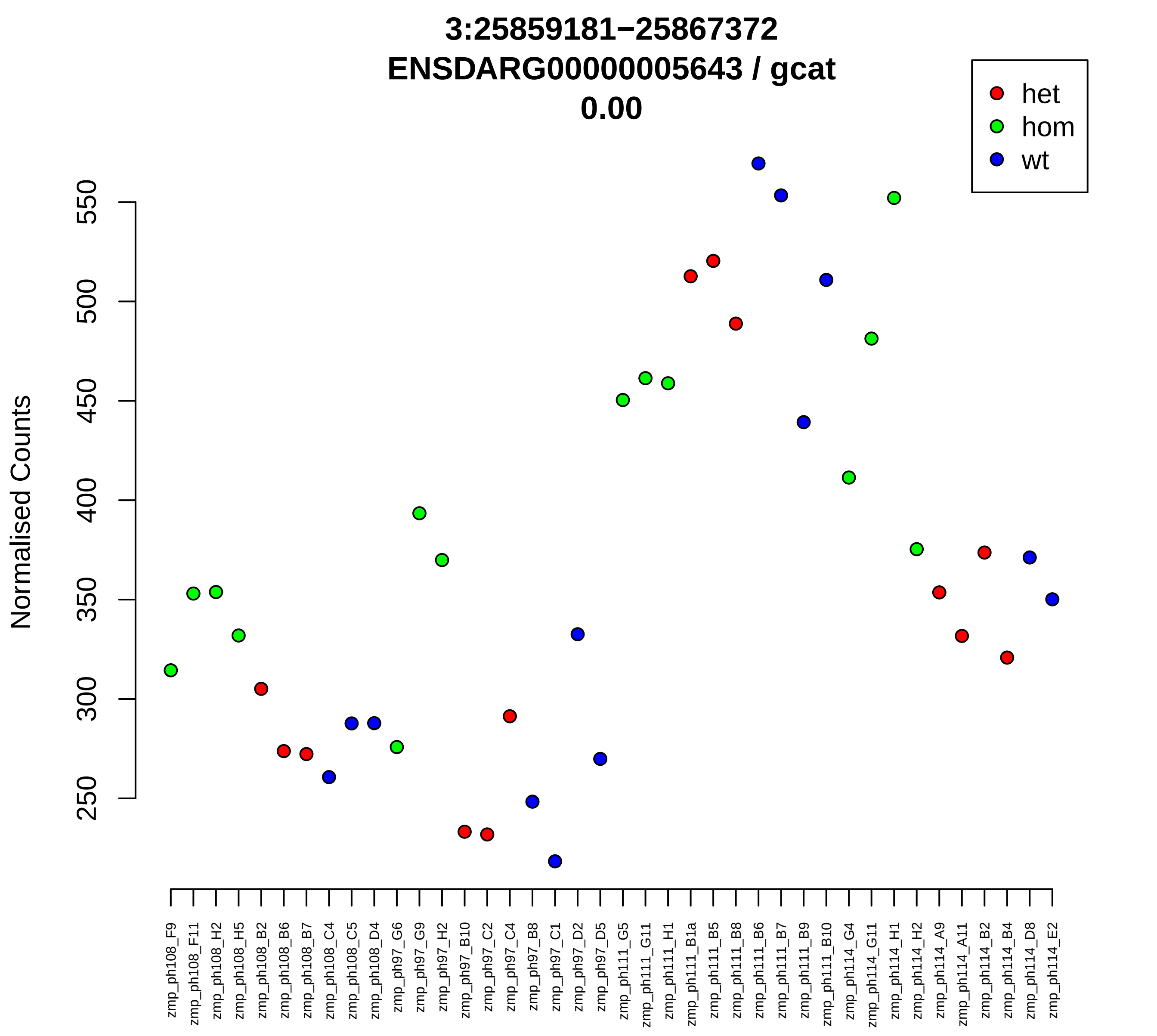
3:25859181−25867372
ENSDARG00000005643 / gcat
0.00
het
hom
wt
Normalised Counts
250
300
350
400
450
500
550
zmp_ph108_F9
zmp_ph108_F11
zmp_ph108_H2
zmp_ph108_H5
zmp_ph108_B2
zmp_ph108_B6
zmp_ph108_B7
zmp_ph108_C4
zmp_ph108_C5
zmp_ph108_D4
zmp_ph97_G6
zmp_ph97_G9
zmp_ph97_H2
zmp_ph97_B10
zmp_ph97_C2
zmp_ph97_C4
zmp_ph97_B8
zmp_ph97_C1
zmp_ph97_D2
zmp_ph97_D5
zmp_ph111_G5
zmp_ph111_G11
zmp_ph111_H1
zmp_ph111_B1a
zmp_ph111_B5
zmp_ph111_B8
zmp_ph111_B6
zmp_ph111_B7
zmp_ph111_B9
zmp_ph111_B10
zmp_ph114_G4
zmp_ph114_G11
zmp_ph114_H1
zmp_ph114_H2
zmp_ph114_A9
zmp_ph114_A11
zmp_ph114_B2
zmp_ph114_B4
zmp_ph114_D8
zmp_ph114_E2

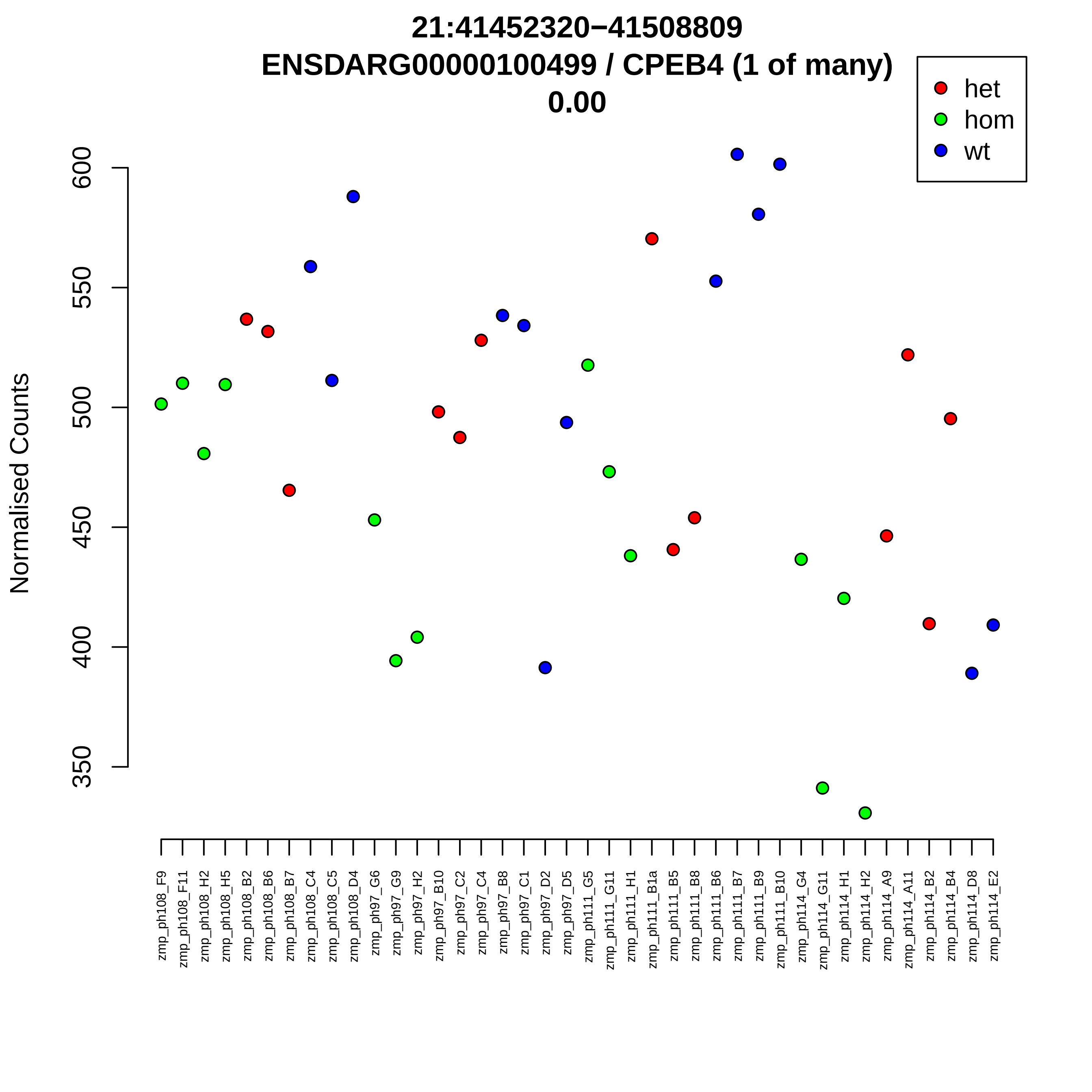

21:41452320−41508809
ENSDARG00000100499 / CPEB4 (1 of many)
0.00
het
hom
wt
Normalised Counts
600
550
500
450
400
350
zmp_ph108_F9
zmp_ph108_F11
zmp_ph108_H2
zmp_ph108_H5
zmp_ph108_B2
zmp_ph108_B6
zmp_ph108_B7
zmp_ph108_C4
zmp_ph108_C5
zmp_ph108_D4
zmp_ph97_G6
zmp_ph97_G9
zmp_ph97_H2
zmp_ph97_B10
zmp_ph97_C2
zmp_ph97_C4
zmp_ph97_B8
zmp_ph97_C1
zmp_ph97_D2
zmp_ph97_D5
zmp_ph111_G5
zmp_ph111_G11
zmp_ph111_H1
zmp_ph111_B1a
zmp_ph111_B5
zmp_ph111_B8
zmp_ph111_B6
zmp_ph111_B7
zmp_ph111_B9
zmp_ph111_B10
zmp_ph114_G4
zmp_ph114_G11
zmp_ph114_H1
zmp_ph114_H2
zmp_ph114_A9
zmp_ph114_A11
zmp_ph114_B2
zmp_ph114_B4
zmp_ph114_D8
zmp_ph114_E2

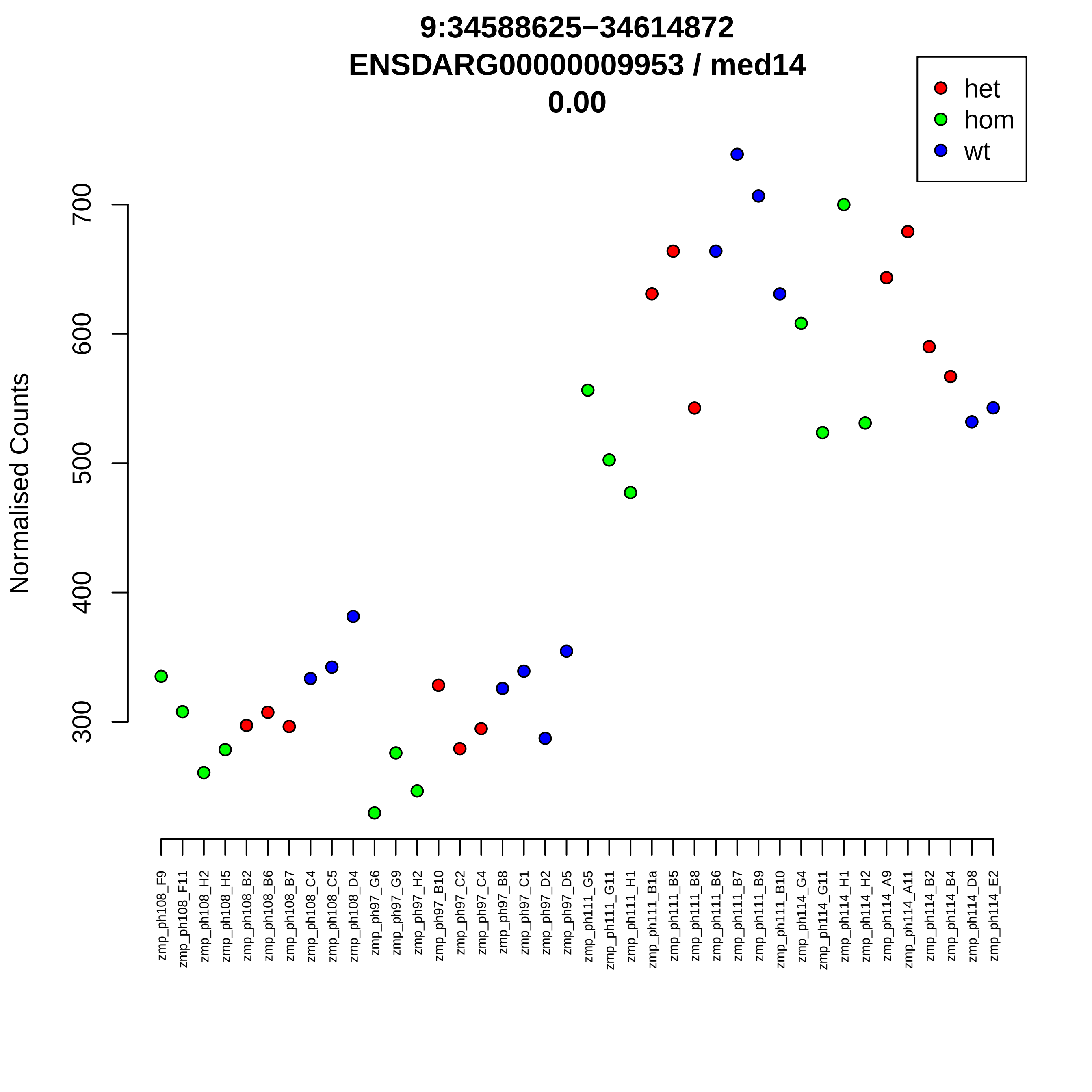
9:34588625−34614872
ENSDARG00000009953 / med14
0.00
het
hom
wt
Normalised Counts
300
400
500
600
700
zmp_ph108_F9
zmp_ph108_F11
zmp_ph108_H2
zmp_ph108_H5
zmp_ph108_B2
zmp_ph108_B6
zmp_ph108_B7
zmp_ph108_C4
zmp_ph108_C5
zmp_ph108_D4
zmp_ph97_G6
zmp_ph97_G9
zmp_ph97_H2
zmp_ph97_B10
zmp_ph97_C2
zmp_ph97_C4
zmp_ph97_B8
zmp_ph97_C1
zmp_ph97_D2
zmp_ph97_D5
zmp_ph111_G5
zmp_ph111_G11
zmp_ph111_H1
zmp_ph111_B1a
zmp_ph111_B5
zmp_ph111_B8
zmp_ph111_B6
zmp_ph111_B7
zmp_ph111_B9
zmp_ph111_B10
zmp_ph114_G4
zmp_ph114_G11
zmp_ph114_H1
zmp_ph114_H2
zmp_ph114_A9
zmp_ph114_A11
zmp_ph114_B2
zmp_ph114_B4
zmp_ph114_D8
zmp_ph114_E2

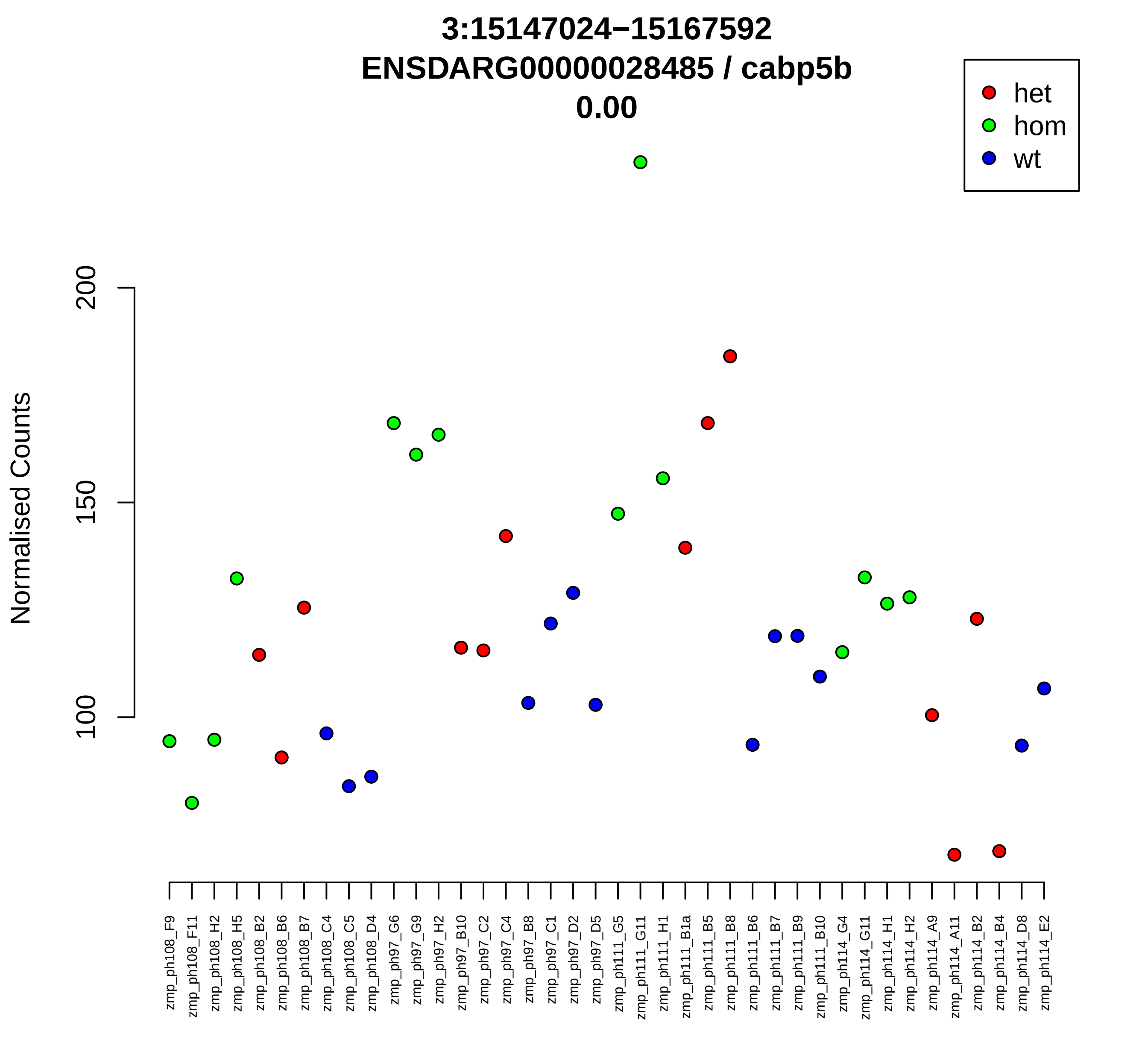

3:15147024−15167592
ENSDARG00000028485 / cabp5b
0.00
het
hom
wt
Normalised Counts
100
150
200
zmp_ph108_F9
zmp_ph108_F11
zmp_ph108_H2
zmp_ph108_H5
zmp_ph108_B2
zmp_ph108_B6
zmp_ph108_B7
zmp_ph108_C4
zmp_ph108_C5
zmp_ph108_D4
zmp_ph97_G6
zmp_ph97_G9
zmp_ph97_H2
zmp_ph97_B10
zmp_ph97_C2
zmp_ph97_C4
zmp_ph97_B8
zmp_ph97_C1
zmp_ph97_D2
zmp_ph97_D5
zmp_ph111_G5
zmp_ph111_G11
zmp_ph111_H1
zmp_ph111_B1a
zmp_ph111_B5
zmp_ph111_B8
zmp_ph111_B6
zmp_ph111_B7
zmp_ph111_B9
zmp_ph111_B10
zmp_ph114_G4
zmp_ph114_G11
zmp_ph114_H1
zmp_ph114_H2
zmp_ph114_A9
zmp_ph114_A11
zmp_ph114_B2
zmp_ph114_B4
zmp_ph114_D8
zmp_ph114_E2

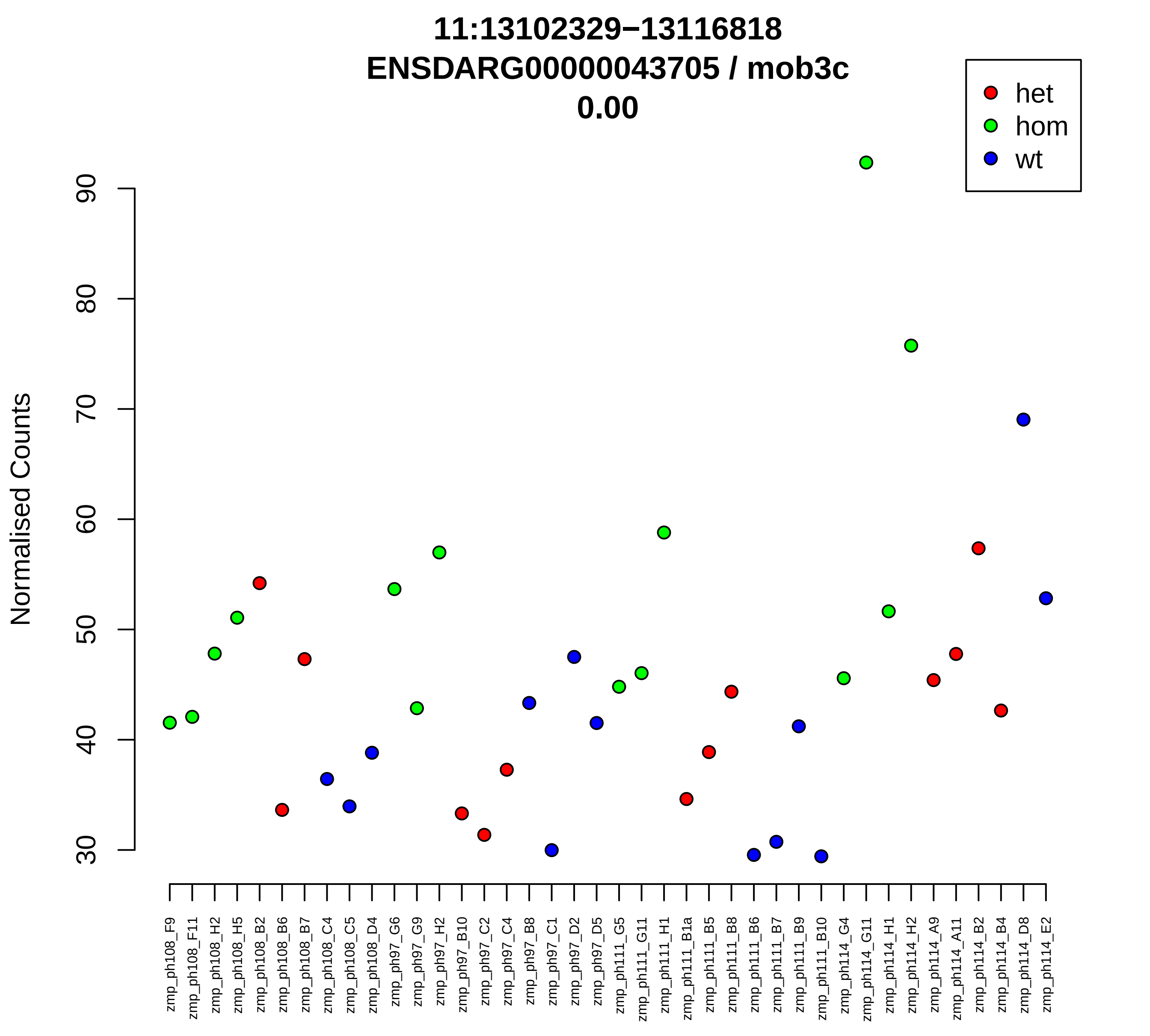
11:13102329−13116818
ENSDARG00000043705 / mob3c
0.00
het
hom
wt
Normalised Counts
30
40
50
60
70
80
90
zmp_ph108_F9
zmp_ph108_F11
zmp_ph108_H2
zmp_ph108_H5
zmp_ph108_B2
zmp_ph108_B6
zmp_ph108_B7
zmp_ph108_C4
zmp_ph108_C5
zmp_ph108_D4
zmp_ph97_G6
zmp_ph97_G9
zmp_ph97_H2
zmp_ph97_B10
zmp_ph97_C2
zmp_ph97_C4
zmp_ph97_B8
zmp_ph97_C1
zmp_ph97_D2
zmp_ph97_D5
zmp_ph111_G5
zmp_ph111_G11
zmp_ph111_H1
zmp_ph111_B1a
zmp_ph111_B5
zmp_ph111_B8
zmp_ph111_B6
zmp_ph111_B7
zmp_ph111_B9
zmp_ph111_B10
zmp_ph114_G4
zmp_ph114_G11
zmp_ph114_H1
zmp_ph114_H2
zmp_ph114_A9
zmp_ph114_A11
zmp_ph114_B2
zmp_ph114_B4
zmp_ph114_D8
zmp_ph114_E2

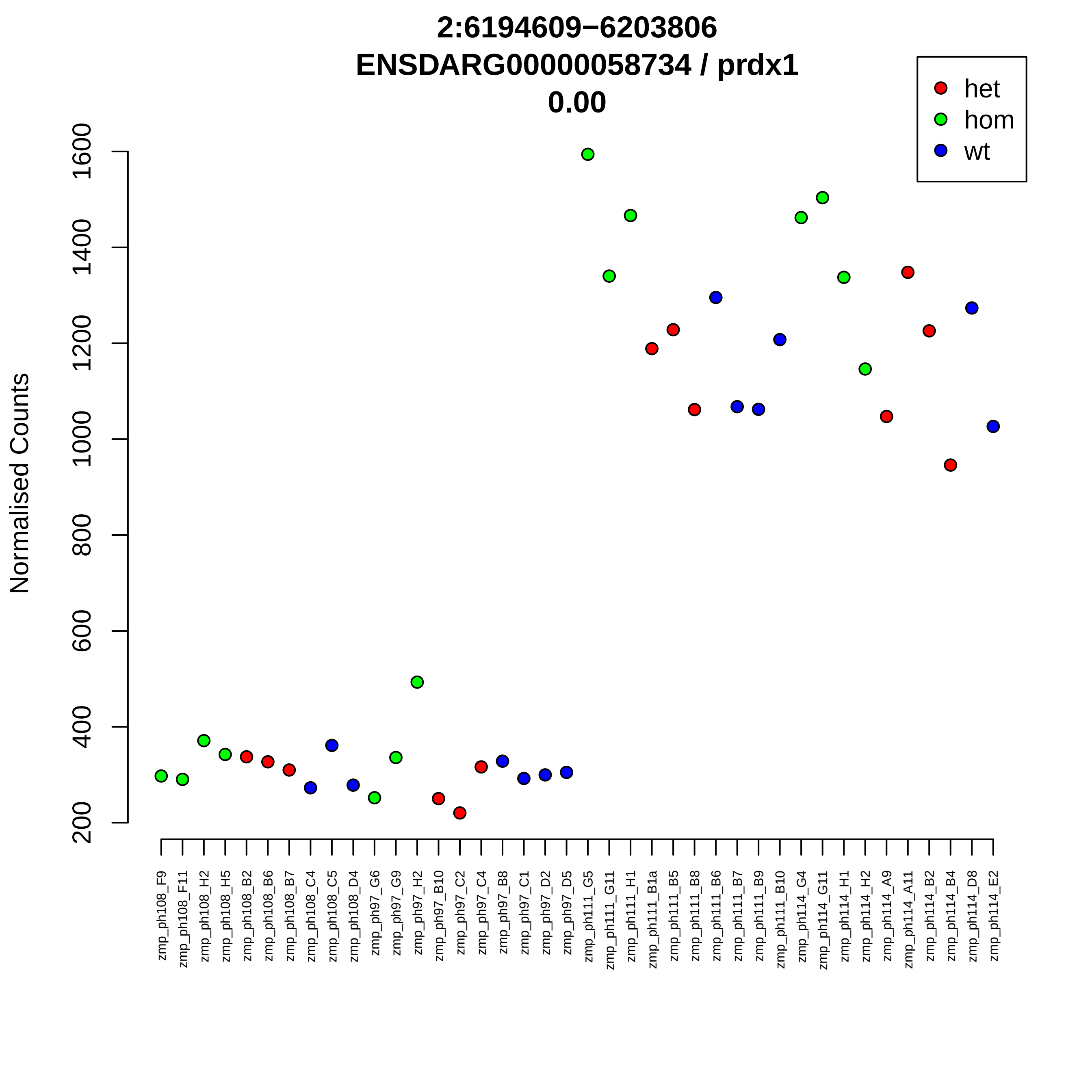
2:6194609−6203806
ENSDARG00000058734 / prdx1
0.00
het
hom
wt
Normalised Counts
200
400
600
800
1000
1200
1400
1600
zmp_ph108_F9
zmp_ph108_F11
zmp_ph108_H2
zmp_ph108_H5
zmp_ph108_B2
zmp_ph108_B6
zmp_ph108_B7
zmp_ph108_C4
zmp_ph108_C5
zmp_ph108_D4
zmp_ph97_G6
zmp_ph97_G9
zmp_ph97_H2
zmp_ph97_B10
zmp_ph97_C2
zmp_ph97_C4
zmp_ph97_B8
zmp_ph97_C1
zmp_ph97_D2
zmp_ph97_D5
zmp_ph111_G5
zmp_ph111_G11
zmp_ph111_H1
zmp_ph111_B1a
zmp_ph111_B5
zmp_ph111_B8
zmp_ph111_B6
zmp_ph111_B7
zmp_ph111_B9
zmp_ph111_B10
zmp_ph114_G4
zmp_ph114_G11
zmp_ph114_H1
zmp_ph114_H2
zmp_ph114_A9
zmp_ph114_A11
zmp_ph114_B2
zmp_ph114_B4
zmp_ph114_D8
zmp_ph114_E2

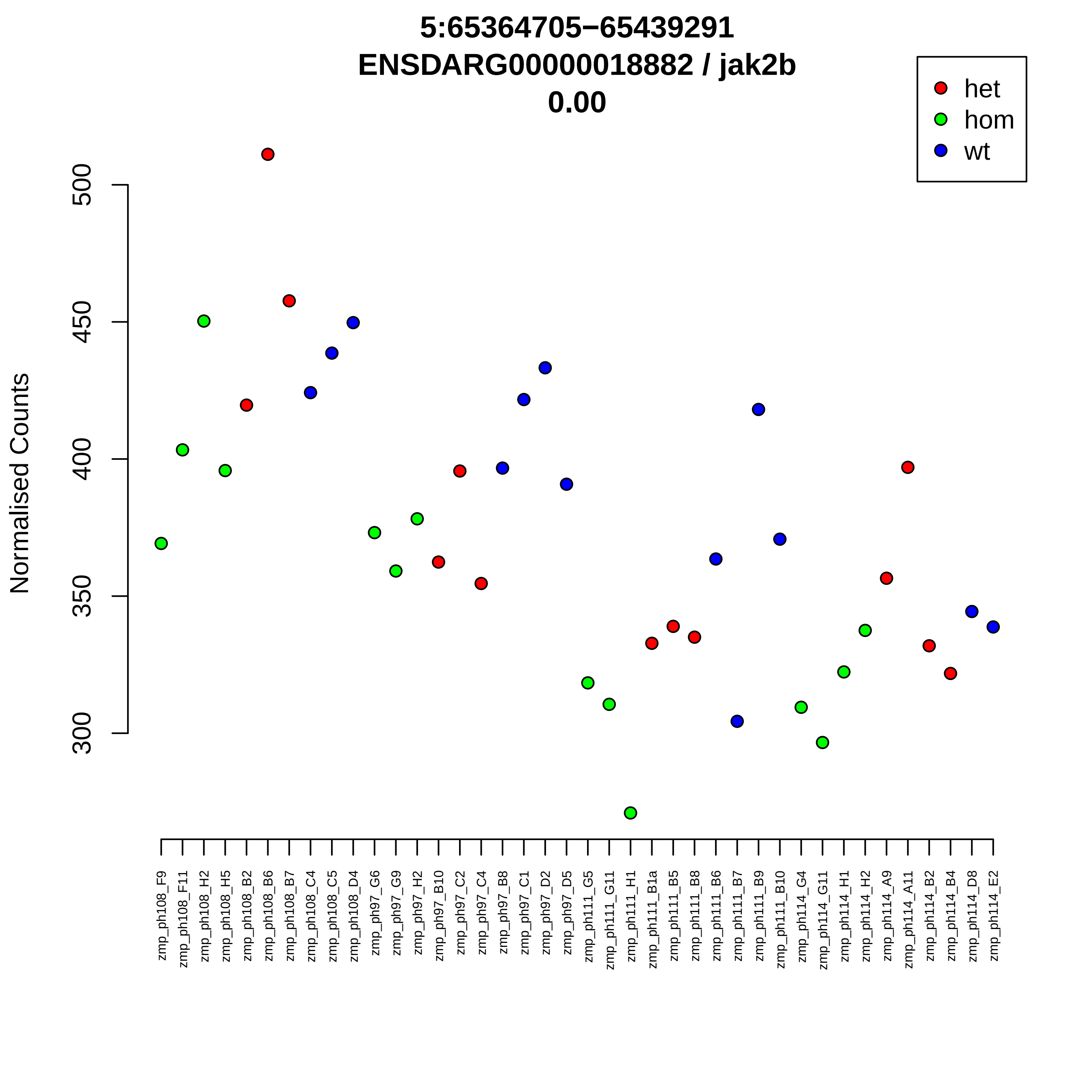

5:65364705−65439291
ENSDARG00000018882 / jak2b
0.00
het
hom
wt
Normalised Counts
300
350
400
450
500
zmp_ph108_F9
zmp_ph108_F11
zmp_ph108_H2
zmp_ph108_H5
zmp_ph108_B2
zmp_ph108_B6
zmp_ph108_B7
zmp_ph108_C4
zmp_ph108_C5
zmp_ph108_D4
zmp_ph97_G6
zmp_ph97_G9
zmp_ph97_H2
zmp_ph97_B10
zmp_ph97_C2
zmp_ph97_C4
zmp_ph97_B8
zmp_ph97_C1
zmp_ph97_D2
zmp_ph97_D5
zmp_ph111_G5
zmp_ph111_G11
zmp_ph111_H1
zmp_ph111_B1a
zmp_ph111_B5
zmp_ph111_B8
zmp_ph111_B6
zmp_ph111_B7
zmp_ph111_B9
zmp_ph111_B10
zmp_ph114_G4
zmp_ph114_G11
zmp_ph114_H1
zmp_ph114_H2
zmp_ph114_A9
zmp_ph114_A11
zmp_ph114_B2
zmp_ph114_B4
zmp_ph114_D8
zmp_ph114_E2

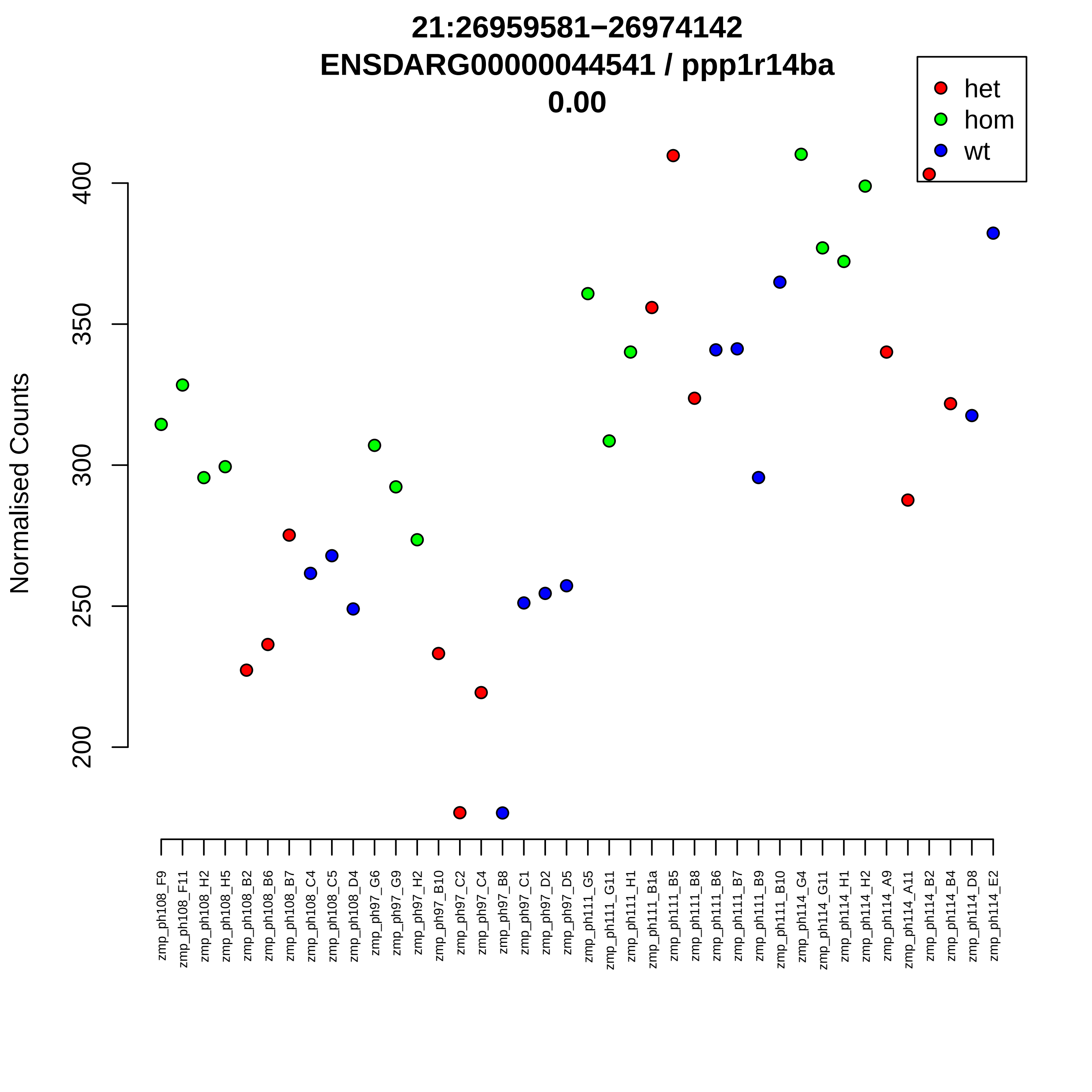

21:26959581−26974142
ENSDARG00000044541 / ppp1r14ba
0.00
het
hom
wt
Normalised Counts
200
250
300
350
400
zmp_ph108_F9
zmp_ph108_F11
zmp_ph108_H2
zmp_ph108_H5
zmp_ph108_B2
zmp_ph108_B6
zmp_ph108_B7
zmp_ph108_C4
zmp_ph108_C5
zmp_ph108_D4
zmp_ph97_G6
zmp_ph97_G9
zmp_ph97_H2
zmp_ph97_B10
zmp_ph97_C2
zmp_ph97_C4
zmp_ph97_B8
zmp_ph97_C1
zmp_ph97_D2
zmp_ph97_D5
zmp_ph111_G5
zmp_ph111_G11
zmp_ph111_H1
zmp_ph111_B1a
zmp_ph111_B5
zmp_ph111_B8
zmp_ph111_B6
zmp_ph111_B7
zmp_ph111_B9
zmp_ph111_B10
zmp_ph114_G4
zmp_ph114_G11
zmp_ph114_H1
zmp_ph114_H2
zmp_ph114_A9
zmp_ph114_A11
zmp_ph114_B2
zmp_ph114_B4
zmp_ph114_D8
zmp_ph114_E2

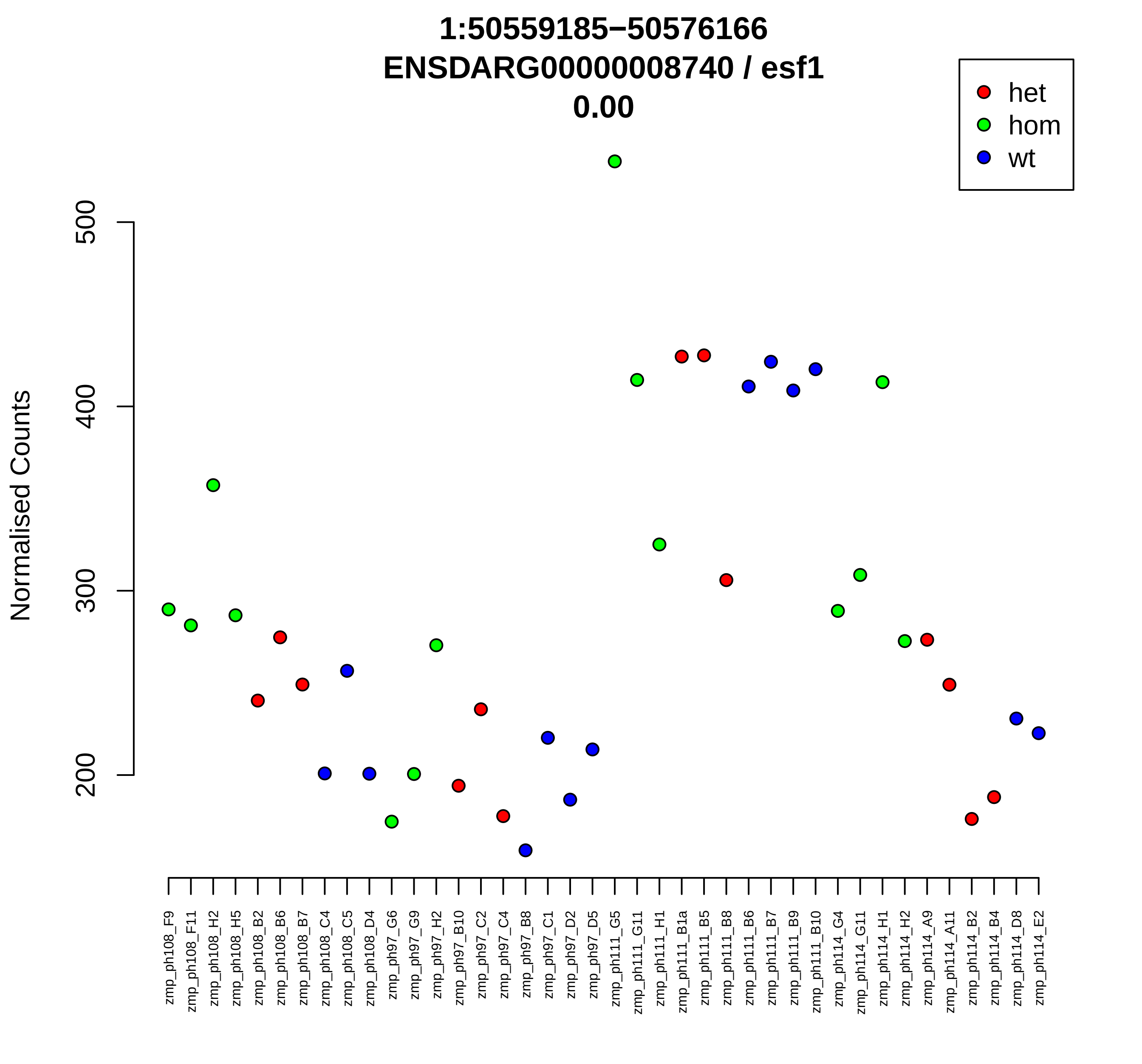
1:50559185−50576166
ENSDARG00000008740 / esf1
0.00
het
hom
wt
Normalised Counts
500
400
300
200
zmp_ph108_F9
zmp_ph108_F11
zmp_ph108_H2
zmp_ph108_H5
zmp_ph108_B2
zmp_ph108_B6
zmp_ph108_B7
zmp_ph108_C4
zmp_ph108_C5
zmp_ph108_D4
zmp_ph97_G6
zmp_ph97_G9
zmp_ph97_H2
zmp_ph97_B10
zmp_ph97_C2
zmp_ph97_C4
zmp_ph97_B8
zmp_ph97_C1
zmp_ph97_D2
zmp_ph97_D5
zmp_ph111_G5
zmp_ph111_G11
zmp_ph111_H1
zmp_ph111_B1a
zmp_ph111_B5
zmp_ph111_B8
zmp_ph111_B6
zmp_ph111_B7
zmp_ph111_B9
zmp_ph111_B10
zmp_ph114_G4
zmp_ph114_G11
zmp_ph114_H1
zmp_ph114_H2
zmp_ph114_A9
zmp_ph114_A11
zmp_ph114_B2
zmp_ph114_B4
zmp_ph114_D8
zmp_ph114_E2

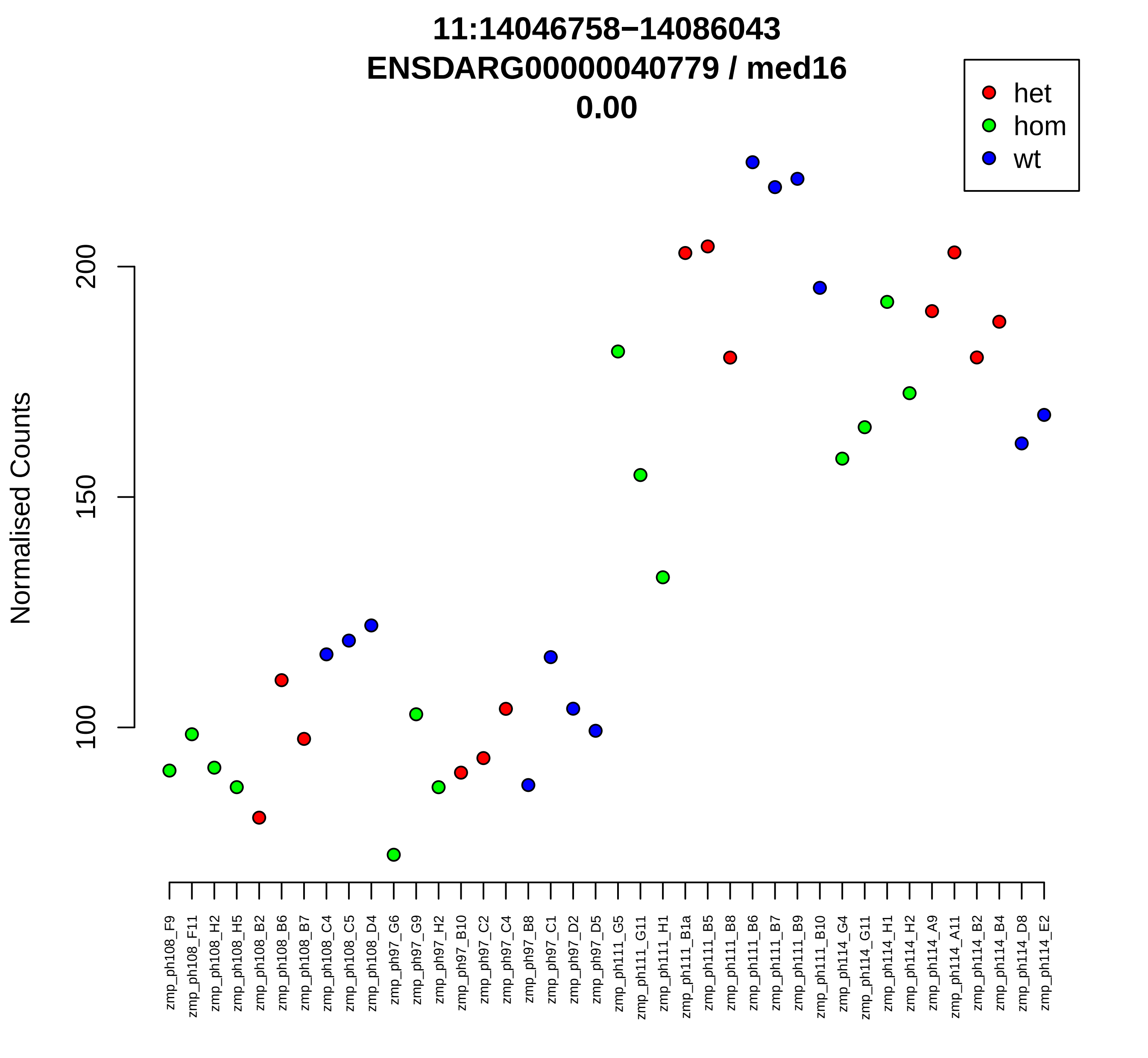
11:14046758−14086043
ENSDARG00000040779 / med16
0.00
het
hom
wt
Normalised Counts
100
150
200
zmp_ph108_F9
zmp_ph108_F11
zmp_ph108_H2
zmp_ph108_H5
zmp_ph108_B2
zmp_ph108_B6
zmp_ph108_B7
zmp_ph108_C4
zmp_ph108_C5
zmp_ph108_D4
zmp_ph97_G6
zmp_ph97_G9
zmp_ph97_H2
zmp_ph97_B10
zmp_ph97_C2
zmp_ph97_C4
zmp_ph97_B8
zmp_ph97_C1
zmp_ph97_D2
zmp_ph97_D5
zmp_ph111_G5
zmp_ph111_G11
zmp_ph111_H1
zmp_ph111_B1a
zmp_ph111_B5
zmp_ph111_B8
zmp_ph111_B6
zmp_ph111_B7
zmp_ph111_B9
zmp_ph111_B10
zmp_ph114_G4
zmp_ph114_G11
zmp_ph114_H1
zmp_ph114_H2
zmp_ph114_A9
zmp_ph114_A11
zmp_ph114_B2
zmp_ph114_B4
zmp_ph114_D8
zmp_ph114_E2

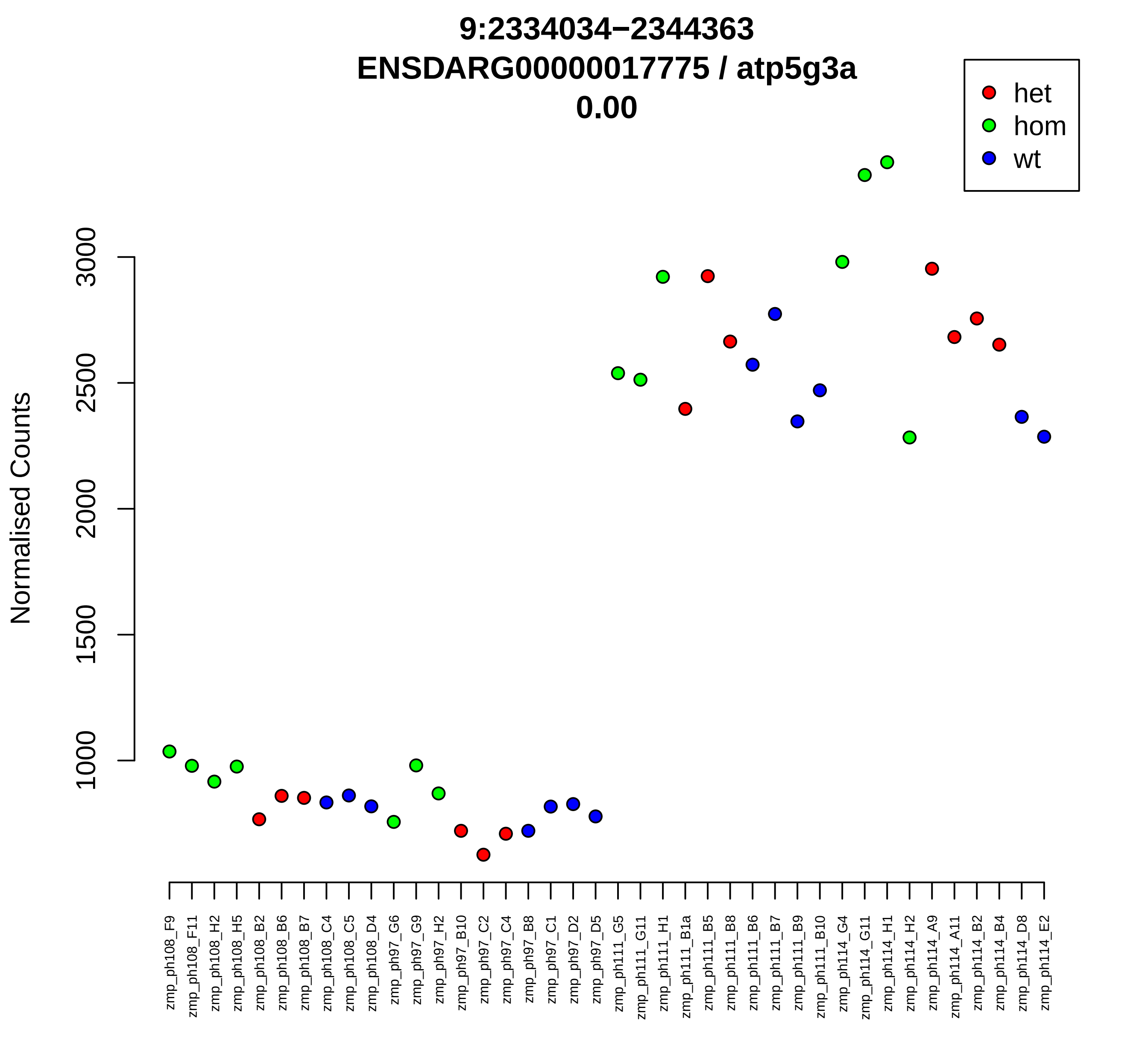
9:2334034−2344363
ENSDARG00000017775 / atp5g3a
0.00
het
hom
wt
Normalised Counts
1000
1500
2000
2500
3000
zmp_ph108_F9
zmp_ph108_F11
zmp_ph108_H2
zmp_ph108_H5
zmp_ph108_B2
zmp_ph108_B6
zmp_ph108_B7
zmp_ph108_C4
zmp_ph108_C5
zmp_ph108_D4
zmp_ph97_G6
zmp_ph97_G9
zmp_ph97_H2
zmp_ph97_B10
zmp_ph97_C2
zmp_ph97_C4
zmp_ph97_B8
zmp_ph97_C1
zmp_ph97_D2
zmp_ph97_D5
zmp_ph111_G5
zmp_ph111_G11
zmp_ph111_H1
zmp_ph111_B1a
zmp_ph111_B5
zmp_ph111_B8
zmp_ph111_B6
zmp_ph111_B7
zmp_ph111_B9
zmp_ph111_B10
zmp_ph114_G4
zmp_ph114_G11
zmp_ph114_H1
zmp_ph114_H2
zmp_ph114_A9
zmp_ph114_A11
zmp_ph114_B2
zmp_ph114_B4
zmp_ph114_D8
zmp_ph114_E2

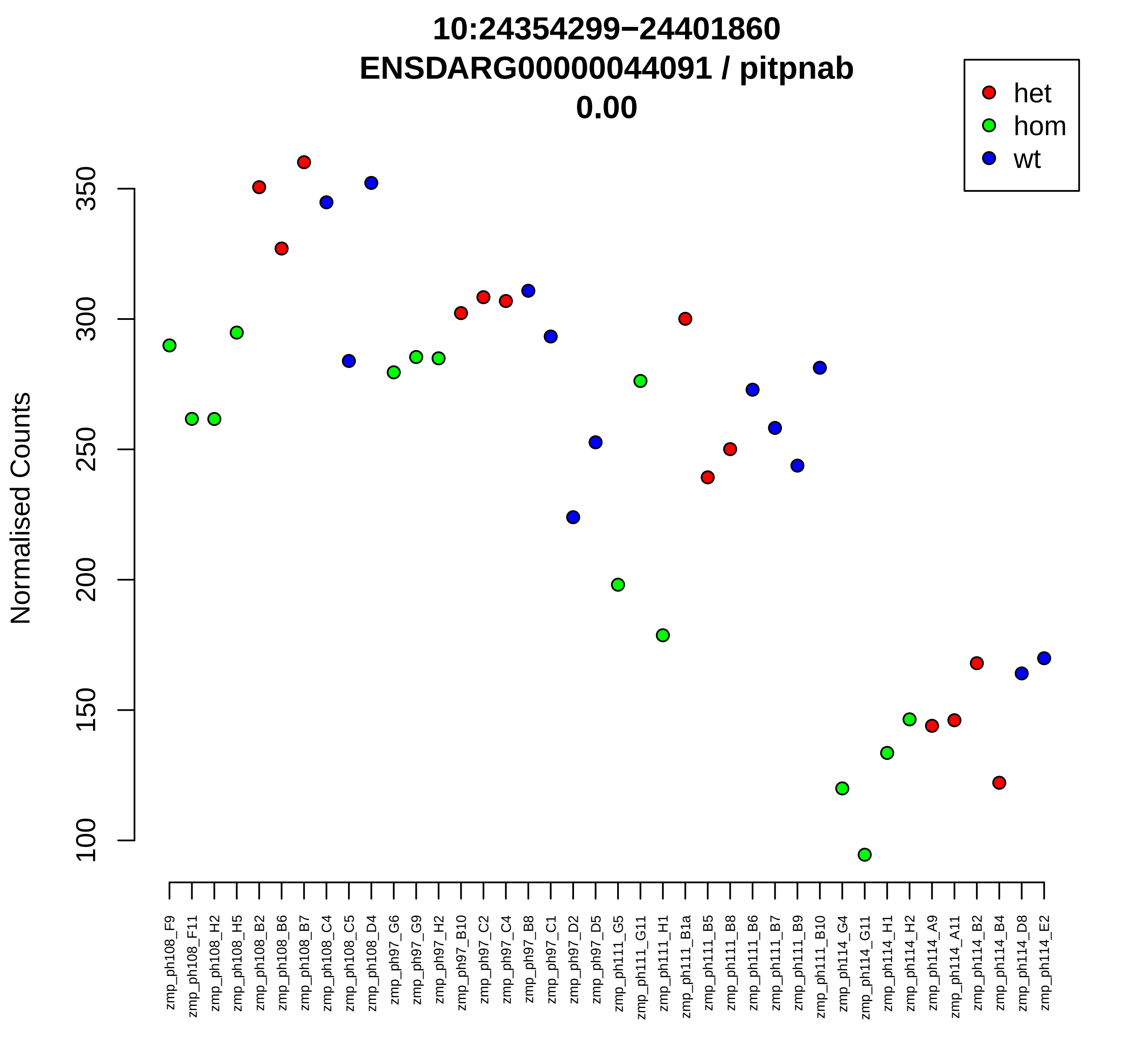
10:24354299−24401860
ENSDARG00000044091 / pitpnab
0.00
het
hom
wt
Normalised Counts
100
150
200
250
300
350
zmp_ph108_F9
zmp_ph108_F11
zmp_ph108_H2
zmp_ph108_H5
zmp_ph108_B2
zmp_ph108_B6
zmp_ph108_B7
zmp_ph108_C4
zmp_ph108_C5
zmp_ph108_D4
zmp_ph97_G6
zmp_ph97_G9
zmp_ph97_H2
zmp_ph97_B10
zmp_ph97_C2
zmp_ph97_C4
zmp_ph97_B8
zmp_ph97_C1
zmp_ph97_D2
zmp_ph97_D5
zmp_ph111_G5
zmp_ph111_G11
zmp_ph111_H1
zmp_ph111_B1a
zmp_ph111_B5
zmp_ph111_B8
zmp_ph111_B6
zmp_ph111_B7
zmp_ph111_B9
zmp_ph111_B10
zmp_ph114_G4
zmp_ph114_G11
zmp_ph114_H1
zmp_ph114_H2
zmp_ph114_A9
zmp_ph114_A11
zmp_ph114_B2
zmp_ph114_B4
zmp_ph114_D8
zmp_ph114_E2

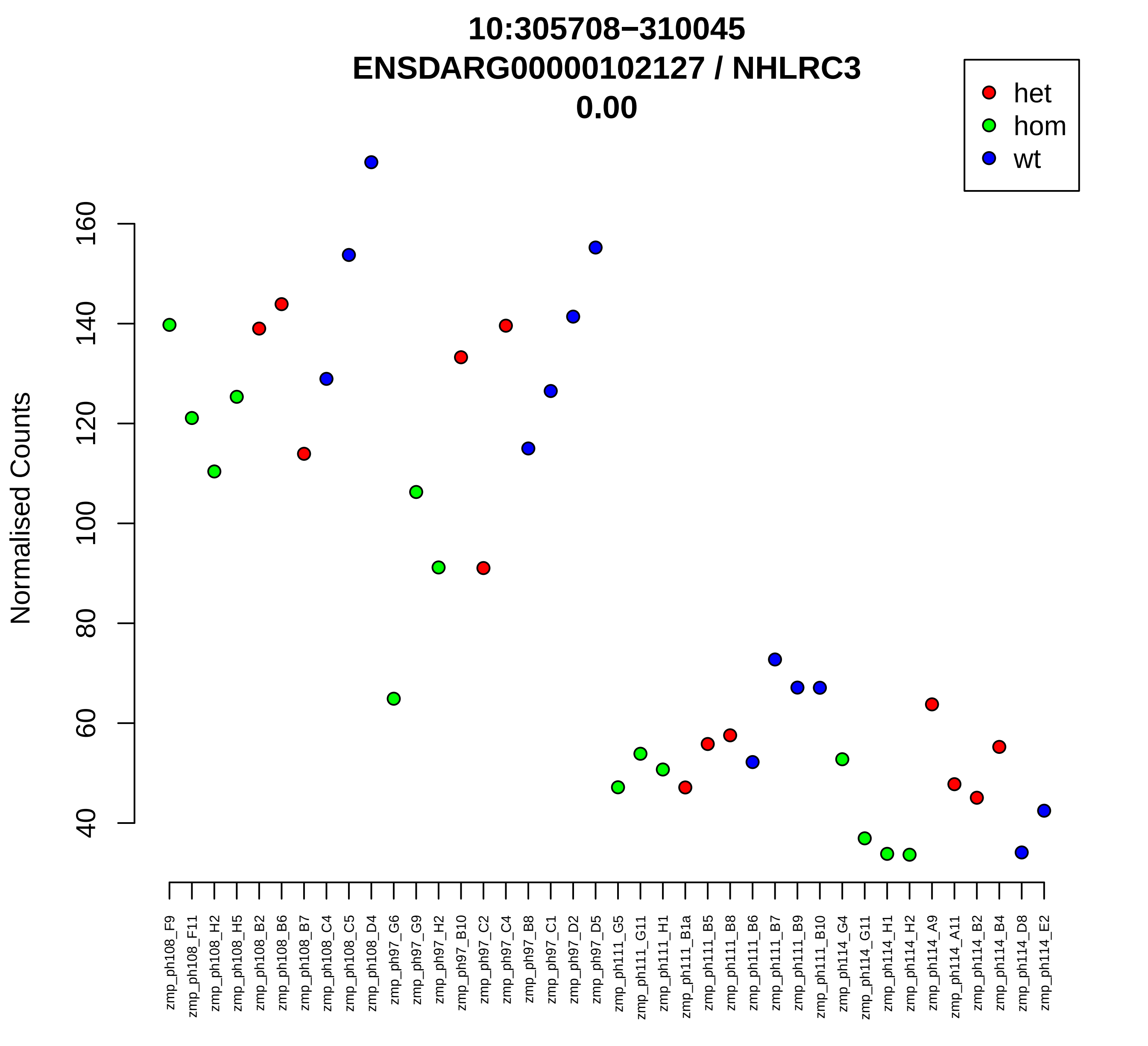
10:305708−310045
ENSDARG00000102127 / NHLRC3
0.00
het
hom
wt
Normalised Counts
40
60
80
100
120
140
160
zmp_ph108_F9
zmp_ph108_F11
zmp_ph108_H2
zmp_ph108_H5
zmp_ph108_B2
zmp_ph108_B6
zmp_ph108_B7
zmp_ph108_C4
zmp_ph108_C5
zmp_ph108_D4
zmp_ph97_G6
zmp_ph97_G9
zmp_ph97_H2
zmp_ph97_B10
zmp_ph97_C2
zmp_ph97_C4
zmp_ph97_B8
zmp_ph97_C1
zmp_ph97_D2
zmp_ph97_D5
zmp_ph111_G5
zmp_ph111_G11
zmp_ph111_H1
zmp_ph111_B1a
zmp_ph111_B5
zmp_ph111_B8
zmp_ph111_B6
zmp_ph111_B7
zmp_ph111_B9
zmp_ph111_B10
zmp_ph114_G4
zmp_ph114_G11
zmp_ph114_H1
zmp_ph114_H2
zmp_ph114_A9
zmp_ph114_A11
zmp_ph114_B2
zmp_ph114_B4
zmp_ph114_D8
zmp_ph114_E2

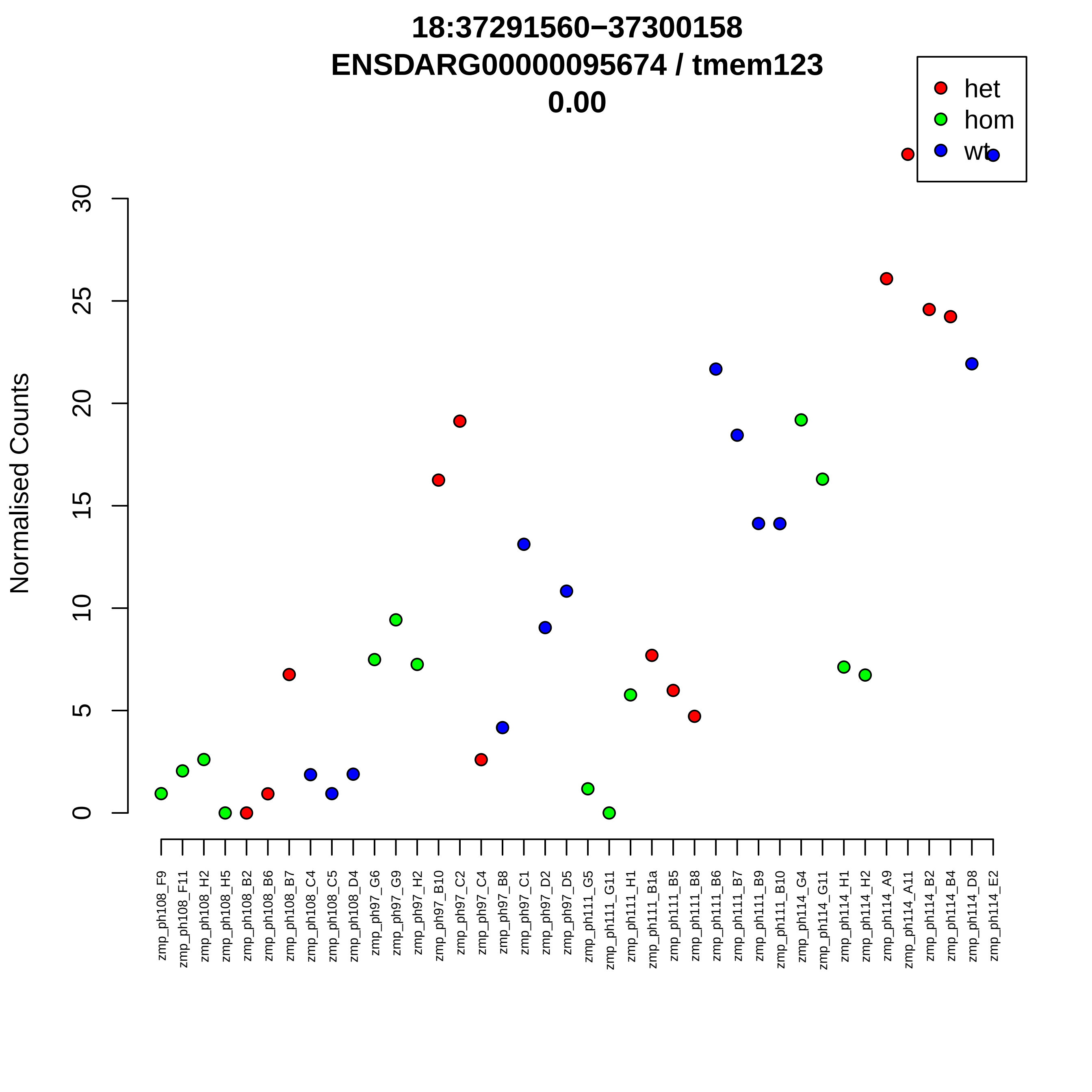

18:37291560−37300158
ENSDARG00000095674 / tmem123
0.00
het
hom
wt
Normalised Counts
0
5
10
15
20
25
30
zmp_ph108_F9
zmp_ph108_F11
zmp_ph108_H2
zmp_ph108_H5
zmp_ph108_B2
zmp_ph108_B6
zmp_ph108_B7
zmp_ph108_C4
zmp_ph108_C5
zmp_ph108_D4
zmp_ph97_G6
zmp_ph97_G9
zmp_ph97_H2
zmp_ph97_B10
zmp_ph97_C2
zmp_ph97_C4
zmp_ph97_B8
zmp_ph97_C1
zmp_ph97_D2
zmp_ph97_D5
zmp_ph111_G5
zmp_ph111_G11
zmp_ph111_H1
zmp_ph111_B1a
zmp_ph111_B5
zmp_ph111_B8
zmp_ph111_B6
zmp_ph111_B7
zmp_ph111_B9
zmp_ph111_B10
zmp_ph114_G4
zmp_ph114_G11
zmp_ph114_H1
zmp_ph114_H2
zmp_ph114_A9
zmp_ph114_A11
zmp_ph114_B2
zmp_ph114_B4
zmp_ph114_D8
zmp_ph114_E2

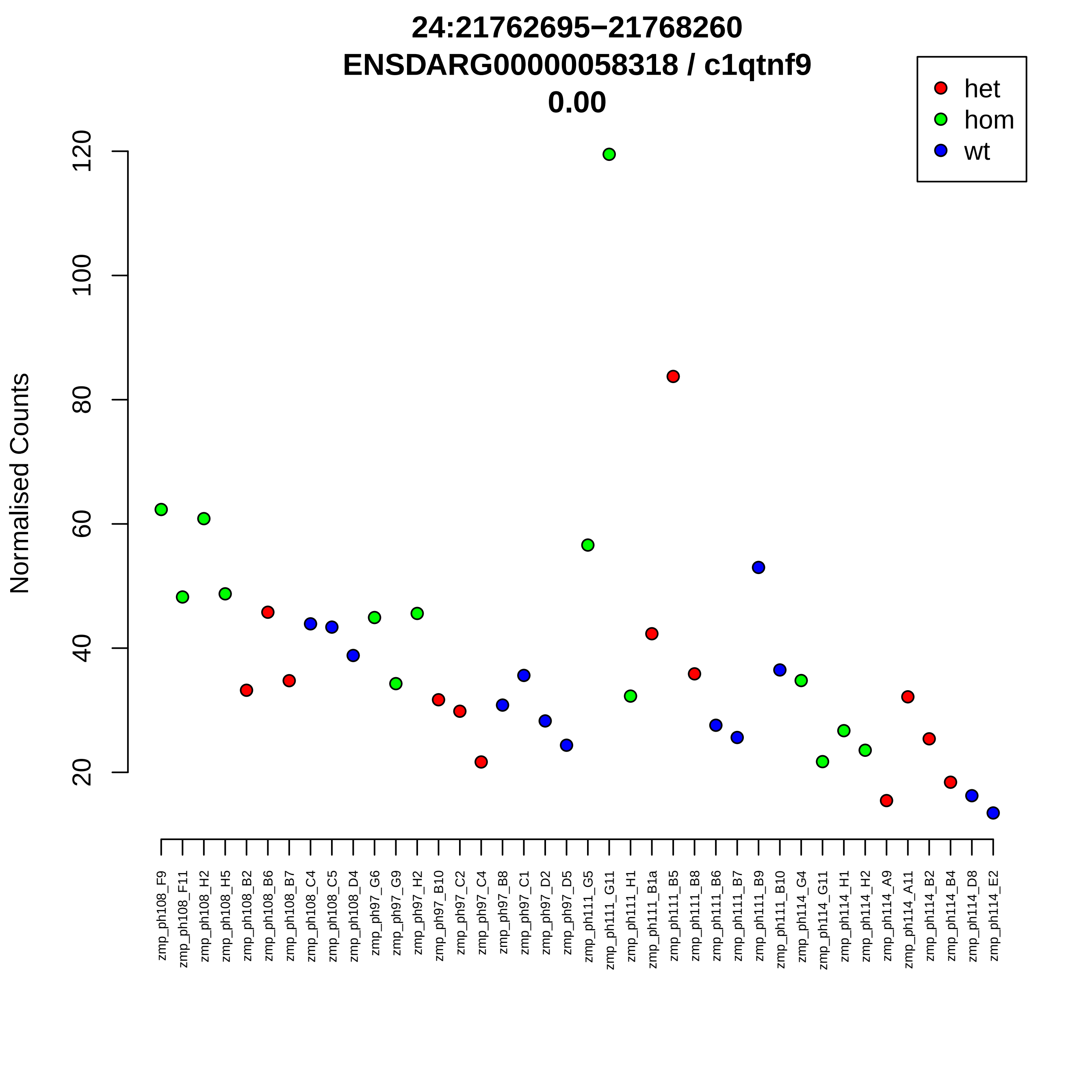

24:21762695−21768260
ENSDARG00000058318 / c1qtnf9
0.00
het
hom
wt
Normalised Counts
20
40
60
80
100
120
zmp_ph108_F9
zmp_ph108_F11
zmp_ph108_H2
zmp_ph108_H5
zmp_ph108_B2
zmp_ph108_B6
zmp_ph108_B7
zmp_ph108_C4
zmp_ph108_C5
zmp_ph108_D4
zmp_ph97_G6
zmp_ph97_G9
zmp_ph97_H2
zmp_ph97_B10
zmp_ph97_C2
zmp_ph97_C4
zmp_ph97_B8
zmp_ph97_C1
zmp_ph97_D2
zmp_ph97_D5
zmp_ph111_G5
zmp_ph111_G11
zmp_ph111_H1
zmp_ph111_B1a
zmp_ph111_B5
zmp_ph111_B8
zmp_ph111_B6
zmp_ph111_B7
zmp_ph111_B9
zmp_ph111_B10
zmp_ph114_G4
zmp_ph114_G11
zmp_ph114_H1
zmp_ph114_H2
zmp_ph114_A9
zmp_ph114_A11
zmp_ph114_B2
zmp_ph114_B4
zmp_ph114_D8
zmp_ph114_E2

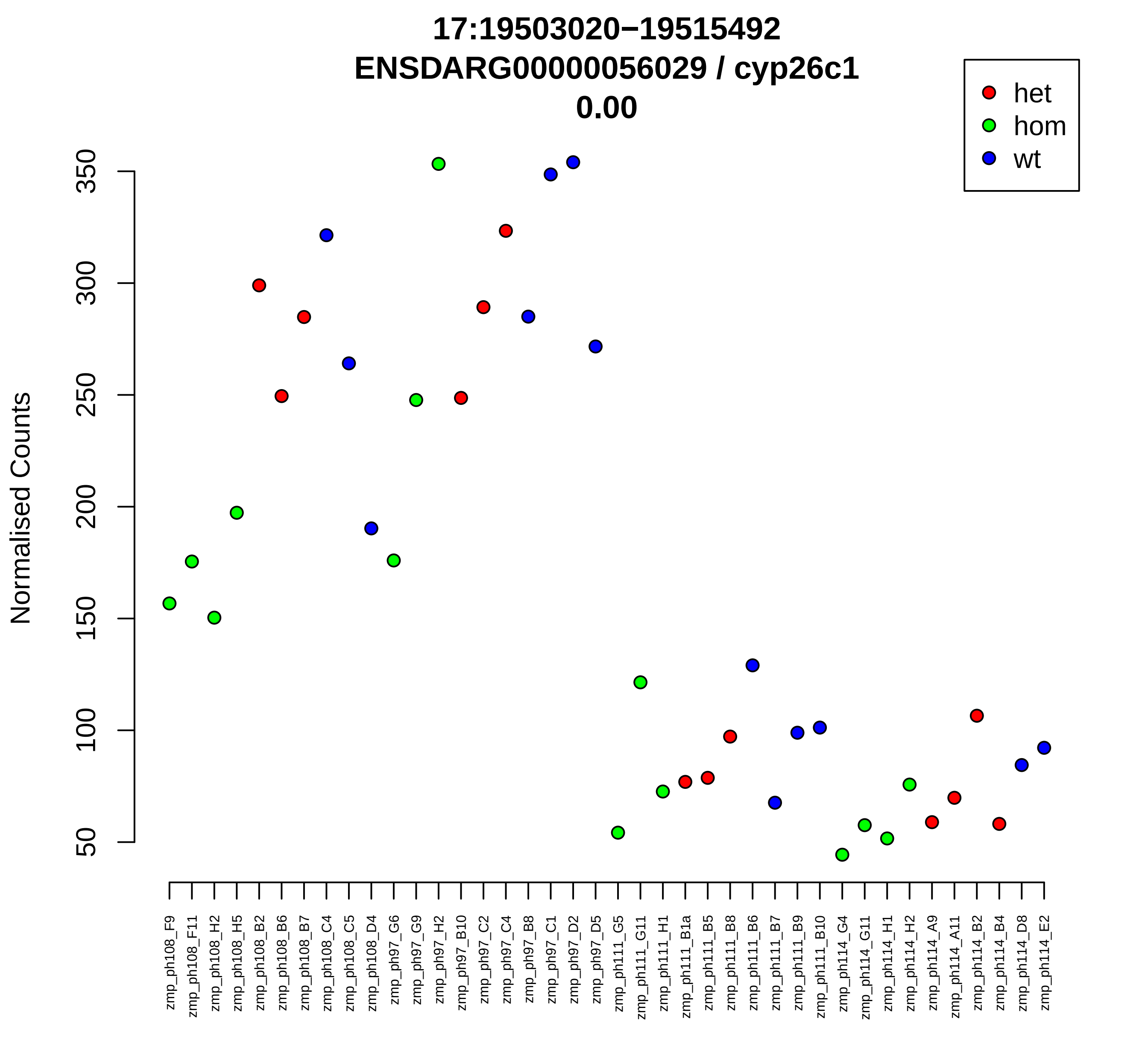

17:19503020−19515492
ENSDARG00000056029 / cyp26c1
0.00
het
hom
wt
Normalised Counts
50
100
150
200
250
300
350
zmp_ph108_F9
zmp_ph108_F11
zmp_ph108_H2
zmp_ph108_H5
zmp_ph108_B2
zmp_ph108_B6
zmp_ph108_B7
zmp_ph108_C4
zmp_ph108_C5
zmp_ph108_D4
zmp_ph97_G6
zmp_ph97_G9
zmp_ph97_H2
zmp_ph97_B10
zmp_ph97_C2
zmp_ph97_C4
zmp_ph97_B8
zmp_ph97_C1
zmp_ph97_D2
zmp_ph97_D5
zmp_ph111_G5
zmp_ph111_G11
zmp_ph111_H1
zmp_ph111_B1a
zmp_ph111_B5
zmp_ph111_B8
zmp_ph111_B6
zmp_ph111_B7
zmp_ph111_B9
zmp_ph111_B10
zmp_ph114_G4
zmp_ph114_G11
zmp_ph114_H1
zmp_ph114_H2
zmp_ph114_A9
zmp_ph114_A11
zmp_ph114_B2
zmp_ph114_B4
zmp_ph114_D8
zmp_ph114_E2

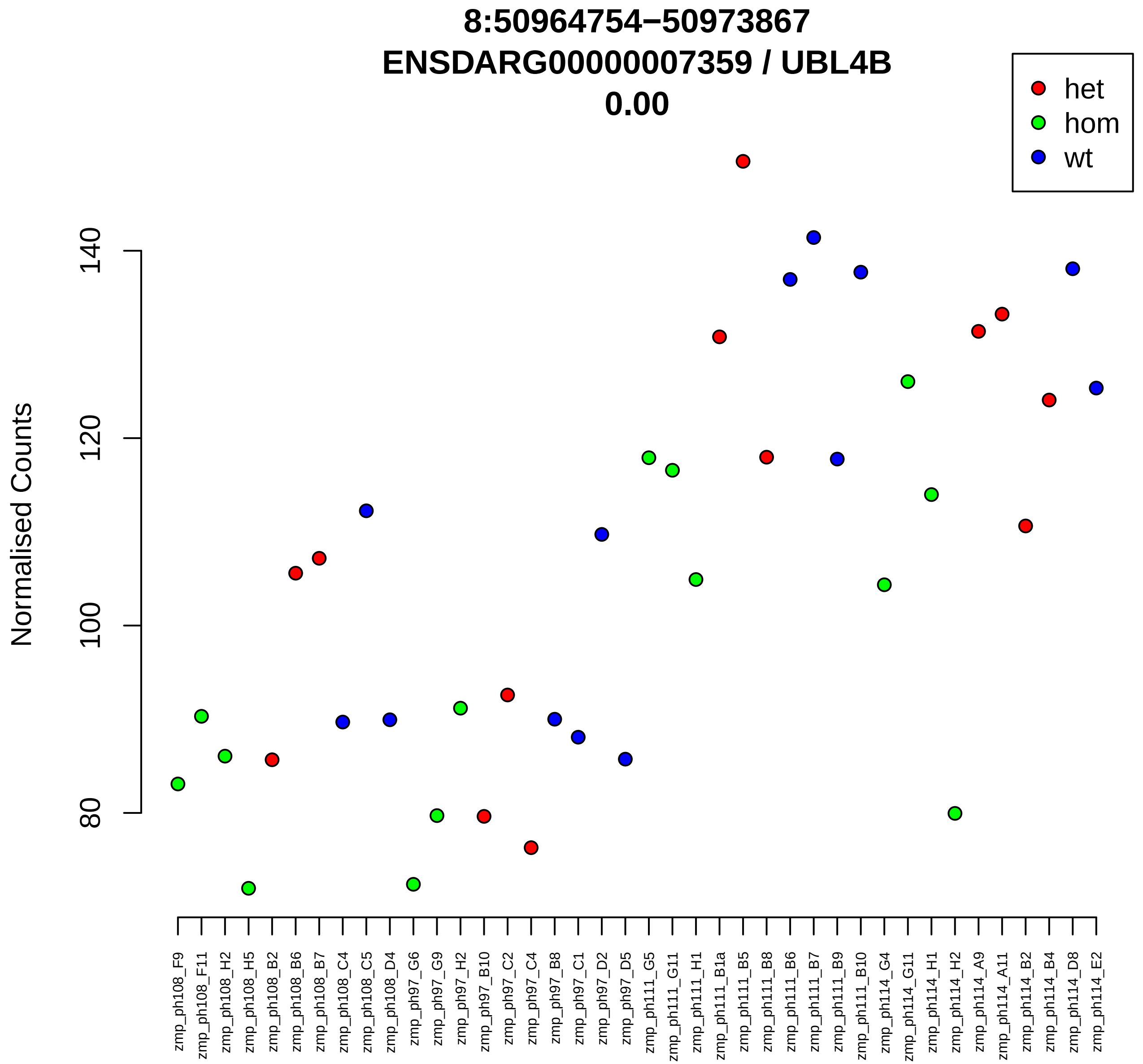

8:50964754−50973867
ENSDARG00000007359 / UBL4B
0.00
het
hom
wt
Normalised Counts
80
100
120
140
zmp_ph108_F9
zmp_ph108_F11
zmp_ph108_H2
zmp_ph108_H5
zmp_ph108_B2
zmp_ph108_B6
zmp_ph108_B7
zmp_ph108_C4
zmp_ph108_C5
zmp_ph108_D4
zmp_ph97_G6
zmp_ph97_G9
zmp_ph97_H2
zmp_ph97_B10
zmp_ph97_C2
zmp_ph97_C4
zmp_ph97_B8
zmp_ph97_C1
zmp_ph97_D2
zmp_ph97_D5
zmp_ph111_G5
zmp_ph111_G11
zmp_ph111_H1
zmp_ph111_B1a
zmp_ph111_B5
zmp_ph111_B8
zmp_ph111_B6
zmp_ph111_B7
zmp_ph111_B9
zmp_ph111_B10
zmp_ph114_G4
zmp_ph114_G11
zmp_ph114_H1
zmp_ph114_H2
zmp_ph114_A9
zmp_ph114_A11
zmp_ph114_B2
zmp_ph114_B4
zmp_ph114_D8
zmp_ph114_E2

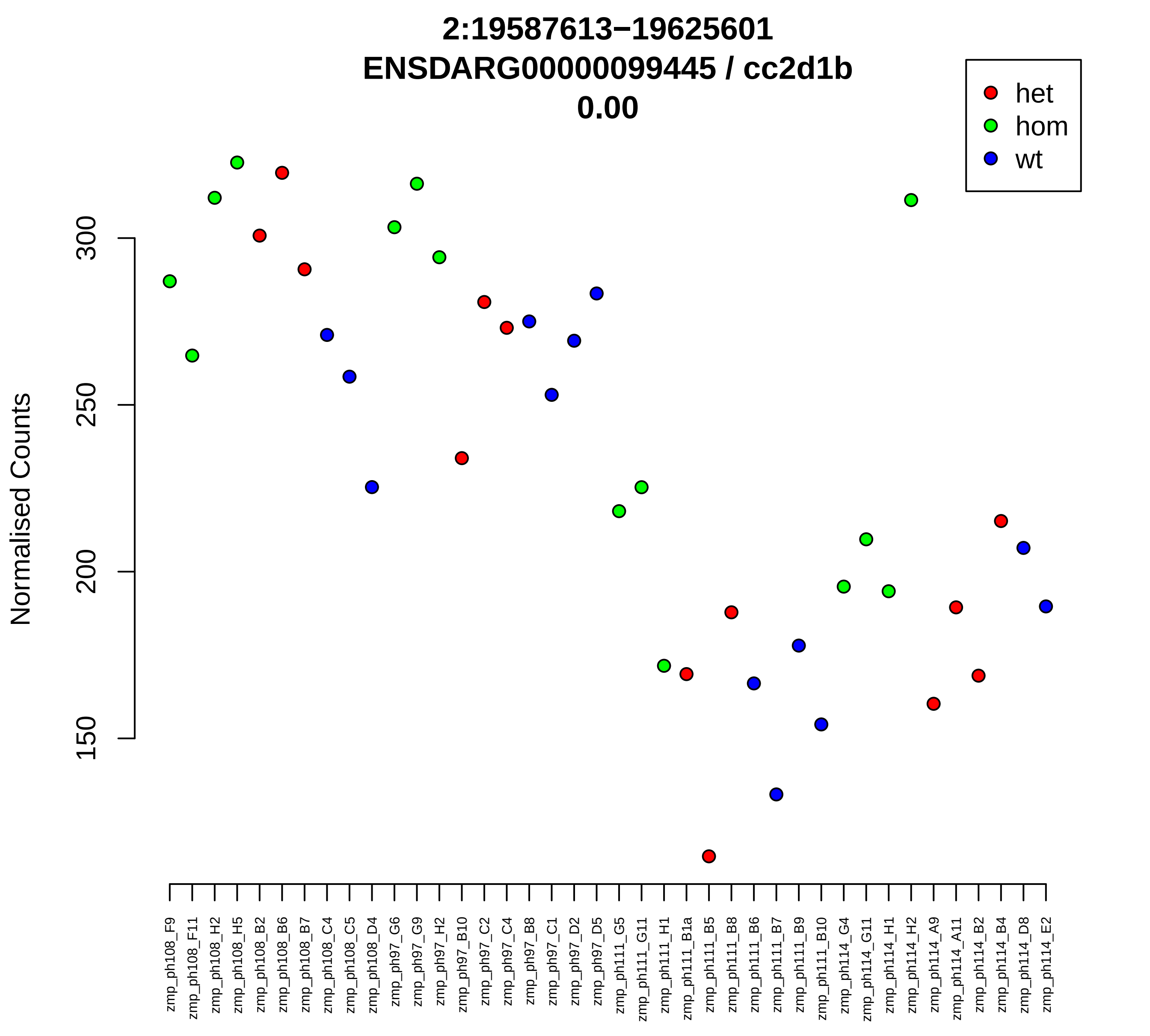

2:19587613−19625601
ENSDARG00000099445 / cc2d1b
0.00
het
hom
wt
Normalised Counts
150
200
250
300
zmp_ph108_F9
zmp_ph108_F11
zmp_ph108_H2
zmp_ph108_H5
zmp_ph108_B2
zmp_ph108_B6
zmp_ph108_B7
zmp_ph108_C4
zmp_ph108_C5
zmp_ph108_D4
zmp_ph97_G6
zmp_ph97_G9
zmp_ph97_H2
zmp_ph97_B10
zmp_ph97_C2
zmp_ph97_C4
zmp_ph97_B8
zmp_ph97_C1
zmp_ph97_D2
zmp_ph97_D5
zmp_ph111_G5
zmp_ph111_G11
zmp_ph111_H1
zmp_ph111_B1a
zmp_ph111_B5
zmp_ph111_B8
zmp_ph111_B6
zmp_ph111_B7
zmp_ph111_B9
zmp_ph111_B10
zmp_ph114_G4
zmp_ph114_G11
zmp_ph114_H1
zmp_ph114_H2
zmp_ph114_A9
zmp_ph114_A11
zmp_ph114_B2
zmp_ph114_B4
zmp_ph114_D8
zmp_ph114_E2

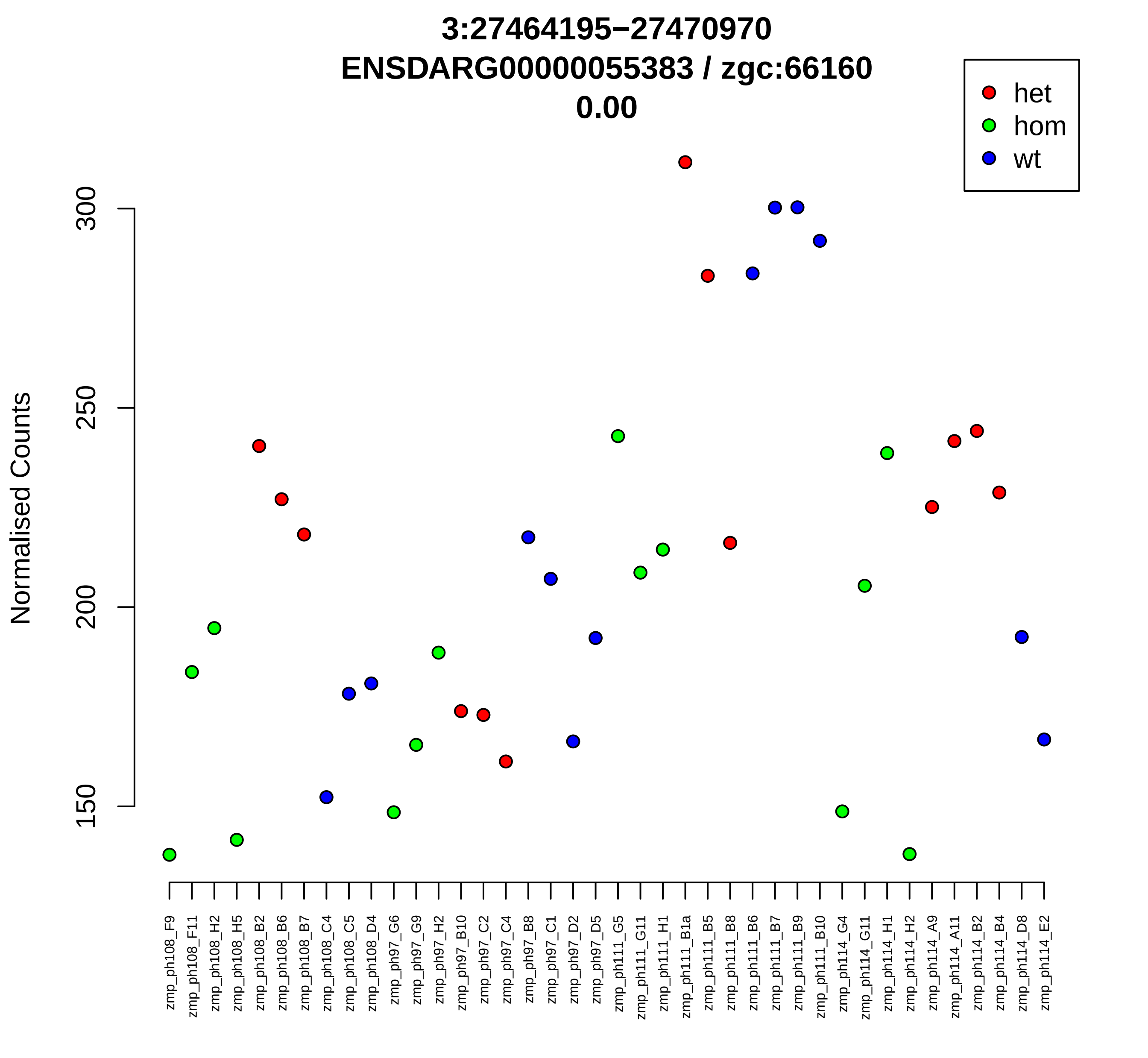

3:27464195−27470970
ENSDARG00000055383 / zgc:66160
0.00
het
hom
wt
Normalised Counts
150
200
250
300
zmp_ph108_F9
zmp_ph108_F11
zmp_ph108_H2
zmp_ph108_H5
zmp_ph108_B2
zmp_ph108_B6
zmp_ph108_B7
zmp_ph108_C4
zmp_ph108_C5
zmp_ph108_D4
zmp_ph97_G6
zmp_ph97_G9
zmp_ph97_H2
zmp_ph97_B10
zmp_ph97_C2
zmp_ph97_C4
zmp_ph97_B8
zmp_ph97_C1
zmp_ph97_D2
zmp_ph97_D5
zmp_ph111_G5
zmp_ph111_G11
zmp_ph111_H1
zmp_ph111_B1a
zmp_ph111_B5
zmp_ph111_B8
zmp_ph111_B6
zmp_ph111_B7
zmp_ph111_B9
zmp_ph111_B10
zmp_ph114_G4
zmp_ph114_G11
zmp_ph114_H1
zmp_ph114_H2
zmp_ph114_A9
zmp_ph114_A11
zmp_ph114_B2
zmp_ph114_B4
zmp_ph114_D8
zmp_ph114_E2

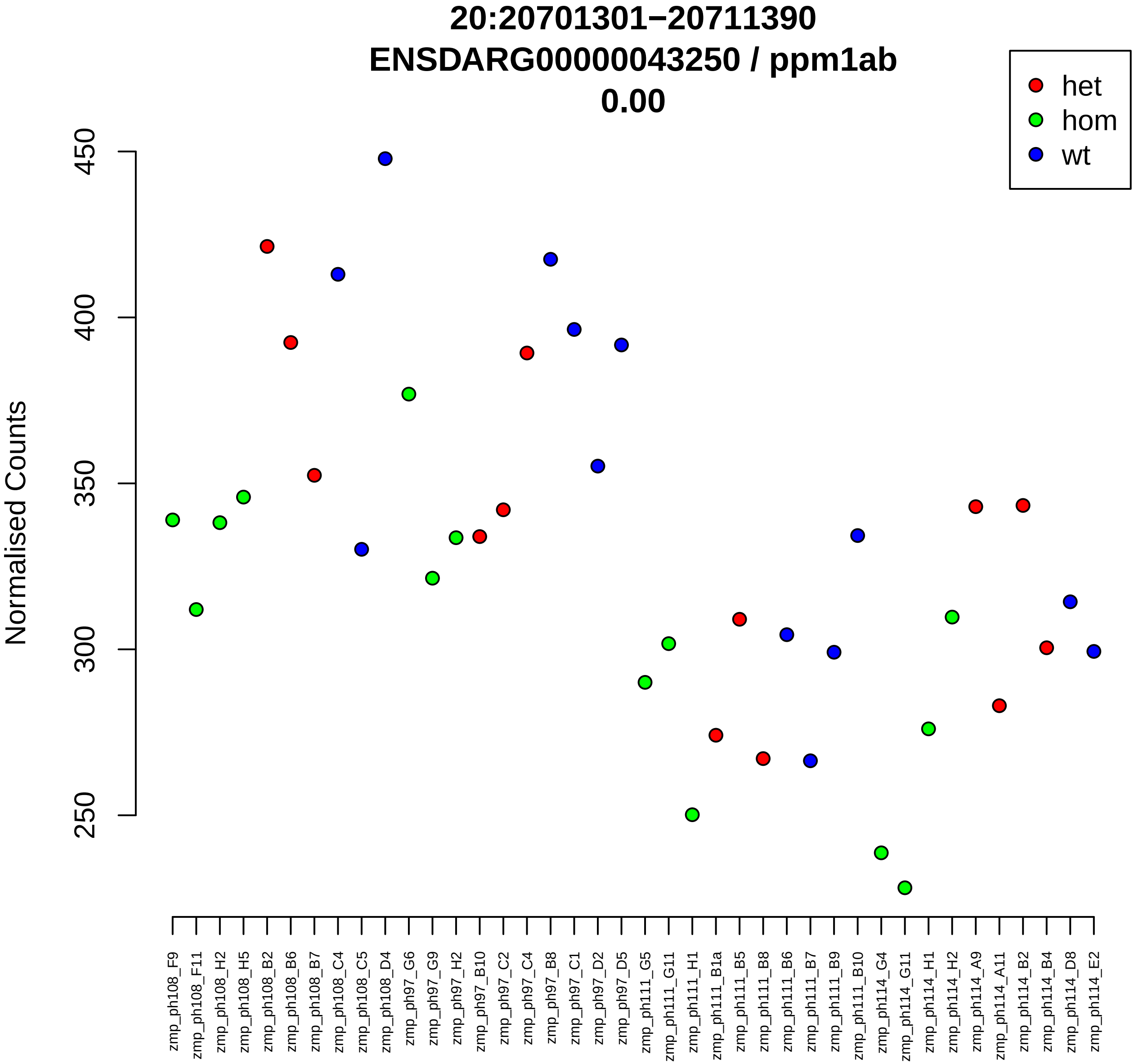

20:20701301−20711390
ENSDARG00000043250 / ppm1ab
0.00
het
hom
wt
Normalised Counts
450
400
350
300
250
zmp_ph108_F9
zmp_ph108_F11
zmp_ph108_H2
zmp_ph108_H5
zmp_ph108_B2
zmp_ph108_B6
zmp_ph108_B7
zmp_ph108_C4
zmp_ph108_C5
zmp_ph108_D4
zmp_ph97_G6
zmp_ph97_G9
zmp_ph97_H2
zmp_ph97_B10
zmp_ph97_C2
zmp_ph97_C4
zmp_ph97_B8
zmp_ph97_C1
zmp_ph97_D2
zmp_ph97_D5
zmp_ph111_G5
zmp_ph111_G11
zmp_ph111_H1
zmp_ph111_B1a
zmp_ph111_B5
zmp_ph111_B8
zmp_ph111_B6
zmp_ph111_B7
zmp_ph111_B9
zmp_ph111_B10
zmp_ph114_G4
zmp_ph114_G11
zmp_ph114_H1
zmp_ph114_H2
zmp_ph114_A9
zmp_ph114_A11
zmp_ph114_B2
zmp_ph114_B4
zmp_ph114_D8
zmp_ph114_E2

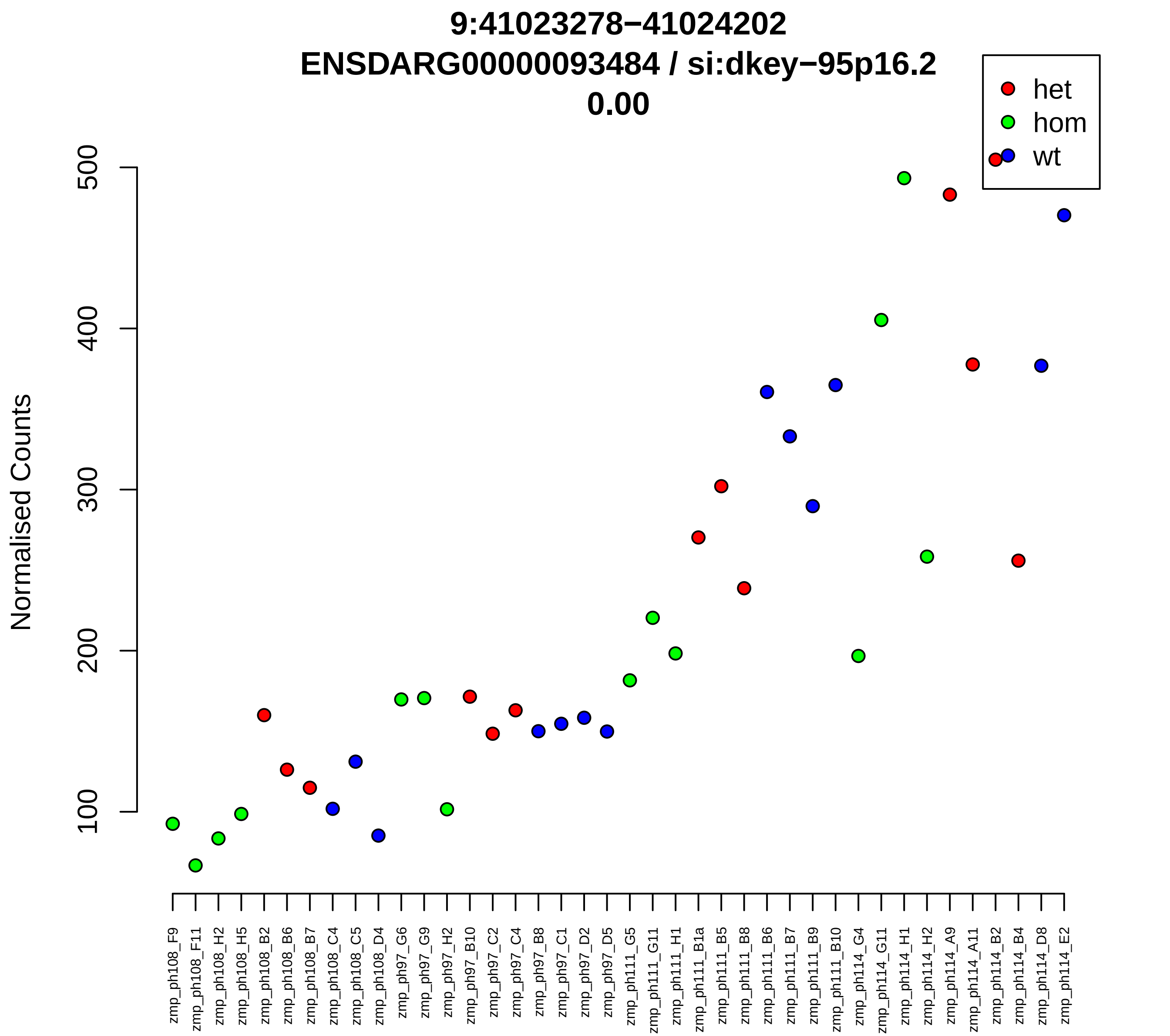

9:41023278−41024202
ENSDARG00000093484 / si:dkey−95p16.2
0.00
het
hom
wt
Normalised Counts
100
200
300
400
500
zmp_ph108_F9
zmp_ph108_F11
zmp_ph108_H2
zmp_ph108_H5
zmp_ph108_B2
zmp_ph108_B6
zmp_ph108_B7
zmp_ph108_C4
zmp_ph108_C5
zmp_ph108_D4
zmp_ph97_G6
zmp_ph97_G9
zmp_ph97_H2
zmp_ph97_B10
zmp_ph97_C2
zmp_ph97_C4
zmp_ph97_B8
zmp_ph97_C1
zmp_ph97_D2
zmp_ph97_D5
zmp_ph111_G5
zmp_ph111_G11
zmp_ph111_H1
zmp_ph111_B1a
zmp_ph111_B5
zmp_ph111_B8
zmp_ph111_B6
zmp_ph111_B7
zmp_ph111_B9
zmp_ph111_B10
zmp_ph114_G4
zmp_ph114_G11
zmp_ph114_H1
zmp_ph114_H2
zmp_ph114_A9
zmp_ph114_A11
zmp_ph114_B2
zmp_ph114_B4
zmp_ph114_D8
zmp_ph114_E2

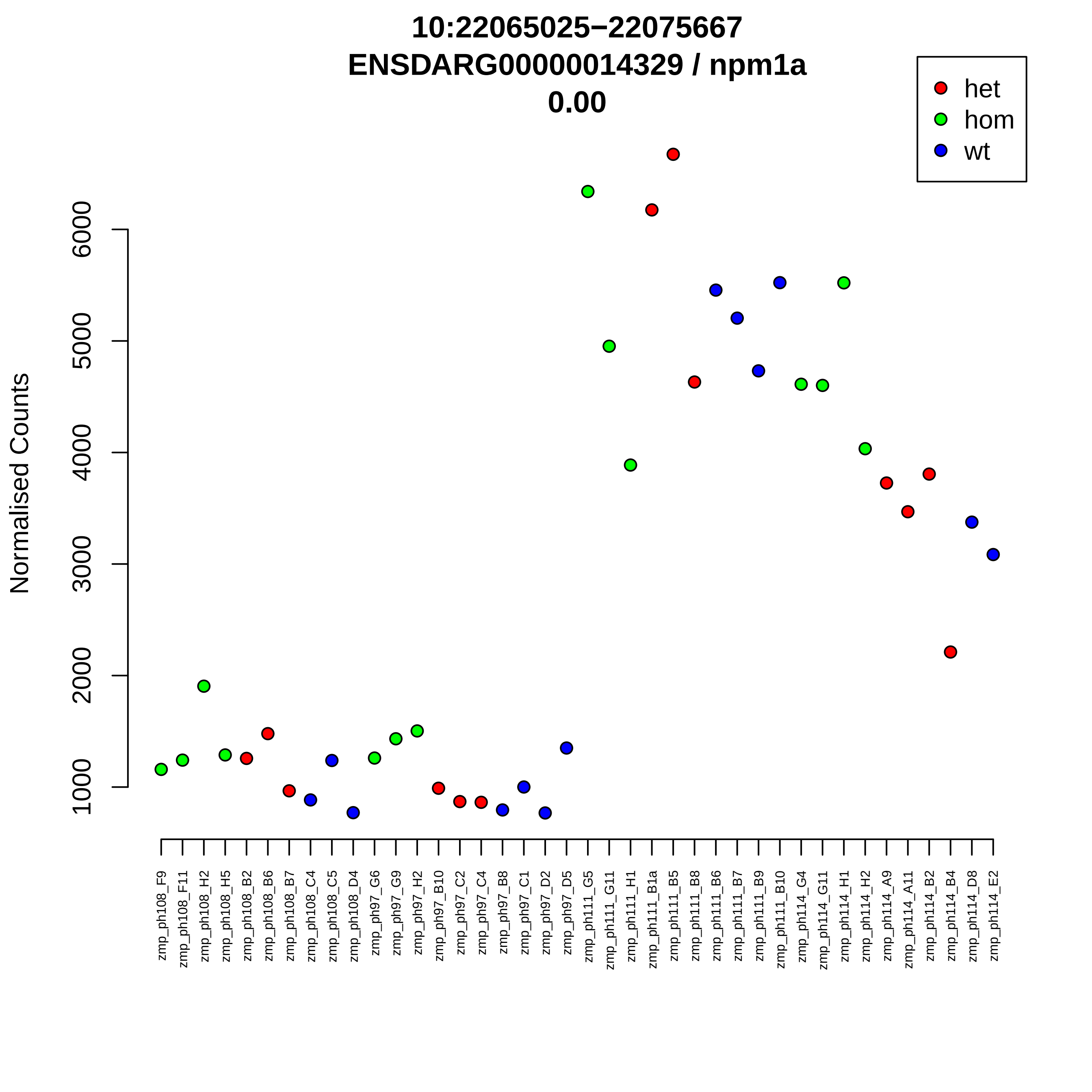

10:22065025−22075667
ENSDARG00000014329 / npm1a
0.00
het
hom
wt
Normalised Counts
1000
2000
3000
4000
5000
6000
zmp_ph108_F9
zmp_ph108_F11
zmp_ph108_H2
zmp_ph108_H5
zmp_ph108_B2
zmp_ph108_B6
zmp_ph108_B7
zmp_ph108_C4
zmp_ph108_C5
zmp_ph108_D4
zmp_ph97_G6
zmp_ph97_G9
zmp_ph97_H2
zmp_ph97_B10
zmp_ph97_C2
zmp_ph97_C4
zmp_ph97_B8
zmp_ph97_C1
zmp_ph97_D2
zmp_ph97_D5
zmp_ph111_G5
zmp_ph111_G11
zmp_ph111_H1
zmp_ph111_B1a
zmp_ph111_B5
zmp_ph111_B8
zmp_ph111_B6
zmp_ph111_B7
zmp_ph111_B9
zmp_ph111_B10
zmp_ph114_G4
zmp_ph114_G11
zmp_ph114_H1
zmp_ph114_H2
zmp_ph114_A9
zmp_ph114_A11
zmp_ph114_B2
zmp_ph114_B4
zmp_ph114_D8
zmp_ph114_E2

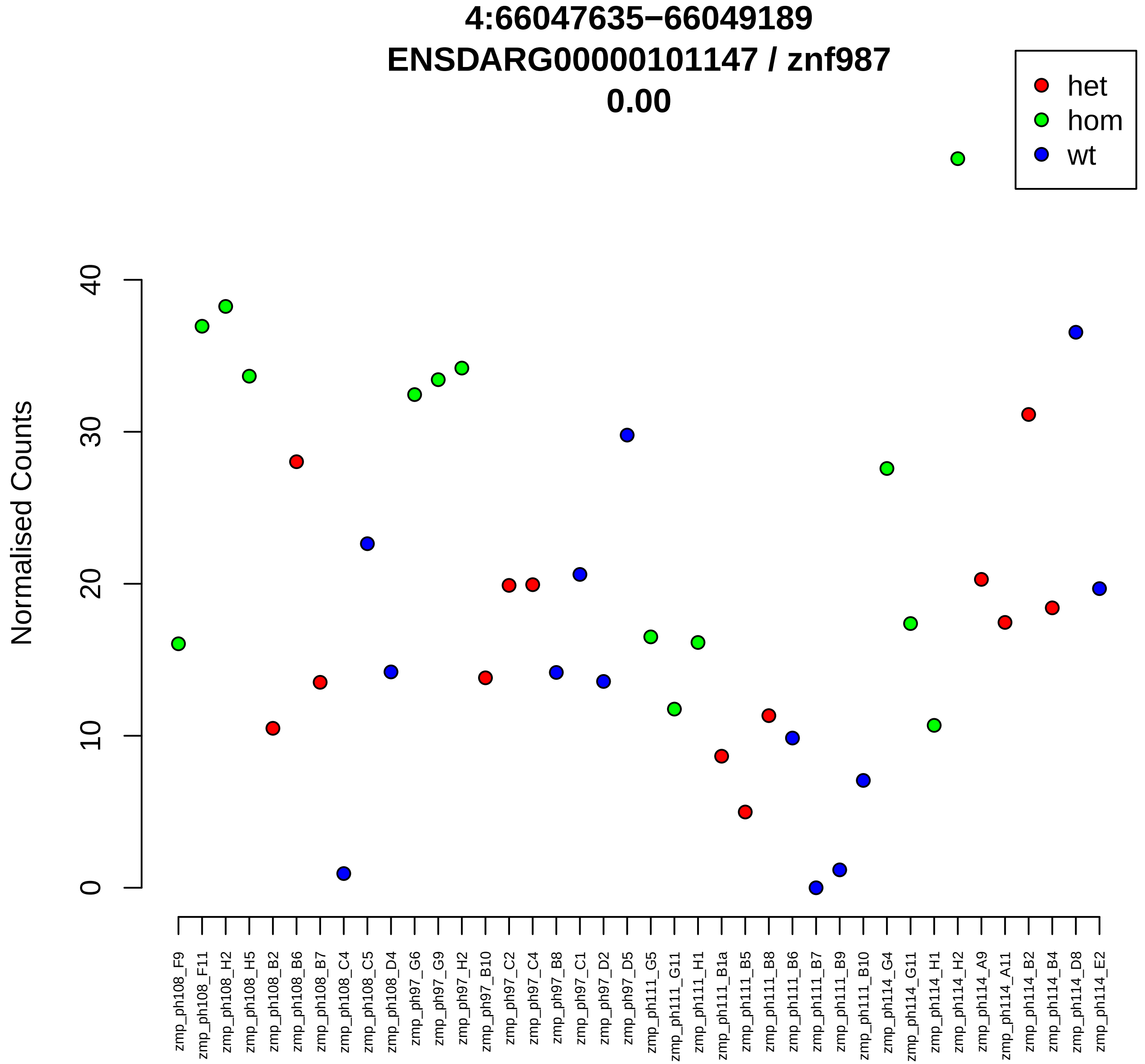
4:66047635−66049189
ENSDARG00000101147 / znf987
0.00
het
hom
wt
Normalised Counts
0
10
20
30
40
zmp_ph108_F9
zmp_ph108_F11
zmp_ph108_H2
zmp_ph108_H5
zmp_ph108_B2
zmp_ph108_B6
zmp_ph108_B7
zmp_ph108_C4
zmp_ph108_C5
zmp_ph108_D4
zmp_ph97_G6
zmp_ph97_G9
zmp_ph97_H2
zmp_ph97_B10
zmp_ph97_C2
zmp_ph97_C4
zmp_ph97_B8
zmp_ph97_C1
zmp_ph97_D2
zmp_ph97_D5
zmp_ph111_G5
zmp_ph111_G11
zmp_ph111_H1
zmp_ph111_B1a
zmp_ph111_B5
zmp_ph111_B8
zmp_ph111_B6
zmp_ph111_B7
zmp_ph111_B9
zmp_ph111_B10
zmp_ph114_G4
zmp_ph114_G11
zmp_ph114_H1
zmp_ph114_H2
zmp_ph114_A9
zmp_ph114_A11
zmp_ph114_B2
zmp_ph114_B4
zmp_ph114_D8
zmp_ph114_E2

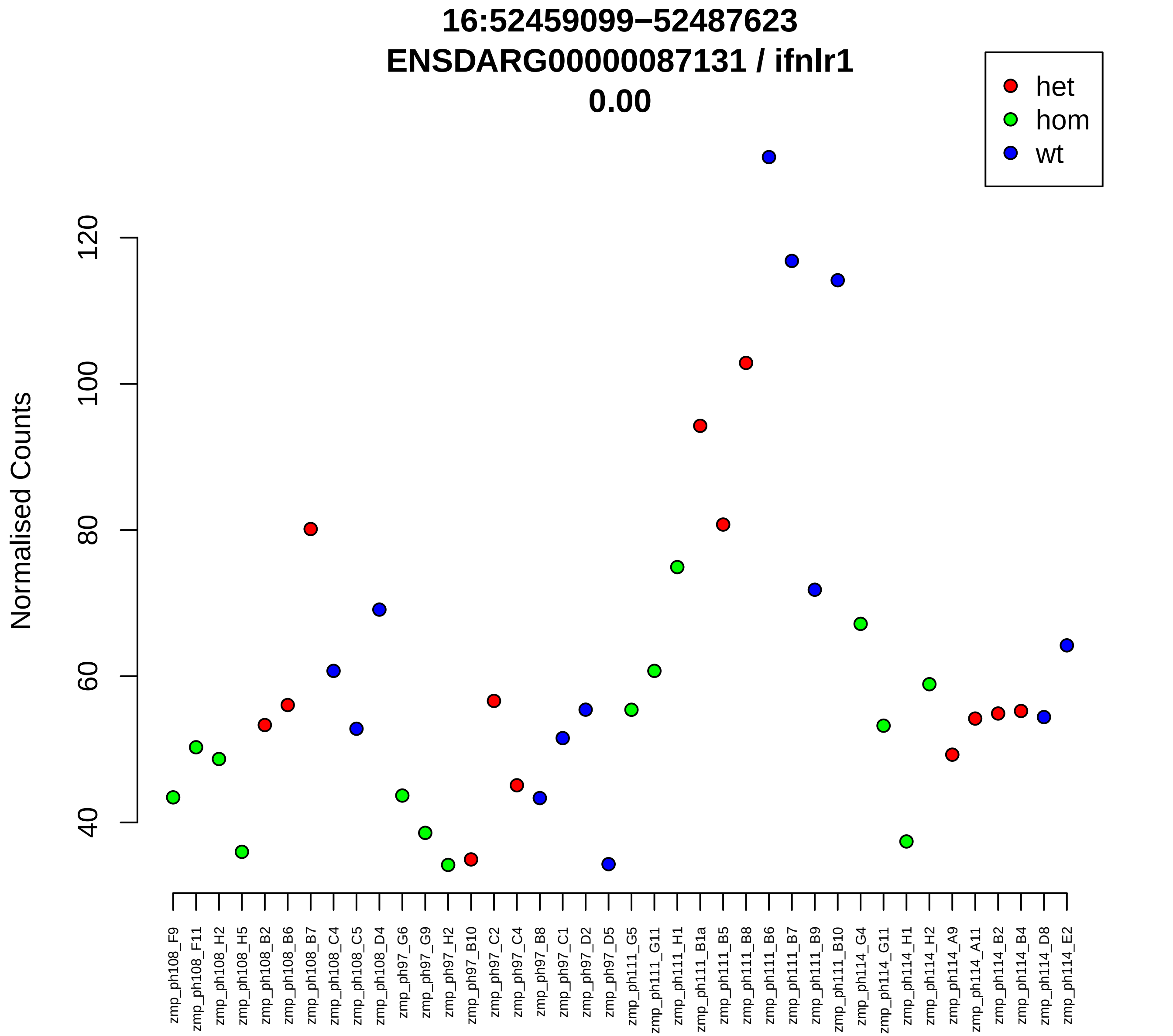
16:52459099−52487623
ENSDARG00000087131 / ifnlr1
0.00
het
hom
wt
Normalised Counts
40
60
80
100
120
zmp_ph108_F9
zmp_ph108_F11
zmp_ph108_H2
zmp_ph108_H5
zmp_ph108_B2
zmp_ph108_B6
zmp_ph108_B7
zmp_ph108_C4
zmp_ph108_C5
zmp_ph108_D4
zmp_ph97_G6
zmp_ph97_G9
zmp_ph97_H2
zmp_ph97_B10
zmp_ph97_C2
zmp_ph97_C4
zmp_ph97_B8
zmp_ph97_C1
zmp_ph97_D2
zmp_ph97_D5
zmp_ph111_G5
zmp_ph111_G11
zmp_ph111_H1
zmp_ph111_B1a
zmp_ph111_B5
zmp_ph111_B8
zmp_ph111_B6
zmp_ph111_B7
zmp_ph111_B9
zmp_ph111_B10
zmp_ph114_G4
zmp_ph114_G11
zmp_ph114_H1
zmp_ph114_H2
zmp_ph114_A9
zmp_ph114_A11
zmp_ph114_B2
zmp_ph114_B4
zmp_ph114_D8
zmp_ph114_E2

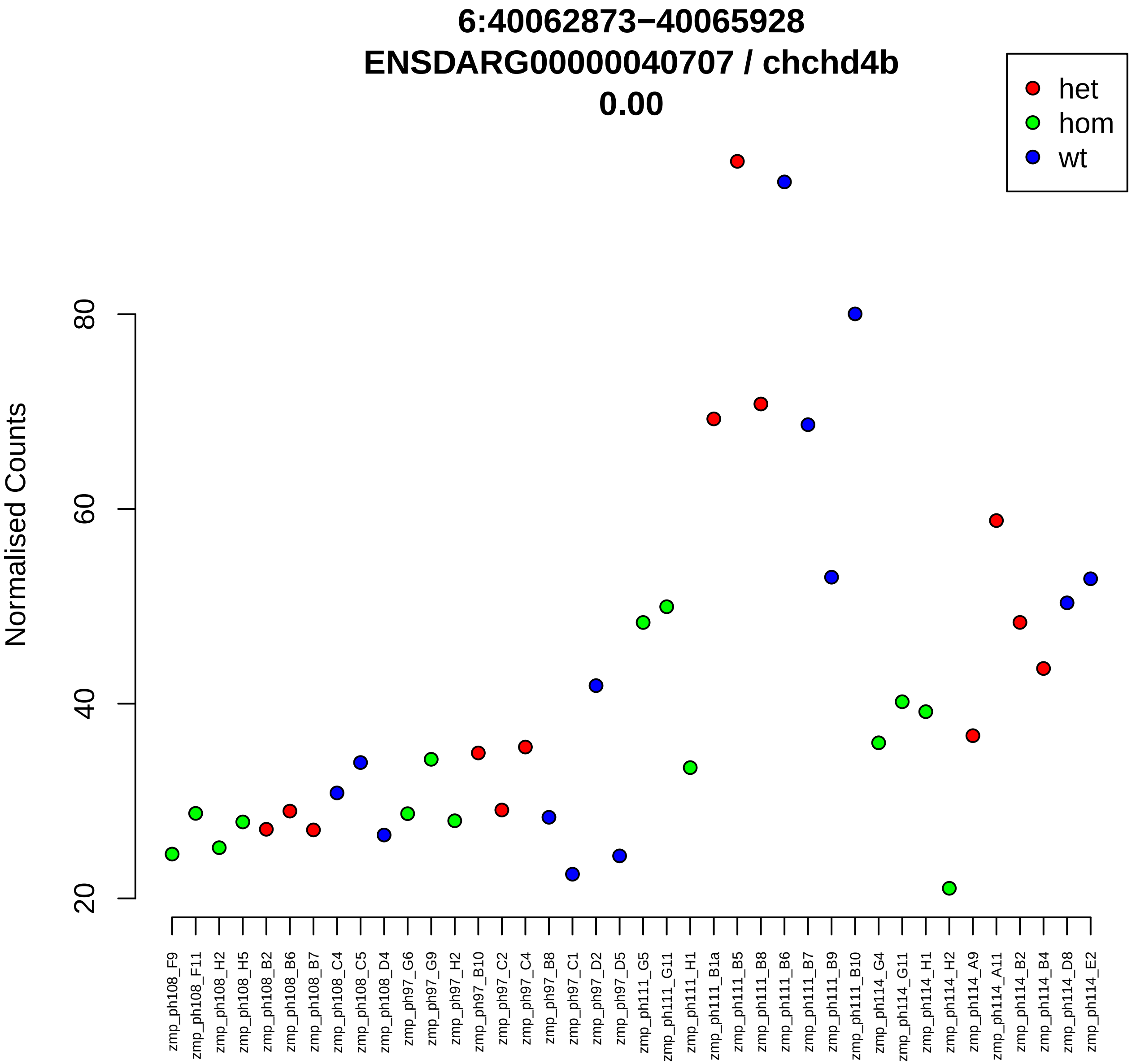

6:40062873−40065928
ENSDARG00000040707 / chchd4b
0.00
het
hom
wt
Normalised Counts
20
40
60
80
zmp_ph108_F9
zmp_ph108_F11
zmp_ph108_H2
zmp_ph108_H5
zmp_ph108_B2
zmp_ph108_B6
zmp_ph108_B7
zmp_ph108_C4
zmp_ph108_C5
zmp_ph108_D4
zmp_ph97_G6
zmp_ph97_G9
zmp_ph97_H2
zmp_ph97_B10
zmp_ph97_C2
zmp_ph97_C4
zmp_ph97_B8
zmp_ph97_C1
zmp_ph97_D2
zmp_ph97_D5
zmp_ph111_G5
zmp_ph111_G11
zmp_ph111_H1
zmp_ph111_B1a
zmp_ph111_B5
zmp_ph111_B8
zmp_ph111_B6
zmp_ph111_B7
zmp_ph111_B9
zmp_ph111_B10
zmp_ph114_G4
zmp_ph114_G11
zmp_ph114_H1
zmp_ph114_H2
zmp_ph114_A9
zmp_ph114_A11
zmp_ph114_B2
zmp_ph114_B4
zmp_ph114_D8
zmp_ph114_E2

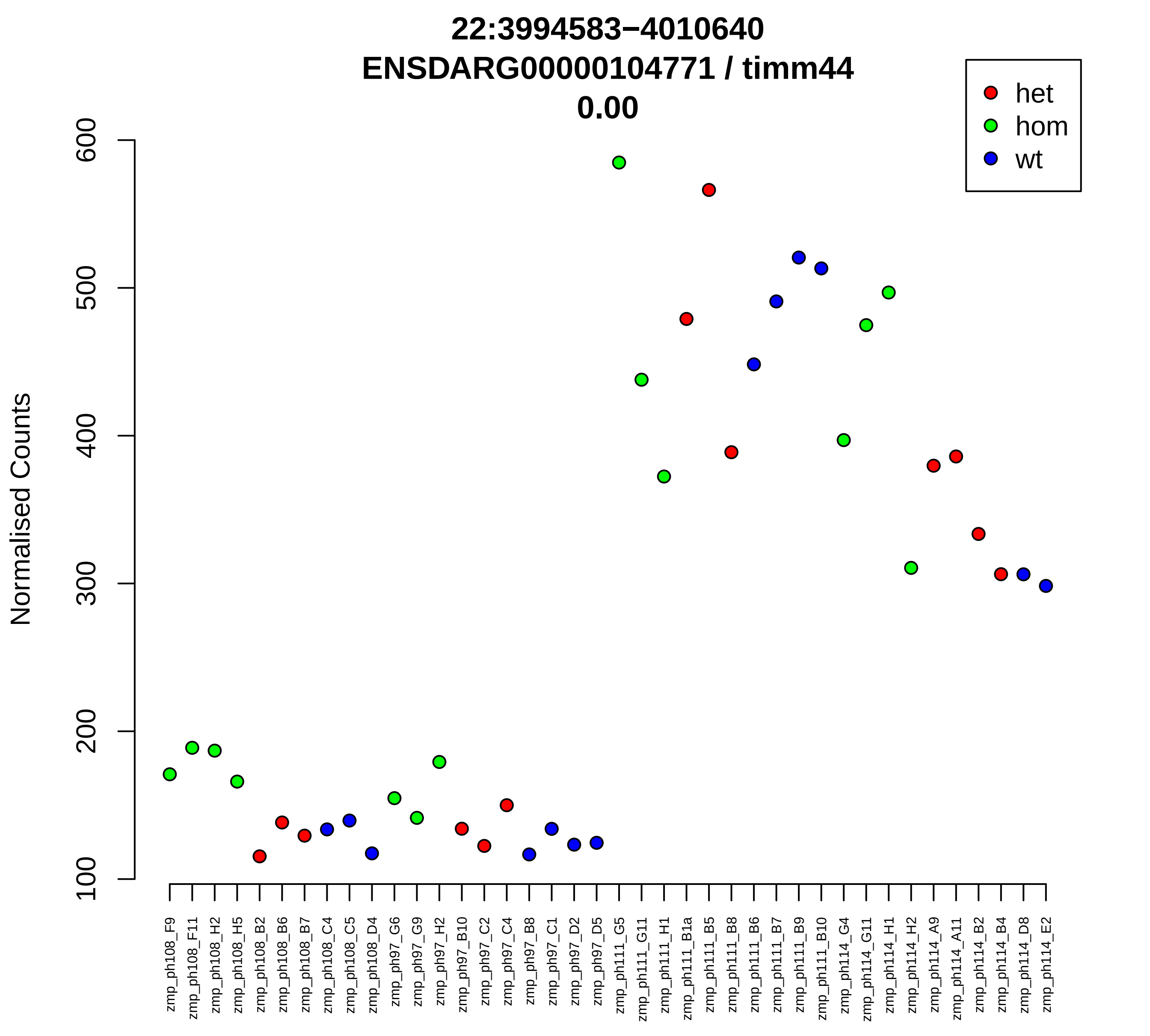

22:3994583−4010640
ENSDARG00000104771 / timm44
0.00
het
hom
wt
Normalised Counts
100
200
300
400
500
600
zmp_ph108_F9
zmp_ph108_F11
zmp_ph108_H2
zmp_ph108_H5
zmp_ph108_B2
zmp_ph108_B6
zmp_ph108_B7
zmp_ph108_C4
zmp_ph108_C5
zmp_ph108_D4
zmp_ph97_G6
zmp_ph97_G9
zmp_ph97_H2
zmp_ph97_B10
zmp_ph97_C2
zmp_ph97_C4
zmp_ph97_B8
zmp_ph97_C1
zmp_ph97_D2
zmp_ph97_D5
zmp_ph111_G5
zmp_ph111_G11
zmp_ph111_H1
zmp_ph111_B1a
zmp_ph111_B5
zmp_ph111_B8
zmp_ph111_B6
zmp_ph111_B7
zmp_ph111_B9
zmp_ph111_B10
zmp_ph114_G4
zmp_ph114_G11
zmp_ph114_H1
zmp_ph114_H2
zmp_ph114_A9
zmp_ph114_A11
zmp_ph114_B2
zmp_ph114_B4
zmp_ph114_D8
zmp_ph114_E2

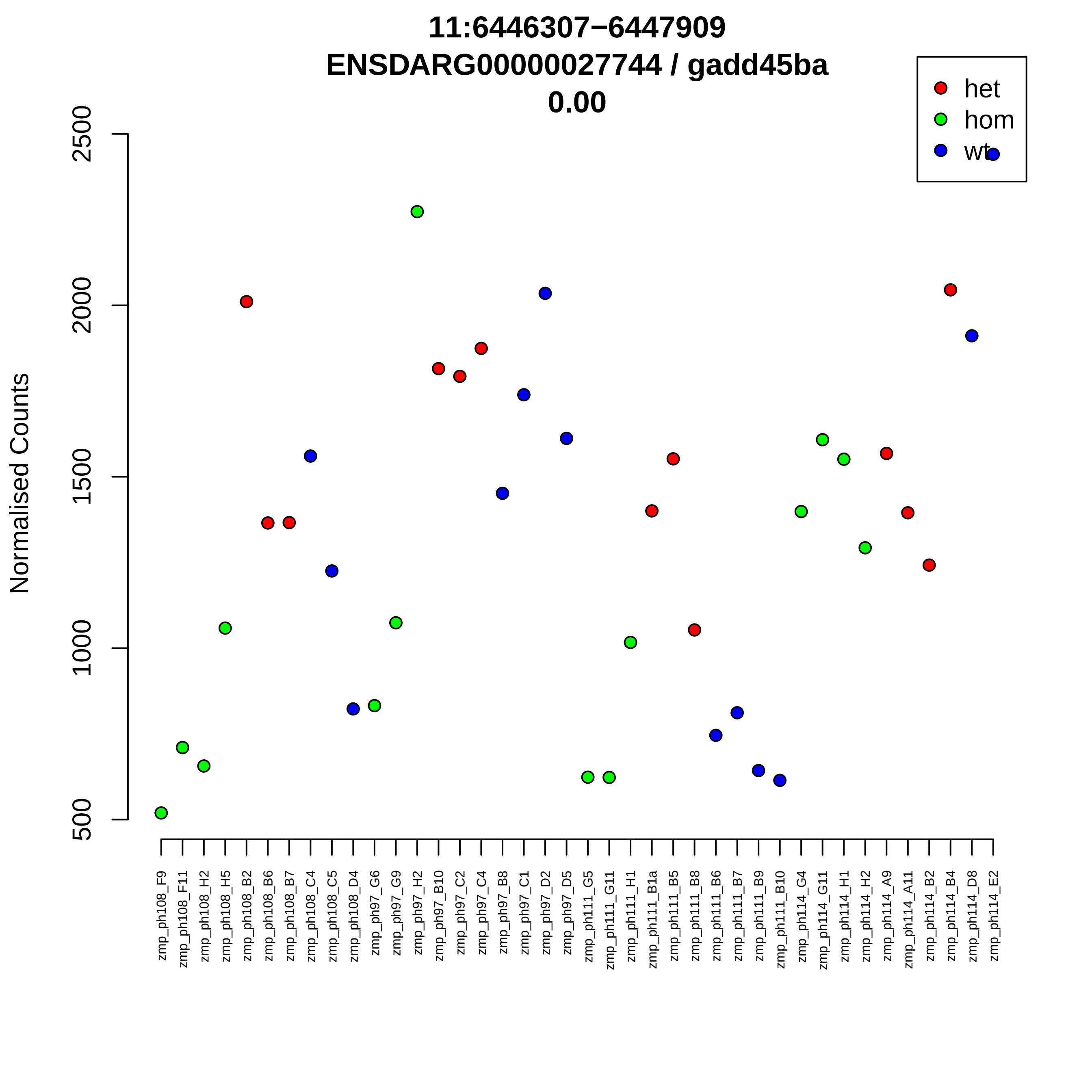

11:6446307−6447909
ENSDARG00000027744 / gadd45ba
0.00
Normalised Counts
500
1000
1500
2000
2500
het
hom
wt
zmp_ph108_F9
zmp_ph108_F11
zmp_ph108_H2
zmp_ph108_H5
zmp_ph108_B2
zmp_ph108_B6
zmp_ph108_B7
zmp_ph108_C4
zmp_ph108_C5
zmp_ph108_D4
zmp_ph97_G6
zmp_ph97_G9
zmp_ph97_H2
zmp_ph97_B10
zmp_ph97_C2
zmp_ph97_C4
zmp_ph97_B8
zmp_ph97_C1
zmp_ph97_D2
zmp_ph97_D5
zmp_ph111_G5
zmp_ph111_G11
zmp_ph111_H1
zmp_ph111_B1a
zmp_ph111_B5
zmp_ph111_B8
zmp_ph111_B6
zmp_ph111_B7
zmp_ph111_B9
zmp_ph111_B10
zmp_ph114_G4
zmp_ph114_G11
zmp_ph114_H1
zmp_ph114_H2
zmp_ph114_A9
zmp_ph114_A11
zmp_ph114_B2
zmp_ph114_B4
zmp_ph114_D8
zmp_ph114_E2

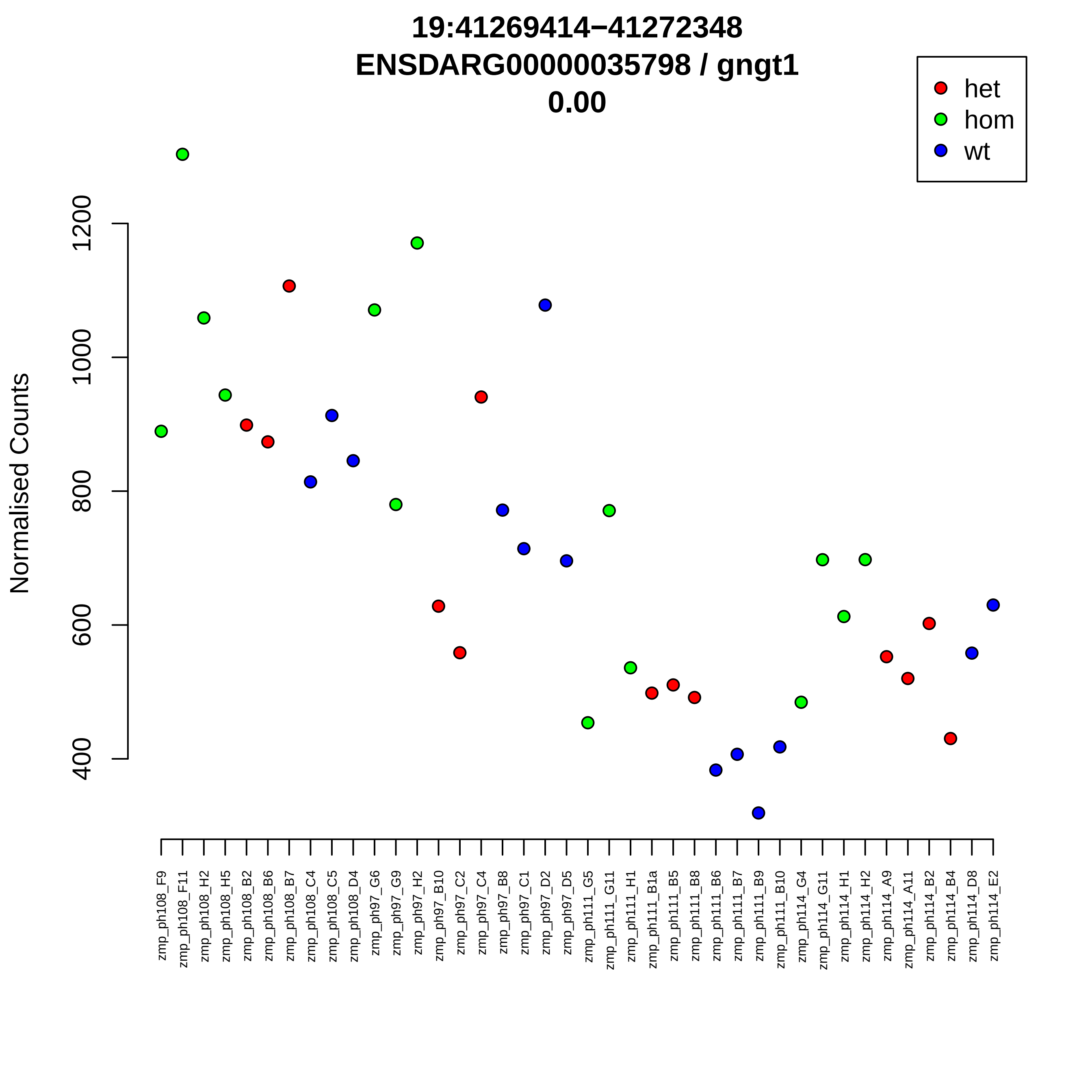
19:41269414−41272348
ENSDARG00000035798 / gngt1
0.00
het
hom
wt
Normalised Counts
400
600
800
1000
1200
zmp_ph108_F9
zmp_ph108_F11
zmp_ph108_H2
zmp_ph108_H5
zmp_ph108_B2
zmp_ph108_B6
zmp_ph108_B7
zmp_ph108_C4
zmp_ph108_C5
zmp_ph108_D4
zmp_ph97_G6
zmp_ph97_G9
zmp_ph97_H2
zmp_ph97_B10
zmp_ph97_C2
zmp_ph97_C4
zmp_ph97_B8
zmp_ph97_C1
zmp_ph97_D2
zmp_ph97_D5
zmp_ph111_G5
zmp_ph111_G11
zmp_ph111_H1
zmp_ph111_B1a
zmp_ph111_B5
zmp_ph111_B8
zmp_ph111_B6
zmp_ph111_B7
zmp_ph111_B9
zmp_ph111_B10
zmp_ph114_G4
zmp_ph114_G11
zmp_ph114_H1
zmp_ph114_H2
zmp_ph114_A9
zmp_ph114_A11
zmp_ph114_B2
zmp_ph114_B4
zmp_ph114_D8
zmp_ph114_E2

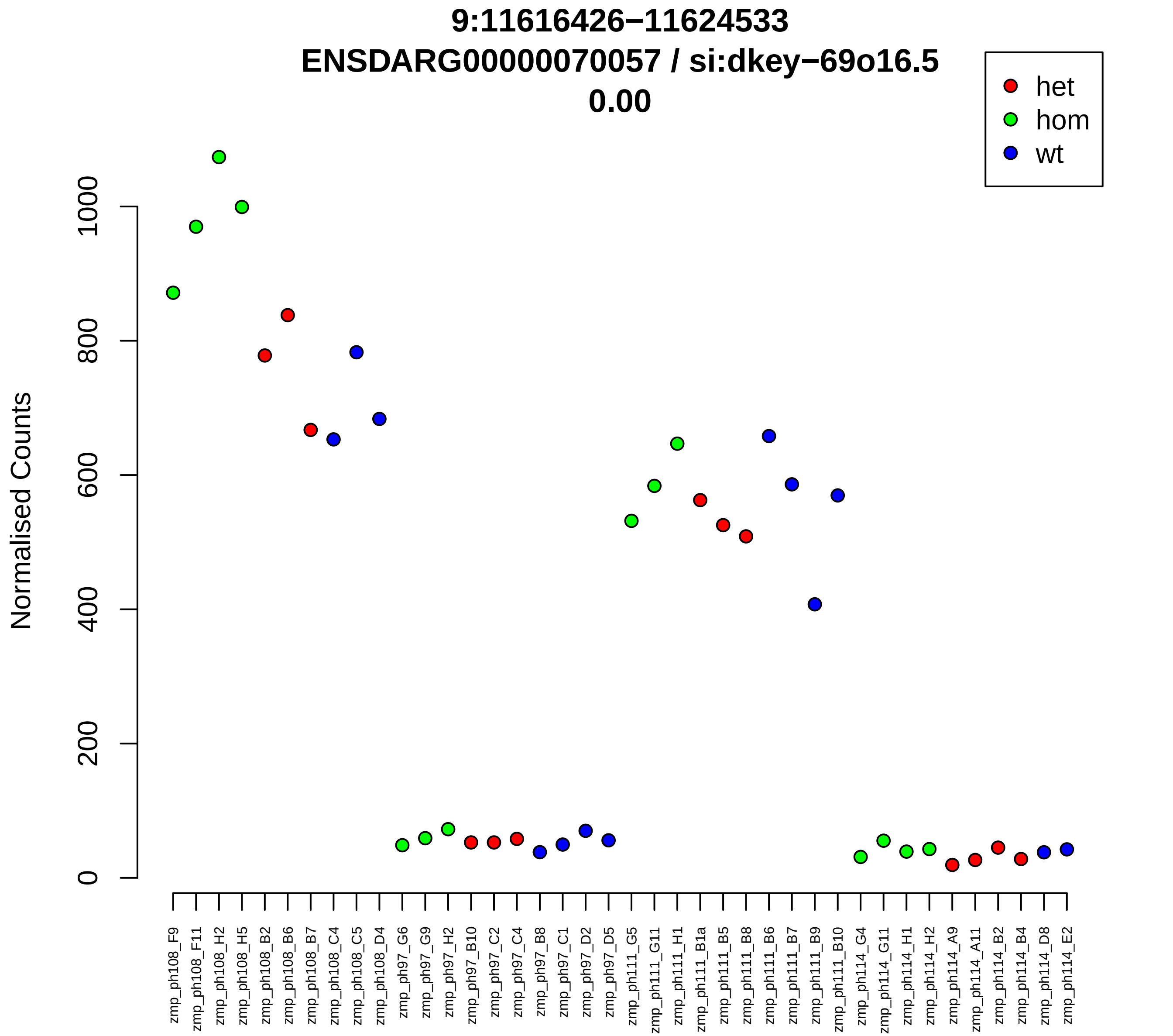

9:11616426−11624533
ENSDARG00000070057 / si:dkey−69o16.5
0.00
het
hom
wt
Normalised Counts
0
200
400
600
800
1000
zmp_ph108_F9
zmp_ph108_F11
zmp_ph108_H2
zmp_ph108_H5
zmp_ph108_B2
zmp_ph108_B6
zmp_ph108_B7
zmp_ph108_C4
zmp_ph108_C5
zmp_ph108_D4
zmp_ph97_G6
zmp_ph97_G9
zmp_ph97_H2
zmp_ph97_B10
zmp_ph97_C2
zmp_ph97_C4
zmp_ph97_B8
zmp_ph97_C1
zmp_ph97_D2
zmp_ph97_D5
zmp_ph111_G5
zmp_ph111_G11
zmp_ph111_H1
zmp_ph111_B1a
zmp_ph111_B5
zmp_ph111_B8
zmp_ph111_B6
zmp_ph111_B7
zmp_ph111_B9
zmp_ph111_B10
zmp_ph114_G4
zmp_ph114_G11
zmp_ph114_H1
zmp_ph114_H2
zmp_ph114_A9
zmp_ph114_A11
zmp_ph114_B2
zmp_ph114_B4
zmp_ph114_D8
zmp_ph114_E2

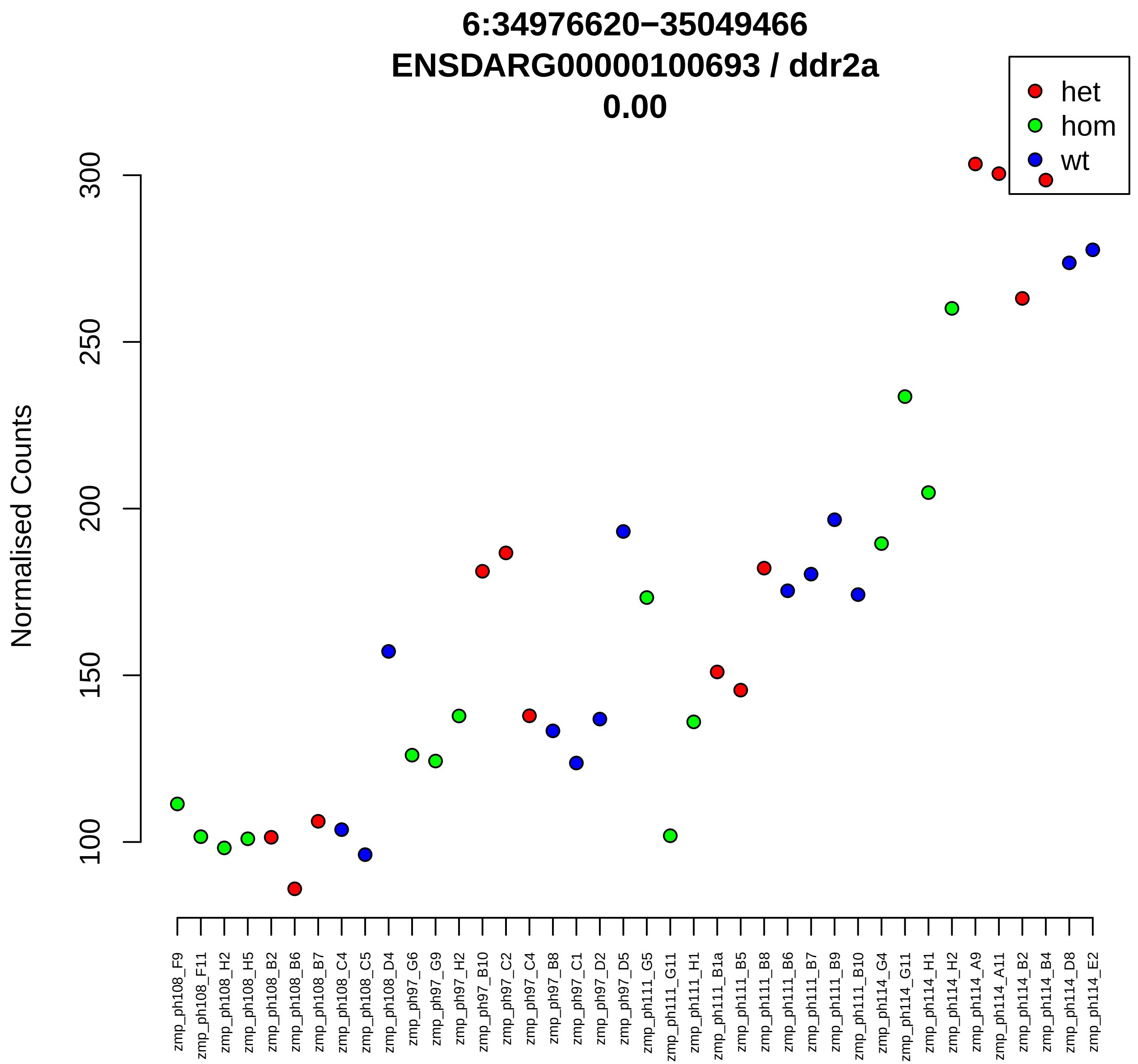

6:34976620−35049466
ENSDARG00000100693 / ddr2a
0.00
het
hom
wt
Normalised Counts
100
150
200
250
300
zmp_ph108_F9
zmp_ph108_F11
zmp_ph108_H2
zmp_ph108_H5
zmp_ph108_B2
zmp_ph108_B6
zmp_ph108_B7
zmp_ph108_C4
zmp_ph108_C5
zmp_ph108_D4
zmp_ph97_G6
zmp_ph97_G9
zmp_ph97_H2
zmp_ph97_B10
zmp_ph97_C2
zmp_ph97_C4
zmp_ph97_B8
zmp_ph97_C1
zmp_ph97_D2
zmp_ph97_D5
zmp_ph111_G5
zmp_ph111_G11
zmp_ph111_H1
zmp_ph111_B1a
zmp_ph111_B5
zmp_ph111_B8
zmp_ph111_B6
zmp_ph111_B7
zmp_ph111_B9
zmp_ph111_B10
zmp_ph114_G4
zmp_ph114_G11
zmp_ph114_H1
zmp_ph114_H2
zmp_ph114_A9
zmp_ph114_A11
zmp_ph114_B2
zmp_ph114_B4
zmp_ph114_D8
zmp_ph114_E2

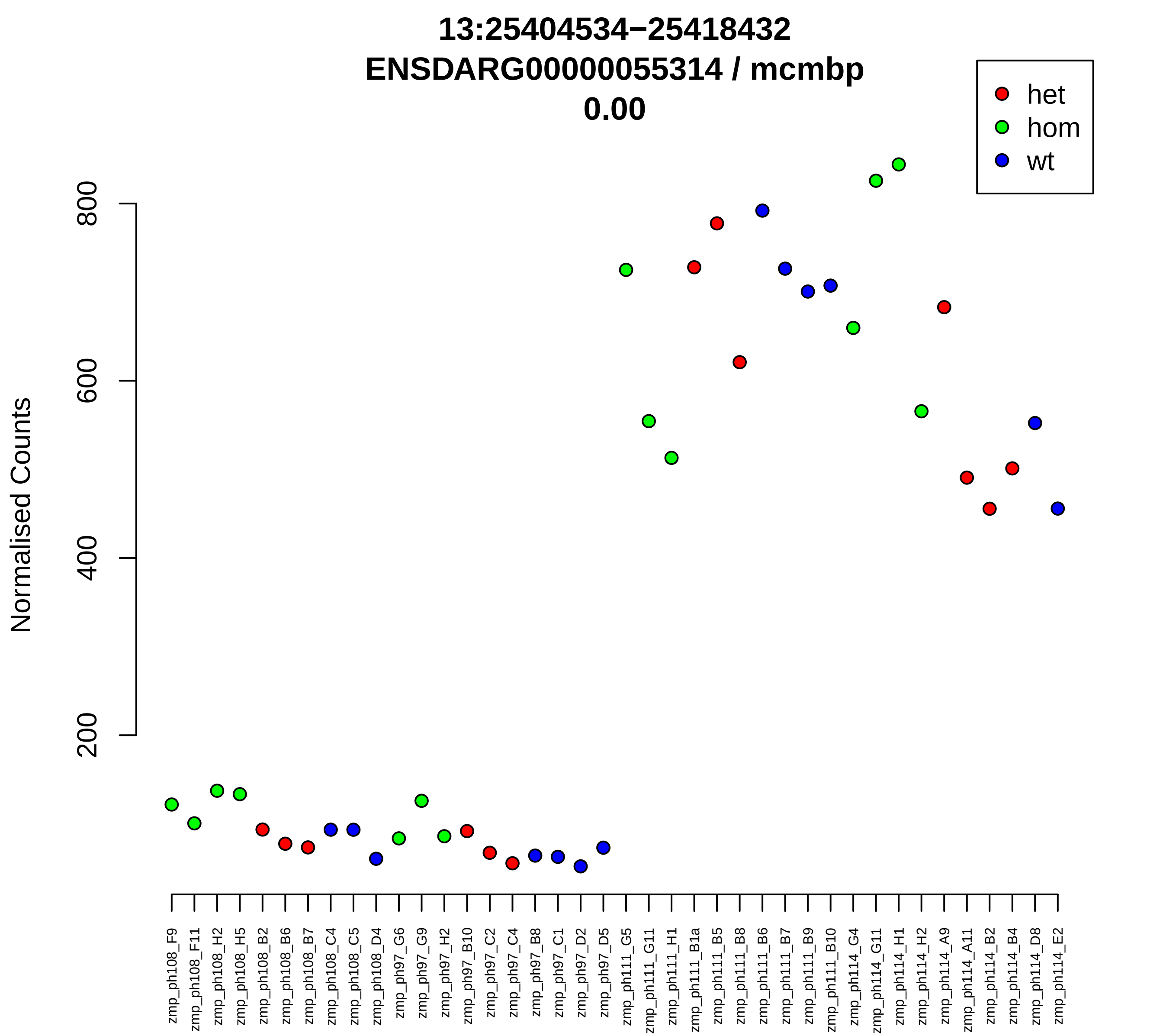

13:25404534−25418432
ENSDARG00000055314 / mcmbp
0.00
het
hom
wt
Normalised Counts
200
400
600
800
zmp_ph108_F9
zmp_ph108_F11
zmp_ph108_H2
zmp_ph108_H5
zmp_ph108_B2
zmp_ph108_B6
zmp_ph108_B7
zmp_ph108_C4
zmp_ph108_C5
zmp_ph108_D4
zmp_ph97_G6
zmp_ph97_G9
zmp_ph97_H2
zmp_ph97_B10
zmp_ph97_C2
zmp_ph97_C4
zmp_ph97_B8
zmp_ph97_C1
zmp_ph97_D2
zmp_ph97_D5
zmp_ph111_G5
zmp_ph111_G11
zmp_ph111_H1
zmp_ph111_B1a
zmp_ph111_B5
zmp_ph111_B8
zmp_ph111_B6
zmp_ph111_B7
zmp_ph111_B9
zmp_ph111_B10
zmp_ph114_G4
zmp_ph114_G11
zmp_ph114_H1
zmp_ph114_H2
zmp_ph114_A9
zmp_ph114_A11
zmp_ph114_B2
zmp_ph114_B4
zmp_ph114_D8
zmp_ph114_E2

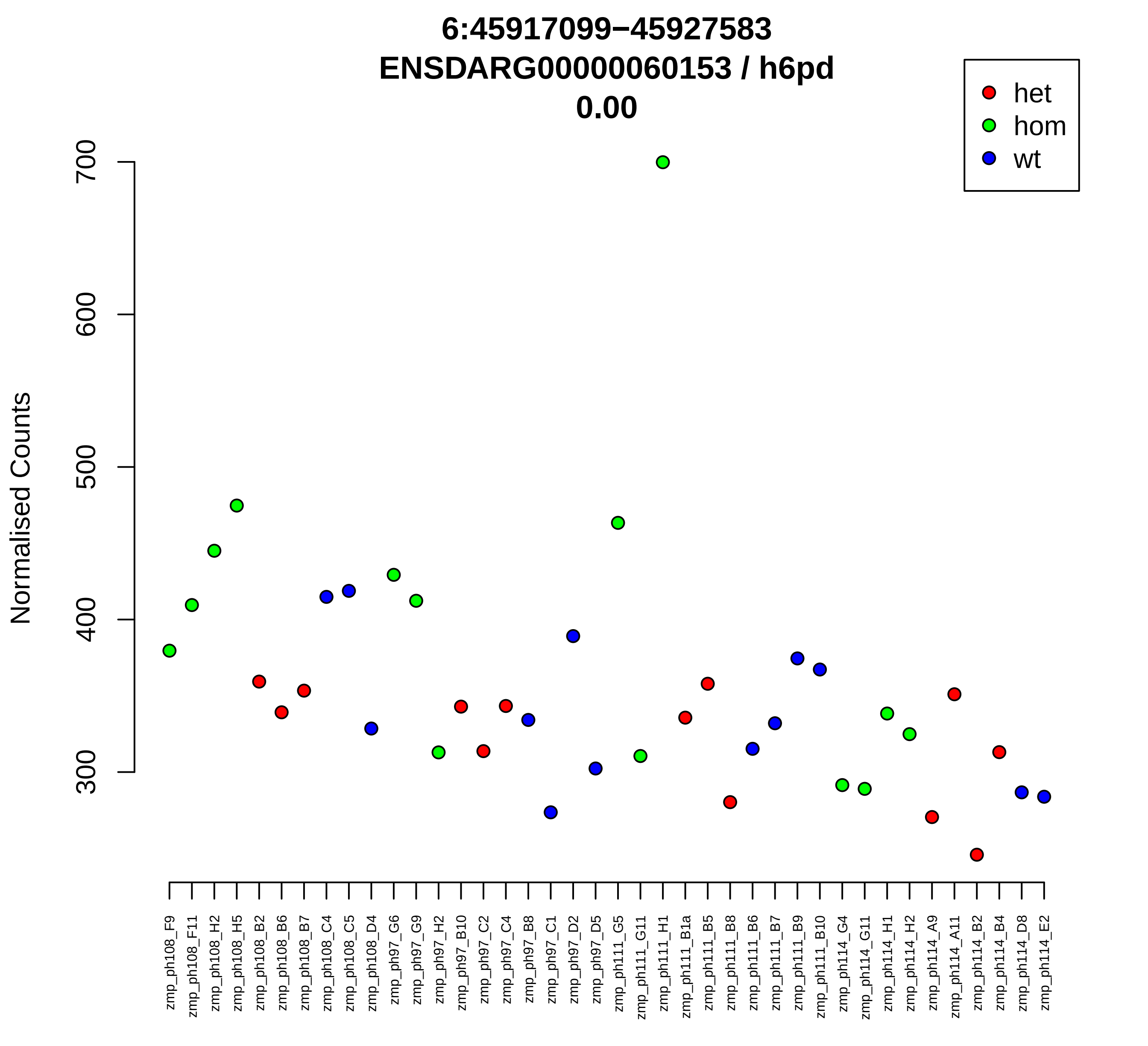

6:45917099−45927583
ENSDARG00000060153 / h6pd
0.00
het
hom
wt
Normalised Counts
300
400
500
600
700
zmp_ph108_F9
zmp_ph108_F11
zmp_ph108_H2
zmp_ph108_H5
zmp_ph108_B2
zmp_ph108_B6
zmp_ph108_B7
zmp_ph108_C4
zmp_ph108_C5
zmp_ph108_D4
zmp_ph97_G6
zmp_ph97_G9
zmp_ph97_H2
zmp_ph97_B10
zmp_ph97_C2
zmp_ph97_C4
zmp_ph97_B8
zmp_ph97_C1
zmp_ph97_D2
zmp_ph97_D5
zmp_ph111_G5
zmp_ph111_G11
zmp_ph111_H1
zmp_ph111_B1a
zmp_ph111_B5
zmp_ph111_B8
zmp_ph111_B6
zmp_ph111_B7
zmp_ph111_B9
zmp_ph111_B10
zmp_ph114_G4
zmp_ph114_G11
zmp_ph114_H1
zmp_ph114_H2
zmp_ph114_A9
zmp_ph114_A11
zmp_ph114_B2
zmp_ph114_B4
zmp_ph114_D8
zmp_ph114_E2

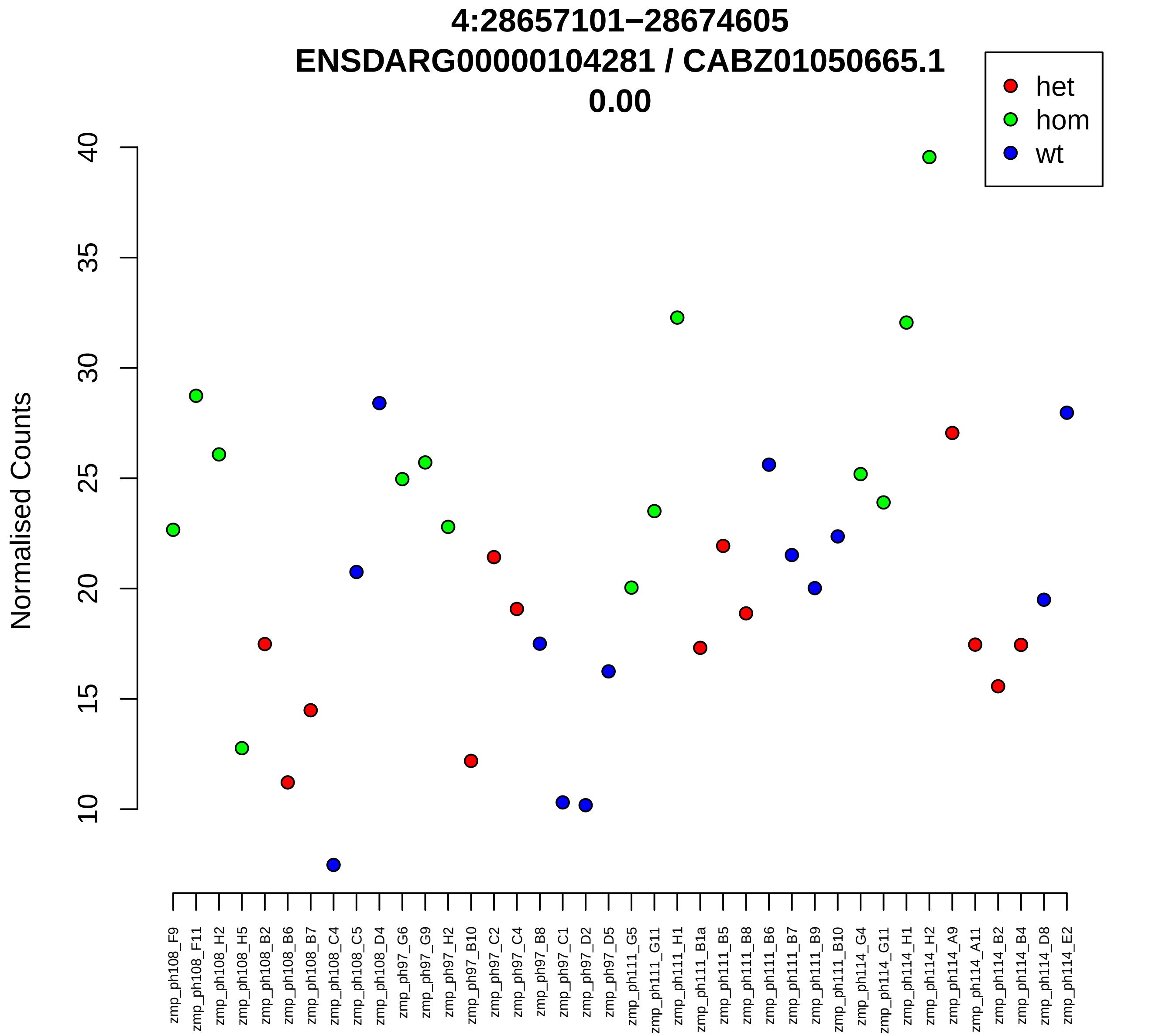
4:28657101−28674605
ENSDARG00000104281 / CABZ01050665.1
0.00
het
hom
wt
Normalised Counts
10
15
20
25
30
35
40
zmp_ph108_F9
zmp_ph108_F11
zmp_ph108_H2
zmp_ph108_H5
zmp_ph108_B2
zmp_ph108_B6
zmp_ph108_B7
zmp_ph108_C4
zmp_ph108_C5
zmp_ph108_D4
zmp_ph97_G6
zmp_ph97_G9
zmp_ph97_H2
zmp_ph97_B10
zmp_ph97_C2
zmp_ph97_C4
zmp_ph97_B8
zmp_ph97_C1
zmp_ph97_D2
zmp_ph97_D5
zmp_ph111_G5
zmp_ph111_G11
zmp_ph111_H1
zmp_ph111_B1a
zmp_ph111_B5
zmp_ph111_B8
zmp_ph111_B6
zmp_ph111_B7
zmp_ph111_B9
zmp_ph111_B10
zmp_ph114_G4
zmp_ph114_G11
zmp_ph114_H1
zmp_ph114_H2
zmp_ph114_A9
zmp_ph114_A11
zmp_ph114_B2
zmp_ph114_B4
zmp_ph114_D8
zmp_ph114_E2

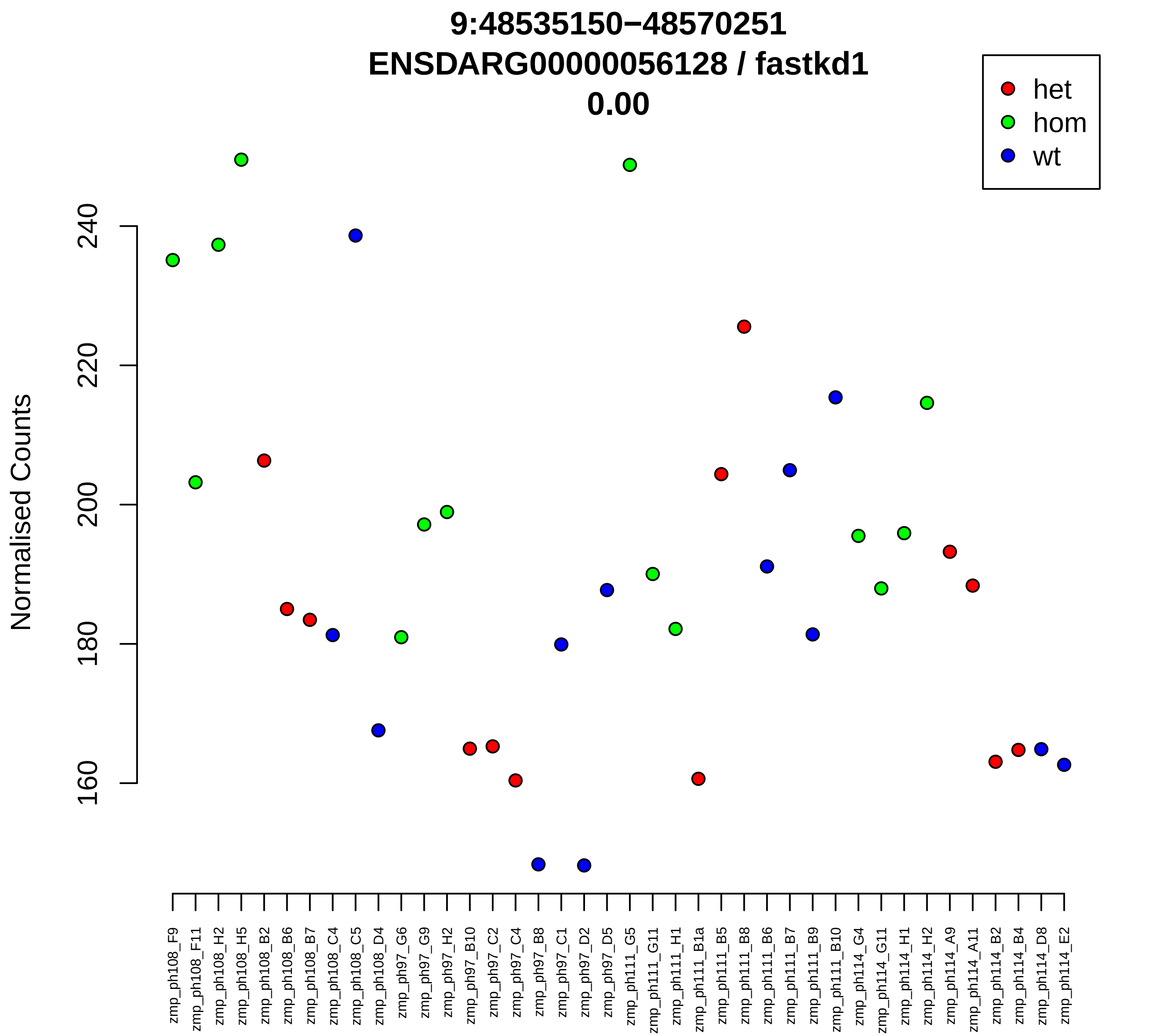

9:48535150−48570251
ENSDARG00000056128 / fastkd1
0.00
het
hom
wt
Normalised Counts
160
180
200
220
240
zmp_ph108_F9
zmp_ph108_F11
zmp_ph108_H2
zmp_ph108_H5
zmp_ph108_B2
zmp_ph108_B6
zmp_ph108_B7
zmp_ph108_C4
zmp_ph108_C5
zmp_ph108_D4
zmp_ph97_G6
zmp_ph97_G9
zmp_ph97_H2
zmp_ph97_B10
zmp_ph97_C2
zmp_ph97_C4
zmp_ph97_B8
zmp_ph97_C1
zmp_ph97_D2
zmp_ph97_D5
zmp_ph111_G5
zmp_ph111_G11
zmp_ph111_H1
zmp_ph111_B1a
zmp_ph111_B5
zmp_ph111_B8
zmp_ph111_B6
zmp_ph111_B7
zmp_ph111_B9
zmp_ph111_B10
zmp_ph114_G4
zmp_ph114_G11
zmp_ph114_H1
zmp_ph114_H2
zmp_ph114_A9
zmp_ph114_A11
zmp_ph114_B2
zmp_ph114_B4
zmp_ph114_D8
zmp_ph114_E2

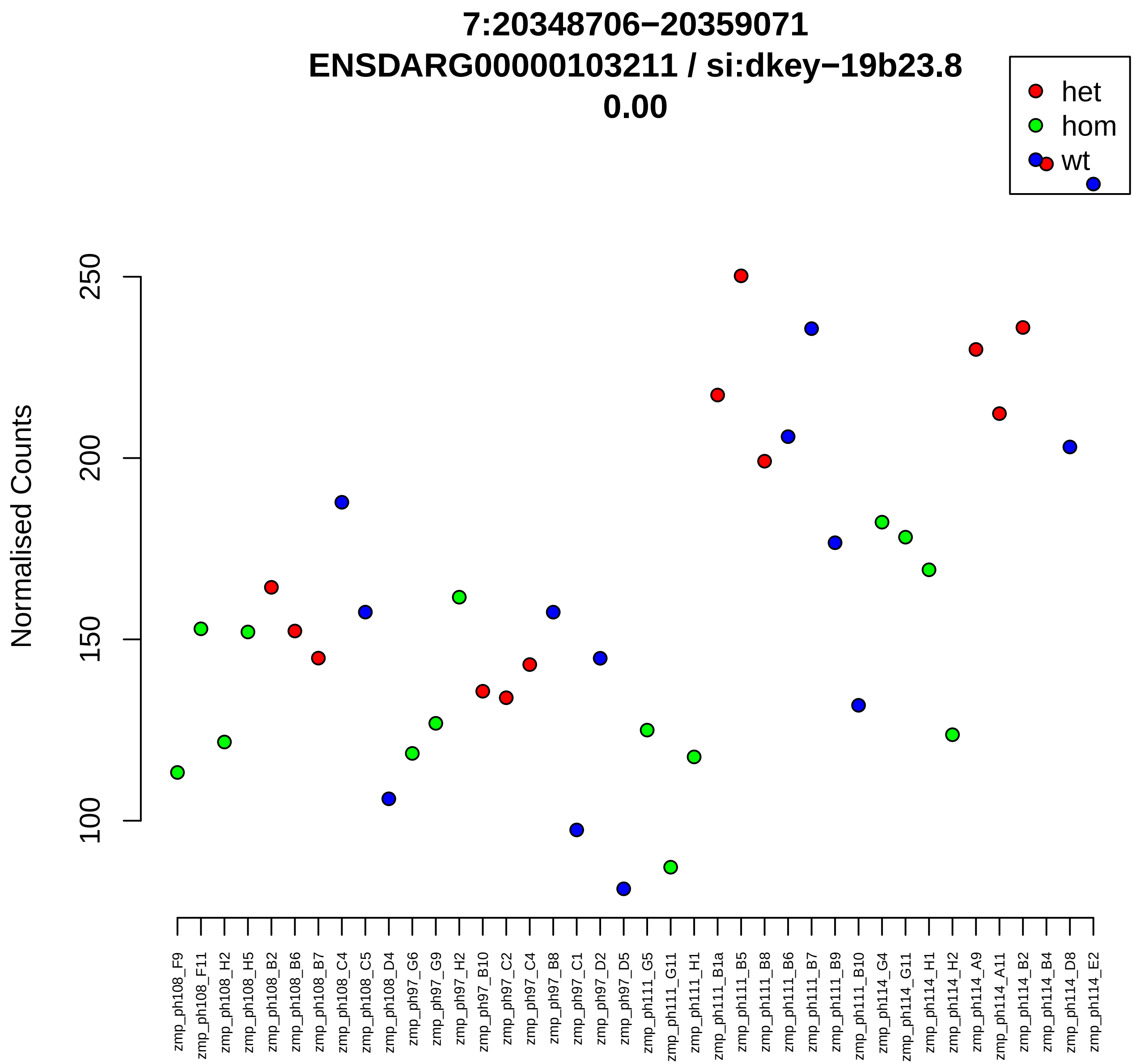
7:20348706−20359071
ENSDARG00000103211 / si:dkey−19b23.8
0.00
het
hom
wt
Normalised Counts
100
150
200
250
zmp_ph108_F9
zmp_ph108_F11
zmp_ph108_H2
zmp_ph108_H5
zmp_ph108_B2
zmp_ph108_B6
zmp_ph108_B7
zmp_ph108_C4
zmp_ph108_C5
zmp_ph108_D4
zmp_ph97_G6
zmp_ph97_G9
zmp_ph97_H2
zmp_ph97_B10
zmp_ph97_C2
zmp_ph97_C4
zmp_ph97_B8
zmp_ph97_C1
zmp_ph97_D2
zmp_ph97_D5
zmp_ph111_G5
zmp_ph111_G11
zmp_ph111_H1
zmp_ph111_B1a
zmp_ph111_B5
zmp_ph111_B8
zmp_ph111_B6
zmp_ph111_B7
zmp_ph111_B9
zmp_ph111_B10
zmp_ph114_G4
zmp_ph114_G11
zmp_ph114_H1
zmp_ph114_H2
zmp_ph114_A9
zmp_ph114_A11
zmp_ph114_B2
zmp_ph114_B4
zmp_ph114_D8
zmp_ph114_E2

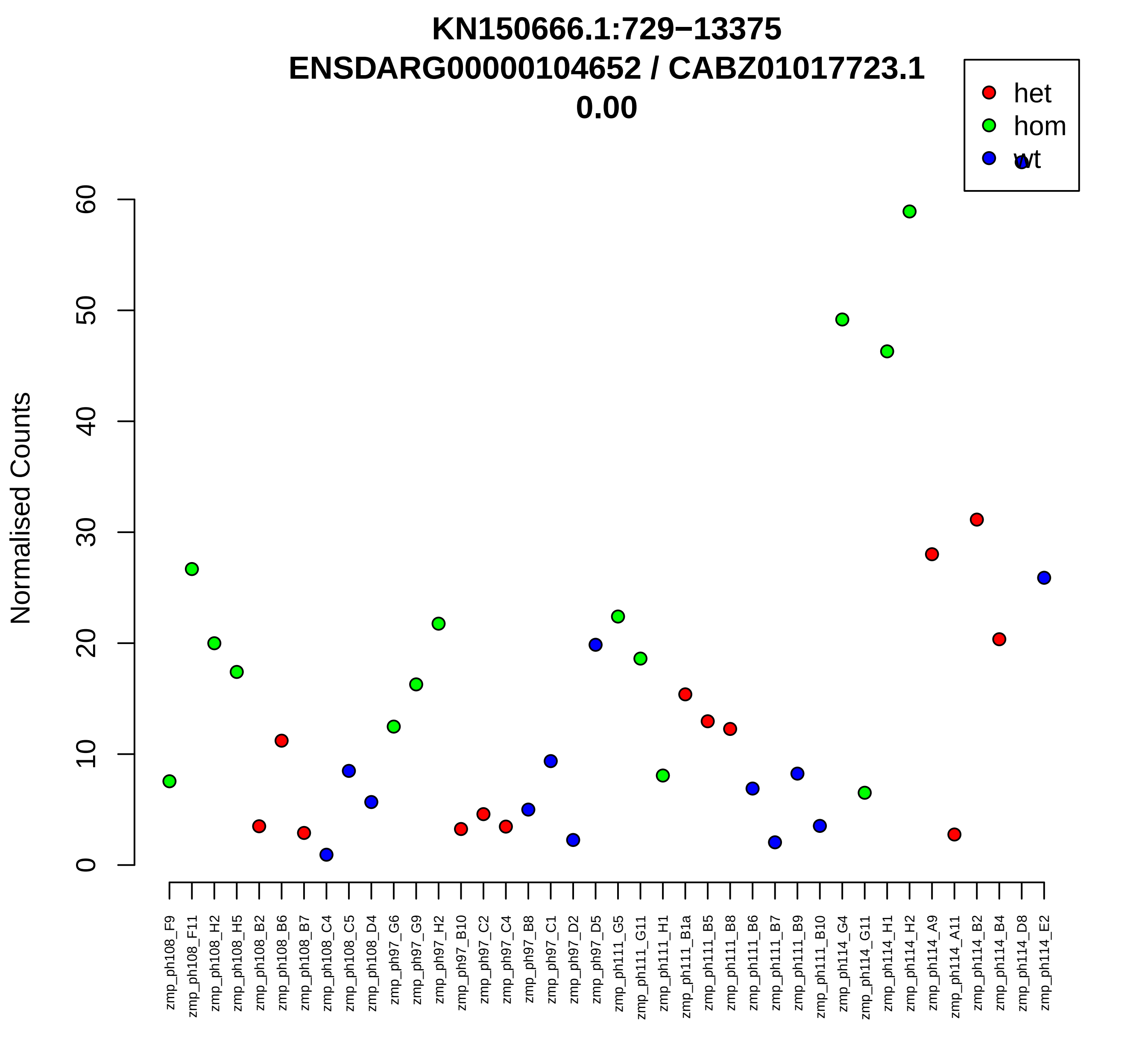
KN150666.1:729−13375
ENSDARG00000104652 / CABZ01017723.1
0.00
het
hom
wt
Normalised Counts
0
10
20
30
40
50
60
zmp_ph108_F9
zmp_ph108_F11
zmp_ph108_H2
zmp_ph108_H5
zmp_ph108_B2
zmp_ph108_B6
zmp_ph108_B7
zmp_ph108_C4
zmp_ph108_C5
zmp_ph108_D4
zmp_ph97_G6
zmp_ph97_G9
zmp_ph97_H2
zmp_ph97_B10
zmp_ph97_C2
zmp_ph97_C4
zmp_ph97_B8
zmp_ph97_C1
zmp_ph97_D2
zmp_ph97_D5
zmp_ph111_G5
zmp_ph111_G11
zmp_ph111_H1
zmp_ph111_B1a
zmp_ph111_B5
zmp_ph111_B8
zmp_ph111_B6
zmp_ph111_B7
zmp_ph111_B9
zmp_ph111_B10
zmp_ph114_G4
zmp_ph114_G11
zmp_ph114_H1
zmp_ph114_H2
zmp_ph114_A9
zmp_ph114_A11
zmp_ph114_B2
zmp_ph114_B4
zmp_ph114_D8
zmp_ph114_E2

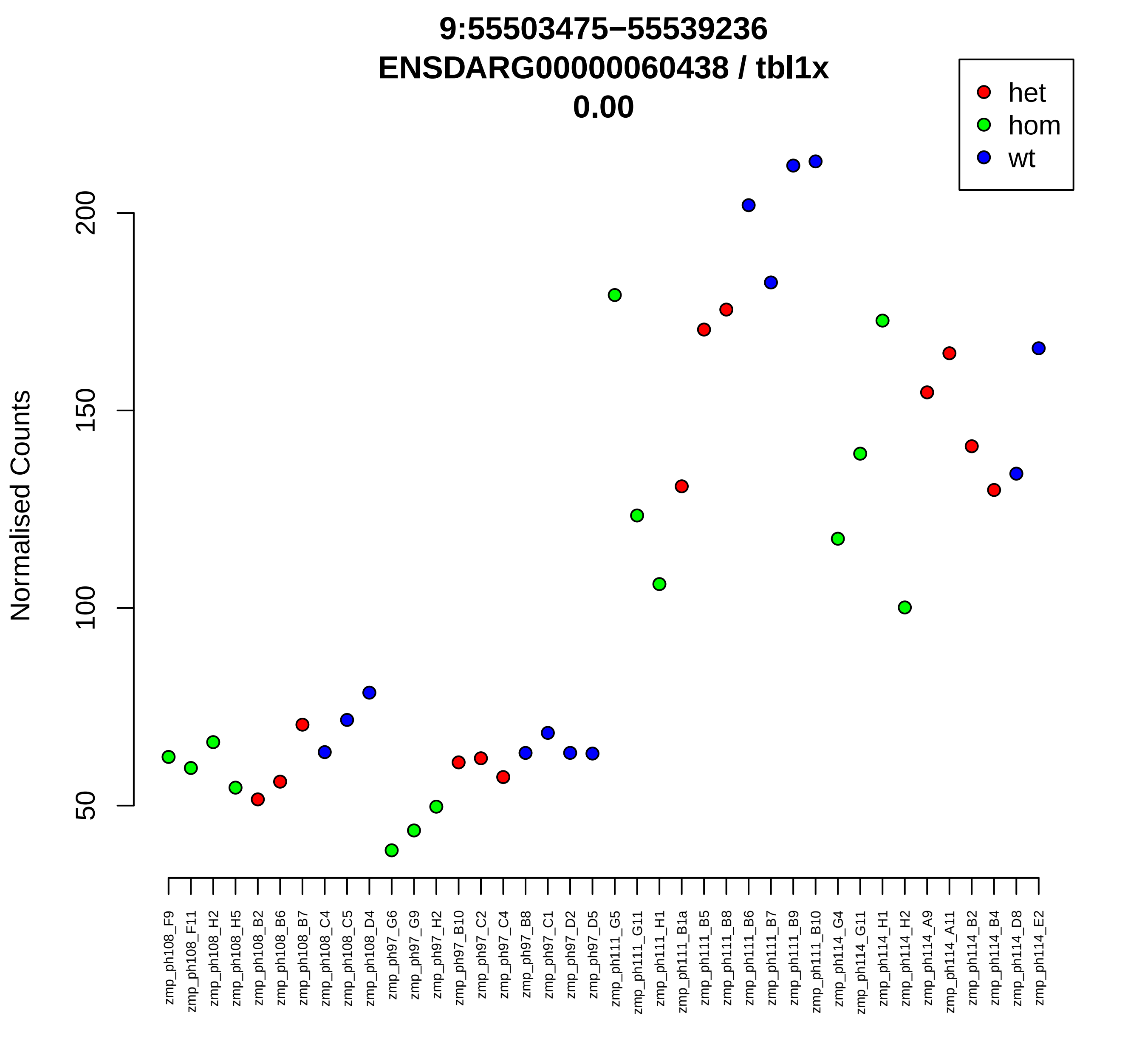

9:55503475−55539236
ENSDARG00000060438 / tbl1x
0.00
het
hom
wt
Normalised Counts
50
100
150
200
zmp_ph108_F9
zmp_ph108_F11
zmp_ph108_H2
zmp_ph108_H5
zmp_ph108_B2
zmp_ph108_B6
zmp_ph108_B7
zmp_ph108_C4
zmp_ph108_C5
zmp_ph108_D4
zmp_ph97_G6
zmp_ph97_G9
zmp_ph97_H2
zmp_ph97_B10
zmp_ph97_C2
zmp_ph97_C4
zmp_ph97_B8
zmp_ph97_C1
zmp_ph97_D2
zmp_ph97_D5
zmp_ph111_G5
zmp_ph111_G11
zmp_ph111_H1
zmp_ph111_B1a
zmp_ph111_B5
zmp_ph111_B8
zmp_ph111_B6
zmp_ph111_B7
zmp_ph111_B9
zmp_ph111_B10
zmp_ph114_G4
zmp_ph114_G11
zmp_ph114_H1
zmp_ph114_H2
zmp_ph114_A9
zmp_ph114_A11
zmp_ph114_B2
zmp_ph114_B4
zmp_ph114_D8
zmp_ph114_E2

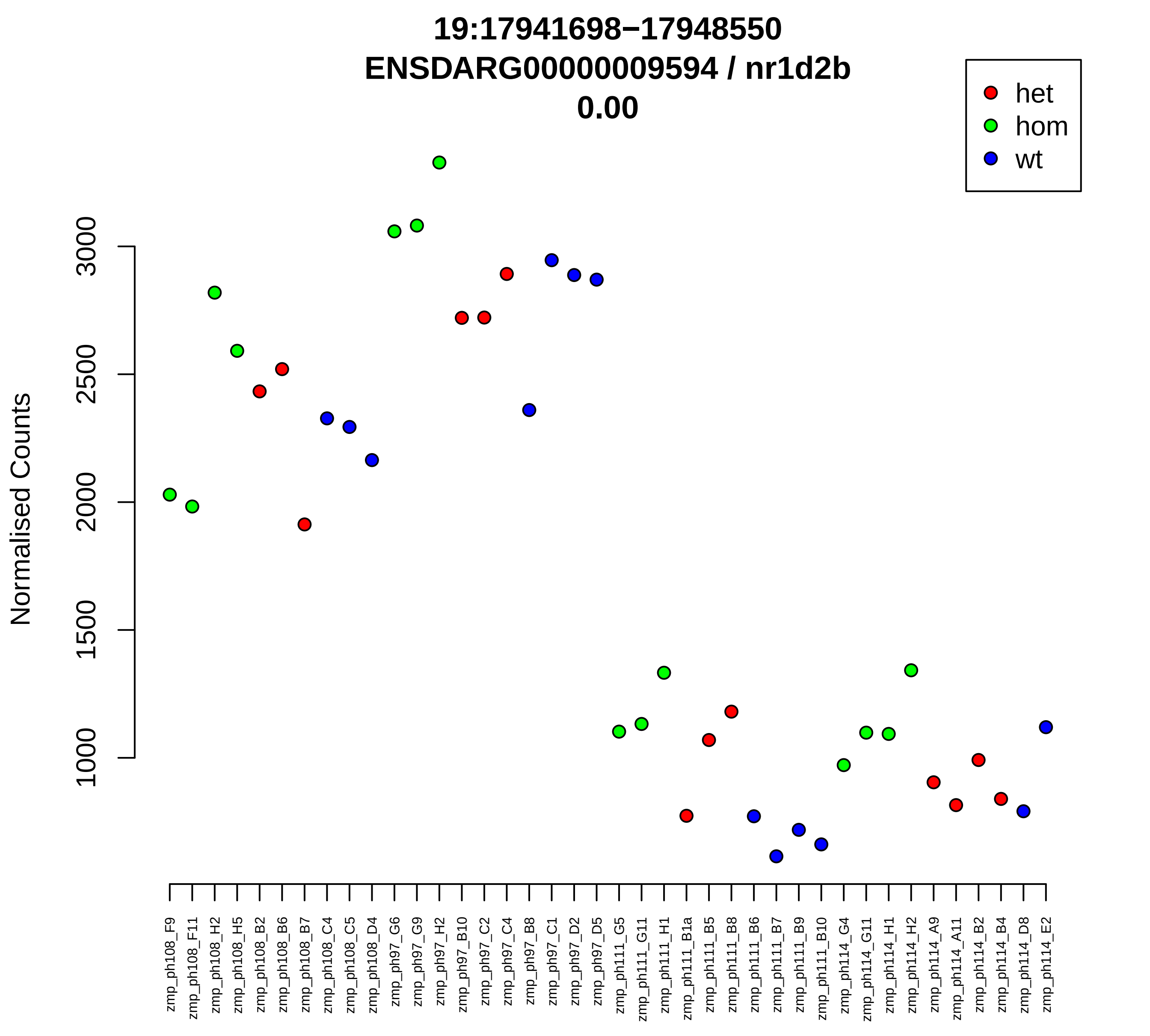

19:17941698−17948550
ENSDARG00000009594 / nr1d2b
0.00
het
hom
wt
Normalised Counts
1000
1500
2000
2500
3000
zmp_ph108_F9
zmp_ph108_F11
zmp_ph108_H2
zmp_ph108_H5
zmp_ph108_B2
zmp_ph108_B6
zmp_ph108_B7
zmp_ph108_C4
zmp_ph108_C5
zmp_ph108_D4
zmp_ph97_G6
zmp_ph97_G9
zmp_ph97_H2
zmp_ph97_B10
zmp_ph97_C2
zmp_ph97_C4
zmp_ph97_B8
zmp_ph97_C1
zmp_ph97_D2
zmp_ph97_D5
zmp_ph111_G5
zmp_ph111_G11
zmp_ph111_H1
zmp_ph111_B1a
zmp_ph111_B5
zmp_ph111_B8
zmp_ph111_B6
zmp_ph111_B7
zmp_ph111_B9
zmp_ph111_B10
zmp_ph114_G4
zmp_ph114_G11
zmp_ph114_H1
zmp_ph114_H2
zmp_ph114_A9
zmp_ph114_A11
zmp_ph114_B2
zmp_ph114_B4
zmp_ph114_D8
zmp_ph114_E2

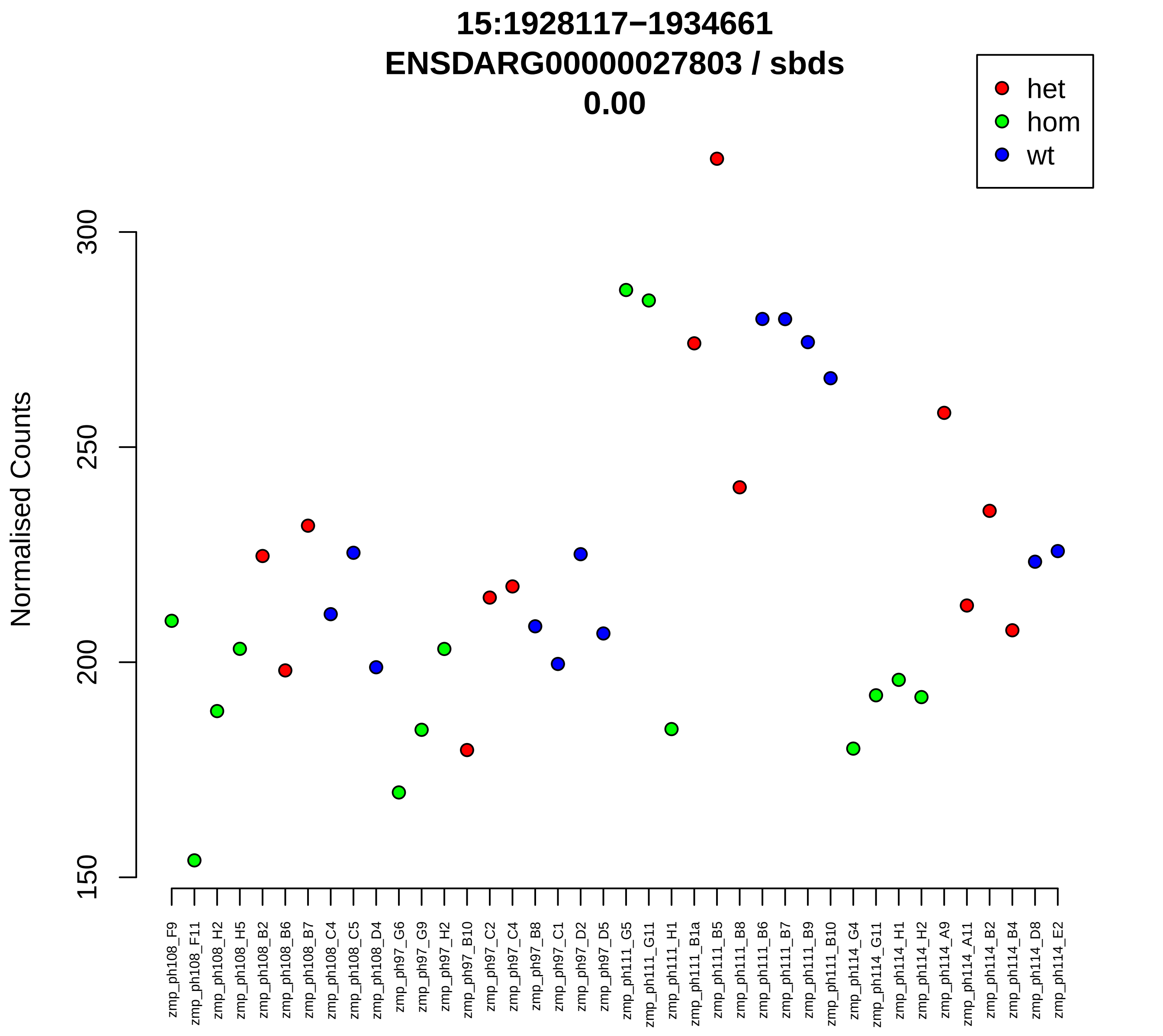
15:1928117−1934661
ENSDARG00000027803 / sbds
0.00
het
hom
wt
Normalised Counts
150
200
250
300
zmp_ph108_F9
zmp_ph108_F11
zmp_ph108_H2
zmp_ph108_H5
zmp_ph108_B2
zmp_ph108_B6
zmp_ph108_B7
zmp_ph108_C4
zmp_ph108_C5
zmp_ph108_D4
zmp_ph97_G6
zmp_ph97_G9
zmp_ph97_H2
zmp_ph97_B10
zmp_ph97_C2
zmp_ph97_C4
zmp_ph97_B8
zmp_ph97_C1
zmp_ph97_D2
zmp_ph97_D5
zmp_ph111_G5
zmp_ph111_G11
zmp_ph111_H1
zmp_ph111_B1a
zmp_ph111_B5
zmp_ph111_B8
zmp_ph111_B6
zmp_ph111_B7
zmp_ph111_B9
zmp_ph111_B10
zmp_ph114_G4
zmp_ph114_G11
zmp_ph114_H1
zmp_ph114_H2
zmp_ph114_A9
zmp_ph114_A11
zmp_ph114_B2
zmp_ph114_B4
zmp_ph114_D8
zmp_ph114_E2

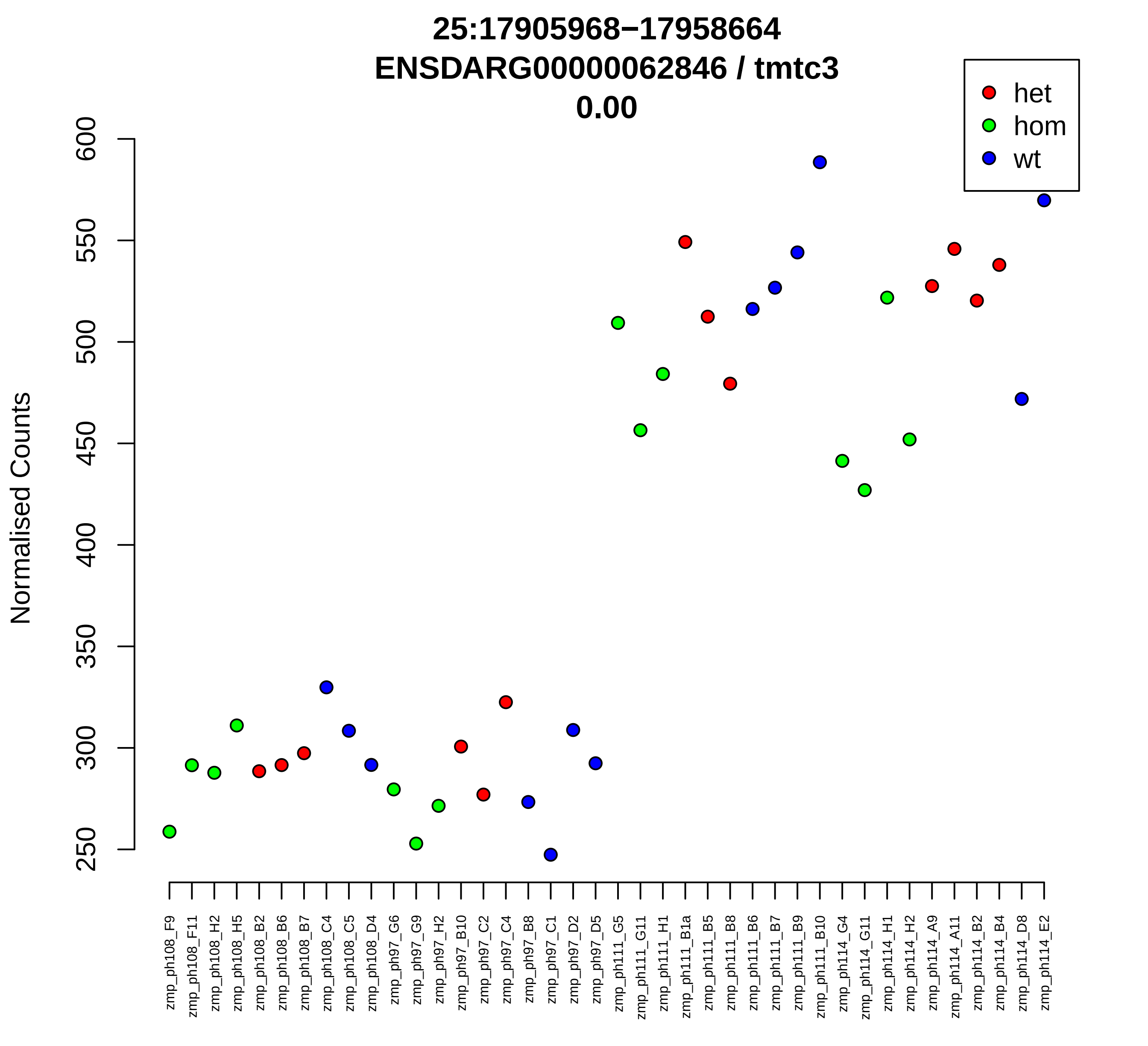
25:17905968−17958664
ENSDARG00000062846 / tmtc3
0.00
het
hom
wt
Normalised Counts
250
300
350
400
450
500
550
600
zmp_ph108_F9
zmp_ph108_F11
zmp_ph108_H2
zmp_ph108_H5
zmp_ph108_B2
zmp_ph108_B6
zmp_ph108_B7
zmp_ph108_C4
zmp_ph108_C5
zmp_ph108_D4
zmp_ph97_G6
zmp_ph97_G9
zmp_ph97_H2
zmp_ph97_B10
zmp_ph97_C2
zmp_ph97_C4
zmp_ph97_B8
zmp_ph97_C1
zmp_ph97_D2
zmp_ph97_D5
zmp_ph111_G5
zmp_ph111_G11
zmp_ph111_H1
zmp_ph111_B1a
zmp_ph111_B5
zmp_ph111_B8
zmp_ph111_B6
zmp_ph111_B7
zmp_ph111_B9
zmp_ph111_B10
zmp_ph114_G4
zmp_ph114_G11
zmp_ph114_H1
zmp_ph114_H2
zmp_ph114_A9
zmp_ph114_A11
zmp_ph114_B2
zmp_ph114_B4
zmp_ph114_D8
zmp_ph114_E2

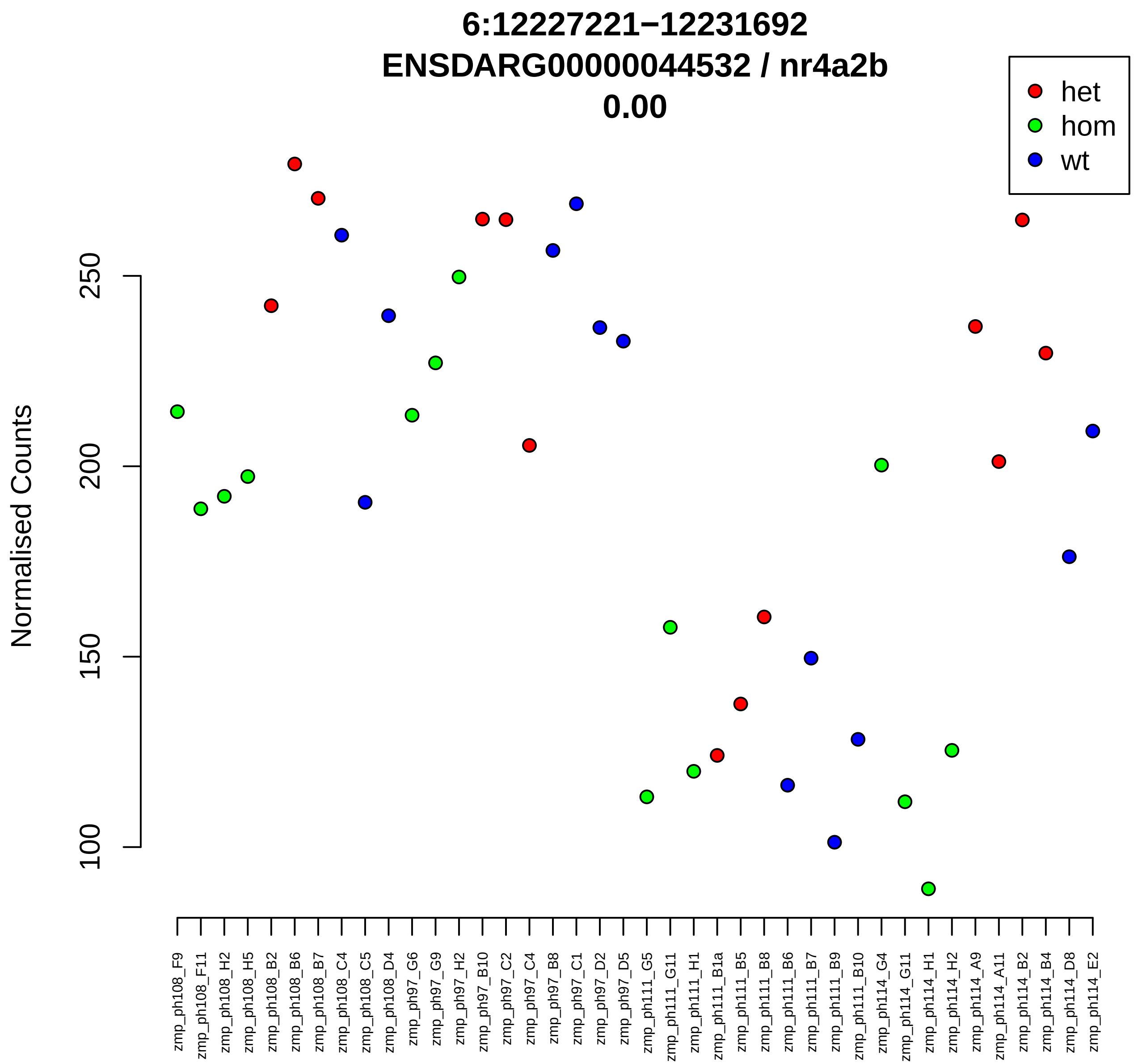
6:12227221−12231692
ENSDARG00000044532 / nr4a2b
0.00
het
hom
wt
Normalised Counts
100
150
200
250
zmp_ph108_F9
zmp_ph108_F11
zmp_ph108_H2
zmp_ph108_H5
zmp_ph108_B2
zmp_ph108_B6
zmp_ph108_B7
zmp_ph108_C4
zmp_ph108_C5
zmp_ph108_D4
zmp_ph97_G6
zmp_ph97_G9
zmp_ph97_H2
zmp_ph97_B10
zmp_ph97_C2
zmp_ph97_C4
zmp_ph97_B8
zmp_ph97_C1
zmp_ph97_D2
zmp_ph97_D5
zmp_ph111_G5
zmp_ph111_G11
zmp_ph111_H1
zmp_ph111_B1a
zmp_ph111_B5
zmp_ph111_B8
zmp_ph111_B6
zmp_ph111_B7
zmp_ph111_B9
zmp_ph111_B10
zmp_ph114_G4
zmp_ph114_G11
zmp_ph114_H1
zmp_ph114_H2
zmp_ph114_A9
zmp_ph114_A11
zmp_ph114_B2
zmp_ph114_B4
zmp_ph114_D8
zmp_ph114_E2

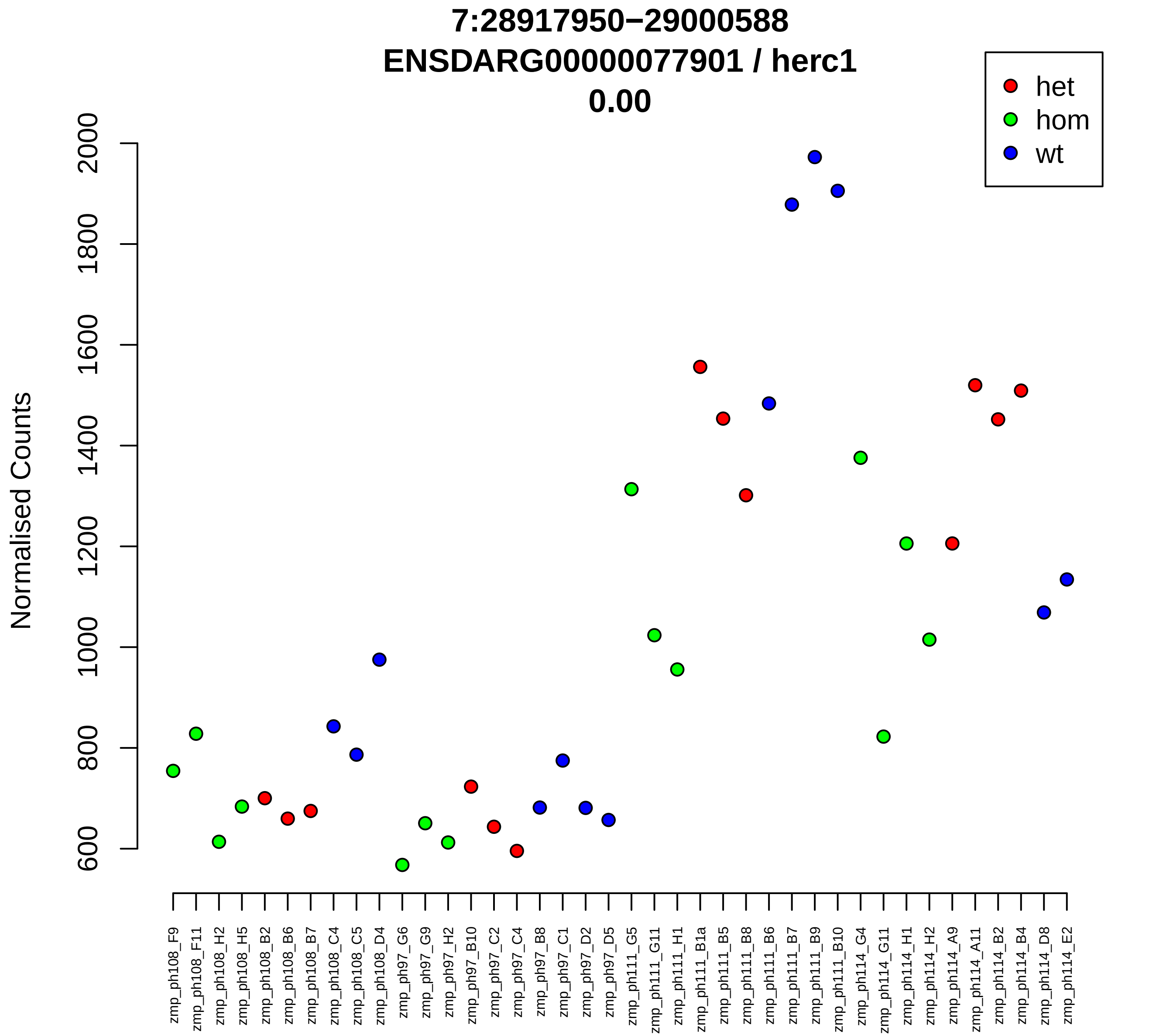
7:28917950−29000588
ENSDARG00000077901 / herc1
0.00
het
hom
wt
Normalised Counts
600
800
1000
1200
1400
1600
1800
2000
zmp_ph108_F9
zmp_ph108_F11
zmp_ph108_H2
zmp_ph108_H5
zmp_ph108_B2
zmp_ph108_B6
zmp_ph108_B7
zmp_ph108_C4
zmp_ph108_C5
zmp_ph108_D4
zmp_ph97_G6
zmp_ph97_G9
zmp_ph97_H2
zmp_ph97_B10
zmp_ph97_C2
zmp_ph97_C4
zmp_ph97_B8
zmp_ph97_C1
zmp_ph97_D2
zmp_ph97_D5
zmp_ph111_G5
zmp_ph111_G11
zmp_ph111_H1
zmp_ph111_B1a
zmp_ph111_B5
zmp_ph111_B8
zmp_ph111_B6
zmp_ph111_B7
zmp_ph111_B9
zmp_ph111_B10
zmp_ph114_G4
zmp_ph114_G11
zmp_ph114_H1
zmp_ph114_H2
zmp_ph114_A9
zmp_ph114_A11
zmp_ph114_B2
zmp_ph114_B4
zmp_ph114_D8
zmp_ph114_E2

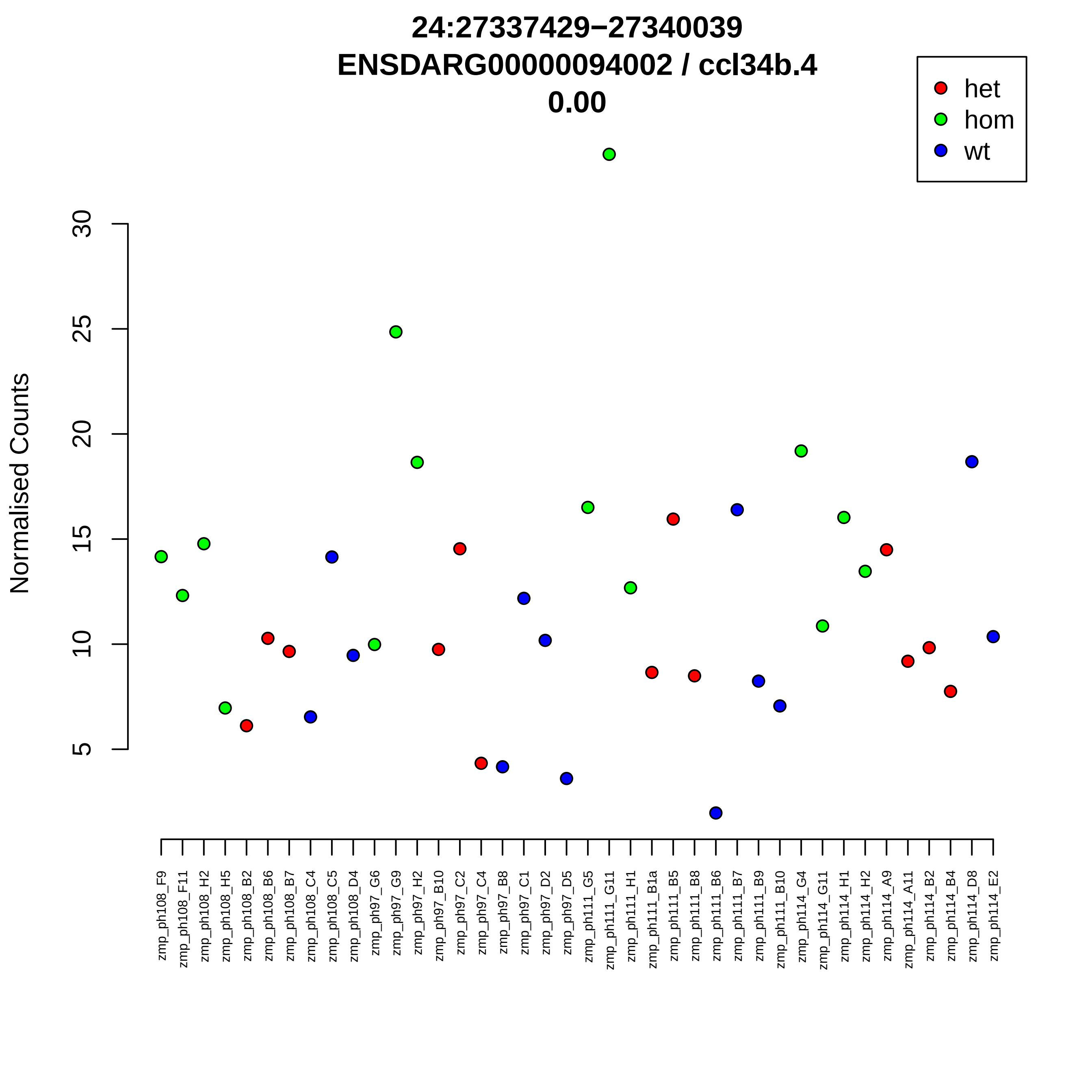

24:27337429−27340039
ENSDARG00000094002 / ccl34b.4
0.00
het
hom
wt
Normalised Counts
5
10
15
20
25
30
zmp_ph108_F9
zmp_ph108_F11
zmp_ph108_H2
zmp_ph108_H5
zmp_ph108_B2
zmp_ph108_B6
zmp_ph108_B7
zmp_ph108_C4
zmp_ph108_C5
zmp_ph108_D4
zmp_ph97_G6
zmp_ph97_G9
zmp_ph97_H2
zmp_ph97_B10
zmp_ph97_C2
zmp_ph97_C4
zmp_ph97_B8
zmp_ph97_C1
zmp_ph97_D2
zmp_ph97_D5
zmp_ph111_G5
zmp_ph111_G11
zmp_ph111_H1
zmp_ph111_B1a
zmp_ph111_B5
zmp_ph111_B8
zmp_ph111_B6
zmp_ph111_B7
zmp_ph111_B9
zmp_ph111_B10
zmp_ph114_G4
zmp_ph114_G11
zmp_ph114_H1
zmp_ph114_H2
zmp_ph114_A9
zmp_ph114_A11
zmp_ph114_B2
zmp_ph114_B4
zmp_ph114_D8
zmp_ph114_E2

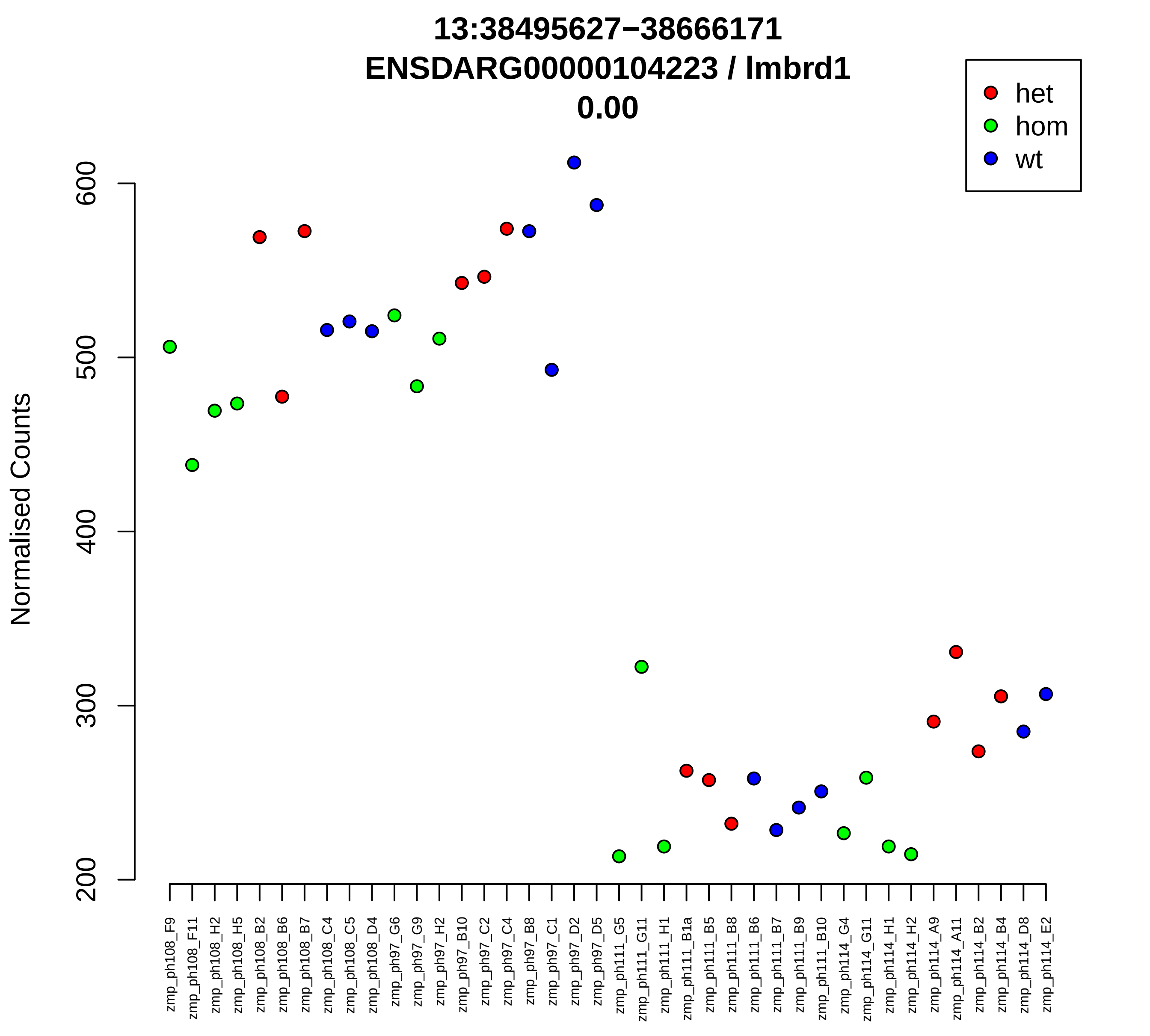
13:38495627−38666171
ENSDARG00000104223 / lmbrd1
0.00
het
hom
wt
Normalised Counts
200
300
400
500
600
zmp_ph108_F9
zmp_ph108_F11
zmp_ph108_H2
zmp_ph108_H5
zmp_ph108_B2
zmp_ph108_B6
zmp_ph108_B7
zmp_ph108_C4
zmp_ph108_C5
zmp_ph108_D4
zmp_ph97_G6
zmp_ph97_G9
zmp_ph97_H2
zmp_ph97_B10
zmp_ph97_C2
zmp_ph97_C4
zmp_ph97_B8
zmp_ph97_C1
zmp_ph97_D2
zmp_ph97_D5
zmp_ph111_G5
zmp_ph111_G11
zmp_ph111_H1
zmp_ph111_B1a
zmp_ph111_B5
zmp_ph111_B8
zmp_ph111_B6
zmp_ph111_B7
zmp_ph111_B9
zmp_ph111_B10
zmp_ph114_G4
zmp_ph114_G11
zmp_ph114_H1
zmp_ph114_H2
zmp_ph114_A9
zmp_ph114_A11
zmp_ph114_B2
zmp_ph114_B4
zmp_ph114_D8
zmp_ph114_E2

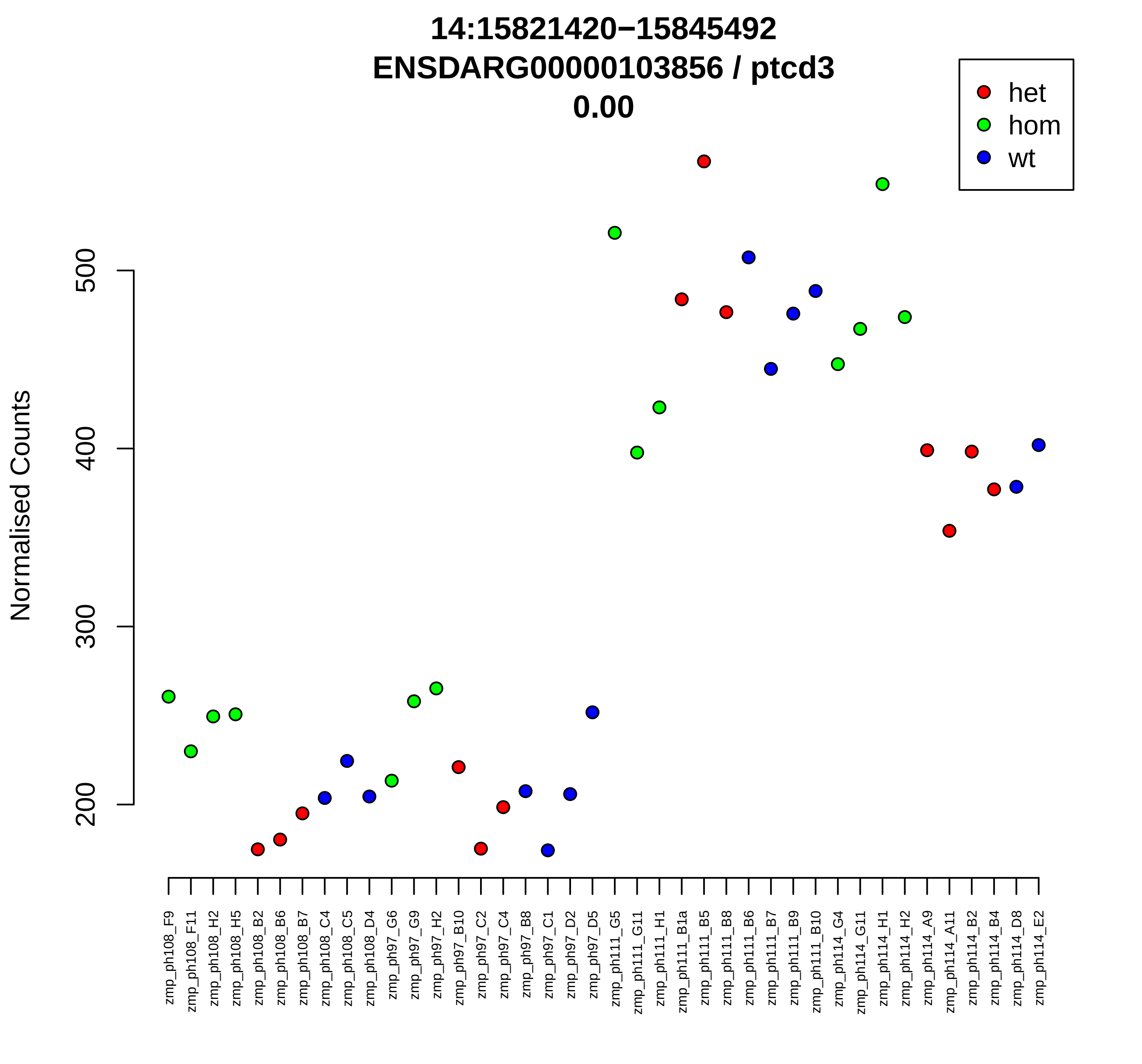
14:15821420−15845492
ENSDARG00000103856 / ptcd3
0.00
het
hom
wt
Normalised Counts
200
300
400
500
zmp_ph108_F9
zmp_ph108_F11
zmp_ph108_H2
zmp_ph108_H5
zmp_ph108_B2
zmp_ph108_B6
zmp_ph108_B7
zmp_ph108_C4
zmp_ph108_C5
zmp_ph108_D4
zmp_ph97_G6
zmp_ph97_G9
zmp_ph97_H2
zmp_ph97_B10
zmp_ph97_C2
zmp_ph97_C4
zmp_ph97_B8
zmp_ph97_C1
zmp_ph97_D2
zmp_ph97_D5
zmp_ph111_G5
zmp_ph111_G11
zmp_ph111_H1
zmp_ph111_B1a
zmp_ph111_B5
zmp_ph111_B8
zmp_ph111_B6
zmp_ph111_B7
zmp_ph111_B9
zmp_ph111_B10
zmp_ph114_G4
zmp_ph114_G11
zmp_ph114_H1
zmp_ph114_H2
zmp_ph114_A9
zmp_ph114_A11
zmp_ph114_B2
zmp_ph114_B4
zmp_ph114_D8
zmp_ph114_E2

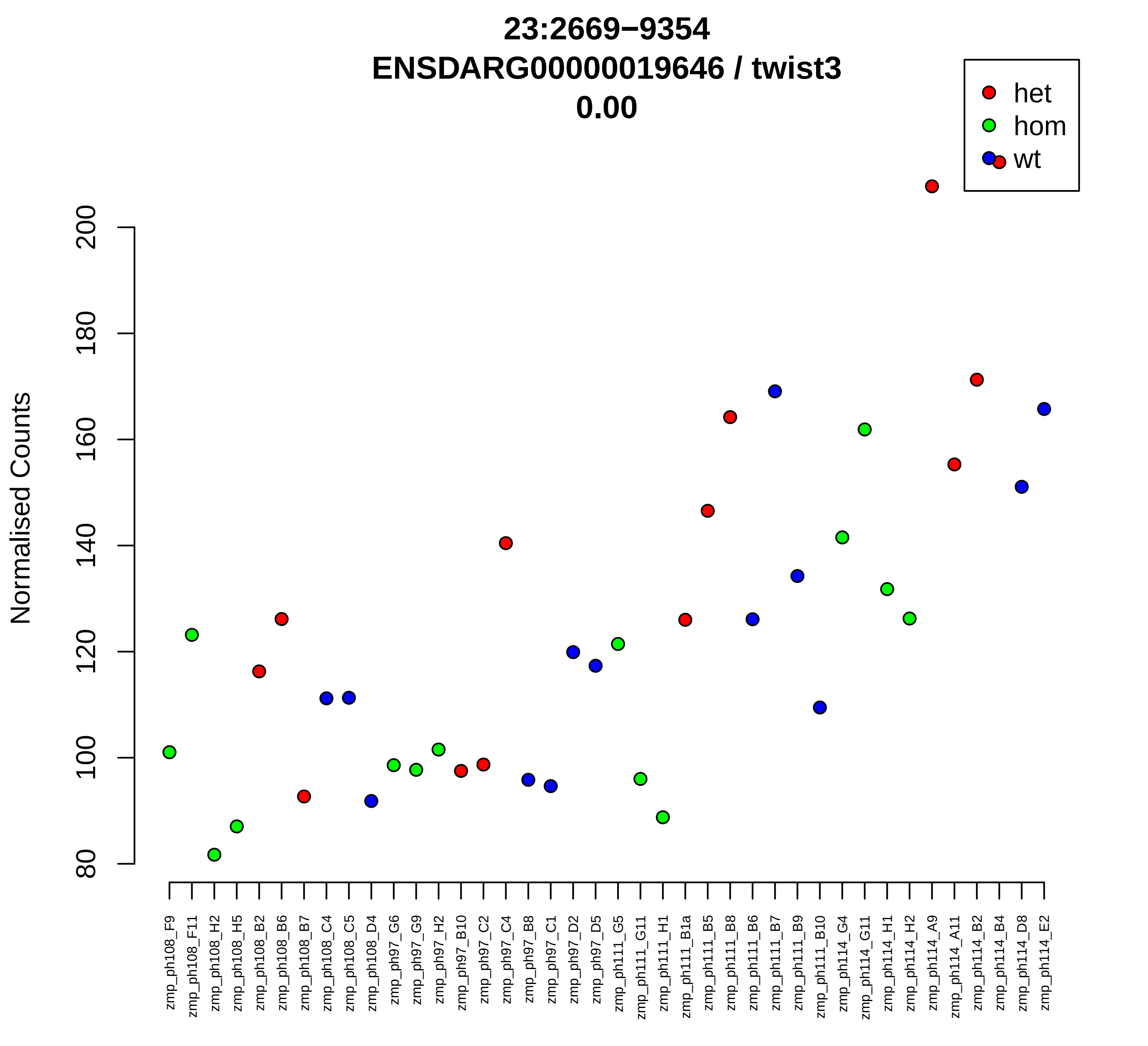

23:2669−9354
ENSDARG00000019646 / twist3
0.00
het
hom
wt
Normalised Counts
80
100
120
140
160
180
200
zmp_ph108_F9
zmp_ph108_F11
zmp_ph108_H2
zmp_ph108_H5
zmp_ph108_B2
zmp_ph108_B6
zmp_ph108_B7
zmp_ph108_C4
zmp_ph108_C5
zmp_ph108_D4
zmp_ph97_G6
zmp_ph97_G9
zmp_ph97_H2
zmp_ph97_B10
zmp_ph97_C2
zmp_ph97_C4
zmp_ph97_B8
zmp_ph97_C1
zmp_ph97_D2
zmp_ph97_D5
zmp_ph111_G5
zmp_ph111_G11
zmp_ph111_H1
zmp_ph111_B1a
zmp_ph111_B5
zmp_ph111_B8
zmp_ph111_B6
zmp_ph111_B7
zmp_ph111_B9
zmp_ph111_B10
zmp_ph114_G4
zmp_ph114_G11
zmp_ph114_H1
zmp_ph114_H2
zmp_ph114_A9
zmp_ph114_A11
zmp_ph114_B2
zmp_ph114_B4
zmp_ph114_D8
zmp_ph114_E2

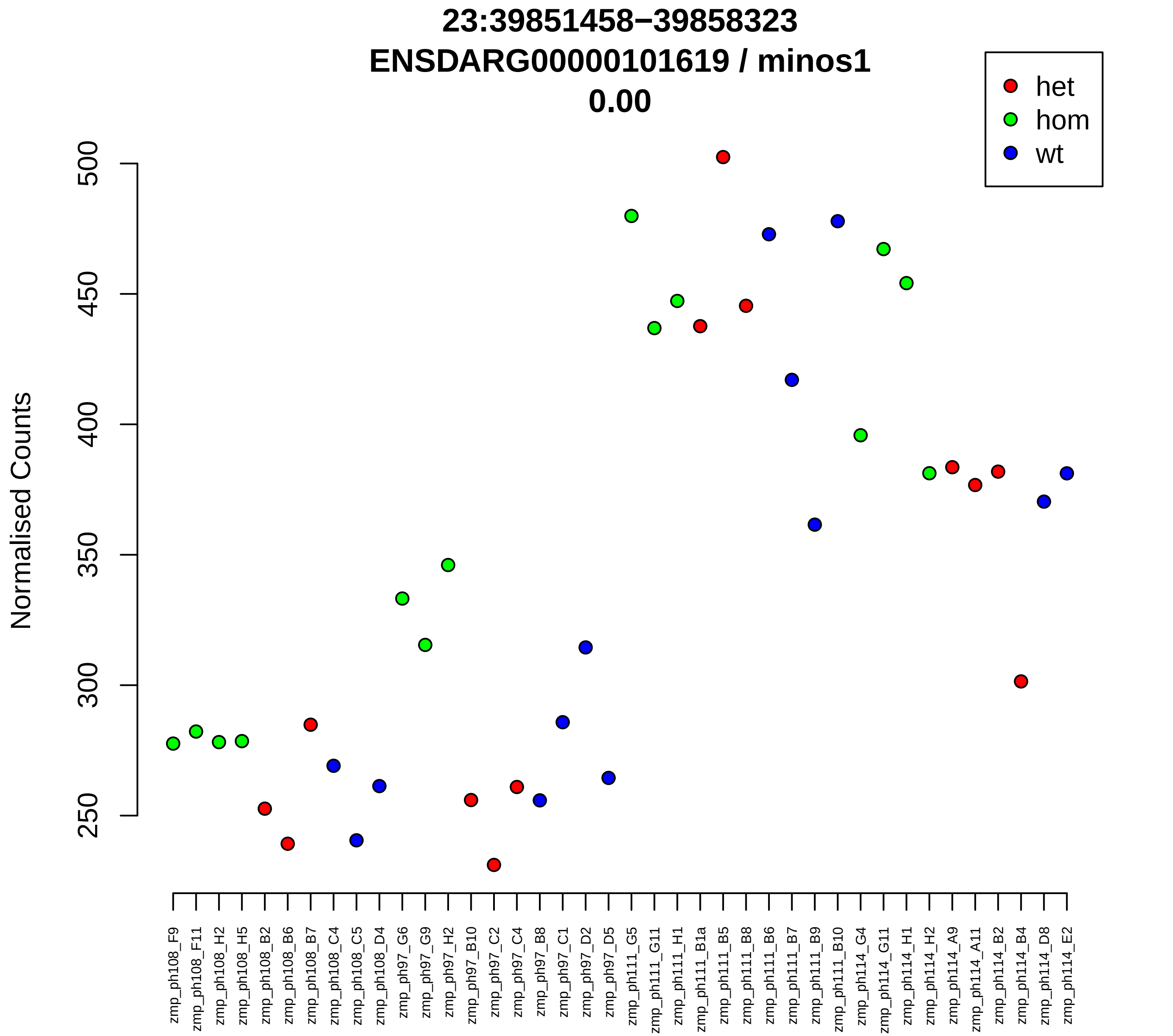
23:39851458−39858323
ENSDARG00000101619 / minos1
0.00
het
hom
wt
Normalised Counts
250
300
350
400
450
500
zmp_ph108_F9
zmp_ph108_F11
zmp_ph108_H2
zmp_ph108_H5
zmp_ph108_B2
zmp_ph108_B6
zmp_ph108_B7
zmp_ph108_C4
zmp_ph108_C5
zmp_ph108_D4
zmp_ph97_G6
zmp_ph97_G9
zmp_ph97_H2
zmp_ph97_B10
zmp_ph97_C2
zmp_ph97_C4
zmp_ph97_B8
zmp_ph97_C1
zmp_ph97_D2
zmp_ph97_D5
zmp_ph111_G5
zmp_ph111_G11
zmp_ph111_H1
zmp_ph111_B1a
zmp_ph111_B5
zmp_ph111_B8
zmp_ph111_B6
zmp_ph111_B7
zmp_ph111_B9
zmp_ph111_B10
zmp_ph114_G4
zmp_ph114_G11
zmp_ph114_H1
zmp_ph114_H2
zmp_ph114_A9
zmp_ph114_A11
zmp_ph114_B2
zmp_ph114_B4
zmp_ph114_D8
zmp_ph114_E2

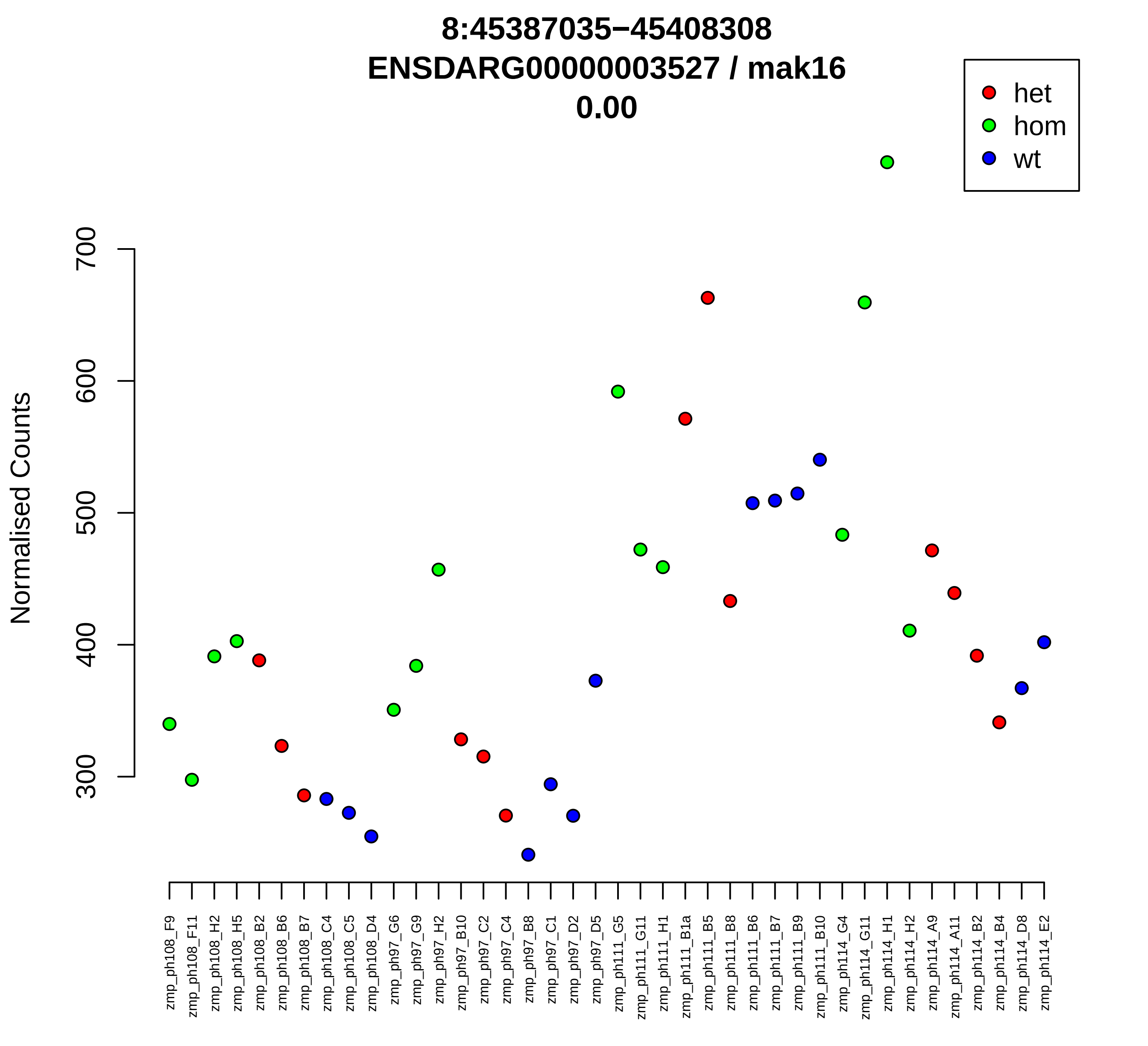
8:45387035−45408308
ENSDARG00000003527 / mak16
0.00
het
hom
wt
Normalised Counts
300
400
500
600
700
zmp_ph108_F9
zmp_ph108_F11
zmp_ph108_H2
zmp_ph108_H5
zmp_ph108_B2
zmp_ph108_B6
zmp_ph108_B7
zmp_ph108_C4
zmp_ph108_C5
zmp_ph108_D4
zmp_ph97_G6
zmp_ph97_G9
zmp_ph97_H2
zmp_ph97_B10
zmp_ph97_C2
zmp_ph97_C4
zmp_ph97_B8
zmp_ph97_C1
zmp_ph97_D2
zmp_ph97_D5
zmp_ph111_G5
zmp_ph111_G11
zmp_ph111_H1
zmp_ph111_B1a
zmp_ph111_B5
zmp_ph111_B8
zmp_ph111_B6
zmp_ph111_B7
zmp_ph111_B9
zmp_ph111_B10
zmp_ph114_G4
zmp_ph114_G11
zmp_ph114_H1
zmp_ph114_H2
zmp_ph114_A9
zmp_ph114_A11
zmp_ph114_B2
zmp_ph114_B4
zmp_ph114_D8
zmp_ph114_E2

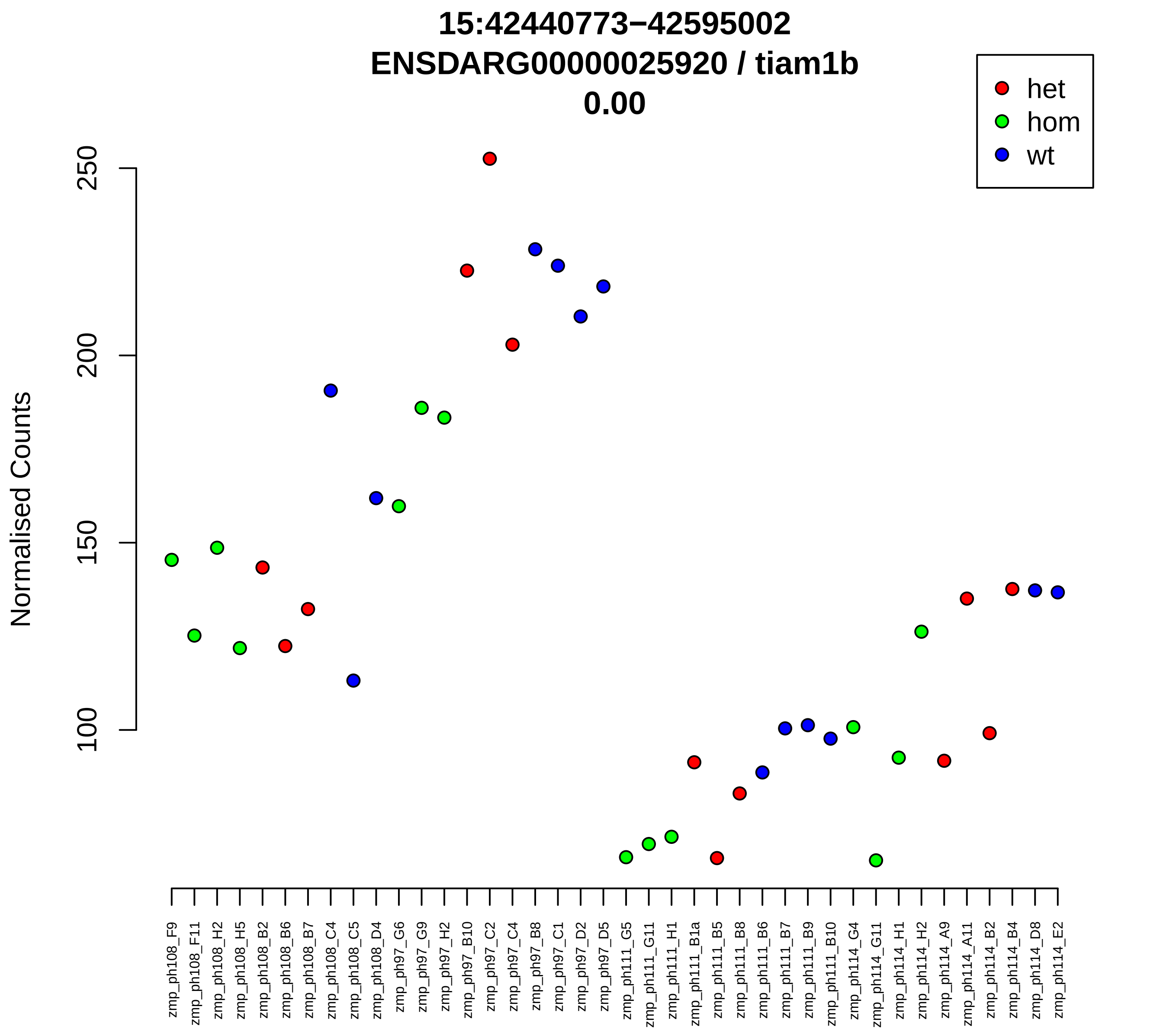
15:42440773−42595002
ENSDARG00000025920 / tiam1b
0.00
het
hom
wt
Normalised Counts
250
200
150
100
zmp_ph108_F9
zmp_ph108_F11
zmp_ph108_H2
zmp_ph108_H5
zmp_ph108_B2
zmp_ph108_B6
zmp_ph108_B7
zmp_ph108_C4
zmp_ph108_C5
zmp_ph108_D4
zmp_ph97_G6
zmp_ph97_G9
zmp_ph97_H2
zmp_ph97_B10
zmp_ph97_C2
zmp_ph97_C4
zmp_ph97_B8
zmp_ph97_C1
zmp_ph97_D2
zmp_ph97_D5
zmp_ph111_G5
zmp_ph111_G11
zmp_ph111_H1
zmp_ph111_B1a
zmp_ph111_B5
zmp_ph111_B8
zmp_ph111_B6
zmp_ph111_B7
zmp_ph111_B9
zmp_ph111_B10
zmp_ph114_G4
zmp_ph114_G11
zmp_ph114_H1
zmp_ph114_H2
zmp_ph114_A9
zmp_ph114_A11
zmp_ph114_B2
zmp_ph114_B4
zmp_ph114_D8
zmp_ph114_E2

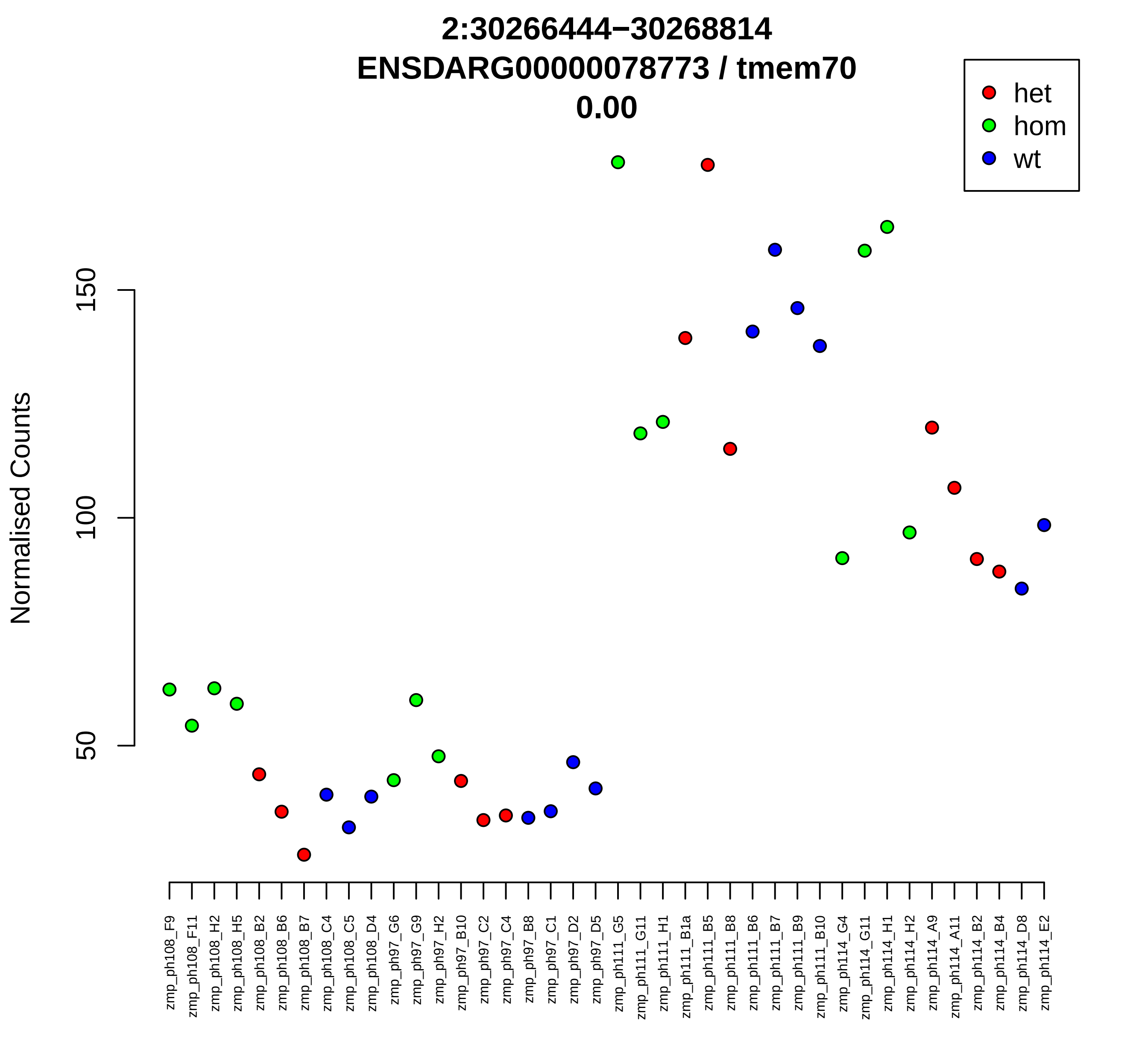
2:30266444−30268814
ENSDARG00000078773 / tmem70
0.00
het
hom
wt
Normalised Counts
50
100
150
zmp_ph108_F9
zmp_ph108_F11
zmp_ph108_H2
zmp_ph108_H5
zmp_ph108_B2
zmp_ph108_B6
zmp_ph108_B7
zmp_ph108_C4
zmp_ph108_C5
zmp_ph108_D4
zmp_ph97_G6
zmp_ph97_G9
zmp_ph97_H2
zmp_ph97_B10
zmp_ph97_C2
zmp_ph97_C4
zmp_ph97_B8
zmp_ph97_C1
zmp_ph97_D2
zmp_ph97_D5
zmp_ph111_G5
zmp_ph111_G11
zmp_ph111_H1
zmp_ph111_B1a
zmp_ph111_B5
zmp_ph111_B8
zmp_ph111_B6
zmp_ph111_B7
zmp_ph111_B9
zmp_ph111_B10
zmp_ph114_G4
zmp_ph114_G11
zmp_ph114_H1
zmp_ph114_H2
zmp_ph114_A9
zmp_ph114_A11
zmp_ph114_B2
zmp_ph114_B4
zmp_ph114_D8
zmp_ph114_E2

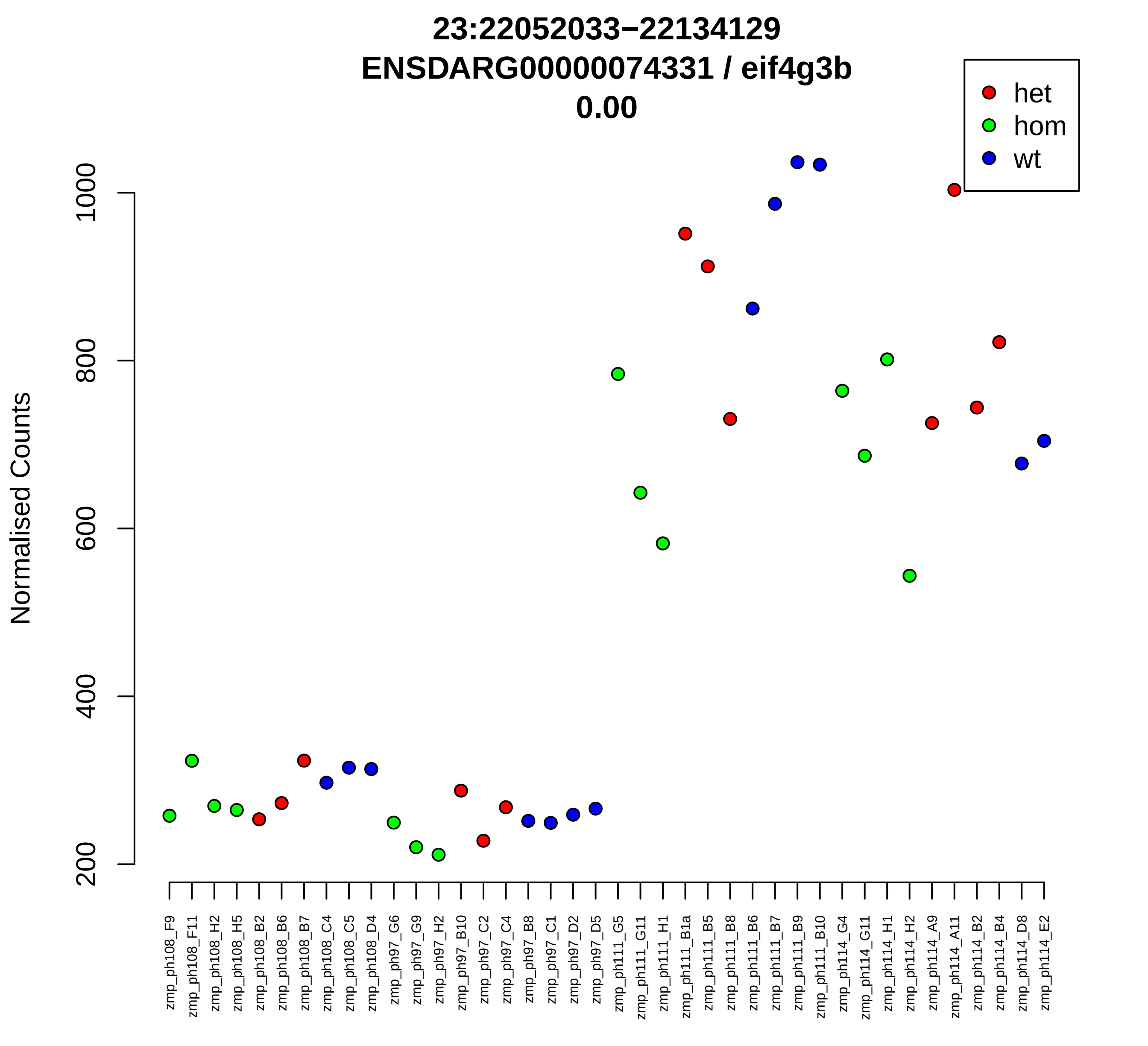

23:22052033−22134129
ENSDARG00000074331 / eif4g3b
0.00
het
hom
wt
Normalised Counts
200
400
600
800
1000
zmp_ph108_F9
zmp_ph108_F11
zmp_ph108_H2
zmp_ph108_H5
zmp_ph108_B2
zmp_ph108_B6
zmp_ph108_B7
zmp_ph108_C4
zmp_ph108_C5
zmp_ph108_D4
zmp_ph97_G6
zmp_ph97_G9
zmp_ph97_H2
zmp_ph97_B10
zmp_ph97_C2
zmp_ph97_C4
zmp_ph97_B8
zmp_ph97_C1
zmp_ph97_D2
zmp_ph97_D5
zmp_ph111_G5
zmp_ph111_G11
zmp_ph111_H1
zmp_ph111_B1a
zmp_ph111_B5
zmp_ph111_B8
zmp_ph111_B6
zmp_ph111_B7
zmp_ph111_B9
zmp_ph111_B10
zmp_ph114_G4
zmp_ph114_G11
zmp_ph114_H1
zmp_ph114_H2
zmp_ph114_A9
zmp_ph114_A11
zmp_ph114_B2
zmp_ph114_B4
zmp_ph114_D8
zmp_ph114_E2

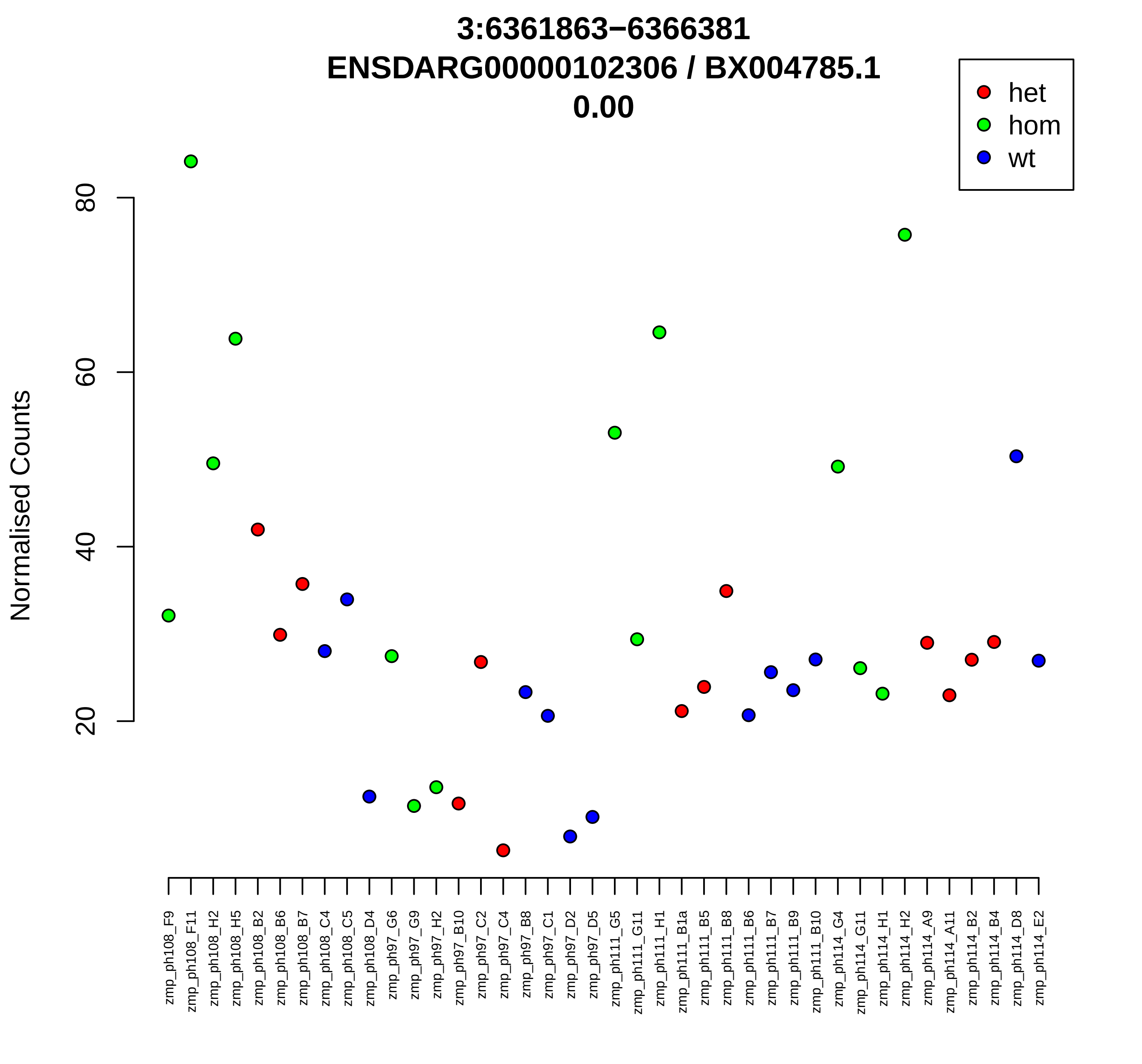
3:6361863−6366381
ENSDARG00000102306 / BX004785.1
0.00
het
hom
wt
Normalised Counts
20
40
60
80
zmp_ph108_F9
zmp_ph108_F11
zmp_ph108_H2
zmp_ph108_H5
zmp_ph108_B2
zmp_ph108_B6
zmp_ph108_B7
zmp_ph108_C4
zmp_ph108_C5
zmp_ph108_D4
zmp_ph97_G6
zmp_ph97_G9
zmp_ph97_H2
zmp_ph97_B10
zmp_ph97_C2
zmp_ph97_C4
zmp_ph97_B8
zmp_ph97_C1
zmp_ph97_D2
zmp_ph97_D5
zmp_ph111_G5
zmp_ph111_G11
zmp_ph111_H1
zmp_ph111_B1a
zmp_ph111_B5
zmp_ph111_B8
zmp_ph111_B6
zmp_ph111_B7
zmp_ph111_B9
zmp_ph111_B10
zmp_ph114_G4
zmp_ph114_G11
zmp_ph114_H1
zmp_ph114_H2
zmp_ph114_A9
zmp_ph114_A11
zmp_ph114_B2
zmp_ph114_B4
zmp_ph114_D8
zmp_ph114_E2

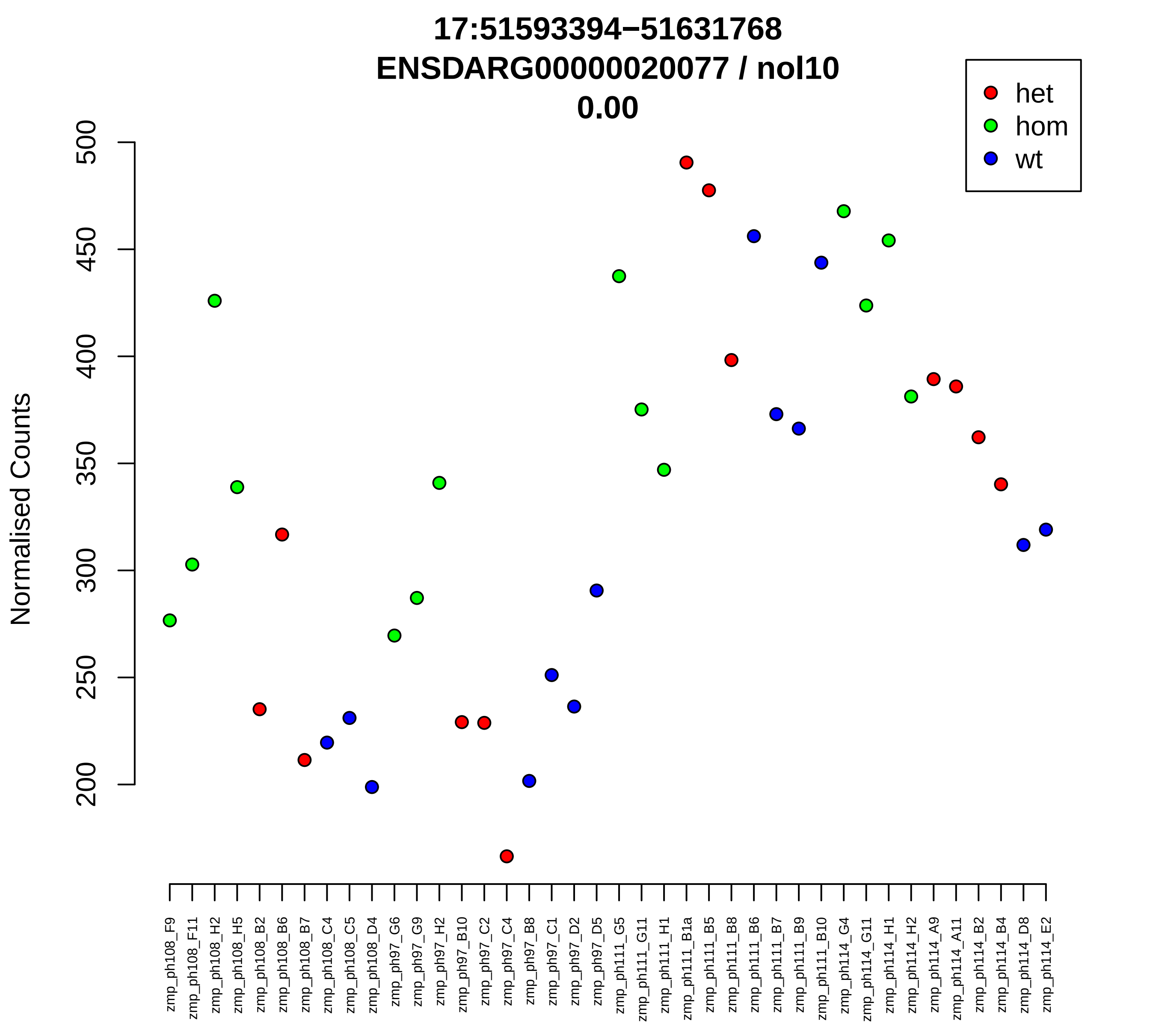

17:51593394−51631768
ENSDARG00000020077 / nol10
0.00
het
hom
wt
Normalised Counts
200
250
300
350
400
450
500
zmp_ph108_F9
zmp_ph108_F11
zmp_ph108_H2
zmp_ph108_H5
zmp_ph108_B2
zmp_ph108_B6
zmp_ph108_B7
zmp_ph108_C4
zmp_ph108_C5
zmp_ph108_D4
zmp_ph97_G6
zmp_ph97_G9
zmp_ph97_H2
zmp_ph97_B10
zmp_ph97_C2
zmp_ph97_C4
zmp_ph97_B8
zmp_ph97_C1
zmp_ph97_D2
zmp_ph97_D5
zmp_ph111_G5
zmp_ph111_G11
zmp_ph111_H1
zmp_ph111_B1a
zmp_ph111_B5
zmp_ph111_B8
zmp_ph111_B6
zmp_ph111_B7
zmp_ph111_B9
zmp_ph111_B10
zmp_ph114_G4
zmp_ph114_G11
zmp_ph114_H1
zmp_ph114_H2
zmp_ph114_A9
zmp_ph114_A11
zmp_ph114_B2
zmp_ph114_B4
zmp_ph114_D8
zmp_ph114_E2

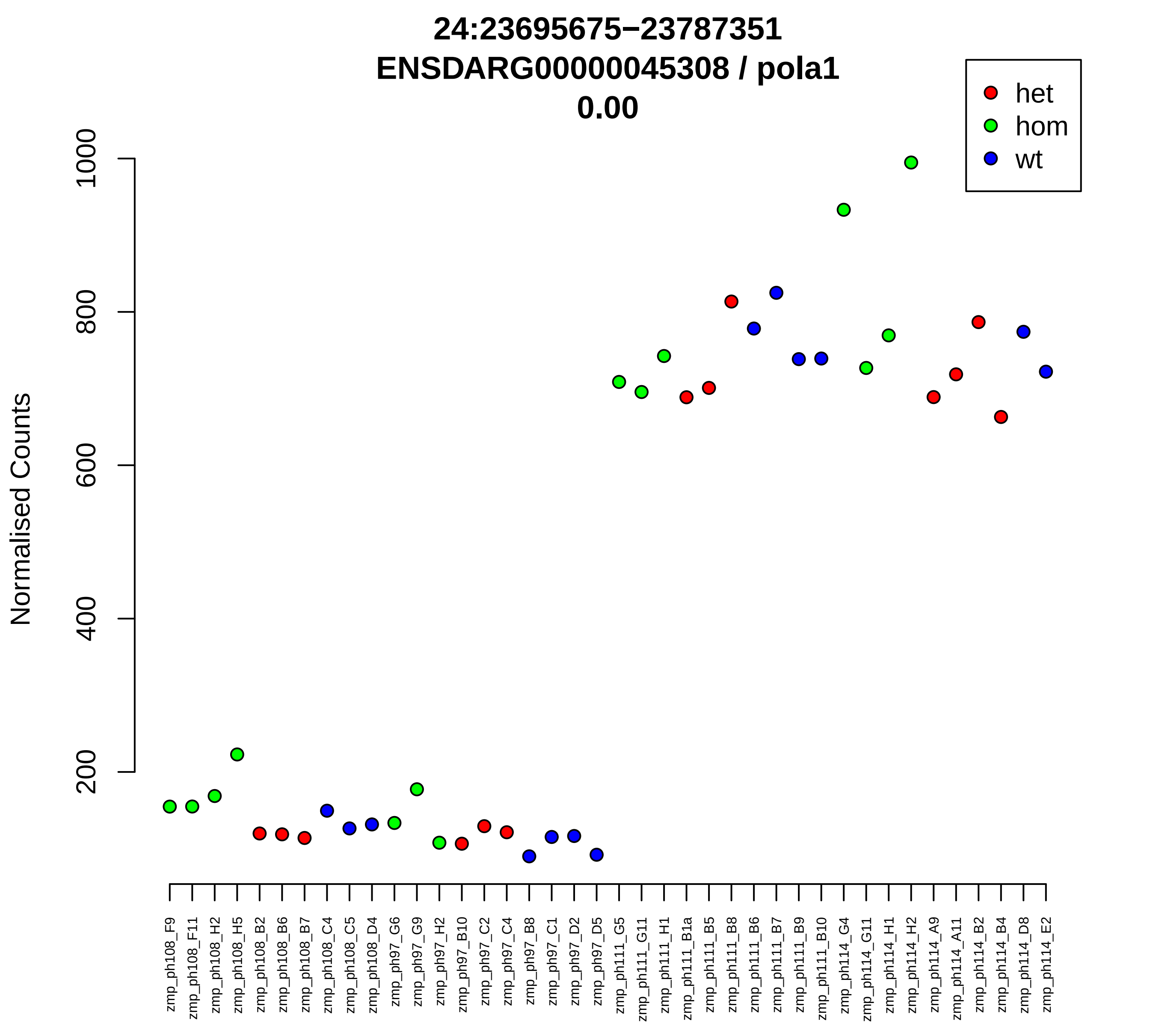
24:23695675−23787351
ENSDARG00000045308 / pola1
0.00
het
hom
wt
Normalised Counts
200
400
600
800
1000
zmp_ph108_F9
zmp_ph108_F11
zmp_ph108_H2
zmp_ph108_H5
zmp_ph108_B2
zmp_ph108_B6
zmp_ph108_B7
zmp_ph108_C4
zmp_ph108_C5
zmp_ph108_D4
zmp_ph97_G6
zmp_ph97_G9
zmp_ph97_H2
zmp_ph97_B10
zmp_ph97_C2
zmp_ph97_C4
zmp_ph97_B8
zmp_ph97_C1
zmp_ph97_D2
zmp_ph97_D5
zmp_ph111_G5
zmp_ph111_G11
zmp_ph111_H1
zmp_ph111_B1a
zmp_ph111_B5
zmp_ph111_B8
zmp_ph111_B6
zmp_ph111_B7
zmp_ph111_B9
zmp_ph111_B10
zmp_ph114_G4
zmp_ph114_G11
zmp_ph114_H1
zmp_ph114_H2
zmp_ph114_A9
zmp_ph114_A11
zmp_ph114_B2
zmp_ph114_B4
zmp_ph114_D8
zmp_ph114_E2

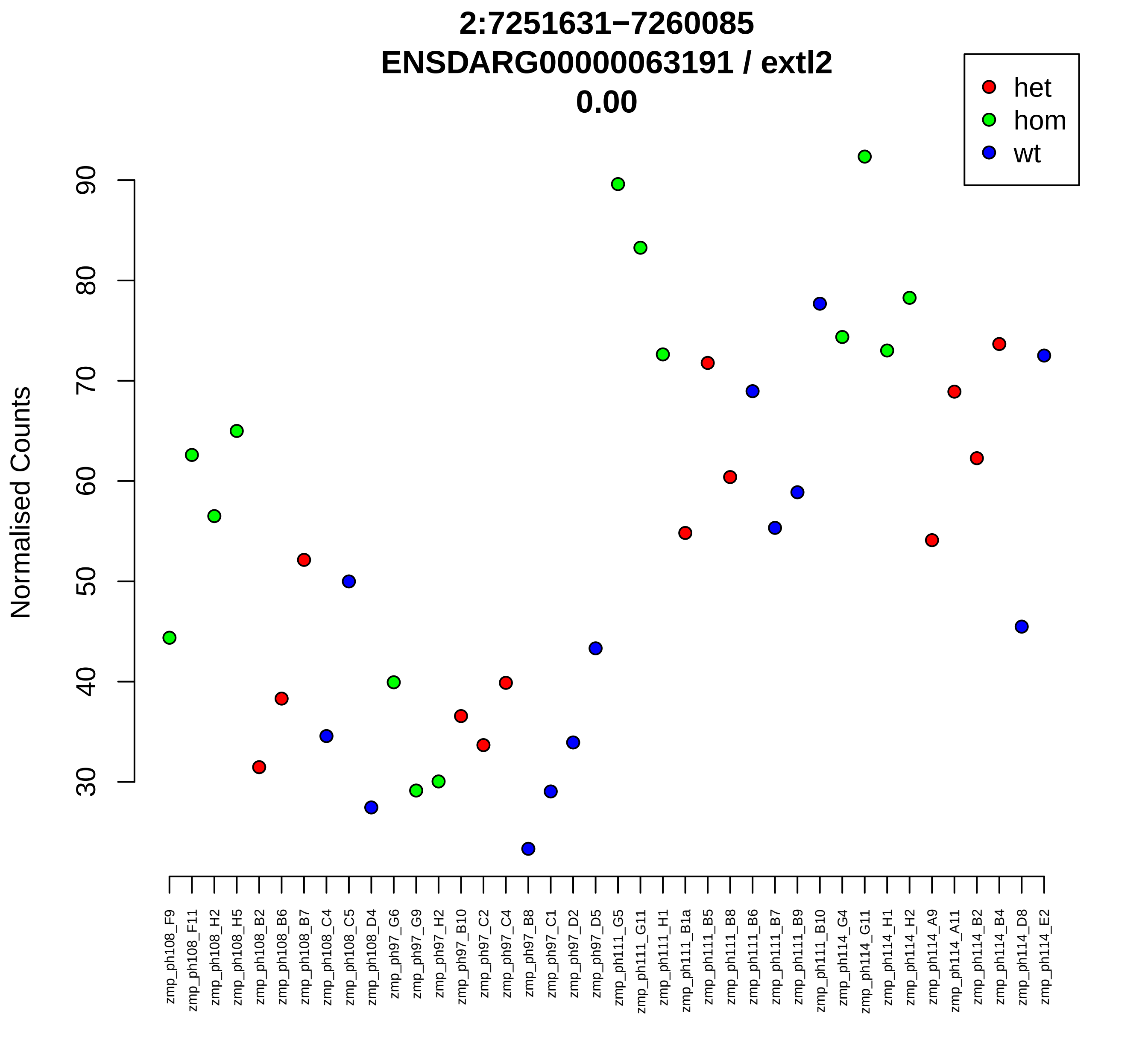

2:7251631−7260085
ENSDARG00000063191 / extl2
0.00
het
hom
wt
Normalised Counts
30
40
50
60
70
80
90
zmp_ph108_F9
zmp_ph108_F11
zmp_ph108_H2
zmp_ph108_H5
zmp_ph108_B2
zmp_ph108_B6
zmp_ph108_B7
zmp_ph108_C4
zmp_ph108_C5
zmp_ph108_D4
zmp_ph97_G6
zmp_ph97_G9
zmp_ph97_H2
zmp_ph97_B10
zmp_ph97_C2
zmp_ph97_C4
zmp_ph97_B8
zmp_ph97_C1
zmp_ph97_D2
zmp_ph97_D5
zmp_ph111_G5
zmp_ph111_G11
zmp_ph111_H1
zmp_ph111_B1a
zmp_ph111_B5
zmp_ph111_B8
zmp_ph111_B6
zmp_ph111_B7
zmp_ph111_B9
zmp_ph111_B10
zmp_ph114_G4
zmp_ph114_G11
zmp_ph114_H1
zmp_ph114_H2
zmp_ph114_A9
zmp_ph114_A11
zmp_ph114_B2
zmp_ph114_B4
zmp_ph114_D8
zmp_ph114_E2

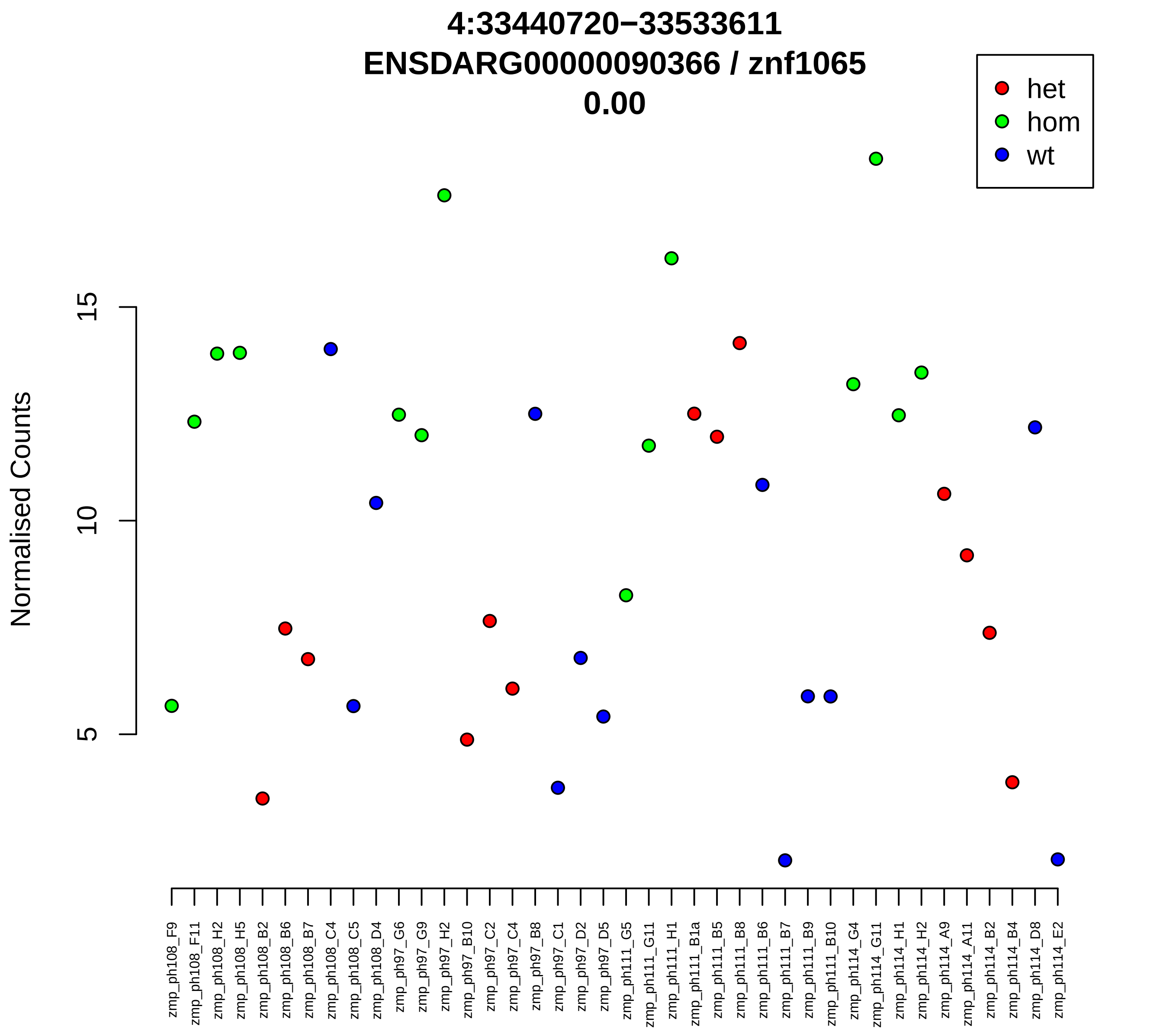
4:33440720−33533611
ENSDARG00000090366 / znf1065
0.00
het
hom
wt
Normalised Counts
5
10
15
zmp_ph108_F9
zmp_ph108_F11
zmp_ph108_H2
zmp_ph108_H5
zmp_ph108_B2
zmp_ph108_B6
zmp_ph108_B7
zmp_ph108_C4
zmp_ph108_C5
zmp_ph108_D4
zmp_ph97_G6
zmp_ph97_G9
zmp_ph97_H2
zmp_ph97_B10
zmp_ph97_C2
zmp_ph97_C4
zmp_ph97_B8
zmp_ph97_C1
zmp_ph97_D2
zmp_ph97_D5
zmp_ph111_G5
zmp_ph111_G11
zmp_ph111_H1
zmp_ph111_B1a
zmp_ph111_B5
zmp_ph111_B8
zmp_ph111_B6
zmp_ph111_B7
zmp_ph111_B9
zmp_ph111_B10
zmp_ph114_G4
zmp_ph114_G11
zmp_ph114_H1
zmp_ph114_H2
zmp_ph114_A9
zmp_ph114_A11
zmp_ph114_B2
zmp_ph114_B4
zmp_ph114_D8
zmp_ph114_E2

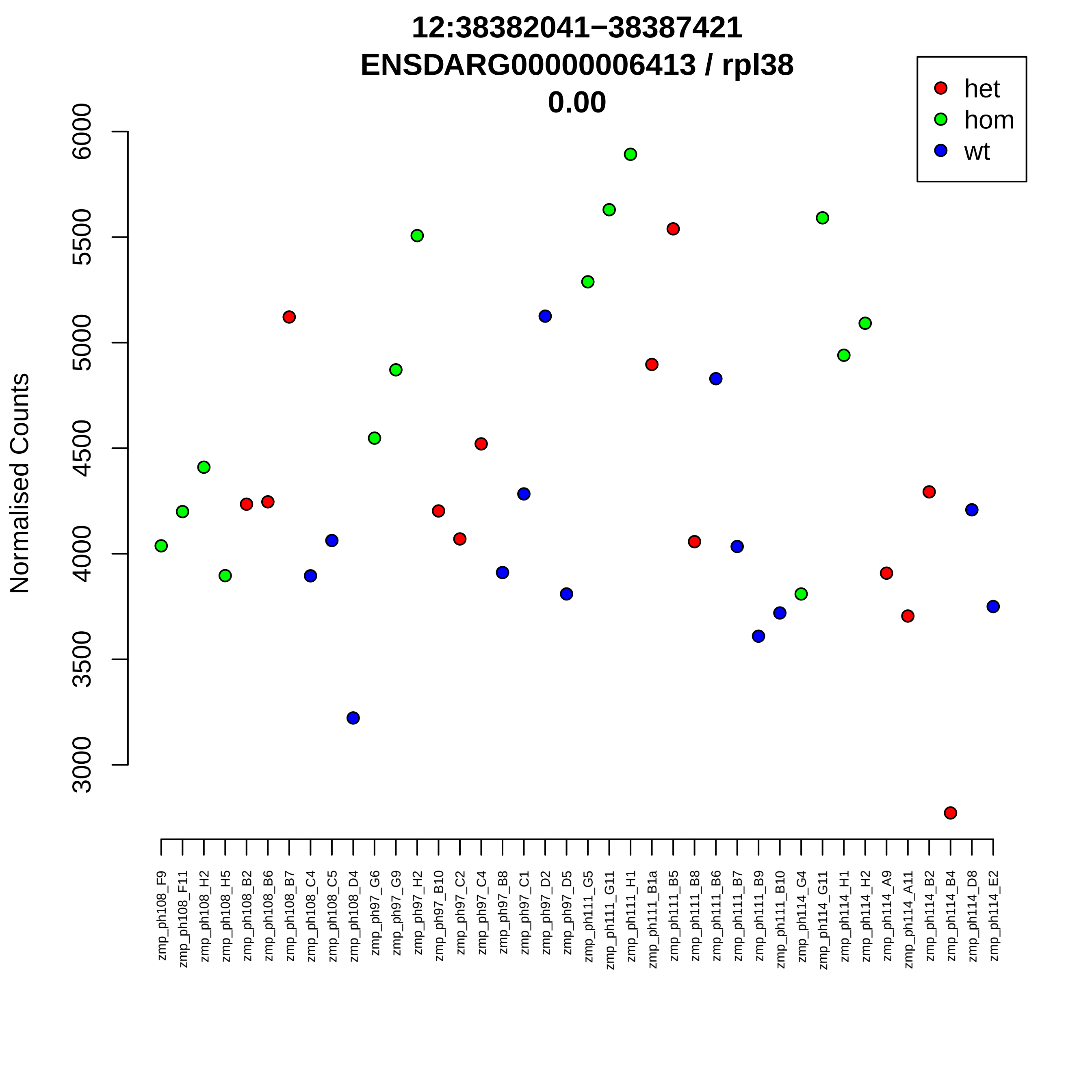
12:38382041−38387421
ENSDARG00000006413 / rpl38
0.00
het
hom
wt
Normalised Counts
3000
3500
4000
4500
5000
5500
6000
zmp_ph108_F9
zmp_ph108_F11
zmp_ph108_H2
zmp_ph108_H5
zmp_ph108_B2
zmp_ph108_B6
zmp_ph108_B7
zmp_ph108_C4
zmp_ph108_C5
zmp_ph108_D4
zmp_ph97_G6
zmp_ph97_G9
zmp_ph97_H2
zmp_ph97_B10
zmp_ph97_C2
zmp_ph97_C4
zmp_ph97_B8
zmp_ph97_C1
zmp_ph97_D2
zmp_ph97_D5
zmp_ph111_G5
zmp_ph111_G11
zmp_ph111_H1
zmp_ph111_B1a
zmp_ph111_B5
zmp_ph111_B8
zmp_ph111_B6
zmp_ph111_B7
zmp_ph111_B9
zmp_ph111_B10
zmp_ph114_G4
zmp_ph114_G11
zmp_ph114_H1
zmp_ph114_H2
zmp_ph114_A9
zmp_ph114_A11
zmp_ph114_B2
zmp_ph114_B4
zmp_ph114_D8
zmp_ph114_E2

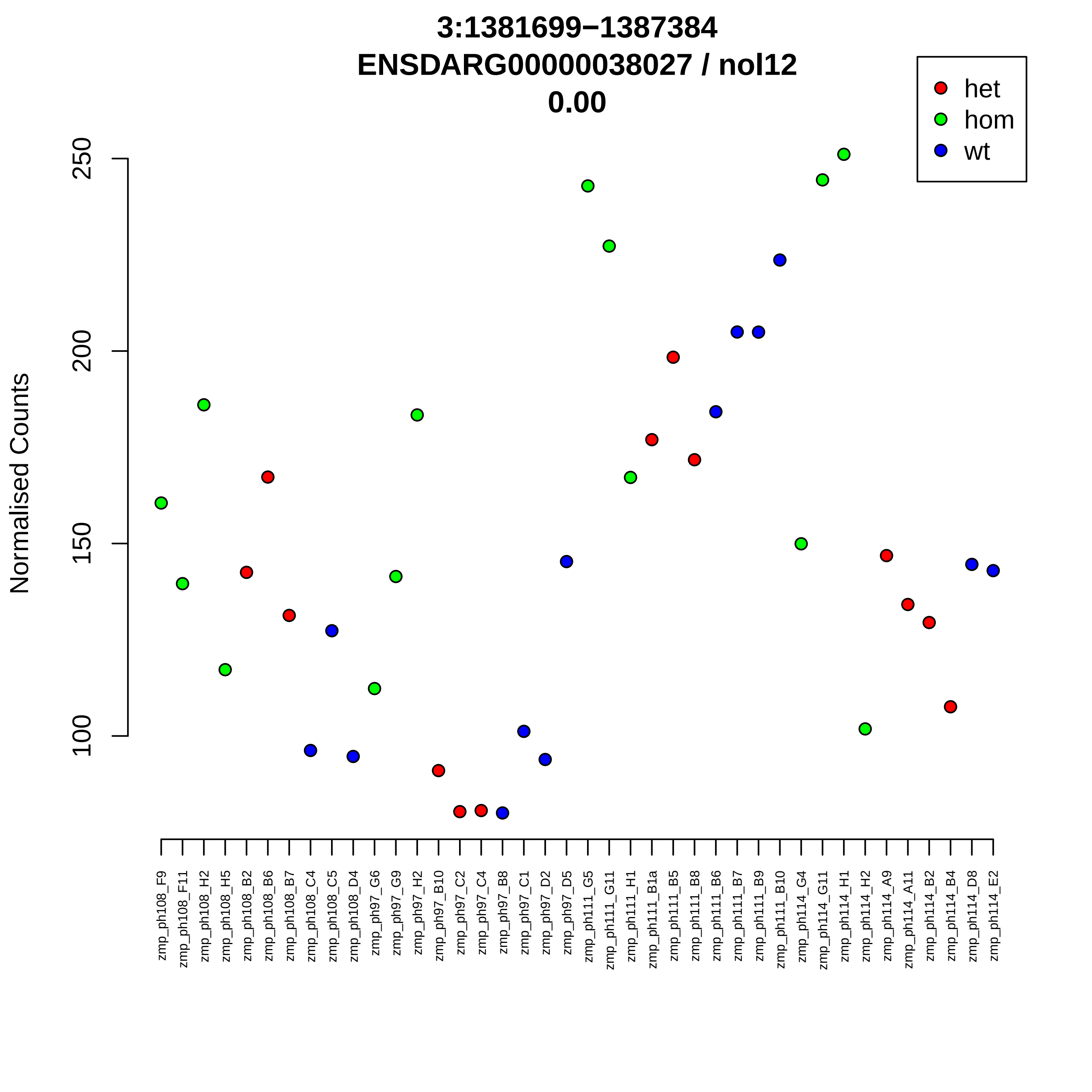

3:1381699−1387384
ENSDARG00000038027 / nol12
0.00
het
hom
wt
Normalised Counts
100
150
200
250
zmp_ph108_F9
zmp_ph108_F11
zmp_ph108_H2
zmp_ph108_H5
zmp_ph108_B2
zmp_ph108_B6
zmp_ph108_B7
zmp_ph108_C4
zmp_ph108_C5
zmp_ph108_D4
zmp_ph97_G6
zmp_ph97_G9
zmp_ph97_H2
zmp_ph97_B10
zmp_ph97_C2
zmp_ph97_C4
zmp_ph97_B8
zmp_ph97_C1
zmp_ph97_D2
zmp_ph97_D5
zmp_ph111_G5
zmp_ph111_G11
zmp_ph111_H1
zmp_ph111_B1a
zmp_ph111_B5
zmp_ph111_B8
zmp_ph111_B6
zmp_ph111_B7
zmp_ph111_B9
zmp_ph111_B10
zmp_ph114_G4
zmp_ph114_G11
zmp_ph114_H1
zmp_ph114_H2
zmp_ph114_A9
zmp_ph114_A11
zmp_ph114_B2
zmp_ph114_B4
zmp_ph114_D8
zmp_ph114_E2

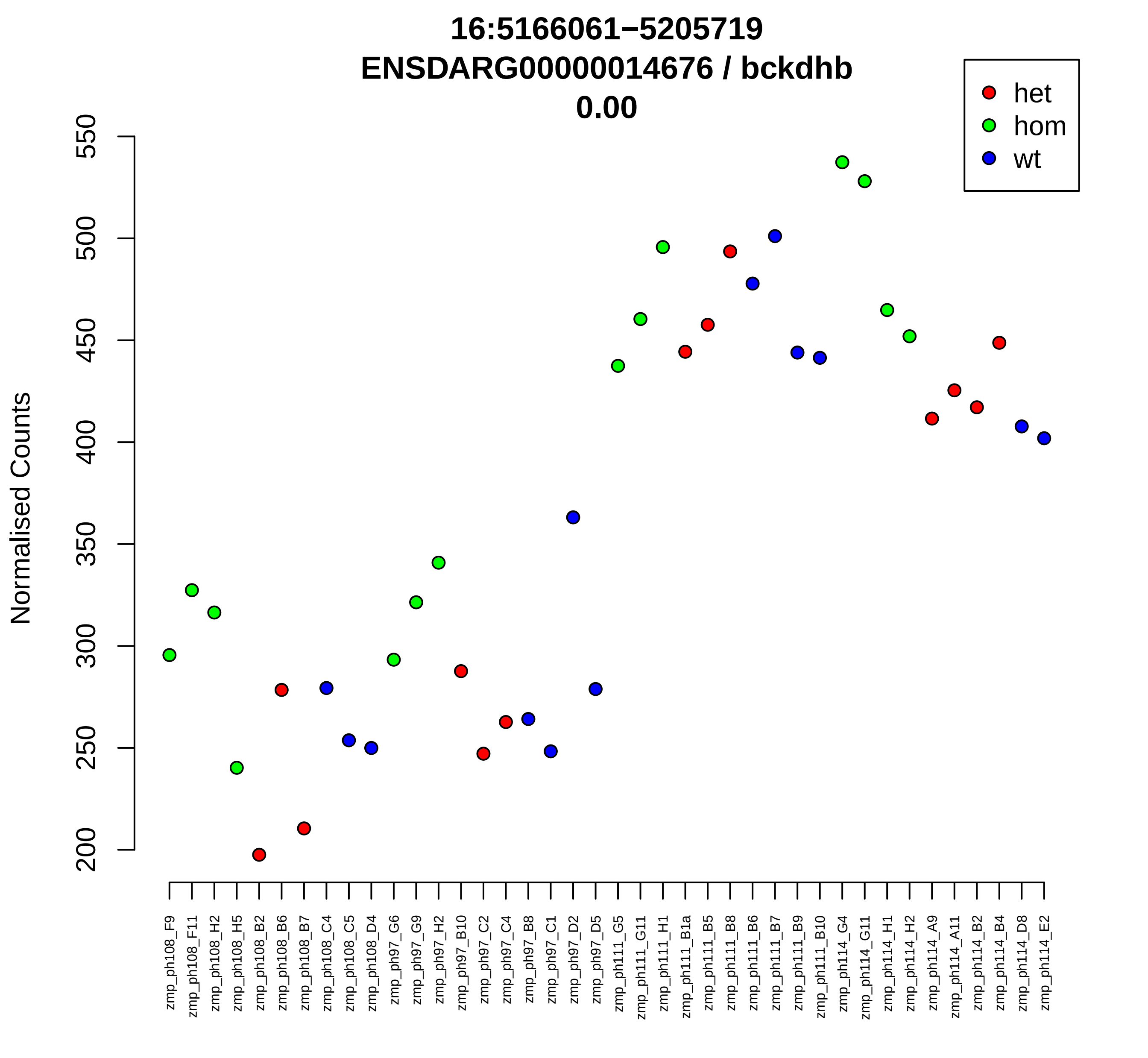

16:5166061−5205719
ENSDARG00000014676 / bckdhb
0.00
het
hom
wt
Normalised Counts
200
250
300
350
400
450
500
550
zmp_ph108_F9
zmp_ph108_F11
zmp_ph108_H2
zmp_ph108_H5
zmp_ph108_B2
zmp_ph108_B6
zmp_ph108_B7
zmp_ph108_C4
zmp_ph108_C5
zmp_ph108_D4
zmp_ph97_G6
zmp_ph97_G9
zmp_ph97_H2
zmp_ph97_B10
zmp_ph97_C2
zmp_ph97_C4
zmp_ph97_B8
zmp_ph97_C1
zmp_ph97_D2
zmp_ph97_D5
zmp_ph111_G5
zmp_ph111_G11
zmp_ph111_H1
zmp_ph111_B1a
zmp_ph111_B5
zmp_ph111_B8
zmp_ph111_B6
zmp_ph111_B7
zmp_ph111_B9
zmp_ph111_B10
zmp_ph114_G4
zmp_ph114_G11
zmp_ph114_H1
zmp_ph114_H2
zmp_ph114_A9
zmp_ph114_A11
zmp_ph114_B2
zmp_ph114_B4
zmp_ph114_D8
zmp_ph114_E2

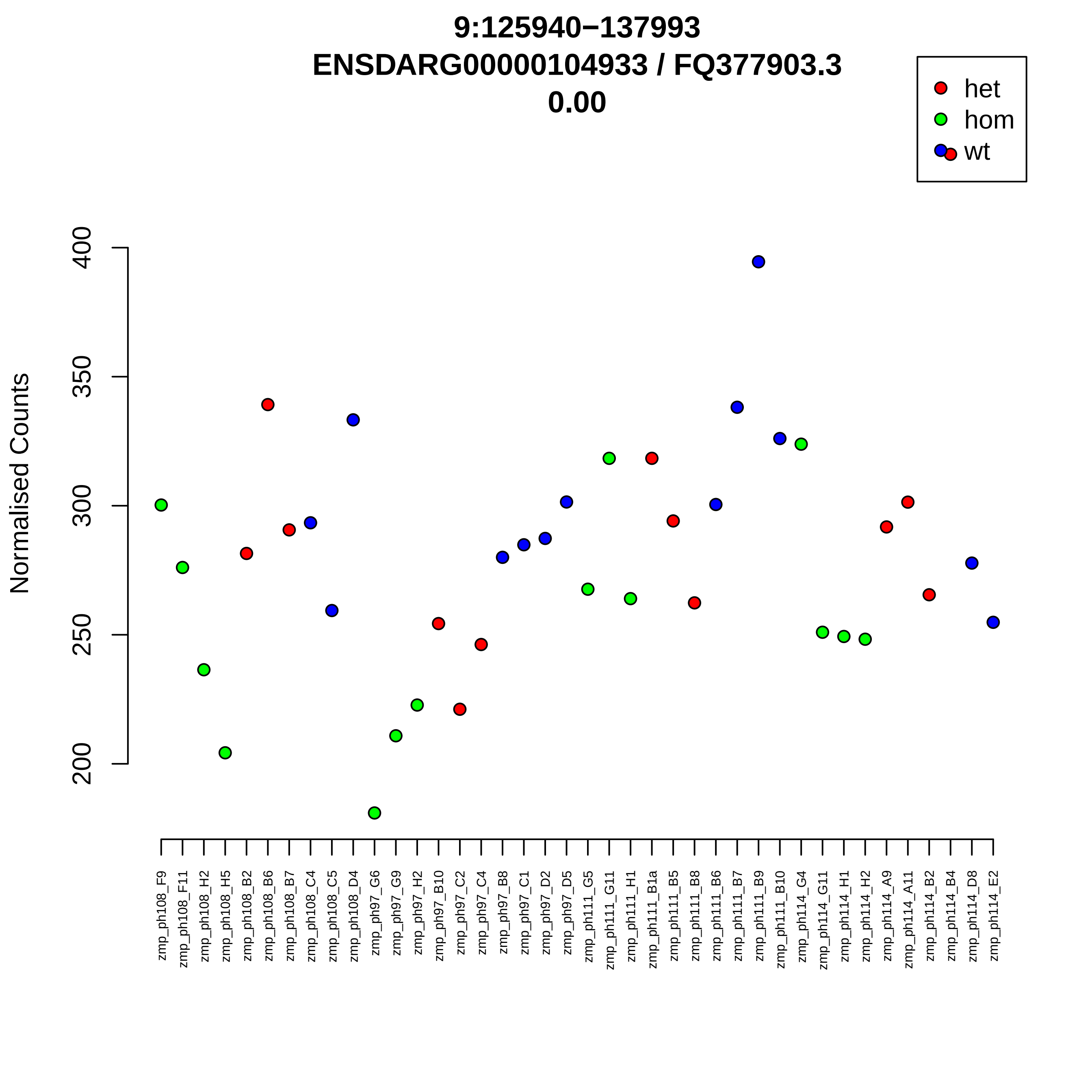

9:125940−137993
ENSDARG00000104933 / FQ377903.3
0.00
het
hom
wt
Normalised Counts
400
350
300
250
200
zmp_ph108_F9
zmp_ph108_F11
zmp_ph108_H2
zmp_ph108_H5
zmp_ph108_B2
zmp_ph108_B6
zmp_ph108_B7
zmp_ph108_C4
zmp_ph108_C5
zmp_ph108_D4
zmp_ph97_G6
zmp_ph97_G9
zmp_ph97_H2
zmp_ph97_B10
zmp_ph97_C2
zmp_ph97_C4
zmp_ph97_B8
zmp_ph97_C1
zmp_ph97_D2
zmp_ph97_D5
zmp_ph111_G5
zmp_ph111_G11
zmp_ph111_H1
zmp_ph111_B1a
zmp_ph111_B5
zmp_ph111_B8
zmp_ph111_B6
zmp_ph111_B7
zmp_ph111_B9
zmp_ph111_B10
zmp_ph114_G4
zmp_ph114_G11
zmp_ph114_H1
zmp_ph114_H2
zmp_ph114_A9
zmp_ph114_A11
zmp_ph114_B2
zmp_ph114_B4
zmp_ph114_D8
zmp_ph114_E2

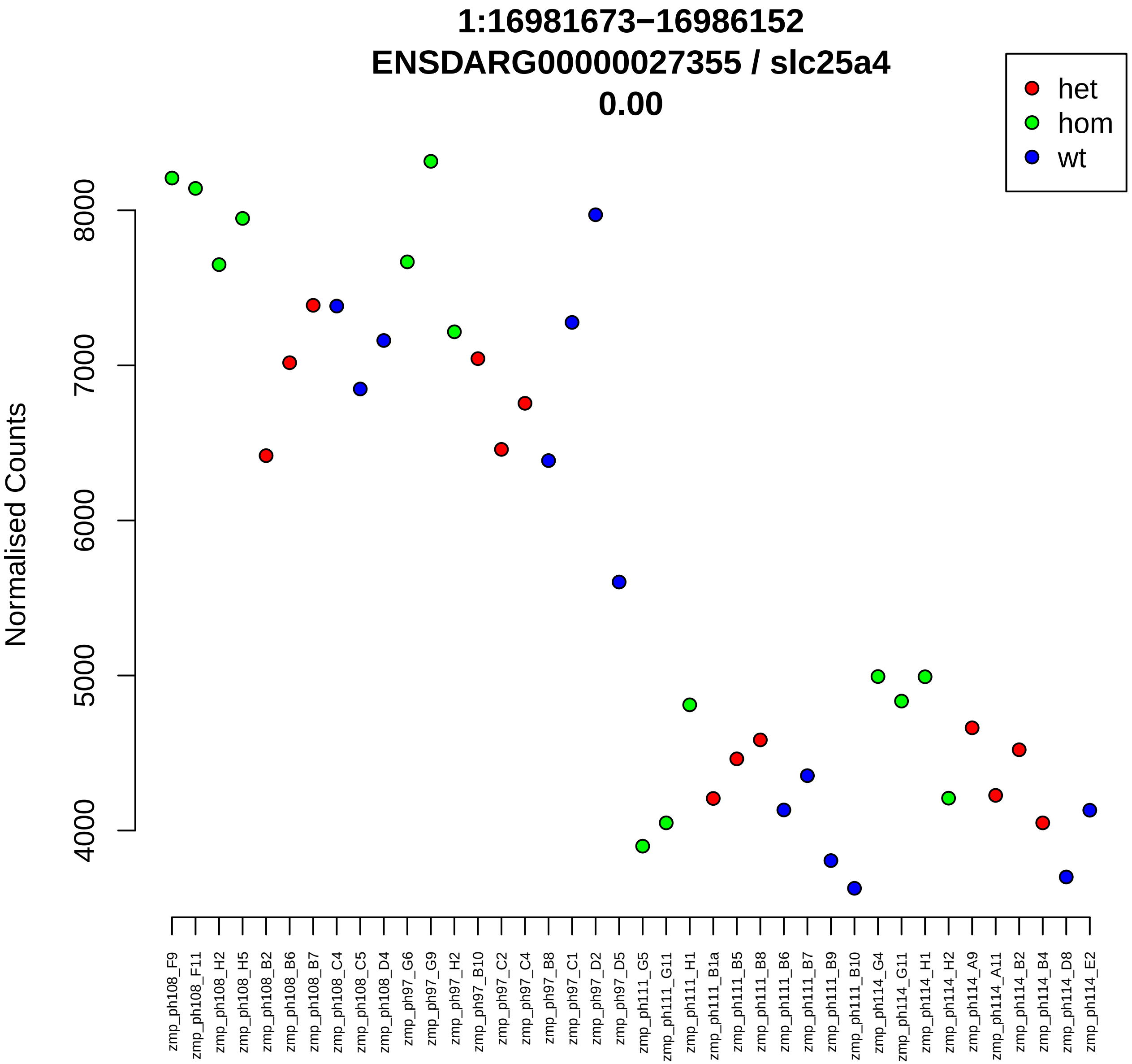
1:16981673−16986152
ENSDARG00000027355 / slc25a4
0.00
het
hom
wt
Normalised Counts
4000
5000
6000
7000
8000
zmp_ph108_F9
zmp_ph108_F11
zmp_ph108_H2
zmp_ph108_H5
zmp_ph108_B2
zmp_ph108_B6
zmp_ph108_B7
zmp_ph108_C4
zmp_ph108_C5
zmp_ph108_D4
zmp_ph97_G6
zmp_ph97_G9
zmp_ph97_H2
zmp_ph97_B10
zmp_ph97_C2
zmp_ph97_C4
zmp_ph97_B8
zmp_ph97_C1
zmp_ph97_D2
zmp_ph97_D5
zmp_ph111_G5
zmp_ph111_G11
zmp_ph111_H1
zmp_ph111_B1a
zmp_ph111_B5
zmp_ph111_B8
zmp_ph111_B6
zmp_ph111_B7
zmp_ph111_B9
zmp_ph111_B10
zmp_ph114_G4
zmp_ph114_G11
zmp_ph114_H1
zmp_ph114_H2
zmp_ph114_A9
zmp_ph114_A11
zmp_ph114_B2
zmp_ph114_B4
zmp_ph114_D8
zmp_ph114_E2

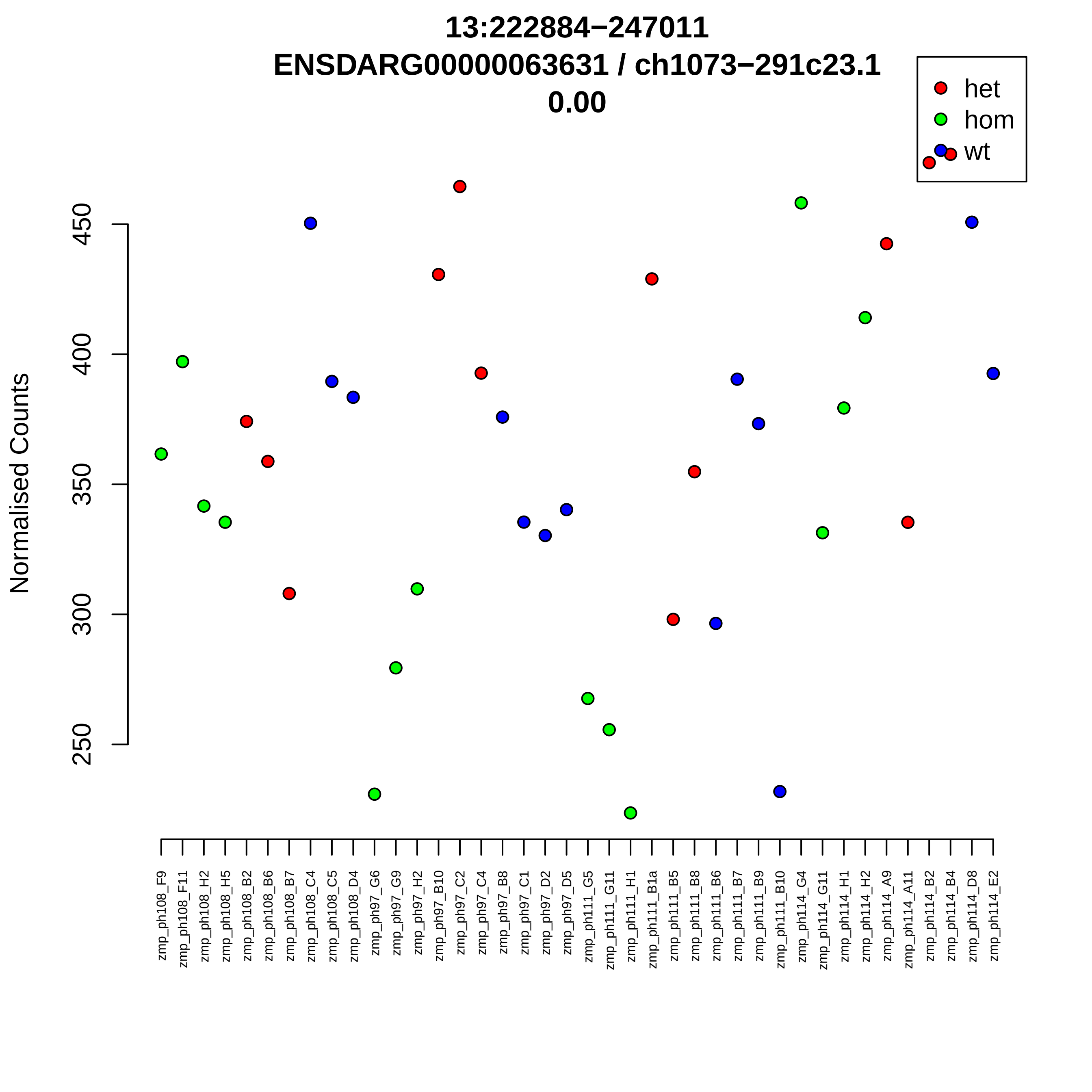
13:222884−247011
ENSDARG00000063631 / ch1073−291c23.1
0.00
het
hom
wt
Normalised Counts
250
300
350
400
450
zmp_ph108_F9
zmp_ph108_F11
zmp_ph108_H2
zmp_ph108_H5
zmp_ph108_B2
zmp_ph108_B6
zmp_ph108_B7
zmp_ph108_C4
zmp_ph108_C5
zmp_ph108_D4
zmp_ph97_G6
zmp_ph97_G9
zmp_ph97_H2
zmp_ph97_B10
zmp_ph97_C2
zmp_ph97_C4
zmp_ph97_B8
zmp_ph97_C1
zmp_ph97_D2
zmp_ph97_D5
zmp_ph111_G5
zmp_ph111_G11
zmp_ph111_H1
zmp_ph111_B1a
zmp_ph111_B5
zmp_ph111_B8
zmp_ph111_B6
zmp_ph111_B7
zmp_ph111_B9
zmp_ph111_B10
zmp_ph114_G4
zmp_ph114_G11
zmp_ph114_H1
zmp_ph114_H2
zmp_ph114_A9
zmp_ph114_A11
zmp_ph114_B2
zmp_ph114_B4
zmp_ph114_D8
zmp_ph114_E2

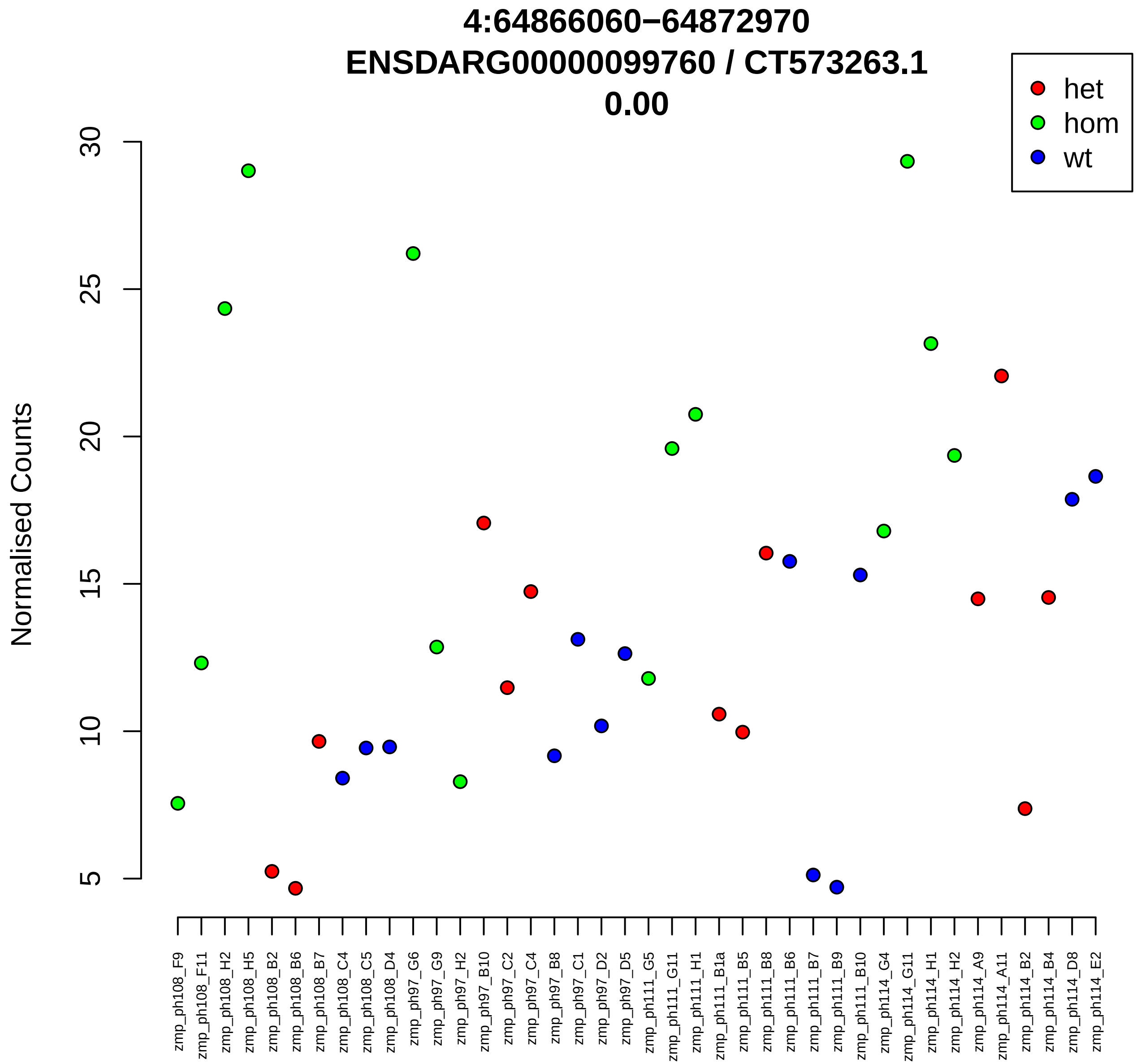

4:64866060−64872970
ENSDARG00000099760 / CT573263.1
0.00
Normalised Counts
het
hom
wt
5
10
15
20
25
30
zmp_ph108_F9
zmp_ph108_F11
zmp_ph108_H2
zmp_ph108_H5
zmp_ph108_B2
zmp_ph108_B6
zmp_ph108_B7
zmp_ph108_C4
zmp_ph108_C5
zmp_ph108_D4
zmp_ph97_G6
zmp_ph97_G9
zmp_ph97_H2
zmp_ph97_B10
zmp_ph97_C2
zmp_ph97_C4
zmp_ph97_B8
zmp_ph97_C1
zmp_ph97_D2
zmp_ph97_D5
zmp_ph111_G5
zmp_ph111_G11
zmp_ph111_H1
zmp_ph111_B1a
zmp_ph111_B5
zmp_ph111_B8
zmp_ph111_B6
zmp_ph111_B7
zmp_ph111_B9
zmp_ph111_B10
zmp_ph114_G4
zmp_ph114_G11
zmp_ph114_H1
zmp_ph114_H2
zmp_ph114_A9
zmp_ph114_A11
zmp_ph114_B2
zmp_ph114_B4
zmp_ph114_D8
zmp_ph114_E2

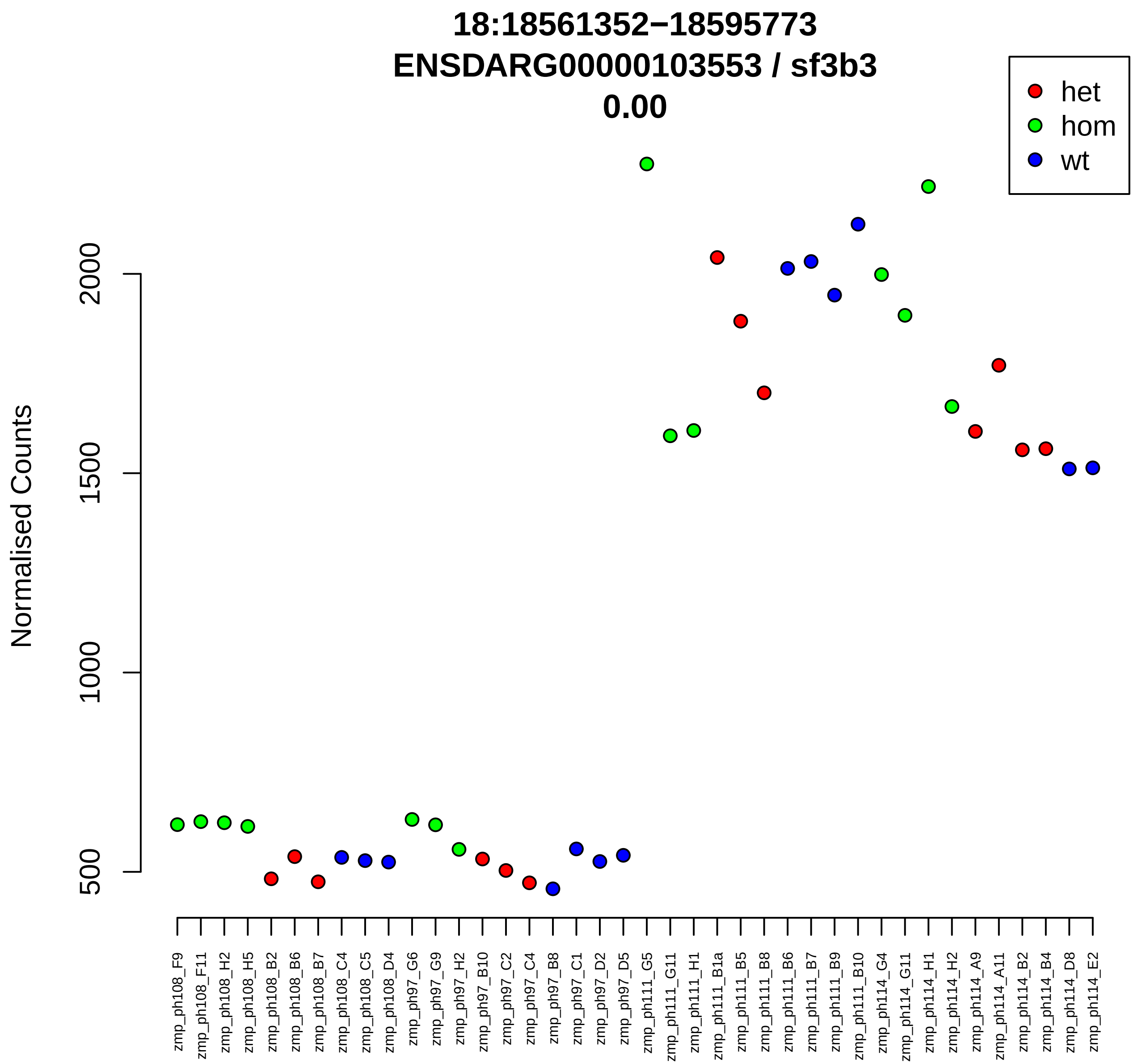

18:18561352−18595773
ENSDARG00000103553 / sf3b3
0.00
het
hom
wt
Normalised Counts
500
1000
1500
2000
zmp_ph108_F9
zmp_ph108_F11
zmp_ph108_H2
zmp_ph108_H5
zmp_ph108_B2
zmp_ph108_B6
zmp_ph108_B7
zmp_ph108_C4
zmp_ph108_C5
zmp_ph108_D4
zmp_ph97_G6
zmp_ph97_G9
zmp_ph97_H2
zmp_ph97_B10
zmp_ph97_C2
zmp_ph97_C4
zmp_ph97_B8
zmp_ph97_C1
zmp_ph97_D2
zmp_ph97_D5
zmp_ph111_G5
zmp_ph111_G11
zmp_ph111_H1
zmp_ph111_B1a
zmp_ph111_B5
zmp_ph111_B8
zmp_ph111_B6
zmp_ph111_B7
zmp_ph111_B9
zmp_ph111_B10
zmp_ph114_G4
zmp_ph114_G11
zmp_ph114_H1
zmp_ph114_H2
zmp_ph114_A9
zmp_ph114_A11
zmp_ph114_B2
zmp_ph114_B4
zmp_ph114_D8
zmp_ph114_E2

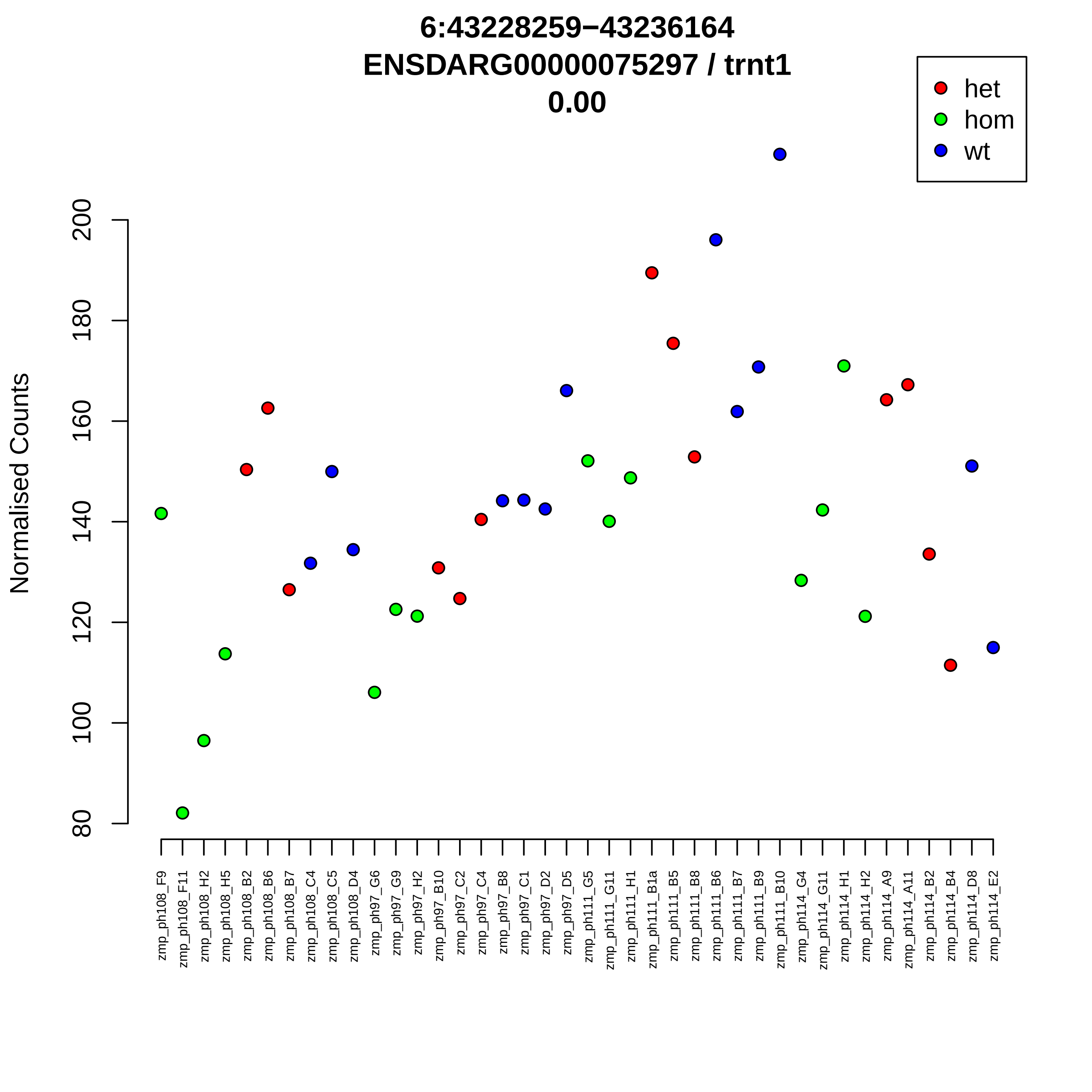

6:43228259−43236164
ENSDARG00000075297 / trnt1
0.00
het
hom
wt
Normalised Counts
80
100
120
140
160
180
200
zmp_ph108_F9
zmp_ph108_F11
zmp_ph108_H2
zmp_ph108_H5
zmp_ph108_B2
zmp_ph108_B6
zmp_ph108_B7
zmp_ph108_C4
zmp_ph108_C5
zmp_ph108_D4
zmp_ph97_G6
zmp_ph97_G9
zmp_ph97_H2
zmp_ph97_B10
zmp_ph97_C2
zmp_ph97_C4
zmp_ph97_B8
zmp_ph97_C1
zmp_ph97_D2
zmp_ph97_D5
zmp_ph111_G5
zmp_ph111_G11
zmp_ph111_H1
zmp_ph111_B1a
zmp_ph111_B5
zmp_ph111_B8
zmp_ph111_B6
zmp_ph111_B7
zmp_ph111_B9
zmp_ph111_B10
zmp_ph114_G4
zmp_ph114_G11
zmp_ph114_H1
zmp_ph114_H2
zmp_ph114_A9
zmp_ph114_A11
zmp_ph114_B2
zmp_ph114_B4
zmp_ph114_D8
zmp_ph114_E2

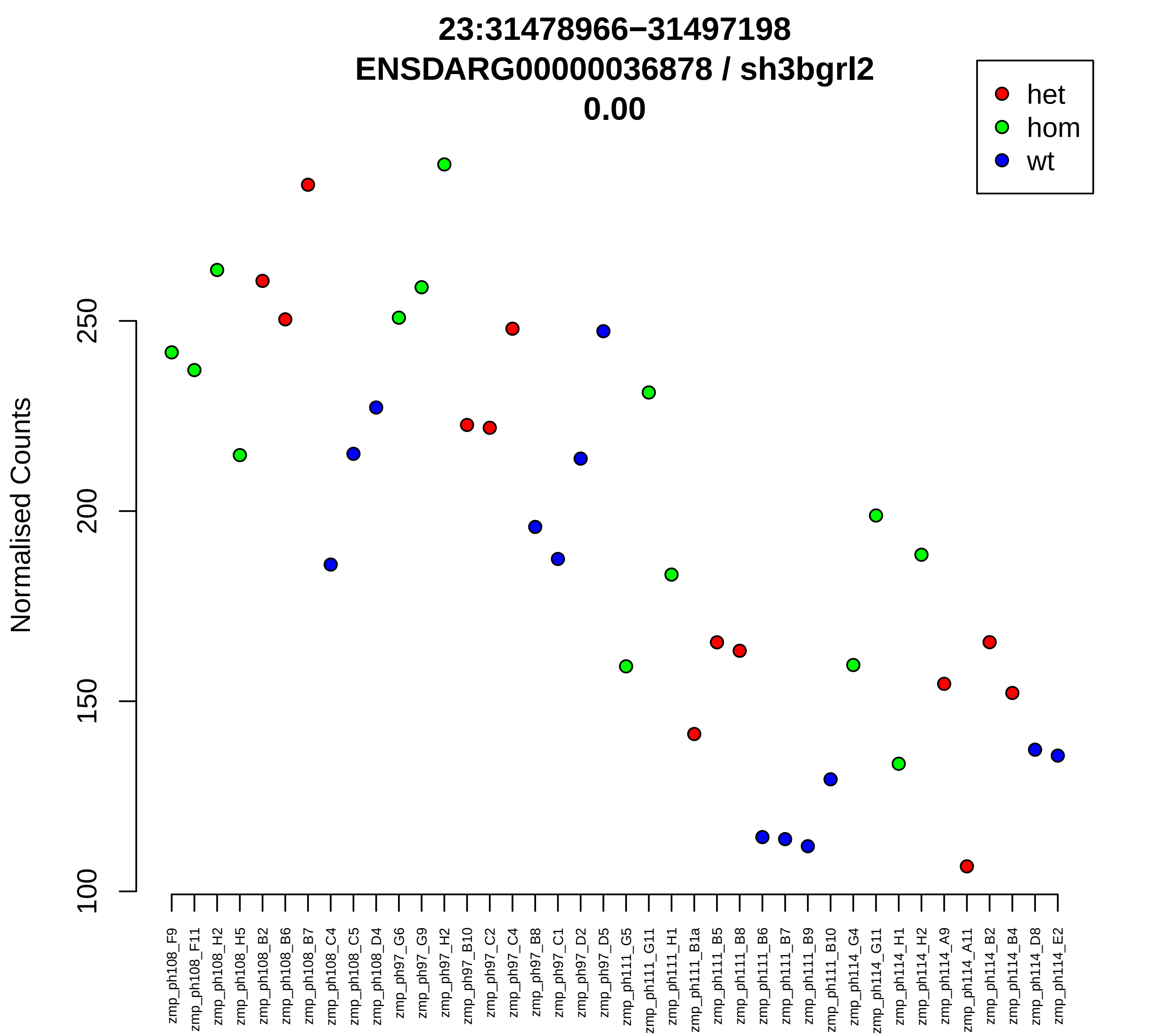

23:31478966−31497198
ENSDARG00000036878 / sh3bgrl2
0.00
het
hom
wt
Normalised Counts
100
150
200
250
zmp_ph108_F9
zmp_ph108_F11
zmp_ph108_H2
zmp_ph108_H5
zmp_ph108_B2
zmp_ph108_B6
zmp_ph108_B7
zmp_ph108_C4
zmp_ph108_C5
zmp_ph108_D4
zmp_ph97_G6
zmp_ph97_G9
zmp_ph97_H2
zmp_ph97_B10
zmp_ph97_C2
zmp_ph97_C4
zmp_ph97_B8
zmp_ph97_C1
zmp_ph97_D2
zmp_ph97_D5
zmp_ph111_G5
zmp_ph111_G11
zmp_ph111_H1
zmp_ph111_B1a
zmp_ph111_B5
zmp_ph111_B8
zmp_ph111_B6
zmp_ph111_B7
zmp_ph111_B9
zmp_ph111_B10
zmp_ph114_G4
zmp_ph114_G11
zmp_ph114_H1
zmp_ph114_H2
zmp_ph114_A9
zmp_ph114_A11
zmp_ph114_B2
zmp_ph114_B4
zmp_ph114_D8
zmp_ph114_E2

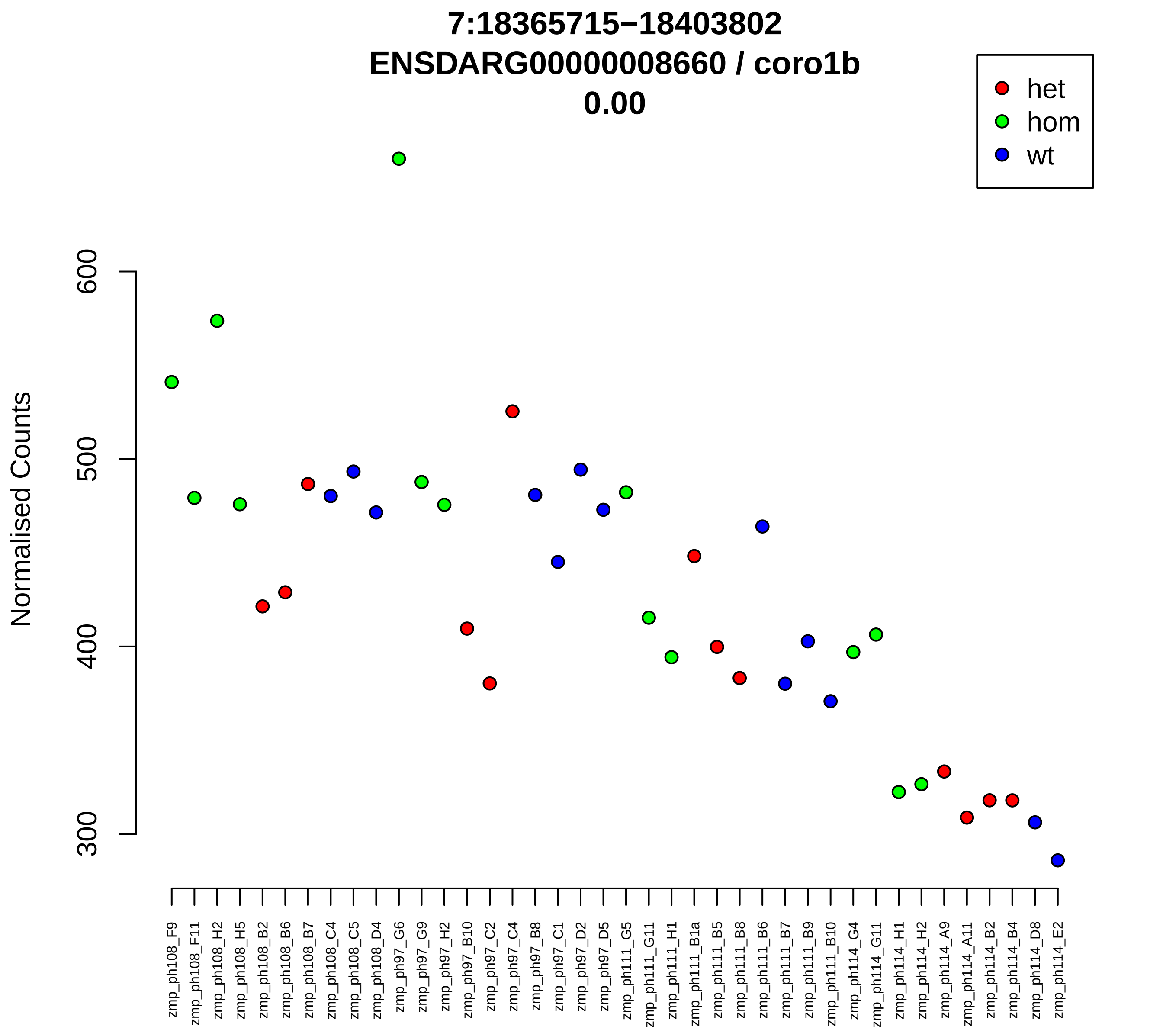

7:18365715−18403802
ENSDARG00000008660 / coro1b
0.00
het
hom
wt
Normalised Counts
600
500
400
300
zmp_ph108_F9
zmp_ph108_F11
zmp_ph108_H2
zmp_ph108_H5
zmp_ph108_B2
zmp_ph108_B6
zmp_ph108_B7
zmp_ph108_C4
zmp_ph108_C5
zmp_ph108_D4
zmp_ph97_G6
zmp_ph97_G9
zmp_ph97_H2
zmp_ph97_B10
zmp_ph97_C2
zmp_ph97_C4
zmp_ph97_B8
zmp_ph97_C1
zmp_ph97_D2
zmp_ph97_D5
zmp_ph111_G5
zmp_ph111_G11
zmp_ph111_H1
zmp_ph111_B1a
zmp_ph111_B5
zmp_ph111_B8
zmp_ph111_B6
zmp_ph111_B7
zmp_ph111_B9
zmp_ph111_B10
zmp_ph114_G4
zmp_ph114_G11
zmp_ph114_H1
zmp_ph114_H2
zmp_ph114_A9
zmp_ph114_A11
zmp_ph114_B2
zmp_ph114_B4
zmp_ph114_D8
zmp_ph114_E2

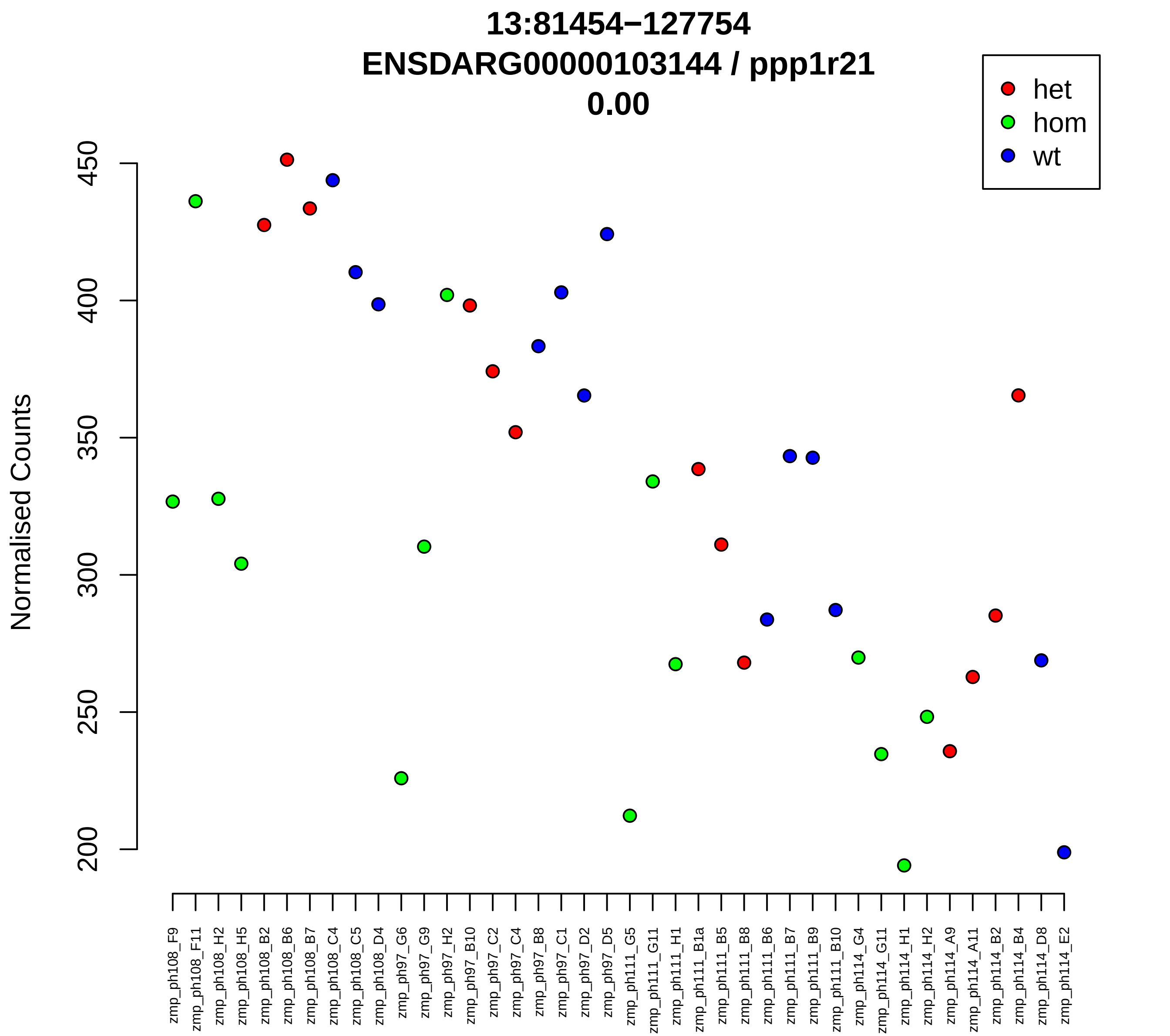

13:81454−127754
ENSDARG00000103144 / ppp1r21
0.00
het
hom
wt
Normalised Counts
200
250
300
350
400
450
zmp_ph108_F9
zmp_ph108_F11
zmp_ph108_H2
zmp_ph108_H5
zmp_ph108_B2
zmp_ph108_B6
zmp_ph108_B7
zmp_ph108_C4
zmp_ph108_C5
zmp_ph108_D4
zmp_ph97_G6
zmp_ph97_G9
zmp_ph97_H2
zmp_ph97_B10
zmp_ph97_C2
zmp_ph97_C4
zmp_ph97_B8
zmp_ph97_C1
zmp_ph97_D2
zmp_ph97_D5
zmp_ph111_G5
zmp_ph111_G11
zmp_ph111_H1
zmp_ph111_B1a
zmp_ph111_B5
zmp_ph111_B8
zmp_ph111_B6
zmp_ph111_B7
zmp_ph111_B9
zmp_ph111_B10
zmp_ph114_G4
zmp_ph114_G11
zmp_ph114_H1
zmp_ph114_H2
zmp_ph114_A9
zmp_ph114_A11
zmp_ph114_B2
zmp_ph114_B4
zmp_ph114_D8
zmp_ph114_E2

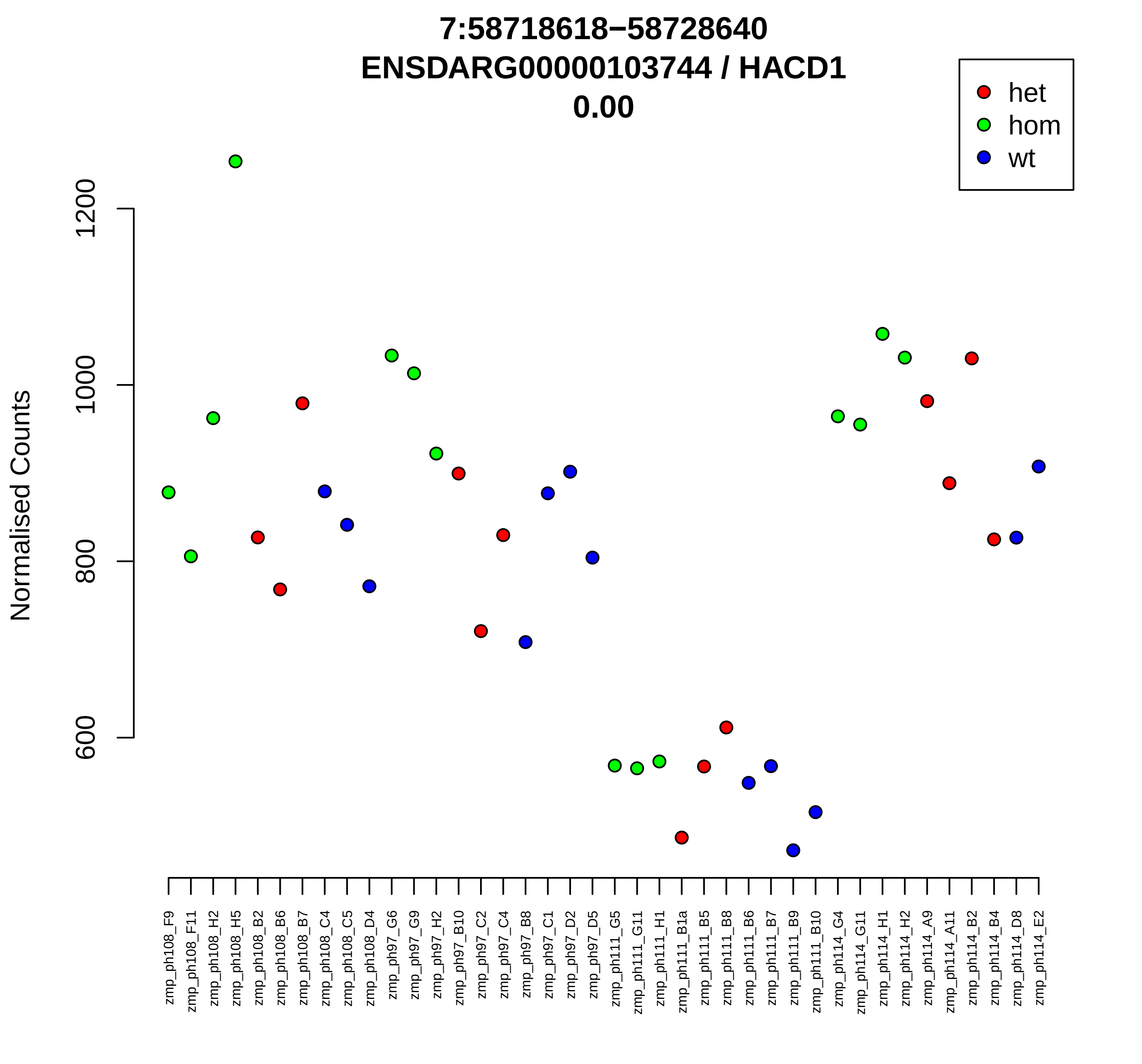

7:58718618−58728640
ENSDARG00000103744 / HACD1
0.00
het
hom
wt
Normalised Counts
600
800
1000
1200
zmp_ph108_F9
zmp_ph108_F11
zmp_ph108_H2
zmp_ph108_H5
zmp_ph108_B2
zmp_ph108_B6
zmp_ph108_B7
zmp_ph108_C4
zmp_ph108_C5
zmp_ph108_D4
zmp_ph97_G6
zmp_ph97_G9
zmp_ph97_H2
zmp_ph97_B10
zmp_ph97_C2
zmp_ph97_C4
zmp_ph97_B8
zmp_ph97_C1
zmp_ph97_D2
zmp_ph97_D5
zmp_ph111_G5
zmp_ph111_G11
zmp_ph111_H1
zmp_ph111_B1a
zmp_ph111_B5
zmp_ph111_B8
zmp_ph111_B6
zmp_ph111_B7
zmp_ph111_B9
zmp_ph111_B10
zmp_ph114_G4
zmp_ph114_G11
zmp_ph114_H1
zmp_ph114_H2
zmp_ph114_A9
zmp_ph114_A11
zmp_ph114_B2
zmp_ph114_B4
zmp_ph114_D8
zmp_ph114_E2

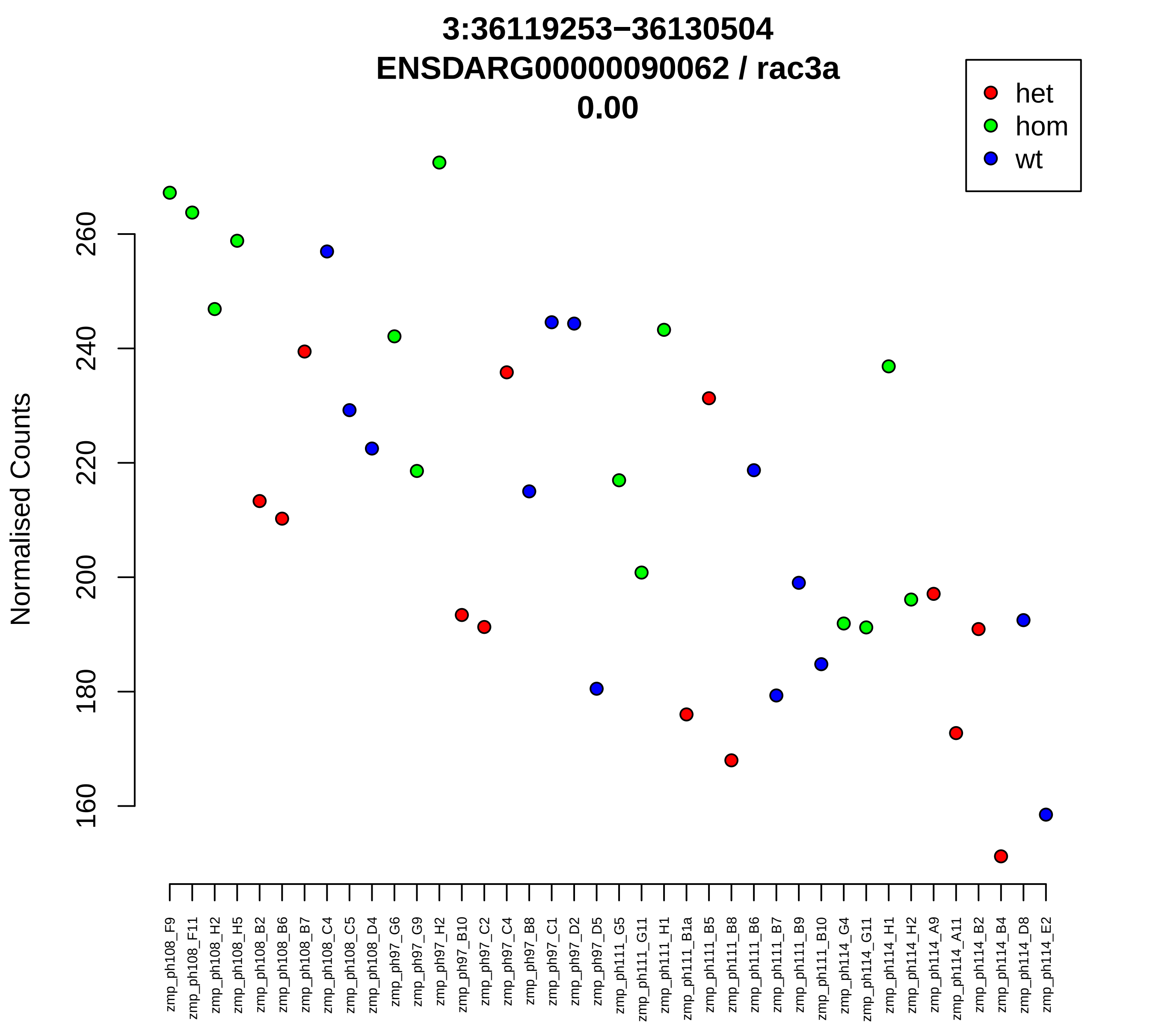

3:36119253−36130504
ENSDARG00000090062 / rac3a
0.00
het
hom
wt
Normalised Counts
160
180
200
220
240
260
zmp_ph108_F9
zmp_ph108_F11
zmp_ph108_H2
zmp_ph108_H5
zmp_ph108_B2
zmp_ph108_B6
zmp_ph108_B7
zmp_ph108_C4
zmp_ph108_C5
zmp_ph108_D4
zmp_ph97_G6
zmp_ph97_G9
zmp_ph97_H2
zmp_ph97_B10
zmp_ph97_C2
zmp_ph97_C4
zmp_ph97_B8
zmp_ph97_C1
zmp_ph97_D2
zmp_ph97_D5
zmp_ph111_G5
zmp_ph111_G11
zmp_ph111_H1
zmp_ph111_B1a
zmp_ph111_B5
zmp_ph111_B8
zmp_ph111_B6
zmp_ph111_B7
zmp_ph111_B9
zmp_ph111_B10
zmp_ph114_G4
zmp_ph114_G11
zmp_ph114_H1
zmp_ph114_H2
zmp_ph114_A9
zmp_ph114_A11
zmp_ph114_B2
zmp_ph114_B4
zmp_ph114_D8
zmp_ph114_E2

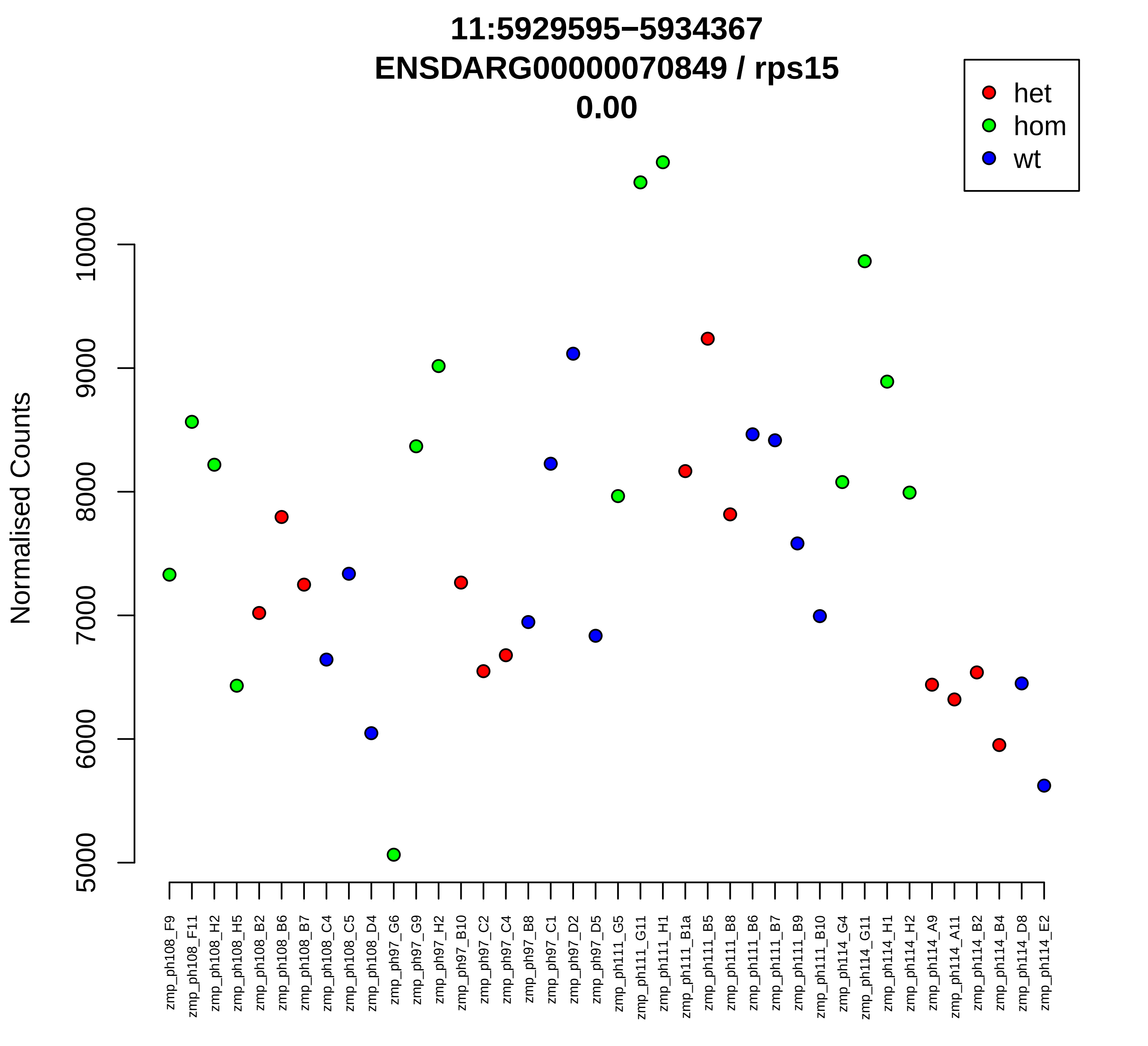

11:5929595−5934367
ENSDARG00000070849 / rps15
0.00
het
hom
wt
Normalised Counts
5000
6000
7000
8000
9000
10000
zmp_ph108_F9
zmp_ph108_F11
zmp_ph108_H2
zmp_ph108_H5
zmp_ph108_B2
zmp_ph108_B6
zmp_ph108_B7
zmp_ph108_C4
zmp_ph108_C5
zmp_ph108_D4
zmp_ph97_G6
zmp_ph97_G9
zmp_ph97_H2
zmp_ph97_B10
zmp_ph97_C2
zmp_ph97_C4
zmp_ph97_B8
zmp_ph97_C1
zmp_ph97_D2
zmp_ph97_D5
zmp_ph111_G5
zmp_ph111_G11
zmp_ph111_H1
zmp_ph111_B1a
zmp_ph111_B5
zmp_ph111_B8
zmp_ph111_B6
zmp_ph111_B7
zmp_ph111_B9
zmp_ph111_B10
zmp_ph114_G4
zmp_ph114_G11
zmp_ph114_H1
zmp_ph114_H2
zmp_ph114_A9
zmp_ph114_A11
zmp_ph114_B2
zmp_ph114_B4
zmp_ph114_D8
zmp_ph114_E2

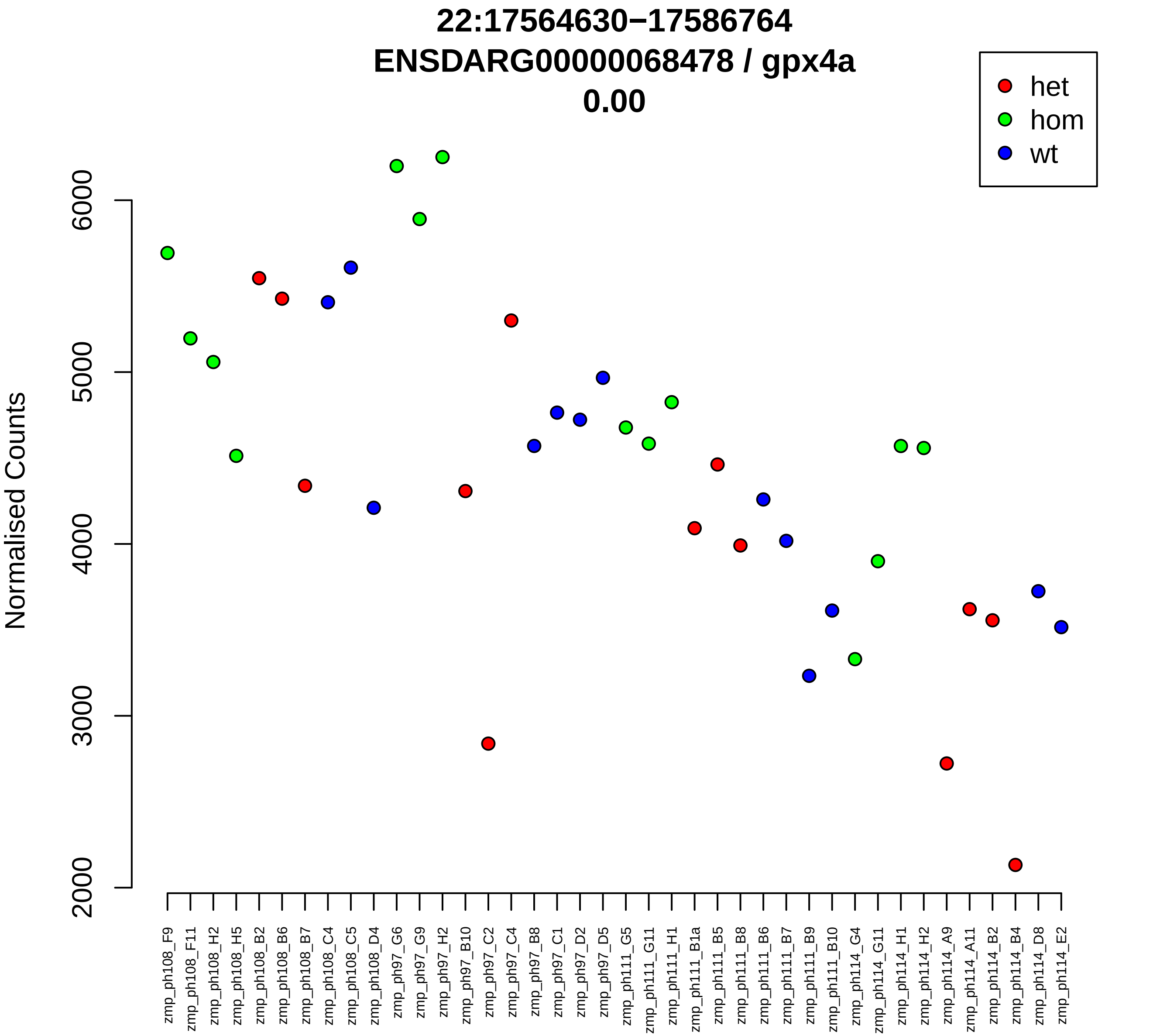

22:17564630−17586764
ENSDARG00000068478 / gpx4a
0.00
het
hom
wt
Normalised Counts
2000
3000
4000
5000
6000
zmp_ph108_F9
zmp_ph108_F11
zmp_ph108_H2
zmp_ph108_H5
zmp_ph108_B2
zmp_ph108_B6
zmp_ph108_B7
zmp_ph108_C4
zmp_ph108_C5
zmp_ph108_D4
zmp_ph97_G6
zmp_ph97_G9
zmp_ph97_H2
zmp_ph97_B10
zmp_ph97_C2
zmp_ph97_C4
zmp_ph97_B8
zmp_ph97_C1
zmp_ph97_D2
zmp_ph97_D5
zmp_ph111_G5
zmp_ph111_G11
zmp_ph111_H1
zmp_ph111_B1a
zmp_ph111_B5
zmp_ph111_B8
zmp_ph111_B6
zmp_ph111_B7
zmp_ph111_B9
zmp_ph111_B10
zmp_ph114_G4
zmp_ph114_G11
zmp_ph114_H1
zmp_ph114_H2
zmp_ph114_A9
zmp_ph114_A11
zmp_ph114_B2
zmp_ph114_B4
zmp_ph114_D8
zmp_ph114_E2

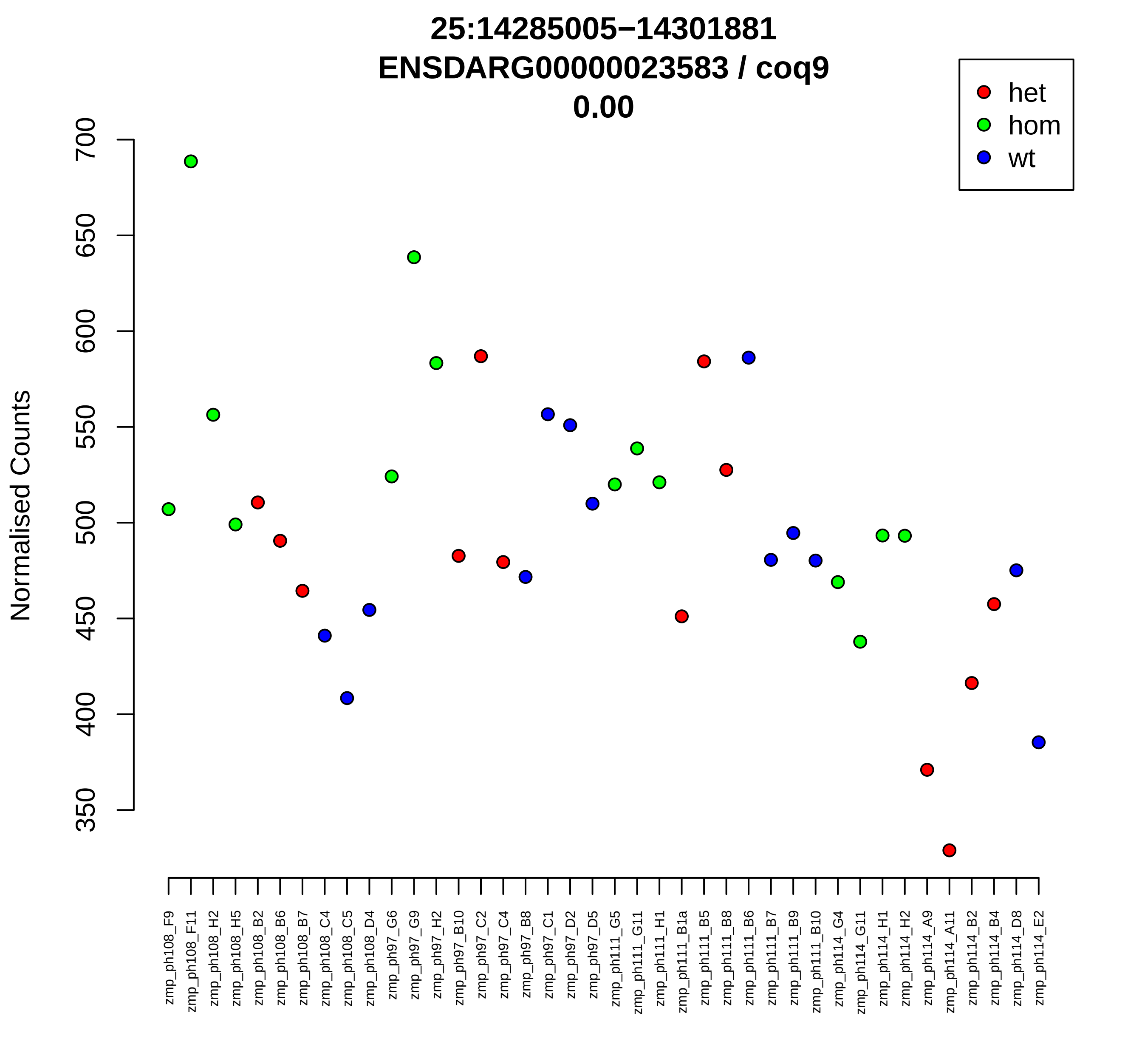

25:14285005−14301881
ENSDARG00000023583 / coq9
0.00
Normalised Counts
350
400
450
500
550
600
650
700
het
hom
wt
zmp_ph108_F9
zmp_ph108_F11
zmp_ph108_H2
zmp_ph108_H5
zmp_ph108_B2
zmp_ph108_B6
zmp_ph108_B7
zmp_ph108_C4
zmp_ph108_C5
zmp_ph108_D4
zmp_ph97_G6
zmp_ph97_G9
zmp_ph97_H2
zmp_ph97_B10
zmp_ph97_C2
zmp_ph97_C4
zmp_ph97_B8
zmp_ph97_C1
zmp_ph97_D2
zmp_ph97_D5
zmp_ph111_G5
zmp_ph111_G11
zmp_ph111_H1
zmp_ph111_B1a
zmp_ph111_B5
zmp_ph111_B8
zmp_ph111_B6
zmp_ph111_B7
zmp_ph111_B9
zmp_ph111_B10
zmp_ph114_G4
zmp_ph114_G11
zmp_ph114_H1
zmp_ph114_H2
zmp_ph114_A9
zmp_ph114_A11
zmp_ph114_B2
zmp_ph114_B4
zmp_ph114_D8
zmp_ph114_E2

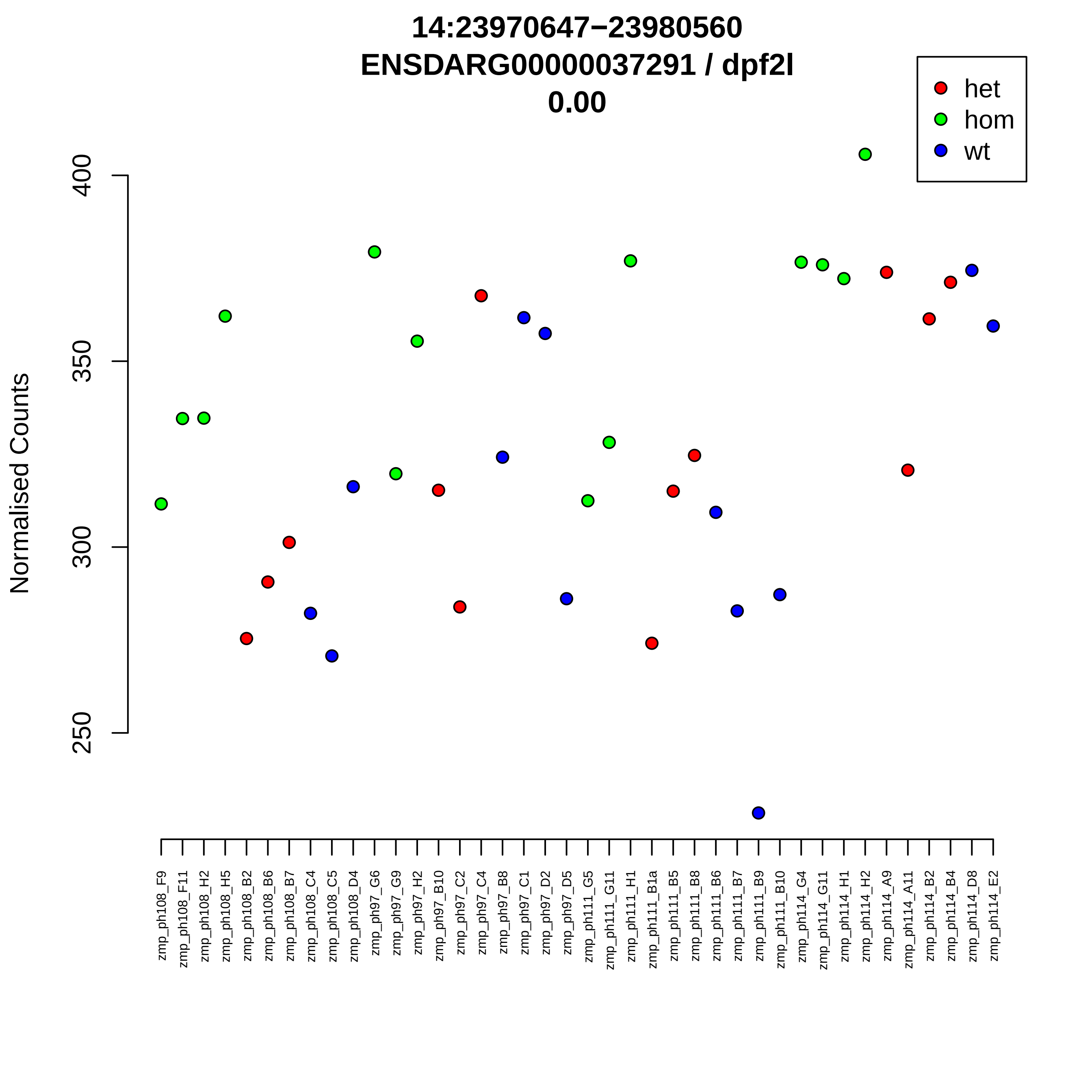

14:23970647−23980560
ENSDARG00000037291 / dpf2l
0.00
het
hom
wt
Normalised Counts
250
300
350
400
zmp_ph108_F9
zmp_ph108_F11
zmp_ph108_H2
zmp_ph108_H5
zmp_ph108_B2
zmp_ph108_B6
zmp_ph108_B7
zmp_ph108_C4
zmp_ph108_C5
zmp_ph108_D4
zmp_ph97_G6
zmp_ph97_G9
zmp_ph97_H2
zmp_ph97_B10
zmp_ph97_C2
zmp_ph97_C4
zmp_ph97_B8
zmp_ph97_C1
zmp_ph97_D2
zmp_ph97_D5
zmp_ph111_G5
zmp_ph111_G11
zmp_ph111_H1
zmp_ph111_B1a
zmp_ph111_B5
zmp_ph111_B8
zmp_ph111_B6
zmp_ph111_B7
zmp_ph111_B9
zmp_ph111_B10
zmp_ph114_G4
zmp_ph114_G11
zmp_ph114_H1
zmp_ph114_H2
zmp_ph114_A9
zmp_ph114_A11
zmp_ph114_B2
zmp_ph114_B4
zmp_ph114_D8
zmp_ph114_E2

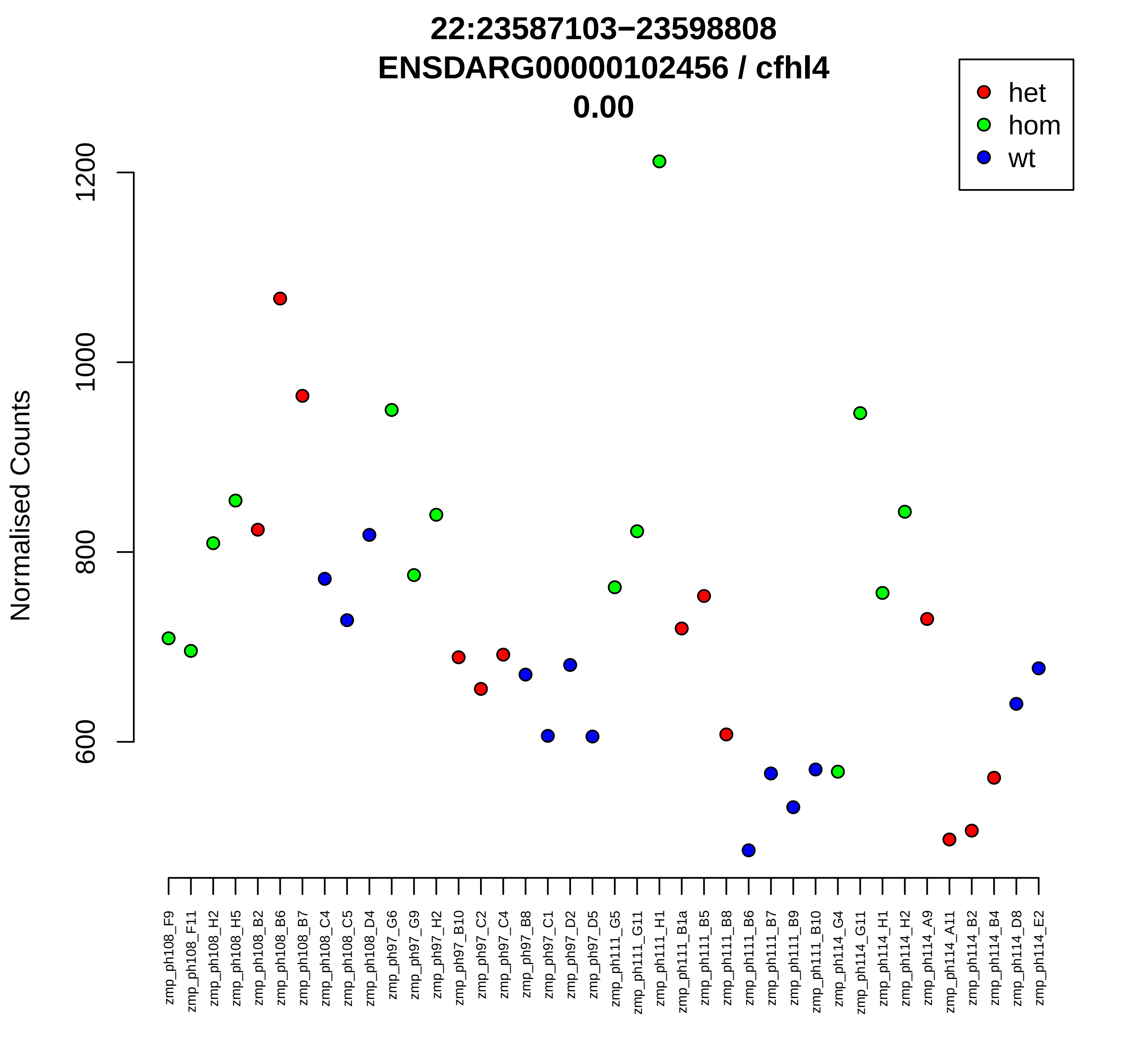

22:23587103−23598808
ENSDARG00000102456 / cfhl4
0.00
het
hom
wt
Normalised Counts
600
800
1000
1200
zmp_ph108_F9
zmp_ph108_F11
zmp_ph108_H2
zmp_ph108_H5
zmp_ph108_B2
zmp_ph108_B6
zmp_ph108_B7
zmp_ph108_C4
zmp_ph108_C5
zmp_ph108_D4
zmp_ph97_G6
zmp_ph97_G9
zmp_ph97_H2
zmp_ph97_B10
zmp_ph97_C2
zmp_ph97_C4
zmp_ph97_B8
zmp_ph97_C1
zmp_ph97_D2
zmp_ph97_D5
zmp_ph111_G5
zmp_ph111_G11
zmp_ph111_H1
zmp_ph111_B1a
zmp_ph111_B5
zmp_ph111_B8
zmp_ph111_B6
zmp_ph111_B7
zmp_ph111_B9
zmp_ph111_B10
zmp_ph114_G4
zmp_ph114_G11
zmp_ph114_H1
zmp_ph114_H2
zmp_ph114_A9
zmp_ph114_A11
zmp_ph114_B2
zmp_ph114_B4
zmp_ph114_D8
zmp_ph114_E2

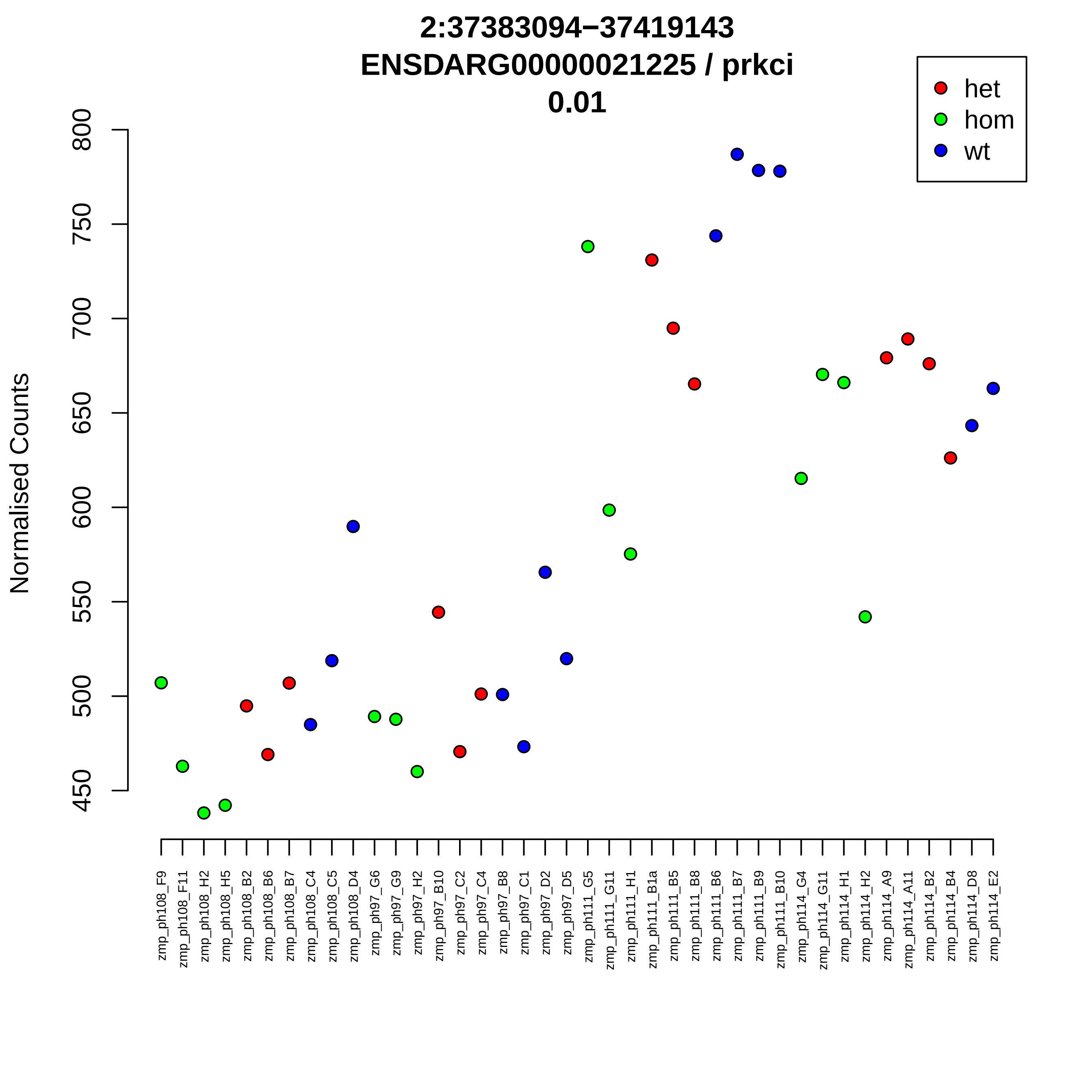

2:37383094−37419143
ENSDARG00000021225 / prkci
0.01
Normalised Counts
450
500
550
600
650
700
750
800
het
hom
wt
zmp_ph108_F9
zmp_ph108_F11
zmp_ph108_H2
zmp_ph108_H5
zmp_ph108_B2
zmp_ph108_B6
zmp_ph108_B7
zmp_ph108_C4
zmp_ph108_C5
zmp_ph108_D4
zmp_ph97_G6
zmp_ph97_G9
zmp_ph97_H2
zmp_ph97_B10
zmp_ph97_C2
zmp_ph97_C4
zmp_ph97_B8
zmp_ph97_C1
zmp_ph97_D2
zmp_ph97_D5
zmp_ph111_G5
zmp_ph111_G11
zmp_ph111_H1
zmp_ph111_B1a
zmp_ph111_B5
zmp_ph111_B8
zmp_ph111_B6
zmp_ph111_B7
zmp_ph111_B9
zmp_ph111_B10
zmp_ph114_G4
zmp_ph114_G11
zmp_ph114_H1
zmp_ph114_H2
zmp_ph114_A9
zmp_ph114_A11
zmp_ph114_B2
zmp_ph114_B4
zmp_ph114_D8
zmp_ph114_E2

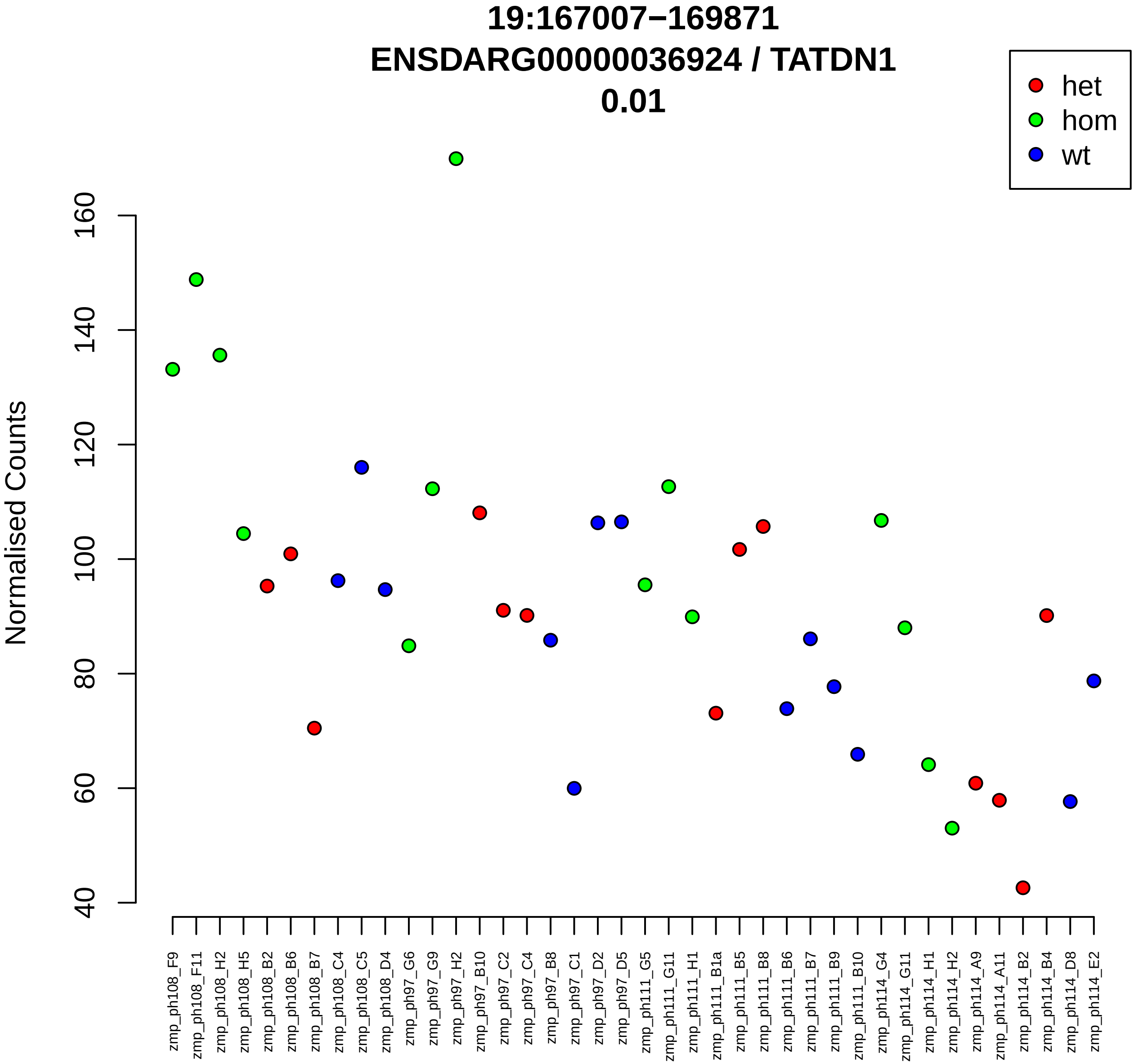
19:167007−169871
ENSDARG00000036924 / TATDN1
0.01
het
hom
wt
Normalised Counts
40
60
80
100
120
140
160
zmp_ph108_F9
zmp_ph108_F11
zmp_ph108_H2
zmp_ph108_H5
zmp_ph108_B2
zmp_ph108_B6
zmp_ph108_B7
zmp_ph108_C4
zmp_ph108_C5
zmp_ph108_D4
zmp_ph97_G6
zmp_ph97_G9
zmp_ph97_H2
zmp_ph97_B10
zmp_ph97_C2
zmp_ph97_C4
zmp_ph97_B8
zmp_ph97_C1
zmp_ph97_D2
zmp_ph97_D5
zmp_ph111_G5
zmp_ph111_G11
zmp_ph111_H1
zmp_ph111_B1a
zmp_ph111_B5
zmp_ph111_B8
zmp_ph111_B6
zmp_ph111_B7
zmp_ph111_B9
zmp_ph111_B10
zmp_ph114_G4
zmp_ph114_G11
zmp_ph114_H1
zmp_ph114_H2
zmp_ph114_A9
zmp_ph114_A11
zmp_ph114_B2
zmp_ph114_B4
zmp_ph114_D8
zmp_ph114_E2

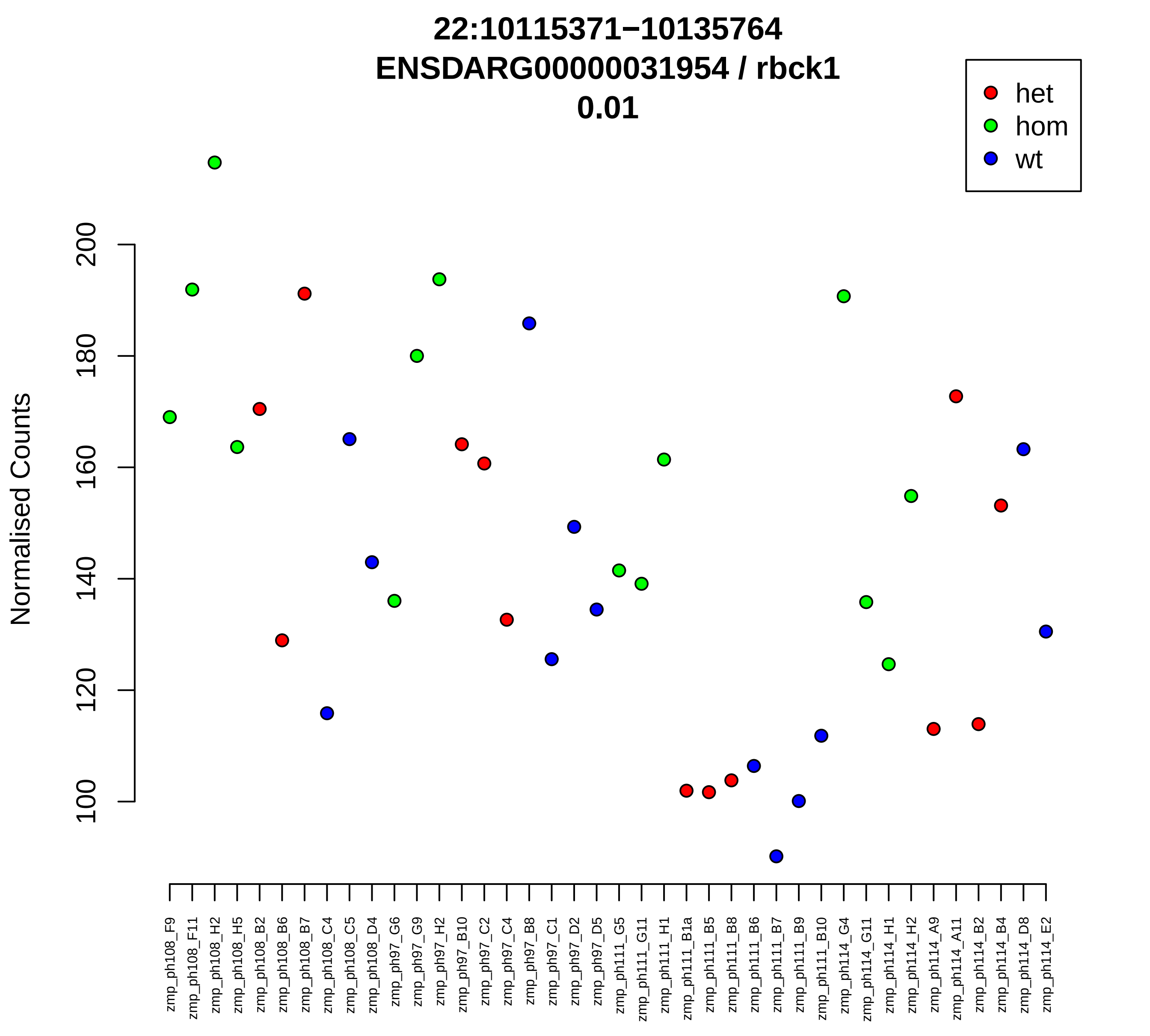
22:10115371−10135764
ENSDARG00000031954 / rbck1
0.01
het
hom
wt
Normalised Counts
100
120
140
160
180
200
zmp_ph108_F9
zmp_ph108_F11
zmp_ph108_H2
zmp_ph108_H5
zmp_ph108_B2
zmp_ph108_B6
zmp_ph108_B7
zmp_ph108_C4
zmp_ph108_C5
zmp_ph108_D4
zmp_ph97_G6
zmp_ph97_G9
zmp_ph97_H2
zmp_ph97_B10
zmp_ph97_C2
zmp_ph97_C4
zmp_ph97_B8
zmp_ph97_C1
zmp_ph97_D2
zmp_ph97_D5
zmp_ph111_G5
zmp_ph111_G11
zmp_ph111_H1
zmp_ph111_B1a
zmp_ph111_B5
zmp_ph111_B8
zmp_ph111_B6
zmp_ph111_B7
zmp_ph111_B9
zmp_ph111_B10
zmp_ph114_G4
zmp_ph114_G11
zmp_ph114_H1
zmp_ph114_H2
zmp_ph114_A9
zmp_ph114_A11
zmp_ph114_B2
zmp_ph114_B4
zmp_ph114_D8
zmp_ph114_E2

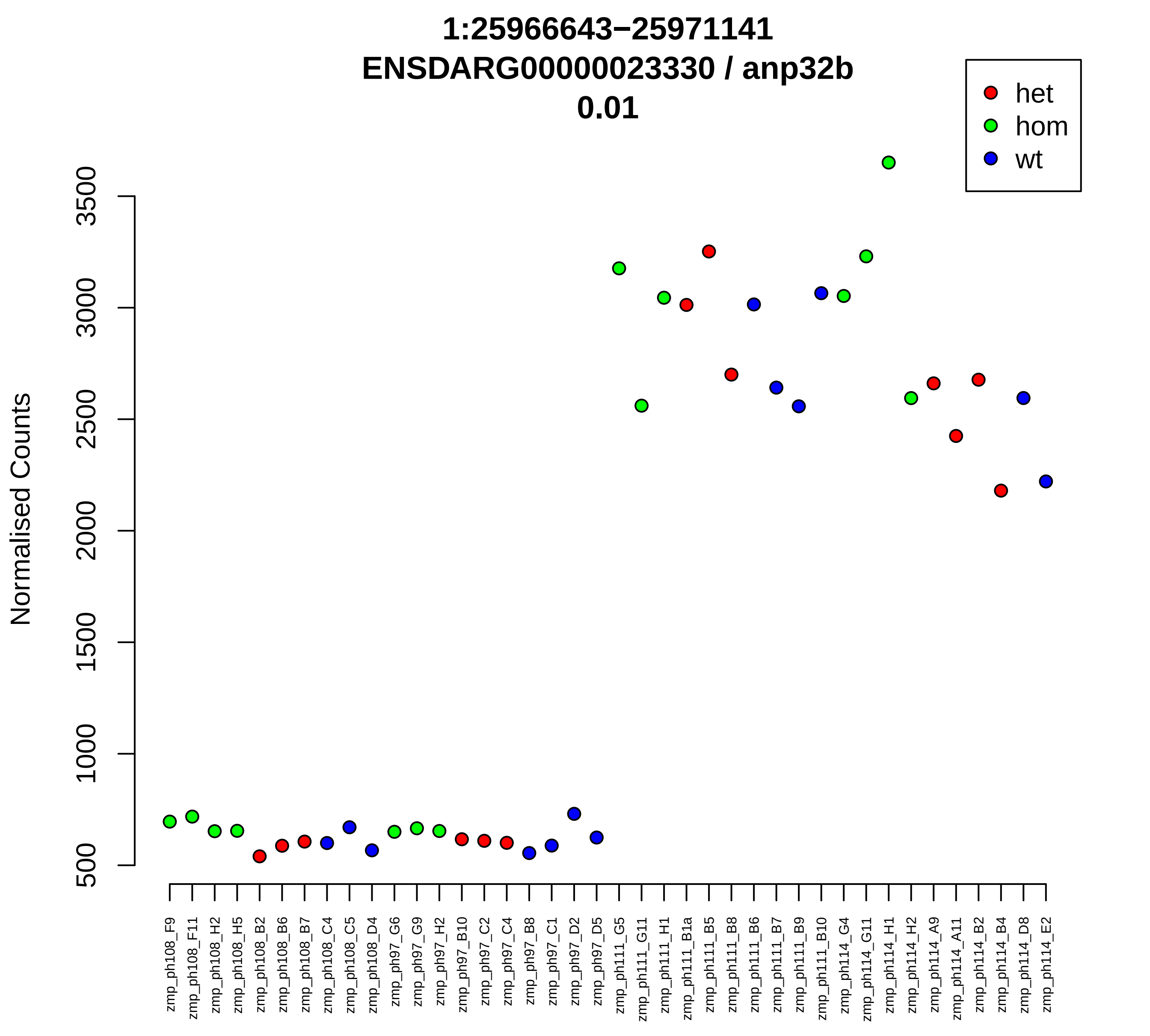
1:25966643−25971141
ENSDARG00000023330 / anp32b
0.01
het
hom
wt
Normalised Counts
500
1000
1500
2000
2500
3000
3500
zmp_ph108_F9
zmp_ph108_F11
zmp_ph108_H2
zmp_ph108_H5
zmp_ph108_B2
zmp_ph108_B6
zmp_ph108_B7
zmp_ph108_C4
zmp_ph108_C5
zmp_ph108_D4
zmp_ph97_G6
zmp_ph97_G9
zmp_ph97_H2
zmp_ph97_B10
zmp_ph97_C2
zmp_ph97_C4
zmp_ph97_B8
zmp_ph97_C1
zmp_ph97_D2
zmp_ph97_D5
zmp_ph111_G5
zmp_ph111_G11
zmp_ph111_H1
zmp_ph111_B1a
zmp_ph111_B5
zmp_ph111_B8
zmp_ph111_B6
zmp_ph111_B7
zmp_ph111_B9
zmp_ph111_B10
zmp_ph114_G4
zmp_ph114_G11
zmp_ph114_H1
zmp_ph114_H2
zmp_ph114_A9
zmp_ph114_A11
zmp_ph114_B2
zmp_ph114_B4
zmp_ph114_D8
zmp_ph114_E2

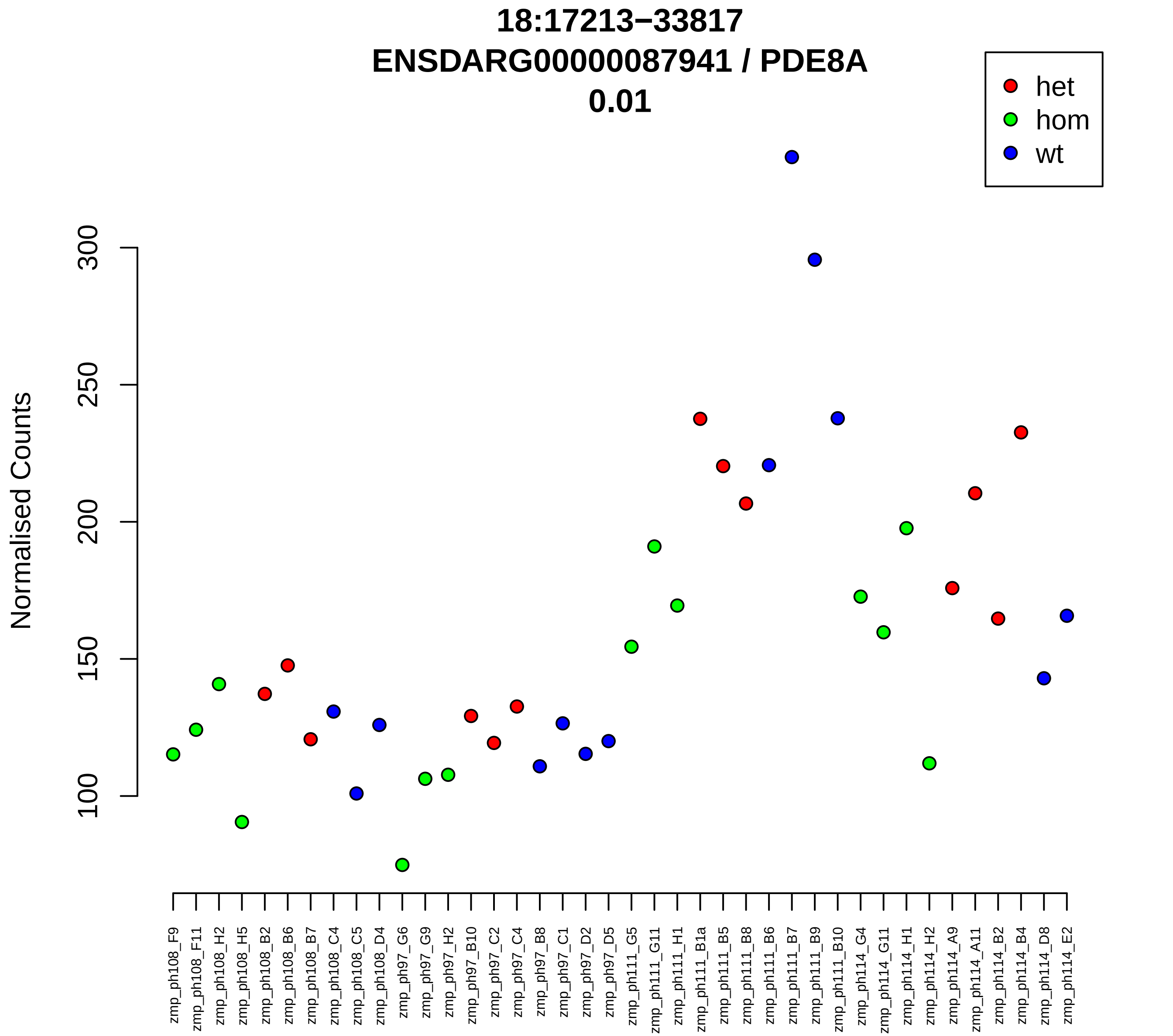
18:17213−33817
ENSDARG00000087941 / PDE8A
0.01
het
hom
wt
Normalised Counts
100
150
200
250
300
zmp_ph108_F9
zmp_ph108_F11
zmp_ph108_H2
zmp_ph108_H5
zmp_ph108_B2
zmp_ph108_B6
zmp_ph108_B7
zmp_ph108_C4
zmp_ph108_C5
zmp_ph108_D4
zmp_ph97_G6
zmp_ph97_G9
zmp_ph97_H2
zmp_ph97_B10
zmp_ph97_C2
zmp_ph97_C4
zmp_ph97_B8
zmp_ph97_C1
zmp_ph97_D2
zmp_ph97_D5
zmp_ph111_G5
zmp_ph111_G11
zmp_ph111_H1
zmp_ph111_B1a
zmp_ph111_B5
zmp_ph111_B8
zmp_ph111_B6
zmp_ph111_B7
zmp_ph111_B9
zmp_ph111_B10
zmp_ph114_G4
zmp_ph114_G11
zmp_ph114_H1
zmp_ph114_H2
zmp_ph114_A9
zmp_ph114_A11
zmp_ph114_B2
zmp_ph114_B4
zmp_ph114_D8
zmp_ph114_E2

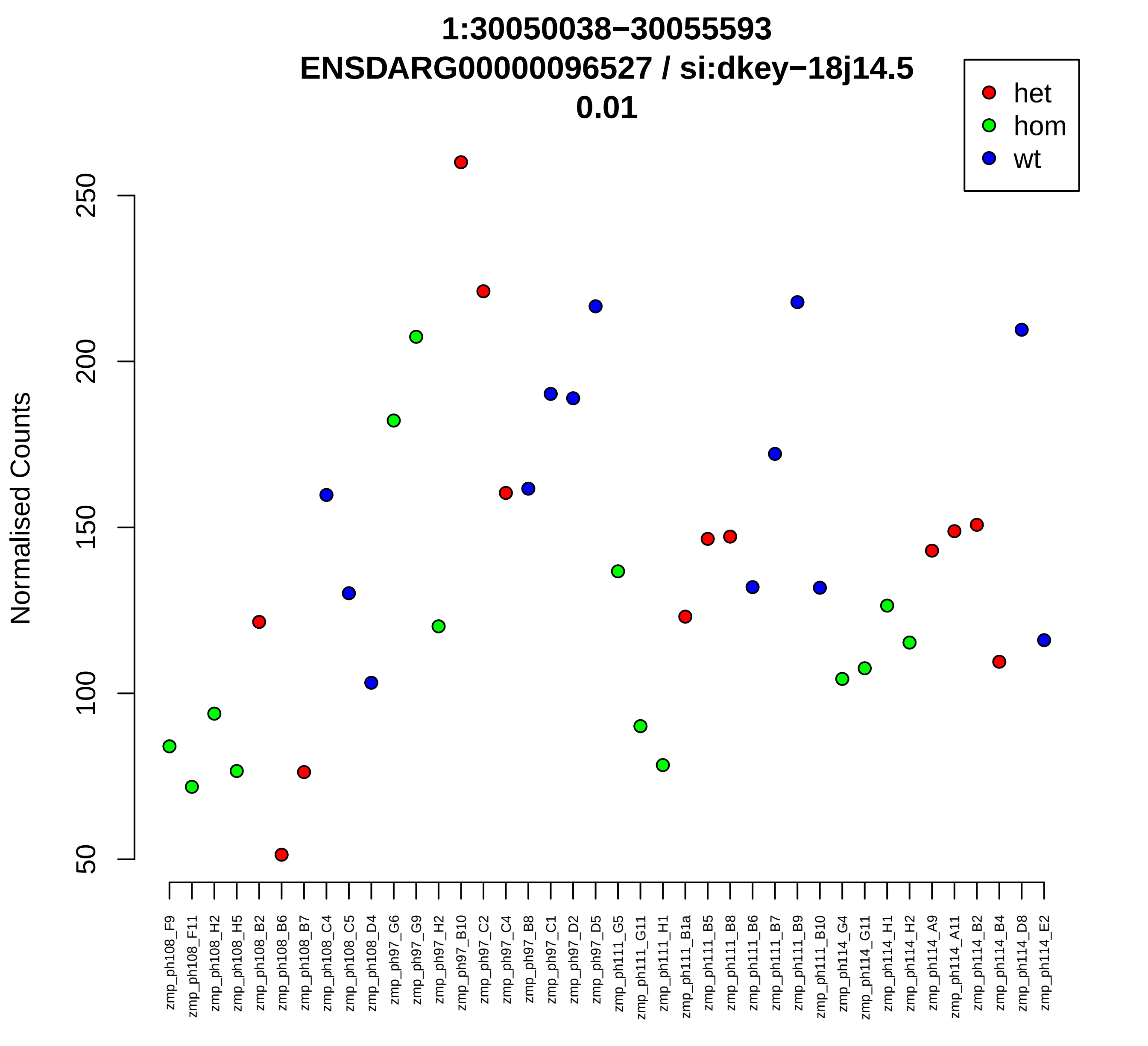

1:30050038−30055593
ENSDARG00000096527 / si:dkey−18j14.5
0.01
het
hom
wt
Normalised Counts
50
100
150
200
250
zmp_ph108_F9
zmp_ph108_F11
zmp_ph108_H2
zmp_ph108_H5
zmp_ph108_B2
zmp_ph108_B6
zmp_ph108_B7
zmp_ph108_C4
zmp_ph108_C5
zmp_ph108_D4
zmp_ph97_G6
zmp_ph97_G9
zmp_ph97_H2
zmp_ph97_B10
zmp_ph97_C2
zmp_ph97_C4
zmp_ph97_B8
zmp_ph97_C1
zmp_ph97_D2
zmp_ph97_D5
zmp_ph111_G5
zmp_ph111_G11
zmp_ph111_H1
zmp_ph111_B1a
zmp_ph111_B5
zmp_ph111_B8
zmp_ph111_B6
zmp_ph111_B7
zmp_ph111_B9
zmp_ph111_B10
zmp_ph114_G4
zmp_ph114_G11
zmp_ph114_H1
zmp_ph114_H2
zmp_ph114_A9
zmp_ph114_A11
zmp_ph114_B2
zmp_ph114_B4
zmp_ph114_D8
zmp_ph114_E2

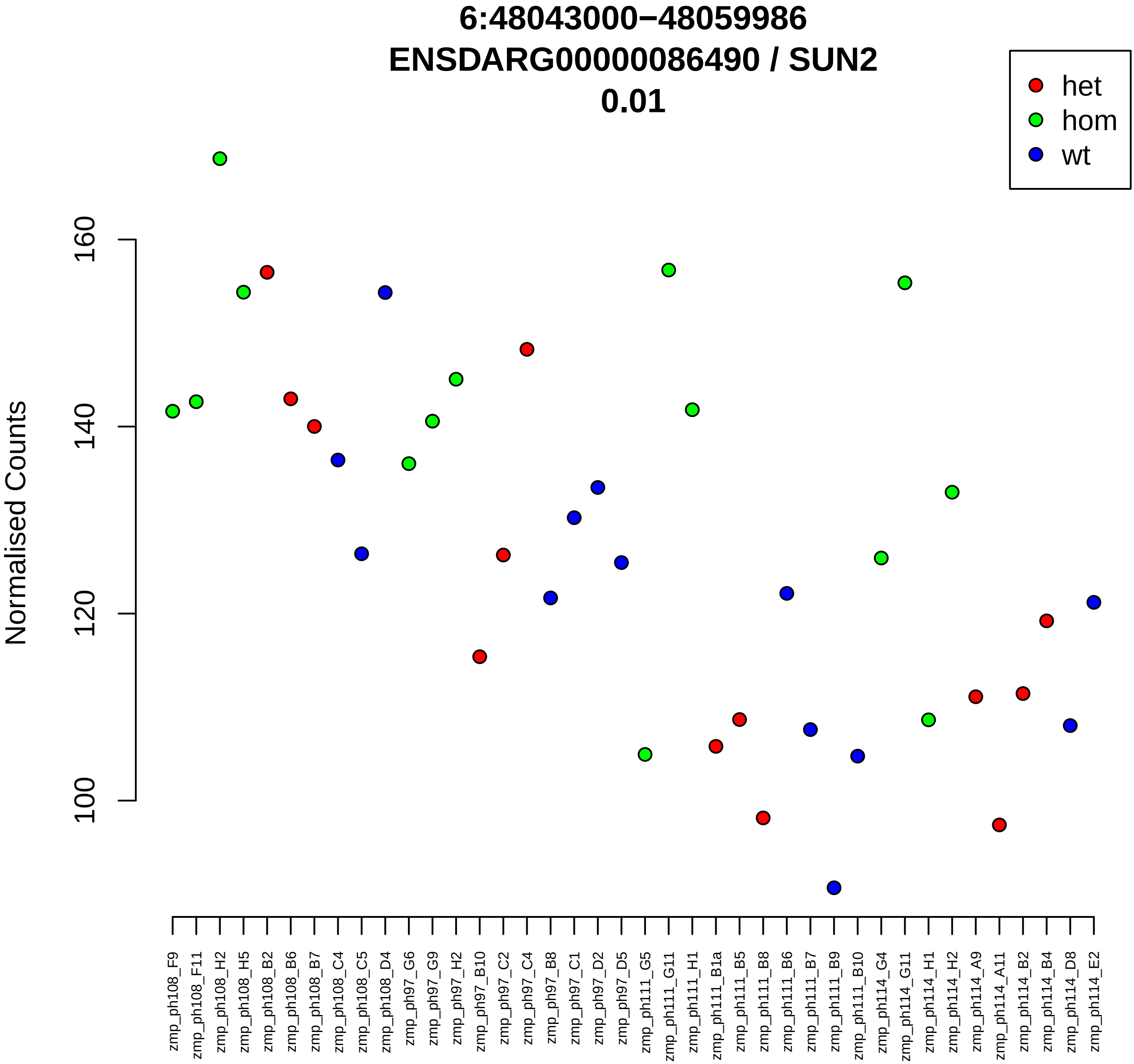

6:48043000−48059986
ENSDARG00000086490 / SUN2
0.01
het
hom
wt
Normalised Counts
100
120
140
160
zmp_ph108_F9
zmp_ph108_F11
zmp_ph108_H2
zmp_ph108_H5
zmp_ph108_B2
zmp_ph108_B6
zmp_ph108_B7
zmp_ph108_C4
zmp_ph108_C5
zmp_ph108_D4
zmp_ph97_G6
zmp_ph97_G9
zmp_ph97_H2
zmp_ph97_B10
zmp_ph97_C2
zmp_ph97_C4
zmp_ph97_B8
zmp_ph97_C1
zmp_ph97_D2
zmp_ph97_D5
zmp_ph111_G5
zmp_ph111_G11
zmp_ph111_H1
zmp_ph111_B1a
zmp_ph111_B5
zmp_ph111_B8
zmp_ph111_B6
zmp_ph111_B7
zmp_ph111_B9
zmp_ph111_B10
zmp_ph114_G4
zmp_ph114_G11
zmp_ph114_H1
zmp_ph114_H2
zmp_ph114_A9
zmp_ph114_A11
zmp_ph114_B2
zmp_ph114_B4
zmp_ph114_D8
zmp_ph114_E2

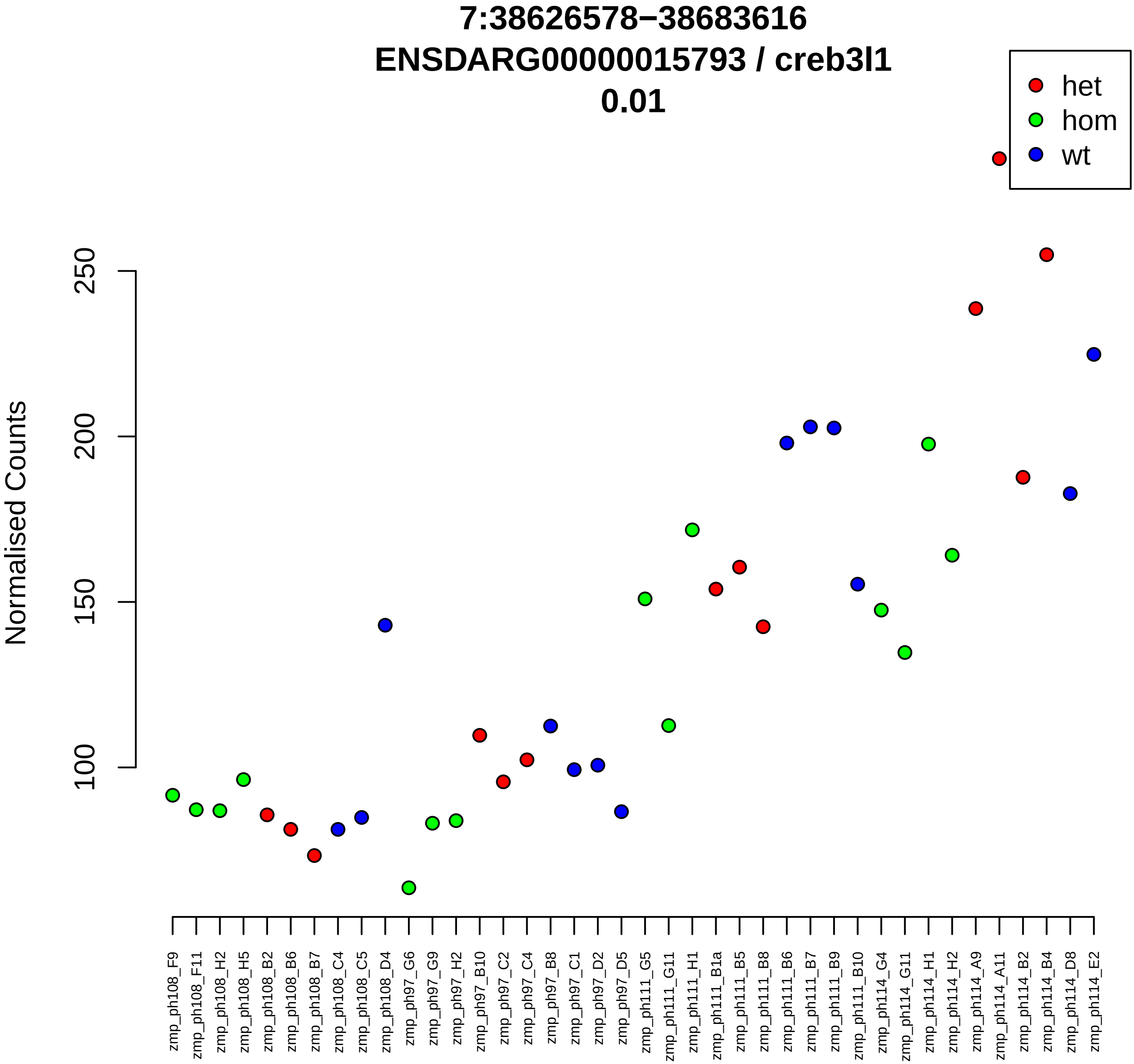
7:38626578−38683616
ENSDARG00000015793 / creb3l1
0.01
het
hom
wt
Normalised Counts
100
150
200
250
zmp_ph108_F9
zmp_ph108_F11
zmp_ph108_H2
zmp_ph108_H5
zmp_ph108_B2
zmp_ph108_B6
zmp_ph108_B7
zmp_ph108_C4
zmp_ph108_C5
zmp_ph108_D4
zmp_ph97_G6
zmp_ph97_G9
zmp_ph97_H2
zmp_ph97_B10
zmp_ph97_C2
zmp_ph97_C4
zmp_ph97_B8
zmp_ph97_C1
zmp_ph97_D2
zmp_ph97_D5
zmp_ph111_G5
zmp_ph111_G11
zmp_ph111_H1
zmp_ph111_B1a
zmp_ph111_B5
zmp_ph111_B8
zmp_ph111_B6
zmp_ph111_B7
zmp_ph111_B9
zmp_ph111_B10
zmp_ph114_G4
zmp_ph114_G11
zmp_ph114_H1
zmp_ph114_H2
zmp_ph114_A9
zmp_ph114_A11
zmp_ph114_B2
zmp_ph114_B4
zmp_ph114_D8
zmp_ph114_E2

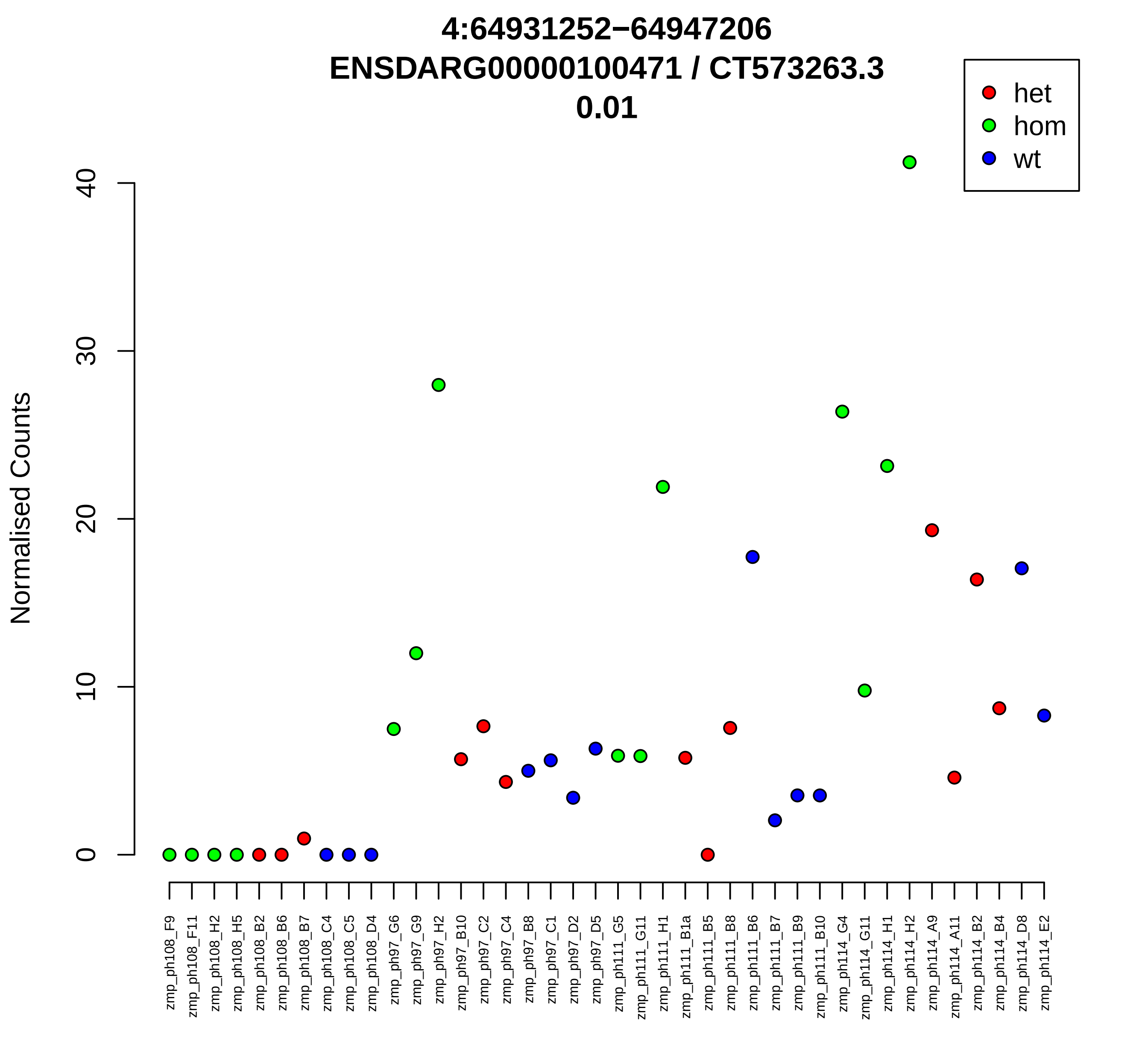
4:64931252−64947206
ENSDARG00000100471 / CT573263.3
0.01
het
hom
wt
Normalised Counts
0
10
20
30
40
zmp_ph108_F9
zmp_ph108_F11
zmp_ph108_H2
zmp_ph108_H5
zmp_ph108_B2
zmp_ph108_B6
zmp_ph108_B7
zmp_ph108_C4
zmp_ph108_C5
zmp_ph108_D4
zmp_ph97_G6
zmp_ph97_G9
zmp_ph97_H2
zmp_ph97_B10
zmp_ph97_C2
zmp_ph97_C4
zmp_ph97_B8
zmp_ph97_C1
zmp_ph97_D2
zmp_ph97_D5
zmp_ph111_G5
zmp_ph111_G11
zmp_ph111_H1
zmp_ph111_B1a
zmp_ph111_B5
zmp_ph111_B8
zmp_ph111_B6
zmp_ph111_B7
zmp_ph111_B9
zmp_ph111_B10
zmp_ph114_G4
zmp_ph114_G11
zmp_ph114_H1
zmp_ph114_H2
zmp_ph114_A9
zmp_ph114_A11
zmp_ph114_B2
zmp_ph114_B4
zmp_ph114_D8
zmp_ph114_E2

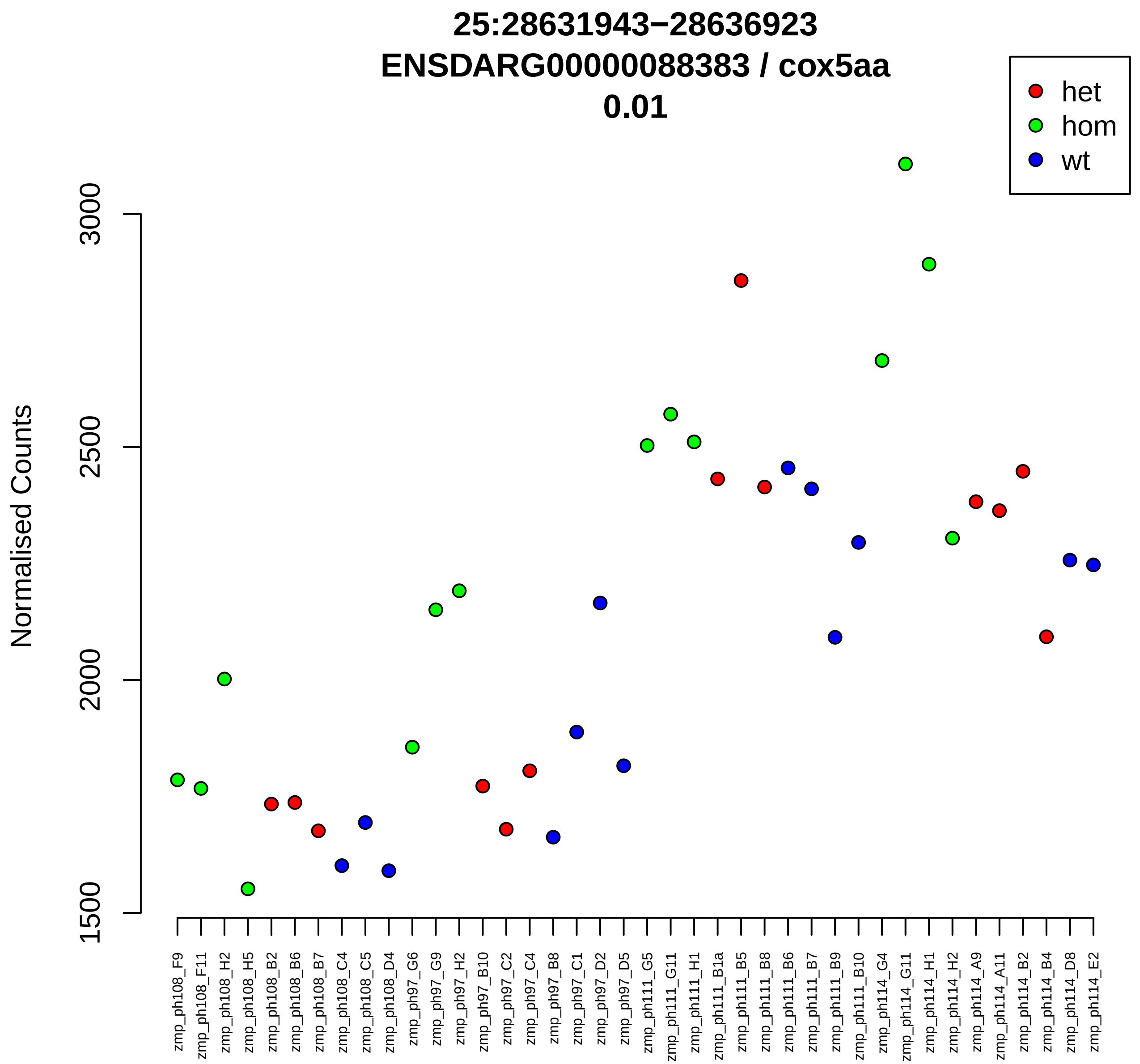

25:28631943−28636923
ENSDARG00000088383 / cox5aa
0.01
het
hom
wt
Normalised Counts
1500
2000
2500
3000
zmp_ph108_F9
zmp_ph108_F11
zmp_ph108_H2
zmp_ph108_H5
zmp_ph108_B2
zmp_ph108_B6
zmp_ph108_B7
zmp_ph108_C4
zmp_ph108_C5
zmp_ph108_D4
zmp_ph97_G6
zmp_ph97_G9
zmp_ph97_H2
zmp_ph97_B10
zmp_ph97_C2
zmp_ph97_C4
zmp_ph97_B8
zmp_ph97_C1
zmp_ph97_D2
zmp_ph97_D5
zmp_ph111_G5
zmp_ph111_G11
zmp_ph111_H1
zmp_ph111_B1a
zmp_ph111_B5
zmp_ph111_B8
zmp_ph111_B6
zmp_ph111_B7
zmp_ph111_B9
zmp_ph111_B10
zmp_ph114_G4
zmp_ph114_G11
zmp_ph114_H1
zmp_ph114_H2
zmp_ph114_A9
zmp_ph114_A11
zmp_ph114_B2
zmp_ph114_B4
zmp_ph114_D8
zmp_ph114_E2

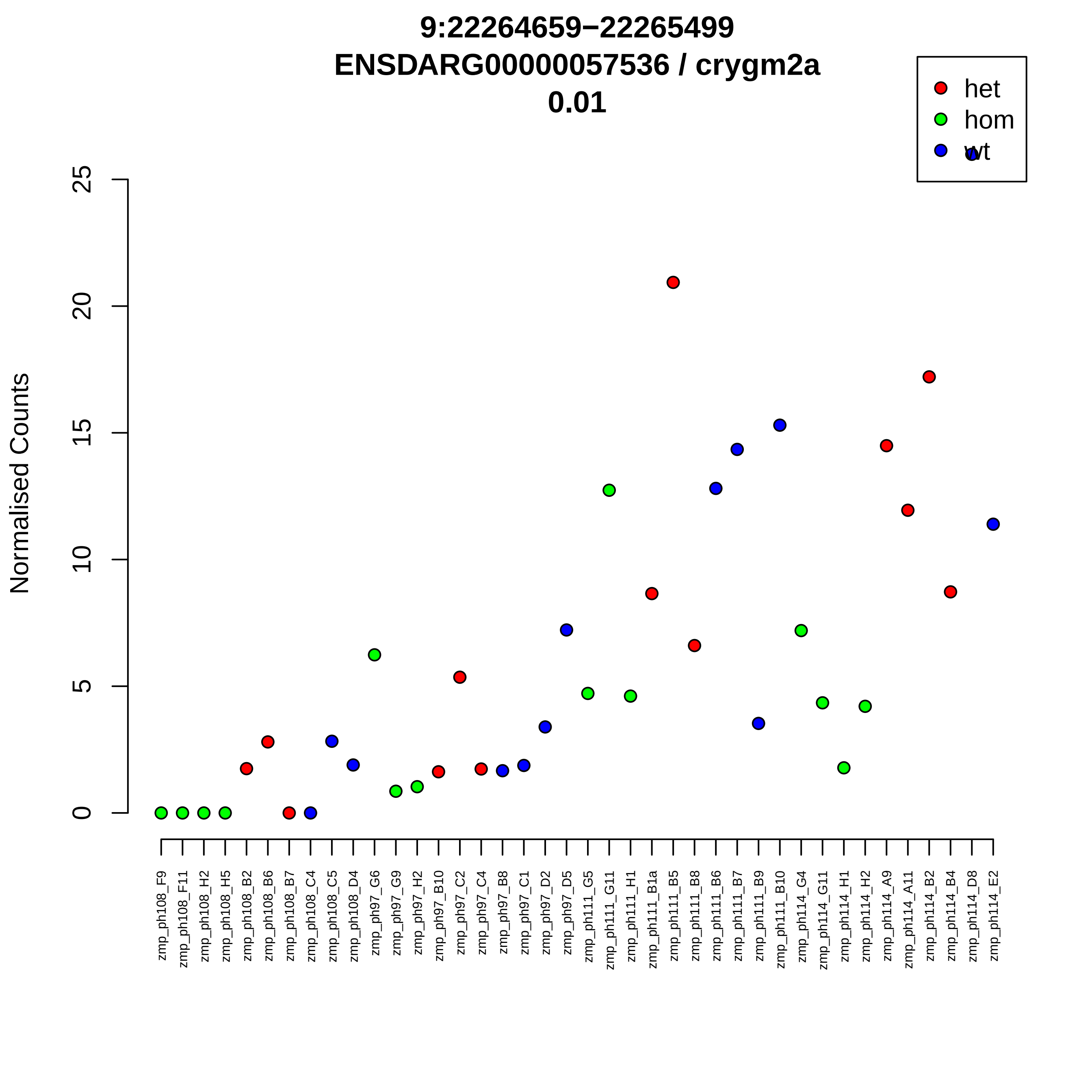

9:22264659−22265499
ENSDARG00000057536 / crygm2a
0.01
het
hom
wt
Normalised Counts
0
5
10
15
20
25
zmp_ph108_F9
zmp_ph108_F11
zmp_ph108_H2
zmp_ph108_H5
zmp_ph108_B2
zmp_ph108_B6
zmp_ph108_B7
zmp_ph108_C4
zmp_ph108_C5
zmp_ph108_D4
zmp_ph97_G6
zmp_ph97_G9
zmp_ph97_H2
zmp_ph97_B10
zmp_ph97_C2
zmp_ph97_C4
zmp_ph97_B8
zmp_ph97_C1
zmp_ph97_D2
zmp_ph97_D5
zmp_ph111_G5
zmp_ph111_G11
zmp_ph111_H1
zmp_ph111_B1a
zmp_ph111_B5
zmp_ph111_B8
zmp_ph111_B6
zmp_ph111_B7
zmp_ph111_B9
zmp_ph111_B10
zmp_ph114_G4
zmp_ph114_G11
zmp_ph114_H1
zmp_ph114_H2
zmp_ph114_A9
zmp_ph114_A11
zmp_ph114_B2
zmp_ph114_B4
zmp_ph114_D8
zmp_ph114_E2

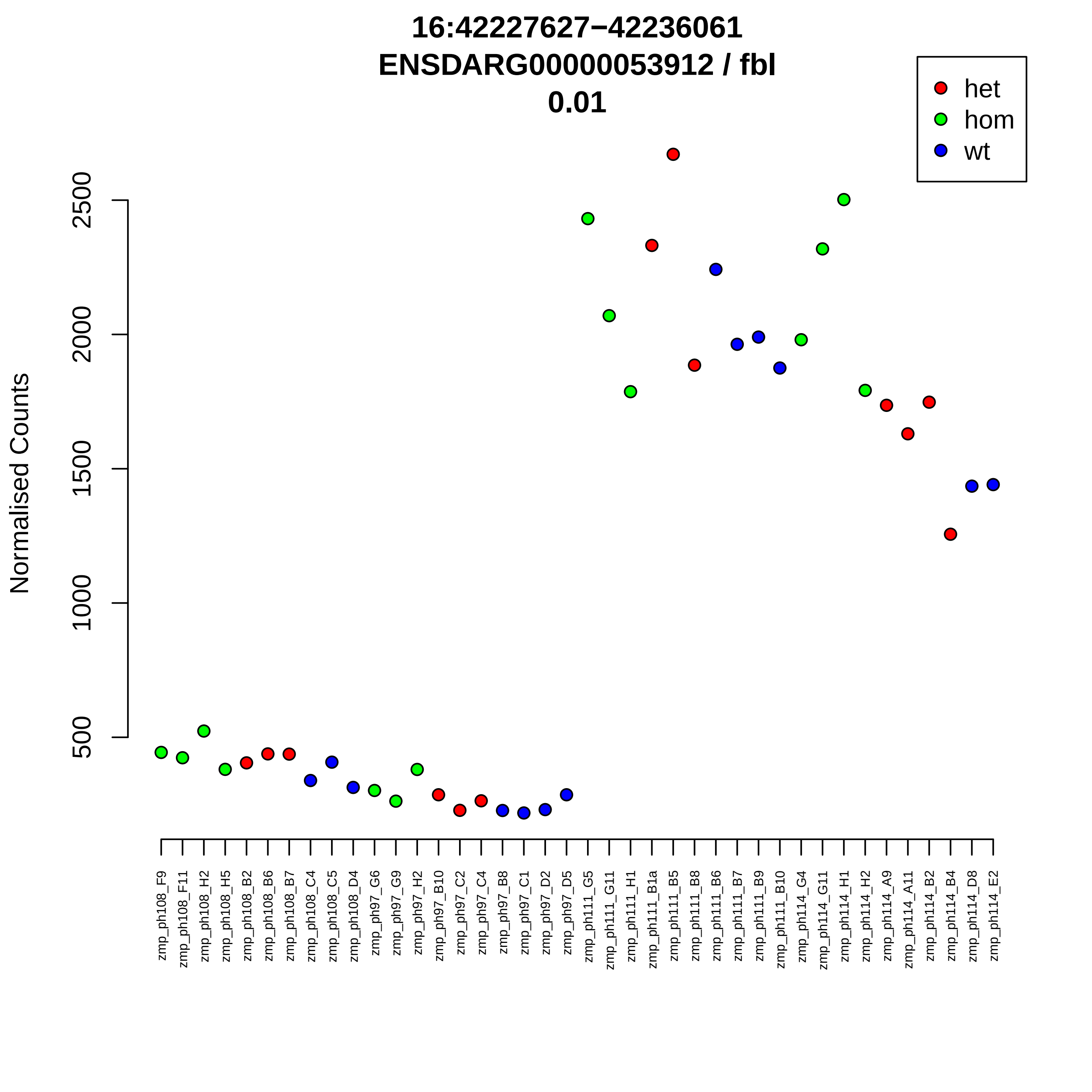
16:42227627−42236061
ENSDARG00000053912 / fbl
0.01
het
hom
wt
Normalised Counts
500
1000
1500
2000
2500
zmp_ph108_F9
zmp_ph108_F11
zmp_ph108_H2
zmp_ph108_H5
zmp_ph108_B2
zmp_ph108_B6
zmp_ph108_B7
zmp_ph108_C4
zmp_ph108_C5
zmp_ph108_D4
zmp_ph97_G6
zmp_ph97_G9
zmp_ph97_H2
zmp_ph97_B10
zmp_ph97_C2
zmp_ph97_C4
zmp_ph97_B8
zmp_ph97_C1
zmp_ph97_D2
zmp_ph97_D5
zmp_ph111_G5
zmp_ph111_G11
zmp_ph111_H1
zmp_ph111_B1a
zmp_ph111_B5
zmp_ph111_B8
zmp_ph111_B6
zmp_ph111_B7
zmp_ph111_B9
zmp_ph111_B10
zmp_ph114_G4
zmp_ph114_G11
zmp_ph114_H1
zmp_ph114_H2
zmp_ph114_A9
zmp_ph114_A11
zmp_ph114_B2
zmp_ph114_B4
zmp_ph114_D8
zmp_ph114_E2

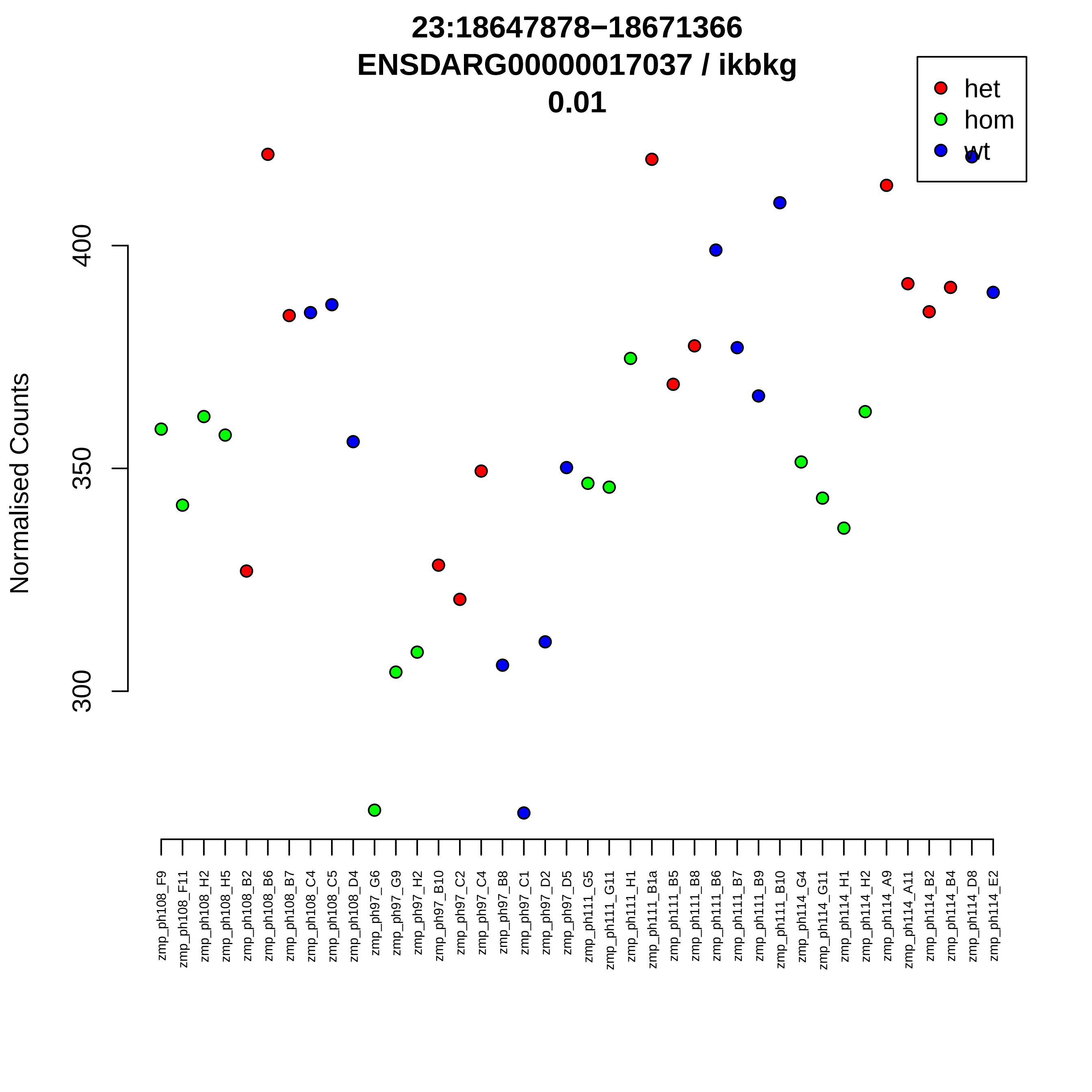
23:18647878−18671366
ENSDARG00000017037 / ikbkg
0.01
het
hom
wt
Normalised Counts
300
350
400
zmp_ph108_F9
zmp_ph108_F11
zmp_ph108_H2
zmp_ph108_H5
zmp_ph108_B2
zmp_ph108_B6
zmp_ph108_B7
zmp_ph108_C4
zmp_ph108_C5
zmp_ph108_D4
zmp_ph97_G6
zmp_ph97_G9
zmp_ph97_H2
zmp_ph97_B10
zmp_ph97_C2
zmp_ph97_C4
zmp_ph97_B8
zmp_ph97_C1
zmp_ph97_D2
zmp_ph97_D5
zmp_ph111_G5
zmp_ph111_G11
zmp_ph111_H1
zmp_ph111_B1a
zmp_ph111_B5
zmp_ph111_B8
zmp_ph111_B6
zmp_ph111_B7
zmp_ph111_B9
zmp_ph111_B10
zmp_ph114_G4
zmp_ph114_G11
zmp_ph114_H1
zmp_ph114_H2
zmp_ph114_A9
zmp_ph114_A11
zmp_ph114_B2
zmp_ph114_B4
zmp_ph114_D8
zmp_ph114_E2

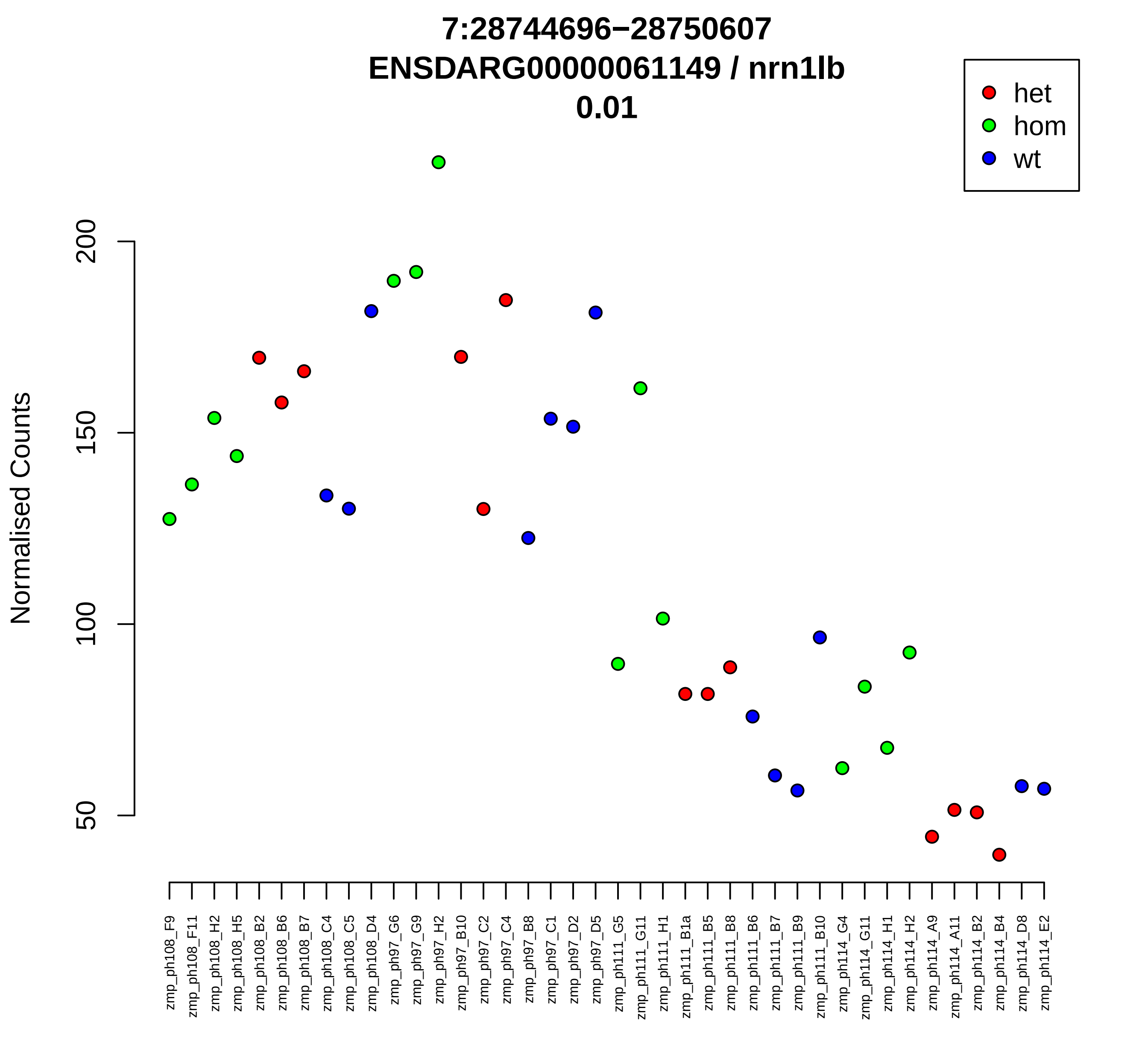
7:28744696−28750607
ENSDARG00000061149 / nrn1lb
0.01
het
hom
wt
Normalised Counts
50
100
150
200
zmp_ph108_F9
zmp_ph108_F11
zmp_ph108_H2
zmp_ph108_H5
zmp_ph108_B2
zmp_ph108_B6
zmp_ph108_B7
zmp_ph108_C4
zmp_ph108_C5
zmp_ph108_D4
zmp_ph97_G6
zmp_ph97_G9
zmp_ph97_H2
zmp_ph97_B10
zmp_ph97_C2
zmp_ph97_C4
zmp_ph97_B8
zmp_ph97_C1
zmp_ph97_D2
zmp_ph97_D5
zmp_ph111_G5
zmp_ph111_G11
zmp_ph111_H1
zmp_ph111_B1a
zmp_ph111_B5
zmp_ph111_B8
zmp_ph111_B6
zmp_ph111_B7
zmp_ph111_B9
zmp_ph111_B10
zmp_ph114_G4
zmp_ph114_G11
zmp_ph114_H1
zmp_ph114_H2
zmp_ph114_A9
zmp_ph114_A11
zmp_ph114_B2
zmp_ph114_B4
zmp_ph114_D8
zmp_ph114_E2

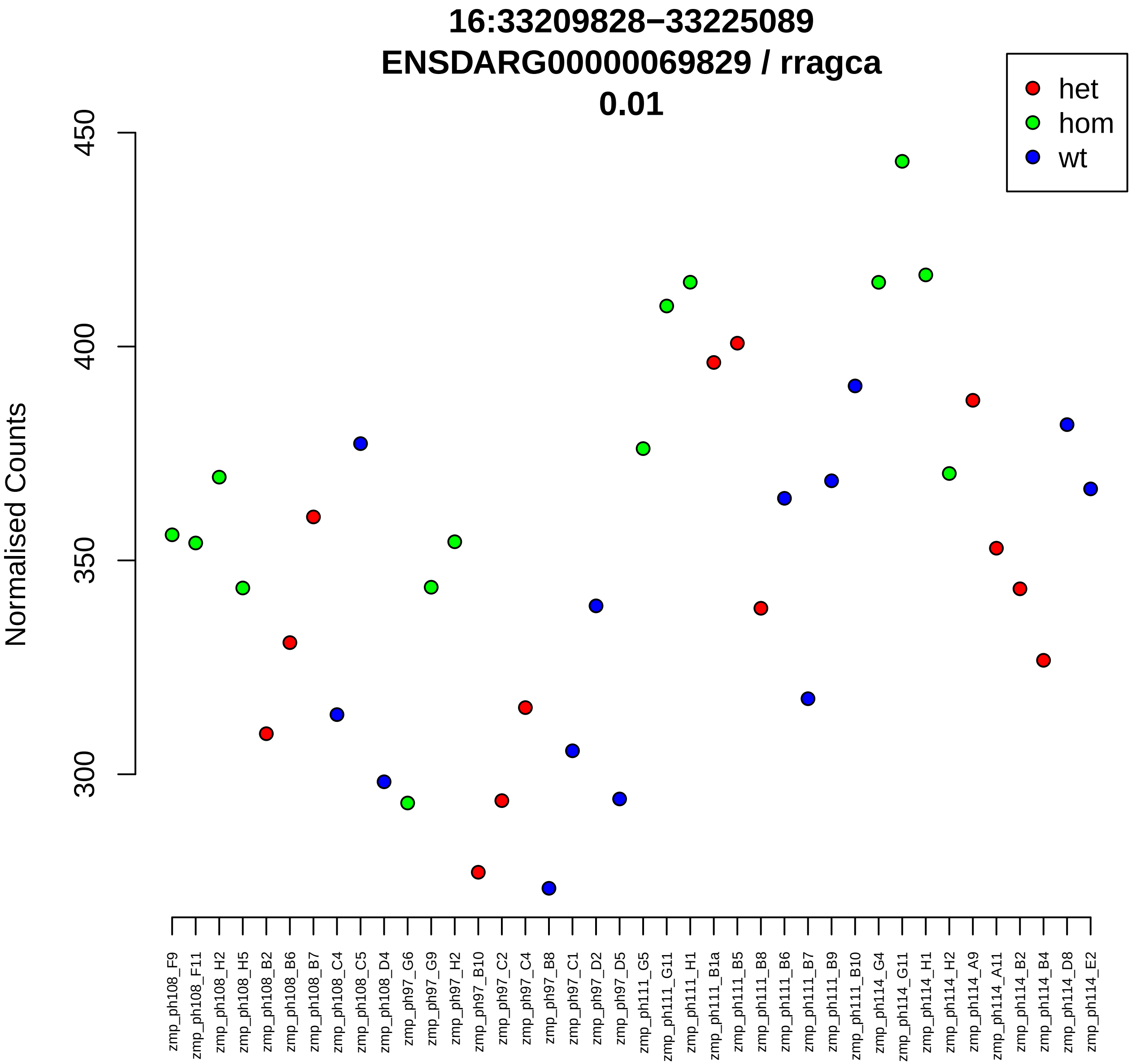

16:33209828−33225089
ENSDARG00000069829 / rragca
0.01
het
hom
wt
Normalised Counts
300
350
400
450
zmp_ph108_F9
zmp_ph108_F11
zmp_ph108_H2
zmp_ph108_H5
zmp_ph108_B2
zmp_ph108_B6
zmp_ph108_B7
zmp_ph108_C4
zmp_ph108_C5
zmp_ph108_D4
zmp_ph97_G6
zmp_ph97_G9
zmp_ph97_H2
zmp_ph97_B10
zmp_ph97_C2
zmp_ph97_C4
zmp_ph97_B8
zmp_ph97_C1
zmp_ph97_D2
zmp_ph97_D5
zmp_ph111_G5
zmp_ph111_G11
zmp_ph111_H1
zmp_ph111_B1a
zmp_ph111_B5
zmp_ph111_B8
zmp_ph111_B6
zmp_ph111_B7
zmp_ph111_B9
zmp_ph111_B10
zmp_ph114_G4
zmp_ph114_G11
zmp_ph114_H1
zmp_ph114_H2
zmp_ph114_A9
zmp_ph114_A11
zmp_ph114_B2
zmp_ph114_B4
zmp_ph114_D8
zmp_ph114_E2

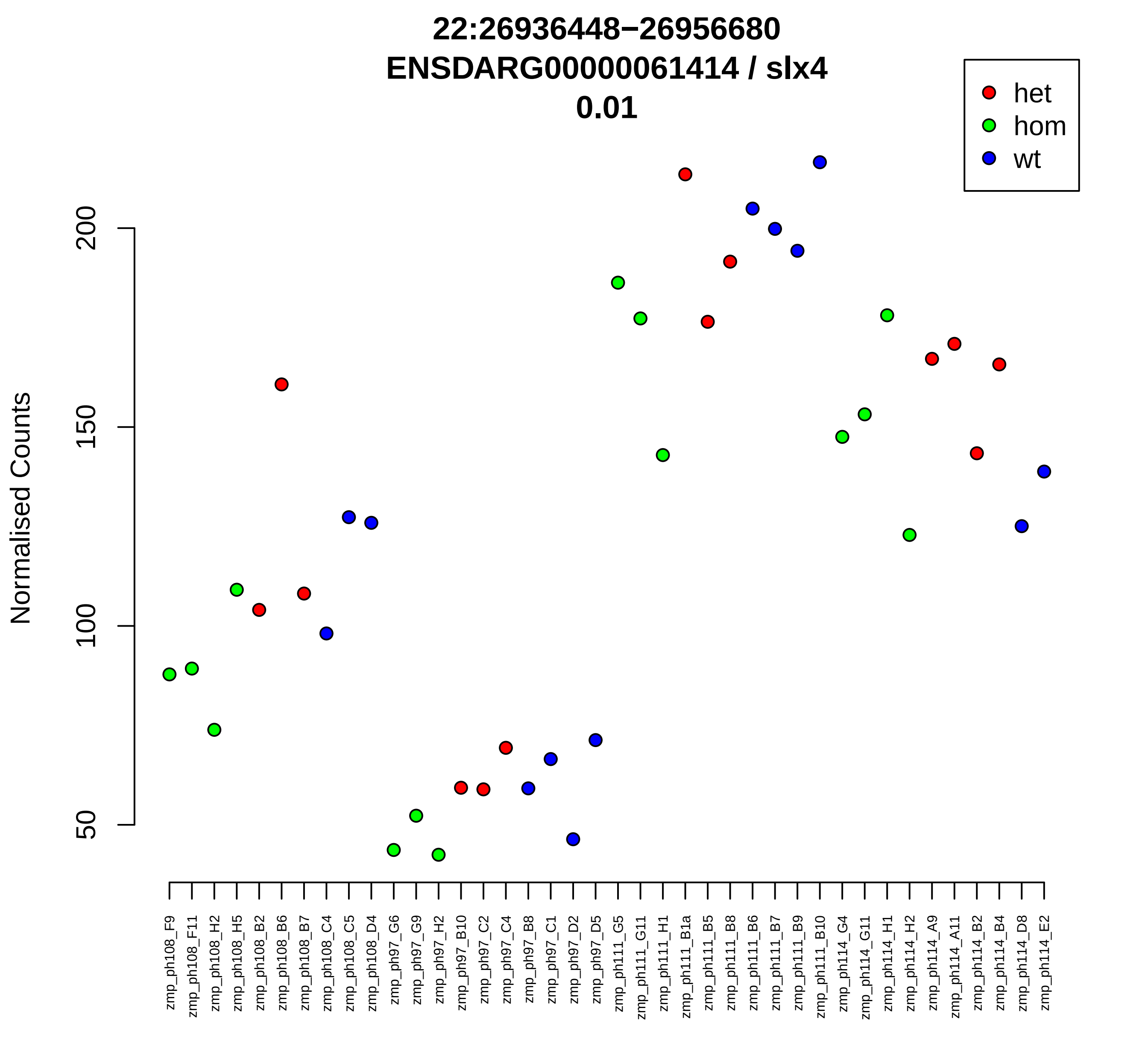

22:26936448−26956680
ENSDARG00000061414 / slx4
0.01
het
hom
wt
Normalised Counts
50
100
150
200
zmp_ph108_F9
zmp_ph108_F11
zmp_ph108_H2
zmp_ph108_H5
zmp_ph108_B2
zmp_ph108_B6
zmp_ph108_B7
zmp_ph108_C4
zmp_ph108_C5
zmp_ph108_D4
zmp_ph97_G6
zmp_ph97_G9
zmp_ph97_H2
zmp_ph97_B10
zmp_ph97_C2
zmp_ph97_C4
zmp_ph97_B8
zmp_ph97_C1
zmp_ph97_D2
zmp_ph97_D5
zmp_ph111_G5
zmp_ph111_G11
zmp_ph111_H1
zmp_ph111_B1a
zmp_ph111_B5
zmp_ph111_B8
zmp_ph111_B6
zmp_ph111_B7
zmp_ph111_B9
zmp_ph111_B10
zmp_ph114_G4
zmp_ph114_G11
zmp_ph114_H1
zmp_ph114_H2
zmp_ph114_A9
zmp_ph114_A11
zmp_ph114_B2
zmp_ph114_B4
zmp_ph114_D8
zmp_ph114_E2

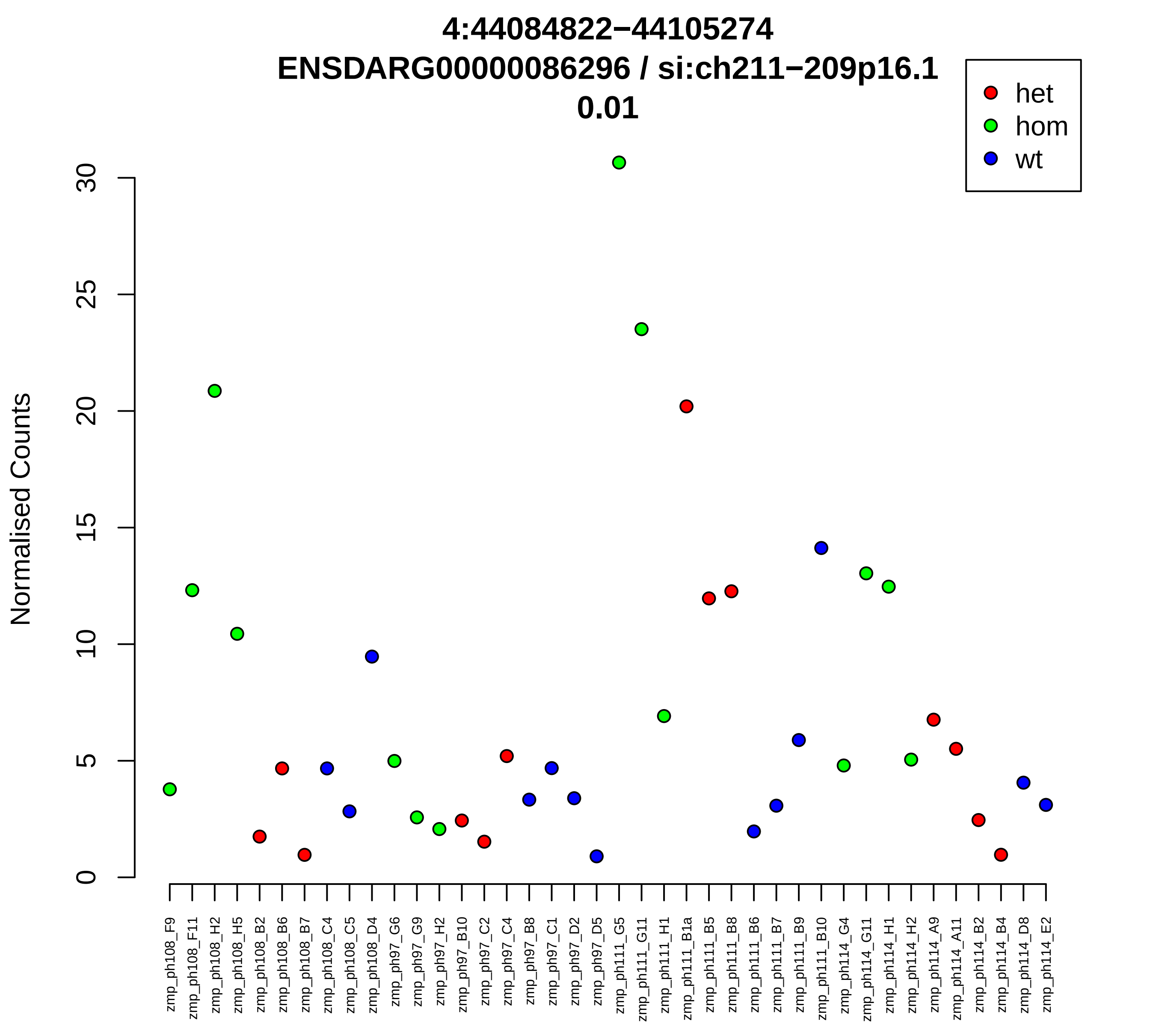
4:44084822−44105274
ENSDARG00000086296 / si:ch211−209p16.1
0.01
het
hom
wt
Normalised Counts
0
5
10
15
20
25
30
zmp_ph108_F9
zmp_ph108_F11
zmp_ph108_H2
zmp_ph108_H5
zmp_ph108_B2
zmp_ph108_B6
zmp_ph108_B7
zmp_ph108_C4
zmp_ph108_C5
zmp_ph108_D4
zmp_ph97_G6
zmp_ph97_G9
zmp_ph97_H2
zmp_ph97_B10
zmp_ph97_C2
zmp_ph97_C4
zmp_ph97_B8
zmp_ph97_C1
zmp_ph97_D2
zmp_ph97_D5
zmp_ph111_G5
zmp_ph111_G11
zmp_ph111_H1
zmp_ph111_B1a
zmp_ph111_B5
zmp_ph111_B8
zmp_ph111_B6
zmp_ph111_B7
zmp_ph111_B9
zmp_ph111_B10
zmp_ph114_G4
zmp_ph114_G11
zmp_ph114_H1
zmp_ph114_H2
zmp_ph114_A9
zmp_ph114_A11
zmp_ph114_B2
zmp_ph114_B4
zmp_ph114_D8
zmp_ph114_E2

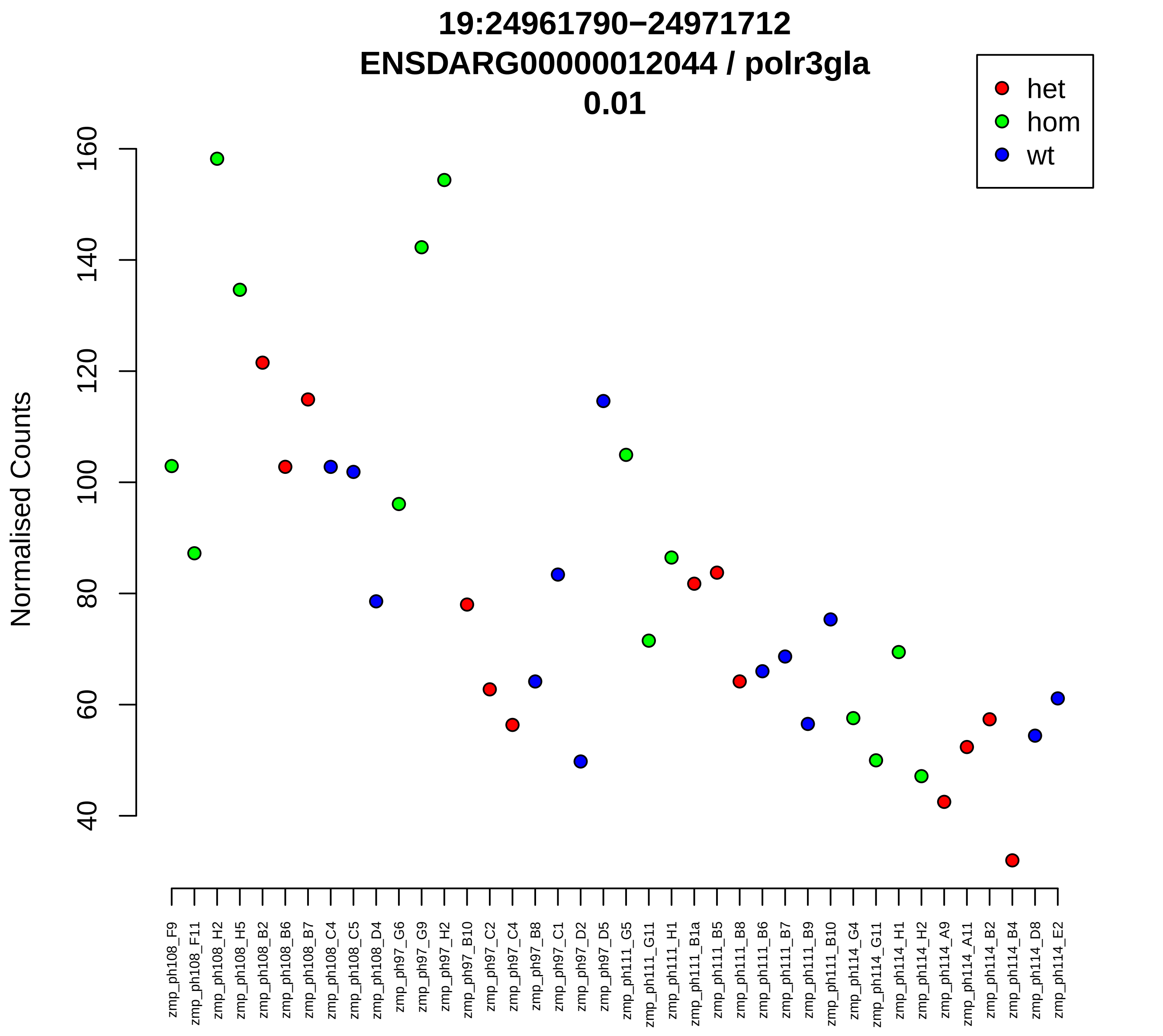

19:24961790−24971712
ENSDARG00000012044 / polr3gla
0.01
het
hom
wt
Normalised Counts
40
60
80
100
120
140
160
zmp_ph108_F9
zmp_ph108_F11
zmp_ph108_H2
zmp_ph108_H5
zmp_ph108_B2
zmp_ph108_B6
zmp_ph108_B7
zmp_ph108_C4
zmp_ph108_C5
zmp_ph108_D4
zmp_ph97_G6
zmp_ph97_G9
zmp_ph97_H2
zmp_ph97_B10
zmp_ph97_C2
zmp_ph97_C4
zmp_ph97_B8
zmp_ph97_C1
zmp_ph97_D2
zmp_ph97_D5
zmp_ph111_G5
zmp_ph111_G11
zmp_ph111_H1
zmp_ph111_B1a
zmp_ph111_B5
zmp_ph111_B8
zmp_ph111_B6
zmp_ph111_B7
zmp_ph111_B9
zmp_ph111_B10
zmp_ph114_G4
zmp_ph114_G11
zmp_ph114_H1
zmp_ph114_H2
zmp_ph114_A9
zmp_ph114_A11
zmp_ph114_B2
zmp_ph114_B4
zmp_ph114_D8
zmp_ph114_E2

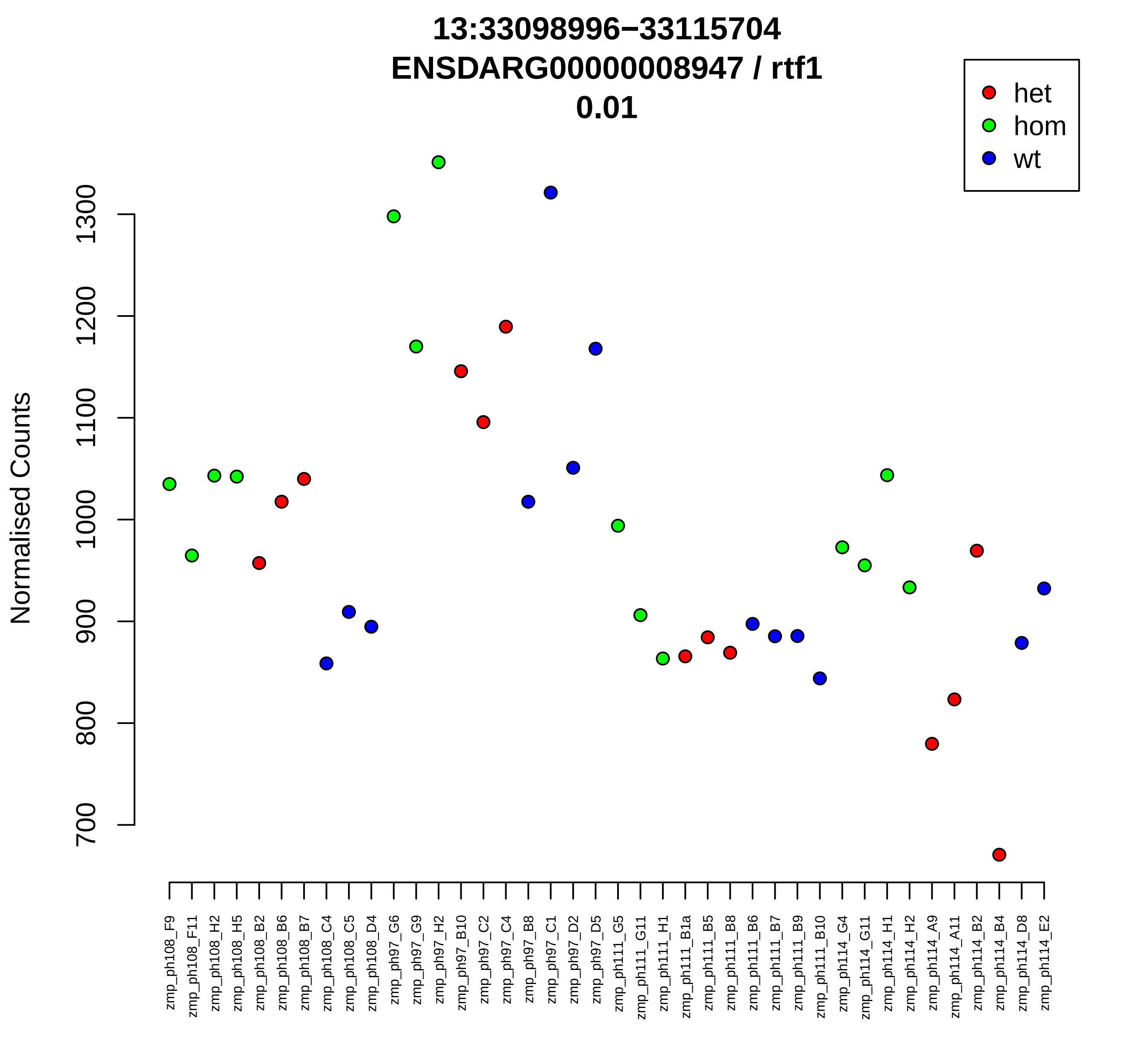
13:33098996−33115704
ENSDARG00000008947 / rtf1
0.01
het
hom
wt
Normalised Counts
700
800
900
1000
1100
1200
1300
zmp_ph108_F9
zmp_ph108_F11
zmp_ph108_H2
zmp_ph108_H5
zmp_ph108_B2
zmp_ph108_B6
zmp_ph108_B7
zmp_ph108_C4
zmp_ph108_C5
zmp_ph108_D4
zmp_ph97_G6
zmp_ph97_G9
zmp_ph97_H2
zmp_ph97_B10
zmp_ph97_C2
zmp_ph97_C4
zmp_ph97_B8
zmp_ph97_C1
zmp_ph97_D2
zmp_ph97_D5
zmp_ph111_G5
zmp_ph111_G11
zmp_ph111_H1
zmp_ph111_B1a
zmp_ph111_B5
zmp_ph111_B8
zmp_ph111_B6
zmp_ph111_B7
zmp_ph111_B9
zmp_ph111_B10
zmp_ph114_G4
zmp_ph114_G11
zmp_ph114_H1
zmp_ph114_H2
zmp_ph114_A9
zmp_ph114_A11
zmp_ph114_B2
zmp_ph114_B4
zmp_ph114_D8
zmp_ph114_E2

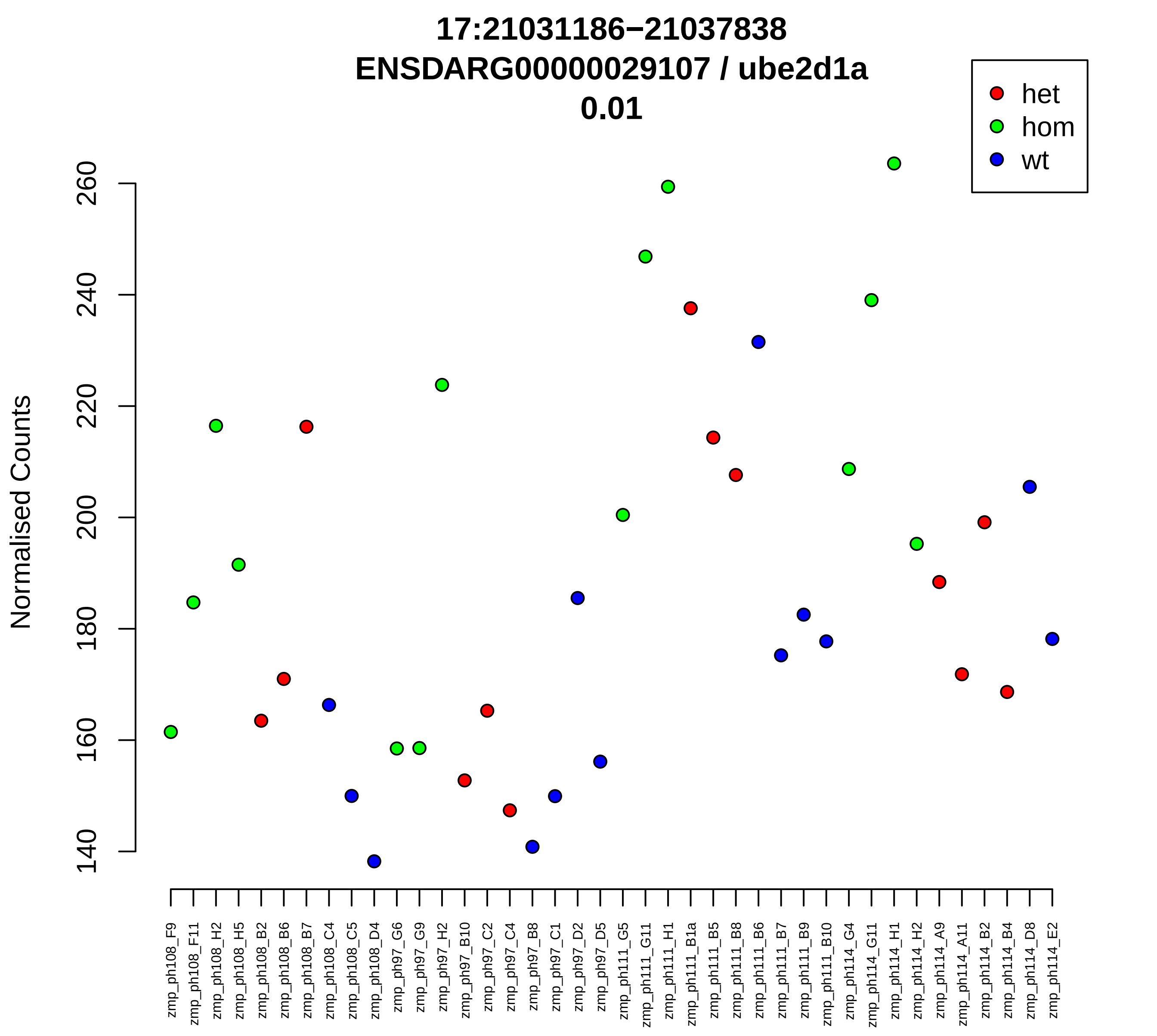

17:21031186−21037838
ENSDARG00000029107 / ube2d1a
0.01
het
hom
wt
Normalised Counts
140
160
180
200
220
240
260
zmp_ph108_F9
zmp_ph108_F11
zmp_ph108_H2
zmp_ph108_H5
zmp_ph108_B2
zmp_ph108_B6
zmp_ph108_B7
zmp_ph108_C4
zmp_ph108_C5
zmp_ph108_D4
zmp_ph97_G6
zmp_ph97_G9
zmp_ph97_H2
zmp_ph97_B10
zmp_ph97_C2
zmp_ph97_C4
zmp_ph97_B8
zmp_ph97_C1
zmp_ph97_D2
zmp_ph97_D5
zmp_ph111_G5
zmp_ph111_G11
zmp_ph111_H1
zmp_ph111_B1a
zmp_ph111_B5
zmp_ph111_B8
zmp_ph111_B6
zmp_ph111_B7
zmp_ph111_B9
zmp_ph111_B10
zmp_ph114_G4
zmp_ph114_G11
zmp_ph114_H1
zmp_ph114_H2
zmp_ph114_A9
zmp_ph114_A11
zmp_ph114_B2
zmp_ph114_B4
zmp_ph114_D8
zmp_ph114_E2

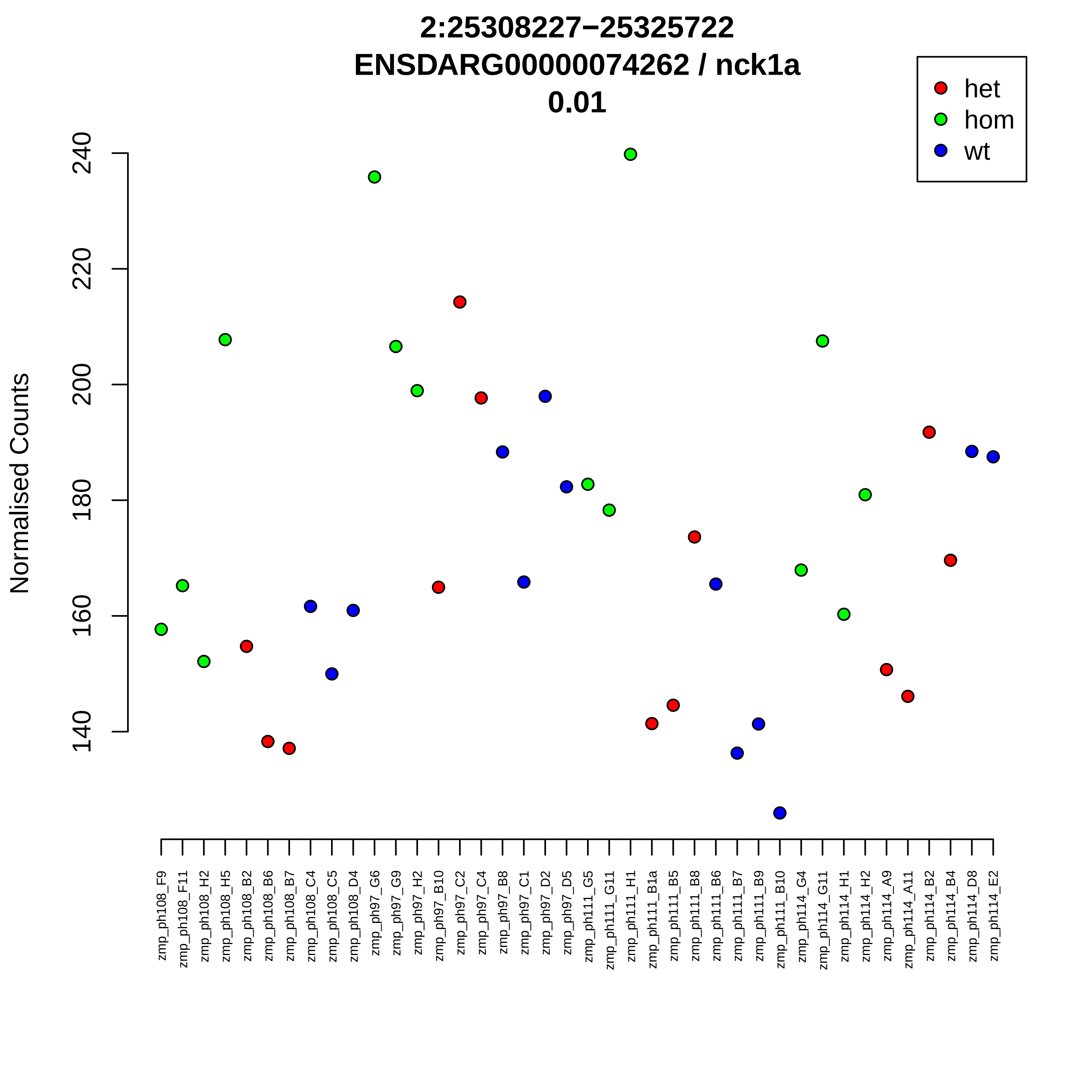

2:25308227−25325722
ENSDARG00000074262 / nck1a
0.01
het
hom
wt
Normalised Counts
140
160
180
200
220
240
zmp_ph108_F9
zmp_ph108_F11
zmp_ph108_H2
zmp_ph108_H5
zmp_ph108_B2
zmp_ph108_B6
zmp_ph108_B7
zmp_ph108_C4
zmp_ph108_C5
zmp_ph108_D4
zmp_ph97_G6
zmp_ph97_G9
zmp_ph97_H2
zmp_ph97_B10
zmp_ph97_C2
zmp_ph97_C4
zmp_ph97_B8
zmp_ph97_C1
zmp_ph97_D2
zmp_ph97_D5
zmp_ph111_G5
zmp_ph111_G11
zmp_ph111_H1
zmp_ph111_B1a
zmp_ph111_B5
zmp_ph111_B8
zmp_ph111_B6
zmp_ph111_B7
zmp_ph111_B9
zmp_ph111_B10
zmp_ph114_G4
zmp_ph114_G11
zmp_ph114_H1
zmp_ph114_H2
zmp_ph114_A9
zmp_ph114_A11
zmp_ph114_B2
zmp_ph114_B4
zmp_ph114_D8
zmp_ph114_E2

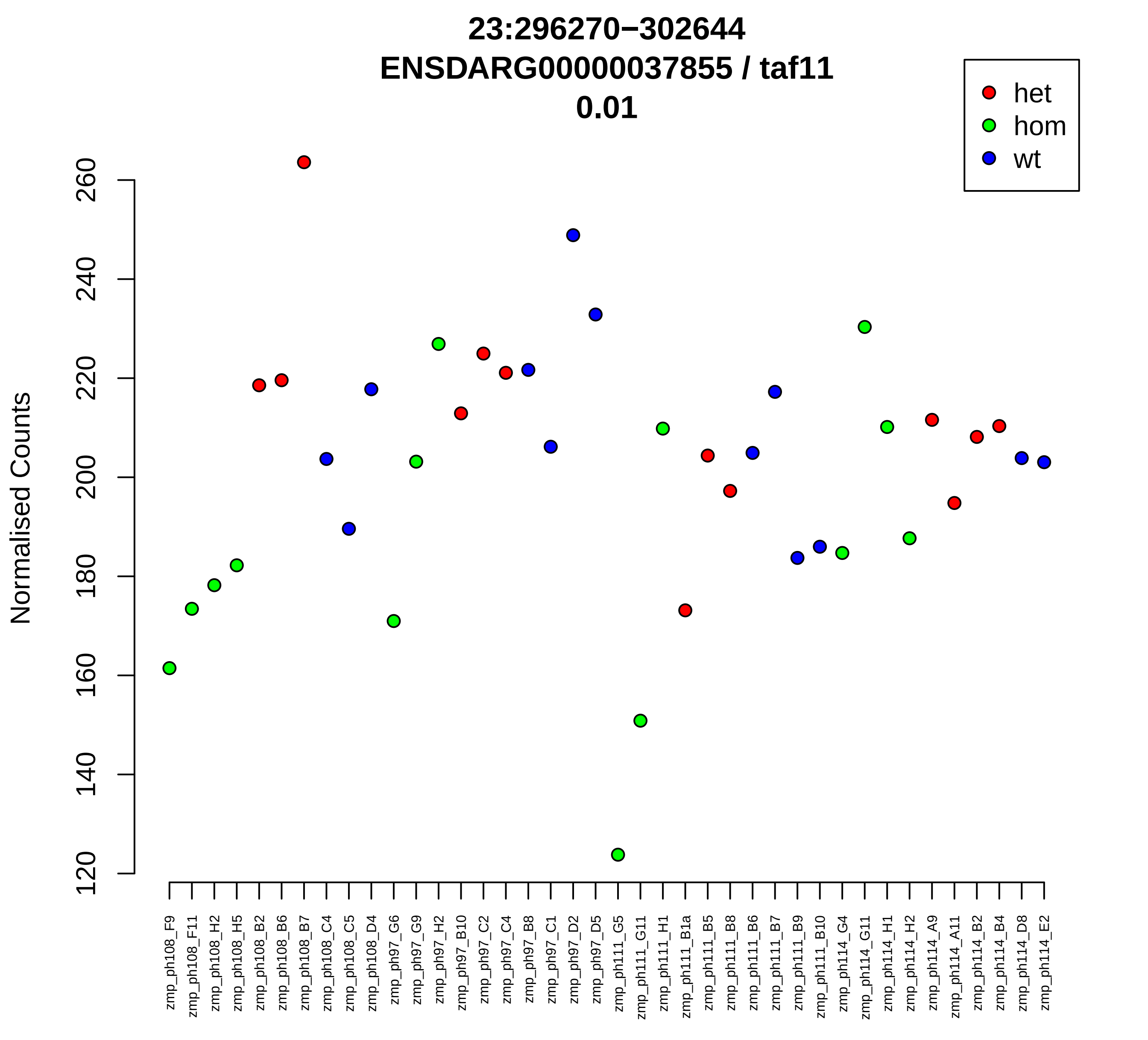

23:296270−302644
ENSDARG00000037855 / taf11
0.01
het
hom
wt
Normalised Counts
120
140
160
180
200
220
240
260
zmp_ph108_F9
zmp_ph108_F11
zmp_ph108_H2
zmp_ph108_H5
zmp_ph108_B2
zmp_ph108_B6
zmp_ph108_B7
zmp_ph108_C4
zmp_ph108_C5
zmp_ph108_D4
zmp_ph97_G6
zmp_ph97_G9
zmp_ph97_H2
zmp_ph97_B10
zmp_ph97_C2
zmp_ph97_C4
zmp_ph97_B8
zmp_ph97_C1
zmp_ph97_D2
zmp_ph97_D5
zmp_ph111_G5
zmp_ph111_G11
zmp_ph111_H1
zmp_ph111_B1a
zmp_ph111_B5
zmp_ph111_B8
zmp_ph111_B6
zmp_ph111_B7
zmp_ph111_B9
zmp_ph111_B10
zmp_ph114_G4
zmp_ph114_G11
zmp_ph114_H1
zmp_ph114_H2
zmp_ph114_A9
zmp_ph114_A11
zmp_ph114_B2
zmp_ph114_B4
zmp_ph114_D8
zmp_ph114_E2

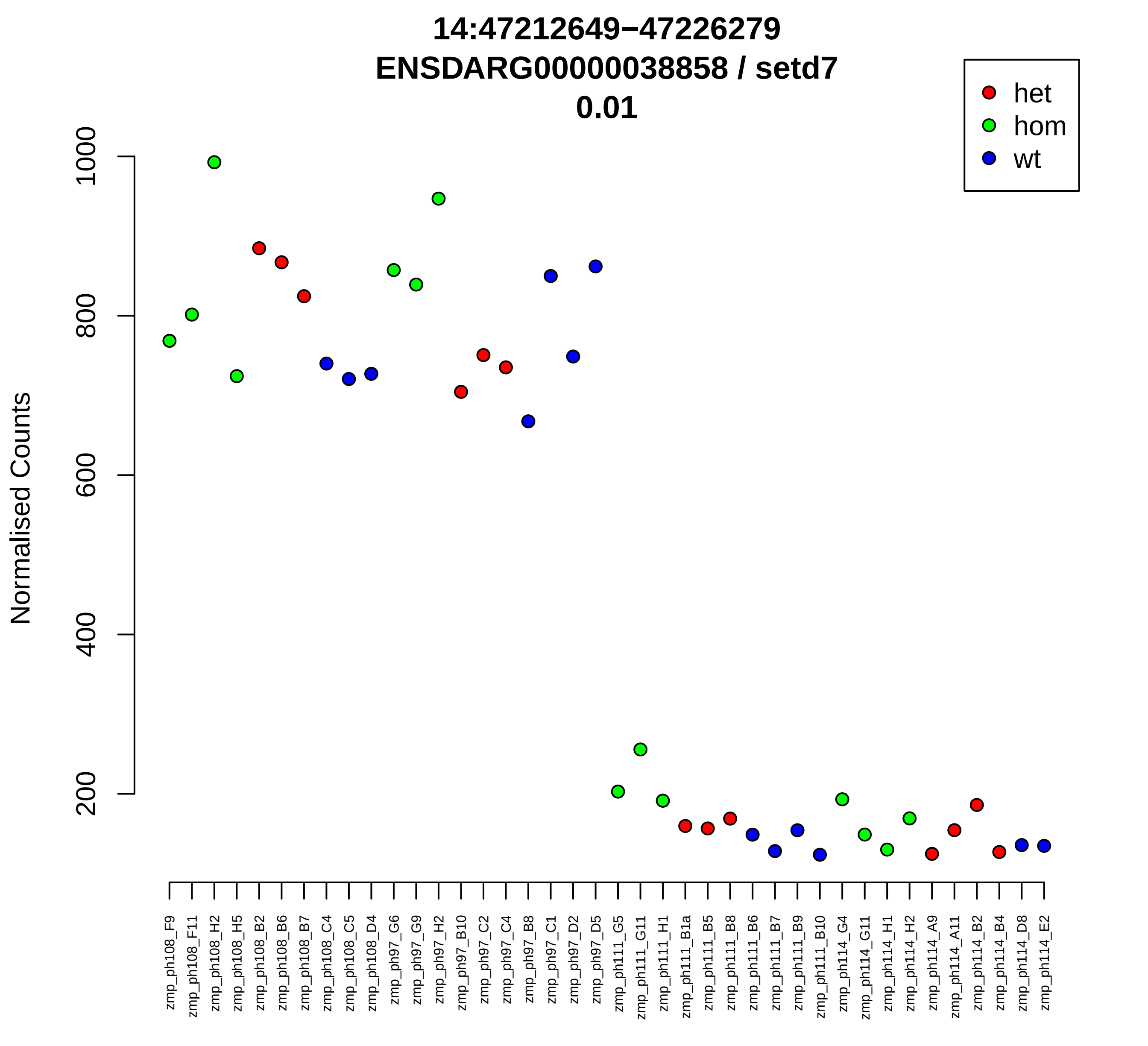
14:47212649−47226279
ENSDARG00000038858 / setd7
0.01
het
hom
wt
Normalised Counts
200
400
600
800
1000
zmp_ph108_F9
zmp_ph108_F11
zmp_ph108_H2
zmp_ph108_H5
zmp_ph108_B2
zmp_ph108_B6
zmp_ph108_B7
zmp_ph108_C4
zmp_ph108_C5
zmp_ph108_D4
zmp_ph97_G6
zmp_ph97_G9
zmp_ph97_H2
zmp_ph97_B10
zmp_ph97_C2
zmp_ph97_C4
zmp_ph97_B8
zmp_ph97_C1
zmp_ph97_D2
zmp_ph97_D5
zmp_ph111_G5
zmp_ph111_G11
zmp_ph111_H1
zmp_ph111_B1a
zmp_ph111_B5
zmp_ph111_B8
zmp_ph111_B6
zmp_ph111_B7
zmp_ph111_B9
zmp_ph111_B10
zmp_ph114_G4
zmp_ph114_G11
zmp_ph114_H1
zmp_ph114_H2
zmp_ph114_A9
zmp_ph114_A11
zmp_ph114_B2
zmp_ph114_B4
zmp_ph114_D8
zmp_ph114_E2

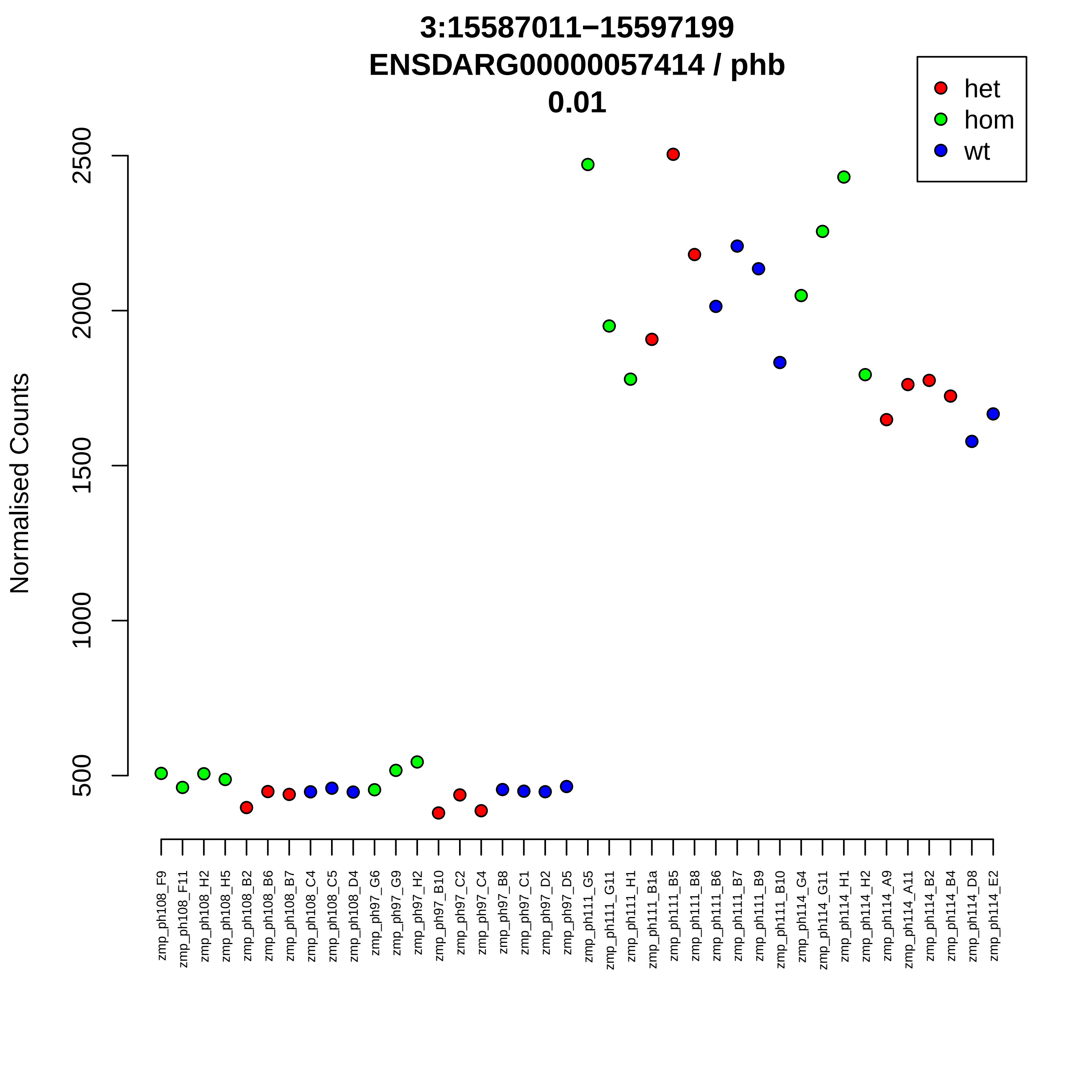
3:15587011−15597199
ENSDARG00000057414 / phb
0.01
het
hom
wt
Normalised Counts
500
1000
1500
2000
2500
zmp_ph108_F9
zmp_ph108_F11
zmp_ph108_H2
zmp_ph108_H5
zmp_ph108_B2
zmp_ph108_B6
zmp_ph108_B7
zmp_ph108_C4
zmp_ph108_C5
zmp_ph108_D4
zmp_ph97_G6
zmp_ph97_G9
zmp_ph97_H2
zmp_ph97_B10
zmp_ph97_C2
zmp_ph97_C4
zmp_ph97_B8
zmp_ph97_C1
zmp_ph97_D2
zmp_ph97_D5
zmp_ph111_G5
zmp_ph111_G11
zmp_ph111_H1
zmp_ph111_B1a
zmp_ph111_B5
zmp_ph111_B8
zmp_ph111_B6
zmp_ph111_B7
zmp_ph111_B9
zmp_ph111_B10
zmp_ph114_G4
zmp_ph114_G11
zmp_ph114_H1
zmp_ph114_H2
zmp_ph114_A9
zmp_ph114_A11
zmp_ph114_B2
zmp_ph114_B4
zmp_ph114_D8
zmp_ph114_E2

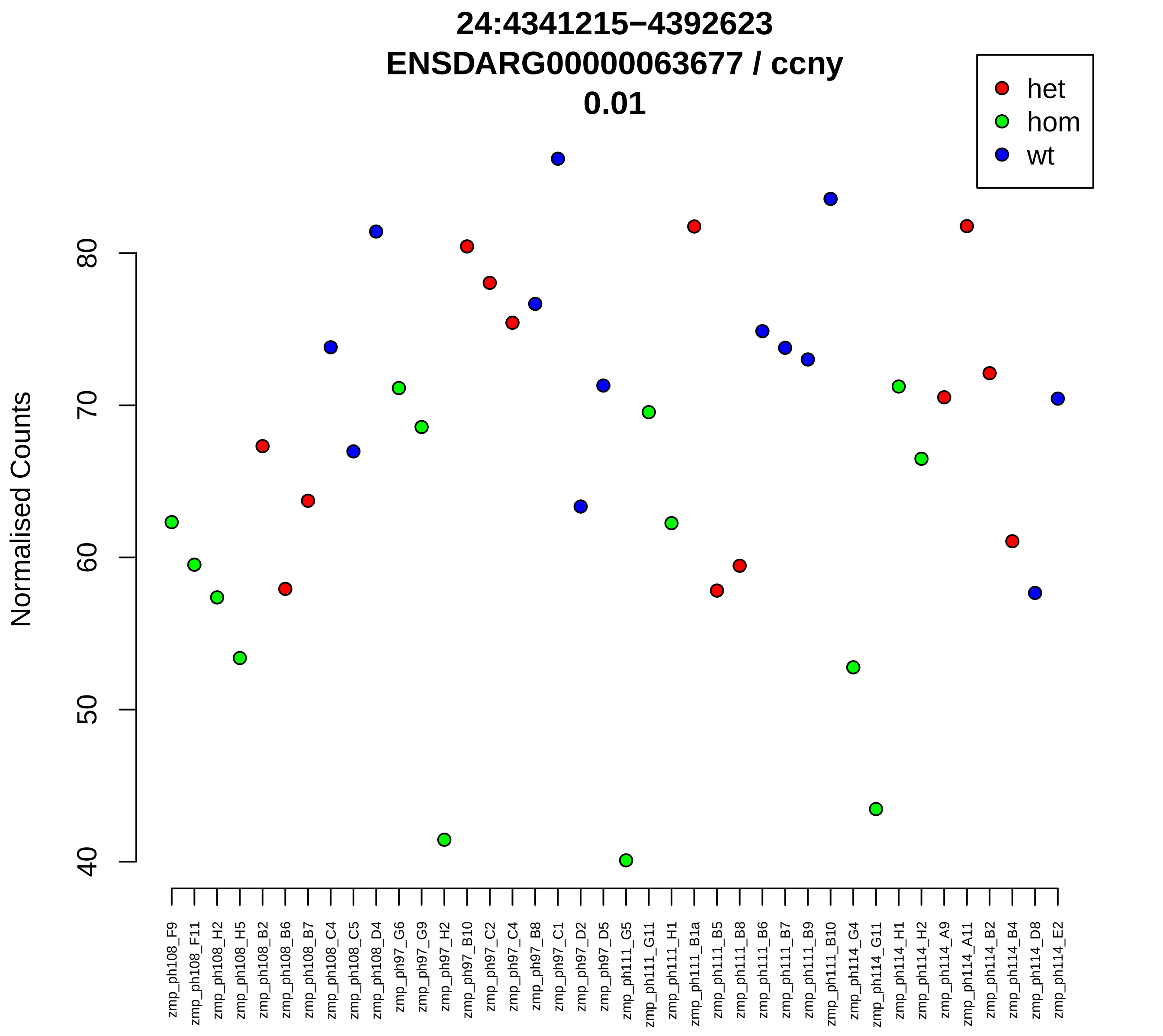
24:4341215−4392623
ENSDARG00000063677 / ccny
0.01
het
hom
wt
Normalised Counts
40
50
60
70
80
zmp_ph108_F9
zmp_ph108_F11
zmp_ph108_H2
zmp_ph108_H5
zmp_ph108_B2
zmp_ph108_B6
zmp_ph108_B7
zmp_ph108_C4
zmp_ph108_C5
zmp_ph108_D4
zmp_ph97_G6
zmp_ph97_G9
zmp_ph97_H2
zmp_ph97_B10
zmp_ph97_C2
zmp_ph97_C4
zmp_ph97_B8
zmp_ph97_C1
zmp_ph97_D2
zmp_ph97_D5
zmp_ph111_G5
zmp_ph111_G11
zmp_ph111_H1
zmp_ph111_B1a
zmp_ph111_B5
zmp_ph111_B8
zmp_ph111_B6
zmp_ph111_B7
zmp_ph111_B9
zmp_ph111_B10
zmp_ph114_G4
zmp_ph114_G11
zmp_ph114_H1
zmp_ph114_H2
zmp_ph114_A9
zmp_ph114_A11
zmp_ph114_B2
zmp_ph114_B4
zmp_ph114_D8
zmp_ph114_E2

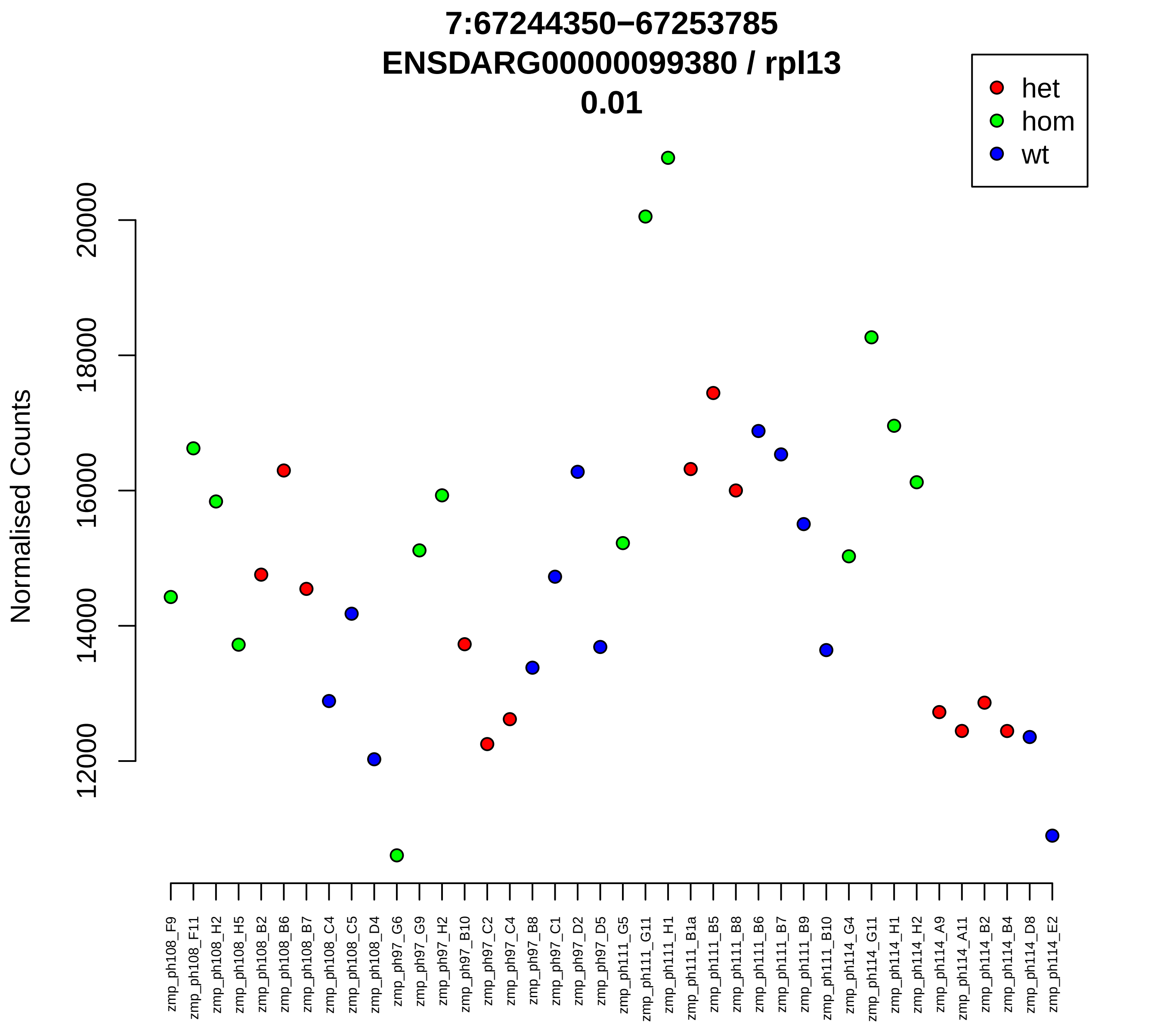

7:67244350−67253785
ENSDARG00000099380 / rpl13
0.01
het
hom
wt
Normalised Counts
12000
14000
16000
18000
20000
zmp_ph108_F9
zmp_ph108_F11
zmp_ph108_H2
zmp_ph108_H5
zmp_ph108_B2
zmp_ph108_B6
zmp_ph108_B7
zmp_ph108_C4
zmp_ph108_C5
zmp_ph108_D4
zmp_ph97_G6
zmp_ph97_G9
zmp_ph97_H2
zmp_ph97_B10
zmp_ph97_C2
zmp_ph97_C4
zmp_ph97_B8
zmp_ph97_C1
zmp_ph97_D2
zmp_ph97_D5
zmp_ph111_G5
zmp_ph111_G11
zmp_ph111_H1
zmp_ph111_B1a
zmp_ph111_B5
zmp_ph111_B8
zmp_ph111_B6
zmp_ph111_B7
zmp_ph111_B9
zmp_ph111_B10
zmp_ph114_G4
zmp_ph114_G11
zmp_ph114_H1
zmp_ph114_H2
zmp_ph114_A9
zmp_ph114_A11
zmp_ph114_B2
zmp_ph114_B4
zmp_ph114_D8
zmp_ph114_E2

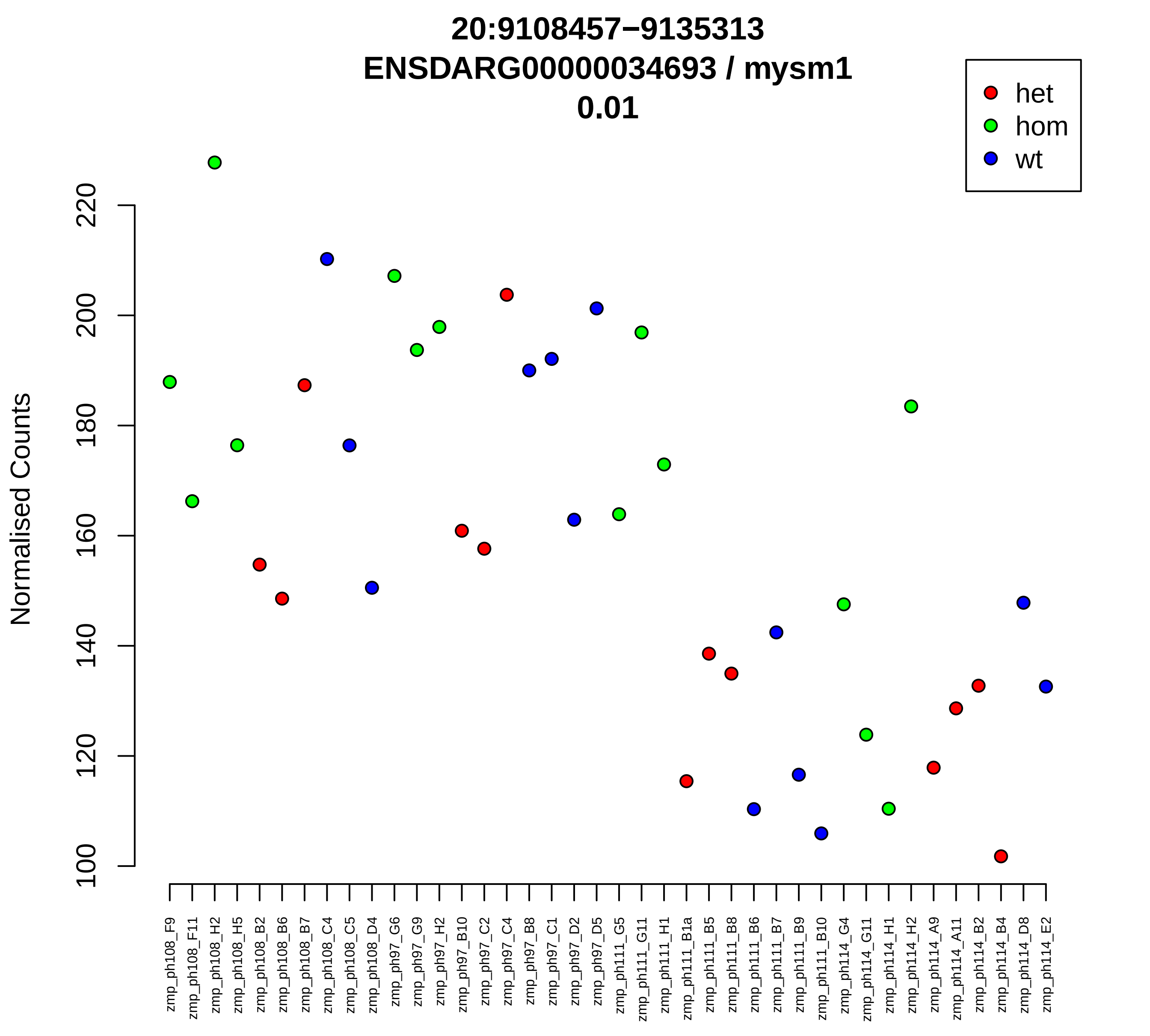
20:9108457−9135313
ENSDARG00000034693 / mysm1
0.01
het
hom
wt
Normalised Counts
100
120
140
160
180
200
220
zmp_ph108_F9
zmp_ph108_F11
zmp_ph108_H2
zmp_ph108_H5
zmp_ph108_B2
zmp_ph108_B6
zmp_ph108_B7
zmp_ph108_C4
zmp_ph108_C5
zmp_ph108_D4
zmp_ph97_G6
zmp_ph97_G9
zmp_ph97_H2
zmp_ph97_B10
zmp_ph97_C2
zmp_ph97_C4
zmp_ph97_B8
zmp_ph97_C1
zmp_ph97_D2
zmp_ph97_D5
zmp_ph111_G5
zmp_ph111_G11
zmp_ph111_H1
zmp_ph111_B1a
zmp_ph111_B5
zmp_ph111_B8
zmp_ph111_B6
zmp_ph111_B7
zmp_ph111_B9
zmp_ph111_B10
zmp_ph114_G4
zmp_ph114_G11
zmp_ph114_H1
zmp_ph114_H2
zmp_ph114_A9
zmp_ph114_A11
zmp_ph114_B2
zmp_ph114_B4
zmp_ph114_D8
zmp_ph114_E2

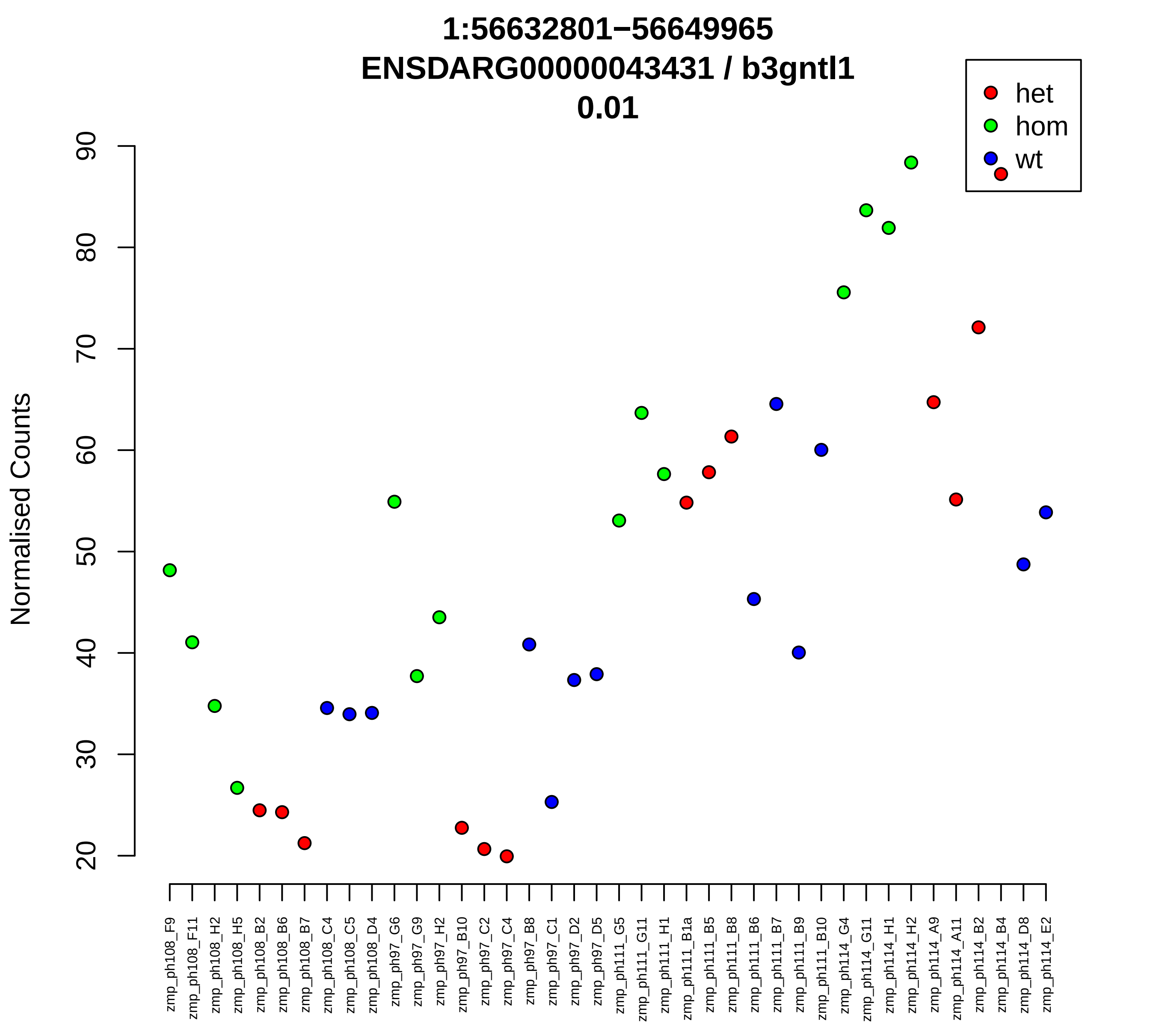
1:56632801−56649965
ENSDARG00000043431 / b3gntl1
0.01
het
hom
wt
Normalised Counts
20
30
40
50
60
70
80
90
zmp_ph108_F9
zmp_ph108_F11
zmp_ph108_H2
zmp_ph108_H5
zmp_ph108_B2
zmp_ph108_B6
zmp_ph108_B7
zmp_ph108_C4
zmp_ph108_C5
zmp_ph108_D4
zmp_ph97_G6
zmp_ph97_G9
zmp_ph97_H2
zmp_ph97_B10
zmp_ph97_C2
zmp_ph97_C4
zmp_ph97_B8
zmp_ph97_C1
zmp_ph97_D2
zmp_ph97_D5
zmp_ph111_G5
zmp_ph111_G11
zmp_ph111_H1
zmp_ph111_B1a
zmp_ph111_B5
zmp_ph111_B8
zmp_ph111_B6
zmp_ph111_B7
zmp_ph111_B9
zmp_ph111_B10
zmp_ph114_G4
zmp_ph114_G11
zmp_ph114_H1
zmp_ph114_H2
zmp_ph114_A9
zmp_ph114_A11
zmp_ph114_B2
zmp_ph114_B4
zmp_ph114_D8
zmp_ph114_E2

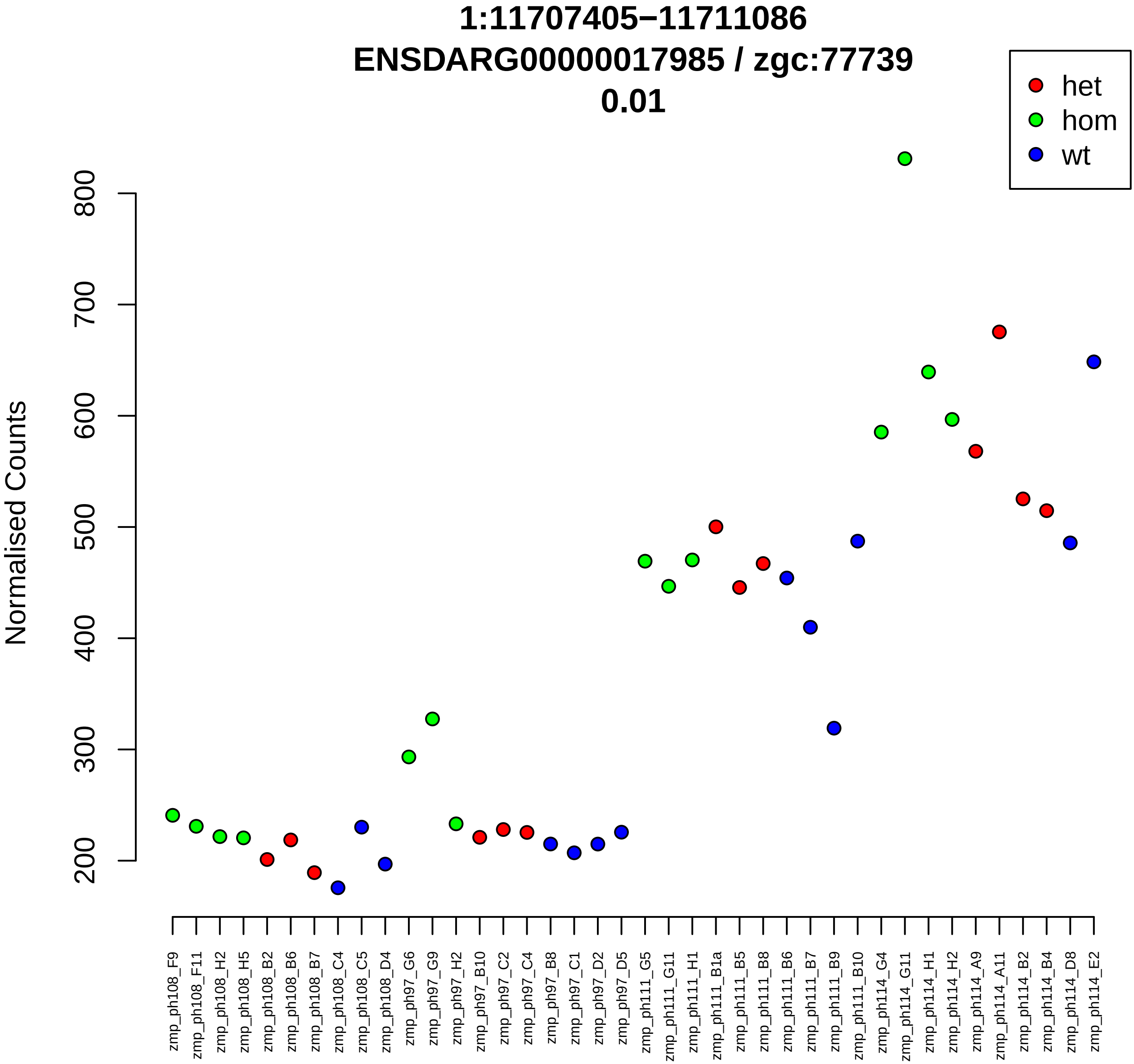
1:11707405−11711086
ENSDARG00000017985 / zgc:77739
0.01
het
hom
wt
Normalised Counts
200
300
400
500
600
700
800
zmp_ph108_F9
zmp_ph108_F11
zmp_ph108_H2
zmp_ph108_H5
zmp_ph108_B2
zmp_ph108_B6
zmp_ph108_B7
zmp_ph108_C4
zmp_ph108_C5
zmp_ph108_D4
zmp_ph97_G6
zmp_ph97_G9
zmp_ph97_H2
zmp_ph97_B10
zmp_ph97_C2
zmp_ph97_C4
zmp_ph97_B8
zmp_ph97_C1
zmp_ph97_D2
zmp_ph97_D5
zmp_ph111_G5
zmp_ph111_G11
zmp_ph111_H1
zmp_ph111_B1a
zmp_ph111_B5
zmp_ph111_B8
zmp_ph111_B6
zmp_ph111_B7
zmp_ph111_B9
zmp_ph111_B10
zmp_ph114_G4
zmp_ph114_G11
zmp_ph114_H1
zmp_ph114_H2
zmp_ph114_A9
zmp_ph114_A11
zmp_ph114_B2
zmp_ph114_B4
zmp_ph114_D8
zmp_ph114_E2

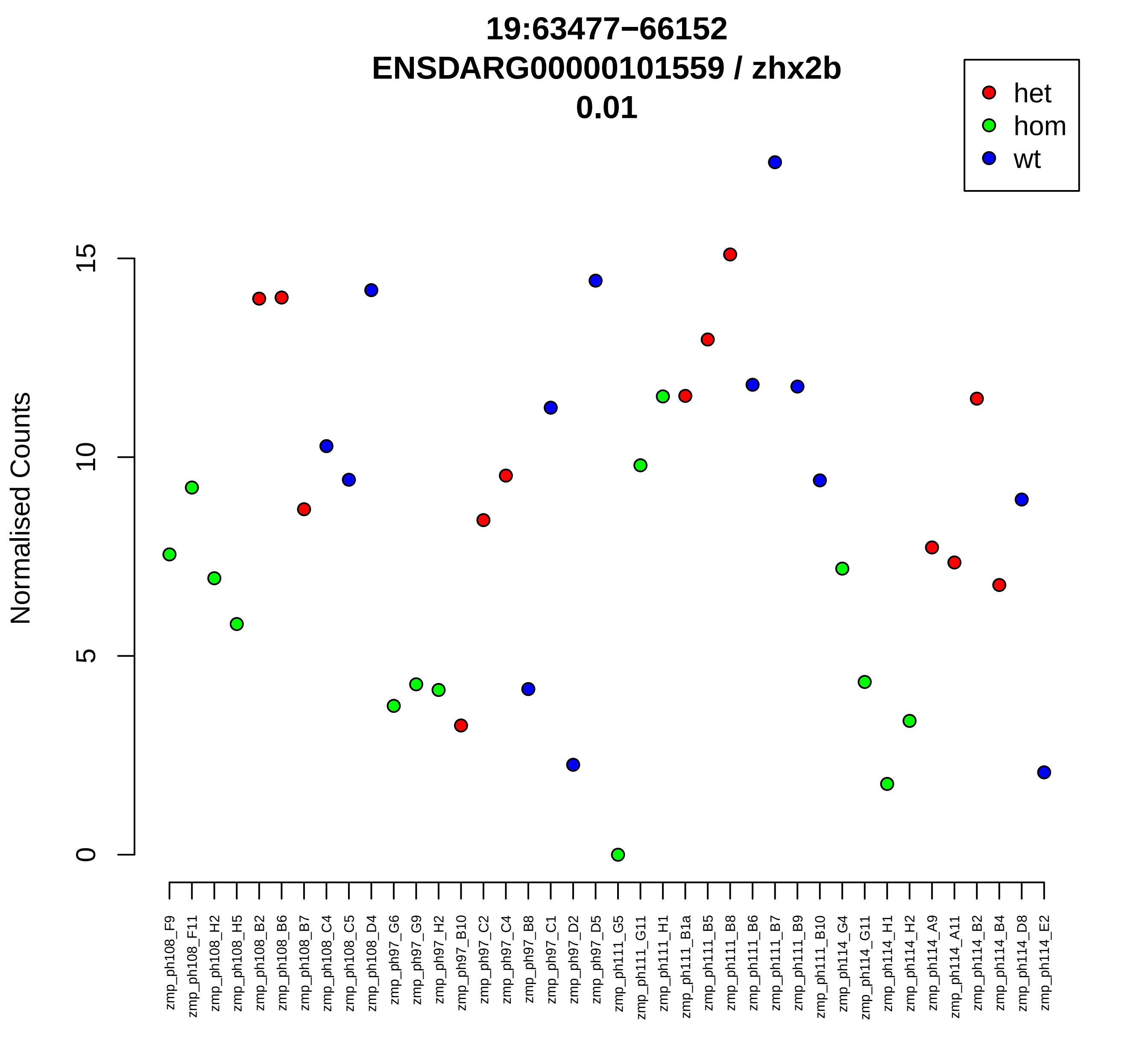
19:63477−66152
ENSDARG00000101559 / zhx2b
0.01
het
hom
wt
Normalised Counts
0
5
10
15
zmp_ph108_F9
zmp_ph108_F11
zmp_ph108_H2
zmp_ph108_H5
zmp_ph108_B2
zmp_ph108_B6
zmp_ph108_B7
zmp_ph108_C4
zmp_ph108_C5
zmp_ph108_D4
zmp_ph97_G6
zmp_ph97_G9
zmp_ph97_H2
zmp_ph97_B10
zmp_ph97_C2
zmp_ph97_C4
zmp_ph97_B8
zmp_ph97_C1
zmp_ph97_D2
zmp_ph97_D5
zmp_ph111_G5
zmp_ph111_G11
zmp_ph111_H1
zmp_ph111_B1a
zmp_ph111_B5
zmp_ph111_B8
zmp_ph111_B6
zmp_ph111_B7
zmp_ph111_B9
zmp_ph111_B10
zmp_ph114_G4
zmp_ph114_G11
zmp_ph114_H1
zmp_ph114_H2
zmp_ph114_A9
zmp_ph114_A11
zmp_ph114_B2
zmp_ph114_B4
zmp_ph114_D8
zmp_ph114_E2

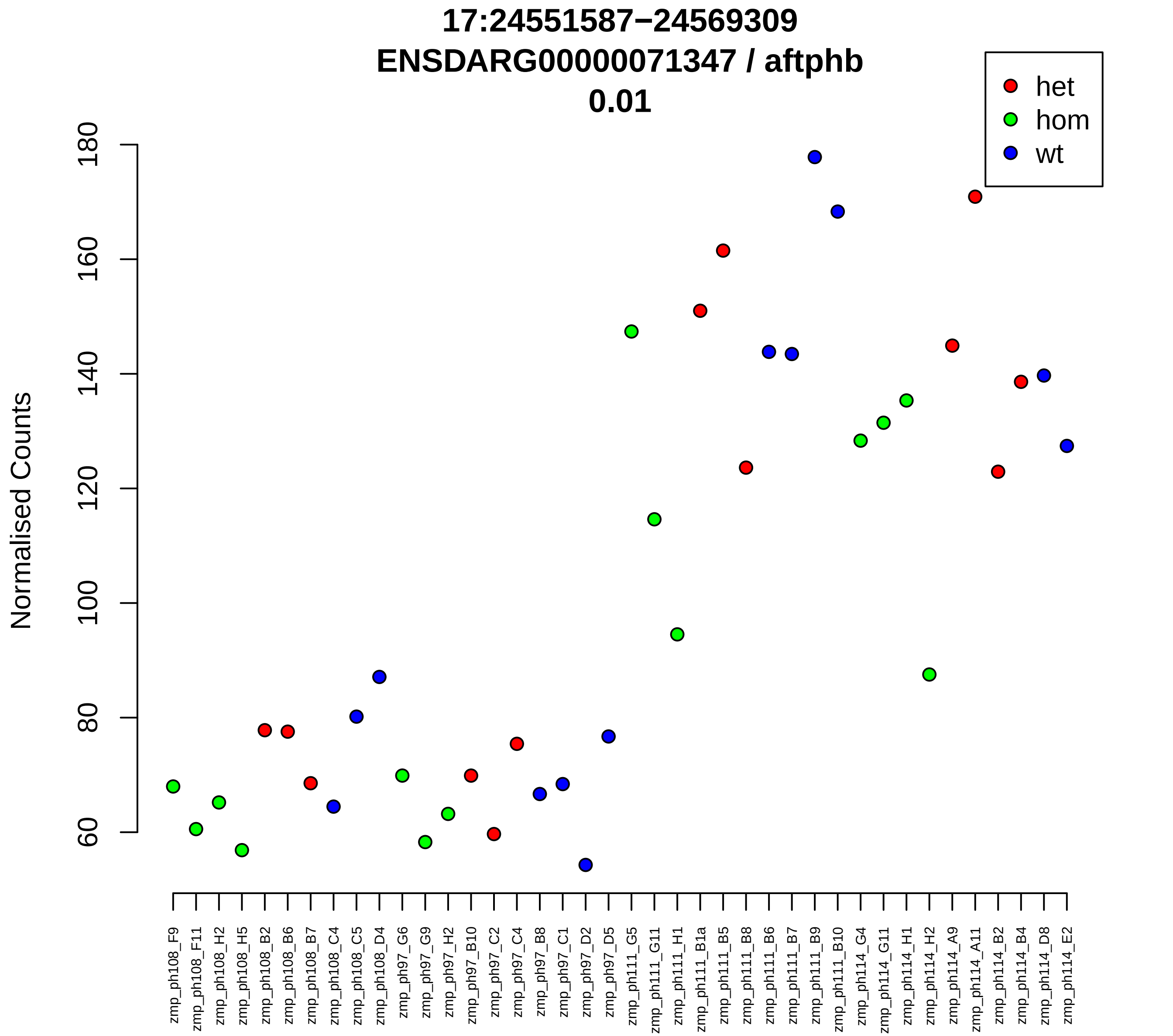
17:24551587−24569309
ENSDARG00000071347 / aftphb
0.01
het
hom
wt
Normalised Counts
60
80
100
120
140
160
180
zmp_ph108_F9
zmp_ph108_F11
zmp_ph108_H2
zmp_ph108_H5
zmp_ph108_B2
zmp_ph108_B6
zmp_ph108_B7
zmp_ph108_C4
zmp_ph108_C5
zmp_ph108_D4
zmp_ph97_G6
zmp_ph97_G9
zmp_ph97_H2
zmp_ph97_B10
zmp_ph97_C2
zmp_ph97_C4
zmp_ph97_B8
zmp_ph97_C1
zmp_ph97_D2
zmp_ph97_D5
zmp_ph111_G5
zmp_ph111_G11
zmp_ph111_H1
zmp_ph111_B1a
zmp_ph111_B5
zmp_ph111_B8
zmp_ph111_B6
zmp_ph111_B7
zmp_ph111_B9
zmp_ph111_B10
zmp_ph114_G4
zmp_ph114_G11
zmp_ph114_H1
zmp_ph114_H2
zmp_ph114_A9
zmp_ph114_A11
zmp_ph114_B2
zmp_ph114_B4
zmp_ph114_D8
zmp_ph114_E2

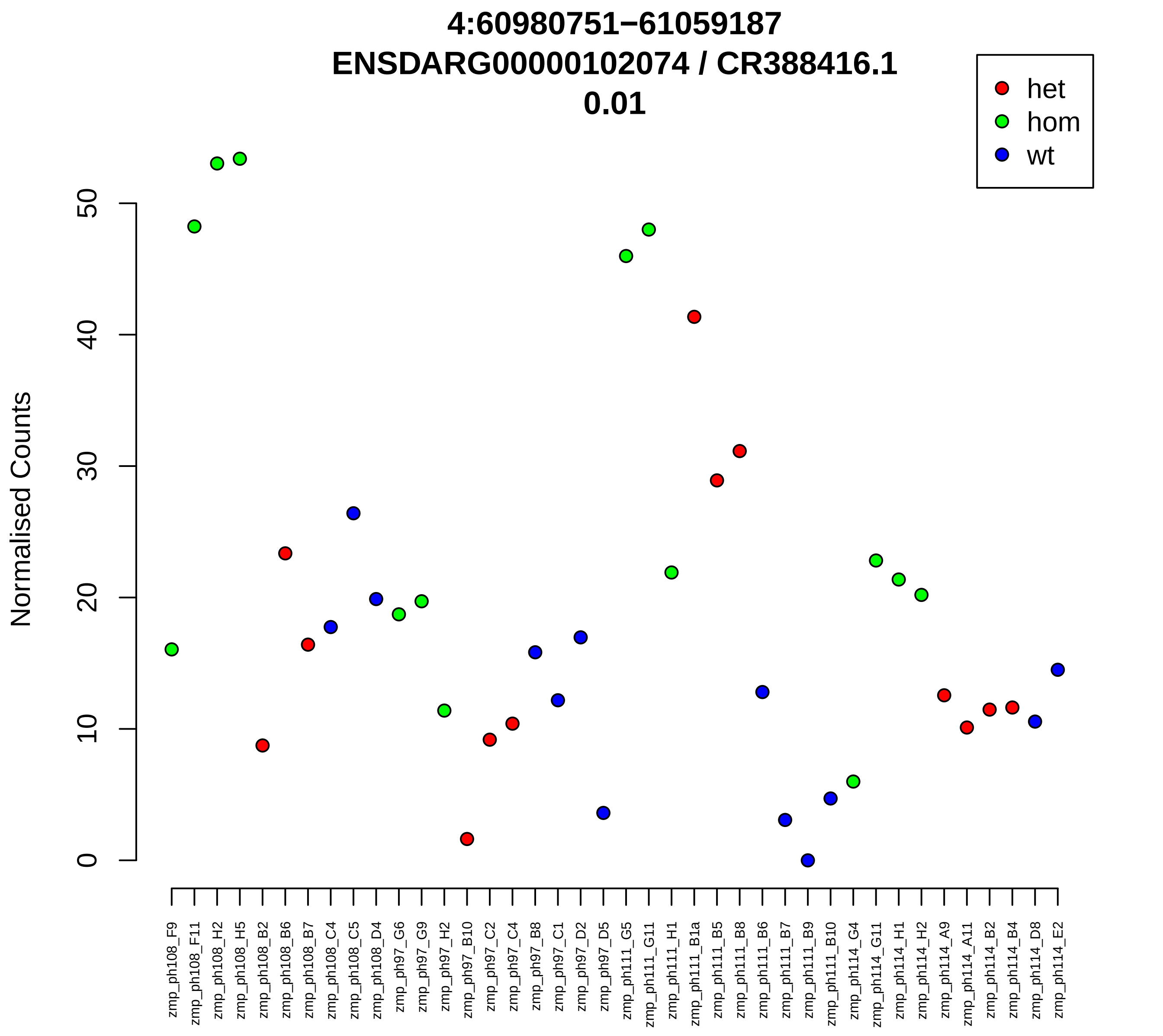
4:60980751−61059187
ENSDARG00000102074 / CR388416.1
0.01
het
hom
wt
Normalised Counts
0
10
20
30
40
50
zmp_ph108_F9
zmp_ph108_F11
zmp_ph108_H2
zmp_ph108_H5
zmp_ph108_B2
zmp_ph108_B6
zmp_ph108_B7
zmp_ph108_C4
zmp_ph108_C5
zmp_ph108_D4
zmp_ph97_G6
zmp_ph97_G9
zmp_ph97_H2
zmp_ph97_B10
zmp_ph97_C2
zmp_ph97_C4
zmp_ph97_B8
zmp_ph97_C1
zmp_ph97_D2
zmp_ph97_D5
zmp_ph111_G5
zmp_ph111_G11
zmp_ph111_H1
zmp_ph111_B1a
zmp_ph111_B5
zmp_ph111_B8
zmp_ph111_B6
zmp_ph111_B7
zmp_ph111_B9
zmp_ph111_B10
zmp_ph114_G4
zmp_ph114_G11
zmp_ph114_H1
zmp_ph114_H2
zmp_ph114_A9
zmp_ph114_A11
zmp_ph114_B2
zmp_ph114_B4
zmp_ph114_D8
zmp_ph114_E2

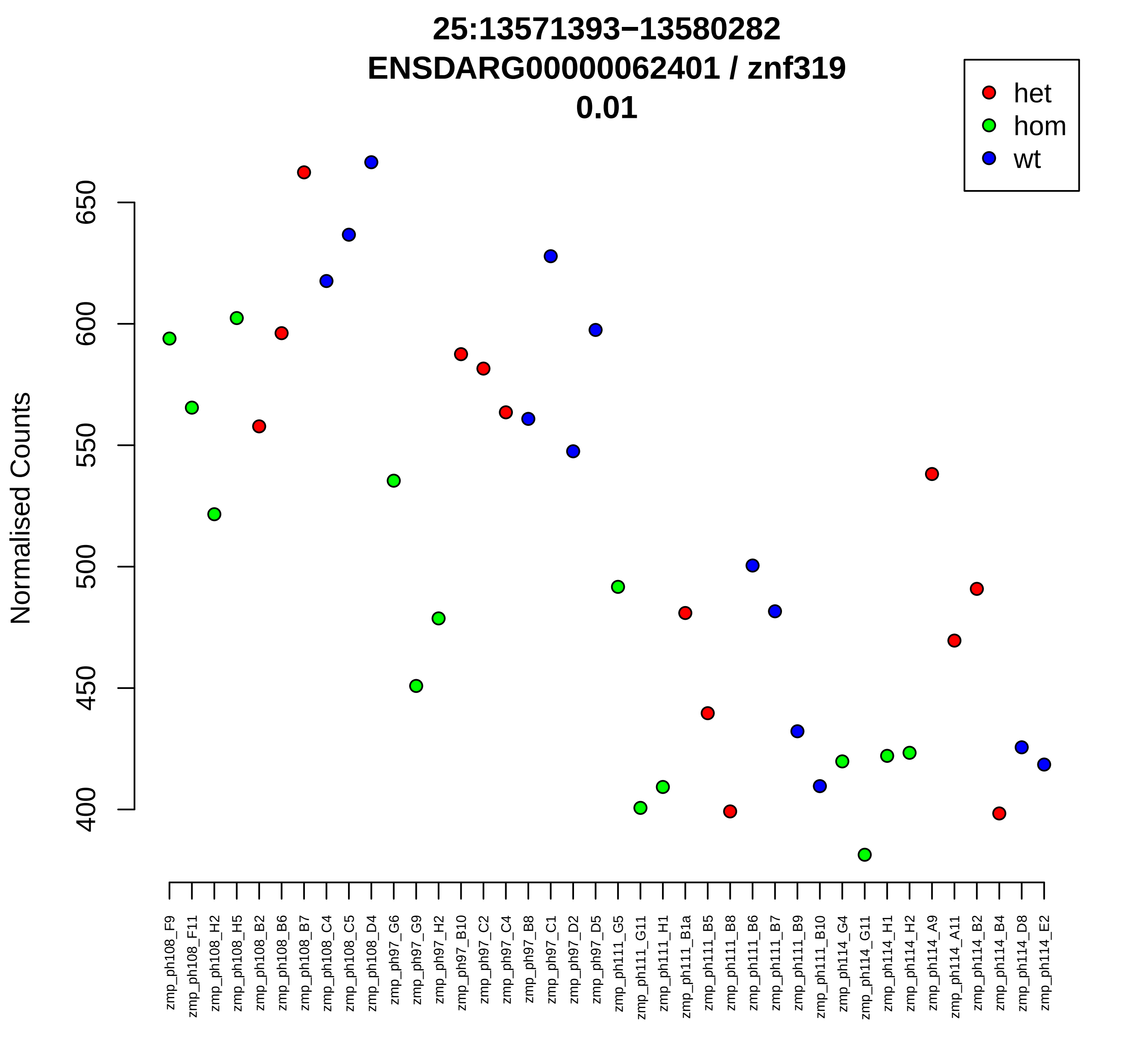
25:13571393−13580282
ENSDARG00000062401 / znf319
0.01
het
hom
wt
Normalised Counts
400
450
500
550
600
650
zmp_ph108_F9
zmp_ph108_F11
zmp_ph108_H2
zmp_ph108_H5
zmp_ph108_B2
zmp_ph108_B6
zmp_ph108_B7
zmp_ph108_C4
zmp_ph108_C5
zmp_ph108_D4
zmp_ph97_G6
zmp_ph97_G9
zmp_ph97_H2
zmp_ph97_B10
zmp_ph97_C2
zmp_ph97_C4
zmp_ph97_B8
zmp_ph97_C1
zmp_ph97_D2
zmp_ph97_D5
zmp_ph111_G5
zmp_ph111_G11
zmp_ph111_H1
zmp_ph111_B1a
zmp_ph111_B5
zmp_ph111_B8
zmp_ph111_B6
zmp_ph111_B7
zmp_ph111_B9
zmp_ph111_B10
zmp_ph114_G4
zmp_ph114_G11
zmp_ph114_H1
zmp_ph114_H2
zmp_ph114_A9
zmp_ph114_A11
zmp_ph114_B2
zmp_ph114_B4
zmp_ph114_D8
zmp_ph114_E2

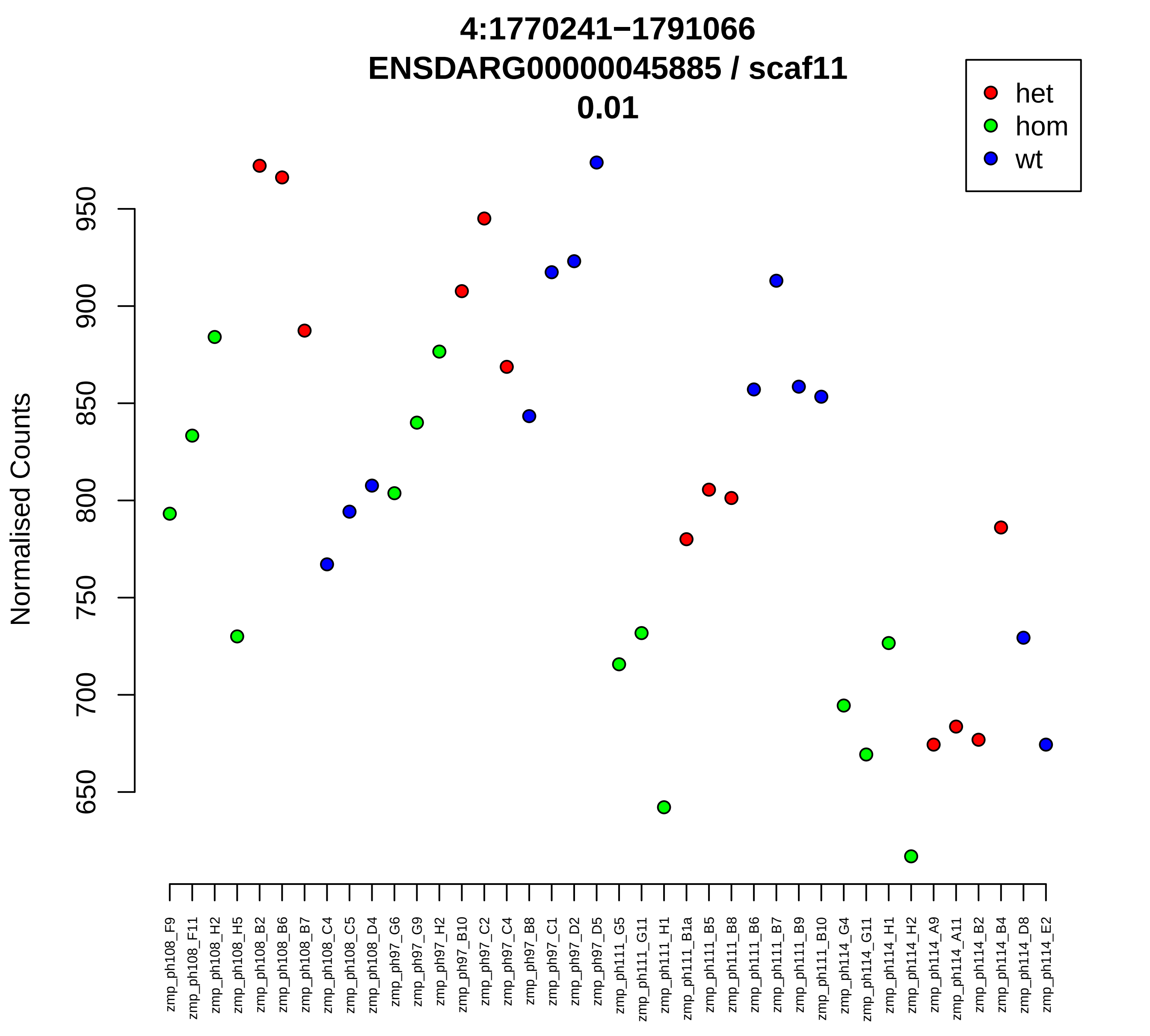

4:1770241−1791066
ENSDARG00000045885 / scaf11
0.01
het
hom
wt
Normalised Counts
950
900
850
800
750
700
650
zmp_ph108_F9
zmp_ph108_F11
zmp_ph108_H2
zmp_ph108_H5
zmp_ph108_B2
zmp_ph108_B6
zmp_ph108_B7
zmp_ph108_C4
zmp_ph108_C5
zmp_ph108_D4
zmp_ph97_G6
zmp_ph97_G9
zmp_ph97_H2
zmp_ph97_B10
zmp_ph97_C2
zmp_ph97_C4
zmp_ph97_B8
zmp_ph97_C1
zmp_ph97_D2
zmp_ph97_D5
zmp_ph111_G5
zmp_ph111_G11
zmp_ph111_H1
zmp_ph111_B1a
zmp_ph111_B5
zmp_ph111_B8
zmp_ph111_B6
zmp_ph111_B7
zmp_ph111_B9
zmp_ph111_B10
zmp_ph114_G4
zmp_ph114_G11
zmp_ph114_H1
zmp_ph114_H2
zmp_ph114_A9
zmp_ph114_A11
zmp_ph114_B2
zmp_ph114_B4
zmp_ph114_D8
zmp_ph114_E2

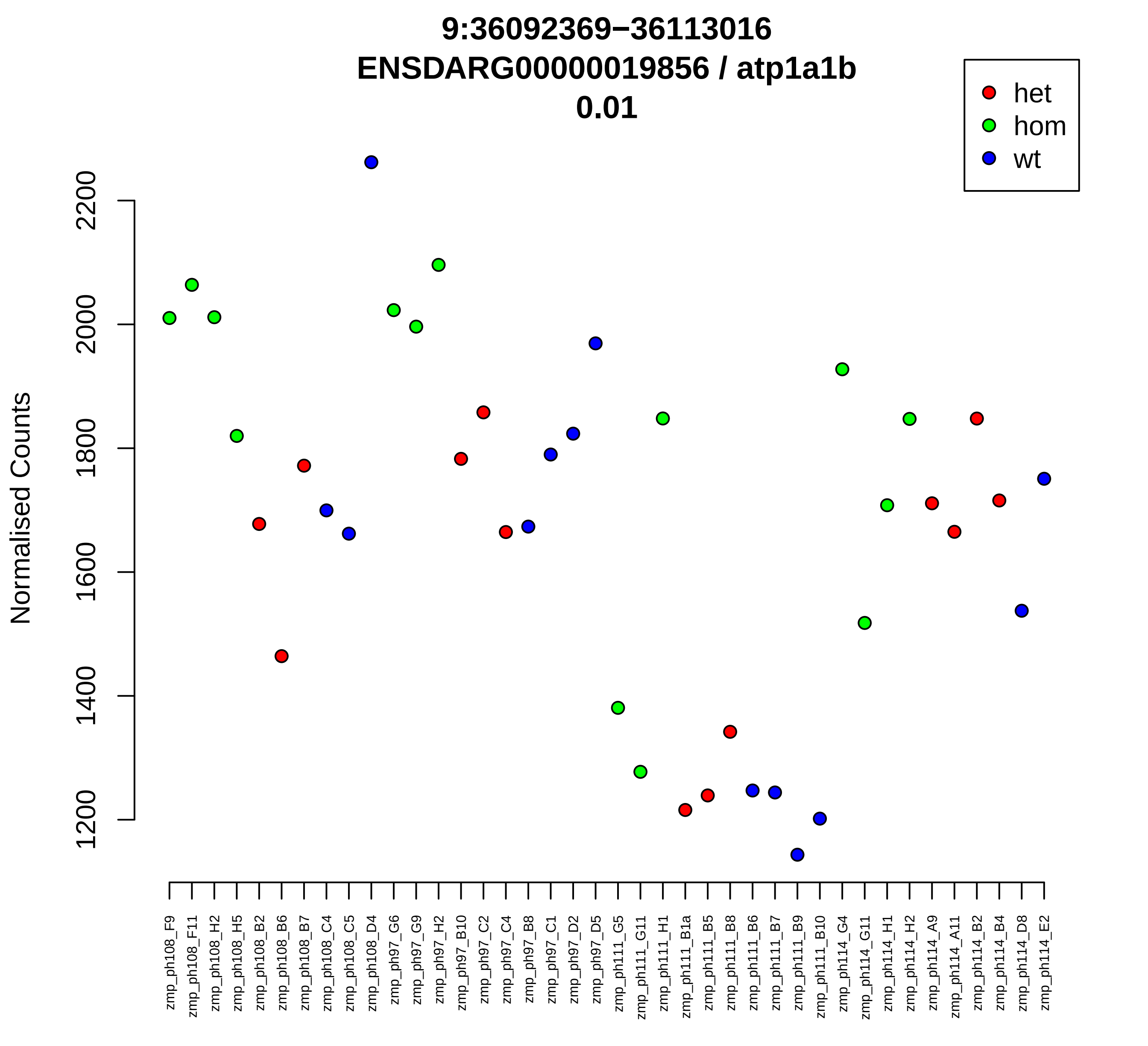

9:36092369−36113016
ENSDARG00000019856 / atp1a1b
0.01
het
hom
wt
Normalised Counts
1200
1400
1600
1800
2000
2200
zmp_ph108_F9
zmp_ph108_F11
zmp_ph108_H2
zmp_ph108_H5
zmp_ph108_B2
zmp_ph108_B6
zmp_ph108_B7
zmp_ph108_C4
zmp_ph108_C5
zmp_ph108_D4
zmp_ph97_G6
zmp_ph97_G9
zmp_ph97_H2
zmp_ph97_B10
zmp_ph97_C2
zmp_ph97_C4
zmp_ph97_B8
zmp_ph97_C1
zmp_ph97_D2
zmp_ph97_D5
zmp_ph111_G5
zmp_ph111_G11
zmp_ph111_H1
zmp_ph111_B1a
zmp_ph111_B5
zmp_ph111_B8
zmp_ph111_B6
zmp_ph111_B7
zmp_ph111_B9
zmp_ph111_B10
zmp_ph114_G4
zmp_ph114_G11
zmp_ph114_H1
zmp_ph114_H2
zmp_ph114_A9
zmp_ph114_A11
zmp_ph114_B2
zmp_ph114_B4
zmp_ph114_D8
zmp_ph114_E2

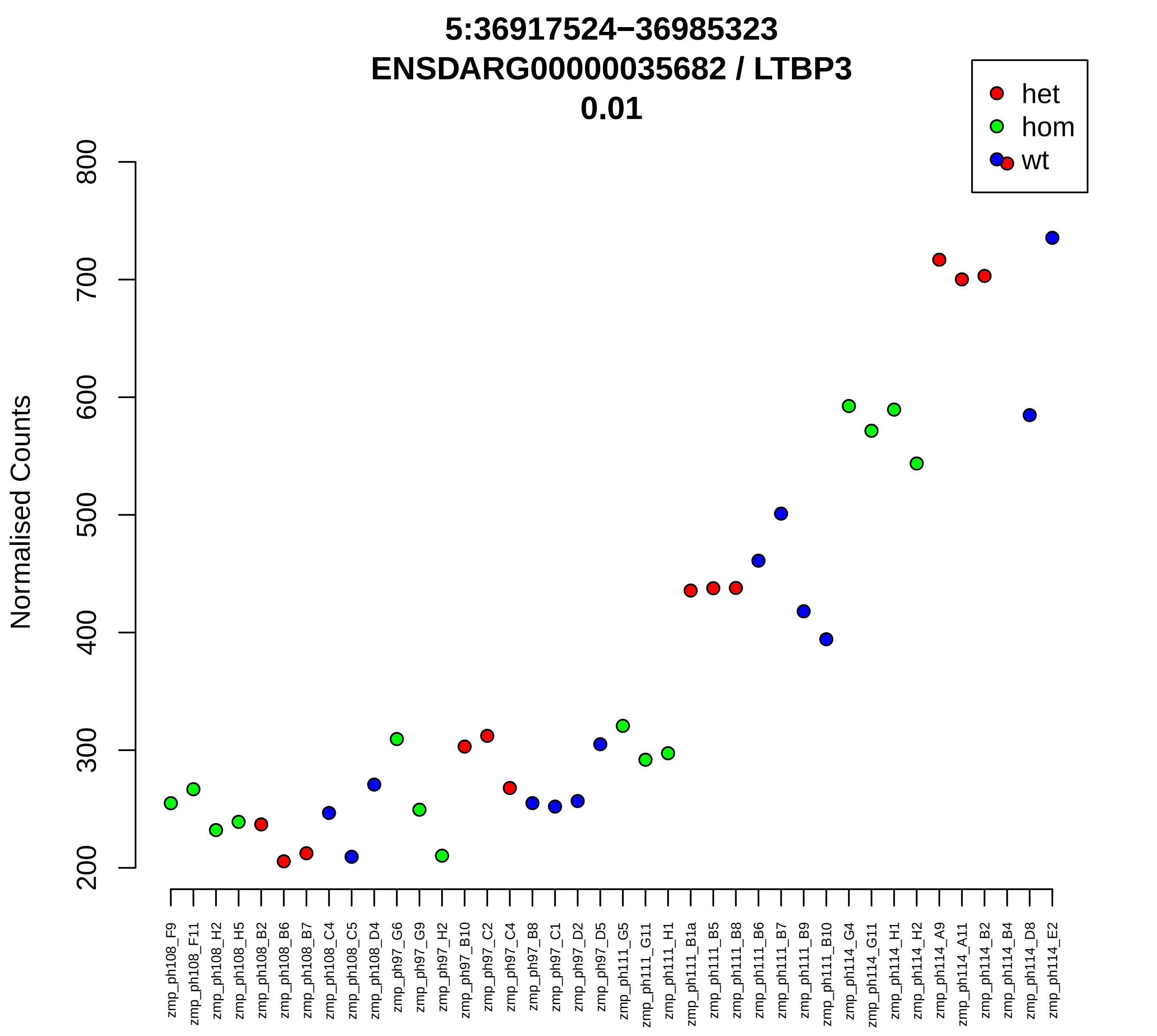
5:36917524−36985323
ENSDARG00000035682 / LTBP3
0.01
het
hom
wt
Normalised Counts
200
300
400
500
600
700
800
zmp_ph108_F9
zmp_ph108_F11
zmp_ph108_H2
zmp_ph108_H5
zmp_ph108_B2
zmp_ph108_B6
zmp_ph108_B7
zmp_ph108_C4
zmp_ph108_C5
zmp_ph108_D4
zmp_ph97_G6
zmp_ph97_G9
zmp_ph97_H2
zmp_ph97_B10
zmp_ph97_C2
zmp_ph97_C4
zmp_ph97_B8
zmp_ph97_C1
zmp_ph97_D2
zmp_ph97_D5
zmp_ph111_G5
zmp_ph111_G11
zmp_ph111_H1
zmp_ph111_B1a
zmp_ph111_B5
zmp_ph111_B8
zmp_ph111_B6
zmp_ph111_B7
zmp_ph111_B9
zmp_ph111_B10
zmp_ph114_G4
zmp_ph114_G11
zmp_ph114_H1
zmp_ph114_H2
zmp_ph114_A9
zmp_ph114_A11
zmp_ph114_B2
zmp_ph114_B4
zmp_ph114_D8
zmp_ph114_E2

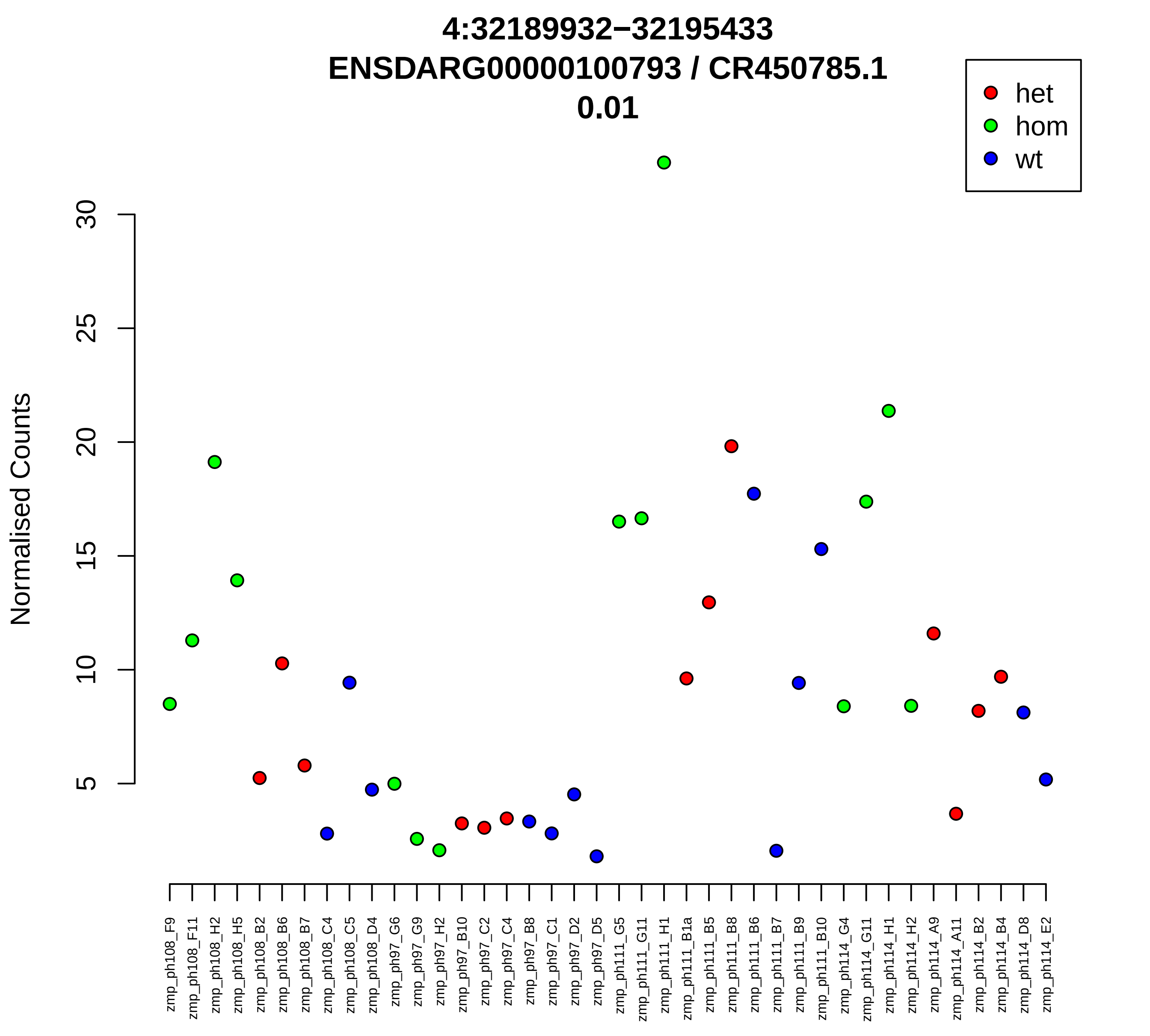

4:32189932−32195433
ENSDARG00000100793 / CR450785.1
0.01
het
hom
wt
Normalised Counts
5
10
15
20
25
30
zmp_ph108_F9
zmp_ph108_F11
zmp_ph108_H2
zmp_ph108_H5
zmp_ph108_B2
zmp_ph108_B6
zmp_ph108_B7
zmp_ph108_C4
zmp_ph108_C5
zmp_ph108_D4
zmp_ph97_G6
zmp_ph97_G9
zmp_ph97_H2
zmp_ph97_B10
zmp_ph97_C2
zmp_ph97_C4
zmp_ph97_B8
zmp_ph97_C1
zmp_ph97_D2
zmp_ph97_D5
zmp_ph111_G5
zmp_ph111_G11
zmp_ph111_H1
zmp_ph111_B1a
zmp_ph111_B5
zmp_ph111_B8
zmp_ph111_B6
zmp_ph111_B7
zmp_ph111_B9
zmp_ph111_B10
zmp_ph114_G4
zmp_ph114_G11
zmp_ph114_H1
zmp_ph114_H2
zmp_ph114_A9
zmp_ph114_A11
zmp_ph114_B2
zmp_ph114_B4
zmp_ph114_D8
zmp_ph114_E2

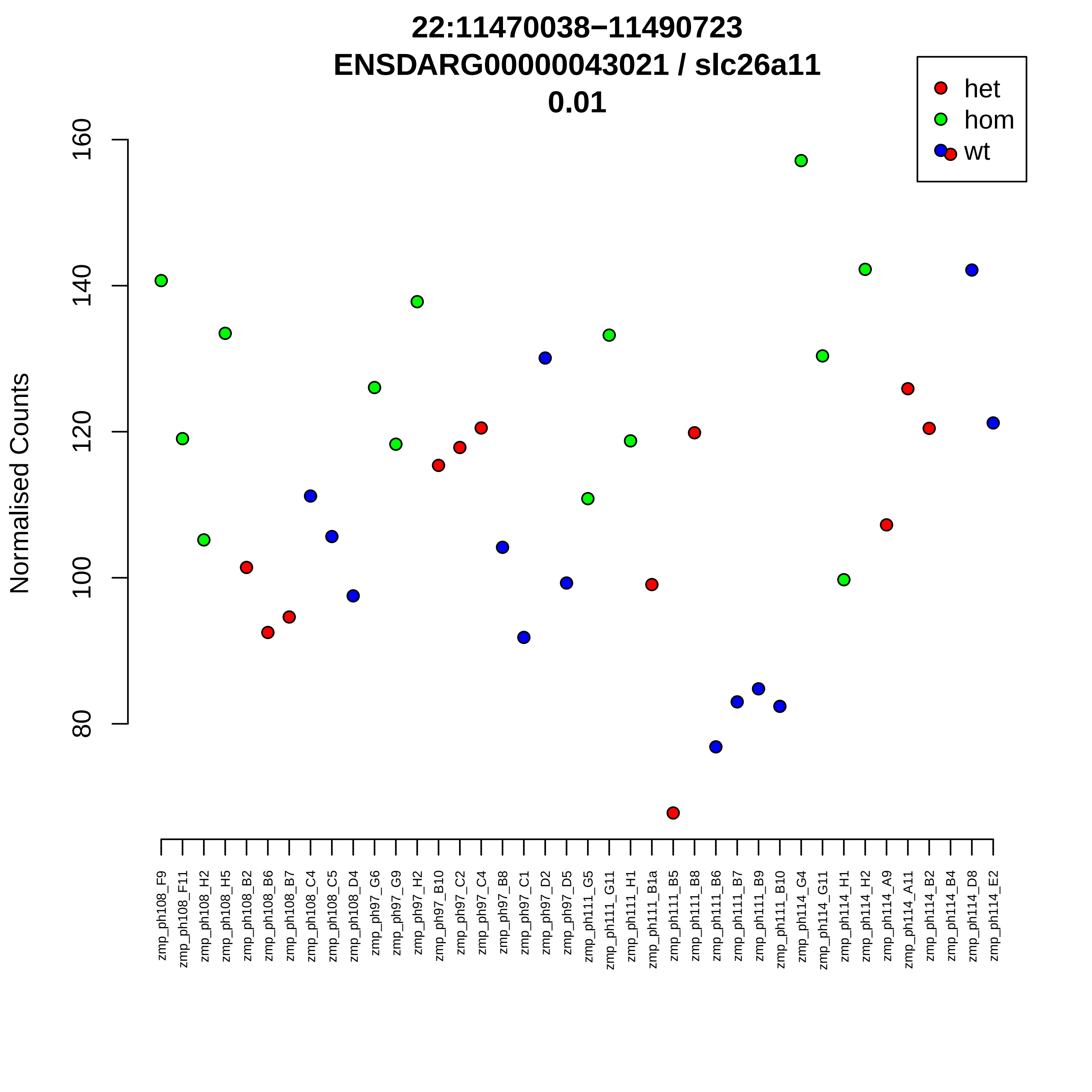

22:11470038−11490723
ENSDARG00000043021 / slc26a11
0.01
Normalised Counts
80
100
120
140
160
het
hom
wt
zmp_ph108_F9
zmp_ph108_F11
zmp_ph108_H2
zmp_ph108_H5
zmp_ph108_B2
zmp_ph108_B6
zmp_ph108_B7
zmp_ph108_C4
zmp_ph108_C5
zmp_ph108_D4
zmp_ph97_G6
zmp_ph97_G9
zmp_ph97_H2
zmp_ph97_B10
zmp_ph97_C2
zmp_ph97_C4
zmp_ph97_B8
zmp_ph97_C1
zmp_ph97_D2
zmp_ph97_D5
zmp_ph111_G5
zmp_ph111_G11
zmp_ph111_H1
zmp_ph111_B1a
zmp_ph111_B5
zmp_ph111_B8
zmp_ph111_B6
zmp_ph111_B7
zmp_ph111_B9
zmp_ph111_B10
zmp_ph114_G4
zmp_ph114_G11
zmp_ph114_H1
zmp_ph114_H2
zmp_ph114_A9
zmp_ph114_A11
zmp_ph114_B2
zmp_ph114_B4
zmp_ph114_D8
zmp_ph114_E2

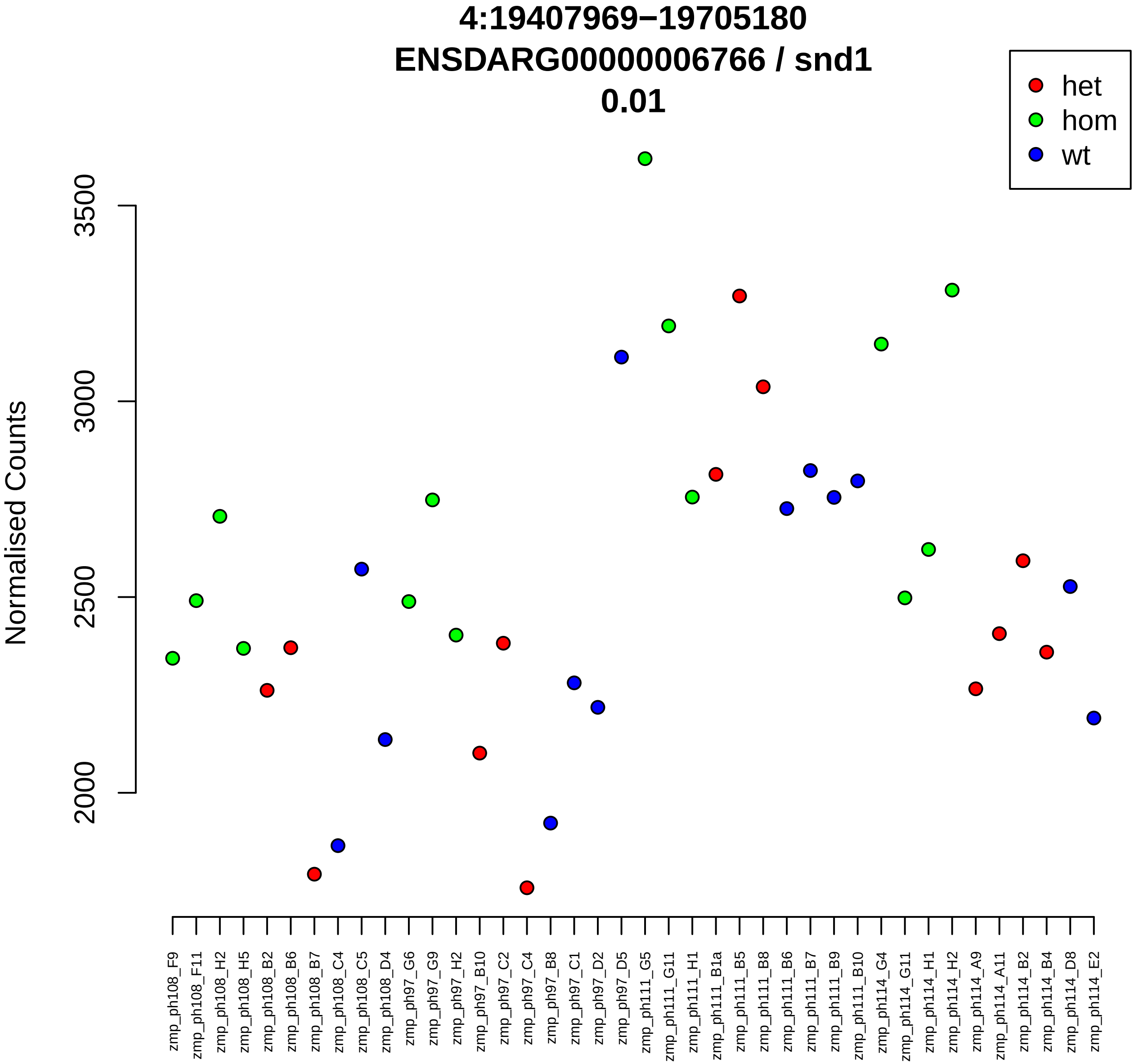
4:19407969−19705180
ENSDARG00000006766 / snd1
0.01
het
hom
wt
Normalised Counts
2000
2500
3000
3500
zmp_ph108_F9
zmp_ph108_F11
zmp_ph108_H2
zmp_ph108_H5
zmp_ph108_B2
zmp_ph108_B6
zmp_ph108_B7
zmp_ph108_C4
zmp_ph108_C5
zmp_ph108_D4
zmp_ph97_G6
zmp_ph97_G9
zmp_ph97_H2
zmp_ph97_B10
zmp_ph97_C2
zmp_ph97_C4
zmp_ph97_B8
zmp_ph97_C1
zmp_ph97_D2
zmp_ph97_D5
zmp_ph111_G5
zmp_ph111_G11
zmp_ph111_H1
zmp_ph111_B1a
zmp_ph111_B5
zmp_ph111_B8
zmp_ph111_B6
zmp_ph111_B7
zmp_ph111_B9
zmp_ph111_B10
zmp_ph114_G4
zmp_ph114_G11
zmp_ph114_H1
zmp_ph114_H2
zmp_ph114_A9
zmp_ph114_A11
zmp_ph114_B2
zmp_ph114_B4
zmp_ph114_D8
zmp_ph114_E2

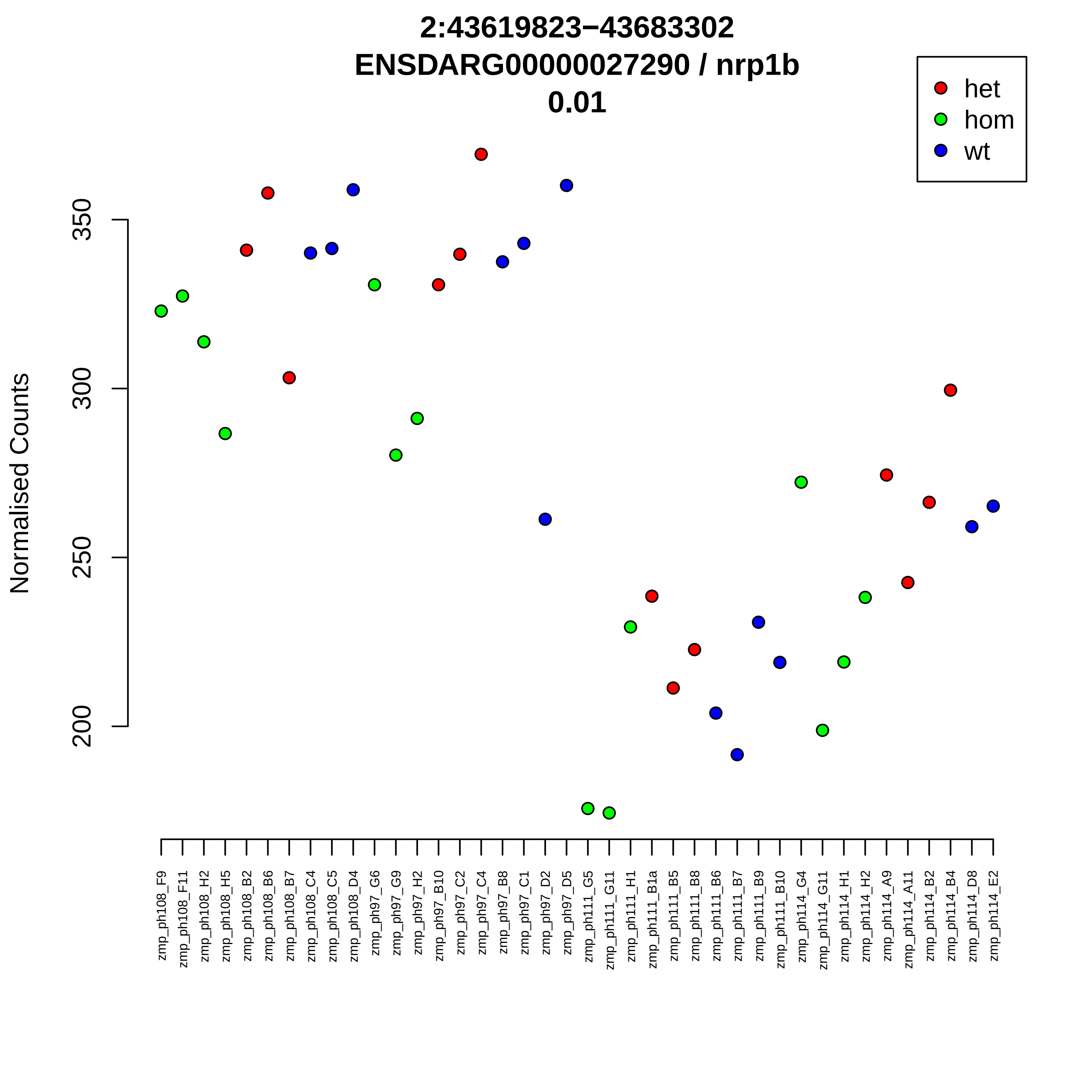

2:43619823−43683302
ENSDARG00000027290 / nrp1b
0.01
het
hom
wt
Normalised Counts
200
250
300
350
zmp_ph108_F9
zmp_ph108_F11
zmp_ph108_H2
zmp_ph108_H5
zmp_ph108_B2
zmp_ph108_B6
zmp_ph108_B7
zmp_ph108_C4
zmp_ph108_C5
zmp_ph108_D4
zmp_ph97_G6
zmp_ph97_G9
zmp_ph97_H2
zmp_ph97_B10
zmp_ph97_C2
zmp_ph97_C4
zmp_ph97_B8
zmp_ph97_C1
zmp_ph97_D2
zmp_ph97_D5
zmp_ph111_G5
zmp_ph111_G11
zmp_ph111_H1
zmp_ph111_B1a
zmp_ph111_B5
zmp_ph111_B8
zmp_ph111_B6
zmp_ph111_B7
zmp_ph111_B9
zmp_ph111_B10
zmp_ph114_G4
zmp_ph114_G11
zmp_ph114_H1
zmp_ph114_H2
zmp_ph114_A9
zmp_ph114_A11
zmp_ph114_B2
zmp_ph114_B4
zmp_ph114_D8
zmp_ph114_E2

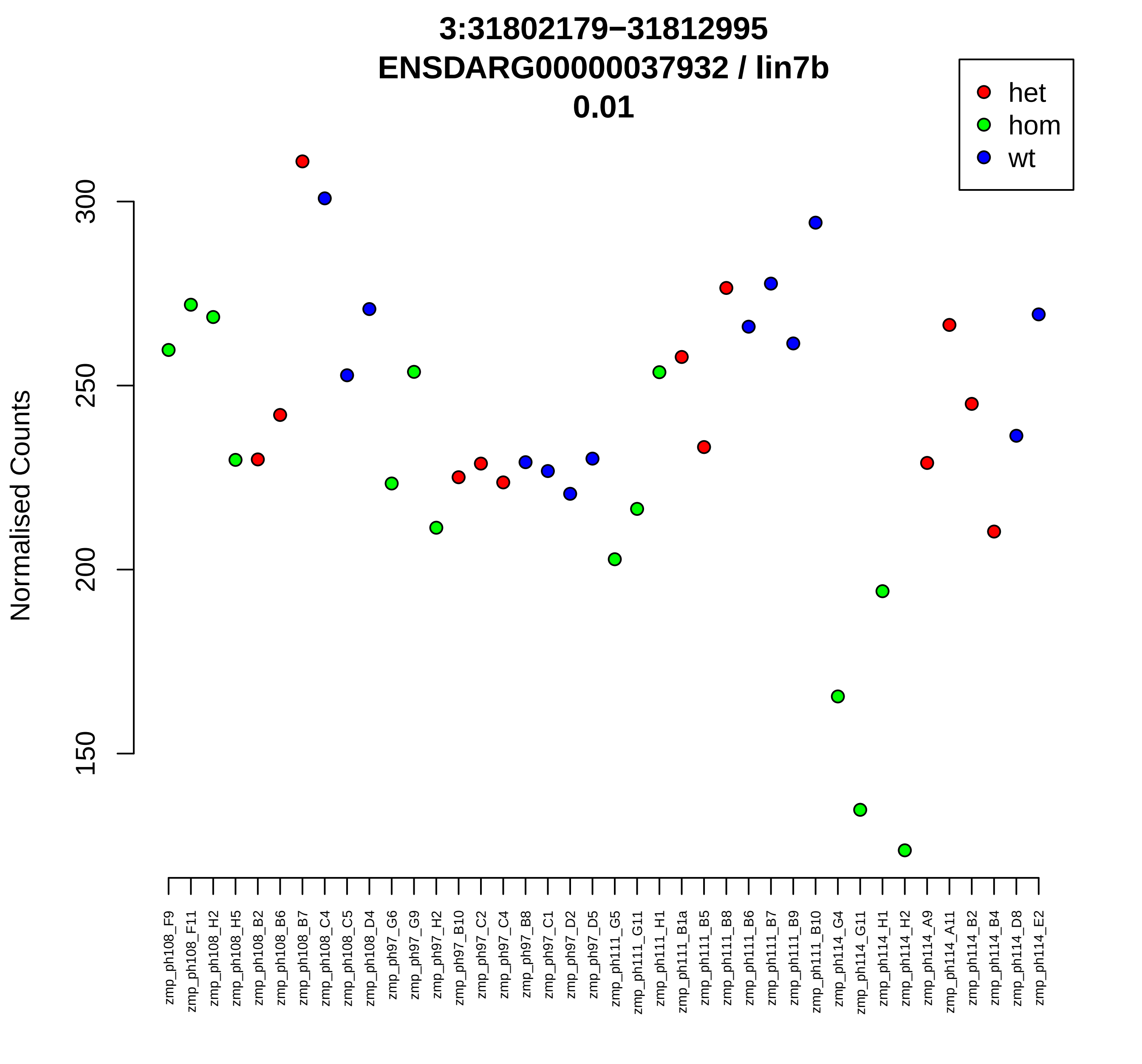

3:31802179−31812995
ENSDARG00000037932 / lin7b
0.01
het
hom
wt
Normalised Counts
300
250
200
150
zmp_ph108_F9
zmp_ph108_F11
zmp_ph108_H2
zmp_ph108_H5
zmp_ph108_B2
zmp_ph108_B6
zmp_ph108_B7
zmp_ph108_C4
zmp_ph108_C5
zmp_ph108_D4
zmp_ph97_G6
zmp_ph97_G9
zmp_ph97_H2
zmp_ph97_B10
zmp_ph97_C2
zmp_ph97_C4
zmp_ph97_B8
zmp_ph97_C1
zmp_ph97_D2
zmp_ph97_D5
zmp_ph111_G5
zmp_ph111_G11
zmp_ph111_H1
zmp_ph111_B1a
zmp_ph111_B5
zmp_ph111_B8
zmp_ph111_B6
zmp_ph111_B7
zmp_ph111_B9
zmp_ph111_B10
zmp_ph114_G4
zmp_ph114_G11
zmp_ph114_H1
zmp_ph114_H2
zmp_ph114_A9
zmp_ph114_A11
zmp_ph114_B2
zmp_ph114_B4
zmp_ph114_D8
zmp_ph114_E2

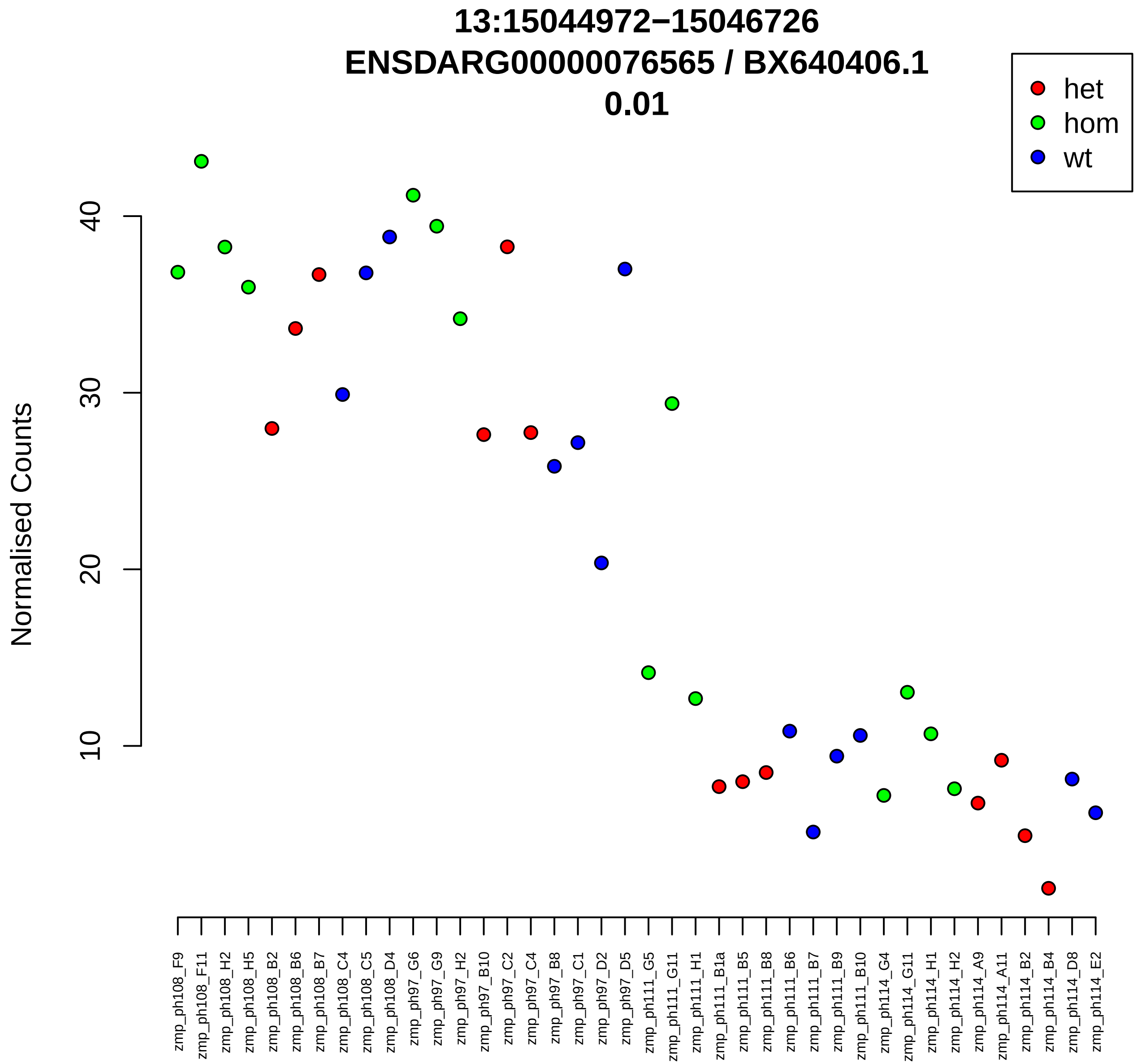

13:15044972−15046726
ENSDARG00000076565 / BX640406.1
0.01
het
hom
wt
Normalised Counts
10
20
30
40
zmp_ph108_F9
zmp_ph108_F11
zmp_ph108_H2
zmp_ph108_H5
zmp_ph108_B2
zmp_ph108_B6
zmp_ph108_B7
zmp_ph108_C4
zmp_ph108_C5
zmp_ph108_D4
zmp_ph97_G6
zmp_ph97_G9
zmp_ph97_H2
zmp_ph97_B10
zmp_ph97_C2
zmp_ph97_C4
zmp_ph97_B8
zmp_ph97_C1
zmp_ph97_D2
zmp_ph97_D5
zmp_ph111_G5
zmp_ph111_G11
zmp_ph111_H1
zmp_ph111_B1a
zmp_ph111_B5
zmp_ph111_B8
zmp_ph111_B6
zmp_ph111_B7
zmp_ph111_B9
zmp_ph111_B10
zmp_ph114_G4
zmp_ph114_G11
zmp_ph114_H1
zmp_ph114_H2
zmp_ph114_A9
zmp_ph114_A11
zmp_ph114_B2
zmp_ph114_B4
zmp_ph114_D8
zmp_ph114_E2

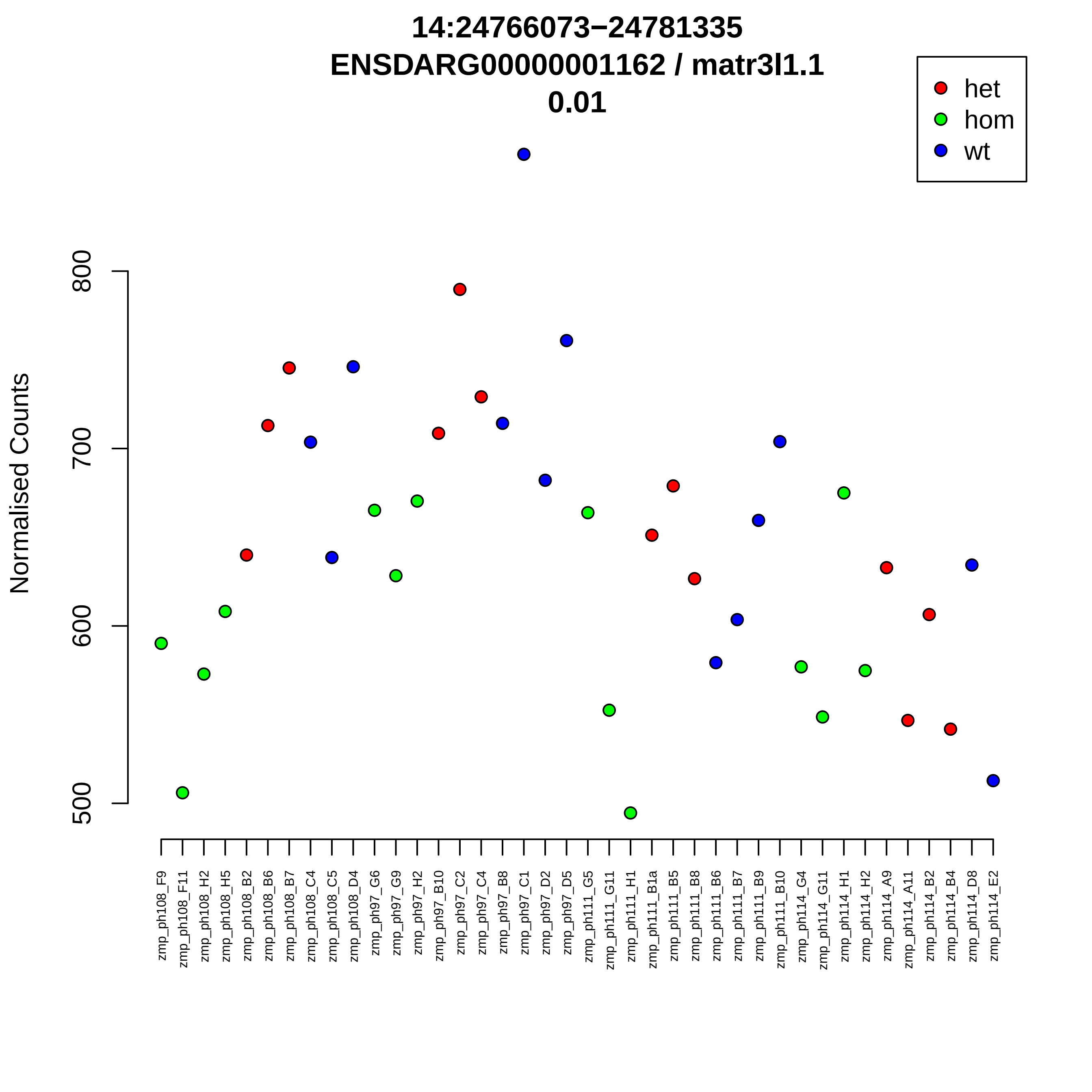

14:24766073−24781335
ENSDARG00000001162 / matr3l1.1
0.01
het
hom
wt
Normalised Counts
500
600
700
800
zmp_ph108_F9
zmp_ph108_F11
zmp_ph108_H2
zmp_ph108_H5
zmp_ph108_B2
zmp_ph108_B6
zmp_ph108_B7
zmp_ph108_C4
zmp_ph108_C5
zmp_ph108_D4
zmp_ph97_G6
zmp_ph97_G9
zmp_ph97_H2
zmp_ph97_B10
zmp_ph97_C2
zmp_ph97_C4
zmp_ph97_B8
zmp_ph97_C1
zmp_ph97_D2
zmp_ph97_D5
zmp_ph111_G5
zmp_ph111_G11
zmp_ph111_H1
zmp_ph111_B1a
zmp_ph111_B5
zmp_ph111_B8
zmp_ph111_B6
zmp_ph111_B7
zmp_ph111_B9
zmp_ph111_B10
zmp_ph114_G4
zmp_ph114_G11
zmp_ph114_H1
zmp_ph114_H2
zmp_ph114_A9
zmp_ph114_A11
zmp_ph114_B2
zmp_ph114_B4
zmp_ph114_D8
zmp_ph114_E2

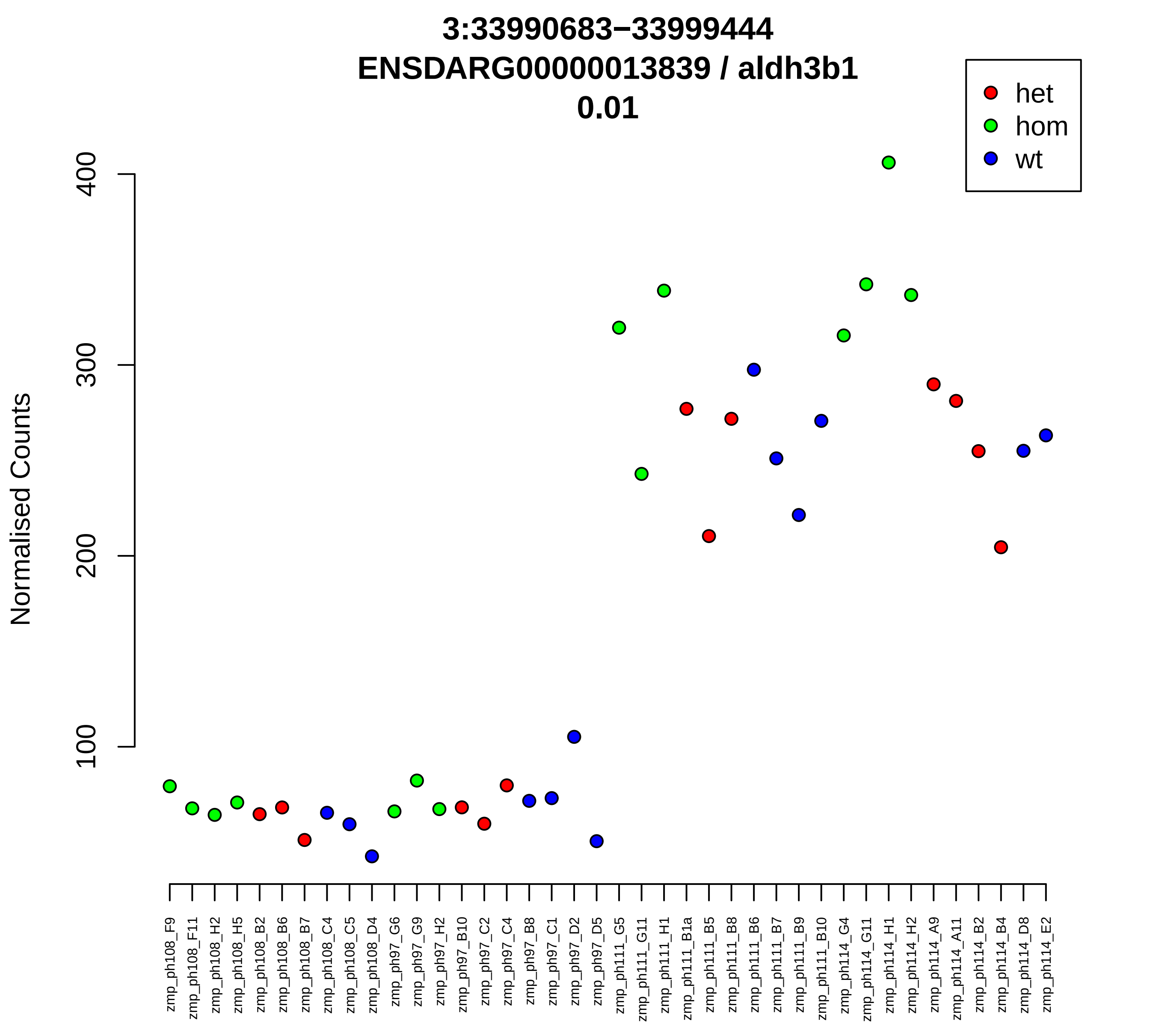
3:33990683−33999444
ENSDARG00000013839 / aldh3b1
0.01
het
hom
wt
Normalised Counts
100
200
300
400
zmp_ph108_F9
zmp_ph108_F11
zmp_ph108_H2
zmp_ph108_H5
zmp_ph108_B2
zmp_ph108_B6
zmp_ph108_B7
zmp_ph108_C4
zmp_ph108_C5
zmp_ph108_D4
zmp_ph97_G6
zmp_ph97_G9
zmp_ph97_H2
zmp_ph97_B10
zmp_ph97_C2
zmp_ph97_C4
zmp_ph97_B8
zmp_ph97_C1
zmp_ph97_D2
zmp_ph97_D5
zmp_ph111_G5
zmp_ph111_G11
zmp_ph111_H1
zmp_ph111_B1a
zmp_ph111_B5
zmp_ph111_B8
zmp_ph111_B6
zmp_ph111_B7
zmp_ph111_B9
zmp_ph111_B10
zmp_ph114_G4
zmp_ph114_G11
zmp_ph114_H1
zmp_ph114_H2
zmp_ph114_A9
zmp_ph114_A11
zmp_ph114_B2
zmp_ph114_B4
zmp_ph114_D8
zmp_ph114_E2

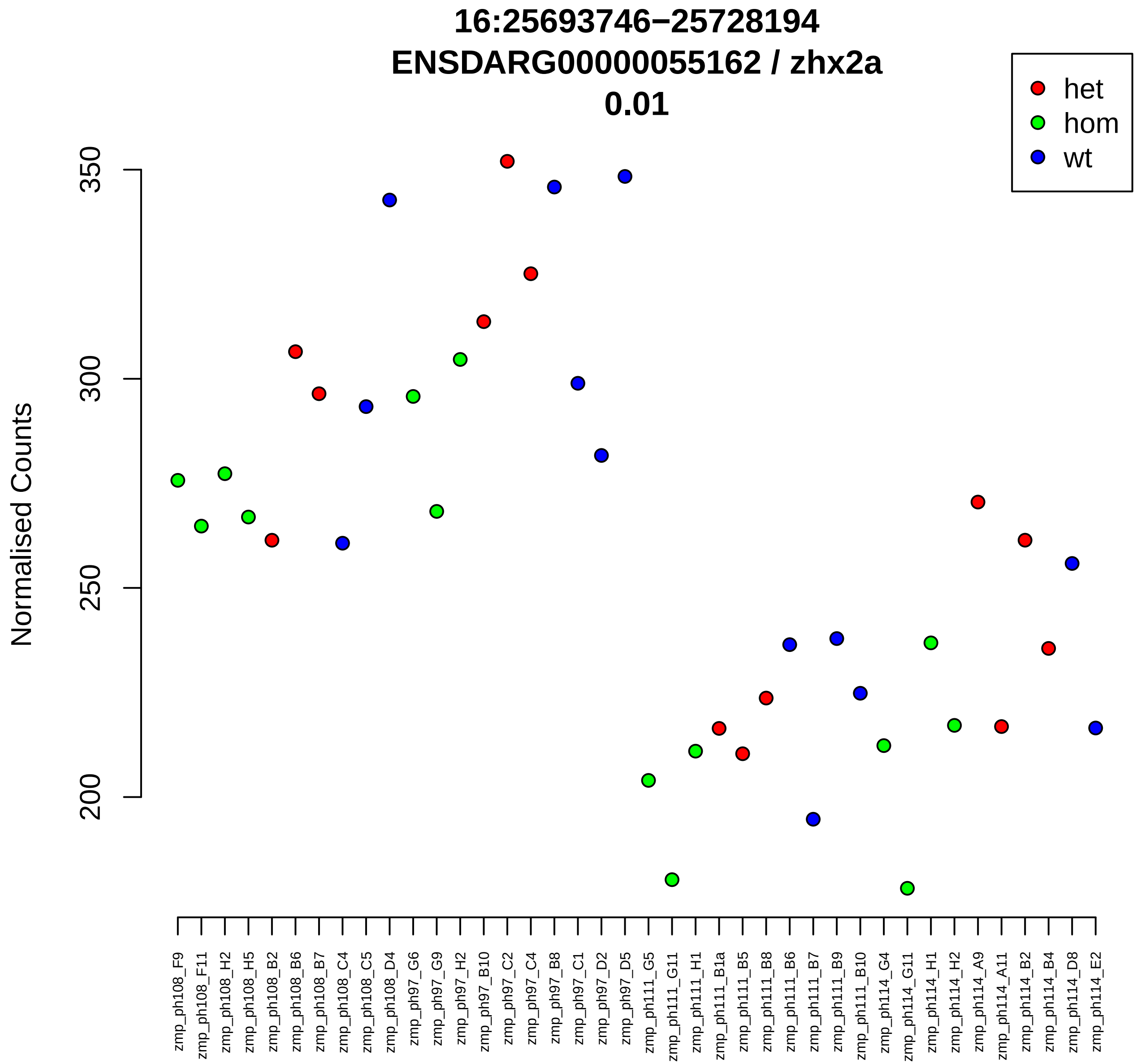
16:25693746−25728194
ENSDARG00000055162 / zhx2a
0.01
het
hom
wt
Normalised Counts
350
300
250
200
zmp_ph108_F9
zmp_ph108_F11
zmp_ph108_H2
zmp_ph108_H5
zmp_ph108_B2
zmp_ph108_B6
zmp_ph108_B7
zmp_ph108_C4
zmp_ph108_C5
zmp_ph108_D4
zmp_ph97_G6
zmp_ph97_G9
zmp_ph97_H2
zmp_ph97_B10
zmp_ph97_C2
zmp_ph97_C4
zmp_ph97_B8
zmp_ph97_C1
zmp_ph97_D2
zmp_ph97_D5
zmp_ph111_G5
zmp_ph111_G11
zmp_ph111_H1
zmp_ph111_B1a
zmp_ph111_B5
zmp_ph111_B8
zmp_ph111_B6
zmp_ph111_B7
zmp_ph111_B9
zmp_ph111_B10
zmp_ph114_G4
zmp_ph114_G11
zmp_ph114_H1
zmp_ph114_H2
zmp_ph114_A9
zmp_ph114_A11
zmp_ph114_B2
zmp_ph114_B4
zmp_ph114_D8
zmp_ph114_E2

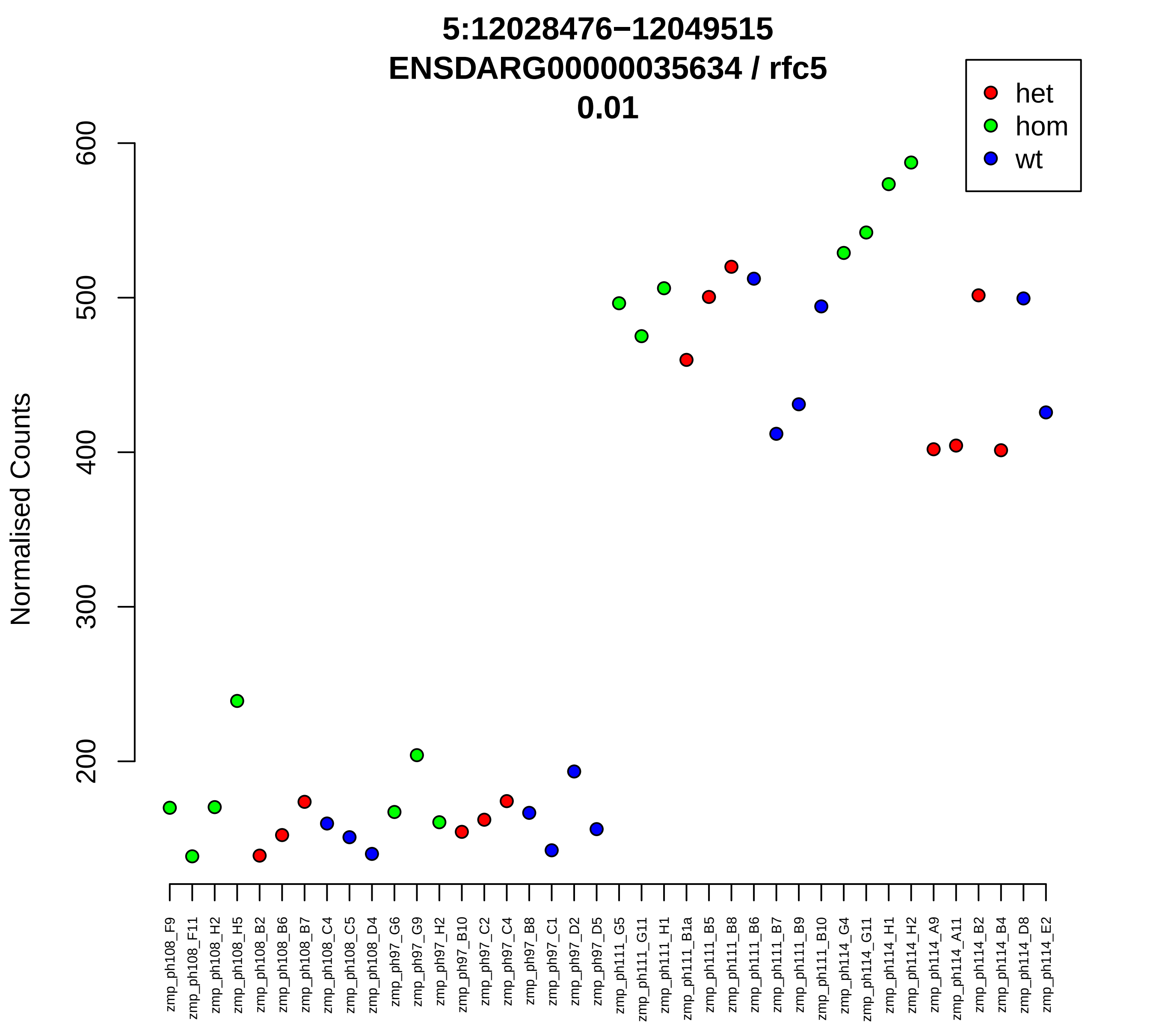
5:12028476−12049515
ENSDARG00000035634 / rfc5
0.01
het
hom
wt
Normalised Counts
200
300
400
500
600
zmp_ph108_F9
zmp_ph108_F11
zmp_ph108_H2
zmp_ph108_H5
zmp_ph108_B2
zmp_ph108_B6
zmp_ph108_B7
zmp_ph108_C4
zmp_ph108_C5
zmp_ph108_D4
zmp_ph97_G6
zmp_ph97_G9
zmp_ph97_H2
zmp_ph97_B10
zmp_ph97_C2
zmp_ph97_C4
zmp_ph97_B8
zmp_ph97_C1
zmp_ph97_D2
zmp_ph97_D5
zmp_ph111_G5
zmp_ph111_G11
zmp_ph111_H1
zmp_ph111_B1a
zmp_ph111_B5
zmp_ph111_B8
zmp_ph111_B6
zmp_ph111_B7
zmp_ph111_B9
zmp_ph111_B10
zmp_ph114_G4
zmp_ph114_G11
zmp_ph114_H1
zmp_ph114_H2
zmp_ph114_A9
zmp_ph114_A11
zmp_ph114_B2
zmp_ph114_B4
zmp_ph114_D8
zmp_ph114_E2

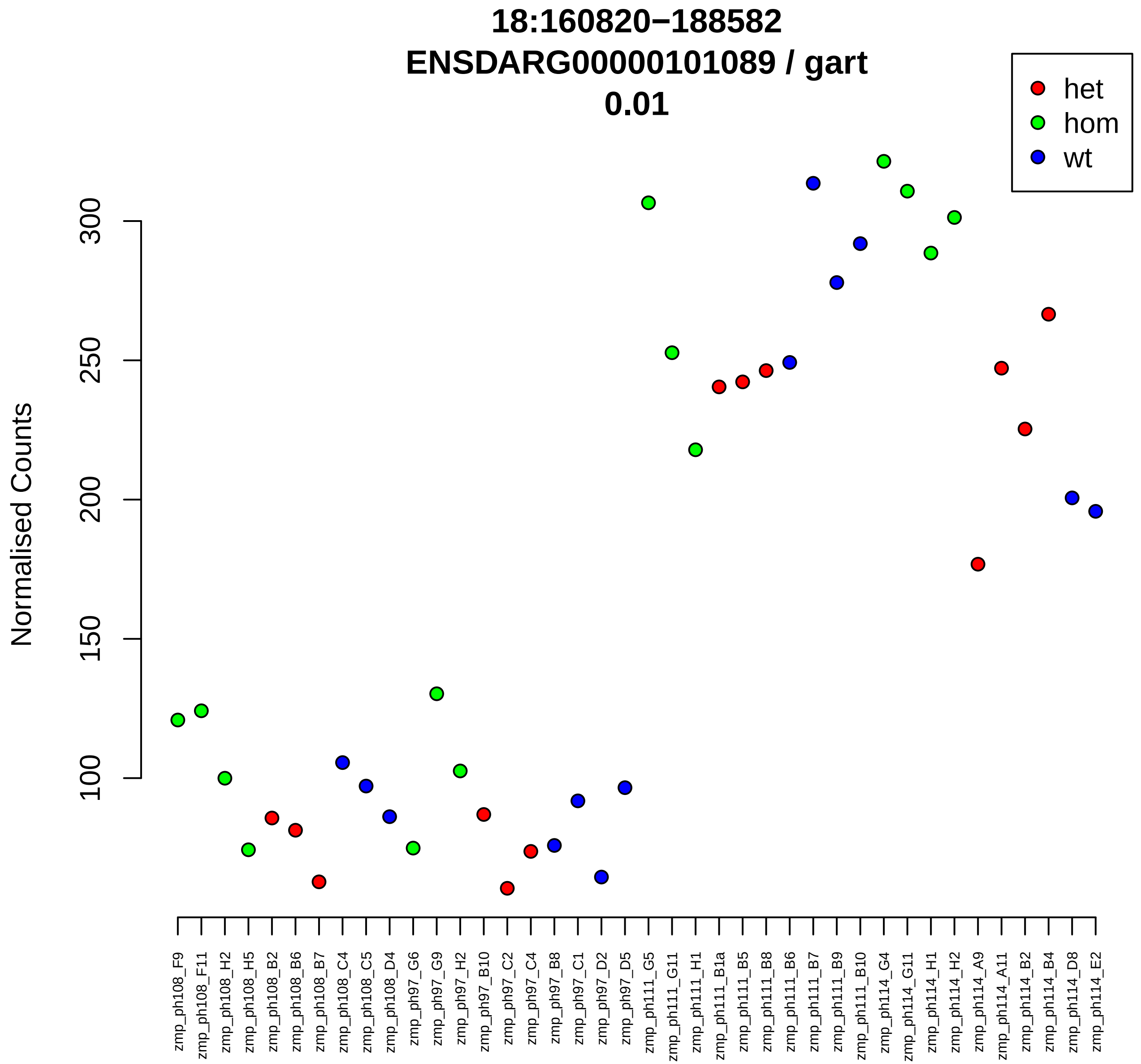
18:160820−188582
ENSDARG00000101089 / gart
0.01
het
hom
wt
Normalised Counts
100
150
200
250
300
zmp_ph108_F9
zmp_ph108_F11
zmp_ph108_H2
zmp_ph108_H5
zmp_ph108_B2
zmp_ph108_B6
zmp_ph108_B7
zmp_ph108_C4
zmp_ph108_C5
zmp_ph108_D4
zmp_ph97_G6
zmp_ph97_G9
zmp_ph97_H2
zmp_ph97_B10
zmp_ph97_C2
zmp_ph97_C4
zmp_ph97_B8
zmp_ph97_C1
zmp_ph97_D2
zmp_ph97_D5
zmp_ph111_G5
zmp_ph111_G11
zmp_ph111_H1
zmp_ph111_B1a
zmp_ph111_B5
zmp_ph111_B8
zmp_ph111_B6
zmp_ph111_B7
zmp_ph111_B9
zmp_ph111_B10
zmp_ph114_G4
zmp_ph114_G11
zmp_ph114_H1
zmp_ph114_H2
zmp_ph114_A9
zmp_ph114_A11
zmp_ph114_B2
zmp_ph114_B4
zmp_ph114_D8
zmp_ph114_E2

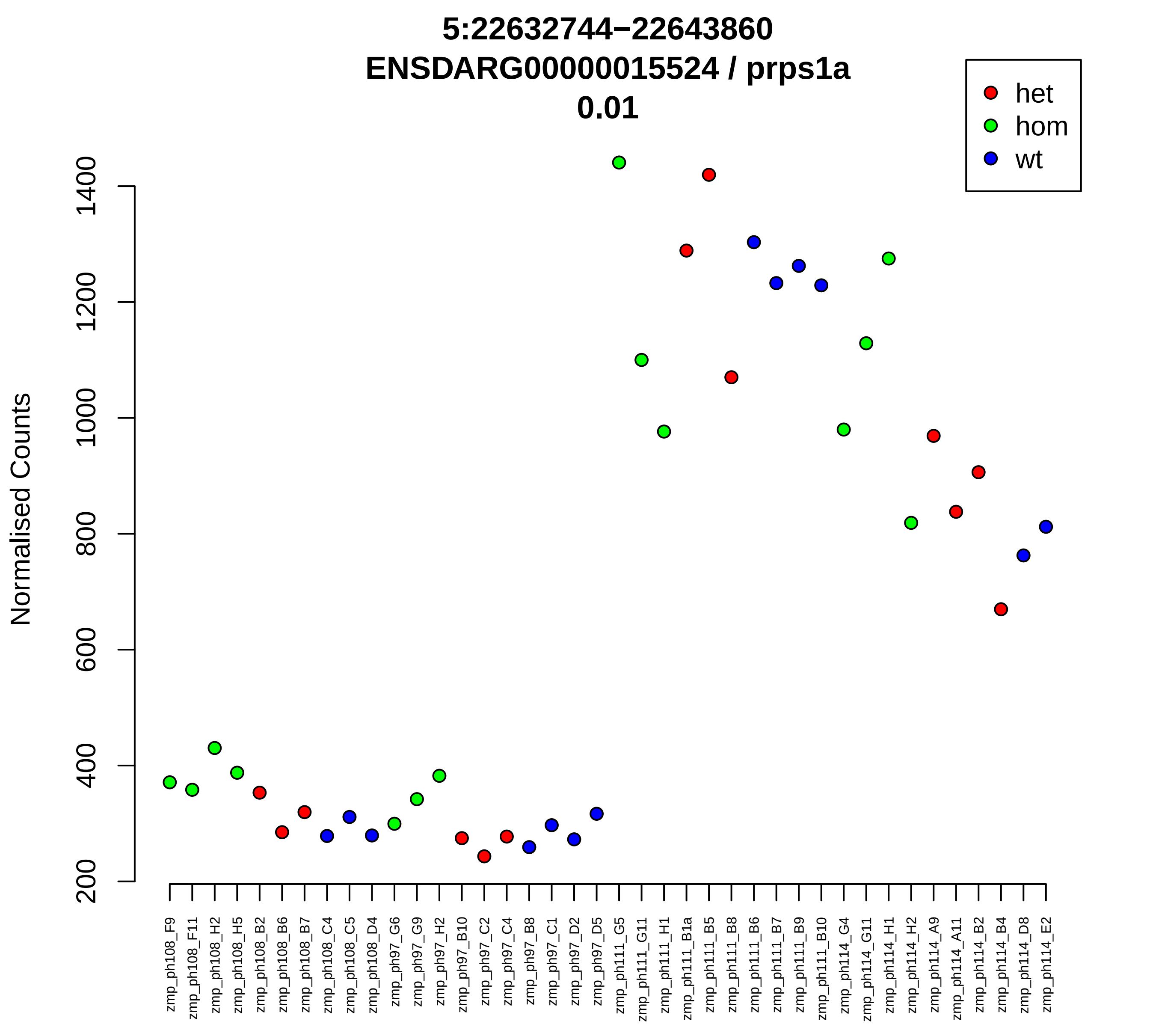

5:22632744−22643860
ENSDARG00000015524 / prps1a
0.01
het
hom
wt
Normalised Counts
200
400
600
800
1000
1200
1400
zmp_ph108_F9
zmp_ph108_F11
zmp_ph108_H2
zmp_ph108_H5
zmp_ph108_B2
zmp_ph108_B6
zmp_ph108_B7
zmp_ph108_C4
zmp_ph108_C5
zmp_ph108_D4
zmp_ph97_G6
zmp_ph97_G9
zmp_ph97_H2
zmp_ph97_B10
zmp_ph97_C2
zmp_ph97_C4
zmp_ph97_B8
zmp_ph97_C1
zmp_ph97_D2
zmp_ph97_D5
zmp_ph111_G5
zmp_ph111_G11
zmp_ph111_H1
zmp_ph111_B1a
zmp_ph111_B5
zmp_ph111_B8
zmp_ph111_B6
zmp_ph111_B7
zmp_ph111_B9
zmp_ph111_B10
zmp_ph114_G4
zmp_ph114_G11
zmp_ph114_H1
zmp_ph114_H2
zmp_ph114_A9
zmp_ph114_A11
zmp_ph114_B2
zmp_ph114_B4
zmp_ph114_D8
zmp_ph114_E2

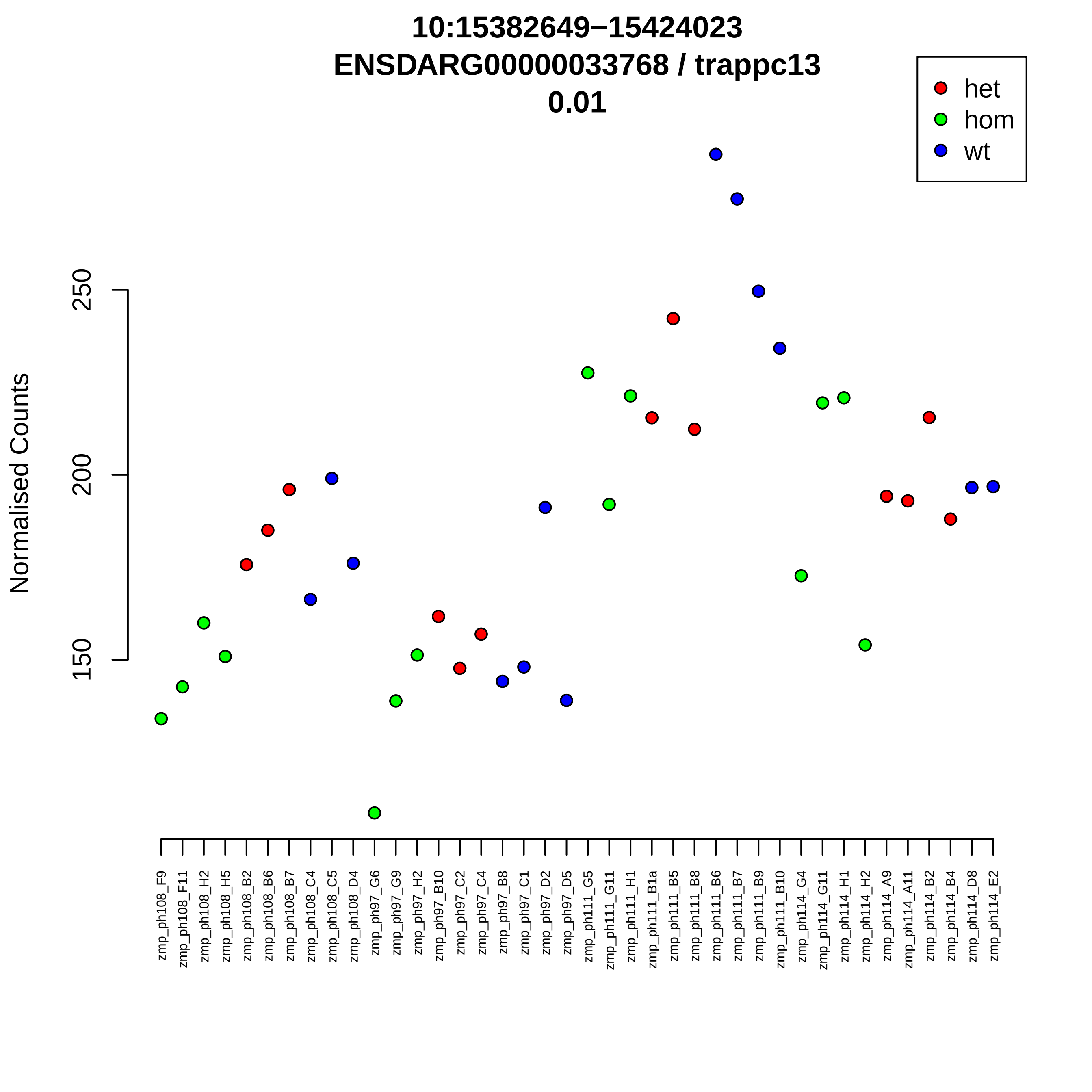
10:15382649−15424023
ENSDARG00000033768 / trappc13
0.01
het
hom
wt
Normalised Counts
150
200
250
zmp_ph108_F9
zmp_ph108_F11
zmp_ph108_H2
zmp_ph108_H5
zmp_ph108_B2
zmp_ph108_B6
zmp_ph108_B7
zmp_ph108_C4
zmp_ph108_C5
zmp_ph108_D4
zmp_ph97_G6
zmp_ph97_G9
zmp_ph97_H2
zmp_ph97_B10
zmp_ph97_C2
zmp_ph97_C4
zmp_ph97_B8
zmp_ph97_C1
zmp_ph97_D2
zmp_ph97_D5
zmp_ph111_G5
zmp_ph111_G11
zmp_ph111_H1
zmp_ph111_B1a
zmp_ph111_B5
zmp_ph111_B8
zmp_ph111_B6
zmp_ph111_B7
zmp_ph111_B9
zmp_ph111_B10
zmp_ph114_G4
zmp_ph114_G11
zmp_ph114_H1
zmp_ph114_H2
zmp_ph114_A9
zmp_ph114_A11
zmp_ph114_B2
zmp_ph114_B4
zmp_ph114_D8
zmp_ph114_E2

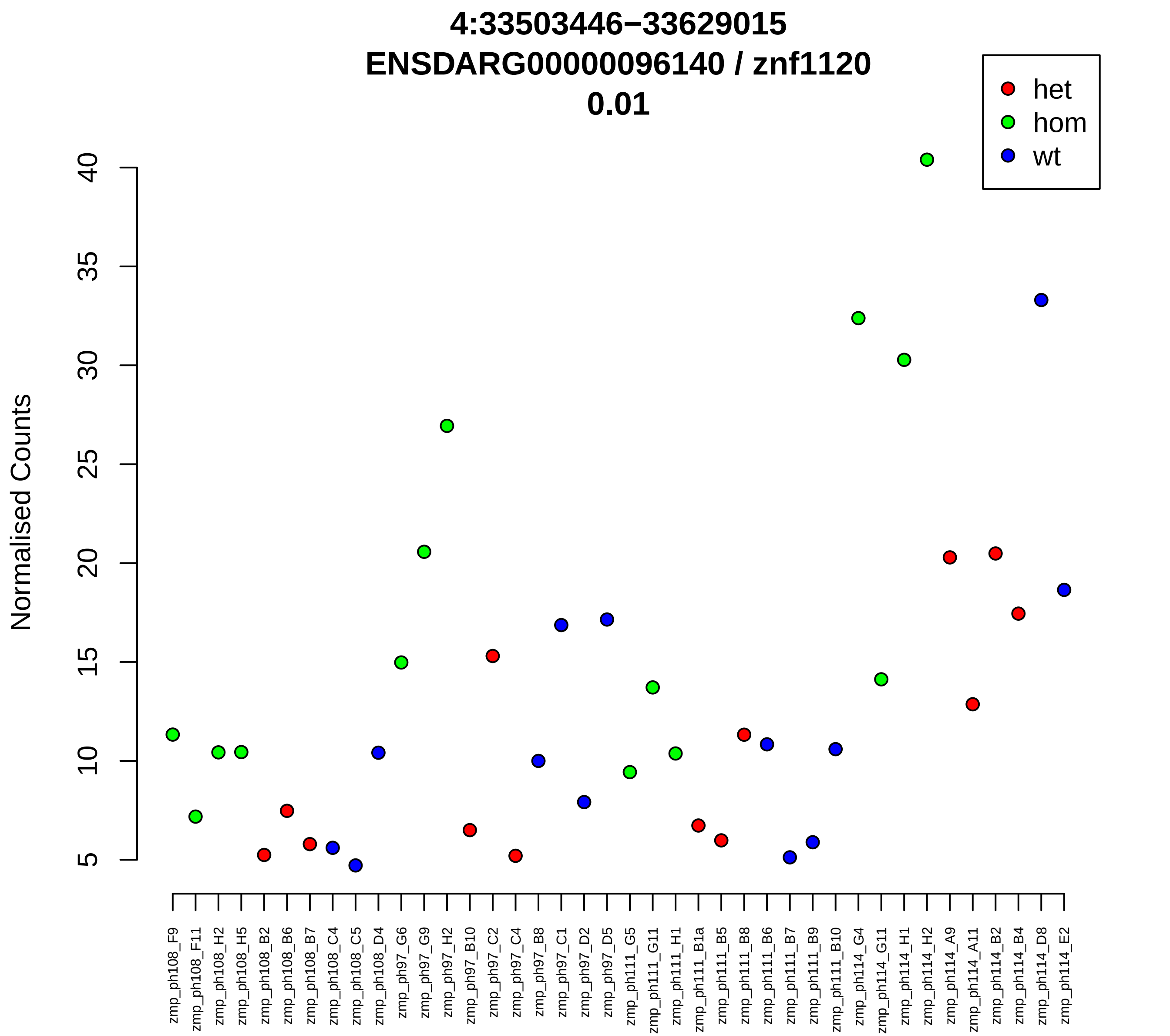

4:33503446−33629015
ENSDARG00000096140 / znf1120
0.01
het
hom
wt
Normalised Counts
5
10
15
20
25
30
35
40
zmp_ph108_F9
zmp_ph108_F11
zmp_ph108_H2
zmp_ph108_H5
zmp_ph108_B2
zmp_ph108_B6
zmp_ph108_B7
zmp_ph108_C4
zmp_ph108_C5
zmp_ph108_D4
zmp_ph97_G6
zmp_ph97_G9
zmp_ph97_H2
zmp_ph97_B10
zmp_ph97_C2
zmp_ph97_C4
zmp_ph97_B8
zmp_ph97_C1
zmp_ph97_D2
zmp_ph97_D5
zmp_ph111_G5
zmp_ph111_G11
zmp_ph111_H1
zmp_ph111_B1a
zmp_ph111_B5
zmp_ph111_B8
zmp_ph111_B6
zmp_ph111_B7
zmp_ph111_B9
zmp_ph111_B10
zmp_ph114_G4
zmp_ph114_G11
zmp_ph114_H1
zmp_ph114_H2
zmp_ph114_A9
zmp_ph114_A11
zmp_ph114_B2
zmp_ph114_B4
zmp_ph114_D8
zmp_ph114_E2

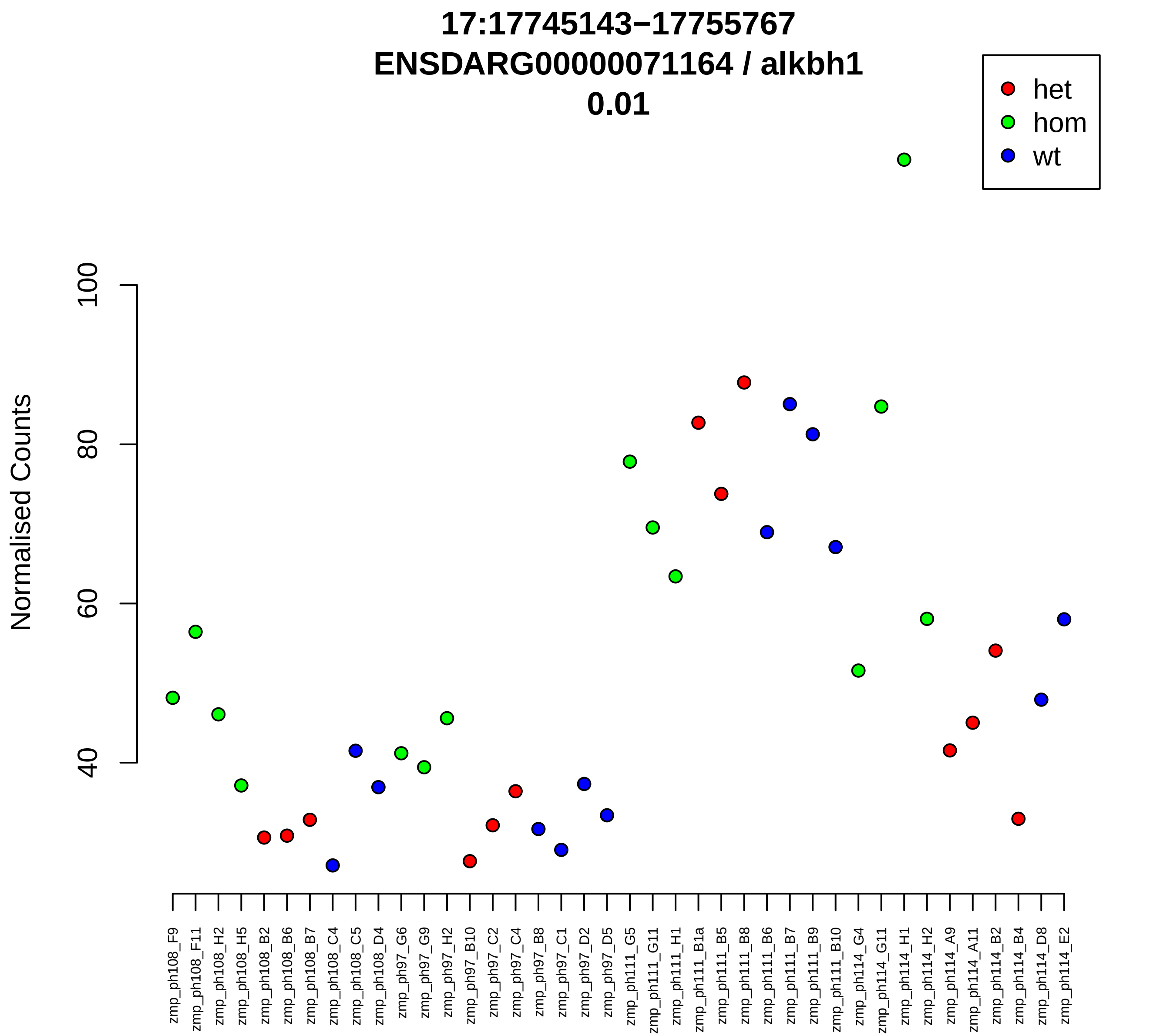

17:17745143−17755767
ENSDARG00000071164 / alkbh1
0.01
het
hom
wt
Normalised Counts
40
60
80
100
zmp_ph108_F9
zmp_ph108_F11
zmp_ph108_H2
zmp_ph108_H5
zmp_ph108_B2
zmp_ph108_B6
zmp_ph108_B7
zmp_ph108_C4
zmp_ph108_C5
zmp_ph108_D4
zmp_ph97_G6
zmp_ph97_G9
zmp_ph97_H2
zmp_ph97_B10
zmp_ph97_C2
zmp_ph97_C4
zmp_ph97_B8
zmp_ph97_C1
zmp_ph97_D2
zmp_ph97_D5
zmp_ph111_G5
zmp_ph111_G11
zmp_ph111_H1
zmp_ph111_B1a
zmp_ph111_B5
zmp_ph111_B8
zmp_ph111_B6
zmp_ph111_B7
zmp_ph111_B9
zmp_ph111_B10
zmp_ph114_G4
zmp_ph114_G11
zmp_ph114_H1
zmp_ph114_H2
zmp_ph114_A9
zmp_ph114_A11
zmp_ph114_B2
zmp_ph114_B4
zmp_ph114_D8
zmp_ph114_E2

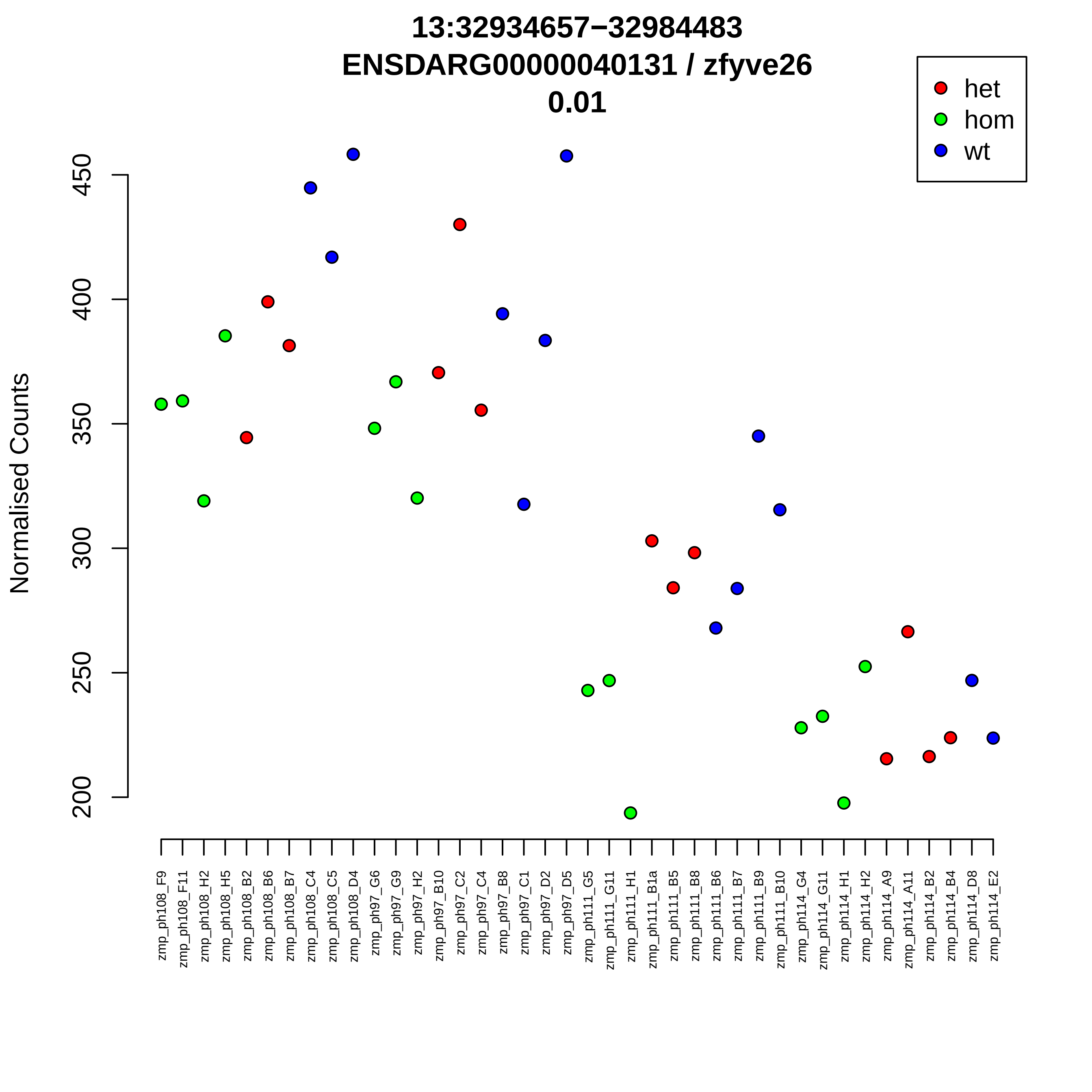

13:32934657−32984483
ENSDARG00000040131 / zfyve26
0.01
het
hom
wt
Normalised Counts
200
250
300
350
400
450
zmp_ph108_F9
zmp_ph108_F11
zmp_ph108_H2
zmp_ph108_H5
zmp_ph108_B2
zmp_ph108_B6
zmp_ph108_B7
zmp_ph108_C4
zmp_ph108_C5
zmp_ph108_D4
zmp_ph97_G6
zmp_ph97_G9
zmp_ph97_H2
zmp_ph97_B10
zmp_ph97_C2
zmp_ph97_C4
zmp_ph97_B8
zmp_ph97_C1
zmp_ph97_D2
zmp_ph97_D5
zmp_ph111_G5
zmp_ph111_G11
zmp_ph111_H1
zmp_ph111_B1a
zmp_ph111_B5
zmp_ph111_B8
zmp_ph111_B6
zmp_ph111_B7
zmp_ph111_B9
zmp_ph111_B10
zmp_ph114_G4
zmp_ph114_G11
zmp_ph114_H1
zmp_ph114_H2
zmp_ph114_A9
zmp_ph114_A11
zmp_ph114_B2
zmp_ph114_B4
zmp_ph114_D8
zmp_ph114_E2

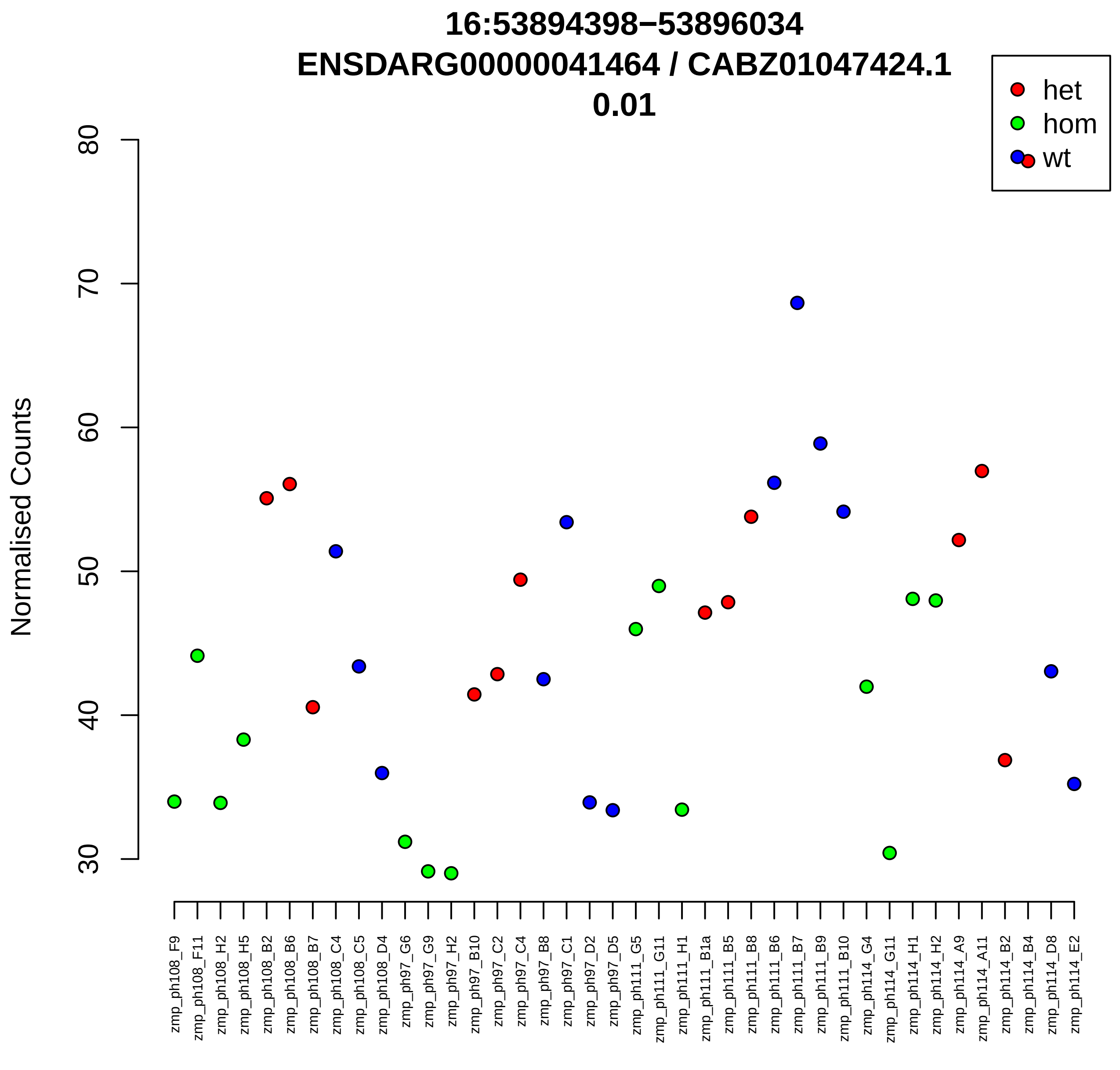
16:53894398−53896034
ENSDARG00000041464 / CABZ01047424.1
0.01
het
hom
wt
Normalised Counts
30
40
50
60
70
80
zmp_ph108_F9
zmp_ph108_F11
zmp_ph108_H2
zmp_ph108_H5
zmp_ph108_B2
zmp_ph108_B6
zmp_ph108_B7
zmp_ph108_C4
zmp_ph108_C5
zmp_ph108_D4
zmp_ph97_G6
zmp_ph97_G9
zmp_ph97_H2
zmp_ph97_B10
zmp_ph97_C2
zmp_ph97_C4
zmp_ph97_B8
zmp_ph97_C1
zmp_ph97_D2
zmp_ph97_D5
zmp_ph111_G5
zmp_ph111_G11
zmp_ph111_H1
zmp_ph111_B1a
zmp_ph111_B5
zmp_ph111_B8
zmp_ph111_B6
zmp_ph111_B7
zmp_ph111_B9
zmp_ph111_B10
zmp_ph114_G4
zmp_ph114_G11
zmp_ph114_H1
zmp_ph114_H2
zmp_ph114_A9
zmp_ph114_A11
zmp_ph114_B2
zmp_ph114_B4
zmp_ph114_D8
zmp_ph114_E2

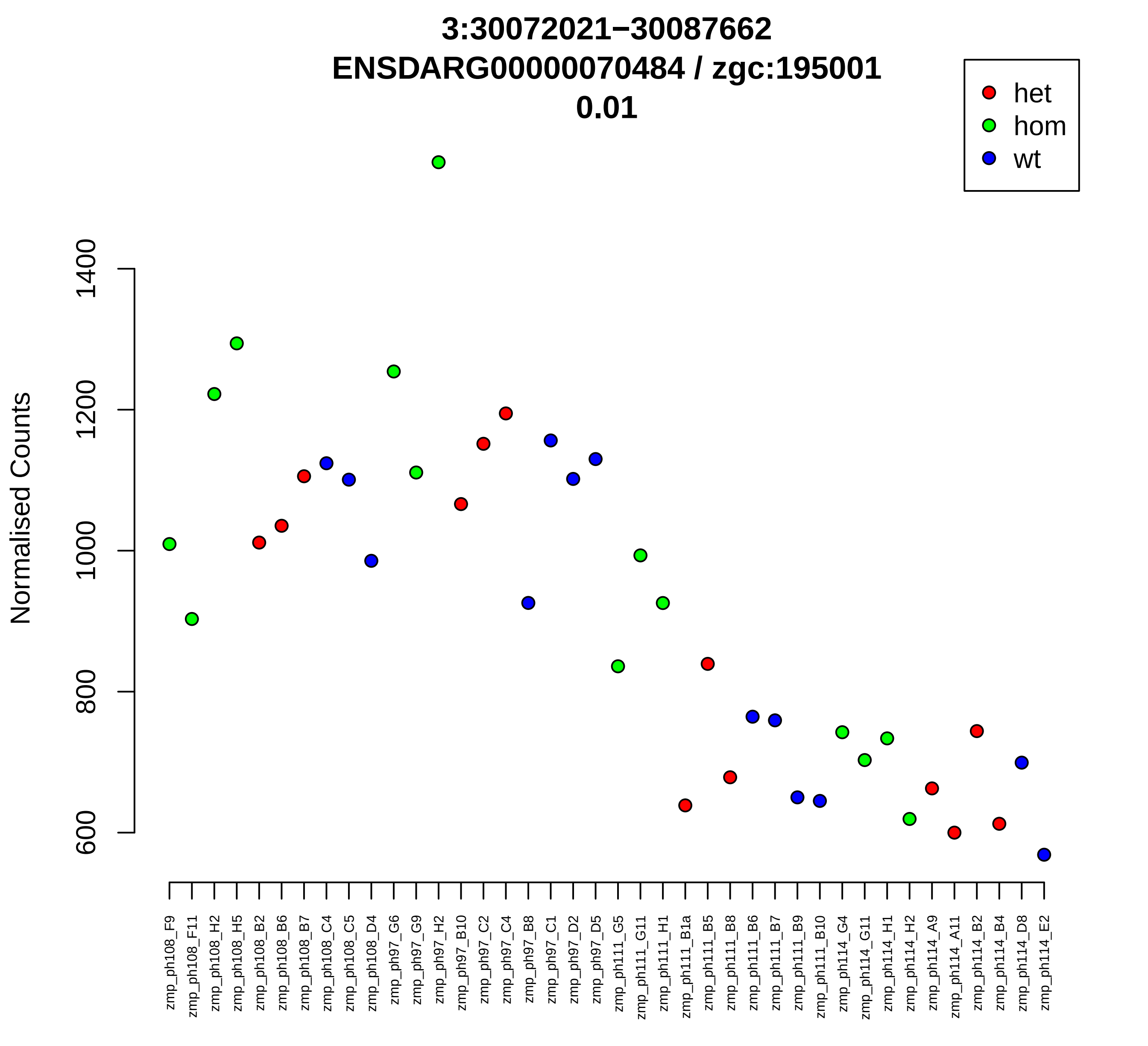
3:30072021−30087662
ENSDARG00000070484 / zgc:195001
0.01
het
hom
wt
Normalised Counts
600
800
1000
1200
1400
zmp_ph108_F9
zmp_ph108_F11
zmp_ph108_H2
zmp_ph108_H5
zmp_ph108_B2
zmp_ph108_B6
zmp_ph108_B7
zmp_ph108_C4
zmp_ph108_C5
zmp_ph108_D4
zmp_ph97_G6
zmp_ph97_G9
zmp_ph97_H2
zmp_ph97_B10
zmp_ph97_C2
zmp_ph97_C4
zmp_ph97_B8
zmp_ph97_C1
zmp_ph97_D2
zmp_ph97_D5
zmp_ph111_G5
zmp_ph111_G11
zmp_ph111_H1
zmp_ph111_B1a
zmp_ph111_B5
zmp_ph111_B8
zmp_ph111_B6
zmp_ph111_B7
zmp_ph111_B9
zmp_ph111_B10
zmp_ph114_G4
zmp_ph114_G11
zmp_ph114_H1
zmp_ph114_H2
zmp_ph114_A9
zmp_ph114_A11
zmp_ph114_B2
zmp_ph114_B4
zmp_ph114_D8
zmp_ph114_E2

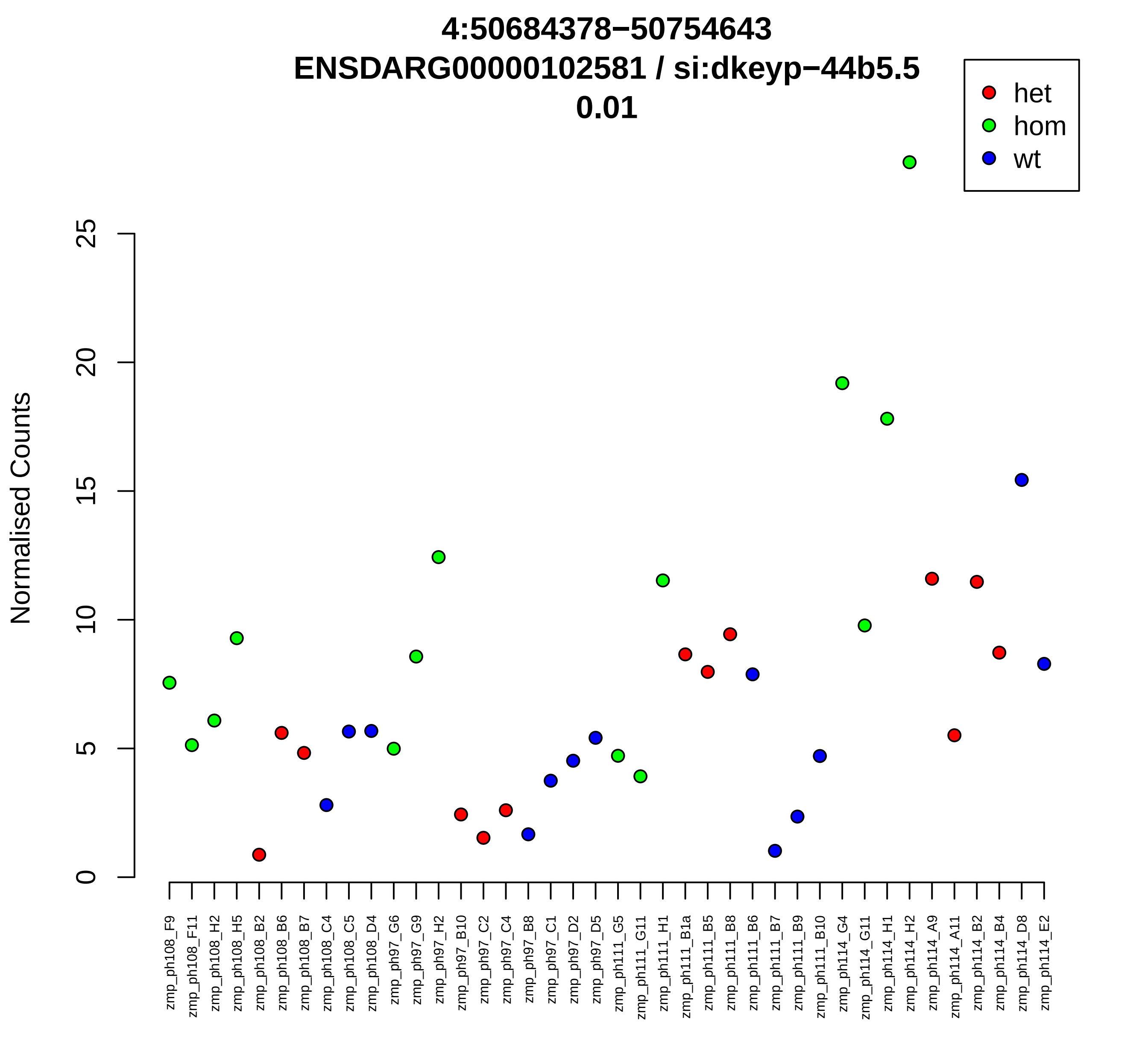

4:50684378−50754643
ENSDARG00000102581 / si:dkeyp−44b5.5
0.01
het
hom
wt
Normalised Counts
0
5
10
15
20
25
zmp_ph108_F9
zmp_ph108_F11
zmp_ph108_H2
zmp_ph108_H5
zmp_ph108_B2
zmp_ph108_B6
zmp_ph108_B7
zmp_ph108_C4
zmp_ph108_C5
zmp_ph108_D4
zmp_ph97_G6
zmp_ph97_G9
zmp_ph97_H2
zmp_ph97_B10
zmp_ph97_C2
zmp_ph97_C4
zmp_ph97_B8
zmp_ph97_C1
zmp_ph97_D2
zmp_ph97_D5
zmp_ph111_G5
zmp_ph111_G11
zmp_ph111_H1
zmp_ph111_B1a
zmp_ph111_B5
zmp_ph111_B8
zmp_ph111_B6
zmp_ph111_B7
zmp_ph111_B9
zmp_ph111_B10
zmp_ph114_G4
zmp_ph114_G11
zmp_ph114_H1
zmp_ph114_H2
zmp_ph114_A9
zmp_ph114_A11
zmp_ph114_B2
zmp_ph114_B4
zmp_ph114_D8
zmp_ph114_E2

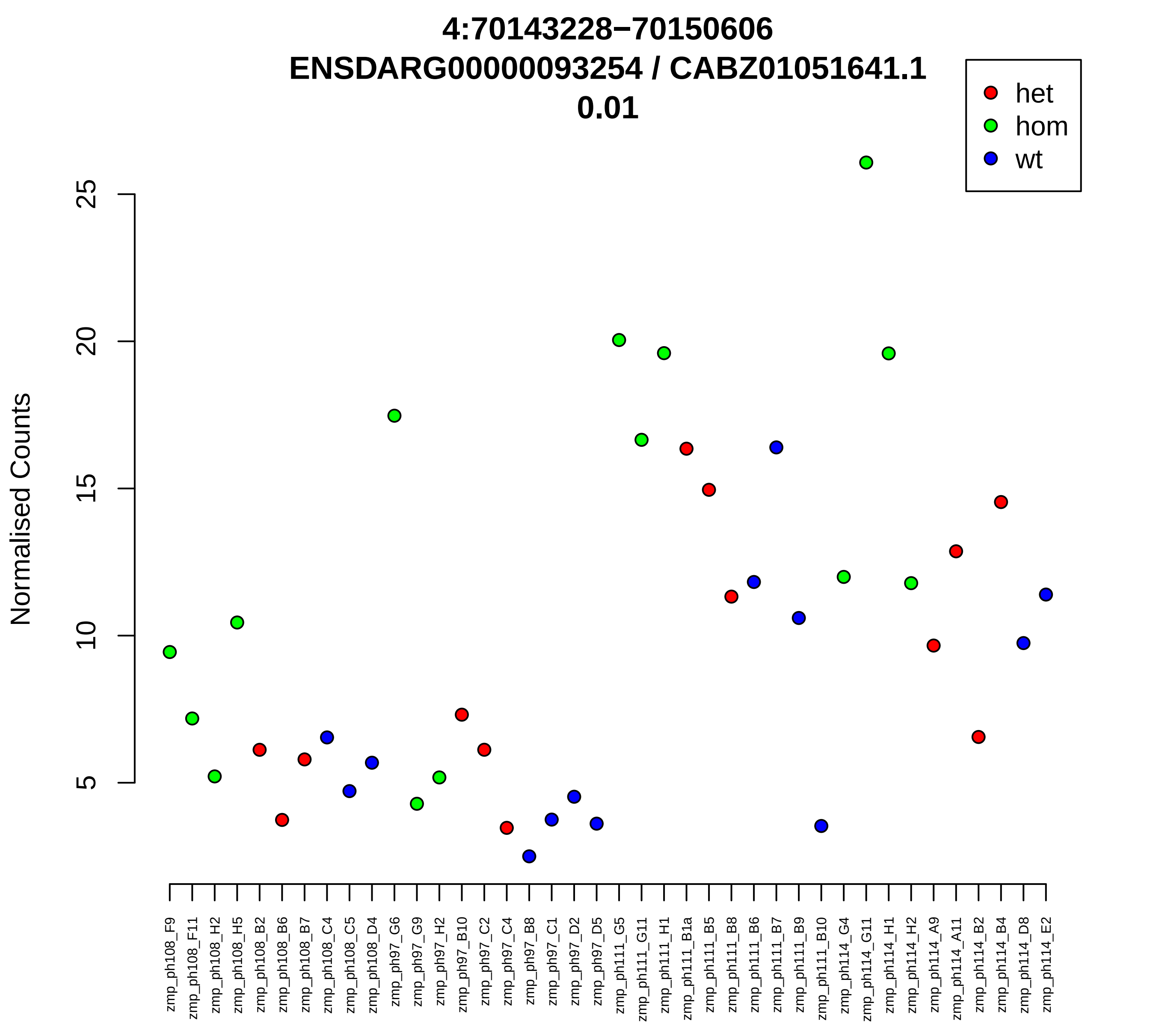
4:70143228−70150606
ENSDARG00000093254 / CABZ01051641.1
0.01
het
hom
wt
Normalised Counts
5
10
15
20
25
zmp_ph108_F9
zmp_ph108_F11
zmp_ph108_H2
zmp_ph108_H5
zmp_ph108_B2
zmp_ph108_B6
zmp_ph108_B7
zmp_ph108_C4
zmp_ph108_C5
zmp_ph108_D4
zmp_ph97_G6
zmp_ph97_G9
zmp_ph97_H2
zmp_ph97_B10
zmp_ph97_C2
zmp_ph97_C4
zmp_ph97_B8
zmp_ph97_C1
zmp_ph97_D2
zmp_ph97_D5
zmp_ph111_G5
zmp_ph111_G11
zmp_ph111_H1
zmp_ph111_B1a
zmp_ph111_B5
zmp_ph111_B8
zmp_ph111_B6
zmp_ph111_B7
zmp_ph111_B9
zmp_ph111_B10
zmp_ph114_G4
zmp_ph114_G11
zmp_ph114_H1
zmp_ph114_H2
zmp_ph114_A9
zmp_ph114_A11
zmp_ph114_B2
zmp_ph114_B4
zmp_ph114_D8
zmp_ph114_E2

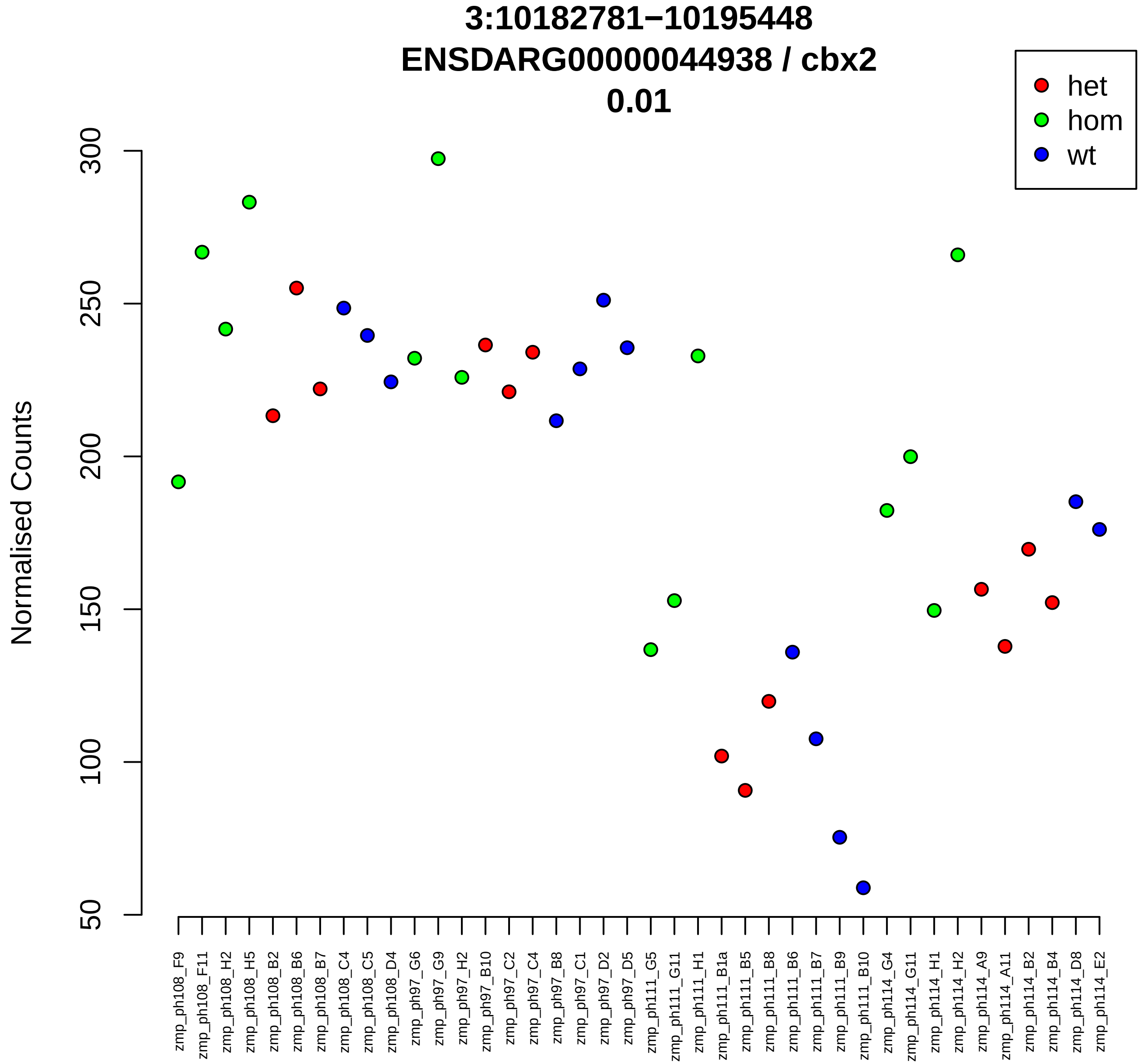

3:10182781−10195448
ENSDARG00000044938 / cbx2
0.01
het
hom
wt
Normalised Counts
50
100
150
200
250
300
zmp_ph108_F9
zmp_ph108_F11
zmp_ph108_H2
zmp_ph108_H5
zmp_ph108_B2
zmp_ph108_B6
zmp_ph108_B7
zmp_ph108_C4
zmp_ph108_C5
zmp_ph108_D4
zmp_ph97_G6
zmp_ph97_G9
zmp_ph97_H2
zmp_ph97_B10
zmp_ph97_C2
zmp_ph97_C4
zmp_ph97_B8
zmp_ph97_C1
zmp_ph97_D2
zmp_ph97_D5
zmp_ph111_G5
zmp_ph111_G11
zmp_ph111_H1
zmp_ph111_B1a
zmp_ph111_B5
zmp_ph111_B8
zmp_ph111_B6
zmp_ph111_B7
zmp_ph111_B9
zmp_ph111_B10
zmp_ph114_G4
zmp_ph114_G11
zmp_ph114_H1
zmp_ph114_H2
zmp_ph114_A9
zmp_ph114_A11
zmp_ph114_B2
zmp_ph114_B4
zmp_ph114_D8
zmp_ph114_E2

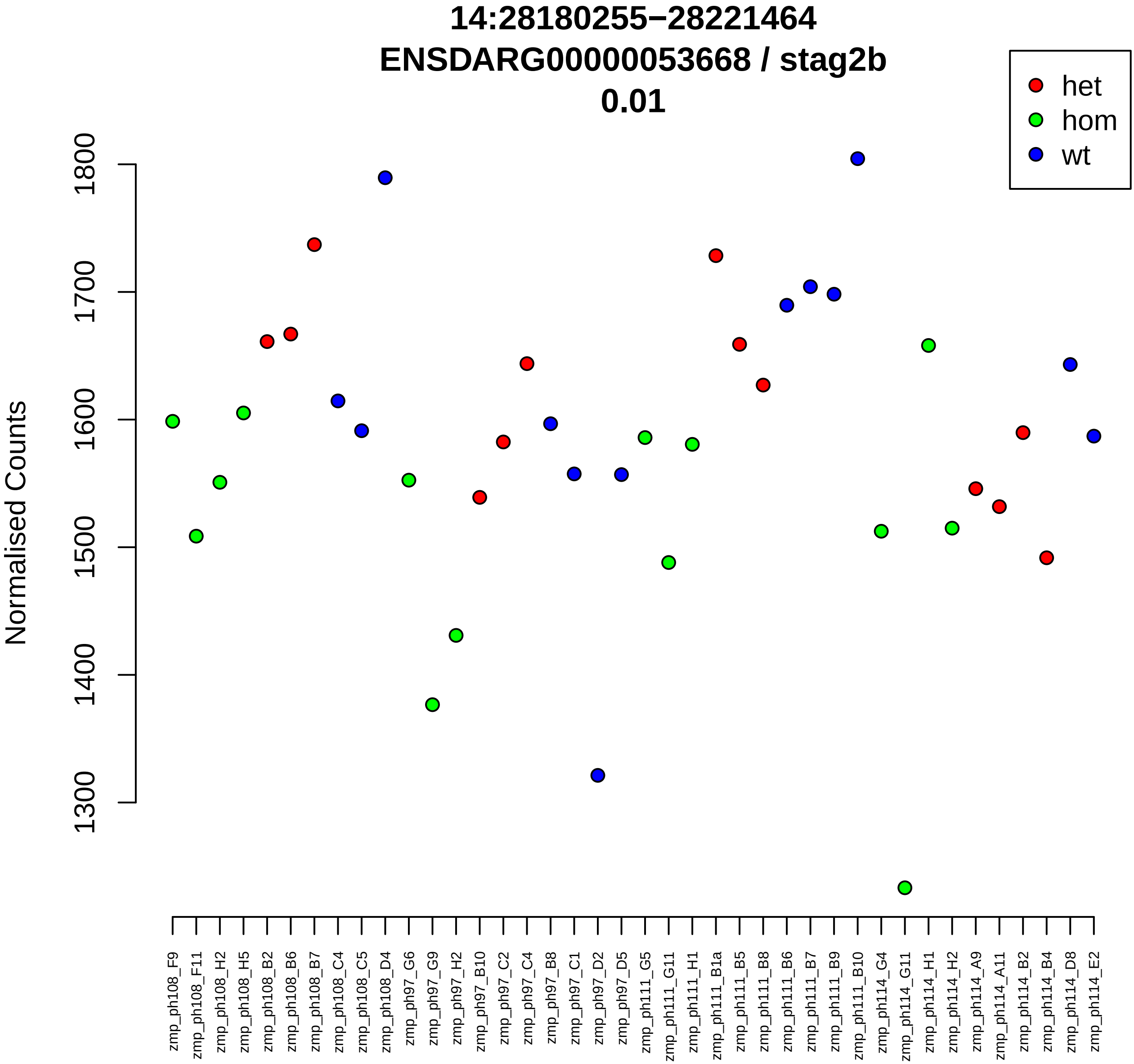

14:28180255−28221464
ENSDARG00000053668 / stag2b
0.01
het
hom
wt
Normalised Counts
1300
1400
1500
1600
1700
1800
zmp_ph108_F9
zmp_ph108_F11
zmp_ph108_H2
zmp_ph108_H5
zmp_ph108_B2
zmp_ph108_B6
zmp_ph108_B7
zmp_ph108_C4
zmp_ph108_C5
zmp_ph108_D4
zmp_ph97_G6
zmp_ph97_G9
zmp_ph97_H2
zmp_ph97_B10
zmp_ph97_C2
zmp_ph97_C4
zmp_ph97_B8
zmp_ph97_C1
zmp_ph97_D2
zmp_ph97_D5
zmp_ph111_G5
zmp_ph111_G11
zmp_ph111_H1
zmp_ph111_B1a
zmp_ph111_B5
zmp_ph111_B8
zmp_ph111_B6
zmp_ph111_B7
zmp_ph111_B9
zmp_ph111_B10
zmp_ph114_G4
zmp_ph114_G11
zmp_ph114_H1
zmp_ph114_H2
zmp_ph114_A9
zmp_ph114_A11
zmp_ph114_B2
zmp_ph114_B4
zmp_ph114_D8
zmp_ph114_E2

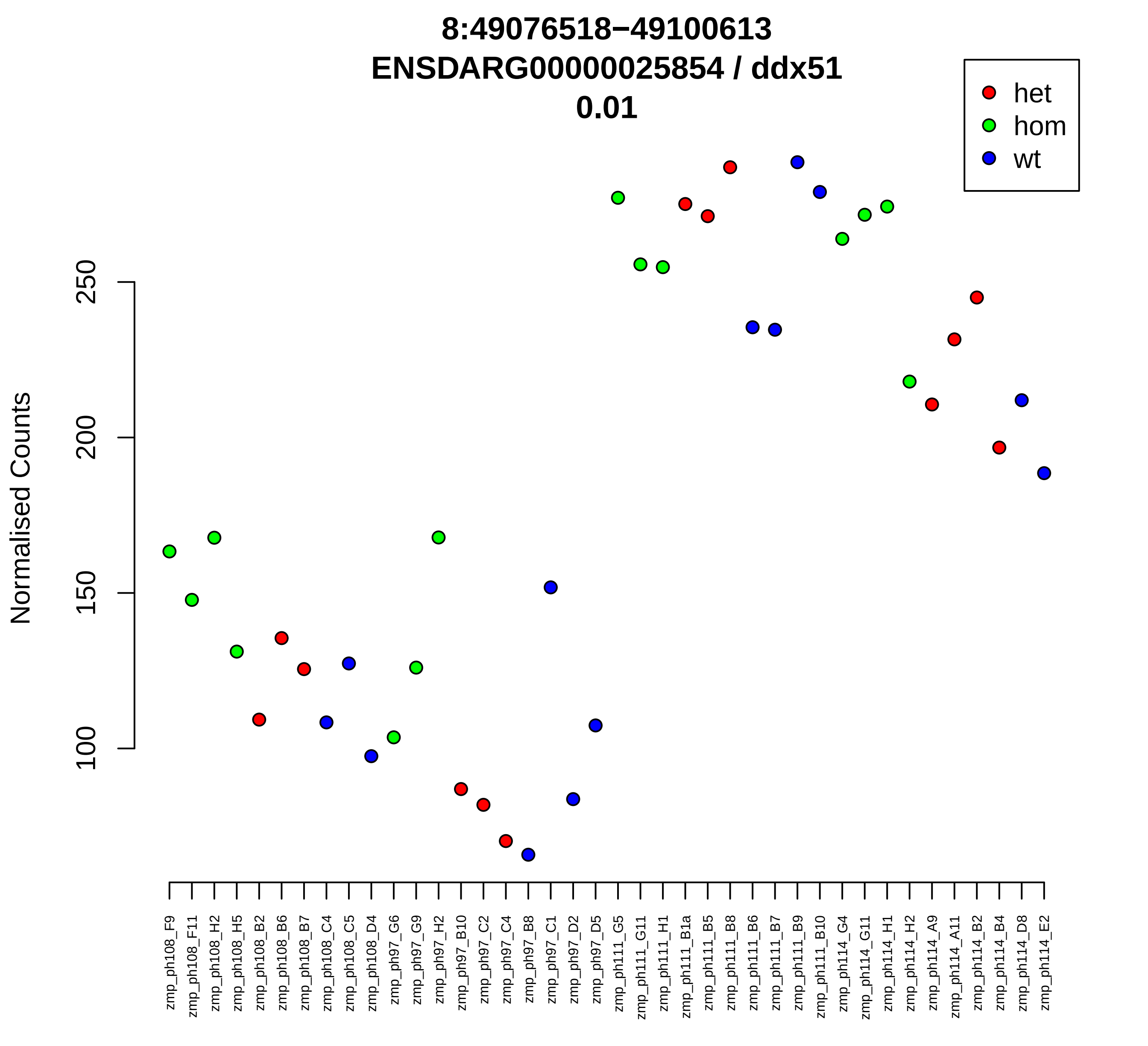

8:49076518−49100613
ENSDARG00000025854 / ddx51
0.01
het
hom
wt
Normalised Counts
100
150
200
250
zmp_ph108_F9
zmp_ph108_F11
zmp_ph108_H2
zmp_ph108_H5
zmp_ph108_B2
zmp_ph108_B6
zmp_ph108_B7
zmp_ph108_C4
zmp_ph108_C5
zmp_ph108_D4
zmp_ph97_G6
zmp_ph97_G9
zmp_ph97_H2
zmp_ph97_B10
zmp_ph97_C2
zmp_ph97_C4
zmp_ph97_B8
zmp_ph97_C1
zmp_ph97_D2
zmp_ph97_D5
zmp_ph111_G5
zmp_ph111_G11
zmp_ph111_H1
zmp_ph111_B1a
zmp_ph111_B5
zmp_ph111_B8
zmp_ph111_B6
zmp_ph111_B7
zmp_ph111_B9
zmp_ph111_B10
zmp_ph114_G4
zmp_ph114_G11
zmp_ph114_H1
zmp_ph114_H2
zmp_ph114_A9
zmp_ph114_A11
zmp_ph114_B2
zmp_ph114_B4
zmp_ph114_D8
zmp_ph114_E2

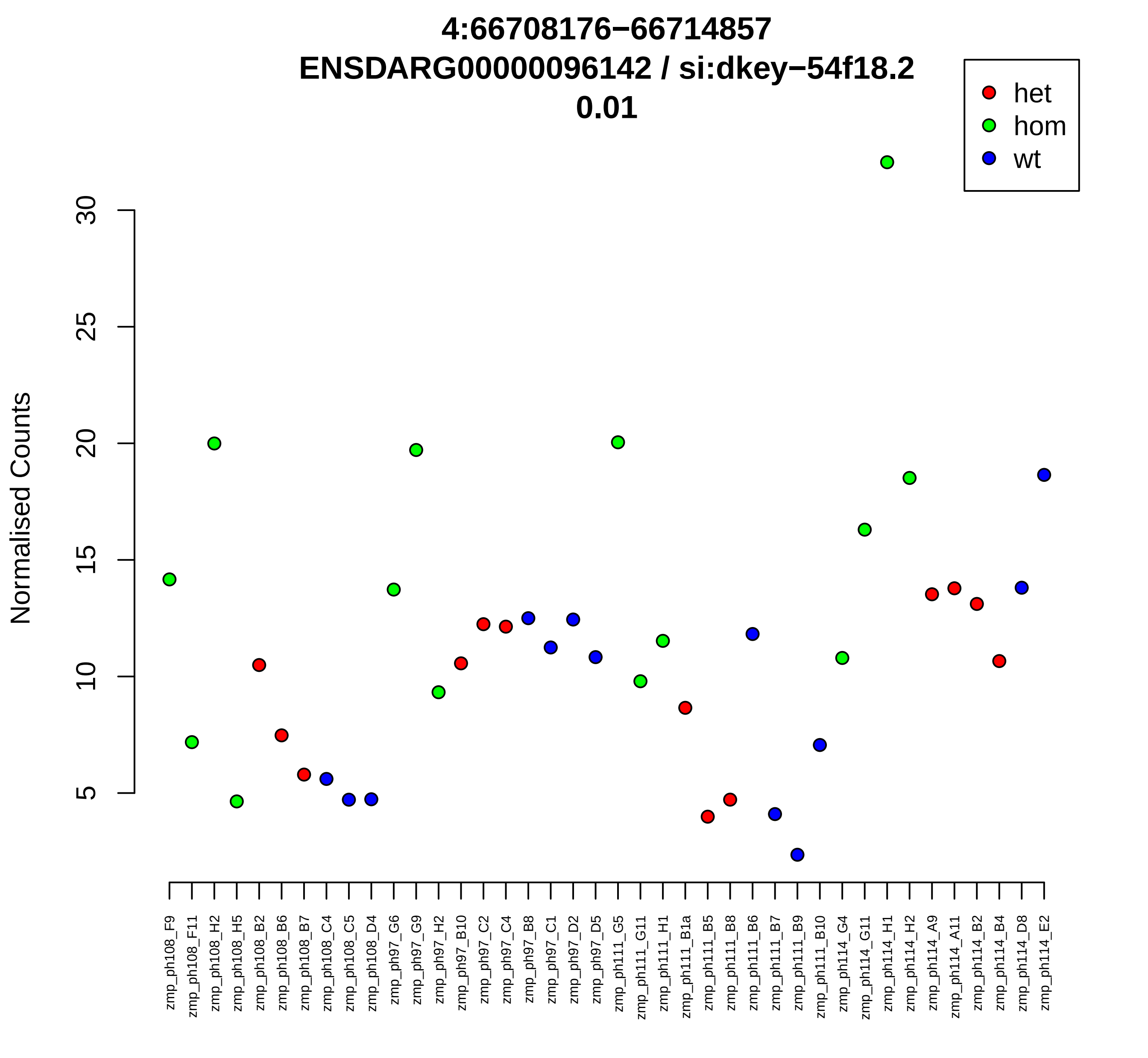
4:66708176−66714857
ENSDARG00000096142 / si:dkey−54f18.2
0.01
het
hom
wt
Normalised Counts
5
10
15
20
25
30
zmp_ph108_F9
zmp_ph108_F11
zmp_ph108_H2
zmp_ph108_H5
zmp_ph108_B2
zmp_ph108_B6
zmp_ph108_B7
zmp_ph108_C4
zmp_ph108_C5
zmp_ph108_D4
zmp_ph97_G6
zmp_ph97_G9
zmp_ph97_H2
zmp_ph97_B10
zmp_ph97_C2
zmp_ph97_C4
zmp_ph97_B8
zmp_ph97_C1
zmp_ph97_D2
zmp_ph97_D5
zmp_ph111_G5
zmp_ph111_G11
zmp_ph111_H1
zmp_ph111_B1a
zmp_ph111_B5
zmp_ph111_B8
zmp_ph111_B6
zmp_ph111_B7
zmp_ph111_B9
zmp_ph111_B10
zmp_ph114_G4
zmp_ph114_G11
zmp_ph114_H1
zmp_ph114_H2
zmp_ph114_A9
zmp_ph114_A11
zmp_ph114_B2
zmp_ph114_B4
zmp_ph114_D8
zmp_ph114_E2

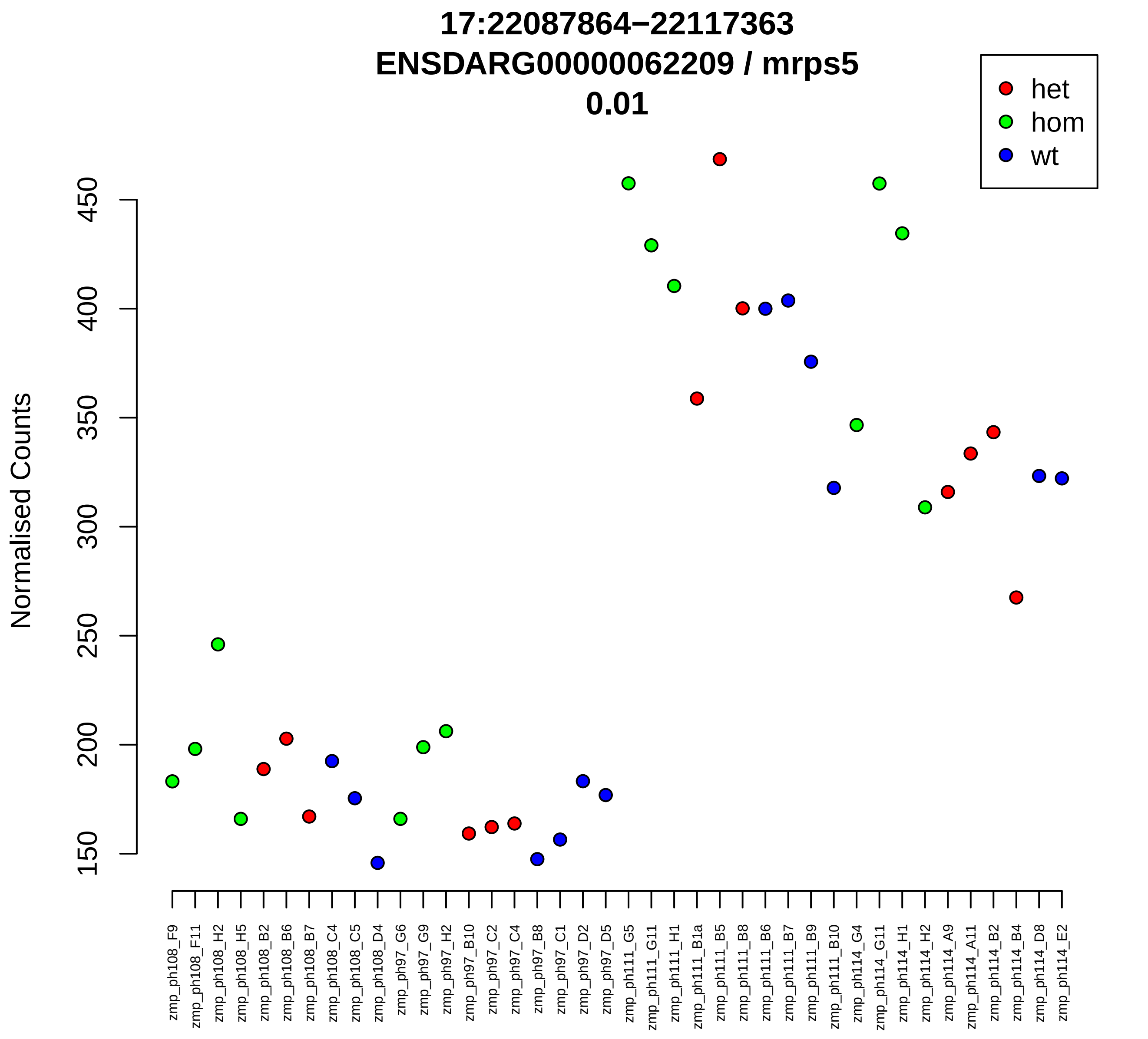
17:22087864−22117363
ENSDARG00000062209 / mrps5
0.01
het
hom
wt
Normalised Counts
150
200
250
300
350
400
450
zmp_ph108_F9
zmp_ph108_F11
zmp_ph108_H2
zmp_ph108_H5
zmp_ph108_B2
zmp_ph108_B6
zmp_ph108_B7
zmp_ph108_C4
zmp_ph108_C5
zmp_ph108_D4
zmp_ph97_G6
zmp_ph97_G9
zmp_ph97_H2
zmp_ph97_B10
zmp_ph97_C2
zmp_ph97_C4
zmp_ph97_B8
zmp_ph97_C1
zmp_ph97_D2
zmp_ph97_D5
zmp_ph111_G5
zmp_ph111_G11
zmp_ph111_H1
zmp_ph111_B1a
zmp_ph111_B5
zmp_ph111_B8
zmp_ph111_B6
zmp_ph111_B7
zmp_ph111_B9
zmp_ph111_B10
zmp_ph114_G4
zmp_ph114_G11
zmp_ph114_H1
zmp_ph114_H2
zmp_ph114_A9
zmp_ph114_A11
zmp_ph114_B2
zmp_ph114_B4
zmp_ph114_D8
zmp_ph114_E2

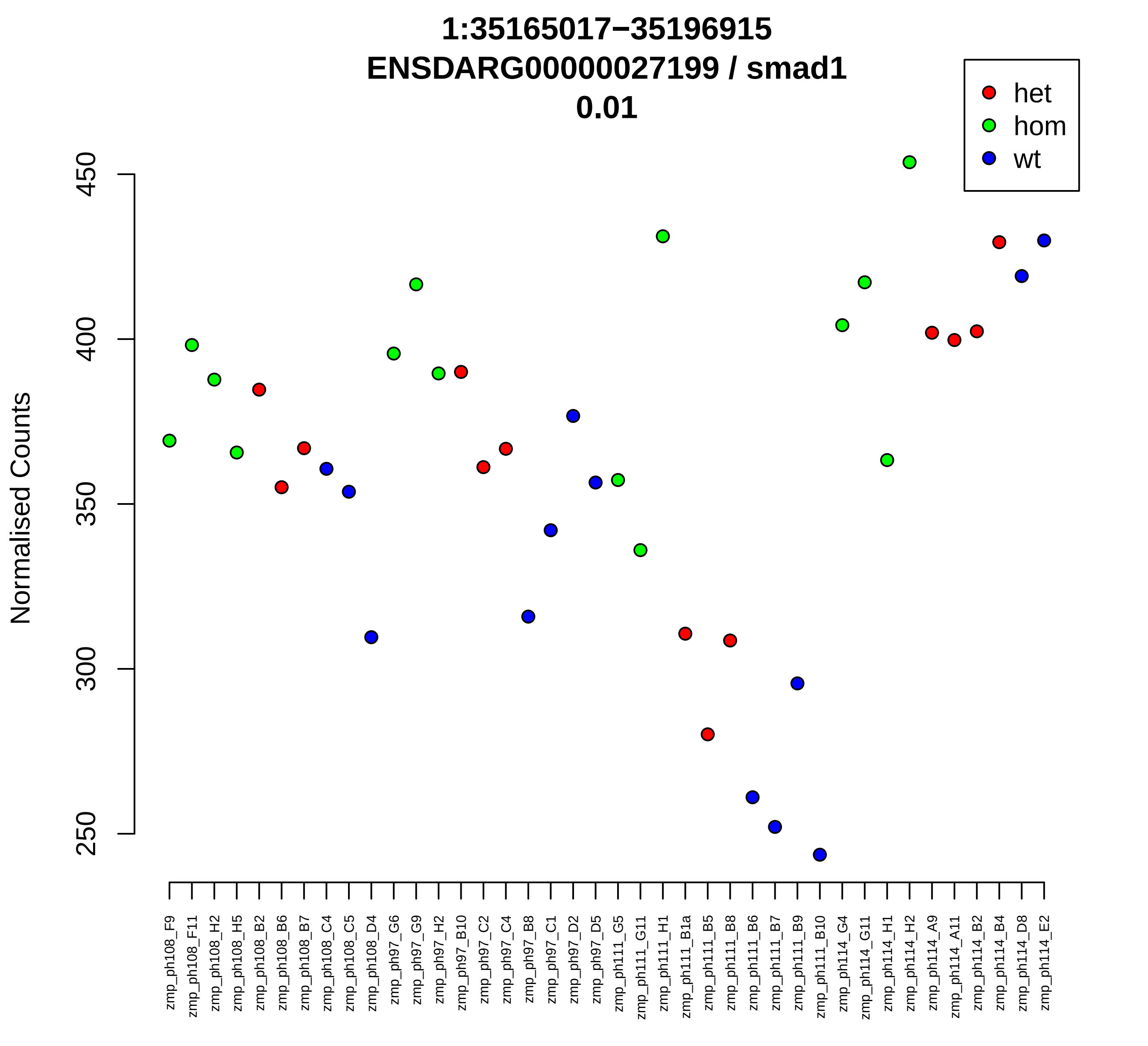

1:35165017−35196915
ENSDARG00000027199 / smad1
0.01
het
hom
wt
Normalised Counts
250
300
350
400
450
zmp_ph108_F9
zmp_ph108_F11
zmp_ph108_H2
zmp_ph108_H5
zmp_ph108_B2
zmp_ph108_B6
zmp_ph108_B7
zmp_ph108_C4
zmp_ph108_C5
zmp_ph108_D4
zmp_ph97_G6
zmp_ph97_G9
zmp_ph97_H2
zmp_ph97_B10
zmp_ph97_C2
zmp_ph97_C4
zmp_ph97_B8
zmp_ph97_C1
zmp_ph97_D2
zmp_ph97_D5
zmp_ph111_G5
zmp_ph111_G11
zmp_ph111_H1
zmp_ph111_B1a
zmp_ph111_B5
zmp_ph111_B8
zmp_ph111_B6
zmp_ph111_B7
zmp_ph111_B9
zmp_ph111_B10
zmp_ph114_G4
zmp_ph114_G11
zmp_ph114_H1
zmp_ph114_H2
zmp_ph114_A9
zmp_ph114_A11
zmp_ph114_B2
zmp_ph114_B4
zmp_ph114_D8
zmp_ph114_E2

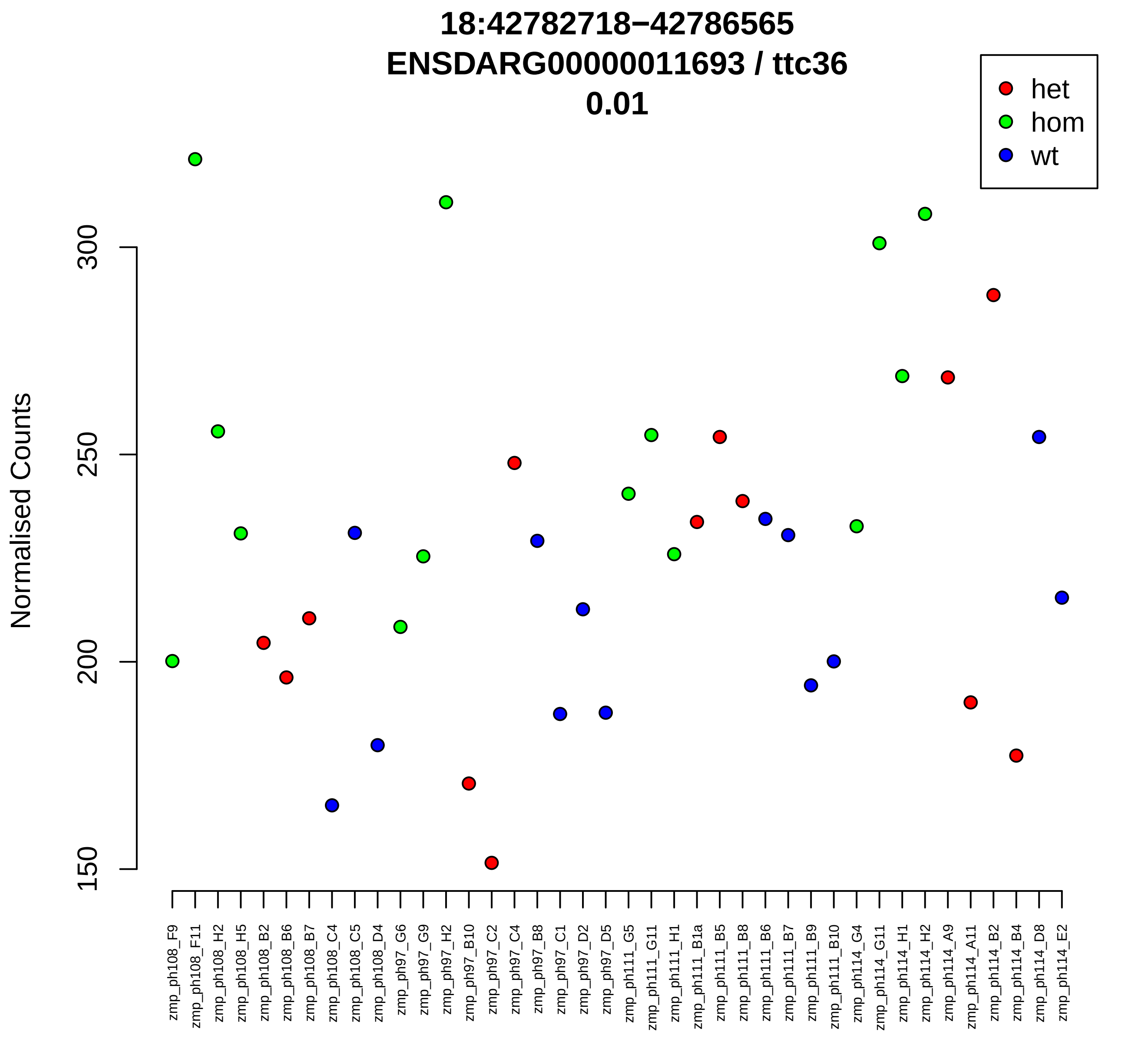

18:42782718−42786565
ENSDARG00000011693 / ttc36
0.01
het
hom
wt
Normalised Counts
150
200
250
300
zmp_ph108_F9
zmp_ph108_F11
zmp_ph108_H2
zmp_ph108_H5
zmp_ph108_B2
zmp_ph108_B6
zmp_ph108_B7
zmp_ph108_C4
zmp_ph108_C5
zmp_ph108_D4
zmp_ph97_G6
zmp_ph97_G9
zmp_ph97_H2
zmp_ph97_B10
zmp_ph97_C2
zmp_ph97_C4
zmp_ph97_B8
zmp_ph97_C1
zmp_ph97_D2
zmp_ph97_D5
zmp_ph111_G5
zmp_ph111_G11
zmp_ph111_H1
zmp_ph111_B1a
zmp_ph111_B5
zmp_ph111_B8
zmp_ph111_B6
zmp_ph111_B7
zmp_ph111_B9
zmp_ph111_B10
zmp_ph114_G4
zmp_ph114_G11
zmp_ph114_H1
zmp_ph114_H2
zmp_ph114_A9
zmp_ph114_A11
zmp_ph114_B2
zmp_ph114_B4
zmp_ph114_D8
zmp_ph114_E2

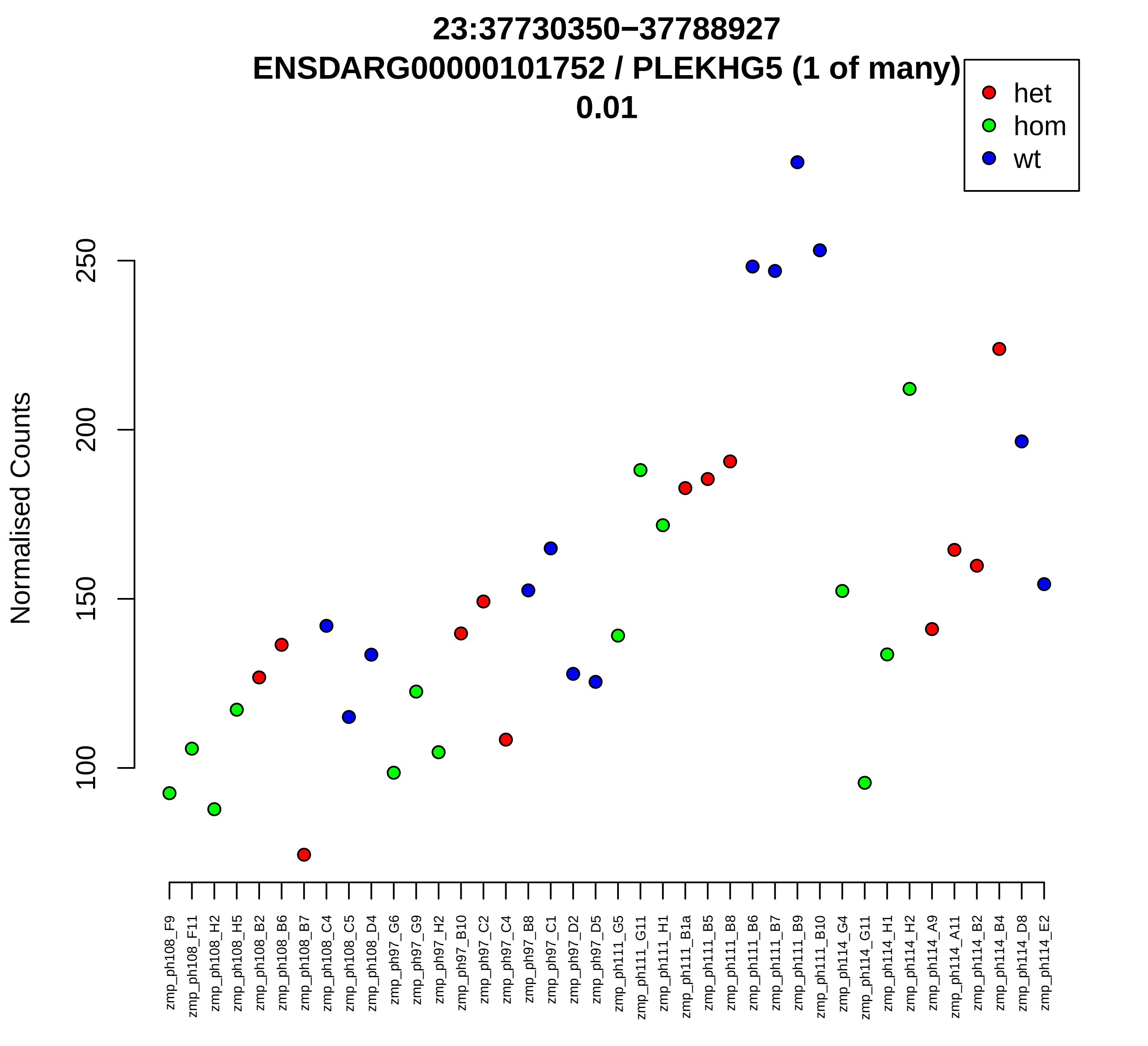

23:37730350−37788927
ENSDARG00000101752 / PLEKHG5 (1 of many)
0.01
het
hom
wt
Normalised Counts
100
150
200
250
zmp_ph108_F9
zmp_ph108_F11
zmp_ph108_H2
zmp_ph108_H5
zmp_ph108_B2
zmp_ph108_B6
zmp_ph108_B7
zmp_ph108_C4
zmp_ph108_C5
zmp_ph108_D4
zmp_ph97_G6
zmp_ph97_G9
zmp_ph97_H2
zmp_ph97_B10
zmp_ph97_C2
zmp_ph97_C4
zmp_ph97_B8
zmp_ph97_C1
zmp_ph97_D2
zmp_ph97_D5
zmp_ph111_G5
zmp_ph111_G11
zmp_ph111_H1
zmp_ph111_B1a
zmp_ph111_B5
zmp_ph111_B8
zmp_ph111_B6
zmp_ph111_B7
zmp_ph111_B9
zmp_ph111_B10
zmp_ph114_G4
zmp_ph114_G11
zmp_ph114_H1
zmp_ph114_H2
zmp_ph114_A9
zmp_ph114_A11
zmp_ph114_B2
zmp_ph114_B4
zmp_ph114_D8
zmp_ph114_E2

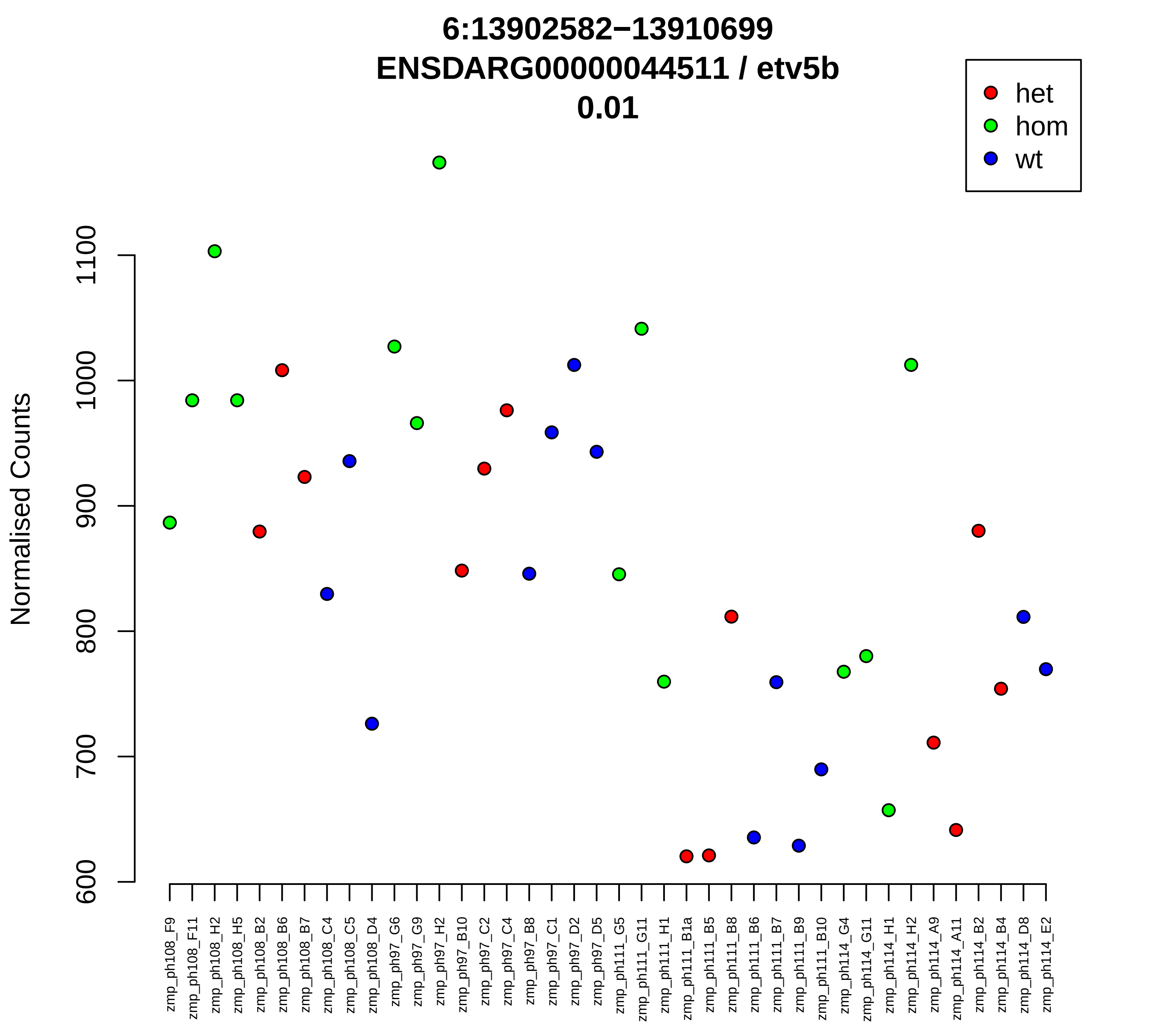
6:13902582−13910699
ENSDARG00000044511 / etv5b
0.01
het
hom
wt
Normalised Counts
600
700
800
900
1000
1100
zmp_ph108_F9
zmp_ph108_F11
zmp_ph108_H2
zmp_ph108_H5
zmp_ph108_B2
zmp_ph108_B6
zmp_ph108_B7
zmp_ph108_C4
zmp_ph108_C5
zmp_ph108_D4
zmp_ph97_G6
zmp_ph97_G9
zmp_ph97_H2
zmp_ph97_B10
zmp_ph97_C2
zmp_ph97_C4
zmp_ph97_B8
zmp_ph97_C1
zmp_ph97_D2
zmp_ph97_D5
zmp_ph111_G5
zmp_ph111_G11
zmp_ph111_H1
zmp_ph111_B1a
zmp_ph111_B5
zmp_ph111_B8
zmp_ph111_B6
zmp_ph111_B7
zmp_ph111_B9
zmp_ph111_B10
zmp_ph114_G4
zmp_ph114_G11
zmp_ph114_H1
zmp_ph114_H2
zmp_ph114_A9
zmp_ph114_A11
zmp_ph114_B2
zmp_ph114_B4
zmp_ph114_D8
zmp_ph114_E2

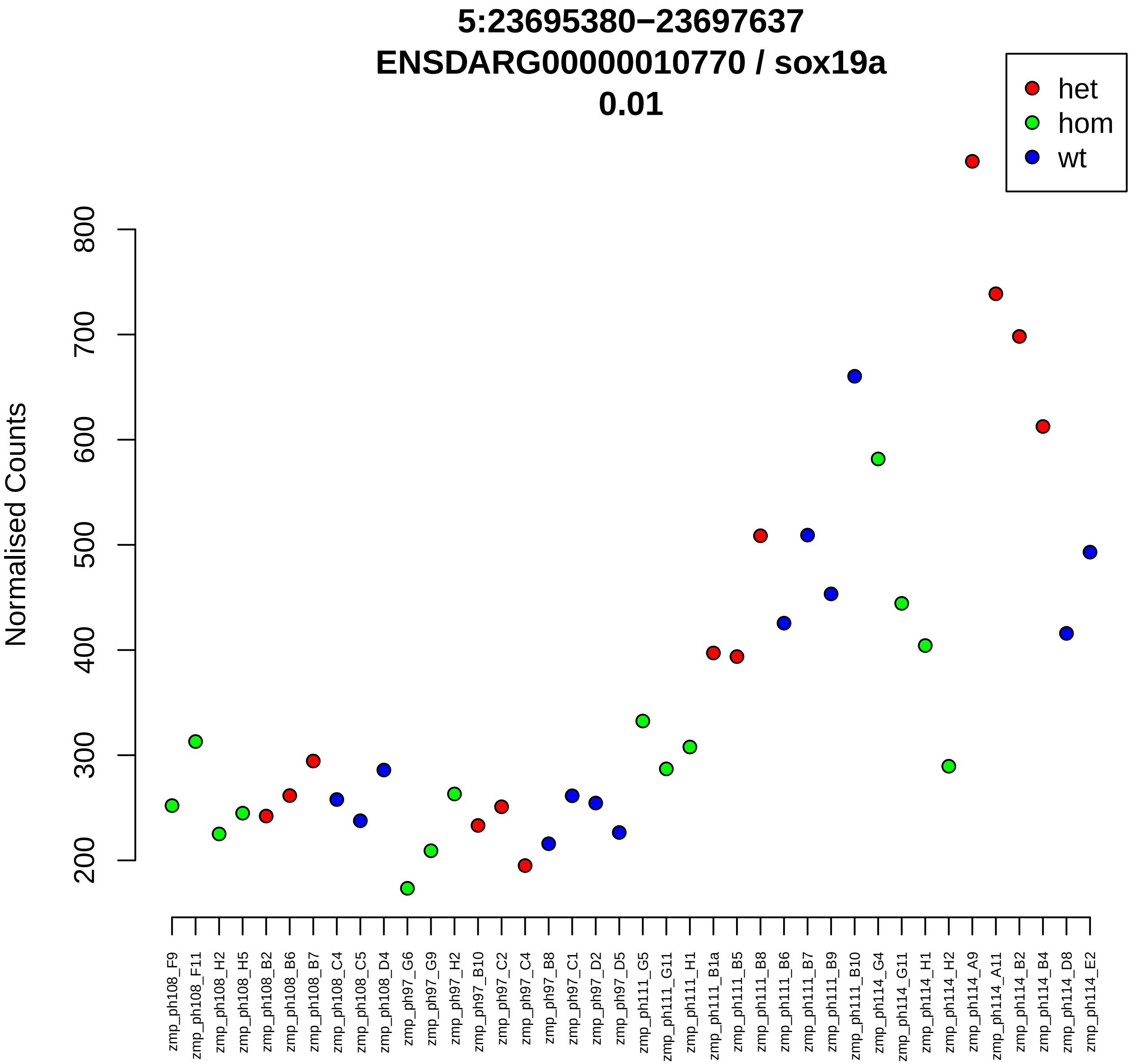

5:23695380−23697637
ENSDARG00000010770 / sox19a
0.01
het
hom
wt
Normalised Counts
200
300
400
500
600
700
800
zmp_ph108_F9
zmp_ph108_F11
zmp_ph108_H2
zmp_ph108_H5
zmp_ph108_B2
zmp_ph108_B6
zmp_ph108_B7
zmp_ph108_C4
zmp_ph108_C5
zmp_ph108_D4
zmp_ph97_G6
zmp_ph97_G9
zmp_ph97_H2
zmp_ph97_B10
zmp_ph97_C2
zmp_ph97_C4
zmp_ph97_B8
zmp_ph97_C1
zmp_ph97_D2
zmp_ph97_D5
zmp_ph111_G5
zmp_ph111_G11
zmp_ph111_H1
zmp_ph111_B1a
zmp_ph111_B5
zmp_ph111_B8
zmp_ph111_B6
zmp_ph111_B7
zmp_ph111_B9
zmp_ph111_B10
zmp_ph114_G4
zmp_ph114_G11
zmp_ph114_H1
zmp_ph114_H2
zmp_ph114_A9
zmp_ph114_A11
zmp_ph114_B2
zmp_ph114_B4
zmp_ph114_D8
zmp_ph114_E2

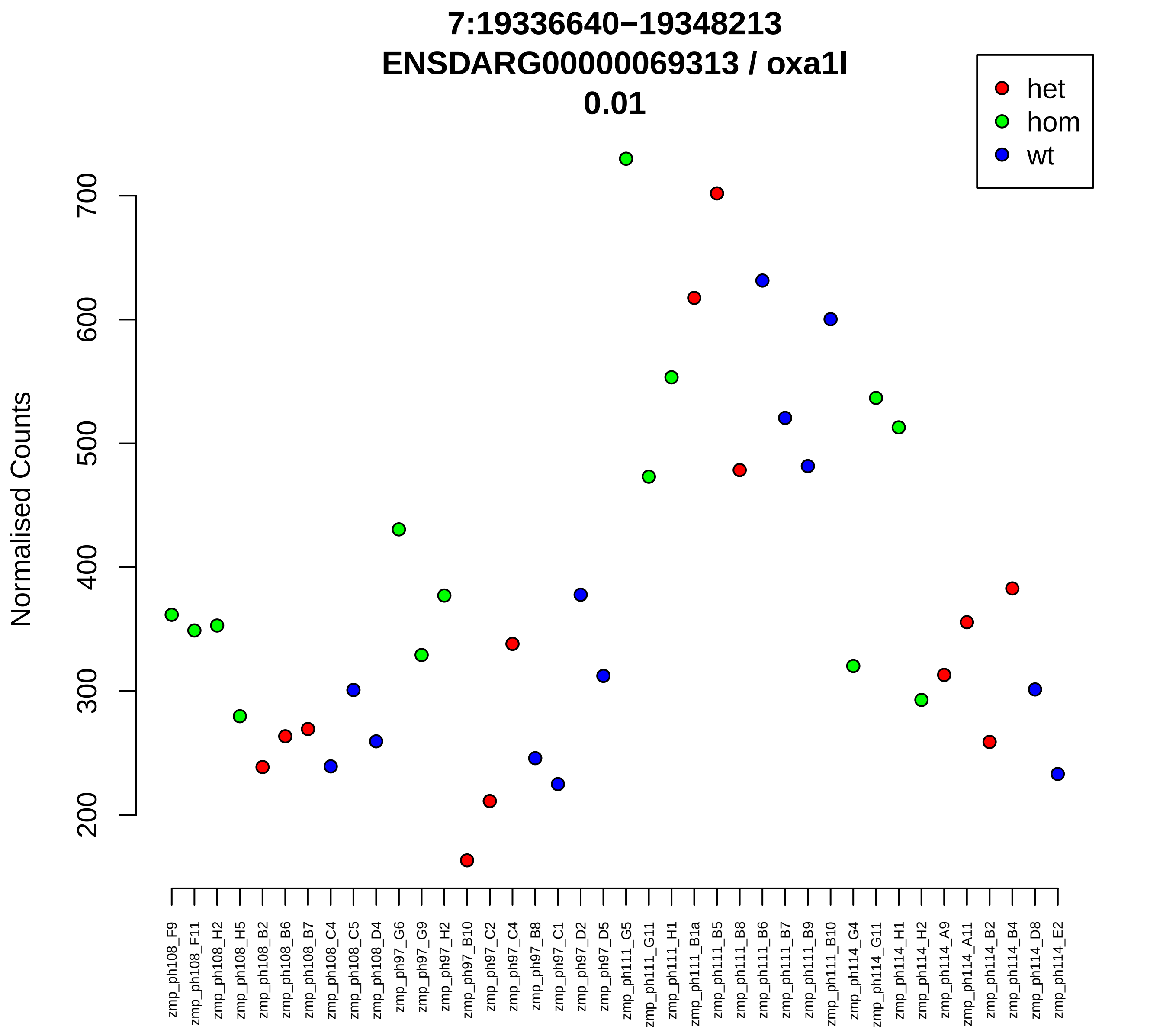
7:19336640−19348213
ENSDARG00000069313 / oxa1l
0.01
het
hom
wt
Normalised Counts
200
300
400
500
600
700
zmp_ph108_F9
zmp_ph108_F11
zmp_ph108_H2
zmp_ph108_H5
zmp_ph108_B2
zmp_ph108_B6
zmp_ph108_B7
zmp_ph108_C4
zmp_ph108_C5
zmp_ph108_D4
zmp_ph97_G6
zmp_ph97_G9
zmp_ph97_H2
zmp_ph97_B10
zmp_ph97_C2
zmp_ph97_C4
zmp_ph97_B8
zmp_ph97_C1
zmp_ph97_D2
zmp_ph97_D5
zmp_ph111_G5
zmp_ph111_G11
zmp_ph111_H1
zmp_ph111_B1a
zmp_ph111_B5
zmp_ph111_B8
zmp_ph111_B6
zmp_ph111_B7
zmp_ph111_B9
zmp_ph111_B10
zmp_ph114_G4
zmp_ph114_G11
zmp_ph114_H1
zmp_ph114_H2
zmp_ph114_A9
zmp_ph114_A11
zmp_ph114_B2
zmp_ph114_B4
zmp_ph114_D8
zmp_ph114_E2

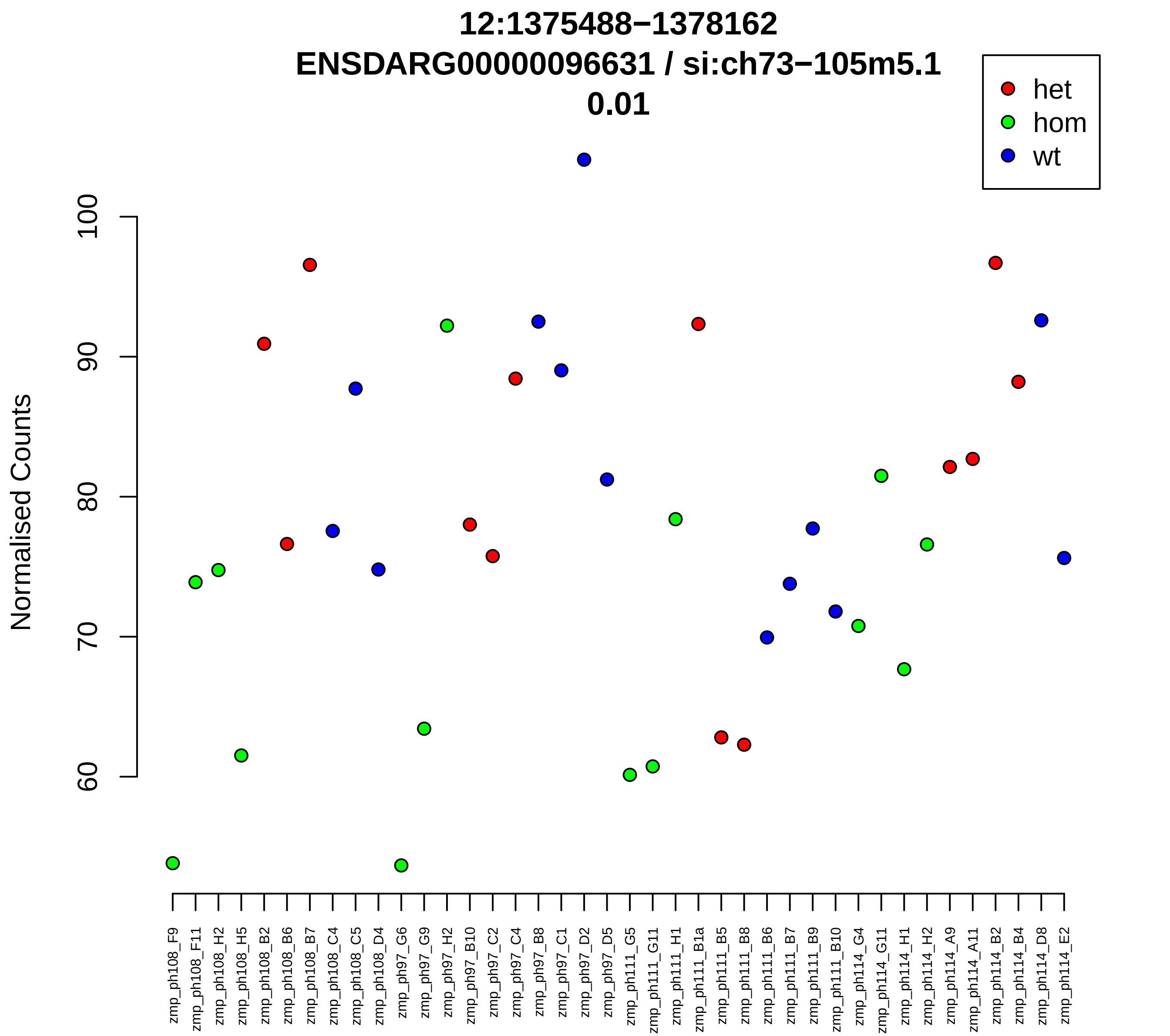

12:1375488−1378162
ENSDARG00000096631 / si:ch73−105m5.1
0.01
het
hom
wt
Normalised Counts
100
90
80
70
60
zmp_ph108_F9
zmp_ph108_F11
zmp_ph108_H2
zmp_ph108_H5
zmp_ph108_B2
zmp_ph108_B6
zmp_ph108_B7
zmp_ph108_C4
zmp_ph108_C5
zmp_ph108_D4
zmp_ph97_G6
zmp_ph97_G9
zmp_ph97_H2
zmp_ph97_B10
zmp_ph97_C2
zmp_ph97_C4
zmp_ph97_B8
zmp_ph97_C1
zmp_ph97_D2
zmp_ph97_D5
zmp_ph111_G5
zmp_ph111_G11
zmp_ph111_H1
zmp_ph111_B1a
zmp_ph111_B5
zmp_ph111_B8
zmp_ph111_B6
zmp_ph111_B7
zmp_ph111_B9
zmp_ph111_B10
zmp_ph114_G4
zmp_ph114_G11
zmp_ph114_H1
zmp_ph114_H2
zmp_ph114_A9
zmp_ph114_A11
zmp_ph114_B2
zmp_ph114_B4
zmp_ph114_D8
zmp_ph114_E2

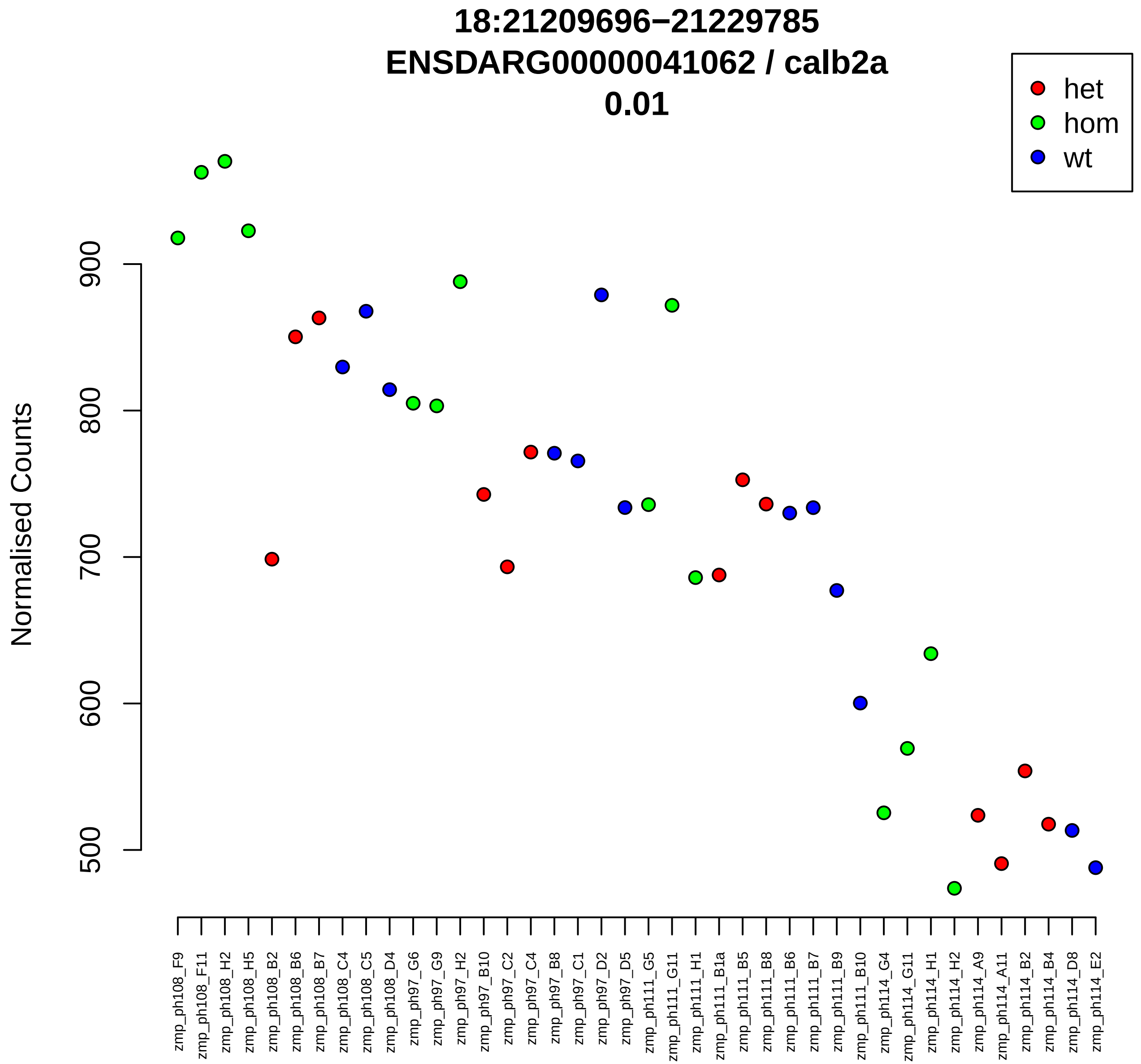
18:21209696−21229785
ENSDARG00000041062 / calb2a
0.01
het
hom
wt
Normalised Counts
500
600
700
800
900
zmp_ph108_F9
zmp_ph108_F11
zmp_ph108_H2
zmp_ph108_H5
zmp_ph108_B2
zmp_ph108_B6
zmp_ph108_B7
zmp_ph108_C4
zmp_ph108_C5
zmp_ph108_D4
zmp_ph97_G6
zmp_ph97_G9
zmp_ph97_H2
zmp_ph97_B10
zmp_ph97_C2
zmp_ph97_C4
zmp_ph97_B8
zmp_ph97_C1
zmp_ph97_D2
zmp_ph97_D5
zmp_ph111_G5
zmp_ph111_G11
zmp_ph111_H1
zmp_ph111_B1a
zmp_ph111_B5
zmp_ph111_B8
zmp_ph111_B6
zmp_ph111_B7
zmp_ph111_B9
zmp_ph111_B10
zmp_ph114_G4
zmp_ph114_G11
zmp_ph114_H1
zmp_ph114_H2
zmp_ph114_A9
zmp_ph114_A11
zmp_ph114_B2
zmp_ph114_B4
zmp_ph114_D8
zmp_ph114_E2

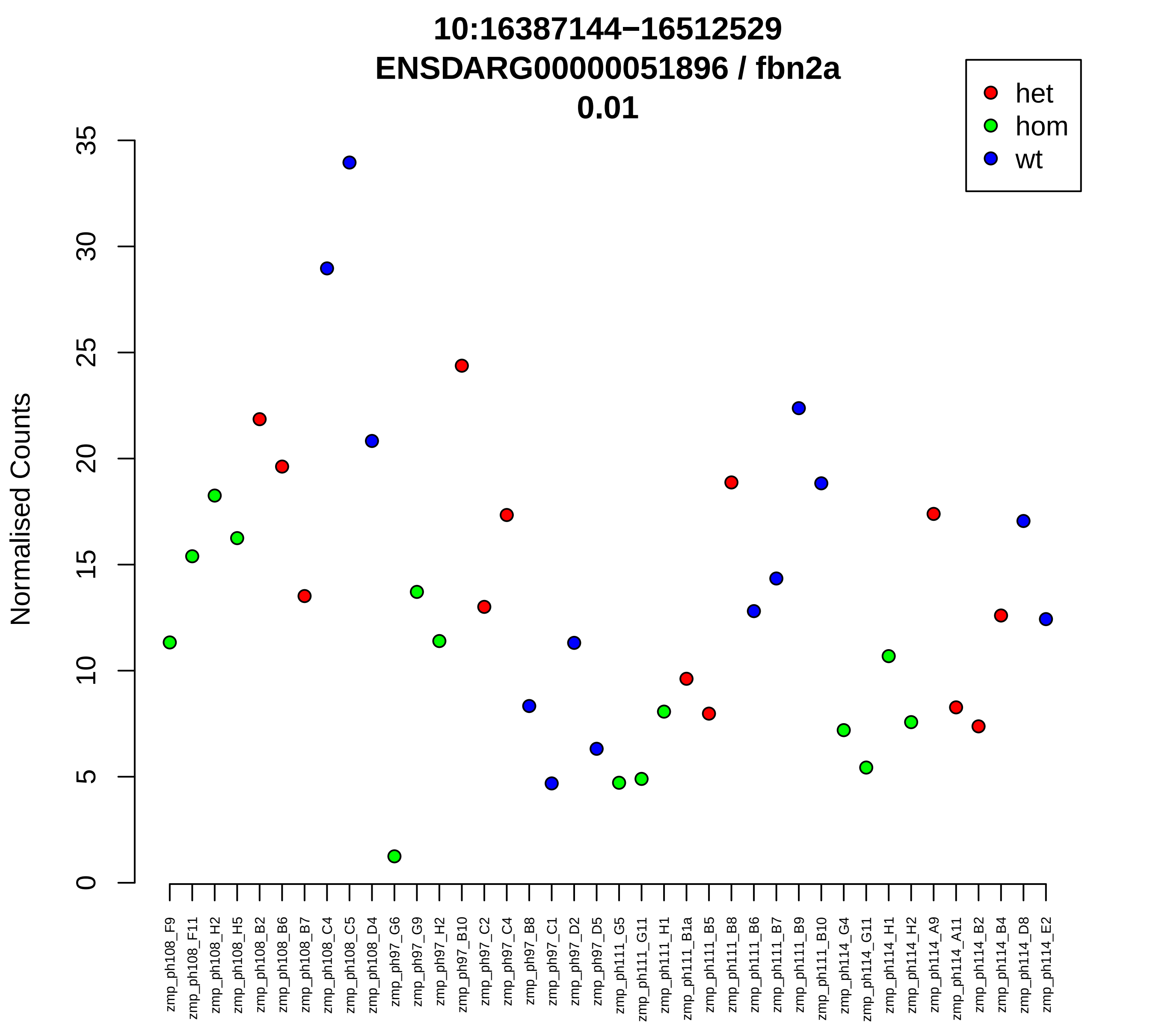

10:16387144−16512529
ENSDARG00000051896 / fbn2a
0.01
het
hom
wt
Normalised Counts
0
5
10
15
20
25
30
35
zmp_ph108_F9
zmp_ph108_F11
zmp_ph108_H2
zmp_ph108_H5
zmp_ph108_B2
zmp_ph108_B6
zmp_ph108_B7
zmp_ph108_C4
zmp_ph108_C5
zmp_ph108_D4
zmp_ph97_G6
zmp_ph97_G9
zmp_ph97_H2
zmp_ph97_B10
zmp_ph97_C2
zmp_ph97_C4
zmp_ph97_B8
zmp_ph97_C1
zmp_ph97_D2
zmp_ph97_D5
zmp_ph111_G5
zmp_ph111_G11
zmp_ph111_H1
zmp_ph111_B1a
zmp_ph111_B5
zmp_ph111_B8
zmp_ph111_B6
zmp_ph111_B7
zmp_ph111_B9
zmp_ph111_B10
zmp_ph114_G4
zmp_ph114_G11
zmp_ph114_H1
zmp_ph114_H2
zmp_ph114_A9
zmp_ph114_A11
zmp_ph114_B2
zmp_ph114_B4
zmp_ph114_D8
zmp_ph114_E2

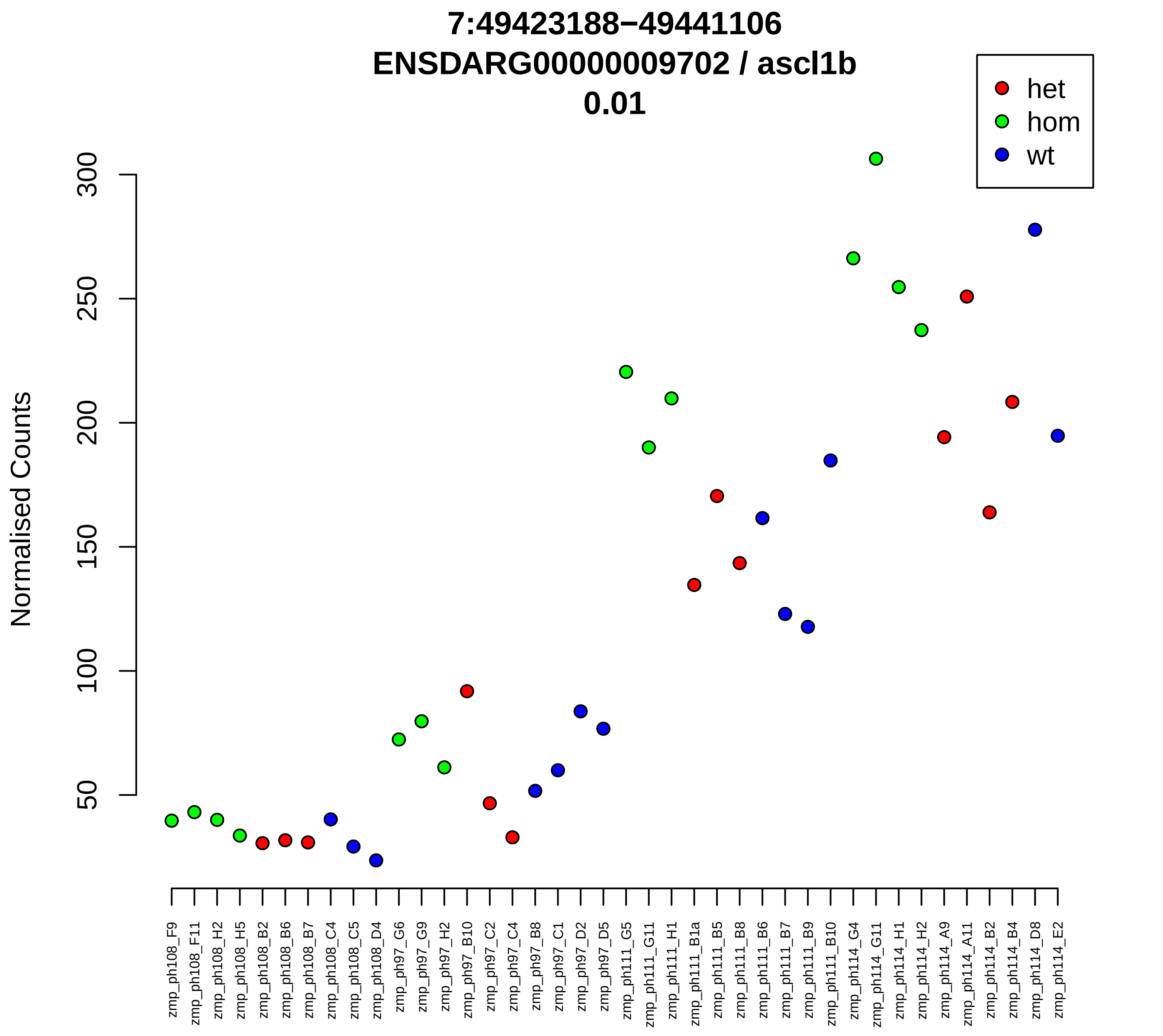
7:49423188−49441106
ENSDARG00000009702 / ascl1b
0.01
het
hom
wt
Normalised Counts
50
100
150
200
250
300
zmp_ph108_F9
zmp_ph108_F11
zmp_ph108_H2
zmp_ph108_H5
zmp_ph108_B2
zmp_ph108_B6
zmp_ph108_B7
zmp_ph108_C4
zmp_ph108_C5
zmp_ph108_D4
zmp_ph97_G6
zmp_ph97_G9
zmp_ph97_H2
zmp_ph97_B10
zmp_ph97_C2
zmp_ph97_C4
zmp_ph97_B8
zmp_ph97_C1
zmp_ph97_D2
zmp_ph97_D5
zmp_ph111_G5
zmp_ph111_G11
zmp_ph111_H1
zmp_ph111_B1a
zmp_ph111_B5
zmp_ph111_B8
zmp_ph111_B6
zmp_ph111_B7
zmp_ph111_B9
zmp_ph111_B10
zmp_ph114_G4
zmp_ph114_G11
zmp_ph114_H1
zmp_ph114_H2
zmp_ph114_A9
zmp_ph114_A11
zmp_ph114_B2
zmp_ph114_B4
zmp_ph114_D8
zmp_ph114_E2

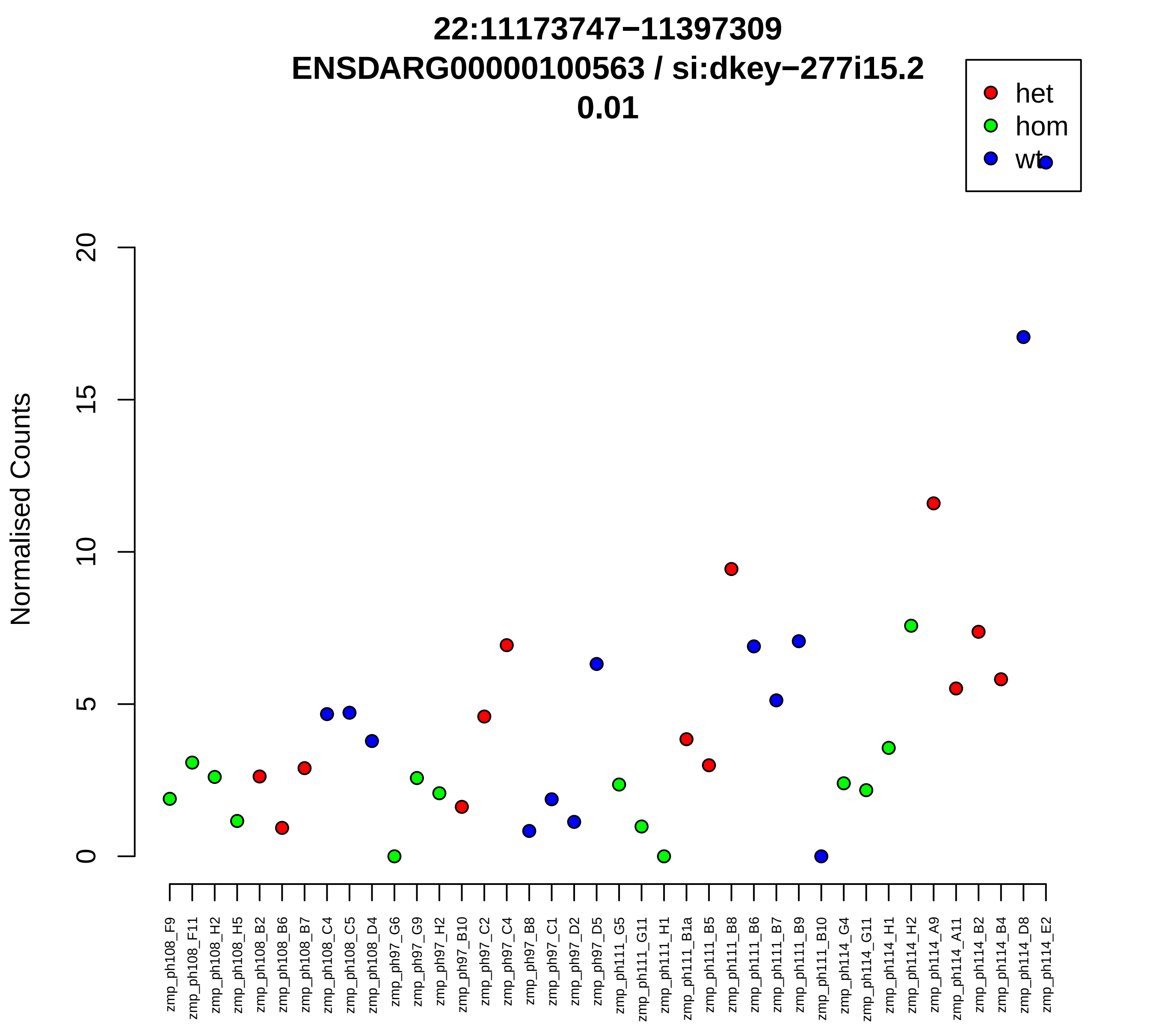

22:11173747−11397309
ENSDARG00000100563 / si:dkey−277i15.2
0.01
het
hom
wt
Normalised Counts
0
5
10
15
20
zmp_ph108_F9
zmp_ph108_F11
zmp_ph108_H2
zmp_ph108_H5
zmp_ph108_B2
zmp_ph108_B6
zmp_ph108_B7
zmp_ph108_C4
zmp_ph108_C5
zmp_ph108_D4
zmp_ph97_G6
zmp_ph97_G9
zmp_ph97_H2
zmp_ph97_B10
zmp_ph97_C2
zmp_ph97_C4
zmp_ph97_B8
zmp_ph97_C1
zmp_ph97_D2
zmp_ph97_D5
zmp_ph111_G5
zmp_ph111_G11
zmp_ph111_H1
zmp_ph111_B1a
zmp_ph111_B5
zmp_ph111_B8
zmp_ph111_B6
zmp_ph111_B7
zmp_ph111_B9
zmp_ph111_B10
zmp_ph114_G4
zmp_ph114_G11
zmp_ph114_H1
zmp_ph114_H2
zmp_ph114_A9
zmp_ph114_A11
zmp_ph114_B2
zmp_ph114_B4
zmp_ph114_D8
zmp_ph114_E2

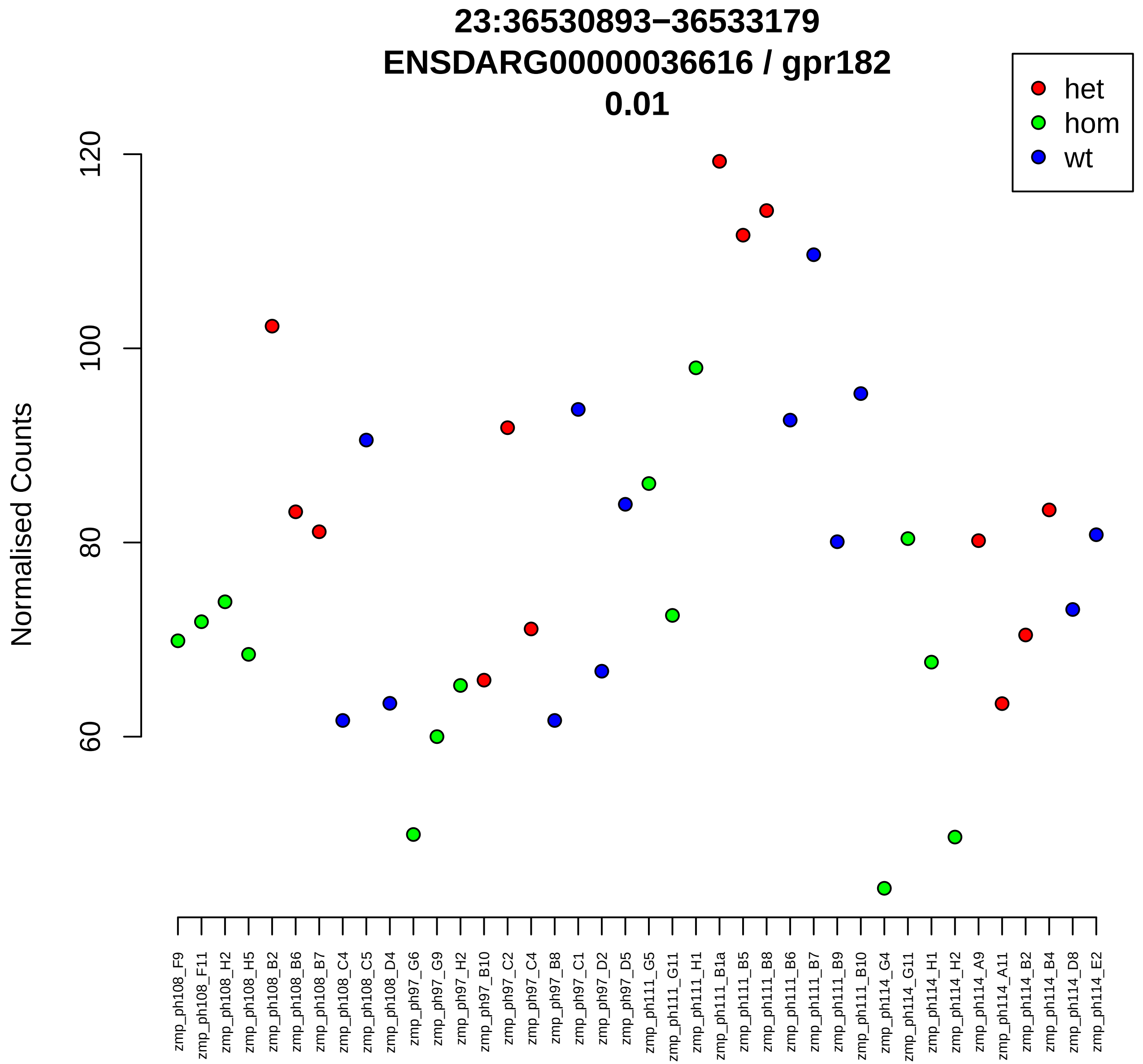

23:36530893−36533179
ENSDARG00000036616 / gpr182
0.01
het
hom
wt
Normalised Counts
60
80
100
120
zmp_ph108_F9
zmp_ph108_F11
zmp_ph108_H2
zmp_ph108_H5
zmp_ph108_B2
zmp_ph108_B6
zmp_ph108_B7
zmp_ph108_C4
zmp_ph108_C5
zmp_ph108_D4
zmp_ph97_G6
zmp_ph97_G9
zmp_ph97_H2
zmp_ph97_B10
zmp_ph97_C2
zmp_ph97_C4
zmp_ph97_B8
zmp_ph97_C1
zmp_ph97_D2
zmp_ph97_D5
zmp_ph111_G5
zmp_ph111_G11
zmp_ph111_H1
zmp_ph111_B1a
zmp_ph111_B5
zmp_ph111_B8
zmp_ph111_B6
zmp_ph111_B7
zmp_ph111_B9
zmp_ph111_B10
zmp_ph114_G4
zmp_ph114_G11
zmp_ph114_H1
zmp_ph114_H2
zmp_ph114_A9
zmp_ph114_A11
zmp_ph114_B2
zmp_ph114_B4
zmp_ph114_D8
zmp_ph114_E2

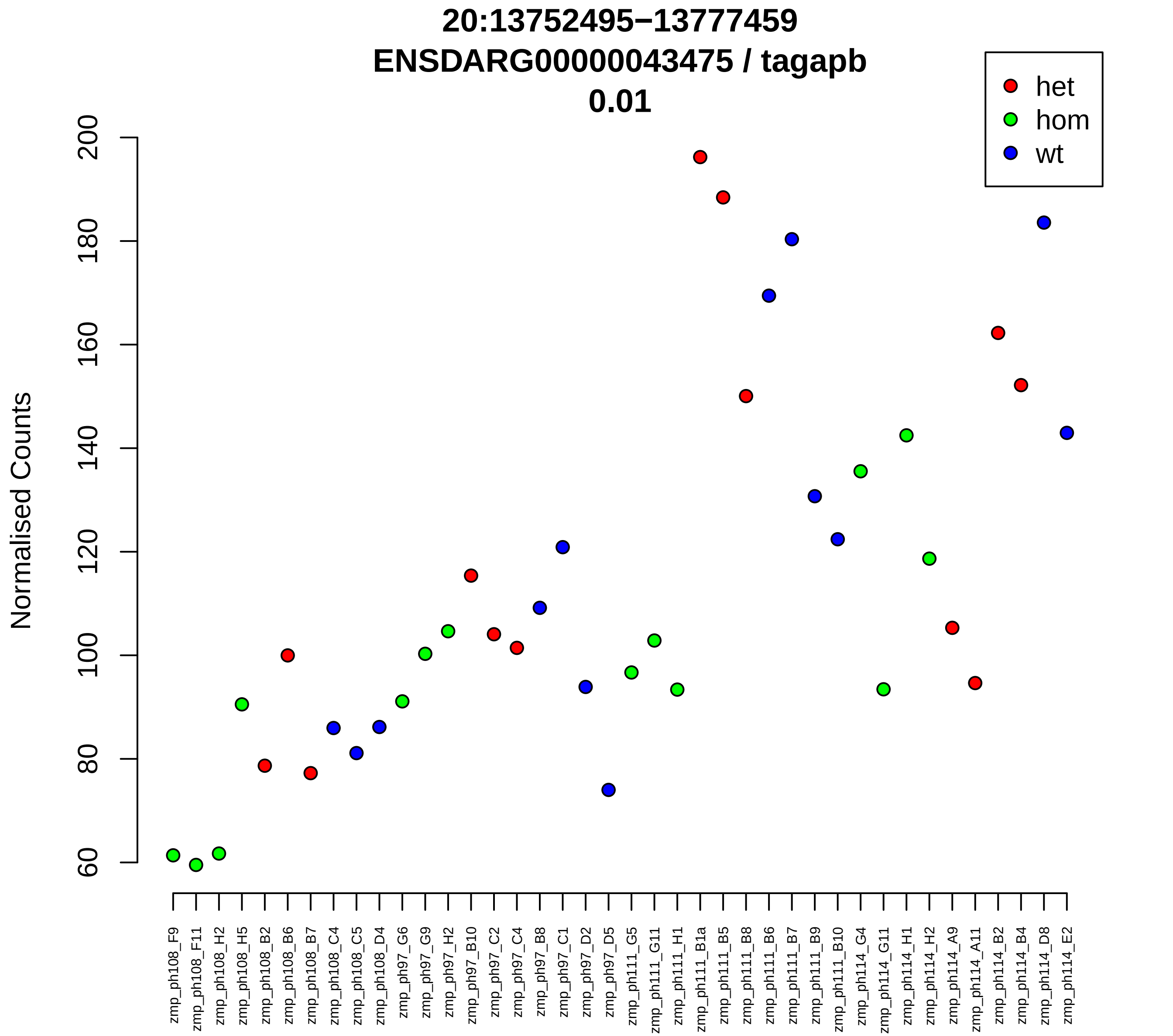
20:13752495−13777459
ENSDARG00000043475 / tagapb
0.01
Normalised Counts
het
hom
wt
60
80
100
120
140
160
180
200
zmp_ph108_F9
zmp_ph108_F11
zmp_ph108_H2
zmp_ph108_H5
zmp_ph108_B2
zmp_ph108_B6
zmp_ph108_B7
zmp_ph108_C4
zmp_ph108_C5
zmp_ph108_D4
zmp_ph97_G6
zmp_ph97_G9
zmp_ph97_H2
zmp_ph97_B10
zmp_ph97_C2
zmp_ph97_C4
zmp_ph97_B8
zmp_ph97_C1
zmp_ph97_D2
zmp_ph97_D5
zmp_ph111_G5
zmp_ph111_G11
zmp_ph111_H1
zmp_ph111_B1a
zmp_ph111_B5
zmp_ph111_B8
zmp_ph111_B6
zmp_ph111_B7
zmp_ph111_B9
zmp_ph111_B10
zmp_ph114_G4
zmp_ph114_G11
zmp_ph114_H1
zmp_ph114_H2
zmp_ph114_A9
zmp_ph114_A11
zmp_ph114_B2
zmp_ph114_B4
zmp_ph114_D8
zmp_ph114_E2

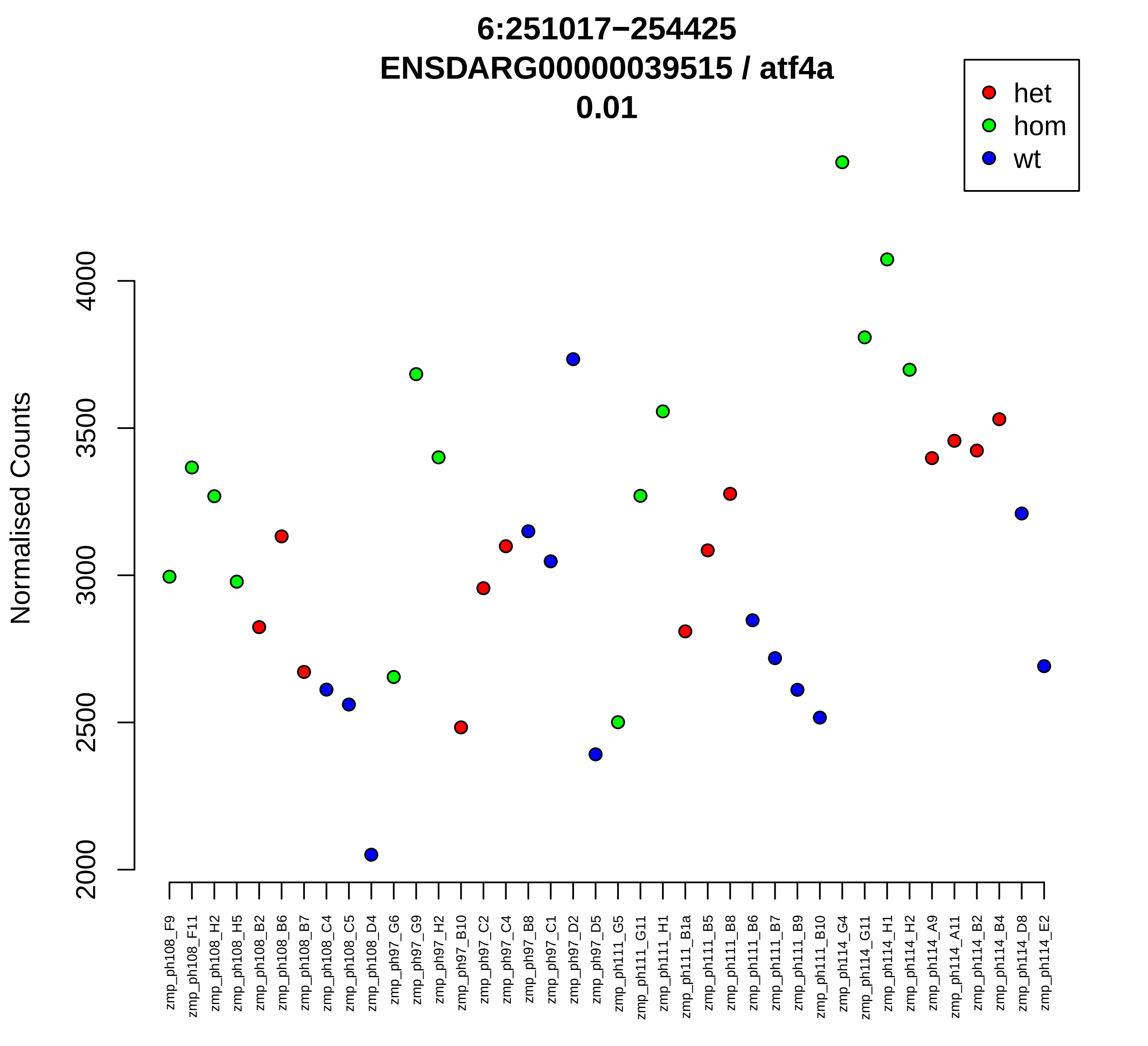
6:251017−254425
ENSDARG00000039515 / atf4a
0.01
het
hom
wt
Normalised Counts
2000
2500
3000
3500
4000
zmp_ph108_F9
zmp_ph108_F11
zmp_ph108_H2
zmp_ph108_H5
zmp_ph108_B2
zmp_ph108_B6
zmp_ph108_B7
zmp_ph108_C4
zmp_ph108_C5
zmp_ph108_D4
zmp_ph97_G6
zmp_ph97_G9
zmp_ph97_H2
zmp_ph97_B10
zmp_ph97_C2
zmp_ph97_C4
zmp_ph97_B8
zmp_ph97_C1
zmp_ph97_D2
zmp_ph97_D5
zmp_ph111_G5
zmp_ph111_G11
zmp_ph111_H1
zmp_ph111_B1a
zmp_ph111_B5
zmp_ph111_B8
zmp_ph111_B6
zmp_ph111_B7
zmp_ph111_B9
zmp_ph111_B10
zmp_ph114_G4
zmp_ph114_G11
zmp_ph114_H1
zmp_ph114_H2
zmp_ph114_A9
zmp_ph114_A11
zmp_ph114_B2
zmp_ph114_B4
zmp_ph114_D8
zmp_ph114_E2

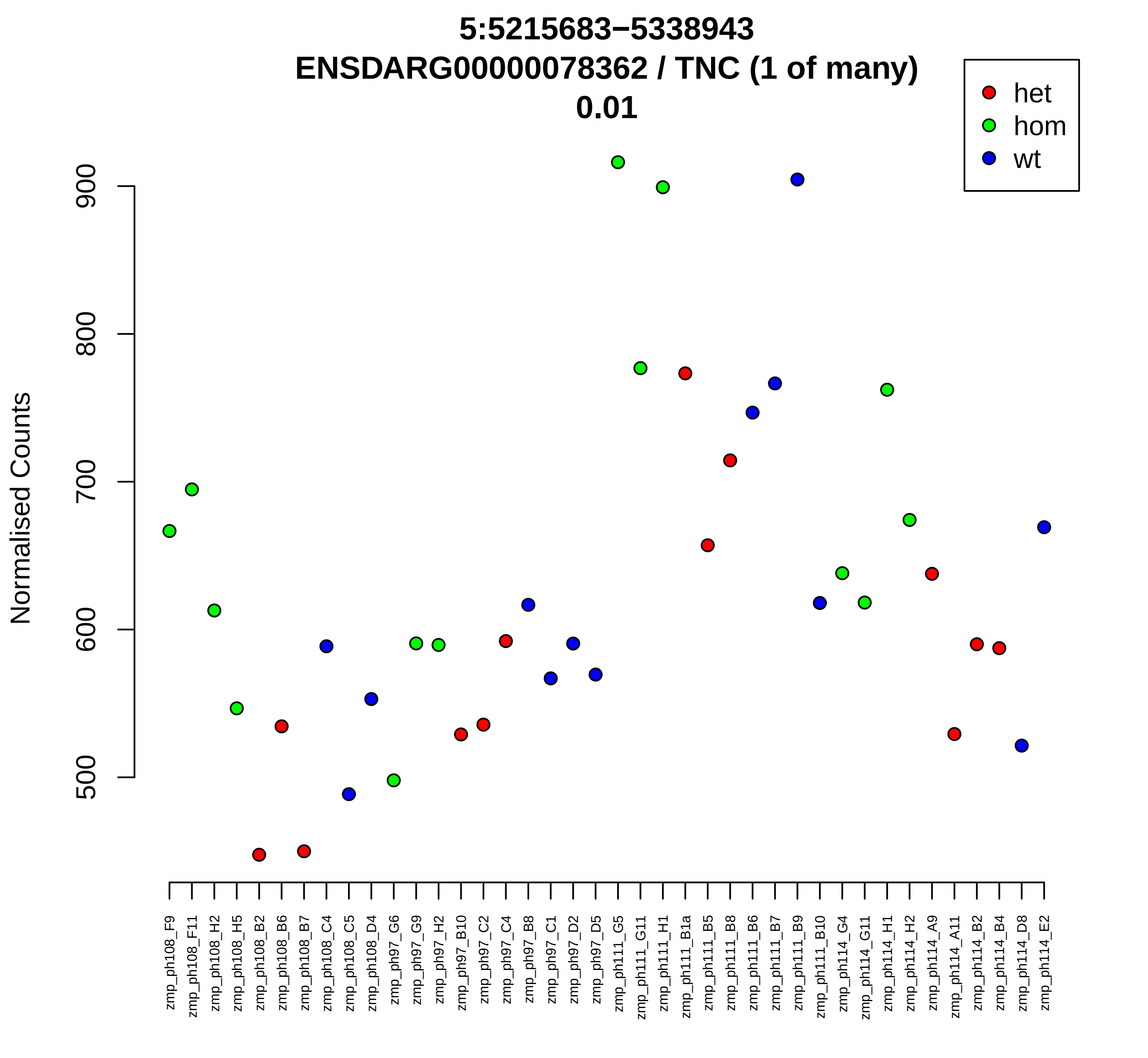

5:5215683−5338943
ENSDARG00000078362 / TNC (1 of many)
0.01
het
hom
wt
Normalised Counts
900
800
700
600
500
zmp_ph108_F9
zmp_ph108_F11
zmp_ph108_H2
zmp_ph108_H5
zmp_ph108_B2
zmp_ph108_B6
zmp_ph108_B7
zmp_ph108_C4
zmp_ph108_C5
zmp_ph108_D4
zmp_ph97_G6
zmp_ph97_G9
zmp_ph97_H2
zmp_ph97_B10
zmp_ph97_C2
zmp_ph97_C4
zmp_ph97_B8
zmp_ph97_C1
zmp_ph97_D2
zmp_ph97_D5
zmp_ph111_G5
zmp_ph111_G11
zmp_ph111_H1
zmp_ph111_B1a
zmp_ph111_B5
zmp_ph111_B8
zmp_ph111_B6
zmp_ph111_B7
zmp_ph111_B9
zmp_ph111_B10
zmp_ph114_G4
zmp_ph114_G11
zmp_ph114_H1
zmp_ph114_H2
zmp_ph114_A9
zmp_ph114_A11
zmp_ph114_B2
zmp_ph114_B4
zmp_ph114_D8
zmp_ph114_E2

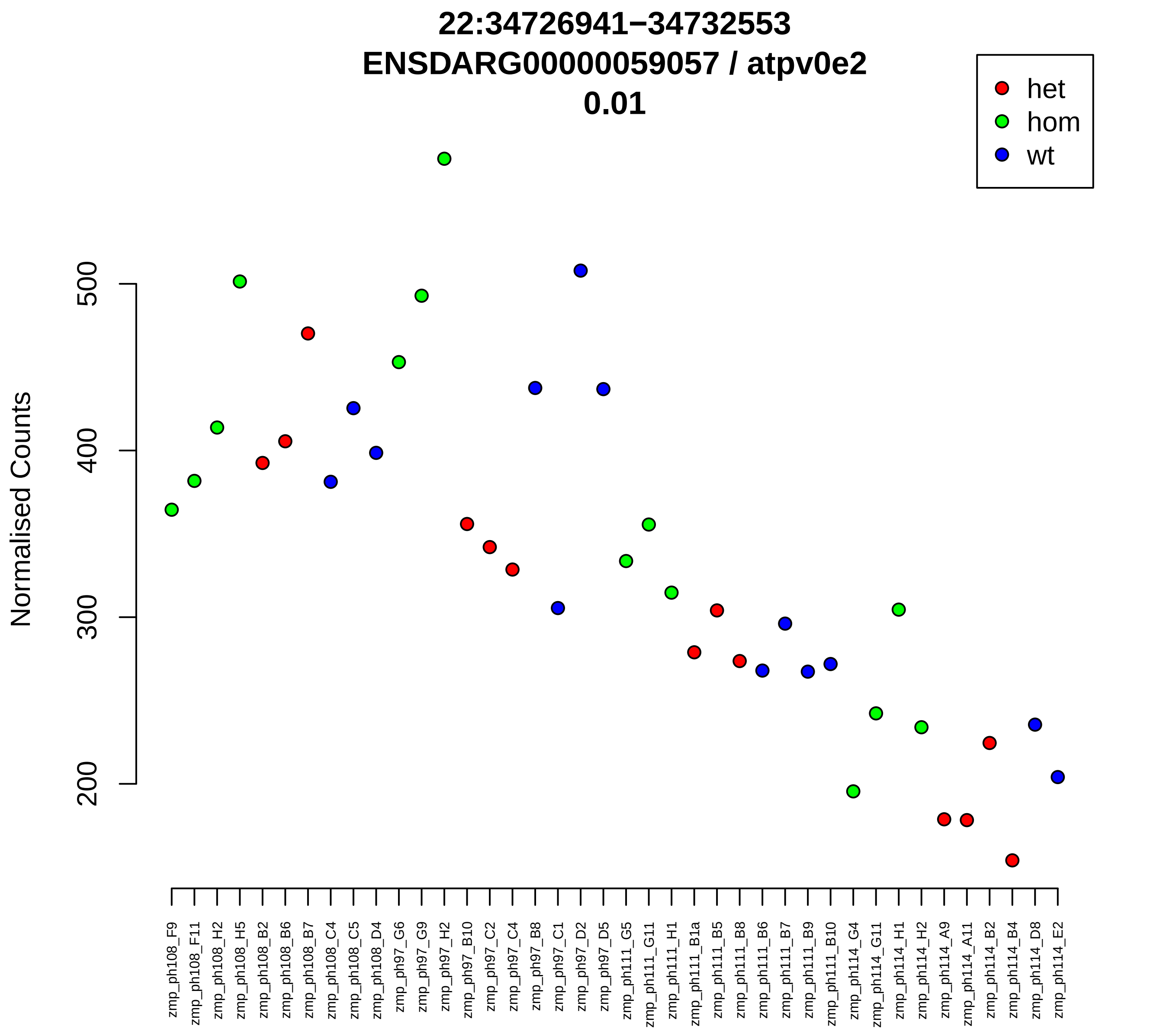

22:34726941−34732553
ENSDARG00000059057 / atpv0e2
0.01
het
hom
wt
Normalised Counts
200
300
400
500
zmp_ph108_F9
zmp_ph108_F11
zmp_ph108_H2
zmp_ph108_H5
zmp_ph108_B2
zmp_ph108_B6
zmp_ph108_B7
zmp_ph108_C4
zmp_ph108_C5
zmp_ph108_D4
zmp_ph97_G6
zmp_ph97_G9
zmp_ph97_H2
zmp_ph97_B10
zmp_ph97_C2
zmp_ph97_C4
zmp_ph97_B8
zmp_ph97_C1
zmp_ph97_D2
zmp_ph97_D5
zmp_ph111_G5
zmp_ph111_G11
zmp_ph111_H1
zmp_ph111_B1a
zmp_ph111_B5
zmp_ph111_B8
zmp_ph111_B6
zmp_ph111_B7
zmp_ph111_B9
zmp_ph111_B10
zmp_ph114_G4
zmp_ph114_G11
zmp_ph114_H1
zmp_ph114_H2
zmp_ph114_A9
zmp_ph114_A11
zmp_ph114_B2
zmp_ph114_B4
zmp_ph114_D8
zmp_ph114_E2

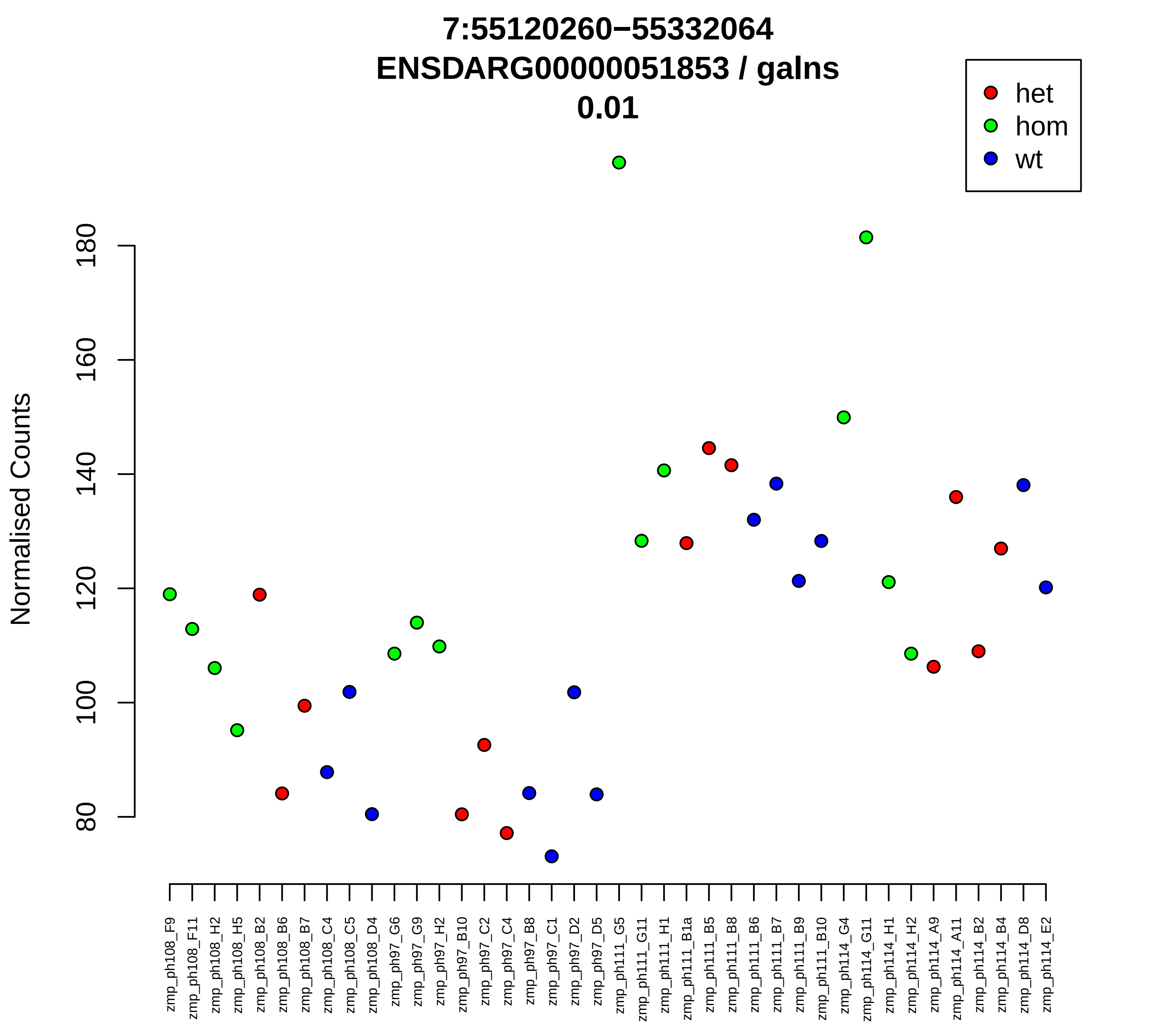
7:55120260−55332064
ENSDARG00000051853 / galns
0.01
het
hom
wt
Normalised Counts
80
100
120
140
160
180
zmp_ph108_F9
zmp_ph108_F11
zmp_ph108_H2
zmp_ph108_H5
zmp_ph108_B2
zmp_ph108_B6
zmp_ph108_B7
zmp_ph108_C4
zmp_ph108_C5
zmp_ph108_D4
zmp_ph97_G6
zmp_ph97_G9
zmp_ph97_H2
zmp_ph97_B10
zmp_ph97_C2
zmp_ph97_C4
zmp_ph97_B8
zmp_ph97_C1
zmp_ph97_D2
zmp_ph97_D5
zmp_ph111_G5
zmp_ph111_G11
zmp_ph111_H1
zmp_ph111_B1a
zmp_ph111_B5
zmp_ph111_B8
zmp_ph111_B6
zmp_ph111_B7
zmp_ph111_B9
zmp_ph111_B10
zmp_ph114_G4
zmp_ph114_G11
zmp_ph114_H1
zmp_ph114_H2
zmp_ph114_A9
zmp_ph114_A11
zmp_ph114_B2
zmp_ph114_B4
zmp_ph114_D8
zmp_ph114_E2

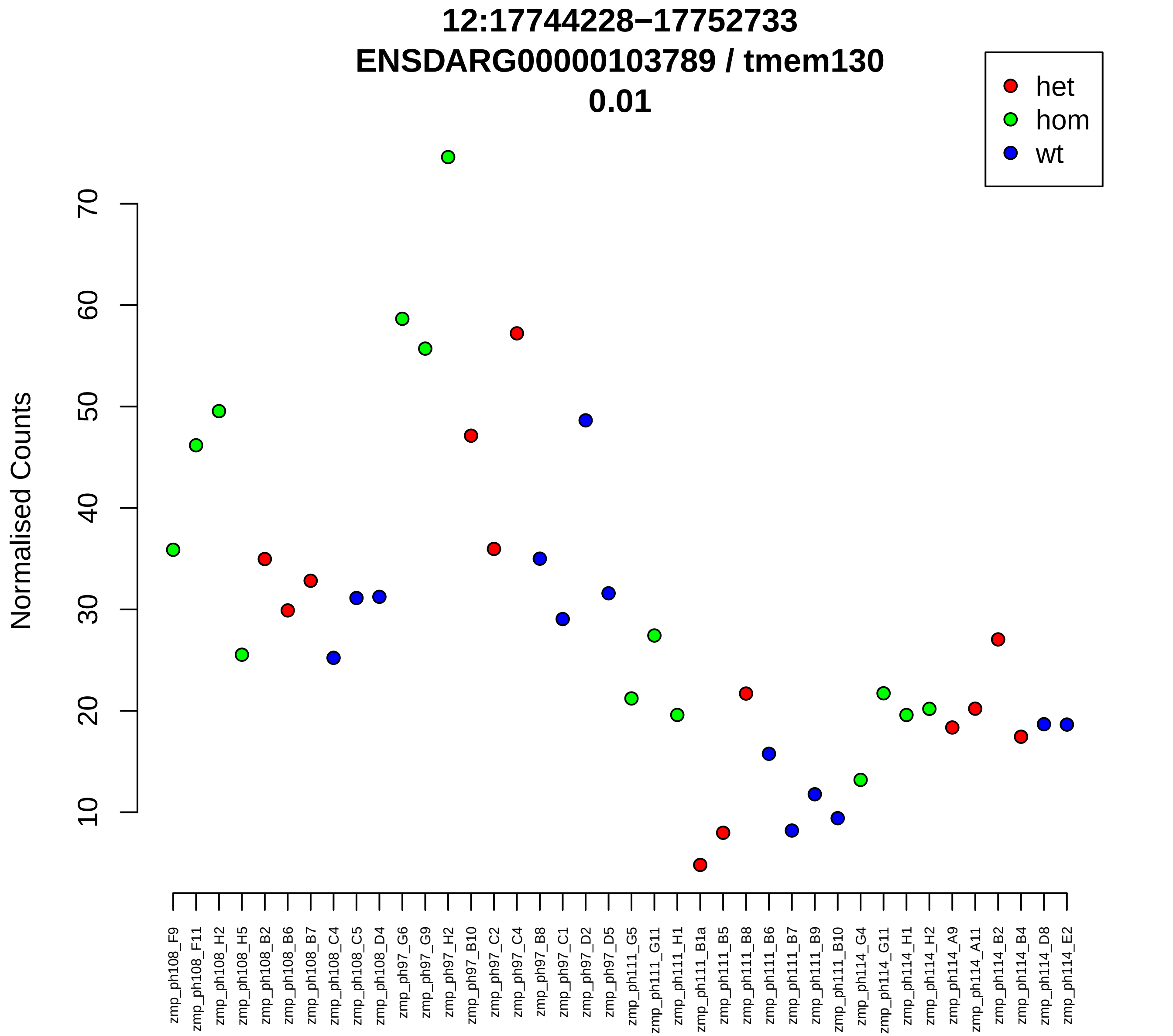

12:17744228−17752733
ENSDARG00000103789 / tmem130
0.01
het
hom
wt
Normalised Counts
10
20
30
40
50
60
70
zmp_ph108_F9
zmp_ph108_F11
zmp_ph108_H2
zmp_ph108_H5
zmp_ph108_B2
zmp_ph108_B6
zmp_ph108_B7
zmp_ph108_C4
zmp_ph108_C5
zmp_ph108_D4
zmp_ph97_G6
zmp_ph97_G9
zmp_ph97_H2
zmp_ph97_B10
zmp_ph97_C2
zmp_ph97_C4
zmp_ph97_B8
zmp_ph97_C1
zmp_ph97_D2
zmp_ph97_D5
zmp_ph111_G5
zmp_ph111_G11
zmp_ph111_H1
zmp_ph111_B1a
zmp_ph111_B5
zmp_ph111_B8
zmp_ph111_B6
zmp_ph111_B7
zmp_ph111_B9
zmp_ph111_B10
zmp_ph114_G4
zmp_ph114_G11
zmp_ph114_H1
zmp_ph114_H2
zmp_ph114_A9
zmp_ph114_A11
zmp_ph114_B2
zmp_ph114_B4
zmp_ph114_D8
zmp_ph114_E2

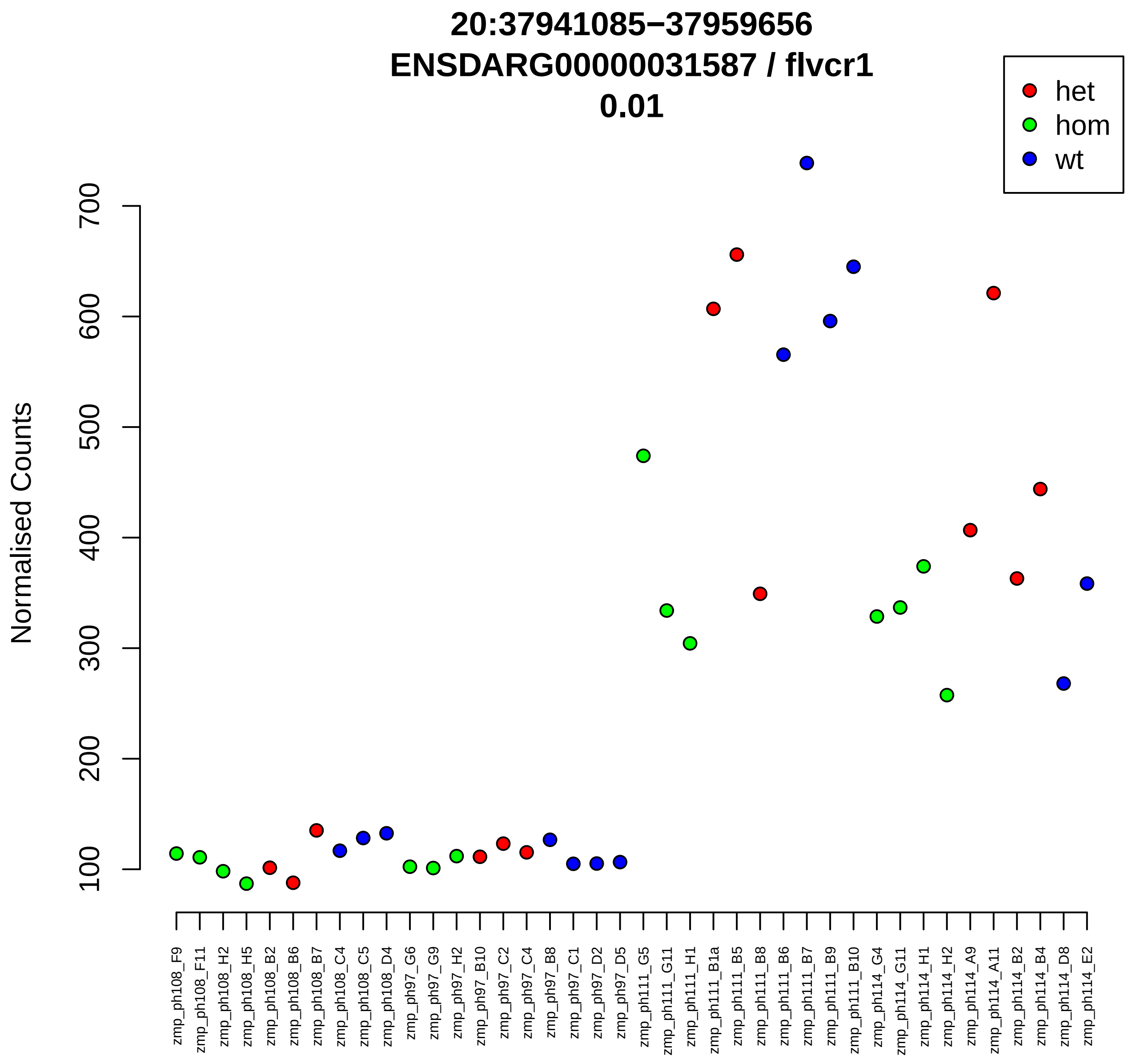
20:37941085−37959656
ENSDARG00000031587 / flvcr1
0.01
het
hom
wt
Normalised Counts
100
200
300
400
500
600
700
zmp_ph108_F9
zmp_ph108_F11
zmp_ph108_H2
zmp_ph108_H5
zmp_ph108_B2
zmp_ph108_B6
zmp_ph108_B7
zmp_ph108_C4
zmp_ph108_C5
zmp_ph108_D4
zmp_ph97_G6
zmp_ph97_G9
zmp_ph97_H2
zmp_ph97_B10
zmp_ph97_C2
zmp_ph97_C4
zmp_ph97_B8
zmp_ph97_C1
zmp_ph97_D2
zmp_ph97_D5
zmp_ph111_G5
zmp_ph111_G11
zmp_ph111_H1
zmp_ph111_B1a
zmp_ph111_B5
zmp_ph111_B8
zmp_ph111_B6
zmp_ph111_B7
zmp_ph111_B9
zmp_ph111_B10
zmp_ph114_G4
zmp_ph114_G11
zmp_ph114_H1
zmp_ph114_H2
zmp_ph114_A9
zmp_ph114_A11
zmp_ph114_B2
zmp_ph114_B4
zmp_ph114_D8
zmp_ph114_E2

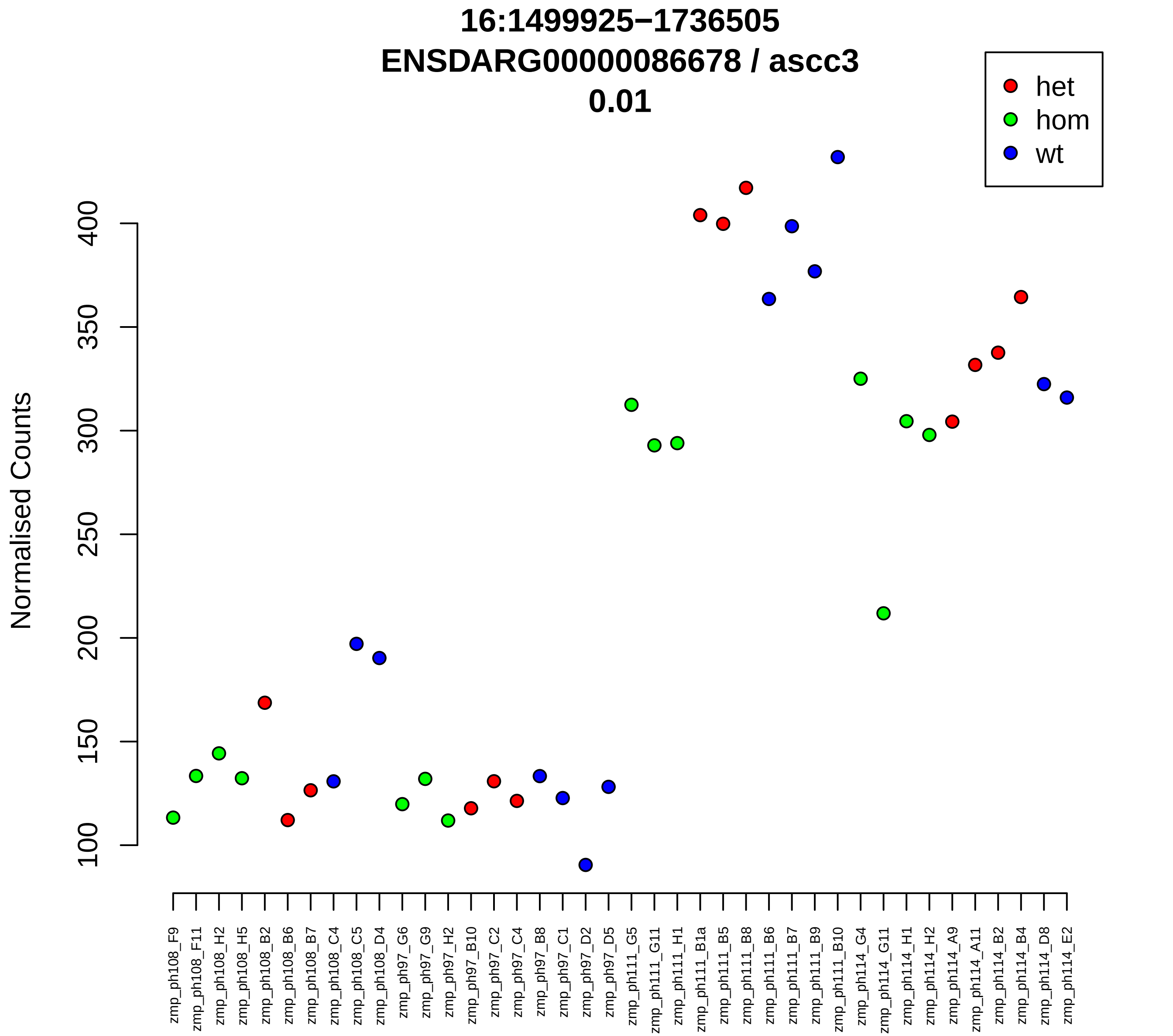
16:1499925−1736505
ENSDARG00000086678 / ascc3
0.01
het
hom
wt
Normalised Counts
100
150
200
250
300
350
400
zmp_ph108_F9
zmp_ph108_F11
zmp_ph108_H2
zmp_ph108_H5
zmp_ph108_B2
zmp_ph108_B6
zmp_ph108_B7
zmp_ph108_C4
zmp_ph108_C5
zmp_ph108_D4
zmp_ph97_G6
zmp_ph97_G9
zmp_ph97_H2
zmp_ph97_B10
zmp_ph97_C2
zmp_ph97_C4
zmp_ph97_B8
zmp_ph97_C1
zmp_ph97_D2
zmp_ph97_D5
zmp_ph111_G5
zmp_ph111_G11
zmp_ph111_H1
zmp_ph111_B1a
zmp_ph111_B5
zmp_ph111_B8
zmp_ph111_B6
zmp_ph111_B7
zmp_ph111_B9
zmp_ph111_B10
zmp_ph114_G4
zmp_ph114_G11
zmp_ph114_H1
zmp_ph114_H2
zmp_ph114_A9
zmp_ph114_A11
zmp_ph114_B2
zmp_ph114_B4
zmp_ph114_D8
zmp_ph114_E2

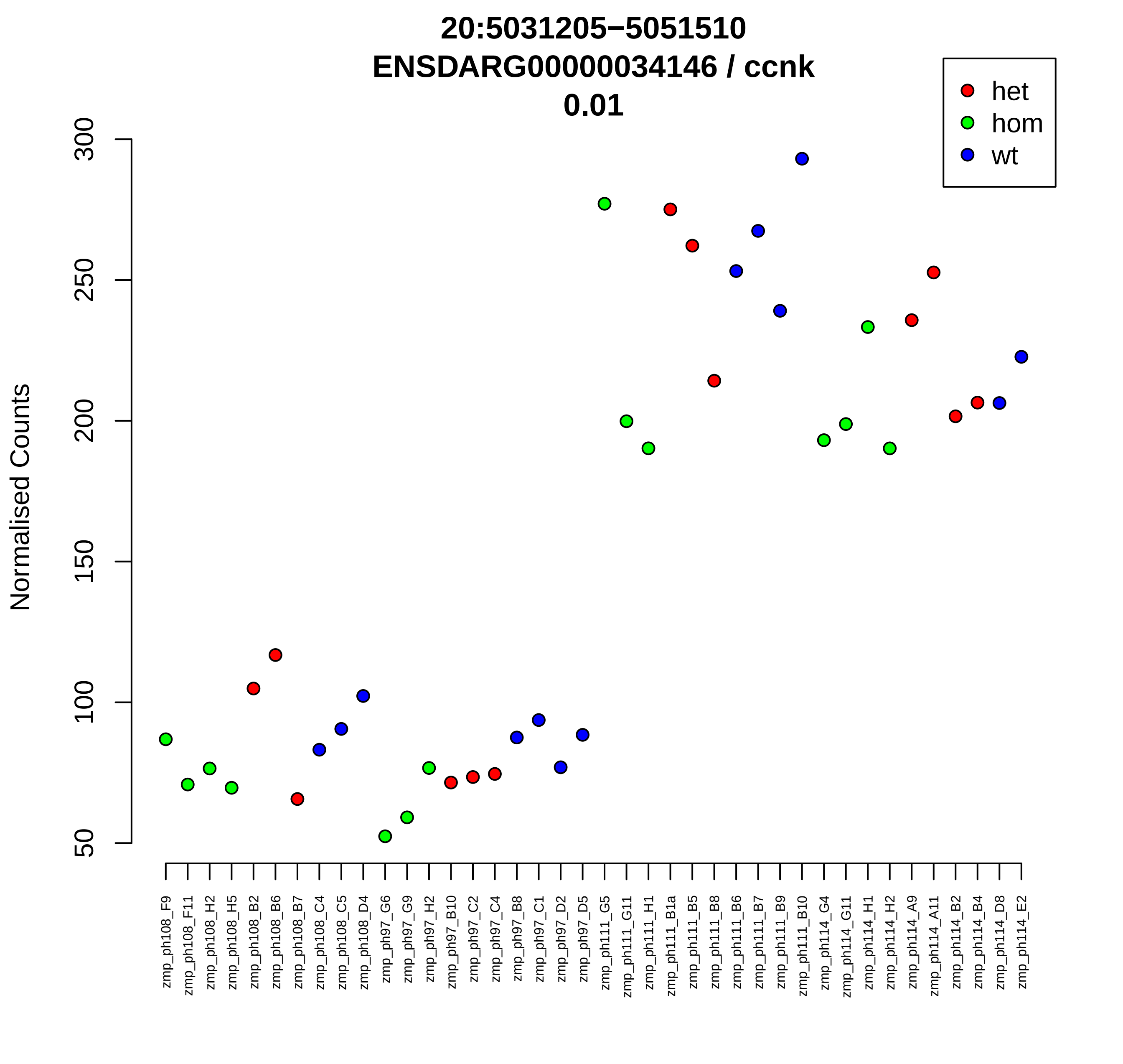
20:5031205−5051510
ENSDARG00000034146 / ccnk
0.01
het
hom
wt
Normalised Counts
300
250
200
150
100
50
zmp_ph108_F9
zmp_ph108_F11
zmp_ph108_H2
zmp_ph108_H5
zmp_ph108_B2
zmp_ph108_B6
zmp_ph108_B7
zmp_ph108_C4
zmp_ph108_C5
zmp_ph108_D4
zmp_ph97_G6
zmp_ph97_G9
zmp_ph97_H2
zmp_ph97_B10
zmp_ph97_C2
zmp_ph97_C4
zmp_ph97_B8
zmp_ph97_C1
zmp_ph97_D2
zmp_ph97_D5
zmp_ph111_G5
zmp_ph111_G11
zmp_ph111_H1
zmp_ph111_B1a
zmp_ph111_B5
zmp_ph111_B8
zmp_ph111_B6
zmp_ph111_B7
zmp_ph111_B9
zmp_ph111_B10
zmp_ph114_G4
zmp_ph114_G11
zmp_ph114_H1
zmp_ph114_H2
zmp_ph114_A9
zmp_ph114_A11
zmp_ph114_B2
zmp_ph114_B4
zmp_ph114_D8
zmp_ph114_E2

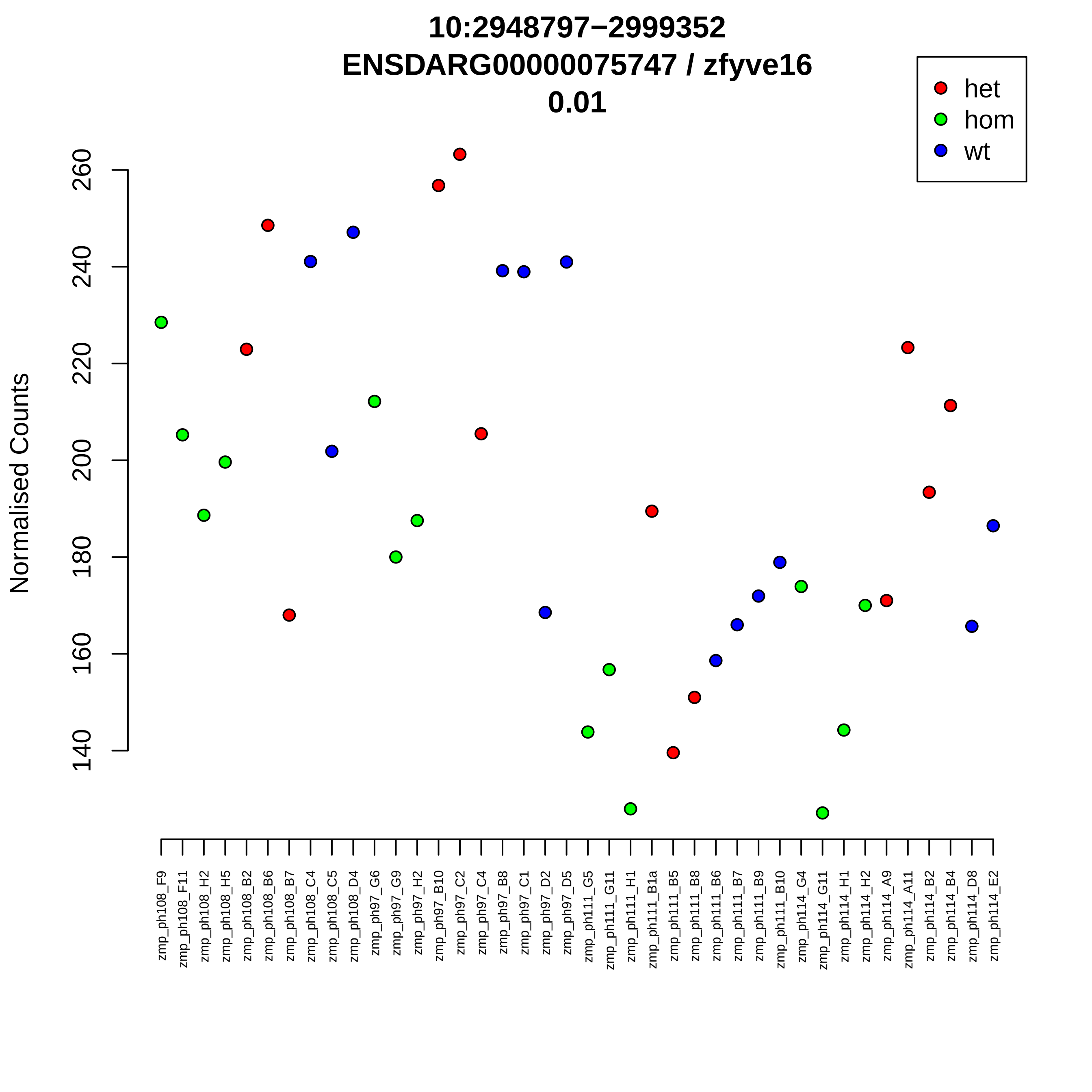

10:2948797−2999352
ENSDARG00000075747 / zfyve16
0.01
het
hom
wt
Normalised Counts
140
160
180
200
220
240
260
zmp_ph108_F9
zmp_ph108_F11
zmp_ph108_H2
zmp_ph108_H5
zmp_ph108_B2
zmp_ph108_B6
zmp_ph108_B7
zmp_ph108_C4
zmp_ph108_C5
zmp_ph108_D4
zmp_ph97_G6
zmp_ph97_G9
zmp_ph97_H2
zmp_ph97_B10
zmp_ph97_C2
zmp_ph97_C4
zmp_ph97_B8
zmp_ph97_C1
zmp_ph97_D2
zmp_ph97_D5
zmp_ph111_G5
zmp_ph111_G11
zmp_ph111_H1
zmp_ph111_B1a
zmp_ph111_B5
zmp_ph111_B8
zmp_ph111_B6
zmp_ph111_B7
zmp_ph111_B9
zmp_ph111_B10
zmp_ph114_G4
zmp_ph114_G11
zmp_ph114_H1
zmp_ph114_H2
zmp_ph114_A9
zmp_ph114_A11
zmp_ph114_B2
zmp_ph114_B4
zmp_ph114_D8
zmp_ph114_E2

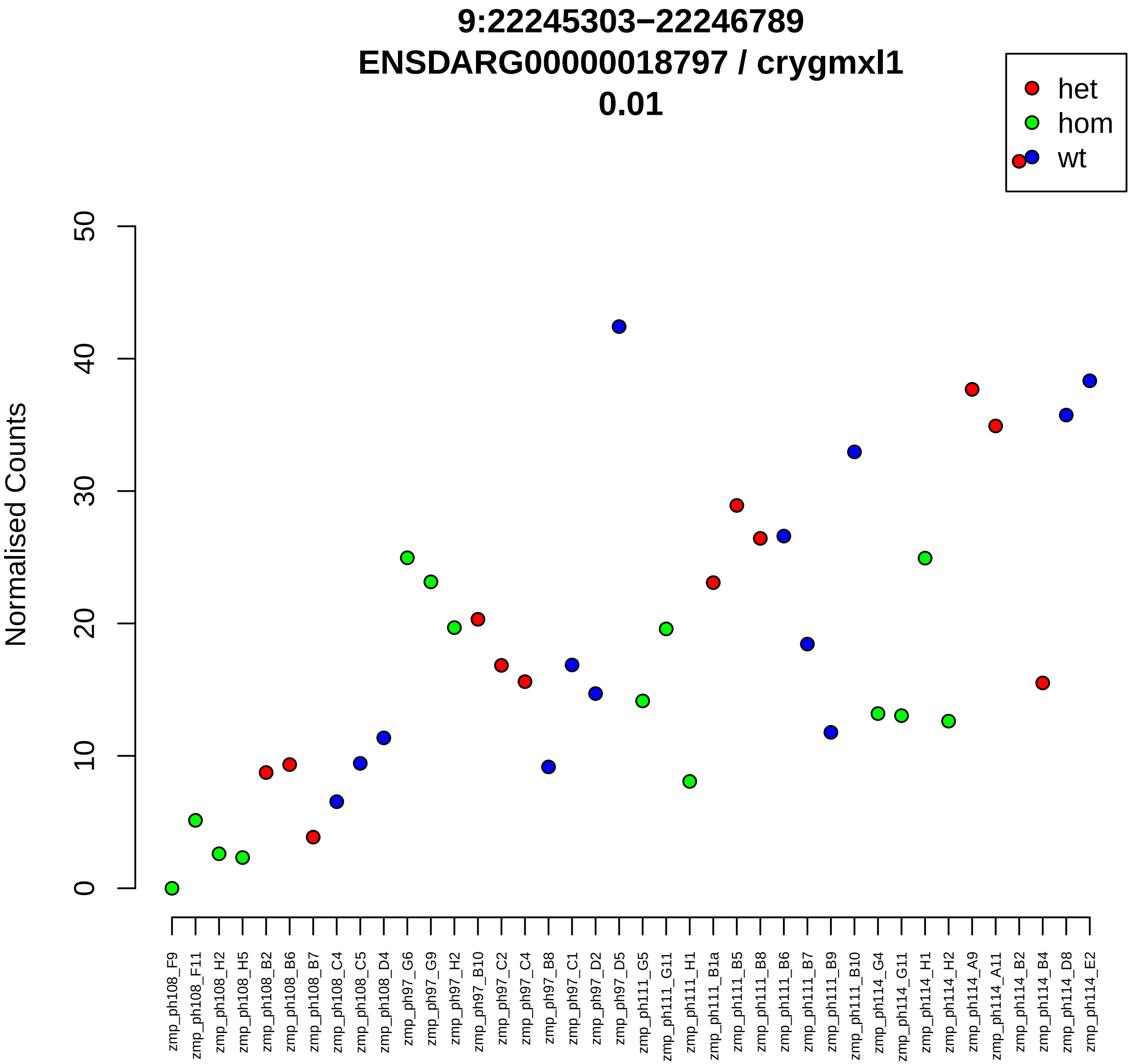

9:22245303−22246789
ENSDARG00000018797 / crygmxl1
0.01
het
hom
wt
Normalised Counts
0
10
20
30
40
50
zmp_ph108_F9
zmp_ph108_F11
zmp_ph108_H2
zmp_ph108_H5
zmp_ph108_B2
zmp_ph108_B6
zmp_ph108_B7
zmp_ph108_C4
zmp_ph108_C5
zmp_ph108_D4
zmp_ph97_G6
zmp_ph97_G9
zmp_ph97_H2
zmp_ph97_B10
zmp_ph97_C2
zmp_ph97_C4
zmp_ph97_B8
zmp_ph97_C1
zmp_ph97_D2
zmp_ph97_D5
zmp_ph111_G5
zmp_ph111_G11
zmp_ph111_H1
zmp_ph111_B1a
zmp_ph111_B5
zmp_ph111_B8
zmp_ph111_B6
zmp_ph111_B7
zmp_ph111_B9
zmp_ph111_B10
zmp_ph114_G4
zmp_ph114_G11
zmp_ph114_H1
zmp_ph114_H2
zmp_ph114_A9
zmp_ph114_A11
zmp_ph114_B2
zmp_ph114_B4
zmp_ph114_D8
zmp_ph114_E2

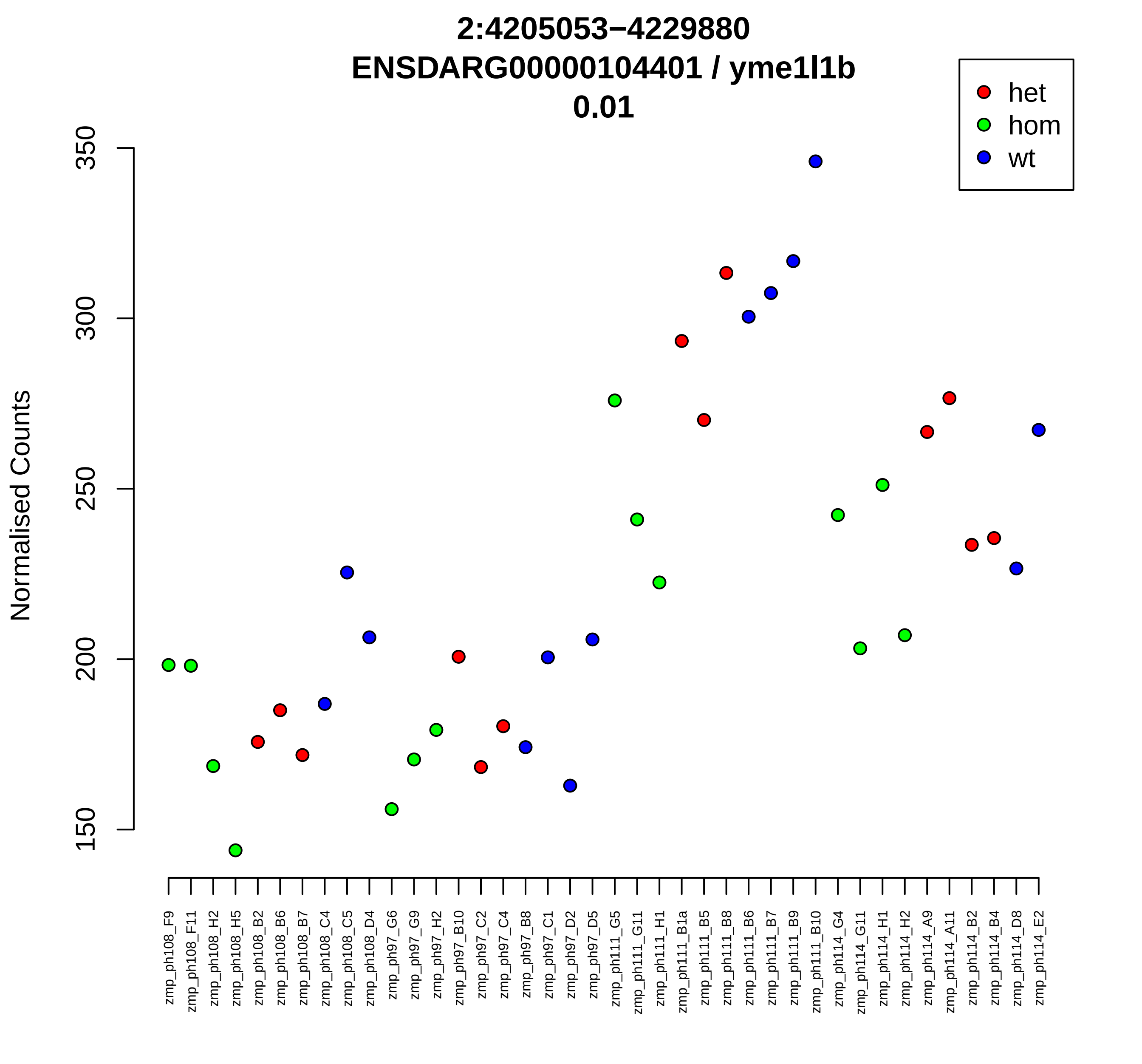
2:4205053−4229880
ENSDARG00000104401 / yme1l1b
0.01
het
hom
wt
Normalised Counts
150
200
250
300
350
zmp_ph108_F9
zmp_ph108_F11
zmp_ph108_H2
zmp_ph108_H5
zmp_ph108_B2
zmp_ph108_B6
zmp_ph108_B7
zmp_ph108_C4
zmp_ph108_C5
zmp_ph108_D4
zmp_ph97_G6
zmp_ph97_G9
zmp_ph97_H2
zmp_ph97_B10
zmp_ph97_C2
zmp_ph97_C4
zmp_ph97_B8
zmp_ph97_C1
zmp_ph97_D2
zmp_ph97_D5
zmp_ph111_G5
zmp_ph111_G11
zmp_ph111_H1
zmp_ph111_B1a
zmp_ph111_B5
zmp_ph111_B8
zmp_ph111_B6
zmp_ph111_B7
zmp_ph111_B9
zmp_ph111_B10
zmp_ph114_G4
zmp_ph114_G11
zmp_ph114_H1
zmp_ph114_H2
zmp_ph114_A9
zmp_ph114_A11
zmp_ph114_B2
zmp_ph114_B4
zmp_ph114_D8
zmp_ph114_E2

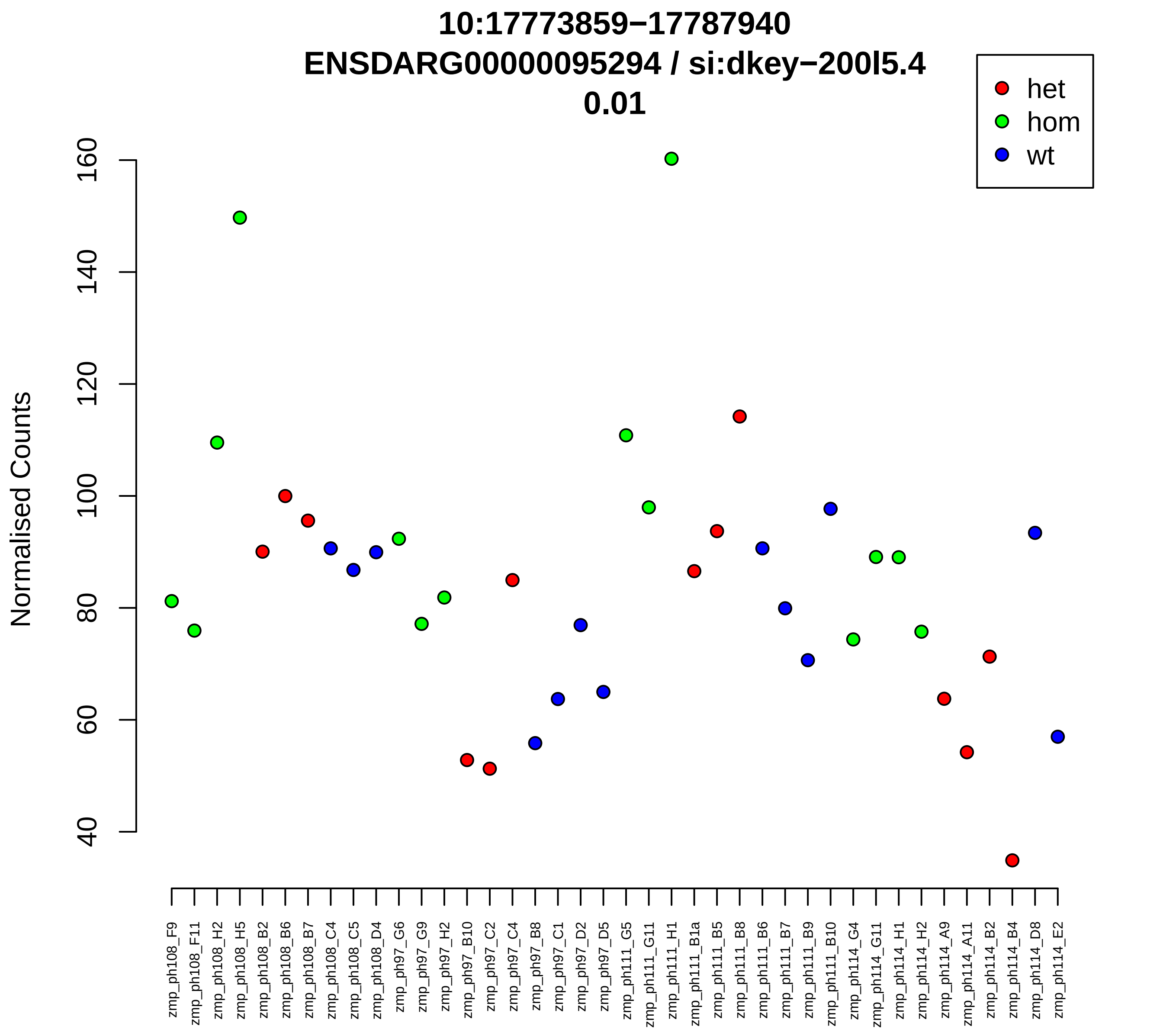

10:17773859−17787940
ENSDARG00000095294 / si:dkey−200l5.4
0.01
het
hom
wt
Normalised Counts
40
60
80
100
120
140
160
zmp_ph108_F9
zmp_ph108_F11
zmp_ph108_H2
zmp_ph108_H5
zmp_ph108_B2
zmp_ph108_B6
zmp_ph108_B7
zmp_ph108_C4
zmp_ph108_C5
zmp_ph108_D4
zmp_ph97_G6
zmp_ph97_G9
zmp_ph97_H2
zmp_ph97_B10
zmp_ph97_C2
zmp_ph97_C4
zmp_ph97_B8
zmp_ph97_C1
zmp_ph97_D2
zmp_ph97_D5
zmp_ph111_G5
zmp_ph111_G11
zmp_ph111_H1
zmp_ph111_B1a
zmp_ph111_B5
zmp_ph111_B8
zmp_ph111_B6
zmp_ph111_B7
zmp_ph111_B9
zmp_ph111_B10
zmp_ph114_G4
zmp_ph114_G11
zmp_ph114_H1
zmp_ph114_H2
zmp_ph114_A9
zmp_ph114_A11
zmp_ph114_B2
zmp_ph114_B4
zmp_ph114_D8
zmp_ph114_E2

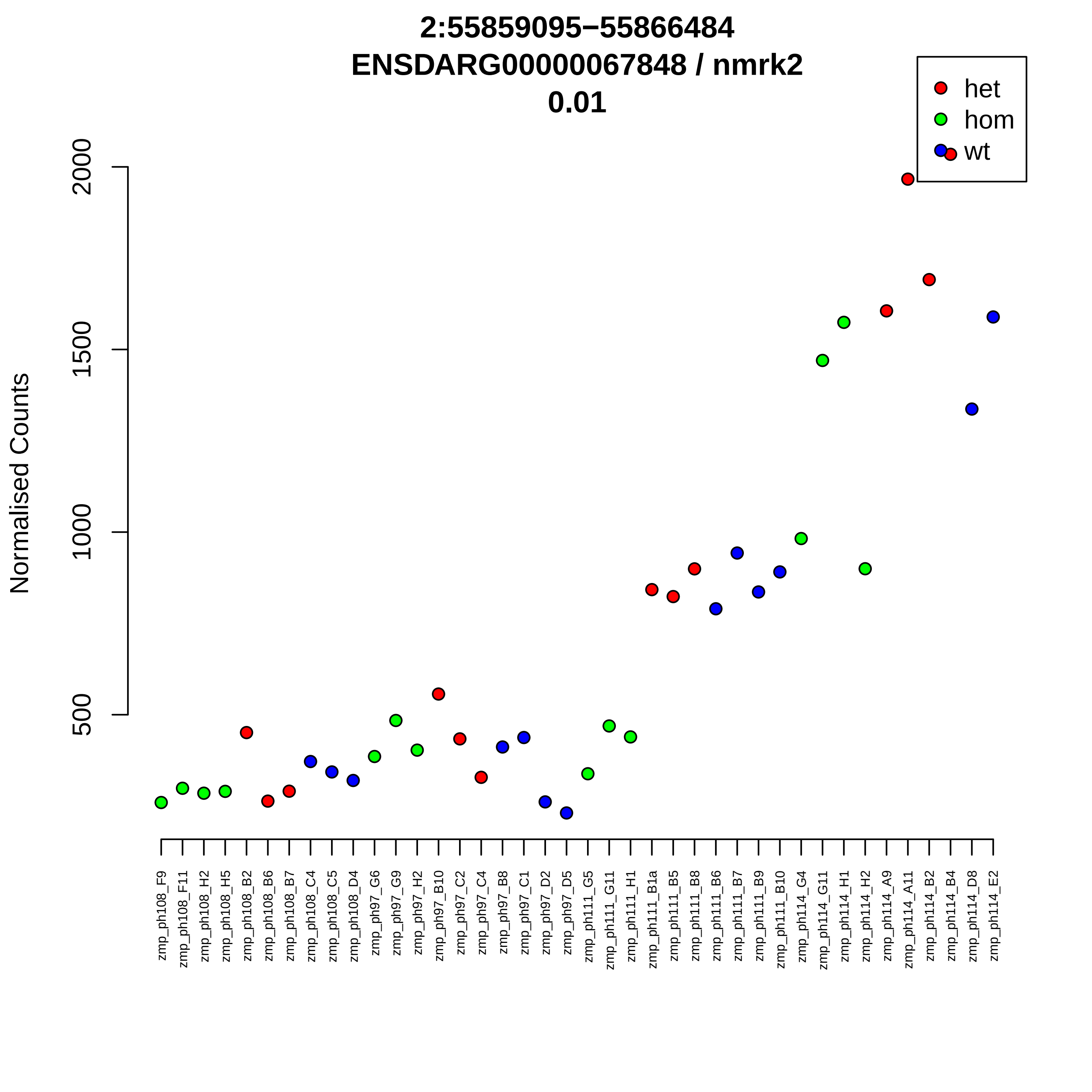
2:55859095−55866484
ENSDARG00000067848 / nmrk2
0.01
het
hom
wt
Normalised Counts
500
1000
1500
2000
zmp_ph108_F9
zmp_ph108_F11
zmp_ph108_H2
zmp_ph108_H5
zmp_ph108_B2
zmp_ph108_B6
zmp_ph108_B7
zmp_ph108_C4
zmp_ph108_C5
zmp_ph108_D4
zmp_ph97_G6
zmp_ph97_G9
zmp_ph97_H2
zmp_ph97_B10
zmp_ph97_C2
zmp_ph97_C4
zmp_ph97_B8
zmp_ph97_C1
zmp_ph97_D2
zmp_ph97_D5
zmp_ph111_G5
zmp_ph111_G11
zmp_ph111_H1
zmp_ph111_B1a
zmp_ph111_B5
zmp_ph111_B8
zmp_ph111_B6
zmp_ph111_B7
zmp_ph111_B9
zmp_ph111_B10
zmp_ph114_G4
zmp_ph114_G11
zmp_ph114_H1
zmp_ph114_H2
zmp_ph114_A9
zmp_ph114_A11
zmp_ph114_B2
zmp_ph114_B4
zmp_ph114_D8
zmp_ph114_E2

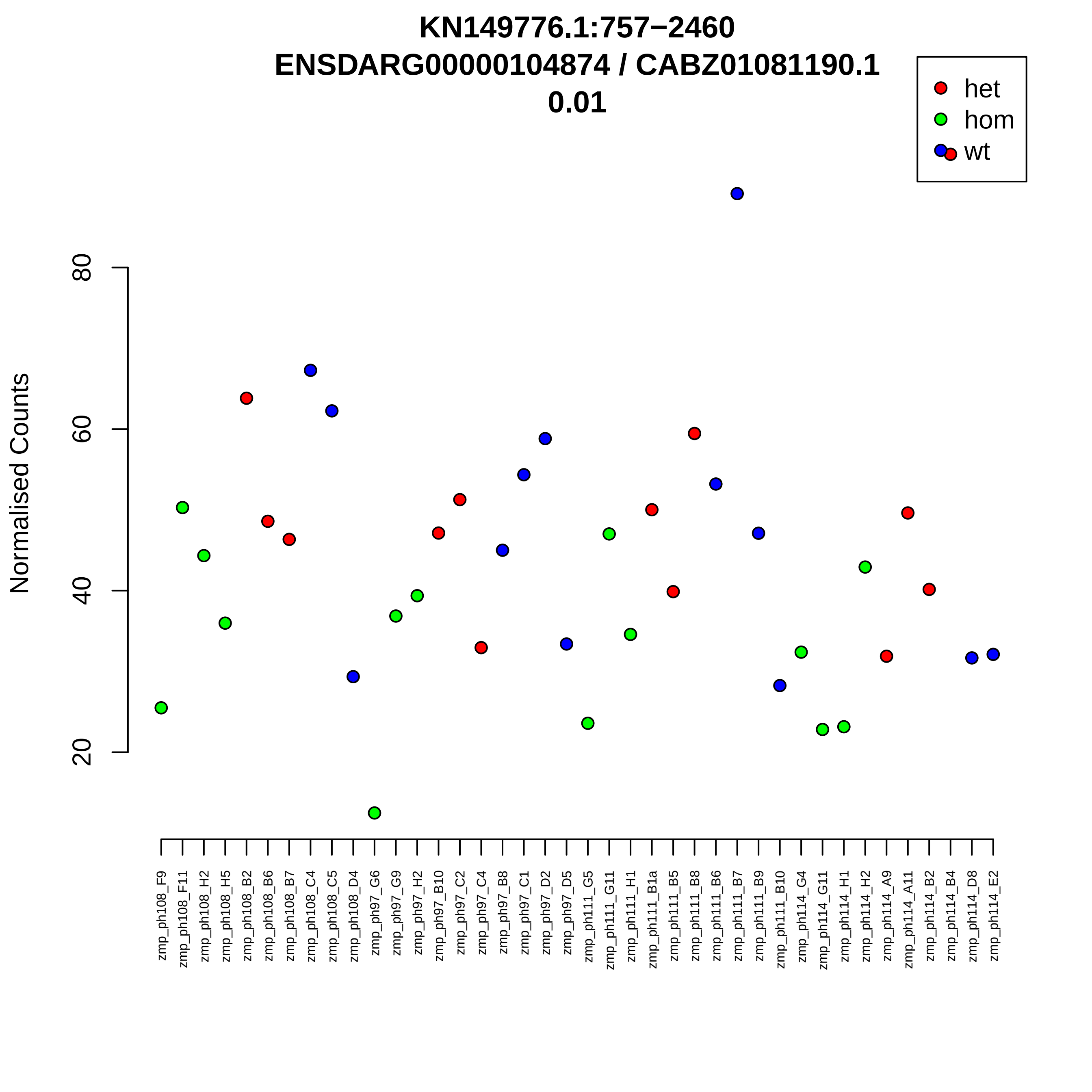

KN149776.1:757−2460
ENSDARG00000104874 / CABZ01081190.1
0.01
het
hom
wt
Normalised Counts
20
40
60
80
zmp_ph108_F9
zmp_ph108_F11
zmp_ph108_H2
zmp_ph108_H5
zmp_ph108_B2
zmp_ph108_B6
zmp_ph108_B7
zmp_ph108_C4
zmp_ph108_C5
zmp_ph108_D4
zmp_ph97_G6
zmp_ph97_G9
zmp_ph97_H2
zmp_ph97_B10
zmp_ph97_C2
zmp_ph97_C4
zmp_ph97_B8
zmp_ph97_C1
zmp_ph97_D2
zmp_ph97_D5
zmp_ph111_G5
zmp_ph111_G11
zmp_ph111_H1
zmp_ph111_B1a
zmp_ph111_B5
zmp_ph111_B8
zmp_ph111_B6
zmp_ph111_B7
zmp_ph111_B9
zmp_ph111_B10
zmp_ph114_G4
zmp_ph114_G11
zmp_ph114_H1
zmp_ph114_H2
zmp_ph114_A9
zmp_ph114_A11
zmp_ph114_B2
zmp_ph114_B4
zmp_ph114_D8
zmp_ph114_E2

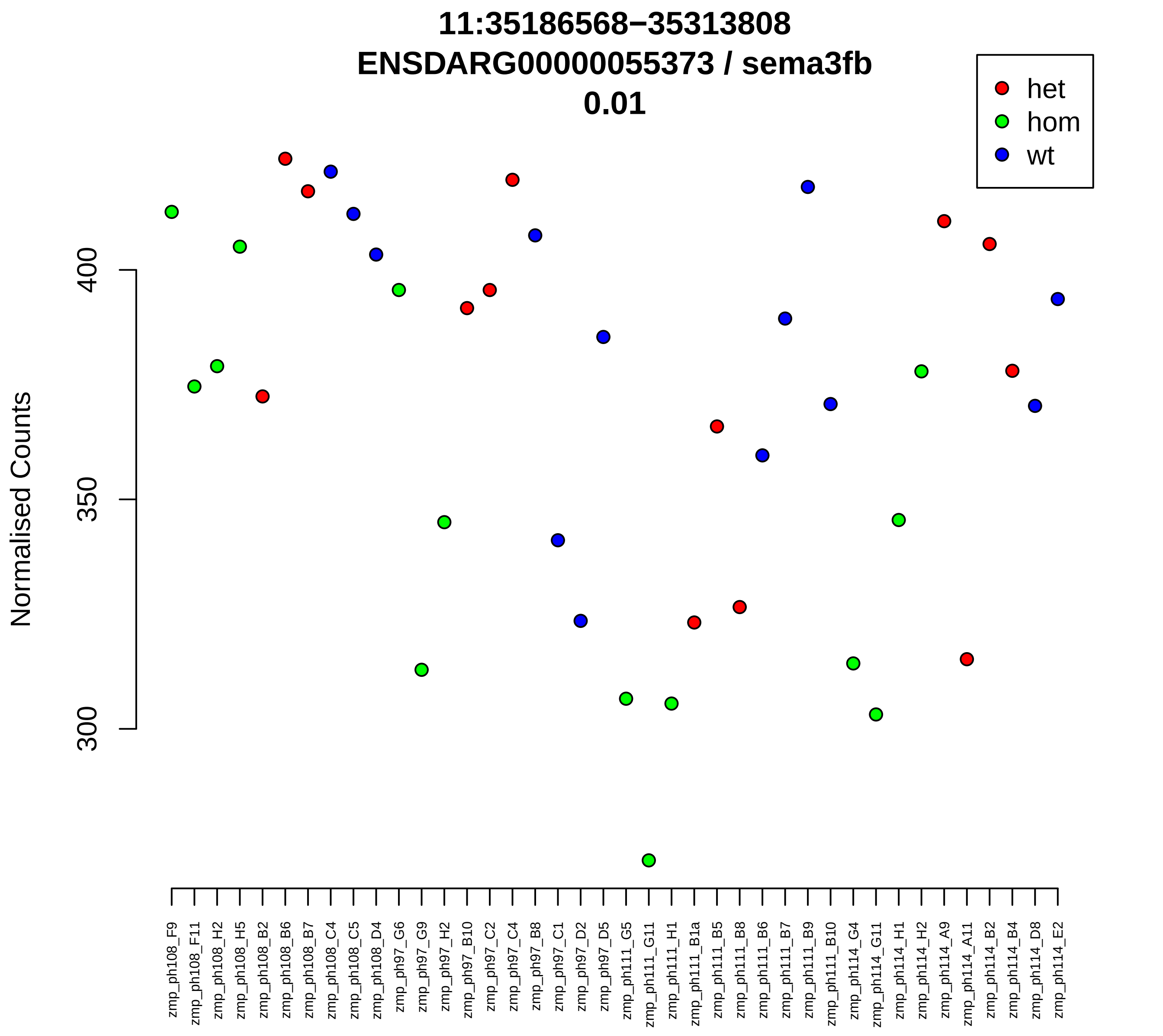

11:35186568−35313808
ENSDARG00000055373 / sema3fb
0.01
het
hom
wt
Normalised Counts
300
350
400
zmp_ph108_F9
zmp_ph108_F11
zmp_ph108_H2
zmp_ph108_H5
zmp_ph108_B2
zmp_ph108_B6
zmp_ph108_B7
zmp_ph108_C4
zmp_ph108_C5
zmp_ph108_D4
zmp_ph97_G6
zmp_ph97_G9
zmp_ph97_H2
zmp_ph97_B10
zmp_ph97_C2
zmp_ph97_C4
zmp_ph97_B8
zmp_ph97_C1
zmp_ph97_D2
zmp_ph97_D5
zmp_ph111_G5
zmp_ph111_G11
zmp_ph111_H1
zmp_ph111_B1a
zmp_ph111_B5
zmp_ph111_B8
zmp_ph111_B6
zmp_ph111_B7
zmp_ph111_B9
zmp_ph111_B10
zmp_ph114_G4
zmp_ph114_G11
zmp_ph114_H1
zmp_ph114_H2
zmp_ph114_A9
zmp_ph114_A11
zmp_ph114_B2
zmp_ph114_B4
zmp_ph114_D8
zmp_ph114_E2

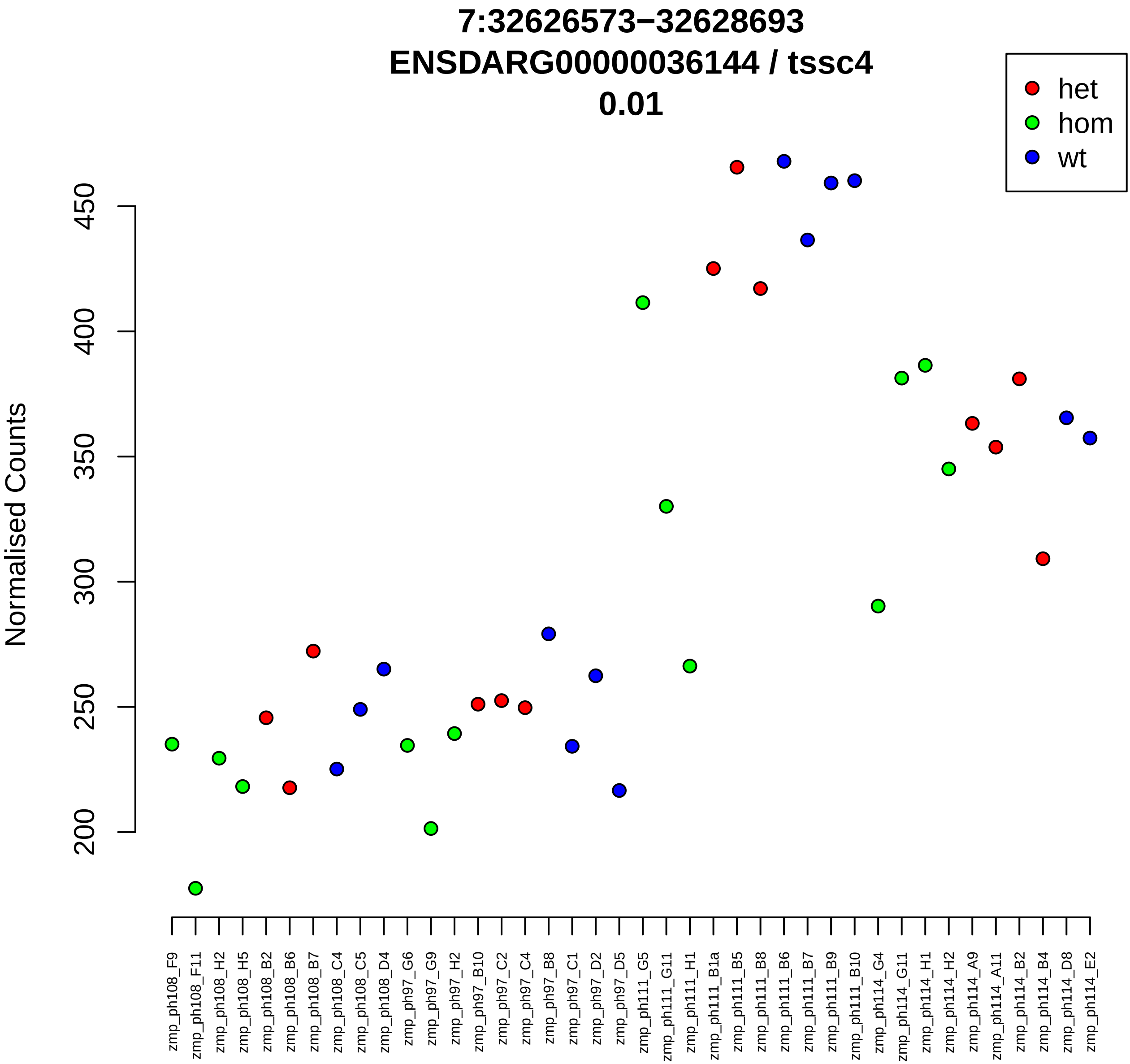

7:32626573−32628693
ENSDARG00000036144 / tssc4
0.01
het
hom
wt
Normalised Counts
200
250
300
350
400
450
zmp_ph108_F9
zmp_ph108_F11
zmp_ph108_H2
zmp_ph108_H5
zmp_ph108_B2
zmp_ph108_B6
zmp_ph108_B7
zmp_ph108_C4
zmp_ph108_C5
zmp_ph108_D4
zmp_ph97_G6
zmp_ph97_G9
zmp_ph97_H2
zmp_ph97_B10
zmp_ph97_C2
zmp_ph97_C4
zmp_ph97_B8
zmp_ph97_C1
zmp_ph97_D2
zmp_ph97_D5
zmp_ph111_G5
zmp_ph111_G11
zmp_ph111_H1
zmp_ph111_B1a
zmp_ph111_B5
zmp_ph111_B8
zmp_ph111_B6
zmp_ph111_B7
zmp_ph111_B9
zmp_ph111_B10
zmp_ph114_G4
zmp_ph114_G11
zmp_ph114_H1
zmp_ph114_H2
zmp_ph114_A9
zmp_ph114_A11
zmp_ph114_B2
zmp_ph114_B4
zmp_ph114_D8
zmp_ph114_E2

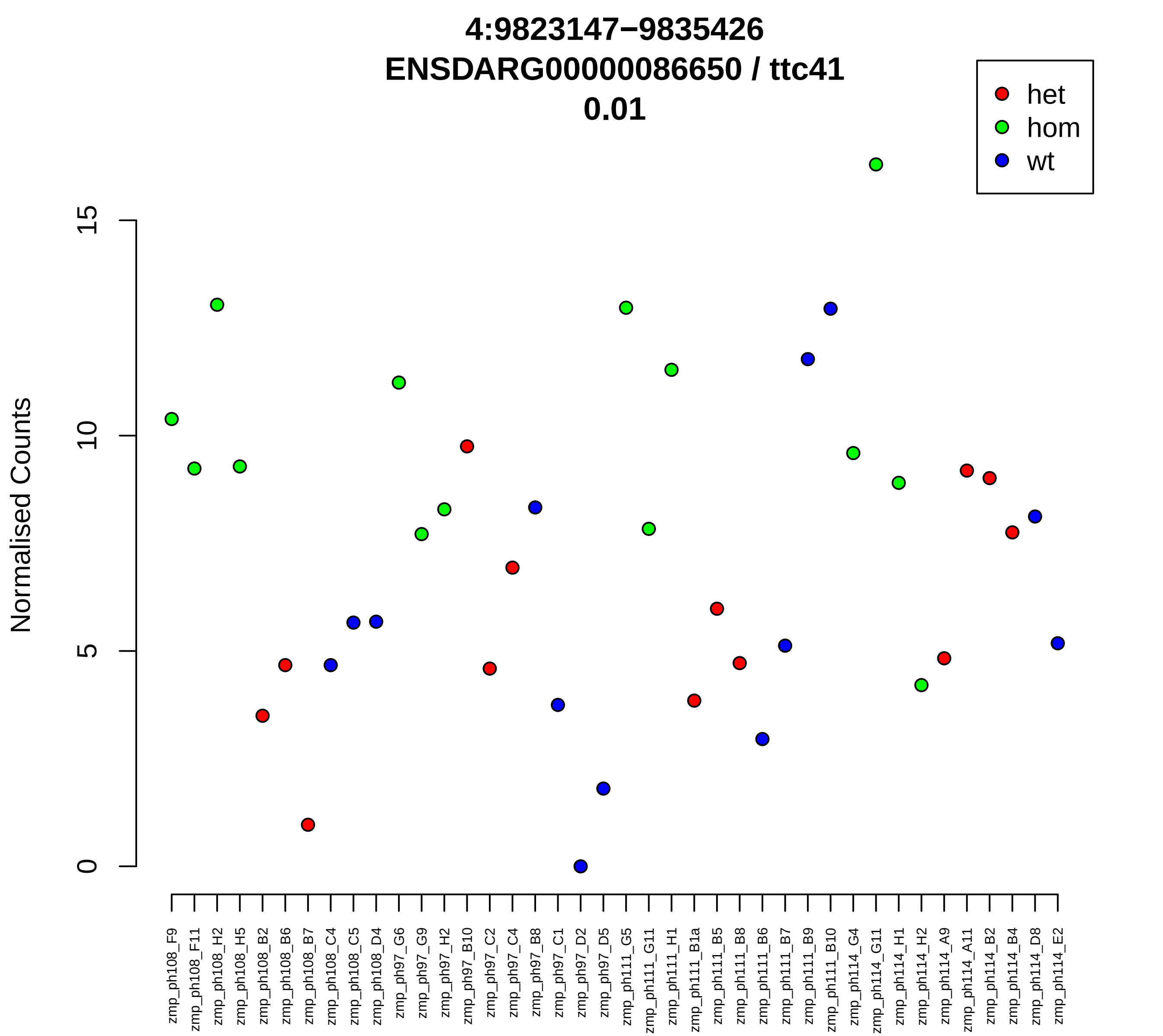
4:9823147−9835426
ENSDARG00000086650 / ttc41
0.01
het
hom
wt
Normalised Counts
0
5
10
15
zmp_ph108_F9
zmp_ph108_F11
zmp_ph108_H2
zmp_ph108_H5
zmp_ph108_B2
zmp_ph108_B6
zmp_ph108_B7
zmp_ph108_C4
zmp_ph108_C5
zmp_ph108_D4
zmp_ph97_G6
zmp_ph97_G9
zmp_ph97_H2
zmp_ph97_B10
zmp_ph97_C2
zmp_ph97_C4
zmp_ph97_B8
zmp_ph97_C1
zmp_ph97_D2
zmp_ph97_D5
zmp_ph111_G5
zmp_ph111_G11
zmp_ph111_H1
zmp_ph111_B1a
zmp_ph111_B5
zmp_ph111_B8
zmp_ph111_B6
zmp_ph111_B7
zmp_ph111_B9
zmp_ph111_B10
zmp_ph114_G4
zmp_ph114_G11
zmp_ph114_H1
zmp_ph114_H2
zmp_ph114_A9
zmp_ph114_A11
zmp_ph114_B2
zmp_ph114_B4
zmp_ph114_D8
zmp_ph114_E2

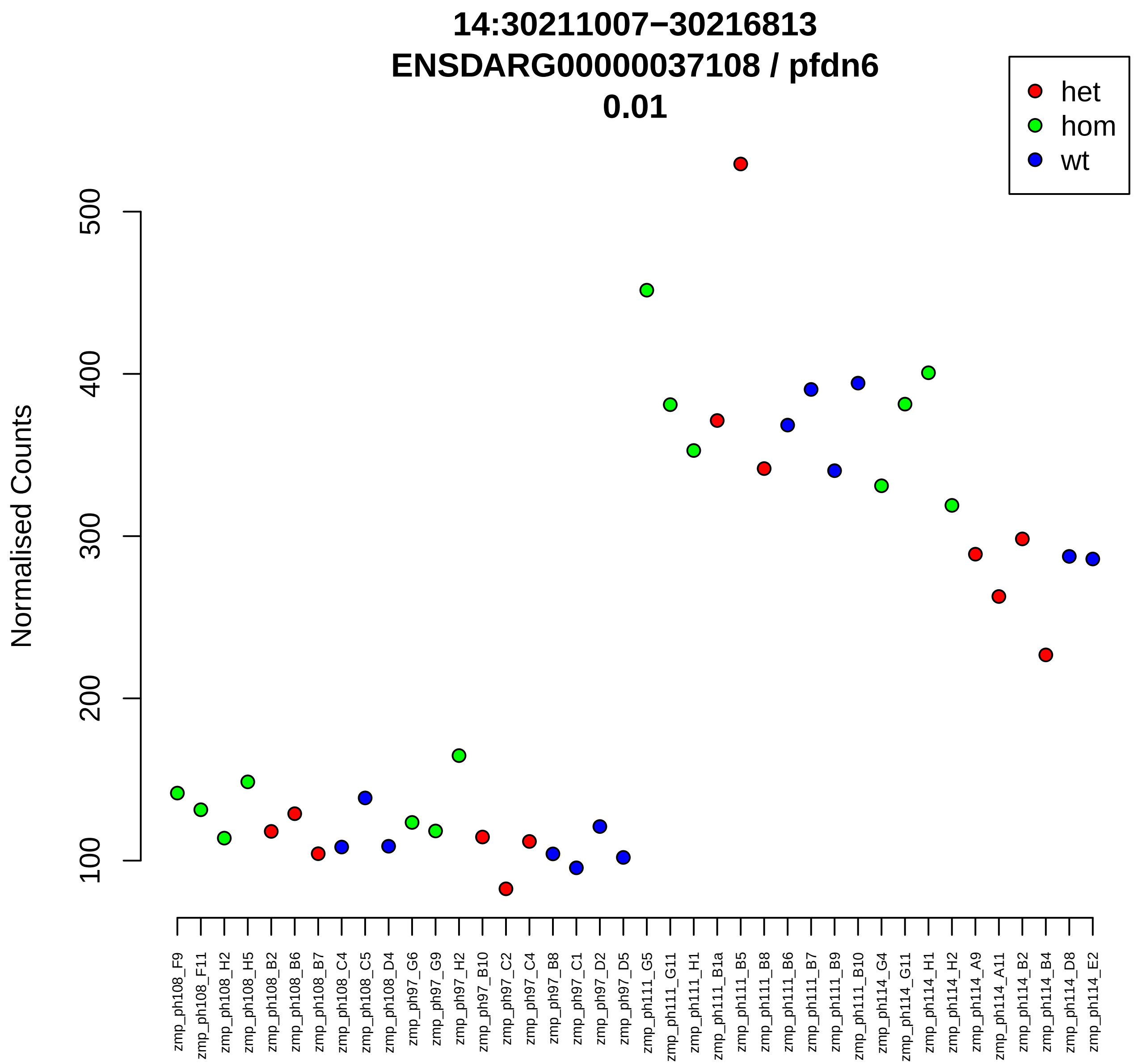
14:30211007−30216813
ENSDARG00000037108 / pfdn6
0.01
het
hom
wt
Normalised Counts
100
200
300
400
500
zmp_ph108_F9
zmp_ph108_F11
zmp_ph108_H2
zmp_ph108_H5
zmp_ph108_B2
zmp_ph108_B6
zmp_ph108_B7
zmp_ph108_C4
zmp_ph108_C5
zmp_ph108_D4
zmp_ph97_G6
zmp_ph97_G9
zmp_ph97_H2
zmp_ph97_B10
zmp_ph97_C2
zmp_ph97_C4
zmp_ph97_B8
zmp_ph97_C1
zmp_ph97_D2
zmp_ph97_D5
zmp_ph111_G5
zmp_ph111_G11
zmp_ph111_H1
zmp_ph111_B1a
zmp_ph111_B5
zmp_ph111_B8
zmp_ph111_B6
zmp_ph111_B7
zmp_ph111_B9
zmp_ph111_B10
zmp_ph114_G4
zmp_ph114_G11
zmp_ph114_H1
zmp_ph114_H2
zmp_ph114_A9
zmp_ph114_A11
zmp_ph114_B2
zmp_ph114_B4
zmp_ph114_D8
zmp_ph114_E2

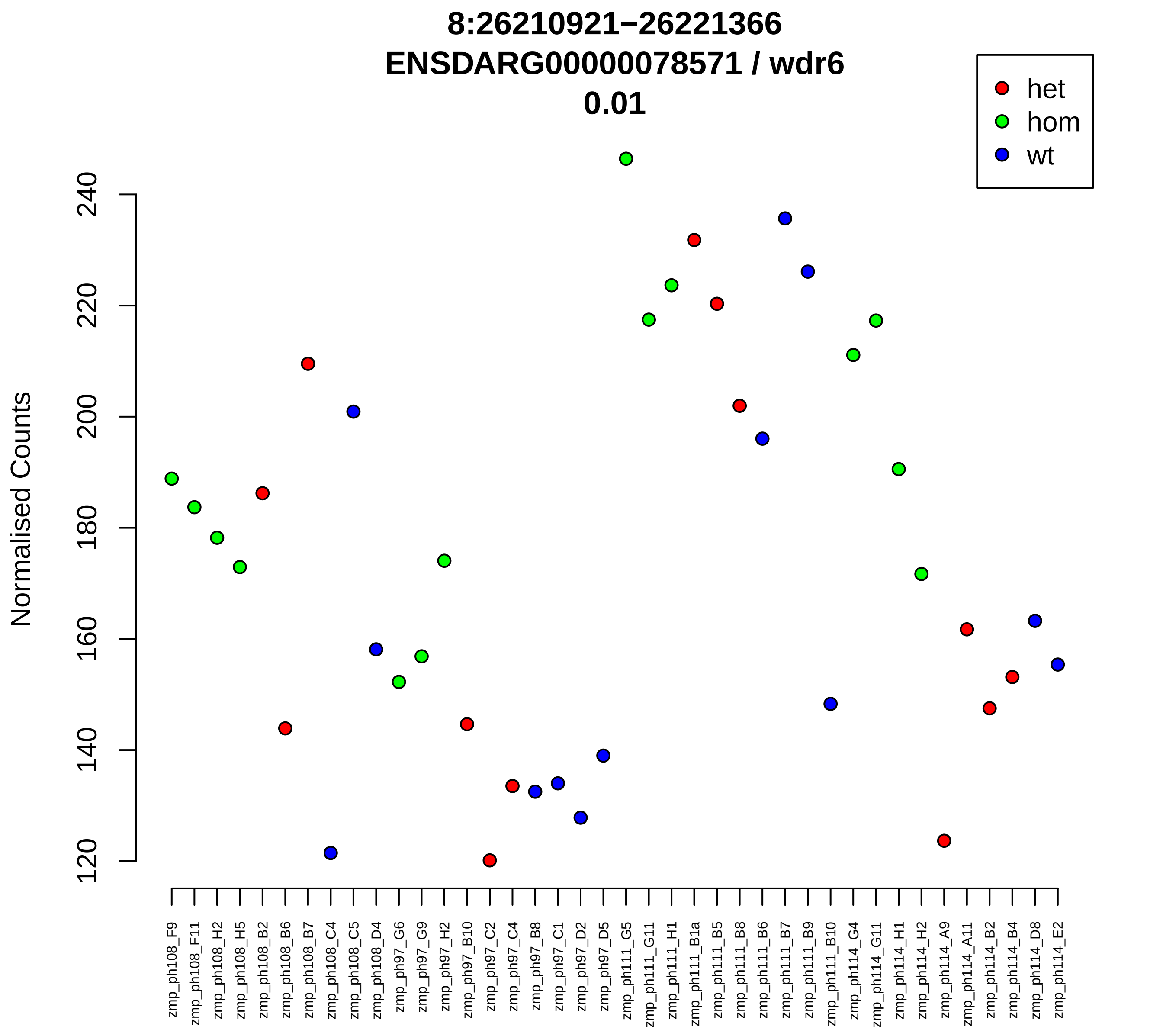

8:26210921−26221366
ENSDARG00000078571 / wdr6
0.01
het
hom
wt
Normalised Counts
120
140
160
180
200
220
240
zmp_ph108_F9
zmp_ph108_F11
zmp_ph108_H2
zmp_ph108_H5
zmp_ph108_B2
zmp_ph108_B6
zmp_ph108_B7
zmp_ph108_C4
zmp_ph108_C5
zmp_ph108_D4
zmp_ph97_G6
zmp_ph97_G9
zmp_ph97_H2
zmp_ph97_B10
zmp_ph97_C2
zmp_ph97_C4
zmp_ph97_B8
zmp_ph97_C1
zmp_ph97_D2
zmp_ph97_D5
zmp_ph111_G5
zmp_ph111_G11
zmp_ph111_H1
zmp_ph111_B1a
zmp_ph111_B5
zmp_ph111_B8
zmp_ph111_B6
zmp_ph111_B7
zmp_ph111_B9
zmp_ph111_B10
zmp_ph114_G4
zmp_ph114_G11
zmp_ph114_H1
zmp_ph114_H2
zmp_ph114_A9
zmp_ph114_A11
zmp_ph114_B2
zmp_ph114_B4
zmp_ph114_D8
zmp_ph114_E2

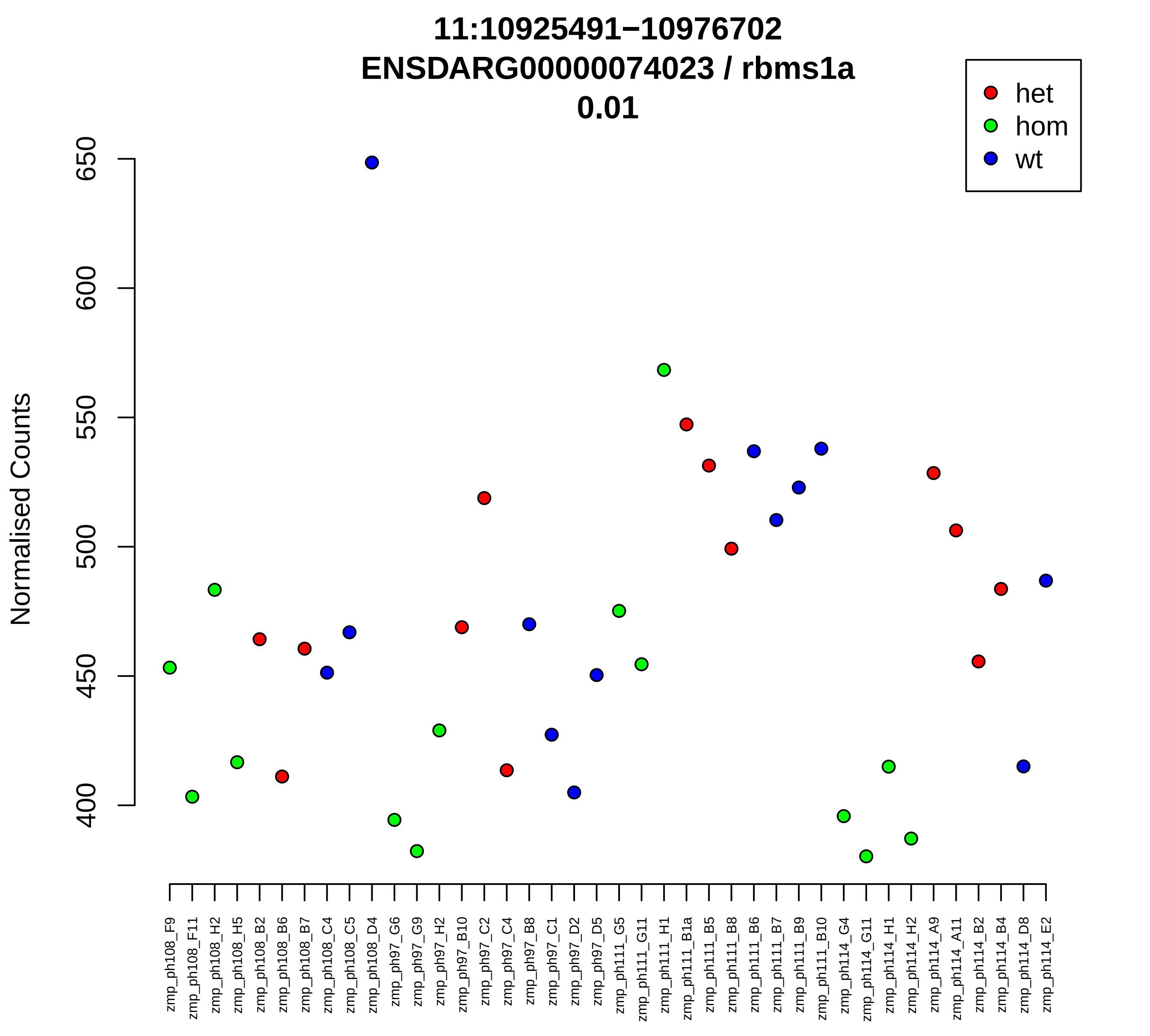

11:10925491−10976702
ENSDARG00000074023 / rbms1a
0.01
het
hom
wt
Normalised Counts
400
450
500
550
600
650
zmp_ph108_F9
zmp_ph108_F11
zmp_ph108_H2
zmp_ph108_H5
zmp_ph108_B2
zmp_ph108_B6
zmp_ph108_B7
zmp_ph108_C4
zmp_ph108_C5
zmp_ph108_D4
zmp_ph97_G6
zmp_ph97_G9
zmp_ph97_H2
zmp_ph97_B10
zmp_ph97_C2
zmp_ph97_C4
zmp_ph97_B8
zmp_ph97_C1
zmp_ph97_D2
zmp_ph97_D5
zmp_ph111_G5
zmp_ph111_G11
zmp_ph111_H1
zmp_ph111_B1a
zmp_ph111_B5
zmp_ph111_B8
zmp_ph111_B6
zmp_ph111_B7
zmp_ph111_B9
zmp_ph111_B10
zmp_ph114_G4
zmp_ph114_G11
zmp_ph114_H1
zmp_ph114_H2
zmp_ph114_A9
zmp_ph114_A11
zmp_ph114_B2
zmp_ph114_B4
zmp_ph114_D8
zmp_ph114_E2

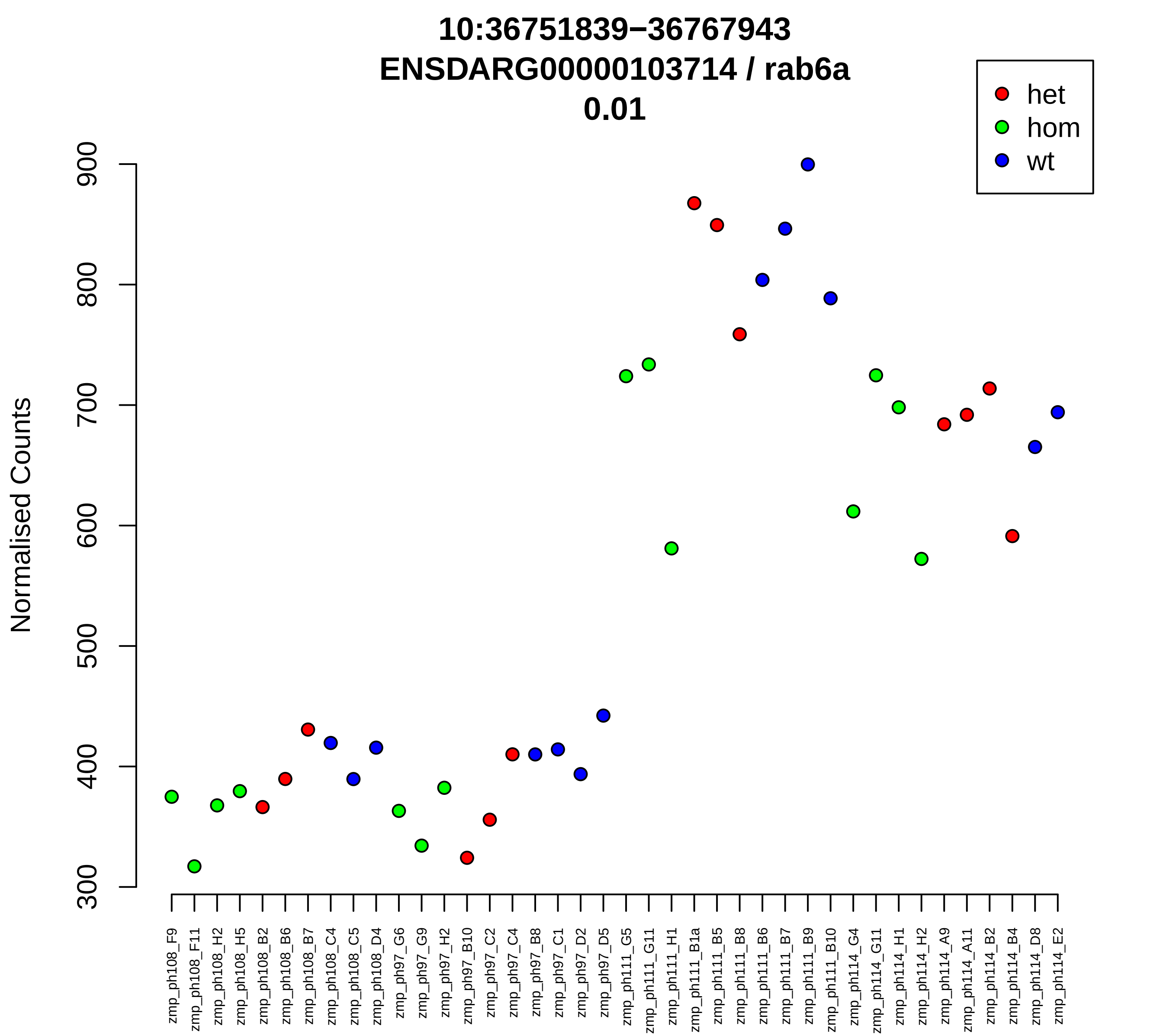
10:36751839−36767943
ENSDARG00000103714 / rab6a
0.01
het
hom
wt
Normalised Counts
300
400
500
600
700
800
900
zmp_ph108_F9
zmp_ph108_F11
zmp_ph108_H2
zmp_ph108_H5
zmp_ph108_B2
zmp_ph108_B6
zmp_ph108_B7
zmp_ph108_C4
zmp_ph108_C5
zmp_ph108_D4
zmp_ph97_G6
zmp_ph97_G9
zmp_ph97_H2
zmp_ph97_B10
zmp_ph97_C2
zmp_ph97_C4
zmp_ph97_B8
zmp_ph97_C1
zmp_ph97_D2
zmp_ph97_D5
zmp_ph111_G5
zmp_ph111_G11
zmp_ph111_H1
zmp_ph111_B1a
zmp_ph111_B5
zmp_ph111_B8
zmp_ph111_B6
zmp_ph111_B7
zmp_ph111_B9
zmp_ph111_B10
zmp_ph114_G4
zmp_ph114_G11
zmp_ph114_H1
zmp_ph114_H2
zmp_ph114_A9
zmp_ph114_A11
zmp_ph114_B2
zmp_ph114_B4
zmp_ph114_D8
zmp_ph114_E2

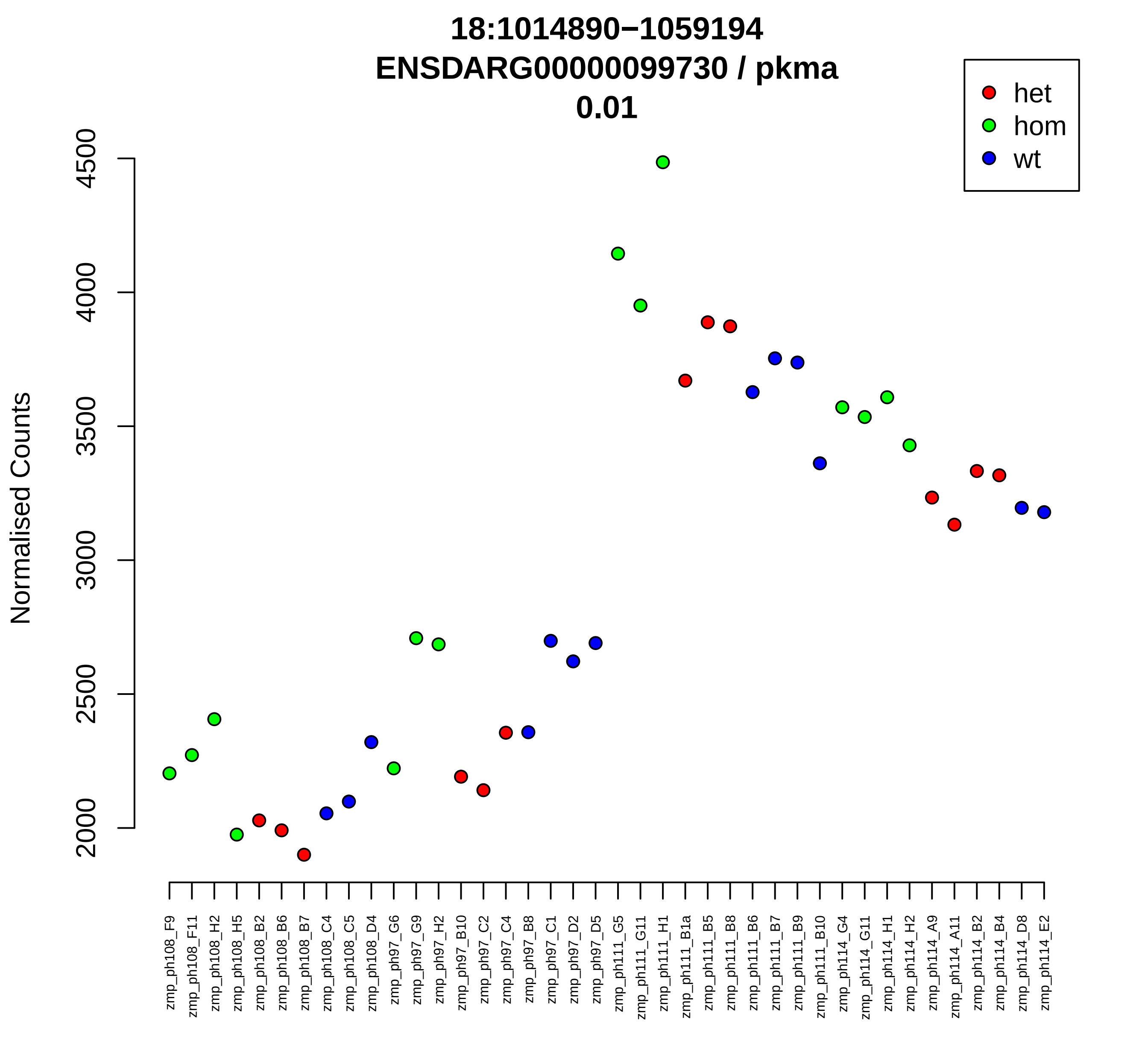
18:1014890−1059194
ENSDARG00000099730 / pkma
0.01
het
hom
wt
Normalised Counts
2000
2500
3000
3500
4000
4500
zmp_ph108_F9
zmp_ph108_F11
zmp_ph108_H2
zmp_ph108_H5
zmp_ph108_B2
zmp_ph108_B6
zmp_ph108_B7
zmp_ph108_C4
zmp_ph108_C5
zmp_ph108_D4
zmp_ph97_G6
zmp_ph97_G9
zmp_ph97_H2
zmp_ph97_B10
zmp_ph97_C2
zmp_ph97_C4
zmp_ph97_B8
zmp_ph97_C1
zmp_ph97_D2
zmp_ph97_D5
zmp_ph111_G5
zmp_ph111_G11
zmp_ph111_H1
zmp_ph111_B1a
zmp_ph111_B5
zmp_ph111_B8
zmp_ph111_B6
zmp_ph111_B7
zmp_ph111_B9
zmp_ph111_B10
zmp_ph114_G4
zmp_ph114_G11
zmp_ph114_H1
zmp_ph114_H2
zmp_ph114_A9
zmp_ph114_A11
zmp_ph114_B2
zmp_ph114_B4
zmp_ph114_D8
zmp_ph114_E2

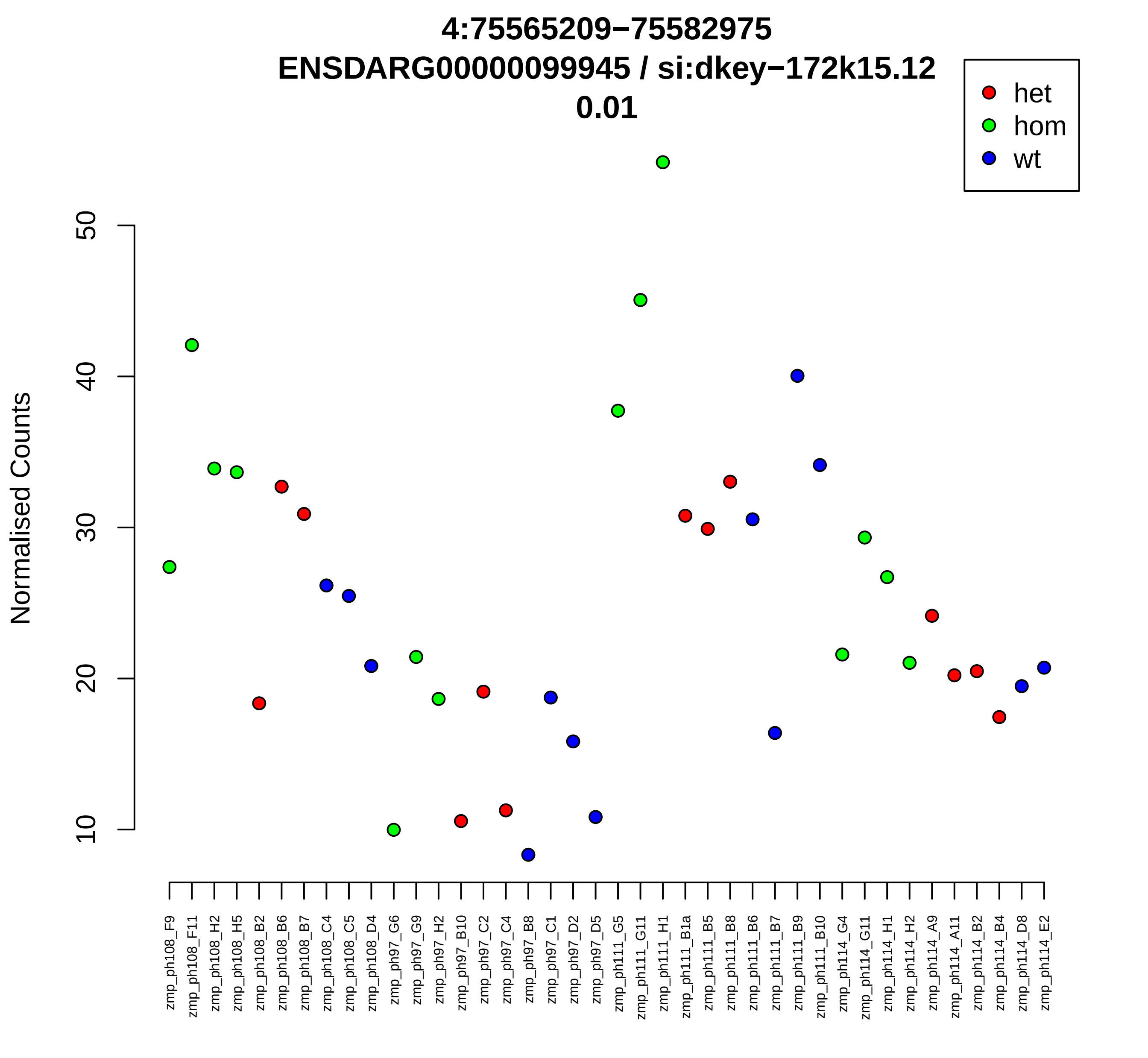

4:75565209−75582975
ENSDARG00000099945 / si:dkey−172k15.12
0.01
het
hom
wt
Normalised Counts
10
20
30
40
50
zmp_ph108_F9
zmp_ph108_F11
zmp_ph108_H2
zmp_ph108_H5
zmp_ph108_B2
zmp_ph108_B6
zmp_ph108_B7
zmp_ph108_C4
zmp_ph108_C5
zmp_ph108_D4
zmp_ph97_G6
zmp_ph97_G9
zmp_ph97_H2
zmp_ph97_B10
zmp_ph97_C2
zmp_ph97_C4
zmp_ph97_B8
zmp_ph97_C1
zmp_ph97_D2
zmp_ph97_D5
zmp_ph111_G5
zmp_ph111_G11
zmp_ph111_H1
zmp_ph111_B1a
zmp_ph111_B5
zmp_ph111_B8
zmp_ph111_B6
zmp_ph111_B7
zmp_ph111_B9
zmp_ph111_B10
zmp_ph114_G4
zmp_ph114_G11
zmp_ph114_H1
zmp_ph114_H2
zmp_ph114_A9
zmp_ph114_A11
zmp_ph114_B2
zmp_ph114_B4
zmp_ph114_D8
zmp_ph114_E2

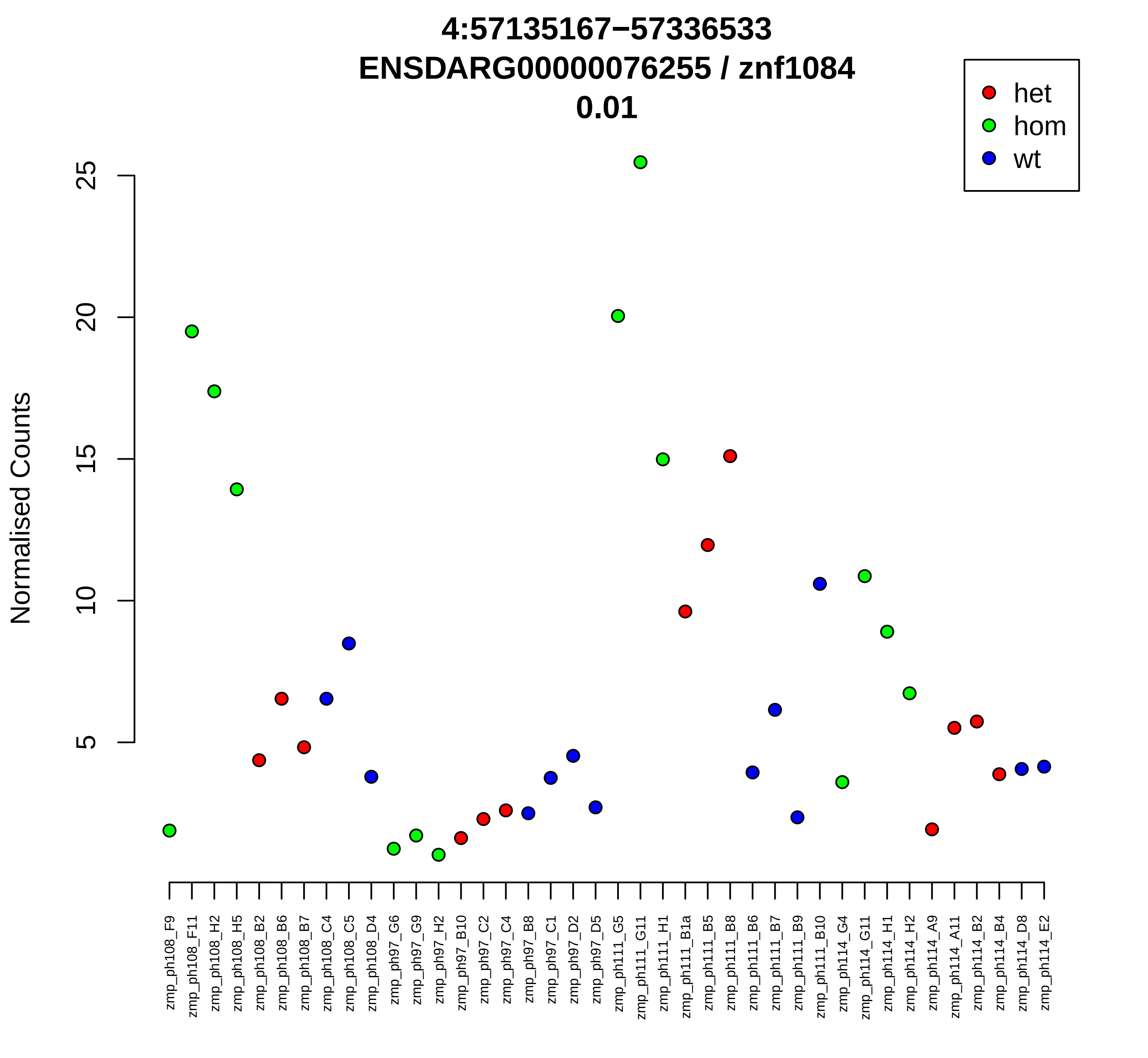
4:57135167−57336533
ENSDARG00000076255 / znf1084
0.01
het
hom
wt
Normalised Counts
5
10
15
20
25
zmp_ph108_F9
zmp_ph108_F11
zmp_ph108_H2
zmp_ph108_H5
zmp_ph108_B2
zmp_ph108_B6
zmp_ph108_B7
zmp_ph108_C4
zmp_ph108_C5
zmp_ph108_D4
zmp_ph97_G6
zmp_ph97_G9
zmp_ph97_H2
zmp_ph97_B10
zmp_ph97_C2
zmp_ph97_C4
zmp_ph97_B8
zmp_ph97_C1
zmp_ph97_D2
zmp_ph97_D5
zmp_ph111_G5
zmp_ph111_G11
zmp_ph111_H1
zmp_ph111_B1a
zmp_ph111_B5
zmp_ph111_B8
zmp_ph111_B6
zmp_ph111_B7
zmp_ph111_B9
zmp_ph111_B10
zmp_ph114_G4
zmp_ph114_G11
zmp_ph114_H1
zmp_ph114_H2
zmp_ph114_A9
zmp_ph114_A11
zmp_ph114_B2
zmp_ph114_B4
zmp_ph114_D8
zmp_ph114_E2

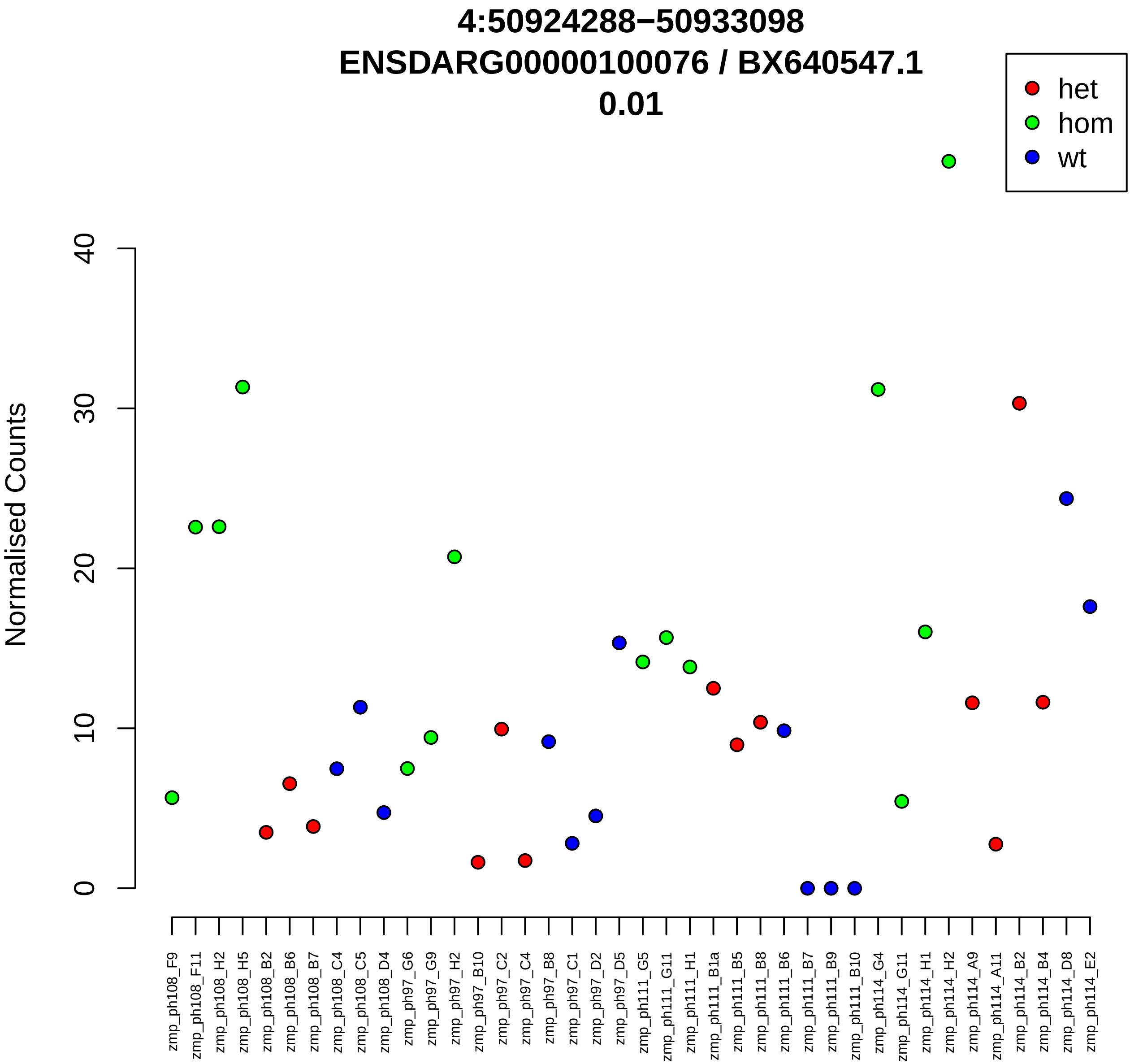
4:50924288−50933098
ENSDARG00000100076 / BX640547.1
0.01
het
hom
wt
Normalised Counts
0
10
20
30
40
zmp_ph108_F9
zmp_ph108_F11
zmp_ph108_H2
zmp_ph108_H5
zmp_ph108_B2
zmp_ph108_B6
zmp_ph108_B7
zmp_ph108_C4
zmp_ph108_C5
zmp_ph108_D4
zmp_ph97_G6
zmp_ph97_G9
zmp_ph97_H2
zmp_ph97_B10
zmp_ph97_C2
zmp_ph97_C4
zmp_ph97_B8
zmp_ph97_C1
zmp_ph97_D2
zmp_ph97_D5
zmp_ph111_G5
zmp_ph111_G11
zmp_ph111_H1
zmp_ph111_B1a
zmp_ph111_B5
zmp_ph111_B8
zmp_ph111_B6
zmp_ph111_B7
zmp_ph111_B9
zmp_ph111_B10
zmp_ph114_G4
zmp_ph114_G11
zmp_ph114_H1
zmp_ph114_H2
zmp_ph114_A9
zmp_ph114_A11
zmp_ph114_B2
zmp_ph114_B4
zmp_ph114_D8
zmp_ph114_E2

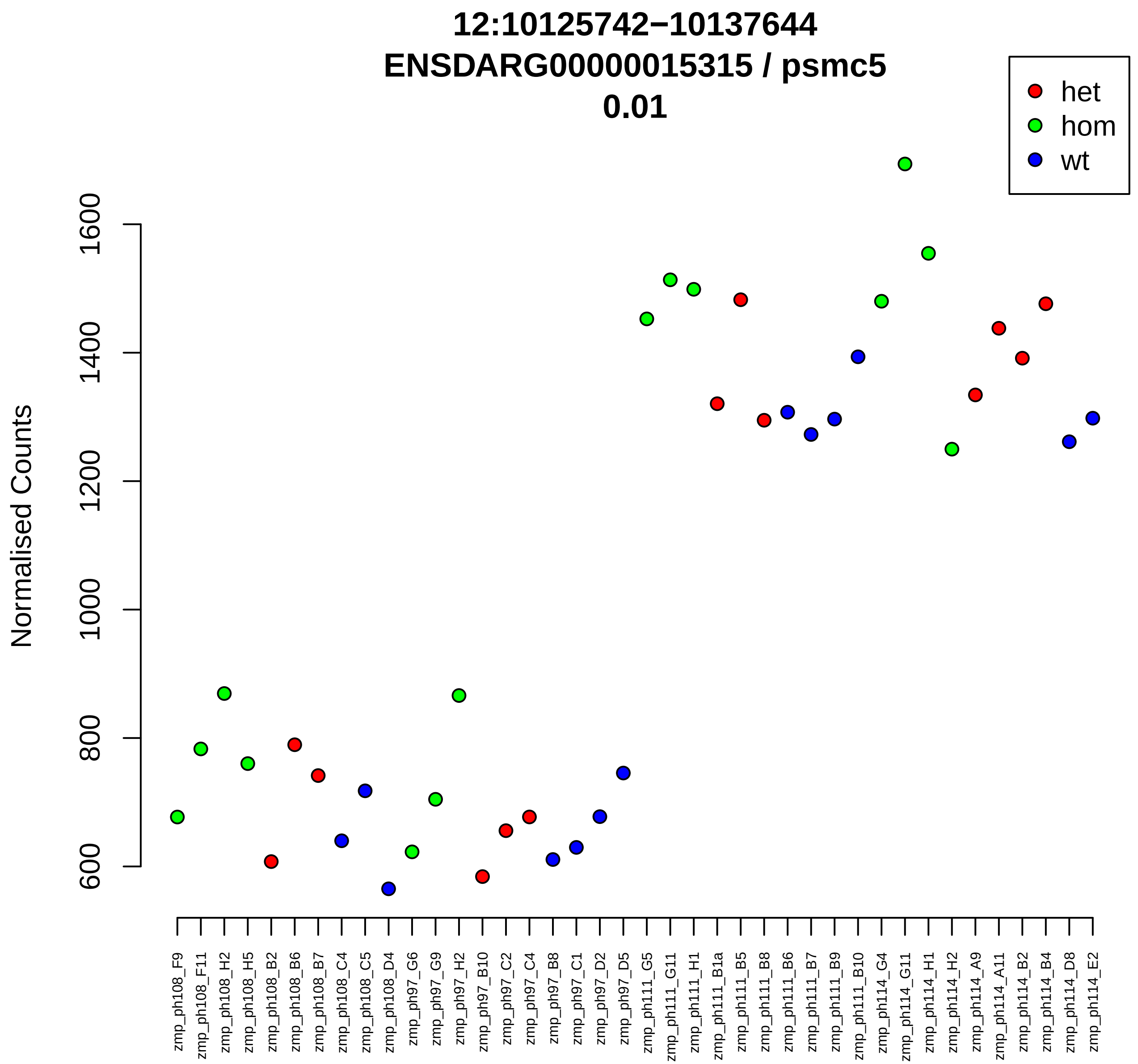

12:10125742−10137644
ENSDARG00000015315 / psmc5
0.01
het
hom
wt
Normalised Counts
600
800
1000
1200
1400
1600
zmp_ph108_F9
zmp_ph108_F11
zmp_ph108_H2
zmp_ph108_H5
zmp_ph108_B2
zmp_ph108_B6
zmp_ph108_B7
zmp_ph108_C4
zmp_ph108_C5
zmp_ph108_D4
zmp_ph97_G6
zmp_ph97_G9
zmp_ph97_H2
zmp_ph97_B10
zmp_ph97_C2
zmp_ph97_C4
zmp_ph97_B8
zmp_ph97_C1
zmp_ph97_D2
zmp_ph97_D5
zmp_ph111_G5
zmp_ph111_G11
zmp_ph111_H1
zmp_ph111_B1a
zmp_ph111_B5
zmp_ph111_B8
zmp_ph111_B6
zmp_ph111_B7
zmp_ph111_B9
zmp_ph111_B10
zmp_ph114_G4
zmp_ph114_G11
zmp_ph114_H1
zmp_ph114_H2
zmp_ph114_A9
zmp_ph114_A11
zmp_ph114_B2
zmp_ph114_B4
zmp_ph114_D8
zmp_ph114_E2

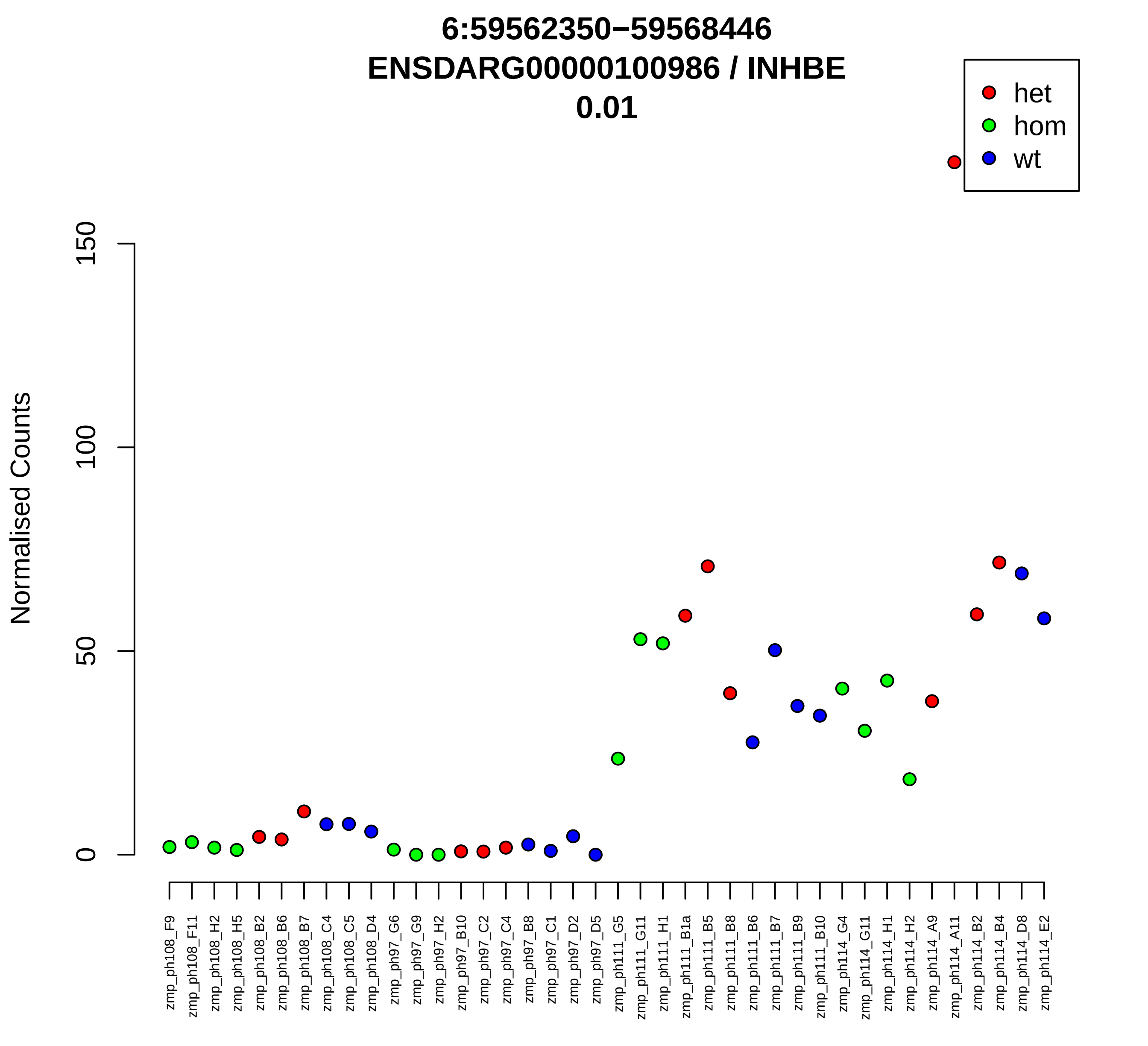

6:59562350−59568446
ENSDARG00000100986 / INHBE
0.01
het
hom
wt
Normalised Counts
0
50
100
150
zmp_ph108_F9
zmp_ph108_F11
zmp_ph108_H2
zmp_ph108_H5
zmp_ph108_B2
zmp_ph108_B6
zmp_ph108_B7
zmp_ph108_C4
zmp_ph108_C5
zmp_ph108_D4
zmp_ph97_G6
zmp_ph97_G9
zmp_ph97_H2
zmp_ph97_B10
zmp_ph97_C2
zmp_ph97_C4
zmp_ph97_B8
zmp_ph97_C1
zmp_ph97_D2
zmp_ph97_D5
zmp_ph111_G5
zmp_ph111_G11
zmp_ph111_H1
zmp_ph111_B1a
zmp_ph111_B5
zmp_ph111_B8
zmp_ph111_B6
zmp_ph111_B7
zmp_ph111_B9
zmp_ph111_B10
zmp_ph114_G4
zmp_ph114_G11
zmp_ph114_H1
zmp_ph114_H2
zmp_ph114_A9
zmp_ph114_A11
zmp_ph114_B2
zmp_ph114_B4
zmp_ph114_D8
zmp_ph114_E2

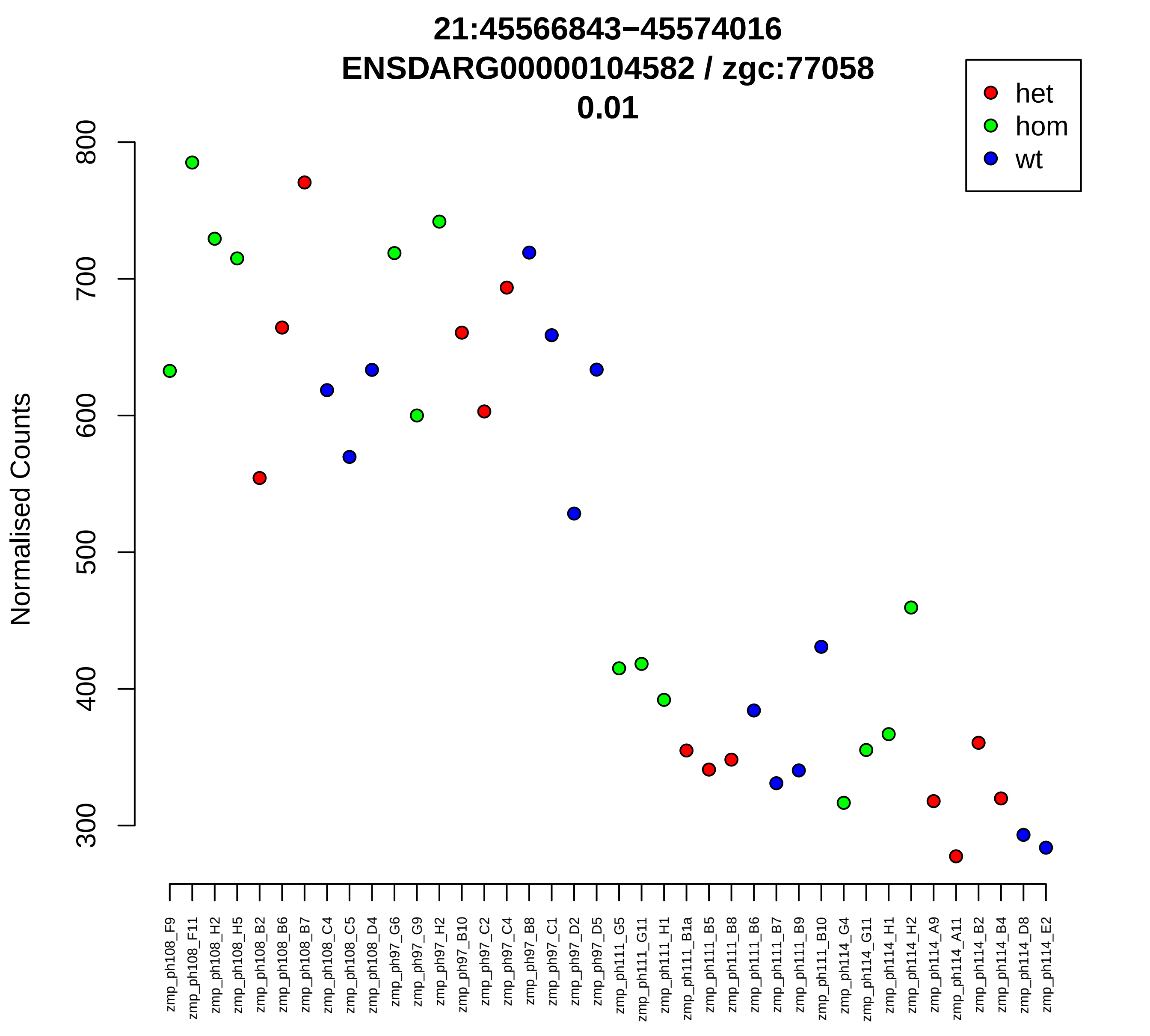

21:45566843−45574016
ENSDARG00000104582 / zgc:77058
0.01
het
hom
wt
Normalised Counts
800
700
600
500
400
300
zmp_ph108_F9
zmp_ph108_F11
zmp_ph108_H2
zmp_ph108_H5
zmp_ph108_B2
zmp_ph108_B6
zmp_ph108_B7
zmp_ph108_C4
zmp_ph108_C5
zmp_ph108_D4
zmp_ph97_G6
zmp_ph97_G9
zmp_ph97_H2
zmp_ph97_B10
zmp_ph97_C2
zmp_ph97_C4
zmp_ph97_B8
zmp_ph97_C1
zmp_ph97_D2
zmp_ph97_D5
zmp_ph111_G5
zmp_ph111_G11
zmp_ph111_H1
zmp_ph111_B1a
zmp_ph111_B5
zmp_ph111_B8
zmp_ph111_B6
zmp_ph111_B7
zmp_ph111_B9
zmp_ph111_B10
zmp_ph114_G4
zmp_ph114_G11
zmp_ph114_H1
zmp_ph114_H2
zmp_ph114_A9
zmp_ph114_A11
zmp_ph114_B2
zmp_ph114_B4
zmp_ph114_D8
zmp_ph114_E2

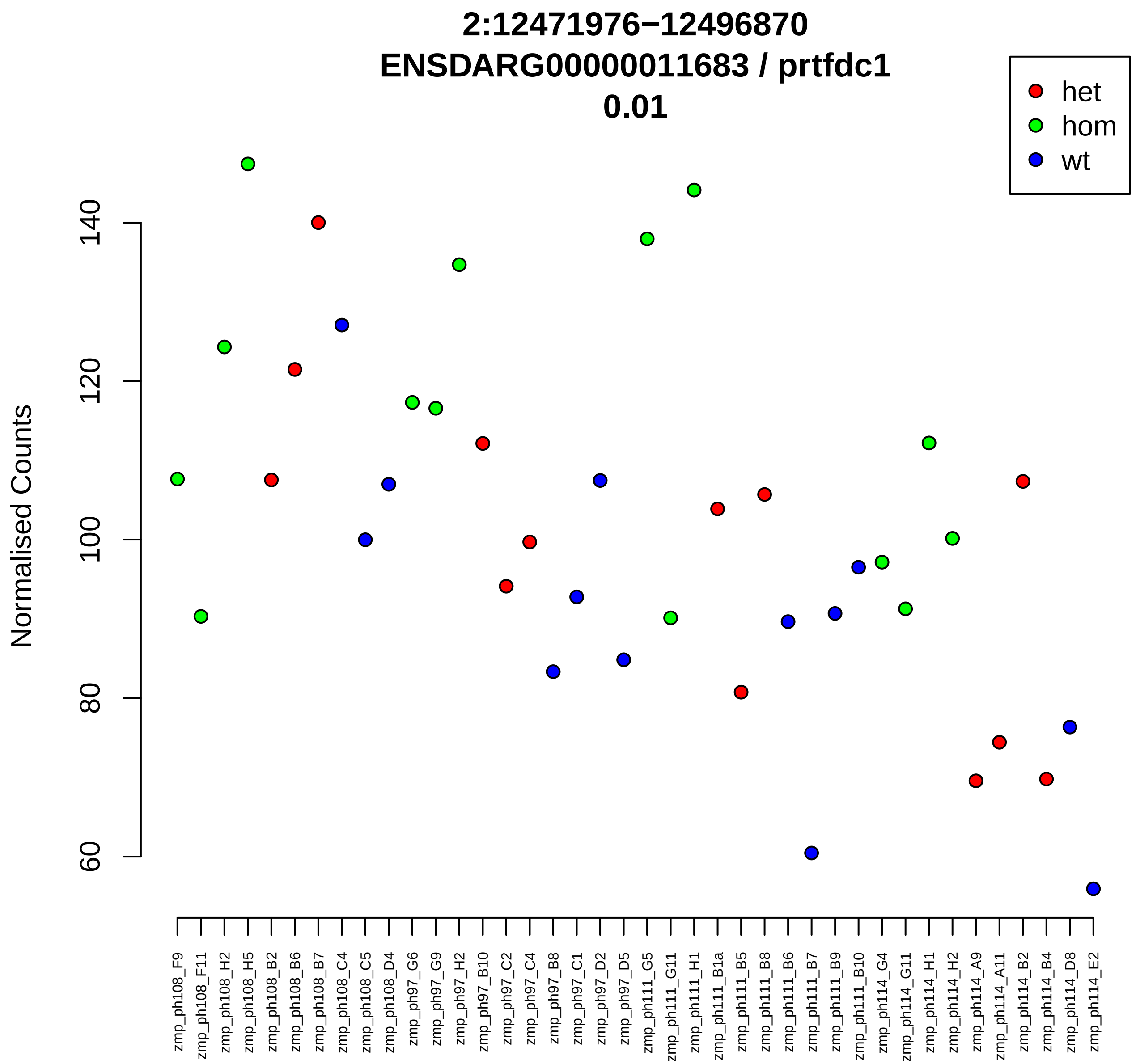

2:12471976−12496870
ENSDARG00000011683 / prtfdc1
0.01
het
hom
wt
Normalised Counts
60
80
100
120
140
zmp_ph108_F9
zmp_ph108_F11
zmp_ph108_H2
zmp_ph108_H5
zmp_ph108_B2
zmp_ph108_B6
zmp_ph108_B7
zmp_ph108_C4
zmp_ph108_C5
zmp_ph108_D4
zmp_ph97_G6
zmp_ph97_G9
zmp_ph97_H2
zmp_ph97_B10
zmp_ph97_C2
zmp_ph97_C4
zmp_ph97_B8
zmp_ph97_C1
zmp_ph97_D2
zmp_ph97_D5
zmp_ph111_G5
zmp_ph111_G11
zmp_ph111_H1
zmp_ph111_B1a
zmp_ph111_B5
zmp_ph111_B8
zmp_ph111_B6
zmp_ph111_B7
zmp_ph111_B9
zmp_ph111_B10
zmp_ph114_G4
zmp_ph114_G11
zmp_ph114_H1
zmp_ph114_H2
zmp_ph114_A9
zmp_ph114_A11
zmp_ph114_B2
zmp_ph114_B4
zmp_ph114_D8
zmp_ph114_E2

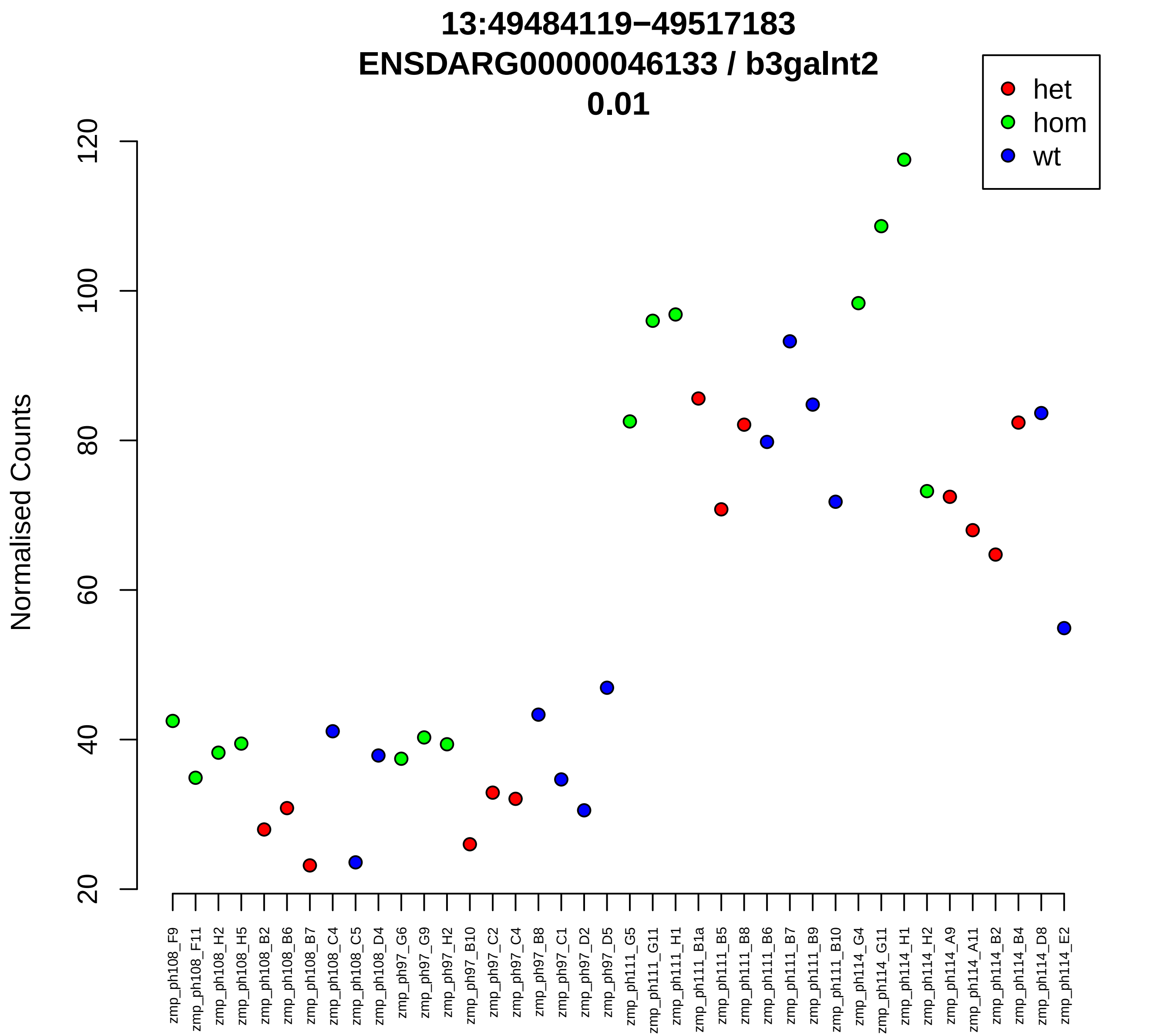

13:49484119−49517183
ENSDARG00000046133 / b3galnt2
0.01
het
hom
wt
Normalised Counts
20
40
60
80
100
120
zmp_ph108_F9
zmp_ph108_F11
zmp_ph108_H2
zmp_ph108_H5
zmp_ph108_B2
zmp_ph108_B6
zmp_ph108_B7
zmp_ph108_C4
zmp_ph108_C5
zmp_ph108_D4
zmp_ph97_G6
zmp_ph97_G9
zmp_ph97_H2
zmp_ph97_B10
zmp_ph97_C2
zmp_ph97_C4
zmp_ph97_B8
zmp_ph97_C1
zmp_ph97_D2
zmp_ph97_D5
zmp_ph111_G5
zmp_ph111_G11
zmp_ph111_H1
zmp_ph111_B1a
zmp_ph111_B5
zmp_ph111_B8
zmp_ph111_B6
zmp_ph111_B7
zmp_ph111_B9
zmp_ph111_B10
zmp_ph114_G4
zmp_ph114_G11
zmp_ph114_H1
zmp_ph114_H2
zmp_ph114_A9
zmp_ph114_A11
zmp_ph114_B2
zmp_ph114_B4
zmp_ph114_D8
zmp_ph114_E2

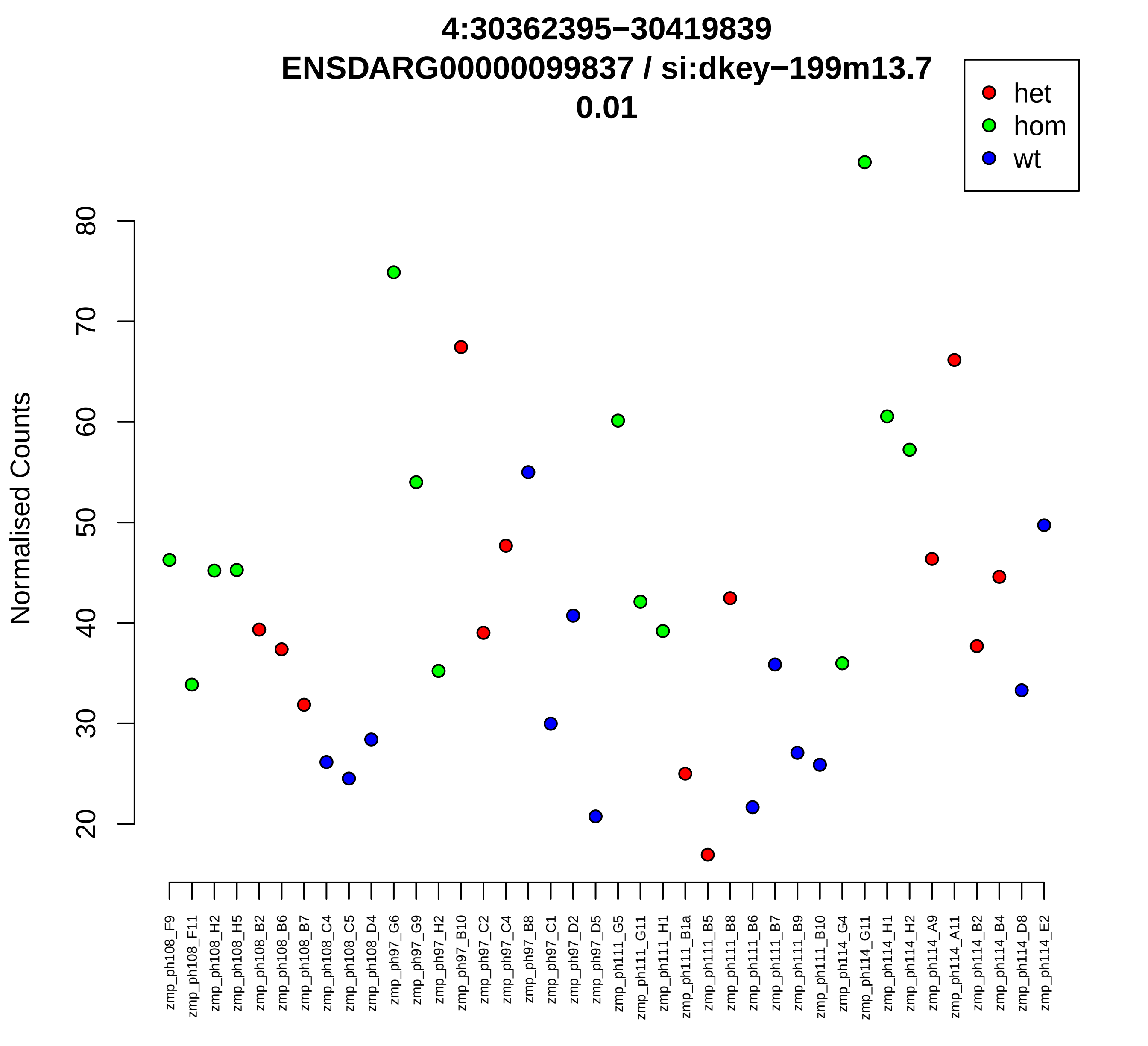
4:30362395−30419839
ENSDARG00000099837 / si:dkey−199m13.7
0.01
het
hom
wt
Normalised Counts
20
30
40
50
60
70
80
zmp_ph108_F9
zmp_ph108_F11
zmp_ph108_H2
zmp_ph108_H5
zmp_ph108_B2
zmp_ph108_B6
zmp_ph108_B7
zmp_ph108_C4
zmp_ph108_C5
zmp_ph108_D4
zmp_ph97_G6
zmp_ph97_G9
zmp_ph97_H2
zmp_ph97_B10
zmp_ph97_C2
zmp_ph97_C4
zmp_ph97_B8
zmp_ph97_C1
zmp_ph97_D2
zmp_ph97_D5
zmp_ph111_G5
zmp_ph111_G11
zmp_ph111_H1
zmp_ph111_B1a
zmp_ph111_B5
zmp_ph111_B8
zmp_ph111_B6
zmp_ph111_B7
zmp_ph111_B9
zmp_ph111_B10
zmp_ph114_G4
zmp_ph114_G11
zmp_ph114_H1
zmp_ph114_H2
zmp_ph114_A9
zmp_ph114_A11
zmp_ph114_B2
zmp_ph114_B4
zmp_ph114_D8
zmp_ph114_E2

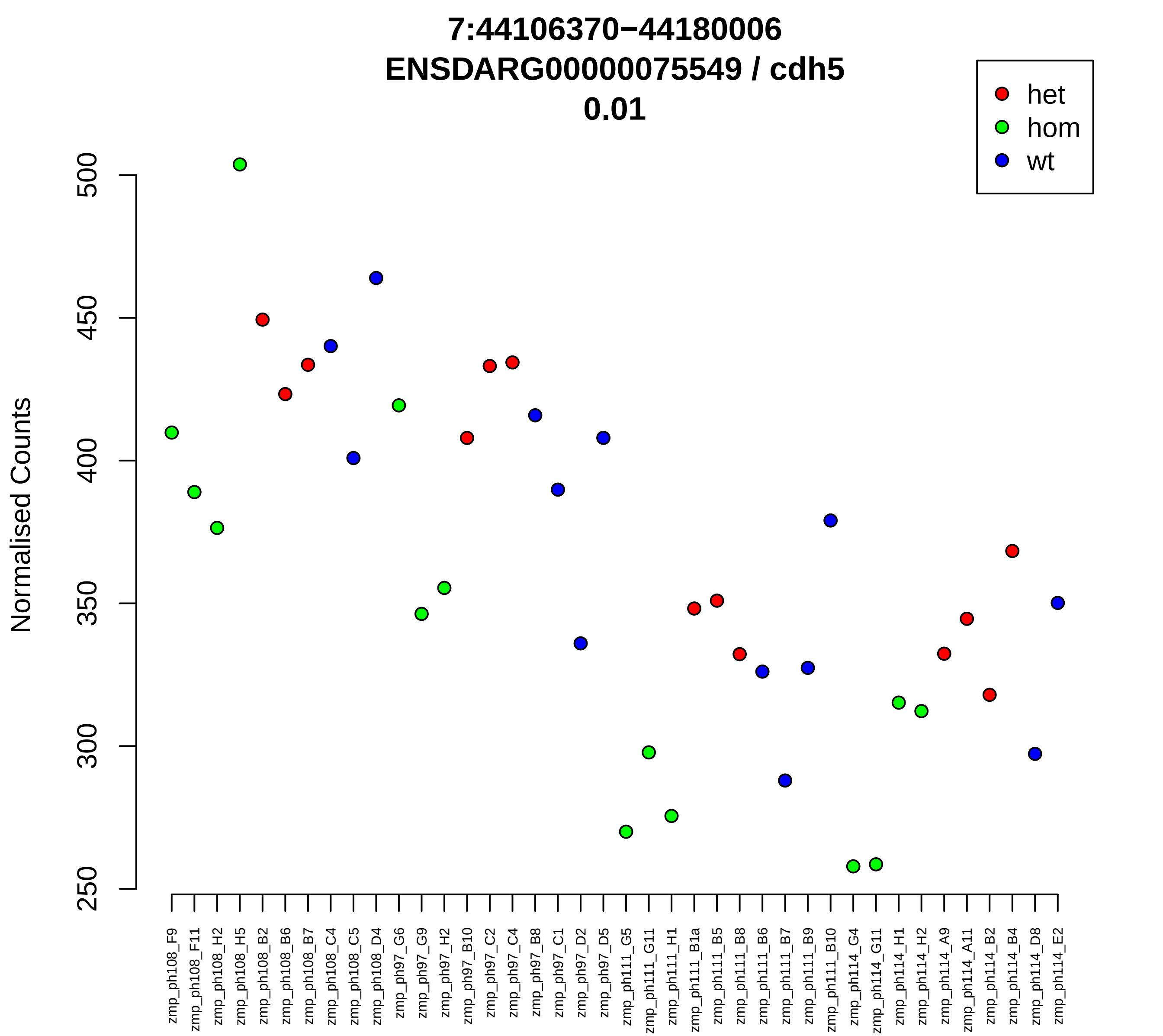
7:44106370−44180006
ENSDARG00000075549 / cdh5
0.01
het
hom
wt
Normalised Counts
250
300
350
400
450
500
zmp_ph108_F9
zmp_ph108_F11
zmp_ph108_H2
zmp_ph108_H5
zmp_ph108_B2
zmp_ph108_B6
zmp_ph108_B7
zmp_ph108_C4
zmp_ph108_C5
zmp_ph108_D4
zmp_ph97_G6
zmp_ph97_G9
zmp_ph97_H2
zmp_ph97_B10
zmp_ph97_C2
zmp_ph97_C4
zmp_ph97_B8
zmp_ph97_C1
zmp_ph97_D2
zmp_ph97_D5
zmp_ph111_G5
zmp_ph111_G11
zmp_ph111_H1
zmp_ph111_B1a
zmp_ph111_B5
zmp_ph111_B8
zmp_ph111_B6
zmp_ph111_B7
zmp_ph111_B9
zmp_ph111_B10
zmp_ph114_G4
zmp_ph114_G11
zmp_ph114_H1
zmp_ph114_H2
zmp_ph114_A9
zmp_ph114_A11
zmp_ph114_B2
zmp_ph114_B4
zmp_ph114_D8
zmp_ph114_E2

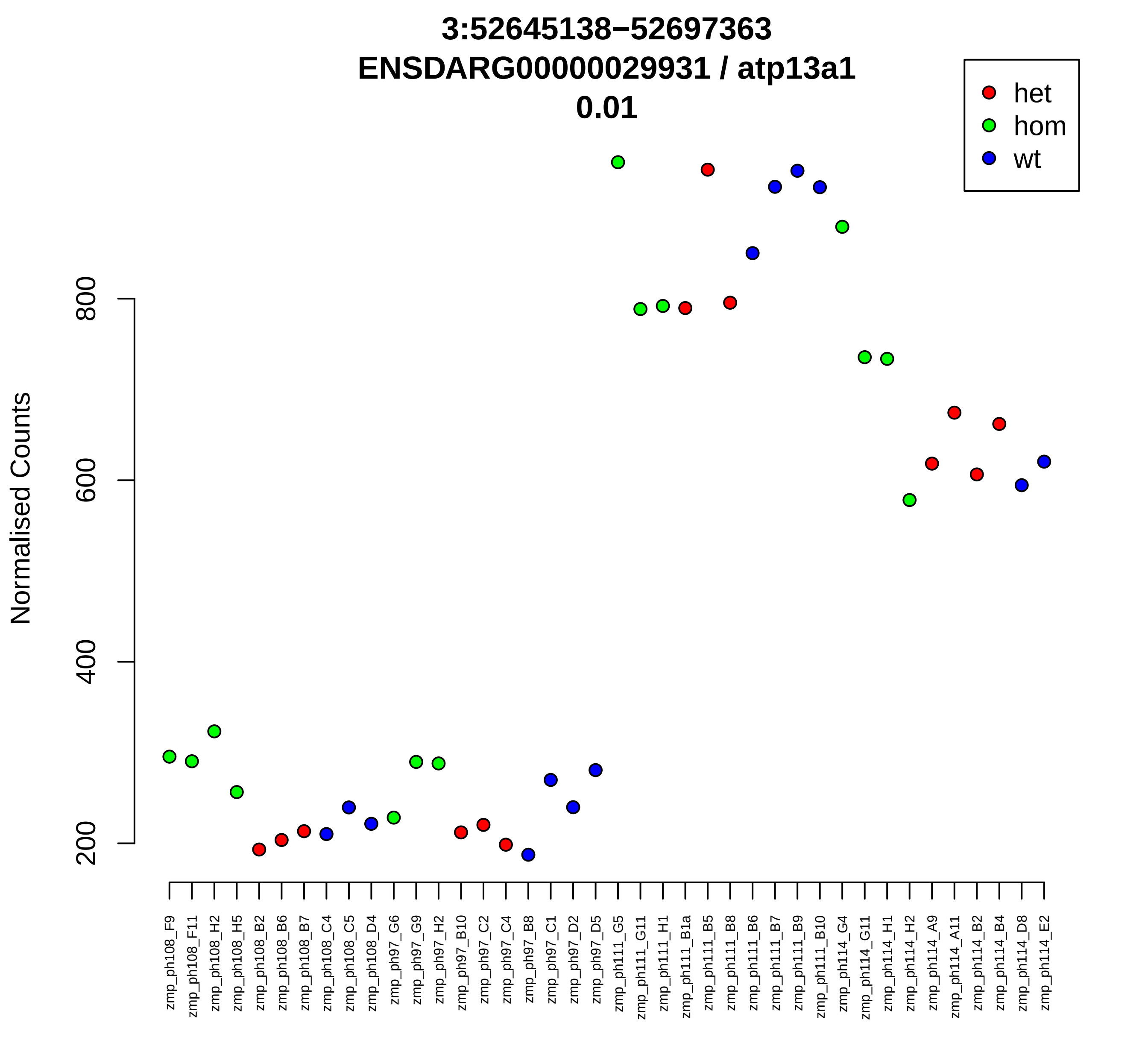
3:52645138−52697363
ENSDARG00000029931 / atp13a1
0.01
het
hom
wt
Normalised Counts
200
400
600
800
zmp_ph108_F9
zmp_ph108_F11
zmp_ph108_H2
zmp_ph108_H5
zmp_ph108_B2
zmp_ph108_B6
zmp_ph108_B7
zmp_ph108_C4
zmp_ph108_C5
zmp_ph108_D4
zmp_ph97_G6
zmp_ph97_G9
zmp_ph97_H2
zmp_ph97_B10
zmp_ph97_C2
zmp_ph97_C4
zmp_ph97_B8
zmp_ph97_C1
zmp_ph97_D2
zmp_ph97_D5
zmp_ph111_G5
zmp_ph111_G11
zmp_ph111_H1
zmp_ph111_B1a
zmp_ph111_B5
zmp_ph111_B8
zmp_ph111_B6
zmp_ph111_B7
zmp_ph111_B9
zmp_ph111_B10
zmp_ph114_G4
zmp_ph114_G11
zmp_ph114_H1
zmp_ph114_H2
zmp_ph114_A9
zmp_ph114_A11
zmp_ph114_B2
zmp_ph114_B4
zmp_ph114_D8
zmp_ph114_E2

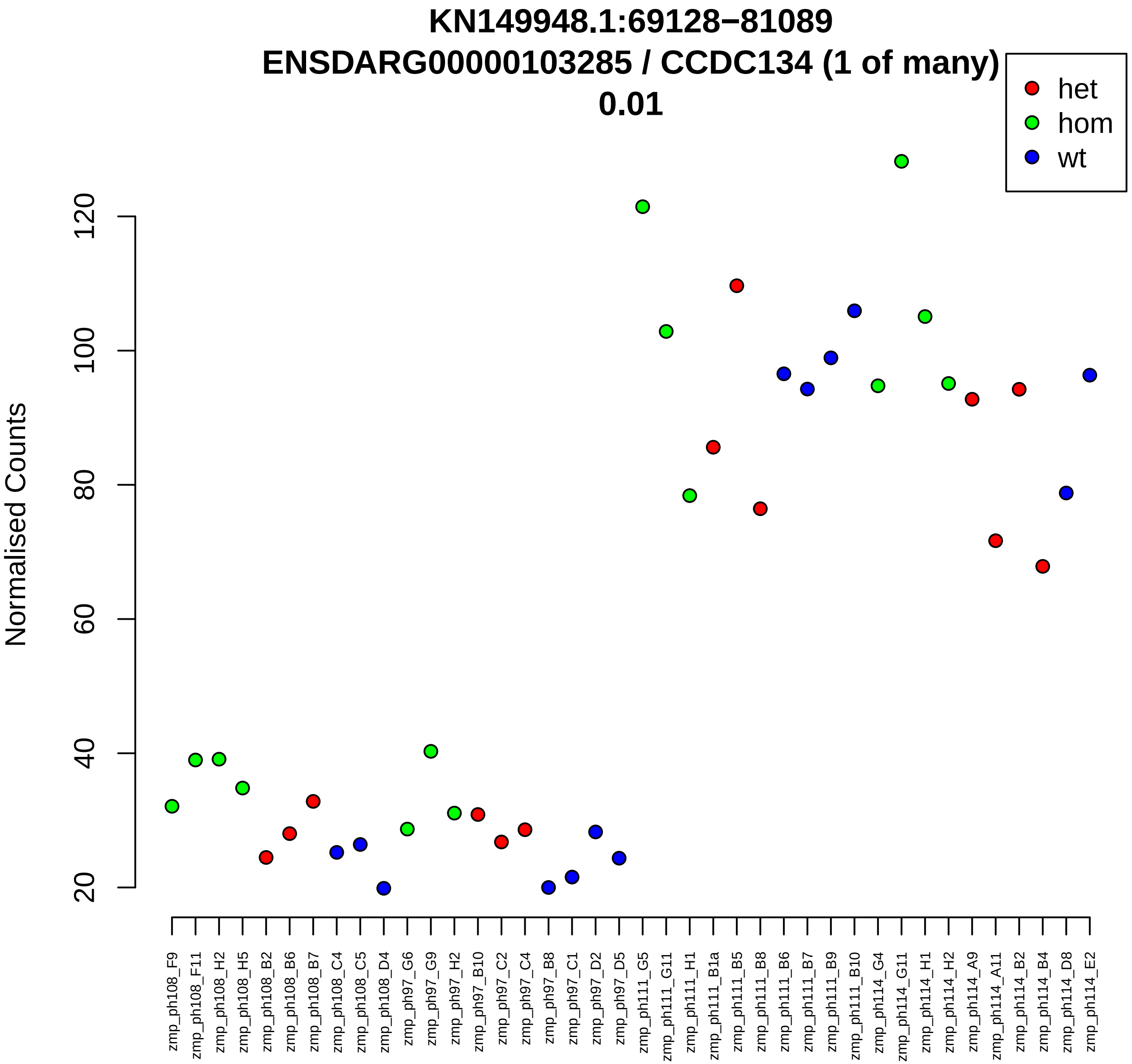
KN149948.1:69128−81089
ENSDARG00000103285 / CCDC134 (1 of many)
0.01
het
hom
wt
Normalised Counts
20
40
60
80
100
120
zmp_ph108_F9
zmp_ph108_F11
zmp_ph108_H2
zmp_ph108_H5
zmp_ph108_B2
zmp_ph108_B6
zmp_ph108_B7
zmp_ph108_C4
zmp_ph108_C5
zmp_ph108_D4
zmp_ph97_G6
zmp_ph97_G9
zmp_ph97_H2
zmp_ph97_B10
zmp_ph97_C2
zmp_ph97_C4
zmp_ph97_B8
zmp_ph97_C1
zmp_ph97_D2
zmp_ph97_D5
zmp_ph111_G5
zmp_ph111_G11
zmp_ph111_H1
zmp_ph111_B1a
zmp_ph111_B5
zmp_ph111_B8
zmp_ph111_B6
zmp_ph111_B7
zmp_ph111_B9
zmp_ph111_B10
zmp_ph114_G4
zmp_ph114_G11
zmp_ph114_H1
zmp_ph114_H2
zmp_ph114_A9
zmp_ph114_A11
zmp_ph114_B2
zmp_ph114_B4
zmp_ph114_D8
zmp_ph114_E2

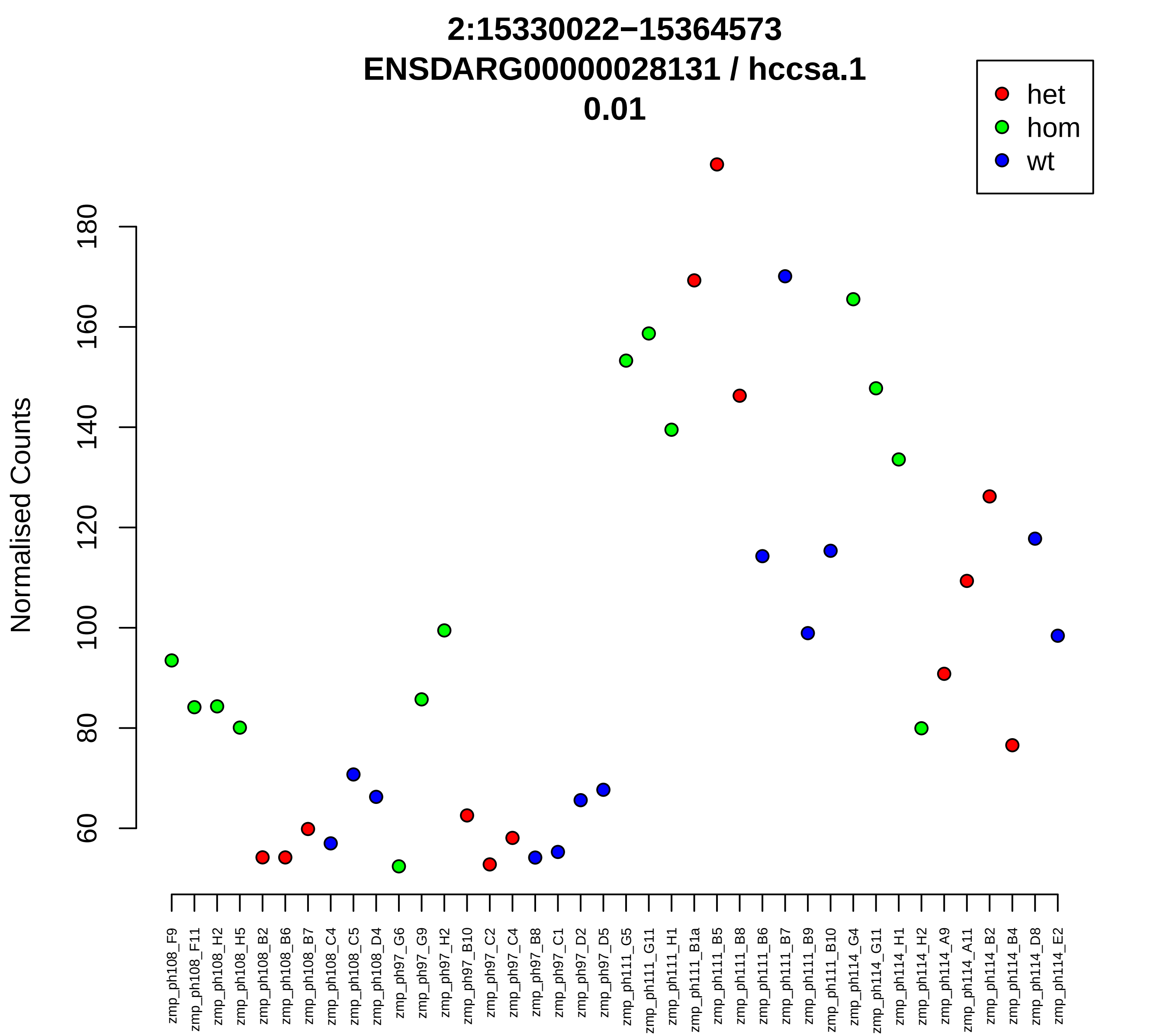
2:15330022−15364573
ENSDARG00000028131 / hccsa.1
0.01
het
hom
wt
Normalised Counts
60
80
100
120
140
160
180
zmp_ph108_F9
zmp_ph108_F11
zmp_ph108_H2
zmp_ph108_H5
zmp_ph108_B2
zmp_ph108_B6
zmp_ph108_B7
zmp_ph108_C4
zmp_ph108_C5
zmp_ph108_D4
zmp_ph97_G6
zmp_ph97_G9
zmp_ph97_H2
zmp_ph97_B10
zmp_ph97_C2
zmp_ph97_C4
zmp_ph97_B8
zmp_ph97_C1
zmp_ph97_D2
zmp_ph97_D5
zmp_ph111_G5
zmp_ph111_G11
zmp_ph111_H1
zmp_ph111_B1a
zmp_ph111_B5
zmp_ph111_B8
zmp_ph111_B6
zmp_ph111_B7
zmp_ph111_B9
zmp_ph111_B10
zmp_ph114_G4
zmp_ph114_G11
zmp_ph114_H1
zmp_ph114_H2
zmp_ph114_A9
zmp_ph114_A11
zmp_ph114_B2
zmp_ph114_B4
zmp_ph114_D8
zmp_ph114_E2

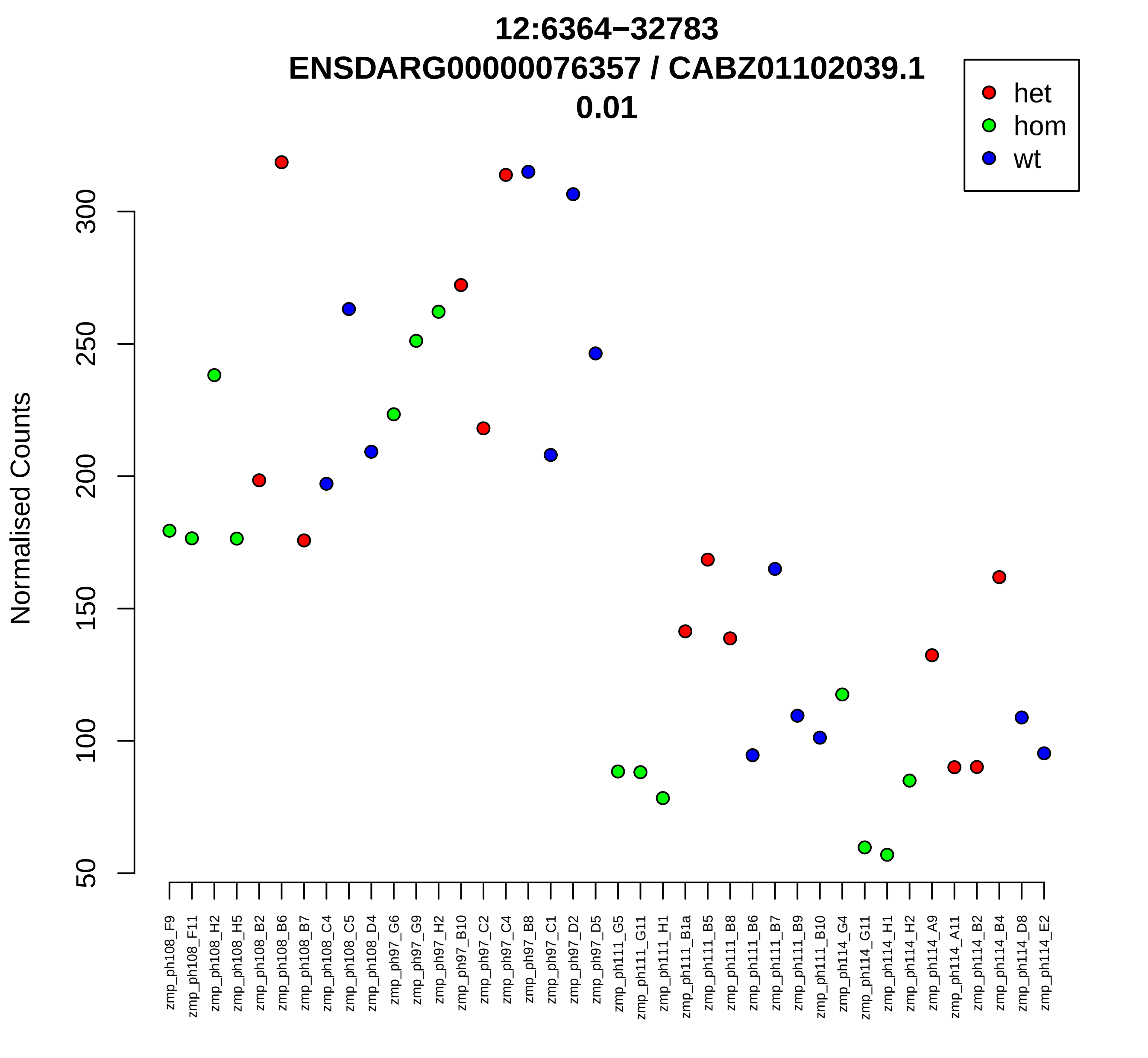

12:6364−32783
ENSDARG00000076357 / CABZ01102039.1
0.01
het
hom
wt
Normalised Counts
50
100
150
200
250
300
zmp_ph108_F9
zmp_ph108_F11
zmp_ph108_H2
zmp_ph108_H5
zmp_ph108_B2
zmp_ph108_B6
zmp_ph108_B7
zmp_ph108_C4
zmp_ph108_C5
zmp_ph108_D4
zmp_ph97_G6
zmp_ph97_G9
zmp_ph97_H2
zmp_ph97_B10
zmp_ph97_C2
zmp_ph97_C4
zmp_ph97_B8
zmp_ph97_C1
zmp_ph97_D2
zmp_ph97_D5
zmp_ph111_G5
zmp_ph111_G11
zmp_ph111_H1
zmp_ph111_B1a
zmp_ph111_B5
zmp_ph111_B8
zmp_ph111_B6
zmp_ph111_B7
zmp_ph111_B9
zmp_ph111_B10
zmp_ph114_G4
zmp_ph114_G11
zmp_ph114_H1
zmp_ph114_H2
zmp_ph114_A9
zmp_ph114_A11
zmp_ph114_B2
zmp_ph114_B4
zmp_ph114_D8
zmp_ph114_E2

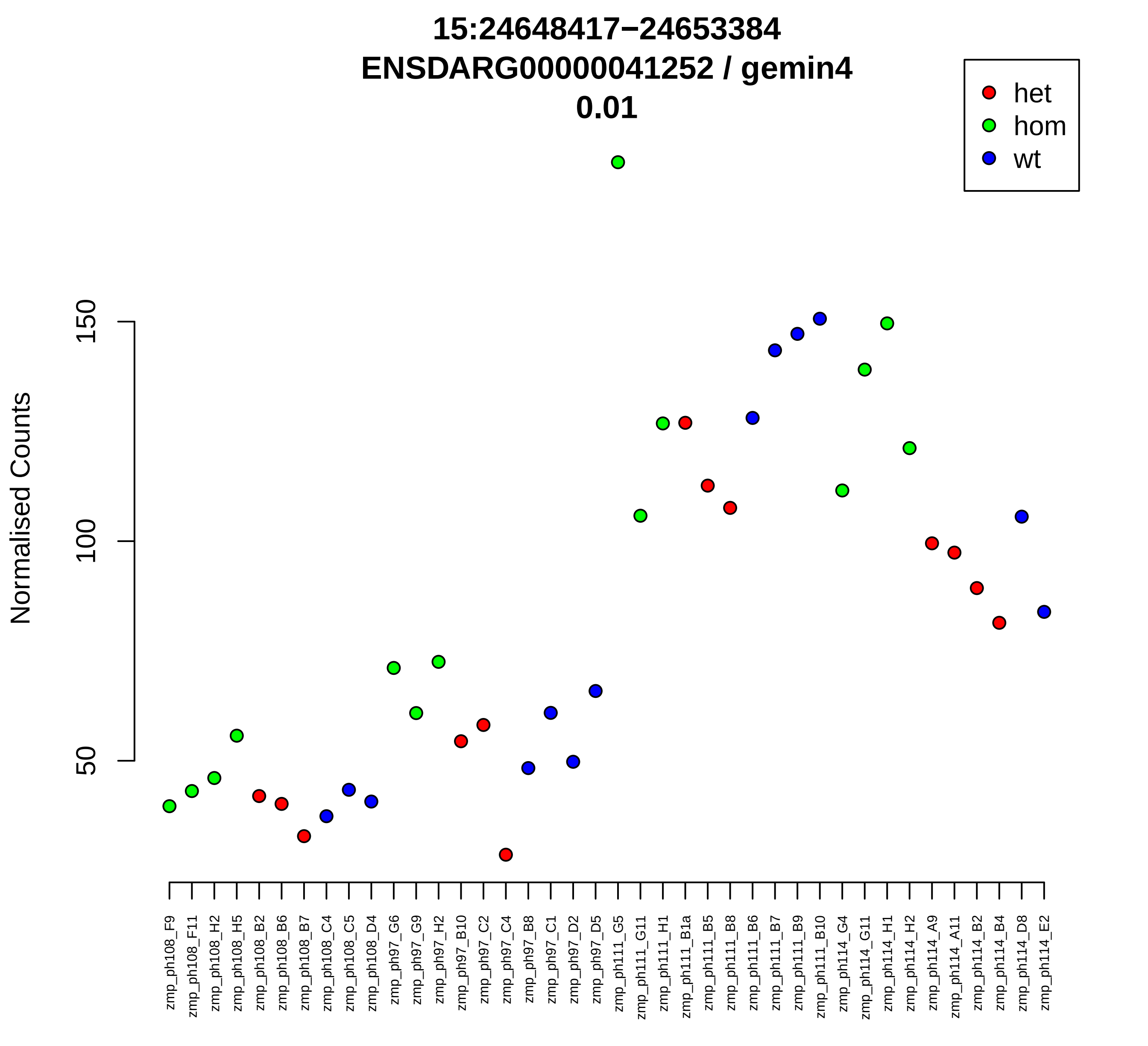
15:24648417−24653384
ENSDARG00000041252 / gemin4
0.01
het
hom
wt
Normalised Counts
50
100
150
zmp_ph108_F9
zmp_ph108_F11
zmp_ph108_H2
zmp_ph108_H5
zmp_ph108_B2
zmp_ph108_B6
zmp_ph108_B7
zmp_ph108_C4
zmp_ph108_C5
zmp_ph108_D4
zmp_ph97_G6
zmp_ph97_G9
zmp_ph97_H2
zmp_ph97_B10
zmp_ph97_C2
zmp_ph97_C4
zmp_ph97_B8
zmp_ph97_C1
zmp_ph97_D2
zmp_ph97_D5
zmp_ph111_G5
zmp_ph111_G11
zmp_ph111_H1
zmp_ph111_B1a
zmp_ph111_B5
zmp_ph111_B8
zmp_ph111_B6
zmp_ph111_B7
zmp_ph111_B9
zmp_ph111_B10
zmp_ph114_G4
zmp_ph114_G11
zmp_ph114_H1
zmp_ph114_H2
zmp_ph114_A9
zmp_ph114_A11
zmp_ph114_B2
zmp_ph114_B4
zmp_ph114_D8
zmp_ph114_E2

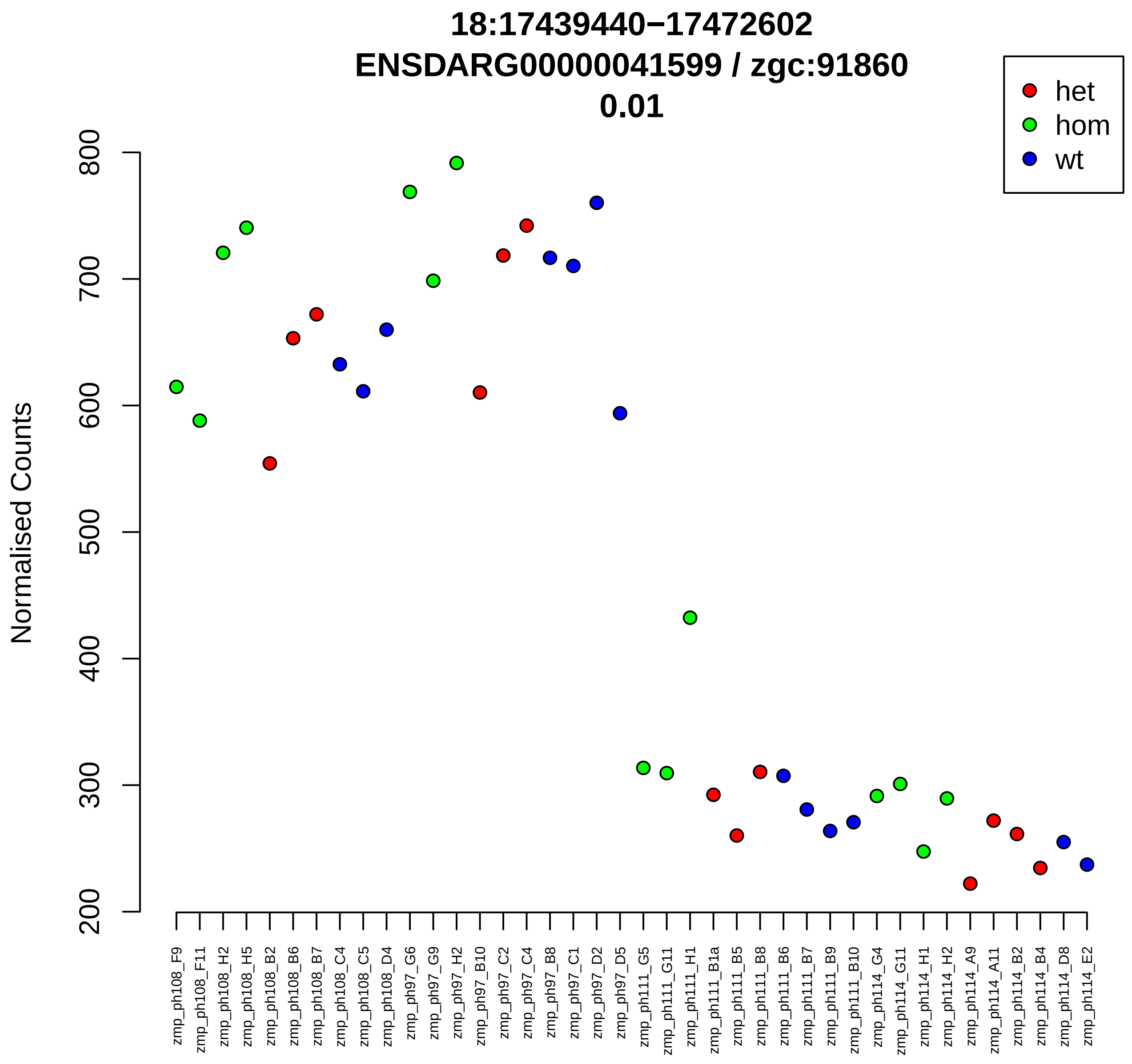
18:17439440−17472602
ENSDARG00000041599 / zgc:91860
0.01
het
hom
wt
Normalised Counts
200
300
400
500
600
700
800
zmp_ph108_F9
zmp_ph108_F11
zmp_ph108_H2
zmp_ph108_H5
zmp_ph108_B2
zmp_ph108_B6
zmp_ph108_B7
zmp_ph108_C4
zmp_ph108_C5
zmp_ph108_D4
zmp_ph97_G6
zmp_ph97_G9
zmp_ph97_H2
zmp_ph97_B10
zmp_ph97_C2
zmp_ph97_C4
zmp_ph97_B8
zmp_ph97_C1
zmp_ph97_D2
zmp_ph97_D5
zmp_ph111_G5
zmp_ph111_G11
zmp_ph111_H1
zmp_ph111_B1a
zmp_ph111_B5
zmp_ph111_B8
zmp_ph111_B6
zmp_ph111_B7
zmp_ph111_B9
zmp_ph111_B10
zmp_ph114_G4
zmp_ph114_G11
zmp_ph114_H1
zmp_ph114_H2
zmp_ph114_A9
zmp_ph114_A11
zmp_ph114_B2
zmp_ph114_B4
zmp_ph114_D8
zmp_ph114_E2

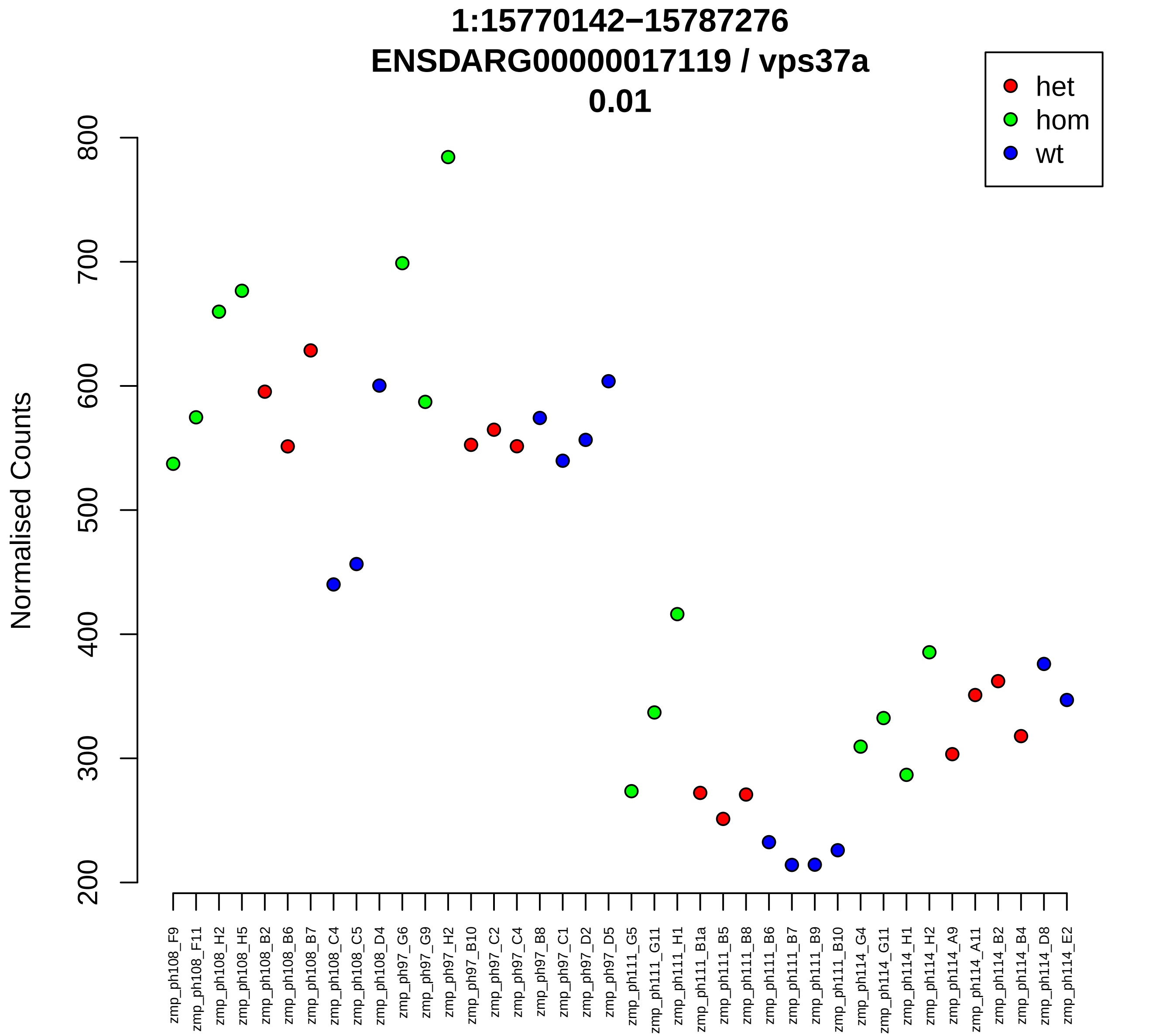

1:15770142−15787276
ENSDARG00000017119 / vps37a
0.01
het
hom
wt
Normalised Counts
200
300
400
500
600
700
800
zmp_ph108_F9
zmp_ph108_F11
zmp_ph108_H2
zmp_ph108_H5
zmp_ph108_B2
zmp_ph108_B6
zmp_ph108_B7
zmp_ph108_C4
zmp_ph108_C5
zmp_ph108_D4
zmp_ph97_G6
zmp_ph97_G9
zmp_ph97_H2
zmp_ph97_B10
zmp_ph97_C2
zmp_ph97_C4
zmp_ph97_B8
zmp_ph97_C1
zmp_ph97_D2
zmp_ph97_D5
zmp_ph111_G5
zmp_ph111_G11
zmp_ph111_H1
zmp_ph111_B1a
zmp_ph111_B5
zmp_ph111_B8
zmp_ph111_B6
zmp_ph111_B7
zmp_ph111_B9
zmp_ph111_B10
zmp_ph114_G4
zmp_ph114_G11
zmp_ph114_H1
zmp_ph114_H2
zmp_ph114_A9
zmp_ph114_A11
zmp_ph114_B2
zmp_ph114_B4
zmp_ph114_D8
zmp_ph114_E2

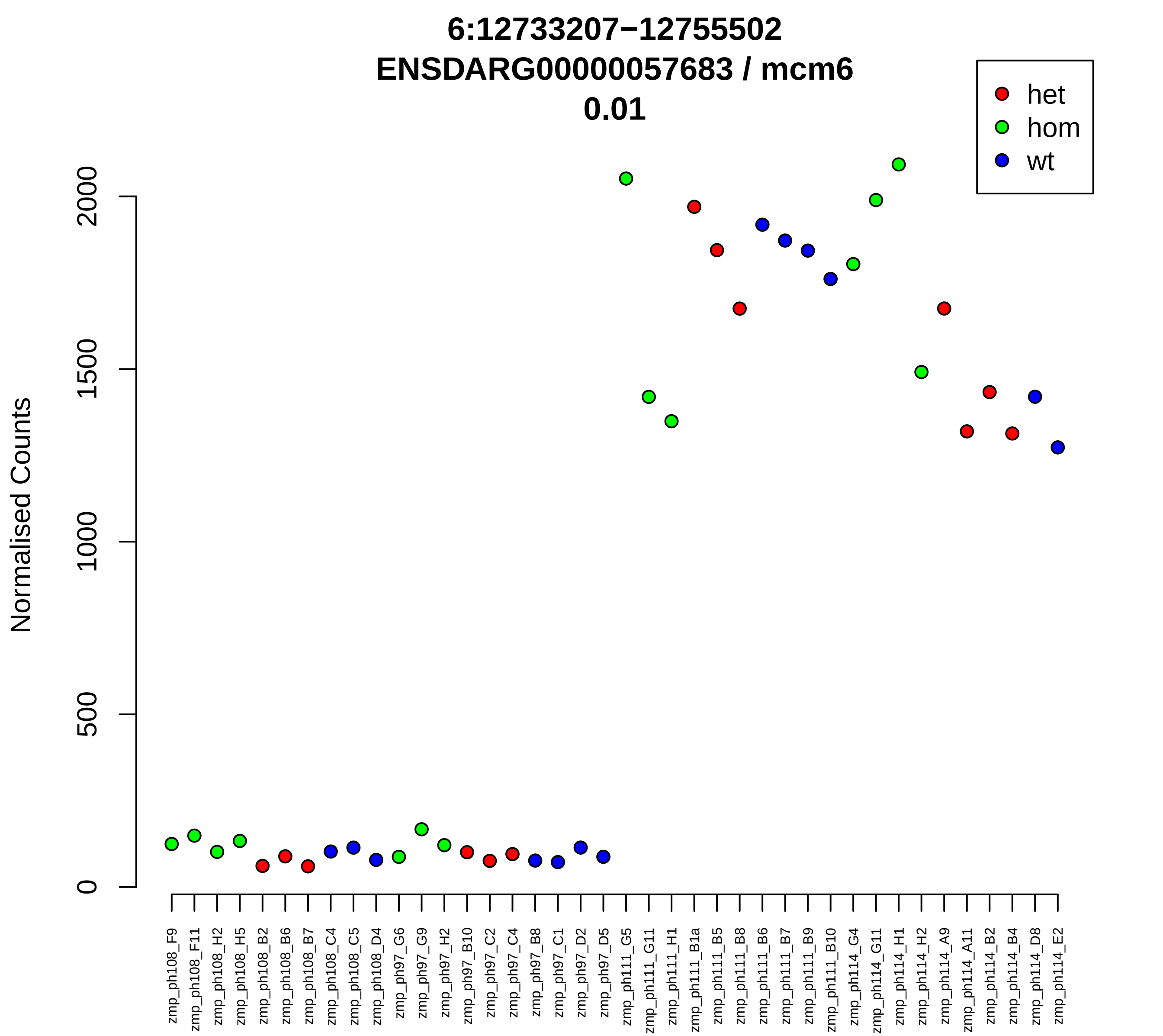

6:12733207−12755502
ENSDARG00000057683 / mcm6
0.01
het
hom
wt
Normalised Counts
0
500
1000
1500
2000
zmp_ph108_F9
zmp_ph108_F11
zmp_ph108_H2
zmp_ph108_H5
zmp_ph108_B2
zmp_ph108_B6
zmp_ph108_B7
zmp_ph108_C4
zmp_ph108_C5
zmp_ph108_D4
zmp_ph97_G6
zmp_ph97_G9
zmp_ph97_H2
zmp_ph97_B10
zmp_ph97_C2
zmp_ph97_C4
zmp_ph97_B8
zmp_ph97_C1
zmp_ph97_D2
zmp_ph97_D5
zmp_ph111_G5
zmp_ph111_G11
zmp_ph111_H1
zmp_ph111_B1a
zmp_ph111_B5
zmp_ph111_B8
zmp_ph111_B6
zmp_ph111_B7
zmp_ph111_B9
zmp_ph111_B10
zmp_ph114_G4
zmp_ph114_G11
zmp_ph114_H1
zmp_ph114_H2
zmp_ph114_A9
zmp_ph114_A11
zmp_ph114_B2
zmp_ph114_B4
zmp_ph114_D8
zmp_ph114_E2

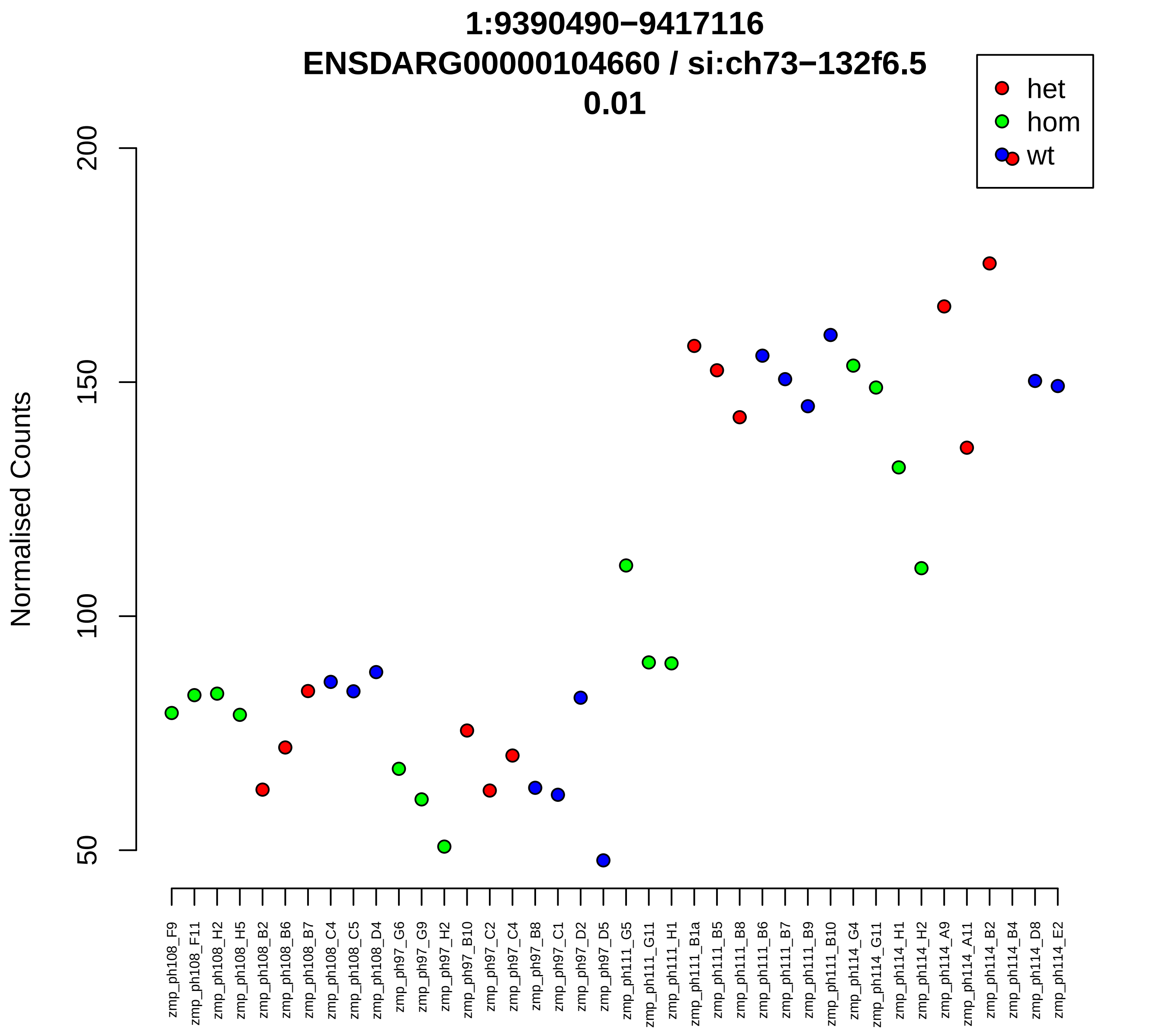
1:9390490−9417116
ENSDARG00000104660 / si:ch73−132f6.5
0.01
het
hom
wt
Normalised Counts
50
100
150
200
zmp_ph108_F9
zmp_ph108_F11
zmp_ph108_H2
zmp_ph108_H5
zmp_ph108_B2
zmp_ph108_B6
zmp_ph108_B7
zmp_ph108_C4
zmp_ph108_C5
zmp_ph108_D4
zmp_ph97_G6
zmp_ph97_G9
zmp_ph97_H2
zmp_ph97_B10
zmp_ph97_C2
zmp_ph97_C4
zmp_ph97_B8
zmp_ph97_C1
zmp_ph97_D2
zmp_ph97_D5
zmp_ph111_G5
zmp_ph111_G11
zmp_ph111_H1
zmp_ph111_B1a
zmp_ph111_B5
zmp_ph111_B8
zmp_ph111_B6
zmp_ph111_B7
zmp_ph111_B9
zmp_ph111_B10
zmp_ph114_G4
zmp_ph114_G11
zmp_ph114_H1
zmp_ph114_H2
zmp_ph114_A9
zmp_ph114_A11
zmp_ph114_B2
zmp_ph114_B4
zmp_ph114_D8
zmp_ph114_E2

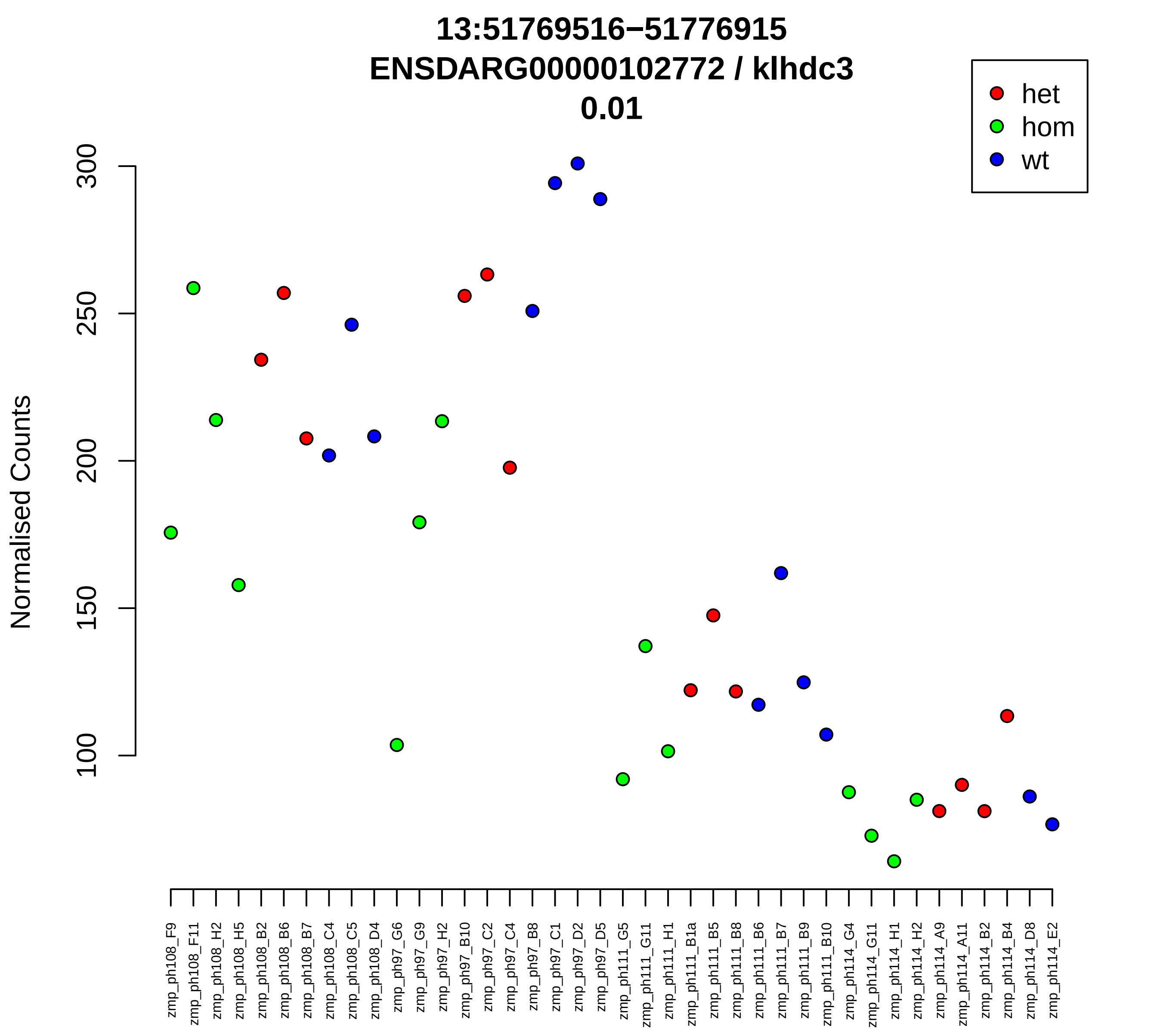

13:51769516−51776915
ENSDARG00000102772 / klhdc3
0.01
het
hom
wt
Normalised Counts
100
150
200
250
300
zmp_ph108_F9
zmp_ph108_F11
zmp_ph108_H2
zmp_ph108_H5
zmp_ph108_B2
zmp_ph108_B6
zmp_ph108_B7
zmp_ph108_C4
zmp_ph108_C5
zmp_ph108_D4
zmp_ph97_G6
zmp_ph97_G9
zmp_ph97_H2
zmp_ph97_B10
zmp_ph97_C2
zmp_ph97_C4
zmp_ph97_B8
zmp_ph97_C1
zmp_ph97_D2
zmp_ph97_D5
zmp_ph111_G5
zmp_ph111_G11
zmp_ph111_H1
zmp_ph111_B1a
zmp_ph111_B5
zmp_ph111_B8
zmp_ph111_B6
zmp_ph111_B7
zmp_ph111_B9
zmp_ph111_B10
zmp_ph114_G4
zmp_ph114_G11
zmp_ph114_H1
zmp_ph114_H2
zmp_ph114_A9
zmp_ph114_A11
zmp_ph114_B2
zmp_ph114_B4
zmp_ph114_D8
zmp_ph114_E2

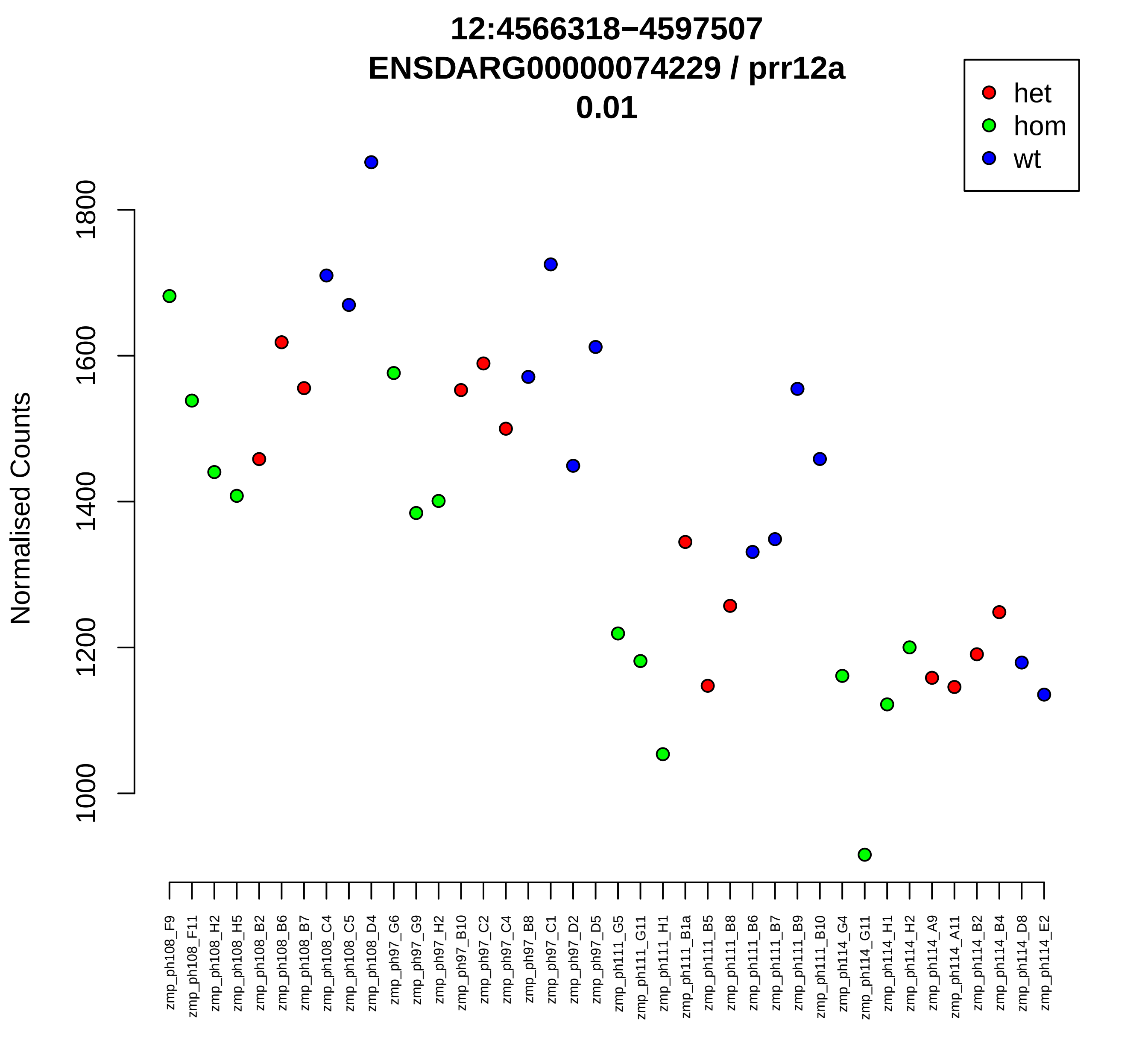

12:4566318−4597507
ENSDARG00000074229 / prr12a
0.01
het
hom
wt
Normalised Counts
1000
1200
1400
1600
1800
zmp_ph108_F9
zmp_ph108_F11
zmp_ph108_H2
zmp_ph108_H5
zmp_ph108_B2
zmp_ph108_B6
zmp_ph108_B7
zmp_ph108_C4
zmp_ph108_C5
zmp_ph108_D4
zmp_ph97_G6
zmp_ph97_G9
zmp_ph97_H2
zmp_ph97_B10
zmp_ph97_C2
zmp_ph97_C4
zmp_ph97_B8
zmp_ph97_C1
zmp_ph97_D2
zmp_ph97_D5
zmp_ph111_G5
zmp_ph111_G11
zmp_ph111_H1
zmp_ph111_B1a
zmp_ph111_B5
zmp_ph111_B8
zmp_ph111_B6
zmp_ph111_B7
zmp_ph111_B9
zmp_ph111_B10
zmp_ph114_G4
zmp_ph114_G11
zmp_ph114_H1
zmp_ph114_H2
zmp_ph114_A9
zmp_ph114_A11
zmp_ph114_B2
zmp_ph114_B4
zmp_ph114_D8
zmp_ph114_E2

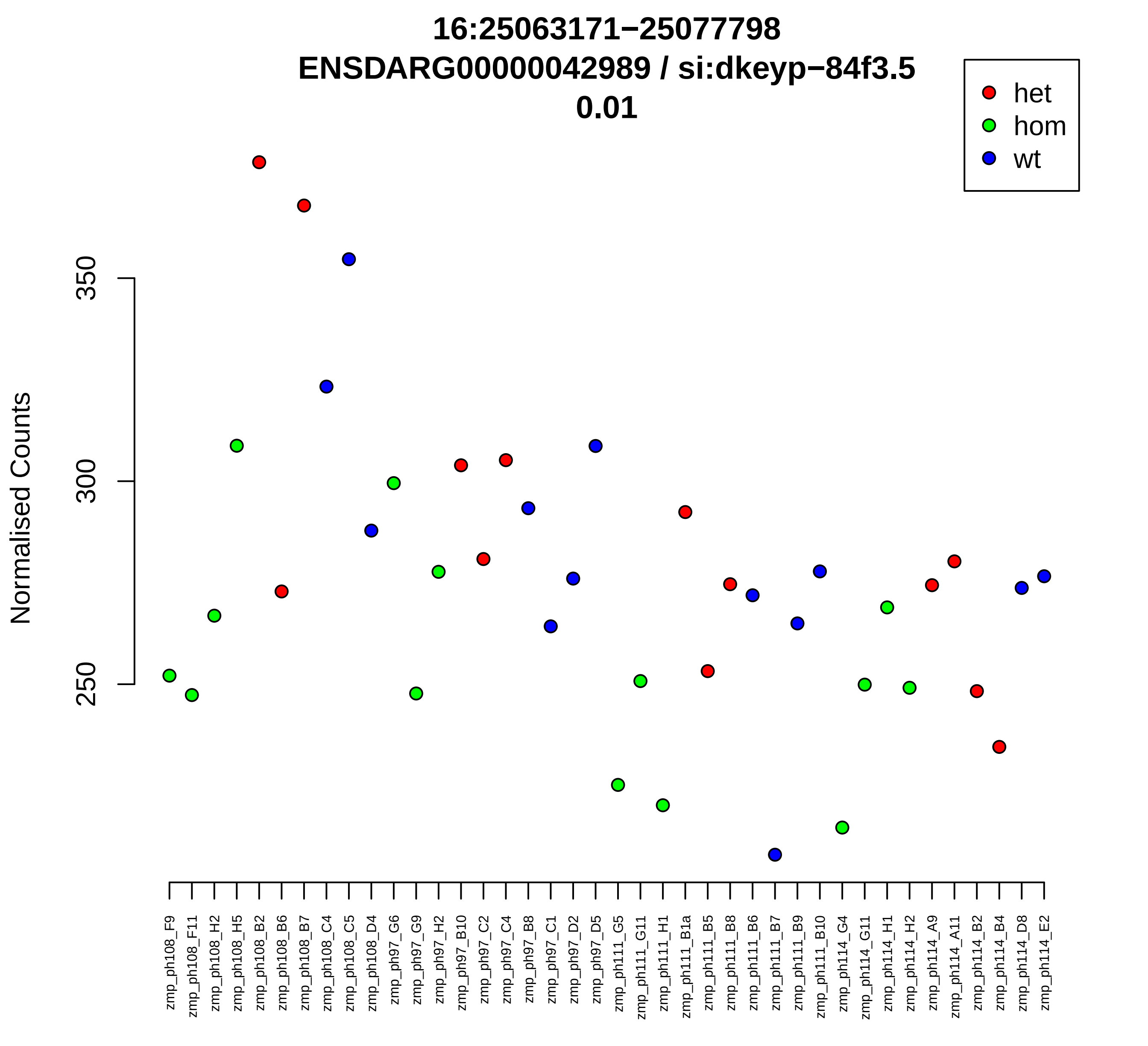

16:25063171−25077798
ENSDARG00000042989 / si:dkeyp−84f3.5
0.01
het
hom
wt
Normalised Counts
250
300
350
zmp_ph108_F9
zmp_ph108_F11
zmp_ph108_H2
zmp_ph108_H5
zmp_ph108_B2
zmp_ph108_B6
zmp_ph108_B7
zmp_ph108_C4
zmp_ph108_C5
zmp_ph108_D4
zmp_ph97_G6
zmp_ph97_G9
zmp_ph97_H2
zmp_ph97_B10
zmp_ph97_C2
zmp_ph97_C4
zmp_ph97_B8
zmp_ph97_C1
zmp_ph97_D2
zmp_ph97_D5
zmp_ph111_G5
zmp_ph111_G11
zmp_ph111_H1
zmp_ph111_B1a
zmp_ph111_B5
zmp_ph111_B8
zmp_ph111_B6
zmp_ph111_B7
zmp_ph111_B9
zmp_ph111_B10
zmp_ph114_G4
zmp_ph114_G11
zmp_ph114_H1
zmp_ph114_H2
zmp_ph114_A9
zmp_ph114_A11
zmp_ph114_B2
zmp_ph114_B4
zmp_ph114_D8
zmp_ph114_E2

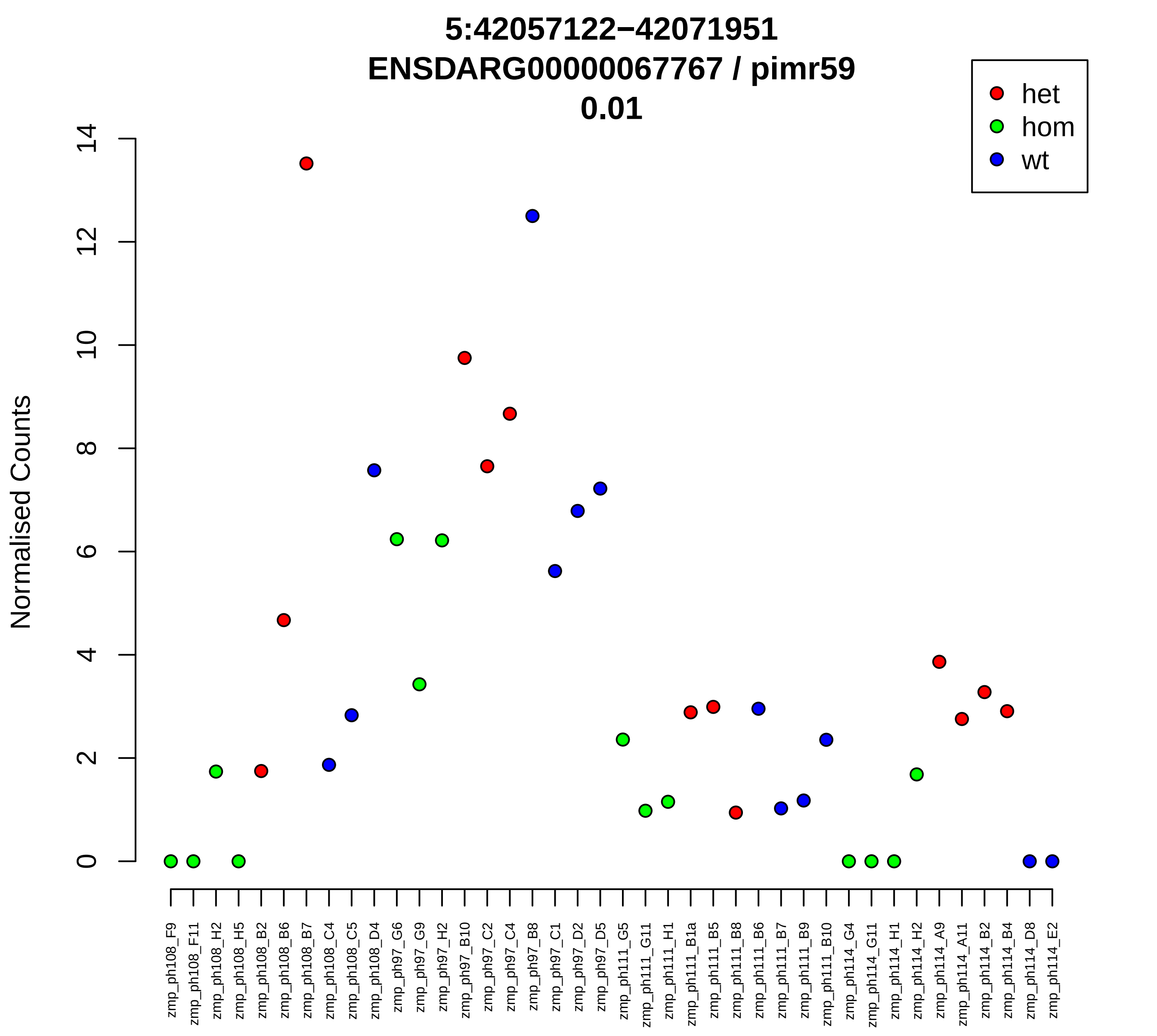

5:42057122−42071951
ENSDARG00000067767 / pimr59
0.01
het
hom
wt
Normalised Counts
0
2
4
6
8
10
12
14
zmp_ph108_F9
zmp_ph108_F11
zmp_ph108_H2
zmp_ph108_H5
zmp_ph108_B2
zmp_ph108_B6
zmp_ph108_B7
zmp_ph108_C4
zmp_ph108_C5
zmp_ph108_D4
zmp_ph97_G6
zmp_ph97_G9
zmp_ph97_H2
zmp_ph97_B10
zmp_ph97_C2
zmp_ph97_C4
zmp_ph97_B8
zmp_ph97_C1
zmp_ph97_D2
zmp_ph97_D5
zmp_ph111_G5
zmp_ph111_G11
zmp_ph111_H1
zmp_ph111_B1a
zmp_ph111_B5
zmp_ph111_B8
zmp_ph111_B6
zmp_ph111_B7
zmp_ph111_B9
zmp_ph111_B10
zmp_ph114_G4
zmp_ph114_G11
zmp_ph114_H1
zmp_ph114_H2
zmp_ph114_A9
zmp_ph114_A11
zmp_ph114_B2
zmp_ph114_B4
zmp_ph114_D8
zmp_ph114_E2

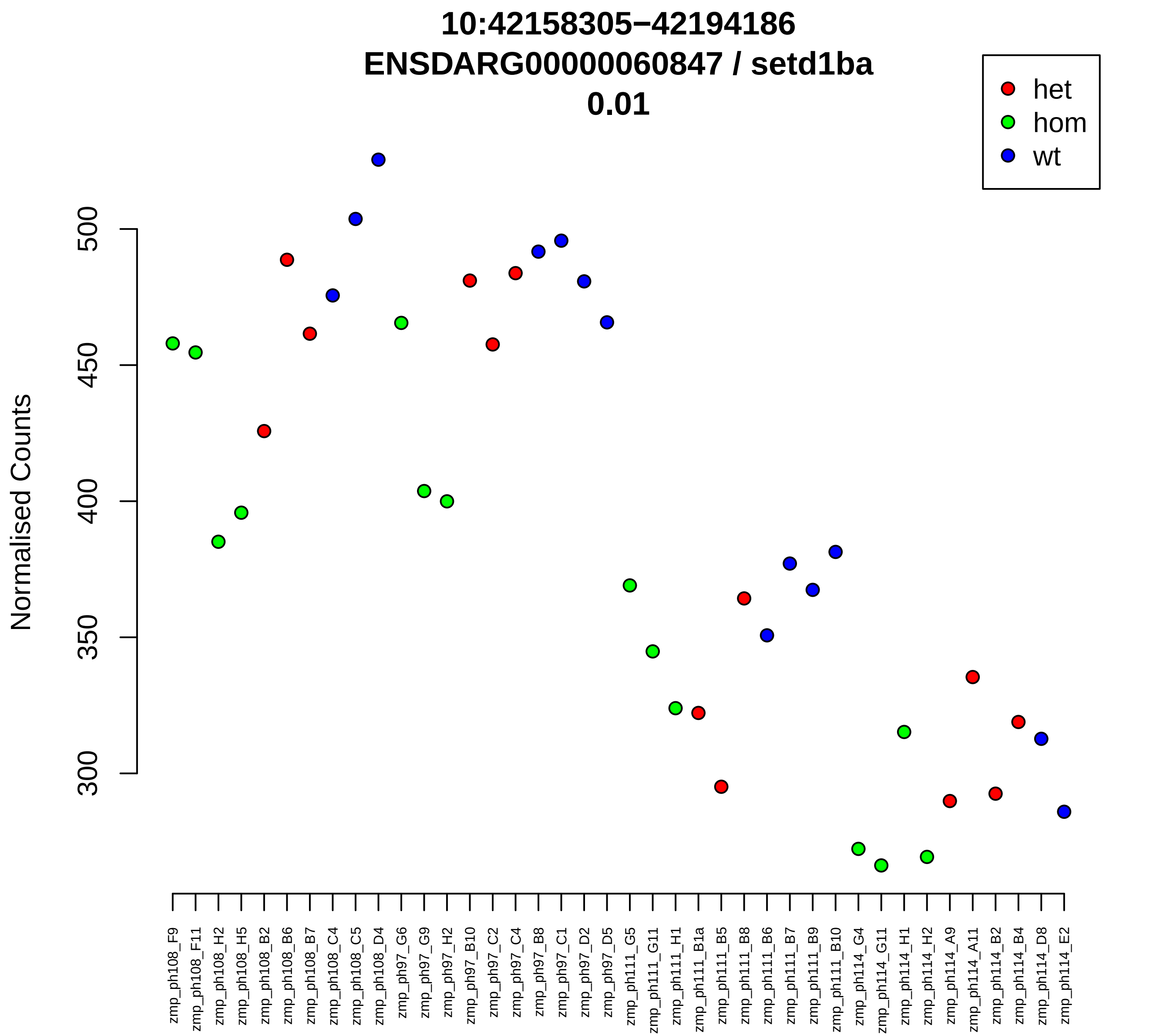
10:42158305−42194186
ENSDARG00000060847 / setd1ba
0.01
het
hom
wt
Normalised Counts
300
350
400
450
500
zmp_ph108_F9
zmp_ph108_F11
zmp_ph108_H2
zmp_ph108_H5
zmp_ph108_B2
zmp_ph108_B6
zmp_ph108_B7
zmp_ph108_C4
zmp_ph108_C5
zmp_ph108_D4
zmp_ph97_G6
zmp_ph97_G9
zmp_ph97_H2
zmp_ph97_B10
zmp_ph97_C2
zmp_ph97_C4
zmp_ph97_B8
zmp_ph97_C1
zmp_ph97_D2
zmp_ph97_D5
zmp_ph111_G5
zmp_ph111_G11
zmp_ph111_H1
zmp_ph111_B1a
zmp_ph111_B5
zmp_ph111_B8
zmp_ph111_B6
zmp_ph111_B7
zmp_ph111_B9
zmp_ph111_B10
zmp_ph114_G4
zmp_ph114_G11
zmp_ph114_H1
zmp_ph114_H2
zmp_ph114_A9
zmp_ph114_A11
zmp_ph114_B2
zmp_ph114_B4
zmp_ph114_D8
zmp_ph114_E2

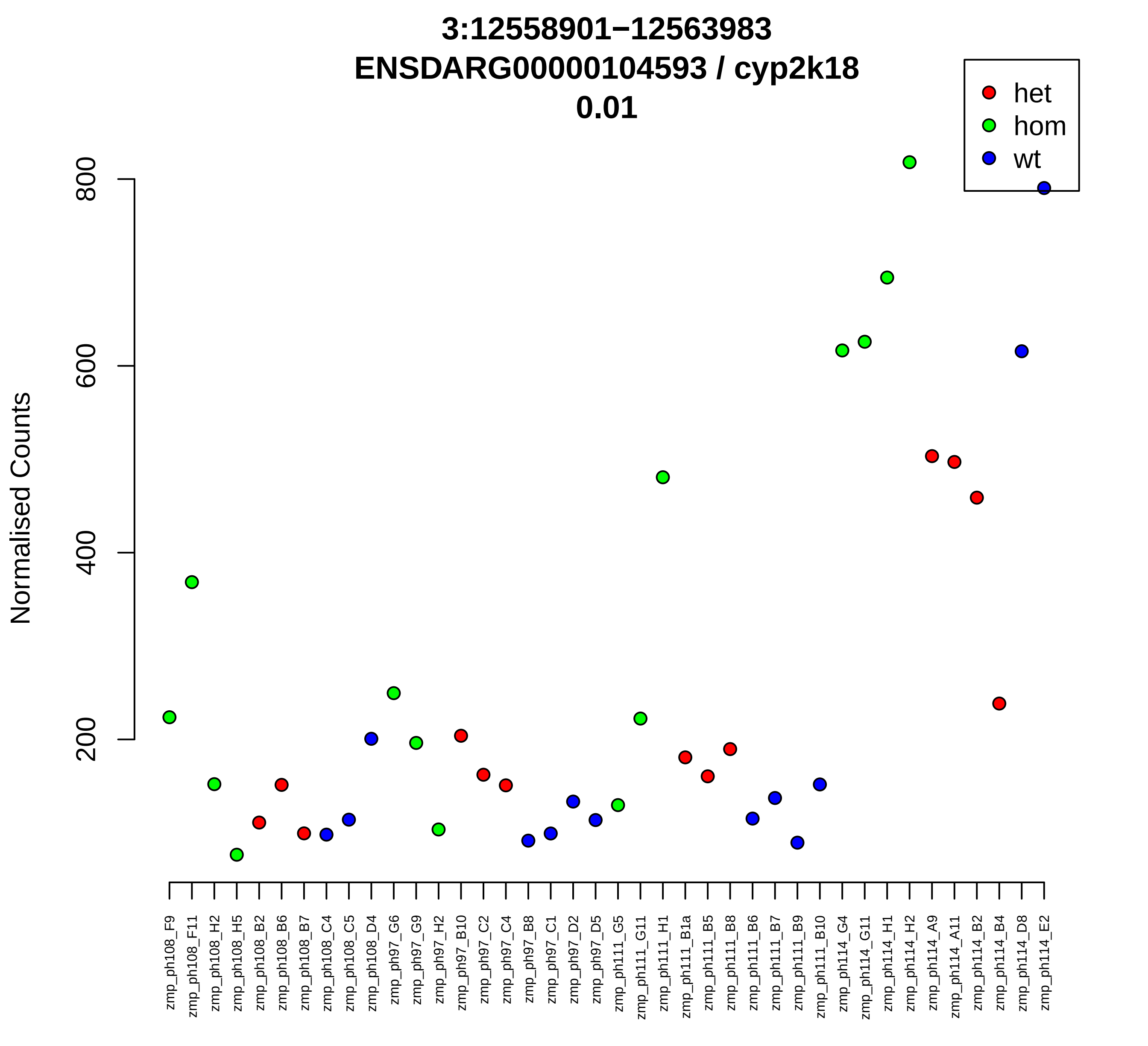
3:12558901−12563983
ENSDARG00000104593 / cyp2k18
0.01
het
hom
wt
Normalised Counts
200
400
600
800
zmp_ph108_F9
zmp_ph108_F11
zmp_ph108_H2
zmp_ph108_H5
zmp_ph108_B2
zmp_ph108_B6
zmp_ph108_B7
zmp_ph108_C4
zmp_ph108_C5
zmp_ph108_D4
zmp_ph97_G6
zmp_ph97_G9
zmp_ph97_H2
zmp_ph97_B10
zmp_ph97_C2
zmp_ph97_C4
zmp_ph97_B8
zmp_ph97_C1
zmp_ph97_D2
zmp_ph97_D5
zmp_ph111_G5
zmp_ph111_G11
zmp_ph111_H1
zmp_ph111_B1a
zmp_ph111_B5
zmp_ph111_B8
zmp_ph111_B6
zmp_ph111_B7
zmp_ph111_B9
zmp_ph111_B10
zmp_ph114_G4
zmp_ph114_G11
zmp_ph114_H1
zmp_ph114_H2
zmp_ph114_A9
zmp_ph114_A11
zmp_ph114_B2
zmp_ph114_B4
zmp_ph114_D8
zmp_ph114_E2

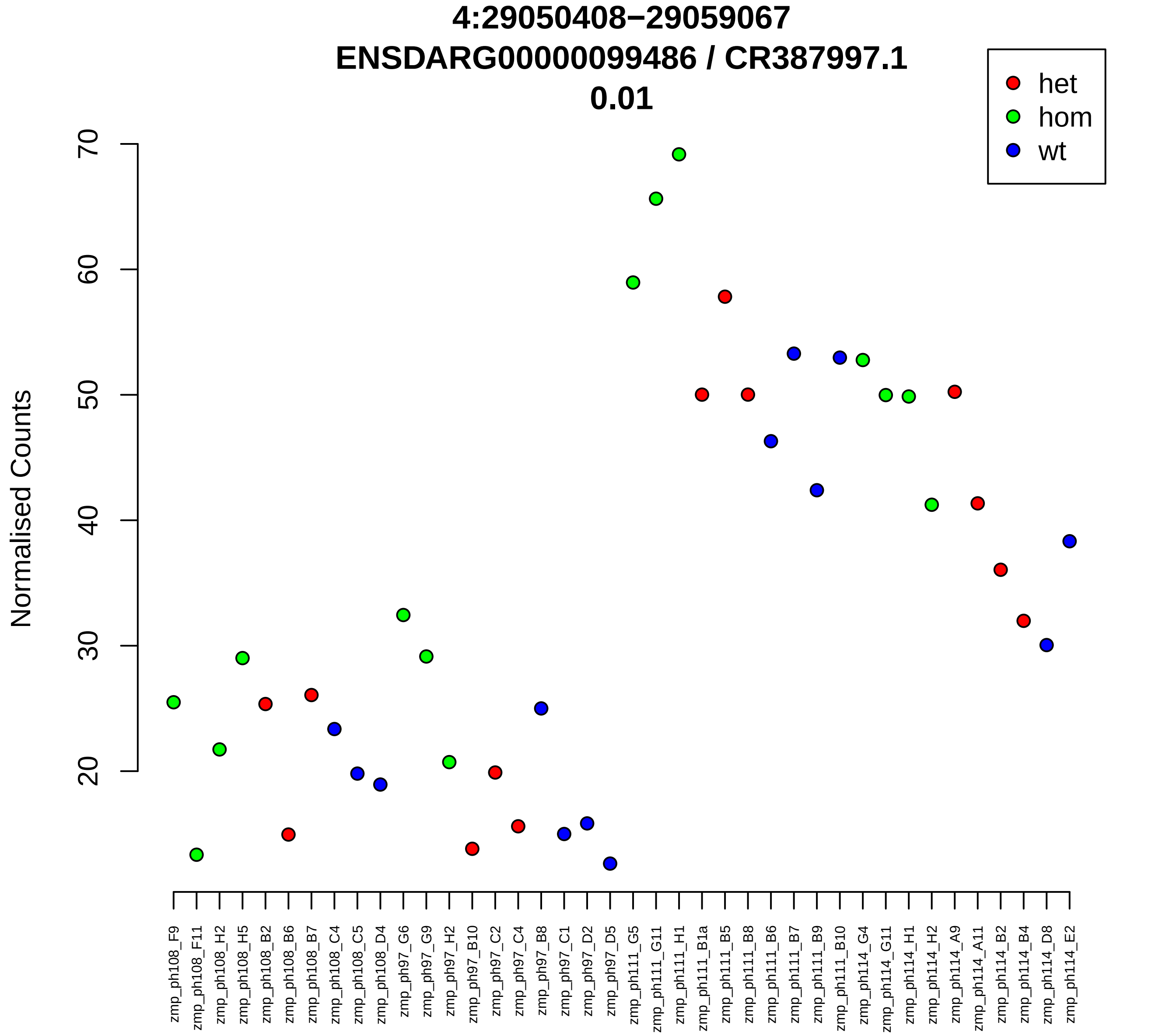
4:29050408−29059067
ENSDARG00000099486 / CR387997.1
0.01
het
hom
wt
Normalised Counts
20
30
40
50
60
70
zmp_ph108_F9
zmp_ph108_F11
zmp_ph108_H2
zmp_ph108_H5
zmp_ph108_B2
zmp_ph108_B6
zmp_ph108_B7
zmp_ph108_C4
zmp_ph108_C5
zmp_ph108_D4
zmp_ph97_G6
zmp_ph97_G9
zmp_ph97_H2
zmp_ph97_B10
zmp_ph97_C2
zmp_ph97_C4
zmp_ph97_B8
zmp_ph97_C1
zmp_ph97_D2
zmp_ph97_D5
zmp_ph111_G5
zmp_ph111_G11
zmp_ph111_H1
zmp_ph111_B1a
zmp_ph111_B5
zmp_ph111_B8
zmp_ph111_B6
zmp_ph111_B7
zmp_ph111_B9
zmp_ph111_B10
zmp_ph114_G4
zmp_ph114_G11
zmp_ph114_H1
zmp_ph114_H2
zmp_ph114_A9
zmp_ph114_A11
zmp_ph114_B2
zmp_ph114_B4
zmp_ph114_D8
zmp_ph114_E2

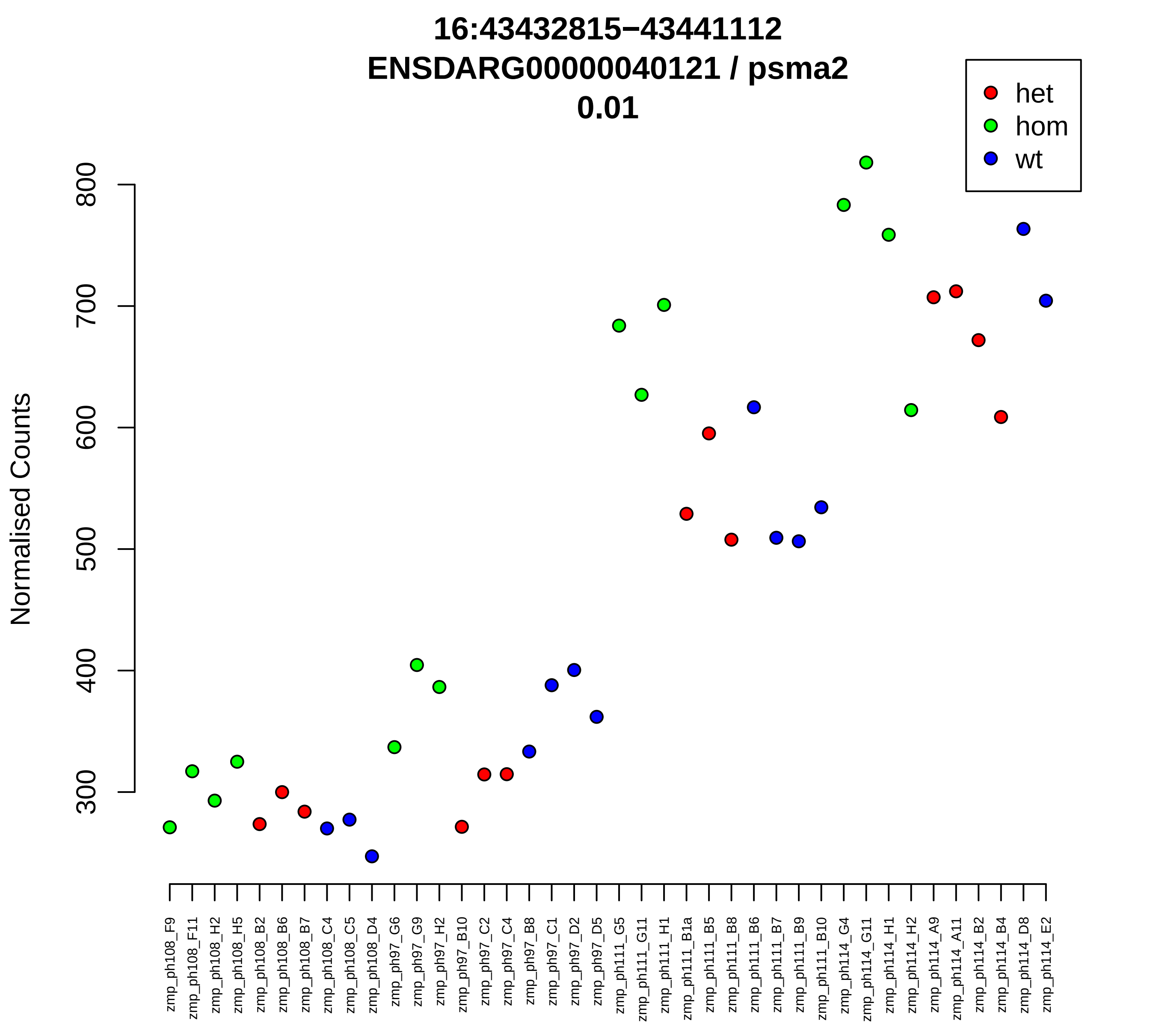

16:43432815−43441112
ENSDARG00000040121 / psma2
0.01
het
hom
wt
Normalised Counts
300
400
500
600
700
800
zmp_ph108_F9
zmp_ph108_F11
zmp_ph108_H2
zmp_ph108_H5
zmp_ph108_B2
zmp_ph108_B6
zmp_ph108_B7
zmp_ph108_C4
zmp_ph108_C5
zmp_ph108_D4
zmp_ph97_G6
zmp_ph97_G9
zmp_ph97_H2
zmp_ph97_B10
zmp_ph97_C2
zmp_ph97_C4
zmp_ph97_B8
zmp_ph97_C1
zmp_ph97_D2
zmp_ph97_D5
zmp_ph111_G5
zmp_ph111_G11
zmp_ph111_H1
zmp_ph111_B1a
zmp_ph111_B5
zmp_ph111_B8
zmp_ph111_B6
zmp_ph111_B7
zmp_ph111_B9
zmp_ph111_B10
zmp_ph114_G4
zmp_ph114_G11
zmp_ph114_H1
zmp_ph114_H2
zmp_ph114_A9
zmp_ph114_A11
zmp_ph114_B2
zmp_ph114_B4
zmp_ph114_D8
zmp_ph114_E2

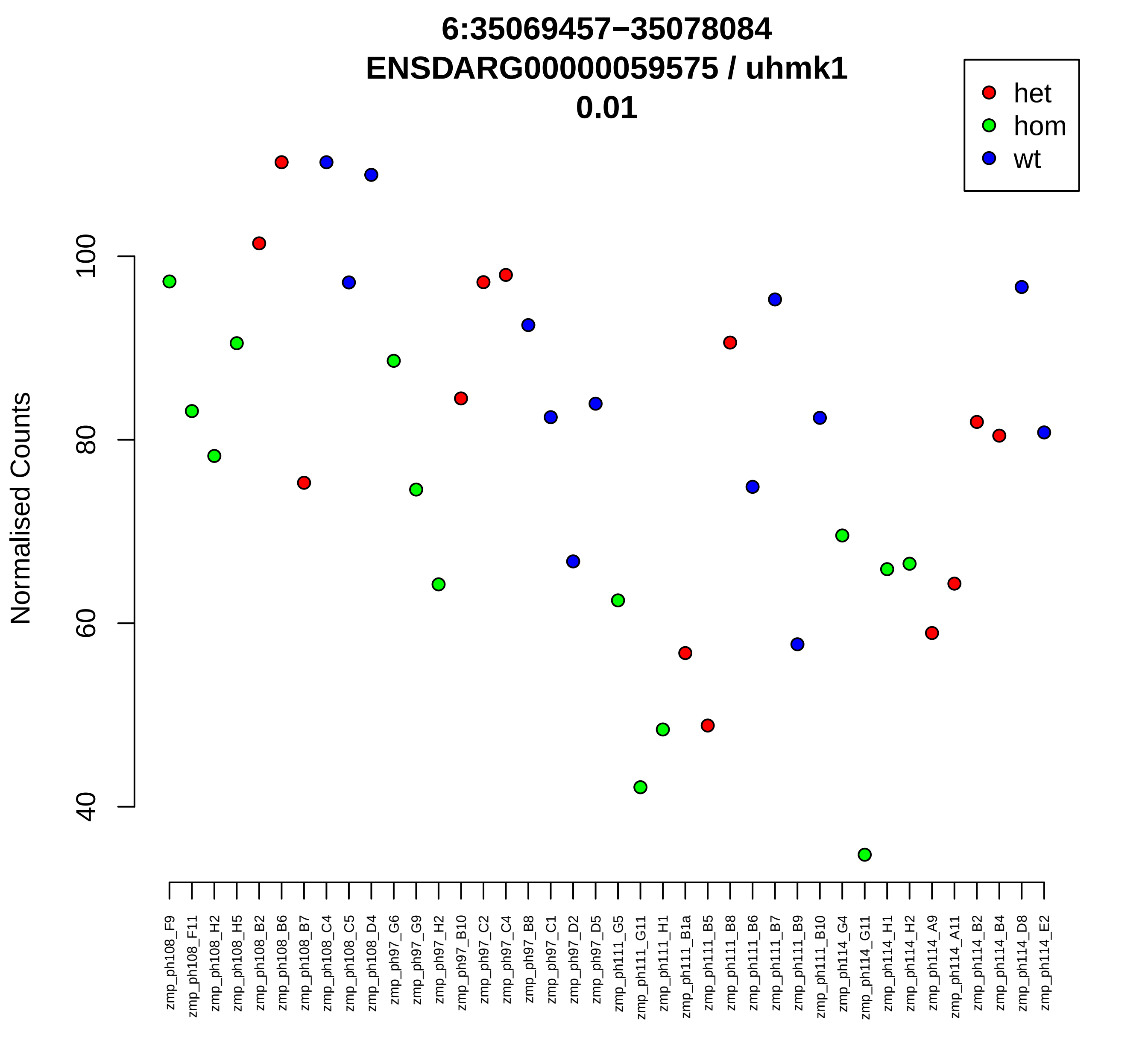

6:35069457−35078084
ENSDARG00000059575 / uhmk1
0.01
het
hom
wt
Normalised Counts
40
60
80
100
zmp_ph108_F9
zmp_ph108_F11
zmp_ph108_H2
zmp_ph108_H5
zmp_ph108_B2
zmp_ph108_B6
zmp_ph108_B7
zmp_ph108_C4
zmp_ph108_C5
zmp_ph108_D4
zmp_ph97_G6
zmp_ph97_G9
zmp_ph97_H2
zmp_ph97_B10
zmp_ph97_C2
zmp_ph97_C4
zmp_ph97_B8
zmp_ph97_C1
zmp_ph97_D2
zmp_ph97_D5
zmp_ph111_G5
zmp_ph111_G11
zmp_ph111_H1
zmp_ph111_B1a
zmp_ph111_B5
zmp_ph111_B8
zmp_ph111_B6
zmp_ph111_B7
zmp_ph111_B9
zmp_ph111_B10
zmp_ph114_G4
zmp_ph114_G11
zmp_ph114_H1
zmp_ph114_H2
zmp_ph114_A9
zmp_ph114_A11
zmp_ph114_B2
zmp_ph114_B4
zmp_ph114_D8
zmp_ph114_E2

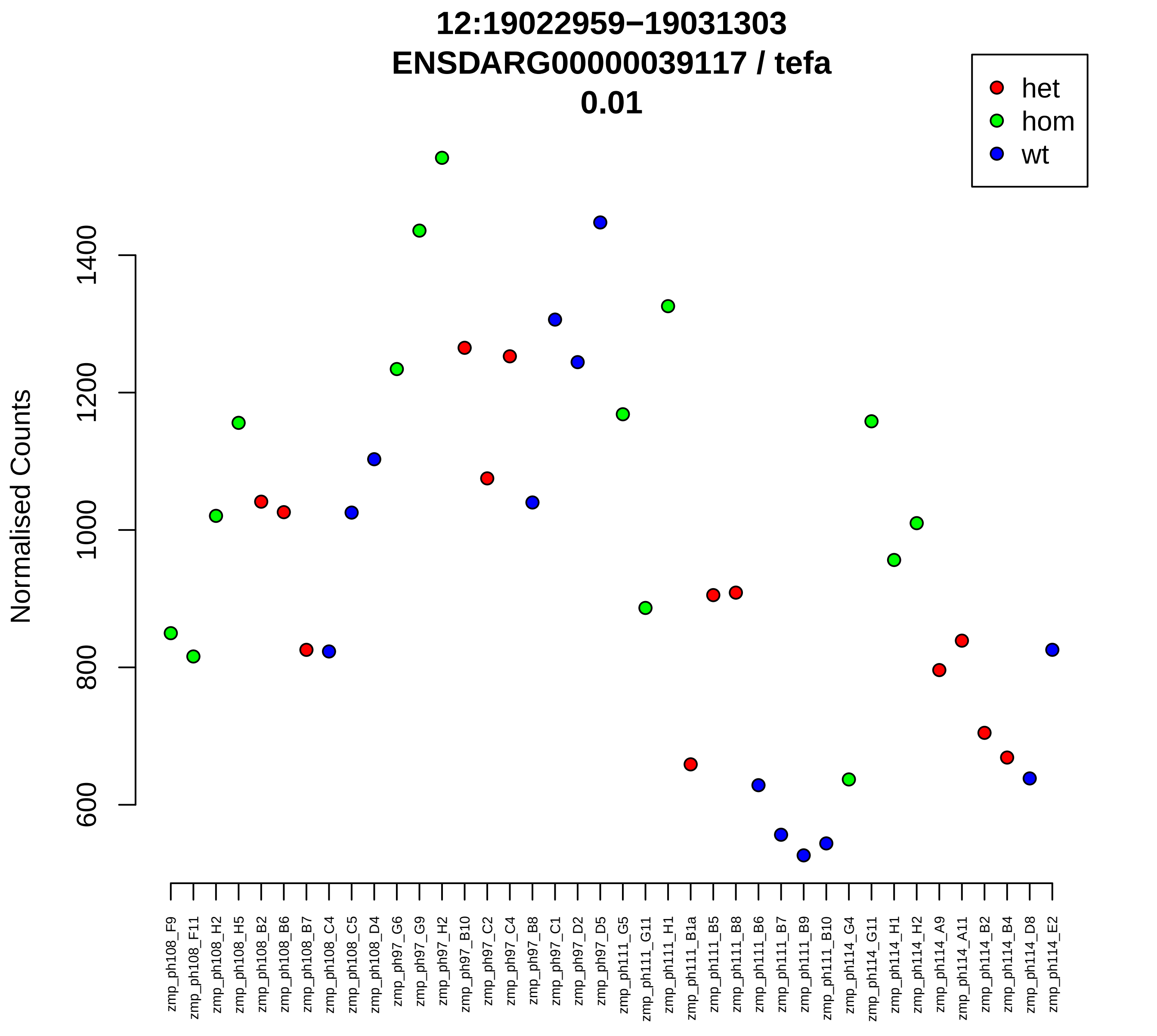
12:19022959−19031303
ENSDARG00000039117 / tefa
0.01
het
hom
wt
Normalised Counts
600
800
1000
1200
1400
zmp_ph108_F9
zmp_ph108_F11
zmp_ph108_H2
zmp_ph108_H5
zmp_ph108_B2
zmp_ph108_B6
zmp_ph108_B7
zmp_ph108_C4
zmp_ph108_C5
zmp_ph108_D4
zmp_ph97_G6
zmp_ph97_G9
zmp_ph97_H2
zmp_ph97_B10
zmp_ph97_C2
zmp_ph97_C4
zmp_ph97_B8
zmp_ph97_C1
zmp_ph97_D2
zmp_ph97_D5
zmp_ph111_G5
zmp_ph111_G11
zmp_ph111_H1
zmp_ph111_B1a
zmp_ph111_B5
zmp_ph111_B8
zmp_ph111_B6
zmp_ph111_B7
zmp_ph111_B9
zmp_ph111_B10
zmp_ph114_G4
zmp_ph114_G11
zmp_ph114_H1
zmp_ph114_H2
zmp_ph114_A9
zmp_ph114_A11
zmp_ph114_B2
zmp_ph114_B4
zmp_ph114_D8
zmp_ph114_E2

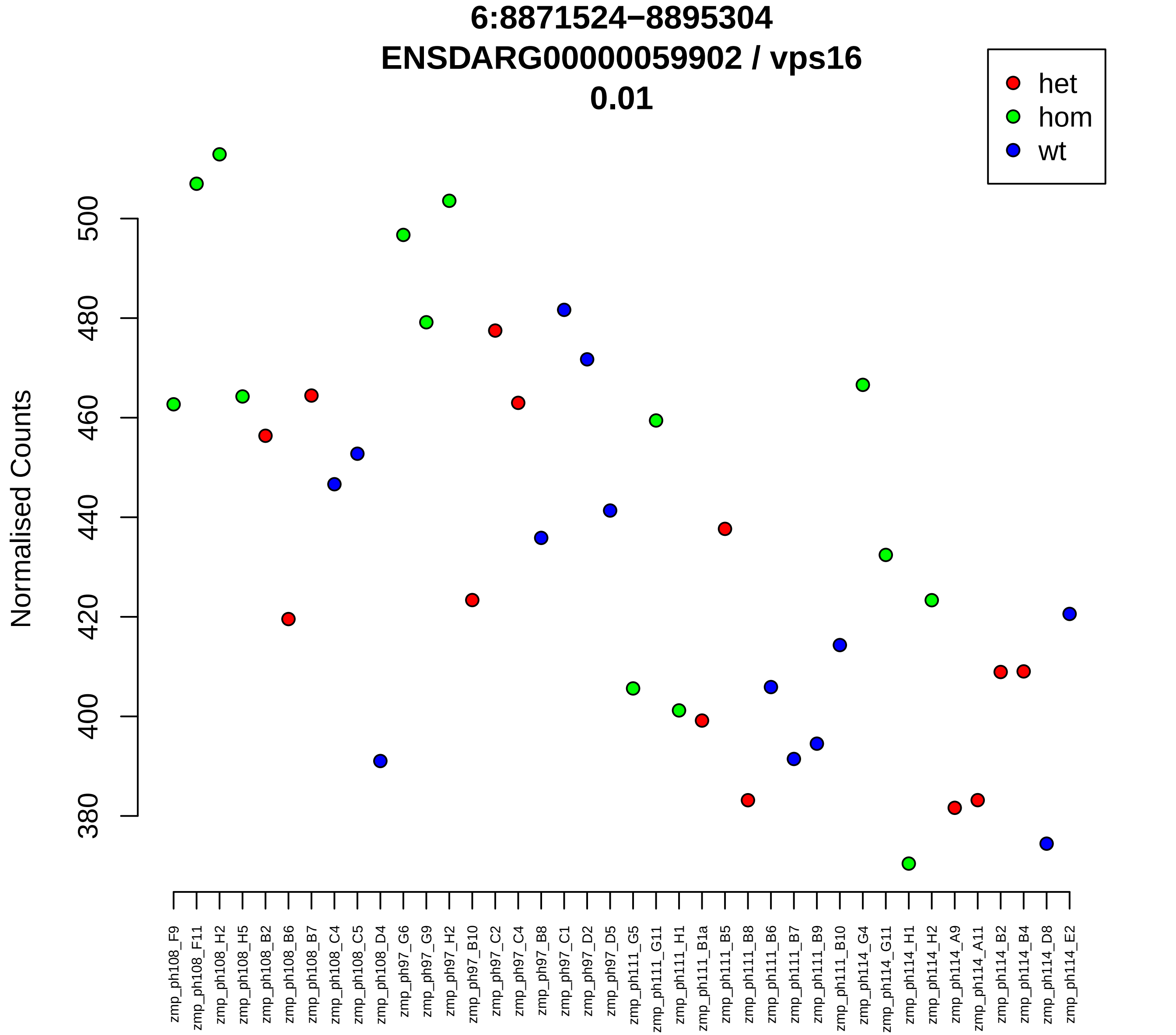

6:8871524−8895304
ENSDARG00000059902 / vps16
0.01
het
hom
wt
Normalised Counts
380
400
420
440
460
480
500
zmp_ph108_F9
zmp_ph108_F11
zmp_ph108_H2
zmp_ph108_H5
zmp_ph108_B2
zmp_ph108_B6
zmp_ph108_B7
zmp_ph108_C4
zmp_ph108_C5
zmp_ph108_D4
zmp_ph97_G6
zmp_ph97_G9
zmp_ph97_H2
zmp_ph97_B10
zmp_ph97_C2
zmp_ph97_C4
zmp_ph97_B8
zmp_ph97_C1
zmp_ph97_D2
zmp_ph97_D5
zmp_ph111_G5
zmp_ph111_G11
zmp_ph111_H1
zmp_ph111_B1a
zmp_ph111_B5
zmp_ph111_B8
zmp_ph111_B6
zmp_ph111_B7
zmp_ph111_B9
zmp_ph111_B10
zmp_ph114_G4
zmp_ph114_G11
zmp_ph114_H1
zmp_ph114_H2
zmp_ph114_A9
zmp_ph114_A11
zmp_ph114_B2
zmp_ph114_B4
zmp_ph114_D8
zmp_ph114_E2

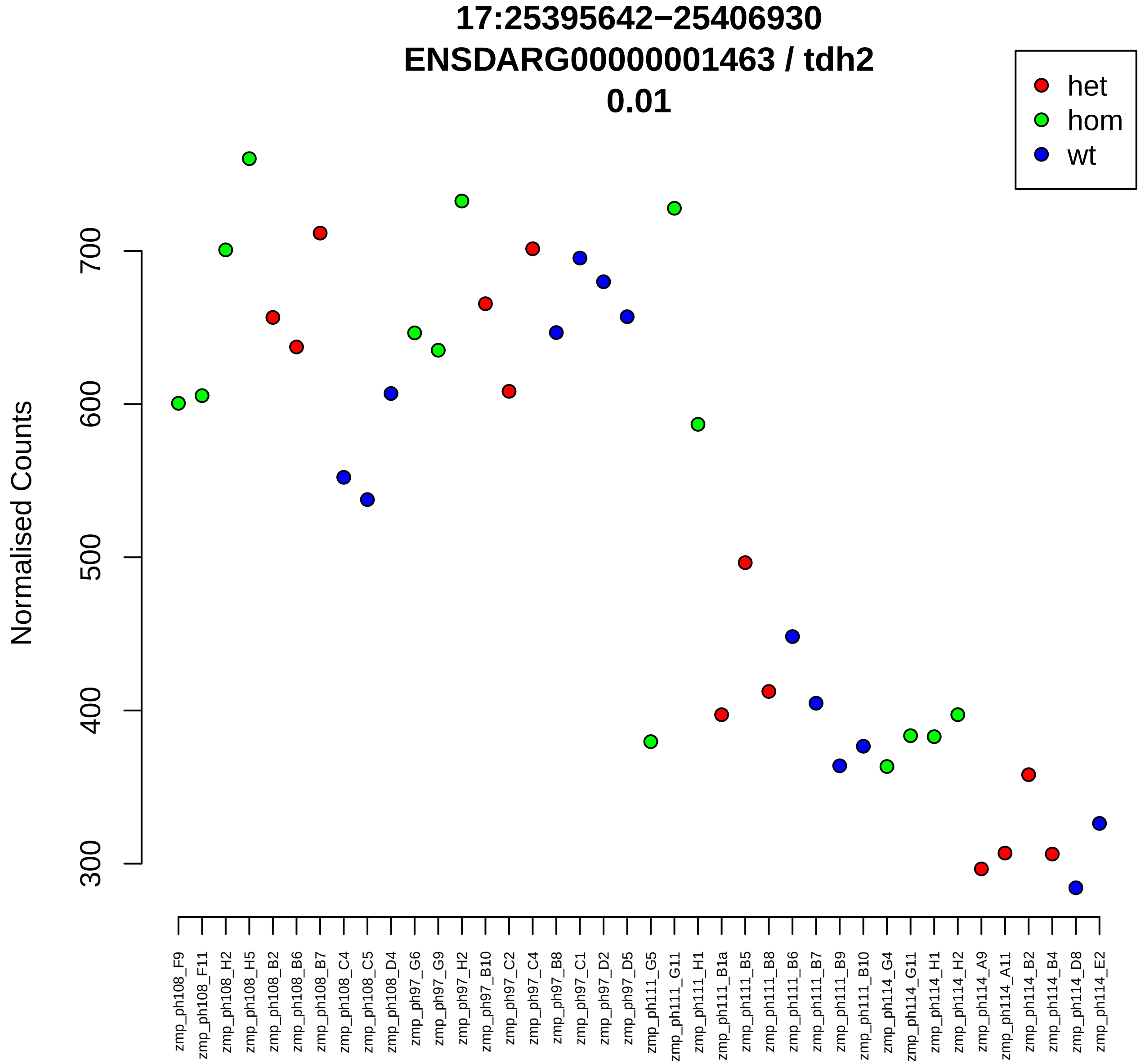

17:25395642−25406930
ENSDARG00000001463 / tdh2
0.01
het
hom
wt
Normalised Counts
300
400
500
600
700
zmp_ph108_F9
zmp_ph108_F11
zmp_ph108_H2
zmp_ph108_H5
zmp_ph108_B2
zmp_ph108_B6
zmp_ph108_B7
zmp_ph108_C4
zmp_ph108_C5
zmp_ph108_D4
zmp_ph97_G6
zmp_ph97_G9
zmp_ph97_H2
zmp_ph97_B10
zmp_ph97_C2
zmp_ph97_C4
zmp_ph97_B8
zmp_ph97_C1
zmp_ph97_D2
zmp_ph97_D5
zmp_ph111_G5
zmp_ph111_G11
zmp_ph111_H1
zmp_ph111_B1a
zmp_ph111_B5
zmp_ph111_B8
zmp_ph111_B6
zmp_ph111_B7
zmp_ph111_B9
zmp_ph111_B10
zmp_ph114_G4
zmp_ph114_G11
zmp_ph114_H1
zmp_ph114_H2
zmp_ph114_A9
zmp_ph114_A11
zmp_ph114_B2
zmp_ph114_B4
zmp_ph114_D8
zmp_ph114_E2

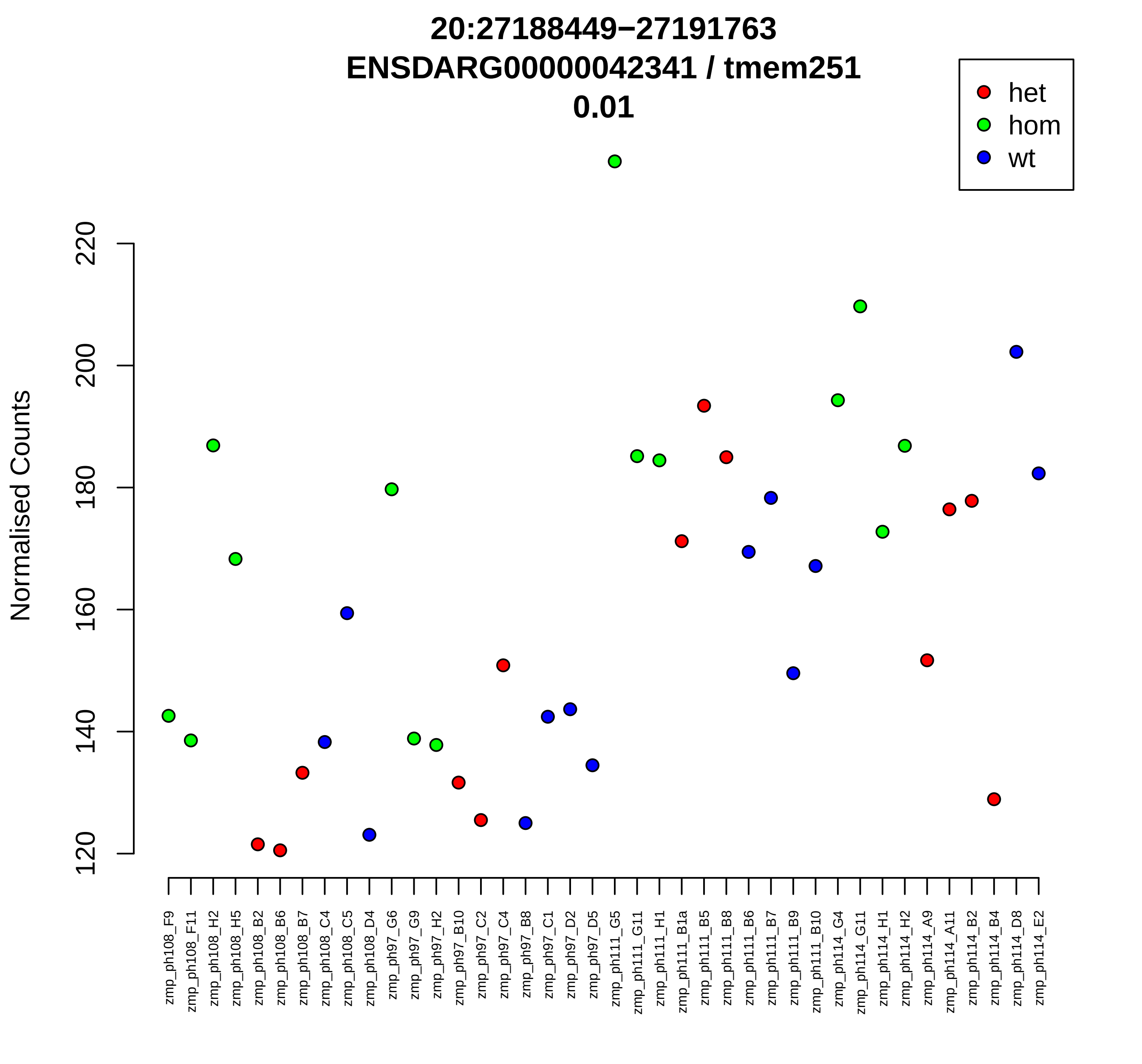

20:27188449−27191763
ENSDARG00000042341 / tmem251
0.01
het
hom
wt
Normalised Counts
120
140
160
180
200
220
zmp_ph108_F9
zmp_ph108_F11
zmp_ph108_H2
zmp_ph108_H5
zmp_ph108_B2
zmp_ph108_B6
zmp_ph108_B7
zmp_ph108_C4
zmp_ph108_C5
zmp_ph108_D4
zmp_ph97_G6
zmp_ph97_G9
zmp_ph97_H2
zmp_ph97_B10
zmp_ph97_C2
zmp_ph97_C4
zmp_ph97_B8
zmp_ph97_C1
zmp_ph97_D2
zmp_ph97_D5
zmp_ph111_G5
zmp_ph111_G11
zmp_ph111_H1
zmp_ph111_B1a
zmp_ph111_B5
zmp_ph111_B8
zmp_ph111_B6
zmp_ph111_B7
zmp_ph111_B9
zmp_ph111_B10
zmp_ph114_G4
zmp_ph114_G11
zmp_ph114_H1
zmp_ph114_H2
zmp_ph114_A9
zmp_ph114_A11
zmp_ph114_B2
zmp_ph114_B4
zmp_ph114_D8
zmp_ph114_E2

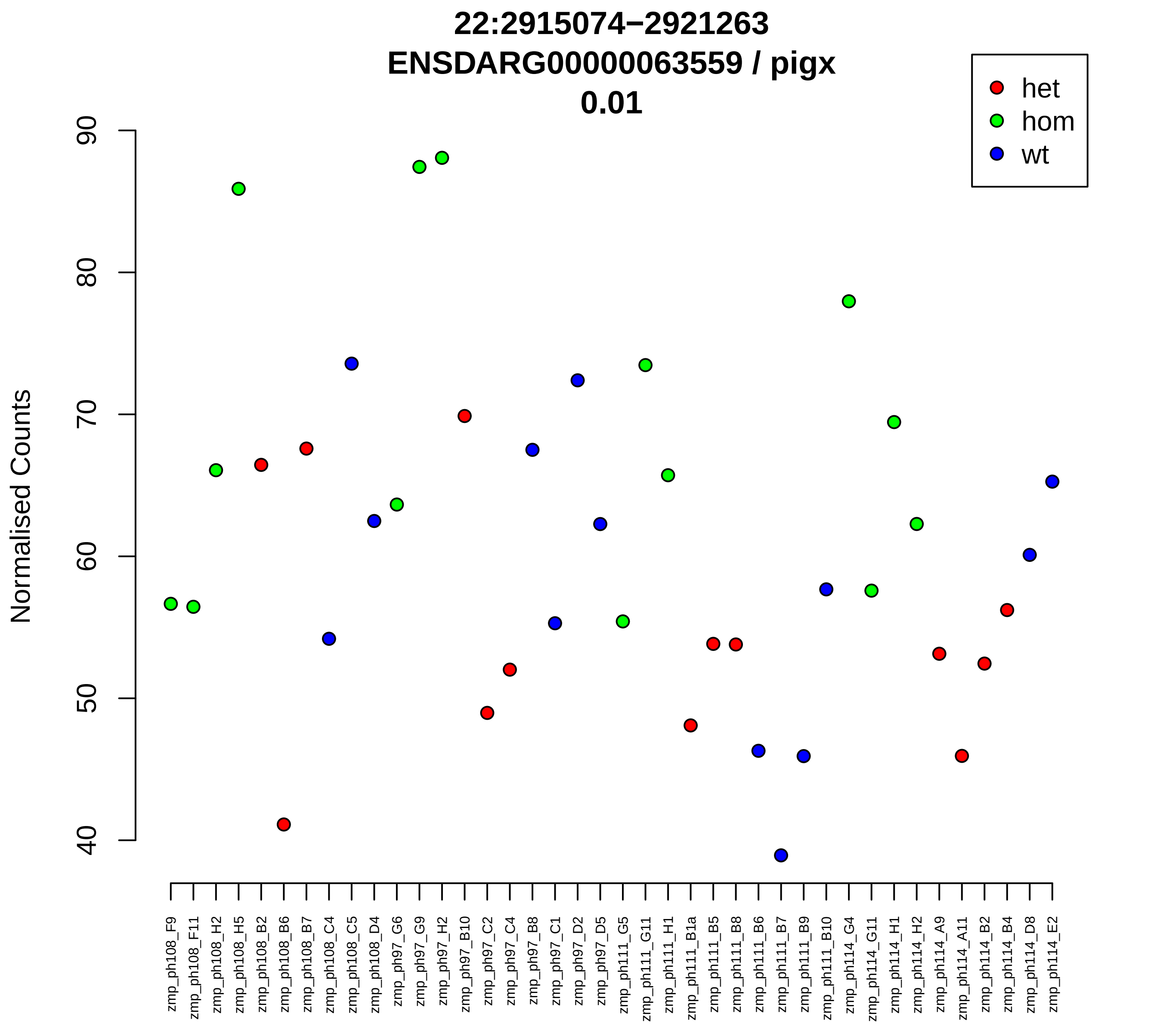
22:2915074−2921263
ENSDARG00000063559 / pigx
0.01
het
hom
wt
Normalised Counts
40
50
60
70
80
90
zmp_ph108_F9
zmp_ph108_F11
zmp_ph108_H2
zmp_ph108_H5
zmp_ph108_B2
zmp_ph108_B6
zmp_ph108_B7
zmp_ph108_C4
zmp_ph108_C5
zmp_ph108_D4
zmp_ph97_G6
zmp_ph97_G9
zmp_ph97_H2
zmp_ph97_B10
zmp_ph97_C2
zmp_ph97_C4
zmp_ph97_B8
zmp_ph97_C1
zmp_ph97_D2
zmp_ph97_D5
zmp_ph111_G5
zmp_ph111_G11
zmp_ph111_H1
zmp_ph111_B1a
zmp_ph111_B5
zmp_ph111_B8
zmp_ph111_B6
zmp_ph111_B7
zmp_ph111_B9
zmp_ph111_B10
zmp_ph114_G4
zmp_ph114_G11
zmp_ph114_H1
zmp_ph114_H2
zmp_ph114_A9
zmp_ph114_A11
zmp_ph114_B2
zmp_ph114_B4
zmp_ph114_D8
zmp_ph114_E2

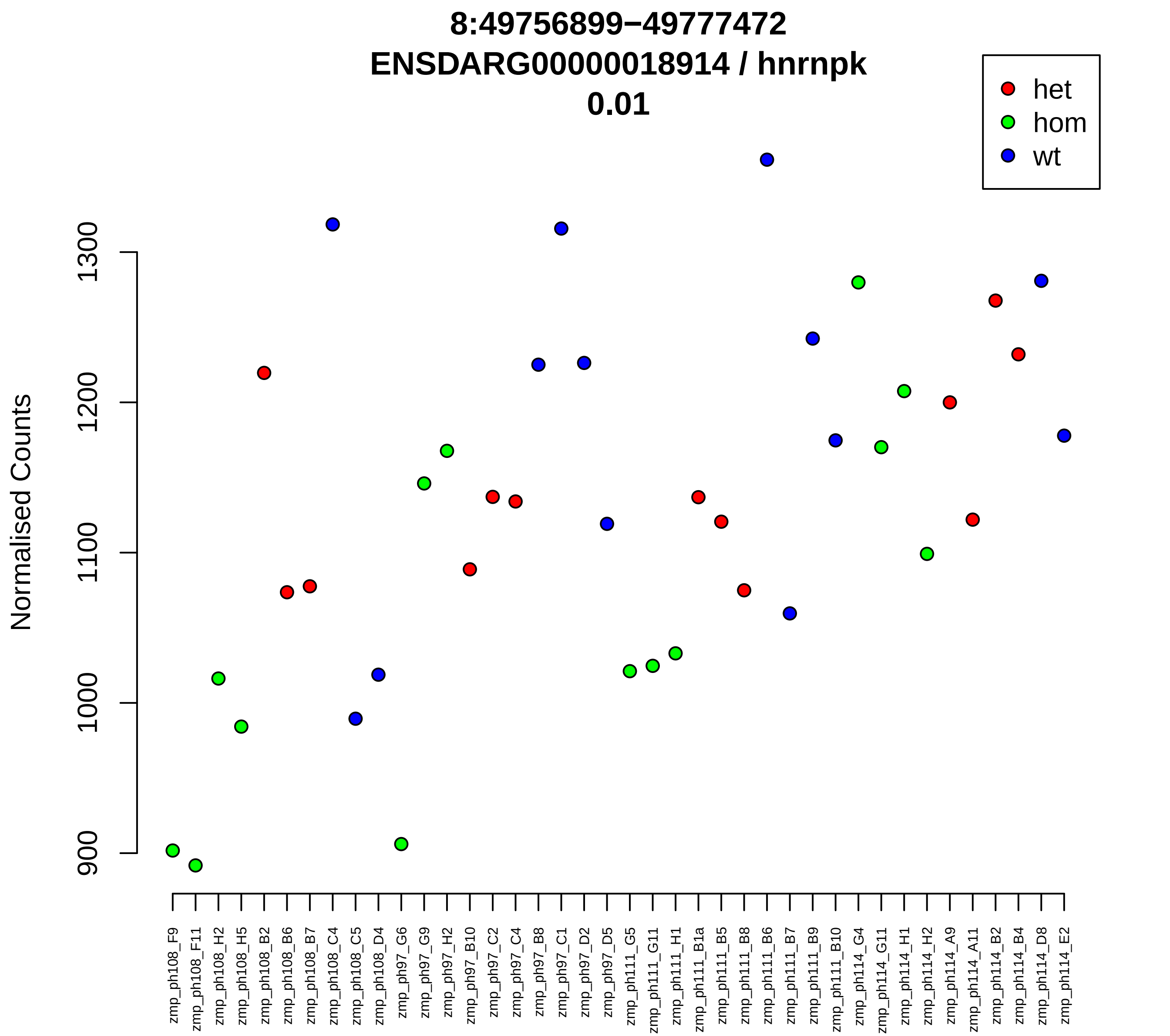

8:49756899−49777472
ENSDARG00000018914 / hnrnpk
0.01
het
hom
wt
Normalised Counts
900
1000
1100
1200
1300
zmp_ph108_F9
zmp_ph108_F11
zmp_ph108_H2
zmp_ph108_H5
zmp_ph108_B2
zmp_ph108_B6
zmp_ph108_B7
zmp_ph108_C4
zmp_ph108_C5
zmp_ph108_D4
zmp_ph97_G6
zmp_ph97_G9
zmp_ph97_H2
zmp_ph97_B10
zmp_ph97_C2
zmp_ph97_C4
zmp_ph97_B8
zmp_ph97_C1
zmp_ph97_D2
zmp_ph97_D5
zmp_ph111_G5
zmp_ph111_G11
zmp_ph111_H1
zmp_ph111_B1a
zmp_ph111_B5
zmp_ph111_B8
zmp_ph111_B6
zmp_ph111_B7
zmp_ph111_B9
zmp_ph111_B10
zmp_ph114_G4
zmp_ph114_G11
zmp_ph114_H1
zmp_ph114_H2
zmp_ph114_A9
zmp_ph114_A11
zmp_ph114_B2
zmp_ph114_B4
zmp_ph114_D8
zmp_ph114_E2

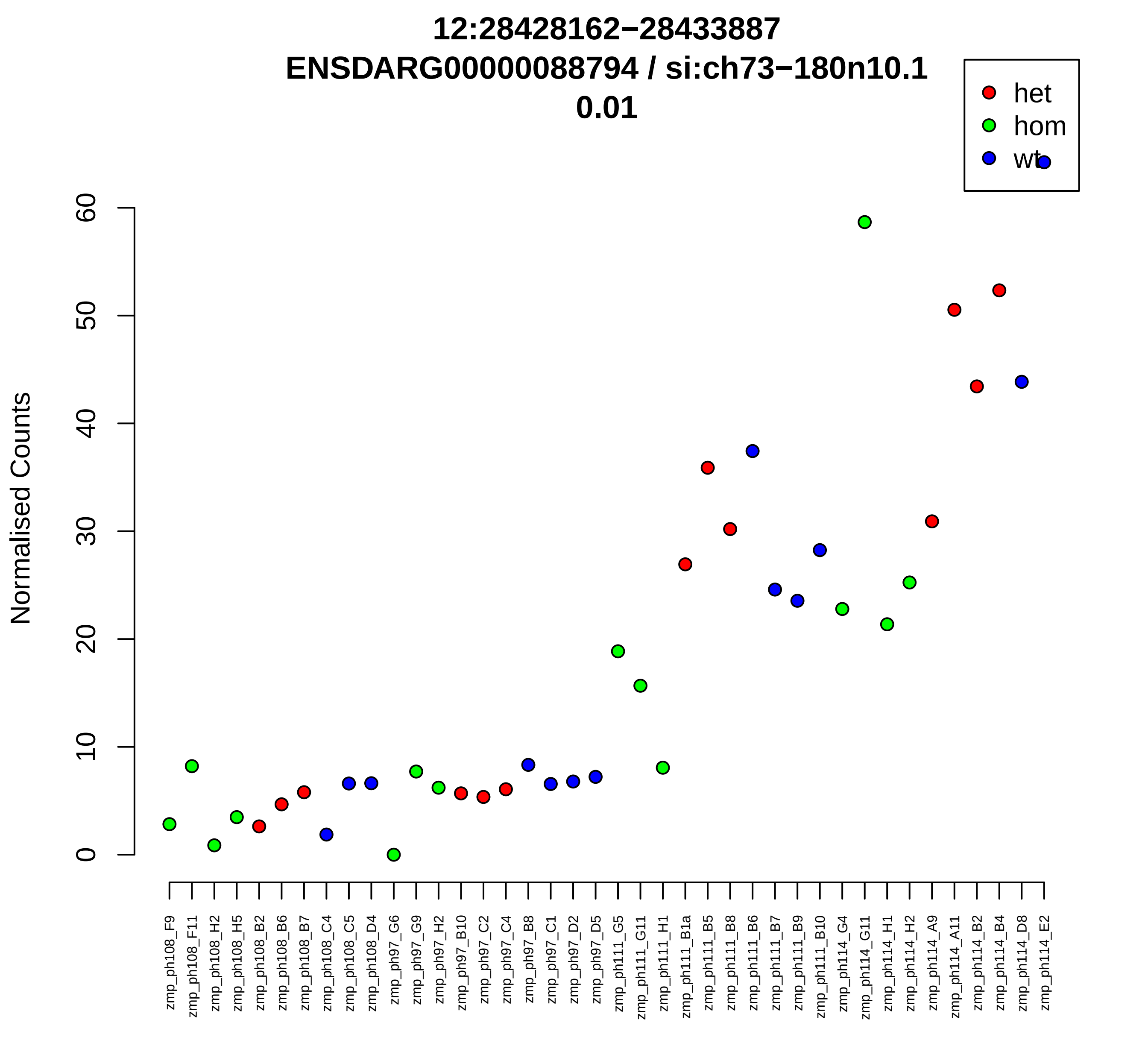
12:28428162−28433887
ENSDARG00000088794 / si:ch73−180n10.1
0.01
het
hom
wt
Normalised Counts
0
10
20
30
40
50
60
zmp_ph108_F9
zmp_ph108_F11
zmp_ph108_H2
zmp_ph108_H5
zmp_ph108_B2
zmp_ph108_B6
zmp_ph108_B7
zmp_ph108_C4
zmp_ph108_C5
zmp_ph108_D4
zmp_ph97_G6
zmp_ph97_G9
zmp_ph97_H2
zmp_ph97_B10
zmp_ph97_C2
zmp_ph97_C4
zmp_ph97_B8
zmp_ph97_C1
zmp_ph97_D2
zmp_ph97_D5
zmp_ph111_G5
zmp_ph111_G11
zmp_ph111_H1
zmp_ph111_B1a
zmp_ph111_B5
zmp_ph111_B8
zmp_ph111_B6
zmp_ph111_B7
zmp_ph111_B9
zmp_ph111_B10
zmp_ph114_G4
zmp_ph114_G11
zmp_ph114_H1
zmp_ph114_H2
zmp_ph114_A9
zmp_ph114_A11
zmp_ph114_B2
zmp_ph114_B4
zmp_ph114_D8
zmp_ph114_E2

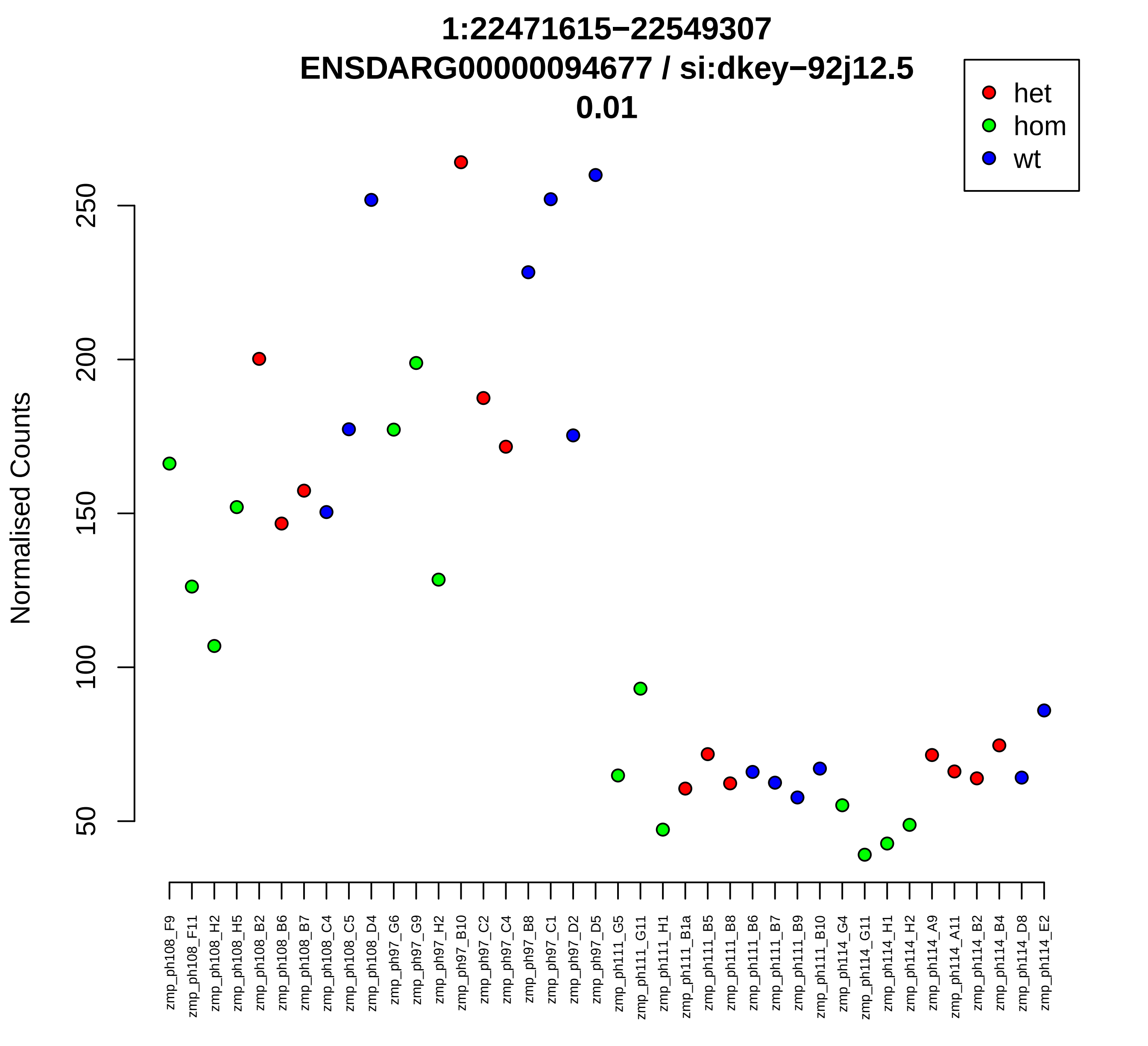

1:22471615−22549307
ENSDARG00000094677 / si:dkey−92j12.5
0.01
het
hom
wt
Normalised Counts
50
100
150
200
250
zmp_ph108_F9
zmp_ph108_F11
zmp_ph108_H2
zmp_ph108_H5
zmp_ph108_B2
zmp_ph108_B6
zmp_ph108_B7
zmp_ph108_C4
zmp_ph108_C5
zmp_ph108_D4
zmp_ph97_G6
zmp_ph97_G9
zmp_ph97_H2
zmp_ph97_B10
zmp_ph97_C2
zmp_ph97_C4
zmp_ph97_B8
zmp_ph97_C1
zmp_ph97_D2
zmp_ph97_D5
zmp_ph111_G5
zmp_ph111_G11
zmp_ph111_H1
zmp_ph111_B1a
zmp_ph111_B5
zmp_ph111_B8
zmp_ph111_B6
zmp_ph111_B7
zmp_ph111_B9
zmp_ph111_B10
zmp_ph114_G4
zmp_ph114_G11
zmp_ph114_H1
zmp_ph114_H2
zmp_ph114_A9
zmp_ph114_A11
zmp_ph114_B2
zmp_ph114_B4
zmp_ph114_D8
zmp_ph114_E2

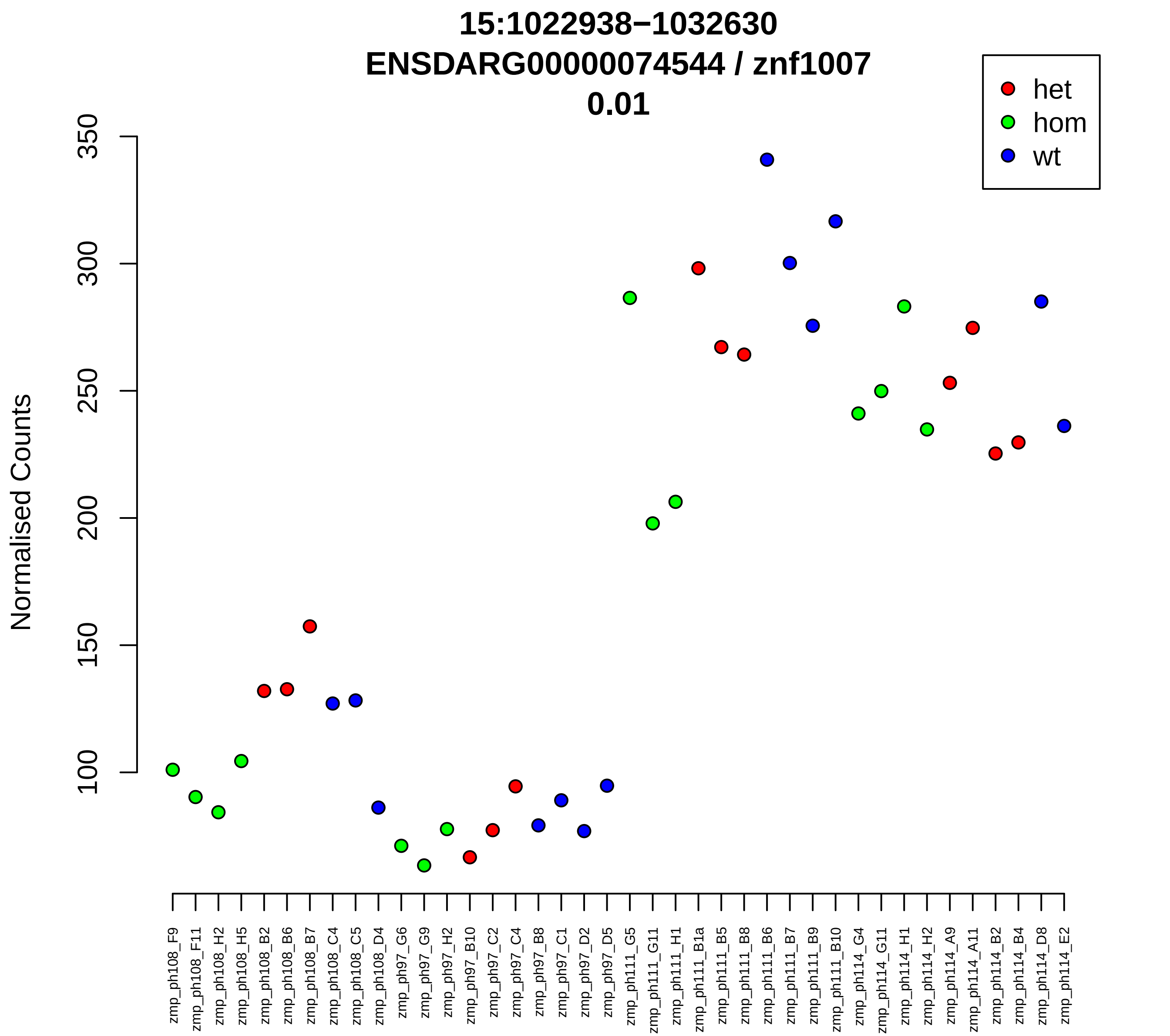

15:1022938−1032630
ENSDARG00000074544 / znf1007
0.01
het
hom
wt
Normalised Counts
100
150
200
250
300
350
zmp_ph108_F9
zmp_ph108_F11
zmp_ph108_H2
zmp_ph108_H5
zmp_ph108_B2
zmp_ph108_B6
zmp_ph108_B7
zmp_ph108_C4
zmp_ph108_C5
zmp_ph108_D4
zmp_ph97_G6
zmp_ph97_G9
zmp_ph97_H2
zmp_ph97_B10
zmp_ph97_C2
zmp_ph97_C4
zmp_ph97_B8
zmp_ph97_C1
zmp_ph97_D2
zmp_ph97_D5
zmp_ph111_G5
zmp_ph111_G11
zmp_ph111_H1
zmp_ph111_B1a
zmp_ph111_B5
zmp_ph111_B8
zmp_ph111_B6
zmp_ph111_B7
zmp_ph111_B9
zmp_ph111_B10
zmp_ph114_G4
zmp_ph114_G11
zmp_ph114_H1
zmp_ph114_H2
zmp_ph114_A9
zmp_ph114_A11
zmp_ph114_B2
zmp_ph114_B4
zmp_ph114_D8
zmp_ph114_E2

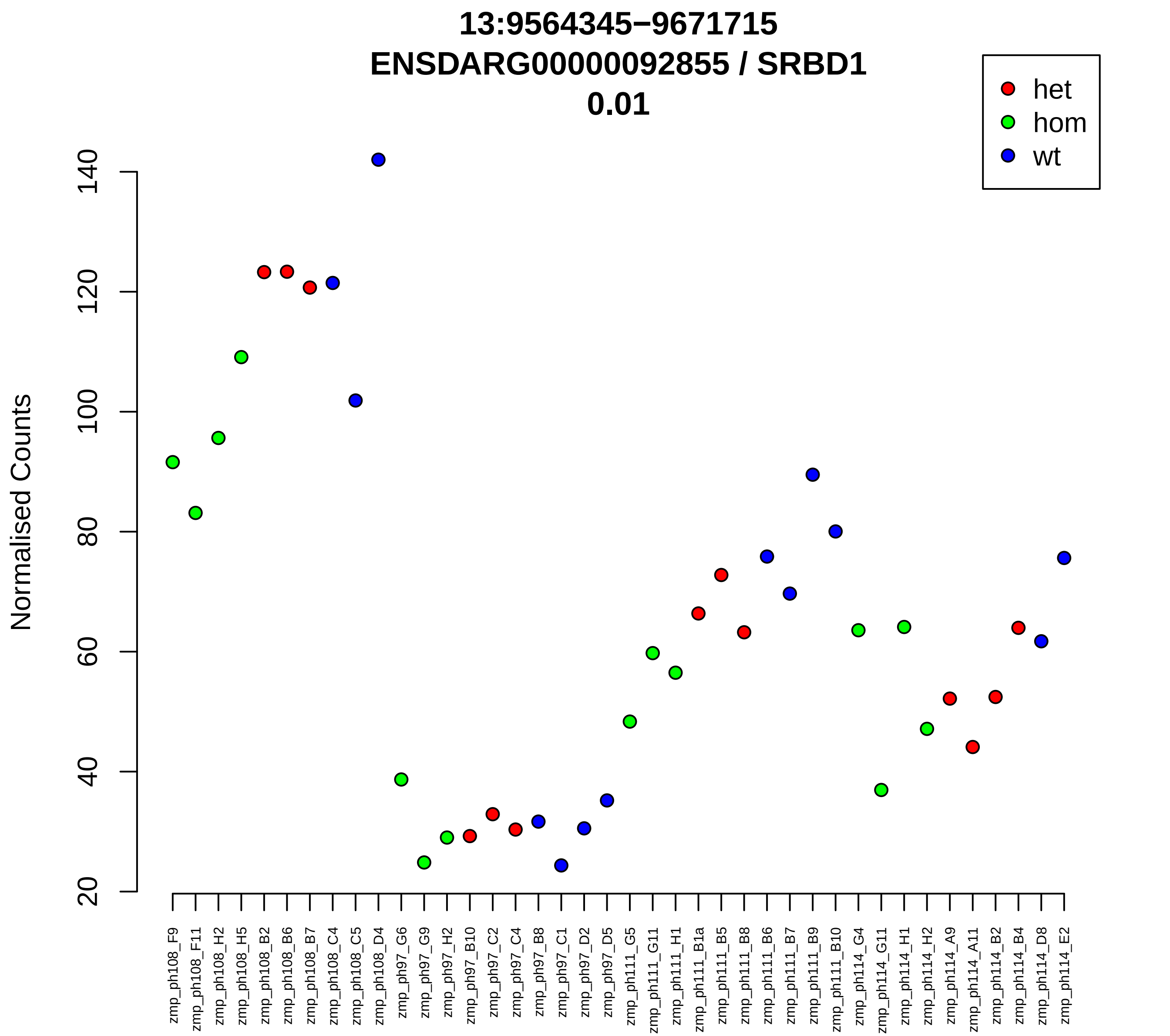
13:9564345−9671715
ENSDARG00000092855 / SRBD1
0.01
het
hom
wt
Normalised Counts
20
40
60
80
100
120
140
zmp_ph108_F9
zmp_ph108_F11
zmp_ph108_H2
zmp_ph108_H5
zmp_ph108_B2
zmp_ph108_B6
zmp_ph108_B7
zmp_ph108_C4
zmp_ph108_C5
zmp_ph108_D4
zmp_ph97_G6
zmp_ph97_G9
zmp_ph97_H2
zmp_ph97_B10
zmp_ph97_C2
zmp_ph97_C4
zmp_ph97_B8
zmp_ph97_C1
zmp_ph97_D2
zmp_ph97_D5
zmp_ph111_G5
zmp_ph111_G11
zmp_ph111_H1
zmp_ph111_B1a
zmp_ph111_B5
zmp_ph111_B8
zmp_ph111_B6
zmp_ph111_B7
zmp_ph111_B9
zmp_ph111_B10
zmp_ph114_G4
zmp_ph114_G11
zmp_ph114_H1
zmp_ph114_H2
zmp_ph114_A9
zmp_ph114_A11
zmp_ph114_B2
zmp_ph114_B4
zmp_ph114_D8
zmp_ph114_E2

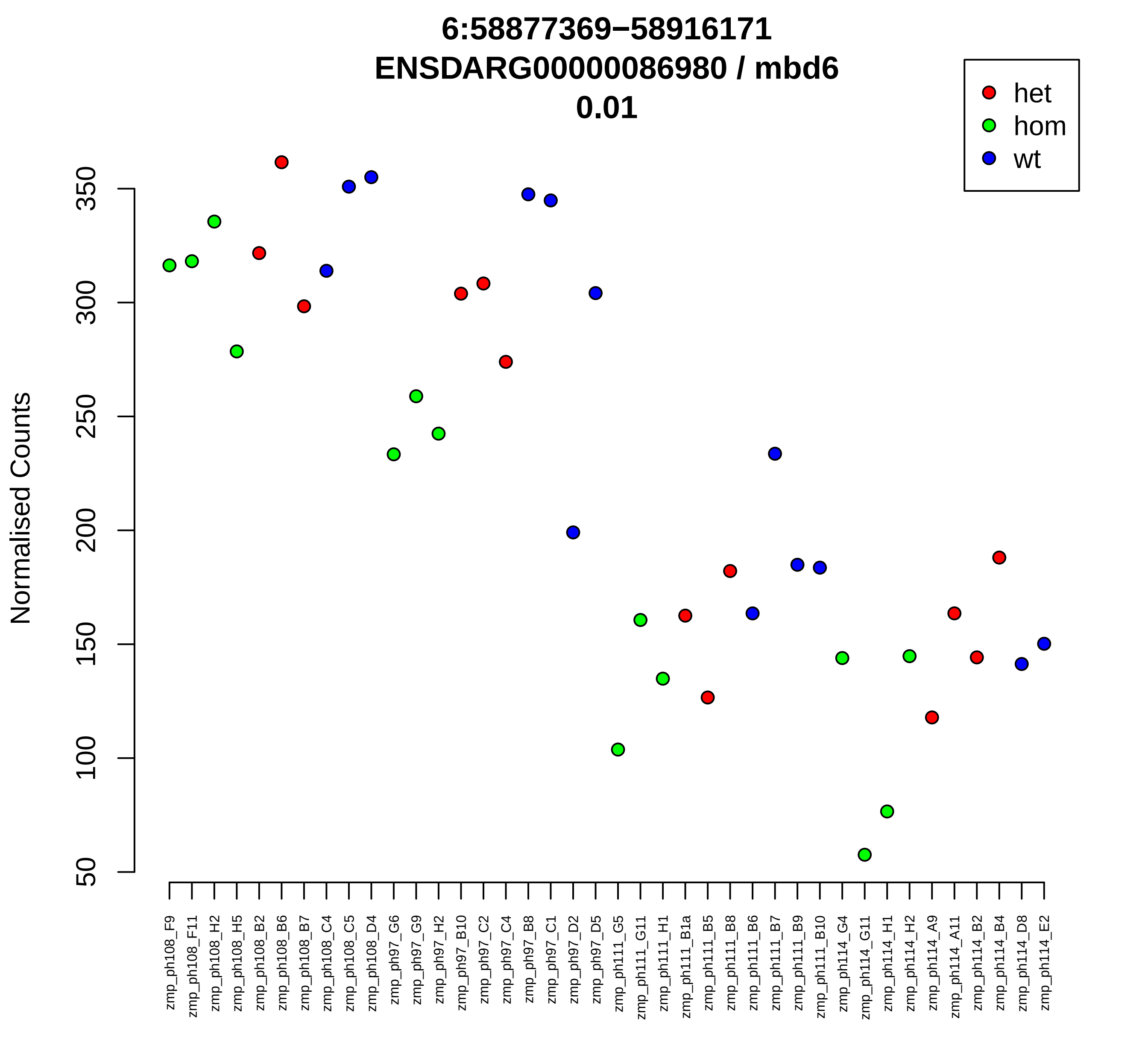
6:58877369−58916171
ENSDARG00000086980 / mbd6
0.01
het
hom
wt
Normalised Counts
50
100
150
200
250
300
350
zmp_ph108_F9
zmp_ph108_F11
zmp_ph108_H2
zmp_ph108_H5
zmp_ph108_B2
zmp_ph108_B6
zmp_ph108_B7
zmp_ph108_C4
zmp_ph108_C5
zmp_ph108_D4
zmp_ph97_G6
zmp_ph97_G9
zmp_ph97_H2
zmp_ph97_B10
zmp_ph97_C2
zmp_ph97_C4
zmp_ph97_B8
zmp_ph97_C1
zmp_ph97_D2
zmp_ph97_D5
zmp_ph111_G5
zmp_ph111_G11
zmp_ph111_H1
zmp_ph111_B1a
zmp_ph111_B5
zmp_ph111_B8
zmp_ph111_B6
zmp_ph111_B7
zmp_ph111_B9
zmp_ph111_B10
zmp_ph114_G4
zmp_ph114_G11
zmp_ph114_H1
zmp_ph114_H2
zmp_ph114_A9
zmp_ph114_A11
zmp_ph114_B2
zmp_ph114_B4
zmp_ph114_D8
zmp_ph114_E2

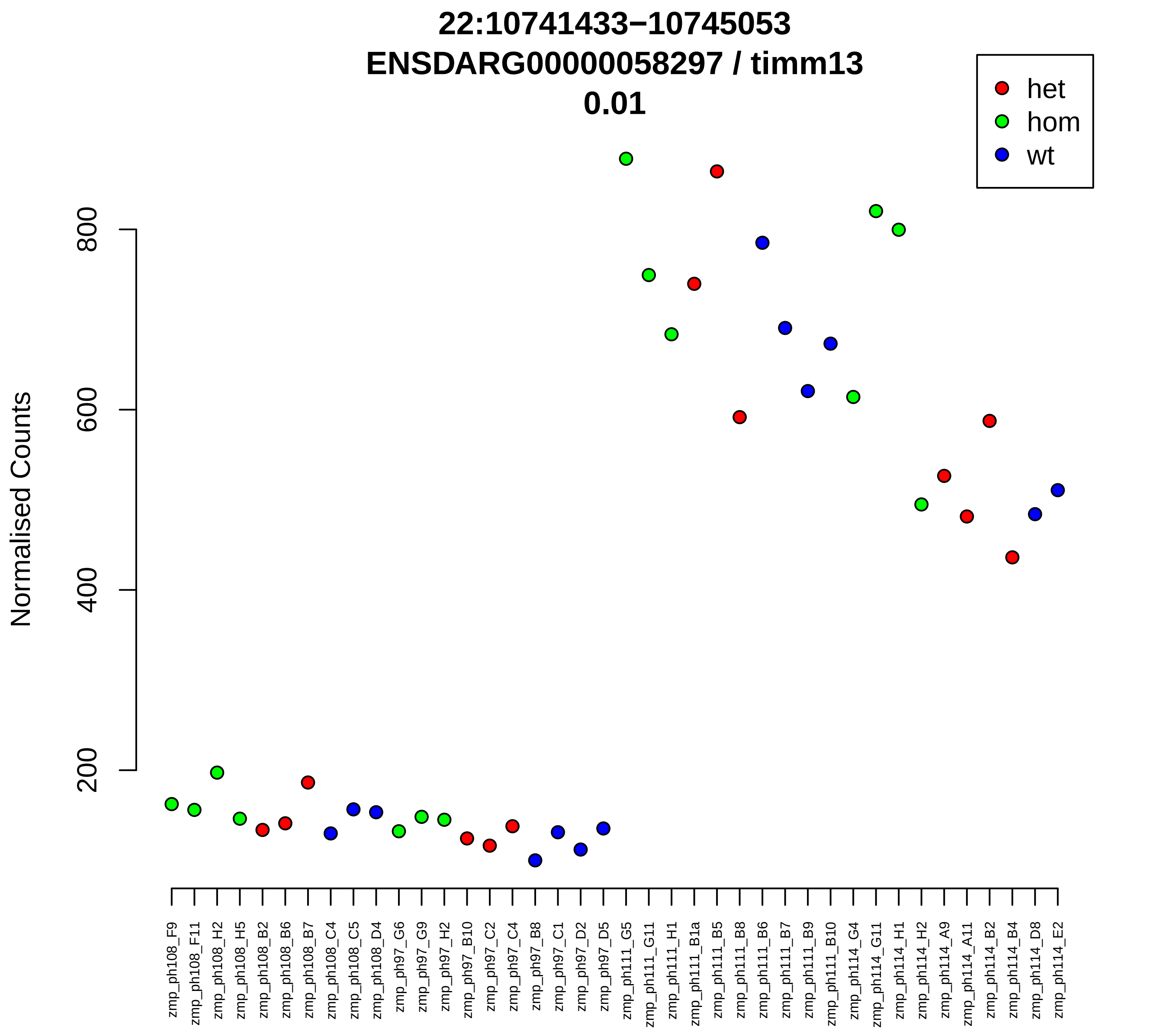

22:10741433−10745053
ENSDARG00000058297 / timm13
0.01
het
hom
wt
Normalised Counts
200
400
600
800
zmp_ph108_F9
zmp_ph108_F11
zmp_ph108_H2
zmp_ph108_H5
zmp_ph108_B2
zmp_ph108_B6
zmp_ph108_B7
zmp_ph108_C4
zmp_ph108_C5
zmp_ph108_D4
zmp_ph97_G6
zmp_ph97_G9
zmp_ph97_H2
zmp_ph97_B10
zmp_ph97_C2
zmp_ph97_C4
zmp_ph97_B8
zmp_ph97_C1
zmp_ph97_D2
zmp_ph97_D5
zmp_ph111_G5
zmp_ph111_G11
zmp_ph111_H1
zmp_ph111_B1a
zmp_ph111_B5
zmp_ph111_B8
zmp_ph111_B6
zmp_ph111_B7
zmp_ph111_B9
zmp_ph111_B10
zmp_ph114_G4
zmp_ph114_G11
zmp_ph114_H1
zmp_ph114_H2
zmp_ph114_A9
zmp_ph114_A11
zmp_ph114_B2
zmp_ph114_B4
zmp_ph114_D8
zmp_ph114_E2

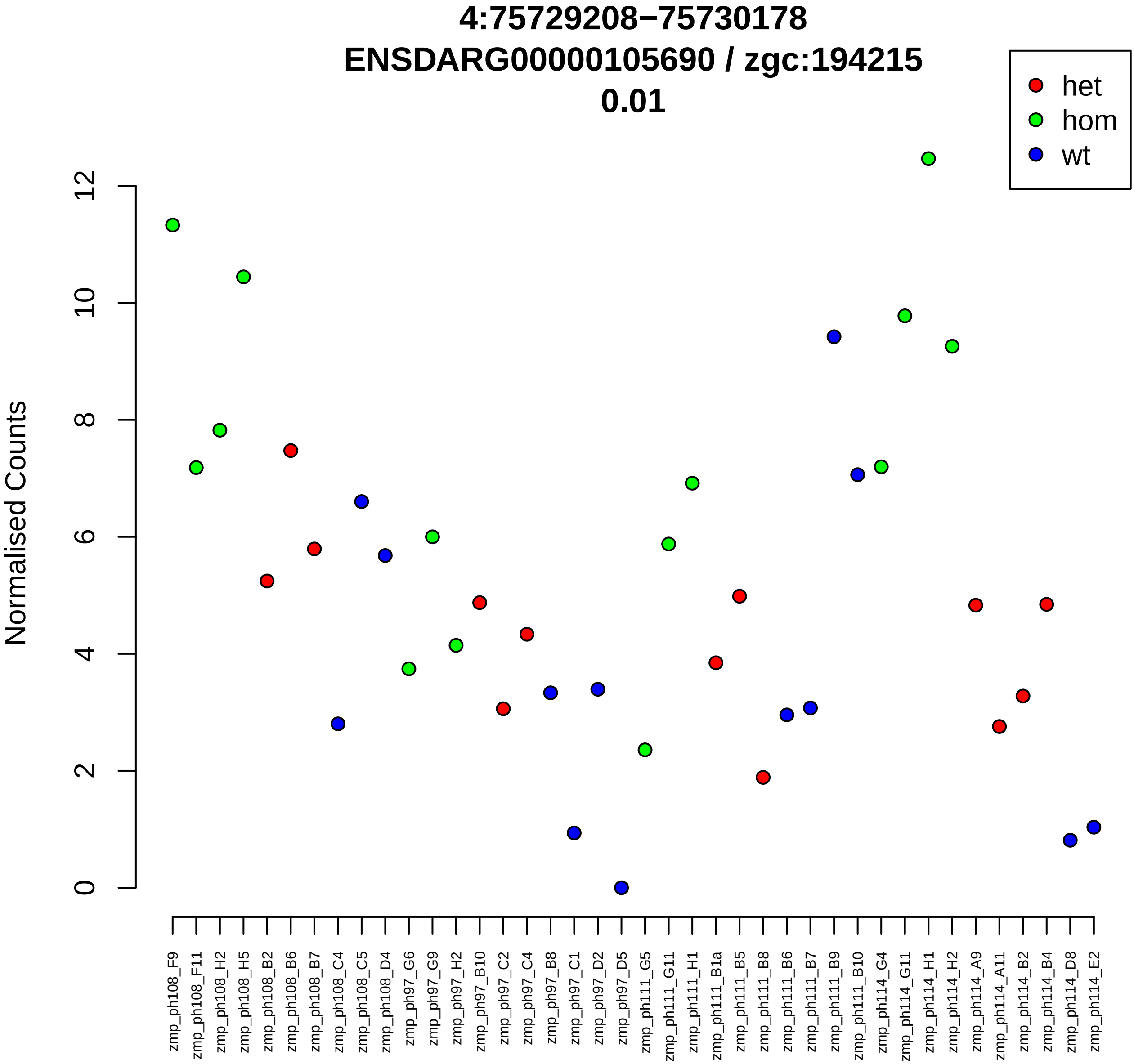
4:75729208−75730178
ENSDARG00000105690 / zgc:194215
0.01
het
hom
wt
Normalised Counts
0
2
4
6
8
10
12
zmp_ph108_F9
zmp_ph108_F11
zmp_ph108_H2
zmp_ph108_H5
zmp_ph108_B2
zmp_ph108_B6
zmp_ph108_B7
zmp_ph108_C4
zmp_ph108_C5
zmp_ph108_D4
zmp_ph97_G6
zmp_ph97_G9
zmp_ph97_H2
zmp_ph97_B10
zmp_ph97_C2
zmp_ph97_C4
zmp_ph97_B8
zmp_ph97_C1
zmp_ph97_D2
zmp_ph97_D5
zmp_ph111_G5
zmp_ph111_G11
zmp_ph111_H1
zmp_ph111_B1a
zmp_ph111_B5
zmp_ph111_B8
zmp_ph111_B6
zmp_ph111_B7
zmp_ph111_B9
zmp_ph111_B10
zmp_ph114_G4
zmp_ph114_G11
zmp_ph114_H1
zmp_ph114_H2
zmp_ph114_A9
zmp_ph114_A11
zmp_ph114_B2
zmp_ph114_B4
zmp_ph114_D8
zmp_ph114_E2

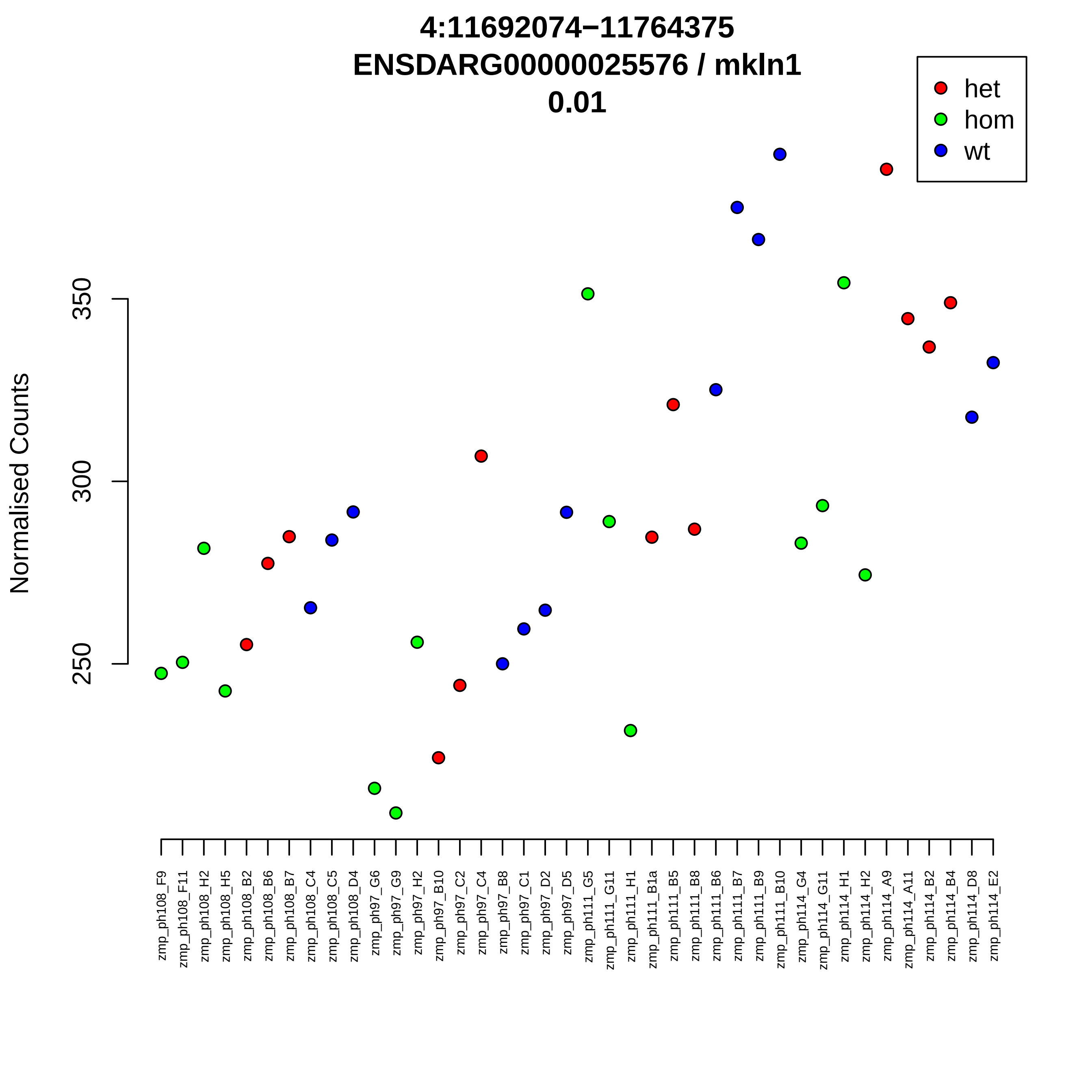

4:11692074−11764375
ENSDARG00000025576 / mkln1
0.01
het
hom
wt
Normalised Counts
250
300
350
zmp_ph108_F9
zmp_ph108_F11
zmp_ph108_H2
zmp_ph108_H5
zmp_ph108_B2
zmp_ph108_B6
zmp_ph108_B7
zmp_ph108_C4
zmp_ph108_C5
zmp_ph108_D4
zmp_ph97_G6
zmp_ph97_G9
zmp_ph97_H2
zmp_ph97_B10
zmp_ph97_C2
zmp_ph97_C4
zmp_ph97_B8
zmp_ph97_C1
zmp_ph97_D2
zmp_ph97_D5
zmp_ph111_G5
zmp_ph111_G11
zmp_ph111_H1
zmp_ph111_B1a
zmp_ph111_B5
zmp_ph111_B8
zmp_ph111_B6
zmp_ph111_B7
zmp_ph111_B9
zmp_ph111_B10
zmp_ph114_G4
zmp_ph114_G11
zmp_ph114_H1
zmp_ph114_H2
zmp_ph114_A9
zmp_ph114_A11
zmp_ph114_B2
zmp_ph114_B4
zmp_ph114_D8
zmp_ph114_E2

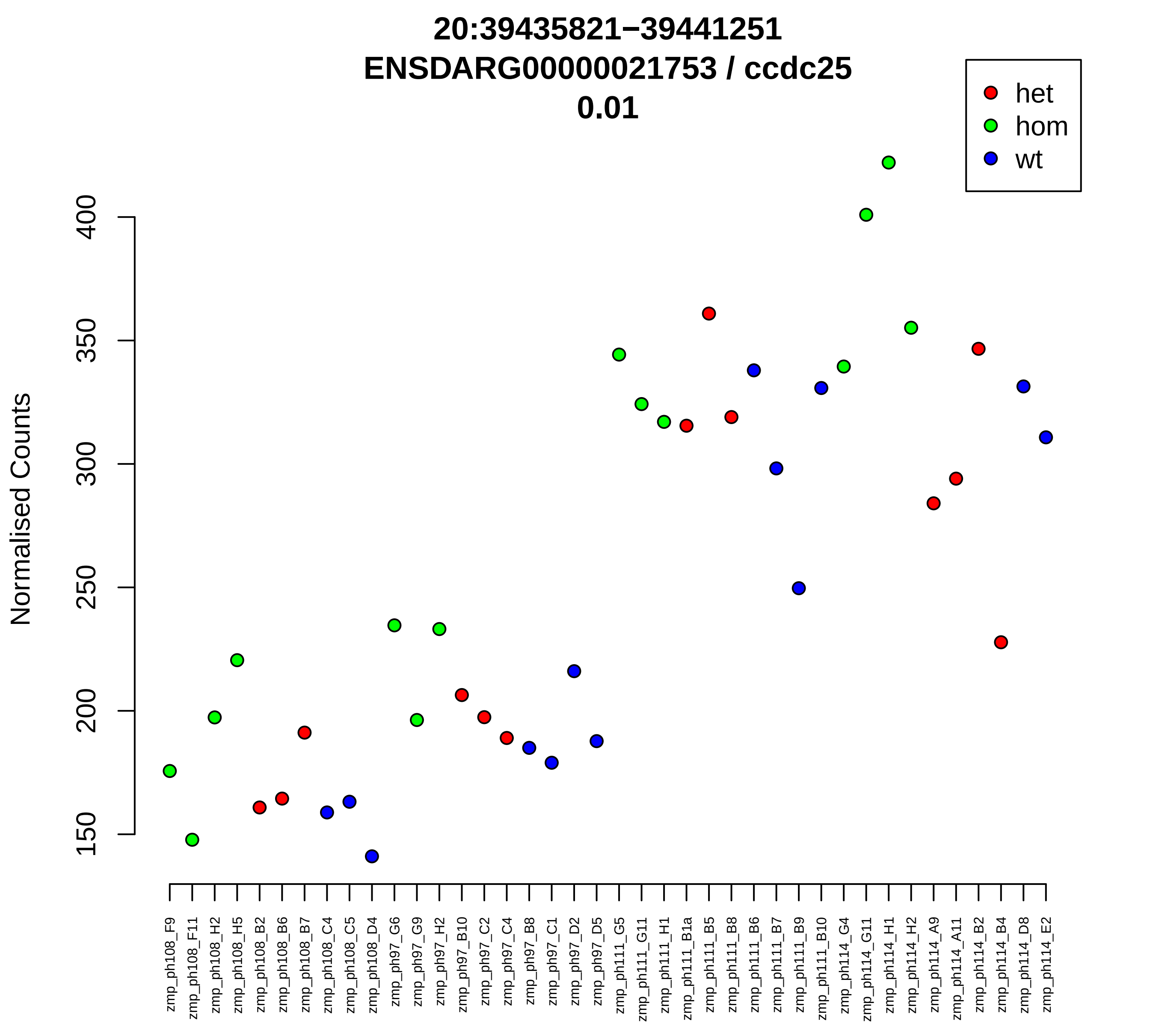

20:39435821−39441251
ENSDARG00000021753 / ccdc25
0.01
het
hom
wt
Normalised Counts
150
200
250
300
350
400
zmp_ph108_F9
zmp_ph108_F11
zmp_ph108_H2
zmp_ph108_H5
zmp_ph108_B2
zmp_ph108_B6
zmp_ph108_B7
zmp_ph108_C4
zmp_ph108_C5
zmp_ph108_D4
zmp_ph97_G6
zmp_ph97_G9
zmp_ph97_H2
zmp_ph97_B10
zmp_ph97_C2
zmp_ph97_C4
zmp_ph97_B8
zmp_ph97_C1
zmp_ph97_D2
zmp_ph97_D5
zmp_ph111_G5
zmp_ph111_G11
zmp_ph111_H1
zmp_ph111_B1a
zmp_ph111_B5
zmp_ph111_B8
zmp_ph111_B6
zmp_ph111_B7
zmp_ph111_B9
zmp_ph111_B10
zmp_ph114_G4
zmp_ph114_G11
zmp_ph114_H1
zmp_ph114_H2
zmp_ph114_A9
zmp_ph114_A11
zmp_ph114_B2
zmp_ph114_B4
zmp_ph114_D8
zmp_ph114_E2

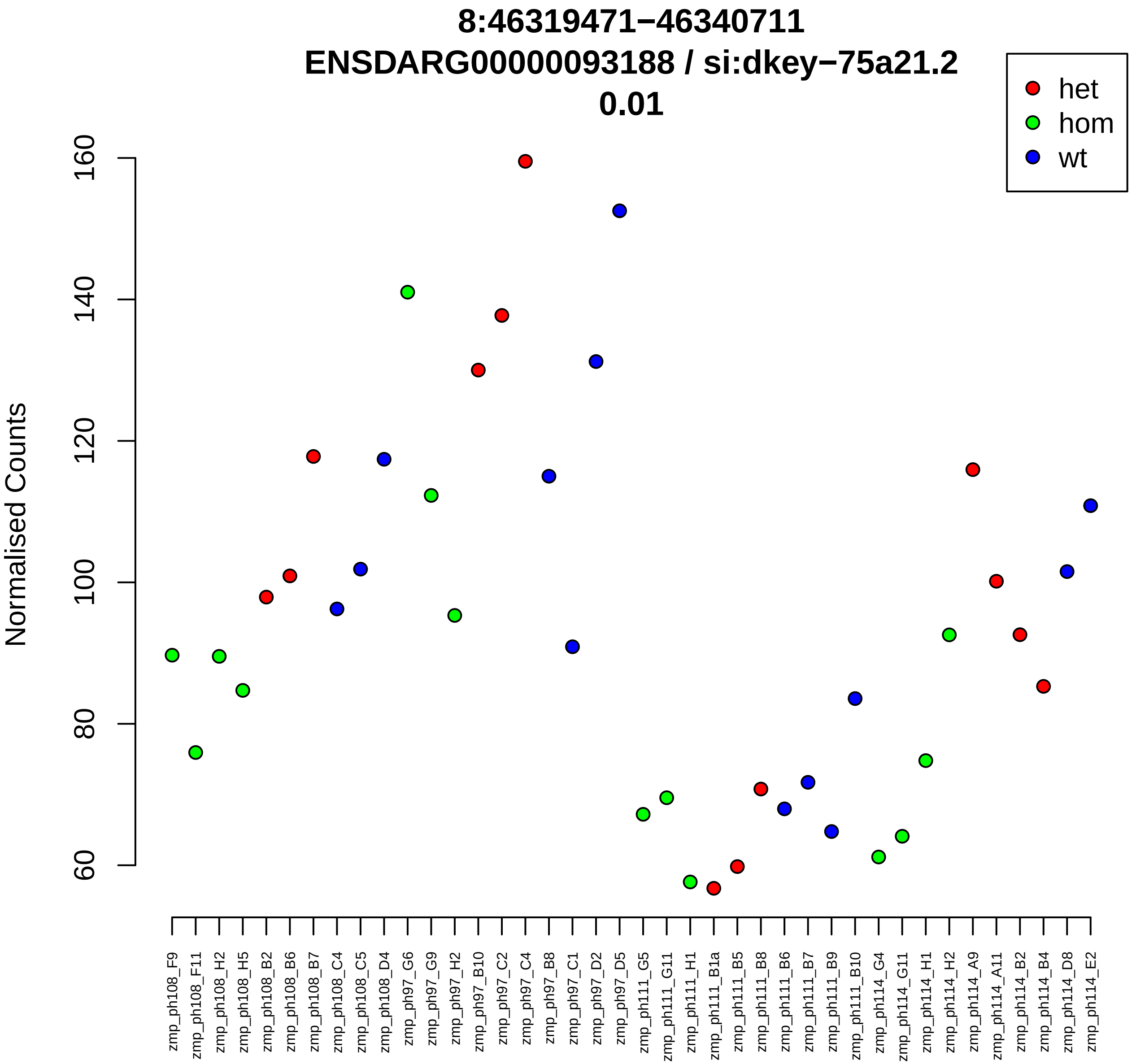
8:46319471−46340711
ENSDARG00000093188 / si:dkey−75a21.2
0.01
het
hom
wt
Normalised Counts
60
80
100
120
140
160
zmp_ph108_F9
zmp_ph108_F11
zmp_ph108_H2
zmp_ph108_H5
zmp_ph108_B2
zmp_ph108_B6
zmp_ph108_B7
zmp_ph108_C4
zmp_ph108_C5
zmp_ph108_D4
zmp_ph97_G6
zmp_ph97_G9
zmp_ph97_H2
zmp_ph97_B10
zmp_ph97_C2
zmp_ph97_C4
zmp_ph97_B8
zmp_ph97_C1
zmp_ph97_D2
zmp_ph97_D5
zmp_ph111_G5
zmp_ph111_G11
zmp_ph111_H1
zmp_ph111_B1a
zmp_ph111_B5
zmp_ph111_B8
zmp_ph111_B6
zmp_ph111_B7
zmp_ph111_B9
zmp_ph111_B10
zmp_ph114_G4
zmp_ph114_G11
zmp_ph114_H1
zmp_ph114_H2
zmp_ph114_A9
zmp_ph114_A11
zmp_ph114_B2
zmp_ph114_B4
zmp_ph114_D8
zmp_ph114_E2

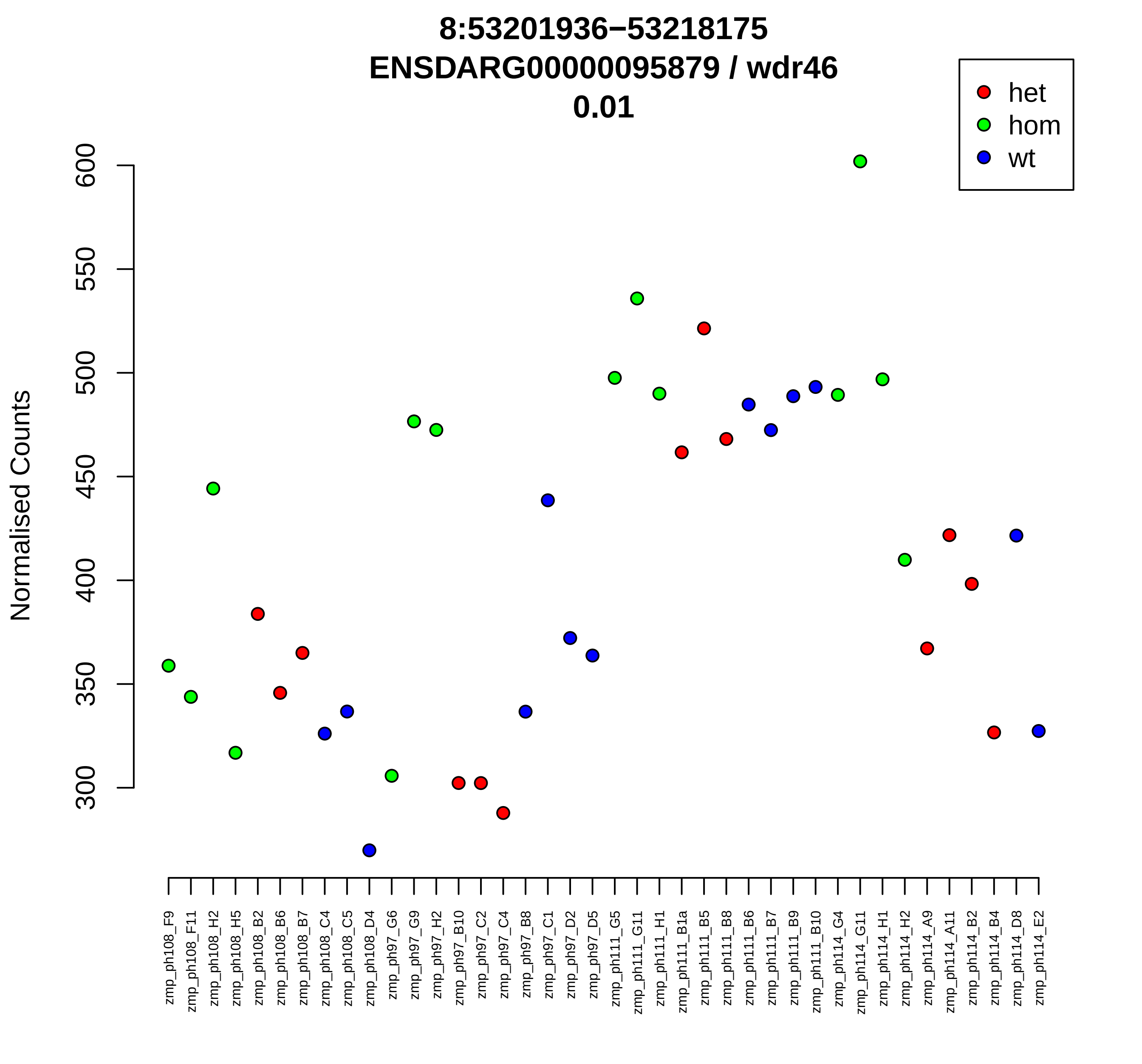
8:53201936−53218175
ENSDARG00000095879 / wdr46
0.01
het
hom
wt
Normalised Counts
300
350
400
450
500
550
600
zmp_ph108_F9
zmp_ph108_F11
zmp_ph108_H2
zmp_ph108_H5
zmp_ph108_B2
zmp_ph108_B6
zmp_ph108_B7
zmp_ph108_C4
zmp_ph108_C5
zmp_ph108_D4
zmp_ph97_G6
zmp_ph97_G9
zmp_ph97_H2
zmp_ph97_B10
zmp_ph97_C2
zmp_ph97_C4
zmp_ph97_B8
zmp_ph97_C1
zmp_ph97_D2
zmp_ph97_D5
zmp_ph111_G5
zmp_ph111_G11
zmp_ph111_H1
zmp_ph111_B1a
zmp_ph111_B5
zmp_ph111_B8
zmp_ph111_B6
zmp_ph111_B7
zmp_ph111_B9
zmp_ph111_B10
zmp_ph114_G4
zmp_ph114_G11
zmp_ph114_H1
zmp_ph114_H2
zmp_ph114_A9
zmp_ph114_A11
zmp_ph114_B2
zmp_ph114_B4
zmp_ph114_D8
zmp_ph114_E2

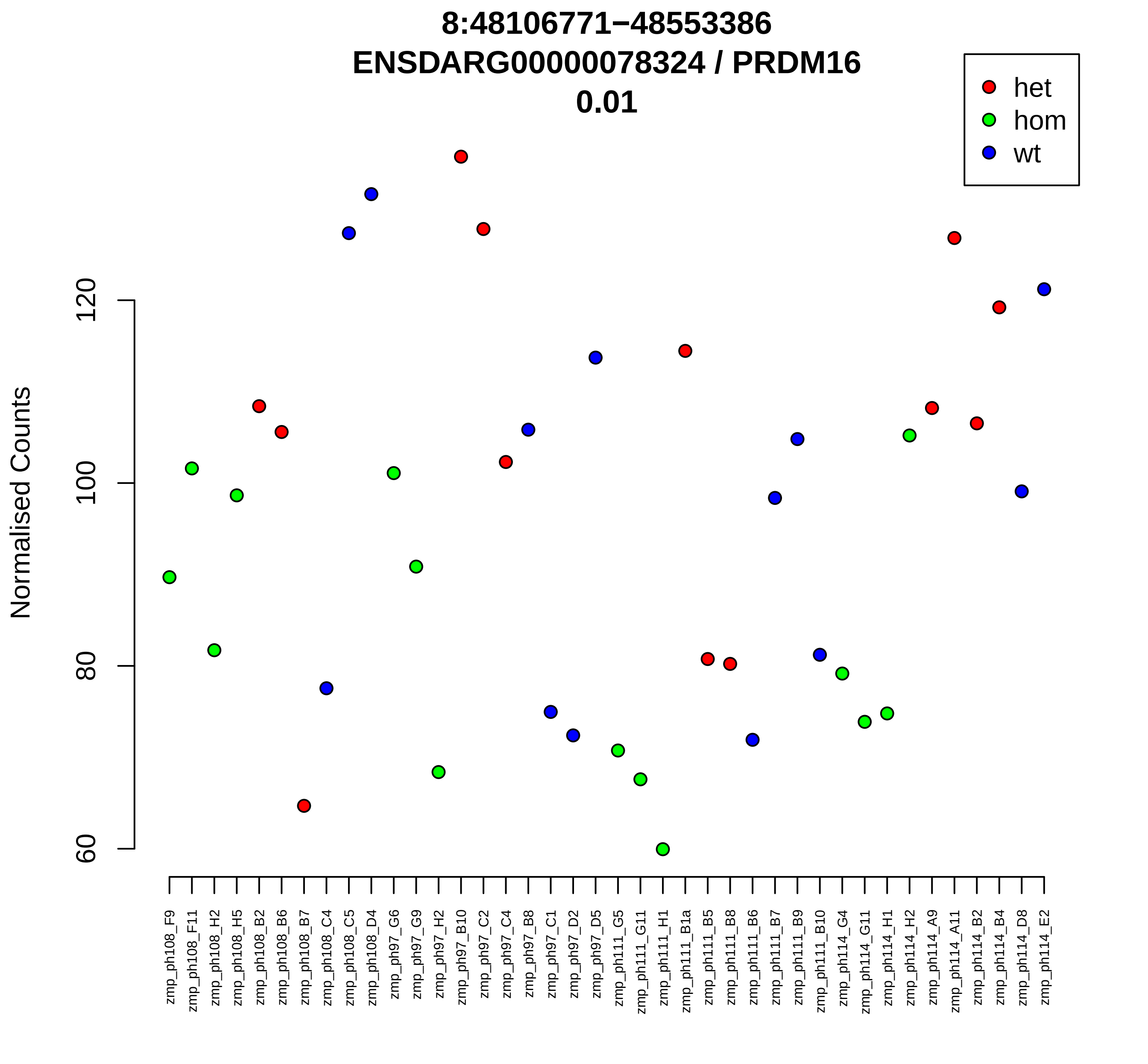
8:48106771−48553386
ENSDARG00000078324 / PRDM16
0.01
het
hom
wt
Normalised Counts
60
80
100
120
zmp_ph108_F9
zmp_ph108_F11
zmp_ph108_H2
zmp_ph108_H5
zmp_ph108_B2
zmp_ph108_B6
zmp_ph108_B7
zmp_ph108_C4
zmp_ph108_C5
zmp_ph108_D4
zmp_ph97_G6
zmp_ph97_G9
zmp_ph97_H2
zmp_ph97_B10
zmp_ph97_C2
zmp_ph97_C4
zmp_ph97_B8
zmp_ph97_C1
zmp_ph97_D2
zmp_ph97_D5
zmp_ph111_G5
zmp_ph111_G11
zmp_ph111_H1
zmp_ph111_B1a
zmp_ph111_B5
zmp_ph111_B8
zmp_ph111_B6
zmp_ph111_B7
zmp_ph111_B9
zmp_ph111_B10
zmp_ph114_G4
zmp_ph114_G11
zmp_ph114_H1
zmp_ph114_H2
zmp_ph114_A9
zmp_ph114_A11
zmp_ph114_B2
zmp_ph114_B4
zmp_ph114_D8
zmp_ph114_E2

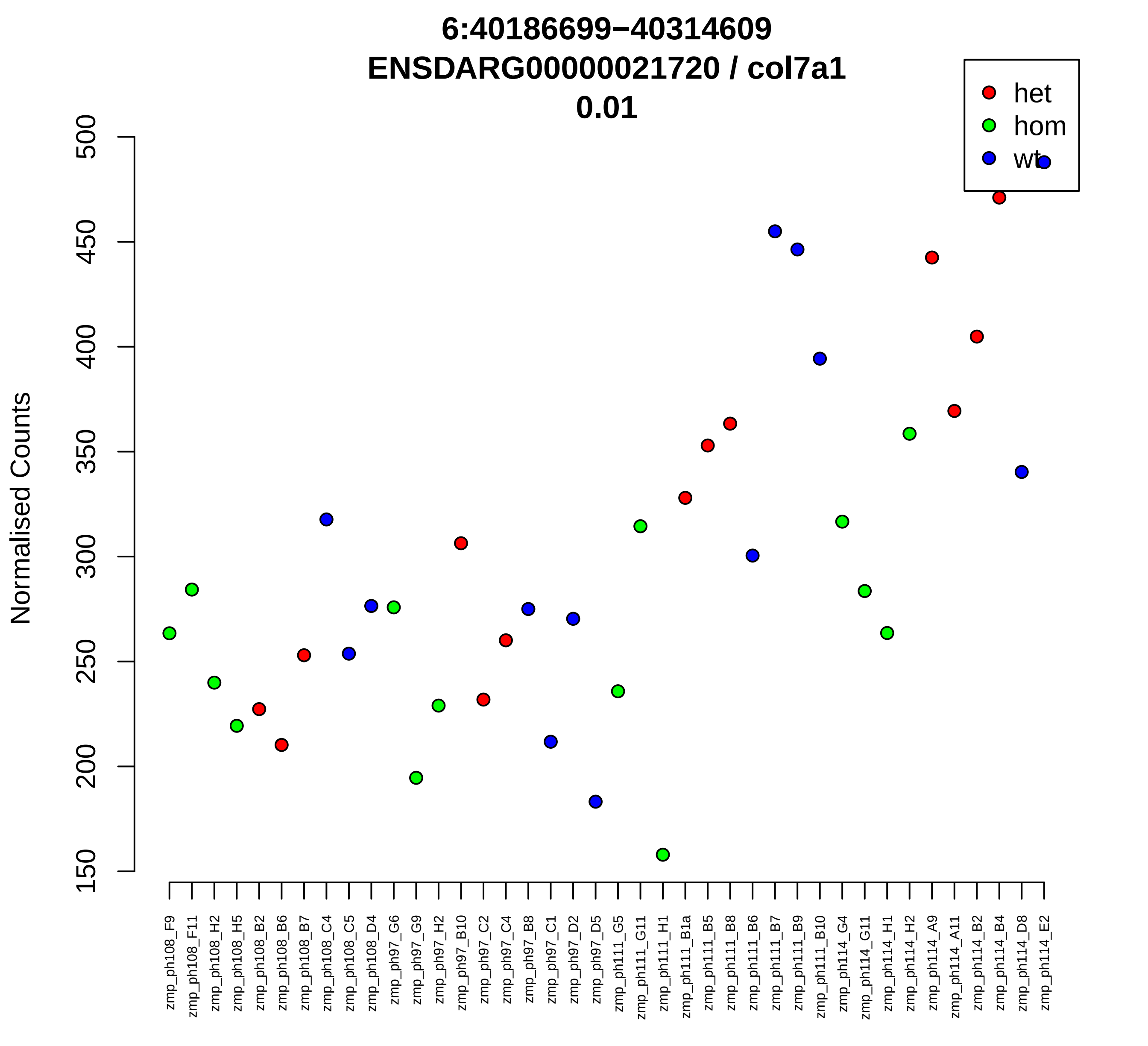

6:40186699−40314609
ENSDARG00000021720 / col7a1
0.01
het
hom
wt
Normalised Counts
150
200
250
300
350
400
450
500
zmp_ph108_F9
zmp_ph108_F11
zmp_ph108_H2
zmp_ph108_H5
zmp_ph108_B2
zmp_ph108_B6
zmp_ph108_B7
zmp_ph108_C4
zmp_ph108_C5
zmp_ph108_D4
zmp_ph97_G6
zmp_ph97_G9
zmp_ph97_H2
zmp_ph97_B10
zmp_ph97_C2
zmp_ph97_C4
zmp_ph97_B8
zmp_ph97_C1
zmp_ph97_D2
zmp_ph97_D5
zmp_ph111_G5
zmp_ph111_G11
zmp_ph111_H1
zmp_ph111_B1a
zmp_ph111_B5
zmp_ph111_B8
zmp_ph111_B6
zmp_ph111_B7
zmp_ph111_B9
zmp_ph111_B10
zmp_ph114_G4
zmp_ph114_G11
zmp_ph114_H1
zmp_ph114_H2
zmp_ph114_A9
zmp_ph114_A11
zmp_ph114_B2
zmp_ph114_B4
zmp_ph114_D8
zmp_ph114_E2

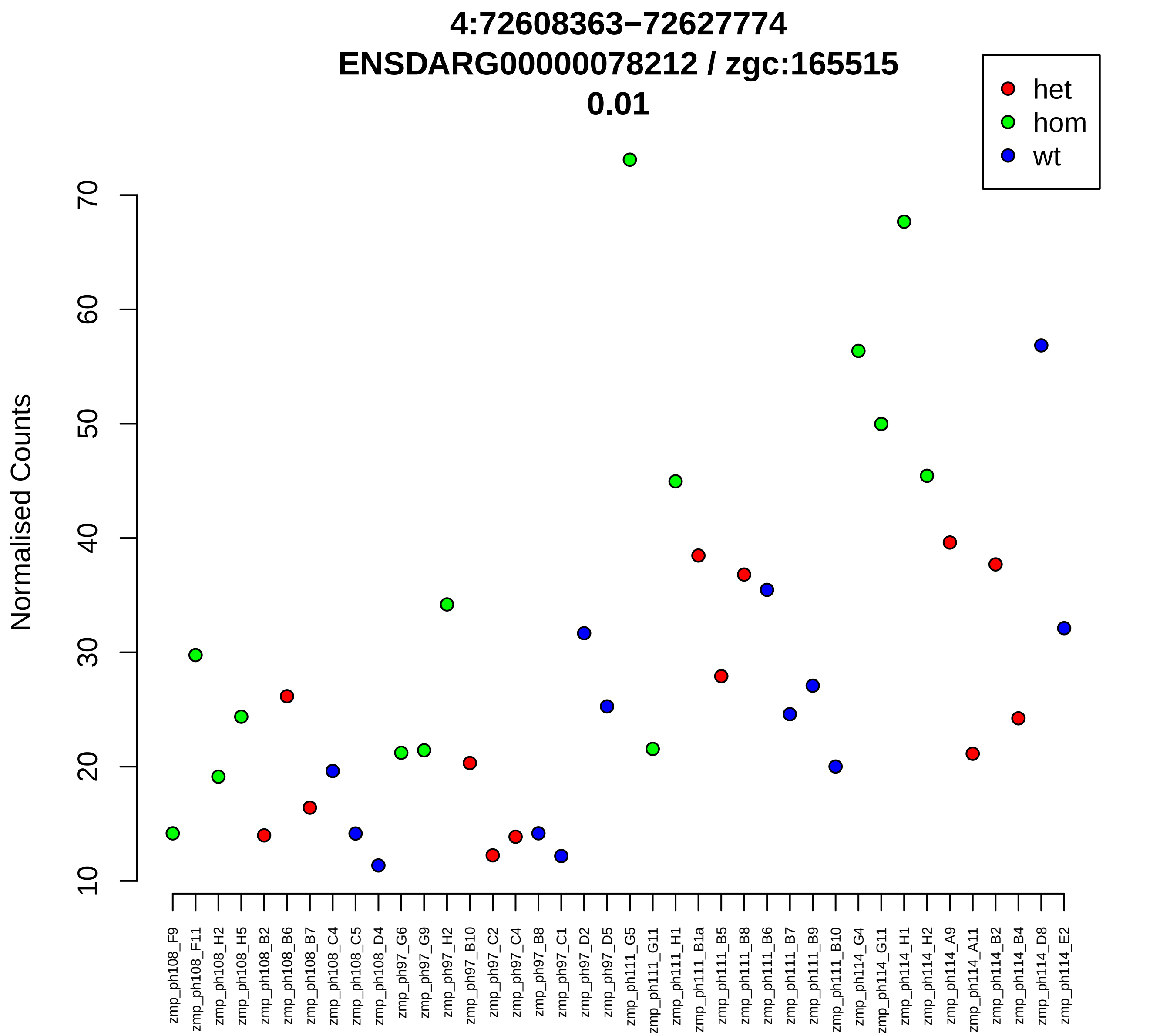

4:72608363−72627774
ENSDARG00000078212 / zgc:165515
0.01
het
hom
wt
Normalised Counts
10
20
30
40
50
60
70
zmp_ph108_F9
zmp_ph108_F11
zmp_ph108_H2
zmp_ph108_H5
zmp_ph108_B2
zmp_ph108_B6
zmp_ph108_B7
zmp_ph108_C4
zmp_ph108_C5
zmp_ph108_D4
zmp_ph97_G6
zmp_ph97_G9
zmp_ph97_H2
zmp_ph97_B10
zmp_ph97_C2
zmp_ph97_C4
zmp_ph97_B8
zmp_ph97_C1
zmp_ph97_D2
zmp_ph97_D5
zmp_ph111_G5
zmp_ph111_G11
zmp_ph111_H1
zmp_ph111_B1a
zmp_ph111_B5
zmp_ph111_B8
zmp_ph111_B6
zmp_ph111_B7
zmp_ph111_B9
zmp_ph111_B10
zmp_ph114_G4
zmp_ph114_G11
zmp_ph114_H1
zmp_ph114_H2
zmp_ph114_A9
zmp_ph114_A11
zmp_ph114_B2
zmp_ph114_B4
zmp_ph114_D8
zmp_ph114_E2

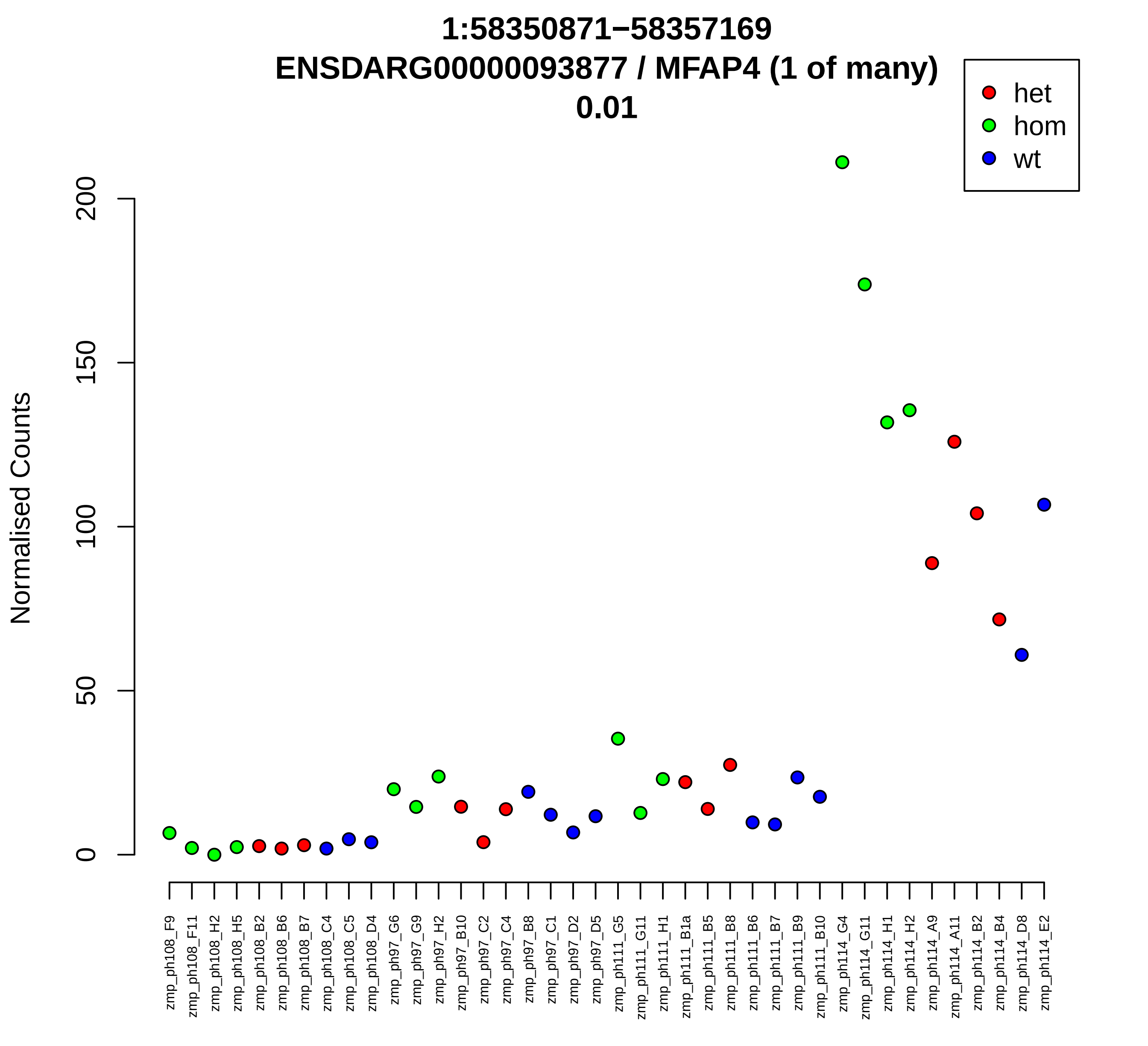
1:58350871−58357169
ENSDARG00000093877 / MFAP4 (1 of many)
0.01
het
hom
wt
Normalised Counts
0
50
100
150
200
zmp_ph108_F9
zmp_ph108_F11
zmp_ph108_H2
zmp_ph108_H5
zmp_ph108_B2
zmp_ph108_B6
zmp_ph108_B7
zmp_ph108_C4
zmp_ph108_C5
zmp_ph108_D4
zmp_ph97_G6
zmp_ph97_G9
zmp_ph97_H2
zmp_ph97_B10
zmp_ph97_C2
zmp_ph97_C4
zmp_ph97_B8
zmp_ph97_C1
zmp_ph97_D2
zmp_ph97_D5
zmp_ph111_G5
zmp_ph111_G11
zmp_ph111_H1
zmp_ph111_B1a
zmp_ph111_B5
zmp_ph111_B8
zmp_ph111_B6
zmp_ph111_B7
zmp_ph111_B9
zmp_ph111_B10
zmp_ph114_G4
zmp_ph114_G11
zmp_ph114_H1
zmp_ph114_H2
zmp_ph114_A9
zmp_ph114_A11
zmp_ph114_B2
zmp_ph114_B4
zmp_ph114_D8
zmp_ph114_E2

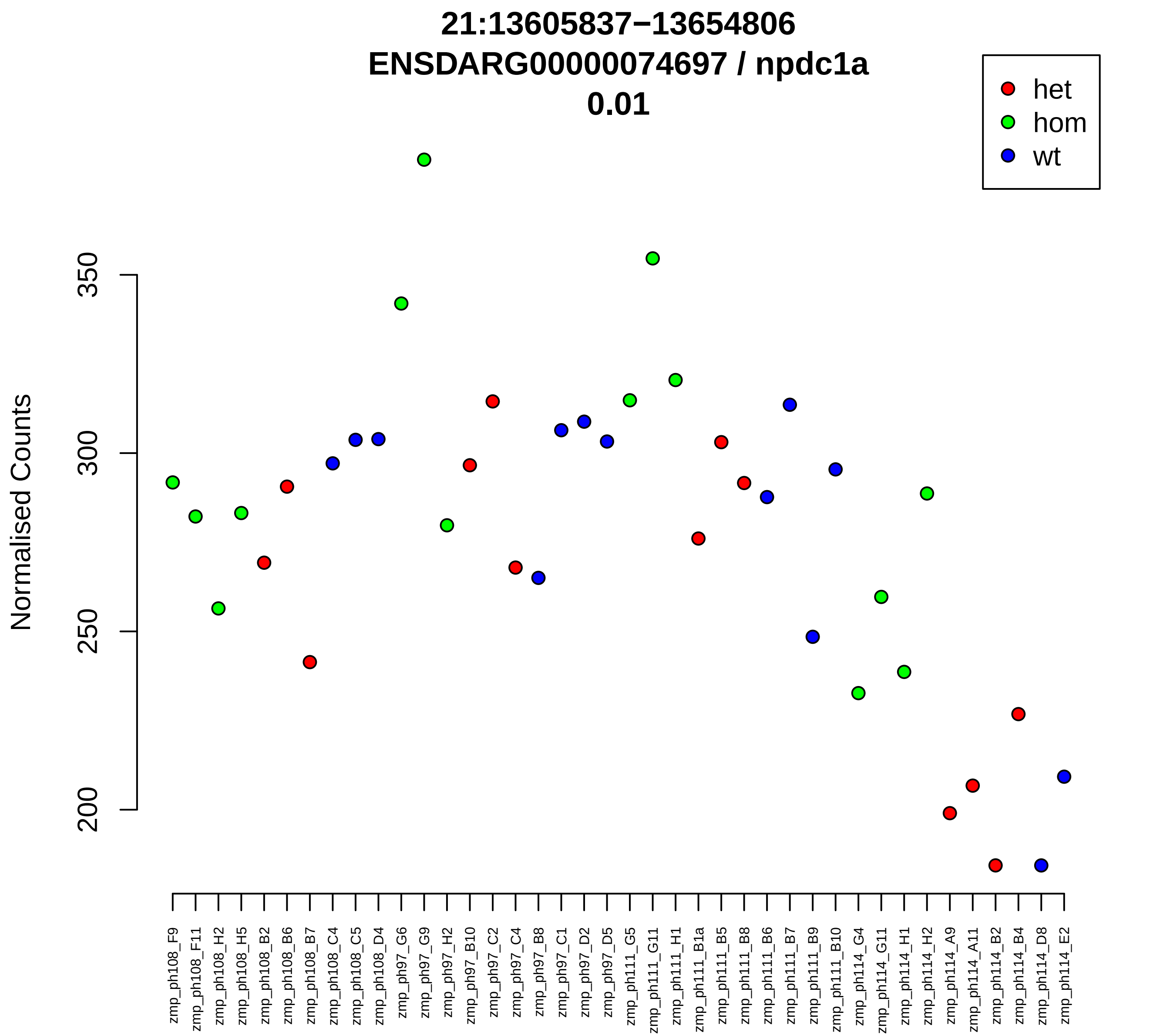

21:13605837−13654806
ENSDARG00000074697 / npdc1a
0.01
het
hom
wt
Normalised Counts
350
300
250
200
zmp_ph108_F9
zmp_ph108_F11
zmp_ph108_H2
zmp_ph108_H5
zmp_ph108_B2
zmp_ph108_B6
zmp_ph108_B7
zmp_ph108_C4
zmp_ph108_C5
zmp_ph108_D4
zmp_ph97_G6
zmp_ph97_G9
zmp_ph97_H2
zmp_ph97_B10
zmp_ph97_C2
zmp_ph97_C4
zmp_ph97_B8
zmp_ph97_C1
zmp_ph97_D2
zmp_ph97_D5
zmp_ph111_G5
zmp_ph111_G11
zmp_ph111_H1
zmp_ph111_B1a
zmp_ph111_B5
zmp_ph111_B8
zmp_ph111_B6
zmp_ph111_B7
zmp_ph111_B9
zmp_ph111_B10
zmp_ph114_G4
zmp_ph114_G11
zmp_ph114_H1
zmp_ph114_H2
zmp_ph114_A9
zmp_ph114_A11
zmp_ph114_B2
zmp_ph114_B4
zmp_ph114_D8
zmp_ph114_E2

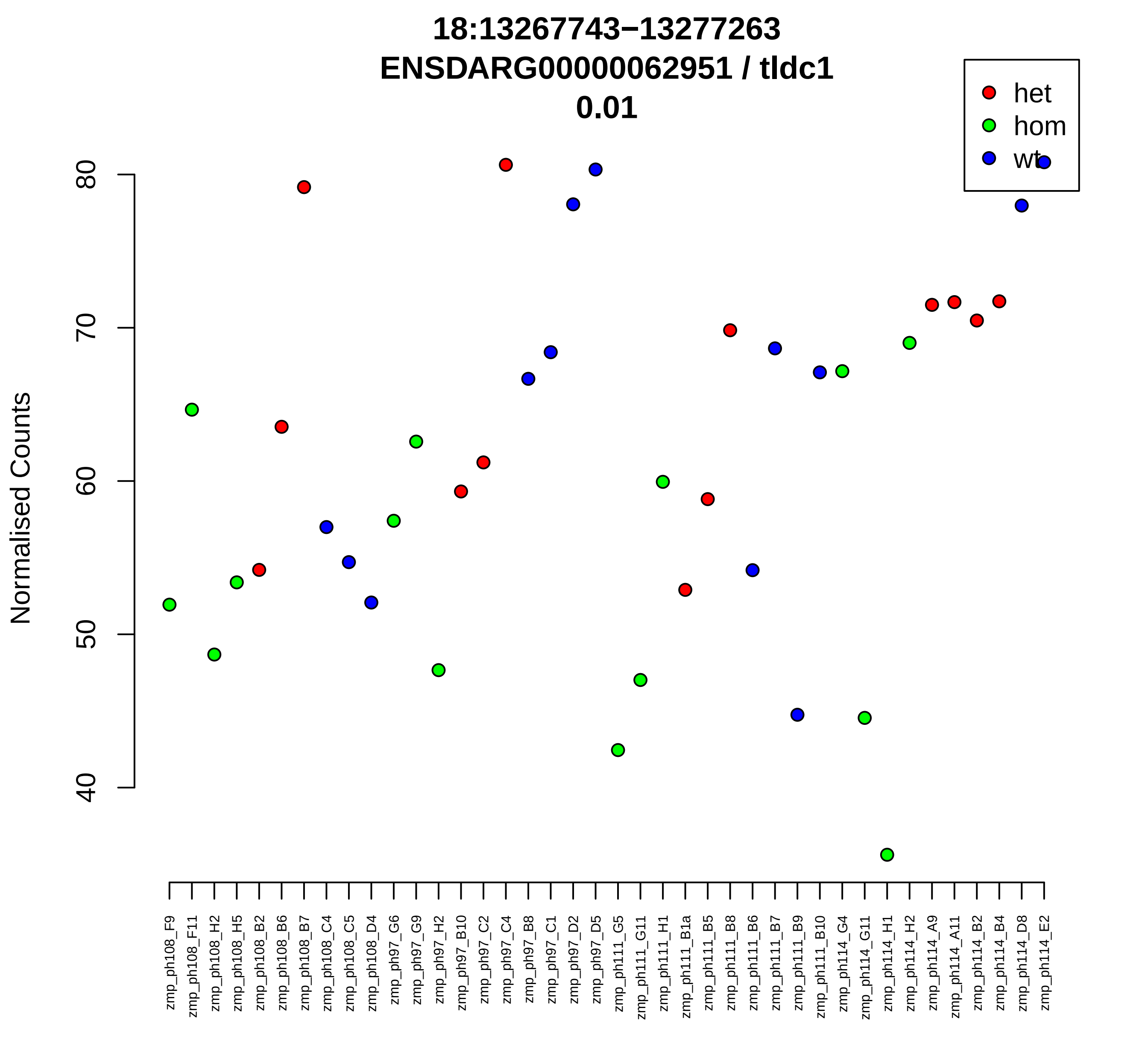
18:13267743−13277263
ENSDARG00000062951 / tldc1
0.01
het
hom
wt
Normalised Counts
40
50
60
70
80
zmp_ph108_F9
zmp_ph108_F11
zmp_ph108_H2
zmp_ph108_H5
zmp_ph108_B2
zmp_ph108_B6
zmp_ph108_B7
zmp_ph108_C4
zmp_ph108_C5
zmp_ph108_D4
zmp_ph97_G6
zmp_ph97_G9
zmp_ph97_H2
zmp_ph97_B10
zmp_ph97_C2
zmp_ph97_C4
zmp_ph97_B8
zmp_ph97_C1
zmp_ph97_D2
zmp_ph97_D5
zmp_ph111_G5
zmp_ph111_G11
zmp_ph111_H1
zmp_ph111_B1a
zmp_ph111_B5
zmp_ph111_B8
zmp_ph111_B6
zmp_ph111_B7
zmp_ph111_B9
zmp_ph111_B10
zmp_ph114_G4
zmp_ph114_G11
zmp_ph114_H1
zmp_ph114_H2
zmp_ph114_A9
zmp_ph114_A11
zmp_ph114_B2
zmp_ph114_B4
zmp_ph114_D8
zmp_ph114_E2

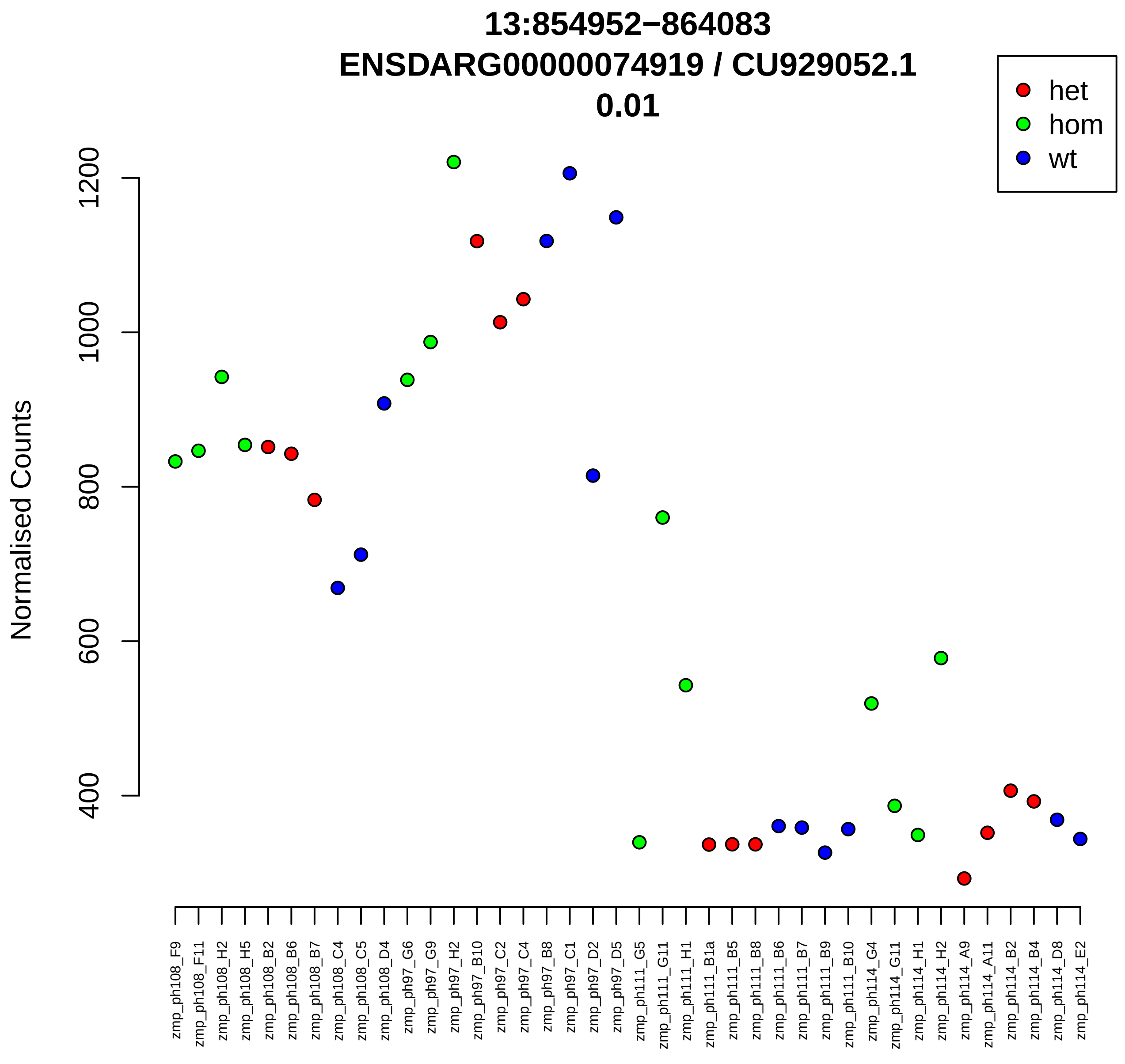
13:854952−864083
ENSDARG00000074919 / CU929052.1
0.01
het
hom
wt
Normalised Counts
400
600
800
1000
1200
zmp_ph108_F9
zmp_ph108_F11
zmp_ph108_H2
zmp_ph108_H5
zmp_ph108_B2
zmp_ph108_B6
zmp_ph108_B7
zmp_ph108_C4
zmp_ph108_C5
zmp_ph108_D4
zmp_ph97_G6
zmp_ph97_G9
zmp_ph97_H2
zmp_ph97_B10
zmp_ph97_C2
zmp_ph97_C4
zmp_ph97_B8
zmp_ph97_C1
zmp_ph97_D2
zmp_ph97_D5
zmp_ph111_G5
zmp_ph111_G11
zmp_ph111_H1
zmp_ph111_B1a
zmp_ph111_B5
zmp_ph111_B8
zmp_ph111_B6
zmp_ph111_B7
zmp_ph111_B9
zmp_ph111_B10
zmp_ph114_G4
zmp_ph114_G11
zmp_ph114_H1
zmp_ph114_H2
zmp_ph114_A9
zmp_ph114_A11
zmp_ph114_B2
zmp_ph114_B4
zmp_ph114_D8
zmp_ph114_E2

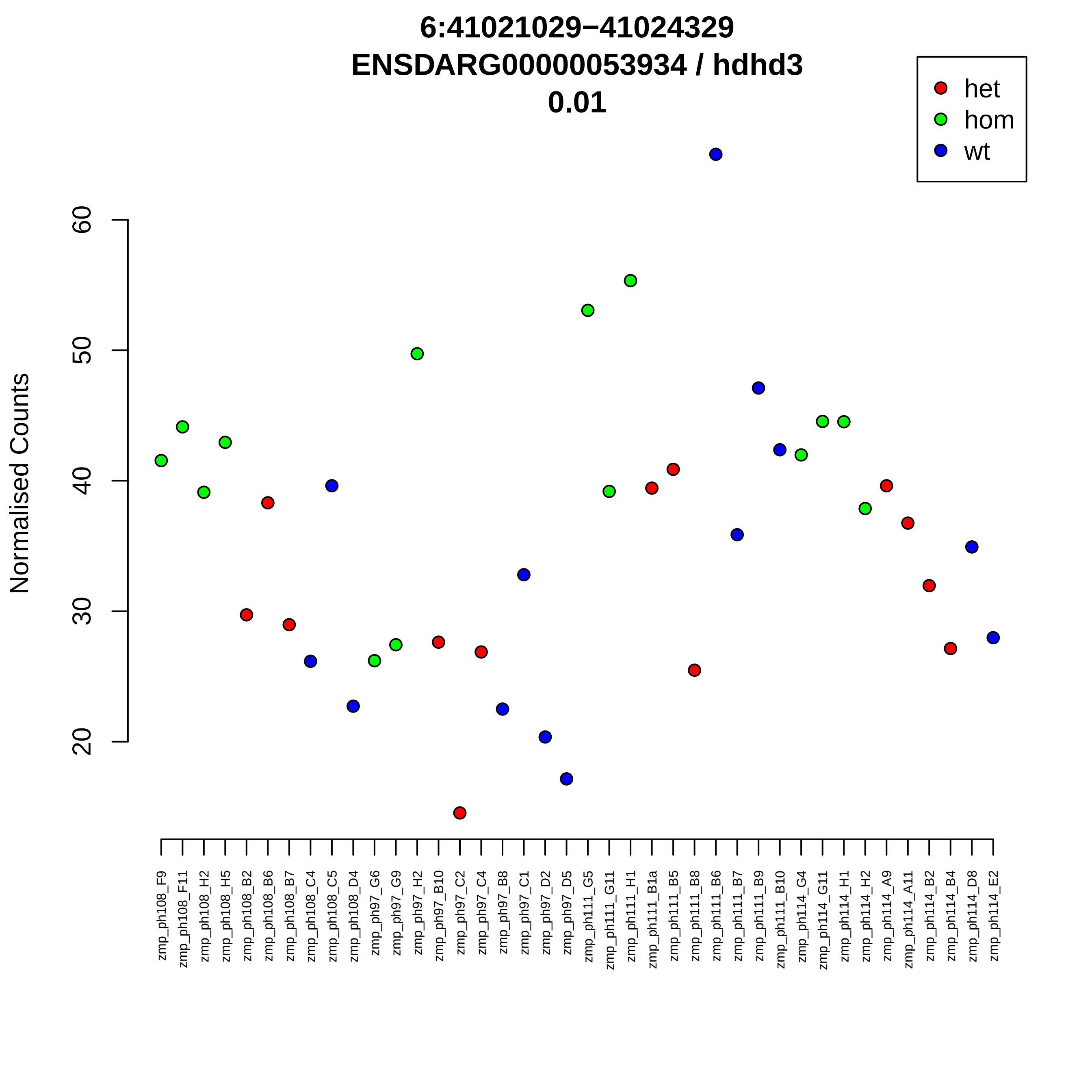

6:41021029−41024329
ENSDARG00000053934 / hdhd3
0.01
het
hom
wt
Normalised Counts
20
30
40
50
60
zmp_ph108_F9
zmp_ph108_F11
zmp_ph108_H2
zmp_ph108_H5
zmp_ph108_B2
zmp_ph108_B6
zmp_ph108_B7
zmp_ph108_C4
zmp_ph108_C5
zmp_ph108_D4
zmp_ph97_G6
zmp_ph97_G9
zmp_ph97_H2
zmp_ph97_B10
zmp_ph97_C2
zmp_ph97_C4
zmp_ph97_B8
zmp_ph97_C1
zmp_ph97_D2
zmp_ph97_D5
zmp_ph111_G5
zmp_ph111_G11
zmp_ph111_H1
zmp_ph111_B1a
zmp_ph111_B5
zmp_ph111_B8
zmp_ph111_B6
zmp_ph111_B7
zmp_ph111_B9
zmp_ph111_B10
zmp_ph114_G4
zmp_ph114_G11
zmp_ph114_H1
zmp_ph114_H2
zmp_ph114_A9
zmp_ph114_A11
zmp_ph114_B2
zmp_ph114_B4
zmp_ph114_D8
zmp_ph114_E2

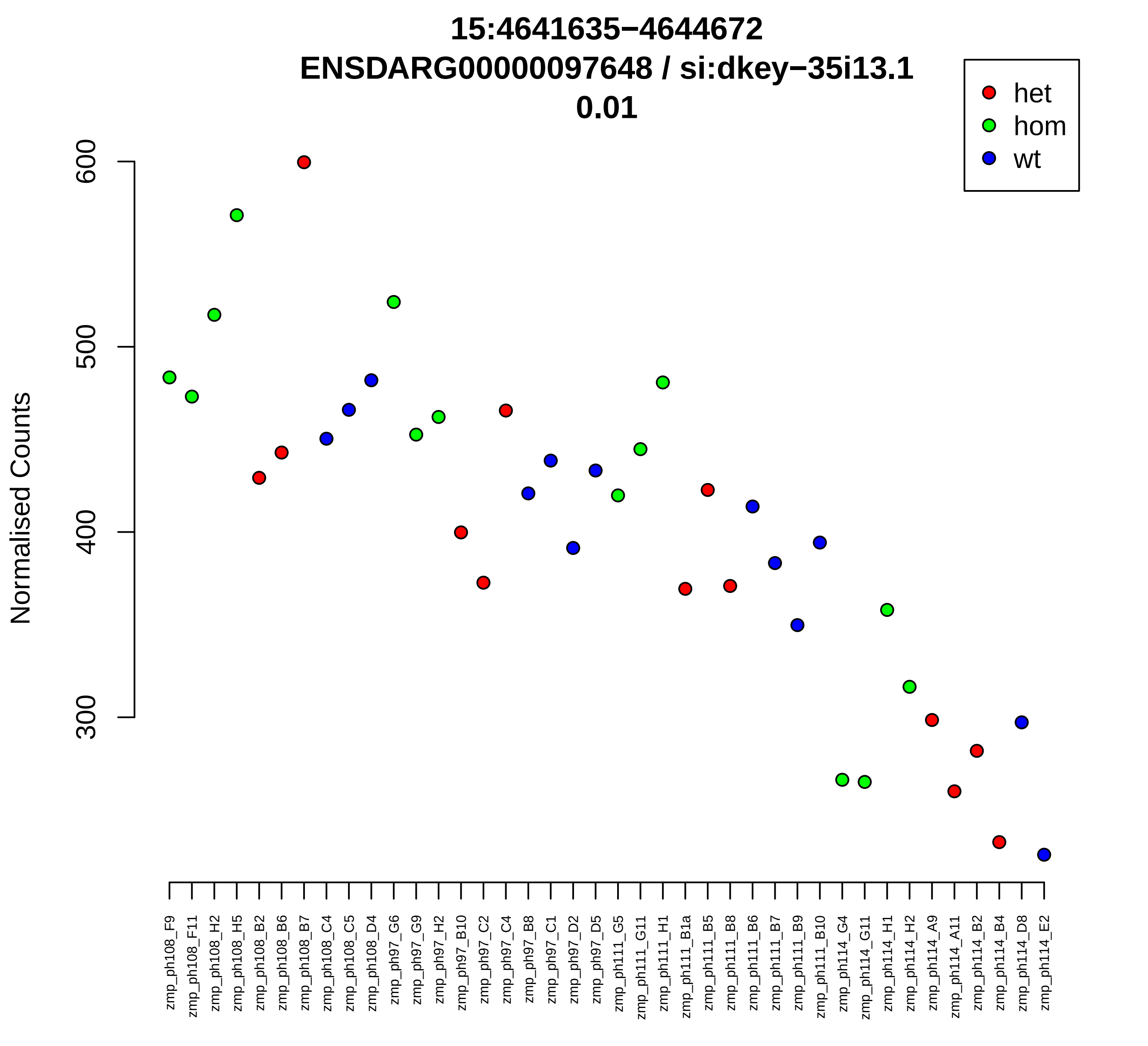

15:4641635−4644672
ENSDARG00000097648 / si:dkey−35i13.1
0.01
het
hom
wt
Normalised Counts
600
500
400
300
zmp_ph108_F9
zmp_ph108_F11
zmp_ph108_H2
zmp_ph108_H5
zmp_ph108_B2
zmp_ph108_B6
zmp_ph108_B7
zmp_ph108_C4
zmp_ph108_C5
zmp_ph108_D4
zmp_ph97_G6
zmp_ph97_G9
zmp_ph97_H2
zmp_ph97_B10
zmp_ph97_C2
zmp_ph97_C4
zmp_ph97_B8
zmp_ph97_C1
zmp_ph97_D2
zmp_ph97_D5
zmp_ph111_G5
zmp_ph111_G11
zmp_ph111_H1
zmp_ph111_B1a
zmp_ph111_B5
zmp_ph111_B8
zmp_ph111_B6
zmp_ph111_B7
zmp_ph111_B9
zmp_ph111_B10
zmp_ph114_G4
zmp_ph114_G11
zmp_ph114_H1
zmp_ph114_H2
zmp_ph114_A9
zmp_ph114_A11
zmp_ph114_B2
zmp_ph114_B4
zmp_ph114_D8
zmp_ph114_E2

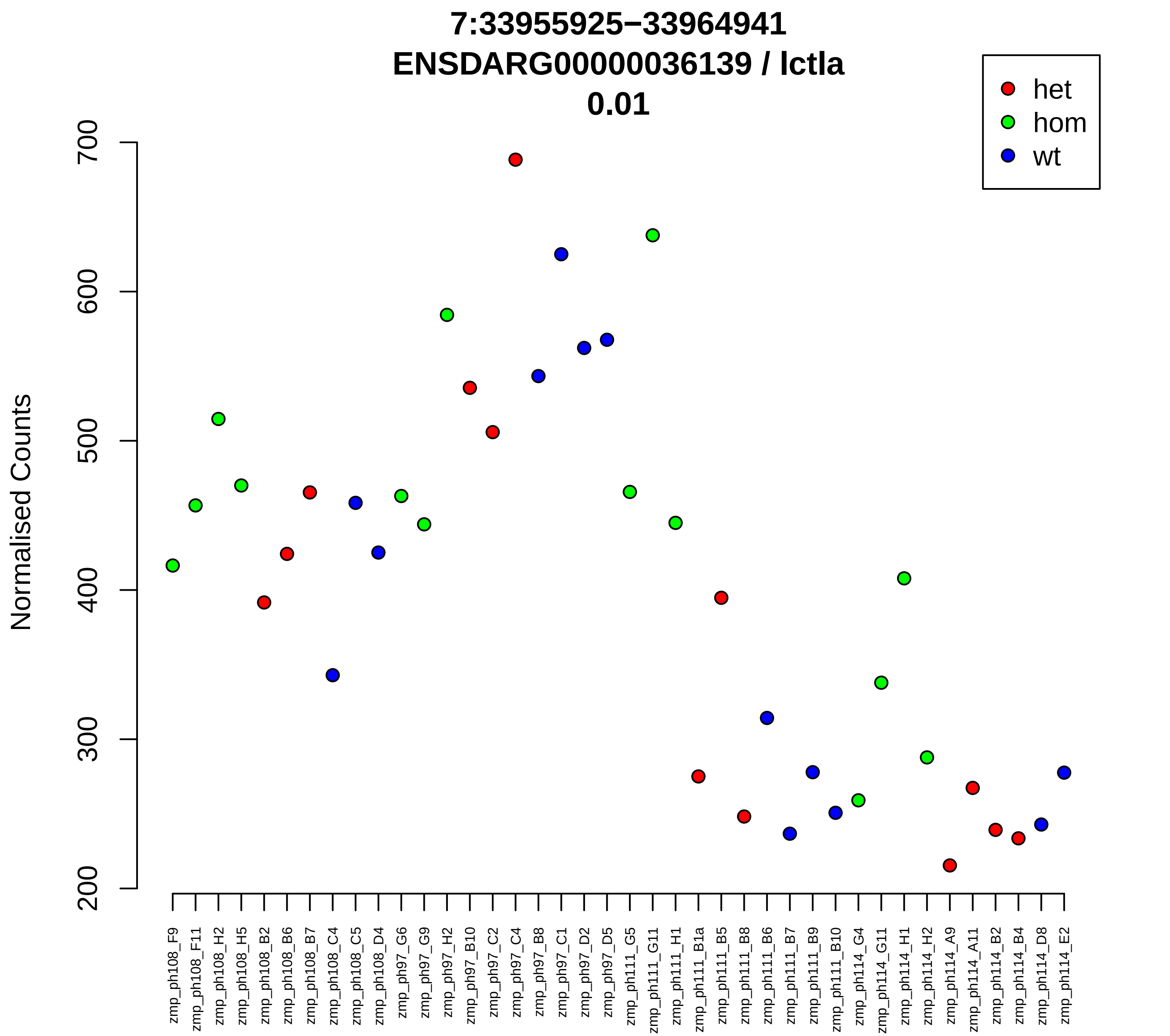
7:33955925−33964941
ENSDARG00000036139 / lctla
0.01
Normalised Counts
200
300
400
500
600
700
het
hom
wt
zmp_ph108_F9
zmp_ph108_F11
zmp_ph108_H2
zmp_ph108_H5
zmp_ph108_B2
zmp_ph108_B6
zmp_ph108_B7
zmp_ph108_C4
zmp_ph108_C5
zmp_ph108_D4
zmp_ph97_G6
zmp_ph97_G9
zmp_ph97_H2
zmp_ph97_B10
zmp_ph97_C2
zmp_ph97_C4
zmp_ph97_B8
zmp_ph97_C1
zmp_ph97_D2
zmp_ph97_D5
zmp_ph111_G5
zmp_ph111_G11
zmp_ph111_H1
zmp_ph111_B1a
zmp_ph111_B5
zmp_ph111_B8
zmp_ph111_B6
zmp_ph111_B7
zmp_ph111_B9
zmp_ph111_B10
zmp_ph114_G4
zmp_ph114_G11
zmp_ph114_H1
zmp_ph114_H2
zmp_ph114_A9
zmp_ph114_A11
zmp_ph114_B2
zmp_ph114_B4
zmp_ph114_D8
zmp_ph114_E2

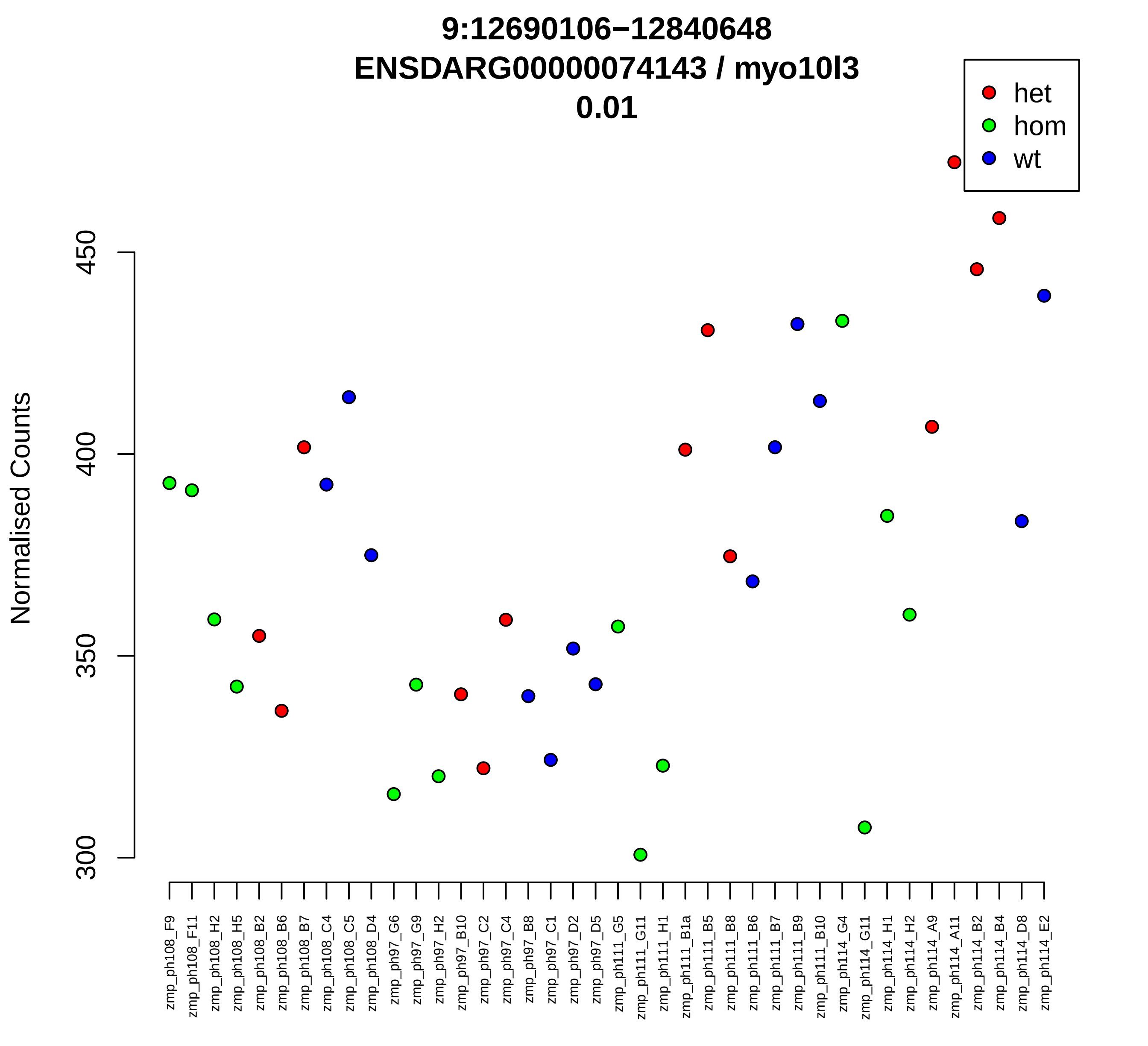
9:12690106−12840648
ENSDARG00000074143 / myo10l3
0.01
het
hom
wt
Normalised Counts
300
350
400
450
zmp_ph108_F9
zmp_ph108_F11
zmp_ph108_H2
zmp_ph108_H5
zmp_ph108_B2
zmp_ph108_B6
zmp_ph108_B7
zmp_ph108_C4
zmp_ph108_C5
zmp_ph108_D4
zmp_ph97_G6
zmp_ph97_G9
zmp_ph97_H2
zmp_ph97_B10
zmp_ph97_C2
zmp_ph97_C4
zmp_ph97_B8
zmp_ph97_C1
zmp_ph97_D2
zmp_ph97_D5
zmp_ph111_G5
zmp_ph111_G11
zmp_ph111_H1
zmp_ph111_B1a
zmp_ph111_B5
zmp_ph111_B8
zmp_ph111_B6
zmp_ph111_B7
zmp_ph111_B9
zmp_ph111_B10
zmp_ph114_G4
zmp_ph114_G11
zmp_ph114_H1
zmp_ph114_H2
zmp_ph114_A9
zmp_ph114_A11
zmp_ph114_B2
zmp_ph114_B4
zmp_ph114_D8
zmp_ph114_E2

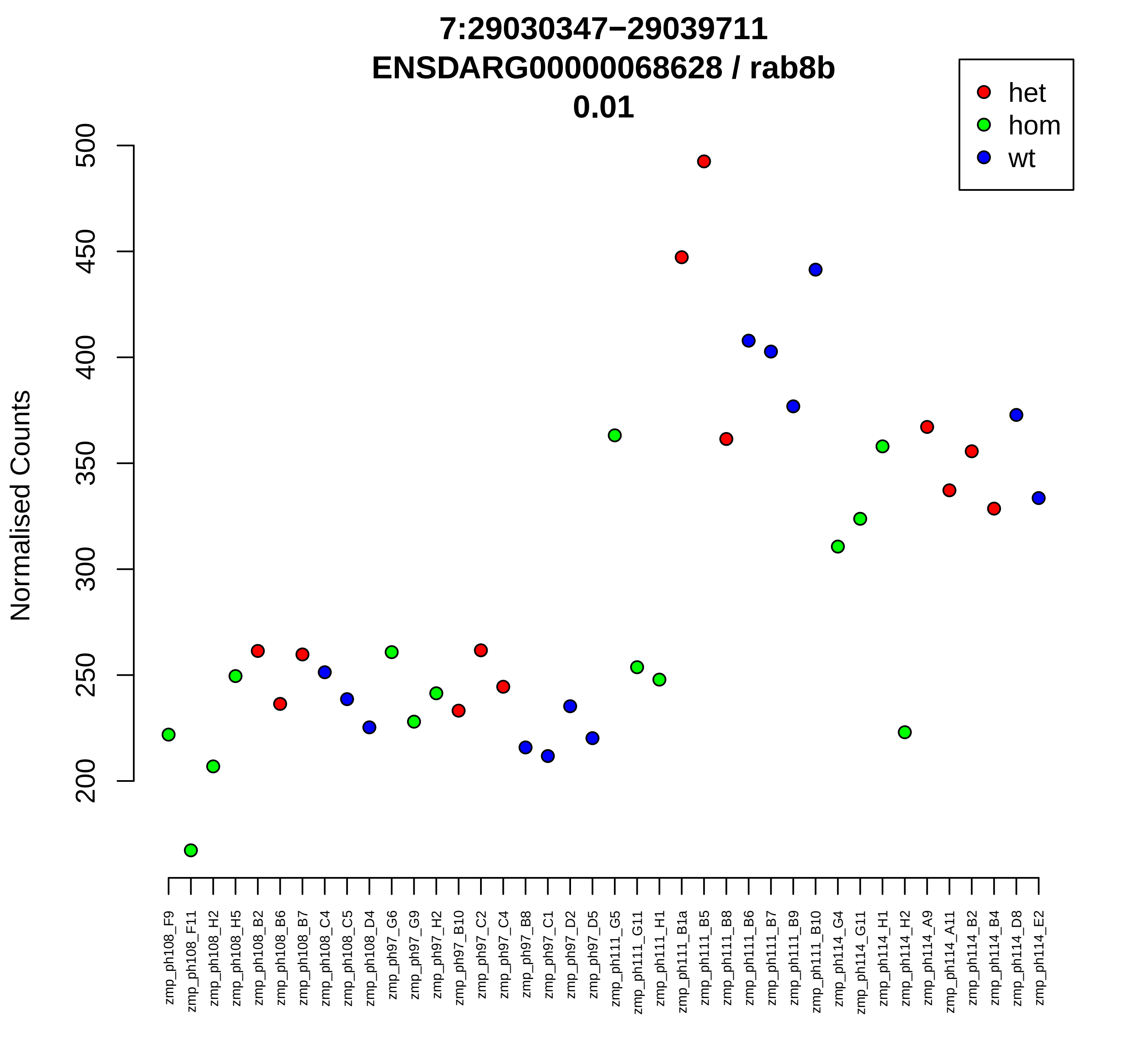

7:29030347−29039711
ENSDARG00000068628 / rab8b
0.01
het
hom
wt
Normalised Counts
200
250
300
350
400
450
500
zmp_ph108_F9
zmp_ph108_F11
zmp_ph108_H2
zmp_ph108_H5
zmp_ph108_B2
zmp_ph108_B6
zmp_ph108_B7
zmp_ph108_C4
zmp_ph108_C5
zmp_ph108_D4
zmp_ph97_G6
zmp_ph97_G9
zmp_ph97_H2
zmp_ph97_B10
zmp_ph97_C2
zmp_ph97_C4
zmp_ph97_B8
zmp_ph97_C1
zmp_ph97_D2
zmp_ph97_D5
zmp_ph111_G5
zmp_ph111_G11
zmp_ph111_H1
zmp_ph111_B1a
zmp_ph111_B5
zmp_ph111_B8
zmp_ph111_B6
zmp_ph111_B7
zmp_ph111_B9
zmp_ph111_B10
zmp_ph114_G4
zmp_ph114_G11
zmp_ph114_H1
zmp_ph114_H2
zmp_ph114_A9
zmp_ph114_A11
zmp_ph114_B2
zmp_ph114_B4
zmp_ph114_D8
zmp_ph114_E2

20:19045474−19084186
ENSDARG00000015860 / mtmr9
0.01

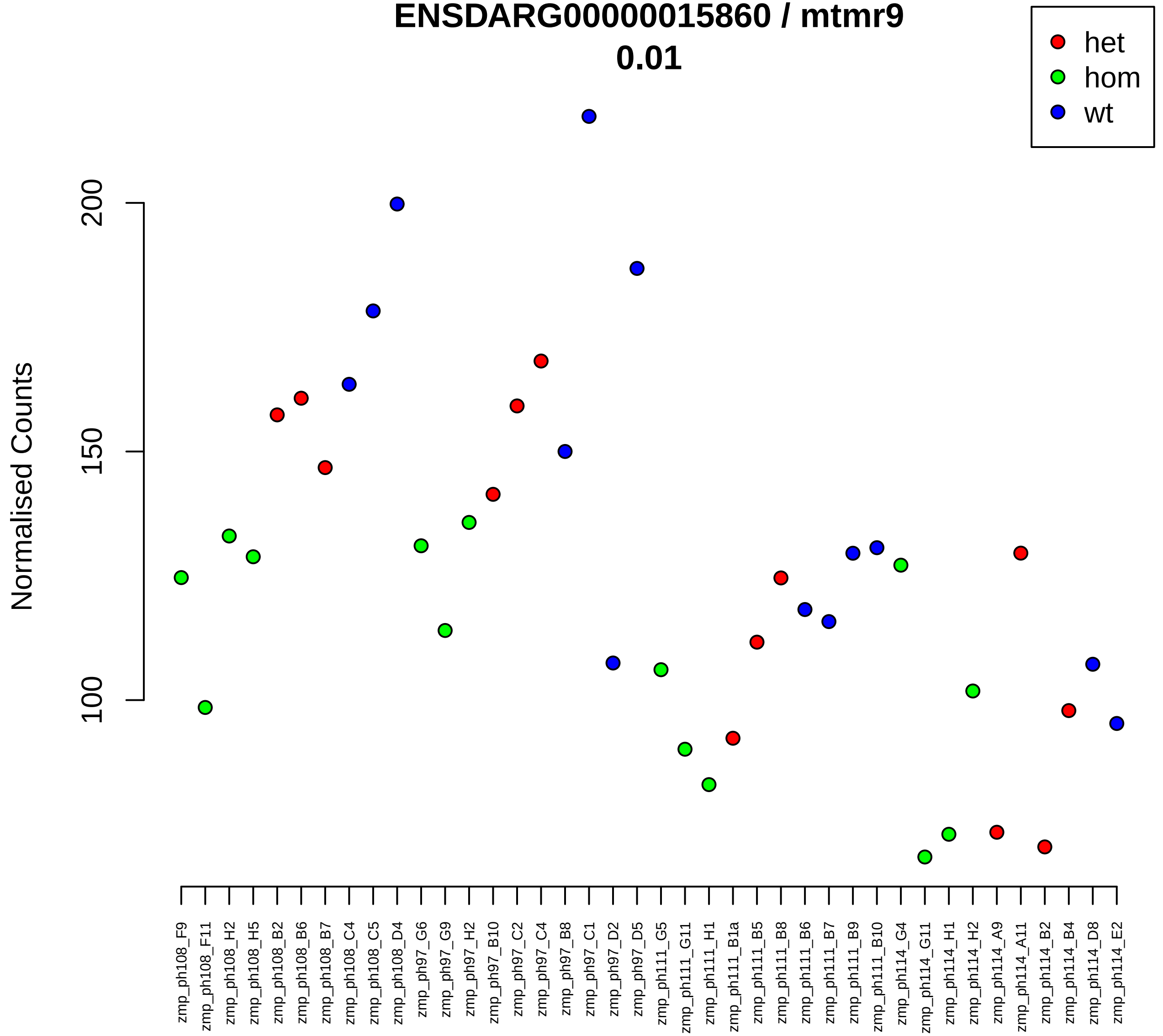

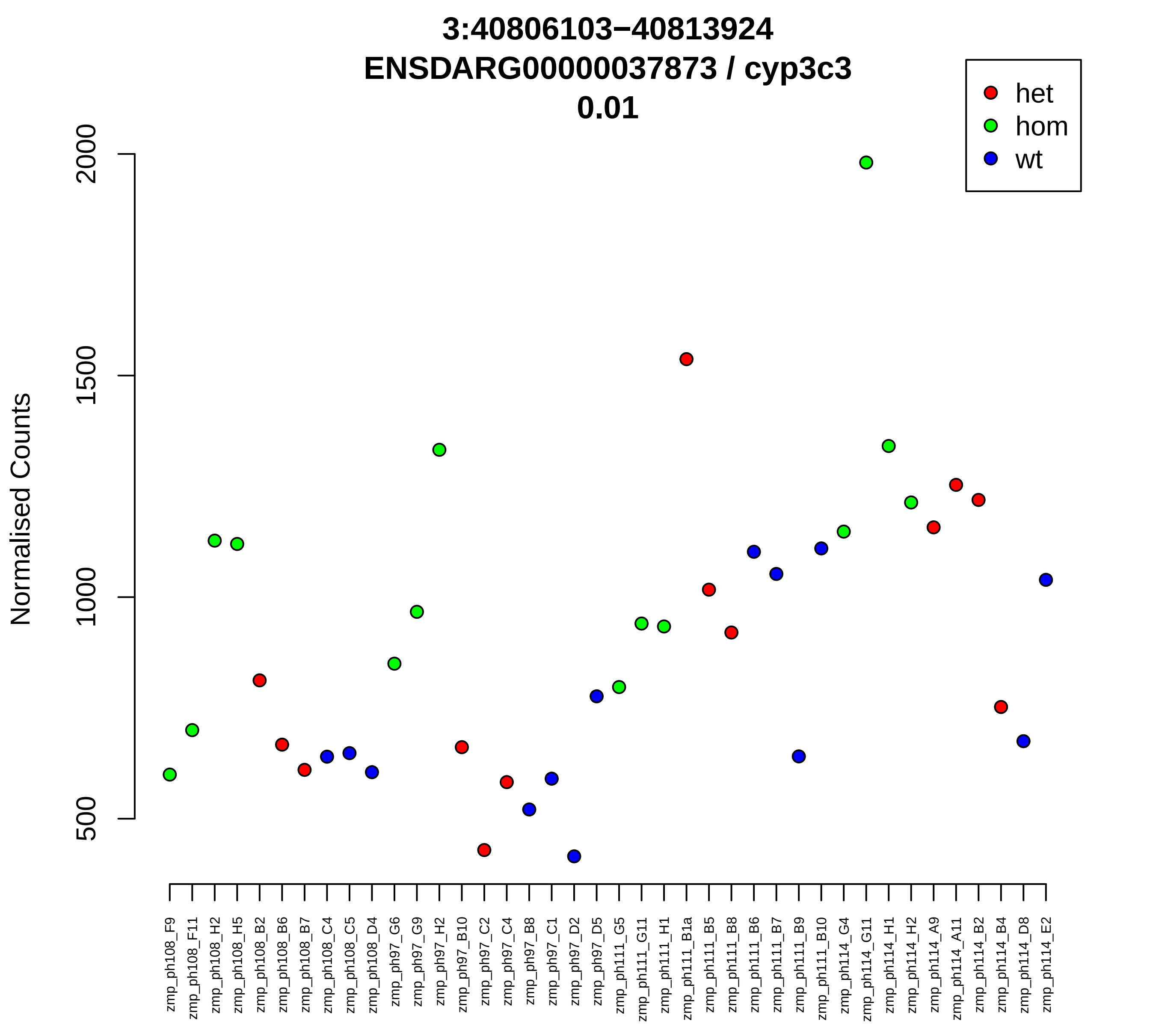
3:40806103−40813924
ENSDARG00000037873 / cyp3c3
0.01
het
hom
wt
Normalised Counts
500
1000
1500
2000
zmp_ph108_F9
zmp_ph108_F11
zmp_ph108_H2
zmp_ph108_H5
zmp_ph108_B2
zmp_ph108_B6
zmp_ph108_B7
zmp_ph108_C4
zmp_ph108_C5
zmp_ph108_D4
zmp_ph97_G6
zmp_ph97_G9
zmp_ph97_H2
zmp_ph97_B10
zmp_ph97_C2
zmp_ph97_C4
zmp_ph97_B8
zmp_ph97_C1
zmp_ph97_D2
zmp_ph97_D5
zmp_ph111_G5
zmp_ph111_G11
zmp_ph111_H1
zmp_ph111_B1a
zmp_ph111_B5
zmp_ph111_B8
zmp_ph111_B6
zmp_ph111_B7
zmp_ph111_B9
zmp_ph111_B10
zmp_ph114_G4
zmp_ph114_G11
zmp_ph114_H1
zmp_ph114_H2
zmp_ph114_A9
zmp_ph114_A11
zmp_ph114_B2
zmp_ph114_B4
zmp_ph114_D8
zmp_ph114_E2

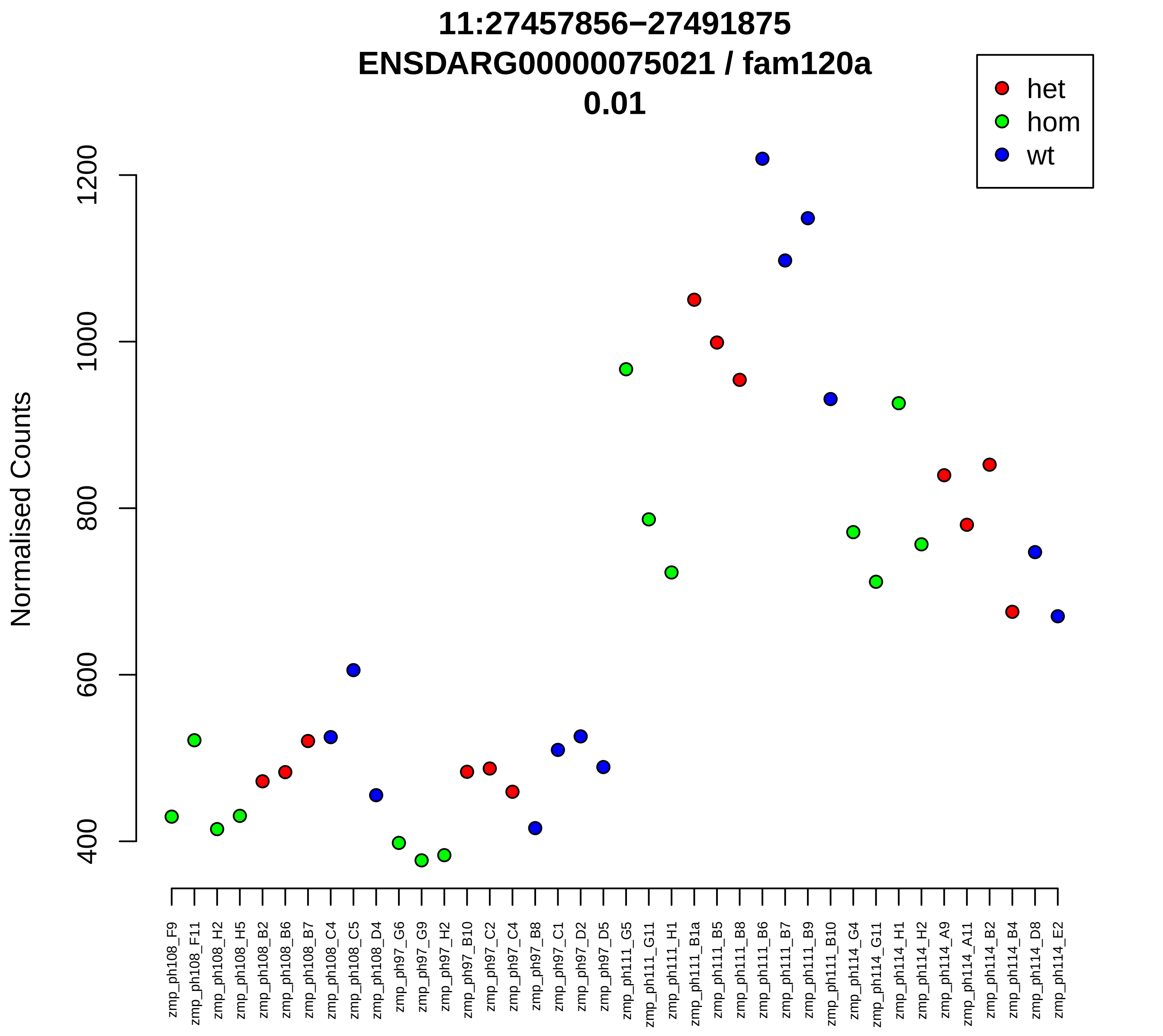
11:27457856−27491875
ENSDARG00000075021 / fam120a
0.01
het
hom
wt
Normalised Counts
400
600
800
1000
1200
zmp_ph108_F9
zmp_ph108_F11
zmp_ph108_H2
zmp_ph108_H5
zmp_ph108_B2
zmp_ph108_B6
zmp_ph108_B7
zmp_ph108_C4
zmp_ph108_C5
zmp_ph108_D4
zmp_ph97_G6
zmp_ph97_G9
zmp_ph97_H2
zmp_ph97_B10
zmp_ph97_C2
zmp_ph97_C4
zmp_ph97_B8
zmp_ph97_C1
zmp_ph97_D2
zmp_ph97_D5
zmp_ph111_G5
zmp_ph111_G11
zmp_ph111_H1
zmp_ph111_B1a
zmp_ph111_B5
zmp_ph111_B8
zmp_ph111_B6
zmp_ph111_B7
zmp_ph111_B9
zmp_ph111_B10
zmp_ph114_G4
zmp_ph114_G11
zmp_ph114_H1
zmp_ph114_H2
zmp_ph114_A9
zmp_ph114_A11
zmp_ph114_B2
zmp_ph114_B4
zmp_ph114_D8
zmp_ph114_E2

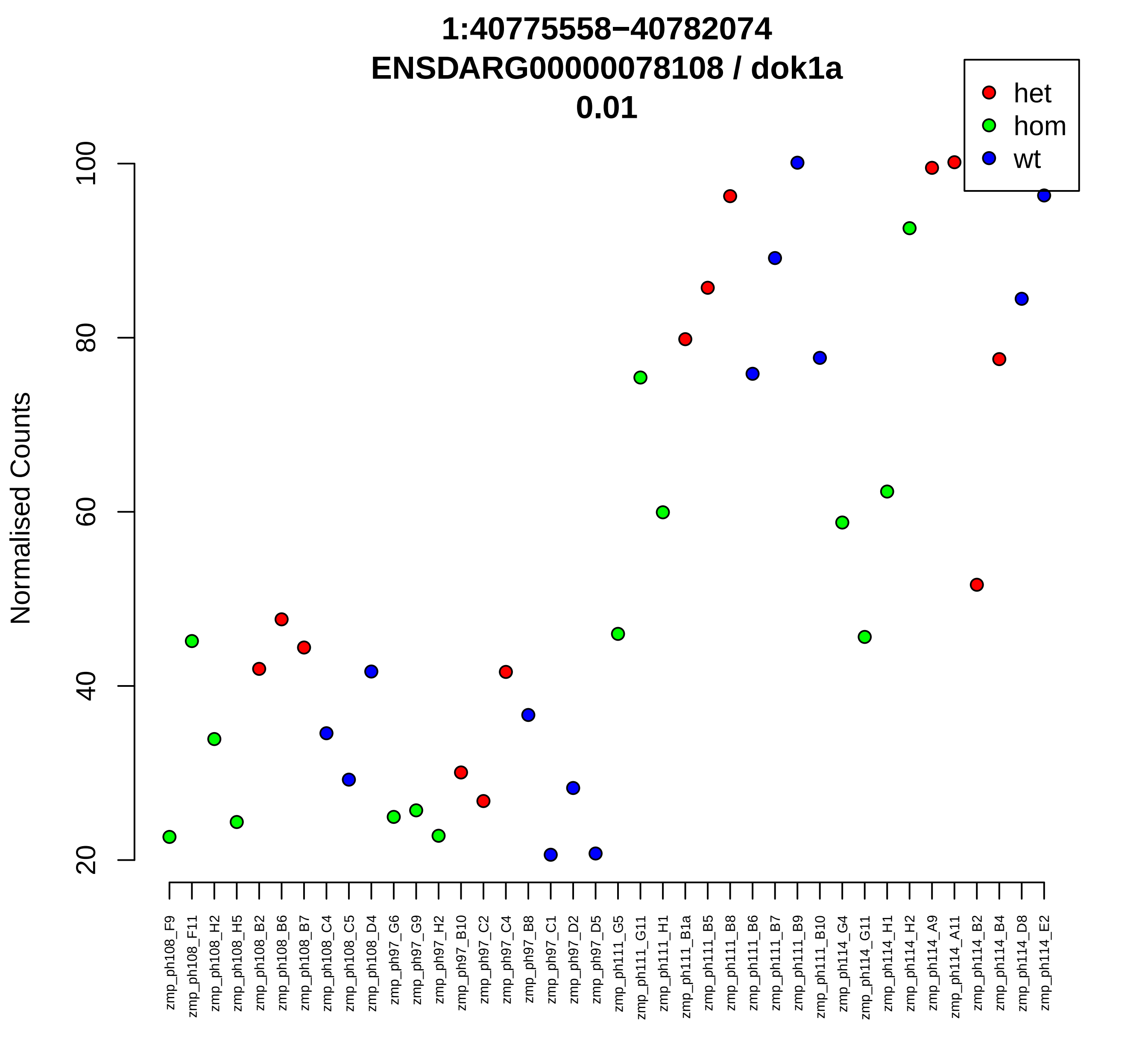

1:40775558−40782074
ENSDARG00000078108 / dok1a
0.01
Normalised Counts
20
40
60
80
100
het
hom
wt
zmp_ph108_F9
zmp_ph108_F11
zmp_ph108_H2
zmp_ph108_H5
zmp_ph108_B2
zmp_ph108_B6
zmp_ph108_B7
zmp_ph108_C4
zmp_ph108_C5
zmp_ph108_D4
zmp_ph97_G6
zmp_ph97_G9
zmp_ph97_H2
zmp_ph97_B10
zmp_ph97_C2
zmp_ph97_C4
zmp_ph97_B8
zmp_ph97_C1
zmp_ph97_D2
zmp_ph97_D5
zmp_ph111_G5
zmp_ph111_G11
zmp_ph111_H1
zmp_ph111_B1a
zmp_ph111_B5
zmp_ph111_B8
zmp_ph111_B6
zmp_ph111_B7
zmp_ph111_B9
zmp_ph111_B10
zmp_ph114_G4
zmp_ph114_G11
zmp_ph114_H1
zmp_ph114_H2
zmp_ph114_A9
zmp_ph114_A11
zmp_ph114_B2
zmp_ph114_B4
zmp_ph114_D8
zmp_ph114_E2

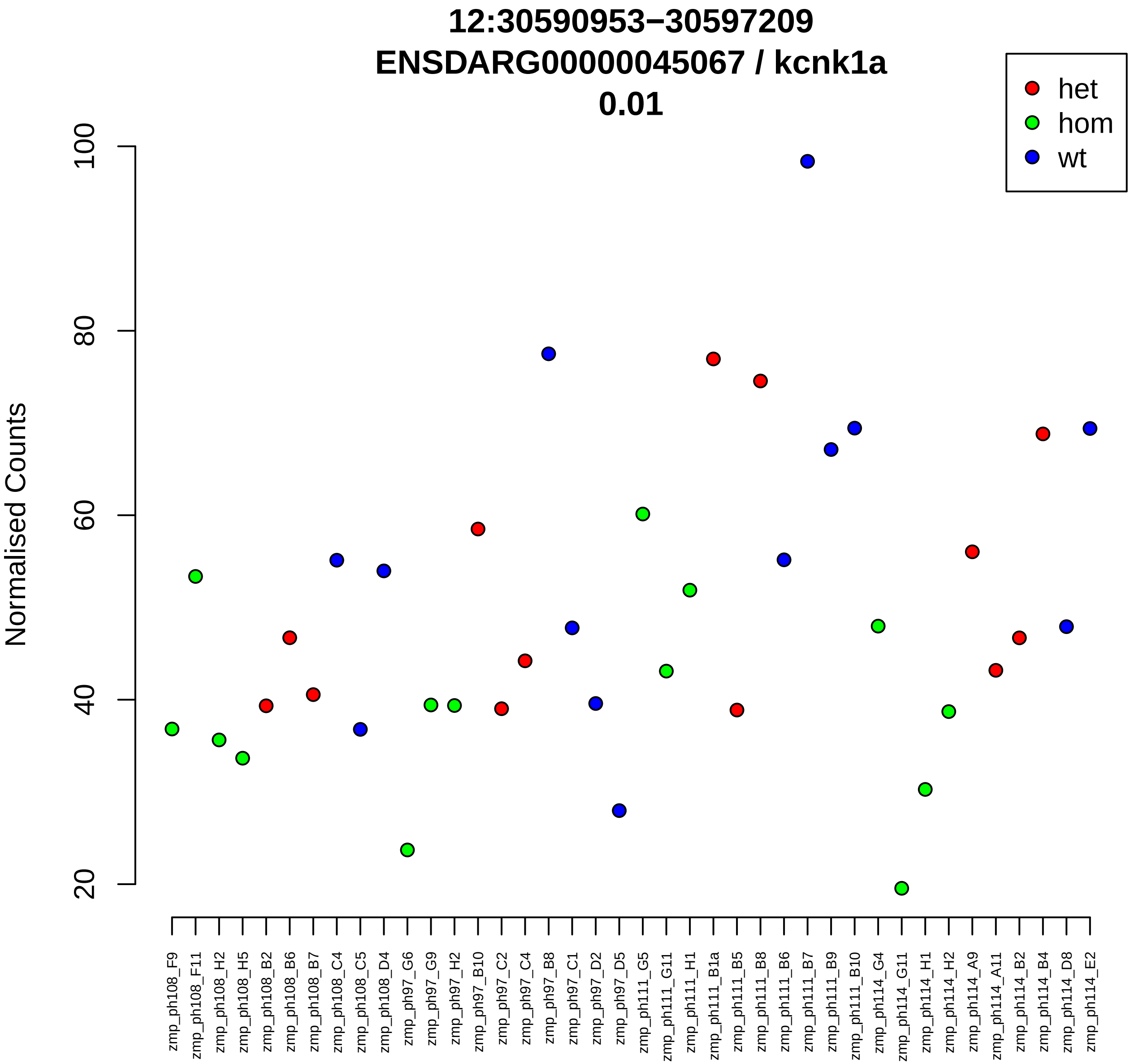
12:30590953−30597209
ENSDARG00000045067 / kcnk1a
0.01
het
hom
wt
Normalised Counts
100
80
60
40
20
zmp_ph108_F9
zmp_ph108_F11
zmp_ph108_H2
zmp_ph108_H5
zmp_ph108_B2
zmp_ph108_B6
zmp_ph108_B7
zmp_ph108_C4
zmp_ph108_C5
zmp_ph108_D4
zmp_ph97_G6
zmp_ph97_G9
zmp_ph97_H2
zmp_ph97_B10
zmp_ph97_C2
zmp_ph97_C4
zmp_ph97_B8
zmp_ph97_C1
zmp_ph97_D2
zmp_ph97_D5
zmp_ph111_G5
zmp_ph111_G11
zmp_ph111_H1
zmp_ph111_B1a
zmp_ph111_B5
zmp_ph111_B8
zmp_ph111_B6
zmp_ph111_B7
zmp_ph111_B9
zmp_ph111_B10
zmp_ph114_G4
zmp_ph114_G11
zmp_ph114_H1
zmp_ph114_H2
zmp_ph114_A9
zmp_ph114_A11
zmp_ph114_B2
zmp_ph114_B4
zmp_ph114_D8
zmp_ph114_E2

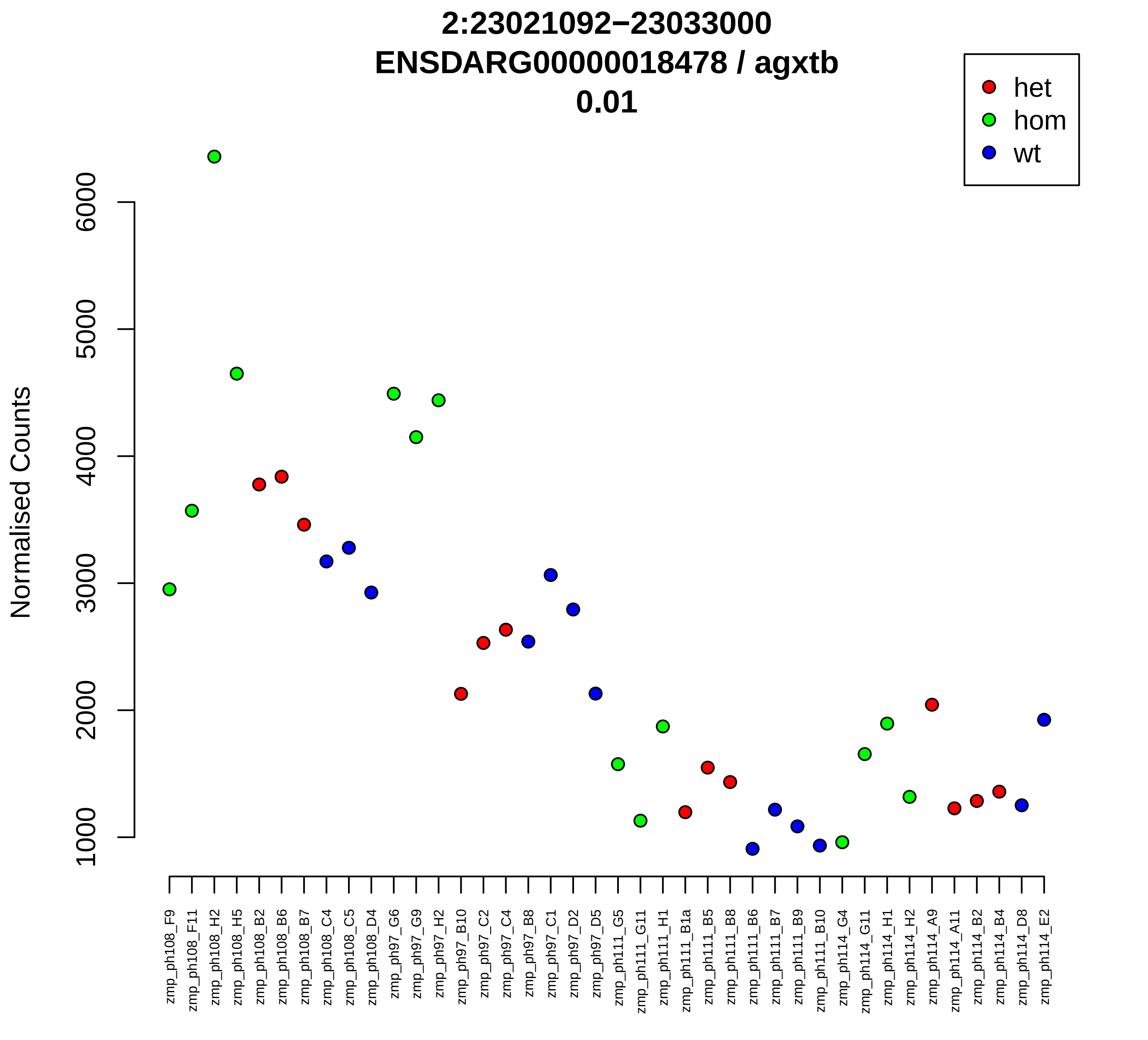

2:23021092−23033000
ENSDARG00000018478 / agxtb
0.01
het
hom
wt
Normalised Counts
1000
2000
3000
4000
5000
6000
zmp_ph108_F9
zmp_ph108_F11
zmp_ph108_H2
zmp_ph108_H5
zmp_ph108_B2
zmp_ph108_B6
zmp_ph108_B7
zmp_ph108_C4
zmp_ph108_C5
zmp_ph108_D4
zmp_ph97_G6
zmp_ph97_G9
zmp_ph97_H2
zmp_ph97_B10
zmp_ph97_C2
zmp_ph97_C4
zmp_ph97_B8
zmp_ph97_C1
zmp_ph97_D2
zmp_ph97_D5
zmp_ph111_G5
zmp_ph111_G11
zmp_ph111_H1
zmp_ph111_B1a
zmp_ph111_B5
zmp_ph111_B8
zmp_ph111_B6
zmp_ph111_B7
zmp_ph111_B9
zmp_ph111_B10
zmp_ph114_G4
zmp_ph114_G11
zmp_ph114_H1
zmp_ph114_H2
zmp_ph114_A9
zmp_ph114_A11
zmp_ph114_B2
zmp_ph114_B4
zmp_ph114_D8
zmp_ph114_E2

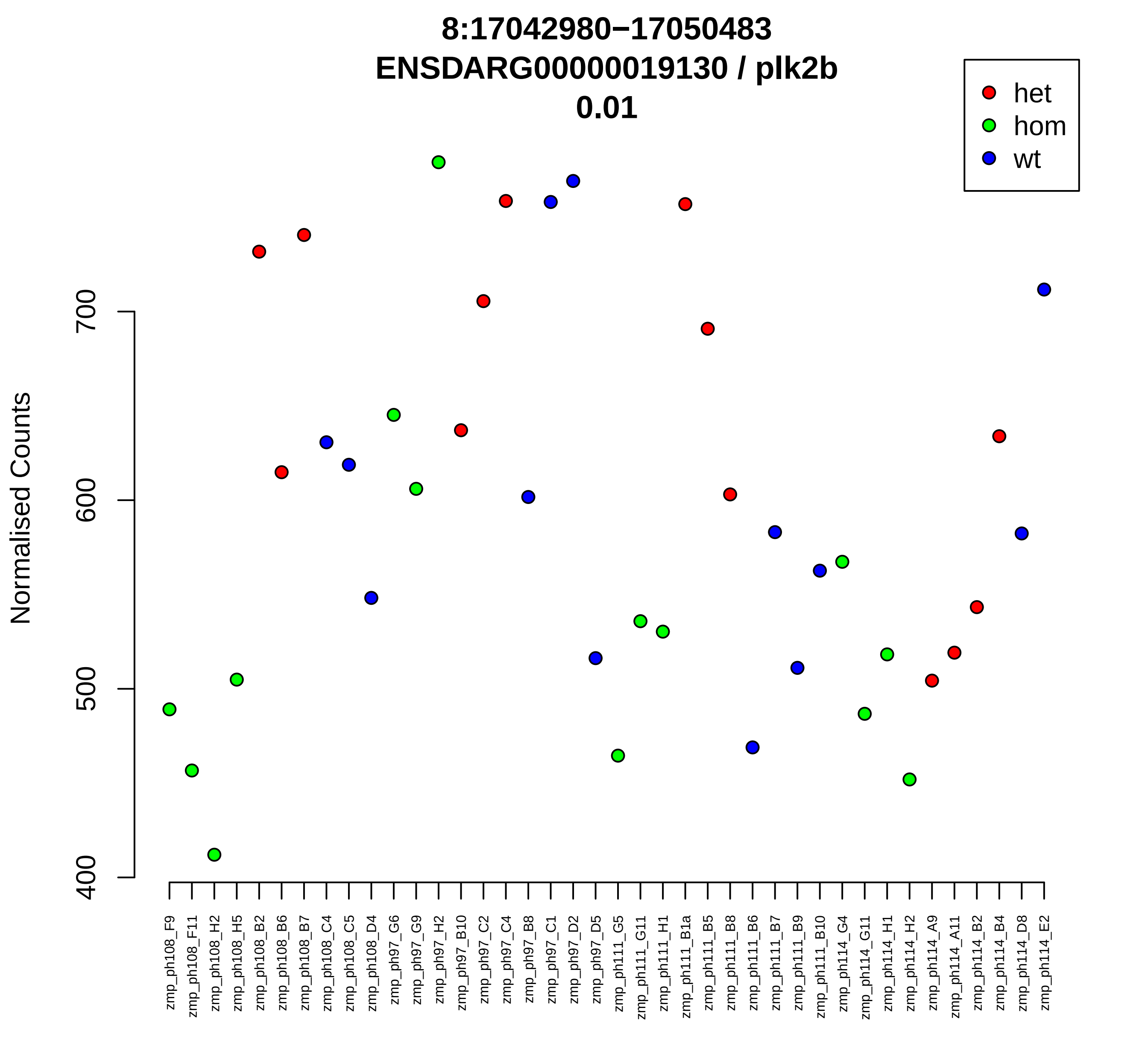

8:17042980−17050483
ENSDARG00000019130 / plk2b
0.01
het
hom
wt
Normalised Counts
400
500
600
700
zmp_ph108_F9
zmp_ph108_F11
zmp_ph108_H2
zmp_ph108_H5
zmp_ph108_B2
zmp_ph108_B6
zmp_ph108_B7
zmp_ph108_C4
zmp_ph108_C5
zmp_ph108_D4
zmp_ph97_G6
zmp_ph97_G9
zmp_ph97_H2
zmp_ph97_B10
zmp_ph97_C2
zmp_ph97_C4
zmp_ph97_B8
zmp_ph97_C1
zmp_ph97_D2
zmp_ph97_D5
zmp_ph111_G5
zmp_ph111_G11
zmp_ph111_H1
zmp_ph111_B1a
zmp_ph111_B5
zmp_ph111_B8
zmp_ph111_B6
zmp_ph111_B7
zmp_ph111_B9
zmp_ph111_B10
zmp_ph114_G4
zmp_ph114_G11
zmp_ph114_H1
zmp_ph114_H2
zmp_ph114_A9
zmp_ph114_A11
zmp_ph114_B2
zmp_ph114_B4
zmp_ph114_D8
zmp_ph114_E2

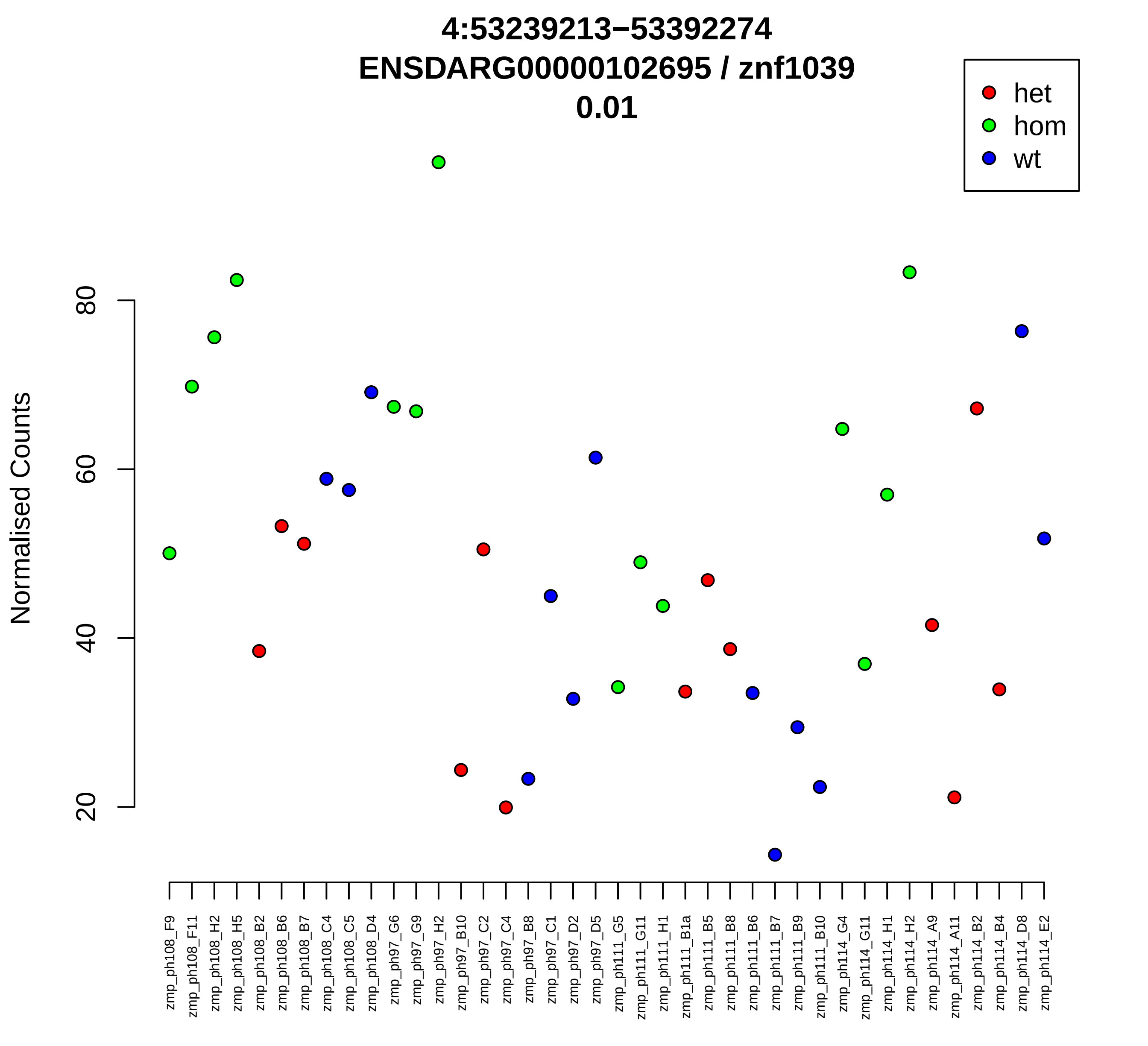
4:53239213−53392274
ENSDARG00000102695 / znf1039
0.01
het
hom
wt
Normalised Counts
20
40
60
80
zmp_ph108_F9
zmp_ph108_F11
zmp_ph108_H2
zmp_ph108_H5
zmp_ph108_B2
zmp_ph108_B6
zmp_ph108_B7
zmp_ph108_C4
zmp_ph108_C5
zmp_ph108_D4
zmp_ph97_G6
zmp_ph97_G9
zmp_ph97_H2
zmp_ph97_B10
zmp_ph97_C2
zmp_ph97_C4
zmp_ph97_B8
zmp_ph97_C1
zmp_ph97_D2
zmp_ph97_D5
zmp_ph111_G5
zmp_ph111_G11
zmp_ph111_H1
zmp_ph111_B1a
zmp_ph111_B5
zmp_ph111_B8
zmp_ph111_B6
zmp_ph111_B7
zmp_ph111_B9
zmp_ph111_B10
zmp_ph114_G4
zmp_ph114_G11
zmp_ph114_H1
zmp_ph114_H2
zmp_ph114_A9
zmp_ph114_A11
zmp_ph114_B2
zmp_ph114_B4
zmp_ph114_D8
zmp_ph114_E2

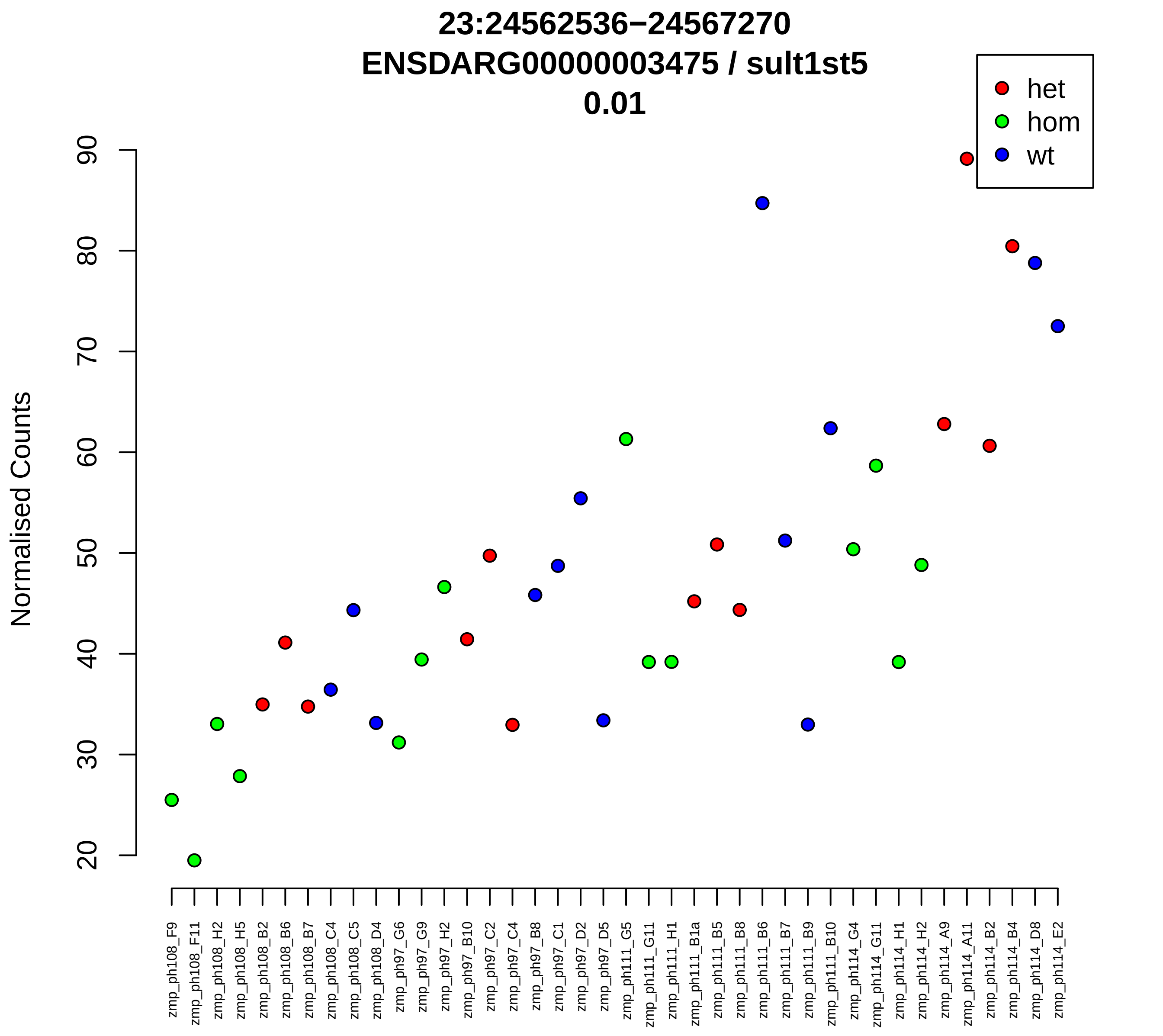

23:24562536−24567270
ENSDARG00000003475 / sult1st5
0.01
het
hom
wt
Normalised Counts
20
30
40
50
60
70
80
90
zmp_ph108_F9
zmp_ph108_F11
zmp_ph108_H2
zmp_ph108_H5
zmp_ph108_B2
zmp_ph108_B6
zmp_ph108_B7
zmp_ph108_C4
zmp_ph108_C5
zmp_ph108_D4
zmp_ph97_G6
zmp_ph97_G9
zmp_ph97_H2
zmp_ph97_B10
zmp_ph97_C2
zmp_ph97_C4
zmp_ph97_B8
zmp_ph97_C1
zmp_ph97_D2
zmp_ph97_D5
zmp_ph111_G5
zmp_ph111_G11
zmp_ph111_H1
zmp_ph111_B1a
zmp_ph111_B5
zmp_ph111_B8
zmp_ph111_B6
zmp_ph111_B7
zmp_ph111_B9
zmp_ph111_B10
zmp_ph114_G4
zmp_ph114_G11
zmp_ph114_H1
zmp_ph114_H2
zmp_ph114_A9
zmp_ph114_A11
zmp_ph114_B2
zmp_ph114_B4
zmp_ph114_D8
zmp_ph114_E2

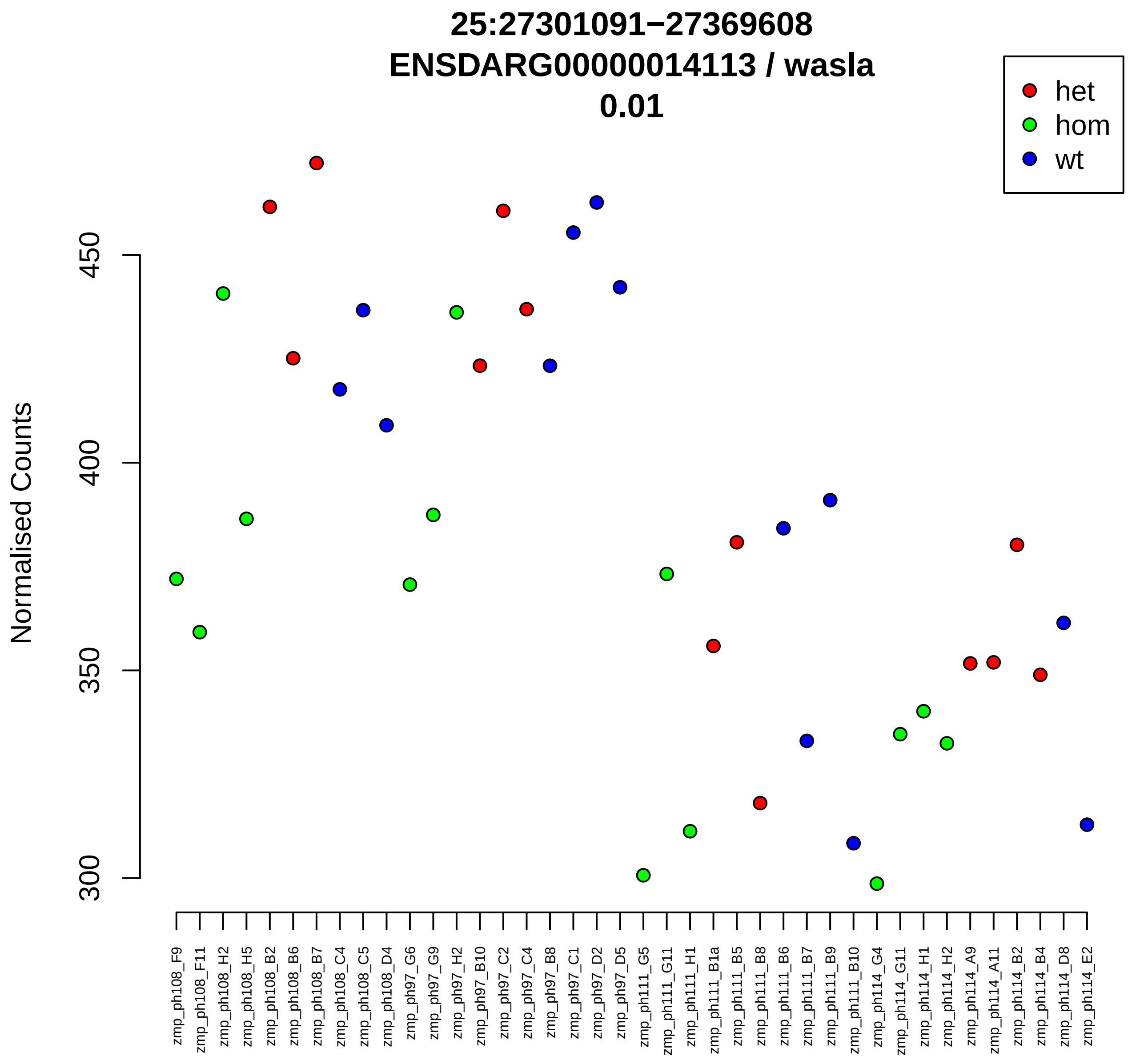
25:27301091−27369608
ENSDARG00000014113 / wasla
0.01
het
hom
wt
Normalised Counts
300
350
400
450
zmp_ph108_F9
zmp_ph108_F11
zmp_ph108_H2
zmp_ph108_H5
zmp_ph108_B2
zmp_ph108_B6
zmp_ph108_B7
zmp_ph108_C4
zmp_ph108_C5
zmp_ph108_D4
zmp_ph97_G6
zmp_ph97_G9
zmp_ph97_H2
zmp_ph97_B10
zmp_ph97_C2
zmp_ph97_C4
zmp_ph97_B8
zmp_ph97_C1
zmp_ph97_D2
zmp_ph97_D5
zmp_ph111_G5
zmp_ph111_G11
zmp_ph111_H1
zmp_ph111_B1a
zmp_ph111_B5
zmp_ph111_B8
zmp_ph111_B6
zmp_ph111_B7
zmp_ph111_B9
zmp_ph111_B10
zmp_ph114_G4
zmp_ph114_G11
zmp_ph114_H1
zmp_ph114_H2
zmp_ph114_A9
zmp_ph114_A11
zmp_ph114_B2
zmp_ph114_B4
zmp_ph114_D8
zmp_ph114_E2

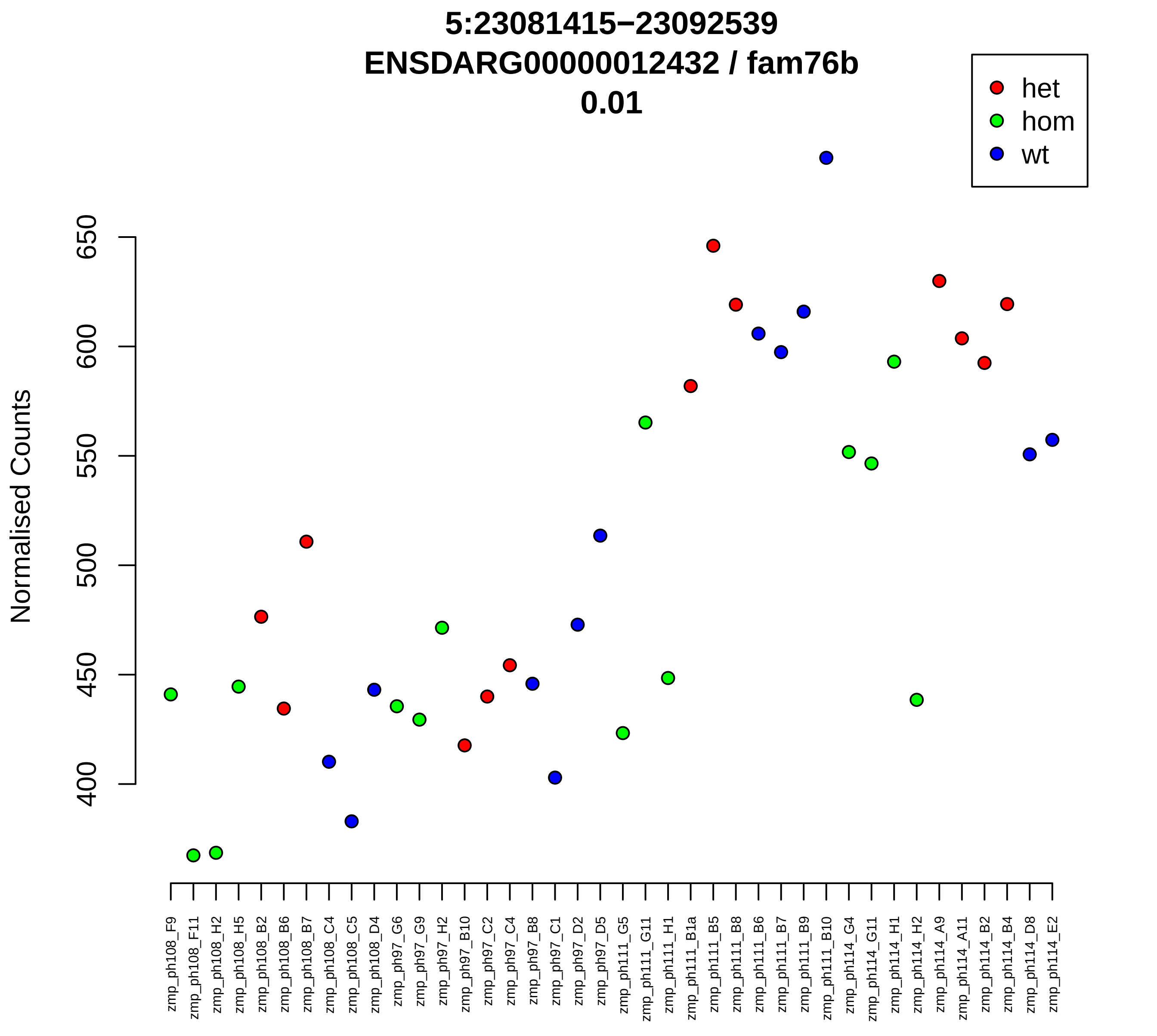
5:23081415−23092539
ENSDARG00000012432 / fam76b
0.01
het
hom
wt
Normalised Counts
400
450
500
550
600
650
zmp_ph108_F9
zmp_ph108_F11
zmp_ph108_H2
zmp_ph108_H5
zmp_ph108_B2
zmp_ph108_B6
zmp_ph108_B7
zmp_ph108_C4
zmp_ph108_C5
zmp_ph108_D4
zmp_ph97_G6
zmp_ph97_G9
zmp_ph97_H2
zmp_ph97_B10
zmp_ph97_C2
zmp_ph97_C4
zmp_ph97_B8
zmp_ph97_C1
zmp_ph97_D2
zmp_ph97_D5
zmp_ph111_G5
zmp_ph111_G11
zmp_ph111_H1
zmp_ph111_B1a
zmp_ph111_B5
zmp_ph111_B8
zmp_ph111_B6
zmp_ph111_B7
zmp_ph111_B9
zmp_ph111_B10
zmp_ph114_G4
zmp_ph114_G11
zmp_ph114_H1
zmp_ph114_H2
zmp_ph114_A9
zmp_ph114_A11
zmp_ph114_B2
zmp_ph114_B4
zmp_ph114_D8
zmp_ph114_E2

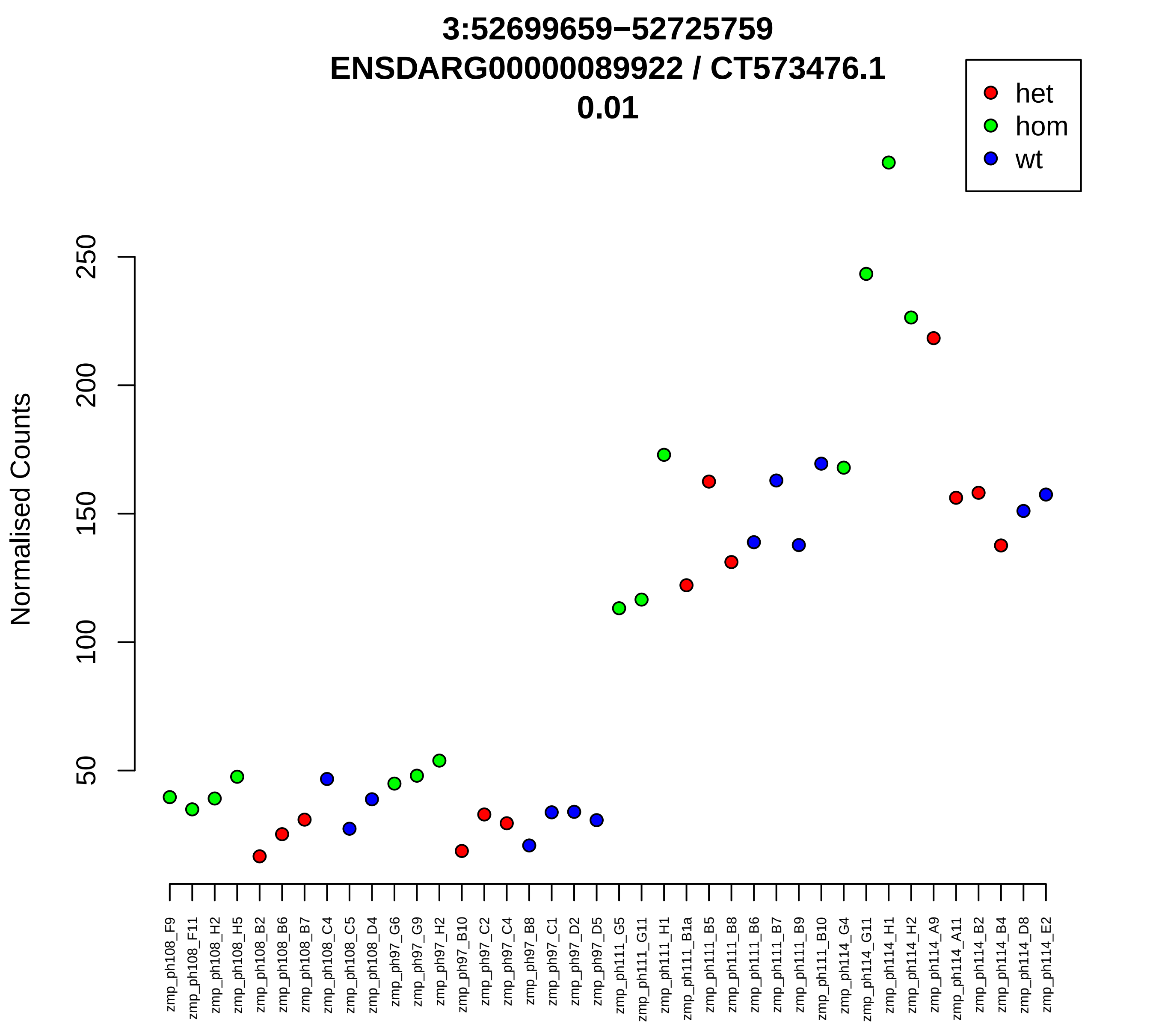

3:52699659−52725759
ENSDARG00000089922 / CT573476.1
0.01
het
hom
wt
Normalised Counts
50
100
150
200
250
zmp_ph108_F9
zmp_ph108_F11
zmp_ph108_H2
zmp_ph108_H5
zmp_ph108_B2
zmp_ph108_B6
zmp_ph108_B7
zmp_ph108_C4
zmp_ph108_C5
zmp_ph108_D4
zmp_ph97_G6
zmp_ph97_G9
zmp_ph97_H2
zmp_ph97_B10
zmp_ph97_C2
zmp_ph97_C4
zmp_ph97_B8
zmp_ph97_C1
zmp_ph97_D2
zmp_ph97_D5
zmp_ph111_G5
zmp_ph111_G11
zmp_ph111_H1
zmp_ph111_B1a
zmp_ph111_B5
zmp_ph111_B8
zmp_ph111_B6
zmp_ph111_B7
zmp_ph111_B9
zmp_ph111_B10
zmp_ph114_G4
zmp_ph114_G11
zmp_ph114_H1
zmp_ph114_H2
zmp_ph114_A9
zmp_ph114_A11
zmp_ph114_B2
zmp_ph114_B4
zmp_ph114_D8
zmp_ph114_E2

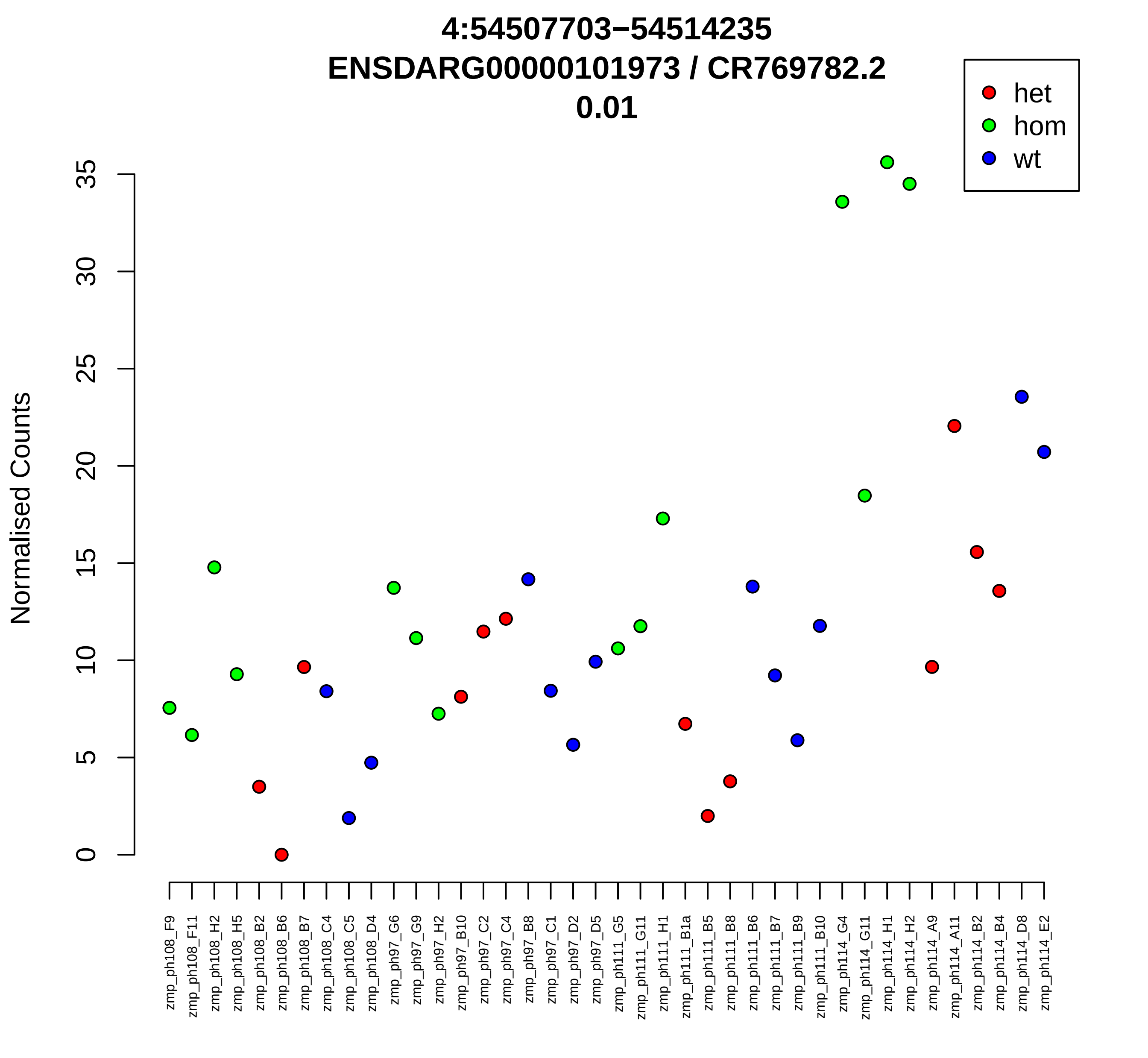
4:54507703−54514235
ENSDARG00000101973 / CR769782.2
0.01
het
hom
wt
Normalised Counts
0
5
10
15
20
25
30
35
zmp_ph108_F9
zmp_ph108_F11
zmp_ph108_H2
zmp_ph108_H5
zmp_ph108_B2
zmp_ph108_B6
zmp_ph108_B7
zmp_ph108_C4
zmp_ph108_C5
zmp_ph108_D4
zmp_ph97_G6
zmp_ph97_G9
zmp_ph97_H2
zmp_ph97_B10
zmp_ph97_C2
zmp_ph97_C4
zmp_ph97_B8
zmp_ph97_C1
zmp_ph97_D2
zmp_ph97_D5
zmp_ph111_G5
zmp_ph111_G11
zmp_ph111_H1
zmp_ph111_B1a
zmp_ph111_B5
zmp_ph111_B8
zmp_ph111_B6
zmp_ph111_B7
zmp_ph111_B9
zmp_ph111_B10
zmp_ph114_G4
zmp_ph114_G11
zmp_ph114_H1
zmp_ph114_H2
zmp_ph114_A9
zmp_ph114_A11
zmp_ph114_B2
zmp_ph114_B4
zmp_ph114_D8
zmp_ph114_E2

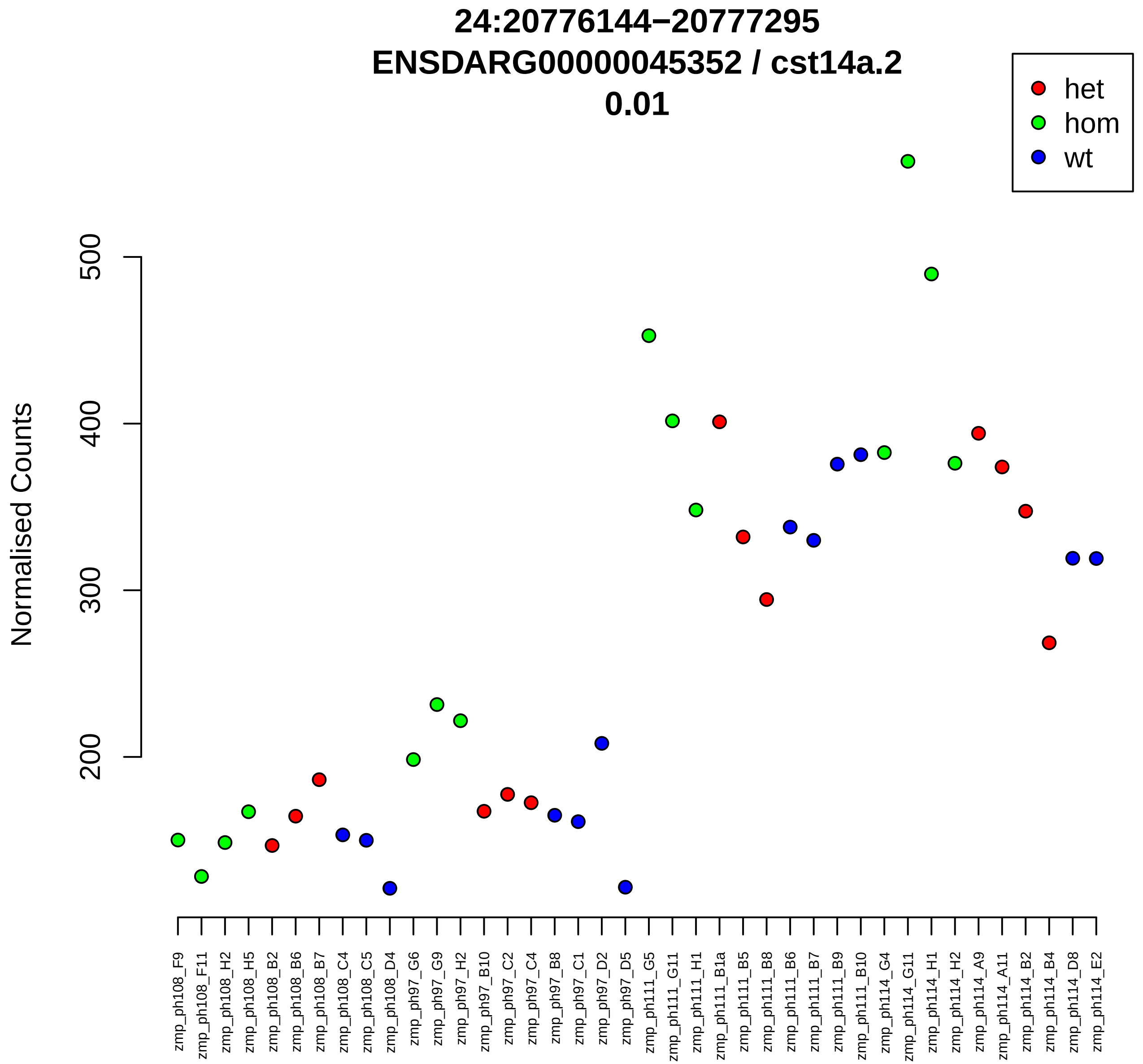

24:20776144−20777295
ENSDARG00000045352 / cst14a.2
0.01
het
hom
wt
Normalised Counts
200
300
400
500
zmp_ph108_F9
zmp_ph108_F11
zmp_ph108_H2
zmp_ph108_H5
zmp_ph108_B2
zmp_ph108_B6
zmp_ph108_B7
zmp_ph108_C4
zmp_ph108_C5
zmp_ph108_D4
zmp_ph97_G6
zmp_ph97_G9
zmp_ph97_H2
zmp_ph97_B10
zmp_ph97_C2
zmp_ph97_C4
zmp_ph97_B8
zmp_ph97_C1
zmp_ph97_D2
zmp_ph97_D5
zmp_ph111_G5
zmp_ph111_G11
zmp_ph111_H1
zmp_ph111_B1a
zmp_ph111_B5
zmp_ph111_B8
zmp_ph111_B6
zmp_ph111_B7
zmp_ph111_B9
zmp_ph111_B10
zmp_ph114_G4
zmp_ph114_G11
zmp_ph114_H1
zmp_ph114_H2
zmp_ph114_A9
zmp_ph114_A11
zmp_ph114_B2
zmp_ph114_B4
zmp_ph114_D8
zmp_ph114_E2

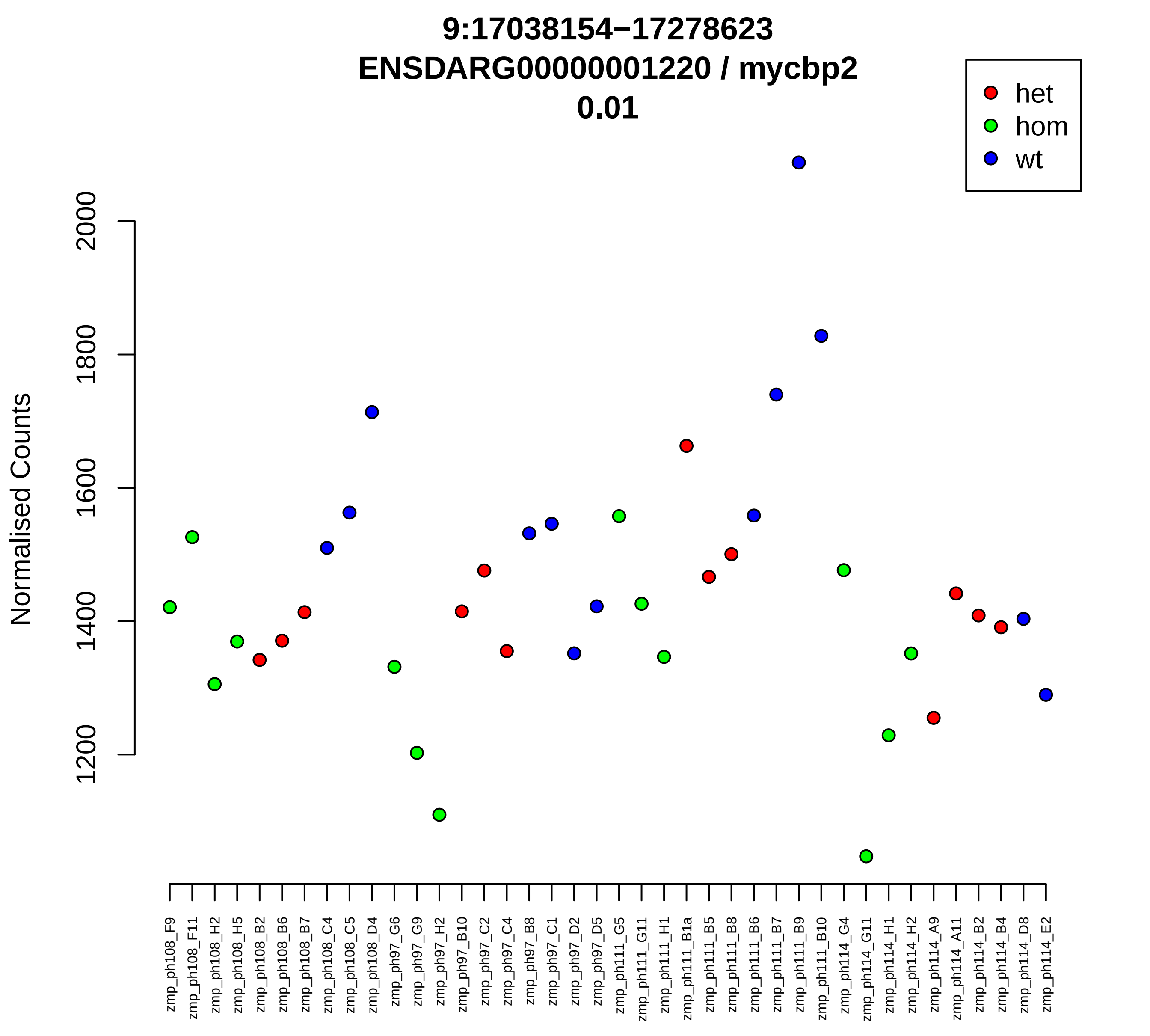

9:17038154−17278623
ENSDARG00000001220 / mycbp2
0.01
het
hom
wt
Normalised Counts
1200
1400
1600
1800
2000
zmp_ph108_F9
zmp_ph108_F11
zmp_ph108_H2
zmp_ph108_H5
zmp_ph108_B2
zmp_ph108_B6
zmp_ph108_B7
zmp_ph108_C4
zmp_ph108_C5
zmp_ph108_D4
zmp_ph97_G6
zmp_ph97_G9
zmp_ph97_H2
zmp_ph97_B10
zmp_ph97_C2
zmp_ph97_C4
zmp_ph97_B8
zmp_ph97_C1
zmp_ph97_D2
zmp_ph97_D5
zmp_ph111_G5
zmp_ph111_G11
zmp_ph111_H1
zmp_ph111_B1a
zmp_ph111_B5
zmp_ph111_B8
zmp_ph111_B6
zmp_ph111_B7
zmp_ph111_B9
zmp_ph111_B10
zmp_ph114_G4
zmp_ph114_G11
zmp_ph114_H1
zmp_ph114_H2
zmp_ph114_A9
zmp_ph114_A11
zmp_ph114_B2
zmp_ph114_B4
zmp_ph114_D8
zmp_ph114_E2

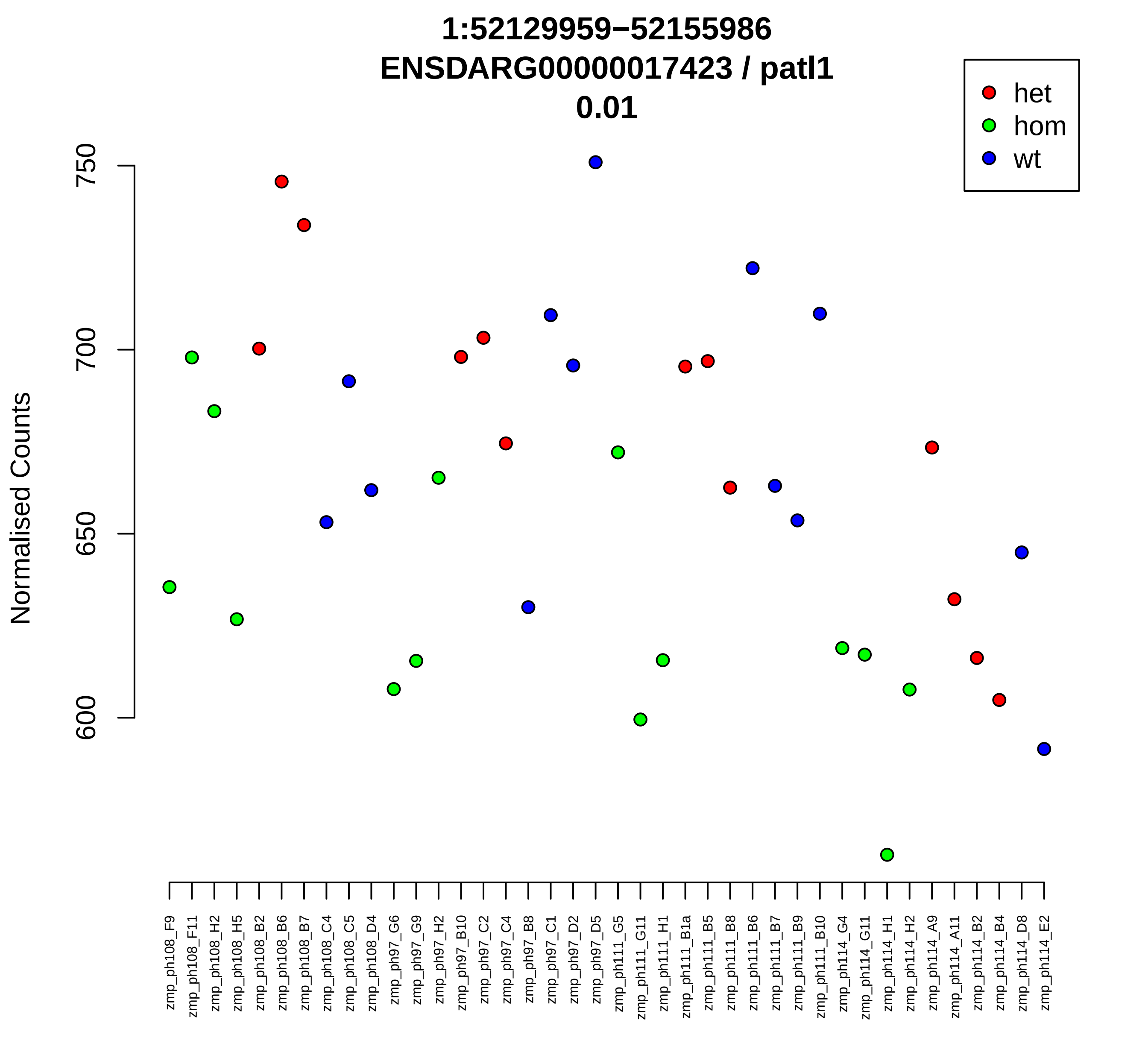

1:52129959−52155986
ENSDARG00000017423 / patl1
0.01
het
hom
wt
Normalised Counts
750
700
650
600
zmp_ph108_F9
zmp_ph108_F11
zmp_ph108_H2
zmp_ph108_H5
zmp_ph108_B2
zmp_ph108_B6
zmp_ph108_B7
zmp_ph108_C4
zmp_ph108_C5
zmp_ph108_D4
zmp_ph97_G6
zmp_ph97_G9
zmp_ph97_H2
zmp_ph97_B10
zmp_ph97_C2
zmp_ph97_C4
zmp_ph97_B8
zmp_ph97_C1
zmp_ph97_D2
zmp_ph97_D5
zmp_ph111_G5
zmp_ph111_G11
zmp_ph111_H1
zmp_ph111_B1a
zmp_ph111_B5
zmp_ph111_B8
zmp_ph111_B6
zmp_ph111_B7
zmp_ph111_B9
zmp_ph111_B10
zmp_ph114_G4
zmp_ph114_G11
zmp_ph114_H1
zmp_ph114_H2
zmp_ph114_A9
zmp_ph114_A11
zmp_ph114_B2
zmp_ph114_B4
zmp_ph114_D8
zmp_ph114_E2

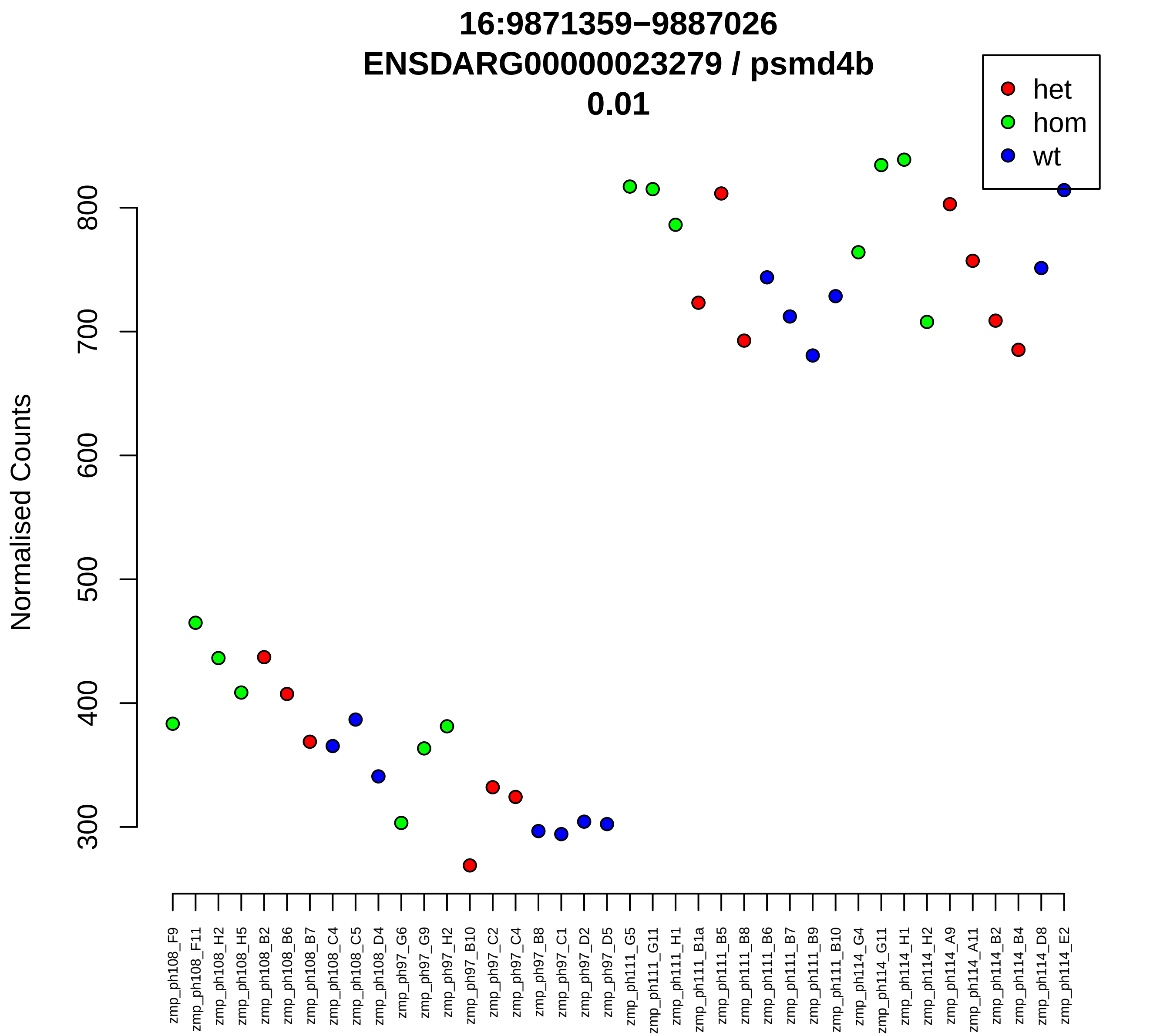
16:9871359−9887026
ENSDARG00000023279 / psmd4b
0.01
het
hom
wt
Normalised Counts
300
400
500
600
700
800
zmp_ph108_F9
zmp_ph108_F11
zmp_ph108_H2
zmp_ph108_H5
zmp_ph108_B2
zmp_ph108_B6
zmp_ph108_B7
zmp_ph108_C4
zmp_ph108_C5
zmp_ph108_D4
zmp_ph97_G6
zmp_ph97_G9
zmp_ph97_H2
zmp_ph97_B10
zmp_ph97_C2
zmp_ph97_C4
zmp_ph97_B8
zmp_ph97_C1
zmp_ph97_D2
zmp_ph97_D5
zmp_ph111_G5
zmp_ph111_G11
zmp_ph111_H1
zmp_ph111_B1a
zmp_ph111_B5
zmp_ph111_B8
zmp_ph111_B6
zmp_ph111_B7
zmp_ph111_B9
zmp_ph111_B10
zmp_ph114_G4
zmp_ph114_G11
zmp_ph114_H1
zmp_ph114_H2
zmp_ph114_A9
zmp_ph114_A11
zmp_ph114_B2
zmp_ph114_B4
zmp_ph114_D8
zmp_ph114_E2

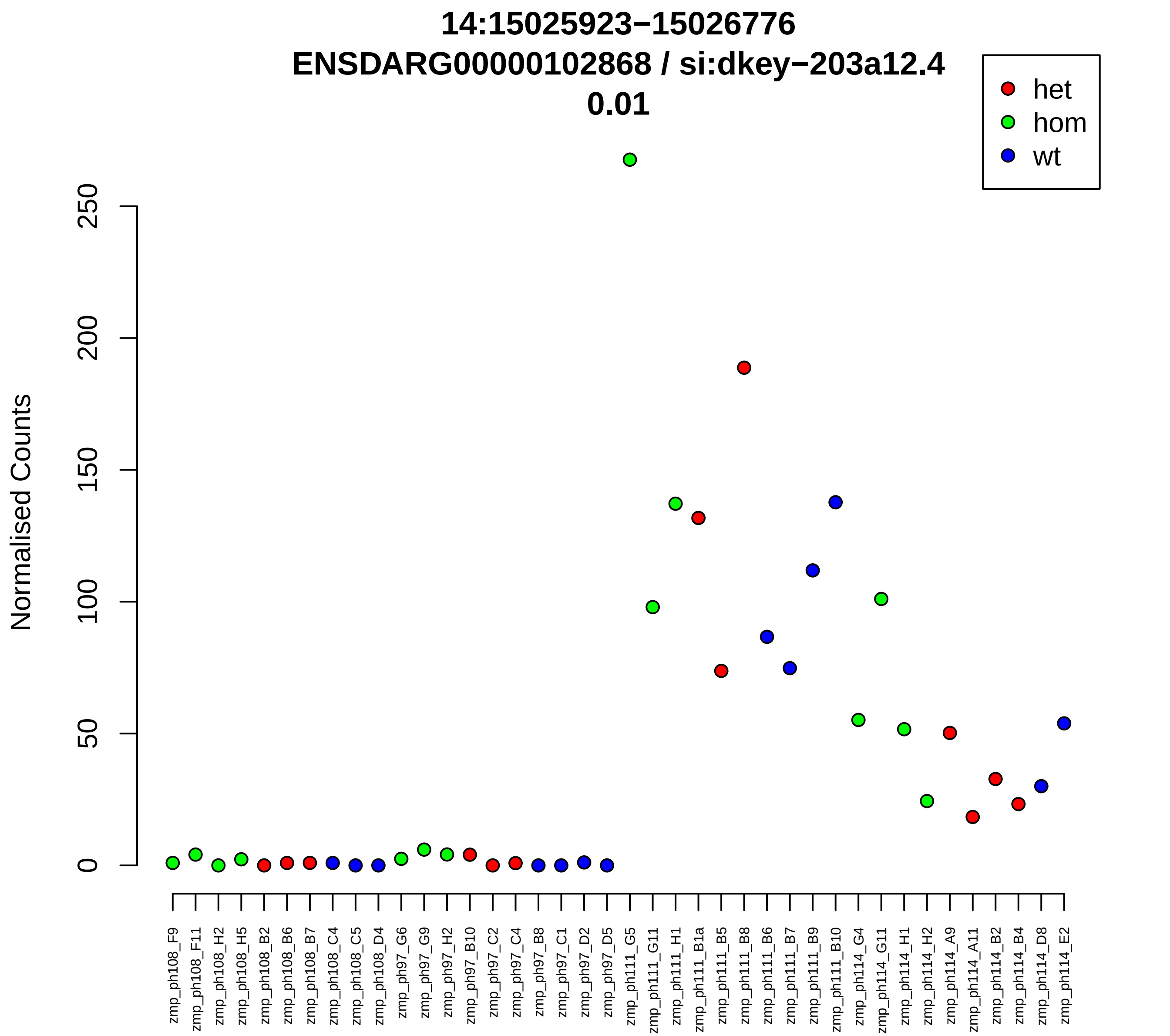

14:15025923−15026776
ENSDARG00000102868 / si:dkey−203a12.4
0.01
het
hom
wt
Normalised Counts
0
50
100
150
200
250
zmp_ph108_F9
zmp_ph108_F11
zmp_ph108_H2
zmp_ph108_H5
zmp_ph108_B2
zmp_ph108_B6
zmp_ph108_B7
zmp_ph108_C4
zmp_ph108_C5
zmp_ph108_D4
zmp_ph97_G6
zmp_ph97_G9
zmp_ph97_H2
zmp_ph97_B10
zmp_ph97_C2
zmp_ph97_C4
zmp_ph97_B8
zmp_ph97_C1
zmp_ph97_D2
zmp_ph97_D5
zmp_ph111_G5
zmp_ph111_G11
zmp_ph111_H1
zmp_ph111_B1a
zmp_ph111_B5
zmp_ph111_B8
zmp_ph111_B6
zmp_ph111_B7
zmp_ph111_B9
zmp_ph111_B10
zmp_ph114_G4
zmp_ph114_G11
zmp_ph114_H1
zmp_ph114_H2
zmp_ph114_A9
zmp_ph114_A11
zmp_ph114_B2
zmp_ph114_B4
zmp_ph114_D8
zmp_ph114_E2

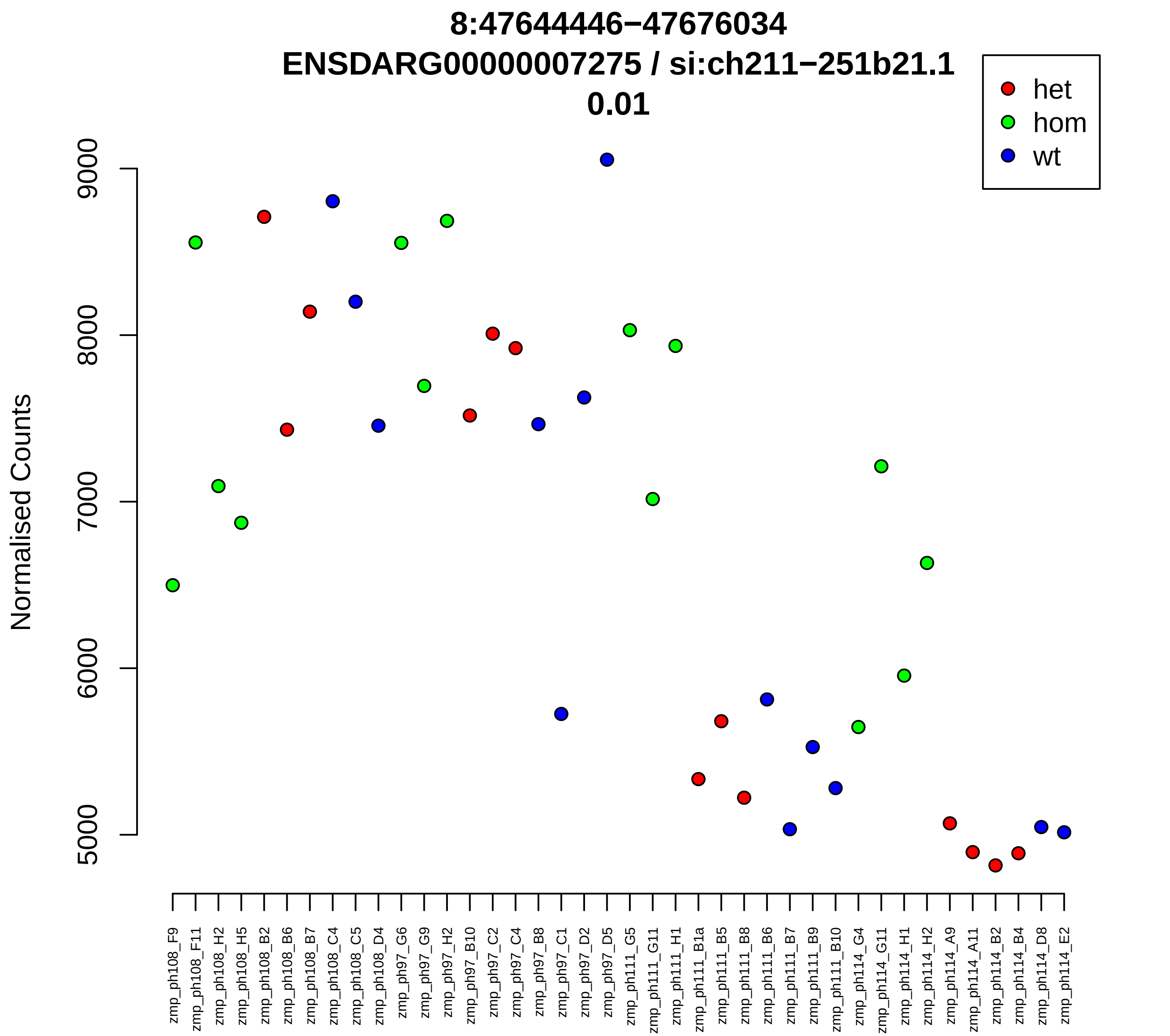
8:47644446−47676034
ENSDARG00000007275 / si:ch211−251b21.1
0.01
het
hom
wt
Normalised Counts
5000
6000
7000
8000
9000
zmp_ph108_F9
zmp_ph108_F11
zmp_ph108_H2
zmp_ph108_H5
zmp_ph108_B2
zmp_ph108_B6
zmp_ph108_B7
zmp_ph108_C4
zmp_ph108_C5
zmp_ph108_D4
zmp_ph97_G6
zmp_ph97_G9
zmp_ph97_H2
zmp_ph97_B10
zmp_ph97_C2
zmp_ph97_C4
zmp_ph97_B8
zmp_ph97_C1
zmp_ph97_D2
zmp_ph97_D5
zmp_ph111_G5
zmp_ph111_G11
zmp_ph111_H1
zmp_ph111_B1a
zmp_ph111_B5
zmp_ph111_B8
zmp_ph111_B6
zmp_ph111_B7
zmp_ph111_B9
zmp_ph111_B10
zmp_ph114_G4
zmp_ph114_G11
zmp_ph114_H1
zmp_ph114_H2
zmp_ph114_A9
zmp_ph114_A11
zmp_ph114_B2
zmp_ph114_B4
zmp_ph114_D8
zmp_ph114_E2

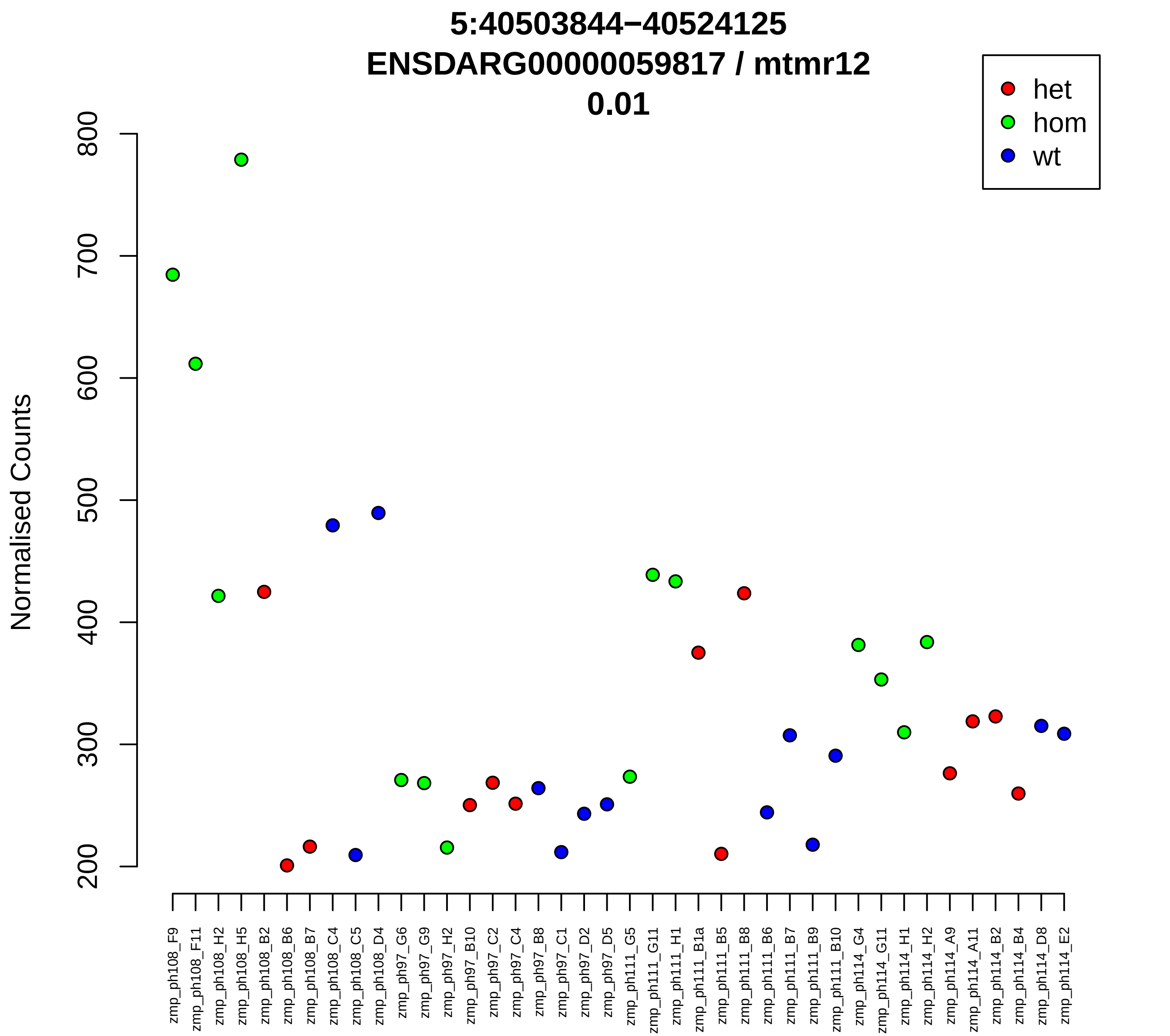

5:40503844−40524125
ENSDARG00000059817 / mtmr12
0.01
het
hom
wt
Normalised Counts
200
300
400
500
600
700
800
zmp_ph108_F9
zmp_ph108_F11
zmp_ph108_H2
zmp_ph108_H5
zmp_ph108_B2
zmp_ph108_B6
zmp_ph108_B7
zmp_ph108_C4
zmp_ph108_C5
zmp_ph108_D4
zmp_ph97_G6
zmp_ph97_G9
zmp_ph97_H2
zmp_ph97_B10
zmp_ph97_C2
zmp_ph97_C4
zmp_ph97_B8
zmp_ph97_C1
zmp_ph97_D2
zmp_ph97_D5
zmp_ph111_G5
zmp_ph111_G11
zmp_ph111_H1
zmp_ph111_B1a
zmp_ph111_B5
zmp_ph111_B8
zmp_ph111_B6
zmp_ph111_B7
zmp_ph111_B9
zmp_ph111_B10
zmp_ph114_G4
zmp_ph114_G11
zmp_ph114_H1
zmp_ph114_H2
zmp_ph114_A9
zmp_ph114_A11
zmp_ph114_B2
zmp_ph114_B4
zmp_ph114_D8
zmp_ph114_E2

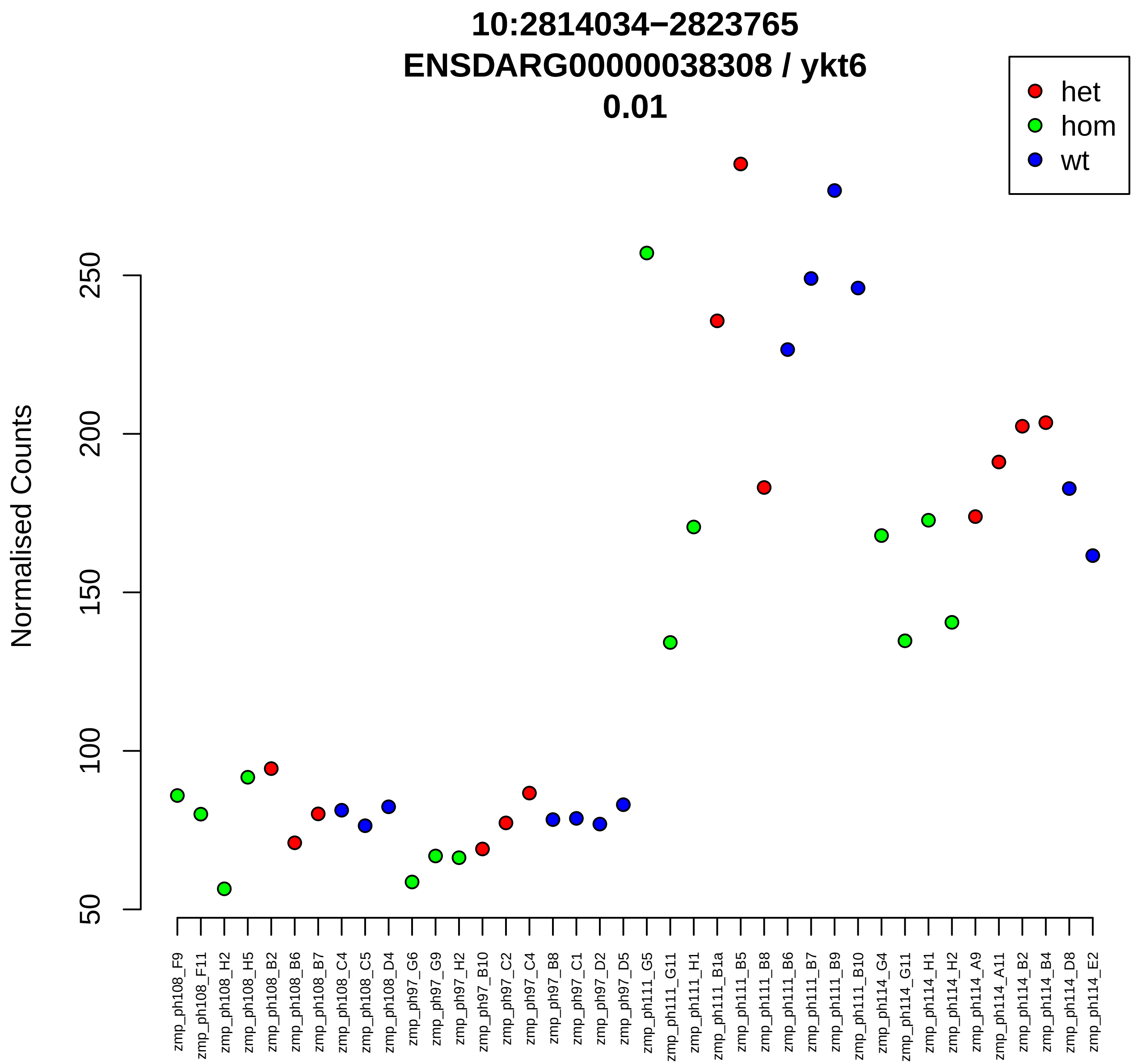

10:2814034−2823765
ENSDARG00000038308 / ykt6
0.01
het
hom
wt
Normalised Counts
50
100
150
200
250
zmp_ph108_F9
zmp_ph108_F11
zmp_ph108_H2
zmp_ph108_H5
zmp_ph108_B2
zmp_ph108_B6
zmp_ph108_B7
zmp_ph108_C4
zmp_ph108_C5
zmp_ph108_D4
zmp_ph97_G6
zmp_ph97_G9
zmp_ph97_H2
zmp_ph97_B10
zmp_ph97_C2
zmp_ph97_C4
zmp_ph97_B8
zmp_ph97_C1
zmp_ph97_D2
zmp_ph97_D5
zmp_ph111_G5
zmp_ph111_G11
zmp_ph111_H1
zmp_ph111_B1a
zmp_ph111_B5
zmp_ph111_B8
zmp_ph111_B6
zmp_ph111_B7
zmp_ph111_B9
zmp_ph111_B10
zmp_ph114_G4
zmp_ph114_G11
zmp_ph114_H1
zmp_ph114_H2
zmp_ph114_A9
zmp_ph114_A11
zmp_ph114_B2
zmp_ph114_B4
zmp_ph114_D8
zmp_ph114_E2

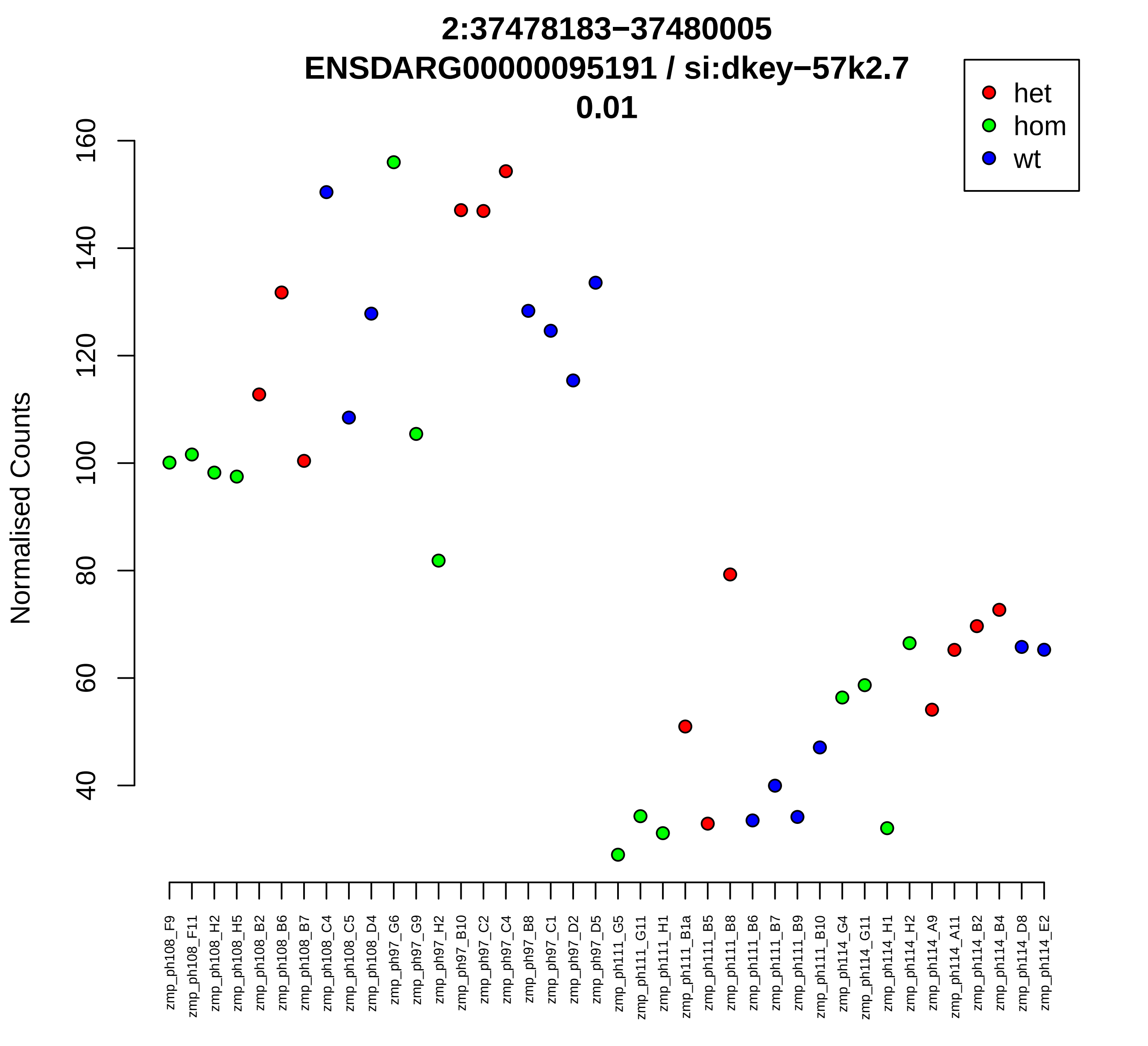
2:37478183−37480005
ENSDARG00000095191 / si:dkey−57k2.7
0.01
het
hom
wt
Normalised Counts
160
140
120
100
80
60
40
zmp_ph108_F9
zmp_ph108_F11
zmp_ph108_H2
zmp_ph108_H5
zmp_ph108_B2
zmp_ph108_B6
zmp_ph108_B7
zmp_ph108_C4
zmp_ph108_C5
zmp_ph108_D4
zmp_ph97_G6
zmp_ph97_G9
zmp_ph97_H2
zmp_ph97_B10
zmp_ph97_C2
zmp_ph97_C4
zmp_ph97_B8
zmp_ph97_C1
zmp_ph97_D2
zmp_ph97_D5
zmp_ph111_G5
zmp_ph111_G11
zmp_ph111_H1
zmp_ph111_B1a
zmp_ph111_B5
zmp_ph111_B8
zmp_ph111_B6
zmp_ph111_B7
zmp_ph111_B9
zmp_ph111_B10
zmp_ph114_G4
zmp_ph114_G11
zmp_ph114_H1
zmp_ph114_H2
zmp_ph114_A9
zmp_ph114_A11
zmp_ph114_B2
zmp_ph114_B4
zmp_ph114_D8
zmp_ph114_E2

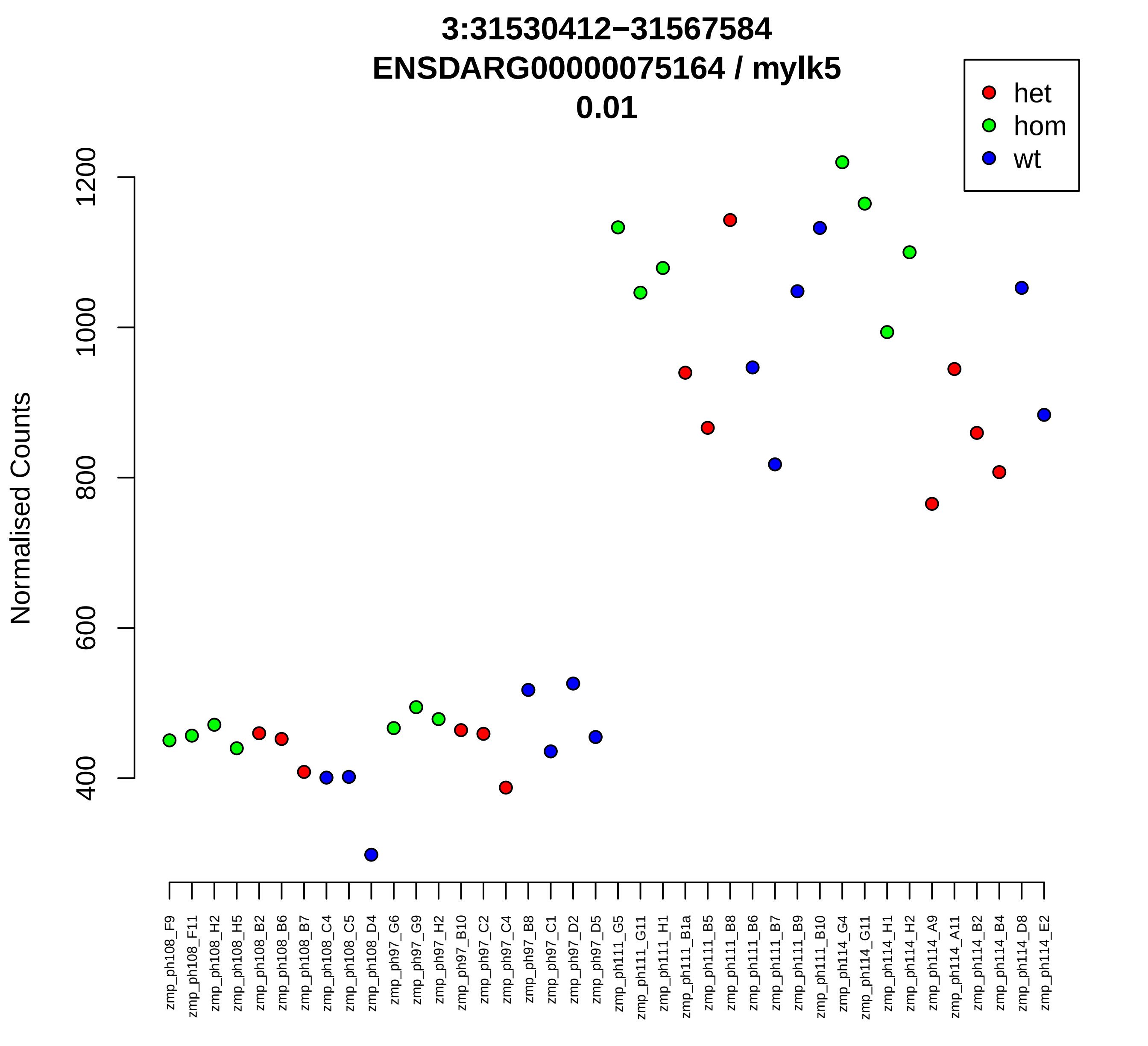

3:31530412−31567584
ENSDARG00000075164 / mylk5
0.01
het
hom
wt
Normalised Counts
400
600
800
1000
1200
zmp_ph108_F9
zmp_ph108_F11
zmp_ph108_H2
zmp_ph108_H5
zmp_ph108_B2
zmp_ph108_B6
zmp_ph108_B7
zmp_ph108_C4
zmp_ph108_C5
zmp_ph108_D4
zmp_ph97_G6
zmp_ph97_G9
zmp_ph97_H2
zmp_ph97_B10
zmp_ph97_C2
zmp_ph97_C4
zmp_ph97_B8
zmp_ph97_C1
zmp_ph97_D2
zmp_ph97_D5
zmp_ph111_G5
zmp_ph111_G11
zmp_ph111_H1
zmp_ph111_B1a
zmp_ph111_B5
zmp_ph111_B8
zmp_ph111_B6
zmp_ph111_B7
zmp_ph111_B9
zmp_ph111_B10
zmp_ph114_G4
zmp_ph114_G11
zmp_ph114_H1
zmp_ph114_H2
zmp_ph114_A9
zmp_ph114_A11
zmp_ph114_B2
zmp_ph114_B4
zmp_ph114_D8
zmp_ph114_E2

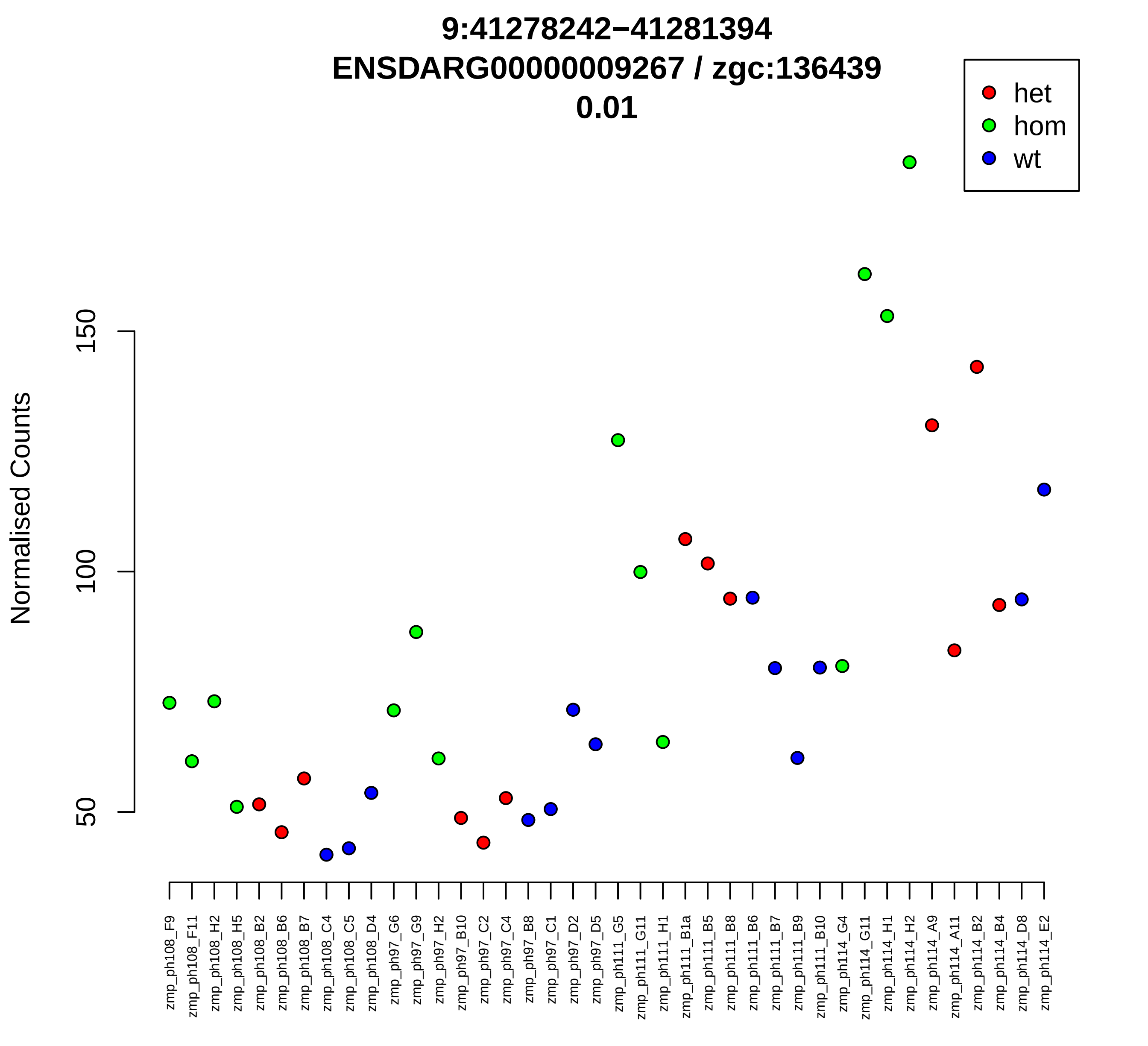
9:41278242−41281394
ENSDARG00000009267 / zgc:136439
0.01
het
hom
wt
Normalised Counts
50
100
150
zmp_ph108_F9
zmp_ph108_F11
zmp_ph108_H2
zmp_ph108_H5
zmp_ph108_B2
zmp_ph108_B6
zmp_ph108_B7
zmp_ph108_C4
zmp_ph108_C5
zmp_ph108_D4
zmp_ph97_G6
zmp_ph97_G9
zmp_ph97_H2
zmp_ph97_B10
zmp_ph97_C2
zmp_ph97_C4
zmp_ph97_B8
zmp_ph97_C1
zmp_ph97_D2
zmp_ph97_D5
zmp_ph111_G5
zmp_ph111_G11
zmp_ph111_H1
zmp_ph111_B1a
zmp_ph111_B5
zmp_ph111_B8
zmp_ph111_B6
zmp_ph111_B7
zmp_ph111_B9
zmp_ph111_B10
zmp_ph114_G4
zmp_ph114_G11
zmp_ph114_H1
zmp_ph114_H2
zmp_ph114_A9
zmp_ph114_A11
zmp_ph114_B2
zmp_ph114_B4
zmp_ph114_D8
zmp_ph114_E2

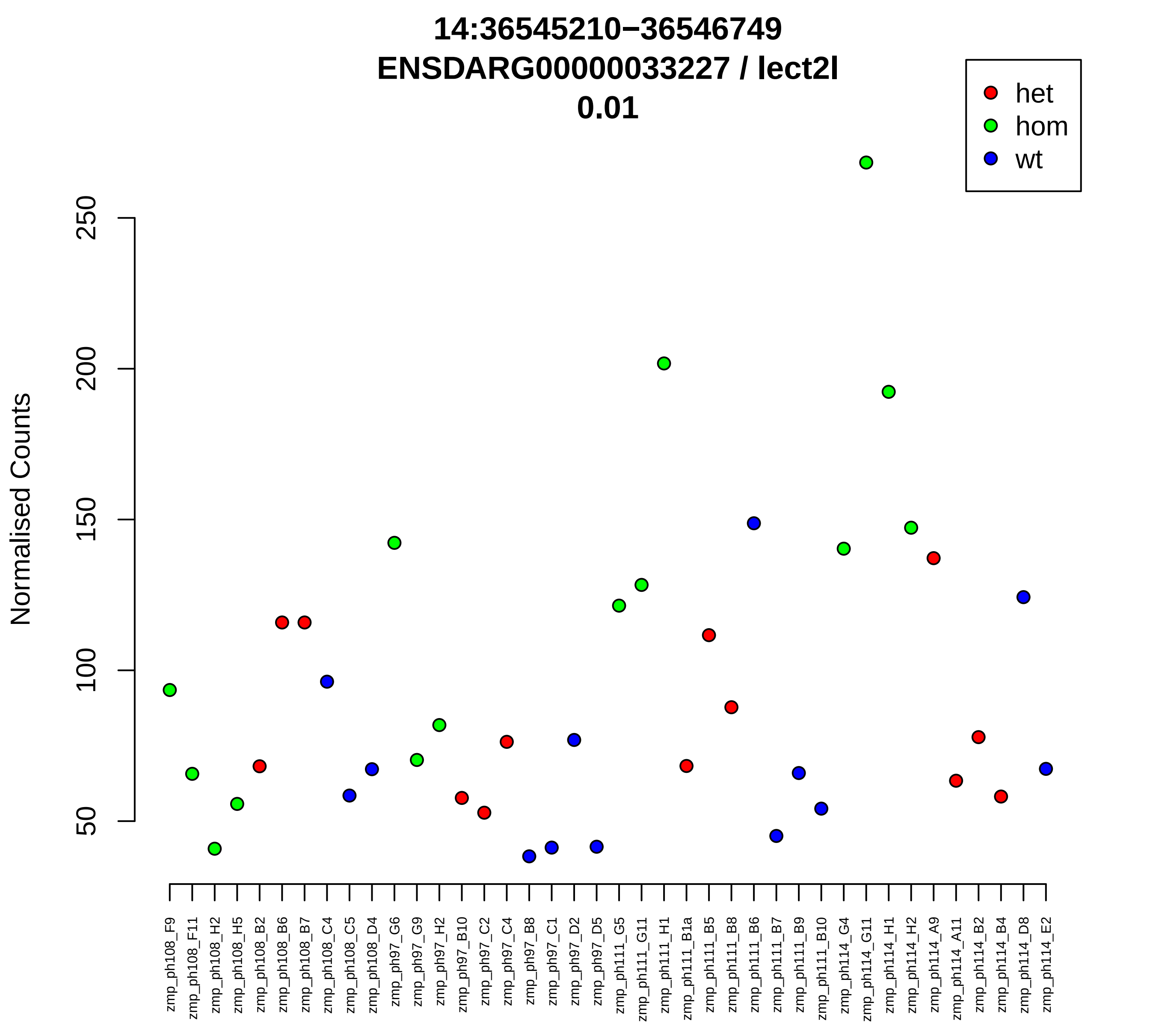
14:36545210−36546749
ENSDARG00000033227 / lect2l
0.01
het
hom
wt
Normalised Counts
50
100
150
200
250
zmp_ph108_F9
zmp_ph108_F11
zmp_ph108_H2
zmp_ph108_H5
zmp_ph108_B2
zmp_ph108_B6
zmp_ph108_B7
zmp_ph108_C4
zmp_ph108_C5
zmp_ph108_D4
zmp_ph97_G6
zmp_ph97_G9
zmp_ph97_H2
zmp_ph97_B10
zmp_ph97_C2
zmp_ph97_C4
zmp_ph97_B8
zmp_ph97_C1
zmp_ph97_D2
zmp_ph97_D5
zmp_ph111_G5
zmp_ph111_G11
zmp_ph111_H1
zmp_ph111_B1a
zmp_ph111_B5
zmp_ph111_B8
zmp_ph111_B6
zmp_ph111_B7
zmp_ph111_B9
zmp_ph111_B10
zmp_ph114_G4
zmp_ph114_G11
zmp_ph114_H1
zmp_ph114_H2
zmp_ph114_A9
zmp_ph114_A11
zmp_ph114_B2
zmp_ph114_B4
zmp_ph114_D8
zmp_ph114_E2

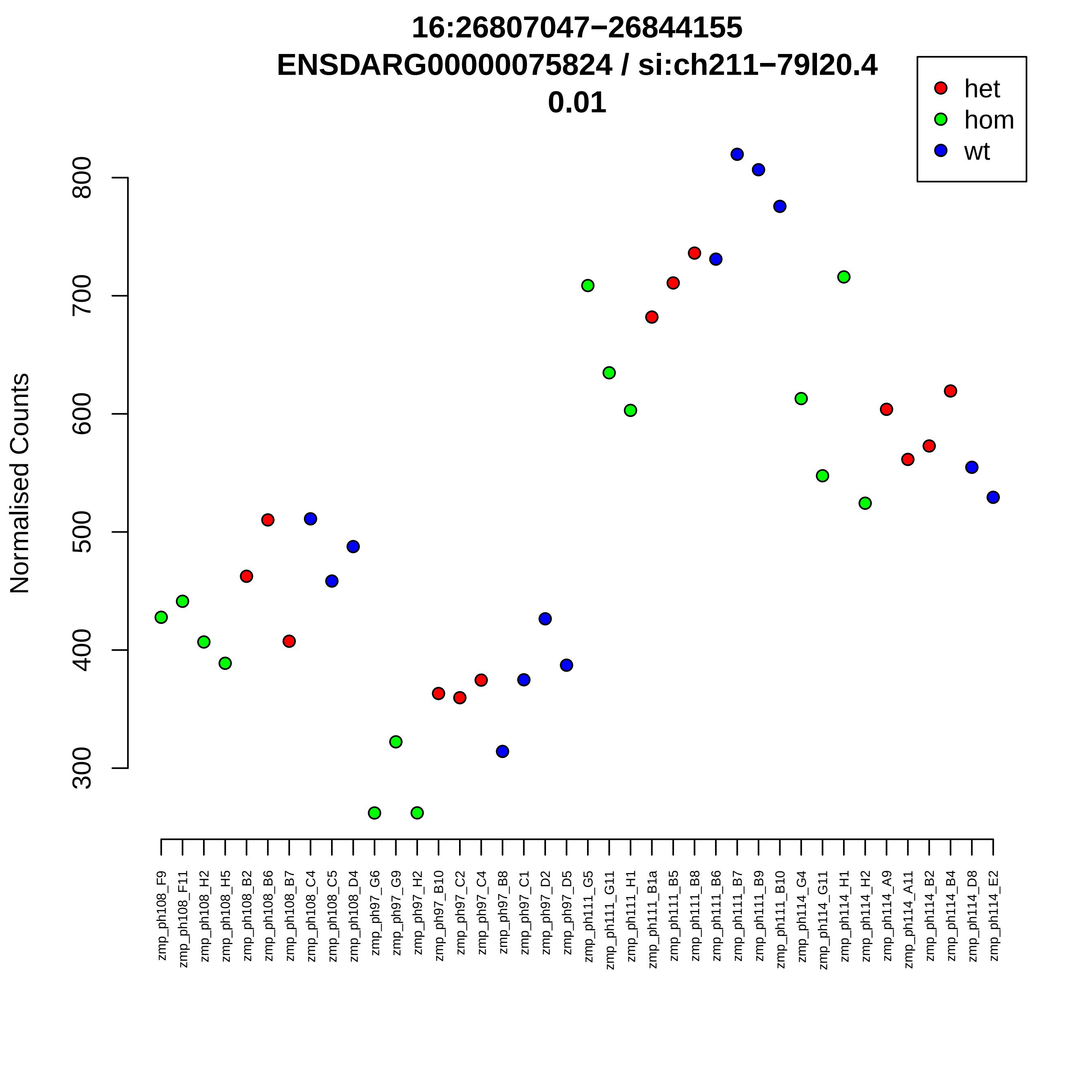
16:26807047−26844155
ENSDARG00000075824 / si:ch211−79l20.4
0.01
het
hom
wt
Normalised Counts
300
400
500
600
700
800
zmp_ph108_F9
zmp_ph108_F11
zmp_ph108_H2
zmp_ph108_H5
zmp_ph108_B2
zmp_ph108_B6
zmp_ph108_B7
zmp_ph108_C4
zmp_ph108_C5
zmp_ph108_D4
zmp_ph97_G6
zmp_ph97_G9
zmp_ph97_H2
zmp_ph97_B10
zmp_ph97_C2
zmp_ph97_C4
zmp_ph97_B8
zmp_ph97_C1
zmp_ph97_D2
zmp_ph97_D5
zmp_ph111_G5
zmp_ph111_G11
zmp_ph111_H1
zmp_ph111_B1a
zmp_ph111_B5
zmp_ph111_B8
zmp_ph111_B6
zmp_ph111_B7
zmp_ph111_B9
zmp_ph111_B10
zmp_ph114_G4
zmp_ph114_G11
zmp_ph114_H1
zmp_ph114_H2
zmp_ph114_A9
zmp_ph114_A11
zmp_ph114_B2
zmp_ph114_B4
zmp_ph114_D8
zmp_ph114_E2

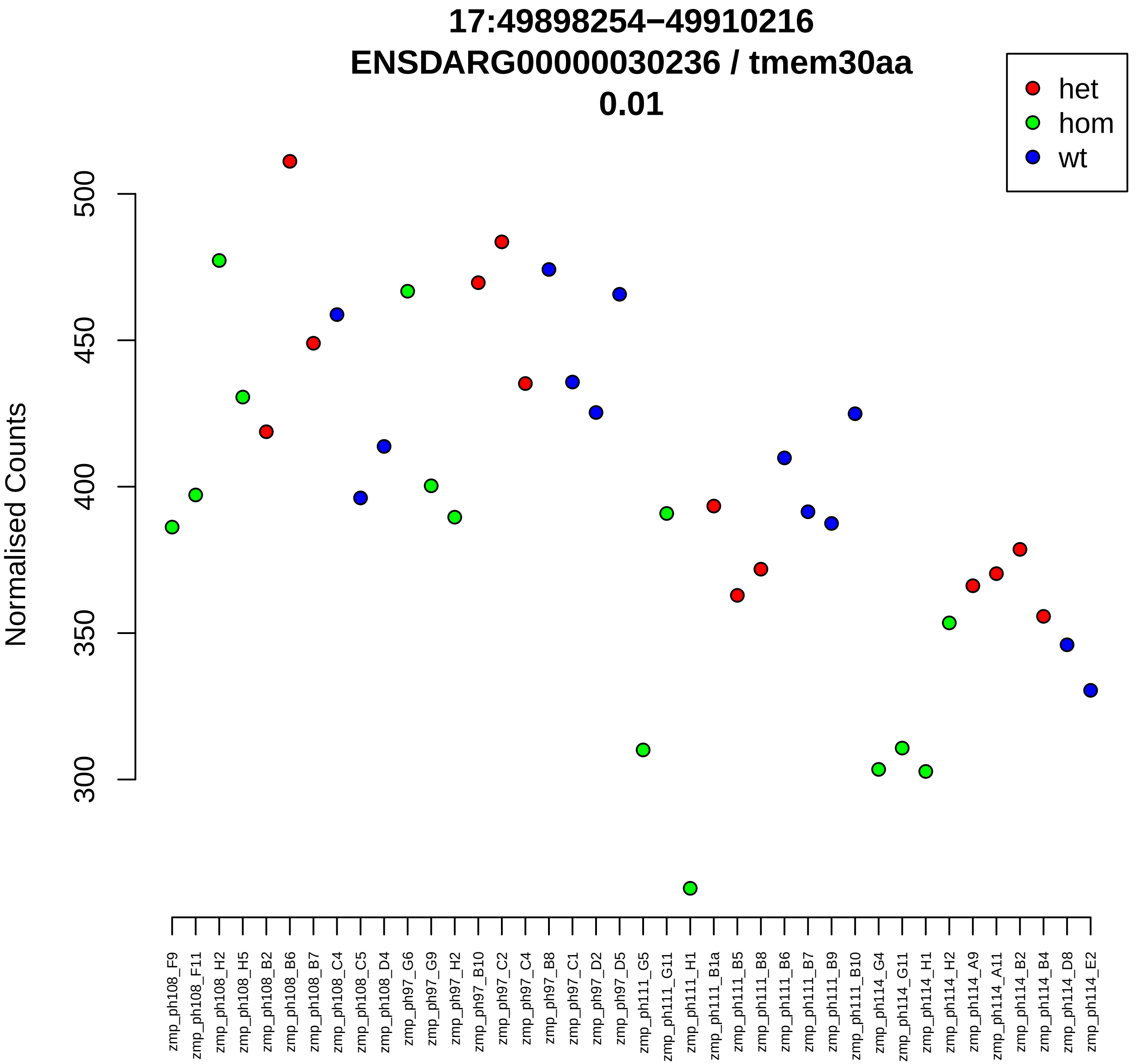
17:49898254−49910216
ENSDARG00000030236 / tmem30aa
0.01
het
hom
wt
Normalised Counts
300
350
400
450
500
zmp_ph108_F9
zmp_ph108_F11
zmp_ph108_H2
zmp_ph108_H5
zmp_ph108_B2
zmp_ph108_B6
zmp_ph108_B7
zmp_ph108_C4
zmp_ph108_C5
zmp_ph108_D4
zmp_ph97_G6
zmp_ph97_G9
zmp_ph97_H2
zmp_ph97_B10
zmp_ph97_C2
zmp_ph97_C4
zmp_ph97_B8
zmp_ph97_C1
zmp_ph97_D2
zmp_ph97_D5
zmp_ph111_G5
zmp_ph111_G11
zmp_ph111_H1
zmp_ph111_B1a
zmp_ph111_B5
zmp_ph111_B8
zmp_ph111_B6
zmp_ph111_B7
zmp_ph111_B9
zmp_ph111_B10
zmp_ph114_G4
zmp_ph114_G11
zmp_ph114_H1
zmp_ph114_H2
zmp_ph114_A9
zmp_ph114_A11
zmp_ph114_B2
zmp_ph114_B4
zmp_ph114_D8
zmp_ph114_E2

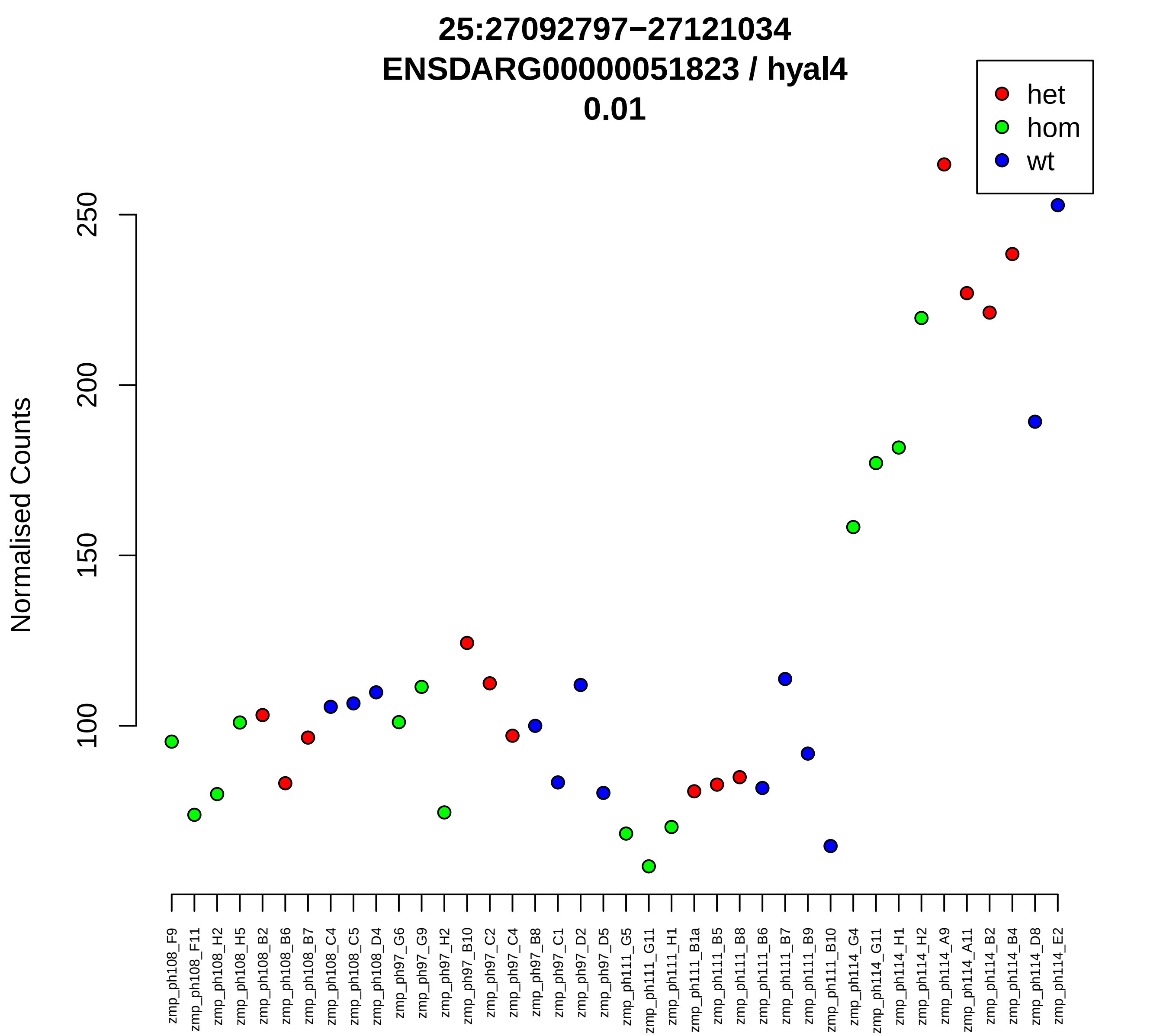

25:27092797−27121034
ENSDARG00000051823 / hyal4
0.01
het
hom
wt
Normalised Counts
100
150
200
250
zmp_ph108_F9
zmp_ph108_F11
zmp_ph108_H2
zmp_ph108_H5
zmp_ph108_B2
zmp_ph108_B6
zmp_ph108_B7
zmp_ph108_C4
zmp_ph108_C5
zmp_ph108_D4
zmp_ph97_G6
zmp_ph97_G9
zmp_ph97_H2
zmp_ph97_B10
zmp_ph97_C2
zmp_ph97_C4
zmp_ph97_B8
zmp_ph97_C1
zmp_ph97_D2
zmp_ph97_D5
zmp_ph111_G5
zmp_ph111_G11
zmp_ph111_H1
zmp_ph111_B1a
zmp_ph111_B5
zmp_ph111_B8
zmp_ph111_B6
zmp_ph111_B7
zmp_ph111_B9
zmp_ph111_B10
zmp_ph114_G4
zmp_ph114_G11
zmp_ph114_H1
zmp_ph114_H2
zmp_ph114_A9
zmp_ph114_A11
zmp_ph114_B2
zmp_ph114_B4
zmp_ph114_D8
zmp_ph114_E2

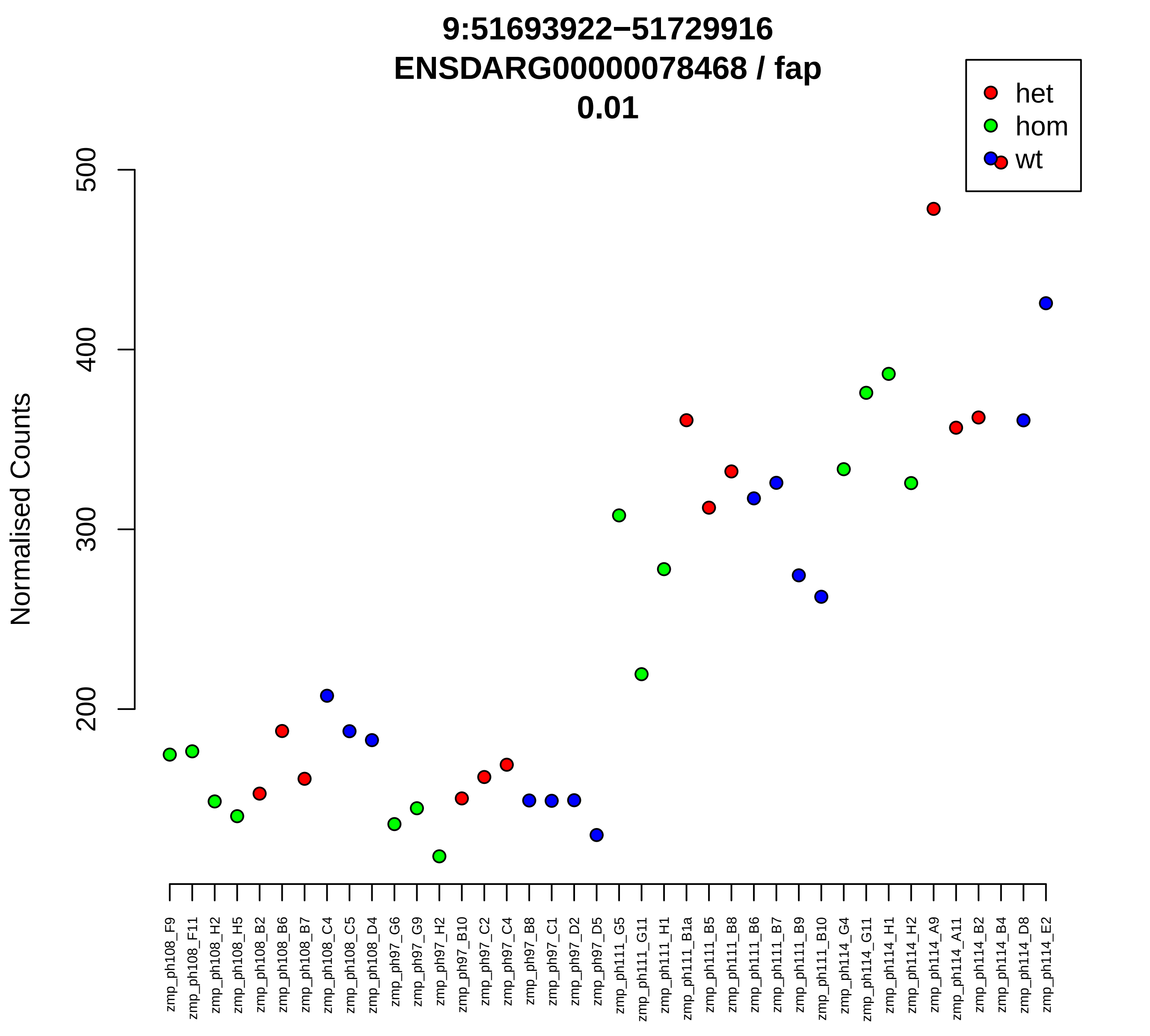

9:51693922−51729916
ENSDARG00000078468 / fap
0.01
het
hom
wt
Normalised Counts
500
400
300
200
zmp_ph108_F9
zmp_ph108_F11
zmp_ph108_H2
zmp_ph108_H5
zmp_ph108_B2
zmp_ph108_B6
zmp_ph108_B7
zmp_ph108_C4
zmp_ph108_C5
zmp_ph108_D4
zmp_ph97_G6
zmp_ph97_G9
zmp_ph97_H2
zmp_ph97_B10
zmp_ph97_C2
zmp_ph97_C4
zmp_ph97_B8
zmp_ph97_C1
zmp_ph97_D2
zmp_ph97_D5
zmp_ph111_G5
zmp_ph111_G11
zmp_ph111_H1
zmp_ph111_B1a
zmp_ph111_B5
zmp_ph111_B8
zmp_ph111_B6
zmp_ph111_B7
zmp_ph111_B9
zmp_ph111_B10
zmp_ph114_G4
zmp_ph114_G11
zmp_ph114_H1
zmp_ph114_H2
zmp_ph114_A9
zmp_ph114_A11
zmp_ph114_B2
zmp_ph114_B4
zmp_ph114_D8
zmp_ph114_E2

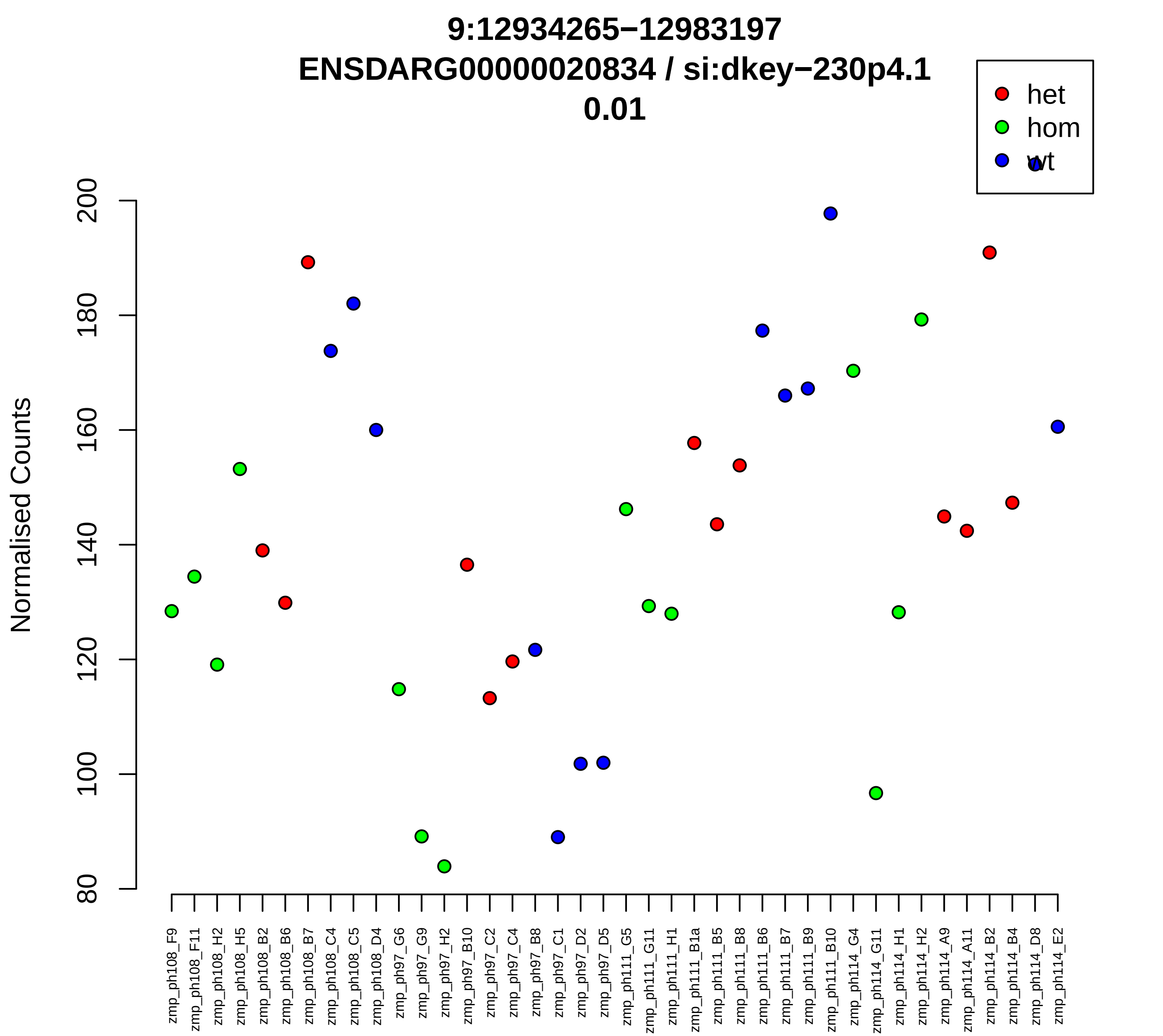

9:12934265−12983197
ENSDARG00000020834 / si:dkey−230p4.1
0.01
het
hom
wt
Normalised Counts
80
100
120
140
160
180
200
zmp_ph108_F9
zmp_ph108_F11
zmp_ph108_H2
zmp_ph108_H5
zmp_ph108_B2
zmp_ph108_B6
zmp_ph108_B7
zmp_ph108_C4
zmp_ph108_C5
zmp_ph108_D4
zmp_ph97_G6
zmp_ph97_G9
zmp_ph97_H2
zmp_ph97_B10
zmp_ph97_C2
zmp_ph97_C4
zmp_ph97_B8
zmp_ph97_C1
zmp_ph97_D2
zmp_ph97_D5
zmp_ph111_G5
zmp_ph111_G11
zmp_ph111_H1
zmp_ph111_B1a
zmp_ph111_B5
zmp_ph111_B8
zmp_ph111_B6
zmp_ph111_B7
zmp_ph111_B9
zmp_ph111_B10
zmp_ph114_G4
zmp_ph114_G11
zmp_ph114_H1
zmp_ph114_H2
zmp_ph114_A9
zmp_ph114_A11
zmp_ph114_B2
zmp_ph114_B4
zmp_ph114_D8
zmp_ph114_E2

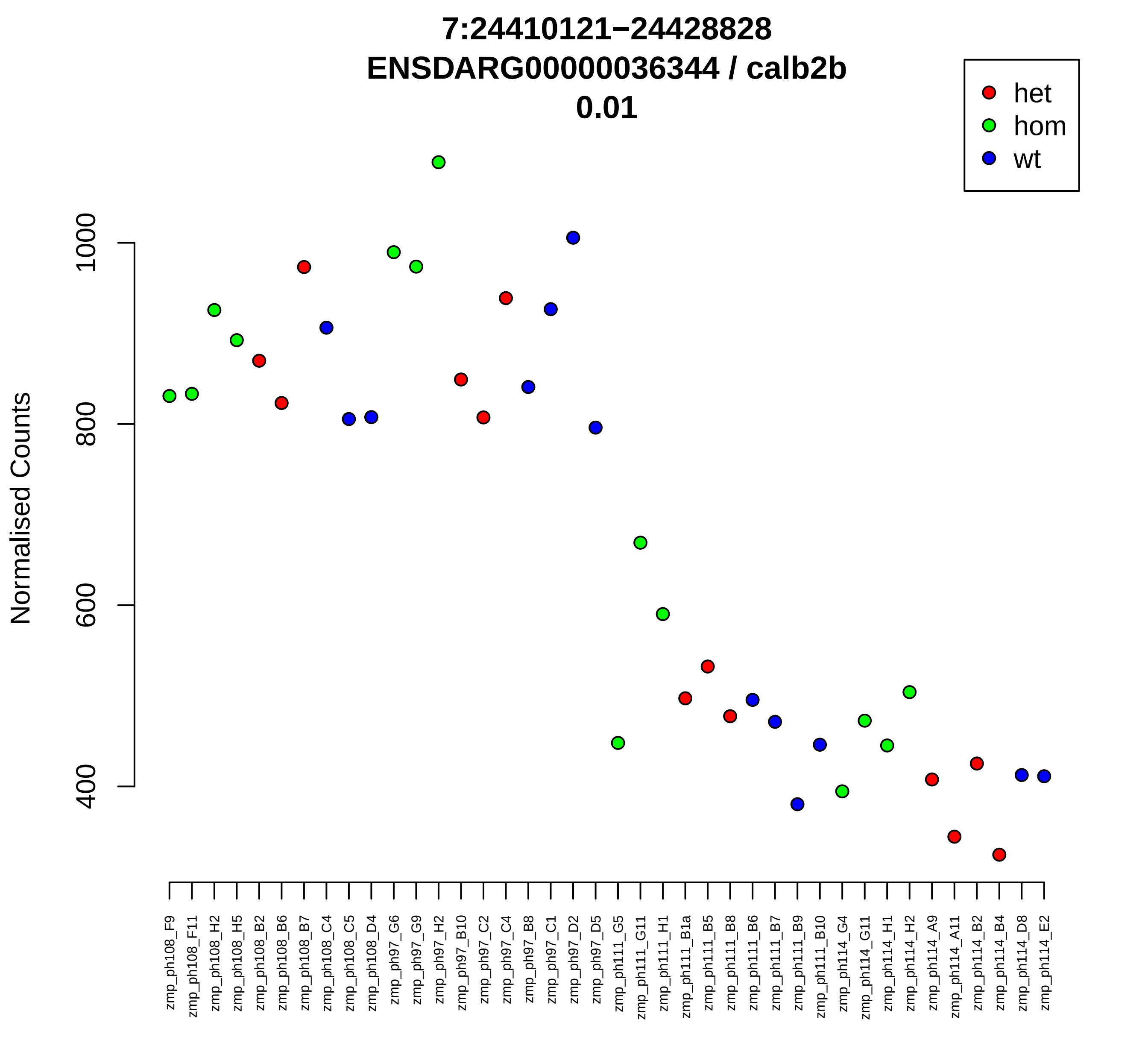
7:24410121−24428828
ENSDARG00000036344 / calb2b
0.01
het
hom
wt
Normalised Counts
400
600
800
1000
zmp_ph108_F9
zmp_ph108_F11
zmp_ph108_H2
zmp_ph108_H5
zmp_ph108_B2
zmp_ph108_B6
zmp_ph108_B7
zmp_ph108_C4
zmp_ph108_C5
zmp_ph108_D4
zmp_ph97_G6
zmp_ph97_G9
zmp_ph97_H2
zmp_ph97_B10
zmp_ph97_C2
zmp_ph97_C4
zmp_ph97_B8
zmp_ph97_C1
zmp_ph97_D2
zmp_ph97_D5
zmp_ph111_G5
zmp_ph111_G11
zmp_ph111_H1
zmp_ph111_B1a
zmp_ph111_B5
zmp_ph111_B8
zmp_ph111_B6
zmp_ph111_B7
zmp_ph111_B9
zmp_ph111_B10
zmp_ph114_G4
zmp_ph114_G11
zmp_ph114_H1
zmp_ph114_H2
zmp_ph114_A9
zmp_ph114_A11
zmp_ph114_B2
zmp_ph114_B4
zmp_ph114_D8
zmp_ph114_E2

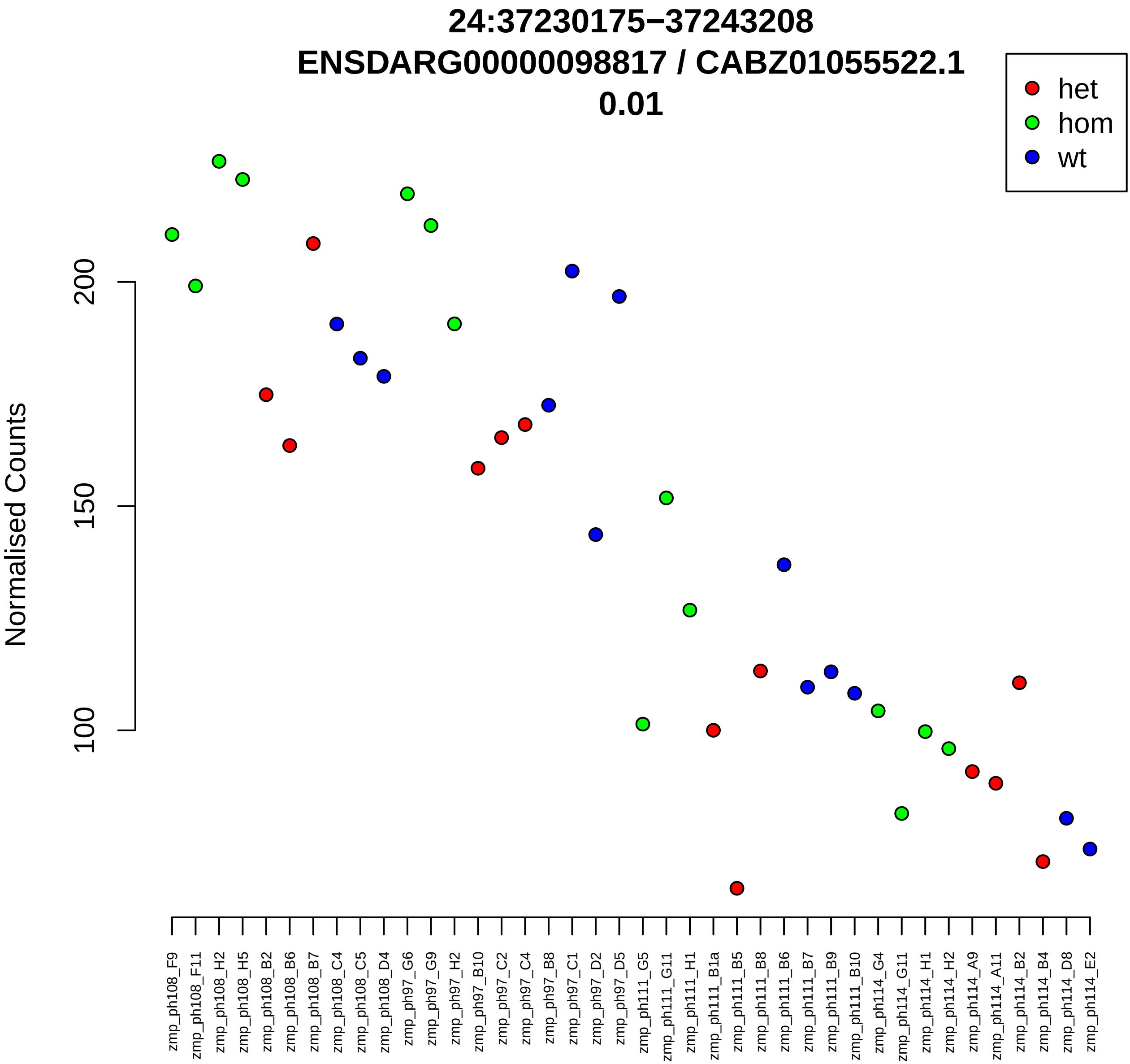

24:37230175−37243208
ENSDARG00000098817 / CABZ01055522.1
0.01
het
hom
wt
Normalised Counts
100
150
200
zmp_ph108_F9
zmp_ph108_F11
zmp_ph108_H2
zmp_ph108_H5
zmp_ph108_B2
zmp_ph108_B6
zmp_ph108_B7
zmp_ph108_C4
zmp_ph108_C5
zmp_ph108_D4
zmp_ph97_G6
zmp_ph97_G9
zmp_ph97_H2
zmp_ph97_B10
zmp_ph97_C2
zmp_ph97_C4
zmp_ph97_B8
zmp_ph97_C1
zmp_ph97_D2
zmp_ph97_D5
zmp_ph111_G5
zmp_ph111_G11
zmp_ph111_H1
zmp_ph111_B1a
zmp_ph111_B5
zmp_ph111_B8
zmp_ph111_B6
zmp_ph111_B7
zmp_ph111_B9
zmp_ph111_B10
zmp_ph114_G4
zmp_ph114_G11
zmp_ph114_H1
zmp_ph114_H2
zmp_ph114_A9
zmp_ph114_A11
zmp_ph114_B2
zmp_ph114_B4
zmp_ph114_D8
zmp_ph114_E2

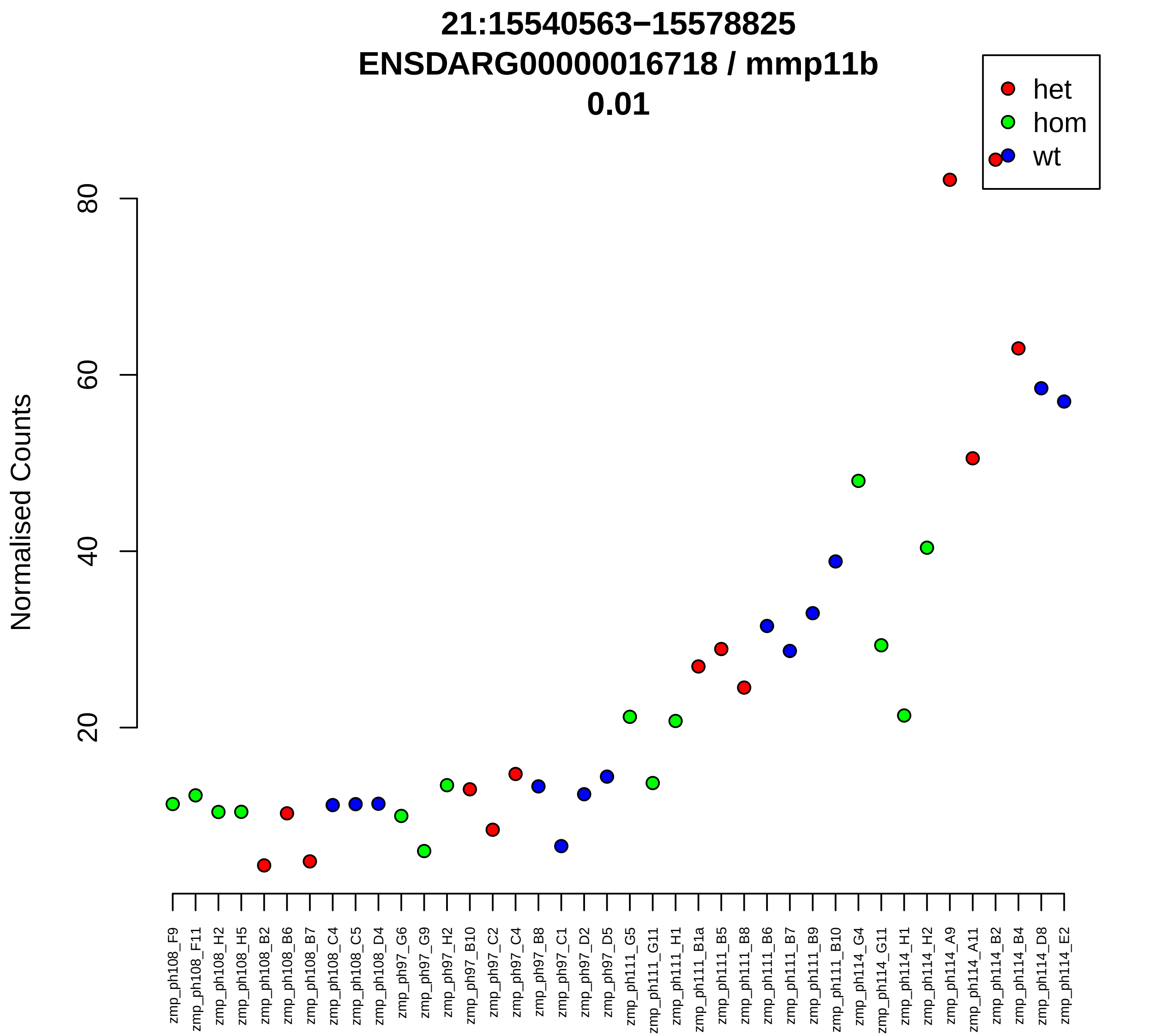

21:15540563−15578825
ENSDARG00000016718 / mmp11b
0.01
het
hom
wt
Normalised Counts
20
40
60
80
zmp_ph108_F9
zmp_ph108_F11
zmp_ph108_H2
zmp_ph108_H5
zmp_ph108_B2
zmp_ph108_B6
zmp_ph108_B7
zmp_ph108_C4
zmp_ph108_C5
zmp_ph108_D4
zmp_ph97_G6
zmp_ph97_G9
zmp_ph97_H2
zmp_ph97_B10
zmp_ph97_C2
zmp_ph97_C4
zmp_ph97_B8
zmp_ph97_C1
zmp_ph97_D2
zmp_ph97_D5
zmp_ph111_G5
zmp_ph111_G11
zmp_ph111_H1
zmp_ph111_B1a
zmp_ph111_B5
zmp_ph111_B8
zmp_ph111_B6
zmp_ph111_B7
zmp_ph111_B9
zmp_ph111_B10
zmp_ph114_G4
zmp_ph114_G11
zmp_ph114_H1
zmp_ph114_H2
zmp_ph114_A9
zmp_ph114_A11
zmp_ph114_B2
zmp_ph114_B4
zmp_ph114_D8
zmp_ph114_E2

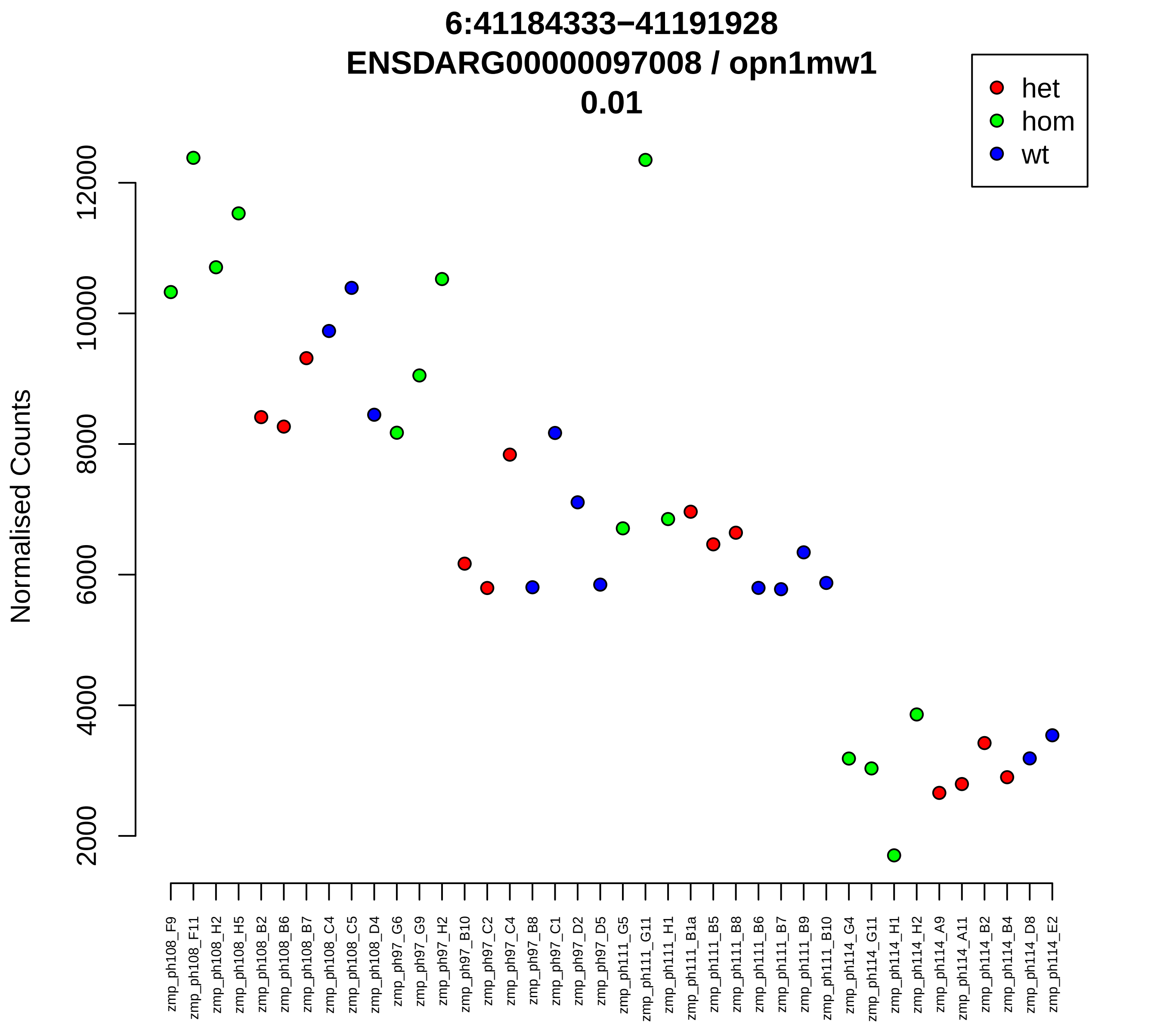

6:41184333−41191928
ENSDARG00000097008 / opn1mw1
0.01
het
hom
wt
Normalised Counts
2000
4000
6000
8000
10000
12000
zmp_ph108_F9
zmp_ph108_F11
zmp_ph108_H2
zmp_ph108_H5
zmp_ph108_B2
zmp_ph108_B6
zmp_ph108_B7
zmp_ph108_C4
zmp_ph108_C5
zmp_ph108_D4
zmp_ph97_G6
zmp_ph97_G9
zmp_ph97_H2
zmp_ph97_B10
zmp_ph97_C2
zmp_ph97_C4
zmp_ph97_B8
zmp_ph97_C1
zmp_ph97_D2
zmp_ph97_D5
zmp_ph111_G5
zmp_ph111_G11
zmp_ph111_H1
zmp_ph111_B1a
zmp_ph111_B5
zmp_ph111_B8
zmp_ph111_B6
zmp_ph111_B7
zmp_ph111_B9
zmp_ph111_B10
zmp_ph114_G4
zmp_ph114_G11
zmp_ph114_H1
zmp_ph114_H2
zmp_ph114_A9
zmp_ph114_A11
zmp_ph114_B2
zmp_ph114_B4
zmp_ph114_D8
zmp_ph114_E2

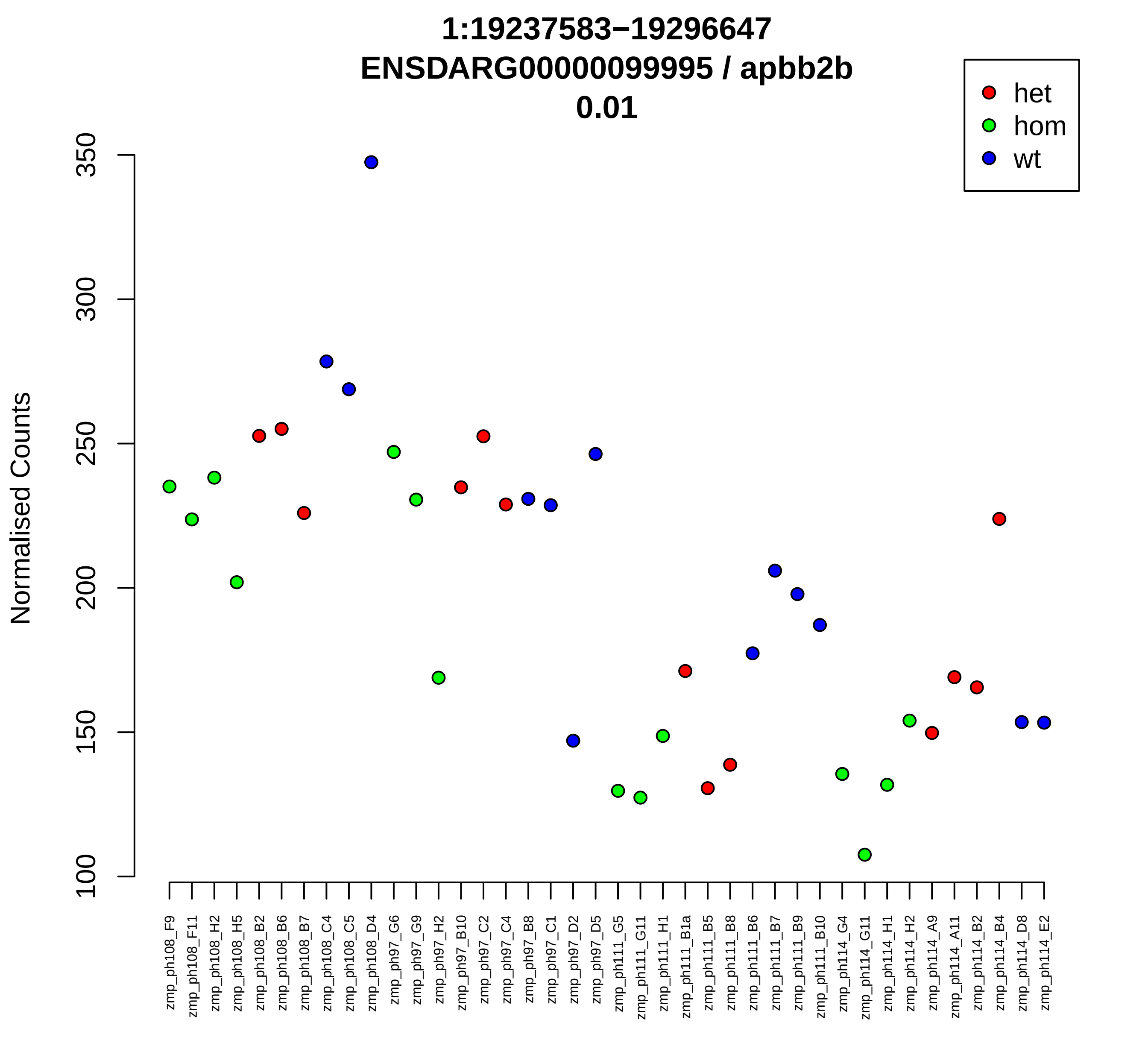
1:19237583−19296647
ENSDARG00000099995 / apbb2b
0.01
het
hom
wt
Normalised Counts
100
150
200
250
300
350
zmp_ph108_F9
zmp_ph108_F11
zmp_ph108_H2
zmp_ph108_H5
zmp_ph108_B2
zmp_ph108_B6
zmp_ph108_B7
zmp_ph108_C4
zmp_ph108_C5
zmp_ph108_D4
zmp_ph97_G6
zmp_ph97_G9
zmp_ph97_H2
zmp_ph97_B10
zmp_ph97_C2
zmp_ph97_C4
zmp_ph97_B8
zmp_ph97_C1
zmp_ph97_D2
zmp_ph97_D5
zmp_ph111_G5
zmp_ph111_G11
zmp_ph111_H1
zmp_ph111_B1a
zmp_ph111_B5
zmp_ph111_B8
zmp_ph111_B6
zmp_ph111_B7
zmp_ph111_B9
zmp_ph111_B10
zmp_ph114_G4
zmp_ph114_G11
zmp_ph114_H1
zmp_ph114_H2
zmp_ph114_A9
zmp_ph114_A11
zmp_ph114_B2
zmp_ph114_B4
zmp_ph114_D8
zmp_ph114_E2

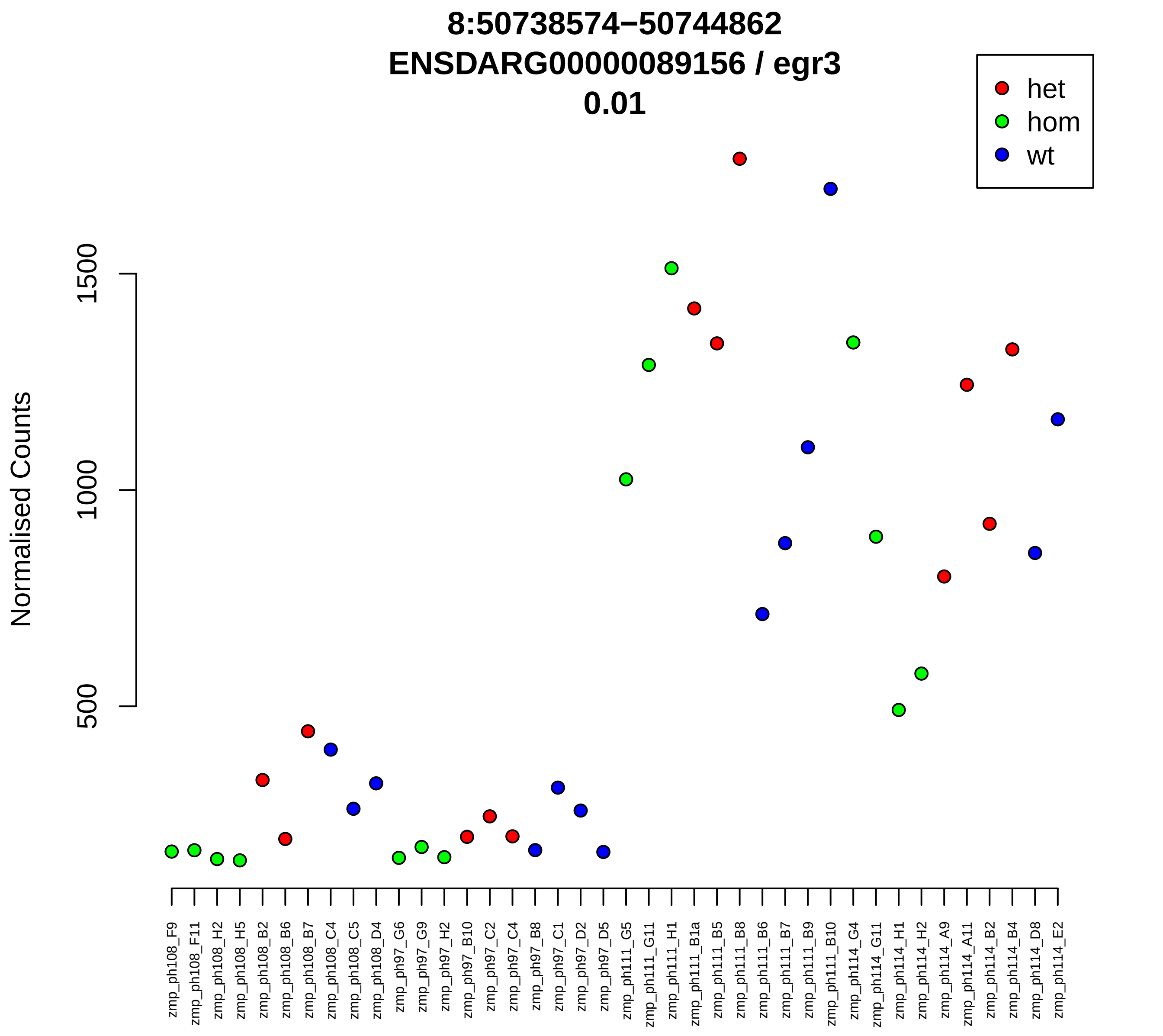

8:50738574−50744862
ENSDARG00000089156 / egr3
0.01
het
hom
wt
Normalised Counts
500
1000
1500
zmp_ph108_F9
zmp_ph108_F11
zmp_ph108_H2
zmp_ph108_H5
zmp_ph108_B2
zmp_ph108_B6
zmp_ph108_B7
zmp_ph108_C4
zmp_ph108_C5
zmp_ph108_D4
zmp_ph97_G6
zmp_ph97_G9
zmp_ph97_H2
zmp_ph97_B10
zmp_ph97_C2
zmp_ph97_C4
zmp_ph97_B8
zmp_ph97_C1
zmp_ph97_D2
zmp_ph97_D5
zmp_ph111_G5
zmp_ph111_G11
zmp_ph111_H1
zmp_ph111_B1a
zmp_ph111_B5
zmp_ph111_B8
zmp_ph111_B6
zmp_ph111_B7
zmp_ph111_B9
zmp_ph111_B10
zmp_ph114_G4
zmp_ph114_G11
zmp_ph114_H1
zmp_ph114_H2
zmp_ph114_A9
zmp_ph114_A11
zmp_ph114_B2
zmp_ph114_B4
zmp_ph114_D8
zmp_ph114_E2

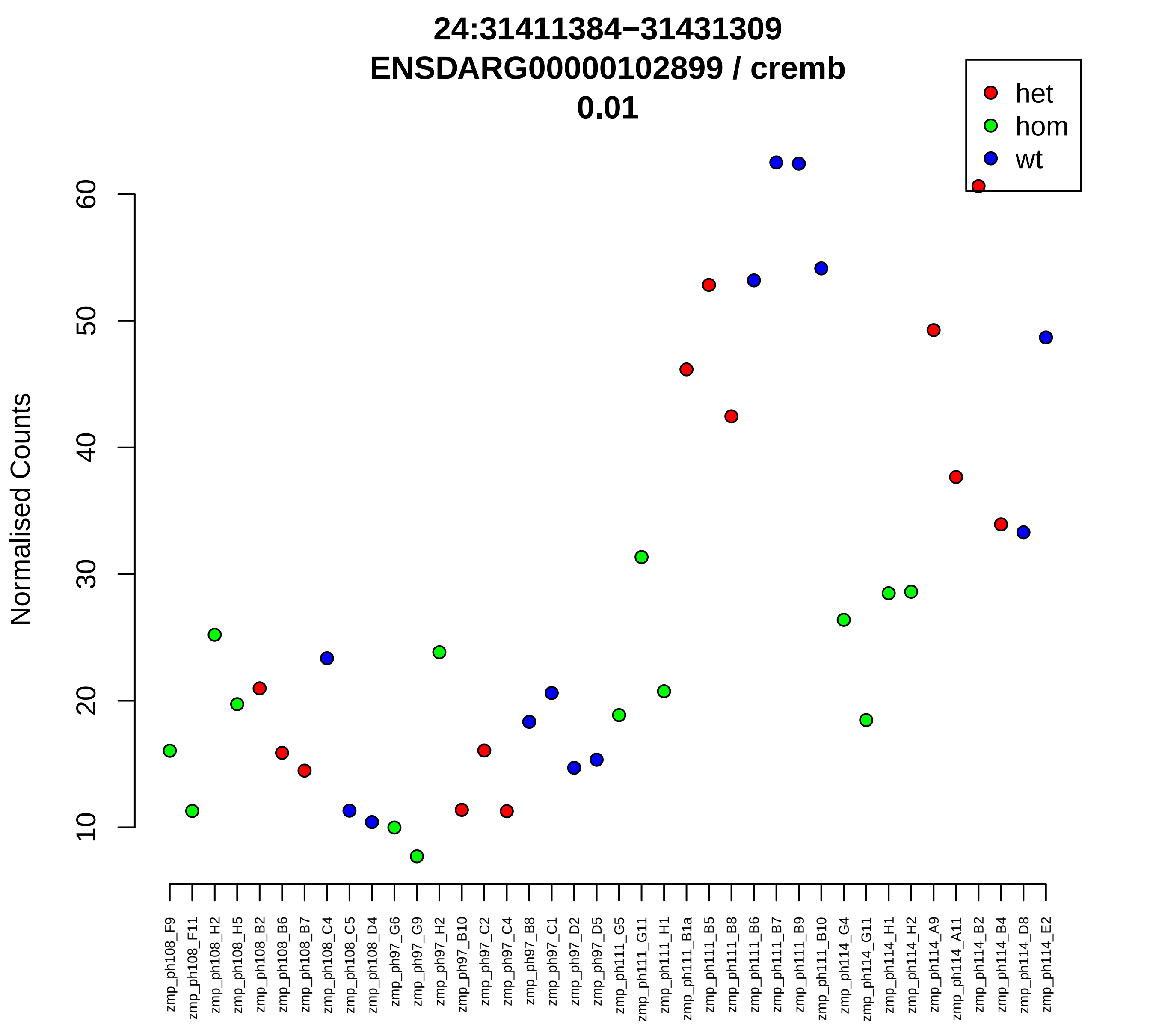
24:31411384−31431309
ENSDARG00000102899 / cremb
0.01
het
hom
wt
Normalised Counts
10
20
30
40
50
60
zmp_ph108_F9
zmp_ph108_F11
zmp_ph108_H2
zmp_ph108_H5
zmp_ph108_B2
zmp_ph108_B6
zmp_ph108_B7
zmp_ph108_C4
zmp_ph108_C5
zmp_ph108_D4
zmp_ph97_G6
zmp_ph97_G9
zmp_ph97_H2
zmp_ph97_B10
zmp_ph97_C2
zmp_ph97_C4
zmp_ph97_B8
zmp_ph97_C1
zmp_ph97_D2
zmp_ph97_D5
zmp_ph111_G5
zmp_ph111_G11
zmp_ph111_H1
zmp_ph111_B1a
zmp_ph111_B5
zmp_ph111_B8
zmp_ph111_B6
zmp_ph111_B7
zmp_ph111_B9
zmp_ph111_B10
zmp_ph114_G4
zmp_ph114_G11
zmp_ph114_H1
zmp_ph114_H2
zmp_ph114_A9
zmp_ph114_A11
zmp_ph114_B2
zmp_ph114_B4
zmp_ph114_D8
zmp_ph114_E2

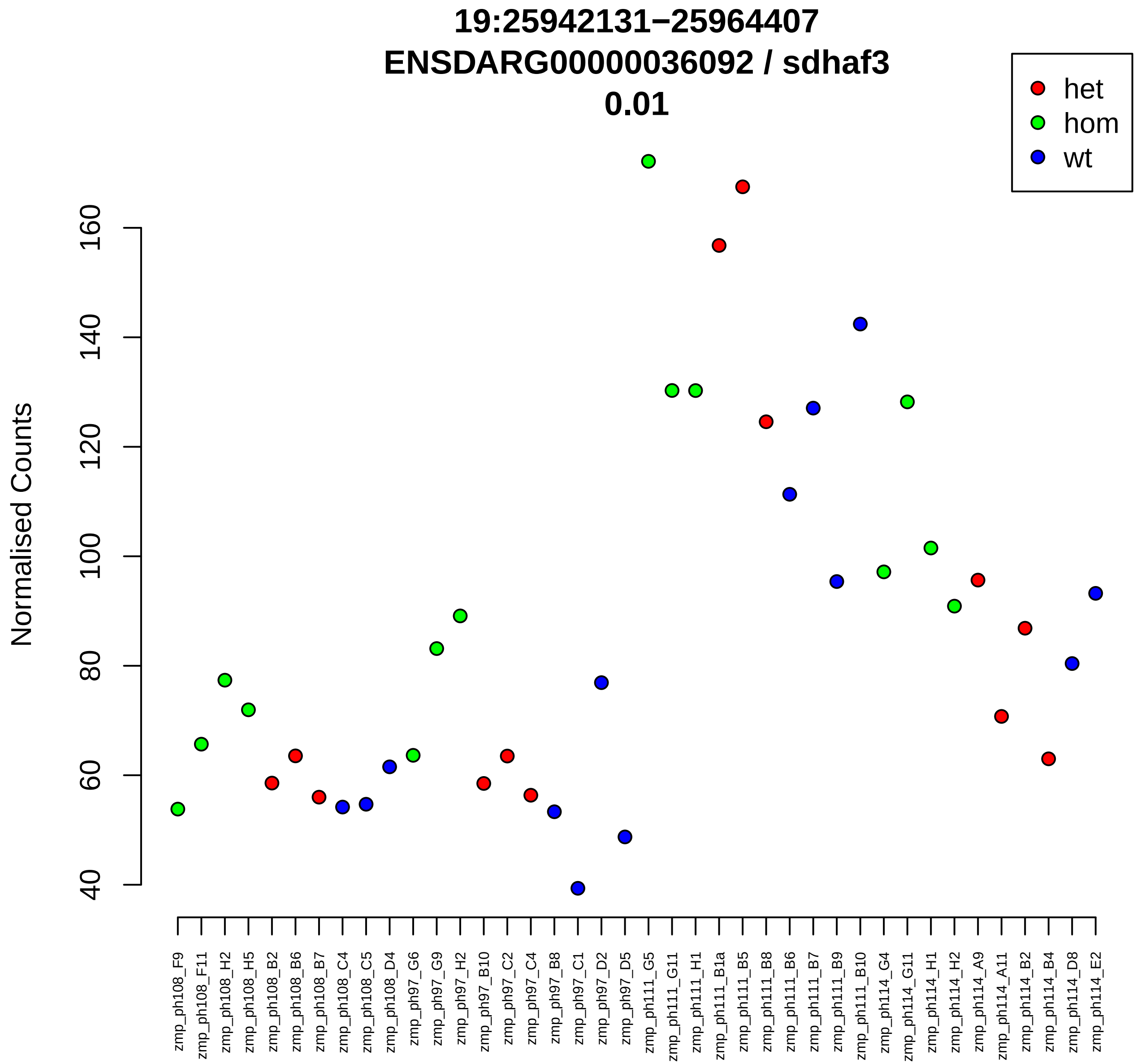

19:25942131−25964407
ENSDARG00000036092 / sdhaf3
0.01
het
hom
wt
Normalised Counts
40
60
80
100
120
140
160
zmp_ph108_F9
zmp_ph108_F11
zmp_ph108_H2
zmp_ph108_H5
zmp_ph108_B2
zmp_ph108_B6
zmp_ph108_B7
zmp_ph108_C4
zmp_ph108_C5
zmp_ph108_D4
zmp_ph97_G6
zmp_ph97_G9
zmp_ph97_H2
zmp_ph97_B10
zmp_ph97_C2
zmp_ph97_C4
zmp_ph97_B8
zmp_ph97_C1
zmp_ph97_D2
zmp_ph97_D5
zmp_ph111_G5
zmp_ph111_G11
zmp_ph111_H1
zmp_ph111_B1a
zmp_ph111_B5
zmp_ph111_B8
zmp_ph111_B6
zmp_ph111_B7
zmp_ph111_B9
zmp_ph111_B10
zmp_ph114_G4
zmp_ph114_G11
zmp_ph114_H1
zmp_ph114_H2
zmp_ph114_A9
zmp_ph114_A11
zmp_ph114_B2
zmp_ph114_B4
zmp_ph114_D8
zmp_ph114_E2

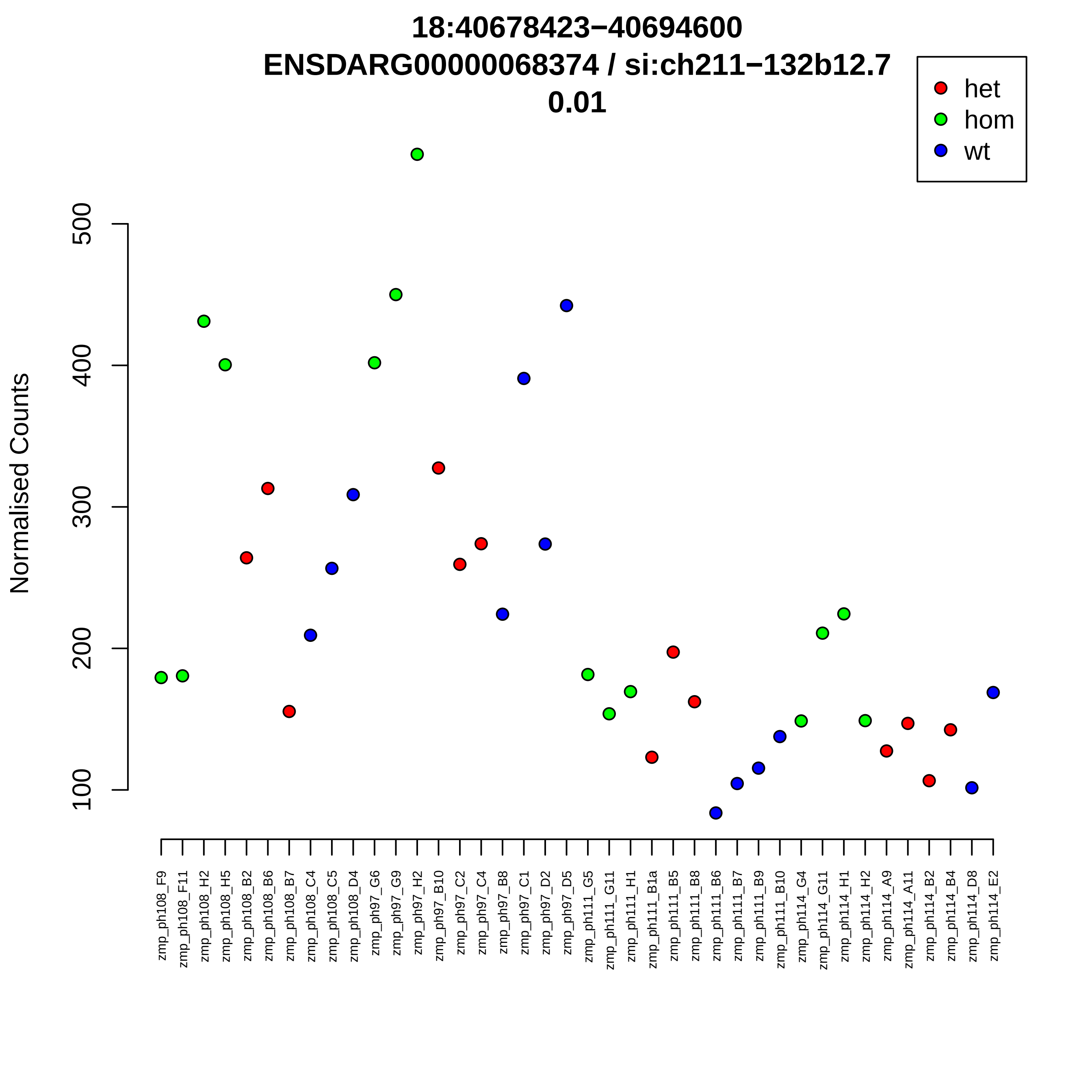

18:40678423−40694600
ENSDARG00000068374 / si:ch211−132b12.7
0.01
het
hom
wt
Normalised Counts
100
200
300
400
500
zmp_ph108_F9
zmp_ph108_F11
zmp_ph108_H2
zmp_ph108_H5
zmp_ph108_B2
zmp_ph108_B6
zmp_ph108_B7
zmp_ph108_C4
zmp_ph108_C5
zmp_ph108_D4
zmp_ph97_G6
zmp_ph97_G9
zmp_ph97_H2
zmp_ph97_B10
zmp_ph97_C2
zmp_ph97_C4
zmp_ph97_B8
zmp_ph97_C1
zmp_ph97_D2
zmp_ph97_D5
zmp_ph111_G5
zmp_ph111_G11
zmp_ph111_H1
zmp_ph111_B1a
zmp_ph111_B5
zmp_ph111_B8
zmp_ph111_B6
zmp_ph111_B7
zmp_ph111_B9
zmp_ph111_B10
zmp_ph114_G4
zmp_ph114_G11
zmp_ph114_H1
zmp_ph114_H2
zmp_ph114_A9
zmp_ph114_A11
zmp_ph114_B2
zmp_ph114_B4
zmp_ph114_D8
zmp_ph114_E2

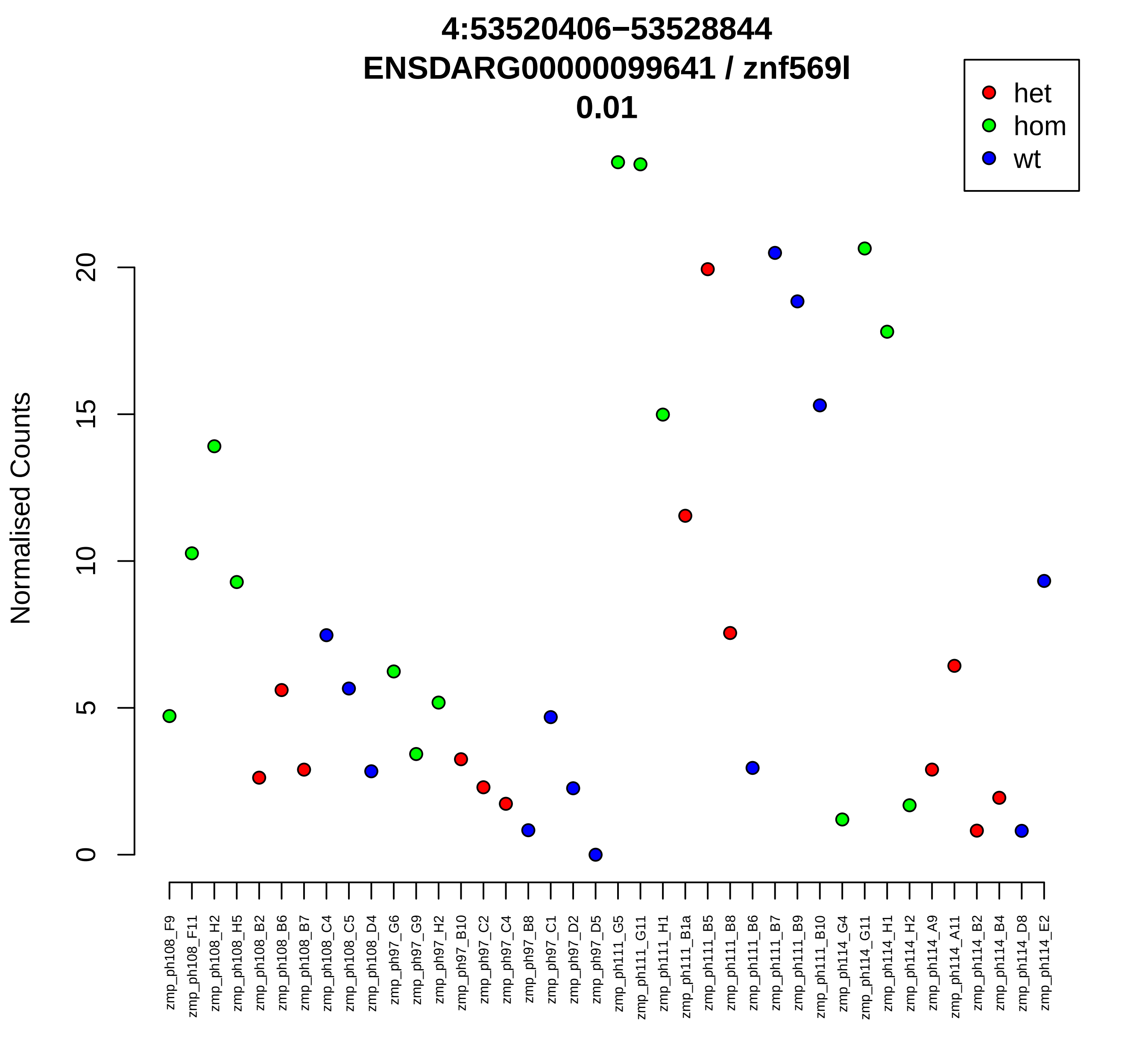

4:53520406−53528844
ENSDARG00000099641 / znf569l
0.01
het
hom
wt
Normalised Counts
0
5
10
15
20
zmp_ph108_F9
zmp_ph108_F11
zmp_ph108_H2
zmp_ph108_H5
zmp_ph108_B2
zmp_ph108_B6
zmp_ph108_B7
zmp_ph108_C4
zmp_ph108_C5
zmp_ph108_D4
zmp_ph97_G6
zmp_ph97_G9
zmp_ph97_H2
zmp_ph97_B10
zmp_ph97_C2
zmp_ph97_C4
zmp_ph97_B8
zmp_ph97_C1
zmp_ph97_D2
zmp_ph97_D5
zmp_ph111_G5
zmp_ph111_G11
zmp_ph111_H1
zmp_ph111_B1a
zmp_ph111_B5
zmp_ph111_B8
zmp_ph111_B6
zmp_ph111_B7
zmp_ph111_B9
zmp_ph111_B10
zmp_ph114_G4
zmp_ph114_G11
zmp_ph114_H1
zmp_ph114_H2
zmp_ph114_A9
zmp_ph114_A11
zmp_ph114_B2
zmp_ph114_B4
zmp_ph114_D8
zmp_ph114_E2

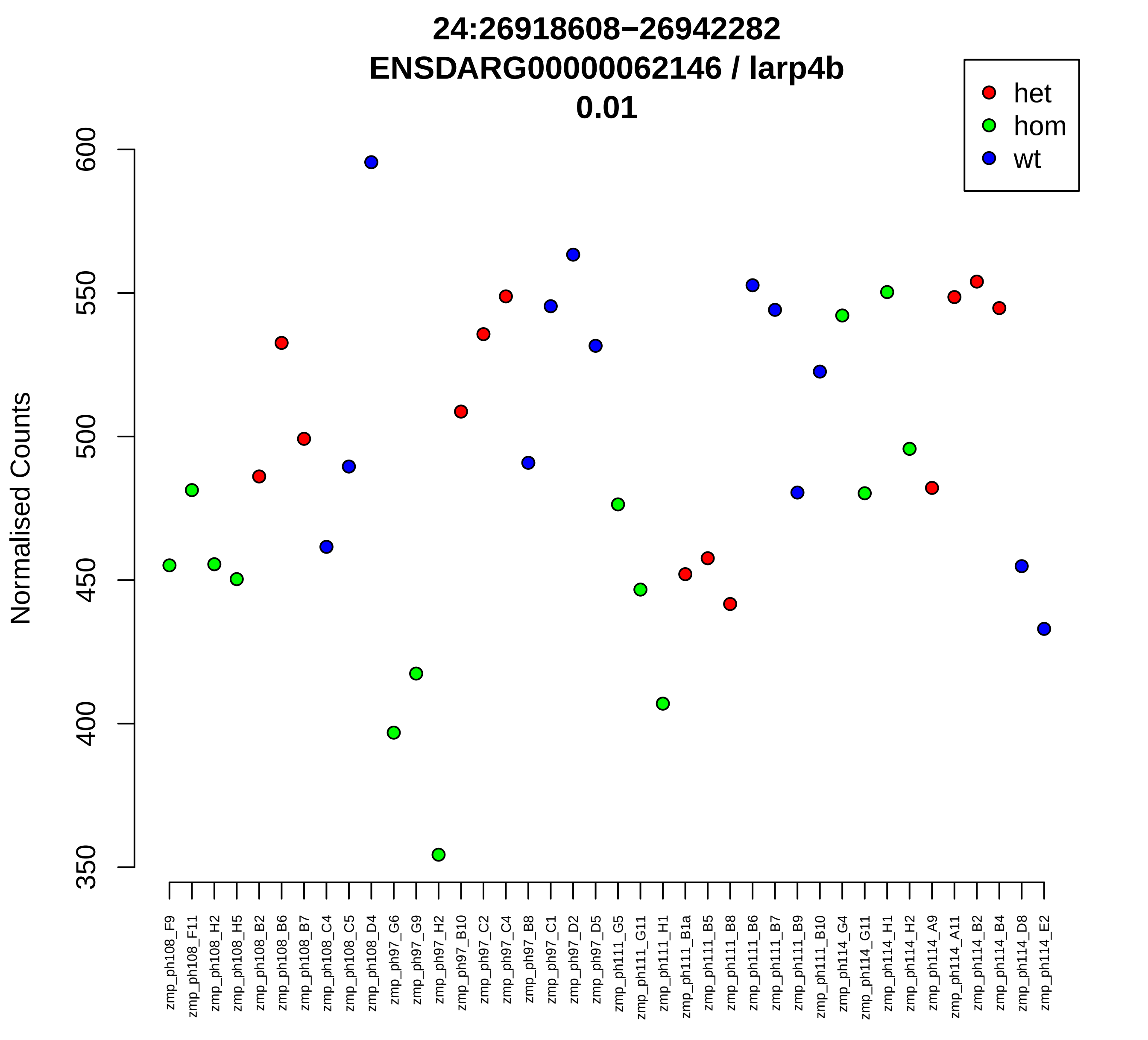
24:26918608−26942282
ENSDARG00000062146 / larp4b
0.01
het
hom
wt
Normalised Counts
350
400
450
500
550
600
zmp_ph108_F9
zmp_ph108_F11
zmp_ph108_H2
zmp_ph108_H5
zmp_ph108_B2
zmp_ph108_B6
zmp_ph108_B7
zmp_ph108_C4
zmp_ph108_C5
zmp_ph108_D4
zmp_ph97_G6
zmp_ph97_G9
zmp_ph97_H2
zmp_ph97_B10
zmp_ph97_C2
zmp_ph97_C4
zmp_ph97_B8
zmp_ph97_C1
zmp_ph97_D2
zmp_ph97_D5
zmp_ph111_G5
zmp_ph111_G11
zmp_ph111_H1
zmp_ph111_B1a
zmp_ph111_B5
zmp_ph111_B8
zmp_ph111_B6
zmp_ph111_B7
zmp_ph111_B9
zmp_ph111_B10
zmp_ph114_G4
zmp_ph114_G11
zmp_ph114_H1
zmp_ph114_H2
zmp_ph114_A9
zmp_ph114_A11
zmp_ph114_B2
zmp_ph114_B4
zmp_ph114_D8
zmp_ph114_E2

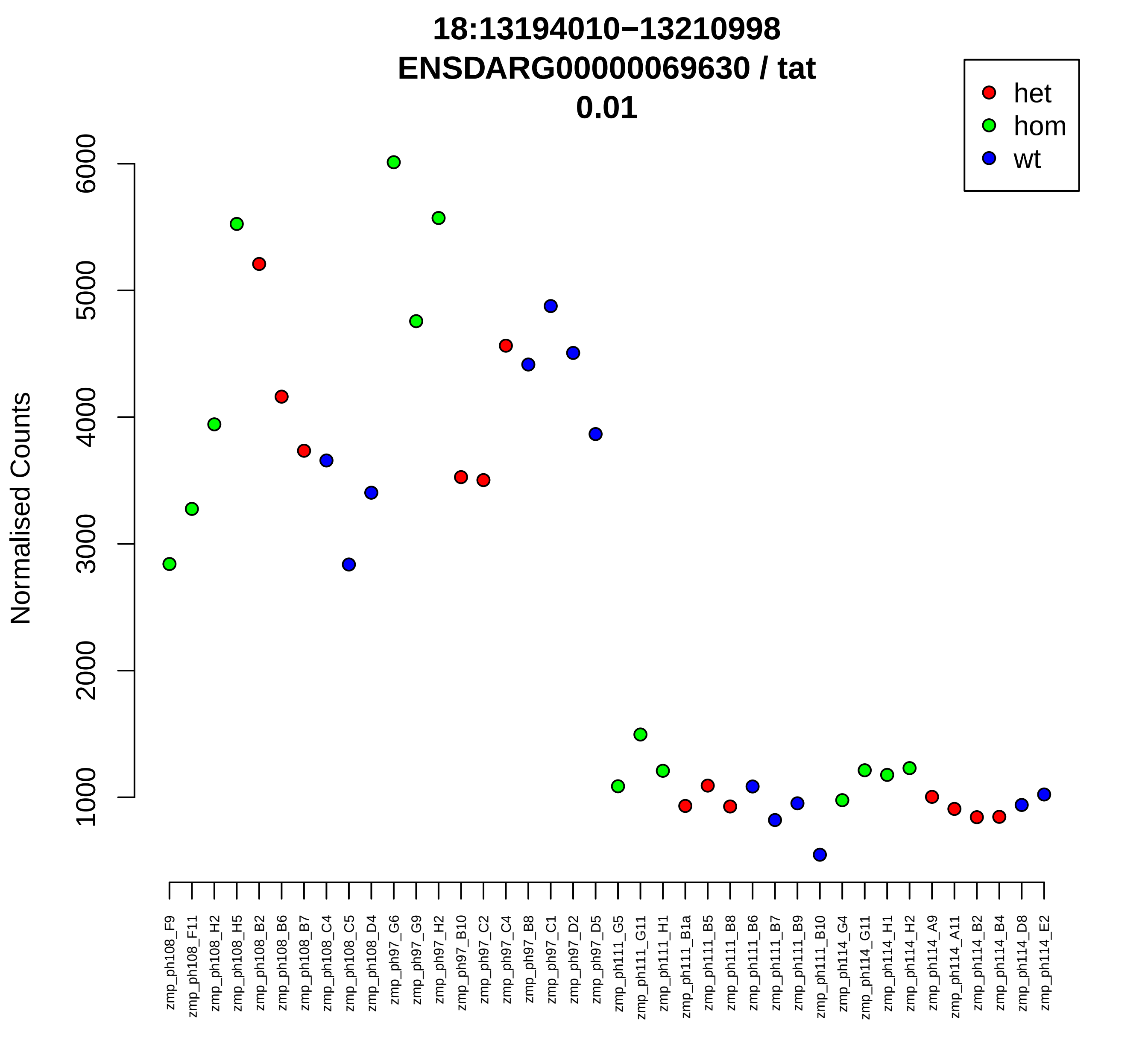

18:13194010−13210998
ENSDARG00000069630 / tat
0.01
het
hom
wt
Normalised Counts
1000
2000
3000
4000
5000
6000
zmp_ph108_F9
zmp_ph108_F11
zmp_ph108_H2
zmp_ph108_H5
zmp_ph108_B2
zmp_ph108_B6
zmp_ph108_B7
zmp_ph108_C4
zmp_ph108_C5
zmp_ph108_D4
zmp_ph97_G6
zmp_ph97_G9
zmp_ph97_H2
zmp_ph97_B10
zmp_ph97_C2
zmp_ph97_C4
zmp_ph97_B8
zmp_ph97_C1
zmp_ph97_D2
zmp_ph97_D5
zmp_ph111_G5
zmp_ph111_G11
zmp_ph111_H1
zmp_ph111_B1a
zmp_ph111_B5
zmp_ph111_B8
zmp_ph111_B6
zmp_ph111_B7
zmp_ph111_B9
zmp_ph111_B10
zmp_ph114_G4
zmp_ph114_G11
zmp_ph114_H1
zmp_ph114_H2
zmp_ph114_A9
zmp_ph114_A11
zmp_ph114_B2
zmp_ph114_B4
zmp_ph114_D8
zmp_ph114_E2

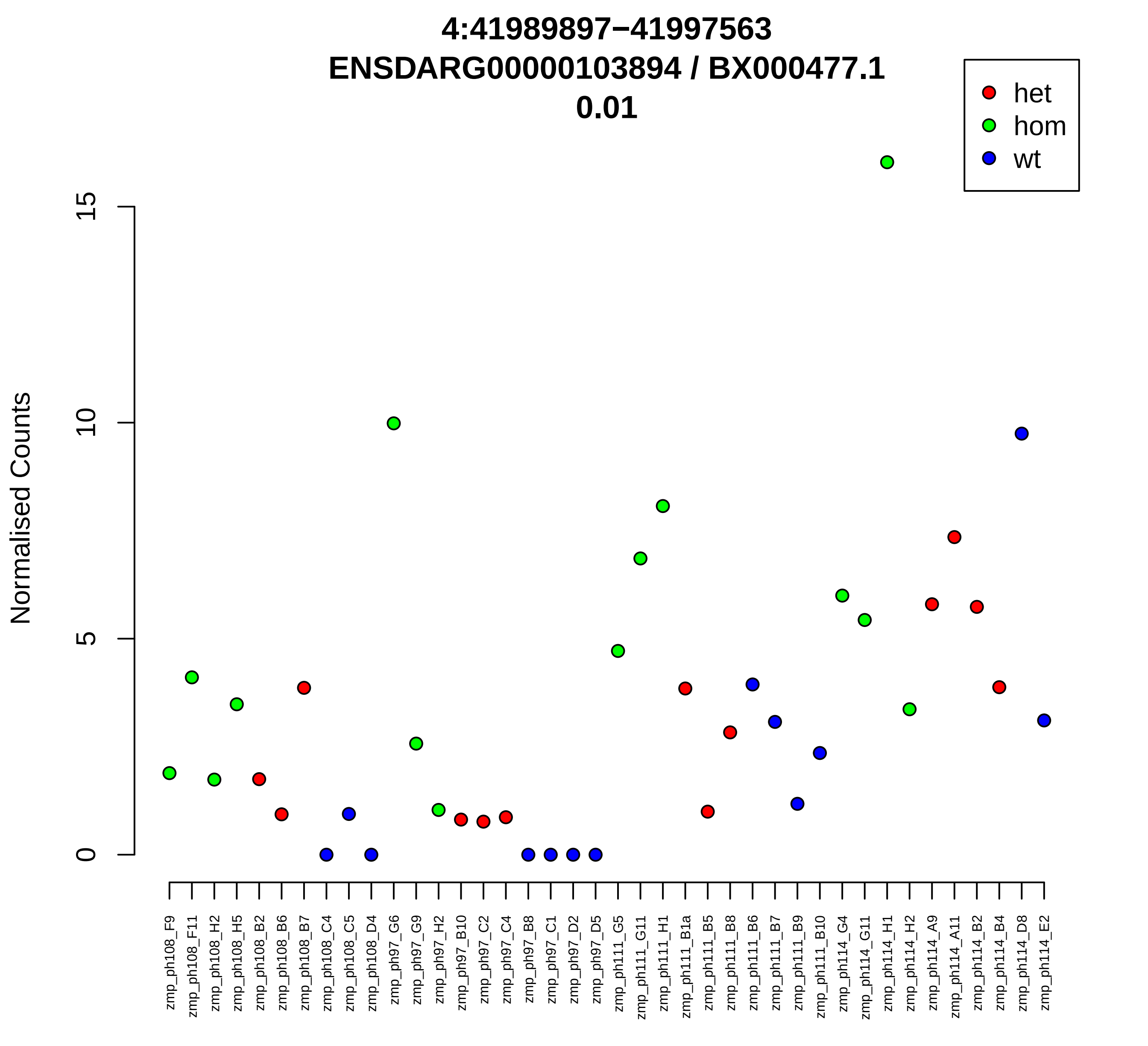

4:41989897−41997563
ENSDARG00000103894 / BX000477.1
0.01
het
hom
wt
Normalised Counts
0
5
10
15
zmp_ph108_F9
zmp_ph108_F11
zmp_ph108_H2
zmp_ph108_H5
zmp_ph108_B2
zmp_ph108_B6
zmp_ph108_B7
zmp_ph108_C4
zmp_ph108_C5
zmp_ph108_D4
zmp_ph97_G6
zmp_ph97_G9
zmp_ph97_H2
zmp_ph97_B10
zmp_ph97_C2
zmp_ph97_C4
zmp_ph97_B8
zmp_ph97_C1
zmp_ph97_D2
zmp_ph97_D5
zmp_ph111_G5
zmp_ph111_G11
zmp_ph111_H1
zmp_ph111_B1a
zmp_ph111_B5
zmp_ph111_B8
zmp_ph111_B6
zmp_ph111_B7
zmp_ph111_B9
zmp_ph111_B10
zmp_ph114_G4
zmp_ph114_G11
zmp_ph114_H1
zmp_ph114_H2
zmp_ph114_A9
zmp_ph114_A11
zmp_ph114_B2
zmp_ph114_B4
zmp_ph114_D8
zmp_ph114_E2

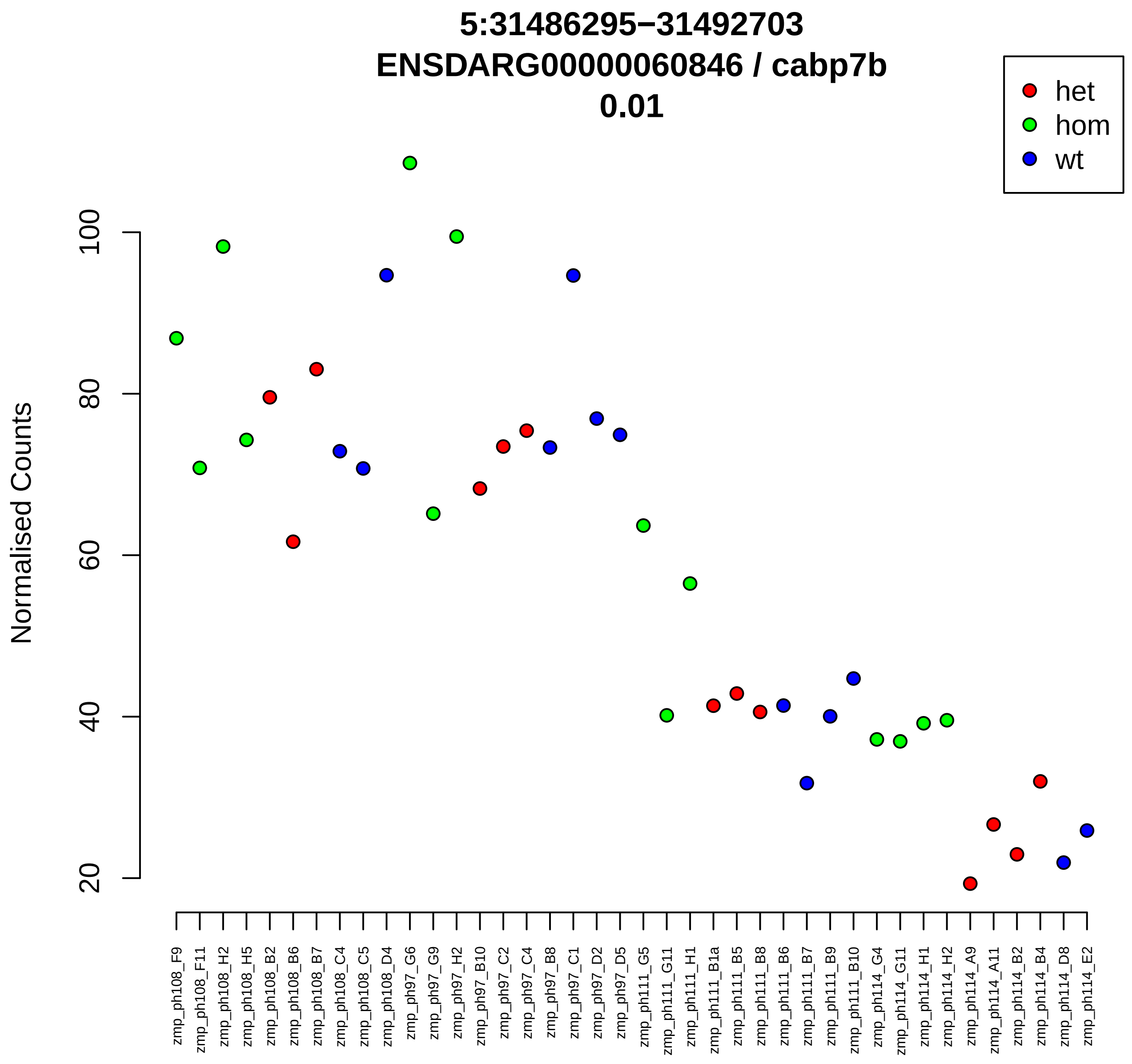

5:31486295−31492703
ENSDARG00000060846 / cabp7b
0.01
het
hom
wt
Normalised Counts
20
40
60
80
100
zmp_ph108_F9
zmp_ph108_F11
zmp_ph108_H2
zmp_ph108_H5
zmp_ph108_B2
zmp_ph108_B6
zmp_ph108_B7
zmp_ph108_C4
zmp_ph108_C5
zmp_ph108_D4
zmp_ph97_G6
zmp_ph97_G9
zmp_ph97_H2
zmp_ph97_B10
zmp_ph97_C2
zmp_ph97_C4
zmp_ph97_B8
zmp_ph97_C1
zmp_ph97_D2
zmp_ph97_D5
zmp_ph111_G5
zmp_ph111_G11
zmp_ph111_H1
zmp_ph111_B1a
zmp_ph111_B5
zmp_ph111_B8
zmp_ph111_B6
zmp_ph111_B7
zmp_ph111_B9
zmp_ph111_B10
zmp_ph114_G4
zmp_ph114_G11
zmp_ph114_H1
zmp_ph114_H2
zmp_ph114_A9
zmp_ph114_A11
zmp_ph114_B2
zmp_ph114_B4
zmp_ph114_D8
zmp_ph114_E2

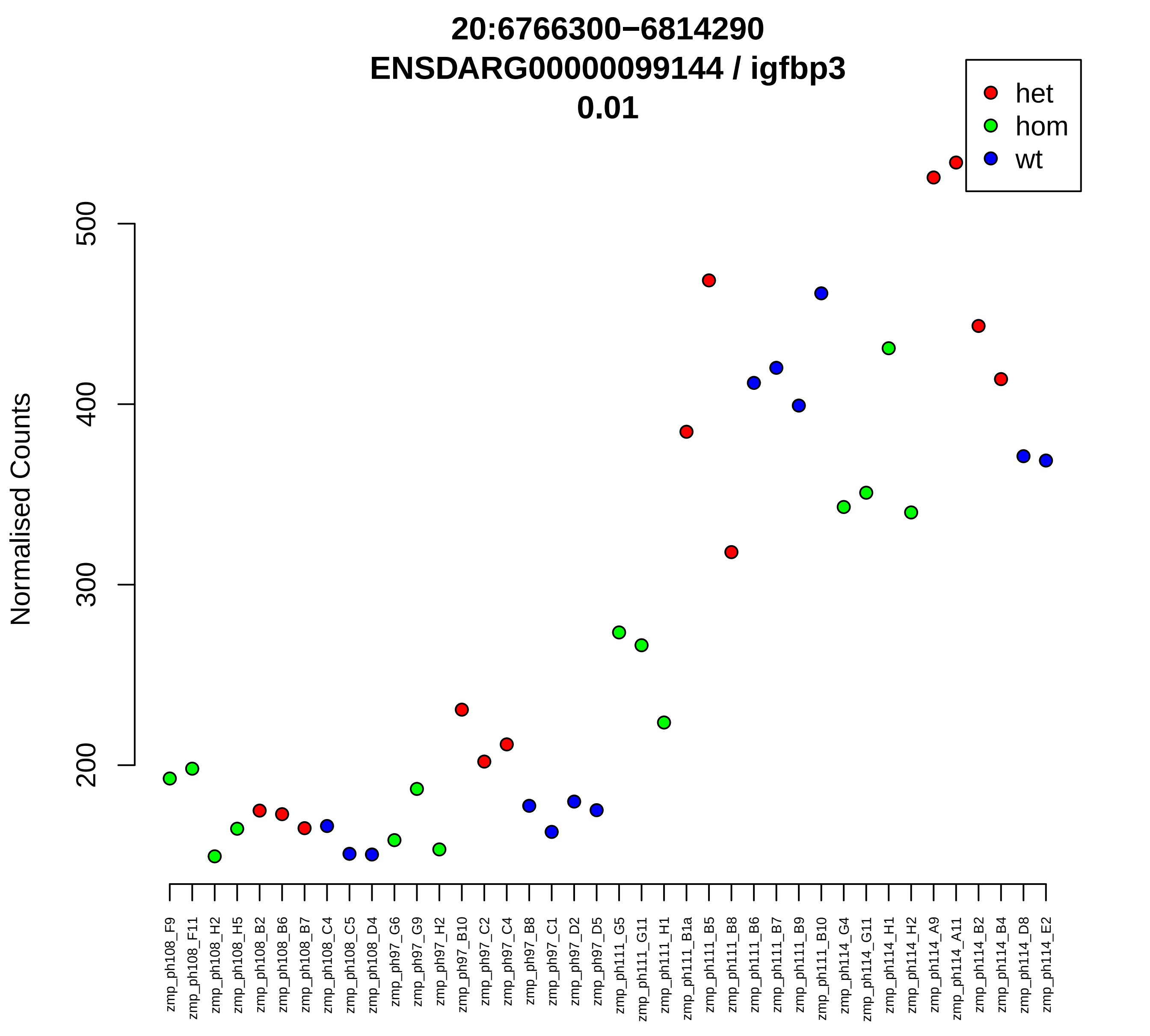
20:6766300−6814290
ENSDARG00000099144 / igfbp3
0.01
het
hom
wt
Normalised Counts
200
300
400
500
zmp_ph108_F9
zmp_ph108_F11
zmp_ph108_H2
zmp_ph108_H5
zmp_ph108_B2
zmp_ph108_B6
zmp_ph108_B7
zmp_ph108_C4
zmp_ph108_C5
zmp_ph108_D4
zmp_ph97_G6
zmp_ph97_G9
zmp_ph97_H2
zmp_ph97_B10
zmp_ph97_C2
zmp_ph97_C4
zmp_ph97_B8
zmp_ph97_C1
zmp_ph97_D2
zmp_ph97_D5
zmp_ph111_G5
zmp_ph111_G11
zmp_ph111_H1
zmp_ph111_B1a
zmp_ph111_B5
zmp_ph111_B8
zmp_ph111_B6
zmp_ph111_B7
zmp_ph111_B9
zmp_ph111_B10
zmp_ph114_G4
zmp_ph114_G11
zmp_ph114_H1
zmp_ph114_H2
zmp_ph114_A9
zmp_ph114_A11
zmp_ph114_B2
zmp_ph114_B4
zmp_ph114_D8
zmp_ph114_E2

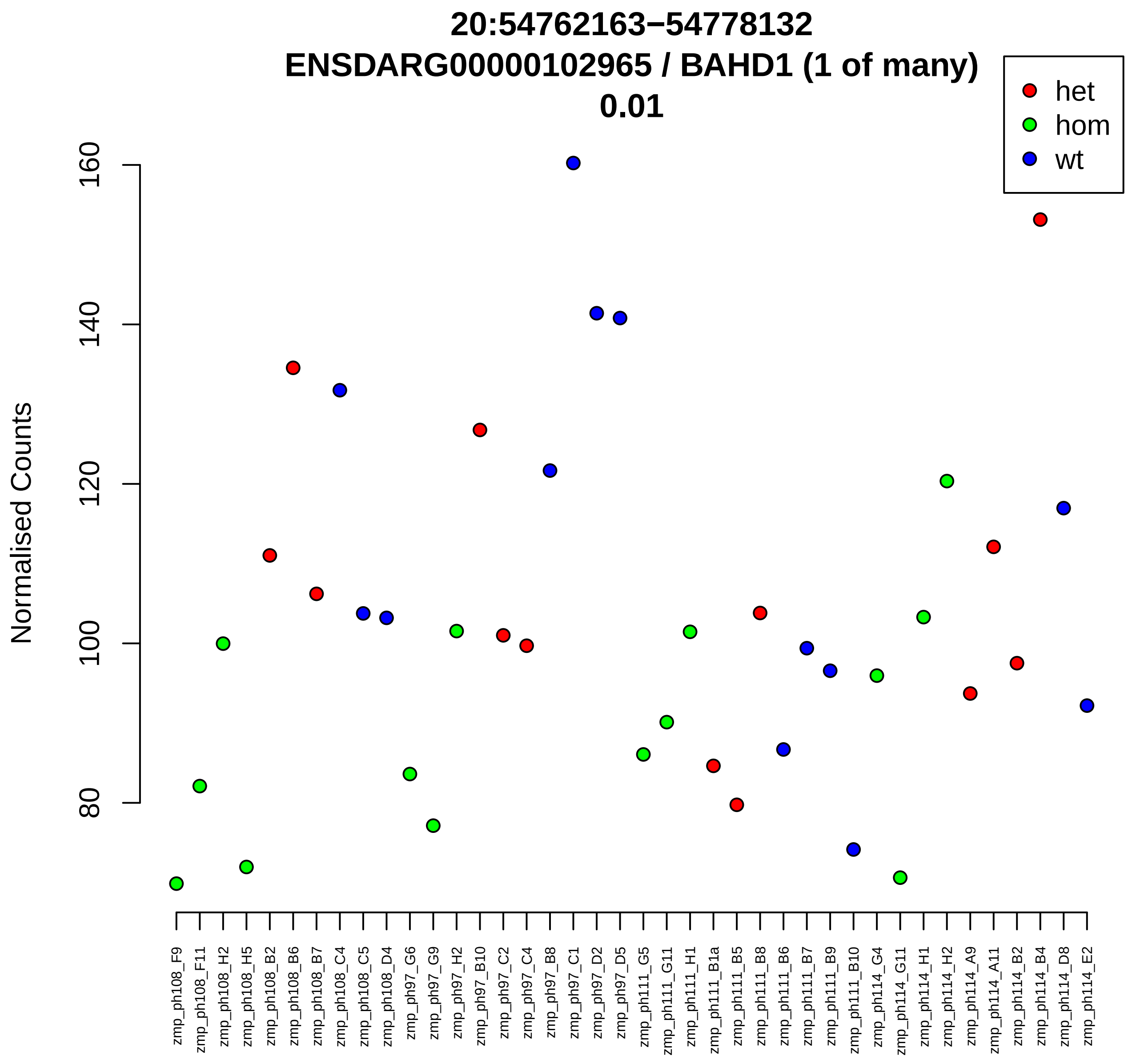
20:54762163−54778132
ENSDARG00000102965 / BAHD1 (1 of many)
0.01
het
hom
wt
Normalised Counts
80
100
120
140
160
zmp_ph108_F9
zmp_ph108_F11
zmp_ph108_H2
zmp_ph108_H5
zmp_ph108_B2
zmp_ph108_B6
zmp_ph108_B7
zmp_ph108_C4
zmp_ph108_C5
zmp_ph108_D4
zmp_ph97_G6
zmp_ph97_G9
zmp_ph97_H2
zmp_ph97_B10
zmp_ph97_C2
zmp_ph97_C4
zmp_ph97_B8
zmp_ph97_C1
zmp_ph97_D2
zmp_ph97_D5
zmp_ph111_G5
zmp_ph111_G11
zmp_ph111_H1
zmp_ph111_B1a
zmp_ph111_B5
zmp_ph111_B8
zmp_ph111_B6
zmp_ph111_B7
zmp_ph111_B9
zmp_ph111_B10
zmp_ph114_G4
zmp_ph114_G11
zmp_ph114_H1
zmp_ph114_H2
zmp_ph114_A9
zmp_ph114_A11
zmp_ph114_B2
zmp_ph114_B4
zmp_ph114_D8
zmp_ph114_E2

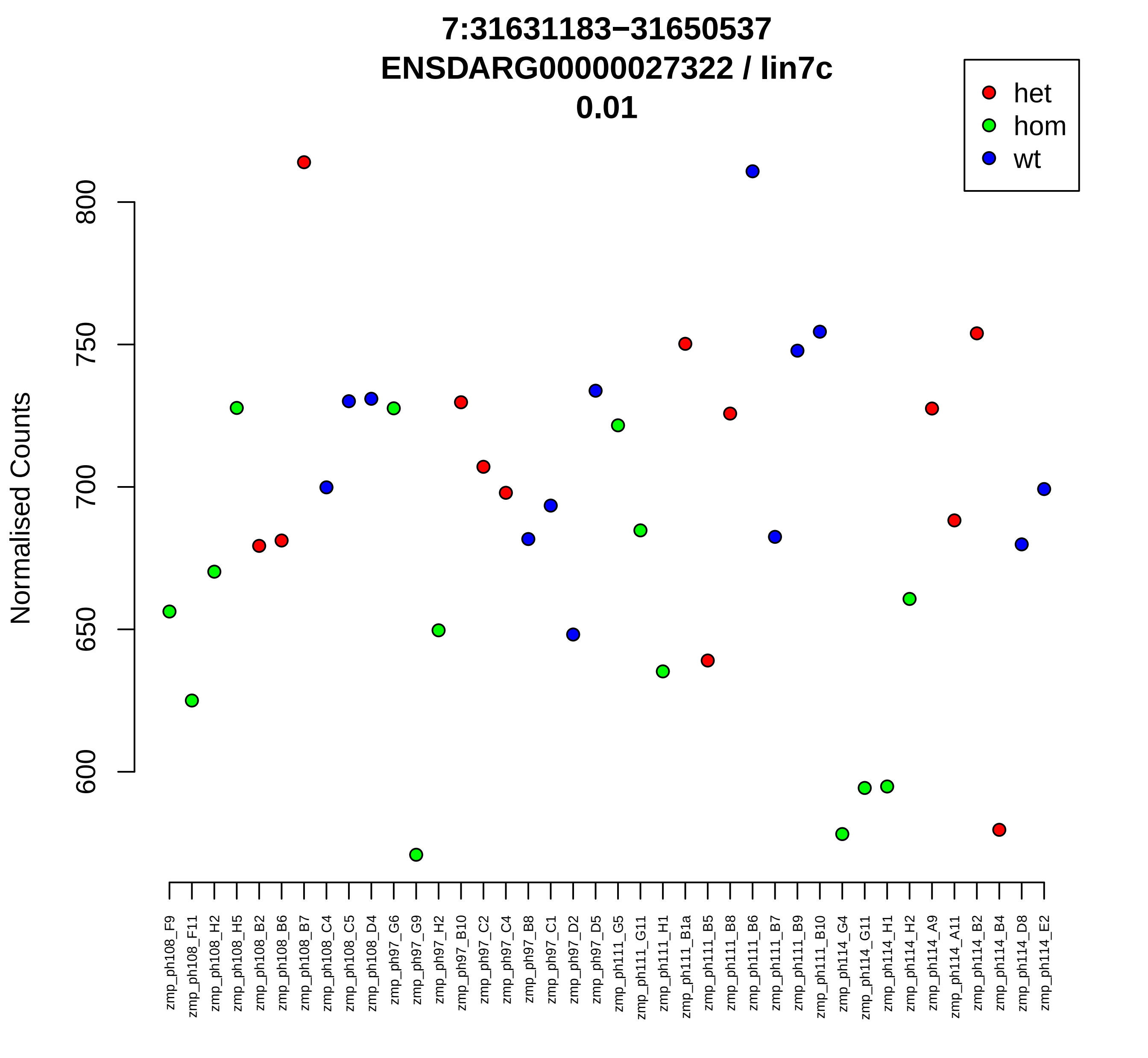
7:31631183−31650537
ENSDARG00000027322 / lin7c
0.01
het
hom
wt
Normalised Counts
800
750
700
650
600
zmp_ph108_F9
zmp_ph108_F11
zmp_ph108_H2
zmp_ph108_H5
zmp_ph108_B2
zmp_ph108_B6
zmp_ph108_B7
zmp_ph108_C4
zmp_ph108_C5
zmp_ph108_D4
zmp_ph97_G6
zmp_ph97_G9
zmp_ph97_H2
zmp_ph97_B10
zmp_ph97_C2
zmp_ph97_C4
zmp_ph97_B8
zmp_ph97_C1
zmp_ph97_D2
zmp_ph97_D5
zmp_ph111_G5
zmp_ph111_G11
zmp_ph111_H1
zmp_ph111_B1a
zmp_ph111_B5
zmp_ph111_B8
zmp_ph111_B6
zmp_ph111_B7
zmp_ph111_B9
zmp_ph111_B10
zmp_ph114_G4
zmp_ph114_G11
zmp_ph114_H1
zmp_ph114_H2
zmp_ph114_A9
zmp_ph114_A11
zmp_ph114_B2
zmp_ph114_B4
zmp_ph114_D8
zmp_ph114_E2

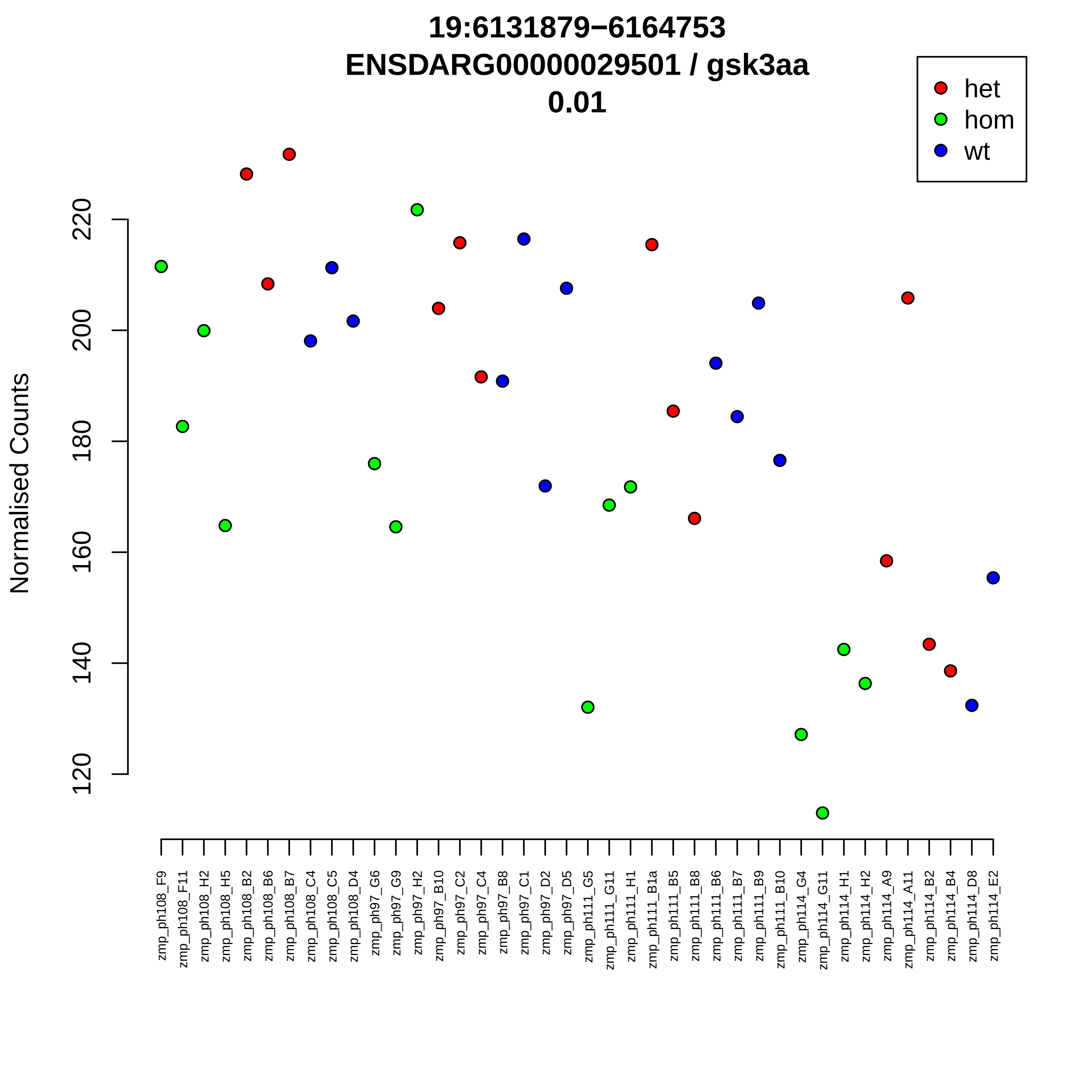
19:6131879−6164753
ENSDARG00000029501 / gsk3aa
0.01
het
hom
wt
Normalised Counts
120
140
160
180
200
220
zmp_ph108_F9
zmp_ph108_F11
zmp_ph108_H2
zmp_ph108_H5
zmp_ph108_B2
zmp_ph108_B6
zmp_ph108_B7
zmp_ph108_C4
zmp_ph108_C5
zmp_ph108_D4
zmp_ph97_G6
zmp_ph97_G9
zmp_ph97_H2
zmp_ph97_B10
zmp_ph97_C2
zmp_ph97_C4
zmp_ph97_B8
zmp_ph97_C1
zmp_ph97_D2
zmp_ph97_D5
zmp_ph111_G5
zmp_ph111_G11
zmp_ph111_H1
zmp_ph111_B1a
zmp_ph111_B5
zmp_ph111_B8
zmp_ph111_B6
zmp_ph111_B7
zmp_ph111_B9
zmp_ph111_B10
zmp_ph114_G4
zmp_ph114_G11
zmp_ph114_H1
zmp_ph114_H2
zmp_ph114_A9
zmp_ph114_A11
zmp_ph114_B2
zmp_ph114_B4
zmp_ph114_D8
zmp_ph114_E2

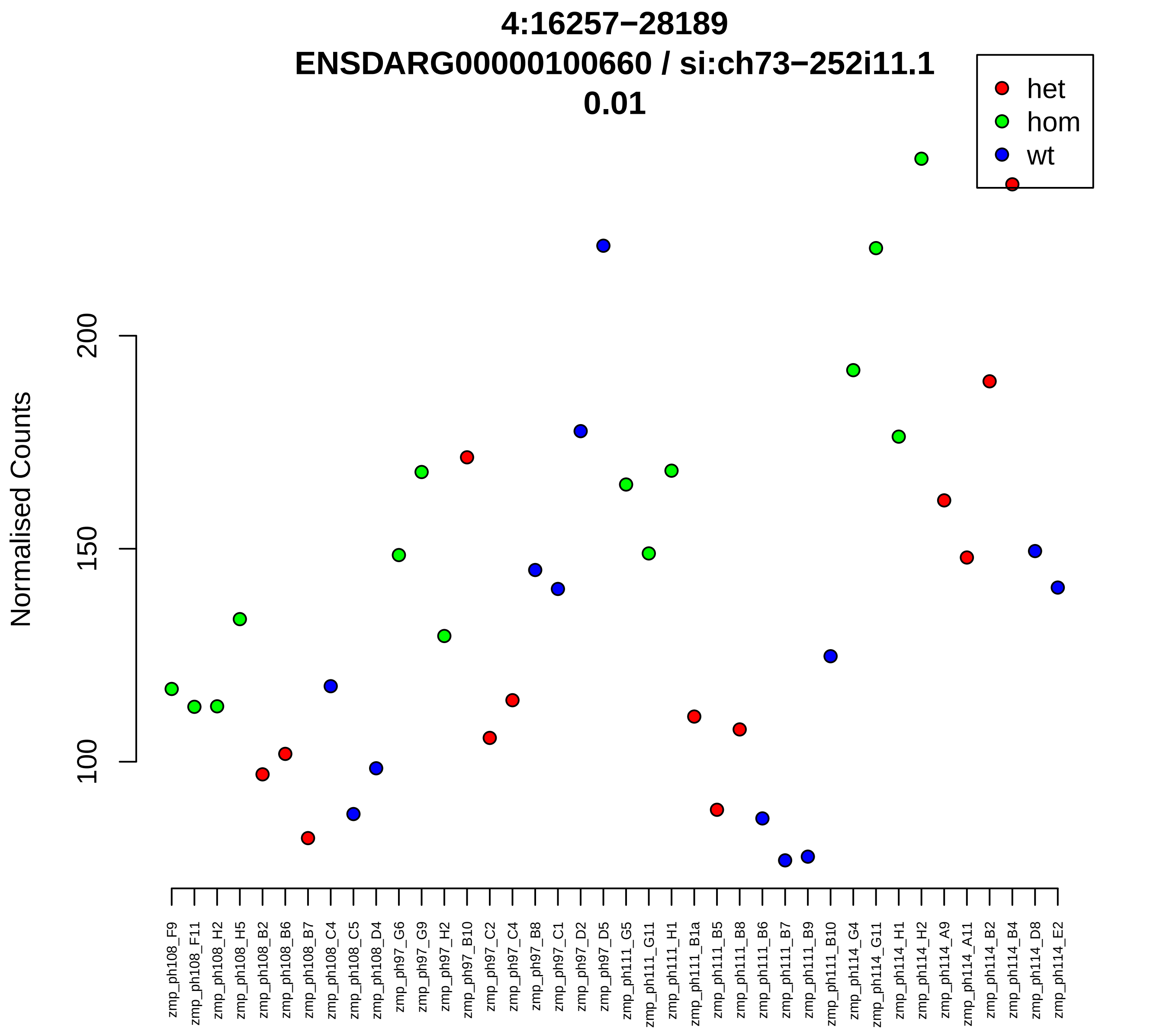
4:16257−28189
ENSDARG00000100660 / si:ch73−252i11.1
0.01
het
hom
wt
Normalised Counts
100
150
200
zmp_ph108_F9
zmp_ph108_F11
zmp_ph108_H2
zmp_ph108_H5
zmp_ph108_B2
zmp_ph108_B6
zmp_ph108_B7
zmp_ph108_C4
zmp_ph108_C5
zmp_ph108_D4
zmp_ph97_G6
zmp_ph97_G9
zmp_ph97_H2
zmp_ph97_B10
zmp_ph97_C2
zmp_ph97_C4
zmp_ph97_B8
zmp_ph97_C1
zmp_ph97_D2
zmp_ph97_D5
zmp_ph111_G5
zmp_ph111_G11
zmp_ph111_H1
zmp_ph111_B1a
zmp_ph111_B5
zmp_ph111_B8
zmp_ph111_B6
zmp_ph111_B7
zmp_ph111_B9
zmp_ph111_B10
zmp_ph114_G4
zmp_ph114_G11
zmp_ph114_H1
zmp_ph114_H2
zmp_ph114_A9
zmp_ph114_A11
zmp_ph114_B2
zmp_ph114_B4
zmp_ph114_D8
zmp_ph114_E2

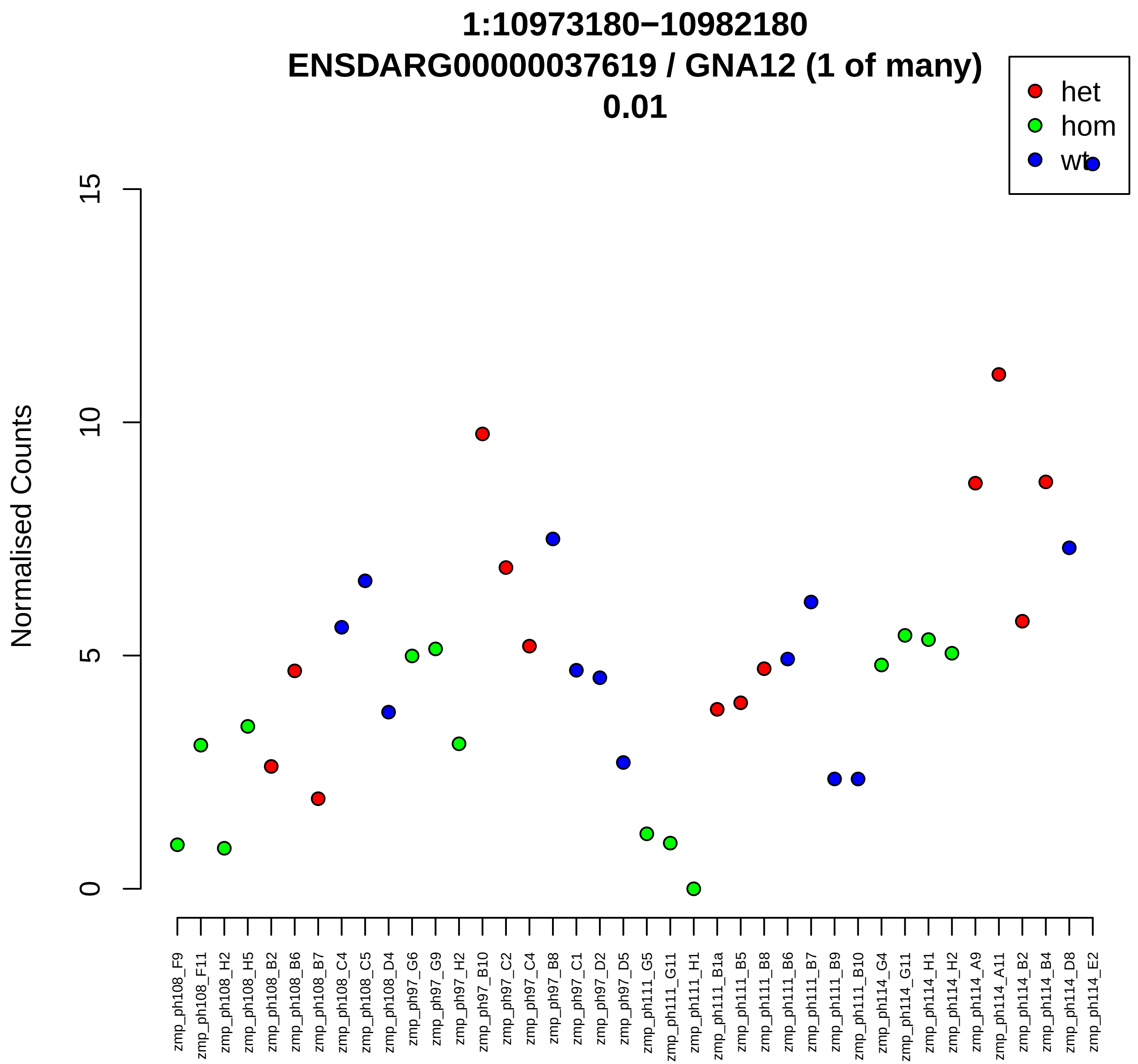

1:10973180−10982180
ENSDARG00000037619 / GNA12 (1 of many)
0.01
het
hom
wt
Normalised Counts
0
5
10
15
zmp_ph108_F9
zmp_ph108_F11
zmp_ph108_H2
zmp_ph108_H5
zmp_ph108_B2
zmp_ph108_B6
zmp_ph108_B7
zmp_ph108_C4
zmp_ph108_C5
zmp_ph108_D4
zmp_ph97_G6
zmp_ph97_G9
zmp_ph97_H2
zmp_ph97_B10
zmp_ph97_C2
zmp_ph97_C4
zmp_ph97_B8
zmp_ph97_C1
zmp_ph97_D2
zmp_ph97_D5
zmp_ph111_G5
zmp_ph111_G11
zmp_ph111_H1
zmp_ph111_B1a
zmp_ph111_B5
zmp_ph111_B8
zmp_ph111_B6
zmp_ph111_B7
zmp_ph111_B9
zmp_ph111_B10
zmp_ph114_G4
zmp_ph114_G11
zmp_ph114_H1
zmp_ph114_H2
zmp_ph114_A9
zmp_ph114_A11
zmp_ph114_B2
zmp_ph114_B4
zmp_ph114_D8
zmp_ph114_E2

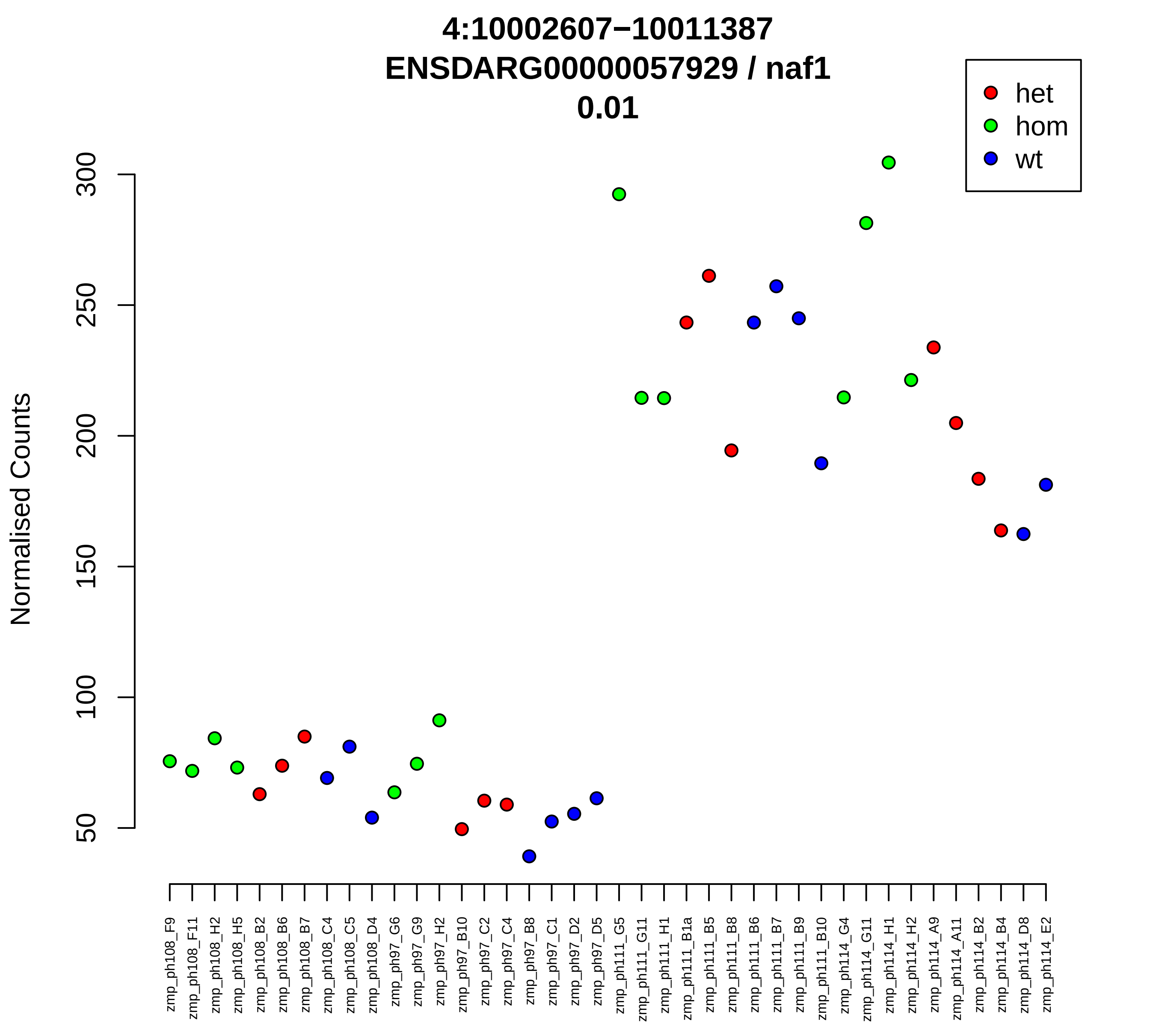

4:10002607−10011387
ENSDARG00000057929 / naf1
0.01
het
hom
wt
Normalised Counts
50
100
150
200
250
300
zmp_ph108_F9
zmp_ph108_F11
zmp_ph108_H2
zmp_ph108_H5
zmp_ph108_B2
zmp_ph108_B6
zmp_ph108_B7
zmp_ph108_C4
zmp_ph108_C5
zmp_ph108_D4
zmp_ph97_G6
zmp_ph97_G9
zmp_ph97_H2
zmp_ph97_B10
zmp_ph97_C2
zmp_ph97_C4
zmp_ph97_B8
zmp_ph97_C1
zmp_ph97_D2
zmp_ph97_D5
zmp_ph111_G5
zmp_ph111_G11
zmp_ph111_H1
zmp_ph111_B1a
zmp_ph111_B5
zmp_ph111_B8
zmp_ph111_B6
zmp_ph111_B7
zmp_ph111_B9
zmp_ph111_B10
zmp_ph114_G4
zmp_ph114_G11
zmp_ph114_H1
zmp_ph114_H2
zmp_ph114_A9
zmp_ph114_A11
zmp_ph114_B2
zmp_ph114_B4
zmp_ph114_D8
zmp_ph114_E2

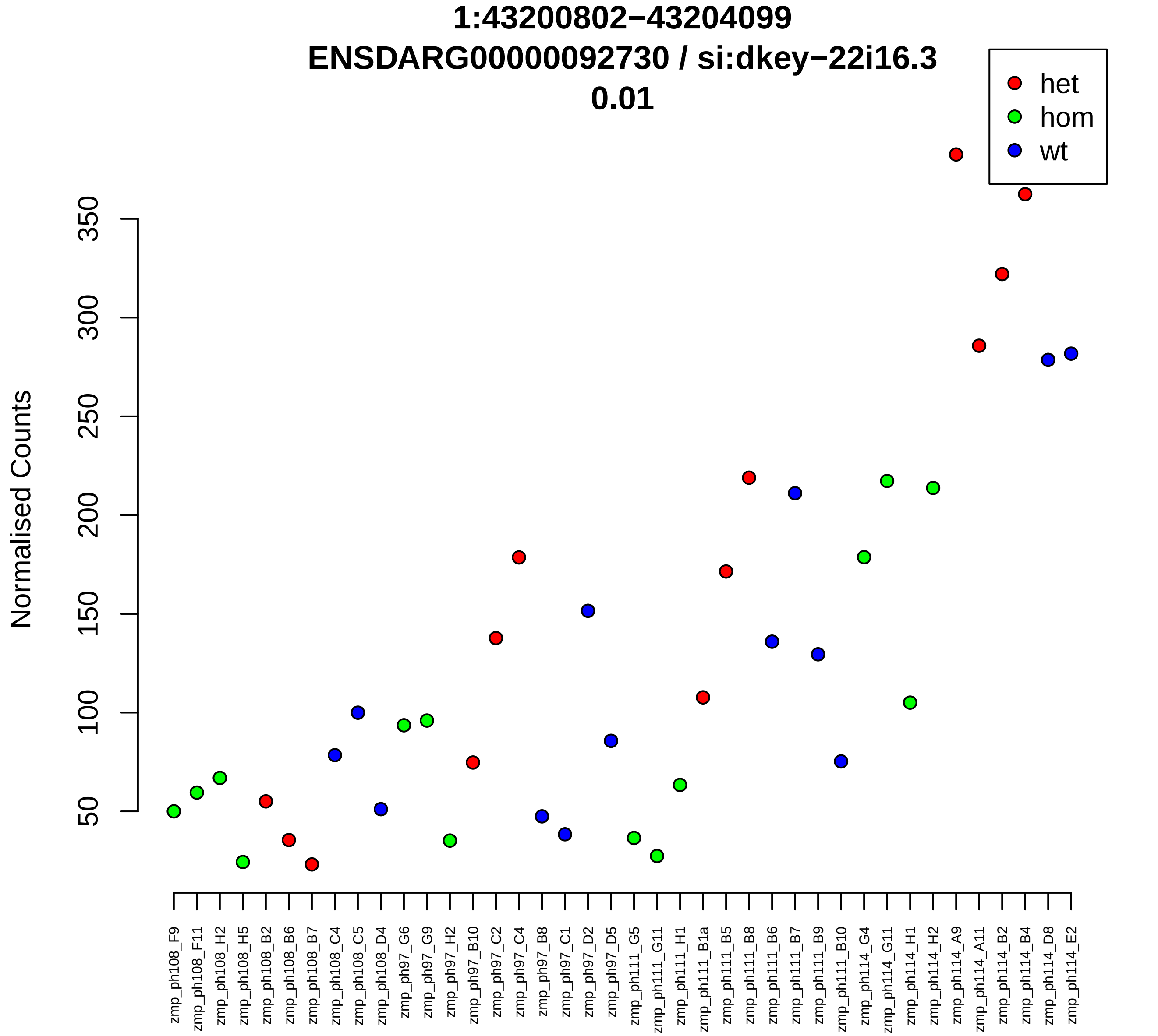
1:43200802−43204099
ENSDARG00000092730 / si:dkey−22i16.3
0.01
het
hom
wt
Normalised Counts
50
100
150
200
250
300
350
zmp_ph108_F9
zmp_ph108_F11
zmp_ph108_H2
zmp_ph108_H5
zmp_ph108_B2
zmp_ph108_B6
zmp_ph108_B7
zmp_ph108_C4
zmp_ph108_C5
zmp_ph108_D4
zmp_ph97_G6
zmp_ph97_G9
zmp_ph97_H2
zmp_ph97_B10
zmp_ph97_C2
zmp_ph97_C4
zmp_ph97_B8
zmp_ph97_C1
zmp_ph97_D2
zmp_ph97_D5
zmp_ph111_G5
zmp_ph111_G11
zmp_ph111_H1
zmp_ph111_B1a
zmp_ph111_B5
zmp_ph111_B8
zmp_ph111_B6
zmp_ph111_B7
zmp_ph111_B9
zmp_ph111_B10
zmp_ph114_G4
zmp_ph114_G11
zmp_ph114_H1
zmp_ph114_H2
zmp_ph114_A9
zmp_ph114_A11
zmp_ph114_B2
zmp_ph114_B4
zmp_ph114_D8
zmp_ph114_E2

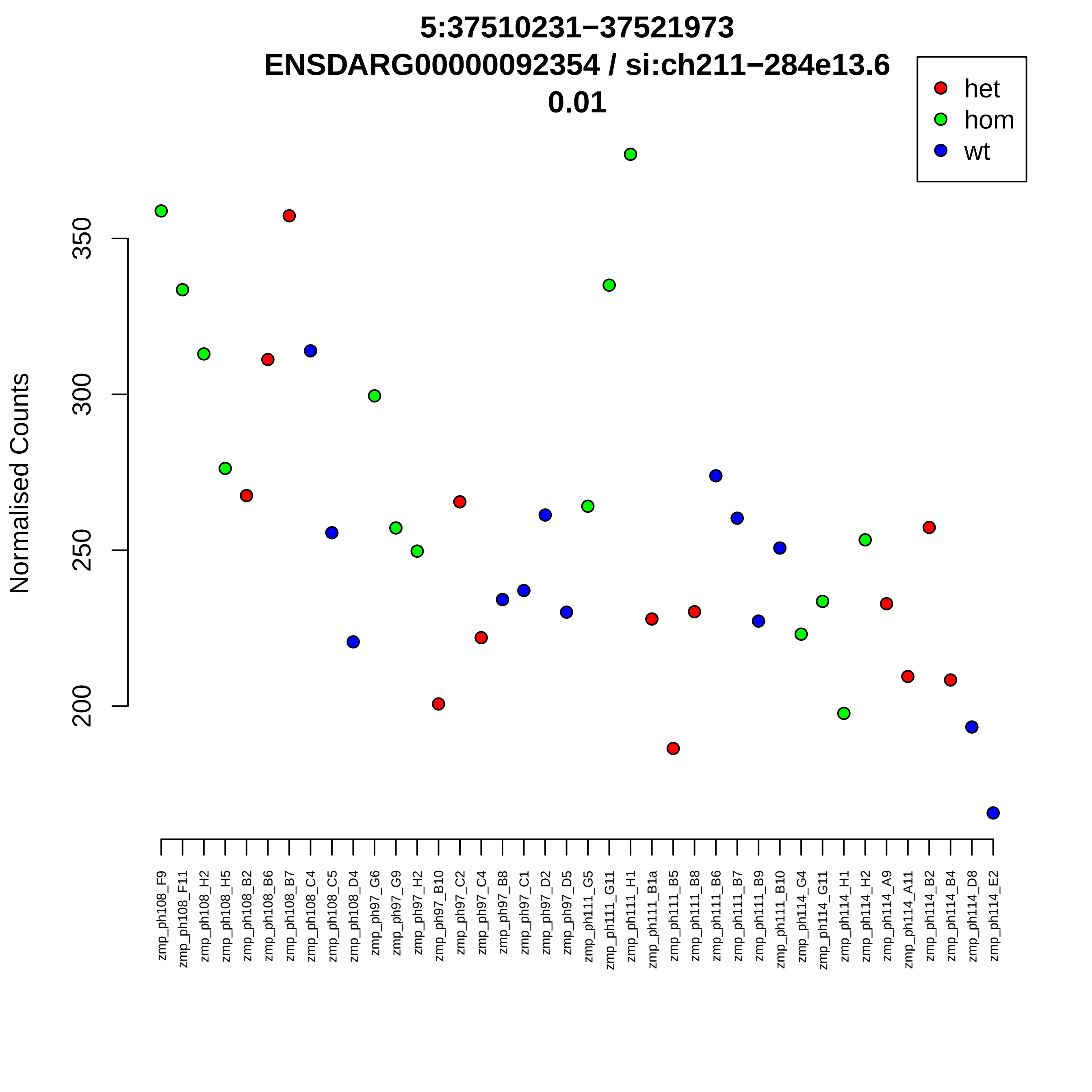
5:37510231−37521973
ENSDARG00000092354 / si:ch211−284e13.6
0.01
het
hom
wt
Normalised Counts
200
250
300
350
zmp_ph108_F9
zmp_ph108_F11
zmp_ph108_H2
zmp_ph108_H5
zmp_ph108_B2
zmp_ph108_B6
zmp_ph108_B7
zmp_ph108_C4
zmp_ph108_C5
zmp_ph108_D4
zmp_ph97_G6
zmp_ph97_G9
zmp_ph97_H2
zmp_ph97_B10
zmp_ph97_C2
zmp_ph97_C4
zmp_ph97_B8
zmp_ph97_C1
zmp_ph97_D2
zmp_ph97_D5
zmp_ph111_G5
zmp_ph111_G11
zmp_ph111_H1
zmp_ph111_B1a
zmp_ph111_B5
zmp_ph111_B8
zmp_ph111_B6
zmp_ph111_B7
zmp_ph111_B9
zmp_ph111_B10
zmp_ph114_G4
zmp_ph114_G11
zmp_ph114_H1
zmp_ph114_H2
zmp_ph114_A9
zmp_ph114_A11
zmp_ph114_B2
zmp_ph114_B4
zmp_ph114_D8
zmp_ph114_E2

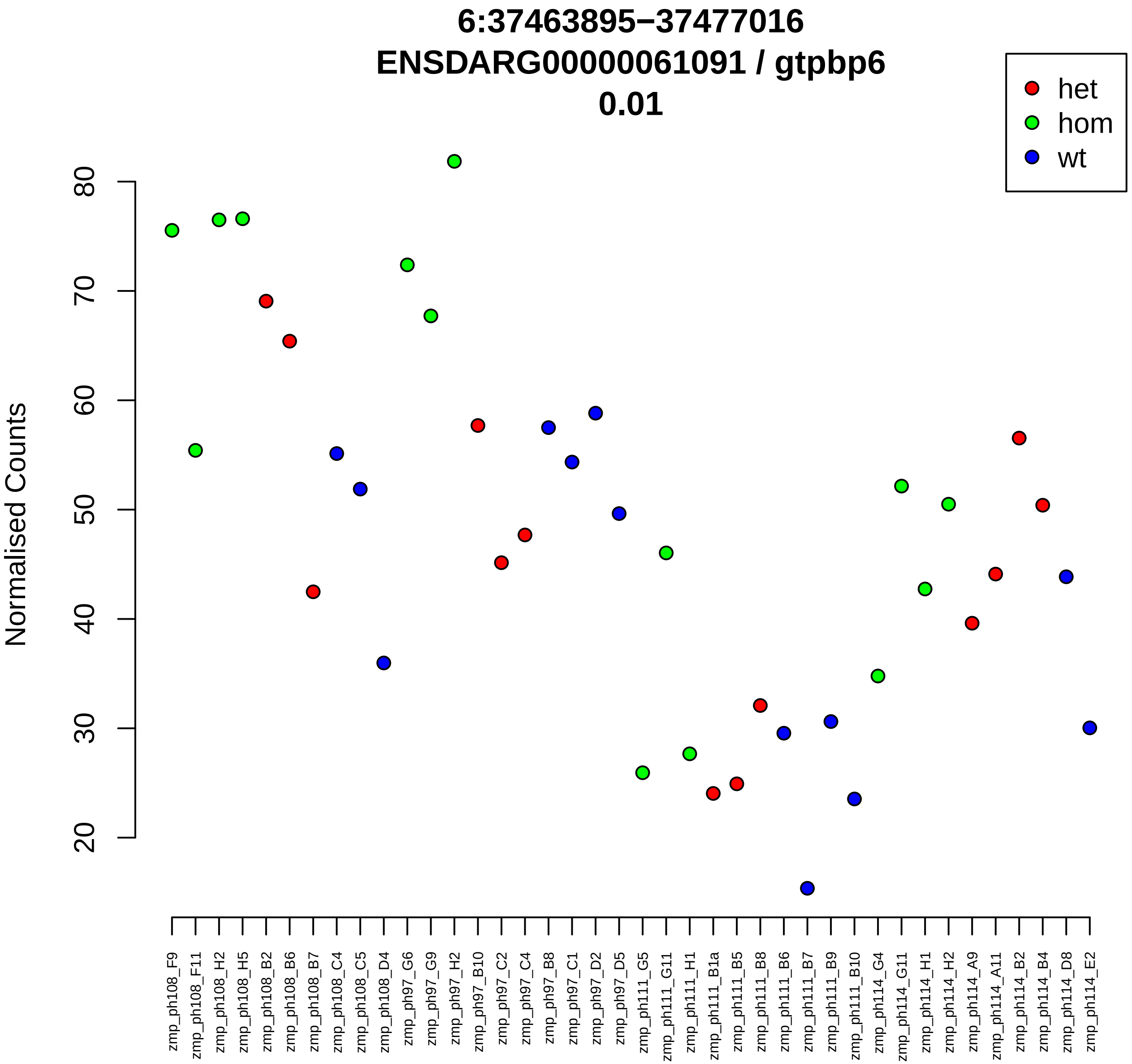
6:37463895−37477016
ENSDARG00000061091 / gtpbp6
0.01
het
hom
wt
Normalised Counts
20
30
40
50
60
70
80
zmp_ph108_F9
zmp_ph108_F11
zmp_ph108_H2
zmp_ph108_H5
zmp_ph108_B2
zmp_ph108_B6
zmp_ph108_B7
zmp_ph108_C4
zmp_ph108_C5
zmp_ph108_D4
zmp_ph97_G6
zmp_ph97_G9
zmp_ph97_H2
zmp_ph97_B10
zmp_ph97_C2
zmp_ph97_C4
zmp_ph97_B8
zmp_ph97_C1
zmp_ph97_D2
zmp_ph97_D5
zmp_ph111_G5
zmp_ph111_G11
zmp_ph111_H1
zmp_ph111_B1a
zmp_ph111_B5
zmp_ph111_B8
zmp_ph111_B6
zmp_ph111_B7
zmp_ph111_B9
zmp_ph111_B10
zmp_ph114_G4
zmp_ph114_G11
zmp_ph114_H1
zmp_ph114_H2
zmp_ph114_A9
zmp_ph114_A11
zmp_ph114_B2
zmp_ph114_B4
zmp_ph114_D8
zmp_ph114_E2

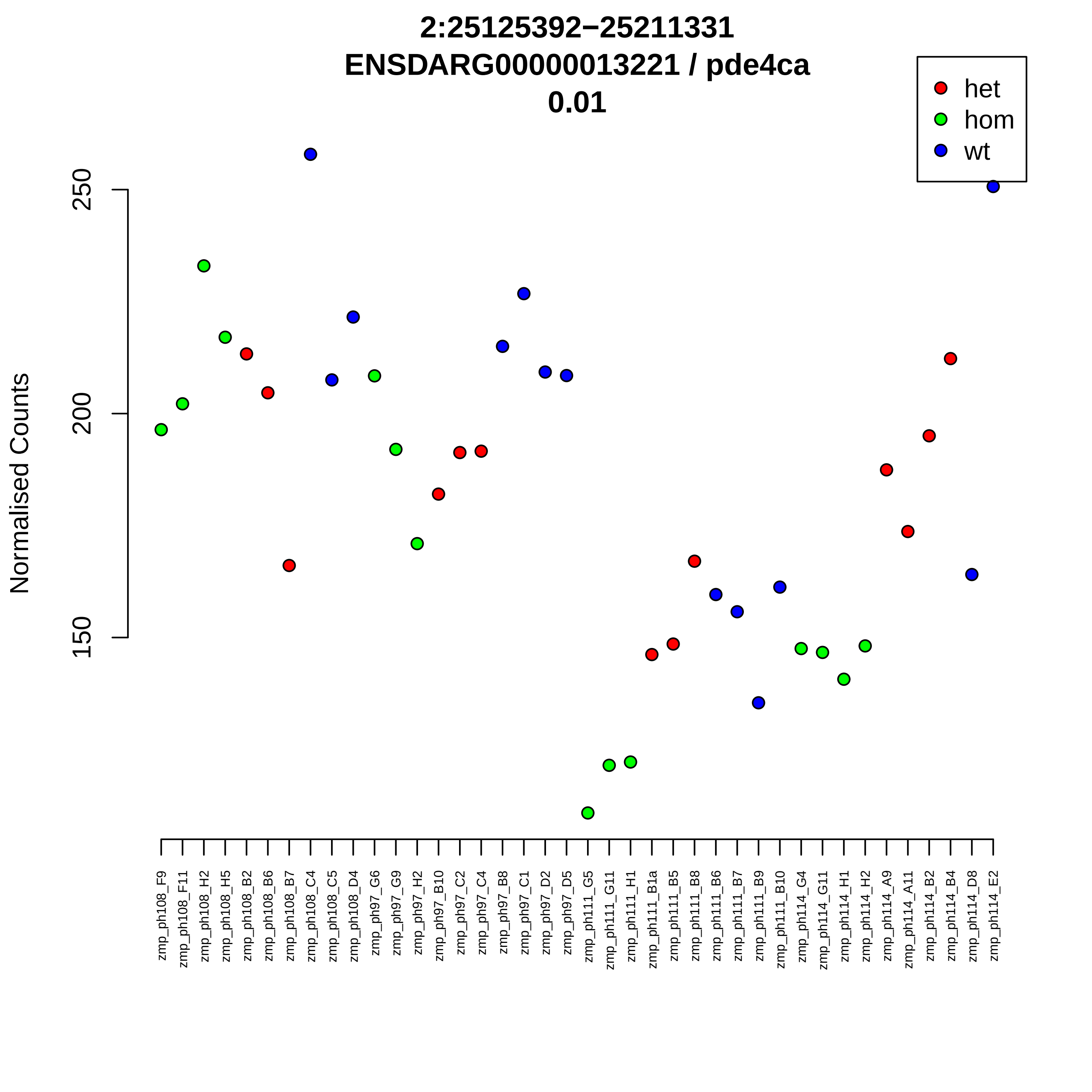

2:25125392−25211331
ENSDARG00000013221 / pde4ca
0.01
het
hom
wt
Normalised Counts
250
200
150
zmp_ph108_F9
zmp_ph108_F11
zmp_ph108_H2
zmp_ph108_H5
zmp_ph108_B2
zmp_ph108_B6
zmp_ph108_B7
zmp_ph108_C4
zmp_ph108_C5
zmp_ph108_D4
zmp_ph97_G6
zmp_ph97_G9
zmp_ph97_H2
zmp_ph97_B10
zmp_ph97_C2
zmp_ph97_C4
zmp_ph97_B8
zmp_ph97_C1
zmp_ph97_D2
zmp_ph97_D5
zmp_ph111_G5
zmp_ph111_G11
zmp_ph111_H1
zmp_ph111_B1a
zmp_ph111_B5
zmp_ph111_B8
zmp_ph111_B6
zmp_ph111_B7
zmp_ph111_B9
zmp_ph111_B10
zmp_ph114_G4
zmp_ph114_G11
zmp_ph114_H1
zmp_ph114_H2
zmp_ph114_A9
zmp_ph114_A11
zmp_ph114_B2
zmp_ph114_B4
zmp_ph114_D8
zmp_ph114_E2

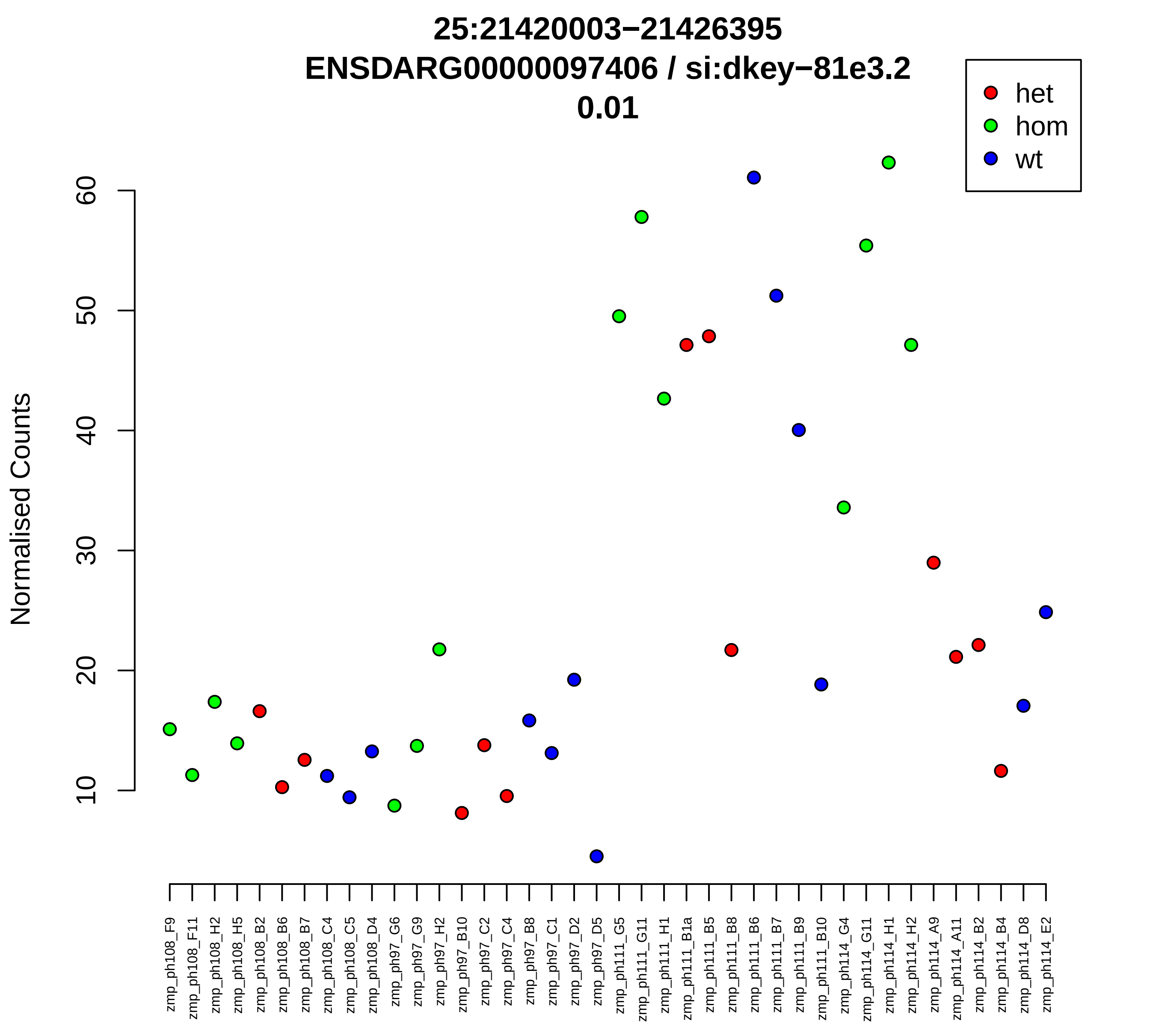

25:21420003−21426395
ENSDARG00000097406 / si:dkey−81e3.2
0.01
het
hom
wt
Normalised Counts
10
20
30
40
50
60
zmp_ph108_F9
zmp_ph108_F11
zmp_ph108_H2
zmp_ph108_H5
zmp_ph108_B2
zmp_ph108_B6
zmp_ph108_B7
zmp_ph108_C4
zmp_ph108_C5
zmp_ph108_D4
zmp_ph97_G6
zmp_ph97_G9
zmp_ph97_H2
zmp_ph97_B10
zmp_ph97_C2
zmp_ph97_C4
zmp_ph97_B8
zmp_ph97_C1
zmp_ph97_D2
zmp_ph97_D5
zmp_ph111_G5
zmp_ph111_G11
zmp_ph111_H1
zmp_ph111_B1a
zmp_ph111_B5
zmp_ph111_B8
zmp_ph111_B6
zmp_ph111_B7
zmp_ph111_B9
zmp_ph111_B10
zmp_ph114_G4
zmp_ph114_G11
zmp_ph114_H1
zmp_ph114_H2
zmp_ph114_A9
zmp_ph114_A11
zmp_ph114_B2
zmp_ph114_B4
zmp_ph114_D8
zmp_ph114_E2

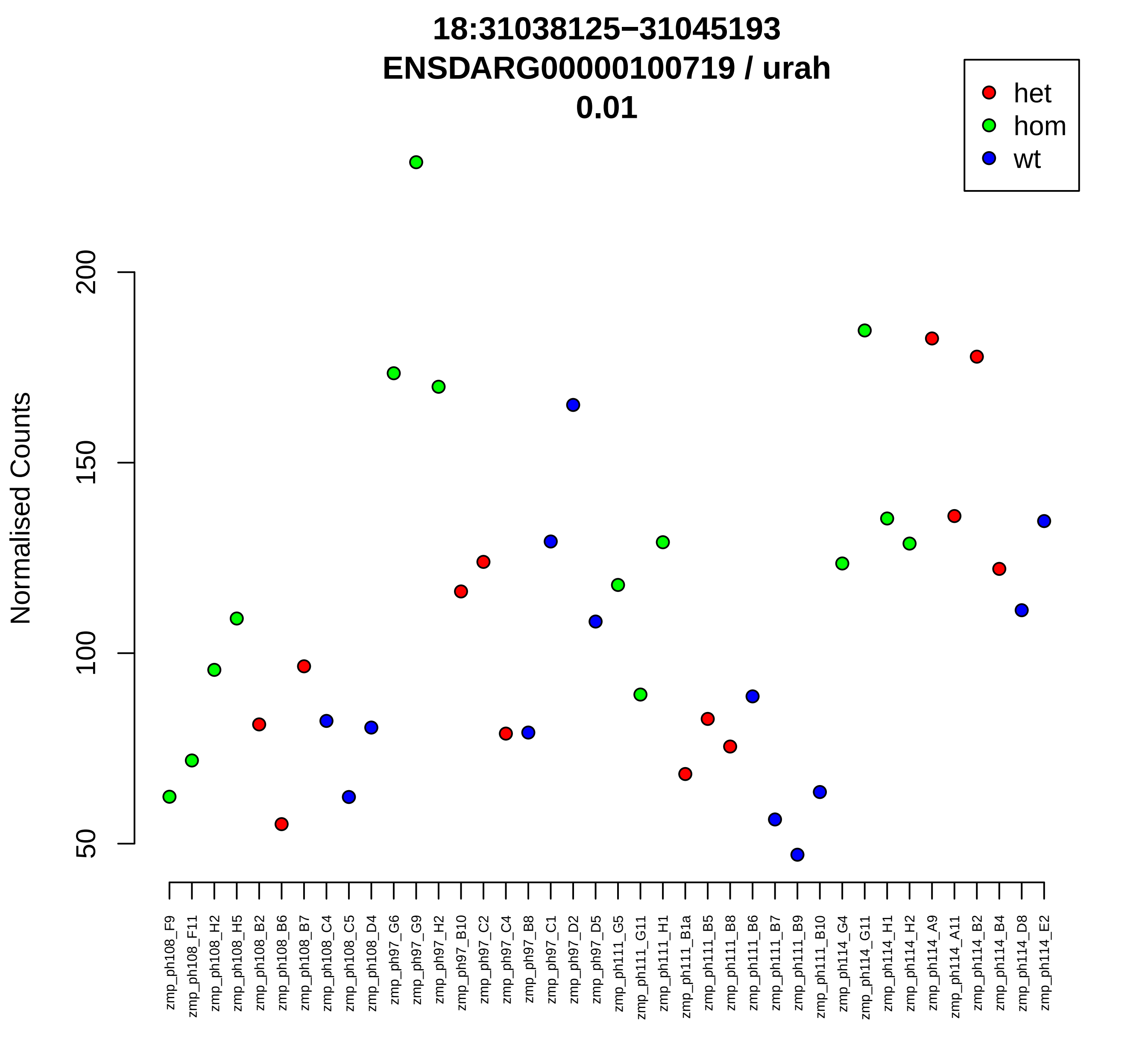

18:31038125−31045193
ENSDARG00000100719 / urah
0.01
het
hom
wt
Normalised Counts
50
100
150
200
zmp_ph108_F9
zmp_ph108_F11
zmp_ph108_H2
zmp_ph108_H5
zmp_ph108_B2
zmp_ph108_B6
zmp_ph108_B7
zmp_ph108_C4
zmp_ph108_C5
zmp_ph108_D4
zmp_ph97_G6
zmp_ph97_G9
zmp_ph97_H2
zmp_ph97_B10
zmp_ph97_C2
zmp_ph97_C4
zmp_ph97_B8
zmp_ph97_C1
zmp_ph97_D2
zmp_ph97_D5
zmp_ph111_G5
zmp_ph111_G11
zmp_ph111_H1
zmp_ph111_B1a
zmp_ph111_B5
zmp_ph111_B8
zmp_ph111_B6
zmp_ph111_B7
zmp_ph111_B9
zmp_ph111_B10
zmp_ph114_G4
zmp_ph114_G11
zmp_ph114_H1
zmp_ph114_H2
zmp_ph114_A9
zmp_ph114_A11
zmp_ph114_B2
zmp_ph114_B4
zmp_ph114_D8
zmp_ph114_E2

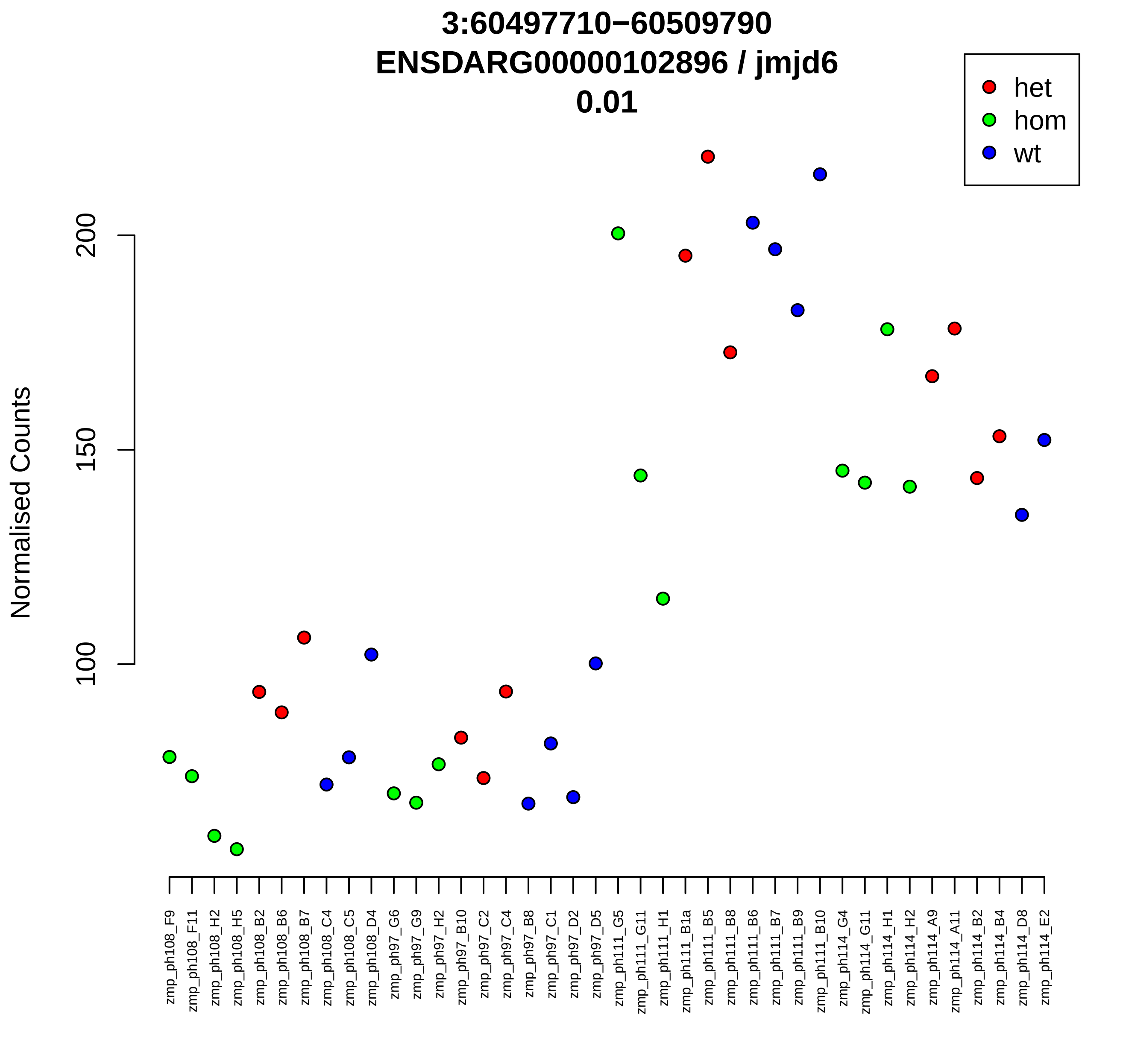

3:60497710−60509790
ENSDARG00000102896 / jmjd6
0.01
het
hom
wt
Normalised Counts
100
150
200
zmp_ph108_F9
zmp_ph108_F11
zmp_ph108_H2
zmp_ph108_H5
zmp_ph108_B2
zmp_ph108_B6
zmp_ph108_B7
zmp_ph108_C4
zmp_ph108_C5
zmp_ph108_D4
zmp_ph97_G6
zmp_ph97_G9
zmp_ph97_H2
zmp_ph97_B10
zmp_ph97_C2
zmp_ph97_C4
zmp_ph97_B8
zmp_ph97_C1
zmp_ph97_D2
zmp_ph97_D5
zmp_ph111_G5
zmp_ph111_G11
zmp_ph111_H1
zmp_ph111_B1a
zmp_ph111_B5
zmp_ph111_B8
zmp_ph111_B6
zmp_ph111_B7
zmp_ph111_B9
zmp_ph111_B10
zmp_ph114_G4
zmp_ph114_G11
zmp_ph114_H1
zmp_ph114_H2
zmp_ph114_A9
zmp_ph114_A11
zmp_ph114_B2
zmp_ph114_B4
zmp_ph114_D8
zmp_ph114_E2

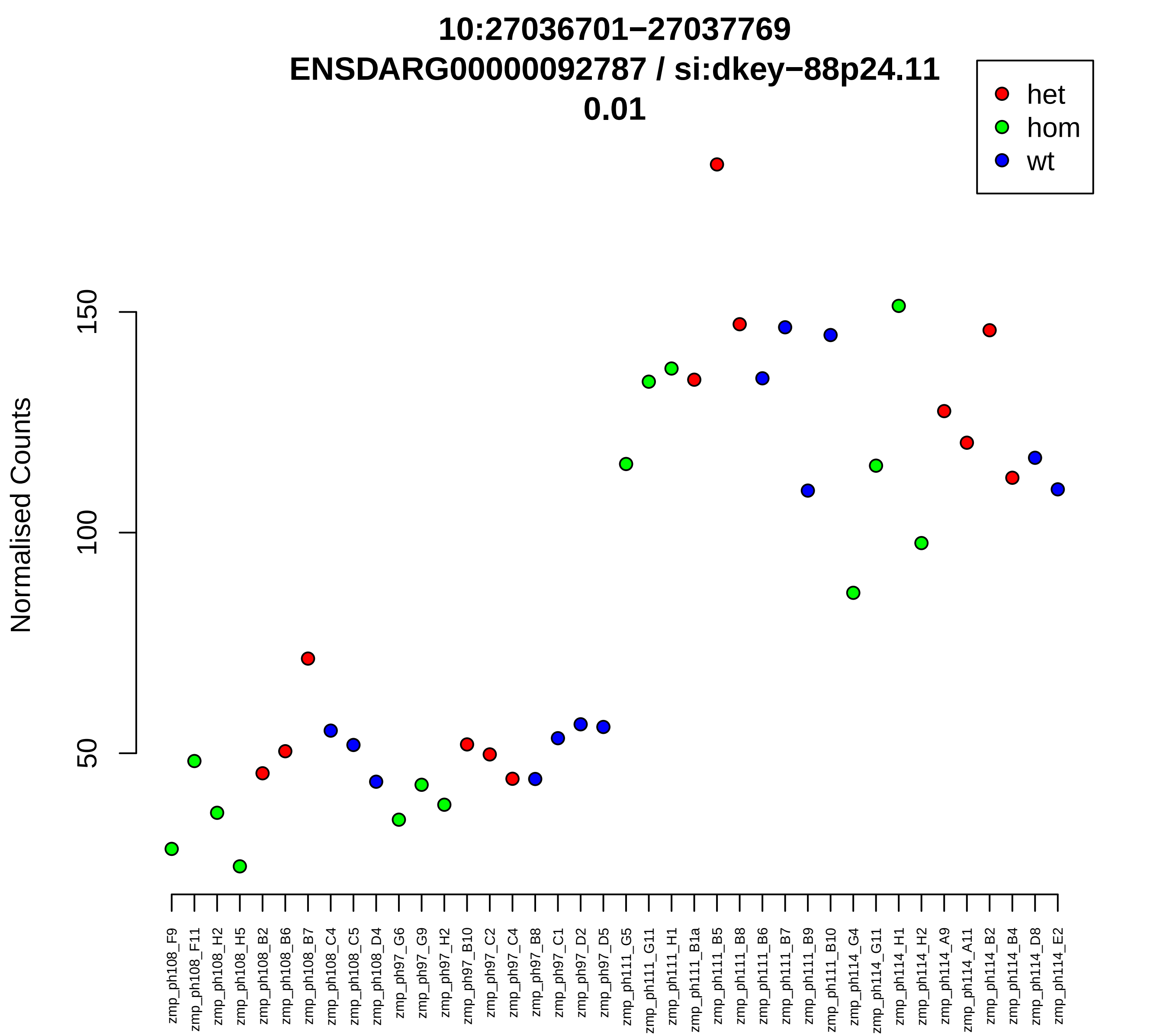

10:27036701−27037769
ENSDARG00000092787 / si:dkey−88p24.11
0.01
het
hom
wt
Normalised Counts
50
100
150
zmp_ph108_F9
zmp_ph108_F11
zmp_ph108_H2
zmp_ph108_H5
zmp_ph108_B2
zmp_ph108_B6
zmp_ph108_B7
zmp_ph108_C4
zmp_ph108_C5
zmp_ph108_D4
zmp_ph97_G6
zmp_ph97_G9
zmp_ph97_H2
zmp_ph97_B10
zmp_ph97_C2
zmp_ph97_C4
zmp_ph97_B8
zmp_ph97_C1
zmp_ph97_D2
zmp_ph97_D5
zmp_ph111_G5
zmp_ph111_G11
zmp_ph111_H1
zmp_ph111_B1a
zmp_ph111_B5
zmp_ph111_B8
zmp_ph111_B6
zmp_ph111_B7
zmp_ph111_B9
zmp_ph111_B10
zmp_ph114_G4
zmp_ph114_G11
zmp_ph114_H1
zmp_ph114_H2
zmp_ph114_A9
zmp_ph114_A11
zmp_ph114_B2
zmp_ph114_B4
zmp_ph114_D8
zmp_ph114_E2

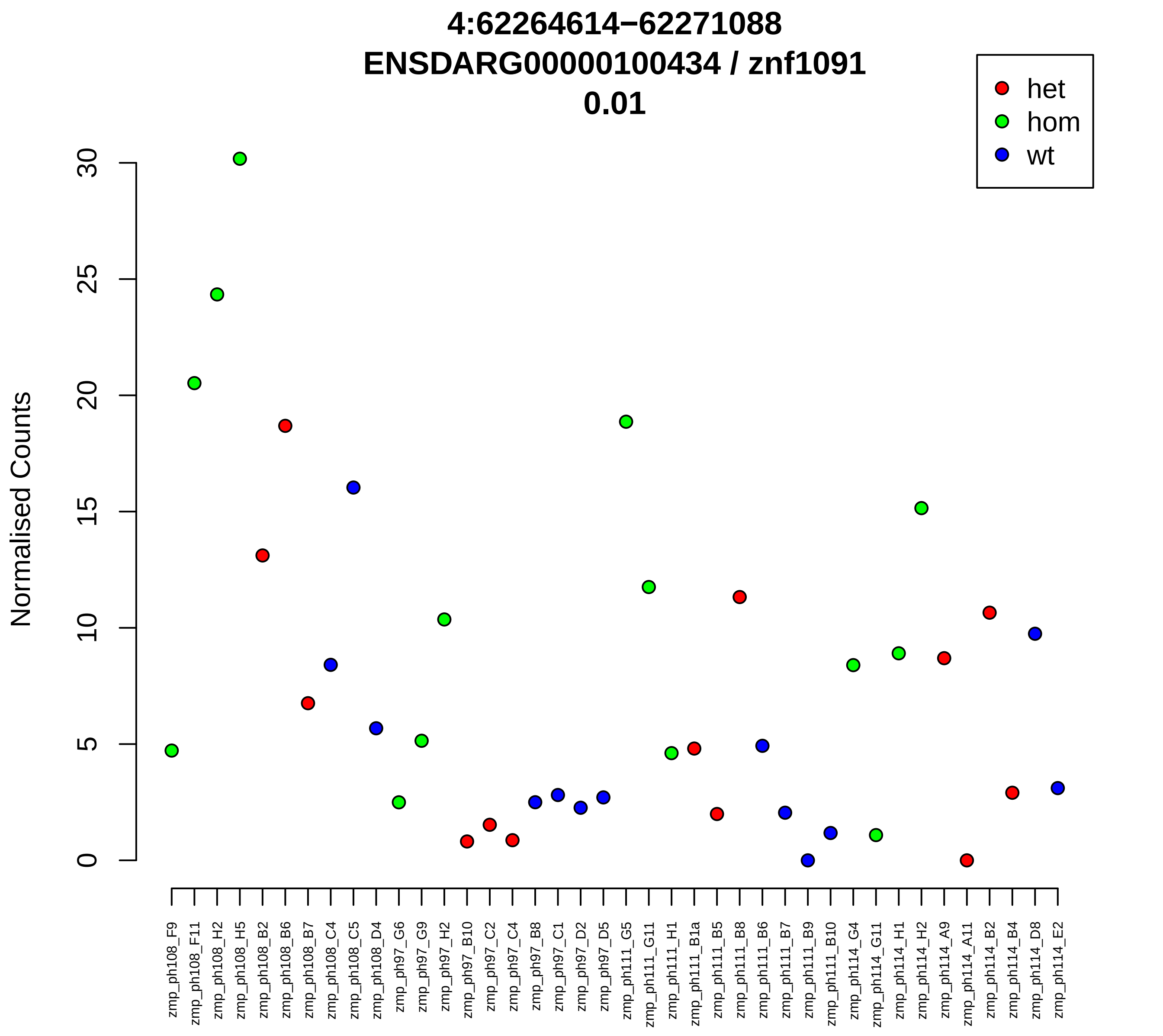
4:62264614−62271088
ENSDARG00000100434 / znf1091
0.01
het
hom
wt
Normalised Counts
0
5
10
15
20
25
30
zmp_ph108_F9
zmp_ph108_F11
zmp_ph108_H2
zmp_ph108_H5
zmp_ph108_B2
zmp_ph108_B6
zmp_ph108_B7
zmp_ph108_C4
zmp_ph108_C5
zmp_ph108_D4
zmp_ph97_G6
zmp_ph97_G9
zmp_ph97_H2
zmp_ph97_B10
zmp_ph97_C2
zmp_ph97_C4
zmp_ph97_B8
zmp_ph97_C1
zmp_ph97_D2
zmp_ph97_D5
zmp_ph111_G5
zmp_ph111_G11
zmp_ph111_H1
zmp_ph111_B1a
zmp_ph111_B5
zmp_ph111_B8
zmp_ph111_B6
zmp_ph111_B7
zmp_ph111_B9
zmp_ph111_B10
zmp_ph114_G4
zmp_ph114_G11
zmp_ph114_H1
zmp_ph114_H2
zmp_ph114_A9
zmp_ph114_A11
zmp_ph114_B2
zmp_ph114_B4
zmp_ph114_D8
zmp_ph114_E2

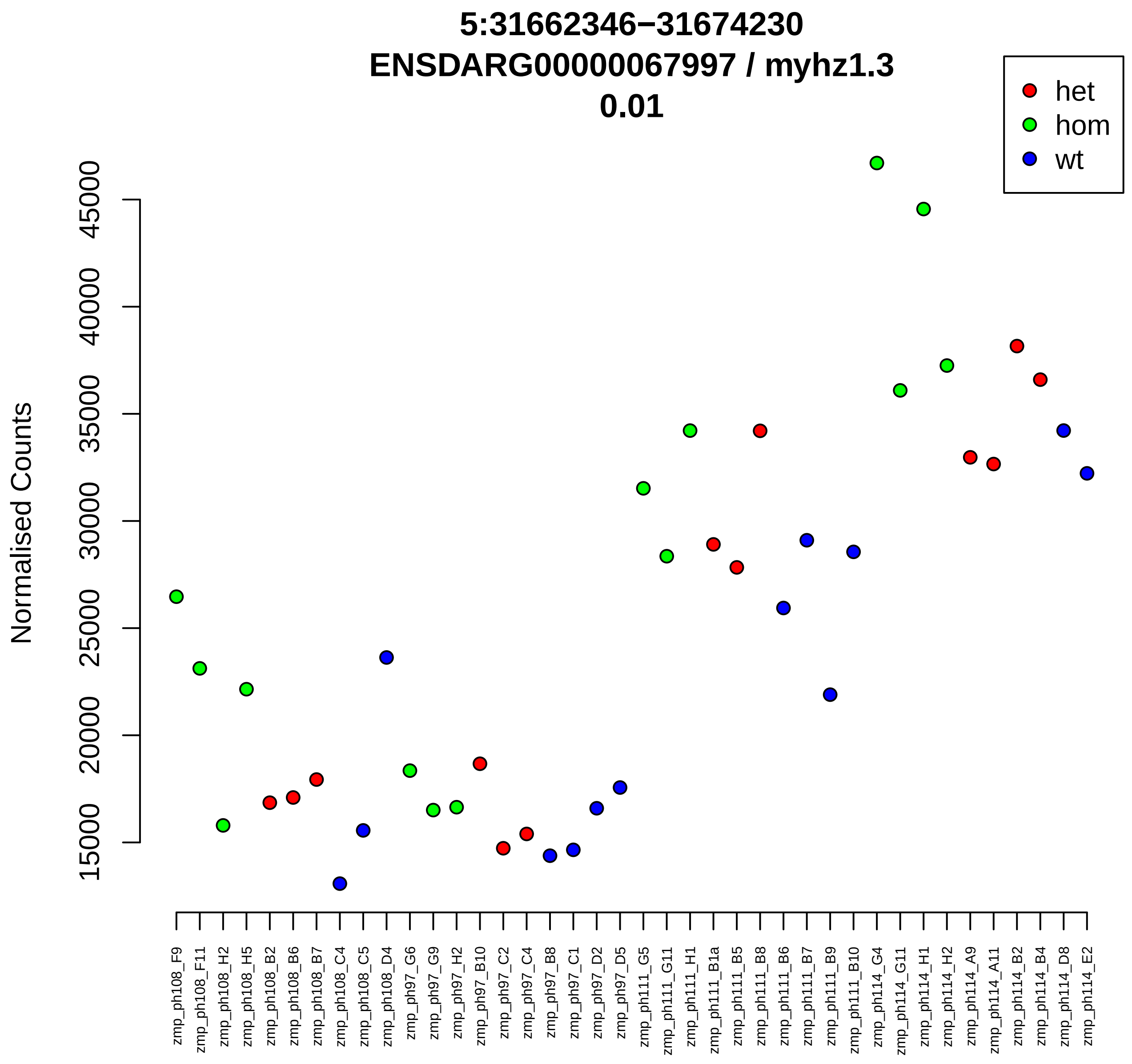
5:31662346−31674230
ENSDARG00000067997 / myhz1.3
0.01
het
hom
wt
Normalised Counts
15000
20000
25000
30000
35000
40000
45000
zmp_ph108_F9
zmp_ph108_F11
zmp_ph108_H2
zmp_ph108_H5
zmp_ph108_B2
zmp_ph108_B6
zmp_ph108_B7
zmp_ph108_C4
zmp_ph108_C5
zmp_ph108_D4
zmp_ph97_G6
zmp_ph97_G9
zmp_ph97_H2
zmp_ph97_B10
zmp_ph97_C2
zmp_ph97_C4
zmp_ph97_B8
zmp_ph97_C1
zmp_ph97_D2
zmp_ph97_D5
zmp_ph111_G5
zmp_ph111_G11
zmp_ph111_H1
zmp_ph111_B1a
zmp_ph111_B5
zmp_ph111_B8
zmp_ph111_B6
zmp_ph111_B7
zmp_ph111_B9
zmp_ph111_B10
zmp_ph114_G4
zmp_ph114_G11
zmp_ph114_H1
zmp_ph114_H2
zmp_ph114_A9
zmp_ph114_A11
zmp_ph114_B2
zmp_ph114_B4
zmp_ph114_D8
zmp_ph114_E2

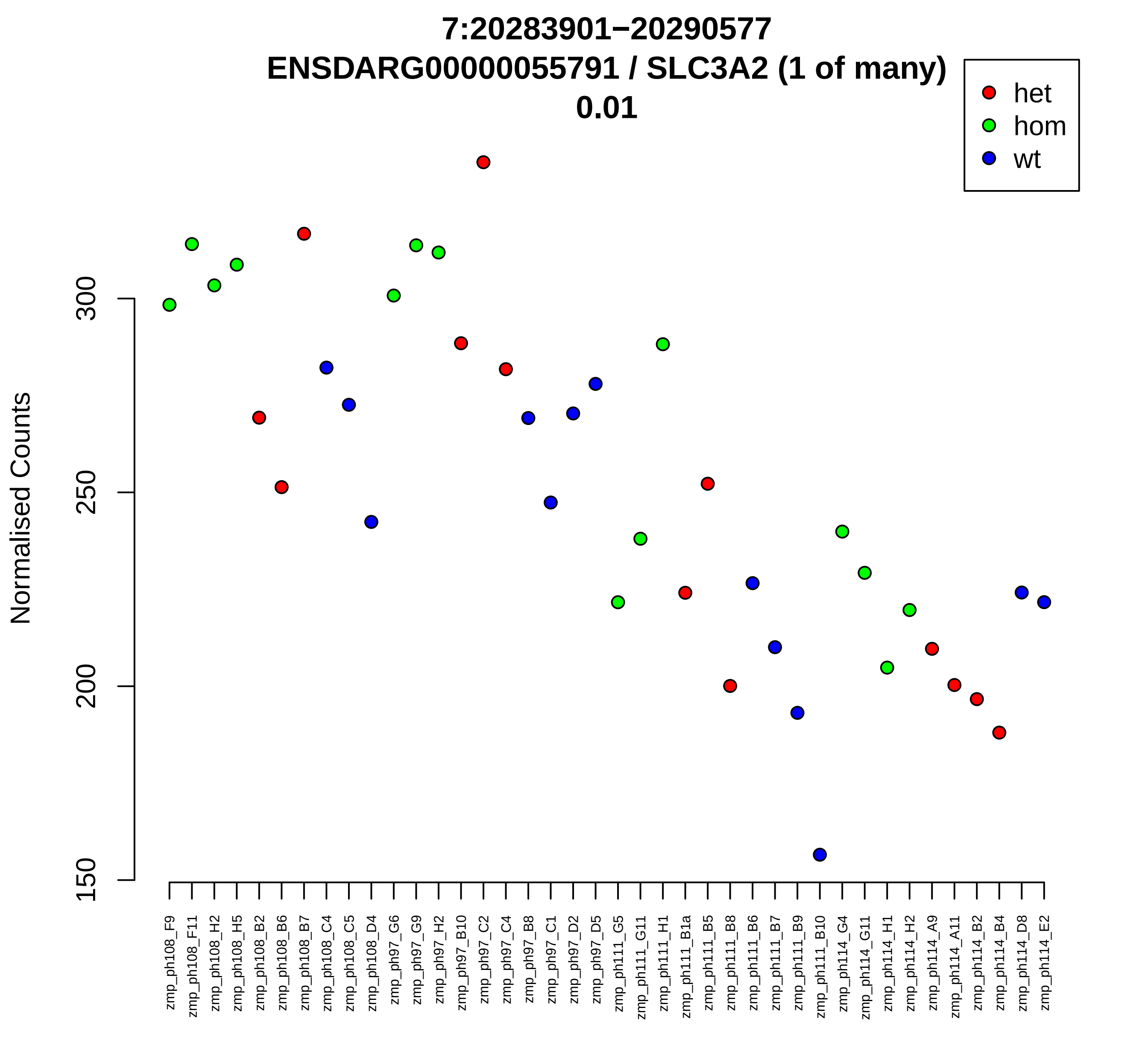

7:20283901−20290577
ENSDARG00000055791 / SLC3A2 (1 of many)
0.01
het
hom
wt
Normalised Counts
150
200
250
300
zmp_ph108_F9
zmp_ph108_F11
zmp_ph108_H2
zmp_ph108_H5
zmp_ph108_B2
zmp_ph108_B6
zmp_ph108_B7
zmp_ph108_C4
zmp_ph108_C5
zmp_ph108_D4
zmp_ph97_G6
zmp_ph97_G9
zmp_ph97_H2
zmp_ph97_B10
zmp_ph97_C2
zmp_ph97_C4
zmp_ph97_B8
zmp_ph97_C1
zmp_ph97_D2
zmp_ph97_D5
zmp_ph111_G5
zmp_ph111_G11
zmp_ph111_H1
zmp_ph111_B1a
zmp_ph111_B5
zmp_ph111_B8
zmp_ph111_B6
zmp_ph111_B7
zmp_ph111_B9
zmp_ph111_B10
zmp_ph114_G4
zmp_ph114_G11
zmp_ph114_H1
zmp_ph114_H2
zmp_ph114_A9
zmp_ph114_A11
zmp_ph114_B2
zmp_ph114_B4
zmp_ph114_D8
zmp_ph114_E2

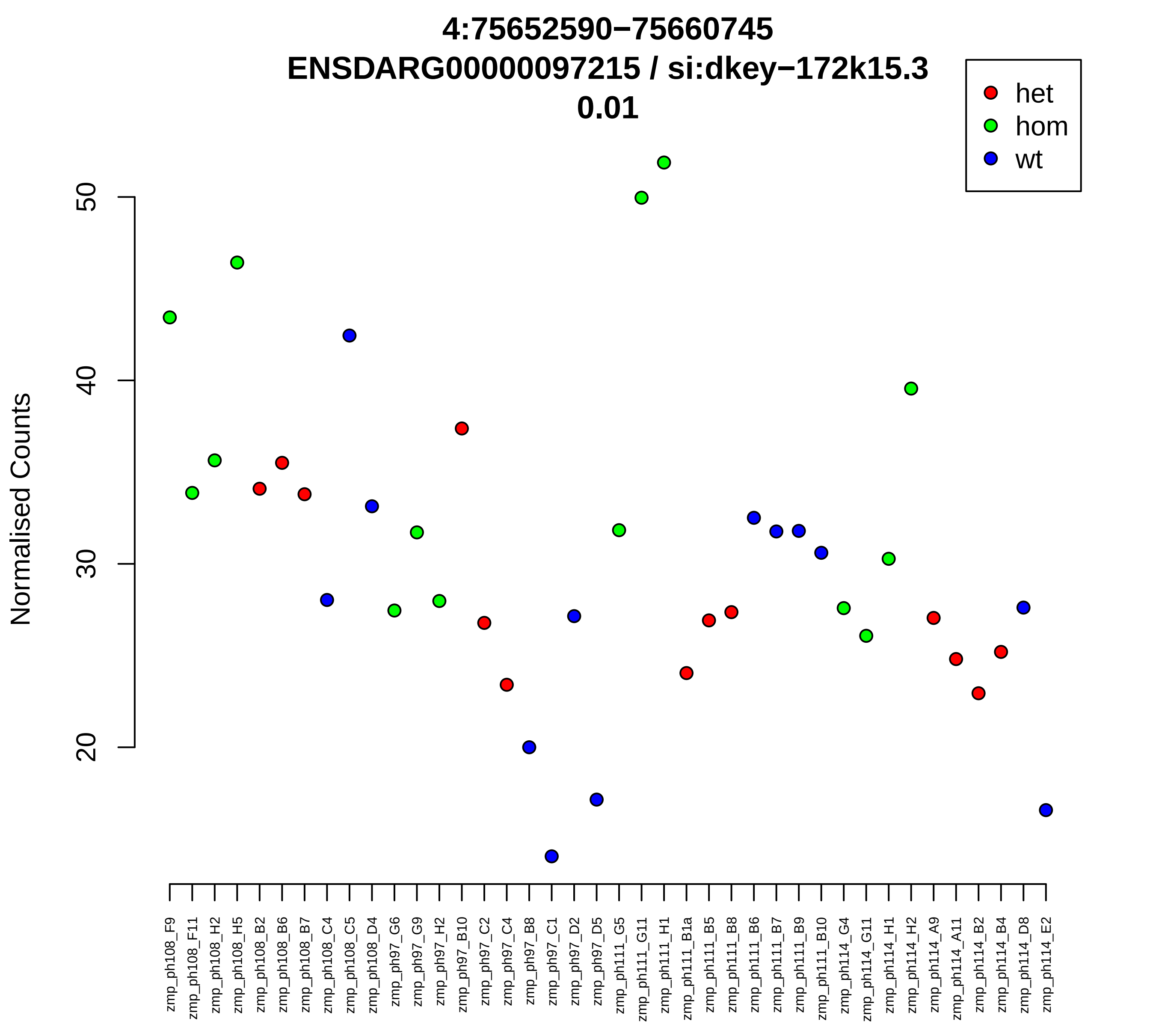

4:75652590−75660745
ENSDARG00000097215 / si:dkey−172k15.3
0.01
het
hom
wt
Normalised Counts
20
30
40
50
zmp_ph108_F9
zmp_ph108_F11
zmp_ph108_H2
zmp_ph108_H5
zmp_ph108_B2
zmp_ph108_B6
zmp_ph108_B7
zmp_ph108_C4
zmp_ph108_C5
zmp_ph108_D4
zmp_ph97_G6
zmp_ph97_G9
zmp_ph97_H2
zmp_ph97_B10
zmp_ph97_C2
zmp_ph97_C4
zmp_ph97_B8
zmp_ph97_C1
zmp_ph97_D2
zmp_ph97_D5
zmp_ph111_G5
zmp_ph111_G11
zmp_ph111_H1
zmp_ph111_B1a
zmp_ph111_B5
zmp_ph111_B8
zmp_ph111_B6
zmp_ph111_B7
zmp_ph111_B9
zmp_ph111_B10
zmp_ph114_G4
zmp_ph114_G11
zmp_ph114_H1
zmp_ph114_H2
zmp_ph114_A9
zmp_ph114_A11
zmp_ph114_B2
zmp_ph114_B4
zmp_ph114_D8
zmp_ph114_E2

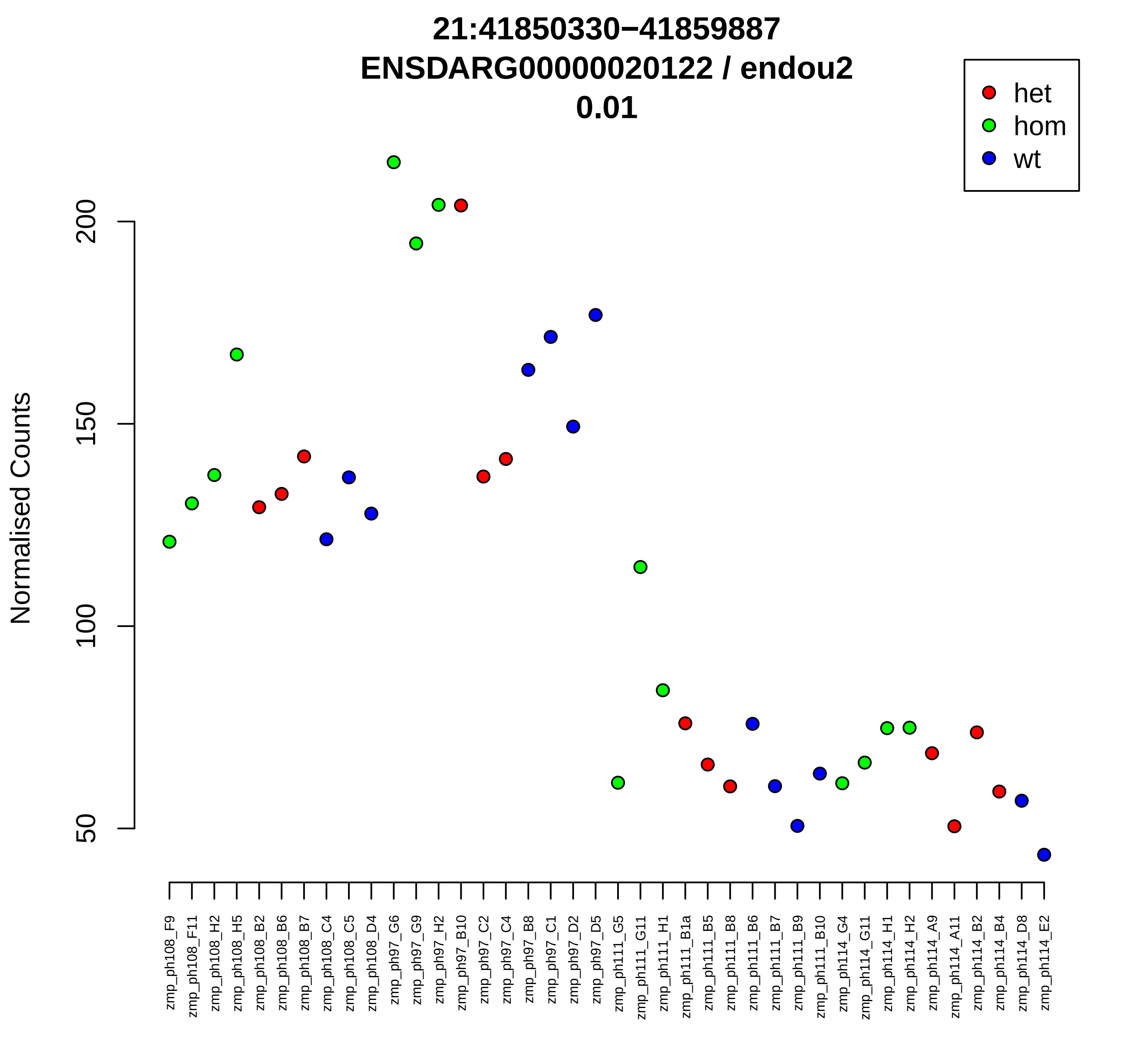

21:41850330−41859887
ENSDARG00000020122 / endou2
0.01
het
hom
wt
Normalised Counts
50
100
150
200
zmp_ph108_F9
zmp_ph108_F11
zmp_ph108_H2
zmp_ph108_H5
zmp_ph108_B2
zmp_ph108_B6
zmp_ph108_B7
zmp_ph108_C4
zmp_ph108_C5
zmp_ph108_D4
zmp_ph97_G6
zmp_ph97_G9
zmp_ph97_H2
zmp_ph97_B10
zmp_ph97_C2
zmp_ph97_C4
zmp_ph97_B8
zmp_ph97_C1
zmp_ph97_D2
zmp_ph97_D5
zmp_ph111_G5
zmp_ph111_G11
zmp_ph111_H1
zmp_ph111_B1a
zmp_ph111_B5
zmp_ph111_B8
zmp_ph111_B6
zmp_ph111_B7
zmp_ph111_B9
zmp_ph111_B10
zmp_ph114_G4
zmp_ph114_G11
zmp_ph114_H1
zmp_ph114_H2
zmp_ph114_A9
zmp_ph114_A11
zmp_ph114_B2
zmp_ph114_B4
zmp_ph114_D8
zmp_ph114_E2

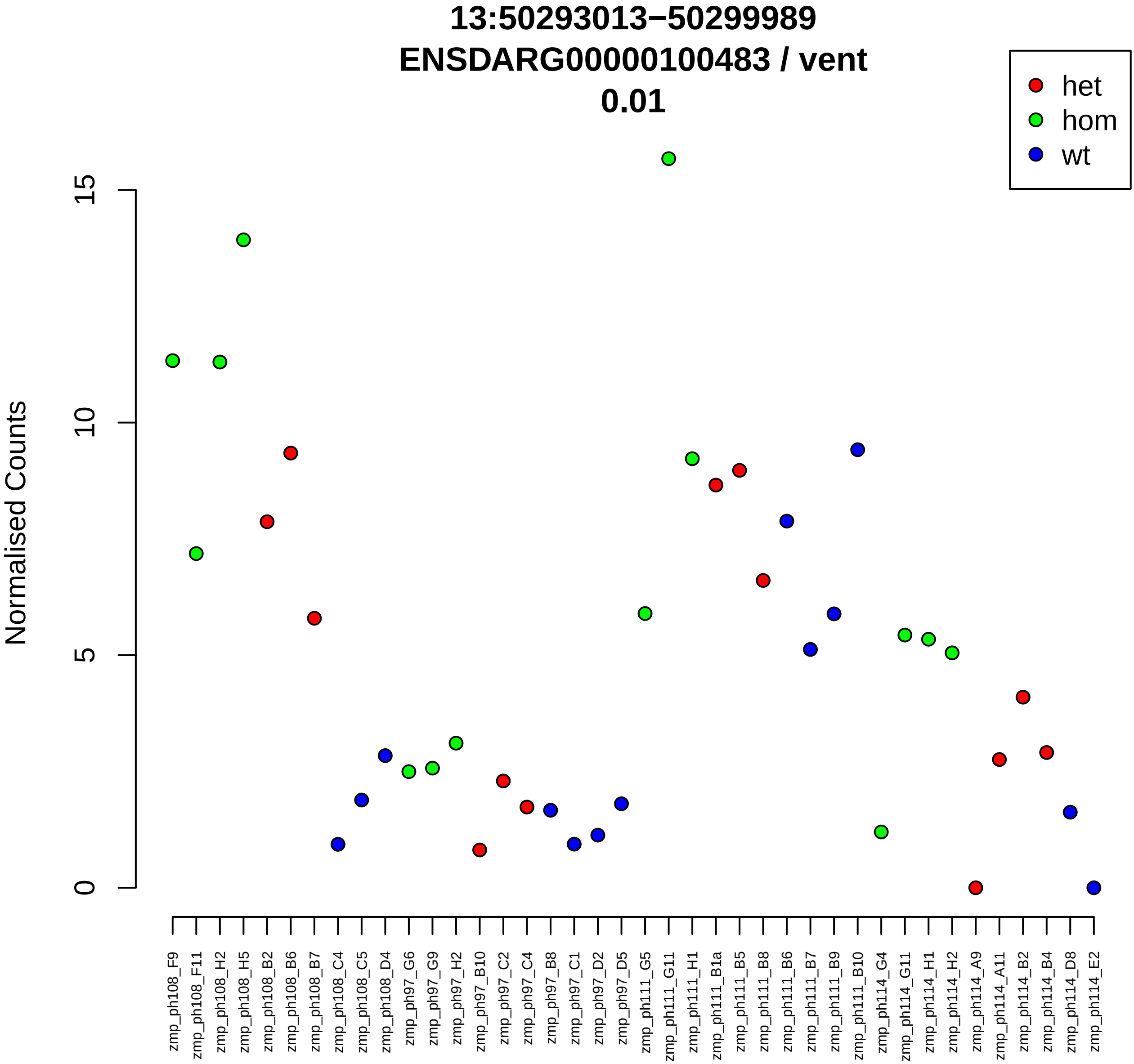
13:50293013−50299989
ENSDARG00000100483 / vent
0.01
het
hom
wt
Normalised Counts
0
5
10
15
zmp_ph108_F9
zmp_ph108_F11
zmp_ph108_H2
zmp_ph108_H5
zmp_ph108_B2
zmp_ph108_B6
zmp_ph108_B7
zmp_ph108_C4
zmp_ph108_C5
zmp_ph108_D4
zmp_ph97_G6
zmp_ph97_G9
zmp_ph97_H2
zmp_ph97_B10
zmp_ph97_C2
zmp_ph97_C4
zmp_ph97_B8
zmp_ph97_C1
zmp_ph97_D2
zmp_ph97_D5
zmp_ph111_G5
zmp_ph111_G11
zmp_ph111_H1
zmp_ph111_B1a
zmp_ph111_B5
zmp_ph111_B8
zmp_ph111_B6
zmp_ph111_B7
zmp_ph111_B9
zmp_ph111_B10
zmp_ph114_G4
zmp_ph114_G11
zmp_ph114_H1
zmp_ph114_H2
zmp_ph114_A9
zmp_ph114_A11
zmp_ph114_B2
zmp_ph114_B4
zmp_ph114_D8
zmp_ph114_E2

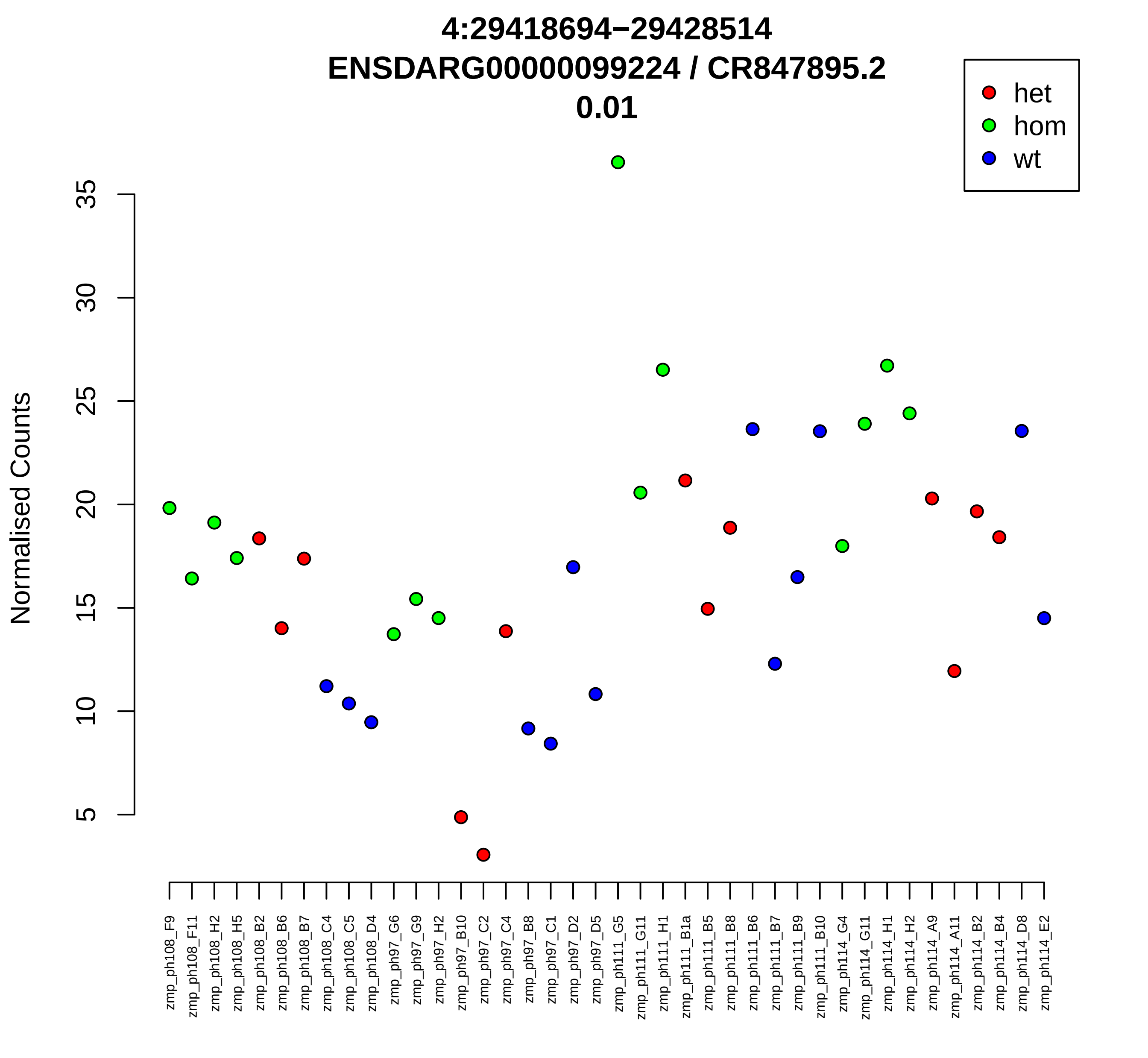

4:29418694−29428514
ENSDARG00000099224 / CR847895.2
0.01
het
hom
wt
Normalised Counts
5
10
15
20
25
30
35
zmp_ph108_F9
zmp_ph108_F11
zmp_ph108_H2
zmp_ph108_H5
zmp_ph108_B2
zmp_ph108_B6
zmp_ph108_B7
zmp_ph108_C4
zmp_ph108_C5
zmp_ph108_D4
zmp_ph97_G6
zmp_ph97_G9
zmp_ph97_H2
zmp_ph97_B10
zmp_ph97_C2
zmp_ph97_C4
zmp_ph97_B8
zmp_ph97_C1
zmp_ph97_D2
zmp_ph97_D5
zmp_ph111_G5
zmp_ph111_G11
zmp_ph111_H1
zmp_ph111_B1a
zmp_ph111_B5
zmp_ph111_B8
zmp_ph111_B6
zmp_ph111_B7
zmp_ph111_B9
zmp_ph111_B10
zmp_ph114_G4
zmp_ph114_G11
zmp_ph114_H1
zmp_ph114_H2
zmp_ph114_A9
zmp_ph114_A11
zmp_ph114_B2
zmp_ph114_B4
zmp_ph114_D8
zmp_ph114_E2

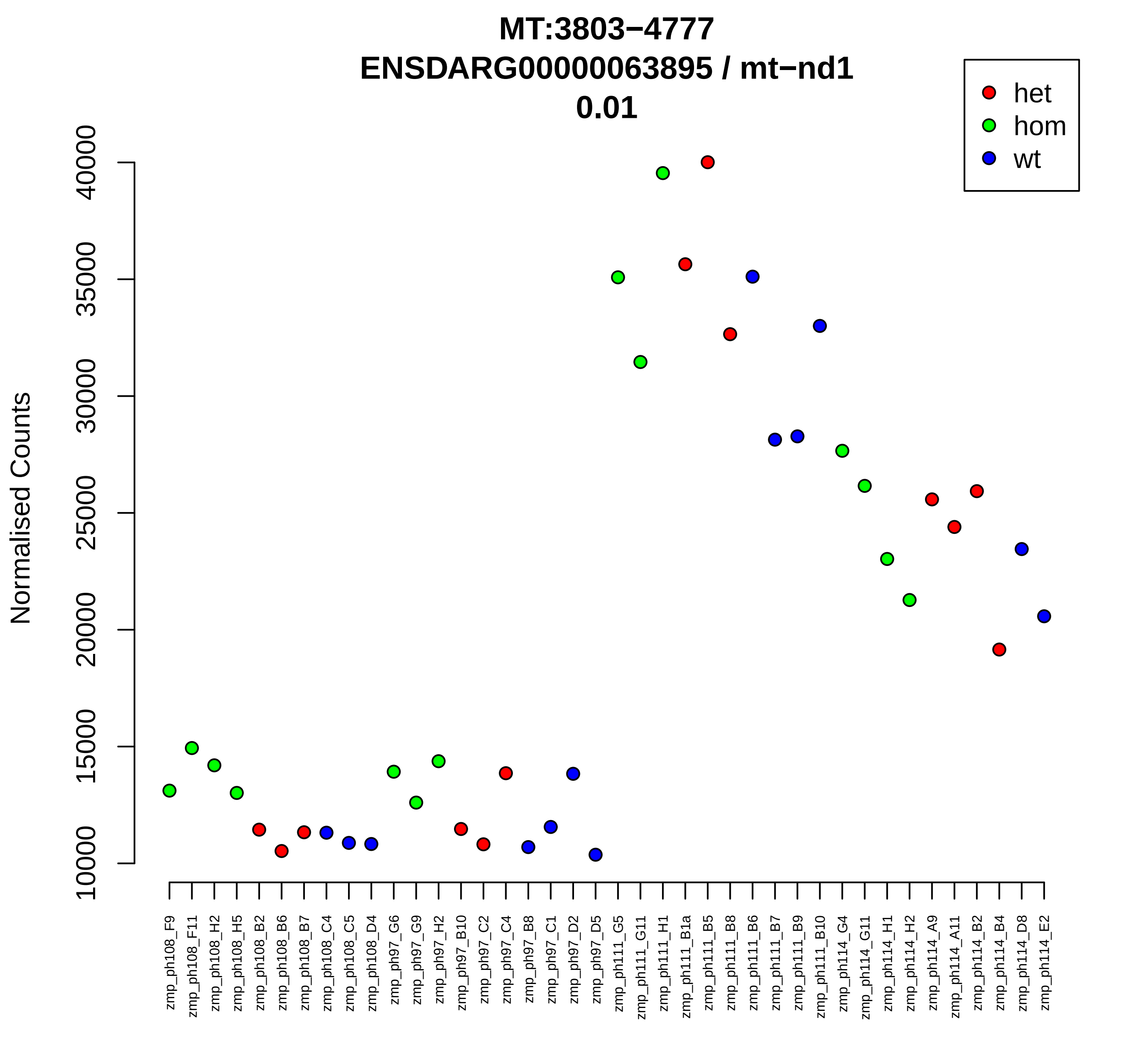
MT:3803−4777
ENSDARG00000063895 / mt−nd1
0.01
het
hom
wt
Normalised Counts
10000
15000
20000
25000
30000
35000
40000
zmp_ph108_F9
zmp_ph108_F11
zmp_ph108_H2
zmp_ph108_H5
zmp_ph108_B2
zmp_ph108_B6
zmp_ph108_B7
zmp_ph108_C4
zmp_ph108_C5
zmp_ph108_D4
zmp_ph97_G6
zmp_ph97_G9
zmp_ph97_H2
zmp_ph97_B10
zmp_ph97_C2
zmp_ph97_C4
zmp_ph97_B8
zmp_ph97_C1
zmp_ph97_D2
zmp_ph97_D5
zmp_ph111_G5
zmp_ph111_G11
zmp_ph111_H1
zmp_ph111_B1a
zmp_ph111_B5
zmp_ph111_B8
zmp_ph111_B6
zmp_ph111_B7
zmp_ph111_B9
zmp_ph111_B10
zmp_ph114_G4
zmp_ph114_G11
zmp_ph114_H1
zmp_ph114_H2
zmp_ph114_A9
zmp_ph114_A11
zmp_ph114_B2
zmp_ph114_B4
zmp_ph114_D8
zmp_ph114_E2

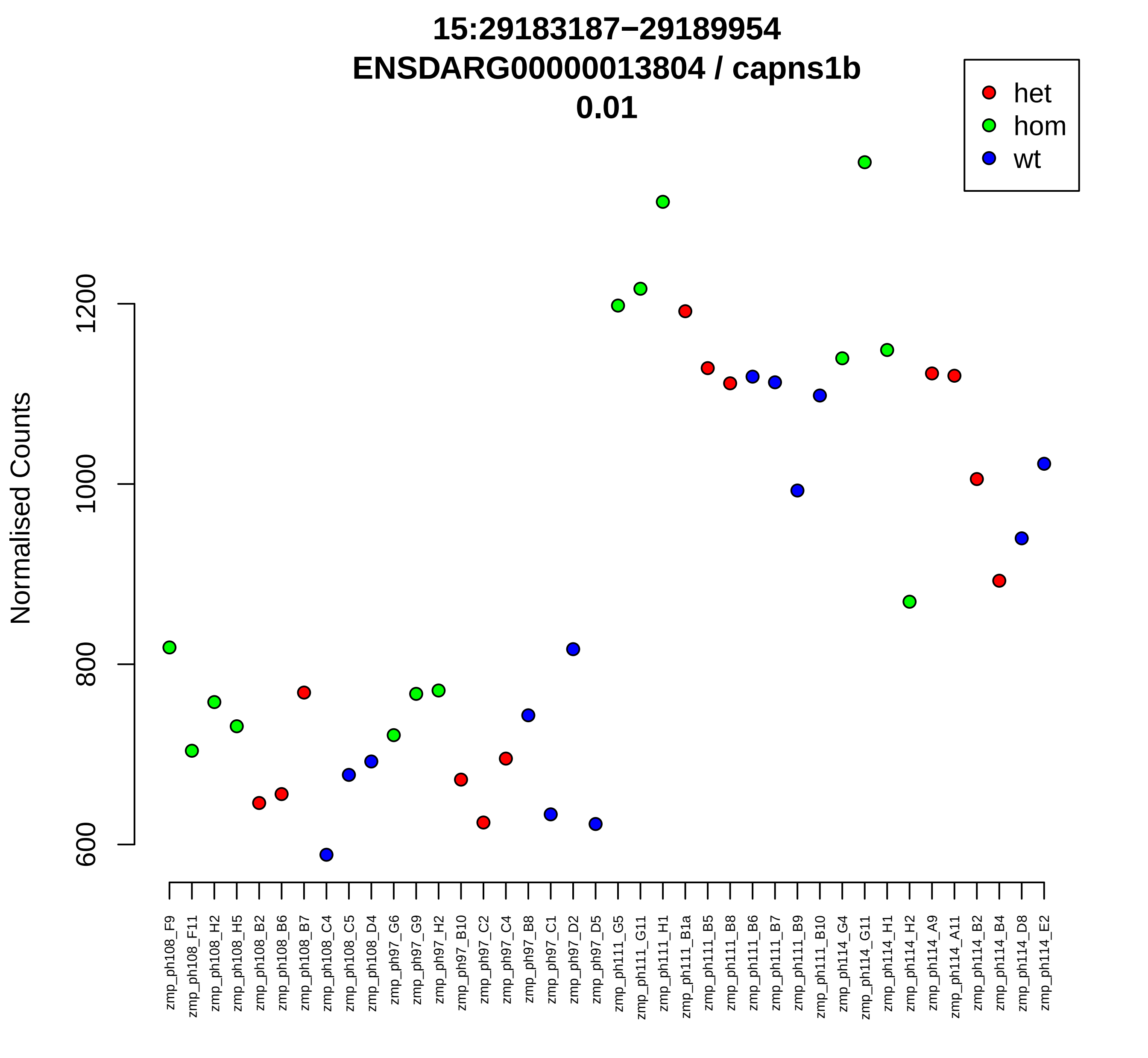

15:29183187−29189954
ENSDARG00000013804 / capns1b
0.01
het
hom
wt
Normalised Counts
600
800
1000
1200
zmp_ph108_F9
zmp_ph108_F11
zmp_ph108_H2
zmp_ph108_H5
zmp_ph108_B2
zmp_ph108_B6
zmp_ph108_B7
zmp_ph108_C4
zmp_ph108_C5
zmp_ph108_D4
zmp_ph97_G6
zmp_ph97_G9
zmp_ph97_H2
zmp_ph97_B10
zmp_ph97_C2
zmp_ph97_C4
zmp_ph97_B8
zmp_ph97_C1
zmp_ph97_D2
zmp_ph97_D5
zmp_ph111_G5
zmp_ph111_G11
zmp_ph111_H1
zmp_ph111_B1a
zmp_ph111_B5
zmp_ph111_B8
zmp_ph111_B6
zmp_ph111_B7
zmp_ph111_B9
zmp_ph111_B10
zmp_ph114_G4
zmp_ph114_G11
zmp_ph114_H1
zmp_ph114_H2
zmp_ph114_A9
zmp_ph114_A11
zmp_ph114_B2
zmp_ph114_B4
zmp_ph114_D8
zmp_ph114_E2

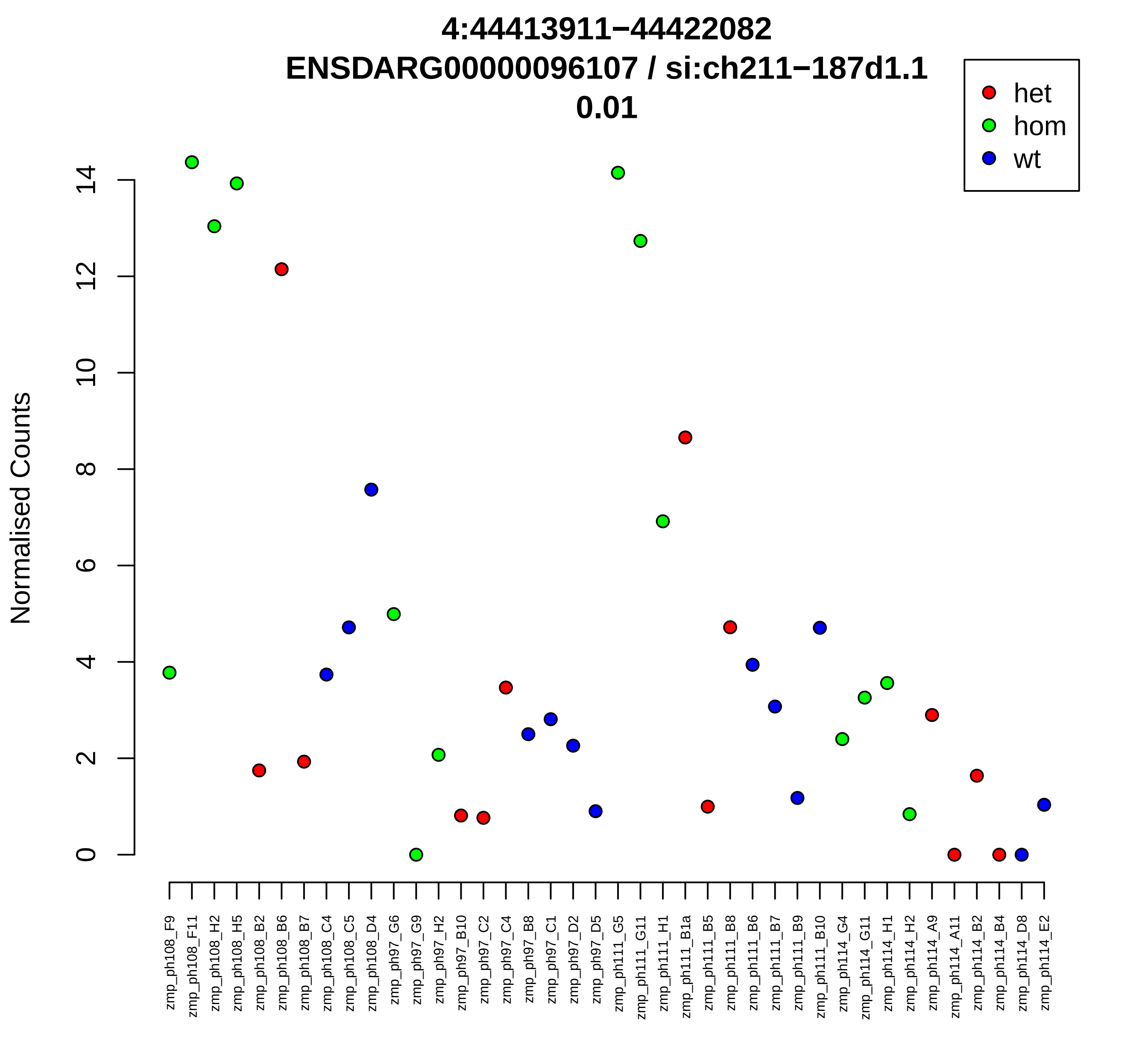
4:44413911−44422082
ENSDARG00000096107 / si:ch211−187d1.1
0.01
het
hom
wt
Normalised Counts
0
2
4
6
8
10
12
14
zmp_ph108_F9
zmp_ph108_F11
zmp_ph108_H2
zmp_ph108_H5
zmp_ph108_B2
zmp_ph108_B6
zmp_ph108_B7
zmp_ph108_C4
zmp_ph108_C5
zmp_ph108_D4
zmp_ph97_G6
zmp_ph97_G9
zmp_ph97_H2
zmp_ph97_B10
zmp_ph97_C2
zmp_ph97_C4
zmp_ph97_B8
zmp_ph97_C1
zmp_ph97_D2
zmp_ph97_D5
zmp_ph111_G5
zmp_ph111_G11
zmp_ph111_H1
zmp_ph111_B1a
zmp_ph111_B5
zmp_ph111_B8
zmp_ph111_B6
zmp_ph111_B7
zmp_ph111_B9
zmp_ph111_B10
zmp_ph114_G4
zmp_ph114_G11
zmp_ph114_H1
zmp_ph114_H2
zmp_ph114_A9
zmp_ph114_A11
zmp_ph114_B2
zmp_ph114_B4
zmp_ph114_D8
zmp_ph114_E2

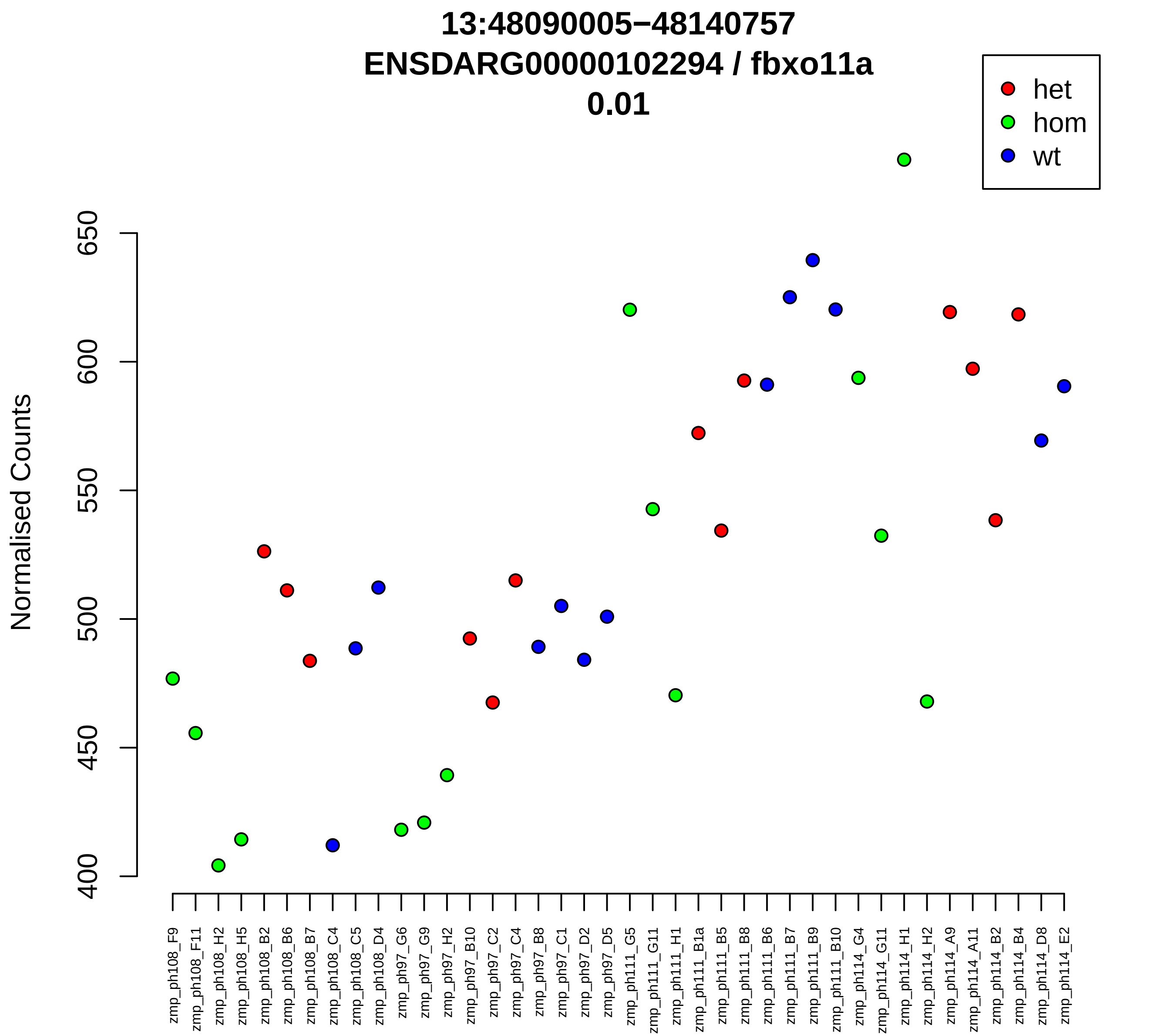
13:48090005−48140757
ENSDARG00000102294 / fbxo11a
0.01
het
hom
wt
Normalised Counts
400
450
500
550
600
650
zmp_ph108_F9
zmp_ph108_F11
zmp_ph108_H2
zmp_ph108_H5
zmp_ph108_B2
zmp_ph108_B6
zmp_ph108_B7
zmp_ph108_C4
zmp_ph108_C5
zmp_ph108_D4
zmp_ph97_G6
zmp_ph97_G9
zmp_ph97_H2
zmp_ph97_B10
zmp_ph97_C2
zmp_ph97_C4
zmp_ph97_B8
zmp_ph97_C1
zmp_ph97_D2
zmp_ph97_D5
zmp_ph111_G5
zmp_ph111_G11
zmp_ph111_H1
zmp_ph111_B1a
zmp_ph111_B5
zmp_ph111_B8
zmp_ph111_B6
zmp_ph111_B7
zmp_ph111_B9
zmp_ph111_B10
zmp_ph114_G4
zmp_ph114_G11
zmp_ph114_H1
zmp_ph114_H2
zmp_ph114_A9
zmp_ph114_A11
zmp_ph114_B2
zmp_ph114_B4
zmp_ph114_D8
zmp_ph114_E2

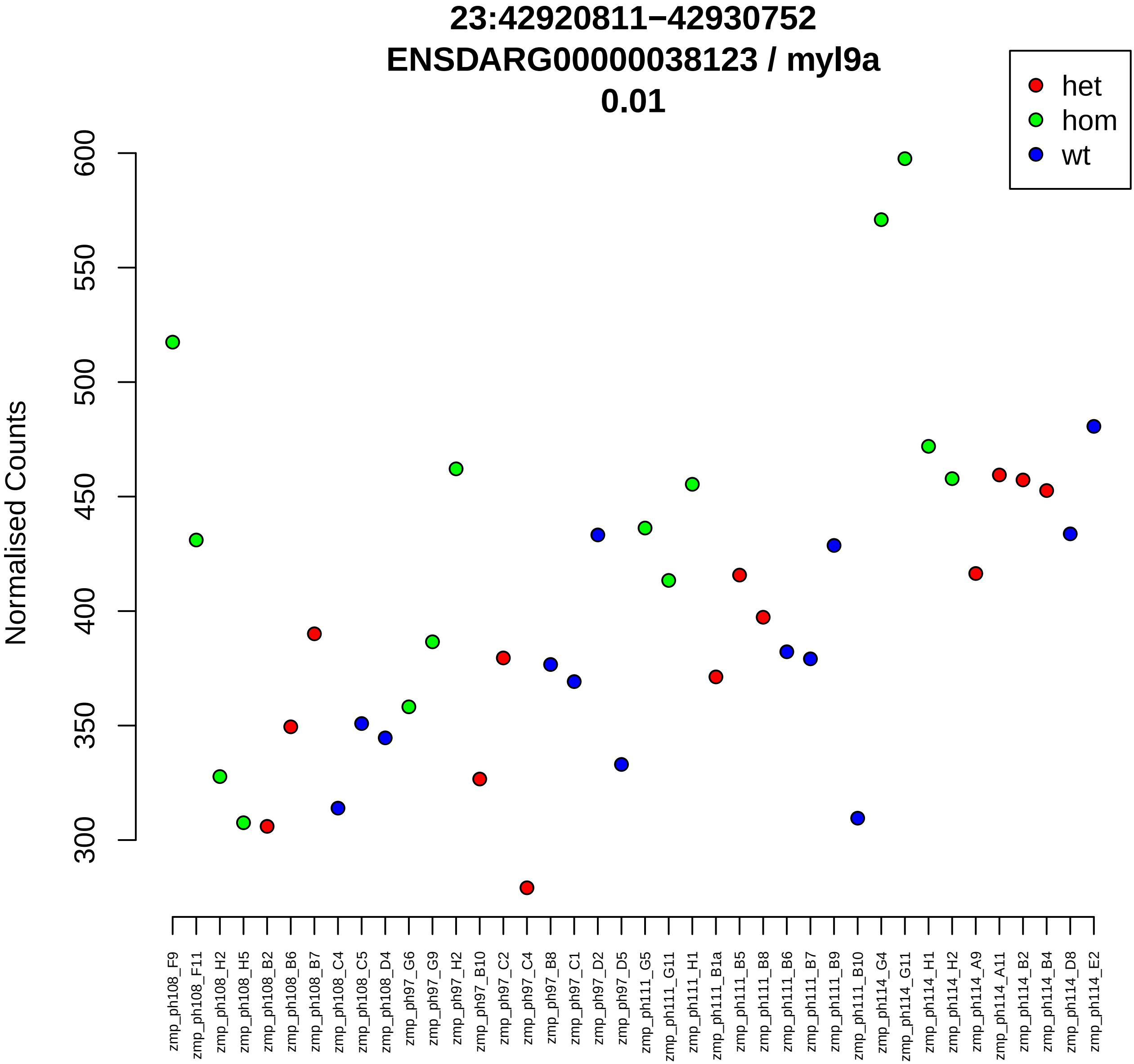

23:42920811−42930752
ENSDARG00000038123 / myl9a
0.01
het
hom
wt
Normalised Counts
300
350
400
450
500
550
600
zmp_ph108_F9
zmp_ph108_F11
zmp_ph108_H2
zmp_ph108_H5
zmp_ph108_B2
zmp_ph108_B6
zmp_ph108_B7
zmp_ph108_C4
zmp_ph108_C5
zmp_ph108_D4
zmp_ph97_G6
zmp_ph97_G9
zmp_ph97_H2
zmp_ph97_B10
zmp_ph97_C2
zmp_ph97_C4
zmp_ph97_B8
zmp_ph97_C1
zmp_ph97_D2
zmp_ph97_D5
zmp_ph111_G5
zmp_ph111_G11
zmp_ph111_H1
zmp_ph111_B1a
zmp_ph111_B5
zmp_ph111_B8
zmp_ph111_B6
zmp_ph111_B7
zmp_ph111_B9
zmp_ph111_B10
zmp_ph114_G4
zmp_ph114_G11
zmp_ph114_H1
zmp_ph114_H2
zmp_ph114_A9
zmp_ph114_A11
zmp_ph114_B2
zmp_ph114_B4
zmp_ph114_D8
zmp_ph114_E2

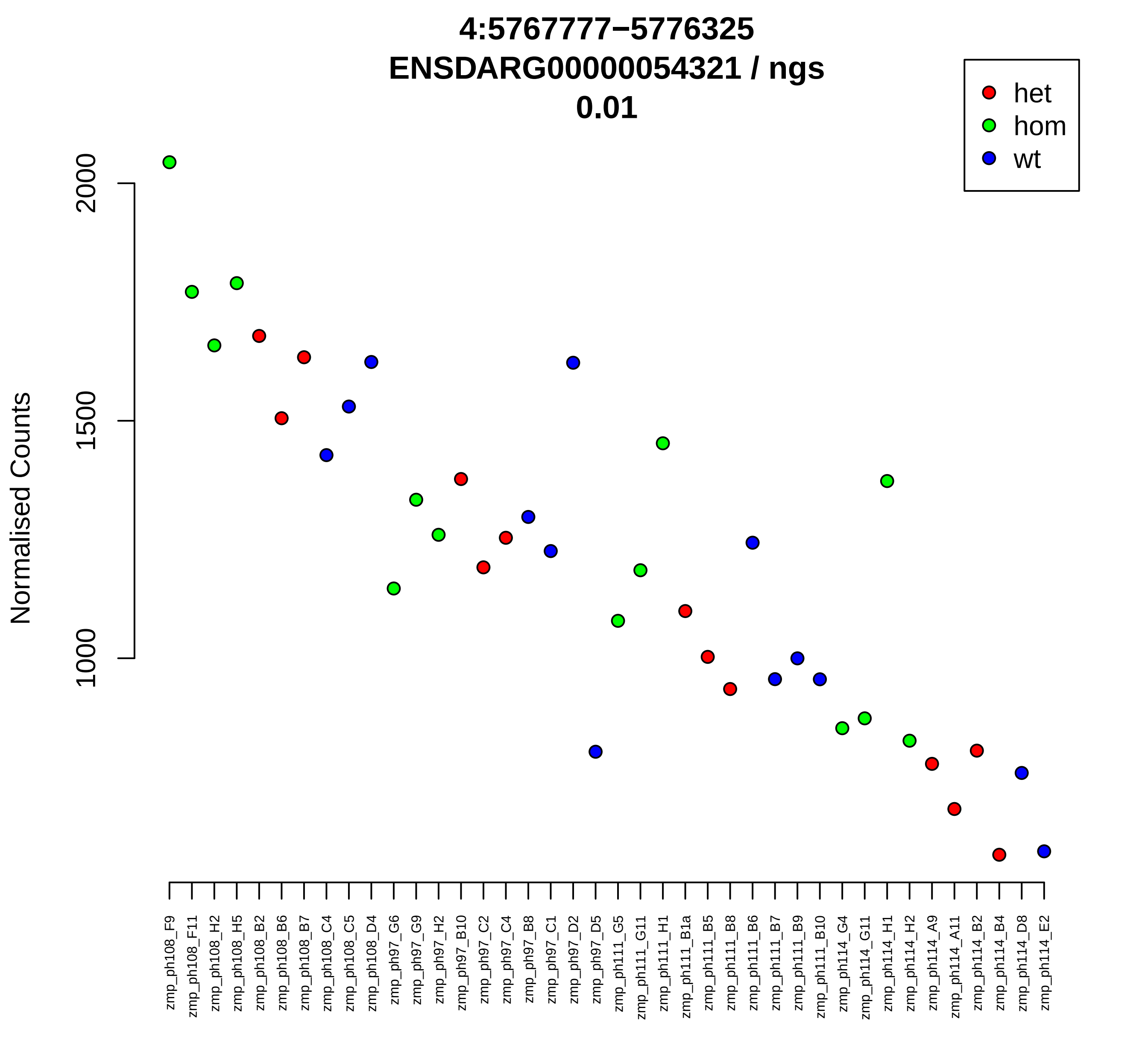
4:5767777−5776325
ENSDARG00000054321 / ngs
0.01
het
hom
wt
Normalised Counts
2000
1500
1000
zmp_ph108_F9
zmp_ph108_F11
zmp_ph108_H2
zmp_ph108_H5
zmp_ph108_B2
zmp_ph108_B6
zmp_ph108_B7
zmp_ph108_C4
zmp_ph108_C5
zmp_ph108_D4
zmp_ph97_G6
zmp_ph97_G9
zmp_ph97_H2
zmp_ph97_B10
zmp_ph97_C2
zmp_ph97_C4
zmp_ph97_B8
zmp_ph97_C1
zmp_ph97_D2
zmp_ph97_D5
zmp_ph111_G5
zmp_ph111_G11
zmp_ph111_H1
zmp_ph111_B1a
zmp_ph111_B5
zmp_ph111_B8
zmp_ph111_B6
zmp_ph111_B7
zmp_ph111_B9
zmp_ph111_B10
zmp_ph114_G4
zmp_ph114_G11
zmp_ph114_H1
zmp_ph114_H2
zmp_ph114_A9
zmp_ph114_A11
zmp_ph114_B2
zmp_ph114_B4
zmp_ph114_D8
zmp_ph114_E2

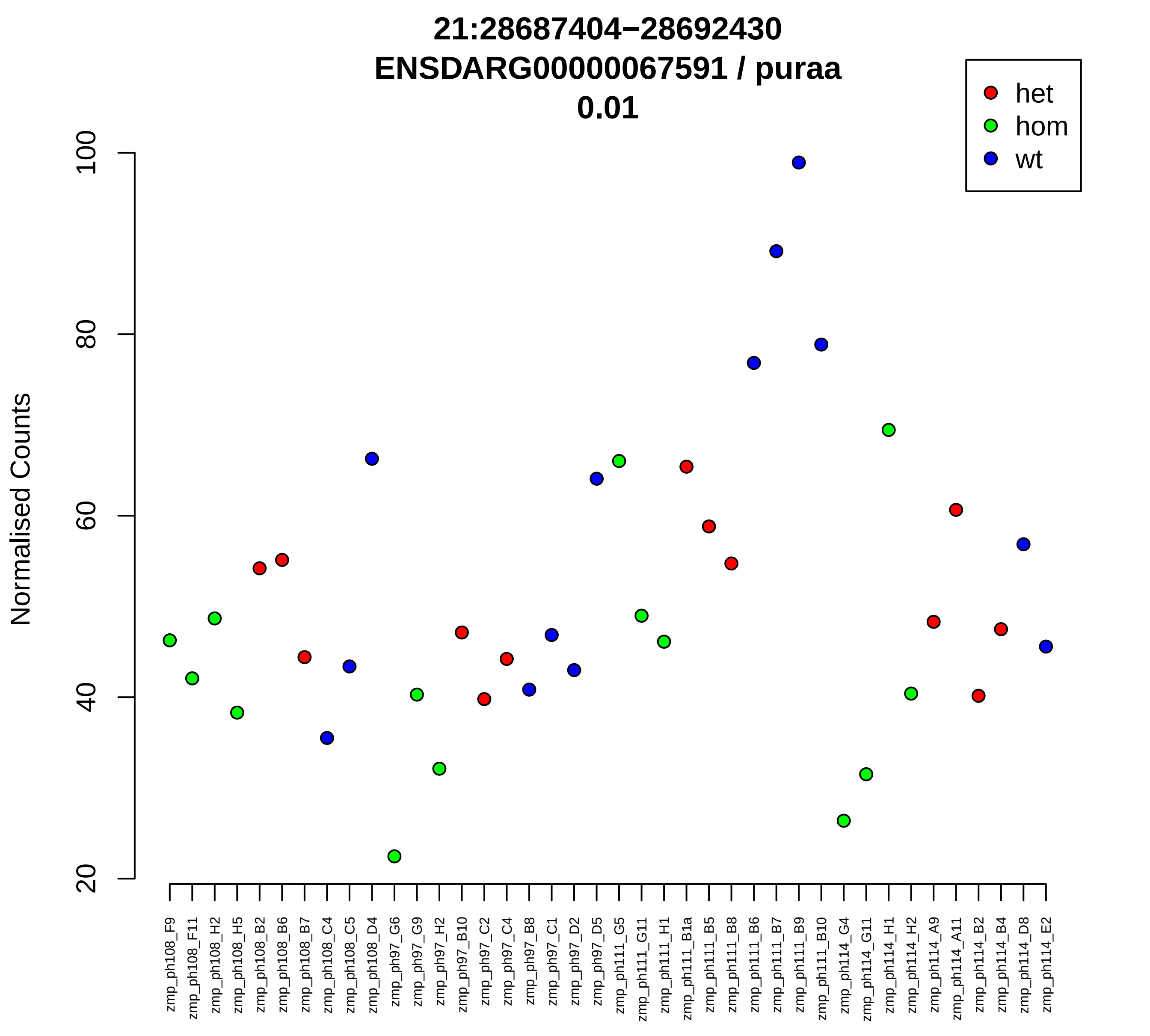
21:28687404−28692430
ENSDARG00000067591 / puraa
0.01
het
hom
wt
Normalised Counts
20
40
60
80
100
zmp_ph108_F9
zmp_ph108_F11
zmp_ph108_H2
zmp_ph108_H5
zmp_ph108_B2
zmp_ph108_B6
zmp_ph108_B7
zmp_ph108_C4
zmp_ph108_C5
zmp_ph108_D4
zmp_ph97_G6
zmp_ph97_G9
zmp_ph97_H2
zmp_ph97_B10
zmp_ph97_C2
zmp_ph97_C4
zmp_ph97_B8
zmp_ph97_C1
zmp_ph97_D2
zmp_ph97_D5
zmp_ph111_G5
zmp_ph111_G11
zmp_ph111_H1
zmp_ph111_B1a
zmp_ph111_B5
zmp_ph111_B8
zmp_ph111_B6
zmp_ph111_B7
zmp_ph111_B9
zmp_ph111_B10
zmp_ph114_G4
zmp_ph114_G11
zmp_ph114_H1
zmp_ph114_H2
zmp_ph114_A9
zmp_ph114_A11
zmp_ph114_B2
zmp_ph114_B4
zmp_ph114_D8
zmp_ph114_E2

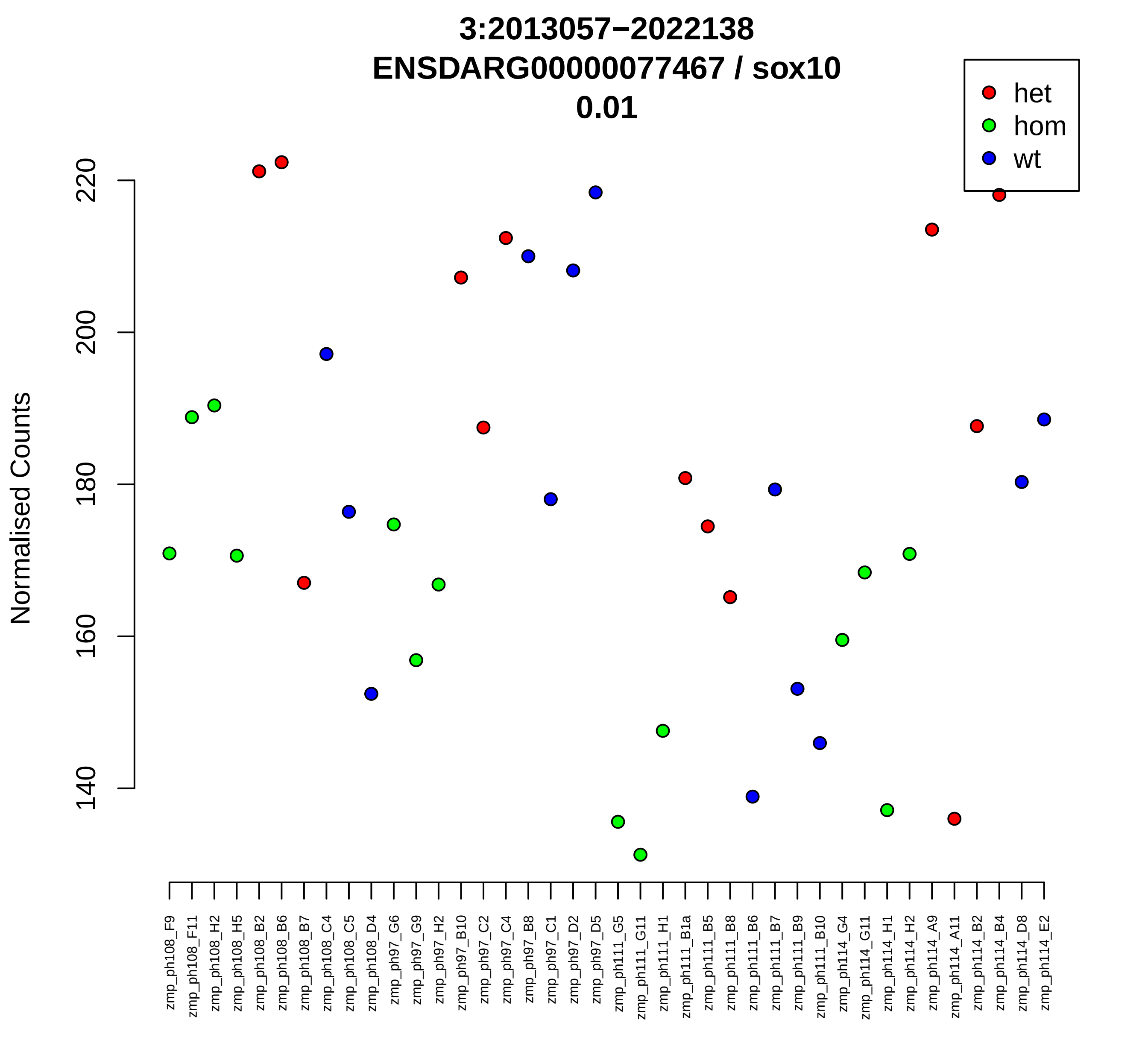

3:2013057−2022138
ENSDARG00000077467 / sox10
0.01
het
hom
wt
Normalised Counts
140
160
180
200
220
zmp_ph108_F9
zmp_ph108_F11
zmp_ph108_H2
zmp_ph108_H5
zmp_ph108_B2
zmp_ph108_B6
zmp_ph108_B7
zmp_ph108_C4
zmp_ph108_C5
zmp_ph108_D4
zmp_ph97_G6
zmp_ph97_G9
zmp_ph97_H2
zmp_ph97_B10
zmp_ph97_C2
zmp_ph97_C4
zmp_ph97_B8
zmp_ph97_C1
zmp_ph97_D2
zmp_ph97_D5
zmp_ph111_G5
zmp_ph111_G11
zmp_ph111_H1
zmp_ph111_B1a
zmp_ph111_B5
zmp_ph111_B8
zmp_ph111_B6
zmp_ph111_B7
zmp_ph111_B9
zmp_ph111_B10
zmp_ph114_G4
zmp_ph114_G11
zmp_ph114_H1
zmp_ph114_H2
zmp_ph114_A9
zmp_ph114_A11
zmp_ph114_B2
zmp_ph114_B4
zmp_ph114_D8
zmp_ph114_E2

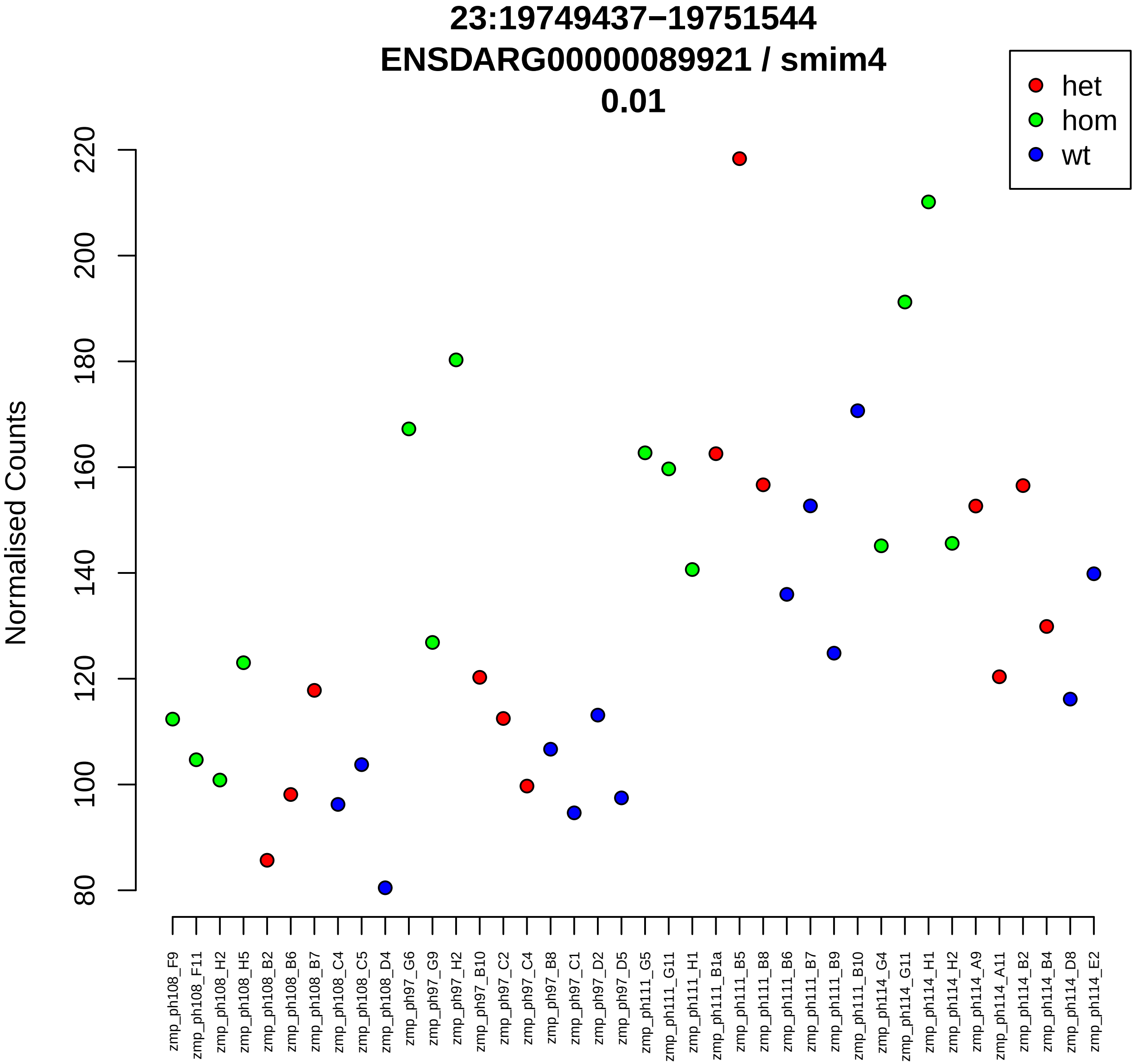
23:19749437−19751544
ENSDARG00000089921 / smim4
0.01
het
hom
wt
Normalised Counts
80
100
120
140
160
180
200
220
zmp_ph108_F9
zmp_ph108_F11
zmp_ph108_H2
zmp_ph108_H5
zmp_ph108_B2
zmp_ph108_B6
zmp_ph108_B7
zmp_ph108_C4
zmp_ph108_C5
zmp_ph108_D4
zmp_ph97_G6
zmp_ph97_G9
zmp_ph97_H2
zmp_ph97_B10
zmp_ph97_C2
zmp_ph97_C4
zmp_ph97_B8
zmp_ph97_C1
zmp_ph97_D2
zmp_ph97_D5
zmp_ph111_G5
zmp_ph111_G11
zmp_ph111_H1
zmp_ph111_B1a
zmp_ph111_B5
zmp_ph111_B8
zmp_ph111_B6
zmp_ph111_B7
zmp_ph111_B9
zmp_ph111_B10
zmp_ph114_G4
zmp_ph114_G11
zmp_ph114_H1
zmp_ph114_H2
zmp_ph114_A9
zmp_ph114_A11
zmp_ph114_B2
zmp_ph114_B4
zmp_ph114_D8
zmp_ph114_E2

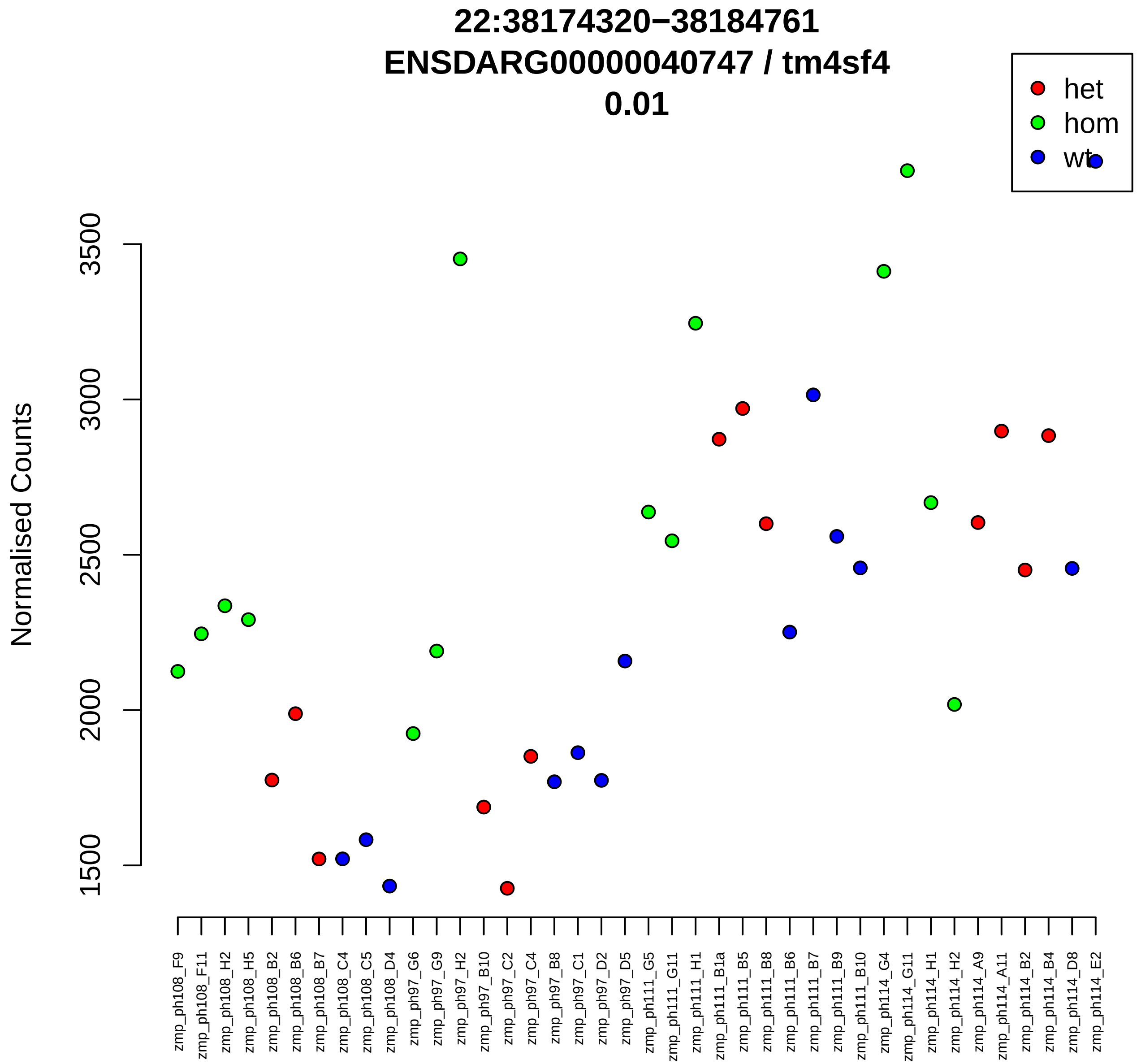
22:38174320−38184761
ENSDARG00000040747 / tm4sf4
0.01
het
hom
wt
Normalised Counts
1500
2000
2500
3000
3500
zmp_ph108_F9
zmp_ph108_F11
zmp_ph108_H2
zmp_ph108_H5
zmp_ph108_B2
zmp_ph108_B6
zmp_ph108_B7
zmp_ph108_C4
zmp_ph108_C5
zmp_ph108_D4
zmp_ph97_G6
zmp_ph97_G9
zmp_ph97_H2
zmp_ph97_B10
zmp_ph97_C2
zmp_ph97_C4
zmp_ph97_B8
zmp_ph97_C1
zmp_ph97_D2
zmp_ph97_D5
zmp_ph111_G5
zmp_ph111_G11
zmp_ph111_H1
zmp_ph111_B1a
zmp_ph111_B5
zmp_ph111_B8
zmp_ph111_B6
zmp_ph111_B7
zmp_ph111_B9
zmp_ph111_B10
zmp_ph114_G4
zmp_ph114_G11
zmp_ph114_H1
zmp_ph114_H2
zmp_ph114_A9
zmp_ph114_A11
zmp_ph114_B2
zmp_ph114_B4
zmp_ph114_D8
zmp_ph114_E2

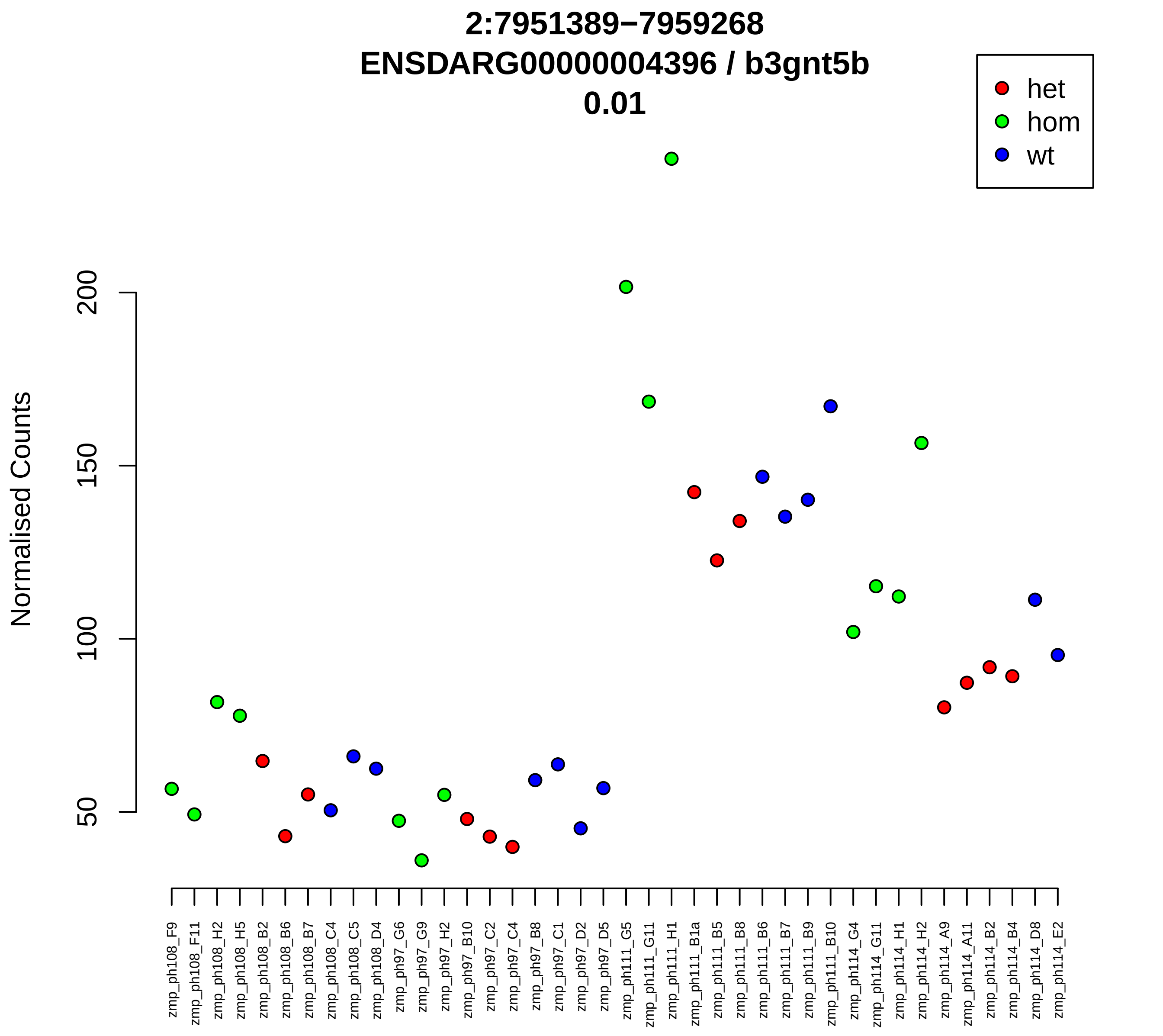

2:7951389−7959268
ENSDARG00000004396 / b3gnt5b
0.01
het
hom
wt
Normalised Counts
50
100
150
200
zmp_ph108_F9
zmp_ph108_F11
zmp_ph108_H2
zmp_ph108_H5
zmp_ph108_B2
zmp_ph108_B6
zmp_ph108_B7
zmp_ph108_C4
zmp_ph108_C5
zmp_ph108_D4
zmp_ph97_G6
zmp_ph97_G9
zmp_ph97_H2
zmp_ph97_B10
zmp_ph97_C2
zmp_ph97_C4
zmp_ph97_B8
zmp_ph97_C1
zmp_ph97_D2
zmp_ph97_D5
zmp_ph111_G5
zmp_ph111_G11
zmp_ph111_H1
zmp_ph111_B1a
zmp_ph111_B5
zmp_ph111_B8
zmp_ph111_B6
zmp_ph111_B7
zmp_ph111_B9
zmp_ph111_B10
zmp_ph114_G4
zmp_ph114_G11
zmp_ph114_H1
zmp_ph114_H2
zmp_ph114_A9
zmp_ph114_A11
zmp_ph114_B2
zmp_ph114_B4
zmp_ph114_D8
zmp_ph114_E2

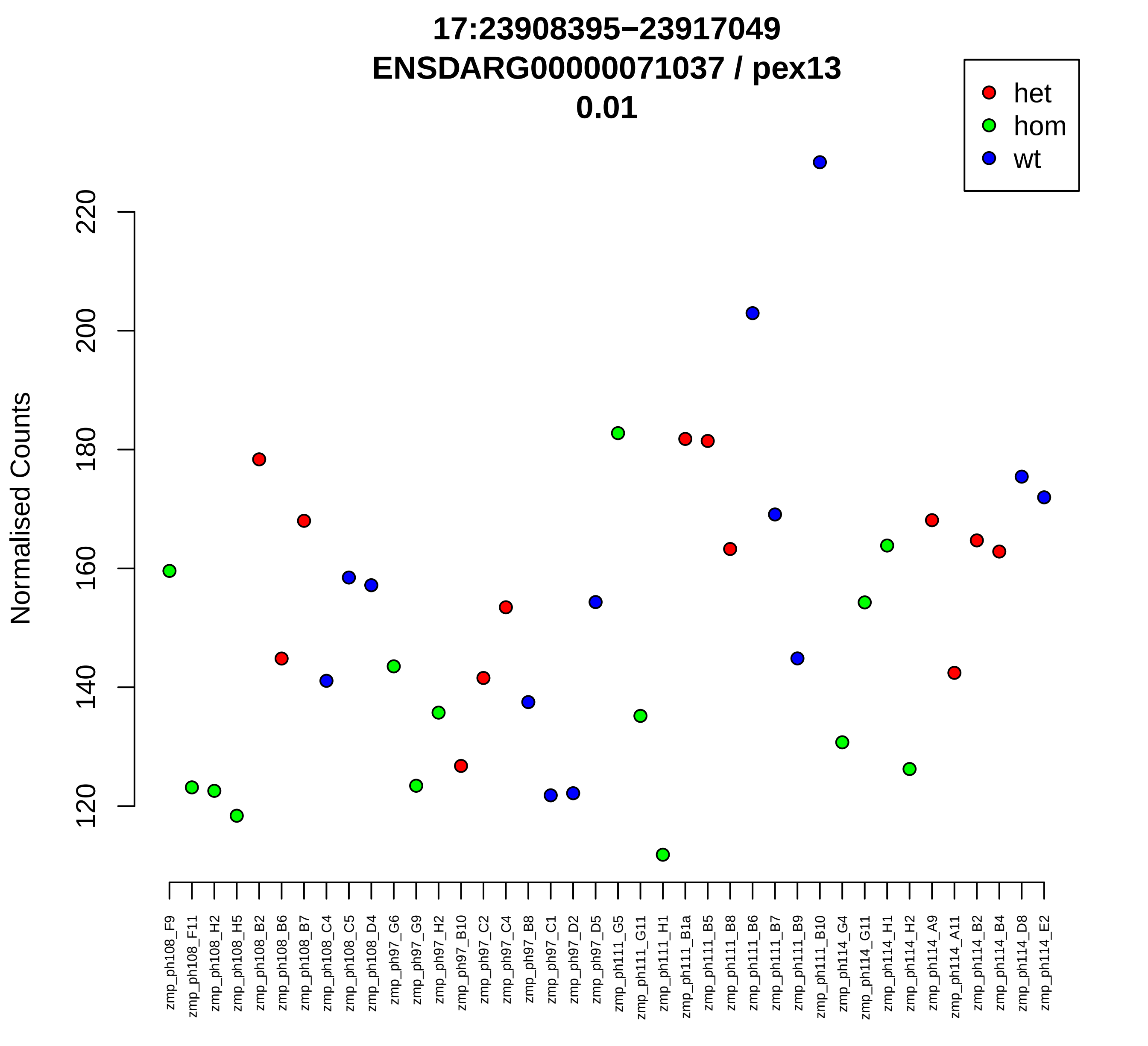

17:23908395−23917049
ENSDARG00000071037 / pex13
0.01
het
hom
wt
Normalised Counts
120
140
160
180
200
220
zmp_ph108_F9
zmp_ph108_F11
zmp_ph108_H2
zmp_ph108_H5
zmp_ph108_B2
zmp_ph108_B6
zmp_ph108_B7
zmp_ph108_C4
zmp_ph108_C5
zmp_ph108_D4
zmp_ph97_G6
zmp_ph97_G9
zmp_ph97_H2
zmp_ph97_B10
zmp_ph97_C2
zmp_ph97_C4
zmp_ph97_B8
zmp_ph97_C1
zmp_ph97_D2
zmp_ph97_D5
zmp_ph111_G5
zmp_ph111_G11
zmp_ph111_H1
zmp_ph111_B1a
zmp_ph111_B5
zmp_ph111_B8
zmp_ph111_B6
zmp_ph111_B7
zmp_ph111_B9
zmp_ph111_B10
zmp_ph114_G4
zmp_ph114_G11
zmp_ph114_H1
zmp_ph114_H2
zmp_ph114_A9
zmp_ph114_A11
zmp_ph114_B2
zmp_ph114_B4
zmp_ph114_D8
zmp_ph114_E2

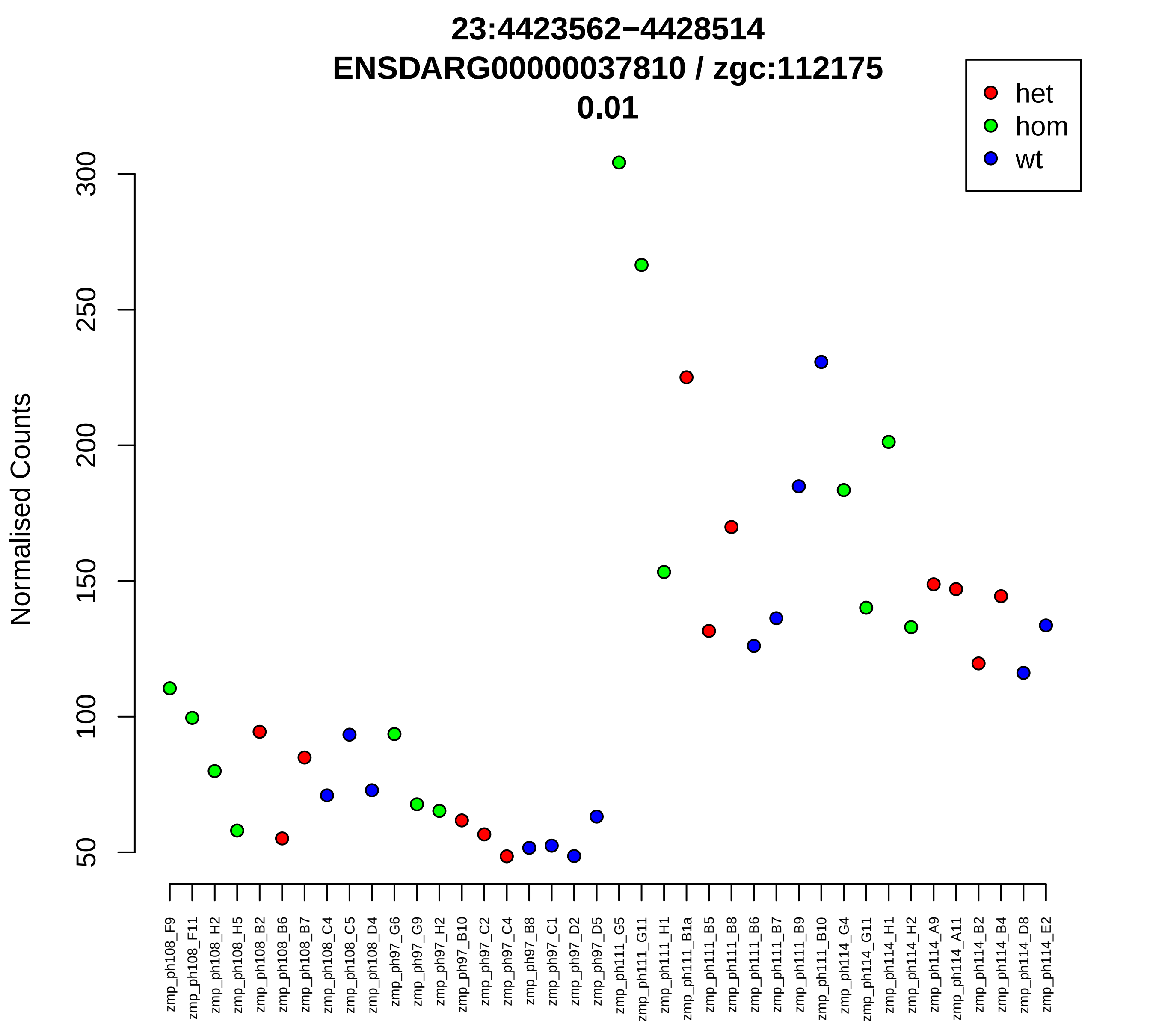

23:4423562−4428514
ENSDARG00000037810 / zgc:112175
0.01
het
hom
wt
Normalised Counts
50
100
150
200
250
300
zmp_ph108_F9
zmp_ph108_F11
zmp_ph108_H2
zmp_ph108_H5
zmp_ph108_B2
zmp_ph108_B6
zmp_ph108_B7
zmp_ph108_C4
zmp_ph108_C5
zmp_ph108_D4
zmp_ph97_G6
zmp_ph97_G9
zmp_ph97_H2
zmp_ph97_B10
zmp_ph97_C2
zmp_ph97_C4
zmp_ph97_B8
zmp_ph97_C1
zmp_ph97_D2
zmp_ph97_D5
zmp_ph111_G5
zmp_ph111_G11
zmp_ph111_H1
zmp_ph111_B1a
zmp_ph111_B5
zmp_ph111_B8
zmp_ph111_B6
zmp_ph111_B7
zmp_ph111_B9
zmp_ph111_B10
zmp_ph114_G4
zmp_ph114_G11
zmp_ph114_H1
zmp_ph114_H2
zmp_ph114_A9
zmp_ph114_A11
zmp_ph114_B2
zmp_ph114_B4
zmp_ph114_D8
zmp_ph114_E2

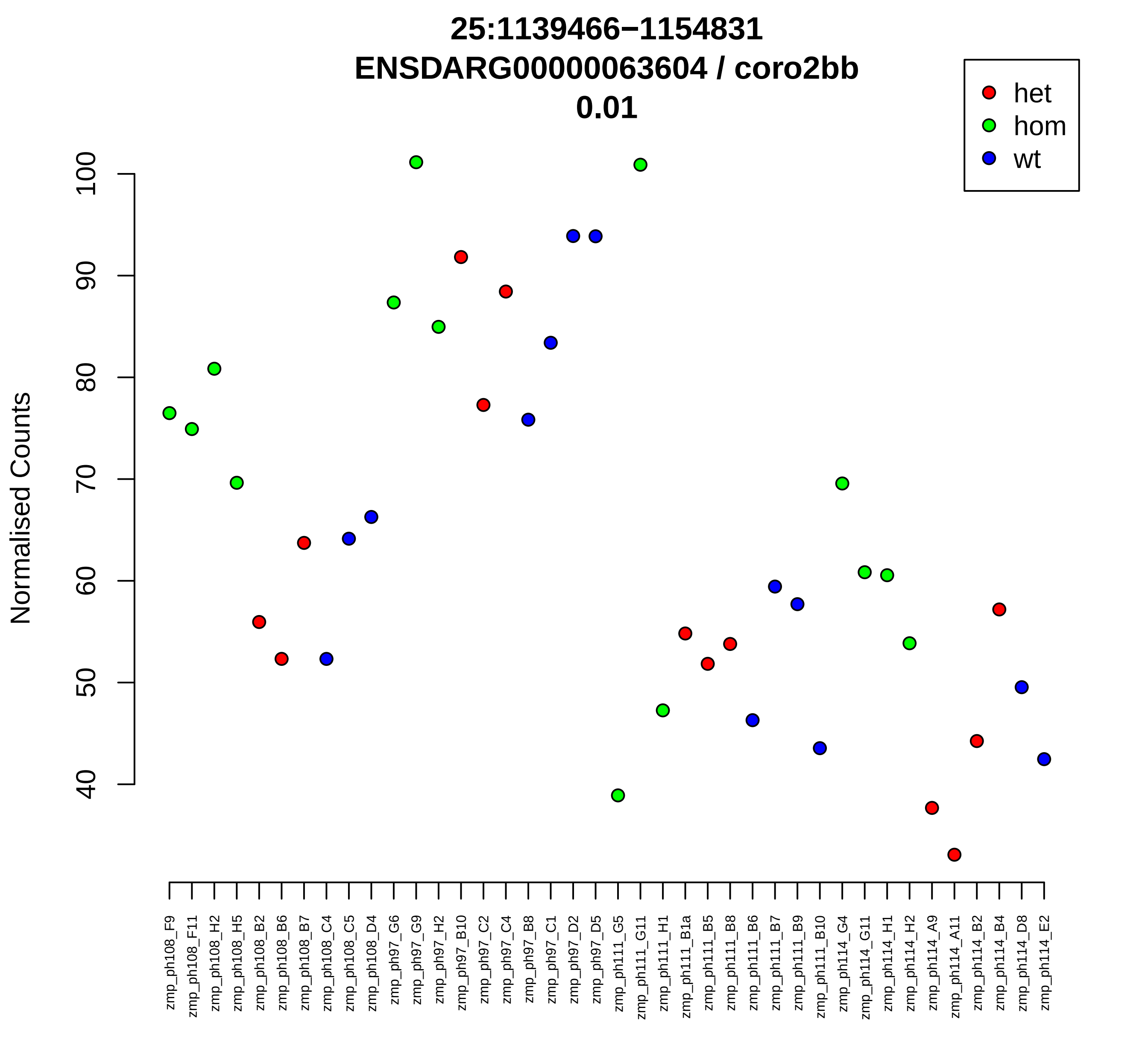

25:1139466−1154831
ENSDARG00000063604 / coro2bb
0.01
het
hom
wt
Normalised Counts
100
90
80
70
60
50
40
zmp_ph108_F9
zmp_ph108_F11
zmp_ph108_H2
zmp_ph108_H5
zmp_ph108_B2
zmp_ph108_B6
zmp_ph108_B7
zmp_ph108_C4
zmp_ph108_C5
zmp_ph108_D4
zmp_ph97_G6
zmp_ph97_G9
zmp_ph97_H2
zmp_ph97_B10
zmp_ph97_C2
zmp_ph97_C4
zmp_ph97_B8
zmp_ph97_C1
zmp_ph97_D2
zmp_ph97_D5
zmp_ph111_G5
zmp_ph111_G11
zmp_ph111_H1
zmp_ph111_B1a
zmp_ph111_B5
zmp_ph111_B8
zmp_ph111_B6
zmp_ph111_B7
zmp_ph111_B9
zmp_ph111_B10
zmp_ph114_G4
zmp_ph114_G11
zmp_ph114_H1
zmp_ph114_H2
zmp_ph114_A9
zmp_ph114_A11
zmp_ph114_B2
zmp_ph114_B4
zmp_ph114_D8
zmp_ph114_E2

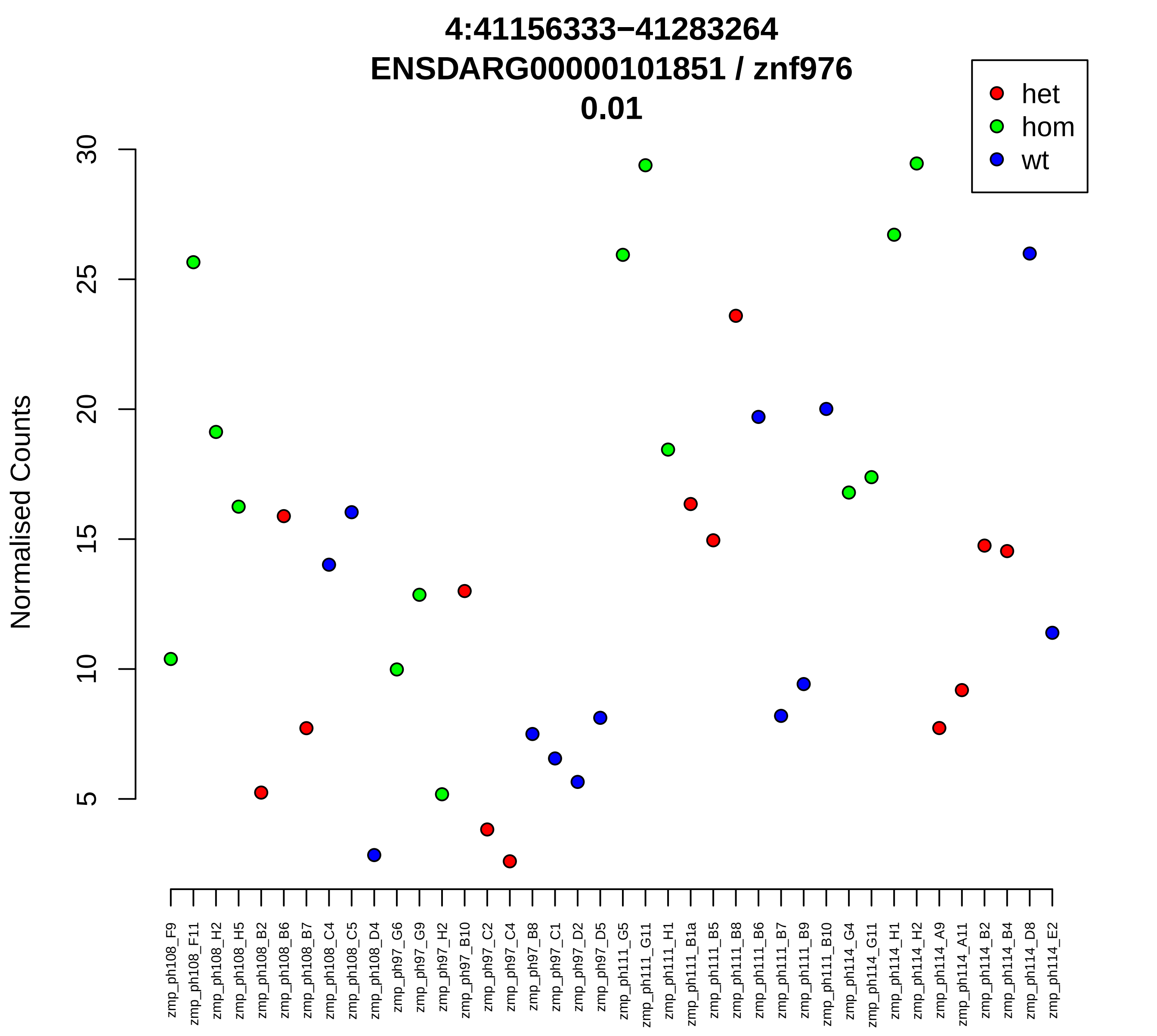

4:41156333−41283264
ENSDARG00000101851 / znf976
0.01
het
hom
wt
Normalised Counts
5
10
15
20
25
30
zmp_ph108_F9
zmp_ph108_F11
zmp_ph108_H2
zmp_ph108_H5
zmp_ph108_B2
zmp_ph108_B6
zmp_ph108_B7
zmp_ph108_C4
zmp_ph108_C5
zmp_ph108_D4
zmp_ph97_G6
zmp_ph97_G9
zmp_ph97_H2
zmp_ph97_B10
zmp_ph97_C2
zmp_ph97_C4
zmp_ph97_B8
zmp_ph97_C1
zmp_ph97_D2
zmp_ph97_D5
zmp_ph111_G5
zmp_ph111_G11
zmp_ph111_H1
zmp_ph111_B1a
zmp_ph111_B5
zmp_ph111_B8
zmp_ph111_B6
zmp_ph111_B7
zmp_ph111_B9
zmp_ph111_B10
zmp_ph114_G4
zmp_ph114_G11
zmp_ph114_H1
zmp_ph114_H2
zmp_ph114_A9
zmp_ph114_A11
zmp_ph114_B2
zmp_ph114_B4
zmp_ph114_D8
zmp_ph114_E2

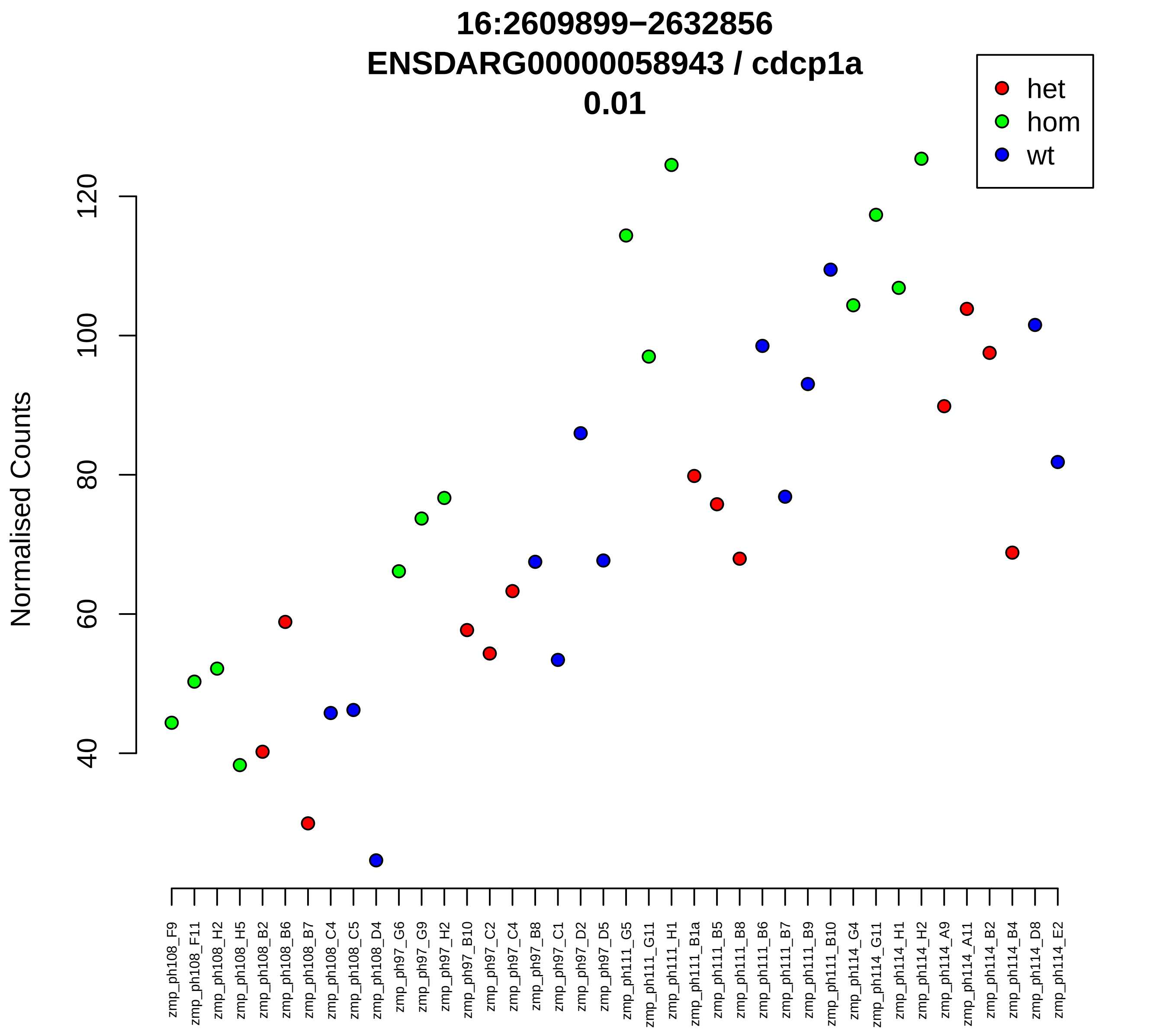
16:2609899−2632856
ENSDARG00000058943 / cdcp1a
0.01
het
hom
wt
Normalised Counts
40
60
80
100
120
zmp_ph108_F9
zmp_ph108_F11
zmp_ph108_H2
zmp_ph108_H5
zmp_ph108_B2
zmp_ph108_B6
zmp_ph108_B7
zmp_ph108_C4
zmp_ph108_C5
zmp_ph108_D4
zmp_ph97_G6
zmp_ph97_G9
zmp_ph97_H2
zmp_ph97_B10
zmp_ph97_C2
zmp_ph97_C4
zmp_ph97_B8
zmp_ph97_C1
zmp_ph97_D2
zmp_ph97_D5
zmp_ph111_G5
zmp_ph111_G11
zmp_ph111_H1
zmp_ph111_B1a
zmp_ph111_B5
zmp_ph111_B8
zmp_ph111_B6
zmp_ph111_B7
zmp_ph111_B9
zmp_ph111_B10
zmp_ph114_G4
zmp_ph114_G11
zmp_ph114_H1
zmp_ph114_H2
zmp_ph114_A9
zmp_ph114_A11
zmp_ph114_B2
zmp_ph114_B4
zmp_ph114_D8
zmp_ph114_E2

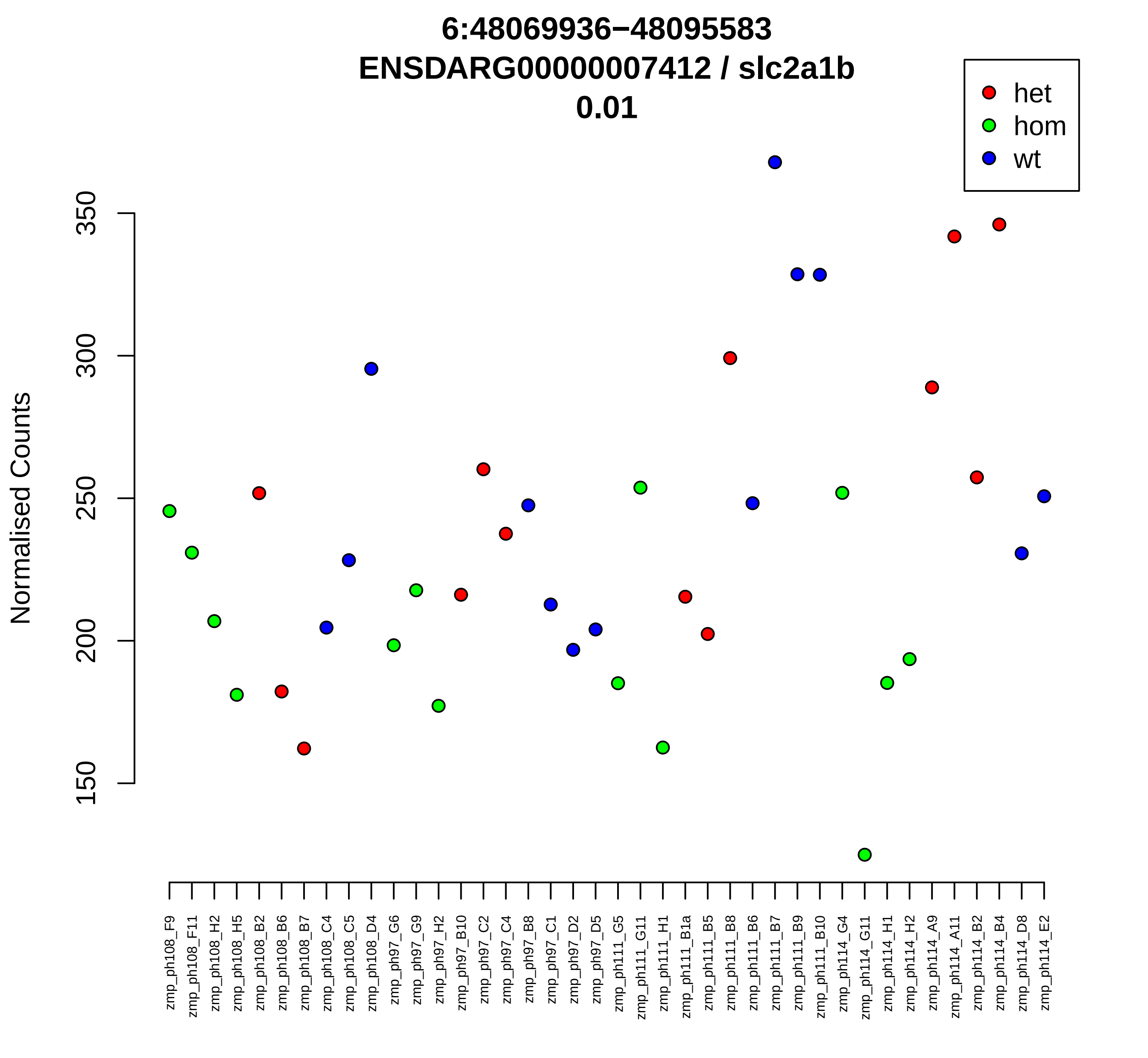

6:48069936−48095583
ENSDARG00000007412 / slc2a1b
0.01
het
hom
wt
Normalised Counts
150
200
250
300
350
zmp_ph108_F9
zmp_ph108_F11
zmp_ph108_H2
zmp_ph108_H5
zmp_ph108_B2
zmp_ph108_B6
zmp_ph108_B7
zmp_ph108_C4
zmp_ph108_C5
zmp_ph108_D4
zmp_ph97_G6
zmp_ph97_G9
zmp_ph97_H2
zmp_ph97_B10
zmp_ph97_C2
zmp_ph97_C4
zmp_ph97_B8
zmp_ph97_C1
zmp_ph97_D2
zmp_ph97_D5
zmp_ph111_G5
zmp_ph111_G11
zmp_ph111_H1
zmp_ph111_B1a
zmp_ph111_B5
zmp_ph111_B8
zmp_ph111_B6
zmp_ph111_B7
zmp_ph111_B9
zmp_ph111_B10
zmp_ph114_G4
zmp_ph114_G11
zmp_ph114_H1
zmp_ph114_H2
zmp_ph114_A9
zmp_ph114_A11
zmp_ph114_B2
zmp_ph114_B4
zmp_ph114_D8
zmp_ph114_E2

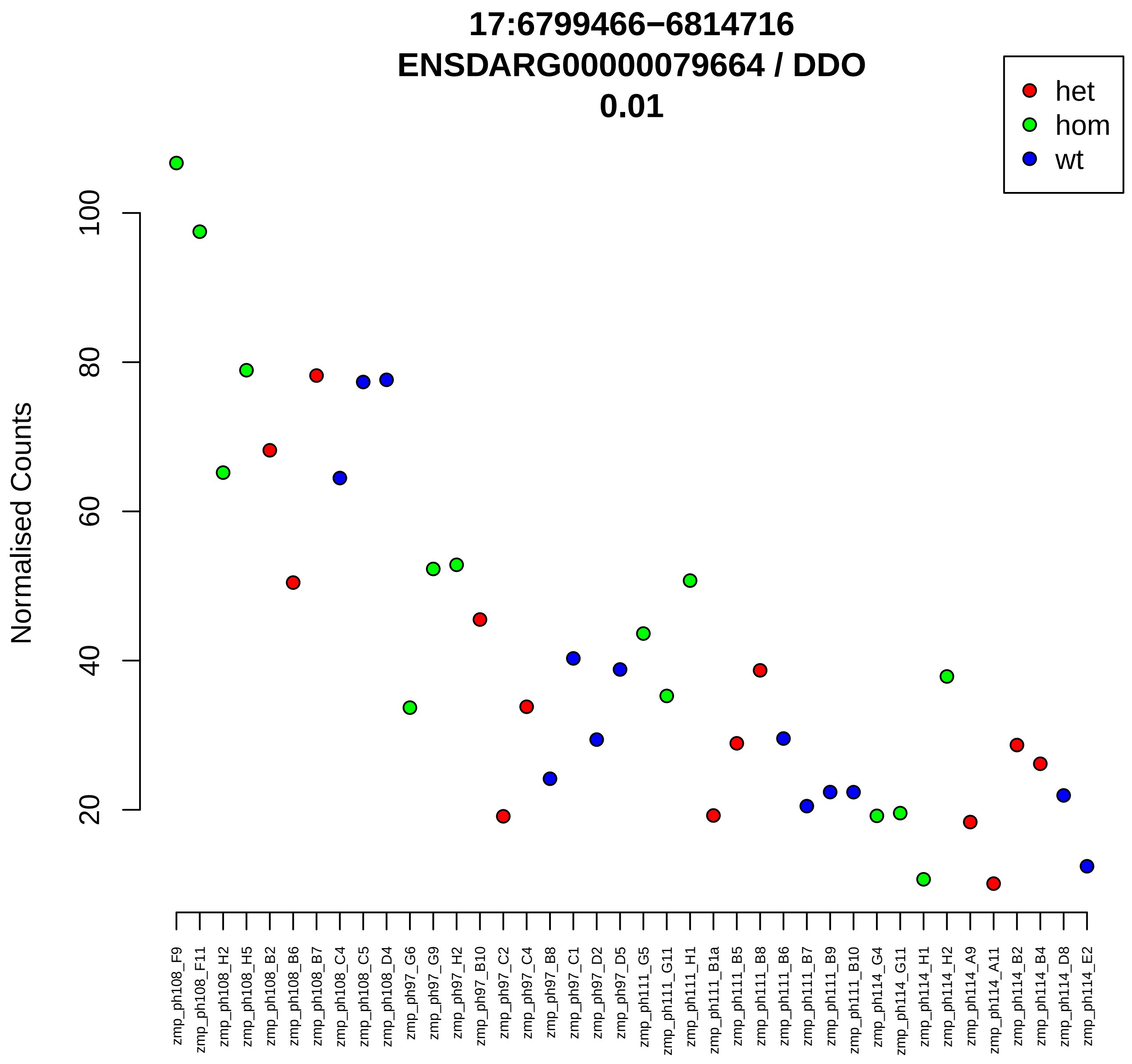

17:6799466−6814716
ENSDARG00000079664 / DDO
0.01
het
hom
wt
Normalised Counts
100
80
60
40
20
zmp_ph108_F9
zmp_ph108_F11
zmp_ph108_H2
zmp_ph108_H5
zmp_ph108_B2
zmp_ph108_B6
zmp_ph108_B7
zmp_ph108_C4
zmp_ph108_C5
zmp_ph108_D4
zmp_ph97_G6
zmp_ph97_G9
zmp_ph97_H2
zmp_ph97_B10
zmp_ph97_C2
zmp_ph97_C4
zmp_ph97_B8
zmp_ph97_C1
zmp_ph97_D2
zmp_ph97_D5
zmp_ph111_G5
zmp_ph111_G11
zmp_ph111_H1
zmp_ph111_B1a
zmp_ph111_B5
zmp_ph111_B8
zmp_ph111_B6
zmp_ph111_B7
zmp_ph111_B9
zmp_ph111_B10
zmp_ph114_G4
zmp_ph114_G11
zmp_ph114_H1
zmp_ph114_H2
zmp_ph114_A9
zmp_ph114_A11
zmp_ph114_B2
zmp_ph114_B4
zmp_ph114_D8
zmp_ph114_E2

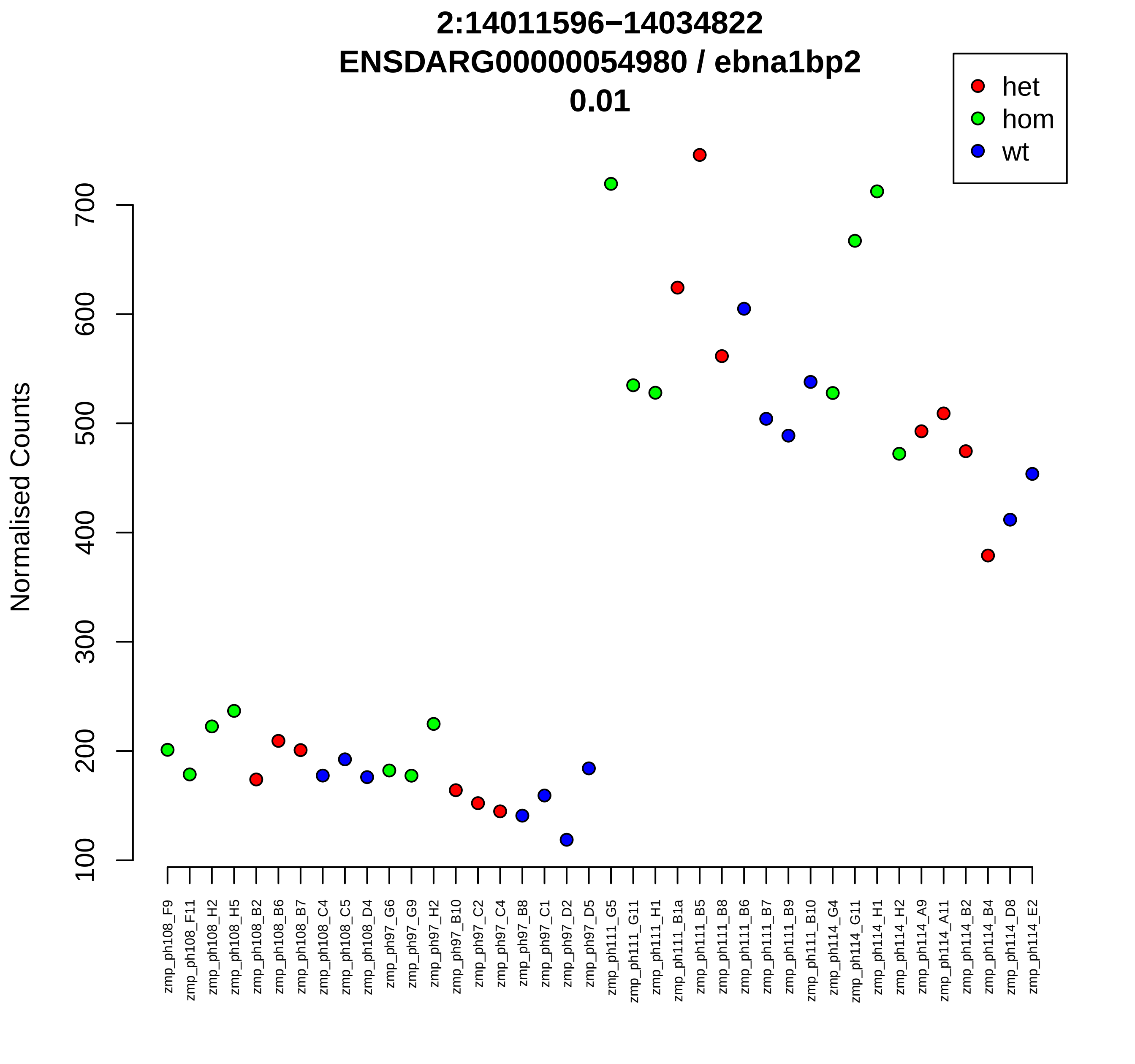
2:14011596−14034822
ENSDARG00000054980 / ebna1bp2
0.01
het
hom
wt
Normalised Counts
100
200
300
400
500
600
700
zmp_ph108_F9
zmp_ph108_F11
zmp_ph108_H2
zmp_ph108_H5
zmp_ph108_B2
zmp_ph108_B6
zmp_ph108_B7
zmp_ph108_C4
zmp_ph108_C5
zmp_ph108_D4
zmp_ph97_G6
zmp_ph97_G9
zmp_ph97_H2
zmp_ph97_B10
zmp_ph97_C2
zmp_ph97_C4
zmp_ph97_B8
zmp_ph97_C1
zmp_ph97_D2
zmp_ph97_D5
zmp_ph111_G5
zmp_ph111_G11
zmp_ph111_H1
zmp_ph111_B1a
zmp_ph111_B5
zmp_ph111_B8
zmp_ph111_B6
zmp_ph111_B7
zmp_ph111_B9
zmp_ph111_B10
zmp_ph114_G4
zmp_ph114_G11
zmp_ph114_H1
zmp_ph114_H2
zmp_ph114_A9
zmp_ph114_A11
zmp_ph114_B2
zmp_ph114_B4
zmp_ph114_D8
zmp_ph114_E2

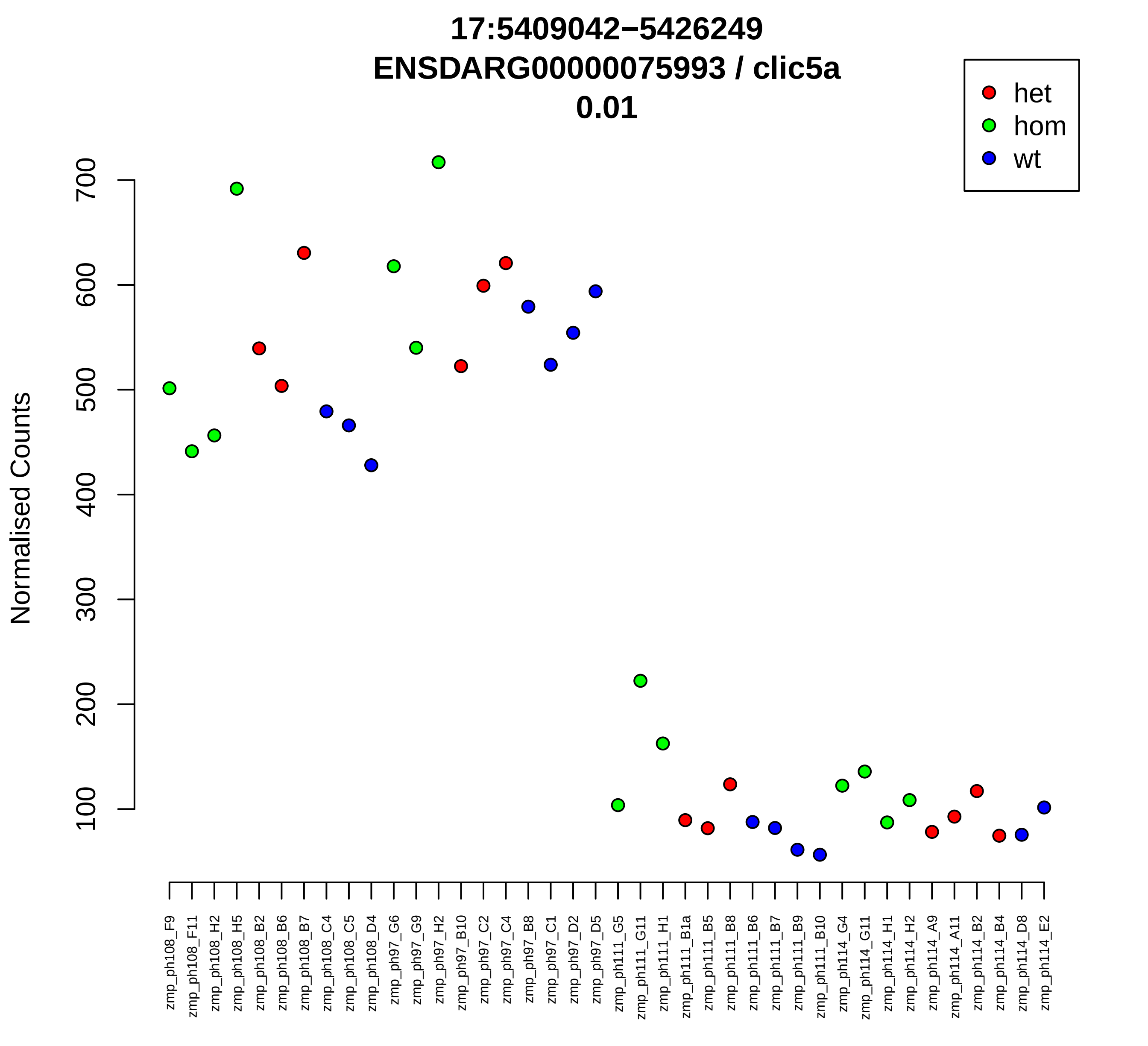

17:5409042−5426249
ENSDARG00000075993 / clic5a
0.01
het
hom
wt
Normalised Counts
100
200
300
400
500
600
700
zmp_ph108_F9
zmp_ph108_F11
zmp_ph108_H2
zmp_ph108_H5
zmp_ph108_B2
zmp_ph108_B6
zmp_ph108_B7
zmp_ph108_C4
zmp_ph108_C5
zmp_ph108_D4
zmp_ph97_G6
zmp_ph97_G9
zmp_ph97_H2
zmp_ph97_B10
zmp_ph97_C2
zmp_ph97_C4
zmp_ph97_B8
zmp_ph97_C1
zmp_ph97_D2
zmp_ph97_D5
zmp_ph111_G5
zmp_ph111_G11
zmp_ph111_H1
zmp_ph111_B1a
zmp_ph111_B5
zmp_ph111_B8
zmp_ph111_B6
zmp_ph111_B7
zmp_ph111_B9
zmp_ph111_B10
zmp_ph114_G4
zmp_ph114_G11
zmp_ph114_H1
zmp_ph114_H2
zmp_ph114_A9
zmp_ph114_A11
zmp_ph114_B2
zmp_ph114_B4
zmp_ph114_D8
zmp_ph114_E2

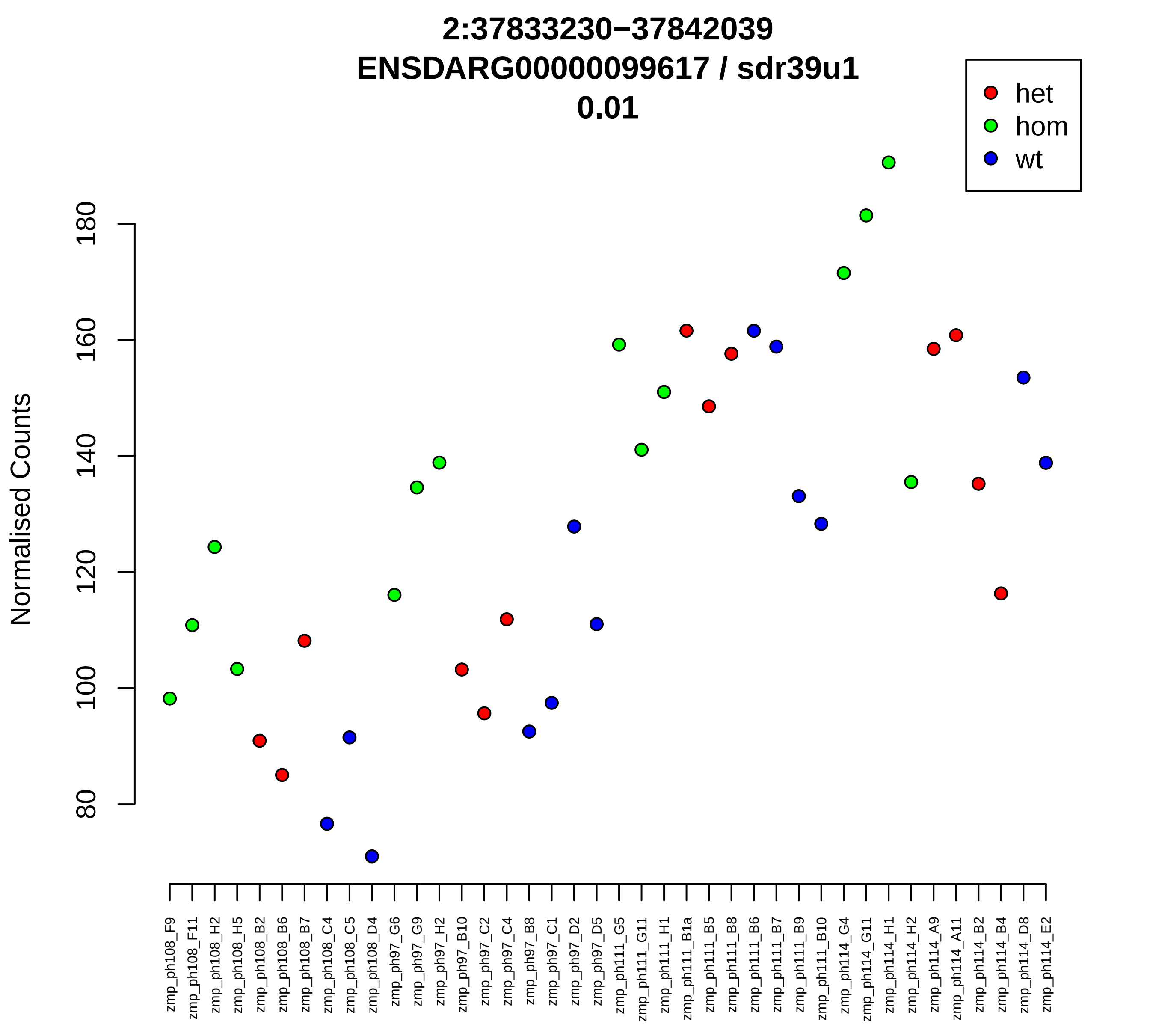

2:37833230−37842039
ENSDARG00000099617 / sdr39u1
0.01
het
hom
wt
Normalised Counts
80
100
120
140
160
180
zmp_ph108_F9
zmp_ph108_F11
zmp_ph108_H2
zmp_ph108_H5
zmp_ph108_B2
zmp_ph108_B6
zmp_ph108_B7
zmp_ph108_C4
zmp_ph108_C5
zmp_ph108_D4
zmp_ph97_G6
zmp_ph97_G9
zmp_ph97_H2
zmp_ph97_B10
zmp_ph97_C2
zmp_ph97_C4
zmp_ph97_B8
zmp_ph97_C1
zmp_ph97_D2
zmp_ph97_D5
zmp_ph111_G5
zmp_ph111_G11
zmp_ph111_H1
zmp_ph111_B1a
zmp_ph111_B5
zmp_ph111_B8
zmp_ph111_B6
zmp_ph111_B7
zmp_ph111_B9
zmp_ph111_B10
zmp_ph114_G4
zmp_ph114_G11
zmp_ph114_H1
zmp_ph114_H2
zmp_ph114_A9
zmp_ph114_A11
zmp_ph114_B2
zmp_ph114_B4
zmp_ph114_D8
zmp_ph114_E2

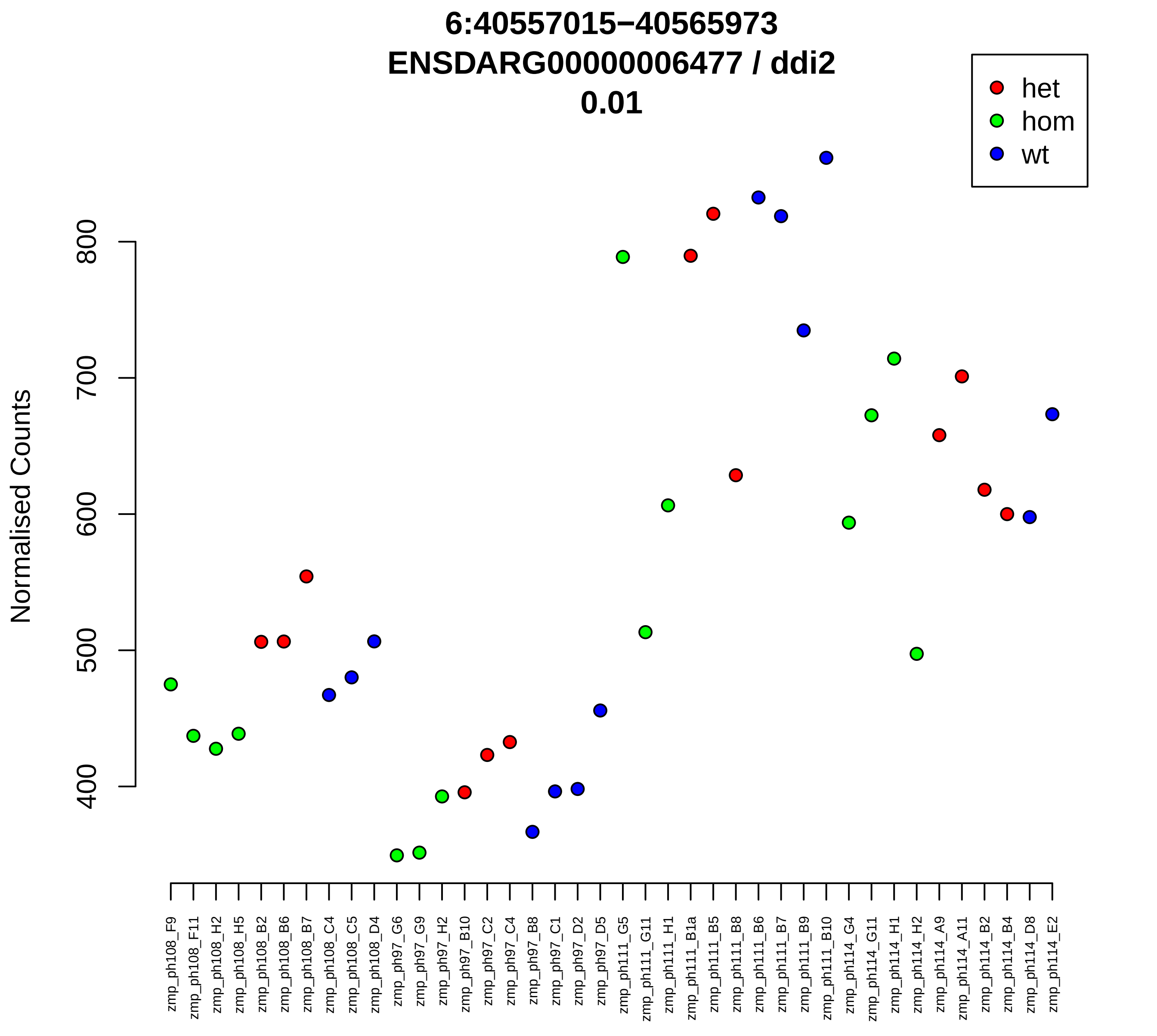
6:40557015−40565973
ENSDARG00000006477 / ddi2
0.01
het
hom
wt
Normalised Counts
400
500
600
700
800
zmp_ph108_F9
zmp_ph108_F11
zmp_ph108_H2
zmp_ph108_H5
zmp_ph108_B2
zmp_ph108_B6
zmp_ph108_B7
zmp_ph108_C4
zmp_ph108_C5
zmp_ph108_D4
zmp_ph97_G6
zmp_ph97_G9
zmp_ph97_H2
zmp_ph97_B10
zmp_ph97_C2
zmp_ph97_C4
zmp_ph97_B8
zmp_ph97_C1
zmp_ph97_D2
zmp_ph97_D5
zmp_ph111_G5
zmp_ph111_G11
zmp_ph111_H1
zmp_ph111_B1a
zmp_ph111_B5
zmp_ph111_B8
zmp_ph111_B6
zmp_ph111_B7
zmp_ph111_B9
zmp_ph111_B10
zmp_ph114_G4
zmp_ph114_G11
zmp_ph114_H1
zmp_ph114_H2
zmp_ph114_A9
zmp_ph114_A11
zmp_ph114_B2
zmp_ph114_B4
zmp_ph114_D8
zmp_ph114_E2

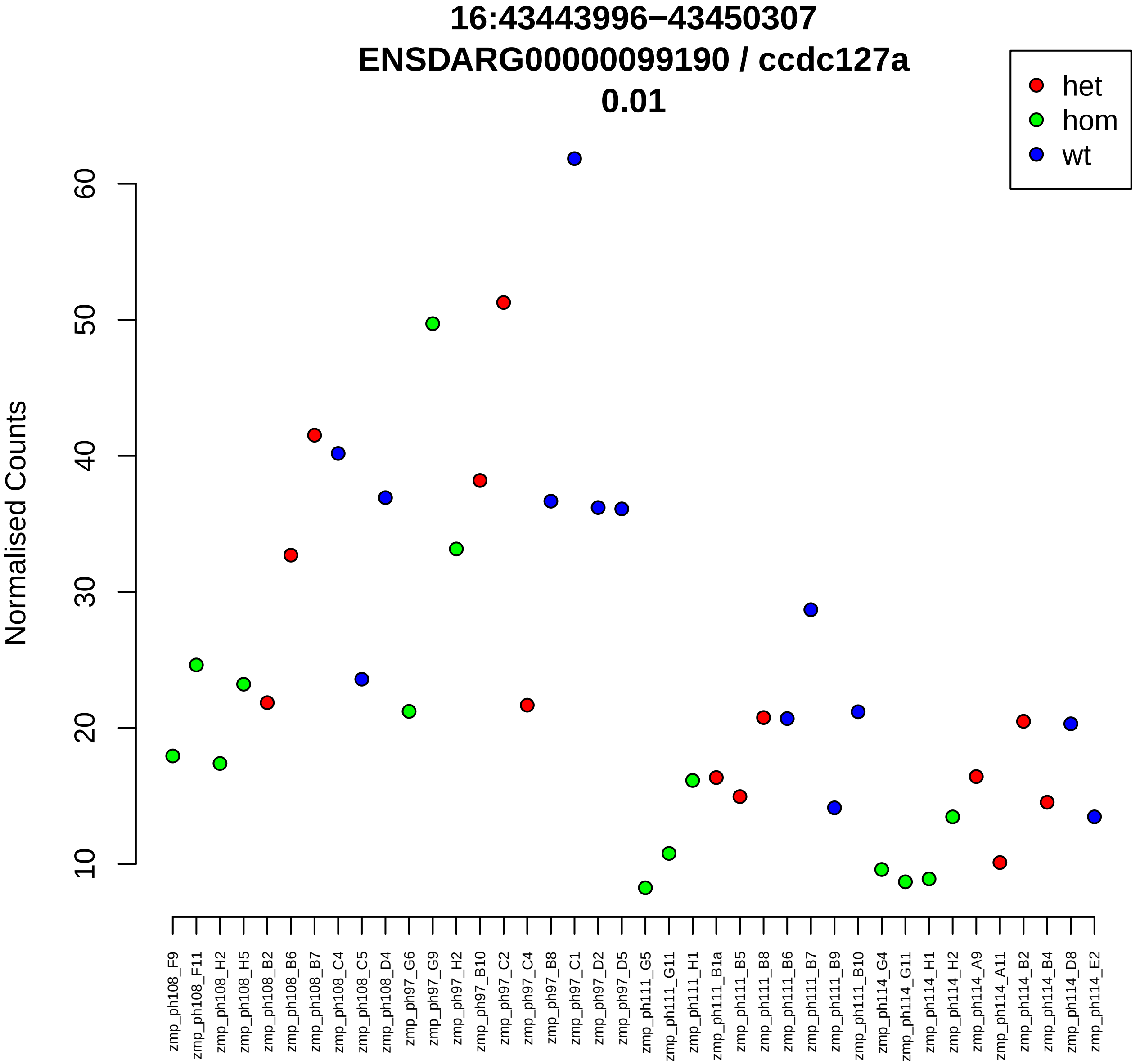
16:43443996−43450307
ENSDARG00000099190 / ccdc127a
0.01
het
hom
wt
Normalised Counts
10
20
30
40
50
60
zmp_ph108_F9
zmp_ph108_F11
zmp_ph108_H2
zmp_ph108_H5
zmp_ph108_B2
zmp_ph108_B6
zmp_ph108_B7
zmp_ph108_C4
zmp_ph108_C5
zmp_ph108_D4
zmp_ph97_G6
zmp_ph97_G9
zmp_ph97_H2
zmp_ph97_B10
zmp_ph97_C2
zmp_ph97_C4
zmp_ph97_B8
zmp_ph97_C1
zmp_ph97_D2
zmp_ph97_D5
zmp_ph111_G5
zmp_ph111_G11
zmp_ph111_H1
zmp_ph111_B1a
zmp_ph111_B5
zmp_ph111_B8
zmp_ph111_B6
zmp_ph111_B7
zmp_ph111_B9
zmp_ph111_B10
zmp_ph114_G4
zmp_ph114_G11
zmp_ph114_H1
zmp_ph114_H2
zmp_ph114_A9
zmp_ph114_A11
zmp_ph114_B2
zmp_ph114_B4
zmp_ph114_D8
zmp_ph114_E2

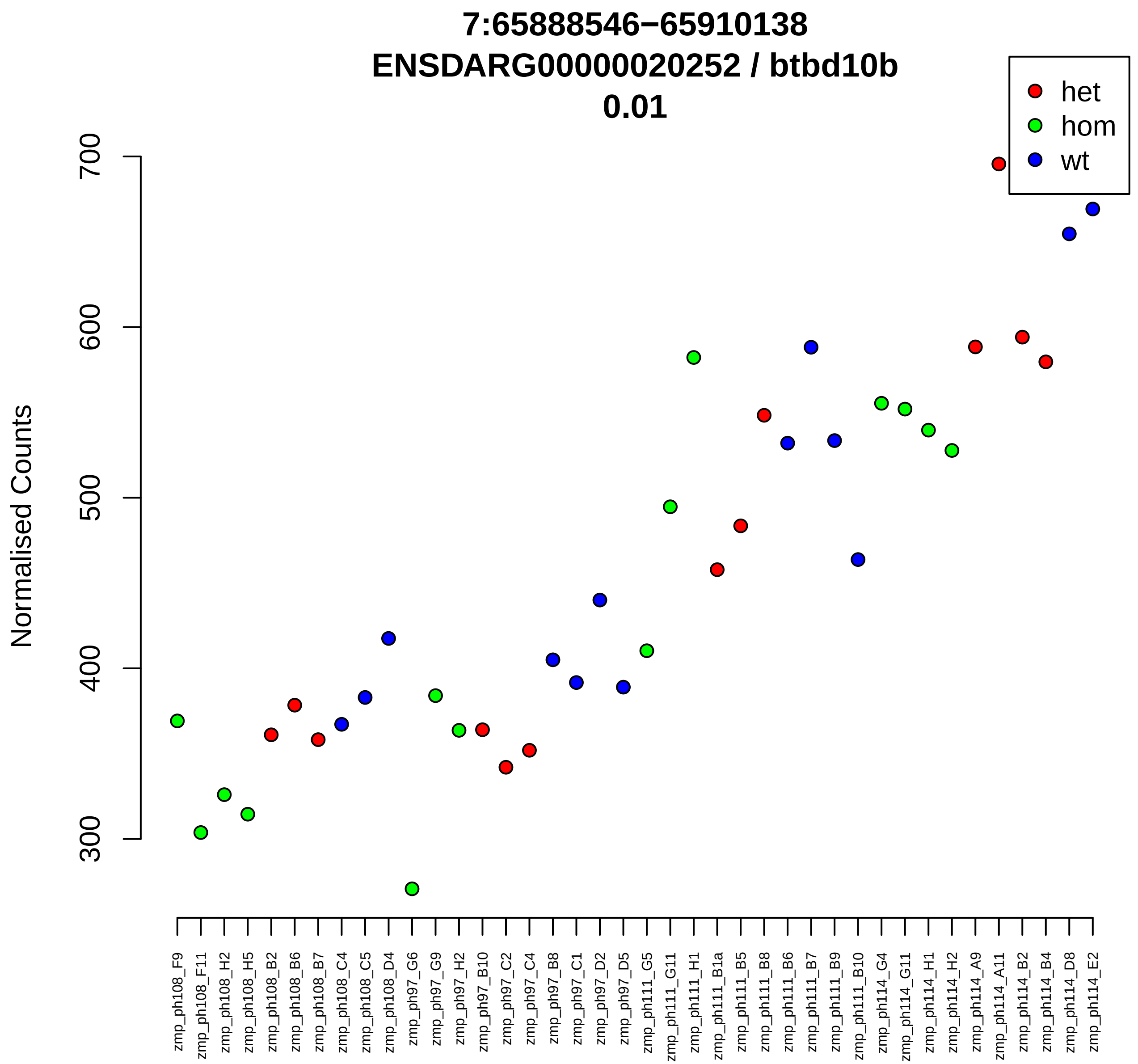
7:65888546−65910138
ENSDARG00000020252 / btbd10b
0.01
het
hom
wt
Normalised Counts
300
400
500
600
700
zmp_ph108_F9
zmp_ph108_F11
zmp_ph108_H2
zmp_ph108_H5
zmp_ph108_B2
zmp_ph108_B6
zmp_ph108_B7
zmp_ph108_C4
zmp_ph108_C5
zmp_ph108_D4
zmp_ph97_G6
zmp_ph97_G9
zmp_ph97_H2
zmp_ph97_B10
zmp_ph97_C2
zmp_ph97_C4
zmp_ph97_B8
zmp_ph97_C1
zmp_ph97_D2
zmp_ph97_D5
zmp_ph111_G5
zmp_ph111_G11
zmp_ph111_H1
zmp_ph111_B1a
zmp_ph111_B5
zmp_ph111_B8
zmp_ph111_B6
zmp_ph111_B7
zmp_ph111_B9
zmp_ph111_B10
zmp_ph114_G4
zmp_ph114_G11
zmp_ph114_H1
zmp_ph114_H2
zmp_ph114_A9
zmp_ph114_A11
zmp_ph114_B2
zmp_ph114_B4
zmp_ph114_D8
zmp_ph114_E2

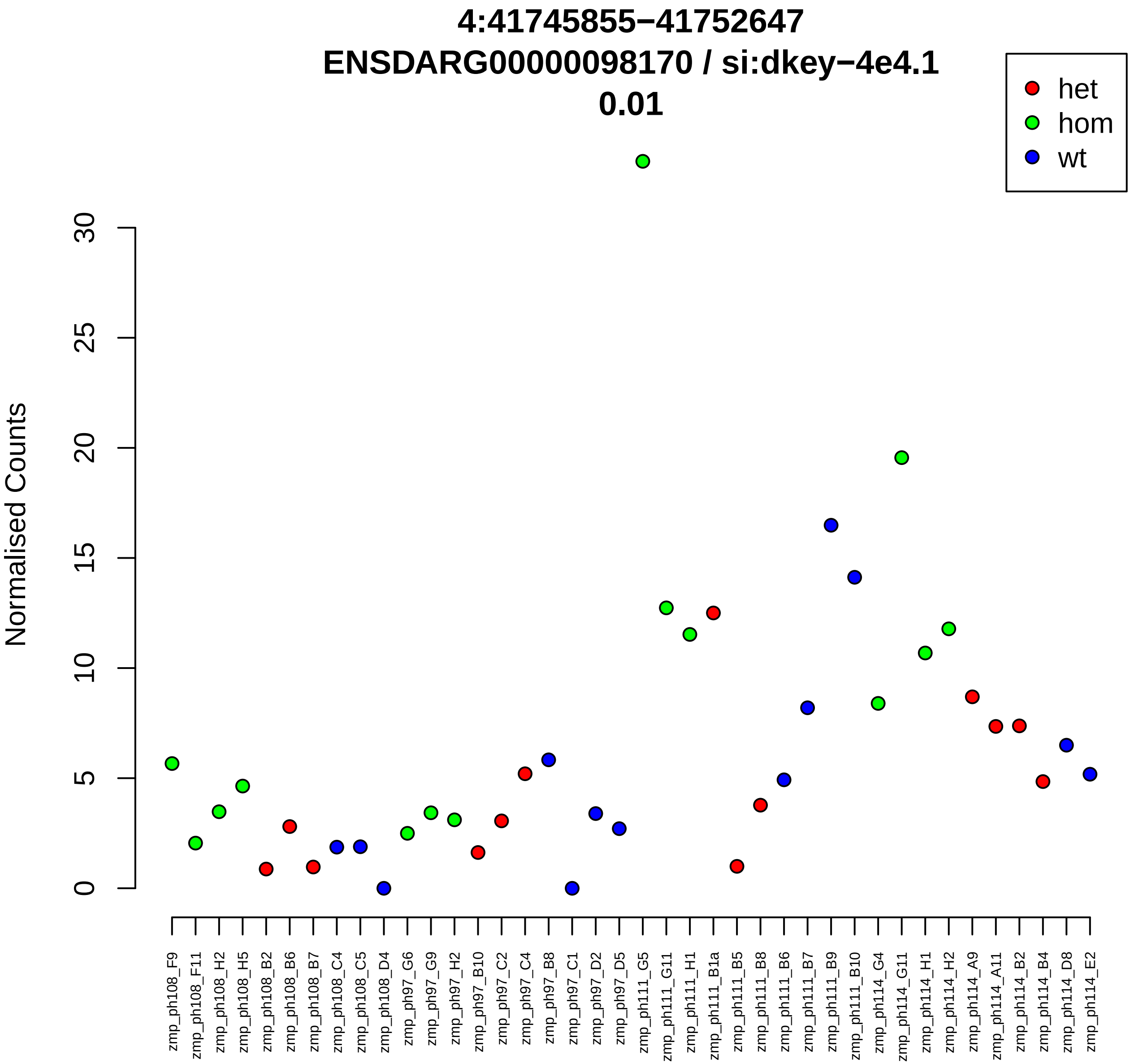

4:41745855−41752647
ENSDARG00000098170 / si:dkey−4e4.1
0.01
het
hom
wt
Normalised Counts
0
5
10
15
20
25
30
zmp_ph108_F9
zmp_ph108_F11
zmp_ph108_H2
zmp_ph108_H5
zmp_ph108_B2
zmp_ph108_B6
zmp_ph108_B7
zmp_ph108_C4
zmp_ph108_C5
zmp_ph108_D4
zmp_ph97_G6
zmp_ph97_G9
zmp_ph97_H2
zmp_ph97_B10
zmp_ph97_C2
zmp_ph97_C4
zmp_ph97_B8
zmp_ph97_C1
zmp_ph97_D2
zmp_ph97_D5
zmp_ph111_G5
zmp_ph111_G11
zmp_ph111_H1
zmp_ph111_B1a
zmp_ph111_B5
zmp_ph111_B8
zmp_ph111_B6
zmp_ph111_B7
zmp_ph111_B9
zmp_ph111_B10
zmp_ph114_G4
zmp_ph114_G11
zmp_ph114_H1
zmp_ph114_H2
zmp_ph114_A9
zmp_ph114_A11
zmp_ph114_B2
zmp_ph114_B4
zmp_ph114_D8
zmp_ph114_E2

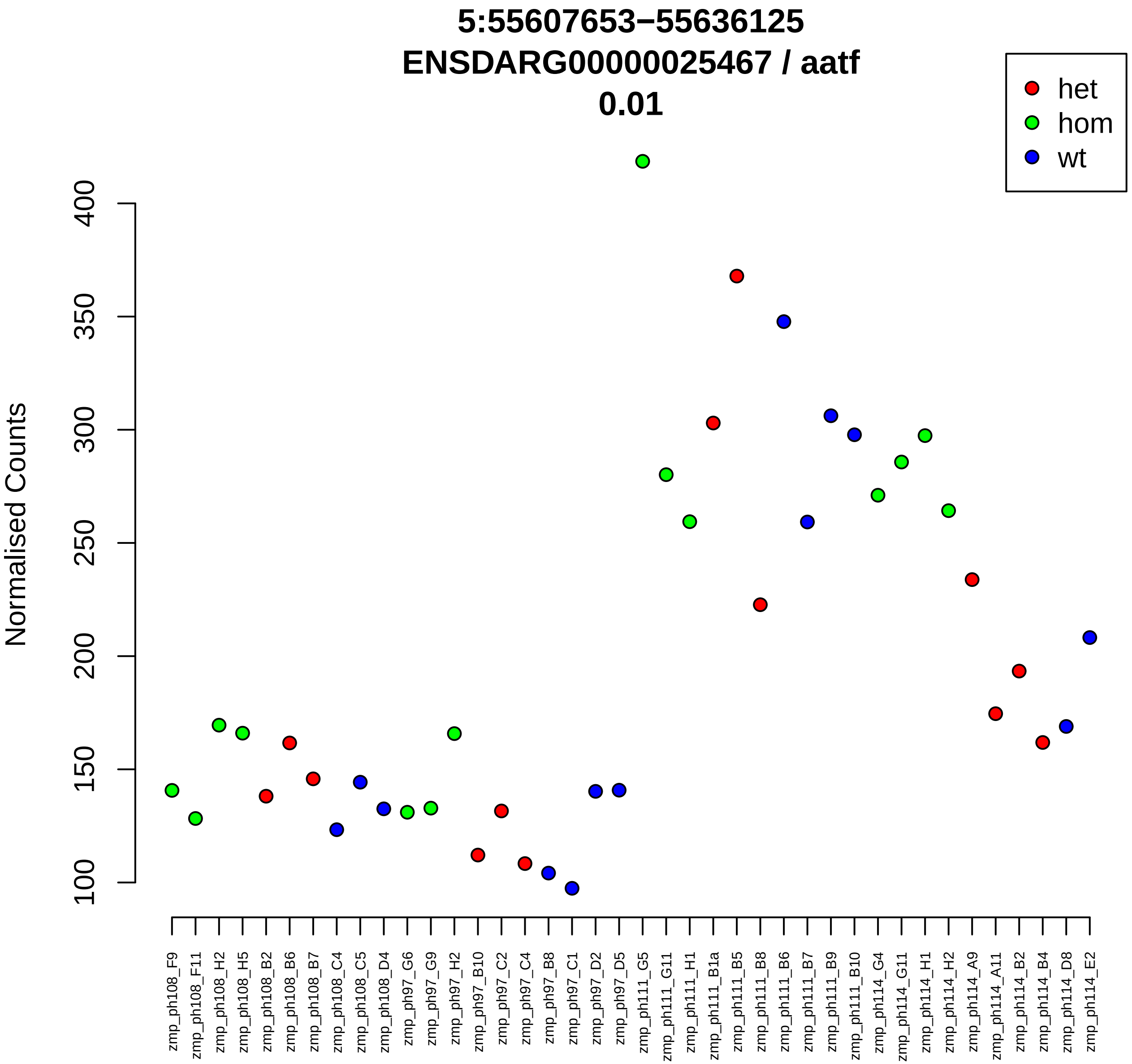

5:55607653−55636125
ENSDARG00000025467 / aatf
0.01
het
hom
wt
Normalised Counts
100
150
200
250
300
350
400
zmp_ph108_F9
zmp_ph108_F11
zmp_ph108_H2
zmp_ph108_H5
zmp_ph108_B2
zmp_ph108_B6
zmp_ph108_B7
zmp_ph108_C4
zmp_ph108_C5
zmp_ph108_D4
zmp_ph97_G6
zmp_ph97_G9
zmp_ph97_H2
zmp_ph97_B10
zmp_ph97_C2
zmp_ph97_C4
zmp_ph97_B8
zmp_ph97_C1
zmp_ph97_D2
zmp_ph97_D5
zmp_ph111_G5
zmp_ph111_G11
zmp_ph111_H1
zmp_ph111_B1a
zmp_ph111_B5
zmp_ph111_B8
zmp_ph111_B6
zmp_ph111_B7
zmp_ph111_B9
zmp_ph111_B10
zmp_ph114_G4
zmp_ph114_G11
zmp_ph114_H1
zmp_ph114_H2
zmp_ph114_A9
zmp_ph114_A11
zmp_ph114_B2
zmp_ph114_B4
zmp_ph114_D8
zmp_ph114_E2

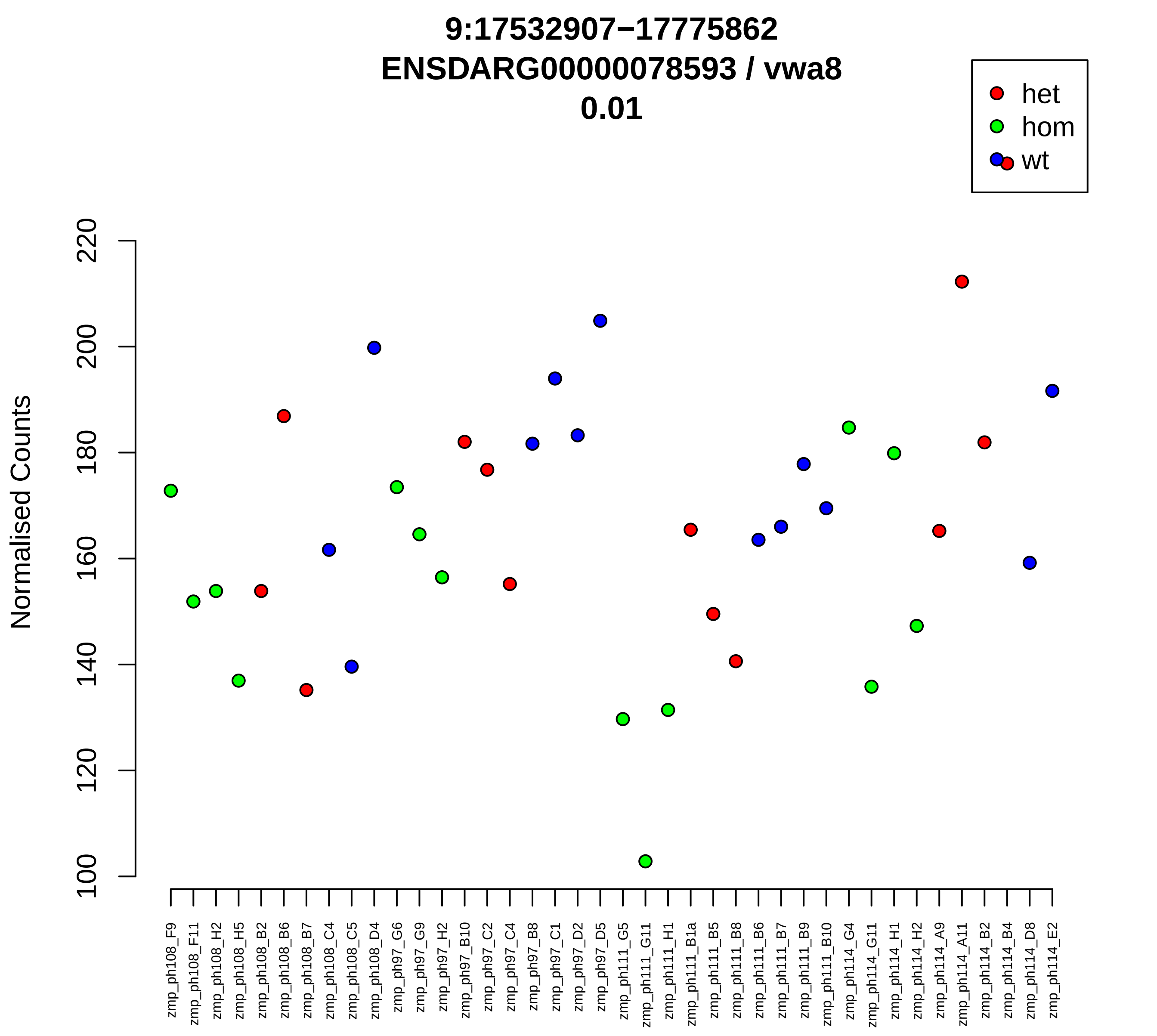
9:17532907−17775862
ENSDARG00000078593 / vwa8
0.01
het
hom
wt
Normalised Counts
100
120
140
160
180
200
220
zmp_ph108_F9
zmp_ph108_F11
zmp_ph108_H2
zmp_ph108_H5
zmp_ph108_B2
zmp_ph108_B6
zmp_ph108_B7
zmp_ph108_C4
zmp_ph108_C5
zmp_ph108_D4
zmp_ph97_G6
zmp_ph97_G9
zmp_ph97_H2
zmp_ph97_B10
zmp_ph97_C2
zmp_ph97_C4
zmp_ph97_B8
zmp_ph97_C1
zmp_ph97_D2
zmp_ph97_D5
zmp_ph111_G5
zmp_ph111_G11
zmp_ph111_H1
zmp_ph111_B1a
zmp_ph111_B5
zmp_ph111_B8
zmp_ph111_B6
zmp_ph111_B7
zmp_ph111_B9
zmp_ph111_B10
zmp_ph114_G4
zmp_ph114_G11
zmp_ph114_H1
zmp_ph114_H2
zmp_ph114_A9
zmp_ph114_A11
zmp_ph114_B2
zmp_ph114_B4
zmp_ph114_D8
zmp_ph114_E2

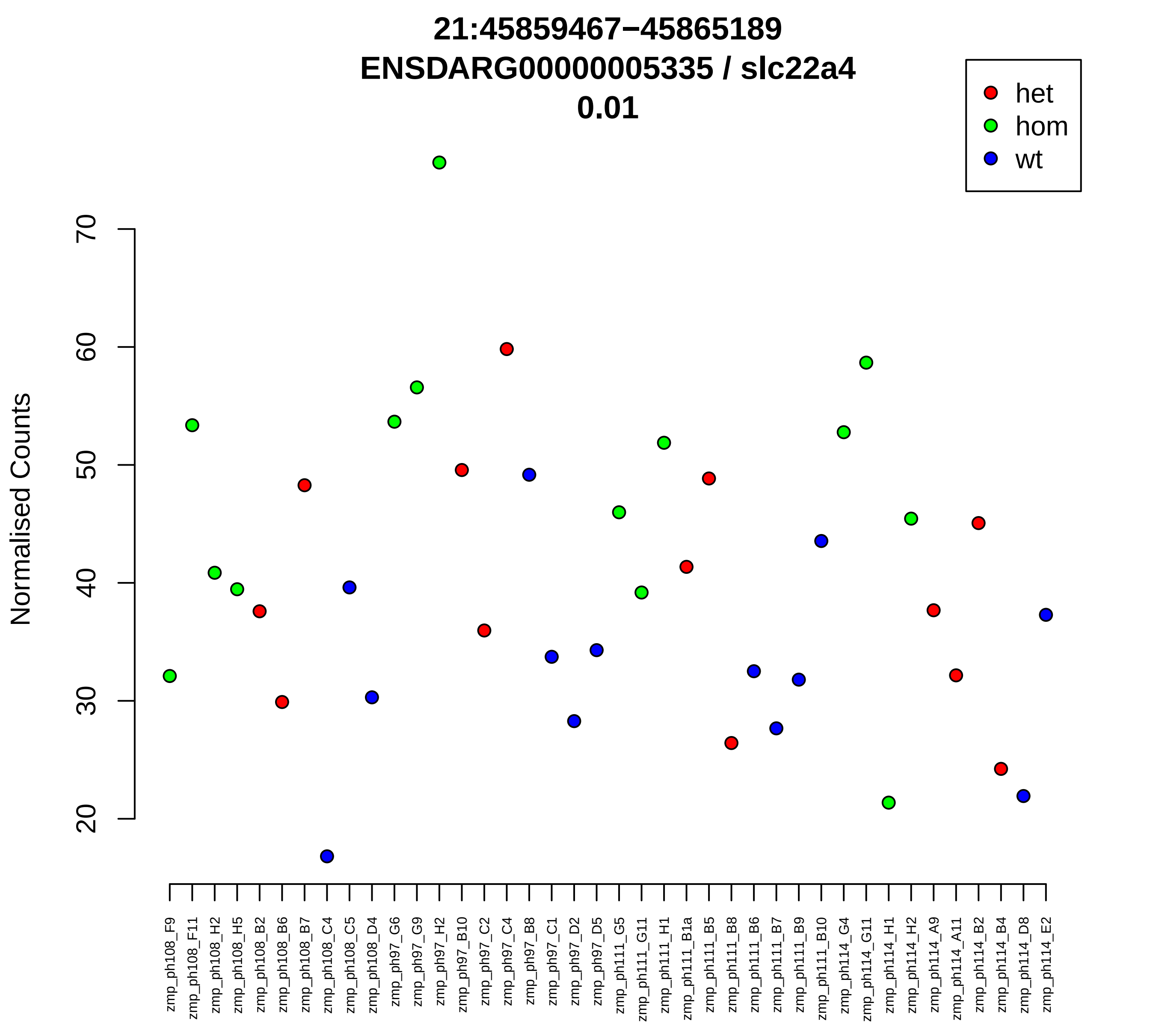
21:45859467−45865189
ENSDARG00000005335 / slc22a4
0.01
het
hom
wt
Normalised Counts
20
30
40
50
60
70
zmp_ph108_F9
zmp_ph108_F11
zmp_ph108_H2
zmp_ph108_H5
zmp_ph108_B2
zmp_ph108_B6
zmp_ph108_B7
zmp_ph108_C4
zmp_ph108_C5
zmp_ph108_D4
zmp_ph97_G6
zmp_ph97_G9
zmp_ph97_H2
zmp_ph97_B10
zmp_ph97_C2
zmp_ph97_C4
zmp_ph97_B8
zmp_ph97_C1
zmp_ph97_D2
zmp_ph97_D5
zmp_ph111_G5
zmp_ph111_G11
zmp_ph111_H1
zmp_ph111_B1a
zmp_ph111_B5
zmp_ph111_B8
zmp_ph111_B6
zmp_ph111_B7
zmp_ph111_B9
zmp_ph111_B10
zmp_ph114_G4
zmp_ph114_G11
zmp_ph114_H1
zmp_ph114_H2
zmp_ph114_A9
zmp_ph114_A11
zmp_ph114_B2
zmp_ph114_B4
zmp_ph114_D8
zmp_ph114_E2

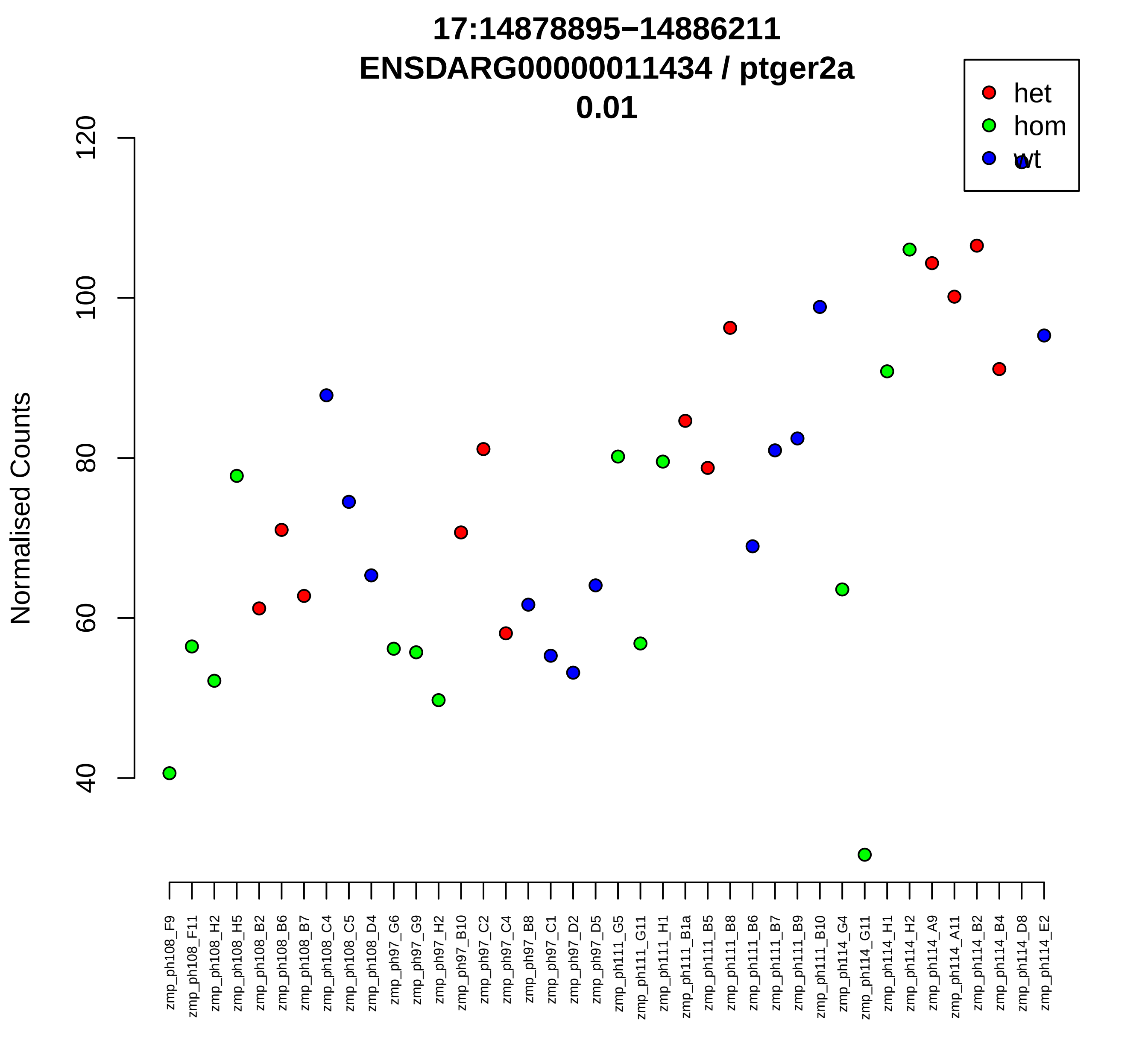

17:14878895−14886211
ENSDARG00000011434 / ptger2a
0.01
het
hom
wt
Normalised Counts
120
100
80
60
40
zmp_ph108_F9
zmp_ph108_F11
zmp_ph108_H2
zmp_ph108_H5
zmp_ph108_B2
zmp_ph108_B6
zmp_ph108_B7
zmp_ph108_C4
zmp_ph108_C5
zmp_ph108_D4
zmp_ph97_G6
zmp_ph97_G9
zmp_ph97_H2
zmp_ph97_B10
zmp_ph97_C2
zmp_ph97_C4
zmp_ph97_B8
zmp_ph97_C1
zmp_ph97_D2
zmp_ph97_D5
zmp_ph111_G5
zmp_ph111_G11
zmp_ph111_H1
zmp_ph111_B1a
zmp_ph111_B5
zmp_ph111_B8
zmp_ph111_B6
zmp_ph111_B7
zmp_ph111_B9
zmp_ph111_B10
zmp_ph114_G4
zmp_ph114_G11
zmp_ph114_H1
zmp_ph114_H2
zmp_ph114_A9
zmp_ph114_A11
zmp_ph114_B2
zmp_ph114_B4
zmp_ph114_D8
zmp_ph114_E2

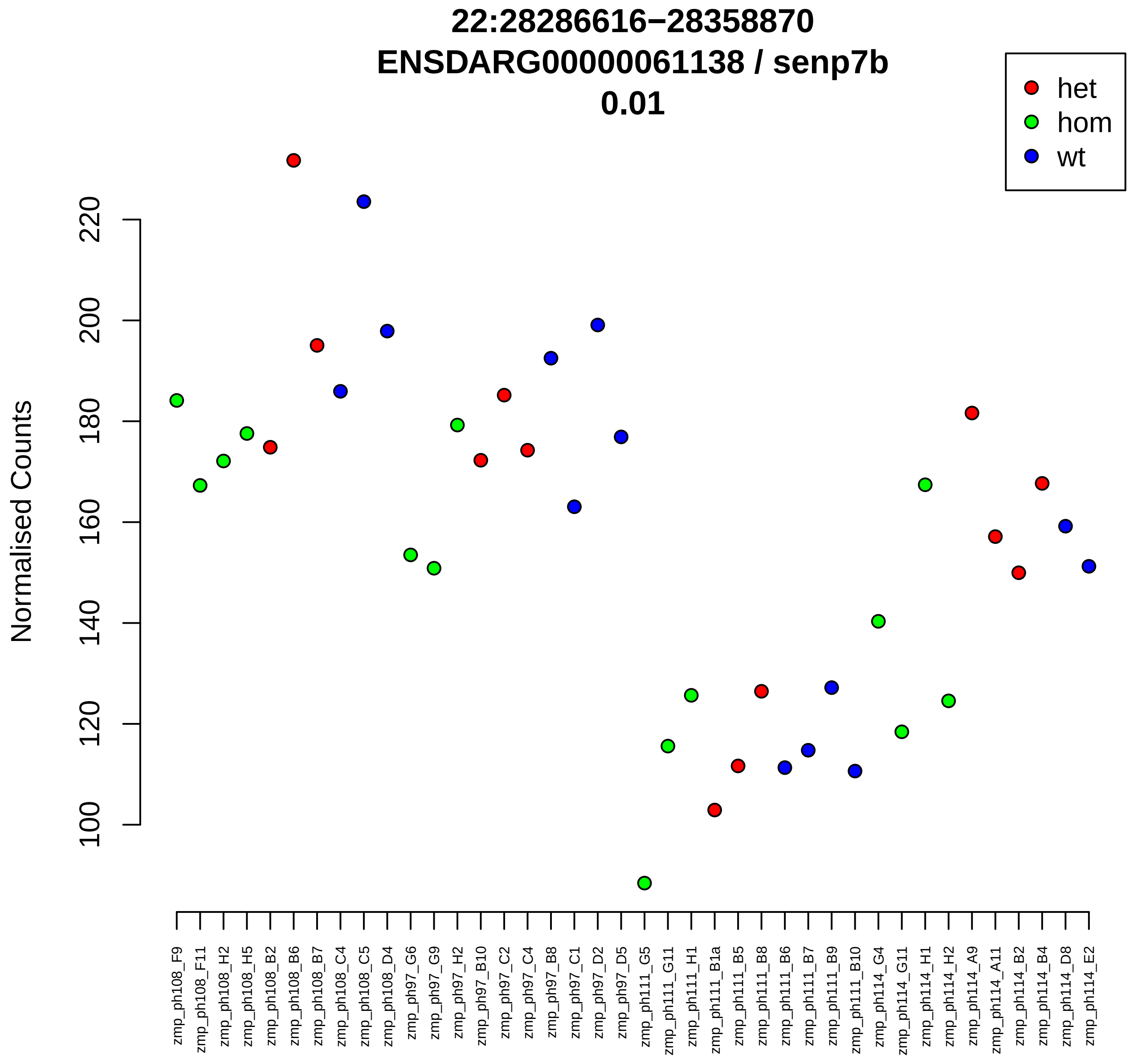
22:28286616−28358870
ENSDARG00000061138 / senp7b
0.01
het
hom
wt
Normalised Counts
100
120
140
160
180
200
220
zmp_ph108_F9
zmp_ph108_F11
zmp_ph108_H2
zmp_ph108_H5
zmp_ph108_B2
zmp_ph108_B6
zmp_ph108_B7
zmp_ph108_C4
zmp_ph108_C5
zmp_ph108_D4
zmp_ph97_G6
zmp_ph97_G9
zmp_ph97_H2
zmp_ph97_B10
zmp_ph97_C2
zmp_ph97_C4
zmp_ph97_B8
zmp_ph97_C1
zmp_ph97_D2
zmp_ph97_D5
zmp_ph111_G5
zmp_ph111_G11
zmp_ph111_H1
zmp_ph111_B1a
zmp_ph111_B5
zmp_ph111_B8
zmp_ph111_B6
zmp_ph111_B7
zmp_ph111_B9
zmp_ph111_B10
zmp_ph114_G4
zmp_ph114_G11
zmp_ph114_H1
zmp_ph114_H2
zmp_ph114_A9
zmp_ph114_A11
zmp_ph114_B2
zmp_ph114_B4
zmp_ph114_D8
zmp_ph114_E2

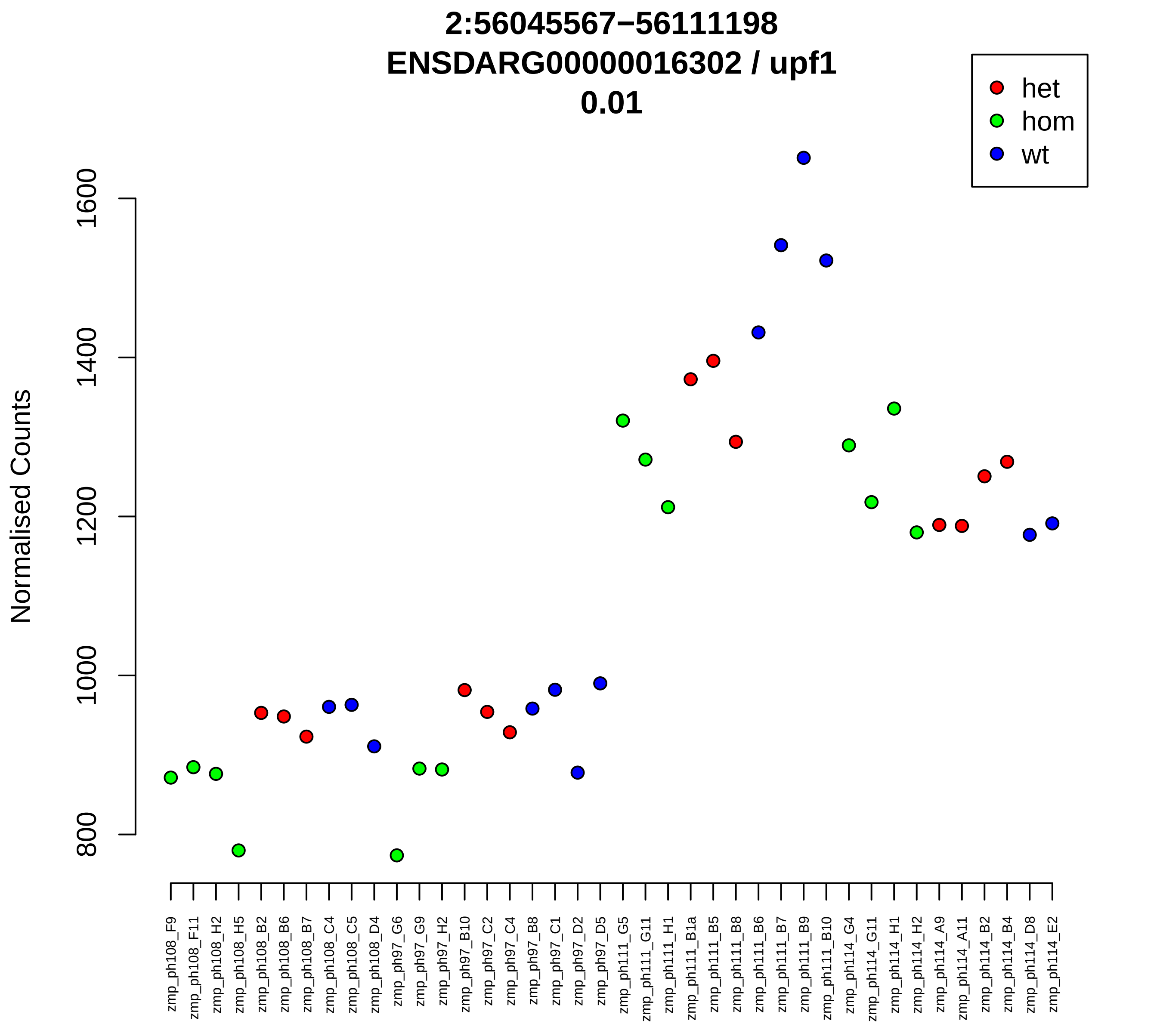
2:56045567−56111198
ENSDARG00000016302 / upf1
0.01
het
hom
wt
Normalised Counts
800
1000
1200
1400
1600
zmp_ph108_F9
zmp_ph108_F11
zmp_ph108_H2
zmp_ph108_H5
zmp_ph108_B2
zmp_ph108_B6
zmp_ph108_B7
zmp_ph108_C4
zmp_ph108_C5
zmp_ph108_D4
zmp_ph97_G6
zmp_ph97_G9
zmp_ph97_H2
zmp_ph97_B10
zmp_ph97_C2
zmp_ph97_C4
zmp_ph97_B8
zmp_ph97_C1
zmp_ph97_D2
zmp_ph97_D5
zmp_ph111_G5
zmp_ph111_G11
zmp_ph111_H1
zmp_ph111_B1a
zmp_ph111_B5
zmp_ph111_B8
zmp_ph111_B6
zmp_ph111_B7
zmp_ph111_B9
zmp_ph111_B10
zmp_ph114_G4
zmp_ph114_G11
zmp_ph114_H1
zmp_ph114_H2
zmp_ph114_A9
zmp_ph114_A11
zmp_ph114_B2
zmp_ph114_B4
zmp_ph114_D8
zmp_ph114_E2

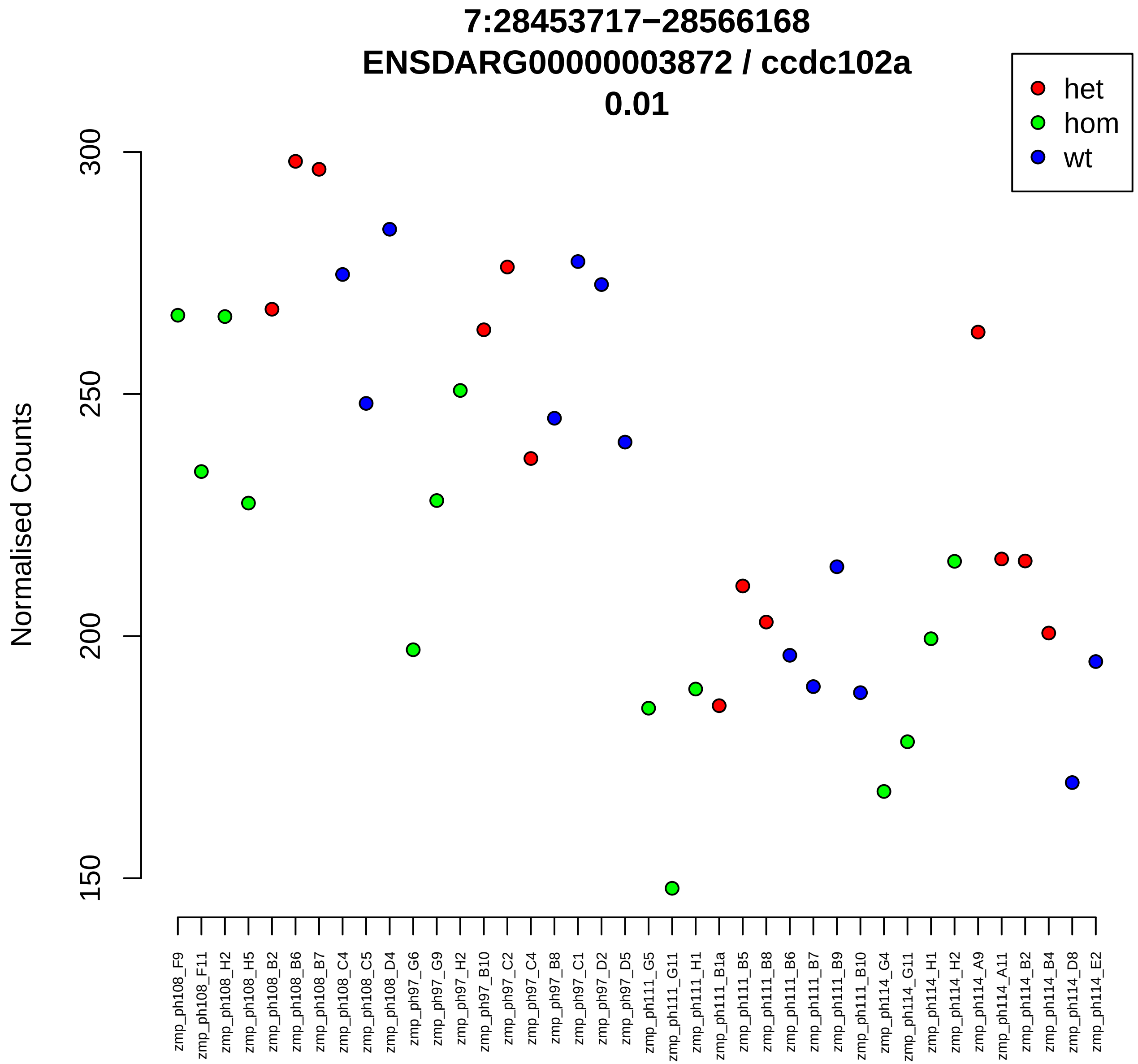

7:28453717−28566168
ENSDARG00000003872 / ccdc102a
0.01
het
hom
wt
Normalised Counts
300
250
200
150
zmp_ph108_F9
zmp_ph108_F11
zmp_ph108_H2
zmp_ph108_H5
zmp_ph108_B2
zmp_ph108_B6
zmp_ph108_B7
zmp_ph108_C4
zmp_ph108_C5
zmp_ph108_D4
zmp_ph97_G6
zmp_ph97_G9
zmp_ph97_H2
zmp_ph97_B10
zmp_ph97_C2
zmp_ph97_C4
zmp_ph97_B8
zmp_ph97_C1
zmp_ph97_D2
zmp_ph97_D5
zmp_ph111_G5
zmp_ph111_G11
zmp_ph111_H1
zmp_ph111_B1a
zmp_ph111_B5
zmp_ph111_B8
zmp_ph111_B6
zmp_ph111_B7
zmp_ph111_B9
zmp_ph111_B10
zmp_ph114_G4
zmp_ph114_G11
zmp_ph114_H1
zmp_ph114_H2
zmp_ph114_A9
zmp_ph114_A11
zmp_ph114_B2
zmp_ph114_B4
zmp_ph114_D8
zmp_ph114_E2

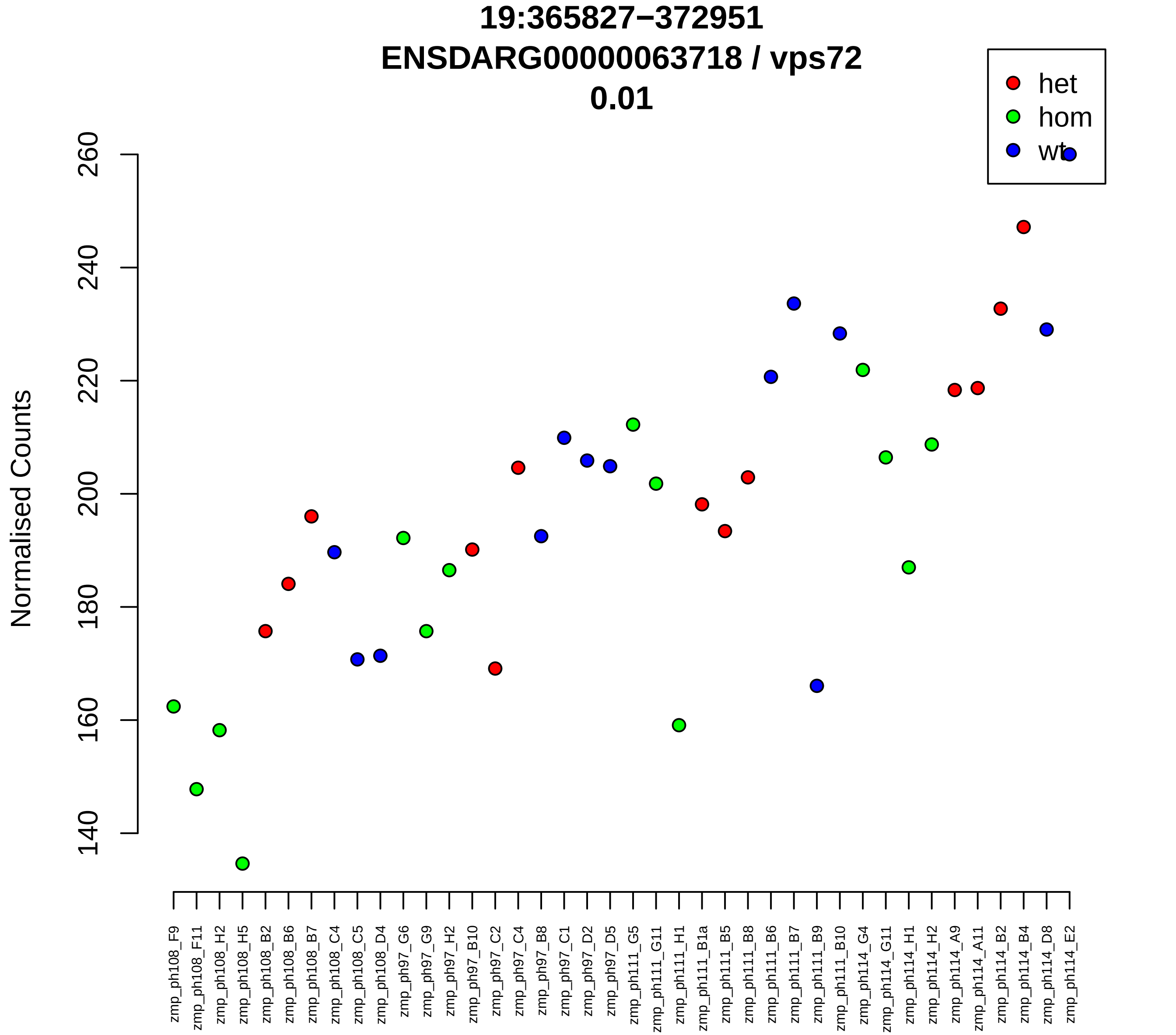
19:365827−372951
ENSDARG00000063718 / vps72
0.01
het
hom
wt
Normalised Counts
140
160
180
200
220
240
260
zmp_ph108_F9
zmp_ph108_F11
zmp_ph108_H2
zmp_ph108_H5
zmp_ph108_B2
zmp_ph108_B6
zmp_ph108_B7
zmp_ph108_C4
zmp_ph108_C5
zmp_ph108_D4
zmp_ph97_G6
zmp_ph97_G9
zmp_ph97_H2
zmp_ph97_B10
zmp_ph97_C2
zmp_ph97_C4
zmp_ph97_B8
zmp_ph97_C1
zmp_ph97_D2
zmp_ph97_D5
zmp_ph111_G5
zmp_ph111_G11
zmp_ph111_H1
zmp_ph111_B1a
zmp_ph111_B5
zmp_ph111_B8
zmp_ph111_B6
zmp_ph111_B7
zmp_ph111_B9
zmp_ph111_B10
zmp_ph114_G4
zmp_ph114_G11
zmp_ph114_H1
zmp_ph114_H2
zmp_ph114_A9
zmp_ph114_A11
zmp_ph114_B2
zmp_ph114_B4
zmp_ph114_D8
zmp_ph114_E2

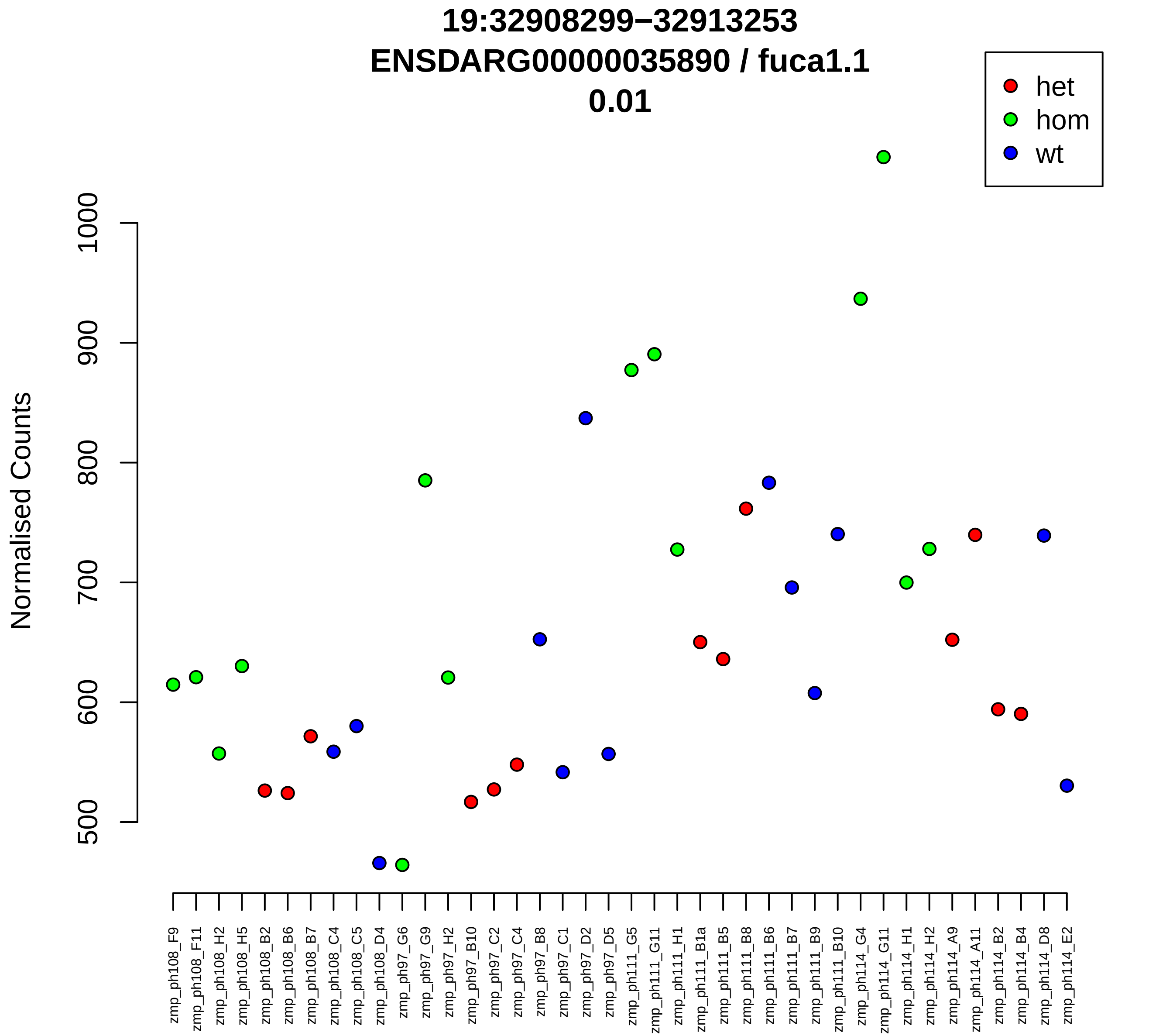
19:32908299−32913253
ENSDARG00000035890 / fuca1.1
0.01
het
hom
wt
Normalised Counts
500
600
700
800
900
1000
zmp_ph108_F9
zmp_ph108_F11
zmp_ph108_H2
zmp_ph108_H5
zmp_ph108_B2
zmp_ph108_B6
zmp_ph108_B7
zmp_ph108_C4
zmp_ph108_C5
zmp_ph108_D4
zmp_ph97_G6
zmp_ph97_G9
zmp_ph97_H2
zmp_ph97_B10
zmp_ph97_C2
zmp_ph97_C4
zmp_ph97_B8
zmp_ph97_C1
zmp_ph97_D2
zmp_ph97_D5
zmp_ph111_G5
zmp_ph111_G11
zmp_ph111_H1
zmp_ph111_B1a
zmp_ph111_B5
zmp_ph111_B8
zmp_ph111_B6
zmp_ph111_B7
zmp_ph111_B9
zmp_ph111_B10
zmp_ph114_G4
zmp_ph114_G11
zmp_ph114_H1
zmp_ph114_H2
zmp_ph114_A9
zmp_ph114_A11
zmp_ph114_B2
zmp_ph114_B4
zmp_ph114_D8
zmp_ph114_E2

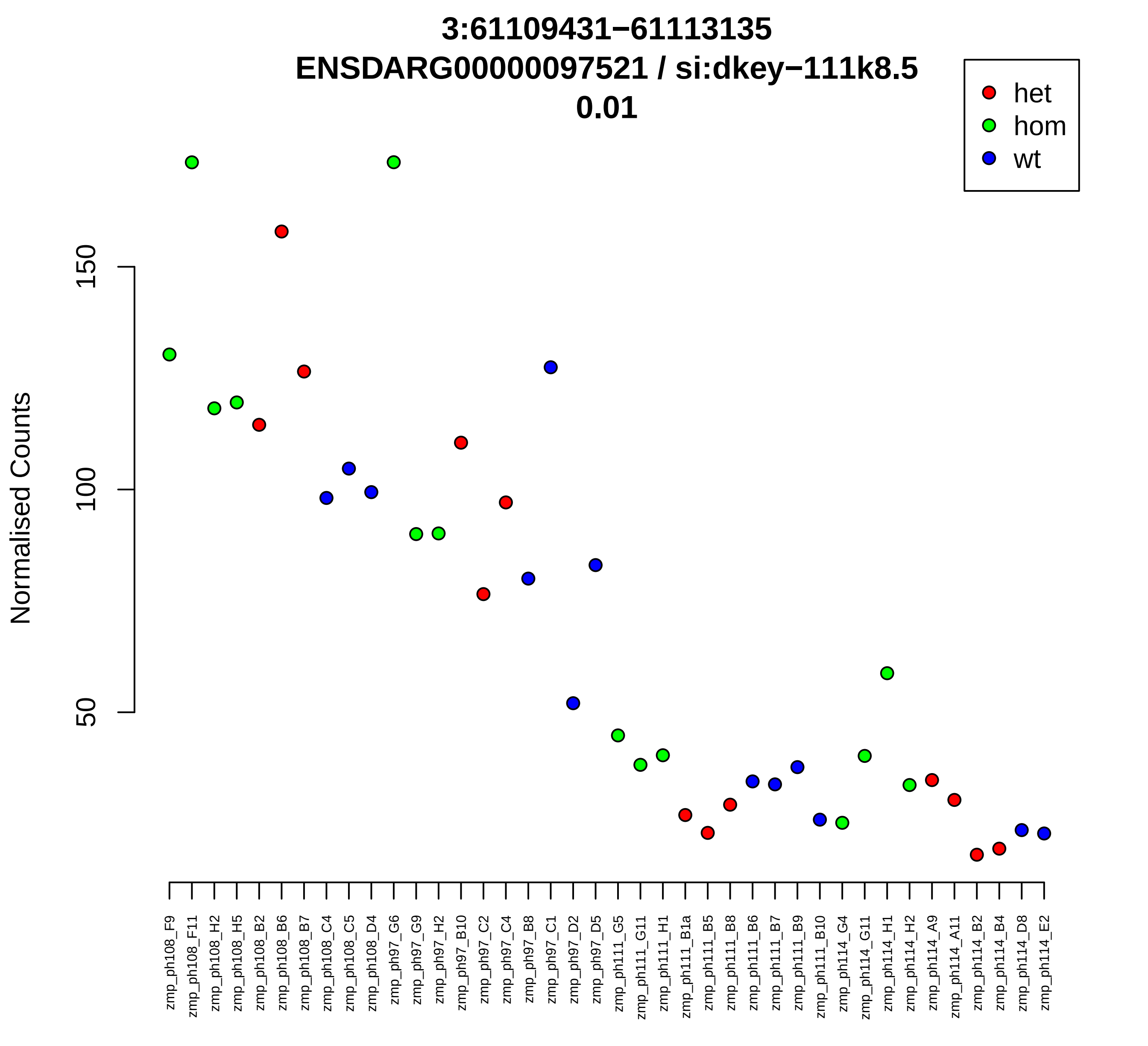

3:61109431−61113135
ENSDARG00000097521 / si:dkey−111k8.5
0.01
het
hom
wt
Normalised Counts
50
100
150
zmp_ph108_F9
zmp_ph108_F11
zmp_ph108_H2
zmp_ph108_H5
zmp_ph108_B2
zmp_ph108_B6
zmp_ph108_B7
zmp_ph108_C4
zmp_ph108_C5
zmp_ph108_D4
zmp_ph97_G6
zmp_ph97_G9
zmp_ph97_H2
zmp_ph97_B10
zmp_ph97_C2
zmp_ph97_C4
zmp_ph97_B8
zmp_ph97_C1
zmp_ph97_D2
zmp_ph97_D5
zmp_ph111_G5
zmp_ph111_G11
zmp_ph111_H1
zmp_ph111_B1a
zmp_ph111_B5
zmp_ph111_B8
zmp_ph111_B6
zmp_ph111_B7
zmp_ph111_B9
zmp_ph111_B10
zmp_ph114_G4
zmp_ph114_G11
zmp_ph114_H1
zmp_ph114_H2
zmp_ph114_A9
zmp_ph114_A11
zmp_ph114_B2
zmp_ph114_B4
zmp_ph114_D8
zmp_ph114_E2

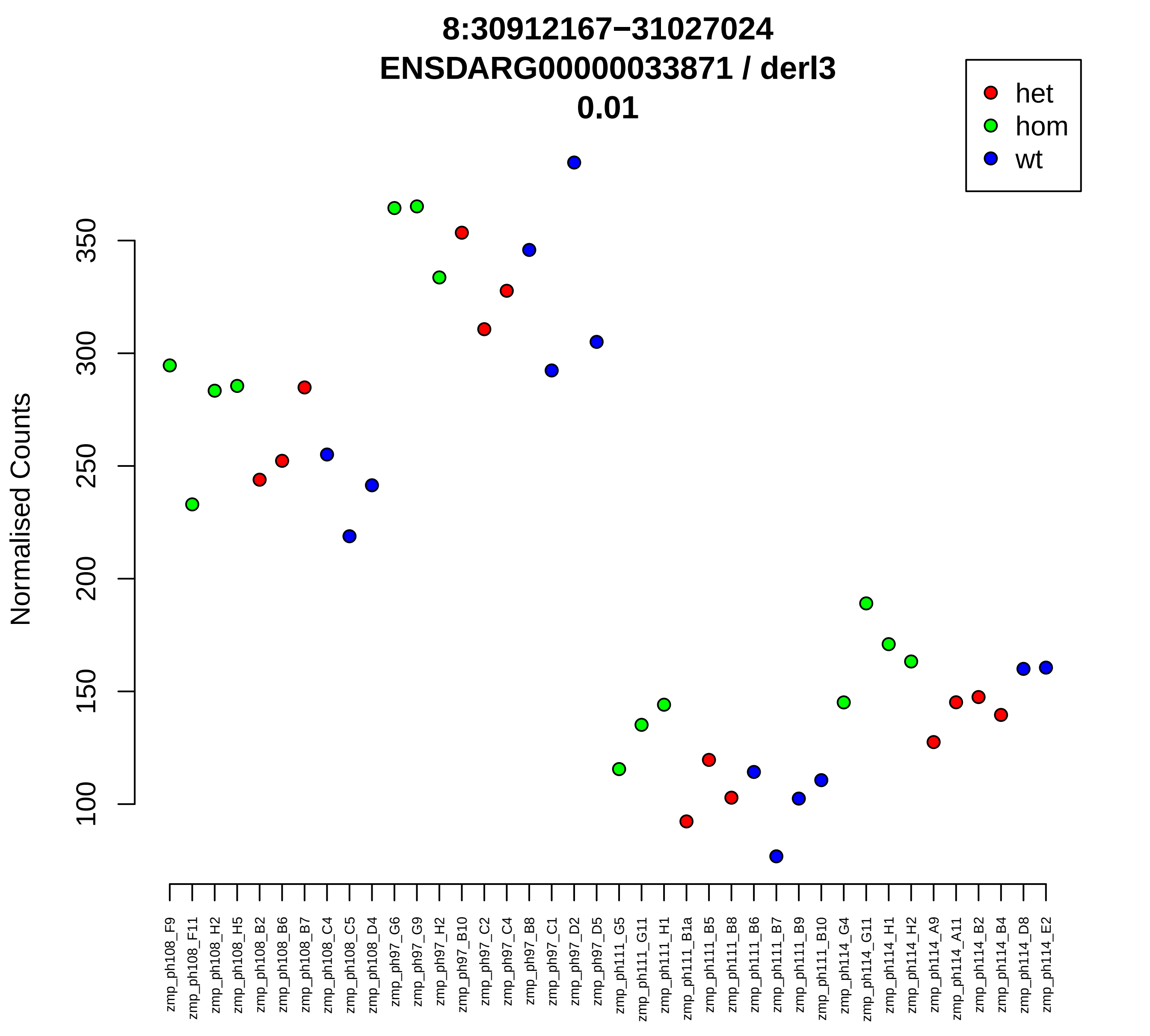

8:30912167−31027024
ENSDARG00000033871 / derl3
0.01
het
hom
wt
Normalised Counts
100
150
200
250
300
350
zmp_ph108_F9
zmp_ph108_F11
zmp_ph108_H2
zmp_ph108_H5
zmp_ph108_B2
zmp_ph108_B6
zmp_ph108_B7
zmp_ph108_C4
zmp_ph108_C5
zmp_ph108_D4
zmp_ph97_G6
zmp_ph97_G9
zmp_ph97_H2
zmp_ph97_B10
zmp_ph97_C2
zmp_ph97_C4
zmp_ph97_B8
zmp_ph97_C1
zmp_ph97_D2
zmp_ph97_D5
zmp_ph111_G5
zmp_ph111_G11
zmp_ph111_H1
zmp_ph111_B1a
zmp_ph111_B5
zmp_ph111_B8
zmp_ph111_B6
zmp_ph111_B7
zmp_ph111_B9
zmp_ph111_B10
zmp_ph114_G4
zmp_ph114_G11
zmp_ph114_H1
zmp_ph114_H2
zmp_ph114_A9
zmp_ph114_A11
zmp_ph114_B2
zmp_ph114_B4
zmp_ph114_D8
zmp_ph114_E2

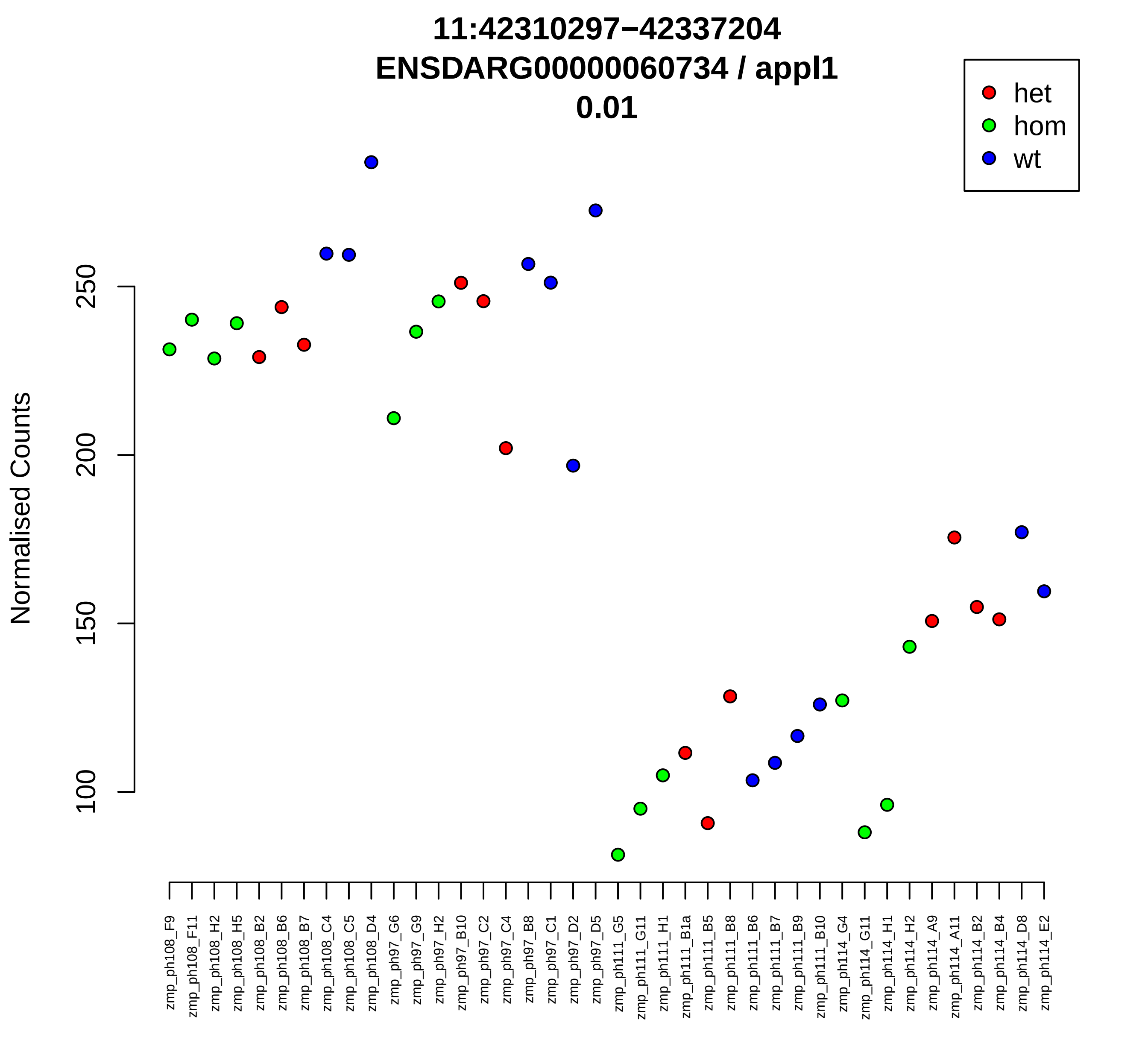
11:42310297−42337204
ENSDARG00000060734 / appl1
0.01
het
hom
wt
Normalised Counts
100
150
200
250
zmp_ph108_F9
zmp_ph108_F11
zmp_ph108_H2
zmp_ph108_H5
zmp_ph108_B2
zmp_ph108_B6
zmp_ph108_B7
zmp_ph108_C4
zmp_ph108_C5
zmp_ph108_D4
zmp_ph97_G6
zmp_ph97_G9
zmp_ph97_H2
zmp_ph97_B10
zmp_ph97_C2
zmp_ph97_C4
zmp_ph97_B8
zmp_ph97_C1
zmp_ph97_D2
zmp_ph97_D5
zmp_ph111_G5
zmp_ph111_G11
zmp_ph111_H1
zmp_ph111_B1a
zmp_ph111_B5
zmp_ph111_B8
zmp_ph111_B6
zmp_ph111_B7
zmp_ph111_B9
zmp_ph111_B10
zmp_ph114_G4
zmp_ph114_G11
zmp_ph114_H1
zmp_ph114_H2
zmp_ph114_A9
zmp_ph114_A11
zmp_ph114_B2
zmp_ph114_B4
zmp_ph114_D8
zmp_ph114_E2

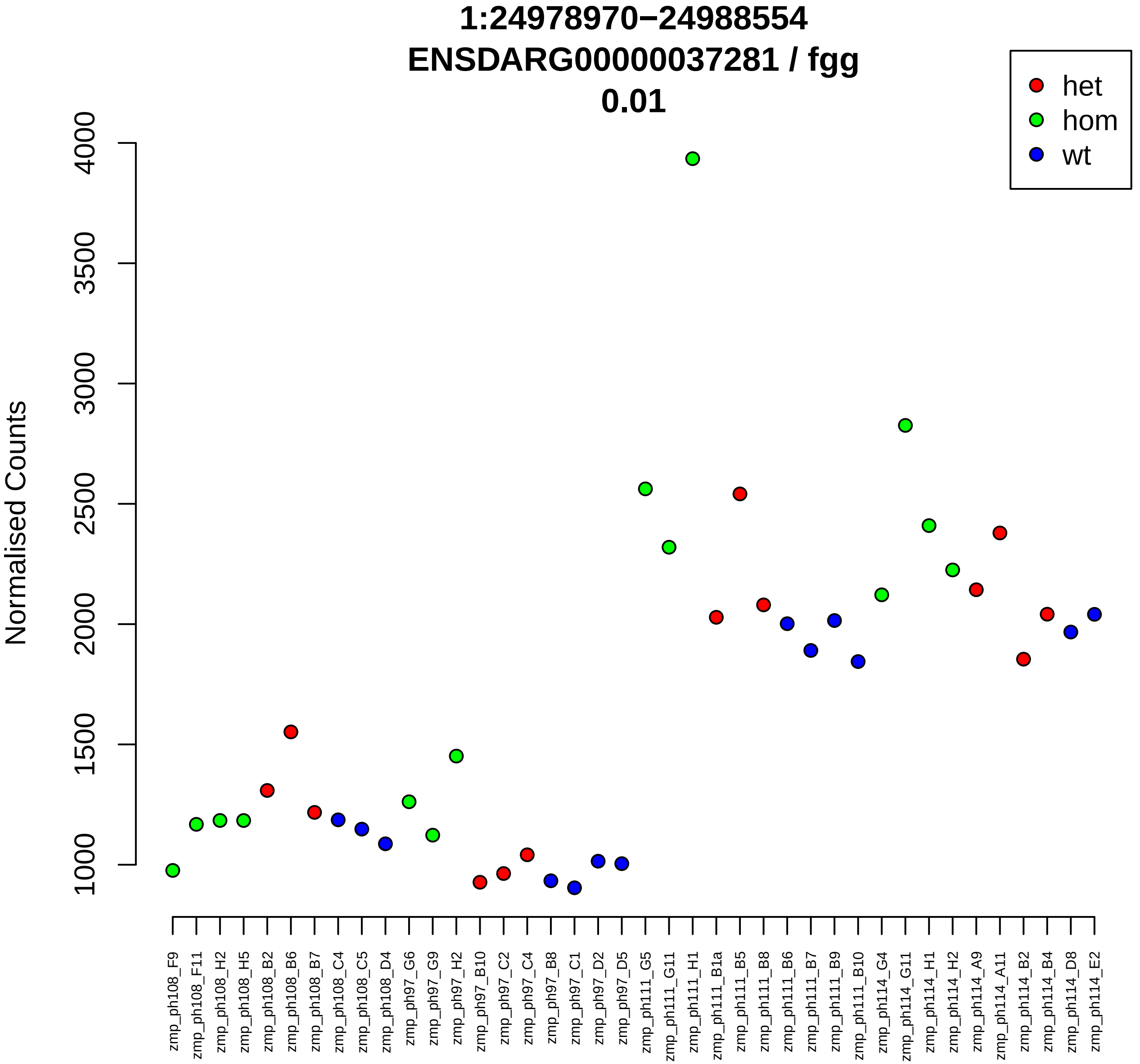

1:24978970−24988554
ENSDARG00000037281 / fgg
0.01
het
hom
wt
Normalised Counts
1000
1500
2000
2500
3000
3500
4000
zmp_ph108_F9
zmp_ph108_F11
zmp_ph108_H2
zmp_ph108_H5
zmp_ph108_B2
zmp_ph108_B6
zmp_ph108_B7
zmp_ph108_C4
zmp_ph108_C5
zmp_ph108_D4
zmp_ph97_G6
zmp_ph97_G9
zmp_ph97_H2
zmp_ph97_B10
zmp_ph97_C2
zmp_ph97_C4
zmp_ph97_B8
zmp_ph97_C1
zmp_ph97_D2
zmp_ph97_D5
zmp_ph111_G5
zmp_ph111_G11
zmp_ph111_H1
zmp_ph111_B1a
zmp_ph111_B5
zmp_ph111_B8
zmp_ph111_B6
zmp_ph111_B7
zmp_ph111_B9
zmp_ph111_B10
zmp_ph114_G4
zmp_ph114_G11
zmp_ph114_H1
zmp_ph114_H2
zmp_ph114_A9
zmp_ph114_A11
zmp_ph114_B2
zmp_ph114_B4
zmp_ph114_D8
zmp_ph114_E2

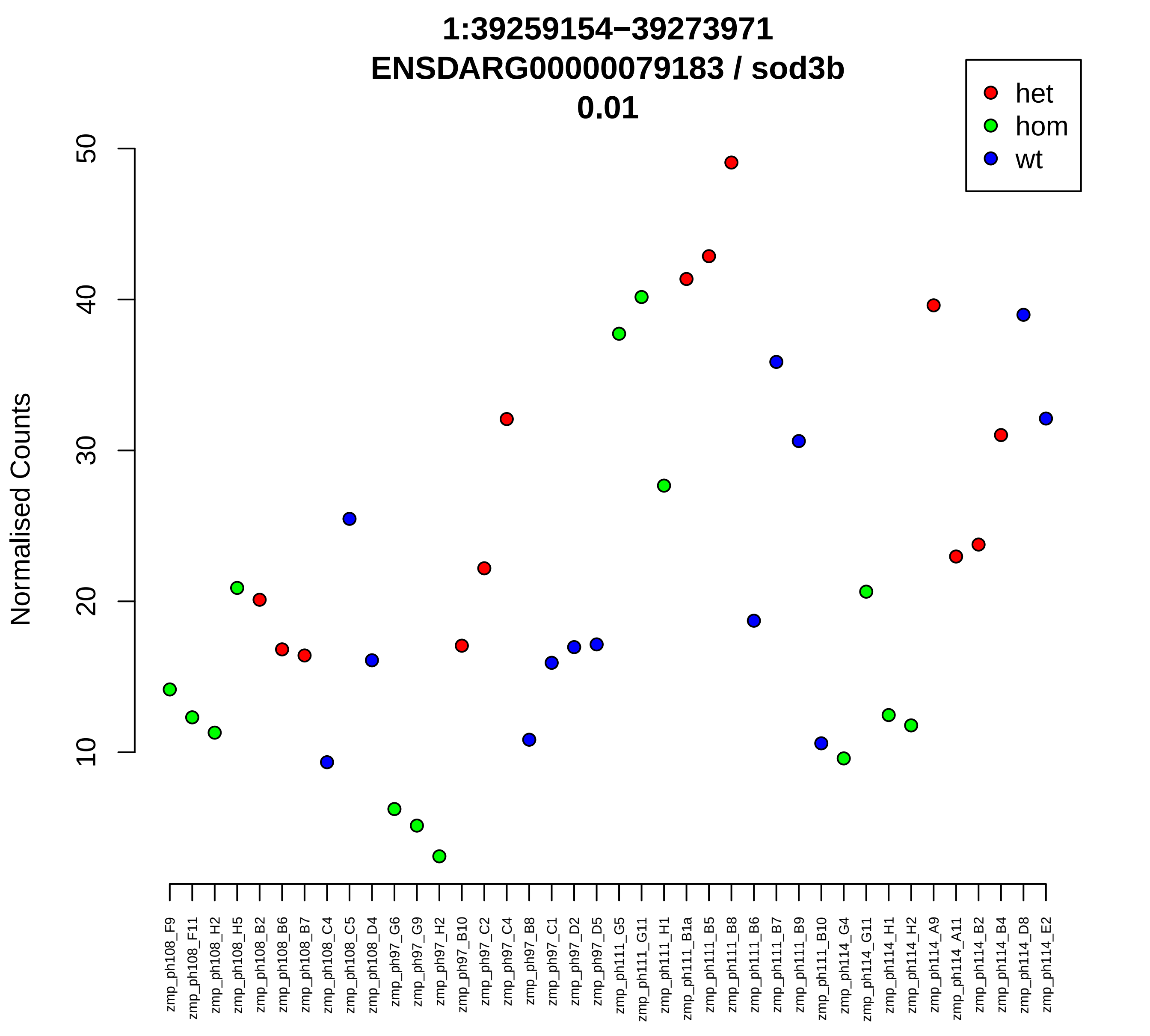
1:39259154−39273971
ENSDARG00000079183 / sod3b
0.01
het
hom
wt
Normalised Counts
10
20
30
40
50
zmp_ph108_F9
zmp_ph108_F11
zmp_ph108_H2
zmp_ph108_H5
zmp_ph108_B2
zmp_ph108_B6
zmp_ph108_B7
zmp_ph108_C4
zmp_ph108_C5
zmp_ph108_D4
zmp_ph97_G6
zmp_ph97_G9
zmp_ph97_H2
zmp_ph97_B10
zmp_ph97_C2
zmp_ph97_C4
zmp_ph97_B8
zmp_ph97_C1
zmp_ph97_D2
zmp_ph97_D5
zmp_ph111_G5
zmp_ph111_G11
zmp_ph111_H1
zmp_ph111_B1a
zmp_ph111_B5
zmp_ph111_B8
zmp_ph111_B6
zmp_ph111_B7
zmp_ph111_B9
zmp_ph111_B10
zmp_ph114_G4
zmp_ph114_G11
zmp_ph114_H1
zmp_ph114_H2
zmp_ph114_A9
zmp_ph114_A11
zmp_ph114_B2
zmp_ph114_B4
zmp_ph114_D8
zmp_ph114_E2

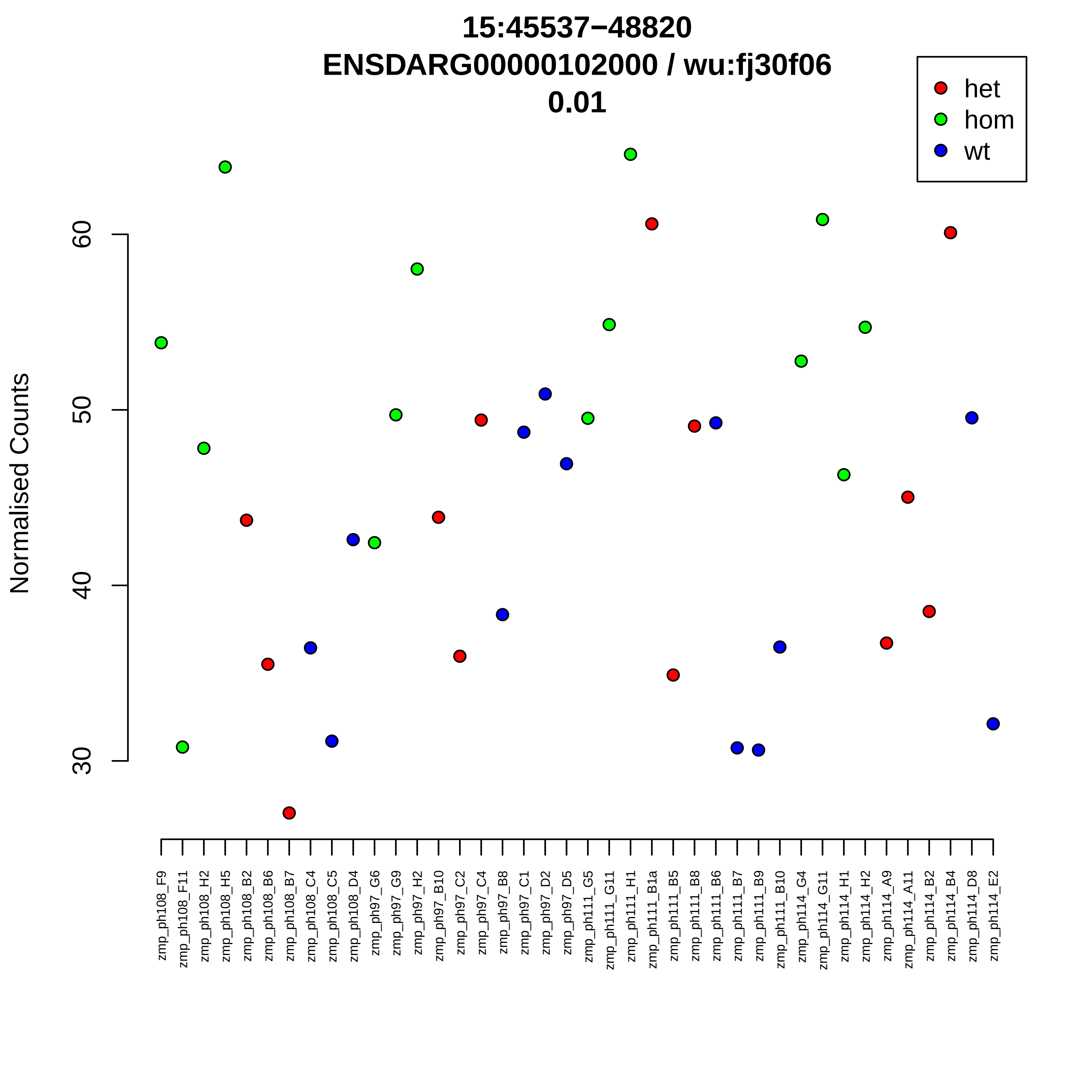
15:45537−48820
ENSDARG00000102000 / wu:fj30f06
0.01
het
hom
wt
Normalised Counts
30
40
50
60
zmp_ph108_F9
zmp_ph108_F11
zmp_ph108_H2
zmp_ph108_H5
zmp_ph108_B2
zmp_ph108_B6
zmp_ph108_B7
zmp_ph108_C4
zmp_ph108_C5
zmp_ph108_D4
zmp_ph97_G6
zmp_ph97_G9
zmp_ph97_H2
zmp_ph97_B10
zmp_ph97_C2
zmp_ph97_C4
zmp_ph97_B8
zmp_ph97_C1
zmp_ph97_D2
zmp_ph97_D5
zmp_ph111_G5
zmp_ph111_G11
zmp_ph111_H1
zmp_ph111_B1a
zmp_ph111_B5
zmp_ph111_B8
zmp_ph111_B6
zmp_ph111_B7
zmp_ph111_B9
zmp_ph111_B10
zmp_ph114_G4
zmp_ph114_G11
zmp_ph114_H1
zmp_ph114_H2
zmp_ph114_A9
zmp_ph114_A11
zmp_ph114_B2
zmp_ph114_B4
zmp_ph114_D8
zmp_ph114_E2

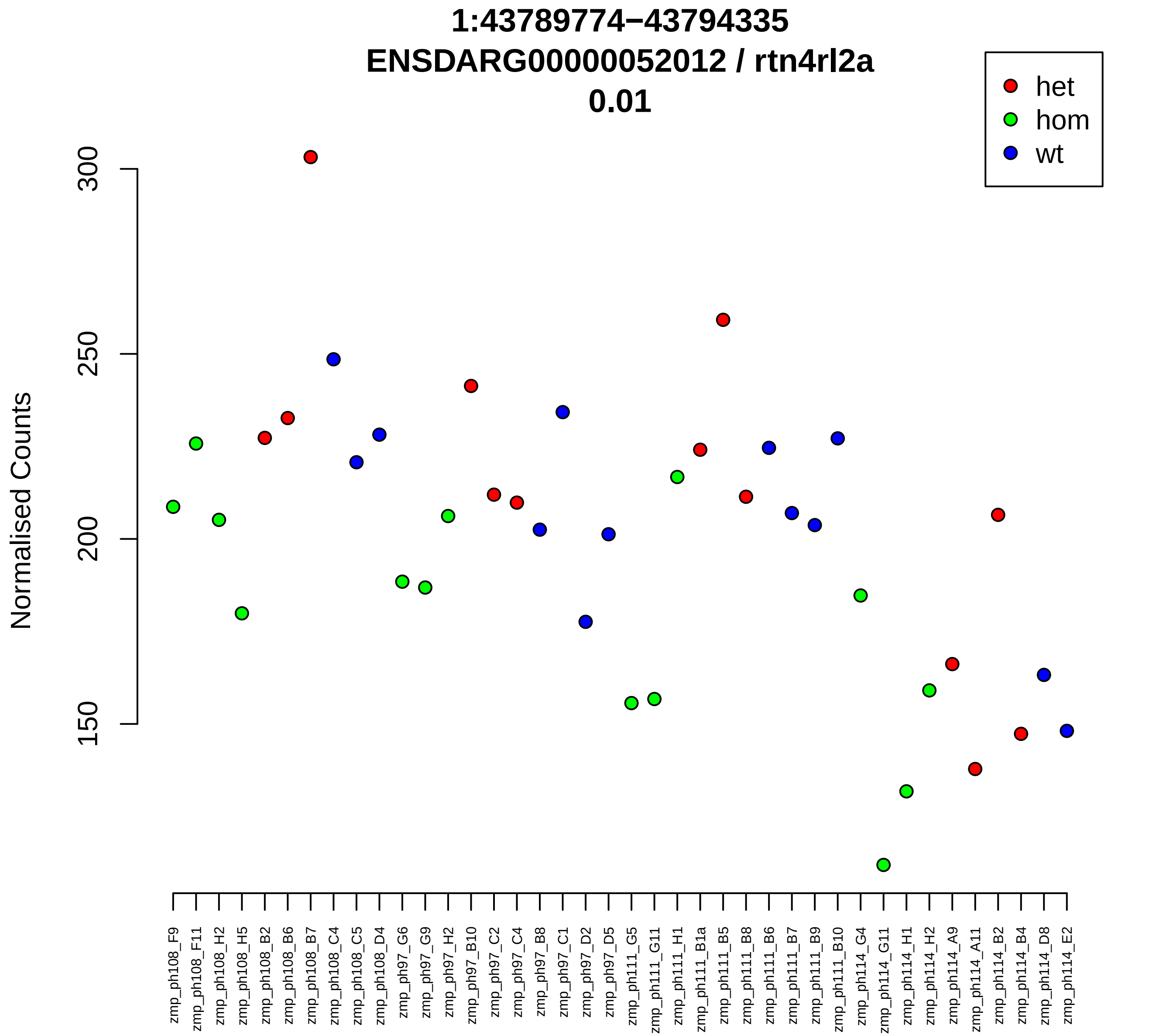
1:43789774−43794335
ENSDARG00000052012 / rtn4rl2a
0.01
het
hom
wt
Normalised Counts
150
200
250
300
zmp_ph108_F9
zmp_ph108_F11
zmp_ph108_H2
zmp_ph108_H5
zmp_ph108_B2
zmp_ph108_B6
zmp_ph108_B7
zmp_ph108_C4
zmp_ph108_C5
zmp_ph108_D4
zmp_ph97_G6
zmp_ph97_G9
zmp_ph97_H2
zmp_ph97_B10
zmp_ph97_C2
zmp_ph97_C4
zmp_ph97_B8
zmp_ph97_C1
zmp_ph97_D2
zmp_ph97_D5
zmp_ph111_G5
zmp_ph111_G11
zmp_ph111_H1
zmp_ph111_B1a
zmp_ph111_B5
zmp_ph111_B8
zmp_ph111_B6
zmp_ph111_B7
zmp_ph111_B9
zmp_ph111_B10
zmp_ph114_G4
zmp_ph114_G11
zmp_ph114_H1
zmp_ph114_H2
zmp_ph114_A9
zmp_ph114_A11
zmp_ph114_B2
zmp_ph114_B4
zmp_ph114_D8
zmp_ph114_E2

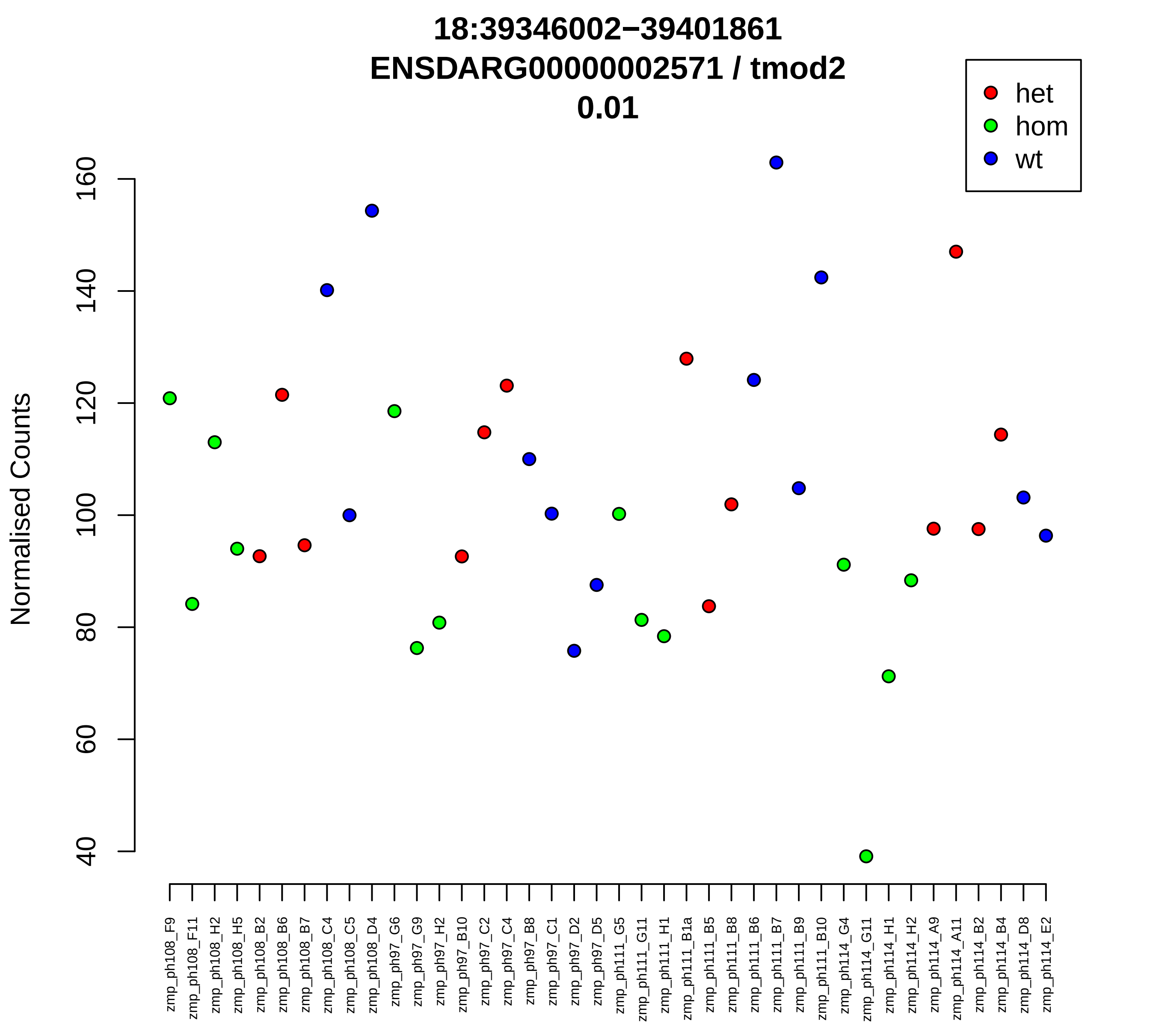

18:39346002−39401861
ENSDARG00000002571 / tmod2
0.01
het
hom
wt
Normalised Counts
40
60
80
100
120
140
160
zmp_ph108_F9
zmp_ph108_F11
zmp_ph108_H2
zmp_ph108_H5
zmp_ph108_B2
zmp_ph108_B6
zmp_ph108_B7
zmp_ph108_C4
zmp_ph108_C5
zmp_ph108_D4
zmp_ph97_G6
zmp_ph97_G9
zmp_ph97_H2
zmp_ph97_B10
zmp_ph97_C2
zmp_ph97_C4
zmp_ph97_B8
zmp_ph97_C1
zmp_ph97_D2
zmp_ph97_D5
zmp_ph111_G5
zmp_ph111_G11
zmp_ph111_H1
zmp_ph111_B1a
zmp_ph111_B5
zmp_ph111_B8
zmp_ph111_B6
zmp_ph111_B7
zmp_ph111_B9
zmp_ph111_B10
zmp_ph114_G4
zmp_ph114_G11
zmp_ph114_H1
zmp_ph114_H2
zmp_ph114_A9
zmp_ph114_A11
zmp_ph114_B2
zmp_ph114_B4
zmp_ph114_D8
zmp_ph114_E2

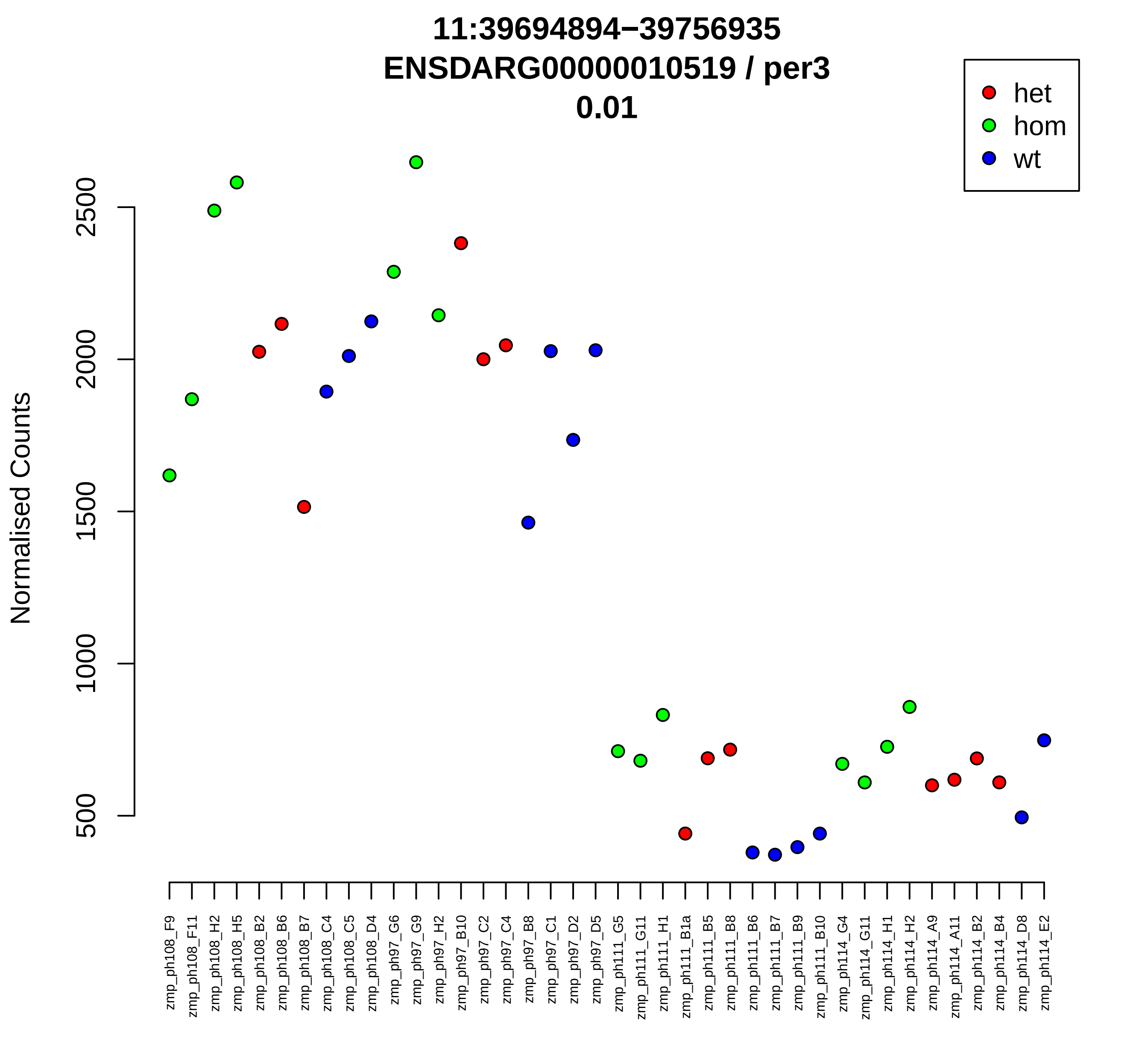
11:39694894−39756935
ENSDARG00000010519 / per3
0.01
het
hom
wt
Normalised Counts
500
1000
1500
2000
2500
zmp_ph108_F9
zmp_ph108_F11
zmp_ph108_H2
zmp_ph108_H5
zmp_ph108_B2
zmp_ph108_B6
zmp_ph108_B7
zmp_ph108_C4
zmp_ph108_C5
zmp_ph108_D4
zmp_ph97_G6
zmp_ph97_G9
zmp_ph97_H2
zmp_ph97_B10
zmp_ph97_C2
zmp_ph97_C4
zmp_ph97_B8
zmp_ph97_C1
zmp_ph97_D2
zmp_ph97_D5
zmp_ph111_G5
zmp_ph111_G11
zmp_ph111_H1
zmp_ph111_B1a
zmp_ph111_B5
zmp_ph111_B8
zmp_ph111_B6
zmp_ph111_B7
zmp_ph111_B9
zmp_ph111_B10
zmp_ph114_G4
zmp_ph114_G11
zmp_ph114_H1
zmp_ph114_H2
zmp_ph114_A9
zmp_ph114_A11
zmp_ph114_B2
zmp_ph114_B4
zmp_ph114_D8
zmp_ph114_E2

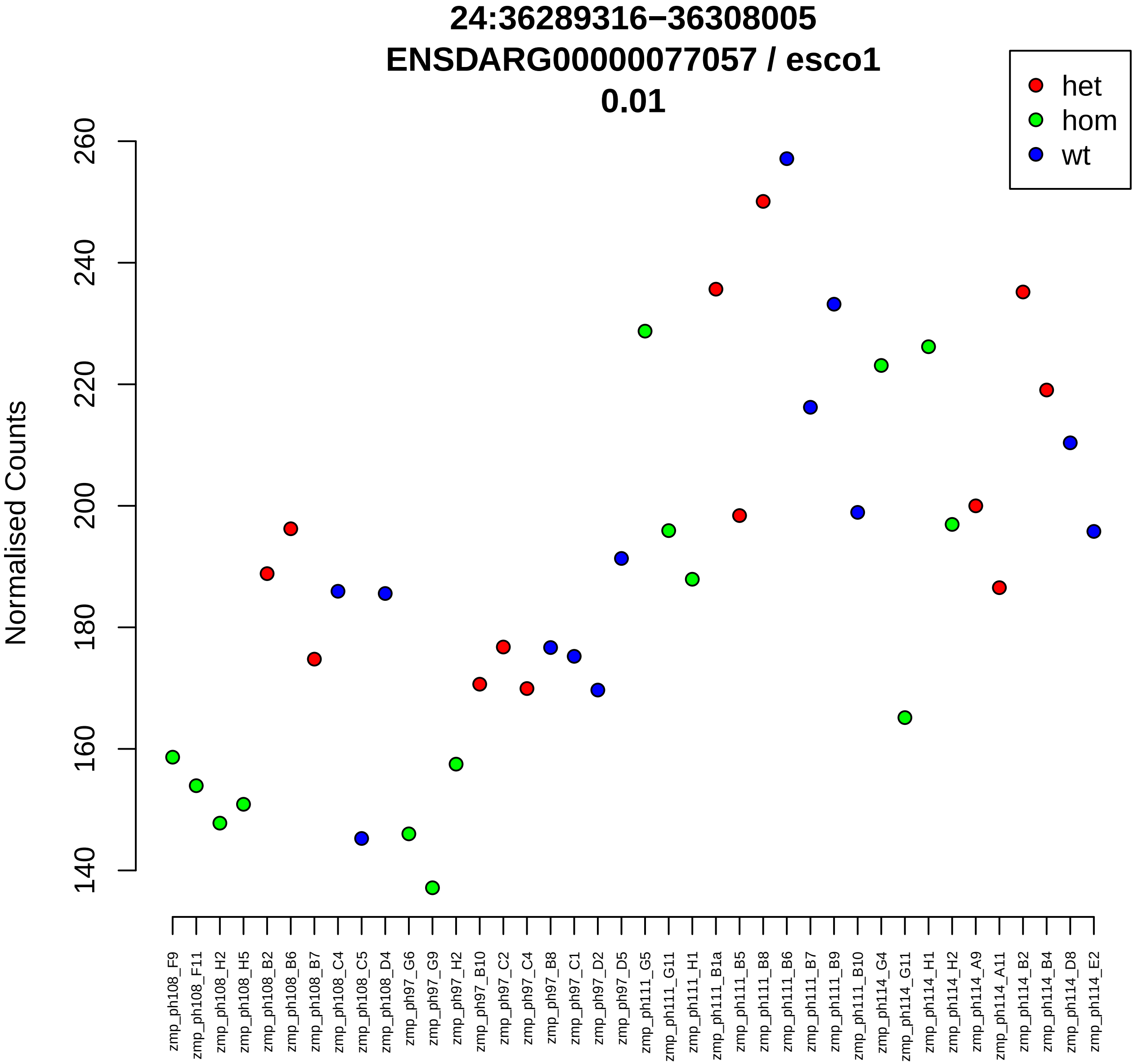
24:36289316−36308005
ENSDARG00000077057 / esco1
0.01
het
hom
wt
Normalised Counts
140
160
180
200
220
240
260
zmp_ph108_F9
zmp_ph108_F11
zmp_ph108_H2
zmp_ph108_H5
zmp_ph108_B2
zmp_ph108_B6
zmp_ph108_B7
zmp_ph108_C4
zmp_ph108_C5
zmp_ph108_D4
zmp_ph97_G6
zmp_ph97_G9
zmp_ph97_H2
zmp_ph97_B10
zmp_ph97_C2
zmp_ph97_C4
zmp_ph97_B8
zmp_ph97_C1
zmp_ph97_D2
zmp_ph97_D5
zmp_ph111_G5
zmp_ph111_G11
zmp_ph111_H1
zmp_ph111_B1a
zmp_ph111_B5
zmp_ph111_B8
zmp_ph111_B6
zmp_ph111_B7
zmp_ph111_B9
zmp_ph111_B10
zmp_ph114_G4
zmp_ph114_G11
zmp_ph114_H1
zmp_ph114_H2
zmp_ph114_A9
zmp_ph114_A11
zmp_ph114_B2
zmp_ph114_B4
zmp_ph114_D8
zmp_ph114_E2

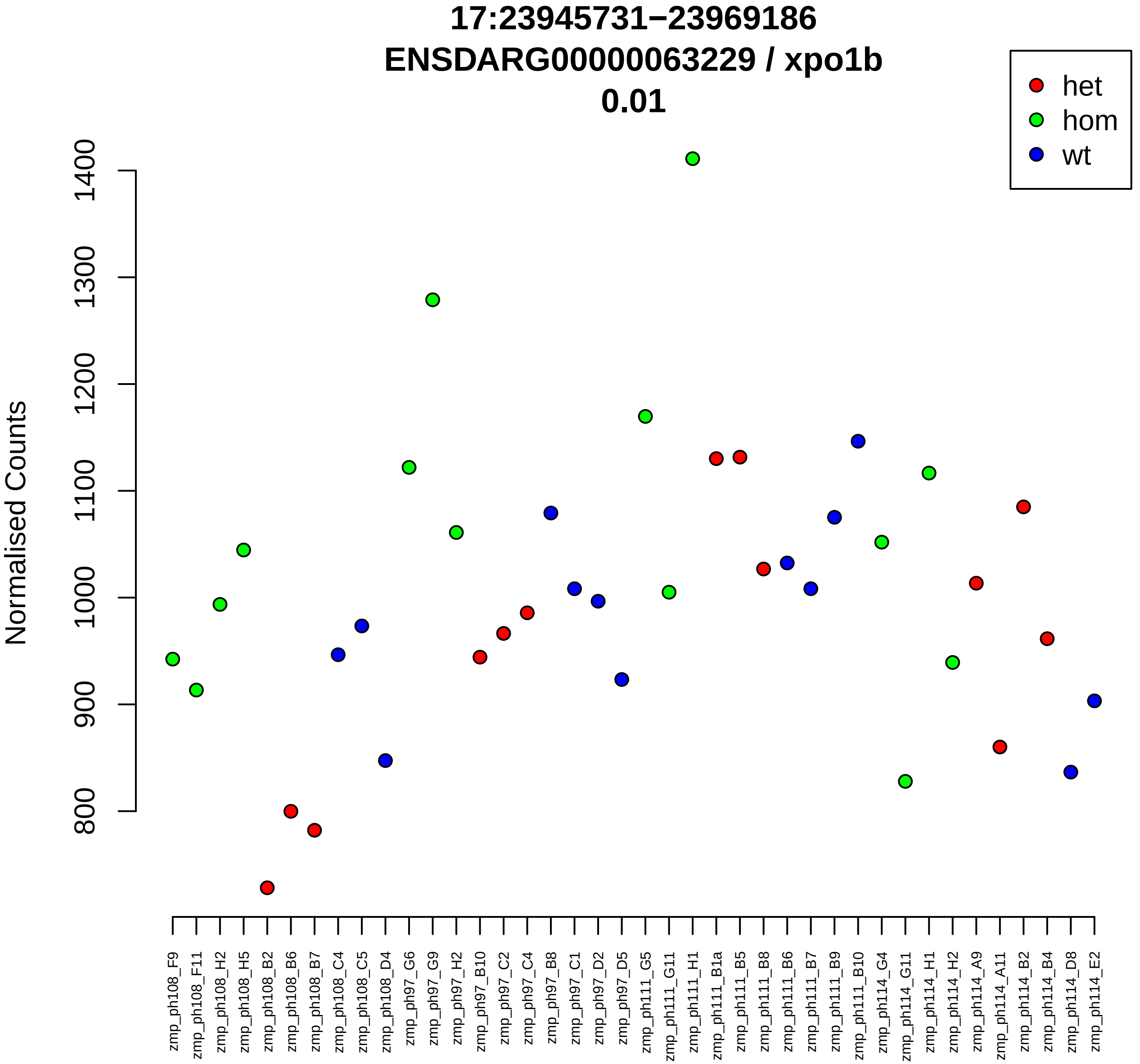

17:23945731−23969186
ENSDARG00000063229 / xpo1b
0.01
het
hom
wt
Normalised Counts
800
900
1000
1100
1200
1300
1400
zmp_ph108_F9
zmp_ph108_F11
zmp_ph108_H2
zmp_ph108_H5
zmp_ph108_B2
zmp_ph108_B6
zmp_ph108_B7
zmp_ph108_C4
zmp_ph108_C5
zmp_ph108_D4
zmp_ph97_G6
zmp_ph97_G9
zmp_ph97_H2
zmp_ph97_B10
zmp_ph97_C2
zmp_ph97_C4
zmp_ph97_B8
zmp_ph97_C1
zmp_ph97_D2
zmp_ph97_D5
zmp_ph111_G5
zmp_ph111_G11
zmp_ph111_H1
zmp_ph111_B1a
zmp_ph111_B5
zmp_ph111_B8
zmp_ph111_B6
zmp_ph111_B7
zmp_ph111_B9
zmp_ph111_B10
zmp_ph114_G4
zmp_ph114_G11
zmp_ph114_H1
zmp_ph114_H2
zmp_ph114_A9
zmp_ph114_A11
zmp_ph114_B2
zmp_ph114_B4
zmp_ph114_D8
zmp_ph114_E2

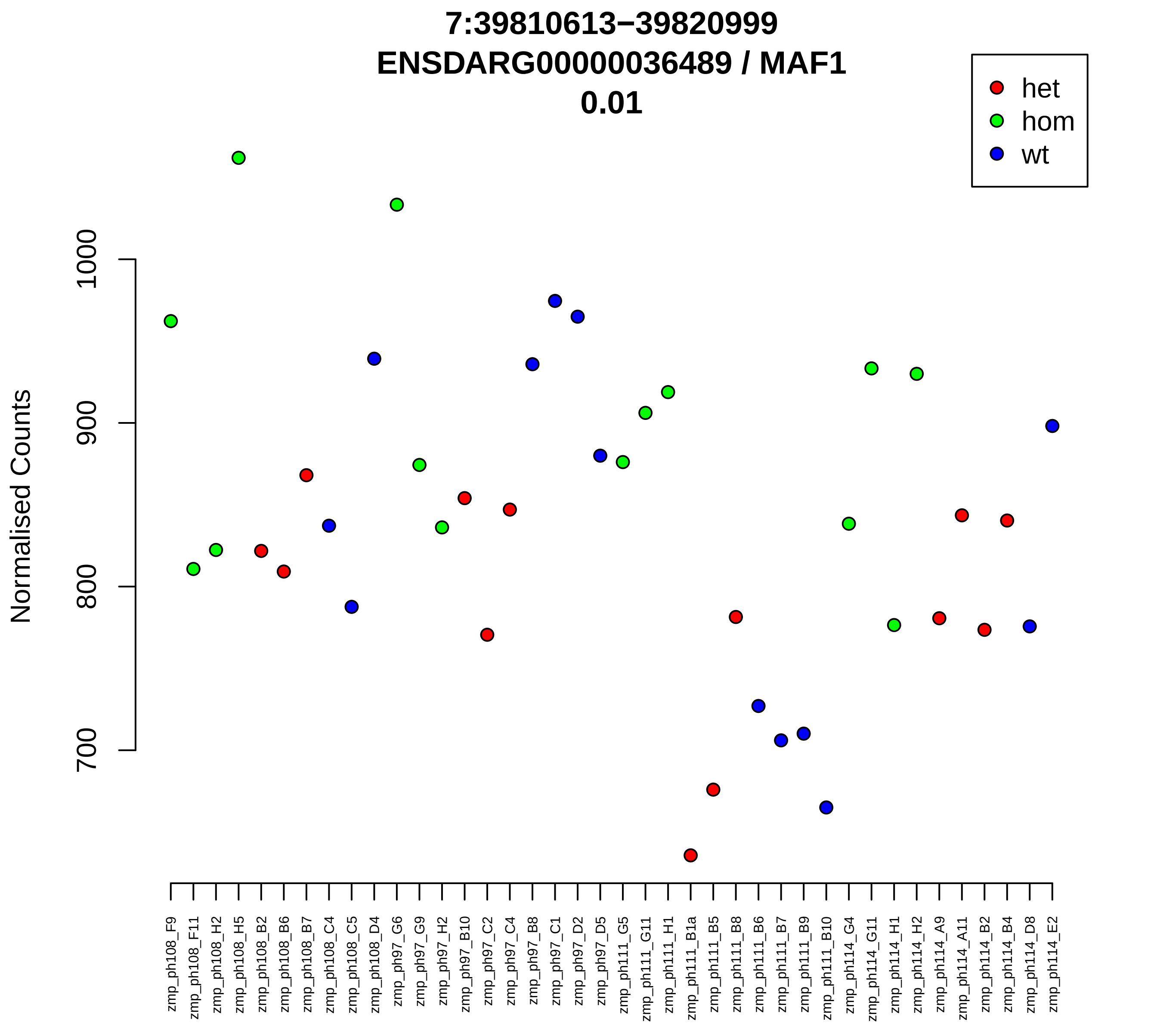
7:39810613−39820999
ENSDARG00000036489 / MAF1
0.01
het
hom
wt
Normalised Counts
700
800
900
1000
zmp_ph108_F9
zmp_ph108_F11
zmp_ph108_H2
zmp_ph108_H5
zmp_ph108_B2
zmp_ph108_B6
zmp_ph108_B7
zmp_ph108_C4
zmp_ph108_C5
zmp_ph108_D4
zmp_ph97_G6
zmp_ph97_G9
zmp_ph97_H2
zmp_ph97_B10
zmp_ph97_C2
zmp_ph97_C4
zmp_ph97_B8
zmp_ph97_C1
zmp_ph97_D2
zmp_ph97_D5
zmp_ph111_G5
zmp_ph111_G11
zmp_ph111_H1
zmp_ph111_B1a
zmp_ph111_B5
zmp_ph111_B8
zmp_ph111_B6
zmp_ph111_B7
zmp_ph111_B9
zmp_ph111_B10
zmp_ph114_G4
zmp_ph114_G11
zmp_ph114_H1
zmp_ph114_H2
zmp_ph114_A9
zmp_ph114_A11
zmp_ph114_B2
zmp_ph114_B4
zmp_ph114_D8
zmp_ph114_E2

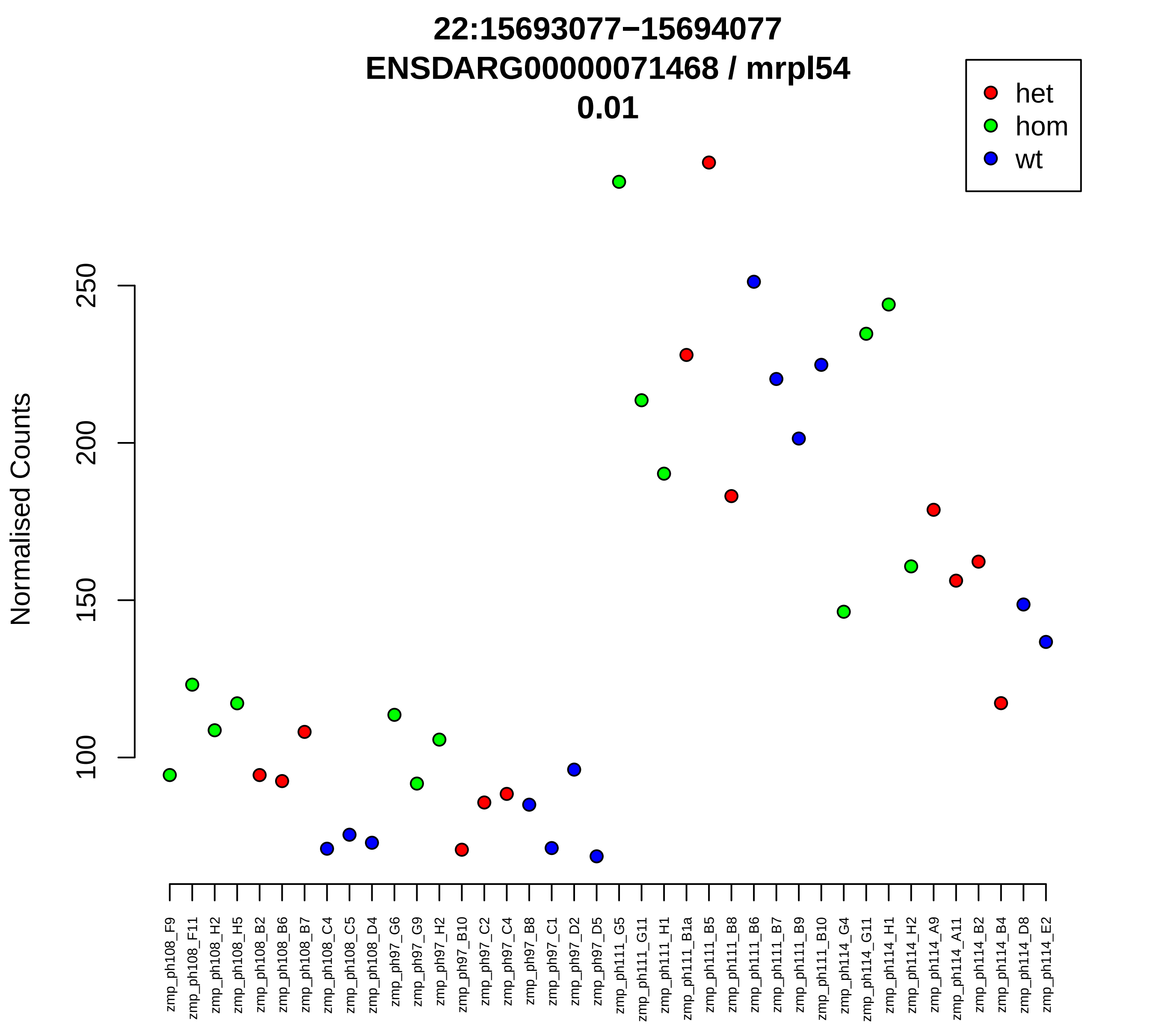
22:15693077−15694077
ENSDARG00000071468 / mrpl54
0.01
het
hom
wt
Normalised Counts
100
150
200
250
zmp_ph108_F9
zmp_ph108_F11
zmp_ph108_H2
zmp_ph108_H5
zmp_ph108_B2
zmp_ph108_B6
zmp_ph108_B7
zmp_ph108_C4
zmp_ph108_C5
zmp_ph108_D4
zmp_ph97_G6
zmp_ph97_G9
zmp_ph97_H2
zmp_ph97_B10
zmp_ph97_C2
zmp_ph97_C4
zmp_ph97_B8
zmp_ph97_C1
zmp_ph97_D2
zmp_ph97_D5
zmp_ph111_G5
zmp_ph111_G11
zmp_ph111_H1
zmp_ph111_B1a
zmp_ph111_B5
zmp_ph111_B8
zmp_ph111_B6
zmp_ph111_B7
zmp_ph111_B9
zmp_ph111_B10
zmp_ph114_G4
zmp_ph114_G11
zmp_ph114_H1
zmp_ph114_H2
zmp_ph114_A9
zmp_ph114_A11
zmp_ph114_B2
zmp_ph114_B4
zmp_ph114_D8
zmp_ph114_E2

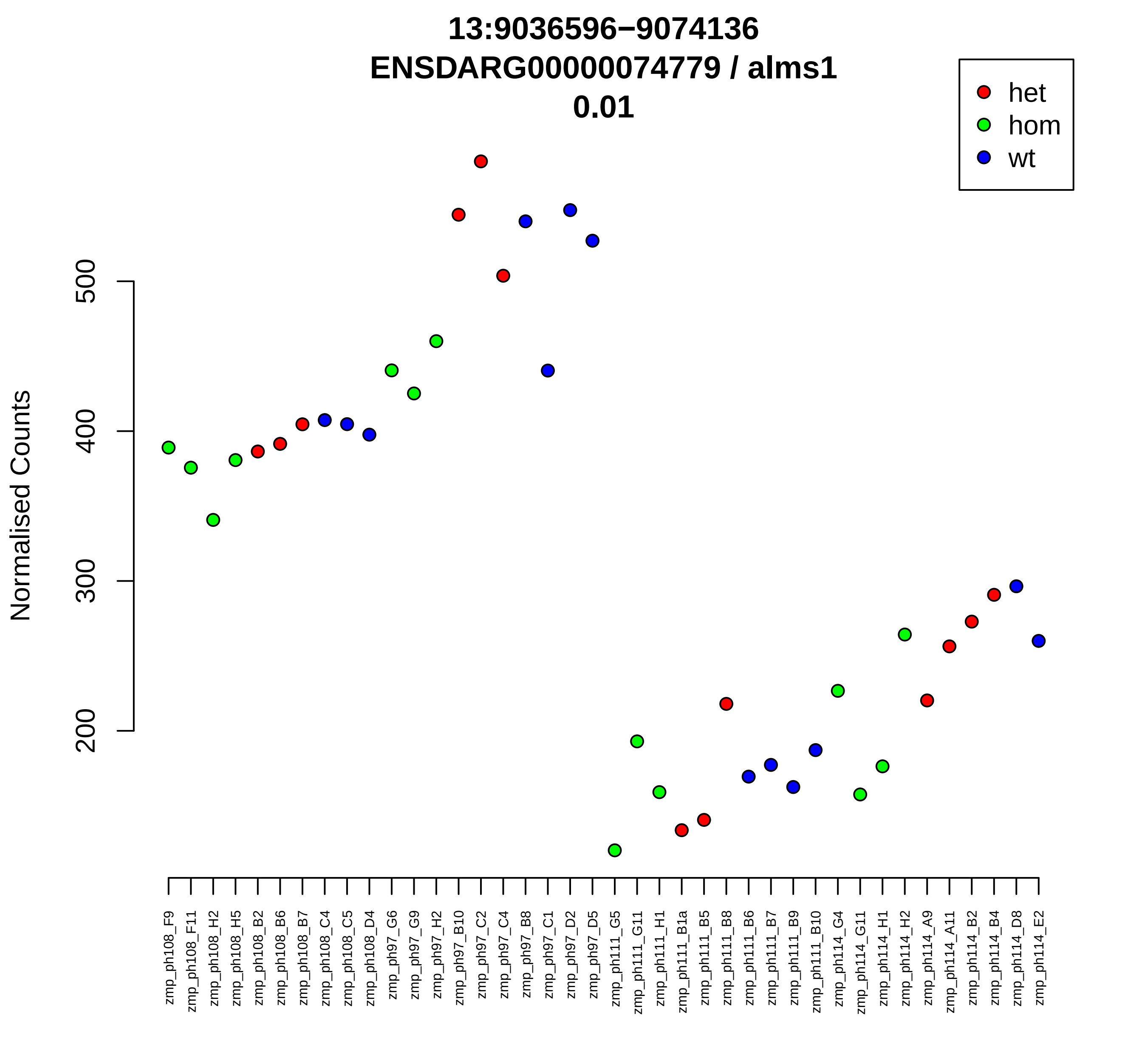
13:9036596−9074136
ENSDARG00000074779 / alms1
0.01
het
hom
wt
Normalised Counts
200
300
400
500
zmp_ph108_F9
zmp_ph108_F11
zmp_ph108_H2
zmp_ph108_H5
zmp_ph108_B2
zmp_ph108_B6
zmp_ph108_B7
zmp_ph108_C4
zmp_ph108_C5
zmp_ph108_D4
zmp_ph97_G6
zmp_ph97_G9
zmp_ph97_H2
zmp_ph97_B10
zmp_ph97_C2
zmp_ph97_C4
zmp_ph97_B8
zmp_ph97_C1
zmp_ph97_D2
zmp_ph97_D5
zmp_ph111_G5
zmp_ph111_G11
zmp_ph111_H1
zmp_ph111_B1a
zmp_ph111_B5
zmp_ph111_B8
zmp_ph111_B6
zmp_ph111_B7
zmp_ph111_B9
zmp_ph111_B10
zmp_ph114_G4
zmp_ph114_G11
zmp_ph114_H1
zmp_ph114_H2
zmp_ph114_A9
zmp_ph114_A11
zmp_ph114_B2
zmp_ph114_B4
zmp_ph114_D8
zmp_ph114_E2

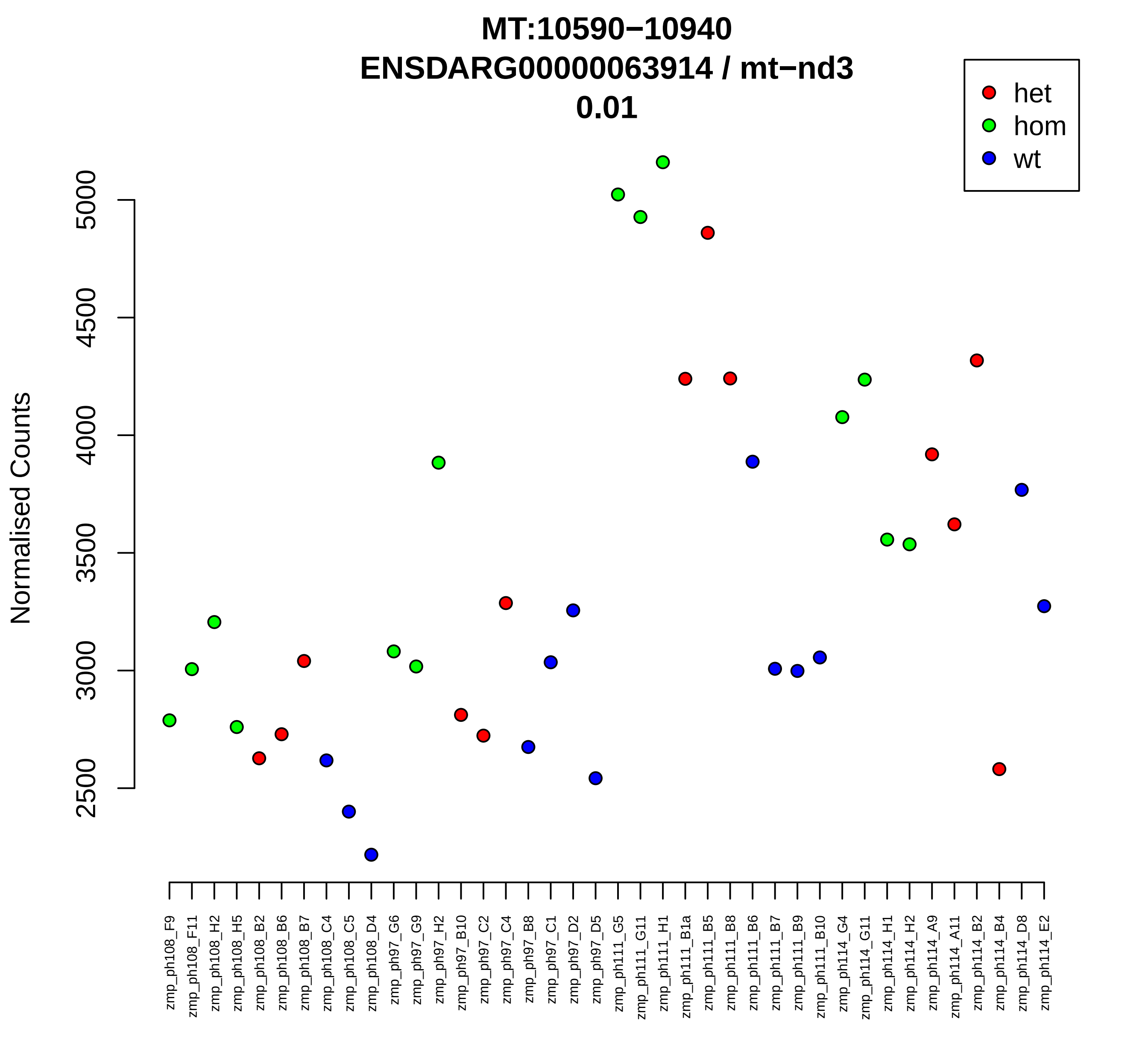
MT:10590−10940
ENSDARG00000063914 / mt−nd3
0.01
het
hom
wt
Normalised Counts
2500
3000
3500
4000
4500
5000
zmp_ph108_F9
zmp_ph108_F11
zmp_ph108_H2
zmp_ph108_H5
zmp_ph108_B2
zmp_ph108_B6
zmp_ph108_B7
zmp_ph108_C4
zmp_ph108_C5
zmp_ph108_D4
zmp_ph97_G6
zmp_ph97_G9
zmp_ph97_H2
zmp_ph97_B10
zmp_ph97_C2
zmp_ph97_C4
zmp_ph97_B8
zmp_ph97_C1
zmp_ph97_D2
zmp_ph97_D5
zmp_ph111_G5
zmp_ph111_G11
zmp_ph111_H1
zmp_ph111_B1a
zmp_ph111_B5
zmp_ph111_B8
zmp_ph111_B6
zmp_ph111_B7
zmp_ph111_B9
zmp_ph111_B10
zmp_ph114_G4
zmp_ph114_G11
zmp_ph114_H1
zmp_ph114_H2
zmp_ph114_A9
zmp_ph114_A11
zmp_ph114_B2
zmp_ph114_B4
zmp_ph114_D8
zmp_ph114_E2

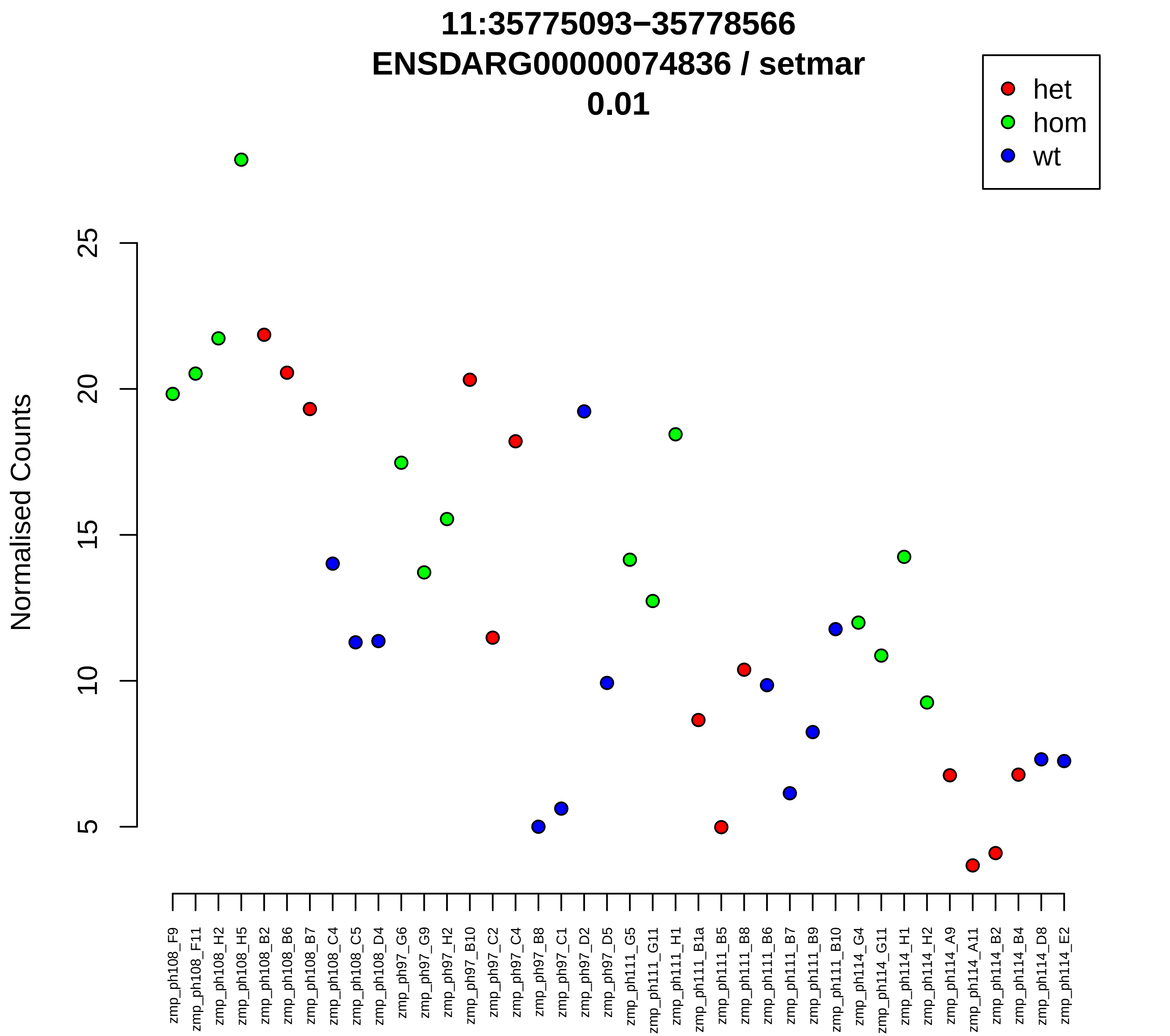

11:35775093−35778566
ENSDARG00000074836 / setmar
0.01
het
hom
wt
Normalised Counts
5
10
15
20
25
zmp_ph108_F9
zmp_ph108_F11
zmp_ph108_H2
zmp_ph108_H5
zmp_ph108_B2
zmp_ph108_B6
zmp_ph108_B7
zmp_ph108_C4
zmp_ph108_C5
zmp_ph108_D4
zmp_ph97_G6
zmp_ph97_G9
zmp_ph97_H2
zmp_ph97_B10
zmp_ph97_C2
zmp_ph97_C4
zmp_ph97_B8
zmp_ph97_C1
zmp_ph97_D2
zmp_ph97_D5
zmp_ph111_G5
zmp_ph111_G11
zmp_ph111_H1
zmp_ph111_B1a
zmp_ph111_B5
zmp_ph111_B8
zmp_ph111_B6
zmp_ph111_B7
zmp_ph111_B9
zmp_ph111_B10
zmp_ph114_G4
zmp_ph114_G11
zmp_ph114_H1
zmp_ph114_H2
zmp_ph114_A9
zmp_ph114_A11
zmp_ph114_B2
zmp_ph114_B4
zmp_ph114_D8
zmp_ph114_E2

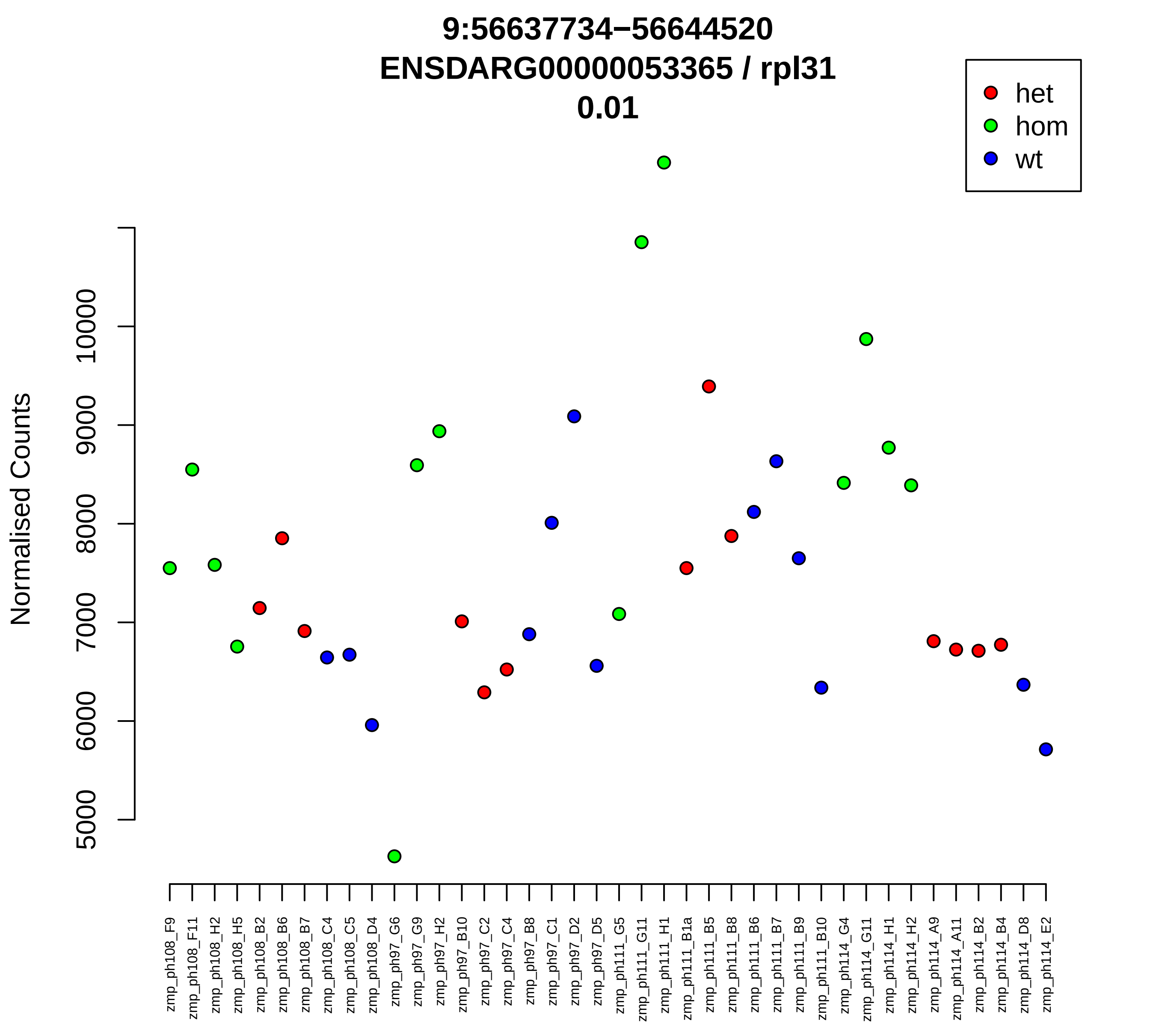
9:56637734−56644520
ENSDARG00000053365 / rpl31
0.01
het
hom
wt
Normalised Counts
5000
6000
7000
8000
9000
10000
zmp_ph108_F9
zmp_ph108_F11
zmp_ph108_H2
zmp_ph108_H5
zmp_ph108_B2
zmp_ph108_B6
zmp_ph108_B7
zmp_ph108_C4
zmp_ph108_C5
zmp_ph108_D4
zmp_ph97_G6
zmp_ph97_G9
zmp_ph97_H2
zmp_ph97_B10
zmp_ph97_C2
zmp_ph97_C4
zmp_ph97_B8
zmp_ph97_C1
zmp_ph97_D2
zmp_ph97_D5
zmp_ph111_G5
zmp_ph111_G11
zmp_ph111_H1
zmp_ph111_B1a
zmp_ph111_B5
zmp_ph111_B8
zmp_ph111_B6
zmp_ph111_B7
zmp_ph111_B9
zmp_ph111_B10
zmp_ph114_G4
zmp_ph114_G11
zmp_ph114_H1
zmp_ph114_H2
zmp_ph114_A9
zmp_ph114_A11
zmp_ph114_B2
zmp_ph114_B4
zmp_ph114_D8
zmp_ph114_E2

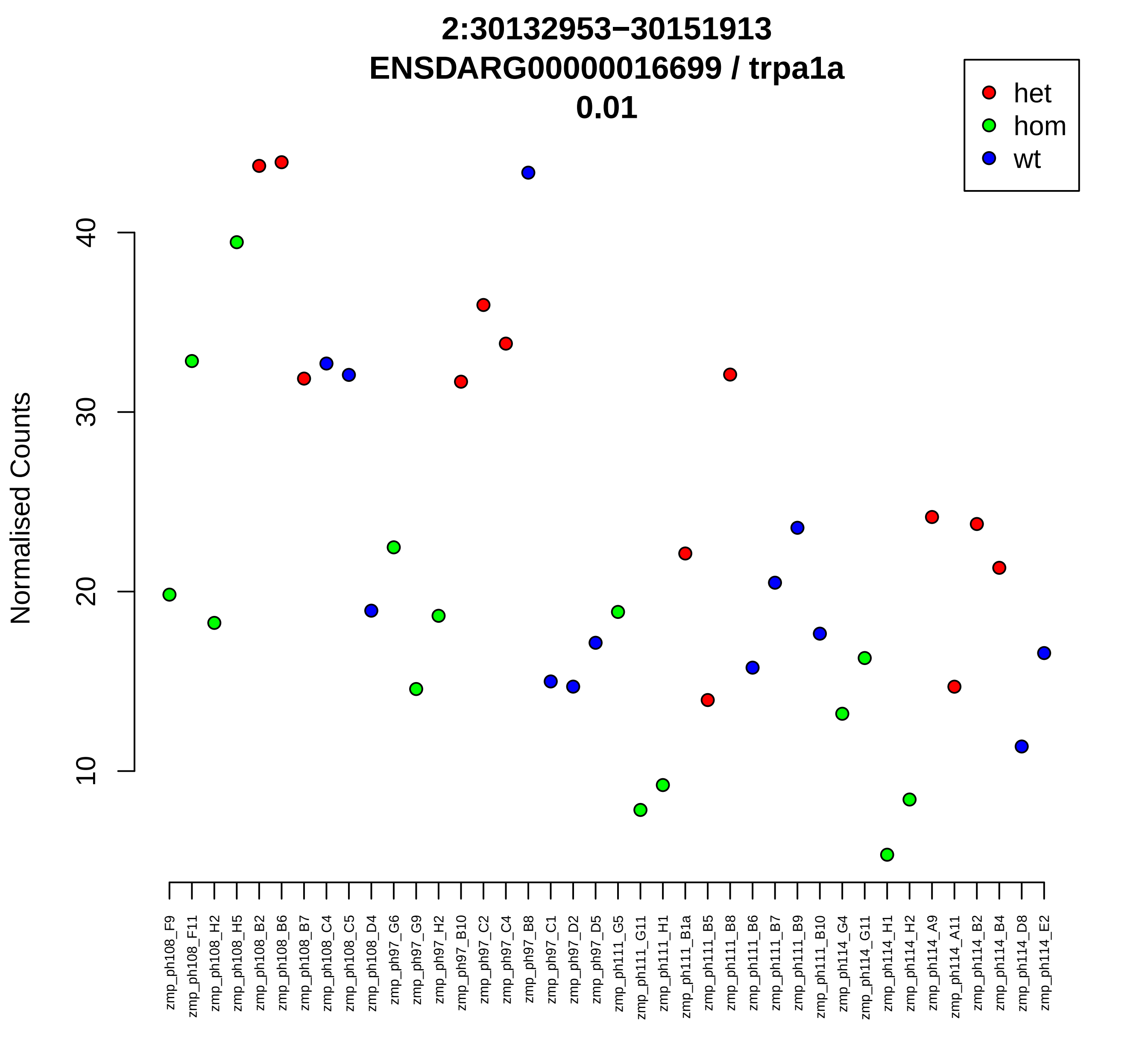

2:30132953−30151913
ENSDARG00000016699 / trpa1a
0.01
het
hom
wt
Normalised Counts
10
20
30
40
zmp_ph108_F9
zmp_ph108_F11
zmp_ph108_H2
zmp_ph108_H5
zmp_ph108_B2
zmp_ph108_B6
zmp_ph108_B7
zmp_ph108_C4
zmp_ph108_C5
zmp_ph108_D4
zmp_ph97_G6
zmp_ph97_G9
zmp_ph97_H2
zmp_ph97_B10
zmp_ph97_C2
zmp_ph97_C4
zmp_ph97_B8
zmp_ph97_C1
zmp_ph97_D2
zmp_ph97_D5
zmp_ph111_G5
zmp_ph111_G11
zmp_ph111_H1
zmp_ph111_B1a
zmp_ph111_B5
zmp_ph111_B8
zmp_ph111_B6
zmp_ph111_B7
zmp_ph111_B9
zmp_ph111_B10
zmp_ph114_G4
zmp_ph114_G11
zmp_ph114_H1
zmp_ph114_H2
zmp_ph114_A9
zmp_ph114_A11
zmp_ph114_B2
zmp_ph114_B4
zmp_ph114_D8
zmp_ph114_E2

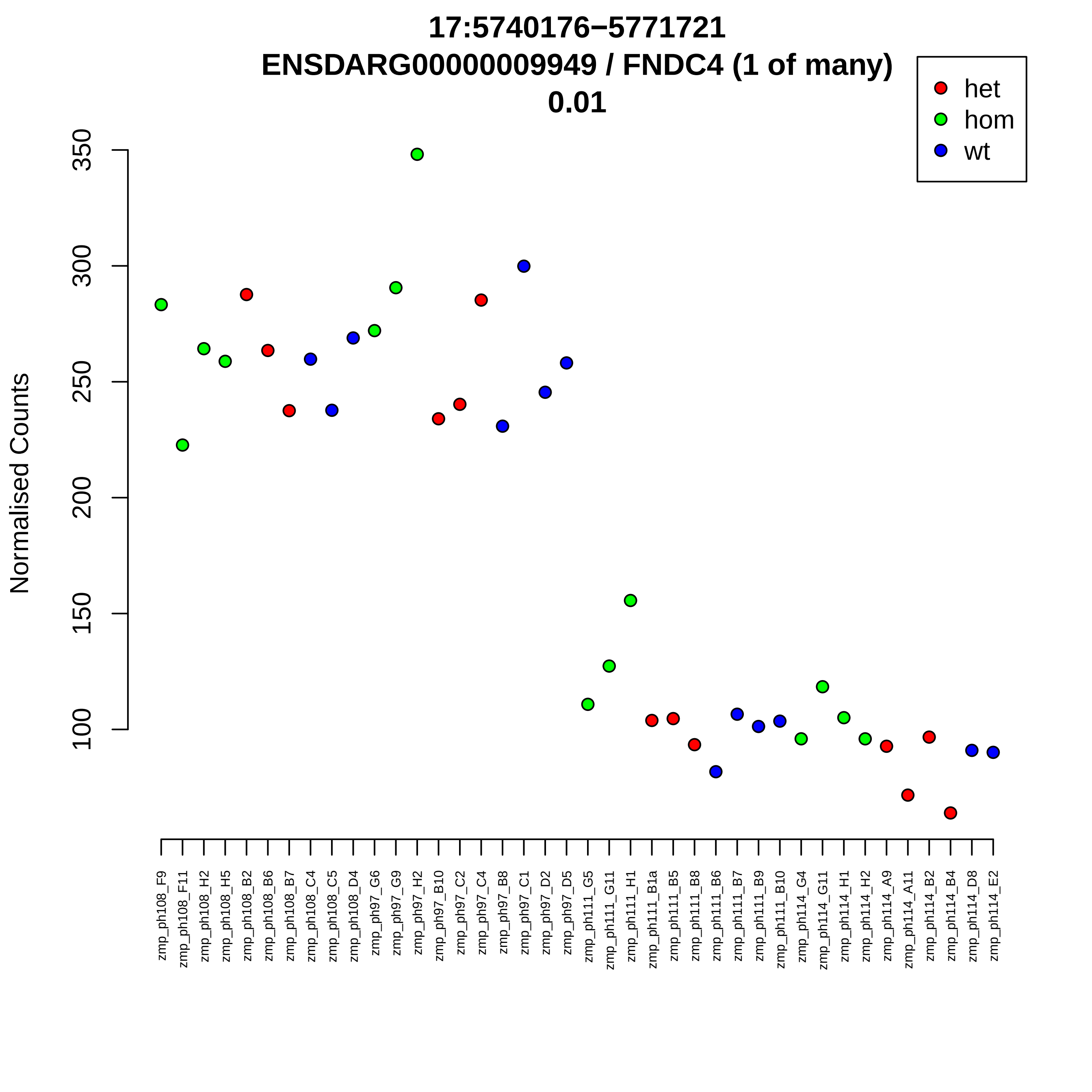

17:5740176−5771721
ENSDARG00000009949 / FNDC4 (1 of many)
0.01
het
hom
wt
Normalised Counts
100
150
200
250
300
350
zmp_ph108_F9
zmp_ph108_F11
zmp_ph108_H2
zmp_ph108_H5
zmp_ph108_B2
zmp_ph108_B6
zmp_ph108_B7
zmp_ph108_C4
zmp_ph108_C5
zmp_ph108_D4
zmp_ph97_G6
zmp_ph97_G9
zmp_ph97_H2
zmp_ph97_B10
zmp_ph97_C2
zmp_ph97_C4
zmp_ph97_B8
zmp_ph97_C1
zmp_ph97_D2
zmp_ph97_D5
zmp_ph111_G5
zmp_ph111_G11
zmp_ph111_H1
zmp_ph111_B1a
zmp_ph111_B5
zmp_ph111_B8
zmp_ph111_B6
zmp_ph111_B7
zmp_ph111_B9
zmp_ph111_B10
zmp_ph114_G4
zmp_ph114_G11
zmp_ph114_H1
zmp_ph114_H2
zmp_ph114_A9
zmp_ph114_A11
zmp_ph114_B2
zmp_ph114_B4
zmp_ph114_D8
zmp_ph114_E2

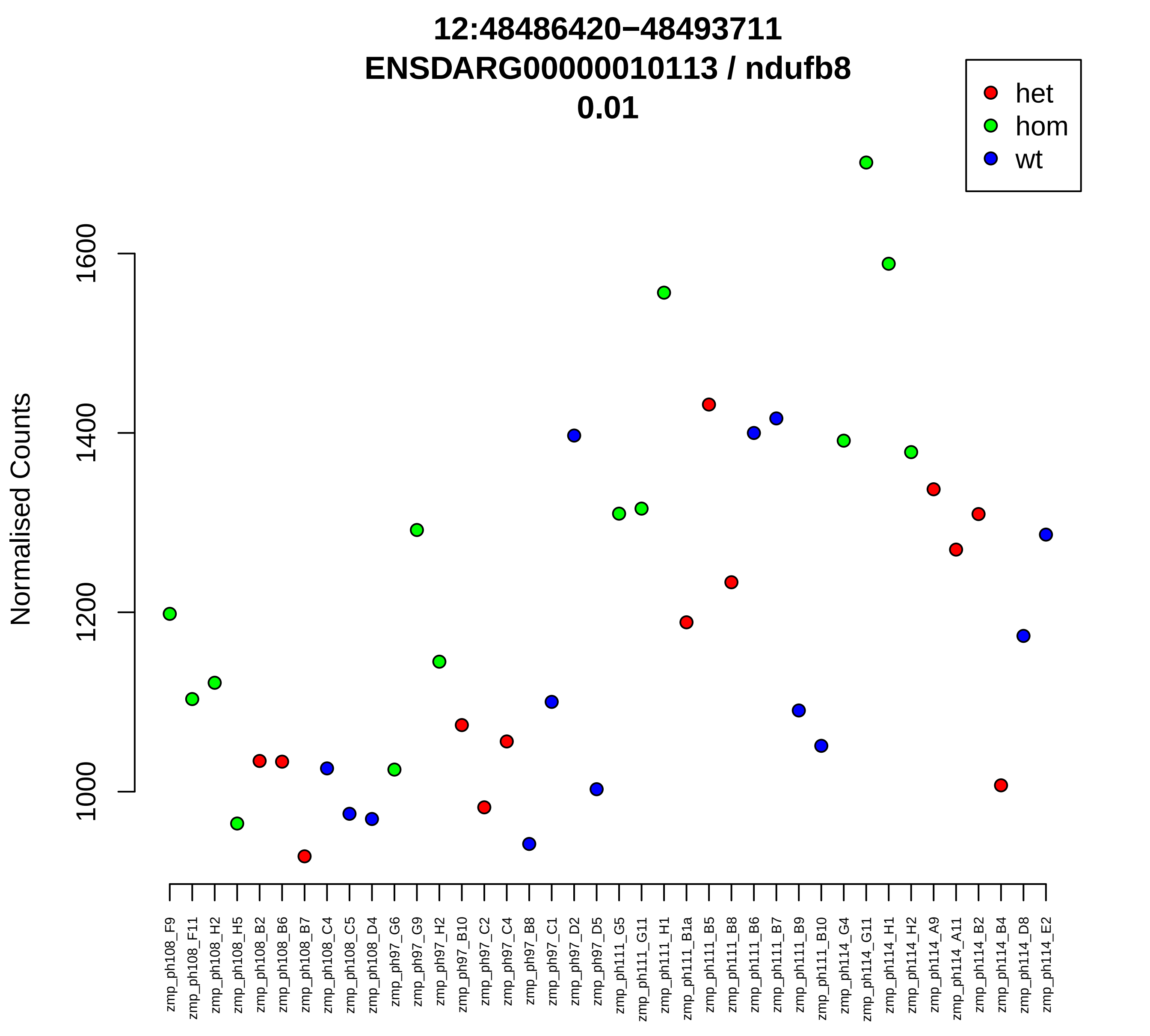

12:48486420−48493711
ENSDARG00000010113 / ndufb8
0.01
het
hom
wt
Normalised Counts
1000
1200
1400
1600
zmp_ph108_F9
zmp_ph108_F11
zmp_ph108_H2
zmp_ph108_H5
zmp_ph108_B2
zmp_ph108_B6
zmp_ph108_B7
zmp_ph108_C4
zmp_ph108_C5
zmp_ph108_D4
zmp_ph97_G6
zmp_ph97_G9
zmp_ph97_H2
zmp_ph97_B10
zmp_ph97_C2
zmp_ph97_C4
zmp_ph97_B8
zmp_ph97_C1
zmp_ph97_D2
zmp_ph97_D5
zmp_ph111_G5
zmp_ph111_G11
zmp_ph111_H1
zmp_ph111_B1a
zmp_ph111_B5
zmp_ph111_B8
zmp_ph111_B6
zmp_ph111_B7
zmp_ph111_B9
zmp_ph111_B10
zmp_ph114_G4
zmp_ph114_G11
zmp_ph114_H1
zmp_ph114_H2
zmp_ph114_A9
zmp_ph114_A11
zmp_ph114_B2
zmp_ph114_B4
zmp_ph114_D8
zmp_ph114_E2

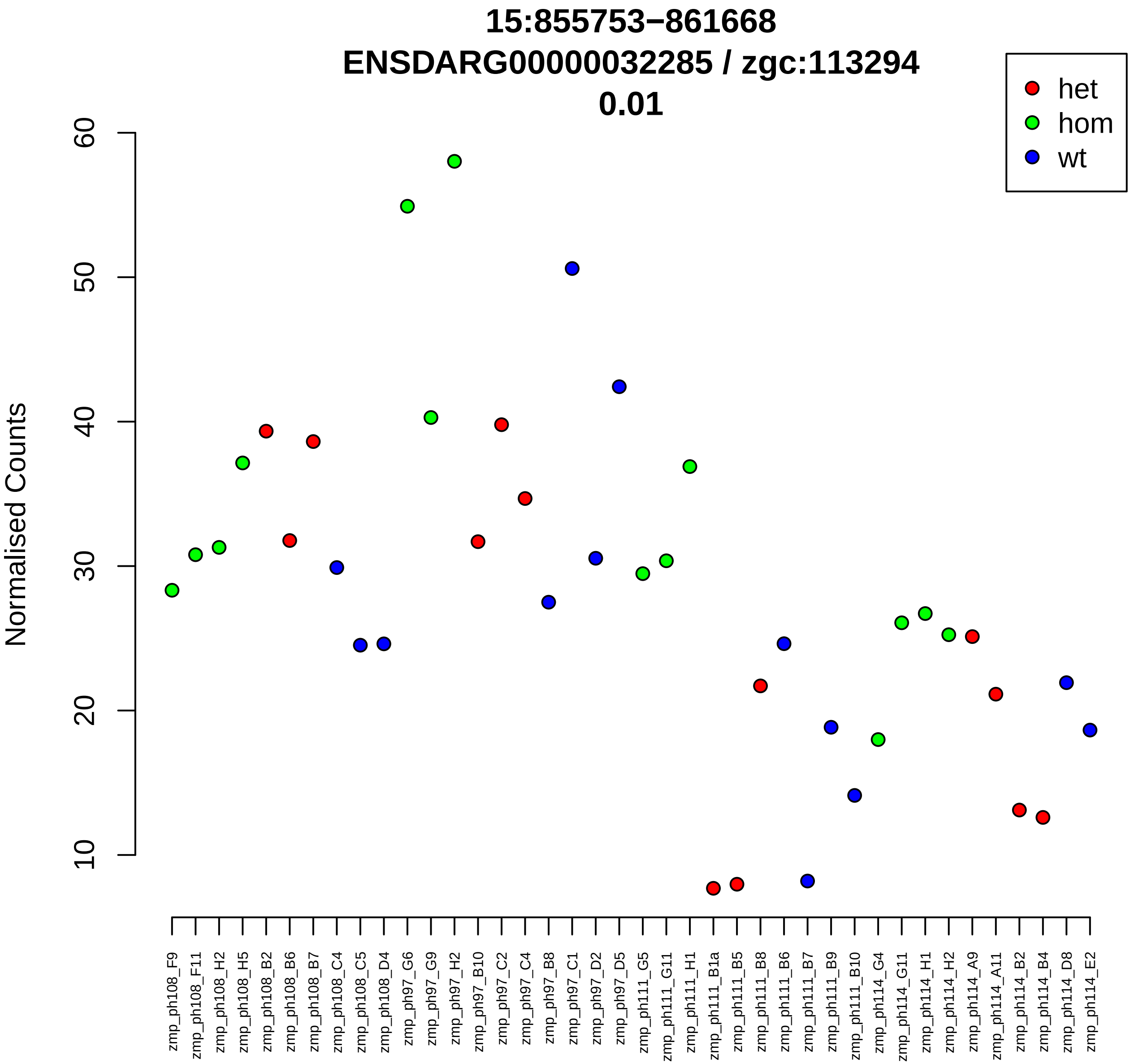
15:855753−861668
ENSDARG00000032285 / zgc:113294
0.01
het
hom
wt
Normalised Counts
10
20
30
40
50
60
zmp_ph108_F9
zmp_ph108_F11
zmp_ph108_H2
zmp_ph108_H5
zmp_ph108_B2
zmp_ph108_B6
zmp_ph108_B7
zmp_ph108_C4
zmp_ph108_C5
zmp_ph108_D4
zmp_ph97_G6
zmp_ph97_G9
zmp_ph97_H2
zmp_ph97_B10
zmp_ph97_C2
zmp_ph97_C4
zmp_ph97_B8
zmp_ph97_C1
zmp_ph97_D2
zmp_ph97_D5
zmp_ph111_G5
zmp_ph111_G11
zmp_ph111_H1
zmp_ph111_B1a
zmp_ph111_B5
zmp_ph111_B8
zmp_ph111_B6
zmp_ph111_B7
zmp_ph111_B9
zmp_ph111_B10
zmp_ph114_G4
zmp_ph114_G11
zmp_ph114_H1
zmp_ph114_H2
zmp_ph114_A9
zmp_ph114_A11
zmp_ph114_B2
zmp_ph114_B4
zmp_ph114_D8
zmp_ph114_E2

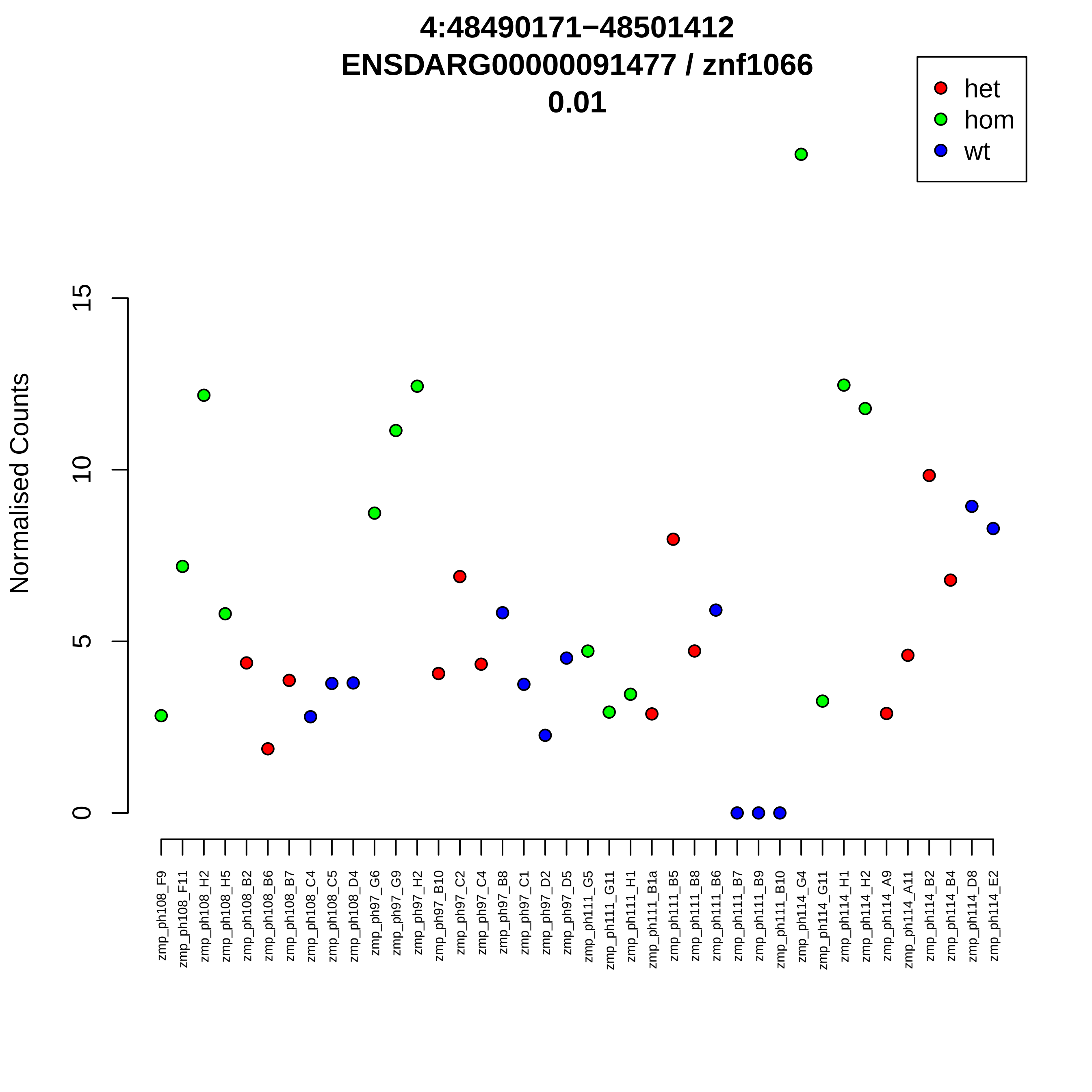
4:48490171−48501412
ENSDARG00000091477 / znf1066
0.01
het
hom
wt
Normalised Counts
0
5
10
15
zmp_ph108_F9
zmp_ph108_F11
zmp_ph108_H2
zmp_ph108_H5
zmp_ph108_B2
zmp_ph108_B6
zmp_ph108_B7
zmp_ph108_C4
zmp_ph108_C5
zmp_ph108_D4
zmp_ph97_G6
zmp_ph97_G9
zmp_ph97_H2
zmp_ph97_B10
zmp_ph97_C2
zmp_ph97_C4
zmp_ph97_B8
zmp_ph97_C1
zmp_ph97_D2
zmp_ph97_D5
zmp_ph111_G5
zmp_ph111_G11
zmp_ph111_H1
zmp_ph111_B1a
zmp_ph111_B5
zmp_ph111_B8
zmp_ph111_B6
zmp_ph111_B7
zmp_ph111_B9
zmp_ph111_B10
zmp_ph114_G4
zmp_ph114_G11
zmp_ph114_H1
zmp_ph114_H2
zmp_ph114_A9
zmp_ph114_A11
zmp_ph114_B2
zmp_ph114_B4
zmp_ph114_D8
zmp_ph114_E2

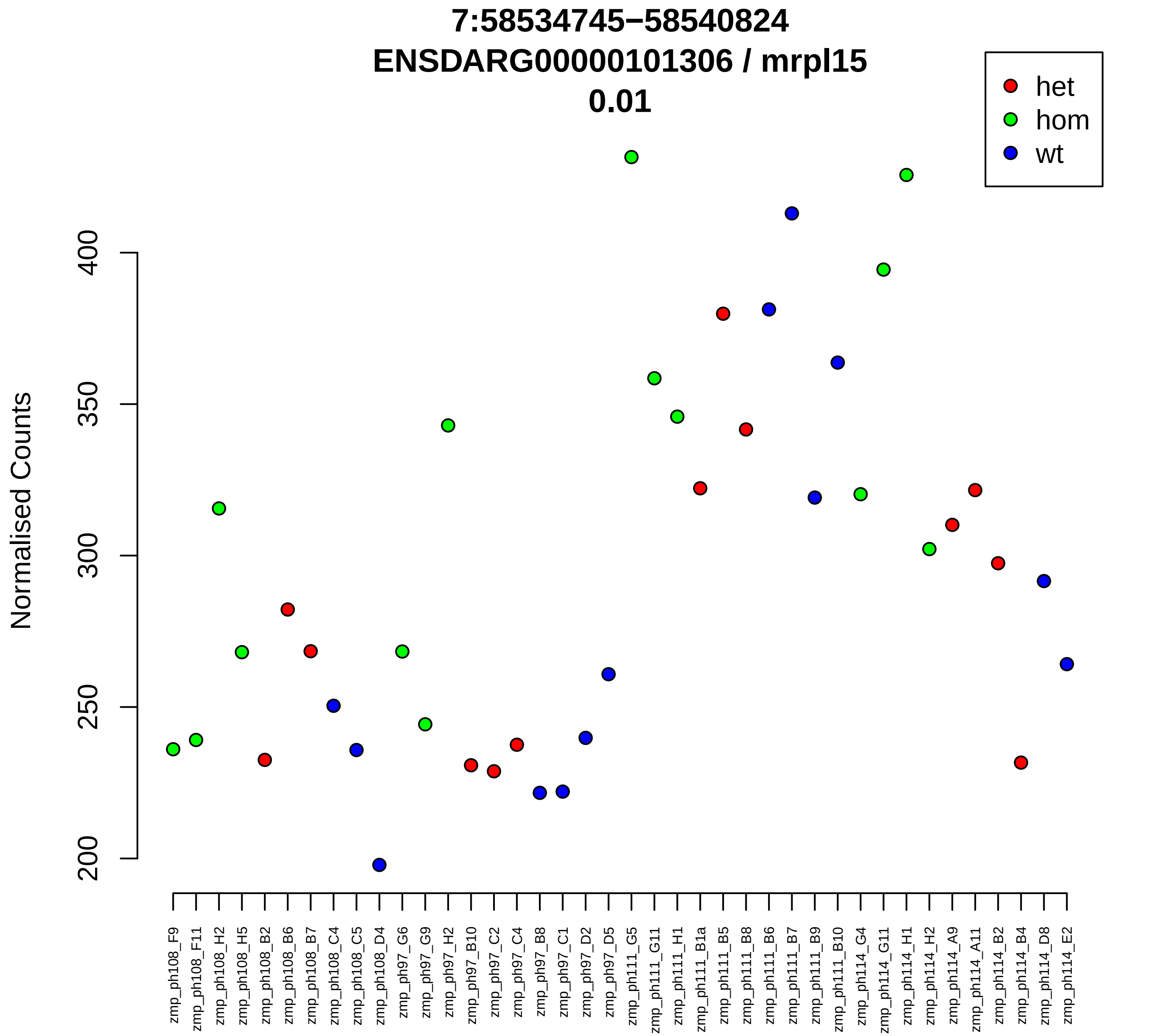

7:58534745−58540824
ENSDARG00000101306 / mrpl15
0.01
het
hom
wt
Normalised Counts
200
250
300
350
400
zmp_ph108_F9
zmp_ph108_F11
zmp_ph108_H2
zmp_ph108_H5
zmp_ph108_B2
zmp_ph108_B6
zmp_ph108_B7
zmp_ph108_C4
zmp_ph108_C5
zmp_ph108_D4
zmp_ph97_G6
zmp_ph97_G9
zmp_ph97_H2
zmp_ph97_B10
zmp_ph97_C2
zmp_ph97_C4
zmp_ph97_B8
zmp_ph97_C1
zmp_ph97_D2
zmp_ph97_D5
zmp_ph111_G5
zmp_ph111_G11
zmp_ph111_H1
zmp_ph111_B1a
zmp_ph111_B5
zmp_ph111_B8
zmp_ph111_B6
zmp_ph111_B7
zmp_ph111_B9
zmp_ph111_B10
zmp_ph114_G4
zmp_ph114_G11
zmp_ph114_H1
zmp_ph114_H2
zmp_ph114_A9
zmp_ph114_A11
zmp_ph114_B2
zmp_ph114_B4
zmp_ph114_D8
zmp_ph114_E2

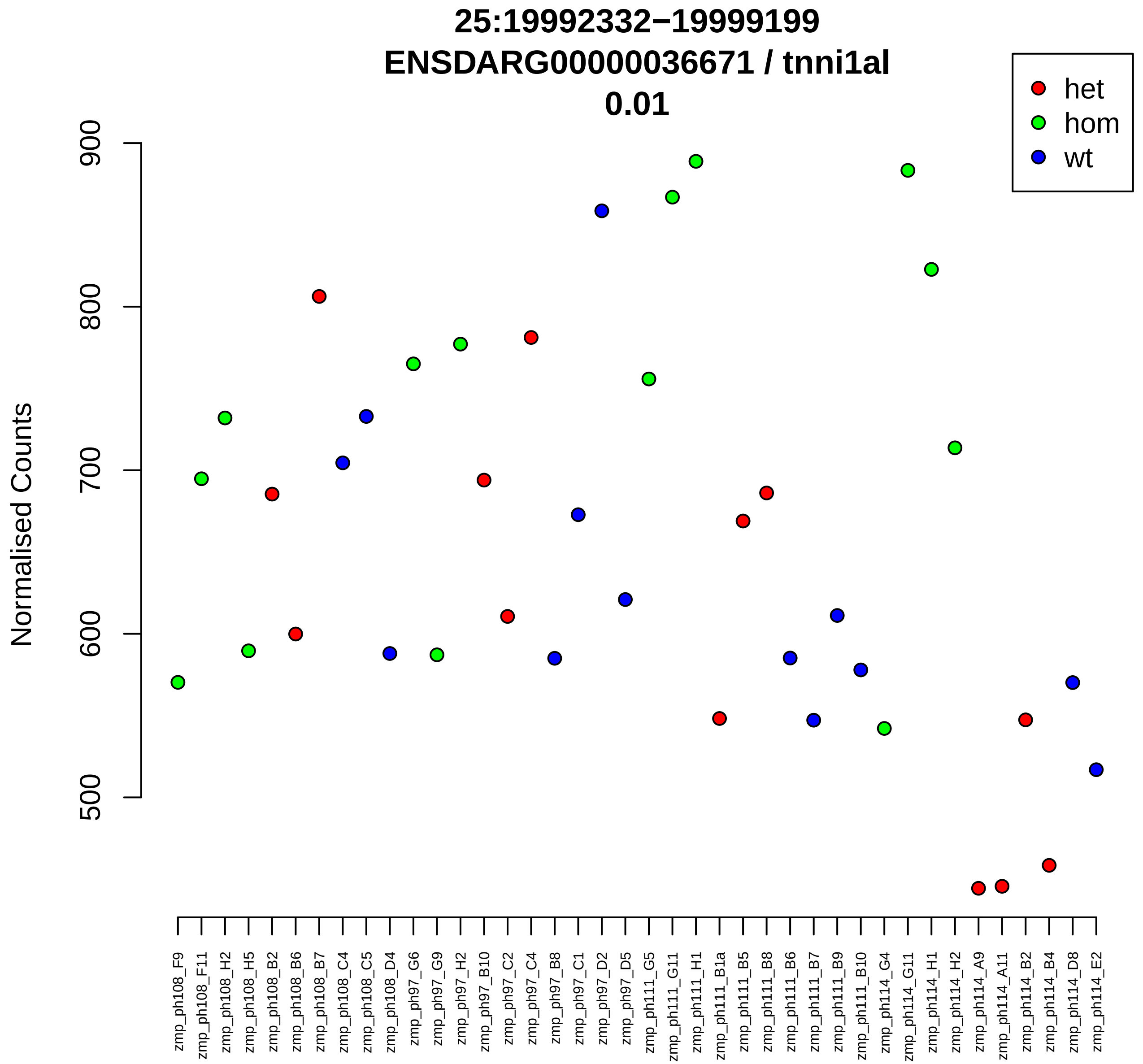
25:19992332−19999199
ENSDARG00000036671 / tnni1al
0.01
het
hom
wt
Normalised Counts
900
800
700
600
500
zmp_ph108_F9
zmp_ph108_F11
zmp_ph108_H2
zmp_ph108_H5
zmp_ph108_B2
zmp_ph108_B6
zmp_ph108_B7
zmp_ph108_C4
zmp_ph108_C5
zmp_ph108_D4
zmp_ph97_G6
zmp_ph97_G9
zmp_ph97_H2
zmp_ph97_B10
zmp_ph97_C2
zmp_ph97_C4
zmp_ph97_B8
zmp_ph97_C1
zmp_ph97_D2
zmp_ph97_D5
zmp_ph111_G5
zmp_ph111_G11
zmp_ph111_H1
zmp_ph111_B1a
zmp_ph111_B5
zmp_ph111_B8
zmp_ph111_B6
zmp_ph111_B7
zmp_ph111_B9
zmp_ph111_B10
zmp_ph114_G4
zmp_ph114_G11
zmp_ph114_H1
zmp_ph114_H2
zmp_ph114_A9
zmp_ph114_A11
zmp_ph114_B2
zmp_ph114_B4
zmp_ph114_D8
zmp_ph114_E2

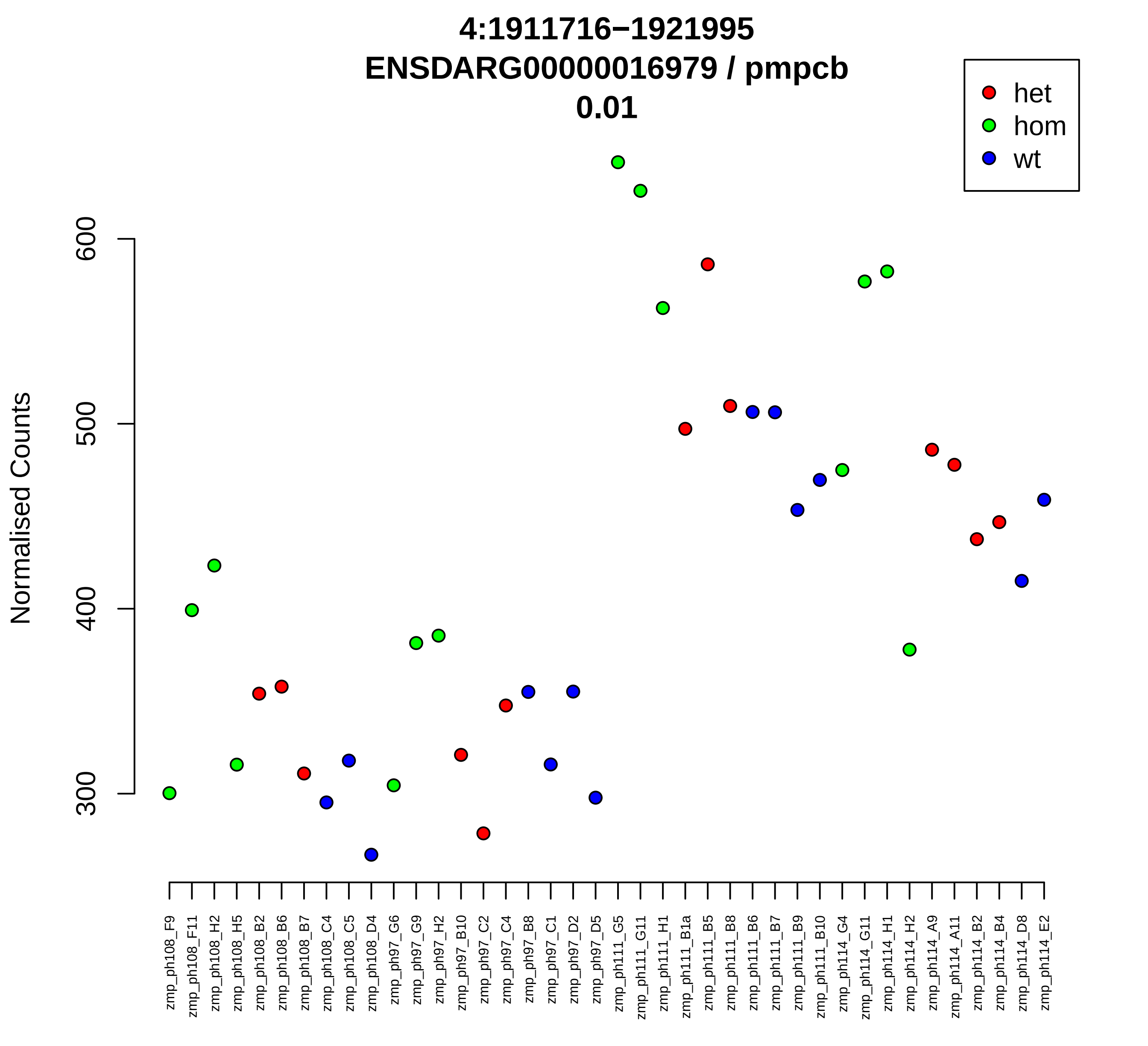

4:1911716−1921995
ENSDARG00000016979 / pmpcb
0.01
het
hom
wt
Normalised Counts
300
400
500
600
zmp_ph108_F9
zmp_ph108_F11
zmp_ph108_H2
zmp_ph108_H5
zmp_ph108_B2
zmp_ph108_B6
zmp_ph108_B7
zmp_ph108_C4
zmp_ph108_C5
zmp_ph108_D4
zmp_ph97_G6
zmp_ph97_G9
zmp_ph97_H2
zmp_ph97_B10
zmp_ph97_C2
zmp_ph97_C4
zmp_ph97_B8
zmp_ph97_C1
zmp_ph97_D2
zmp_ph97_D5
zmp_ph111_G5
zmp_ph111_G11
zmp_ph111_H1
zmp_ph111_B1a
zmp_ph111_B5
zmp_ph111_B8
zmp_ph111_B6
zmp_ph111_B7
zmp_ph111_B9
zmp_ph111_B10
zmp_ph114_G4
zmp_ph114_G11
zmp_ph114_H1
zmp_ph114_H2
zmp_ph114_A9
zmp_ph114_A11
zmp_ph114_B2
zmp_ph114_B4
zmp_ph114_D8
zmp_ph114_E2

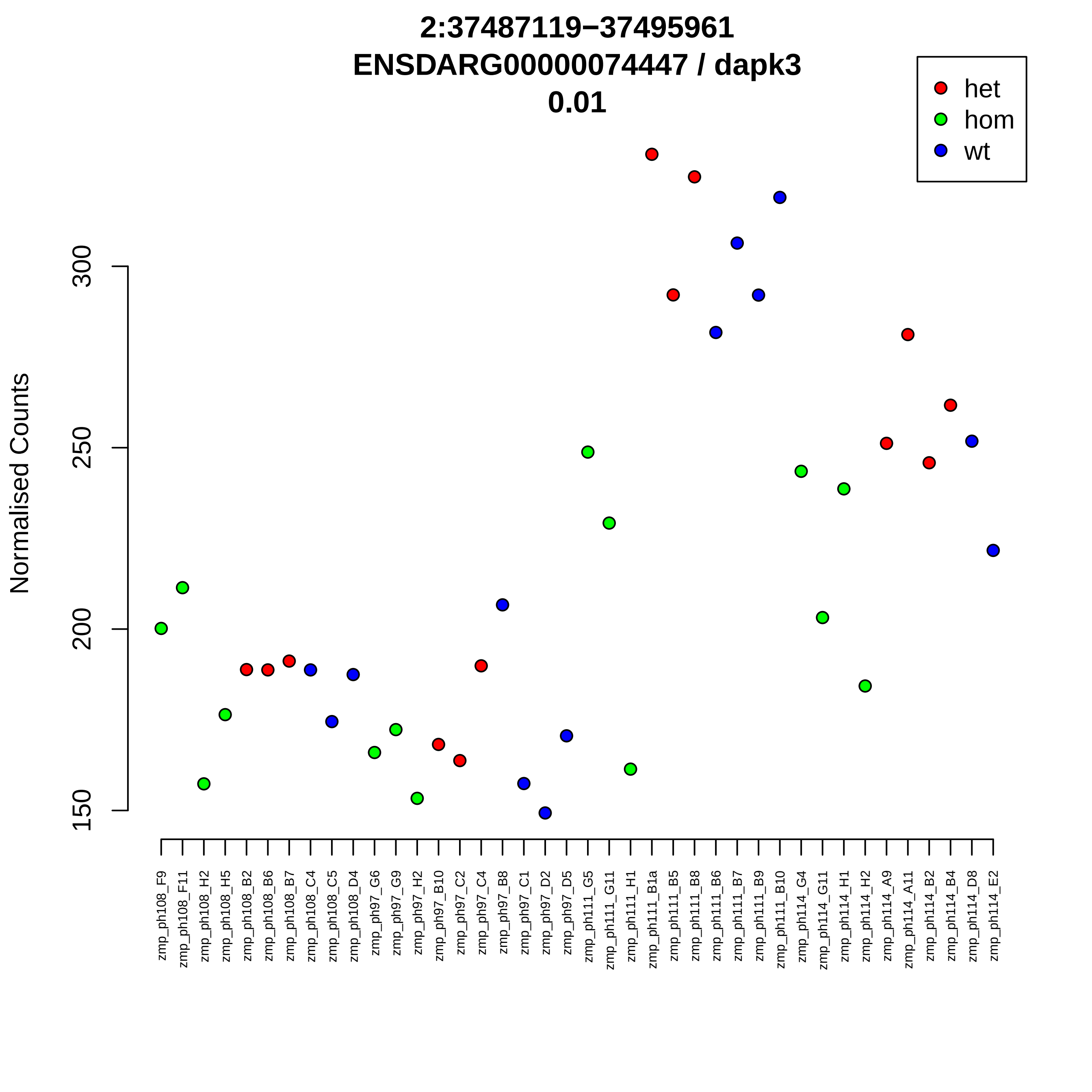

2:37487119−37495961
ENSDARG00000074447 / dapk3
0.01
het
hom
wt
Normalised Counts
150
200
250
300
zmp_ph108_F9
zmp_ph108_F11
zmp_ph108_H2
zmp_ph108_H5
zmp_ph108_B2
zmp_ph108_B6
zmp_ph108_B7
zmp_ph108_C4
zmp_ph108_C5
zmp_ph108_D4
zmp_ph97_G6
zmp_ph97_G9
zmp_ph97_H2
zmp_ph97_B10
zmp_ph97_C2
zmp_ph97_C4
zmp_ph97_B8
zmp_ph97_C1
zmp_ph97_D2
zmp_ph97_D5
zmp_ph111_G5
zmp_ph111_G11
zmp_ph111_H1
zmp_ph111_B1a
zmp_ph111_B5
zmp_ph111_B8
zmp_ph111_B6
zmp_ph111_B7
zmp_ph111_B9
zmp_ph111_B10
zmp_ph114_G4
zmp_ph114_G11
zmp_ph114_H1
zmp_ph114_H2
zmp_ph114_A9
zmp_ph114_A11
zmp_ph114_B2
zmp_ph114_B4
zmp_ph114_D8
zmp_ph114_E2

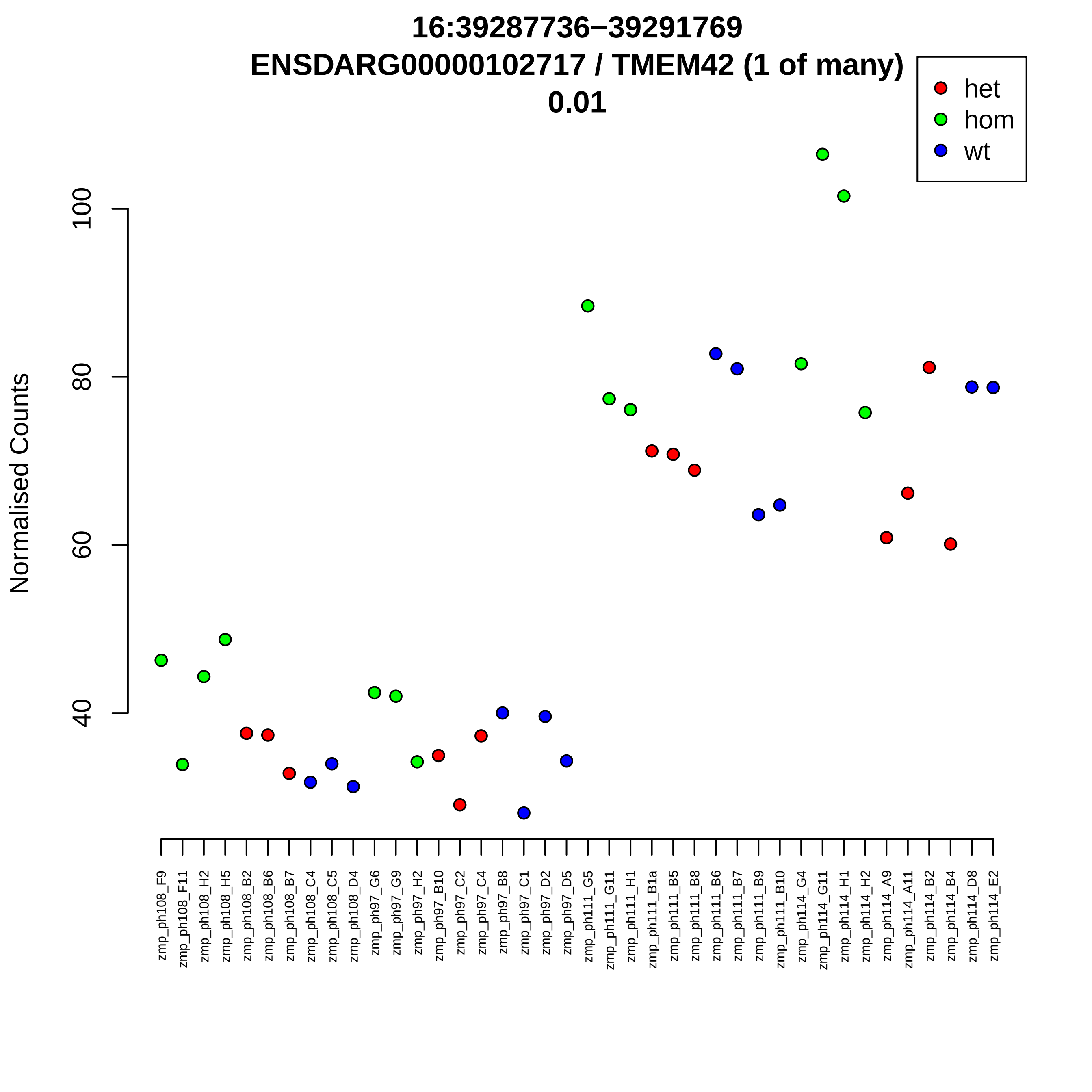
16:39287736−39291769
ENSDARG00000102717 / TMEM42 (1 of many)
0.01
het
hom
wt
Normalised Counts
40
60
80
100
zmp_ph108_F9
zmp_ph108_F11
zmp_ph108_H2
zmp_ph108_H5
zmp_ph108_B2
zmp_ph108_B6
zmp_ph108_B7
zmp_ph108_C4
zmp_ph108_C5
zmp_ph108_D4
zmp_ph97_G6
zmp_ph97_G9
zmp_ph97_H2
zmp_ph97_B10
zmp_ph97_C2
zmp_ph97_C4
zmp_ph97_B8
zmp_ph97_C1
zmp_ph97_D2
zmp_ph97_D5
zmp_ph111_G5
zmp_ph111_G11
zmp_ph111_H1
zmp_ph111_B1a
zmp_ph111_B5
zmp_ph111_B8
zmp_ph111_B6
zmp_ph111_B7
zmp_ph111_B9
zmp_ph111_B10
zmp_ph114_G4
zmp_ph114_G11
zmp_ph114_H1
zmp_ph114_H2
zmp_ph114_A9
zmp_ph114_A11
zmp_ph114_B2
zmp_ph114_B4
zmp_ph114_D8
zmp_ph114_E2

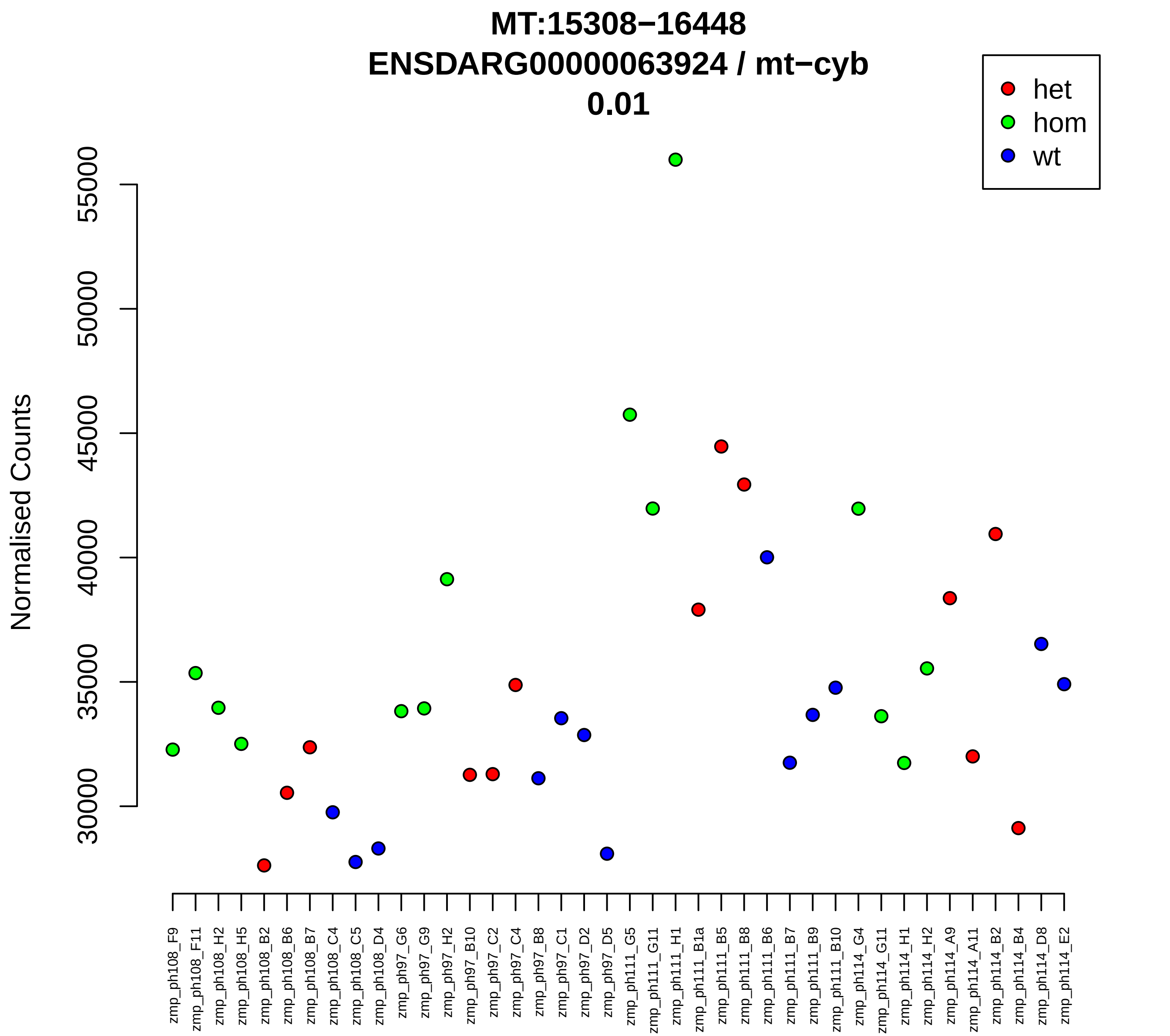

MT:15308−16448
ENSDARG00000063924 / mt−cyb
0.01
het
hom
wt
Normalised Counts
30000
35000
40000
45000
50000
55000
zmp_ph108_F9
zmp_ph108_F11
zmp_ph108_H2
zmp_ph108_H5
zmp_ph108_B2
zmp_ph108_B6
zmp_ph108_B7
zmp_ph108_C4
zmp_ph108_C5
zmp_ph108_D4
zmp_ph97_G6
zmp_ph97_G9
zmp_ph97_H2
zmp_ph97_B10
zmp_ph97_C2
zmp_ph97_C4
zmp_ph97_B8
zmp_ph97_C1
zmp_ph97_D2
zmp_ph97_D5
zmp_ph111_G5
zmp_ph111_G11
zmp_ph111_H1
zmp_ph111_B1a
zmp_ph111_B5
zmp_ph111_B8
zmp_ph111_B6
zmp_ph111_B7
zmp_ph111_B9
zmp_ph111_B10
zmp_ph114_G4
zmp_ph114_G11
zmp_ph114_H1
zmp_ph114_H2
zmp_ph114_A9
zmp_ph114_A11
zmp_ph114_B2
zmp_ph114_B4
zmp_ph114_D8
zmp_ph114_E2

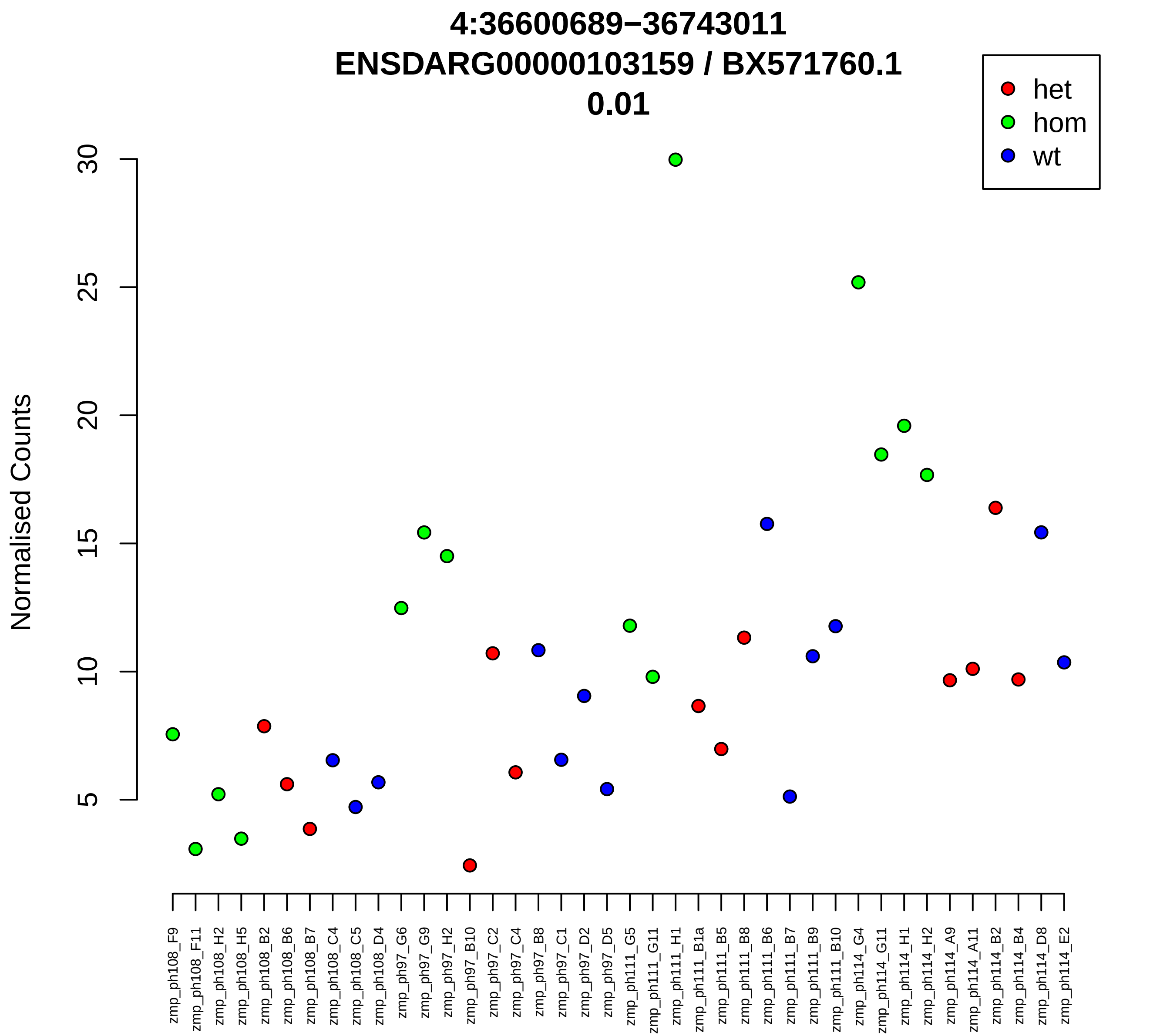
4:36600689−36743011
ENSDARG00000103159 / BX571760.1
0.01
het
hom
wt
Normalised Counts
5
10
15
20
25
30
zmp_ph108_F9
zmp_ph108_F11
zmp_ph108_H2
zmp_ph108_H5
zmp_ph108_B2
zmp_ph108_B6
zmp_ph108_B7
zmp_ph108_C4
zmp_ph108_C5
zmp_ph108_D4
zmp_ph97_G6
zmp_ph97_G9
zmp_ph97_H2
zmp_ph97_B10
zmp_ph97_C2
zmp_ph97_C4
zmp_ph97_B8
zmp_ph97_C1
zmp_ph97_D2
zmp_ph97_D5
zmp_ph111_G5
zmp_ph111_G11
zmp_ph111_H1
zmp_ph111_B1a
zmp_ph111_B5
zmp_ph111_B8
zmp_ph111_B6
zmp_ph111_B7
zmp_ph111_B9
zmp_ph111_B10
zmp_ph114_G4
zmp_ph114_G11
zmp_ph114_H1
zmp_ph114_H2
zmp_ph114_A9
zmp_ph114_A11
zmp_ph114_B2
zmp_ph114_B4
zmp_ph114_D8
zmp_ph114_E2

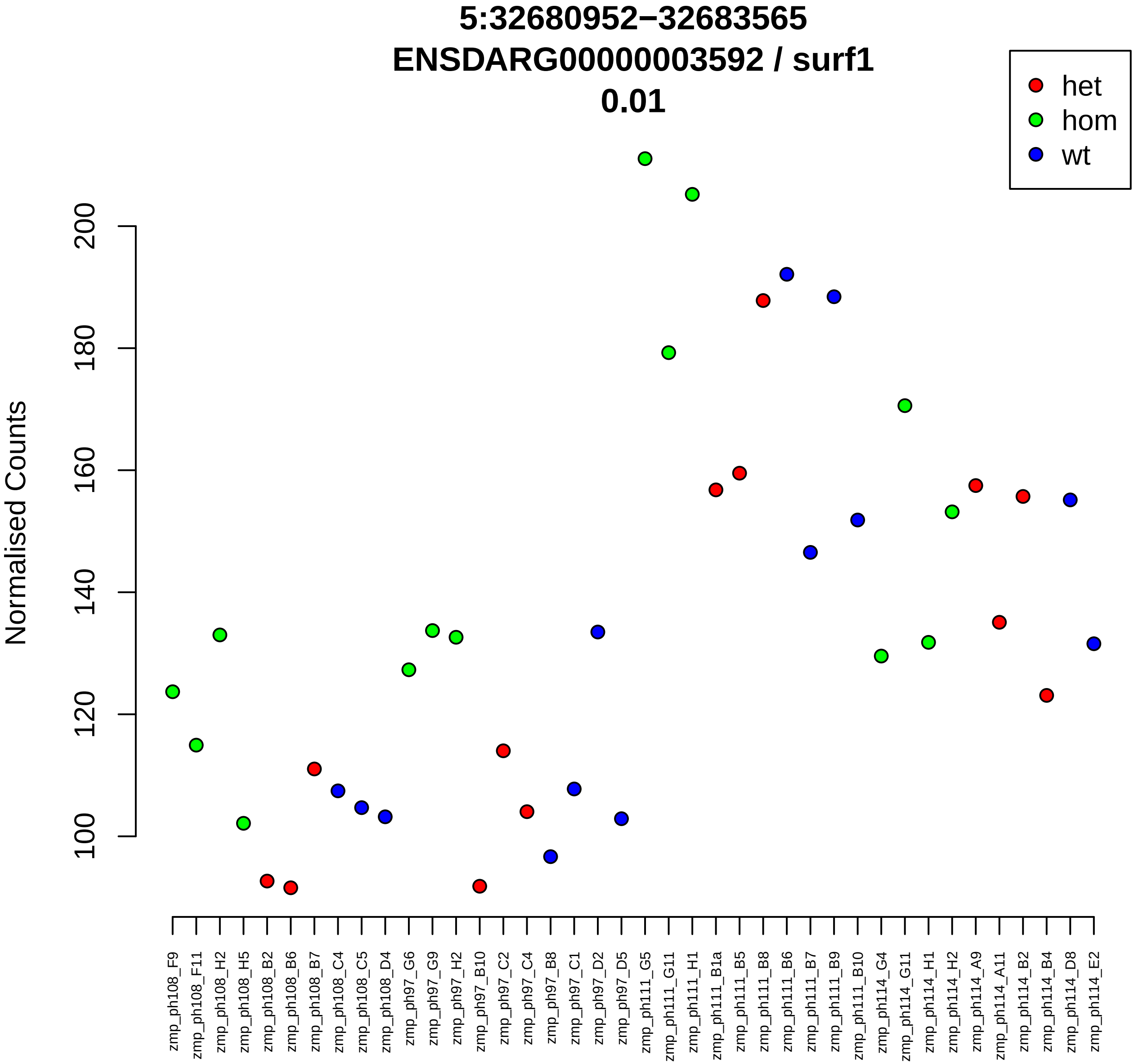
5:32680952−32683565
ENSDARG00000003592 / surf1
0.01
het
hom
wt
Normalised Counts
100
120
140
160
180
200
zmp_ph108_F9
zmp_ph108_F11
zmp_ph108_H2
zmp_ph108_H5
zmp_ph108_B2
zmp_ph108_B6
zmp_ph108_B7
zmp_ph108_C4
zmp_ph108_C5
zmp_ph108_D4
zmp_ph97_G6
zmp_ph97_G9
zmp_ph97_H2
zmp_ph97_B10
zmp_ph97_C2
zmp_ph97_C4
zmp_ph97_B8
zmp_ph97_C1
zmp_ph97_D2
zmp_ph97_D5
zmp_ph111_G5
zmp_ph111_G11
zmp_ph111_H1
zmp_ph111_B1a
zmp_ph111_B5
zmp_ph111_B8
zmp_ph111_B6
zmp_ph111_B7
zmp_ph111_B9
zmp_ph111_B10
zmp_ph114_G4
zmp_ph114_G11
zmp_ph114_H1
zmp_ph114_H2
zmp_ph114_A9
zmp_ph114_A11
zmp_ph114_B2
zmp_ph114_B4
zmp_ph114_D8
zmp_ph114_E2

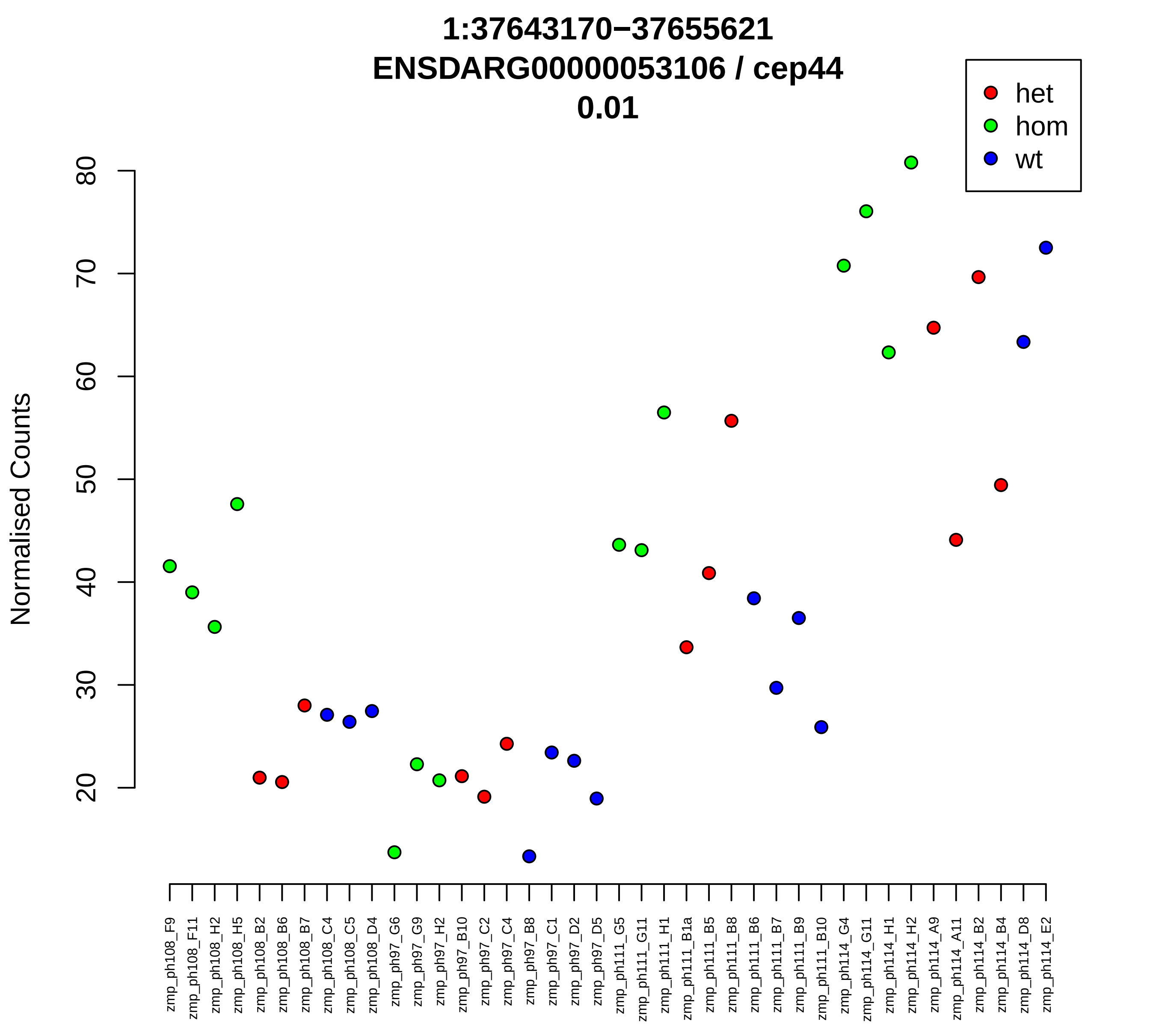

1:37643170−37655621
ENSDARG00000053106 / cep44
0.01
het
hom
wt
Normalised Counts
20
30
40
50
60
70
80
zmp_ph108_F9
zmp_ph108_F11
zmp_ph108_H2
zmp_ph108_H5
zmp_ph108_B2
zmp_ph108_B6
zmp_ph108_B7
zmp_ph108_C4
zmp_ph108_C5
zmp_ph108_D4
zmp_ph97_G6
zmp_ph97_G9
zmp_ph97_H2
zmp_ph97_B10
zmp_ph97_C2
zmp_ph97_C4
zmp_ph97_B8
zmp_ph97_C1
zmp_ph97_D2
zmp_ph97_D5
zmp_ph111_G5
zmp_ph111_G11
zmp_ph111_H1
zmp_ph111_B1a
zmp_ph111_B5
zmp_ph111_B8
zmp_ph111_B6
zmp_ph111_B7
zmp_ph111_B9
zmp_ph111_B10
zmp_ph114_G4
zmp_ph114_G11
zmp_ph114_H1
zmp_ph114_H2
zmp_ph114_A9
zmp_ph114_A11
zmp_ph114_B2
zmp_ph114_B4
zmp_ph114_D8
zmp_ph114_E2

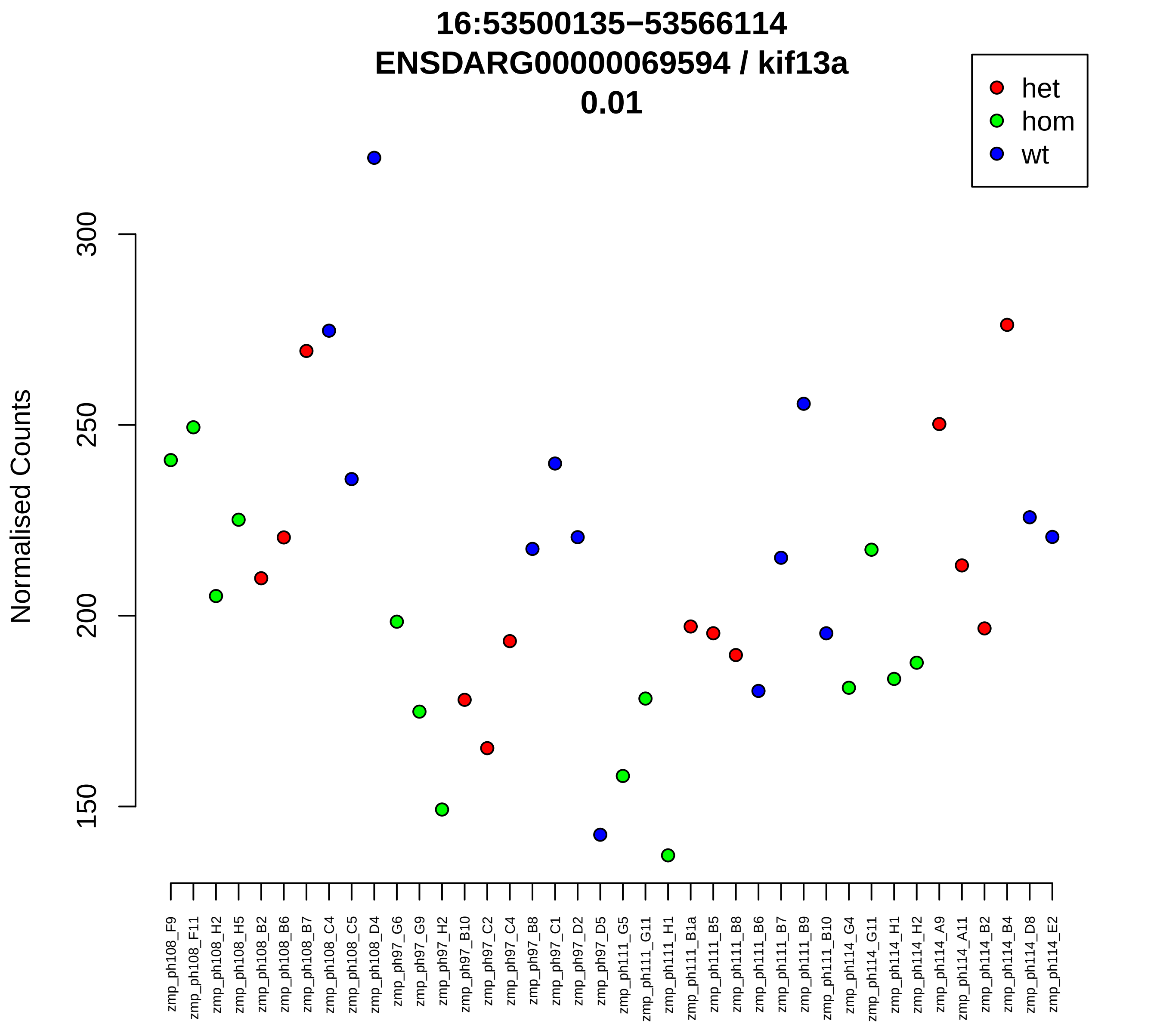
16:53500135−53566114
ENSDARG00000069594 / kif13a
0.01
het
hom
wt
Normalised Counts
300
250
200
150
zmp_ph108_F9
zmp_ph108_F11
zmp_ph108_H2
zmp_ph108_H5
zmp_ph108_B2
zmp_ph108_B6
zmp_ph108_B7
zmp_ph108_C4
zmp_ph108_C5
zmp_ph108_D4
zmp_ph97_G6
zmp_ph97_G9
zmp_ph97_H2
zmp_ph97_B10
zmp_ph97_C2
zmp_ph97_C4
zmp_ph97_B8
zmp_ph97_C1
zmp_ph97_D2
zmp_ph97_D5
zmp_ph111_G5
zmp_ph111_G11
zmp_ph111_H1
zmp_ph111_B1a
zmp_ph111_B5
zmp_ph111_B8
zmp_ph111_B6
zmp_ph111_B7
zmp_ph111_B9
zmp_ph111_B10
zmp_ph114_G4
zmp_ph114_G11
zmp_ph114_H1
zmp_ph114_H2
zmp_ph114_A9
zmp_ph114_A11
zmp_ph114_B2
zmp_ph114_B4
zmp_ph114_D8
zmp_ph114_E2

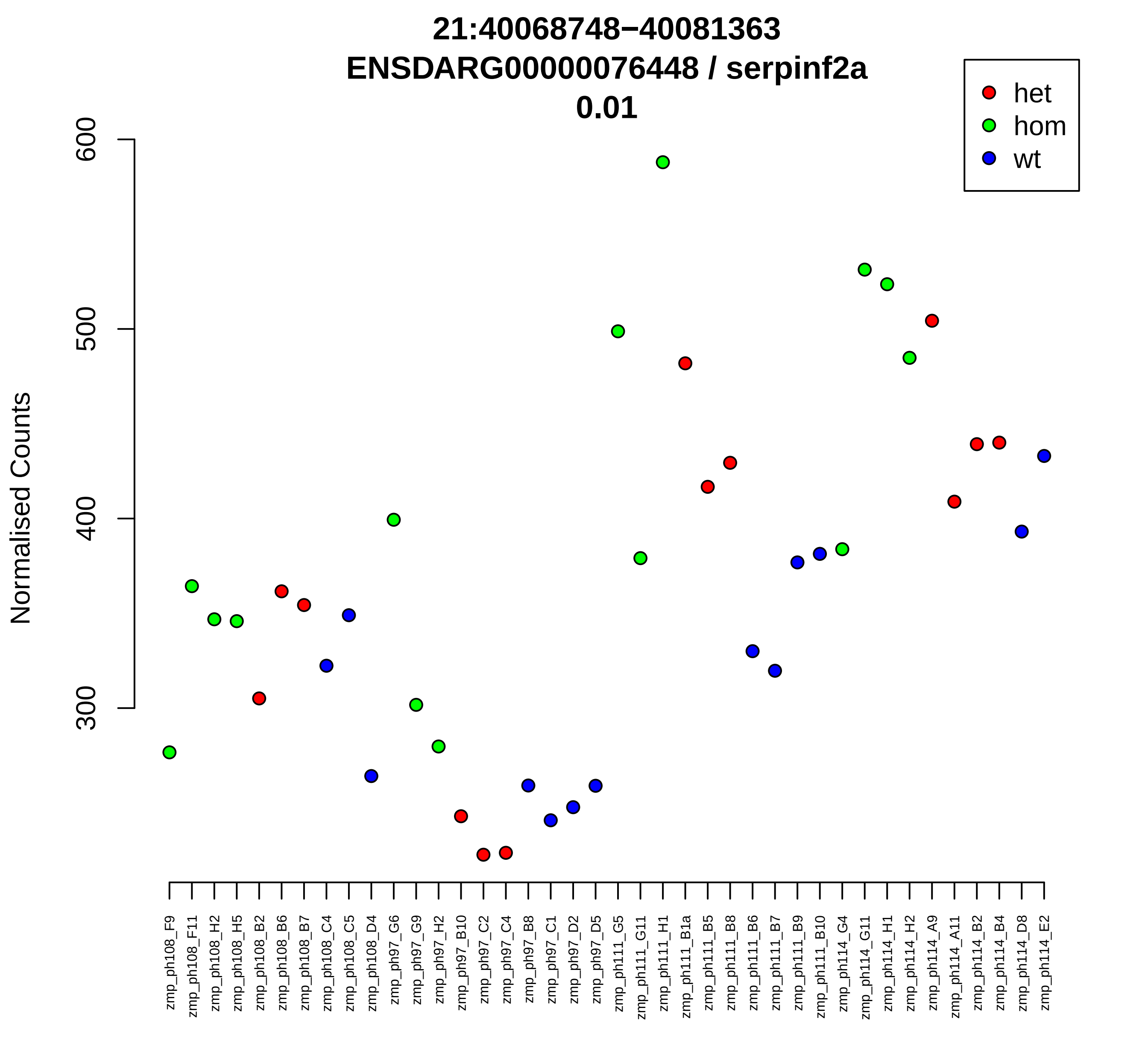

21:40068748−40081363
ENSDARG00000076448 / serpinf2a
0.01
het
hom
wt
Normalised Counts
600
500
400
300
zmp_ph108_F9
zmp_ph108_F11
zmp_ph108_H2
zmp_ph108_H5
zmp_ph108_B2
zmp_ph108_B6
zmp_ph108_B7
zmp_ph108_C4
zmp_ph108_C5
zmp_ph108_D4
zmp_ph97_G6
zmp_ph97_G9
zmp_ph97_H2
zmp_ph97_B10
zmp_ph97_C2
zmp_ph97_C4
zmp_ph97_B8
zmp_ph97_C1
zmp_ph97_D2
zmp_ph97_D5
zmp_ph111_G5
zmp_ph111_G11
zmp_ph111_H1
zmp_ph111_B1a
zmp_ph111_B5
zmp_ph111_B8
zmp_ph111_B6
zmp_ph111_B7
zmp_ph111_B9
zmp_ph111_B10
zmp_ph114_G4
zmp_ph114_G11
zmp_ph114_H1
zmp_ph114_H2
zmp_ph114_A9
zmp_ph114_A11
zmp_ph114_B2
zmp_ph114_B4
zmp_ph114_D8
zmp_ph114_E2

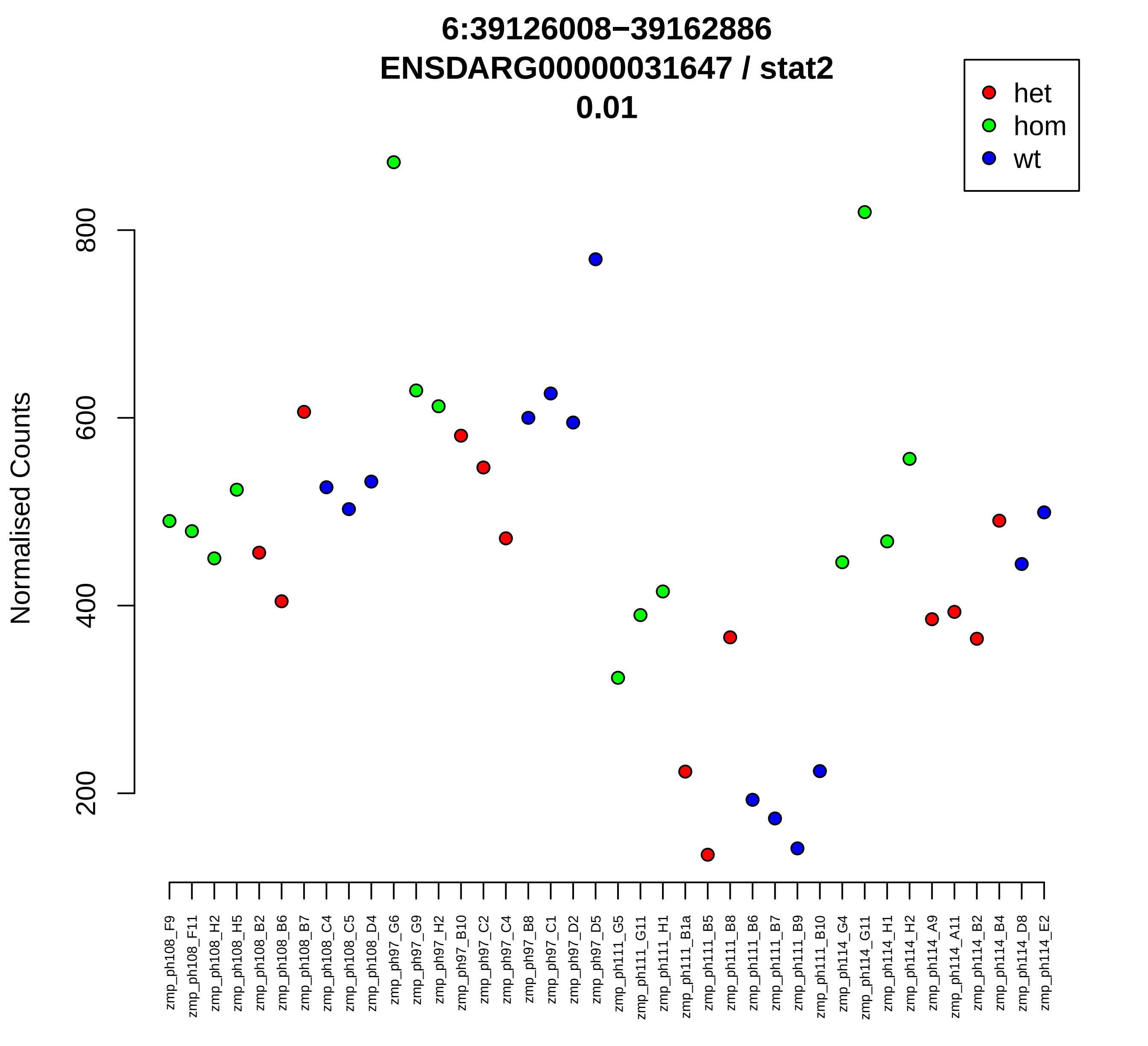

6:39126008−39162886
ENSDARG00000031647 / stat2
0.01
het
hom
wt
Normalised Counts
200
400
600
800
zmp_ph108_F9
zmp_ph108_F11
zmp_ph108_H2
zmp_ph108_H5
zmp_ph108_B2
zmp_ph108_B6
zmp_ph108_B7
zmp_ph108_C4
zmp_ph108_C5
zmp_ph108_D4
zmp_ph97_G6
zmp_ph97_G9
zmp_ph97_H2
zmp_ph97_B10
zmp_ph97_C2
zmp_ph97_C4
zmp_ph97_B8
zmp_ph97_C1
zmp_ph97_D2
zmp_ph97_D5
zmp_ph111_G5
zmp_ph111_G11
zmp_ph111_H1
zmp_ph111_B1a
zmp_ph111_B5
zmp_ph111_B8
zmp_ph111_B6
zmp_ph111_B7
zmp_ph111_B9
zmp_ph111_B10
zmp_ph114_G4
zmp_ph114_G11
zmp_ph114_H1
zmp_ph114_H2
zmp_ph114_A9
zmp_ph114_A11
zmp_ph114_B2
zmp_ph114_B4
zmp_ph114_D8
zmp_ph114_E2

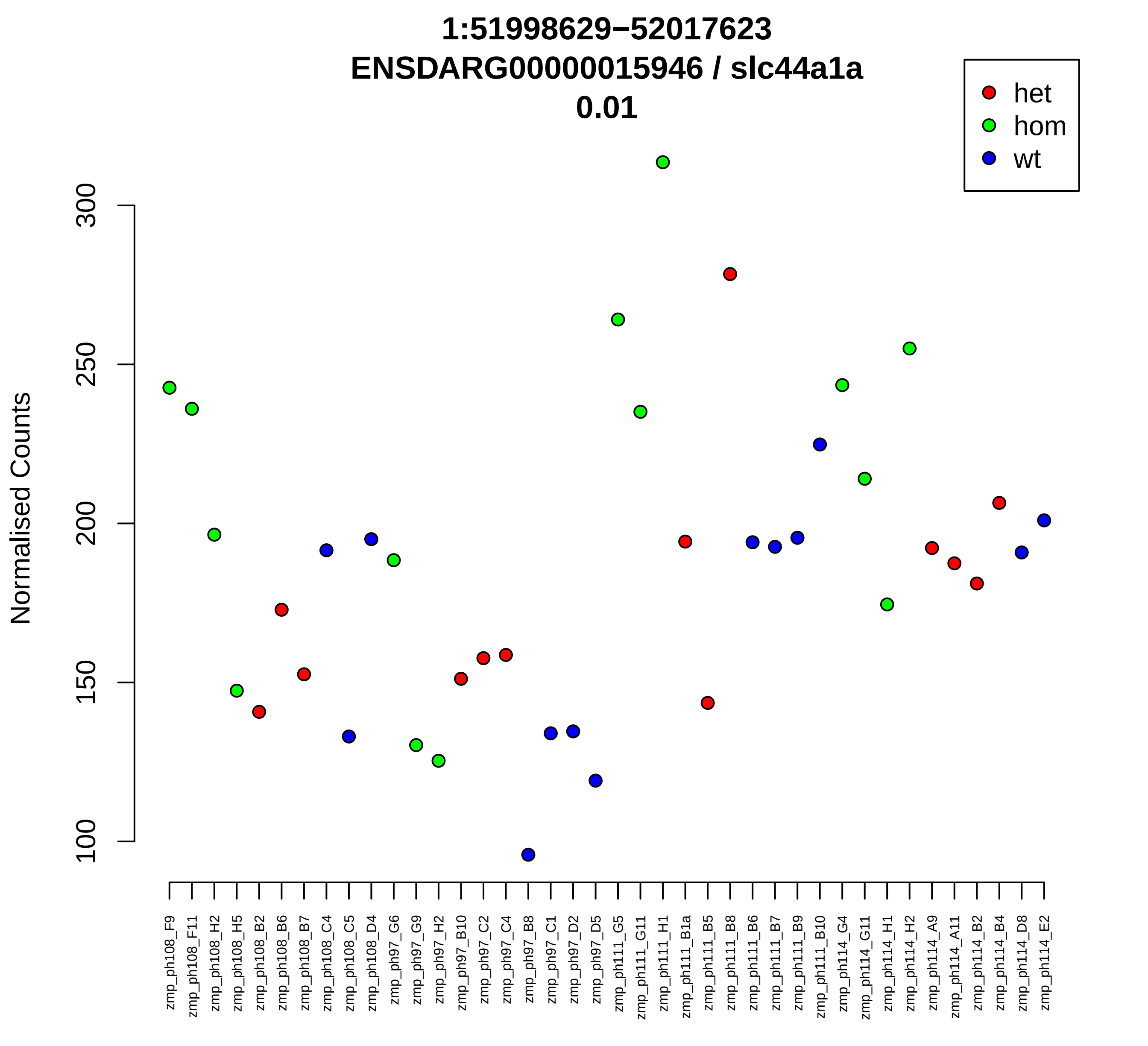
1:51998629−52017623
ENSDARG00000015946 / slc44a1a
0.01
het
hom
wt
Normalised Counts
100
150
200
250
300
zmp_ph108_F9
zmp_ph108_F11
zmp_ph108_H2
zmp_ph108_H5
zmp_ph108_B2
zmp_ph108_B6
zmp_ph108_B7
zmp_ph108_C4
zmp_ph108_C5
zmp_ph108_D4
zmp_ph97_G6
zmp_ph97_G9
zmp_ph97_H2
zmp_ph97_B10
zmp_ph97_C2
zmp_ph97_C4
zmp_ph97_B8
zmp_ph97_C1
zmp_ph97_D2
zmp_ph97_D5
zmp_ph111_G5
zmp_ph111_G11
zmp_ph111_H1
zmp_ph111_B1a
zmp_ph111_B5
zmp_ph111_B8
zmp_ph111_B6
zmp_ph111_B7
zmp_ph111_B9
zmp_ph111_B10
zmp_ph114_G4
zmp_ph114_G11
zmp_ph114_H1
zmp_ph114_H2
zmp_ph114_A9
zmp_ph114_A11
zmp_ph114_B2
zmp_ph114_B4
zmp_ph114_D8
zmp_ph114_E2

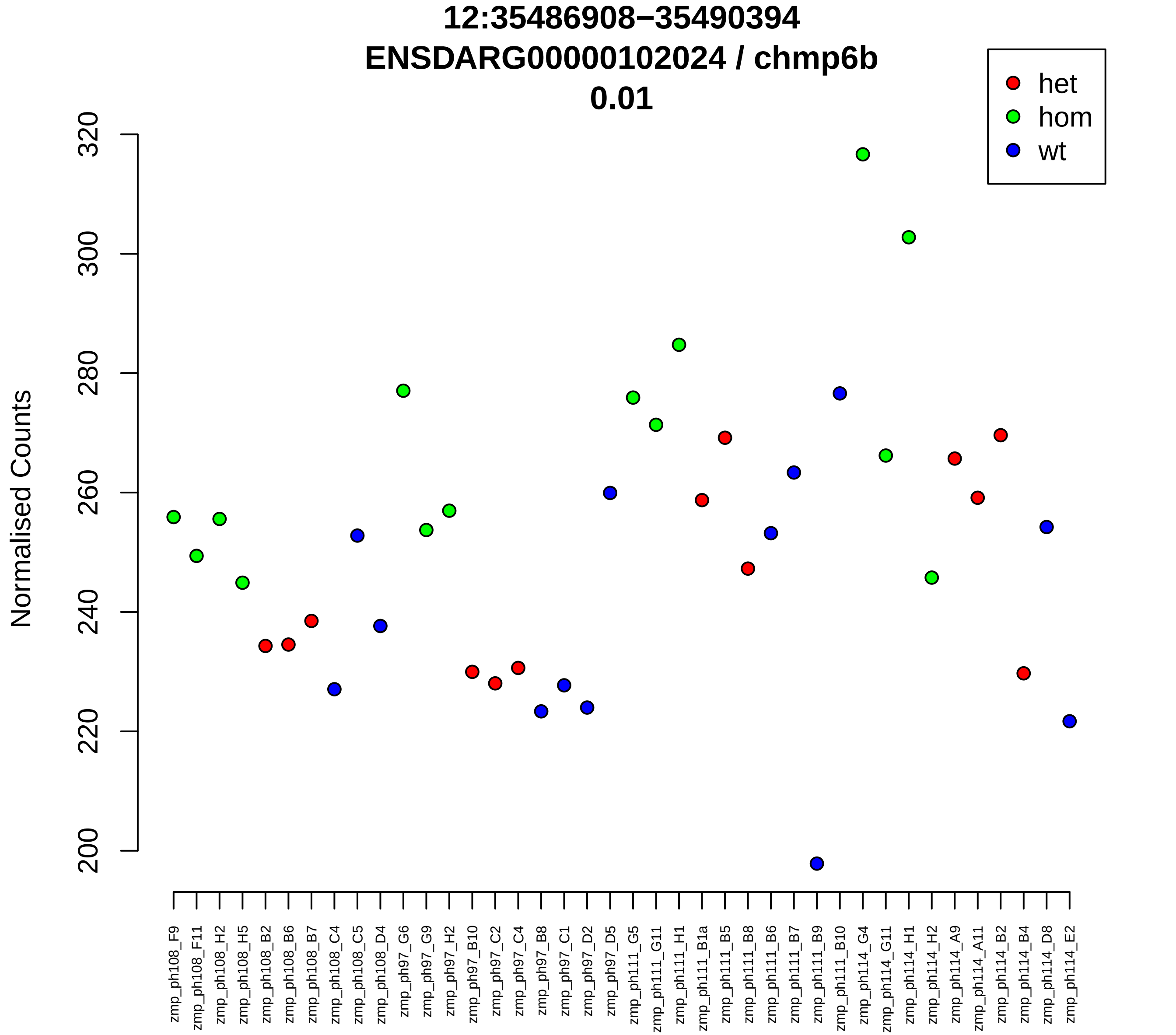

12:35486908−35490394
ENSDARG00000102024 / chmp6b
0.01
het
hom
wt
Normalised Counts
200
220
240
260
280
300
320
zmp_ph108_F9
zmp_ph108_F11
zmp_ph108_H2
zmp_ph108_H5
zmp_ph108_B2
zmp_ph108_B6
zmp_ph108_B7
zmp_ph108_C4
zmp_ph108_C5
zmp_ph108_D4
zmp_ph97_G6
zmp_ph97_G9
zmp_ph97_H2
zmp_ph97_B10
zmp_ph97_C2
zmp_ph97_C4
zmp_ph97_B8
zmp_ph97_C1
zmp_ph97_D2
zmp_ph97_D5
zmp_ph111_G5
zmp_ph111_G11
zmp_ph111_H1
zmp_ph111_B1a
zmp_ph111_B5
zmp_ph111_B8
zmp_ph111_B6
zmp_ph111_B7
zmp_ph111_B9
zmp_ph111_B10
zmp_ph114_G4
zmp_ph114_G11
zmp_ph114_H1
zmp_ph114_H2
zmp_ph114_A9
zmp_ph114_A11
zmp_ph114_B2
zmp_ph114_B4
zmp_ph114_D8
zmp_ph114_E2

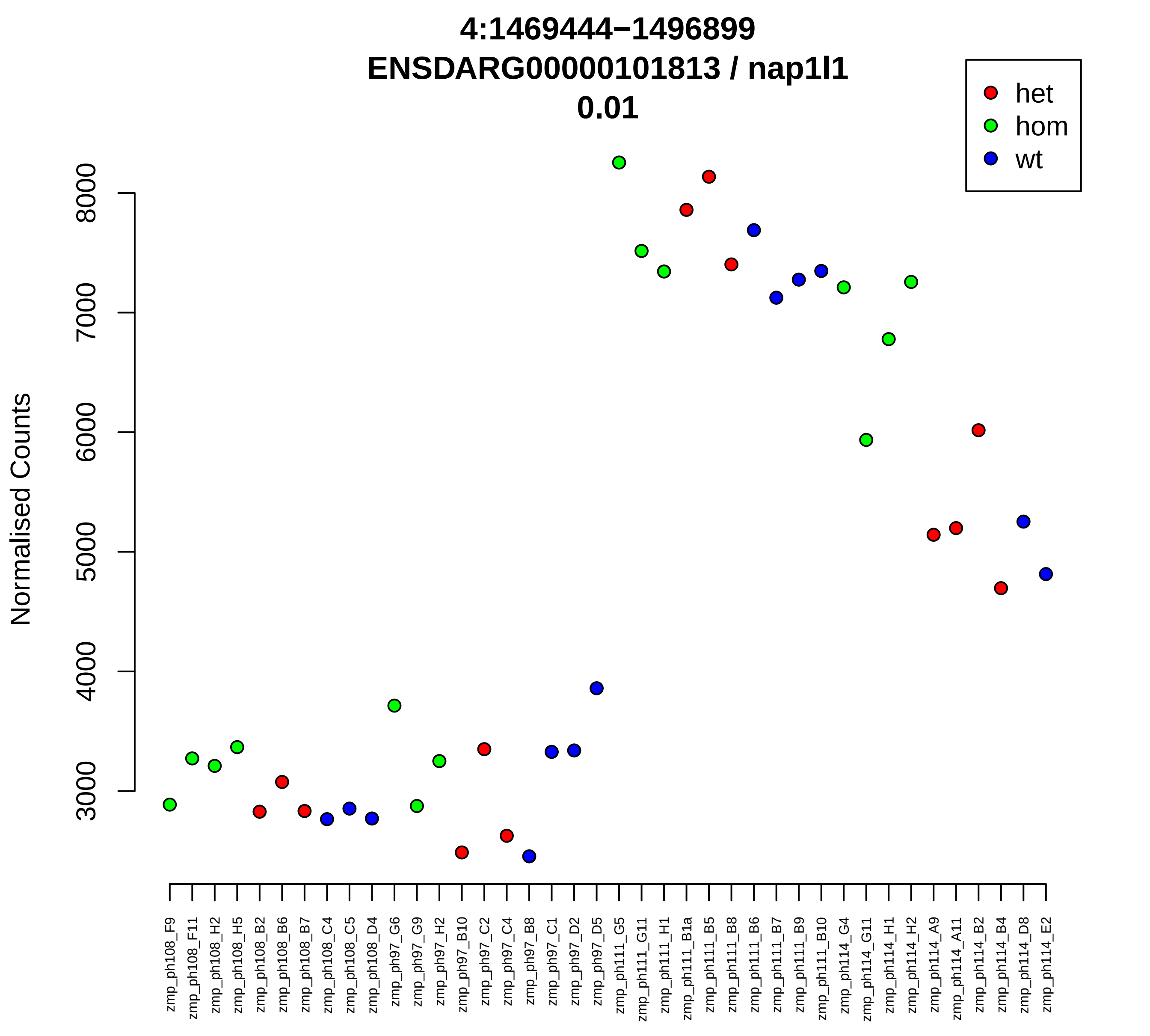

4:1469444−1496899
ENSDARG00000101813 / nap1l1
0.01
het
hom
wt
Normalised Counts
3000
4000
5000
6000
7000
8000
zmp_ph108_F9
zmp_ph108_F11
zmp_ph108_H2
zmp_ph108_H5
zmp_ph108_B2
zmp_ph108_B6
zmp_ph108_B7
zmp_ph108_C4
zmp_ph108_C5
zmp_ph108_D4
zmp_ph97_G6
zmp_ph97_G9
zmp_ph97_H2
zmp_ph97_B10
zmp_ph97_C2
zmp_ph97_C4
zmp_ph97_B8
zmp_ph97_C1
zmp_ph97_D2
zmp_ph97_D5
zmp_ph111_G5
zmp_ph111_G11
zmp_ph111_H1
zmp_ph111_B1a
zmp_ph111_B5
zmp_ph111_B8
zmp_ph111_B6
zmp_ph111_B7
zmp_ph111_B9
zmp_ph111_B10
zmp_ph114_G4
zmp_ph114_G11
zmp_ph114_H1
zmp_ph114_H2
zmp_ph114_A9
zmp_ph114_A11
zmp_ph114_B2
zmp_ph114_B4
zmp_ph114_D8
zmp_ph114_E2

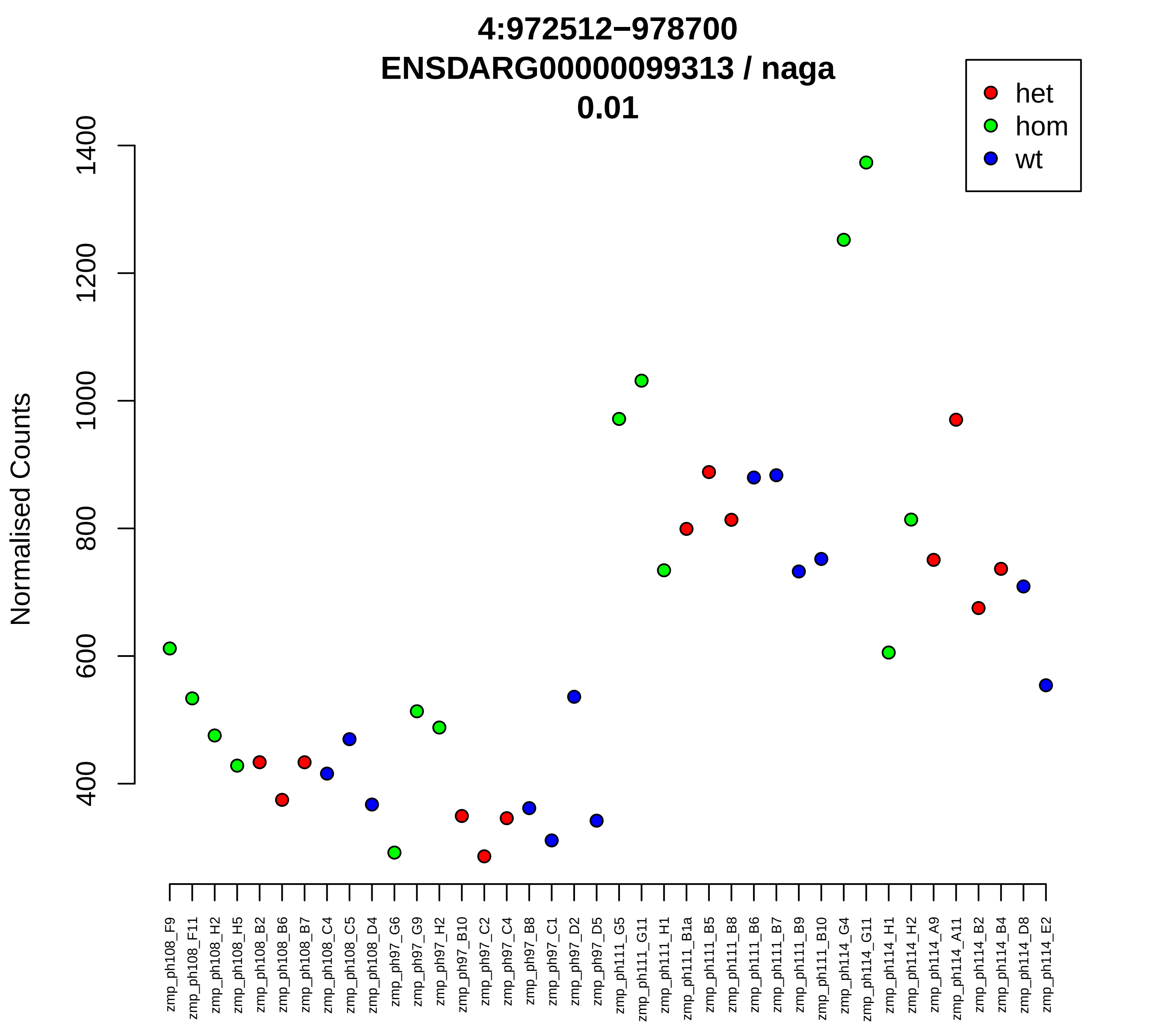

4:972512−978700
ENSDARG00000099313 / naga
0.01
het
hom
wt
Normalised Counts
400
600
800
1000
1200
1400
zmp_ph108_F9
zmp_ph108_F11
zmp_ph108_H2
zmp_ph108_H5
zmp_ph108_B2
zmp_ph108_B6
zmp_ph108_B7
zmp_ph108_C4
zmp_ph108_C5
zmp_ph108_D4
zmp_ph97_G6
zmp_ph97_G9
zmp_ph97_H2
zmp_ph97_B10
zmp_ph97_C2
zmp_ph97_C4
zmp_ph97_B8
zmp_ph97_C1
zmp_ph97_D2
zmp_ph97_D5
zmp_ph111_G5
zmp_ph111_G11
zmp_ph111_H1
zmp_ph111_B1a
zmp_ph111_B5
zmp_ph111_B8
zmp_ph111_B6
zmp_ph111_B7
zmp_ph111_B9
zmp_ph111_B10
zmp_ph114_G4
zmp_ph114_G11
zmp_ph114_H1
zmp_ph114_H2
zmp_ph114_A9
zmp_ph114_A11
zmp_ph114_B2
zmp_ph114_B4
zmp_ph114_D8
zmp_ph114_E2

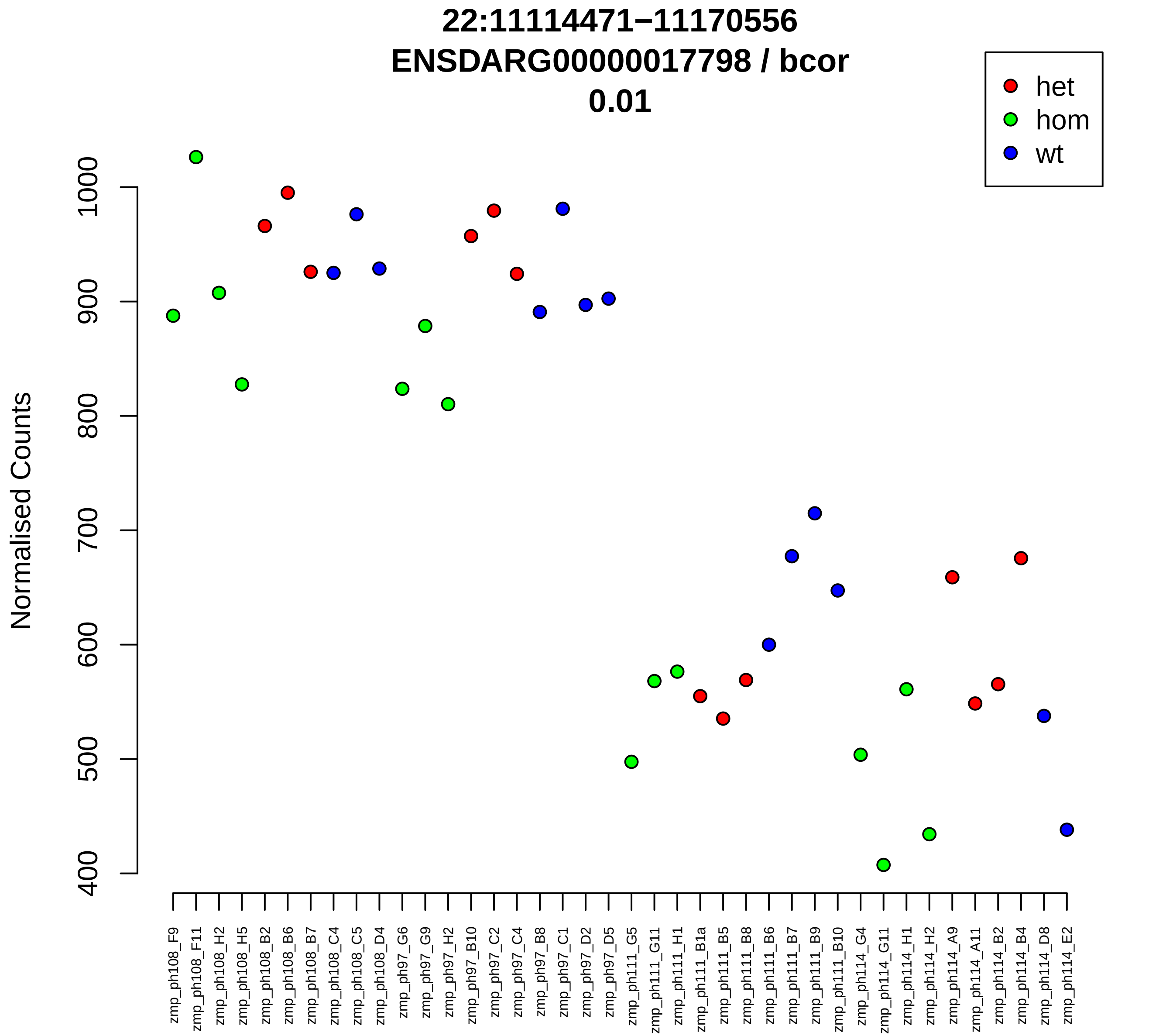
22:11114471−11170556
ENSDARG00000017798 / bcor
0.01
het
hom
wt
Normalised Counts
400
500
600
700
800
900
1000
zmp_ph108_F9
zmp_ph108_F11
zmp_ph108_H2
zmp_ph108_H5
zmp_ph108_B2
zmp_ph108_B6
zmp_ph108_B7
zmp_ph108_C4
zmp_ph108_C5
zmp_ph108_D4
zmp_ph97_G6
zmp_ph97_G9
zmp_ph97_H2
zmp_ph97_B10
zmp_ph97_C2
zmp_ph97_C4
zmp_ph97_B8
zmp_ph97_C1
zmp_ph97_D2
zmp_ph97_D5
zmp_ph111_G5
zmp_ph111_G11
zmp_ph111_H1
zmp_ph111_B1a
zmp_ph111_B5
zmp_ph111_B8
zmp_ph111_B6
zmp_ph111_B7
zmp_ph111_B9
zmp_ph111_B10
zmp_ph114_G4
zmp_ph114_G11
zmp_ph114_H1
zmp_ph114_H2
zmp_ph114_A9
zmp_ph114_A11
zmp_ph114_B2
zmp_ph114_B4
zmp_ph114_D8
zmp_ph114_E2

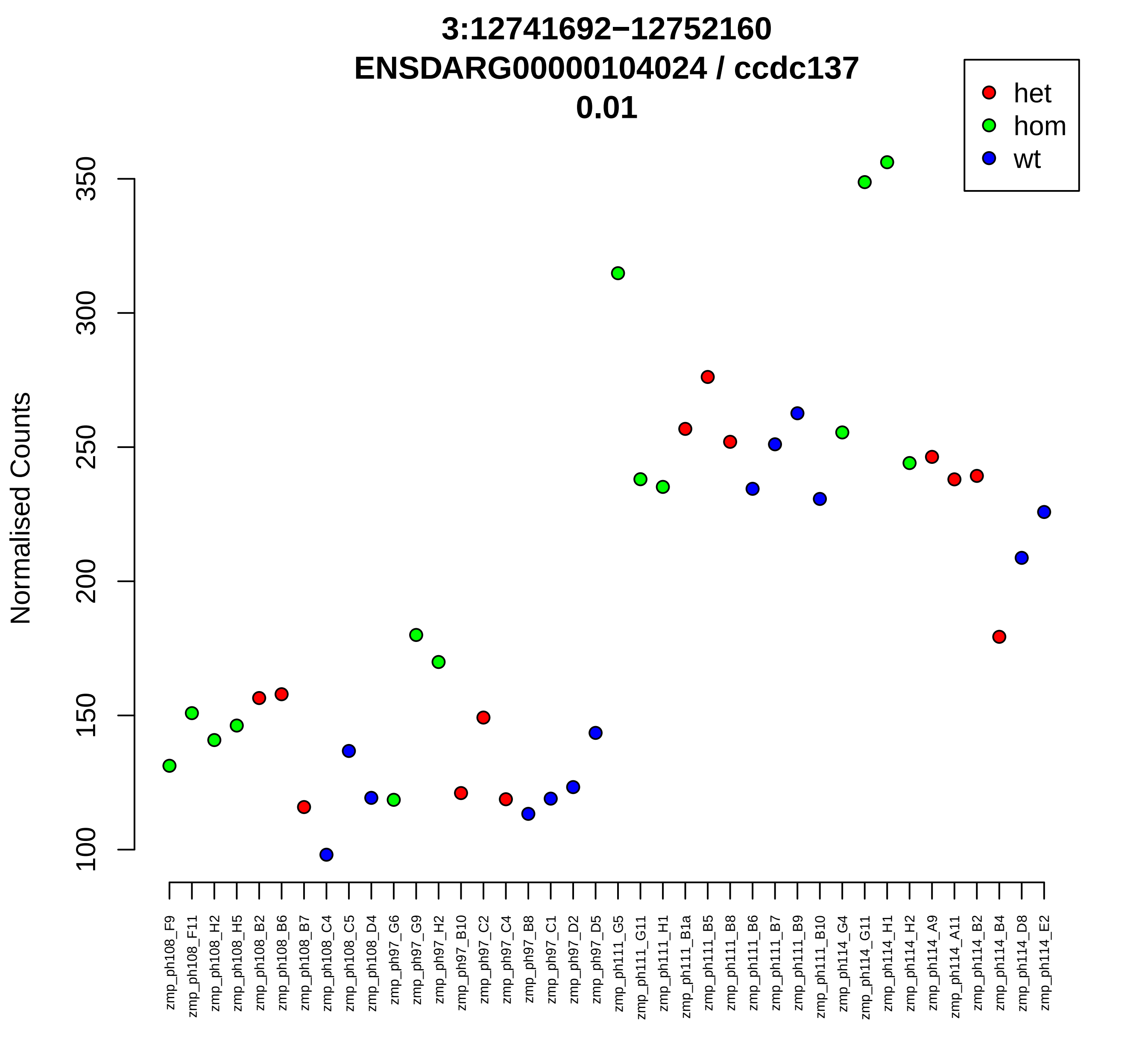
3:12741692−12752160
ENSDARG00000104024 / ccdc137
0.01
het
hom
wt
Normalised Counts
100
150
200
250
300
350
zmp_ph108_F9
zmp_ph108_F11
zmp_ph108_H2
zmp_ph108_H5
zmp_ph108_B2
zmp_ph108_B6
zmp_ph108_B7
zmp_ph108_C4
zmp_ph108_C5
zmp_ph108_D4
zmp_ph97_G6
zmp_ph97_G9
zmp_ph97_H2
zmp_ph97_B10
zmp_ph97_C2
zmp_ph97_C4
zmp_ph97_B8
zmp_ph97_C1
zmp_ph97_D2
zmp_ph97_D5
zmp_ph111_G5
zmp_ph111_G11
zmp_ph111_H1
zmp_ph111_B1a
zmp_ph111_B5
zmp_ph111_B8
zmp_ph111_B6
zmp_ph111_B7
zmp_ph111_B9
zmp_ph111_B10
zmp_ph114_G4
zmp_ph114_G11
zmp_ph114_H1
zmp_ph114_H2
zmp_ph114_A9
zmp_ph114_A11
zmp_ph114_B2
zmp_ph114_B4
zmp_ph114_D8
zmp_ph114_E2

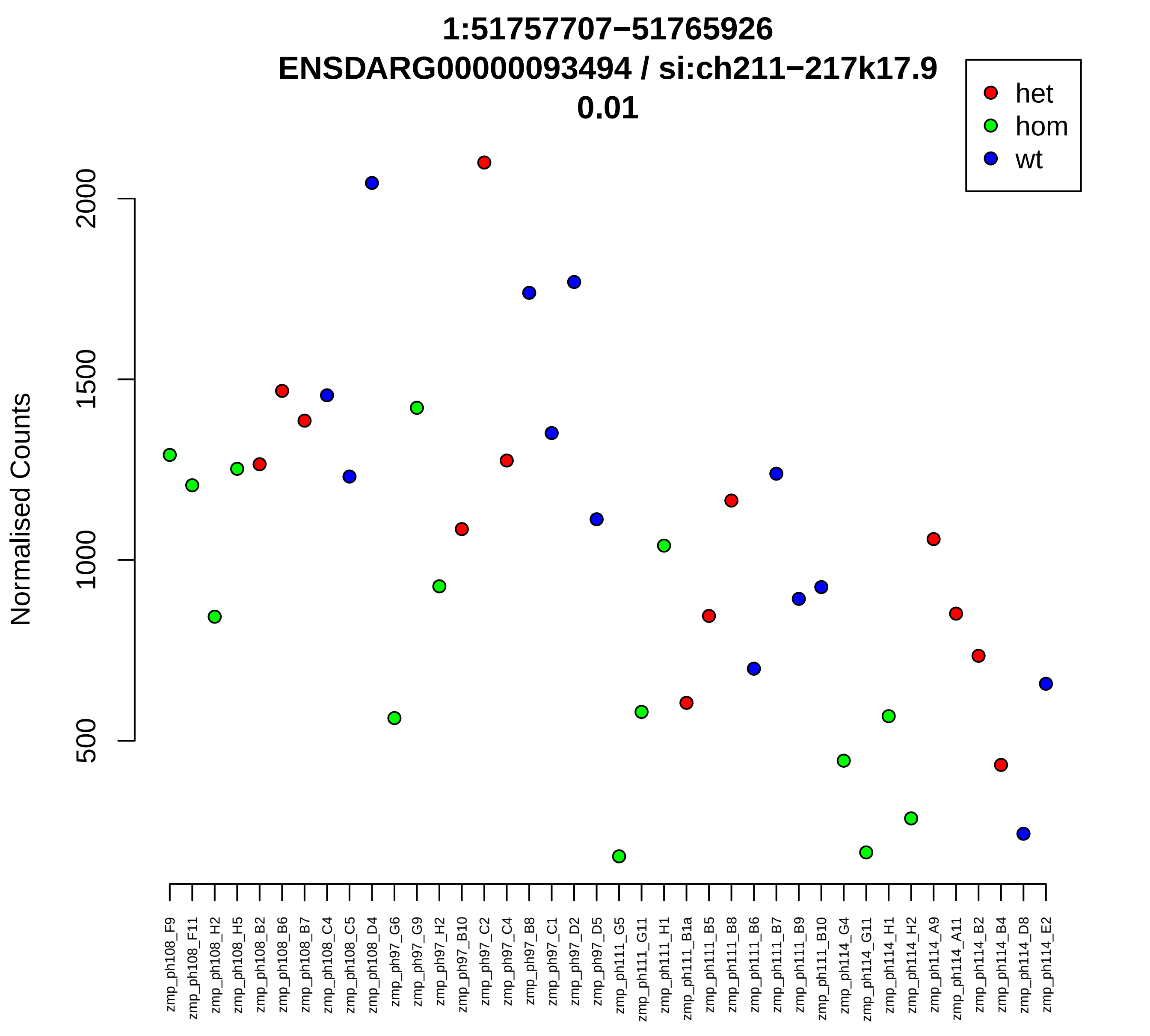
1:51757707−51765926
ENSDARG00000093494 / si:ch211−217k17.9
0.01
het
hom
wt
Normalised Counts
500
1000
1500
2000
zmp_ph108_F9
zmp_ph108_F11
zmp_ph108_H2
zmp_ph108_H5
zmp_ph108_B2
zmp_ph108_B6
zmp_ph108_B7
zmp_ph108_C4
zmp_ph108_C5
zmp_ph108_D4
zmp_ph97_G6
zmp_ph97_G9
zmp_ph97_H2
zmp_ph97_B10
zmp_ph97_C2
zmp_ph97_C4
zmp_ph97_B8
zmp_ph97_C1
zmp_ph97_D2
zmp_ph97_D5
zmp_ph111_G5
zmp_ph111_G11
zmp_ph111_H1
zmp_ph111_B1a
zmp_ph111_B5
zmp_ph111_B8
zmp_ph111_B6
zmp_ph111_B7
zmp_ph111_B9
zmp_ph111_B10
zmp_ph114_G4
zmp_ph114_G11
zmp_ph114_H1
zmp_ph114_H2
zmp_ph114_A9
zmp_ph114_A11
zmp_ph114_B2
zmp_ph114_B4
zmp_ph114_D8
zmp_ph114_E2

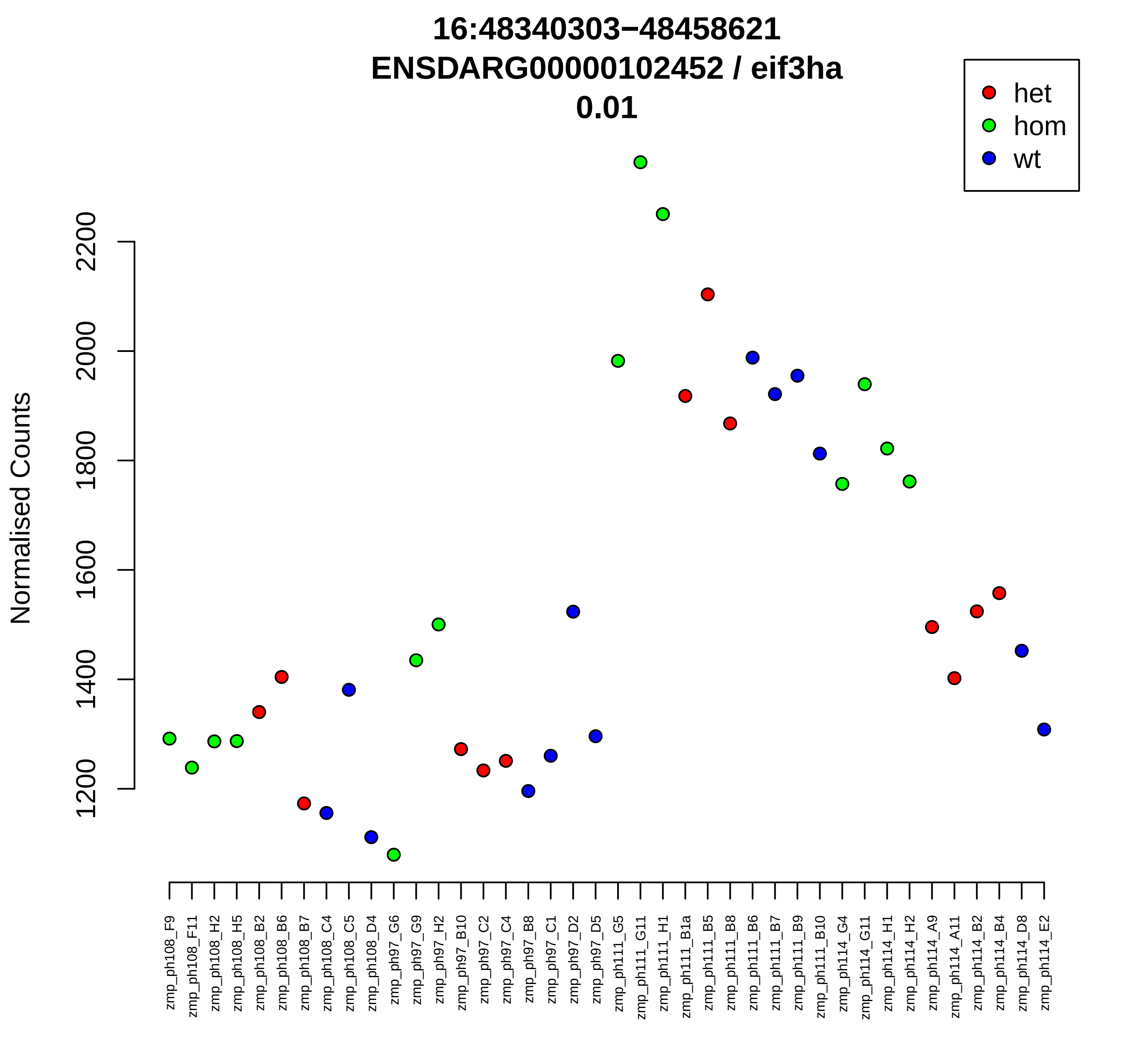

16:48340303−48458621
ENSDARG00000102452 / eif3ha
0.01
het
hom
wt
Normalised Counts
1200
1400
1600
1800
2000
2200
zmp_ph108_F9
zmp_ph108_F11
zmp_ph108_H2
zmp_ph108_H5
zmp_ph108_B2
zmp_ph108_B6
zmp_ph108_B7
zmp_ph108_C4
zmp_ph108_C5
zmp_ph108_D4
zmp_ph97_G6
zmp_ph97_G9
zmp_ph97_H2
zmp_ph97_B10
zmp_ph97_C2
zmp_ph97_C4
zmp_ph97_B8
zmp_ph97_C1
zmp_ph97_D2
zmp_ph97_D5
zmp_ph111_G5
zmp_ph111_G11
zmp_ph111_H1
zmp_ph111_B1a
zmp_ph111_B5
zmp_ph111_B8
zmp_ph111_B6
zmp_ph111_B7
zmp_ph111_B9
zmp_ph111_B10
zmp_ph114_G4
zmp_ph114_G11
zmp_ph114_H1
zmp_ph114_H2
zmp_ph114_A9
zmp_ph114_A11
zmp_ph114_B2
zmp_ph114_B4
zmp_ph114_D8
zmp_ph114_E2

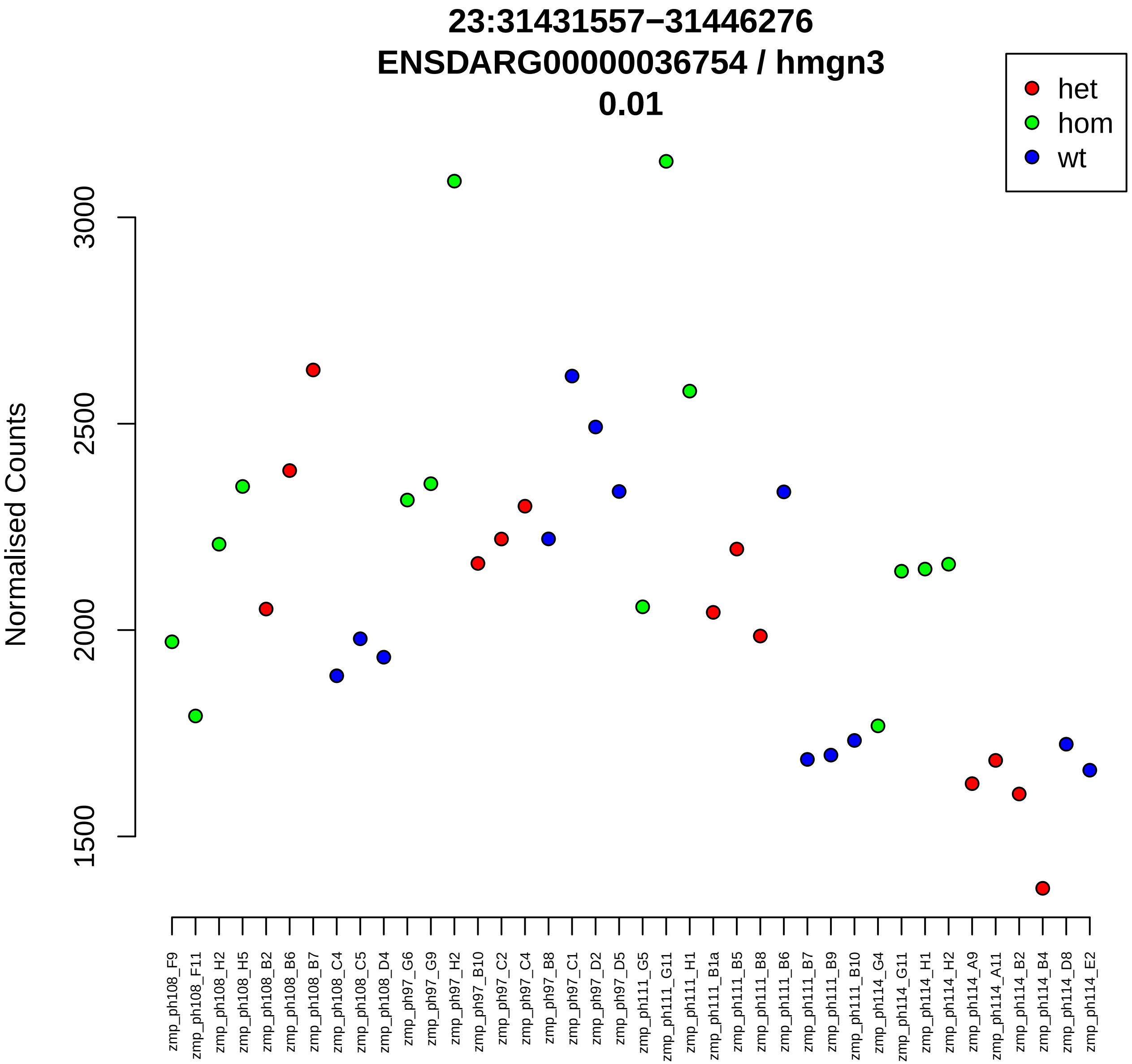
23:31431557−31446276
ENSDARG00000036754 / hmgn3
0.01
het
hom
wt
Normalised Counts
1500
2000
2500
3000
zmp_ph108_F9
zmp_ph108_F11
zmp_ph108_H2
zmp_ph108_H5
zmp_ph108_B2
zmp_ph108_B6
zmp_ph108_B7
zmp_ph108_C4
zmp_ph108_C5
zmp_ph108_D4
zmp_ph97_G6
zmp_ph97_G9
zmp_ph97_H2
zmp_ph97_B10
zmp_ph97_C2
zmp_ph97_C4
zmp_ph97_B8
zmp_ph97_C1
zmp_ph97_D2
zmp_ph97_D5
zmp_ph111_G5
zmp_ph111_G11
zmp_ph111_H1
zmp_ph111_B1a
zmp_ph111_B5
zmp_ph111_B8
zmp_ph111_B6
zmp_ph111_B7
zmp_ph111_B9
zmp_ph111_B10
zmp_ph114_G4
zmp_ph114_G11
zmp_ph114_H1
zmp_ph114_H2
zmp_ph114_A9
zmp_ph114_A11
zmp_ph114_B2
zmp_ph114_B4
zmp_ph114_D8
zmp_ph114_E2

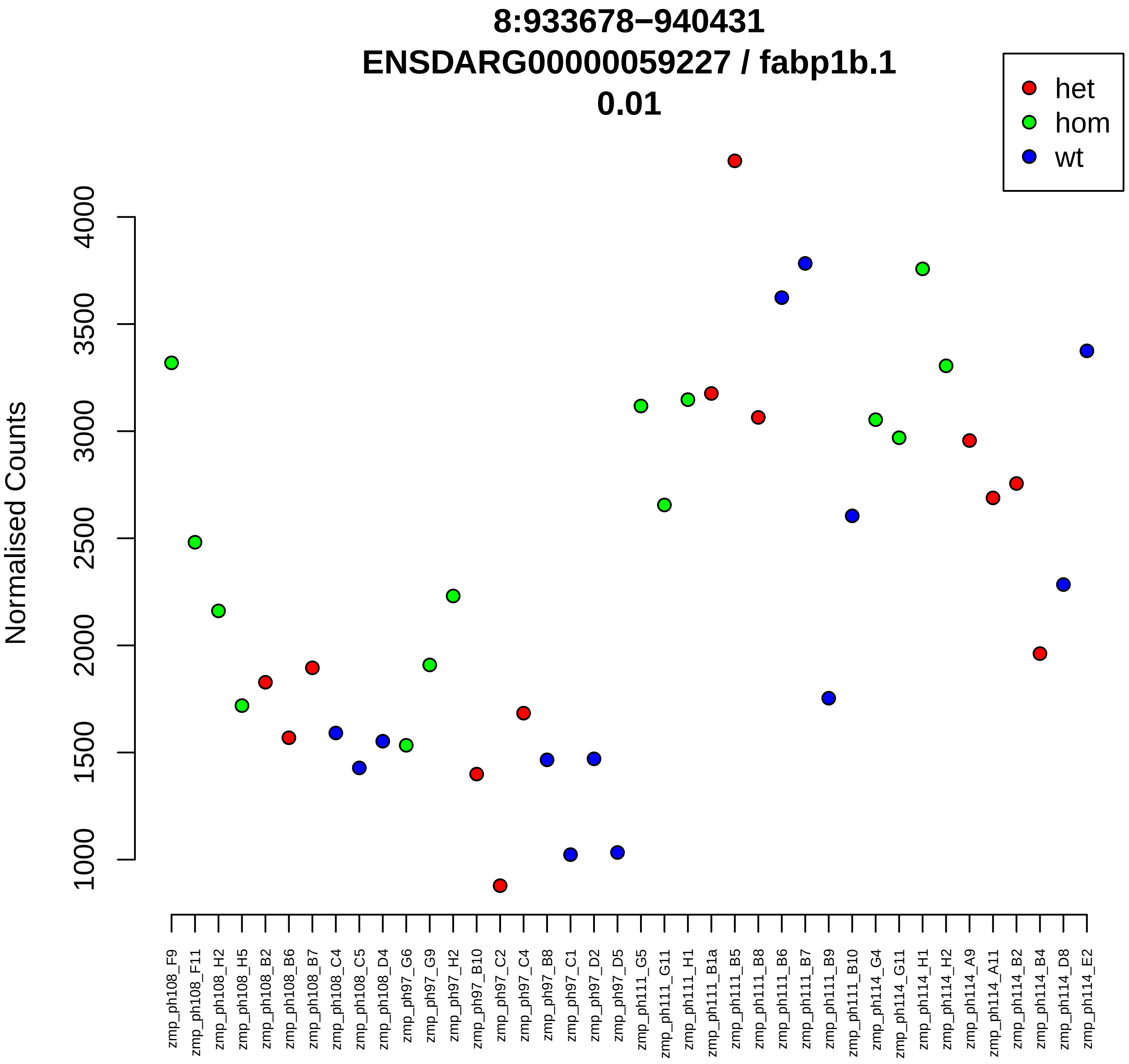

8:933678−940431
ENSDARG00000059227 / fabp1b.1
0.01
het
hom
wt
Normalised Counts
1000
1500
2000
2500
3000
3500
4000
zmp_ph108_F9
zmp_ph108_F11
zmp_ph108_H2
zmp_ph108_H5
zmp_ph108_B2
zmp_ph108_B6
zmp_ph108_B7
zmp_ph108_C4
zmp_ph108_C5
zmp_ph108_D4
zmp_ph97_G6
zmp_ph97_G9
zmp_ph97_H2
zmp_ph97_B10
zmp_ph97_C2
zmp_ph97_C4
zmp_ph97_B8
zmp_ph97_C1
zmp_ph97_D2
zmp_ph97_D5
zmp_ph111_G5
zmp_ph111_G11
zmp_ph111_H1
zmp_ph111_B1a
zmp_ph111_B5
zmp_ph111_B8
zmp_ph111_B6
zmp_ph111_B7
zmp_ph111_B9
zmp_ph111_B10
zmp_ph114_G4
zmp_ph114_G11
zmp_ph114_H1
zmp_ph114_H2
zmp_ph114_A9
zmp_ph114_A11
zmp_ph114_B2
zmp_ph114_B4
zmp_ph114_D8
zmp_ph114_E2

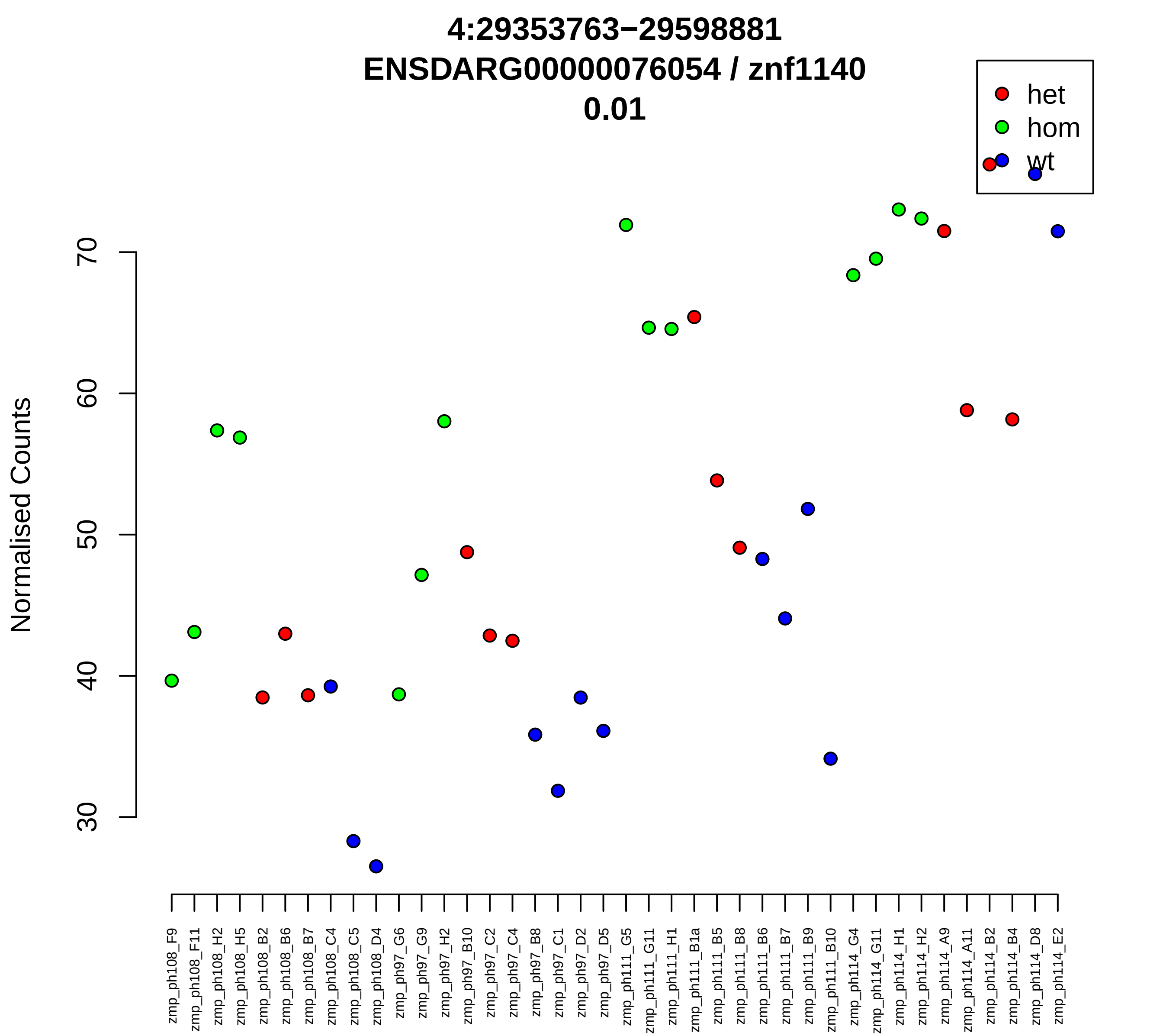
4:29353763−29598881
ENSDARG00000076054 / znf1140
0.01
het
hom
wt
Normalised Counts
30
40
50
60
70
zmp_ph108_F9
zmp_ph108_F11
zmp_ph108_H2
zmp_ph108_H5
zmp_ph108_B2
zmp_ph108_B6
zmp_ph108_B7
zmp_ph108_C4
zmp_ph108_C5
zmp_ph108_D4
zmp_ph97_G6
zmp_ph97_G9
zmp_ph97_H2
zmp_ph97_B10
zmp_ph97_C2
zmp_ph97_C4
zmp_ph97_B8
zmp_ph97_C1
zmp_ph97_D2
zmp_ph97_D5
zmp_ph111_G5
zmp_ph111_G11
zmp_ph111_H1
zmp_ph111_B1a
zmp_ph111_B5
zmp_ph111_B8
zmp_ph111_B6
zmp_ph111_B7
zmp_ph111_B9
zmp_ph111_B10
zmp_ph114_G4
zmp_ph114_G11
zmp_ph114_H1
zmp_ph114_H2
zmp_ph114_A9
zmp_ph114_A11
zmp_ph114_B2
zmp_ph114_B4
zmp_ph114_D8
zmp_ph114_E2

25:5631895−5689334
ENSDARG00000078139 / appl2
0.01

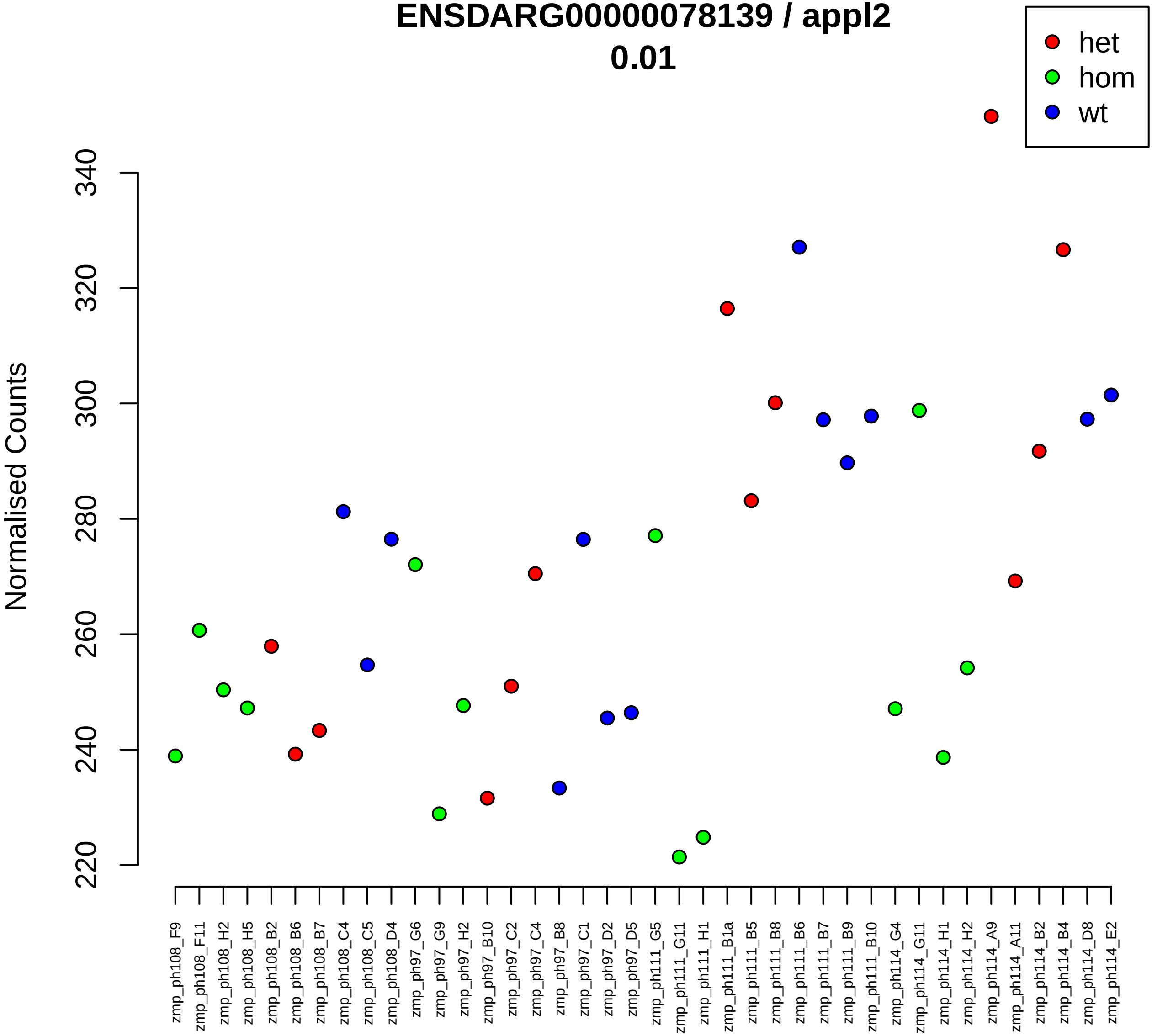

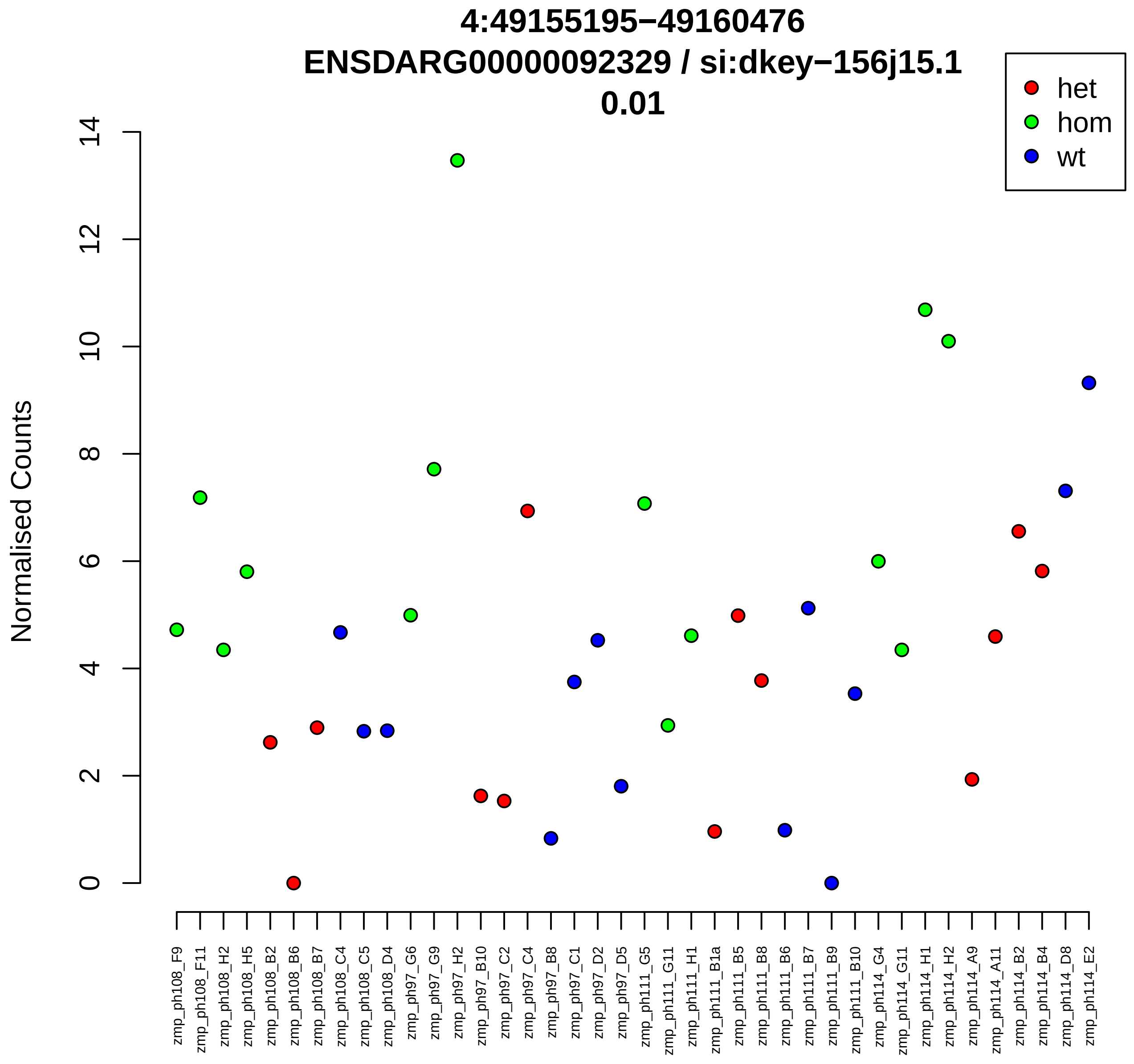

4:49155195−49160476
ENSDARG00000092329 / si:dkey−156j15.1
0.01
het
hom
wt
Normalised Counts
0
2
4
6
8
10
12
14
zmp_ph108_F9
zmp_ph108_F11
zmp_ph108_H2
zmp_ph108_H5
zmp_ph108_B2
zmp_ph108_B6
zmp_ph108_B7
zmp_ph108_C4
zmp_ph108_C5
zmp_ph108_D4
zmp_ph97_G6
zmp_ph97_G9
zmp_ph97_H2
zmp_ph97_B10
zmp_ph97_C2
zmp_ph97_C4
zmp_ph97_B8
zmp_ph97_C1
zmp_ph97_D2
zmp_ph97_D5
zmp_ph111_G5
zmp_ph111_G11
zmp_ph111_H1
zmp_ph111_B1a
zmp_ph111_B5
zmp_ph111_B8
zmp_ph111_B6
zmp_ph111_B7
zmp_ph111_B9
zmp_ph111_B10
zmp_ph114_G4
zmp_ph114_G11
zmp_ph114_H1
zmp_ph114_H2
zmp_ph114_A9
zmp_ph114_A11
zmp_ph114_B2
zmp_ph114_B4
zmp_ph114_D8
zmp_ph114_E2

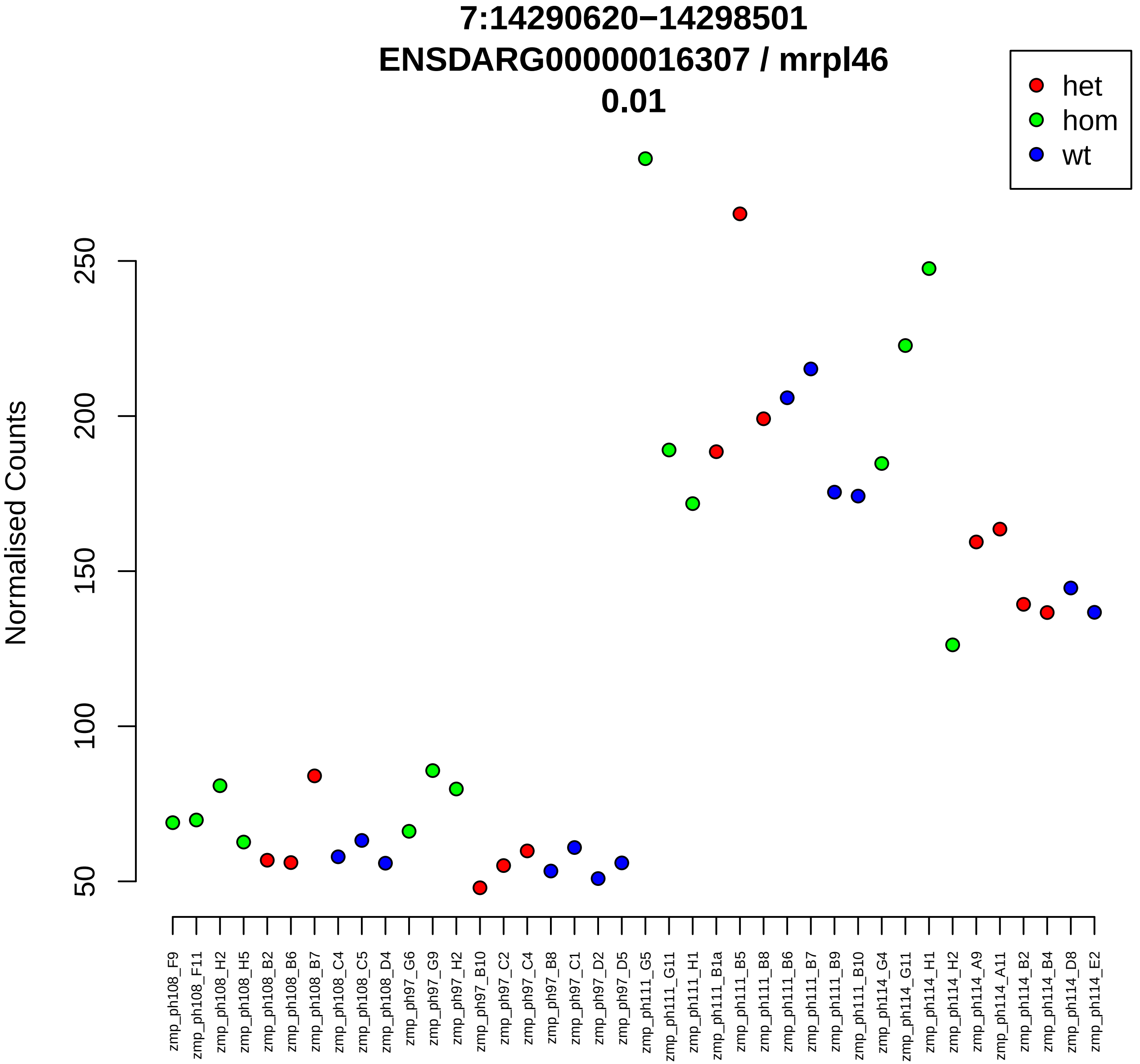

7:14290620−14298501
ENSDARG00000016307 / mrpl46
0.01
het
hom
wt
Normalised Counts
50
100
150
200
250
zmp_ph108_F9
zmp_ph108_F11
zmp_ph108_H2
zmp_ph108_H5
zmp_ph108_B2
zmp_ph108_B6
zmp_ph108_B7
zmp_ph108_C4
zmp_ph108_C5
zmp_ph108_D4
zmp_ph97_G6
zmp_ph97_G9
zmp_ph97_H2
zmp_ph97_B10
zmp_ph97_C2
zmp_ph97_C4
zmp_ph97_B8
zmp_ph97_C1
zmp_ph97_D2
zmp_ph97_D5
zmp_ph111_G5
zmp_ph111_G11
zmp_ph111_H1
zmp_ph111_B1a
zmp_ph111_B5
zmp_ph111_B8
zmp_ph111_B6
zmp_ph111_B7
zmp_ph111_B9
zmp_ph111_B10
zmp_ph114_G4
zmp_ph114_G11
zmp_ph114_H1
zmp_ph114_H2
zmp_ph114_A9
zmp_ph114_A11
zmp_ph114_B2
zmp_ph114_B4
zmp_ph114_D8
zmp_ph114_E2

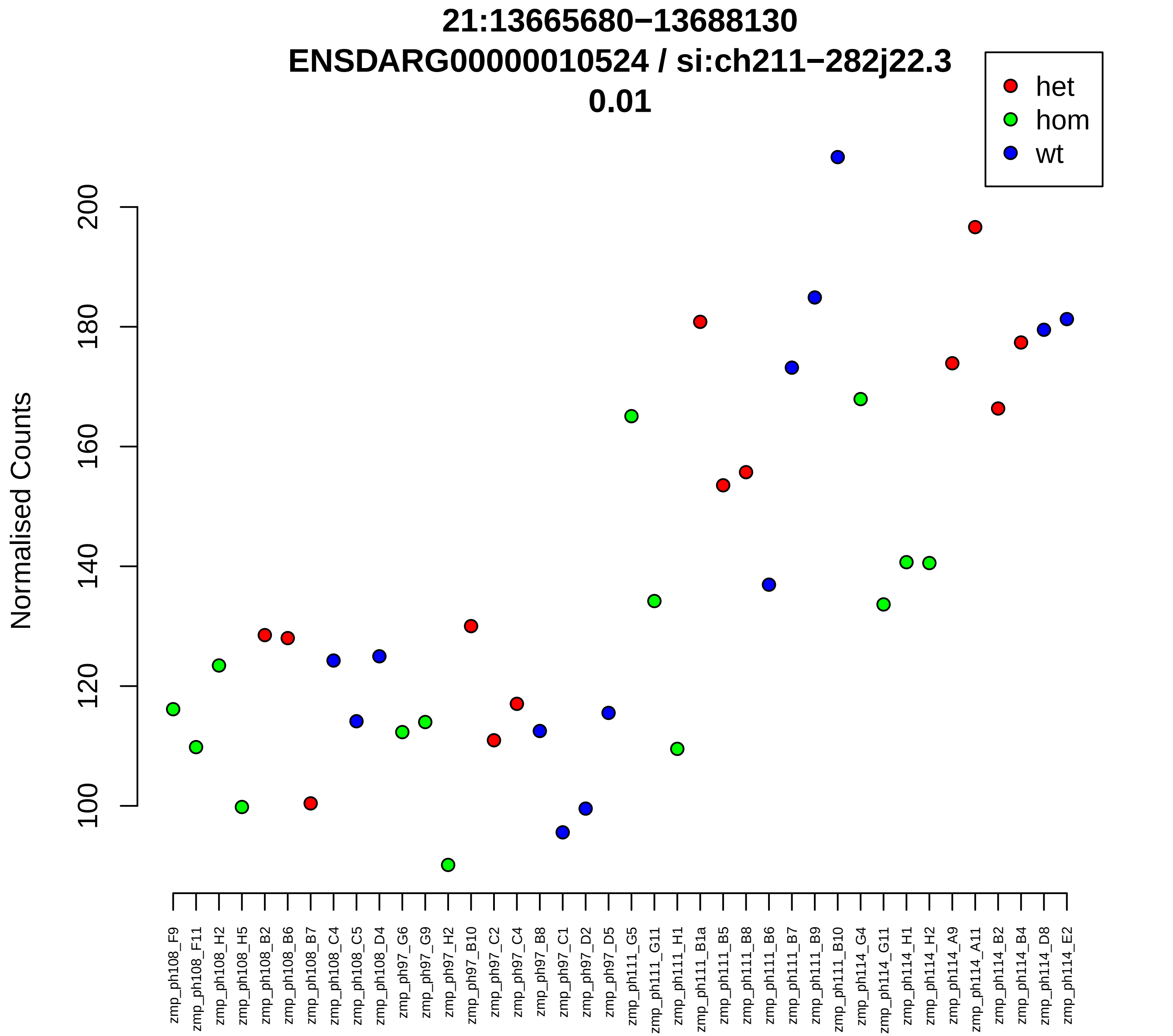

21:13665680−13688130
ENSDARG00000010524 / si:ch211−282j22.3
0.01
het
hom
wt
Normalised Counts
100
120
140
160
180
200
zmp_ph108_F9
zmp_ph108_F11
zmp_ph108_H2
zmp_ph108_H5
zmp_ph108_B2
zmp_ph108_B6
zmp_ph108_B7
zmp_ph108_C4
zmp_ph108_C5
zmp_ph108_D4
zmp_ph97_G6
zmp_ph97_G9
zmp_ph97_H2
zmp_ph97_B10
zmp_ph97_C2
zmp_ph97_C4
zmp_ph97_B8
zmp_ph97_C1
zmp_ph97_D2
zmp_ph97_D5
zmp_ph111_G5
zmp_ph111_G11
zmp_ph111_H1
zmp_ph111_B1a
zmp_ph111_B5
zmp_ph111_B8
zmp_ph111_B6
zmp_ph111_B7
zmp_ph111_B9
zmp_ph111_B10
zmp_ph114_G4
zmp_ph114_G11
zmp_ph114_H1
zmp_ph114_H2
zmp_ph114_A9
zmp_ph114_A11
zmp_ph114_B2
zmp_ph114_B4
zmp_ph114_D8
zmp_ph114_E2

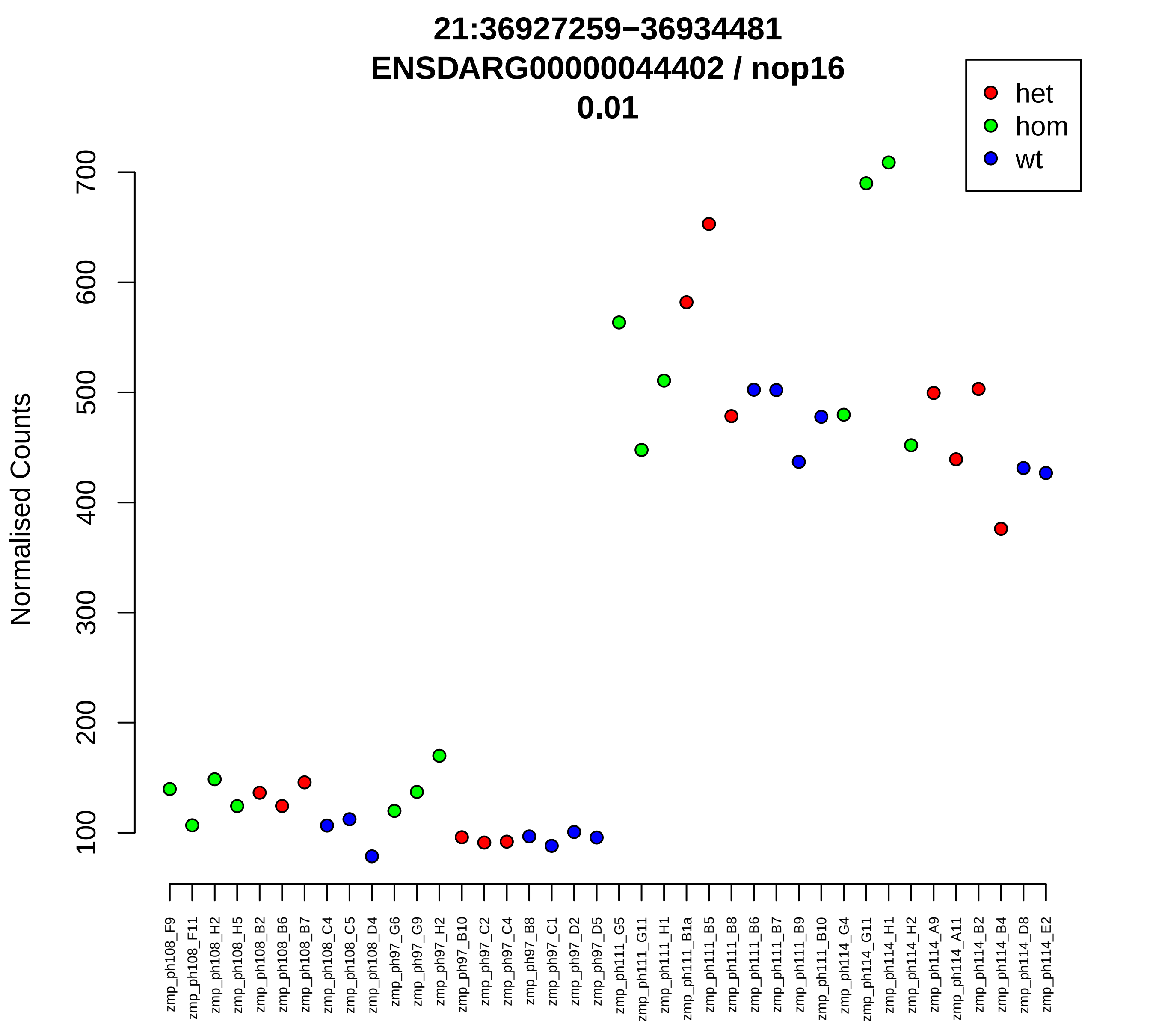

21:36927259−36934481
ENSDARG00000044402 / nop16
0.01
het
hom
wt
Normalised Counts
100
200
300
400
500
600
700
zmp_ph108_F9
zmp_ph108_F11
zmp_ph108_H2
zmp_ph108_H5
zmp_ph108_B2
zmp_ph108_B6
zmp_ph108_B7
zmp_ph108_C4
zmp_ph108_C5
zmp_ph108_D4
zmp_ph97_G6
zmp_ph97_G9
zmp_ph97_H2
zmp_ph97_B10
zmp_ph97_C2
zmp_ph97_C4
zmp_ph97_B8
zmp_ph97_C1
zmp_ph97_D2
zmp_ph97_D5
zmp_ph111_G5
zmp_ph111_G11
zmp_ph111_H1
zmp_ph111_B1a
zmp_ph111_B5
zmp_ph111_B8
zmp_ph111_B6
zmp_ph111_B7
zmp_ph111_B9
zmp_ph111_B10
zmp_ph114_G4
zmp_ph114_G11
zmp_ph114_H1
zmp_ph114_H2
zmp_ph114_A9
zmp_ph114_A11
zmp_ph114_B2
zmp_ph114_B4
zmp_ph114_D8
zmp_ph114_E2

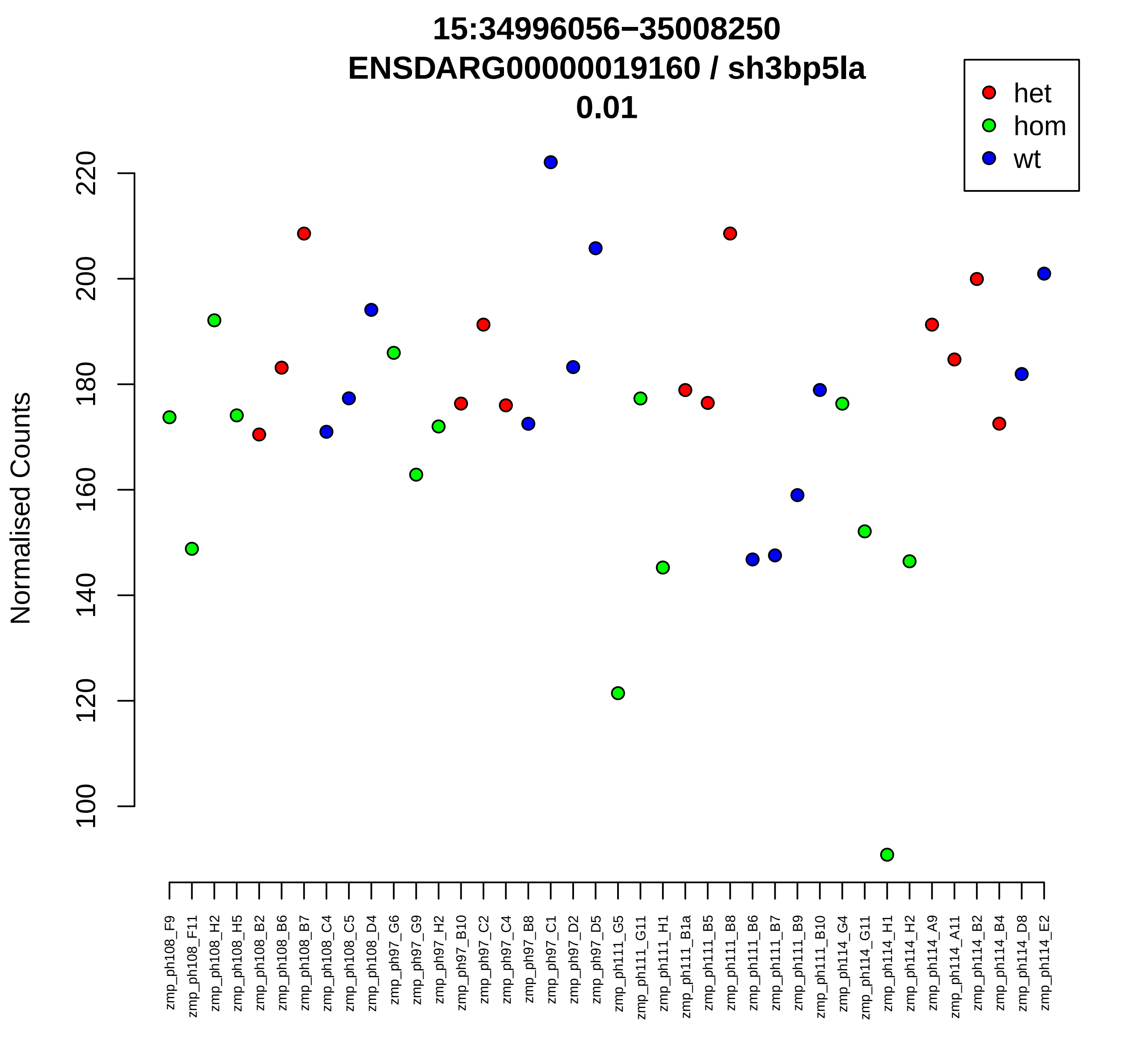

15:34996056−35008250
ENSDARG00000019160 / sh3bp5la
0.01
het
hom
wt
Normalised Counts
100
120
140
160
180
200
220
zmp_ph108_F9
zmp_ph108_F11
zmp_ph108_H2
zmp_ph108_H5
zmp_ph108_B2
zmp_ph108_B6
zmp_ph108_B7
zmp_ph108_C4
zmp_ph108_C5
zmp_ph108_D4
zmp_ph97_G6
zmp_ph97_G9
zmp_ph97_H2
zmp_ph97_B10
zmp_ph97_C2
zmp_ph97_C4
zmp_ph97_B8
zmp_ph97_C1
zmp_ph97_D2
zmp_ph97_D5
zmp_ph111_G5
zmp_ph111_G11
zmp_ph111_H1
zmp_ph111_B1a
zmp_ph111_B5
zmp_ph111_B8
zmp_ph111_B6
zmp_ph111_B7
zmp_ph111_B9
zmp_ph111_B10
zmp_ph114_G4
zmp_ph114_G11
zmp_ph114_H1
zmp_ph114_H2
zmp_ph114_A9
zmp_ph114_A11
zmp_ph114_B2
zmp_ph114_B4
zmp_ph114_D8
zmp_ph114_E2

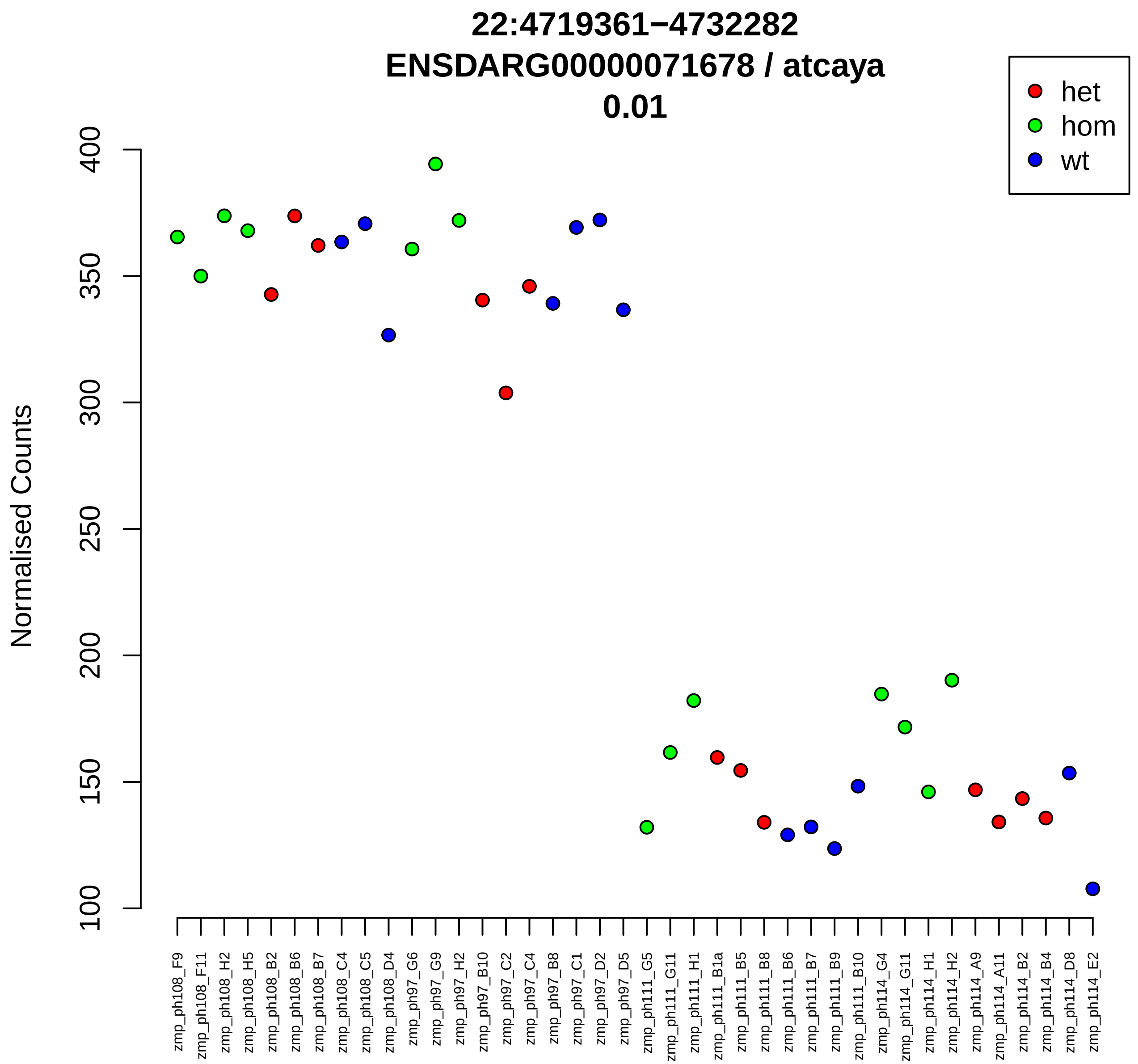
22:4719361−4732282
ENSDARG00000071678 / atcaya
0.01
het
hom
wt
Normalised Counts
100
150
200
250
300
350
400
zmp_ph108_F9
zmp_ph108_F11
zmp_ph108_H2
zmp_ph108_H5
zmp_ph108_B2
zmp_ph108_B6
zmp_ph108_B7
zmp_ph108_C4
zmp_ph108_C5
zmp_ph108_D4
zmp_ph97_G6
zmp_ph97_G9
zmp_ph97_H2
zmp_ph97_B10
zmp_ph97_C2
zmp_ph97_C4
zmp_ph97_B8
zmp_ph97_C1
zmp_ph97_D2
zmp_ph97_D5
zmp_ph111_G5
zmp_ph111_G11
zmp_ph111_H1
zmp_ph111_B1a
zmp_ph111_B5
zmp_ph111_B8
zmp_ph111_B6
zmp_ph111_B7
zmp_ph111_B9
zmp_ph111_B10
zmp_ph114_G4
zmp_ph114_G11
zmp_ph114_H1
zmp_ph114_H2
zmp_ph114_A9
zmp_ph114_A11
zmp_ph114_B2
zmp_ph114_B4
zmp_ph114_D8
zmp_ph114_E2

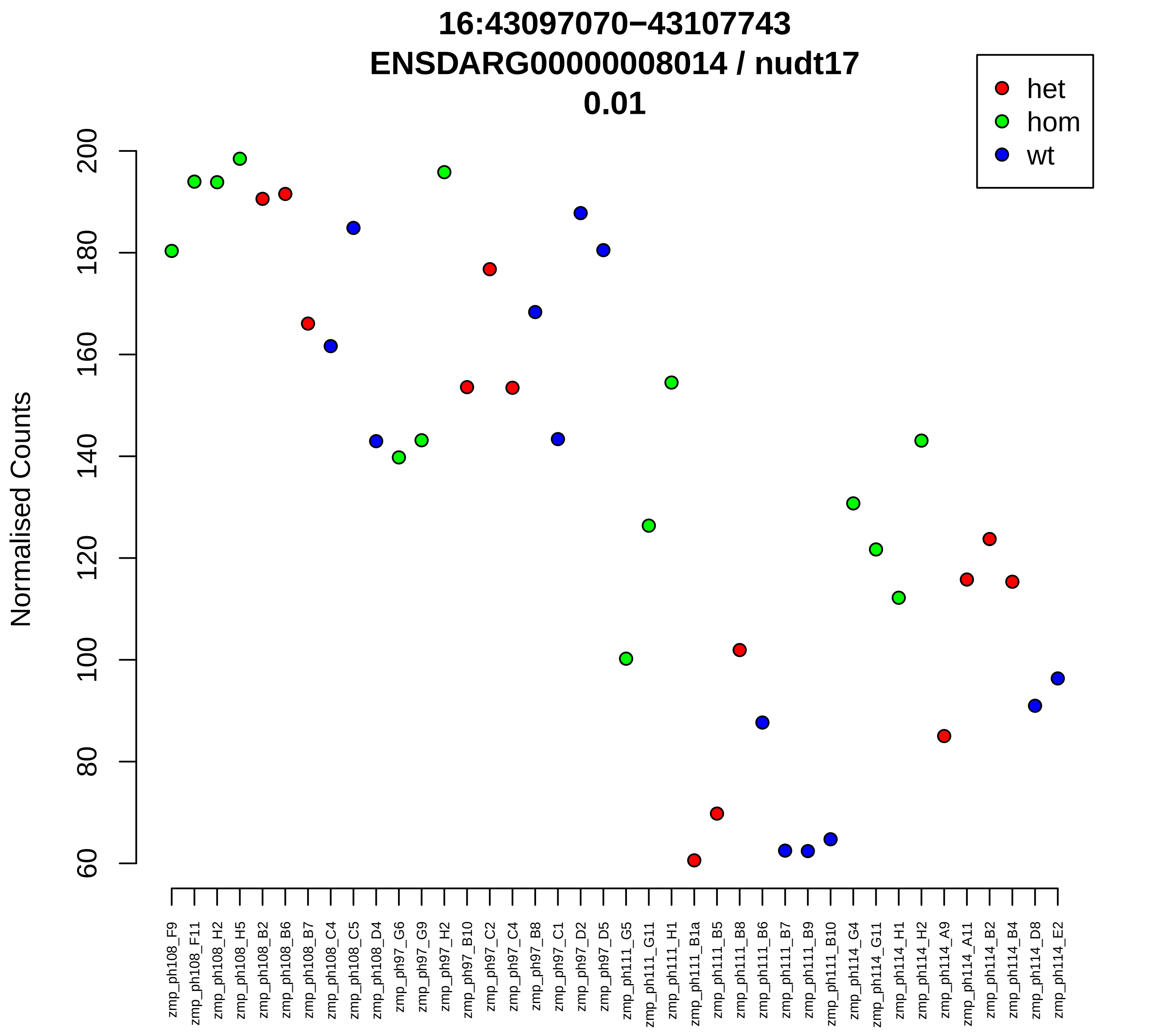
16:43097070−43107743
ENSDARG00000008014 / nudt17
0.01
het
hom
wt
Normalised Counts
60
80
100
120
140
160
180
200
zmp_ph108_F9
zmp_ph108_F11
zmp_ph108_H2
zmp_ph108_H5
zmp_ph108_B2
zmp_ph108_B6
zmp_ph108_B7
zmp_ph108_C4
zmp_ph108_C5
zmp_ph108_D4
zmp_ph97_G6
zmp_ph97_G9
zmp_ph97_H2
zmp_ph97_B10
zmp_ph97_C2
zmp_ph97_C4
zmp_ph97_B8
zmp_ph97_C1
zmp_ph97_D2
zmp_ph97_D5
zmp_ph111_G5
zmp_ph111_G11
zmp_ph111_H1
zmp_ph111_B1a
zmp_ph111_B5
zmp_ph111_B8
zmp_ph111_B6
zmp_ph111_B7
zmp_ph111_B9
zmp_ph111_B10
zmp_ph114_G4
zmp_ph114_G11
zmp_ph114_H1
zmp_ph114_H2
zmp_ph114_A9
zmp_ph114_A11
zmp_ph114_B2
zmp_ph114_B4
zmp_ph114_D8
zmp_ph114_E2

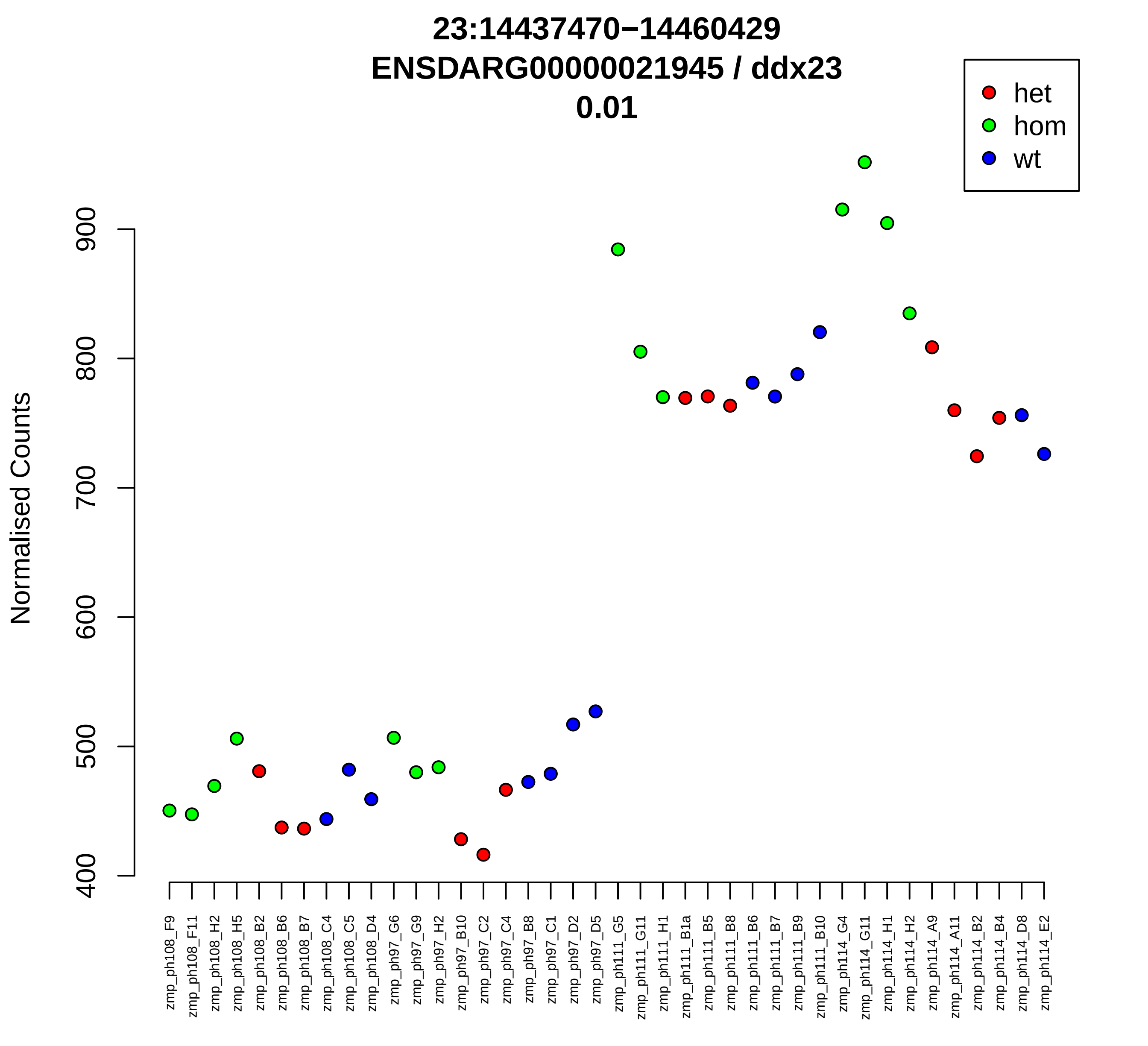

23:14437470−14460429
ENSDARG00000021945 / ddx23
0.01
het
hom
wt
Normalised Counts
400
500
600
700
800
900
zmp_ph108_F9
zmp_ph108_F11
zmp_ph108_H2
zmp_ph108_H5
zmp_ph108_B2
zmp_ph108_B6
zmp_ph108_B7
zmp_ph108_C4
zmp_ph108_C5
zmp_ph108_D4
zmp_ph97_G6
zmp_ph97_G9
zmp_ph97_H2
zmp_ph97_B10
zmp_ph97_C2
zmp_ph97_C4
zmp_ph97_B8
zmp_ph97_C1
zmp_ph97_D2
zmp_ph97_D5
zmp_ph111_G5
zmp_ph111_G11
zmp_ph111_H1
zmp_ph111_B1a
zmp_ph111_B5
zmp_ph111_B8
zmp_ph111_B6
zmp_ph111_B7
zmp_ph111_B9
zmp_ph111_B10
zmp_ph114_G4
zmp_ph114_G11
zmp_ph114_H1
zmp_ph114_H2
zmp_ph114_A9
zmp_ph114_A11
zmp_ph114_B2
zmp_ph114_B4
zmp_ph114_D8
zmp_ph114_E2

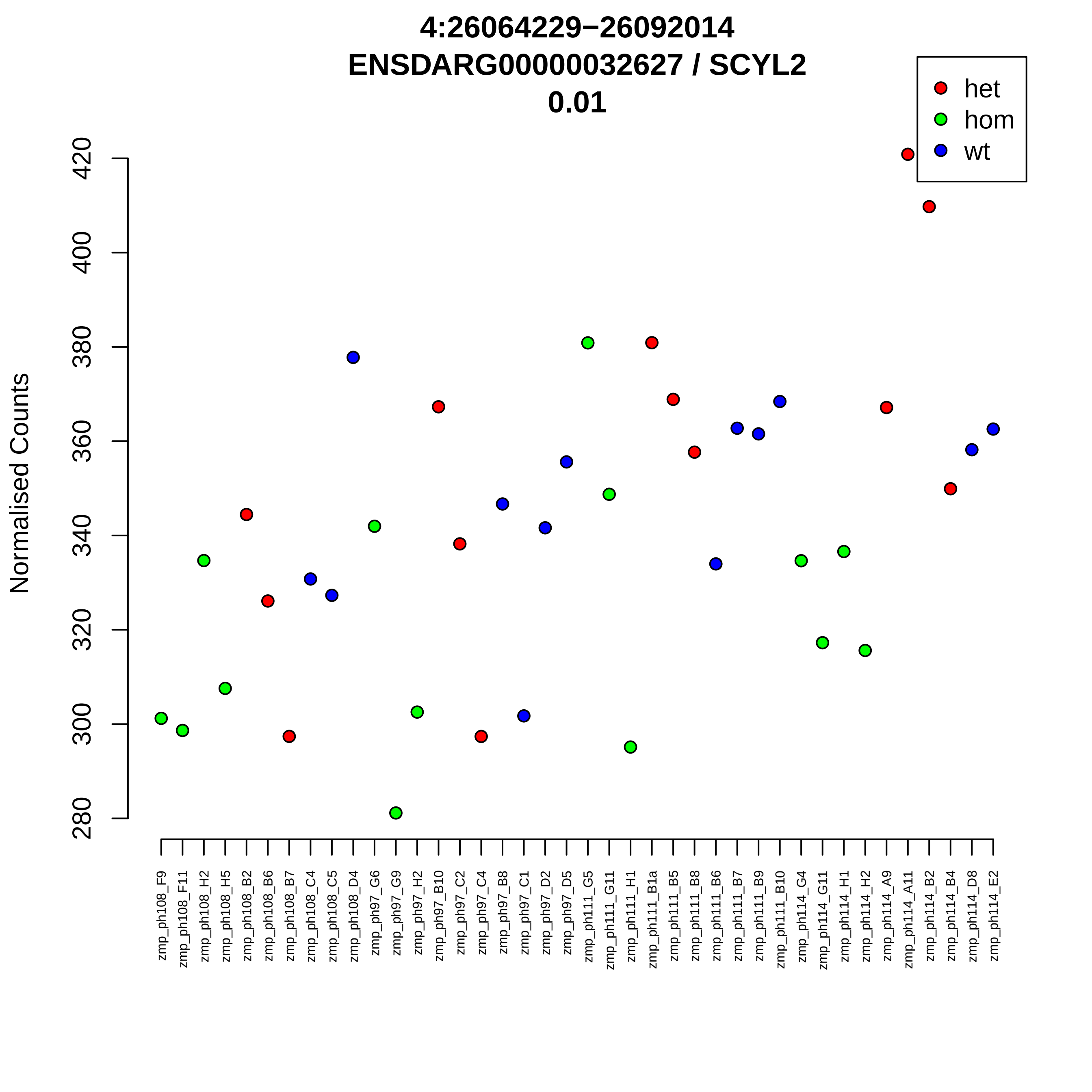

4:26064229−26092014
ENSDARG00000032627 / SCYL2
0.01
het
hom
wt
Normalised Counts
280
300
320
340
360
380
400
420
zmp_ph108_F9
zmp_ph108_F11
zmp_ph108_H2
zmp_ph108_H5
zmp_ph108_B2
zmp_ph108_B6
zmp_ph108_B7
zmp_ph108_C4
zmp_ph108_C5
zmp_ph108_D4
zmp_ph97_G6
zmp_ph97_G9
zmp_ph97_H2
zmp_ph97_B10
zmp_ph97_C2
zmp_ph97_C4
zmp_ph97_B8
zmp_ph97_C1
zmp_ph97_D2
zmp_ph97_D5
zmp_ph111_G5
zmp_ph111_G11
zmp_ph111_H1
zmp_ph111_B1a
zmp_ph111_B5
zmp_ph111_B8
zmp_ph111_B6
zmp_ph111_B7
zmp_ph111_B9
zmp_ph111_B10
zmp_ph114_G4
zmp_ph114_G11
zmp_ph114_H1
zmp_ph114_H2
zmp_ph114_A9
zmp_ph114_A11
zmp_ph114_B2
zmp_ph114_B4
zmp_ph114_D8
zmp_ph114_E2

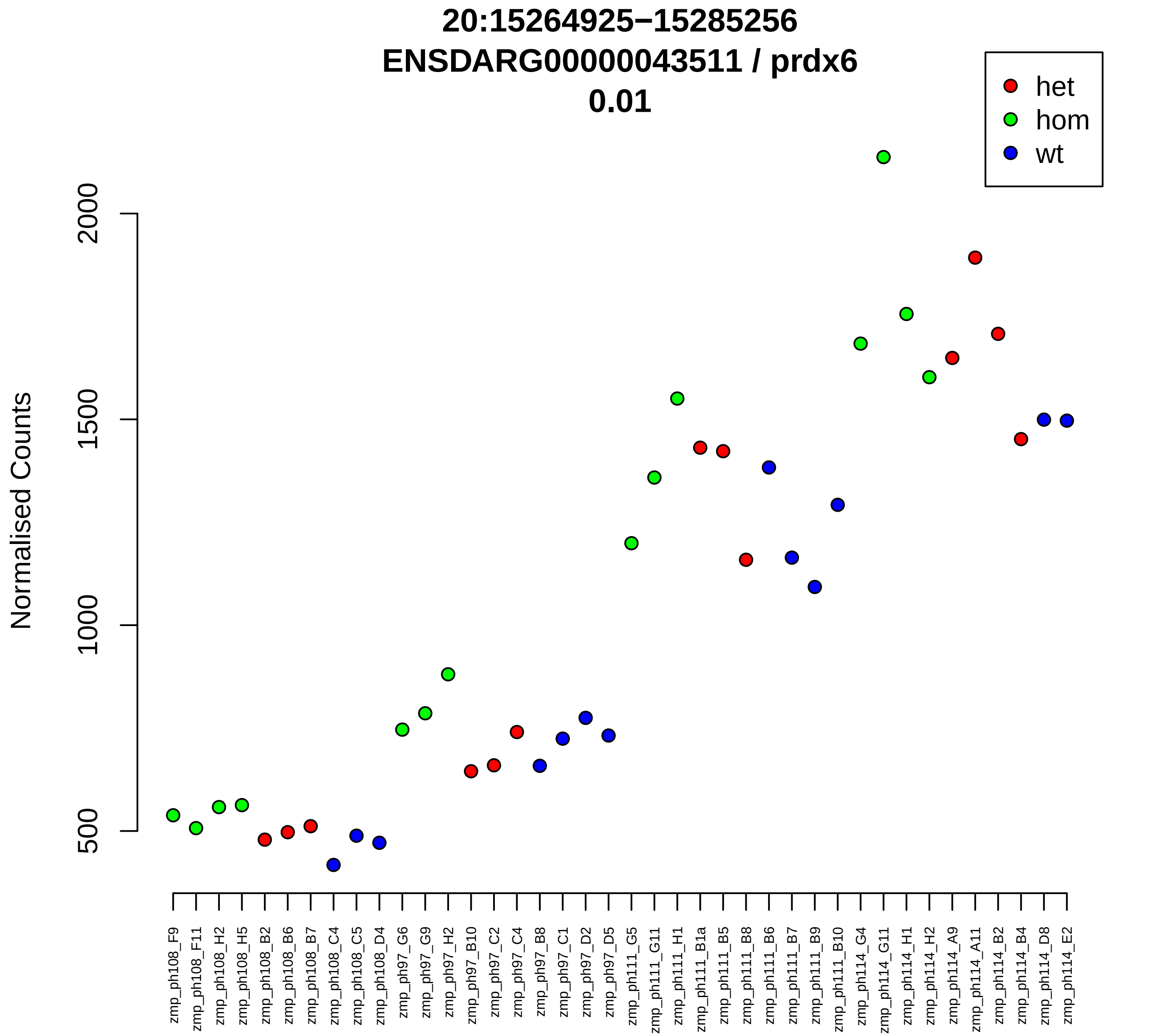

20:15264925−15285256
ENSDARG00000043511 / prdx6
0.01
het
hom
wt
Normalised Counts
500
1000
1500
2000
zmp_ph108_F9
zmp_ph108_F11
zmp_ph108_H2
zmp_ph108_H5
zmp_ph108_B2
zmp_ph108_B6
zmp_ph108_B7
zmp_ph108_C4
zmp_ph108_C5
zmp_ph108_D4
zmp_ph97_G6
zmp_ph97_G9
zmp_ph97_H2
zmp_ph97_B10
zmp_ph97_C2
zmp_ph97_C4
zmp_ph97_B8
zmp_ph97_C1
zmp_ph97_D2
zmp_ph97_D5
zmp_ph111_G5
zmp_ph111_G11
zmp_ph111_H1
zmp_ph111_B1a
zmp_ph111_B5
zmp_ph111_B8
zmp_ph111_B6
zmp_ph111_B7
zmp_ph111_B9
zmp_ph111_B10
zmp_ph114_G4
zmp_ph114_G11
zmp_ph114_H1
zmp_ph114_H2
zmp_ph114_A9
zmp_ph114_A11
zmp_ph114_B2
zmp_ph114_B4
zmp_ph114_D8
zmp_ph114_E2

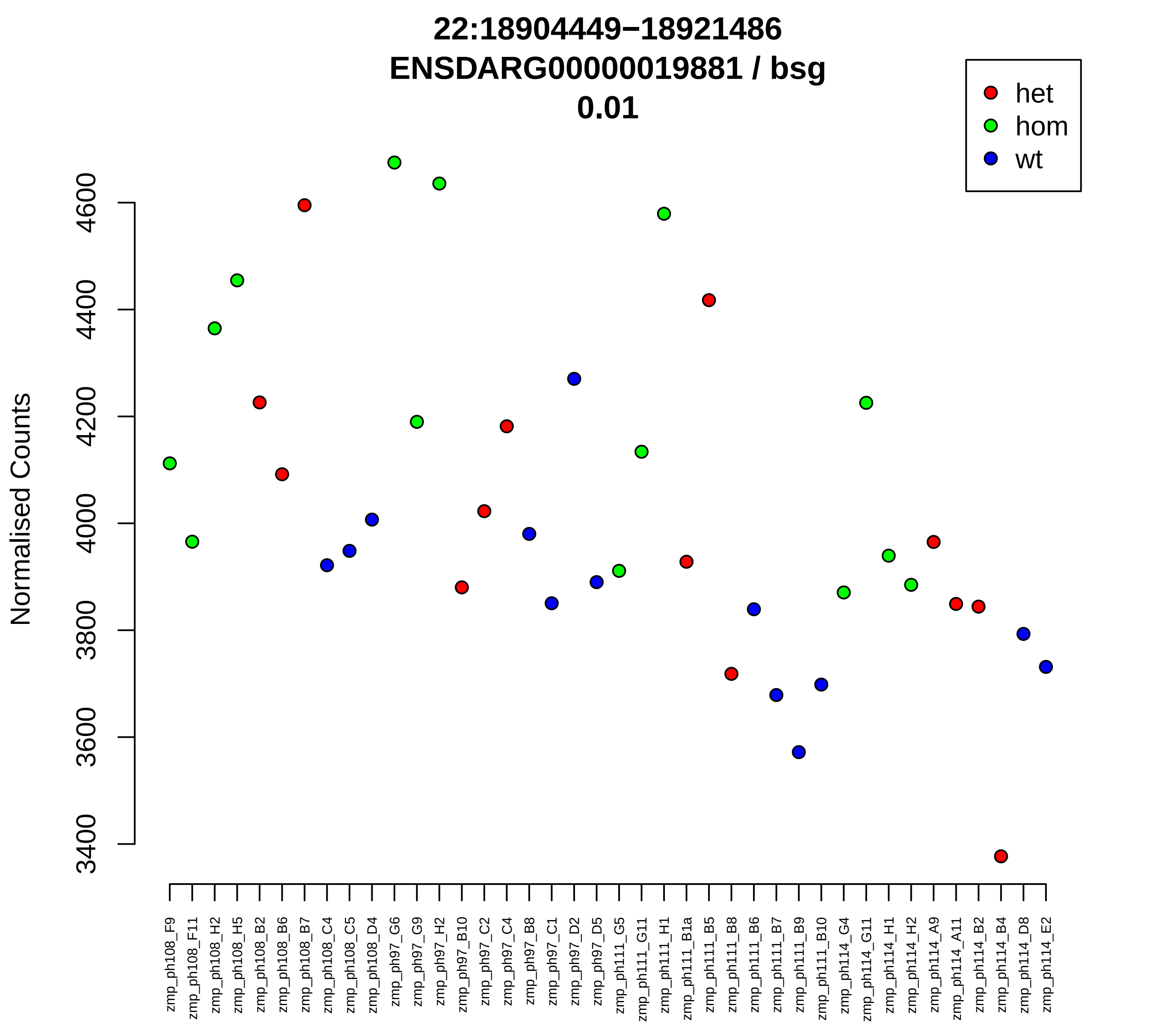

22:18904449−18921486
ENSDARG00000019881 / bsg
0.01
het
hom
wt
Normalised Counts
3400
3600
3800
4000
4200
4400
4600
zmp_ph108_F9
zmp_ph108_F11
zmp_ph108_H2
zmp_ph108_H5
zmp_ph108_B2
zmp_ph108_B6
zmp_ph108_B7
zmp_ph108_C4
zmp_ph108_C5
zmp_ph108_D4
zmp_ph97_G6
zmp_ph97_G9
zmp_ph97_H2
zmp_ph97_B10
zmp_ph97_C2
zmp_ph97_C4
zmp_ph97_B8
zmp_ph97_C1
zmp_ph97_D2
zmp_ph97_D5
zmp_ph111_G5
zmp_ph111_G11
zmp_ph111_H1
zmp_ph111_B1a
zmp_ph111_B5
zmp_ph111_B8
zmp_ph111_B6
zmp_ph111_B7
zmp_ph111_B9
zmp_ph111_B10
zmp_ph114_G4
zmp_ph114_G11
zmp_ph114_H1
zmp_ph114_H2
zmp_ph114_A9
zmp_ph114_A11
zmp_ph114_B2
zmp_ph114_B4
zmp_ph114_D8
zmp_ph114_E2

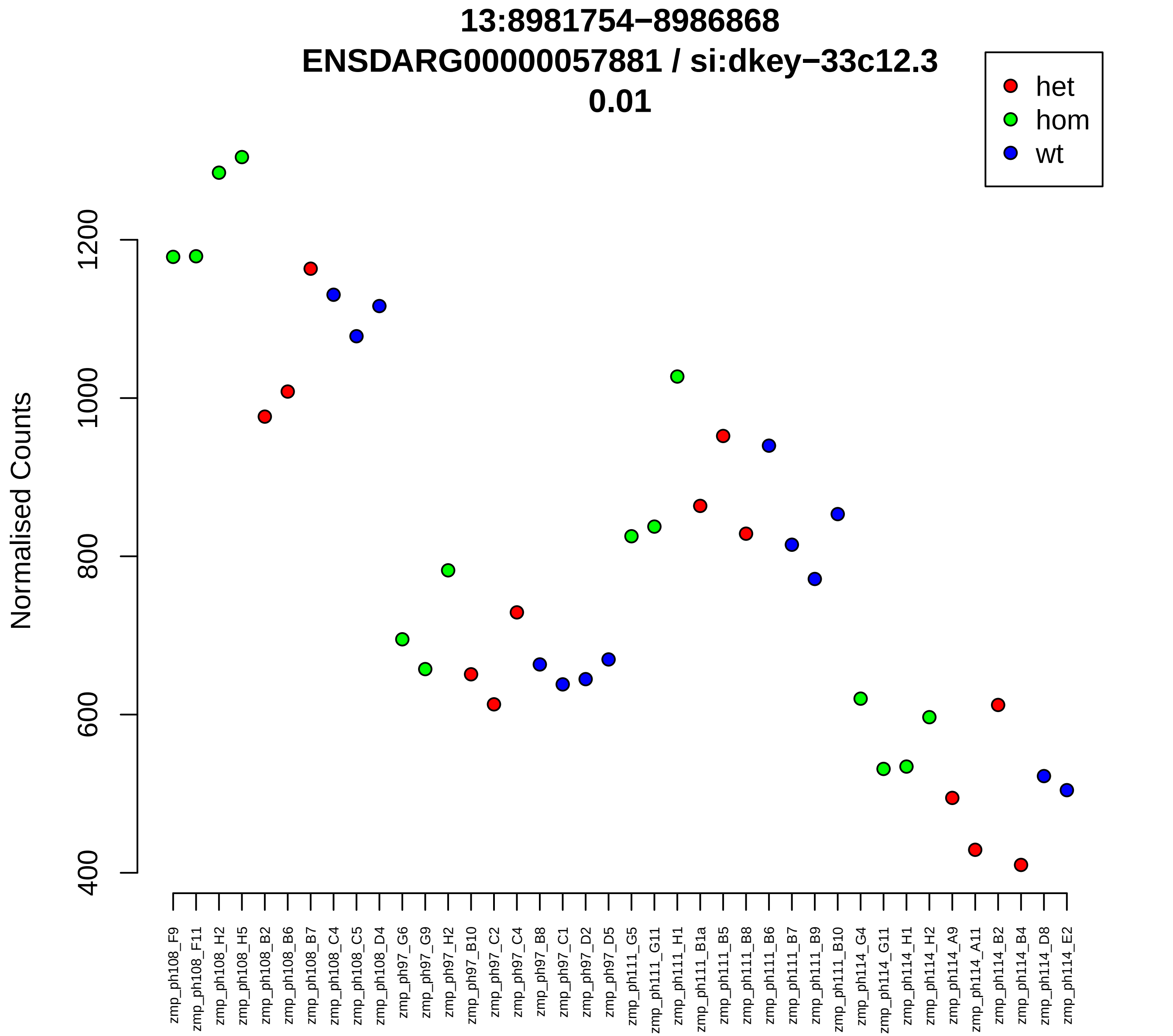
13:8981754−8986868
ENSDARG00000057881 / si:dkey−33c12.3
0.01
het
hom
wt
Normalised Counts
400
600
800
1000
1200
zmp_ph108_F9
zmp_ph108_F11
zmp_ph108_H2
zmp_ph108_H5
zmp_ph108_B2
zmp_ph108_B6
zmp_ph108_B7
zmp_ph108_C4
zmp_ph108_C5
zmp_ph108_D4
zmp_ph97_G6
zmp_ph97_G9
zmp_ph97_H2
zmp_ph97_B10
zmp_ph97_C2
zmp_ph97_C4
zmp_ph97_B8
zmp_ph97_C1
zmp_ph97_D2
zmp_ph97_D5
zmp_ph111_G5
zmp_ph111_G11
zmp_ph111_H1
zmp_ph111_B1a
zmp_ph111_B5
zmp_ph111_B8
zmp_ph111_B6
zmp_ph111_B7
zmp_ph111_B9
zmp_ph111_B10
zmp_ph114_G4
zmp_ph114_G11
zmp_ph114_H1
zmp_ph114_H2
zmp_ph114_A9
zmp_ph114_A11
zmp_ph114_B2
zmp_ph114_B4
zmp_ph114_D8
zmp_ph114_E2

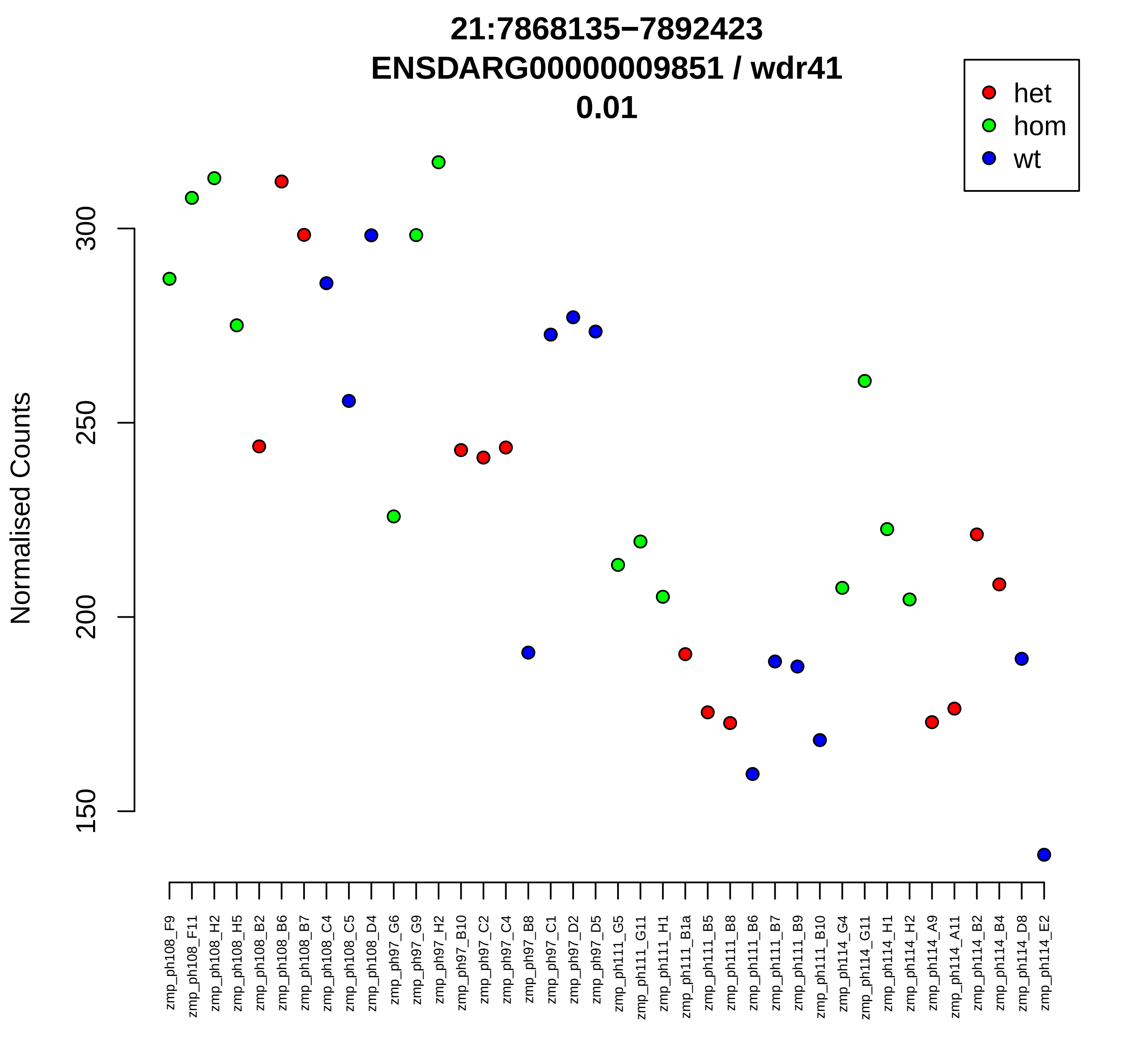
21:7868135−7892423
ENSDARG00000009851 / wdr41
0.01
het
hom
wt
Normalised Counts
150
200
250
300
zmp_ph108_F9
zmp_ph108_F11
zmp_ph108_H2
zmp_ph108_H5
zmp_ph108_B2
zmp_ph108_B6
zmp_ph108_B7
zmp_ph108_C4
zmp_ph108_C5
zmp_ph108_D4
zmp_ph97_G6
zmp_ph97_G9
zmp_ph97_H2
zmp_ph97_B10
zmp_ph97_C2
zmp_ph97_C4
zmp_ph97_B8
zmp_ph97_C1
zmp_ph97_D2
zmp_ph97_D5
zmp_ph111_G5
zmp_ph111_G11
zmp_ph111_H1
zmp_ph111_B1a
zmp_ph111_B5
zmp_ph111_B8
zmp_ph111_B6
zmp_ph111_B7
zmp_ph111_B9
zmp_ph111_B10
zmp_ph114_G4
zmp_ph114_G11
zmp_ph114_H1
zmp_ph114_H2
zmp_ph114_A9
zmp_ph114_A11
zmp_ph114_B2
zmp_ph114_B4
zmp_ph114_D8
zmp_ph114_E2

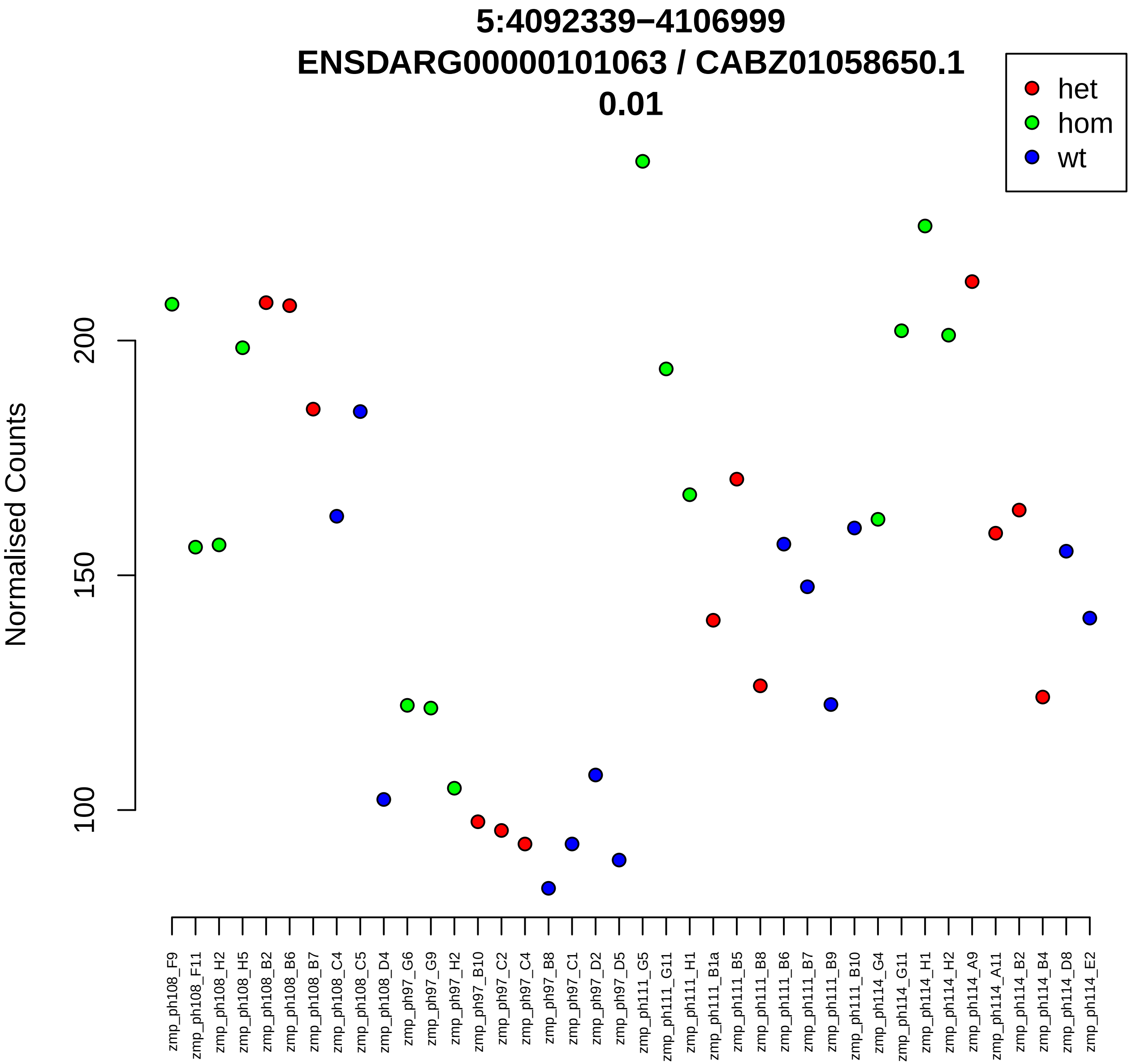

5:4092339−4106999
ENSDARG00000101063 / CABZ01058650.1
0.01
het
hom
wt
Normalised Counts
100
150
200
zmp_ph108_F9
zmp_ph108_F11
zmp_ph108_H2
zmp_ph108_H5
zmp_ph108_B2
zmp_ph108_B6
zmp_ph108_B7
zmp_ph108_C4
zmp_ph108_C5
zmp_ph108_D4
zmp_ph97_G6
zmp_ph97_G9
zmp_ph97_H2
zmp_ph97_B10
zmp_ph97_C2
zmp_ph97_C4
zmp_ph97_B8
zmp_ph97_C1
zmp_ph97_D2
zmp_ph97_D5
zmp_ph111_G5
zmp_ph111_G11
zmp_ph111_H1
zmp_ph111_B1a
zmp_ph111_B5
zmp_ph111_B8
zmp_ph111_B6
zmp_ph111_B7
zmp_ph111_B9
zmp_ph111_B10
zmp_ph114_G4
zmp_ph114_G11
zmp_ph114_H1
zmp_ph114_H2
zmp_ph114_A9
zmp_ph114_A11
zmp_ph114_B2
zmp_ph114_B4
zmp_ph114_D8
zmp_ph114_E2

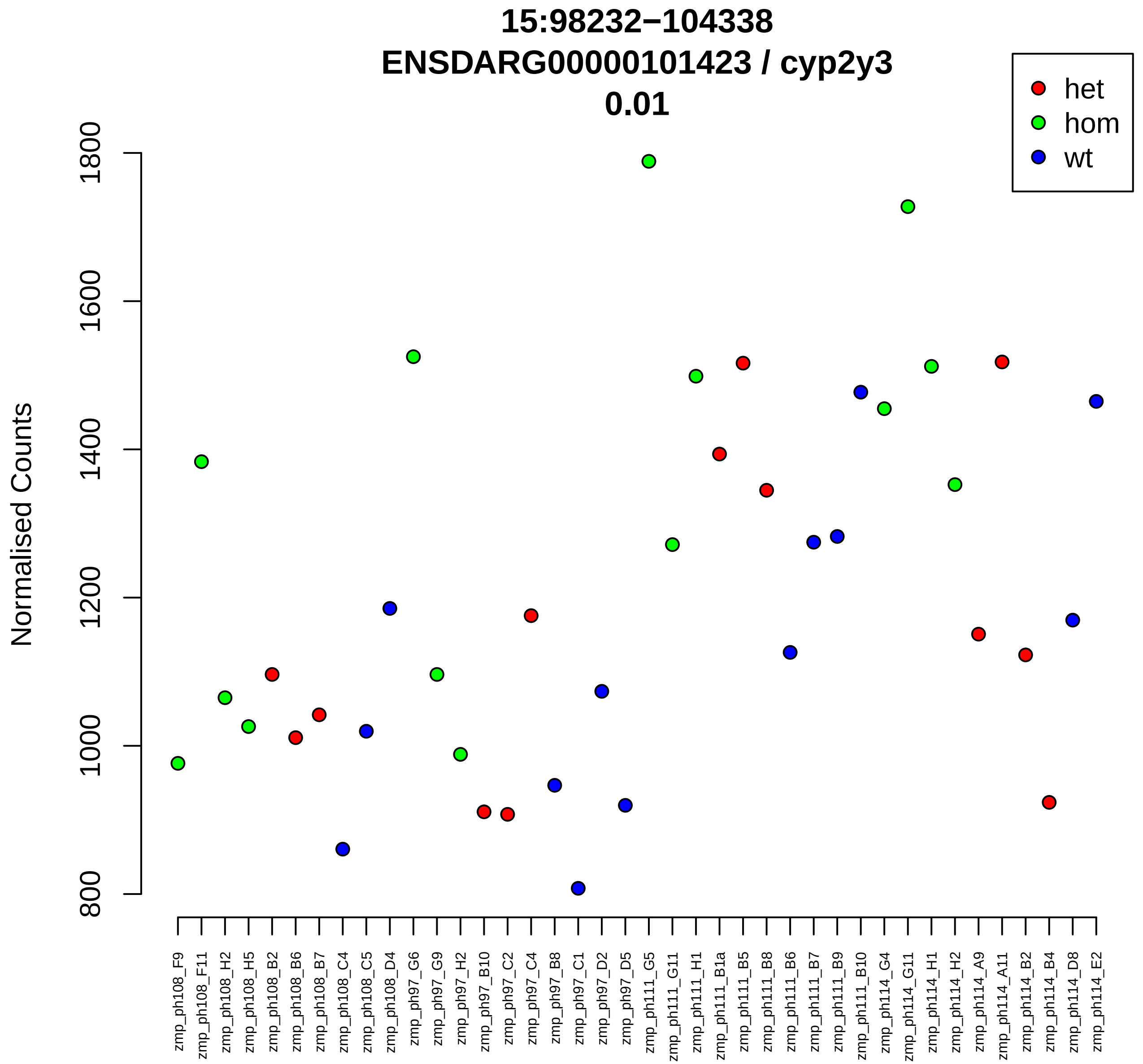

15:98232−104338
ENSDARG00000101423 / cyp2y3
0.01
het
hom
wt
Normalised Counts
800
1000
1200
1400
1600
1800
zmp_ph108_F9
zmp_ph108_F11
zmp_ph108_H2
zmp_ph108_H5
zmp_ph108_B2
zmp_ph108_B6
zmp_ph108_B7
zmp_ph108_C4
zmp_ph108_C5
zmp_ph108_D4
zmp_ph97_G6
zmp_ph97_G9
zmp_ph97_H2
zmp_ph97_B10
zmp_ph97_C2
zmp_ph97_C4
zmp_ph97_B8
zmp_ph97_C1
zmp_ph97_D2
zmp_ph97_D5
zmp_ph111_G5
zmp_ph111_G11
zmp_ph111_H1
zmp_ph111_B1a
zmp_ph111_B5
zmp_ph111_B8
zmp_ph111_B6
zmp_ph111_B7
zmp_ph111_B9
zmp_ph111_B10
zmp_ph114_G4
zmp_ph114_G11
zmp_ph114_H1
zmp_ph114_H2
zmp_ph114_A9
zmp_ph114_A11
zmp_ph114_B2
zmp_ph114_B4
zmp_ph114_D8
zmp_ph114_E2

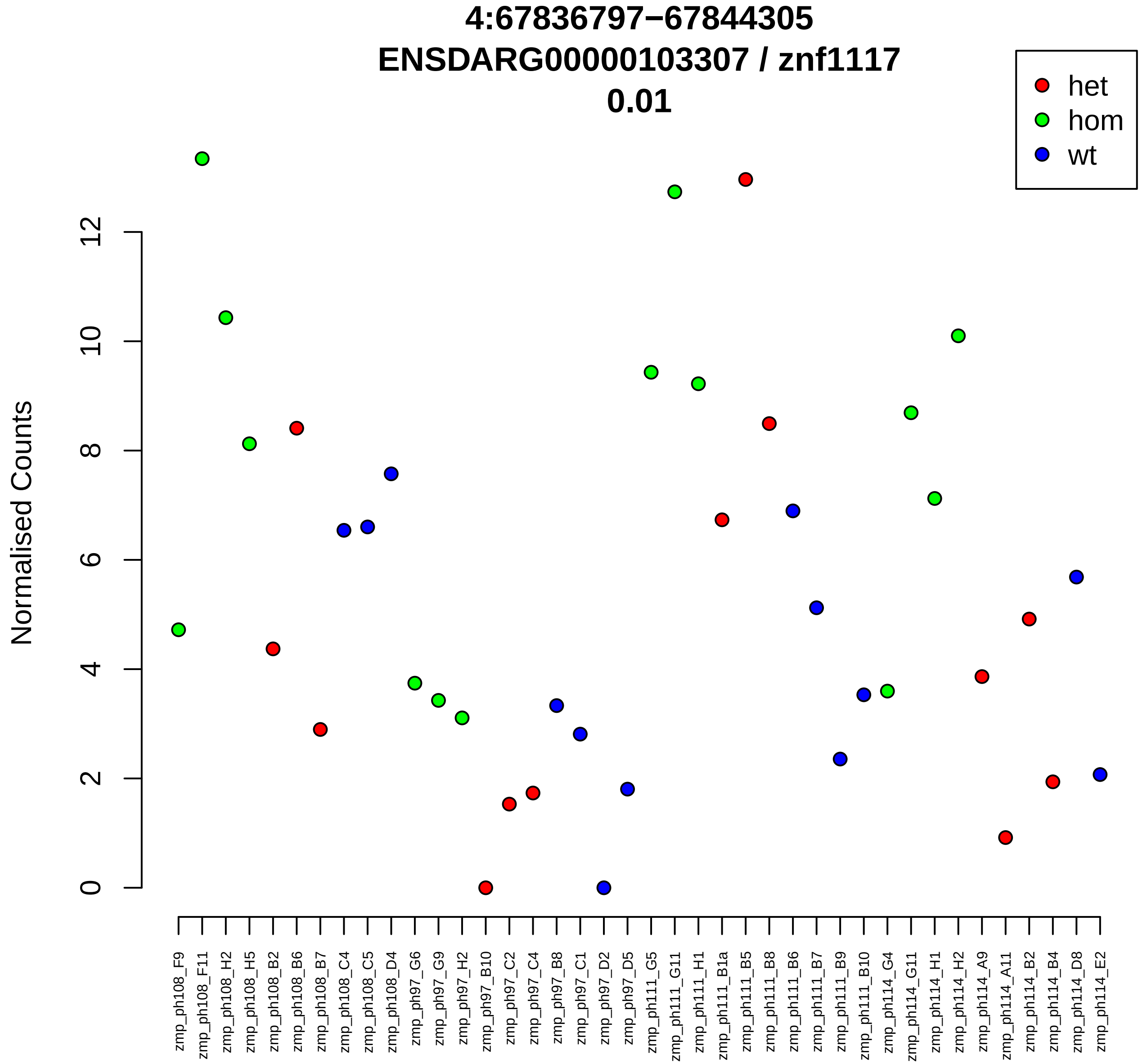
4:67836797−67844305
ENSDARG00000103307 / znf1117
0.01
het
hom
wt
Normalised Counts
0
2
4
6
8
10
12
zmp_ph108_F9
zmp_ph108_F11
zmp_ph108_H2
zmp_ph108_H5
zmp_ph108_B2
zmp_ph108_B6
zmp_ph108_B7
zmp_ph108_C4
zmp_ph108_C5
zmp_ph108_D4
zmp_ph97_G6
zmp_ph97_G9
zmp_ph97_H2
zmp_ph97_B10
zmp_ph97_C2
zmp_ph97_C4
zmp_ph97_B8
zmp_ph97_C1
zmp_ph97_D2
zmp_ph97_D5
zmp_ph111_G5
zmp_ph111_G11
zmp_ph111_H1
zmp_ph111_B1a
zmp_ph111_B5
zmp_ph111_B8
zmp_ph111_B6
zmp_ph111_B7
zmp_ph111_B9
zmp_ph111_B10
zmp_ph114_G4
zmp_ph114_G11
zmp_ph114_H1
zmp_ph114_H2
zmp_ph114_A9
zmp_ph114_A11
zmp_ph114_B2
zmp_ph114_B4
zmp_ph114_D8
zmp_ph114_E2

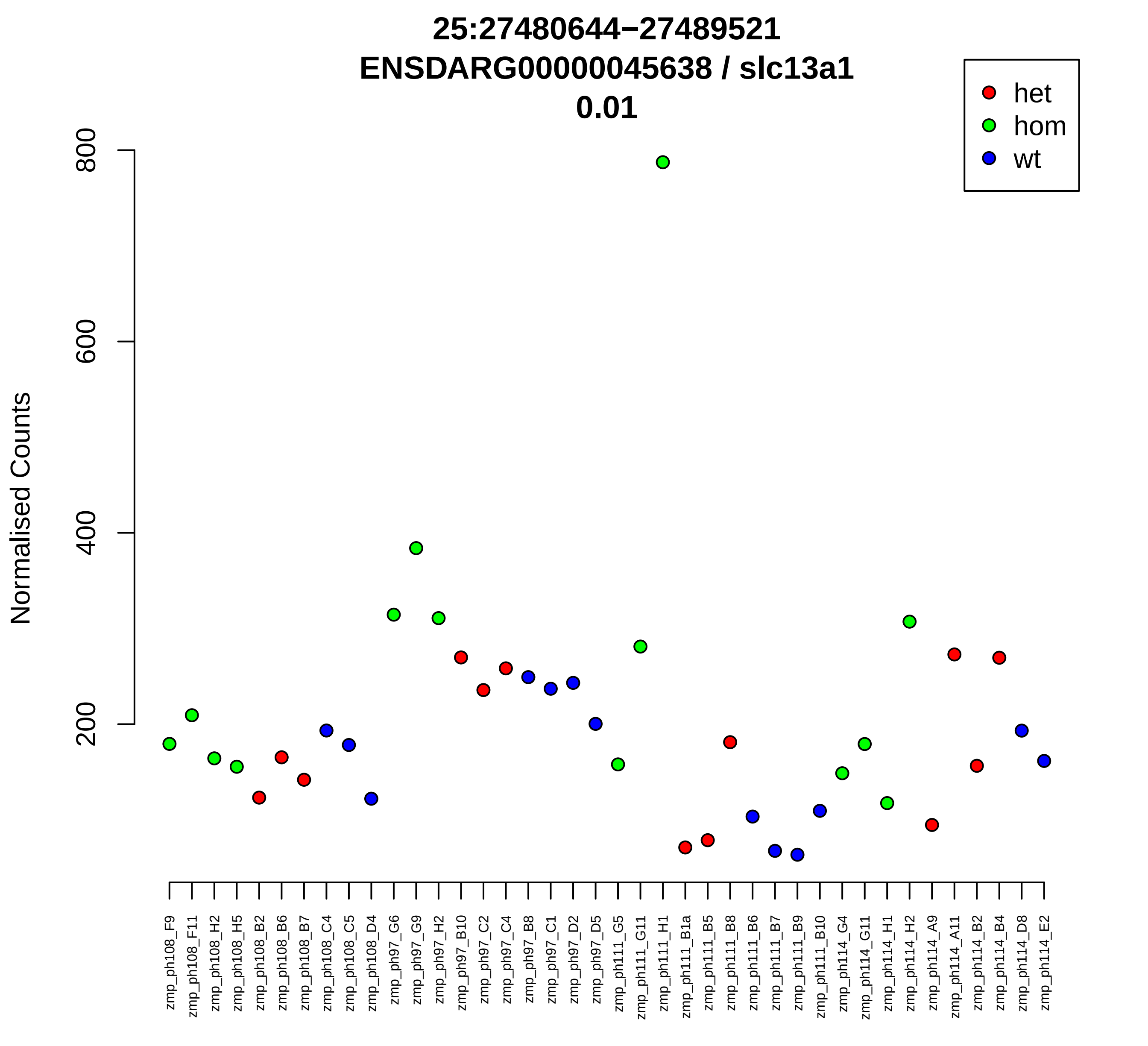
25:27480644−27489521
ENSDARG00000045638 / slc13a1
0.01
het
hom
wt
Normalised Counts
800
600
400
200
zmp_ph108_F9
zmp_ph108_F11
zmp_ph108_H2
zmp_ph108_H5
zmp_ph108_B2
zmp_ph108_B6
zmp_ph108_B7
zmp_ph108_C4
zmp_ph108_C5
zmp_ph108_D4
zmp_ph97_G6
zmp_ph97_G9
zmp_ph97_H2
zmp_ph97_B10
zmp_ph97_C2
zmp_ph97_C4
zmp_ph97_B8
zmp_ph97_C1
zmp_ph97_D2
zmp_ph97_D5
zmp_ph111_G5
zmp_ph111_G11
zmp_ph111_H1
zmp_ph111_B1a
zmp_ph111_B5
zmp_ph111_B8
zmp_ph111_B6
zmp_ph111_B7
zmp_ph111_B9
zmp_ph111_B10
zmp_ph114_G4
zmp_ph114_G11
zmp_ph114_H1
zmp_ph114_H2
zmp_ph114_A9
zmp_ph114_A11
zmp_ph114_B2
zmp_ph114_B4
zmp_ph114_D8
zmp_ph114_E2

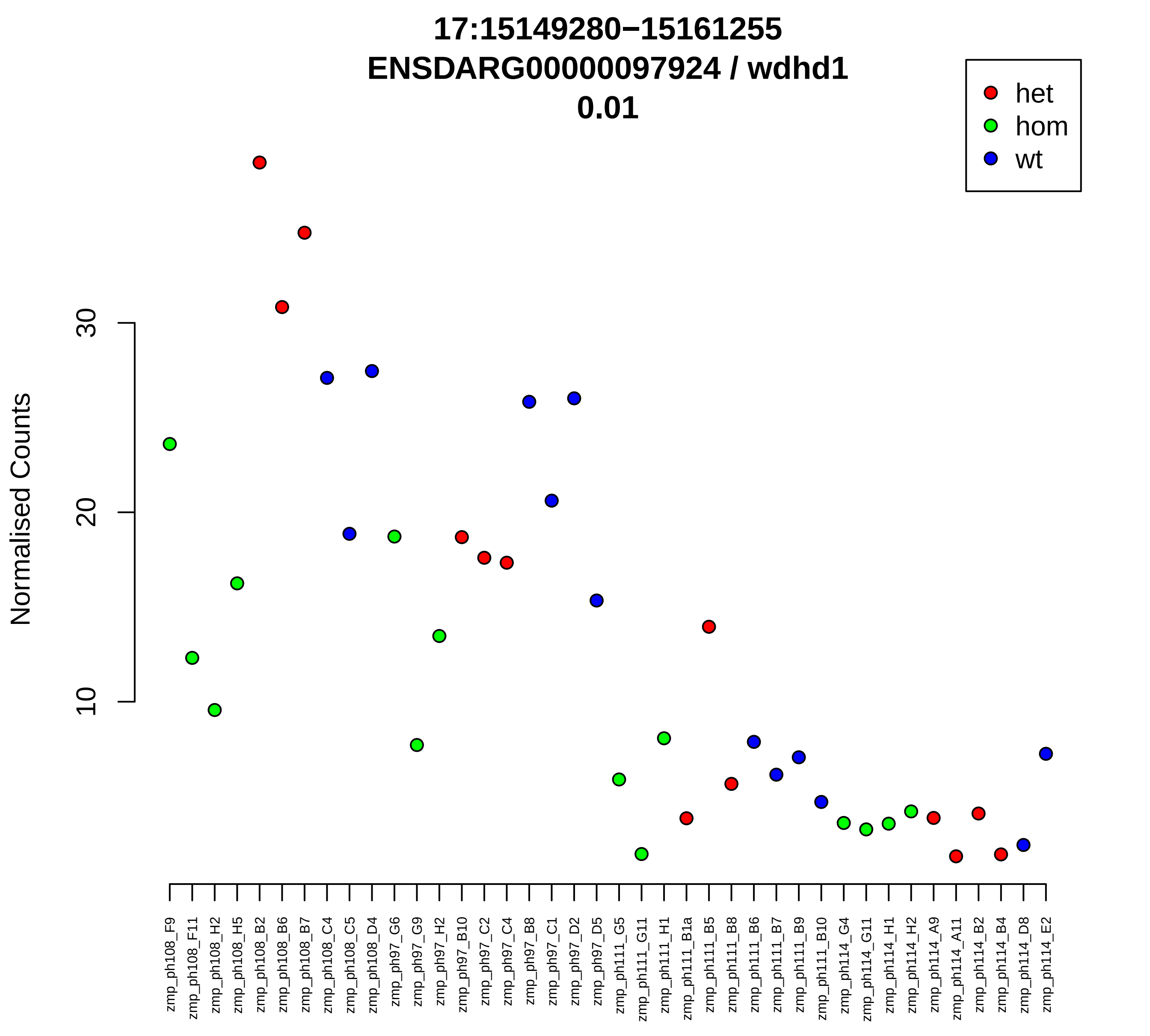

17:15149280−15161255
ENSDARG00000097924 / wdhd1
0.01
het
hom
wt
Normalised Counts
10
20
30
zmp_ph108_F9
zmp_ph108_F11
zmp_ph108_H2
zmp_ph108_H5
zmp_ph108_B2
zmp_ph108_B6
zmp_ph108_B7
zmp_ph108_C4
zmp_ph108_C5
zmp_ph108_D4
zmp_ph97_G6
zmp_ph97_G9
zmp_ph97_H2
zmp_ph97_B10
zmp_ph97_C2
zmp_ph97_C4
zmp_ph97_B8
zmp_ph97_C1
zmp_ph97_D2
zmp_ph97_D5
zmp_ph111_G5
zmp_ph111_G11
zmp_ph111_H1
zmp_ph111_B1a
zmp_ph111_B5
zmp_ph111_B8
zmp_ph111_B6
zmp_ph111_B7
zmp_ph111_B9
zmp_ph111_B10
zmp_ph114_G4
zmp_ph114_G11
zmp_ph114_H1
zmp_ph114_H2
zmp_ph114_A9
zmp_ph114_A11
zmp_ph114_B2
zmp_ph114_B4
zmp_ph114_D8
zmp_ph114_E2

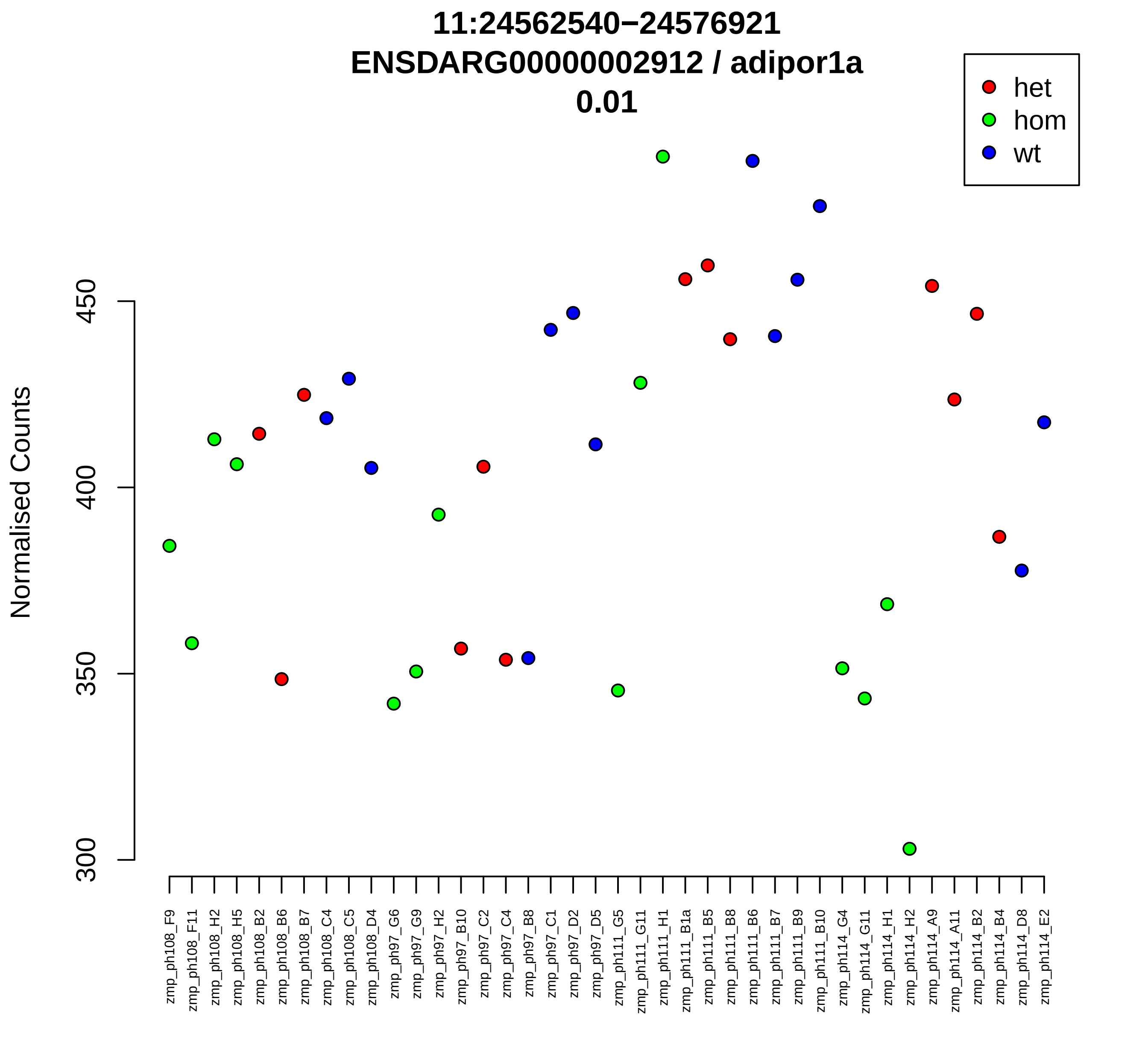
11:24562540−24576921
ENSDARG00000002912 / adipor1a
0.01
het
hom
wt
Normalised Counts
300
350
400
450
zmp_ph108_F9
zmp_ph108_F11
zmp_ph108_H2
zmp_ph108_H5
zmp_ph108_B2
zmp_ph108_B6
zmp_ph108_B7
zmp_ph108_C4
zmp_ph108_C5
zmp_ph108_D4
zmp_ph97_G6
zmp_ph97_G9
zmp_ph97_H2
zmp_ph97_B10
zmp_ph97_C2
zmp_ph97_C4
zmp_ph97_B8
zmp_ph97_C1
zmp_ph97_D2
zmp_ph97_D5
zmp_ph111_G5
zmp_ph111_G11
zmp_ph111_H1
zmp_ph111_B1a
zmp_ph111_B5
zmp_ph111_B8
zmp_ph111_B6
zmp_ph111_B7
zmp_ph111_B9
zmp_ph111_B10
zmp_ph114_G4
zmp_ph114_G11
zmp_ph114_H1
zmp_ph114_H2
zmp_ph114_A9
zmp_ph114_A11
zmp_ph114_B2
zmp_ph114_B4
zmp_ph114_D8
zmp_ph114_E2

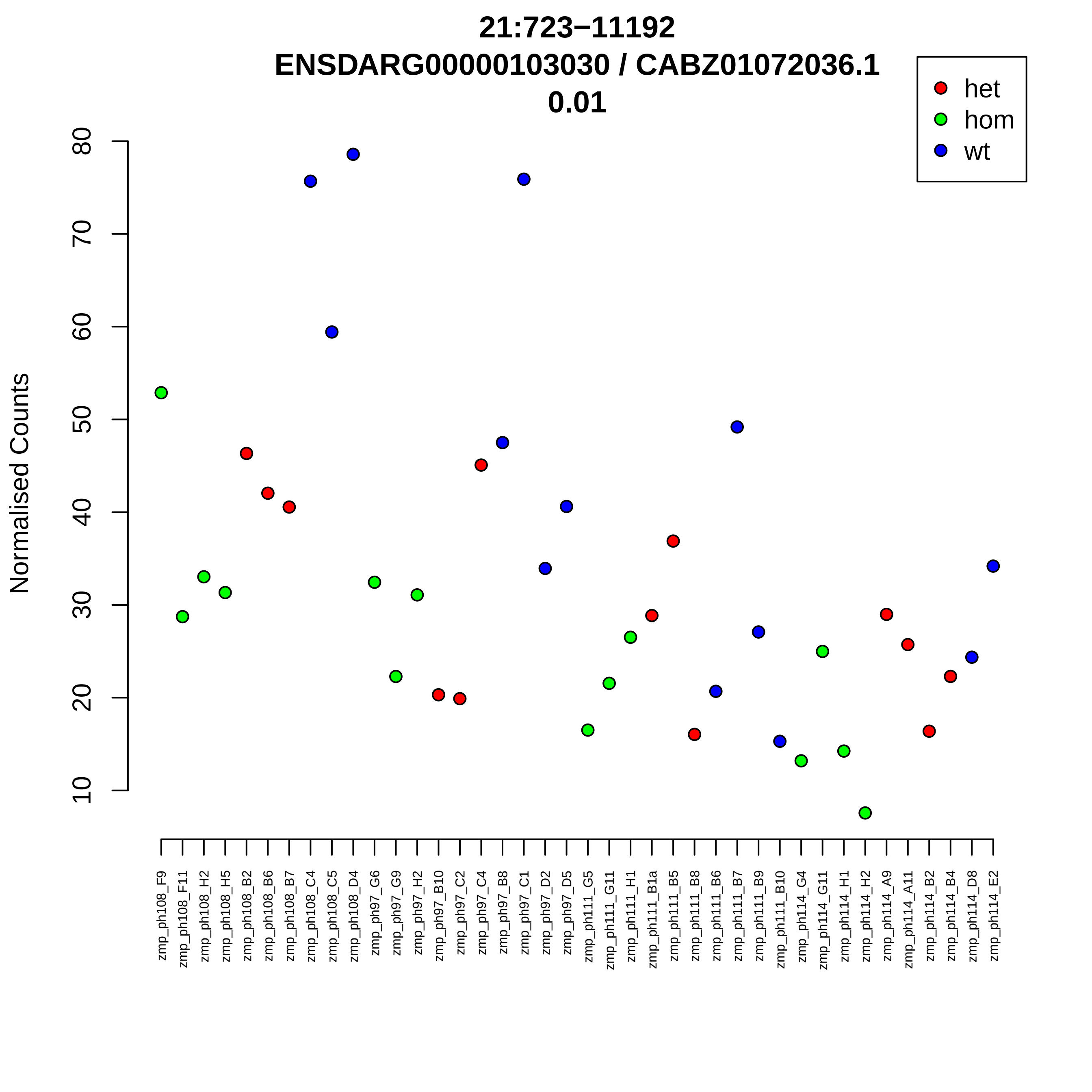

21:723−11192
ENSDARG00000103030 / CABZ01072036.1
0.01
het
hom
wt
Normalised Counts
10
20
30
40
50
60
70
80
zmp_ph108_F9
zmp_ph108_F11
zmp_ph108_H2
zmp_ph108_H5
zmp_ph108_B2
zmp_ph108_B6
zmp_ph108_B7
zmp_ph108_C4
zmp_ph108_C5
zmp_ph108_D4
zmp_ph97_G6
zmp_ph97_G9
zmp_ph97_H2
zmp_ph97_B10
zmp_ph97_C2
zmp_ph97_C4
zmp_ph97_B8
zmp_ph97_C1
zmp_ph97_D2
zmp_ph97_D5
zmp_ph111_G5
zmp_ph111_G11
zmp_ph111_H1
zmp_ph111_B1a
zmp_ph111_B5
zmp_ph111_B8
zmp_ph111_B6
zmp_ph111_B7
zmp_ph111_B9
zmp_ph111_B10
zmp_ph114_G4
zmp_ph114_G11
zmp_ph114_H1
zmp_ph114_H2
zmp_ph114_A9
zmp_ph114_A11
zmp_ph114_B2
zmp_ph114_B4
zmp_ph114_D8
zmp_ph114_E2

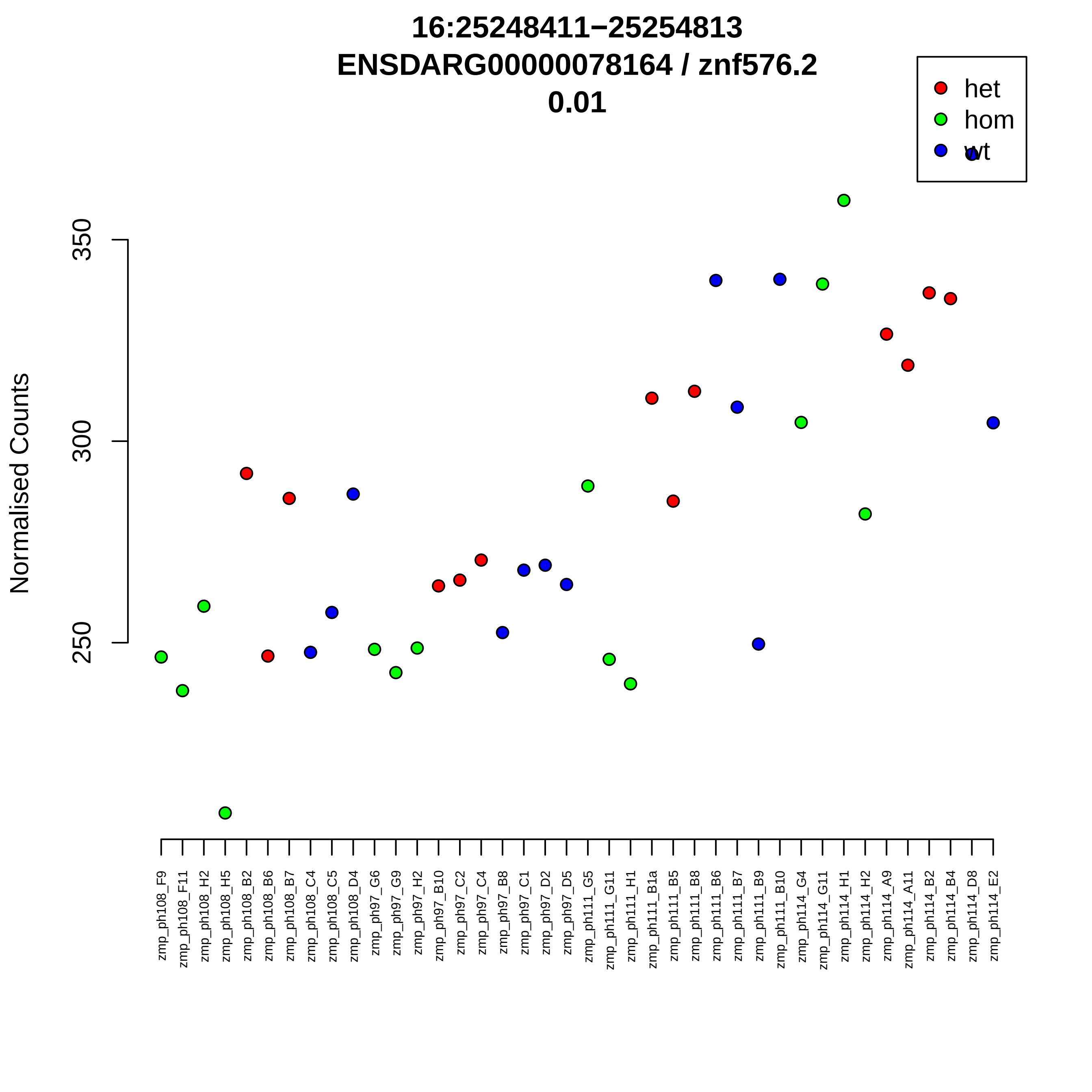

16:25248411−25254813
ENSDARG00000078164 / znf576.2
0.01
het
hom
wt
Normalised Counts
250
300
350
zmp_ph108_F9
zmp_ph108_F11
zmp_ph108_H2
zmp_ph108_H5
zmp_ph108_B2
zmp_ph108_B6
zmp_ph108_B7
zmp_ph108_C4
zmp_ph108_C5
zmp_ph108_D4
zmp_ph97_G6
zmp_ph97_G9
zmp_ph97_H2
zmp_ph97_B10
zmp_ph97_C2
zmp_ph97_C4
zmp_ph97_B8
zmp_ph97_C1
zmp_ph97_D2
zmp_ph97_D5
zmp_ph111_G5
zmp_ph111_G11
zmp_ph111_H1
zmp_ph111_B1a
zmp_ph111_B5
zmp_ph111_B8
zmp_ph111_B6
zmp_ph111_B7
zmp_ph111_B9
zmp_ph111_B10
zmp_ph114_G4
zmp_ph114_G11
zmp_ph114_H1
zmp_ph114_H2
zmp_ph114_A9
zmp_ph114_A11
zmp_ph114_B2
zmp_ph114_B4
zmp_ph114_D8
zmp_ph114_E2

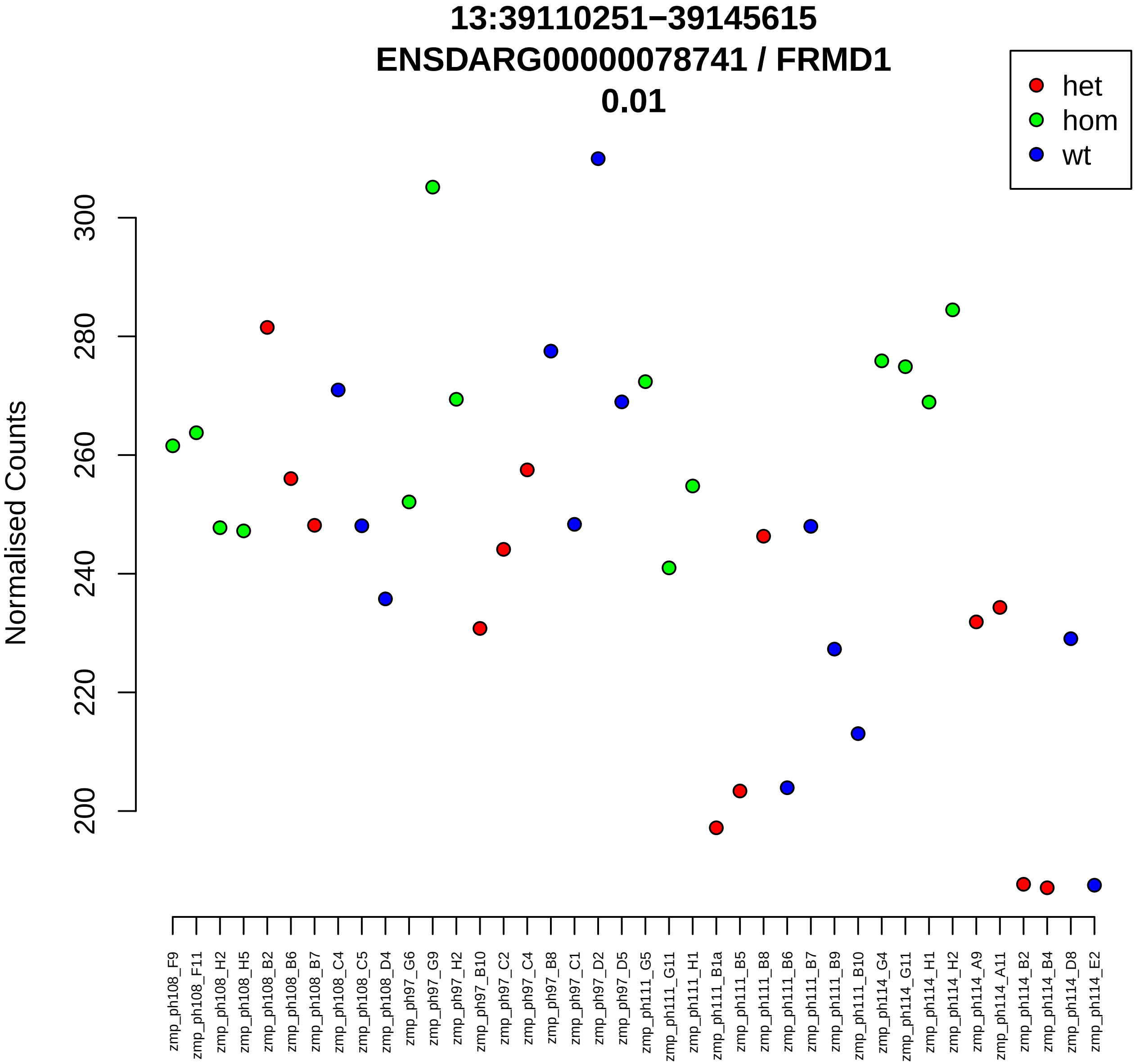

13:39110251−39145615
ENSDARG00000078741 / FRMD1
0.01
het
hom
wt
Normalised Counts
300
280
260
240
220
200
zmp_ph108_F9
zmp_ph108_F11
zmp_ph108_H2
zmp_ph108_H5
zmp_ph108_B2
zmp_ph108_B6
zmp_ph108_B7
zmp_ph108_C4
zmp_ph108_C5
zmp_ph108_D4
zmp_ph97_G6
zmp_ph97_G9
zmp_ph97_H2
zmp_ph97_B10
zmp_ph97_C2
zmp_ph97_C4
zmp_ph97_B8
zmp_ph97_C1
zmp_ph97_D2
zmp_ph97_D5
zmp_ph111_G5
zmp_ph111_G11
zmp_ph111_H1
zmp_ph111_B1a
zmp_ph111_B5
zmp_ph111_B8
zmp_ph111_B6
zmp_ph111_B7
zmp_ph111_B9
zmp_ph111_B10
zmp_ph114_G4
zmp_ph114_G11
zmp_ph114_H1
zmp_ph114_H2
zmp_ph114_A9
zmp_ph114_A11
zmp_ph114_B2
zmp_ph114_B4
zmp_ph114_D8
zmp_ph114_E2

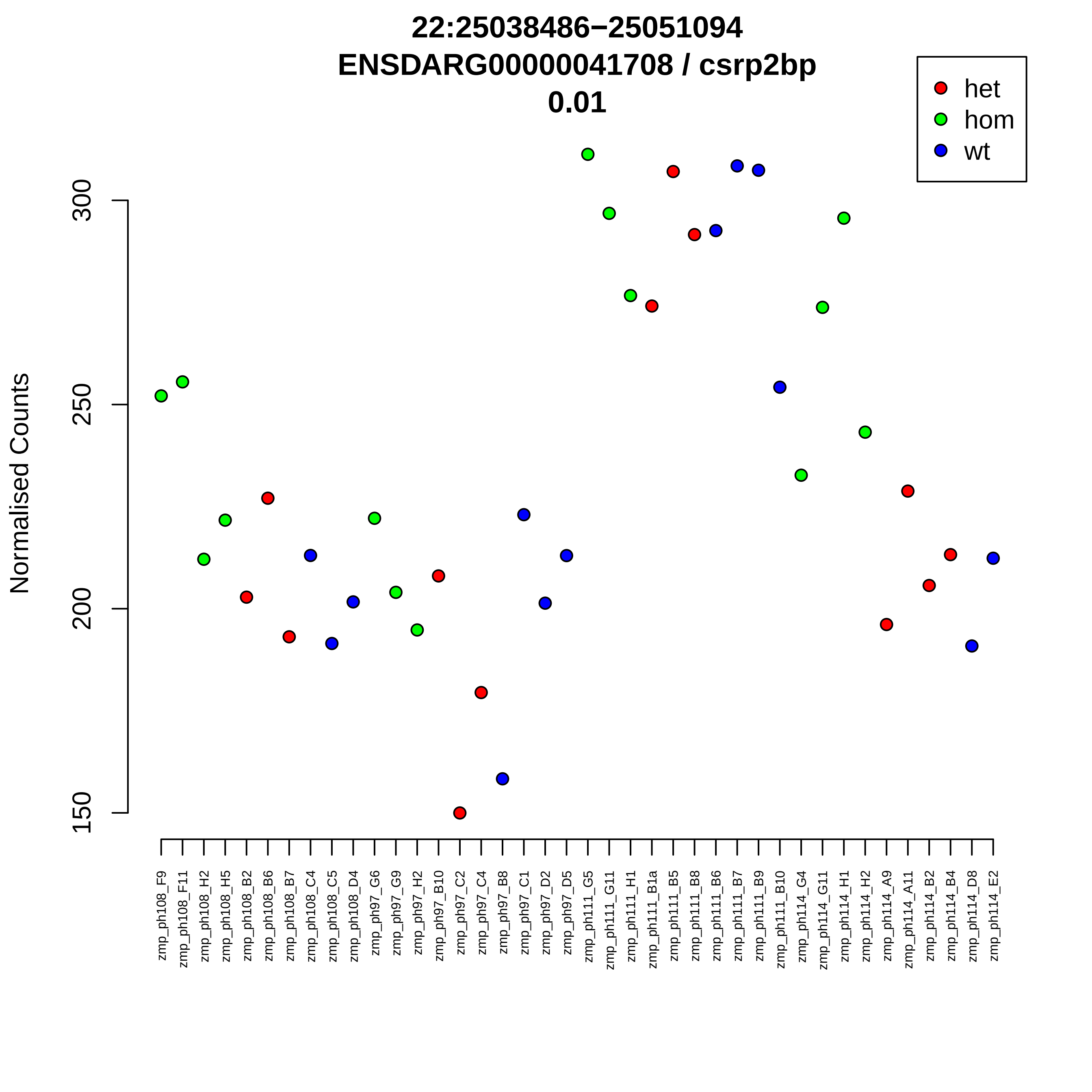

22:25038486−25051094
ENSDARG00000041708 / csrp2bp
0.01
het
hom
wt
Normalised Counts
300
250
200
150
zmp_ph108_F9
zmp_ph108_F11
zmp_ph108_H2
zmp_ph108_H5
zmp_ph108_B2
zmp_ph108_B6
zmp_ph108_B7
zmp_ph108_C4
zmp_ph108_C5
zmp_ph108_D4
zmp_ph97_G6
zmp_ph97_G9
zmp_ph97_H2
zmp_ph97_B10
zmp_ph97_C2
zmp_ph97_C4
zmp_ph97_B8
zmp_ph97_C1
zmp_ph97_D2
zmp_ph97_D5
zmp_ph111_G5
zmp_ph111_G11
zmp_ph111_H1
zmp_ph111_B1a
zmp_ph111_B5
zmp_ph111_B8
zmp_ph111_B6
zmp_ph111_B7
zmp_ph111_B9
zmp_ph111_B10
zmp_ph114_G4
zmp_ph114_G11
zmp_ph114_H1
zmp_ph114_H2
zmp_ph114_A9
zmp_ph114_A11
zmp_ph114_B2
zmp_ph114_B4
zmp_ph114_D8
zmp_ph114_E2

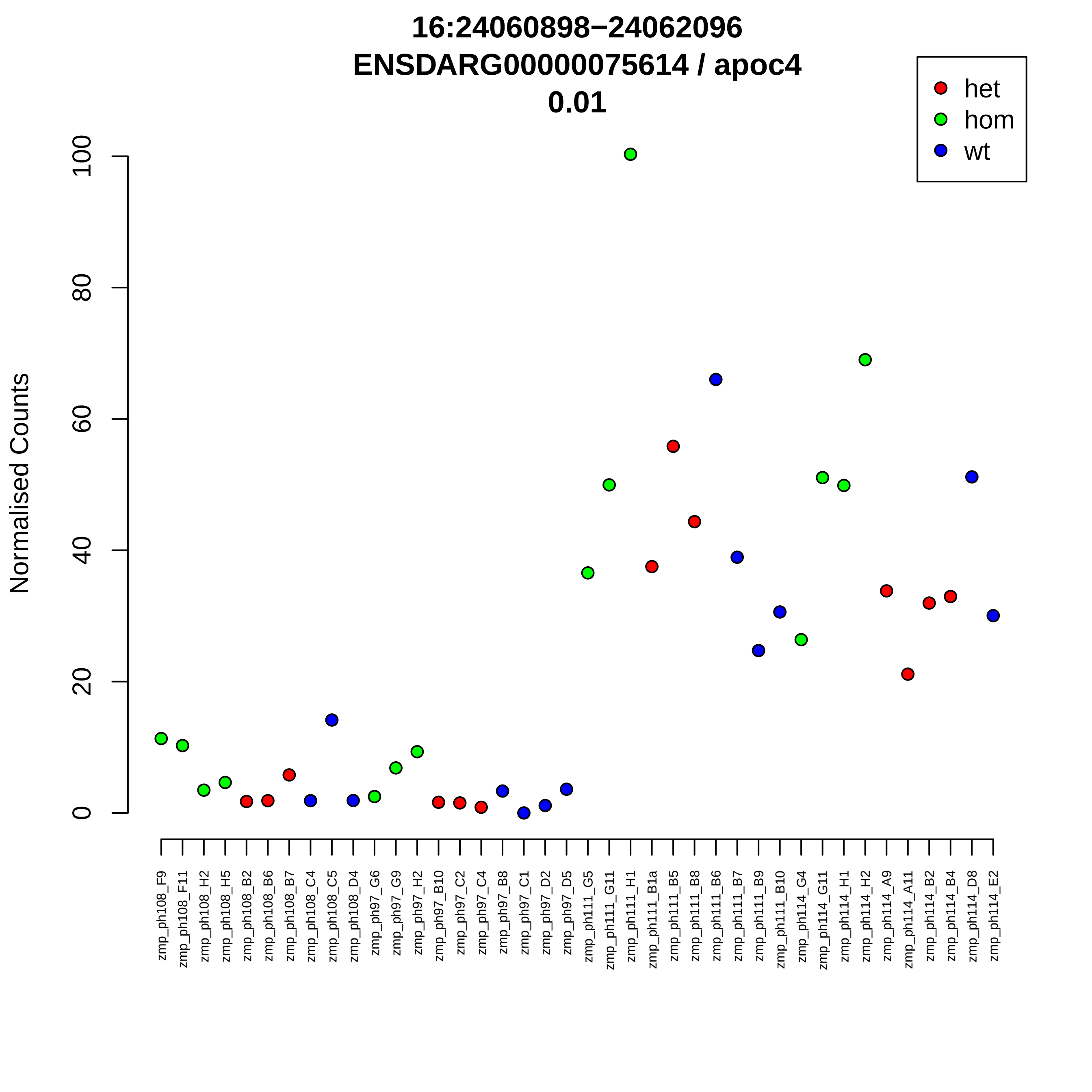

16:24060898−24062096
ENSDARG00000075614 / apoc4
0.01
het
hom
wt
Normalised Counts
0
20
40
60
80
100
zmp_ph108_F9
zmp_ph108_F11
zmp_ph108_H2
zmp_ph108_H5
zmp_ph108_B2
zmp_ph108_B6
zmp_ph108_B7
zmp_ph108_C4
zmp_ph108_C5
zmp_ph108_D4
zmp_ph97_G6
zmp_ph97_G9
zmp_ph97_H2
zmp_ph97_B10
zmp_ph97_C2
zmp_ph97_C4
zmp_ph97_B8
zmp_ph97_C1
zmp_ph97_D2
zmp_ph97_D5
zmp_ph111_G5
zmp_ph111_G11
zmp_ph111_H1
zmp_ph111_B1a
zmp_ph111_B5
zmp_ph111_B8
zmp_ph111_B6
zmp_ph111_B7
zmp_ph111_B9
zmp_ph111_B10
zmp_ph114_G4
zmp_ph114_G11
zmp_ph114_H1
zmp_ph114_H2
zmp_ph114_A9
zmp_ph114_A11
zmp_ph114_B2
zmp_ph114_B4
zmp_ph114_D8
zmp_ph114_E2

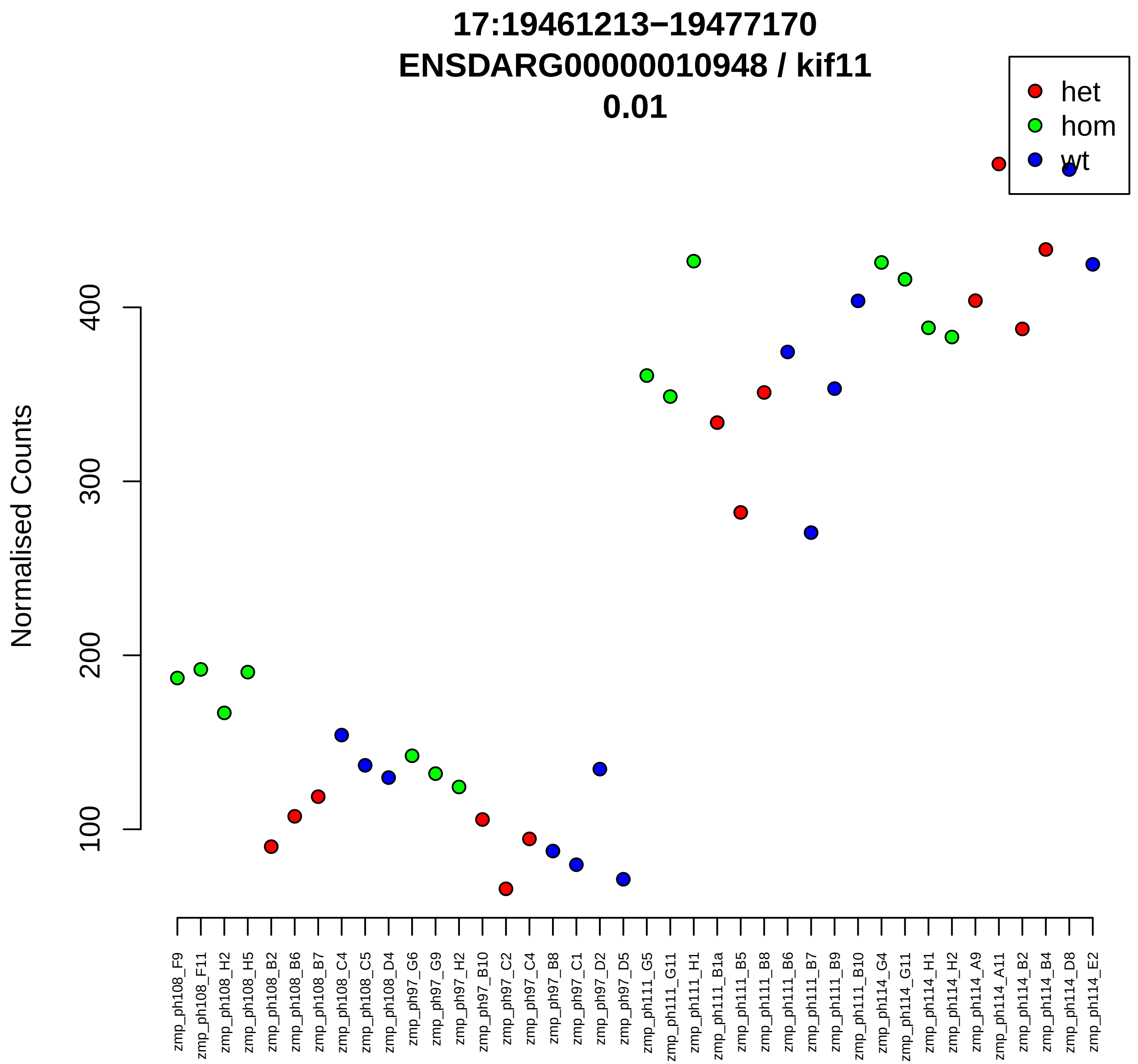
17:19461213−19477170
ENSDARG00000010948 / kif11
0.01
het
hom
wt
Normalised Counts
100
200
300
400
zmp_ph108_F9
zmp_ph108_F11
zmp_ph108_H2
zmp_ph108_H5
zmp_ph108_B2
zmp_ph108_B6
zmp_ph108_B7
zmp_ph108_C4
zmp_ph108_C5
zmp_ph108_D4
zmp_ph97_G6
zmp_ph97_G9
zmp_ph97_H2
zmp_ph97_B10
zmp_ph97_C2
zmp_ph97_C4
zmp_ph97_B8
zmp_ph97_C1
zmp_ph97_D2
zmp_ph97_D5
zmp_ph111_G5
zmp_ph111_G11
zmp_ph111_H1
zmp_ph111_B1a
zmp_ph111_B5
zmp_ph111_B8
zmp_ph111_B6
zmp_ph111_B7
zmp_ph111_B9
zmp_ph111_B10
zmp_ph114_G4
zmp_ph114_G11
zmp_ph114_H1
zmp_ph114_H2
zmp_ph114_A9
zmp_ph114_A11
zmp_ph114_B2
zmp_ph114_B4
zmp_ph114_D8
zmp_ph114_E2

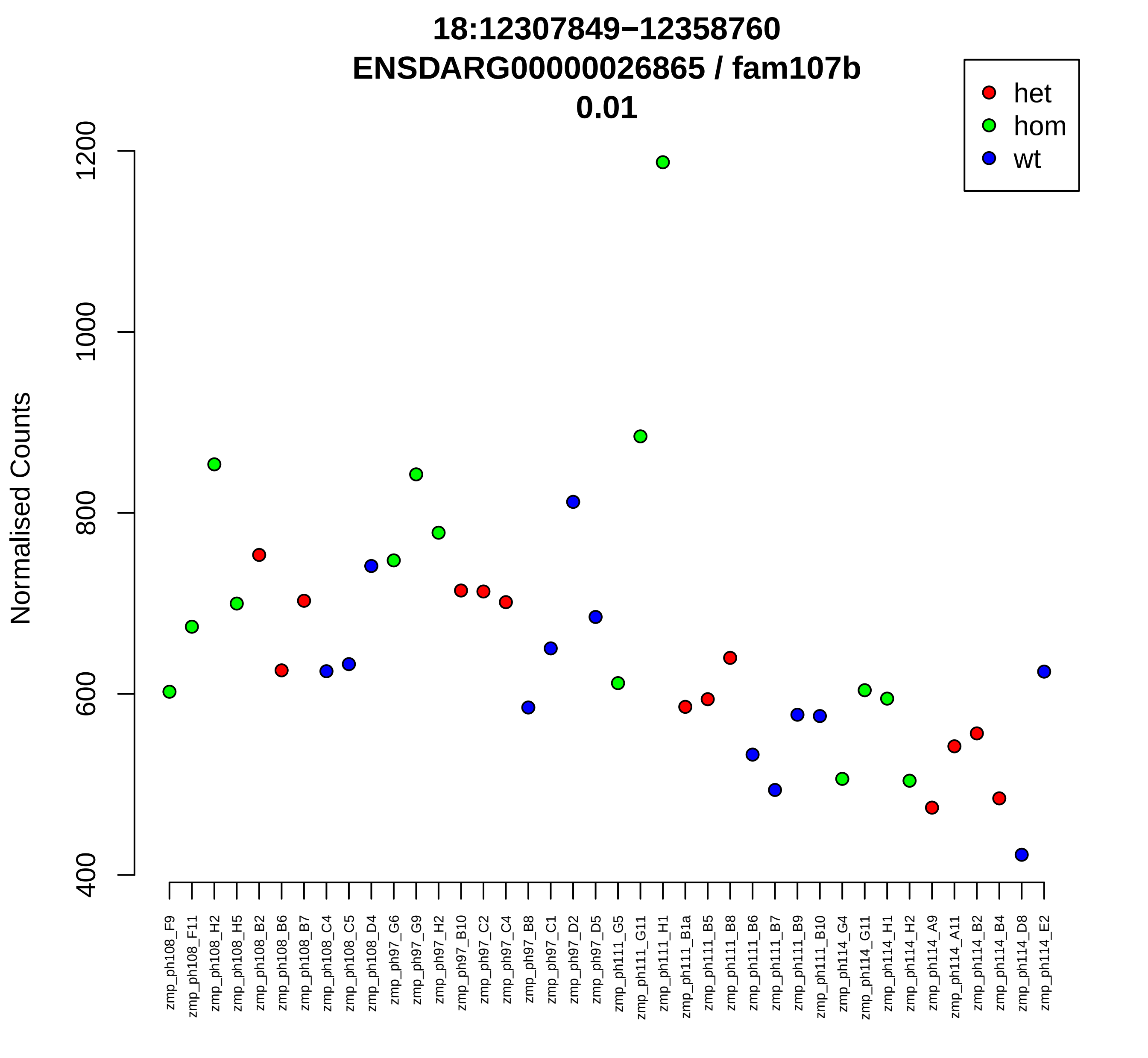

18:12307849−12358760
ENSDARG00000026865 / fam107b
0.01
het
hom
wt
Normalised Counts
400
600
800
1000
1200
zmp_ph108_F9
zmp_ph108_F11
zmp_ph108_H2
zmp_ph108_H5
zmp_ph108_B2
zmp_ph108_B6
zmp_ph108_B7
zmp_ph108_C4
zmp_ph108_C5
zmp_ph108_D4
zmp_ph97_G6
zmp_ph97_G9
zmp_ph97_H2
zmp_ph97_B10
zmp_ph97_C2
zmp_ph97_C4
zmp_ph97_B8
zmp_ph97_C1
zmp_ph97_D2
zmp_ph97_D5
zmp_ph111_G5
zmp_ph111_G11
zmp_ph111_H1
zmp_ph111_B1a
zmp_ph111_B5
zmp_ph111_B8
zmp_ph111_B6
zmp_ph111_B7
zmp_ph111_B9
zmp_ph111_B10
zmp_ph114_G4
zmp_ph114_G11
zmp_ph114_H1
zmp_ph114_H2
zmp_ph114_A9
zmp_ph114_A11
zmp_ph114_B2
zmp_ph114_B4
zmp_ph114_D8
zmp_ph114_E2

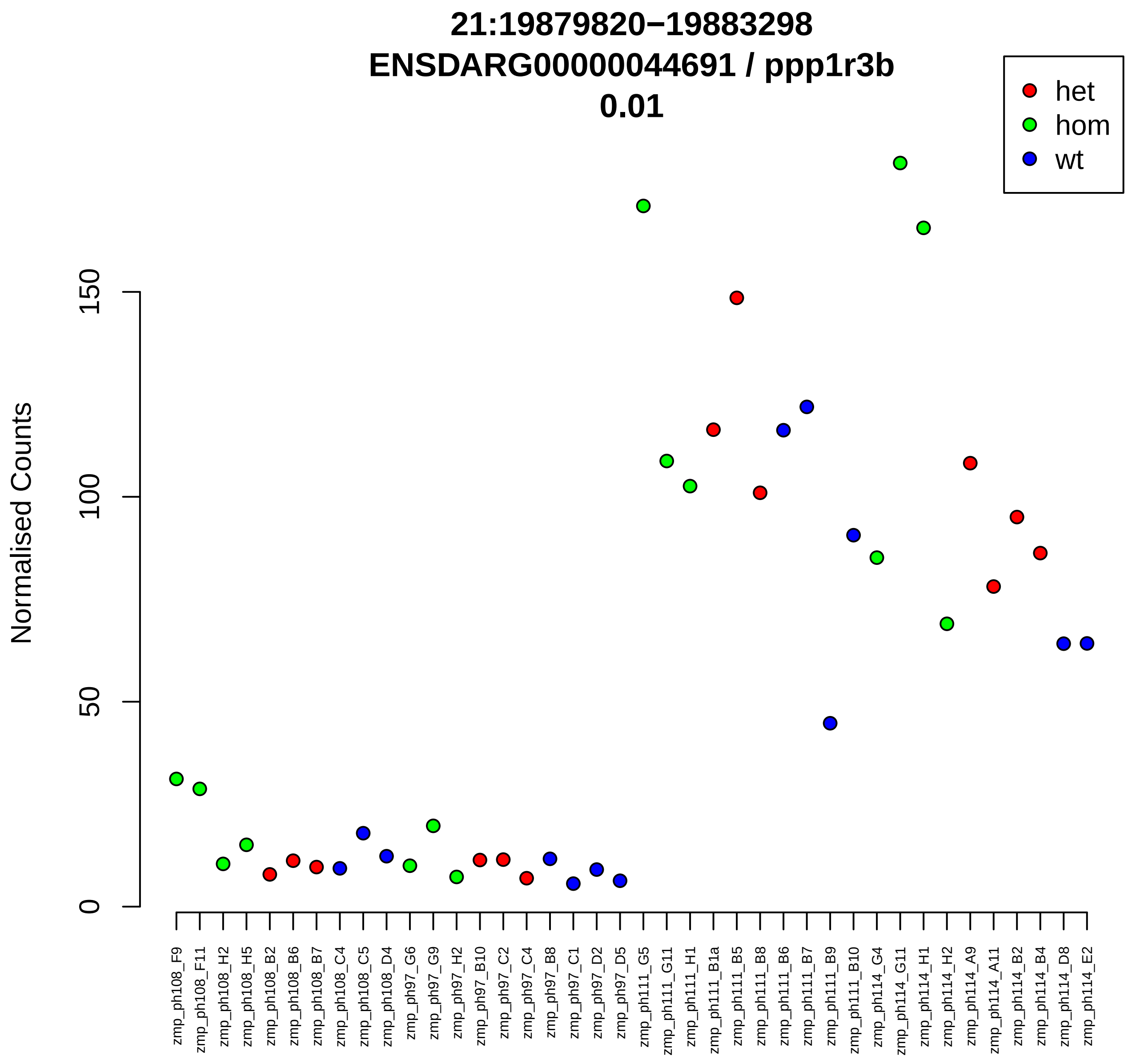
21:19879820−19883298
ENSDARG00000044691 / ppp1r3b
0.01
het
hom
wt
Normalised Counts
0
50
100
150
zmp_ph108_F9
zmp_ph108_F11
zmp_ph108_H2
zmp_ph108_H5
zmp_ph108_B2
zmp_ph108_B6
zmp_ph108_B7
zmp_ph108_C4
zmp_ph108_C5
zmp_ph108_D4
zmp_ph97_G6
zmp_ph97_G9
zmp_ph97_H2
zmp_ph97_B10
zmp_ph97_C2
zmp_ph97_C4
zmp_ph97_B8
zmp_ph97_C1
zmp_ph97_D2
zmp_ph97_D5
zmp_ph111_G5
zmp_ph111_G11
zmp_ph111_H1
zmp_ph111_B1a
zmp_ph111_B5
zmp_ph111_B8
zmp_ph111_B6
zmp_ph111_B7
zmp_ph111_B9
zmp_ph111_B10
zmp_ph114_G4
zmp_ph114_G11
zmp_ph114_H1
zmp_ph114_H2
zmp_ph114_A9
zmp_ph114_A11
zmp_ph114_B2
zmp_ph114_B4
zmp_ph114_D8
zmp_ph114_E2

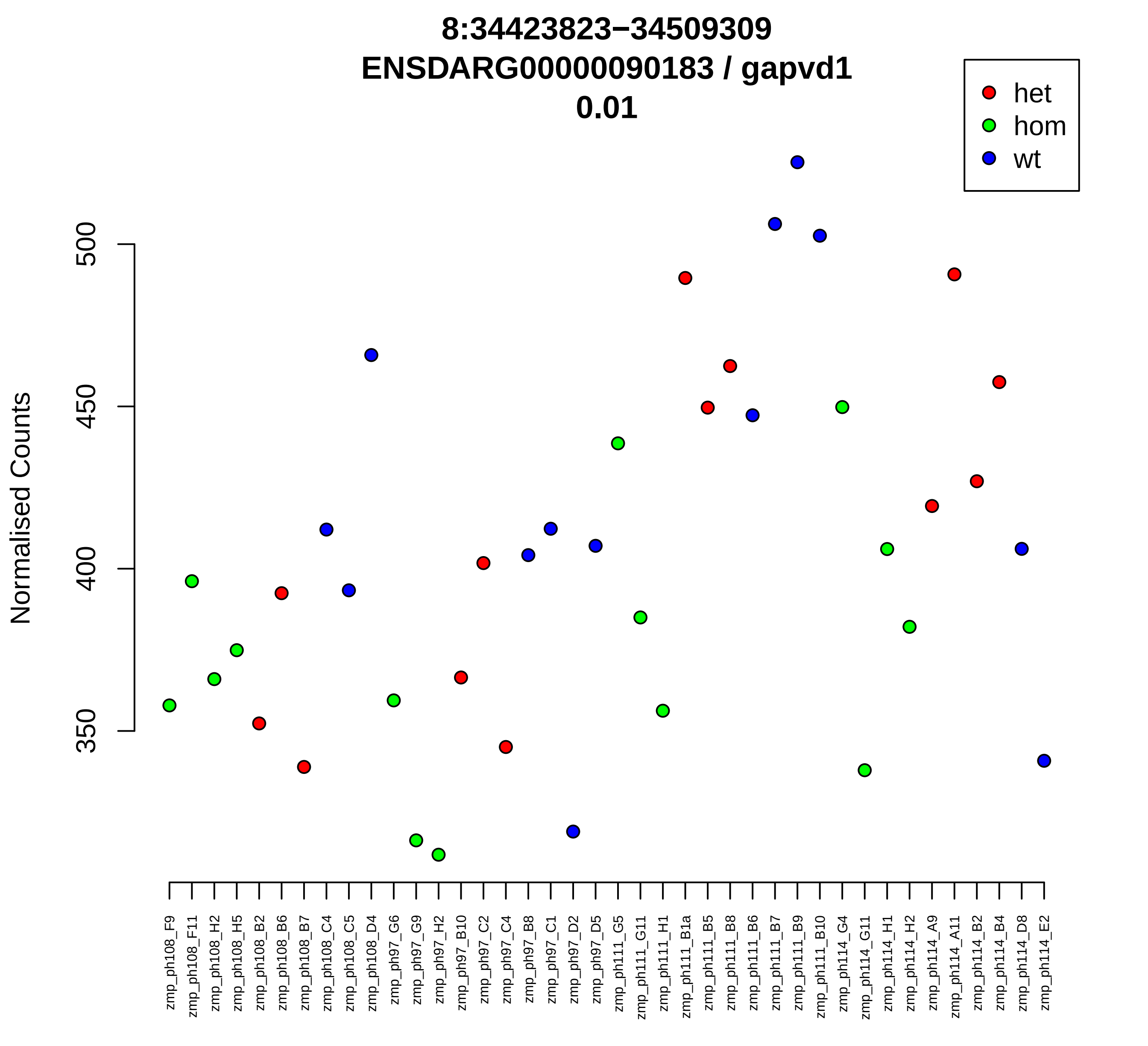

8:34423823−34509309
ENSDARG00000090183 / gapvd1
0.01
het
hom
wt
Normalised Counts
350
400
450
500
zmp_ph108_F9
zmp_ph108_F11
zmp_ph108_H2
zmp_ph108_H5
zmp_ph108_B2
zmp_ph108_B6
zmp_ph108_B7
zmp_ph108_C4
zmp_ph108_C5
zmp_ph108_D4
zmp_ph97_G6
zmp_ph97_G9
zmp_ph97_H2
zmp_ph97_B10
zmp_ph97_C2
zmp_ph97_C4
zmp_ph97_B8
zmp_ph97_C1
zmp_ph97_D2
zmp_ph97_D5
zmp_ph111_G5
zmp_ph111_G11
zmp_ph111_H1
zmp_ph111_B1a
zmp_ph111_B5
zmp_ph111_B8
zmp_ph111_B6
zmp_ph111_B7
zmp_ph111_B9
zmp_ph111_B10
zmp_ph114_G4
zmp_ph114_G11
zmp_ph114_H1
zmp_ph114_H2
zmp_ph114_A9
zmp_ph114_A11
zmp_ph114_B2
zmp_ph114_B4
zmp_ph114_D8
zmp_ph114_E2

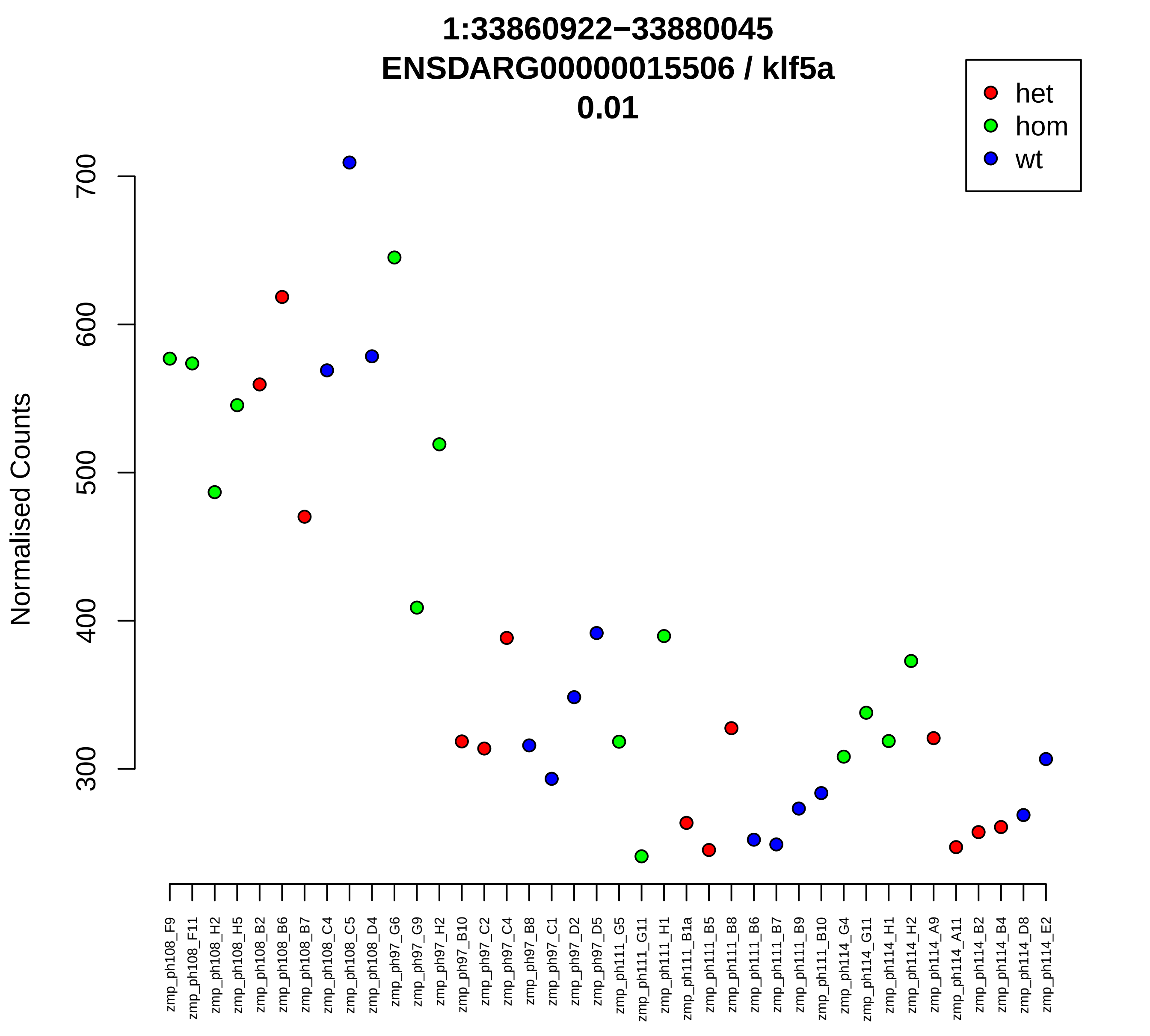

1:33860922−33880045
ENSDARG00000015506 / klf5a
0.01
het
hom
wt
Normalised Counts
300
400
500
600
700
zmp_ph108_F9
zmp_ph108_F11
zmp_ph108_H2
zmp_ph108_H5
zmp_ph108_B2
zmp_ph108_B6
zmp_ph108_B7
zmp_ph108_C4
zmp_ph108_C5
zmp_ph108_D4
zmp_ph97_G6
zmp_ph97_G9
zmp_ph97_H2
zmp_ph97_B10
zmp_ph97_C2
zmp_ph97_C4
zmp_ph97_B8
zmp_ph97_C1
zmp_ph97_D2
zmp_ph97_D5
zmp_ph111_G5
zmp_ph111_G11
zmp_ph111_H1
zmp_ph111_B1a
zmp_ph111_B5
zmp_ph111_B8
zmp_ph111_B6
zmp_ph111_B7
zmp_ph111_B9
zmp_ph111_B10
zmp_ph114_G4
zmp_ph114_G11
zmp_ph114_H1
zmp_ph114_H2
zmp_ph114_A9
zmp_ph114_A11
zmp_ph114_B2
zmp_ph114_B4
zmp_ph114_D8
zmp_ph114_E2

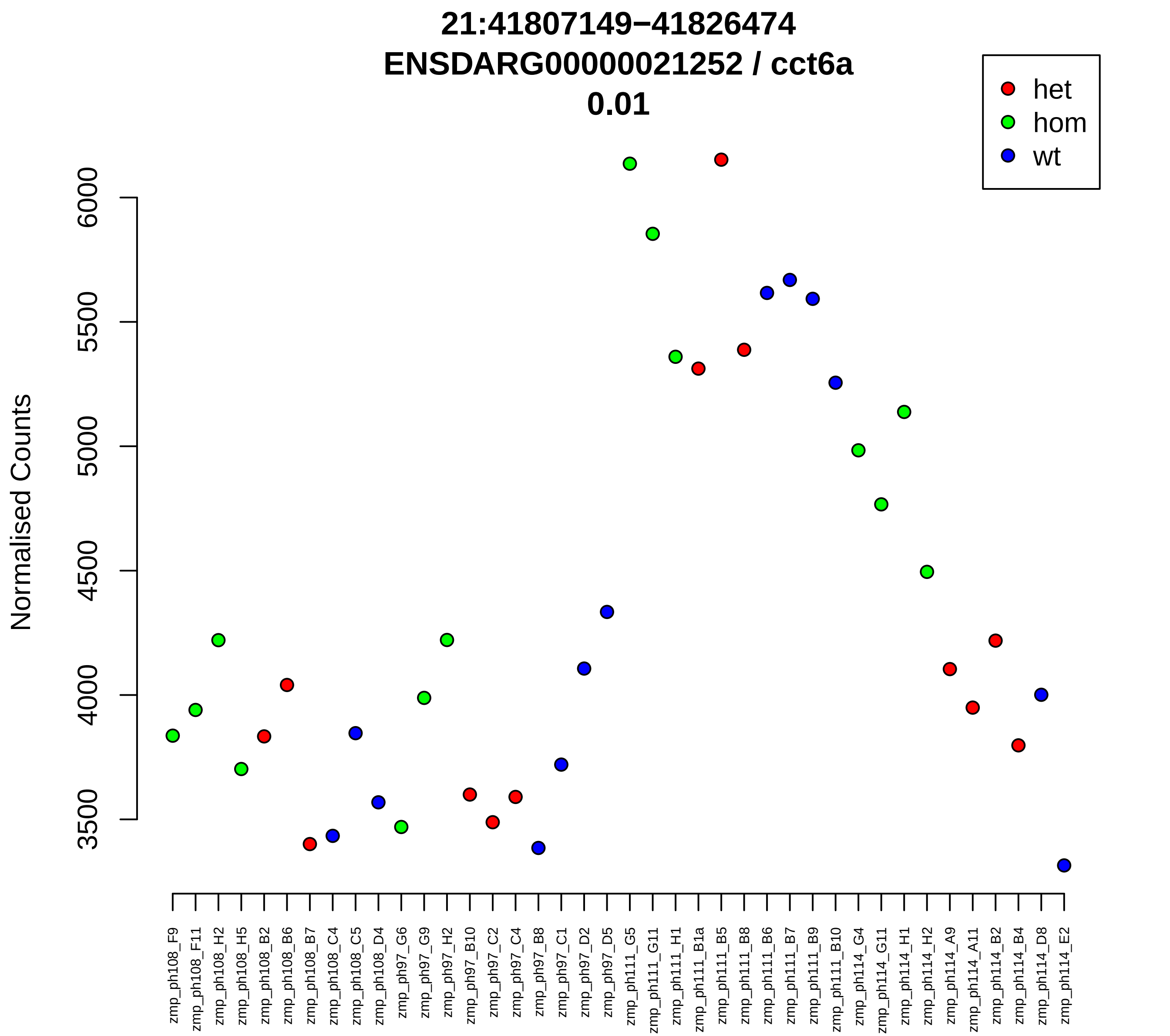
21:41807149−41826474
ENSDARG00000021252 / cct6a
0.01
het
hom
wt
Normalised Counts
3500
4000
4500
5000
5500
6000
zmp_ph108_F9
zmp_ph108_F11
zmp_ph108_H2
zmp_ph108_H5
zmp_ph108_B2
zmp_ph108_B6
zmp_ph108_B7
zmp_ph108_C4
zmp_ph108_C5
zmp_ph108_D4
zmp_ph97_G6
zmp_ph97_G9
zmp_ph97_H2
zmp_ph97_B10
zmp_ph97_C2
zmp_ph97_C4
zmp_ph97_B8
zmp_ph97_C1
zmp_ph97_D2
zmp_ph97_D5
zmp_ph111_G5
zmp_ph111_G11
zmp_ph111_H1
zmp_ph111_B1a
zmp_ph111_B5
zmp_ph111_B8
zmp_ph111_B6
zmp_ph111_B7
zmp_ph111_B9
zmp_ph111_B10
zmp_ph114_G4
zmp_ph114_G11
zmp_ph114_H1
zmp_ph114_H2
zmp_ph114_A9
zmp_ph114_A11
zmp_ph114_B2
zmp_ph114_B4
zmp_ph114_D8
zmp_ph114_E2

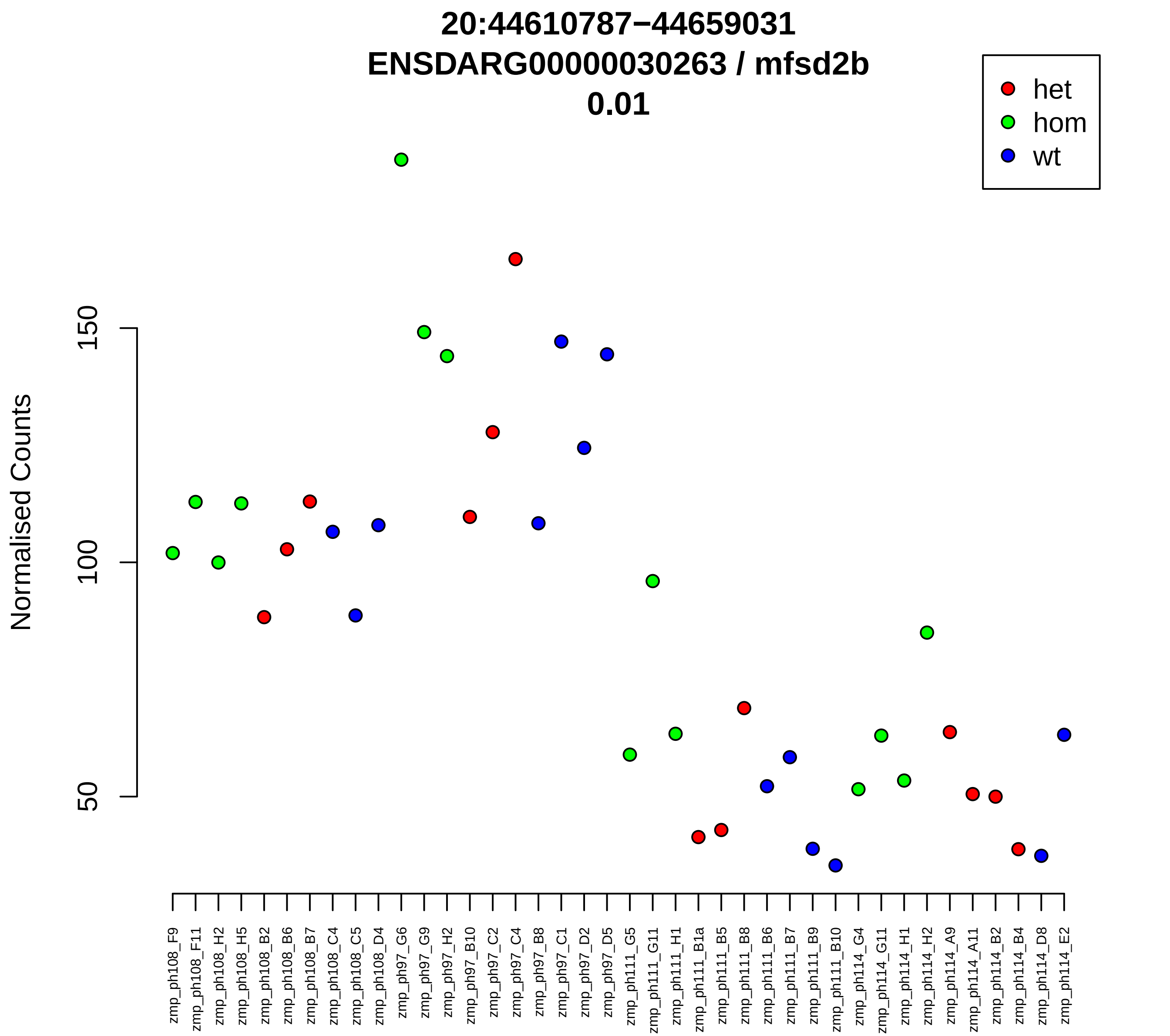

20:44610787−44659031
ENSDARG00000030263 / mfsd2b
0.01
het
hom
wt
Normalised Counts
50
100
150
zmp_ph108_F9
zmp_ph108_F11
zmp_ph108_H2
zmp_ph108_H5
zmp_ph108_B2
zmp_ph108_B6
zmp_ph108_B7
zmp_ph108_C4
zmp_ph108_C5
zmp_ph108_D4
zmp_ph97_G6
zmp_ph97_G9
zmp_ph97_H2
zmp_ph97_B10
zmp_ph97_C2
zmp_ph97_C4
zmp_ph97_B8
zmp_ph97_C1
zmp_ph97_D2
zmp_ph97_D5
zmp_ph111_G5
zmp_ph111_G11
zmp_ph111_H1
zmp_ph111_B1a
zmp_ph111_B5
zmp_ph111_B8
zmp_ph111_B6
zmp_ph111_B7
zmp_ph111_B9
zmp_ph111_B10
zmp_ph114_G4
zmp_ph114_G11
zmp_ph114_H1
zmp_ph114_H2
zmp_ph114_A9
zmp_ph114_A11
zmp_ph114_B2
zmp_ph114_B4
zmp_ph114_D8
zmp_ph114_E2

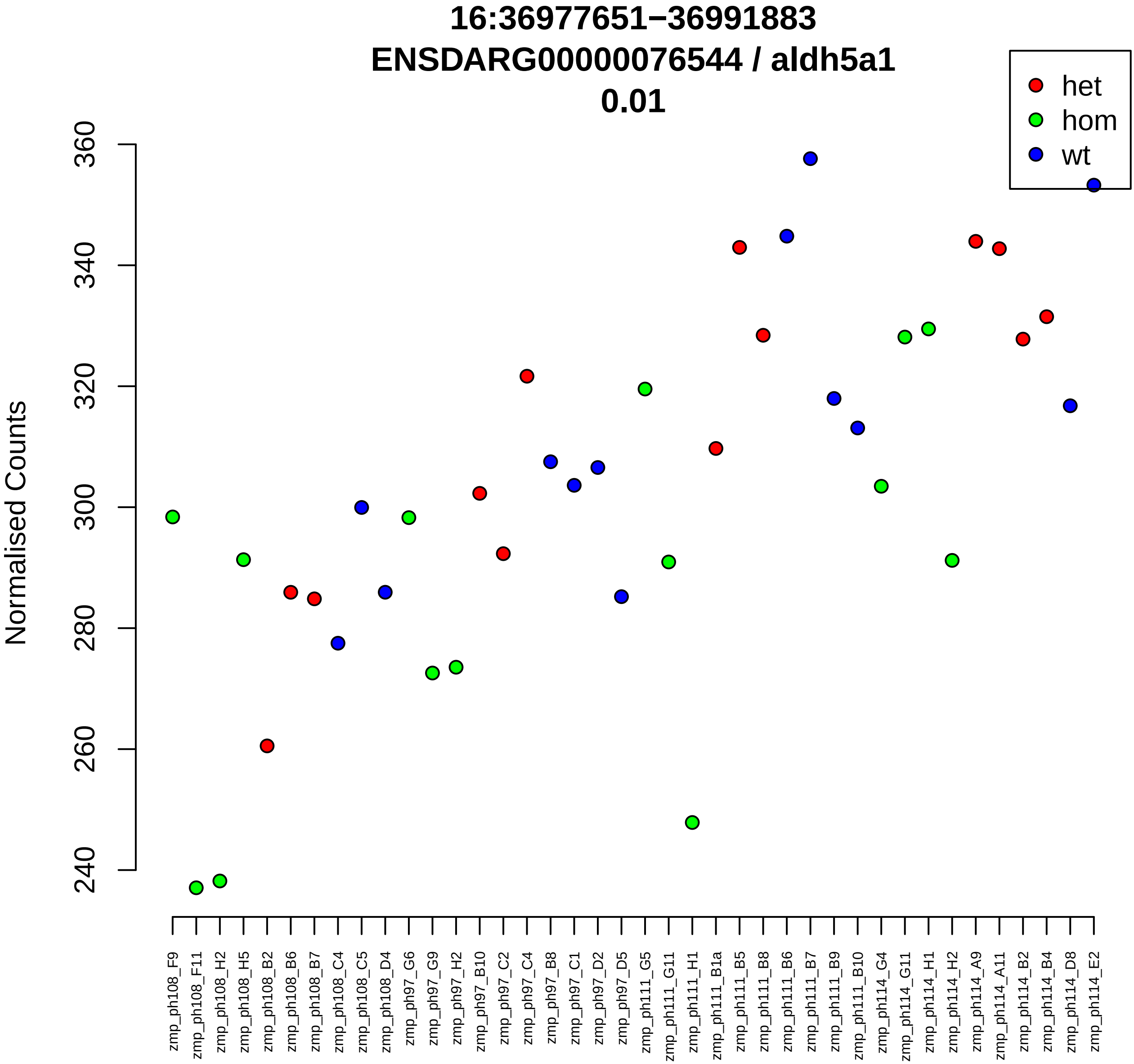
16:36977651−36991883
ENSDARG00000076544 / aldh5a1
0.01
het
hom
wt
Normalised Counts
240
260
280
300
320
340
360
zmp_ph108_F9
zmp_ph108_F11
zmp_ph108_H2
zmp_ph108_H5
zmp_ph108_B2
zmp_ph108_B6
zmp_ph108_B7
zmp_ph108_C4
zmp_ph108_C5
zmp_ph108_D4
zmp_ph97_G6
zmp_ph97_G9
zmp_ph97_H2
zmp_ph97_B10
zmp_ph97_C2
zmp_ph97_C4
zmp_ph97_B8
zmp_ph97_C1
zmp_ph97_D2
zmp_ph97_D5
zmp_ph111_G5
zmp_ph111_G11
zmp_ph111_H1
zmp_ph111_B1a
zmp_ph111_B5
zmp_ph111_B8
zmp_ph111_B6
zmp_ph111_B7
zmp_ph111_B9
zmp_ph111_B10
zmp_ph114_G4
zmp_ph114_G11
zmp_ph114_H1
zmp_ph114_H2
zmp_ph114_A9
zmp_ph114_A11
zmp_ph114_B2
zmp_ph114_B4
zmp_ph114_D8
zmp_ph114_E2

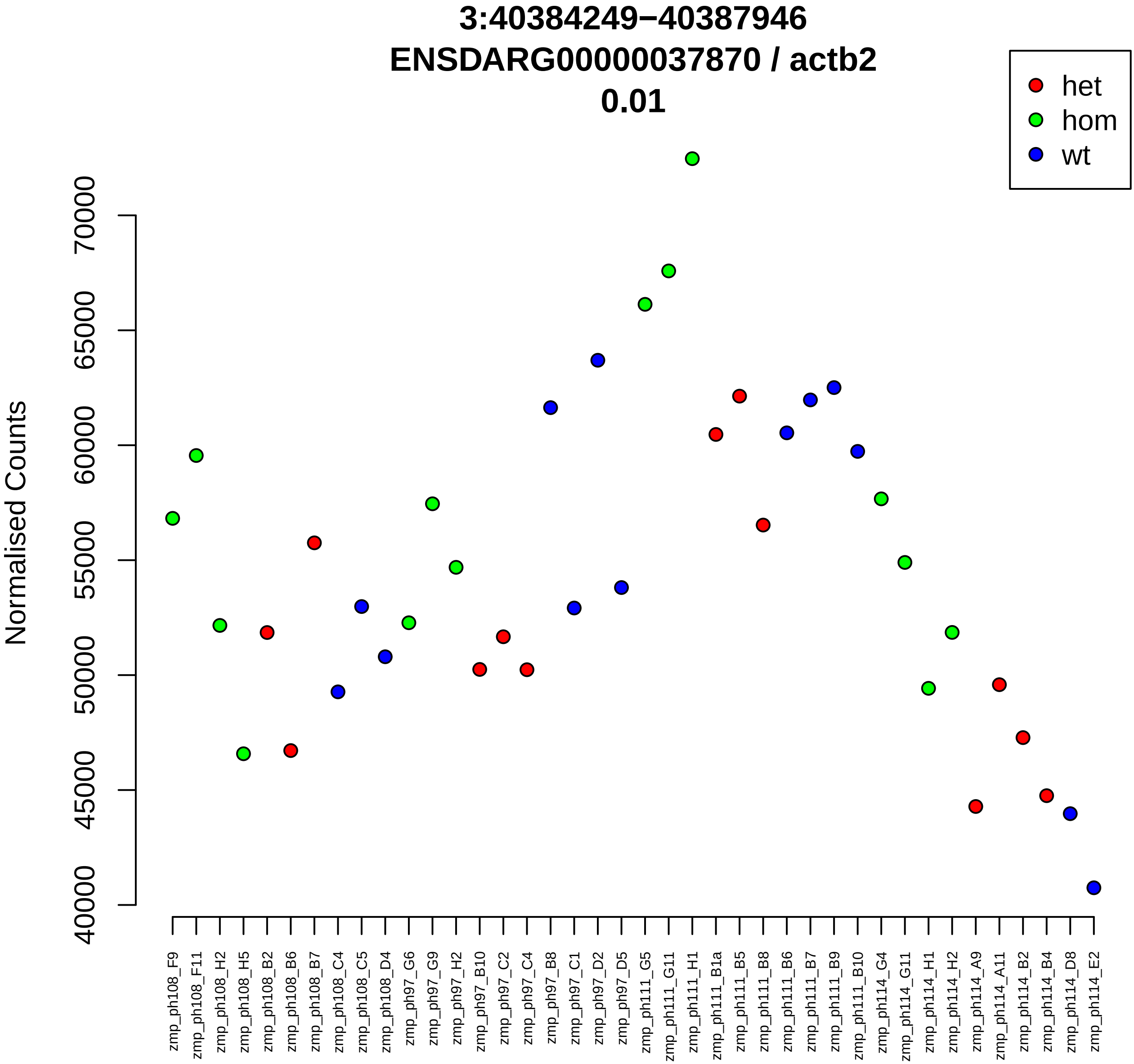
3:40384249−40387946
ENSDARG00000037870 / actb2
0.01
het
hom
wt
Normalised Counts
40000
45000
50000
55000
60000
65000
70000
zmp_ph108_F9
zmp_ph108_F11
zmp_ph108_H2
zmp_ph108_H5
zmp_ph108_B2
zmp_ph108_B6
zmp_ph108_B7
zmp_ph108_C4
zmp_ph108_C5
zmp_ph108_D4
zmp_ph97_G6
zmp_ph97_G9
zmp_ph97_H2
zmp_ph97_B10
zmp_ph97_C2
zmp_ph97_C4
zmp_ph97_B8
zmp_ph97_C1
zmp_ph97_D2
zmp_ph97_D5
zmp_ph111_G5
zmp_ph111_G11
zmp_ph111_H1
zmp_ph111_B1a
zmp_ph111_B5
zmp_ph111_B8
zmp_ph111_B6
zmp_ph111_B7
zmp_ph111_B9
zmp_ph111_B10
zmp_ph114_G4
zmp_ph114_G11
zmp_ph114_H1
zmp_ph114_H2
zmp_ph114_A9
zmp_ph114_A11
zmp_ph114_B2
zmp_ph114_B4
zmp_ph114_D8
zmp_ph114_E2

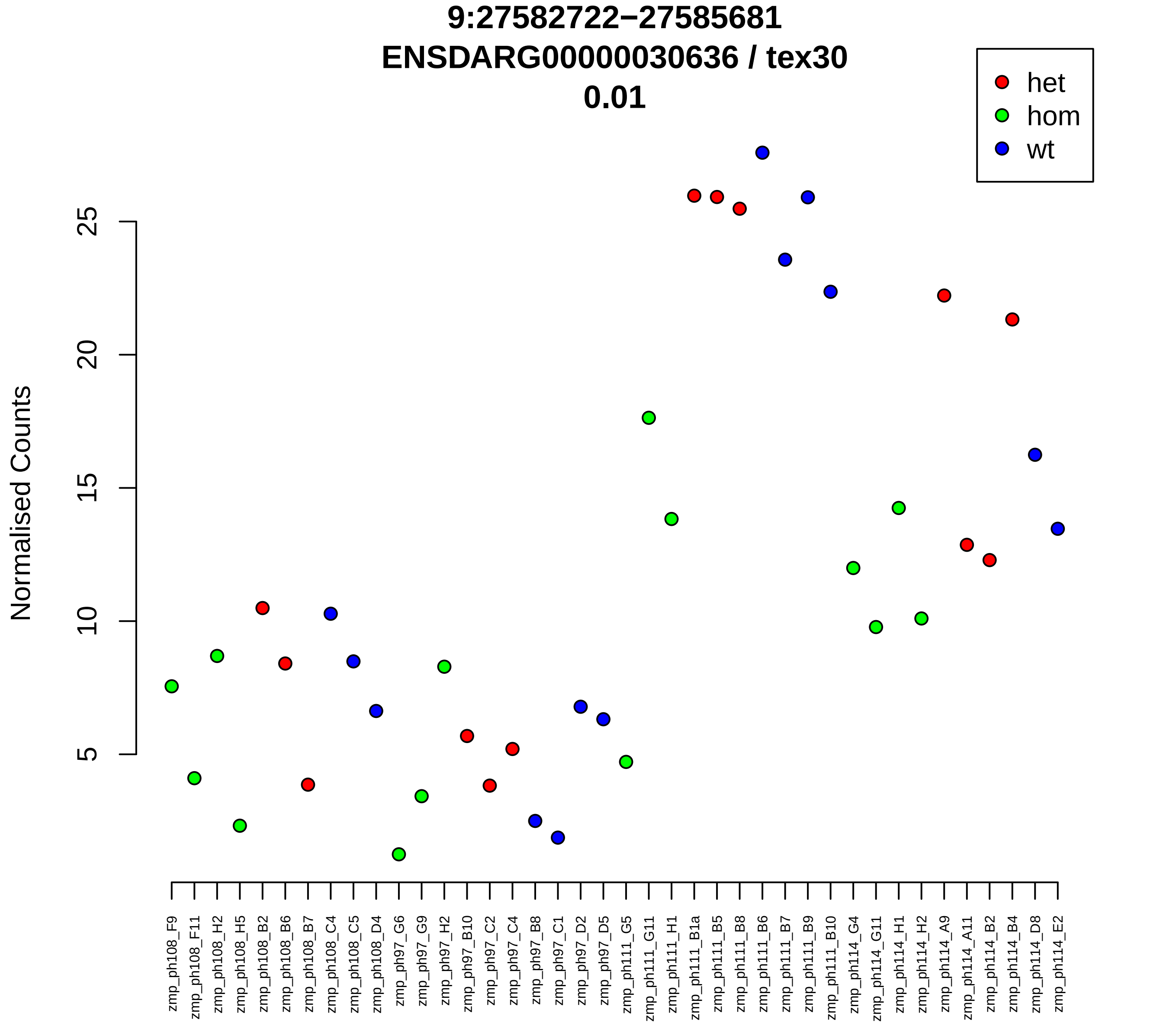

9:27582722−27585681
ENSDARG00000030636 / tex30
0.01
het
hom
wt
Normalised Counts
5
10
15
20
25
zmp_ph108_F9
zmp_ph108_F11
zmp_ph108_H2
zmp_ph108_H5
zmp_ph108_B2
zmp_ph108_B6
zmp_ph108_B7
zmp_ph108_C4
zmp_ph108_C5
zmp_ph108_D4
zmp_ph97_G6
zmp_ph97_G9
zmp_ph97_H2
zmp_ph97_B10
zmp_ph97_C2
zmp_ph97_C4
zmp_ph97_B8
zmp_ph97_C1
zmp_ph97_D2
zmp_ph97_D5
zmp_ph111_G5
zmp_ph111_G11
zmp_ph111_H1
zmp_ph111_B1a
zmp_ph111_B5
zmp_ph111_B8
zmp_ph111_B6
zmp_ph111_B7
zmp_ph111_B9
zmp_ph111_B10
zmp_ph114_G4
zmp_ph114_G11
zmp_ph114_H1
zmp_ph114_H2
zmp_ph114_A9
zmp_ph114_A11
zmp_ph114_B2
zmp_ph114_B4
zmp_ph114_D8
zmp_ph114_E2

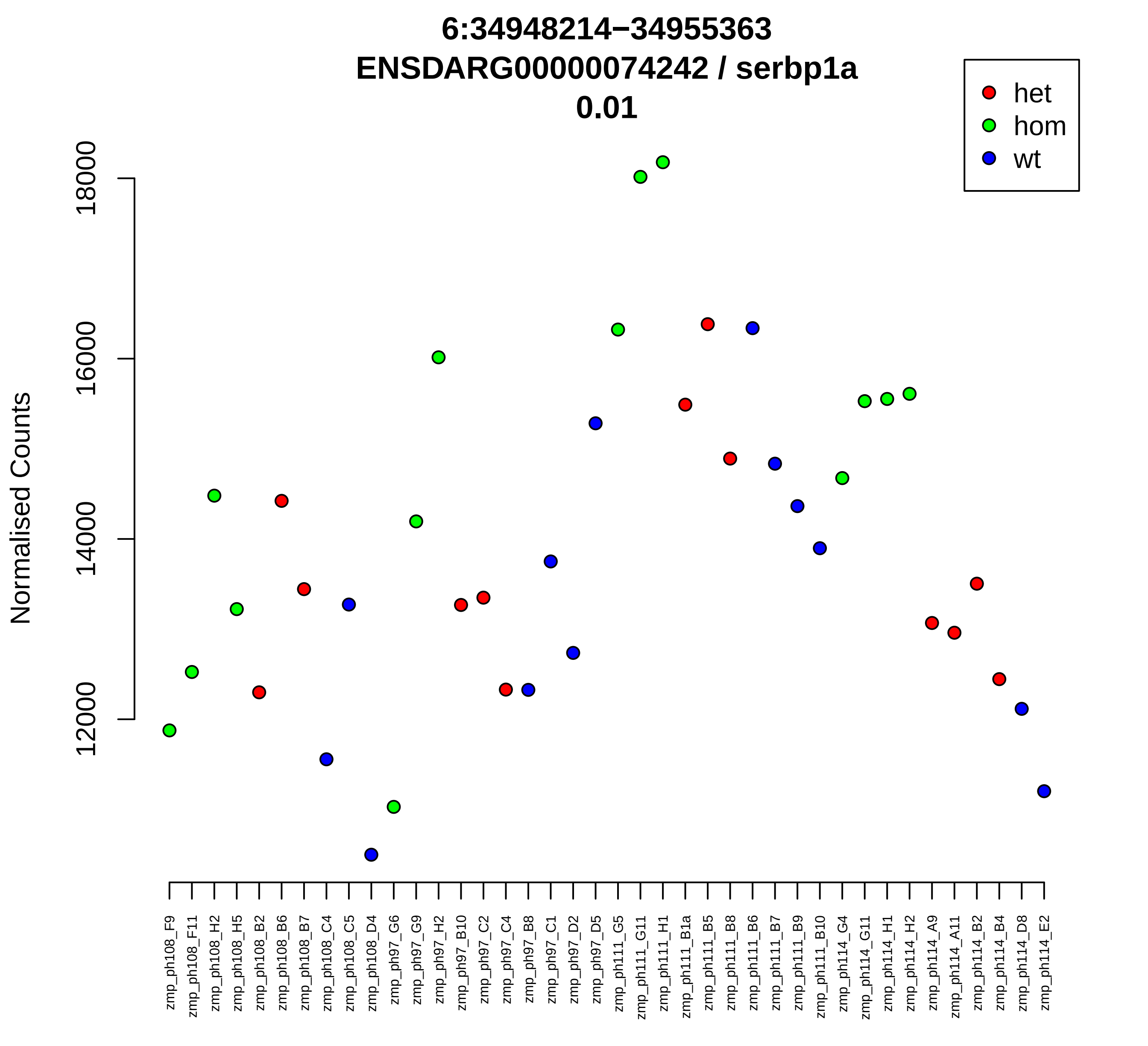
6:34948214−34955363
ENSDARG00000074242 / serbp1a
0.01
het
hom
wt
Normalised Counts
12000
14000
16000
18000
zmp_ph108_F9
zmp_ph108_F11
zmp_ph108_H2
zmp_ph108_H5
zmp_ph108_B2
zmp_ph108_B6
zmp_ph108_B7
zmp_ph108_C4
zmp_ph108_C5
zmp_ph108_D4
zmp_ph97_G6
zmp_ph97_G9
zmp_ph97_H2
zmp_ph97_B10
zmp_ph97_C2
zmp_ph97_C4
zmp_ph97_B8
zmp_ph97_C1
zmp_ph97_D2
zmp_ph97_D5
zmp_ph111_G5
zmp_ph111_G11
zmp_ph111_H1
zmp_ph111_B1a
zmp_ph111_B5
zmp_ph111_B8
zmp_ph111_B6
zmp_ph111_B7
zmp_ph111_B9
zmp_ph111_B10
zmp_ph114_G4
zmp_ph114_G11
zmp_ph114_H1
zmp_ph114_H2
zmp_ph114_A9
zmp_ph114_A11
zmp_ph114_B2
zmp_ph114_B4
zmp_ph114_D8
zmp_ph114_E2

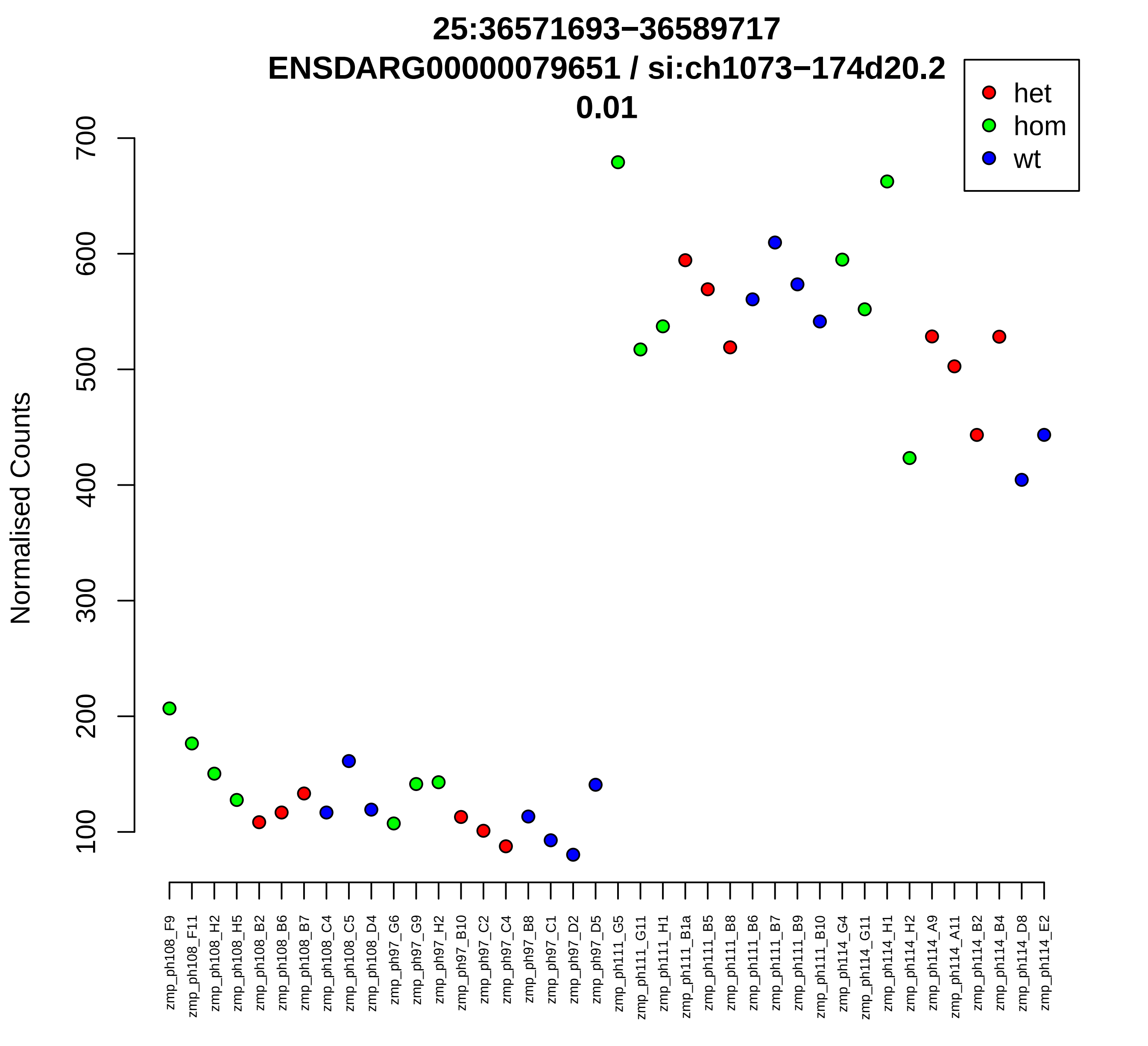

25:36571693−36589717
ENSDARG00000079651 / si:ch1073−174d20.2
0.01
het
hom
wt
Normalised Counts
100
200
300
400
500
600
700
zmp_ph108_F9
zmp_ph108_F11
zmp_ph108_H2
zmp_ph108_H5
zmp_ph108_B2
zmp_ph108_B6
zmp_ph108_B7
zmp_ph108_C4
zmp_ph108_C5
zmp_ph108_D4
zmp_ph97_G6
zmp_ph97_G9
zmp_ph97_H2
zmp_ph97_B10
zmp_ph97_C2
zmp_ph97_C4
zmp_ph97_B8
zmp_ph97_C1
zmp_ph97_D2
zmp_ph97_D5
zmp_ph111_G5
zmp_ph111_G11
zmp_ph111_H1
zmp_ph111_B1a
zmp_ph111_B5
zmp_ph111_B8
zmp_ph111_B6
zmp_ph111_B7
zmp_ph111_B9
zmp_ph111_B10
zmp_ph114_G4
zmp_ph114_G11
zmp_ph114_H1
zmp_ph114_H2
zmp_ph114_A9
zmp_ph114_A11
zmp_ph114_B2
zmp_ph114_B4
zmp_ph114_D8
zmp_ph114_E2

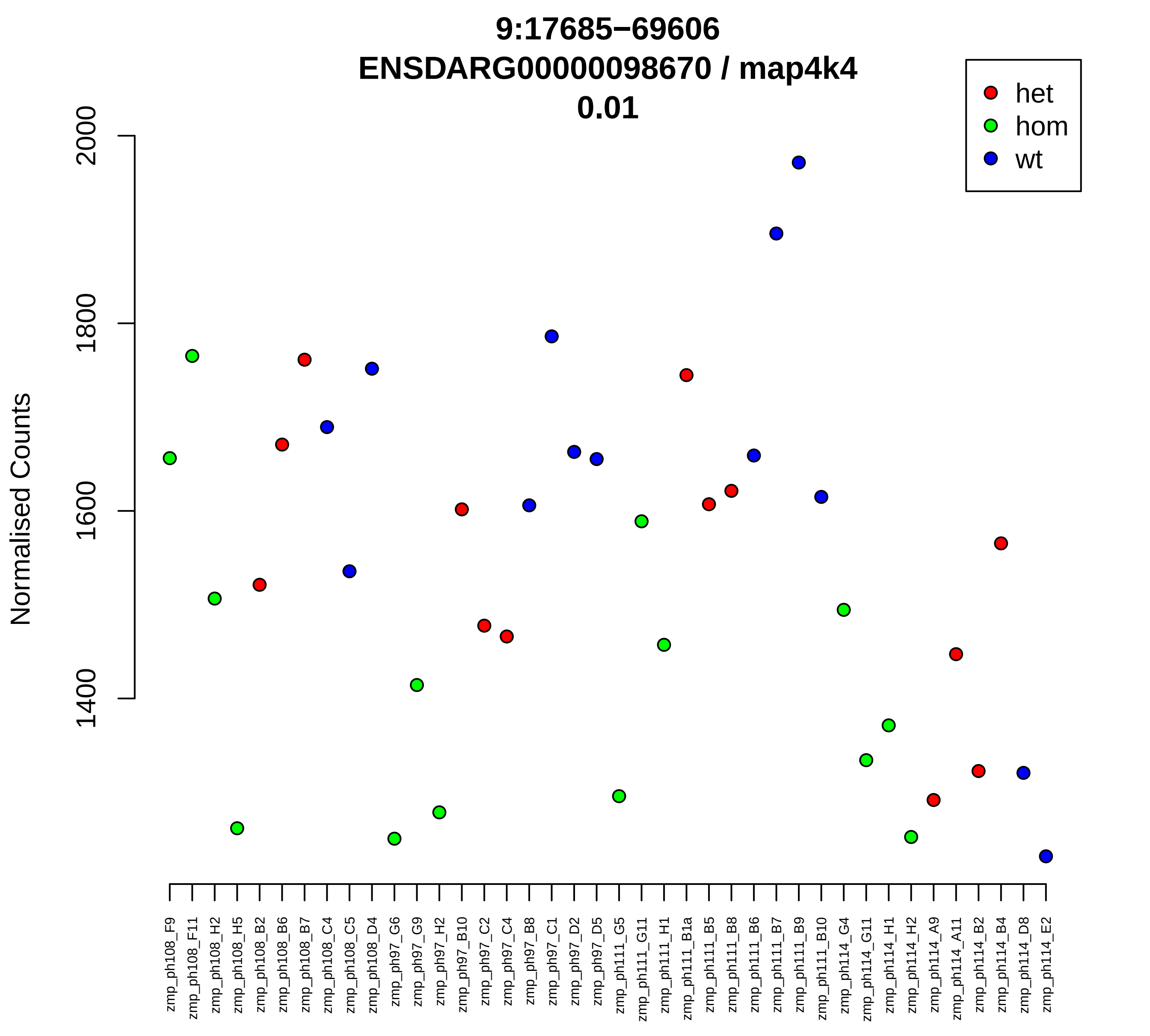
9:17685−69606
ENSDARG00000098670 / map4k4
0.01
het
hom
wt
Normalised Counts
2000
1800
1600
1400
zmp_ph108_F9
zmp_ph108_F11
zmp_ph108_H2
zmp_ph108_H5
zmp_ph108_B2
zmp_ph108_B6
zmp_ph108_B7
zmp_ph108_C4
zmp_ph108_C5
zmp_ph108_D4
zmp_ph97_G6
zmp_ph97_G9
zmp_ph97_H2
zmp_ph97_B10
zmp_ph97_C2
zmp_ph97_C4
zmp_ph97_B8
zmp_ph97_C1
zmp_ph97_D2
zmp_ph97_D5
zmp_ph111_G5
zmp_ph111_G11
zmp_ph111_H1
zmp_ph111_B1a
zmp_ph111_B5
zmp_ph111_B8
zmp_ph111_B6
zmp_ph111_B7
zmp_ph111_B9
zmp_ph111_B10
zmp_ph114_G4
zmp_ph114_G11
zmp_ph114_H1
zmp_ph114_H2
zmp_ph114_A9
zmp_ph114_A11
zmp_ph114_B2
zmp_ph114_B4
zmp_ph114_D8
zmp_ph114_E2

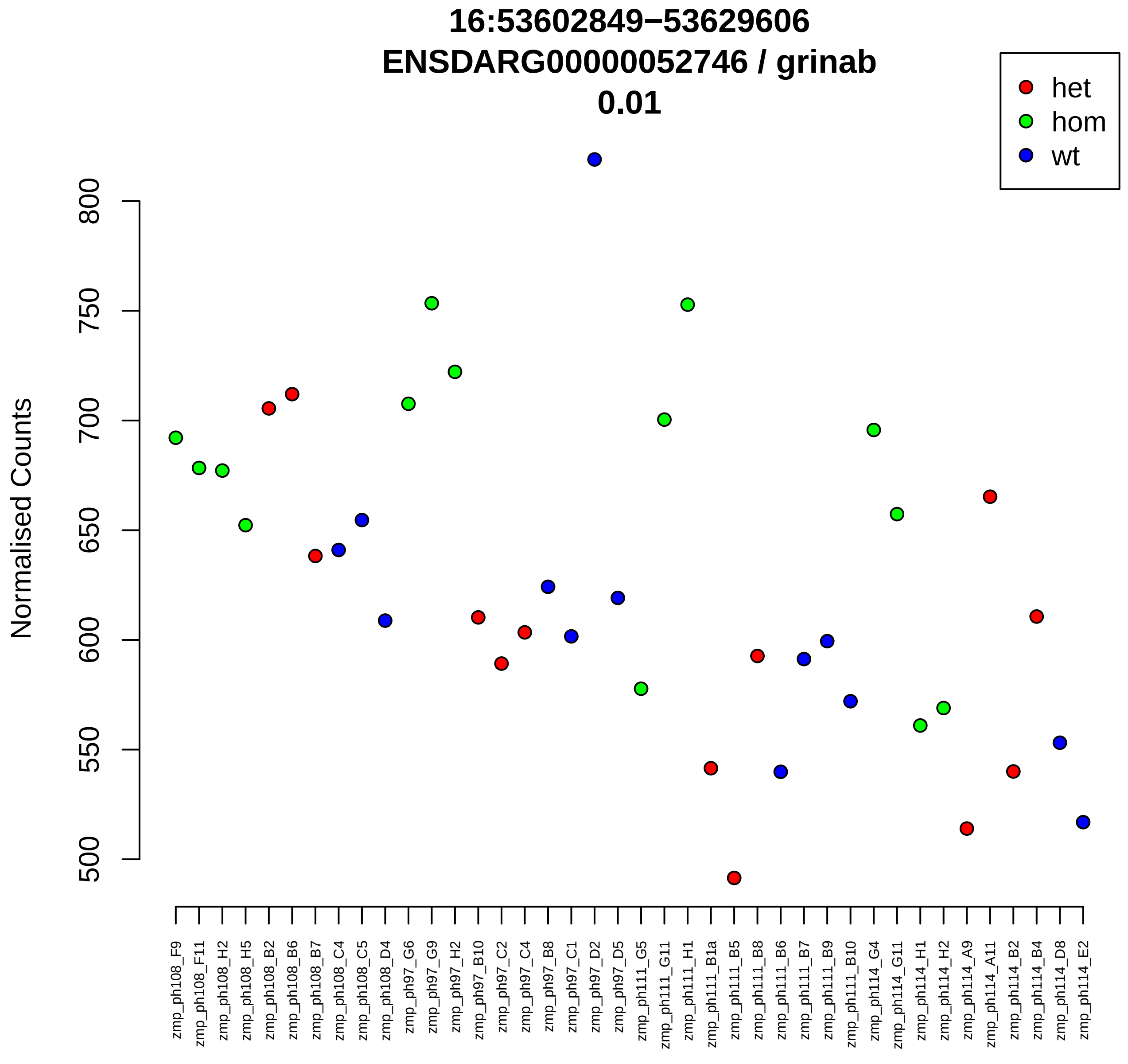
16:53602849−53629606
ENSDARG00000052746 / grinab
0.01
het
hom
wt
Normalised Counts
500
550
600
650
700
750
800
zmp_ph108_F9
zmp_ph108_F11
zmp_ph108_H2
zmp_ph108_H5
zmp_ph108_B2
zmp_ph108_B6
zmp_ph108_B7
zmp_ph108_C4
zmp_ph108_C5
zmp_ph108_D4
zmp_ph97_G6
zmp_ph97_G9
zmp_ph97_H2
zmp_ph97_B10
zmp_ph97_C2
zmp_ph97_C4
zmp_ph97_B8
zmp_ph97_C1
zmp_ph97_D2
zmp_ph97_D5
zmp_ph111_G5
zmp_ph111_G11
zmp_ph111_H1
zmp_ph111_B1a
zmp_ph111_B5
zmp_ph111_B8
zmp_ph111_B6
zmp_ph111_B7
zmp_ph111_B9
zmp_ph111_B10
zmp_ph114_G4
zmp_ph114_G11
zmp_ph114_H1
zmp_ph114_H2
zmp_ph114_A9
zmp_ph114_A11
zmp_ph114_B2
zmp_ph114_B4
zmp_ph114_D8
zmp_ph114_E2

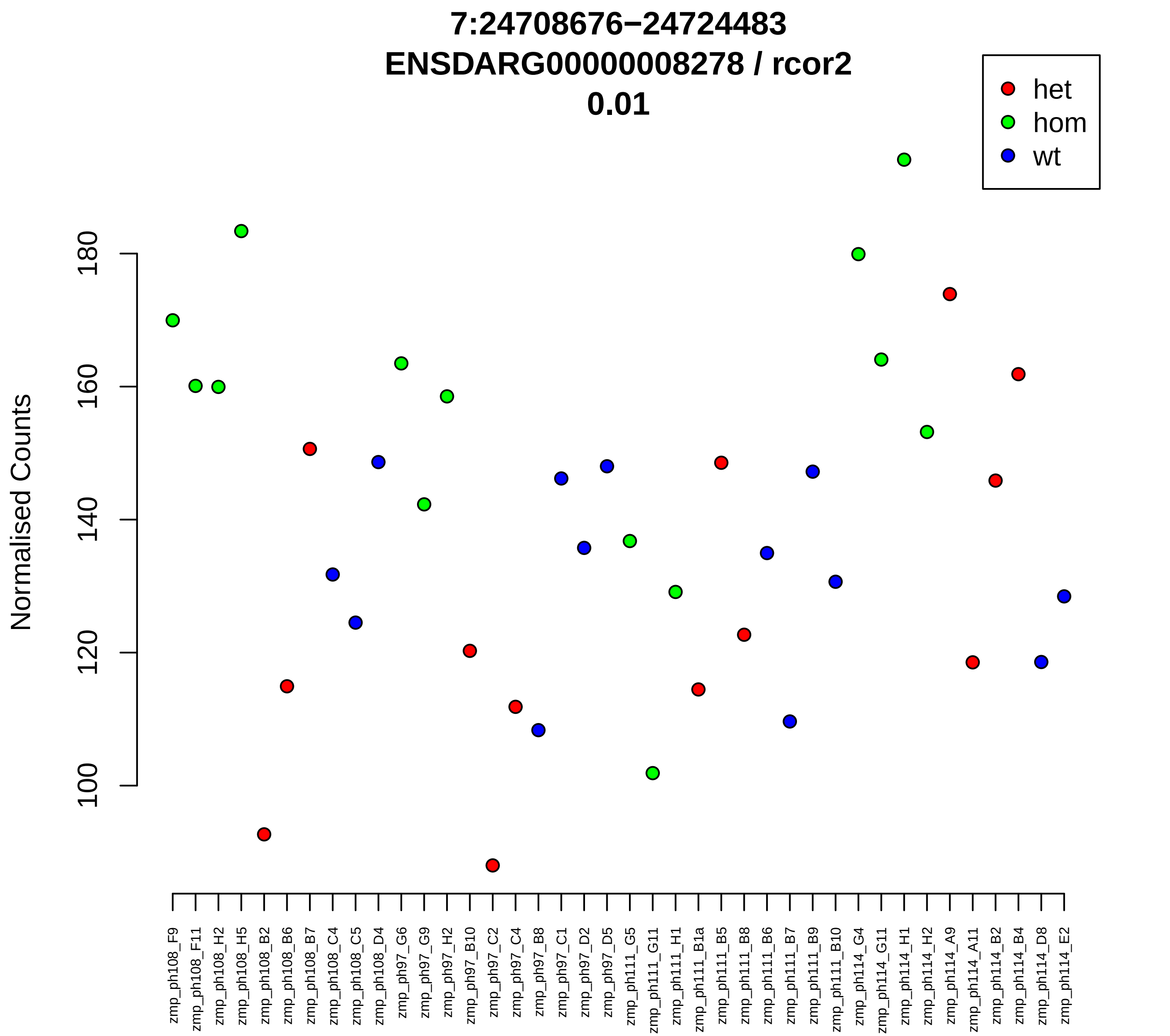

7:24708676−24724483
ENSDARG00000008278 / rcor2
0.01
het
hom
wt
Normalised Counts
100
120
140
160
180
zmp_ph108_F9
zmp_ph108_F11
zmp_ph108_H2
zmp_ph108_H5
zmp_ph108_B2
zmp_ph108_B6
zmp_ph108_B7
zmp_ph108_C4
zmp_ph108_C5
zmp_ph108_D4
zmp_ph97_G6
zmp_ph97_G9
zmp_ph97_H2
zmp_ph97_B10
zmp_ph97_C2
zmp_ph97_C4
zmp_ph97_B8
zmp_ph97_C1
zmp_ph97_D2
zmp_ph97_D5
zmp_ph111_G5
zmp_ph111_G11
zmp_ph111_H1
zmp_ph111_B1a
zmp_ph111_B5
zmp_ph111_B8
zmp_ph111_B6
zmp_ph111_B7
zmp_ph111_B9
zmp_ph111_B10
zmp_ph114_G4
zmp_ph114_G11
zmp_ph114_H1
zmp_ph114_H2
zmp_ph114_A9
zmp_ph114_A11
zmp_ph114_B2
zmp_ph114_B4
zmp_ph114_D8
zmp_ph114_E2

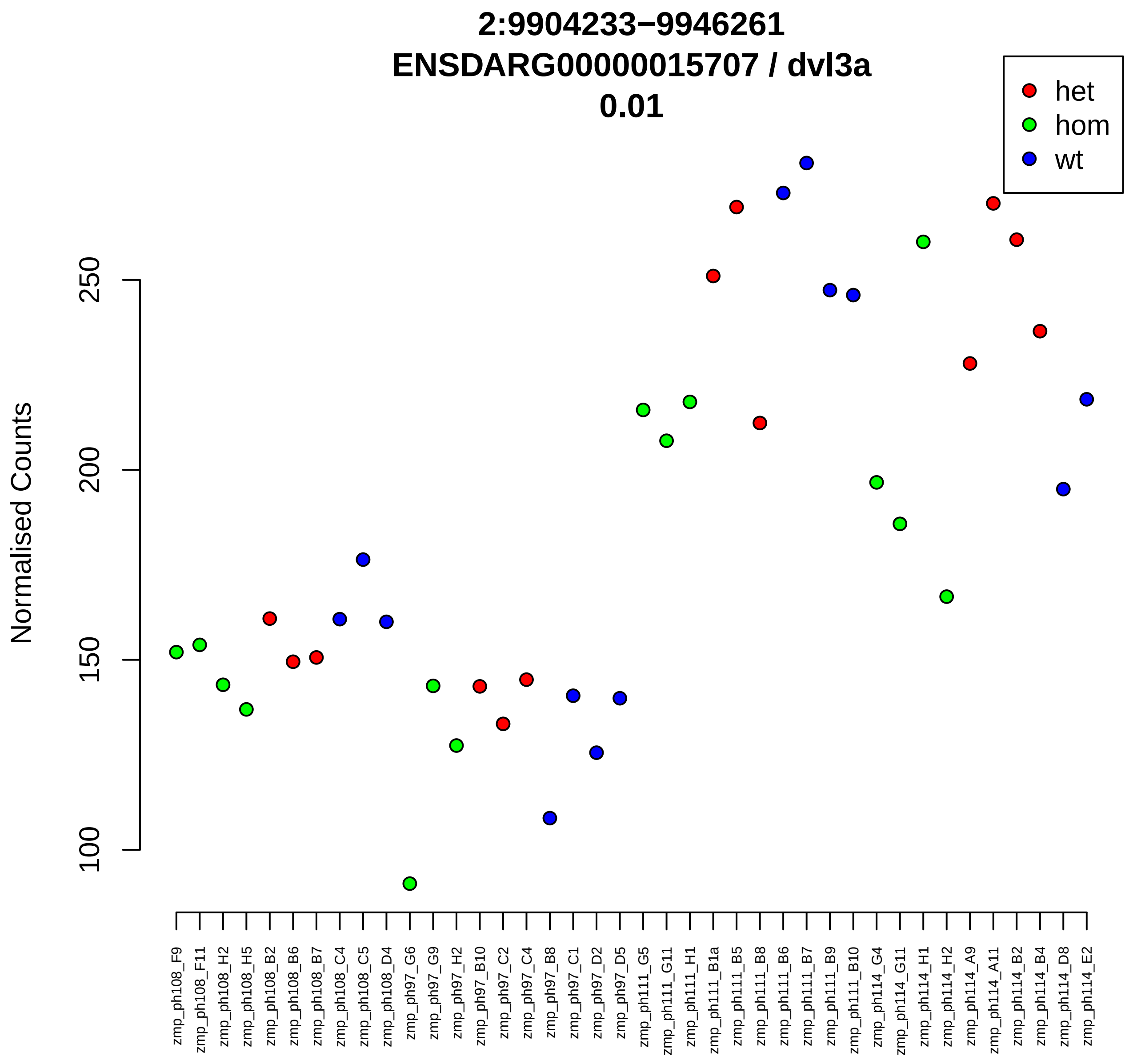

2:9904233−9946261
ENSDARG00000015707 / dvl3a
0.01
Normalised Counts
100
150
200
250
het
hom
wt
zmp_ph108_F9
zmp_ph108_F11
zmp_ph108_H2
zmp_ph108_H5
zmp_ph108_B2
zmp_ph108_B6
zmp_ph108_B7
zmp_ph108_C4
zmp_ph108_C5
zmp_ph108_D4
zmp_ph97_G6
zmp_ph97_G9
zmp_ph97_H2
zmp_ph97_B10
zmp_ph97_C2
zmp_ph97_C4
zmp_ph97_B8
zmp_ph97_C1
zmp_ph97_D2
zmp_ph97_D5
zmp_ph111_G5
zmp_ph111_G11
zmp_ph111_H1
zmp_ph111_B1a
zmp_ph111_B5
zmp_ph111_B8
zmp_ph111_B6
zmp_ph111_B7
zmp_ph111_B9
zmp_ph111_B10
zmp_ph114_G4
zmp_ph114_G11
zmp_ph114_H1
zmp_ph114_H2
zmp_ph114_A9
zmp_ph114_A11
zmp_ph114_B2
zmp_ph114_B4
zmp_ph114_D8
zmp_ph114_E2

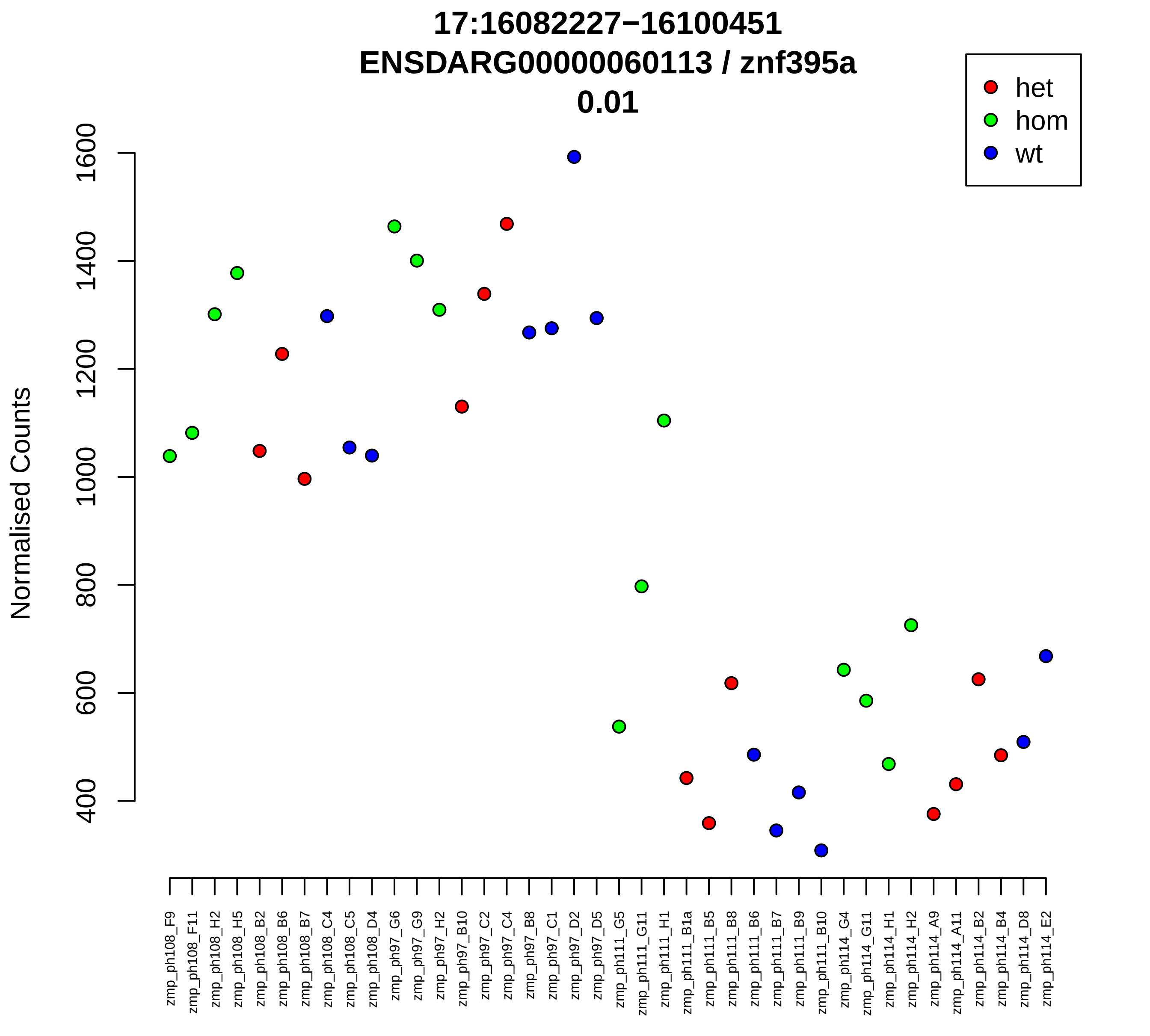

17:16082227−16100451
ENSDARG00000060113 / znf395a
0.01
het
hom
wt
Normalised Counts
400
600
800
1000
1200
1400
1600
zmp_ph108_F9
zmp_ph108_F11
zmp_ph108_H2
zmp_ph108_H5
zmp_ph108_B2
zmp_ph108_B6
zmp_ph108_B7
zmp_ph108_C4
zmp_ph108_C5
zmp_ph108_D4
zmp_ph97_G6
zmp_ph97_G9
zmp_ph97_H2
zmp_ph97_B10
zmp_ph97_C2
zmp_ph97_C4
zmp_ph97_B8
zmp_ph97_C1
zmp_ph97_D2
zmp_ph97_D5
zmp_ph111_G5
zmp_ph111_G11
zmp_ph111_H1
zmp_ph111_B1a
zmp_ph111_B5
zmp_ph111_B8
zmp_ph111_B6
zmp_ph111_B7
zmp_ph111_B9
zmp_ph111_B10
zmp_ph114_G4
zmp_ph114_G11
zmp_ph114_H1
zmp_ph114_H2
zmp_ph114_A9
zmp_ph114_A11
zmp_ph114_B2
zmp_ph114_B4
zmp_ph114_D8
zmp_ph114_E2

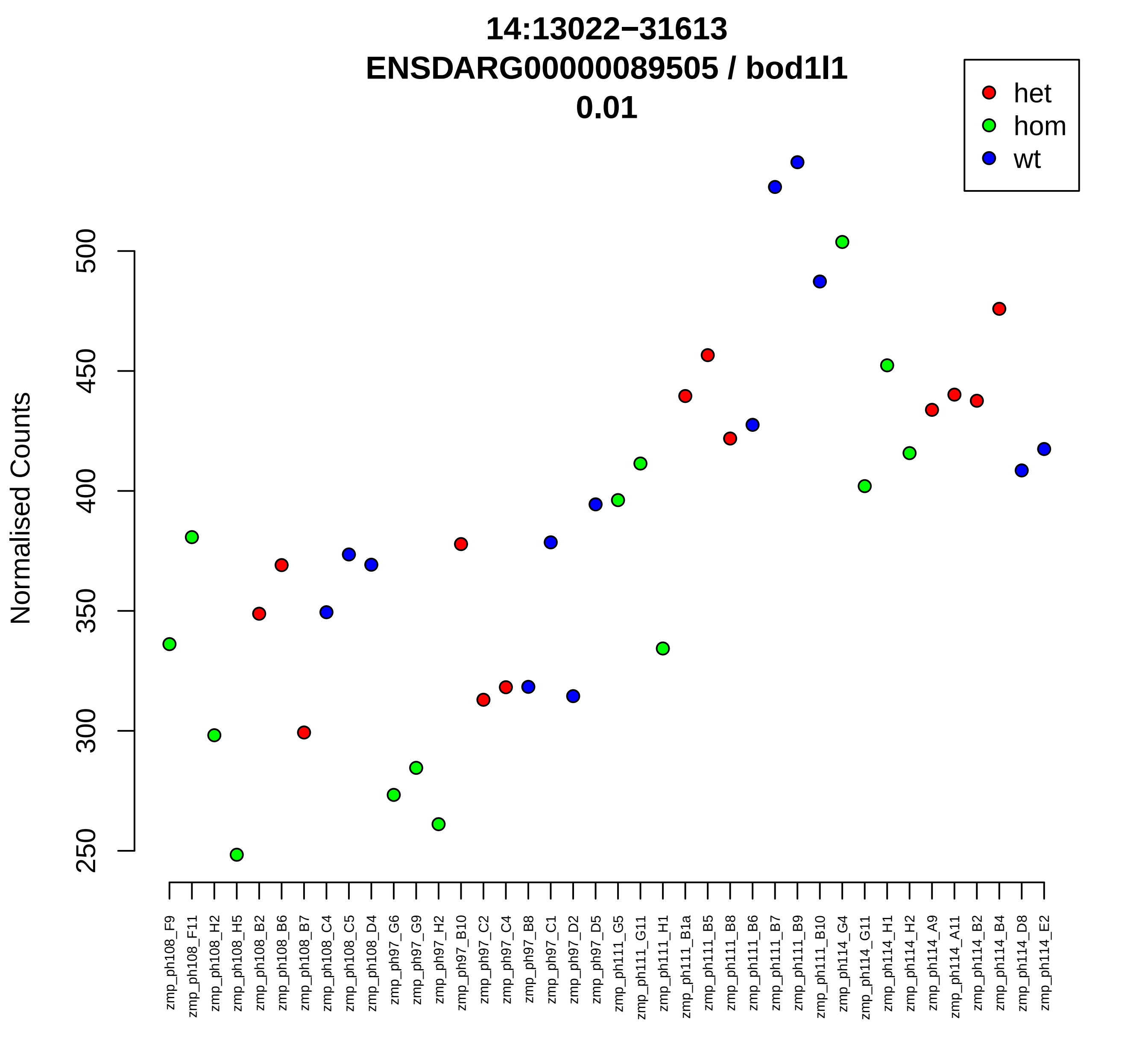
14:13022−31613
ENSDARG00000089505 / bod1l1
0.01
het
hom
wt
Normalised Counts
250
300
350
400
450
500
zmp_ph108_F9
zmp_ph108_F11
zmp_ph108_H2
zmp_ph108_H5
zmp_ph108_B2
zmp_ph108_B6
zmp_ph108_B7
zmp_ph108_C4
zmp_ph108_C5
zmp_ph108_D4
zmp_ph97_G6
zmp_ph97_G9
zmp_ph97_H2
zmp_ph97_B10
zmp_ph97_C2
zmp_ph97_C4
zmp_ph97_B8
zmp_ph97_C1
zmp_ph97_D2
zmp_ph97_D5
zmp_ph111_G5
zmp_ph111_G11
zmp_ph111_H1
zmp_ph111_B1a
zmp_ph111_B5
zmp_ph111_B8
zmp_ph111_B6
zmp_ph111_B7
zmp_ph111_B9
zmp_ph111_B10
zmp_ph114_G4
zmp_ph114_G11
zmp_ph114_H1
zmp_ph114_H2
zmp_ph114_A9
zmp_ph114_A11
zmp_ph114_B2
zmp_ph114_B4
zmp_ph114_D8
zmp_ph114_E2

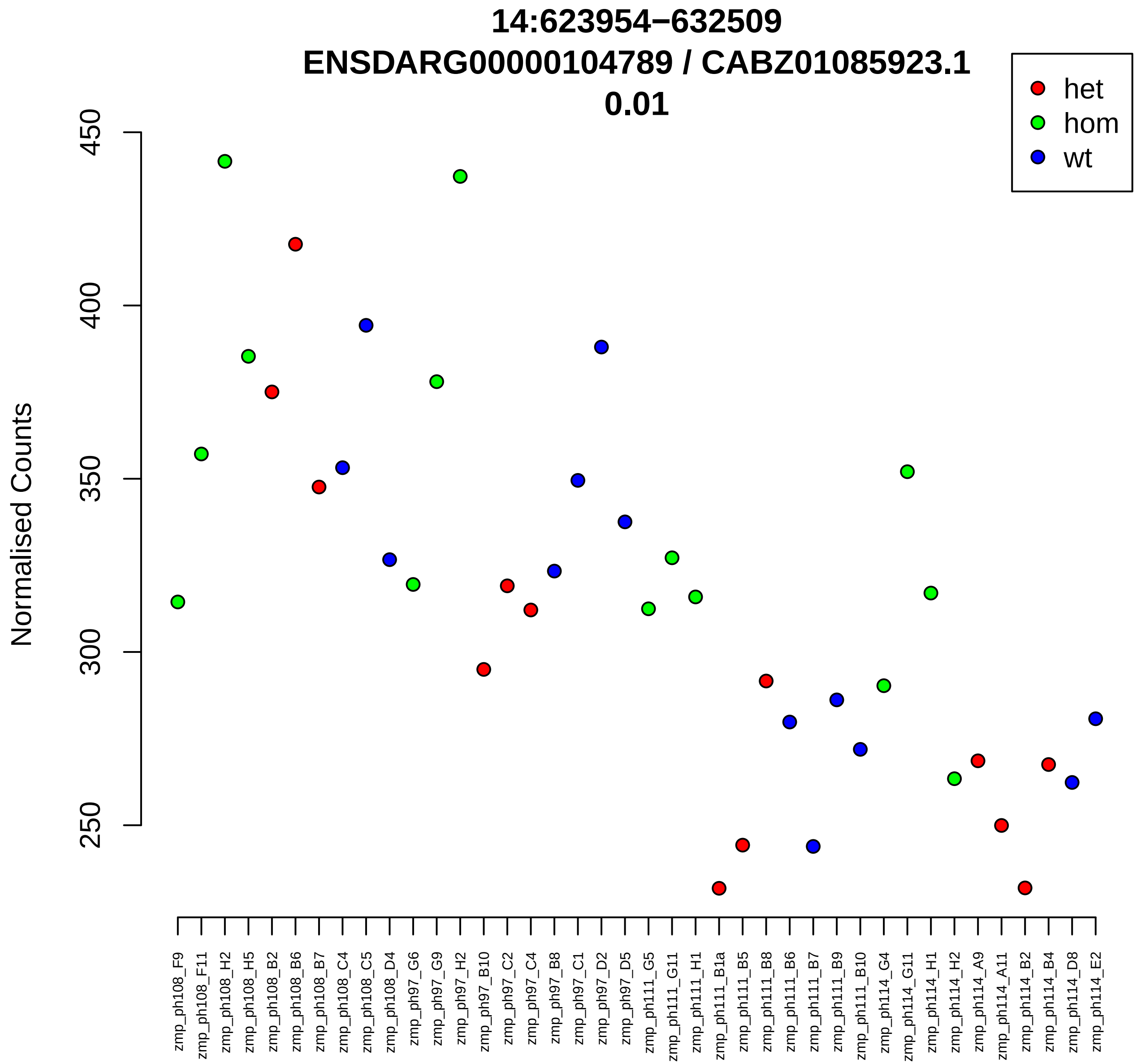
14:623954−632509
ENSDARG00000104789 / CABZ01085923.1
0.01
het
hom
wt
Normalised Counts
450
400
350
300
250
zmp_ph108_F9
zmp_ph108_F11
zmp_ph108_H2
zmp_ph108_H5
zmp_ph108_B2
zmp_ph108_B6
zmp_ph108_B7
zmp_ph108_C4
zmp_ph108_C5
zmp_ph108_D4
zmp_ph97_G6
zmp_ph97_G9
zmp_ph97_H2
zmp_ph97_B10
zmp_ph97_C2
zmp_ph97_C4
zmp_ph97_B8
zmp_ph97_C1
zmp_ph97_D2
zmp_ph97_D5
zmp_ph111_G5
zmp_ph111_G11
zmp_ph111_H1
zmp_ph111_B1a
zmp_ph111_B5
zmp_ph111_B8
zmp_ph111_B6
zmp_ph111_B7
zmp_ph111_B9
zmp_ph111_B10
zmp_ph114_G4
zmp_ph114_G11
zmp_ph114_H1
zmp_ph114_H2
zmp_ph114_A9
zmp_ph114_A11
zmp_ph114_B2
zmp_ph114_B4
zmp_ph114_D8
zmp_ph114_E2

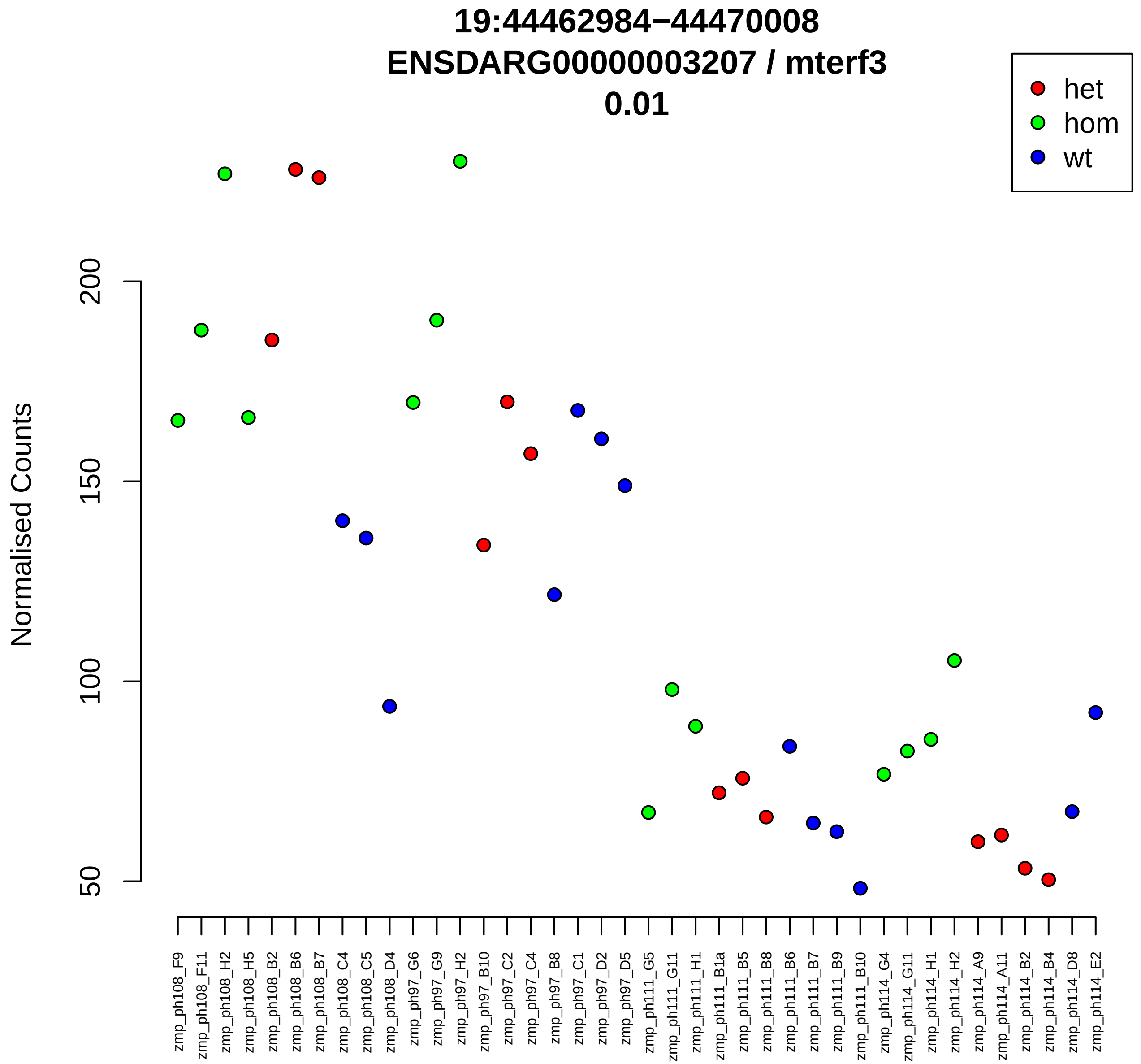

19:44462984−44470008
ENSDARG00000003207 / mterf3
0.01
het
hom
wt
Normalised Counts
200
150
100
50
zmp_ph108_F9
zmp_ph108_F11
zmp_ph108_H2
zmp_ph108_H5
zmp_ph108_B2
zmp_ph108_B6
zmp_ph108_B7
zmp_ph108_C4
zmp_ph108_C5
zmp_ph108_D4
zmp_ph97_G6
zmp_ph97_G9
zmp_ph97_H2
zmp_ph97_B10
zmp_ph97_C2
zmp_ph97_C4
zmp_ph97_B8
zmp_ph97_C1
zmp_ph97_D2
zmp_ph97_D5
zmp_ph111_G5
zmp_ph111_G11
zmp_ph111_H1
zmp_ph111_B1a
zmp_ph111_B5
zmp_ph111_B8
zmp_ph111_B6
zmp_ph111_B7
zmp_ph111_B9
zmp_ph111_B10
zmp_ph114_G4
zmp_ph114_G11
zmp_ph114_H1
zmp_ph114_H2
zmp_ph114_A9
zmp_ph114_A11
zmp_ph114_B2
zmp_ph114_B4
zmp_ph114_D8
zmp_ph114_E2

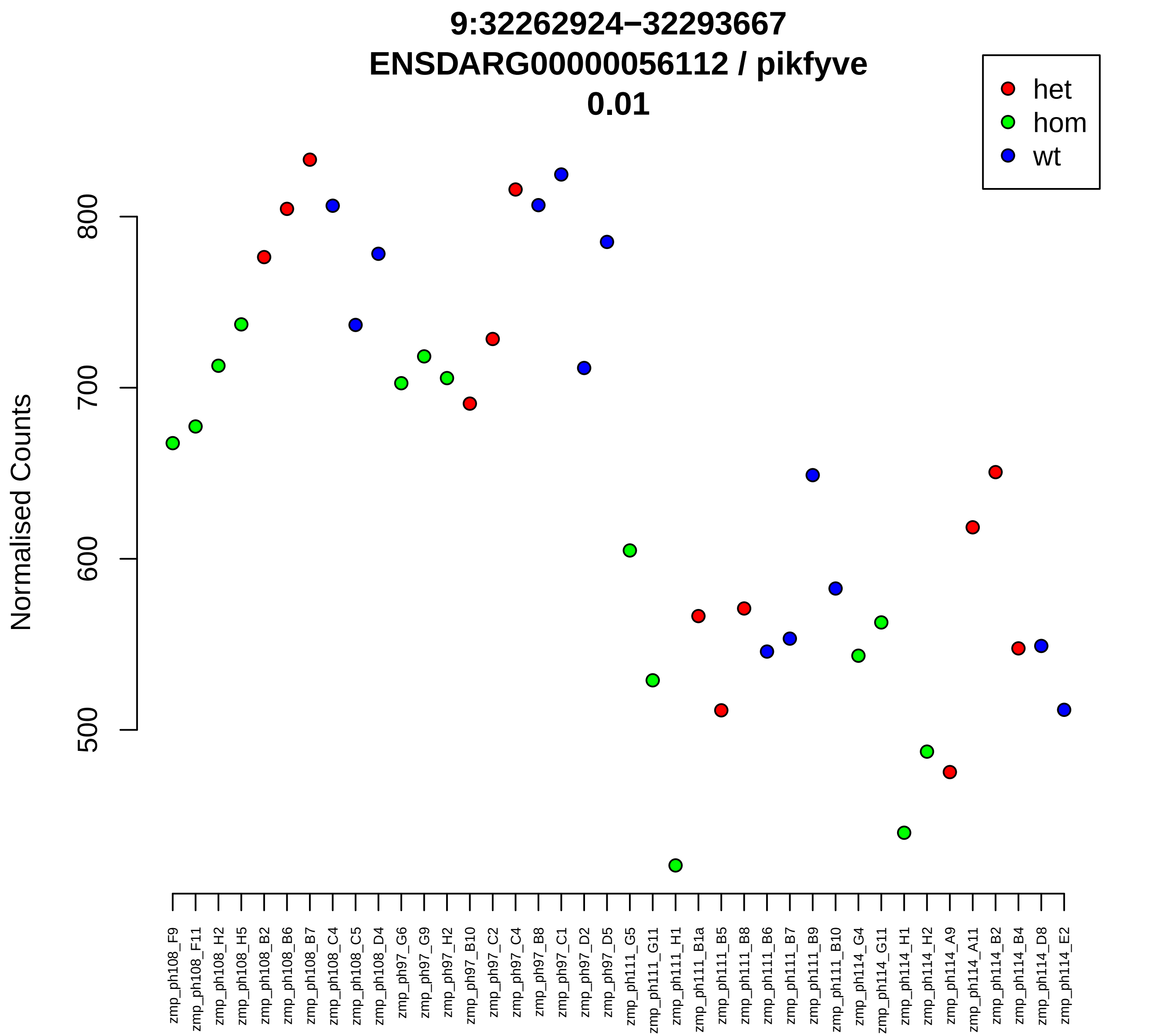
9:32262924−32293667
ENSDARG00000056112 / pikfyve
0.01
het
hom
wt
Normalised Counts
500
600
700
800
zmp_ph108_F9
zmp_ph108_F11
zmp_ph108_H2
zmp_ph108_H5
zmp_ph108_B2
zmp_ph108_B6
zmp_ph108_B7
zmp_ph108_C4
zmp_ph108_C5
zmp_ph108_D4
zmp_ph97_G6
zmp_ph97_G9
zmp_ph97_H2
zmp_ph97_B10
zmp_ph97_C2
zmp_ph97_C4
zmp_ph97_B8
zmp_ph97_C1
zmp_ph97_D2
zmp_ph97_D5
zmp_ph111_G5
zmp_ph111_G11
zmp_ph111_H1
zmp_ph111_B1a
zmp_ph111_B5
zmp_ph111_B8
zmp_ph111_B6
zmp_ph111_B7
zmp_ph111_B9
zmp_ph111_B10
zmp_ph114_G4
zmp_ph114_G11
zmp_ph114_H1
zmp_ph114_H2
zmp_ph114_A9
zmp_ph114_A11
zmp_ph114_B2
zmp_ph114_B4
zmp_ph114_D8
zmp_ph114_E2

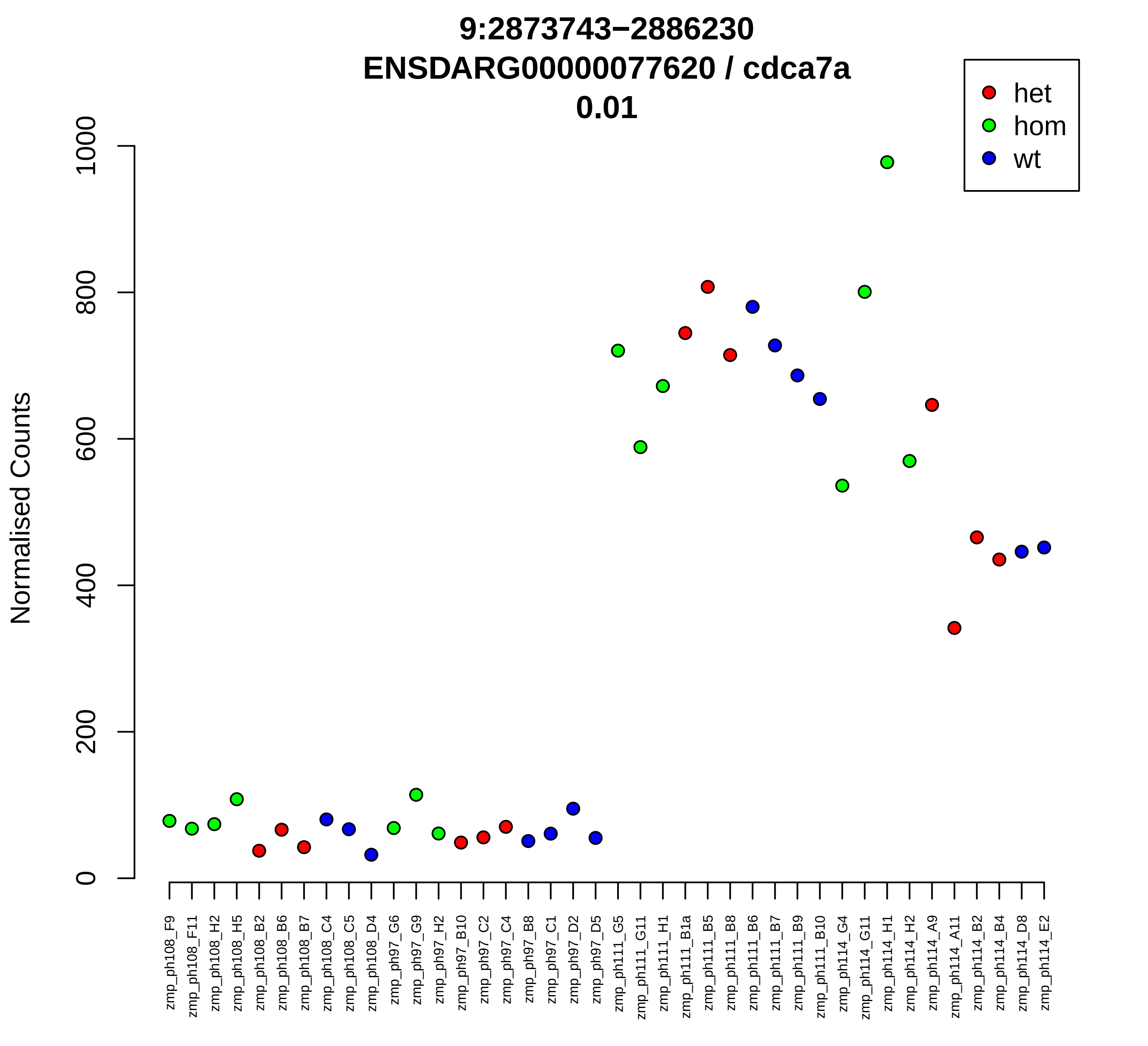

9:2873743−2886230
ENSDARG00000077620 / cdca7a
0.01
het
hom
wt
Normalised Counts
0
200
400
600
800
1000
zmp_ph108_F9
zmp_ph108_F11
zmp_ph108_H2
zmp_ph108_H5
zmp_ph108_B2
zmp_ph108_B6
zmp_ph108_B7
zmp_ph108_C4
zmp_ph108_C5
zmp_ph108_D4
zmp_ph97_G6
zmp_ph97_G9
zmp_ph97_H2
zmp_ph97_B10
zmp_ph97_C2
zmp_ph97_C4
zmp_ph97_B8
zmp_ph97_C1
zmp_ph97_D2
zmp_ph97_D5
zmp_ph111_G5
zmp_ph111_G11
zmp_ph111_H1
zmp_ph111_B1a
zmp_ph111_B5
zmp_ph111_B8
zmp_ph111_B6
zmp_ph111_B7
zmp_ph111_B9
zmp_ph111_B10
zmp_ph114_G4
zmp_ph114_G11
zmp_ph114_H1
zmp_ph114_H2
zmp_ph114_A9
zmp_ph114_A11
zmp_ph114_B2
zmp_ph114_B4
zmp_ph114_D8
zmp_ph114_E2

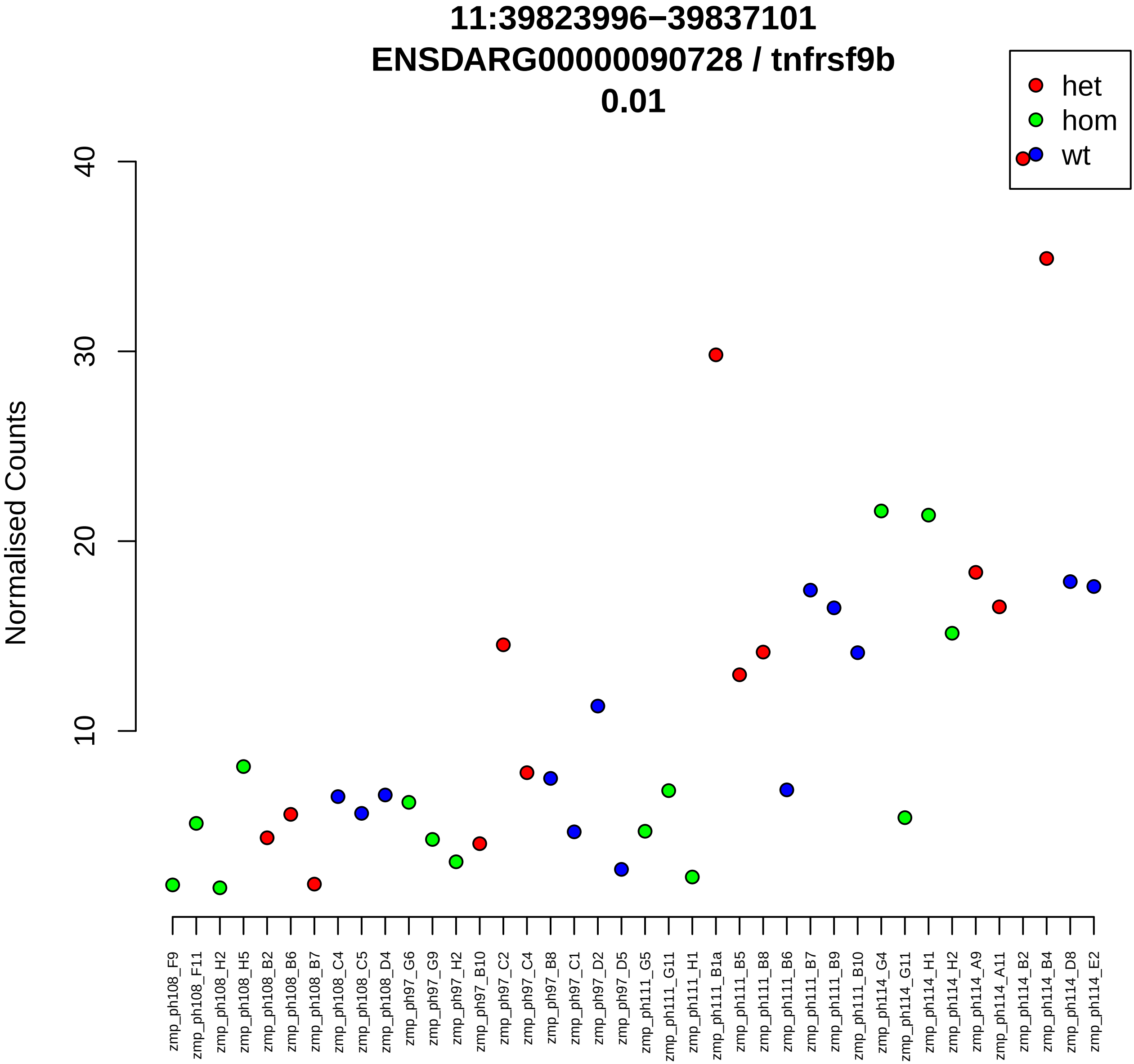

11:39823996−39837101
ENSDARG00000090728 / tnfrsf9b
0.01
het
hom
wt
Normalised Counts
10
20
30
40
zmp_ph108_F9
zmp_ph108_F11
zmp_ph108_H2
zmp_ph108_H5
zmp_ph108_B2
zmp_ph108_B6
zmp_ph108_B7
zmp_ph108_C4
zmp_ph108_C5
zmp_ph108_D4
zmp_ph97_G6
zmp_ph97_G9
zmp_ph97_H2
zmp_ph97_B10
zmp_ph97_C2
zmp_ph97_C4
zmp_ph97_B8
zmp_ph97_C1
zmp_ph97_D2
zmp_ph97_D5
zmp_ph111_G5
zmp_ph111_G11
zmp_ph111_H1
zmp_ph111_B1a
zmp_ph111_B5
zmp_ph111_B8
zmp_ph111_B6
zmp_ph111_B7
zmp_ph111_B9
zmp_ph111_B10
zmp_ph114_G4
zmp_ph114_G11
zmp_ph114_H1
zmp_ph114_H2
zmp_ph114_A9
zmp_ph114_A11
zmp_ph114_B2
zmp_ph114_B4
zmp_ph114_D8
zmp_ph114_E2

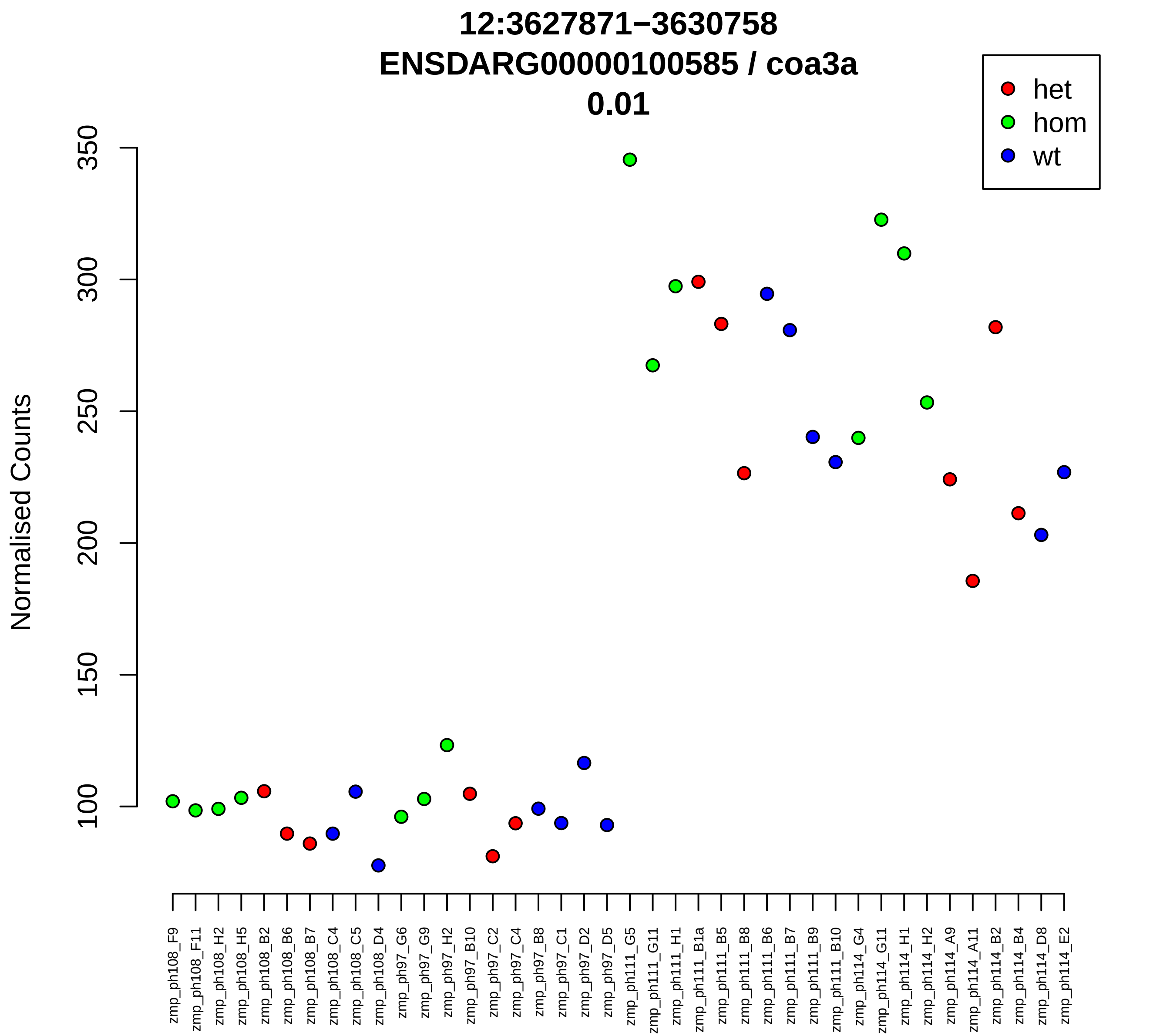

12:3627871−3630758
ENSDARG00000100585 / coa3a
0.01
het
hom
wt
Normalised Counts
100
150
200
250
300
350
zmp_ph108_F9
zmp_ph108_F11
zmp_ph108_H2
zmp_ph108_H5
zmp_ph108_B2
zmp_ph108_B6
zmp_ph108_B7
zmp_ph108_C4
zmp_ph108_C5
zmp_ph108_D4
zmp_ph97_G6
zmp_ph97_G9
zmp_ph97_H2
zmp_ph97_B10
zmp_ph97_C2
zmp_ph97_C4
zmp_ph97_B8
zmp_ph97_C1
zmp_ph97_D2
zmp_ph97_D5
zmp_ph111_G5
zmp_ph111_G11
zmp_ph111_H1
zmp_ph111_B1a
zmp_ph111_B5
zmp_ph111_B8
zmp_ph111_B6
zmp_ph111_B7
zmp_ph111_B9
zmp_ph111_B10
zmp_ph114_G4
zmp_ph114_G11
zmp_ph114_H1
zmp_ph114_H2
zmp_ph114_A9
zmp_ph114_A11
zmp_ph114_B2
zmp_ph114_B4
zmp_ph114_D8
zmp_ph114_E2

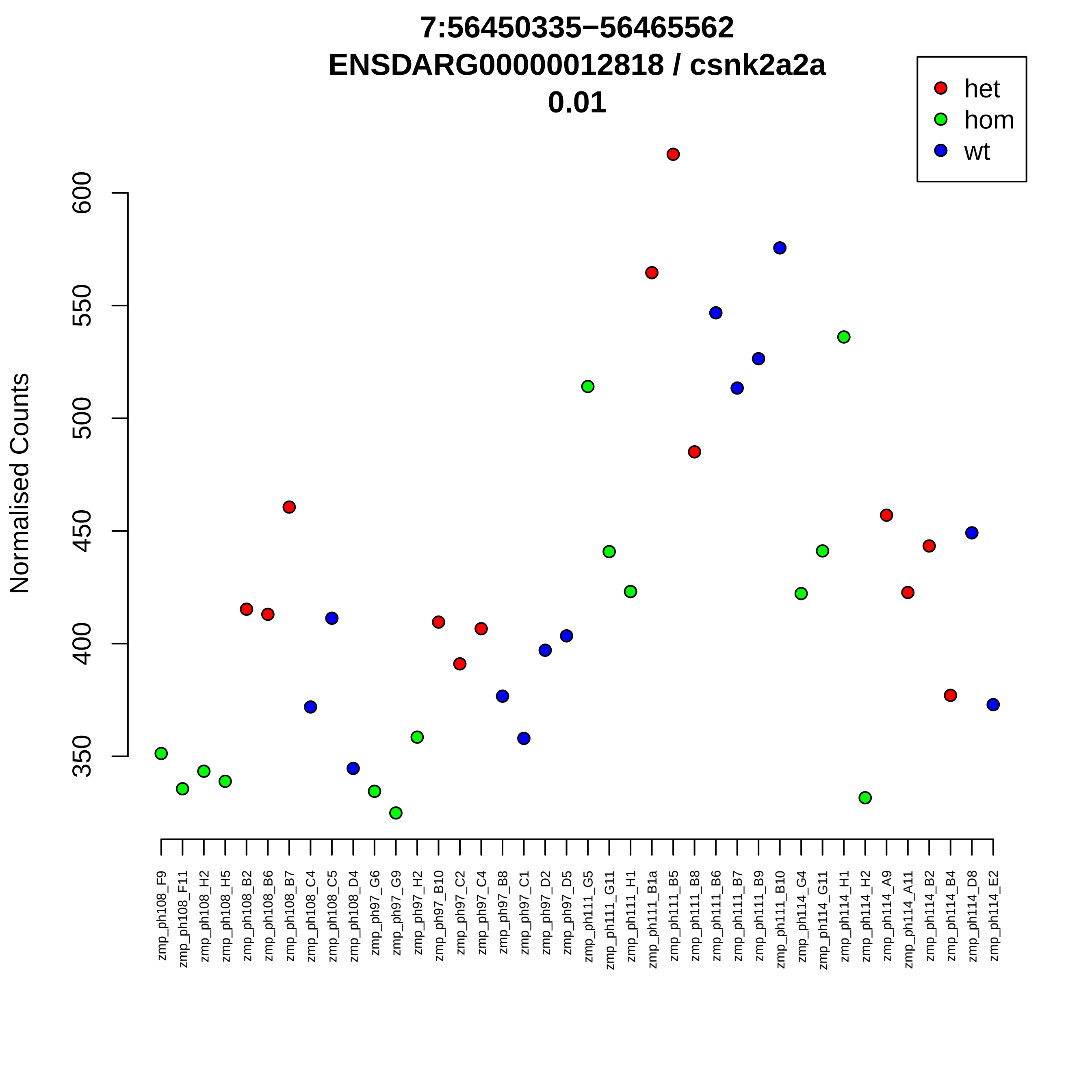
7:56450335−56465562
ENSDARG00000012818 / csnk2a2a
0.01
het
hom
wt
Normalised Counts
350
400
450
500
550
600
zmp_ph108_F9
zmp_ph108_F11
zmp_ph108_H2
zmp_ph108_H5
zmp_ph108_B2
zmp_ph108_B6
zmp_ph108_B7
zmp_ph108_C4
zmp_ph108_C5
zmp_ph108_D4
zmp_ph97_G6
zmp_ph97_G9
zmp_ph97_H2
zmp_ph97_B10
zmp_ph97_C2
zmp_ph97_C4
zmp_ph97_B8
zmp_ph97_C1
zmp_ph97_D2
zmp_ph97_D5
zmp_ph111_G5
zmp_ph111_G11
zmp_ph111_H1
zmp_ph111_B1a
zmp_ph111_B5
zmp_ph111_B8
zmp_ph111_B6
zmp_ph111_B7
zmp_ph111_B9
zmp_ph111_B10
zmp_ph114_G4
zmp_ph114_G11
zmp_ph114_H1
zmp_ph114_H2
zmp_ph114_A9
zmp_ph114_A11
zmp_ph114_B2
zmp_ph114_B4
zmp_ph114_D8
zmp_ph114_E2

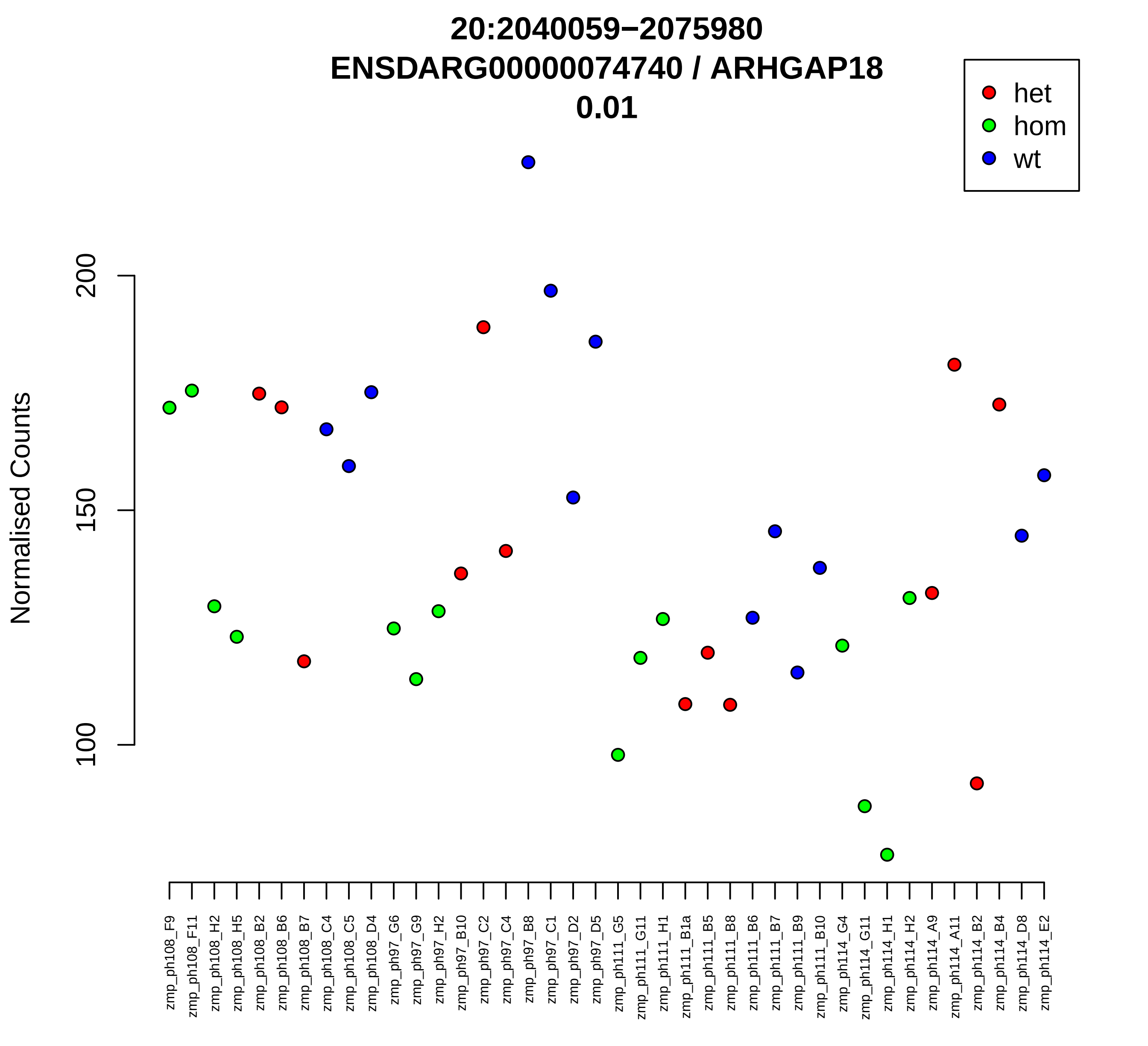
20:2040059−2075980
ENSDARG00000074740 / ARHGAP18
0.01
het
hom
wt
Normalised Counts
200
150
100
zmp_ph108_F9
zmp_ph108_F11
zmp_ph108_H2
zmp_ph108_H5
zmp_ph108_B2
zmp_ph108_B6
zmp_ph108_B7
zmp_ph108_C4
zmp_ph108_C5
zmp_ph108_D4
zmp_ph97_G6
zmp_ph97_G9
zmp_ph97_H2
zmp_ph97_B10
zmp_ph97_C2
zmp_ph97_C4
zmp_ph97_B8
zmp_ph97_C1
zmp_ph97_D2
zmp_ph97_D5
zmp_ph111_G5
zmp_ph111_G11
zmp_ph111_H1
zmp_ph111_B1a
zmp_ph111_B5
zmp_ph111_B8
zmp_ph111_B6
zmp_ph111_B7
zmp_ph111_B9
zmp_ph111_B10
zmp_ph114_G4
zmp_ph114_G11
zmp_ph114_H1
zmp_ph114_H2
zmp_ph114_A9
zmp_ph114_A11
zmp_ph114_B2
zmp_ph114_B4
zmp_ph114_D8
zmp_ph114_E2

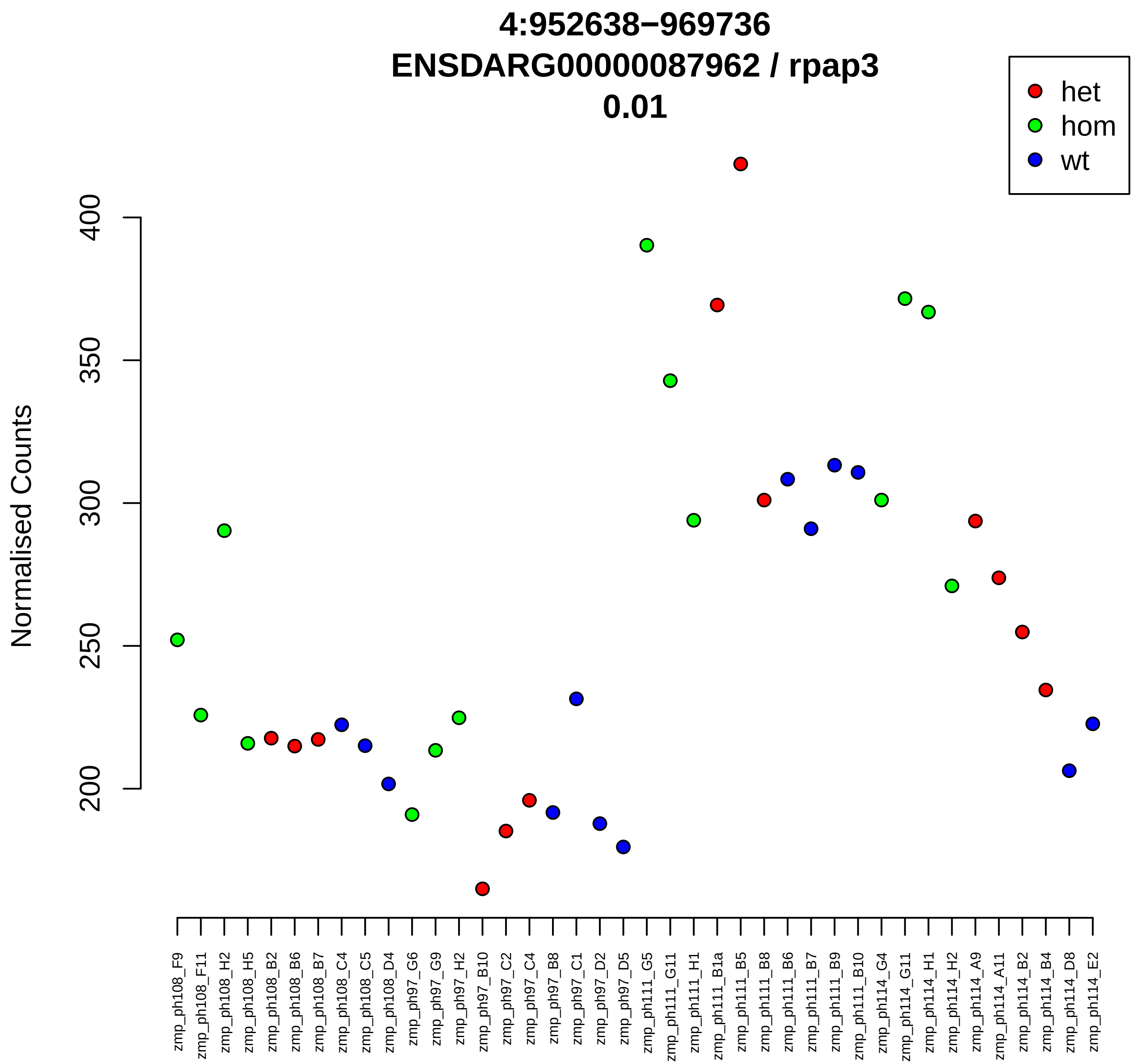

4:952638−969736
ENSDARG00000087962 / rpap3
0.01
het
hom
wt
Normalised Counts
200
250
300
350
400
zmp_ph108_F9
zmp_ph108_F11
zmp_ph108_H2
zmp_ph108_H5
zmp_ph108_B2
zmp_ph108_B6
zmp_ph108_B7
zmp_ph108_C4
zmp_ph108_C5
zmp_ph108_D4
zmp_ph97_G6
zmp_ph97_G9
zmp_ph97_H2
zmp_ph97_B10
zmp_ph97_C2
zmp_ph97_C4
zmp_ph97_B8
zmp_ph97_C1
zmp_ph97_D2
zmp_ph97_D5
zmp_ph111_G5
zmp_ph111_G11
zmp_ph111_H1
zmp_ph111_B1a
zmp_ph111_B5
zmp_ph111_B8
zmp_ph111_B6
zmp_ph111_B7
zmp_ph111_B9
zmp_ph111_B10
zmp_ph114_G4
zmp_ph114_G11
zmp_ph114_H1
zmp_ph114_H2
zmp_ph114_A9
zmp_ph114_A11
zmp_ph114_B2
zmp_ph114_B4
zmp_ph114_D8
zmp_ph114_E2

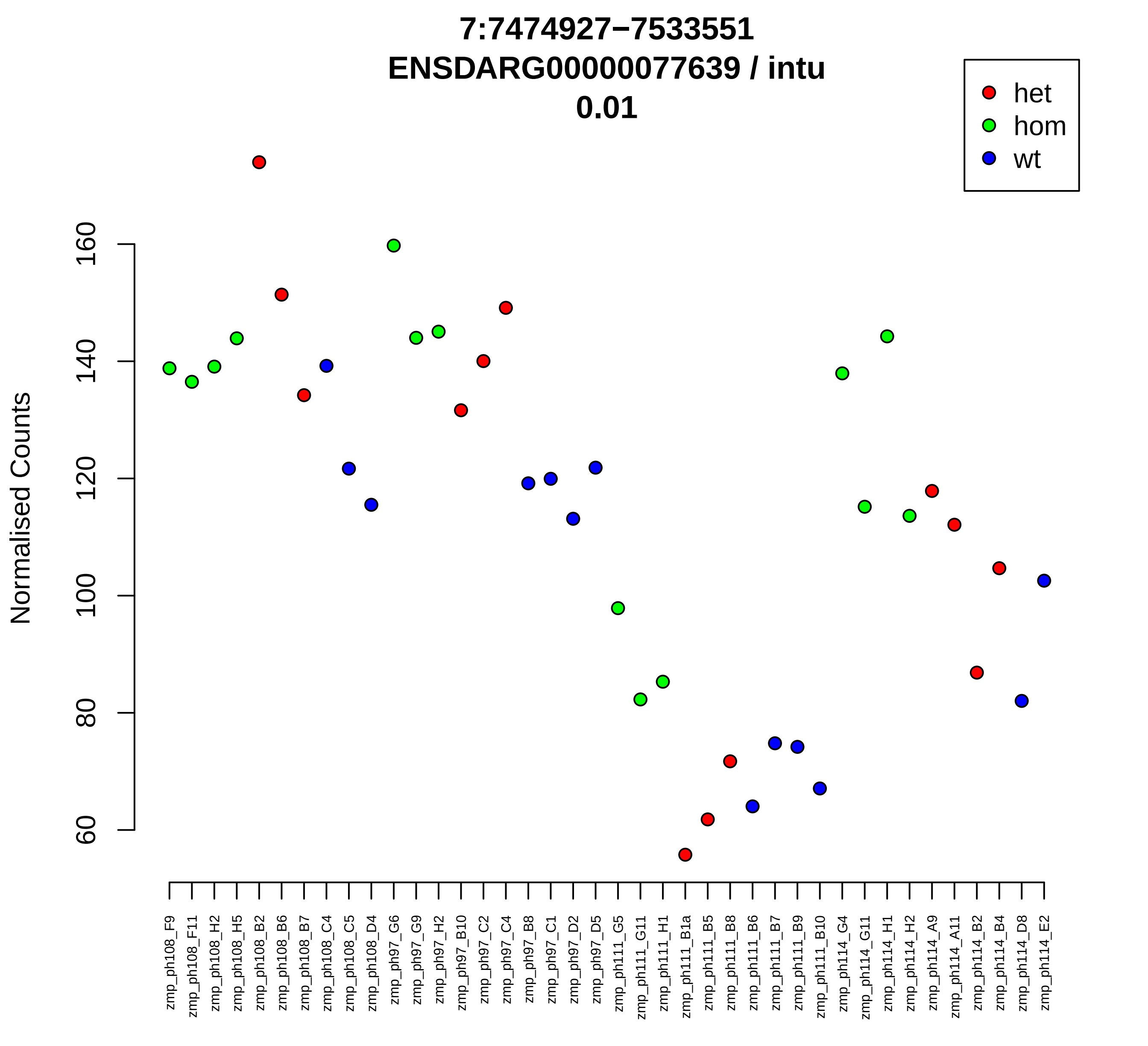
7:7474927−7533551
ENSDARG00000077639 / intu
0.01
het
hom
wt
Normalised Counts
60
80
100
120
140
160
zmp_ph108_F9
zmp_ph108_F11
zmp_ph108_H2
zmp_ph108_H5
zmp_ph108_B2
zmp_ph108_B6
zmp_ph108_B7
zmp_ph108_C4
zmp_ph108_C5
zmp_ph108_D4
zmp_ph97_G6
zmp_ph97_G9
zmp_ph97_H2
zmp_ph97_B10
zmp_ph97_C2
zmp_ph97_C4
zmp_ph97_B8
zmp_ph97_C1
zmp_ph97_D2
zmp_ph97_D5
zmp_ph111_G5
zmp_ph111_G11
zmp_ph111_H1
zmp_ph111_B1a
zmp_ph111_B5
zmp_ph111_B8
zmp_ph111_B6
zmp_ph111_B7
zmp_ph111_B9
zmp_ph111_B10
zmp_ph114_G4
zmp_ph114_G11
zmp_ph114_H1
zmp_ph114_H2
zmp_ph114_A9
zmp_ph114_A11
zmp_ph114_B2
zmp_ph114_B4
zmp_ph114_D8
zmp_ph114_E2

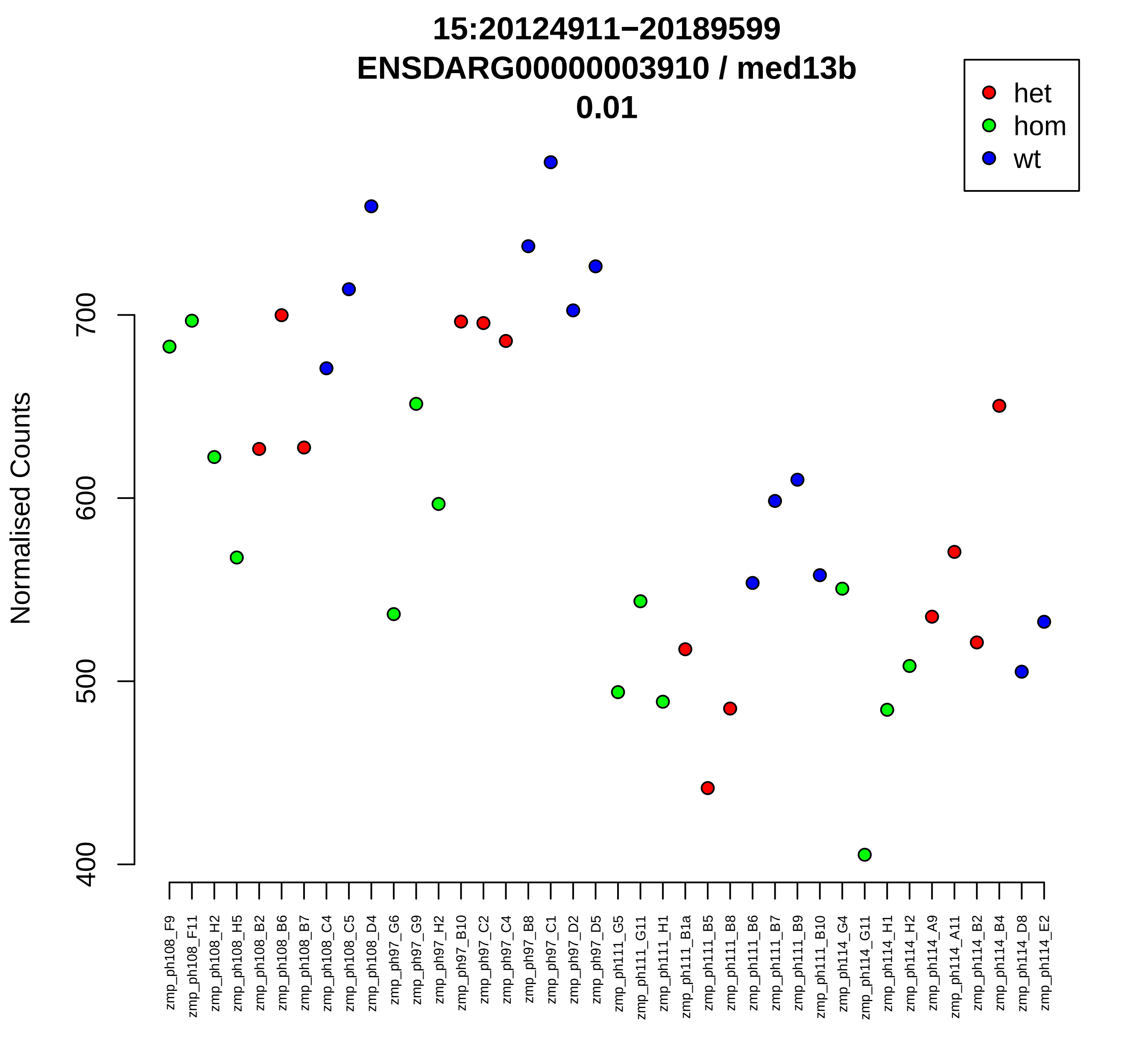

15:20124911−20189599
ENSDARG00000003910 / med13b
0.01
het
hom
wt
Normalised Counts
400
500
600
700
zmp_ph108_F9
zmp_ph108_F11
zmp_ph108_H2
zmp_ph108_H5
zmp_ph108_B2
zmp_ph108_B6
zmp_ph108_B7
zmp_ph108_C4
zmp_ph108_C5
zmp_ph108_D4
zmp_ph97_G6
zmp_ph97_G9
zmp_ph97_H2
zmp_ph97_B10
zmp_ph97_C2
zmp_ph97_C4
zmp_ph97_B8
zmp_ph97_C1
zmp_ph97_D2
zmp_ph97_D5
zmp_ph111_G5
zmp_ph111_G11
zmp_ph111_H1
zmp_ph111_B1a
zmp_ph111_B5
zmp_ph111_B8
zmp_ph111_B6
zmp_ph111_B7
zmp_ph111_B9
zmp_ph111_B10
zmp_ph114_G4
zmp_ph114_G11
zmp_ph114_H1
zmp_ph114_H2
zmp_ph114_A9
zmp_ph114_A11
zmp_ph114_B2
zmp_ph114_B4
zmp_ph114_D8
zmp_ph114_E2

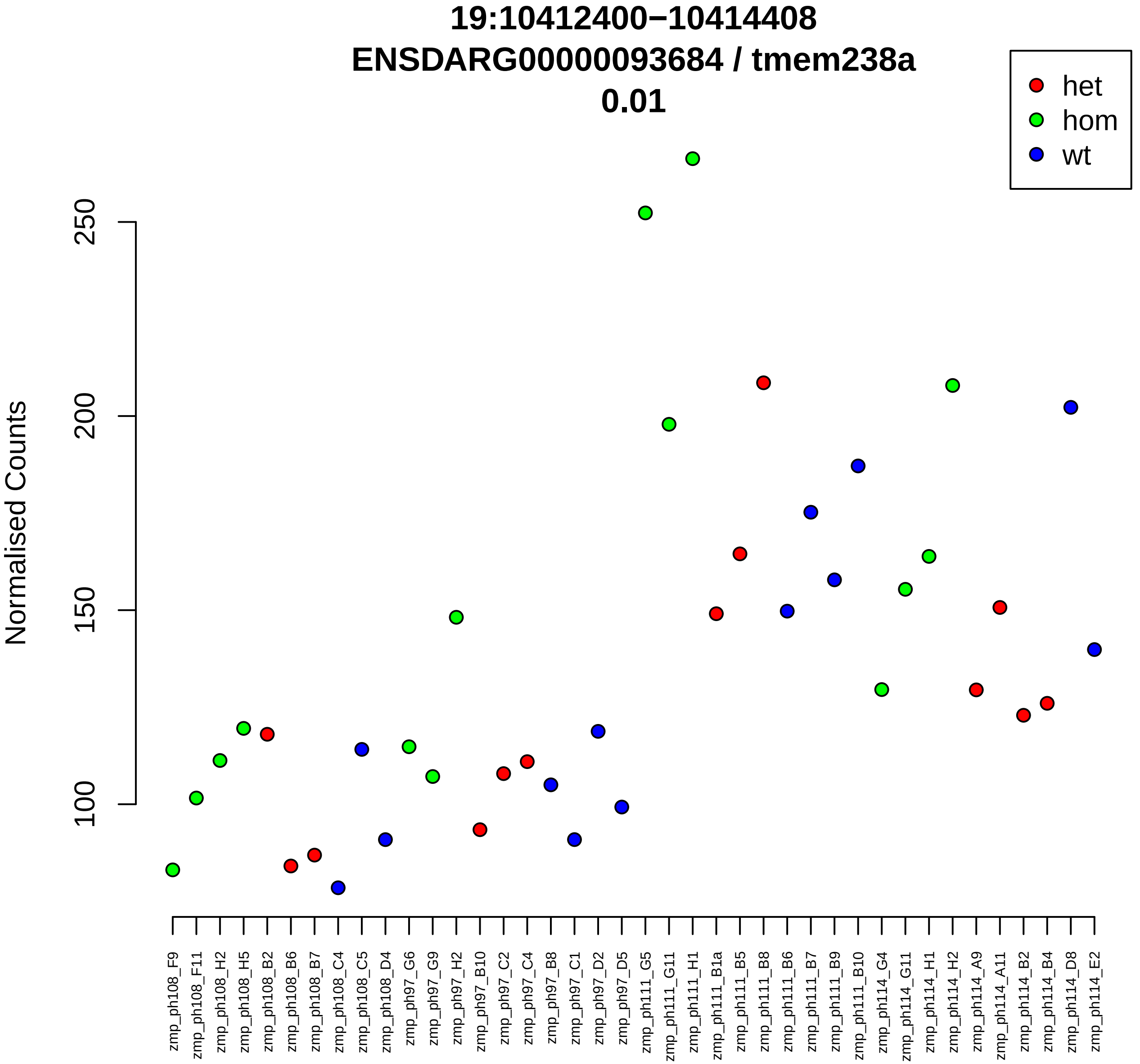

19:10412400−10414408
ENSDARG00000093684 / tmem238a
0.01
het
hom
wt
Normalised Counts
100
150
200
250
zmp_ph108_F9
zmp_ph108_F11
zmp_ph108_H2
zmp_ph108_H5
zmp_ph108_B2
zmp_ph108_B6
zmp_ph108_B7
zmp_ph108_C4
zmp_ph108_C5
zmp_ph108_D4
zmp_ph97_G6
zmp_ph97_G9
zmp_ph97_H2
zmp_ph97_B10
zmp_ph97_C2
zmp_ph97_C4
zmp_ph97_B8
zmp_ph97_C1
zmp_ph97_D2
zmp_ph97_D5
zmp_ph111_G5
zmp_ph111_G11
zmp_ph111_H1
zmp_ph111_B1a
zmp_ph111_B5
zmp_ph111_B8
zmp_ph111_B6
zmp_ph111_B7
zmp_ph111_B9
zmp_ph111_B10
zmp_ph114_G4
zmp_ph114_G11
zmp_ph114_H1
zmp_ph114_H2
zmp_ph114_A9
zmp_ph114_A11
zmp_ph114_B2
zmp_ph114_B4
zmp_ph114_D8
zmp_ph114_E2

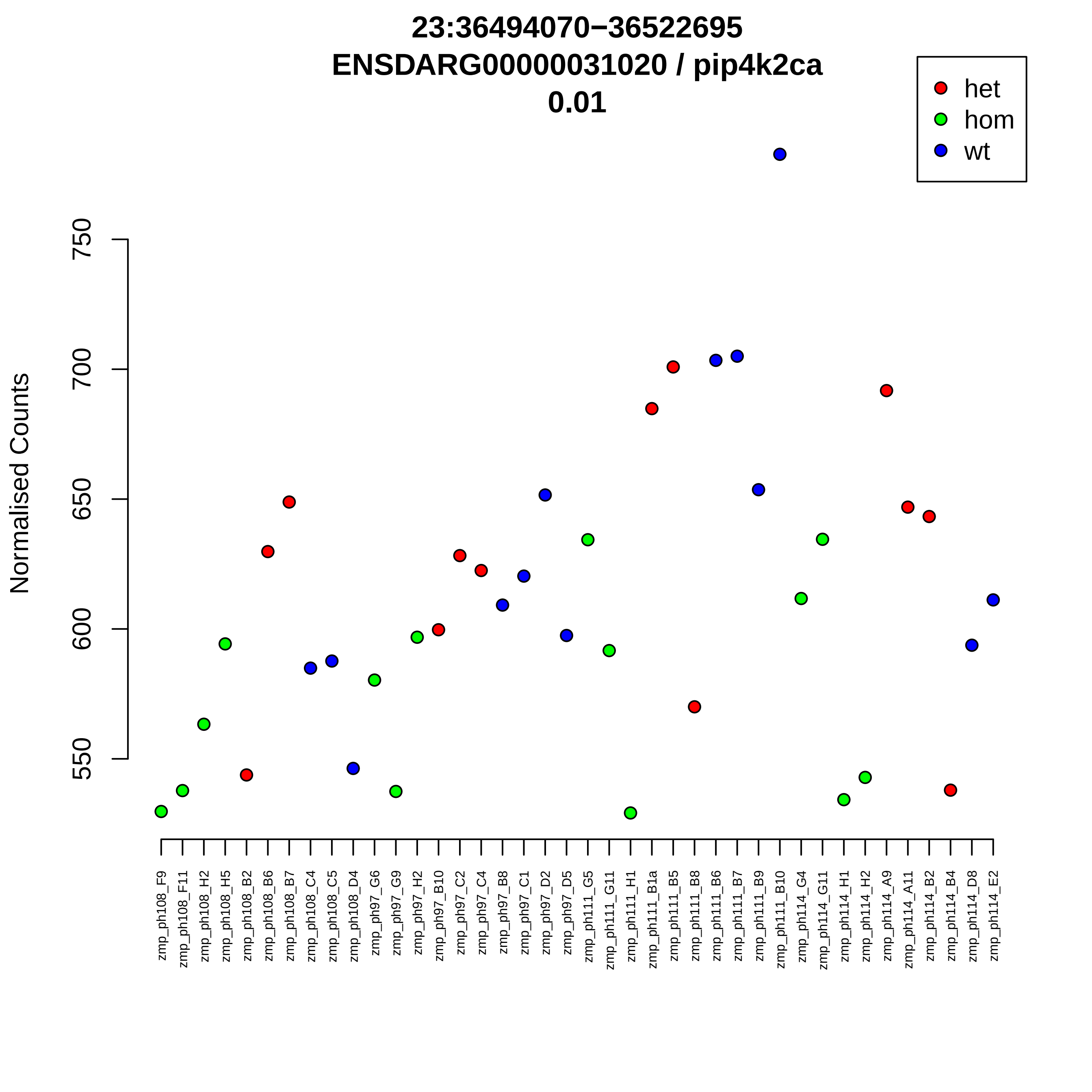
23:36494070−36522695
ENSDARG00000031020 / pip4k2ca
0.01
het
hom
wt
Normalised Counts
550
600
650
700
750
zmp_ph108_F9
zmp_ph108_F11
zmp_ph108_H2
zmp_ph108_H5
zmp_ph108_B2
zmp_ph108_B6
zmp_ph108_B7
zmp_ph108_C4
zmp_ph108_C5
zmp_ph108_D4
zmp_ph97_G6
zmp_ph97_G9
zmp_ph97_H2
zmp_ph97_B10
zmp_ph97_C2
zmp_ph97_C4
zmp_ph97_B8
zmp_ph97_C1
zmp_ph97_D2
zmp_ph97_D5
zmp_ph111_G5
zmp_ph111_G11
zmp_ph111_H1
zmp_ph111_B1a
zmp_ph111_B5
zmp_ph111_B8
zmp_ph111_B6
zmp_ph111_B7
zmp_ph111_B9
zmp_ph111_B10
zmp_ph114_G4
zmp_ph114_G11
zmp_ph114_H1
zmp_ph114_H2
zmp_ph114_A9
zmp_ph114_A11
zmp_ph114_B2
zmp_ph114_B4
zmp_ph114_D8
zmp_ph114_E2

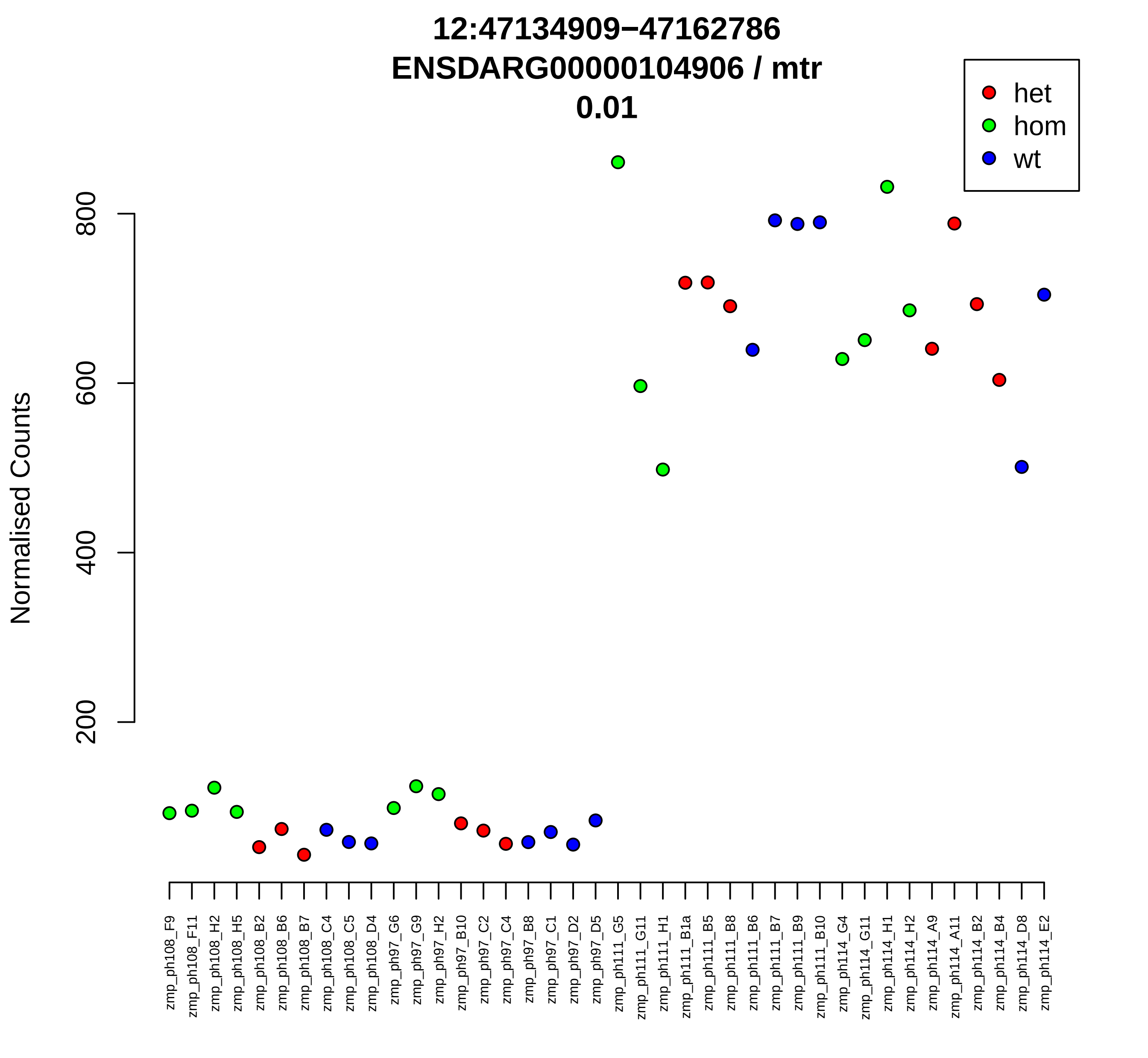
12:47134909−47162786
ENSDARG00000104906 / mtr
0.01
het
hom
wt
Normalised Counts
200
400
600
800
zmp_ph108_F9
zmp_ph108_F11
zmp_ph108_H2
zmp_ph108_H5
zmp_ph108_B2
zmp_ph108_B6
zmp_ph108_B7
zmp_ph108_C4
zmp_ph108_C5
zmp_ph108_D4
zmp_ph97_G6
zmp_ph97_G9
zmp_ph97_H2
zmp_ph97_B10
zmp_ph97_C2
zmp_ph97_C4
zmp_ph97_B8
zmp_ph97_C1
zmp_ph97_D2
zmp_ph97_D5
zmp_ph111_G5
zmp_ph111_G11
zmp_ph111_H1
zmp_ph111_B1a
zmp_ph111_B5
zmp_ph111_B8
zmp_ph111_B6
zmp_ph111_B7
zmp_ph111_B9
zmp_ph111_B10
zmp_ph114_G4
zmp_ph114_G11
zmp_ph114_H1
zmp_ph114_H2
zmp_ph114_A9
zmp_ph114_A11
zmp_ph114_B2
zmp_ph114_B4
zmp_ph114_D8
zmp_ph114_E2

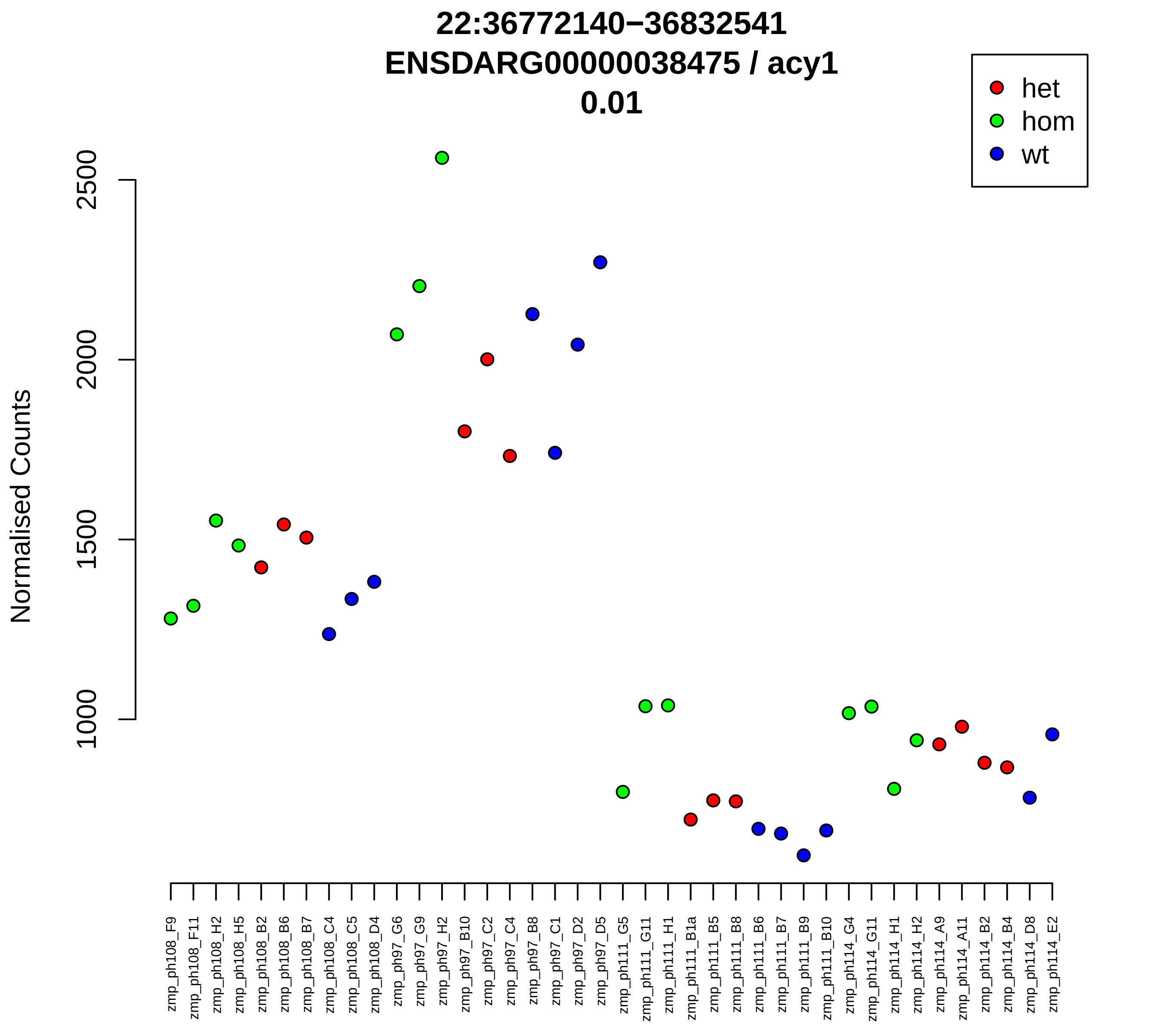
22:36772140−36832541
ENSDARG00000038475 / acy1
0.01
het
hom
wt
Normalised Counts
2500
2000
1500
1000
zmp_ph108_F9
zmp_ph108_F11
zmp_ph108_H2
zmp_ph108_H5
zmp_ph108_B2
zmp_ph108_B6
zmp_ph108_B7
zmp_ph108_C4
zmp_ph108_C5
zmp_ph108_D4
zmp_ph97_G6
zmp_ph97_G9
zmp_ph97_H2
zmp_ph97_B10
zmp_ph97_C2
zmp_ph97_C4
zmp_ph97_B8
zmp_ph97_C1
zmp_ph97_D2
zmp_ph97_D5
zmp_ph111_G5
zmp_ph111_G11
zmp_ph111_H1
zmp_ph111_B1a
zmp_ph111_B5
zmp_ph111_B8
zmp_ph111_B6
zmp_ph111_B7
zmp_ph111_B9
zmp_ph111_B10
zmp_ph114_G4
zmp_ph114_G11
zmp_ph114_H1
zmp_ph114_H2
zmp_ph114_A9
zmp_ph114_A11
zmp_ph114_B2
zmp_ph114_B4
zmp_ph114_D8
zmp_ph114_E2

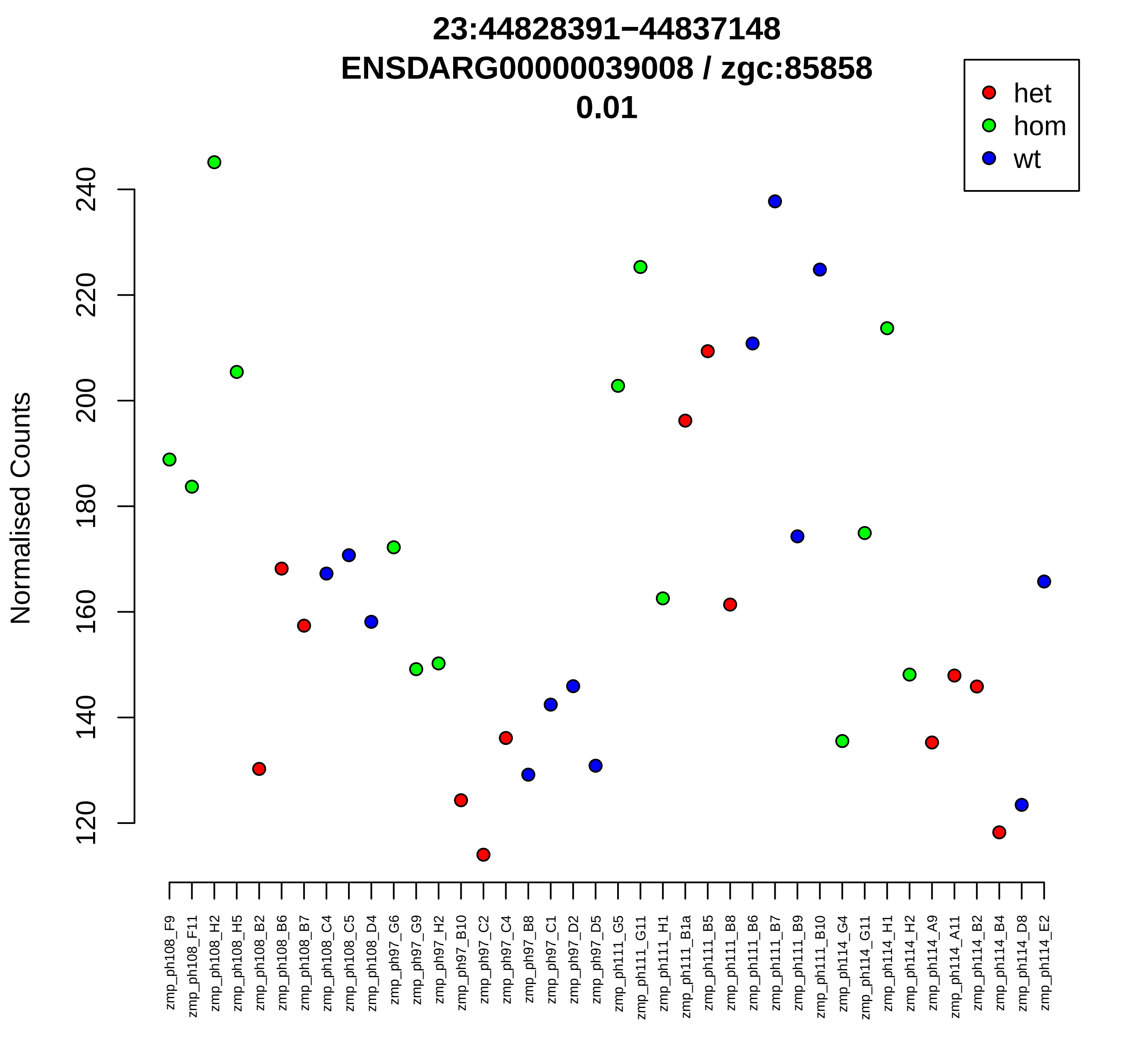
23:44828391−44837148
ENSDARG00000039008 / zgc:85858
0.01
het
hom
wt
Normalised Counts
120
140
160
180
200
220
240
zmp_ph108_F9
zmp_ph108_F11
zmp_ph108_H2
zmp_ph108_H5
zmp_ph108_B2
zmp_ph108_B6
zmp_ph108_B7
zmp_ph108_C4
zmp_ph108_C5
zmp_ph108_D4
zmp_ph97_G6
zmp_ph97_G9
zmp_ph97_H2
zmp_ph97_B10
zmp_ph97_C2
zmp_ph97_C4
zmp_ph97_B8
zmp_ph97_C1
zmp_ph97_D2
zmp_ph97_D5
zmp_ph111_G5
zmp_ph111_G11
zmp_ph111_H1
zmp_ph111_B1a
zmp_ph111_B5
zmp_ph111_B8
zmp_ph111_B6
zmp_ph111_B7
zmp_ph111_B9
zmp_ph111_B10
zmp_ph114_G4
zmp_ph114_G11
zmp_ph114_H1
zmp_ph114_H2
zmp_ph114_A9
zmp_ph114_A11
zmp_ph114_B2
zmp_ph114_B4
zmp_ph114_D8
zmp_ph114_E2

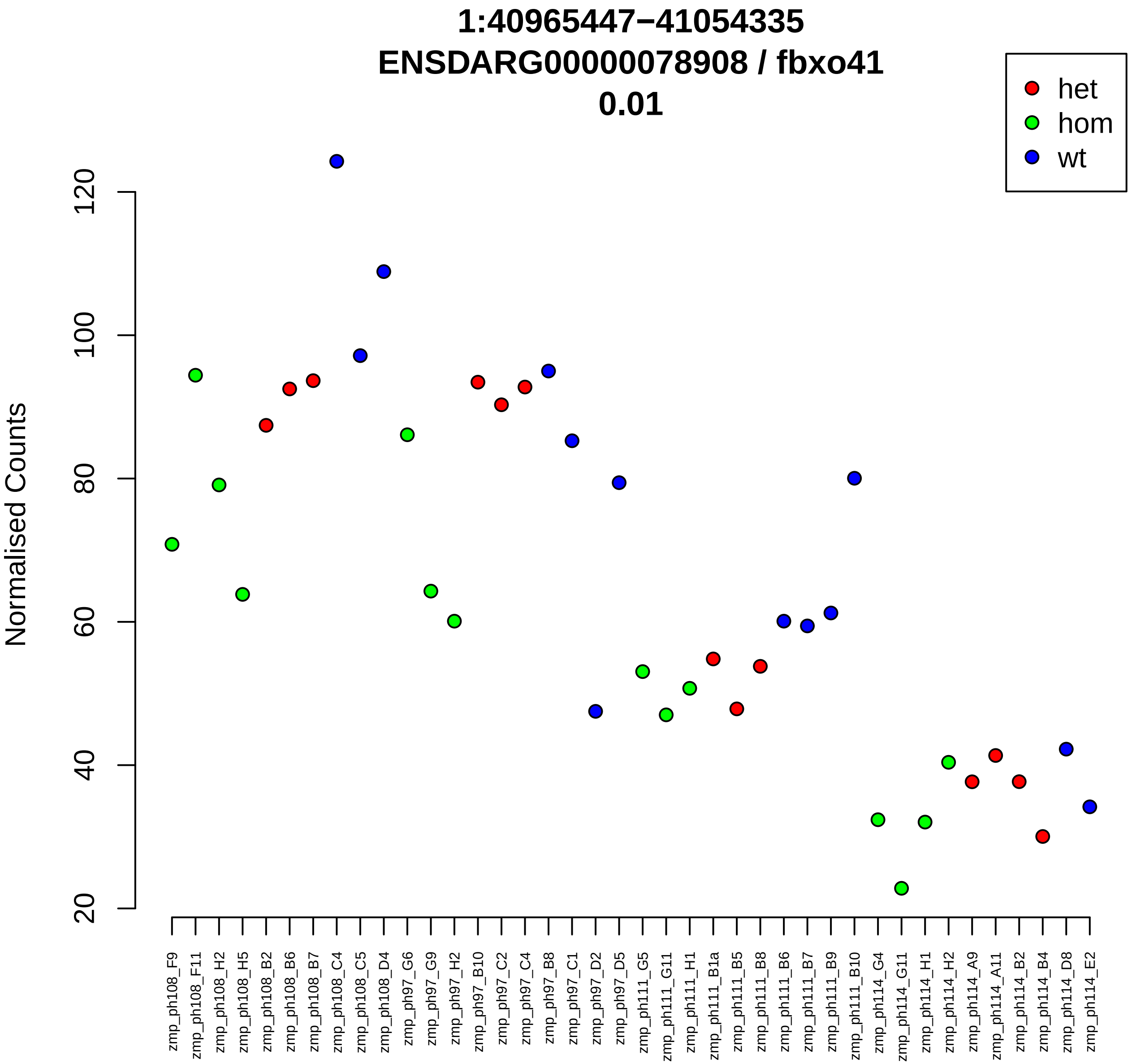
1:40965447−41054335
ENSDARG00000078908 / fbxo41
0.01
het
hom
wt
Normalised Counts
20
40
60
80
100
120
zmp_ph108_F9
zmp_ph108_F11
zmp_ph108_H2
zmp_ph108_H5
zmp_ph108_B2
zmp_ph108_B6
zmp_ph108_B7
zmp_ph108_C4
zmp_ph108_C5
zmp_ph108_D4
zmp_ph97_G6
zmp_ph97_G9
zmp_ph97_H2
zmp_ph97_B10
zmp_ph97_C2
zmp_ph97_C4
zmp_ph97_B8
zmp_ph97_C1
zmp_ph97_D2
zmp_ph97_D5
zmp_ph111_G5
zmp_ph111_G11
zmp_ph111_H1
zmp_ph111_B1a
zmp_ph111_B5
zmp_ph111_B8
zmp_ph111_B6
zmp_ph111_B7
zmp_ph111_B9
zmp_ph111_B10
zmp_ph114_G4
zmp_ph114_G11
zmp_ph114_H1
zmp_ph114_H2
zmp_ph114_A9
zmp_ph114_A11
zmp_ph114_B2
zmp_ph114_B4
zmp_ph114_D8
zmp_ph114_E2

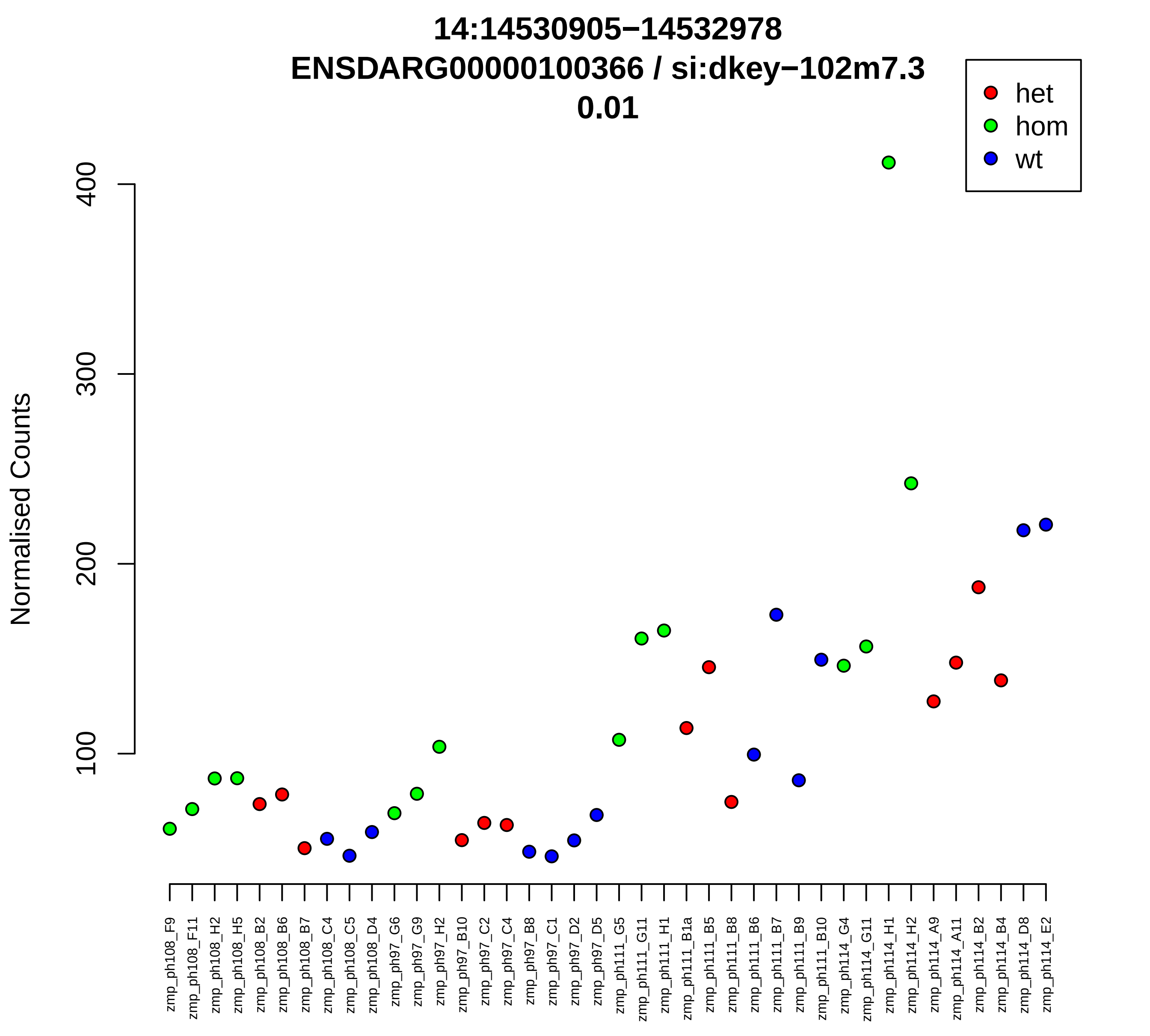

14:14530905−14532978
ENSDARG00000100366 / si:dkey−102m7.3
0.01
het
hom
wt
Normalised Counts
100
200
300
400
zmp_ph108_F9
zmp_ph108_F11
zmp_ph108_H2
zmp_ph108_H5
zmp_ph108_B2
zmp_ph108_B6
zmp_ph108_B7
zmp_ph108_C4
zmp_ph108_C5
zmp_ph108_D4
zmp_ph97_G6
zmp_ph97_G9
zmp_ph97_H2
zmp_ph97_B10
zmp_ph97_C2
zmp_ph97_C4
zmp_ph97_B8
zmp_ph97_C1
zmp_ph97_D2
zmp_ph97_D5
zmp_ph111_G5
zmp_ph111_G11
zmp_ph111_H1
zmp_ph111_B1a
zmp_ph111_B5
zmp_ph111_B8
zmp_ph111_B6
zmp_ph111_B7
zmp_ph111_B9
zmp_ph111_B10
zmp_ph114_G4
zmp_ph114_G11
zmp_ph114_H1
zmp_ph114_H2
zmp_ph114_A9
zmp_ph114_A11
zmp_ph114_B2
zmp_ph114_B4
zmp_ph114_D8
zmp_ph114_E2

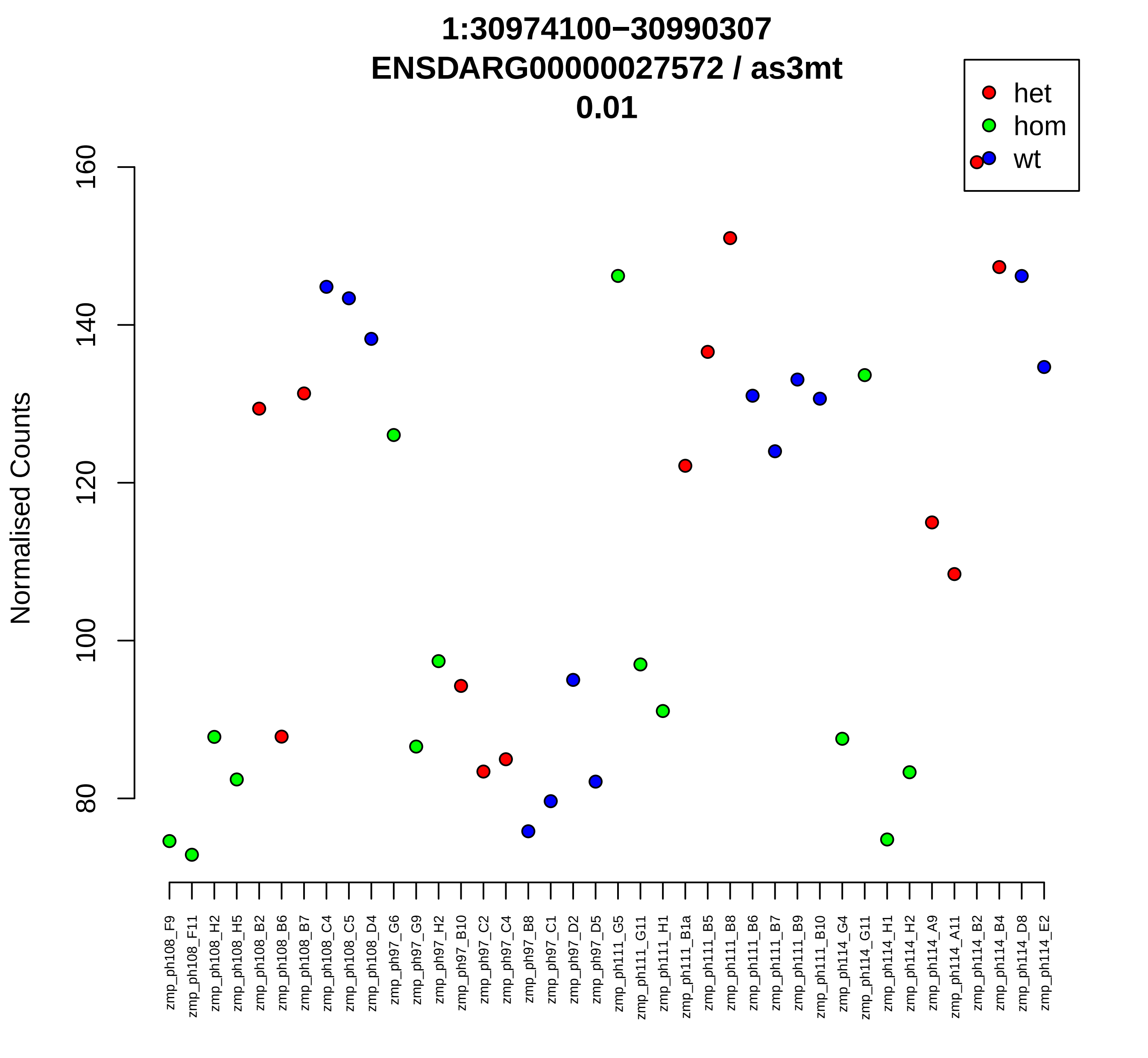

1:30974100−30990307
ENSDARG00000027572 / as3mt
0.01
het
hom
wt
Normalised Counts
80
100
120
140
160
zmp_ph108_F9
zmp_ph108_F11
zmp_ph108_H2
zmp_ph108_H5
zmp_ph108_B2
zmp_ph108_B6
zmp_ph108_B7
zmp_ph108_C4
zmp_ph108_C5
zmp_ph108_D4
zmp_ph97_G6
zmp_ph97_G9
zmp_ph97_H2
zmp_ph97_B10
zmp_ph97_C2
zmp_ph97_C4
zmp_ph97_B8
zmp_ph97_C1
zmp_ph97_D2
zmp_ph97_D5
zmp_ph111_G5
zmp_ph111_G11
zmp_ph111_H1
zmp_ph111_B1a
zmp_ph111_B5
zmp_ph111_B8
zmp_ph111_B6
zmp_ph111_B7
zmp_ph111_B9
zmp_ph111_B10
zmp_ph114_G4
zmp_ph114_G11
zmp_ph114_H1
zmp_ph114_H2
zmp_ph114_A9
zmp_ph114_A11
zmp_ph114_B2
zmp_ph114_B4
zmp_ph114_D8
zmp_ph114_E2

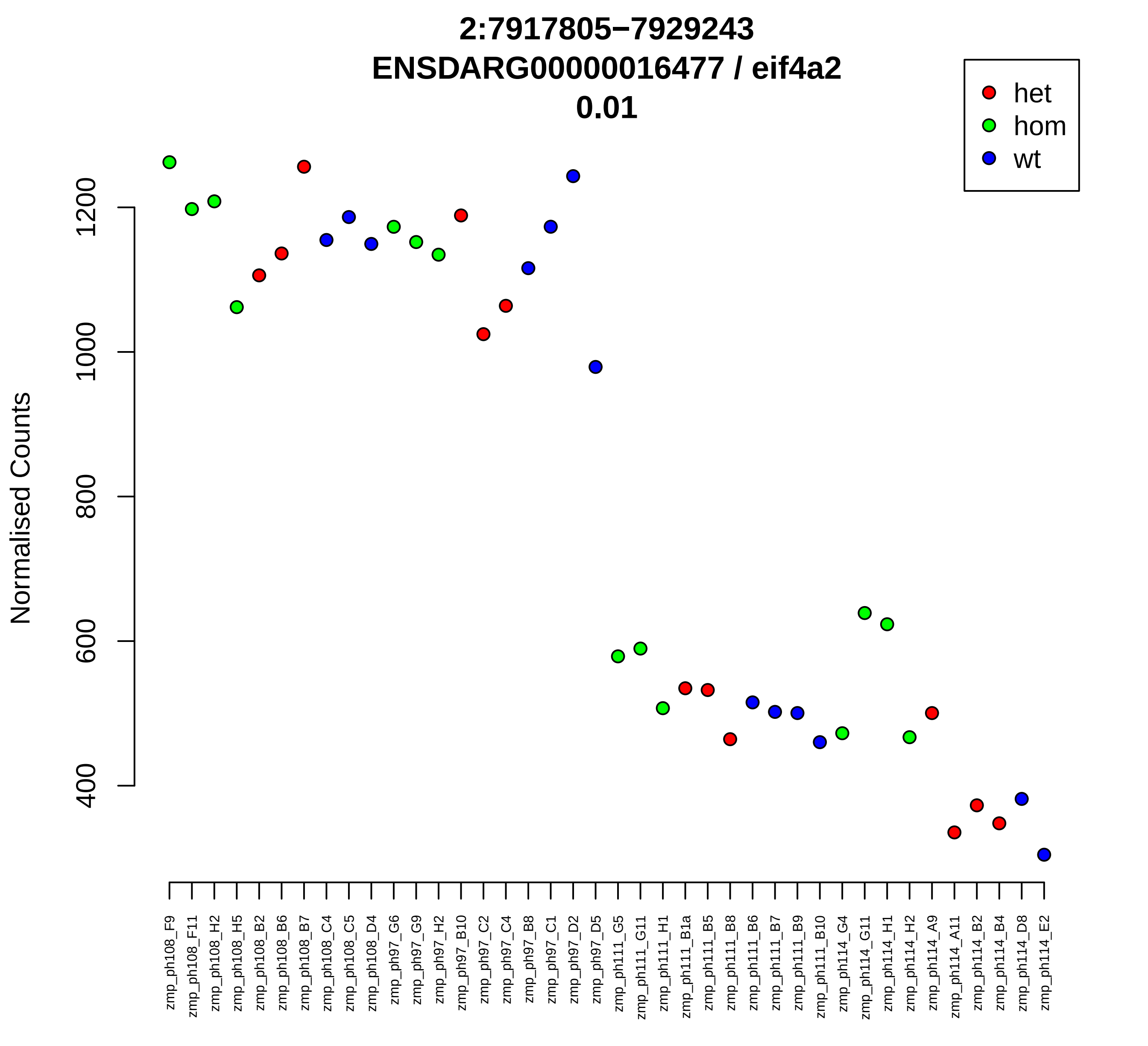
2:7917805−7929243
ENSDARG00000016477 / eif4a2
0.01
het
hom
wt
Normalised Counts
400
600
800
1000
1200
zmp_ph108_F9
zmp_ph108_F11
zmp_ph108_H2
zmp_ph108_H5
zmp_ph108_B2
zmp_ph108_B6
zmp_ph108_B7
zmp_ph108_C4
zmp_ph108_C5
zmp_ph108_D4
zmp_ph97_G6
zmp_ph97_G9
zmp_ph97_H2
zmp_ph97_B10
zmp_ph97_C2
zmp_ph97_C4
zmp_ph97_B8
zmp_ph97_C1
zmp_ph97_D2
zmp_ph97_D5
zmp_ph111_G5
zmp_ph111_G11
zmp_ph111_H1
zmp_ph111_B1a
zmp_ph111_B5
zmp_ph111_B8
zmp_ph111_B6
zmp_ph111_B7
zmp_ph111_B9
zmp_ph111_B10
zmp_ph114_G4
zmp_ph114_G11
zmp_ph114_H1
zmp_ph114_H2
zmp_ph114_A9
zmp_ph114_A11
zmp_ph114_B2
zmp_ph114_B4
zmp_ph114_D8
zmp_ph114_E2

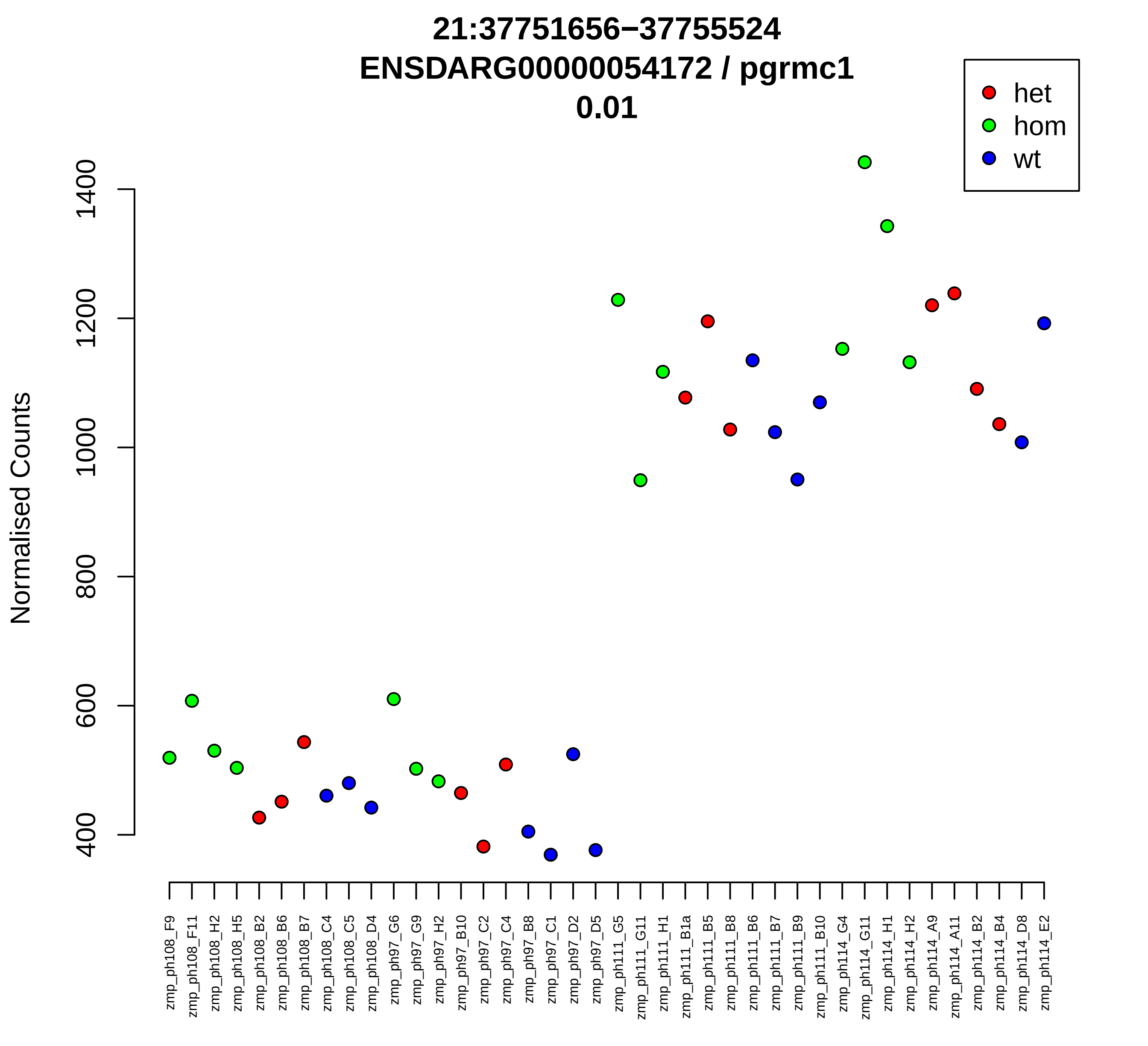

21:37751656−37755524
ENSDARG00000054172 / pgrmc1
0.01
het
hom
wt
Normalised Counts
400
600
800
1000
1200
1400
zmp_ph108_F9
zmp_ph108_F11
zmp_ph108_H2
zmp_ph108_H5
zmp_ph108_B2
zmp_ph108_B6
zmp_ph108_B7
zmp_ph108_C4
zmp_ph108_C5
zmp_ph108_D4
zmp_ph97_G6
zmp_ph97_G9
zmp_ph97_H2
zmp_ph97_B10
zmp_ph97_C2
zmp_ph97_C4
zmp_ph97_B8
zmp_ph97_C1
zmp_ph97_D2
zmp_ph97_D5
zmp_ph111_G5
zmp_ph111_G11
zmp_ph111_H1
zmp_ph111_B1a
zmp_ph111_B5
zmp_ph111_B8
zmp_ph111_B6
zmp_ph111_B7
zmp_ph111_B9
zmp_ph111_B10
zmp_ph114_G4
zmp_ph114_G11
zmp_ph114_H1
zmp_ph114_H2
zmp_ph114_A9
zmp_ph114_A11
zmp_ph114_B2
zmp_ph114_B4
zmp_ph114_D8
zmp_ph114_E2

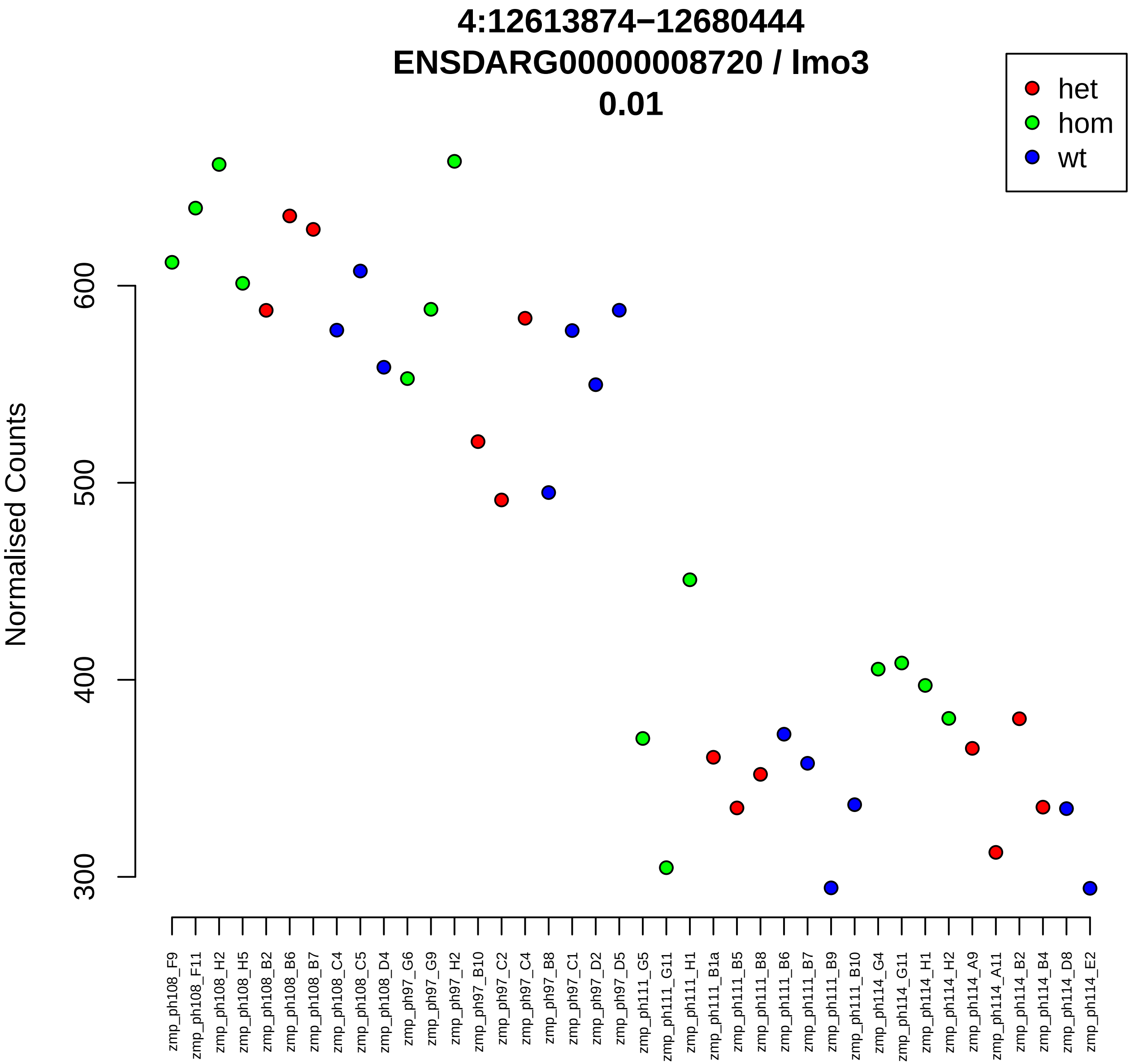

4:12613874−12680444
ENSDARG00000008720 / lmo3
0.01
het
hom
wt
Normalised Counts
300
400
500
600
zmp_ph108_F9
zmp_ph108_F11
zmp_ph108_H2
zmp_ph108_H5
zmp_ph108_B2
zmp_ph108_B6
zmp_ph108_B7
zmp_ph108_C4
zmp_ph108_C5
zmp_ph108_D4
zmp_ph97_G6
zmp_ph97_G9
zmp_ph97_H2
zmp_ph97_B10
zmp_ph97_C2
zmp_ph97_C4
zmp_ph97_B8
zmp_ph97_C1
zmp_ph97_D2
zmp_ph97_D5
zmp_ph111_G5
zmp_ph111_G11
zmp_ph111_H1
zmp_ph111_B1a
zmp_ph111_B5
zmp_ph111_B8
zmp_ph111_B6
zmp_ph111_B7
zmp_ph111_B9
zmp_ph111_B10
zmp_ph114_G4
zmp_ph114_G11
zmp_ph114_H1
zmp_ph114_H2
zmp_ph114_A9
zmp_ph114_A11
zmp_ph114_B2
zmp_ph114_B4
zmp_ph114_D8
zmp_ph114_E2

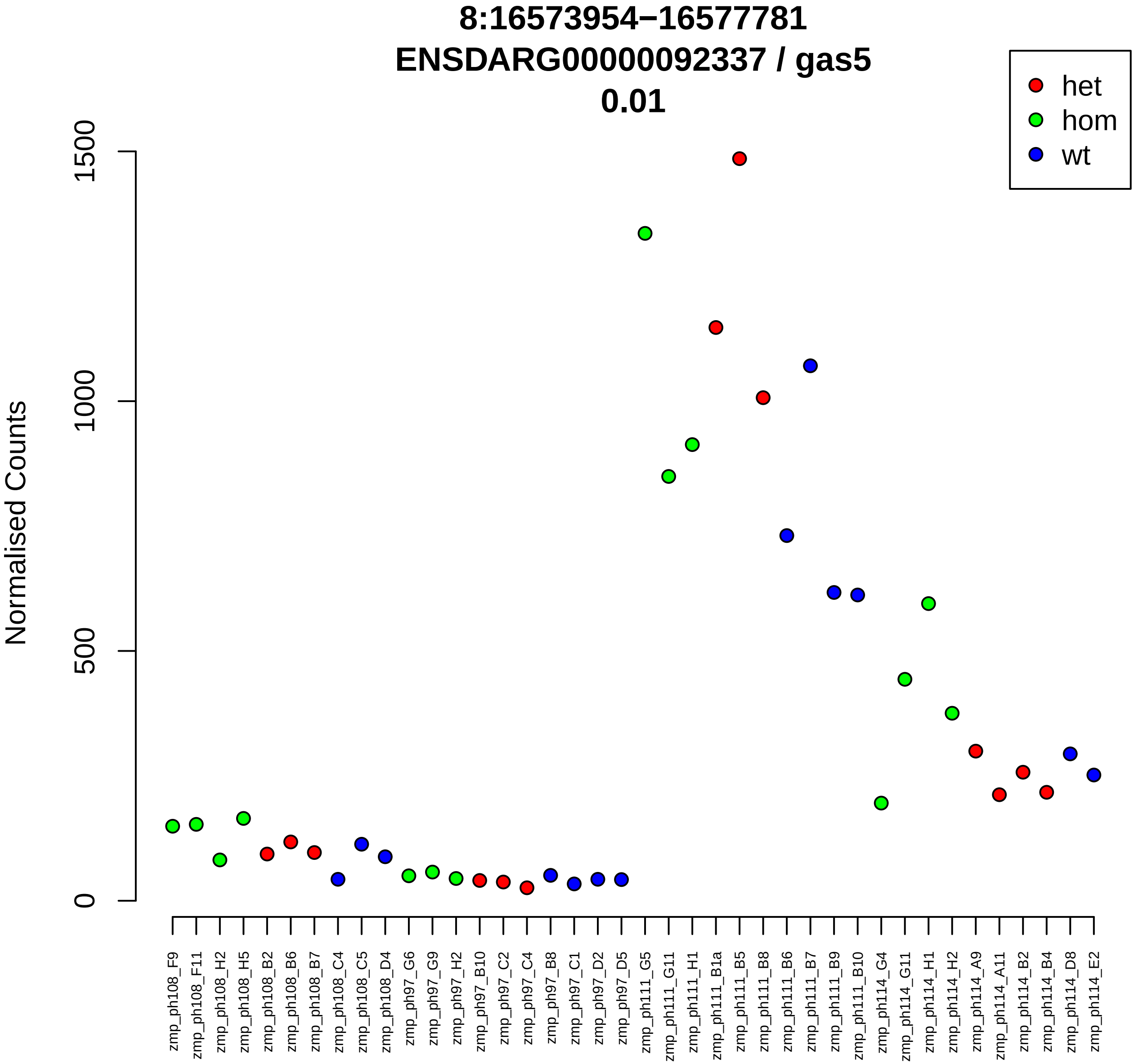
8:16573954−16577781
ENSDARG00000092337 / gas5
0.01
het
hom
wt
Normalised Counts
0
500
1000
1500
zmp_ph108_F9
zmp_ph108_F11
zmp_ph108_H2
zmp_ph108_H5
zmp_ph108_B2
zmp_ph108_B6
zmp_ph108_B7
zmp_ph108_C4
zmp_ph108_C5
zmp_ph108_D4
zmp_ph97_G6
zmp_ph97_G9
zmp_ph97_H2
zmp_ph97_B10
zmp_ph97_C2
zmp_ph97_C4
zmp_ph97_B8
zmp_ph97_C1
zmp_ph97_D2
zmp_ph97_D5
zmp_ph111_G5
zmp_ph111_G11
zmp_ph111_H1
zmp_ph111_B1a
zmp_ph111_B5
zmp_ph111_B8
zmp_ph111_B6
zmp_ph111_B7
zmp_ph111_B9
zmp_ph111_B10
zmp_ph114_G4
zmp_ph114_G11
zmp_ph114_H1
zmp_ph114_H2
zmp_ph114_A9
zmp_ph114_A11
zmp_ph114_B2
zmp_ph114_B4
zmp_ph114_D8
zmp_ph114_E2

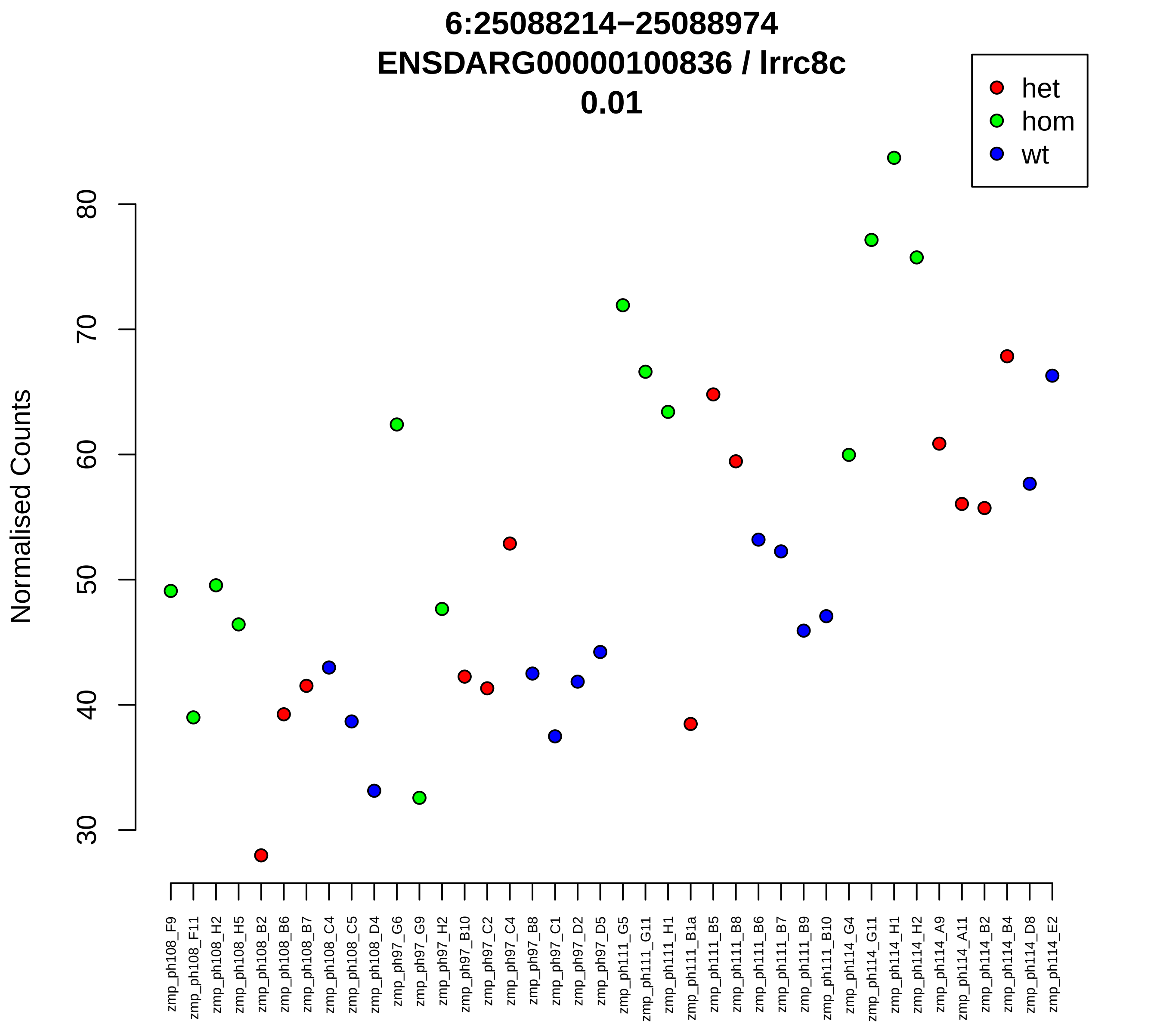

6:25088214−25088974
ENSDARG00000100836 / lrrc8c
0.01
het
hom
wt
Normalised Counts
30
40
50
60
70
80
zmp_ph108_F9
zmp_ph108_F11
zmp_ph108_H2
zmp_ph108_H5
zmp_ph108_B2
zmp_ph108_B6
zmp_ph108_B7
zmp_ph108_C4
zmp_ph108_C5
zmp_ph108_D4
zmp_ph97_G6
zmp_ph97_G9
zmp_ph97_H2
zmp_ph97_B10
zmp_ph97_C2
zmp_ph97_C4
zmp_ph97_B8
zmp_ph97_C1
zmp_ph97_D2
zmp_ph97_D5
zmp_ph111_G5
zmp_ph111_G11
zmp_ph111_H1
zmp_ph111_B1a
zmp_ph111_B5
zmp_ph111_B8
zmp_ph111_B6
zmp_ph111_B7
zmp_ph111_B9
zmp_ph111_B10
zmp_ph114_G4
zmp_ph114_G11
zmp_ph114_H1
zmp_ph114_H2
zmp_ph114_A9
zmp_ph114_A11
zmp_ph114_B2
zmp_ph114_B4
zmp_ph114_D8
zmp_ph114_E2

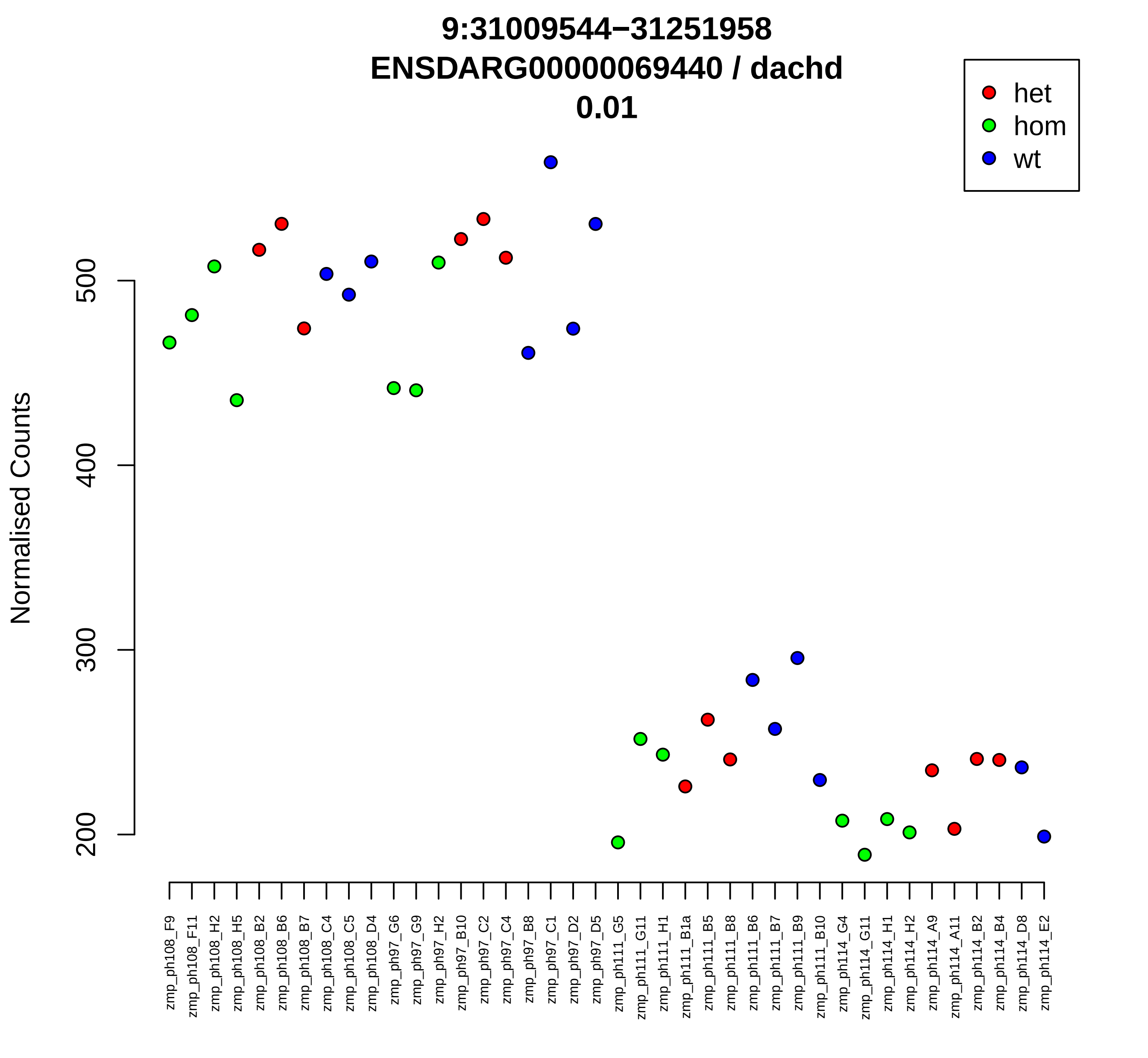

9:31009544−31251958
ENSDARG00000069440 / dachd
0.01
het
hom
wt
Normalised Counts
200
300
400
500
zmp_ph108_F9
zmp_ph108_F11
zmp_ph108_H2
zmp_ph108_H5
zmp_ph108_B2
zmp_ph108_B6
zmp_ph108_B7
zmp_ph108_C4
zmp_ph108_C5
zmp_ph108_D4
zmp_ph97_G6
zmp_ph97_G9
zmp_ph97_H2
zmp_ph97_B10
zmp_ph97_C2
zmp_ph97_C4
zmp_ph97_B8
zmp_ph97_C1
zmp_ph97_D2
zmp_ph97_D5
zmp_ph111_G5
zmp_ph111_G11
zmp_ph111_H1
zmp_ph111_B1a
zmp_ph111_B5
zmp_ph111_B8
zmp_ph111_B6
zmp_ph111_B7
zmp_ph111_B9
zmp_ph111_B10
zmp_ph114_G4
zmp_ph114_G11
zmp_ph114_H1
zmp_ph114_H2
zmp_ph114_A9
zmp_ph114_A11
zmp_ph114_B2
zmp_ph114_B4
zmp_ph114_D8
zmp_ph114_E2

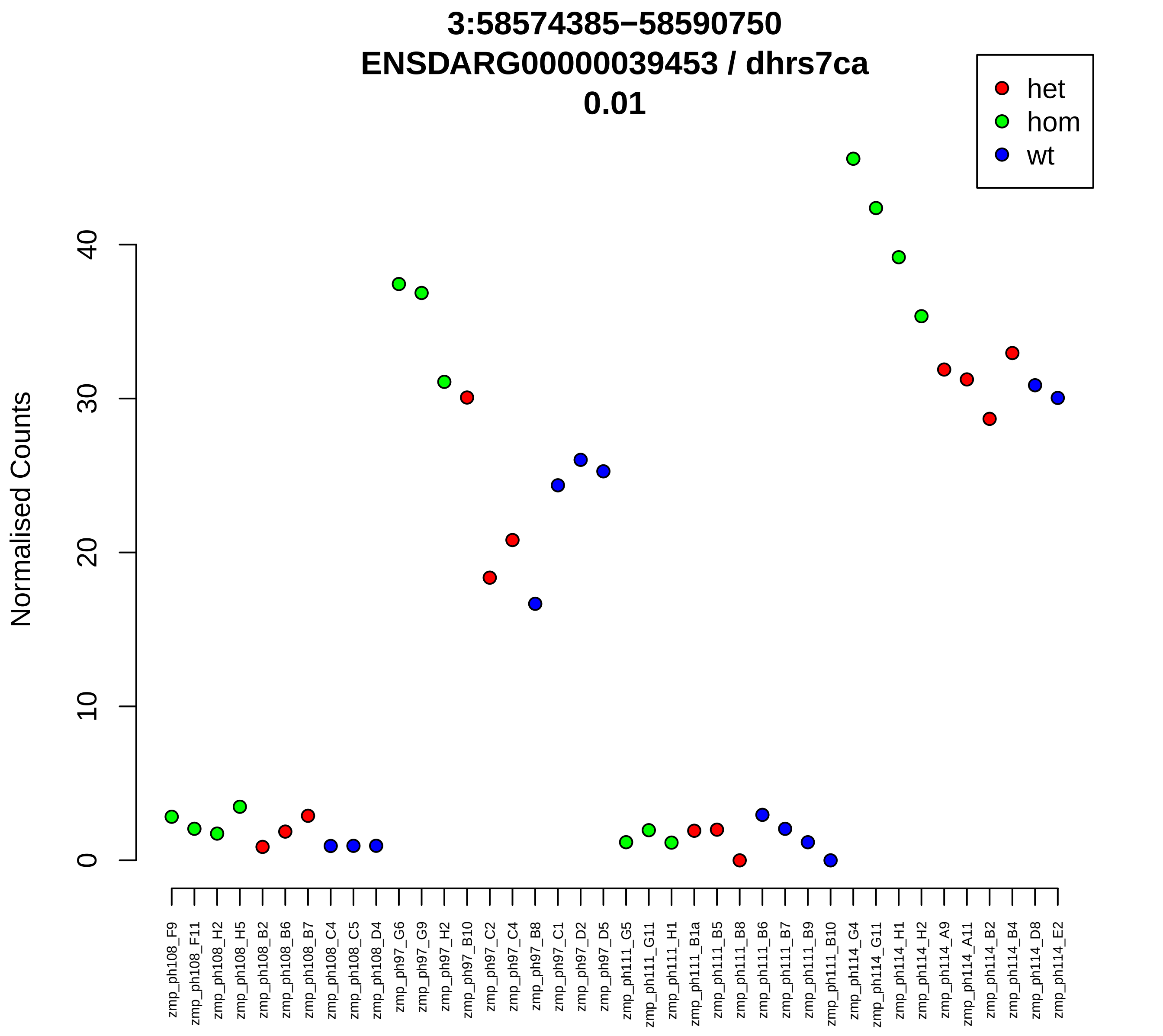
3:58574385−58590750
ENSDARG00000039453 / dhrs7ca
0.01
het
hom
wt
Normalised Counts
0
10
20
30
40
zmp_ph108_F9
zmp_ph108_F11
zmp_ph108_H2
zmp_ph108_H5
zmp_ph108_B2
zmp_ph108_B6
zmp_ph108_B7
zmp_ph108_C4
zmp_ph108_C5
zmp_ph108_D4
zmp_ph97_G6
zmp_ph97_G9
zmp_ph97_H2
zmp_ph97_B10
zmp_ph97_C2
zmp_ph97_C4
zmp_ph97_B8
zmp_ph97_C1
zmp_ph97_D2
zmp_ph97_D5
zmp_ph111_G5
zmp_ph111_G11
zmp_ph111_H1
zmp_ph111_B1a
zmp_ph111_B5
zmp_ph111_B8
zmp_ph111_B6
zmp_ph111_B7
zmp_ph111_B9
zmp_ph111_B10
zmp_ph114_G4
zmp_ph114_G11
zmp_ph114_H1
zmp_ph114_H2
zmp_ph114_A9
zmp_ph114_A11
zmp_ph114_B2
zmp_ph114_B4
zmp_ph114_D8
zmp_ph114_E2

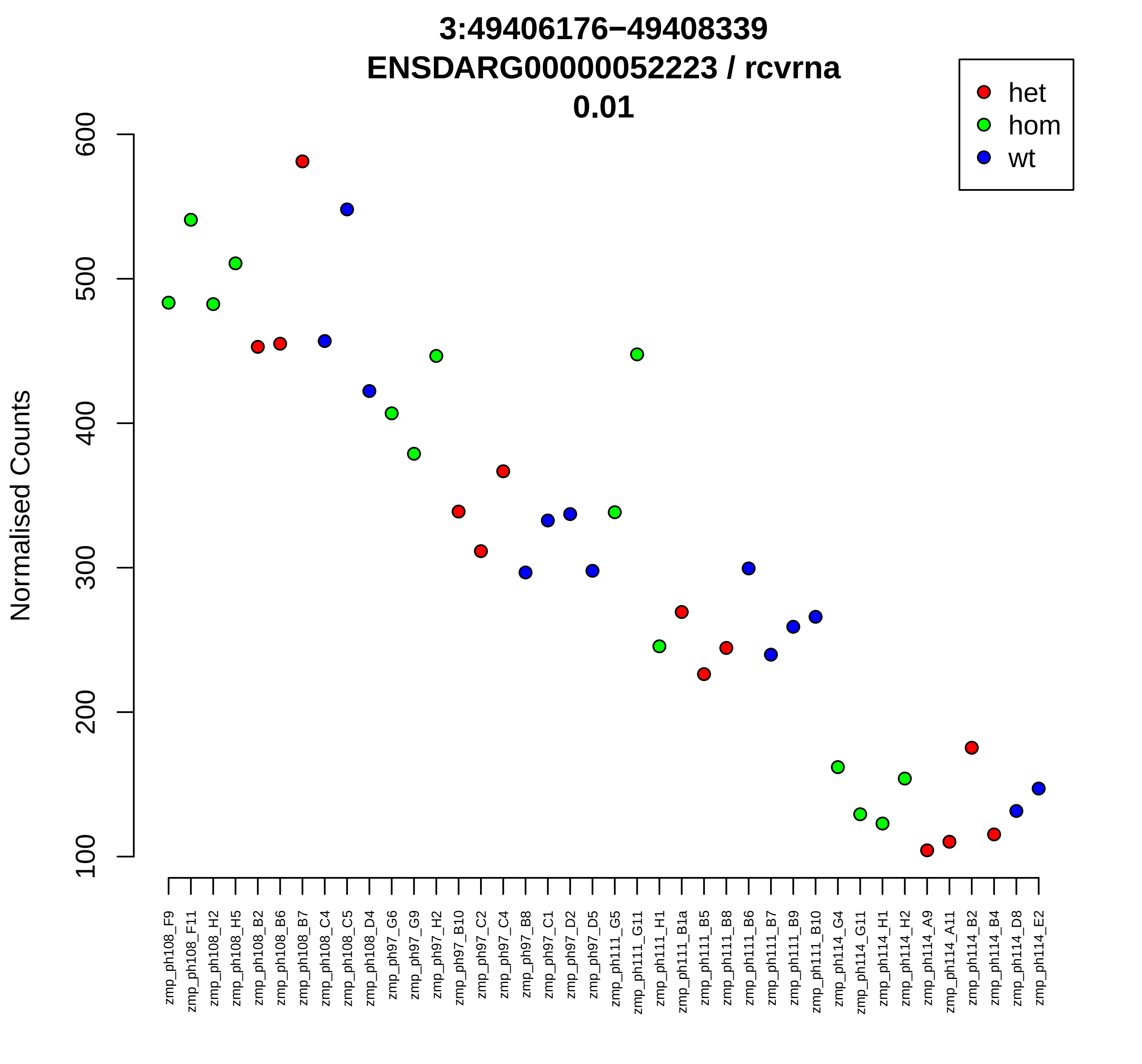

3:49406176−49408339
ENSDARG00000052223 / rcvrna
0.01
het
hom
wt
Normalised Counts
100
200
300
400
500
600
zmp_ph108_F9
zmp_ph108_F11
zmp_ph108_H2
zmp_ph108_H5
zmp_ph108_B2
zmp_ph108_B6
zmp_ph108_B7
zmp_ph108_C4
zmp_ph108_C5
zmp_ph108_D4
zmp_ph97_G6
zmp_ph97_G9
zmp_ph97_H2
zmp_ph97_B10
zmp_ph97_C2
zmp_ph97_C4
zmp_ph97_B8
zmp_ph97_C1
zmp_ph97_D2
zmp_ph97_D5
zmp_ph111_G5
zmp_ph111_G11
zmp_ph111_H1
zmp_ph111_B1a
zmp_ph111_B5
zmp_ph111_B8
zmp_ph111_B6
zmp_ph111_B7
zmp_ph111_B9
zmp_ph111_B10
zmp_ph114_G4
zmp_ph114_G11
zmp_ph114_H1
zmp_ph114_H2
zmp_ph114_A9
zmp_ph114_A11
zmp_ph114_B2
zmp_ph114_B4
zmp_ph114_D8
zmp_ph114_E2

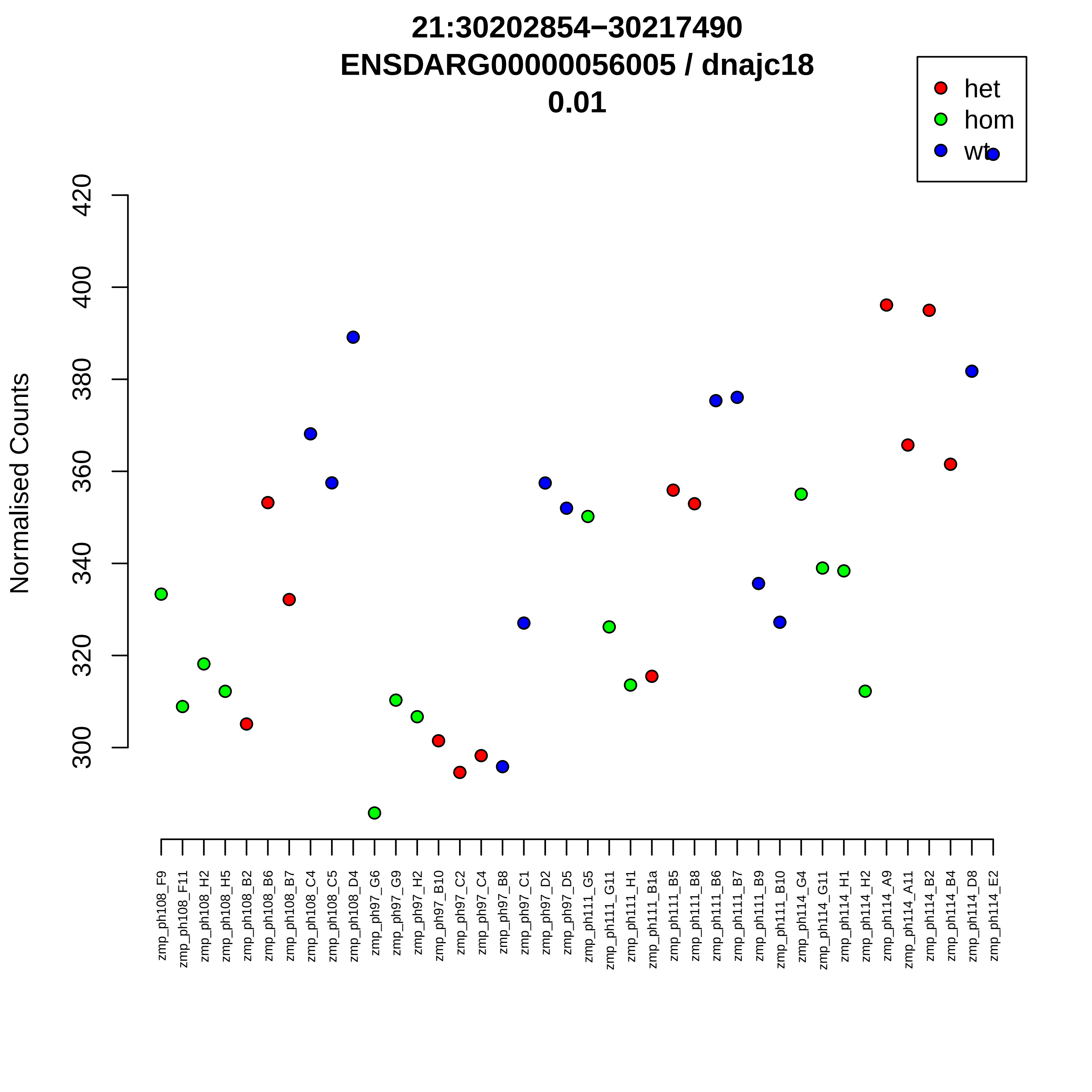

21:30202854−30217490
ENSDARG00000056005 / dnajc18
0.01
het
hom
wt
Normalised Counts
300
320
340
360
380
400
420
zmp_ph108_F9
zmp_ph108_F11
zmp_ph108_H2
zmp_ph108_H5
zmp_ph108_B2
zmp_ph108_B6
zmp_ph108_B7
zmp_ph108_C4
zmp_ph108_C5
zmp_ph108_D4
zmp_ph97_G6
zmp_ph97_G9
zmp_ph97_H2
zmp_ph97_B10
zmp_ph97_C2
zmp_ph97_C4
zmp_ph97_B8
zmp_ph97_C1
zmp_ph97_D2
zmp_ph97_D5
zmp_ph111_G5
zmp_ph111_G11
zmp_ph111_H1
zmp_ph111_B1a
zmp_ph111_B5
zmp_ph111_B8
zmp_ph111_B6
zmp_ph111_B7
zmp_ph111_B9
zmp_ph111_B10
zmp_ph114_G4
zmp_ph114_G11
zmp_ph114_H1
zmp_ph114_H2
zmp_ph114_A9
zmp_ph114_A11
zmp_ph114_B2
zmp_ph114_B4
zmp_ph114_D8
zmp_ph114_E2

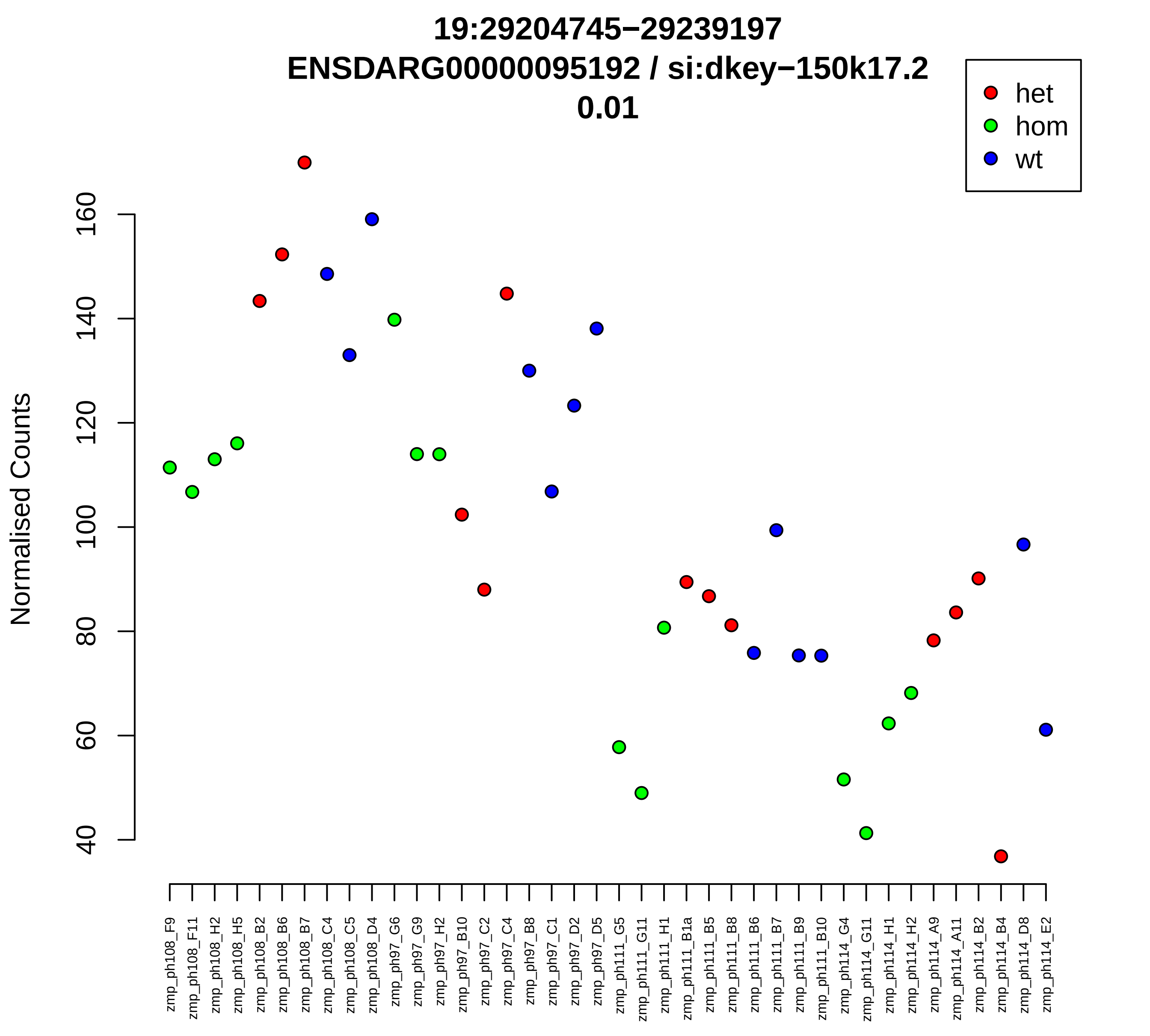

19:29204745−29239197
ENSDARG00000095192 / si:dkey−150k17.2
0.01
het
hom
wt
Normalised Counts
40
60
80
100
120
140
160
zmp_ph108_F9
zmp_ph108_F11
zmp_ph108_H2
zmp_ph108_H5
zmp_ph108_B2
zmp_ph108_B6
zmp_ph108_B7
zmp_ph108_C4
zmp_ph108_C5
zmp_ph108_D4
zmp_ph97_G6
zmp_ph97_G9
zmp_ph97_H2
zmp_ph97_B10
zmp_ph97_C2
zmp_ph97_C4
zmp_ph97_B8
zmp_ph97_C1
zmp_ph97_D2
zmp_ph97_D5
zmp_ph111_G5
zmp_ph111_G11
zmp_ph111_H1
zmp_ph111_B1a
zmp_ph111_B5
zmp_ph111_B8
zmp_ph111_B6
zmp_ph111_B7
zmp_ph111_B9
zmp_ph111_B10
zmp_ph114_G4
zmp_ph114_G11
zmp_ph114_H1
zmp_ph114_H2
zmp_ph114_A9
zmp_ph114_A11
zmp_ph114_B2
zmp_ph114_B4
zmp_ph114_D8
zmp_ph114_E2

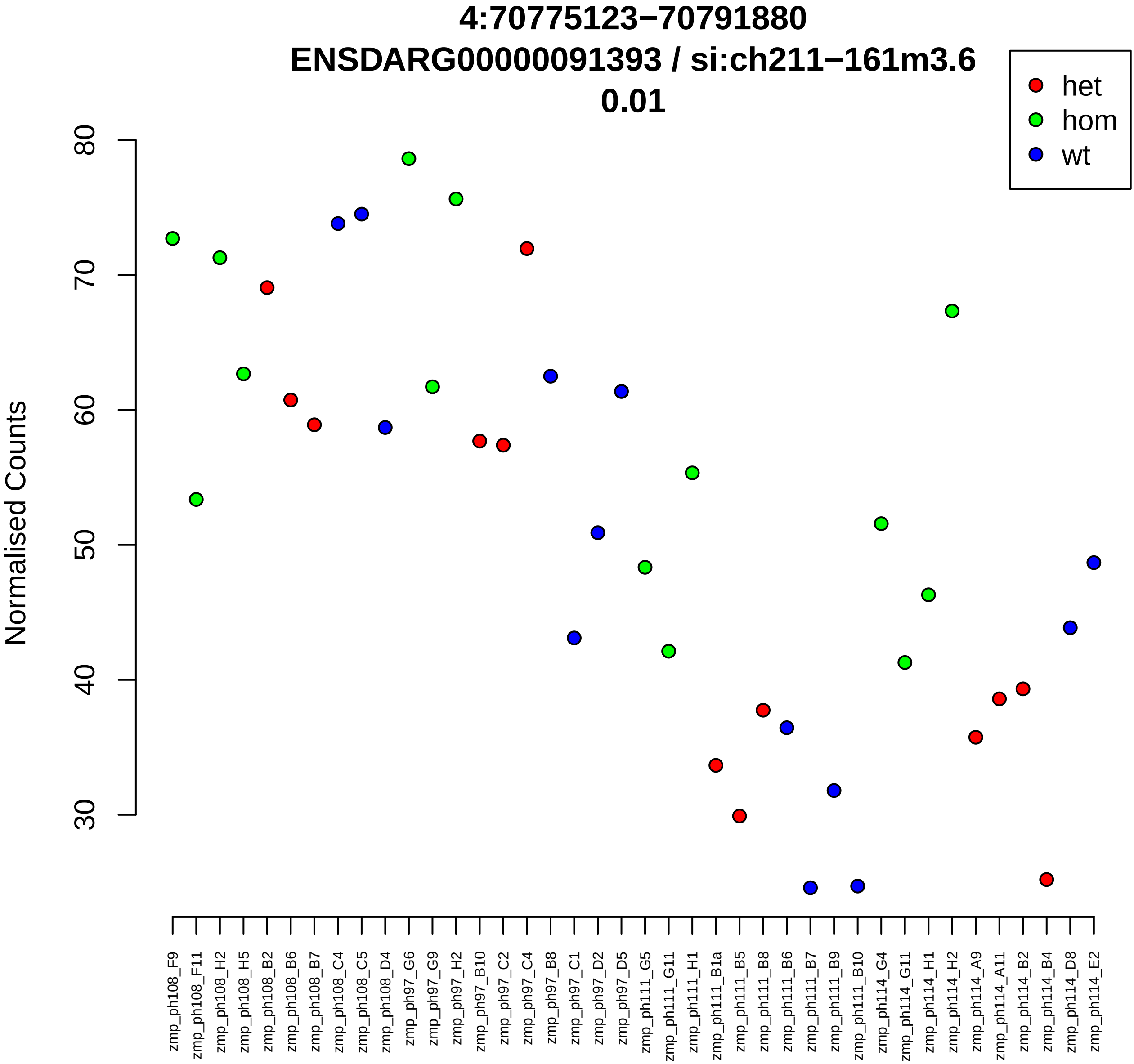

4:70775123−70791880
ENSDARG00000091393 / si:ch211−161m3.6
0.01
het
hom
wt
Normalised Counts
30
40
50
60
70
80
zmp_ph108_F9
zmp_ph108_F11
zmp_ph108_H2
zmp_ph108_H5
zmp_ph108_B2
zmp_ph108_B6
zmp_ph108_B7
zmp_ph108_C4
zmp_ph108_C5
zmp_ph108_D4
zmp_ph97_G6
zmp_ph97_G9
zmp_ph97_H2
zmp_ph97_B10
zmp_ph97_C2
zmp_ph97_C4
zmp_ph97_B8
zmp_ph97_C1
zmp_ph97_D2
zmp_ph97_D5
zmp_ph111_G5
zmp_ph111_G11
zmp_ph111_H1
zmp_ph111_B1a
zmp_ph111_B5
zmp_ph111_B8
zmp_ph111_B6
zmp_ph111_B7
zmp_ph111_B9
zmp_ph111_B10
zmp_ph114_G4
zmp_ph114_G11
zmp_ph114_H1
zmp_ph114_H2
zmp_ph114_A9
zmp_ph114_A11
zmp_ph114_B2
zmp_ph114_B4
zmp_ph114_D8
zmp_ph114_E2

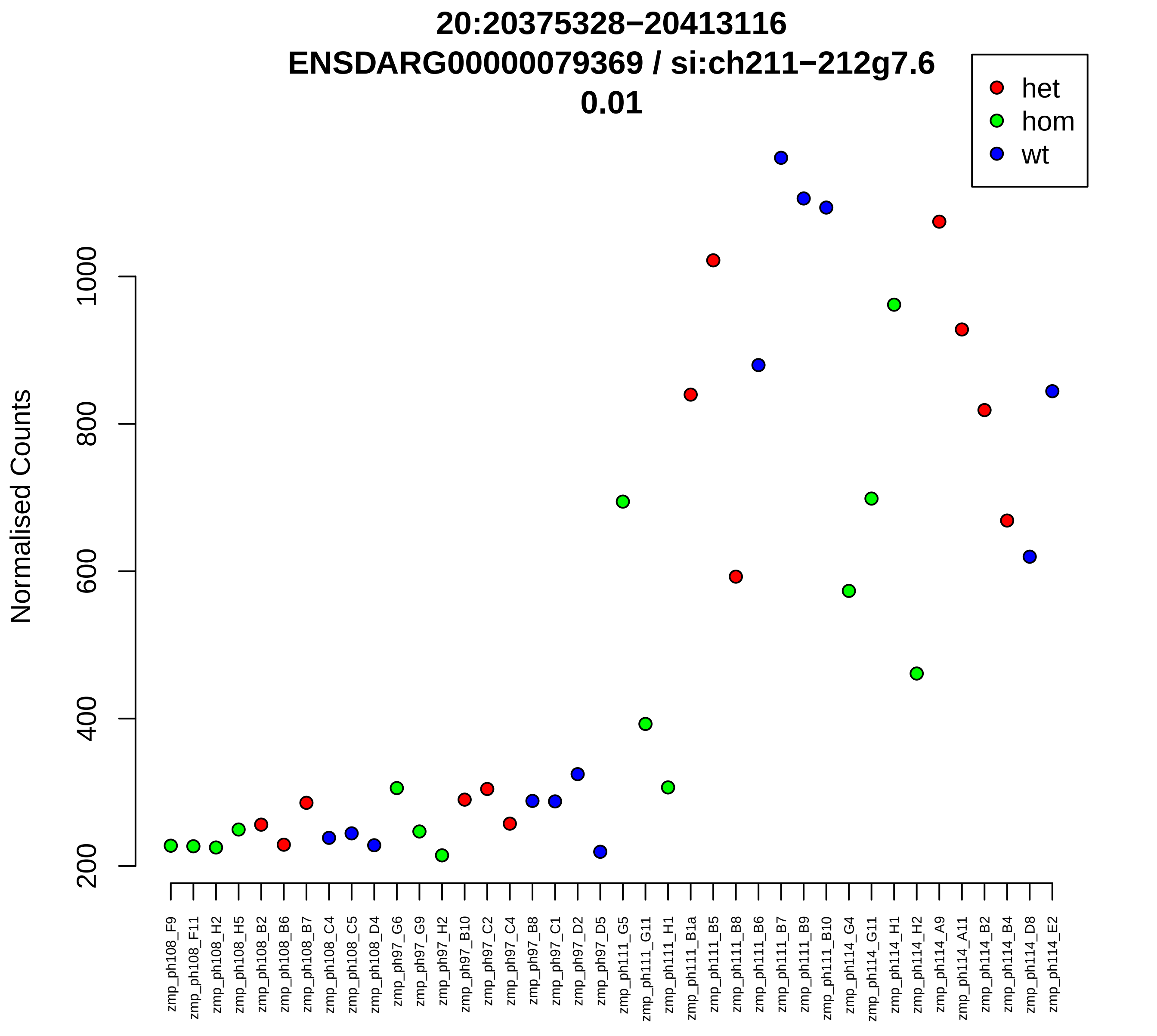

20:20375328−20413116
ENSDARG00000079369 / si:ch211−212g7.6
0.01
het
hom
wt
Normalised Counts
200
400
600
800
1000
zmp_ph108_F9
zmp_ph108_F11
zmp_ph108_H2
zmp_ph108_H5
zmp_ph108_B2
zmp_ph108_B6
zmp_ph108_B7
zmp_ph108_C4
zmp_ph108_C5
zmp_ph108_D4
zmp_ph97_G6
zmp_ph97_G9
zmp_ph97_H2
zmp_ph97_B10
zmp_ph97_C2
zmp_ph97_C4
zmp_ph97_B8
zmp_ph97_C1
zmp_ph97_D2
zmp_ph97_D5
zmp_ph111_G5
zmp_ph111_G11
zmp_ph111_H1
zmp_ph111_B1a
zmp_ph111_B5
zmp_ph111_B8
zmp_ph111_B6
zmp_ph111_B7
zmp_ph111_B9
zmp_ph111_B10
zmp_ph114_G4
zmp_ph114_G11
zmp_ph114_H1
zmp_ph114_H2
zmp_ph114_A9
zmp_ph114_A11
zmp_ph114_B2
zmp_ph114_B4
zmp_ph114_D8
zmp_ph114_E2

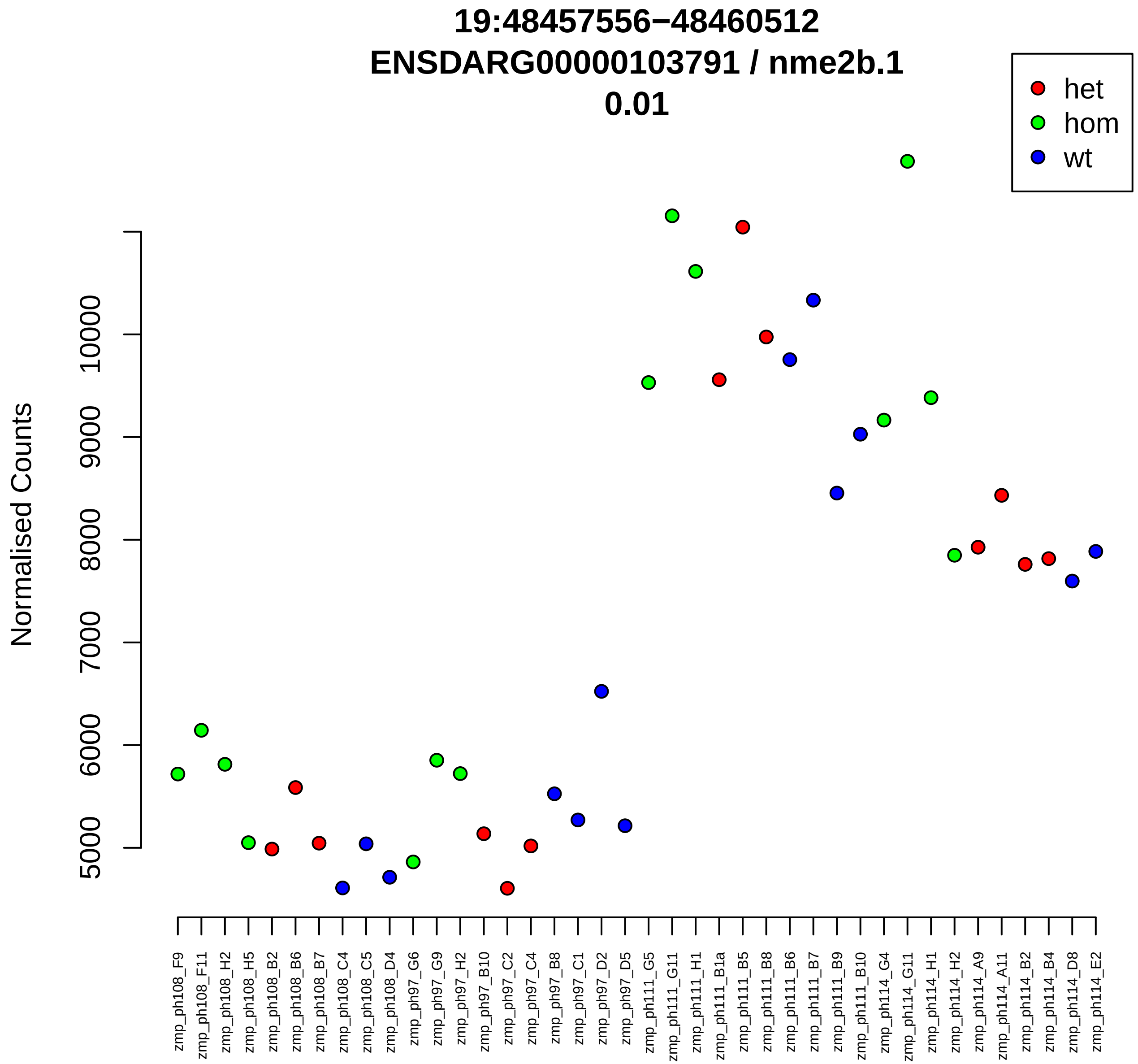

19:48457556−48460512
ENSDARG00000103791 / nme2b.1
0.01
het
hom
wt
Normalised Counts
5000
6000
7000
8000
9000
10000
zmp_ph108_F9
zmp_ph108_F11
zmp_ph108_H2
zmp_ph108_H5
zmp_ph108_B2
zmp_ph108_B6
zmp_ph108_B7
zmp_ph108_C4
zmp_ph108_C5
zmp_ph108_D4
zmp_ph97_G6
zmp_ph97_G9
zmp_ph97_H2
zmp_ph97_B10
zmp_ph97_C2
zmp_ph97_C4
zmp_ph97_B8
zmp_ph97_C1
zmp_ph97_D2
zmp_ph97_D5
zmp_ph111_G5
zmp_ph111_G11
zmp_ph111_H1
zmp_ph111_B1a
zmp_ph111_B5
zmp_ph111_B8
zmp_ph111_B6
zmp_ph111_B7
zmp_ph111_B9
zmp_ph111_B10
zmp_ph114_G4
zmp_ph114_G11
zmp_ph114_H1
zmp_ph114_H2
zmp_ph114_A9
zmp_ph114_A11
zmp_ph114_B2
zmp_ph114_B4
zmp_ph114_D8
zmp_ph114_E2

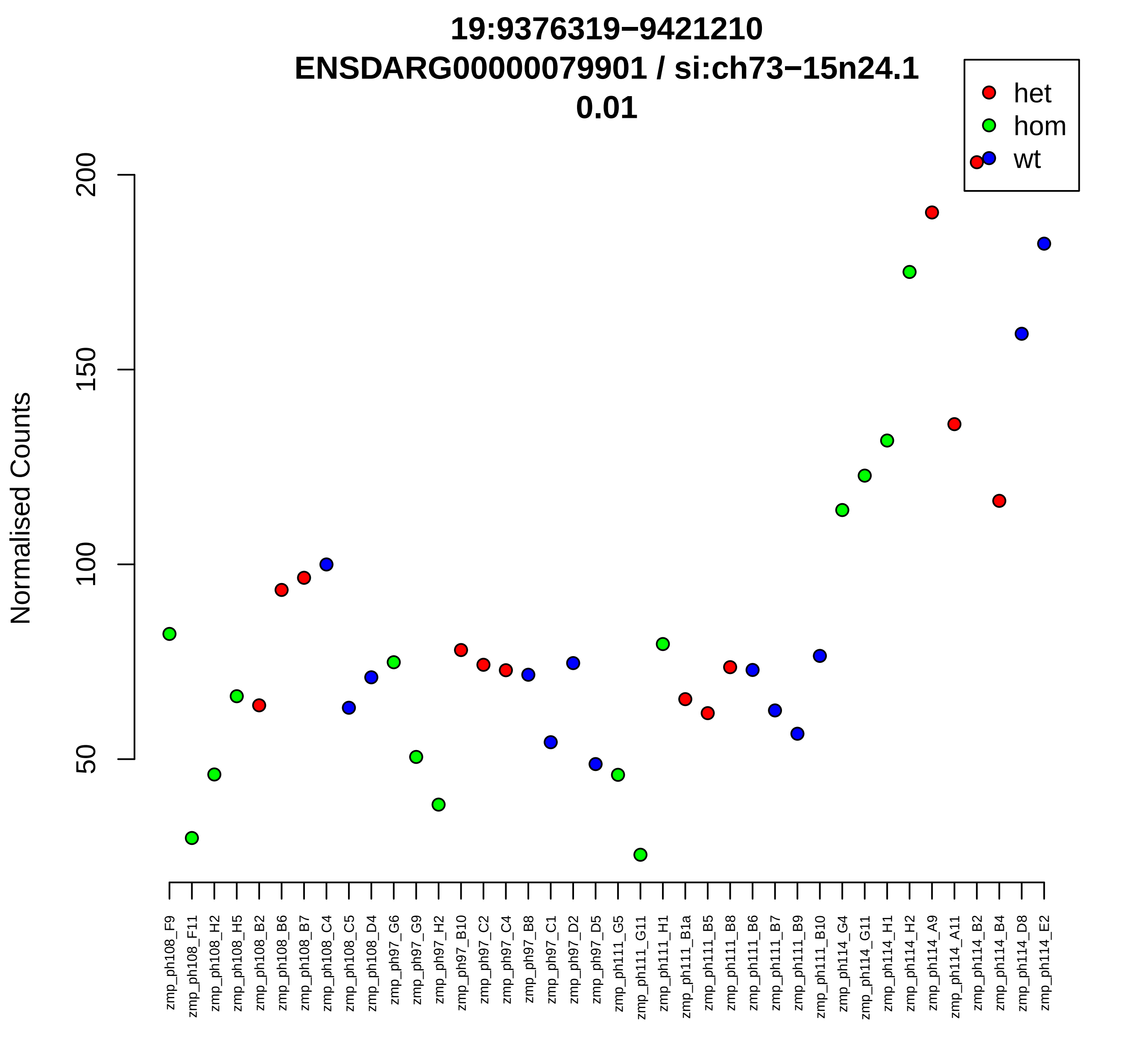
19:9376319−9421210
ENSDARG00000079901 / si:ch73−15n24.1
0.01
het
hom
wt
Normalised Counts
200
150
100
50
zmp_ph108_F9
zmp_ph108_F11
zmp_ph108_H2
zmp_ph108_H5
zmp_ph108_B2
zmp_ph108_B6
zmp_ph108_B7
zmp_ph108_C4
zmp_ph108_C5
zmp_ph108_D4
zmp_ph97_G6
zmp_ph97_G9
zmp_ph97_H2
zmp_ph97_B10
zmp_ph97_C2
zmp_ph97_C4
zmp_ph97_B8
zmp_ph97_C1
zmp_ph97_D2
zmp_ph97_D5
zmp_ph111_G5
zmp_ph111_G11
zmp_ph111_H1
zmp_ph111_B1a
zmp_ph111_B5
zmp_ph111_B8
zmp_ph111_B6
zmp_ph111_B7
zmp_ph111_B9
zmp_ph111_B10
zmp_ph114_G4
zmp_ph114_G11
zmp_ph114_H1
zmp_ph114_H2
zmp_ph114_A9
zmp_ph114_A11
zmp_ph114_B2
zmp_ph114_B4
zmp_ph114_D8
zmp_ph114_E2

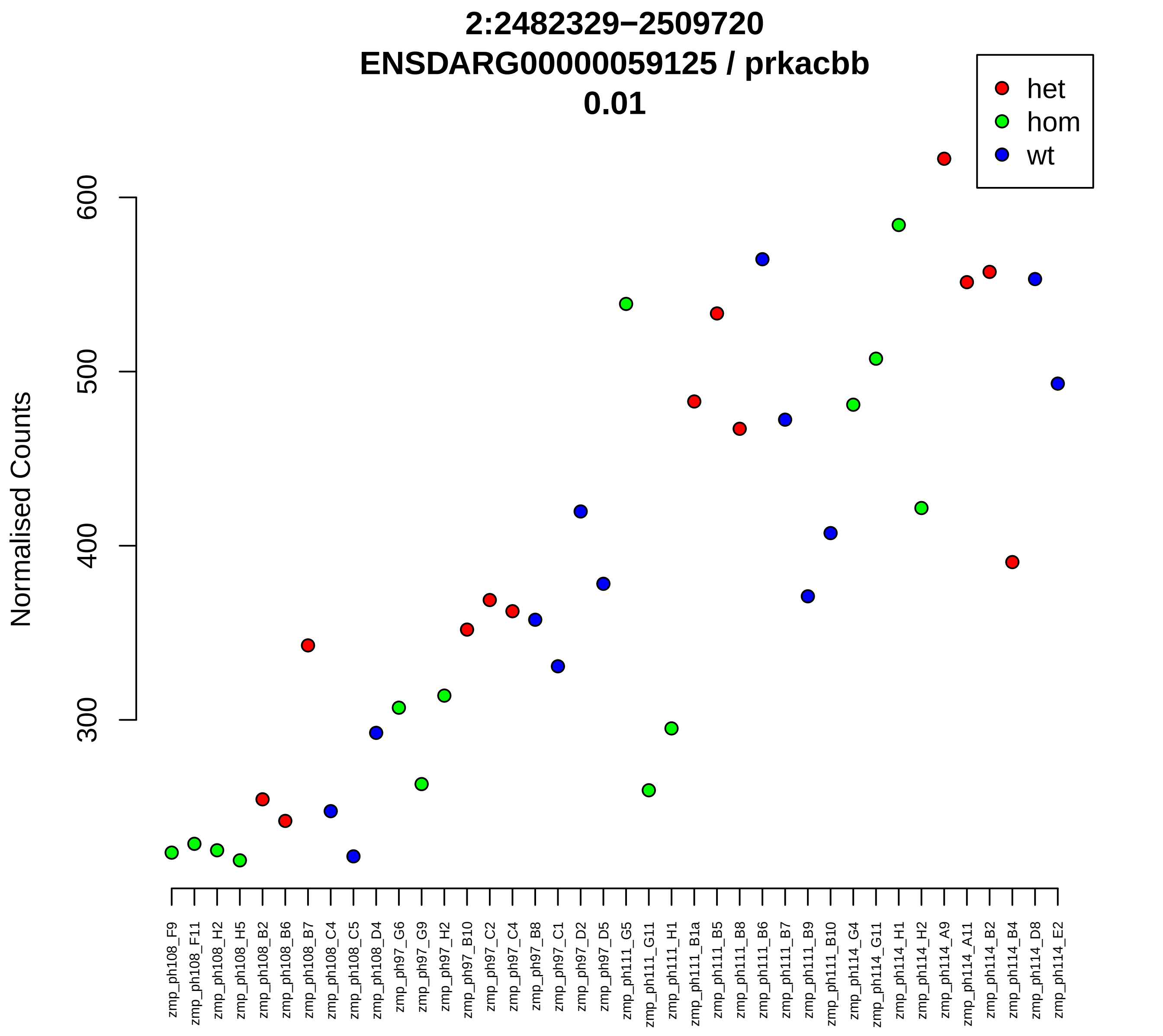

2:2482329−2509720
ENSDARG00000059125 / prkacbb
0.01
het
hom
wt
Normalised Counts
600
500
400
300
zmp_ph108_F9
zmp_ph108_F11
zmp_ph108_H2
zmp_ph108_H5
zmp_ph108_B2
zmp_ph108_B6
zmp_ph108_B7
zmp_ph108_C4
zmp_ph108_C5
zmp_ph108_D4
zmp_ph97_G6
zmp_ph97_G9
zmp_ph97_H2
zmp_ph97_B10
zmp_ph97_C2
zmp_ph97_C4
zmp_ph97_B8
zmp_ph97_C1
zmp_ph97_D2
zmp_ph97_D5
zmp_ph111_G5
zmp_ph111_G11
zmp_ph111_H1
zmp_ph111_B1a
zmp_ph111_B5
zmp_ph111_B8
zmp_ph111_B6
zmp_ph111_B7
zmp_ph111_B9
zmp_ph111_B10
zmp_ph114_G4
zmp_ph114_G11
zmp_ph114_H1
zmp_ph114_H2
zmp_ph114_A9
zmp_ph114_A11
zmp_ph114_B2
zmp_ph114_B4
zmp_ph114_D8
zmp_ph114_E2

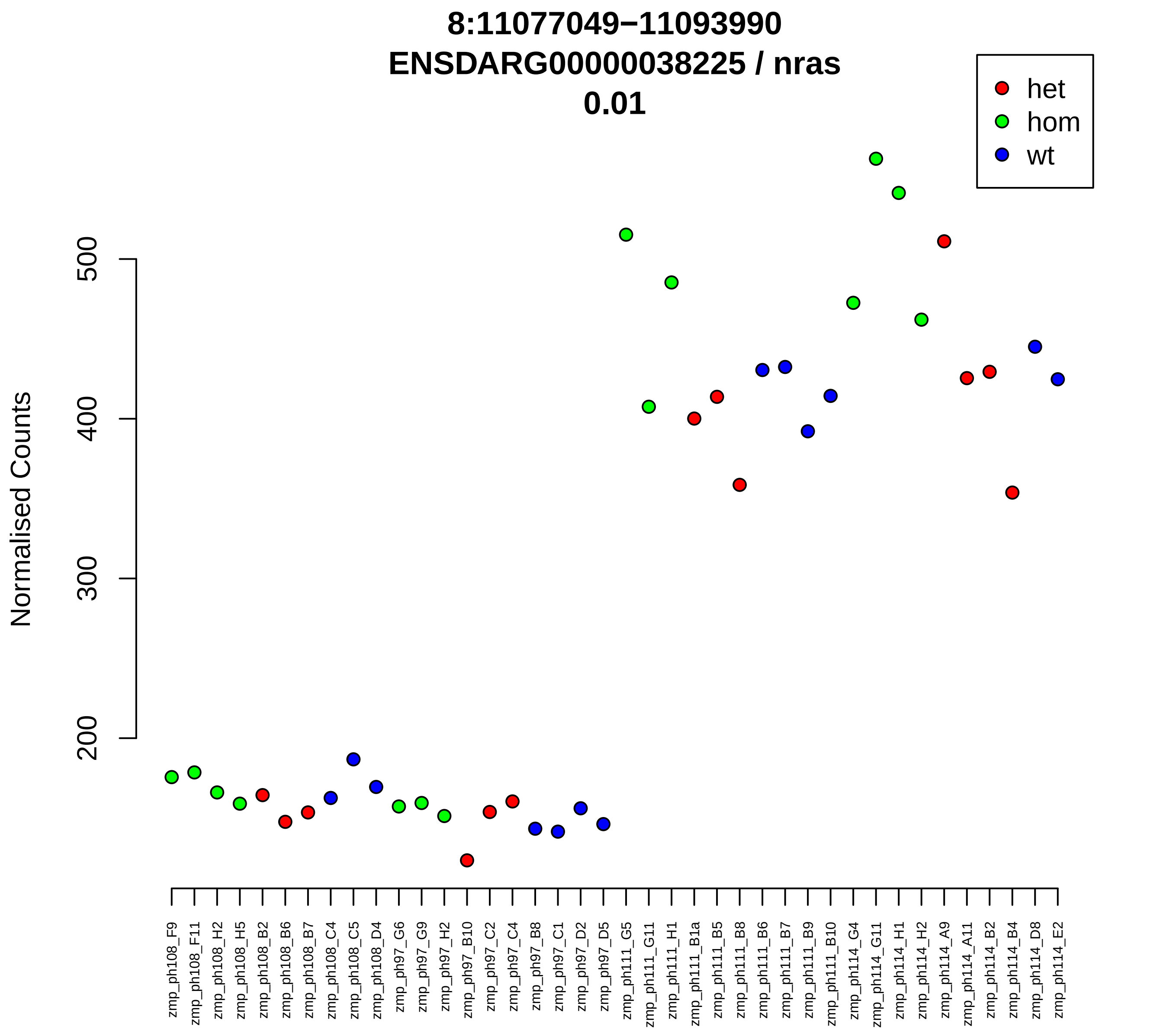

8:11077049−11093990
ENSDARG00000038225 / nras
0.01
het
hom
wt
Normalised Counts
200
300
400
500
zmp_ph108_F9
zmp_ph108_F11
zmp_ph108_H2
zmp_ph108_H5
zmp_ph108_B2
zmp_ph108_B6
zmp_ph108_B7
zmp_ph108_C4
zmp_ph108_C5
zmp_ph108_D4
zmp_ph97_G6
zmp_ph97_G9
zmp_ph97_H2
zmp_ph97_B10
zmp_ph97_C2
zmp_ph97_C4
zmp_ph97_B8
zmp_ph97_C1
zmp_ph97_D2
zmp_ph97_D5
zmp_ph111_G5
zmp_ph111_G11
zmp_ph111_H1
zmp_ph111_B1a
zmp_ph111_B5
zmp_ph111_B8
zmp_ph111_B6
zmp_ph111_B7
zmp_ph111_B9
zmp_ph111_B10
zmp_ph114_G4
zmp_ph114_G11
zmp_ph114_H1
zmp_ph114_H2
zmp_ph114_A9
zmp_ph114_A11
zmp_ph114_B2
zmp_ph114_B4
zmp_ph114_D8
zmp_ph114_E2

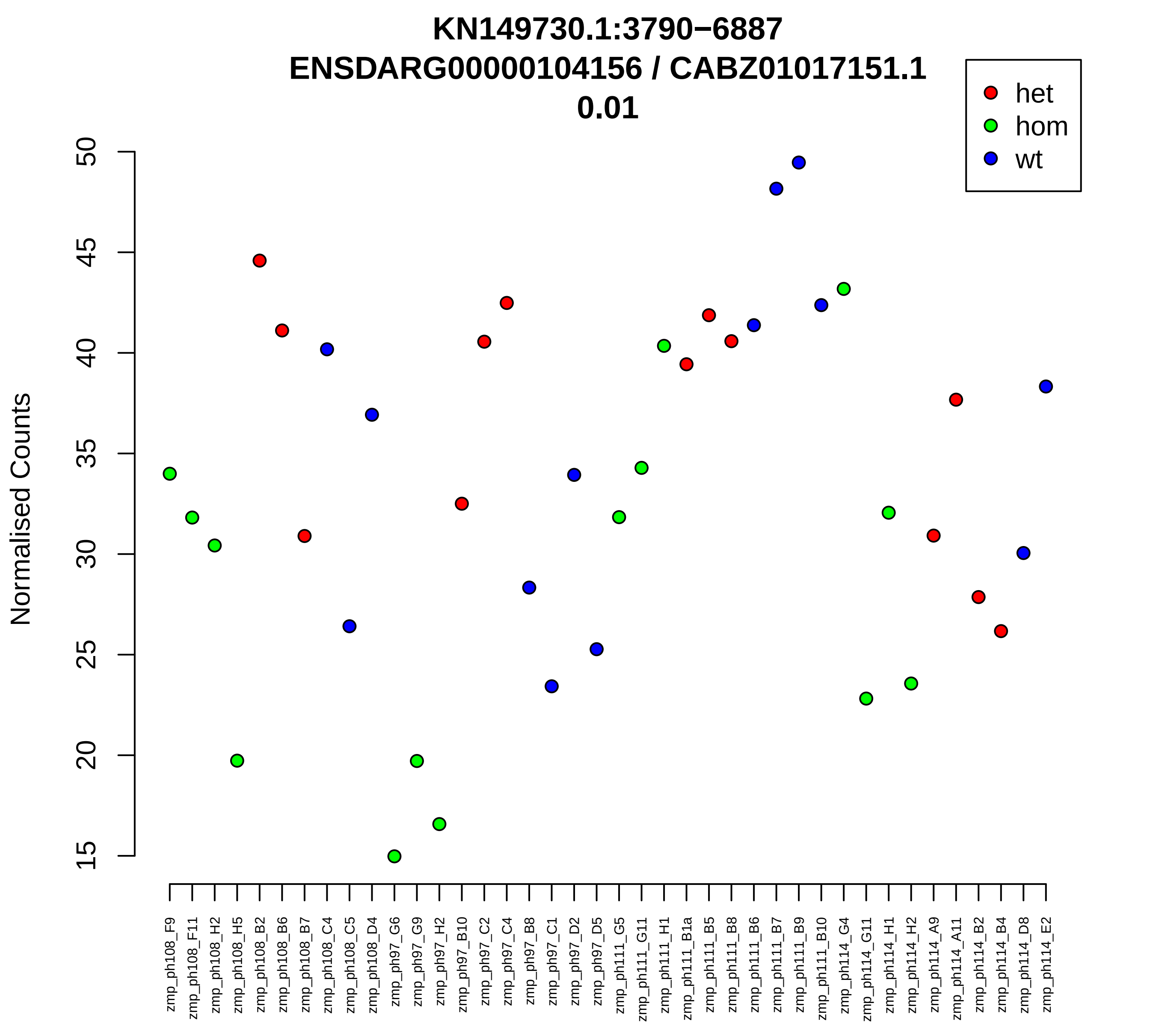
KN149730.1:3790−6887
ENSDARG00000104156 / CABZ01017151.1
0.01
het
hom
wt
Normalised Counts
15
20
25
30
35
40
45
50
zmp_ph108_F9
zmp_ph108_F11
zmp_ph108_H2
zmp_ph108_H5
zmp_ph108_B2
zmp_ph108_B6
zmp_ph108_B7
zmp_ph108_C4
zmp_ph108_C5
zmp_ph108_D4
zmp_ph97_G6
zmp_ph97_G9
zmp_ph97_H2
zmp_ph97_B10
zmp_ph97_C2
zmp_ph97_C4
zmp_ph97_B8
zmp_ph97_C1
zmp_ph97_D2
zmp_ph97_D5
zmp_ph111_G5
zmp_ph111_G11
zmp_ph111_H1
zmp_ph111_B1a
zmp_ph111_B5
zmp_ph111_B8
zmp_ph111_B6
zmp_ph111_B7
zmp_ph111_B9
zmp_ph111_B10
zmp_ph114_G4
zmp_ph114_G11
zmp_ph114_H1
zmp_ph114_H2
zmp_ph114_A9
zmp_ph114_A11
zmp_ph114_B2
zmp_ph114_B4
zmp_ph114_D8
zmp_ph114_E2

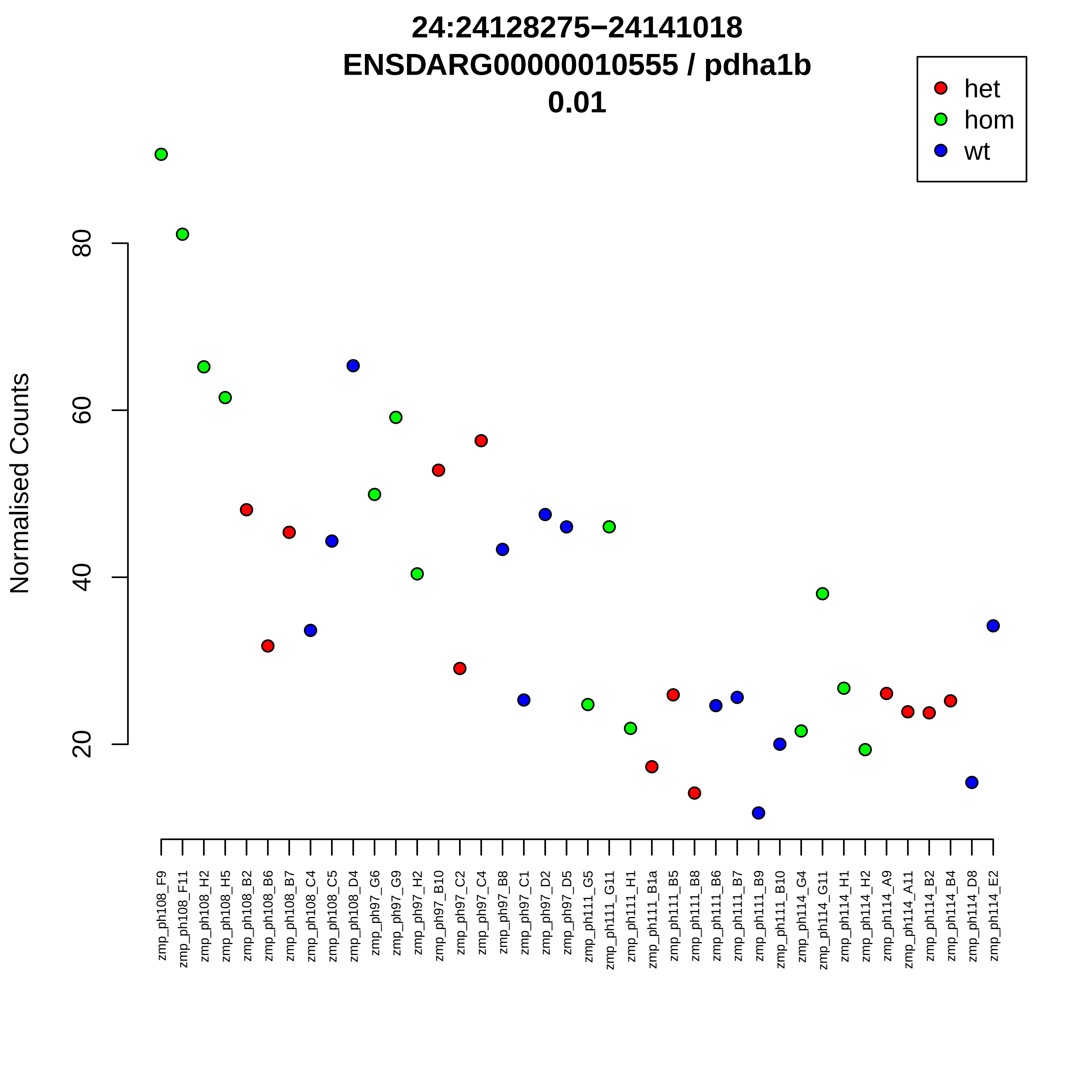

24:24128275−24141018
ENSDARG00000010555 / pdha1b
0.01
het
hom
wt
Normalised Counts
20
40
60
80
zmp_ph108_F9
zmp_ph108_F11
zmp_ph108_H2
zmp_ph108_H5
zmp_ph108_B2
zmp_ph108_B6
zmp_ph108_B7
zmp_ph108_C4
zmp_ph108_C5
zmp_ph108_D4
zmp_ph97_G6
zmp_ph97_G9
zmp_ph97_H2
zmp_ph97_B10
zmp_ph97_C2
zmp_ph97_C4
zmp_ph97_B8
zmp_ph97_C1
zmp_ph97_D2
zmp_ph97_D5
zmp_ph111_G5
zmp_ph111_G11
zmp_ph111_H1
zmp_ph111_B1a
zmp_ph111_B5
zmp_ph111_B8
zmp_ph111_B6
zmp_ph111_B7
zmp_ph111_B9
zmp_ph111_B10
zmp_ph114_G4
zmp_ph114_G11
zmp_ph114_H1
zmp_ph114_H2
zmp_ph114_A9
zmp_ph114_A11
zmp_ph114_B2
zmp_ph114_B4
zmp_ph114_D8
zmp_ph114_E2

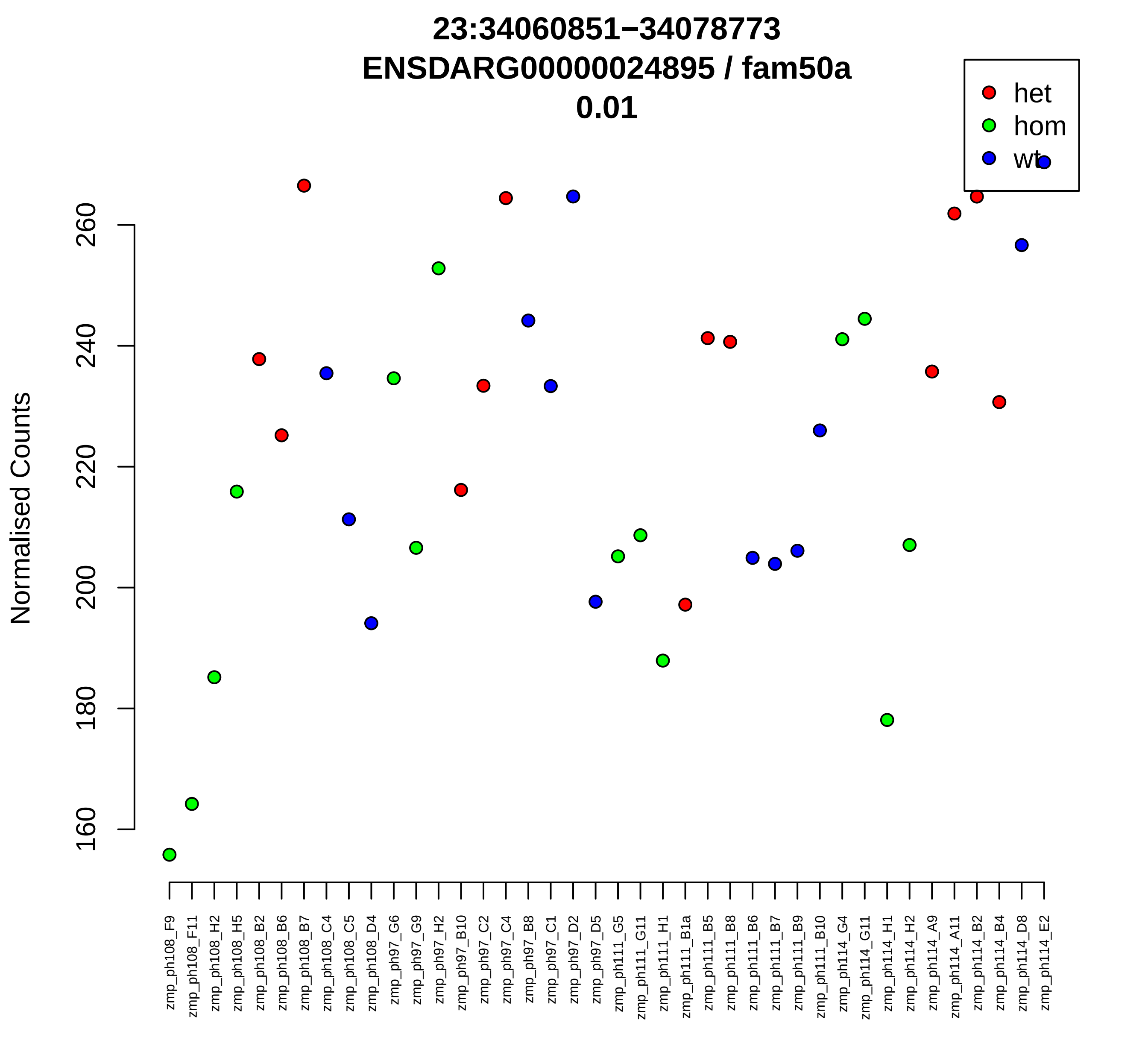

23:34060851−34078773
ENSDARG00000024895 / fam50a
0.01
het
hom
wt
Normalised Counts
160
180
200
220
240
260
zmp_ph108_F9
zmp_ph108_F11
zmp_ph108_H2
zmp_ph108_H5
zmp_ph108_B2
zmp_ph108_B6
zmp_ph108_B7
zmp_ph108_C4
zmp_ph108_C5
zmp_ph108_D4
zmp_ph97_G6
zmp_ph97_G9
zmp_ph97_H2
zmp_ph97_B10
zmp_ph97_C2
zmp_ph97_C4
zmp_ph97_B8
zmp_ph97_C1
zmp_ph97_D2
zmp_ph97_D5
zmp_ph111_G5
zmp_ph111_G11
zmp_ph111_H1
zmp_ph111_B1a
zmp_ph111_B5
zmp_ph111_B8
zmp_ph111_B6
zmp_ph111_B7
zmp_ph111_B9
zmp_ph111_B10
zmp_ph114_G4
zmp_ph114_G11
zmp_ph114_H1
zmp_ph114_H2
zmp_ph114_A9
zmp_ph114_A11
zmp_ph114_B2
zmp_ph114_B4
zmp_ph114_D8
zmp_ph114_E2

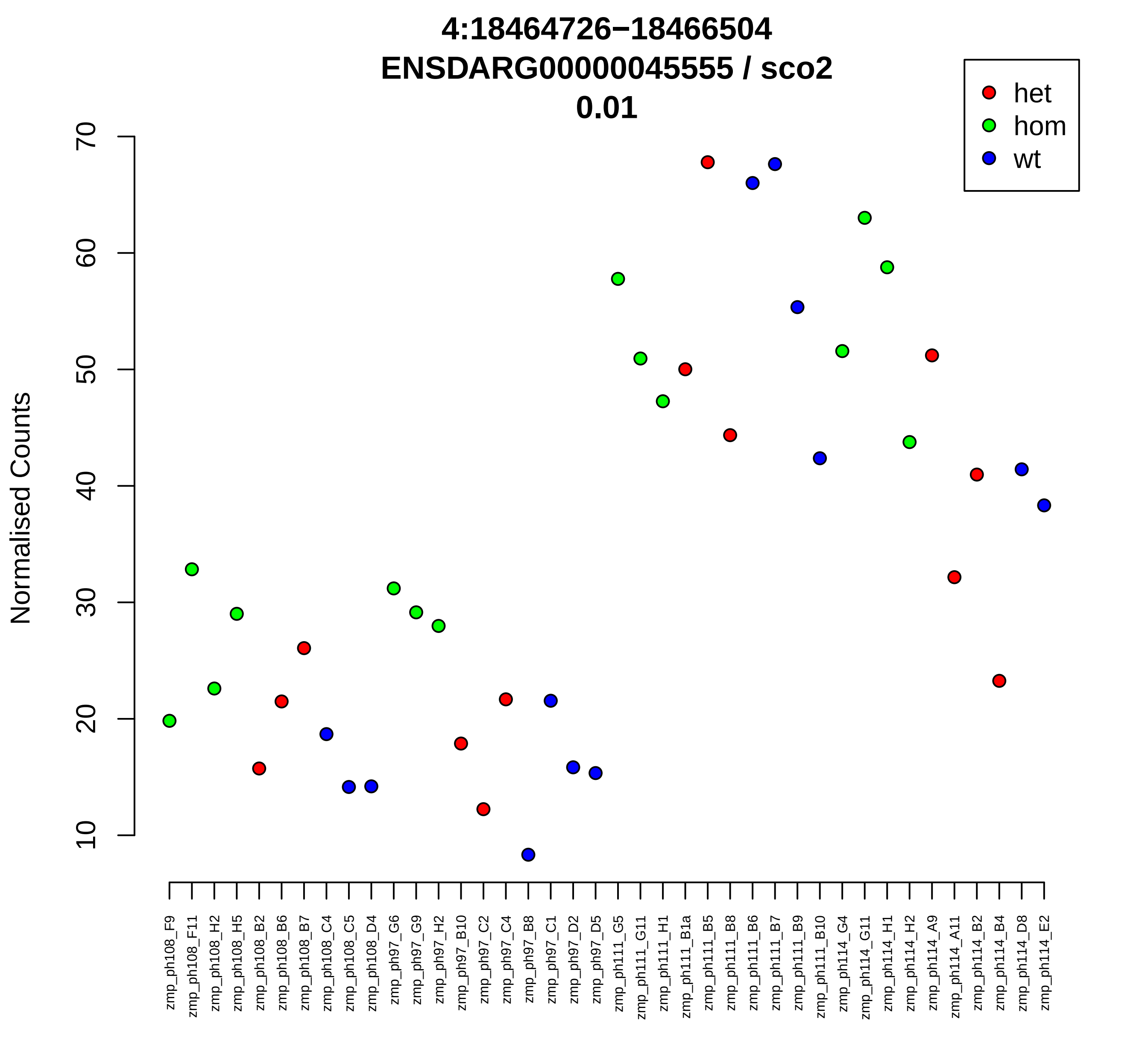

4:18464726−18466504
ENSDARG00000045555 / sco2
0.01
het
hom
wt
Normalised Counts
10
20
30
40
50
60
70
zmp_ph108_F9
zmp_ph108_F11
zmp_ph108_H2
zmp_ph108_H5
zmp_ph108_B2
zmp_ph108_B6
zmp_ph108_B7
zmp_ph108_C4
zmp_ph108_C5
zmp_ph108_D4
zmp_ph97_G6
zmp_ph97_G9
zmp_ph97_H2
zmp_ph97_B10
zmp_ph97_C2
zmp_ph97_C4
zmp_ph97_B8
zmp_ph97_C1
zmp_ph97_D2
zmp_ph97_D5
zmp_ph111_G5
zmp_ph111_G11
zmp_ph111_H1
zmp_ph111_B1a
zmp_ph111_B5
zmp_ph111_B8
zmp_ph111_B6
zmp_ph111_B7
zmp_ph111_B9
zmp_ph111_B10
zmp_ph114_G4
zmp_ph114_G11
zmp_ph114_H1
zmp_ph114_H2
zmp_ph114_A9
zmp_ph114_A11
zmp_ph114_B2
zmp_ph114_B4
zmp_ph114_D8
zmp_ph114_E2

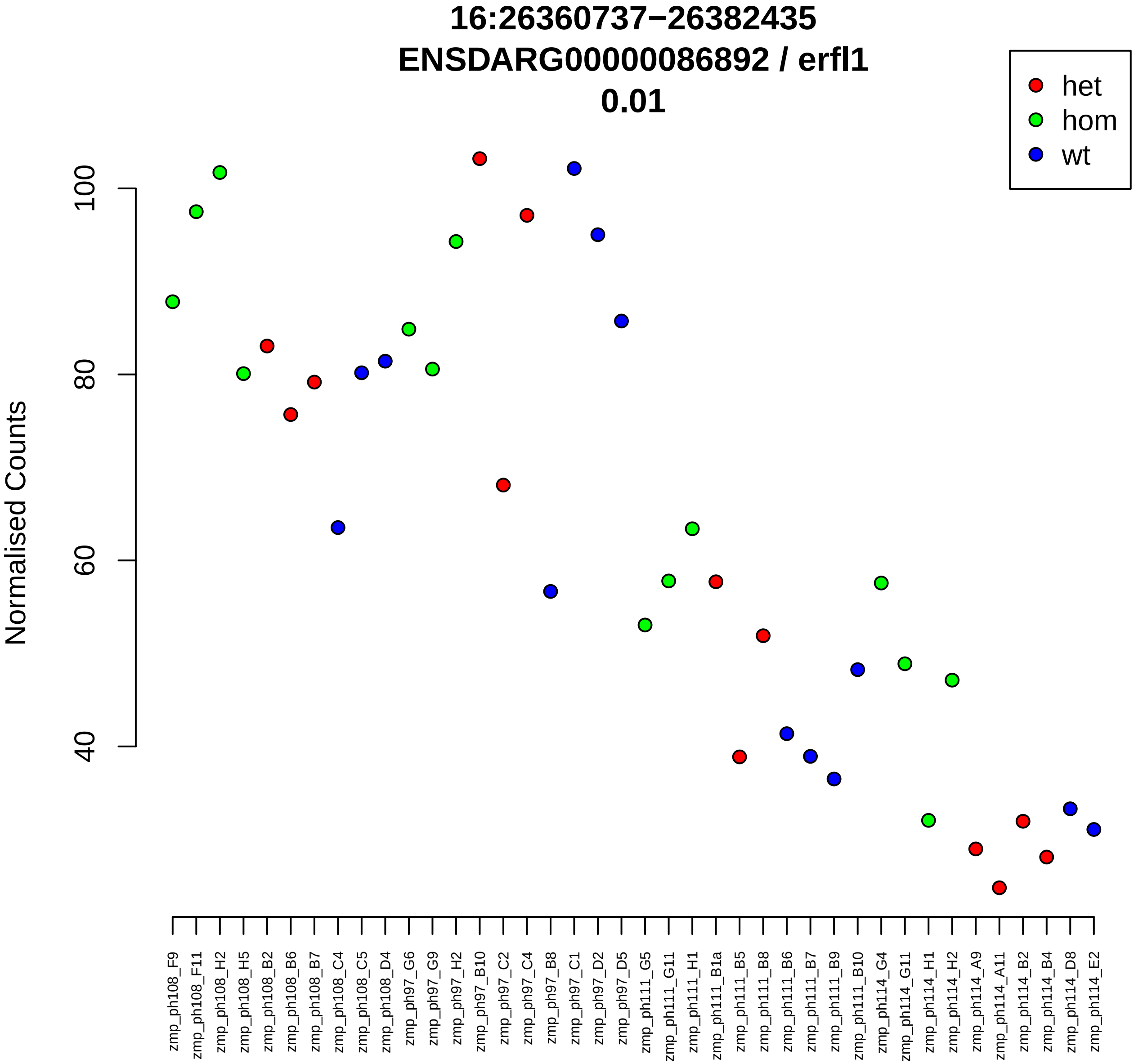
16:26360737−26382435
ENSDARG00000086892 / erfl1
0.01
het
hom
wt
Normalised Counts
40
60
80
100
zmp_ph108_F9
zmp_ph108_F11
zmp_ph108_H2
zmp_ph108_H5
zmp_ph108_B2
zmp_ph108_B6
zmp_ph108_B7
zmp_ph108_C4
zmp_ph108_C5
zmp_ph108_D4
zmp_ph97_G6
zmp_ph97_G9
zmp_ph97_H2
zmp_ph97_B10
zmp_ph97_C2
zmp_ph97_C4
zmp_ph97_B8
zmp_ph97_C1
zmp_ph97_D2
zmp_ph97_D5
zmp_ph111_G5
zmp_ph111_G11
zmp_ph111_H1
zmp_ph111_B1a
zmp_ph111_B5
zmp_ph111_B8
zmp_ph111_B6
zmp_ph111_B7
zmp_ph111_B9
zmp_ph111_B10
zmp_ph114_G4
zmp_ph114_G11
zmp_ph114_H1
zmp_ph114_H2
zmp_ph114_A9
zmp_ph114_A11
zmp_ph114_B2
zmp_ph114_B4
zmp_ph114_D8
zmp_ph114_E2

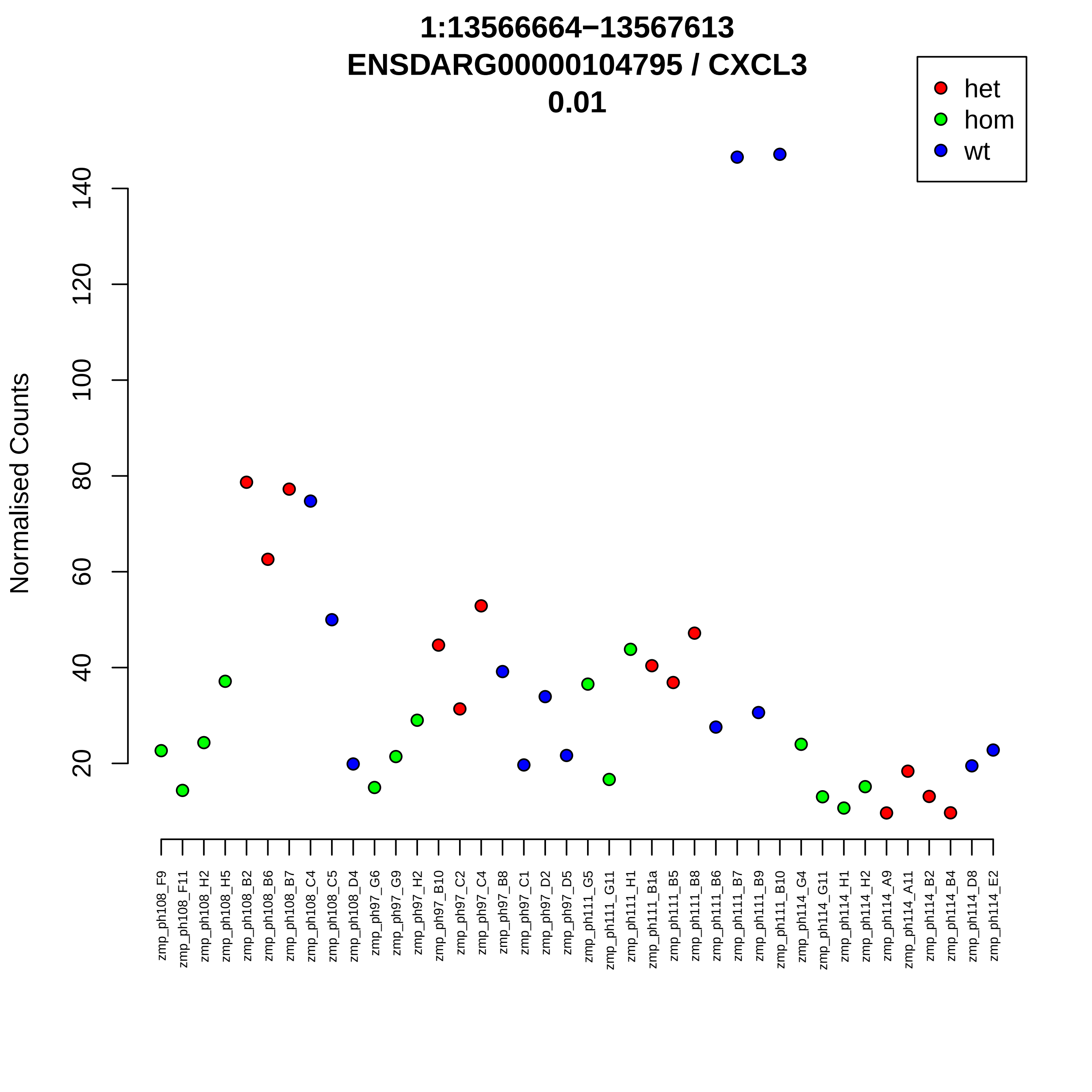
1:13566664−13567613
ENSDARG00000104795 / CXCL3
0.01
het
hom
wt
Normalised Counts
20
40
60
80
100
120
140
zmp_ph108_F9
zmp_ph108_F11
zmp_ph108_H2
zmp_ph108_H5
zmp_ph108_B2
zmp_ph108_B6
zmp_ph108_B7
zmp_ph108_C4
zmp_ph108_C5
zmp_ph108_D4
zmp_ph97_G6
zmp_ph97_G9
zmp_ph97_H2
zmp_ph97_B10
zmp_ph97_C2
zmp_ph97_C4
zmp_ph97_B8
zmp_ph97_C1
zmp_ph97_D2
zmp_ph97_D5
zmp_ph111_G5
zmp_ph111_G11
zmp_ph111_H1
zmp_ph111_B1a
zmp_ph111_B5
zmp_ph111_B8
zmp_ph111_B6
zmp_ph111_B7
zmp_ph111_B9
zmp_ph111_B10
zmp_ph114_G4
zmp_ph114_G11
zmp_ph114_H1
zmp_ph114_H2
zmp_ph114_A9
zmp_ph114_A11
zmp_ph114_B2
zmp_ph114_B4
zmp_ph114_D8
zmp_ph114_E2

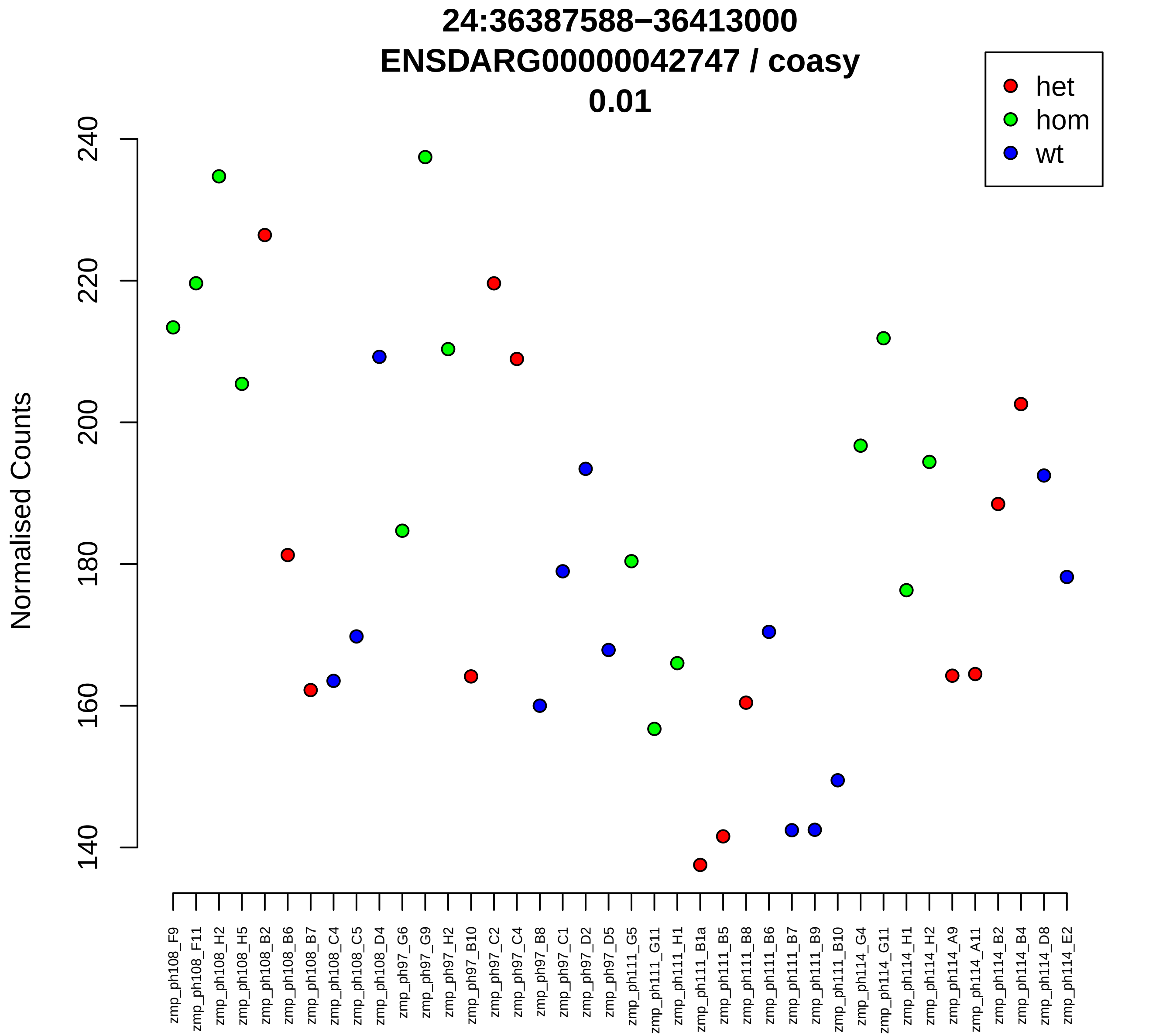

24:36387588−36413000
ENSDARG00000042747 / coasy
0.01
het
hom
wt
Normalised Counts
240
220
200
180
160
140
zmp_ph108_F9
zmp_ph108_F11
zmp_ph108_H2
zmp_ph108_H5
zmp_ph108_B2
zmp_ph108_B6
zmp_ph108_B7
zmp_ph108_C4
zmp_ph108_C5
zmp_ph108_D4
zmp_ph97_G6
zmp_ph97_G9
zmp_ph97_H2
zmp_ph97_B10
zmp_ph97_C2
zmp_ph97_C4
zmp_ph97_B8
zmp_ph97_C1
zmp_ph97_D2
zmp_ph97_D5
zmp_ph111_G5
zmp_ph111_G11
zmp_ph111_H1
zmp_ph111_B1a
zmp_ph111_B5
zmp_ph111_B8
zmp_ph111_B6
zmp_ph111_B7
zmp_ph111_B9
zmp_ph111_B10
zmp_ph114_G4
zmp_ph114_G11
zmp_ph114_H1
zmp_ph114_H2
zmp_ph114_A9
zmp_ph114_A11
zmp_ph114_B2
zmp_ph114_B4
zmp_ph114_D8
zmp_ph114_E2

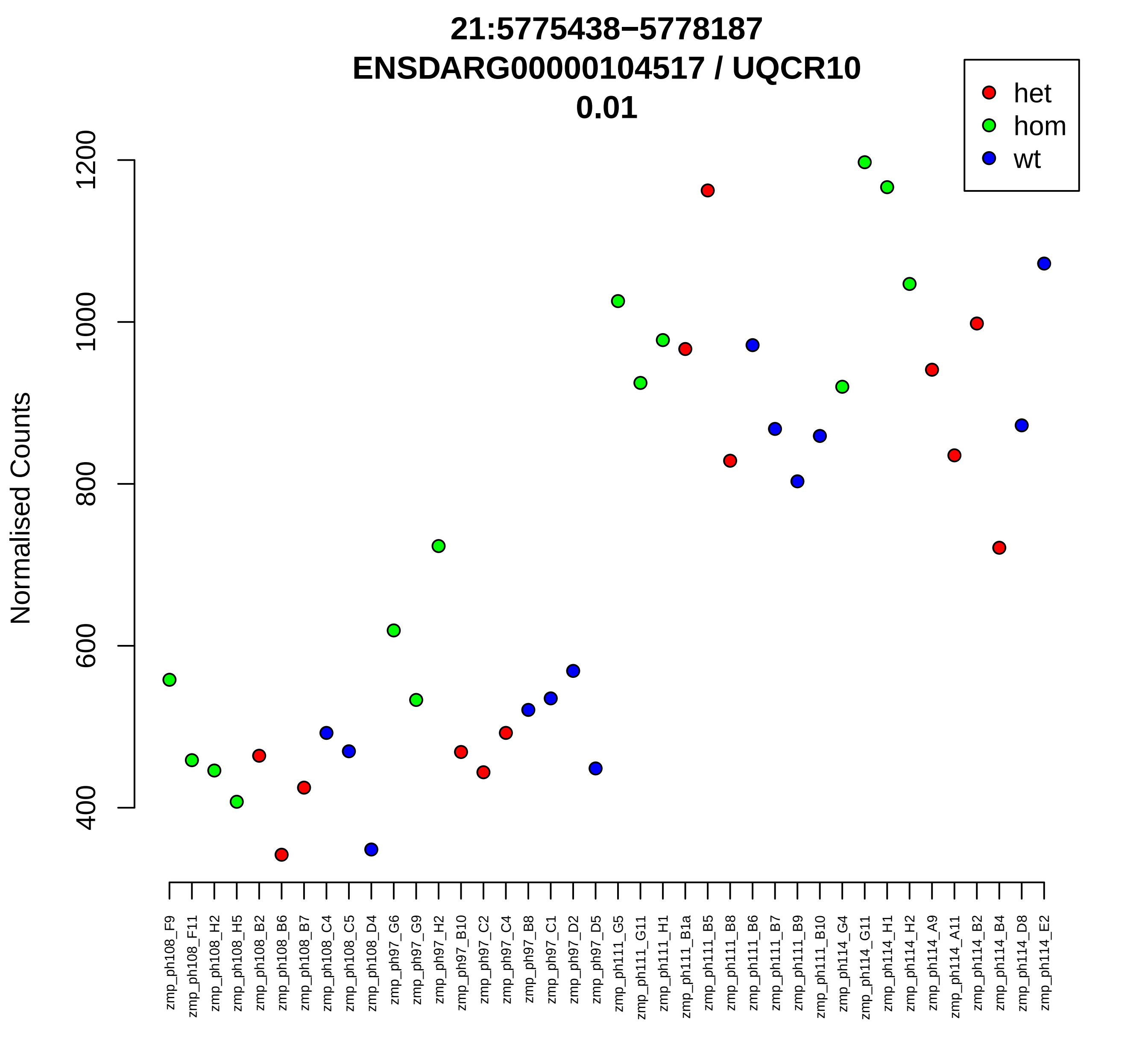
21:5775438−5778187
ENSDARG00000104517 / UQCR10
0.01
het
hom
wt
Normalised Counts
400
600
800
1000
1200
zmp_ph108_F9
zmp_ph108_F11
zmp_ph108_H2
zmp_ph108_H5
zmp_ph108_B2
zmp_ph108_B6
zmp_ph108_B7
zmp_ph108_C4
zmp_ph108_C5
zmp_ph108_D4
zmp_ph97_G6
zmp_ph97_G9
zmp_ph97_H2
zmp_ph97_B10
zmp_ph97_C2
zmp_ph97_C4
zmp_ph97_B8
zmp_ph97_C1
zmp_ph97_D2
zmp_ph97_D5
zmp_ph111_G5
zmp_ph111_G11
zmp_ph111_H1
zmp_ph111_B1a
zmp_ph111_B5
zmp_ph111_B8
zmp_ph111_B6
zmp_ph111_B7
zmp_ph111_B9
zmp_ph111_B10
zmp_ph114_G4
zmp_ph114_G11
zmp_ph114_H1
zmp_ph114_H2
zmp_ph114_A9
zmp_ph114_A11
zmp_ph114_B2
zmp_ph114_B4
zmp_ph114_D8
zmp_ph114_E2

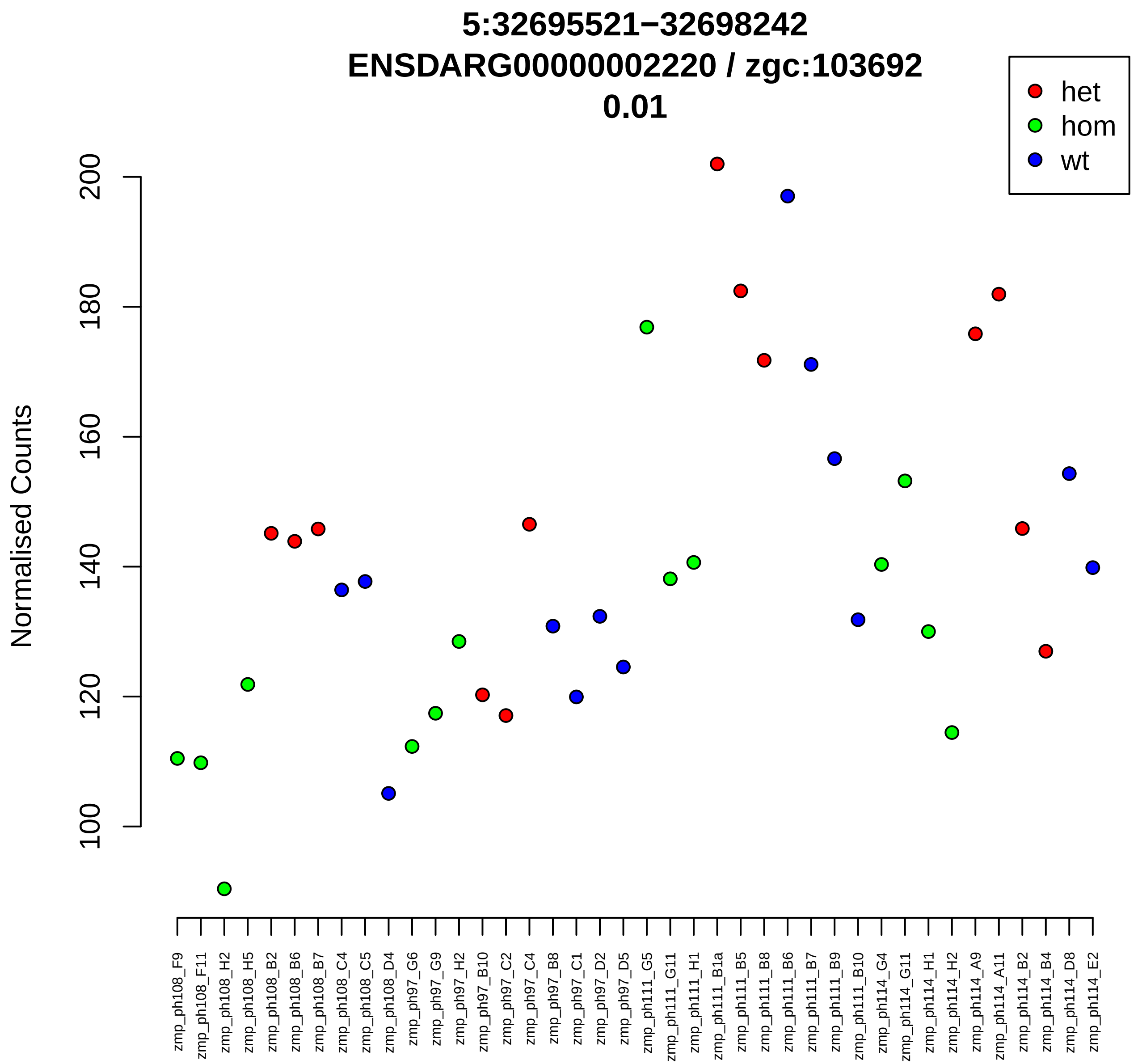

5:32695521−32698242
ENSDARG00000002220 / zgc:103692
0.01
het
hom
wt
Normalised Counts
100
120
140
160
180
200
zmp_ph108_F9
zmp_ph108_F11
zmp_ph108_H2
zmp_ph108_H5
zmp_ph108_B2
zmp_ph108_B6
zmp_ph108_B7
zmp_ph108_C4
zmp_ph108_C5
zmp_ph108_D4
zmp_ph97_G6
zmp_ph97_G9
zmp_ph97_H2
zmp_ph97_B10
zmp_ph97_C2
zmp_ph97_C4
zmp_ph97_B8
zmp_ph97_C1
zmp_ph97_D2
zmp_ph97_D5
zmp_ph111_G5
zmp_ph111_G11
zmp_ph111_H1
zmp_ph111_B1a
zmp_ph111_B5
zmp_ph111_B8
zmp_ph111_B6
zmp_ph111_B7
zmp_ph111_B9
zmp_ph111_B10
zmp_ph114_G4
zmp_ph114_G11
zmp_ph114_H1
zmp_ph114_H2
zmp_ph114_A9
zmp_ph114_A11
zmp_ph114_B2
zmp_ph114_B4
zmp_ph114_D8
zmp_ph114_E2

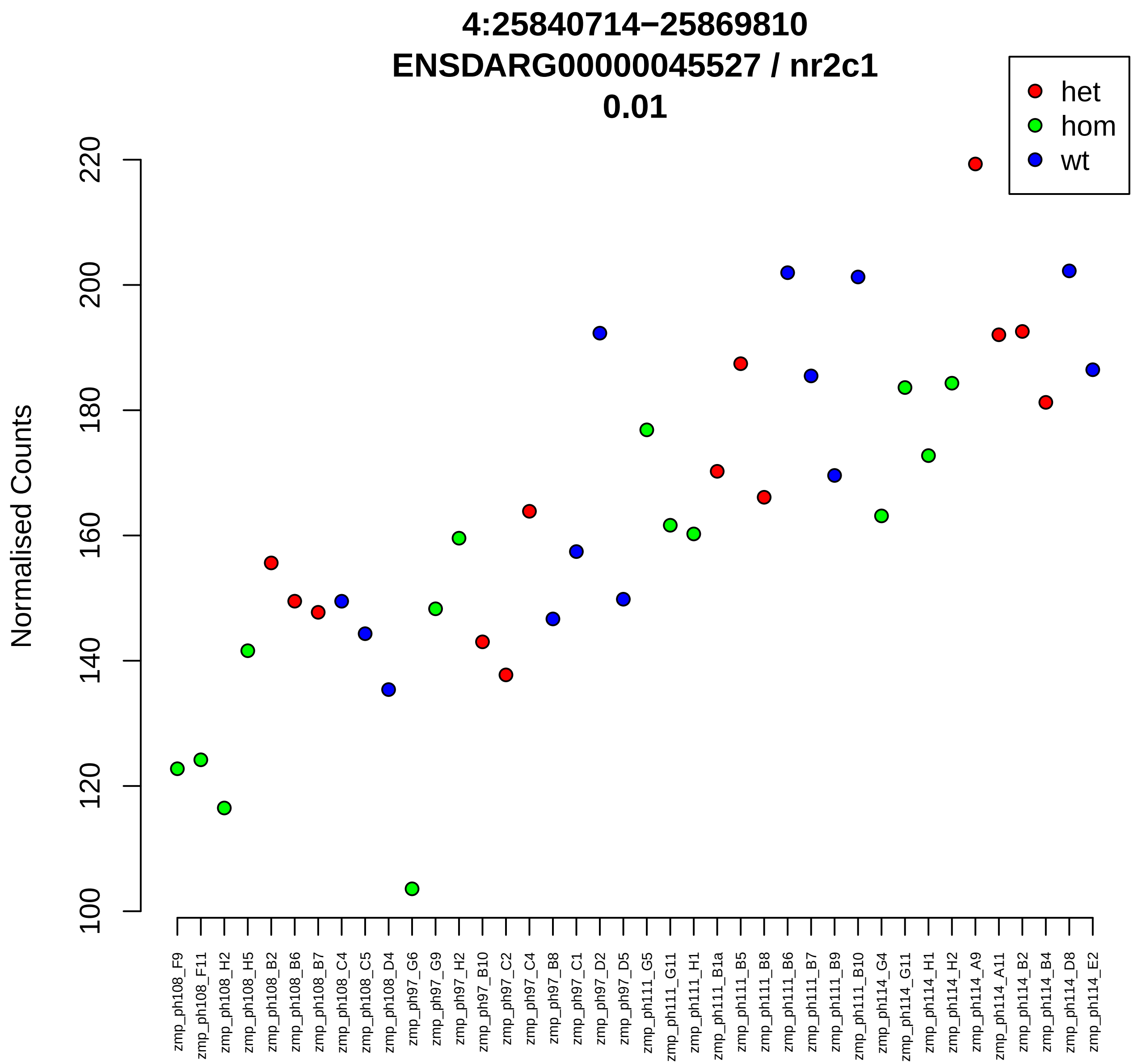
4:25840714−25869810
ENSDARG00000045527 / nr2c1
0.01
het
hom
wt
Normalised Counts
100
120
140
160
180
200
220
zmp_ph108_F9
zmp_ph108_F11
zmp_ph108_H2
zmp_ph108_H5
zmp_ph108_B2
zmp_ph108_B6
zmp_ph108_B7
zmp_ph108_C4
zmp_ph108_C5
zmp_ph108_D4
zmp_ph97_G6
zmp_ph97_G9
zmp_ph97_H2
zmp_ph97_B10
zmp_ph97_C2
zmp_ph97_C4
zmp_ph97_B8
zmp_ph97_C1
zmp_ph97_D2
zmp_ph97_D5
zmp_ph111_G5
zmp_ph111_G11
zmp_ph111_H1
zmp_ph111_B1a
zmp_ph111_B5
zmp_ph111_B8
zmp_ph111_B6
zmp_ph111_B7
zmp_ph111_B9
zmp_ph111_B10
zmp_ph114_G4
zmp_ph114_G11
zmp_ph114_H1
zmp_ph114_H2
zmp_ph114_A9
zmp_ph114_A11
zmp_ph114_B2
zmp_ph114_B4
zmp_ph114_D8
zmp_ph114_E2

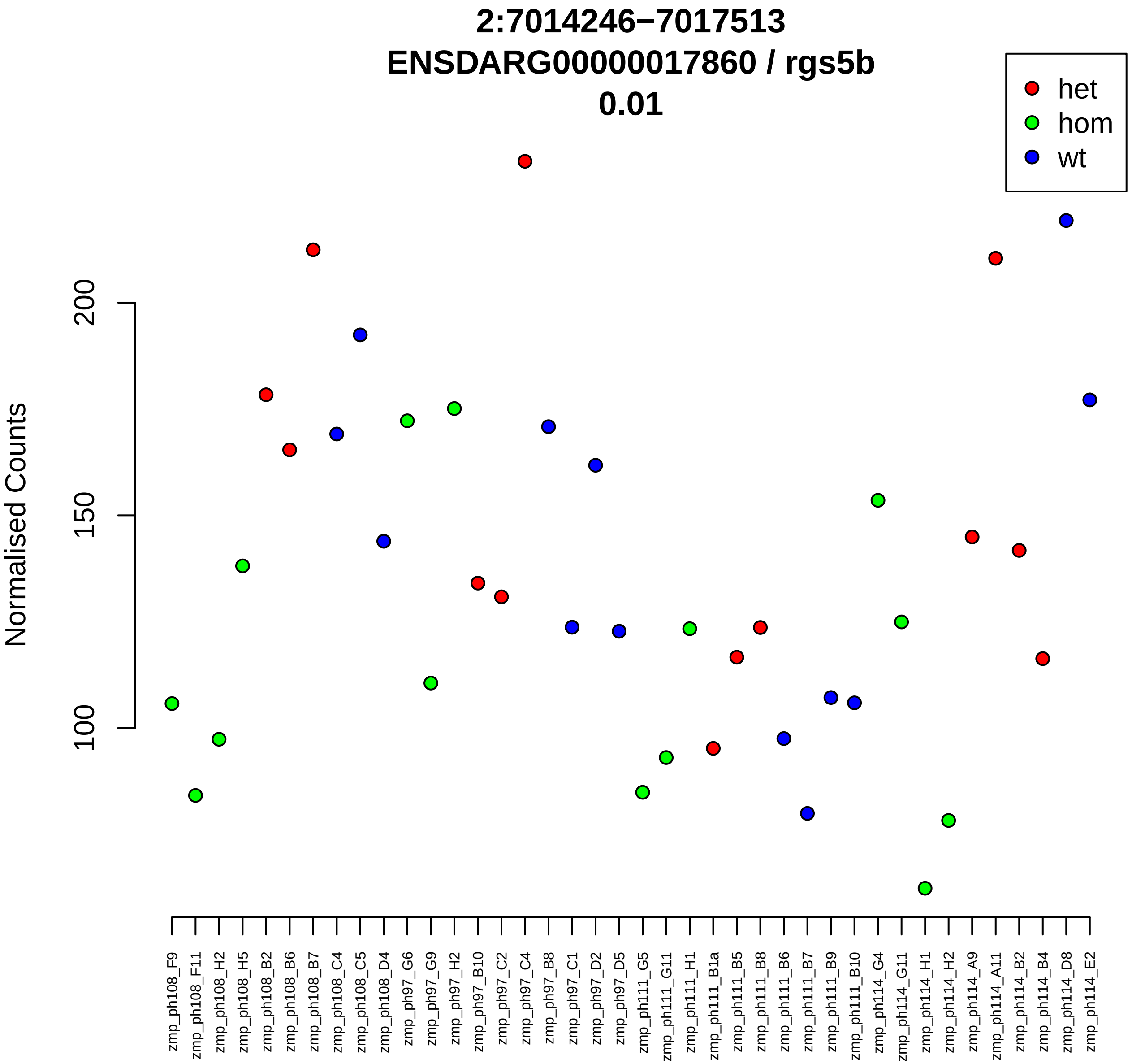
2:7014246−7017513
ENSDARG00000017860 / rgs5b
0.01
het
hom
wt
Normalised Counts
100
150
200
zmp_ph108_F9
zmp_ph108_F11
zmp_ph108_H2
zmp_ph108_H5
zmp_ph108_B2
zmp_ph108_B6
zmp_ph108_B7
zmp_ph108_C4
zmp_ph108_C5
zmp_ph108_D4
zmp_ph97_G6
zmp_ph97_G9
zmp_ph97_H2
zmp_ph97_B10
zmp_ph97_C2
zmp_ph97_C4
zmp_ph97_B8
zmp_ph97_C1
zmp_ph97_D2
zmp_ph97_D5
zmp_ph111_G5
zmp_ph111_G11
zmp_ph111_H1
zmp_ph111_B1a
zmp_ph111_B5
zmp_ph111_B8
zmp_ph111_B6
zmp_ph111_B7
zmp_ph111_B9
zmp_ph111_B10
zmp_ph114_G4
zmp_ph114_G11
zmp_ph114_H1
zmp_ph114_H2
zmp_ph114_A9
zmp_ph114_A11
zmp_ph114_B2
zmp_ph114_B4
zmp_ph114_D8
zmp_ph114_E2

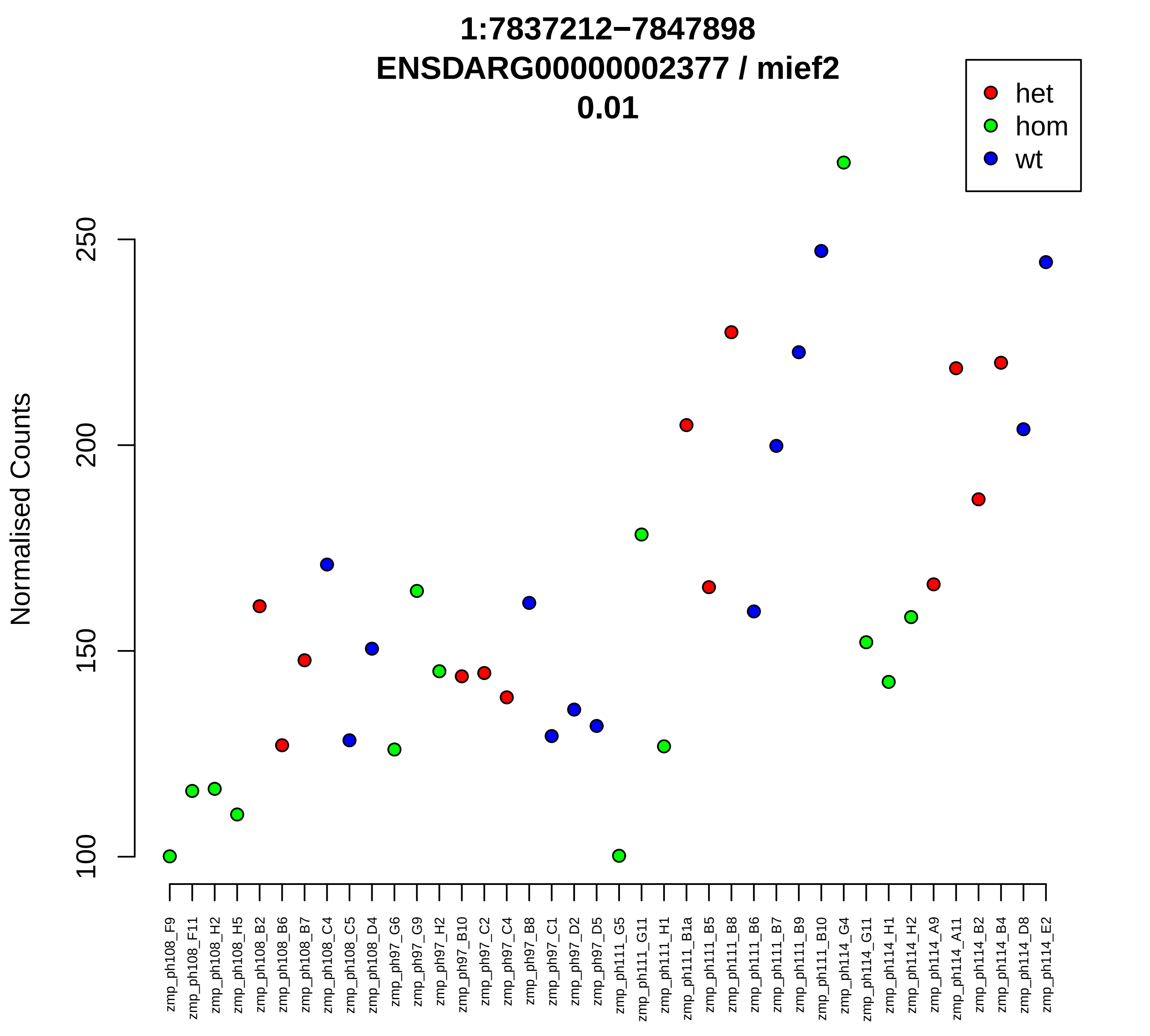
1:7837212−7847898
ENSDARG00000002377 / mief2
0.01
het
hom
wt
Normalised Counts
100
150
200
250
zmp_ph108_F9
zmp_ph108_F11
zmp_ph108_H2
zmp_ph108_H5
zmp_ph108_B2
zmp_ph108_B6
zmp_ph108_B7
zmp_ph108_C4
zmp_ph108_C5
zmp_ph108_D4
zmp_ph97_G6
zmp_ph97_G9
zmp_ph97_H2
zmp_ph97_B10
zmp_ph97_C2
zmp_ph97_C4
zmp_ph97_B8
zmp_ph97_C1
zmp_ph97_D2
zmp_ph97_D5
zmp_ph111_G5
zmp_ph111_G11
zmp_ph111_H1
zmp_ph111_B1a
zmp_ph111_B5
zmp_ph111_B8
zmp_ph111_B6
zmp_ph111_B7
zmp_ph111_B9
zmp_ph111_B10
zmp_ph114_G4
zmp_ph114_G11
zmp_ph114_H1
zmp_ph114_H2
zmp_ph114_A9
zmp_ph114_A11
zmp_ph114_B2
zmp_ph114_B4
zmp_ph114_D8
zmp_ph114_E2

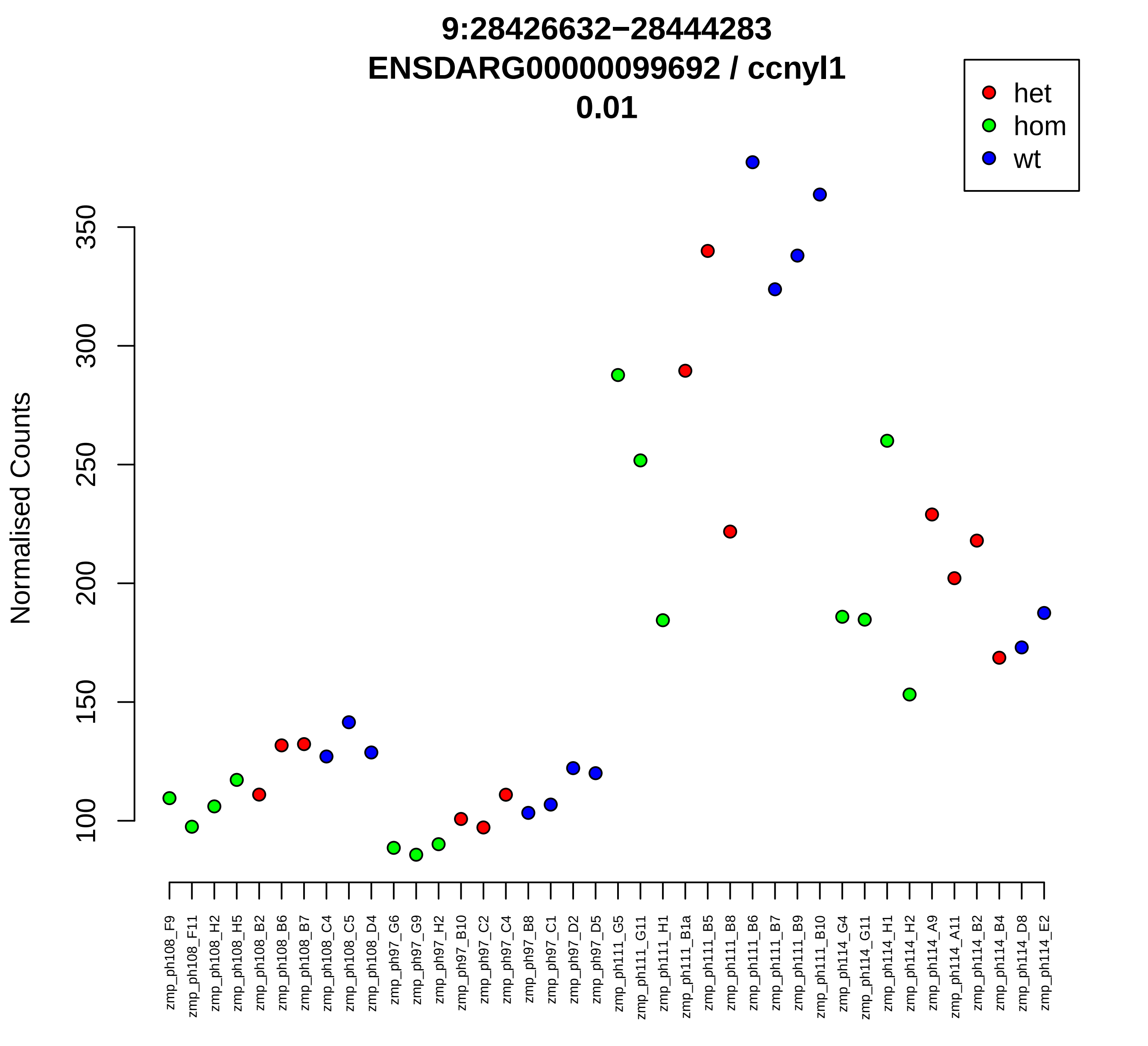
9:28426632−28444283
ENSDARG00000099692 / ccnyl1
0.01
het
hom
wt
Normalised Counts
100
150
200
250
300
350
zmp_ph108_F9
zmp_ph108_F11
zmp_ph108_H2
zmp_ph108_H5
zmp_ph108_B2
zmp_ph108_B6
zmp_ph108_B7
zmp_ph108_C4
zmp_ph108_C5
zmp_ph108_D4
zmp_ph97_G6
zmp_ph97_G9
zmp_ph97_H2
zmp_ph97_B10
zmp_ph97_C2
zmp_ph97_C4
zmp_ph97_B8
zmp_ph97_C1
zmp_ph97_D2
zmp_ph97_D5
zmp_ph111_G5
zmp_ph111_G11
zmp_ph111_H1
zmp_ph111_B1a
zmp_ph111_B5
zmp_ph111_B8
zmp_ph111_B6
zmp_ph111_B7
zmp_ph111_B9
zmp_ph111_B10
zmp_ph114_G4
zmp_ph114_G11
zmp_ph114_H1
zmp_ph114_H2
zmp_ph114_A9
zmp_ph114_A11
zmp_ph114_B2
zmp_ph114_B4
zmp_ph114_D8
zmp_ph114_E2

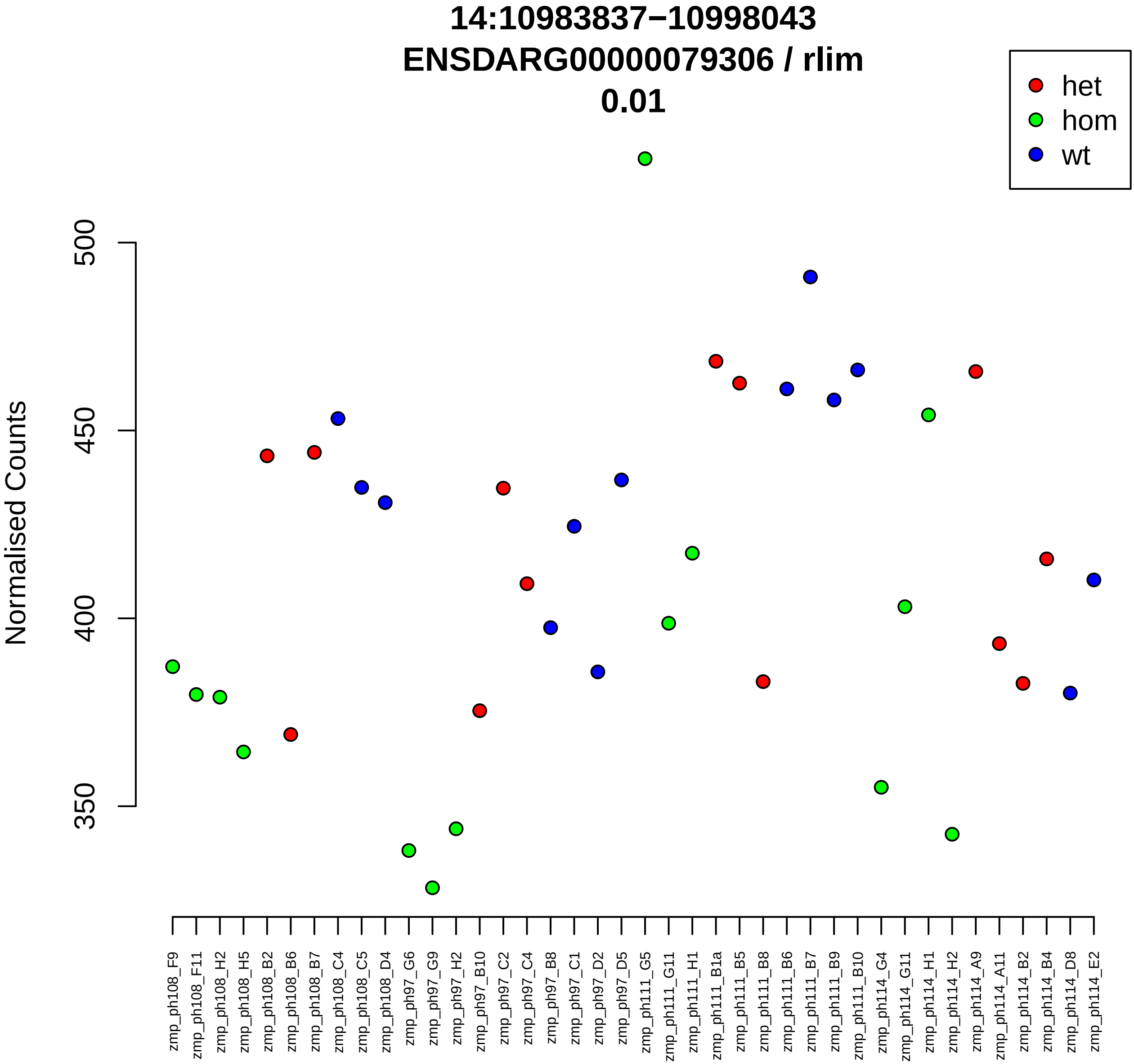

14:10983837−10998043
ENSDARG00000079306 / rlim
0.01
het
hom
wt
Normalised Counts
350
400
450
500
zmp_ph108_F9
zmp_ph108_F11
zmp_ph108_H2
zmp_ph108_H5
zmp_ph108_B2
zmp_ph108_B6
zmp_ph108_B7
zmp_ph108_C4
zmp_ph108_C5
zmp_ph108_D4
zmp_ph97_G6
zmp_ph97_G9
zmp_ph97_H2
zmp_ph97_B10
zmp_ph97_C2
zmp_ph97_C4
zmp_ph97_B8
zmp_ph97_C1
zmp_ph97_D2
zmp_ph97_D5
zmp_ph111_G5
zmp_ph111_G11
zmp_ph111_H1
zmp_ph111_B1a
zmp_ph111_B5
zmp_ph111_B8
zmp_ph111_B6
zmp_ph111_B7
zmp_ph111_B9
zmp_ph111_B10
zmp_ph114_G4
zmp_ph114_G11
zmp_ph114_H1
zmp_ph114_H2
zmp_ph114_A9
zmp_ph114_A11
zmp_ph114_B2
zmp_ph114_B4
zmp_ph114_D8
zmp_ph114_E2

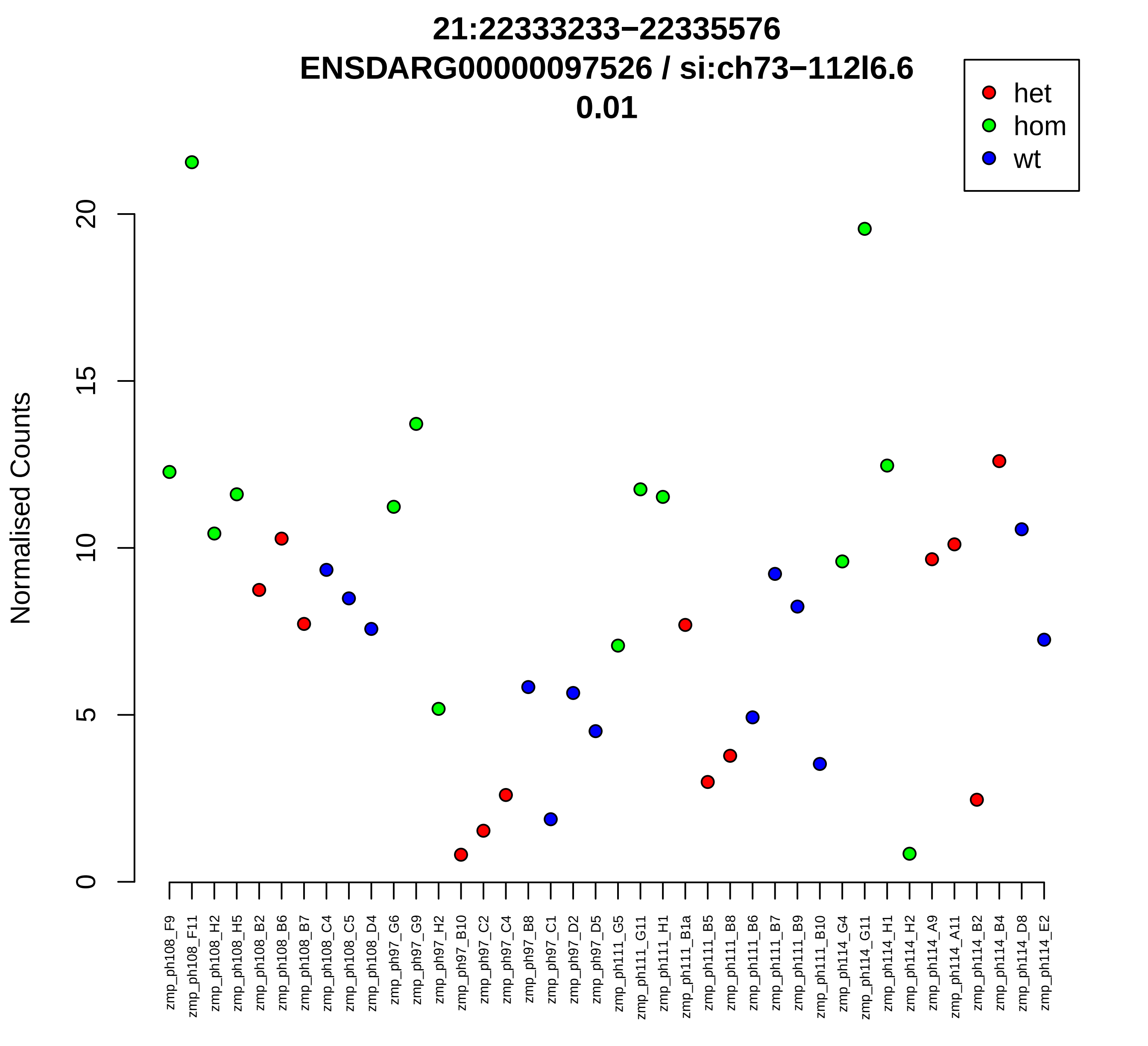
21:22333233−22335576
ENSDARG00000097526 / si:ch73−112l6.6
0.01
het
hom
wt
Normalised Counts
0
5
10
15
20
zmp_ph108_F9
zmp_ph108_F11
zmp_ph108_H2
zmp_ph108_H5
zmp_ph108_B2
zmp_ph108_B6
zmp_ph108_B7
zmp_ph108_C4
zmp_ph108_C5
zmp_ph108_D4
zmp_ph97_G6
zmp_ph97_G9
zmp_ph97_H2
zmp_ph97_B10
zmp_ph97_C2
zmp_ph97_C4
zmp_ph97_B8
zmp_ph97_C1
zmp_ph97_D2
zmp_ph97_D5
zmp_ph111_G5
zmp_ph111_G11
zmp_ph111_H1
zmp_ph111_B1a
zmp_ph111_B5
zmp_ph111_B8
zmp_ph111_B6
zmp_ph111_B7
zmp_ph111_B9
zmp_ph111_B10
zmp_ph114_G4
zmp_ph114_G11
zmp_ph114_H1
zmp_ph114_H2
zmp_ph114_A9
zmp_ph114_A11
zmp_ph114_B2
zmp_ph114_B4
zmp_ph114_D8
zmp_ph114_E2

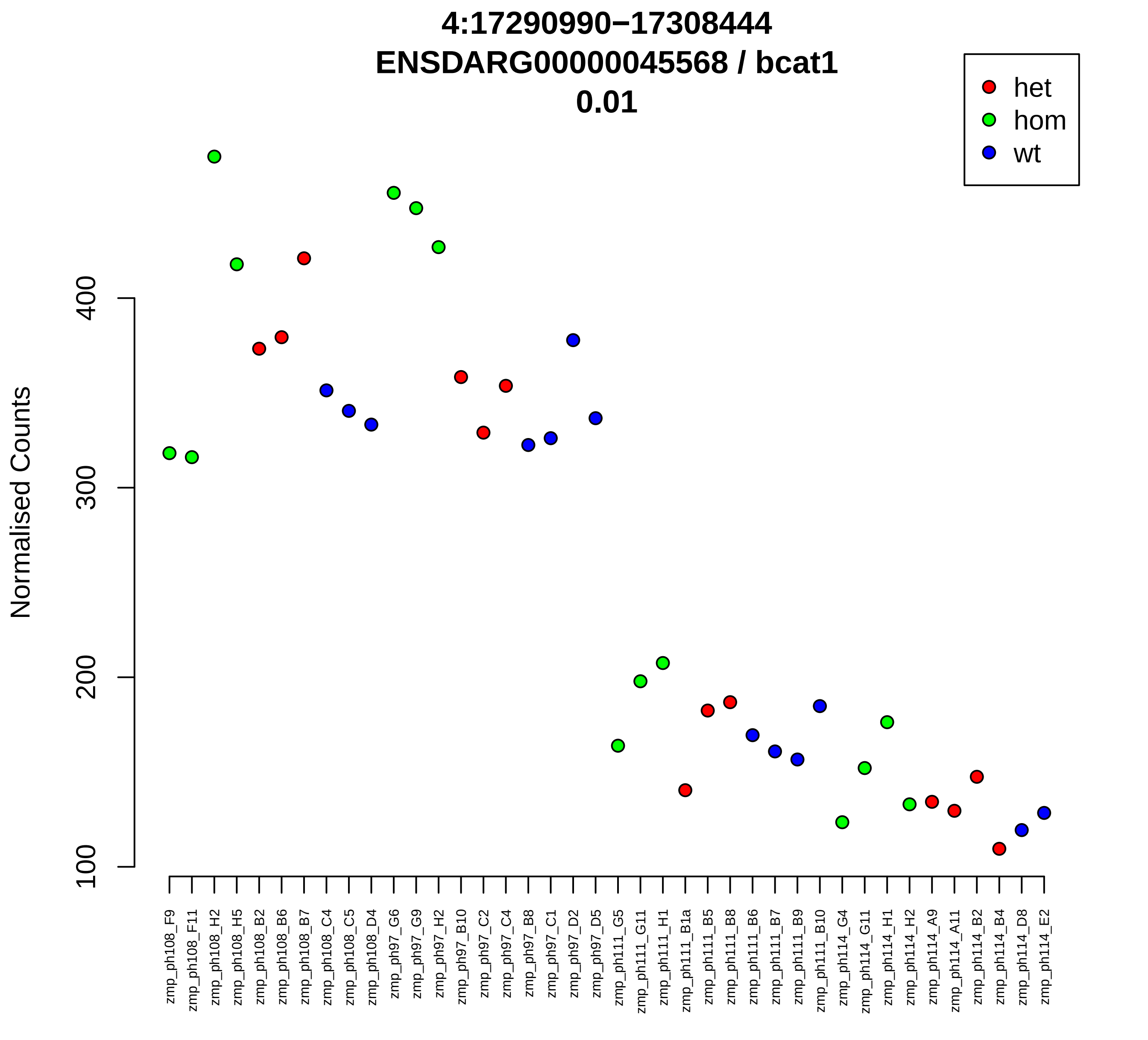

4:17290990−17308444
ENSDARG00000045568 / bcat1
0.01
het
hom
wt
Normalised Counts
100
200
300
400
zmp_ph108_F9
zmp_ph108_F11
zmp_ph108_H2
zmp_ph108_H5
zmp_ph108_B2
zmp_ph108_B6
zmp_ph108_B7
zmp_ph108_C4
zmp_ph108_C5
zmp_ph108_D4
zmp_ph97_G6
zmp_ph97_G9
zmp_ph97_H2
zmp_ph97_B10
zmp_ph97_C2
zmp_ph97_C4
zmp_ph97_B8
zmp_ph97_C1
zmp_ph97_D2
zmp_ph97_D5
zmp_ph111_G5
zmp_ph111_G11
zmp_ph111_H1
zmp_ph111_B1a
zmp_ph111_B5
zmp_ph111_B8
zmp_ph111_B6
zmp_ph111_B7
zmp_ph111_B9
zmp_ph111_B10
zmp_ph114_G4
zmp_ph114_G11
zmp_ph114_H1
zmp_ph114_H2
zmp_ph114_A9
zmp_ph114_A11
zmp_ph114_B2
zmp_ph114_B4
zmp_ph114_D8
zmp_ph114_E2

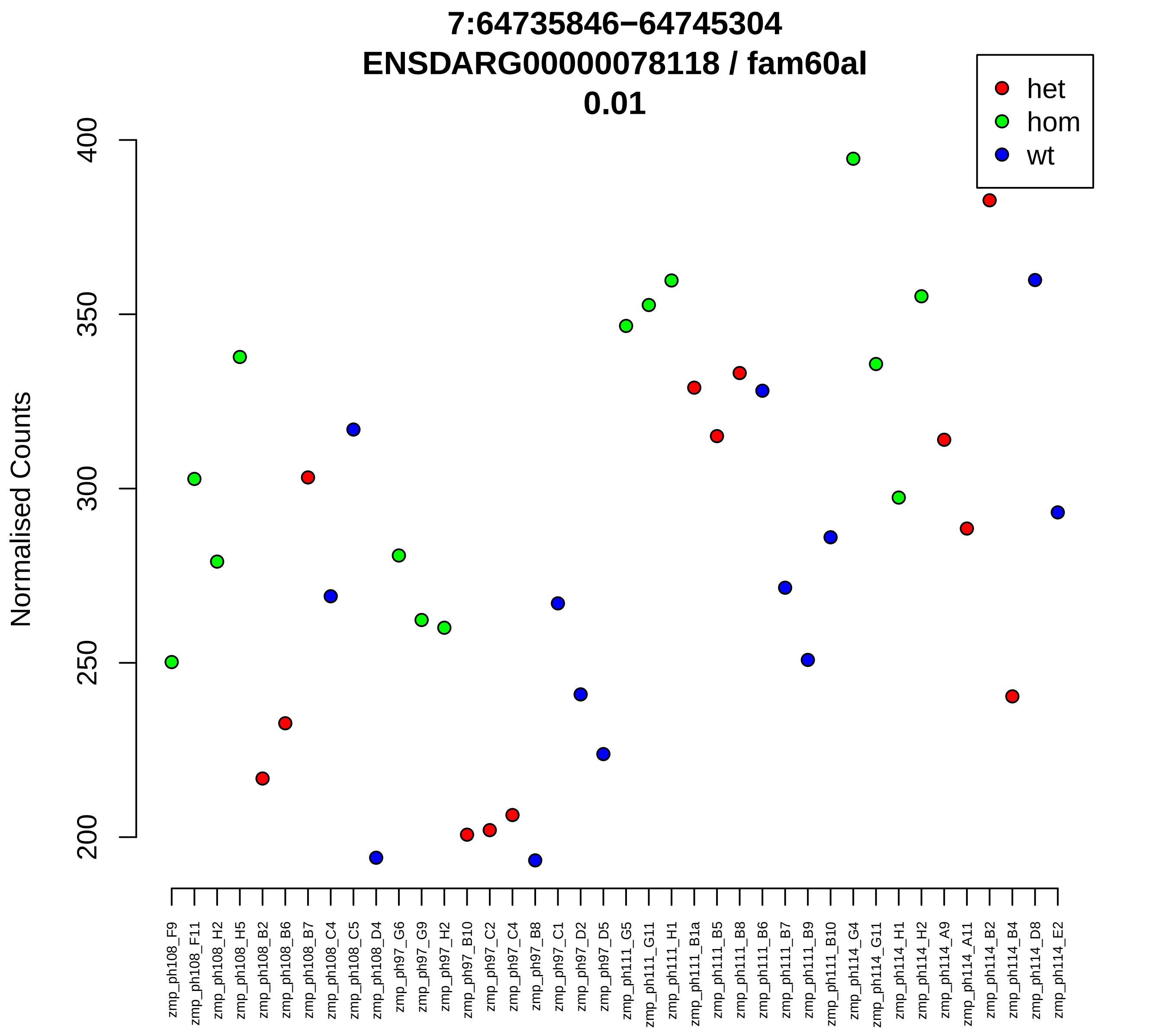
7:64735846−64745304
ENSDARG00000078118 / fam60al
0.01
het
hom
wt
Normalised Counts
200
250
300
350
400
zmp_ph108_F9
zmp_ph108_F11
zmp_ph108_H2
zmp_ph108_H5
zmp_ph108_B2
zmp_ph108_B6
zmp_ph108_B7
zmp_ph108_C4
zmp_ph108_C5
zmp_ph108_D4
zmp_ph97_G6
zmp_ph97_G9
zmp_ph97_H2
zmp_ph97_B10
zmp_ph97_C2
zmp_ph97_C4
zmp_ph97_B8
zmp_ph97_C1
zmp_ph97_D2
zmp_ph97_D5
zmp_ph111_G5
zmp_ph111_G11
zmp_ph111_H1
zmp_ph111_B1a
zmp_ph111_B5
zmp_ph111_B8
zmp_ph111_B6
zmp_ph111_B7
zmp_ph111_B9
zmp_ph111_B10
zmp_ph114_G4
zmp_ph114_G11
zmp_ph114_H1
zmp_ph114_H2
zmp_ph114_A9
zmp_ph114_A11
zmp_ph114_B2
zmp_ph114_B4
zmp_ph114_D8
zmp_ph114_E2

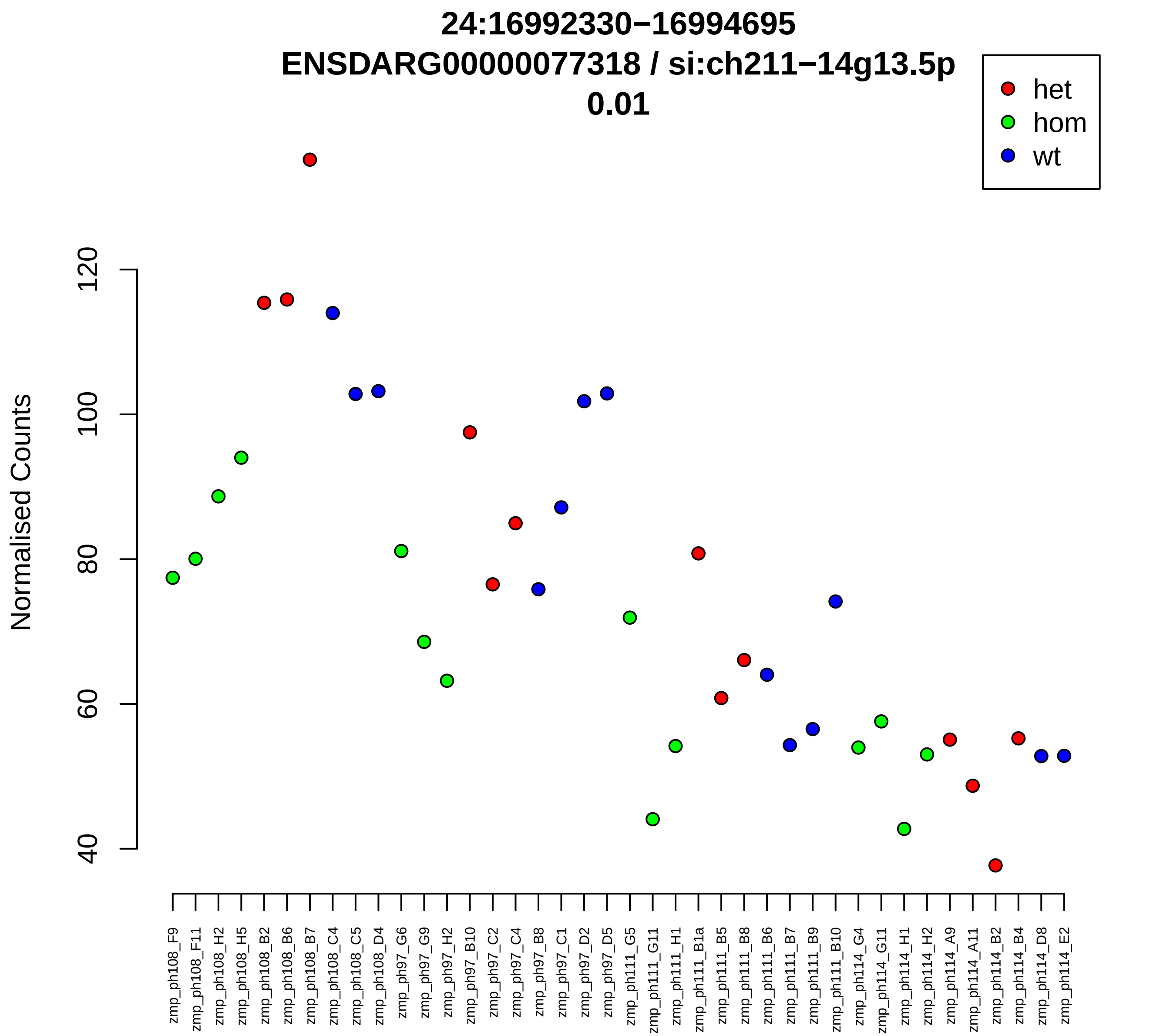
24:16992330−16994695
ENSDARG00000077318 / si:ch211−14g13.5p
0.01
het
hom
wt
Normalised Counts
120
100
80
60
40
zmp_ph108_F9
zmp_ph108_F11
zmp_ph108_H2
zmp_ph108_H5
zmp_ph108_B2
zmp_ph108_B6
zmp_ph108_B7
zmp_ph108_C4
zmp_ph108_C5
zmp_ph108_D4
zmp_ph97_G6
zmp_ph97_G9
zmp_ph97_H2
zmp_ph97_B10
zmp_ph97_C2
zmp_ph97_C4
zmp_ph97_B8
zmp_ph97_C1
zmp_ph97_D2
zmp_ph97_D5
zmp_ph111_G5
zmp_ph111_G11
zmp_ph111_H1
zmp_ph111_B1a
zmp_ph111_B5
zmp_ph111_B8
zmp_ph111_B6
zmp_ph111_B7
zmp_ph111_B9
zmp_ph111_B10
zmp_ph114_G4
zmp_ph114_G11
zmp_ph114_H1
zmp_ph114_H2
zmp_ph114_A9
zmp_ph114_A11
zmp_ph114_B2
zmp_ph114_B4
zmp_ph114_D8
zmp_ph114_E2

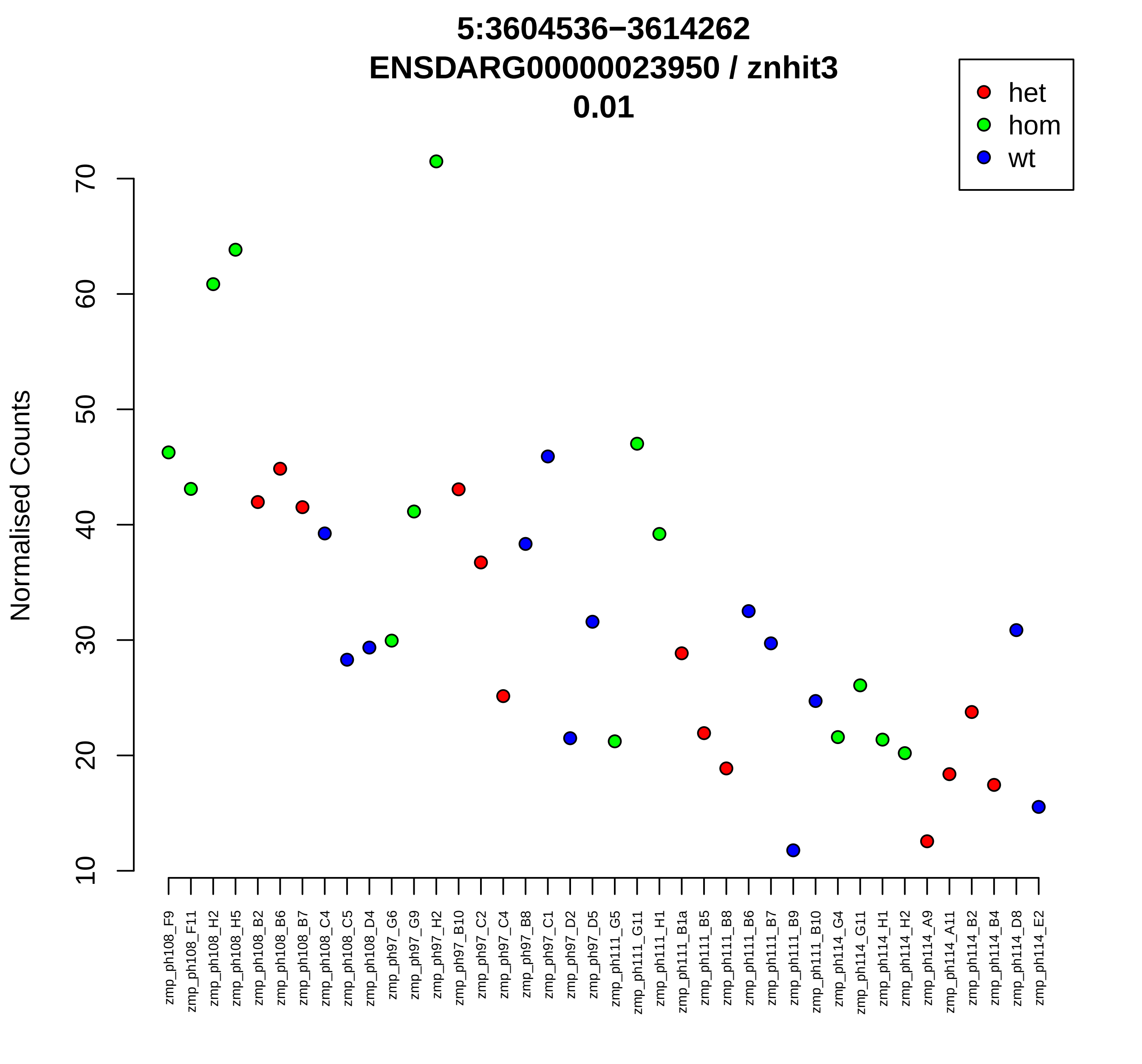

5:3604536−3614262
ENSDARG00000023950 / znhit3
0.01
het
hom
wt
Normalised Counts
10
20
30
40
50
60
70
zmp_ph108_F9
zmp_ph108_F11
zmp_ph108_H2
zmp_ph108_H5
zmp_ph108_B2
zmp_ph108_B6
zmp_ph108_B7
zmp_ph108_C4
zmp_ph108_C5
zmp_ph108_D4
zmp_ph97_G6
zmp_ph97_G9
zmp_ph97_H2
zmp_ph97_B10
zmp_ph97_C2
zmp_ph97_C4
zmp_ph97_B8
zmp_ph97_C1
zmp_ph97_D2
zmp_ph97_D5
zmp_ph111_G5
zmp_ph111_G11
zmp_ph111_H1
zmp_ph111_B1a
zmp_ph111_B5
zmp_ph111_B8
zmp_ph111_B6
zmp_ph111_B7
zmp_ph111_B9
zmp_ph111_B10
zmp_ph114_G4
zmp_ph114_G11
zmp_ph114_H1
zmp_ph114_H2
zmp_ph114_A9
zmp_ph114_A11
zmp_ph114_B2
zmp_ph114_B4
zmp_ph114_D8
zmp_ph114_E2

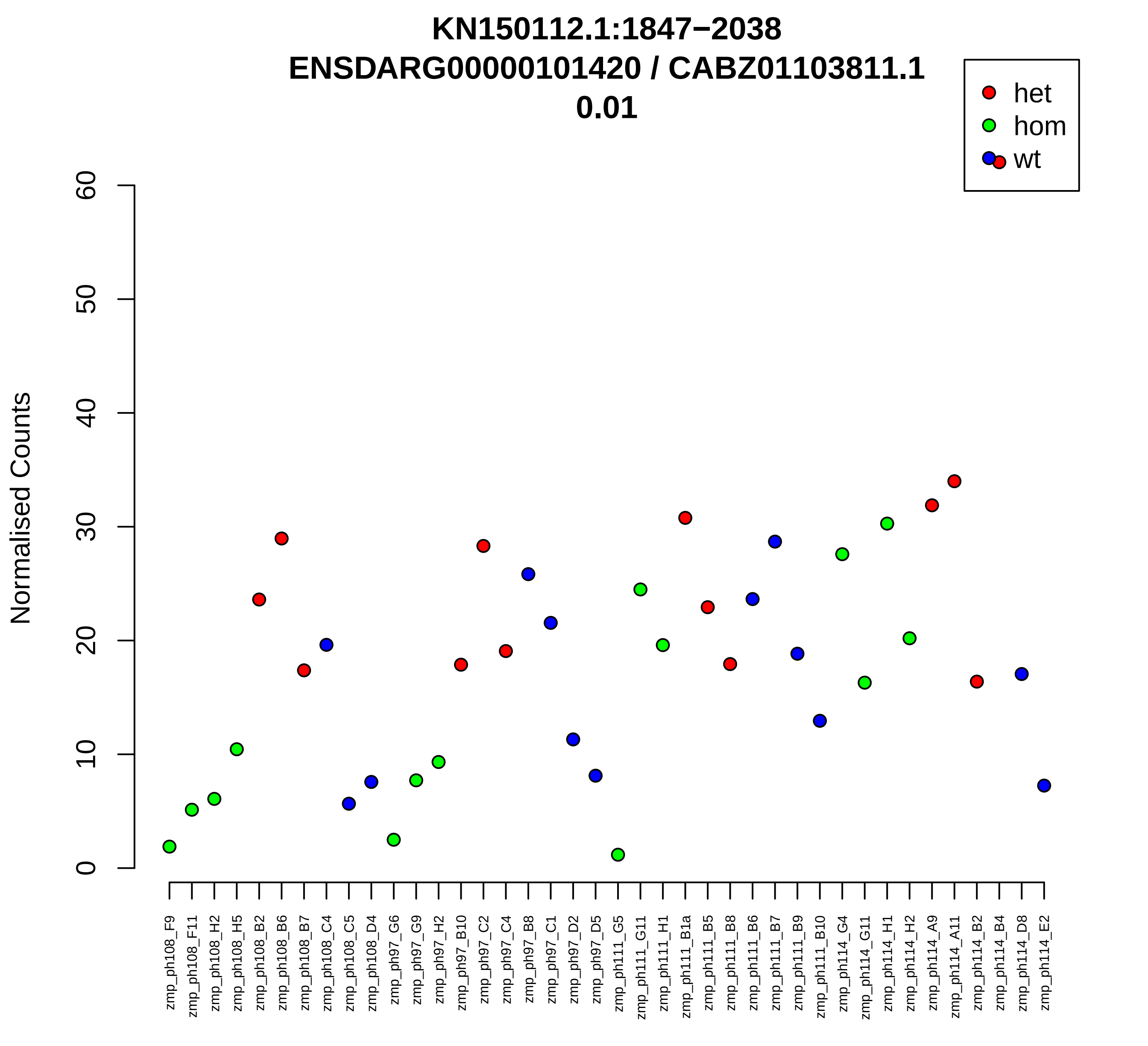

KN150112.1:1847−2038
ENSDARG00000101420 / CABZ01103811.1
0.01
het
hom
wt
Normalised Counts
0
10
20
30
40
50
60
zmp_ph108_F9
zmp_ph108_F11
zmp_ph108_H2
zmp_ph108_H5
zmp_ph108_B2
zmp_ph108_B6
zmp_ph108_B7
zmp_ph108_C4
zmp_ph108_C5
zmp_ph108_D4
zmp_ph97_G6
zmp_ph97_G9
zmp_ph97_H2
zmp_ph97_B10
zmp_ph97_C2
zmp_ph97_C4
zmp_ph97_B8
zmp_ph97_C1
zmp_ph97_D2
zmp_ph97_D5
zmp_ph111_G5
zmp_ph111_G11
zmp_ph111_H1
zmp_ph111_B1a
zmp_ph111_B5
zmp_ph111_B8
zmp_ph111_B6
zmp_ph111_B7
zmp_ph111_B9
zmp_ph111_B10
zmp_ph114_G4
zmp_ph114_G11
zmp_ph114_H1
zmp_ph114_H2
zmp_ph114_A9
zmp_ph114_A11
zmp_ph114_B2
zmp_ph114_B4
zmp_ph114_D8
zmp_ph114_E2

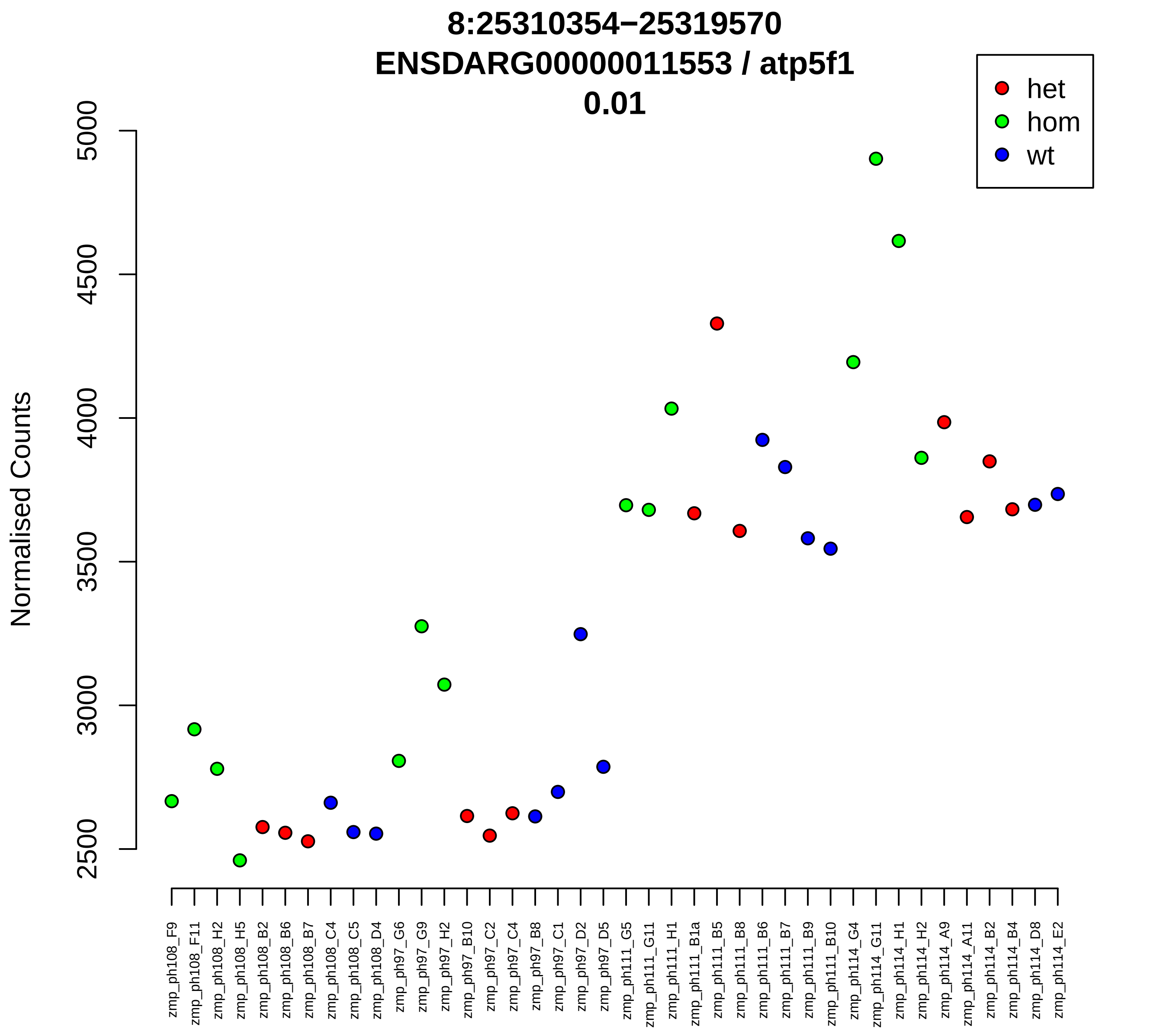
8:25310354−25319570
ENSDARG00000011553 / atp5f1
0.01
het
hom
wt
Normalised Counts
5000
4500
4000
3500
3000
2500
zmp_ph108_F9
zmp_ph108_F11
zmp_ph108_H2
zmp_ph108_H5
zmp_ph108_B2
zmp_ph108_B6
zmp_ph108_B7
zmp_ph108_C4
zmp_ph108_C5
zmp_ph108_D4
zmp_ph97_G6
zmp_ph97_G9
zmp_ph97_H2
zmp_ph97_B10
zmp_ph97_C2
zmp_ph97_C4
zmp_ph97_B8
zmp_ph97_C1
zmp_ph97_D2
zmp_ph97_D5
zmp_ph111_G5
zmp_ph111_G11
zmp_ph111_H1
zmp_ph111_B1a
zmp_ph111_B5
zmp_ph111_B8
zmp_ph111_B6
zmp_ph111_B7
zmp_ph111_B9
zmp_ph111_B10
zmp_ph114_G4
zmp_ph114_G11
zmp_ph114_H1
zmp_ph114_H2
zmp_ph114_A9
zmp_ph114_A11
zmp_ph114_B2
zmp_ph114_B4
zmp_ph114_D8
zmp_ph114_E2

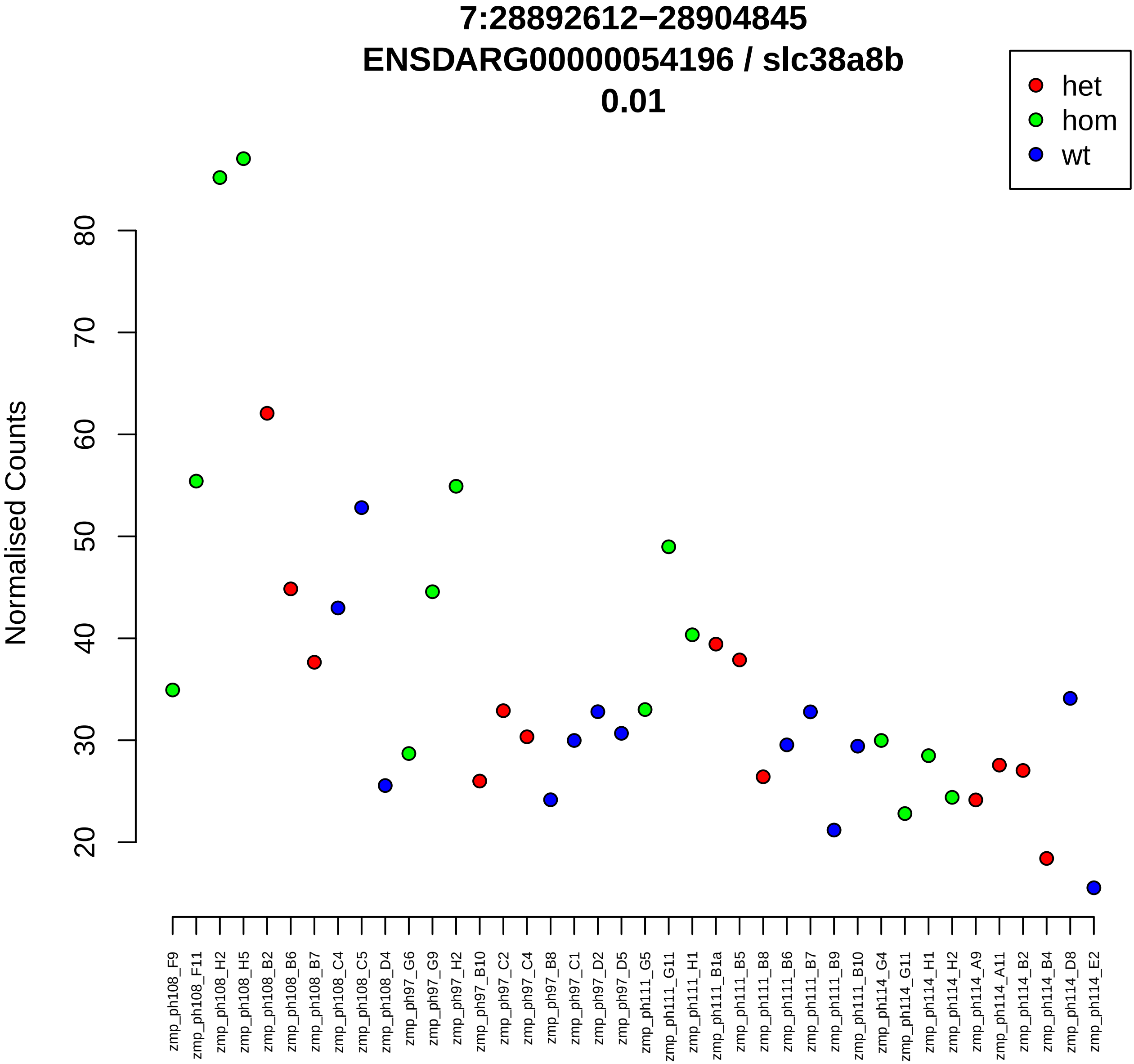

7:28892612−28904845
ENSDARG00000054196 / slc38a8b
0.01
het
hom
wt
Normalised Counts
20
30
40
50
60
70
80
zmp_ph108_F9
zmp_ph108_F11
zmp_ph108_H2
zmp_ph108_H5
zmp_ph108_B2
zmp_ph108_B6
zmp_ph108_B7
zmp_ph108_C4
zmp_ph108_C5
zmp_ph108_D4
zmp_ph97_G6
zmp_ph97_G9
zmp_ph97_H2
zmp_ph97_B10
zmp_ph97_C2
zmp_ph97_C4
zmp_ph97_B8
zmp_ph97_C1
zmp_ph97_D2
zmp_ph97_D5
zmp_ph111_G5
zmp_ph111_G11
zmp_ph111_H1
zmp_ph111_B1a
zmp_ph111_B5
zmp_ph111_B8
zmp_ph111_B6
zmp_ph111_B7
zmp_ph111_B9
zmp_ph111_B10
zmp_ph114_G4
zmp_ph114_G11
zmp_ph114_H1
zmp_ph114_H2
zmp_ph114_A9
zmp_ph114_A11
zmp_ph114_B2
zmp_ph114_B4
zmp_ph114_D8
zmp_ph114_E2

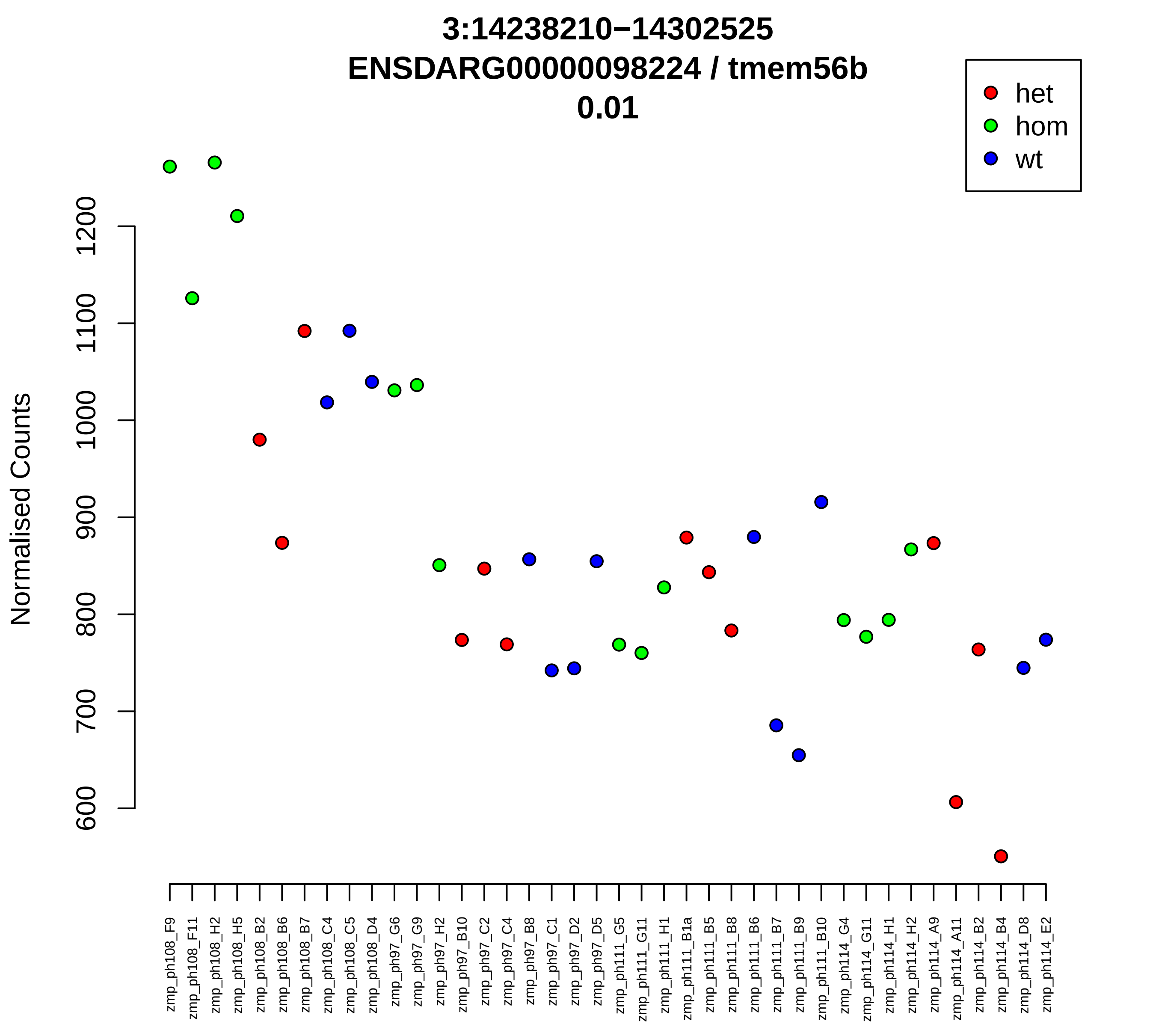
3:14238210−14302525
ENSDARG00000098224 / tmem56b
0.01
het
hom
wt
Normalised Counts
600
700
800
900
1000
1100
1200
zmp_ph108_F9
zmp_ph108_F11
zmp_ph108_H2
zmp_ph108_H5
zmp_ph108_B2
zmp_ph108_B6
zmp_ph108_B7
zmp_ph108_C4
zmp_ph108_C5
zmp_ph108_D4
zmp_ph97_G6
zmp_ph97_G9
zmp_ph97_H2
zmp_ph97_B10
zmp_ph97_C2
zmp_ph97_C4
zmp_ph97_B8
zmp_ph97_C1
zmp_ph97_D2
zmp_ph97_D5
zmp_ph111_G5
zmp_ph111_G11
zmp_ph111_H1
zmp_ph111_B1a
zmp_ph111_B5
zmp_ph111_B8
zmp_ph111_B6
zmp_ph111_B7
zmp_ph111_B9
zmp_ph111_B10
zmp_ph114_G4
zmp_ph114_G11
zmp_ph114_H1
zmp_ph114_H2
zmp_ph114_A9
zmp_ph114_A11
zmp_ph114_B2
zmp_ph114_B4
zmp_ph114_D8
zmp_ph114_E2

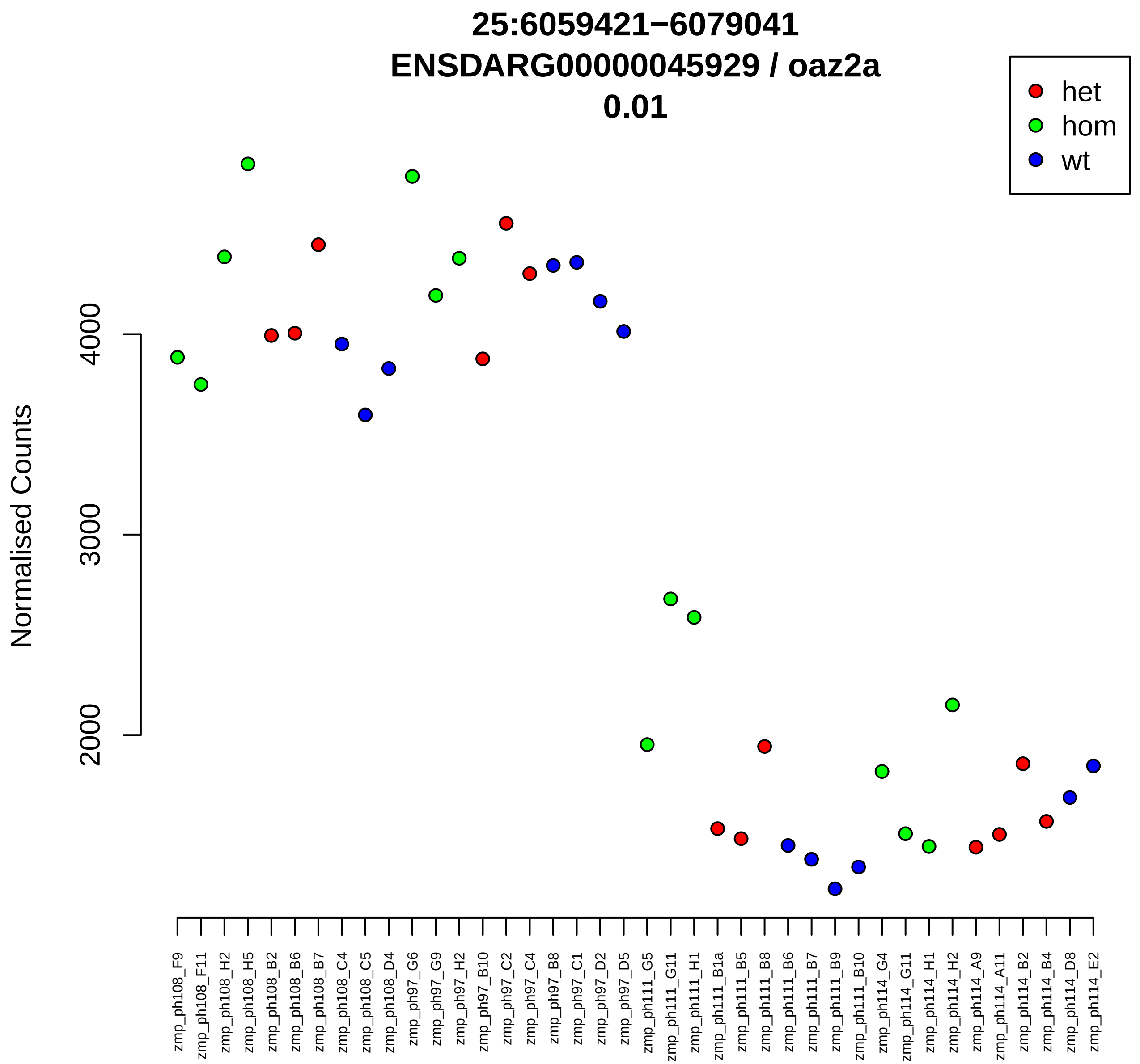

25:6059421−6079041
ENSDARG00000045929 / oaz2a
0.01
het
hom
wt
Normalised Counts
2000
3000
4000
zmp_ph108_F9
zmp_ph108_F11
zmp_ph108_H2
zmp_ph108_H5
zmp_ph108_B2
zmp_ph108_B6
zmp_ph108_B7
zmp_ph108_C4
zmp_ph108_C5
zmp_ph108_D4
zmp_ph97_G6
zmp_ph97_G9
zmp_ph97_H2
zmp_ph97_B10
zmp_ph97_C2
zmp_ph97_C4
zmp_ph97_B8
zmp_ph97_C1
zmp_ph97_D2
zmp_ph97_D5
zmp_ph111_G5
zmp_ph111_G11
zmp_ph111_H1
zmp_ph111_B1a
zmp_ph111_B5
zmp_ph111_B8
zmp_ph111_B6
zmp_ph111_B7
zmp_ph111_B9
zmp_ph111_B10
zmp_ph114_G4
zmp_ph114_G11
zmp_ph114_H1
zmp_ph114_H2
zmp_ph114_A9
zmp_ph114_A11
zmp_ph114_B2
zmp_ph114_B4
zmp_ph114_D8
zmp_ph114_E2

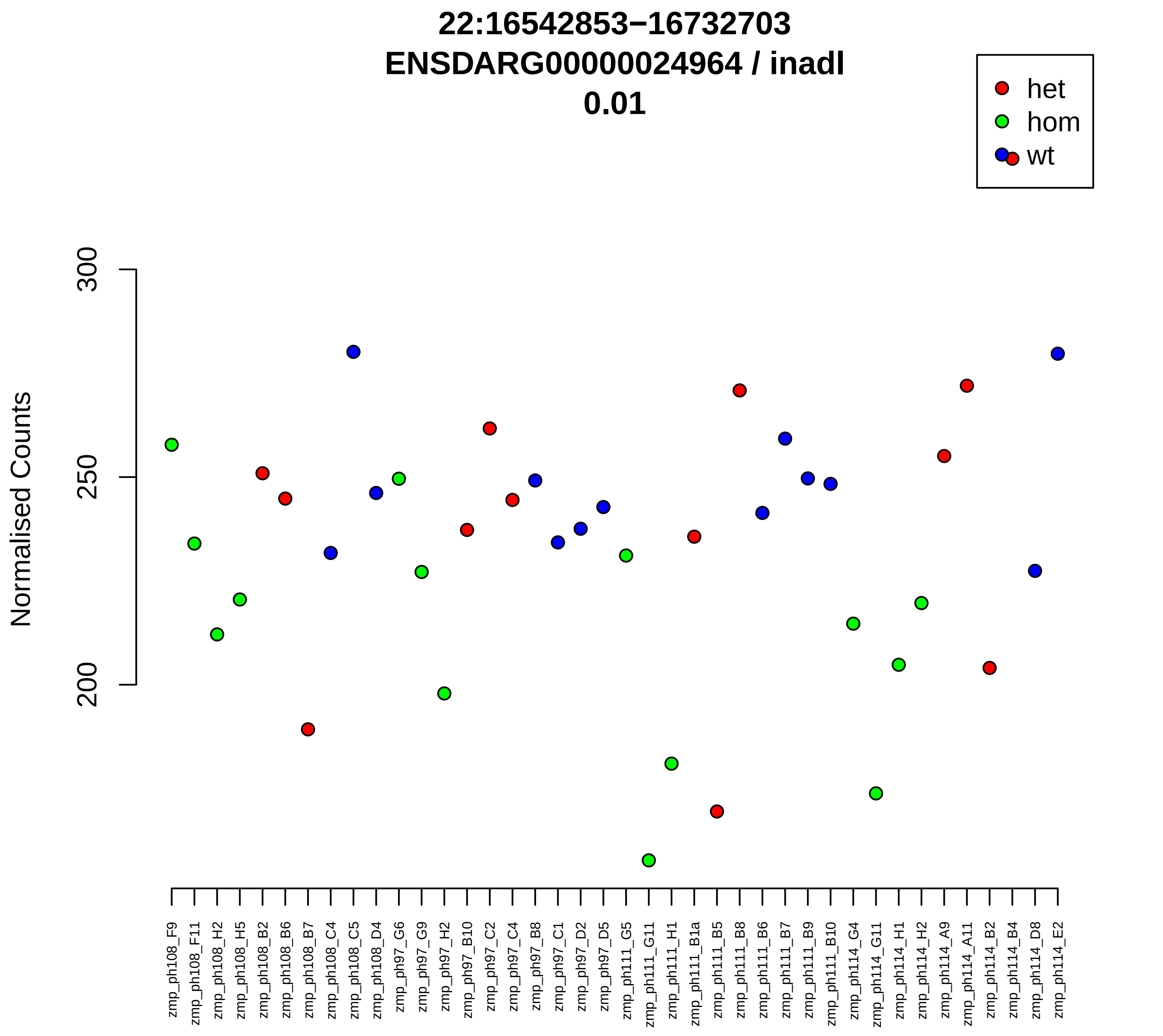

22:16542853−16732703
ENSDARG00000024964 / inadl
0.01
het
hom
wt
Normalised Counts
300
250
200
zmp_ph108_F9
zmp_ph108_F11
zmp_ph108_H2
zmp_ph108_H5
zmp_ph108_B2
zmp_ph108_B6
zmp_ph108_B7
zmp_ph108_C4
zmp_ph108_C5
zmp_ph108_D4
zmp_ph97_G6
zmp_ph97_G9
zmp_ph97_H2
zmp_ph97_B10
zmp_ph97_C2
zmp_ph97_C4
zmp_ph97_B8
zmp_ph97_C1
zmp_ph97_D2
zmp_ph97_D5
zmp_ph111_G5
zmp_ph111_G11
zmp_ph111_H1
zmp_ph111_B1a
zmp_ph111_B5
zmp_ph111_B8
zmp_ph111_B6
zmp_ph111_B7
zmp_ph111_B9
zmp_ph111_B10
zmp_ph114_G4
zmp_ph114_G11
zmp_ph114_H1
zmp_ph114_H2
zmp_ph114_A9
zmp_ph114_A11
zmp_ph114_B2
zmp_ph114_B4
zmp_ph114_D8
zmp_ph114_E2

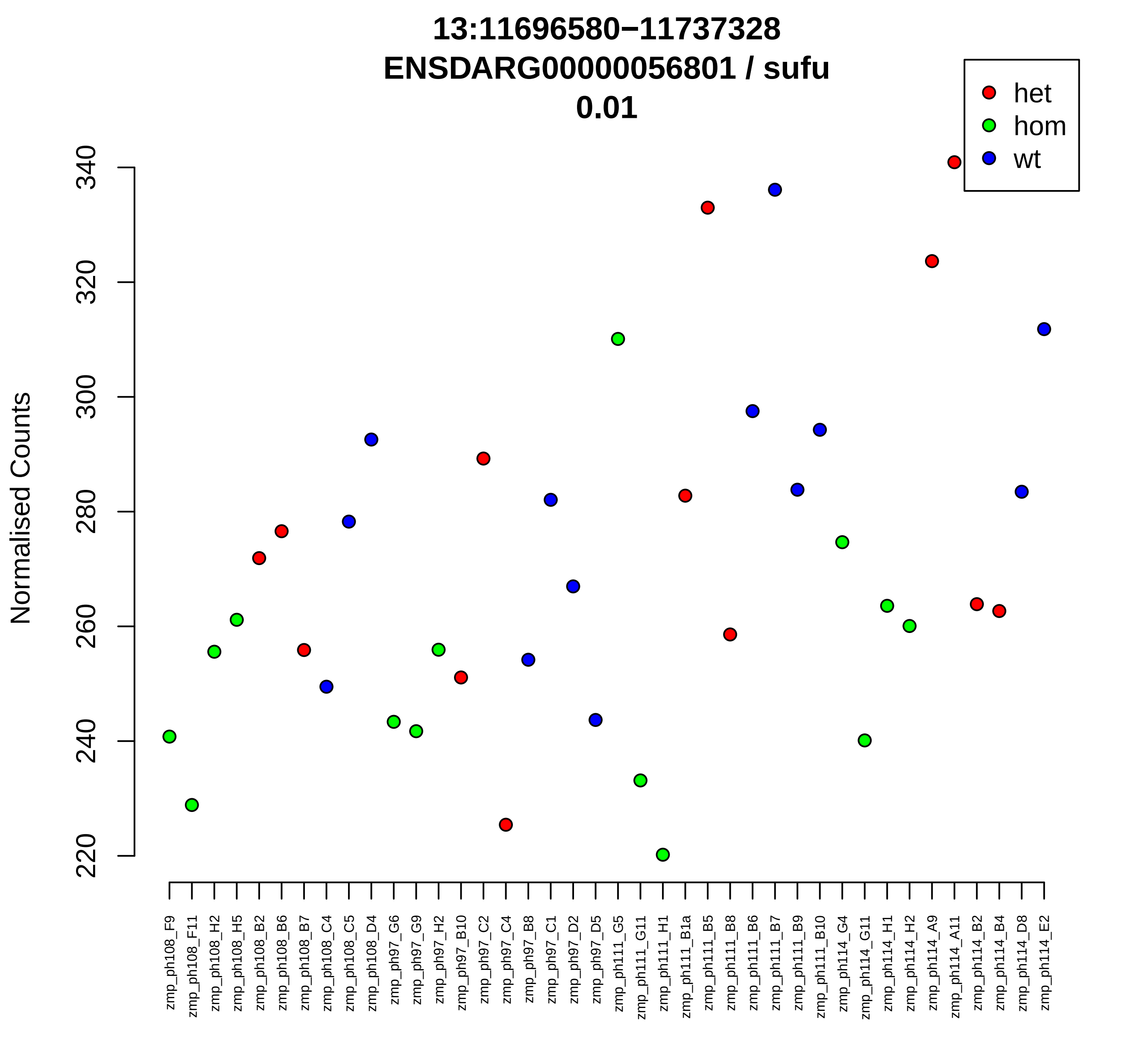

13:11696580−11737328
ENSDARG00000056801 / sufu
0.01
het
hom
wt
Normalised Counts
220
240
260
280
300
320
340
zmp_ph108_F9
zmp_ph108_F11
zmp_ph108_H2
zmp_ph108_H5
zmp_ph108_B2
zmp_ph108_B6
zmp_ph108_B7
zmp_ph108_C4
zmp_ph108_C5
zmp_ph108_D4
zmp_ph97_G6
zmp_ph97_G9
zmp_ph97_H2
zmp_ph97_B10
zmp_ph97_C2
zmp_ph97_C4
zmp_ph97_B8
zmp_ph97_C1
zmp_ph97_D2
zmp_ph97_D5
zmp_ph111_G5
zmp_ph111_G11
zmp_ph111_H1
zmp_ph111_B1a
zmp_ph111_B5
zmp_ph111_B8
zmp_ph111_B6
zmp_ph111_B7
zmp_ph111_B9
zmp_ph111_B10
zmp_ph114_G4
zmp_ph114_G11
zmp_ph114_H1
zmp_ph114_H2
zmp_ph114_A9
zmp_ph114_A11
zmp_ph114_B2
zmp_ph114_B4
zmp_ph114_D8
zmp_ph114_E2

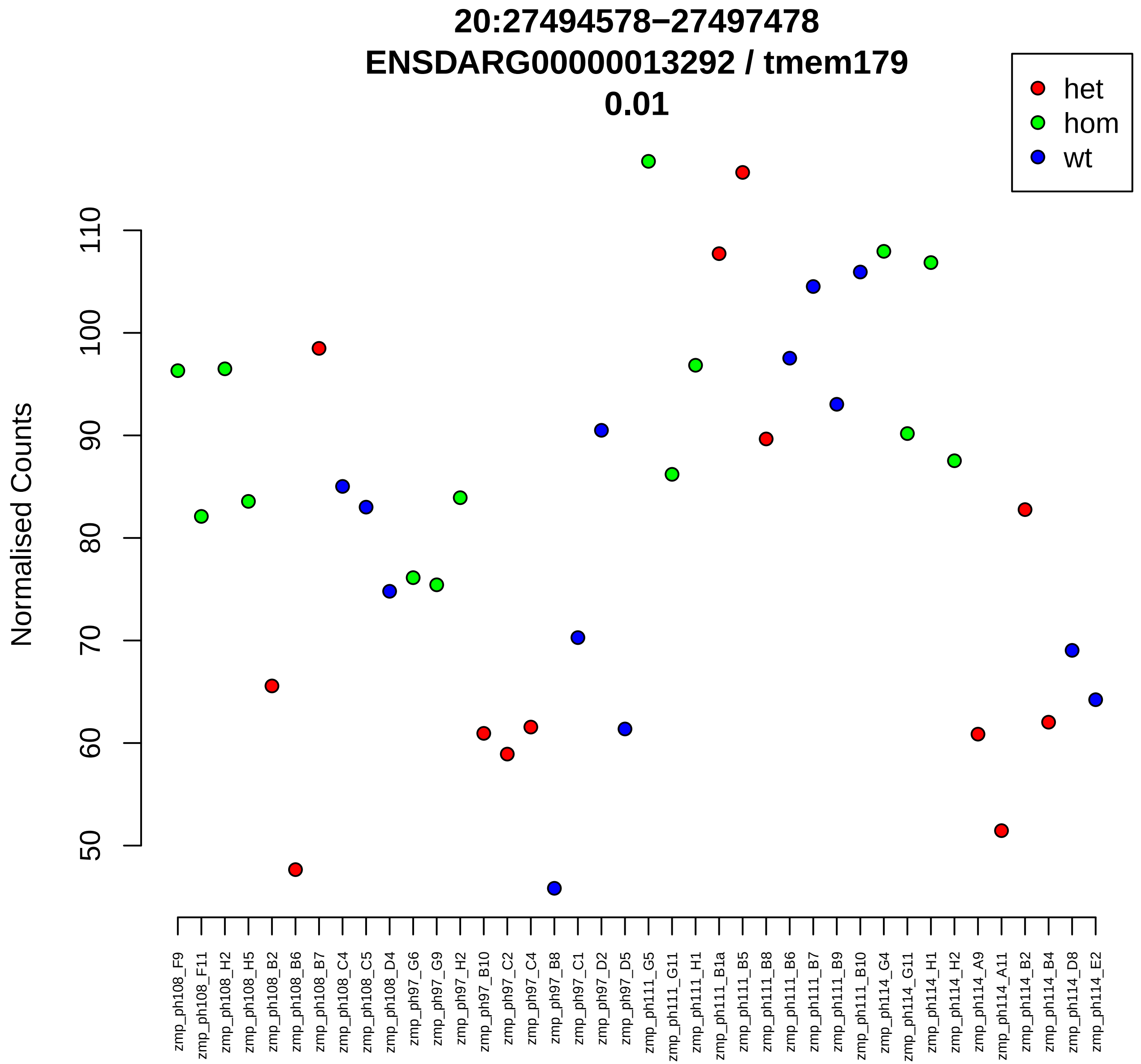
20:27494578−27497478
ENSDARG00000013292 / tmem179
0.01
het
hom
wt
Normalised Counts
50
60
70
80
90
100
110
zmp_ph108_F9
zmp_ph108_F11
zmp_ph108_H2
zmp_ph108_H5
zmp_ph108_B2
zmp_ph108_B6
zmp_ph108_B7
zmp_ph108_C4
zmp_ph108_C5
zmp_ph108_D4
zmp_ph97_G6
zmp_ph97_G9
zmp_ph97_H2
zmp_ph97_B10
zmp_ph97_C2
zmp_ph97_C4
zmp_ph97_B8
zmp_ph97_C1
zmp_ph97_D2
zmp_ph97_D5
zmp_ph111_G5
zmp_ph111_G11
zmp_ph111_H1
zmp_ph111_B1a
zmp_ph111_B5
zmp_ph111_B8
zmp_ph111_B6
zmp_ph111_B7
zmp_ph111_B9
zmp_ph111_B10
zmp_ph114_G4
zmp_ph114_G11
zmp_ph114_H1
zmp_ph114_H2
zmp_ph114_A9
zmp_ph114_A11
zmp_ph114_B2
zmp_ph114_B4
zmp_ph114_D8
zmp_ph114_E2

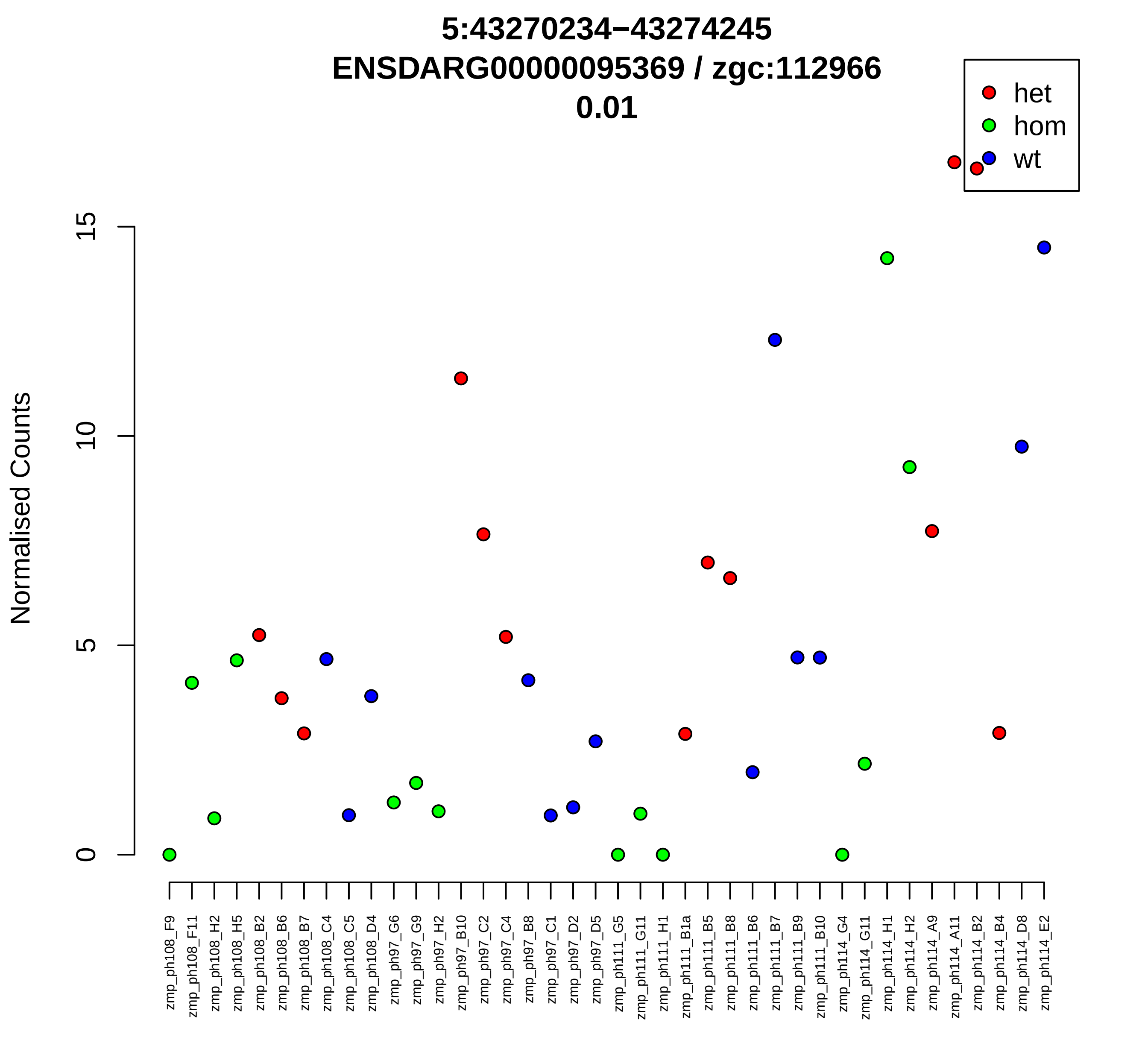
5:43270234−43274245
ENSDARG00000095369 / zgc:112966
0.01
het
hom
wt
Normalised Counts
0
5
10
15
zmp_ph108_F9
zmp_ph108_F11
zmp_ph108_H2
zmp_ph108_H5
zmp_ph108_B2
zmp_ph108_B6
zmp_ph108_B7
zmp_ph108_C4
zmp_ph108_C5
zmp_ph108_D4
zmp_ph97_G6
zmp_ph97_G9
zmp_ph97_H2
zmp_ph97_B10
zmp_ph97_C2
zmp_ph97_C4
zmp_ph97_B8
zmp_ph97_C1
zmp_ph97_D2
zmp_ph97_D5
zmp_ph111_G5
zmp_ph111_G11
zmp_ph111_H1
zmp_ph111_B1a
zmp_ph111_B5
zmp_ph111_B8
zmp_ph111_B6
zmp_ph111_B7
zmp_ph111_B9
zmp_ph111_B10
zmp_ph114_G4
zmp_ph114_G11
zmp_ph114_H1
zmp_ph114_H2
zmp_ph114_A9
zmp_ph114_A11
zmp_ph114_B2
zmp_ph114_B4
zmp_ph114_D8
zmp_ph114_E2

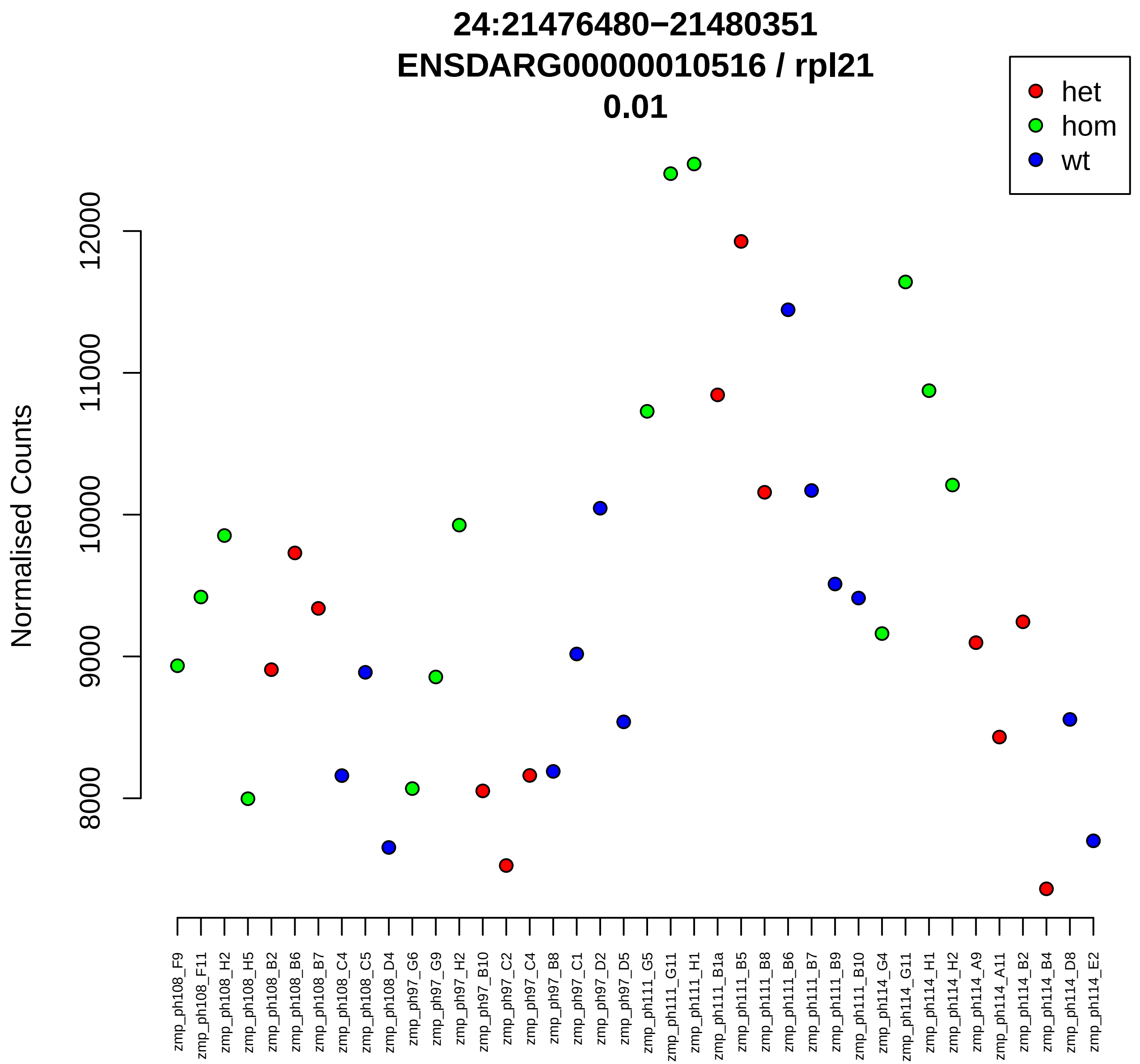

24:21476480−21480351
ENSDARG00000010516 / rpl21
0.01
het
hom
wt
Normalised Counts
8000
9000
10000
11000
12000
zmp_ph108_F9
zmp_ph108_F11
zmp_ph108_H2
zmp_ph108_H5
zmp_ph108_B2
zmp_ph108_B6
zmp_ph108_B7
zmp_ph108_C4
zmp_ph108_C5
zmp_ph108_D4
zmp_ph97_G6
zmp_ph97_G9
zmp_ph97_H2
zmp_ph97_B10
zmp_ph97_C2
zmp_ph97_C4
zmp_ph97_B8
zmp_ph97_C1
zmp_ph97_D2
zmp_ph97_D5
zmp_ph111_G5
zmp_ph111_G11
zmp_ph111_H1
zmp_ph111_B1a
zmp_ph111_B5
zmp_ph111_B8
zmp_ph111_B6
zmp_ph111_B7
zmp_ph111_B9
zmp_ph111_B10
zmp_ph114_G4
zmp_ph114_G11
zmp_ph114_H1
zmp_ph114_H2
zmp_ph114_A9
zmp_ph114_A11
zmp_ph114_B2
zmp_ph114_B4
zmp_ph114_D8
zmp_ph114_E2

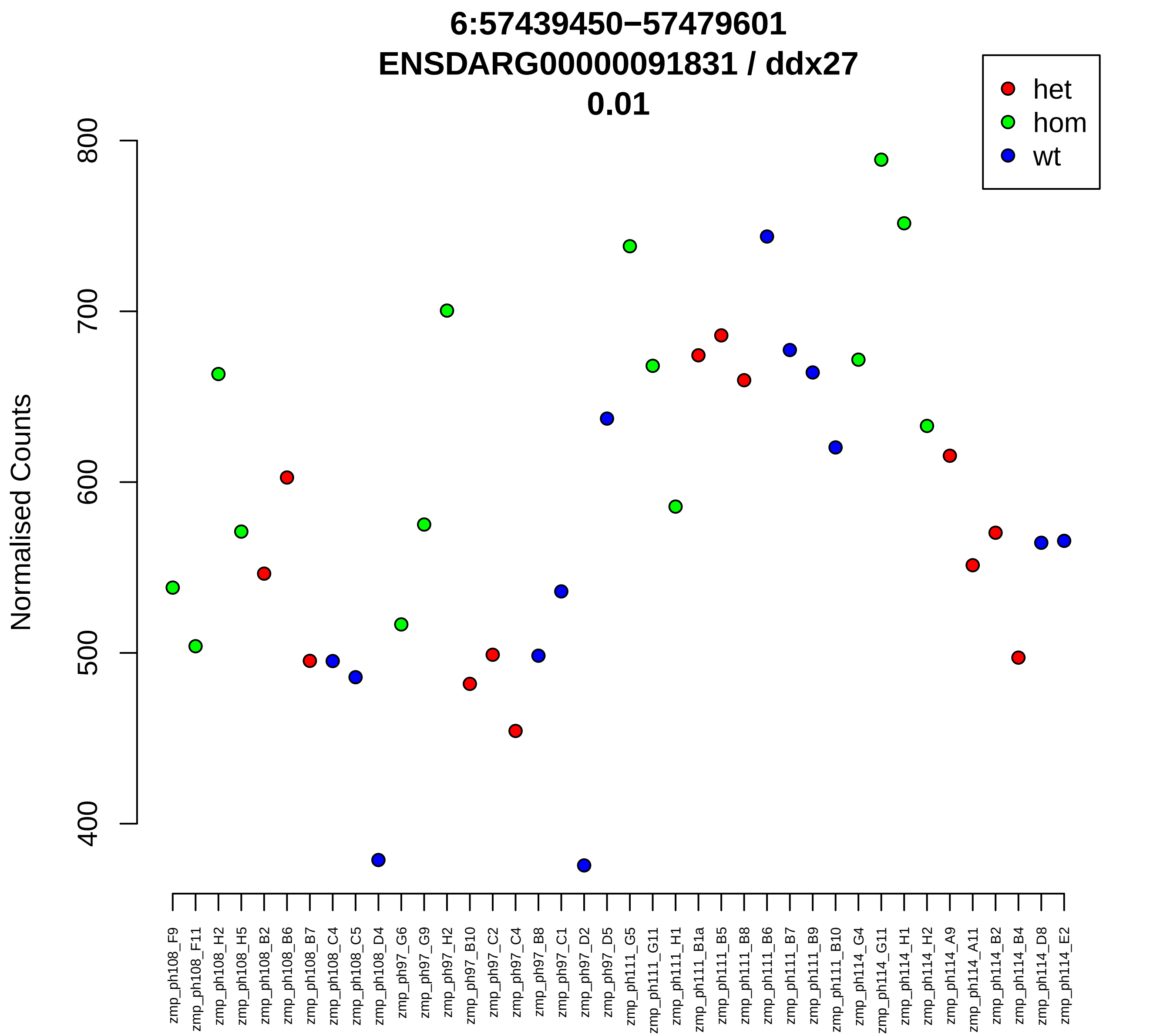
6:57439450−57479601
ENSDARG00000091831 / ddx27
0.01
het
hom
wt
Normalised Counts
400
500
600
700
800
zmp_ph108_F9
zmp_ph108_F11
zmp_ph108_H2
zmp_ph108_H5
zmp_ph108_B2
zmp_ph108_B6
zmp_ph108_B7
zmp_ph108_C4
zmp_ph108_C5
zmp_ph108_D4
zmp_ph97_G6
zmp_ph97_G9
zmp_ph97_H2
zmp_ph97_B10
zmp_ph97_C2
zmp_ph97_C4
zmp_ph97_B8
zmp_ph97_C1
zmp_ph97_D2
zmp_ph97_D5
zmp_ph111_G5
zmp_ph111_G11
zmp_ph111_H1
zmp_ph111_B1a
zmp_ph111_B5
zmp_ph111_B8
zmp_ph111_B6
zmp_ph111_B7
zmp_ph111_B9
zmp_ph111_B10
zmp_ph114_G4
zmp_ph114_G11
zmp_ph114_H1
zmp_ph114_H2
zmp_ph114_A9
zmp_ph114_A11
zmp_ph114_B2
zmp_ph114_B4
zmp_ph114_D8
zmp_ph114_E2

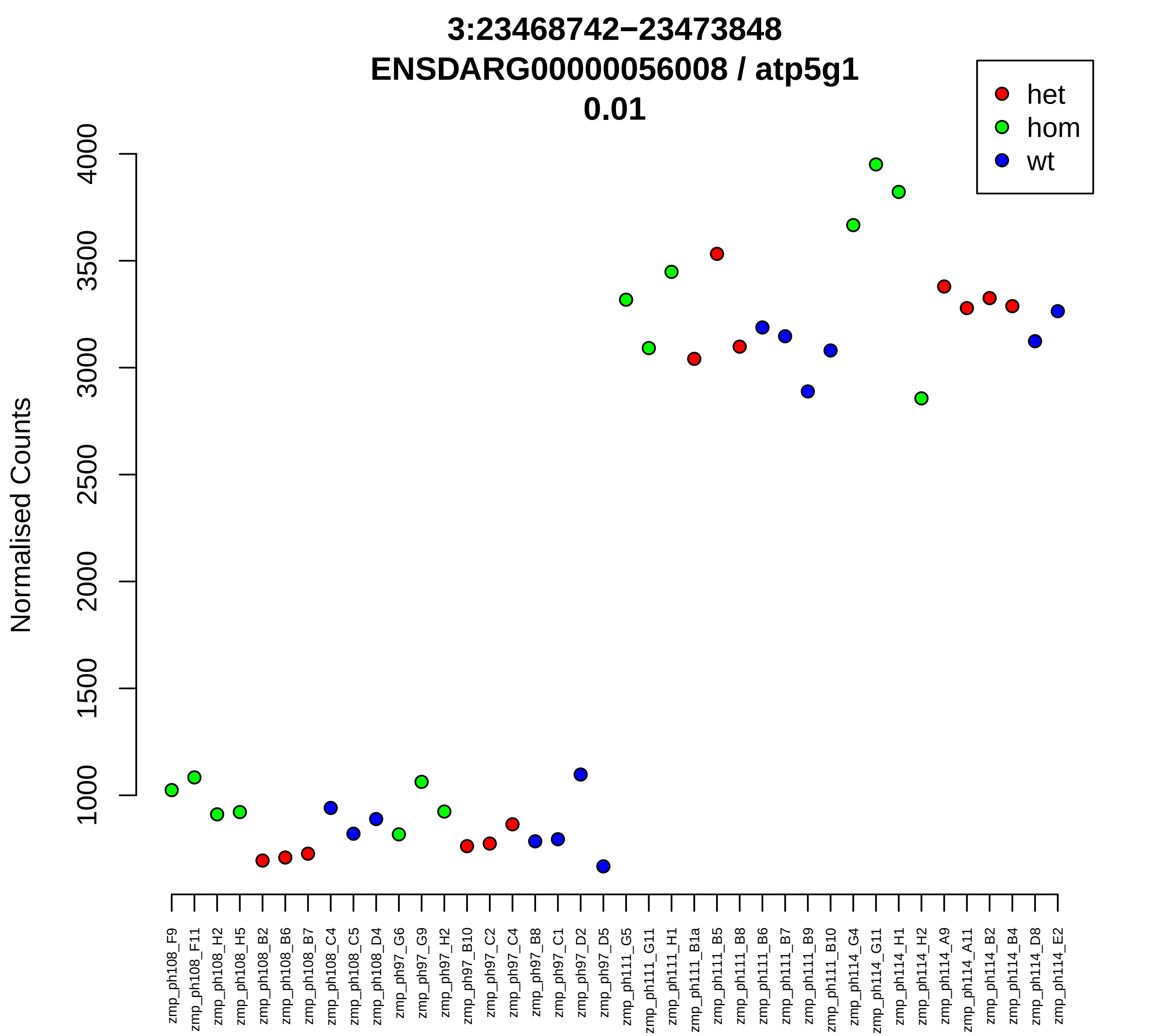
3:23468742−23473848
ENSDARG00000056008 / atp5g1
0.01
het
hom
wt
Normalised Counts
1000
1500
2000
2500
3000
3500
4000
zmp_ph108_F9
zmp_ph108_F11
zmp_ph108_H2
zmp_ph108_H5
zmp_ph108_B2
zmp_ph108_B6
zmp_ph108_B7
zmp_ph108_C4
zmp_ph108_C5
zmp_ph108_D4
zmp_ph97_G6
zmp_ph97_G9
zmp_ph97_H2
zmp_ph97_B10
zmp_ph97_C2
zmp_ph97_C4
zmp_ph97_B8
zmp_ph97_C1
zmp_ph97_D2
zmp_ph97_D5
zmp_ph111_G5
zmp_ph111_G11
zmp_ph111_H1
zmp_ph111_B1a
zmp_ph111_B5
zmp_ph111_B8
zmp_ph111_B6
zmp_ph111_B7
zmp_ph111_B9
zmp_ph111_B10
zmp_ph114_G4
zmp_ph114_G11
zmp_ph114_H1
zmp_ph114_H2
zmp_ph114_A9
zmp_ph114_A11
zmp_ph114_B2
zmp_ph114_B4
zmp_ph114_D8
zmp_ph114_E2

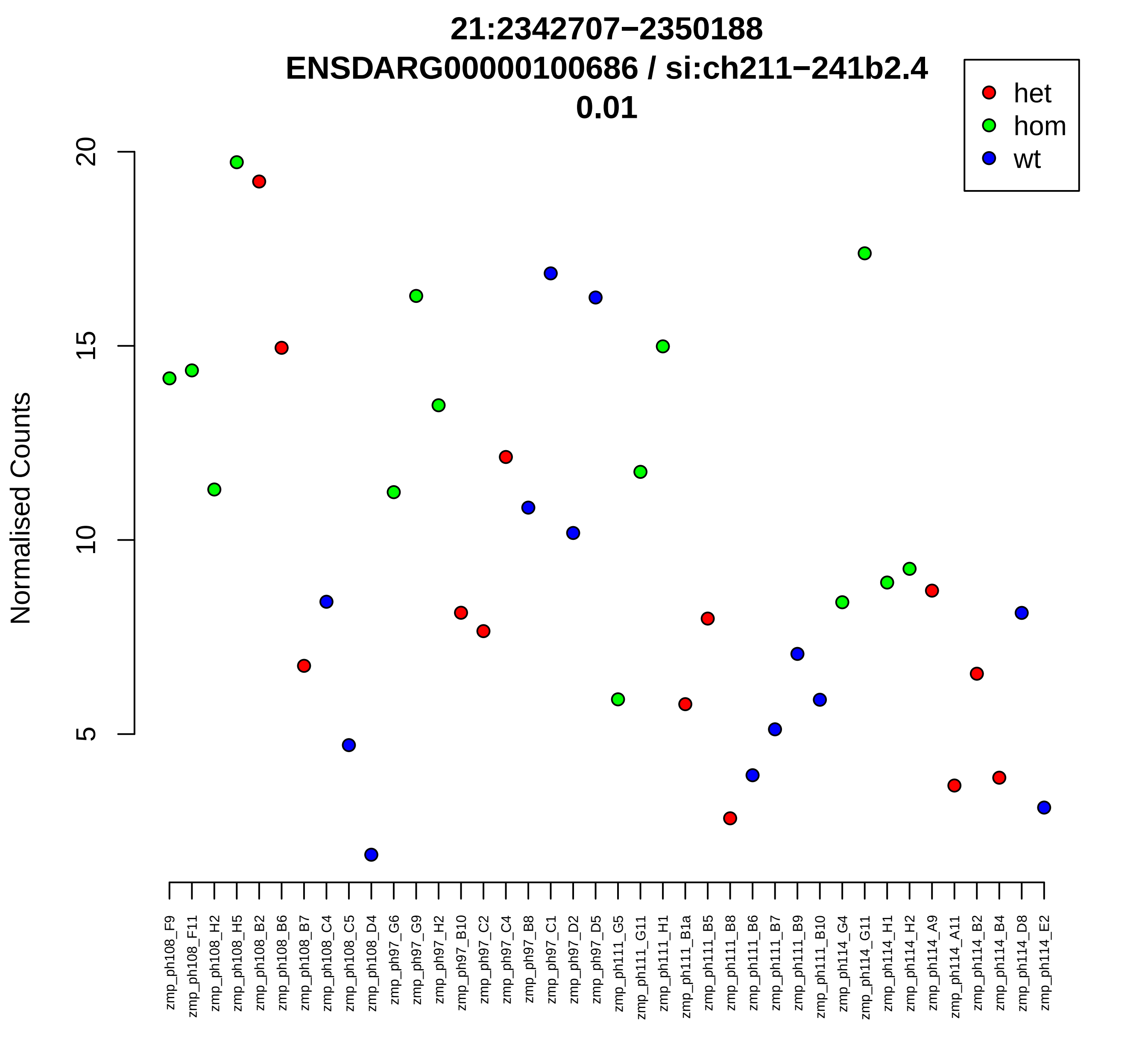
21:2342707−2350188
ENSDARG00000100686 / si:ch211−241b2.4
0.01
het
hom
wt
Normalised Counts
5
10
15
20
zmp_ph108_F9
zmp_ph108_F11
zmp_ph108_H2
zmp_ph108_H5
zmp_ph108_B2
zmp_ph108_B6
zmp_ph108_B7
zmp_ph108_C4
zmp_ph108_C5
zmp_ph108_D4
zmp_ph97_G6
zmp_ph97_G9
zmp_ph97_H2
zmp_ph97_B10
zmp_ph97_C2
zmp_ph97_C4
zmp_ph97_B8
zmp_ph97_C1
zmp_ph97_D2
zmp_ph97_D5
zmp_ph111_G5
zmp_ph111_G11
zmp_ph111_H1
zmp_ph111_B1a
zmp_ph111_B5
zmp_ph111_B8
zmp_ph111_B6
zmp_ph111_B7
zmp_ph111_B9
zmp_ph111_B10
zmp_ph114_G4
zmp_ph114_G11
zmp_ph114_H1
zmp_ph114_H2
zmp_ph114_A9
zmp_ph114_A11
zmp_ph114_B2
zmp_ph114_B4
zmp_ph114_D8
zmp_ph114_E2

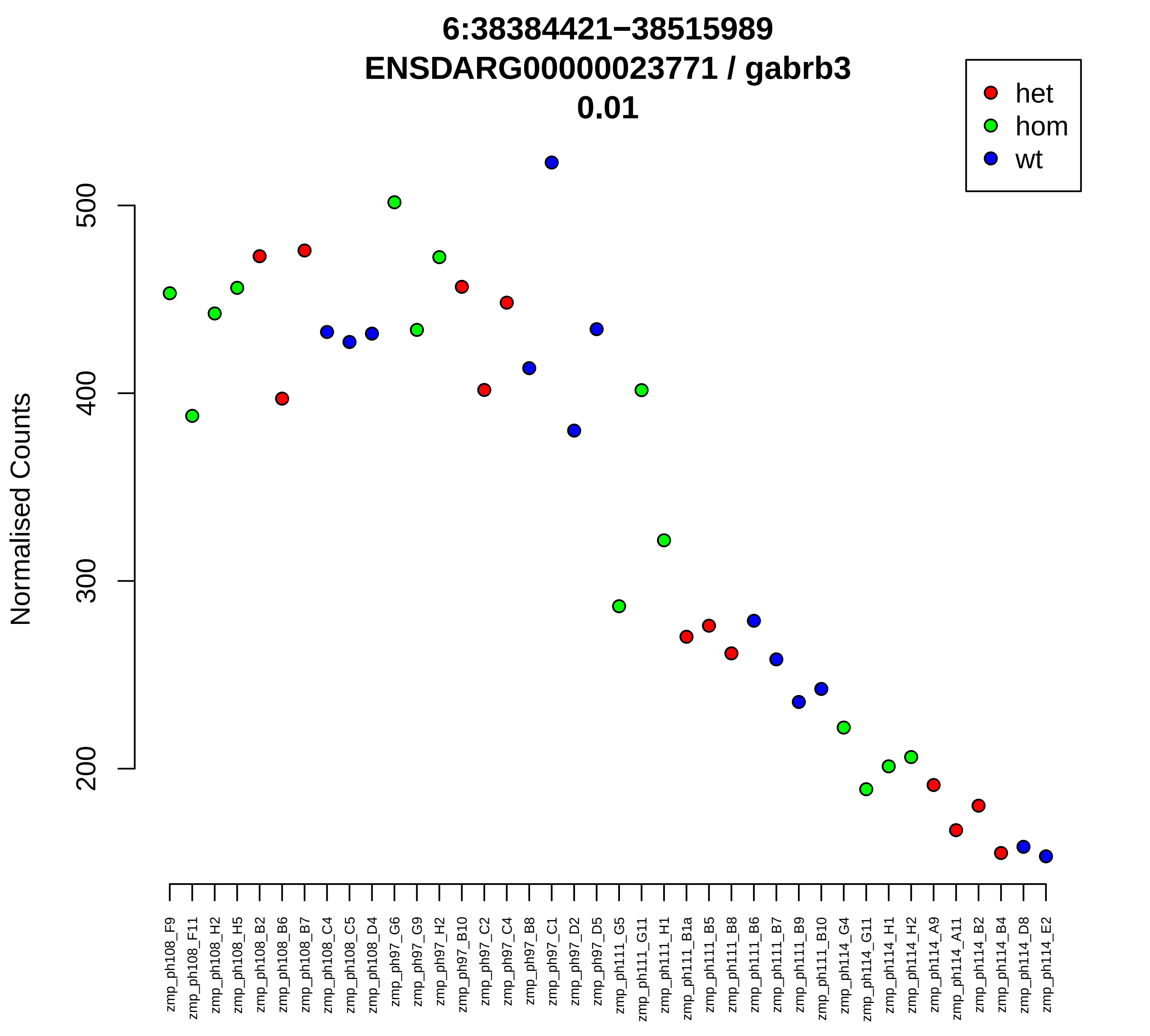

6:38384421−38515989
ENSDARG00000023771 / gabrb3
0.01
het
hom
wt
Normalised Counts
500
400
300
200
zmp_ph108_F9
zmp_ph108_F11
zmp_ph108_H2
zmp_ph108_H5
zmp_ph108_B2
zmp_ph108_B6
zmp_ph108_B7
zmp_ph108_C4
zmp_ph108_C5
zmp_ph108_D4
zmp_ph97_G6
zmp_ph97_G9
zmp_ph97_H2
zmp_ph97_B10
zmp_ph97_C2
zmp_ph97_C4
zmp_ph97_B8
zmp_ph97_C1
zmp_ph97_D2
zmp_ph97_D5
zmp_ph111_G5
zmp_ph111_G11
zmp_ph111_H1
zmp_ph111_B1a
zmp_ph111_B5
zmp_ph111_B8
zmp_ph111_B6
zmp_ph111_B7
zmp_ph111_B9
zmp_ph111_B10
zmp_ph114_G4
zmp_ph114_G11
zmp_ph114_H1
zmp_ph114_H2
zmp_ph114_A9
zmp_ph114_A11
zmp_ph114_B2
zmp_ph114_B4
zmp_ph114_D8
zmp_ph114_E2

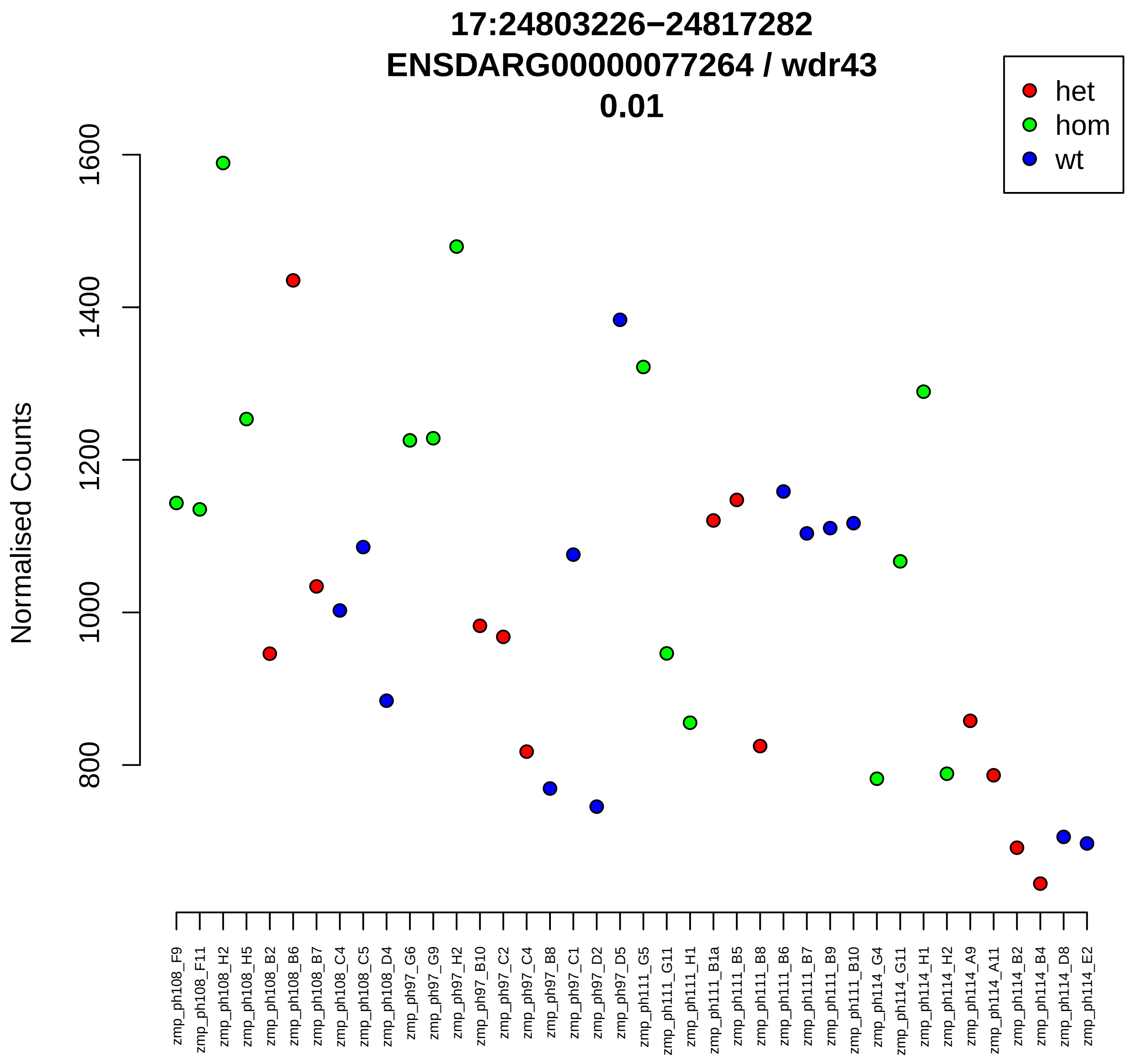

17:24803226−24817282
ENSDARG00000077264 / wdr43
0.01
het
hom
wt
Normalised Counts
800
1000
1200
1400
1600
zmp_ph108_F9
zmp_ph108_F11
zmp_ph108_H2
zmp_ph108_H5
zmp_ph108_B2
zmp_ph108_B6
zmp_ph108_B7
zmp_ph108_C4
zmp_ph108_C5
zmp_ph108_D4
zmp_ph97_G6
zmp_ph97_G9
zmp_ph97_H2
zmp_ph97_B10
zmp_ph97_C2
zmp_ph97_C4
zmp_ph97_B8
zmp_ph97_C1
zmp_ph97_D2
zmp_ph97_D5
zmp_ph111_G5
zmp_ph111_G11
zmp_ph111_H1
zmp_ph111_B1a
zmp_ph111_B5
zmp_ph111_B8
zmp_ph111_B6
zmp_ph111_B7
zmp_ph111_B9
zmp_ph111_B10
zmp_ph114_G4
zmp_ph114_G11
zmp_ph114_H1
zmp_ph114_H2
zmp_ph114_A9
zmp_ph114_A11
zmp_ph114_B2
zmp_ph114_B4
zmp_ph114_D8
zmp_ph114_E2

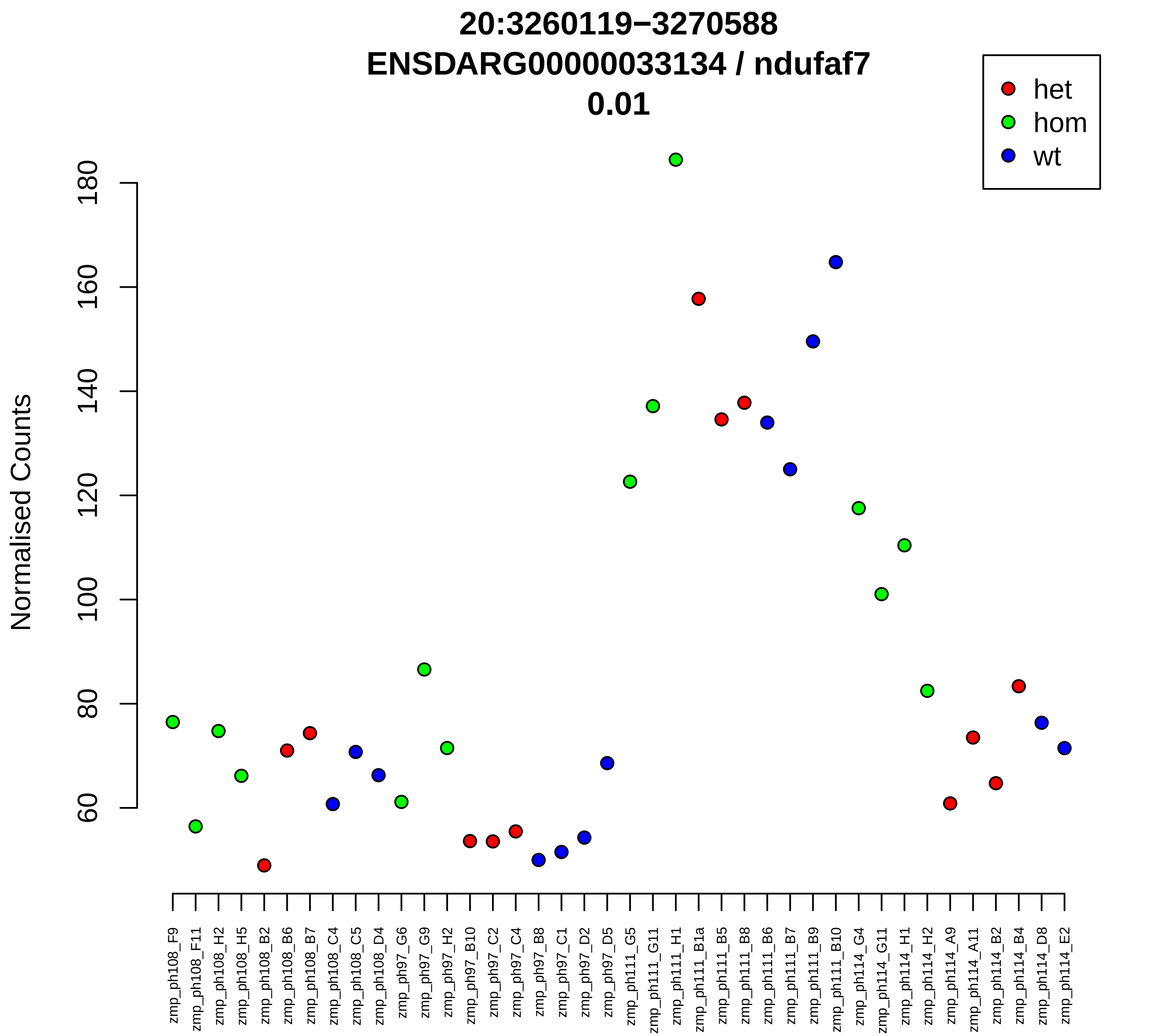

20:3260119−3270588
ENSDARG00000033134 / ndufaf7
0.01
het
hom
wt
Normalised Counts
60
80
100
120
140
160
180
zmp_ph108_F9
zmp_ph108_F11
zmp_ph108_H2
zmp_ph108_H5
zmp_ph108_B2
zmp_ph108_B6
zmp_ph108_B7
zmp_ph108_C4
zmp_ph108_C5
zmp_ph108_D4
zmp_ph97_G6
zmp_ph97_G9
zmp_ph97_H2
zmp_ph97_B10
zmp_ph97_C2
zmp_ph97_C4
zmp_ph97_B8
zmp_ph97_C1
zmp_ph97_D2
zmp_ph97_D5
zmp_ph111_G5
zmp_ph111_G11
zmp_ph111_H1
zmp_ph111_B1a
zmp_ph111_B5
zmp_ph111_B8
zmp_ph111_B6
zmp_ph111_B7
zmp_ph111_B9
zmp_ph111_B10
zmp_ph114_G4
zmp_ph114_G11
zmp_ph114_H1
zmp_ph114_H2
zmp_ph114_A9
zmp_ph114_A11
zmp_ph114_B2
zmp_ph114_B4
zmp_ph114_D8
zmp_ph114_E2

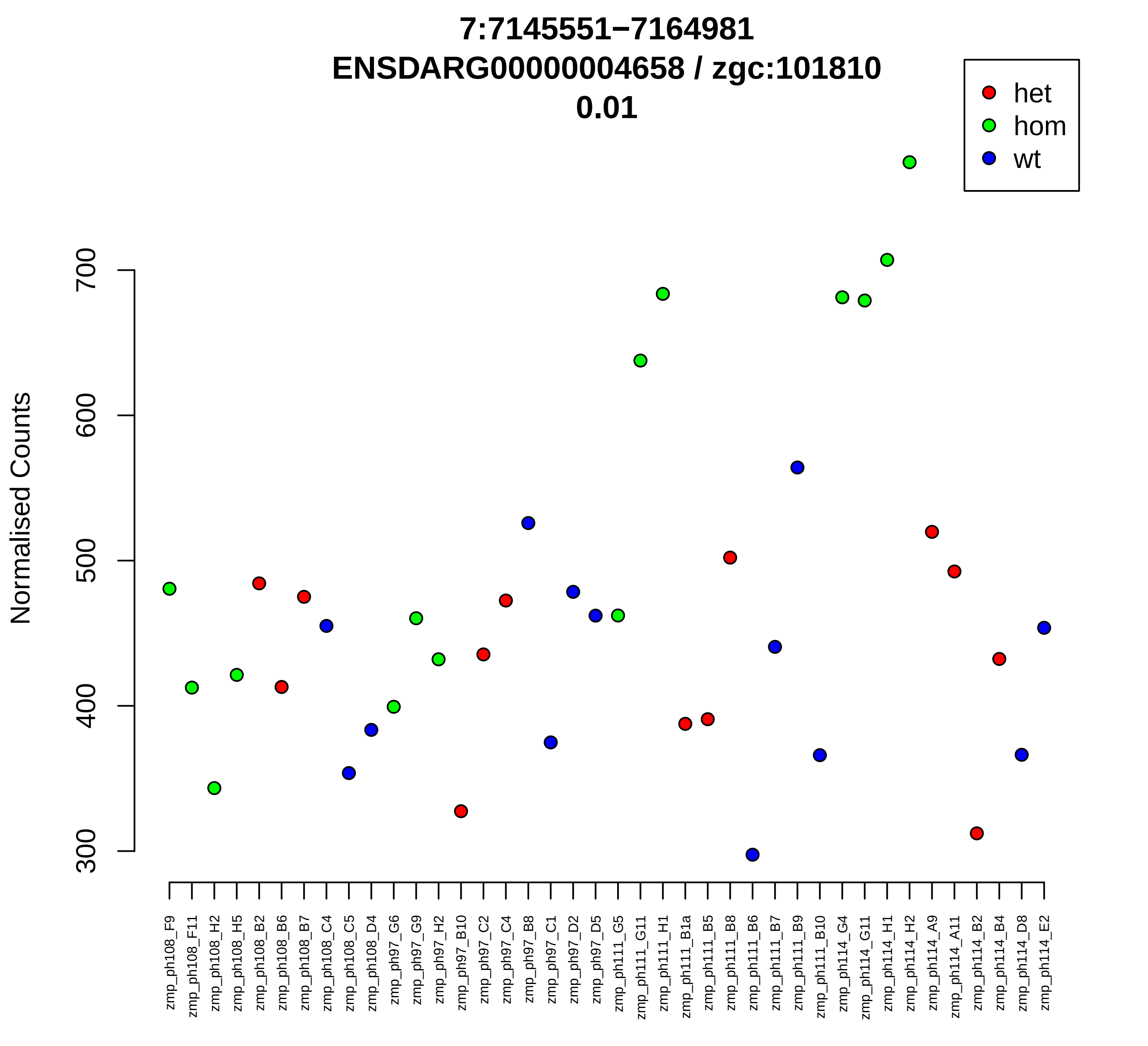
7:7145551−7164981
ENSDARG00000004658 / zgc:101810
0.01
het
hom
wt
Normalised Counts
300
400
500
600
700
zmp_ph108_F9
zmp_ph108_F11
zmp_ph108_H2
zmp_ph108_H5
zmp_ph108_B2
zmp_ph108_B6
zmp_ph108_B7
zmp_ph108_C4
zmp_ph108_C5
zmp_ph108_D4
zmp_ph97_G6
zmp_ph97_G9
zmp_ph97_H2
zmp_ph97_B10
zmp_ph97_C2
zmp_ph97_C4
zmp_ph97_B8
zmp_ph97_C1
zmp_ph97_D2
zmp_ph97_D5
zmp_ph111_G5
zmp_ph111_G11
zmp_ph111_H1
zmp_ph111_B1a
zmp_ph111_B5
zmp_ph111_B8
zmp_ph111_B6
zmp_ph111_B7
zmp_ph111_B9
zmp_ph111_B10
zmp_ph114_G4
zmp_ph114_G11
zmp_ph114_H1
zmp_ph114_H2
zmp_ph114_A9
zmp_ph114_A11
zmp_ph114_B2
zmp_ph114_B4
zmp_ph114_D8
zmp_ph114_E2

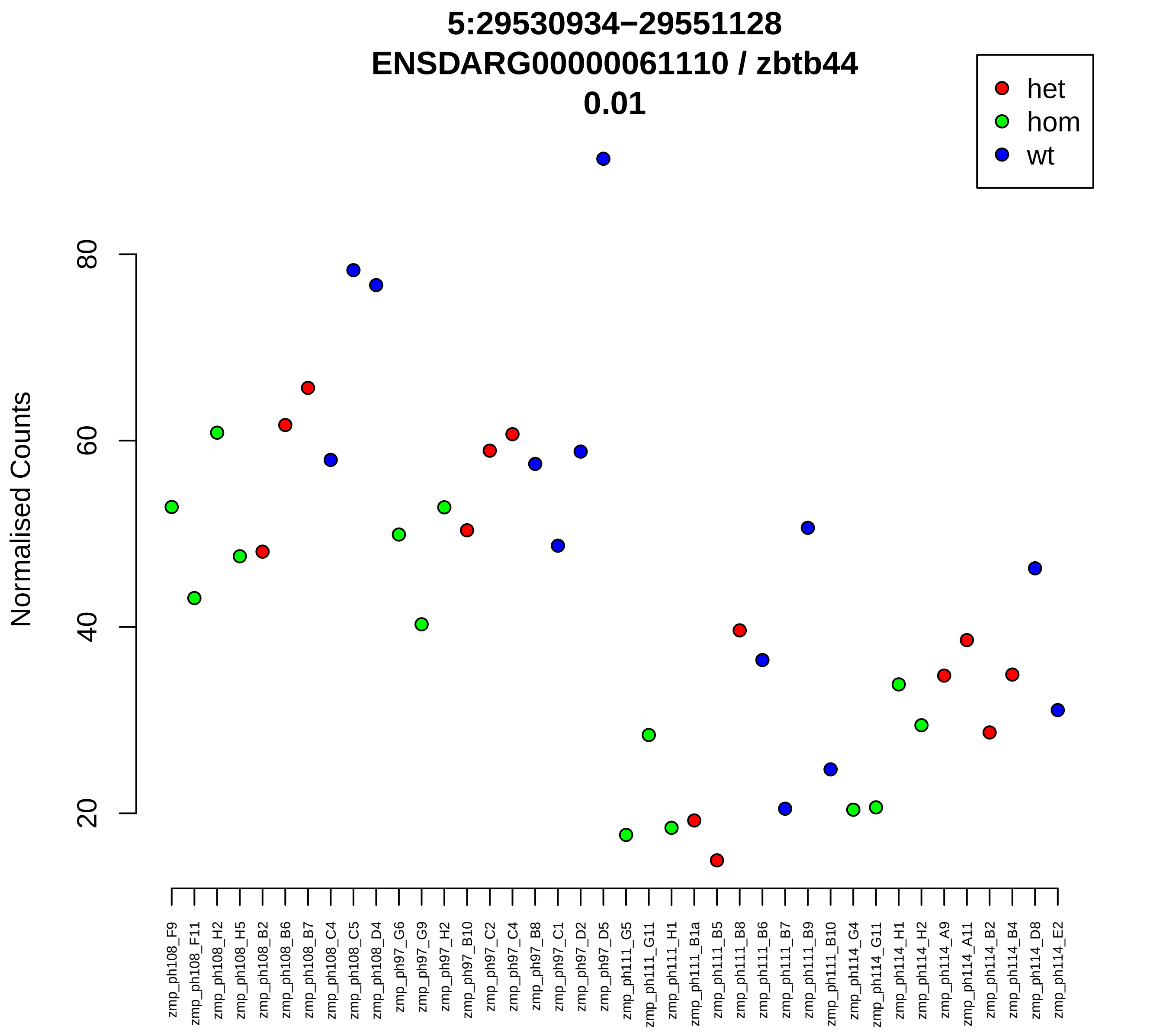

5:29530934−29551128
ENSDARG00000061110 / zbtb44
0.01
het
hom
wt
Normalised Counts
20
40
60
80
zmp_ph108_F9
zmp_ph108_F11
zmp_ph108_H2
zmp_ph108_H5
zmp_ph108_B2
zmp_ph108_B6
zmp_ph108_B7
zmp_ph108_C4
zmp_ph108_C5
zmp_ph108_D4
zmp_ph97_G6
zmp_ph97_G9
zmp_ph97_H2
zmp_ph97_B10
zmp_ph97_C2
zmp_ph97_C4
zmp_ph97_B8
zmp_ph97_C1
zmp_ph97_D2
zmp_ph97_D5
zmp_ph111_G5
zmp_ph111_G11
zmp_ph111_H1
zmp_ph111_B1a
zmp_ph111_B5
zmp_ph111_B8
zmp_ph111_B6
zmp_ph111_B7
zmp_ph111_B9
zmp_ph111_B10
zmp_ph114_G4
zmp_ph114_G11
zmp_ph114_H1
zmp_ph114_H2
zmp_ph114_A9
zmp_ph114_A11
zmp_ph114_B2
zmp_ph114_B4
zmp_ph114_D8
zmp_ph114_E2

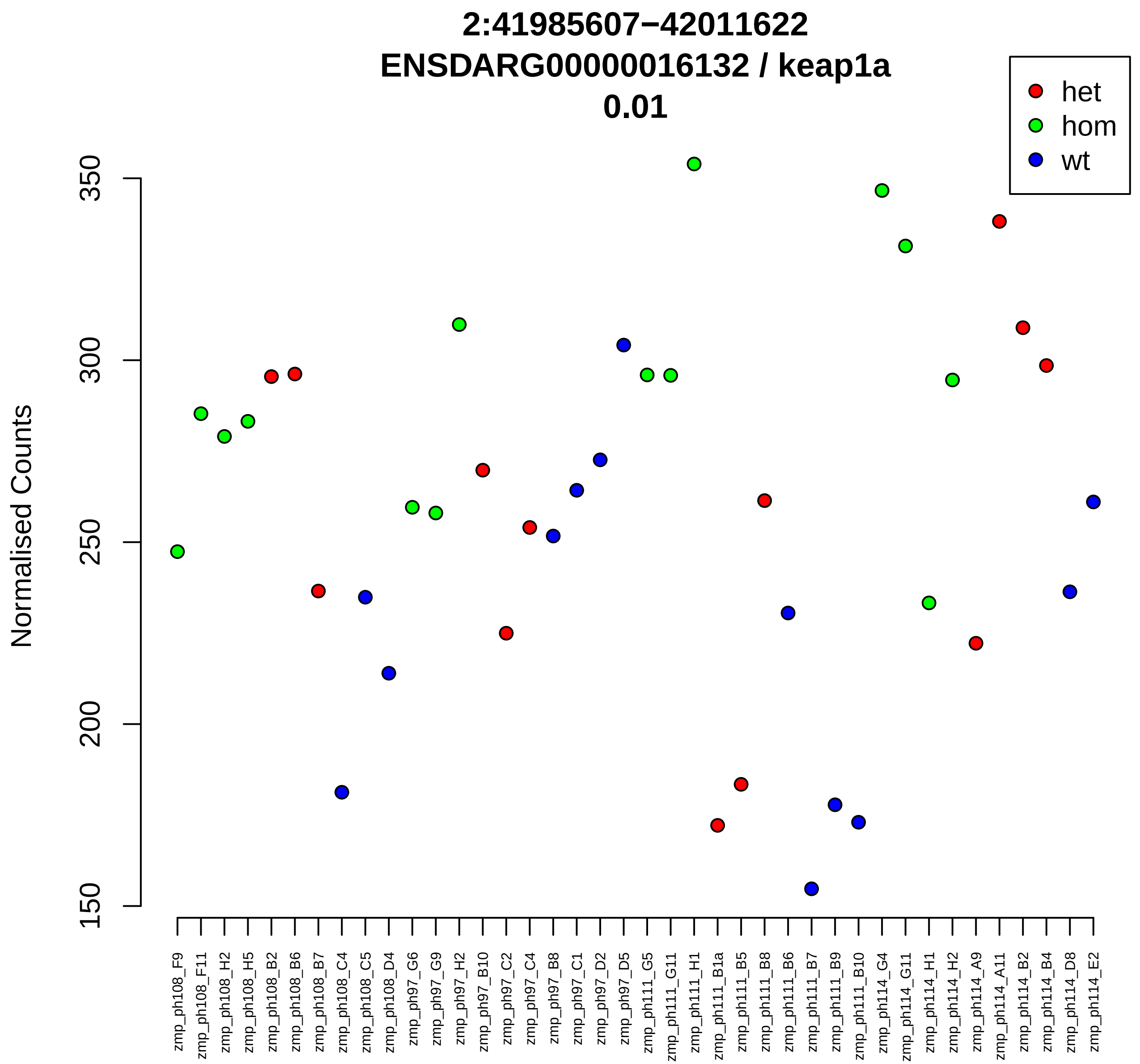

2:41985607−42011622
ENSDARG00000016132 / keap1a
0.01
het
hom
wt
Normalised Counts
150
200
250
300
350
zmp_ph108_F9
zmp_ph108_F11
zmp_ph108_H2
zmp_ph108_H5
zmp_ph108_B2
zmp_ph108_B6
zmp_ph108_B7
zmp_ph108_C4
zmp_ph108_C5
zmp_ph108_D4
zmp_ph97_G6
zmp_ph97_G9
zmp_ph97_H2
zmp_ph97_B10
zmp_ph97_C2
zmp_ph97_C4
zmp_ph97_B8
zmp_ph97_C1
zmp_ph97_D2
zmp_ph97_D5
zmp_ph111_G5
zmp_ph111_G11
zmp_ph111_H1
zmp_ph111_B1a
zmp_ph111_B5
zmp_ph111_B8
zmp_ph111_B6
zmp_ph111_B7
zmp_ph111_B9
zmp_ph111_B10
zmp_ph114_G4
zmp_ph114_G11
zmp_ph114_H1
zmp_ph114_H2
zmp_ph114_A9
zmp_ph114_A11
zmp_ph114_B2
zmp_ph114_B4
zmp_ph114_D8
zmp_ph114_E2

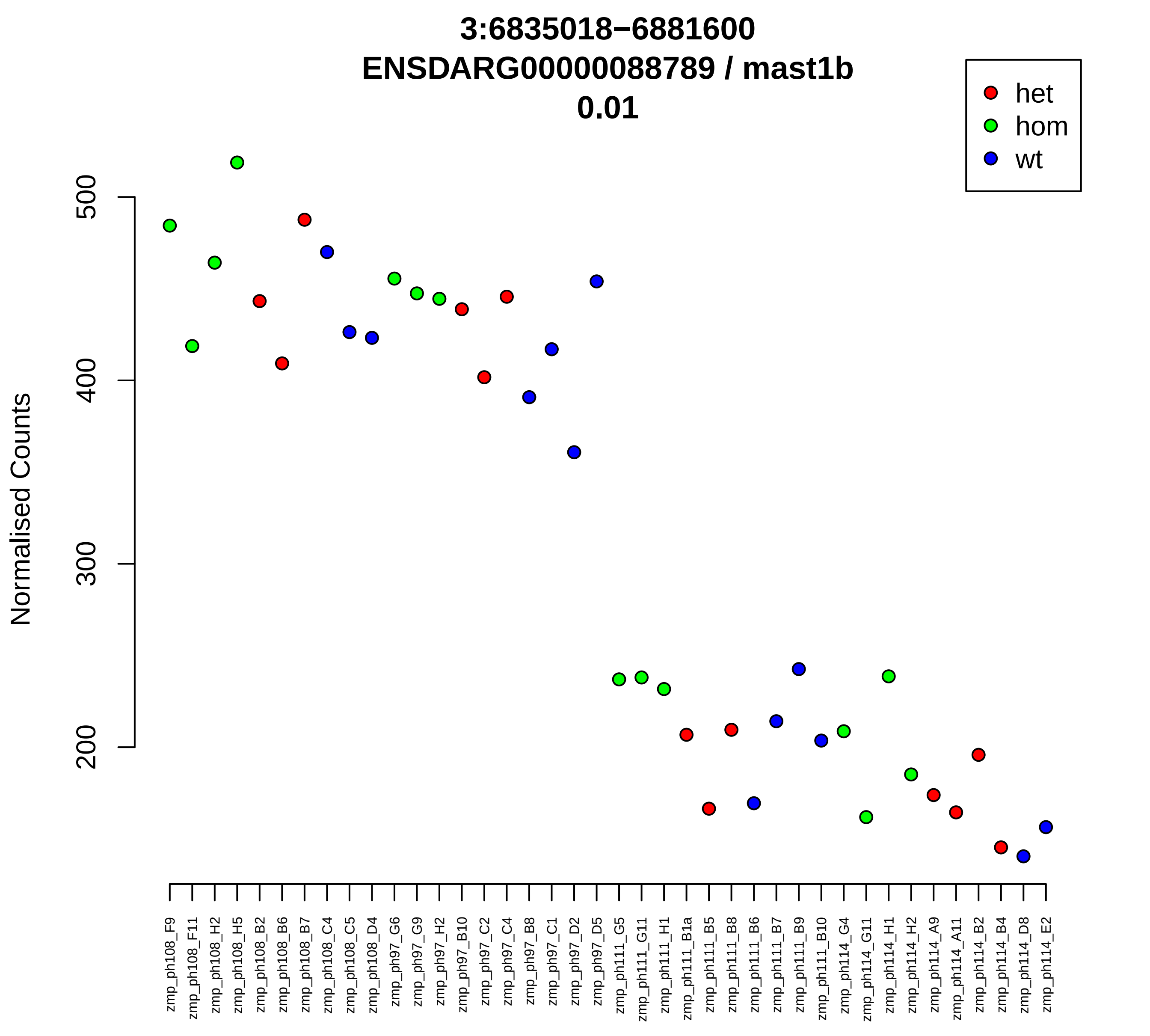
3:6835018−6881600
ENSDARG00000088789 / mast1b
0.01
het
hom
wt
Normalised Counts
200
300
400
500
zmp_ph108_F9
zmp_ph108_F11
zmp_ph108_H2
zmp_ph108_H5
zmp_ph108_B2
zmp_ph108_B6
zmp_ph108_B7
zmp_ph108_C4
zmp_ph108_C5
zmp_ph108_D4
zmp_ph97_G6
zmp_ph97_G9
zmp_ph97_H2
zmp_ph97_B10
zmp_ph97_C2
zmp_ph97_C4
zmp_ph97_B8
zmp_ph97_C1
zmp_ph97_D2
zmp_ph97_D5
zmp_ph111_G5
zmp_ph111_G11
zmp_ph111_H1
zmp_ph111_B1a
zmp_ph111_B5
zmp_ph111_B8
zmp_ph111_B6
zmp_ph111_B7
zmp_ph111_B9
zmp_ph111_B10
zmp_ph114_G4
zmp_ph114_G11
zmp_ph114_H1
zmp_ph114_H2
zmp_ph114_A9
zmp_ph114_A11
zmp_ph114_B2
zmp_ph114_B4
zmp_ph114_D8
zmp_ph114_E2

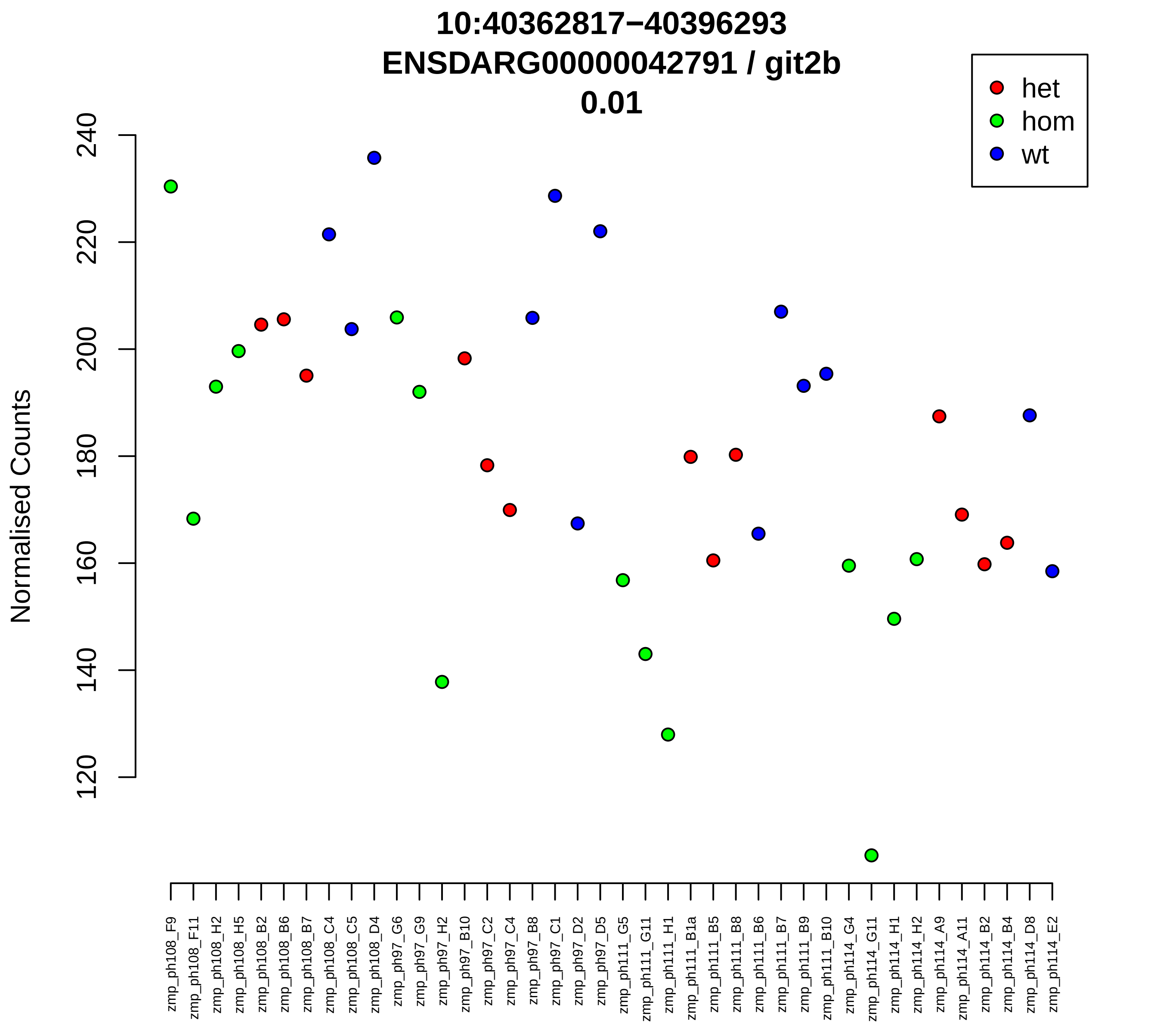

10:40362817−40396293
ENSDARG00000042791 / git2b
0.01
het
hom
wt
Normalised Counts
120
140
160
180
200
220
240
zmp_ph108_F9
zmp_ph108_F11
zmp_ph108_H2
zmp_ph108_H5
zmp_ph108_B2
zmp_ph108_B6
zmp_ph108_B7
zmp_ph108_C4
zmp_ph108_C5
zmp_ph108_D4
zmp_ph97_G6
zmp_ph97_G9
zmp_ph97_H2
zmp_ph97_B10
zmp_ph97_C2
zmp_ph97_C4
zmp_ph97_B8
zmp_ph97_C1
zmp_ph97_D2
zmp_ph97_D5
zmp_ph111_G5
zmp_ph111_G11
zmp_ph111_H1
zmp_ph111_B1a
zmp_ph111_B5
zmp_ph111_B8
zmp_ph111_B6
zmp_ph111_B7
zmp_ph111_B9
zmp_ph111_B10
zmp_ph114_G4
zmp_ph114_G11
zmp_ph114_H1
zmp_ph114_H2
zmp_ph114_A9
zmp_ph114_A11
zmp_ph114_B2
zmp_ph114_B4
zmp_ph114_D8
zmp_ph114_E2

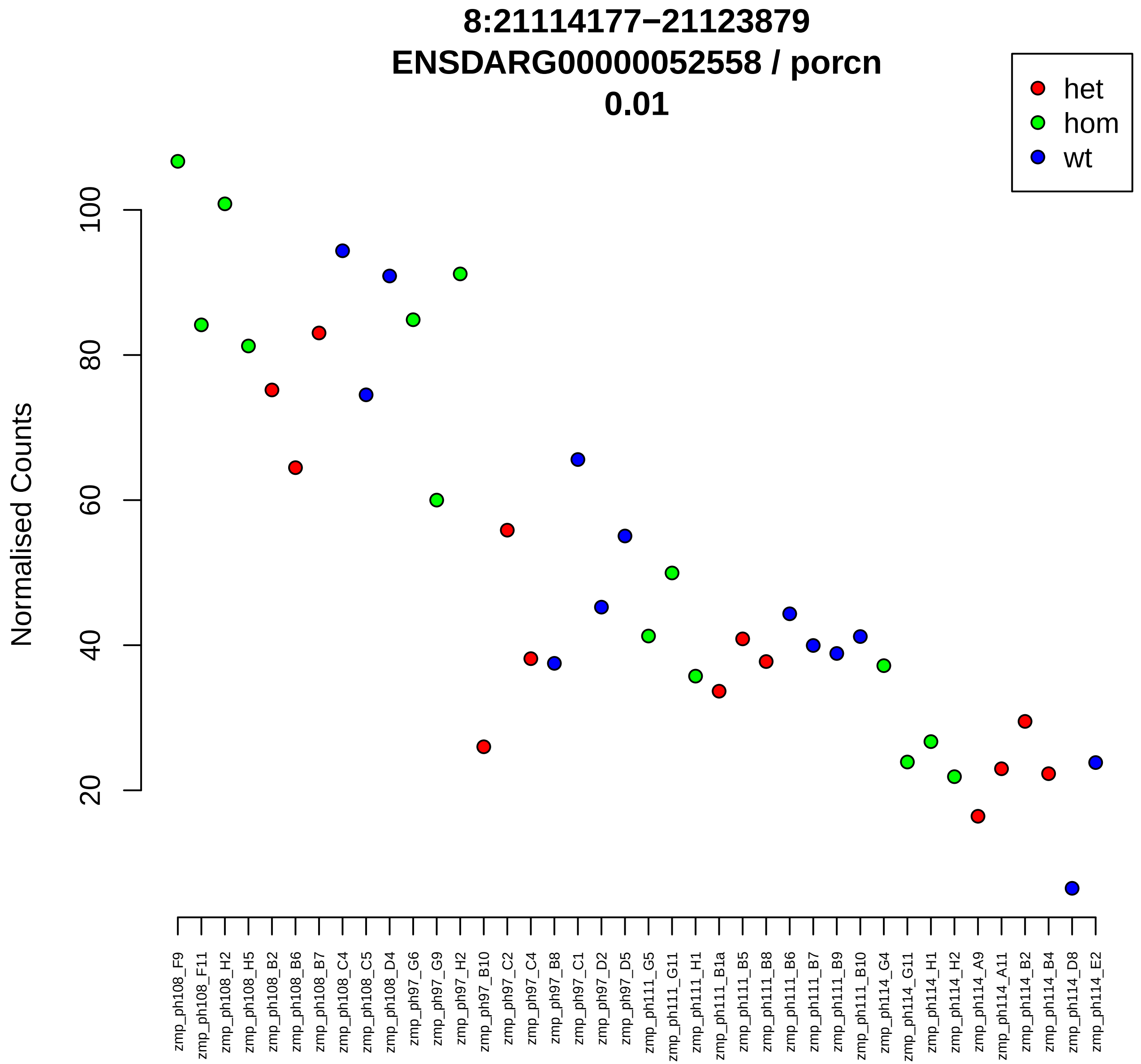

8:21114177−21123879
ENSDARG00000052558 / porcn
0.01
het
hom
wt
Normalised Counts
20
40
60
80
100
zmp_ph108_F9
zmp_ph108_F11
zmp_ph108_H2
zmp_ph108_H5
zmp_ph108_B2
zmp_ph108_B6
zmp_ph108_B7
zmp_ph108_C4
zmp_ph108_C5
zmp_ph108_D4
zmp_ph97_G6
zmp_ph97_G9
zmp_ph97_H2
zmp_ph97_B10
zmp_ph97_C2
zmp_ph97_C4
zmp_ph97_B8
zmp_ph97_C1
zmp_ph97_D2
zmp_ph97_D5
zmp_ph111_G5
zmp_ph111_G11
zmp_ph111_H1
zmp_ph111_B1a
zmp_ph111_B5
zmp_ph111_B8
zmp_ph111_B6
zmp_ph111_B7
zmp_ph111_B9
zmp_ph111_B10
zmp_ph114_G4
zmp_ph114_G11
zmp_ph114_H1
zmp_ph114_H2
zmp_ph114_A9
zmp_ph114_A11
zmp_ph114_B2
zmp_ph114_B4
zmp_ph114_D8
zmp_ph114_E2

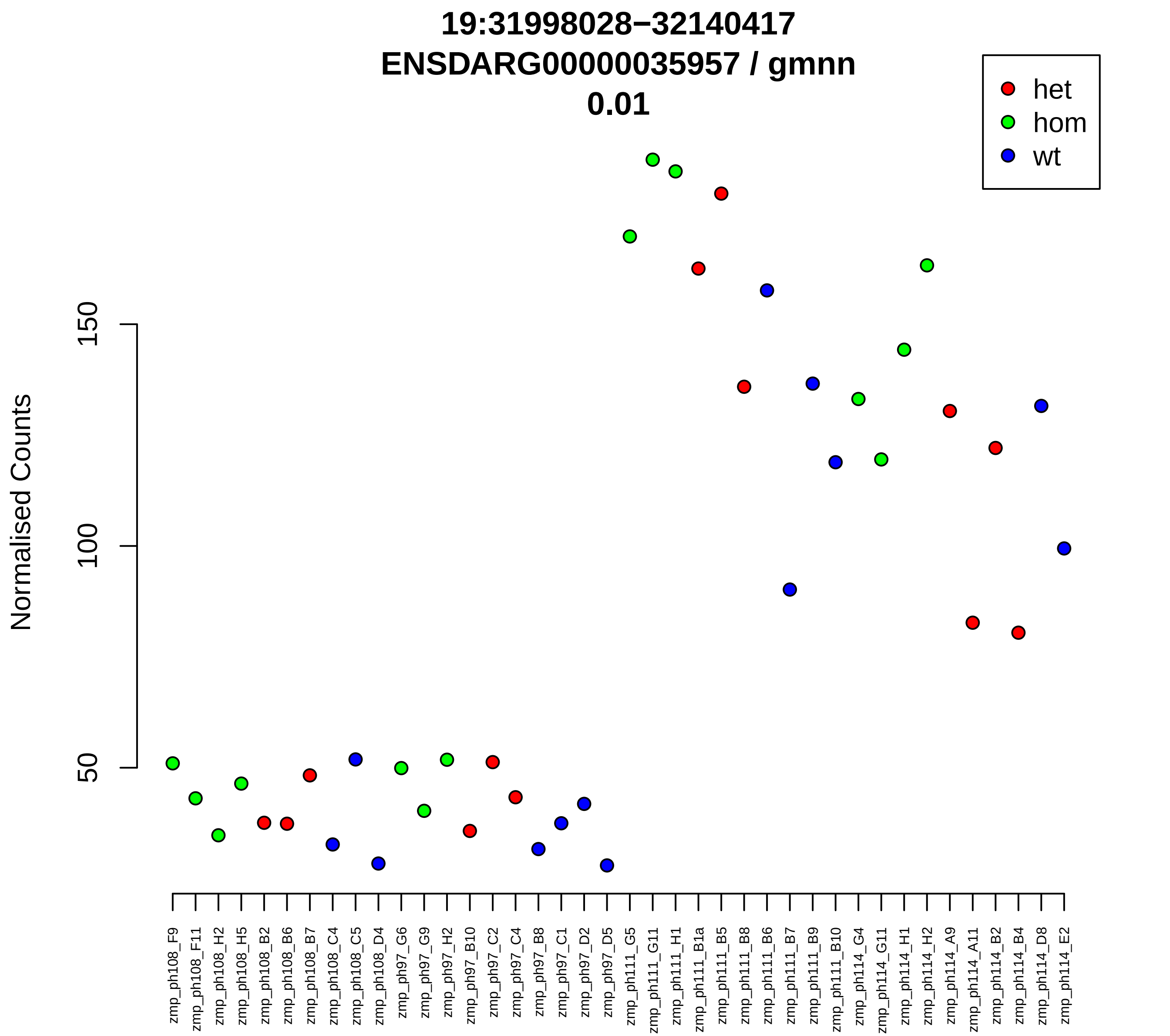

19:31998028−32140417
ENSDARG00000035957 / gmnn
0.01
het
hom
wt
Normalised Counts
50
100
150
zmp_ph108_F9
zmp_ph108_F11
zmp_ph108_H2
zmp_ph108_H5
zmp_ph108_B2
zmp_ph108_B6
zmp_ph108_B7
zmp_ph108_C4
zmp_ph108_C5
zmp_ph108_D4
zmp_ph97_G6
zmp_ph97_G9
zmp_ph97_H2
zmp_ph97_B10
zmp_ph97_C2
zmp_ph97_C4
zmp_ph97_B8
zmp_ph97_C1
zmp_ph97_D2
zmp_ph97_D5
zmp_ph111_G5
zmp_ph111_G11
zmp_ph111_H1
zmp_ph111_B1a
zmp_ph111_B5
zmp_ph111_B8
zmp_ph111_B6
zmp_ph111_B7
zmp_ph111_B9
zmp_ph111_B10
zmp_ph114_G4
zmp_ph114_G11
zmp_ph114_H1
zmp_ph114_H2
zmp_ph114_A9
zmp_ph114_A11
zmp_ph114_B2
zmp_ph114_B4
zmp_ph114_D8
zmp_ph114_E2

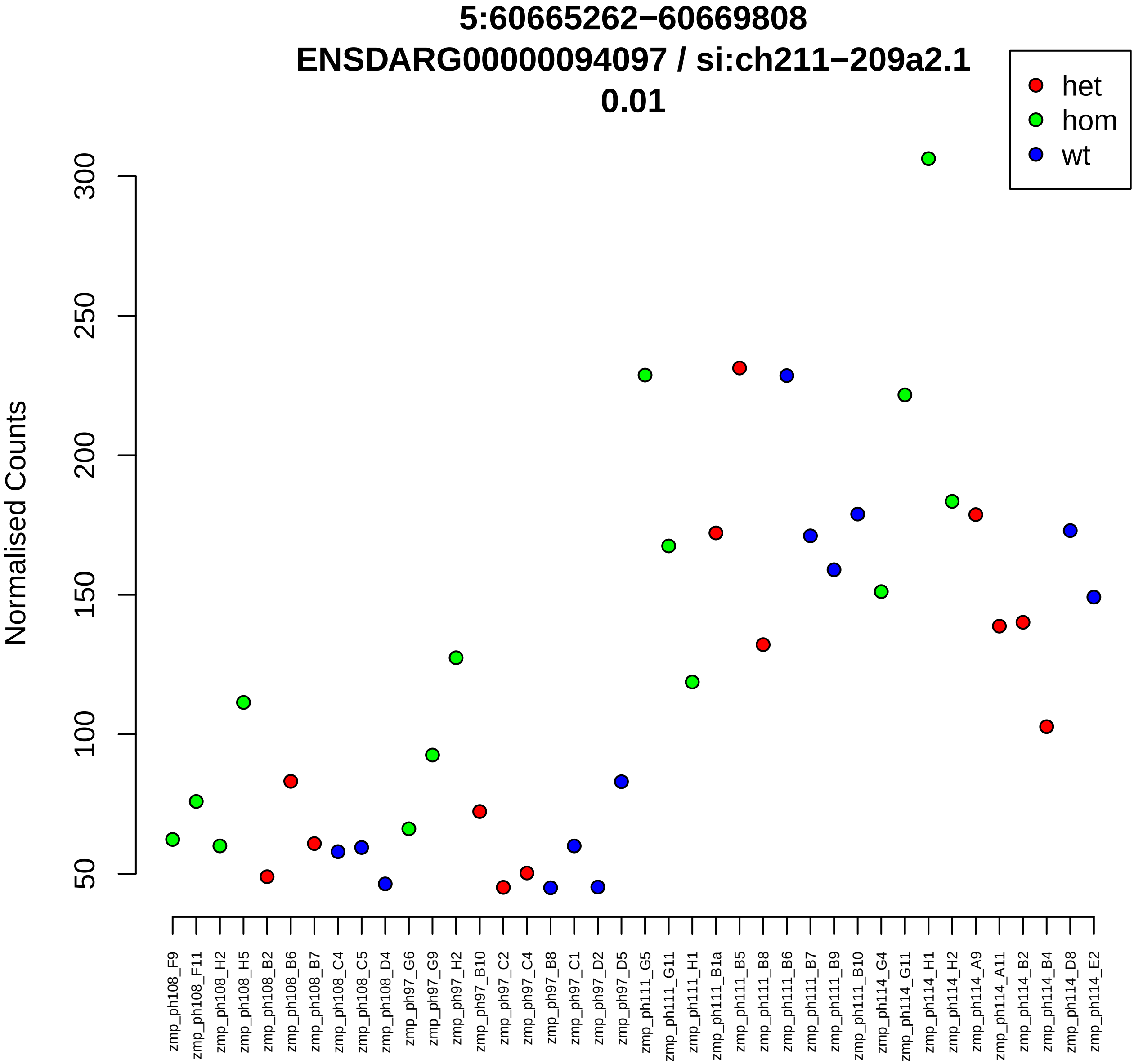

5:60665262−60669808
ENSDARG00000094097 / si:ch211−209a2.1
0.01
het
hom
wt
Normalised Counts
50
100
150
200
250
300
zmp_ph108_F9
zmp_ph108_F11
zmp_ph108_H2
zmp_ph108_H5
zmp_ph108_B2
zmp_ph108_B6
zmp_ph108_B7
zmp_ph108_C4
zmp_ph108_C5
zmp_ph108_D4
zmp_ph97_G6
zmp_ph97_G9
zmp_ph97_H2
zmp_ph97_B10
zmp_ph97_C2
zmp_ph97_C4
zmp_ph97_B8
zmp_ph97_C1
zmp_ph97_D2
zmp_ph97_D5
zmp_ph111_G5
zmp_ph111_G11
zmp_ph111_H1
zmp_ph111_B1a
zmp_ph111_B5
zmp_ph111_B8
zmp_ph111_B6
zmp_ph111_B7
zmp_ph111_B9
zmp_ph111_B10
zmp_ph114_G4
zmp_ph114_G11
zmp_ph114_H1
zmp_ph114_H2
zmp_ph114_A9
zmp_ph114_A11
zmp_ph114_B2
zmp_ph114_B4
zmp_ph114_D8
zmp_ph114_E2

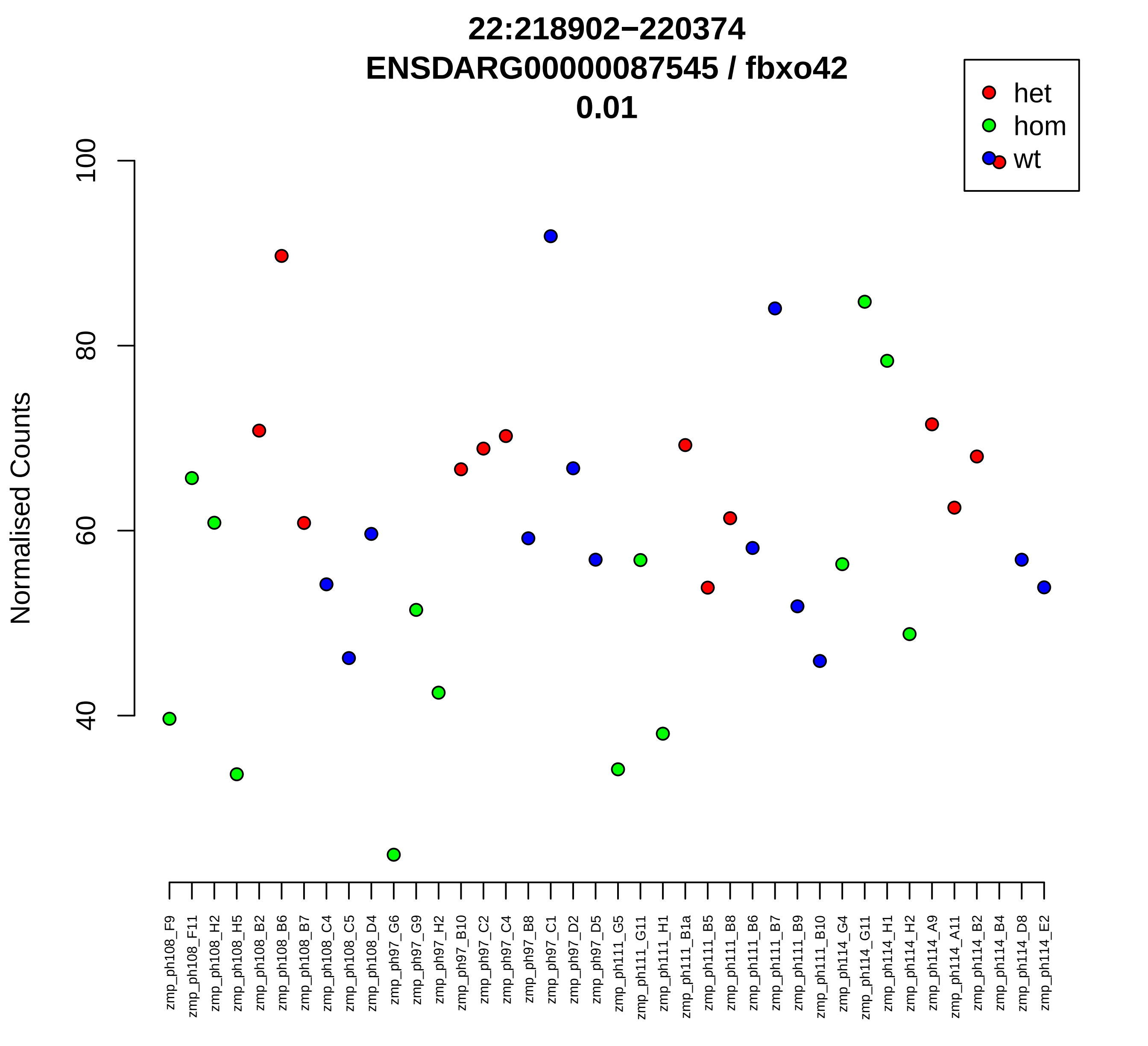

22:218902−220374
ENSDARG00000087545 / fbxo42
0.01
het
hom
wt
Normalised Counts
100
80
60
40
zmp_ph108_F9
zmp_ph108_F11
zmp_ph108_H2
zmp_ph108_H5
zmp_ph108_B2
zmp_ph108_B6
zmp_ph108_B7
zmp_ph108_C4
zmp_ph108_C5
zmp_ph108_D4
zmp_ph97_G6
zmp_ph97_G9
zmp_ph97_H2
zmp_ph97_B10
zmp_ph97_C2
zmp_ph97_C4
zmp_ph97_B8
zmp_ph97_C1
zmp_ph97_D2
zmp_ph97_D5
zmp_ph111_G5
zmp_ph111_G11
zmp_ph111_H1
zmp_ph111_B1a
zmp_ph111_B5
zmp_ph111_B8
zmp_ph111_B6
zmp_ph111_B7
zmp_ph111_B9
zmp_ph111_B10
zmp_ph114_G4
zmp_ph114_G11
zmp_ph114_H1
zmp_ph114_H2
zmp_ph114_A9
zmp_ph114_A11
zmp_ph114_B2
zmp_ph114_B4
zmp_ph114_D8
zmp_ph114_E2

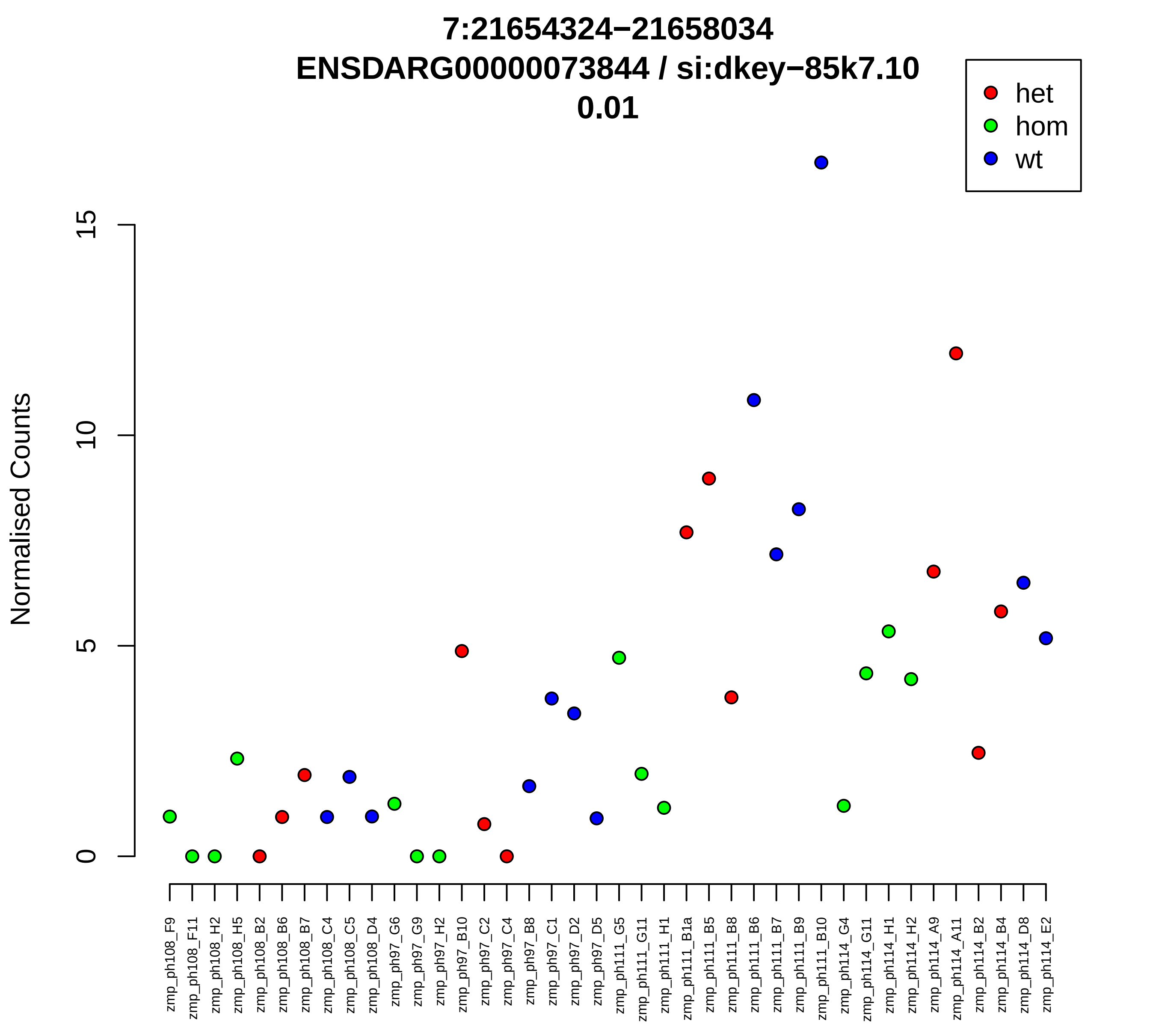
7:21654324−21658034
ENSDARG00000073844 / si:dkey−85k7.10
0.01
het
hom
wt
Normalised Counts
0
5
10
15
zmp_ph108_F9
zmp_ph108_F11
zmp_ph108_H2
zmp_ph108_H5
zmp_ph108_B2
zmp_ph108_B6
zmp_ph108_B7
zmp_ph108_C4
zmp_ph108_C5
zmp_ph108_D4
zmp_ph97_G6
zmp_ph97_G9
zmp_ph97_H2
zmp_ph97_B10
zmp_ph97_C2
zmp_ph97_C4
zmp_ph97_B8
zmp_ph97_C1
zmp_ph97_D2
zmp_ph97_D5
zmp_ph111_G5
zmp_ph111_G11
zmp_ph111_H1
zmp_ph111_B1a
zmp_ph111_B5
zmp_ph111_B8
zmp_ph111_B6
zmp_ph111_B7
zmp_ph111_B9
zmp_ph111_B10
zmp_ph114_G4
zmp_ph114_G11
zmp_ph114_H1
zmp_ph114_H2
zmp_ph114_A9
zmp_ph114_A11
zmp_ph114_B2
zmp_ph114_B4
zmp_ph114_D8
zmp_ph114_E2

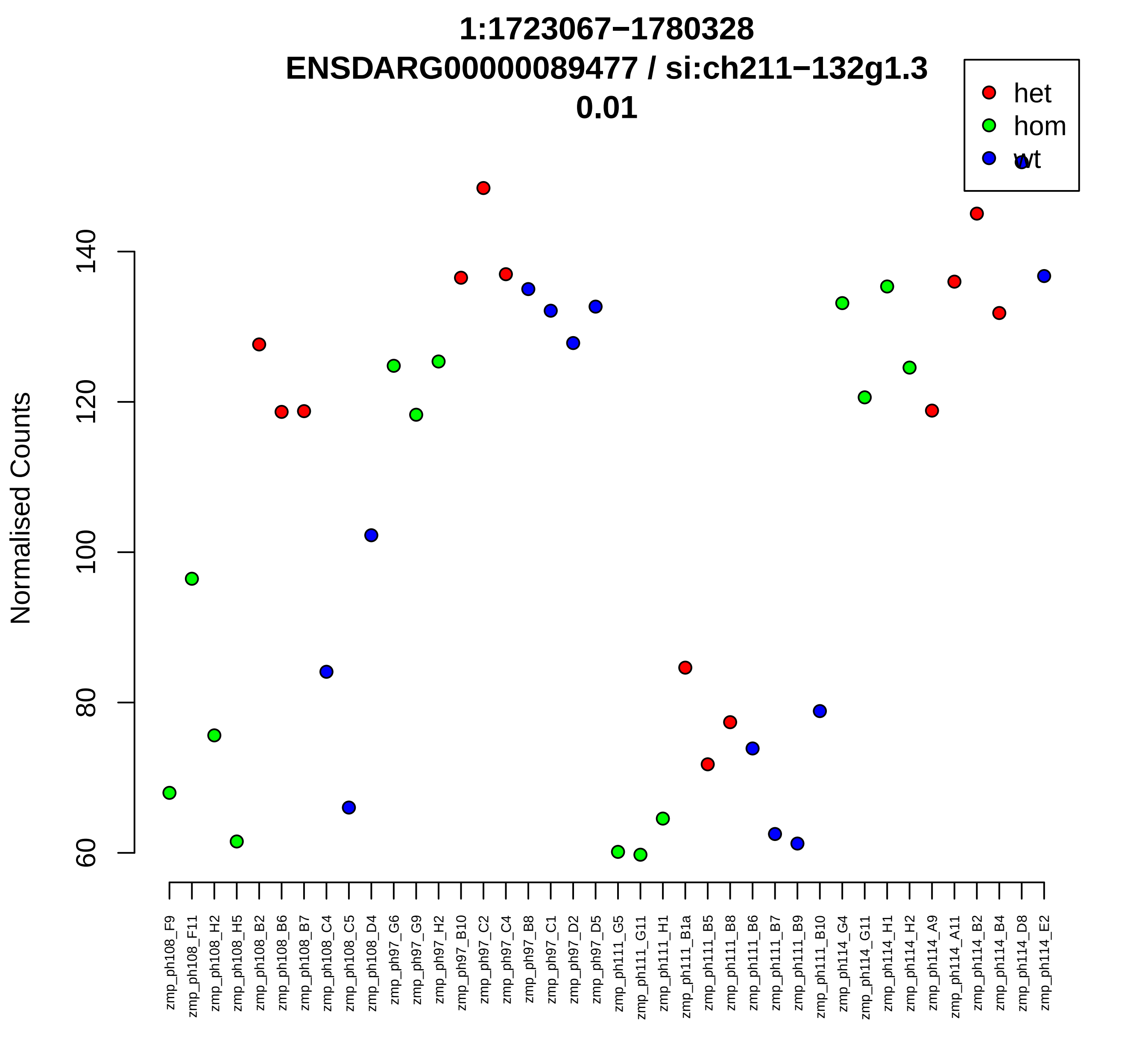
1:1723067−1780328
ENSDARG00000089477 / si:ch211−132g1.3
0.01
het
hom
wt
Normalised Counts
60
80
100
120
140
zmp_ph108_F9
zmp_ph108_F11
zmp_ph108_H2
zmp_ph108_H5
zmp_ph108_B2
zmp_ph108_B6
zmp_ph108_B7
zmp_ph108_C4
zmp_ph108_C5
zmp_ph108_D4
zmp_ph97_G6
zmp_ph97_G9
zmp_ph97_H2
zmp_ph97_B10
zmp_ph97_C2
zmp_ph97_C4
zmp_ph97_B8
zmp_ph97_C1
zmp_ph97_D2
zmp_ph97_D5
zmp_ph111_G5
zmp_ph111_G11
zmp_ph111_H1
zmp_ph111_B1a
zmp_ph111_B5
zmp_ph111_B8
zmp_ph111_B6
zmp_ph111_B7
zmp_ph111_B9
zmp_ph111_B10
zmp_ph114_G4
zmp_ph114_G11
zmp_ph114_H1
zmp_ph114_H2
zmp_ph114_A9
zmp_ph114_A11
zmp_ph114_B2
zmp_ph114_B4
zmp_ph114_D8
zmp_ph114_E2

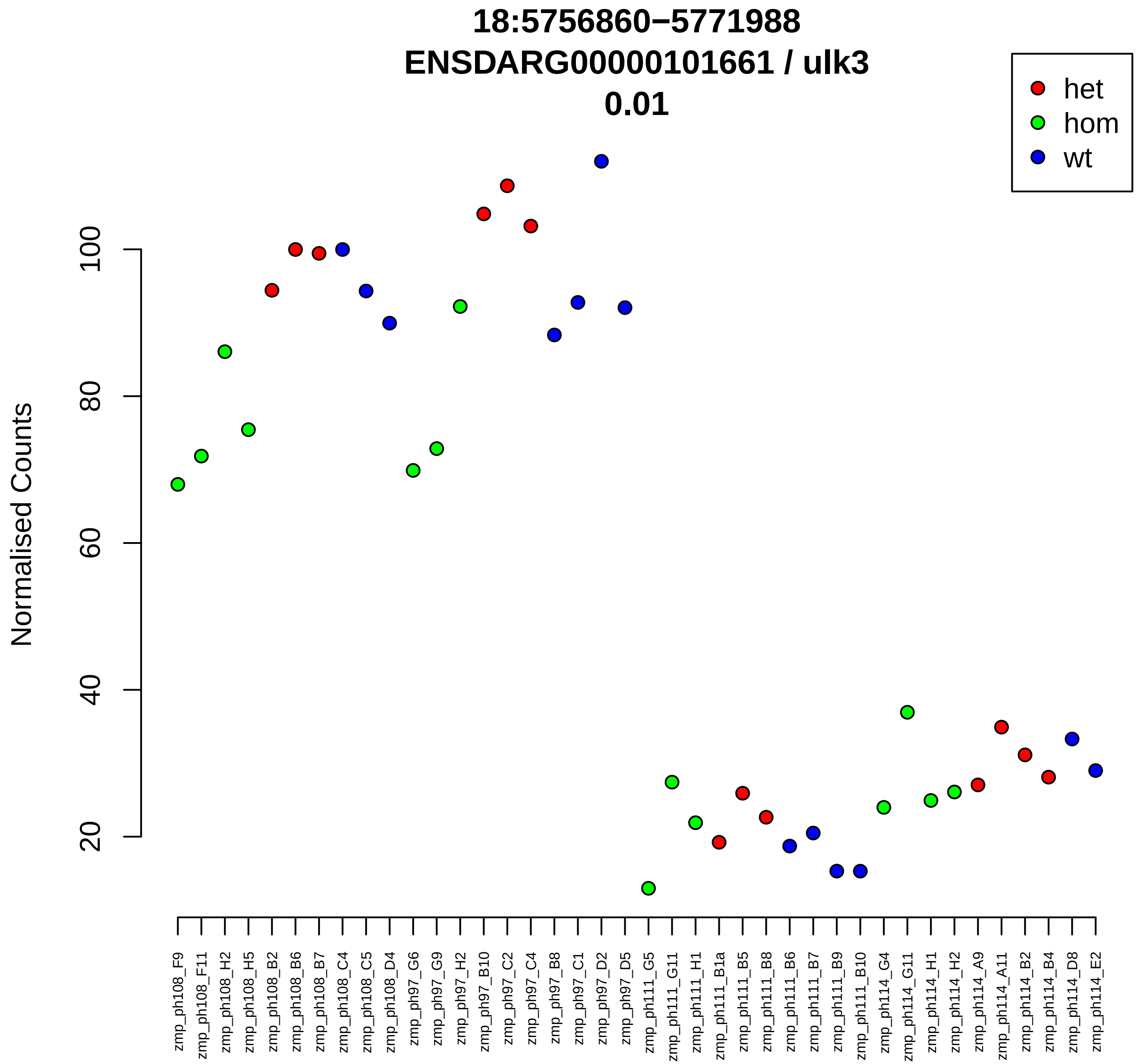

18:5756860−5771988
ENSDARG00000101661 / ulk3
0.01
het
hom
wt
Normalised Counts
20
40
60
80
100
zmp_ph108_F9
zmp_ph108_F11
zmp_ph108_H2
zmp_ph108_H5
zmp_ph108_B2
zmp_ph108_B6
zmp_ph108_B7
zmp_ph108_C4
zmp_ph108_C5
zmp_ph108_D4
zmp_ph97_G6
zmp_ph97_G9
zmp_ph97_H2
zmp_ph97_B10
zmp_ph97_C2
zmp_ph97_C4
zmp_ph97_B8
zmp_ph97_C1
zmp_ph97_D2
zmp_ph97_D5
zmp_ph111_G5
zmp_ph111_G11
zmp_ph111_H1
zmp_ph111_B1a
zmp_ph111_B5
zmp_ph111_B8
zmp_ph111_B6
zmp_ph111_B7
zmp_ph111_B9
zmp_ph111_B10
zmp_ph114_G4
zmp_ph114_G11
zmp_ph114_H1
zmp_ph114_H2
zmp_ph114_A9
zmp_ph114_A11
zmp_ph114_B2
zmp_ph114_B4
zmp_ph114_D8
zmp_ph114_E2

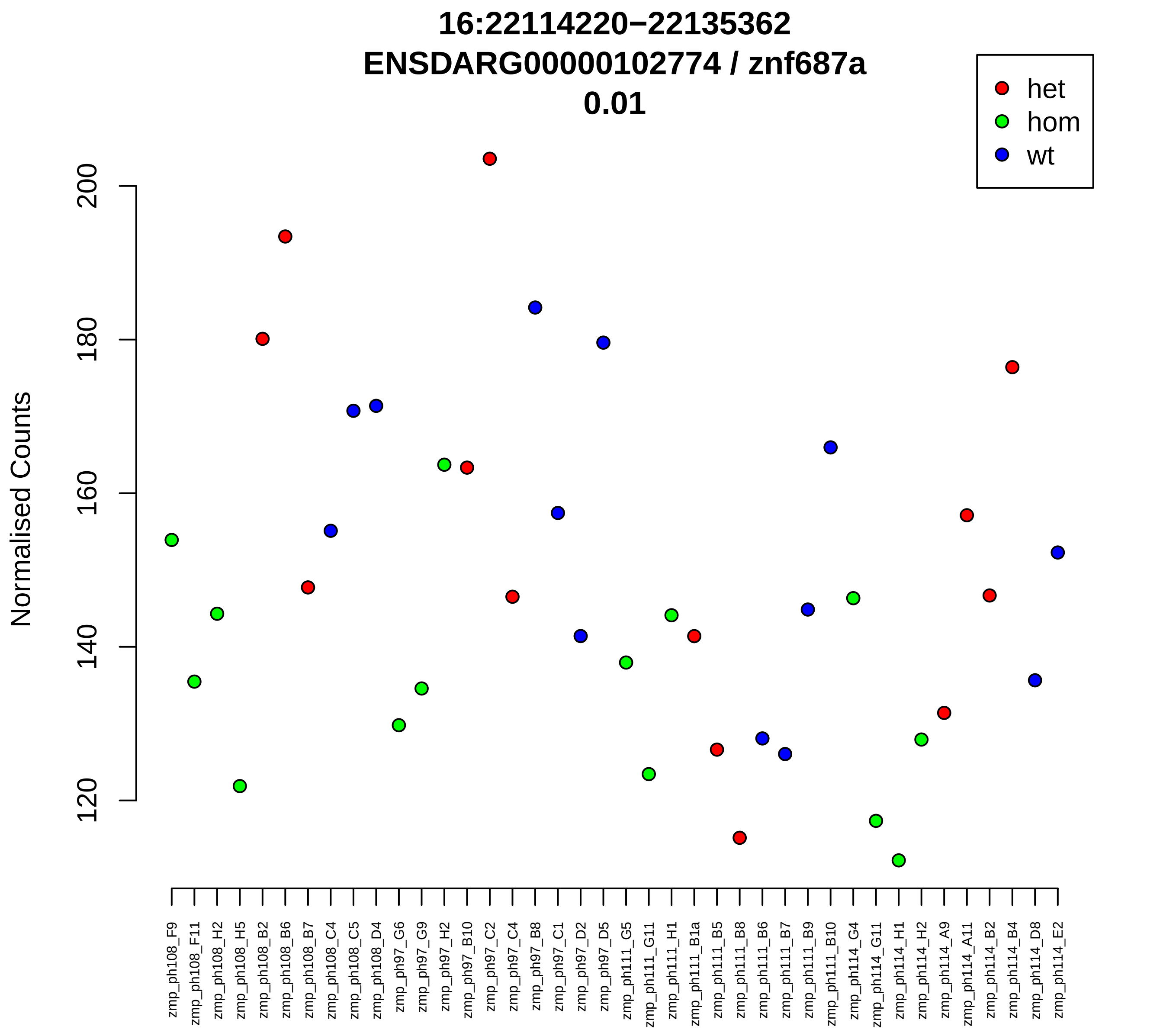

16:22114220−22135362
ENSDARG00000102774 / znf687a
0.01
het
hom
wt
Normalised Counts
120
140
160
180
200
zmp_ph108_F9
zmp_ph108_F11
zmp_ph108_H2
zmp_ph108_H5
zmp_ph108_B2
zmp_ph108_B6
zmp_ph108_B7
zmp_ph108_C4
zmp_ph108_C5
zmp_ph108_D4
zmp_ph97_G6
zmp_ph97_G9
zmp_ph97_H2
zmp_ph97_B10
zmp_ph97_C2
zmp_ph97_C4
zmp_ph97_B8
zmp_ph97_C1
zmp_ph97_D2
zmp_ph97_D5
zmp_ph111_G5
zmp_ph111_G11
zmp_ph111_H1
zmp_ph111_B1a
zmp_ph111_B5
zmp_ph111_B8
zmp_ph111_B6
zmp_ph111_B7
zmp_ph111_B9
zmp_ph111_B10
zmp_ph114_G4
zmp_ph114_G11
zmp_ph114_H1
zmp_ph114_H2
zmp_ph114_A9
zmp_ph114_A11
zmp_ph114_B2
zmp_ph114_B4
zmp_ph114_D8
zmp_ph114_E2

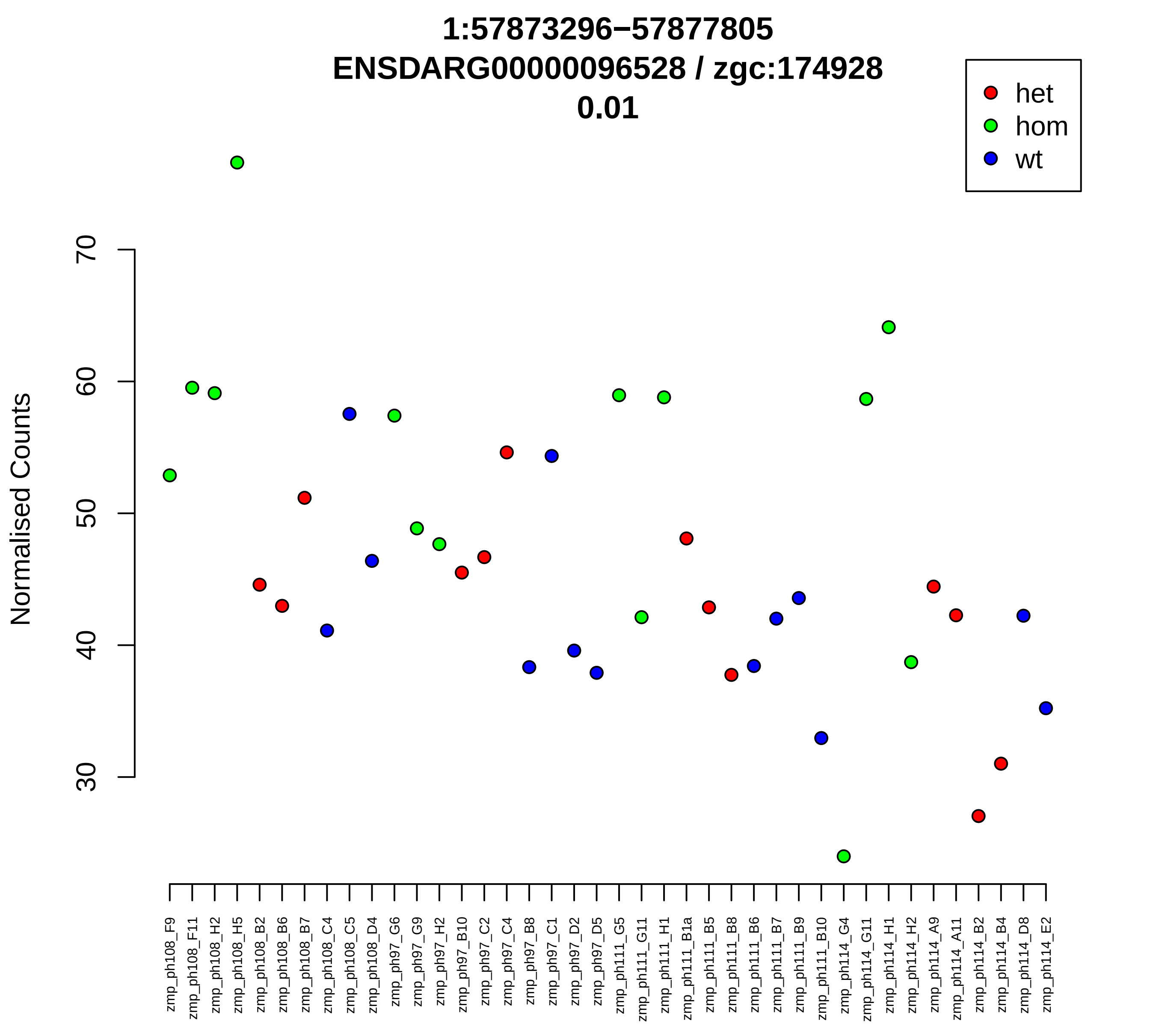
1:57873296−57877805
ENSDARG00000096528 / zgc:174928
0.01
het
hom
wt
Normalised Counts
30
40
50
60
70
zmp_ph108_F9
zmp_ph108_F11
zmp_ph108_H2
zmp_ph108_H5
zmp_ph108_B2
zmp_ph108_B6
zmp_ph108_B7
zmp_ph108_C4
zmp_ph108_C5
zmp_ph108_D4
zmp_ph97_G6
zmp_ph97_G9
zmp_ph97_H2
zmp_ph97_B10
zmp_ph97_C2
zmp_ph97_C4
zmp_ph97_B8
zmp_ph97_C1
zmp_ph97_D2
zmp_ph97_D5
zmp_ph111_G5
zmp_ph111_G11
zmp_ph111_H1
zmp_ph111_B1a
zmp_ph111_B5
zmp_ph111_B8
zmp_ph111_B6
zmp_ph111_B7
zmp_ph111_B9
zmp_ph111_B10
zmp_ph114_G4
zmp_ph114_G11
zmp_ph114_H1
zmp_ph114_H2
zmp_ph114_A9
zmp_ph114_A11
zmp_ph114_B2
zmp_ph114_B4
zmp_ph114_D8
zmp_ph114_E2

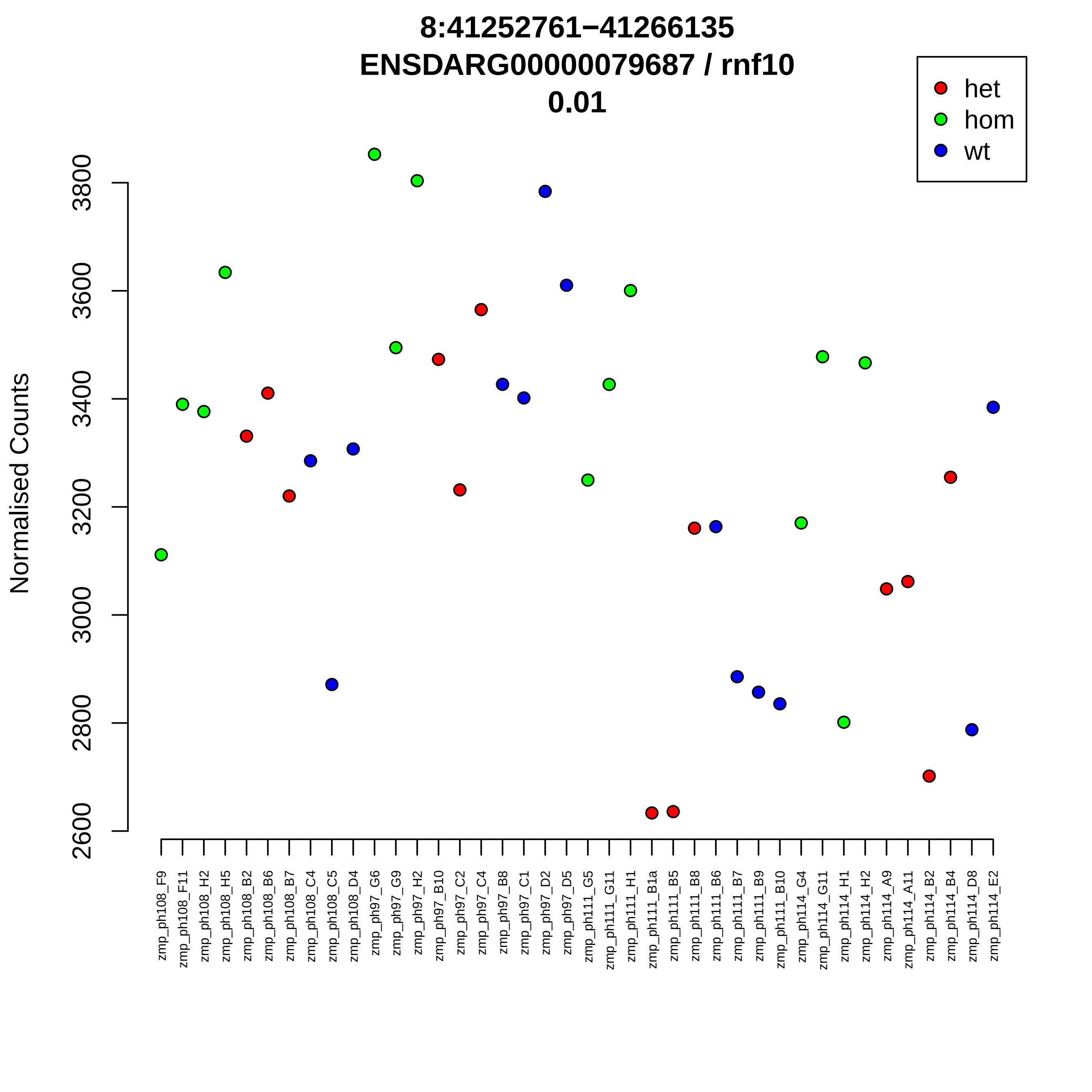

8:41252761−41266135
ENSDARG00000079687 / rnf10
0.01
het
hom
wt
Normalised Counts
2600
2800
3000
3200
3400
3600
3800
zmp_ph108_F9
zmp_ph108_F11
zmp_ph108_H2
zmp_ph108_H5
zmp_ph108_B2
zmp_ph108_B6
zmp_ph108_B7
zmp_ph108_C4
zmp_ph108_C5
zmp_ph108_D4
zmp_ph97_G6
zmp_ph97_G9
zmp_ph97_H2
zmp_ph97_B10
zmp_ph97_C2
zmp_ph97_C4
zmp_ph97_B8
zmp_ph97_C1
zmp_ph97_D2
zmp_ph97_D5
zmp_ph111_G5
zmp_ph111_G11
zmp_ph111_H1
zmp_ph111_B1a
zmp_ph111_B5
zmp_ph111_B8
zmp_ph111_B6
zmp_ph111_B7
zmp_ph111_B9
zmp_ph111_B10
zmp_ph114_G4
zmp_ph114_G11
zmp_ph114_H1
zmp_ph114_H2
zmp_ph114_A9
zmp_ph114_A11
zmp_ph114_B2
zmp_ph114_B4
zmp_ph114_D8
zmp_ph114_E2

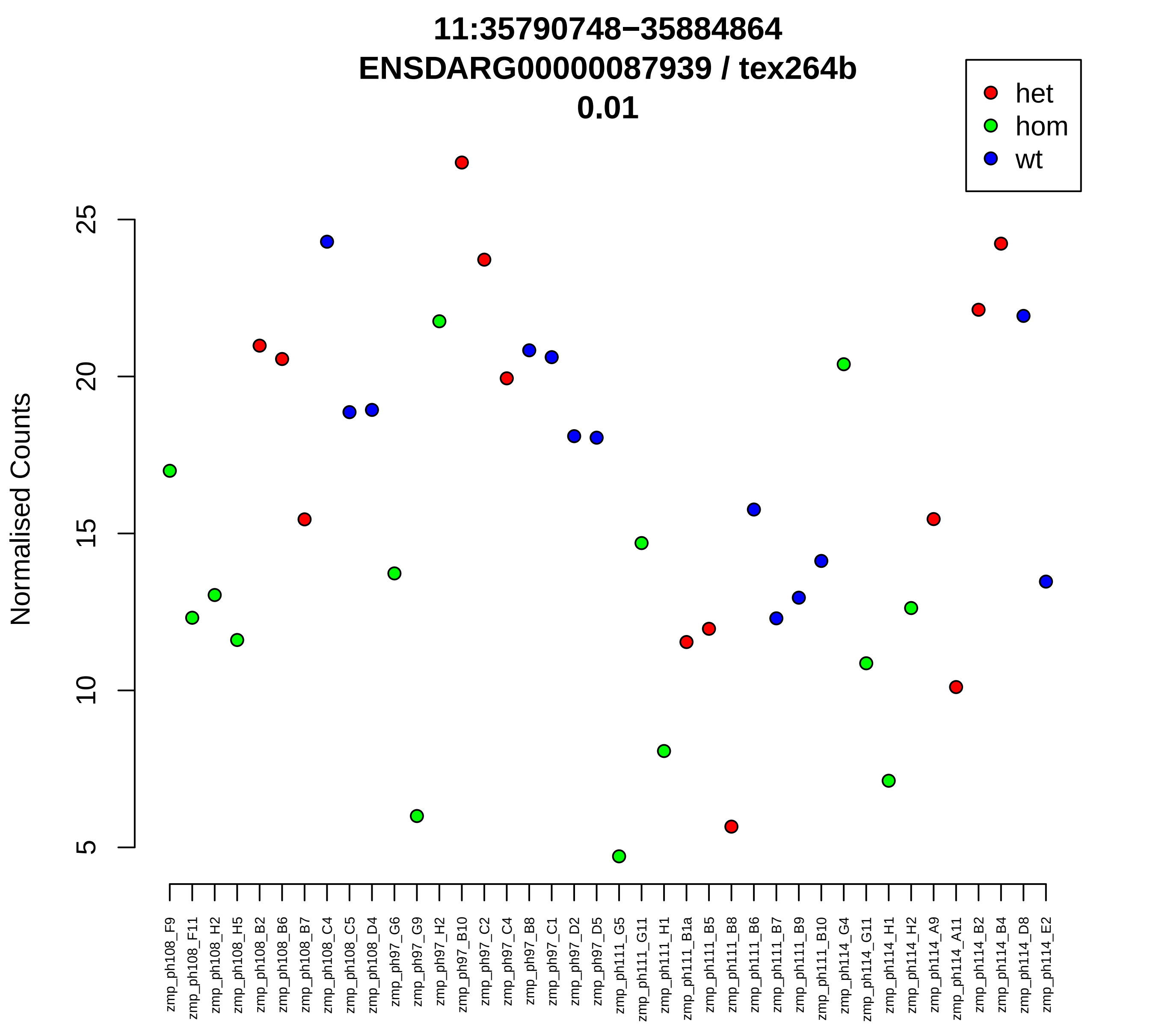

11:35790748−35884864
ENSDARG00000087939 / tex264b
0.01
het
hom
wt
Normalised Counts
5
10
15
20
25
zmp_ph108_F9
zmp_ph108_F11
zmp_ph108_H2
zmp_ph108_H5
zmp_ph108_B2
zmp_ph108_B6
zmp_ph108_B7
zmp_ph108_C4
zmp_ph108_C5
zmp_ph108_D4
zmp_ph97_G6
zmp_ph97_G9
zmp_ph97_H2
zmp_ph97_B10
zmp_ph97_C2
zmp_ph97_C4
zmp_ph97_B8
zmp_ph97_C1
zmp_ph97_D2
zmp_ph97_D5
zmp_ph111_G5
zmp_ph111_G11
zmp_ph111_H1
zmp_ph111_B1a
zmp_ph111_B5
zmp_ph111_B8
zmp_ph111_B6
zmp_ph111_B7
zmp_ph111_B9
zmp_ph111_B10
zmp_ph114_G4
zmp_ph114_G11
zmp_ph114_H1
zmp_ph114_H2
zmp_ph114_A9
zmp_ph114_A11
zmp_ph114_B2
zmp_ph114_B4
zmp_ph114_D8
zmp_ph114_E2

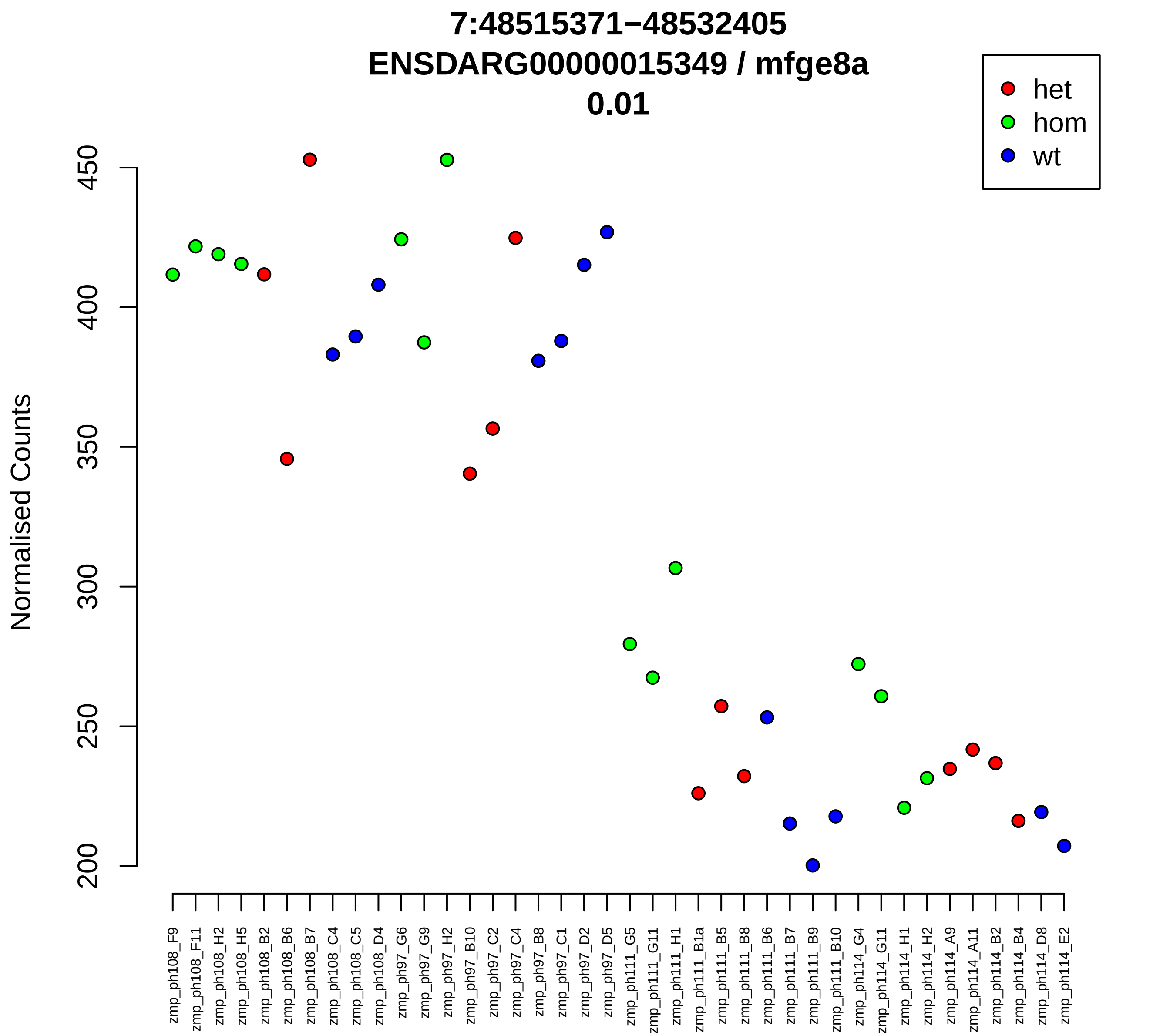

7:48515371−48532405
ENSDARG00000015349 / mfge8a
0.01
het
hom
wt
Normalised Counts
200
250
300
350
400
450
zmp_ph108_F9
zmp_ph108_F11
zmp_ph108_H2
zmp_ph108_H5
zmp_ph108_B2
zmp_ph108_B6
zmp_ph108_B7
zmp_ph108_C4
zmp_ph108_C5
zmp_ph108_D4
zmp_ph97_G6
zmp_ph97_G9
zmp_ph97_H2
zmp_ph97_B10
zmp_ph97_C2
zmp_ph97_C4
zmp_ph97_B8
zmp_ph97_C1
zmp_ph97_D2
zmp_ph97_D5
zmp_ph111_G5
zmp_ph111_G11
zmp_ph111_H1
zmp_ph111_B1a
zmp_ph111_B5
zmp_ph111_B8
zmp_ph111_B6
zmp_ph111_B7
zmp_ph111_B9
zmp_ph111_B10
zmp_ph114_G4
zmp_ph114_G11
zmp_ph114_H1
zmp_ph114_H2
zmp_ph114_A9
zmp_ph114_A11
zmp_ph114_B2
zmp_ph114_B4
zmp_ph114_D8
zmp_ph114_E2

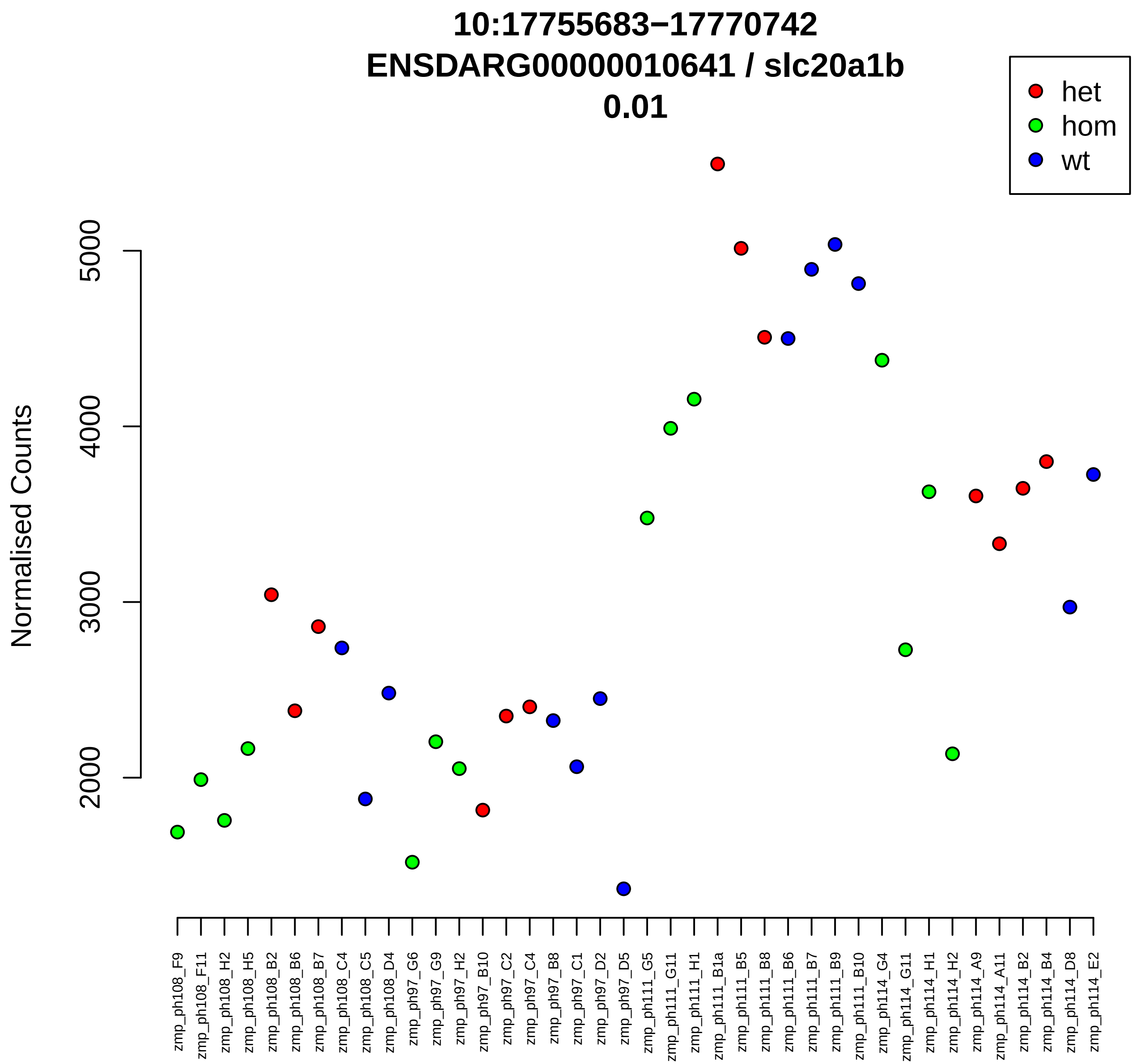

10:17755683−17770742
ENSDARG00000010641 / slc20a1b
0.01
het
hom
wt
Normalised Counts
2000
3000
4000
5000
zmp_ph108_F9
zmp_ph108_F11
zmp_ph108_H2
zmp_ph108_H5
zmp_ph108_B2
zmp_ph108_B6
zmp_ph108_B7
zmp_ph108_C4
zmp_ph108_C5
zmp_ph108_D4
zmp_ph97_G6
zmp_ph97_G9
zmp_ph97_H2
zmp_ph97_B10
zmp_ph97_C2
zmp_ph97_C4
zmp_ph97_B8
zmp_ph97_C1
zmp_ph97_D2
zmp_ph97_D5
zmp_ph111_G5
zmp_ph111_G11
zmp_ph111_H1
zmp_ph111_B1a
zmp_ph111_B5
zmp_ph111_B8
zmp_ph111_B6
zmp_ph111_B7
zmp_ph111_B9
zmp_ph111_B10
zmp_ph114_G4
zmp_ph114_G11
zmp_ph114_H1
zmp_ph114_H2
zmp_ph114_A9
zmp_ph114_A11
zmp_ph114_B2
zmp_ph114_B4
zmp_ph114_D8
zmp_ph114_E2

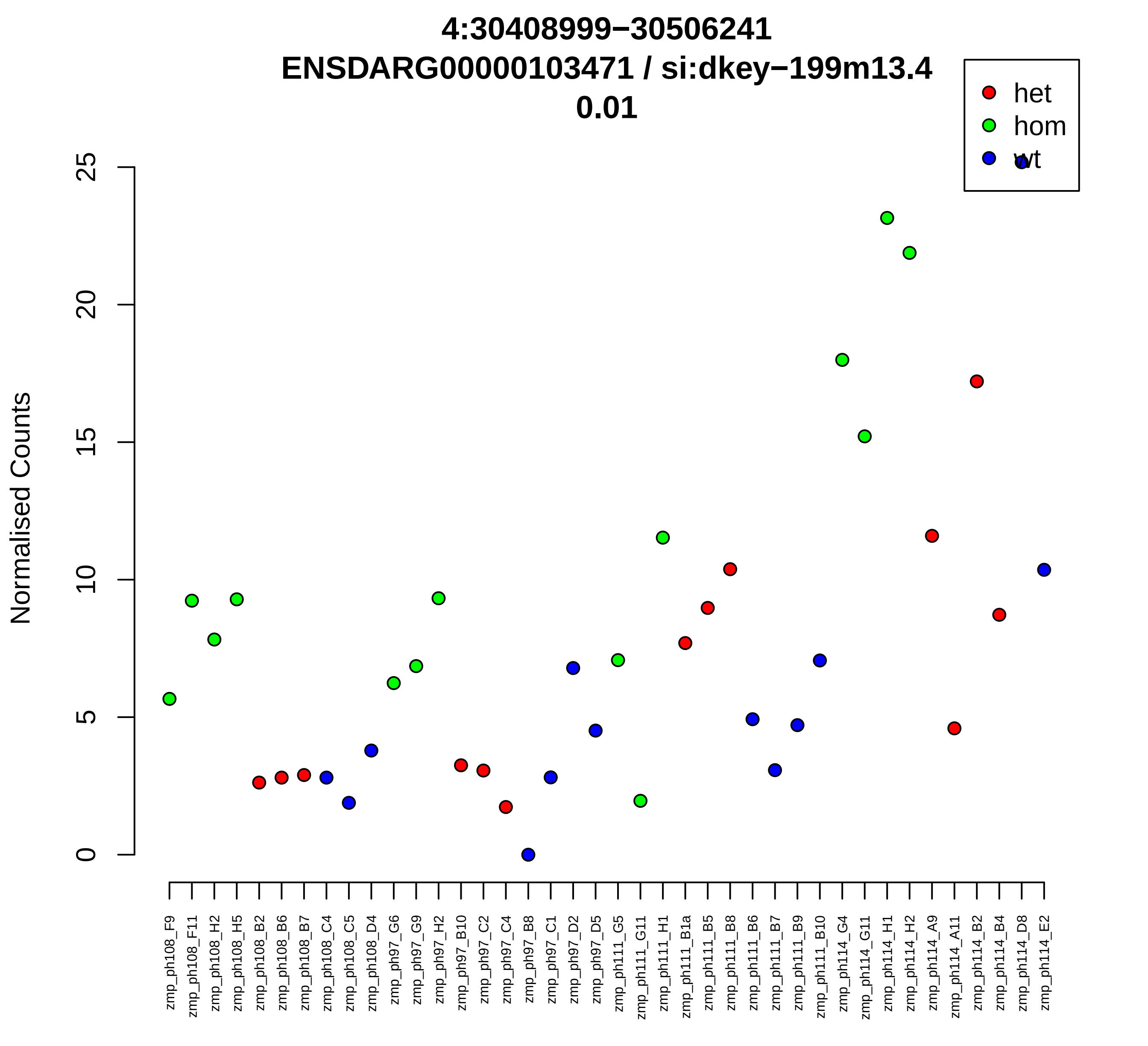
4:30408999−30506241
ENSDARG00000103471 / si:dkey−199m13.4
0.01
het
hom
wt
Normalised Counts
0
5
10
15
20
25
zmp_ph108_F9
zmp_ph108_F11
zmp_ph108_H2
zmp_ph108_H5
zmp_ph108_B2
zmp_ph108_B6
zmp_ph108_B7
zmp_ph108_C4
zmp_ph108_C5
zmp_ph108_D4
zmp_ph97_G6
zmp_ph97_G9
zmp_ph97_H2
zmp_ph97_B10
zmp_ph97_C2
zmp_ph97_C4
zmp_ph97_B8
zmp_ph97_C1
zmp_ph97_D2
zmp_ph97_D5
zmp_ph111_G5
zmp_ph111_G11
zmp_ph111_H1
zmp_ph111_B1a
zmp_ph111_B5
zmp_ph111_B8
zmp_ph111_B6
zmp_ph111_B7
zmp_ph111_B9
zmp_ph111_B10
zmp_ph114_G4
zmp_ph114_G11
zmp_ph114_H1
zmp_ph114_H2
zmp_ph114_A9
zmp_ph114_A11
zmp_ph114_B2
zmp_ph114_B4
zmp_ph114_D8
zmp_ph114_E2

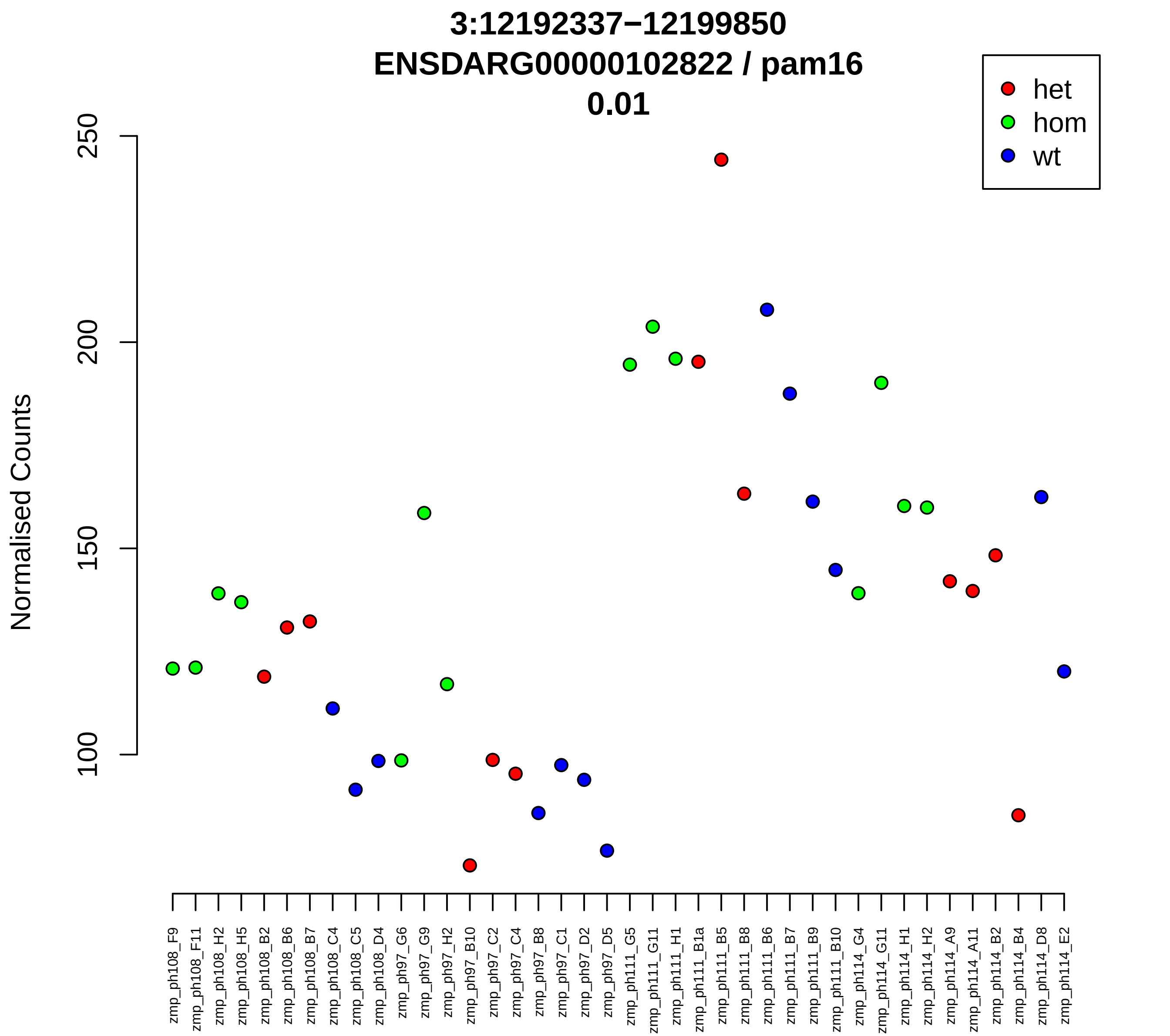

3:12192337−12199850
ENSDARG00000102822 / pam16
0.01
het
hom
wt
Normalised Counts
100
150
200
250
zmp_ph108_F9
zmp_ph108_F11
zmp_ph108_H2
zmp_ph108_H5
zmp_ph108_B2
zmp_ph108_B6
zmp_ph108_B7
zmp_ph108_C4
zmp_ph108_C5
zmp_ph108_D4
zmp_ph97_G6
zmp_ph97_G9
zmp_ph97_H2
zmp_ph97_B10
zmp_ph97_C2
zmp_ph97_C4
zmp_ph97_B8
zmp_ph97_C1
zmp_ph97_D2
zmp_ph97_D5
zmp_ph111_G5
zmp_ph111_G11
zmp_ph111_H1
zmp_ph111_B1a
zmp_ph111_B5
zmp_ph111_B8
zmp_ph111_B6
zmp_ph111_B7
zmp_ph111_B9
zmp_ph111_B10
zmp_ph114_G4
zmp_ph114_G11
zmp_ph114_H1
zmp_ph114_H2
zmp_ph114_A9
zmp_ph114_A11
zmp_ph114_B2
zmp_ph114_B4
zmp_ph114_D8
zmp_ph114_E2

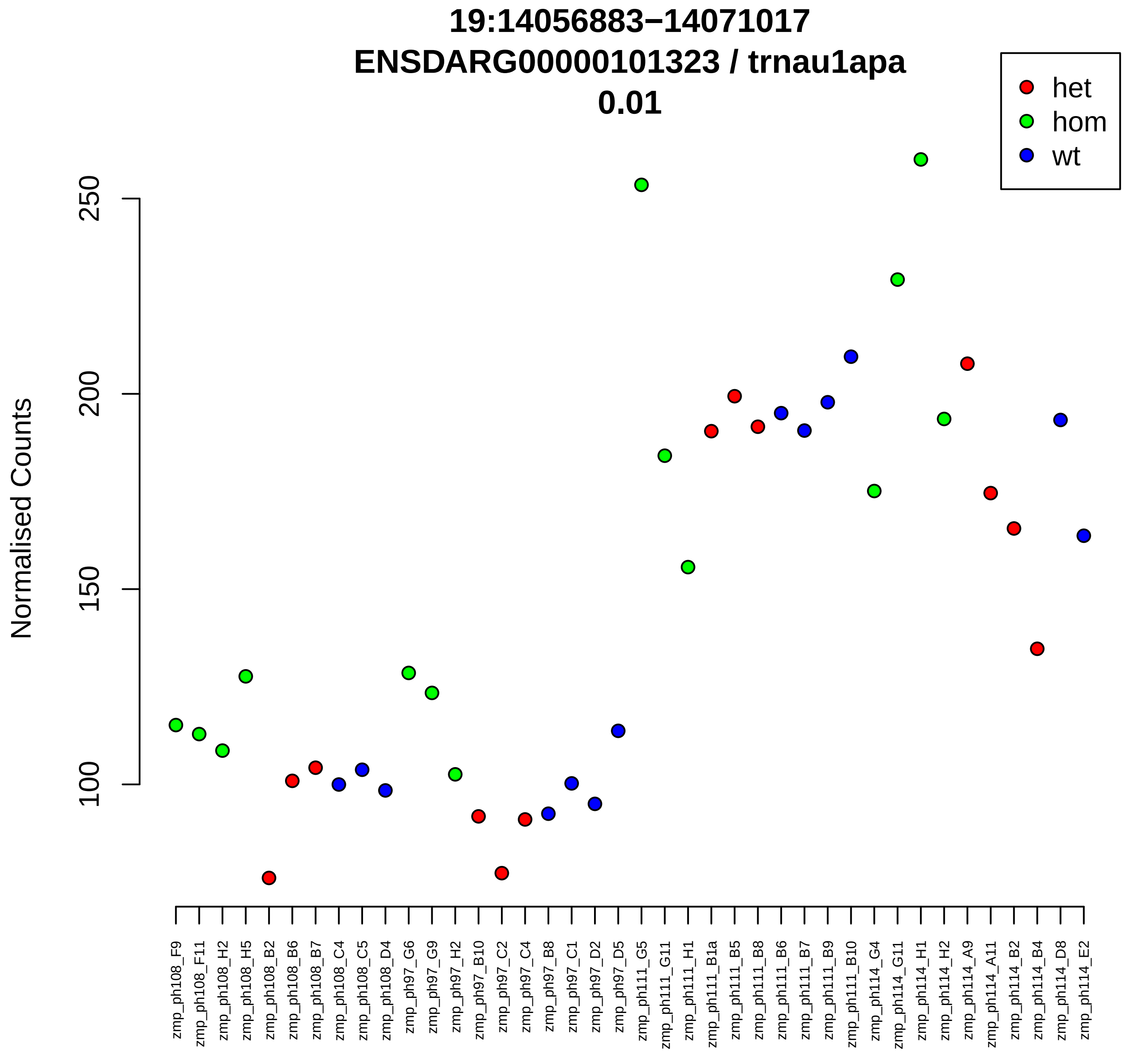
19:14056883−14071017
ENSDARG00000101323 / trnau1apa
0.01
het
hom
wt
Normalised Counts
100
150
200
250
zmp_ph108_F9
zmp_ph108_F11
zmp_ph108_H2
zmp_ph108_H5
zmp_ph108_B2
zmp_ph108_B6
zmp_ph108_B7
zmp_ph108_C4
zmp_ph108_C5
zmp_ph108_D4
zmp_ph97_G6
zmp_ph97_G9
zmp_ph97_H2
zmp_ph97_B10
zmp_ph97_C2
zmp_ph97_C4
zmp_ph97_B8
zmp_ph97_C1
zmp_ph97_D2
zmp_ph97_D5
zmp_ph111_G5
zmp_ph111_G11
zmp_ph111_H1
zmp_ph111_B1a
zmp_ph111_B5
zmp_ph111_B8
zmp_ph111_B6
zmp_ph111_B7
zmp_ph111_B9
zmp_ph111_B10
zmp_ph114_G4
zmp_ph114_G11
zmp_ph114_H1
zmp_ph114_H2
zmp_ph114_A9
zmp_ph114_A11
zmp_ph114_B2
zmp_ph114_B4
zmp_ph114_D8
zmp_ph114_E2

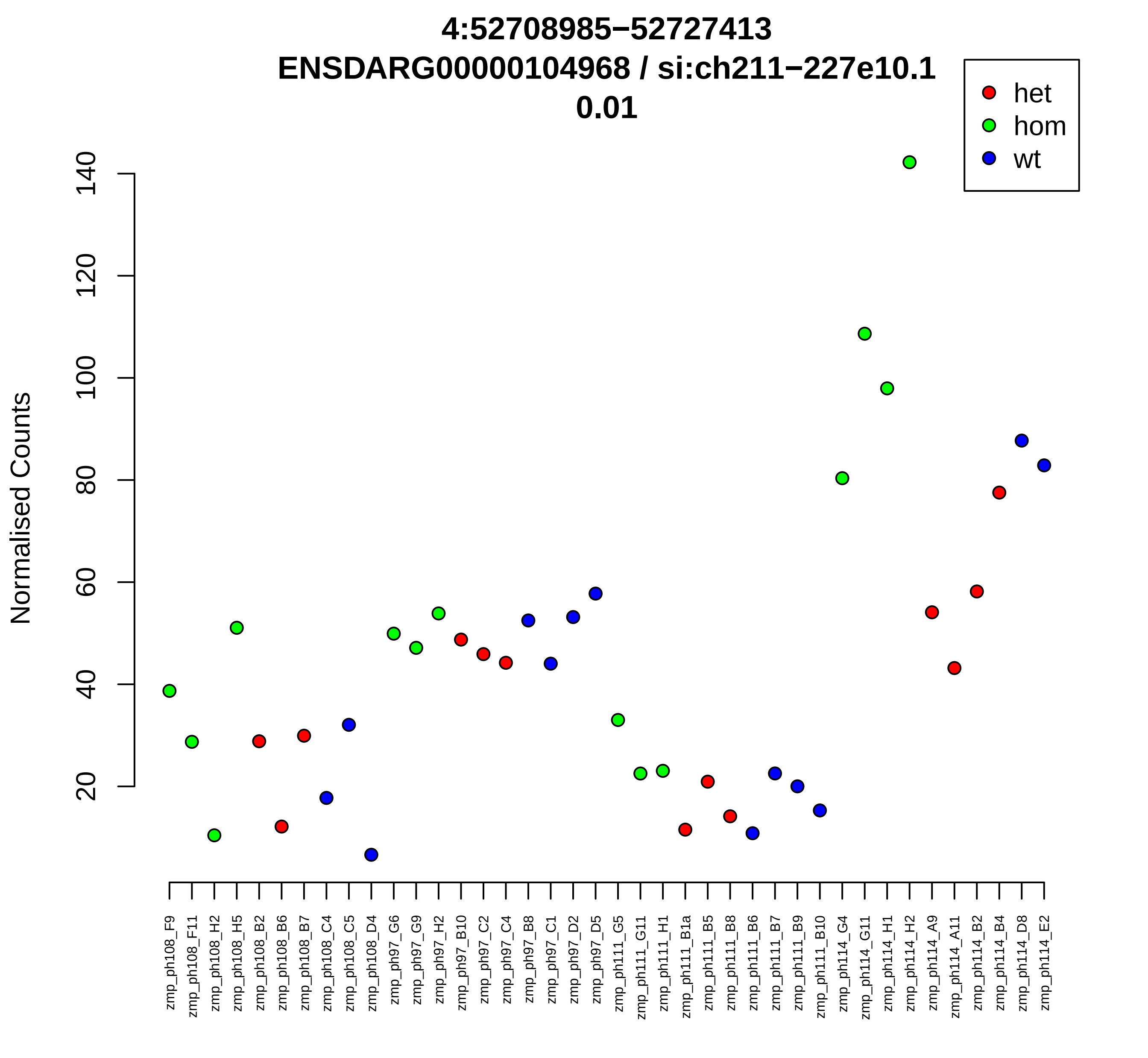
4:52708985−52727413
ENSDARG00000104968 / si:ch211−227e10.1
0.01
het
hom
wt
Normalised Counts
20
40
60
80
100
120
140
zmp_ph108_F9
zmp_ph108_F11
zmp_ph108_H2
zmp_ph108_H5
zmp_ph108_B2
zmp_ph108_B6
zmp_ph108_B7
zmp_ph108_C4
zmp_ph108_C5
zmp_ph108_D4
zmp_ph97_G6
zmp_ph97_G9
zmp_ph97_H2
zmp_ph97_B10
zmp_ph97_C2
zmp_ph97_C4
zmp_ph97_B8
zmp_ph97_C1
zmp_ph97_D2
zmp_ph97_D5
zmp_ph111_G5
zmp_ph111_G11
zmp_ph111_H1
zmp_ph111_B1a
zmp_ph111_B5
zmp_ph111_B8
zmp_ph111_B6
zmp_ph111_B7
zmp_ph111_B9
zmp_ph111_B10
zmp_ph114_G4
zmp_ph114_G11
zmp_ph114_H1
zmp_ph114_H2
zmp_ph114_A9
zmp_ph114_A11
zmp_ph114_B2
zmp_ph114_B4
zmp_ph114_D8
zmp_ph114_E2

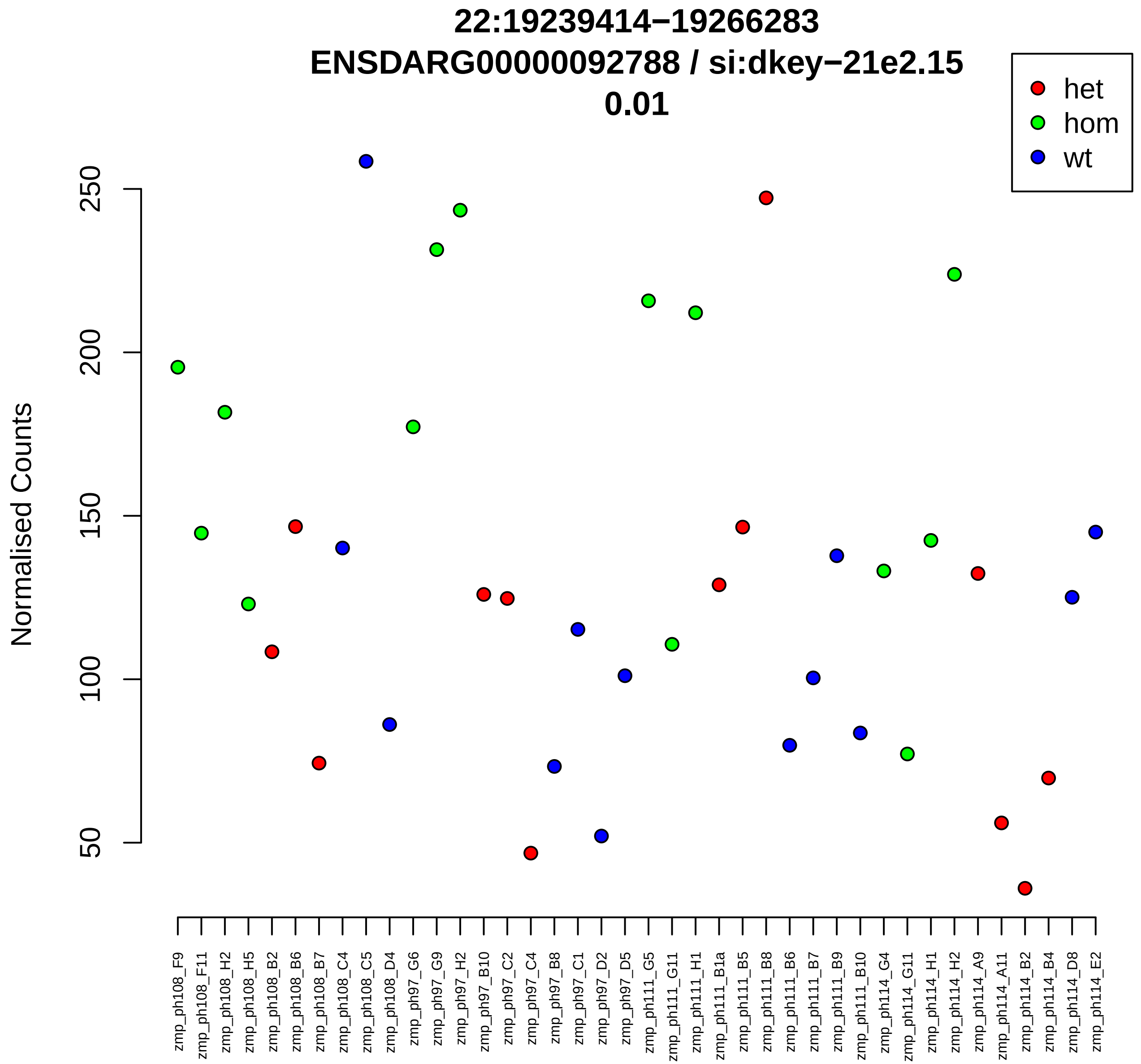
22:19239414−19266283
ENSDARG00000092788 / si:dkey−21e2.15
0.01
het
hom
wt
Normalised Counts
50
100
150
200
250
zmp_ph108_F9
zmp_ph108_F11
zmp_ph108_H2
zmp_ph108_H5
zmp_ph108_B2
zmp_ph108_B6
zmp_ph108_B7
zmp_ph108_C4
zmp_ph108_C5
zmp_ph108_D4
zmp_ph97_G6
zmp_ph97_G9
zmp_ph97_H2
zmp_ph97_B10
zmp_ph97_C2
zmp_ph97_C4
zmp_ph97_B8
zmp_ph97_C1
zmp_ph97_D2
zmp_ph97_D5
zmp_ph111_G5
zmp_ph111_G11
zmp_ph111_H1
zmp_ph111_B1a
zmp_ph111_B5
zmp_ph111_B8
zmp_ph111_B6
zmp_ph111_B7
zmp_ph111_B9
zmp_ph111_B10
zmp_ph114_G4
zmp_ph114_G11
zmp_ph114_H1
zmp_ph114_H2
zmp_ph114_A9
zmp_ph114_A11
zmp_ph114_B2
zmp_ph114_B4
zmp_ph114_D8
zmp_ph114_E2

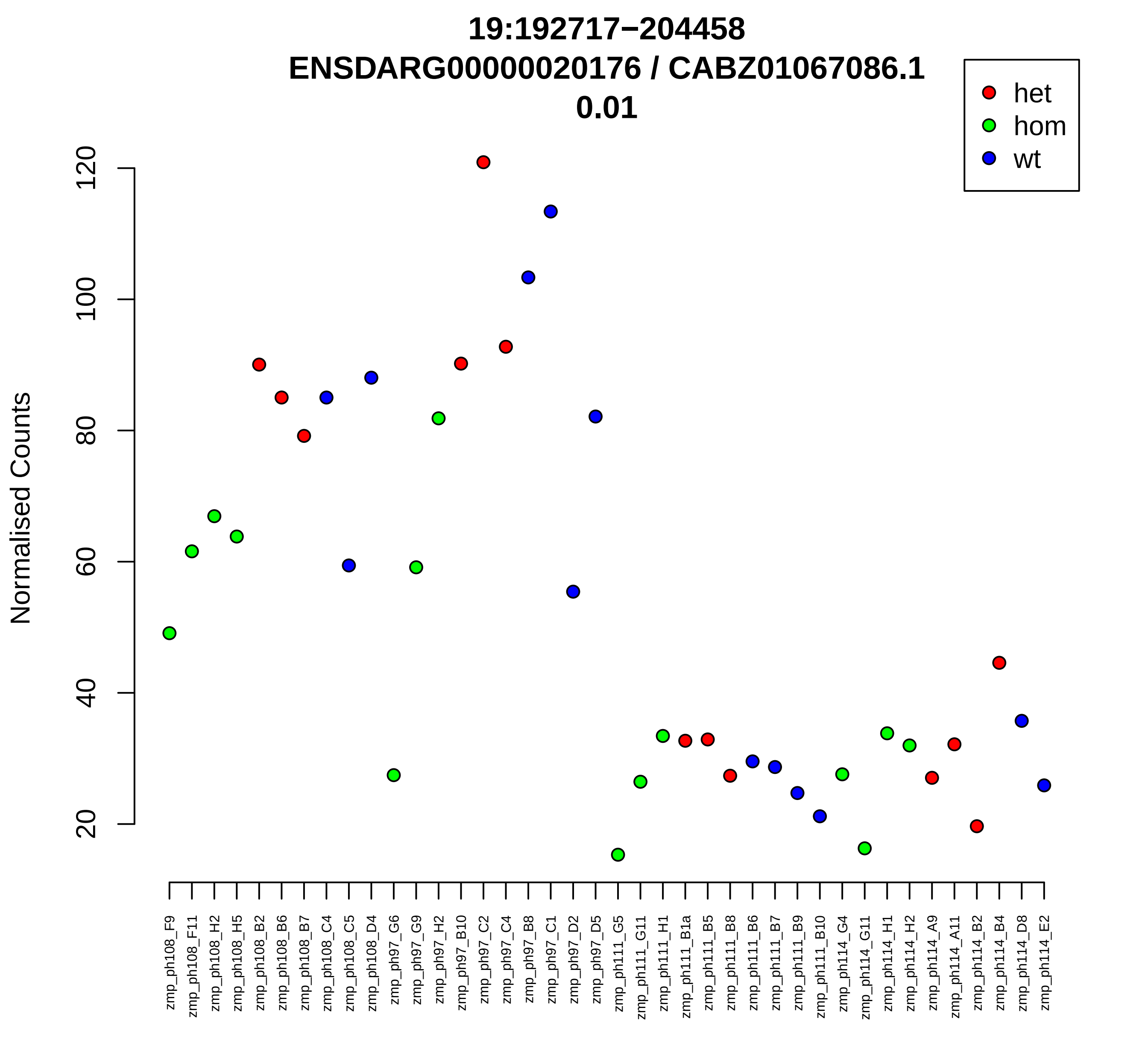

19:192717−204458
ENSDARG00000020176 / CABZ01067086.1
0.01
het
hom
wt
Normalised Counts
20
40
60
80
100
120
zmp_ph108_F9
zmp_ph108_F11
zmp_ph108_H2
zmp_ph108_H5
zmp_ph108_B2
zmp_ph108_B6
zmp_ph108_B7
zmp_ph108_C4
zmp_ph108_C5
zmp_ph108_D4
zmp_ph97_G6
zmp_ph97_G9
zmp_ph97_H2
zmp_ph97_B10
zmp_ph97_C2
zmp_ph97_C4
zmp_ph97_B8
zmp_ph97_C1
zmp_ph97_D2
zmp_ph97_D5
zmp_ph111_G5
zmp_ph111_G11
zmp_ph111_H1
zmp_ph111_B1a
zmp_ph111_B5
zmp_ph111_B8
zmp_ph111_B6
zmp_ph111_B7
zmp_ph111_B9
zmp_ph111_B10
zmp_ph114_G4
zmp_ph114_G11
zmp_ph114_H1
zmp_ph114_H2
zmp_ph114_A9
zmp_ph114_A11
zmp_ph114_B2
zmp_ph114_B4
zmp_ph114_D8
zmp_ph114_E2

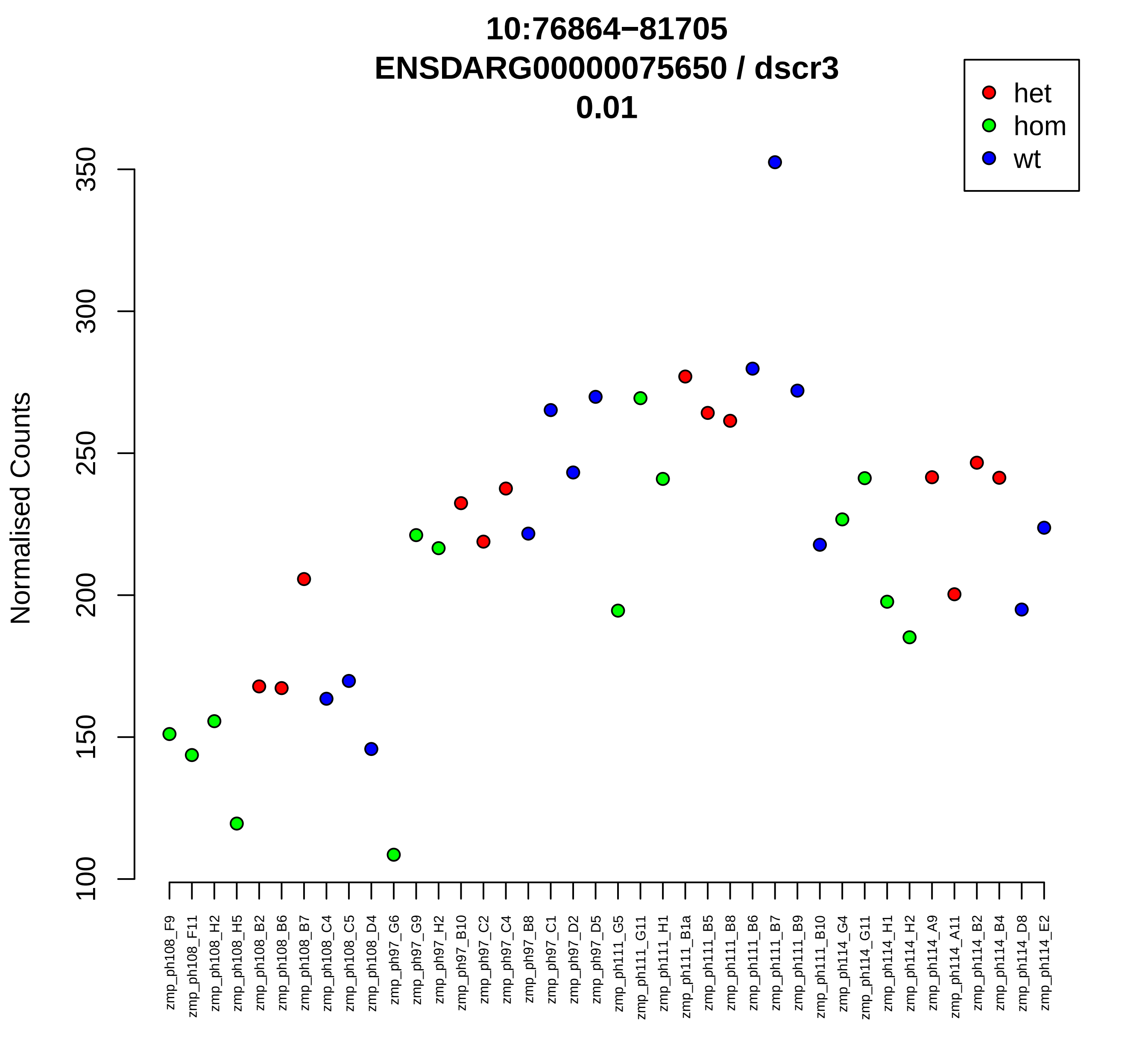

10:76864−81705
ENSDARG00000075650 / dscr3
0.01
het
hom
wt
Normalised Counts
100
150
200
250
300
350
zmp_ph108_F9
zmp_ph108_F11
zmp_ph108_H2
zmp_ph108_H5
zmp_ph108_B2
zmp_ph108_B6
zmp_ph108_B7
zmp_ph108_C4
zmp_ph108_C5
zmp_ph108_D4
zmp_ph97_G6
zmp_ph97_G9
zmp_ph97_H2
zmp_ph97_B10
zmp_ph97_C2
zmp_ph97_C4
zmp_ph97_B8
zmp_ph97_C1
zmp_ph97_D2
zmp_ph97_D5
zmp_ph111_G5
zmp_ph111_G11
zmp_ph111_H1
zmp_ph111_B1a
zmp_ph111_B5
zmp_ph111_B8
zmp_ph111_B6
zmp_ph111_B7
zmp_ph111_B9
zmp_ph111_B10
zmp_ph114_G4
zmp_ph114_G11
zmp_ph114_H1
zmp_ph114_H2
zmp_ph114_A9
zmp_ph114_A11
zmp_ph114_B2
zmp_ph114_B4
zmp_ph114_D8
zmp_ph114_E2

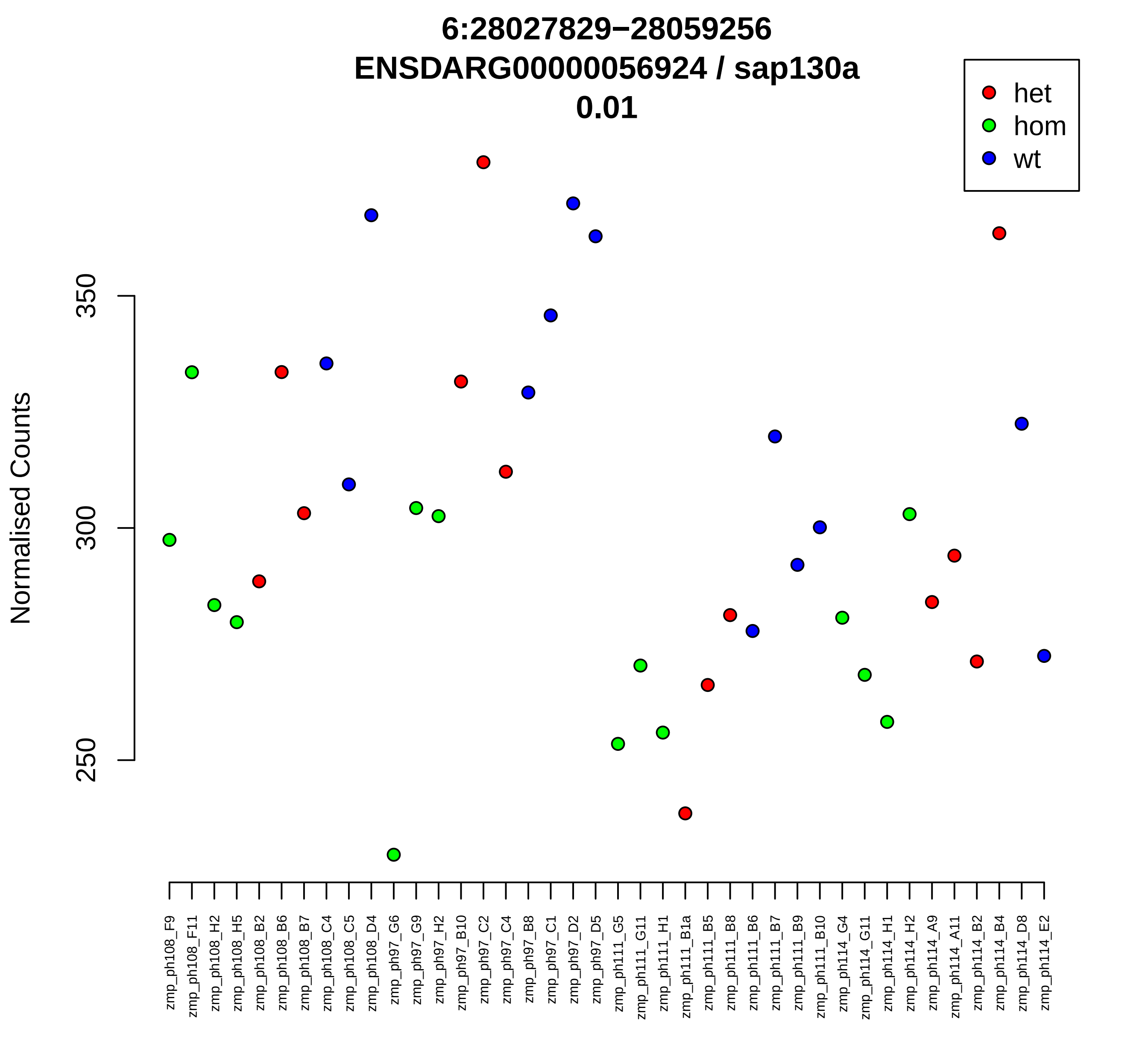

6:28027829−28059256
ENSDARG00000056924 / sap130a
0.01
het
hom
wt
Normalised Counts
250
300
350
zmp_ph108_F9
zmp_ph108_F11
zmp_ph108_H2
zmp_ph108_H5
zmp_ph108_B2
zmp_ph108_B6
zmp_ph108_B7
zmp_ph108_C4
zmp_ph108_C5
zmp_ph108_D4
zmp_ph97_G6
zmp_ph97_G9
zmp_ph97_H2
zmp_ph97_B10
zmp_ph97_C2
zmp_ph97_C4
zmp_ph97_B8
zmp_ph97_C1
zmp_ph97_D2
zmp_ph97_D5
zmp_ph111_G5
zmp_ph111_G11
zmp_ph111_H1
zmp_ph111_B1a
zmp_ph111_B5
zmp_ph111_B8
zmp_ph111_B6
zmp_ph111_B7
zmp_ph111_B9
zmp_ph111_B10
zmp_ph114_G4
zmp_ph114_G11
zmp_ph114_H1
zmp_ph114_H2
zmp_ph114_A9
zmp_ph114_A11
zmp_ph114_B2
zmp_ph114_B4
zmp_ph114_D8
zmp_ph114_E2

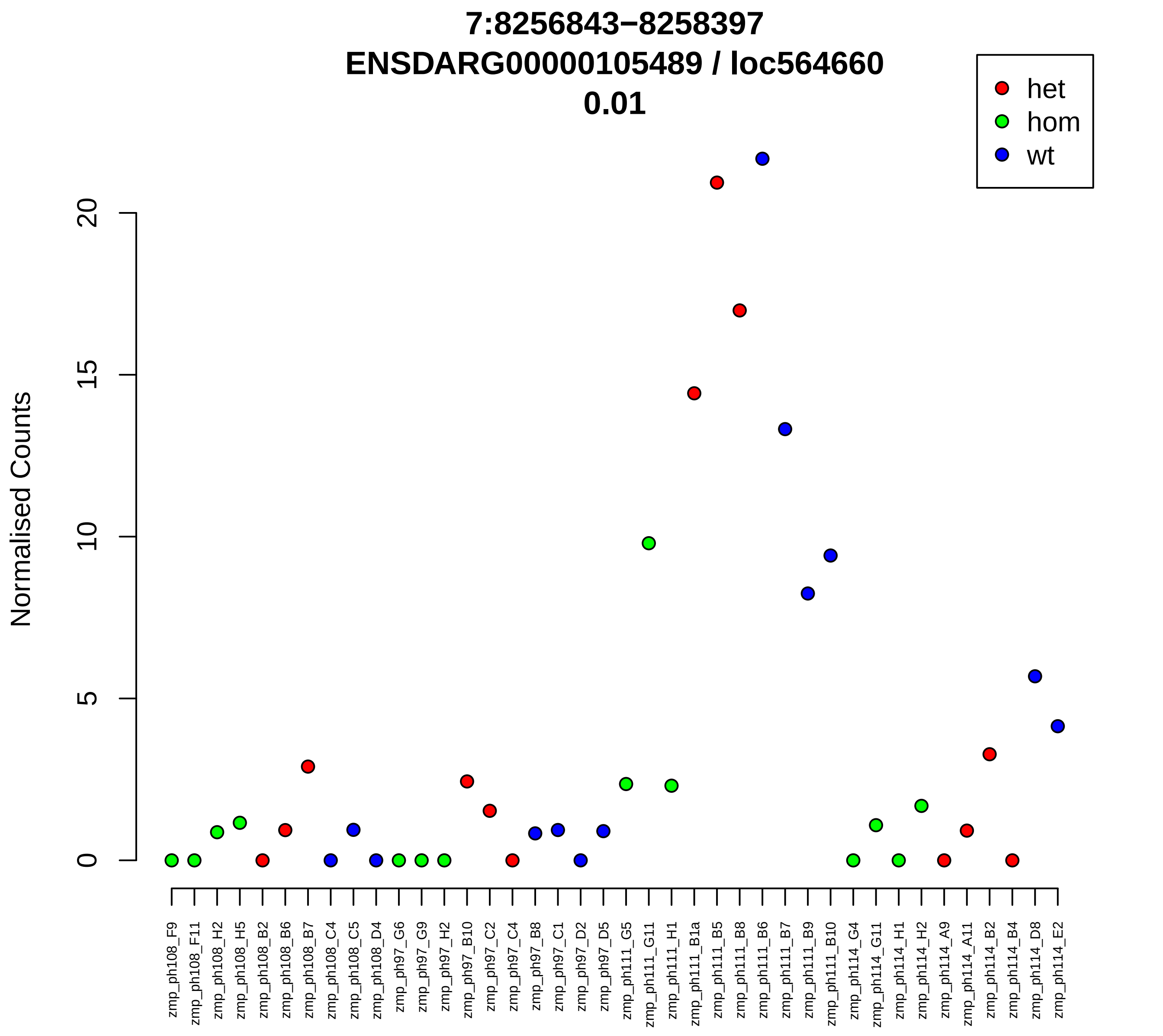

7:8256843−8258397
ENSDARG00000105489 / loc564660
0.01
het
hom
wt
Normalised Counts
0
5
10
15
20
zmp_ph108_F9
zmp_ph108_F11
zmp_ph108_H2
zmp_ph108_H5
zmp_ph108_B2
zmp_ph108_B6
zmp_ph108_B7
zmp_ph108_C4
zmp_ph108_C5
zmp_ph108_D4
zmp_ph97_G6
zmp_ph97_G9
zmp_ph97_H2
zmp_ph97_B10
zmp_ph97_C2
zmp_ph97_C4
zmp_ph97_B8
zmp_ph97_C1
zmp_ph97_D2
zmp_ph97_D5
zmp_ph111_G5
zmp_ph111_G11
zmp_ph111_H1
zmp_ph111_B1a
zmp_ph111_B5
zmp_ph111_B8
zmp_ph111_B6
zmp_ph111_B7
zmp_ph111_B9
zmp_ph111_B10
zmp_ph114_G4
zmp_ph114_G11
zmp_ph114_H1
zmp_ph114_H2
zmp_ph114_A9
zmp_ph114_A11
zmp_ph114_B2
zmp_ph114_B4
zmp_ph114_D8
zmp_ph114_E2

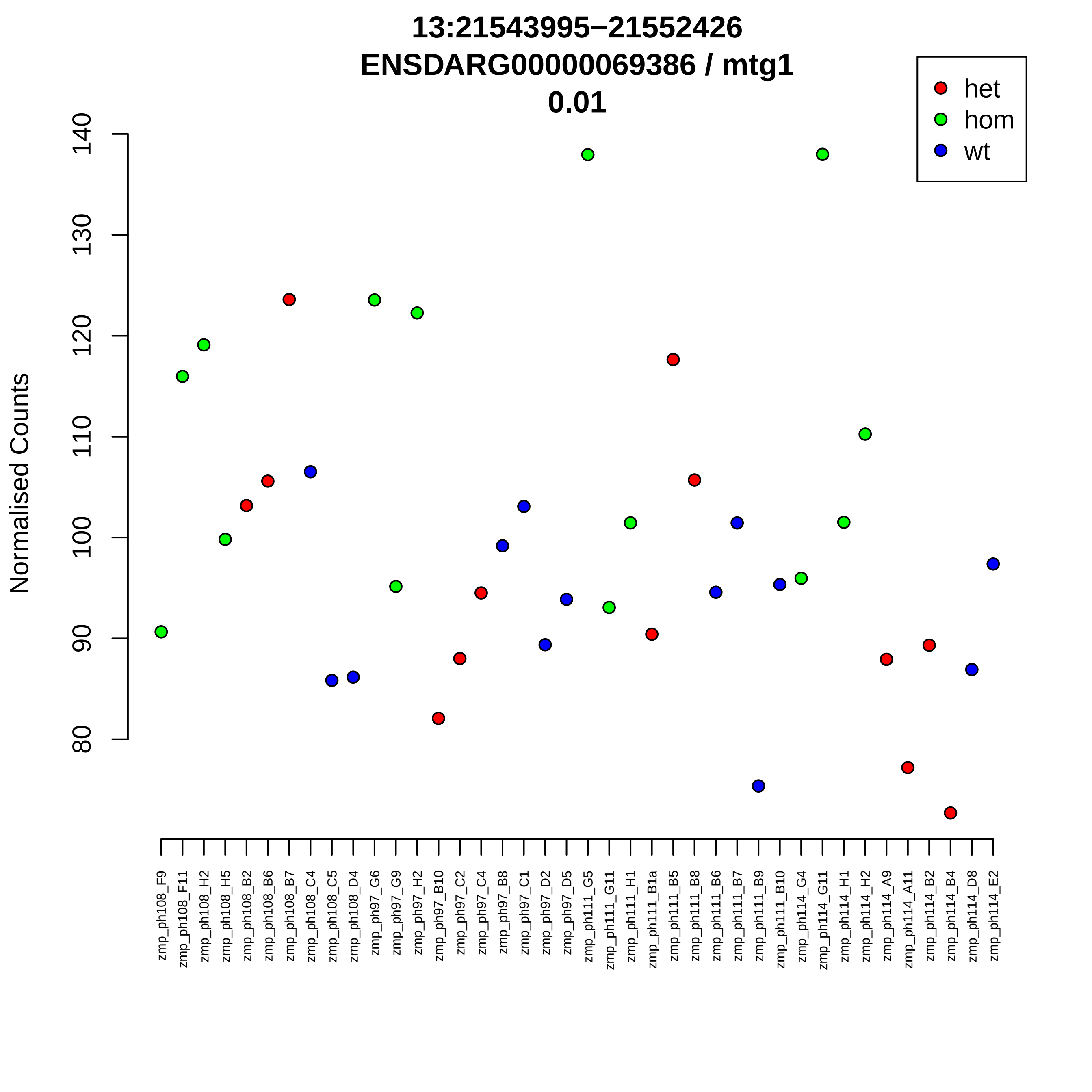

13:21543995−21552426
ENSDARG00000069386 / mtg1
0.01
Normalised Counts
140
130
120
110
100
90
80
het
hom
wt
zmp_ph108_F9
zmp_ph108_F11
zmp_ph108_H2
zmp_ph108_H5
zmp_ph108_B2
zmp_ph108_B6
zmp_ph108_B7
zmp_ph108_C4
zmp_ph108_C5
zmp_ph108_D4
zmp_ph97_G6
zmp_ph97_G9
zmp_ph97_H2
zmp_ph97_B10
zmp_ph97_C2
zmp_ph97_C4
zmp_ph97_B8
zmp_ph97_C1
zmp_ph97_D2
zmp_ph97_D5
zmp_ph111_G5
zmp_ph111_G11
zmp_ph111_H1
zmp_ph111_B1a
zmp_ph111_B5
zmp_ph111_B8
zmp_ph111_B6
zmp_ph111_B7
zmp_ph111_B9
zmp_ph111_B10
zmp_ph114_G4
zmp_ph114_G11
zmp_ph114_H1
zmp_ph114_H2
zmp_ph114_A9
zmp_ph114_A11
zmp_ph114_B2
zmp_ph114_B4
zmp_ph114_D8
zmp_ph114_E2

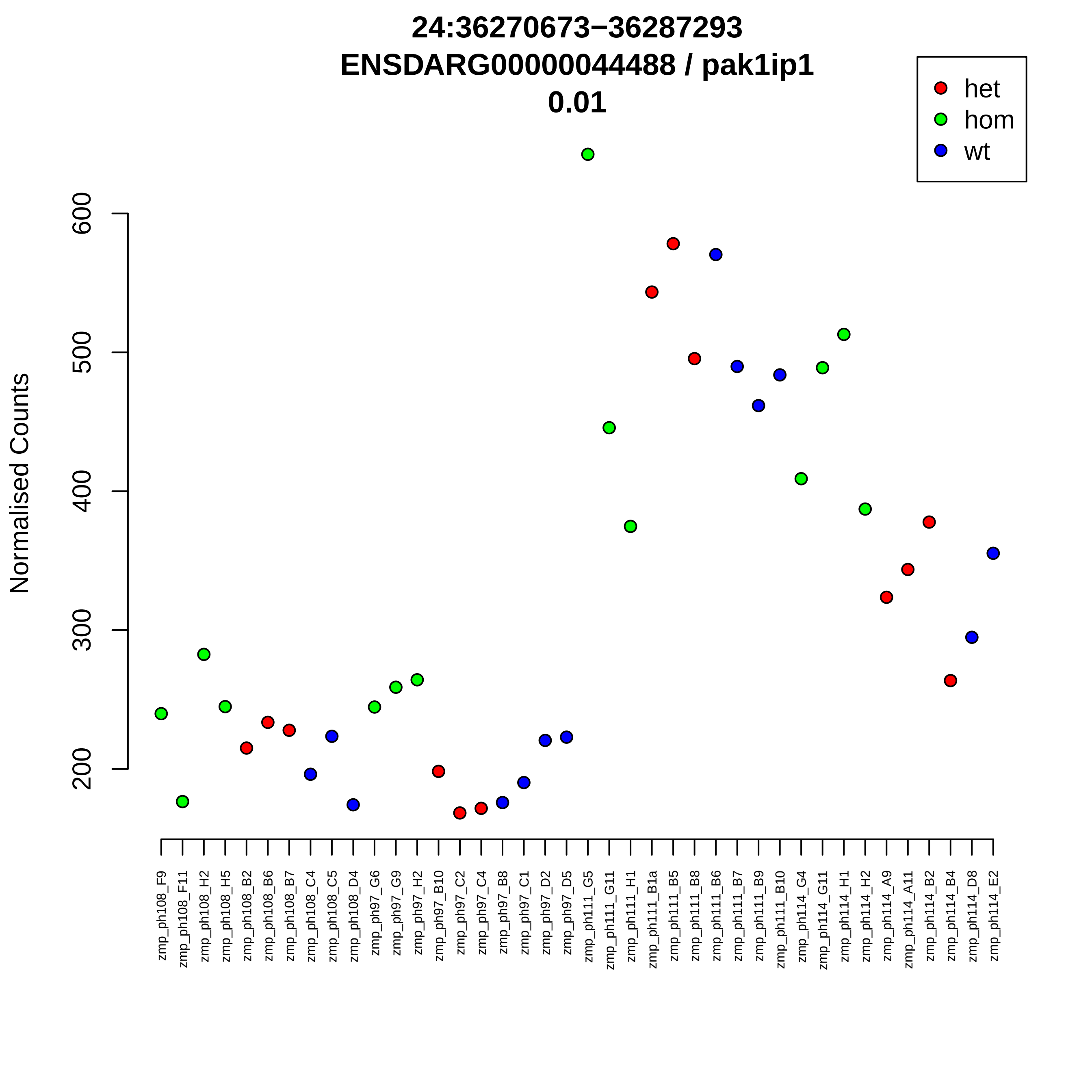
24:36270673−36287293
ENSDARG00000044488 / pak1ip1
0.01
het
hom
wt
Normalised Counts
200
300
400
500
600
zmp_ph108_F9
zmp_ph108_F11
zmp_ph108_H2
zmp_ph108_H5
zmp_ph108_B2
zmp_ph108_B6
zmp_ph108_B7
zmp_ph108_C4
zmp_ph108_C5
zmp_ph108_D4
zmp_ph97_G6
zmp_ph97_G9
zmp_ph97_H2
zmp_ph97_B10
zmp_ph97_C2
zmp_ph97_C4
zmp_ph97_B8
zmp_ph97_C1
zmp_ph97_D2
zmp_ph97_D5
zmp_ph111_G5
zmp_ph111_G11
zmp_ph111_H1
zmp_ph111_B1a
zmp_ph111_B5
zmp_ph111_B8
zmp_ph111_B6
zmp_ph111_B7
zmp_ph111_B9
zmp_ph111_B10
zmp_ph114_G4
zmp_ph114_G11
zmp_ph114_H1
zmp_ph114_H2
zmp_ph114_A9
zmp_ph114_A11
zmp_ph114_B2
zmp_ph114_B4
zmp_ph114_D8
zmp_ph114_E2

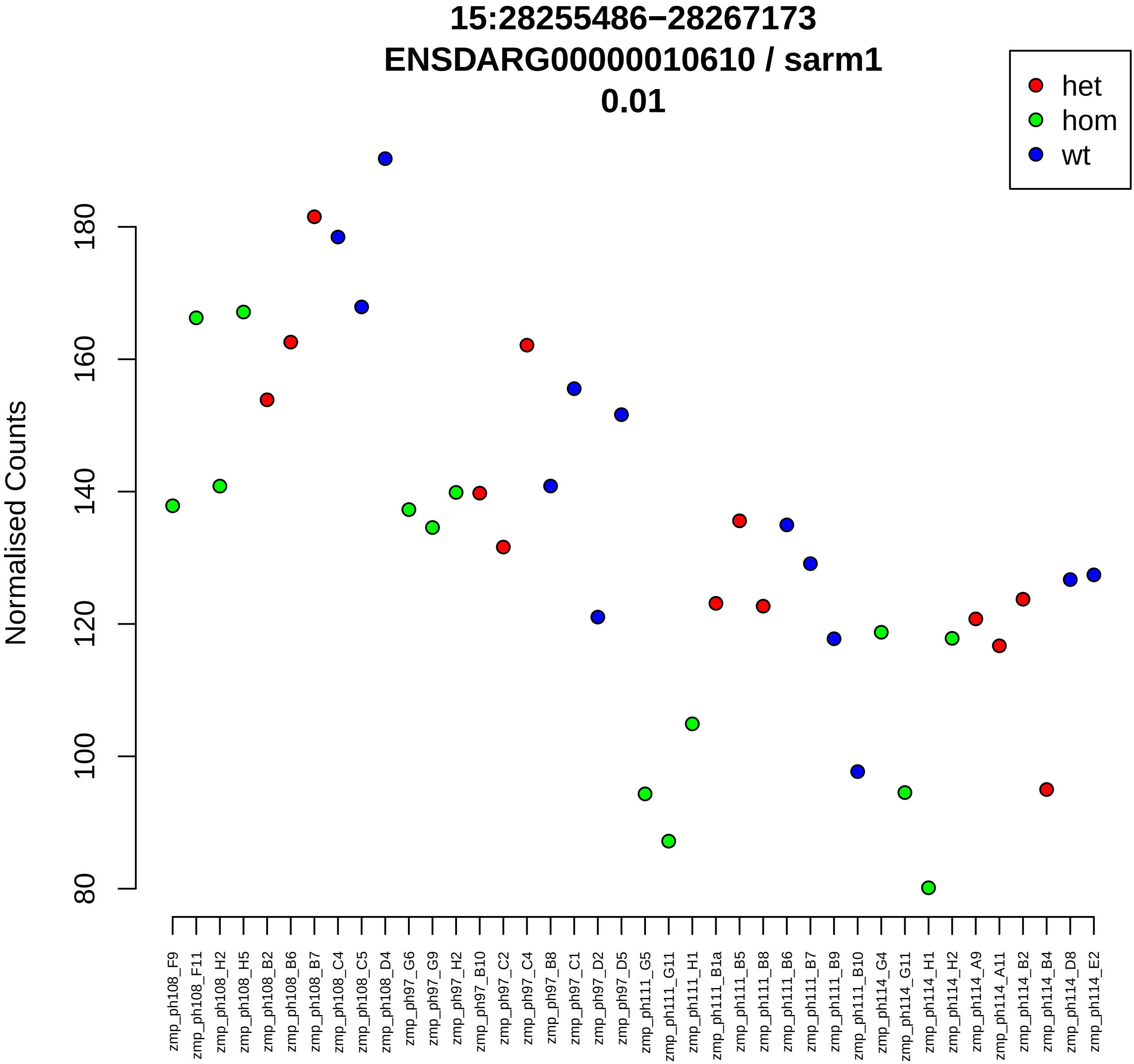

15:28255486−28267173
ENSDARG00000010610 / sarm1
0.01
het
hom
wt
Normalised Counts
80
100
120
140
160
180
zmp_ph108_F9
zmp_ph108_F11
zmp_ph108_H2
zmp_ph108_H5
zmp_ph108_B2
zmp_ph108_B6
zmp_ph108_B7
zmp_ph108_C4
zmp_ph108_C5
zmp_ph108_D4
zmp_ph97_G6
zmp_ph97_G9
zmp_ph97_H2
zmp_ph97_B10
zmp_ph97_C2
zmp_ph97_C4
zmp_ph97_B8
zmp_ph97_C1
zmp_ph97_D2
zmp_ph97_D5
zmp_ph111_G5
zmp_ph111_G11
zmp_ph111_H1
zmp_ph111_B1a
zmp_ph111_B5
zmp_ph111_B8
zmp_ph111_B6
zmp_ph111_B7
zmp_ph111_B9
zmp_ph111_B10
zmp_ph114_G4
zmp_ph114_G11
zmp_ph114_H1
zmp_ph114_H2
zmp_ph114_A9
zmp_ph114_A11
zmp_ph114_B2
zmp_ph114_B4
zmp_ph114_D8
zmp_ph114_E2

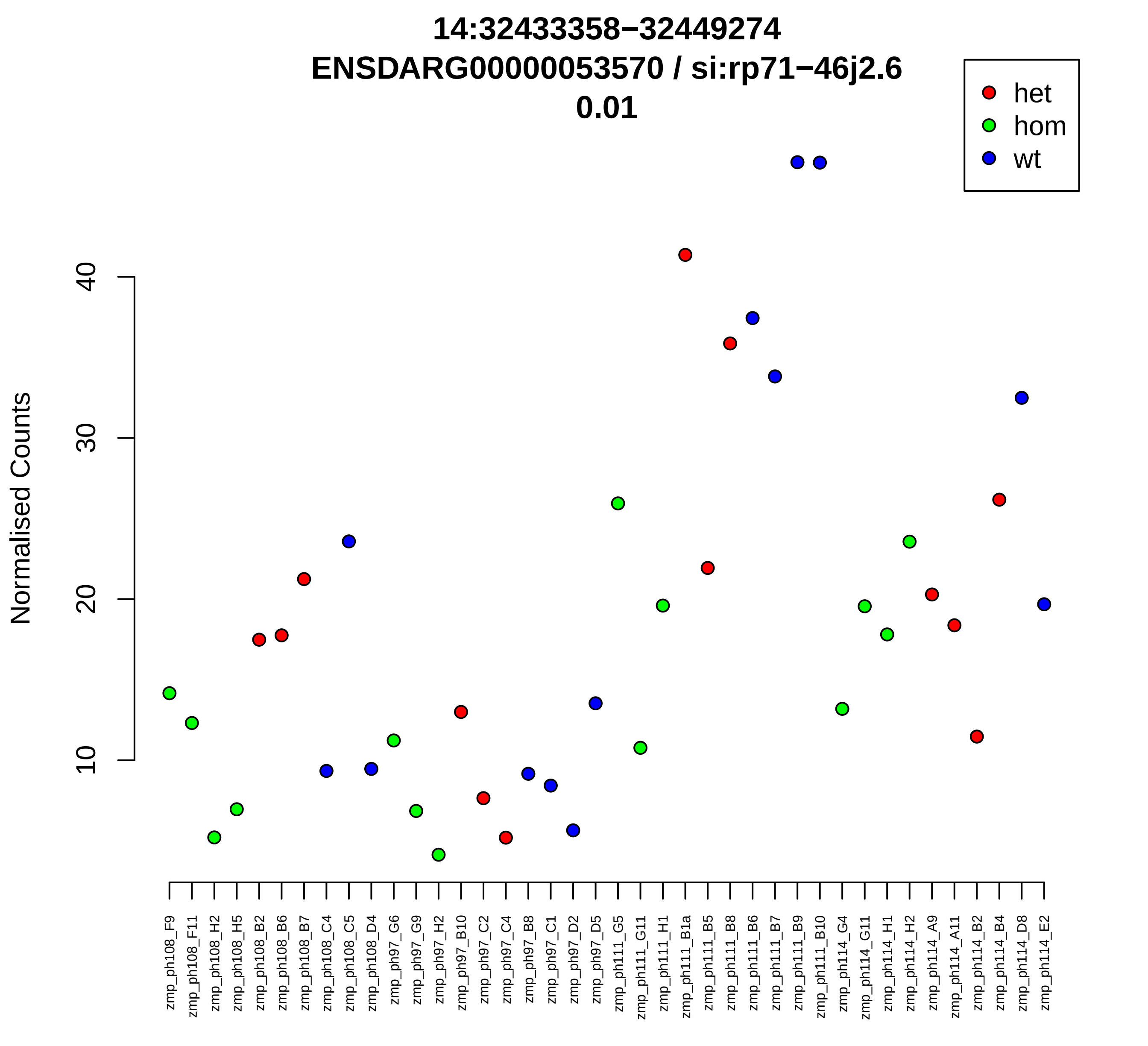
14:32433358−32449274
ENSDARG00000053570 / si:rp71−46j2.6
0.01
het
hom
wt
Normalised Counts
10
20
30
40
zmp_ph108_F9
zmp_ph108_F11
zmp_ph108_H2
zmp_ph108_H5
zmp_ph108_B2
zmp_ph108_B6
zmp_ph108_B7
zmp_ph108_C4
zmp_ph108_C5
zmp_ph108_D4
zmp_ph97_G6
zmp_ph97_G9
zmp_ph97_H2
zmp_ph97_B10
zmp_ph97_C2
zmp_ph97_C4
zmp_ph97_B8
zmp_ph97_C1
zmp_ph97_D2
zmp_ph97_D5
zmp_ph111_G5
zmp_ph111_G11
zmp_ph111_H1
zmp_ph111_B1a
zmp_ph111_B5
zmp_ph111_B8
zmp_ph111_B6
zmp_ph111_B7
zmp_ph111_B9
zmp_ph111_B10
zmp_ph114_G4
zmp_ph114_G11
zmp_ph114_H1
zmp_ph114_H2
zmp_ph114_A9
zmp_ph114_A11
zmp_ph114_B2
zmp_ph114_B4
zmp_ph114_D8
zmp_ph114_E2

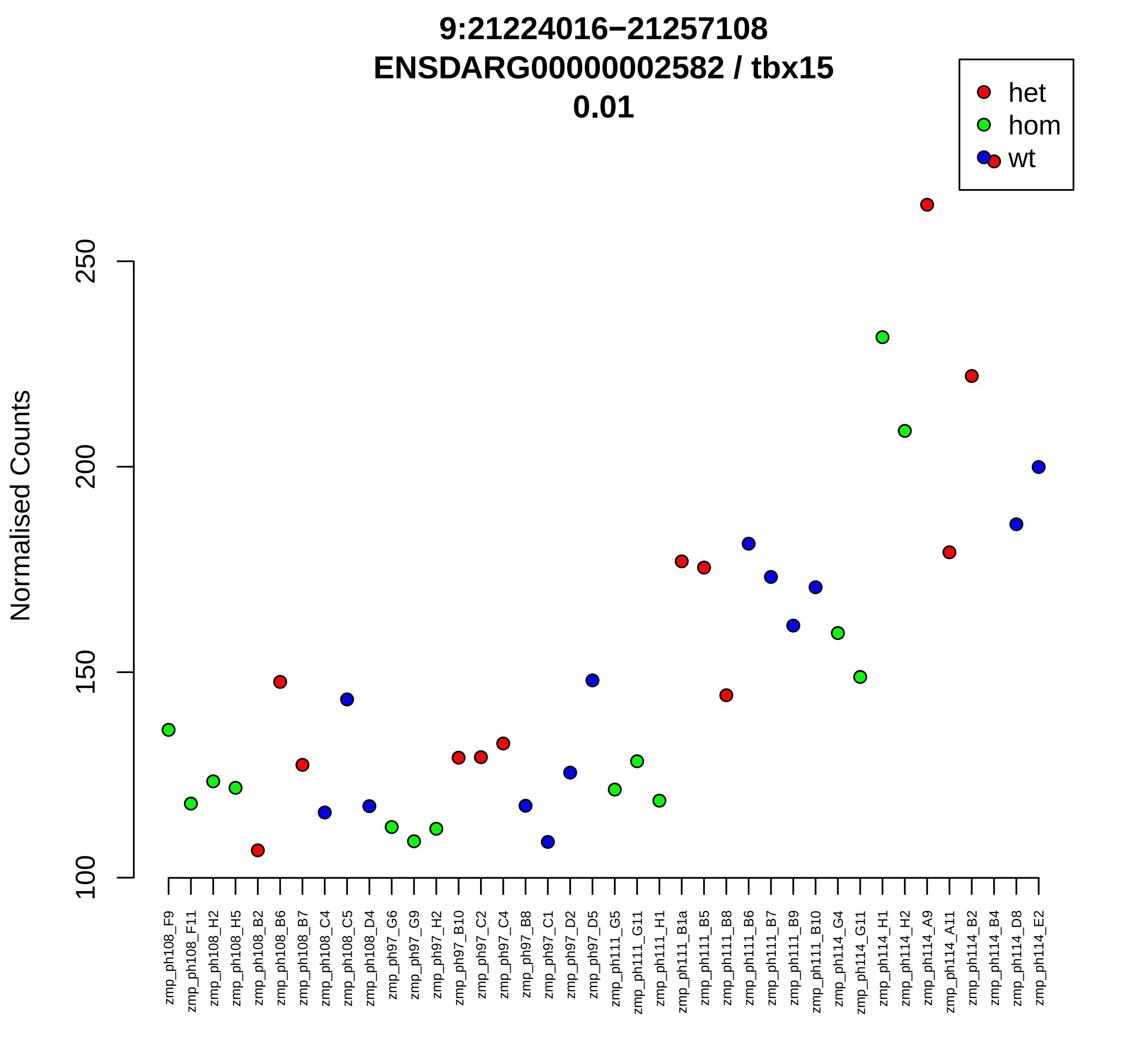
9:21224016−21257108
ENSDARG00000002582 / tbx15
0.01
het
hom
wt
Normalised Counts
100
150
200
250
zmp_ph108_F9
zmp_ph108_F11
zmp_ph108_H2
zmp_ph108_H5
zmp_ph108_B2
zmp_ph108_B6
zmp_ph108_B7
zmp_ph108_C4
zmp_ph108_C5
zmp_ph108_D4
zmp_ph97_G6
zmp_ph97_G9
zmp_ph97_H2
zmp_ph97_B10
zmp_ph97_C2
zmp_ph97_C4
zmp_ph97_B8
zmp_ph97_C1
zmp_ph97_D2
zmp_ph97_D5
zmp_ph111_G5
zmp_ph111_G11
zmp_ph111_H1
zmp_ph111_B1a
zmp_ph111_B5
zmp_ph111_B8
zmp_ph111_B6
zmp_ph111_B7
zmp_ph111_B9
zmp_ph111_B10
zmp_ph114_G4
zmp_ph114_G11
zmp_ph114_H1
zmp_ph114_H2
zmp_ph114_A9
zmp_ph114_A11
zmp_ph114_B2
zmp_ph114_B4
zmp_ph114_D8
zmp_ph114_E2

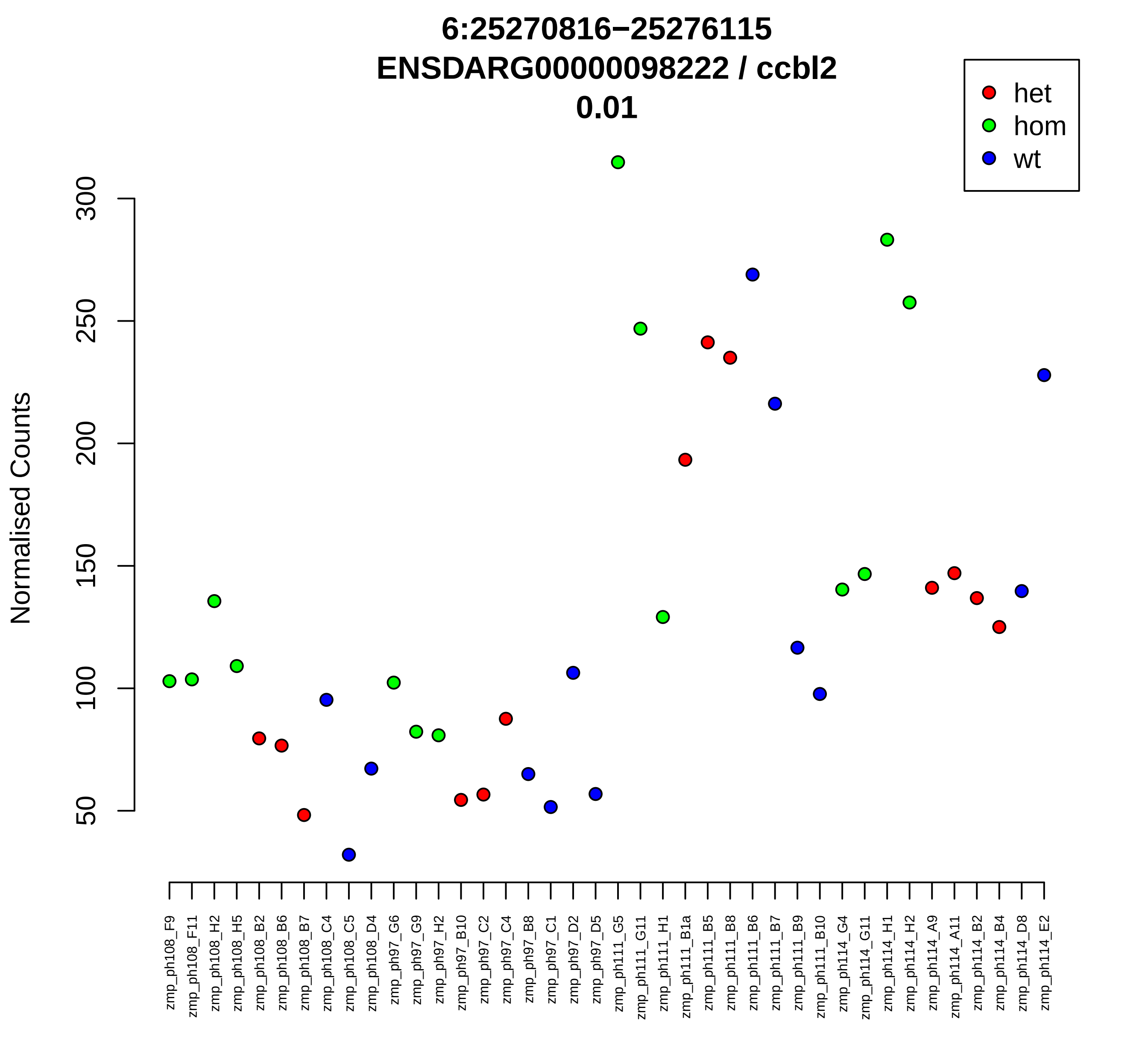
6:25270816−25276115
ENSDARG00000098222 / ccbl2
0.01
het
hom
wt
Normalised Counts
50
100
150
200
250
300
zmp_ph108_F9
zmp_ph108_F11
zmp_ph108_H2
zmp_ph108_H5
zmp_ph108_B2
zmp_ph108_B6
zmp_ph108_B7
zmp_ph108_C4
zmp_ph108_C5
zmp_ph108_D4
zmp_ph97_G6
zmp_ph97_G9
zmp_ph97_H2
zmp_ph97_B10
zmp_ph97_C2
zmp_ph97_C4
zmp_ph97_B8
zmp_ph97_C1
zmp_ph97_D2
zmp_ph97_D5
zmp_ph111_G5
zmp_ph111_G11
zmp_ph111_H1
zmp_ph111_B1a
zmp_ph111_B5
zmp_ph111_B8
zmp_ph111_B6
zmp_ph111_B7
zmp_ph111_B9
zmp_ph111_B10
zmp_ph114_G4
zmp_ph114_G11
zmp_ph114_H1
zmp_ph114_H2
zmp_ph114_A9
zmp_ph114_A11
zmp_ph114_B2
zmp_ph114_B4
zmp_ph114_D8
zmp_ph114_E2

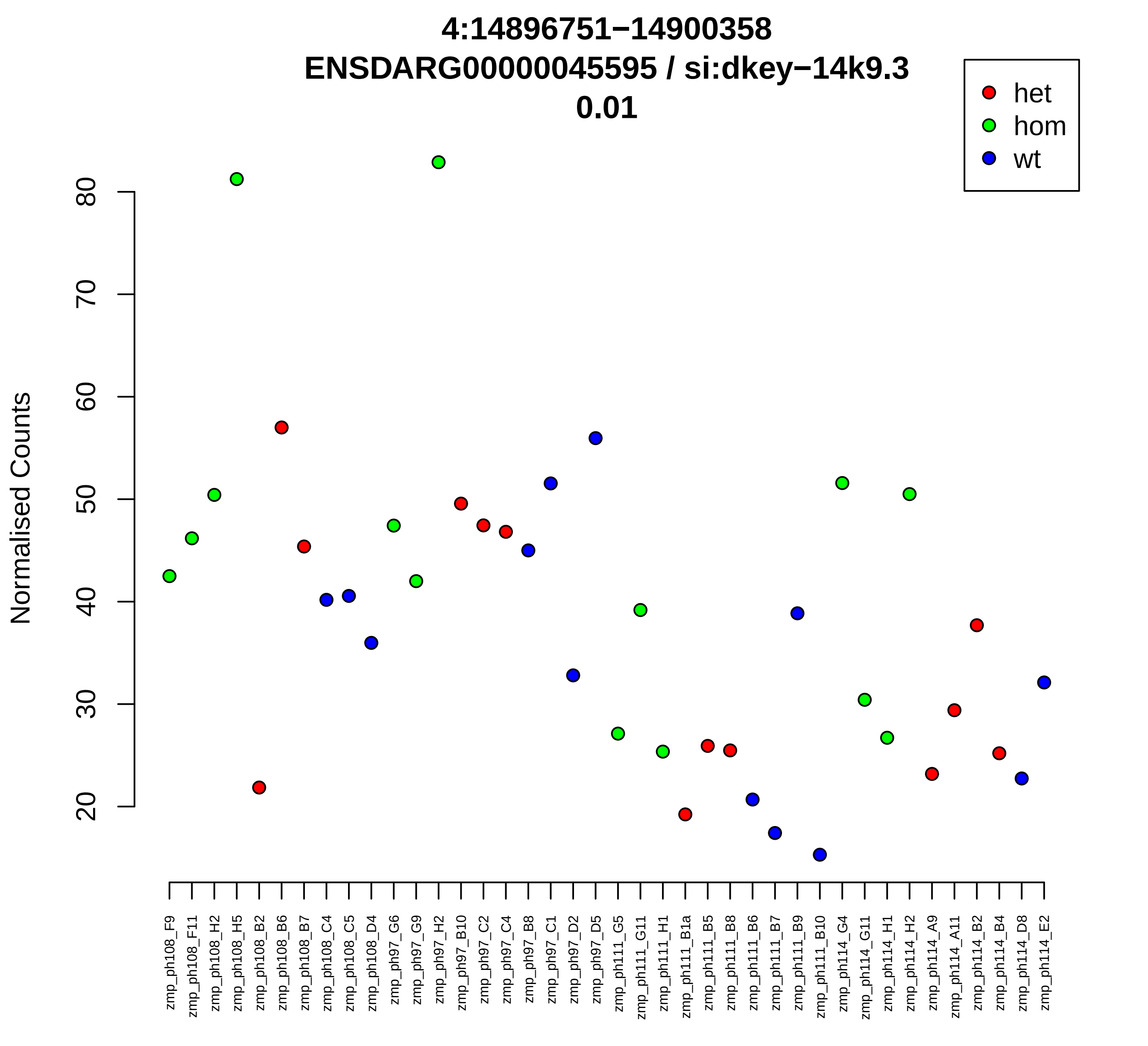
4:14896751−14900358
ENSDARG00000045595 / si:dkey−14k9.3
0.01
het
hom
wt
Normalised Counts
20
30
40
50
60
70
80
zmp_ph108_F9
zmp_ph108_F11
zmp_ph108_H2
zmp_ph108_H5
zmp_ph108_B2
zmp_ph108_B6
zmp_ph108_B7
zmp_ph108_C4
zmp_ph108_C5
zmp_ph108_D4
zmp_ph97_G6
zmp_ph97_G9
zmp_ph97_H2
zmp_ph97_B10
zmp_ph97_C2
zmp_ph97_C4
zmp_ph97_B8
zmp_ph97_C1
zmp_ph97_D2
zmp_ph97_D5
zmp_ph111_G5
zmp_ph111_G11
zmp_ph111_H1
zmp_ph111_B1a
zmp_ph111_B5
zmp_ph111_B8
zmp_ph111_B6
zmp_ph111_B7
zmp_ph111_B9
zmp_ph111_B10
zmp_ph114_G4
zmp_ph114_G11
zmp_ph114_H1
zmp_ph114_H2
zmp_ph114_A9
zmp_ph114_A11
zmp_ph114_B2
zmp_ph114_B4
zmp_ph114_D8
zmp_ph114_E2

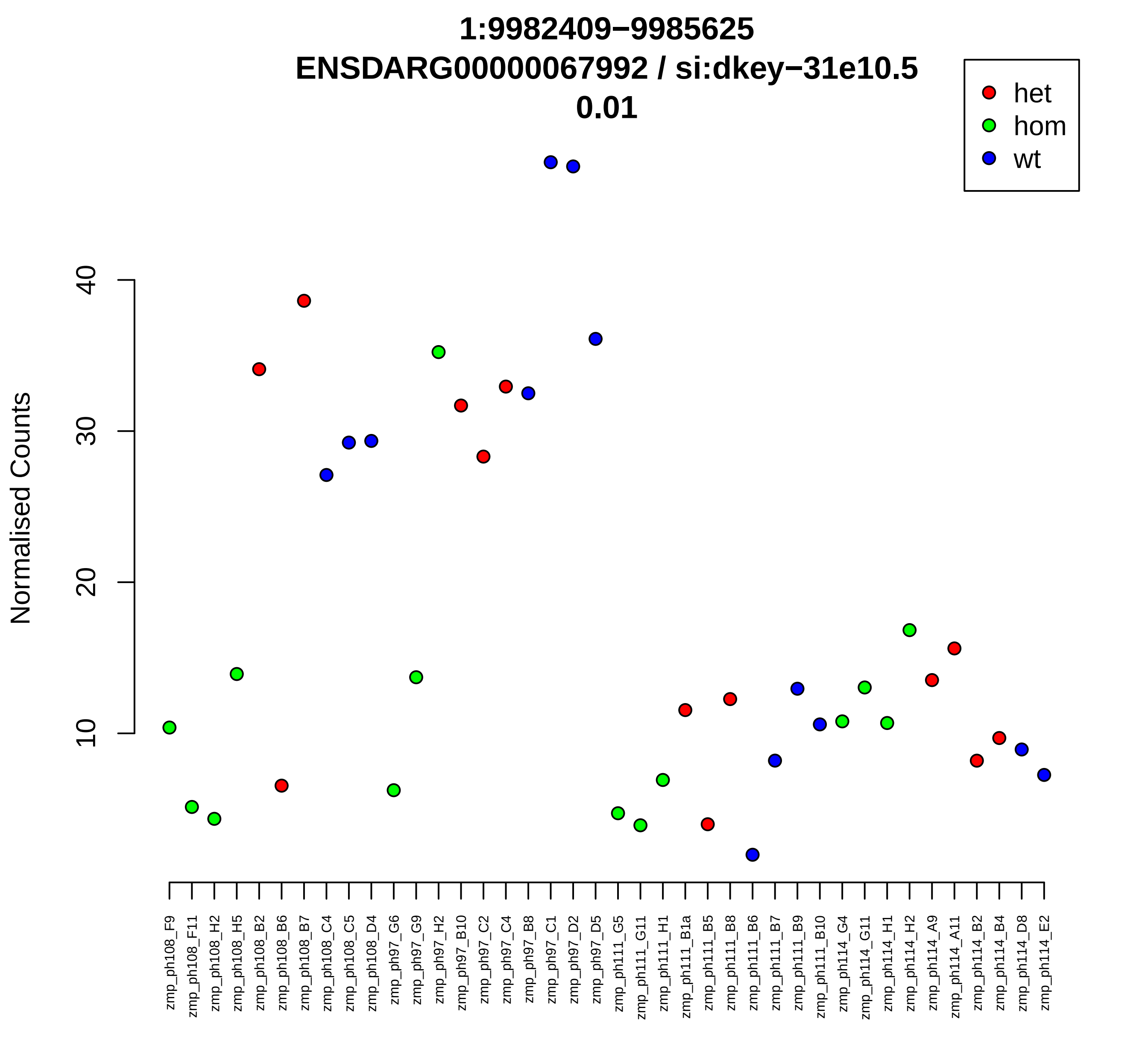

1:9982409−9985625
ENSDARG00000067992 / si:dkey−31e10.5
0.01
het
hom
wt
Normalised Counts
10
20
30
40
zmp_ph108_F9
zmp_ph108_F11
zmp_ph108_H2
zmp_ph108_H5
zmp_ph108_B2
zmp_ph108_B6
zmp_ph108_B7
zmp_ph108_C4
zmp_ph108_C5
zmp_ph108_D4
zmp_ph97_G6
zmp_ph97_G9
zmp_ph97_H2
zmp_ph97_B10
zmp_ph97_C2
zmp_ph97_C4
zmp_ph97_B8
zmp_ph97_C1
zmp_ph97_D2
zmp_ph97_D5
zmp_ph111_G5
zmp_ph111_G11
zmp_ph111_H1
zmp_ph111_B1a
zmp_ph111_B5
zmp_ph111_B8
zmp_ph111_B6
zmp_ph111_B7
zmp_ph111_B9
zmp_ph111_B10
zmp_ph114_G4
zmp_ph114_G11
zmp_ph114_H1
zmp_ph114_H2
zmp_ph114_A9
zmp_ph114_A11
zmp_ph114_B2
zmp_ph114_B4
zmp_ph114_D8
zmp_ph114_E2

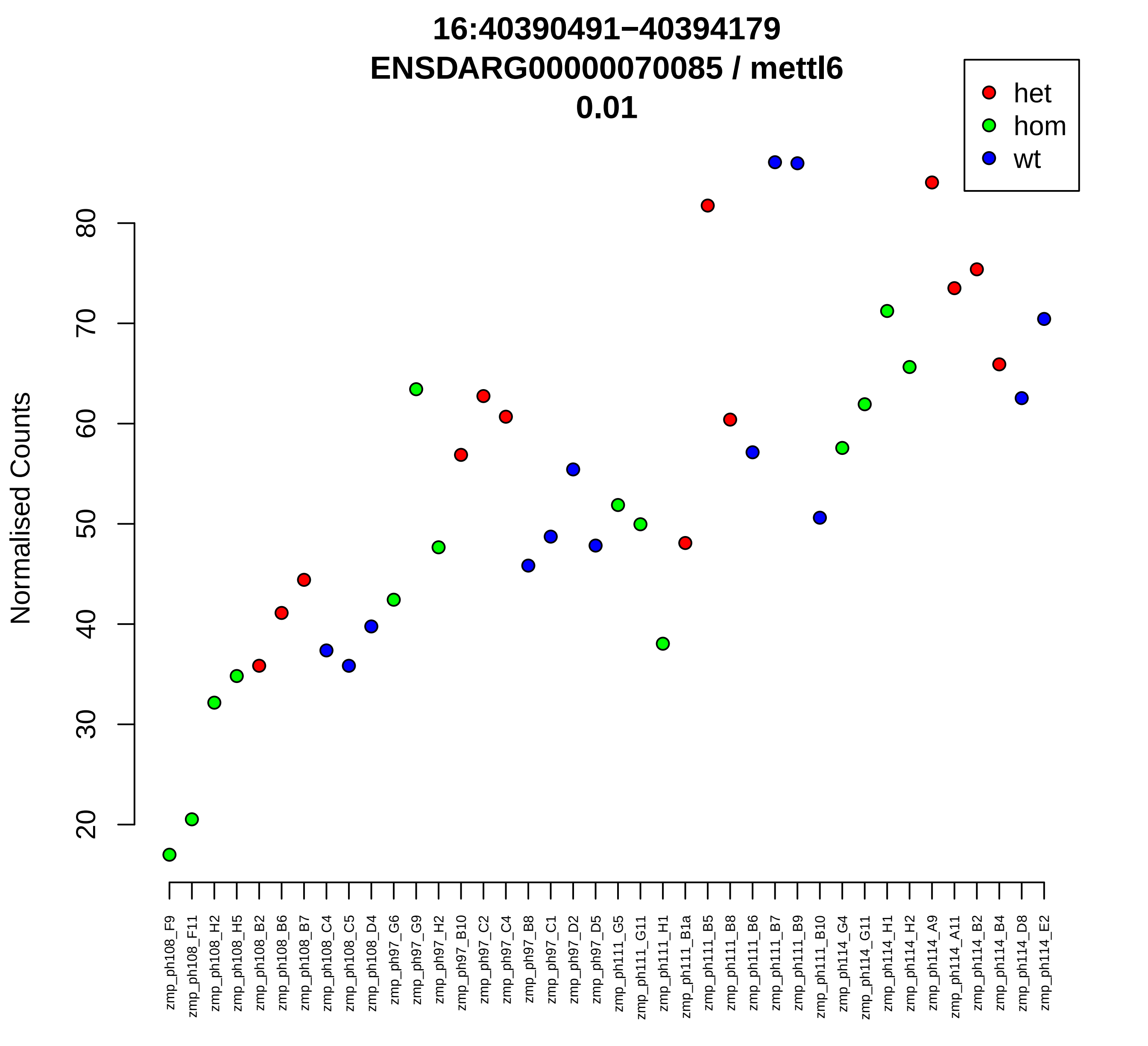

16:40390491−40394179
ENSDARG00000070085 / mettl6
0.01
het
hom
wt
Normalised Counts
20
30
40
50
60
70
80
zmp_ph108_F9
zmp_ph108_F11
zmp_ph108_H2
zmp_ph108_H5
zmp_ph108_B2
zmp_ph108_B6
zmp_ph108_B7
zmp_ph108_C4
zmp_ph108_C5
zmp_ph108_D4
zmp_ph97_G6
zmp_ph97_G9
zmp_ph97_H2
zmp_ph97_B10
zmp_ph97_C2
zmp_ph97_C4
zmp_ph97_B8
zmp_ph97_C1
zmp_ph97_D2
zmp_ph97_D5
zmp_ph111_G5
zmp_ph111_G11
zmp_ph111_H1
zmp_ph111_B1a
zmp_ph111_B5
zmp_ph111_B8
zmp_ph111_B6
zmp_ph111_B7
zmp_ph111_B9
zmp_ph111_B10
zmp_ph114_G4
zmp_ph114_G11
zmp_ph114_H1
zmp_ph114_H2
zmp_ph114_A9
zmp_ph114_A11
zmp_ph114_B2
zmp_ph114_B4
zmp_ph114_D8
zmp_ph114_E2

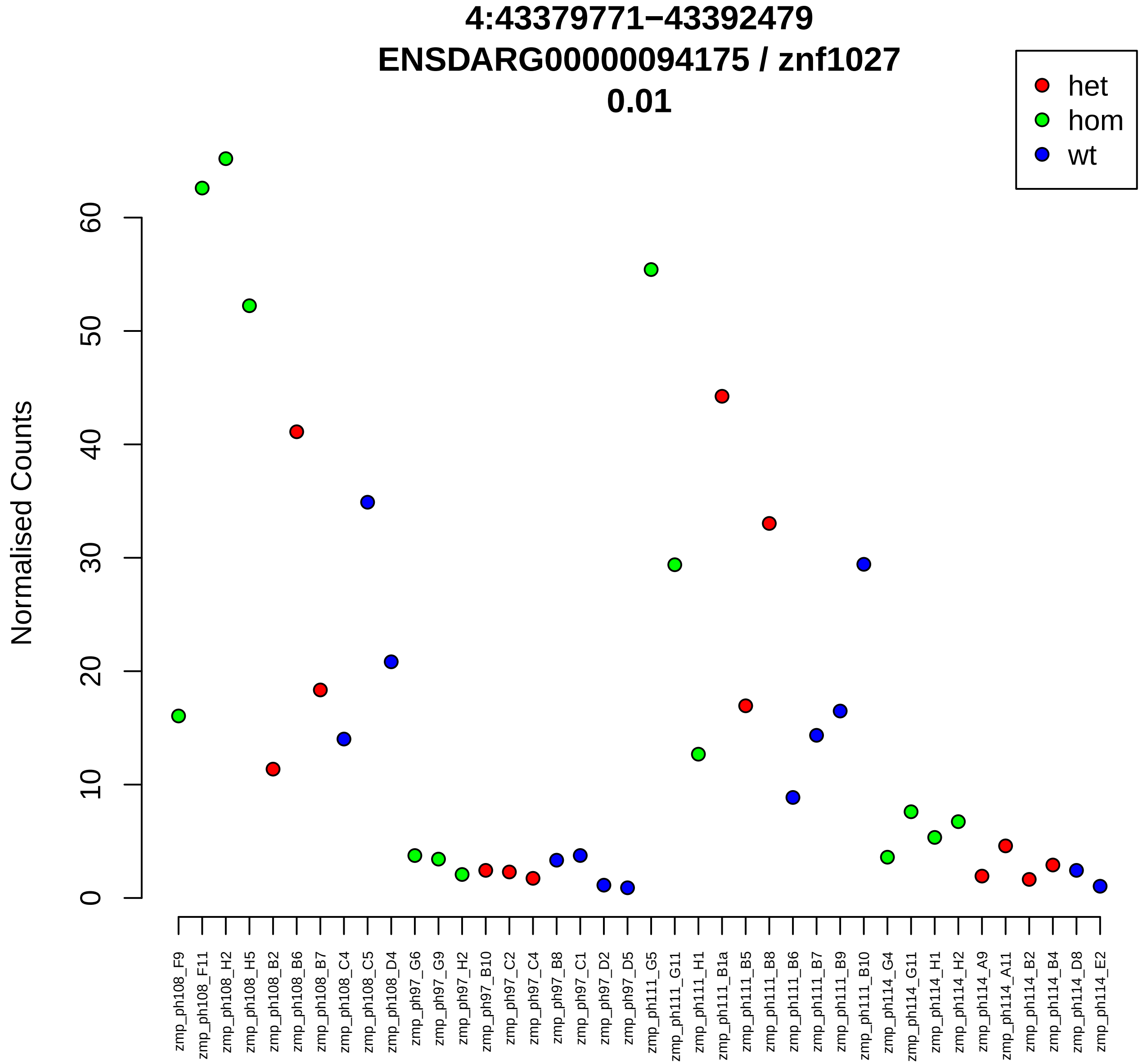
4:43379771−43392479
ENSDARG00000094175 / znf1027
0.01
het
hom
wt
Normalised Counts
0
10
20
30
40
50
60
zmp_ph108_F9
zmp_ph108_F11
zmp_ph108_H2
zmp_ph108_H5
zmp_ph108_B2
zmp_ph108_B6
zmp_ph108_B7
zmp_ph108_C4
zmp_ph108_C5
zmp_ph108_D4
zmp_ph97_G6
zmp_ph97_G9
zmp_ph97_H2
zmp_ph97_B10
zmp_ph97_C2
zmp_ph97_C4
zmp_ph97_B8
zmp_ph97_C1
zmp_ph97_D2
zmp_ph97_D5
zmp_ph111_G5
zmp_ph111_G11
zmp_ph111_H1
zmp_ph111_B1a
zmp_ph111_B5
zmp_ph111_B8
zmp_ph111_B6
zmp_ph111_B7
zmp_ph111_B9
zmp_ph111_B10
zmp_ph114_G4
zmp_ph114_G11
zmp_ph114_H1
zmp_ph114_H2
zmp_ph114_A9
zmp_ph114_A11
zmp_ph114_B2
zmp_ph114_B4
zmp_ph114_D8
zmp_ph114_E2

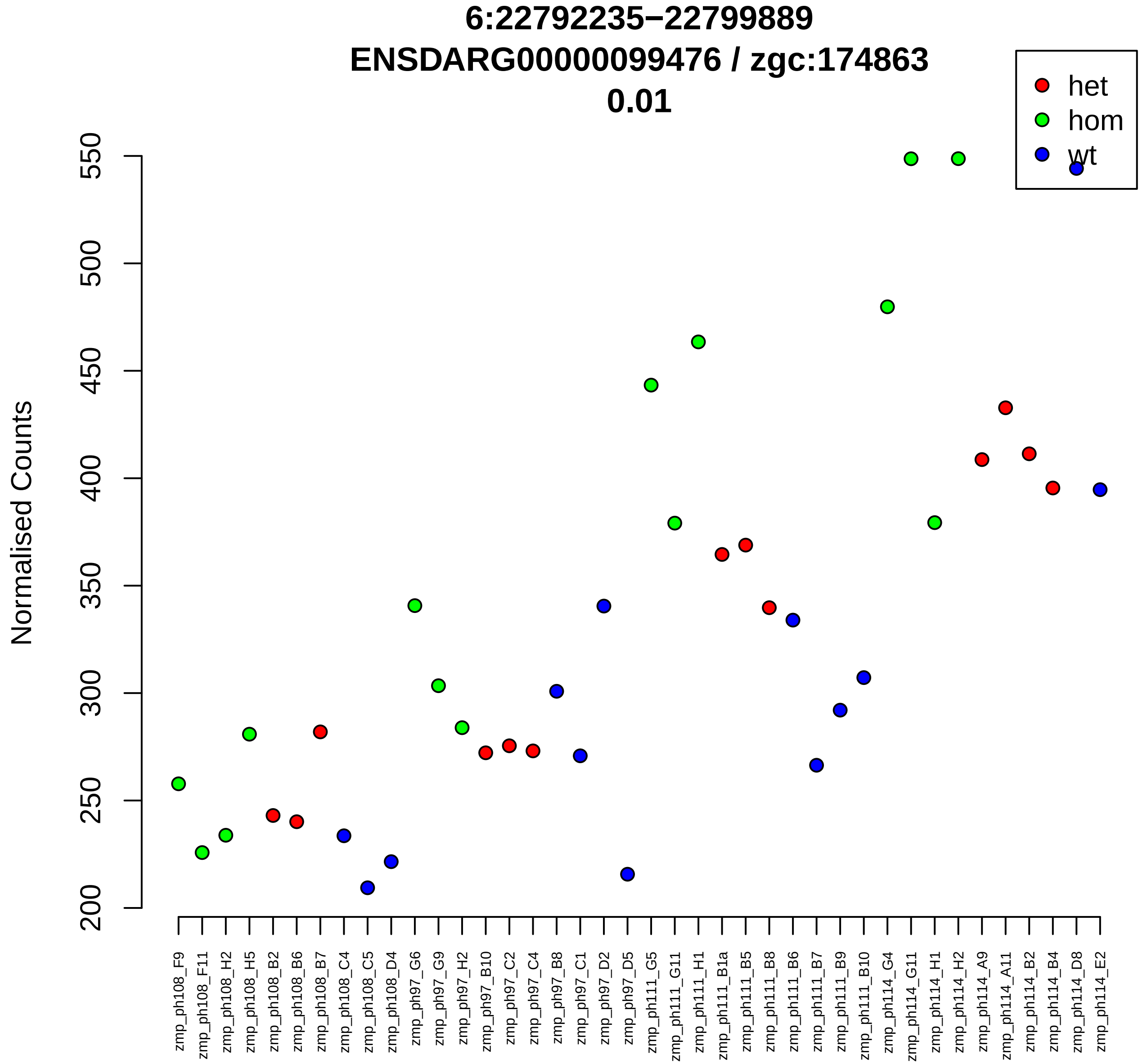
6:22792235−22799889
ENSDARG00000099476 / zgc:174863
0.01
het
hom
wt
Normalised Counts
200
250
300
350
400
450
500
550
zmp_ph108_F9
zmp_ph108_F11
zmp_ph108_H2
zmp_ph108_H5
zmp_ph108_B2
zmp_ph108_B6
zmp_ph108_B7
zmp_ph108_C4
zmp_ph108_C5
zmp_ph108_D4
zmp_ph97_G6
zmp_ph97_G9
zmp_ph97_H2
zmp_ph97_B10
zmp_ph97_C2
zmp_ph97_C4
zmp_ph97_B8
zmp_ph97_C1
zmp_ph97_D2
zmp_ph97_D5
zmp_ph111_G5
zmp_ph111_G11
zmp_ph111_H1
zmp_ph111_B1a
zmp_ph111_B5
zmp_ph111_B8
zmp_ph111_B6
zmp_ph111_B7
zmp_ph111_B9
zmp_ph111_B10
zmp_ph114_G4
zmp_ph114_G11
zmp_ph114_H1
zmp_ph114_H2
zmp_ph114_A9
zmp_ph114_A11
zmp_ph114_B2
zmp_ph114_B4
zmp_ph114_D8
zmp_ph114_E2

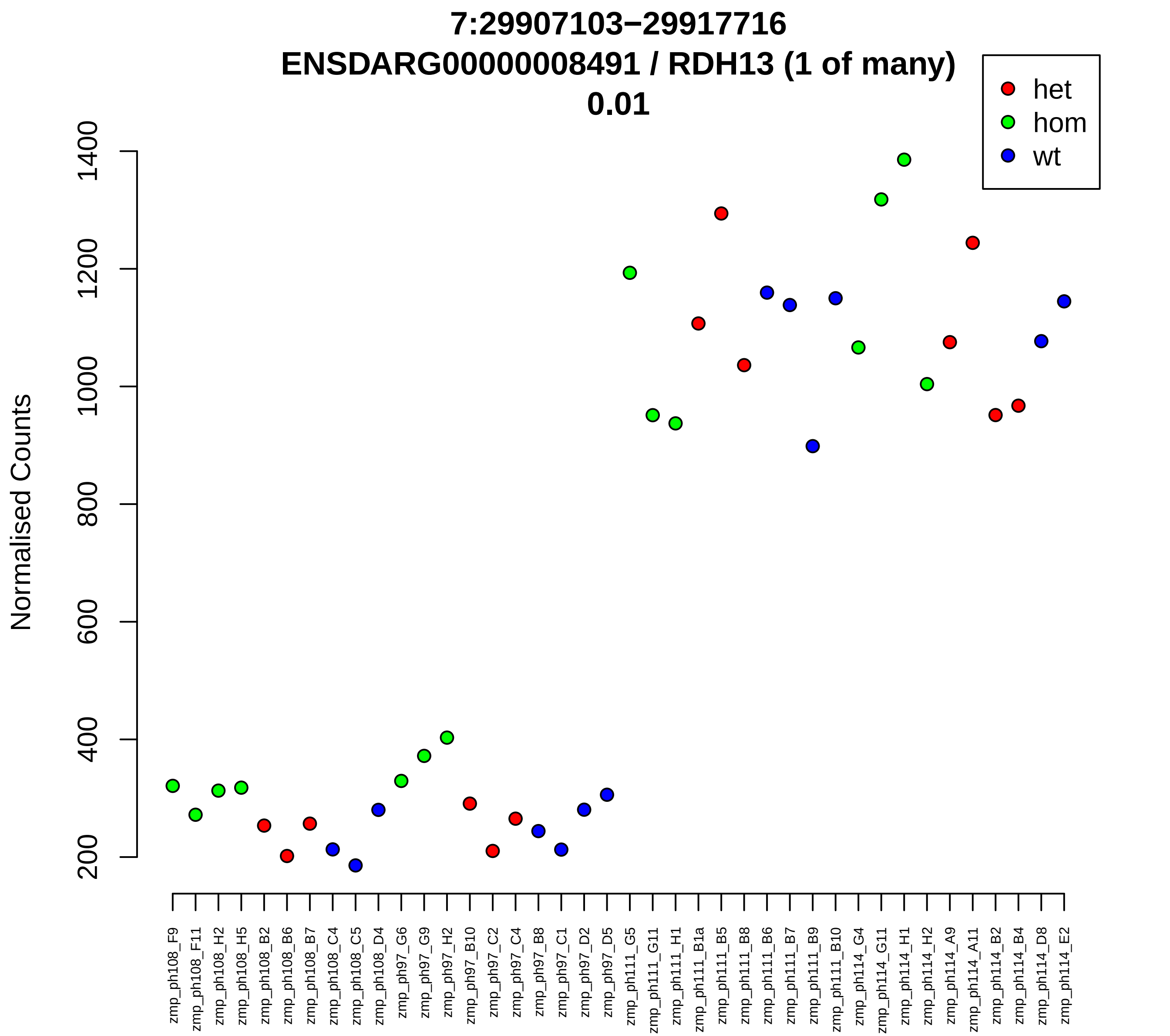
7:29907103−29917716
ENSDARG00000008491 / RDH13 (1 of many)
0.01
het
hom
wt
Normalised Counts
200
400
600
800
1000
1200
1400
zmp_ph108_F9
zmp_ph108_F11
zmp_ph108_H2
zmp_ph108_H5
zmp_ph108_B2
zmp_ph108_B6
zmp_ph108_B7
zmp_ph108_C4
zmp_ph108_C5
zmp_ph108_D4
zmp_ph97_G6
zmp_ph97_G9
zmp_ph97_H2
zmp_ph97_B10
zmp_ph97_C2
zmp_ph97_C4
zmp_ph97_B8
zmp_ph97_C1
zmp_ph97_D2
zmp_ph97_D5
zmp_ph111_G5
zmp_ph111_G11
zmp_ph111_H1
zmp_ph111_B1a
zmp_ph111_B5
zmp_ph111_B8
zmp_ph111_B6
zmp_ph111_B7
zmp_ph111_B9
zmp_ph111_B10
zmp_ph114_G4
zmp_ph114_G11
zmp_ph114_H1
zmp_ph114_H2
zmp_ph114_A9
zmp_ph114_A11
zmp_ph114_B2
zmp_ph114_B4
zmp_ph114_D8
zmp_ph114_E2

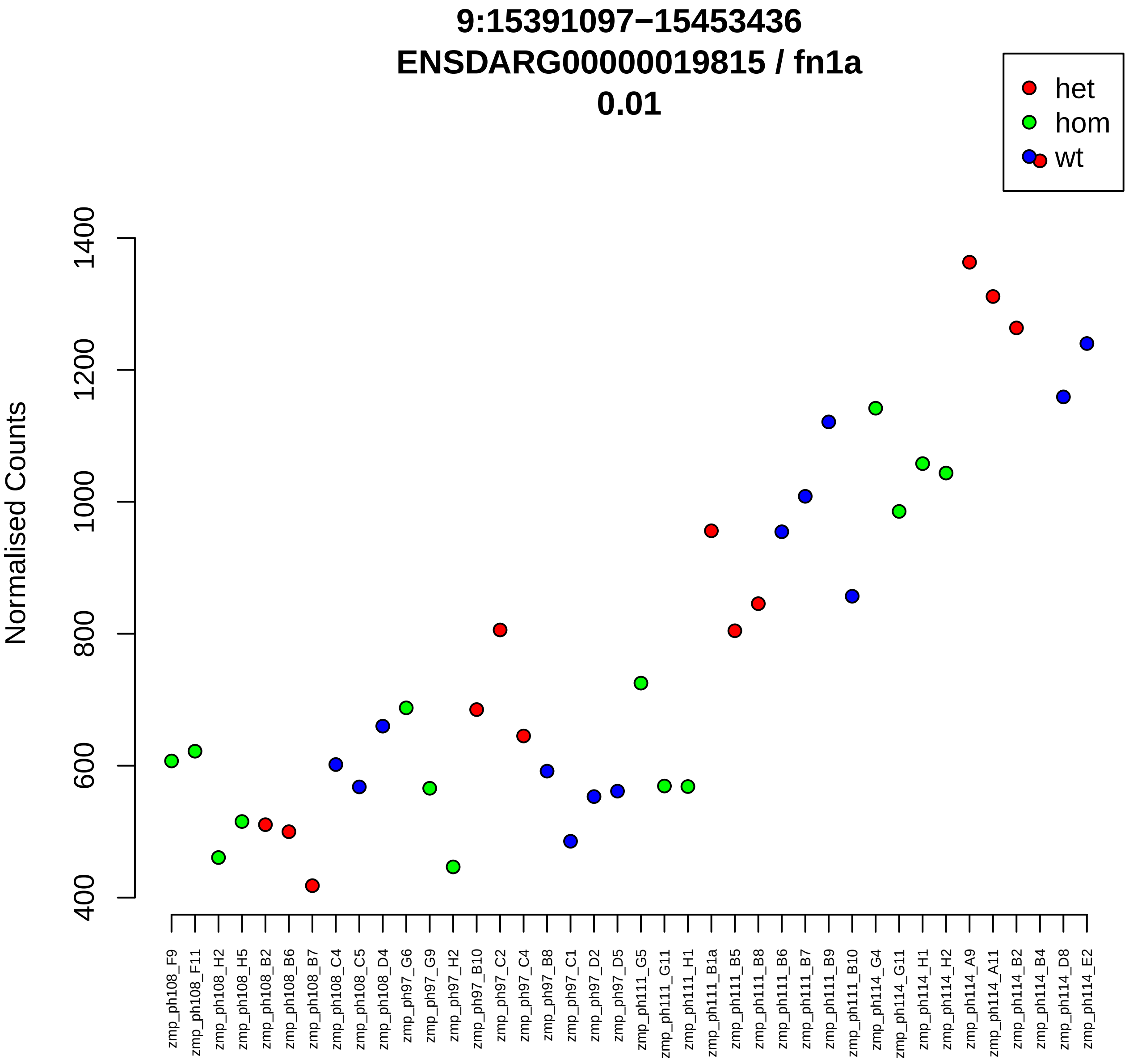
9:15391097−15453436
ENSDARG00000019815 / fn1a
0.01
het
hom
wt
Normalised Counts
400
600
800
1000
1200
1400
zmp_ph108_F9
zmp_ph108_F11
zmp_ph108_H2
zmp_ph108_H5
zmp_ph108_B2
zmp_ph108_B6
zmp_ph108_B7
zmp_ph108_C4
zmp_ph108_C5
zmp_ph108_D4
zmp_ph97_G6
zmp_ph97_G9
zmp_ph97_H2
zmp_ph97_B10
zmp_ph97_C2
zmp_ph97_C4
zmp_ph97_B8
zmp_ph97_C1
zmp_ph97_D2
zmp_ph97_D5
zmp_ph111_G5
zmp_ph111_G11
zmp_ph111_H1
zmp_ph111_B1a
zmp_ph111_B5
zmp_ph111_B8
zmp_ph111_B6
zmp_ph111_B7
zmp_ph111_B9
zmp_ph111_B10
zmp_ph114_G4
zmp_ph114_G11
zmp_ph114_H1
zmp_ph114_H2
zmp_ph114_A9
zmp_ph114_A11
zmp_ph114_B2
zmp_ph114_B4
zmp_ph114_D8
zmp_ph114_E2

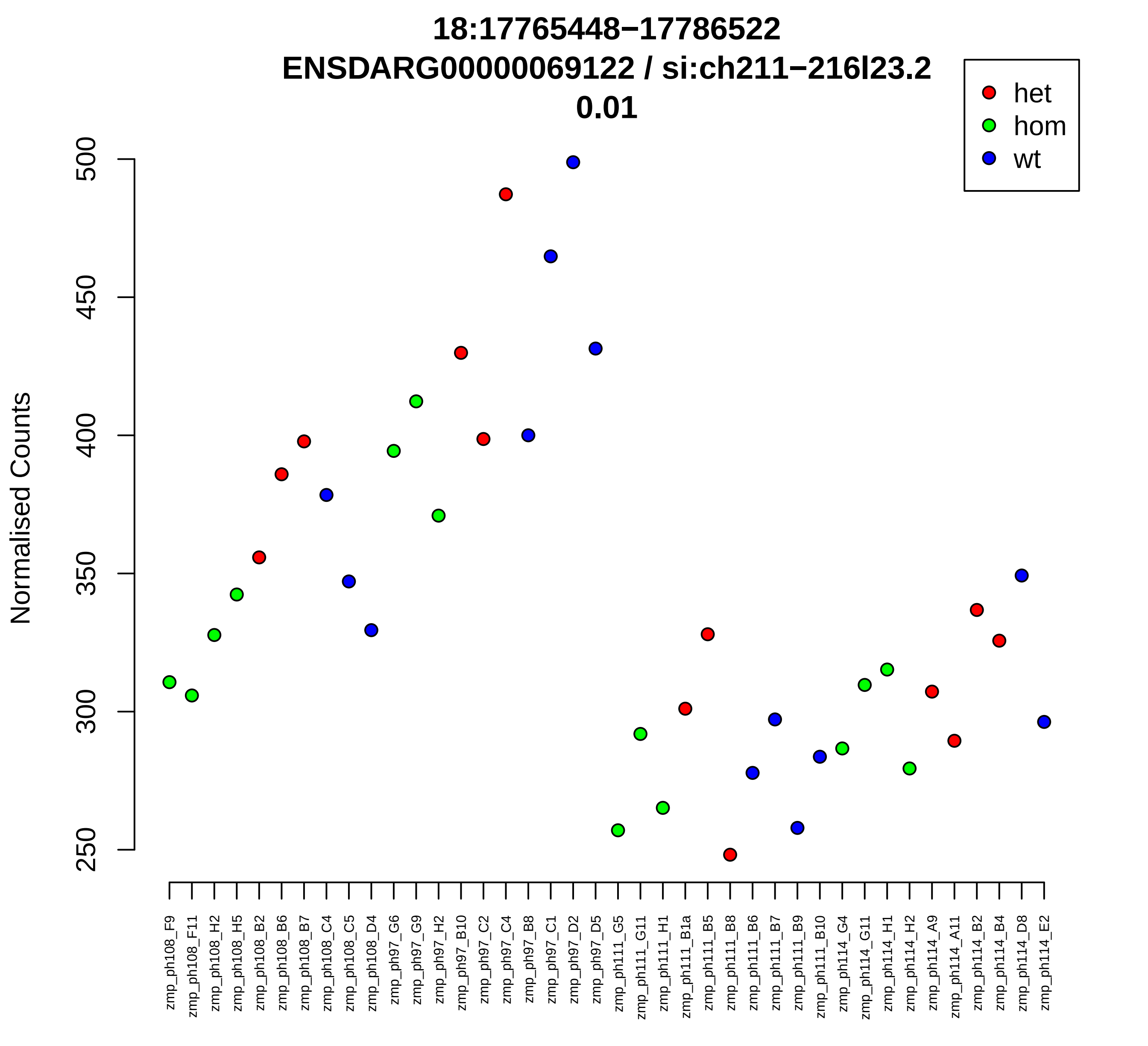

18:17765448−17786522
ENSDARG00000069122 / si:ch211−216l23.2
0.01
het
hom
wt
Normalised Counts
250
300
350
400
450
500
zmp_ph108_F9
zmp_ph108_F11
zmp_ph108_H2
zmp_ph108_H5
zmp_ph108_B2
zmp_ph108_B6
zmp_ph108_B7
zmp_ph108_C4
zmp_ph108_C5
zmp_ph108_D4
zmp_ph97_G6
zmp_ph97_G9
zmp_ph97_H2
zmp_ph97_B10
zmp_ph97_C2
zmp_ph97_C4
zmp_ph97_B8
zmp_ph97_C1
zmp_ph97_D2
zmp_ph97_D5
zmp_ph111_G5
zmp_ph111_G11
zmp_ph111_H1
zmp_ph111_B1a
zmp_ph111_B5
zmp_ph111_B8
zmp_ph111_B6
zmp_ph111_B7
zmp_ph111_B9
zmp_ph111_B10
zmp_ph114_G4
zmp_ph114_G11
zmp_ph114_H1
zmp_ph114_H2
zmp_ph114_A9
zmp_ph114_A11
zmp_ph114_B2
zmp_ph114_B4
zmp_ph114_D8
zmp_ph114_E2

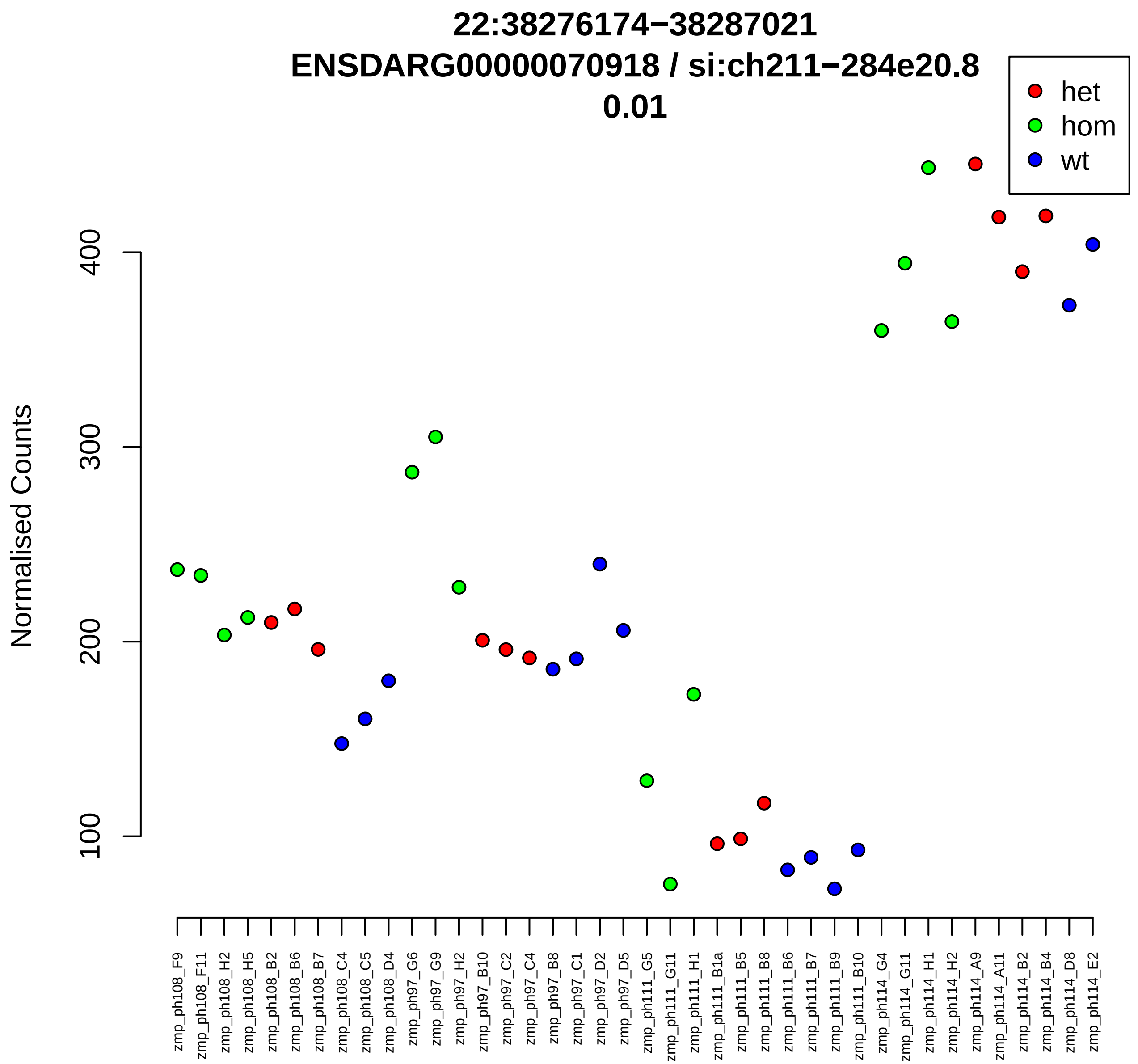
22:38276174−38287021
ENSDARG00000070918 / si:ch211−284e20.8
0.01
het
hom
wt
Normalised Counts
100
200
300
400
zmp_ph108_F9
zmp_ph108_F11
zmp_ph108_H2
zmp_ph108_H5
zmp_ph108_B2
zmp_ph108_B6
zmp_ph108_B7
zmp_ph108_C4
zmp_ph108_C5
zmp_ph108_D4
zmp_ph97_G6
zmp_ph97_G9
zmp_ph97_H2
zmp_ph97_B10
zmp_ph97_C2
zmp_ph97_C4
zmp_ph97_B8
zmp_ph97_C1
zmp_ph97_D2
zmp_ph97_D5
zmp_ph111_G5
zmp_ph111_G11
zmp_ph111_H1
zmp_ph111_B1a
zmp_ph111_B5
zmp_ph111_B8
zmp_ph111_B6
zmp_ph111_B7
zmp_ph111_B9
zmp_ph111_B10
zmp_ph114_G4
zmp_ph114_G11
zmp_ph114_H1
zmp_ph114_H2
zmp_ph114_A9
zmp_ph114_A11
zmp_ph114_B2
zmp_ph114_B4
zmp_ph114_D8
zmp_ph114_E2

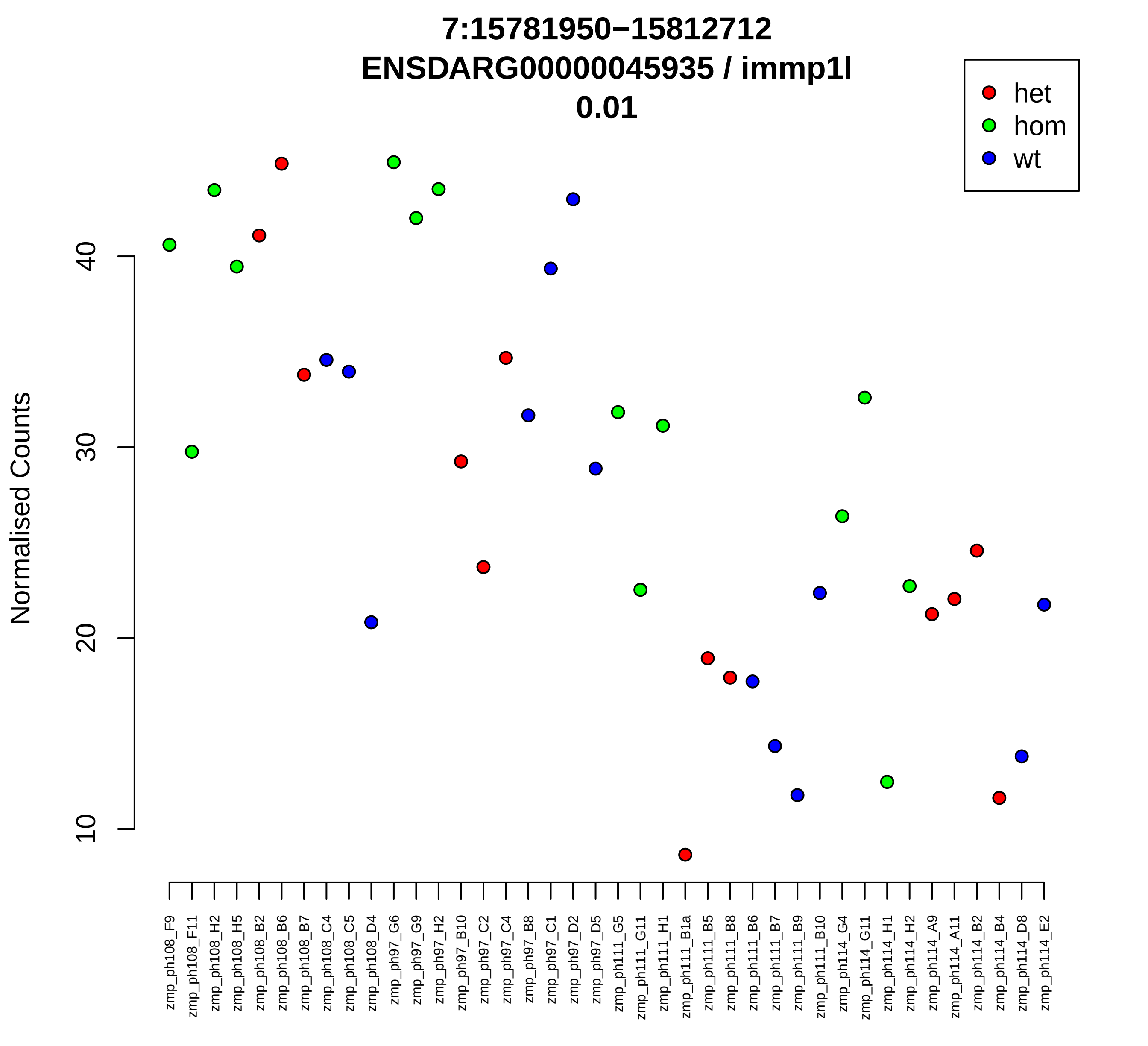
7:15781950−15812712
ENSDARG00000045935 / immp1l
0.01
het
hom
wt
Normalised Counts
10
20
30
40
zmp_ph108_F9
zmp_ph108_F11
zmp_ph108_H2
zmp_ph108_H5
zmp_ph108_B2
zmp_ph108_B6
zmp_ph108_B7
zmp_ph108_C4
zmp_ph108_C5
zmp_ph108_D4
zmp_ph97_G6
zmp_ph97_G9
zmp_ph97_H2
zmp_ph97_B10
zmp_ph97_C2
zmp_ph97_C4
zmp_ph97_B8
zmp_ph97_C1
zmp_ph97_D2
zmp_ph97_D5
zmp_ph111_G5
zmp_ph111_G11
zmp_ph111_H1
zmp_ph111_B1a
zmp_ph111_B5
zmp_ph111_B8
zmp_ph111_B6
zmp_ph111_B7
zmp_ph111_B9
zmp_ph111_B10
zmp_ph114_G4
zmp_ph114_G11
zmp_ph114_H1
zmp_ph114_H2
zmp_ph114_A9
zmp_ph114_A11
zmp_ph114_B2
zmp_ph114_B4
zmp_ph114_D8
zmp_ph114_E2

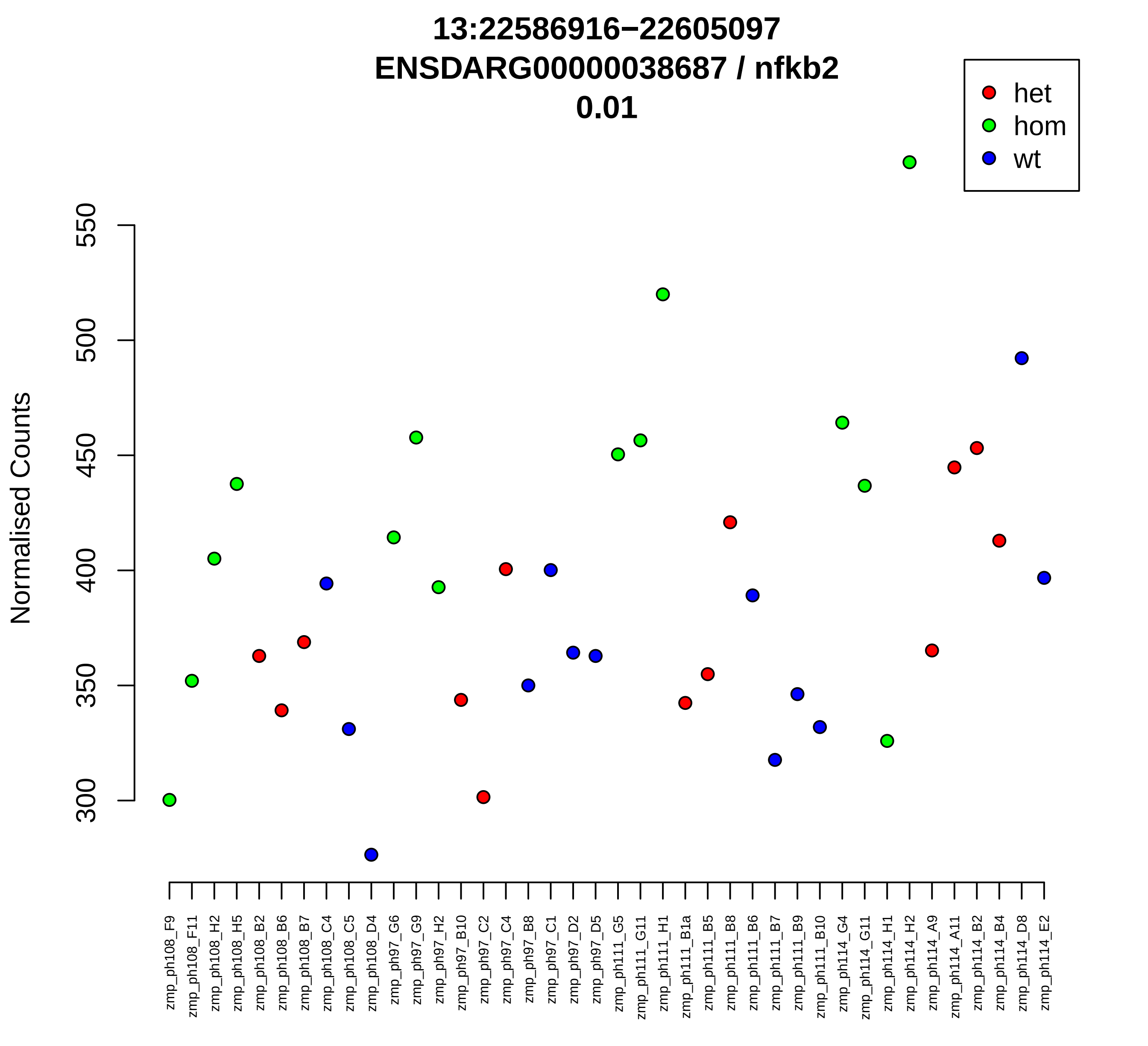
13:22586916−22605097
ENSDARG00000038687 / nfkb2
0.01
het
hom
wt
Normalised Counts
300
350
400
450
500
550
zmp_ph108_F9
zmp_ph108_F11
zmp_ph108_H2
zmp_ph108_H5
zmp_ph108_B2
zmp_ph108_B6
zmp_ph108_B7
zmp_ph108_C4
zmp_ph108_C5
zmp_ph108_D4
zmp_ph97_G6
zmp_ph97_G9
zmp_ph97_H2
zmp_ph97_B10
zmp_ph97_C2
zmp_ph97_C4
zmp_ph97_B8
zmp_ph97_C1
zmp_ph97_D2
zmp_ph97_D5
zmp_ph111_G5
zmp_ph111_G11
zmp_ph111_H1
zmp_ph111_B1a
zmp_ph111_B5
zmp_ph111_B8
zmp_ph111_B6
zmp_ph111_B7
zmp_ph111_B9
zmp_ph111_B10
zmp_ph114_G4
zmp_ph114_G11
zmp_ph114_H1
zmp_ph114_H2
zmp_ph114_A9
zmp_ph114_A11
zmp_ph114_B2
zmp_ph114_B4
zmp_ph114_D8
zmp_ph114_E2

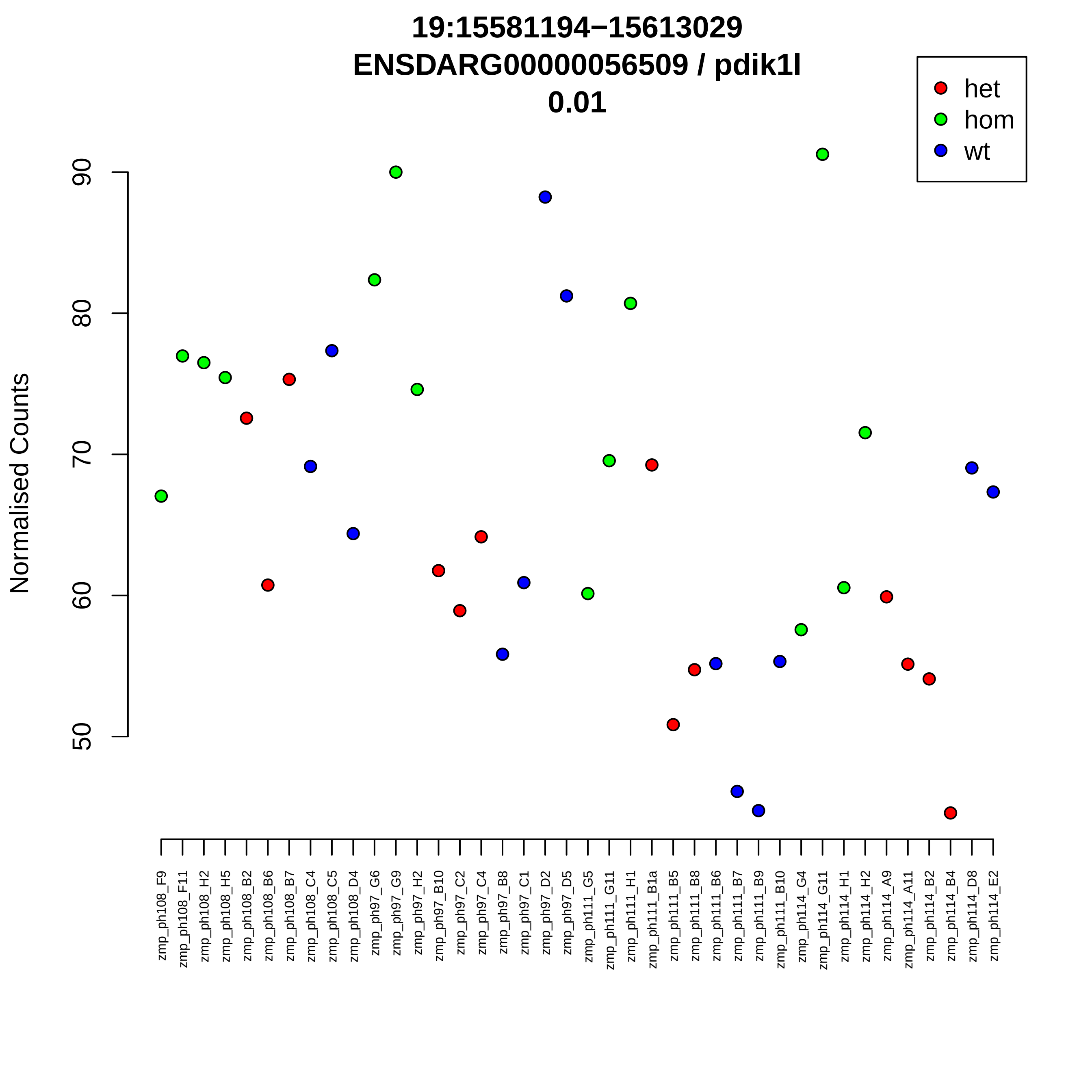

19:15581194−15613029
ENSDARG00000056509 / pdik1l
0.01
het
hom
wt
Normalised Counts
90
80
70
60
50
zmp_ph108_F9
zmp_ph108_F11
zmp_ph108_H2
zmp_ph108_H5
zmp_ph108_B2
zmp_ph108_B6
zmp_ph108_B7
zmp_ph108_C4
zmp_ph108_C5
zmp_ph108_D4
zmp_ph97_G6
zmp_ph97_G9
zmp_ph97_H2
zmp_ph97_B10
zmp_ph97_C2
zmp_ph97_C4
zmp_ph97_B8
zmp_ph97_C1
zmp_ph97_D2
zmp_ph97_D5
zmp_ph111_G5
zmp_ph111_G11
zmp_ph111_H1
zmp_ph111_B1a
zmp_ph111_B5
zmp_ph111_B8
zmp_ph111_B6
zmp_ph111_B7
zmp_ph111_B9
zmp_ph111_B10
zmp_ph114_G4
zmp_ph114_G11
zmp_ph114_H1
zmp_ph114_H2
zmp_ph114_A9
zmp_ph114_A11
zmp_ph114_B2
zmp_ph114_B4
zmp_ph114_D8
zmp_ph114_E2

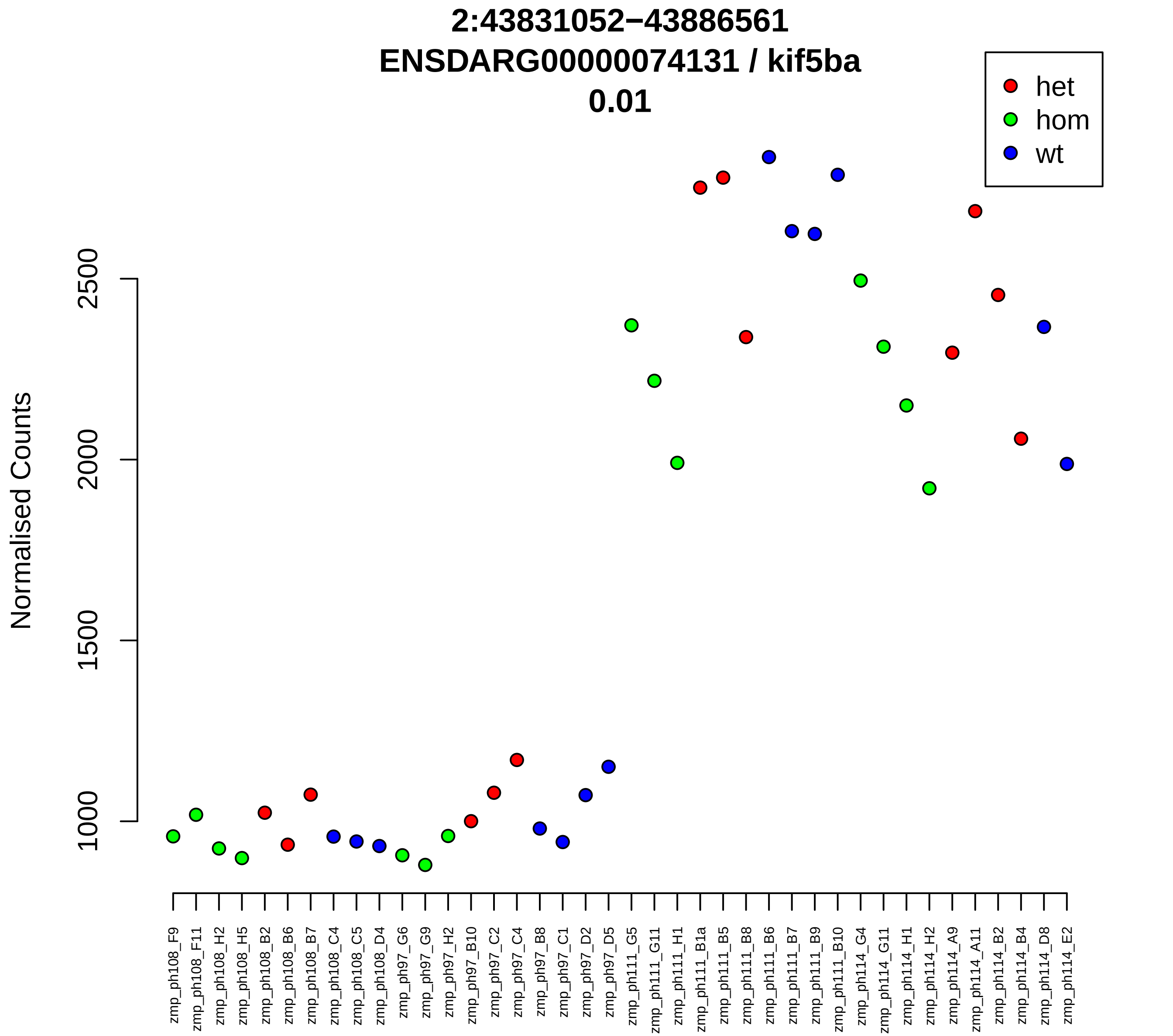

2:43831052−43886561
ENSDARG00000074131 / kif5ba
0.01
het
hom
wt
Normalised Counts
1000
1500
2000
2500
zmp_ph108_F9
zmp_ph108_F11
zmp_ph108_H2
zmp_ph108_H5
zmp_ph108_B2
zmp_ph108_B6
zmp_ph108_B7
zmp_ph108_C4
zmp_ph108_C5
zmp_ph108_D4
zmp_ph97_G6
zmp_ph97_G9
zmp_ph97_H2
zmp_ph97_B10
zmp_ph97_C2
zmp_ph97_C4
zmp_ph97_B8
zmp_ph97_C1
zmp_ph97_D2
zmp_ph97_D5
zmp_ph111_G5
zmp_ph111_G11
zmp_ph111_H1
zmp_ph111_B1a
zmp_ph111_B5
zmp_ph111_B8
zmp_ph111_B6
zmp_ph111_B7
zmp_ph111_B9
zmp_ph111_B10
zmp_ph114_G4
zmp_ph114_G11
zmp_ph114_H1
zmp_ph114_H2
zmp_ph114_A9
zmp_ph114_A11
zmp_ph114_B2
zmp_ph114_B4
zmp_ph114_D8
zmp_ph114_E2

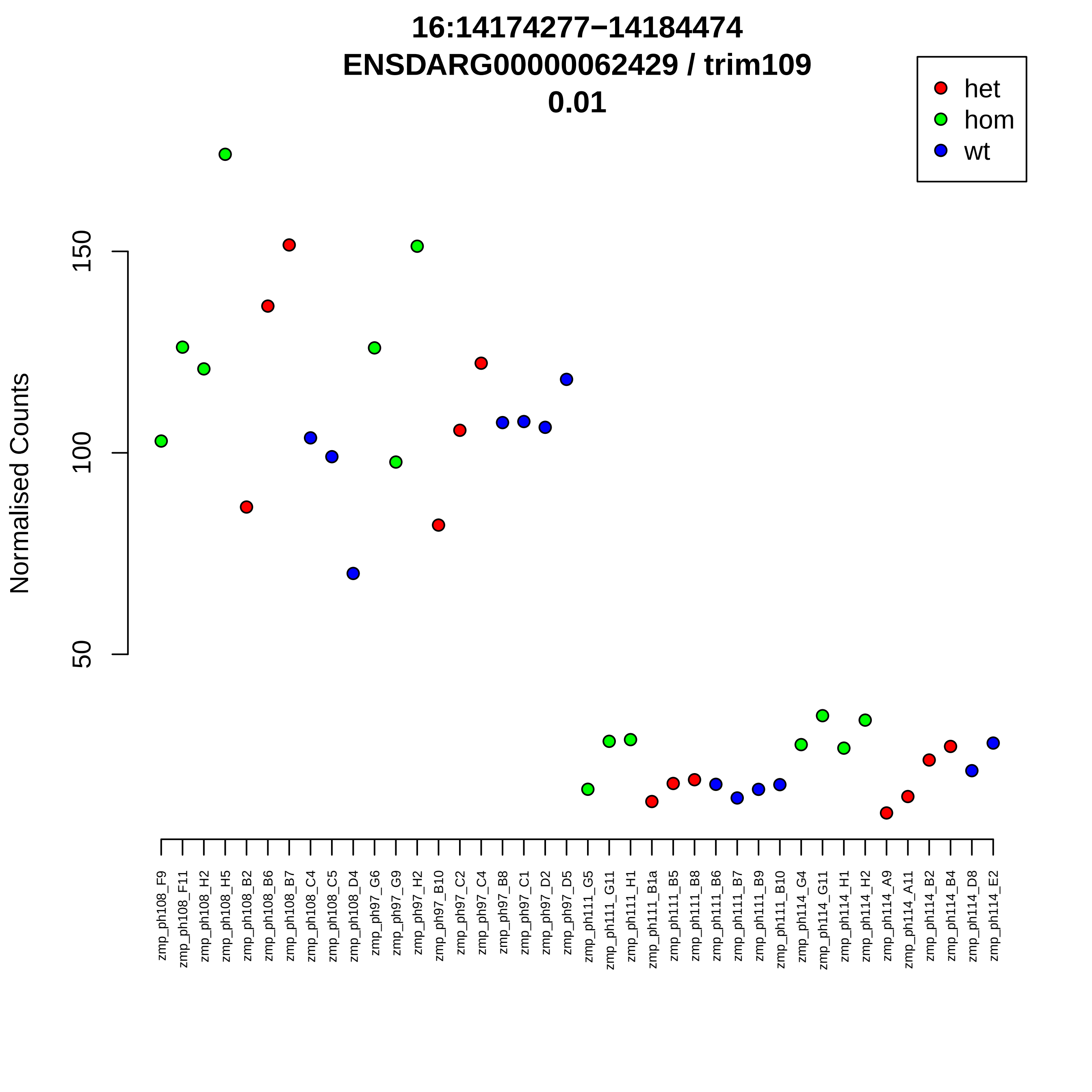

16:14174277−14184474
ENSDARG00000062429 / trim109
0.01
het
hom
wt
Normalised Counts
50
100
150
zmp_ph108_F9
zmp_ph108_F11
zmp_ph108_H2
zmp_ph108_H5
zmp_ph108_B2
zmp_ph108_B6
zmp_ph108_B7
zmp_ph108_C4
zmp_ph108_C5
zmp_ph108_D4
zmp_ph97_G6
zmp_ph97_G9
zmp_ph97_H2
zmp_ph97_B10
zmp_ph97_C2
zmp_ph97_C4
zmp_ph97_B8
zmp_ph97_C1
zmp_ph97_D2
zmp_ph97_D5
zmp_ph111_G5
zmp_ph111_G11
zmp_ph111_H1
zmp_ph111_B1a
zmp_ph111_B5
zmp_ph111_B8
zmp_ph111_B6
zmp_ph111_B7
zmp_ph111_B9
zmp_ph111_B10
zmp_ph114_G4
zmp_ph114_G11
zmp_ph114_H1
zmp_ph114_H2
zmp_ph114_A9
zmp_ph114_A11
zmp_ph114_B2
zmp_ph114_B4
zmp_ph114_D8
zmp_ph114_E2

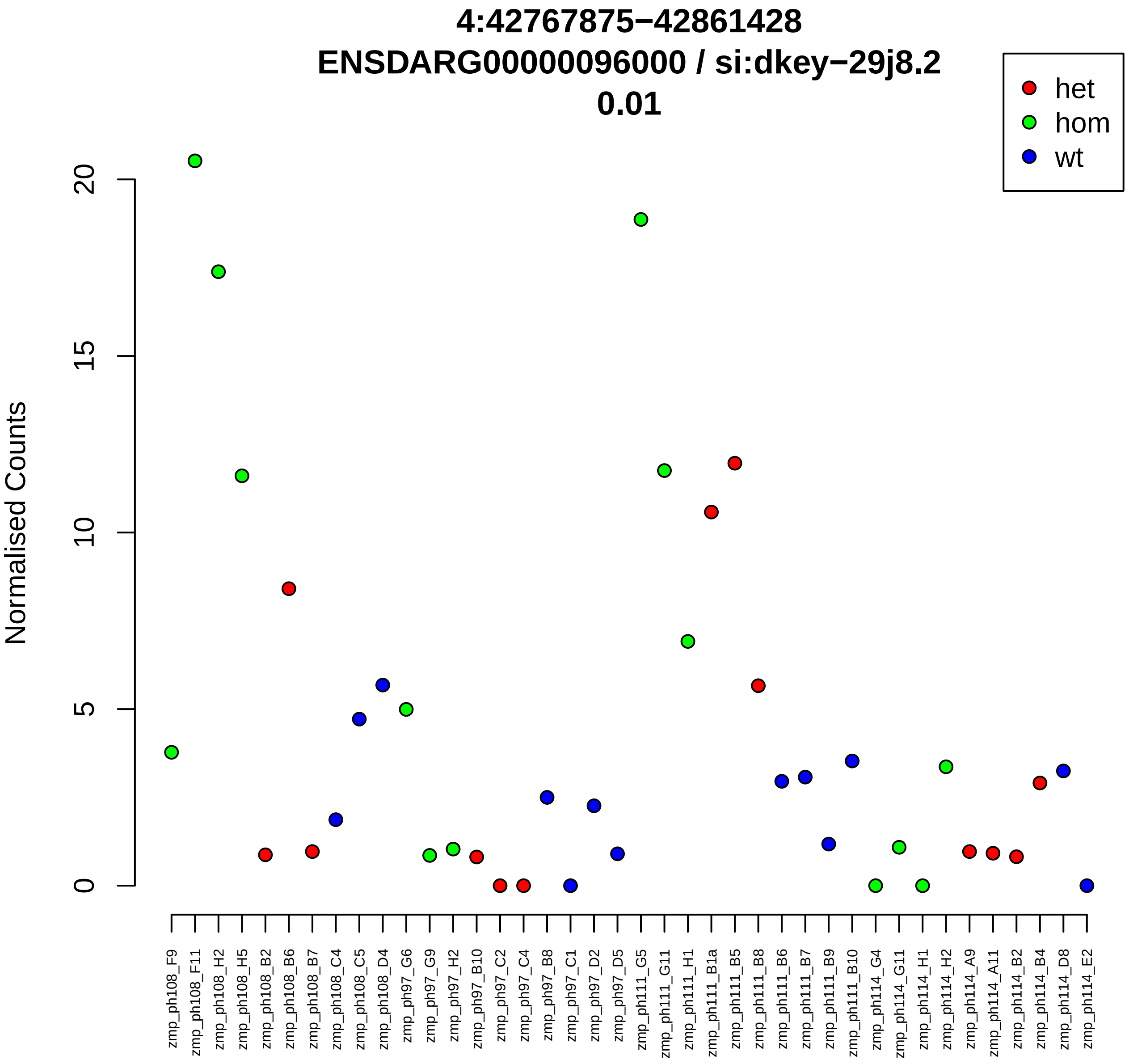
4:42767875−42861428
ENSDARG00000096000 / si:dkey−29j8.2
0.01
het
hom
wt
Normalised Counts
0
5
10
15
20
zmp_ph108_F9
zmp_ph108_F11
zmp_ph108_H2
zmp_ph108_H5
zmp_ph108_B2
zmp_ph108_B6
zmp_ph108_B7
zmp_ph108_C4
zmp_ph108_C5
zmp_ph108_D4
zmp_ph97_G6
zmp_ph97_G9
zmp_ph97_H2
zmp_ph97_B10
zmp_ph97_C2
zmp_ph97_C4
zmp_ph97_B8
zmp_ph97_C1
zmp_ph97_D2
zmp_ph97_D5
zmp_ph111_G5
zmp_ph111_G11
zmp_ph111_H1
zmp_ph111_B1a
zmp_ph111_B5
zmp_ph111_B8
zmp_ph111_B6
zmp_ph111_B7
zmp_ph111_B9
zmp_ph111_B10
zmp_ph114_G4
zmp_ph114_G11
zmp_ph114_H1
zmp_ph114_H2
zmp_ph114_A9
zmp_ph114_A11
zmp_ph114_B2
zmp_ph114_B4
zmp_ph114_D8
zmp_ph114_E2

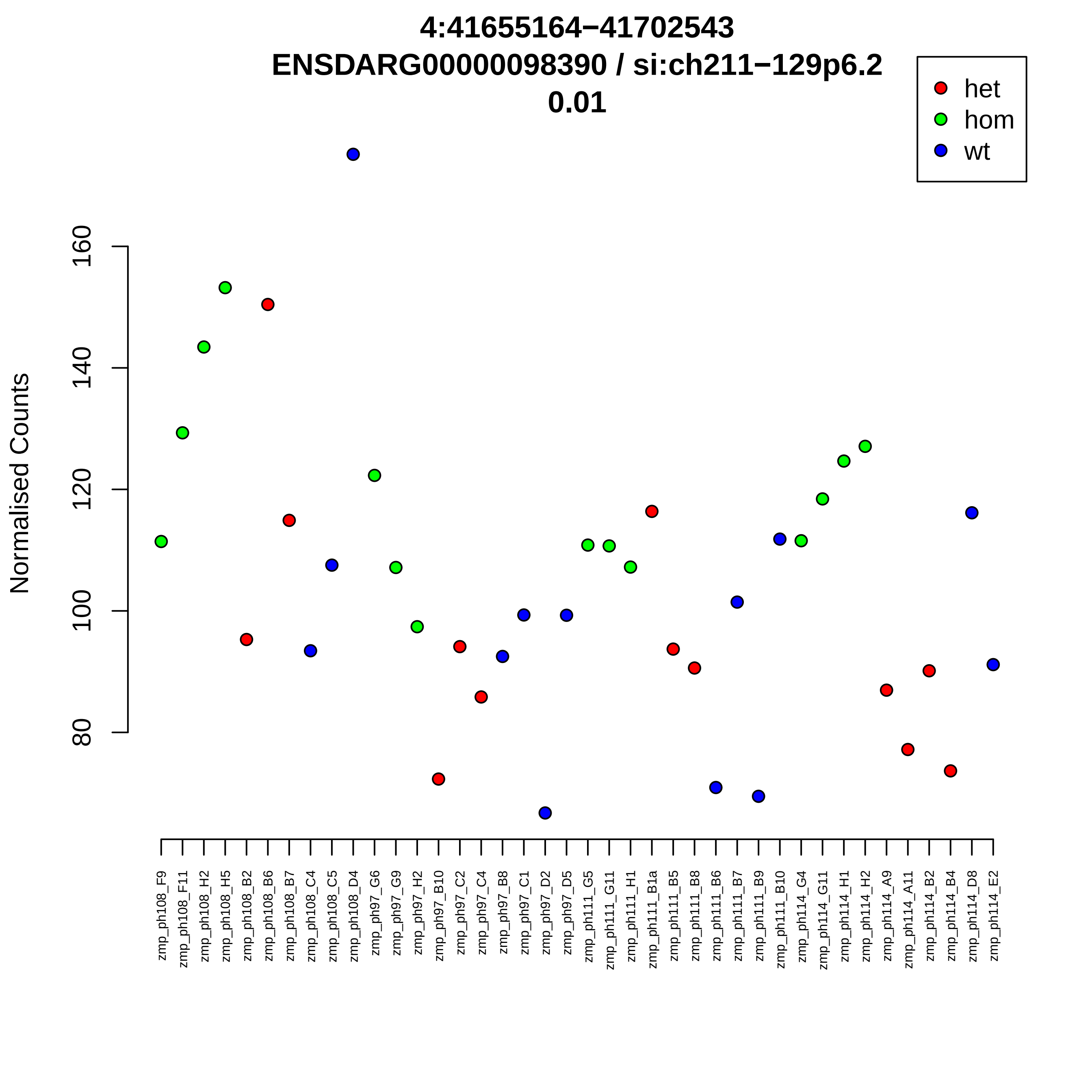
4:41655164−41702543
ENSDARG00000098390 / si:ch211−129p6.2
0.01
het
hom
wt
Normalised Counts
80
100
120
140
160
zmp_ph108_F9
zmp_ph108_F11
zmp_ph108_H2
zmp_ph108_H5
zmp_ph108_B2
zmp_ph108_B6
zmp_ph108_B7
zmp_ph108_C4
zmp_ph108_C5
zmp_ph108_D4
zmp_ph97_G6
zmp_ph97_G9
zmp_ph97_H2
zmp_ph97_B10
zmp_ph97_C2
zmp_ph97_C4
zmp_ph97_B8
zmp_ph97_C1
zmp_ph97_D2
zmp_ph97_D5
zmp_ph111_G5
zmp_ph111_G11
zmp_ph111_H1
zmp_ph111_B1a
zmp_ph111_B5
zmp_ph111_B8
zmp_ph111_B6
zmp_ph111_B7
zmp_ph111_B9
zmp_ph111_B10
zmp_ph114_G4
zmp_ph114_G11
zmp_ph114_H1
zmp_ph114_H2
zmp_ph114_A9
zmp_ph114_A11
zmp_ph114_B2
zmp_ph114_B4
zmp_ph114_D8
zmp_ph114_E2

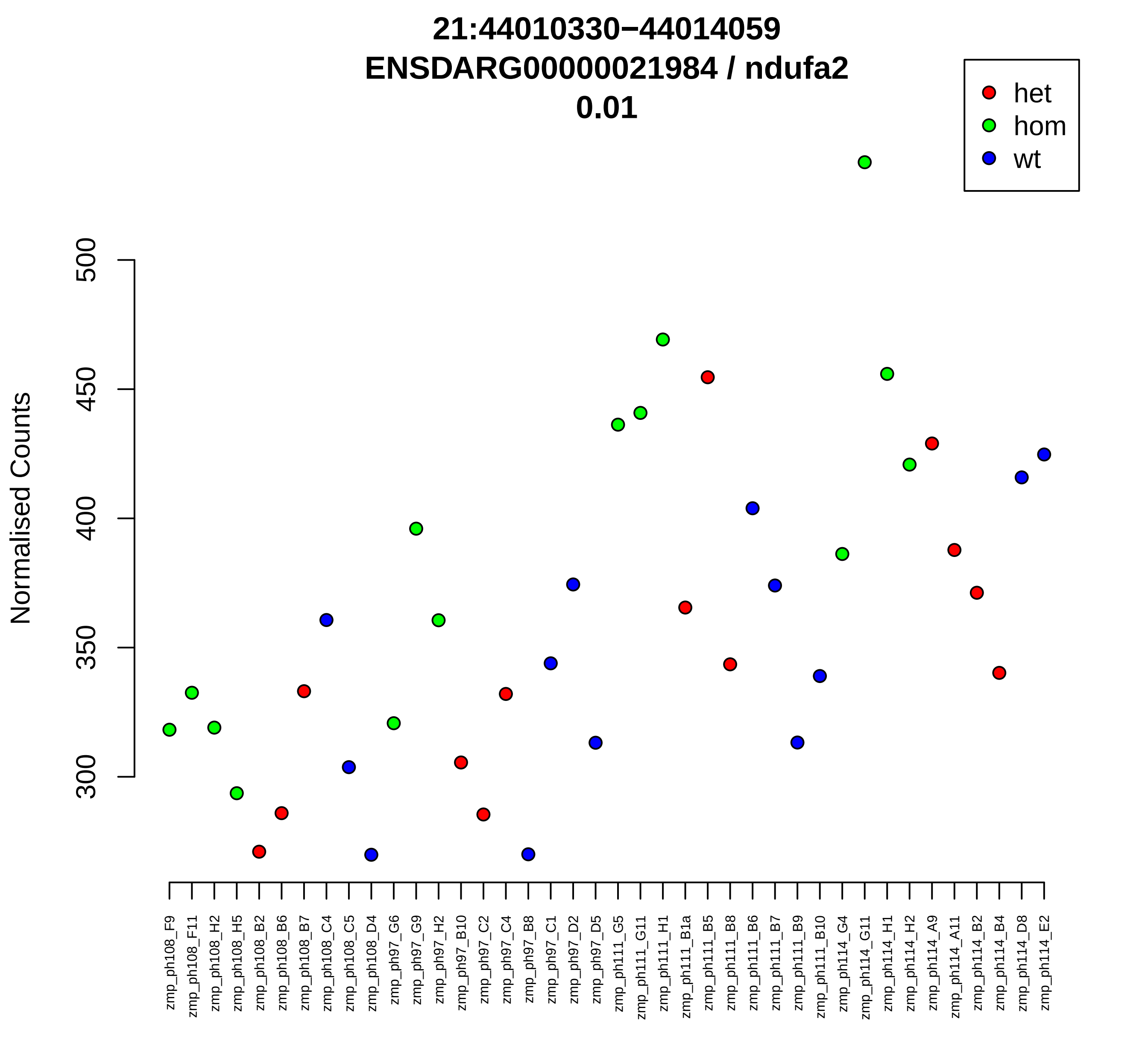
21:44010330−44014059
ENSDARG00000021984 / ndufa2
0.01
het
hom
wt
Normalised Counts
300
350
400
450
500
zmp_ph108_F9
zmp_ph108_F11
zmp_ph108_H2
zmp_ph108_H5
zmp_ph108_B2
zmp_ph108_B6
zmp_ph108_B7
zmp_ph108_C4
zmp_ph108_C5
zmp_ph108_D4
zmp_ph97_G6
zmp_ph97_G9
zmp_ph97_H2
zmp_ph97_B10
zmp_ph97_C2
zmp_ph97_C4
zmp_ph97_B8
zmp_ph97_C1
zmp_ph97_D2
zmp_ph97_D5
zmp_ph111_G5
zmp_ph111_G11
zmp_ph111_H1
zmp_ph111_B1a
zmp_ph111_B5
zmp_ph111_B8
zmp_ph111_B6
zmp_ph111_B7
zmp_ph111_B9
zmp_ph111_B10
zmp_ph114_G4
zmp_ph114_G11
zmp_ph114_H1
zmp_ph114_H2
zmp_ph114_A9
zmp_ph114_A11
zmp_ph114_B2
zmp_ph114_B4
zmp_ph114_D8
zmp_ph114_E2

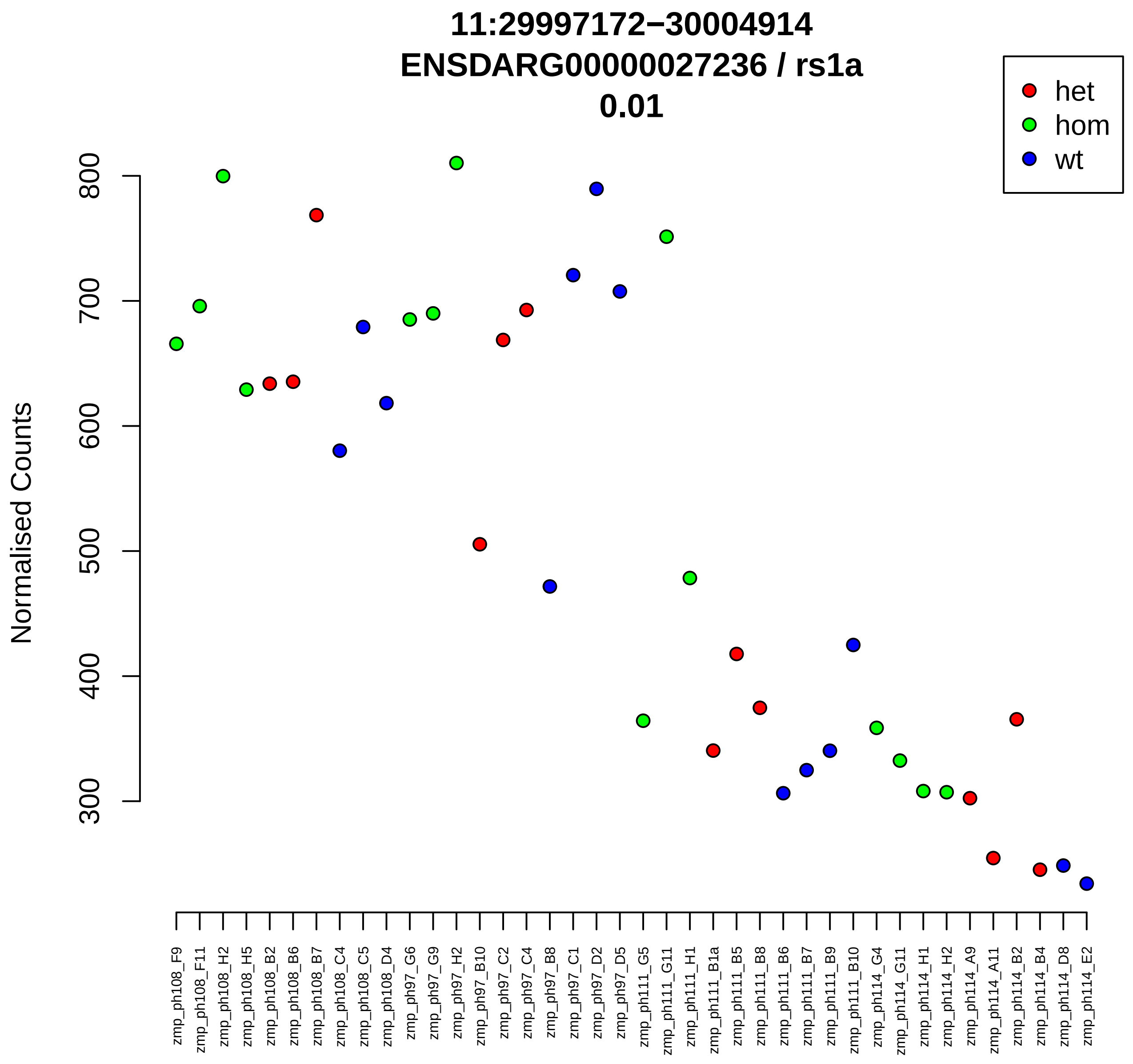

11:29997172−30004914
ENSDARG00000027236 / rs1a
0.01
het
hom
wt
Normalised Counts
300
400
500
600
700
800
zmp_ph108_F9
zmp_ph108_F11
zmp_ph108_H2
zmp_ph108_H5
zmp_ph108_B2
zmp_ph108_B6
zmp_ph108_B7
zmp_ph108_C4
zmp_ph108_C5
zmp_ph108_D4
zmp_ph97_G6
zmp_ph97_G9
zmp_ph97_H2
zmp_ph97_B10
zmp_ph97_C2
zmp_ph97_C4
zmp_ph97_B8
zmp_ph97_C1
zmp_ph97_D2
zmp_ph97_D5
zmp_ph111_G5
zmp_ph111_G11
zmp_ph111_H1
zmp_ph111_B1a
zmp_ph111_B5
zmp_ph111_B8
zmp_ph111_B6
zmp_ph111_B7
zmp_ph111_B9
zmp_ph111_B10
zmp_ph114_G4
zmp_ph114_G11
zmp_ph114_H1
zmp_ph114_H2
zmp_ph114_A9
zmp_ph114_A11
zmp_ph114_B2
zmp_ph114_B4
zmp_ph114_D8
zmp_ph114_E2

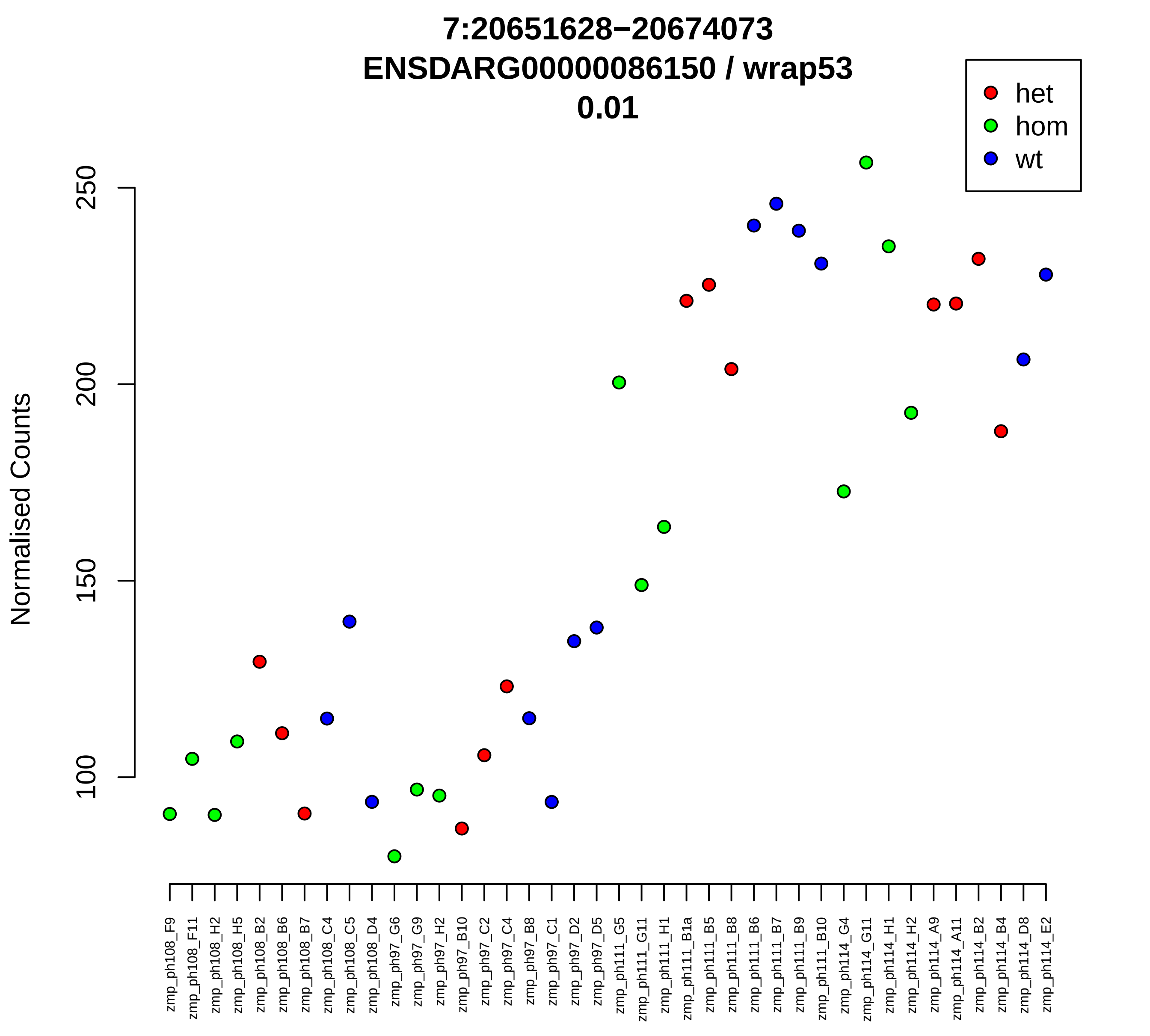
7:20651628−20674073
ENSDARG00000086150 / wrap53
0.01
het
hom
wt
Normalised Counts
250
200
150
100
zmp_ph108_F9
zmp_ph108_F11
zmp_ph108_H2
zmp_ph108_H5
zmp_ph108_B2
zmp_ph108_B6
zmp_ph108_B7
zmp_ph108_C4
zmp_ph108_C5
zmp_ph108_D4
zmp_ph97_G6
zmp_ph97_G9
zmp_ph97_H2
zmp_ph97_B10
zmp_ph97_C2
zmp_ph97_C4
zmp_ph97_B8
zmp_ph97_C1
zmp_ph97_D2
zmp_ph97_D5
zmp_ph111_G5
zmp_ph111_G11
zmp_ph111_H1
zmp_ph111_B1a
zmp_ph111_B5
zmp_ph111_B8
zmp_ph111_B6
zmp_ph111_B7
zmp_ph111_B9
zmp_ph111_B10
zmp_ph114_G4
zmp_ph114_G11
zmp_ph114_H1
zmp_ph114_H2
zmp_ph114_A9
zmp_ph114_A11
zmp_ph114_B2
zmp_ph114_B4
zmp_ph114_D8
zmp_ph114_E2

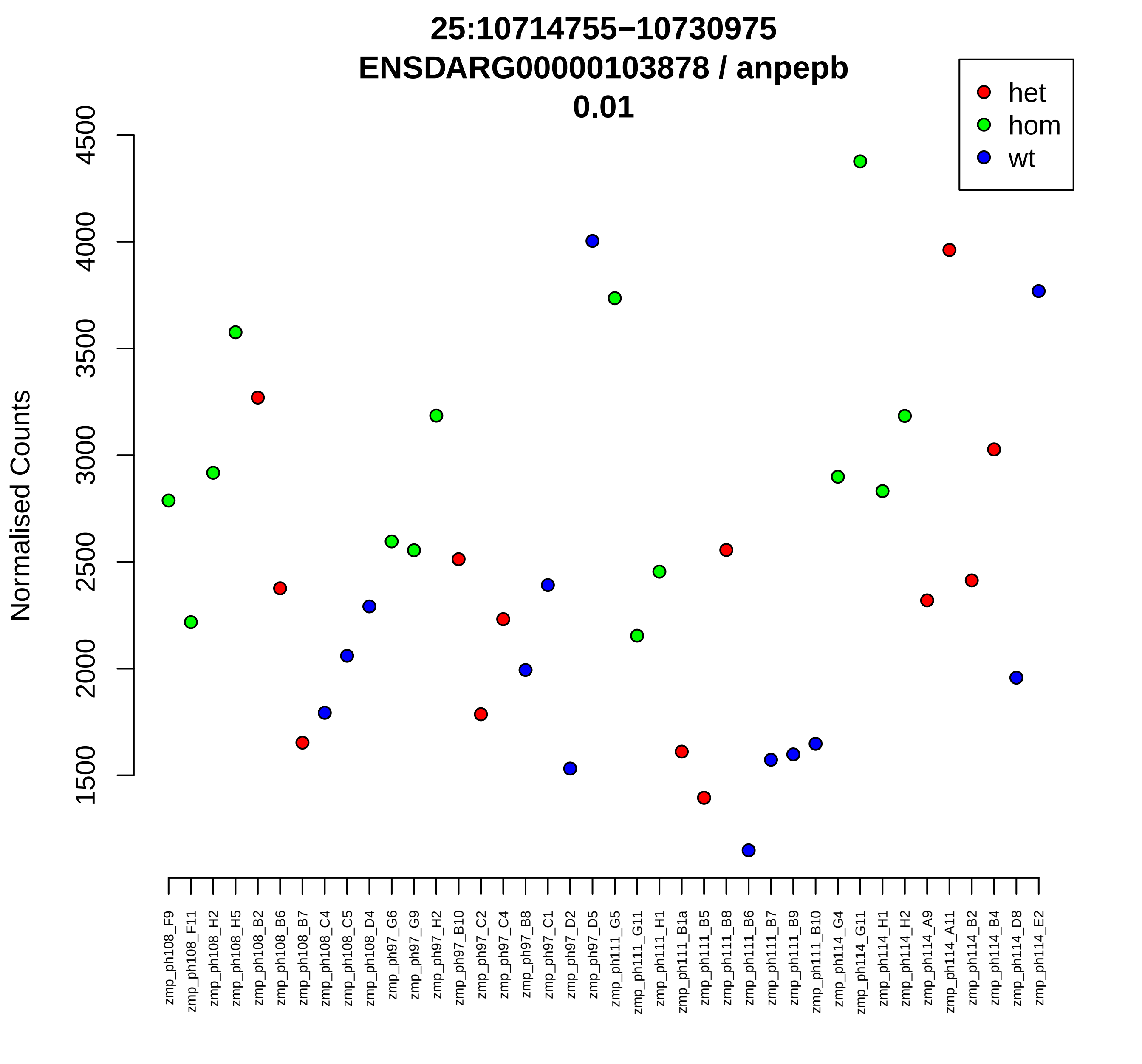
25:10714755−10730975
ENSDARG00000103878 / anpepb
0.01
Normalised Counts
1500
2000
2500
3000
3500
4000
4500
het
hom
wt
zmp_ph108_F9
zmp_ph108_F11
zmp_ph108_H2
zmp_ph108_H5
zmp_ph108_B2
zmp_ph108_B6
zmp_ph108_B7
zmp_ph108_C4
zmp_ph108_C5
zmp_ph108_D4
zmp_ph97_G6
zmp_ph97_G9
zmp_ph97_H2
zmp_ph97_B10
zmp_ph97_C2
zmp_ph97_C4
zmp_ph97_B8
zmp_ph97_C1
zmp_ph97_D2
zmp_ph97_D5
zmp_ph111_G5
zmp_ph111_G11
zmp_ph111_H1
zmp_ph111_B1a
zmp_ph111_B5
zmp_ph111_B8
zmp_ph111_B6
zmp_ph111_B7
zmp_ph111_B9
zmp_ph111_B10
zmp_ph114_G4
zmp_ph114_G11
zmp_ph114_H1
zmp_ph114_H2
zmp_ph114_A9
zmp_ph114_A11
zmp_ph114_B2
zmp_ph114_B4
zmp_ph114_D8
zmp_ph114_E2

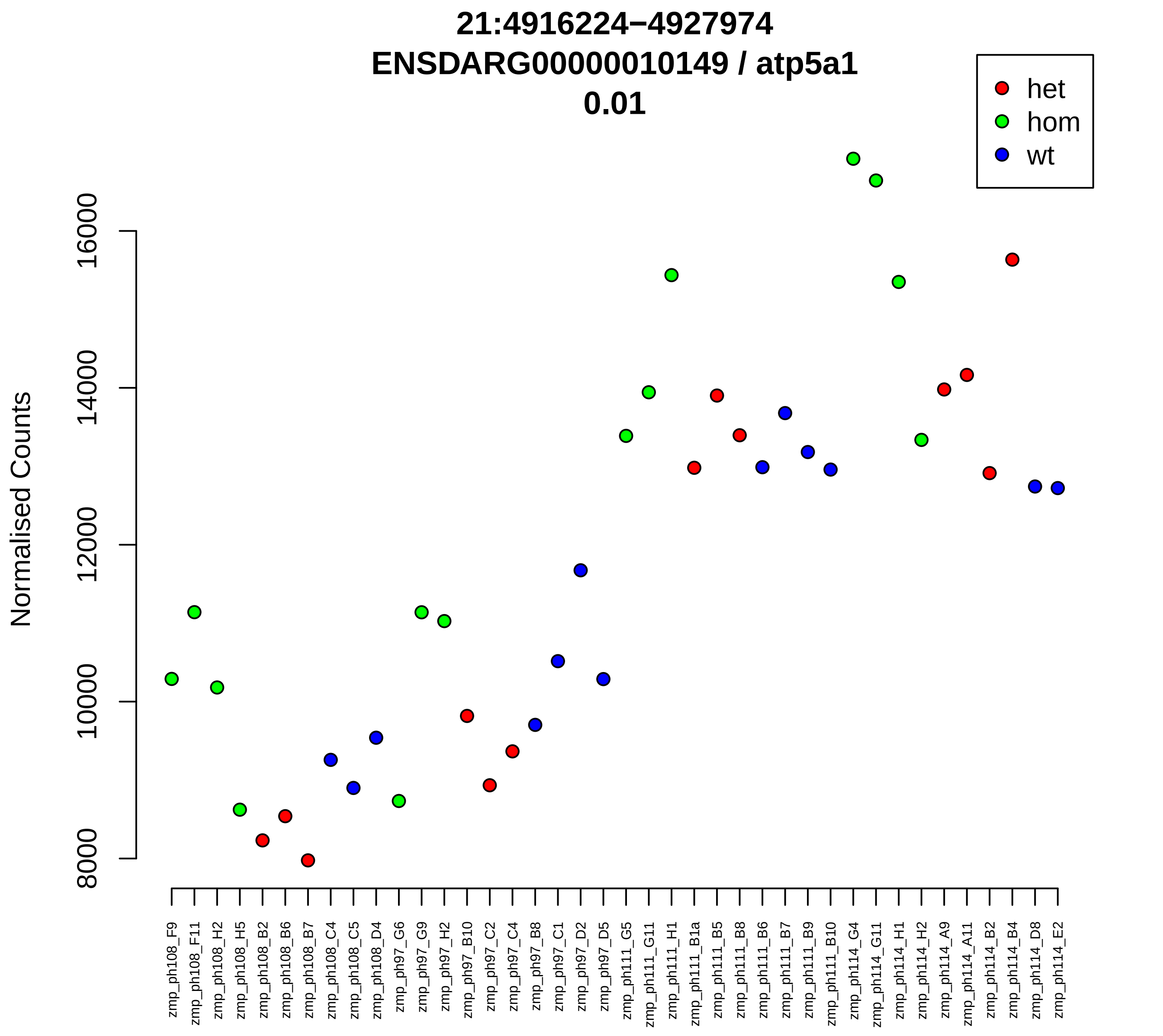
21:4916224−4927974
ENSDARG00000010149 / atp5a1
0.01
het
hom
wt
Normalised Counts
8000
10000
12000
14000
16000
zmp_ph108_F9
zmp_ph108_F11
zmp_ph108_H2
zmp_ph108_H5
zmp_ph108_B2
zmp_ph108_B6
zmp_ph108_B7
zmp_ph108_C4
zmp_ph108_C5
zmp_ph108_D4
zmp_ph97_G6
zmp_ph97_G9
zmp_ph97_H2
zmp_ph97_B10
zmp_ph97_C2
zmp_ph97_C4
zmp_ph97_B8
zmp_ph97_C1
zmp_ph97_D2
zmp_ph97_D5
zmp_ph111_G5
zmp_ph111_G11
zmp_ph111_H1
zmp_ph111_B1a
zmp_ph111_B5
zmp_ph111_B8
zmp_ph111_B6
zmp_ph111_B7
zmp_ph111_B9
zmp_ph111_B10
zmp_ph114_G4
zmp_ph114_G11
zmp_ph114_H1
zmp_ph114_H2
zmp_ph114_A9
zmp_ph114_A11
zmp_ph114_B2
zmp_ph114_B4
zmp_ph114_D8
zmp_ph114_E2

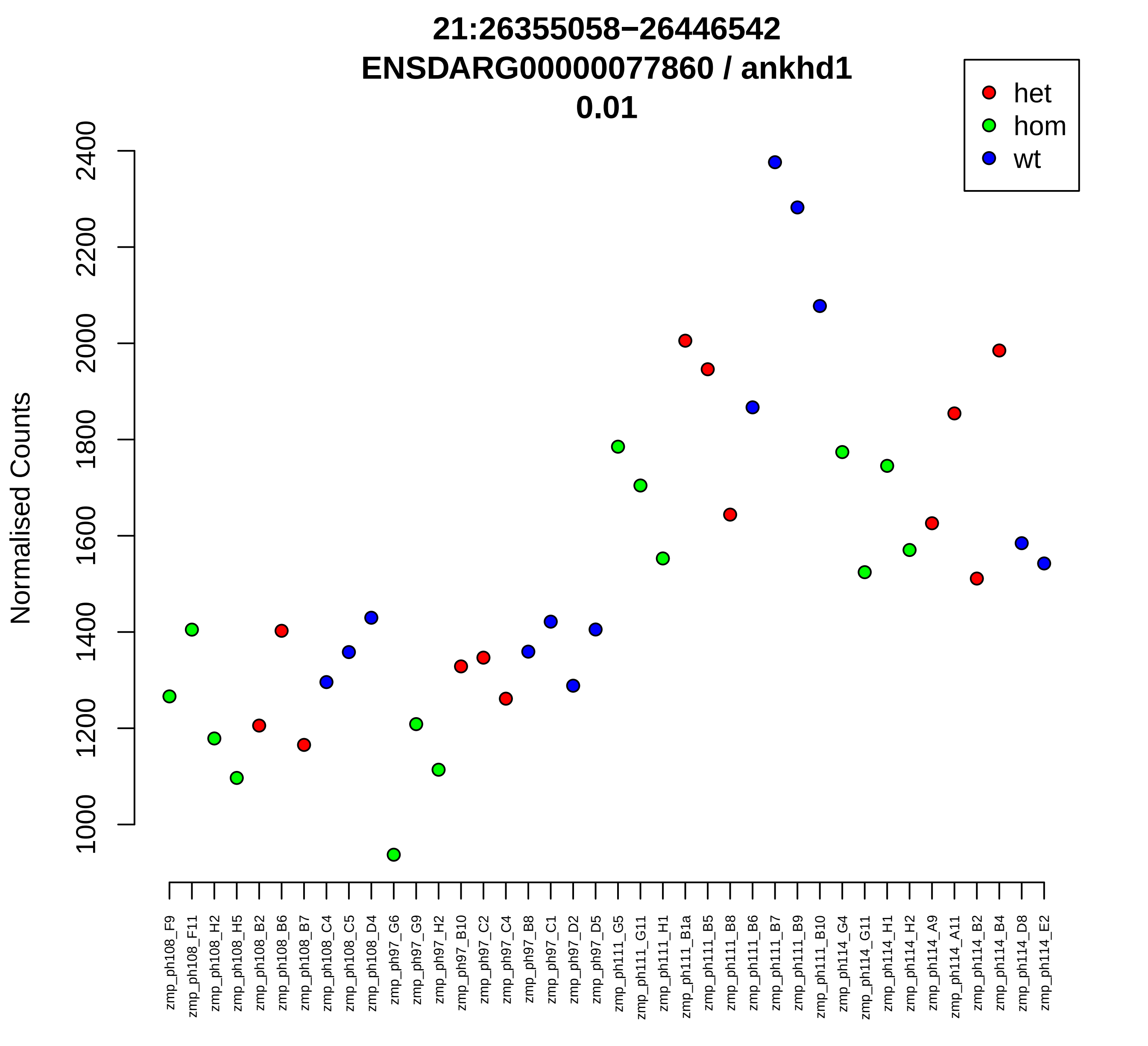

21:26355058−26446542
ENSDARG00000077860 / ankhd1
0.01
Normalised Counts
het
hom
wt
1000
1200
1400
1600
1800
2000
2200
2400
zmp_ph108_F9
zmp_ph108_F11
zmp_ph108_H2
zmp_ph108_H5
zmp_ph108_B2
zmp_ph108_B6
zmp_ph108_B7
zmp_ph108_C4
zmp_ph108_C5
zmp_ph108_D4
zmp_ph97_G6
zmp_ph97_G9
zmp_ph97_H2
zmp_ph97_B10
zmp_ph97_C2
zmp_ph97_C4
zmp_ph97_B8
zmp_ph97_C1
zmp_ph97_D2
zmp_ph97_D5
zmp_ph111_G5
zmp_ph111_G11
zmp_ph111_H1
zmp_ph111_B1a
zmp_ph111_B5
zmp_ph111_B8
zmp_ph111_B6
zmp_ph111_B7
zmp_ph111_B9
zmp_ph111_B10
zmp_ph114_G4
zmp_ph114_G11
zmp_ph114_H1
zmp_ph114_H2
zmp_ph114_A9
zmp_ph114_A11
zmp_ph114_B2
zmp_ph114_B4
zmp_ph114_D8
zmp_ph114_E2

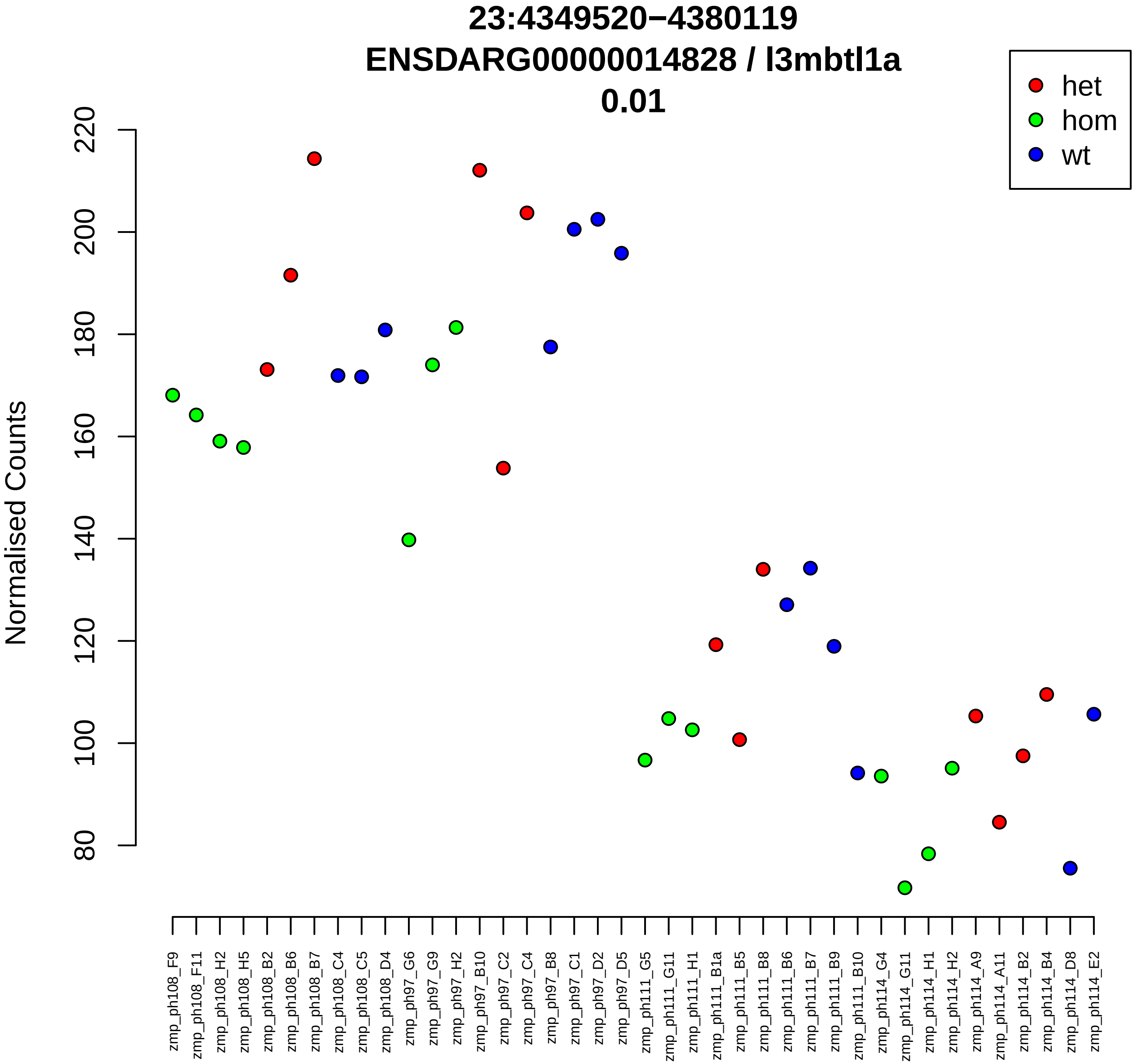

23:4349520−4380119
ENSDARG00000014828 / l3mbtl1a
0.01
het
hom
wt
Normalised Counts
80
100
120
140
160
180
200
220
zmp_ph108_F9
zmp_ph108_F11
zmp_ph108_H2
zmp_ph108_H5
zmp_ph108_B2
zmp_ph108_B6
zmp_ph108_B7
zmp_ph108_C4
zmp_ph108_C5
zmp_ph108_D4
zmp_ph97_G6
zmp_ph97_G9
zmp_ph97_H2
zmp_ph97_B10
zmp_ph97_C2
zmp_ph97_C4
zmp_ph97_B8
zmp_ph97_C1
zmp_ph97_D2
zmp_ph97_D5
zmp_ph111_G5
zmp_ph111_G11
zmp_ph111_H1
zmp_ph111_B1a
zmp_ph111_B5
zmp_ph111_B8
zmp_ph111_B6
zmp_ph111_B7
zmp_ph111_B9
zmp_ph111_B10
zmp_ph114_G4
zmp_ph114_G11
zmp_ph114_H1
zmp_ph114_H2
zmp_ph114_A9
zmp_ph114_A11
zmp_ph114_B2
zmp_ph114_B4
zmp_ph114_D8
zmp_ph114_E2

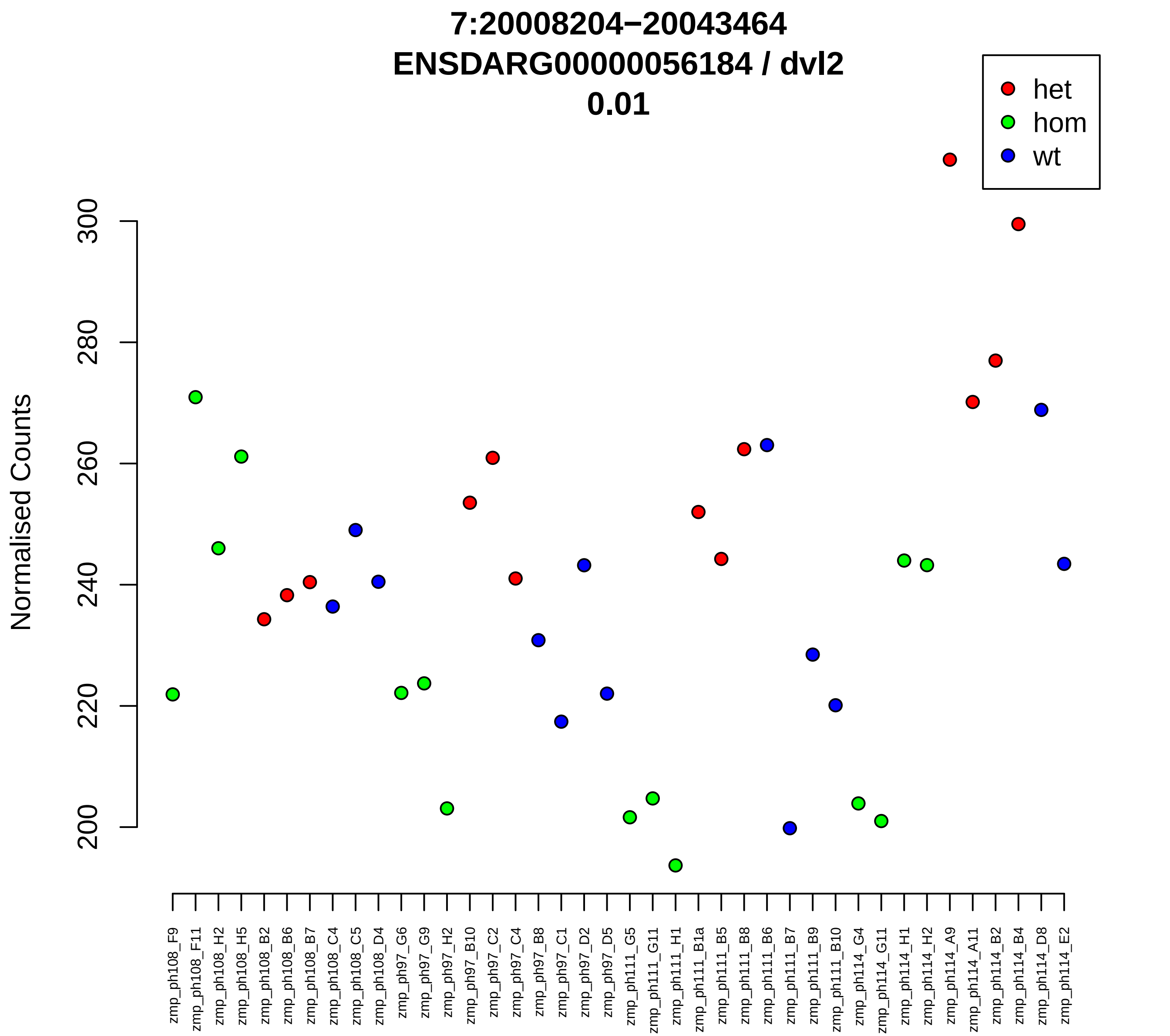
7:20008204−20043464
ENSDARG00000056184 / dvl2
0.01
het
hom
wt
Normalised Counts
200
220
240
260
280
300
zmp_ph108_F9
zmp_ph108_F11
zmp_ph108_H2
zmp_ph108_H5
zmp_ph108_B2
zmp_ph108_B6
zmp_ph108_B7
zmp_ph108_C4
zmp_ph108_C5
zmp_ph108_D4
zmp_ph97_G6
zmp_ph97_G9
zmp_ph97_H2
zmp_ph97_B10
zmp_ph97_C2
zmp_ph97_C4
zmp_ph97_B8
zmp_ph97_C1
zmp_ph97_D2
zmp_ph97_D5
zmp_ph111_G5
zmp_ph111_G11
zmp_ph111_H1
zmp_ph111_B1a
zmp_ph111_B5
zmp_ph111_B8
zmp_ph111_B6
zmp_ph111_B7
zmp_ph111_B9
zmp_ph111_B10
zmp_ph114_G4
zmp_ph114_G11
zmp_ph114_H1
zmp_ph114_H2
zmp_ph114_A9
zmp_ph114_A11
zmp_ph114_B2
zmp_ph114_B4
zmp_ph114_D8
zmp_ph114_E2

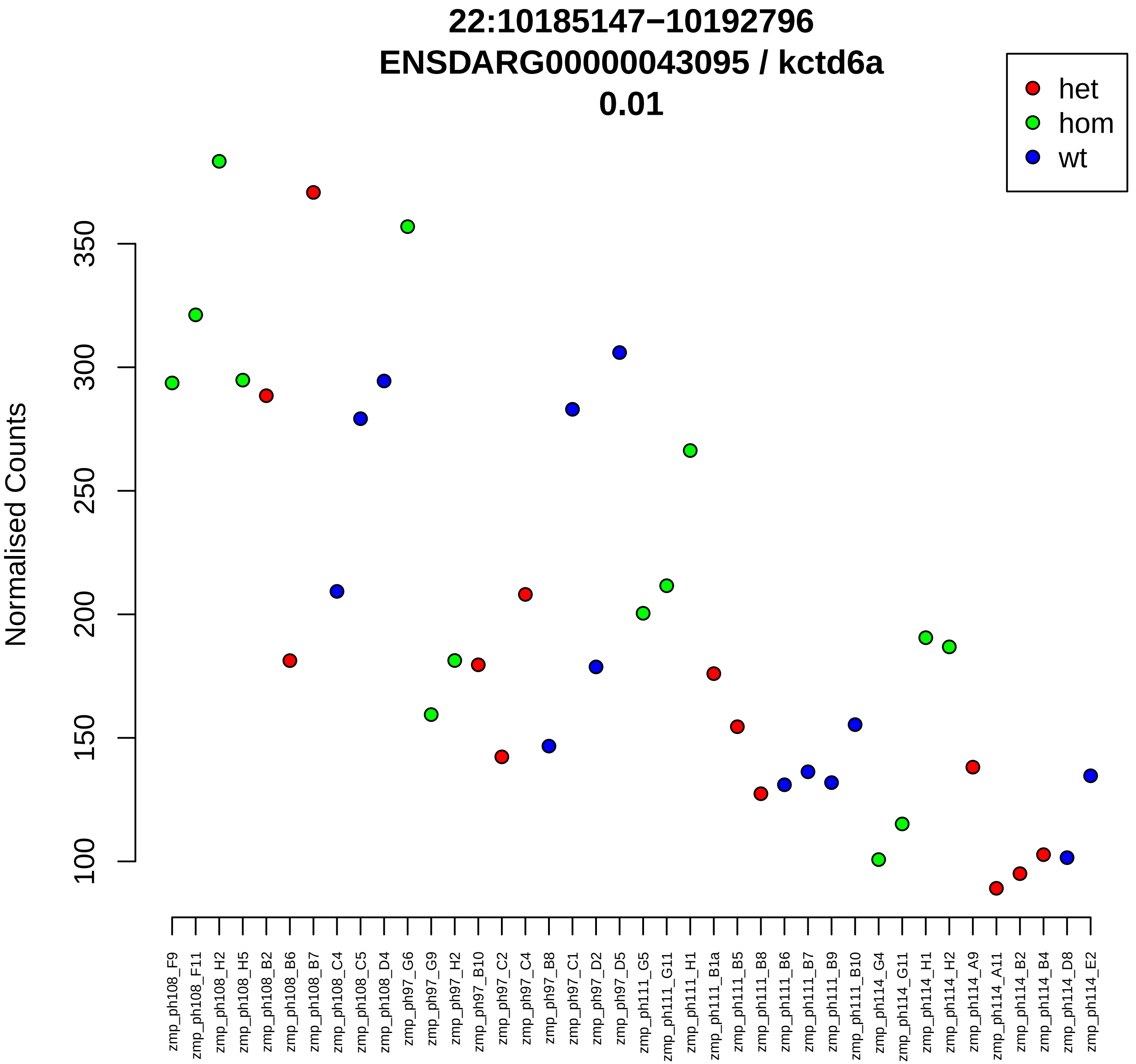

22:10185147−10192796
ENSDARG00000043095 / kctd6a
0.01
het
hom
wt
Normalised Counts
100
150
200
250
300
350
zmp_ph108_F9
zmp_ph108_F11
zmp_ph108_H2
zmp_ph108_H5
zmp_ph108_B2
zmp_ph108_B6
zmp_ph108_B7
zmp_ph108_C4
zmp_ph108_C5
zmp_ph108_D4
zmp_ph97_G6
zmp_ph97_G9
zmp_ph97_H2
zmp_ph97_B10
zmp_ph97_C2
zmp_ph97_C4
zmp_ph97_B8
zmp_ph97_C1
zmp_ph97_D2
zmp_ph97_D5
zmp_ph111_G5
zmp_ph111_G11
zmp_ph111_H1
zmp_ph111_B1a
zmp_ph111_B5
zmp_ph111_B8
zmp_ph111_B6
zmp_ph111_B7
zmp_ph111_B9
zmp_ph111_B10
zmp_ph114_G4
zmp_ph114_G11
zmp_ph114_H1
zmp_ph114_H2
zmp_ph114_A9
zmp_ph114_A11
zmp_ph114_B2
zmp_ph114_B4
zmp_ph114_D8
zmp_ph114_E2

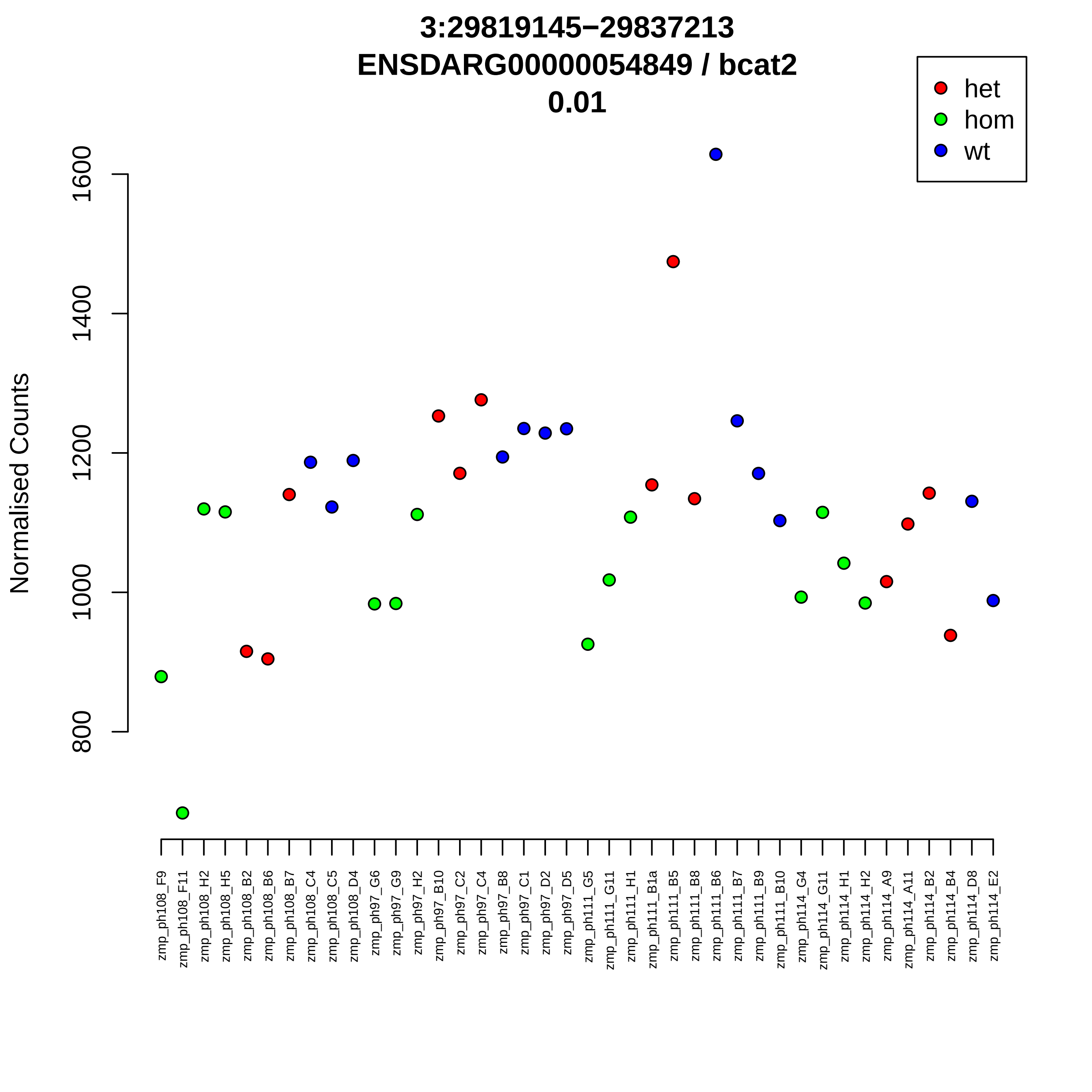
3:29819145−29837213
ENSDARG00000054849 / bcat2
0.01
het
hom
wt
Normalised Counts
800
1000
1200
1400
1600
zmp_ph108_F9
zmp_ph108_F11
zmp_ph108_H2
zmp_ph108_H5
zmp_ph108_B2
zmp_ph108_B6
zmp_ph108_B7
zmp_ph108_C4
zmp_ph108_C5
zmp_ph108_D4
zmp_ph97_G6
zmp_ph97_G9
zmp_ph97_H2
zmp_ph97_B10
zmp_ph97_C2
zmp_ph97_C4
zmp_ph97_B8
zmp_ph97_C1
zmp_ph97_D2
zmp_ph97_D5
zmp_ph111_G5
zmp_ph111_G11
zmp_ph111_H1
zmp_ph111_B1a
zmp_ph111_B5
zmp_ph111_B8
zmp_ph111_B6
zmp_ph111_B7
zmp_ph111_B9
zmp_ph111_B10
zmp_ph114_G4
zmp_ph114_G11
zmp_ph114_H1
zmp_ph114_H2
zmp_ph114_A9
zmp_ph114_A11
zmp_ph114_B2
zmp_ph114_B4
zmp_ph114_D8
zmp_ph114_E2

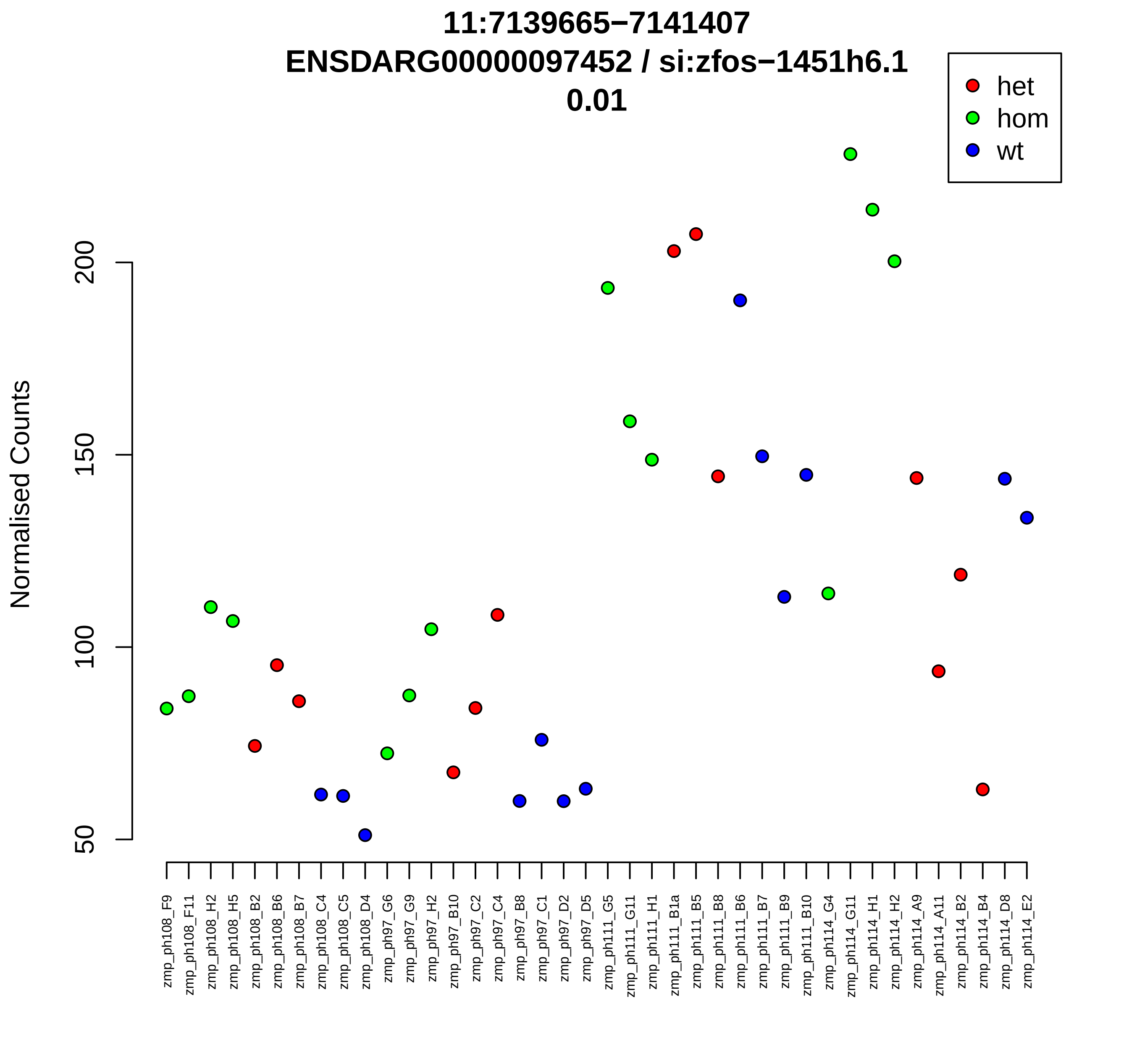

11:7139665−7141407
ENSDARG00000097452 / si:zfos−1451h6.1
0.01
het
hom
wt
Normalised Counts
50
100
150
200
zmp_ph108_F9
zmp_ph108_F11
zmp_ph108_H2
zmp_ph108_H5
zmp_ph108_B2
zmp_ph108_B6
zmp_ph108_B7
zmp_ph108_C4
zmp_ph108_C5
zmp_ph108_D4
zmp_ph97_G6
zmp_ph97_G9
zmp_ph97_H2
zmp_ph97_B10
zmp_ph97_C2
zmp_ph97_C4
zmp_ph97_B8
zmp_ph97_C1
zmp_ph97_D2
zmp_ph97_D5
zmp_ph111_G5
zmp_ph111_G11
zmp_ph111_H1
zmp_ph111_B1a
zmp_ph111_B5
zmp_ph111_B8
zmp_ph111_B6
zmp_ph111_B7
zmp_ph111_B9
zmp_ph111_B10
zmp_ph114_G4
zmp_ph114_G11
zmp_ph114_H1
zmp_ph114_H2
zmp_ph114_A9
zmp_ph114_A11
zmp_ph114_B2
zmp_ph114_B4
zmp_ph114_D8
zmp_ph114_E2

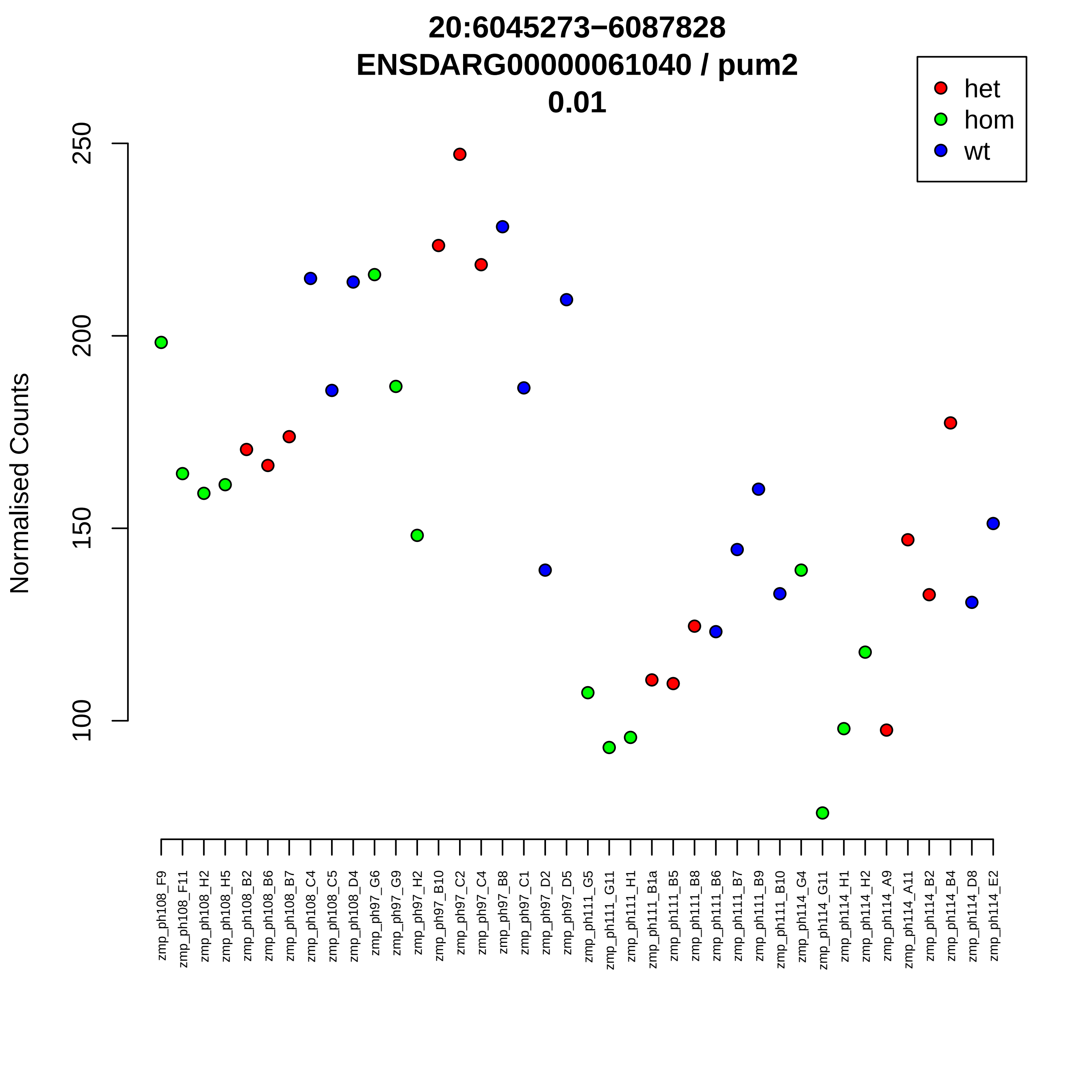
20:6045273−6087828
ENSDARG00000061040 / pum2
0.01
het
hom
wt
Normalised Counts
250
200
150
100
zmp_ph108_F9
zmp_ph108_F11
zmp_ph108_H2
zmp_ph108_H5
zmp_ph108_B2
zmp_ph108_B6
zmp_ph108_B7
zmp_ph108_C4
zmp_ph108_C5
zmp_ph108_D4
zmp_ph97_G6
zmp_ph97_G9
zmp_ph97_H2
zmp_ph97_B10
zmp_ph97_C2
zmp_ph97_C4
zmp_ph97_B8
zmp_ph97_C1
zmp_ph97_D2
zmp_ph97_D5
zmp_ph111_G5
zmp_ph111_G11
zmp_ph111_H1
zmp_ph111_B1a
zmp_ph111_B5
zmp_ph111_B8
zmp_ph111_B6
zmp_ph111_B7
zmp_ph111_B9
zmp_ph111_B10
zmp_ph114_G4
zmp_ph114_G11
zmp_ph114_H1
zmp_ph114_H2
zmp_ph114_A9
zmp_ph114_A11
zmp_ph114_B2
zmp_ph114_B4
zmp_ph114_D8
zmp_ph114_E2

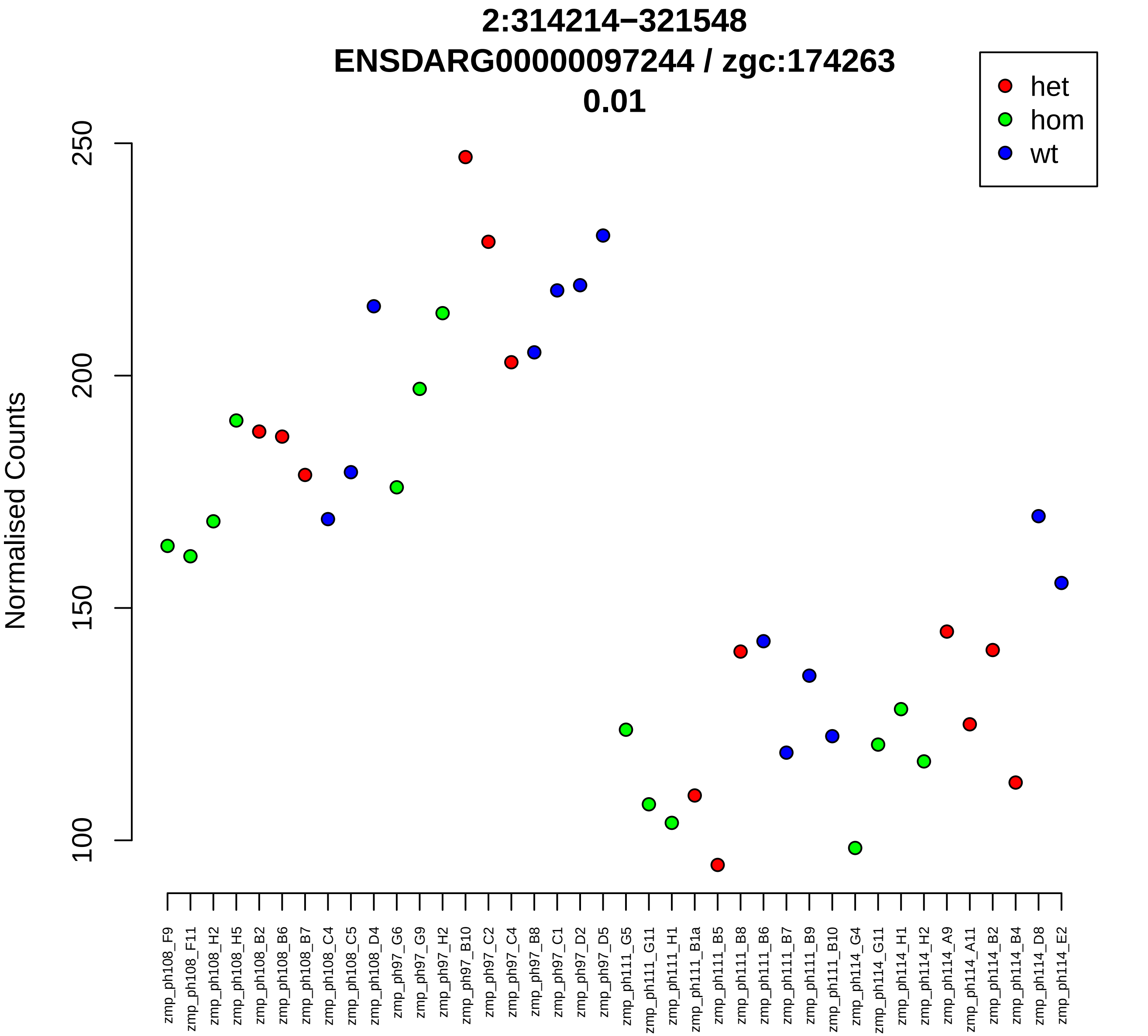

2:314214−321548
ENSDARG00000097244 / zgc:174263
0.01
het
hom
wt
Normalised Counts
250
200
150
100
zmp_ph108_F9
zmp_ph108_F11
zmp_ph108_H2
zmp_ph108_H5
zmp_ph108_B2
zmp_ph108_B6
zmp_ph108_B7
zmp_ph108_C4
zmp_ph108_C5
zmp_ph108_D4
zmp_ph97_G6
zmp_ph97_G9
zmp_ph97_H2
zmp_ph97_B10
zmp_ph97_C2
zmp_ph97_C4
zmp_ph97_B8
zmp_ph97_C1
zmp_ph97_D2
zmp_ph97_D5
zmp_ph111_G5
zmp_ph111_G11
zmp_ph111_H1
zmp_ph111_B1a
zmp_ph111_B5
zmp_ph111_B8
zmp_ph111_B6
zmp_ph111_B7
zmp_ph111_B9
zmp_ph111_B10
zmp_ph114_G4
zmp_ph114_G11
zmp_ph114_H1
zmp_ph114_H2
zmp_ph114_A9
zmp_ph114_A11
zmp_ph114_B2
zmp_ph114_B4
zmp_ph114_D8
zmp_ph114_E2

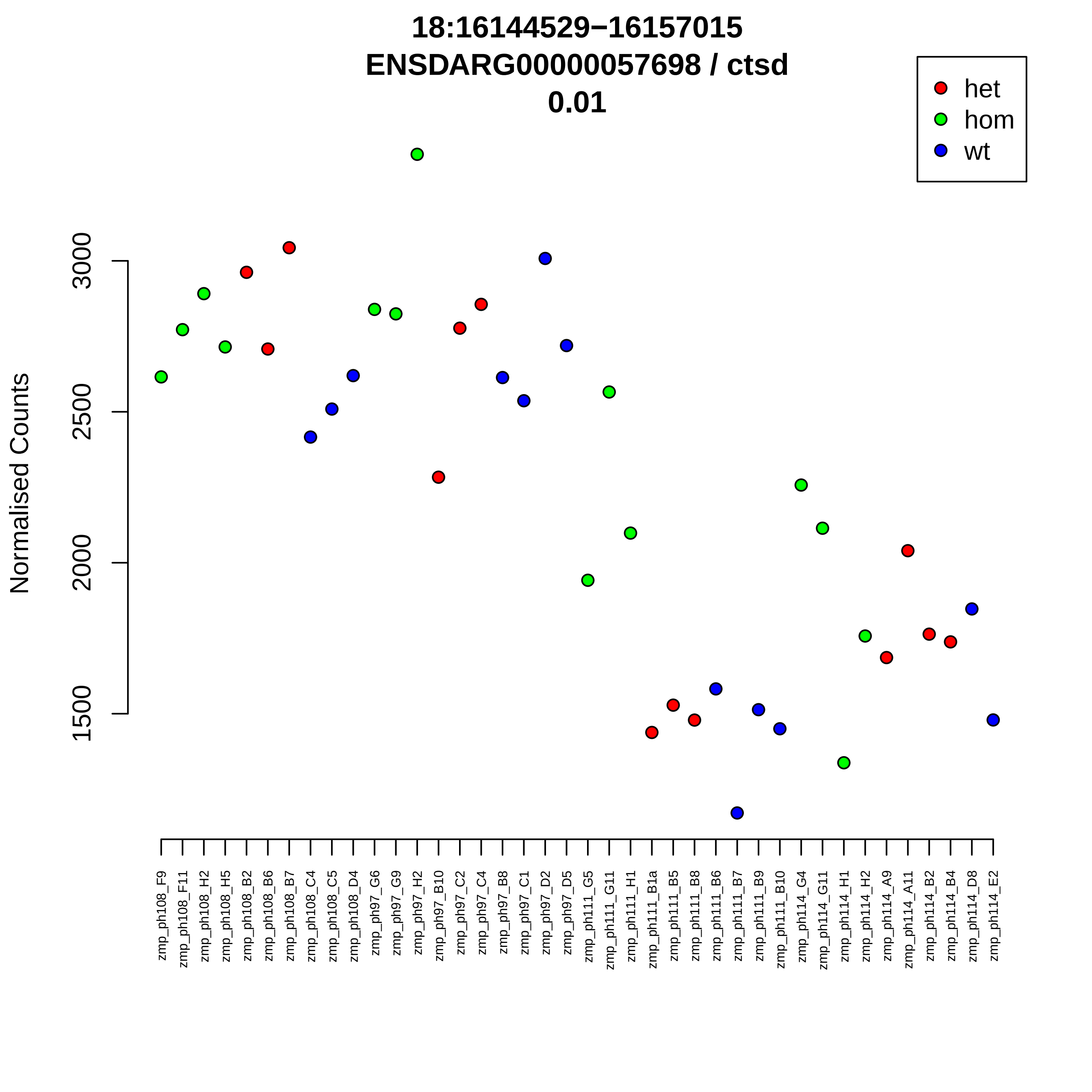

18:16144529−16157015
ENSDARG00000057698 / ctsd
0.01
het
hom
wt
Normalised Counts
3000
2500
2000
1500
zmp_ph108_F9
zmp_ph108_F11
zmp_ph108_H2
zmp_ph108_H5
zmp_ph108_B2
zmp_ph108_B6
zmp_ph108_B7
zmp_ph108_C4
zmp_ph108_C5
zmp_ph108_D4
zmp_ph97_G6
zmp_ph97_G9
zmp_ph97_H2
zmp_ph97_B10
zmp_ph97_C2
zmp_ph97_C4
zmp_ph97_B8
zmp_ph97_C1
zmp_ph97_D2
zmp_ph97_D5
zmp_ph111_G5
zmp_ph111_G11
zmp_ph111_H1
zmp_ph111_B1a
zmp_ph111_B5
zmp_ph111_B8
zmp_ph111_B6
zmp_ph111_B7
zmp_ph111_B9
zmp_ph111_B10
zmp_ph114_G4
zmp_ph114_G11
zmp_ph114_H1
zmp_ph114_H2
zmp_ph114_A9
zmp_ph114_A11
zmp_ph114_B2
zmp_ph114_B4
zmp_ph114_D8
zmp_ph114_E2

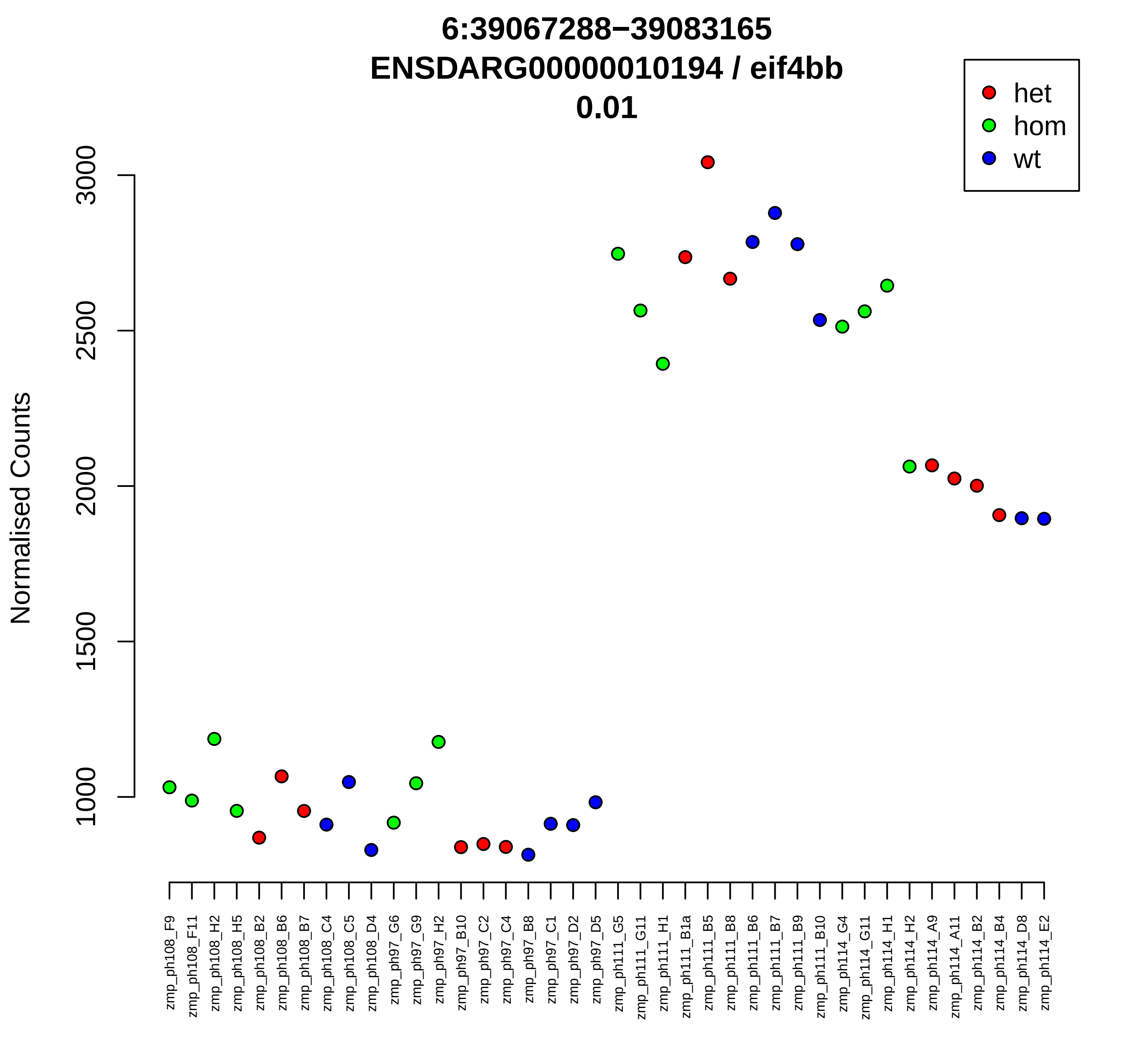

6:39067288−39083165
ENSDARG00000010194 / eif4bb
0.01
het
hom
wt
Normalised Counts
1000
1500
2000
2500
3000
zmp_ph108_F9
zmp_ph108_F11
zmp_ph108_H2
zmp_ph108_H5
zmp_ph108_B2
zmp_ph108_B6
zmp_ph108_B7
zmp_ph108_C4
zmp_ph108_C5
zmp_ph108_D4
zmp_ph97_G6
zmp_ph97_G9
zmp_ph97_H2
zmp_ph97_B10
zmp_ph97_C2
zmp_ph97_C4
zmp_ph97_B8
zmp_ph97_C1
zmp_ph97_D2
zmp_ph97_D5
zmp_ph111_G5
zmp_ph111_G11
zmp_ph111_H1
zmp_ph111_B1a
zmp_ph111_B5
zmp_ph111_B8
zmp_ph111_B6
zmp_ph111_B7
zmp_ph111_B9
zmp_ph111_B10
zmp_ph114_G4
zmp_ph114_G11
zmp_ph114_H1
zmp_ph114_H2
zmp_ph114_A9
zmp_ph114_A11
zmp_ph114_B2
zmp_ph114_B4
zmp_ph114_D8
zmp_ph114_E2

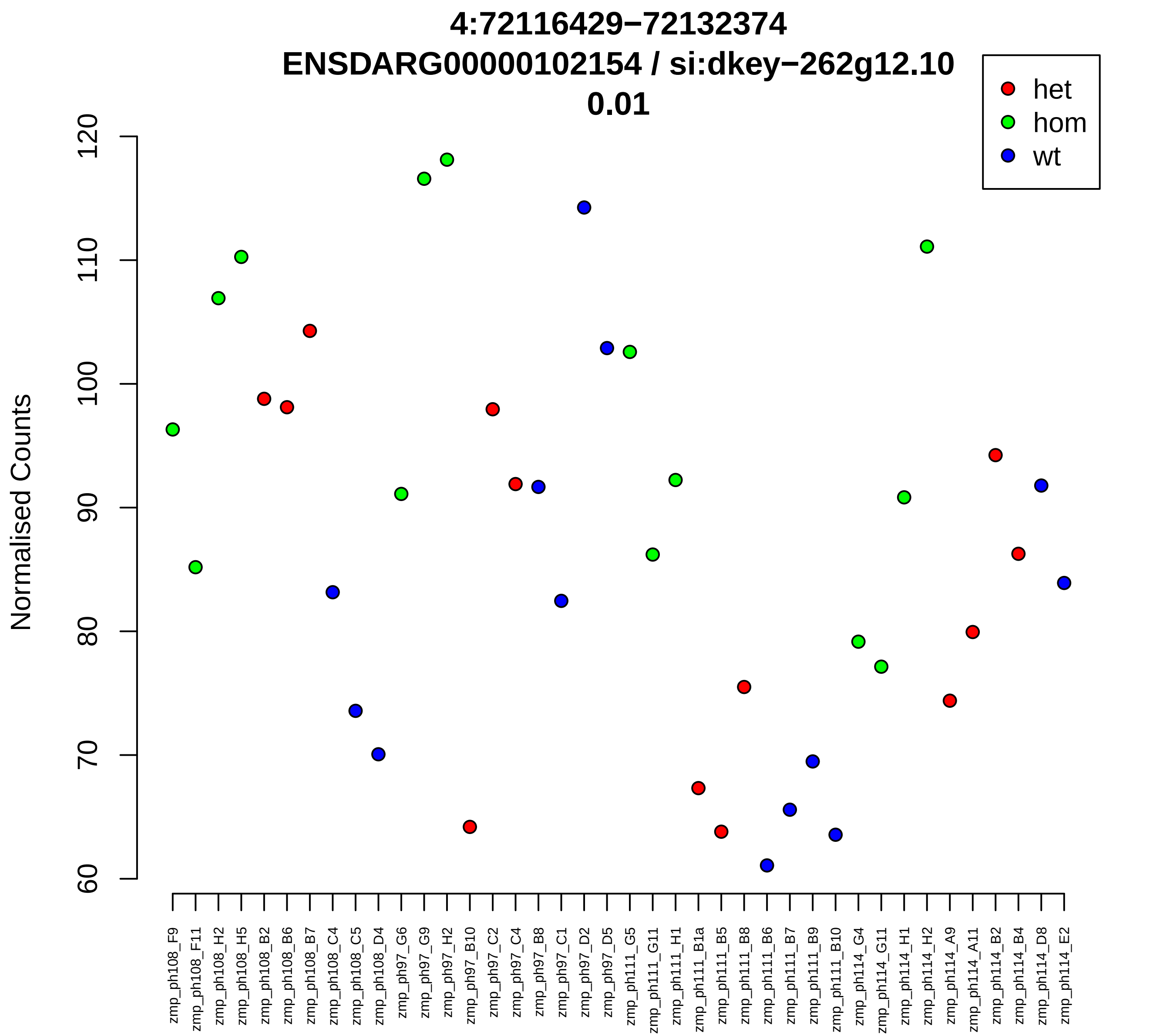

4:72116429−72132374
ENSDARG00000102154 / si:dkey−262g12.10
0.01
Normalised Counts
60
70
80
90
100
110
120
het
hom
wt
zmp_ph108_F9
zmp_ph108_F11
zmp_ph108_H2
zmp_ph108_H5
zmp_ph108_B2
zmp_ph108_B6
zmp_ph108_B7
zmp_ph108_C4
zmp_ph108_C5
zmp_ph108_D4
zmp_ph97_G6
zmp_ph97_G9
zmp_ph97_H2
zmp_ph97_B10
zmp_ph97_C2
zmp_ph97_C4
zmp_ph97_B8
zmp_ph97_C1
zmp_ph97_D2
zmp_ph97_D5
zmp_ph111_G5
zmp_ph111_G11
zmp_ph111_H1
zmp_ph111_B1a
zmp_ph111_B5
zmp_ph111_B8
zmp_ph111_B6
zmp_ph111_B7
zmp_ph111_B9
zmp_ph111_B10
zmp_ph114_G4
zmp_ph114_G11
zmp_ph114_H1
zmp_ph114_H2
zmp_ph114_A9
zmp_ph114_A11
zmp_ph114_B2
zmp_ph114_B4
zmp_ph114_D8
zmp_ph114_E2

4:2418535−2440955
ENSDARG00000053709 / si:ch73−89b15.3
0.01

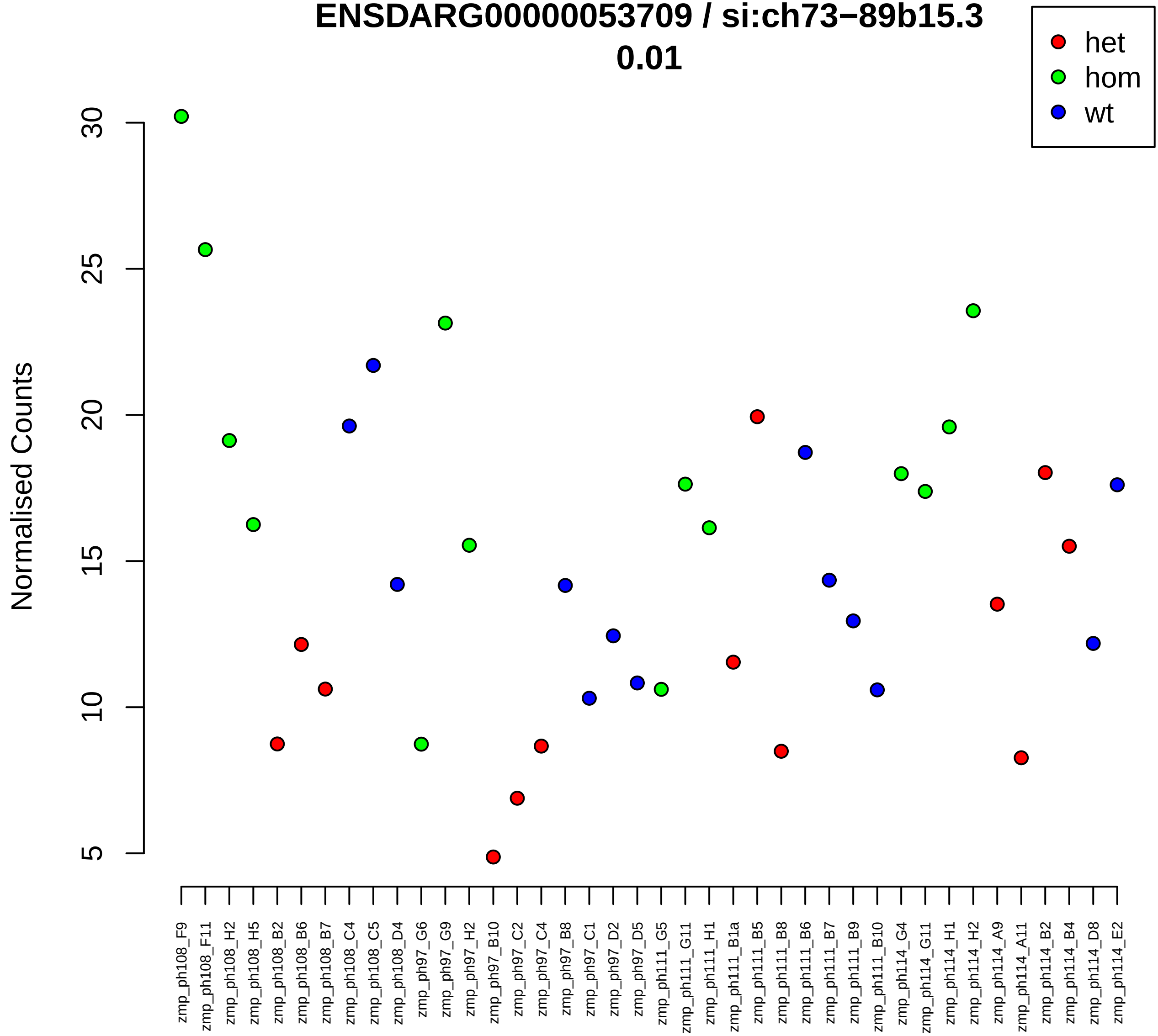

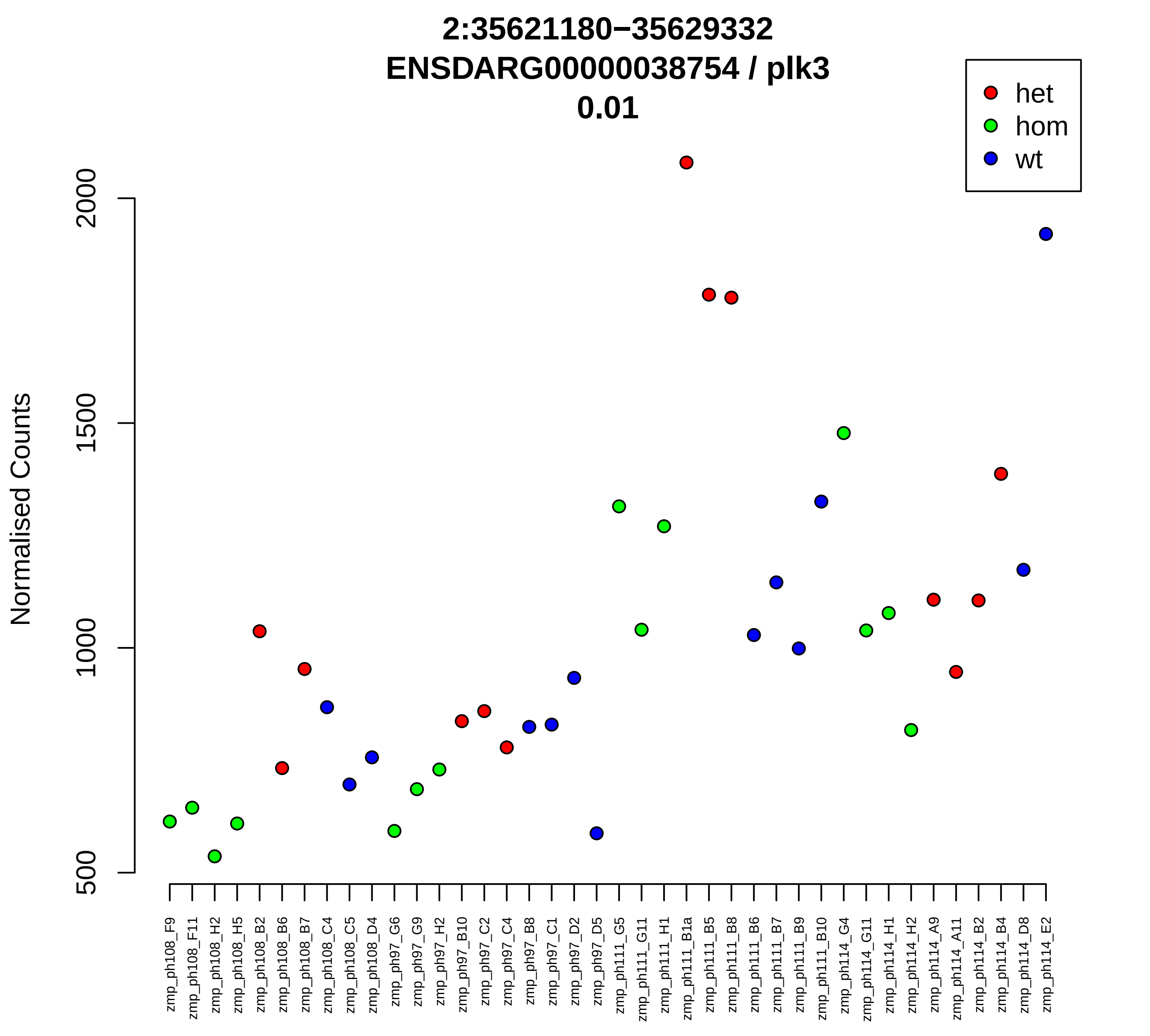

2:35621180−35629332
ENSDARG00000038754 / plk3
0.01
het
hom
wt
Normalised Counts
500
1000
1500
2000
zmp_ph108_F9
zmp_ph108_F11
zmp_ph108_H2
zmp_ph108_H5
zmp_ph108_B2
zmp_ph108_B6
zmp_ph108_B7
zmp_ph108_C4
zmp_ph108_C5
zmp_ph108_D4
zmp_ph97_G6
zmp_ph97_G9
zmp_ph97_H2
zmp_ph97_B10
zmp_ph97_C2
zmp_ph97_C4
zmp_ph97_B8
zmp_ph97_C1
zmp_ph97_D2
zmp_ph97_D5
zmp_ph111_G5
zmp_ph111_G11
zmp_ph111_H1
zmp_ph111_B1a
zmp_ph111_B5
zmp_ph111_B8
zmp_ph111_B6
zmp_ph111_B7
zmp_ph111_B9
zmp_ph111_B10
zmp_ph114_G4
zmp_ph114_G11
zmp_ph114_H1
zmp_ph114_H2
zmp_ph114_A9
zmp_ph114_A11
zmp_ph114_B2
zmp_ph114_B4
zmp_ph114_D8
zmp_ph114_E2

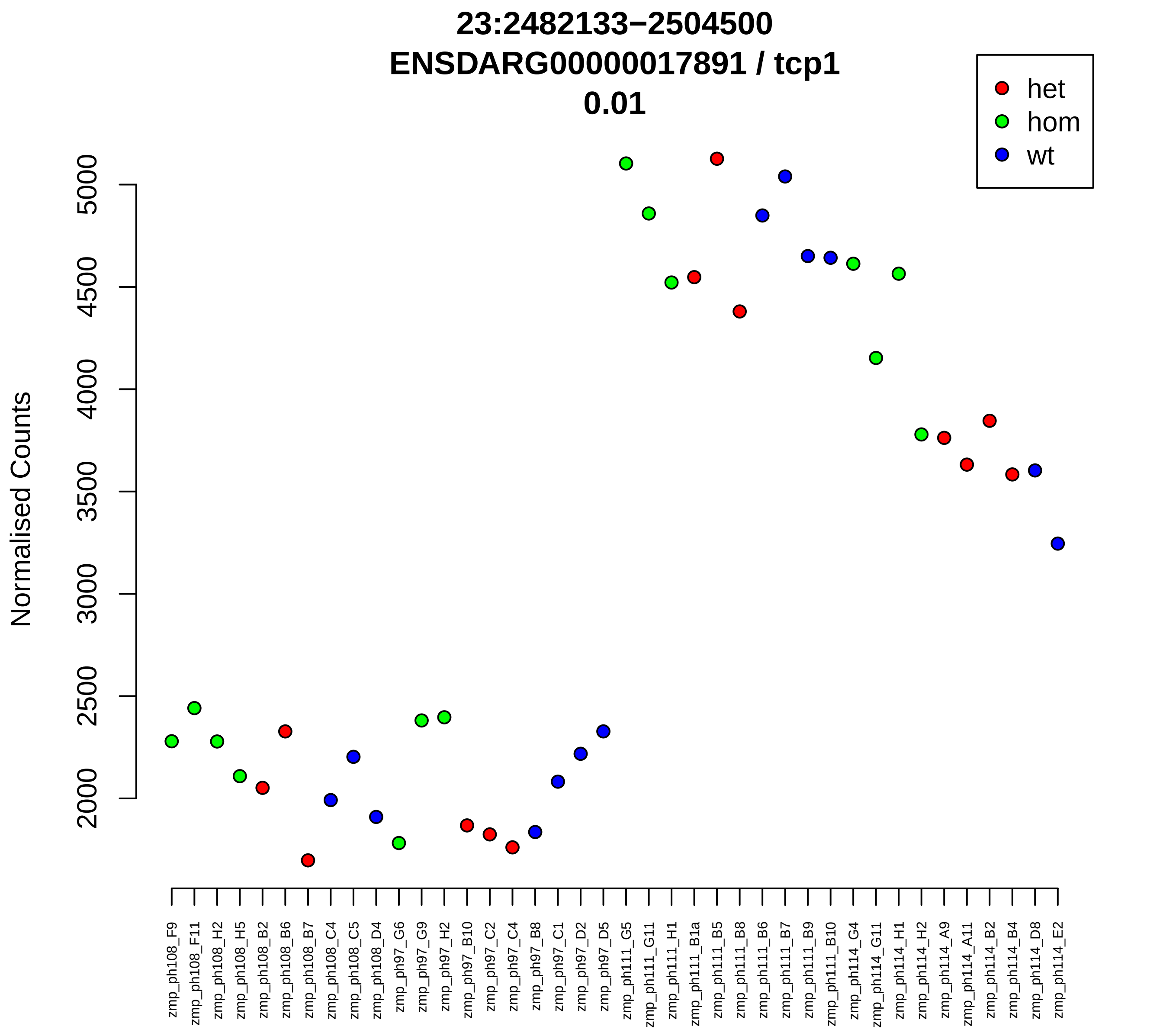

23:2482133−2504500
ENSDARG00000017891 / tcp1
0.01
het
hom
wt
Normalised Counts
2000
2500
3000
3500
4000
4500
5000
zmp_ph108_F9
zmp_ph108_F11
zmp_ph108_H2
zmp_ph108_H5
zmp_ph108_B2
zmp_ph108_B6
zmp_ph108_B7
zmp_ph108_C4
zmp_ph108_C5
zmp_ph108_D4
zmp_ph97_G6
zmp_ph97_G9
zmp_ph97_H2
zmp_ph97_B10
zmp_ph97_C2
zmp_ph97_C4
zmp_ph97_B8
zmp_ph97_C1
zmp_ph97_D2
zmp_ph97_D5
zmp_ph111_G5
zmp_ph111_G11
zmp_ph111_H1
zmp_ph111_B1a
zmp_ph111_B5
zmp_ph111_B8
zmp_ph111_B6
zmp_ph111_B7
zmp_ph111_B9
zmp_ph111_B10
zmp_ph114_G4
zmp_ph114_G11
zmp_ph114_H1
zmp_ph114_H2
zmp_ph114_A9
zmp_ph114_A11
zmp_ph114_B2
zmp_ph114_B4
zmp_ph114_D8
zmp_ph114_E2

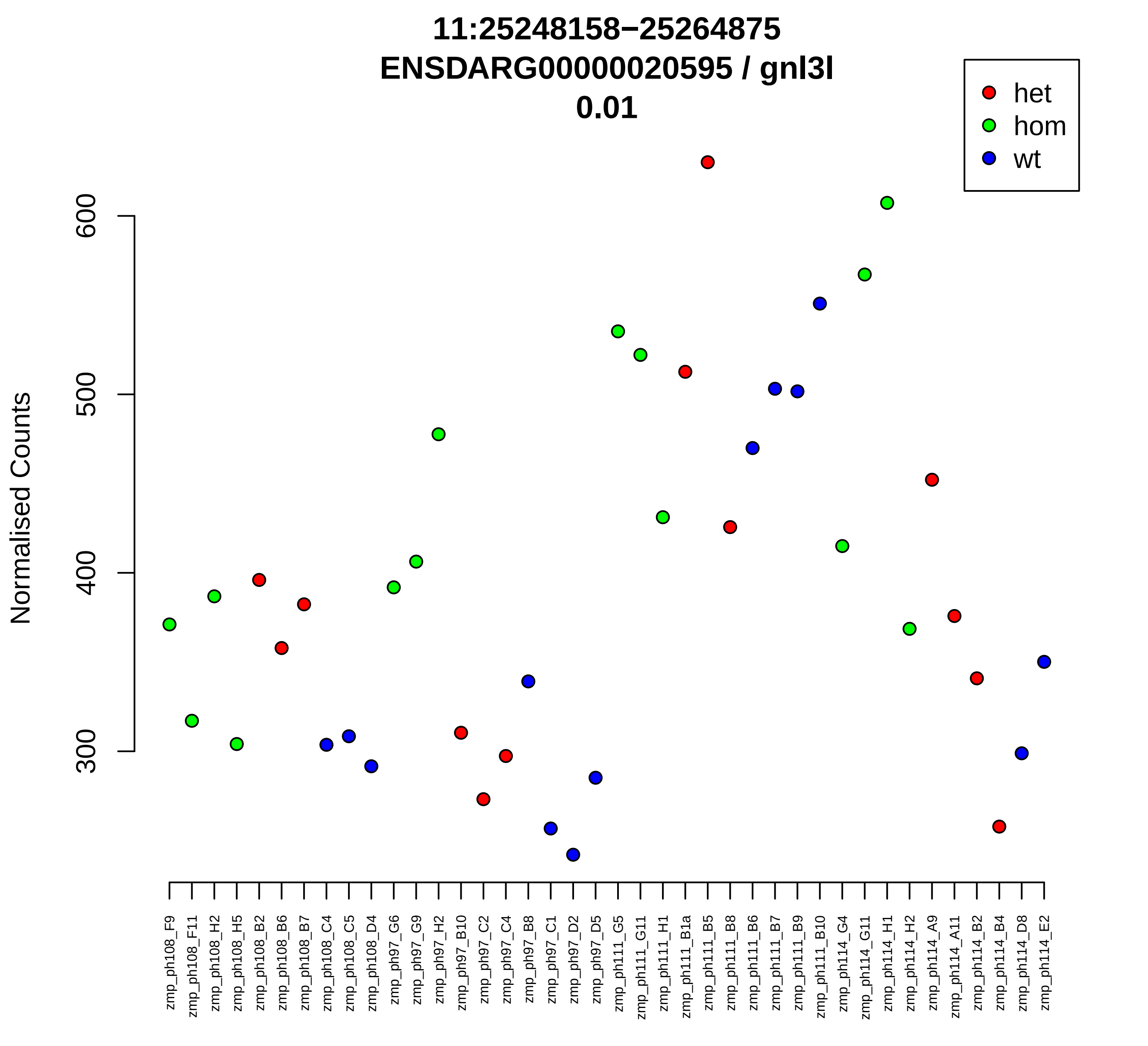
11:25248158−25264875
ENSDARG00000020595 / gnl3l
0.01
het
hom
wt
Normalised Counts
300
400
500
600
zmp_ph108_F9
zmp_ph108_F11
zmp_ph108_H2
zmp_ph108_H5
zmp_ph108_B2
zmp_ph108_B6
zmp_ph108_B7
zmp_ph108_C4
zmp_ph108_C5
zmp_ph108_D4
zmp_ph97_G6
zmp_ph97_G9
zmp_ph97_H2
zmp_ph97_B10
zmp_ph97_C2
zmp_ph97_C4
zmp_ph97_B8
zmp_ph97_C1
zmp_ph97_D2
zmp_ph97_D5
zmp_ph111_G5
zmp_ph111_G11
zmp_ph111_H1
zmp_ph111_B1a
zmp_ph111_B5
zmp_ph111_B8
zmp_ph111_B6
zmp_ph111_B7
zmp_ph111_B9
zmp_ph111_B10
zmp_ph114_G4
zmp_ph114_G11
zmp_ph114_H1
zmp_ph114_H2
zmp_ph114_A9
zmp_ph114_A11
zmp_ph114_B2
zmp_ph114_B4
zmp_ph114_D8
zmp_ph114_E2

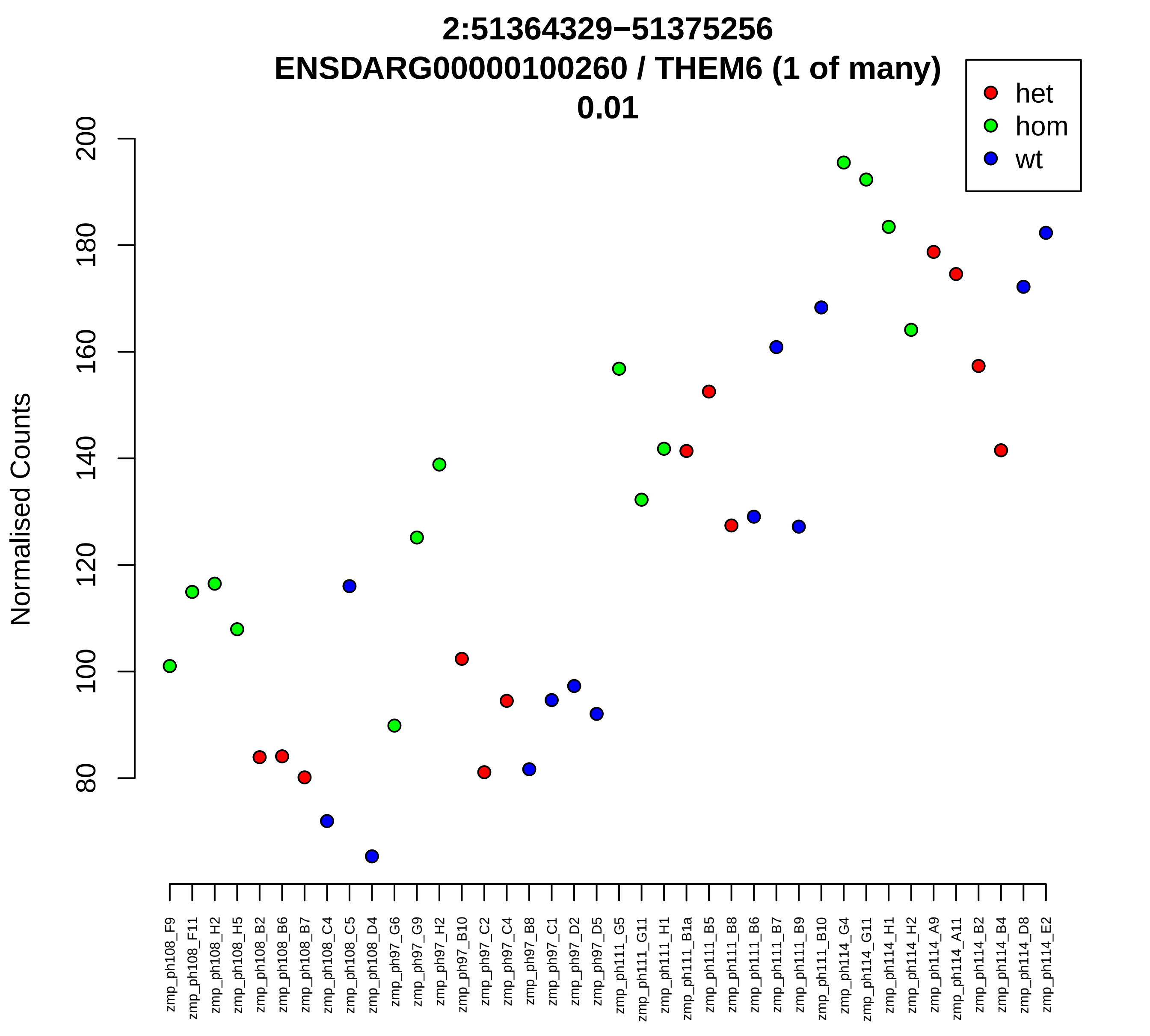
2:51364329−51375256
ENSDARG00000100260 / THEM6 (1 of many)
0.01
het
hom
wt
Normalised Counts
80
100
120
140
160
180
200
zmp_ph108_F9
zmp_ph108_F11
zmp_ph108_H2
zmp_ph108_H5
zmp_ph108_B2
zmp_ph108_B6
zmp_ph108_B7
zmp_ph108_C4
zmp_ph108_C5
zmp_ph108_D4
zmp_ph97_G6
zmp_ph97_G9
zmp_ph97_H2
zmp_ph97_B10
zmp_ph97_C2
zmp_ph97_C4
zmp_ph97_B8
zmp_ph97_C1
zmp_ph97_D2
zmp_ph97_D5
zmp_ph111_G5
zmp_ph111_G11
zmp_ph111_H1
zmp_ph111_B1a
zmp_ph111_B5
zmp_ph111_B8
zmp_ph111_B6
zmp_ph111_B7
zmp_ph111_B9
zmp_ph111_B10
zmp_ph114_G4
zmp_ph114_G11
zmp_ph114_H1
zmp_ph114_H2
zmp_ph114_A9
zmp_ph114_A11
zmp_ph114_B2
zmp_ph114_B4
zmp_ph114_D8
zmp_ph114_E2

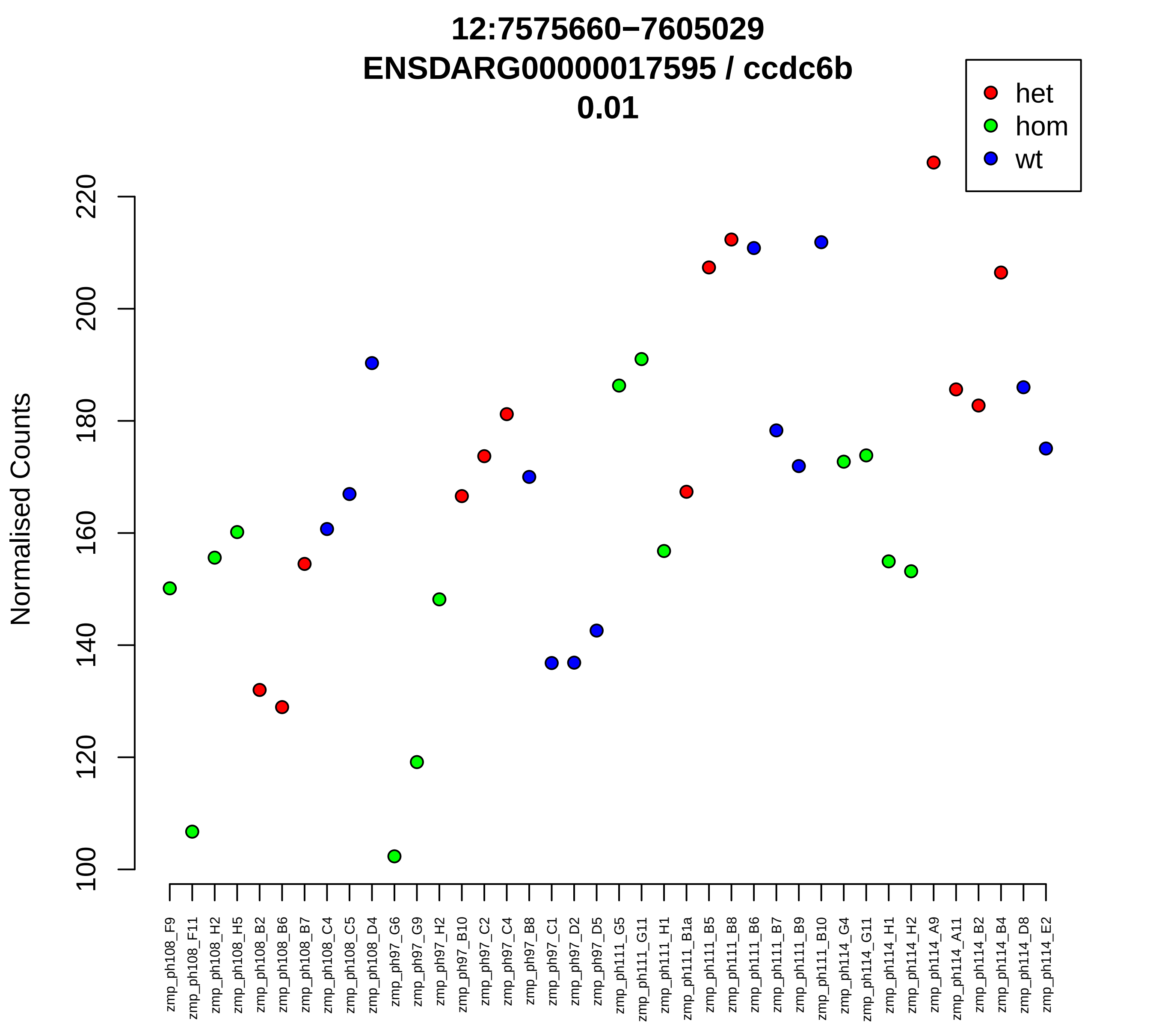
12:7575660−7605029
ENSDARG00000017595 / ccdc6b
0.01
het
hom
wt
Normalised Counts
100
120
140
160
180
200
220
zmp_ph108_F9
zmp_ph108_F11
zmp_ph108_H2
zmp_ph108_H5
zmp_ph108_B2
zmp_ph108_B6
zmp_ph108_B7
zmp_ph108_C4
zmp_ph108_C5
zmp_ph108_D4
zmp_ph97_G6
zmp_ph97_G9
zmp_ph97_H2
zmp_ph97_B10
zmp_ph97_C2
zmp_ph97_C4
zmp_ph97_B8
zmp_ph97_C1
zmp_ph97_D2
zmp_ph97_D5
zmp_ph111_G5
zmp_ph111_G11
zmp_ph111_H1
zmp_ph111_B1a
zmp_ph111_B5
zmp_ph111_B8
zmp_ph111_B6
zmp_ph111_B7
zmp_ph111_B9
zmp_ph111_B10
zmp_ph114_G4
zmp_ph114_G11
zmp_ph114_H1
zmp_ph114_H2
zmp_ph114_A9
zmp_ph114_A11
zmp_ph114_B2
zmp_ph114_B4
zmp_ph114_D8
zmp_ph114_E2

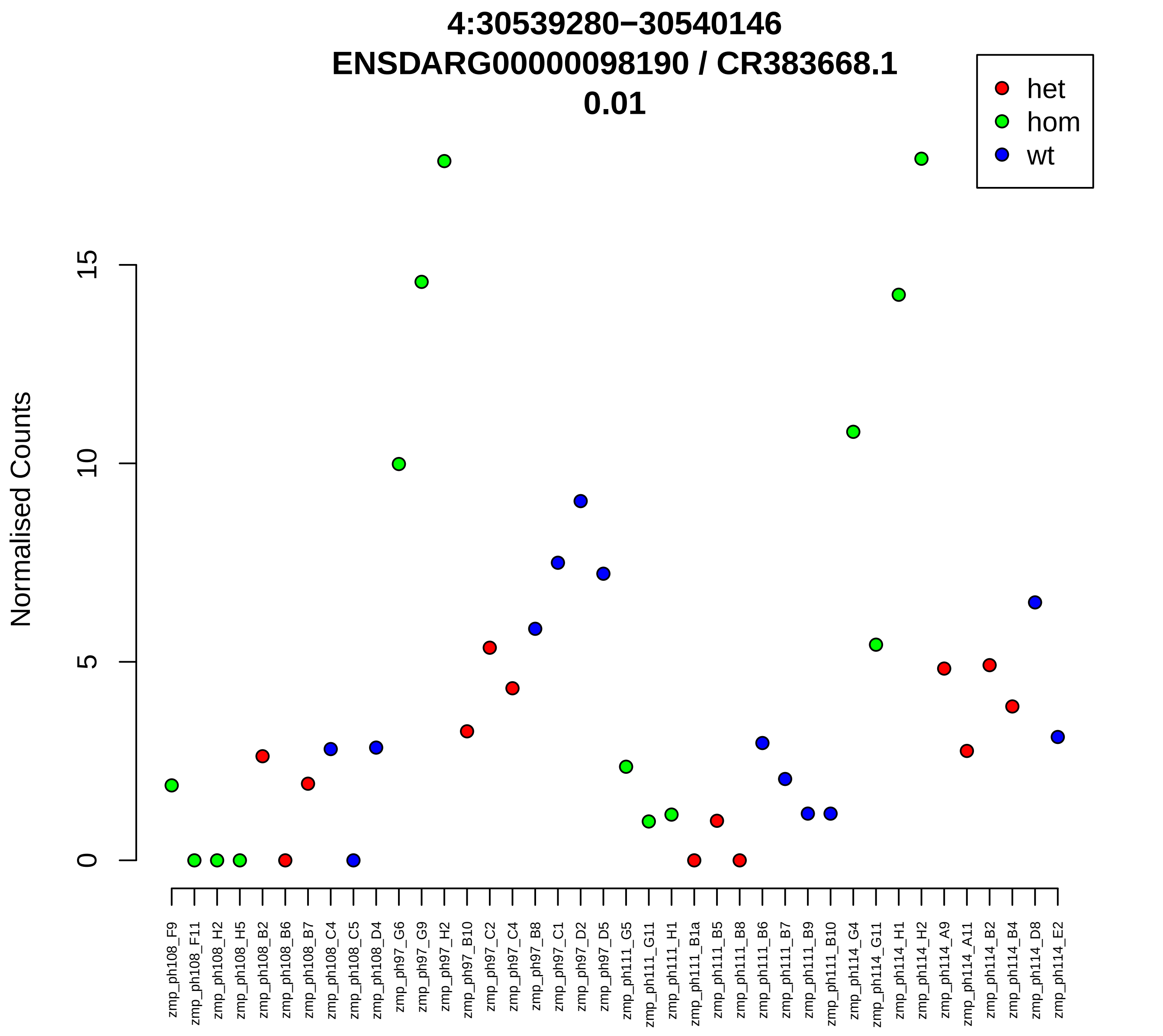
4:30539280−30540146
ENSDARG00000098190 / CR383668.1
0.01
het
hom
wt
Normalised Counts
0
5
10
15
zmp_ph108_F9
zmp_ph108_F11
zmp_ph108_H2
zmp_ph108_H5
zmp_ph108_B2
zmp_ph108_B6
zmp_ph108_B7
zmp_ph108_C4
zmp_ph108_C5
zmp_ph108_D4
zmp_ph97_G6
zmp_ph97_G9
zmp_ph97_H2
zmp_ph97_B10
zmp_ph97_C2
zmp_ph97_C4
zmp_ph97_B8
zmp_ph97_C1
zmp_ph97_D2
zmp_ph97_D5
zmp_ph111_G5
zmp_ph111_G11
zmp_ph111_H1
zmp_ph111_B1a
zmp_ph111_B5
zmp_ph111_B8
zmp_ph111_B6
zmp_ph111_B7
zmp_ph111_B9
zmp_ph111_B10
zmp_ph114_G4
zmp_ph114_G11
zmp_ph114_H1
zmp_ph114_H2
zmp_ph114_A9
zmp_ph114_A11
zmp_ph114_B2
zmp_ph114_B4
zmp_ph114_D8
zmp_ph114_E2

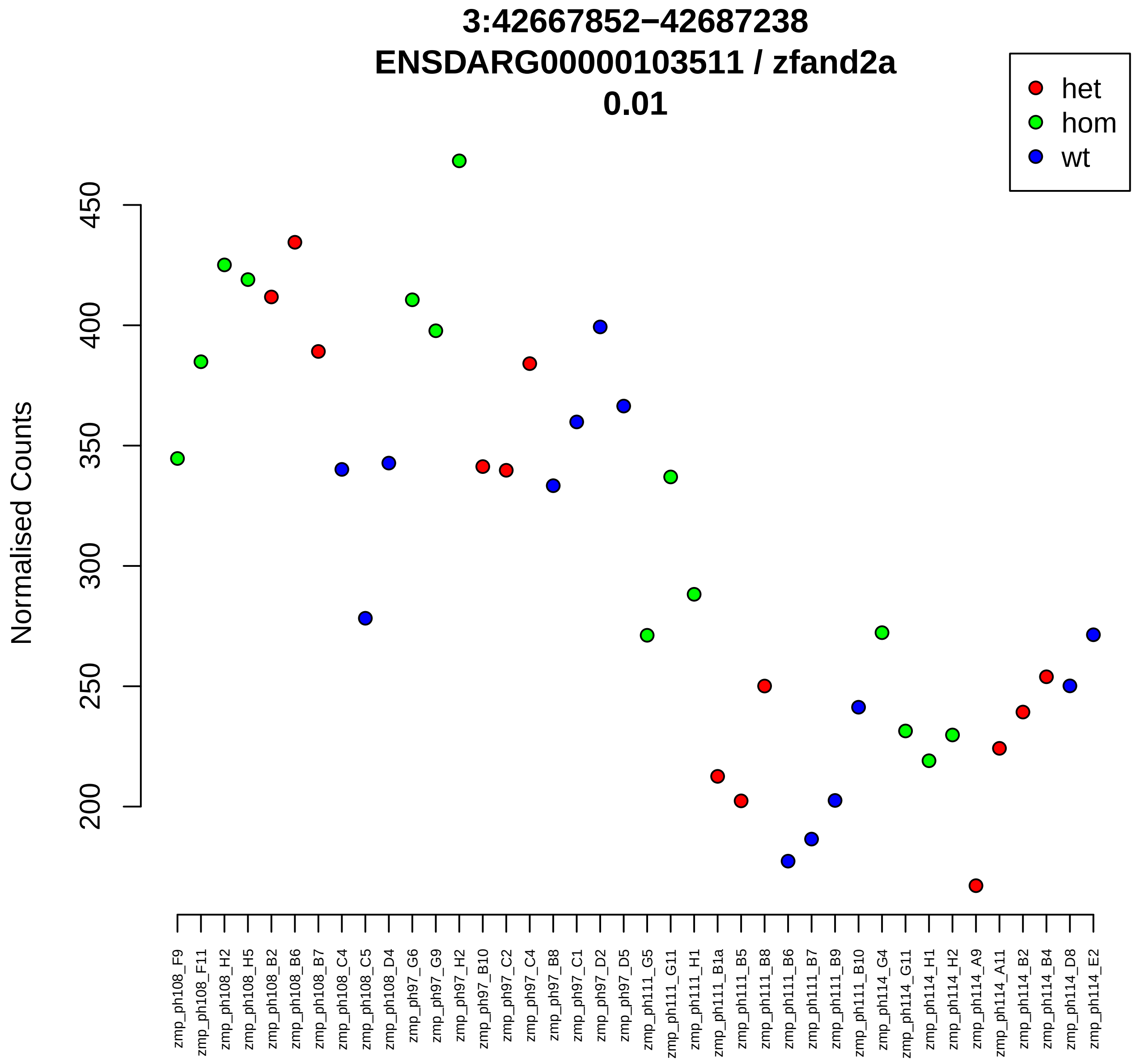
3:42667852−42687238
ENSDARG00000103511 / zfand2a
0.01
het
hom
wt
Normalised Counts
200
250
300
350
400
450
zmp_ph108_F9
zmp_ph108_F11
zmp_ph108_H2
zmp_ph108_H5
zmp_ph108_B2
zmp_ph108_B6
zmp_ph108_B7
zmp_ph108_C4
zmp_ph108_C5
zmp_ph108_D4
zmp_ph97_G6
zmp_ph97_G9
zmp_ph97_H2
zmp_ph97_B10
zmp_ph97_C2
zmp_ph97_C4
zmp_ph97_B8
zmp_ph97_C1
zmp_ph97_D2
zmp_ph97_D5
zmp_ph111_G5
zmp_ph111_G11
zmp_ph111_H1
zmp_ph111_B1a
zmp_ph111_B5
zmp_ph111_B8
zmp_ph111_B6
zmp_ph111_B7
zmp_ph111_B9
zmp_ph111_B10
zmp_ph114_G4
zmp_ph114_G11
zmp_ph114_H1
zmp_ph114_H2
zmp_ph114_A9
zmp_ph114_A11
zmp_ph114_B2
zmp_ph114_B4
zmp_ph114_D8
zmp_ph114_E2

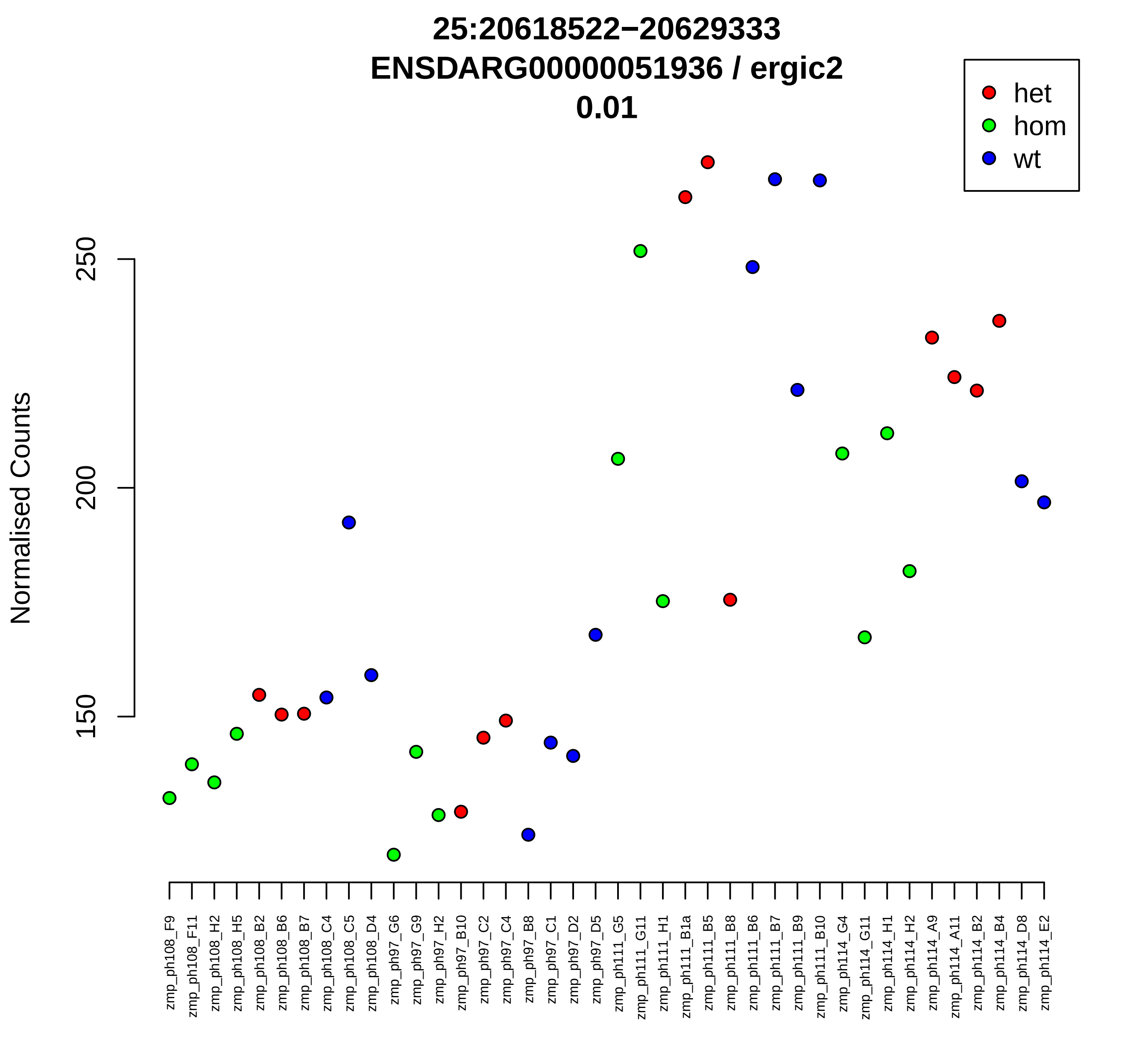

25:20618522−20629333
ENSDARG00000051936 / ergic2
0.01
het
hom
wt
Normalised Counts
150
200
250
zmp_ph108_F9
zmp_ph108_F11
zmp_ph108_H2
zmp_ph108_H5
zmp_ph108_B2
zmp_ph108_B6
zmp_ph108_B7
zmp_ph108_C4
zmp_ph108_C5
zmp_ph108_D4
zmp_ph97_G6
zmp_ph97_G9
zmp_ph97_H2
zmp_ph97_B10
zmp_ph97_C2
zmp_ph97_C4
zmp_ph97_B8
zmp_ph97_C1
zmp_ph97_D2
zmp_ph97_D5
zmp_ph111_G5
zmp_ph111_G11
zmp_ph111_H1
zmp_ph111_B1a
zmp_ph111_B5
zmp_ph111_B8
zmp_ph111_B6
zmp_ph111_B7
zmp_ph111_B9
zmp_ph111_B10
zmp_ph114_G4
zmp_ph114_G11
zmp_ph114_H1
zmp_ph114_H2
zmp_ph114_A9
zmp_ph114_A11
zmp_ph114_B2
zmp_ph114_B4
zmp_ph114_D8
zmp_ph114_E2

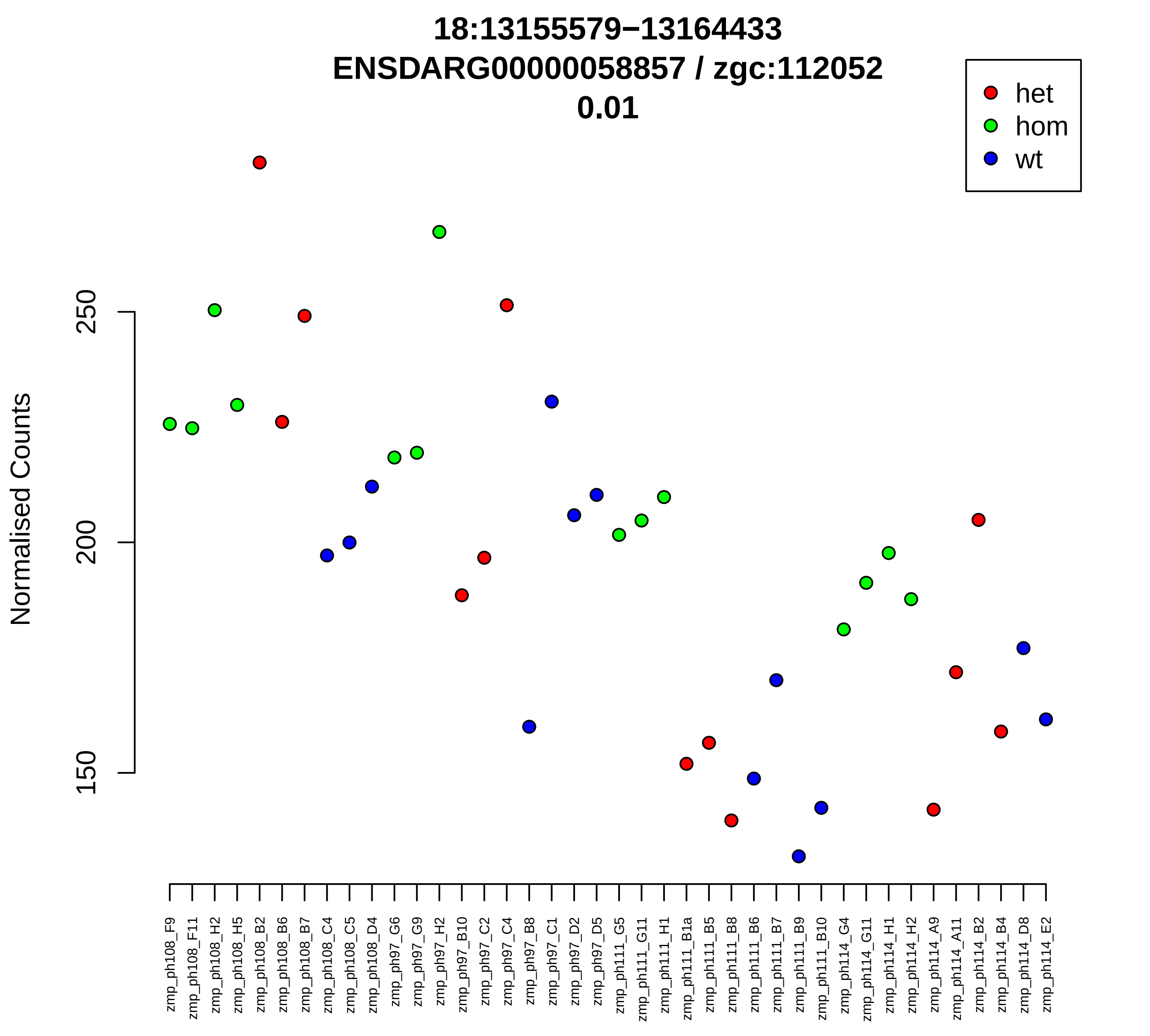
18:13155579−13164433
ENSDARG00000058857 / zgc:112052
0.01
het
hom
wt
Normalised Counts
150
200
250
zmp_ph108_F9
zmp_ph108_F11
zmp_ph108_H2
zmp_ph108_H5
zmp_ph108_B2
zmp_ph108_B6
zmp_ph108_B7
zmp_ph108_C4
zmp_ph108_C5
zmp_ph108_D4
zmp_ph97_G6
zmp_ph97_G9
zmp_ph97_H2
zmp_ph97_B10
zmp_ph97_C2
zmp_ph97_C4
zmp_ph97_B8
zmp_ph97_C1
zmp_ph97_D2
zmp_ph97_D5
zmp_ph111_G5
zmp_ph111_G11
zmp_ph111_H1
zmp_ph111_B1a
zmp_ph111_B5
zmp_ph111_B8
zmp_ph111_B6
zmp_ph111_B7
zmp_ph111_B9
zmp_ph111_B10
zmp_ph114_G4
zmp_ph114_G11
zmp_ph114_H1
zmp_ph114_H2
zmp_ph114_A9
zmp_ph114_A11
zmp_ph114_B2
zmp_ph114_B4
zmp_ph114_D8
zmp_ph114_E2

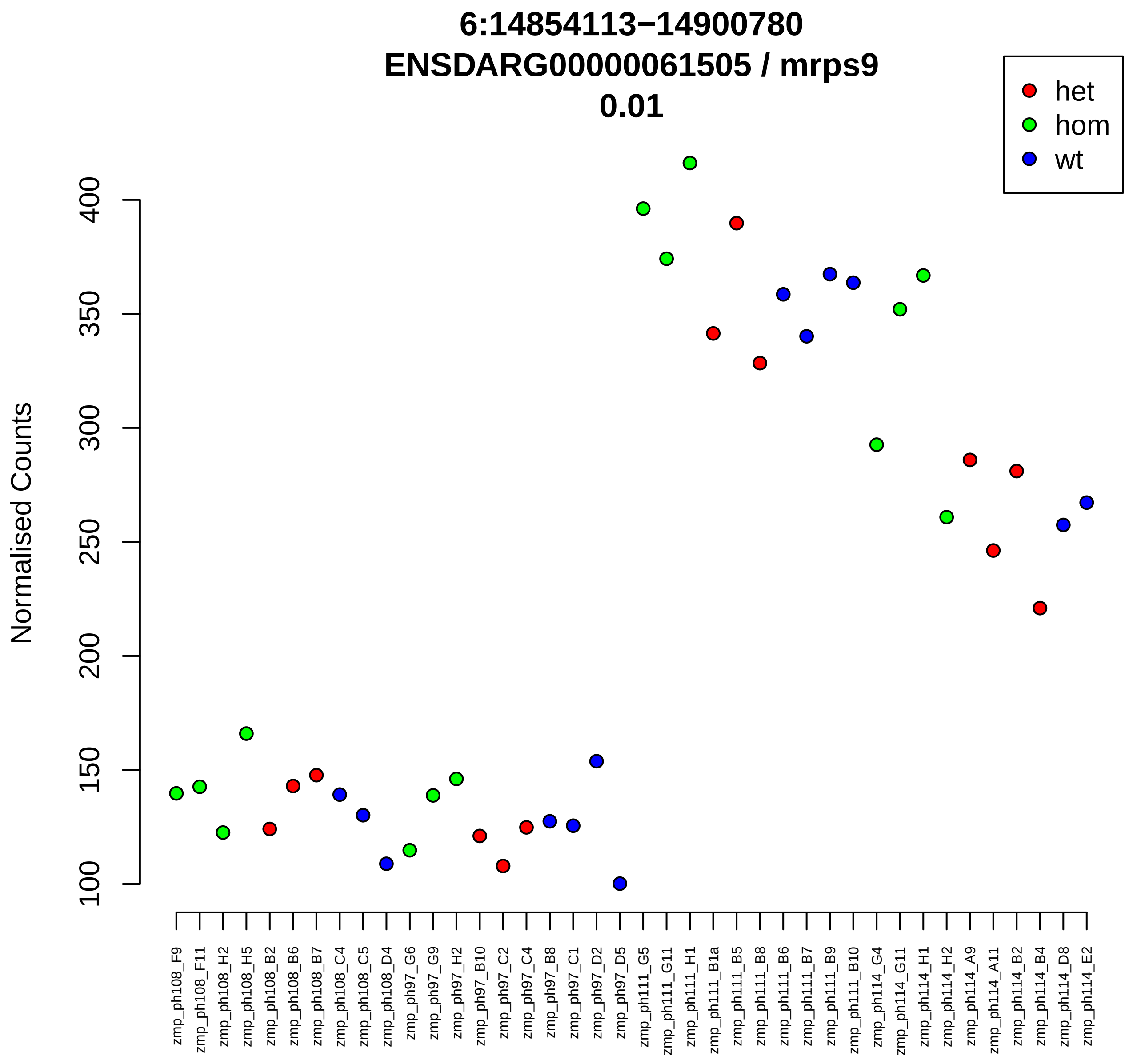
6:14854113−14900780
ENSDARG00000061505 / mrps9
0.01
het
hom
wt
Normalised Counts
100
150
200
250
300
350
400
zmp_ph108_F9
zmp_ph108_F11
zmp_ph108_H2
zmp_ph108_H5
zmp_ph108_B2
zmp_ph108_B6
zmp_ph108_B7
zmp_ph108_C4
zmp_ph108_C5
zmp_ph108_D4
zmp_ph97_G6
zmp_ph97_G9
zmp_ph97_H2
zmp_ph97_B10
zmp_ph97_C2
zmp_ph97_C4
zmp_ph97_B8
zmp_ph97_C1
zmp_ph97_D2
zmp_ph97_D5
zmp_ph111_G5
zmp_ph111_G11
zmp_ph111_H1
zmp_ph111_B1a
zmp_ph111_B5
zmp_ph111_B8
zmp_ph111_B6
zmp_ph111_B7
zmp_ph111_B9
zmp_ph111_B10
zmp_ph114_G4
zmp_ph114_G11
zmp_ph114_H1
zmp_ph114_H2
zmp_ph114_A9
zmp_ph114_A11
zmp_ph114_B2
zmp_ph114_B4
zmp_ph114_D8
zmp_ph114_E2

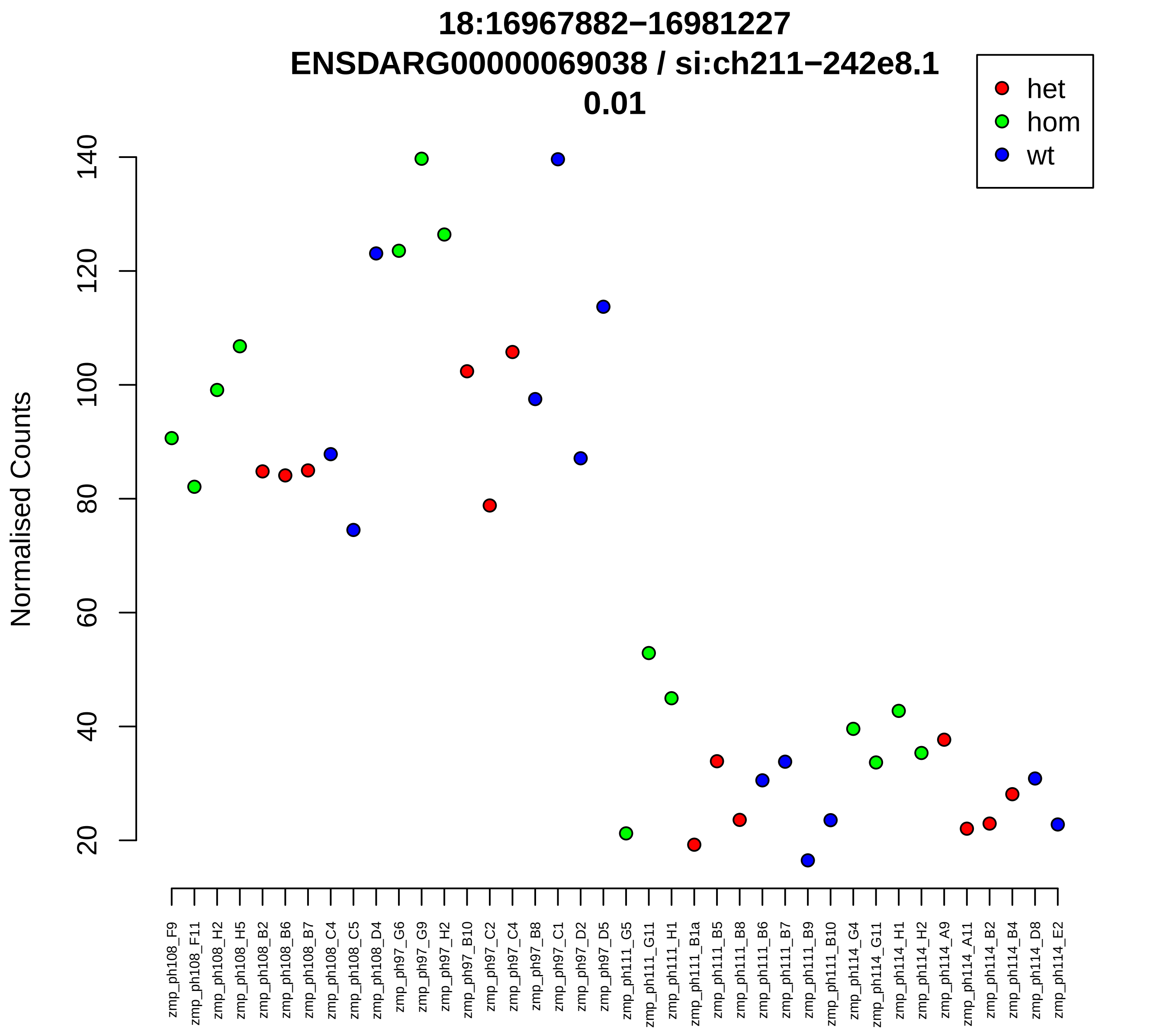

18:16967882−16981227
ENSDARG00000069038 / si:ch211−242e8.1
0.01
het
hom
wt
Normalised Counts
20
40
60
80
100
120
140
zmp_ph108_F9
zmp_ph108_F11
zmp_ph108_H2
zmp_ph108_H5
zmp_ph108_B2
zmp_ph108_B6
zmp_ph108_B7
zmp_ph108_C4
zmp_ph108_C5
zmp_ph108_D4
zmp_ph97_G6
zmp_ph97_G9
zmp_ph97_H2
zmp_ph97_B10
zmp_ph97_C2
zmp_ph97_C4
zmp_ph97_B8
zmp_ph97_C1
zmp_ph97_D2
zmp_ph97_D5
zmp_ph111_G5
zmp_ph111_G11
zmp_ph111_H1
zmp_ph111_B1a
zmp_ph111_B5
zmp_ph111_B8
zmp_ph111_B6
zmp_ph111_B7
zmp_ph111_B9
zmp_ph111_B10
zmp_ph114_G4
zmp_ph114_G11
zmp_ph114_H1
zmp_ph114_H2
zmp_ph114_A9
zmp_ph114_A11
zmp_ph114_B2
zmp_ph114_B4
zmp_ph114_D8
zmp_ph114_E2

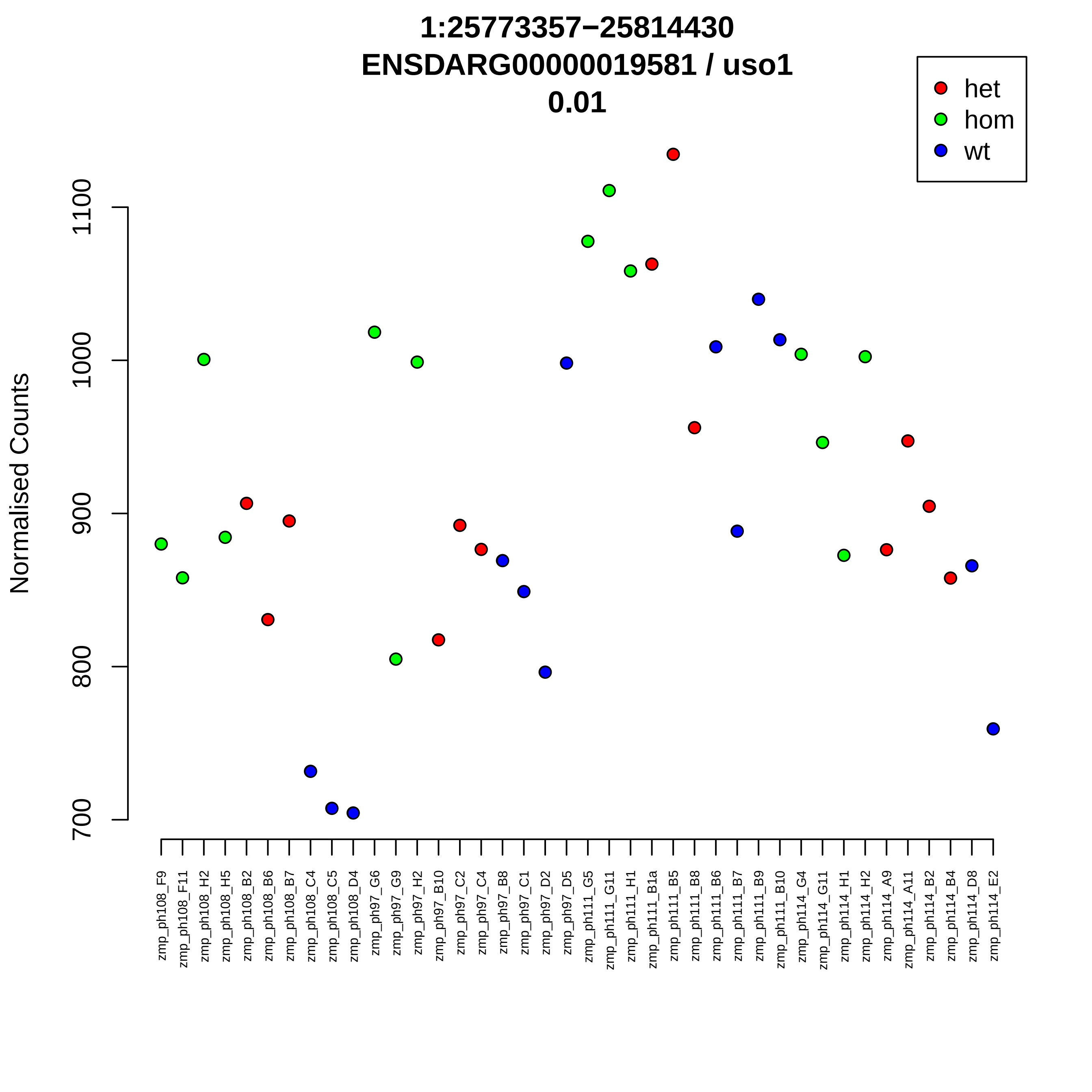
1:25773357−25814430
ENSDARG00000019581 / uso1
0.01
het
hom
wt
Normalised Counts
700
800
900
1000
1100
zmp_ph108_F9
zmp_ph108_F11
zmp_ph108_H2
zmp_ph108_H5
zmp_ph108_B2
zmp_ph108_B6
zmp_ph108_B7
zmp_ph108_C4
zmp_ph108_C5
zmp_ph108_D4
zmp_ph97_G6
zmp_ph97_G9
zmp_ph97_H2
zmp_ph97_B10
zmp_ph97_C2
zmp_ph97_C4
zmp_ph97_B8
zmp_ph97_C1
zmp_ph97_D2
zmp_ph97_D5
zmp_ph111_G5
zmp_ph111_G11
zmp_ph111_H1
zmp_ph111_B1a
zmp_ph111_B5
zmp_ph111_B8
zmp_ph111_B6
zmp_ph111_B7
zmp_ph111_B9
zmp_ph111_B10
zmp_ph114_G4
zmp_ph114_G11
zmp_ph114_H1
zmp_ph114_H2
zmp_ph114_A9
zmp_ph114_A11
zmp_ph114_B2
zmp_ph114_B4
zmp_ph114_D8
zmp_ph114_E2

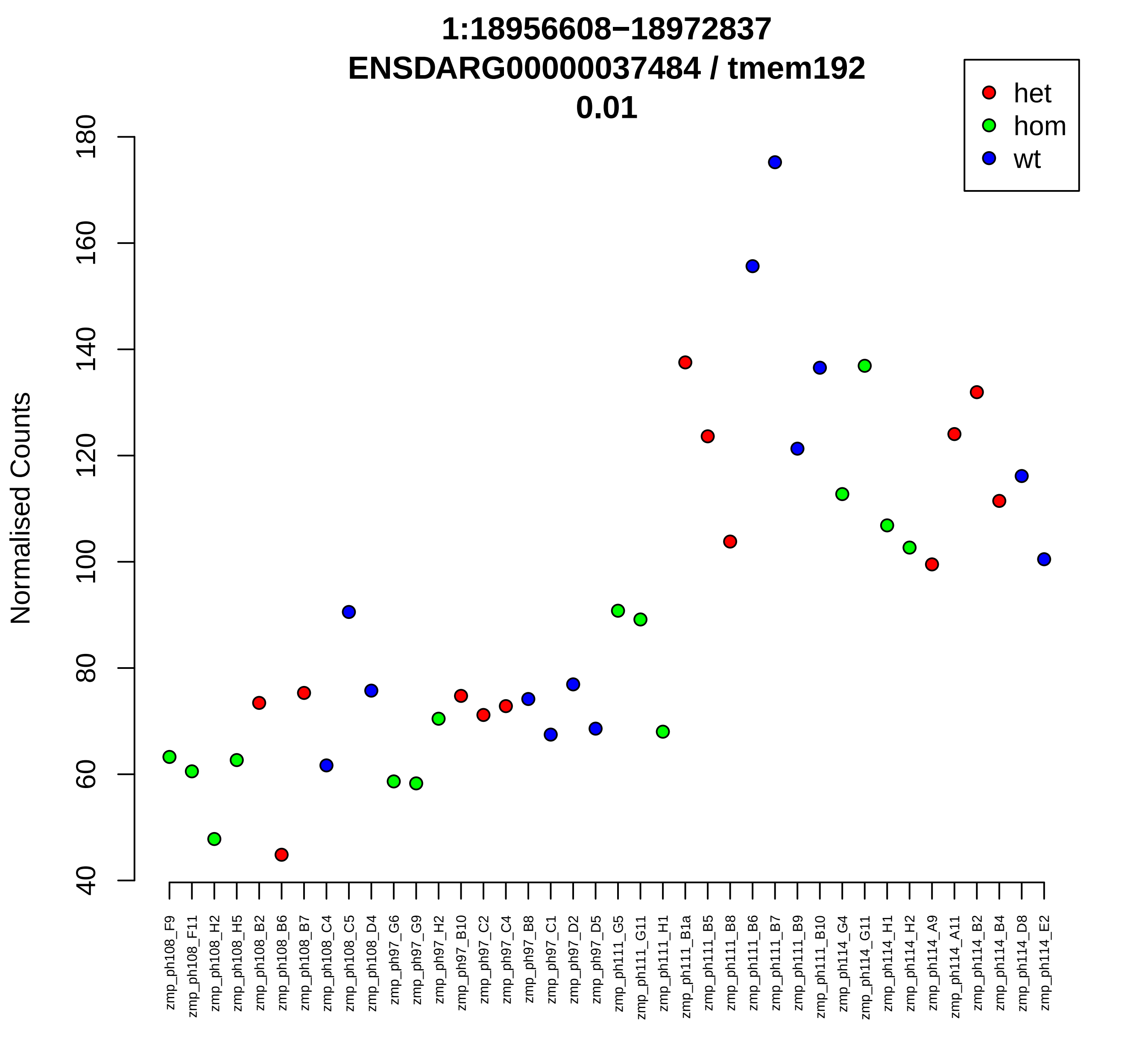
1:18956608−18972837
ENSDARG00000037484 / tmem192
0.01
het
hom
wt
Normalised Counts
40
60
80
100
120
140
160
180
zmp_ph108_F9
zmp_ph108_F11
zmp_ph108_H2
zmp_ph108_H5
zmp_ph108_B2
zmp_ph108_B6
zmp_ph108_B7
zmp_ph108_C4
zmp_ph108_C5
zmp_ph108_D4
zmp_ph97_G6
zmp_ph97_G9
zmp_ph97_H2
zmp_ph97_B10
zmp_ph97_C2
zmp_ph97_C4
zmp_ph97_B8
zmp_ph97_C1
zmp_ph97_D2
zmp_ph97_D5
zmp_ph111_G5
zmp_ph111_G11
zmp_ph111_H1
zmp_ph111_B1a
zmp_ph111_B5
zmp_ph111_B8
zmp_ph111_B6
zmp_ph111_B7
zmp_ph111_B9
zmp_ph111_B10
zmp_ph114_G4
zmp_ph114_G11
zmp_ph114_H1
zmp_ph114_H2
zmp_ph114_A9
zmp_ph114_A11
zmp_ph114_B2
zmp_ph114_B4
zmp_ph114_D8
zmp_ph114_E2

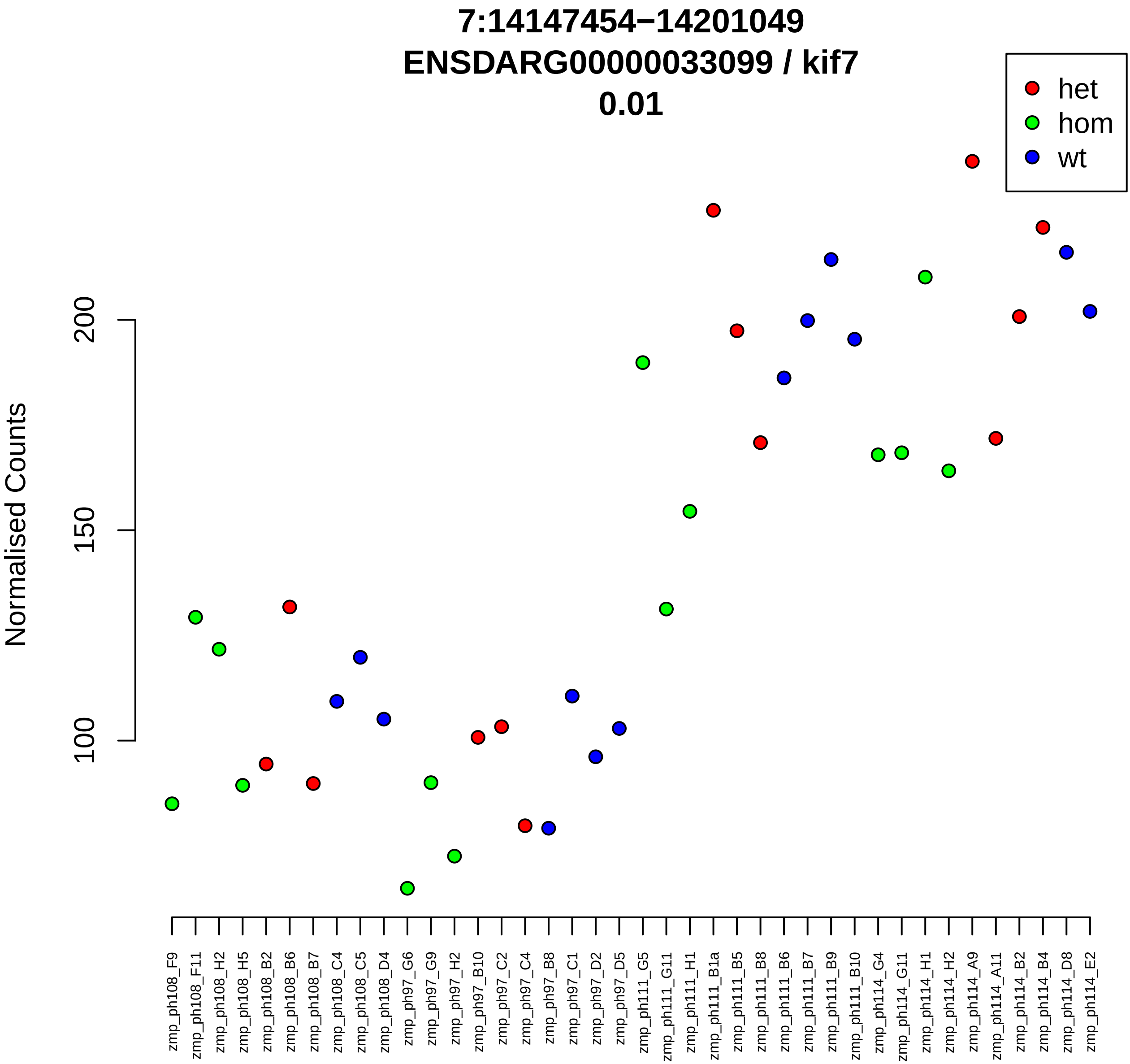
7:14147454−14201049
ENSDARG00000033099 / kif7
0.01
het
hom
wt
Normalised Counts
100
150
200
zmp_ph108_F9
zmp_ph108_F11
zmp_ph108_H2
zmp_ph108_H5
zmp_ph108_B2
zmp_ph108_B6
zmp_ph108_B7
zmp_ph108_C4
zmp_ph108_C5
zmp_ph108_D4
zmp_ph97_G6
zmp_ph97_G9
zmp_ph97_H2
zmp_ph97_B10
zmp_ph97_C2
zmp_ph97_C4
zmp_ph97_B8
zmp_ph97_C1
zmp_ph97_D2
zmp_ph97_D5
zmp_ph111_G5
zmp_ph111_G11
zmp_ph111_H1
zmp_ph111_B1a
zmp_ph111_B5
zmp_ph111_B8
zmp_ph111_B6
zmp_ph111_B7
zmp_ph111_B9
zmp_ph111_B10
zmp_ph114_G4
zmp_ph114_G11
zmp_ph114_H1
zmp_ph114_H2
zmp_ph114_A9
zmp_ph114_A11
zmp_ph114_B2
zmp_ph114_B4
zmp_ph114_D8
zmp_ph114_E2

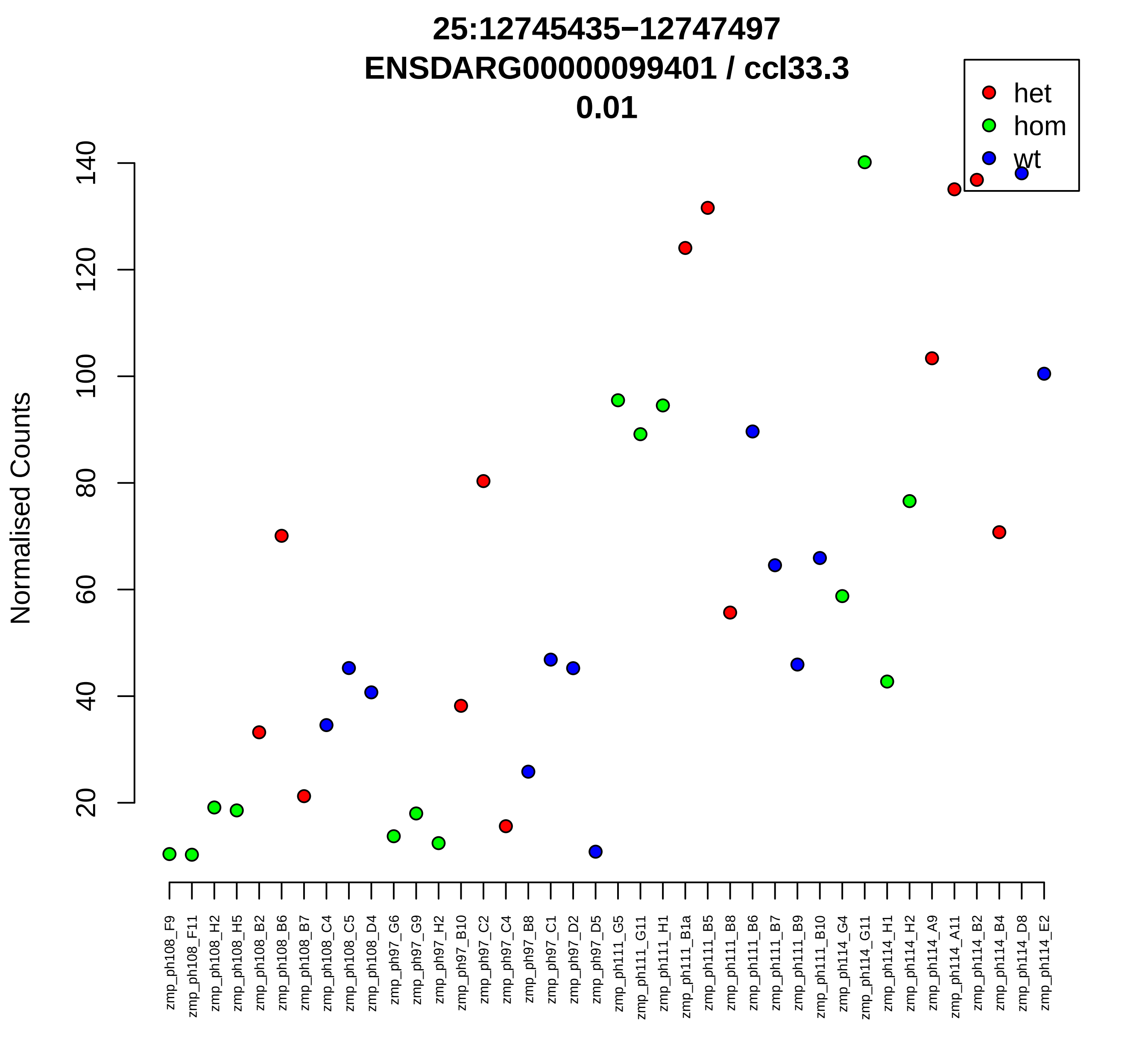

25:12745435−12747497
ENSDARG00000099401 / ccl33.3
0.01
het
hom
wt
Normalised Counts
20
40
60
80
100
120
140
zmp_ph108_F9
zmp_ph108_F11
zmp_ph108_H2
zmp_ph108_H5
zmp_ph108_B2
zmp_ph108_B6
zmp_ph108_B7
zmp_ph108_C4
zmp_ph108_C5
zmp_ph108_D4
zmp_ph97_G6
zmp_ph97_G9
zmp_ph97_H2
zmp_ph97_B10
zmp_ph97_C2
zmp_ph97_C4
zmp_ph97_B8
zmp_ph97_C1
zmp_ph97_D2
zmp_ph97_D5
zmp_ph111_G5
zmp_ph111_G11
zmp_ph111_H1
zmp_ph111_B1a
zmp_ph111_B5
zmp_ph111_B8
zmp_ph111_B6
zmp_ph111_B7
zmp_ph111_B9
zmp_ph111_B10
zmp_ph114_G4
zmp_ph114_G11
zmp_ph114_H1
zmp_ph114_H2
zmp_ph114_A9
zmp_ph114_A11
zmp_ph114_B2
zmp_ph114_B4
zmp_ph114_D8
zmp_ph114_E2

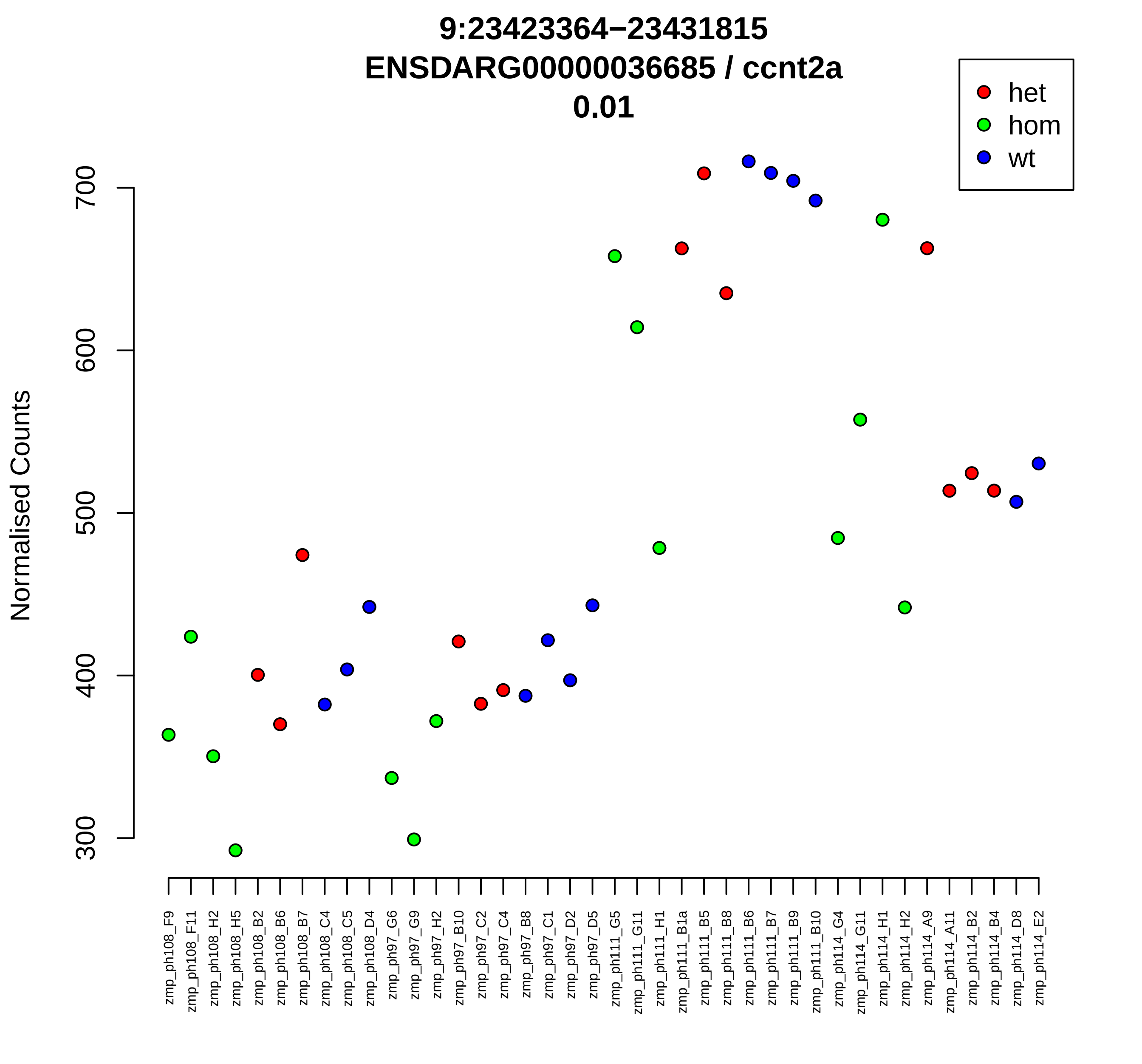

9:23423364−23431815
ENSDARG00000036685 / ccnt2a
0.01
het
hom
wt
Normalised Counts
300
400
500
600
700
zmp_ph108_F9
zmp_ph108_F11
zmp_ph108_H2
zmp_ph108_H5
zmp_ph108_B2
zmp_ph108_B6
zmp_ph108_B7
zmp_ph108_C4
zmp_ph108_C5
zmp_ph108_D4
zmp_ph97_G6
zmp_ph97_G9
zmp_ph97_H2
zmp_ph97_B10
zmp_ph97_C2
zmp_ph97_C4
zmp_ph97_B8
zmp_ph97_C1
zmp_ph97_D2
zmp_ph97_D5
zmp_ph111_G5
zmp_ph111_G11
zmp_ph111_H1
zmp_ph111_B1a
zmp_ph111_B5
zmp_ph111_B8
zmp_ph111_B6
zmp_ph111_B7
zmp_ph111_B9
zmp_ph111_B10
zmp_ph114_G4
zmp_ph114_G11
zmp_ph114_H1
zmp_ph114_H2
zmp_ph114_A9
zmp_ph114_A11
zmp_ph114_B2
zmp_ph114_B4
zmp_ph114_D8
zmp_ph114_E2

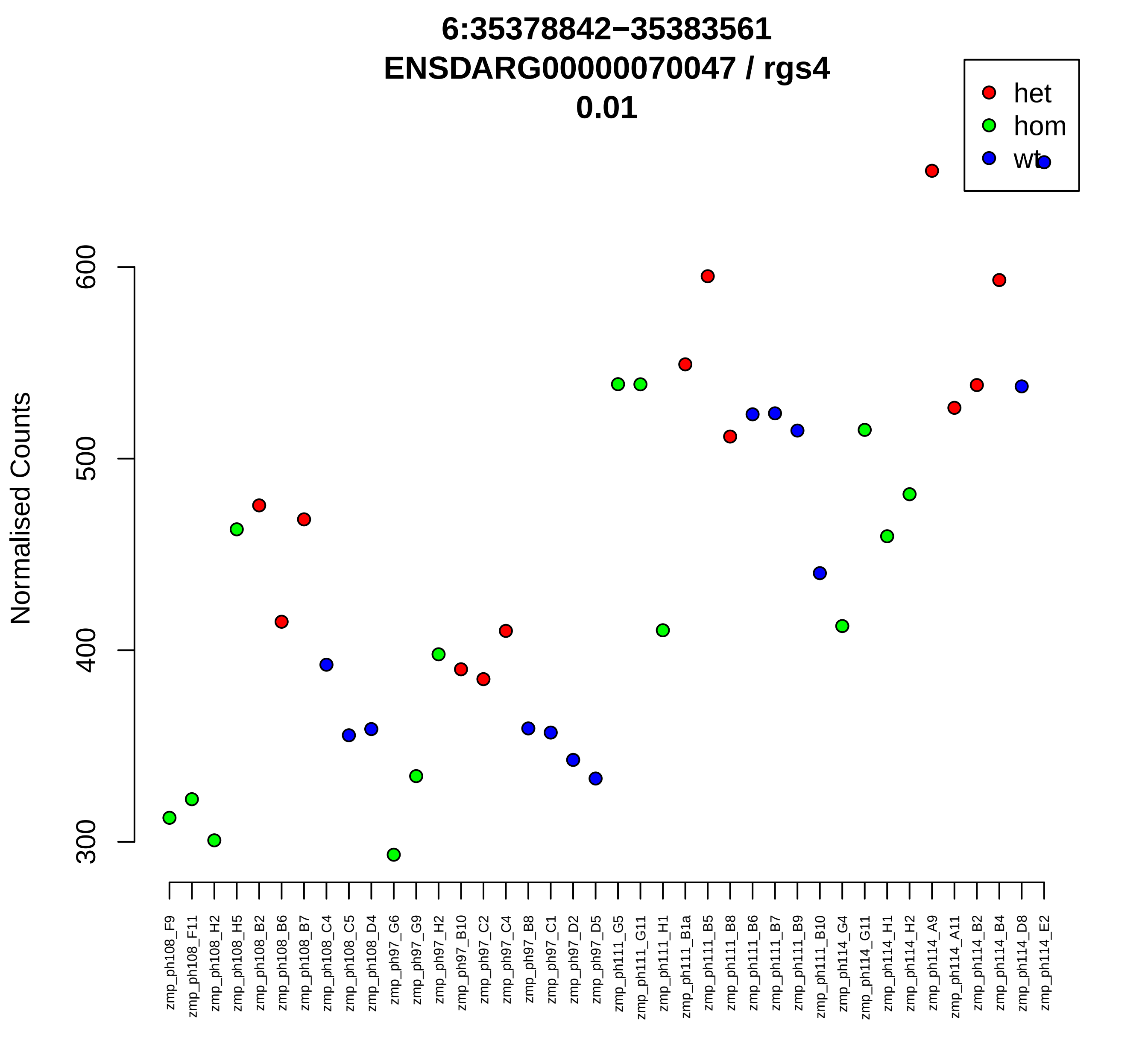
6:35378842−35383561
ENSDARG00000070047 / rgs4
0.01
het
hom
wt
Normalised Counts
300
400
500
600
zmp_ph108_F9
zmp_ph108_F11
zmp_ph108_H2
zmp_ph108_H5
zmp_ph108_B2
zmp_ph108_B6
zmp_ph108_B7
zmp_ph108_C4
zmp_ph108_C5
zmp_ph108_D4
zmp_ph97_G6
zmp_ph97_G9
zmp_ph97_H2
zmp_ph97_B10
zmp_ph97_C2
zmp_ph97_C4
zmp_ph97_B8
zmp_ph97_C1
zmp_ph97_D2
zmp_ph97_D5
zmp_ph111_G5
zmp_ph111_G11
zmp_ph111_H1
zmp_ph111_B1a
zmp_ph111_B5
zmp_ph111_B8
zmp_ph111_B6
zmp_ph111_B7
zmp_ph111_B9
zmp_ph111_B10
zmp_ph114_G4
zmp_ph114_G11
zmp_ph114_H1
zmp_ph114_H2
zmp_ph114_A9
zmp_ph114_A11
zmp_ph114_B2
zmp_ph114_B4
zmp_ph114_D8
zmp_ph114_E2

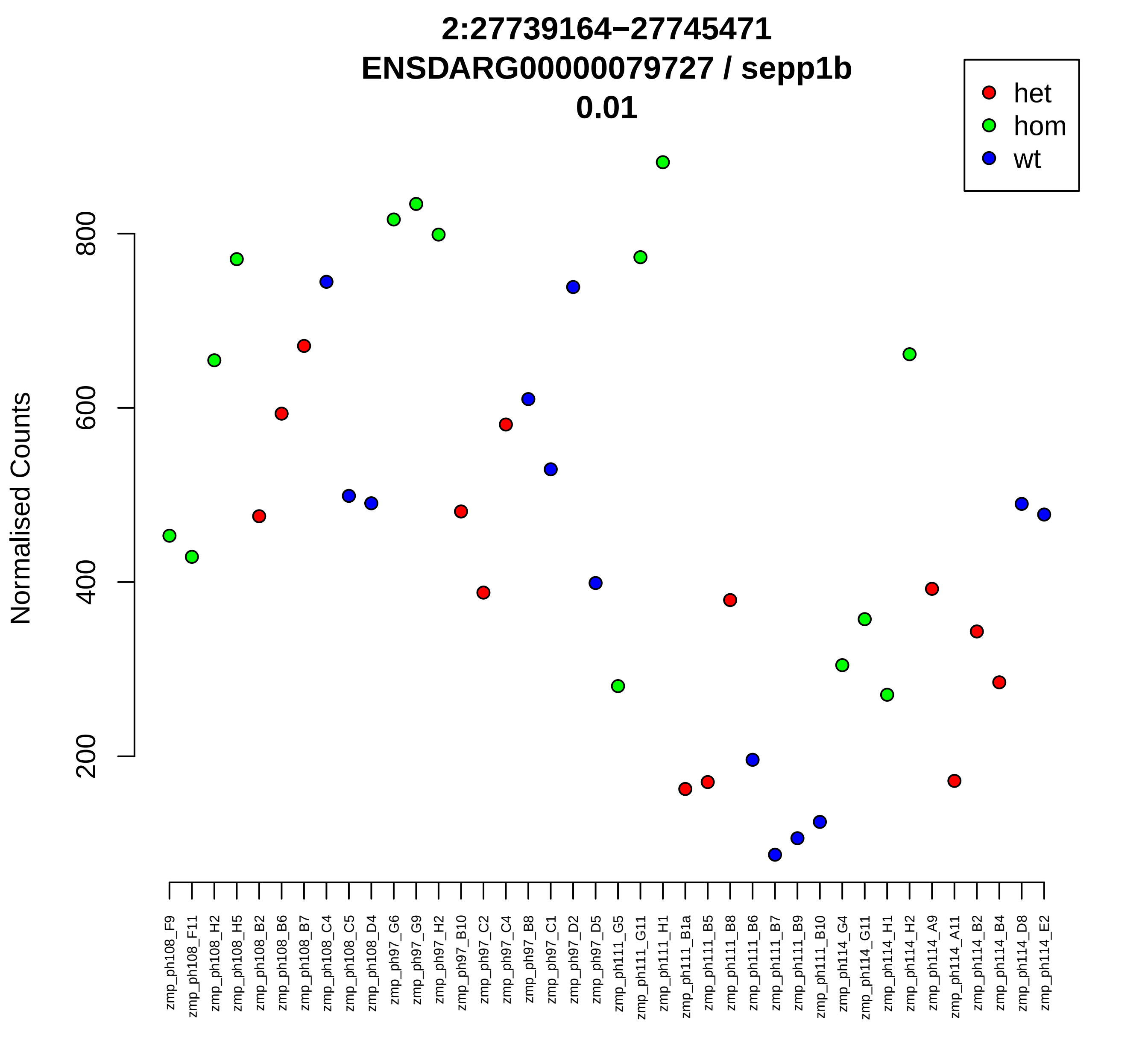

2:27739164−27745471
ENSDARG00000079727 / sepp1b
0.01
het
hom
wt
Normalised Counts
200
400
600
800
zmp_ph108_F9
zmp_ph108_F11
zmp_ph108_H2
zmp_ph108_H5
zmp_ph108_B2
zmp_ph108_B6
zmp_ph108_B7
zmp_ph108_C4
zmp_ph108_C5
zmp_ph108_D4
zmp_ph97_G6
zmp_ph97_G9
zmp_ph97_H2
zmp_ph97_B10
zmp_ph97_C2
zmp_ph97_C4
zmp_ph97_B8
zmp_ph97_C1
zmp_ph97_D2
zmp_ph97_D5
zmp_ph111_G5
zmp_ph111_G11
zmp_ph111_H1
zmp_ph111_B1a
zmp_ph111_B5
zmp_ph111_B8
zmp_ph111_B6
zmp_ph111_B7
zmp_ph111_B9
zmp_ph111_B10
zmp_ph114_G4
zmp_ph114_G11
zmp_ph114_H1
zmp_ph114_H2
zmp_ph114_A9
zmp_ph114_A11
zmp_ph114_B2
zmp_ph114_B4
zmp_ph114_D8
zmp_ph114_E2

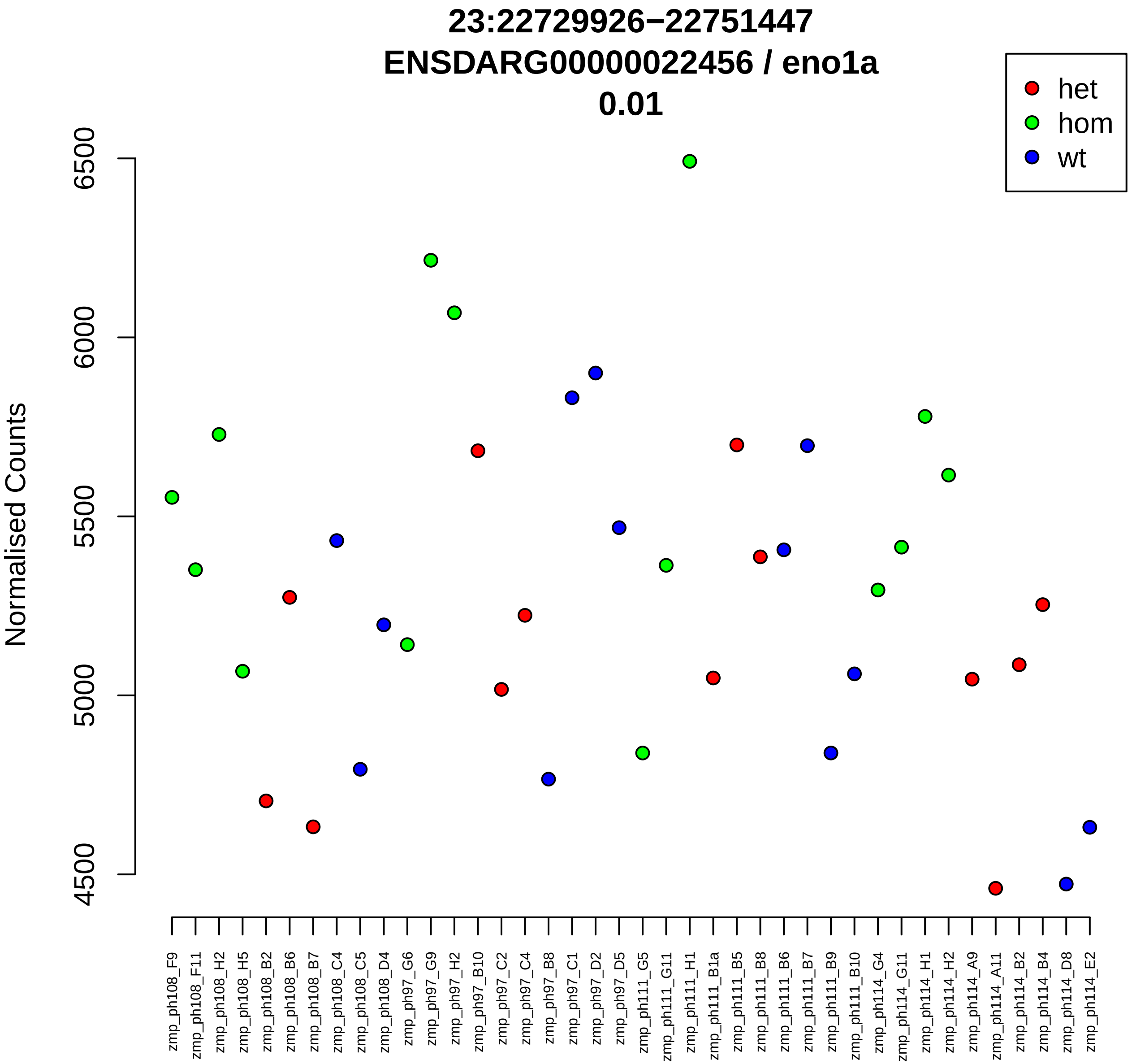

23:22729926−22751447
ENSDARG00000022456 / eno1a
0.01
het
hom
wt
Normalised Counts
6500
6000
5500
5000
4500
zmp_ph108_F9
zmp_ph108_F11
zmp_ph108_H2
zmp_ph108_H5
zmp_ph108_B2
zmp_ph108_B6
zmp_ph108_B7
zmp_ph108_C4
zmp_ph108_C5
zmp_ph108_D4
zmp_ph97_G6
zmp_ph97_G9
zmp_ph97_H2
zmp_ph97_B10
zmp_ph97_C2
zmp_ph97_C4
zmp_ph97_B8
zmp_ph97_C1
zmp_ph97_D2
zmp_ph97_D5
zmp_ph111_G5
zmp_ph111_G11
zmp_ph111_H1
zmp_ph111_B1a
zmp_ph111_B5
zmp_ph111_B8
zmp_ph111_B6
zmp_ph111_B7
zmp_ph111_B9
zmp_ph111_B10
zmp_ph114_G4
zmp_ph114_G11
zmp_ph114_H1
zmp_ph114_H2
zmp_ph114_A9
zmp_ph114_A11
zmp_ph114_B2
zmp_ph114_B4
zmp_ph114_D8
zmp_ph114_E2

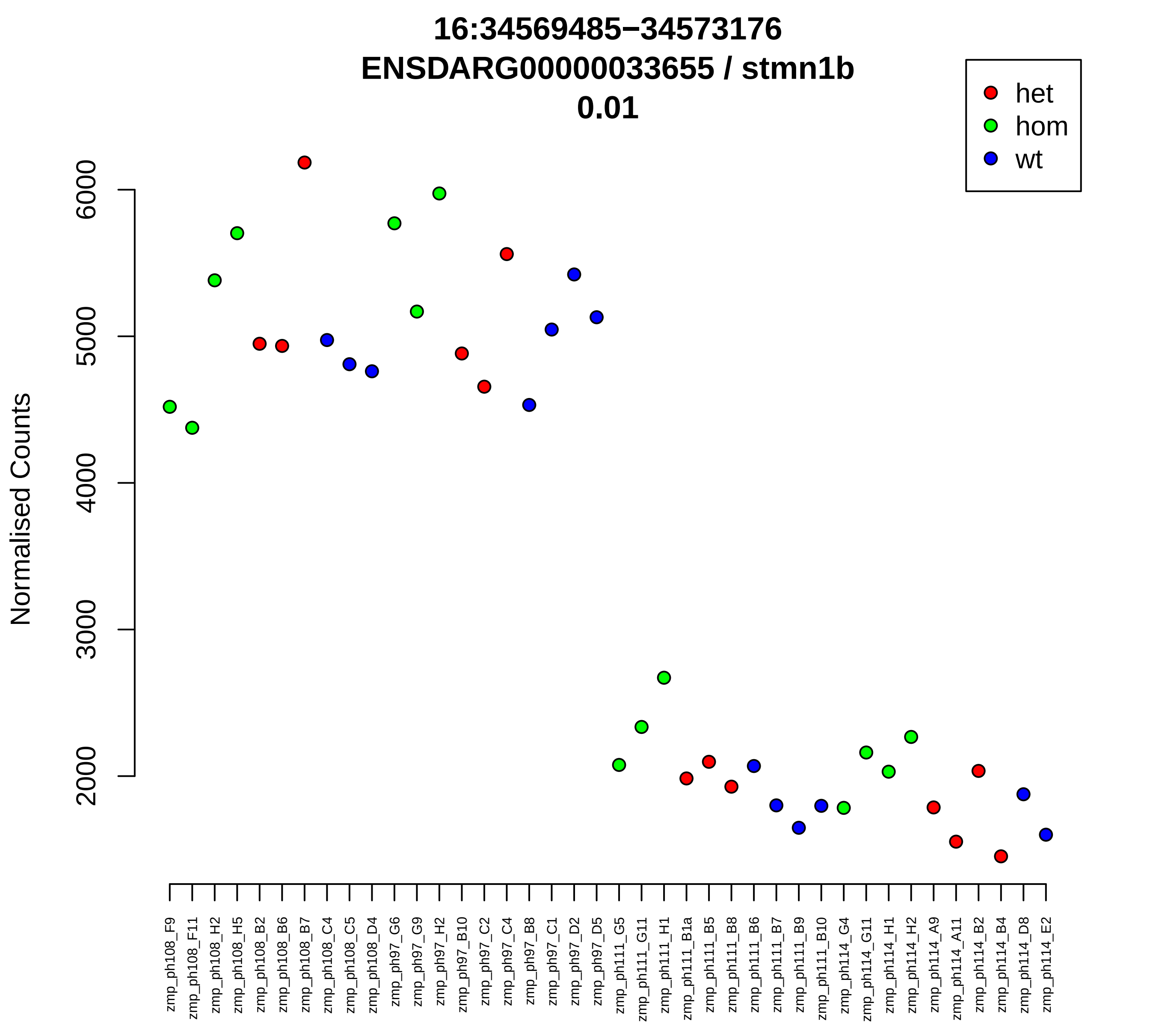
16:34569485−34573176
ENSDARG00000033655 / stmn1b
0.01
het
hom
wt
Normalised Counts
2000
3000
4000
5000
6000
zmp_ph108_F9
zmp_ph108_F11
zmp_ph108_H2
zmp_ph108_H5
zmp_ph108_B2
zmp_ph108_B6
zmp_ph108_B7
zmp_ph108_C4
zmp_ph108_C5
zmp_ph108_D4
zmp_ph97_G6
zmp_ph97_G9
zmp_ph97_H2
zmp_ph97_B10
zmp_ph97_C2
zmp_ph97_C4
zmp_ph97_B8
zmp_ph97_C1
zmp_ph97_D2
zmp_ph97_D5
zmp_ph111_G5
zmp_ph111_G11
zmp_ph111_H1
zmp_ph111_B1a
zmp_ph111_B5
zmp_ph111_B8
zmp_ph111_B6
zmp_ph111_B7
zmp_ph111_B9
zmp_ph111_B10
zmp_ph114_G4
zmp_ph114_G11
zmp_ph114_H1
zmp_ph114_H2
zmp_ph114_A9
zmp_ph114_A11
zmp_ph114_B2
zmp_ph114_B4
zmp_ph114_D8
zmp_ph114_E2

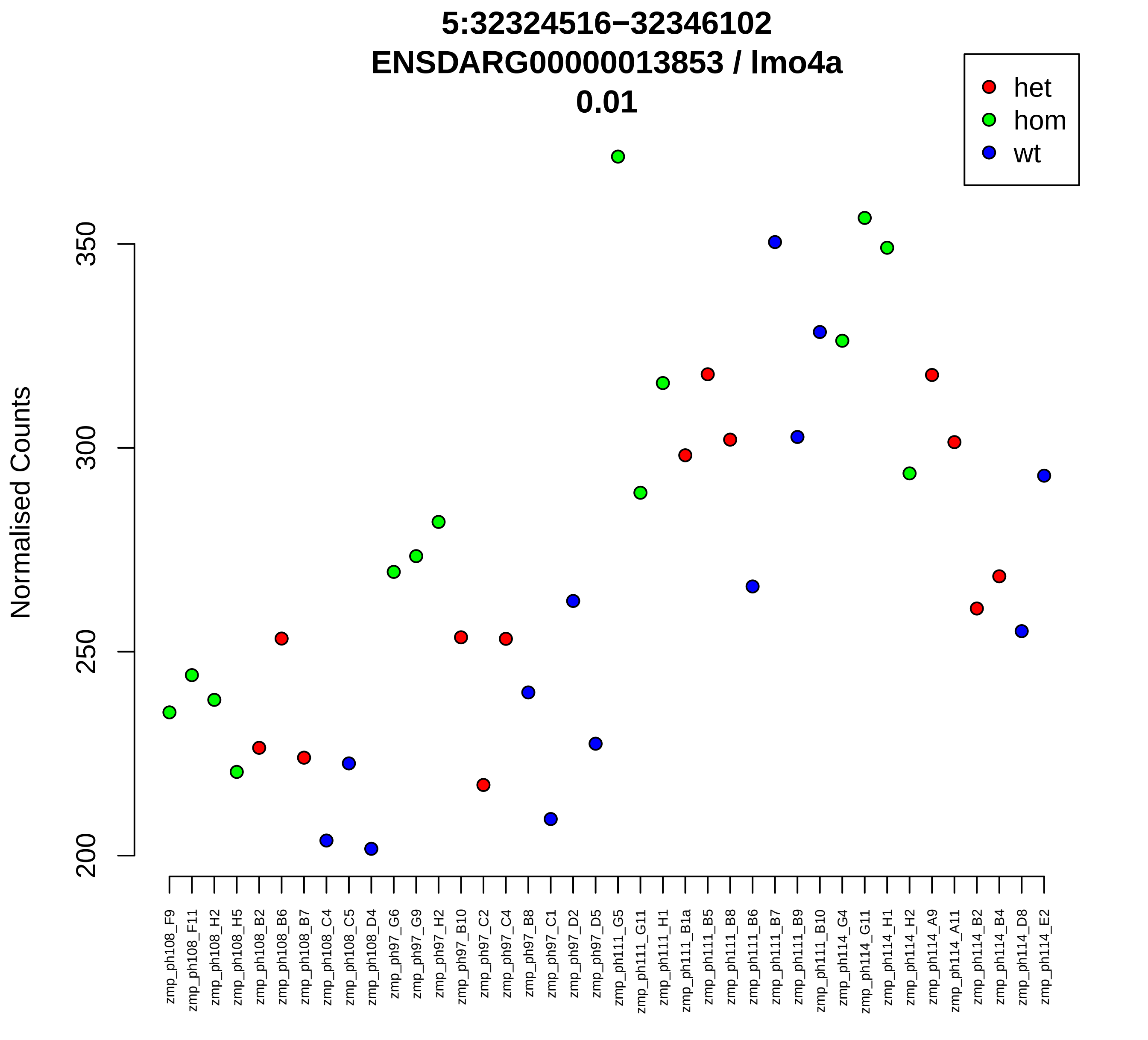

5:32324516−32346102
ENSDARG00000013853 / lmo4a
0.01
het
hom
wt
Normalised Counts
200
250
300
350
zmp_ph108_F9
zmp_ph108_F11
zmp_ph108_H2
zmp_ph108_H5
zmp_ph108_B2
zmp_ph108_B6
zmp_ph108_B7
zmp_ph108_C4
zmp_ph108_C5
zmp_ph108_D4
zmp_ph97_G6
zmp_ph97_G9
zmp_ph97_H2
zmp_ph97_B10
zmp_ph97_C2
zmp_ph97_C4
zmp_ph97_B8
zmp_ph97_C1
zmp_ph97_D2
zmp_ph97_D5
zmp_ph111_G5
zmp_ph111_G11
zmp_ph111_H1
zmp_ph111_B1a
zmp_ph111_B5
zmp_ph111_B8
zmp_ph111_B6
zmp_ph111_B7
zmp_ph111_B9
zmp_ph111_B10
zmp_ph114_G4
zmp_ph114_G11
zmp_ph114_H1
zmp_ph114_H2
zmp_ph114_A9
zmp_ph114_A11
zmp_ph114_B2
zmp_ph114_B4
zmp_ph114_D8
zmp_ph114_E2

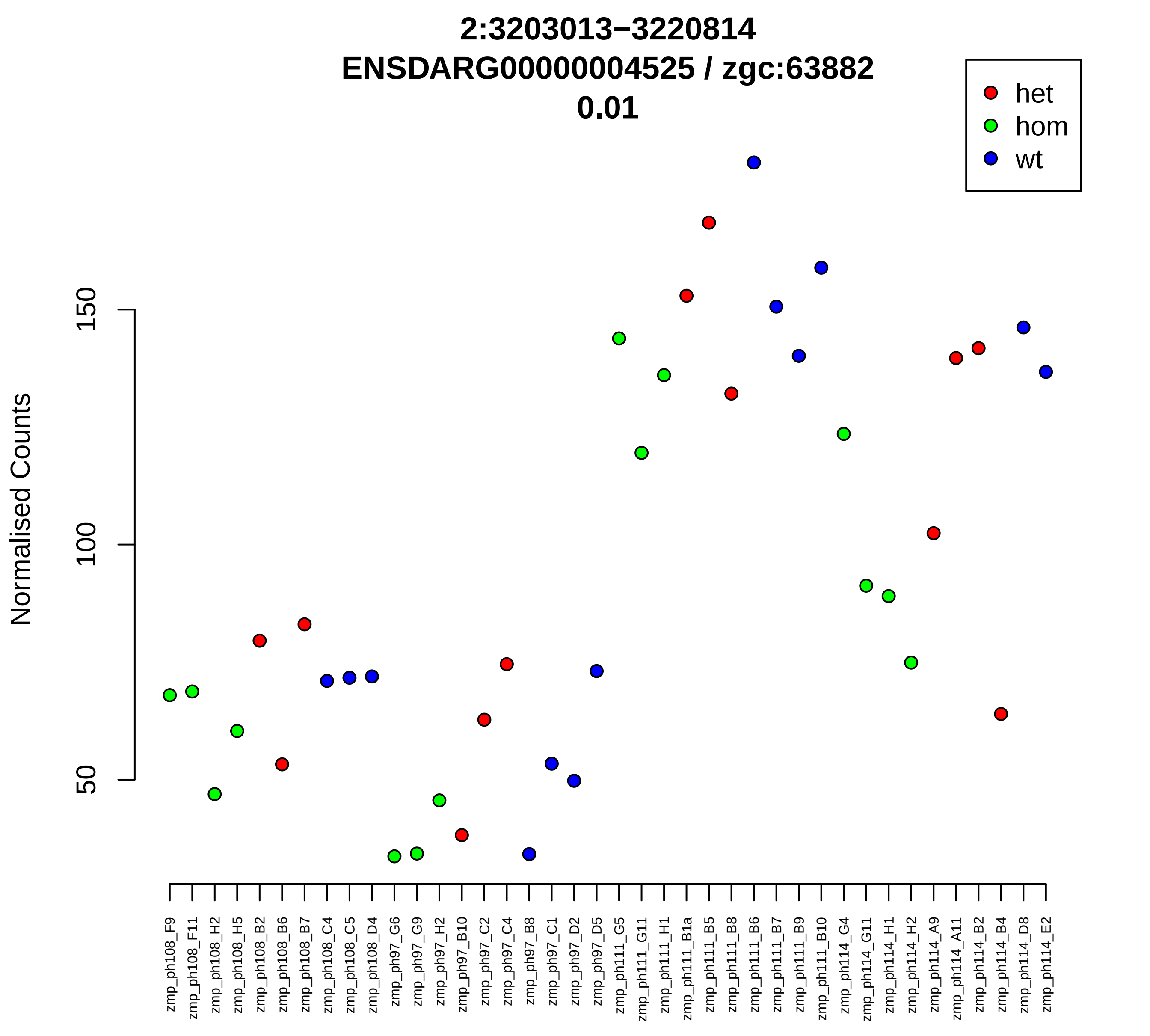
2:3203013−3220814
ENSDARG00000004525 / zgc:63882
0.01
het
hom
wt
Normalised Counts
50
100
150
zmp_ph108_F9
zmp_ph108_F11
zmp_ph108_H2
zmp_ph108_H5
zmp_ph108_B2
zmp_ph108_B6
zmp_ph108_B7
zmp_ph108_C4
zmp_ph108_C5
zmp_ph108_D4
zmp_ph97_G6
zmp_ph97_G9
zmp_ph97_H2
zmp_ph97_B10
zmp_ph97_C2
zmp_ph97_C4
zmp_ph97_B8
zmp_ph97_C1
zmp_ph97_D2
zmp_ph97_D5
zmp_ph111_G5
zmp_ph111_G11
zmp_ph111_H1
zmp_ph111_B1a
zmp_ph111_B5
zmp_ph111_B8
zmp_ph111_B6
zmp_ph111_B7
zmp_ph111_B9
zmp_ph111_B10
zmp_ph114_G4
zmp_ph114_G11
zmp_ph114_H1
zmp_ph114_H2
zmp_ph114_A9
zmp_ph114_A11
zmp_ph114_B2
zmp_ph114_B4
zmp_ph114_D8
zmp_ph114_E2

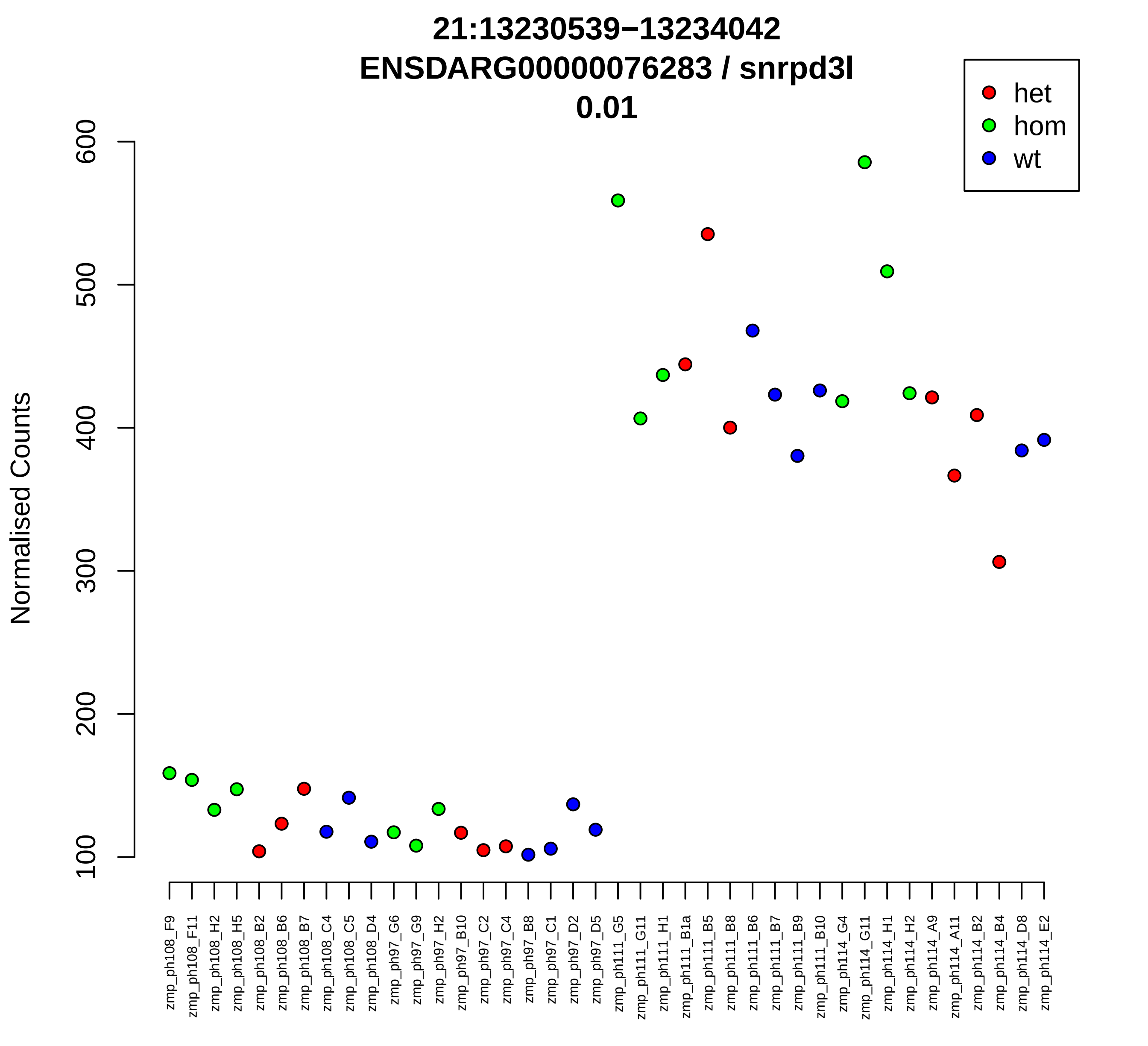
21:13230539−13234042
ENSDARG00000076283 / snrpd3l
0.01
het
hom
wt
Normalised Counts
100
200
300
400
500
600
zmp_ph108_F9
zmp_ph108_F11
zmp_ph108_H2
zmp_ph108_H5
zmp_ph108_B2
zmp_ph108_B6
zmp_ph108_B7
zmp_ph108_C4
zmp_ph108_C5
zmp_ph108_D4
zmp_ph97_G6
zmp_ph97_G9
zmp_ph97_H2
zmp_ph97_B10
zmp_ph97_C2
zmp_ph97_C4
zmp_ph97_B8
zmp_ph97_C1
zmp_ph97_D2
zmp_ph97_D5
zmp_ph111_G5
zmp_ph111_G11
zmp_ph111_H1
zmp_ph111_B1a
zmp_ph111_B5
zmp_ph111_B8
zmp_ph111_B6
zmp_ph111_B7
zmp_ph111_B9
zmp_ph111_B10
zmp_ph114_G4
zmp_ph114_G11
zmp_ph114_H1
zmp_ph114_H2
zmp_ph114_A9
zmp_ph114_A11
zmp_ph114_B2
zmp_ph114_B4
zmp_ph114_D8
zmp_ph114_E2

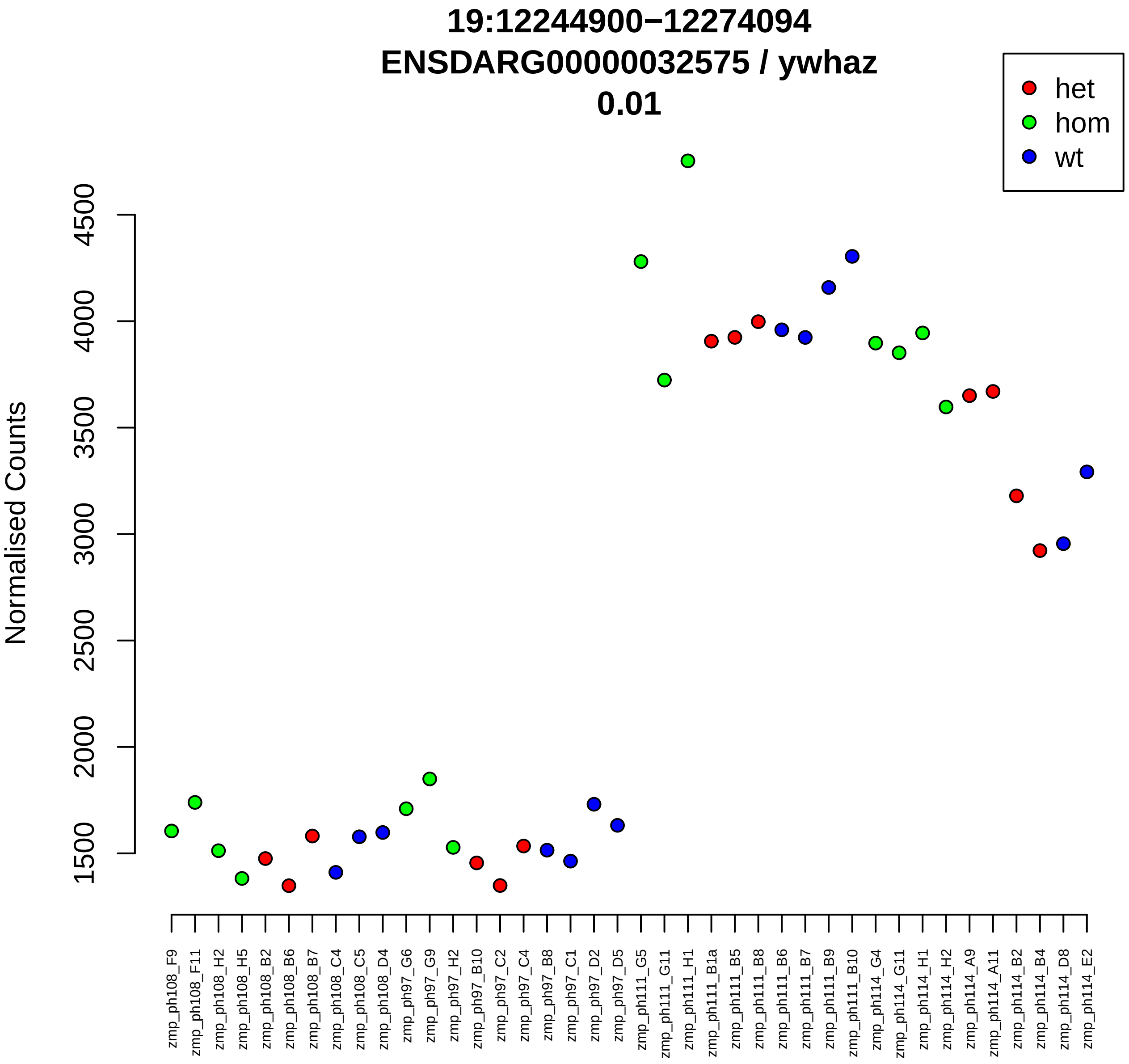
19:12244900−12274094
ENSDARG00000032575 / ywhaz
0.01
het
hom
wt
Normalised Counts
1500
2000
2500
3000
3500
4000
4500
zmp_ph108_F9
zmp_ph108_F11
zmp_ph108_H2
zmp_ph108_H5
zmp_ph108_B2
zmp_ph108_B6
zmp_ph108_B7
zmp_ph108_C4
zmp_ph108_C5
zmp_ph108_D4
zmp_ph97_G6
zmp_ph97_G9
zmp_ph97_H2
zmp_ph97_B10
zmp_ph97_C2
zmp_ph97_C4
zmp_ph97_B8
zmp_ph97_C1
zmp_ph97_D2
zmp_ph97_D5
zmp_ph111_G5
zmp_ph111_G11
zmp_ph111_H1
zmp_ph111_B1a
zmp_ph111_B5
zmp_ph111_B8
zmp_ph111_B6
zmp_ph111_B7
zmp_ph111_B9
zmp_ph111_B10
zmp_ph114_G4
zmp_ph114_G11
zmp_ph114_H1
zmp_ph114_H2
zmp_ph114_A9
zmp_ph114_A11
zmp_ph114_B2
zmp_ph114_B4
zmp_ph114_D8
zmp_ph114_E2

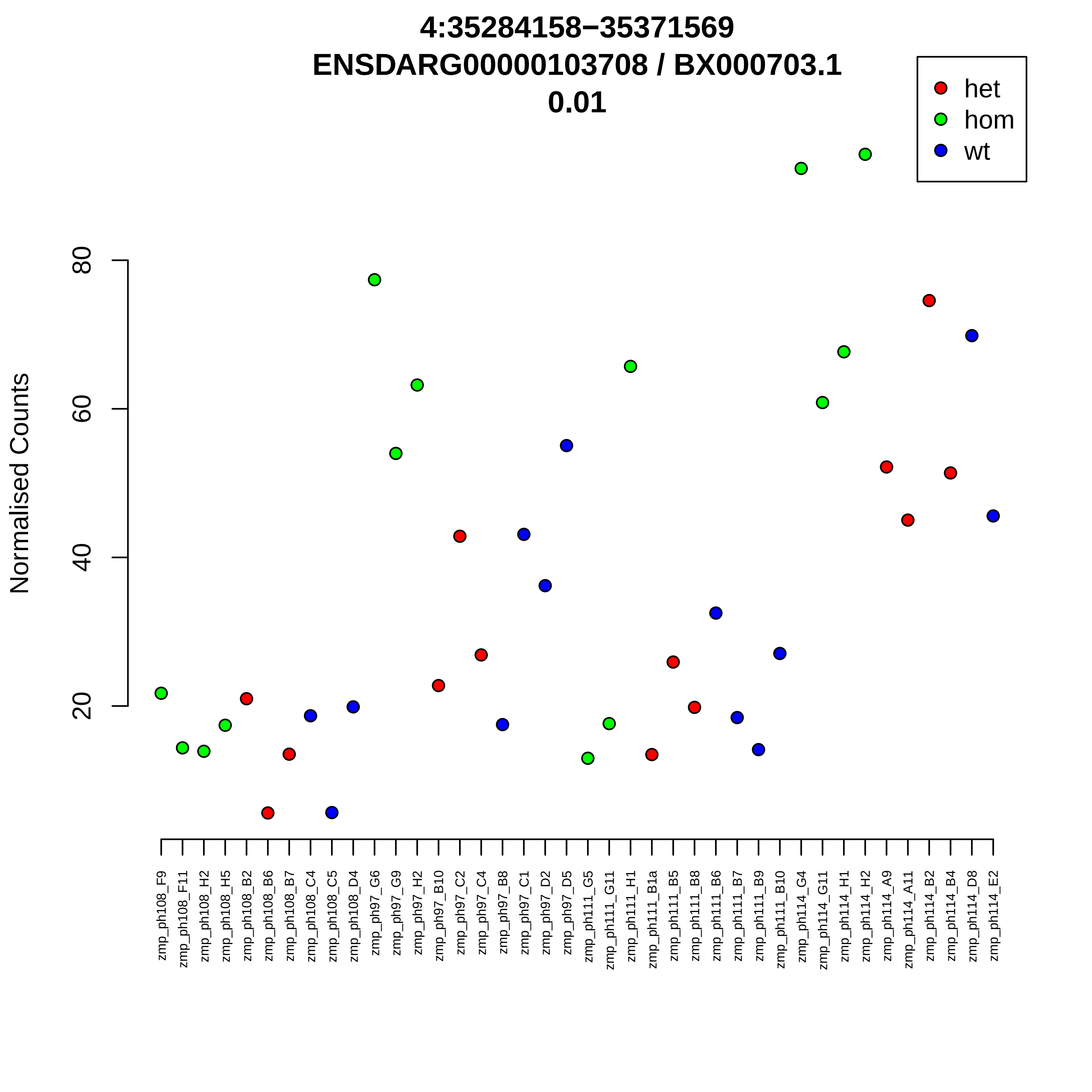
4:35284158−35371569
ENSDARG00000103708 / BX000703.1
0.01
het
hom
wt
Normalised Counts
20
40
60
80
zmp_ph108_F9
zmp_ph108_F11
zmp_ph108_H2
zmp_ph108_H5
zmp_ph108_B2
zmp_ph108_B6
zmp_ph108_B7
zmp_ph108_C4
zmp_ph108_C5
zmp_ph108_D4
zmp_ph97_G6
zmp_ph97_G9
zmp_ph97_H2
zmp_ph97_B10
zmp_ph97_C2
zmp_ph97_C4
zmp_ph97_B8
zmp_ph97_C1
zmp_ph97_D2
zmp_ph97_D5
zmp_ph111_G5
zmp_ph111_G11
zmp_ph111_H1
zmp_ph111_B1a
zmp_ph111_B5
zmp_ph111_B8
zmp_ph111_B6
zmp_ph111_B7
zmp_ph111_B9
zmp_ph111_B10
zmp_ph114_G4
zmp_ph114_G11
zmp_ph114_H1
zmp_ph114_H2
zmp_ph114_A9
zmp_ph114_A11
zmp_ph114_B2
zmp_ph114_B4
zmp_ph114_D8
zmp_ph114_E2

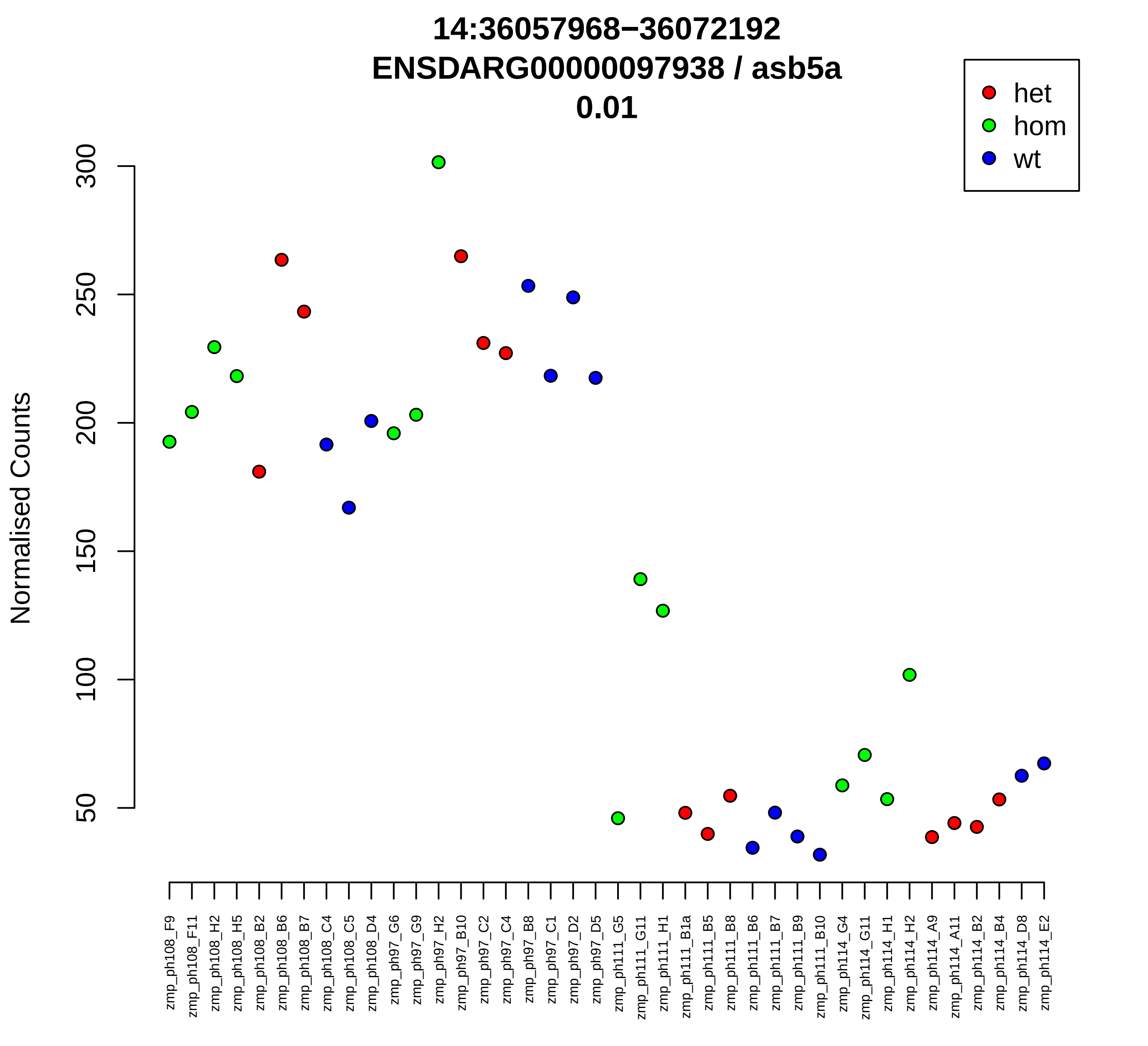
14:36057968−36072192
ENSDARG00000097938 / asb5a
0.01
het
hom
wt
Normalised Counts
50
100
150
200
250
300
zmp_ph108_F9
zmp_ph108_F11
zmp_ph108_H2
zmp_ph108_H5
zmp_ph108_B2
zmp_ph108_B6
zmp_ph108_B7
zmp_ph108_C4
zmp_ph108_C5
zmp_ph108_D4
zmp_ph97_G6
zmp_ph97_G9
zmp_ph97_H2
zmp_ph97_B10
zmp_ph97_C2
zmp_ph97_C4
zmp_ph97_B8
zmp_ph97_C1
zmp_ph97_D2
zmp_ph97_D5
zmp_ph111_G5
zmp_ph111_G11
zmp_ph111_H1
zmp_ph111_B1a
zmp_ph111_B5
zmp_ph111_B8
zmp_ph111_B6
zmp_ph111_B7
zmp_ph111_B9
zmp_ph111_B10
zmp_ph114_G4
zmp_ph114_G11
zmp_ph114_H1
zmp_ph114_H2
zmp_ph114_A9
zmp_ph114_A11
zmp_ph114_B2
zmp_ph114_B4
zmp_ph114_D8
zmp_ph114_E2

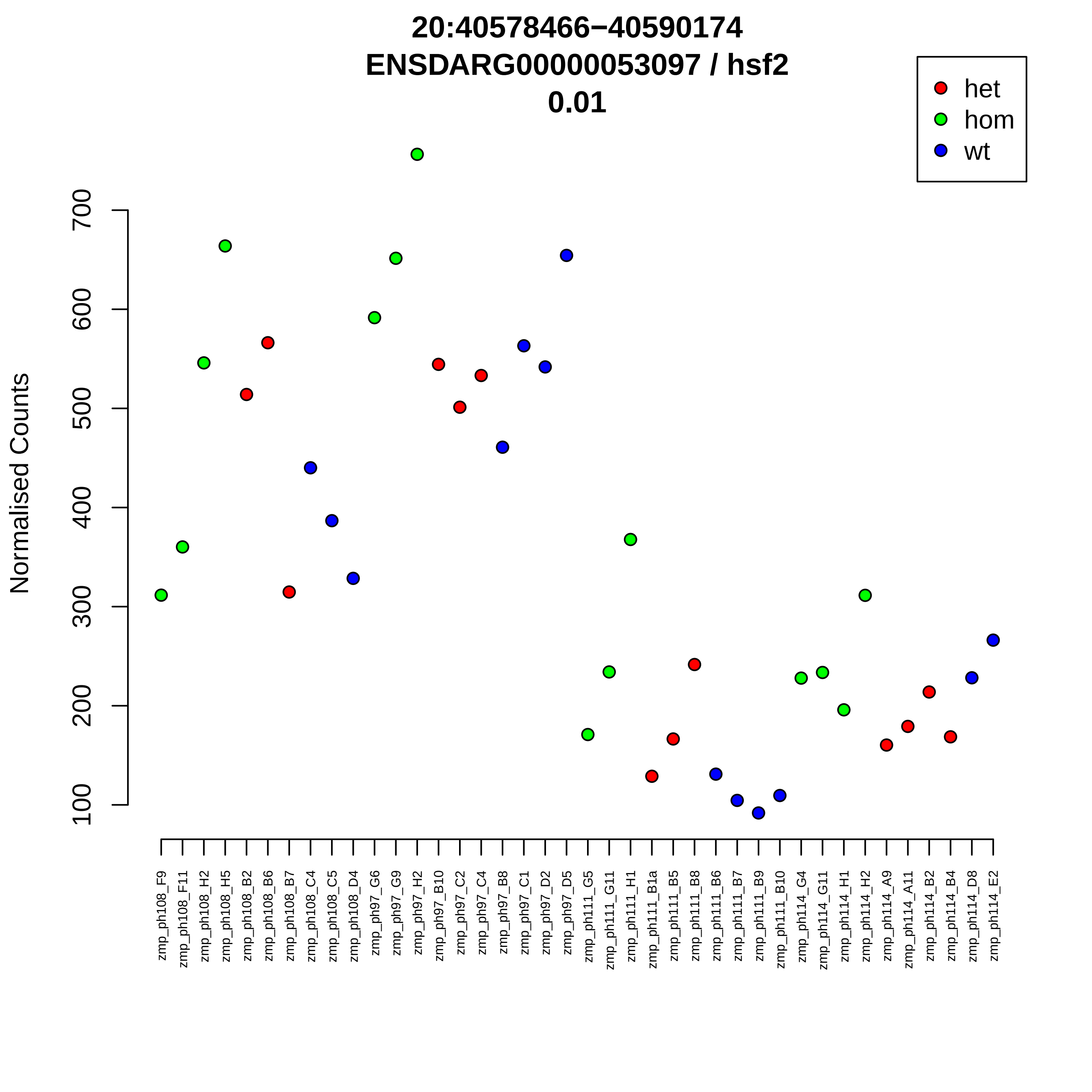

20:40578466−40590174
ENSDARG00000053097 / hsf2
0.01
het
hom
wt
Normalised Counts
100
200
300
400
500
600
700
zmp_ph108_F9
zmp_ph108_F11
zmp_ph108_H2
zmp_ph108_H5
zmp_ph108_B2
zmp_ph108_B6
zmp_ph108_B7
zmp_ph108_C4
zmp_ph108_C5
zmp_ph108_D4
zmp_ph97_G6
zmp_ph97_G9
zmp_ph97_H2
zmp_ph97_B10
zmp_ph97_C2
zmp_ph97_C4
zmp_ph97_B8
zmp_ph97_C1
zmp_ph97_D2
zmp_ph97_D5
zmp_ph111_G5
zmp_ph111_G11
zmp_ph111_H1
zmp_ph111_B1a
zmp_ph111_B5
zmp_ph111_B8
zmp_ph111_B6
zmp_ph111_B7
zmp_ph111_B9
zmp_ph111_B10
zmp_ph114_G4
zmp_ph114_G11
zmp_ph114_H1
zmp_ph114_H2
zmp_ph114_A9
zmp_ph114_A11
zmp_ph114_B2
zmp_ph114_B4
zmp_ph114_D8
zmp_ph114_E2

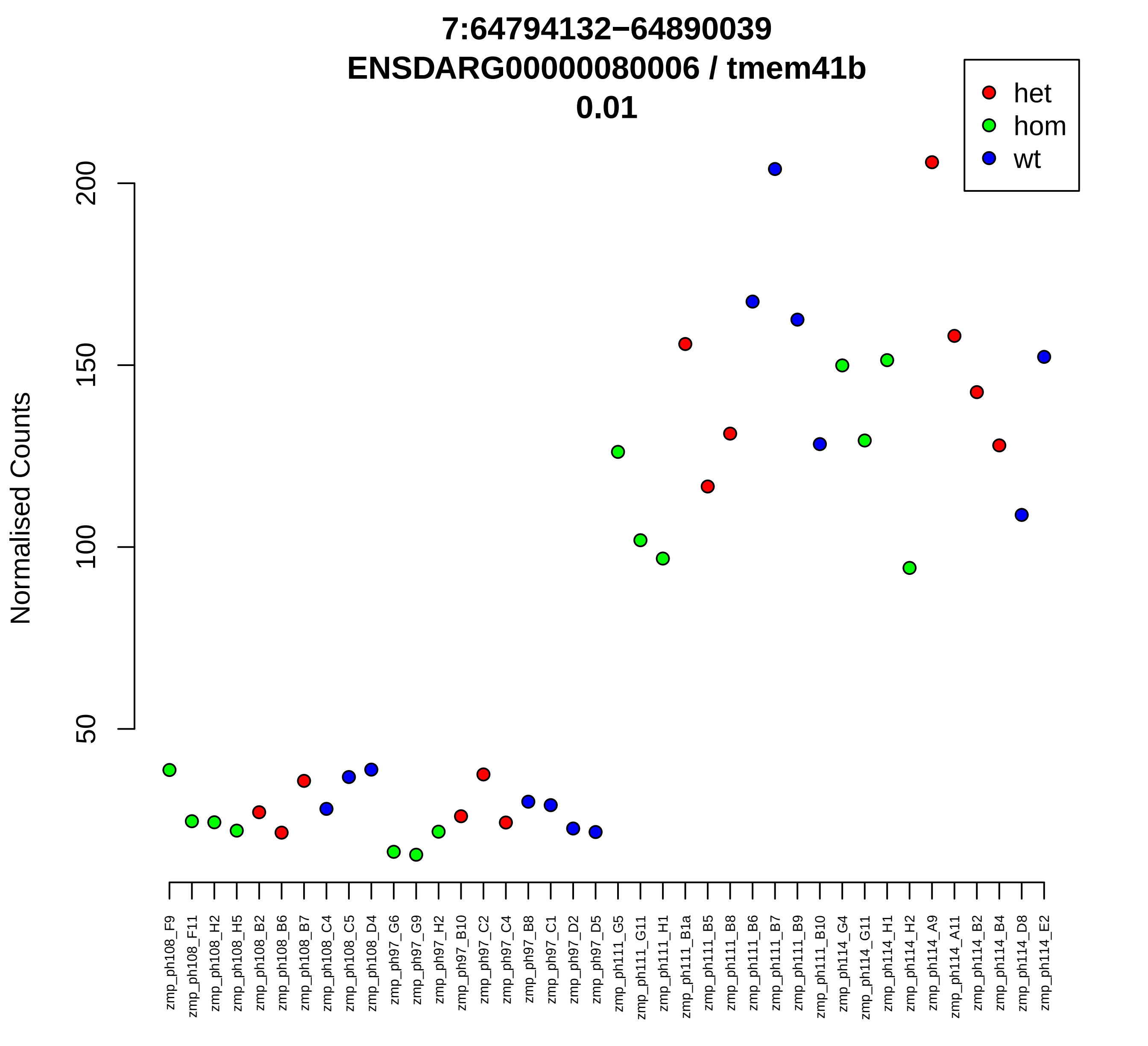
7:64794132−64890039
ENSDARG00000080006 / tmem41b
0.01
het
hom
wt
Normalised Counts
50
100
150
200
zmp_ph108_F9
zmp_ph108_F11
zmp_ph108_H2
zmp_ph108_H5
zmp_ph108_B2
zmp_ph108_B6
zmp_ph108_B7
zmp_ph108_C4
zmp_ph108_C5
zmp_ph108_D4
zmp_ph97_G6
zmp_ph97_G9
zmp_ph97_H2
zmp_ph97_B10
zmp_ph97_C2
zmp_ph97_C4
zmp_ph97_B8
zmp_ph97_C1
zmp_ph97_D2
zmp_ph97_D5
zmp_ph111_G5
zmp_ph111_G11
zmp_ph111_H1
zmp_ph111_B1a
zmp_ph111_B5
zmp_ph111_B8
zmp_ph111_B6
zmp_ph111_B7
zmp_ph111_B9
zmp_ph111_B10
zmp_ph114_G4
zmp_ph114_G11
zmp_ph114_H1
zmp_ph114_H2
zmp_ph114_A9
zmp_ph114_A11
zmp_ph114_B2
zmp_ph114_B4
zmp_ph114_D8
zmp_ph114_E2

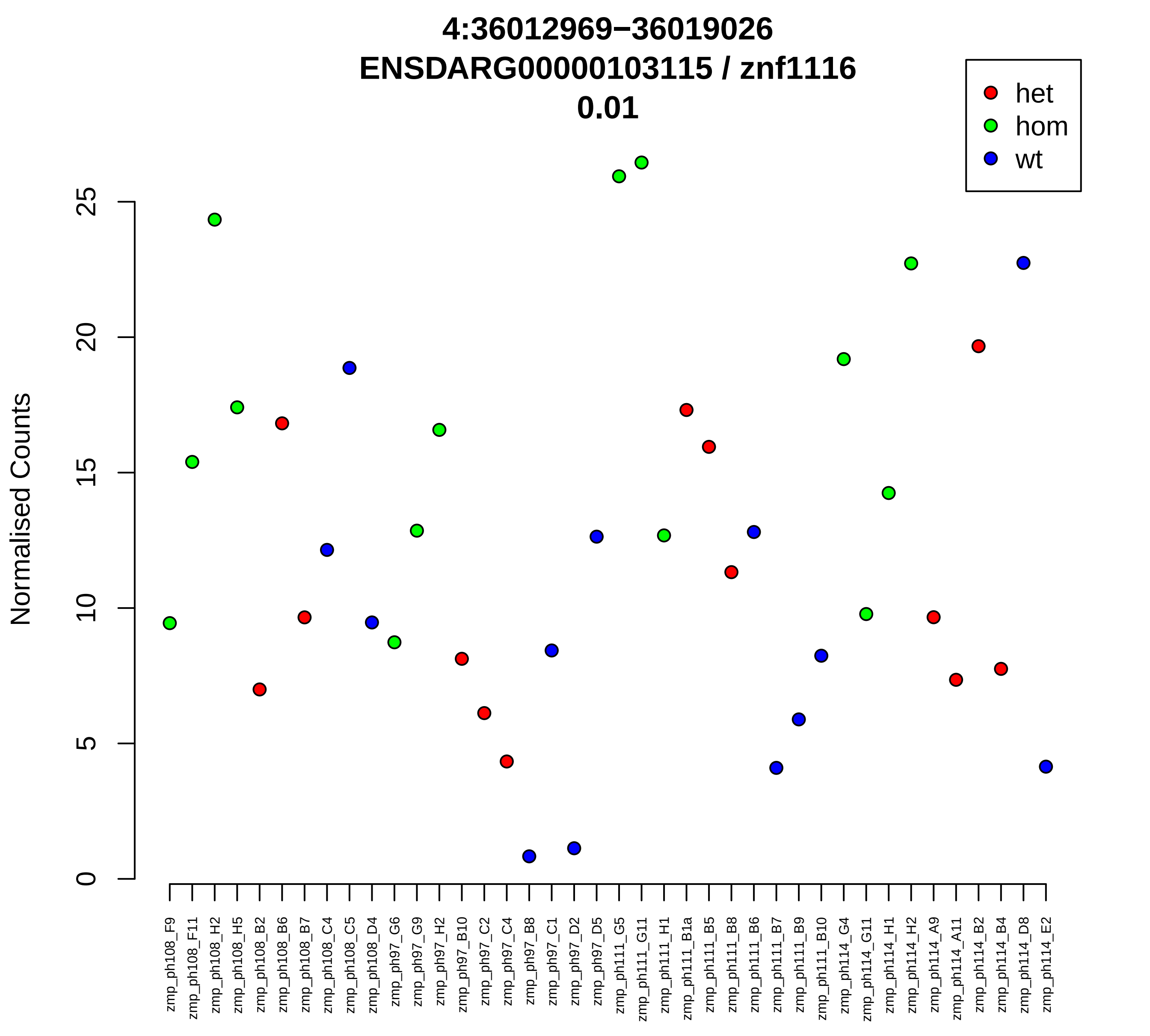

4:36012969−36019026
ENSDARG00000103115 / znf1116
0.01
het
hom
wt
Normalised Counts
0
5
10
15
20
25
zmp_ph108_F9
zmp_ph108_F11
zmp_ph108_H2
zmp_ph108_H5
zmp_ph108_B2
zmp_ph108_B6
zmp_ph108_B7
zmp_ph108_C4
zmp_ph108_C5
zmp_ph108_D4
zmp_ph97_G6
zmp_ph97_G9
zmp_ph97_H2
zmp_ph97_B10
zmp_ph97_C2
zmp_ph97_C4
zmp_ph97_B8
zmp_ph97_C1
zmp_ph97_D2
zmp_ph97_D5
zmp_ph111_G5
zmp_ph111_G11
zmp_ph111_H1
zmp_ph111_B1a
zmp_ph111_B5
zmp_ph111_B8
zmp_ph111_B6
zmp_ph111_B7
zmp_ph111_B9
zmp_ph111_B10
zmp_ph114_G4
zmp_ph114_G11
zmp_ph114_H1
zmp_ph114_H2
zmp_ph114_A9
zmp_ph114_A11
zmp_ph114_B2
zmp_ph114_B4
zmp_ph114_D8
zmp_ph114_E2

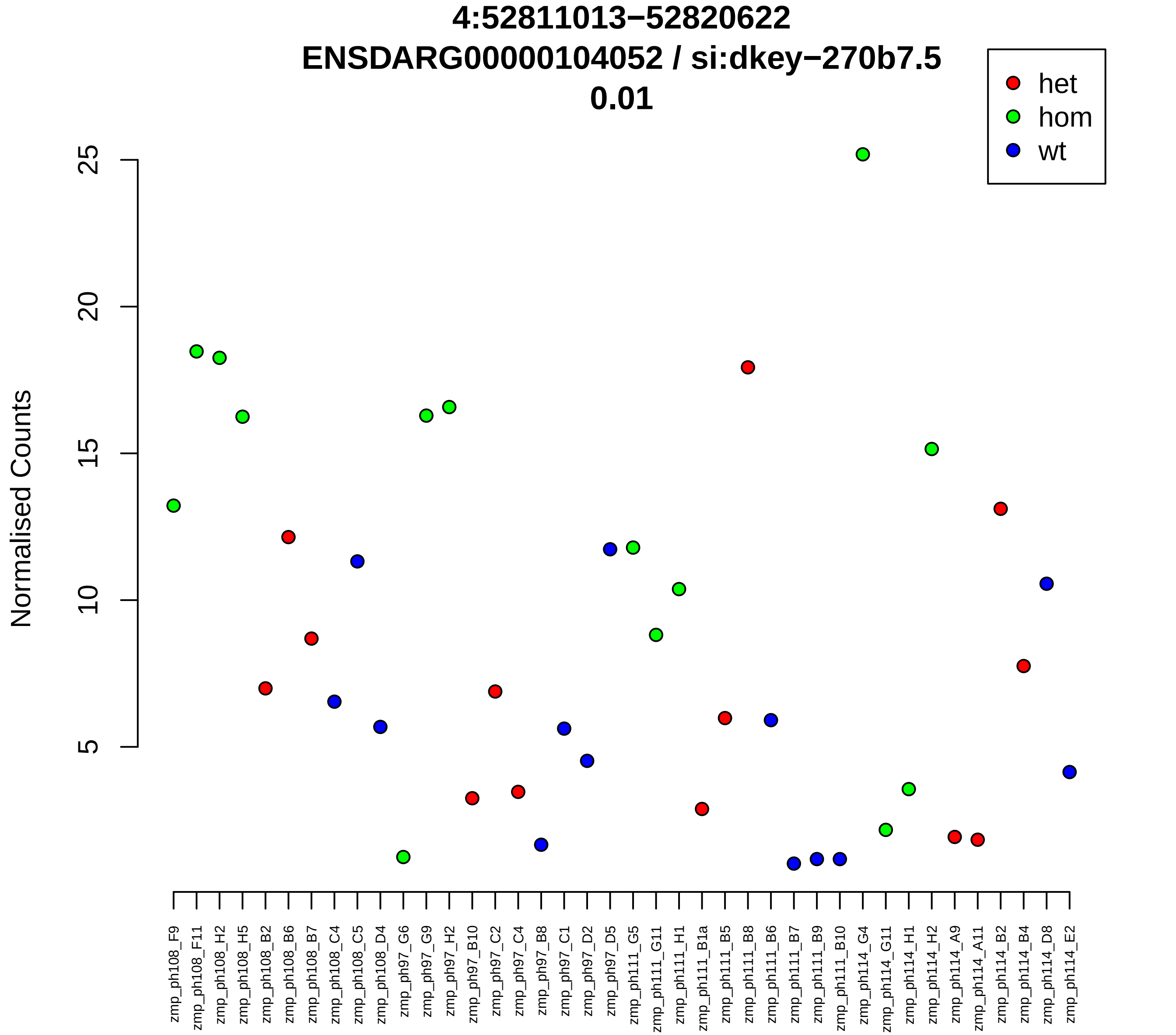

4:52811013−52820622
ENSDARG00000104052 / si:dkey−270b7.5
0.01
het
hom
wt
Normalised Counts
25
20
15
10
5
zmp_ph108_F9
zmp_ph108_F11
zmp_ph108_H2
zmp_ph108_H5
zmp_ph108_B2
zmp_ph108_B6
zmp_ph108_B7
zmp_ph108_C4
zmp_ph108_C5
zmp_ph108_D4
zmp_ph97_G6
zmp_ph97_G9
zmp_ph97_H2
zmp_ph97_B10
zmp_ph97_C2
zmp_ph97_C4
zmp_ph97_B8
zmp_ph97_C1
zmp_ph97_D2
zmp_ph97_D5
zmp_ph111_G5
zmp_ph111_G11
zmp_ph111_H1
zmp_ph111_B1a
zmp_ph111_B5
zmp_ph111_B8
zmp_ph111_B6
zmp_ph111_B7
zmp_ph111_B9
zmp_ph111_B10
zmp_ph114_G4
zmp_ph114_G11
zmp_ph114_H1
zmp_ph114_H2
zmp_ph114_A9
zmp_ph114_A11
zmp_ph114_B2
zmp_ph114_B4
zmp_ph114_D8
zmp_ph114_E2

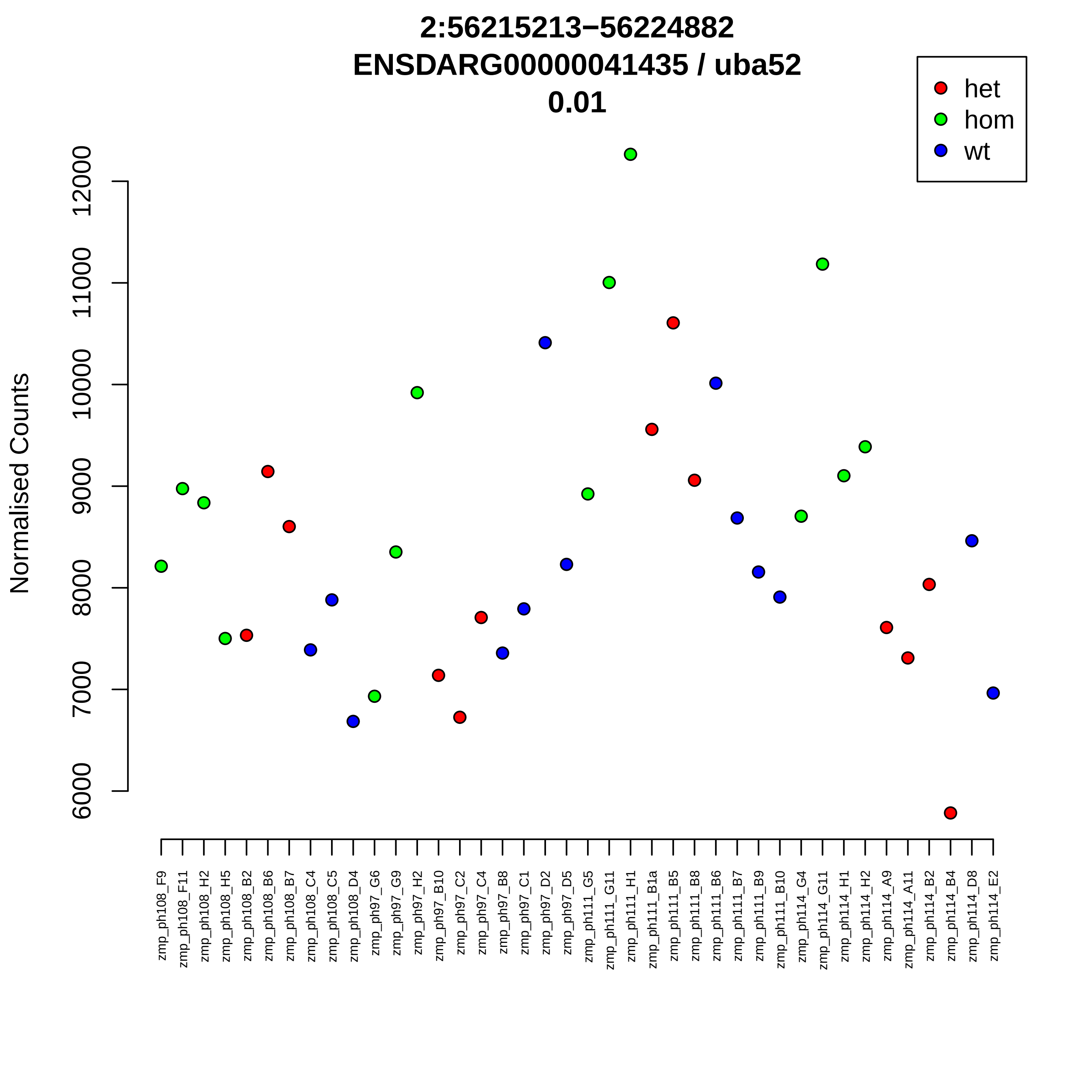

2:56215213−56224882
ENSDARG00000041435 / uba52
0.01
het
hom
wt
Normalised Counts
6000
7000
8000
9000
10000
11000
12000
zmp_ph108_F9
zmp_ph108_F11
zmp_ph108_H2
zmp_ph108_H5
zmp_ph108_B2
zmp_ph108_B6
zmp_ph108_B7
zmp_ph108_C4
zmp_ph108_C5
zmp_ph108_D4
zmp_ph97_G6
zmp_ph97_G9
zmp_ph97_H2
zmp_ph97_B10
zmp_ph97_C2
zmp_ph97_C4
zmp_ph97_B8
zmp_ph97_C1
zmp_ph97_D2
zmp_ph97_D5
zmp_ph111_G5
zmp_ph111_G11
zmp_ph111_H1
zmp_ph111_B1a
zmp_ph111_B5
zmp_ph111_B8
zmp_ph111_B6
zmp_ph111_B7
zmp_ph111_B9
zmp_ph111_B10
zmp_ph114_G4
zmp_ph114_G11
zmp_ph114_H1
zmp_ph114_H2
zmp_ph114_A9
zmp_ph114_A11
zmp_ph114_B2
zmp_ph114_B4
zmp_ph114_D8
zmp_ph114_E2

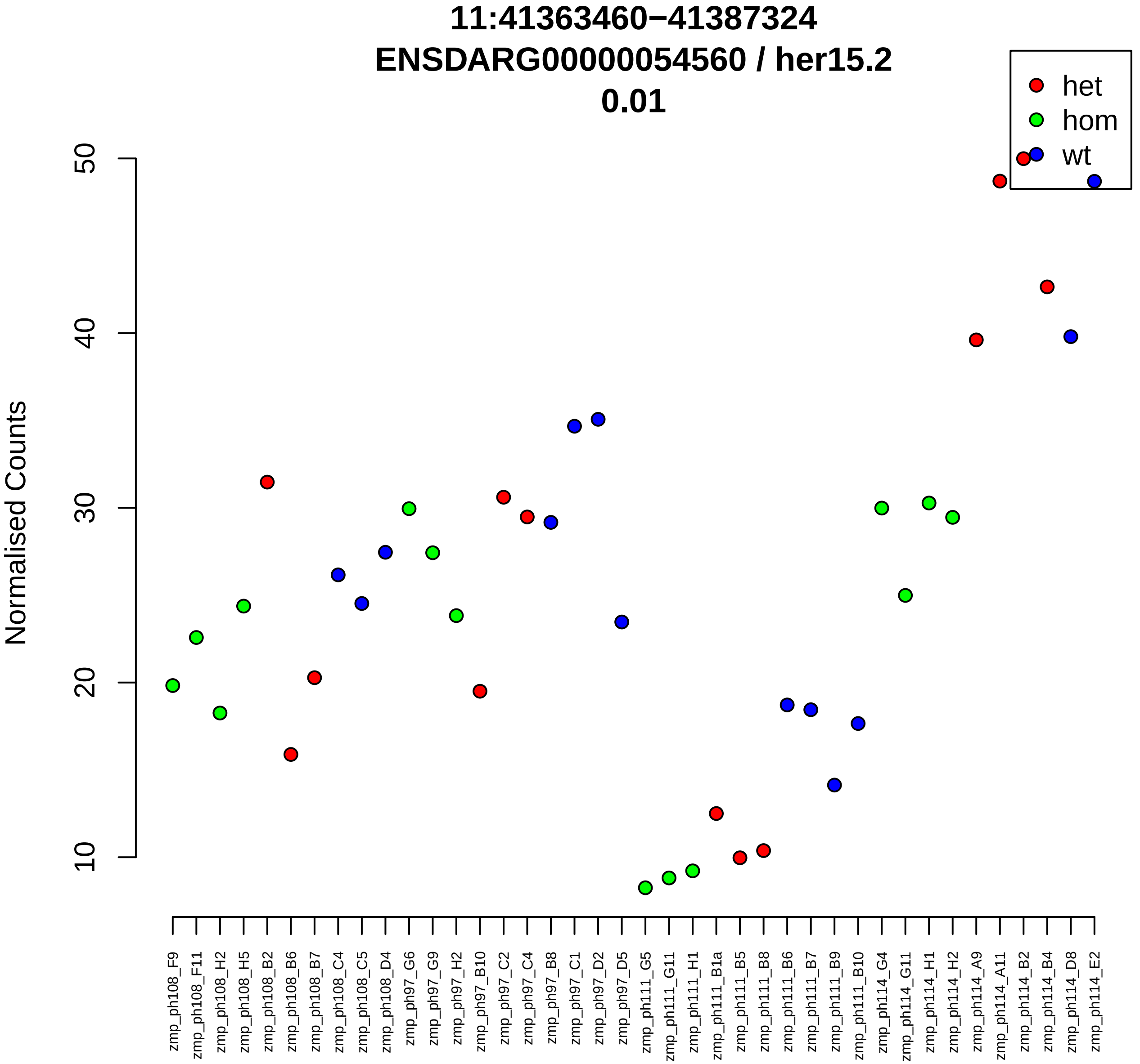
11:41363460−41387324
ENSDARG00000054560 / her15.2
0.01
het
hom
wt
Normalised Counts
10
20
30
40
50
zmp_ph108_F9
zmp_ph108_F11
zmp_ph108_H2
zmp_ph108_H5
zmp_ph108_B2
zmp_ph108_B6
zmp_ph108_B7
zmp_ph108_C4
zmp_ph108_C5
zmp_ph108_D4
zmp_ph97_G6
zmp_ph97_G9
zmp_ph97_H2
zmp_ph97_B10
zmp_ph97_C2
zmp_ph97_C4
zmp_ph97_B8
zmp_ph97_C1
zmp_ph97_D2
zmp_ph97_D5
zmp_ph111_G5
zmp_ph111_G11
zmp_ph111_H1
zmp_ph111_B1a
zmp_ph111_B5
zmp_ph111_B8
zmp_ph111_B6
zmp_ph111_B7
zmp_ph111_B9
zmp_ph111_B10
zmp_ph114_G4
zmp_ph114_G11
zmp_ph114_H1
zmp_ph114_H2
zmp_ph114_A9
zmp_ph114_A11
zmp_ph114_B2
zmp_ph114_B4
zmp_ph114_D8
zmp_ph114_E2

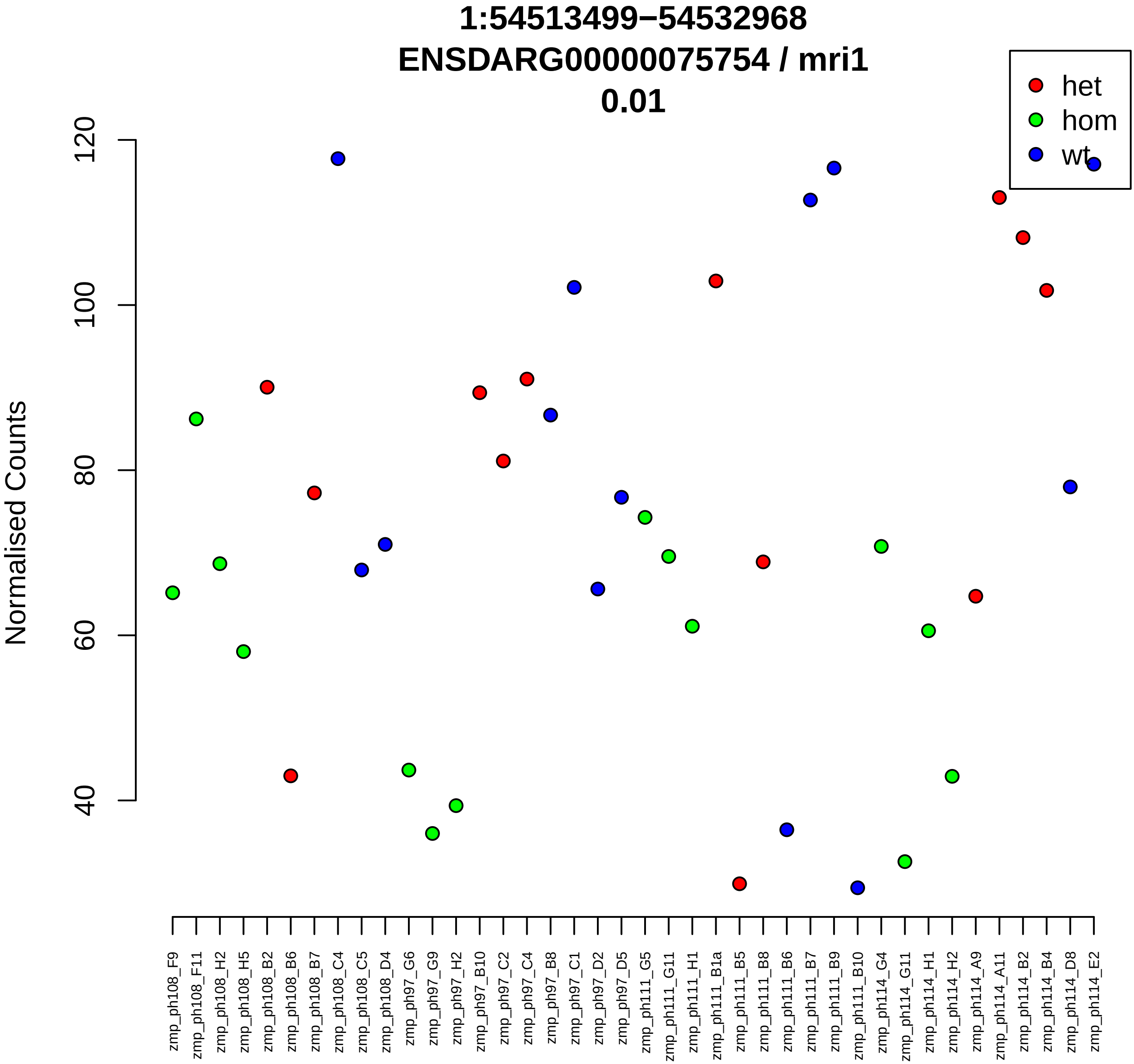

1:54513499−54532968
ENSDARG00000075754 / mri1
0.01
het
hom
wt
Normalised Counts
120
100
80
60
40
zmp_ph108_F9
zmp_ph108_F11
zmp_ph108_H2
zmp_ph108_H5
zmp_ph108_B2
zmp_ph108_B6
zmp_ph108_B7
zmp_ph108_C4
zmp_ph108_C5
zmp_ph108_D4
zmp_ph97_G6
zmp_ph97_G9
zmp_ph97_H2
zmp_ph97_B10
zmp_ph97_C2
zmp_ph97_C4
zmp_ph97_B8
zmp_ph97_C1
zmp_ph97_D2
zmp_ph97_D5
zmp_ph111_G5
zmp_ph111_G11
zmp_ph111_H1
zmp_ph111_B1a
zmp_ph111_B5
zmp_ph111_B8
zmp_ph111_B6
zmp_ph111_B7
zmp_ph111_B9
zmp_ph111_B10
zmp_ph114_G4
zmp_ph114_G11
zmp_ph114_H1
zmp_ph114_H2
zmp_ph114_A9
zmp_ph114_A11
zmp_ph114_B2
zmp_ph114_B4
zmp_ph114_D8
zmp_ph114_E2

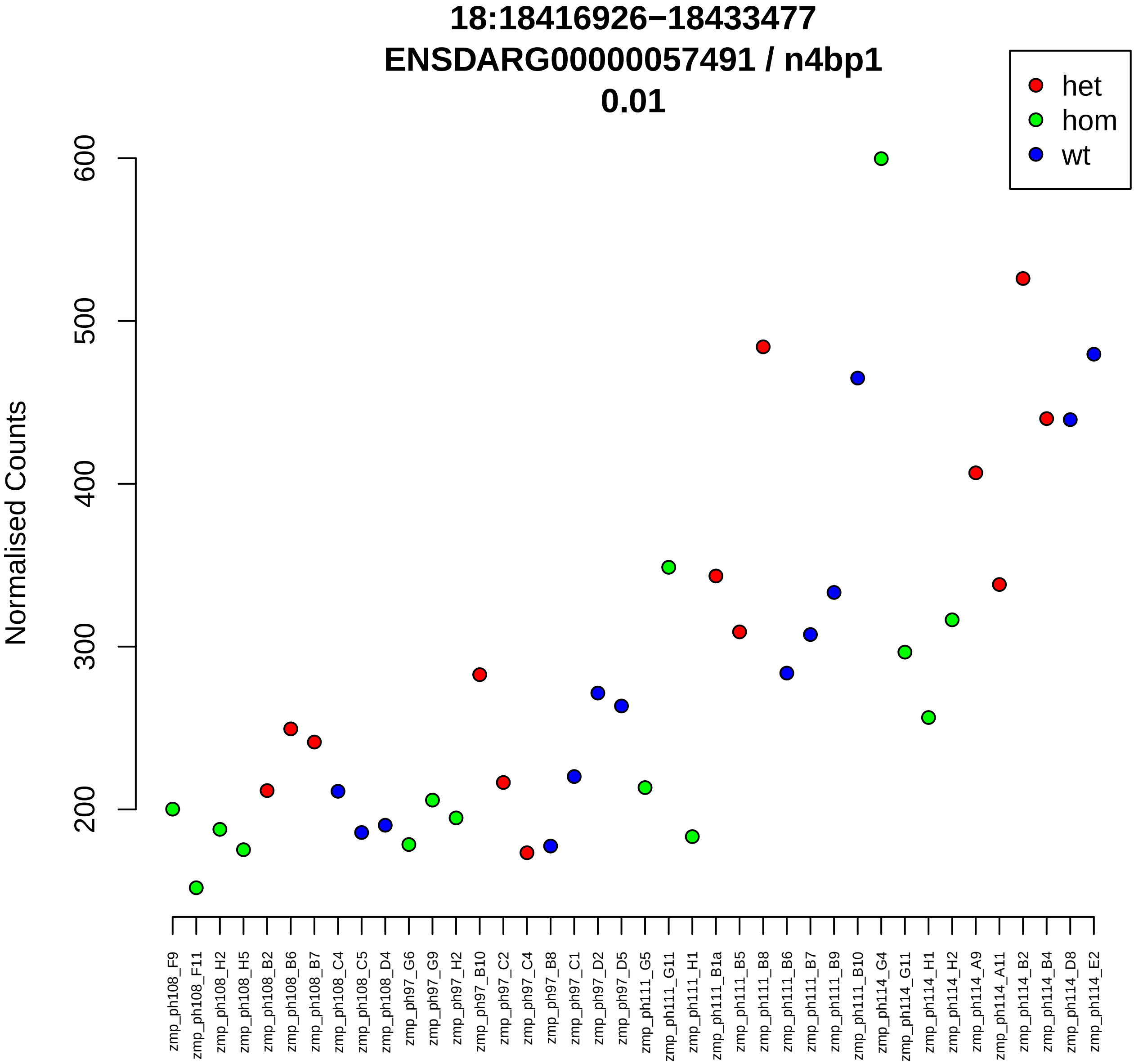

18:18416926−18433477
ENSDARG00000057491 / n4bp1
0.01
het
hom
wt
Normalised Counts
200
300
400
500
600
zmp_ph108_F9
zmp_ph108_F11
zmp_ph108_H2
zmp_ph108_H5
zmp_ph108_B2
zmp_ph108_B6
zmp_ph108_B7
zmp_ph108_C4
zmp_ph108_C5
zmp_ph108_D4
zmp_ph97_G6
zmp_ph97_G9
zmp_ph97_H2
zmp_ph97_B10
zmp_ph97_C2
zmp_ph97_C4
zmp_ph97_B8
zmp_ph97_C1
zmp_ph97_D2
zmp_ph97_D5
zmp_ph111_G5
zmp_ph111_G11
zmp_ph111_H1
zmp_ph111_B1a
zmp_ph111_B5
zmp_ph111_B8
zmp_ph111_B6
zmp_ph111_B7
zmp_ph111_B9
zmp_ph111_B10
zmp_ph114_G4
zmp_ph114_G11
zmp_ph114_H1
zmp_ph114_H2
zmp_ph114_A9
zmp_ph114_A11
zmp_ph114_B2
zmp_ph114_B4
zmp_ph114_D8
zmp_ph114_E2

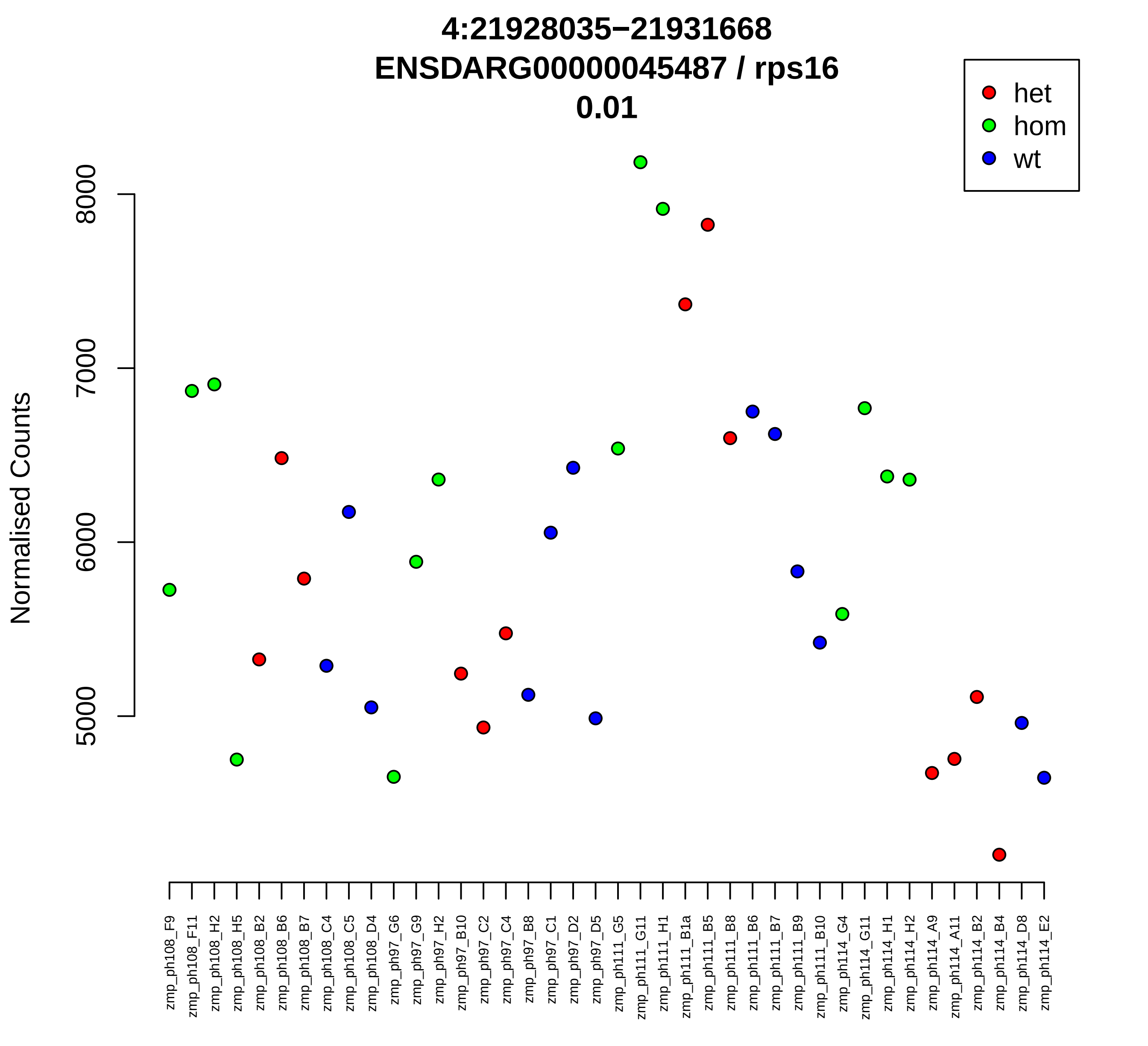
4:21928035−21931668
ENSDARG00000045487 / rps16
0.01
het
hom
wt
Normalised Counts
5000
6000
7000
8000
zmp_ph108_F9
zmp_ph108_F11
zmp_ph108_H2
zmp_ph108_H5
zmp_ph108_B2
zmp_ph108_B6
zmp_ph108_B7
zmp_ph108_C4
zmp_ph108_C5
zmp_ph108_D4
zmp_ph97_G6
zmp_ph97_G9
zmp_ph97_H2
zmp_ph97_B10
zmp_ph97_C2
zmp_ph97_C4
zmp_ph97_B8
zmp_ph97_C1
zmp_ph97_D2
zmp_ph97_D5
zmp_ph111_G5
zmp_ph111_G11
zmp_ph111_H1
zmp_ph111_B1a
zmp_ph111_B5
zmp_ph111_B8
zmp_ph111_B6
zmp_ph111_B7
zmp_ph111_B9
zmp_ph111_B10
zmp_ph114_G4
zmp_ph114_G11
zmp_ph114_H1
zmp_ph114_H2
zmp_ph114_A9
zmp_ph114_A11
zmp_ph114_B2
zmp_ph114_B4
zmp_ph114_D8
zmp_ph114_E2

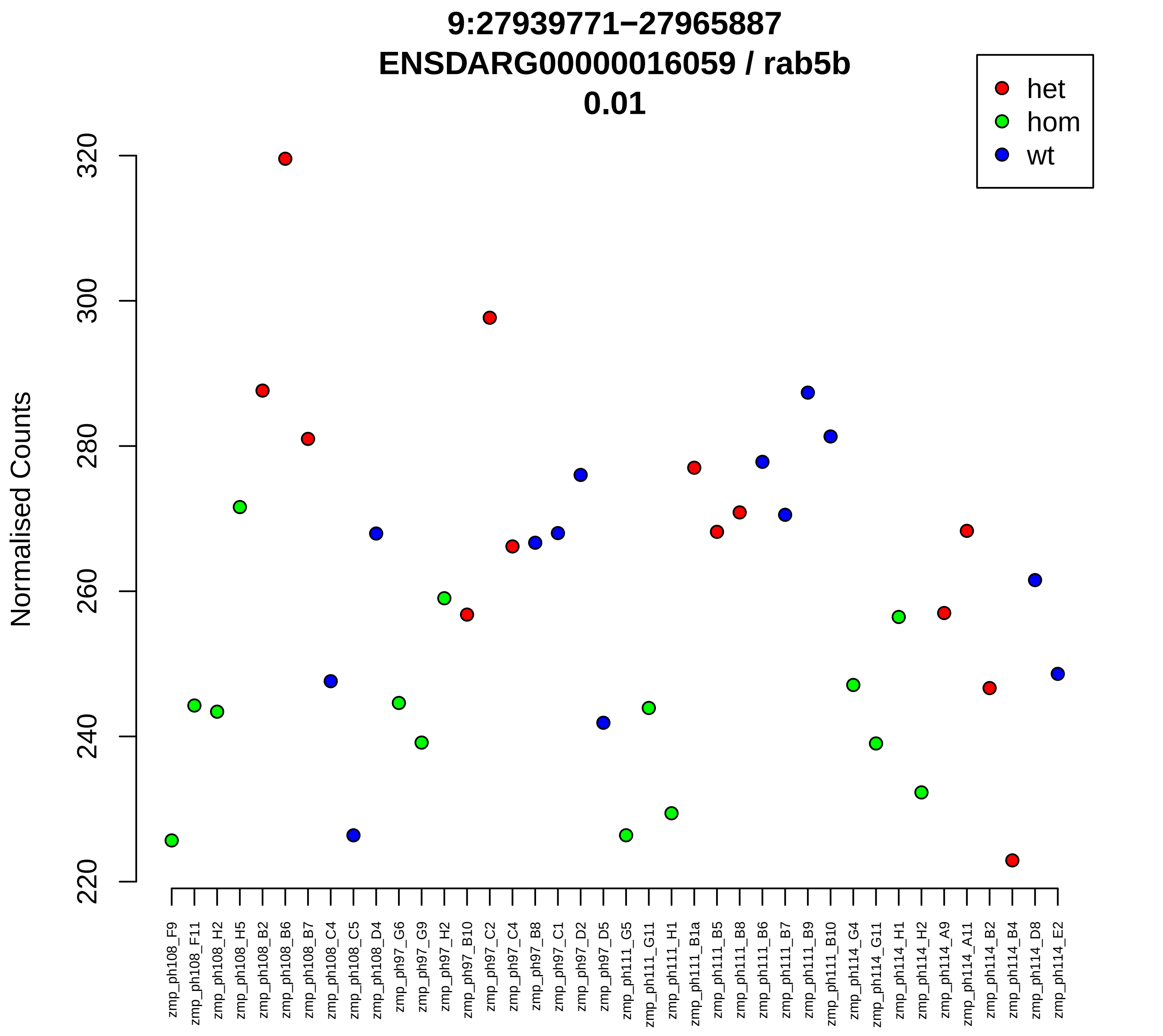

9:27939771−27965887
ENSDARG00000016059 / rab5b
0.01
het
hom
wt
Normalised Counts
220
240
260
280
300
320
zmp_ph108_F9
zmp_ph108_F11
zmp_ph108_H2
zmp_ph108_H5
zmp_ph108_B2
zmp_ph108_B6
zmp_ph108_B7
zmp_ph108_C4
zmp_ph108_C5
zmp_ph108_D4
zmp_ph97_G6
zmp_ph97_G9
zmp_ph97_H2
zmp_ph97_B10
zmp_ph97_C2
zmp_ph97_C4
zmp_ph97_B8
zmp_ph97_C1
zmp_ph97_D2
zmp_ph97_D5
zmp_ph111_G5
zmp_ph111_G11
zmp_ph111_H1
zmp_ph111_B1a
zmp_ph111_B5
zmp_ph111_B8
zmp_ph111_B6
zmp_ph111_B7
zmp_ph111_B9
zmp_ph111_B10
zmp_ph114_G4
zmp_ph114_G11
zmp_ph114_H1
zmp_ph114_H2
zmp_ph114_A9
zmp_ph114_A11
zmp_ph114_B2
zmp_ph114_B4
zmp_ph114_D8
zmp_ph114_E2

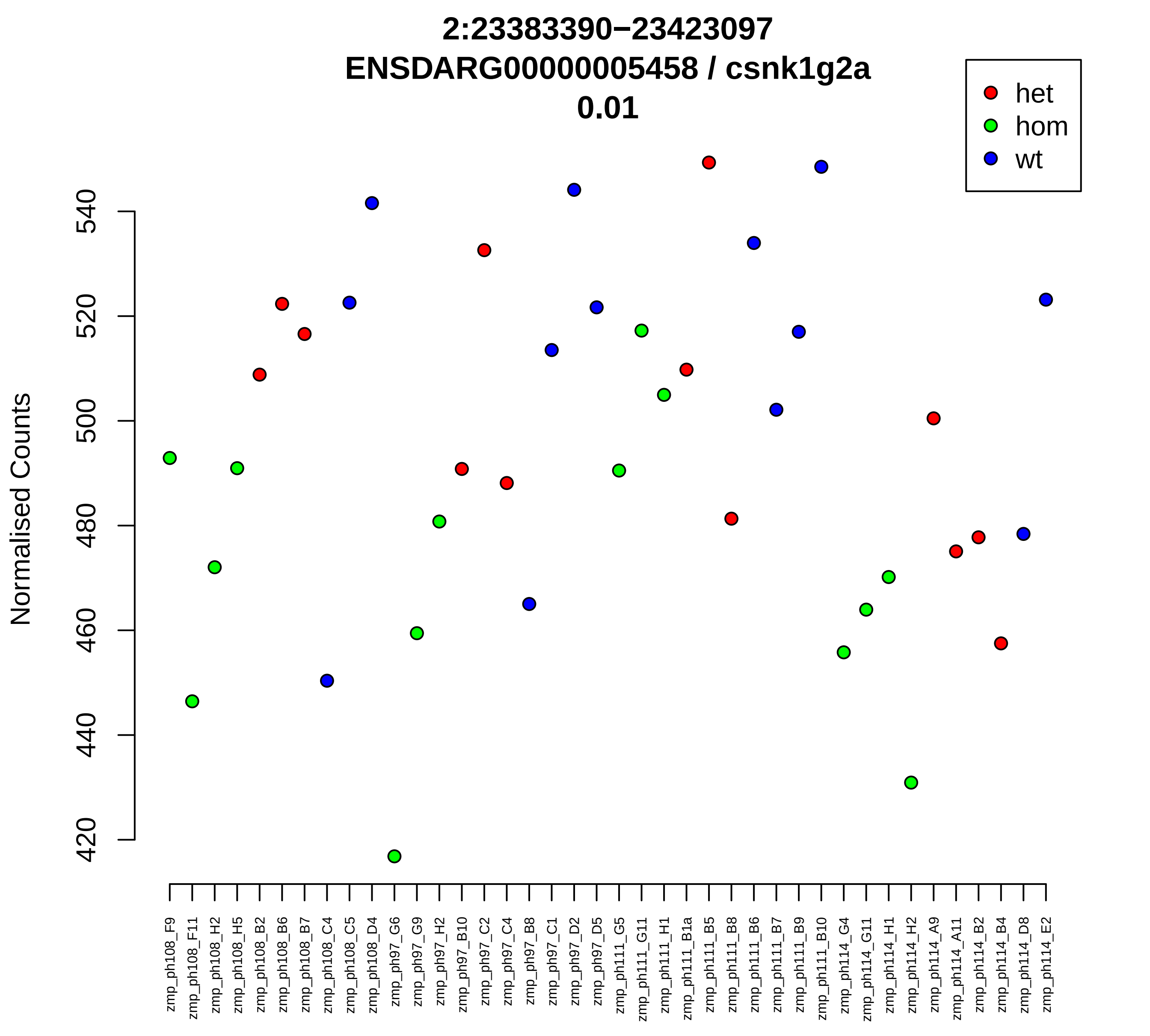

2:23383390−23423097
ENSDARG00000005458 / csnk1g2a
0.01
het
hom
wt
Normalised Counts
420
440
460
480
500
520
540
zmp_ph108_F9
zmp_ph108_F11
zmp_ph108_H2
zmp_ph108_H5
zmp_ph108_B2
zmp_ph108_B6
zmp_ph108_B7
zmp_ph108_C4
zmp_ph108_C5
zmp_ph108_D4
zmp_ph97_G6
zmp_ph97_G9
zmp_ph97_H2
zmp_ph97_B10
zmp_ph97_C2
zmp_ph97_C4
zmp_ph97_B8
zmp_ph97_C1
zmp_ph97_D2
zmp_ph97_D5
zmp_ph111_G5
zmp_ph111_G11
zmp_ph111_H1
zmp_ph111_B1a
zmp_ph111_B5
zmp_ph111_B8
zmp_ph111_B6
zmp_ph111_B7
zmp_ph111_B9
zmp_ph111_B10
zmp_ph114_G4
zmp_ph114_G11
zmp_ph114_H1
zmp_ph114_H2
zmp_ph114_A9
zmp_ph114_A11
zmp_ph114_B2
zmp_ph114_B4
zmp_ph114_D8
zmp_ph114_E2

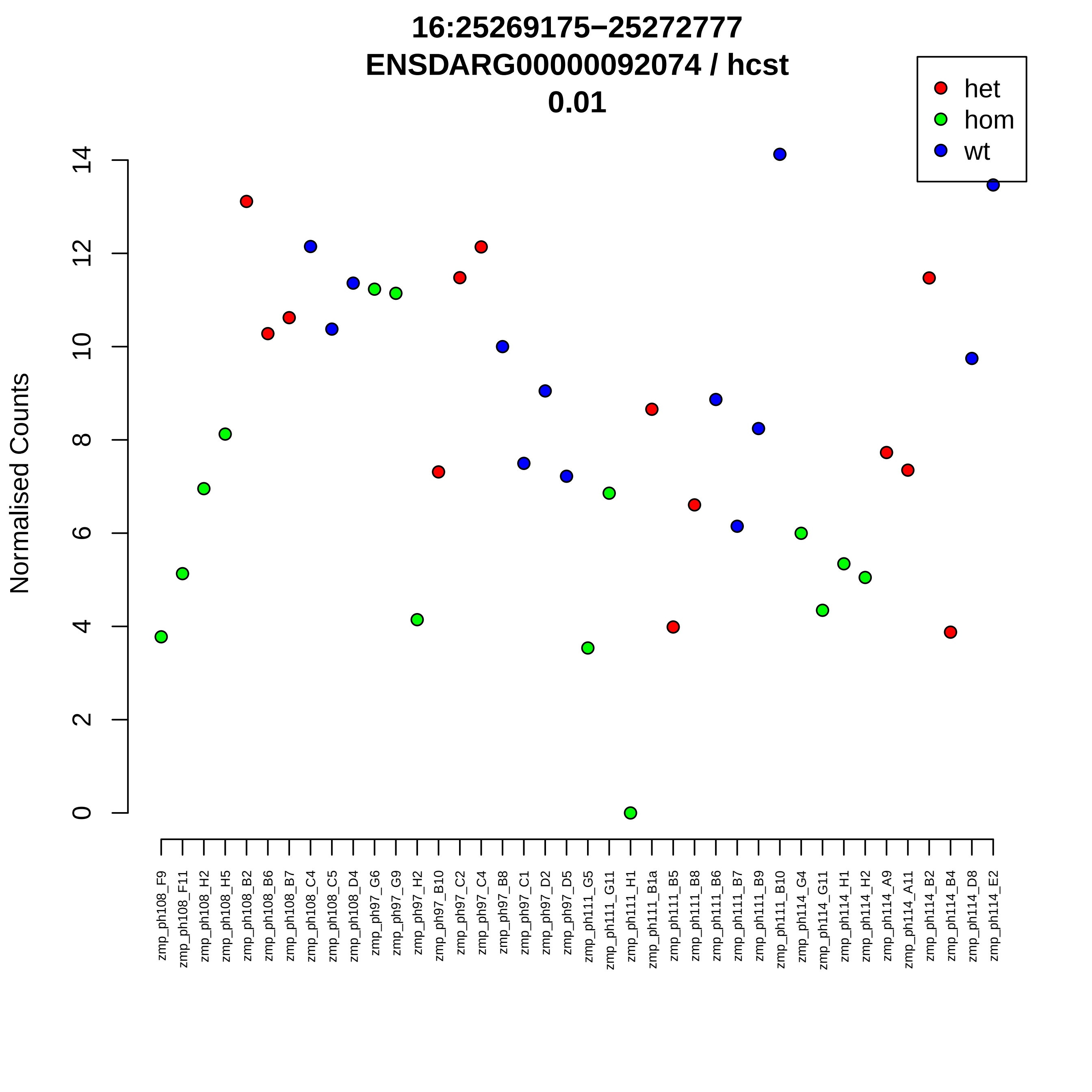
16:25269175−25272777
ENSDARG00000092074 / hcst
0.01
het
hom
wt
Normalised Counts
0
2
4
6
8
10
12
14
zmp_ph108_F9
zmp_ph108_F11
zmp_ph108_H2
zmp_ph108_H5
zmp_ph108_B2
zmp_ph108_B6
zmp_ph108_B7
zmp_ph108_C4
zmp_ph108_C5
zmp_ph108_D4
zmp_ph97_G6
zmp_ph97_G9
zmp_ph97_H2
zmp_ph97_B10
zmp_ph97_C2
zmp_ph97_C4
zmp_ph97_B8
zmp_ph97_C1
zmp_ph97_D2
zmp_ph97_D5
zmp_ph111_G5
zmp_ph111_G11
zmp_ph111_H1
zmp_ph111_B1a
zmp_ph111_B5
zmp_ph111_B8
zmp_ph111_B6
zmp_ph111_B7
zmp_ph111_B9
zmp_ph111_B10
zmp_ph114_G4
zmp_ph114_G11
zmp_ph114_H1
zmp_ph114_H2
zmp_ph114_A9
zmp_ph114_A11
zmp_ph114_B2
zmp_ph114_B4
zmp_ph114_D8
zmp_ph114_E2

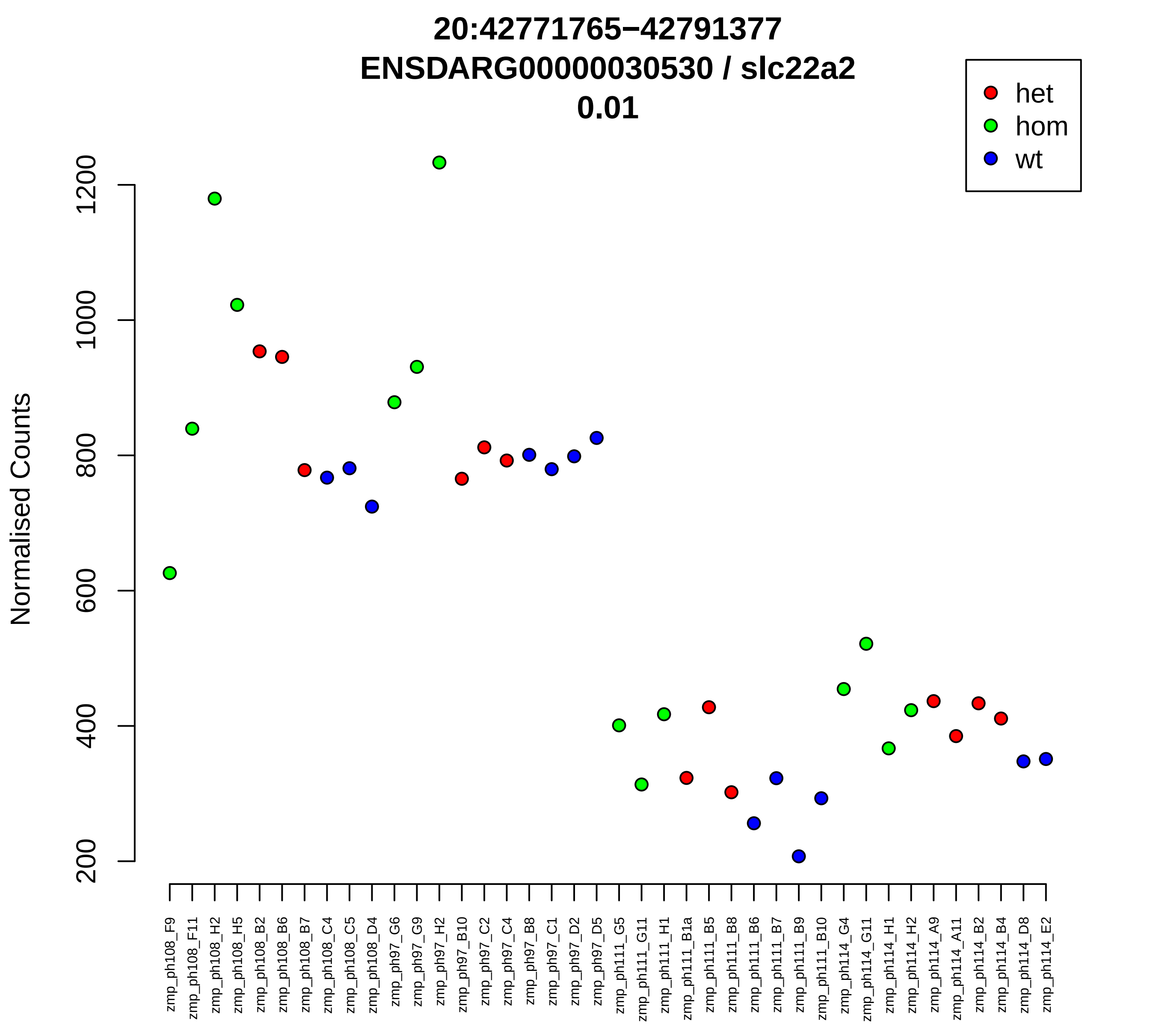
20:42771765−42791377
ENSDARG00000030530 / slc22a2
0.01
het
hom
wt
Normalised Counts
200
400
600
800
1000
1200
zmp_ph108_F9
zmp_ph108_F11
zmp_ph108_H2
zmp_ph108_H5
zmp_ph108_B2
zmp_ph108_B6
zmp_ph108_B7
zmp_ph108_C4
zmp_ph108_C5
zmp_ph108_D4
zmp_ph97_G6
zmp_ph97_G9
zmp_ph97_H2
zmp_ph97_B10
zmp_ph97_C2
zmp_ph97_C4
zmp_ph97_B8
zmp_ph97_C1
zmp_ph97_D2
zmp_ph97_D5
zmp_ph111_G5
zmp_ph111_G11
zmp_ph111_H1
zmp_ph111_B1a
zmp_ph111_B5
zmp_ph111_B8
zmp_ph111_B6
zmp_ph111_B7
zmp_ph111_B9
zmp_ph111_B10
zmp_ph114_G4
zmp_ph114_G11
zmp_ph114_H1
zmp_ph114_H2
zmp_ph114_A9
zmp_ph114_A11
zmp_ph114_B2
zmp_ph114_B4
zmp_ph114_D8
zmp_ph114_E2

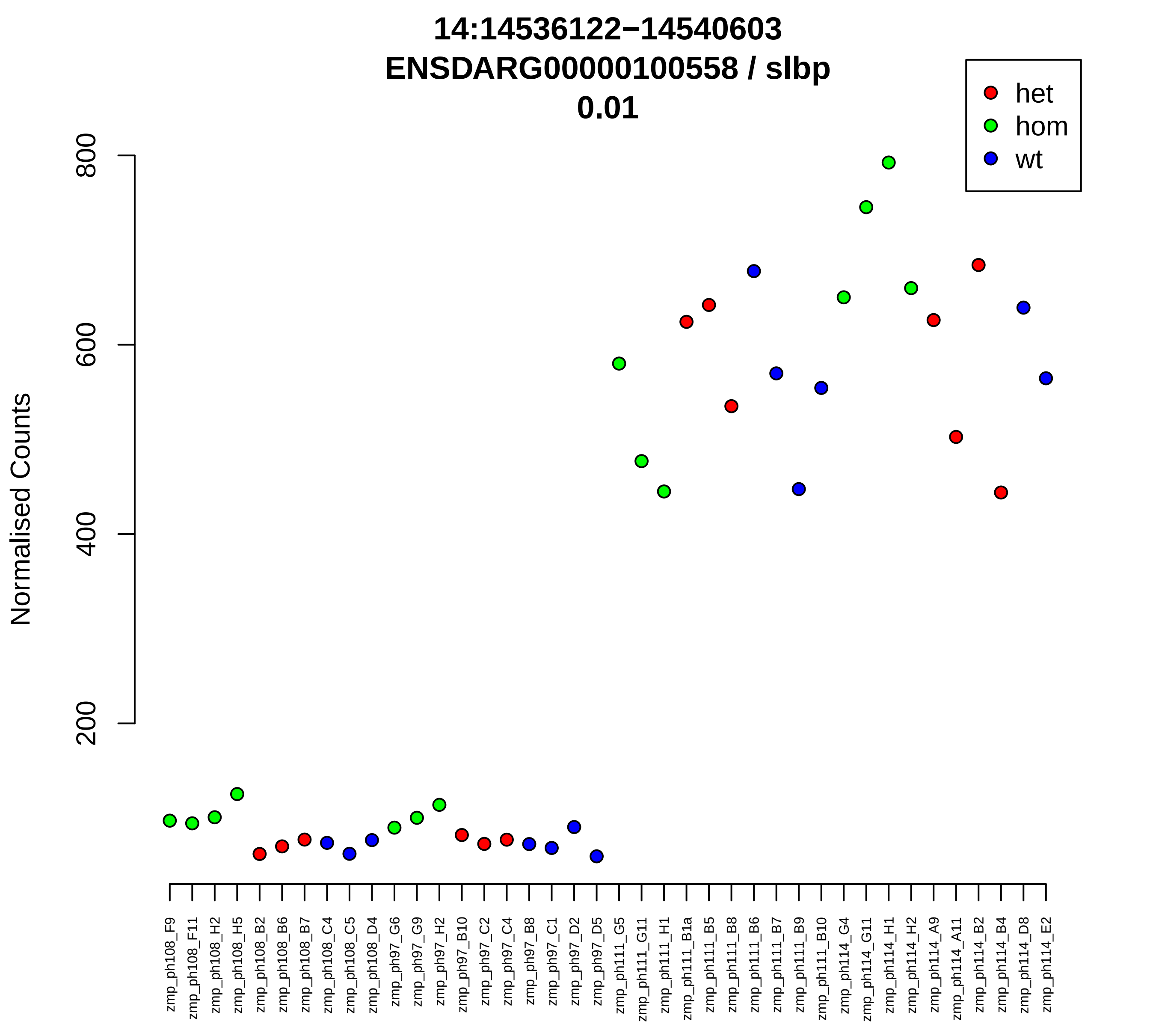
14:14536122−14540603
ENSDARG00000100558 / slbp
0.01
het
hom
wt
Normalised Counts
200
400
600
800
zmp_ph108_F9
zmp_ph108_F11
zmp_ph108_H2
zmp_ph108_H5
zmp_ph108_B2
zmp_ph108_B6
zmp_ph108_B7
zmp_ph108_C4
zmp_ph108_C5
zmp_ph108_D4
zmp_ph97_G6
zmp_ph97_G9
zmp_ph97_H2
zmp_ph97_B10
zmp_ph97_C2
zmp_ph97_C4
zmp_ph97_B8
zmp_ph97_C1
zmp_ph97_D2
zmp_ph97_D5
zmp_ph111_G5
zmp_ph111_G11
zmp_ph111_H1
zmp_ph111_B1a
zmp_ph111_B5
zmp_ph111_B8
zmp_ph111_B6
zmp_ph111_B7
zmp_ph111_B9
zmp_ph111_B10
zmp_ph114_G4
zmp_ph114_G11
zmp_ph114_H1
zmp_ph114_H2
zmp_ph114_A9
zmp_ph114_A11
zmp_ph114_B2
zmp_ph114_B4
zmp_ph114_D8
zmp_ph114_E2

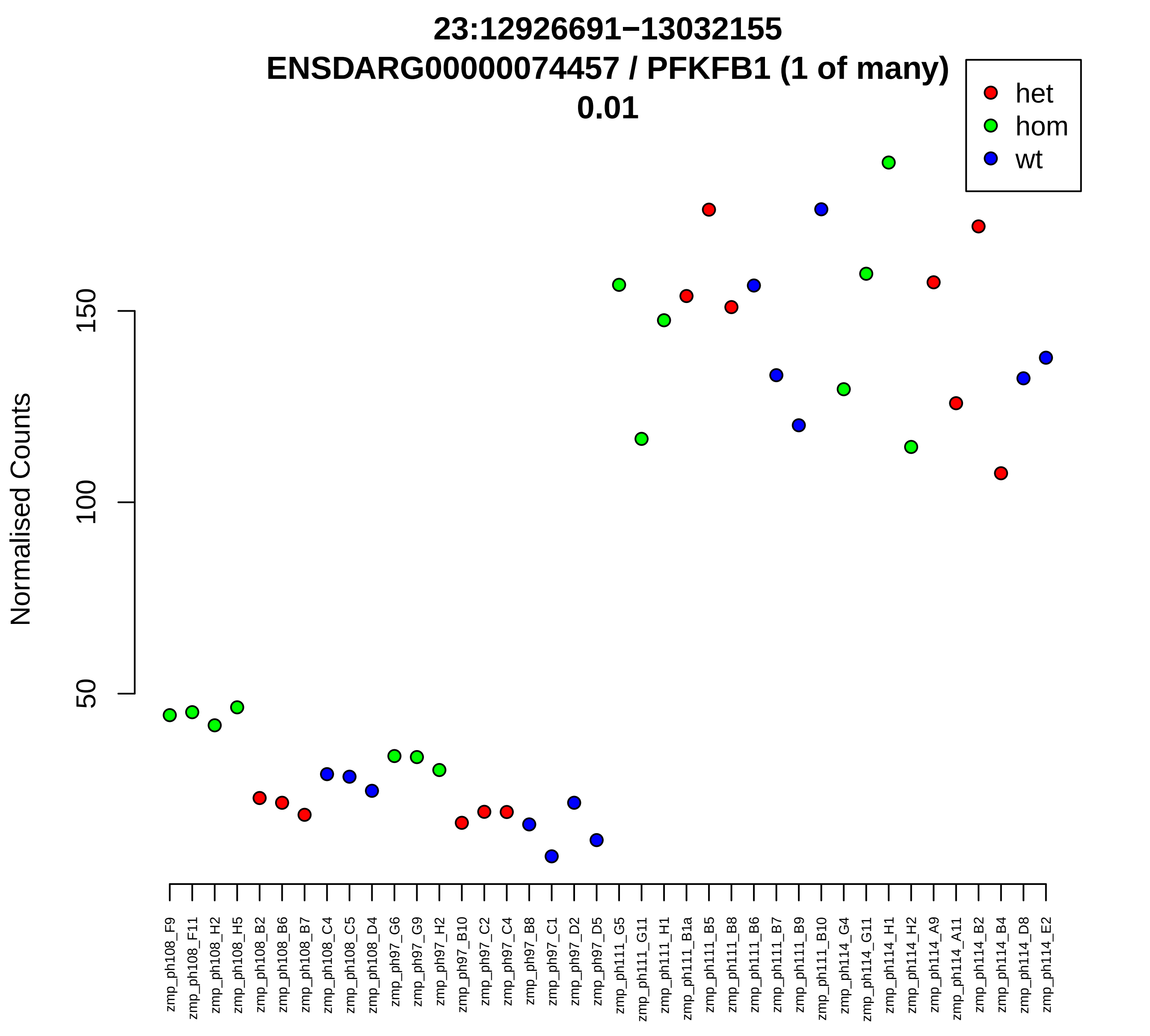

23:12926691−13032155
ENSDARG00000074457 / PFKFB1 (1 of many)
0.01
het
hom
wt
Normalised Counts
50
100
150
zmp_ph108_F9
zmp_ph108_F11
zmp_ph108_H2
zmp_ph108_H5
zmp_ph108_B2
zmp_ph108_B6
zmp_ph108_B7
zmp_ph108_C4
zmp_ph108_C5
zmp_ph108_D4
zmp_ph97_G6
zmp_ph97_G9
zmp_ph97_H2
zmp_ph97_B10
zmp_ph97_C2
zmp_ph97_C4
zmp_ph97_B8
zmp_ph97_C1
zmp_ph97_D2
zmp_ph97_D5
zmp_ph111_G5
zmp_ph111_G11
zmp_ph111_H1
zmp_ph111_B1a
zmp_ph111_B5
zmp_ph111_B8
zmp_ph111_B6
zmp_ph111_B7
zmp_ph111_B9
zmp_ph111_B10
zmp_ph114_G4
zmp_ph114_G11
zmp_ph114_H1
zmp_ph114_H2
zmp_ph114_A9
zmp_ph114_A11
zmp_ph114_B2
zmp_ph114_B4
zmp_ph114_D8
zmp_ph114_E2

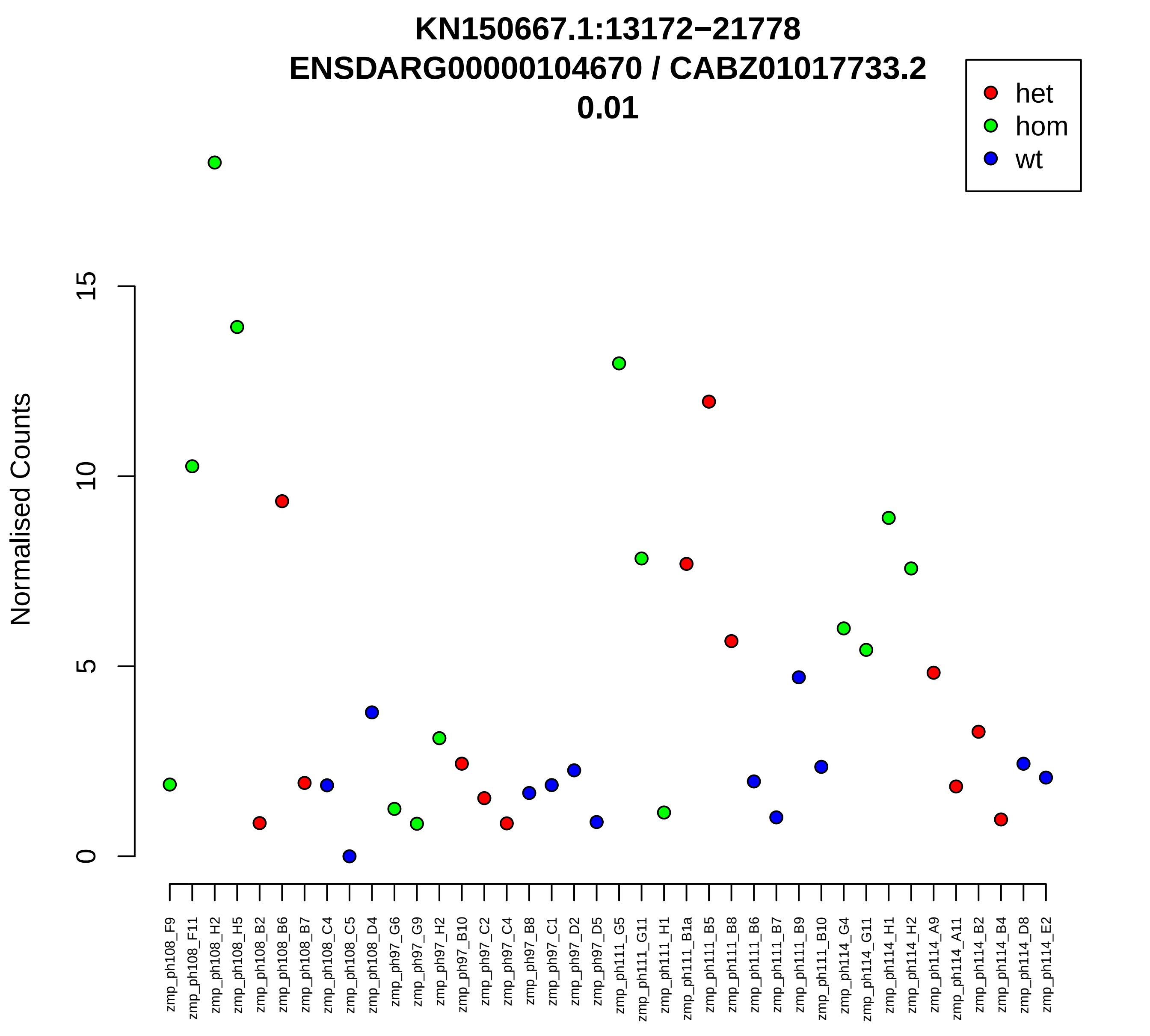
KN150667.1:13172−21778
ENSDARG00000104670 / CABZ01017733.2
0.01
het
hom
wt
Normalised Counts
0
5
10
15
zmp_ph108_F9
zmp_ph108_F11
zmp_ph108_H2
zmp_ph108_H5
zmp_ph108_B2
zmp_ph108_B6
zmp_ph108_B7
zmp_ph108_C4
zmp_ph108_C5
zmp_ph108_D4
zmp_ph97_G6
zmp_ph97_G9
zmp_ph97_H2
zmp_ph97_B10
zmp_ph97_C2
zmp_ph97_C4
zmp_ph97_B8
zmp_ph97_C1
zmp_ph97_D2
zmp_ph97_D5
zmp_ph111_G5
zmp_ph111_G11
zmp_ph111_H1
zmp_ph111_B1a
zmp_ph111_B5
zmp_ph111_B8
zmp_ph111_B6
zmp_ph111_B7
zmp_ph111_B9
zmp_ph111_B10
zmp_ph114_G4
zmp_ph114_G11
zmp_ph114_H1
zmp_ph114_H2
zmp_ph114_A9
zmp_ph114_A11
zmp_ph114_B2
zmp_ph114_B4
zmp_ph114_D8
zmp_ph114_E2

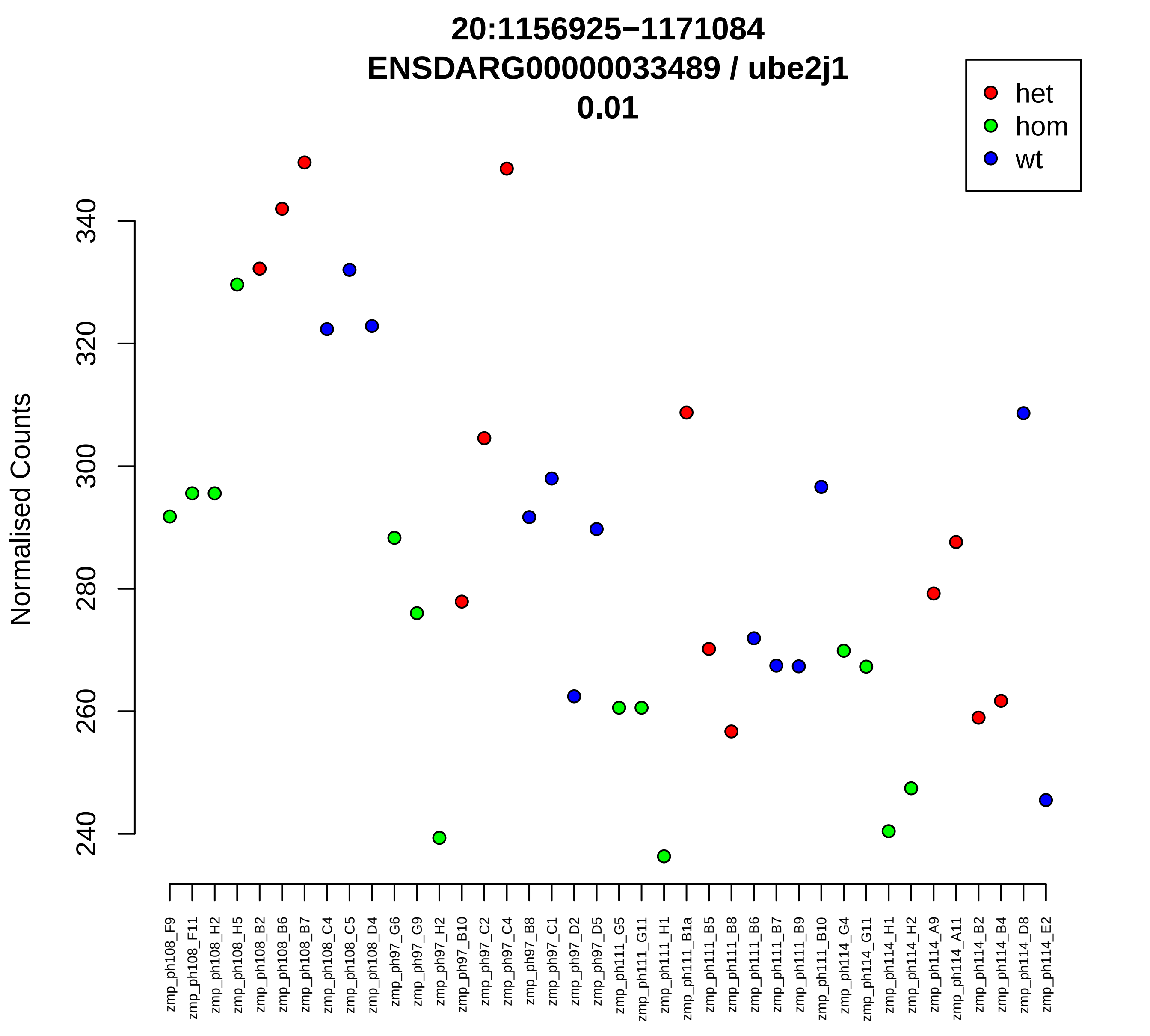
20:1156925−1171084
ENSDARG00000033489 / ube2j1
0.01
het
hom
wt
Normalised Counts
340
320
300
280
260
240
zmp_ph108_F9
zmp_ph108_F11
zmp_ph108_H2
zmp_ph108_H5
zmp_ph108_B2
zmp_ph108_B6
zmp_ph108_B7
zmp_ph108_C4
zmp_ph108_C5
zmp_ph108_D4
zmp_ph97_G6
zmp_ph97_G9
zmp_ph97_H2
zmp_ph97_B10
zmp_ph97_C2
zmp_ph97_C4
zmp_ph97_B8
zmp_ph97_C1
zmp_ph97_D2
zmp_ph97_D5
zmp_ph111_G5
zmp_ph111_G11
zmp_ph111_H1
zmp_ph111_B1a
zmp_ph111_B5
zmp_ph111_B8
zmp_ph111_B6
zmp_ph111_B7
zmp_ph111_B9
zmp_ph111_B10
zmp_ph114_G4
zmp_ph114_G11
zmp_ph114_H1
zmp_ph114_H2
zmp_ph114_A9
zmp_ph114_A11
zmp_ph114_B2
zmp_ph114_B4
zmp_ph114_D8
zmp_ph114_E2

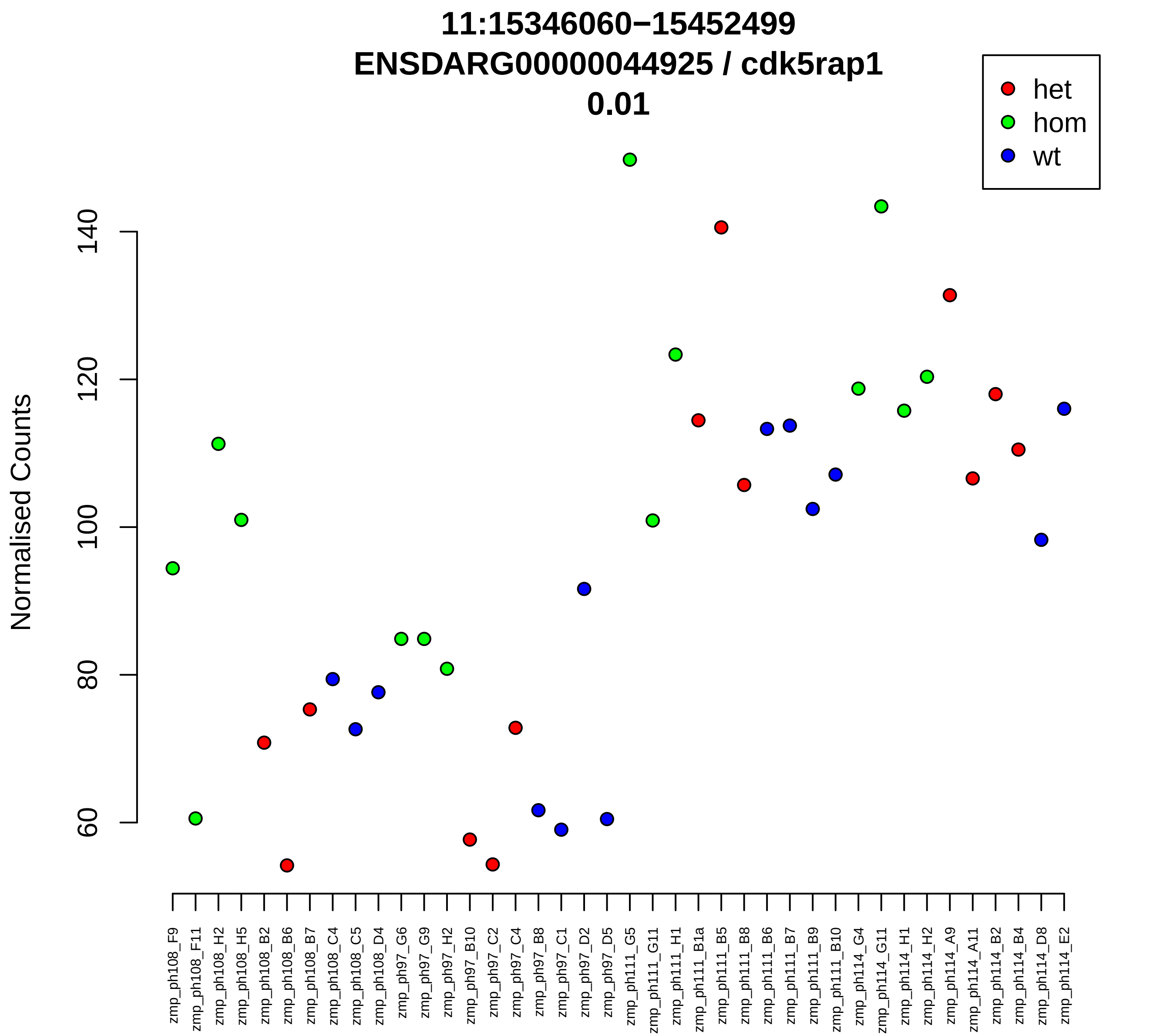

11:15346060−15452499
ENSDARG00000044925 / cdk5rap1
0.01
het
hom
wt
Normalised Counts
60
80
100
120
140
zmp_ph108_F9
zmp_ph108_F11
zmp_ph108_H2
zmp_ph108_H5
zmp_ph108_B2
zmp_ph108_B6
zmp_ph108_B7
zmp_ph108_C4
zmp_ph108_C5
zmp_ph108_D4
zmp_ph97_G6
zmp_ph97_G9
zmp_ph97_H2
zmp_ph97_B10
zmp_ph97_C2
zmp_ph97_C4
zmp_ph97_B8
zmp_ph97_C1
zmp_ph97_D2
zmp_ph97_D5
zmp_ph111_G5
zmp_ph111_G11
zmp_ph111_H1
zmp_ph111_B1a
zmp_ph111_B5
zmp_ph111_B8
zmp_ph111_B6
zmp_ph111_B7
zmp_ph111_B9
zmp_ph111_B10
zmp_ph114_G4
zmp_ph114_G11
zmp_ph114_H1
zmp_ph114_H2
zmp_ph114_A9
zmp_ph114_A11
zmp_ph114_B2
zmp_ph114_B4
zmp_ph114_D8
zmp_ph114_E2

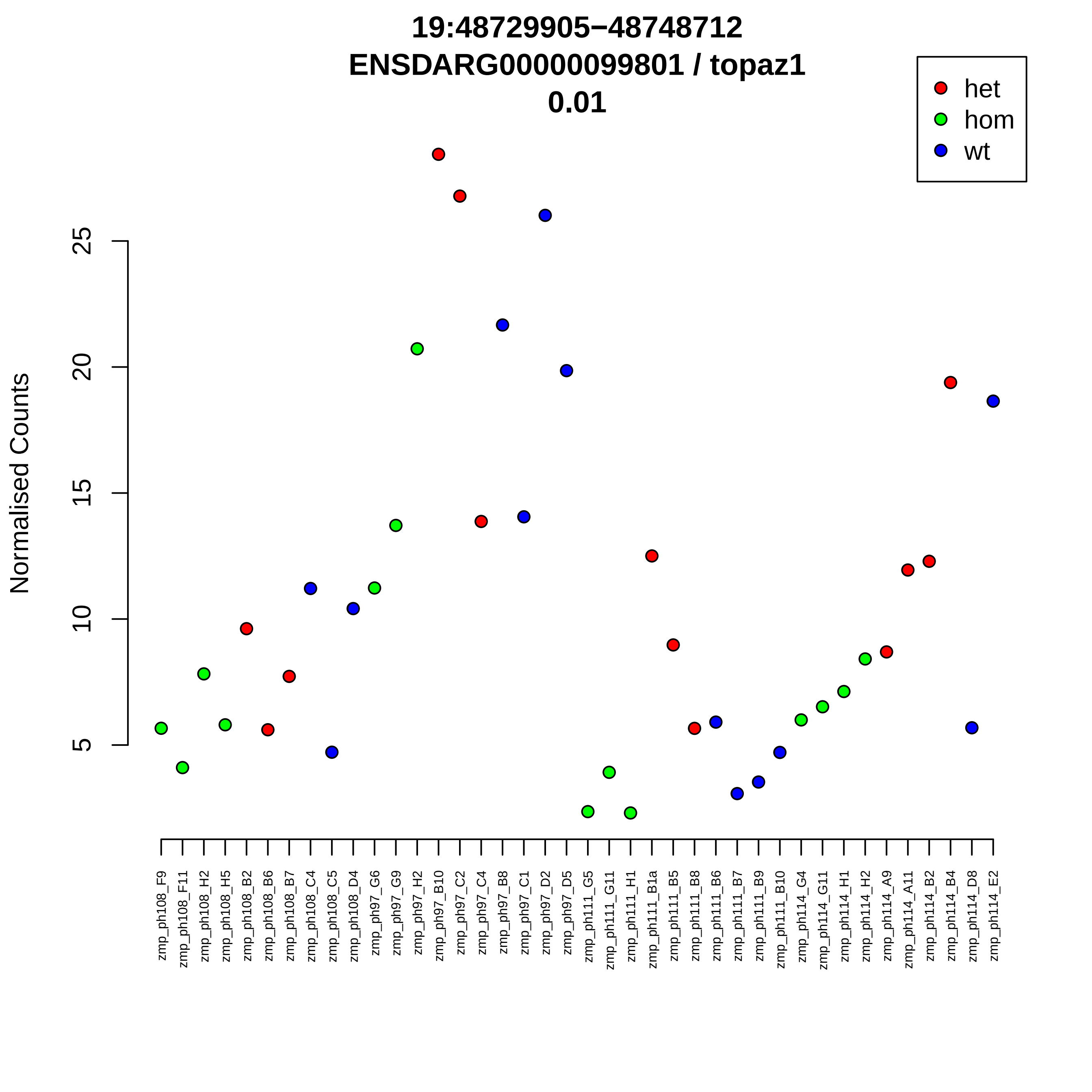

19:48729905−48748712
ENSDARG00000099801 / topaz1
0.01
het
hom
wt
Normalised Counts
5
10
15
20
25
zmp_ph108_F9
zmp_ph108_F11
zmp_ph108_H2
zmp_ph108_H5
zmp_ph108_B2
zmp_ph108_B6
zmp_ph108_B7
zmp_ph108_C4
zmp_ph108_C5
zmp_ph108_D4
zmp_ph97_G6
zmp_ph97_G9
zmp_ph97_H2
zmp_ph97_B10
zmp_ph97_C2
zmp_ph97_C4
zmp_ph97_B8
zmp_ph97_C1
zmp_ph97_D2
zmp_ph97_D5
zmp_ph111_G5
zmp_ph111_G11
zmp_ph111_H1
zmp_ph111_B1a
zmp_ph111_B5
zmp_ph111_B8
zmp_ph111_B6
zmp_ph111_B7
zmp_ph111_B9
zmp_ph111_B10
zmp_ph114_G4
zmp_ph114_G11
zmp_ph114_H1
zmp_ph114_H2
zmp_ph114_A9
zmp_ph114_A11
zmp_ph114_B2
zmp_ph114_B4
zmp_ph114_D8
zmp_ph114_E2

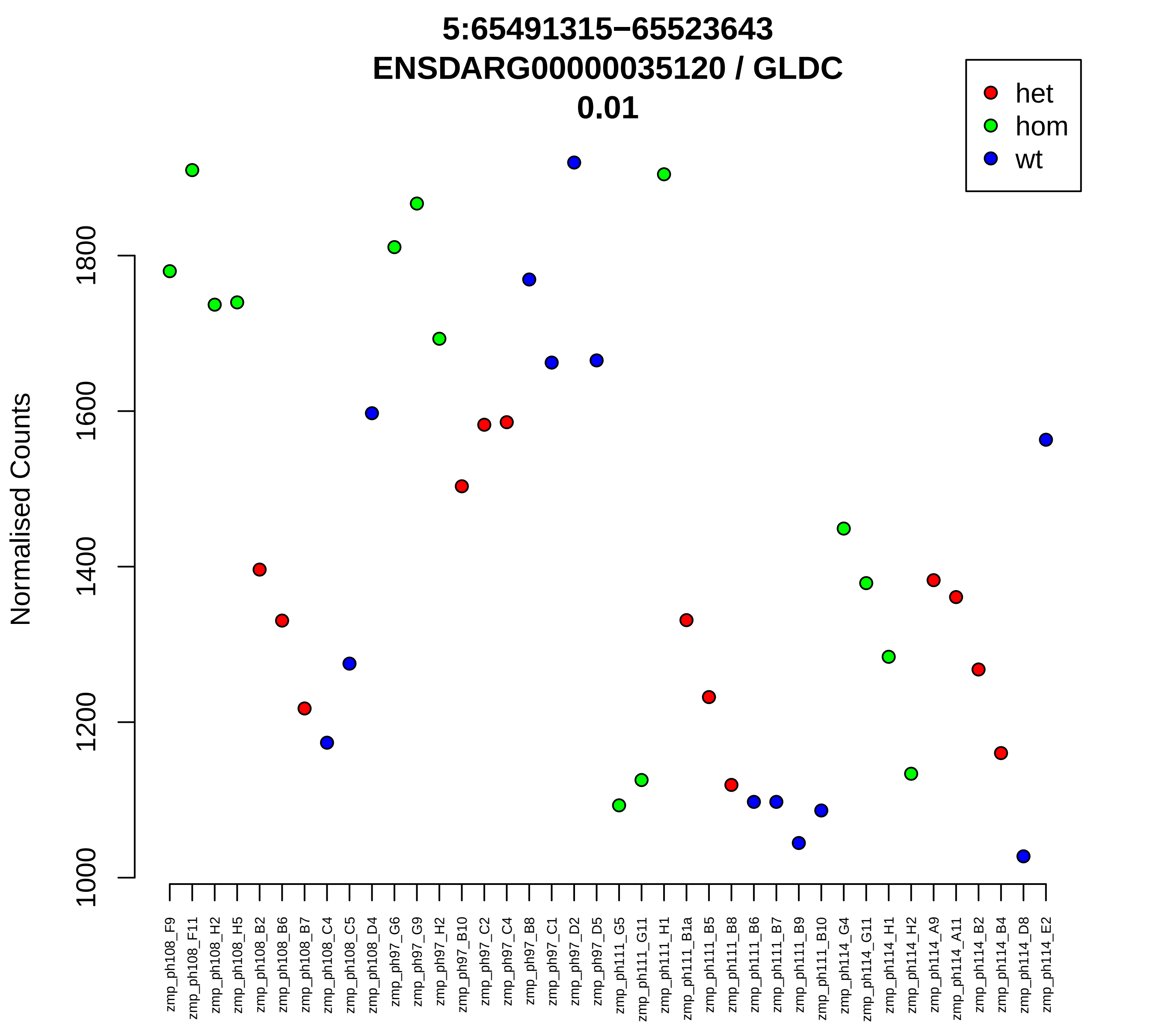

5:65491315−65523643
ENSDARG00000035120 / GLDC
0.01
het
hom
wt
Normalised Counts
1000
1200
1400
1600
1800
zmp_ph108_F9
zmp_ph108_F11
zmp_ph108_H2
zmp_ph108_H5
zmp_ph108_B2
zmp_ph108_B6
zmp_ph108_B7
zmp_ph108_C4
zmp_ph108_C5
zmp_ph108_D4
zmp_ph97_G6
zmp_ph97_G9
zmp_ph97_H2
zmp_ph97_B10
zmp_ph97_C2
zmp_ph97_C4
zmp_ph97_B8
zmp_ph97_C1
zmp_ph97_D2
zmp_ph97_D5
zmp_ph111_G5
zmp_ph111_G11
zmp_ph111_H1
zmp_ph111_B1a
zmp_ph111_B5
zmp_ph111_B8
zmp_ph111_B6
zmp_ph111_B7
zmp_ph111_B9
zmp_ph111_B10
zmp_ph114_G4
zmp_ph114_G11
zmp_ph114_H1
zmp_ph114_H2
zmp_ph114_A9
zmp_ph114_A11
zmp_ph114_B2
zmp_ph114_B4
zmp_ph114_D8
zmp_ph114_E2

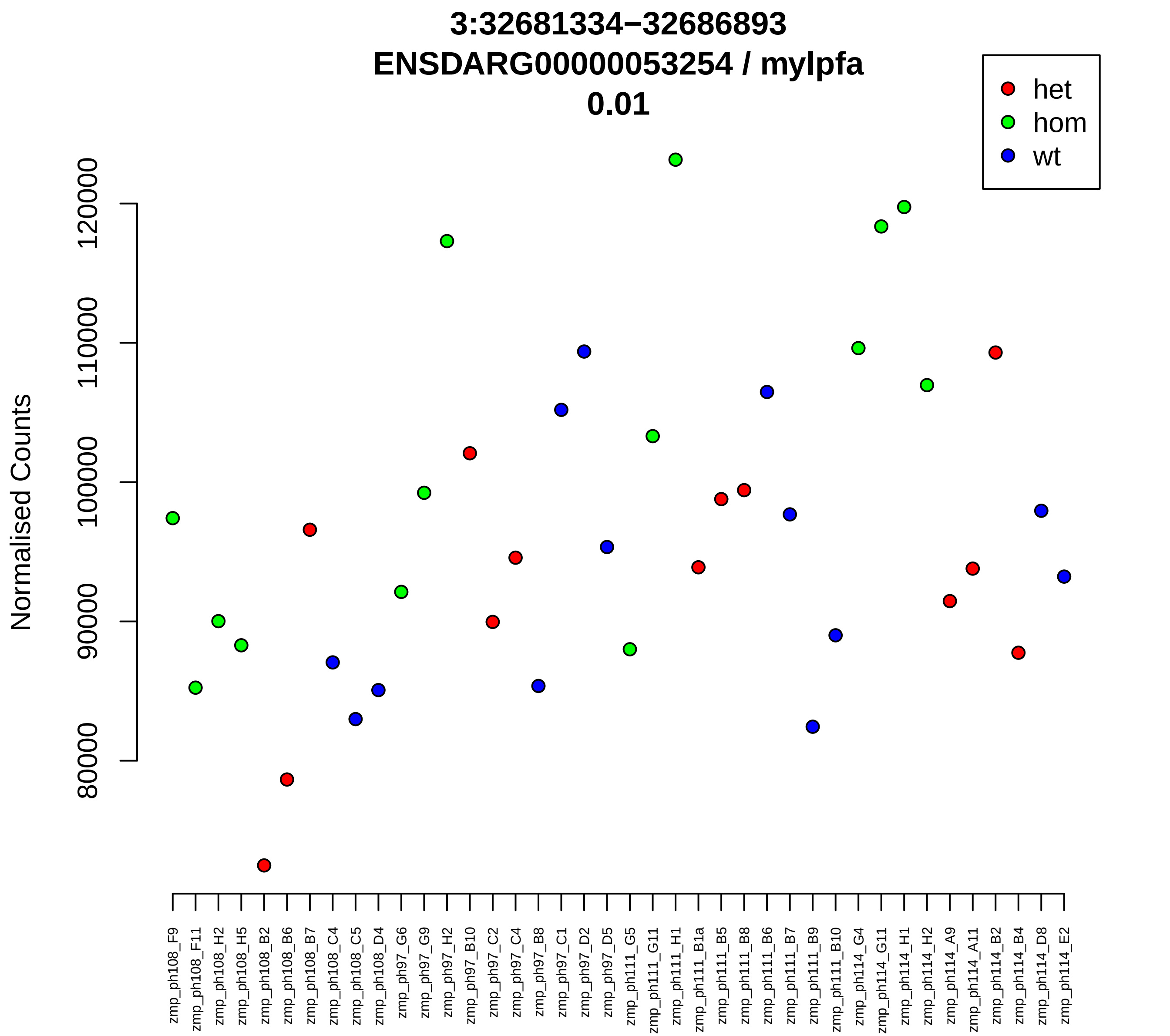
3:32681334−32686893
ENSDARG00000053254 / mylpfa
0.01
het
hom
wt
Normalised Counts
80000
90000
100000
110000
120000
zmp_ph108_F9
zmp_ph108_F11
zmp_ph108_H2
zmp_ph108_H5
zmp_ph108_B2
zmp_ph108_B6
zmp_ph108_B7
zmp_ph108_C4
zmp_ph108_C5
zmp_ph108_D4
zmp_ph97_G6
zmp_ph97_G9
zmp_ph97_H2
zmp_ph97_B10
zmp_ph97_C2
zmp_ph97_C4
zmp_ph97_B8
zmp_ph97_C1
zmp_ph97_D2
zmp_ph97_D5
zmp_ph111_G5
zmp_ph111_G11
zmp_ph111_H1
zmp_ph111_B1a
zmp_ph111_B5
zmp_ph111_B8
zmp_ph111_B6
zmp_ph111_B7
zmp_ph111_B9
zmp_ph111_B10
zmp_ph114_G4
zmp_ph114_G11
zmp_ph114_H1
zmp_ph114_H2
zmp_ph114_A9
zmp_ph114_A11
zmp_ph114_B2
zmp_ph114_B4
zmp_ph114_D8
zmp_ph114_E2

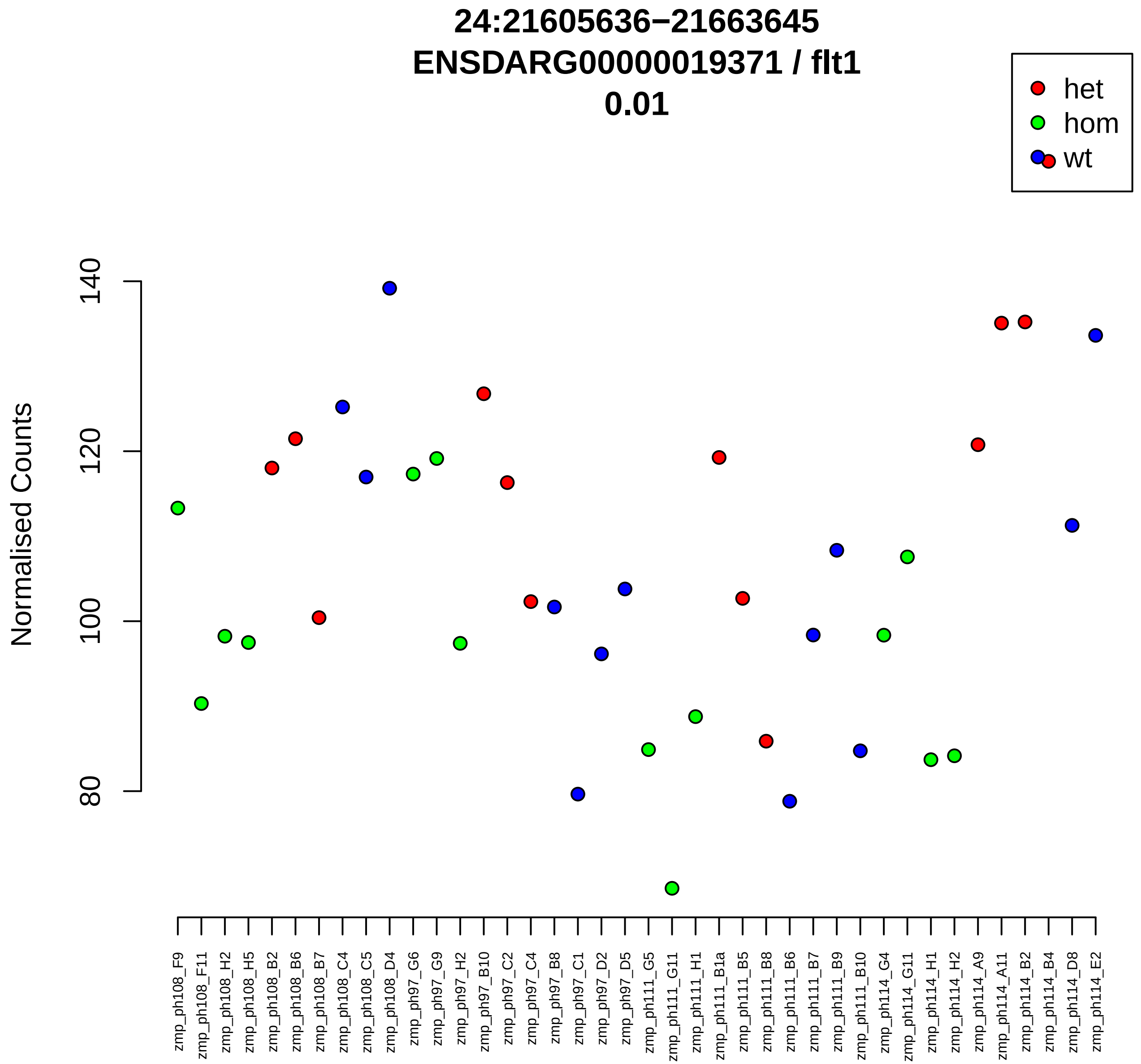

24:21605636−21663645
ENSDARG00000019371 / flt1
0.01
het
hom
wt
Normalised Counts
140
120
100
80
zmp_ph108_F9
zmp_ph108_F11
zmp_ph108_H2
zmp_ph108_H5
zmp_ph108_B2
zmp_ph108_B6
zmp_ph108_B7
zmp_ph108_C4
zmp_ph108_C5
zmp_ph108_D4
zmp_ph97_G6
zmp_ph97_G9
zmp_ph97_H2
zmp_ph97_B10
zmp_ph97_C2
zmp_ph97_C4
zmp_ph97_B8
zmp_ph97_C1
zmp_ph97_D2
zmp_ph97_D5
zmp_ph111_G5
zmp_ph111_G11
zmp_ph111_H1
zmp_ph111_B1a
zmp_ph111_B5
zmp_ph111_B8
zmp_ph111_B6
zmp_ph111_B7
zmp_ph111_B9
zmp_ph111_B10
zmp_ph114_G4
zmp_ph114_G11
zmp_ph114_H1
zmp_ph114_H2
zmp_ph114_A9
zmp_ph114_A11
zmp_ph114_B2
zmp_ph114_B4
zmp_ph114_D8
zmp_ph114_E2

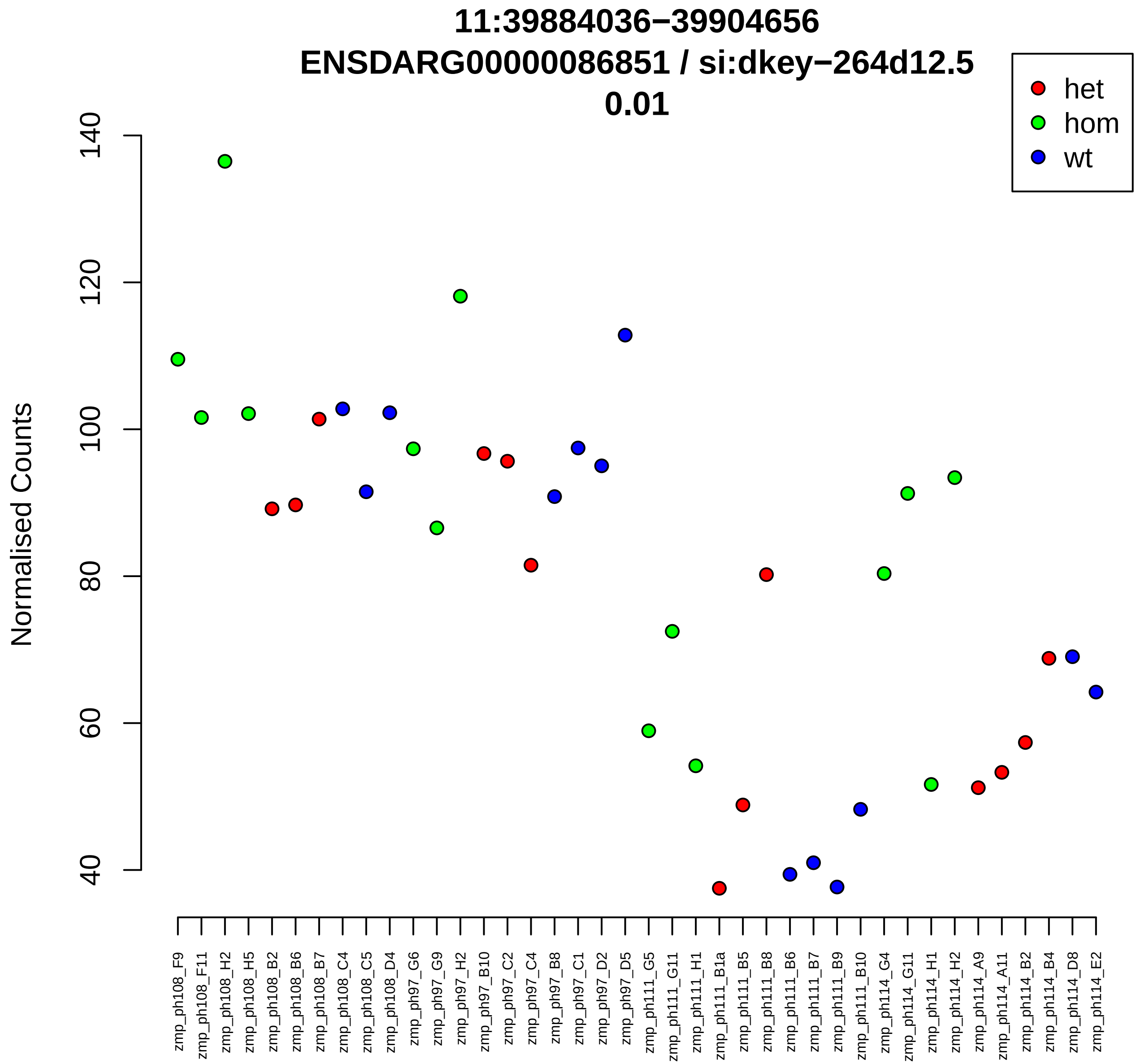

11:39884036−39904656
ENSDARG00000086851 / si:dkey−264d12.5
0.01
het
hom
wt
Normalised Counts
140
120
100
80
60
40
zmp_ph108_F9
zmp_ph108_F11
zmp_ph108_H2
zmp_ph108_H5
zmp_ph108_B2
zmp_ph108_B6
zmp_ph108_B7
zmp_ph108_C4
zmp_ph108_C5
zmp_ph108_D4
zmp_ph97_G6
zmp_ph97_G9
zmp_ph97_H2
zmp_ph97_B10
zmp_ph97_C2
zmp_ph97_C4
zmp_ph97_B8
zmp_ph97_C1
zmp_ph97_D2
zmp_ph97_D5
zmp_ph111_G5
zmp_ph111_G11
zmp_ph111_H1
zmp_ph111_B1a
zmp_ph111_B5
zmp_ph111_B8
zmp_ph111_B6
zmp_ph111_B7
zmp_ph111_B9
zmp_ph111_B10
zmp_ph114_G4
zmp_ph114_G11
zmp_ph114_H1
zmp_ph114_H2
zmp_ph114_A9
zmp_ph114_A11
zmp_ph114_B2
zmp_ph114_B4
zmp_ph114_D8
zmp_ph114_E2

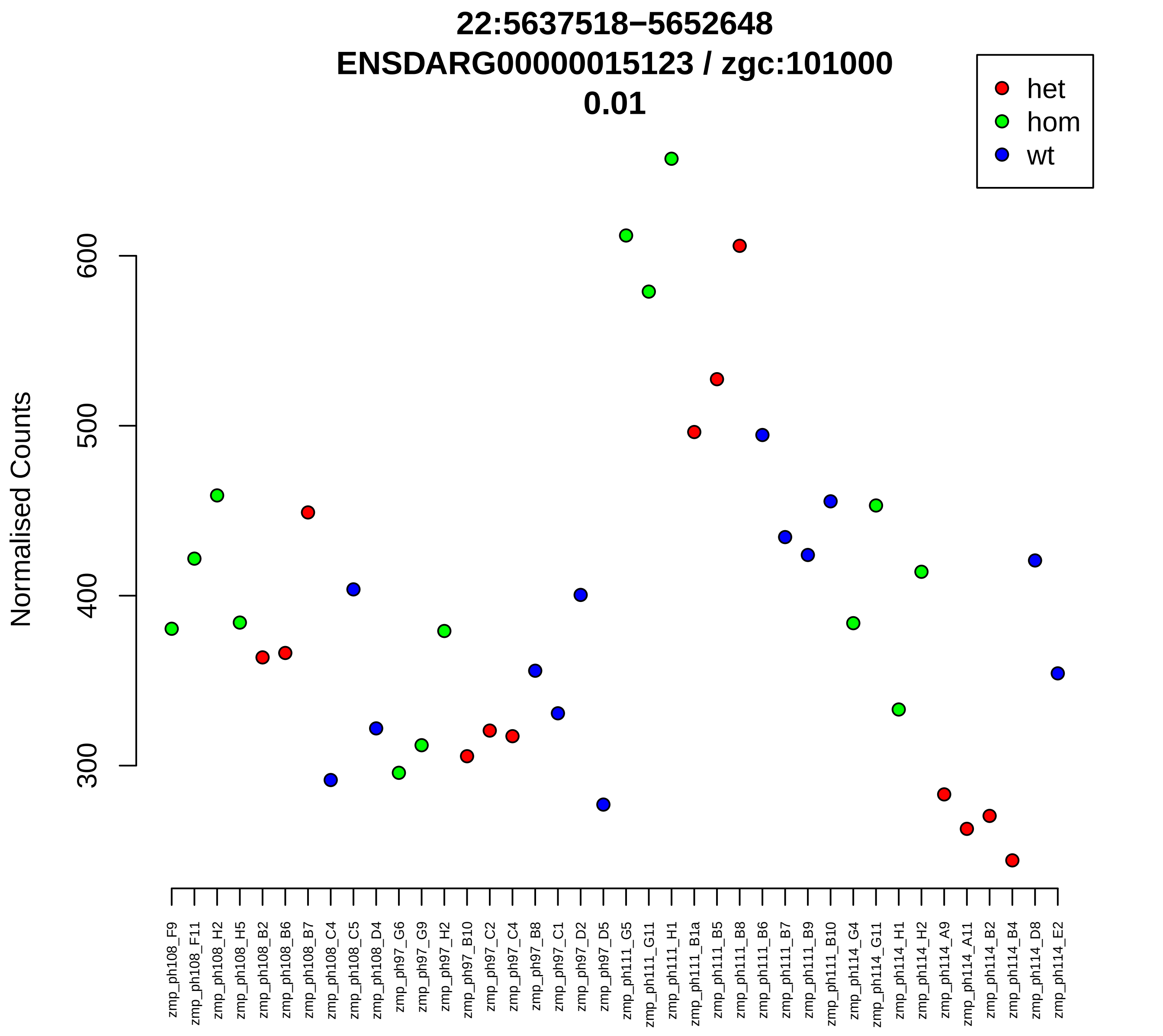

22:5637518−5652648
ENSDARG00000015123 / zgc:101000
0.01
het
hom
wt
Normalised Counts
300
400
500
600
zmp_ph108_F9
zmp_ph108_F11
zmp_ph108_H2
zmp_ph108_H5
zmp_ph108_B2
zmp_ph108_B6
zmp_ph108_B7
zmp_ph108_C4
zmp_ph108_C5
zmp_ph108_D4
zmp_ph97_G6
zmp_ph97_G9
zmp_ph97_H2
zmp_ph97_B10
zmp_ph97_C2
zmp_ph97_C4
zmp_ph97_B8
zmp_ph97_C1
zmp_ph97_D2
zmp_ph97_D5
zmp_ph111_G5
zmp_ph111_G11
zmp_ph111_H1
zmp_ph111_B1a
zmp_ph111_B5
zmp_ph111_B8
zmp_ph111_B6
zmp_ph111_B7
zmp_ph111_B9
zmp_ph111_B10
zmp_ph114_G4
zmp_ph114_G11
zmp_ph114_H1
zmp_ph114_H2
zmp_ph114_A9
zmp_ph114_A11
zmp_ph114_B2
zmp_ph114_B4
zmp_ph114_D8
zmp_ph114_E2

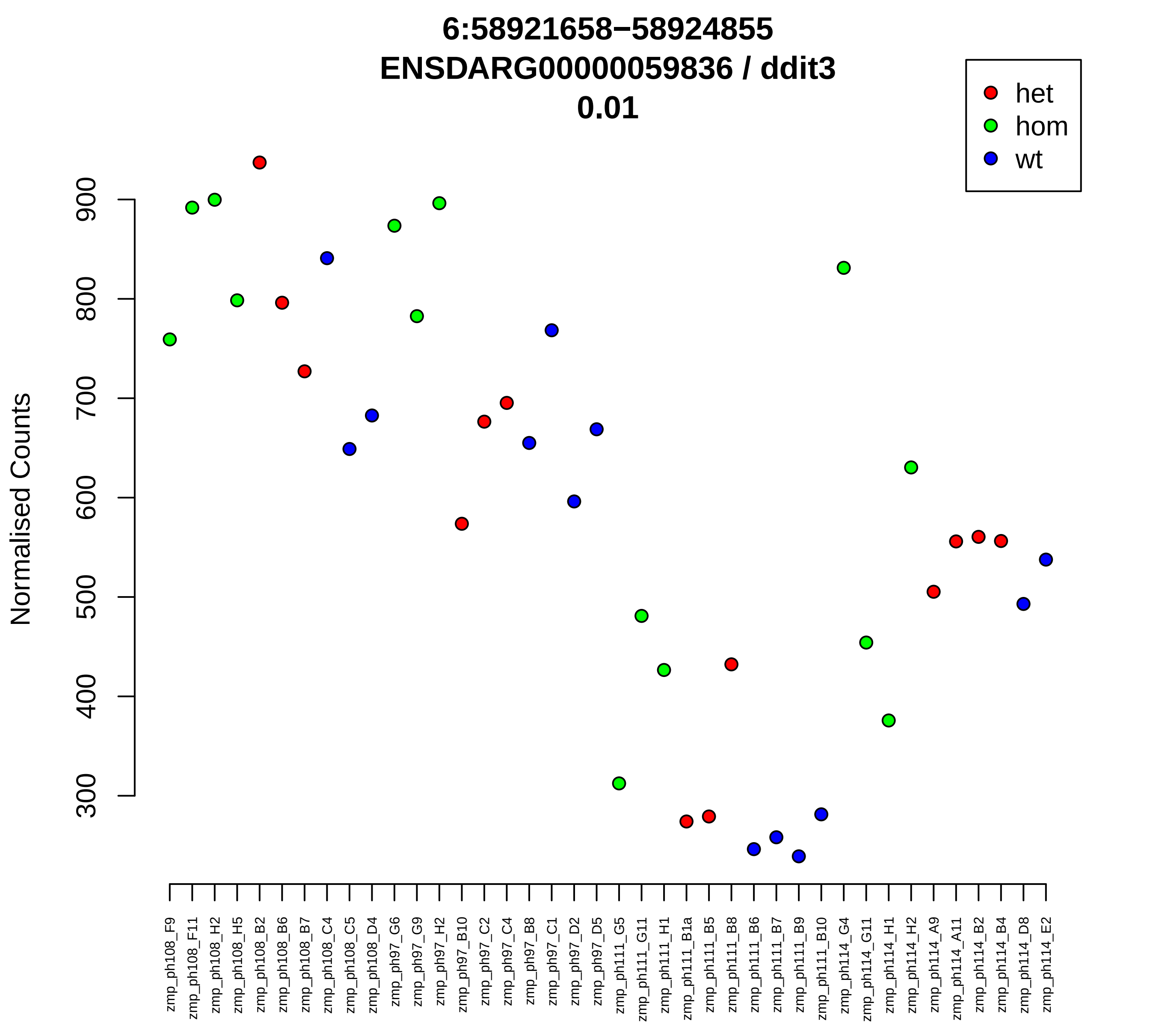

6:58921658−58924855
ENSDARG00000059836 / ddit3
0.01
het
hom
wt
Normalised Counts
300
400
500
600
700
800
900
zmp_ph108_F9
zmp_ph108_F11
zmp_ph108_H2
zmp_ph108_H5
zmp_ph108_B2
zmp_ph108_B6
zmp_ph108_B7
zmp_ph108_C4
zmp_ph108_C5
zmp_ph108_D4
zmp_ph97_G6
zmp_ph97_G9
zmp_ph97_H2
zmp_ph97_B10
zmp_ph97_C2
zmp_ph97_C4
zmp_ph97_B8
zmp_ph97_C1
zmp_ph97_D2
zmp_ph97_D5
zmp_ph111_G5
zmp_ph111_G11
zmp_ph111_H1
zmp_ph111_B1a
zmp_ph111_B5
zmp_ph111_B8
zmp_ph111_B6
zmp_ph111_B7
zmp_ph111_B9
zmp_ph111_B10
zmp_ph114_G4
zmp_ph114_G11
zmp_ph114_H1
zmp_ph114_H2
zmp_ph114_A9
zmp_ph114_A11
zmp_ph114_B2
zmp_ph114_B4
zmp_ph114_D8
zmp_ph114_E2

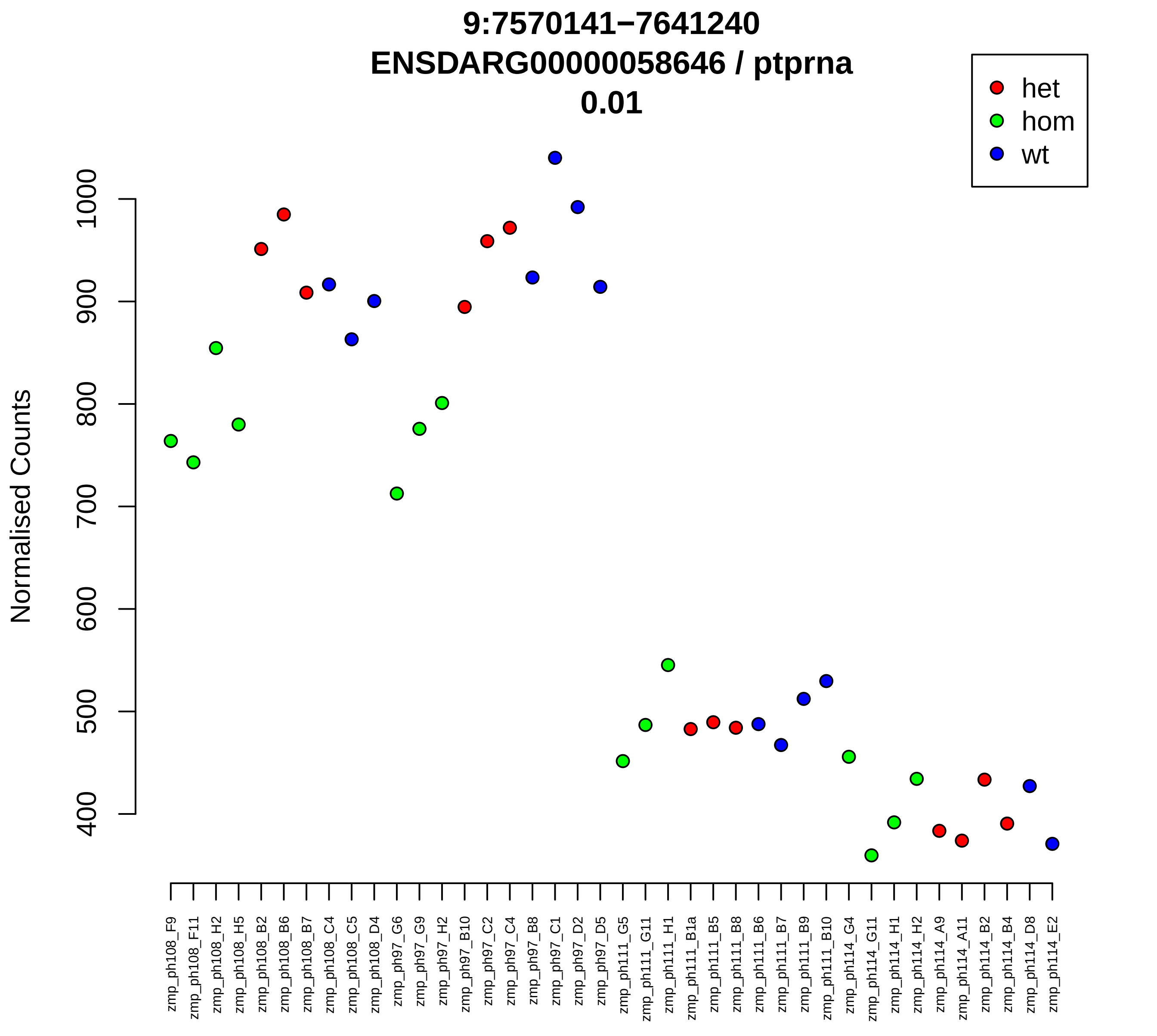

9:7570141−7641240
ENSDARG00000058646 / ptprna
0.01
het
hom
wt
Normalised Counts
400
500
600
700
800
900
1000
zmp_ph108_F9
zmp_ph108_F11
zmp_ph108_H2
zmp_ph108_H5
zmp_ph108_B2
zmp_ph108_B6
zmp_ph108_B7
zmp_ph108_C4
zmp_ph108_C5
zmp_ph108_D4
zmp_ph97_G6
zmp_ph97_G9
zmp_ph97_H2
zmp_ph97_B10
zmp_ph97_C2
zmp_ph97_C4
zmp_ph97_B8
zmp_ph97_C1
zmp_ph97_D2
zmp_ph97_D5
zmp_ph111_G5
zmp_ph111_G11
zmp_ph111_H1
zmp_ph111_B1a
zmp_ph111_B5
zmp_ph111_B8
zmp_ph111_B6
zmp_ph111_B7
zmp_ph111_B9
zmp_ph111_B10
zmp_ph114_G4
zmp_ph114_G11
zmp_ph114_H1
zmp_ph114_H2
zmp_ph114_A9
zmp_ph114_A11
zmp_ph114_B2
zmp_ph114_B4
zmp_ph114_D8
zmp_ph114_E2

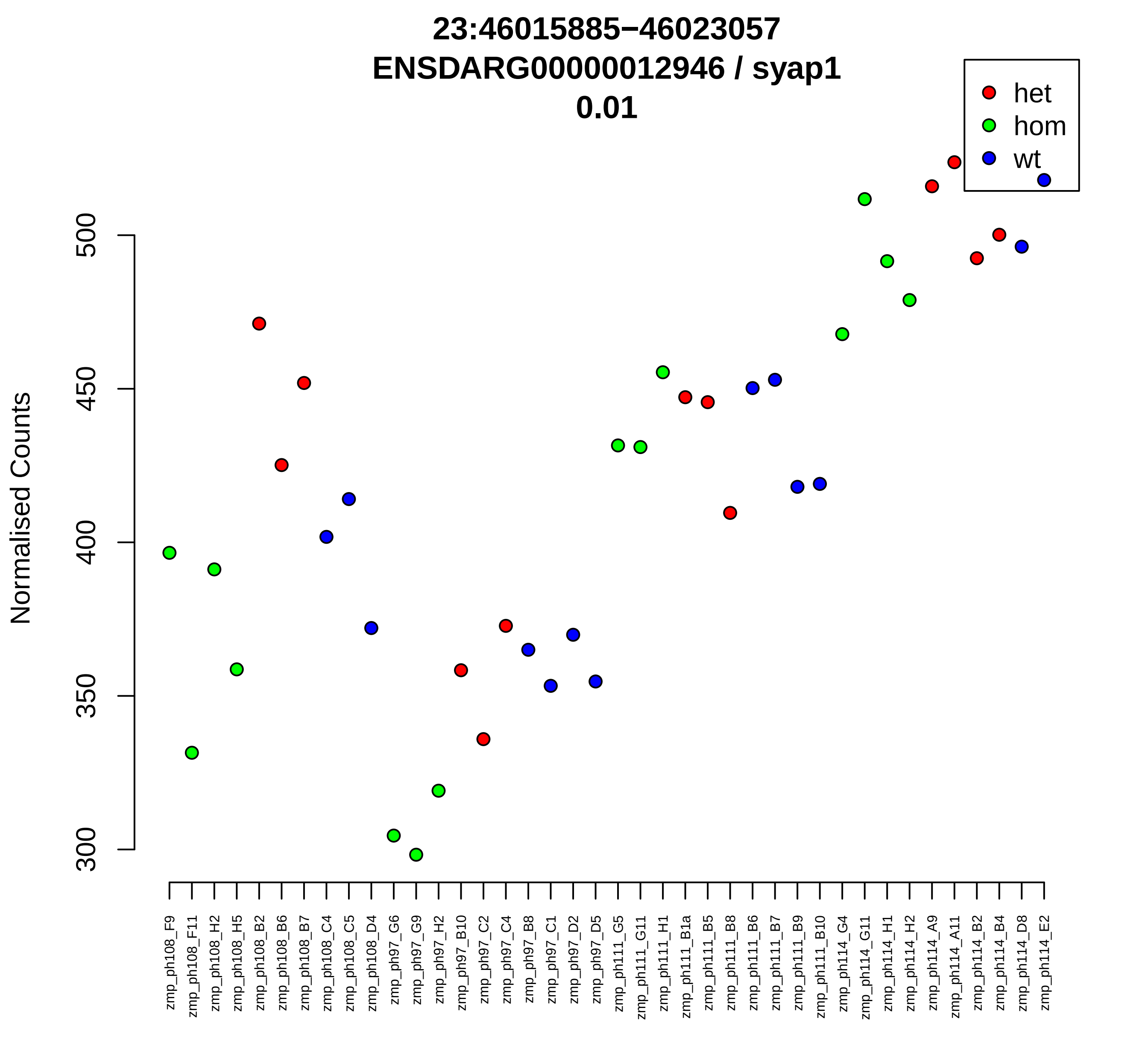
23:46015885−46023057
ENSDARG00000012946 / syap1
0.01
het
hom
wt
Normalised Counts
300
350
400
450
500
zmp_ph108_F9
zmp_ph108_F11
zmp_ph108_H2
zmp_ph108_H5
zmp_ph108_B2
zmp_ph108_B6
zmp_ph108_B7
zmp_ph108_C4
zmp_ph108_C5
zmp_ph108_D4
zmp_ph97_G6
zmp_ph97_G9
zmp_ph97_H2
zmp_ph97_B10
zmp_ph97_C2
zmp_ph97_C4
zmp_ph97_B8
zmp_ph97_C1
zmp_ph97_D2
zmp_ph97_D5
zmp_ph111_G5
zmp_ph111_G11
zmp_ph111_H1
zmp_ph111_B1a
zmp_ph111_B5
zmp_ph111_B8
zmp_ph111_B6
zmp_ph111_B7
zmp_ph111_B9
zmp_ph111_B10
zmp_ph114_G4
zmp_ph114_G11
zmp_ph114_H1
zmp_ph114_H2
zmp_ph114_A9
zmp_ph114_A11
zmp_ph114_B2
zmp_ph114_B4
zmp_ph114_D8
zmp_ph114_E2

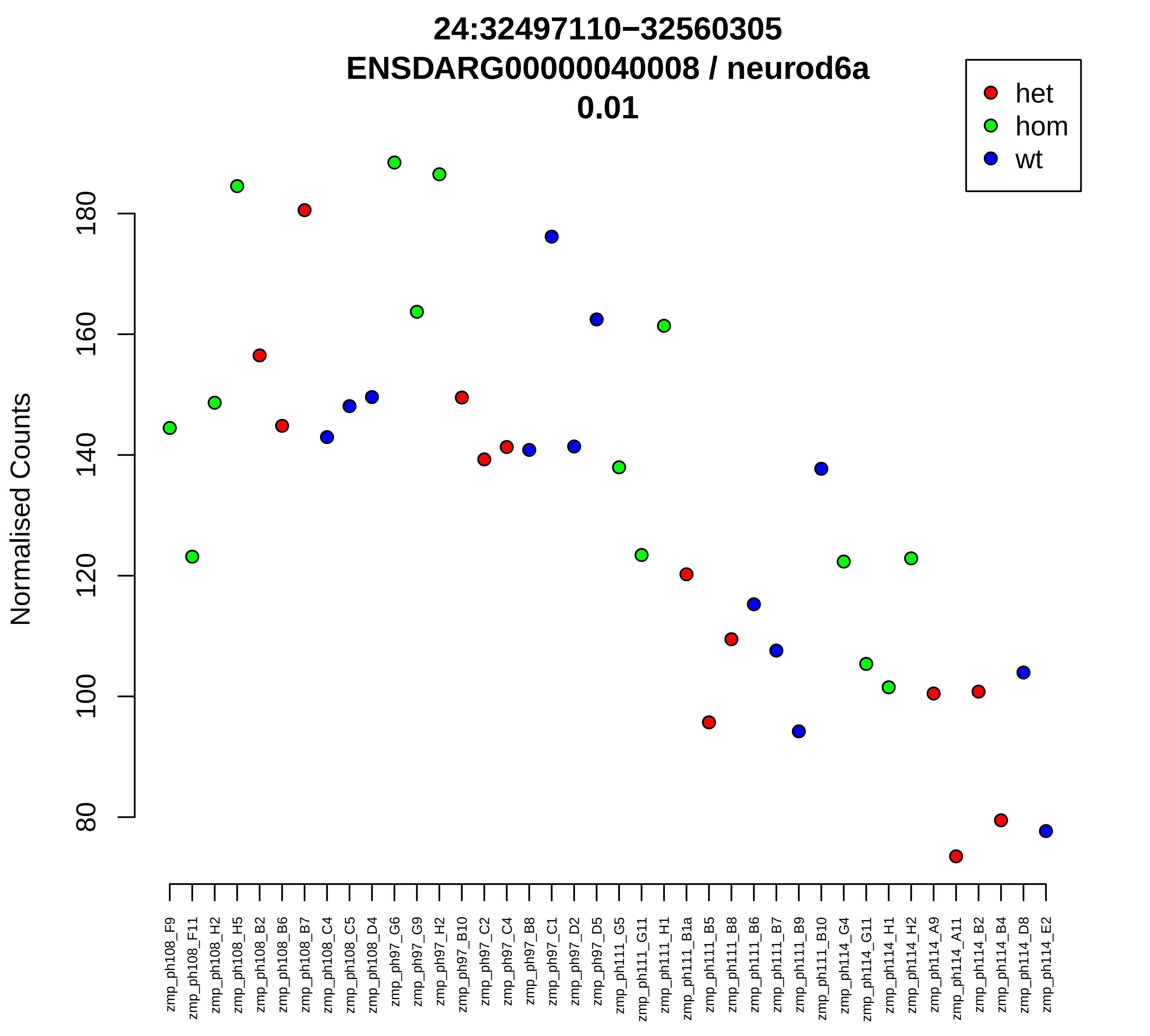
24:32497110−32560305
ENSDARG00000040008 / neurod6a
0.01
het
hom
wt
Normalised Counts
80
100
120
140
160
180
zmp_ph108_F9
zmp_ph108_F11
zmp_ph108_H2
zmp_ph108_H5
zmp_ph108_B2
zmp_ph108_B6
zmp_ph108_B7
zmp_ph108_C4
zmp_ph108_C5
zmp_ph108_D4
zmp_ph97_G6
zmp_ph97_G9
zmp_ph97_H2
zmp_ph97_B10
zmp_ph97_C2
zmp_ph97_C4
zmp_ph97_B8
zmp_ph97_C1
zmp_ph97_D2
zmp_ph97_D5
zmp_ph111_G5
zmp_ph111_G11
zmp_ph111_H1
zmp_ph111_B1a
zmp_ph111_B5
zmp_ph111_B8
zmp_ph111_B6
zmp_ph111_B7
zmp_ph111_B9
zmp_ph111_B10
zmp_ph114_G4
zmp_ph114_G11
zmp_ph114_H1
zmp_ph114_H2
zmp_ph114_A9
zmp_ph114_A11
zmp_ph114_B2
zmp_ph114_B4
zmp_ph114_D8
zmp_ph114_E2

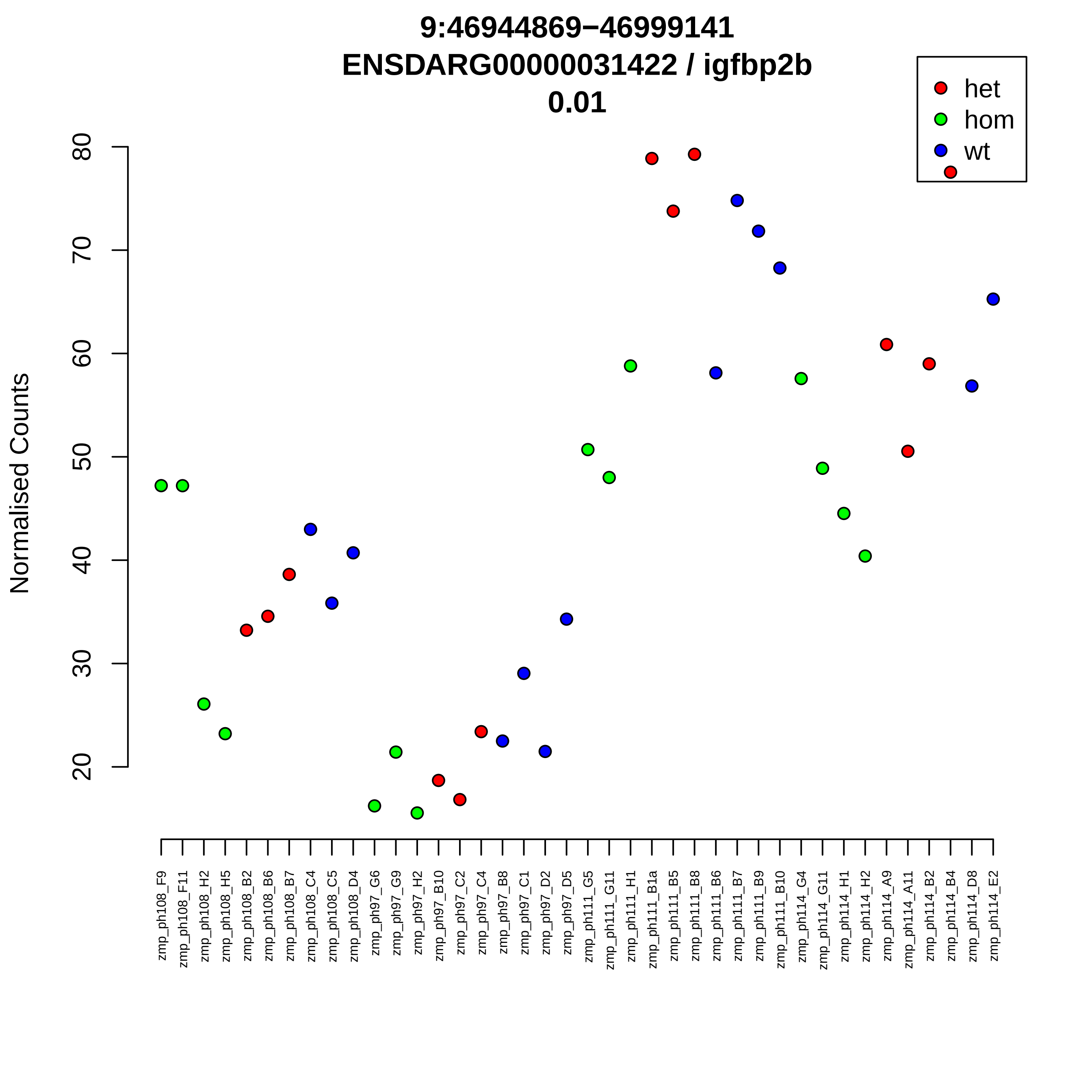

9:46944869−46999141
ENSDARG00000031422 / igfbp2b
0.01
het
hom
wt
Normalised Counts
20
30
40
50
60
70
80
zmp_ph108_F9
zmp_ph108_F11
zmp_ph108_H2
zmp_ph108_H5
zmp_ph108_B2
zmp_ph108_B6
zmp_ph108_B7
zmp_ph108_C4
zmp_ph108_C5
zmp_ph108_D4
zmp_ph97_G6
zmp_ph97_G9
zmp_ph97_H2
zmp_ph97_B10
zmp_ph97_C2
zmp_ph97_C4
zmp_ph97_B8
zmp_ph97_C1
zmp_ph97_D2
zmp_ph97_D5
zmp_ph111_G5
zmp_ph111_G11
zmp_ph111_H1
zmp_ph111_B1a
zmp_ph111_B5
zmp_ph111_B8
zmp_ph111_B6
zmp_ph111_B7
zmp_ph111_B9
zmp_ph111_B10
zmp_ph114_G4
zmp_ph114_G11
zmp_ph114_H1
zmp_ph114_H2
zmp_ph114_A9
zmp_ph114_A11
zmp_ph114_B2
zmp_ph114_B4
zmp_ph114_D8
zmp_ph114_E2

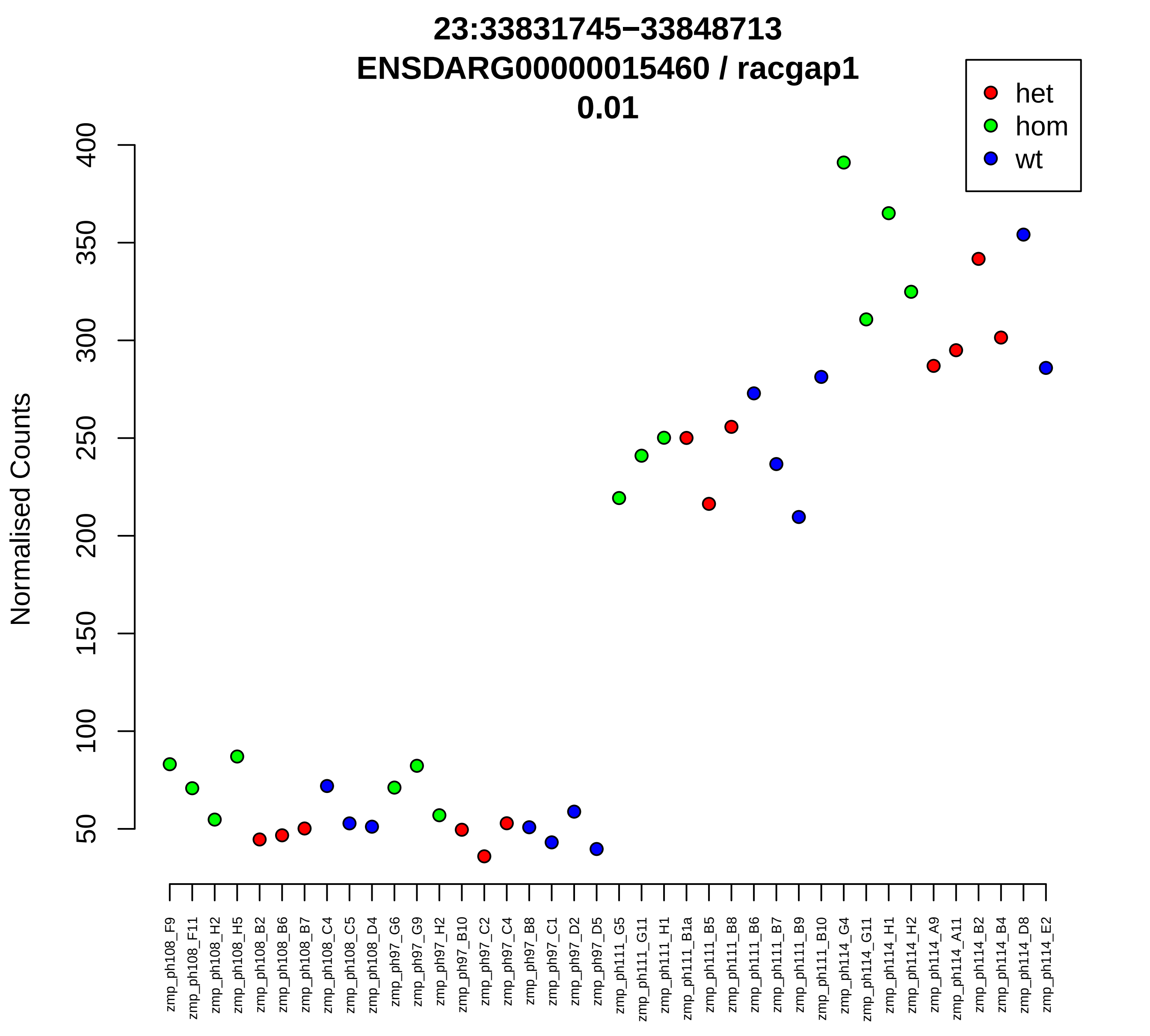
23:33831745−33848713
ENSDARG00000015460 / racgap1
0.01
het
hom
wt
Normalised Counts
50
100
150
200
250
300
350
400
zmp_ph108_F9
zmp_ph108_F11
zmp_ph108_H2
zmp_ph108_H5
zmp_ph108_B2
zmp_ph108_B6
zmp_ph108_B7
zmp_ph108_C4
zmp_ph108_C5
zmp_ph108_D4
zmp_ph97_G6
zmp_ph97_G9
zmp_ph97_H2
zmp_ph97_B10
zmp_ph97_C2
zmp_ph97_C4
zmp_ph97_B8
zmp_ph97_C1
zmp_ph97_D2
zmp_ph97_D5
zmp_ph111_G5
zmp_ph111_G11
zmp_ph111_H1
zmp_ph111_B1a
zmp_ph111_B5
zmp_ph111_B8
zmp_ph111_B6
zmp_ph111_B7
zmp_ph111_B9
zmp_ph111_B10
zmp_ph114_G4
zmp_ph114_G11
zmp_ph114_H1
zmp_ph114_H2
zmp_ph114_A9
zmp_ph114_A11
zmp_ph114_B2
zmp_ph114_B4
zmp_ph114_D8
zmp_ph114_E2

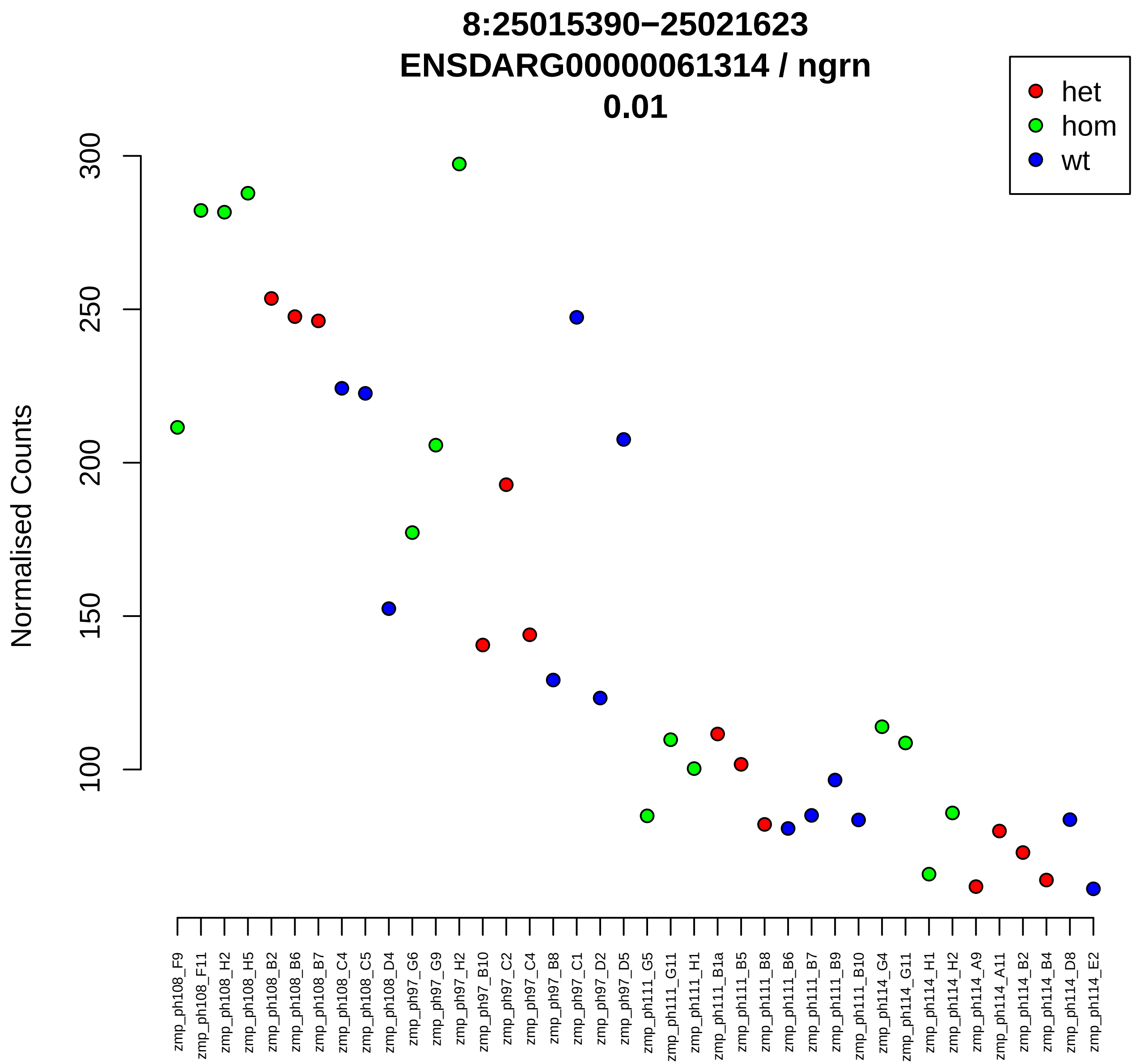
8:25015390−25021623
ENSDARG00000061314 / ngrn
0.01
het
hom
wt
Normalised Counts
100
150
200
250
300
zmp_ph108_F9
zmp_ph108_F11
zmp_ph108_H2
zmp_ph108_H5
zmp_ph108_B2
zmp_ph108_B6
zmp_ph108_B7
zmp_ph108_C4
zmp_ph108_C5
zmp_ph108_D4
zmp_ph97_G6
zmp_ph97_G9
zmp_ph97_H2
zmp_ph97_B10
zmp_ph97_C2
zmp_ph97_C4
zmp_ph97_B8
zmp_ph97_C1
zmp_ph97_D2
zmp_ph97_D5
zmp_ph111_G5
zmp_ph111_G11
zmp_ph111_H1
zmp_ph111_B1a
zmp_ph111_B5
zmp_ph111_B8
zmp_ph111_B6
zmp_ph111_B7
zmp_ph111_B9
zmp_ph111_B10
zmp_ph114_G4
zmp_ph114_G11
zmp_ph114_H1
zmp_ph114_H2
zmp_ph114_A9
zmp_ph114_A11
zmp_ph114_B2
zmp_ph114_B4
zmp_ph114_D8
zmp_ph114_E2

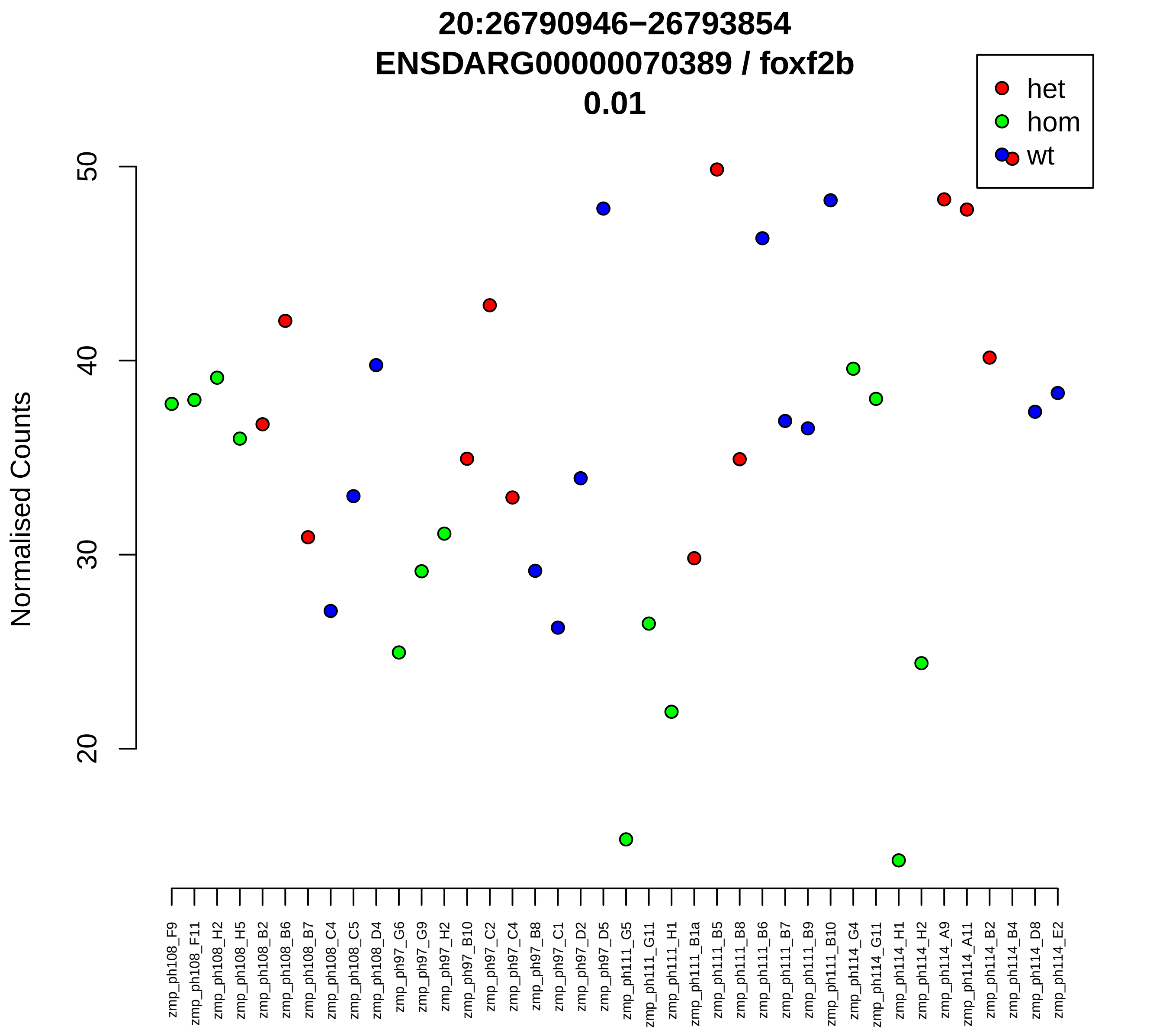
20:26790946−26793854
ENSDARG00000070389 / foxf2b
0.01
het
hom
wt
Normalised Counts
20
30
40
50
zmp_ph108_F9
zmp_ph108_F11
zmp_ph108_H2
zmp_ph108_H5
zmp_ph108_B2
zmp_ph108_B6
zmp_ph108_B7
zmp_ph108_C4
zmp_ph108_C5
zmp_ph108_D4
zmp_ph97_G6
zmp_ph97_G9
zmp_ph97_H2
zmp_ph97_B10
zmp_ph97_C2
zmp_ph97_C4
zmp_ph97_B8
zmp_ph97_C1
zmp_ph97_D2
zmp_ph97_D5
zmp_ph111_G5
zmp_ph111_G11
zmp_ph111_H1
zmp_ph111_B1a
zmp_ph111_B5
zmp_ph111_B8
zmp_ph111_B6
zmp_ph111_B7
zmp_ph111_B9
zmp_ph111_B10
zmp_ph114_G4
zmp_ph114_G11
zmp_ph114_H1
zmp_ph114_H2
zmp_ph114_A9
zmp_ph114_A11
zmp_ph114_B2
zmp_ph114_B4
zmp_ph114_D8
zmp_ph114_E2

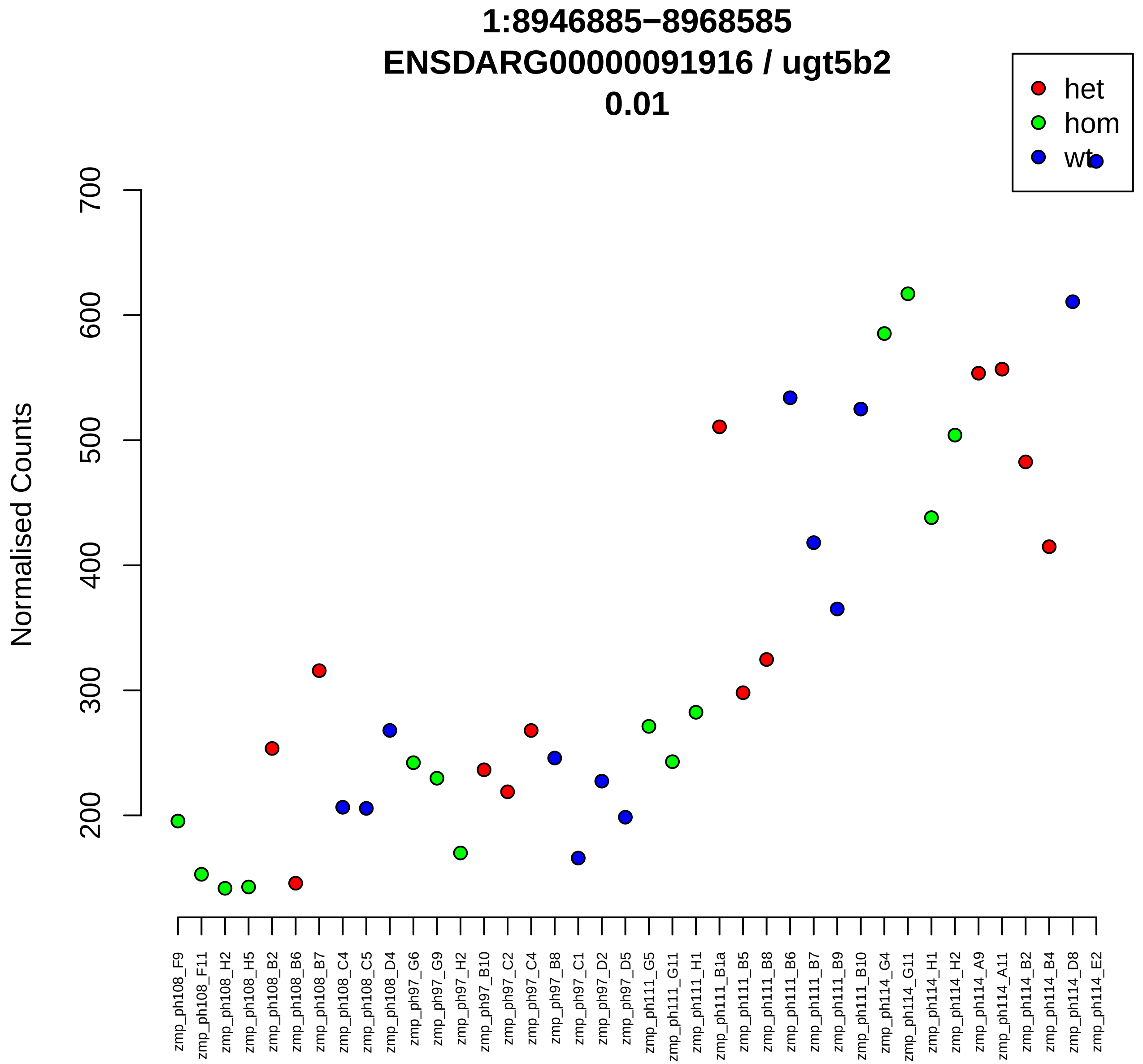
1:8946885−8968585
ENSDARG00000091916 / ugt5b2
0.01
het
hom
wt
Normalised Counts
200
300
400
500
600
700
zmp_ph108_F9
zmp_ph108_F11
zmp_ph108_H2
zmp_ph108_H5
zmp_ph108_B2
zmp_ph108_B6
zmp_ph108_B7
zmp_ph108_C4
zmp_ph108_C5
zmp_ph108_D4
zmp_ph97_G6
zmp_ph97_G9
zmp_ph97_H2
zmp_ph97_B10
zmp_ph97_C2
zmp_ph97_C4
zmp_ph97_B8
zmp_ph97_C1
zmp_ph97_D2
zmp_ph97_D5
zmp_ph111_G5
zmp_ph111_G11
zmp_ph111_H1
zmp_ph111_B1a
zmp_ph111_B5
zmp_ph111_B8
zmp_ph111_B6
zmp_ph111_B7
zmp_ph111_B9
zmp_ph111_B10
zmp_ph114_G4
zmp_ph114_G11
zmp_ph114_H1
zmp_ph114_H2
zmp_ph114_A9
zmp_ph114_A11
zmp_ph114_B2
zmp_ph114_B4
zmp_ph114_D8
zmp_ph114_E2

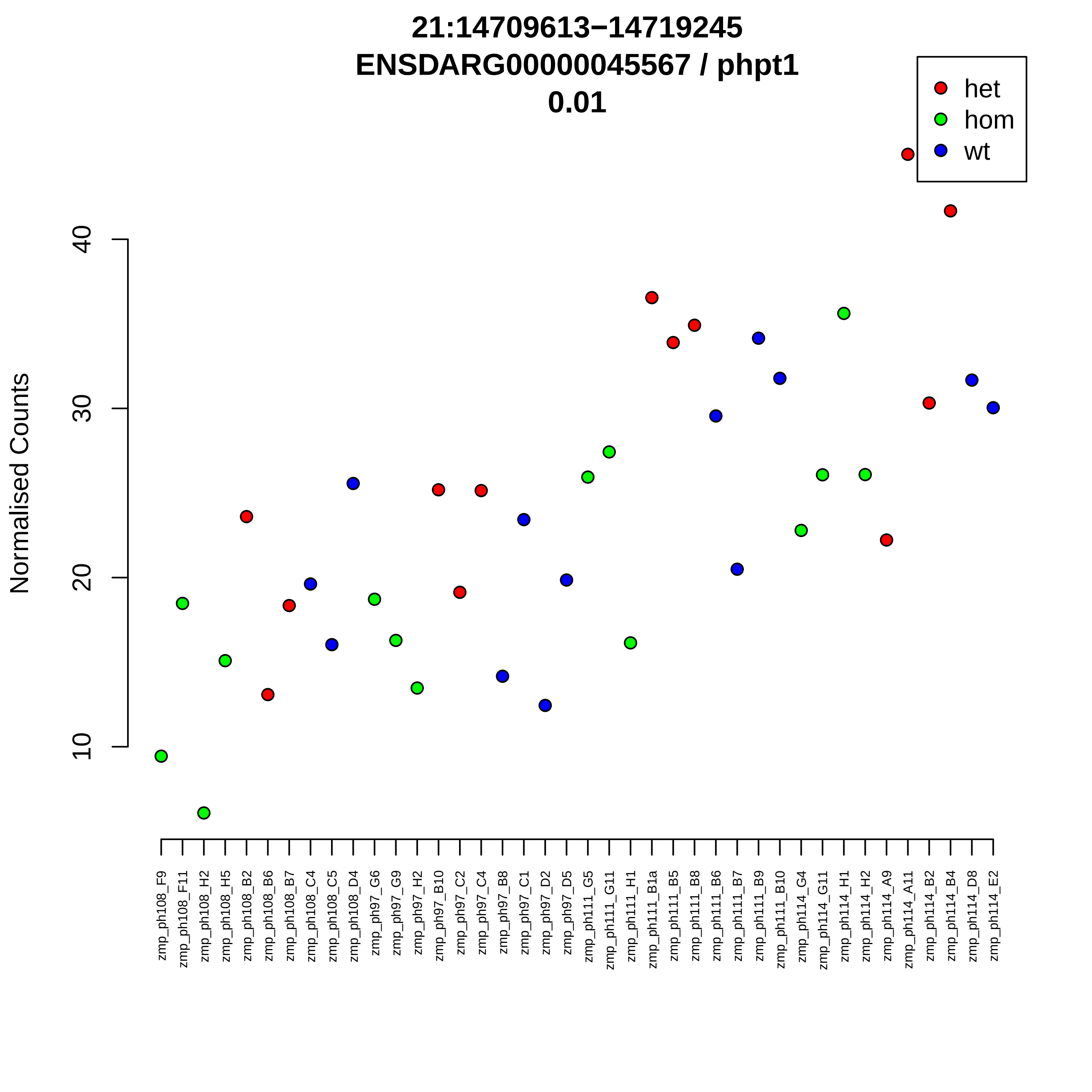

21:14709613−14719245
ENSDARG00000045567 / phpt1
0.01
het
hom
wt
Normalised Counts
10
20
30
40
zmp_ph108_F9
zmp_ph108_F11
zmp_ph108_H2
zmp_ph108_H5
zmp_ph108_B2
zmp_ph108_B6
zmp_ph108_B7
zmp_ph108_C4
zmp_ph108_C5
zmp_ph108_D4
zmp_ph97_G6
zmp_ph97_G9
zmp_ph97_H2
zmp_ph97_B10
zmp_ph97_C2
zmp_ph97_C4
zmp_ph97_B8
zmp_ph97_C1
zmp_ph97_D2
zmp_ph97_D5
zmp_ph111_G5
zmp_ph111_G11
zmp_ph111_H1
zmp_ph111_B1a
zmp_ph111_B5
zmp_ph111_B8
zmp_ph111_B6
zmp_ph111_B7
zmp_ph111_B9
zmp_ph111_B10
zmp_ph114_G4
zmp_ph114_G11
zmp_ph114_H1
zmp_ph114_H2
zmp_ph114_A9
zmp_ph114_A11
zmp_ph114_B2
zmp_ph114_B4
zmp_ph114_D8
zmp_ph114_E2

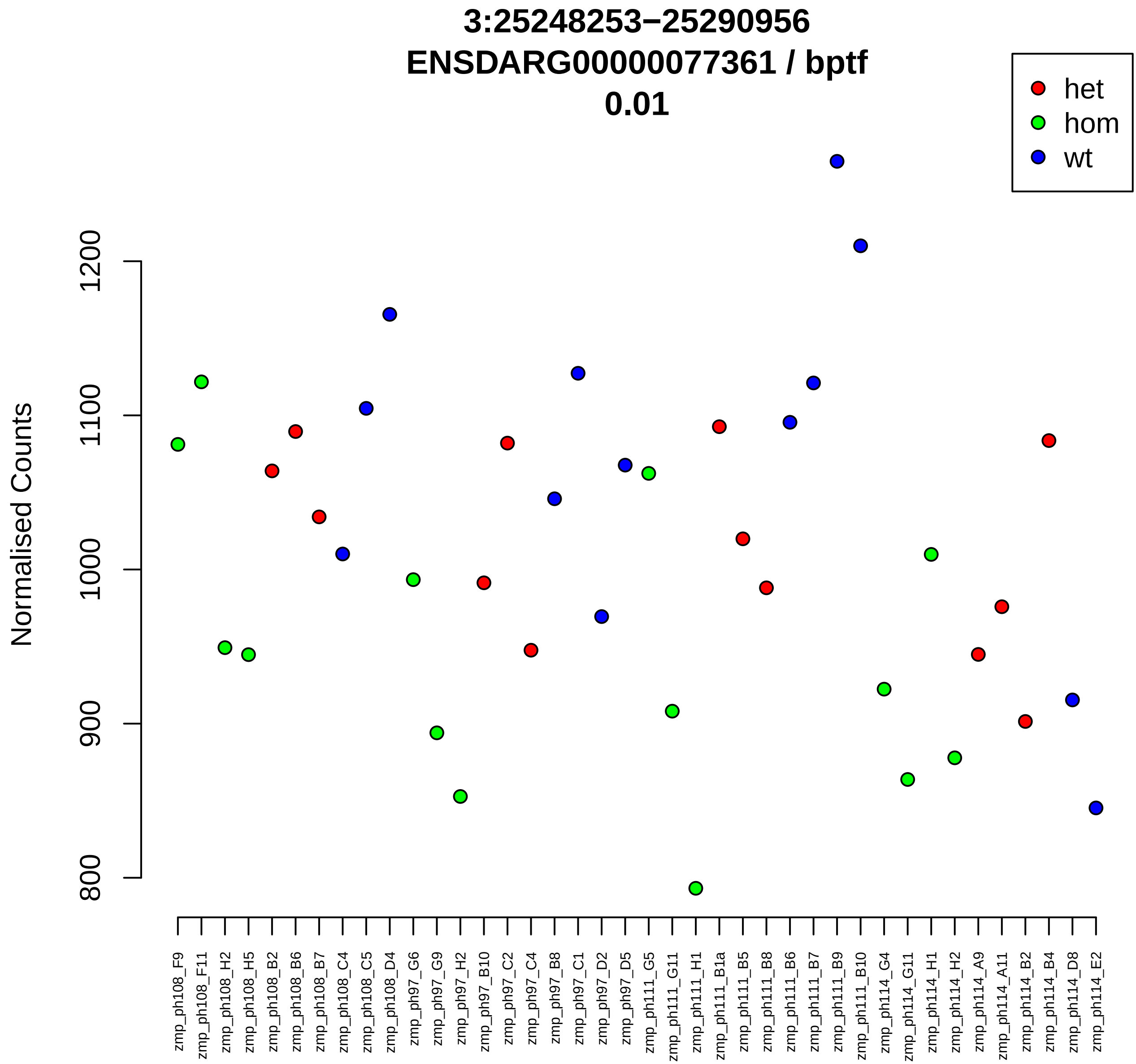
3:25248253−25290956
ENSDARG00000077361 / bptf
0.01
het
hom
wt
Normalised Counts
800
900
1000
1100
1200
zmp_ph108_F9
zmp_ph108_F11
zmp_ph108_H2
zmp_ph108_H5
zmp_ph108_B2
zmp_ph108_B6
zmp_ph108_B7
zmp_ph108_C4
zmp_ph108_C5
zmp_ph108_D4
zmp_ph97_G6
zmp_ph97_G9
zmp_ph97_H2
zmp_ph97_B10
zmp_ph97_C2
zmp_ph97_C4
zmp_ph97_B8
zmp_ph97_C1
zmp_ph97_D2
zmp_ph97_D5
zmp_ph111_G5
zmp_ph111_G11
zmp_ph111_H1
zmp_ph111_B1a
zmp_ph111_B5
zmp_ph111_B8
zmp_ph111_B6
zmp_ph111_B7
zmp_ph111_B9
zmp_ph111_B10
zmp_ph114_G4
zmp_ph114_G11
zmp_ph114_H1
zmp_ph114_H2
zmp_ph114_A9
zmp_ph114_A11
zmp_ph114_B2
zmp_ph114_B4
zmp_ph114_D8
zmp_ph114_E2

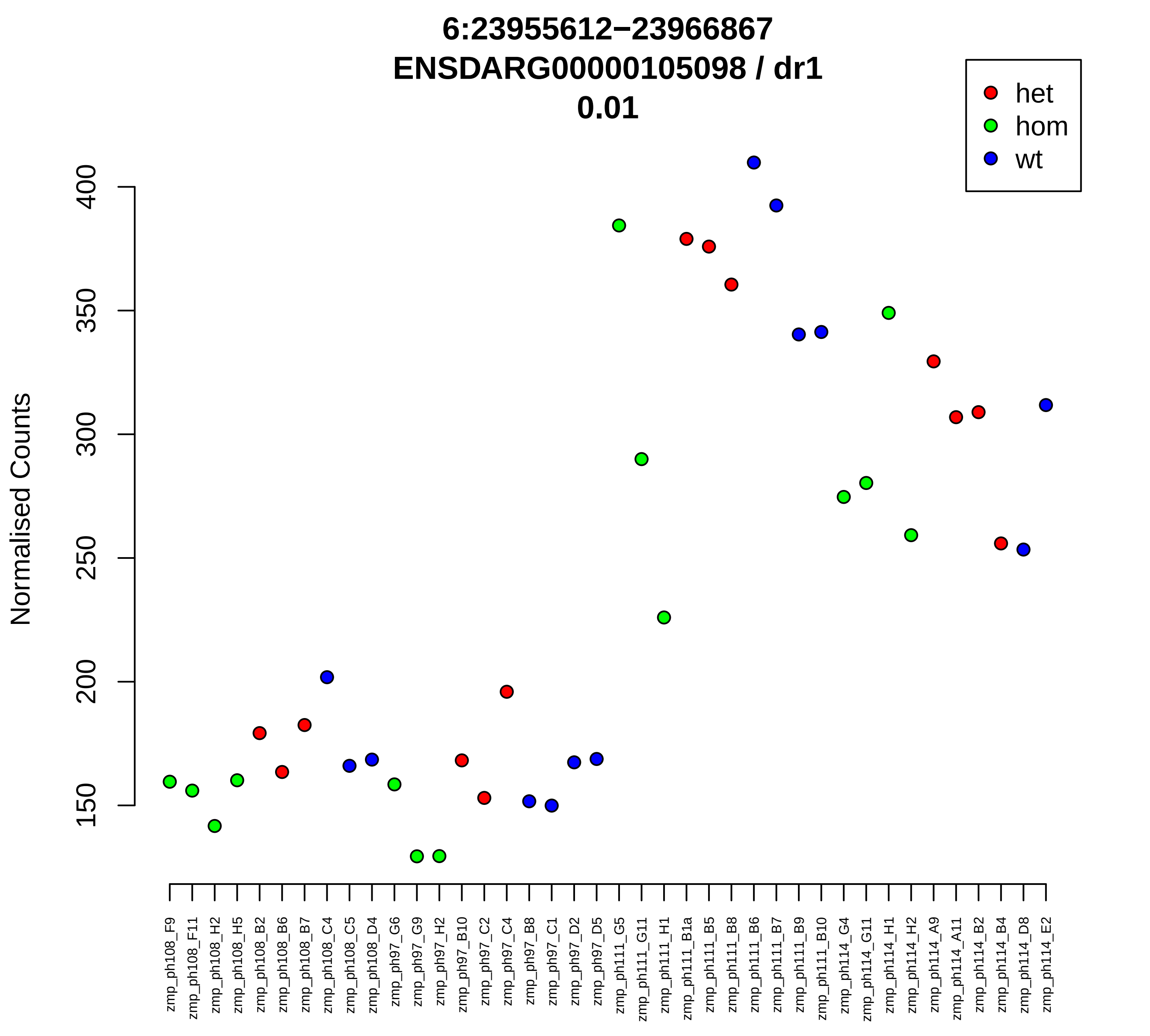

6:23955612−23966867
ENSDARG00000105098 / dr1
0.01
het
hom
wt
Normalised Counts
150
200
250
300
350
400
zmp_ph108_F9
zmp_ph108_F11
zmp_ph108_H2
zmp_ph108_H5
zmp_ph108_B2
zmp_ph108_B6
zmp_ph108_B7
zmp_ph108_C4
zmp_ph108_C5
zmp_ph108_D4
zmp_ph97_G6
zmp_ph97_G9
zmp_ph97_H2
zmp_ph97_B10
zmp_ph97_C2
zmp_ph97_C4
zmp_ph97_B8
zmp_ph97_C1
zmp_ph97_D2
zmp_ph97_D5
zmp_ph111_G5
zmp_ph111_G11
zmp_ph111_H1
zmp_ph111_B1a
zmp_ph111_B5
zmp_ph111_B8
zmp_ph111_B6
zmp_ph111_B7
zmp_ph111_B9
zmp_ph111_B10
zmp_ph114_G4
zmp_ph114_G11
zmp_ph114_H1
zmp_ph114_H2
zmp_ph114_A9
zmp_ph114_A11
zmp_ph114_B2
zmp_ph114_B4
zmp_ph114_D8
zmp_ph114_E2

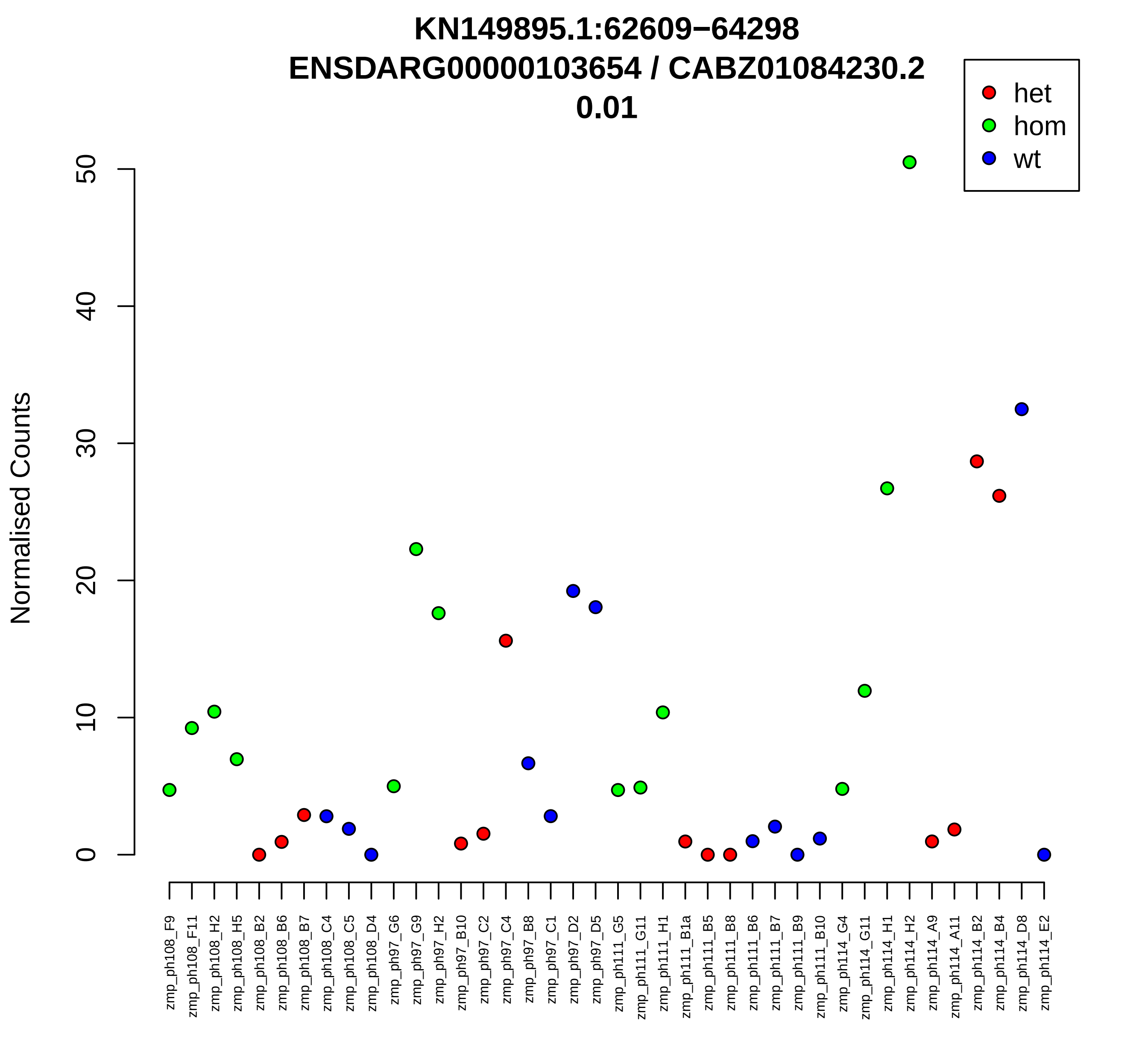
KN149895.1:62609−64298
ENSDARG00000103654 / CABZ01084230.2
0.01
het
hom
wt
Normalised Counts
0
10
20
30
40
50
zmp_ph108_F9
zmp_ph108_F11
zmp_ph108_H2
zmp_ph108_H5
zmp_ph108_B2
zmp_ph108_B6
zmp_ph108_B7
zmp_ph108_C4
zmp_ph108_C5
zmp_ph108_D4
zmp_ph97_G6
zmp_ph97_G9
zmp_ph97_H2
zmp_ph97_B10
zmp_ph97_C2
zmp_ph97_C4
zmp_ph97_B8
zmp_ph97_C1
zmp_ph97_D2
zmp_ph97_D5
zmp_ph111_G5
zmp_ph111_G11
zmp_ph111_H1
zmp_ph111_B1a
zmp_ph111_B5
zmp_ph111_B8
zmp_ph111_B6
zmp_ph111_B7
zmp_ph111_B9
zmp_ph111_B10
zmp_ph114_G4
zmp_ph114_G11
zmp_ph114_H1
zmp_ph114_H2
zmp_ph114_A9
zmp_ph114_A11
zmp_ph114_B2
zmp_ph114_B4
zmp_ph114_D8
zmp_ph114_E2

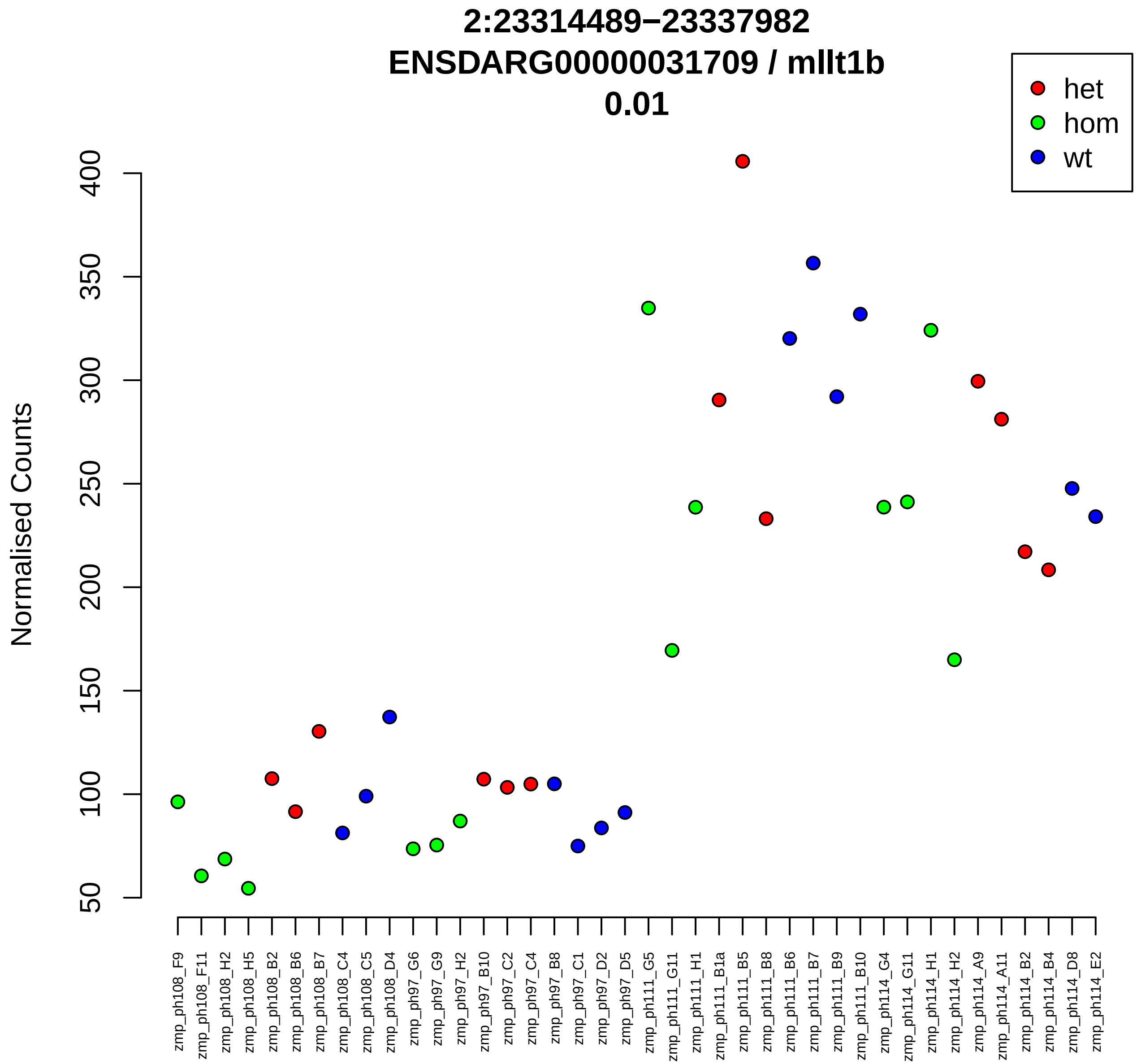
2:23314489−23337982
ENSDARG00000031709 / mllt1b
0.01
het
hom
wt
Normalised Counts
50
100
150
200
250
300
350
400
zmp_ph108_F9
zmp_ph108_F11
zmp_ph108_H2
zmp_ph108_H5
zmp_ph108_B2
zmp_ph108_B6
zmp_ph108_B7
zmp_ph108_C4
zmp_ph108_C5
zmp_ph108_D4
zmp_ph97_G6
zmp_ph97_G9
zmp_ph97_H2
zmp_ph97_B10
zmp_ph97_C2
zmp_ph97_C4
zmp_ph97_B8
zmp_ph97_C1
zmp_ph97_D2
zmp_ph97_D5
zmp_ph111_G5
zmp_ph111_G11
zmp_ph111_H1
zmp_ph111_B1a
zmp_ph111_B5
zmp_ph111_B8
zmp_ph111_B6
zmp_ph111_B7
zmp_ph111_B9
zmp_ph111_B10
zmp_ph114_G4
zmp_ph114_G11
zmp_ph114_H1
zmp_ph114_H2
zmp_ph114_A9
zmp_ph114_A11
zmp_ph114_B2
zmp_ph114_B4
zmp_ph114_D8
zmp_ph114_E2

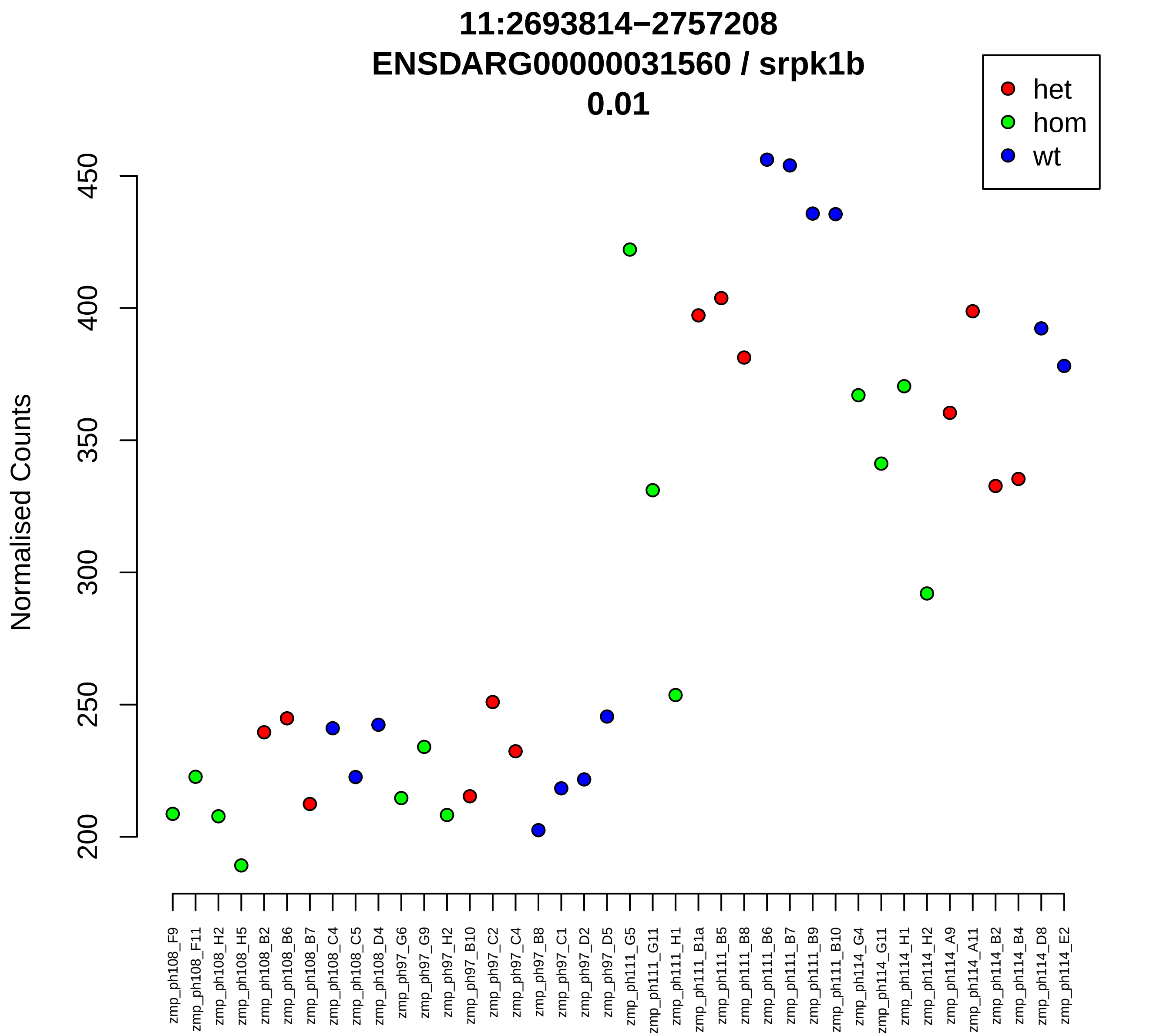
11:2693814−2757208
ENSDARG00000031560 / srpk1b
0.01
Normalised Counts
200
250
300
350
400
450
het
hom
wt
zmp_ph108_F9
zmp_ph108_F11
zmp_ph108_H2
zmp_ph108_H5
zmp_ph108_B2
zmp_ph108_B6
zmp_ph108_B7
zmp_ph108_C4
zmp_ph108_C5
zmp_ph108_D4
zmp_ph97_G6
zmp_ph97_G9
zmp_ph97_H2
zmp_ph97_B10
zmp_ph97_C2
zmp_ph97_C4
zmp_ph97_B8
zmp_ph97_C1
zmp_ph97_D2
zmp_ph97_D5
zmp_ph111_G5
zmp_ph111_G11
zmp_ph111_H1
zmp_ph111_B1a
zmp_ph111_B5
zmp_ph111_B8
zmp_ph111_B6
zmp_ph111_B7
zmp_ph111_B9
zmp_ph111_B10
zmp_ph114_G4
zmp_ph114_G11
zmp_ph114_H1
zmp_ph114_H2
zmp_ph114_A9
zmp_ph114_A11
zmp_ph114_B2
zmp_ph114_B4
zmp_ph114_D8
zmp_ph114_E2

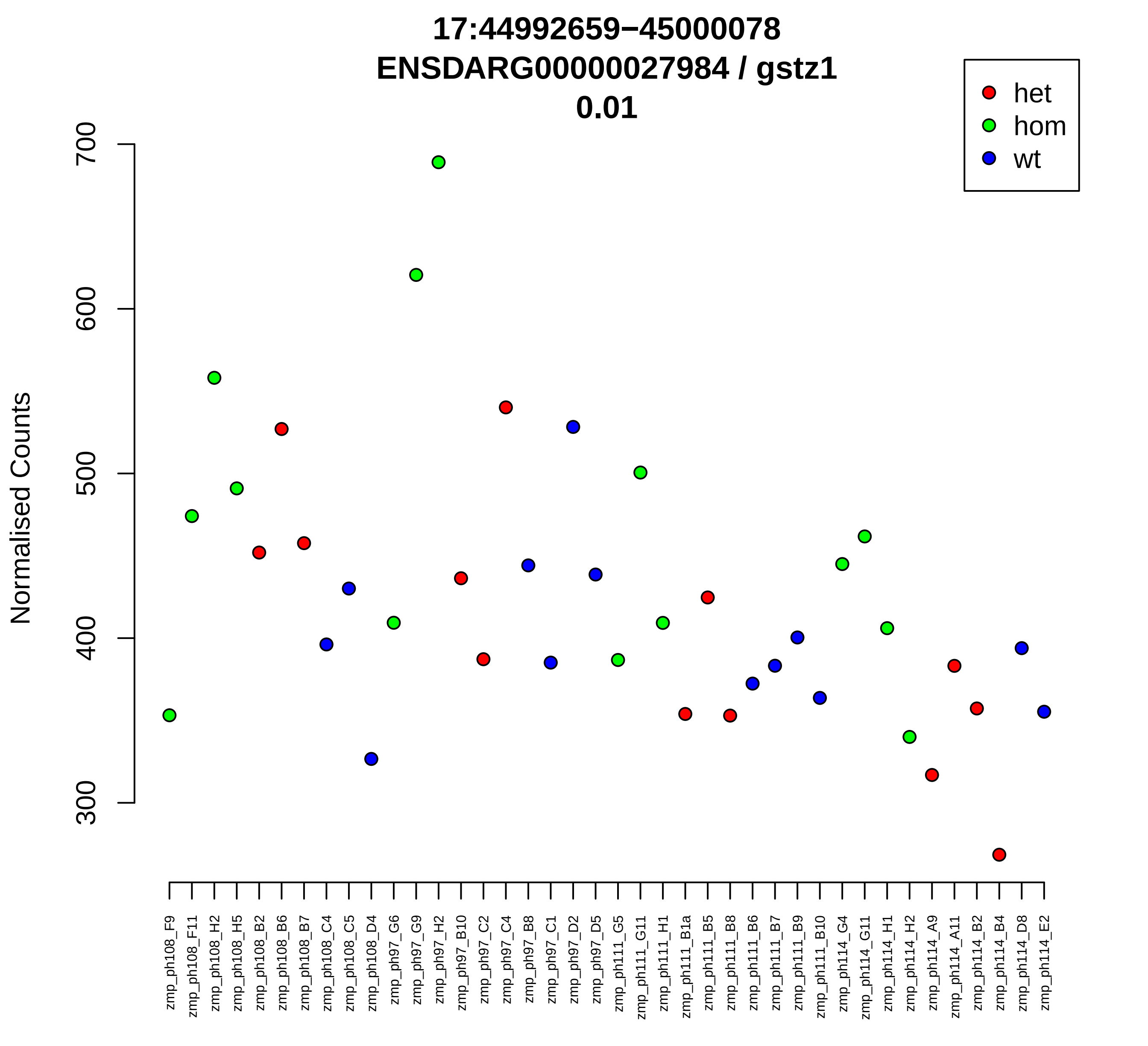
17:44992659−45000078
ENSDARG00000027984 / gstz1
0.01
het
hom
wt
Normalised Counts
300
400
500
600
700
zmp_ph108_F9
zmp_ph108_F11
zmp_ph108_H2
zmp_ph108_H5
zmp_ph108_B2
zmp_ph108_B6
zmp_ph108_B7
zmp_ph108_C4
zmp_ph108_C5
zmp_ph108_D4
zmp_ph97_G6
zmp_ph97_G9
zmp_ph97_H2
zmp_ph97_B10
zmp_ph97_C2
zmp_ph97_C4
zmp_ph97_B8
zmp_ph97_C1
zmp_ph97_D2
zmp_ph97_D5
zmp_ph111_G5
zmp_ph111_G11
zmp_ph111_H1
zmp_ph111_B1a
zmp_ph111_B5
zmp_ph111_B8
zmp_ph111_B6
zmp_ph111_B7
zmp_ph111_B9
zmp_ph111_B10
zmp_ph114_G4
zmp_ph114_G11
zmp_ph114_H1
zmp_ph114_H2
zmp_ph114_A9
zmp_ph114_A11
zmp_ph114_B2
zmp_ph114_B4
zmp_ph114_D8
zmp_ph114_E2

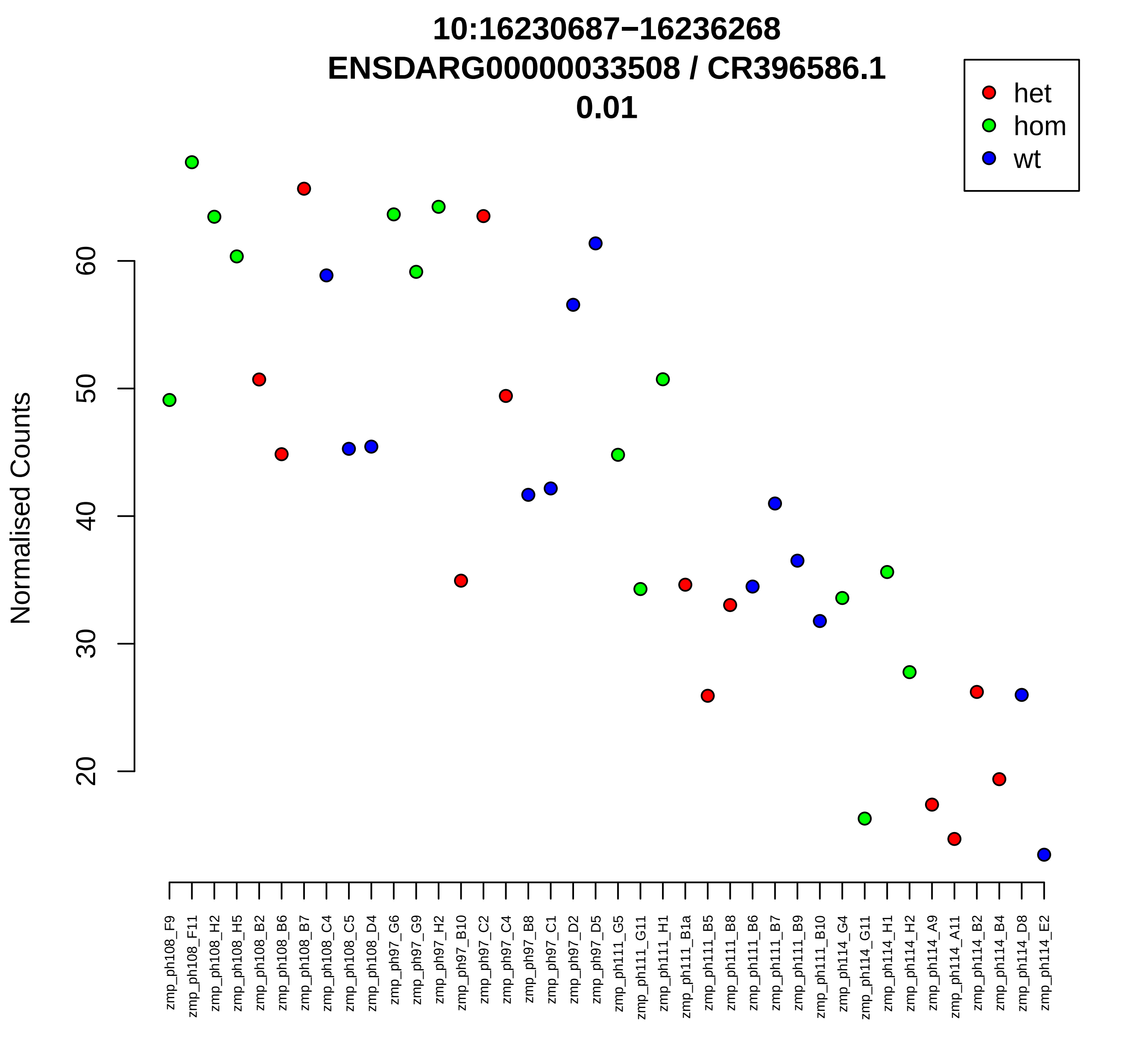

10:16230687−16236268
ENSDARG00000033508 / CR396586.1
0.01
het
hom
wt
Normalised Counts
20
30
40
50
60
zmp_ph108_F9
zmp_ph108_F11
zmp_ph108_H2
zmp_ph108_H5
zmp_ph108_B2
zmp_ph108_B6
zmp_ph108_B7
zmp_ph108_C4
zmp_ph108_C5
zmp_ph108_D4
zmp_ph97_G6
zmp_ph97_G9
zmp_ph97_H2
zmp_ph97_B10
zmp_ph97_C2
zmp_ph97_C4
zmp_ph97_B8
zmp_ph97_C1
zmp_ph97_D2
zmp_ph97_D5
zmp_ph111_G5
zmp_ph111_G11
zmp_ph111_H1
zmp_ph111_B1a
zmp_ph111_B5
zmp_ph111_B8
zmp_ph111_B6
zmp_ph111_B7
zmp_ph111_B9
zmp_ph111_B10
zmp_ph114_G4
zmp_ph114_G11
zmp_ph114_H1
zmp_ph114_H2
zmp_ph114_A9
zmp_ph114_A11
zmp_ph114_B2
zmp_ph114_B4
zmp_ph114_D8
zmp_ph114_E2

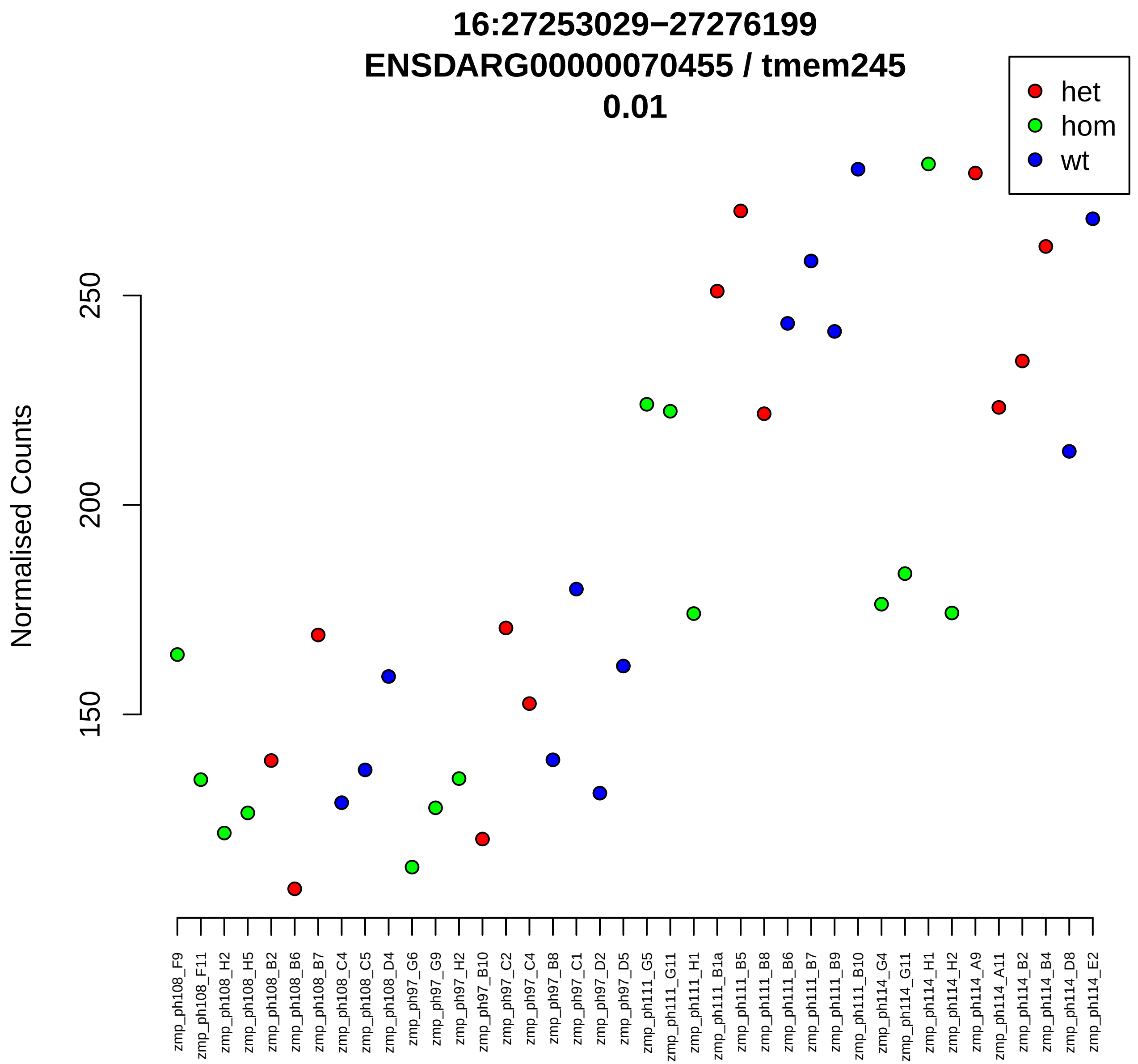
16:27253029−27276199
ENSDARG00000070455 / tmem245
0.01
het
hom
wt
Normalised Counts
150
200
250
zmp_ph108_F9
zmp_ph108_F11
zmp_ph108_H2
zmp_ph108_H5
zmp_ph108_B2
zmp_ph108_B6
zmp_ph108_B7
zmp_ph108_C4
zmp_ph108_C5
zmp_ph108_D4
zmp_ph97_G6
zmp_ph97_G9
zmp_ph97_H2
zmp_ph97_B10
zmp_ph97_C2
zmp_ph97_C4
zmp_ph97_B8
zmp_ph97_C1
zmp_ph97_D2
zmp_ph97_D5
zmp_ph111_G5
zmp_ph111_G11
zmp_ph111_H1
zmp_ph111_B1a
zmp_ph111_B5
zmp_ph111_B8
zmp_ph111_B6
zmp_ph111_B7
zmp_ph111_B9
zmp_ph111_B10
zmp_ph114_G4
zmp_ph114_G11
zmp_ph114_H1
zmp_ph114_H2
zmp_ph114_A9
zmp_ph114_A11
zmp_ph114_B2
zmp_ph114_B4
zmp_ph114_D8
zmp_ph114_E2

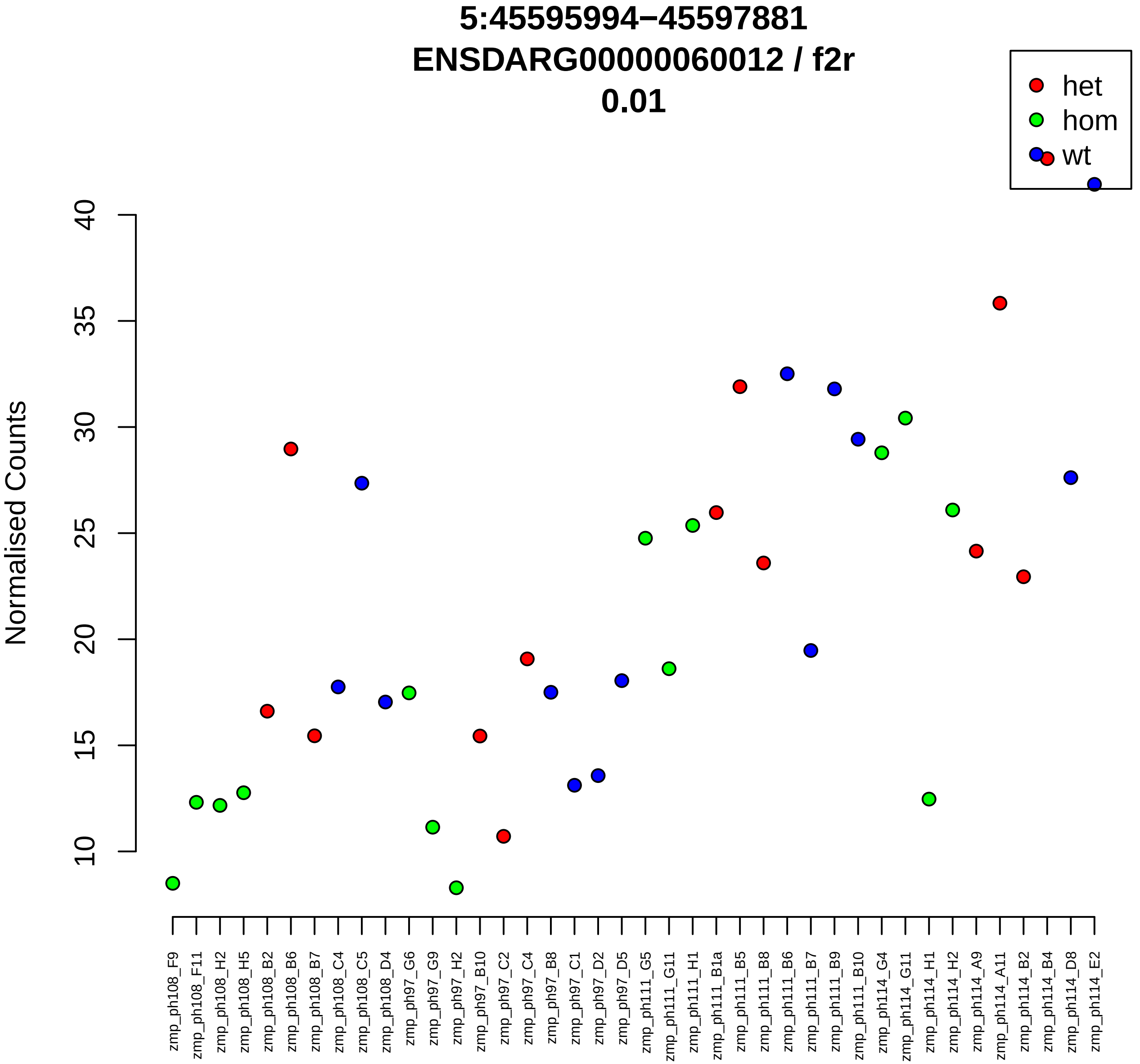
5:45595994−45597881
ENSDARG00000060012 / f2r
0.01
het
hom
wt
Normalised Counts
10
15
20
25
30
35
40
zmp_ph108_F9
zmp_ph108_F11
zmp_ph108_H2
zmp_ph108_H5
zmp_ph108_B2
zmp_ph108_B6
zmp_ph108_B7
zmp_ph108_C4
zmp_ph108_C5
zmp_ph108_D4
zmp_ph97_G6
zmp_ph97_G9
zmp_ph97_H2
zmp_ph97_B10
zmp_ph97_C2
zmp_ph97_C4
zmp_ph97_B8
zmp_ph97_C1
zmp_ph97_D2
zmp_ph97_D5
zmp_ph111_G5
zmp_ph111_G11
zmp_ph111_H1
zmp_ph111_B1a
zmp_ph111_B5
zmp_ph111_B8
zmp_ph111_B6
zmp_ph111_B7
zmp_ph111_B9
zmp_ph111_B10
zmp_ph114_G4
zmp_ph114_G11
zmp_ph114_H1
zmp_ph114_H2
zmp_ph114_A9
zmp_ph114_A11
zmp_ph114_B2
zmp_ph114_B4
zmp_ph114_D8
zmp_ph114_E2

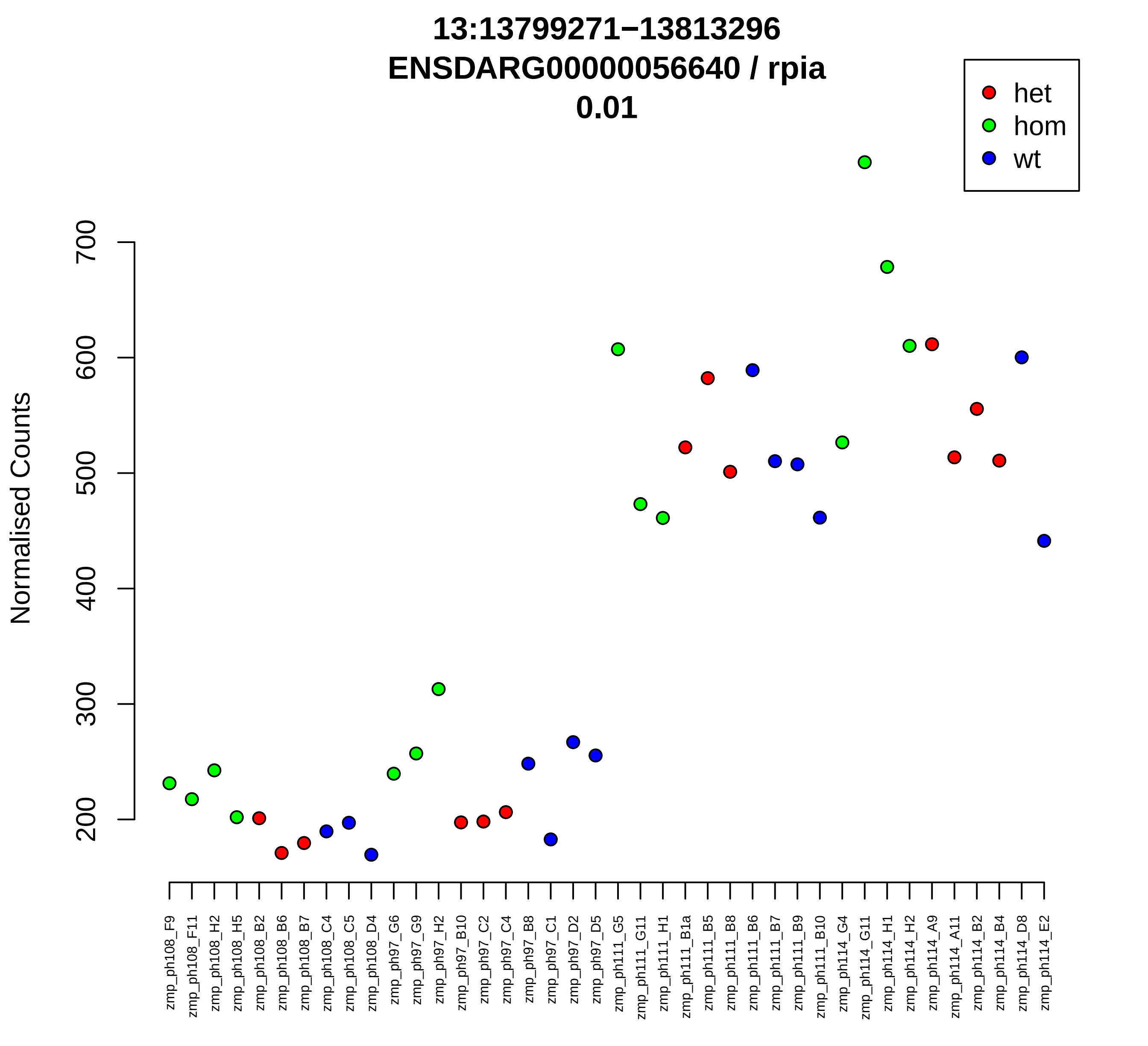

13:13799271−13813296
ENSDARG00000056640 / rpia
0.01
het
hom
wt
Normalised Counts
200
300
400
500
600
700
zmp_ph108_F9
zmp_ph108_F11
zmp_ph108_H2
zmp_ph108_H5
zmp_ph108_B2
zmp_ph108_B6
zmp_ph108_B7
zmp_ph108_C4
zmp_ph108_C5
zmp_ph108_D4
zmp_ph97_G6
zmp_ph97_G9
zmp_ph97_H2
zmp_ph97_B10
zmp_ph97_C2
zmp_ph97_C4
zmp_ph97_B8
zmp_ph97_C1
zmp_ph97_D2
zmp_ph97_D5
zmp_ph111_G5
zmp_ph111_G11
zmp_ph111_H1
zmp_ph111_B1a
zmp_ph111_B5
zmp_ph111_B8
zmp_ph111_B6
zmp_ph111_B7
zmp_ph111_B9
zmp_ph111_B10
zmp_ph114_G4
zmp_ph114_G11
zmp_ph114_H1
zmp_ph114_H2
zmp_ph114_A9
zmp_ph114_A11
zmp_ph114_B2
zmp_ph114_B4
zmp_ph114_D8
zmp_ph114_E2

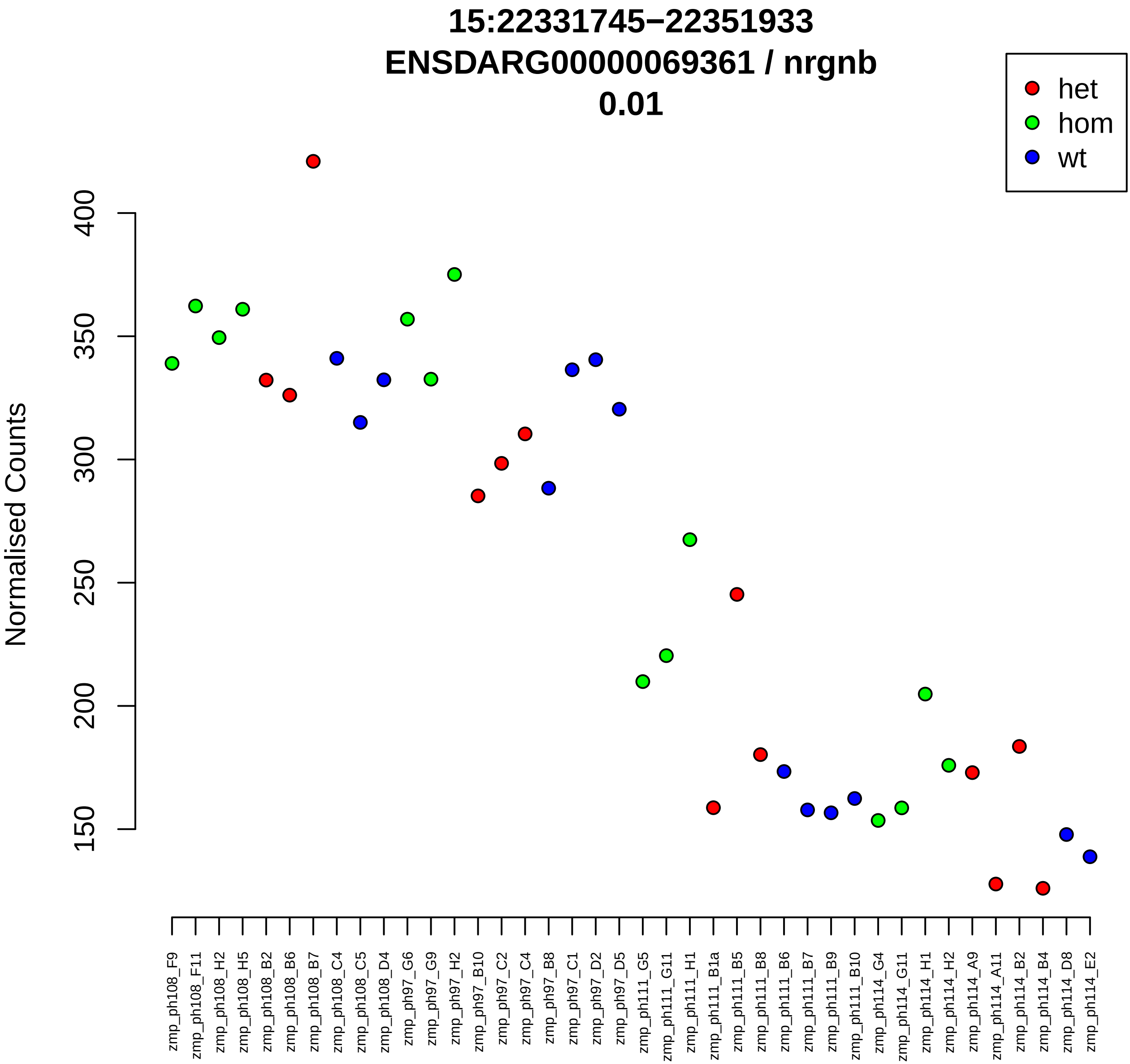

15:22331745−22351933
ENSDARG00000069361 / nrgnb
0.01
het
hom
wt
Normalised Counts
150
200
250
300
350
400
zmp_ph108_F9
zmp_ph108_F11
zmp_ph108_H2
zmp_ph108_H5
zmp_ph108_B2
zmp_ph108_B6
zmp_ph108_B7
zmp_ph108_C4
zmp_ph108_C5
zmp_ph108_D4
zmp_ph97_G6
zmp_ph97_G9
zmp_ph97_H2
zmp_ph97_B10
zmp_ph97_C2
zmp_ph97_C4
zmp_ph97_B8
zmp_ph97_C1
zmp_ph97_D2
zmp_ph97_D5
zmp_ph111_G5
zmp_ph111_G11
zmp_ph111_H1
zmp_ph111_B1a
zmp_ph111_B5
zmp_ph111_B8
zmp_ph111_B6
zmp_ph111_B7
zmp_ph111_B9
zmp_ph111_B10
zmp_ph114_G4
zmp_ph114_G11
zmp_ph114_H1
zmp_ph114_H2
zmp_ph114_A9
zmp_ph114_A11
zmp_ph114_B2
zmp_ph114_B4
zmp_ph114_D8
zmp_ph114_E2

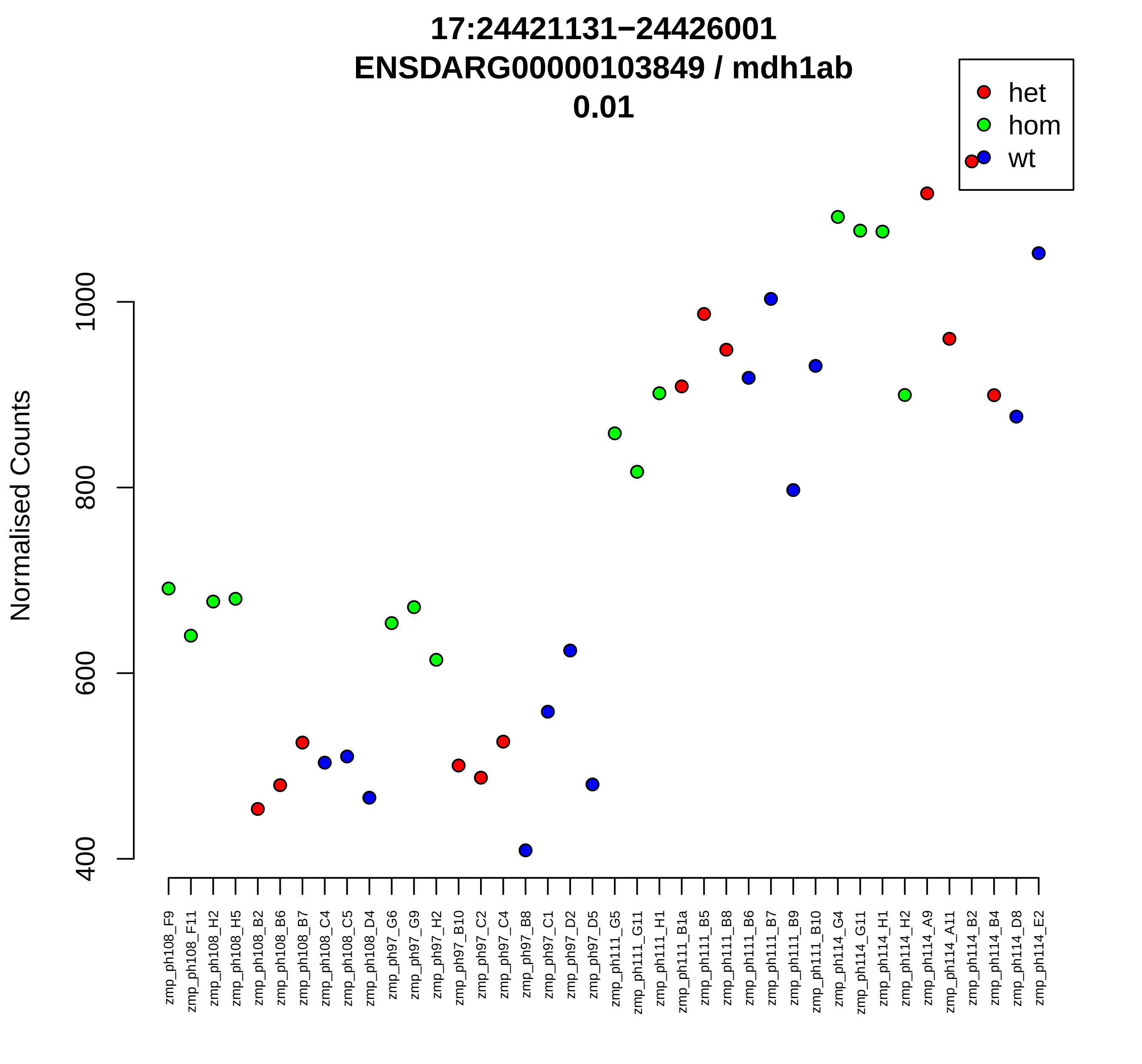

17:24421131−24426001
ENSDARG00000103849 / mdh1ab
0.01
het
hom
wt
Normalised Counts
400
600
800
1000
zmp_ph108_F9
zmp_ph108_F11
zmp_ph108_H2
zmp_ph108_H5
zmp_ph108_B2
zmp_ph108_B6
zmp_ph108_B7
zmp_ph108_C4
zmp_ph108_C5
zmp_ph108_D4
zmp_ph97_G6
zmp_ph97_G9
zmp_ph97_H2
zmp_ph97_B10
zmp_ph97_C2
zmp_ph97_C4
zmp_ph97_B8
zmp_ph97_C1
zmp_ph97_D2
zmp_ph97_D5
zmp_ph111_G5
zmp_ph111_G11
zmp_ph111_H1
zmp_ph111_B1a
zmp_ph111_B5
zmp_ph111_B8
zmp_ph111_B6
zmp_ph111_B7
zmp_ph111_B9
zmp_ph111_B10
zmp_ph114_G4
zmp_ph114_G11
zmp_ph114_H1
zmp_ph114_H2
zmp_ph114_A9
zmp_ph114_A11
zmp_ph114_B2
zmp_ph114_B4
zmp_ph114_D8
zmp_ph114_E2

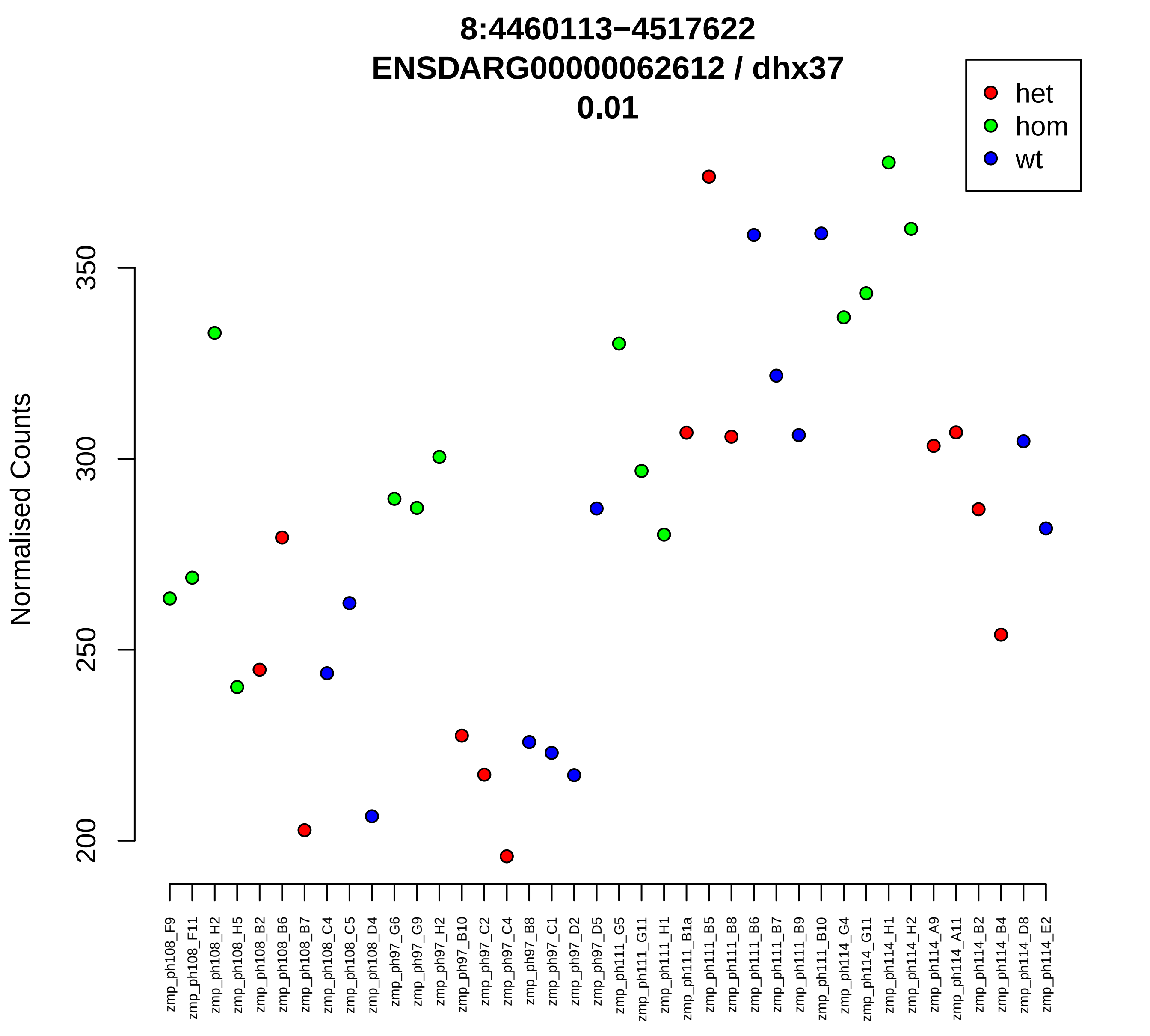

8:4460113−4517622
ENSDARG00000062612 / dhx37
0.01
het
hom
wt
Normalised Counts
200
250
300
350
zmp_ph108_F9
zmp_ph108_F11
zmp_ph108_H2
zmp_ph108_H5
zmp_ph108_B2
zmp_ph108_B6
zmp_ph108_B7
zmp_ph108_C4
zmp_ph108_C5
zmp_ph108_D4
zmp_ph97_G6
zmp_ph97_G9
zmp_ph97_H2
zmp_ph97_B10
zmp_ph97_C2
zmp_ph97_C4
zmp_ph97_B8
zmp_ph97_C1
zmp_ph97_D2
zmp_ph97_D5
zmp_ph111_G5
zmp_ph111_G11
zmp_ph111_H1
zmp_ph111_B1a
zmp_ph111_B5
zmp_ph111_B8
zmp_ph111_B6
zmp_ph111_B7
zmp_ph111_B9
zmp_ph111_B10
zmp_ph114_G4
zmp_ph114_G11
zmp_ph114_H1
zmp_ph114_H2
zmp_ph114_A9
zmp_ph114_A11
zmp_ph114_B2
zmp_ph114_B4
zmp_ph114_D8
zmp_ph114_E2

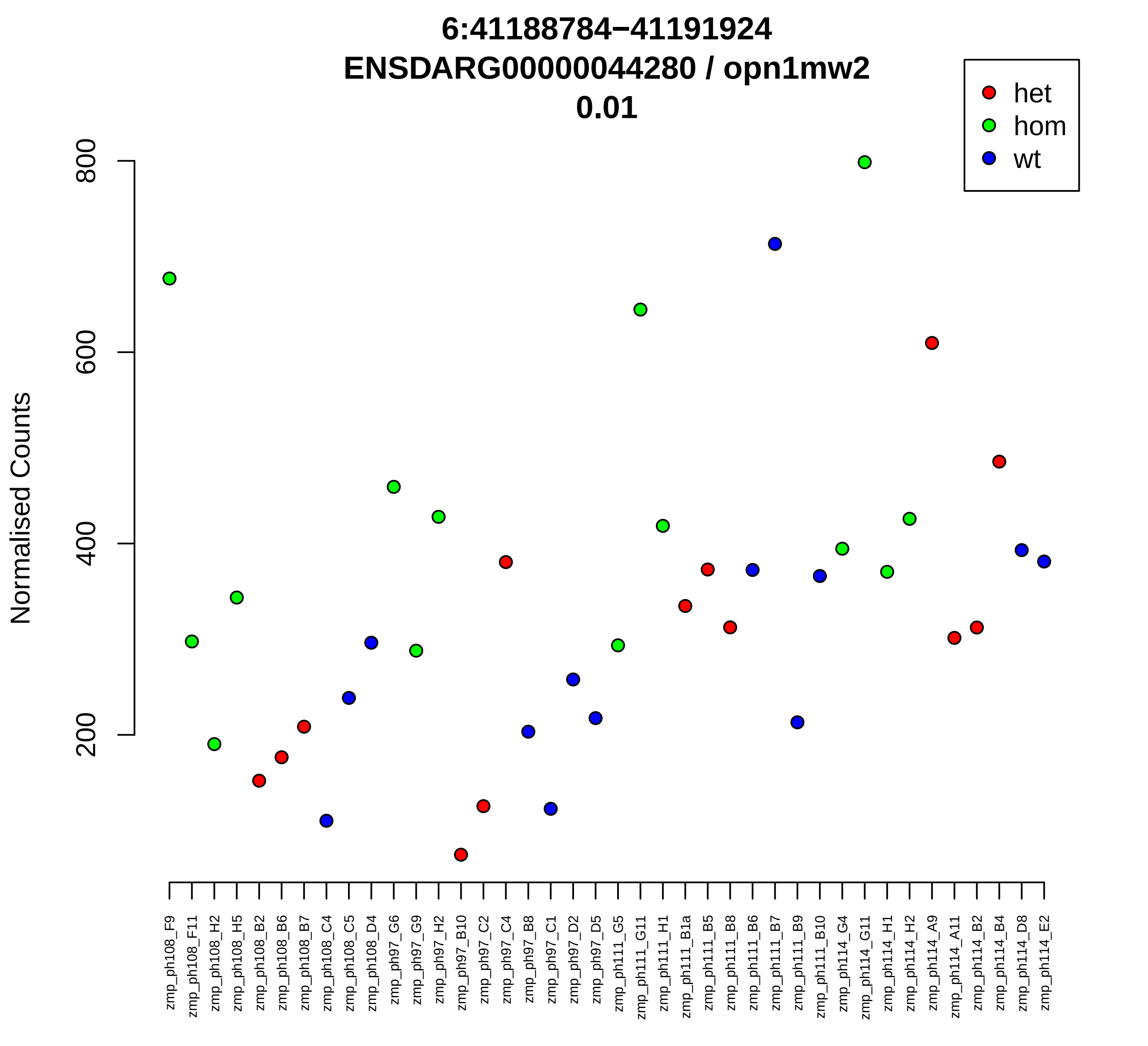
6:41188784−41191924
ENSDARG00000044280 / opn1mw2
0.01
het
hom
wt
Normalised Counts
800
600
400
200
zmp_ph108_F9
zmp_ph108_F11
zmp_ph108_H2
zmp_ph108_H5
zmp_ph108_B2
zmp_ph108_B6
zmp_ph108_B7
zmp_ph108_C4
zmp_ph108_C5
zmp_ph108_D4
zmp_ph97_G6
zmp_ph97_G9
zmp_ph97_H2
zmp_ph97_B10
zmp_ph97_C2
zmp_ph97_C4
zmp_ph97_B8
zmp_ph97_C1
zmp_ph97_D2
zmp_ph97_D5
zmp_ph111_G5
zmp_ph111_G11
zmp_ph111_H1
zmp_ph111_B1a
zmp_ph111_B5
zmp_ph111_B8
zmp_ph111_B6
zmp_ph111_B7
zmp_ph111_B9
zmp_ph111_B10
zmp_ph114_G4
zmp_ph114_G11
zmp_ph114_H1
zmp_ph114_H2
zmp_ph114_A9
zmp_ph114_A11
zmp_ph114_B2
zmp_ph114_B4
zmp_ph114_D8
zmp_ph114_E2

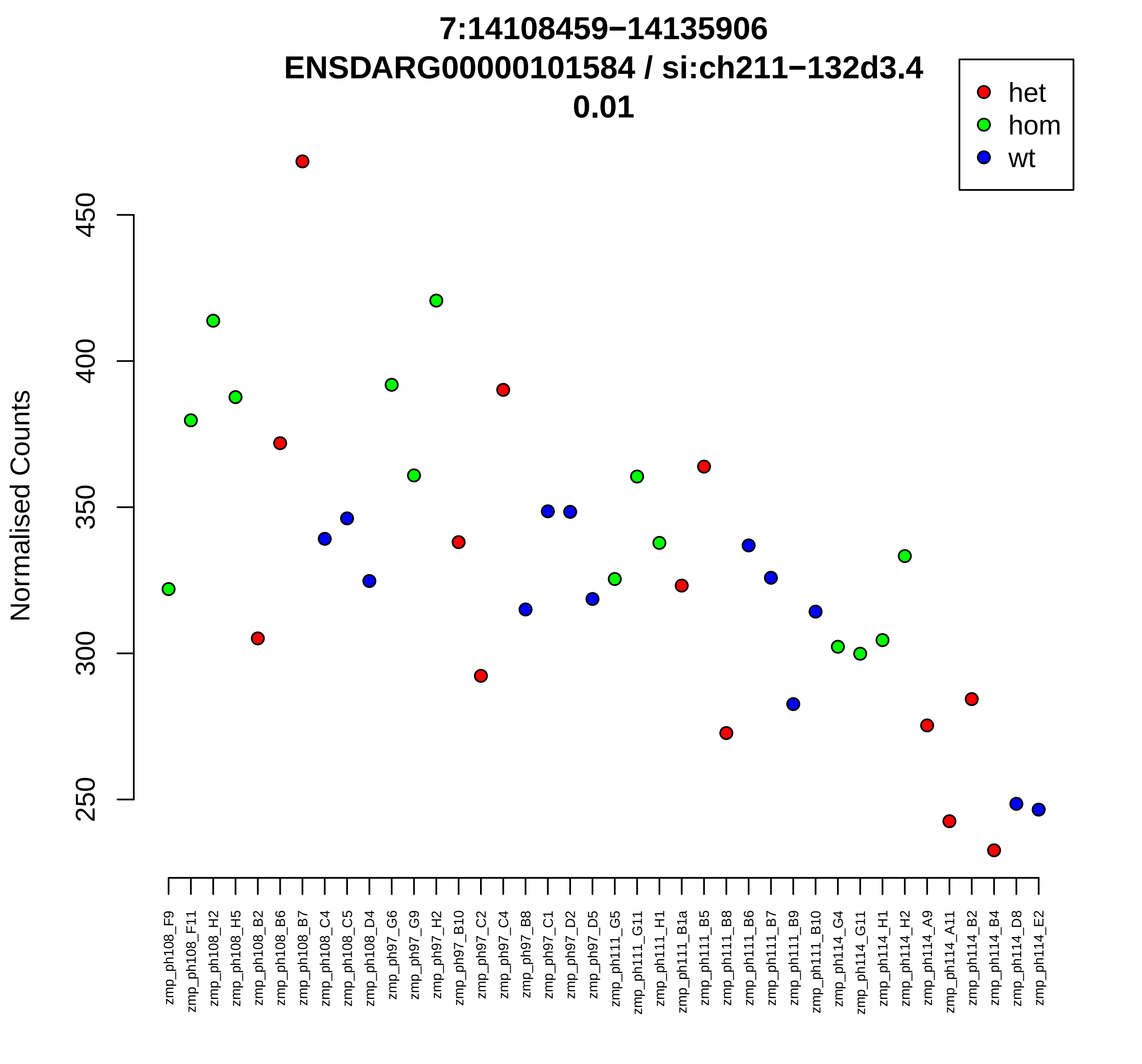

7:14108459−14135906
ENSDARG00000101584 / si:ch211−132d3.4
0.01
het
hom
wt
Normalised Counts
250
300
350
400
450
zmp_ph108_F9
zmp_ph108_F11
zmp_ph108_H2
zmp_ph108_H5
zmp_ph108_B2
zmp_ph108_B6
zmp_ph108_B7
zmp_ph108_C4
zmp_ph108_C5
zmp_ph108_D4
zmp_ph97_G6
zmp_ph97_G9
zmp_ph97_H2
zmp_ph97_B10
zmp_ph97_C2
zmp_ph97_C4
zmp_ph97_B8
zmp_ph97_C1
zmp_ph97_D2
zmp_ph97_D5
zmp_ph111_G5
zmp_ph111_G11
zmp_ph111_H1
zmp_ph111_B1a
zmp_ph111_B5
zmp_ph111_B8
zmp_ph111_B6
zmp_ph111_B7
zmp_ph111_B9
zmp_ph111_B10
zmp_ph114_G4
zmp_ph114_G11
zmp_ph114_H1
zmp_ph114_H2
zmp_ph114_A9
zmp_ph114_A11
zmp_ph114_B2
zmp_ph114_B4
zmp_ph114_D8
zmp_ph114_E2

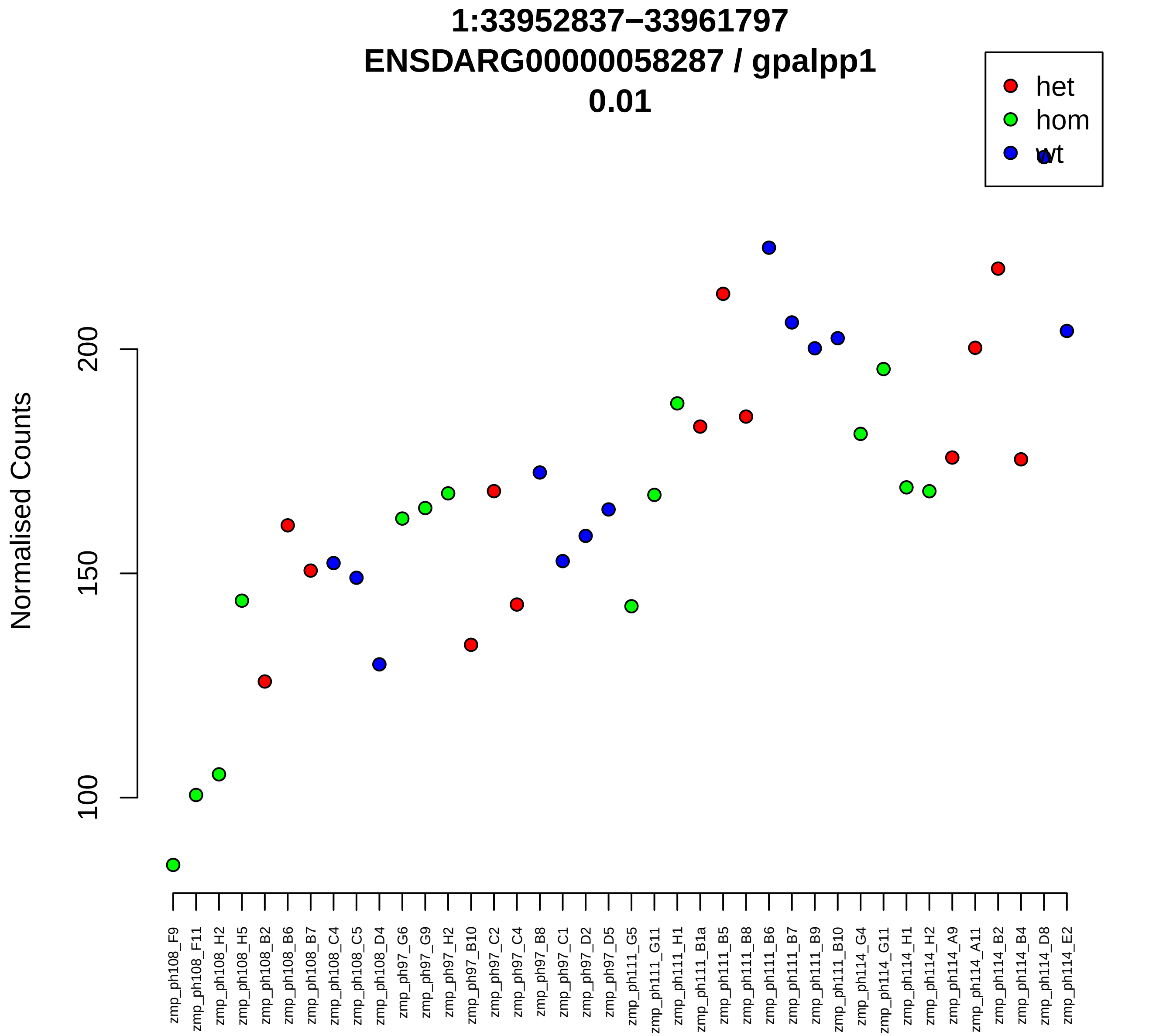
1:33952837−33961797
ENSDARG00000058287 / gpalpp1
0.01
het
hom
wt
Normalised Counts
100
150
200
zmp_ph108_F9
zmp_ph108_F11
zmp_ph108_H2
zmp_ph108_H5
zmp_ph108_B2
zmp_ph108_B6
zmp_ph108_B7
zmp_ph108_C4
zmp_ph108_C5
zmp_ph108_D4
zmp_ph97_G6
zmp_ph97_G9
zmp_ph97_H2
zmp_ph97_B10
zmp_ph97_C2
zmp_ph97_C4
zmp_ph97_B8
zmp_ph97_C1
zmp_ph97_D2
zmp_ph97_D5
zmp_ph111_G5
zmp_ph111_G11
zmp_ph111_H1
zmp_ph111_B1a
zmp_ph111_B5
zmp_ph111_B8
zmp_ph111_B6
zmp_ph111_B7
zmp_ph111_B9
zmp_ph111_B10
zmp_ph114_G4
zmp_ph114_G11
zmp_ph114_H1
zmp_ph114_H2
zmp_ph114_A9
zmp_ph114_A11
zmp_ph114_B2
zmp_ph114_B4
zmp_ph114_D8
zmp_ph114_E2

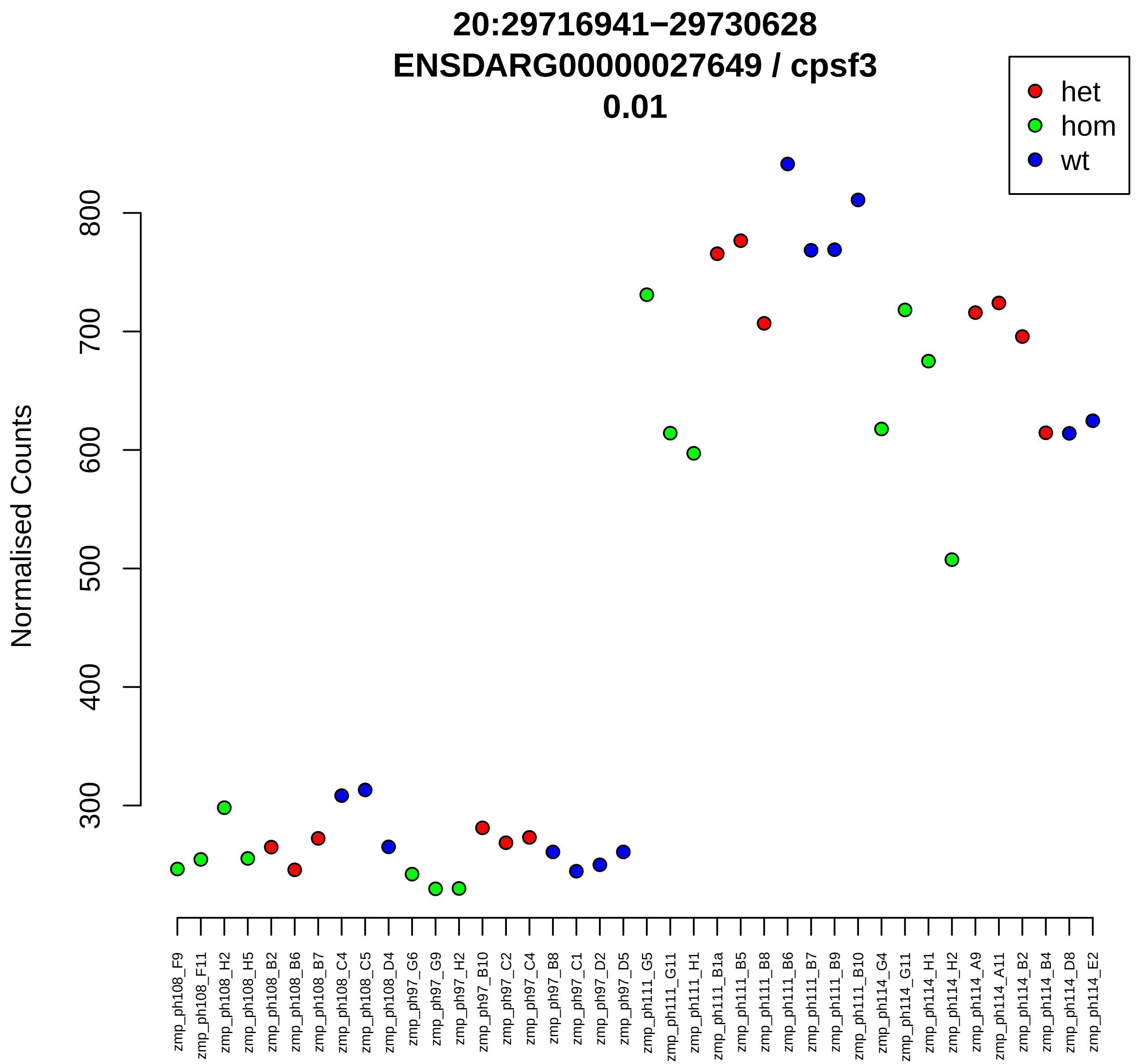

20:29716941−29730628
ENSDARG00000027649 / cpsf3
0.01
het
hom
wt
Normalised Counts
300
400
500
600
700
800
zmp_ph108_F9
zmp_ph108_F11
zmp_ph108_H2
zmp_ph108_H5
zmp_ph108_B2
zmp_ph108_B6
zmp_ph108_B7
zmp_ph108_C4
zmp_ph108_C5
zmp_ph108_D4
zmp_ph97_G6
zmp_ph97_G9
zmp_ph97_H2
zmp_ph97_B10
zmp_ph97_C2
zmp_ph97_C4
zmp_ph97_B8
zmp_ph97_C1
zmp_ph97_D2
zmp_ph97_D5
zmp_ph111_G5
zmp_ph111_G11
zmp_ph111_H1
zmp_ph111_B1a
zmp_ph111_B5
zmp_ph111_B8
zmp_ph111_B6
zmp_ph111_B7
zmp_ph111_B9
zmp_ph111_B10
zmp_ph114_G4
zmp_ph114_G11
zmp_ph114_H1
zmp_ph114_H2
zmp_ph114_A9
zmp_ph114_A11
zmp_ph114_B2
zmp_ph114_B4
zmp_ph114_D8
zmp_ph114_E2

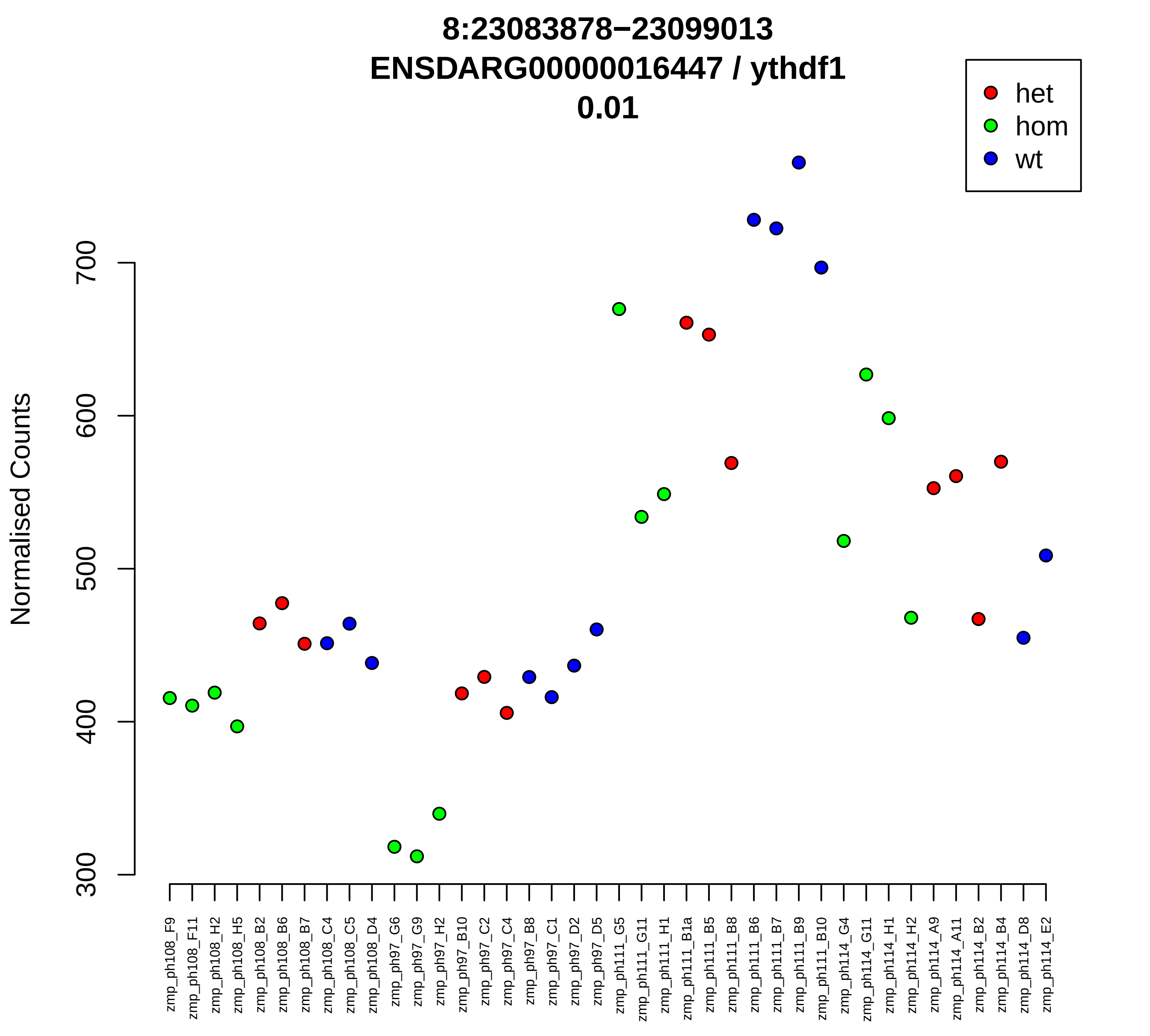

8:23083878−23099013
ENSDARG00000016447 / ythdf1
0.01
het
hom
wt
Normalised Counts
300
400
500
600
700
zmp_ph108_F9
zmp_ph108_F11
zmp_ph108_H2
zmp_ph108_H5
zmp_ph108_B2
zmp_ph108_B6
zmp_ph108_B7
zmp_ph108_C4
zmp_ph108_C5
zmp_ph108_D4
zmp_ph97_G6
zmp_ph97_G9
zmp_ph97_H2
zmp_ph97_B10
zmp_ph97_C2
zmp_ph97_C4
zmp_ph97_B8
zmp_ph97_C1
zmp_ph97_D2
zmp_ph97_D5
zmp_ph111_G5
zmp_ph111_G11
zmp_ph111_H1
zmp_ph111_B1a
zmp_ph111_B5
zmp_ph111_B8
zmp_ph111_B6
zmp_ph111_B7
zmp_ph111_B9
zmp_ph111_B10
zmp_ph114_G4
zmp_ph114_G11
zmp_ph114_H1
zmp_ph114_H2
zmp_ph114_A9
zmp_ph114_A11
zmp_ph114_B2
zmp_ph114_B4
zmp_ph114_D8
zmp_ph114_E2

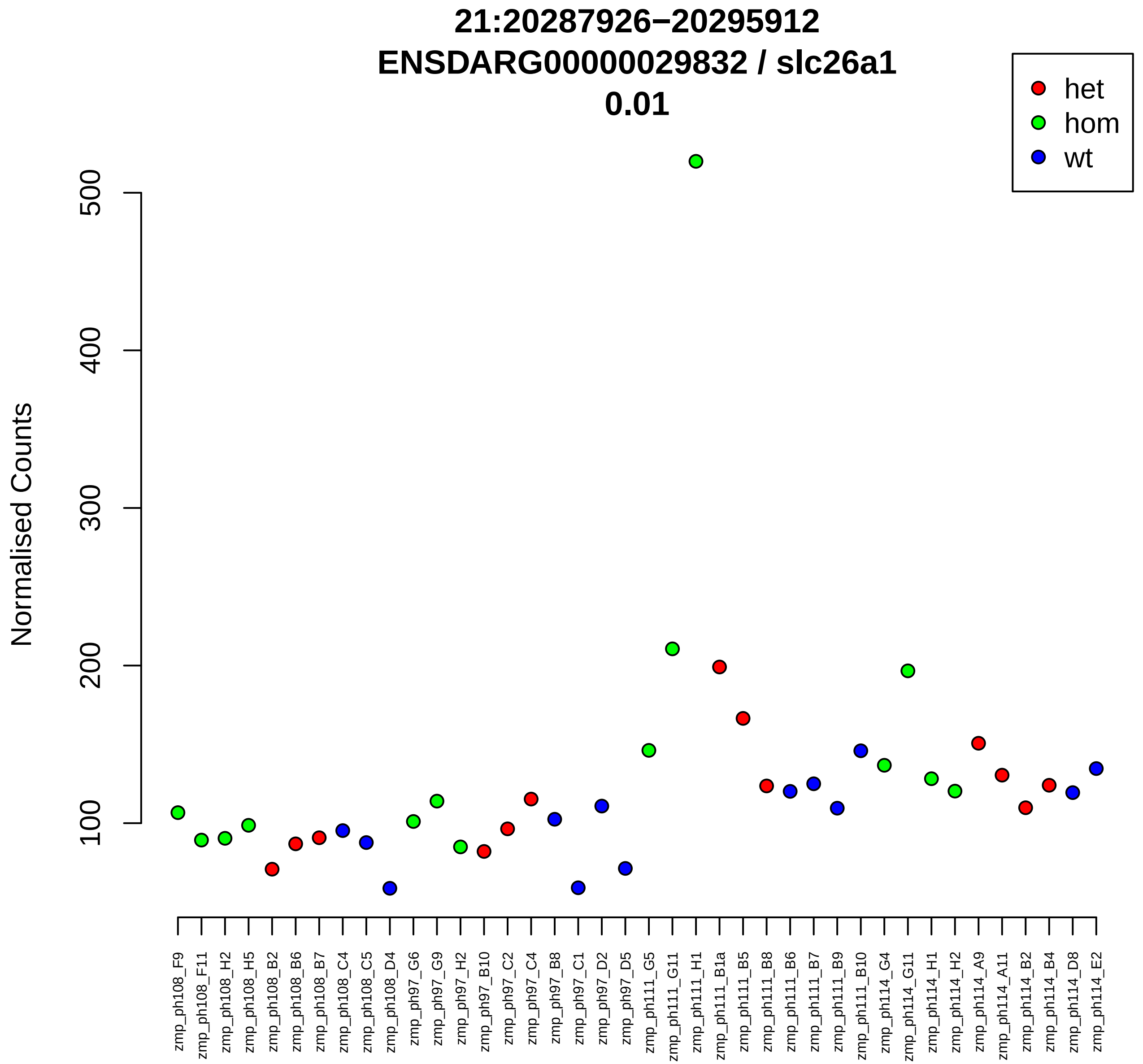
21:20287926−20295912
ENSDARG00000029832 / slc26a1
0.01
het
hom
wt
Normalised Counts
100
200
300
400
500
zmp_ph108_F9
zmp_ph108_F11
zmp_ph108_H2
zmp_ph108_H5
zmp_ph108_B2
zmp_ph108_B6
zmp_ph108_B7
zmp_ph108_C4
zmp_ph108_C5
zmp_ph108_D4
zmp_ph97_G6
zmp_ph97_G9
zmp_ph97_H2
zmp_ph97_B10
zmp_ph97_C2
zmp_ph97_C4
zmp_ph97_B8
zmp_ph97_C1
zmp_ph97_D2
zmp_ph97_D5
zmp_ph111_G5
zmp_ph111_G11
zmp_ph111_H1
zmp_ph111_B1a
zmp_ph111_B5
zmp_ph111_B8
zmp_ph111_B6
zmp_ph111_B7
zmp_ph111_B9
zmp_ph111_B10
zmp_ph114_G4
zmp_ph114_G11
zmp_ph114_H1
zmp_ph114_H2
zmp_ph114_A9
zmp_ph114_A11
zmp_ph114_B2
zmp_ph114_B4
zmp_ph114_D8
zmp_ph114_E2

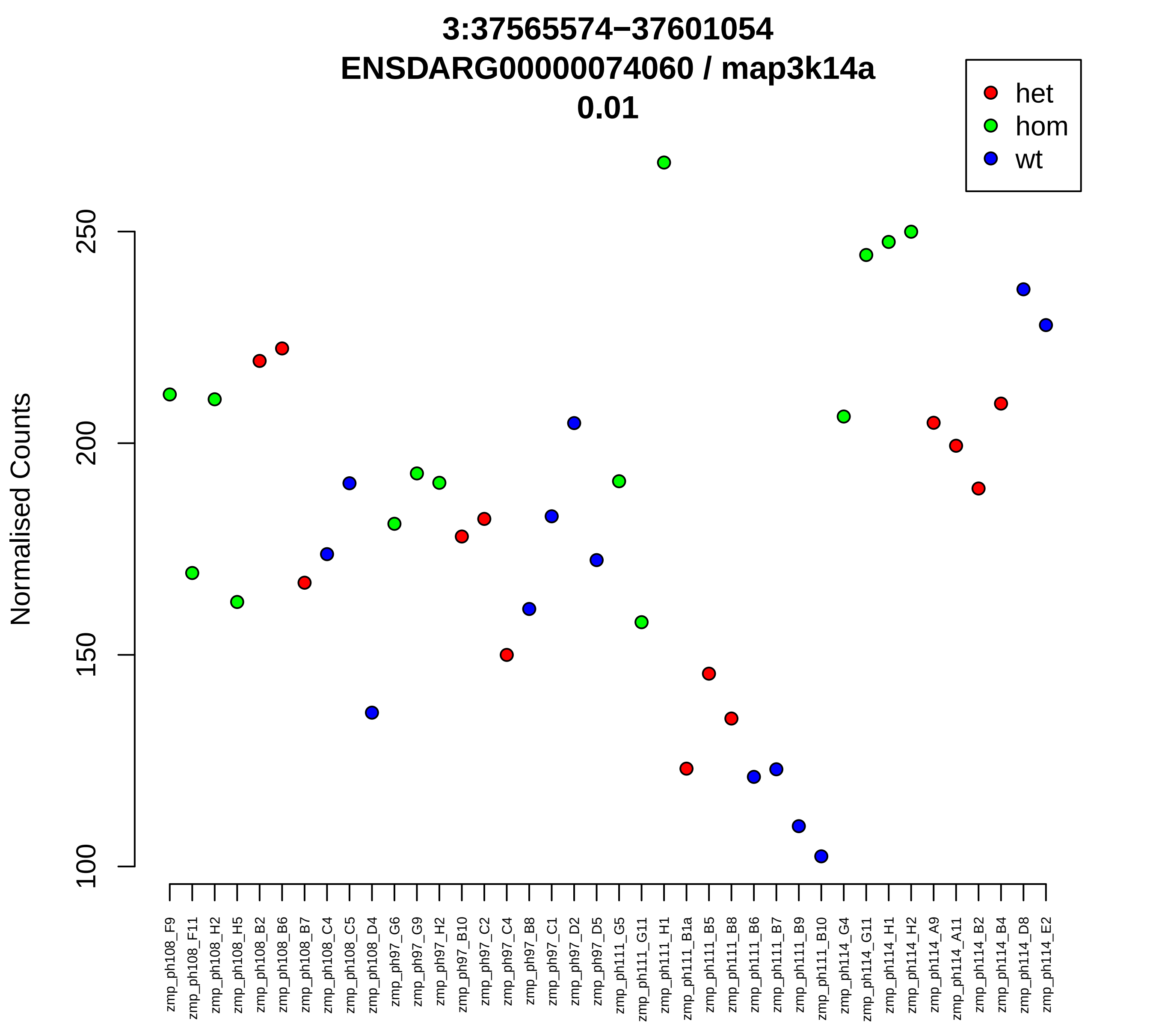

3:37565574−37601054
ENSDARG00000074060 / map3k14a
0.01
het
hom
wt
Normalised Counts
100
150
200
250
zmp_ph108_F9
zmp_ph108_F11
zmp_ph108_H2
zmp_ph108_H5
zmp_ph108_B2
zmp_ph108_B6
zmp_ph108_B7
zmp_ph108_C4
zmp_ph108_C5
zmp_ph108_D4
zmp_ph97_G6
zmp_ph97_G9
zmp_ph97_H2
zmp_ph97_B10
zmp_ph97_C2
zmp_ph97_C4
zmp_ph97_B8
zmp_ph97_C1
zmp_ph97_D2
zmp_ph97_D5
zmp_ph111_G5
zmp_ph111_G11
zmp_ph111_H1
zmp_ph111_B1a
zmp_ph111_B5
zmp_ph111_B8
zmp_ph111_B6
zmp_ph111_B7
zmp_ph111_B9
zmp_ph111_B10
zmp_ph114_G4
zmp_ph114_G11
zmp_ph114_H1
zmp_ph114_H2
zmp_ph114_A9
zmp_ph114_A11
zmp_ph114_B2
zmp_ph114_B4
zmp_ph114_D8
zmp_ph114_E2

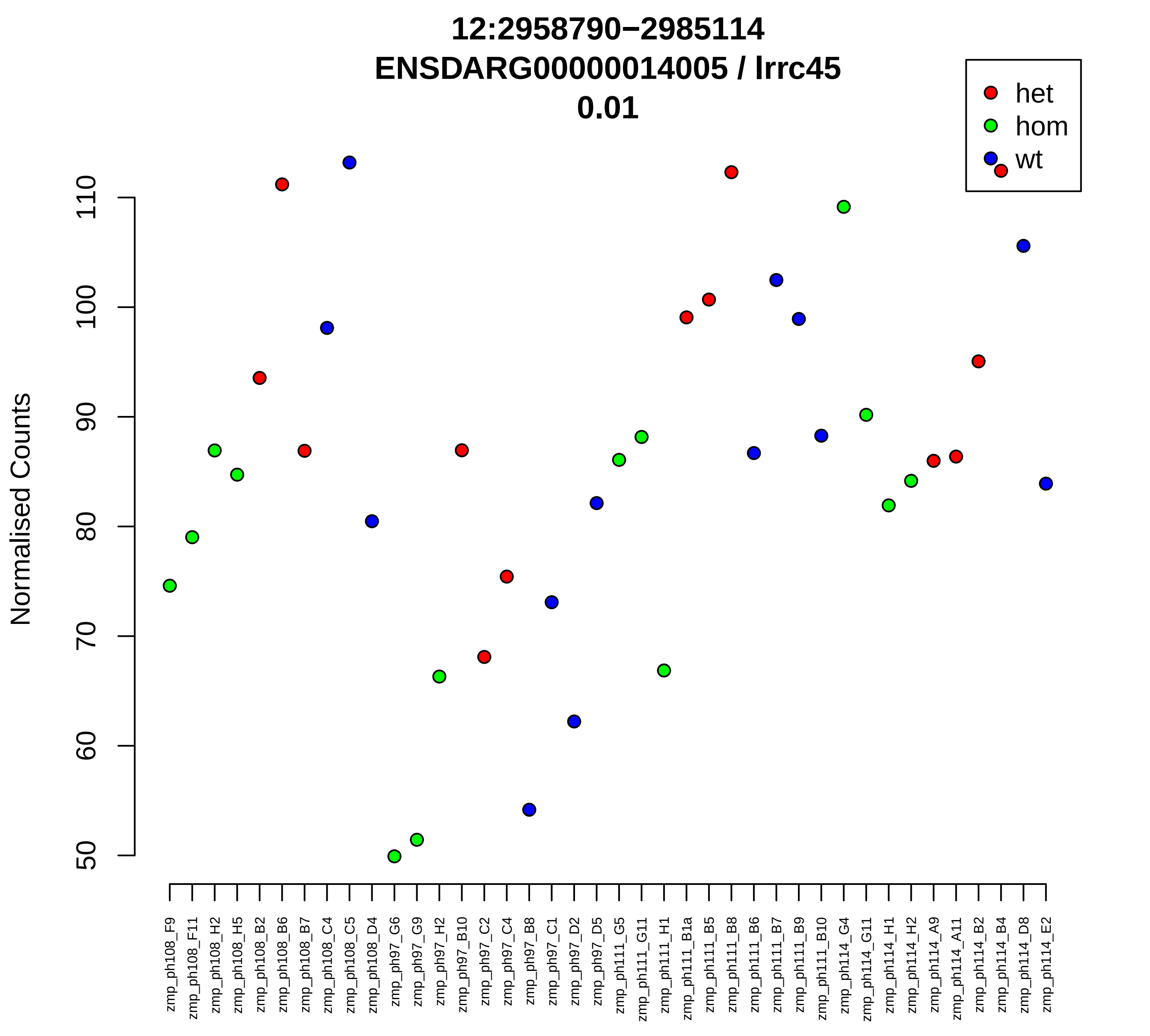
12:2958790−2985114
ENSDARG00000014005 / lrrc45
0.01
het
hom
wt
Normalised Counts
50
60
70
80
90
100
110
zmp_ph108_F9
zmp_ph108_F11
zmp_ph108_H2
zmp_ph108_H5
zmp_ph108_B2
zmp_ph108_B6
zmp_ph108_B7
zmp_ph108_C4
zmp_ph108_C5
zmp_ph108_D4
zmp_ph97_G6
zmp_ph97_G9
zmp_ph97_H2
zmp_ph97_B10
zmp_ph97_C2
zmp_ph97_C4
zmp_ph97_B8
zmp_ph97_C1
zmp_ph97_D2
zmp_ph97_D5
zmp_ph111_G5
zmp_ph111_G11
zmp_ph111_H1
zmp_ph111_B1a
zmp_ph111_B5
zmp_ph111_B8
zmp_ph111_B6
zmp_ph111_B7
zmp_ph111_B9
zmp_ph111_B10
zmp_ph114_G4
zmp_ph114_G11
zmp_ph114_H1
zmp_ph114_H2
zmp_ph114_A9
zmp_ph114_A11
zmp_ph114_B2
zmp_ph114_B4
zmp_ph114_D8
zmp_ph114_E2

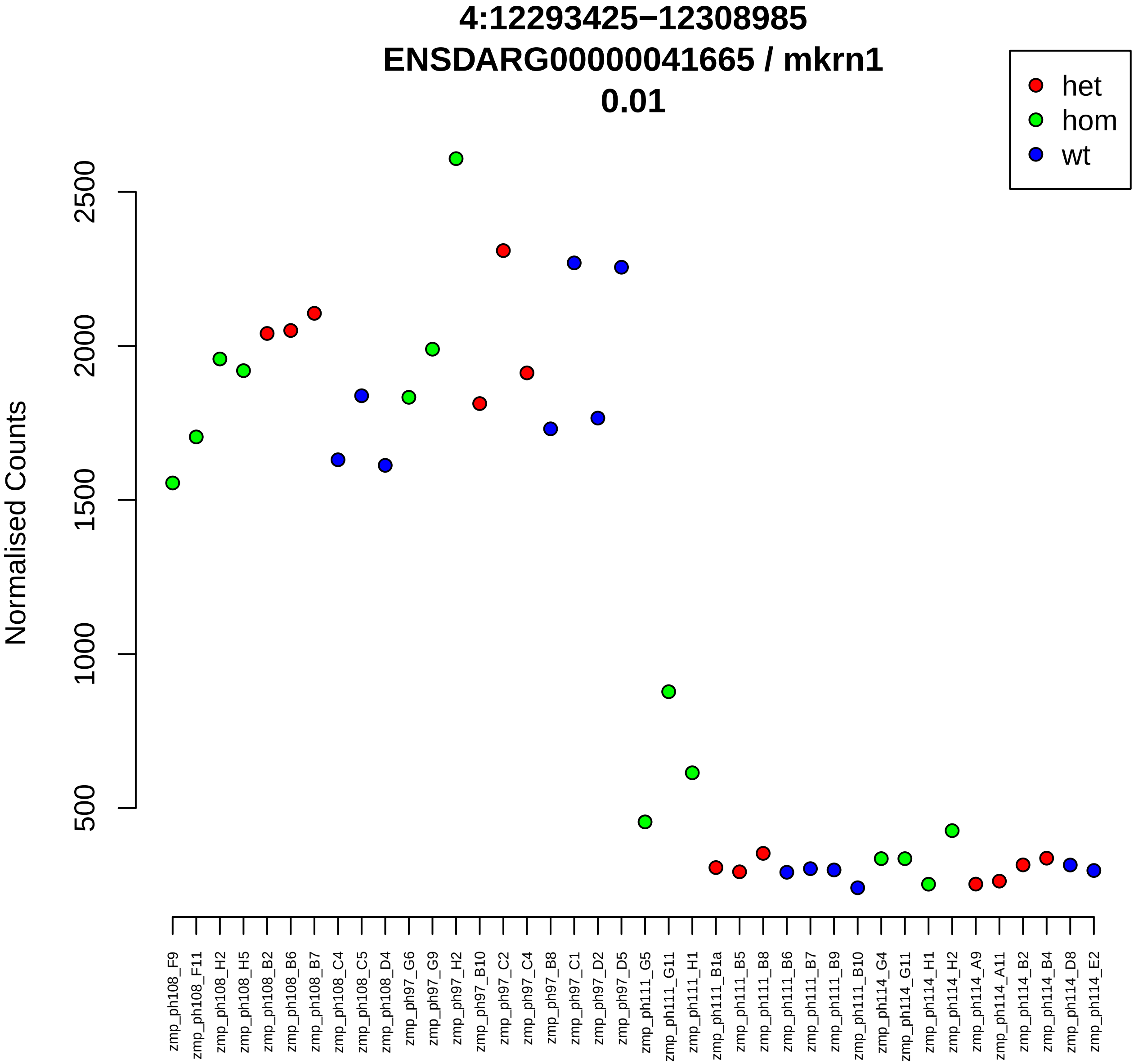

4:12293425−12308985
ENSDARG00000041665 / mkrn1
0.01
het
hom
wt
Normalised Counts
500
1000
1500
2000
2500
zmp_ph108_F9
zmp_ph108_F11
zmp_ph108_H2
zmp_ph108_H5
zmp_ph108_B2
zmp_ph108_B6
zmp_ph108_B7
zmp_ph108_C4
zmp_ph108_C5
zmp_ph108_D4
zmp_ph97_G6
zmp_ph97_G9
zmp_ph97_H2
zmp_ph97_B10
zmp_ph97_C2
zmp_ph97_C4
zmp_ph97_B8
zmp_ph97_C1
zmp_ph97_D2
zmp_ph97_D5
zmp_ph111_G5
zmp_ph111_G11
zmp_ph111_H1
zmp_ph111_B1a
zmp_ph111_B5
zmp_ph111_B8
zmp_ph111_B6
zmp_ph111_B7
zmp_ph111_B9
zmp_ph111_B10
zmp_ph114_G4
zmp_ph114_G11
zmp_ph114_H1
zmp_ph114_H2
zmp_ph114_A9
zmp_ph114_A11
zmp_ph114_B2
zmp_ph114_B4
zmp_ph114_D8
zmp_ph114_E2

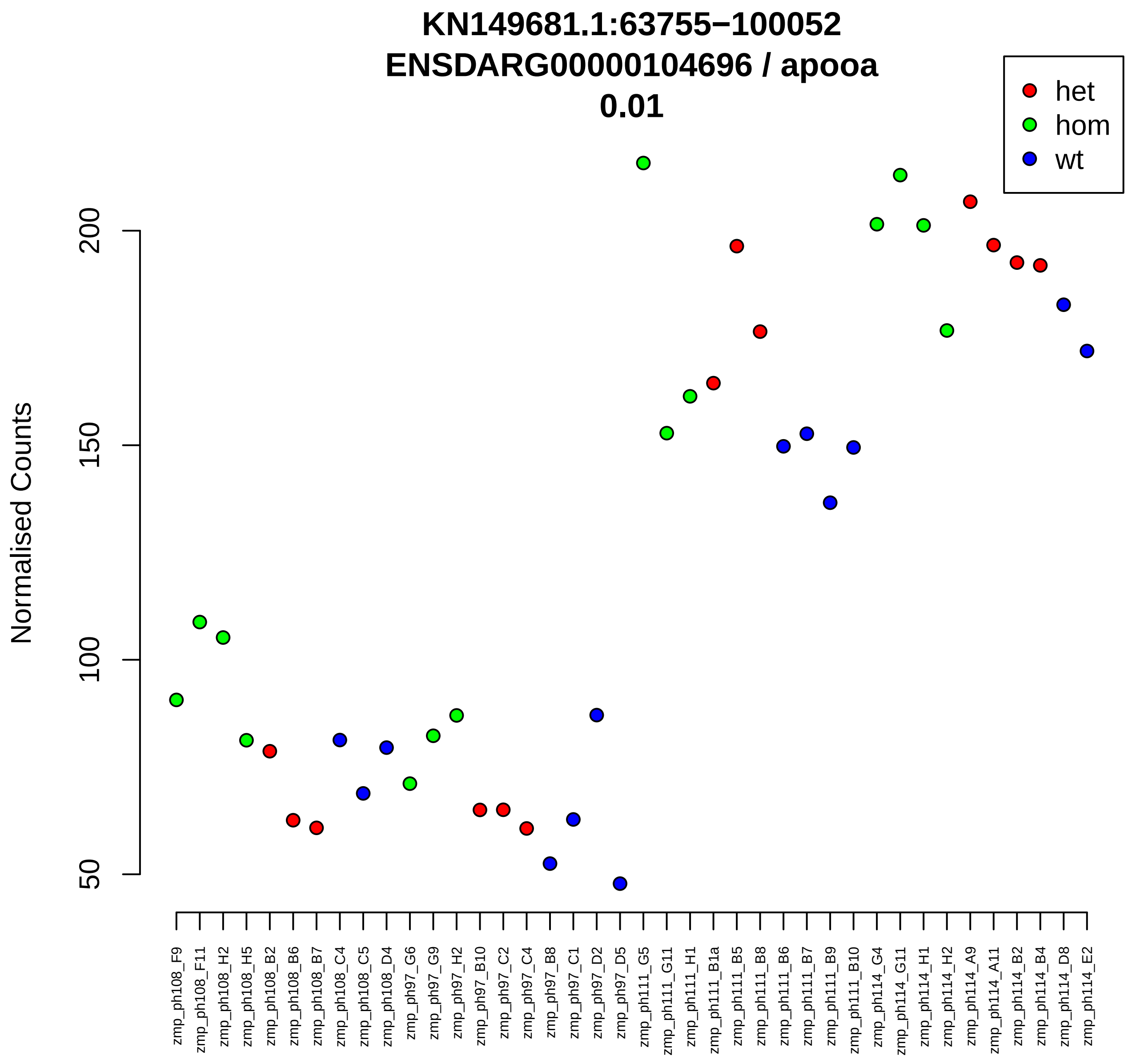
KN149681.1:63755−100052
ENSDARG00000104696 / apooa
0.01
het
hom
wt
Normalised Counts
50
100
150
200
zmp_ph108_F9
zmp_ph108_F11
zmp_ph108_H2
zmp_ph108_H5
zmp_ph108_B2
zmp_ph108_B6
zmp_ph108_B7
zmp_ph108_C4
zmp_ph108_C5
zmp_ph108_D4
zmp_ph97_G6
zmp_ph97_G9
zmp_ph97_H2
zmp_ph97_B10
zmp_ph97_C2
zmp_ph97_C4
zmp_ph97_B8
zmp_ph97_C1
zmp_ph97_D2
zmp_ph97_D5
zmp_ph111_G5
zmp_ph111_G11
zmp_ph111_H1
zmp_ph111_B1a
zmp_ph111_B5
zmp_ph111_B8
zmp_ph111_B6
zmp_ph111_B7
zmp_ph111_B9
zmp_ph111_B10
zmp_ph114_G4
zmp_ph114_G11
zmp_ph114_H1
zmp_ph114_H2
zmp_ph114_A9
zmp_ph114_A11
zmp_ph114_B2
zmp_ph114_B4
zmp_ph114_D8
zmp_ph114_E2

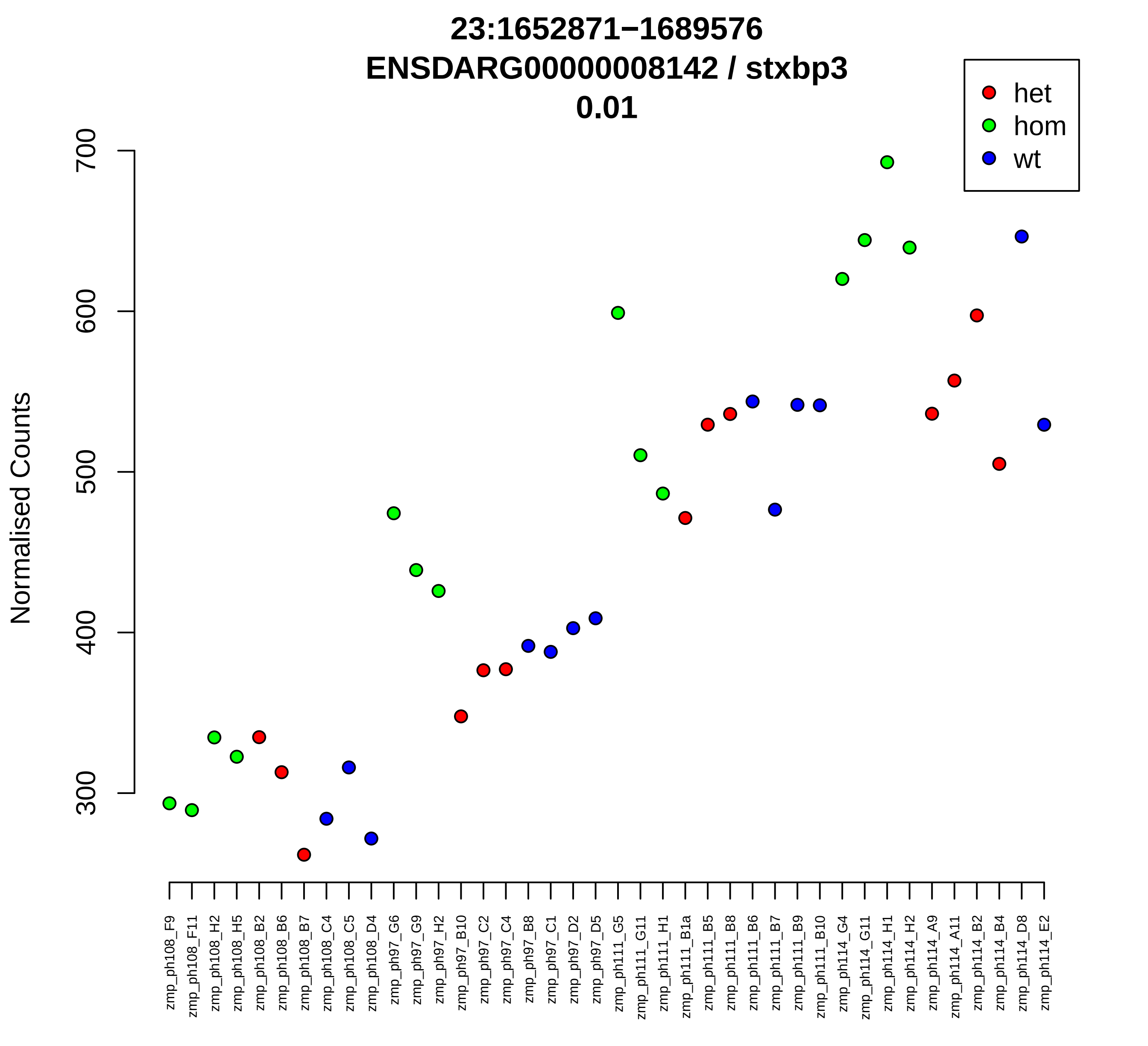
23:1652871−1689576
ENSDARG00000008142 / stxbp3
0.01
het
hom
wt
Normalised Counts
300
400
500
600
700
zmp_ph108_F9
zmp_ph108_F11
zmp_ph108_H2
zmp_ph108_H5
zmp_ph108_B2
zmp_ph108_B6
zmp_ph108_B7
zmp_ph108_C4
zmp_ph108_C5
zmp_ph108_D4
zmp_ph97_G6
zmp_ph97_G9
zmp_ph97_H2
zmp_ph97_B10
zmp_ph97_C2
zmp_ph97_C4
zmp_ph97_B8
zmp_ph97_C1
zmp_ph97_D2
zmp_ph97_D5
zmp_ph111_G5
zmp_ph111_G11
zmp_ph111_H1
zmp_ph111_B1a
zmp_ph111_B5
zmp_ph111_B8
zmp_ph111_B6
zmp_ph111_B7
zmp_ph111_B9
zmp_ph111_B10
zmp_ph114_G4
zmp_ph114_G11
zmp_ph114_H1
zmp_ph114_H2
zmp_ph114_A9
zmp_ph114_A11
zmp_ph114_B2
zmp_ph114_B4
zmp_ph114_D8
zmp_ph114_E2

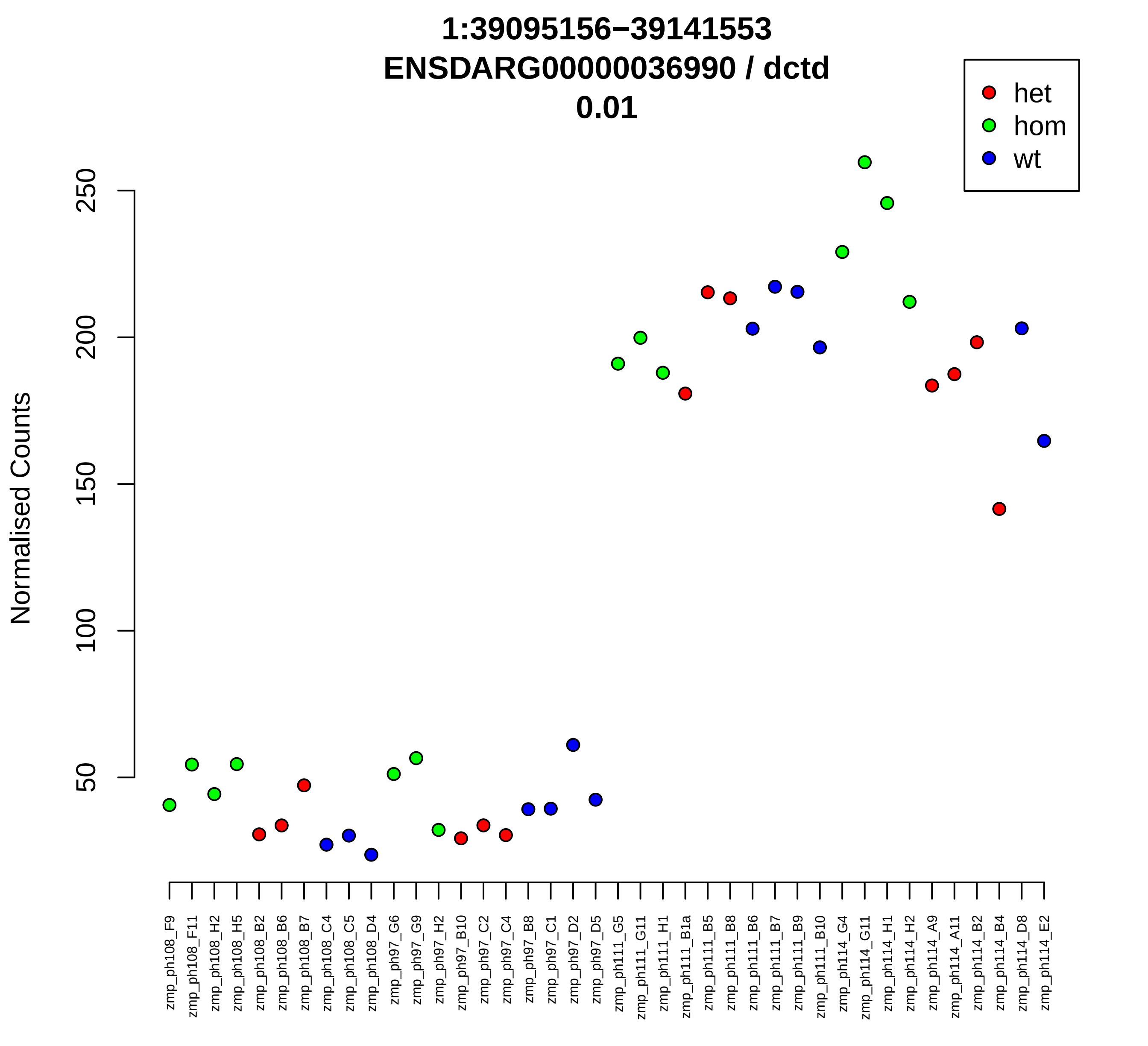

1:39095156−39141553
ENSDARG00000036990 / dctd
0.01
het
hom
wt
Normalised Counts
50
100
150
200
250
zmp_ph108_F9
zmp_ph108_F11
zmp_ph108_H2
zmp_ph108_H5
zmp_ph108_B2
zmp_ph108_B6
zmp_ph108_B7
zmp_ph108_C4
zmp_ph108_C5
zmp_ph108_D4
zmp_ph97_G6
zmp_ph97_G9
zmp_ph97_H2
zmp_ph97_B10
zmp_ph97_C2
zmp_ph97_C4
zmp_ph97_B8
zmp_ph97_C1
zmp_ph97_D2
zmp_ph97_D5
zmp_ph111_G5
zmp_ph111_G11
zmp_ph111_H1
zmp_ph111_B1a
zmp_ph111_B5
zmp_ph111_B8
zmp_ph111_B6
zmp_ph111_B7
zmp_ph111_B9
zmp_ph111_B10
zmp_ph114_G4
zmp_ph114_G11
zmp_ph114_H1
zmp_ph114_H2
zmp_ph114_A9
zmp_ph114_A11
zmp_ph114_B2
zmp_ph114_B4
zmp_ph114_D8
zmp_ph114_E2

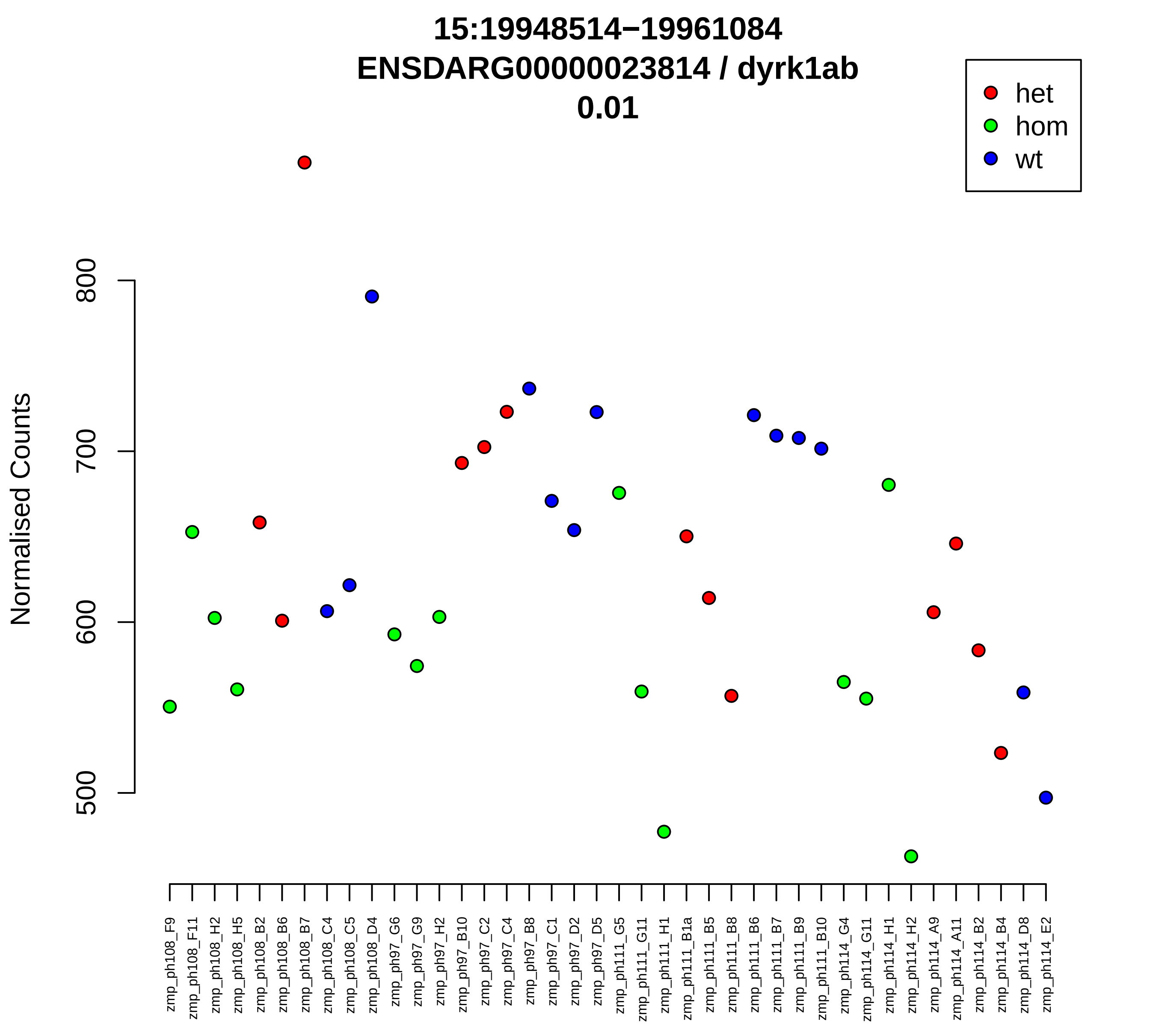

15:19948514−19961084
ENSDARG00000023814 / dyrk1ab
0.01
het
hom
wt
Normalised Counts
500
600
700
800
zmp_ph108_F9
zmp_ph108_F11
zmp_ph108_H2
zmp_ph108_H5
zmp_ph108_B2
zmp_ph108_B6
zmp_ph108_B7
zmp_ph108_C4
zmp_ph108_C5
zmp_ph108_D4
zmp_ph97_G6
zmp_ph97_G9
zmp_ph97_H2
zmp_ph97_B10
zmp_ph97_C2
zmp_ph97_C4
zmp_ph97_B8
zmp_ph97_C1
zmp_ph97_D2
zmp_ph97_D5
zmp_ph111_G5
zmp_ph111_G11
zmp_ph111_H1
zmp_ph111_B1a
zmp_ph111_B5
zmp_ph111_B8
zmp_ph111_B6
zmp_ph111_B7
zmp_ph111_B9
zmp_ph111_B10
zmp_ph114_G4
zmp_ph114_G11
zmp_ph114_H1
zmp_ph114_H2
zmp_ph114_A9
zmp_ph114_A11
zmp_ph114_B2
zmp_ph114_B4
zmp_ph114_D8
zmp_ph114_E2

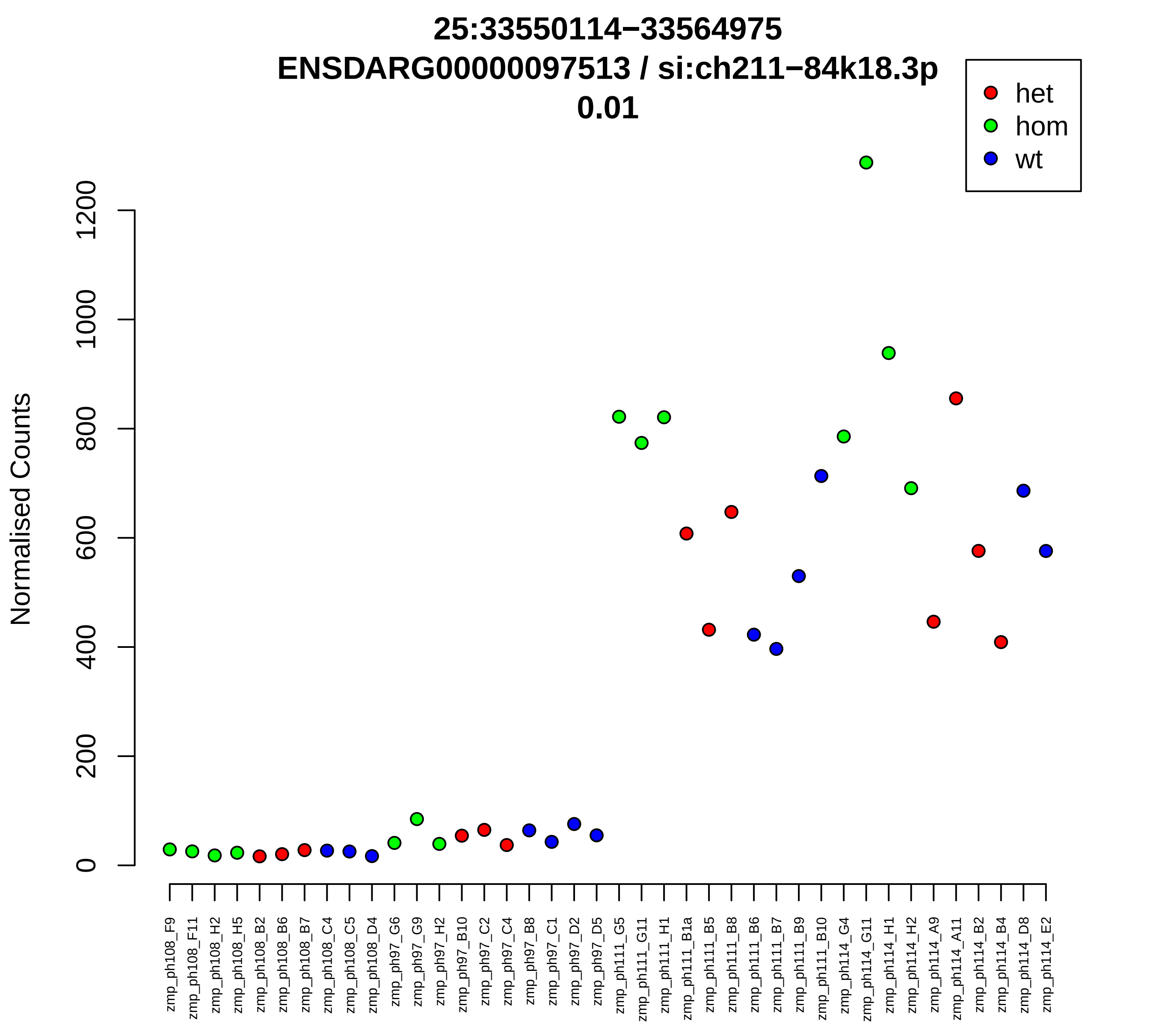

25:33550114−33564975
ENSDARG00000097513 / si:ch211−84k18.3p
0.01
het
hom
wt
Normalised Counts
0
200
400
600
800
1000
1200
zmp_ph108_F9
zmp_ph108_F11
zmp_ph108_H2
zmp_ph108_H5
zmp_ph108_B2
zmp_ph108_B6
zmp_ph108_B7
zmp_ph108_C4
zmp_ph108_C5
zmp_ph108_D4
zmp_ph97_G6
zmp_ph97_G9
zmp_ph97_H2
zmp_ph97_B10
zmp_ph97_C2
zmp_ph97_C4
zmp_ph97_B8
zmp_ph97_C1
zmp_ph97_D2
zmp_ph97_D5
zmp_ph111_G5
zmp_ph111_G11
zmp_ph111_H1
zmp_ph111_B1a
zmp_ph111_B5
zmp_ph111_B8
zmp_ph111_B6
zmp_ph111_B7
zmp_ph111_B9
zmp_ph111_B10
zmp_ph114_G4
zmp_ph114_G11
zmp_ph114_H1
zmp_ph114_H2
zmp_ph114_A9
zmp_ph114_A11
zmp_ph114_B2
zmp_ph114_B4
zmp_ph114_D8
zmp_ph114_E2

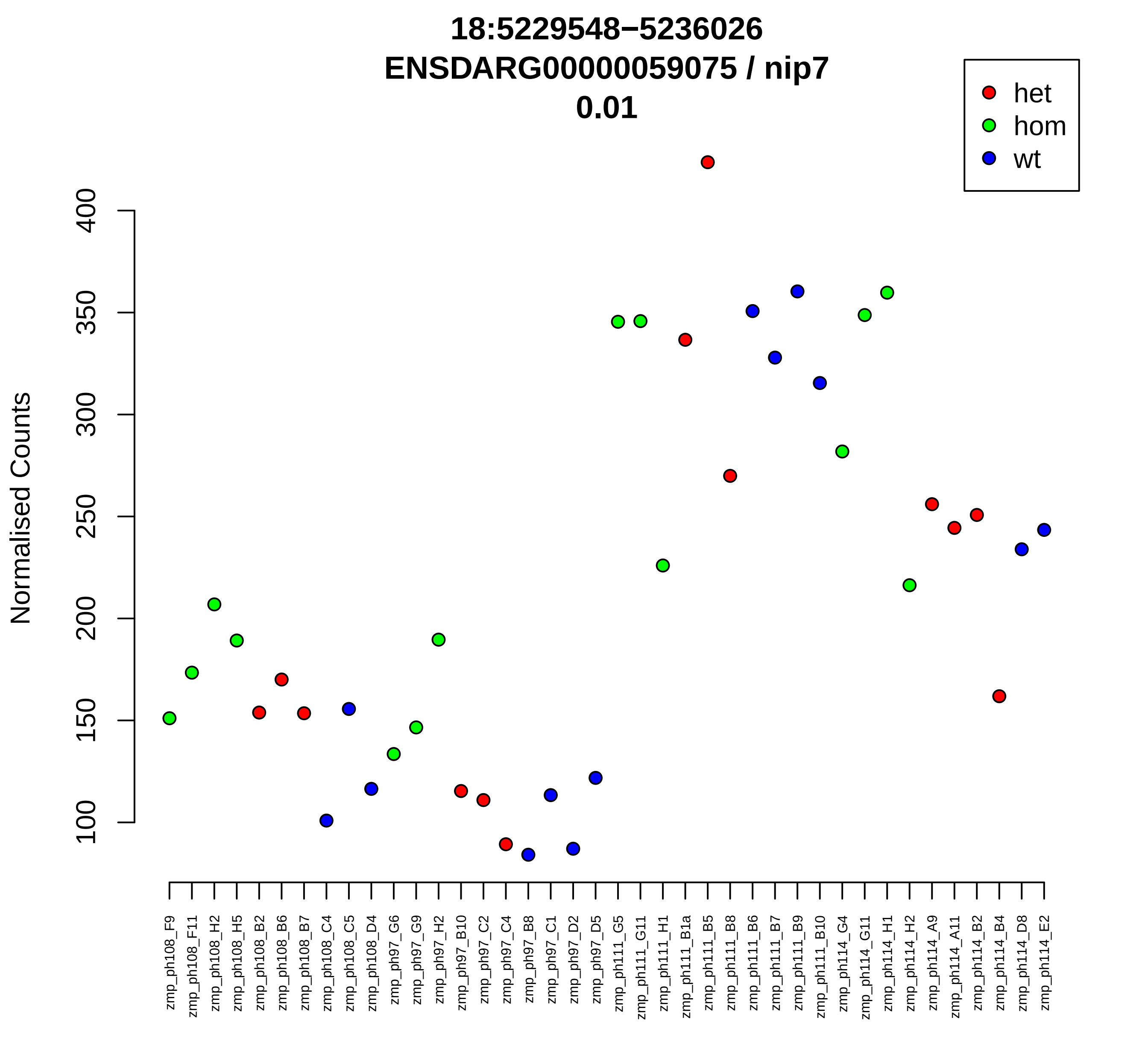
18:5229548−5236026
ENSDARG00000059075 / nip7
0.01
het
hom
wt
Normalised Counts
100
150
200
250
300
350
400
zmp_ph108_F9
zmp_ph108_F11
zmp_ph108_H2
zmp_ph108_H5
zmp_ph108_B2
zmp_ph108_B6
zmp_ph108_B7
zmp_ph108_C4
zmp_ph108_C5
zmp_ph108_D4
zmp_ph97_G6
zmp_ph97_G9
zmp_ph97_H2
zmp_ph97_B10
zmp_ph97_C2
zmp_ph97_C4
zmp_ph97_B8
zmp_ph97_C1
zmp_ph97_D2
zmp_ph97_D5
zmp_ph111_G5
zmp_ph111_G11
zmp_ph111_H1
zmp_ph111_B1a
zmp_ph111_B5
zmp_ph111_B8
zmp_ph111_B6
zmp_ph111_B7
zmp_ph111_B9
zmp_ph111_B10
zmp_ph114_G4
zmp_ph114_G11
zmp_ph114_H1
zmp_ph114_H2
zmp_ph114_A9
zmp_ph114_A11
zmp_ph114_B2
zmp_ph114_B4
zmp_ph114_D8
zmp_ph114_E2

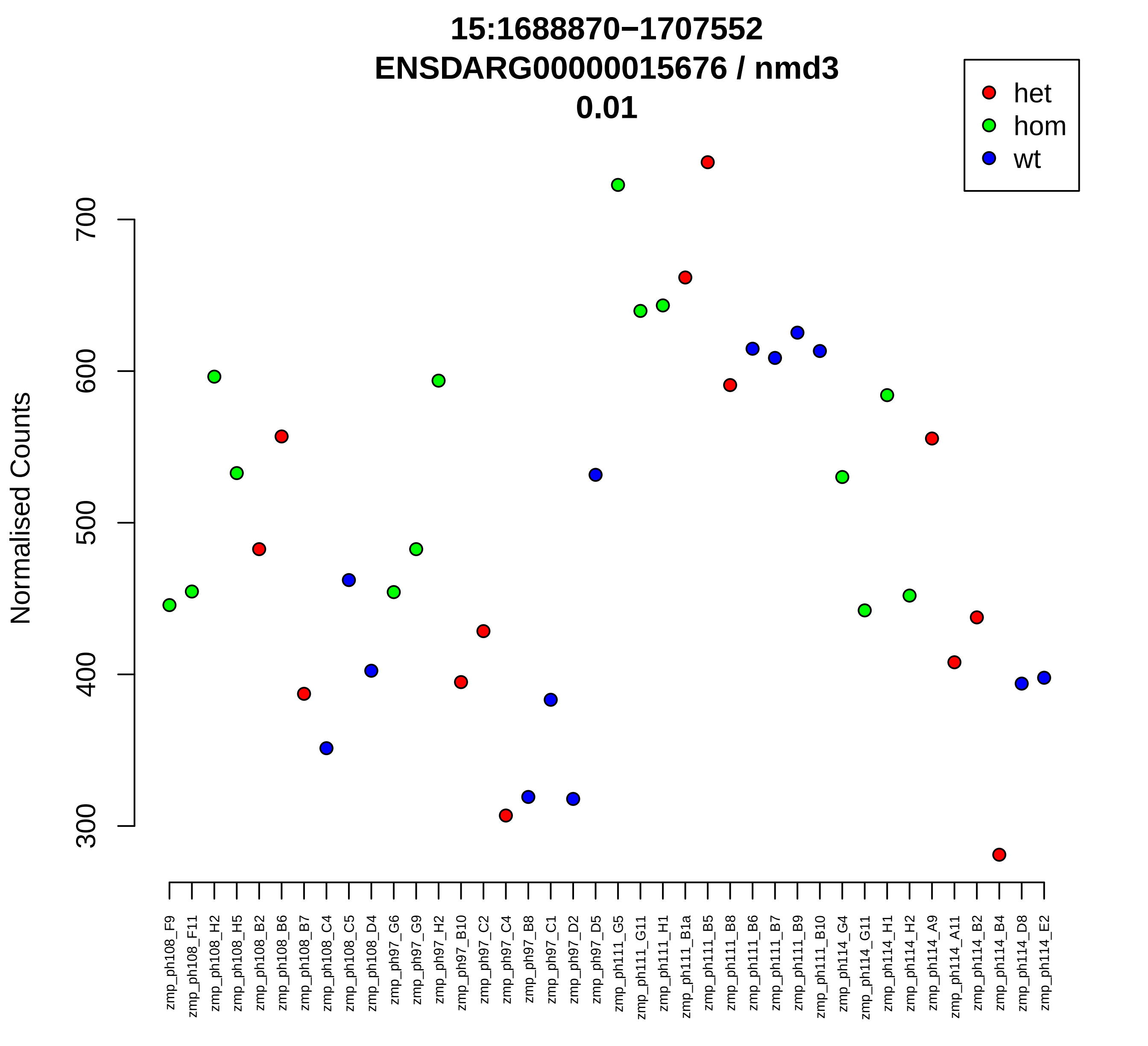

15:1688870−1707552
ENSDARG00000015676 / nmd3
0.01
het
hom
wt
Normalised Counts
300
400
500
600
700
zmp_ph108_F9
zmp_ph108_F11
zmp_ph108_H2
zmp_ph108_H5
zmp_ph108_B2
zmp_ph108_B6
zmp_ph108_B7
zmp_ph108_C4
zmp_ph108_C5
zmp_ph108_D4
zmp_ph97_G6
zmp_ph97_G9
zmp_ph97_H2
zmp_ph97_B10
zmp_ph97_C2
zmp_ph97_C4
zmp_ph97_B8
zmp_ph97_C1
zmp_ph97_D2
zmp_ph97_D5
zmp_ph111_G5
zmp_ph111_G11
zmp_ph111_H1
zmp_ph111_B1a
zmp_ph111_B5
zmp_ph111_B8
zmp_ph111_B6
zmp_ph111_B7
zmp_ph111_B9
zmp_ph111_B10
zmp_ph114_G4
zmp_ph114_G11
zmp_ph114_H1
zmp_ph114_H2
zmp_ph114_A9
zmp_ph114_A11
zmp_ph114_B2
zmp_ph114_B4
zmp_ph114_D8
zmp_ph114_E2

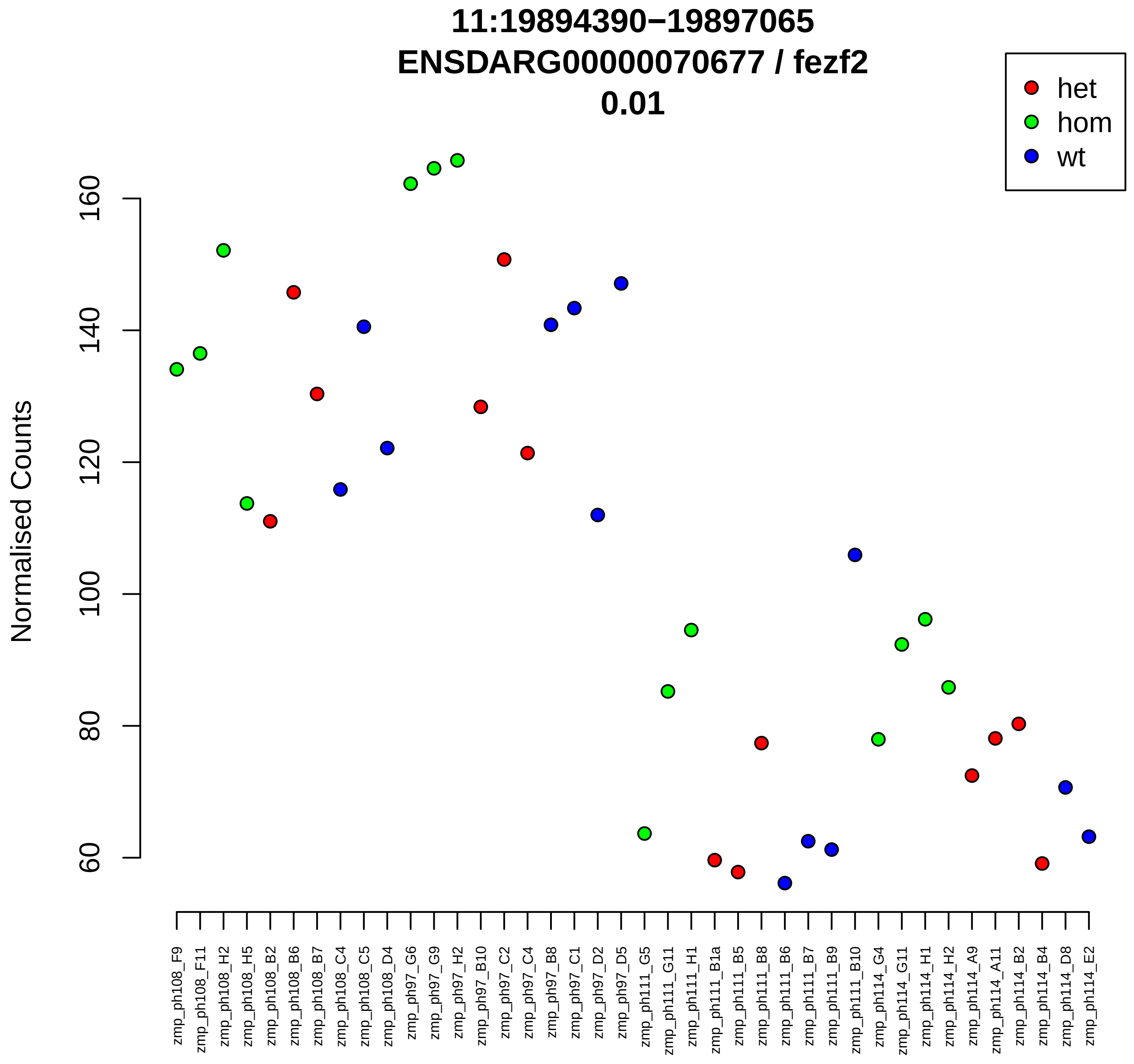
11:19894390−19897065
ENSDARG00000070677 / fezf2
0.01
het
hom
wt
Normalised Counts
60
80
100
120
140
160
zmp_ph108_F9
zmp_ph108_F11
zmp_ph108_H2
zmp_ph108_H5
zmp_ph108_B2
zmp_ph108_B6
zmp_ph108_B7
zmp_ph108_C4
zmp_ph108_C5
zmp_ph108_D4
zmp_ph97_G6
zmp_ph97_G9
zmp_ph97_H2
zmp_ph97_B10
zmp_ph97_C2
zmp_ph97_C4
zmp_ph97_B8
zmp_ph97_C1
zmp_ph97_D2
zmp_ph97_D5
zmp_ph111_G5
zmp_ph111_G11
zmp_ph111_H1
zmp_ph111_B1a
zmp_ph111_B5
zmp_ph111_B8
zmp_ph111_B6
zmp_ph111_B7
zmp_ph111_B9
zmp_ph111_B10
zmp_ph114_G4
zmp_ph114_G11
zmp_ph114_H1
zmp_ph114_H2
zmp_ph114_A9
zmp_ph114_A11
zmp_ph114_B2
zmp_ph114_B4
zmp_ph114_D8
zmp_ph114_E2

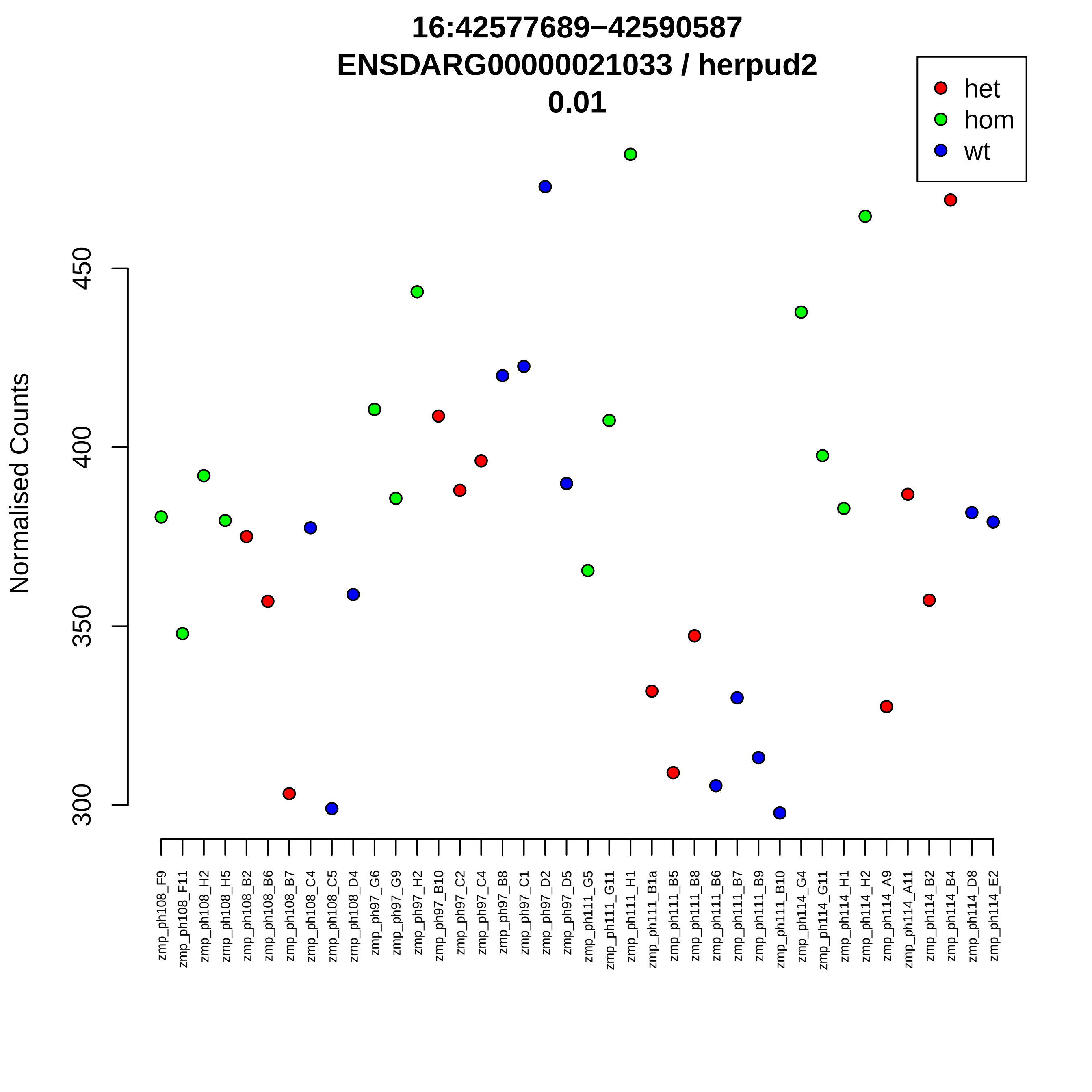
16:42577689−42590587
ENSDARG00000021033 / herpud2
0.01
het
hom
wt
Normalised Counts
300
350
400
450
zmp_ph108_F9
zmp_ph108_F11
zmp_ph108_H2
zmp_ph108_H5
zmp_ph108_B2
zmp_ph108_B6
zmp_ph108_B7
zmp_ph108_C4
zmp_ph108_C5
zmp_ph108_D4
zmp_ph97_G6
zmp_ph97_G9
zmp_ph97_H2
zmp_ph97_B10
zmp_ph97_C2
zmp_ph97_C4
zmp_ph97_B8
zmp_ph97_C1
zmp_ph97_D2
zmp_ph97_D5
zmp_ph111_G5
zmp_ph111_G11
zmp_ph111_H1
zmp_ph111_B1a
zmp_ph111_B5
zmp_ph111_B8
zmp_ph111_B6
zmp_ph111_B7
zmp_ph111_B9
zmp_ph111_B10
zmp_ph114_G4
zmp_ph114_G11
zmp_ph114_H1
zmp_ph114_H2
zmp_ph114_A9
zmp_ph114_A11
zmp_ph114_B2
zmp_ph114_B4
zmp_ph114_D8
zmp_ph114_E2

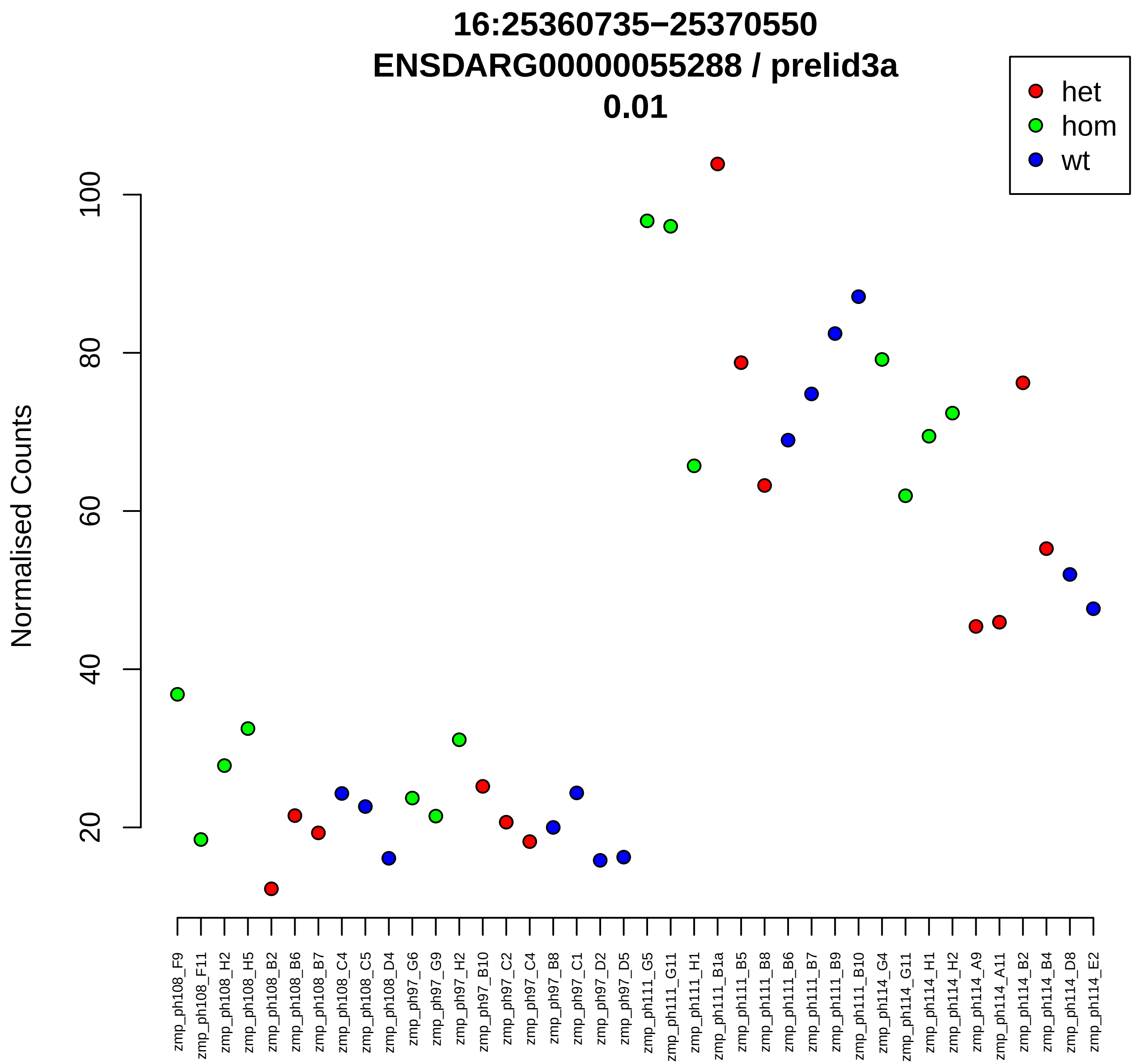
16:25360735−25370550
ENSDARG00000055288 / prelid3a
0.01
het
hom
wt
Normalised Counts
20
40
60
80
100
zmp_ph108_F9
zmp_ph108_F11
zmp_ph108_H2
zmp_ph108_H5
zmp_ph108_B2
zmp_ph108_B6
zmp_ph108_B7
zmp_ph108_C4
zmp_ph108_C5
zmp_ph108_D4
zmp_ph97_G6
zmp_ph97_G9
zmp_ph97_H2
zmp_ph97_B10
zmp_ph97_C2
zmp_ph97_C4
zmp_ph97_B8
zmp_ph97_C1
zmp_ph97_D2
zmp_ph97_D5
zmp_ph111_G5
zmp_ph111_G11
zmp_ph111_H1
zmp_ph111_B1a
zmp_ph111_B5
zmp_ph111_B8
zmp_ph111_B6
zmp_ph111_B7
zmp_ph111_B9
zmp_ph111_B10
zmp_ph114_G4
zmp_ph114_G11
zmp_ph114_H1
zmp_ph114_H2
zmp_ph114_A9
zmp_ph114_A11
zmp_ph114_B2
zmp_ph114_B4
zmp_ph114_D8
zmp_ph114_E2

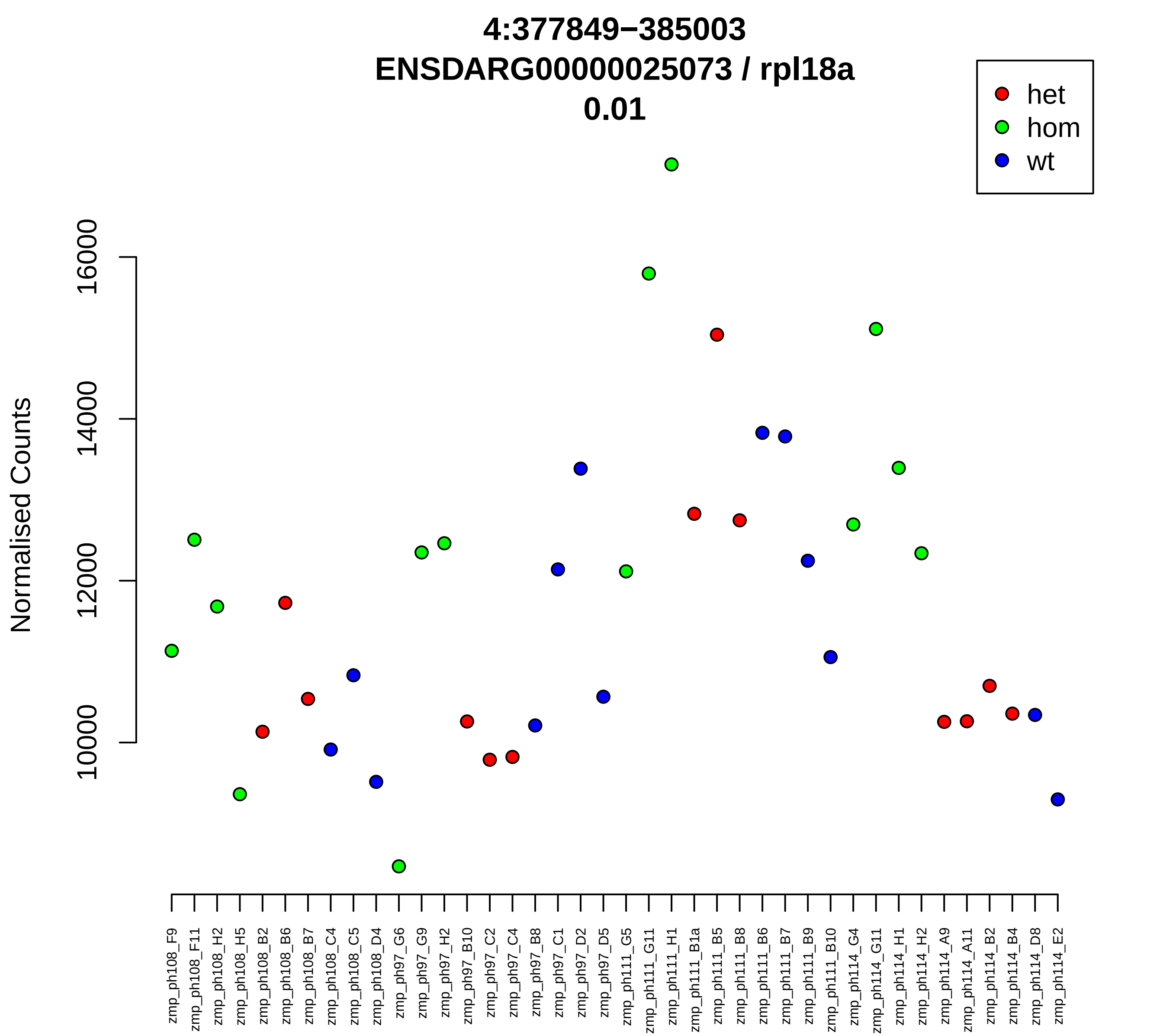

4:377849−385003
ENSDARG00000025073 / rpl18a
0.01
het
hom
wt
Normalised Counts
10000
12000
14000
16000
zmp_ph108_F9
zmp_ph108_F11
zmp_ph108_H2
zmp_ph108_H5
zmp_ph108_B2
zmp_ph108_B6
zmp_ph108_B7
zmp_ph108_C4
zmp_ph108_C5
zmp_ph108_D4
zmp_ph97_G6
zmp_ph97_G9
zmp_ph97_H2
zmp_ph97_B10
zmp_ph97_C2
zmp_ph97_C4
zmp_ph97_B8
zmp_ph97_C1
zmp_ph97_D2
zmp_ph97_D5
zmp_ph111_G5
zmp_ph111_G11
zmp_ph111_H1
zmp_ph111_B1a
zmp_ph111_B5
zmp_ph111_B8
zmp_ph111_B6
zmp_ph111_B7
zmp_ph111_B9
zmp_ph111_B10
zmp_ph114_G4
zmp_ph114_G11
zmp_ph114_H1
zmp_ph114_H2
zmp_ph114_A9
zmp_ph114_A11
zmp_ph114_B2
zmp_ph114_B4
zmp_ph114_D8
zmp_ph114_E2

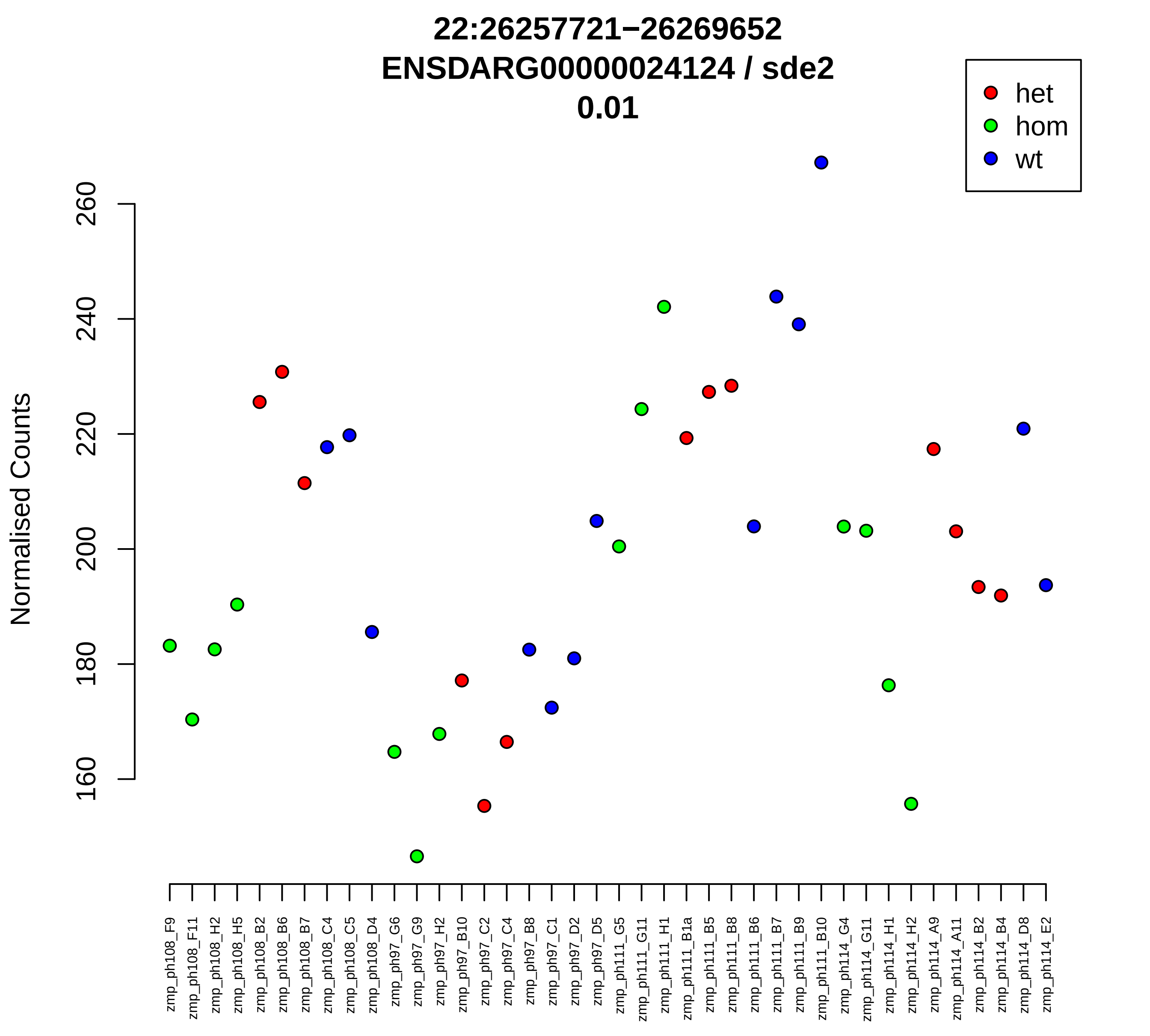

22:26257721−26269652
ENSDARG00000024124 / sde2
0.01
het
hom
wt
Normalised Counts
160
180
200
220
240
260
zmp_ph108_F9
zmp_ph108_F11
zmp_ph108_H2
zmp_ph108_H5
zmp_ph108_B2
zmp_ph108_B6
zmp_ph108_B7
zmp_ph108_C4
zmp_ph108_C5
zmp_ph108_D4
zmp_ph97_G6
zmp_ph97_G9
zmp_ph97_H2
zmp_ph97_B10
zmp_ph97_C2
zmp_ph97_C4
zmp_ph97_B8
zmp_ph97_C1
zmp_ph97_D2
zmp_ph97_D5
zmp_ph111_G5
zmp_ph111_G11
zmp_ph111_H1
zmp_ph111_B1a
zmp_ph111_B5
zmp_ph111_B8
zmp_ph111_B6
zmp_ph111_B7
zmp_ph111_B9
zmp_ph111_B10
zmp_ph114_G4
zmp_ph114_G11
zmp_ph114_H1
zmp_ph114_H2
zmp_ph114_A9
zmp_ph114_A11
zmp_ph114_B2
zmp_ph114_B4
zmp_ph114_D8
zmp_ph114_E2

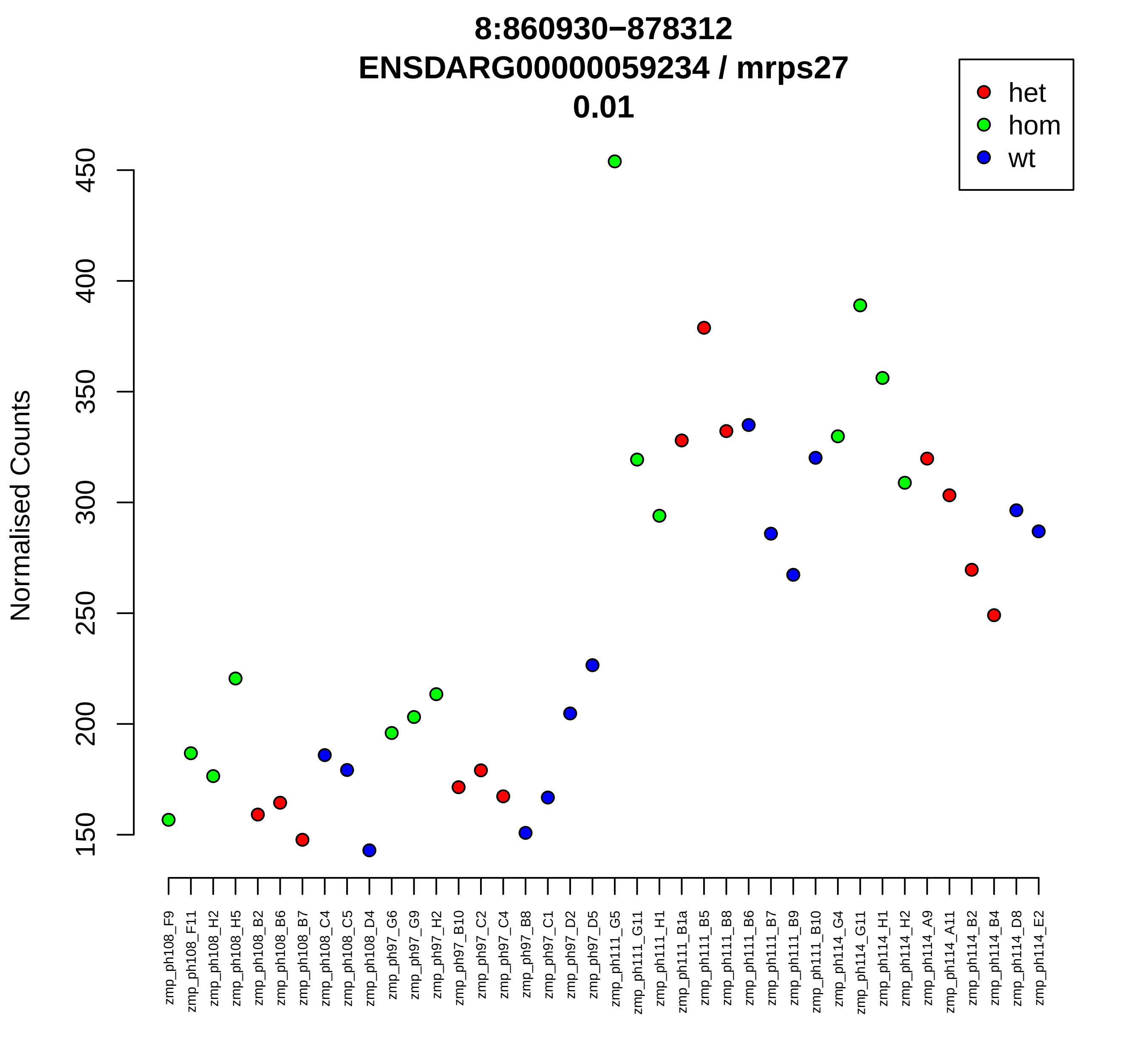
8:860930−878312
ENSDARG00000059234 / mrps27
0.01
het
hom
wt
Normalised Counts
150
200
250
300
350
400
450
zmp_ph108_F9
zmp_ph108_F11
zmp_ph108_H2
zmp_ph108_H5
zmp_ph108_B2
zmp_ph108_B6
zmp_ph108_B7
zmp_ph108_C4
zmp_ph108_C5
zmp_ph108_D4
zmp_ph97_G6
zmp_ph97_G9
zmp_ph97_H2
zmp_ph97_B10
zmp_ph97_C2
zmp_ph97_C4
zmp_ph97_B8
zmp_ph97_C1
zmp_ph97_D2
zmp_ph97_D5
zmp_ph111_G5
zmp_ph111_G11
zmp_ph111_H1
zmp_ph111_B1a
zmp_ph111_B5
zmp_ph111_B8
zmp_ph111_B6
zmp_ph111_B7
zmp_ph111_B9
zmp_ph111_B10
zmp_ph114_G4
zmp_ph114_G11
zmp_ph114_H1
zmp_ph114_H2
zmp_ph114_A9
zmp_ph114_A11
zmp_ph114_B2
zmp_ph114_B4
zmp_ph114_D8
zmp_ph114_E2

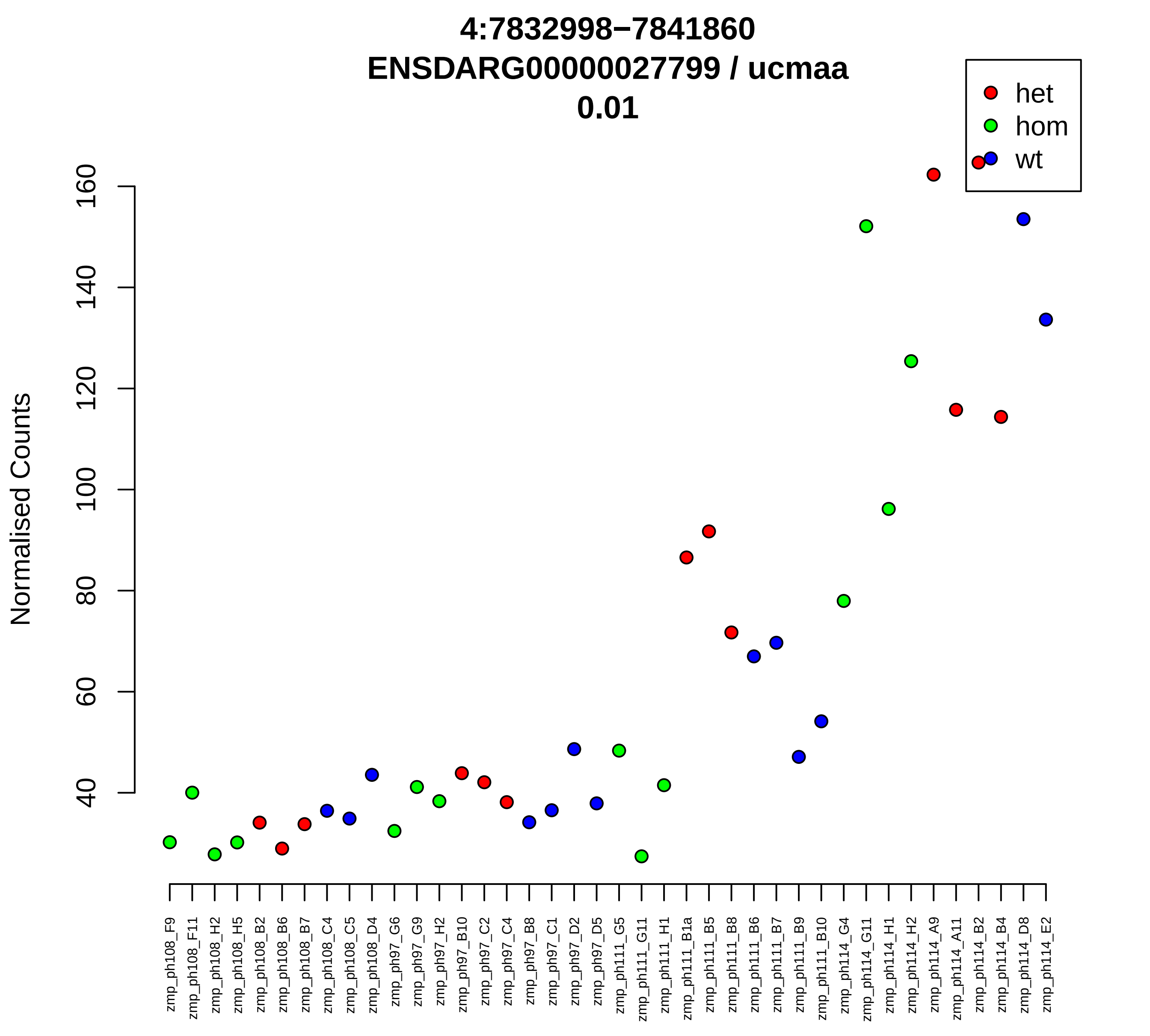

4:7832998−7841860
ENSDARG00000027799 / ucmaa
0.01
het
hom
wt
Normalised Counts
40
60
80
100
120
140
160
zmp_ph108_F9
zmp_ph108_F11
zmp_ph108_H2
zmp_ph108_H5
zmp_ph108_B2
zmp_ph108_B6
zmp_ph108_B7
zmp_ph108_C4
zmp_ph108_C5
zmp_ph108_D4
zmp_ph97_G6
zmp_ph97_G9
zmp_ph97_H2
zmp_ph97_B10
zmp_ph97_C2
zmp_ph97_C4
zmp_ph97_B8
zmp_ph97_C1
zmp_ph97_D2
zmp_ph97_D5
zmp_ph111_G5
zmp_ph111_G11
zmp_ph111_H1
zmp_ph111_B1a
zmp_ph111_B5
zmp_ph111_B8
zmp_ph111_B6
zmp_ph111_B7
zmp_ph111_B9
zmp_ph111_B10
zmp_ph114_G4
zmp_ph114_G11
zmp_ph114_H1
zmp_ph114_H2
zmp_ph114_A9
zmp_ph114_A11
zmp_ph114_B2
zmp_ph114_B4
zmp_ph114_D8
zmp_ph114_E2

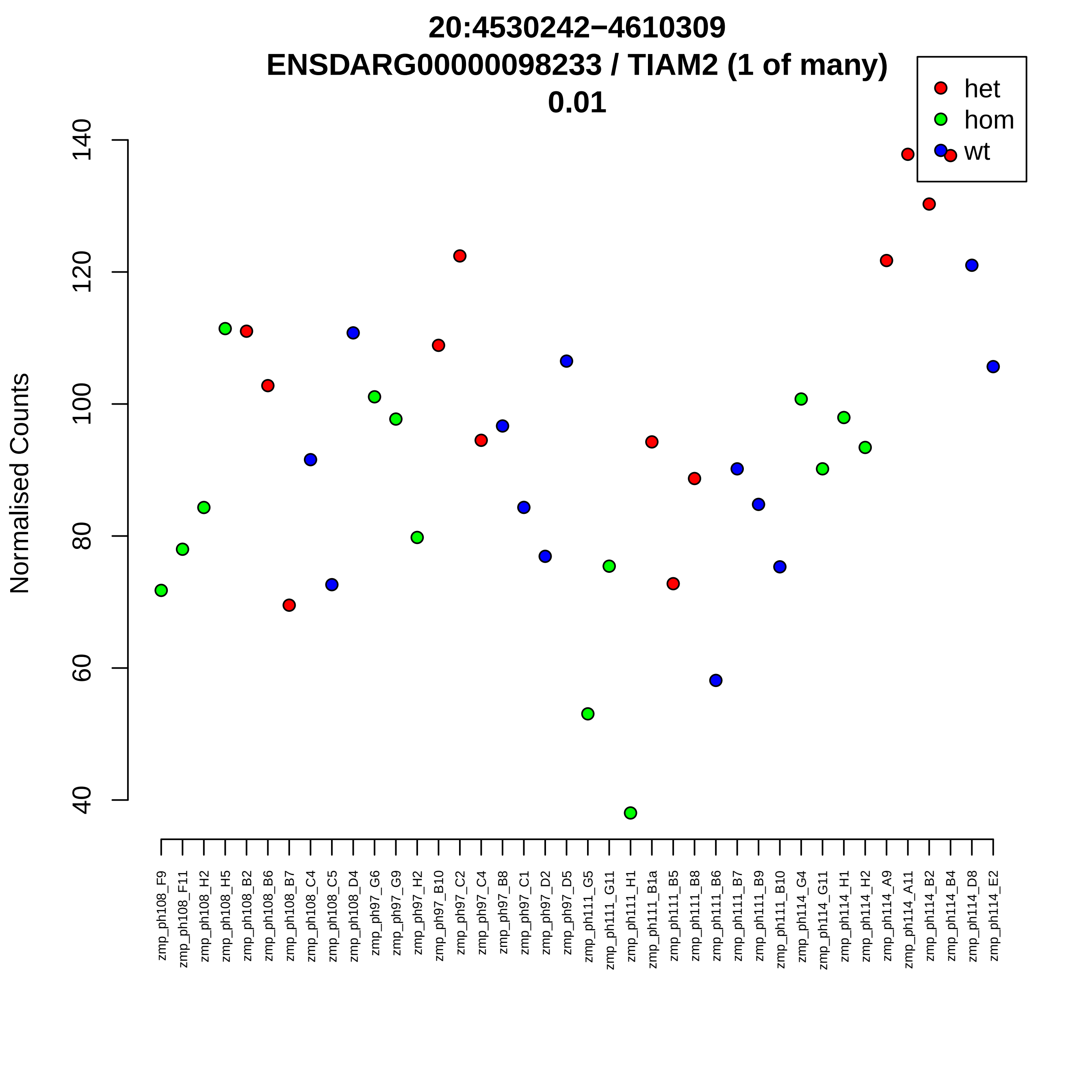

20:4530242−4610309
ENSDARG00000098233 / TIAM2 (1 of many)
0.01
het
hom
wt
Normalised Counts
40
60
80
100
120
140
zmp_ph108_F9
zmp_ph108_F11
zmp_ph108_H2
zmp_ph108_H5
zmp_ph108_B2
zmp_ph108_B6
zmp_ph108_B7
zmp_ph108_C4
zmp_ph108_C5
zmp_ph108_D4
zmp_ph97_G6
zmp_ph97_G9
zmp_ph97_H2
zmp_ph97_B10
zmp_ph97_C2
zmp_ph97_C4
zmp_ph97_B8
zmp_ph97_C1
zmp_ph97_D2
zmp_ph97_D5
zmp_ph111_G5
zmp_ph111_G11
zmp_ph111_H1
zmp_ph111_B1a
zmp_ph111_B5
zmp_ph111_B8
zmp_ph111_B6
zmp_ph111_B7
zmp_ph111_B9
zmp_ph111_B10
zmp_ph114_G4
zmp_ph114_G11
zmp_ph114_H1
zmp_ph114_H2
zmp_ph114_A9
zmp_ph114_A11
zmp_ph114_B2
zmp_ph114_B4
zmp_ph114_D8
zmp_ph114_E2

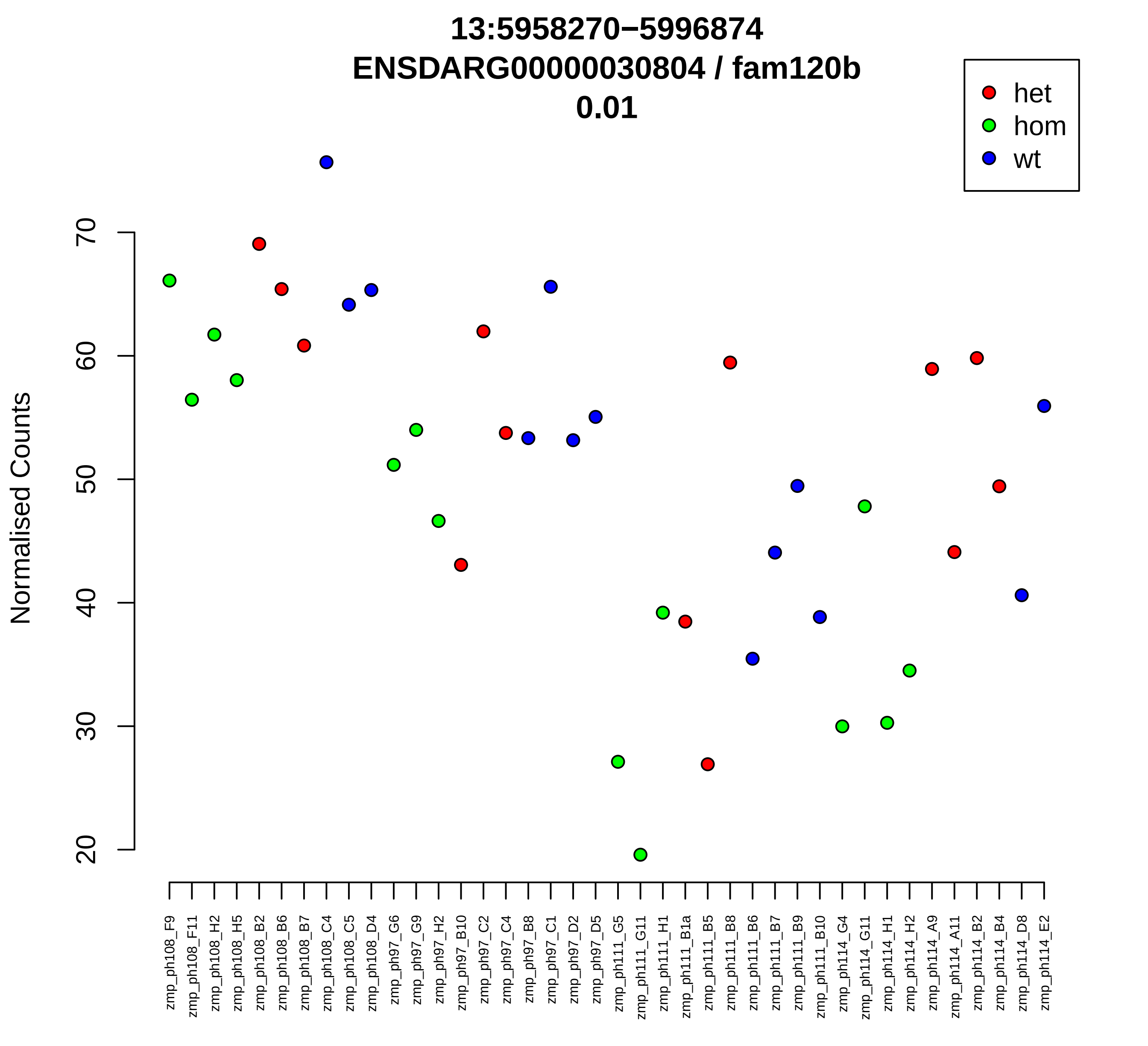
13:5958270−5996874
ENSDARG00000030804 / fam120b
0.01
het
hom
wt
Normalised Counts
20
30
40
50
60
70
zmp_ph108_F9
zmp_ph108_F11
zmp_ph108_H2
zmp_ph108_H5
zmp_ph108_B2
zmp_ph108_B6
zmp_ph108_B7
zmp_ph108_C4
zmp_ph108_C5
zmp_ph108_D4
zmp_ph97_G6
zmp_ph97_G9
zmp_ph97_H2
zmp_ph97_B10
zmp_ph97_C2
zmp_ph97_C4
zmp_ph97_B8
zmp_ph97_C1
zmp_ph97_D2
zmp_ph97_D5
zmp_ph111_G5
zmp_ph111_G11
zmp_ph111_H1
zmp_ph111_B1a
zmp_ph111_B5
zmp_ph111_B8
zmp_ph111_B6
zmp_ph111_B7
zmp_ph111_B9
zmp_ph111_B10
zmp_ph114_G4
zmp_ph114_G11
zmp_ph114_H1
zmp_ph114_H2
zmp_ph114_A9
zmp_ph114_A11
zmp_ph114_B2
zmp_ph114_B4
zmp_ph114_D8
zmp_ph114_E2

10:11439873−11543186
ENSDARG00000043727 / cwc27
0.01

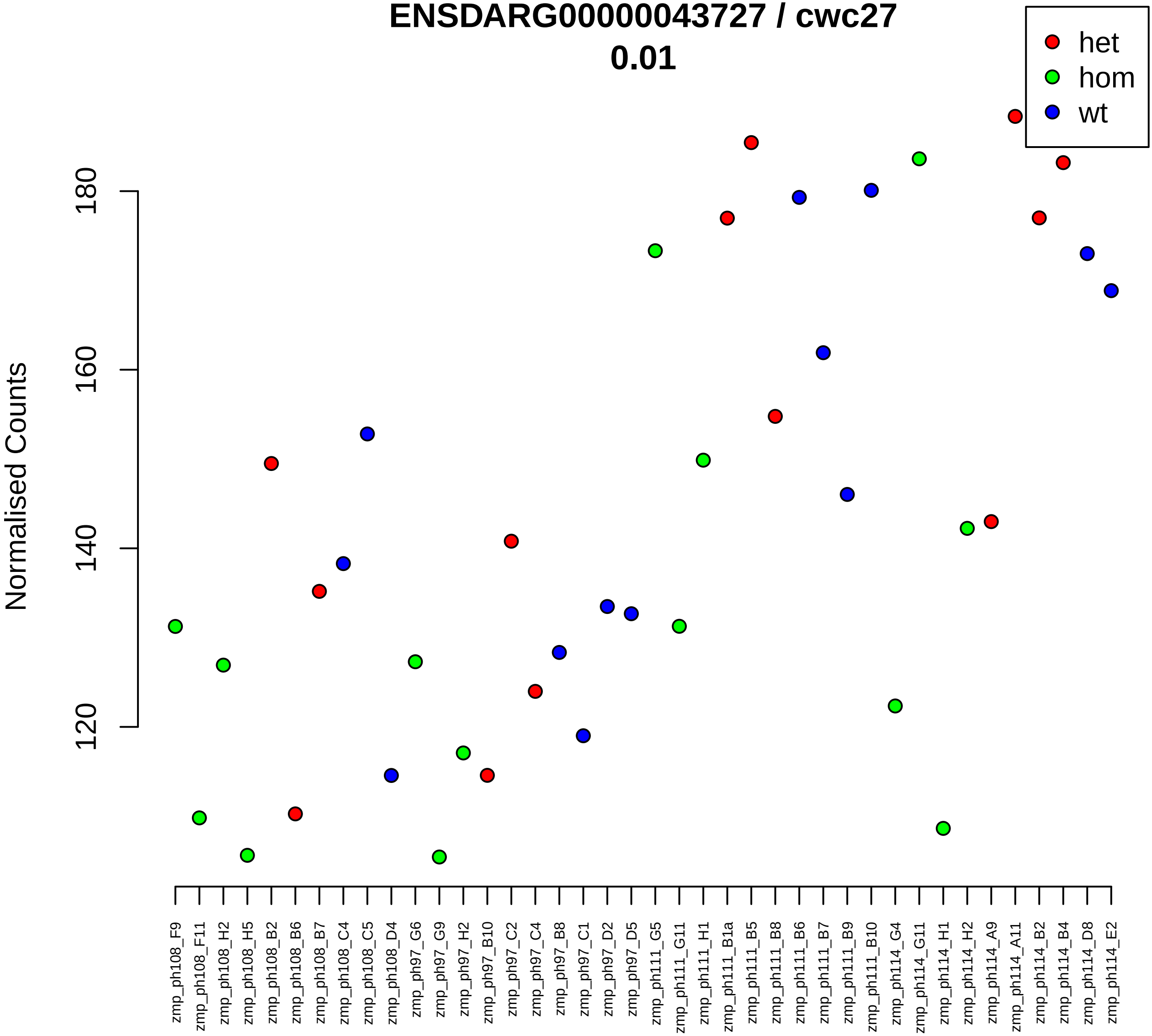

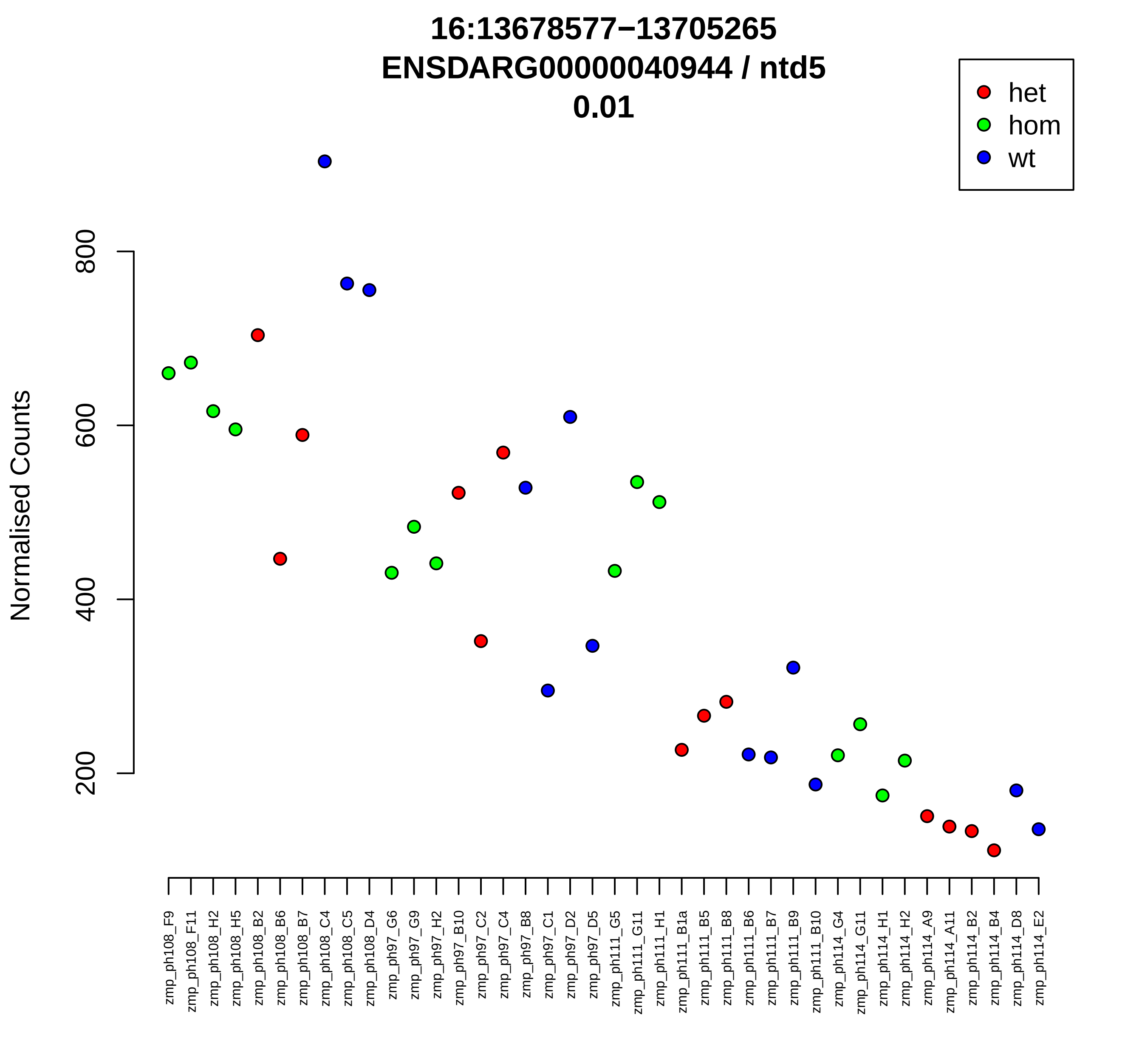

16:13678577−13705265
ENSDARG00000040944 / ntd5
0.01
het
hom
wt
Normalised Counts
200
400
600
800
zmp_ph108_F9
zmp_ph108_F11
zmp_ph108_H2
zmp_ph108_H5
zmp_ph108_B2
zmp_ph108_B6
zmp_ph108_B7
zmp_ph108_C4
zmp_ph108_C5
zmp_ph108_D4
zmp_ph97_G6
zmp_ph97_G9
zmp_ph97_H2
zmp_ph97_B10
zmp_ph97_C2
zmp_ph97_C4
zmp_ph97_B8
zmp_ph97_C1
zmp_ph97_D2
zmp_ph97_D5
zmp_ph111_G5
zmp_ph111_G11
zmp_ph111_H1
zmp_ph111_B1a
zmp_ph111_B5
zmp_ph111_B8
zmp_ph111_B6
zmp_ph111_B7
zmp_ph111_B9
zmp_ph111_B10
zmp_ph114_G4
zmp_ph114_G11
zmp_ph114_H1
zmp_ph114_H2
zmp_ph114_A9
zmp_ph114_A11
zmp_ph114_B2
zmp_ph114_B4
zmp_ph114_D8
zmp_ph114_E2

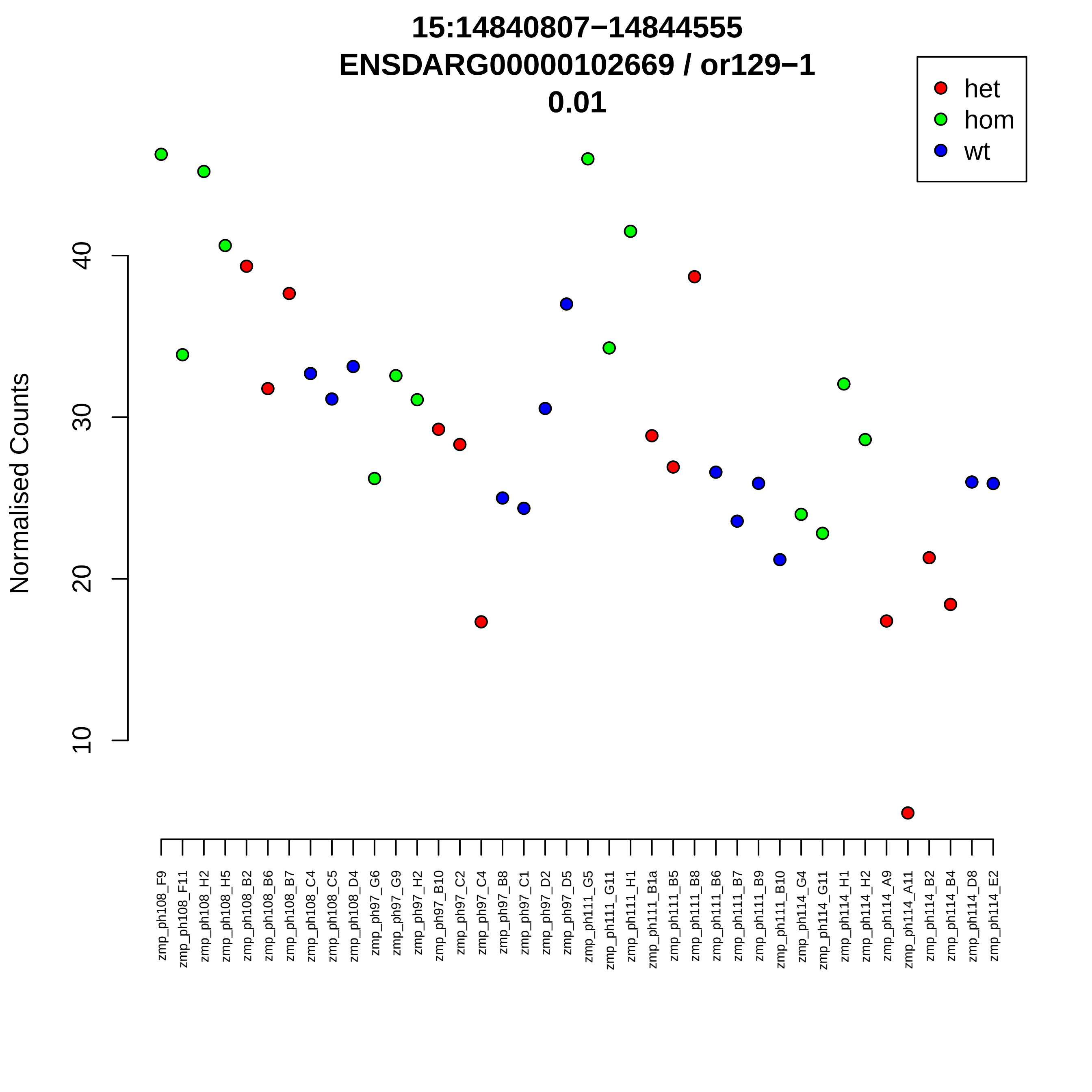
15:14840807−14844555
ENSDARG00000102669 / or129−1
0.01
het
hom
wt
Normalised Counts
10
20
30
40
zmp_ph108_F9
zmp_ph108_F11
zmp_ph108_H2
zmp_ph108_H5
zmp_ph108_B2
zmp_ph108_B6
zmp_ph108_B7
zmp_ph108_C4
zmp_ph108_C5
zmp_ph108_D4
zmp_ph97_G6
zmp_ph97_G9
zmp_ph97_H2
zmp_ph97_B10
zmp_ph97_C2
zmp_ph97_C4
zmp_ph97_B8
zmp_ph97_C1
zmp_ph97_D2
zmp_ph97_D5
zmp_ph111_G5
zmp_ph111_G11
zmp_ph111_H1
zmp_ph111_B1a
zmp_ph111_B5
zmp_ph111_B8
zmp_ph111_B6
zmp_ph111_B7
zmp_ph111_B9
zmp_ph111_B10
zmp_ph114_G4
zmp_ph114_G11
zmp_ph114_H1
zmp_ph114_H2
zmp_ph114_A9
zmp_ph114_A11
zmp_ph114_B2
zmp_ph114_B4
zmp_ph114_D8
zmp_ph114_E2

7:22042376−22053983
ENSDARG00000087162 / si:dkey−11f12.2
0.01

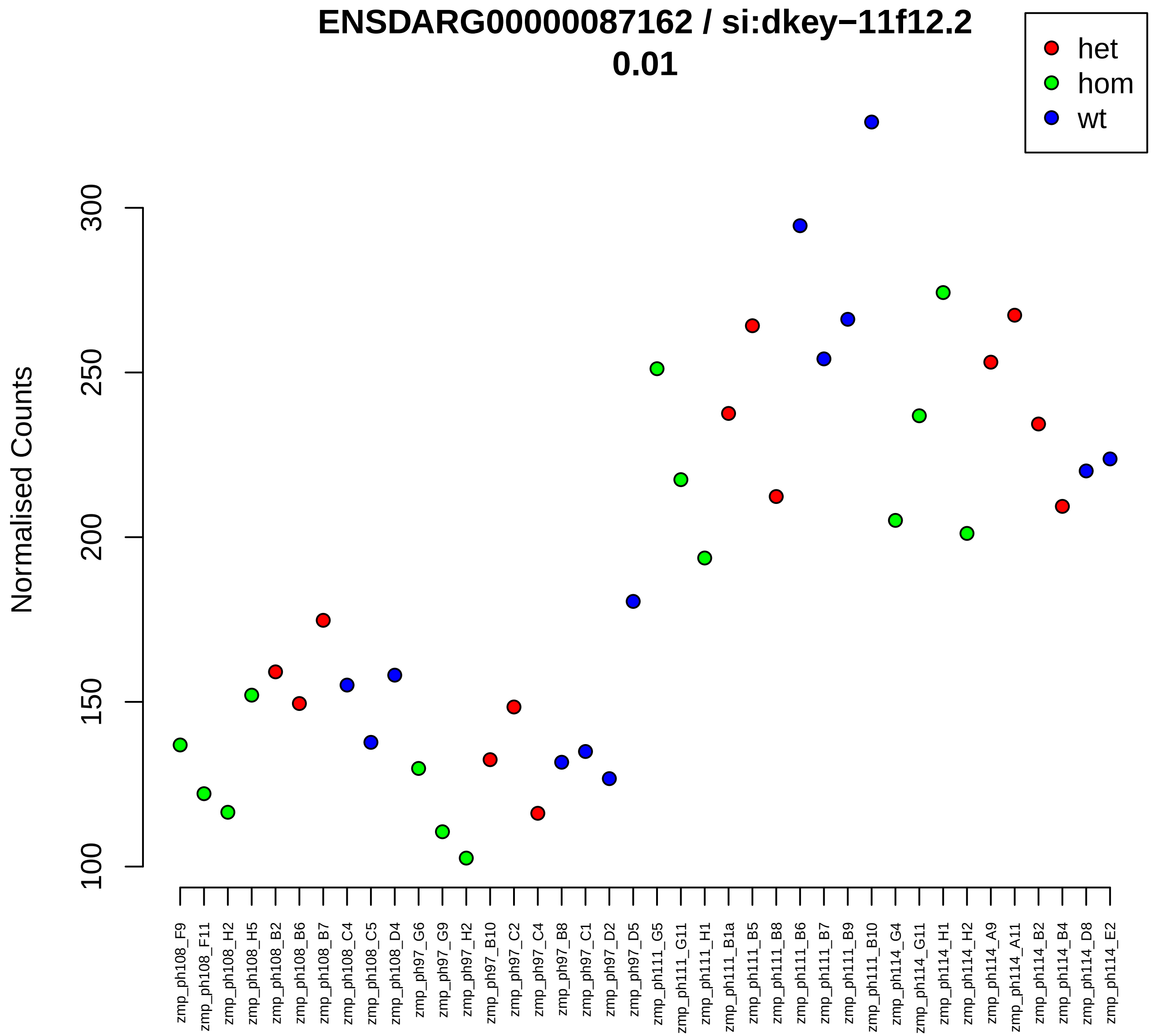

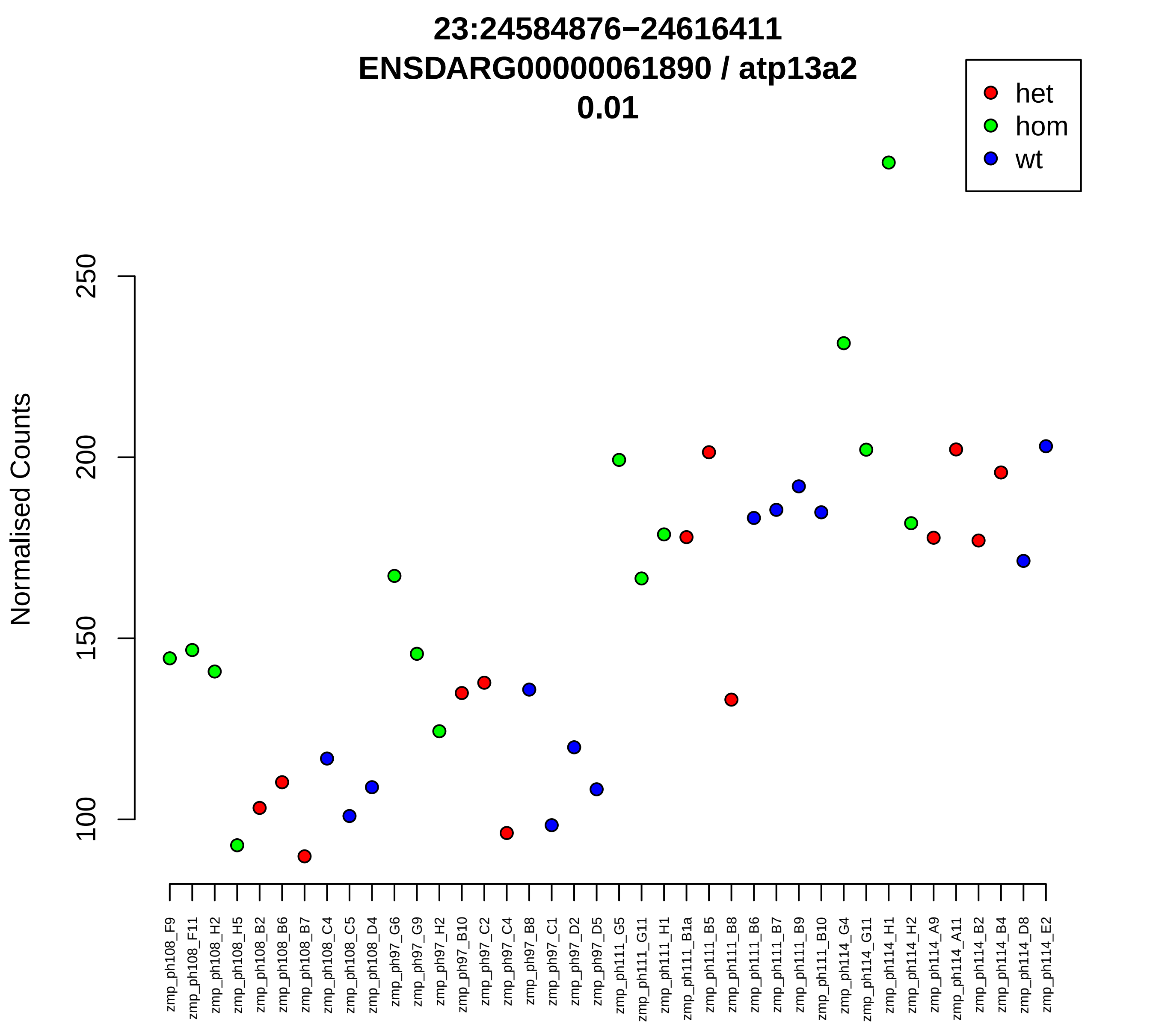
23:24584876−24616411
ENSDARG00000061890 / atp13a2
0.01
het
hom
wt
Normalised Counts
100
150
200
250
zmp_ph108_F9
zmp_ph108_F11
zmp_ph108_H2
zmp_ph108_H5
zmp_ph108_B2
zmp_ph108_B6
zmp_ph108_B7
zmp_ph108_C4
zmp_ph108_C5
zmp_ph108_D4
zmp_ph97_G6
zmp_ph97_G9
zmp_ph97_H2
zmp_ph97_B10
zmp_ph97_C2
zmp_ph97_C4
zmp_ph97_B8
zmp_ph97_C1
zmp_ph97_D2
zmp_ph97_D5
zmp_ph111_G5
zmp_ph111_G11
zmp_ph111_H1
zmp_ph111_B1a
zmp_ph111_B5
zmp_ph111_B8
zmp_ph111_B6
zmp_ph111_B7
zmp_ph111_B9
zmp_ph111_B10
zmp_ph114_G4
zmp_ph114_G11
zmp_ph114_H1
zmp_ph114_H2
zmp_ph114_A9
zmp_ph114_A11
zmp_ph114_B2
zmp_ph114_B4
zmp_ph114_D8
zmp_ph114_E2

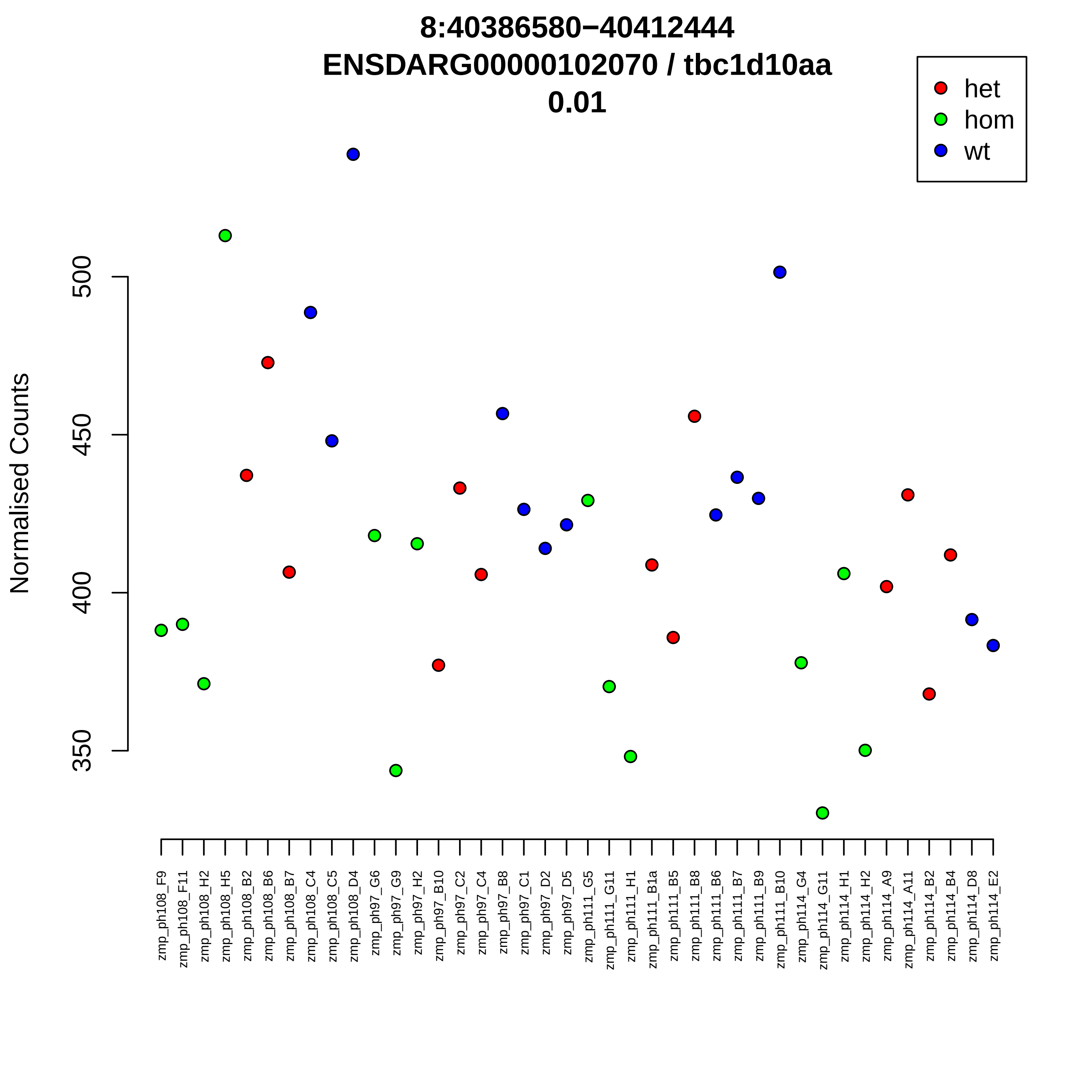

8:40386580−40412444
ENSDARG00000102070 / tbc1d10aa
0.01
het
hom
wt
Normalised Counts
350
400
450
500
zmp_ph108_F9
zmp_ph108_F11
zmp_ph108_H2
zmp_ph108_H5
zmp_ph108_B2
zmp_ph108_B6
zmp_ph108_B7
zmp_ph108_C4
zmp_ph108_C5
zmp_ph108_D4
zmp_ph97_G6
zmp_ph97_G9
zmp_ph97_H2
zmp_ph97_B10
zmp_ph97_C2
zmp_ph97_C4
zmp_ph97_B8
zmp_ph97_C1
zmp_ph97_D2
zmp_ph97_D5
zmp_ph111_G5
zmp_ph111_G11
zmp_ph111_H1
zmp_ph111_B1a
zmp_ph111_B5
zmp_ph111_B8
zmp_ph111_B6
zmp_ph111_B7
zmp_ph111_B9
zmp_ph111_B10
zmp_ph114_G4
zmp_ph114_G11
zmp_ph114_H1
zmp_ph114_H2
zmp_ph114_A9
zmp_ph114_A11
zmp_ph114_B2
zmp_ph114_B4
zmp_ph114_D8
zmp_ph114_E2

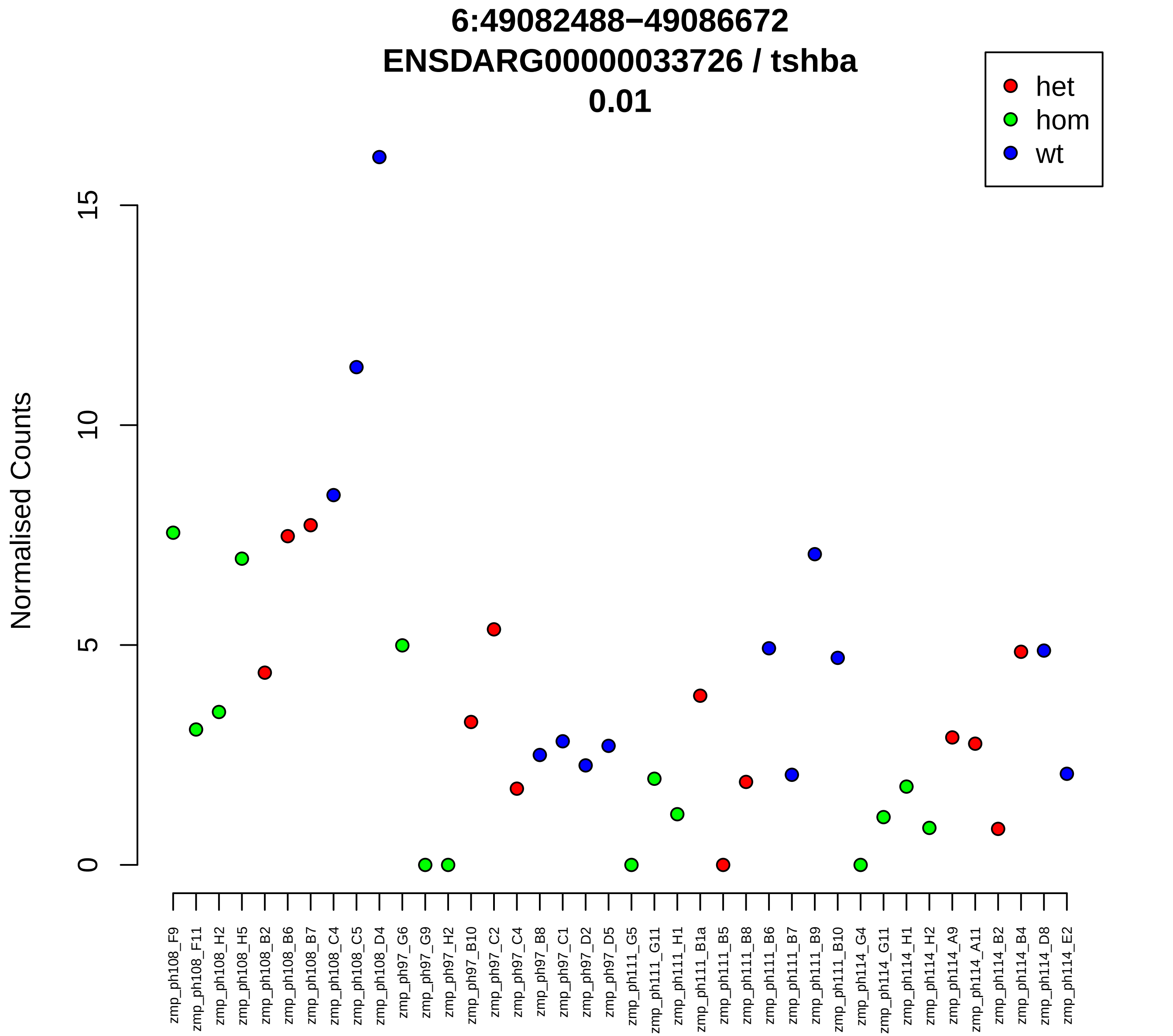
6:49082488−49086672
ENSDARG00000033726 / tshba
0.01
het
hom
wt
Normalised Counts
0
5
10
15
zmp_ph108_F9
zmp_ph108_F11
zmp_ph108_H2
zmp_ph108_H5
zmp_ph108_B2
zmp_ph108_B6
zmp_ph108_B7
zmp_ph108_C4
zmp_ph108_C5
zmp_ph108_D4
zmp_ph97_G6
zmp_ph97_G9
zmp_ph97_H2
zmp_ph97_B10
zmp_ph97_C2
zmp_ph97_C4
zmp_ph97_B8
zmp_ph97_C1
zmp_ph97_D2
zmp_ph97_D5
zmp_ph111_G5
zmp_ph111_G11
zmp_ph111_H1
zmp_ph111_B1a
zmp_ph111_B5
zmp_ph111_B8
zmp_ph111_B6
zmp_ph111_B7
zmp_ph111_B9
zmp_ph111_B10
zmp_ph114_G4
zmp_ph114_G11
zmp_ph114_H1
zmp_ph114_H2
zmp_ph114_A9
zmp_ph114_A11
zmp_ph114_B2
zmp_ph114_B4
zmp_ph114_D8
zmp_ph114_E2

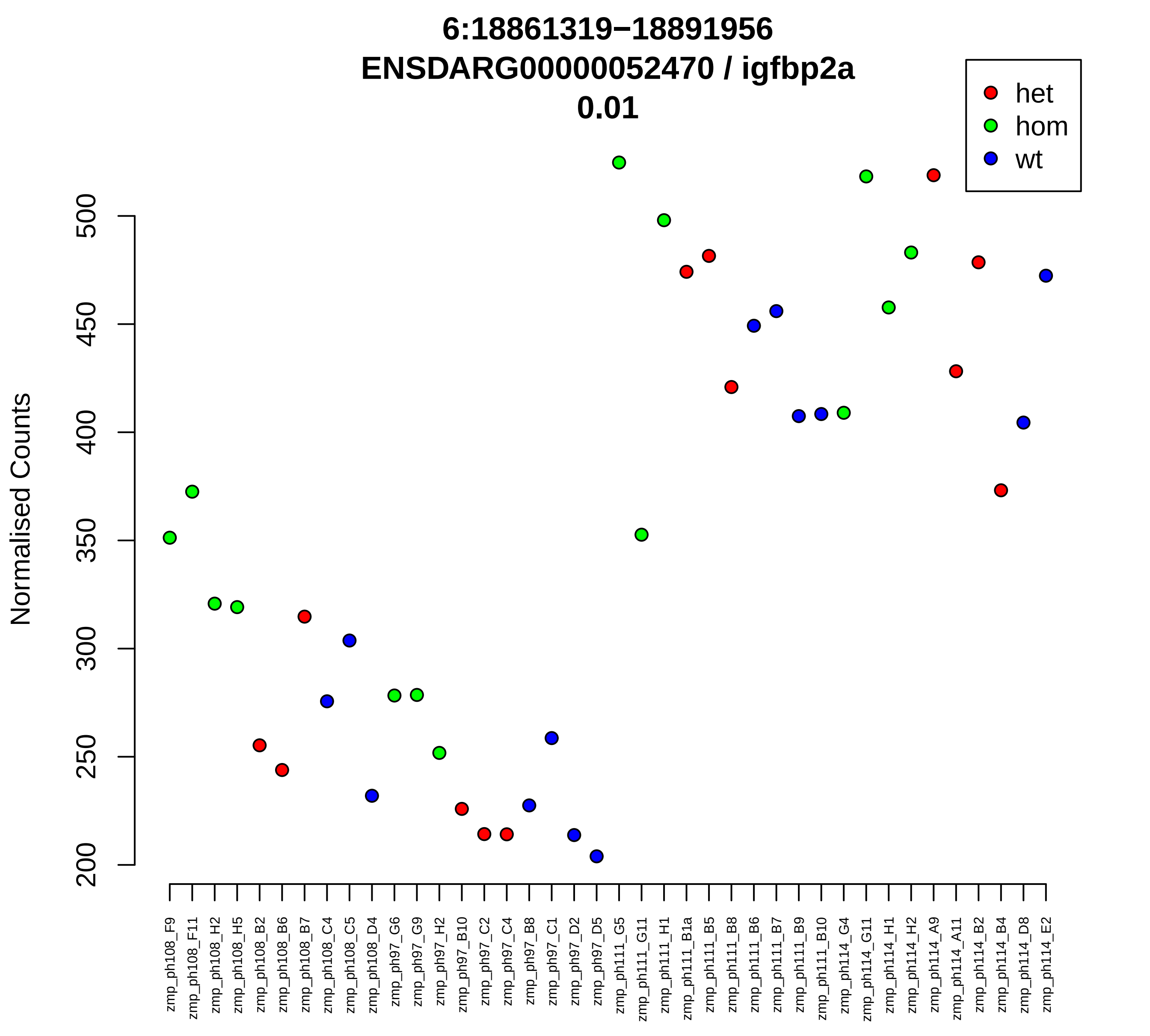

6:18861319−18891956
ENSDARG00000052470 / igfbp2a
0.01
het
hom
wt
Normalised Counts
200
250
300
350
400
450
500
zmp_ph108_F9
zmp_ph108_F11
zmp_ph108_H2
zmp_ph108_H5
zmp_ph108_B2
zmp_ph108_B6
zmp_ph108_B7
zmp_ph108_C4
zmp_ph108_C5
zmp_ph108_D4
zmp_ph97_G6
zmp_ph97_G9
zmp_ph97_H2
zmp_ph97_B10
zmp_ph97_C2
zmp_ph97_C4
zmp_ph97_B8
zmp_ph97_C1
zmp_ph97_D2
zmp_ph97_D5
zmp_ph111_G5
zmp_ph111_G11
zmp_ph111_H1
zmp_ph111_B1a
zmp_ph111_B5
zmp_ph111_B8
zmp_ph111_B6
zmp_ph111_B7
zmp_ph111_B9
zmp_ph111_B10
zmp_ph114_G4
zmp_ph114_G11
zmp_ph114_H1
zmp_ph114_H2
zmp_ph114_A9
zmp_ph114_A11
zmp_ph114_B2
zmp_ph114_B4
zmp_ph114_D8
zmp_ph114_E2

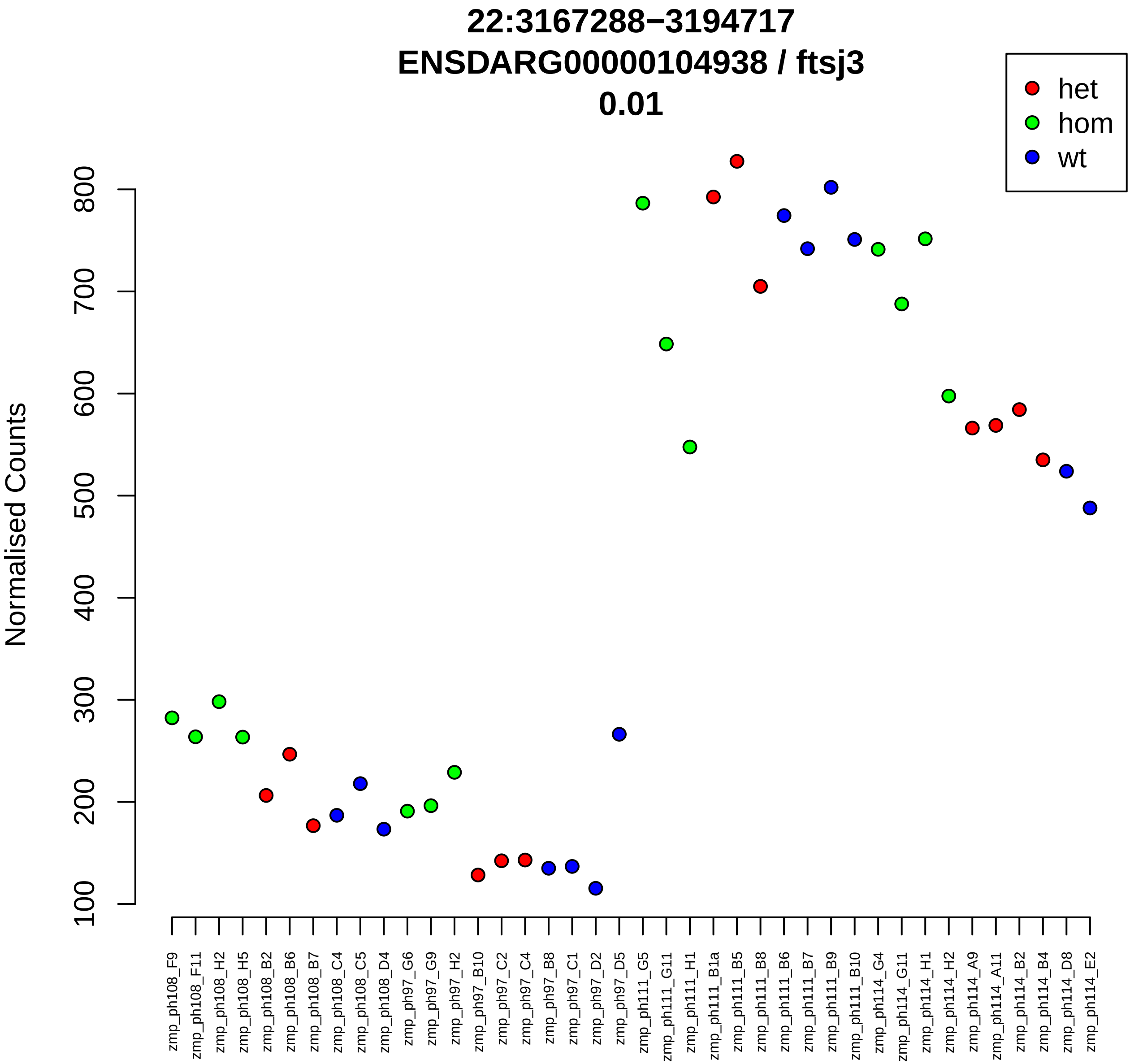

22:3167288−3194717
ENSDARG00000104938 / ftsj3
0.01
het
hom
wt
Normalised Counts
100
200
300
400
500
600
700
800
zmp_ph108_F9
zmp_ph108_F11
zmp_ph108_H2
zmp_ph108_H5
zmp_ph108_B2
zmp_ph108_B6
zmp_ph108_B7
zmp_ph108_C4
zmp_ph108_C5
zmp_ph108_D4
zmp_ph97_G6
zmp_ph97_G9
zmp_ph97_H2
zmp_ph97_B10
zmp_ph97_C2
zmp_ph97_C4
zmp_ph97_B8
zmp_ph97_C1
zmp_ph97_D2
zmp_ph97_D5
zmp_ph111_G5
zmp_ph111_G11
zmp_ph111_H1
zmp_ph111_B1a
zmp_ph111_B5
zmp_ph111_B8
zmp_ph111_B6
zmp_ph111_B7
zmp_ph111_B9
zmp_ph111_B10
zmp_ph114_G4
zmp_ph114_G11
zmp_ph114_H1
zmp_ph114_H2
zmp_ph114_A9
zmp_ph114_A11
zmp_ph114_B2
zmp_ph114_B4
zmp_ph114_D8
zmp_ph114_E2

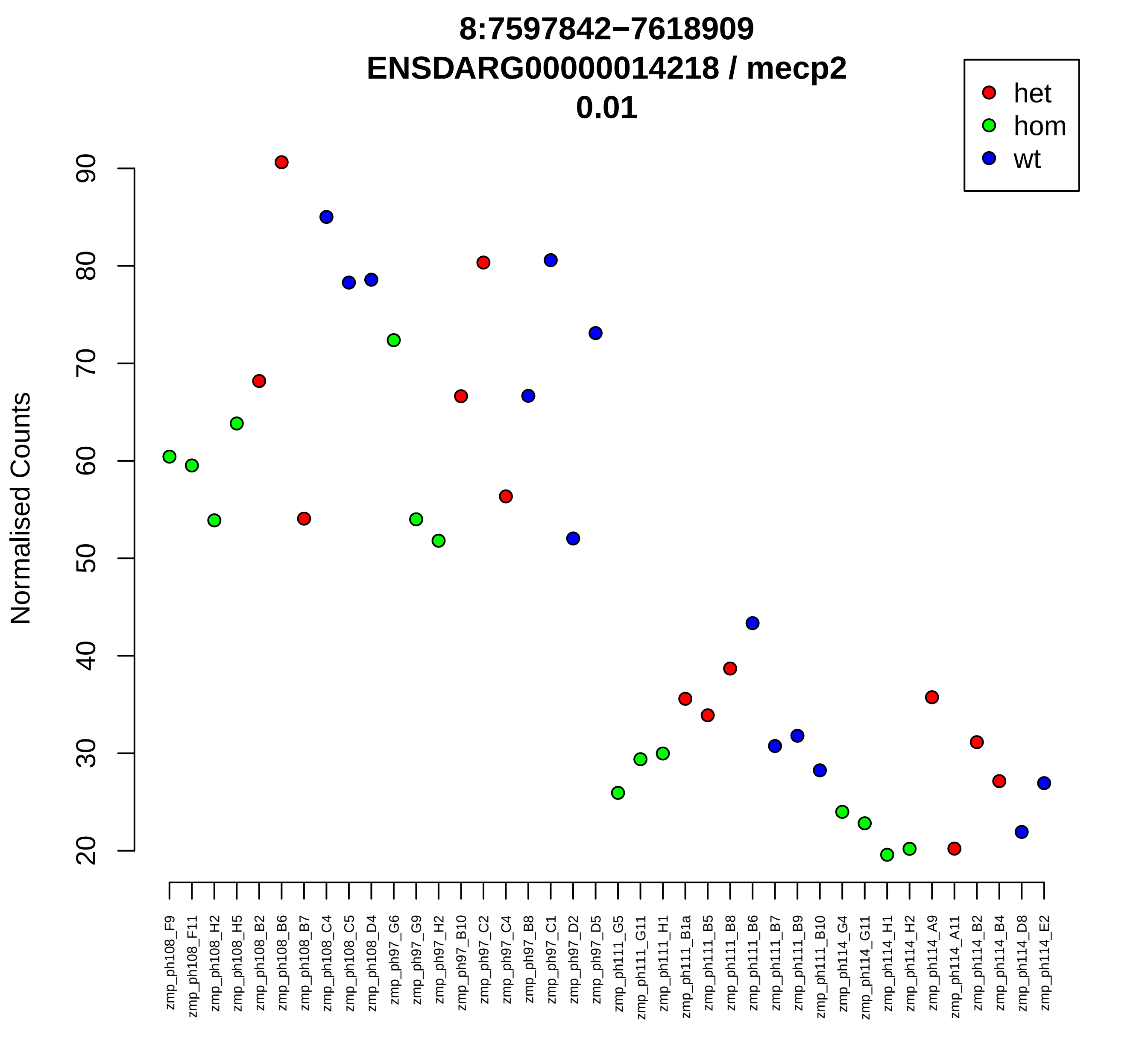
8:7597842−7618909
ENSDARG00000014218 / mecp2
0.01
het
hom
wt
Normalised Counts
20
30
40
50
60
70
80
90
zmp_ph108_F9
zmp_ph108_F11
zmp_ph108_H2
zmp_ph108_H5
zmp_ph108_B2
zmp_ph108_B6
zmp_ph108_B7
zmp_ph108_C4
zmp_ph108_C5
zmp_ph108_D4
zmp_ph97_G6
zmp_ph97_G9
zmp_ph97_H2
zmp_ph97_B10
zmp_ph97_C2
zmp_ph97_C4
zmp_ph97_B8
zmp_ph97_C1
zmp_ph97_D2
zmp_ph97_D5
zmp_ph111_G5
zmp_ph111_G11
zmp_ph111_H1
zmp_ph111_B1a
zmp_ph111_B5
zmp_ph111_B8
zmp_ph111_B6
zmp_ph111_B7
zmp_ph111_B9
zmp_ph111_B10
zmp_ph114_G4
zmp_ph114_G11
zmp_ph114_H1
zmp_ph114_H2
zmp_ph114_A9
zmp_ph114_A11
zmp_ph114_B2
zmp_ph114_B4
zmp_ph114_D8
zmp_ph114_E2

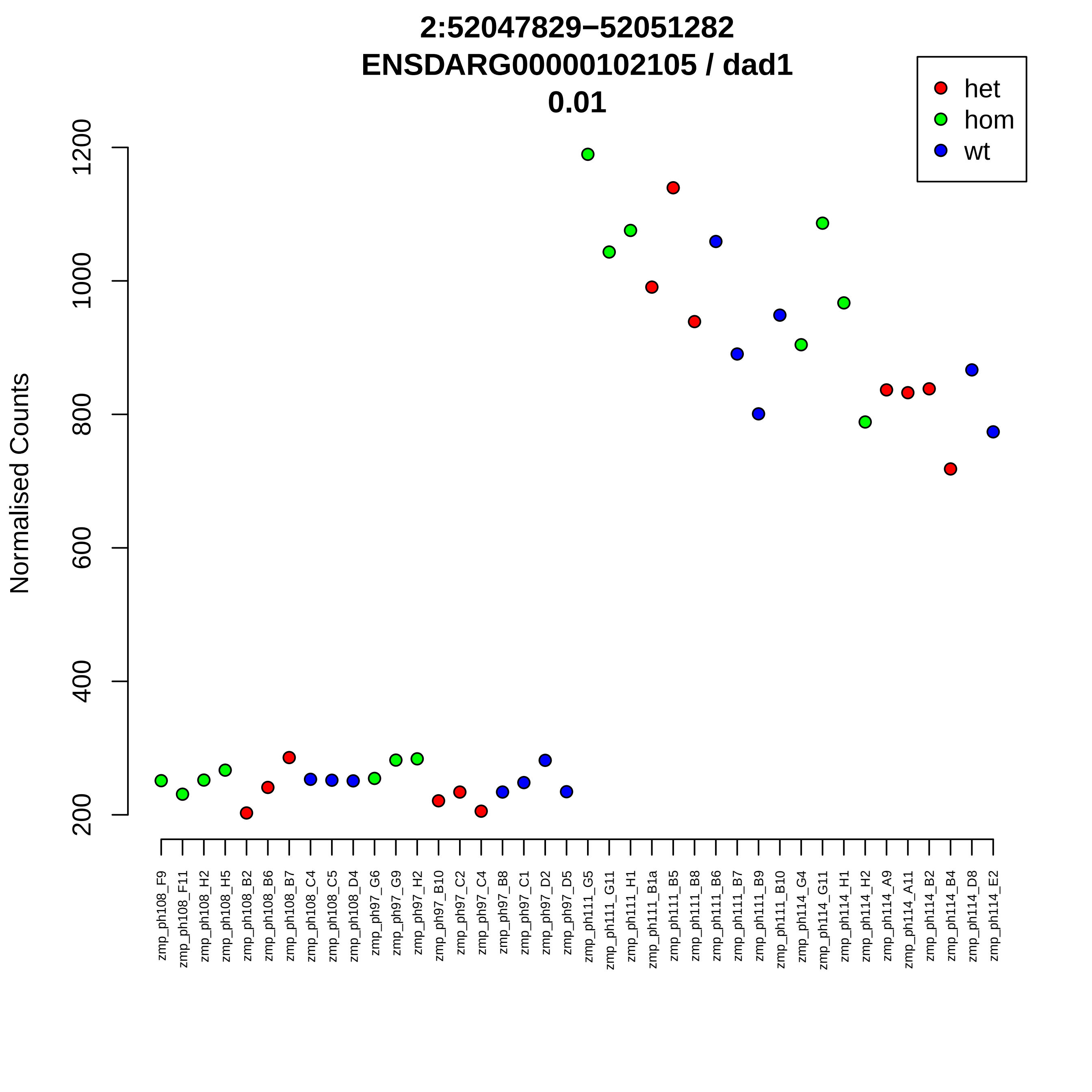
2:52047829−52051282
ENSDARG00000102105 / dad1
0.01
het
hom
wt
Normalised Counts
200
400
600
800
1000
1200
zmp_ph108_F9
zmp_ph108_F11
zmp_ph108_H2
zmp_ph108_H5
zmp_ph108_B2
zmp_ph108_B6
zmp_ph108_B7
zmp_ph108_C4
zmp_ph108_C5
zmp_ph108_D4
zmp_ph97_G6
zmp_ph97_G9
zmp_ph97_H2
zmp_ph97_B10
zmp_ph97_C2
zmp_ph97_C4
zmp_ph97_B8
zmp_ph97_C1
zmp_ph97_D2
zmp_ph97_D5
zmp_ph111_G5
zmp_ph111_G11
zmp_ph111_H1
zmp_ph111_B1a
zmp_ph111_B5
zmp_ph111_B8
zmp_ph111_B6
zmp_ph111_B7
zmp_ph111_B9
zmp_ph111_B10
zmp_ph114_G4
zmp_ph114_G11
zmp_ph114_H1
zmp_ph114_H2
zmp_ph114_A9
zmp_ph114_A11
zmp_ph114_B2
zmp_ph114_B4
zmp_ph114_D8
zmp_ph114_E2

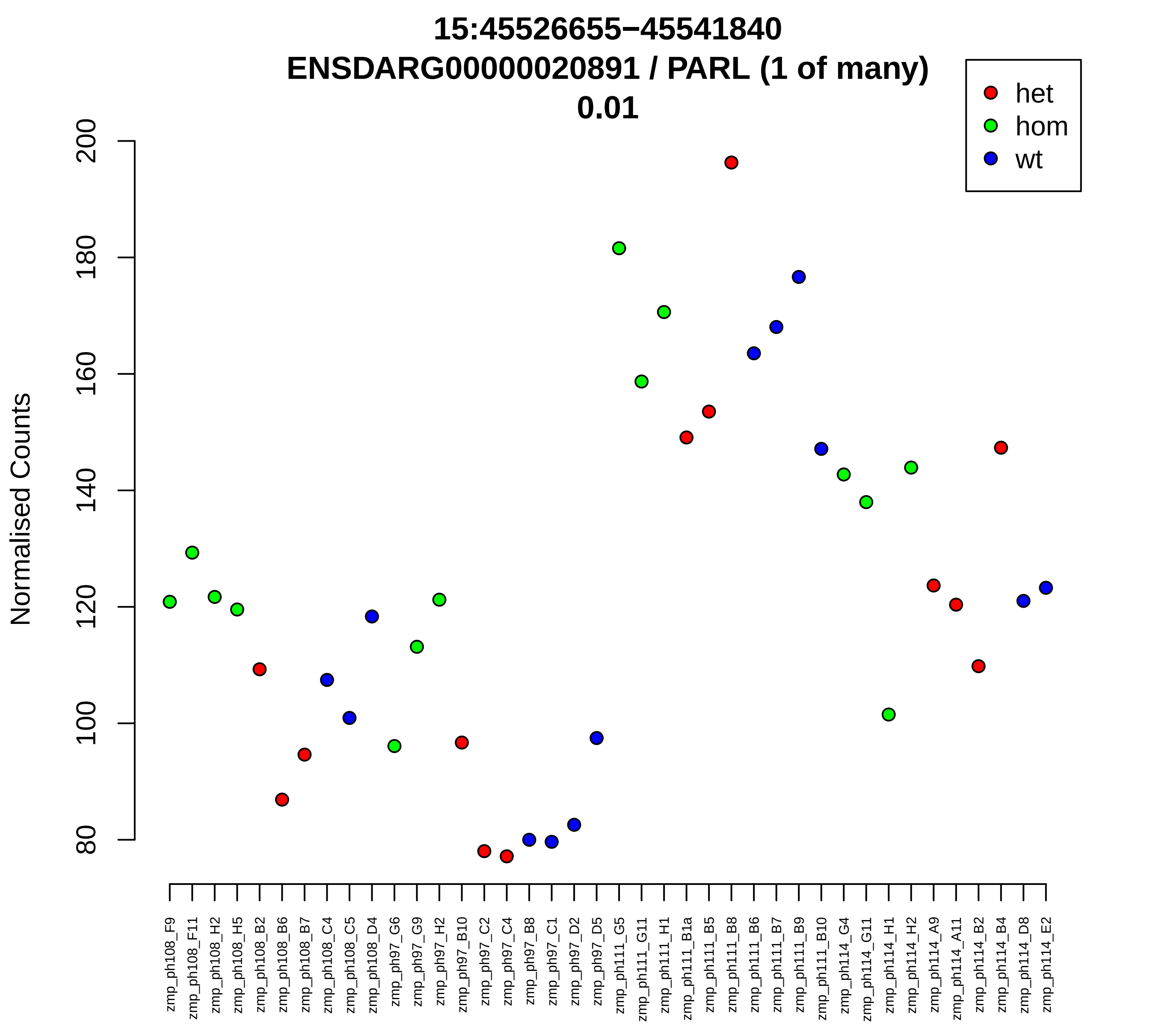
15:45526655−45541840
ENSDARG00000020891 / PARL (1 of many)
0.01
Normalised Counts
80
100
120
140
160
180
200
het
hom
wt
zmp_ph108_F9
zmp_ph108_F11
zmp_ph108_H2
zmp_ph108_H5
zmp_ph108_B2
zmp_ph108_B6
zmp_ph108_B7
zmp_ph108_C4
zmp_ph108_C5
zmp_ph108_D4
zmp_ph97_G6
zmp_ph97_G9
zmp_ph97_H2
zmp_ph97_B10
zmp_ph97_C2
zmp_ph97_C4
zmp_ph97_B8
zmp_ph97_C1
zmp_ph97_D2
zmp_ph97_D5
zmp_ph111_G5
zmp_ph111_G11
zmp_ph111_H1
zmp_ph111_B1a
zmp_ph111_B5
zmp_ph111_B8
zmp_ph111_B6
zmp_ph111_B7
zmp_ph111_B9
zmp_ph111_B10
zmp_ph114_G4
zmp_ph114_G11
zmp_ph114_H1
zmp_ph114_H2
zmp_ph114_A9
zmp_ph114_A11
zmp_ph114_B2
zmp_ph114_B4
zmp_ph114_D8
zmp_ph114_E2

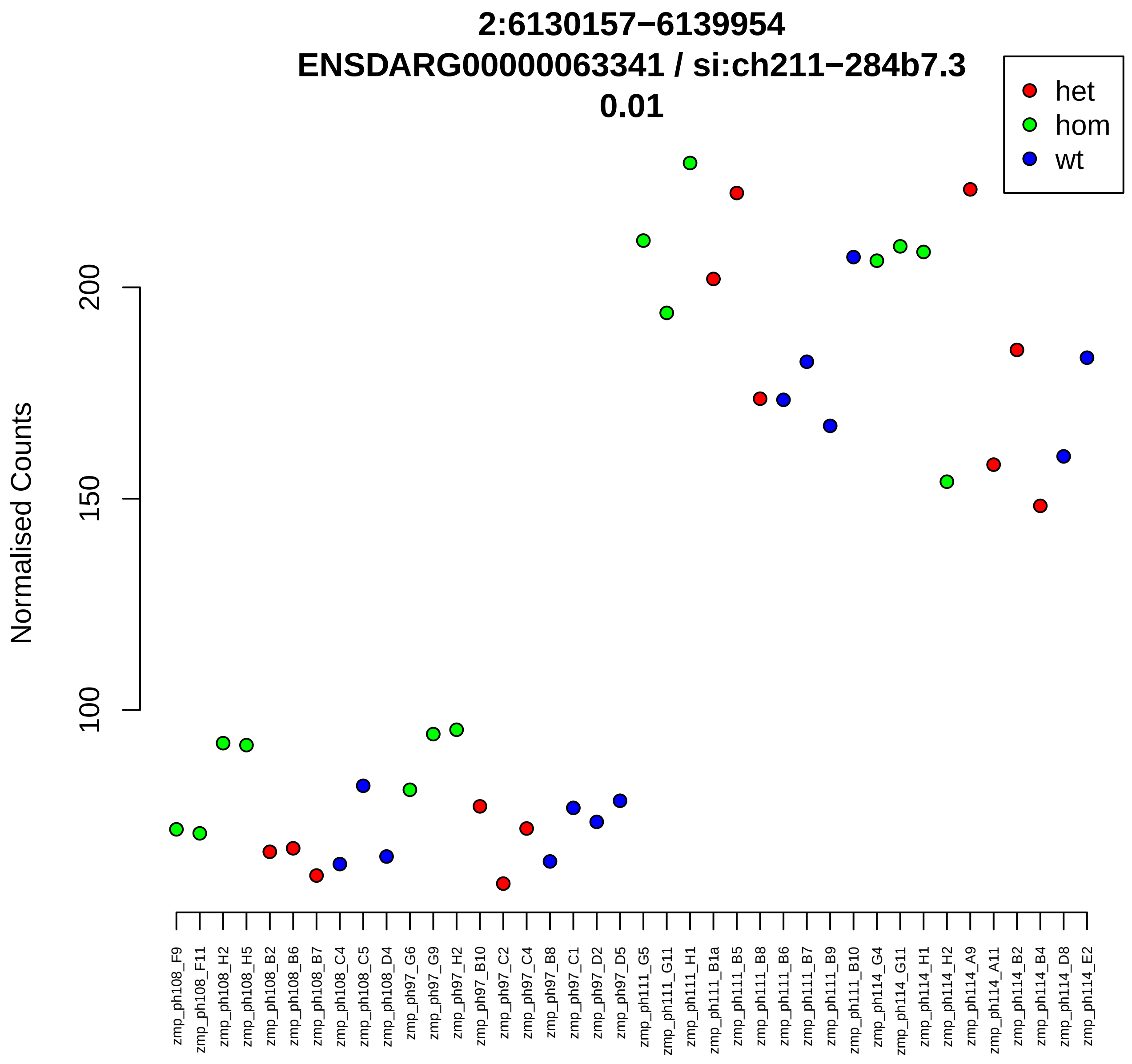
2:6130157−6139954
ENSDARG00000063341 / si:ch211−284b7.3
0.01
het
hom
wt
Normalised Counts
100
150
200
zmp_ph108_F9
zmp_ph108_F11
zmp_ph108_H2
zmp_ph108_H5
zmp_ph108_B2
zmp_ph108_B6
zmp_ph108_B7
zmp_ph108_C4
zmp_ph108_C5
zmp_ph108_D4
zmp_ph97_G6
zmp_ph97_G9
zmp_ph97_H2
zmp_ph97_B10
zmp_ph97_C2
zmp_ph97_C4
zmp_ph97_B8
zmp_ph97_C1
zmp_ph97_D2
zmp_ph97_D5
zmp_ph111_G5
zmp_ph111_G11
zmp_ph111_H1
zmp_ph111_B1a
zmp_ph111_B5
zmp_ph111_B8
zmp_ph111_B6
zmp_ph111_B7
zmp_ph111_B9
zmp_ph111_B10
zmp_ph114_G4
zmp_ph114_G11
zmp_ph114_H1
zmp_ph114_H2
zmp_ph114_A9
zmp_ph114_A11
zmp_ph114_B2
zmp_ph114_B4
zmp_ph114_D8
zmp_ph114_E2

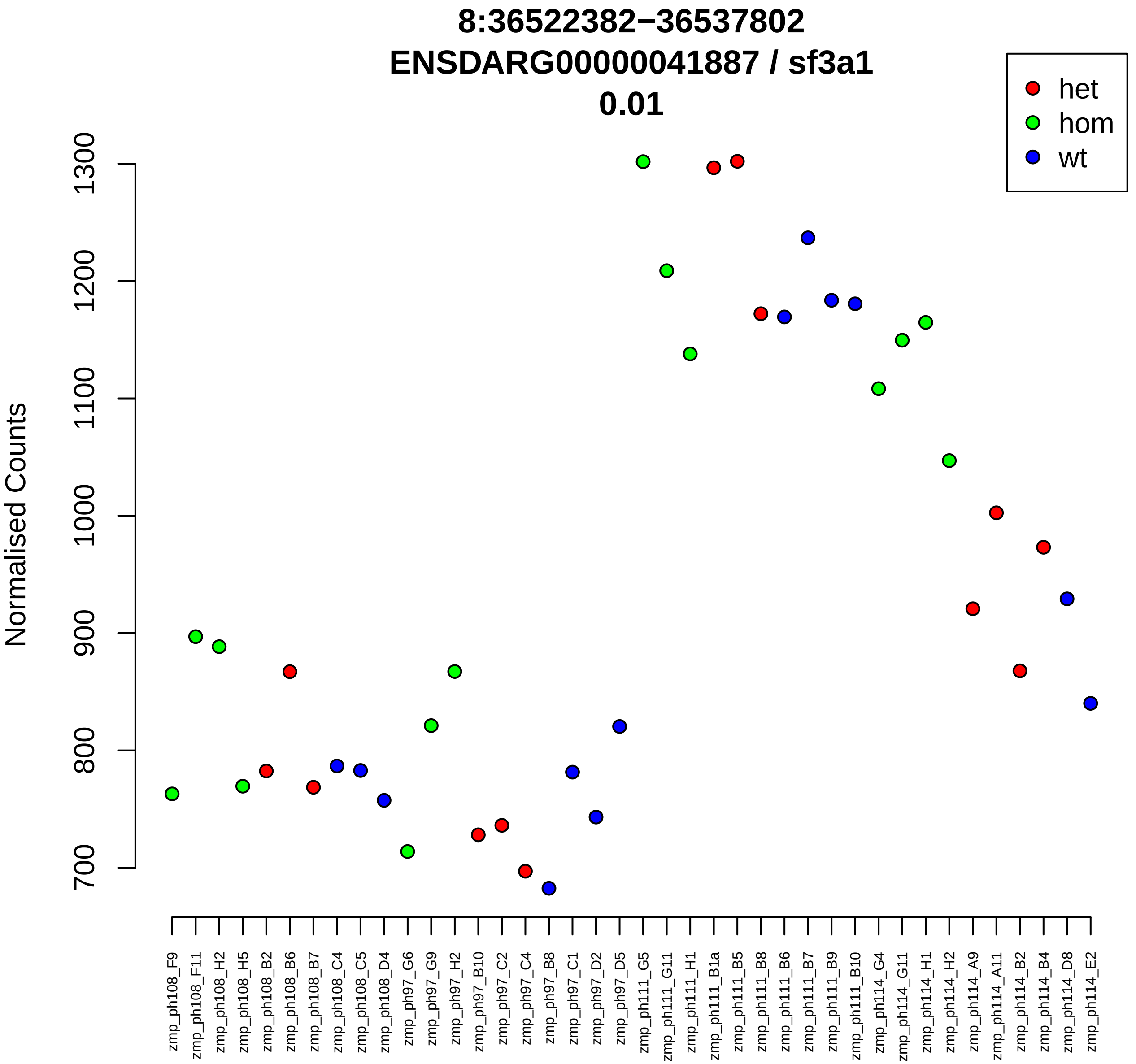

8:36522382−36537802
ENSDARG00000041887 / sf3a1
0.01
het
hom
wt
Normalised Counts
700
800
900
1000
1100
1200
1300
zmp_ph108_F9
zmp_ph108_F11
zmp_ph108_H2
zmp_ph108_H5
zmp_ph108_B2
zmp_ph108_B6
zmp_ph108_B7
zmp_ph108_C4
zmp_ph108_C5
zmp_ph108_D4
zmp_ph97_G6
zmp_ph97_G9
zmp_ph97_H2
zmp_ph97_B10
zmp_ph97_C2
zmp_ph97_C4
zmp_ph97_B8
zmp_ph97_C1
zmp_ph97_D2
zmp_ph97_D5
zmp_ph111_G5
zmp_ph111_G11
zmp_ph111_H1
zmp_ph111_B1a
zmp_ph111_B5
zmp_ph111_B8
zmp_ph111_B6
zmp_ph111_B7
zmp_ph111_B9
zmp_ph111_B10
zmp_ph114_G4
zmp_ph114_G11
zmp_ph114_H1
zmp_ph114_H2
zmp_ph114_A9
zmp_ph114_A11
zmp_ph114_B2
zmp_ph114_B4
zmp_ph114_D8
zmp_ph114_E2

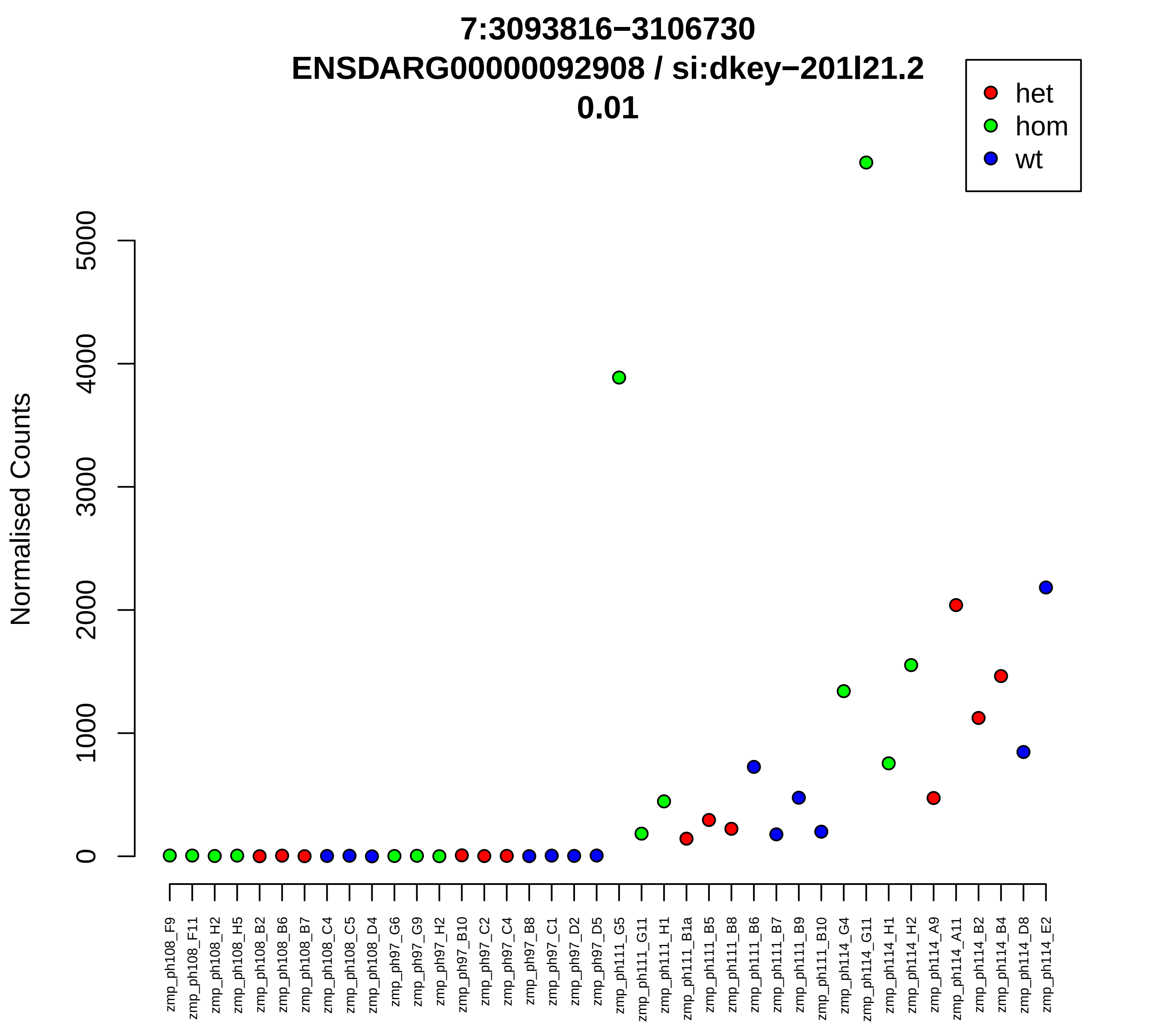
7:3093816−3106730
ENSDARG00000092908 / si:dkey−201l21.2
0.01
het
hom
wt
Normalised Counts
0
1000
2000
3000
4000
5000
zmp_ph108_F9
zmp_ph108_F11
zmp_ph108_H2
zmp_ph108_H5
zmp_ph108_B2
zmp_ph108_B6
zmp_ph108_B7
zmp_ph108_C4
zmp_ph108_C5
zmp_ph108_D4
zmp_ph97_G6
zmp_ph97_G9
zmp_ph97_H2
zmp_ph97_B10
zmp_ph97_C2
zmp_ph97_C4
zmp_ph97_B8
zmp_ph97_C1
zmp_ph97_D2
zmp_ph97_D5
zmp_ph111_G5
zmp_ph111_G11
zmp_ph111_H1
zmp_ph111_B1a
zmp_ph111_B5
zmp_ph111_B8
zmp_ph111_B6
zmp_ph111_B7
zmp_ph111_B9
zmp_ph111_B10
zmp_ph114_G4
zmp_ph114_G11
zmp_ph114_H1
zmp_ph114_H2
zmp_ph114_A9
zmp_ph114_A11
zmp_ph114_B2
zmp_ph114_B4
zmp_ph114_D8
zmp_ph114_E2

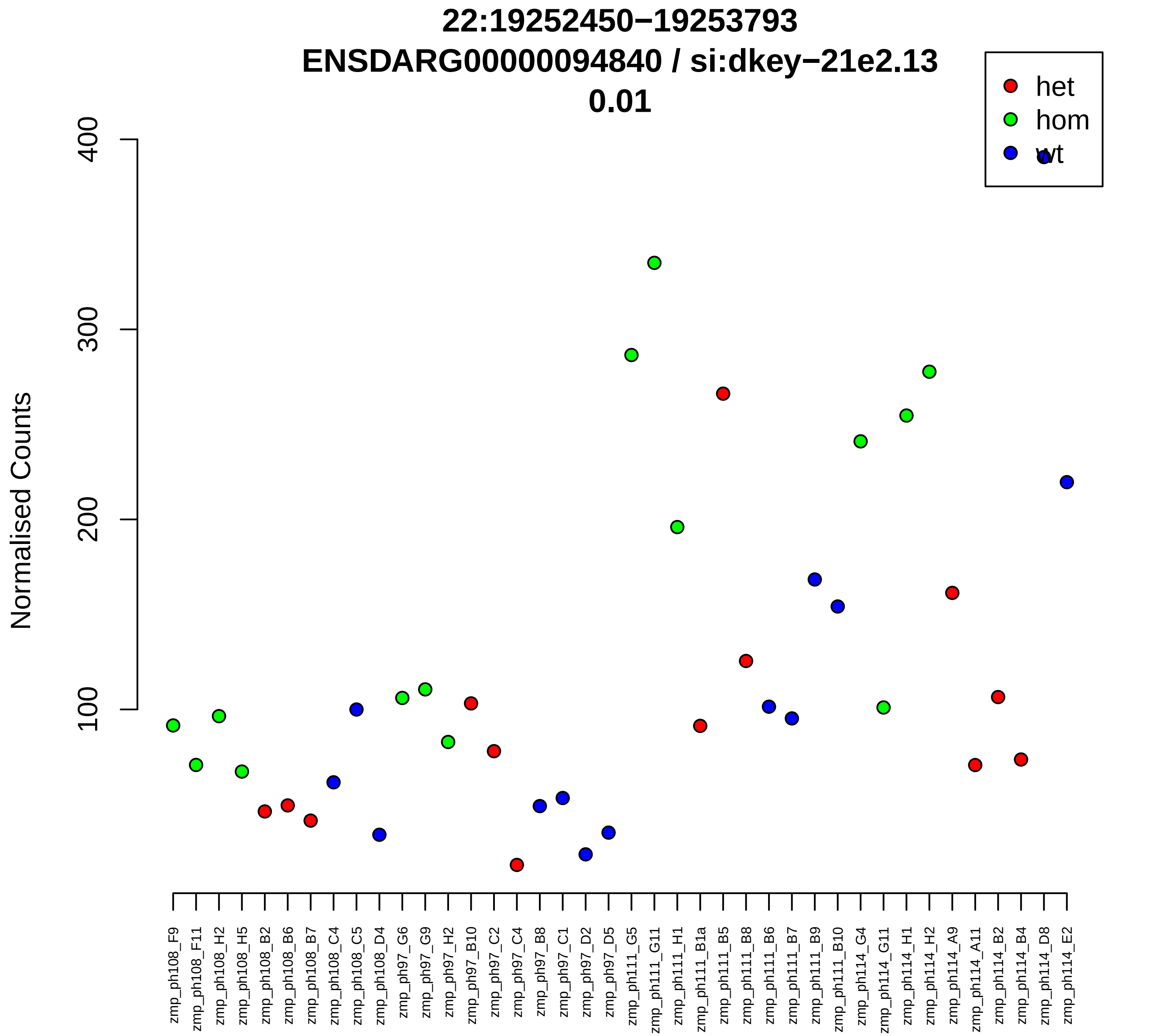
22:19252450−19253793
ENSDARG00000094840 / si:dkey−21e2.13
0.01
het
hom
wt
Normalised Counts
100
200
300
400
zmp_ph108_F9
zmp_ph108_F11
zmp_ph108_H2
zmp_ph108_H5
zmp_ph108_B2
zmp_ph108_B6
zmp_ph108_B7
zmp_ph108_C4
zmp_ph108_C5
zmp_ph108_D4
zmp_ph97_G6
zmp_ph97_G9
zmp_ph97_H2
zmp_ph97_B10
zmp_ph97_C2
zmp_ph97_C4
zmp_ph97_B8
zmp_ph97_C1
zmp_ph97_D2
zmp_ph97_D5
zmp_ph111_G5
zmp_ph111_G11
zmp_ph111_H1
zmp_ph111_B1a
zmp_ph111_B5
zmp_ph111_B8
zmp_ph111_B6
zmp_ph111_B7
zmp_ph111_B9
zmp_ph111_B10
zmp_ph114_G4
zmp_ph114_G11
zmp_ph114_H1
zmp_ph114_H2
zmp_ph114_A9
zmp_ph114_A11
zmp_ph114_B2
zmp_ph114_B4
zmp_ph114_D8
zmp_ph114_E2

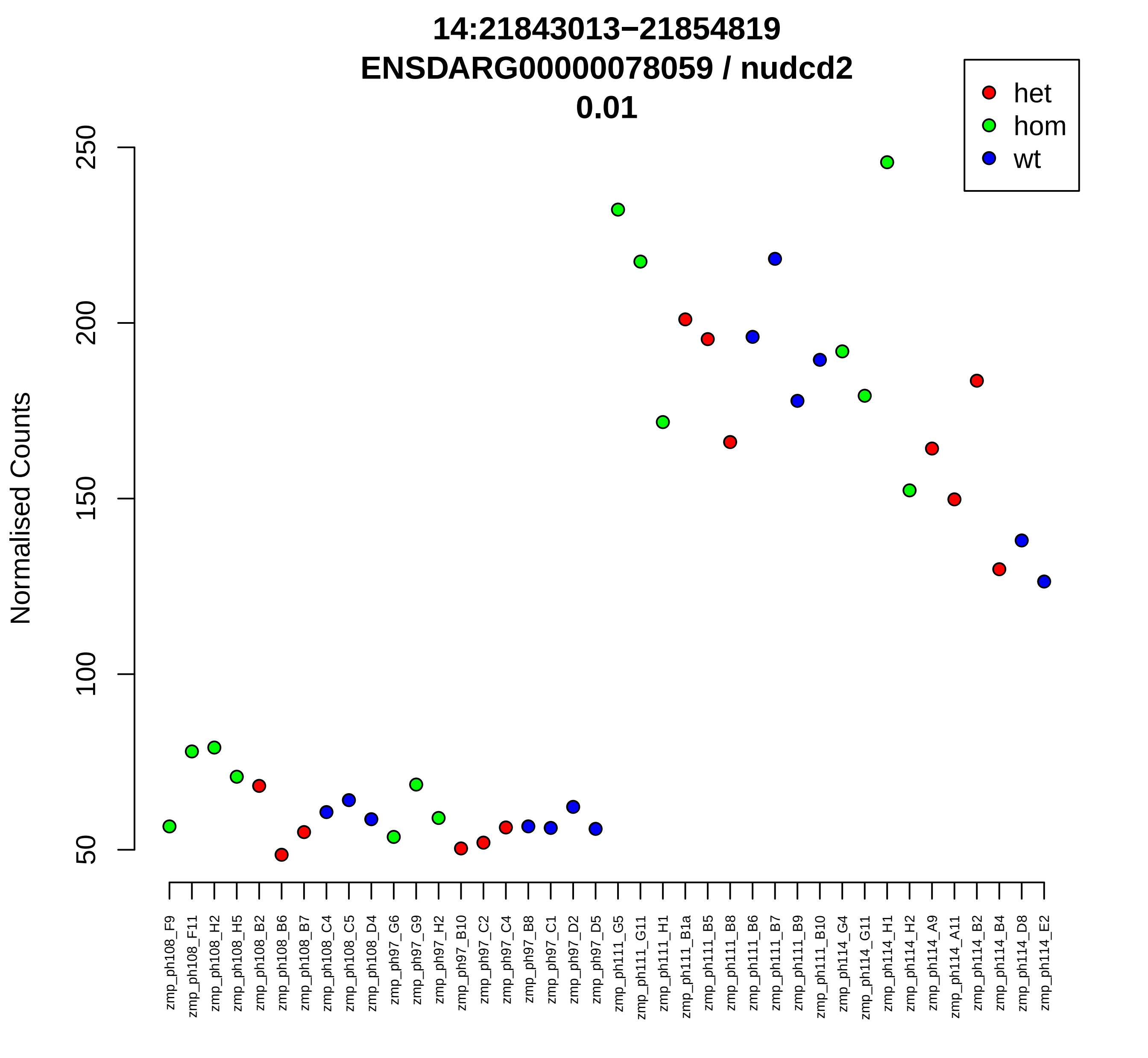
14:21843013−21854819
ENSDARG00000078059 / nudcd2
0.01
het
hom
wt
Normalised Counts
50
100
150
200
250
zmp_ph108_F9
zmp_ph108_F11
zmp_ph108_H2
zmp_ph108_H5
zmp_ph108_B2
zmp_ph108_B6
zmp_ph108_B7
zmp_ph108_C4
zmp_ph108_C5
zmp_ph108_D4
zmp_ph97_G6
zmp_ph97_G9
zmp_ph97_H2
zmp_ph97_B10
zmp_ph97_C2
zmp_ph97_C4
zmp_ph97_B8
zmp_ph97_C1
zmp_ph97_D2
zmp_ph97_D5
zmp_ph111_G5
zmp_ph111_G11
zmp_ph111_H1
zmp_ph111_B1a
zmp_ph111_B5
zmp_ph111_B8
zmp_ph111_B6
zmp_ph111_B7
zmp_ph111_B9
zmp_ph111_B10
zmp_ph114_G4
zmp_ph114_G11
zmp_ph114_H1
zmp_ph114_H2
zmp_ph114_A9
zmp_ph114_A11
zmp_ph114_B2
zmp_ph114_B4
zmp_ph114_D8
zmp_ph114_E2

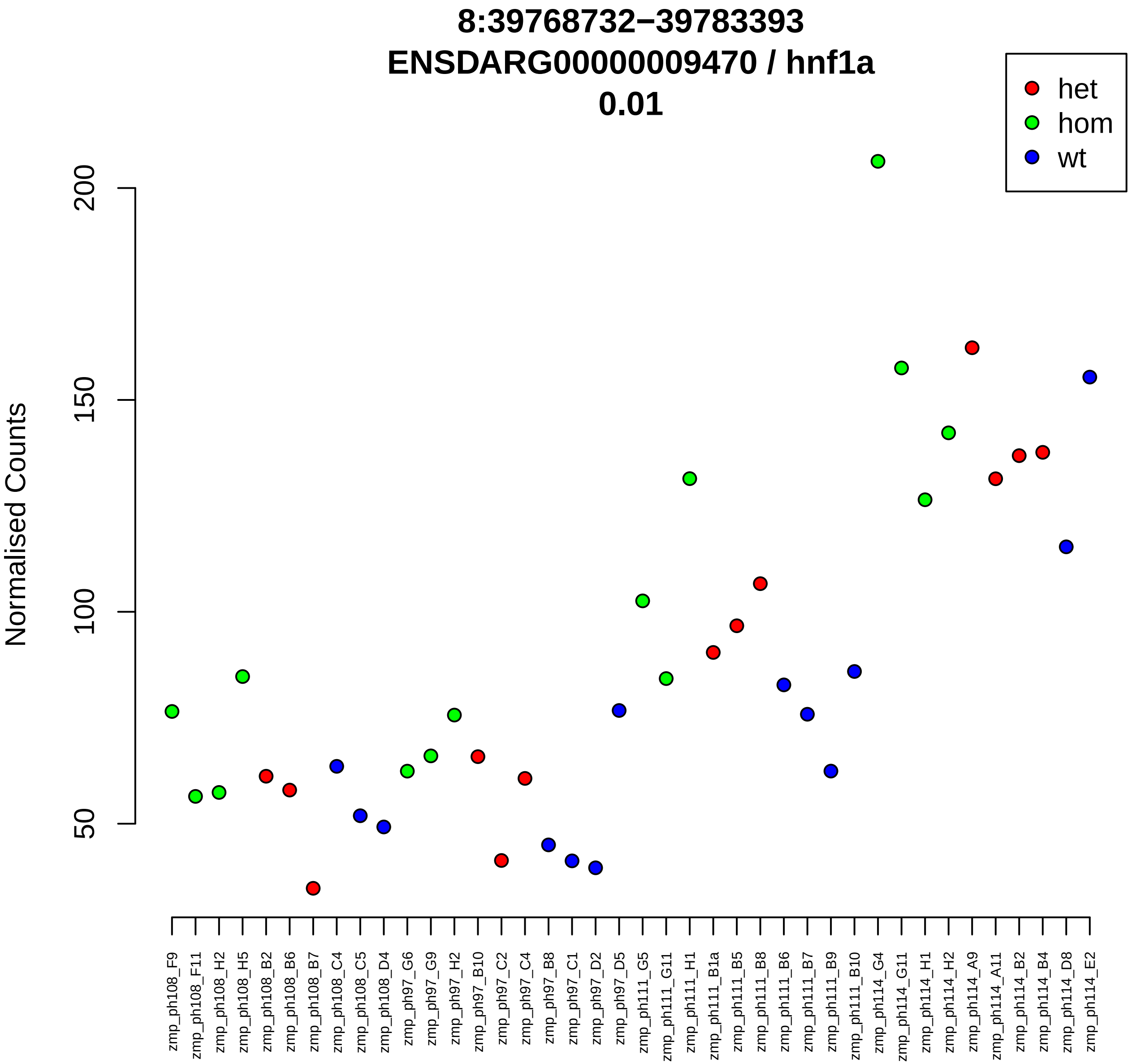
8:39768732−39783393
ENSDARG00000009470 / hnf1a
0.01
het
hom
wt
Normalised Counts
50
100
150
200
zmp_ph108_F9
zmp_ph108_F11
zmp_ph108_H2
zmp_ph108_H5
zmp_ph108_B2
zmp_ph108_B6
zmp_ph108_B7
zmp_ph108_C4
zmp_ph108_C5
zmp_ph108_D4
zmp_ph97_G6
zmp_ph97_G9
zmp_ph97_H2
zmp_ph97_B10
zmp_ph97_C2
zmp_ph97_C4
zmp_ph97_B8
zmp_ph97_C1
zmp_ph97_D2
zmp_ph97_D5
zmp_ph111_G5
zmp_ph111_G11
zmp_ph111_H1
zmp_ph111_B1a
zmp_ph111_B5
zmp_ph111_B8
zmp_ph111_B6
zmp_ph111_B7
zmp_ph111_B9
zmp_ph111_B10
zmp_ph114_G4
zmp_ph114_G11
zmp_ph114_H1
zmp_ph114_H2
zmp_ph114_A9
zmp_ph114_A11
zmp_ph114_B2
zmp_ph114_B4
zmp_ph114_D8
zmp_ph114_E2

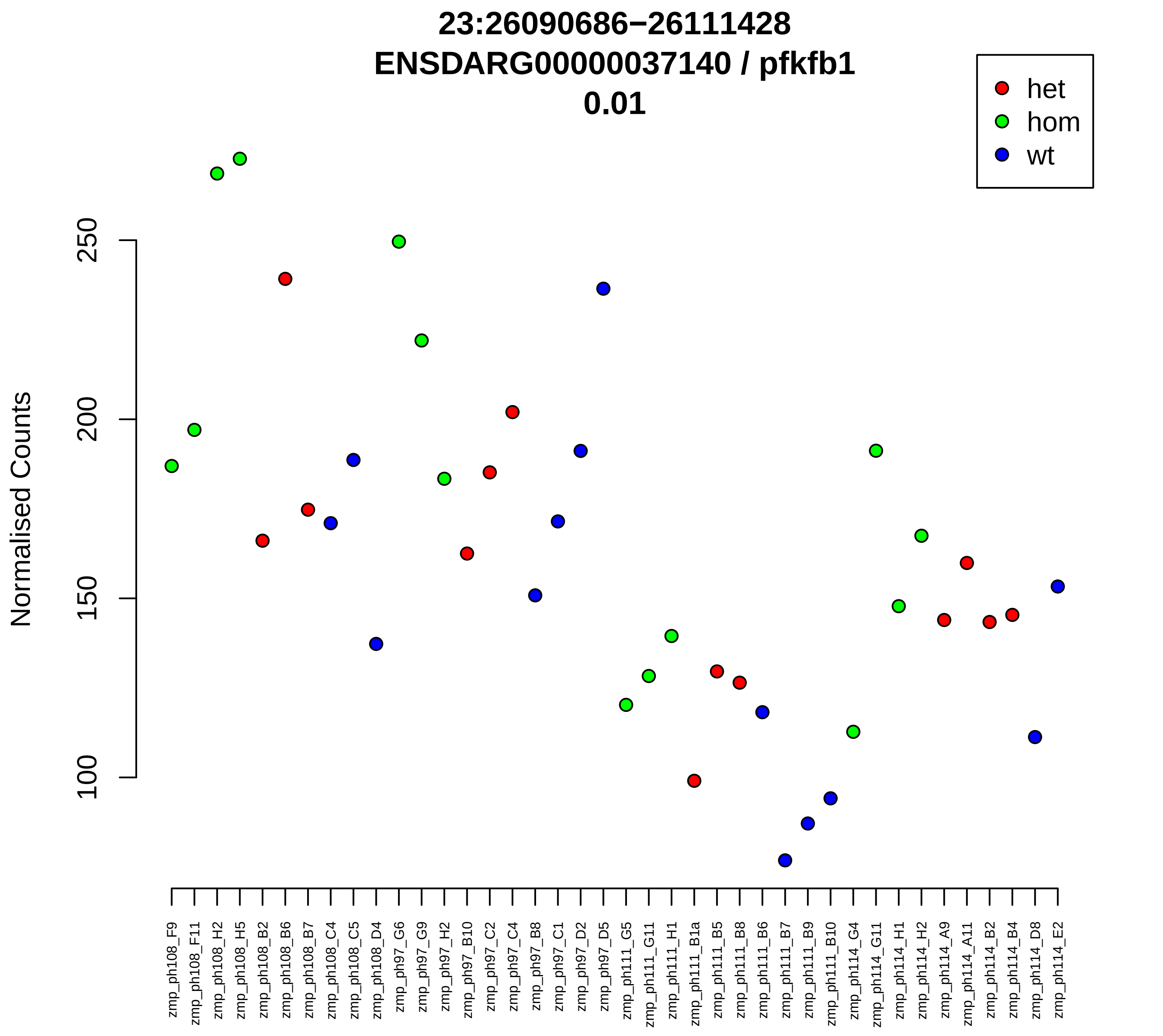
23:26090686−26111428
ENSDARG00000037140 / pfkfb1
0.01
het
hom
wt
Normalised Counts
100
150
200
250
zmp_ph108_F9
zmp_ph108_F11
zmp_ph108_H2
zmp_ph108_H5
zmp_ph108_B2
zmp_ph108_B6
zmp_ph108_B7
zmp_ph108_C4
zmp_ph108_C5
zmp_ph108_D4
zmp_ph97_G6
zmp_ph97_G9
zmp_ph97_H2
zmp_ph97_B10
zmp_ph97_C2
zmp_ph97_C4
zmp_ph97_B8
zmp_ph97_C1
zmp_ph97_D2
zmp_ph97_D5
zmp_ph111_G5
zmp_ph111_G11
zmp_ph111_H1
zmp_ph111_B1a
zmp_ph111_B5
zmp_ph111_B8
zmp_ph111_B6
zmp_ph111_B7
zmp_ph111_B9
zmp_ph111_B10
zmp_ph114_G4
zmp_ph114_G11
zmp_ph114_H1
zmp_ph114_H2
zmp_ph114_A9
zmp_ph114_A11
zmp_ph114_B2
zmp_ph114_B4
zmp_ph114_D8
zmp_ph114_E2

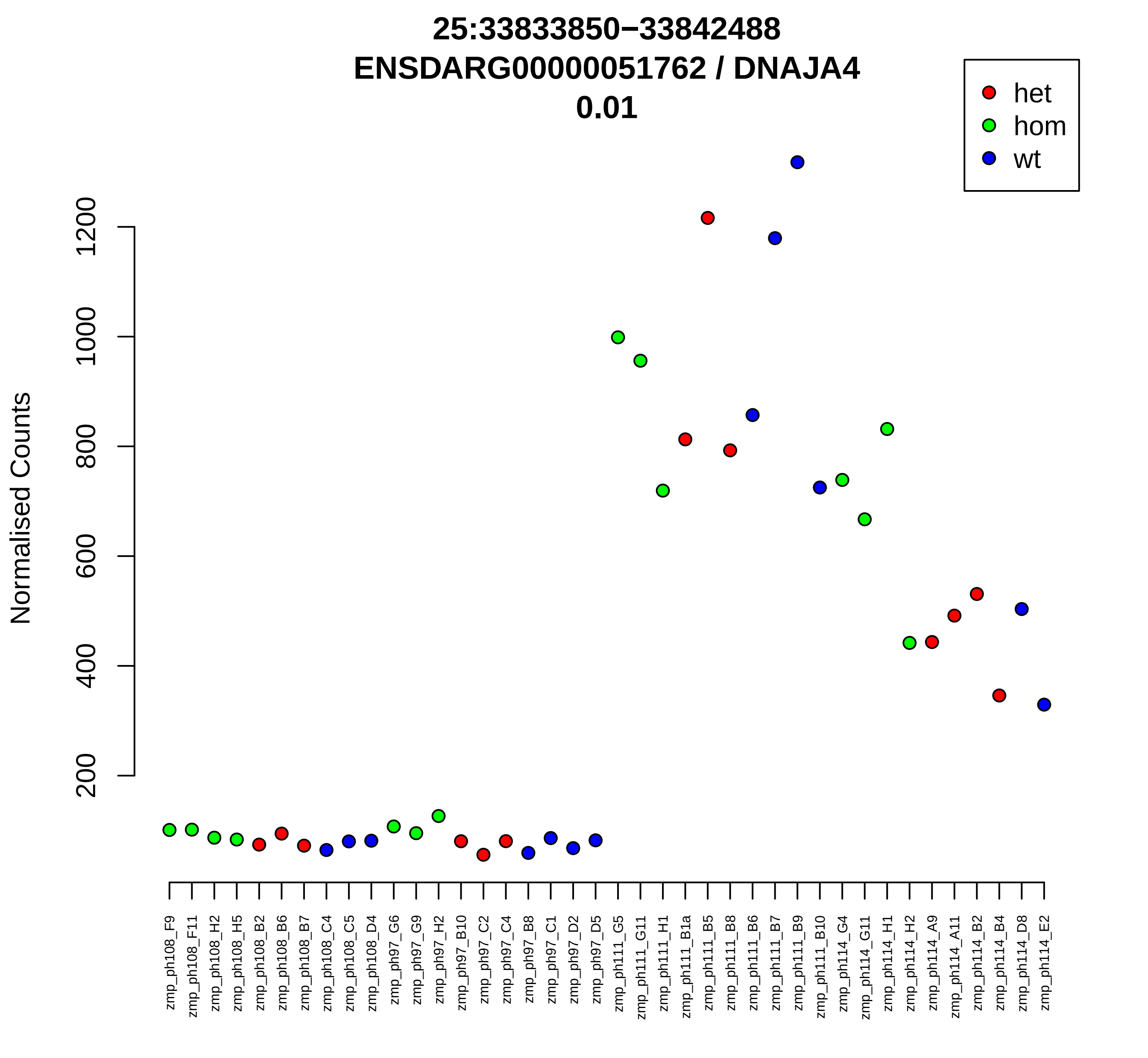
25:33833850−33842488
ENSDARG00000051762 / DNAJA4
0.01
het
hom
wt
Normalised Counts
200
400
600
800
1000
1200
zmp_ph108_F9
zmp_ph108_F11
zmp_ph108_H2
zmp_ph108_H5
zmp_ph108_B2
zmp_ph108_B6
zmp_ph108_B7
zmp_ph108_C4
zmp_ph108_C5
zmp_ph108_D4
zmp_ph97_G6
zmp_ph97_G9
zmp_ph97_H2
zmp_ph97_B10
zmp_ph97_C2
zmp_ph97_C4
zmp_ph97_B8
zmp_ph97_C1
zmp_ph97_D2
zmp_ph97_D5
zmp_ph111_G5
zmp_ph111_G11
zmp_ph111_H1
zmp_ph111_B1a
zmp_ph111_B5
zmp_ph111_B8
zmp_ph111_B6
zmp_ph111_B7
zmp_ph111_B9
zmp_ph111_B10
zmp_ph114_G4
zmp_ph114_G11
zmp_ph114_H1
zmp_ph114_H2
zmp_ph114_A9
zmp_ph114_A11
zmp_ph114_B2
zmp_ph114_B4
zmp_ph114_D8
zmp_ph114_E2

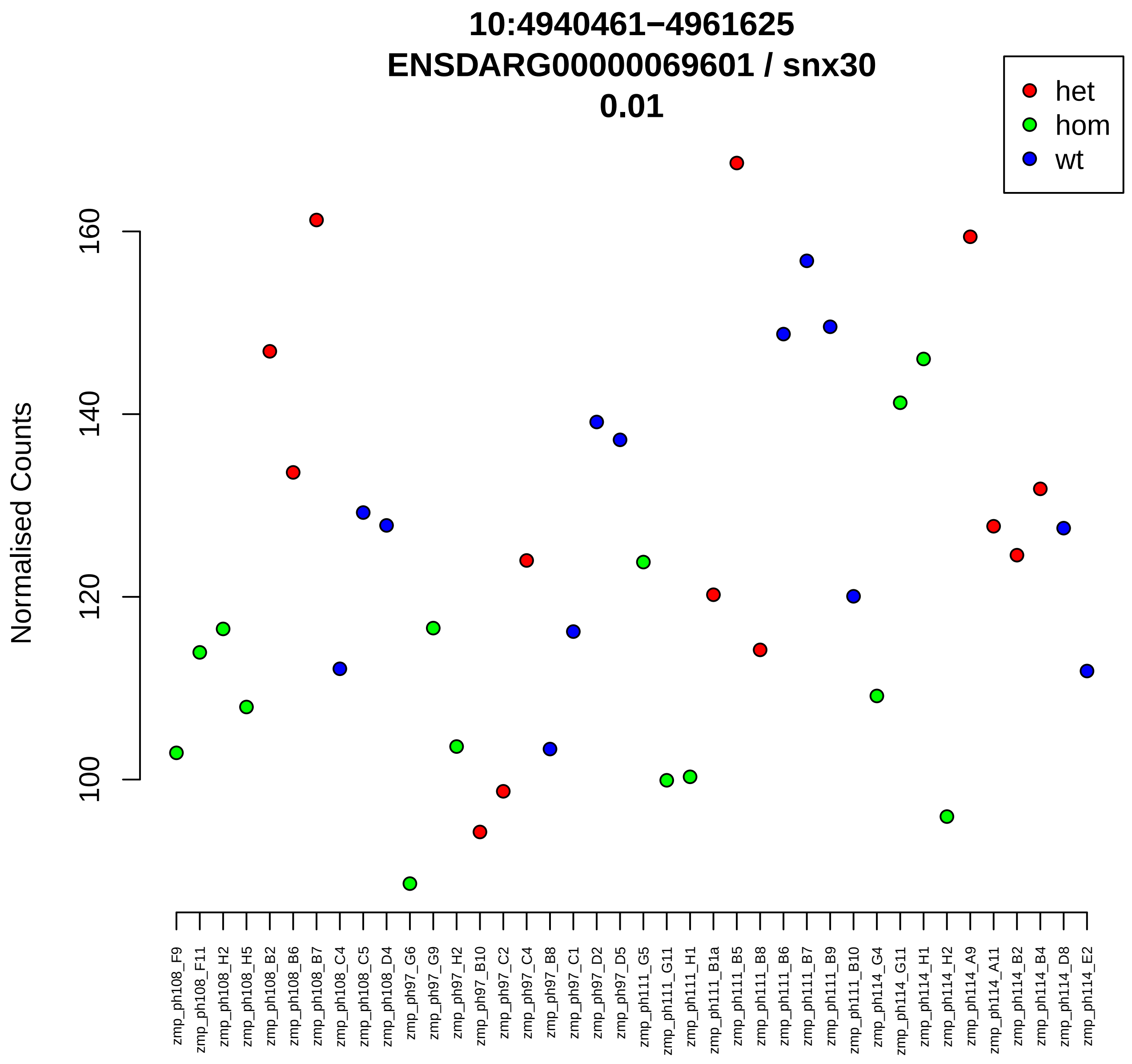
10:4940461−4961625
ENSDARG00000069601 / snx30
0.01
het
hom
wt
Normalised Counts
100
120
140
160
zmp_ph108_F9
zmp_ph108_F11
zmp_ph108_H2
zmp_ph108_H5
zmp_ph108_B2
zmp_ph108_B6
zmp_ph108_B7
zmp_ph108_C4
zmp_ph108_C5
zmp_ph108_D4
zmp_ph97_G6
zmp_ph97_G9
zmp_ph97_H2
zmp_ph97_B10
zmp_ph97_C2
zmp_ph97_C4
zmp_ph97_B8
zmp_ph97_C1
zmp_ph97_D2
zmp_ph97_D5
zmp_ph111_G5
zmp_ph111_G11
zmp_ph111_H1
zmp_ph111_B1a
zmp_ph111_B5
zmp_ph111_B8
zmp_ph111_B6
zmp_ph111_B7
zmp_ph111_B9
zmp_ph111_B10
zmp_ph114_G4
zmp_ph114_G11
zmp_ph114_H1
zmp_ph114_H2
zmp_ph114_A9
zmp_ph114_A11
zmp_ph114_B2
zmp_ph114_B4
zmp_ph114_D8
zmp_ph114_E2

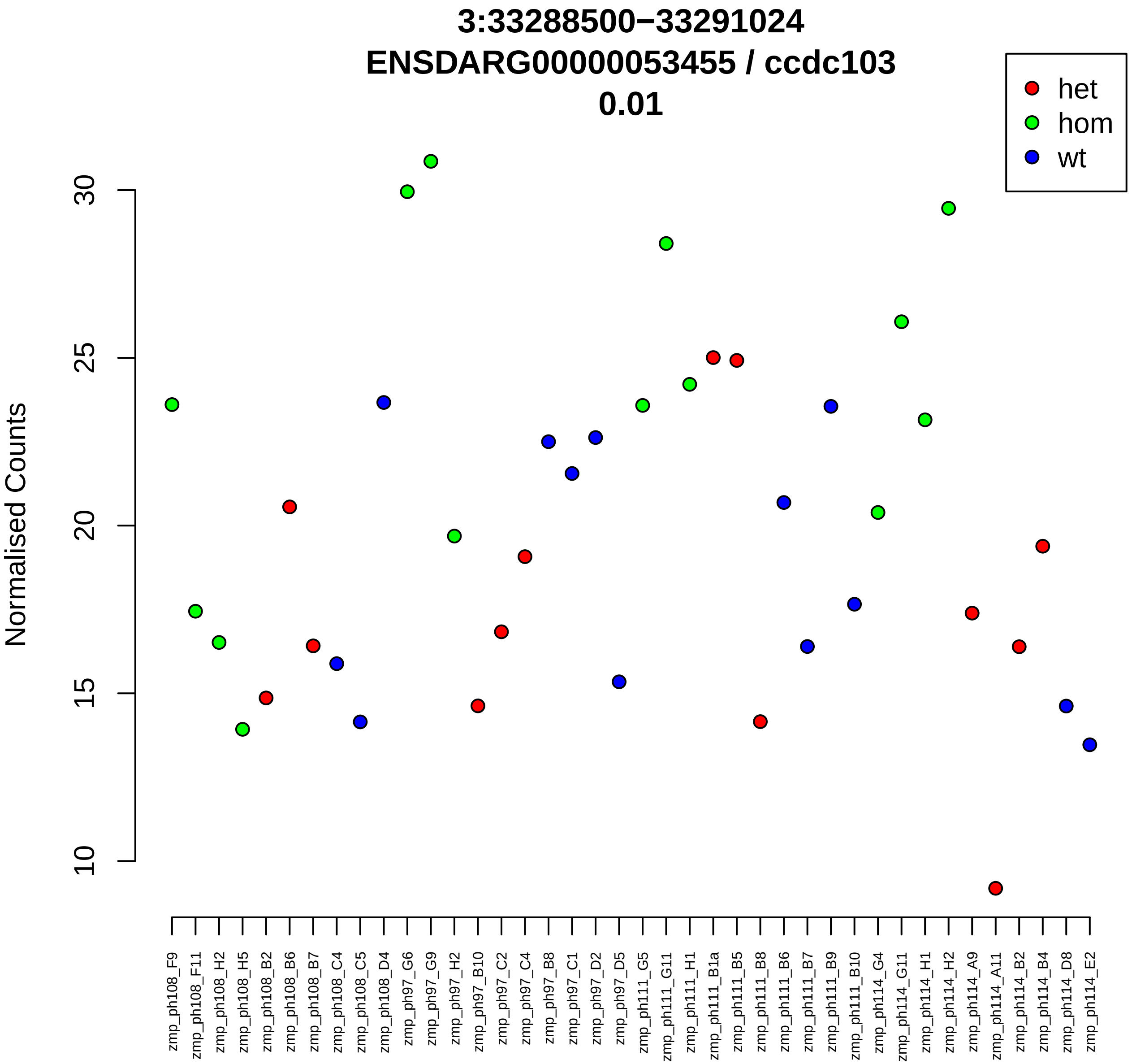
3:33288500−33291024
ENSDARG00000053455 / ccdc103
0.01
het
hom
wt
Normalised Counts
10
15
20
25
30
zmp_ph108_F9
zmp_ph108_F11
zmp_ph108_H2
zmp_ph108_H5
zmp_ph108_B2
zmp_ph108_B6
zmp_ph108_B7
zmp_ph108_C4
zmp_ph108_C5
zmp_ph108_D4
zmp_ph97_G6
zmp_ph97_G9
zmp_ph97_H2
zmp_ph97_B10
zmp_ph97_C2
zmp_ph97_C4
zmp_ph97_B8
zmp_ph97_C1
zmp_ph97_D2
zmp_ph97_D5
zmp_ph111_G5
zmp_ph111_G11
zmp_ph111_H1
zmp_ph111_B1a
zmp_ph111_B5
zmp_ph111_B8
zmp_ph111_B6
zmp_ph111_B7
zmp_ph111_B9
zmp_ph111_B10
zmp_ph114_G4
zmp_ph114_G11
zmp_ph114_H1
zmp_ph114_H2
zmp_ph114_A9
zmp_ph114_A11
zmp_ph114_B2
zmp_ph114_B4
zmp_ph114_D8
zmp_ph114_E2

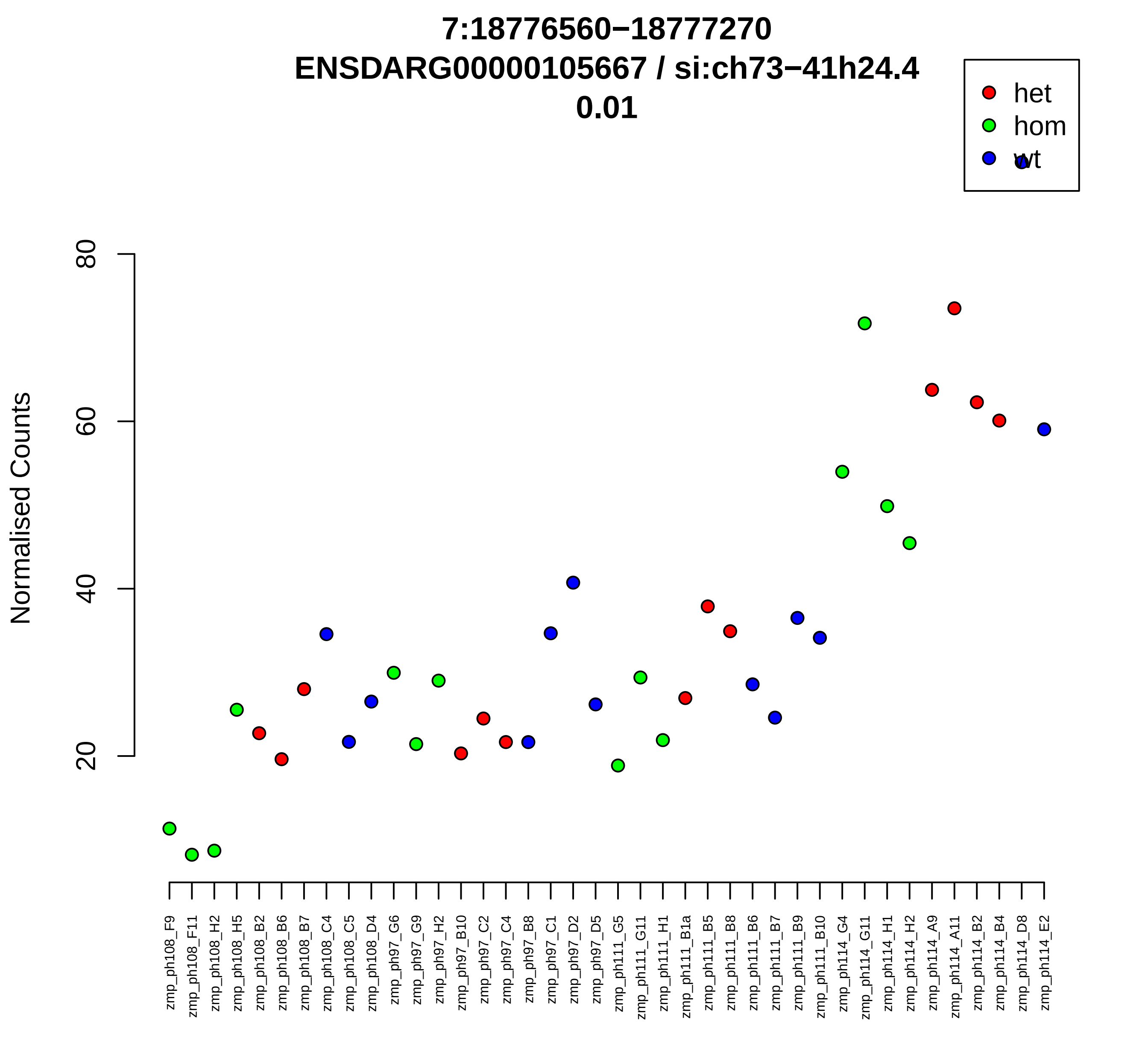

7:18776560−18777270
ENSDARG00000105667 / si:ch73−41h24.4
0.01
het
hom
wt
Normalised Counts
20
40
60
80
zmp_ph108_F9
zmp_ph108_F11
zmp_ph108_H2
zmp_ph108_H5
zmp_ph108_B2
zmp_ph108_B6
zmp_ph108_B7
zmp_ph108_C4
zmp_ph108_C5
zmp_ph108_D4
zmp_ph97_G6
zmp_ph97_G9
zmp_ph97_H2
zmp_ph97_B10
zmp_ph97_C2
zmp_ph97_C4
zmp_ph97_B8
zmp_ph97_C1
zmp_ph97_D2
zmp_ph97_D5
zmp_ph111_G5
zmp_ph111_G11
zmp_ph111_H1
zmp_ph111_B1a
zmp_ph111_B5
zmp_ph111_B8
zmp_ph111_B6
zmp_ph111_B7
zmp_ph111_B9
zmp_ph111_B10
zmp_ph114_G4
zmp_ph114_G11
zmp_ph114_H1
zmp_ph114_H2
zmp_ph114_A9
zmp_ph114_A11
zmp_ph114_B2
zmp_ph114_B4
zmp_ph114_D8
zmp_ph114_E2

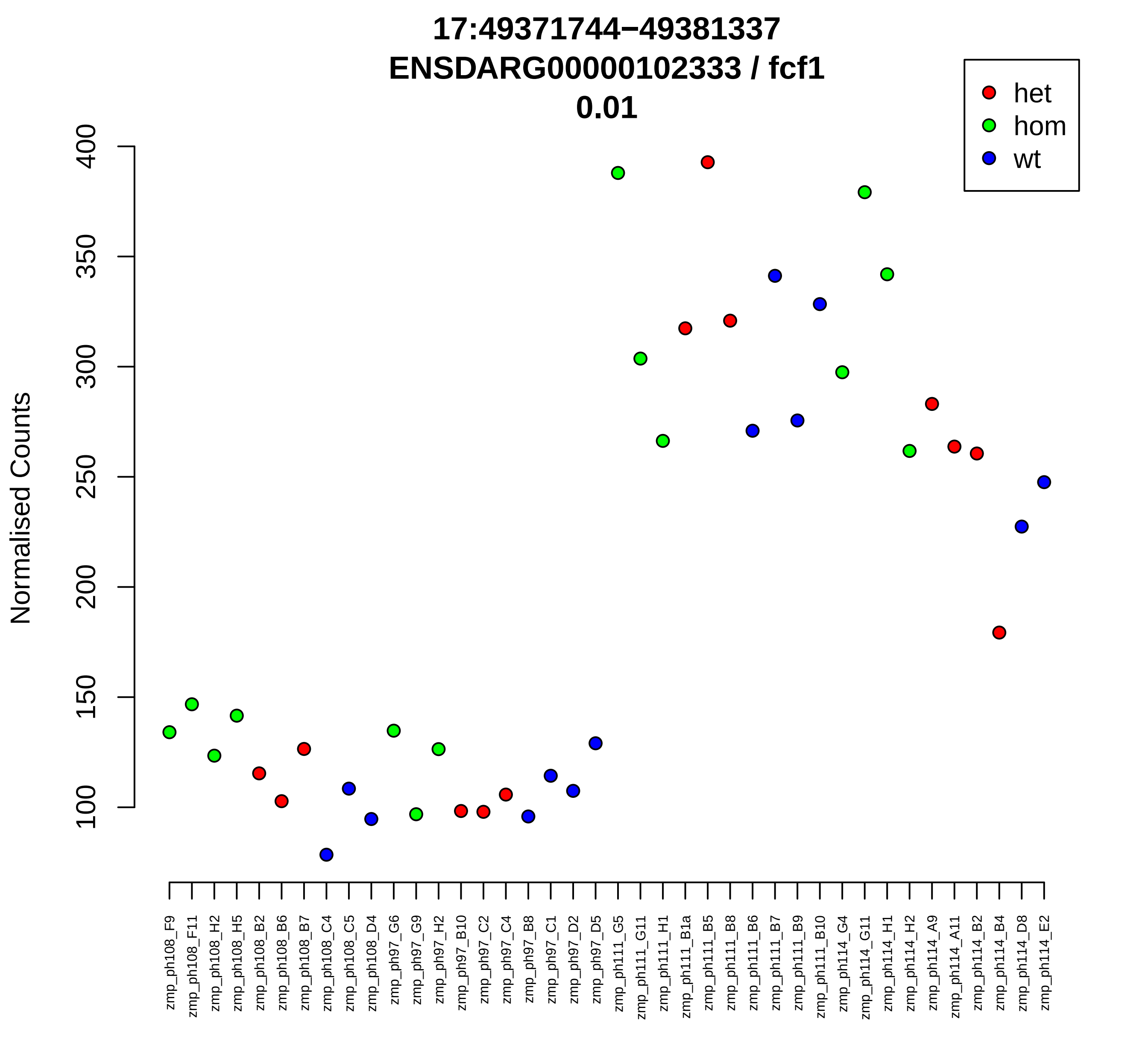

17:49371744−49381337
ENSDARG00000102333 / fcf1
0.01
het
hom
wt
Normalised Counts
100
150
200
250
300
350
400
zmp_ph108_F9
zmp_ph108_F11
zmp_ph108_H2
zmp_ph108_H5
zmp_ph108_B2
zmp_ph108_B6
zmp_ph108_B7
zmp_ph108_C4
zmp_ph108_C5
zmp_ph108_D4
zmp_ph97_G6
zmp_ph97_G9
zmp_ph97_H2
zmp_ph97_B10
zmp_ph97_C2
zmp_ph97_C4
zmp_ph97_B8
zmp_ph97_C1
zmp_ph97_D2
zmp_ph97_D5
zmp_ph111_G5
zmp_ph111_G11
zmp_ph111_H1
zmp_ph111_B1a
zmp_ph111_B5
zmp_ph111_B8
zmp_ph111_B6
zmp_ph111_B7
zmp_ph111_B9
zmp_ph111_B10
zmp_ph114_G4
zmp_ph114_G11
zmp_ph114_H1
zmp_ph114_H2
zmp_ph114_A9
zmp_ph114_A11
zmp_ph114_B2
zmp_ph114_B4
zmp_ph114_D8
zmp_ph114_E2

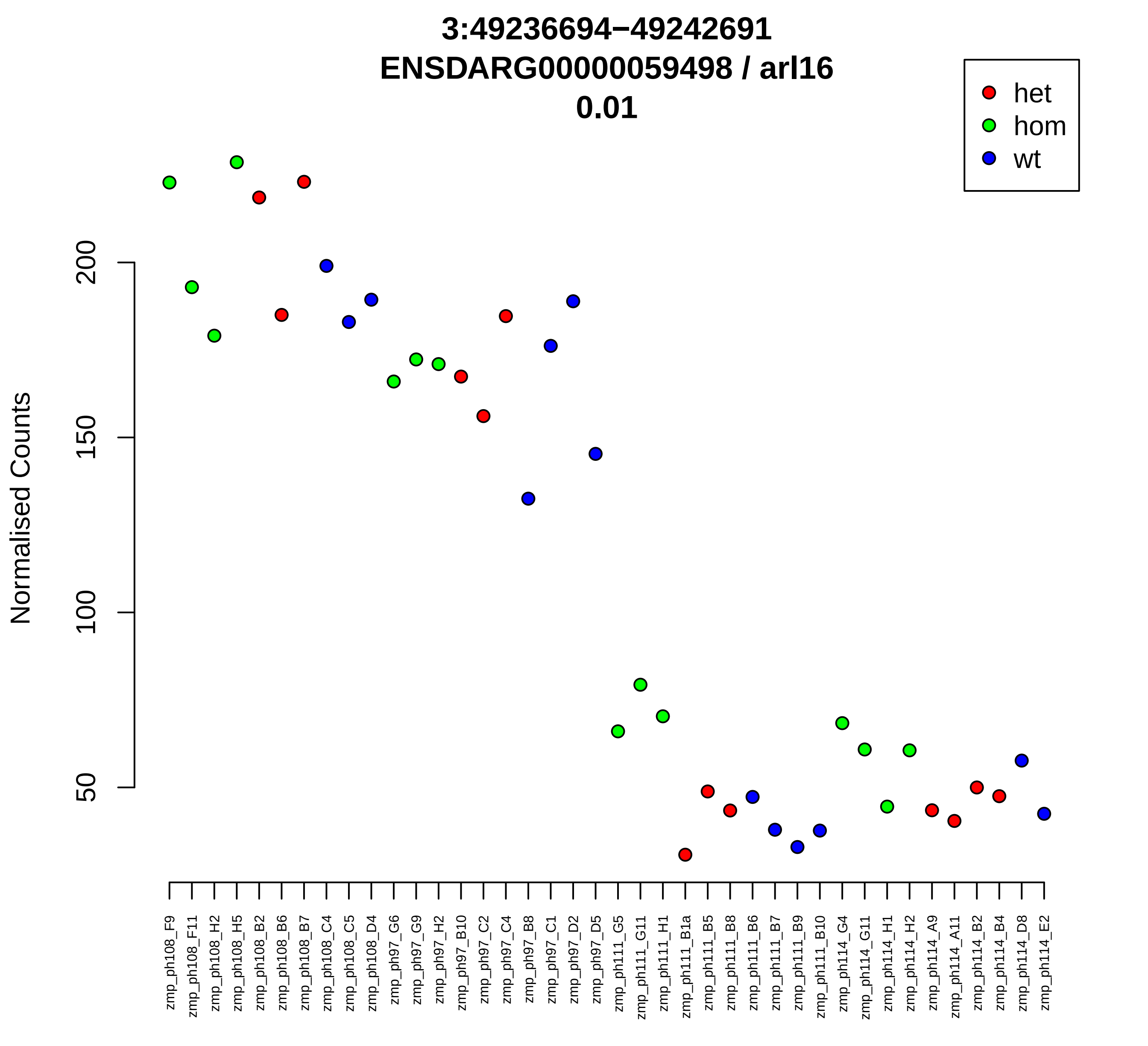
3:49236694−49242691
ENSDARG00000059498 / arl16
0.01
het
hom
wt
Normalised Counts
200
150
100
50
zmp_ph108_F9
zmp_ph108_F11
zmp_ph108_H2
zmp_ph108_H5
zmp_ph108_B2
zmp_ph108_B6
zmp_ph108_B7
zmp_ph108_C4
zmp_ph108_C5
zmp_ph108_D4
zmp_ph97_G6
zmp_ph97_G9
zmp_ph97_H2
zmp_ph97_B10
zmp_ph97_C2
zmp_ph97_C4
zmp_ph97_B8
zmp_ph97_C1
zmp_ph97_D2
zmp_ph97_D5
zmp_ph111_G5
zmp_ph111_G11
zmp_ph111_H1
zmp_ph111_B1a
zmp_ph111_B5
zmp_ph111_B8
zmp_ph111_B6
zmp_ph111_B7
zmp_ph111_B9
zmp_ph111_B10
zmp_ph114_G4
zmp_ph114_G11
zmp_ph114_H1
zmp_ph114_H2
zmp_ph114_A9
zmp_ph114_A11
zmp_ph114_B2
zmp_ph114_B4
zmp_ph114_D8
zmp_ph114_E2

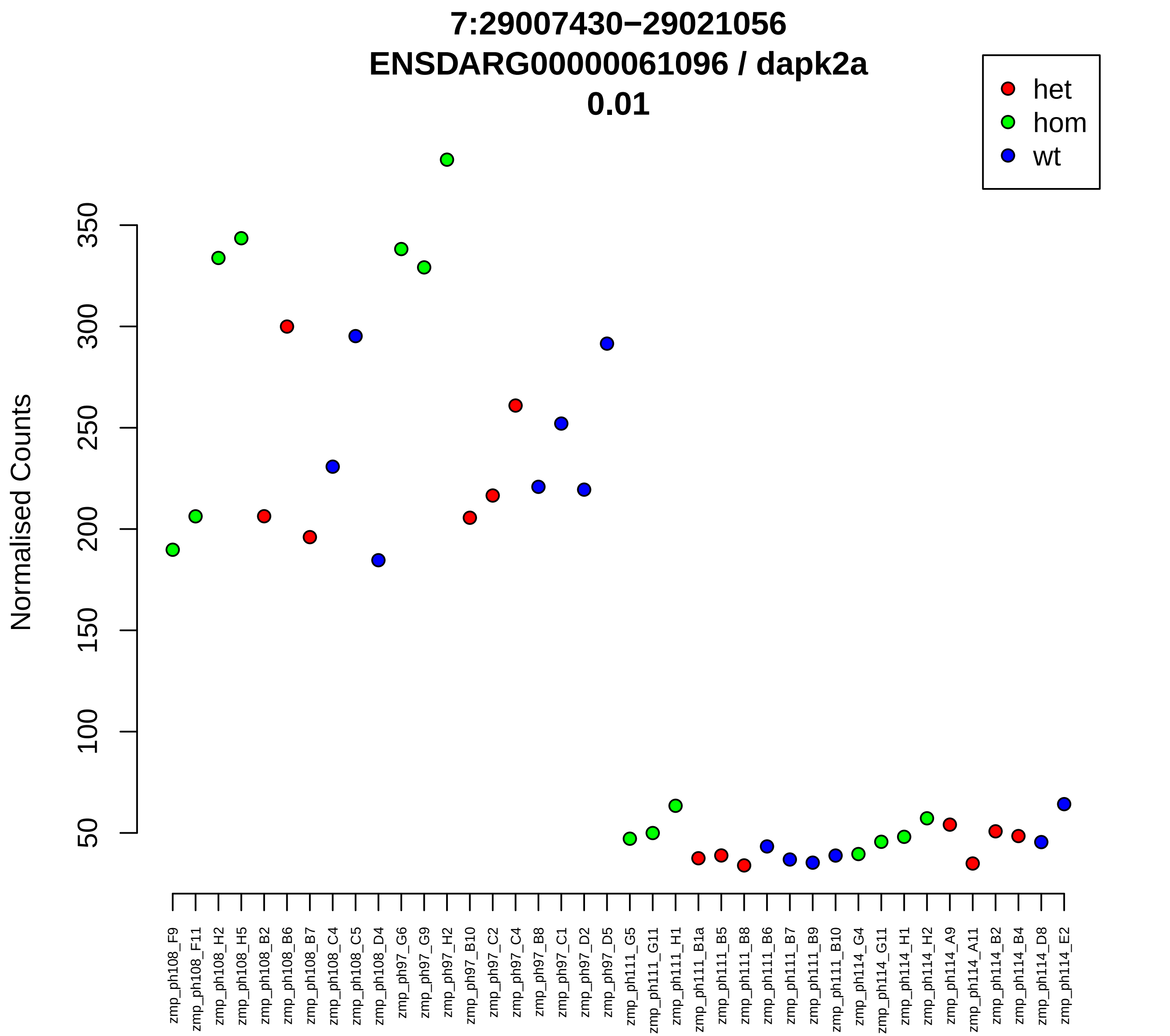

7:29007430−29021056
ENSDARG00000061096 / dapk2a
0.01
het
hom
wt
Normalised Counts
50
100
150
200
250
300
350
zmp_ph108_F9
zmp_ph108_F11
zmp_ph108_H2
zmp_ph108_H5
zmp_ph108_B2
zmp_ph108_B6
zmp_ph108_B7
zmp_ph108_C4
zmp_ph108_C5
zmp_ph108_D4
zmp_ph97_G6
zmp_ph97_G9
zmp_ph97_H2
zmp_ph97_B10
zmp_ph97_C2
zmp_ph97_C4
zmp_ph97_B8
zmp_ph97_C1
zmp_ph97_D2
zmp_ph97_D5
zmp_ph111_G5
zmp_ph111_G11
zmp_ph111_H1
zmp_ph111_B1a
zmp_ph111_B5
zmp_ph111_B8
zmp_ph111_B6
zmp_ph111_B7
zmp_ph111_B9
zmp_ph111_B10
zmp_ph114_G4
zmp_ph114_G11
zmp_ph114_H1
zmp_ph114_H2
zmp_ph114_A9
zmp_ph114_A11
zmp_ph114_B2
zmp_ph114_B4
zmp_ph114_D8
zmp_ph114_E2

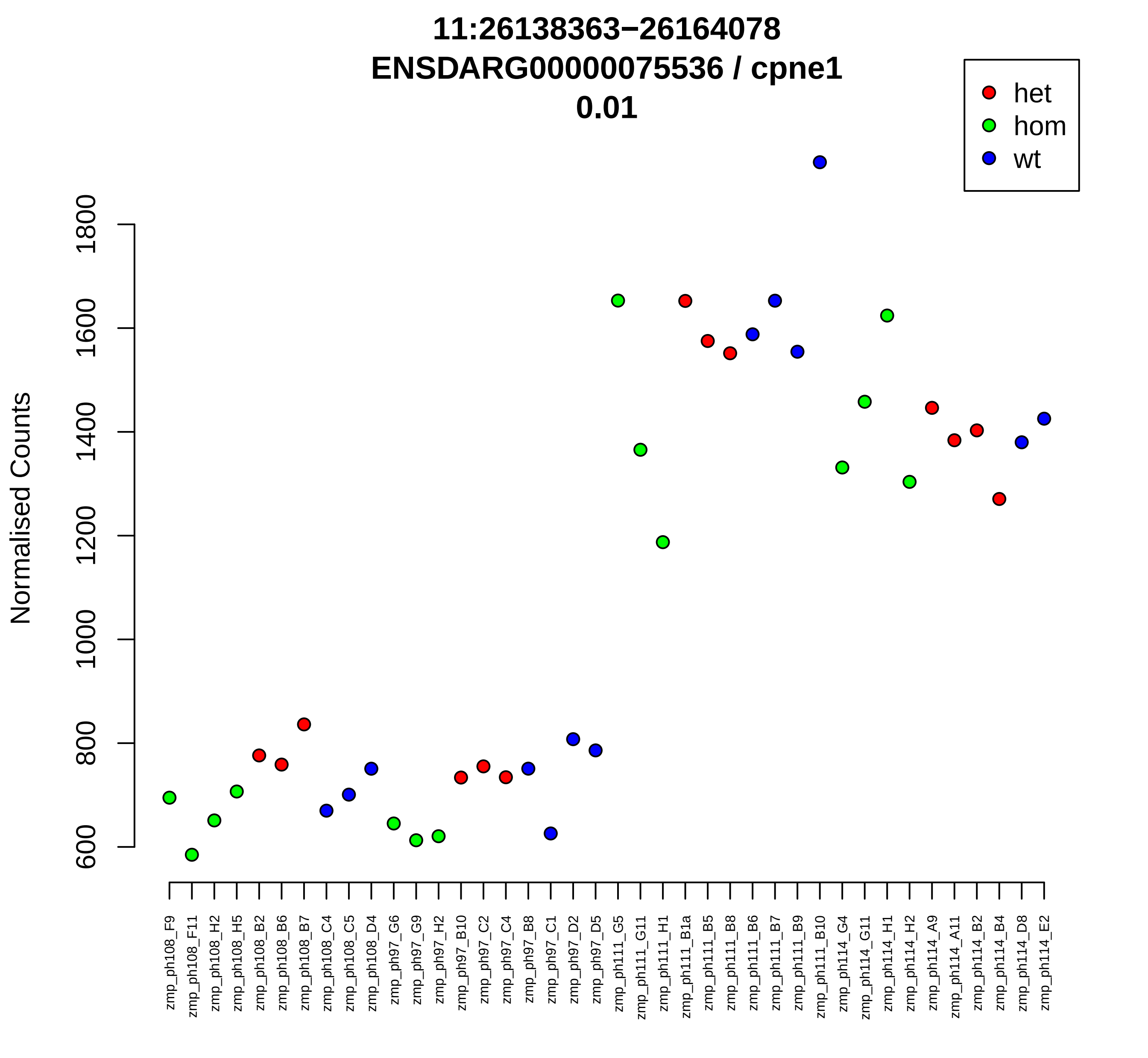
11:26138363−26164078
ENSDARG00000075536 / cpne1
0.01
het
hom
wt
Normalised Counts
600
800
1000
1200
1400
1600
1800
zmp_ph108_F9
zmp_ph108_F11
zmp_ph108_H2
zmp_ph108_H5
zmp_ph108_B2
zmp_ph108_B6
zmp_ph108_B7
zmp_ph108_C4
zmp_ph108_C5
zmp_ph108_D4
zmp_ph97_G6
zmp_ph97_G9
zmp_ph97_H2
zmp_ph97_B10
zmp_ph97_C2
zmp_ph97_C4
zmp_ph97_B8
zmp_ph97_C1
zmp_ph97_D2
zmp_ph97_D5
zmp_ph111_G5
zmp_ph111_G11
zmp_ph111_H1
zmp_ph111_B1a
zmp_ph111_B5
zmp_ph111_B8
zmp_ph111_B6
zmp_ph111_B7
zmp_ph111_B9
zmp_ph111_B10
zmp_ph114_G4
zmp_ph114_G11
zmp_ph114_H1
zmp_ph114_H2
zmp_ph114_A9
zmp_ph114_A11
zmp_ph114_B2
zmp_ph114_B4
zmp_ph114_D8
zmp_ph114_E2

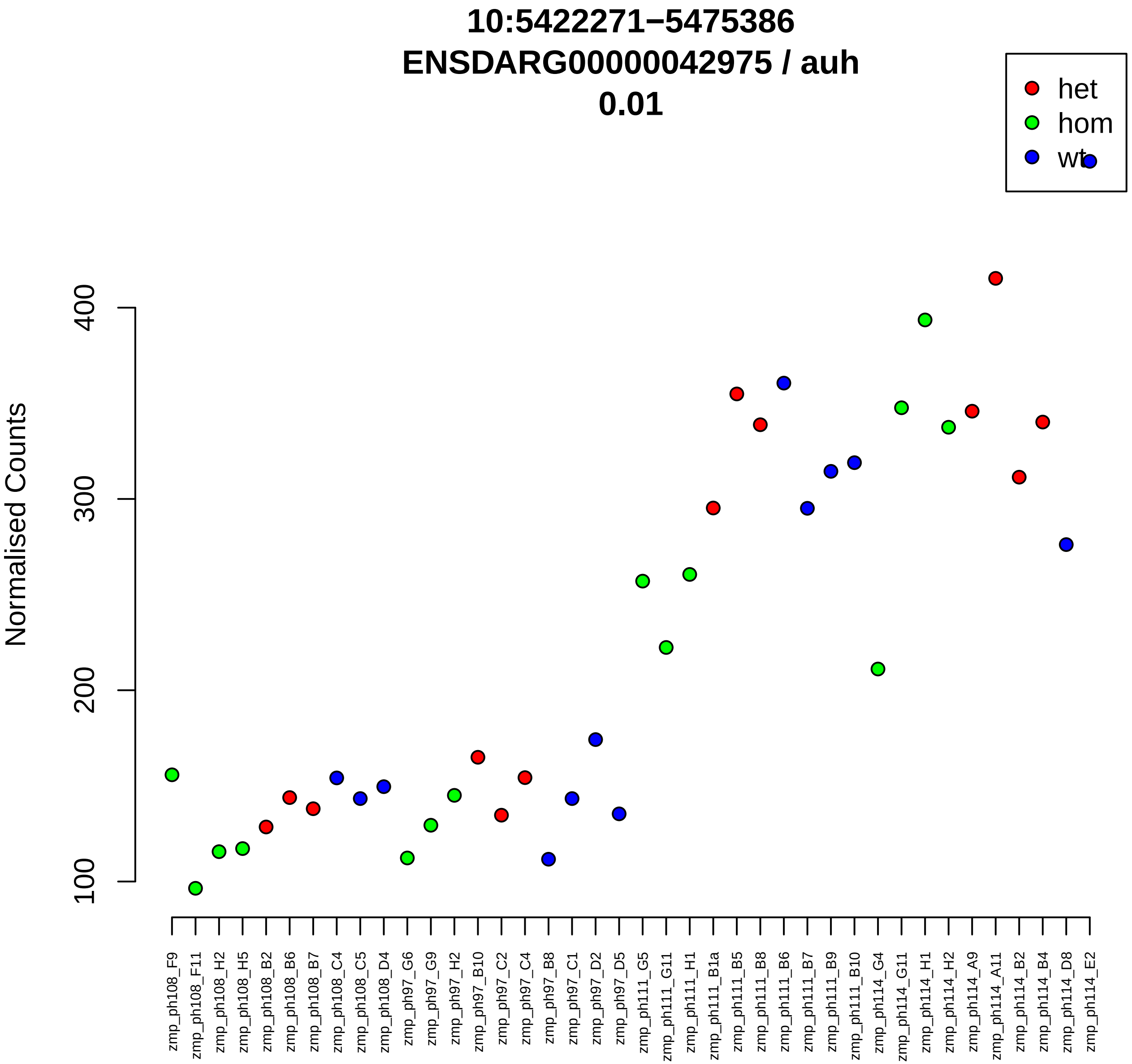

10:5422271−5475386
ENSDARG00000042975 / auh
0.01
het
hom
wt
Normalised Counts
100
200
300
400
zmp_ph108_F9
zmp_ph108_F11
zmp_ph108_H2
zmp_ph108_H5
zmp_ph108_B2
zmp_ph108_B6
zmp_ph108_B7
zmp_ph108_C4
zmp_ph108_C5
zmp_ph108_D4
zmp_ph97_G6
zmp_ph97_G9
zmp_ph97_H2
zmp_ph97_B10
zmp_ph97_C2
zmp_ph97_C4
zmp_ph97_B8
zmp_ph97_C1
zmp_ph97_D2
zmp_ph97_D5
zmp_ph111_G5
zmp_ph111_G11
zmp_ph111_H1
zmp_ph111_B1a
zmp_ph111_B5
zmp_ph111_B8
zmp_ph111_B6
zmp_ph111_B7
zmp_ph111_B9
zmp_ph111_B10
zmp_ph114_G4
zmp_ph114_G11
zmp_ph114_H1
zmp_ph114_H2
zmp_ph114_A9
zmp_ph114_A11
zmp_ph114_B2
zmp_ph114_B4
zmp_ph114_D8
zmp_ph114_E2

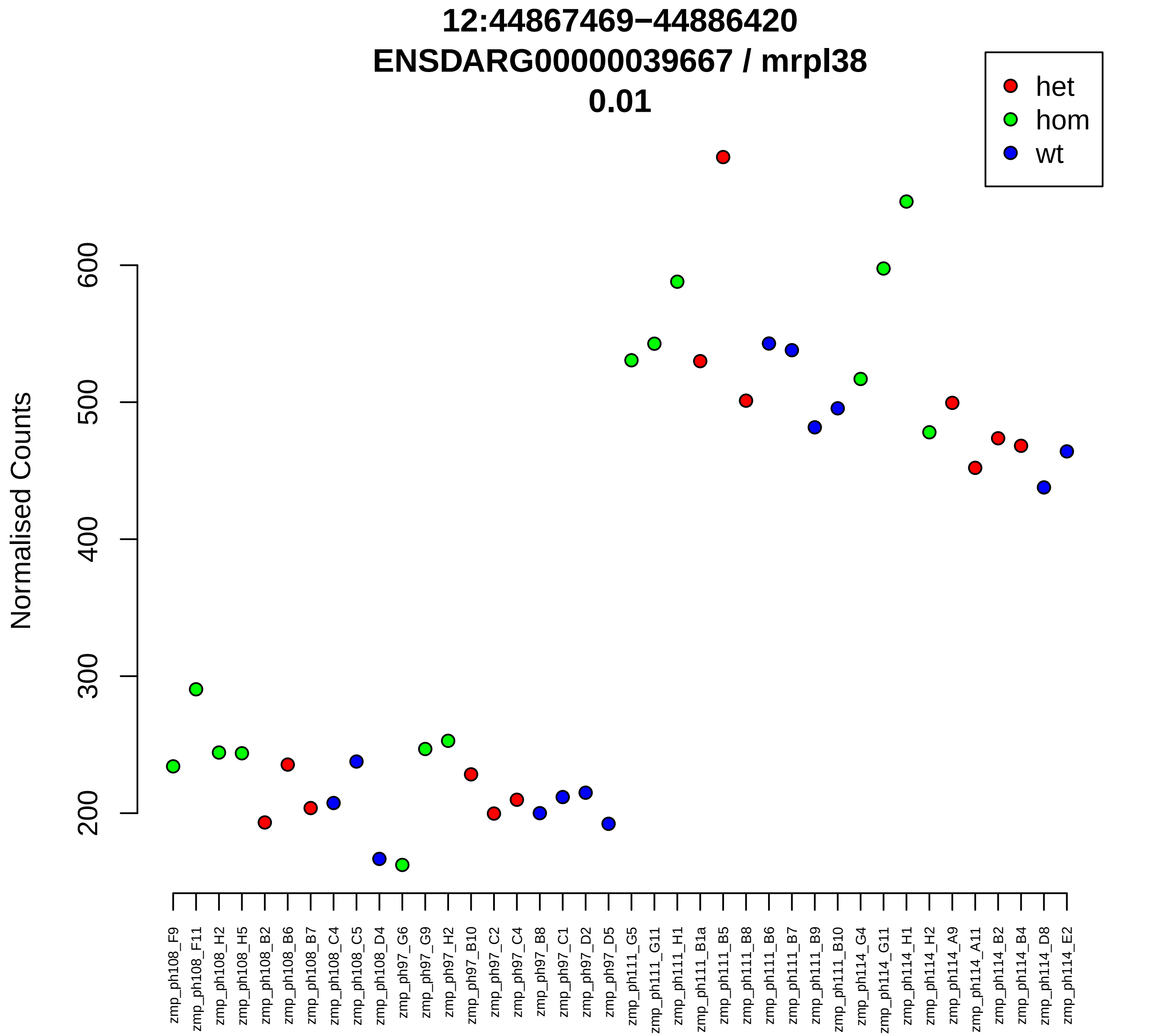

12:44867469−44886420
ENSDARG00000039667 / mrpl38
0.01
het
hom
wt
Normalised Counts
200
300
400
500
600
zmp_ph108_F9
zmp_ph108_F11
zmp_ph108_H2
zmp_ph108_H5
zmp_ph108_B2
zmp_ph108_B6
zmp_ph108_B7
zmp_ph108_C4
zmp_ph108_C5
zmp_ph108_D4
zmp_ph97_G6
zmp_ph97_G9
zmp_ph97_H2
zmp_ph97_B10
zmp_ph97_C2
zmp_ph97_C4
zmp_ph97_B8
zmp_ph97_C1
zmp_ph97_D2
zmp_ph97_D5
zmp_ph111_G5
zmp_ph111_G11
zmp_ph111_H1
zmp_ph111_B1a
zmp_ph111_B5
zmp_ph111_B8
zmp_ph111_B6
zmp_ph111_B7
zmp_ph111_B9
zmp_ph111_B10
zmp_ph114_G4
zmp_ph114_G11
zmp_ph114_H1
zmp_ph114_H2
zmp_ph114_A9
zmp_ph114_A11
zmp_ph114_B2
zmp_ph114_B4
zmp_ph114_D8
zmp_ph114_E2

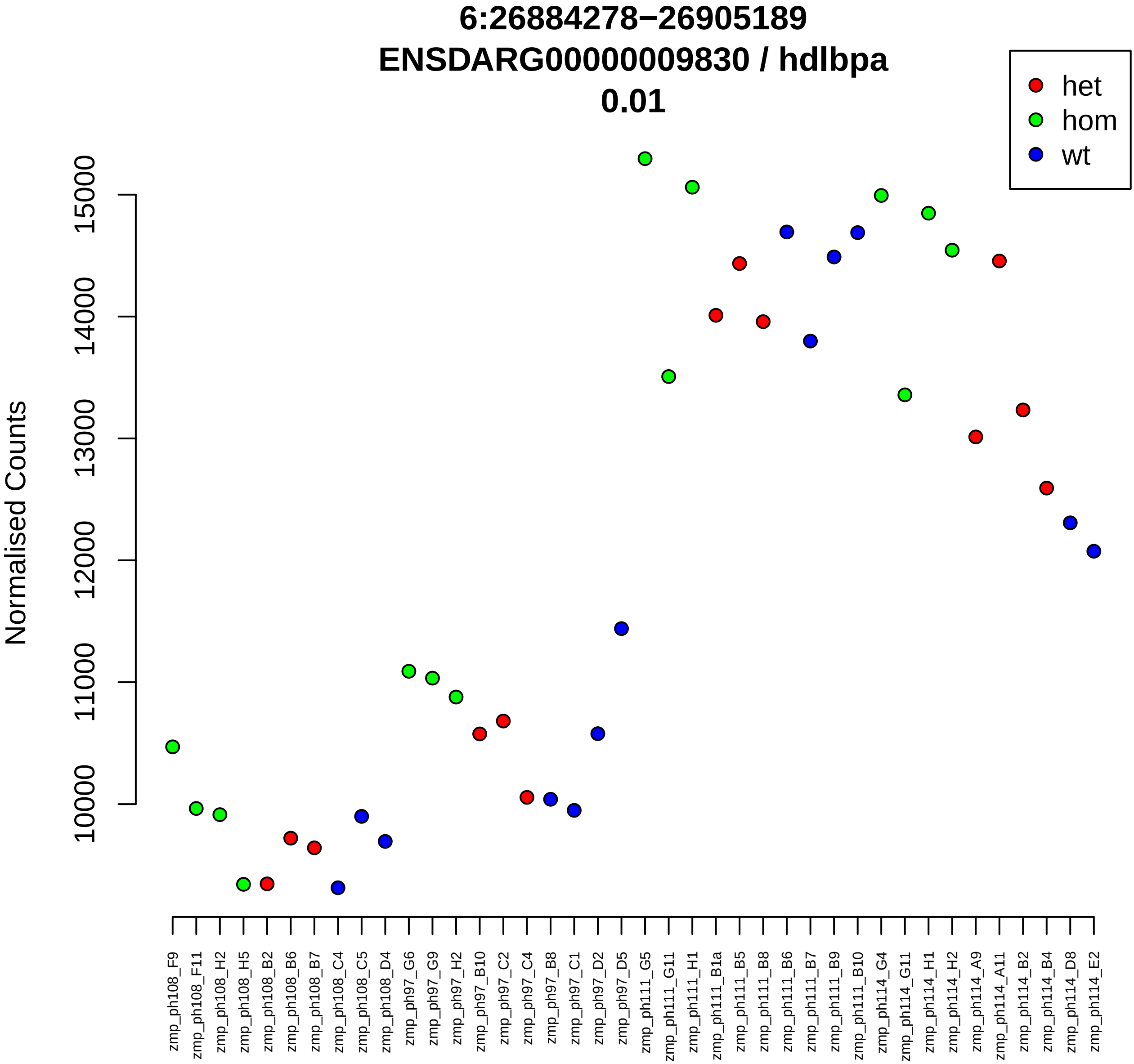
6:26884278−26905189
ENSDARG00000009830 / hdlbpa
0.01
het
hom
wt
Normalised Counts
10000
11000
12000
13000
14000
15000
zmp_ph108_F9
zmp_ph108_F11
zmp_ph108_H2
zmp_ph108_H5
zmp_ph108_B2
zmp_ph108_B6
zmp_ph108_B7
zmp_ph108_C4
zmp_ph108_C5
zmp_ph108_D4
zmp_ph97_G6
zmp_ph97_G9
zmp_ph97_H2
zmp_ph97_B10
zmp_ph97_C2
zmp_ph97_C4
zmp_ph97_B8
zmp_ph97_C1
zmp_ph97_D2
zmp_ph97_D5
zmp_ph111_G5
zmp_ph111_G11
zmp_ph111_H1
zmp_ph111_B1a
zmp_ph111_B5
zmp_ph111_B8
zmp_ph111_B6
zmp_ph111_B7
zmp_ph111_B9
zmp_ph111_B10
zmp_ph114_G4
zmp_ph114_G11
zmp_ph114_H1
zmp_ph114_H2
zmp_ph114_A9
zmp_ph114_A11
zmp_ph114_B2
zmp_ph114_B4
zmp_ph114_D8
zmp_ph114_E2

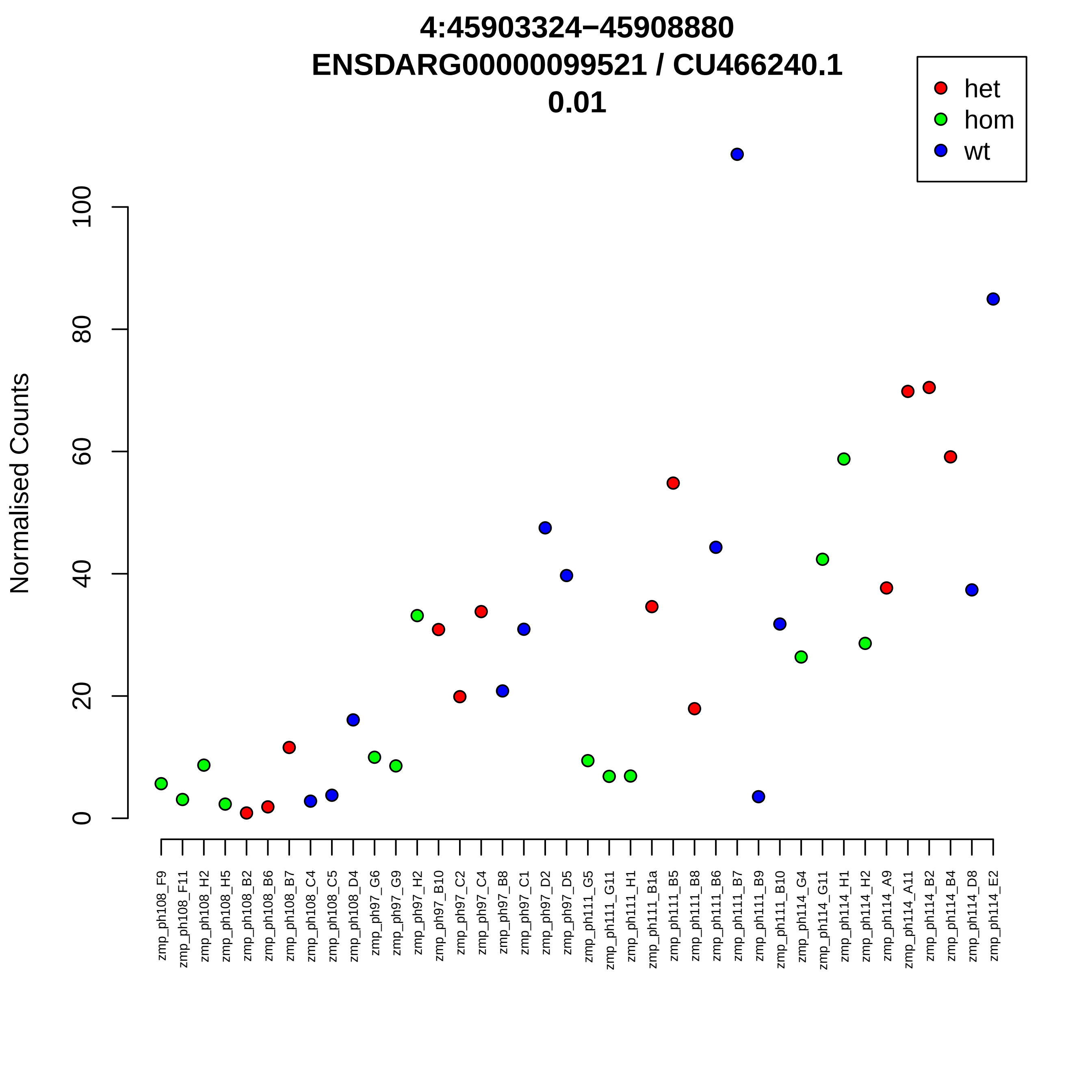
4:45903324−45908880
ENSDARG00000099521 / CU466240.1
0.01
het
hom
wt
Normalised Counts
0
20
40
60
80
100
zmp_ph108_F9
zmp_ph108_F11
zmp_ph108_H2
zmp_ph108_H5
zmp_ph108_B2
zmp_ph108_B6
zmp_ph108_B7
zmp_ph108_C4
zmp_ph108_C5
zmp_ph108_D4
zmp_ph97_G6
zmp_ph97_G9
zmp_ph97_H2
zmp_ph97_B10
zmp_ph97_C2
zmp_ph97_C4
zmp_ph97_B8
zmp_ph97_C1
zmp_ph97_D2
zmp_ph97_D5
zmp_ph111_G5
zmp_ph111_G11
zmp_ph111_H1
zmp_ph111_B1a
zmp_ph111_B5
zmp_ph111_B8
zmp_ph111_B6
zmp_ph111_B7
zmp_ph111_B9
zmp_ph111_B10
zmp_ph114_G4
zmp_ph114_G11
zmp_ph114_H1
zmp_ph114_H2
zmp_ph114_A9
zmp_ph114_A11
zmp_ph114_B2
zmp_ph114_B4
zmp_ph114_D8
zmp_ph114_E2

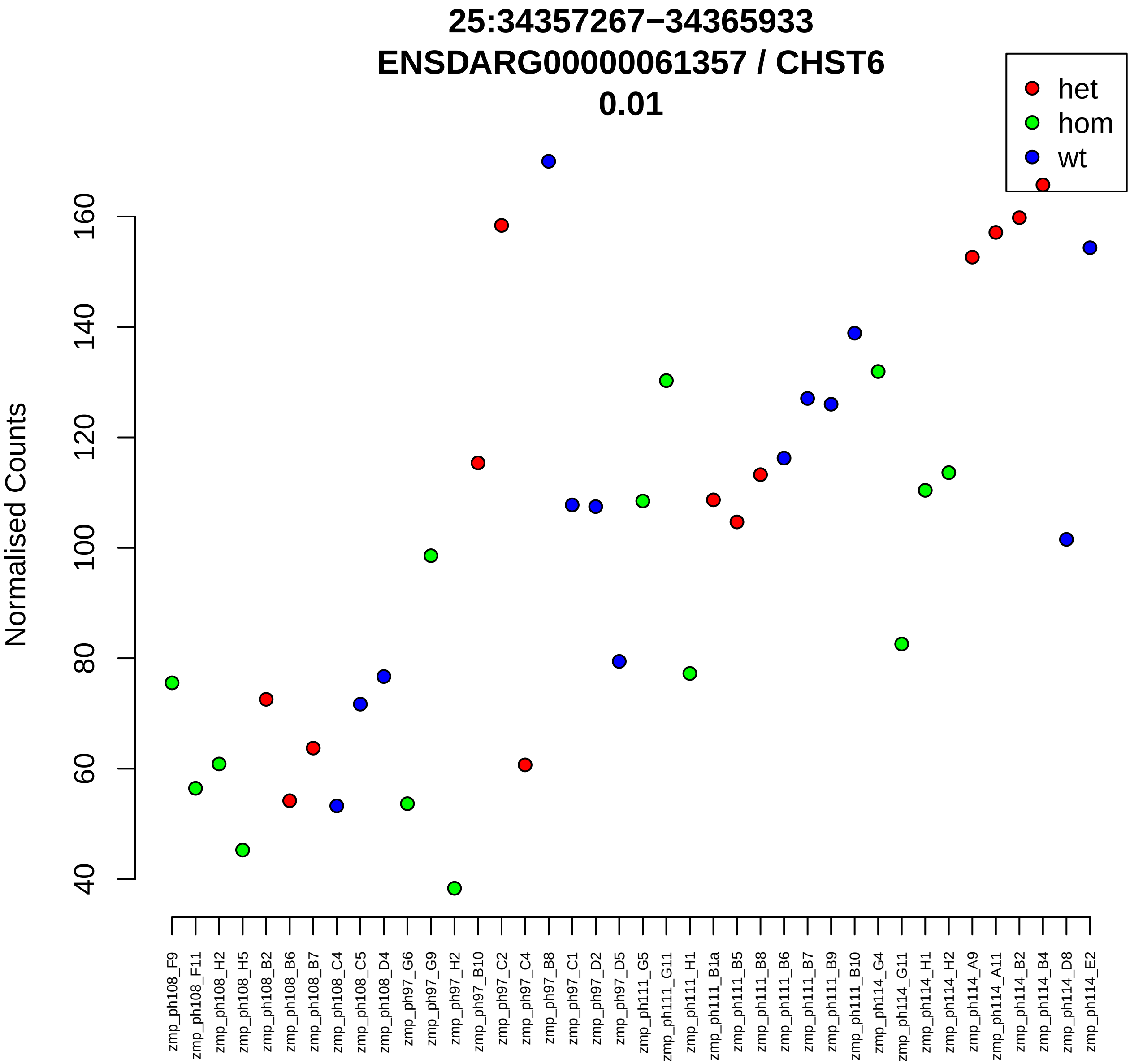
25:34357267−34365933
ENSDARG00000061357 / CHST6
0.01
het
hom
wt
Normalised Counts
40
60
80
100
120
140
160
zmp_ph108_F9
zmp_ph108_F11
zmp_ph108_H2
zmp_ph108_H5
zmp_ph108_B2
zmp_ph108_B6
zmp_ph108_B7
zmp_ph108_C4
zmp_ph108_C5
zmp_ph108_D4
zmp_ph97_G6
zmp_ph97_G9
zmp_ph97_H2
zmp_ph97_B10
zmp_ph97_C2
zmp_ph97_C4
zmp_ph97_B8
zmp_ph97_C1
zmp_ph97_D2
zmp_ph97_D5
zmp_ph111_G5
zmp_ph111_G11
zmp_ph111_H1
zmp_ph111_B1a
zmp_ph111_B5
zmp_ph111_B8
zmp_ph111_B6
zmp_ph111_B7
zmp_ph111_B9
zmp_ph111_B10
zmp_ph114_G4
zmp_ph114_G11
zmp_ph114_H1
zmp_ph114_H2
zmp_ph114_A9
zmp_ph114_A11
zmp_ph114_B2
zmp_ph114_B4
zmp_ph114_D8
zmp_ph114_E2

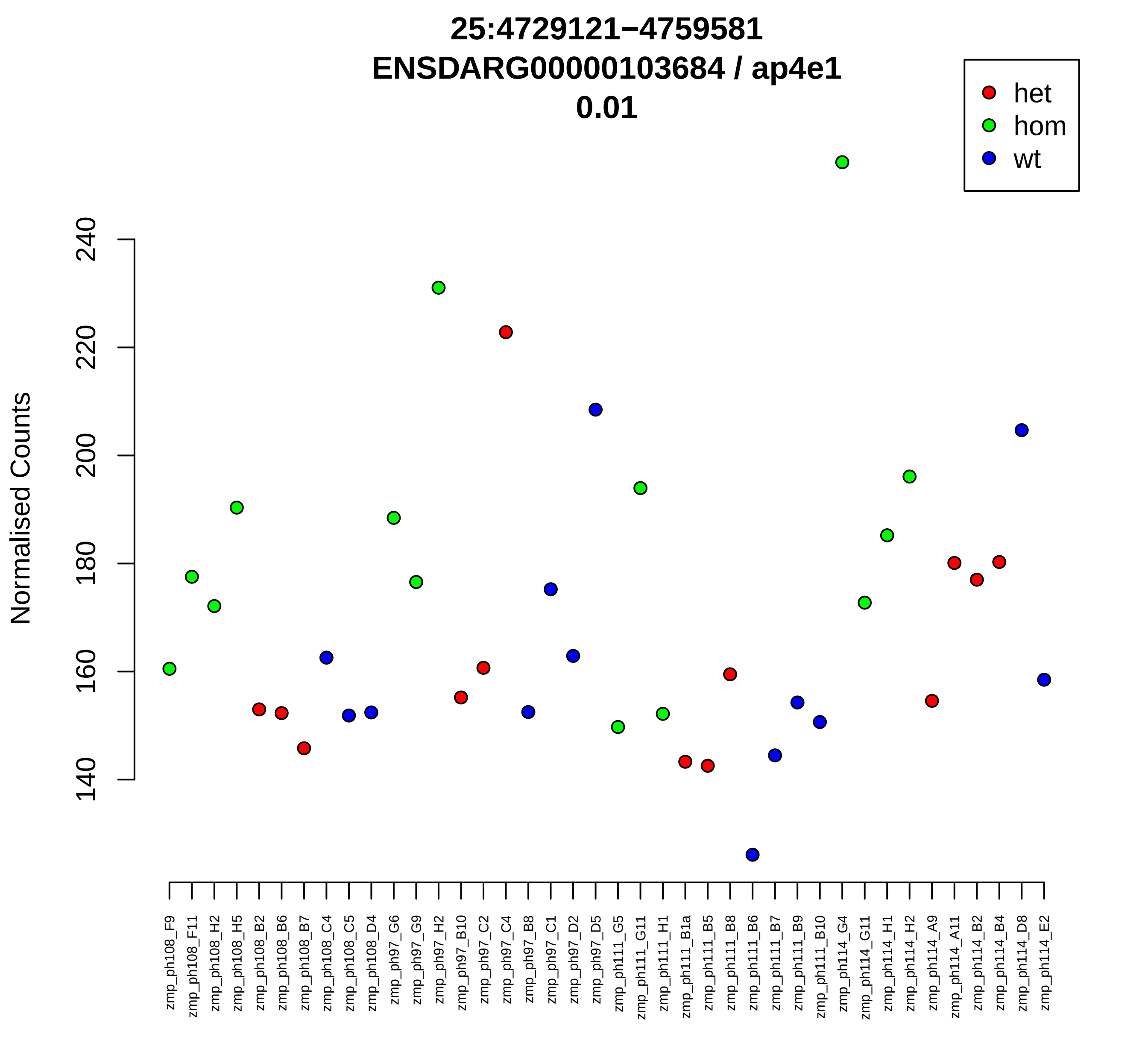

25:4729121−4759581
ENSDARG00000103684 / ap4e1
0.01
het
hom
wt
Normalised Counts
140
160
180
200
220
240
zmp_ph108_F9
zmp_ph108_F11
zmp_ph108_H2
zmp_ph108_H5
zmp_ph108_B2
zmp_ph108_B6
zmp_ph108_B7
zmp_ph108_C4
zmp_ph108_C5
zmp_ph108_D4
zmp_ph97_G6
zmp_ph97_G9
zmp_ph97_H2
zmp_ph97_B10
zmp_ph97_C2
zmp_ph97_C4
zmp_ph97_B8
zmp_ph97_C1
zmp_ph97_D2
zmp_ph97_D5
zmp_ph111_G5
zmp_ph111_G11
zmp_ph111_H1
zmp_ph111_B1a
zmp_ph111_B5
zmp_ph111_B8
zmp_ph111_B6
zmp_ph111_B7
zmp_ph111_B9
zmp_ph111_B10
zmp_ph114_G4
zmp_ph114_G11
zmp_ph114_H1
zmp_ph114_H2
zmp_ph114_A9
zmp_ph114_A11
zmp_ph114_B2
zmp_ph114_B4
zmp_ph114_D8
zmp_ph114_E2

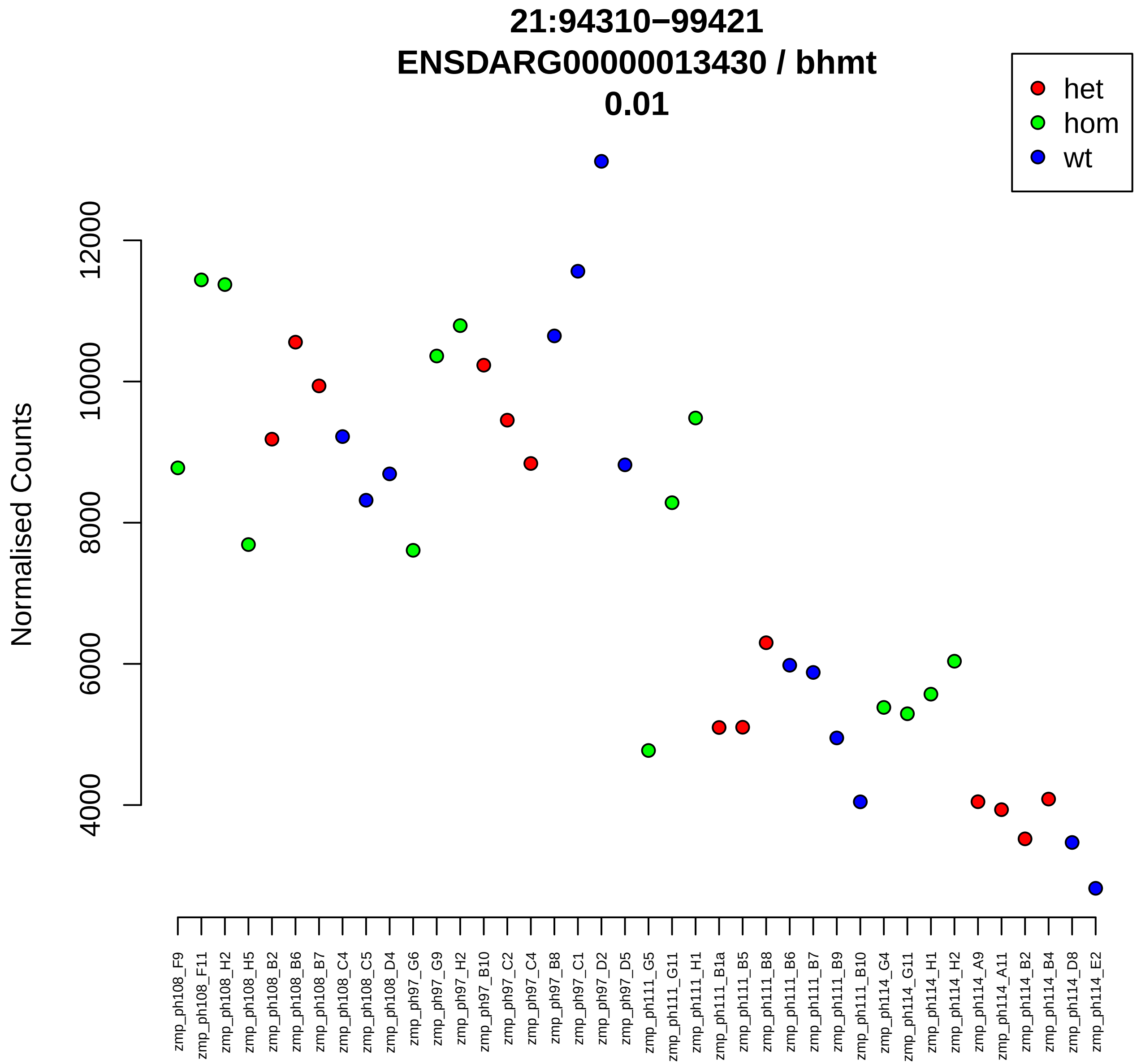
21:94310−99421
ENSDARG00000013430 / bhmt
0.01
het
hom
wt
Normalised Counts
4000
6000
8000
10000
12000
zmp_ph108_F9
zmp_ph108_F11
zmp_ph108_H2
zmp_ph108_H5
zmp_ph108_B2
zmp_ph108_B6
zmp_ph108_B7
zmp_ph108_C4
zmp_ph108_C5
zmp_ph108_D4
zmp_ph97_G6
zmp_ph97_G9
zmp_ph97_H2
zmp_ph97_B10
zmp_ph97_C2
zmp_ph97_C4
zmp_ph97_B8
zmp_ph97_C1
zmp_ph97_D2
zmp_ph97_D5
zmp_ph111_G5
zmp_ph111_G11
zmp_ph111_H1
zmp_ph111_B1a
zmp_ph111_B5
zmp_ph111_B8
zmp_ph111_B6
zmp_ph111_B7
zmp_ph111_B9
zmp_ph111_B10
zmp_ph114_G4
zmp_ph114_G11
zmp_ph114_H1
zmp_ph114_H2
zmp_ph114_A9
zmp_ph114_A11
zmp_ph114_B2
zmp_ph114_B4
zmp_ph114_D8
zmp_ph114_E2

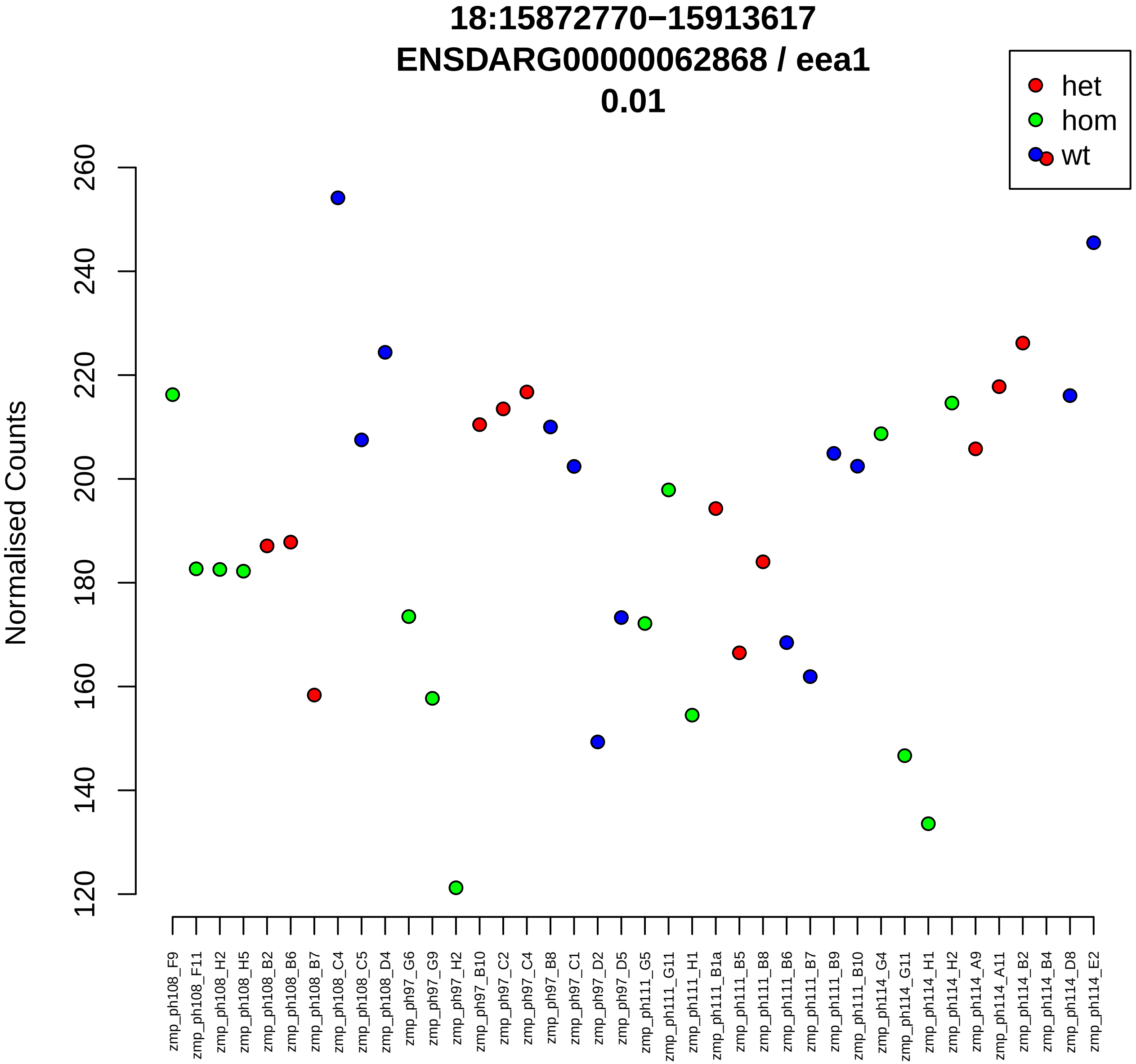

18:15872770−15913617
ENSDARG00000062868 / eea1
0.01
het
hom
wt
Normalised Counts
120
140
160
180
200
220
240
260
zmp_ph108_F9
zmp_ph108_F11
zmp_ph108_H2
zmp_ph108_H5
zmp_ph108_B2
zmp_ph108_B6
zmp_ph108_B7
zmp_ph108_C4
zmp_ph108_C5
zmp_ph108_D4
zmp_ph97_G6
zmp_ph97_G9
zmp_ph97_H2
zmp_ph97_B10
zmp_ph97_C2
zmp_ph97_C4
zmp_ph97_B8
zmp_ph97_C1
zmp_ph97_D2
zmp_ph97_D5
zmp_ph111_G5
zmp_ph111_G11
zmp_ph111_H1
zmp_ph111_B1a
zmp_ph111_B5
zmp_ph111_B8
zmp_ph111_B6
zmp_ph111_B7
zmp_ph111_B9
zmp_ph111_B10
zmp_ph114_G4
zmp_ph114_G11
zmp_ph114_H1
zmp_ph114_H2
zmp_ph114_A9
zmp_ph114_A11
zmp_ph114_B2
zmp_ph114_B4
zmp_ph114_D8
zmp_ph114_E2

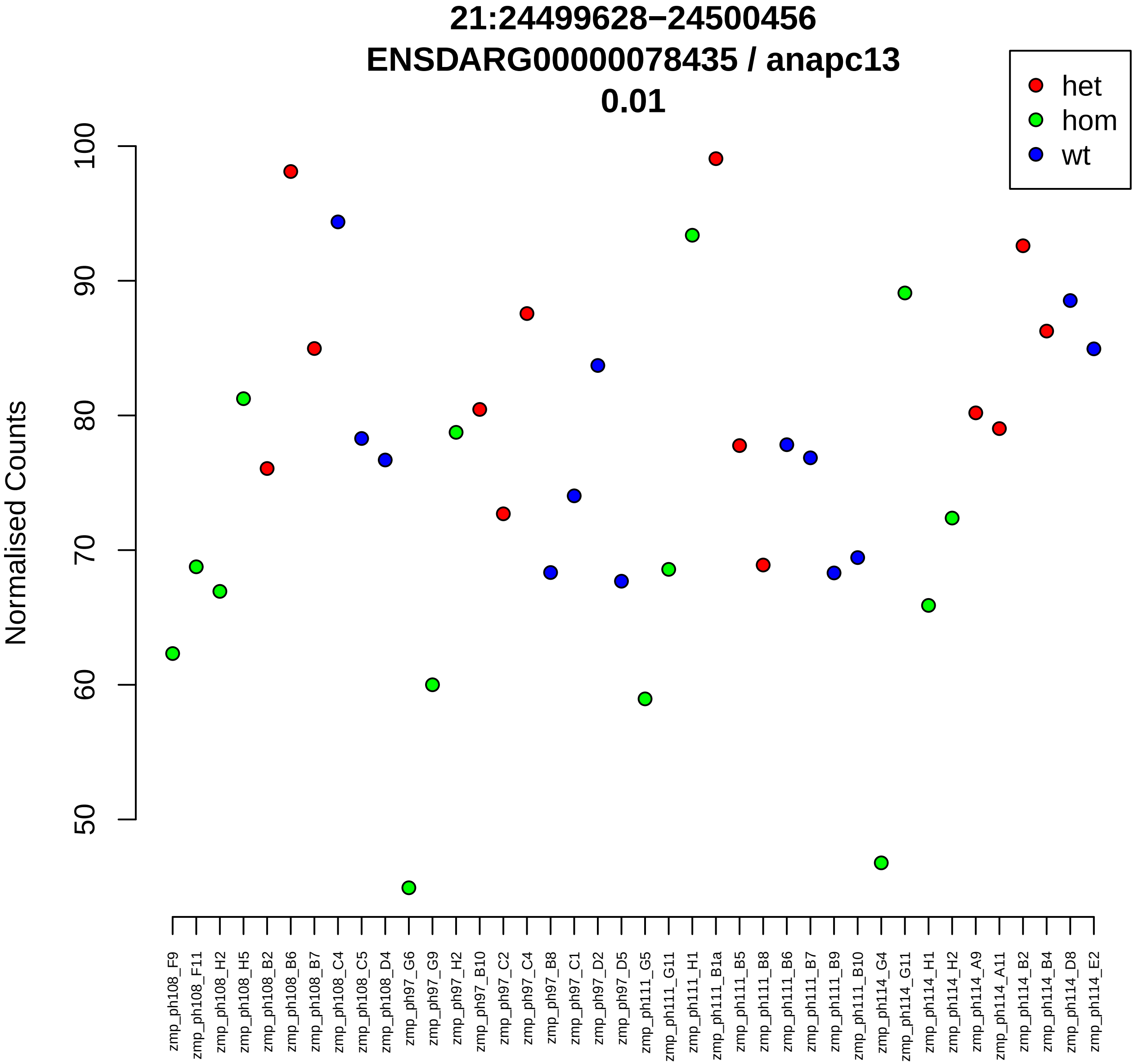

21:24499628−24500456
ENSDARG00000078435 / anapc13
0.01
Normalised Counts
100
90
80
70
60
50
het
hom
wt
zmp_ph108_F9
zmp_ph108_F11
zmp_ph108_H2
zmp_ph108_H5
zmp_ph108_B2
zmp_ph108_B6
zmp_ph108_B7
zmp_ph108_C4
zmp_ph108_C5
zmp_ph108_D4
zmp_ph97_G6
zmp_ph97_G9
zmp_ph97_H2
zmp_ph97_B10
zmp_ph97_C2
zmp_ph97_C4
zmp_ph97_B8
zmp_ph97_C1
zmp_ph97_D2
zmp_ph97_D5
zmp_ph111_G5
zmp_ph111_G11
zmp_ph111_H1
zmp_ph111_B1a
zmp_ph111_B5
zmp_ph111_B8
zmp_ph111_B6
zmp_ph111_B7
zmp_ph111_B9
zmp_ph111_B10
zmp_ph114_G4
zmp_ph114_G11
zmp_ph114_H1
zmp_ph114_H2
zmp_ph114_A9
zmp_ph114_A11
zmp_ph114_B2
zmp_ph114_B4
zmp_ph114_D8
zmp_ph114_E2

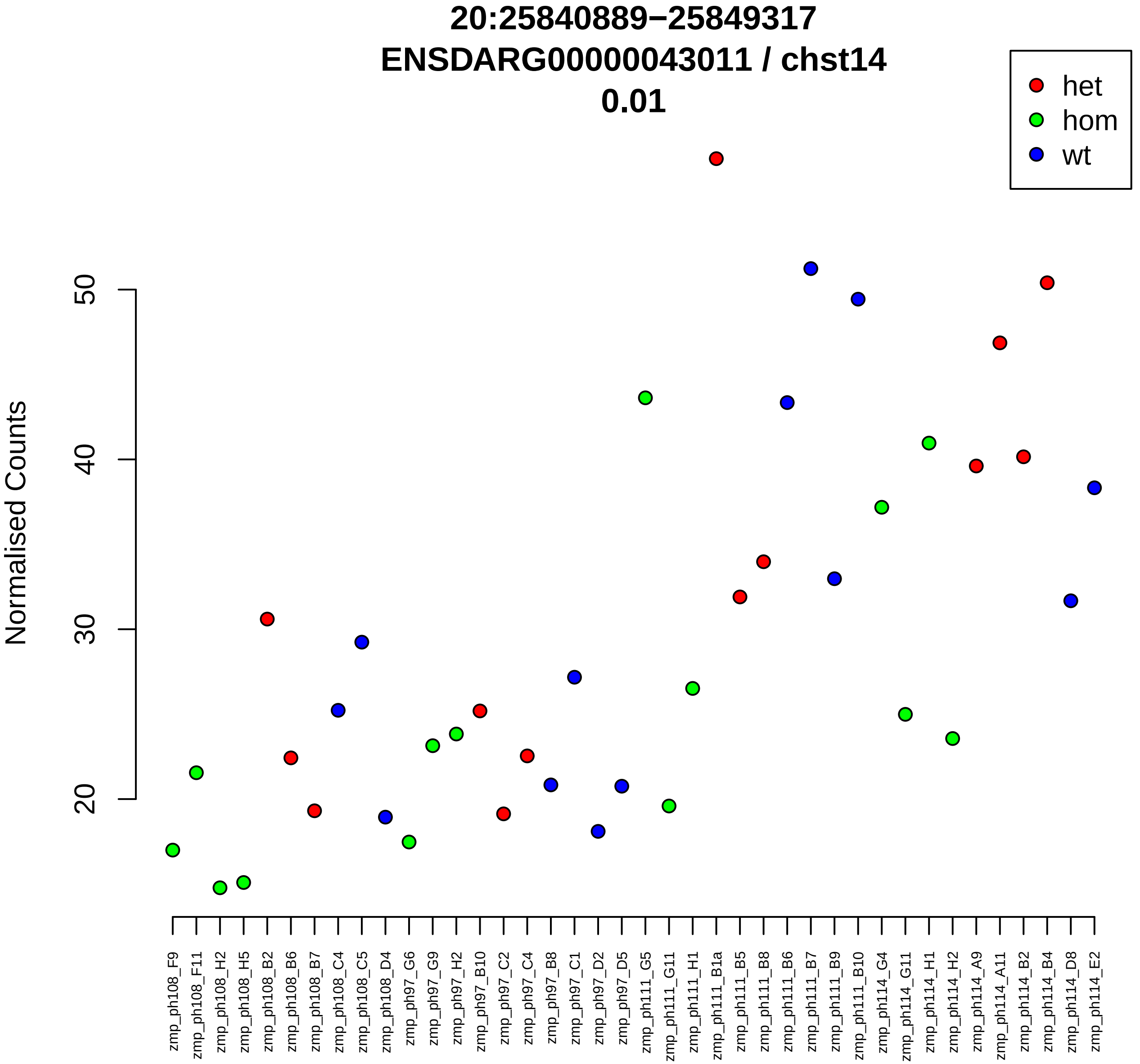

20:25840889−25849317
ENSDARG00000043011 / chst14
0.01
het
hom
wt
Normalised Counts
20
30
40
50
zmp_ph108_F9
zmp_ph108_F11
zmp_ph108_H2
zmp_ph108_H5
zmp_ph108_B2
zmp_ph108_B6
zmp_ph108_B7
zmp_ph108_C4
zmp_ph108_C5
zmp_ph108_D4
zmp_ph97_G6
zmp_ph97_G9
zmp_ph97_H2
zmp_ph97_B10
zmp_ph97_C2
zmp_ph97_C4
zmp_ph97_B8
zmp_ph97_C1
zmp_ph97_D2
zmp_ph97_D5
zmp_ph111_G5
zmp_ph111_G11
zmp_ph111_H1
zmp_ph111_B1a
zmp_ph111_B5
zmp_ph111_B8
zmp_ph111_B6
zmp_ph111_B7
zmp_ph111_B9
zmp_ph111_B10
zmp_ph114_G4
zmp_ph114_G11
zmp_ph114_H1
zmp_ph114_H2
zmp_ph114_A9
zmp_ph114_A11
zmp_ph114_B2
zmp_ph114_B4
zmp_ph114_D8
zmp_ph114_E2

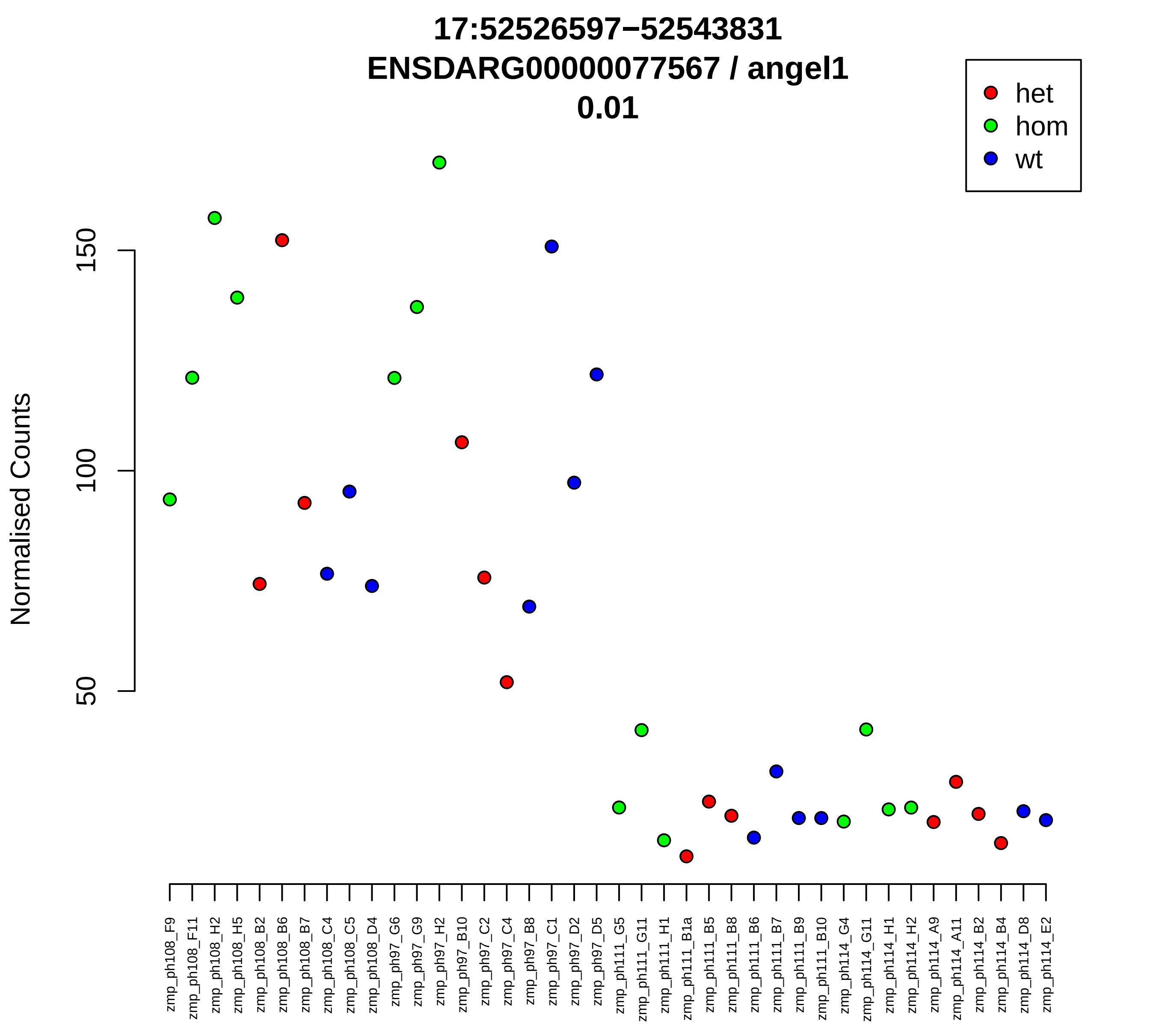

17:52526597−52543831
ENSDARG00000077567 / angel1
0.01
het
hom
wt
Normalised Counts
50
100
150
zmp_ph108_F9
zmp_ph108_F11
zmp_ph108_H2
zmp_ph108_H5
zmp_ph108_B2
zmp_ph108_B6
zmp_ph108_B7
zmp_ph108_C4
zmp_ph108_C5
zmp_ph108_D4
zmp_ph97_G6
zmp_ph97_G9
zmp_ph97_H2
zmp_ph97_B10
zmp_ph97_C2
zmp_ph97_C4
zmp_ph97_B8
zmp_ph97_C1
zmp_ph97_D2
zmp_ph97_D5
zmp_ph111_G5
zmp_ph111_G11
zmp_ph111_H1
zmp_ph111_B1a
zmp_ph111_B5
zmp_ph111_B8
zmp_ph111_B6
zmp_ph111_B7
zmp_ph111_B9
zmp_ph111_B10
zmp_ph114_G4
zmp_ph114_G11
zmp_ph114_H1
zmp_ph114_H2
zmp_ph114_A9
zmp_ph114_A11
zmp_ph114_B2
zmp_ph114_B4
zmp_ph114_D8
zmp_ph114_E2

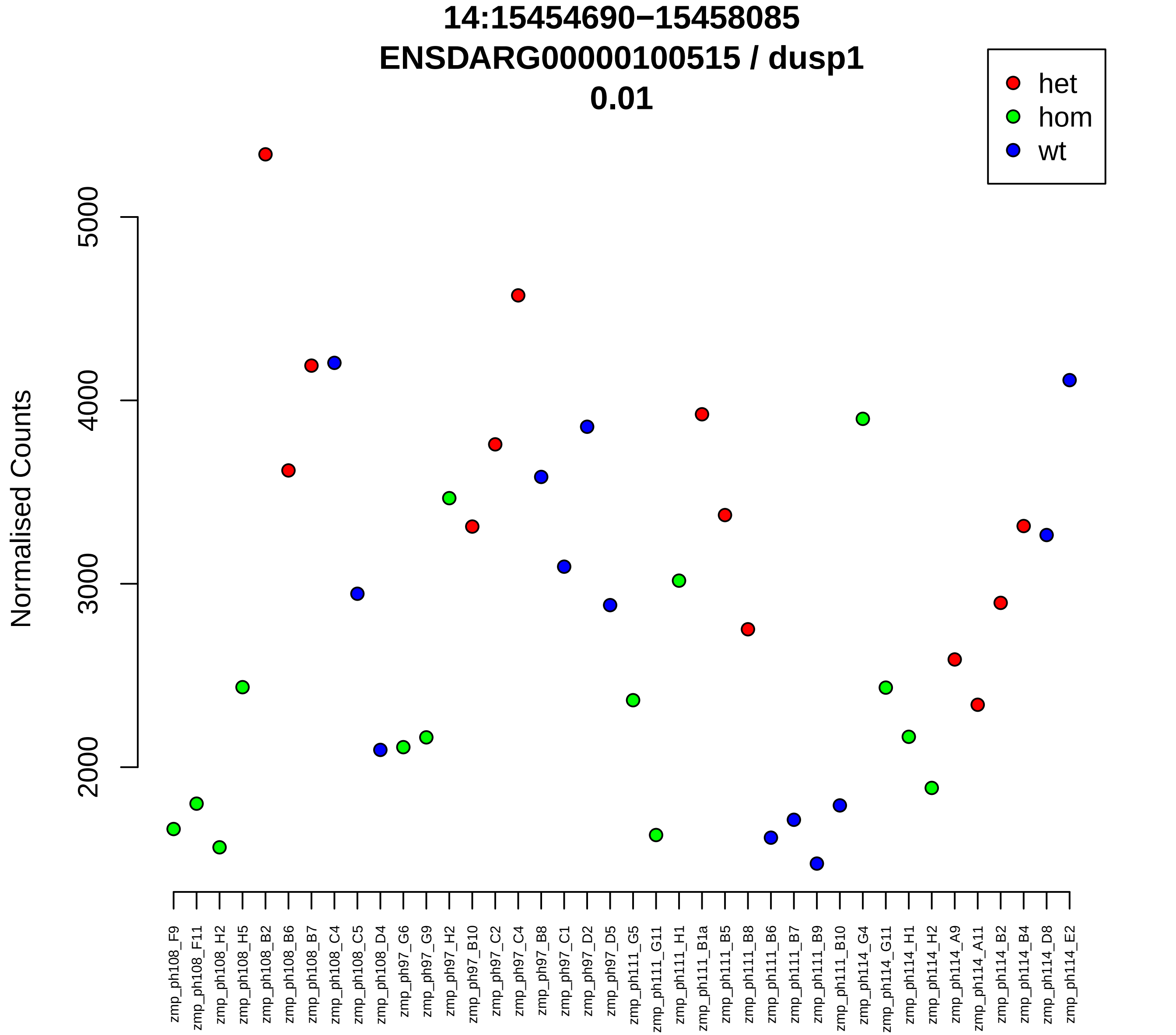
14:15454690−15458085
ENSDARG00000100515 / dusp1
0.01
het
hom
wt
Normalised Counts
2000
3000
4000
5000
zmp_ph108_F9
zmp_ph108_F11
zmp_ph108_H2
zmp_ph108_H5
zmp_ph108_B2
zmp_ph108_B6
zmp_ph108_B7
zmp_ph108_C4
zmp_ph108_C5
zmp_ph108_D4
zmp_ph97_G6
zmp_ph97_G9
zmp_ph97_H2
zmp_ph97_B10
zmp_ph97_C2
zmp_ph97_C4
zmp_ph97_B8
zmp_ph97_C1
zmp_ph97_D2
zmp_ph97_D5
zmp_ph111_G5
zmp_ph111_G11
zmp_ph111_H1
zmp_ph111_B1a
zmp_ph111_B5
zmp_ph111_B8
zmp_ph111_B6
zmp_ph111_B7
zmp_ph111_B9
zmp_ph111_B10
zmp_ph114_G4
zmp_ph114_G11
zmp_ph114_H1
zmp_ph114_H2
zmp_ph114_A9
zmp_ph114_A11
zmp_ph114_B2
zmp_ph114_B4
zmp_ph114_D8
zmp_ph114_E2

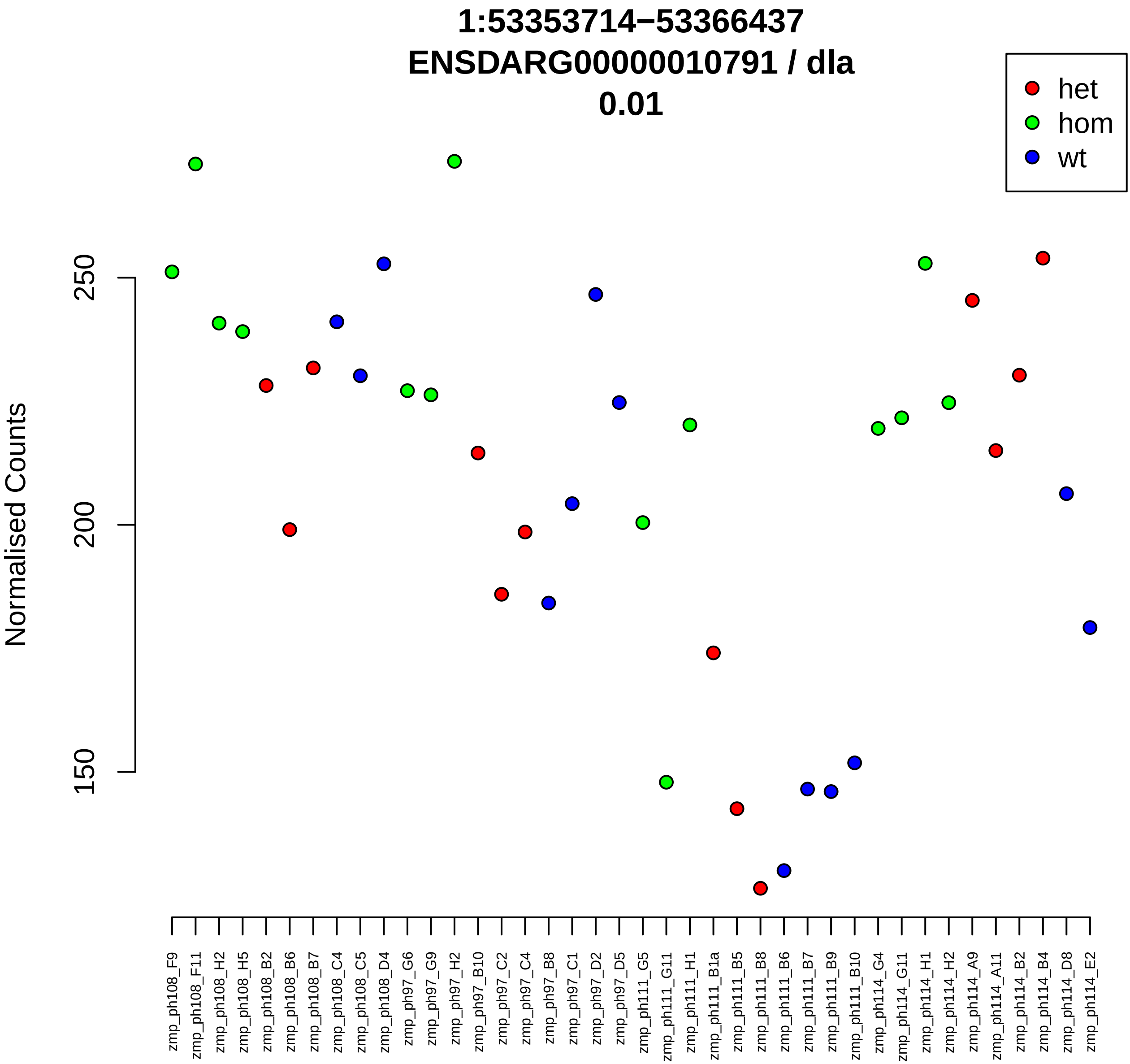

1:53353714−53366437
ENSDARG00000010791 / dla
0.01
het
hom
wt
Normalised Counts
250
200
150
zmp_ph108_F9
zmp_ph108_F11
zmp_ph108_H2
zmp_ph108_H5
zmp_ph108_B2
zmp_ph108_B6
zmp_ph108_B7
zmp_ph108_C4
zmp_ph108_C5
zmp_ph108_D4
zmp_ph97_G6
zmp_ph97_G9
zmp_ph97_H2
zmp_ph97_B10
zmp_ph97_C2
zmp_ph97_C4
zmp_ph97_B8
zmp_ph97_C1
zmp_ph97_D2
zmp_ph97_D5
zmp_ph111_G5
zmp_ph111_G11
zmp_ph111_H1
zmp_ph111_B1a
zmp_ph111_B5
zmp_ph111_B8
zmp_ph111_B6
zmp_ph111_B7
zmp_ph111_B9
zmp_ph111_B10
zmp_ph114_G4
zmp_ph114_G11
zmp_ph114_H1
zmp_ph114_H2
zmp_ph114_A9
zmp_ph114_A11
zmp_ph114_B2
zmp_ph114_B4
zmp_ph114_D8
zmp_ph114_E2

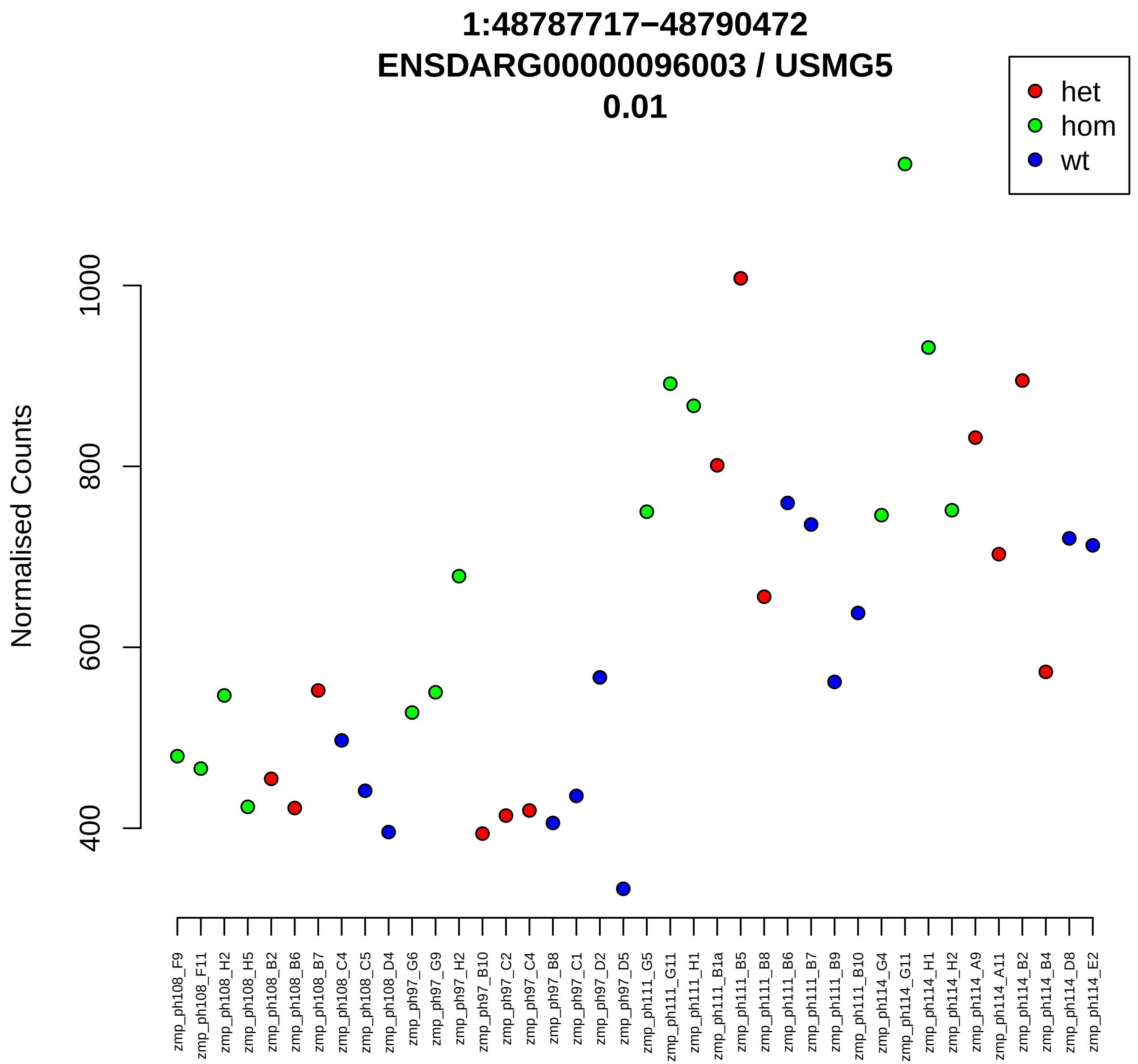
1:48787717−48790472
ENSDARG00000096003 / USMG5
0.01
het
hom
wt
Normalised Counts
400
600
800
1000
zmp_ph108_F9
zmp_ph108_F11
zmp_ph108_H2
zmp_ph108_H5
zmp_ph108_B2
zmp_ph108_B6
zmp_ph108_B7
zmp_ph108_C4
zmp_ph108_C5
zmp_ph108_D4
zmp_ph97_G6
zmp_ph97_G9
zmp_ph97_H2
zmp_ph97_B10
zmp_ph97_C2
zmp_ph97_C4
zmp_ph97_B8
zmp_ph97_C1
zmp_ph97_D2
zmp_ph97_D5
zmp_ph111_G5
zmp_ph111_G11
zmp_ph111_H1
zmp_ph111_B1a
zmp_ph111_B5
zmp_ph111_B8
zmp_ph111_B6
zmp_ph111_B7
zmp_ph111_B9
zmp_ph111_B10
zmp_ph114_G4
zmp_ph114_G11
zmp_ph114_H1
zmp_ph114_H2
zmp_ph114_A9
zmp_ph114_A11
zmp_ph114_B2
zmp_ph114_B4
zmp_ph114_D8
zmp_ph114_E2

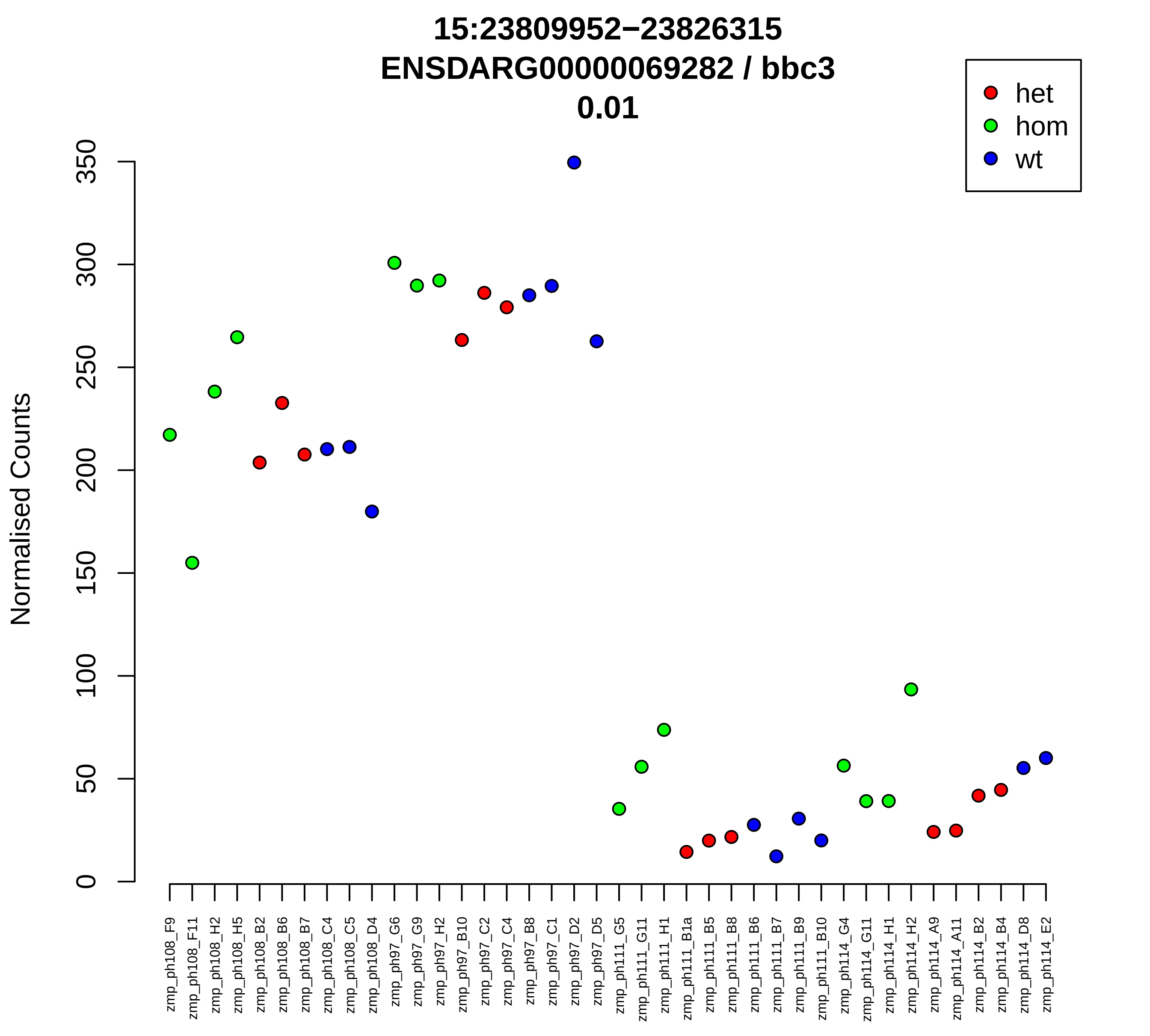
15:23809952−23826315
ENSDARG00000069282 / bbc3
0.01
het
hom
wt
Normalised Counts
0
50
100
150
200
250
300
350
zmp_ph108_F9
zmp_ph108_F11
zmp_ph108_H2
zmp_ph108_H5
zmp_ph108_B2
zmp_ph108_B6
zmp_ph108_B7
zmp_ph108_C4
zmp_ph108_C5
zmp_ph108_D4
zmp_ph97_G6
zmp_ph97_G9
zmp_ph97_H2
zmp_ph97_B10
zmp_ph97_C2
zmp_ph97_C4
zmp_ph97_B8
zmp_ph97_C1
zmp_ph97_D2
zmp_ph97_D5
zmp_ph111_G5
zmp_ph111_G11
zmp_ph111_H1
zmp_ph111_B1a
zmp_ph111_B5
zmp_ph111_B8
zmp_ph111_B6
zmp_ph111_B7
zmp_ph111_B9
zmp_ph111_B10
zmp_ph114_G4
zmp_ph114_G11
zmp_ph114_H1
zmp_ph114_H2
zmp_ph114_A9
zmp_ph114_A11
zmp_ph114_B2
zmp_ph114_B4
zmp_ph114_D8
zmp_ph114_E2

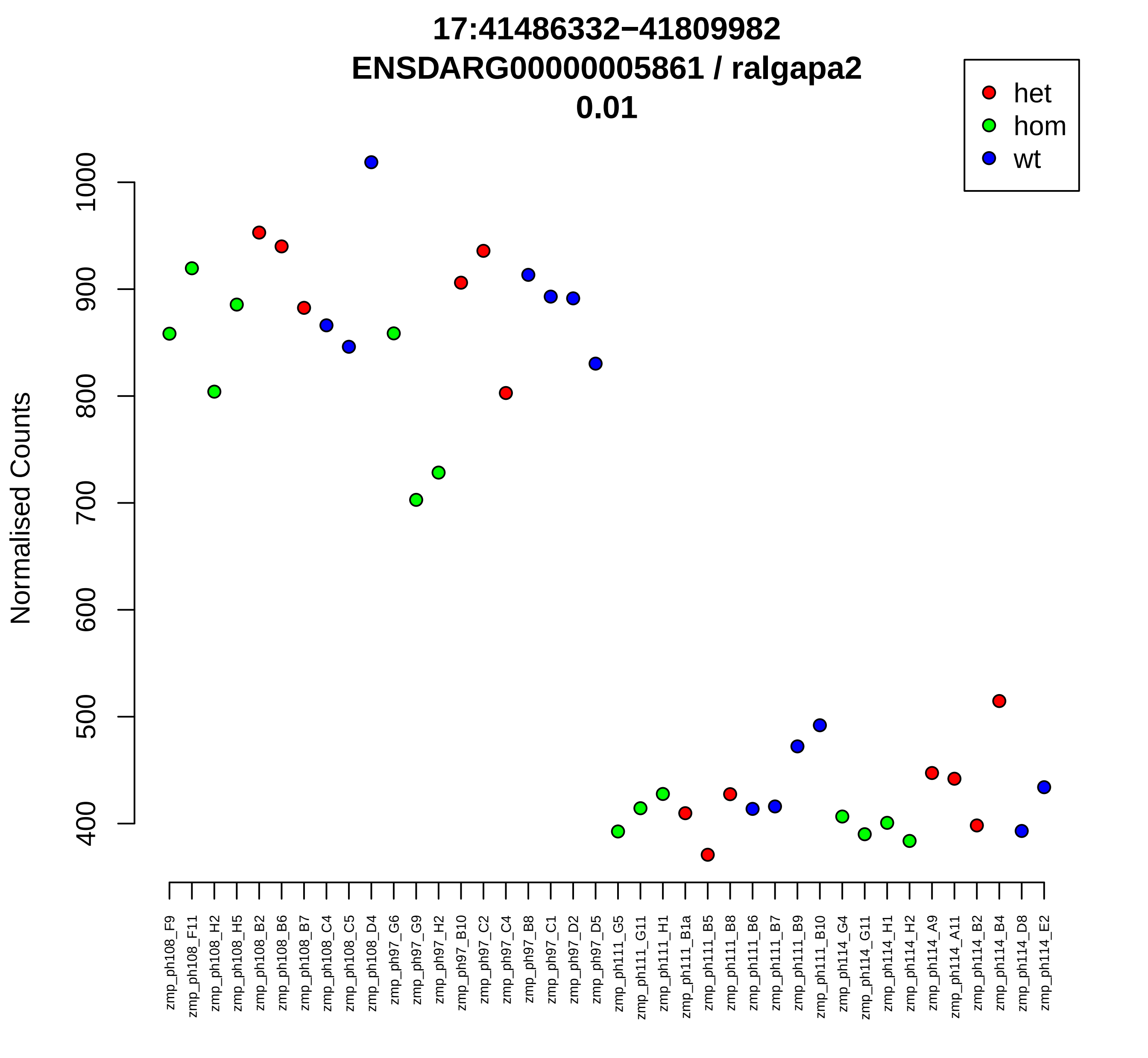

17:41486332−41809982
ENSDARG00000005861 / ralgapa2
0.01
het
hom
wt
Normalised Counts
400
500
600
700
800
900
1000
zmp_ph108_F9
zmp_ph108_F11
zmp_ph108_H2
zmp_ph108_H5
zmp_ph108_B2
zmp_ph108_B6
zmp_ph108_B7
zmp_ph108_C4
zmp_ph108_C5
zmp_ph108_D4
zmp_ph97_G6
zmp_ph97_G9
zmp_ph97_H2
zmp_ph97_B10
zmp_ph97_C2
zmp_ph97_C4
zmp_ph97_B8
zmp_ph97_C1
zmp_ph97_D2
zmp_ph97_D5
zmp_ph111_G5
zmp_ph111_G11
zmp_ph111_H1
zmp_ph111_B1a
zmp_ph111_B5
zmp_ph111_B8
zmp_ph111_B6
zmp_ph111_B7
zmp_ph111_B9
zmp_ph111_B10
zmp_ph114_G4
zmp_ph114_G11
zmp_ph114_H1
zmp_ph114_H2
zmp_ph114_A9
zmp_ph114_A11
zmp_ph114_B2
zmp_ph114_B4
zmp_ph114_D8
zmp_ph114_E2

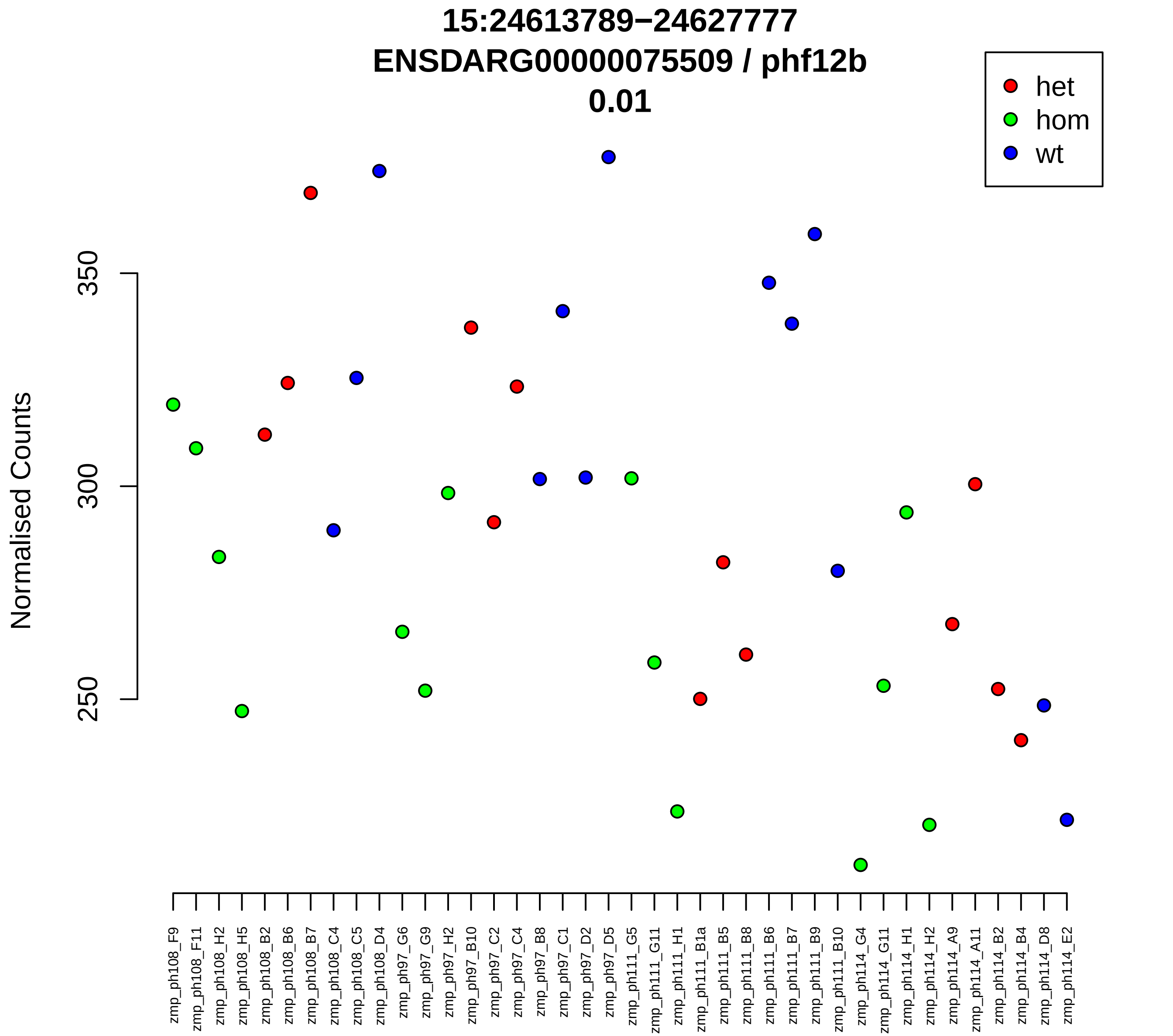

15:24613789−24627777
ENSDARG00000075509 / phf12b
0.01
het
hom
wt
Normalised Counts
250
300
350
zmp_ph108_F9
zmp_ph108_F11
zmp_ph108_H2
zmp_ph108_H5
zmp_ph108_B2
zmp_ph108_B6
zmp_ph108_B7
zmp_ph108_C4
zmp_ph108_C5
zmp_ph108_D4
zmp_ph97_G6
zmp_ph97_G9
zmp_ph97_H2
zmp_ph97_B10
zmp_ph97_C2
zmp_ph97_C4
zmp_ph97_B8
zmp_ph97_C1
zmp_ph97_D2
zmp_ph97_D5
zmp_ph111_G5
zmp_ph111_G11
zmp_ph111_H1
zmp_ph111_B1a
zmp_ph111_B5
zmp_ph111_B8
zmp_ph111_B6
zmp_ph111_B7
zmp_ph111_B9
zmp_ph111_B10
zmp_ph114_G4
zmp_ph114_G11
zmp_ph114_H1
zmp_ph114_H2
zmp_ph114_A9
zmp_ph114_A11
zmp_ph114_B2
zmp_ph114_B4
zmp_ph114_D8
zmp_ph114_E2

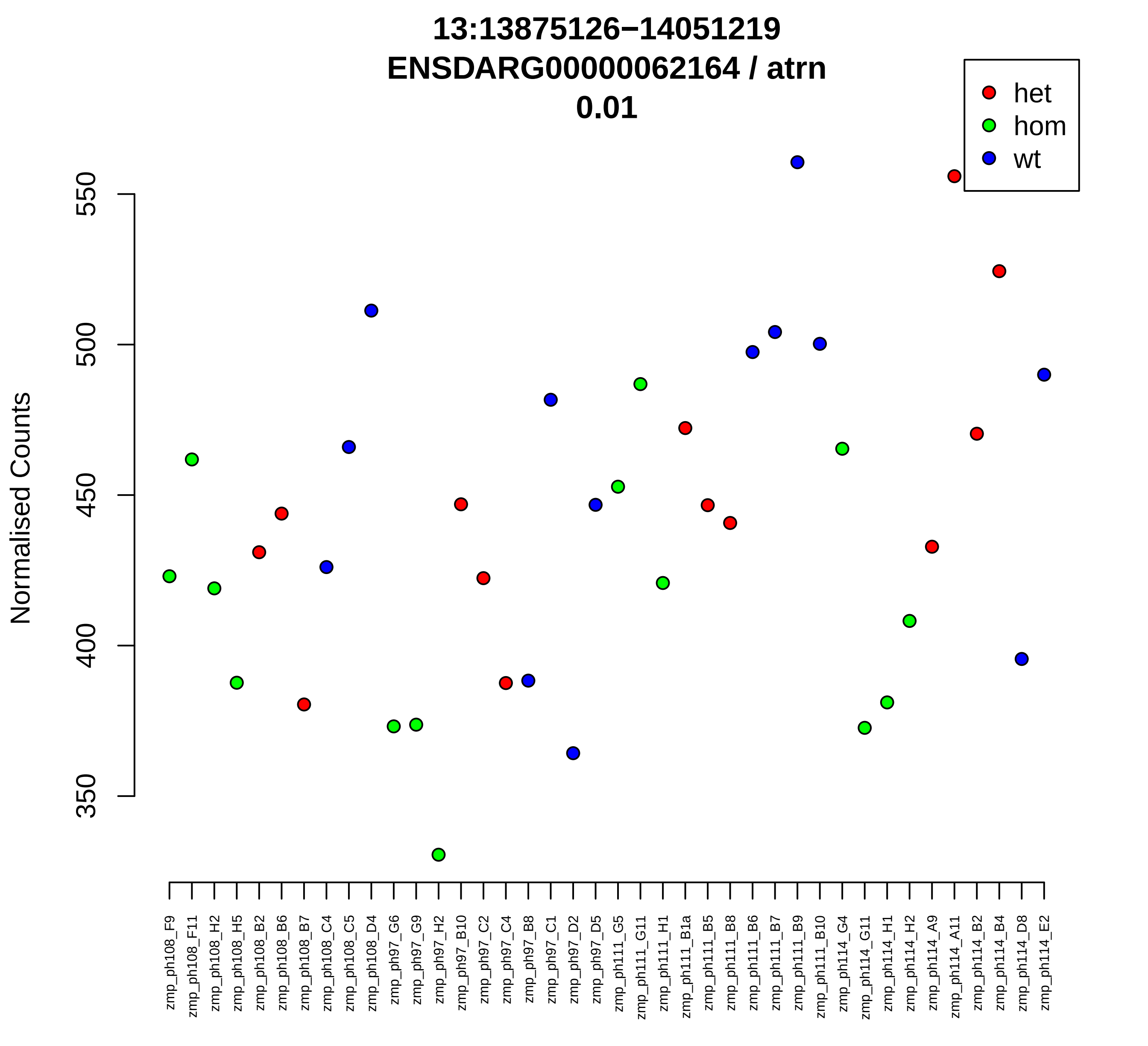
13:13875126−14051219
ENSDARG00000062164 / atrn
0.01
het
hom
wt
Normalised Counts
350
400
450
500
550
zmp_ph108_F9
zmp_ph108_F11
zmp_ph108_H2
zmp_ph108_H5
zmp_ph108_B2
zmp_ph108_B6
zmp_ph108_B7
zmp_ph108_C4
zmp_ph108_C5
zmp_ph108_D4
zmp_ph97_G6
zmp_ph97_G9
zmp_ph97_H2
zmp_ph97_B10
zmp_ph97_C2
zmp_ph97_C4
zmp_ph97_B8
zmp_ph97_C1
zmp_ph97_D2
zmp_ph97_D5
zmp_ph111_G5
zmp_ph111_G11
zmp_ph111_H1
zmp_ph111_B1a
zmp_ph111_B5
zmp_ph111_B8
zmp_ph111_B6
zmp_ph111_B7
zmp_ph111_B9
zmp_ph111_B10
zmp_ph114_G4
zmp_ph114_G11
zmp_ph114_H1
zmp_ph114_H2
zmp_ph114_A9
zmp_ph114_A11
zmp_ph114_B2
zmp_ph114_B4
zmp_ph114_D8
zmp_ph114_E2

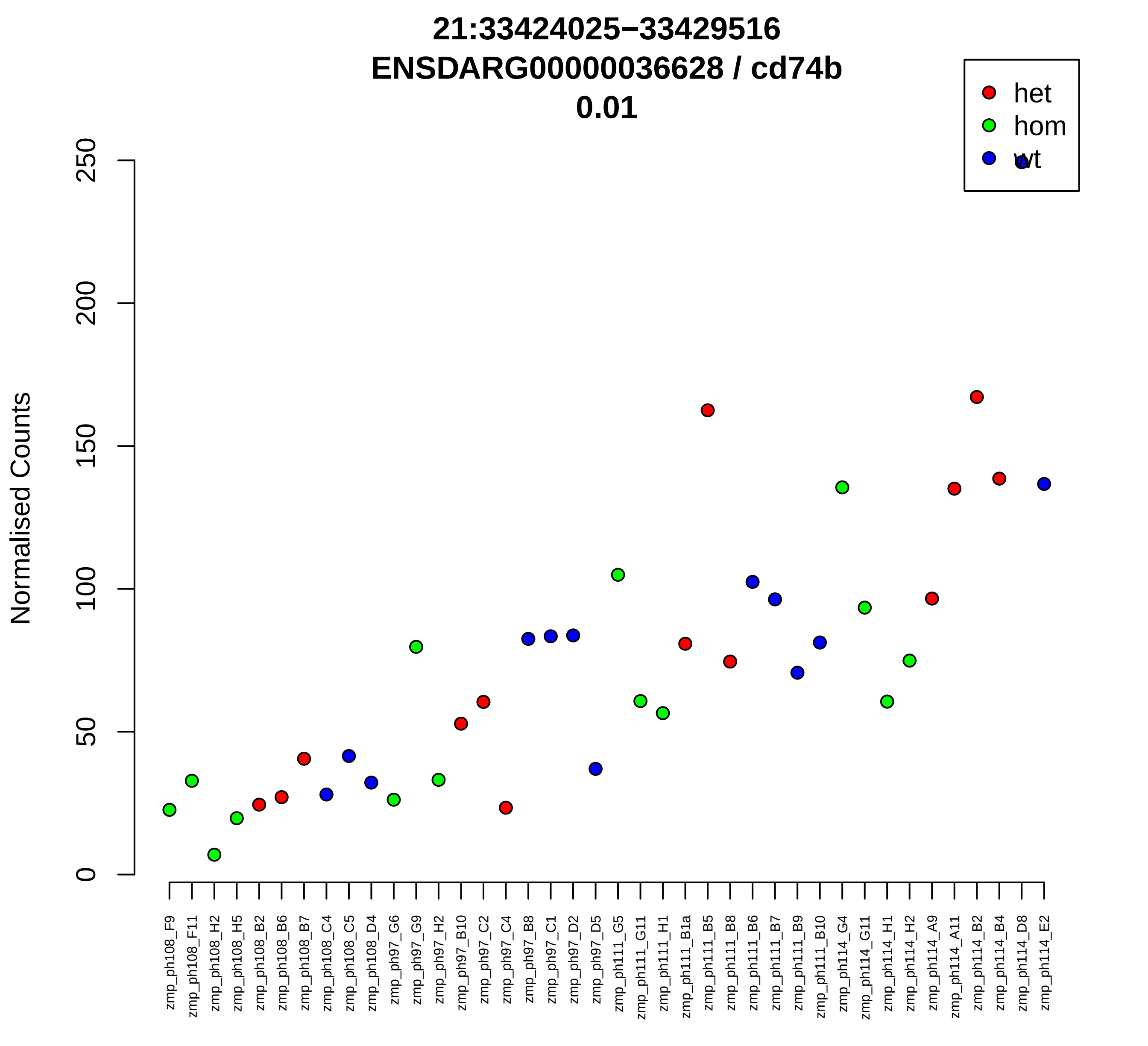

21:33424025−33429516
ENSDARG00000036628 / cd74b
0.01
het
hom
wt
Normalised Counts
0
50
100
150
200
250
zmp_ph108_F9
zmp_ph108_F11
zmp_ph108_H2
zmp_ph108_H5
zmp_ph108_B2
zmp_ph108_B6
zmp_ph108_B7
zmp_ph108_C4
zmp_ph108_C5
zmp_ph108_D4
zmp_ph97_G6
zmp_ph97_G9
zmp_ph97_H2
zmp_ph97_B10
zmp_ph97_C2
zmp_ph97_C4
zmp_ph97_B8
zmp_ph97_C1
zmp_ph97_D2
zmp_ph97_D5
zmp_ph111_G5
zmp_ph111_G11
zmp_ph111_H1
zmp_ph111_B1a
zmp_ph111_B5
zmp_ph111_B8
zmp_ph111_B6
zmp_ph111_B7
zmp_ph111_B9
zmp_ph111_B10
zmp_ph114_G4
zmp_ph114_G11
zmp_ph114_H1
zmp_ph114_H2
zmp_ph114_A9
zmp_ph114_A11
zmp_ph114_B2
zmp_ph114_B4
zmp_ph114_D8
zmp_ph114_E2

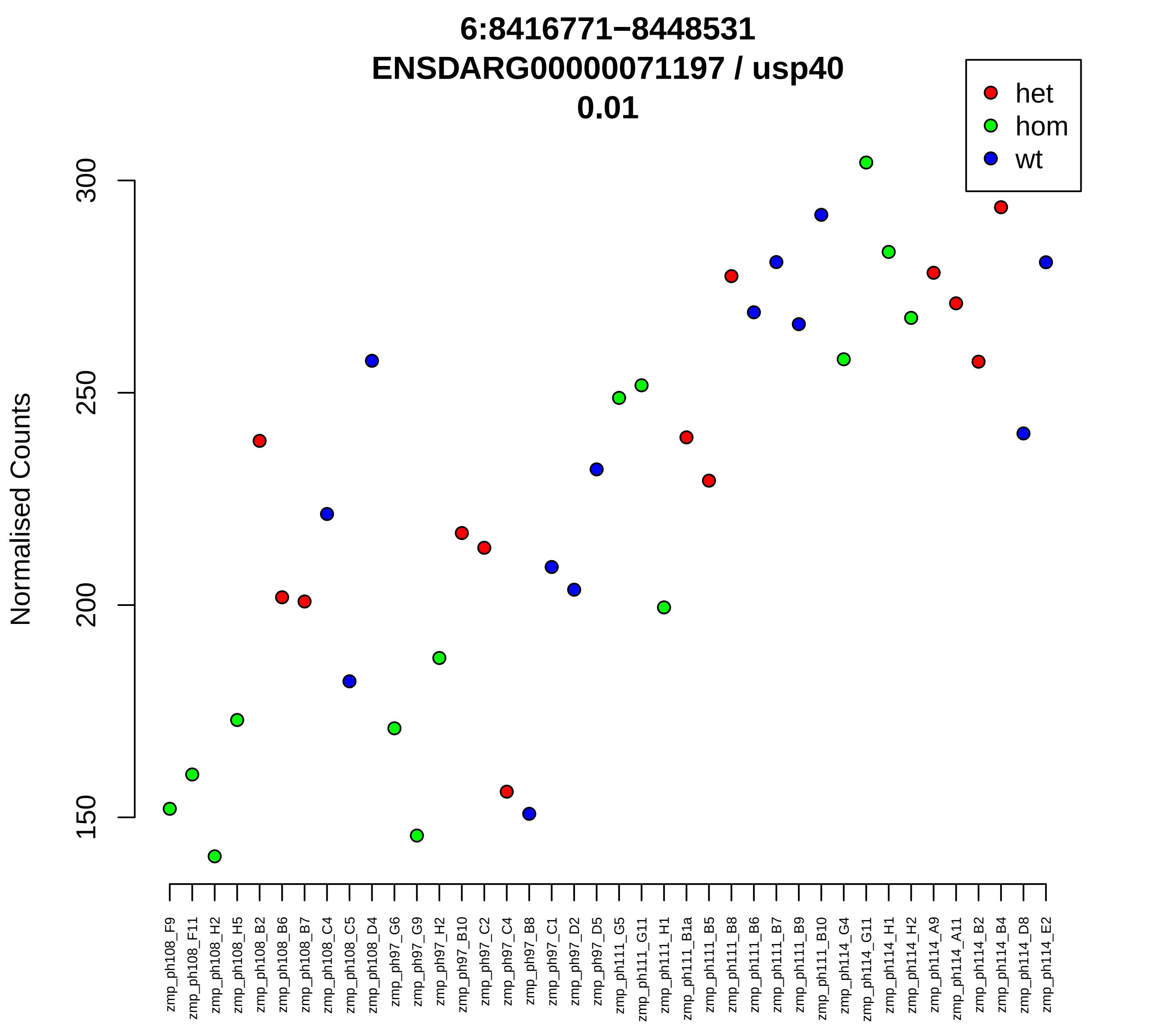

6:8416771−8448531
ENSDARG00000071197 / usp40
0.01
het
hom
wt
Normalised Counts
150
200
250
300
zmp_ph108_F9
zmp_ph108_F11
zmp_ph108_H2
zmp_ph108_H5
zmp_ph108_B2
zmp_ph108_B6
zmp_ph108_B7
zmp_ph108_C4
zmp_ph108_C5
zmp_ph108_D4
zmp_ph97_G6
zmp_ph97_G9
zmp_ph97_H2
zmp_ph97_B10
zmp_ph97_C2
zmp_ph97_C4
zmp_ph97_B8
zmp_ph97_C1
zmp_ph97_D2
zmp_ph97_D5
zmp_ph111_G5
zmp_ph111_G11
zmp_ph111_H1
zmp_ph111_B1a
zmp_ph111_B5
zmp_ph111_B8
zmp_ph111_B6
zmp_ph111_B7
zmp_ph111_B9
zmp_ph111_B10
zmp_ph114_G4
zmp_ph114_G11
zmp_ph114_H1
zmp_ph114_H2
zmp_ph114_A9
zmp_ph114_A11
zmp_ph114_B2
zmp_ph114_B4
zmp_ph114_D8
zmp_ph114_E2

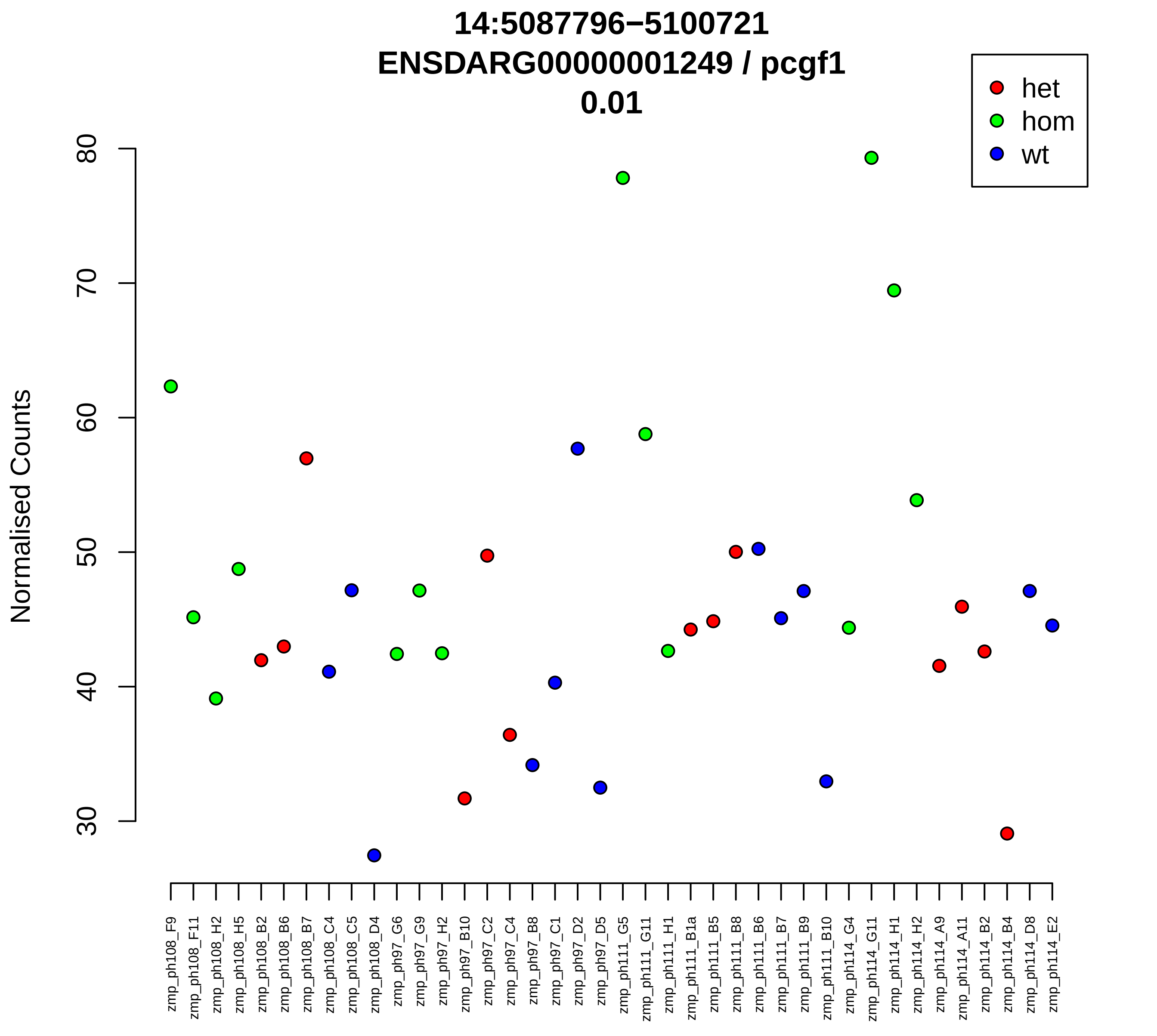
14:5087796−5100721
ENSDARG00000001249 / pcgf1
0.01
het
hom
wt
Normalised Counts
30
40
50
60
70
80
zmp_ph108_F9
zmp_ph108_F11
zmp_ph108_H2
zmp_ph108_H5
zmp_ph108_B2
zmp_ph108_B6
zmp_ph108_B7
zmp_ph108_C4
zmp_ph108_C5
zmp_ph108_D4
zmp_ph97_G6
zmp_ph97_G9
zmp_ph97_H2
zmp_ph97_B10
zmp_ph97_C2
zmp_ph97_C4
zmp_ph97_B8
zmp_ph97_C1
zmp_ph97_D2
zmp_ph97_D5
zmp_ph111_G5
zmp_ph111_G11
zmp_ph111_H1
zmp_ph111_B1a
zmp_ph111_B5
zmp_ph111_B8
zmp_ph111_B6
zmp_ph111_B7
zmp_ph111_B9
zmp_ph111_B10
zmp_ph114_G4
zmp_ph114_G11
zmp_ph114_H1
zmp_ph114_H2
zmp_ph114_A9
zmp_ph114_A11
zmp_ph114_B2
zmp_ph114_B4
zmp_ph114_D8
zmp_ph114_E2

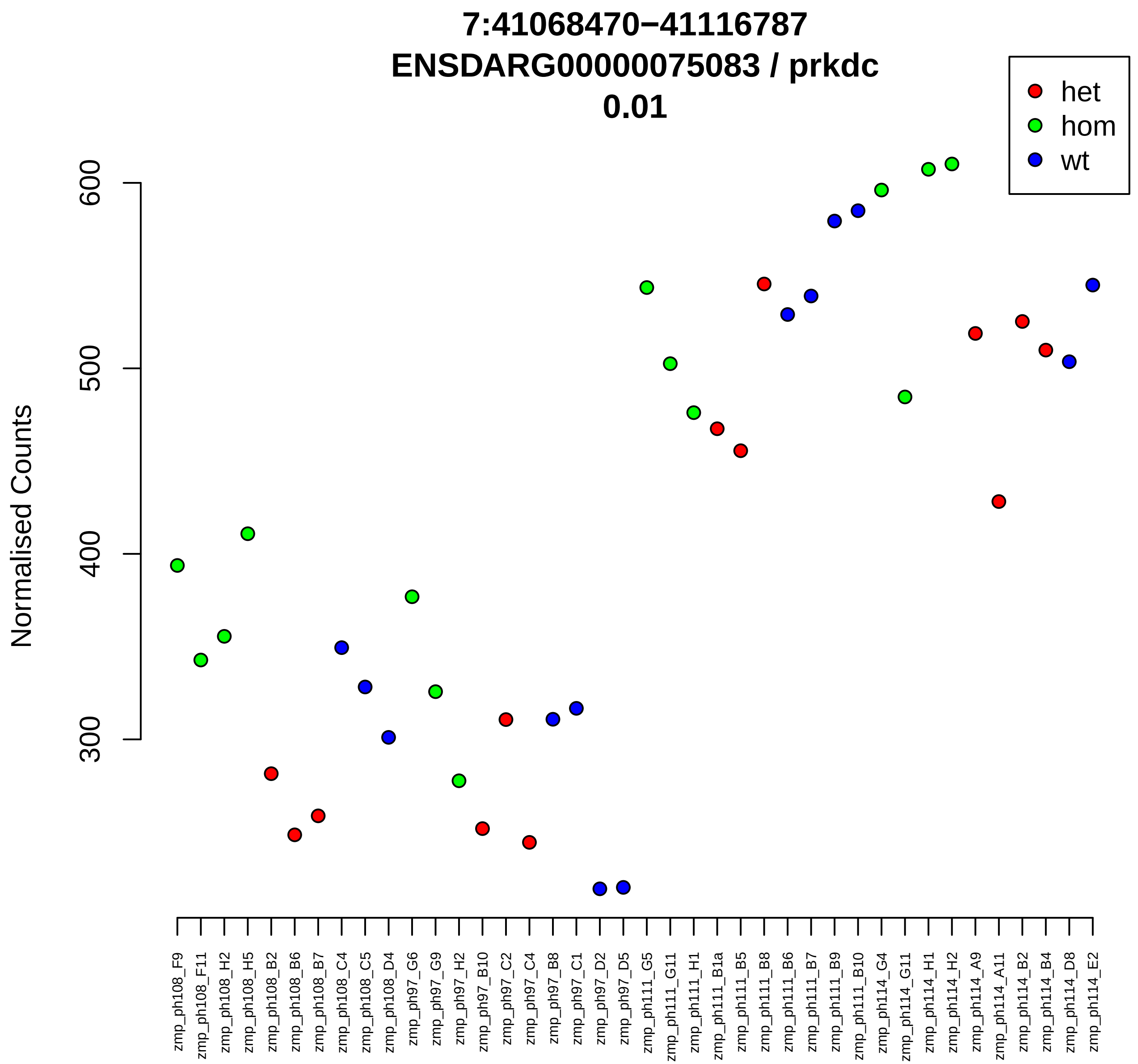

7:41068470−41116787
ENSDARG00000075083 / prkdc
0.01
het
hom
wt
Normalised Counts
300
400
500
600
zmp_ph108_F9
zmp_ph108_F11
zmp_ph108_H2
zmp_ph108_H5
zmp_ph108_B2
zmp_ph108_B6
zmp_ph108_B7
zmp_ph108_C4
zmp_ph108_C5
zmp_ph108_D4
zmp_ph97_G6
zmp_ph97_G9
zmp_ph97_H2
zmp_ph97_B10
zmp_ph97_C2
zmp_ph97_C4
zmp_ph97_B8
zmp_ph97_C1
zmp_ph97_D2
zmp_ph97_D5
zmp_ph111_G5
zmp_ph111_G11
zmp_ph111_H1
zmp_ph111_B1a
zmp_ph111_B5
zmp_ph111_B8
zmp_ph111_B6
zmp_ph111_B7
zmp_ph111_B9
zmp_ph111_B10
zmp_ph114_G4
zmp_ph114_G11
zmp_ph114_H1
zmp_ph114_H2
zmp_ph114_A9
zmp_ph114_A11
zmp_ph114_B2
zmp_ph114_B4
zmp_ph114_D8
zmp_ph114_E2

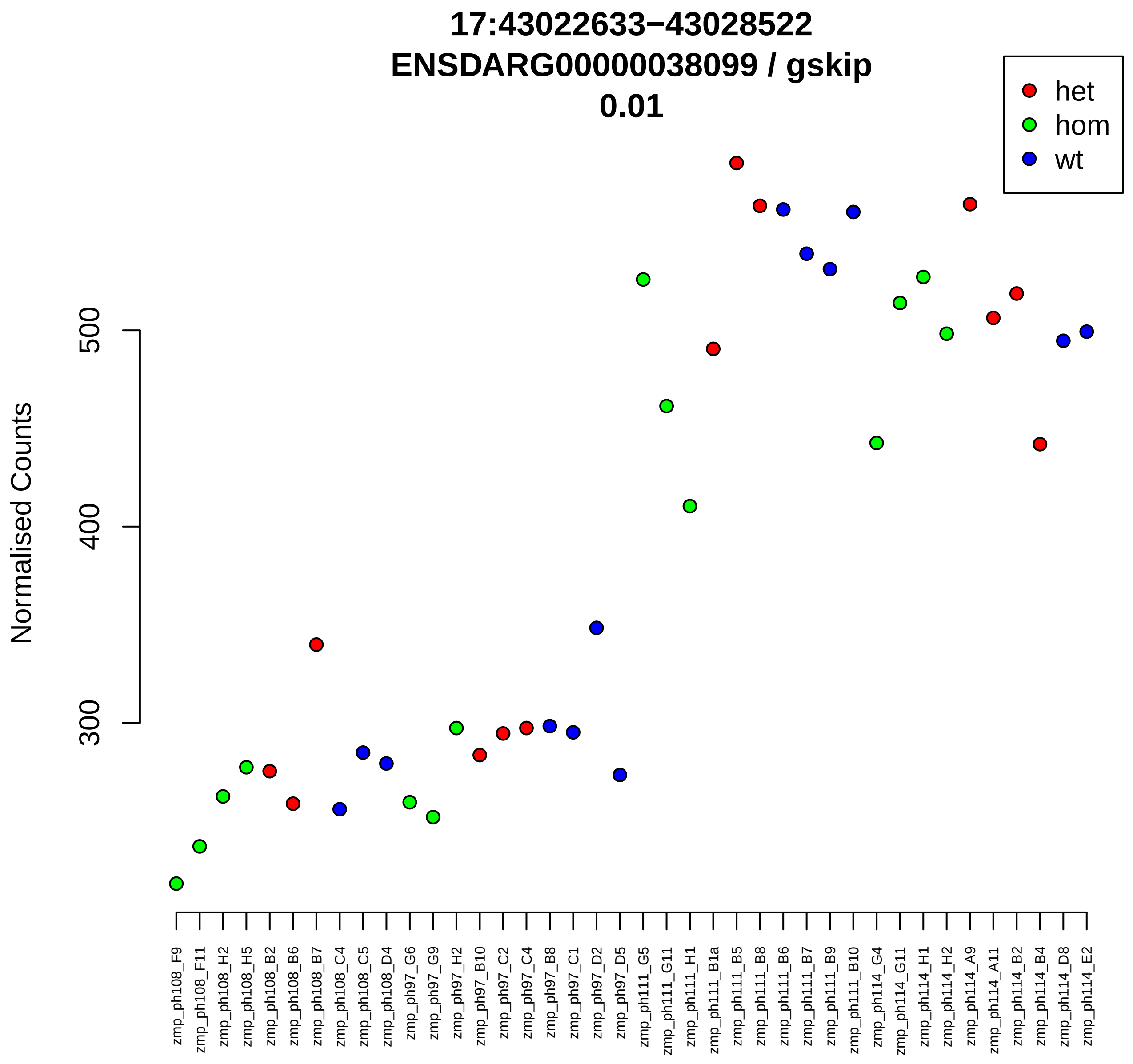
17:43022633−43028522
ENSDARG00000038099 / gskip
0.01
het
hom
wt
Normalised Counts
300
400
500
zmp_ph108_F9
zmp_ph108_F11
zmp_ph108_H2
zmp_ph108_H5
zmp_ph108_B2
zmp_ph108_B6
zmp_ph108_B7
zmp_ph108_C4
zmp_ph108_C5
zmp_ph108_D4
zmp_ph97_G6
zmp_ph97_G9
zmp_ph97_H2
zmp_ph97_B10
zmp_ph97_C2
zmp_ph97_C4
zmp_ph97_B8
zmp_ph97_C1
zmp_ph97_D2
zmp_ph97_D5
zmp_ph111_G5
zmp_ph111_G11
zmp_ph111_H1
zmp_ph111_B1a
zmp_ph111_B5
zmp_ph111_B8
zmp_ph111_B6
zmp_ph111_B7
zmp_ph111_B9
zmp_ph111_B10
zmp_ph114_G4
zmp_ph114_G11
zmp_ph114_H1
zmp_ph114_H2
zmp_ph114_A9
zmp_ph114_A11
zmp_ph114_B2
zmp_ph114_B4
zmp_ph114_D8
zmp_ph114_E2

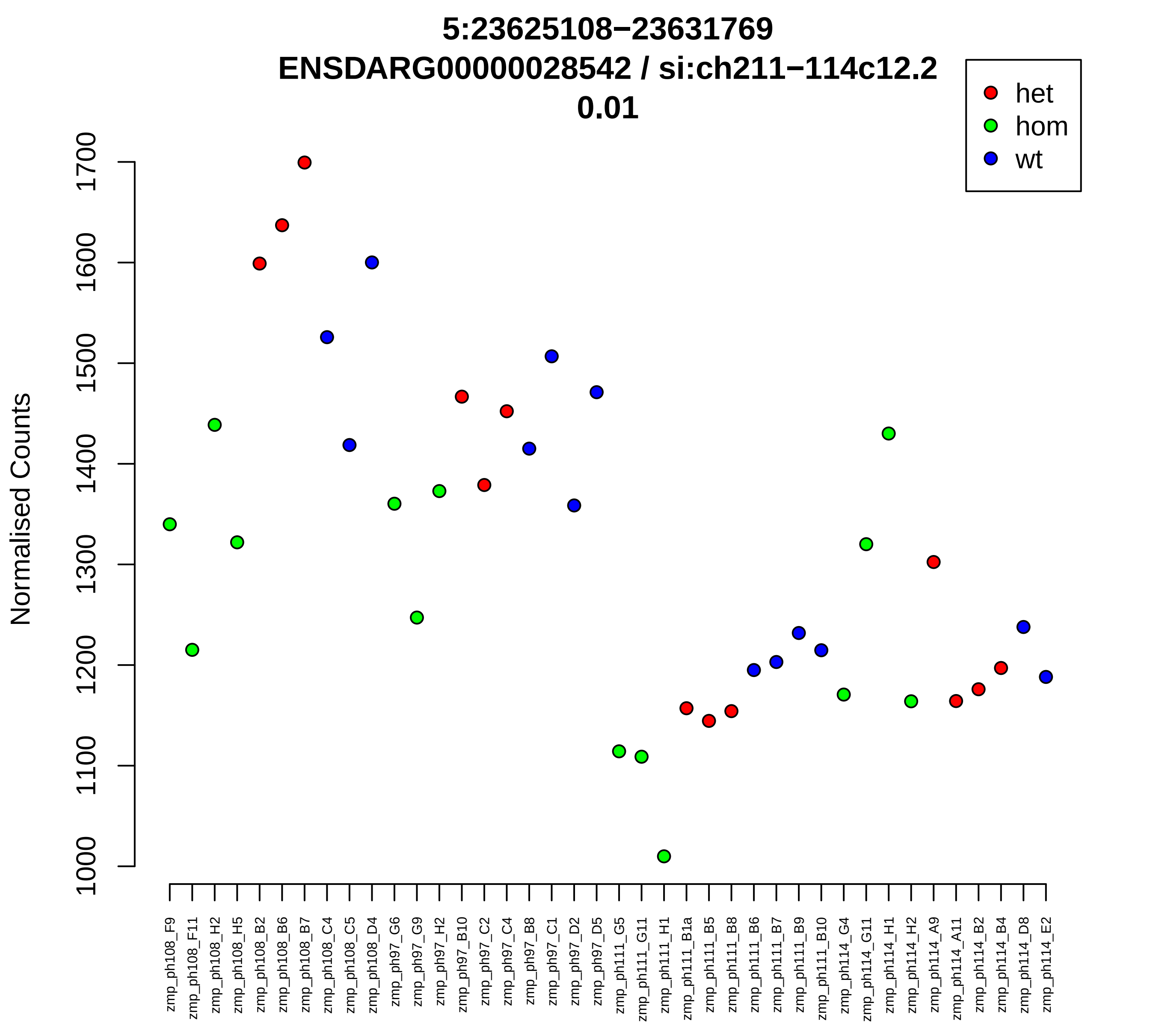
5:23625108−23631769
ENSDARG00000028542 / si:ch211−114c12.2
0.01
het
hom
wt
Normalised Counts
1000
1100
1200
1300
1400
1500
1600
1700
zmp_ph108_F9
zmp_ph108_F11
zmp_ph108_H2
zmp_ph108_H5
zmp_ph108_B2
zmp_ph108_B6
zmp_ph108_B7
zmp_ph108_C4
zmp_ph108_C5
zmp_ph108_D4
zmp_ph97_G6
zmp_ph97_G9
zmp_ph97_H2
zmp_ph97_B10
zmp_ph97_C2
zmp_ph97_C4
zmp_ph97_B8
zmp_ph97_C1
zmp_ph97_D2
zmp_ph97_D5
zmp_ph111_G5
zmp_ph111_G11
zmp_ph111_H1
zmp_ph111_B1a
zmp_ph111_B5
zmp_ph111_B8
zmp_ph111_B6
zmp_ph111_B7
zmp_ph111_B9
zmp_ph111_B10
zmp_ph114_G4
zmp_ph114_G11
zmp_ph114_H1
zmp_ph114_H2
zmp_ph114_A9
zmp_ph114_A11
zmp_ph114_B2
zmp_ph114_B4
zmp_ph114_D8
zmp_ph114_E2

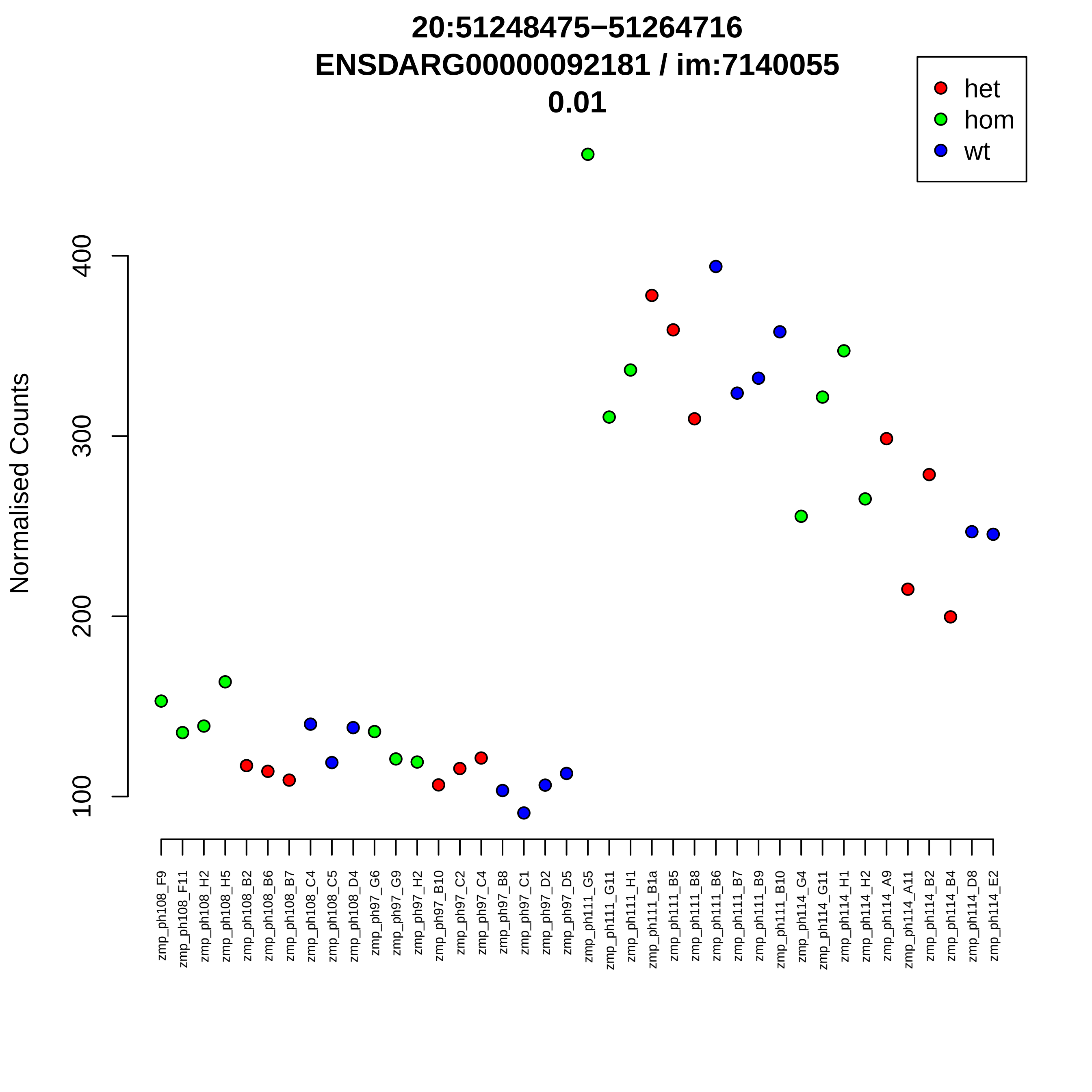
20:51248475−51264716
ENSDARG00000092181 / im:7140055
0.01
het
hom
wt
Normalised Counts
100
200
300
400
zmp_ph108_F9
zmp_ph108_F11
zmp_ph108_H2
zmp_ph108_H5
zmp_ph108_B2
zmp_ph108_B6
zmp_ph108_B7
zmp_ph108_C4
zmp_ph108_C5
zmp_ph108_D4
zmp_ph97_G6
zmp_ph97_G9
zmp_ph97_H2
zmp_ph97_B10
zmp_ph97_C2
zmp_ph97_C4
zmp_ph97_B8
zmp_ph97_C1
zmp_ph97_D2
zmp_ph97_D5
zmp_ph111_G5
zmp_ph111_G11
zmp_ph111_H1
zmp_ph111_B1a
zmp_ph111_B5
zmp_ph111_B8
zmp_ph111_B6
zmp_ph111_B7
zmp_ph111_B9
zmp_ph111_B10
zmp_ph114_G4
zmp_ph114_G11
zmp_ph114_H1
zmp_ph114_H2
zmp_ph114_A9
zmp_ph114_A11
zmp_ph114_B2
zmp_ph114_B4
zmp_ph114_D8
zmp_ph114_E2

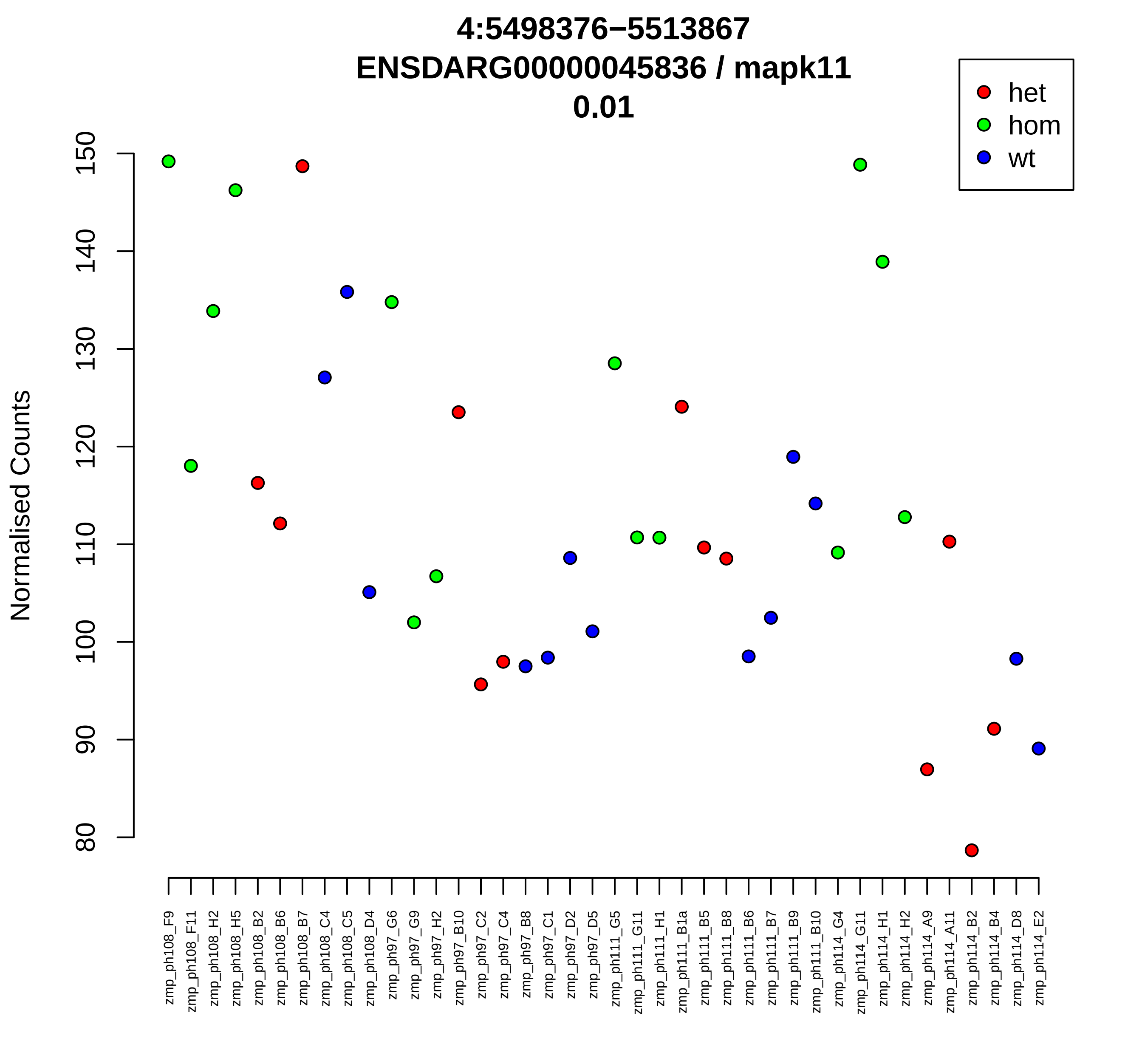

4:5498376−5513867
ENSDARG00000045836 / mapk11
0.01
het
hom
wt
Normalised Counts
150
140
130
120
110
100
90
80
zmp_ph108_F9
zmp_ph108_F11
zmp_ph108_H2
zmp_ph108_H5
zmp_ph108_B2
zmp_ph108_B6
zmp_ph108_B7
zmp_ph108_C4
zmp_ph108_C5
zmp_ph108_D4
zmp_ph97_G6
zmp_ph97_G9
zmp_ph97_H2
zmp_ph97_B10
zmp_ph97_C2
zmp_ph97_C4
zmp_ph97_B8
zmp_ph97_C1
zmp_ph97_D2
zmp_ph97_D5
zmp_ph111_G5
zmp_ph111_G11
zmp_ph111_H1
zmp_ph111_B1a
zmp_ph111_B5
zmp_ph111_B8
zmp_ph111_B6
zmp_ph111_B7
zmp_ph111_B9
zmp_ph111_B10
zmp_ph114_G4
zmp_ph114_G11
zmp_ph114_H1
zmp_ph114_H2
zmp_ph114_A9
zmp_ph114_A11
zmp_ph114_B2
zmp_ph114_B4
zmp_ph114_D8
zmp_ph114_E2

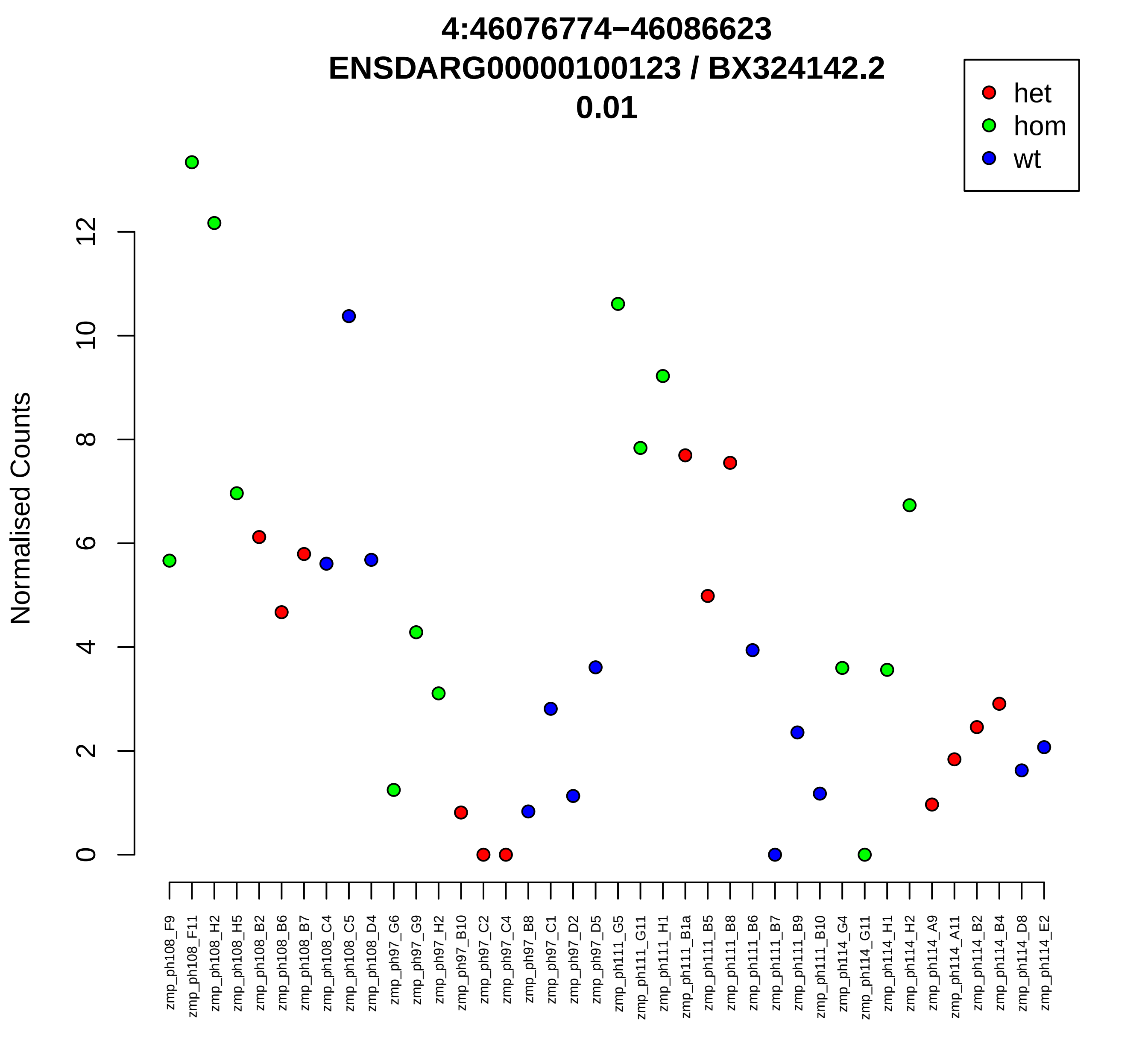

4:46076774−46086623
ENSDARG00000100123 / BX324142.2
0.01
het
hom
wt
Normalised Counts
0
2
4
6
8
10
12
zmp_ph108_F9
zmp_ph108_F11
zmp_ph108_H2
zmp_ph108_H5
zmp_ph108_B2
zmp_ph108_B6
zmp_ph108_B7
zmp_ph108_C4
zmp_ph108_C5
zmp_ph108_D4
zmp_ph97_G6
zmp_ph97_G9
zmp_ph97_H2
zmp_ph97_B10
zmp_ph97_C2
zmp_ph97_C4
zmp_ph97_B8
zmp_ph97_C1
zmp_ph97_D2
zmp_ph97_D5
zmp_ph111_G5
zmp_ph111_G11
zmp_ph111_H1
zmp_ph111_B1a
zmp_ph111_B5
zmp_ph111_B8
zmp_ph111_B6
zmp_ph111_B7
zmp_ph111_B9
zmp_ph111_B10
zmp_ph114_G4
zmp_ph114_G11
zmp_ph114_H1
zmp_ph114_H2
zmp_ph114_A9
zmp_ph114_A11
zmp_ph114_B2
zmp_ph114_B4
zmp_ph114_D8
zmp_ph114_E2

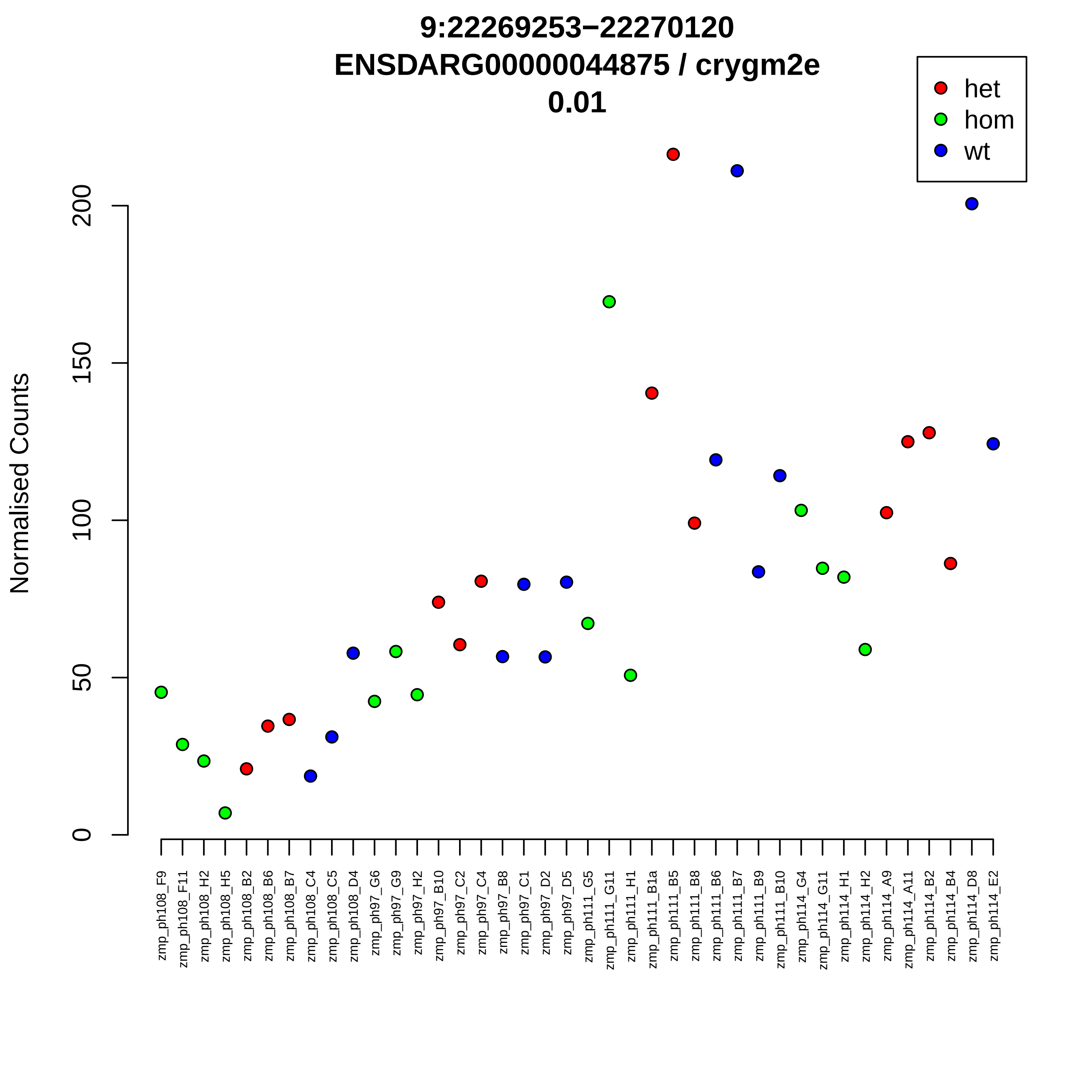
9:22269253−22270120
ENSDARG00000044875 / crygm2e
0.01
het
hom
wt
Normalised Counts
0
50
100
150
200
zmp_ph108_F9
zmp_ph108_F11
zmp_ph108_H2
zmp_ph108_H5
zmp_ph108_B2
zmp_ph108_B6
zmp_ph108_B7
zmp_ph108_C4
zmp_ph108_C5
zmp_ph108_D4
zmp_ph97_G6
zmp_ph97_G9
zmp_ph97_H2
zmp_ph97_B10
zmp_ph97_C2
zmp_ph97_C4
zmp_ph97_B8
zmp_ph97_C1
zmp_ph97_D2
zmp_ph97_D5
zmp_ph111_G5
zmp_ph111_G11
zmp_ph111_H1
zmp_ph111_B1a
zmp_ph111_B5
zmp_ph111_B8
zmp_ph111_B6
zmp_ph111_B7
zmp_ph111_B9
zmp_ph111_B10
zmp_ph114_G4
zmp_ph114_G11
zmp_ph114_H1
zmp_ph114_H2
zmp_ph114_A9
zmp_ph114_A11
zmp_ph114_B2
zmp_ph114_B4
zmp_ph114_D8
zmp_ph114_E2

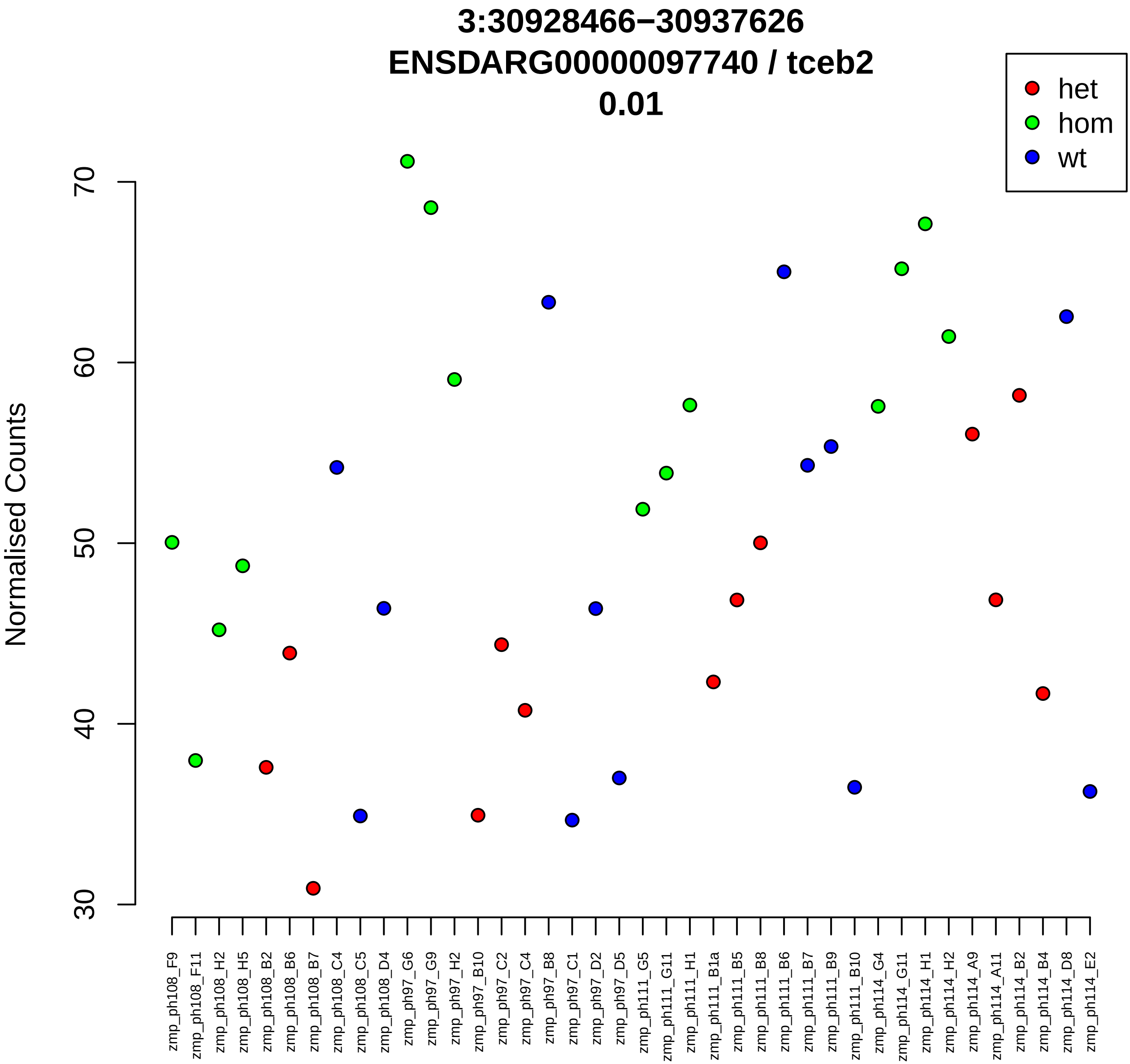

3:30928466−30937626
ENSDARG00000097740 / tceb2
0.01
het
hom
wt
Normalised Counts
30
40
50
60
70
zmp_ph108_F9
zmp_ph108_F11
zmp_ph108_H2
zmp_ph108_H5
zmp_ph108_B2
zmp_ph108_B6
zmp_ph108_B7
zmp_ph108_C4
zmp_ph108_C5
zmp_ph108_D4
zmp_ph97_G6
zmp_ph97_G9
zmp_ph97_H2
zmp_ph97_B10
zmp_ph97_C2
zmp_ph97_C4
zmp_ph97_B8
zmp_ph97_C1
zmp_ph97_D2
zmp_ph97_D5
zmp_ph111_G5
zmp_ph111_G11
zmp_ph111_H1
zmp_ph111_B1a
zmp_ph111_B5
zmp_ph111_B8
zmp_ph111_B6
zmp_ph111_B7
zmp_ph111_B9
zmp_ph111_B10
zmp_ph114_G4
zmp_ph114_G11
zmp_ph114_H1
zmp_ph114_H2
zmp_ph114_A9
zmp_ph114_A11
zmp_ph114_B2
zmp_ph114_B4
zmp_ph114_D8
zmp_ph114_E2

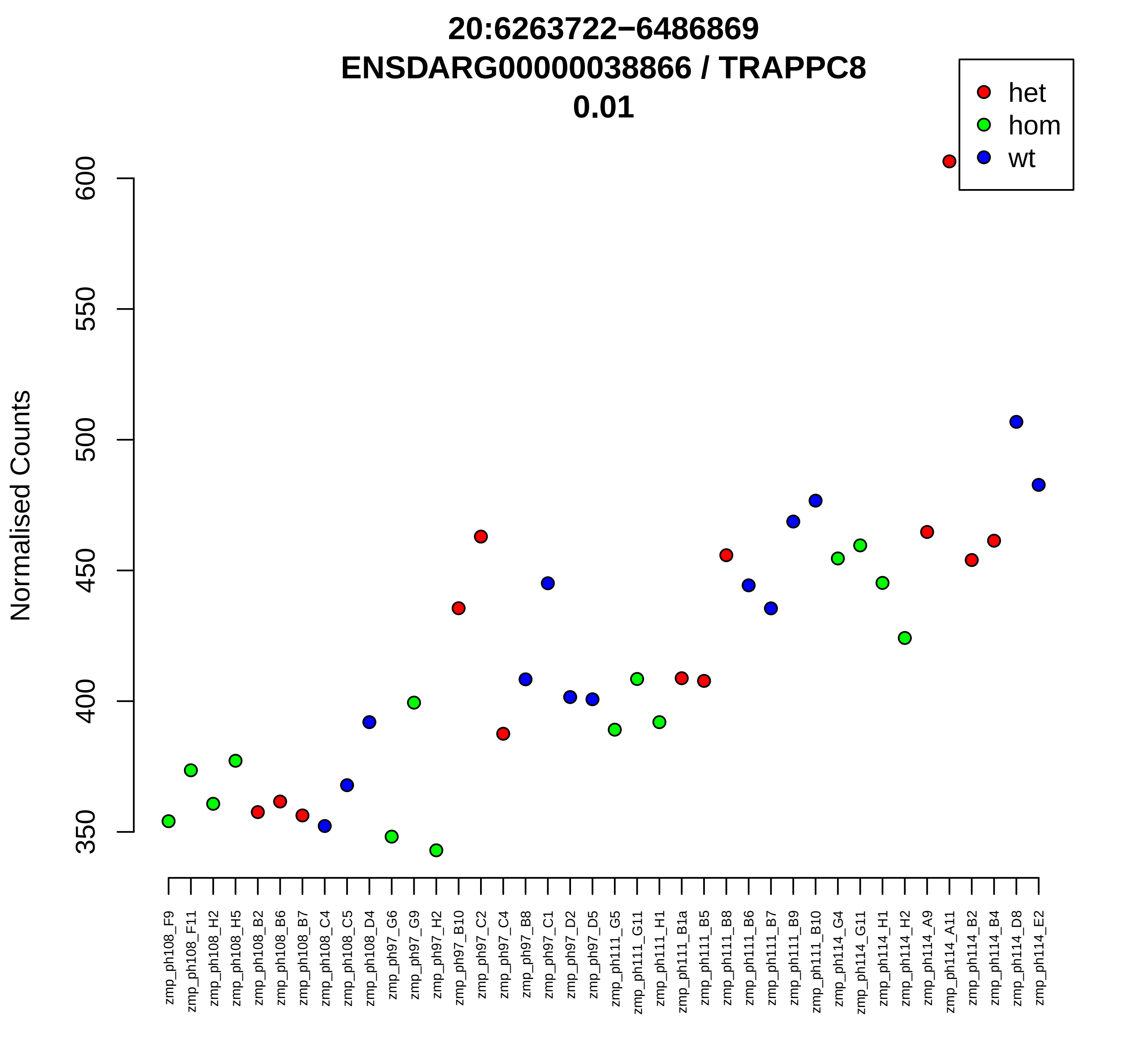
20:6263722−6486869
ENSDARG00000038866 / TRAPPC8
0.01
het
hom
wt
Normalised Counts
350
400
450
500
550
600
zmp_ph108_F9
zmp_ph108_F11
zmp_ph108_H2
zmp_ph108_H5
zmp_ph108_B2
zmp_ph108_B6
zmp_ph108_B7
zmp_ph108_C4
zmp_ph108_C5
zmp_ph108_D4
zmp_ph97_G6
zmp_ph97_G9
zmp_ph97_H2
zmp_ph97_B10
zmp_ph97_C2
zmp_ph97_C4
zmp_ph97_B8
zmp_ph97_C1
zmp_ph97_D2
zmp_ph97_D5
zmp_ph111_G5
zmp_ph111_G11
zmp_ph111_H1
zmp_ph111_B1a
zmp_ph111_B5
zmp_ph111_B8
zmp_ph111_B6
zmp_ph111_B7
zmp_ph111_B9
zmp_ph111_B10
zmp_ph114_G4
zmp_ph114_G11
zmp_ph114_H1
zmp_ph114_H2
zmp_ph114_A9
zmp_ph114_A11
zmp_ph114_B2
zmp_ph114_B4
zmp_ph114_D8
zmp_ph114_E2

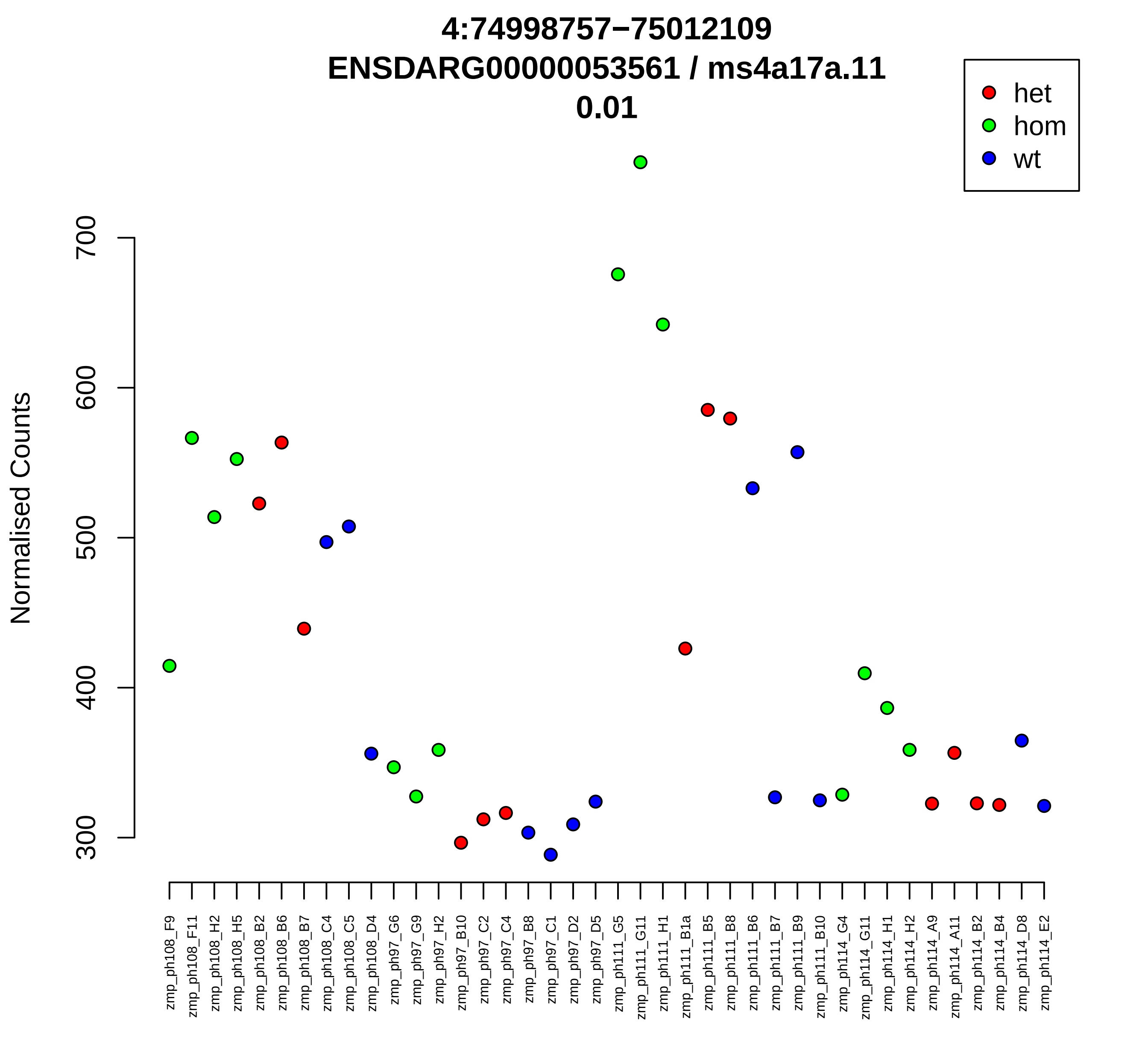

4:74998757−75012109
ENSDARG00000053561 / ms4a17a.11
0.01
het
hom
wt
Normalised Counts
300
400
500
600
700
zmp_ph108_F9
zmp_ph108_F11
zmp_ph108_H2
zmp_ph108_H5
zmp_ph108_B2
zmp_ph108_B6
zmp_ph108_B7
zmp_ph108_C4
zmp_ph108_C5
zmp_ph108_D4
zmp_ph97_G6
zmp_ph97_G9
zmp_ph97_H2
zmp_ph97_B10
zmp_ph97_C2
zmp_ph97_C4
zmp_ph97_B8
zmp_ph97_C1
zmp_ph97_D2
zmp_ph97_D5
zmp_ph111_G5
zmp_ph111_G11
zmp_ph111_H1
zmp_ph111_B1a
zmp_ph111_B5
zmp_ph111_B8
zmp_ph111_B6
zmp_ph111_B7
zmp_ph111_B9
zmp_ph111_B10
zmp_ph114_G4
zmp_ph114_G11
zmp_ph114_H1
zmp_ph114_H2
zmp_ph114_A9
zmp_ph114_A11
zmp_ph114_B2
zmp_ph114_B4
zmp_ph114_D8
zmp_ph114_E2

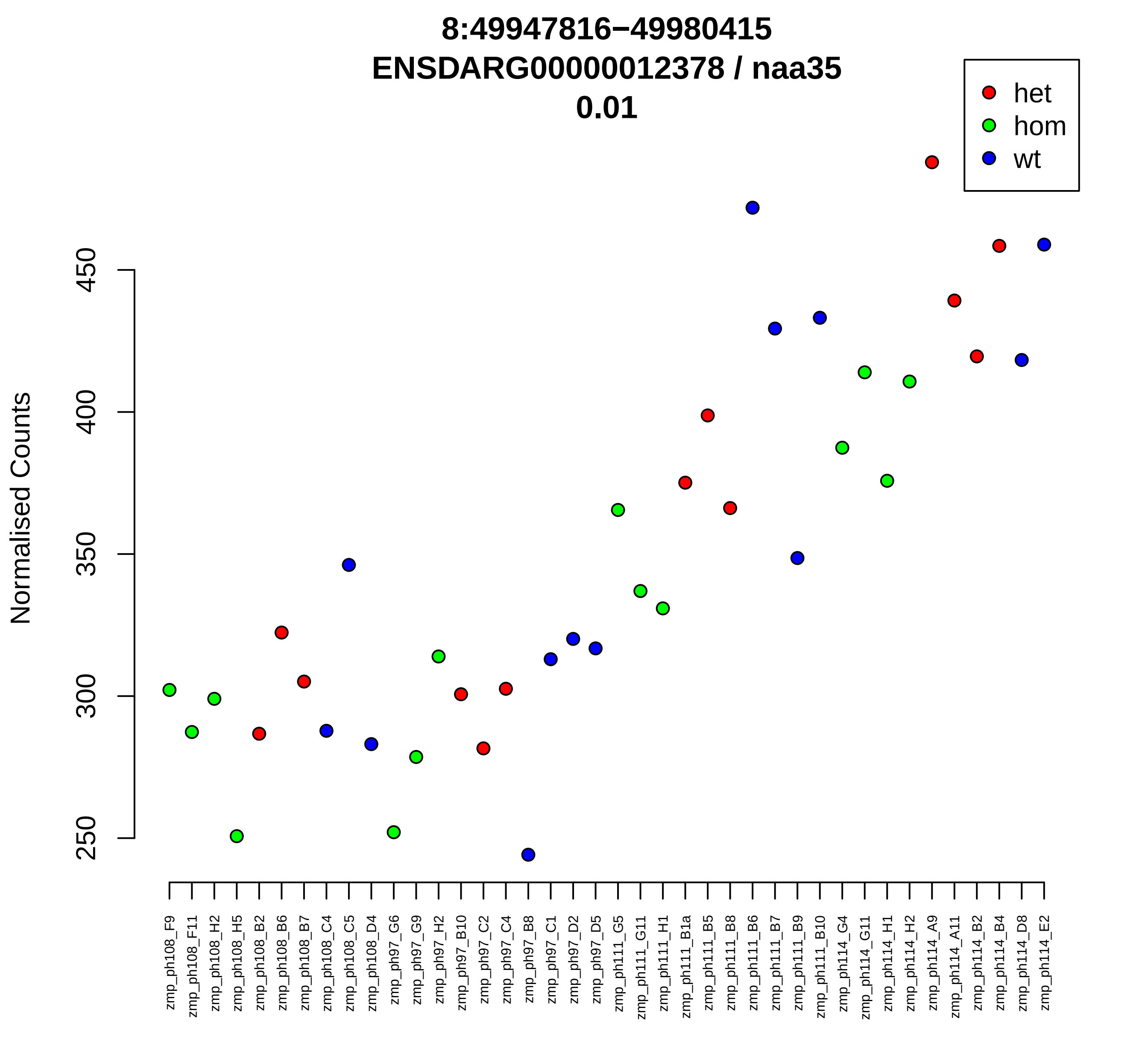

8:49947816−49980415
ENSDARG00000012378 / naa35
0.01
het
hom
wt
Normalised Counts
250
300
350
400
450
zmp_ph108_F9
zmp_ph108_F11
zmp_ph108_H2
zmp_ph108_H5
zmp_ph108_B2
zmp_ph108_B6
zmp_ph108_B7
zmp_ph108_C4
zmp_ph108_C5
zmp_ph108_D4
zmp_ph97_G6
zmp_ph97_G9
zmp_ph97_H2
zmp_ph97_B10
zmp_ph97_C2
zmp_ph97_C4
zmp_ph97_B8
zmp_ph97_C1
zmp_ph97_D2
zmp_ph97_D5
zmp_ph111_G5
zmp_ph111_G11
zmp_ph111_H1
zmp_ph111_B1a
zmp_ph111_B5
zmp_ph111_B8
zmp_ph111_B6
zmp_ph111_B7
zmp_ph111_B9
zmp_ph111_B10
zmp_ph114_G4
zmp_ph114_G11
zmp_ph114_H1
zmp_ph114_H2
zmp_ph114_A9
zmp_ph114_A11
zmp_ph114_B2
zmp_ph114_B4
zmp_ph114_D8
zmp_ph114_E2

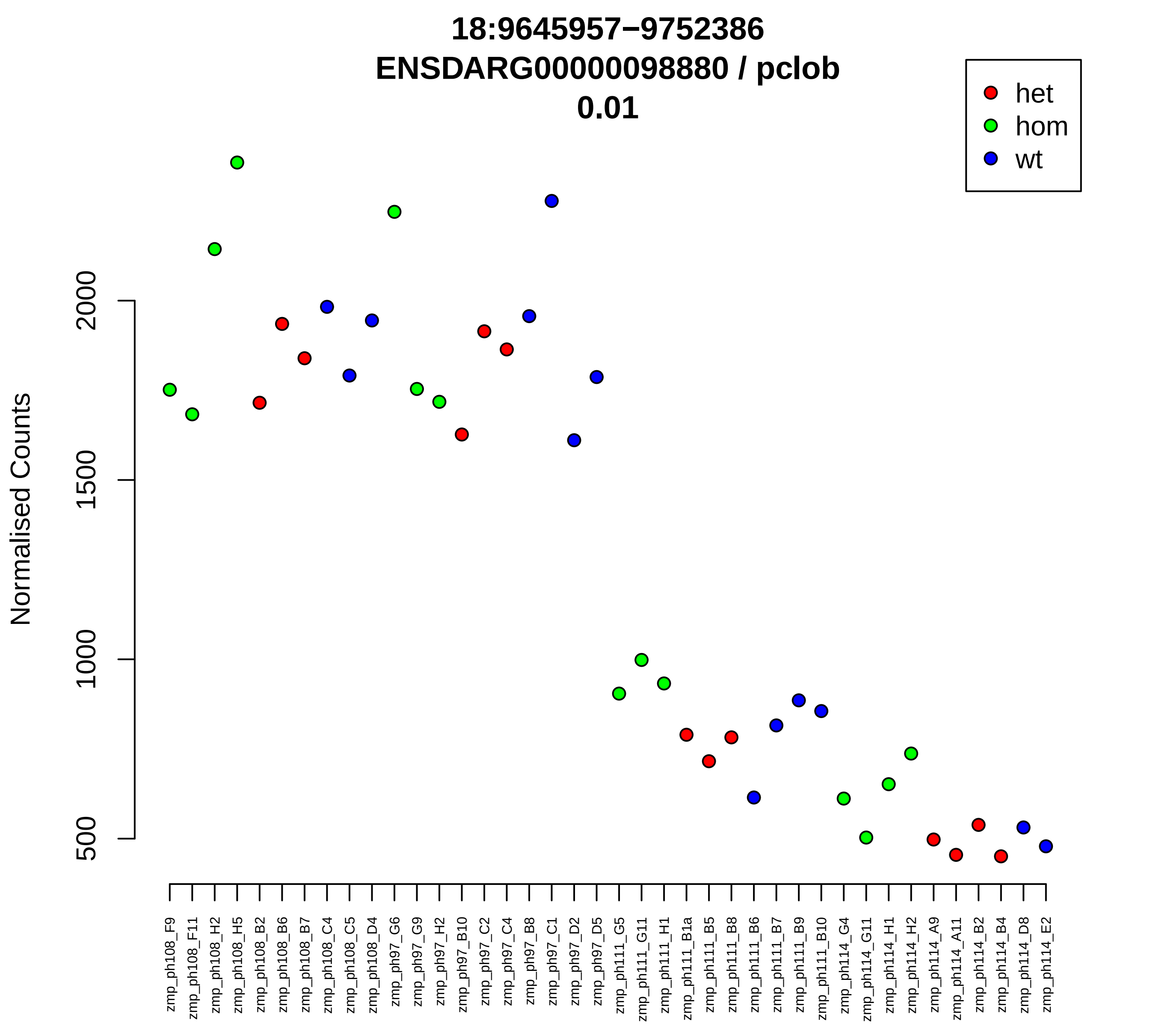

18:9645957−9752386
ENSDARG00000098880 / pclob
0.01
het
hom
wt
Normalised Counts
500
1000
1500
2000
zmp_ph108_F9
zmp_ph108_F11
zmp_ph108_H2
zmp_ph108_H5
zmp_ph108_B2
zmp_ph108_B6
zmp_ph108_B7
zmp_ph108_C4
zmp_ph108_C5
zmp_ph108_D4
zmp_ph97_G6
zmp_ph97_G9
zmp_ph97_H2
zmp_ph97_B10
zmp_ph97_C2
zmp_ph97_C4
zmp_ph97_B8
zmp_ph97_C1
zmp_ph97_D2
zmp_ph97_D5
zmp_ph111_G5
zmp_ph111_G11
zmp_ph111_H1
zmp_ph111_B1a
zmp_ph111_B5
zmp_ph111_B8
zmp_ph111_B6
zmp_ph111_B7
zmp_ph111_B9
zmp_ph111_B10
zmp_ph114_G4
zmp_ph114_G11
zmp_ph114_H1
zmp_ph114_H2
zmp_ph114_A9
zmp_ph114_A11
zmp_ph114_B2
zmp_ph114_B4
zmp_ph114_D8
zmp_ph114_E2

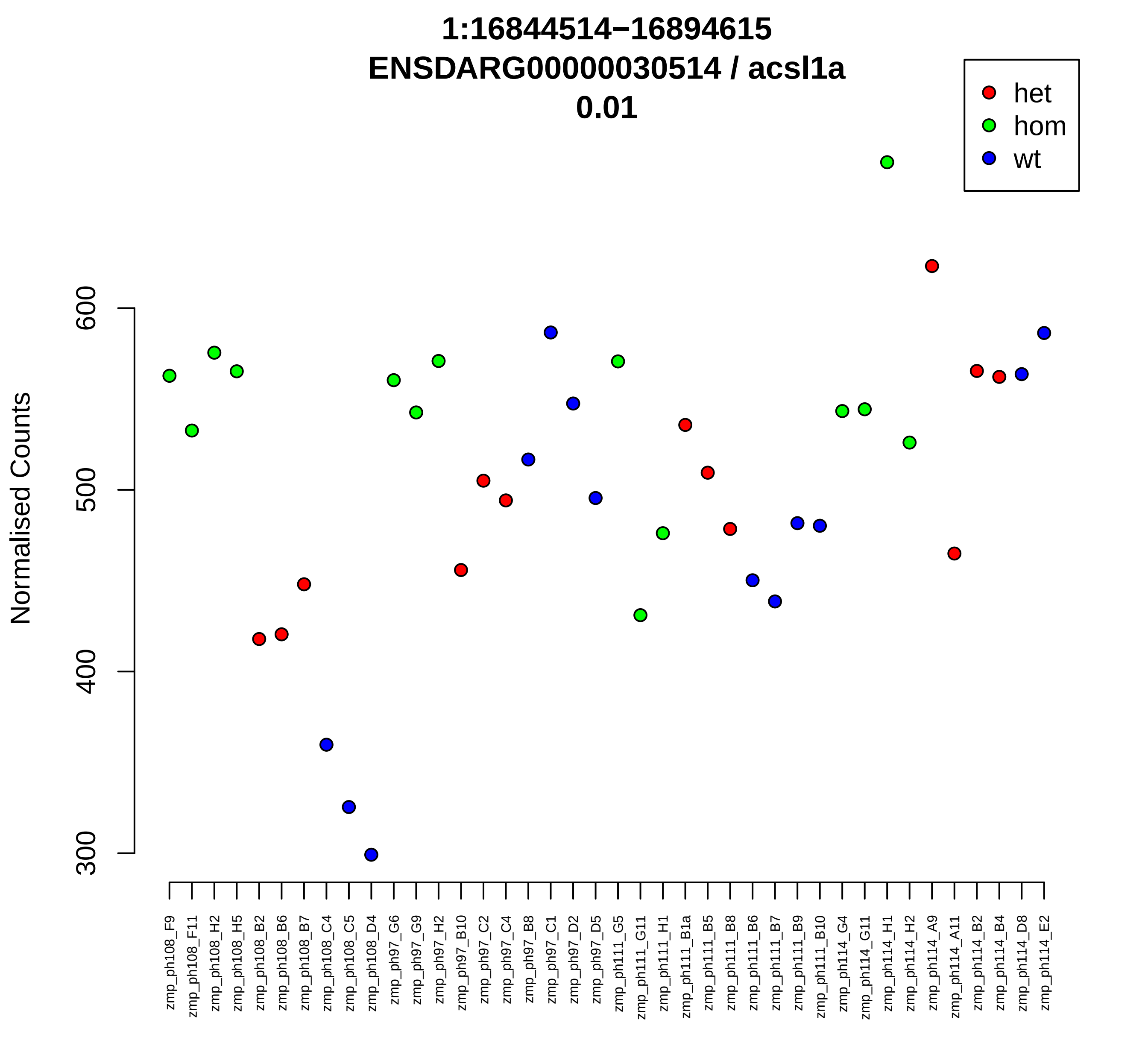
1:16844514−16894615
ENSDARG00000030514 / acsl1a
0.01
het
hom
wt
Normalised Counts
300
400
500
600
zmp_ph108_F9
zmp_ph108_F11
zmp_ph108_H2
zmp_ph108_H5
zmp_ph108_B2
zmp_ph108_B6
zmp_ph108_B7
zmp_ph108_C4
zmp_ph108_C5
zmp_ph108_D4
zmp_ph97_G6
zmp_ph97_G9
zmp_ph97_H2
zmp_ph97_B10
zmp_ph97_C2
zmp_ph97_C4
zmp_ph97_B8
zmp_ph97_C1
zmp_ph97_D2
zmp_ph97_D5
zmp_ph111_G5
zmp_ph111_G11
zmp_ph111_H1
zmp_ph111_B1a
zmp_ph111_B5
zmp_ph111_B8
zmp_ph111_B6
zmp_ph111_B7
zmp_ph111_B9
zmp_ph111_B10
zmp_ph114_G4
zmp_ph114_G11
zmp_ph114_H1
zmp_ph114_H2
zmp_ph114_A9
zmp_ph114_A11
zmp_ph114_B2
zmp_ph114_B4
zmp_ph114_D8
zmp_ph114_E2

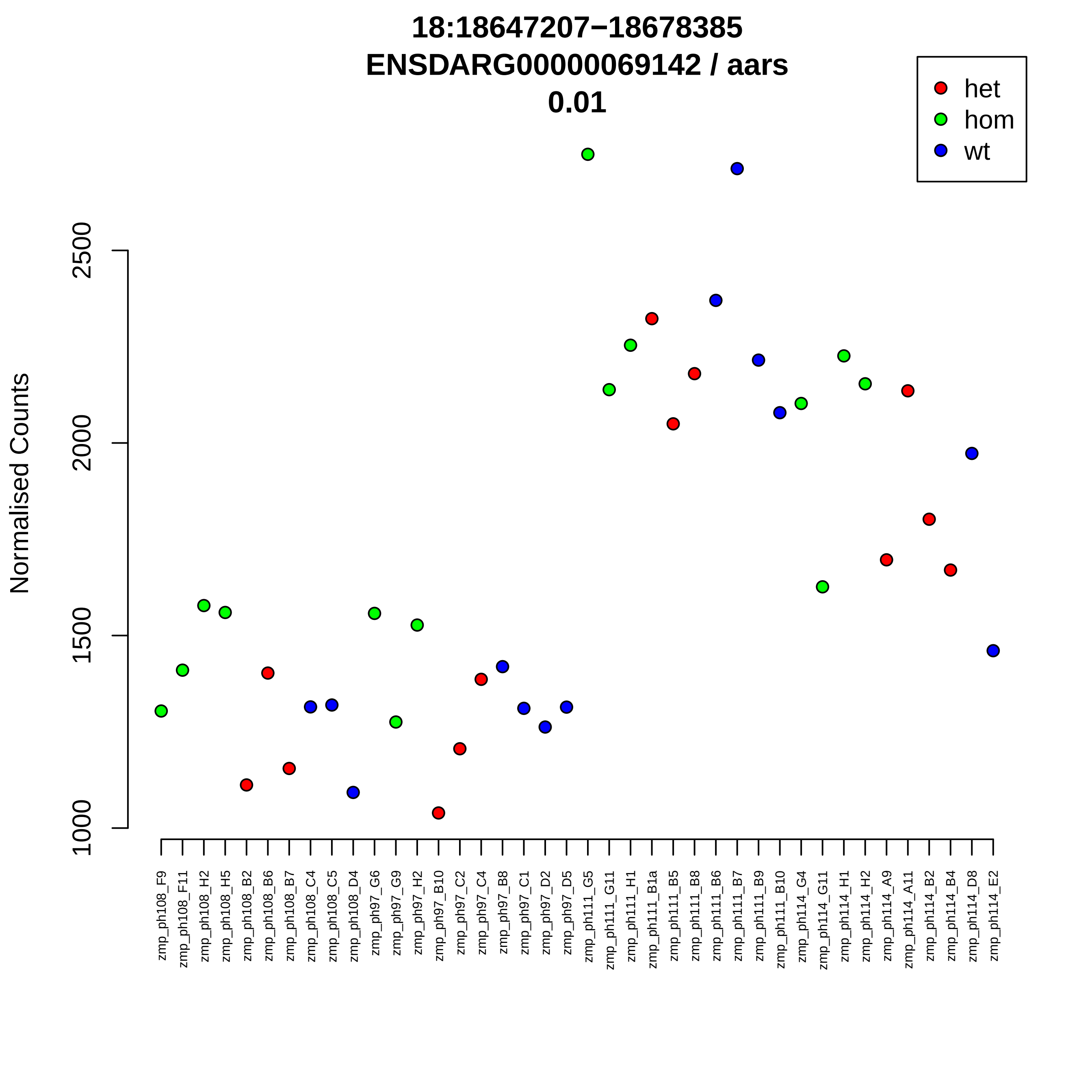
18:18647207−18678385
ENSDARG00000069142 / aars
0.01
het
hom
wt
Normalised Counts
1000
1500
2000
2500
zmp_ph108_F9
zmp_ph108_F11
zmp_ph108_H2
zmp_ph108_H5
zmp_ph108_B2
zmp_ph108_B6
zmp_ph108_B7
zmp_ph108_C4
zmp_ph108_C5
zmp_ph108_D4
zmp_ph97_G6
zmp_ph97_G9
zmp_ph97_H2
zmp_ph97_B10
zmp_ph97_C2
zmp_ph97_C4
zmp_ph97_B8
zmp_ph97_C1
zmp_ph97_D2
zmp_ph97_D5
zmp_ph111_G5
zmp_ph111_G11
zmp_ph111_H1
zmp_ph111_B1a
zmp_ph111_B5
zmp_ph111_B8
zmp_ph111_B6
zmp_ph111_B7
zmp_ph111_B9
zmp_ph111_B10
zmp_ph114_G4
zmp_ph114_G11
zmp_ph114_H1
zmp_ph114_H2
zmp_ph114_A9
zmp_ph114_A11
zmp_ph114_B2
zmp_ph114_B4
zmp_ph114_D8
zmp_ph114_E2

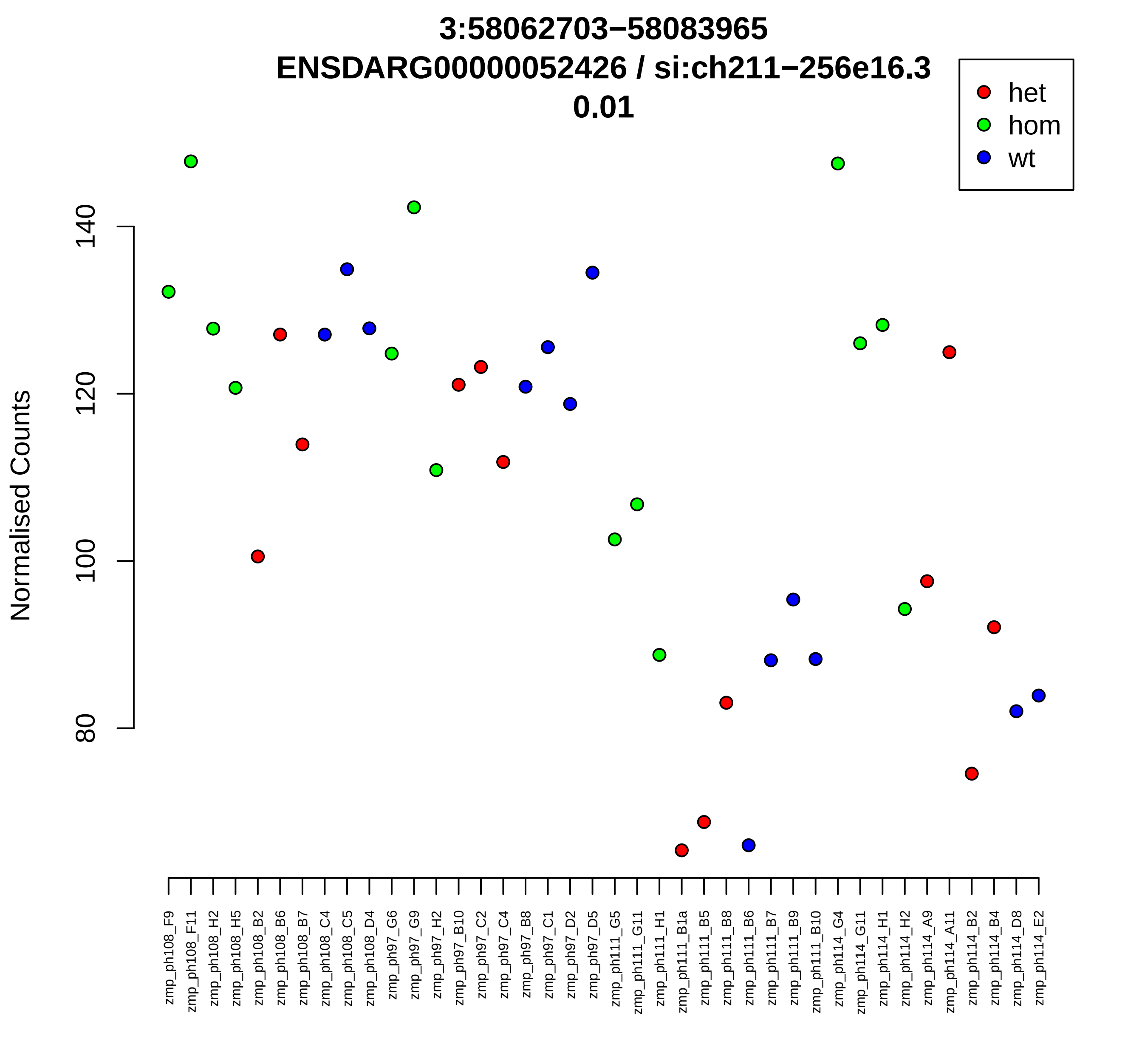
3:58062703−58083965
ENSDARG00000052426 / si:ch211−256e16.3
0.01
het
hom
wt
Normalised Counts
80
100
120
140
zmp_ph108_F9
zmp_ph108_F11
zmp_ph108_H2
zmp_ph108_H5
zmp_ph108_B2
zmp_ph108_B6
zmp_ph108_B7
zmp_ph108_C4
zmp_ph108_C5
zmp_ph108_D4
zmp_ph97_G6
zmp_ph97_G9
zmp_ph97_H2
zmp_ph97_B10
zmp_ph97_C2
zmp_ph97_C4
zmp_ph97_B8
zmp_ph97_C1
zmp_ph97_D2
zmp_ph97_D5
zmp_ph111_G5
zmp_ph111_G11
zmp_ph111_H1
zmp_ph111_B1a
zmp_ph111_B5
zmp_ph111_B8
zmp_ph111_B6
zmp_ph111_B7
zmp_ph111_B9
zmp_ph111_B10
zmp_ph114_G4
zmp_ph114_G11
zmp_ph114_H1
zmp_ph114_H2
zmp_ph114_A9
zmp_ph114_A11
zmp_ph114_B2
zmp_ph114_B4
zmp_ph114_D8
zmp_ph114_E2

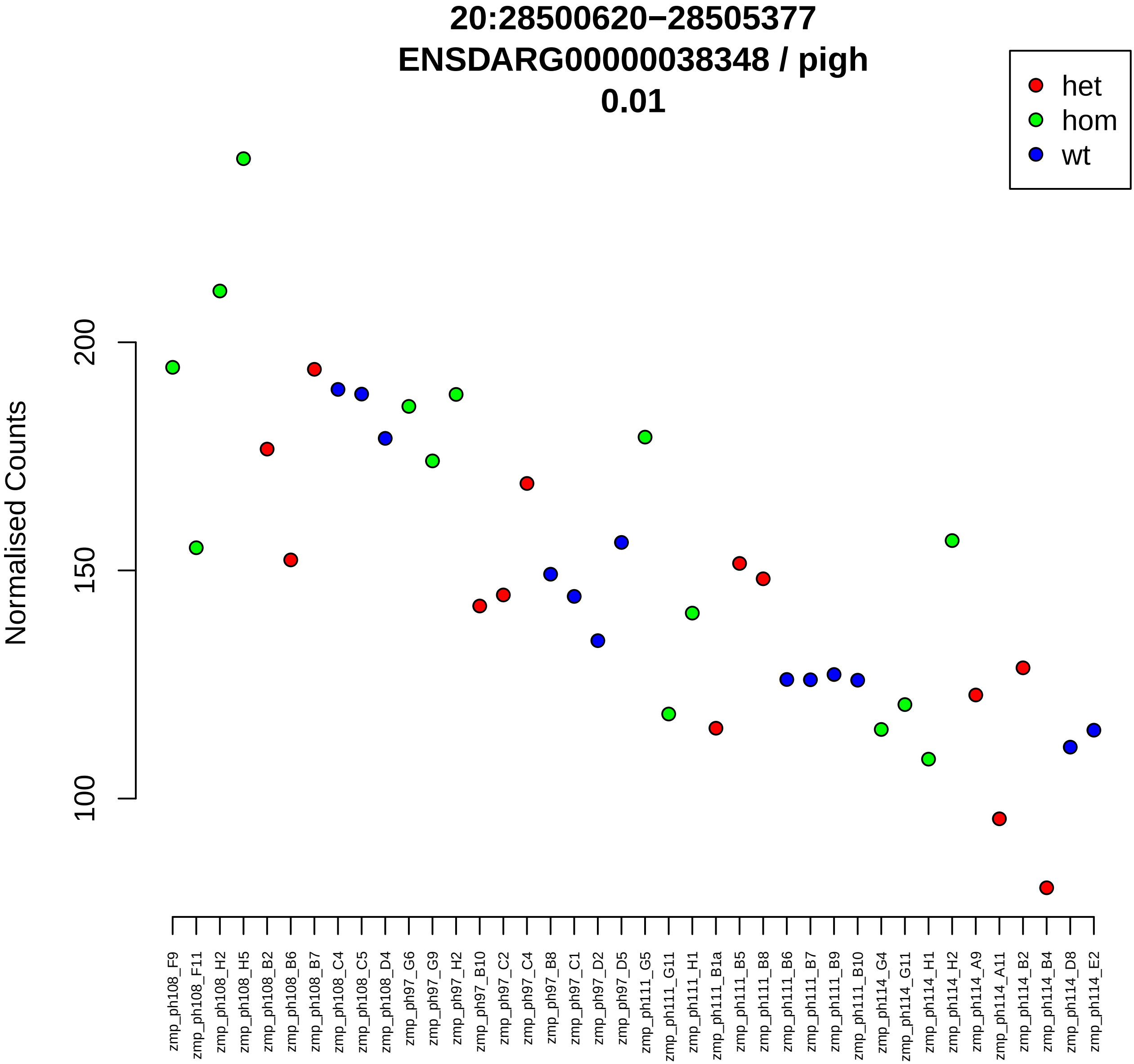
20:28500620−28505377
ENSDARG00000038348 / pigh
0.01
het
hom
wt
Normalised Counts
100
150
200
zmp_ph108_F9
zmp_ph108_F11
zmp_ph108_H2
zmp_ph108_H5
zmp_ph108_B2
zmp_ph108_B6
zmp_ph108_B7
zmp_ph108_C4
zmp_ph108_C5
zmp_ph108_D4
zmp_ph97_G6
zmp_ph97_G9
zmp_ph97_H2
zmp_ph97_B10
zmp_ph97_C2
zmp_ph97_C4
zmp_ph97_B8
zmp_ph97_C1
zmp_ph97_D2
zmp_ph97_D5
zmp_ph111_G5
zmp_ph111_G11
zmp_ph111_H1
zmp_ph111_B1a
zmp_ph111_B5
zmp_ph111_B8
zmp_ph111_B6
zmp_ph111_B7
zmp_ph111_B9
zmp_ph111_B10
zmp_ph114_G4
zmp_ph114_G11
zmp_ph114_H1
zmp_ph114_H2
zmp_ph114_A9
zmp_ph114_A11
zmp_ph114_B2
zmp_ph114_B4
zmp_ph114_D8
zmp_ph114_E2

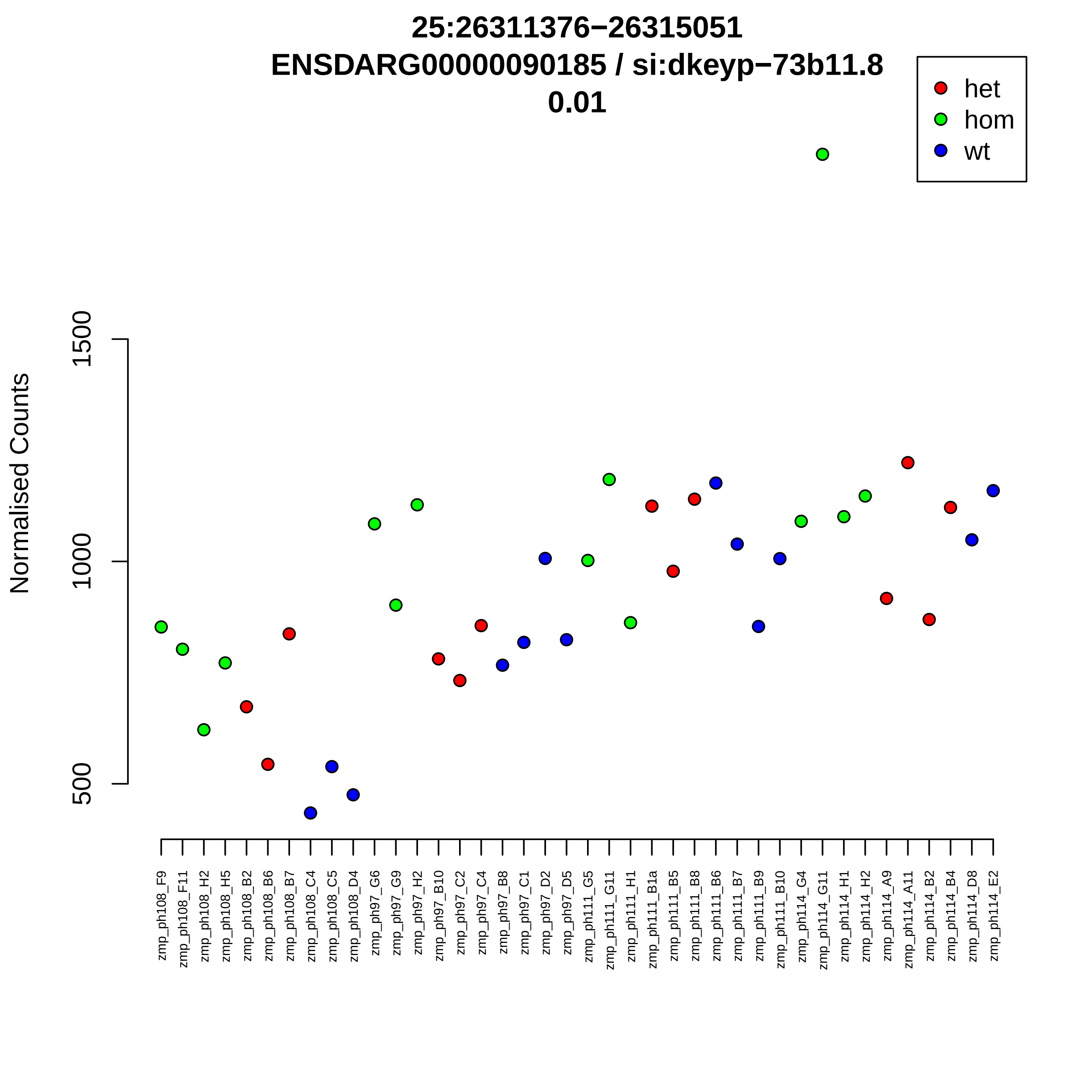

25:26311376−26315051
ENSDARG00000090185 / si:dkeyp−73b11.8
0.01
het
hom
wt
Normalised Counts
500
1000
1500
zmp_ph108_F9
zmp_ph108_F11
zmp_ph108_H2
zmp_ph108_H5
zmp_ph108_B2
zmp_ph108_B6
zmp_ph108_B7
zmp_ph108_C4
zmp_ph108_C5
zmp_ph108_D4
zmp_ph97_G6
zmp_ph97_G9
zmp_ph97_H2
zmp_ph97_B10
zmp_ph97_C2
zmp_ph97_C4
zmp_ph97_B8
zmp_ph97_C1
zmp_ph97_D2
zmp_ph97_D5
zmp_ph111_G5
zmp_ph111_G11
zmp_ph111_H1
zmp_ph111_B1a
zmp_ph111_B5
zmp_ph111_B8
zmp_ph111_B6
zmp_ph111_B7
zmp_ph111_B9
zmp_ph111_B10
zmp_ph114_G4
zmp_ph114_G11
zmp_ph114_H1
zmp_ph114_H2
zmp_ph114_A9
zmp_ph114_A11
zmp_ph114_B2
zmp_ph114_B4
zmp_ph114_D8
zmp_ph114_E2

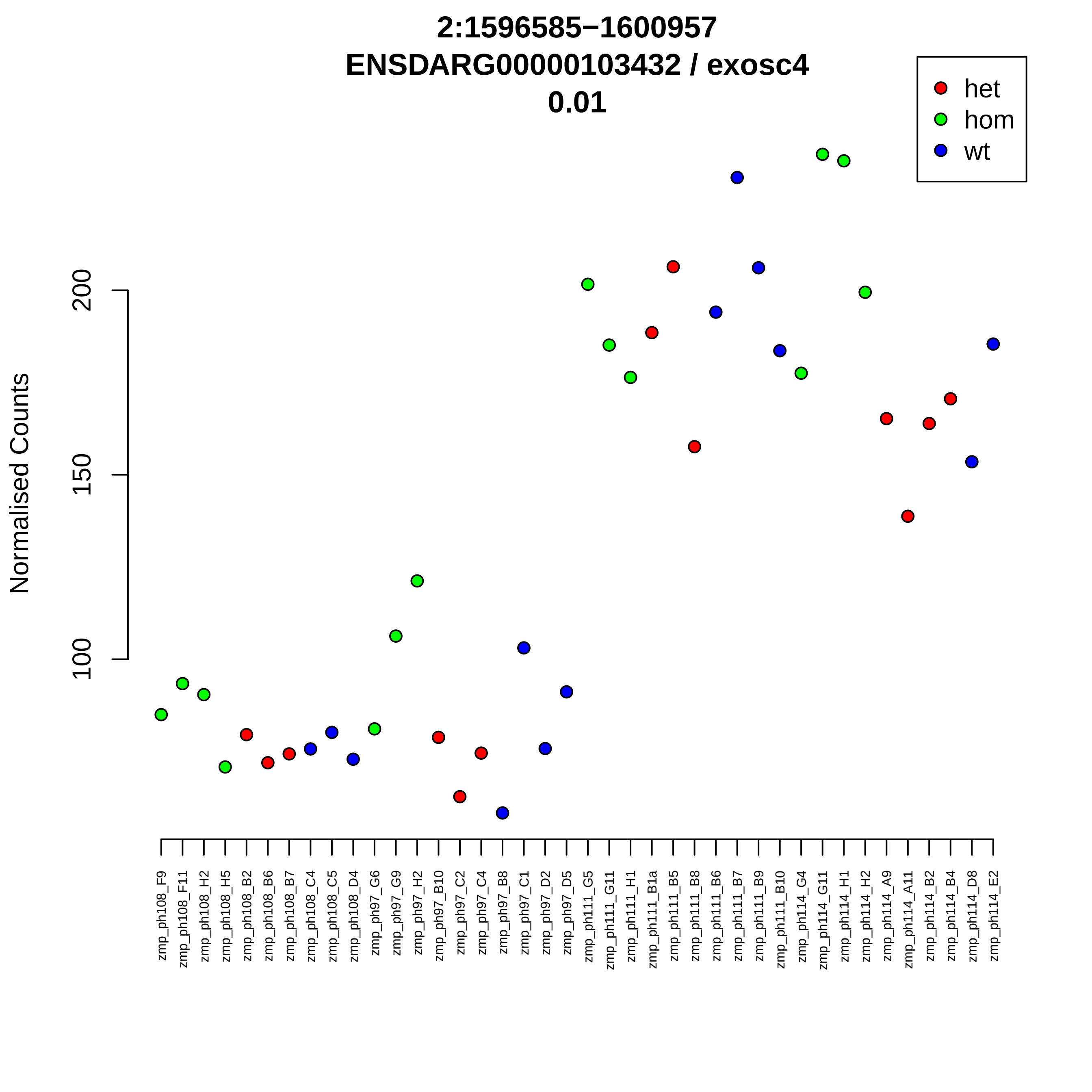
2:1596585−1600957
ENSDARG00000103432 / exosc4
0.01
het
hom
wt
Normalised Counts
100
150
200
zmp_ph108_F9
zmp_ph108_F11
zmp_ph108_H2
zmp_ph108_H5
zmp_ph108_B2
zmp_ph108_B6
zmp_ph108_B7
zmp_ph108_C4
zmp_ph108_C5
zmp_ph108_D4
zmp_ph97_G6
zmp_ph97_G9
zmp_ph97_H2
zmp_ph97_B10
zmp_ph97_C2
zmp_ph97_C4
zmp_ph97_B8
zmp_ph97_C1
zmp_ph97_D2
zmp_ph97_D5
zmp_ph111_G5
zmp_ph111_G11
zmp_ph111_H1
zmp_ph111_B1a
zmp_ph111_B5
zmp_ph111_B8
zmp_ph111_B6
zmp_ph111_B7
zmp_ph111_B9
zmp_ph111_B10
zmp_ph114_G4
zmp_ph114_G11
zmp_ph114_H1
zmp_ph114_H2
zmp_ph114_A9
zmp_ph114_A11
zmp_ph114_B2
zmp_ph114_B4
zmp_ph114_D8
zmp_ph114_E2

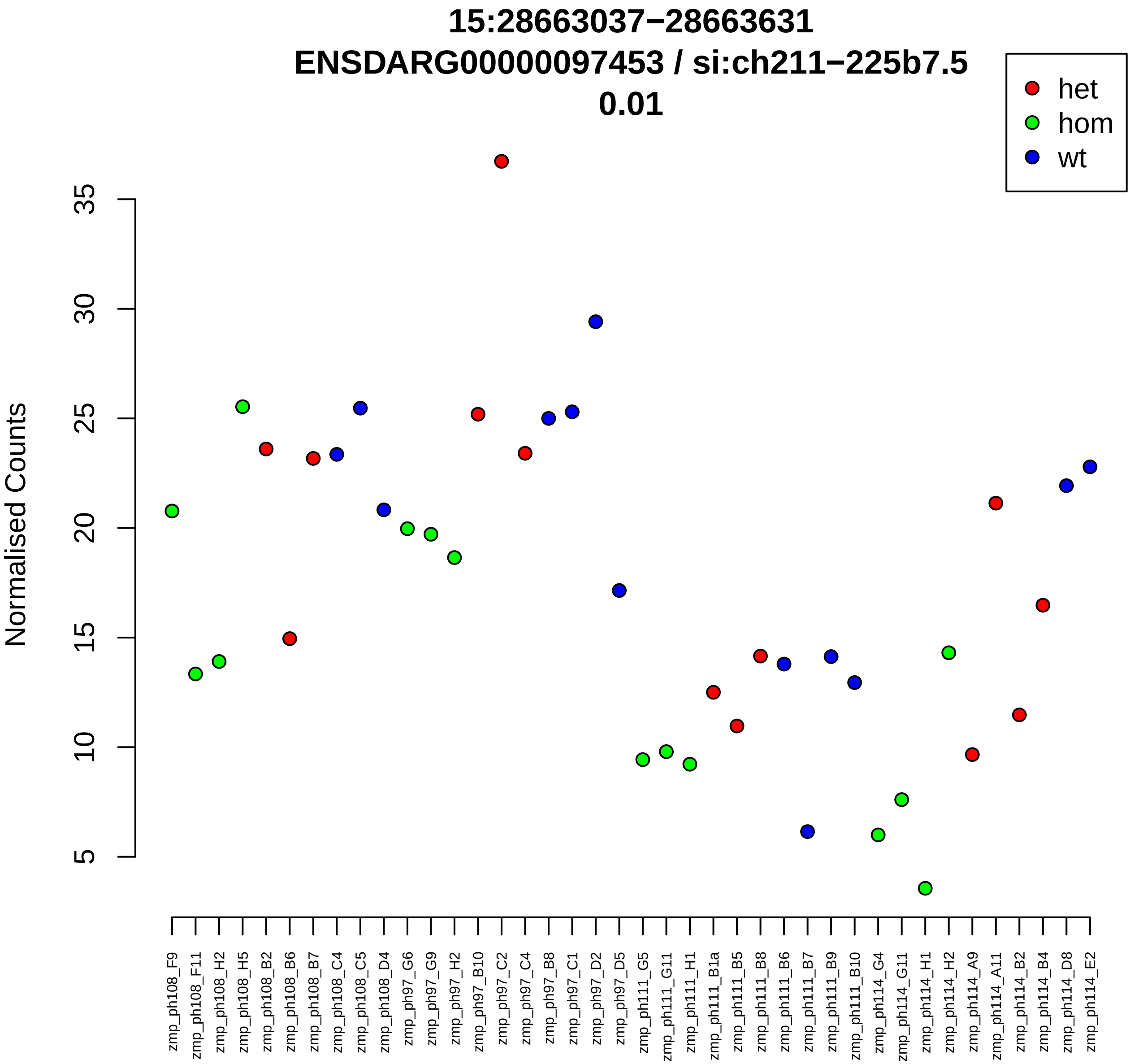

15:28663037−28663631
ENSDARG00000097453 / si:ch211−225b7.5
0.01
het
hom
wt
Normalised Counts
5
10
15
20
25
30
35
zmp_ph108_F9
zmp_ph108_F11
zmp_ph108_H2
zmp_ph108_H5
zmp_ph108_B2
zmp_ph108_B6
zmp_ph108_B7
zmp_ph108_C4
zmp_ph108_C5
zmp_ph108_D4
zmp_ph97_G6
zmp_ph97_G9
zmp_ph97_H2
zmp_ph97_B10
zmp_ph97_C2
zmp_ph97_C4
zmp_ph97_B8
zmp_ph97_C1
zmp_ph97_D2
zmp_ph97_D5
zmp_ph111_G5
zmp_ph111_G11
zmp_ph111_H1
zmp_ph111_B1a
zmp_ph111_B5
zmp_ph111_B8
zmp_ph111_B6
zmp_ph111_B7
zmp_ph111_B9
zmp_ph111_B10
zmp_ph114_G4
zmp_ph114_G11
zmp_ph114_H1
zmp_ph114_H2
zmp_ph114_A9
zmp_ph114_A11
zmp_ph114_B2
zmp_ph114_B4
zmp_ph114_D8
zmp_ph114_E2

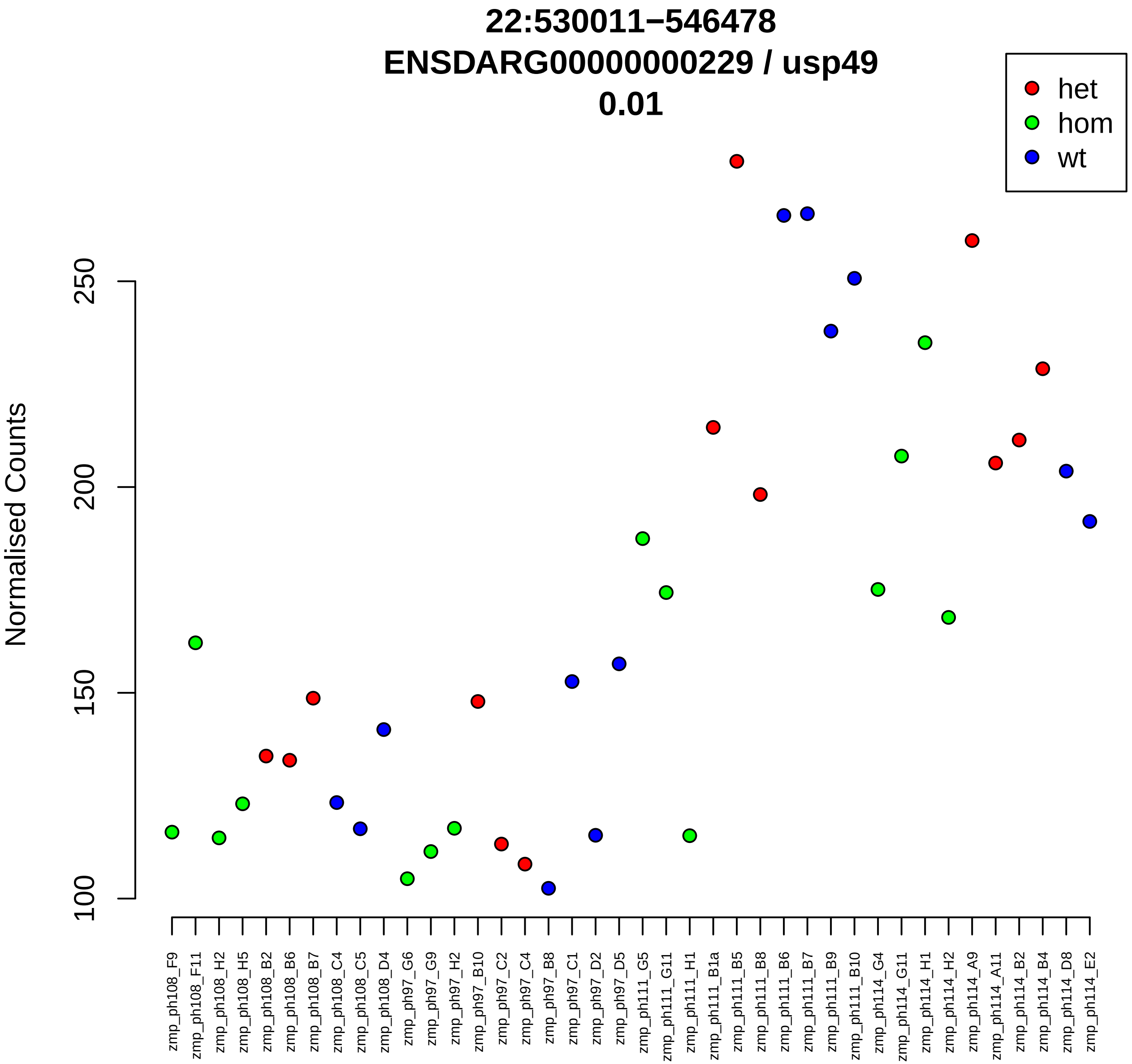
22:530011−546478
ENSDARG00000000229 / usp49
0.01
het
hom
wt
Normalised Counts
100
150
200
250
zmp_ph108_F9
zmp_ph108_F11
zmp_ph108_H2
zmp_ph108_H5
zmp_ph108_B2
zmp_ph108_B6
zmp_ph108_B7
zmp_ph108_C4
zmp_ph108_C5
zmp_ph108_D4
zmp_ph97_G6
zmp_ph97_G9
zmp_ph97_H2
zmp_ph97_B10
zmp_ph97_C2
zmp_ph97_C4
zmp_ph97_B8
zmp_ph97_C1
zmp_ph97_D2
zmp_ph97_D5
zmp_ph111_G5
zmp_ph111_G11
zmp_ph111_H1
zmp_ph111_B1a
zmp_ph111_B5
zmp_ph111_B8
zmp_ph111_B6
zmp_ph111_B7
zmp_ph111_B9
zmp_ph111_B10
zmp_ph114_G4
zmp_ph114_G11
zmp_ph114_H1
zmp_ph114_H2
zmp_ph114_A9
zmp_ph114_A11
zmp_ph114_B2
zmp_ph114_B4
zmp_ph114_D8
zmp_ph114_E2

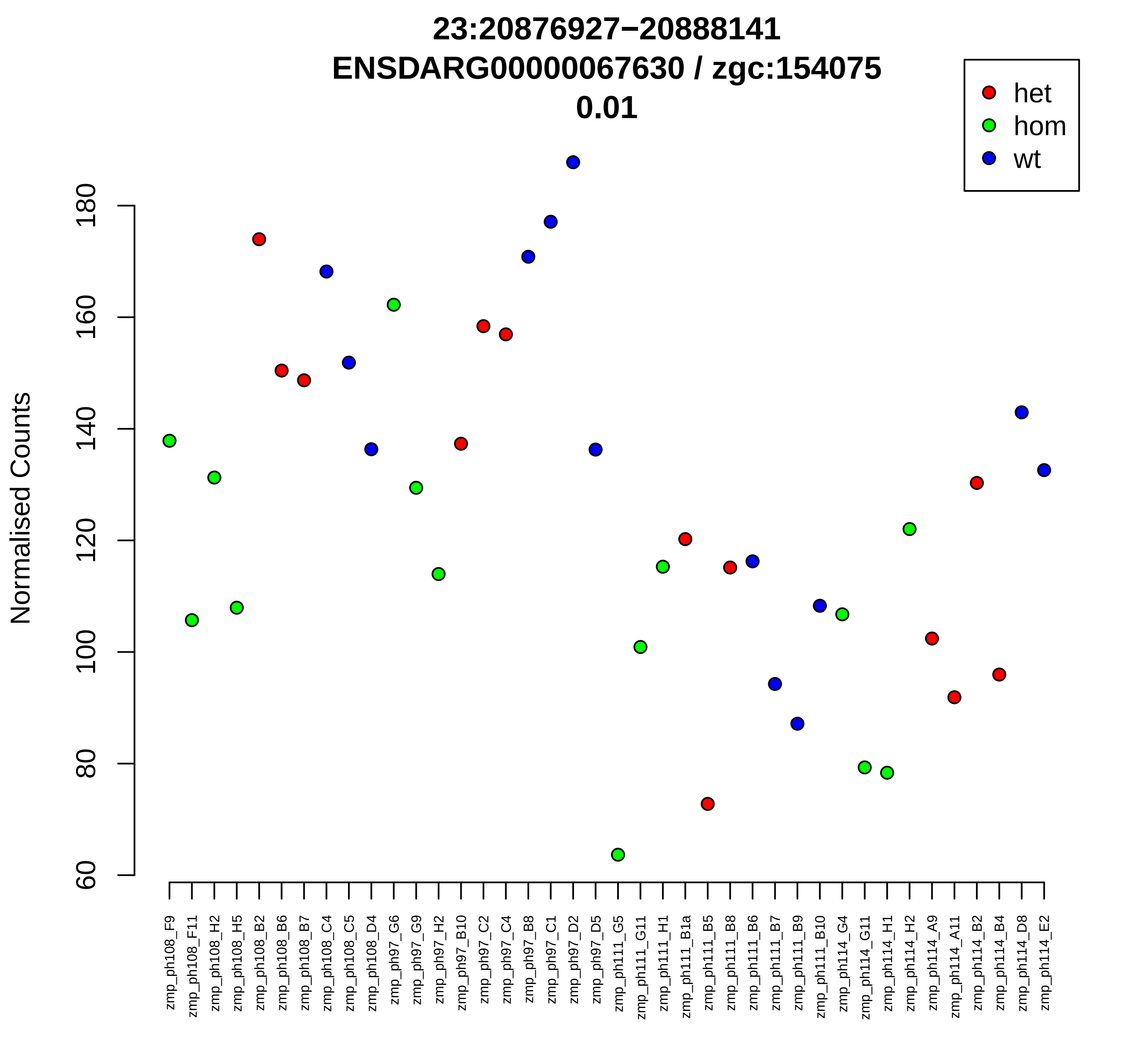
23:20876927−20888141
ENSDARG00000067630 / zgc:154075
0.01
het
hom
wt
Normalised Counts
60
80
100
120
140
160
180
zmp_ph108_F9
zmp_ph108_F11
zmp_ph108_H2
zmp_ph108_H5
zmp_ph108_B2
zmp_ph108_B6
zmp_ph108_B7
zmp_ph108_C4
zmp_ph108_C5
zmp_ph108_D4
zmp_ph97_G6
zmp_ph97_G9
zmp_ph97_H2
zmp_ph97_B10
zmp_ph97_C2
zmp_ph97_C4
zmp_ph97_B8
zmp_ph97_C1
zmp_ph97_D2
zmp_ph97_D5
zmp_ph111_G5
zmp_ph111_G11
zmp_ph111_H1
zmp_ph111_B1a
zmp_ph111_B5
zmp_ph111_B8
zmp_ph111_B6
zmp_ph111_B7
zmp_ph111_B9
zmp_ph111_B10
zmp_ph114_G4
zmp_ph114_G11
zmp_ph114_H1
zmp_ph114_H2
zmp_ph114_A9
zmp_ph114_A11
zmp_ph114_B2
zmp_ph114_B4
zmp_ph114_D8
zmp_ph114_E2

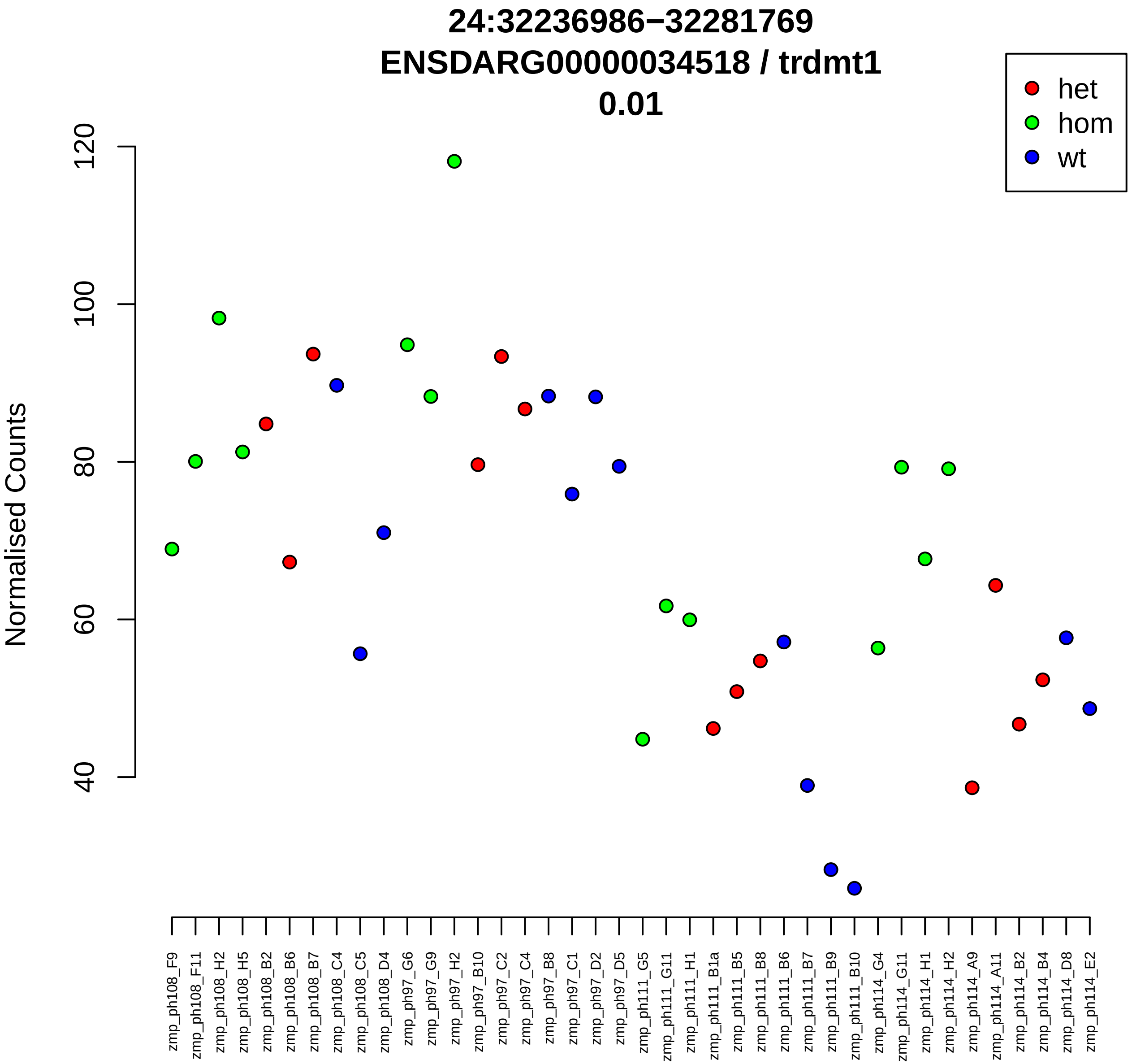

24:32236986−32281769
ENSDARG00000034518 / trdmt1
0.01
het
hom
wt
Normalised Counts
40
60
80
100
120
zmp_ph108_F9
zmp_ph108_F11
zmp_ph108_H2
zmp_ph108_H5
zmp_ph108_B2
zmp_ph108_B6
zmp_ph108_B7
zmp_ph108_C4
zmp_ph108_C5
zmp_ph108_D4
zmp_ph97_G6
zmp_ph97_G9
zmp_ph97_H2
zmp_ph97_B10
zmp_ph97_C2
zmp_ph97_C4
zmp_ph97_B8
zmp_ph97_C1
zmp_ph97_D2
zmp_ph97_D5
zmp_ph111_G5
zmp_ph111_G11
zmp_ph111_H1
zmp_ph111_B1a
zmp_ph111_B5
zmp_ph111_B8
zmp_ph111_B6
zmp_ph111_B7
zmp_ph111_B9
zmp_ph111_B10
zmp_ph114_G4
zmp_ph114_G11
zmp_ph114_H1
zmp_ph114_H2
zmp_ph114_A9
zmp_ph114_A11
zmp_ph114_B2
zmp_ph114_B4
zmp_ph114_D8
zmp_ph114_E2

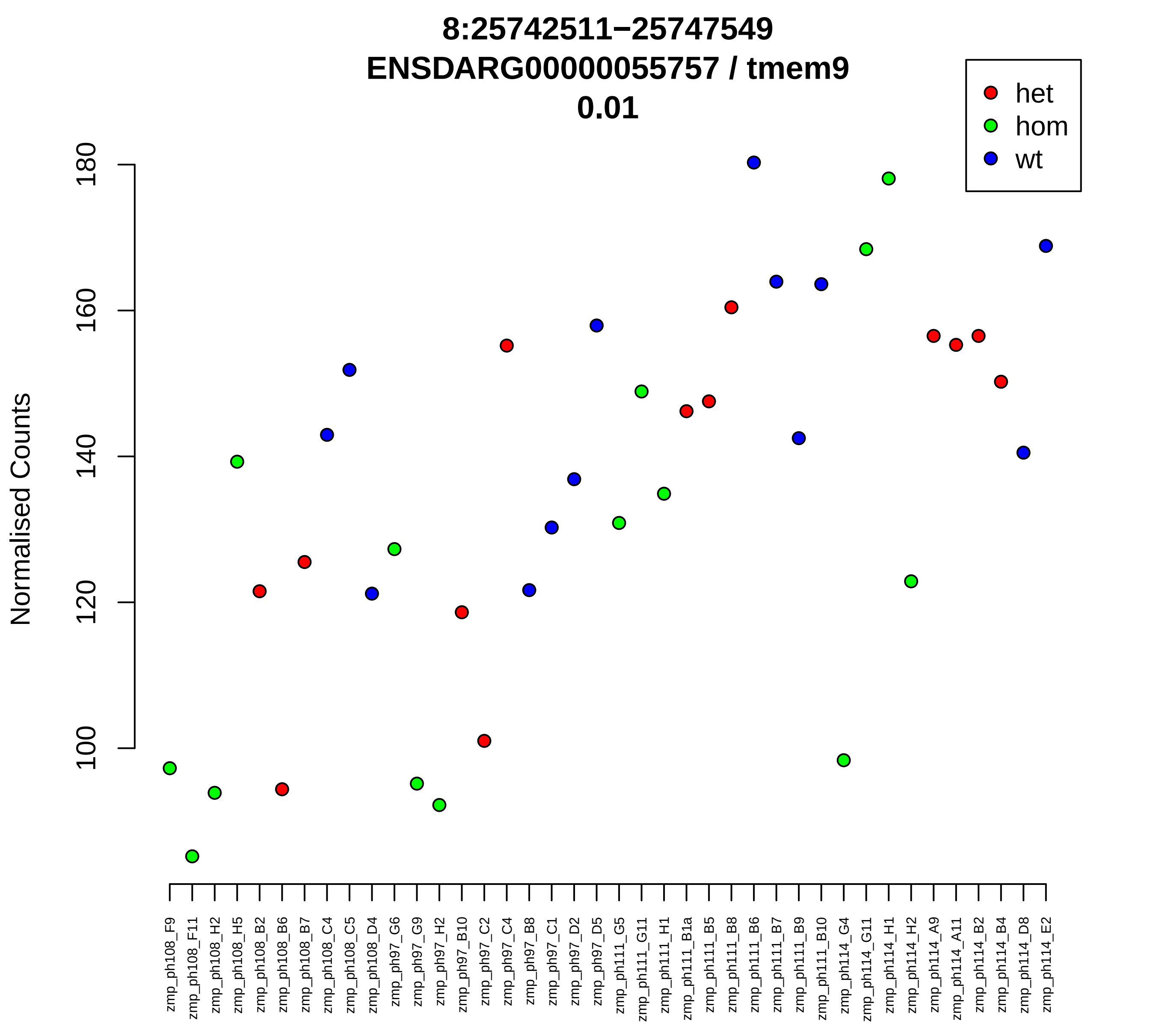

8:25742511−25747549
ENSDARG00000055757 / tmem9
0.01
het
hom
wt
Normalised Counts
100
120
140
160
180
zmp_ph108_F9
zmp_ph108_F11
zmp_ph108_H2
zmp_ph108_H5
zmp_ph108_B2
zmp_ph108_B6
zmp_ph108_B7
zmp_ph108_C4
zmp_ph108_C5
zmp_ph108_D4
zmp_ph97_G6
zmp_ph97_G9
zmp_ph97_H2
zmp_ph97_B10
zmp_ph97_C2
zmp_ph97_C4
zmp_ph97_B8
zmp_ph97_C1
zmp_ph97_D2
zmp_ph97_D5
zmp_ph111_G5
zmp_ph111_G11
zmp_ph111_H1
zmp_ph111_B1a
zmp_ph111_B5
zmp_ph111_B8
zmp_ph111_B6
zmp_ph111_B7
zmp_ph111_B9
zmp_ph111_B10
zmp_ph114_G4
zmp_ph114_G11
zmp_ph114_H1
zmp_ph114_H2
zmp_ph114_A9
zmp_ph114_A11
zmp_ph114_B2
zmp_ph114_B4
zmp_ph114_D8
zmp_ph114_E2

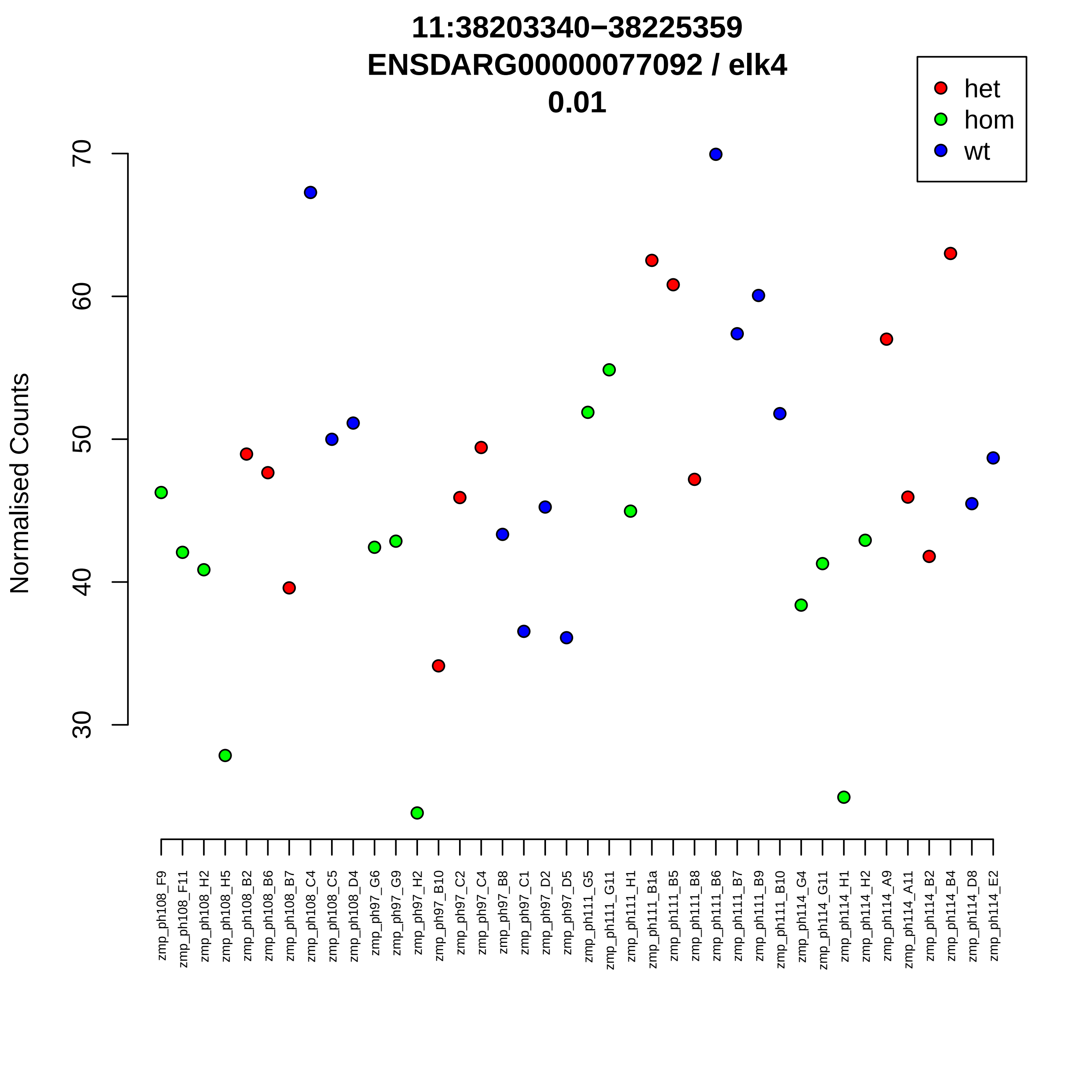
11:38203340−38225359
ENSDARG00000077092 / elk4
0.01
het
hom
wt
Normalised Counts
30
40
50
60
70
zmp_ph108_F9
zmp_ph108_F11
zmp_ph108_H2
zmp_ph108_H5
zmp_ph108_B2
zmp_ph108_B6
zmp_ph108_B7
zmp_ph108_C4
zmp_ph108_C5
zmp_ph108_D4
zmp_ph97_G6
zmp_ph97_G9
zmp_ph97_H2
zmp_ph97_B10
zmp_ph97_C2
zmp_ph97_C4
zmp_ph97_B8
zmp_ph97_C1
zmp_ph97_D2
zmp_ph97_D5
zmp_ph111_G5
zmp_ph111_G11
zmp_ph111_H1
zmp_ph111_B1a
zmp_ph111_B5
zmp_ph111_B8
zmp_ph111_B6
zmp_ph111_B7
zmp_ph111_B9
zmp_ph111_B10
zmp_ph114_G4
zmp_ph114_G11
zmp_ph114_H1
zmp_ph114_H2
zmp_ph114_A9
zmp_ph114_A11
zmp_ph114_B2
zmp_ph114_B4
zmp_ph114_D8
zmp_ph114_E2

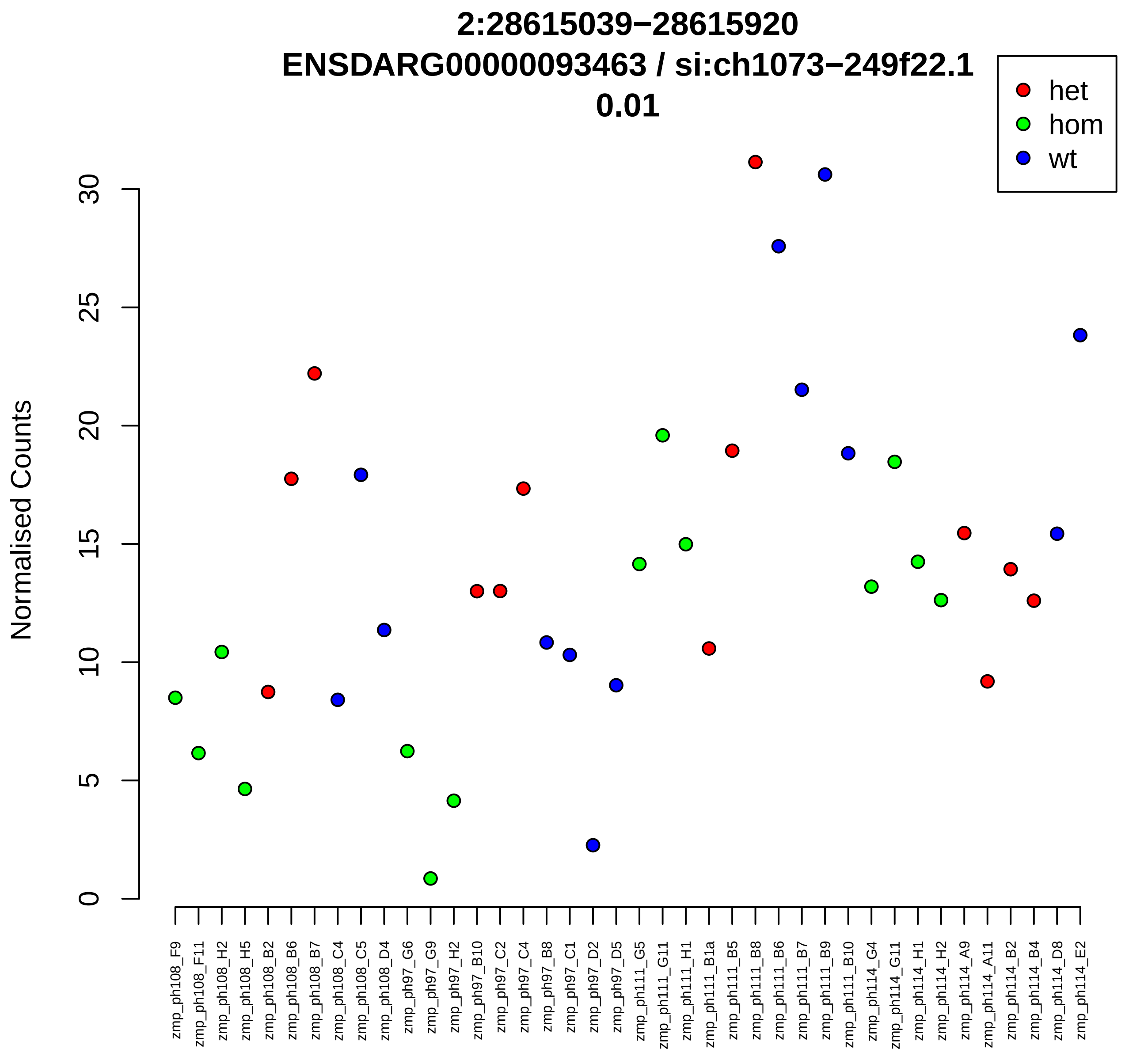

2:28615039−28615920
ENSDARG00000093463 / si:ch1073−249f22.1
0.01
het
hom
wt
Normalised Counts
0
5
10
15
20
25
30
zmp_ph108_F9
zmp_ph108_F11
zmp_ph108_H2
zmp_ph108_H5
zmp_ph108_B2
zmp_ph108_B6
zmp_ph108_B7
zmp_ph108_C4
zmp_ph108_C5
zmp_ph108_D4
zmp_ph97_G6
zmp_ph97_G9
zmp_ph97_H2
zmp_ph97_B10
zmp_ph97_C2
zmp_ph97_C4
zmp_ph97_B8
zmp_ph97_C1
zmp_ph97_D2
zmp_ph97_D5
zmp_ph111_G5
zmp_ph111_G11
zmp_ph111_H1
zmp_ph111_B1a
zmp_ph111_B5
zmp_ph111_B8
zmp_ph111_B6
zmp_ph111_B7
zmp_ph111_B9
zmp_ph111_B10
zmp_ph114_G4
zmp_ph114_G11
zmp_ph114_H1
zmp_ph114_H2
zmp_ph114_A9
zmp_ph114_A11
zmp_ph114_B2
zmp_ph114_B4
zmp_ph114_D8
zmp_ph114_E2

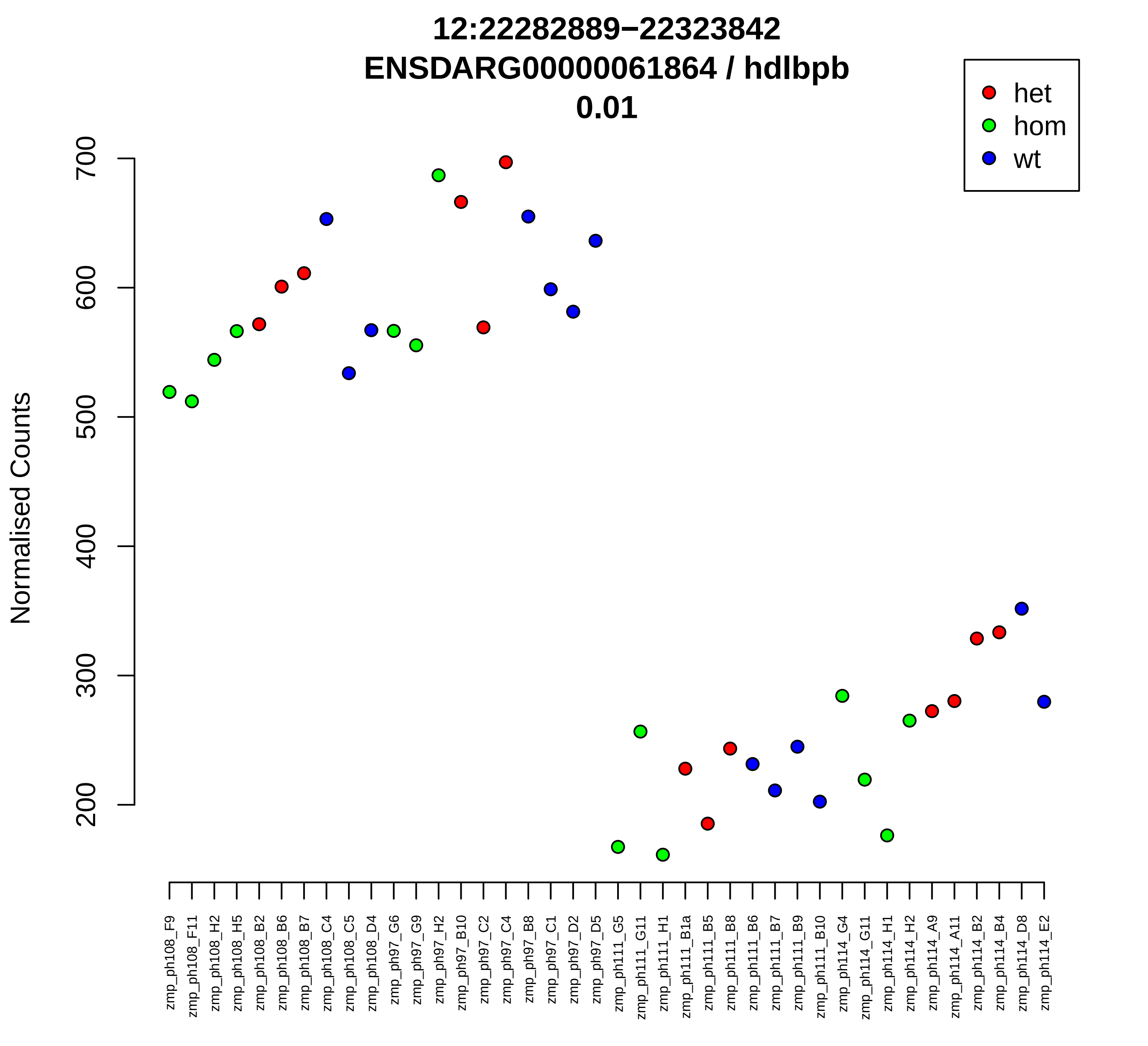

12:22282889−22323842
ENSDARG00000061864 / hdlbpb
0.01
het
hom
wt
Normalised Counts
700
600
500
400
300
200
zmp_ph108_F9
zmp_ph108_F11
zmp_ph108_H2
zmp_ph108_H5
zmp_ph108_B2
zmp_ph108_B6
zmp_ph108_B7
zmp_ph108_C4
zmp_ph108_C5
zmp_ph108_D4
zmp_ph97_G6
zmp_ph97_G9
zmp_ph97_H2
zmp_ph97_B10
zmp_ph97_C2
zmp_ph97_C4
zmp_ph97_B8
zmp_ph97_C1
zmp_ph97_D2
zmp_ph97_D5
zmp_ph111_G5
zmp_ph111_G11
zmp_ph111_H1
zmp_ph111_B1a
zmp_ph111_B5
zmp_ph111_B8
zmp_ph111_B6
zmp_ph111_B7
zmp_ph111_B9
zmp_ph111_B10
zmp_ph114_G4
zmp_ph114_G11
zmp_ph114_H1
zmp_ph114_H2
zmp_ph114_A9
zmp_ph114_A11
zmp_ph114_B2
zmp_ph114_B4
zmp_ph114_D8
zmp_ph114_E2

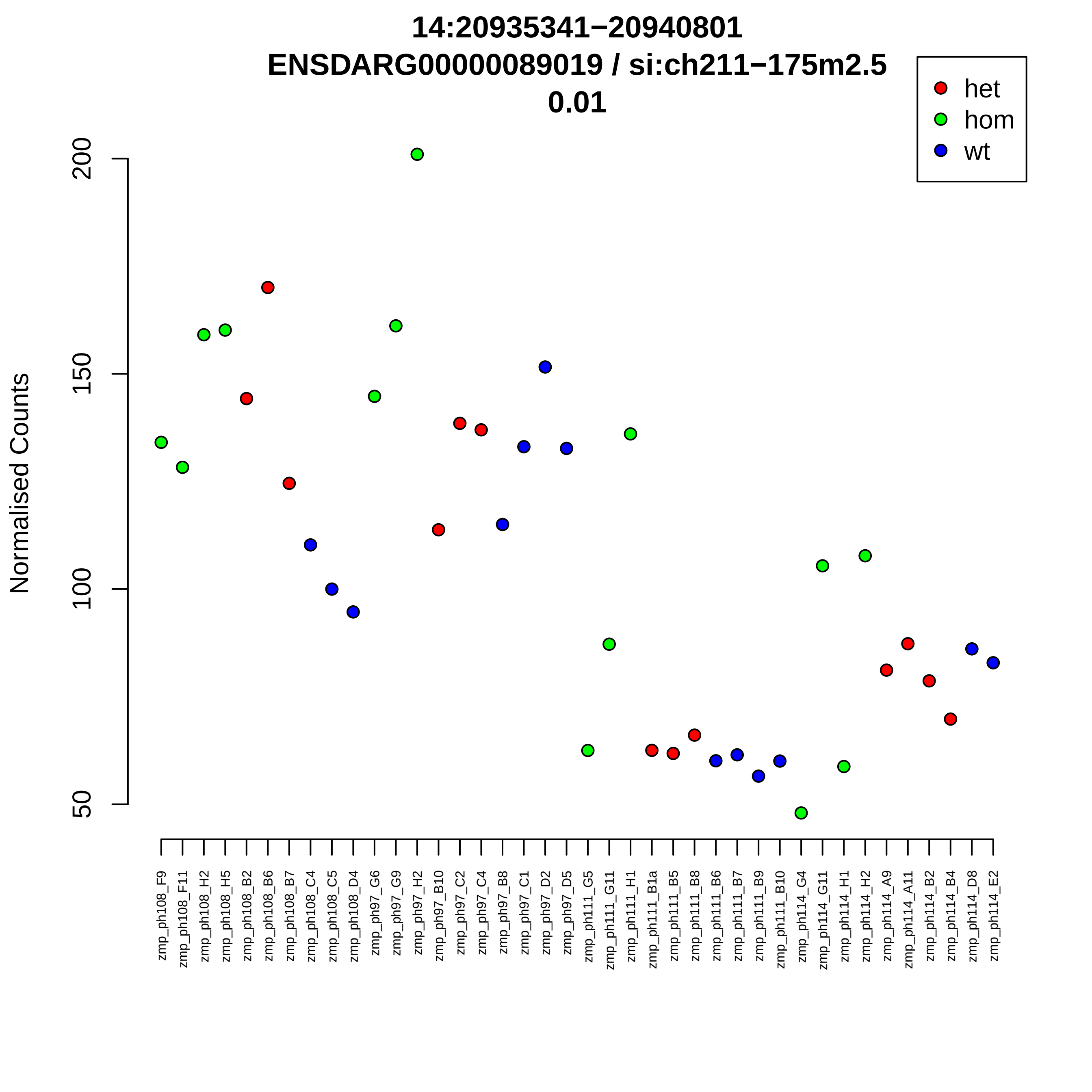

14:20935341−20940801
ENSDARG00000089019 / si:ch211−175m2.5
0.01
het
hom
wt
Normalised Counts
50
100
150
200
zmp_ph108_F9
zmp_ph108_F11
zmp_ph108_H2
zmp_ph108_H5
zmp_ph108_B2
zmp_ph108_B6
zmp_ph108_B7
zmp_ph108_C4
zmp_ph108_C5
zmp_ph108_D4
zmp_ph97_G6
zmp_ph97_G9
zmp_ph97_H2
zmp_ph97_B10
zmp_ph97_C2
zmp_ph97_C4
zmp_ph97_B8
zmp_ph97_C1
zmp_ph97_D2
zmp_ph97_D5
zmp_ph111_G5
zmp_ph111_G11
zmp_ph111_H1
zmp_ph111_B1a
zmp_ph111_B5
zmp_ph111_B8
zmp_ph111_B6
zmp_ph111_B7
zmp_ph111_B9
zmp_ph111_B10
zmp_ph114_G4
zmp_ph114_G11
zmp_ph114_H1
zmp_ph114_H2
zmp_ph114_A9
zmp_ph114_A11
zmp_ph114_B2
zmp_ph114_B4
zmp_ph114_D8
zmp_ph114_E2

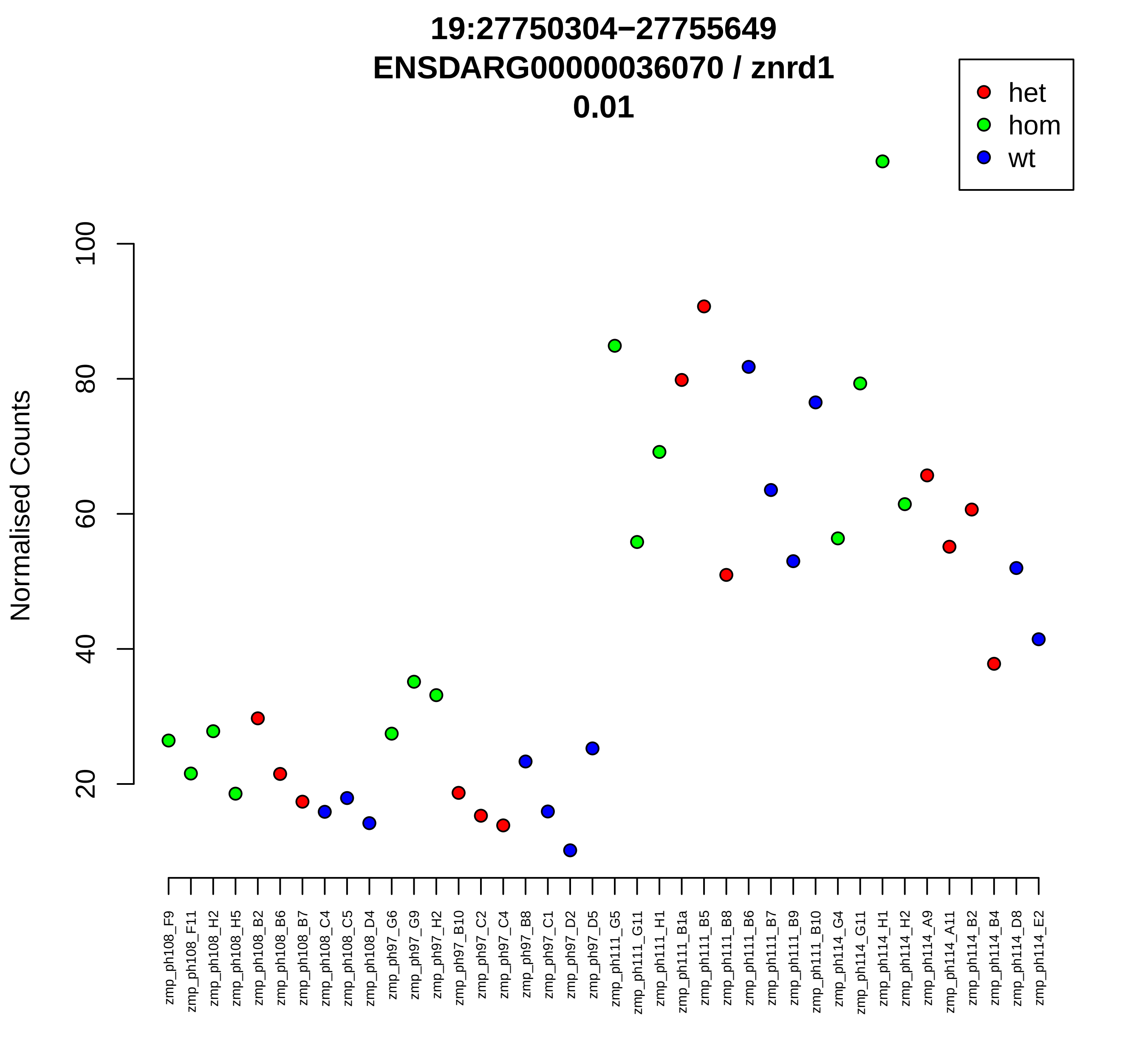

19:27750304−27755649
ENSDARG00000036070 / znrd1
0.01
het
hom
wt
Normalised Counts
20
40
60
80
100
zmp_ph108_F9
zmp_ph108_F11
zmp_ph108_H2
zmp_ph108_H5
zmp_ph108_B2
zmp_ph108_B6
zmp_ph108_B7
zmp_ph108_C4
zmp_ph108_C5
zmp_ph108_D4
zmp_ph97_G6
zmp_ph97_G9
zmp_ph97_H2
zmp_ph97_B10
zmp_ph97_C2
zmp_ph97_C4
zmp_ph97_B8
zmp_ph97_C1
zmp_ph97_D2
zmp_ph97_D5
zmp_ph111_G5
zmp_ph111_G11
zmp_ph111_H1
zmp_ph111_B1a
zmp_ph111_B5
zmp_ph111_B8
zmp_ph111_B6
zmp_ph111_B7
zmp_ph111_B9
zmp_ph111_B10
zmp_ph114_G4
zmp_ph114_G11
zmp_ph114_H1
zmp_ph114_H2
zmp_ph114_A9
zmp_ph114_A11
zmp_ph114_B2
zmp_ph114_B4
zmp_ph114_D8
zmp_ph114_E2

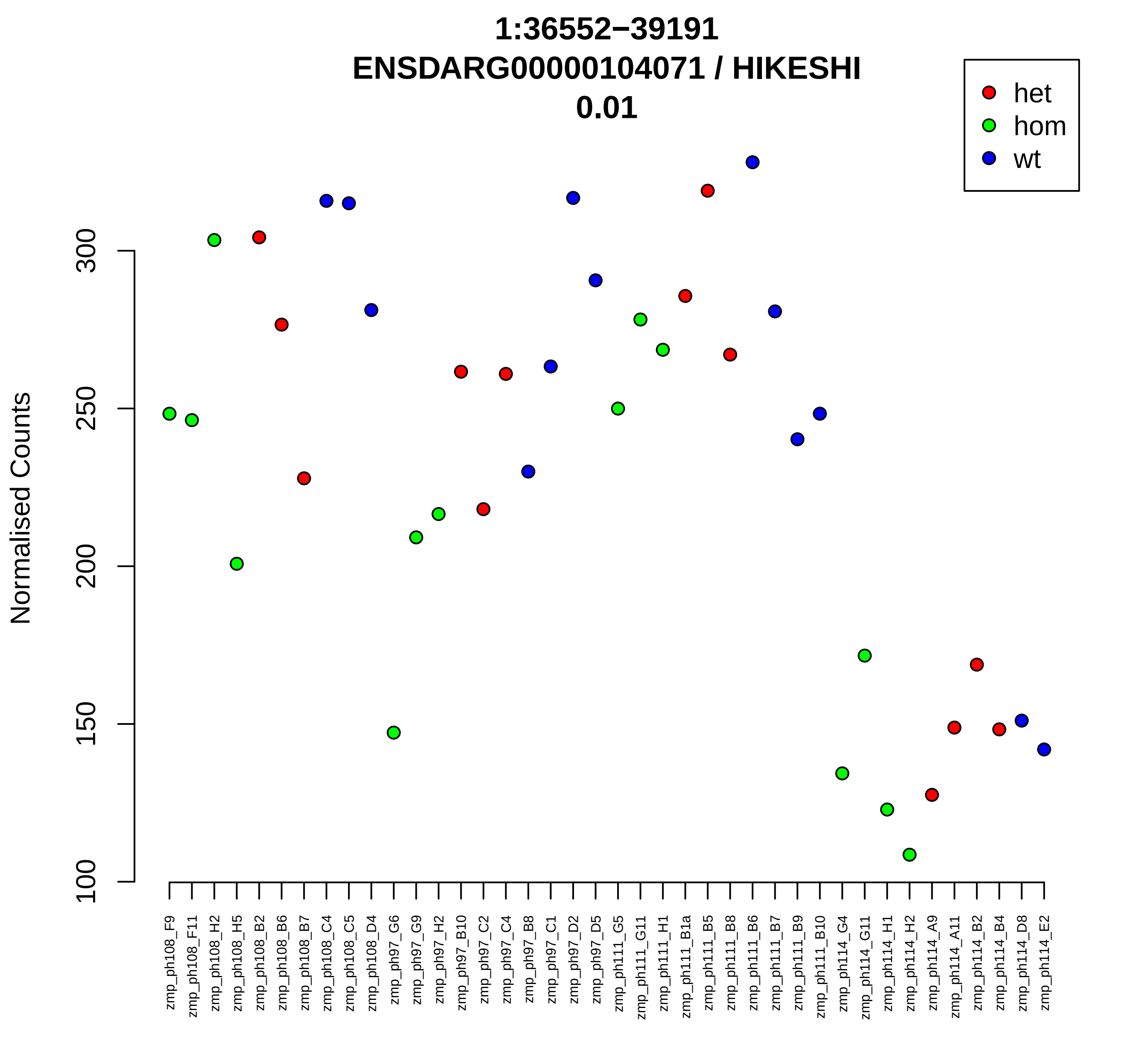
1:36552−39191
ENSDARG00000104071 / HIKESHI
0.01
het
hom
wt
Normalised Counts
100
150
200
250
300
zmp_ph108_F9
zmp_ph108_F11
zmp_ph108_H2
zmp_ph108_H5
zmp_ph108_B2
zmp_ph108_B6
zmp_ph108_B7
zmp_ph108_C4
zmp_ph108_C5
zmp_ph108_D4
zmp_ph97_G6
zmp_ph97_G9
zmp_ph97_H2
zmp_ph97_B10
zmp_ph97_C2
zmp_ph97_C4
zmp_ph97_B8
zmp_ph97_C1
zmp_ph97_D2
zmp_ph97_D5
zmp_ph111_G5
zmp_ph111_G11
zmp_ph111_H1
zmp_ph111_B1a
zmp_ph111_B5
zmp_ph111_B8
zmp_ph111_B6
zmp_ph111_B7
zmp_ph111_B9
zmp_ph111_B10
zmp_ph114_G4
zmp_ph114_G11
zmp_ph114_H1
zmp_ph114_H2
zmp_ph114_A9
zmp_ph114_A11
zmp_ph114_B2
zmp_ph114_B4
zmp_ph114_D8
zmp_ph114_E2

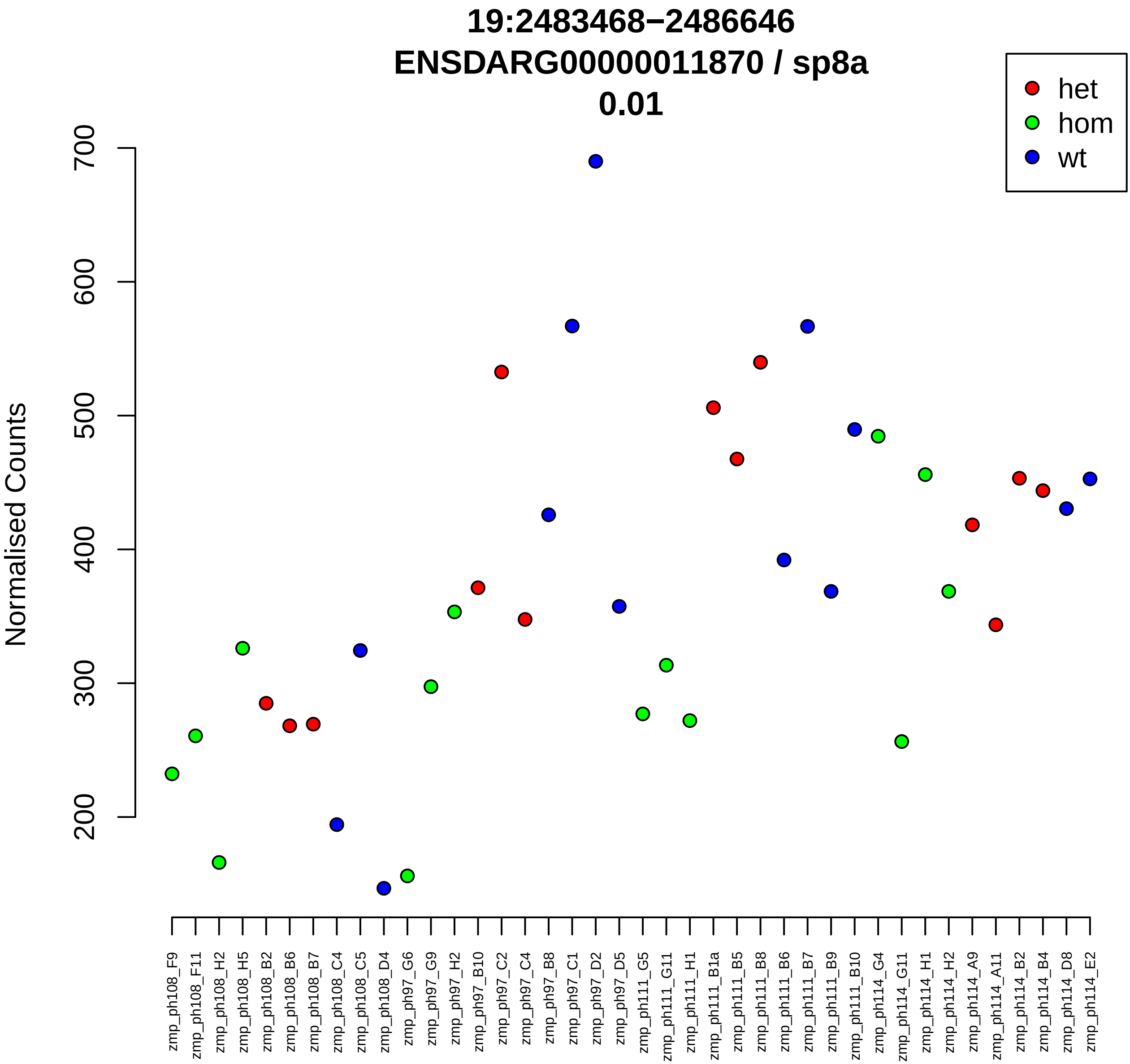

19:2483468−2486646
ENSDARG00000011870 / sp8a
0.01
het
hom
wt
Normalised Counts
200
300
400
500
600
700
zmp_ph108_F9
zmp_ph108_F11
zmp_ph108_H2
zmp_ph108_H5
zmp_ph108_B2
zmp_ph108_B6
zmp_ph108_B7
zmp_ph108_C4
zmp_ph108_C5
zmp_ph108_D4
zmp_ph97_G6
zmp_ph97_G9
zmp_ph97_H2
zmp_ph97_B10
zmp_ph97_C2
zmp_ph97_C4
zmp_ph97_B8
zmp_ph97_C1
zmp_ph97_D2
zmp_ph97_D5
zmp_ph111_G5
zmp_ph111_G11
zmp_ph111_H1
zmp_ph111_B1a
zmp_ph111_B5
zmp_ph111_B8
zmp_ph111_B6
zmp_ph111_B7
zmp_ph111_B9
zmp_ph111_B10
zmp_ph114_G4
zmp_ph114_G11
zmp_ph114_H1
zmp_ph114_H2
zmp_ph114_A9
zmp_ph114_A11
zmp_ph114_B2
zmp_ph114_B4
zmp_ph114_D8
zmp_ph114_E2

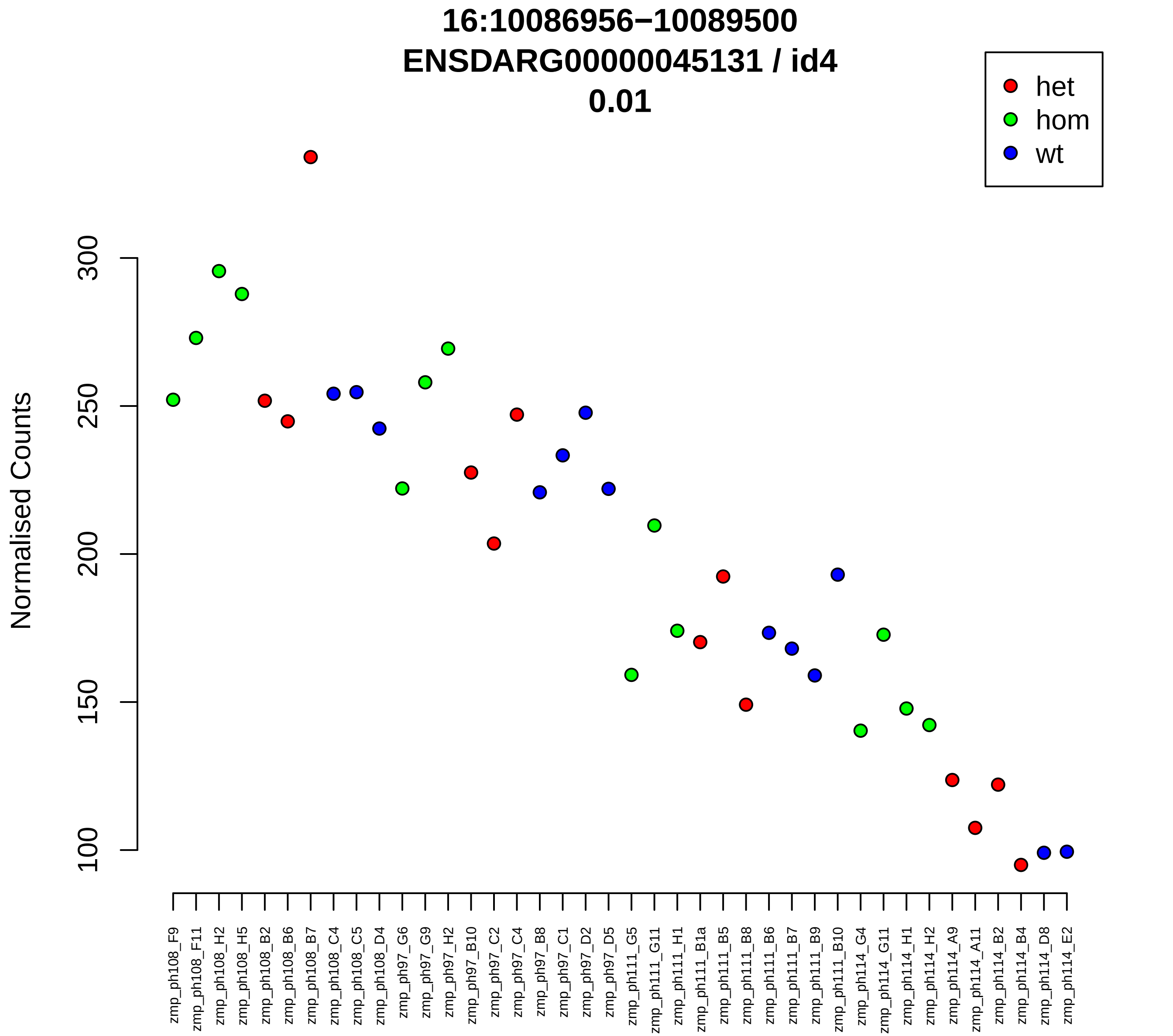

16:10086956−10089500
ENSDARG00000045131 / id4
0.01
het
hom
wt
Normalised Counts
100
150
200
250
300
zmp_ph108_F9
zmp_ph108_F11
zmp_ph108_H2
zmp_ph108_H5
zmp_ph108_B2
zmp_ph108_B6
zmp_ph108_B7
zmp_ph108_C4
zmp_ph108_C5
zmp_ph108_D4
zmp_ph97_G6
zmp_ph97_G9
zmp_ph97_H2
zmp_ph97_B10
zmp_ph97_C2
zmp_ph97_C4
zmp_ph97_B8
zmp_ph97_C1
zmp_ph97_D2
zmp_ph97_D5
zmp_ph111_G5
zmp_ph111_G11
zmp_ph111_H1
zmp_ph111_B1a
zmp_ph111_B5
zmp_ph111_B8
zmp_ph111_B6
zmp_ph111_B7
zmp_ph111_B9
zmp_ph111_B10
zmp_ph114_G4
zmp_ph114_G11
zmp_ph114_H1
zmp_ph114_H2
zmp_ph114_A9
zmp_ph114_A11
zmp_ph114_B2
zmp_ph114_B4
zmp_ph114_D8
zmp_ph114_E2

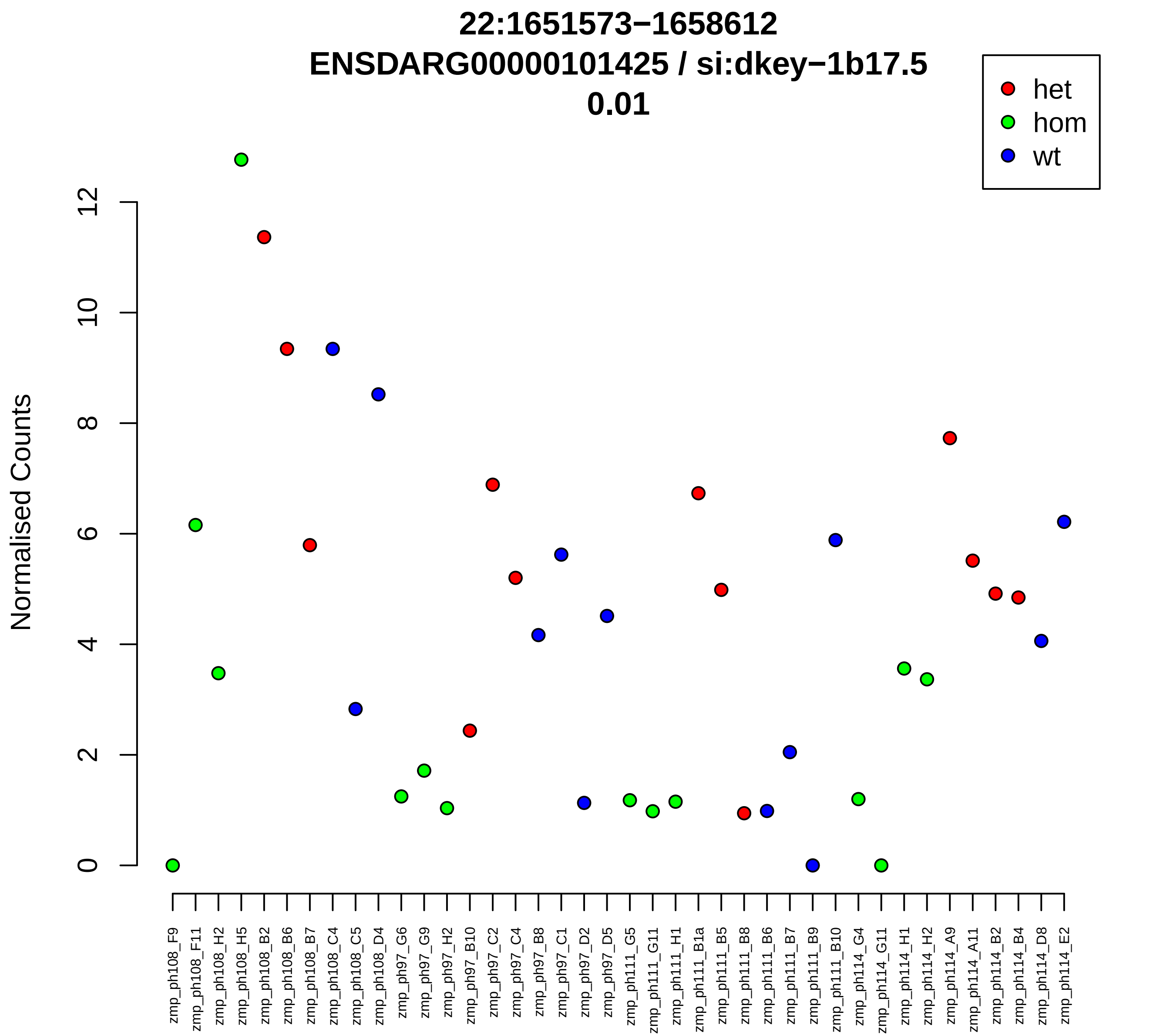
22:1651573−1658612
ENSDARG00000101425 / si:dkey−1b17.5
0.01
het
hom
wt
Normalised Counts
0
2
4
6
8
10
12
zmp_ph108_F9
zmp_ph108_F11
zmp_ph108_H2
zmp_ph108_H5
zmp_ph108_B2
zmp_ph108_B6
zmp_ph108_B7
zmp_ph108_C4
zmp_ph108_C5
zmp_ph108_D4
zmp_ph97_G6
zmp_ph97_G9
zmp_ph97_H2
zmp_ph97_B10
zmp_ph97_C2
zmp_ph97_C4
zmp_ph97_B8
zmp_ph97_C1
zmp_ph97_D2
zmp_ph97_D5
zmp_ph111_G5
zmp_ph111_G11
zmp_ph111_H1
zmp_ph111_B1a
zmp_ph111_B5
zmp_ph111_B8
zmp_ph111_B6
zmp_ph111_B7
zmp_ph111_B9
zmp_ph111_B10
zmp_ph114_G4
zmp_ph114_G11
zmp_ph114_H1
zmp_ph114_H2
zmp_ph114_A9
zmp_ph114_A11
zmp_ph114_B2
zmp_ph114_B4
zmp_ph114_D8
zmp_ph114_E2

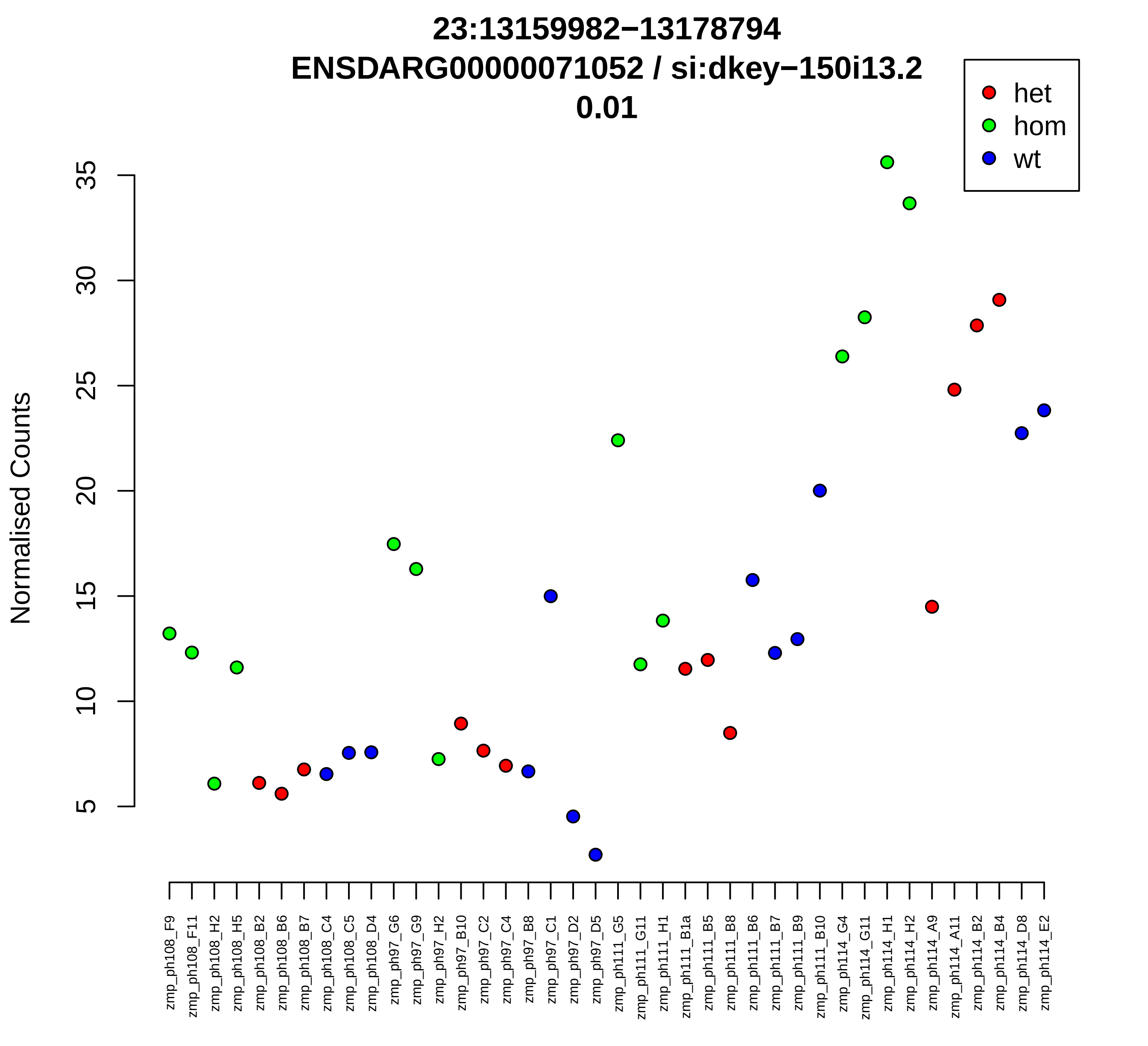

23:13159982−13178794
ENSDARG00000071052 / si:dkey−150i13.2
0.01
het
hom
wt
Normalised Counts
5
10
15
20
25
30
35
zmp_ph108_F9
zmp_ph108_F11
zmp_ph108_H2
zmp_ph108_H5
zmp_ph108_B2
zmp_ph108_B6
zmp_ph108_B7
zmp_ph108_C4
zmp_ph108_C5
zmp_ph108_D4
zmp_ph97_G6
zmp_ph97_G9
zmp_ph97_H2
zmp_ph97_B10
zmp_ph97_C2
zmp_ph97_C4
zmp_ph97_B8
zmp_ph97_C1
zmp_ph97_D2
zmp_ph97_D5
zmp_ph111_G5
zmp_ph111_G11
zmp_ph111_H1
zmp_ph111_B1a
zmp_ph111_B5
zmp_ph111_B8
zmp_ph111_B6
zmp_ph111_B7
zmp_ph111_B9
zmp_ph111_B10
zmp_ph114_G4
zmp_ph114_G11
zmp_ph114_H1
zmp_ph114_H2
zmp_ph114_A9
zmp_ph114_A11
zmp_ph114_B2
zmp_ph114_B4
zmp_ph114_D8
zmp_ph114_E2

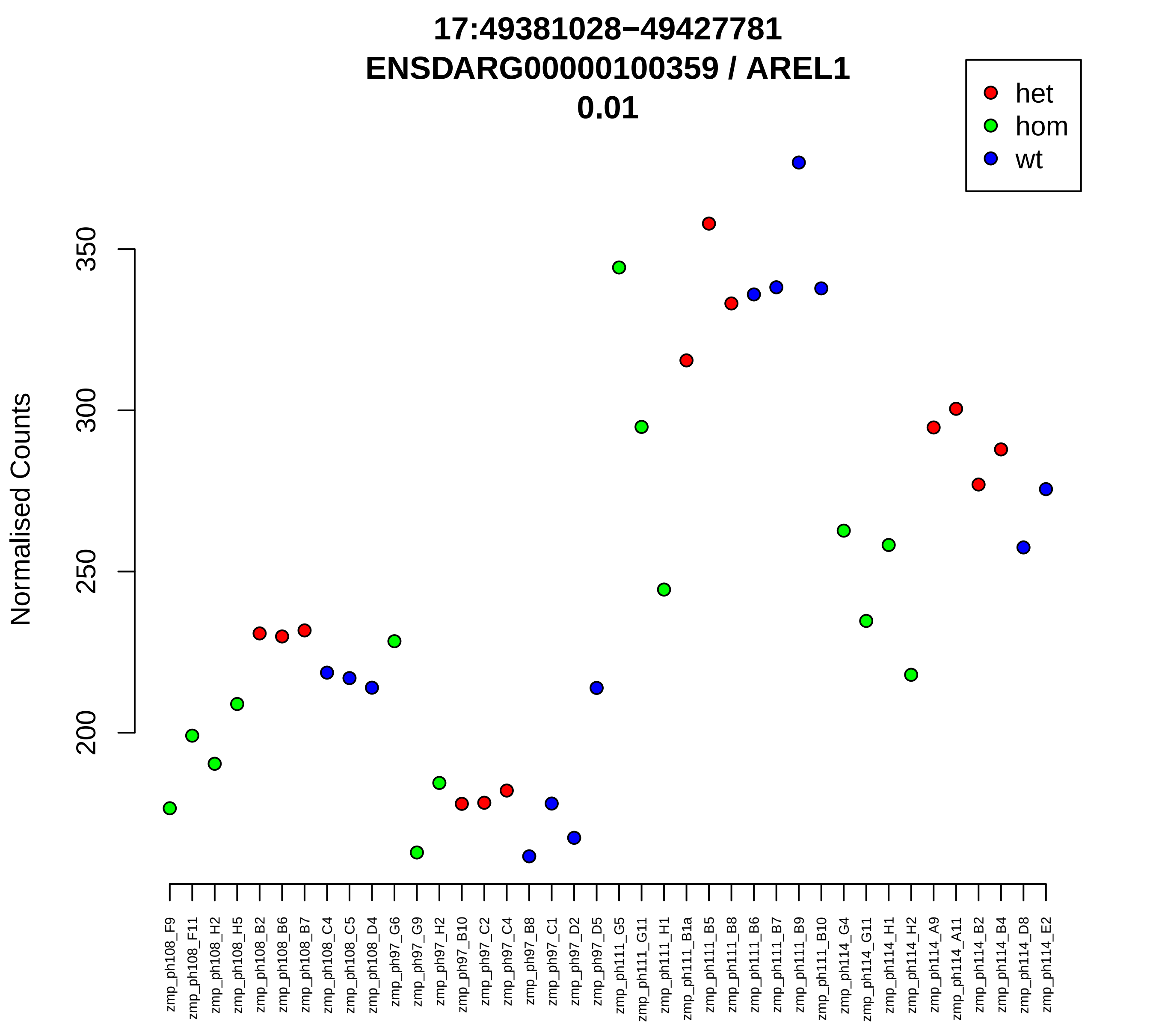
17:49381028−49427781
ENSDARG00000100359 / AREL1
0.01
het
hom
wt
Normalised Counts
200
250
300
350
zmp_ph108_F9
zmp_ph108_F11
zmp_ph108_H2
zmp_ph108_H5
zmp_ph108_B2
zmp_ph108_B6
zmp_ph108_B7
zmp_ph108_C4
zmp_ph108_C5
zmp_ph108_D4
zmp_ph97_G6
zmp_ph97_G9
zmp_ph97_H2
zmp_ph97_B10
zmp_ph97_C2
zmp_ph97_C4
zmp_ph97_B8
zmp_ph97_C1
zmp_ph97_D2
zmp_ph97_D5
zmp_ph111_G5
zmp_ph111_G11
zmp_ph111_H1
zmp_ph111_B1a
zmp_ph111_B5
zmp_ph111_B8
zmp_ph111_B6
zmp_ph111_B7
zmp_ph111_B9
zmp_ph111_B10
zmp_ph114_G4
zmp_ph114_G11
zmp_ph114_H1
zmp_ph114_H2
zmp_ph114_A9
zmp_ph114_A11
zmp_ph114_B2
zmp_ph114_B4
zmp_ph114_D8
zmp_ph114_E2

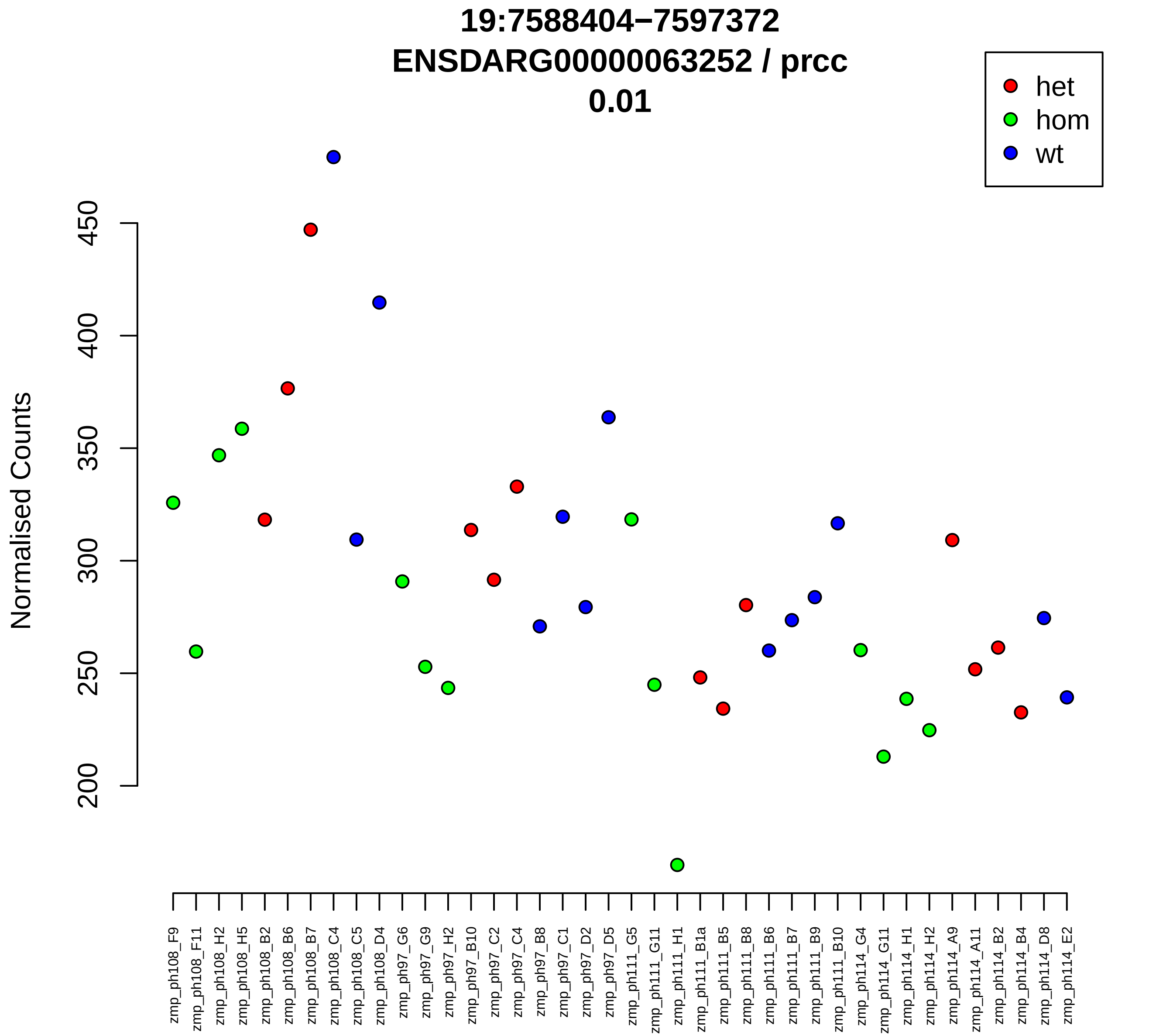

19:7588404−7597372
ENSDARG00000063252 / prcc
0.01
het
hom
wt
Normalised Counts
200
250
300
350
400
450
zmp_ph108_F9
zmp_ph108_F11
zmp_ph108_H2
zmp_ph108_H5
zmp_ph108_B2
zmp_ph108_B6
zmp_ph108_B7
zmp_ph108_C4
zmp_ph108_C5
zmp_ph108_D4
zmp_ph97_G6
zmp_ph97_G9
zmp_ph97_H2
zmp_ph97_B10
zmp_ph97_C2
zmp_ph97_C4
zmp_ph97_B8
zmp_ph97_C1
zmp_ph97_D2
zmp_ph97_D5
zmp_ph111_G5
zmp_ph111_G11
zmp_ph111_H1
zmp_ph111_B1a
zmp_ph111_B5
zmp_ph111_B8
zmp_ph111_B6
zmp_ph111_B7
zmp_ph111_B9
zmp_ph111_B10
zmp_ph114_G4
zmp_ph114_G11
zmp_ph114_H1
zmp_ph114_H2
zmp_ph114_A9
zmp_ph114_A11
zmp_ph114_B2
zmp_ph114_B4
zmp_ph114_D8
zmp_ph114_E2

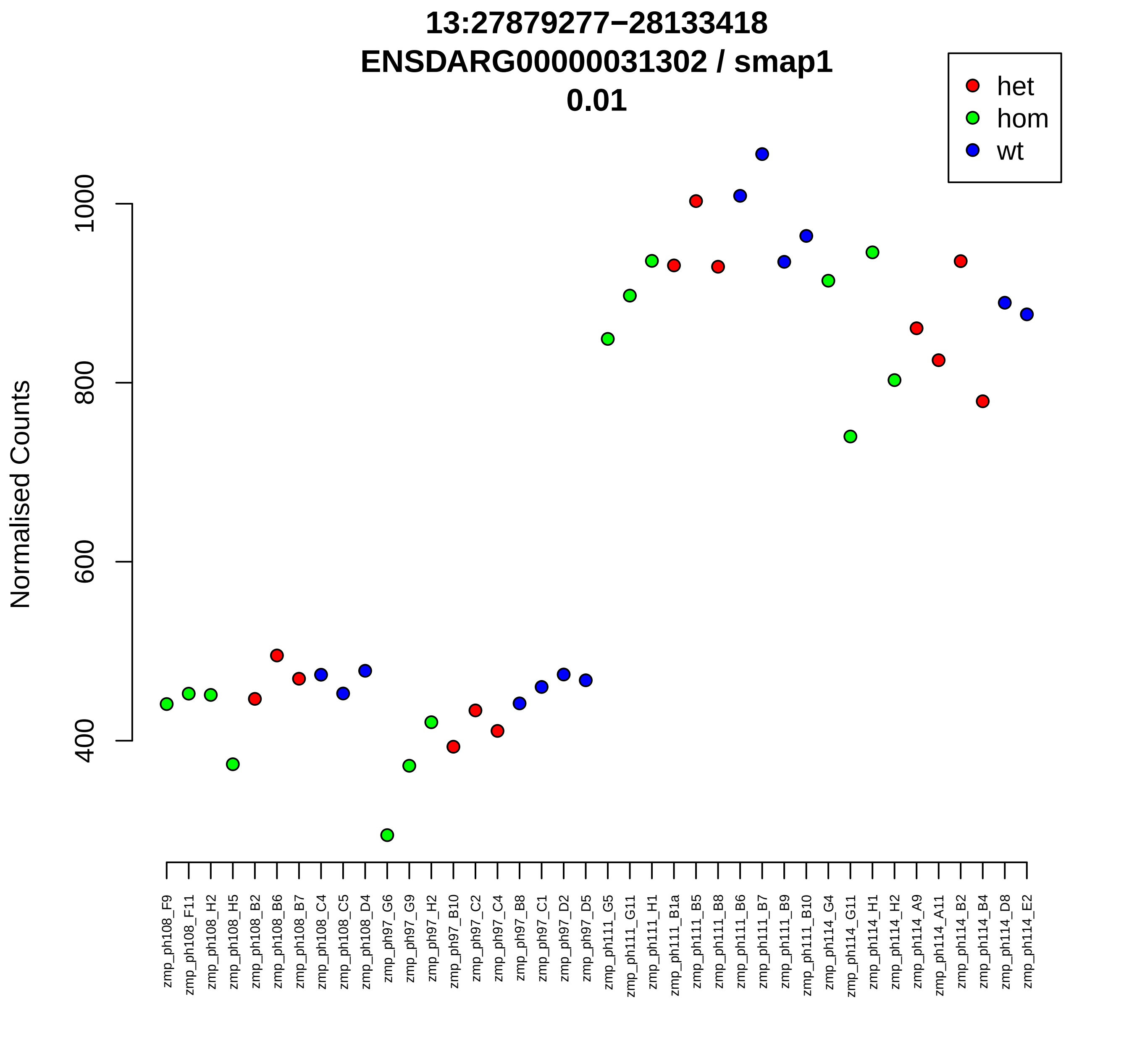
13:27879277−28133418
ENSDARG00000031302 / smap1
0.01
het
hom
wt
Normalised Counts
400
600
800
1000
zmp_ph108_F9
zmp_ph108_F11
zmp_ph108_H2
zmp_ph108_H5
zmp_ph108_B2
zmp_ph108_B6
zmp_ph108_B7
zmp_ph108_C4
zmp_ph108_C5
zmp_ph108_D4
zmp_ph97_G6
zmp_ph97_G9
zmp_ph97_H2
zmp_ph97_B10
zmp_ph97_C2
zmp_ph97_C4
zmp_ph97_B8
zmp_ph97_C1
zmp_ph97_D2
zmp_ph97_D5
zmp_ph111_G5
zmp_ph111_G11
zmp_ph111_H1
zmp_ph111_B1a
zmp_ph111_B5
zmp_ph111_B8
zmp_ph111_B6
zmp_ph111_B7
zmp_ph111_B9
zmp_ph111_B10
zmp_ph114_G4
zmp_ph114_G11
zmp_ph114_H1
zmp_ph114_H2
zmp_ph114_A9
zmp_ph114_A11
zmp_ph114_B2
zmp_ph114_B4
zmp_ph114_D8
zmp_ph114_E2

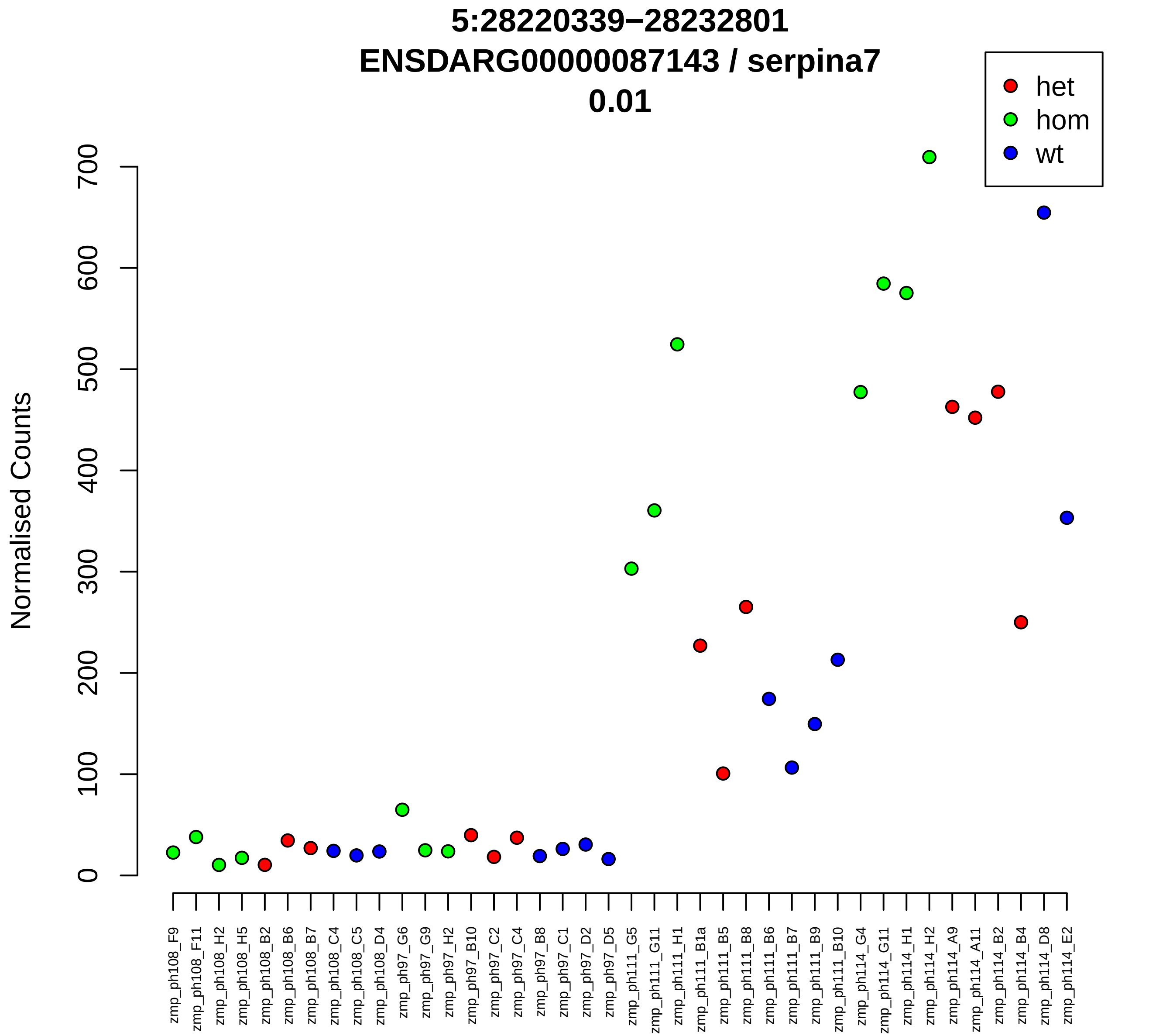

5:28220339−28232801
ENSDARG00000087143 / serpina7
0.01
het
hom
wt
Normalised Counts
0
100
200
300
400
500
600
700
zmp_ph108_F9
zmp_ph108_F11
zmp_ph108_H2
zmp_ph108_H5
zmp_ph108_B2
zmp_ph108_B6
zmp_ph108_B7
zmp_ph108_C4
zmp_ph108_C5
zmp_ph108_D4
zmp_ph97_G6
zmp_ph97_G9
zmp_ph97_H2
zmp_ph97_B10
zmp_ph97_C2
zmp_ph97_C4
zmp_ph97_B8
zmp_ph97_C1
zmp_ph97_D2
zmp_ph97_D5
zmp_ph111_G5
zmp_ph111_G11
zmp_ph111_H1
zmp_ph111_B1a
zmp_ph111_B5
zmp_ph111_B8
zmp_ph111_B6
zmp_ph111_B7
zmp_ph111_B9
zmp_ph111_B10
zmp_ph114_G4
zmp_ph114_G11
zmp_ph114_H1
zmp_ph114_H2
zmp_ph114_A9
zmp_ph114_A11
zmp_ph114_B2
zmp_ph114_B4
zmp_ph114_D8
zmp_ph114_E2

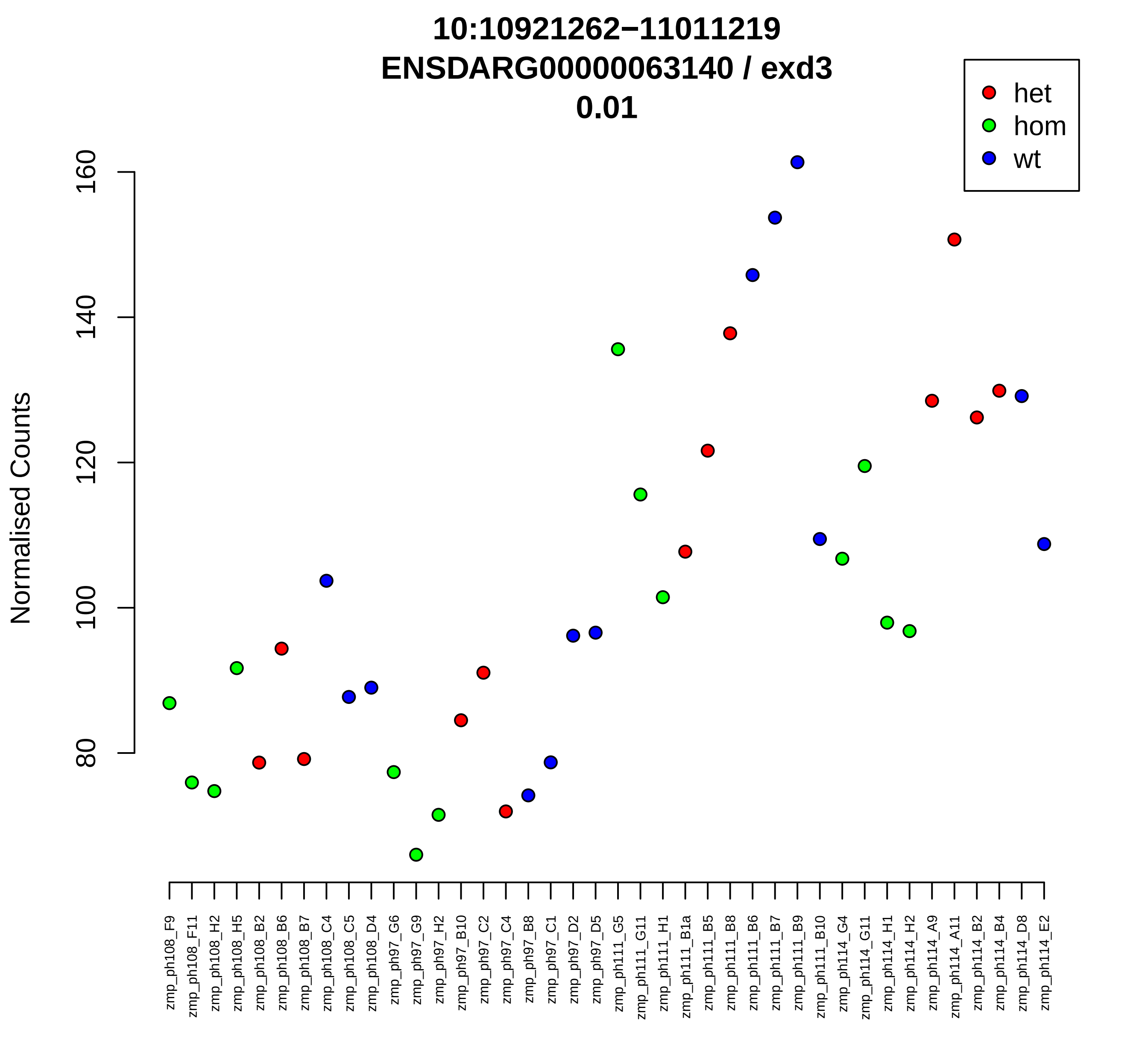
10:10921262−11011219
ENSDARG00000063140 / exd3
0.01
het
hom
wt
Normalised Counts
80
100
120
140
160
zmp_ph108_F9
zmp_ph108_F11
zmp_ph108_H2
zmp_ph108_H5
zmp_ph108_B2
zmp_ph108_B6
zmp_ph108_B7
zmp_ph108_C4
zmp_ph108_C5
zmp_ph108_D4
zmp_ph97_G6
zmp_ph97_G9
zmp_ph97_H2
zmp_ph97_B10
zmp_ph97_C2
zmp_ph97_C4
zmp_ph97_B8
zmp_ph97_C1
zmp_ph97_D2
zmp_ph97_D5
zmp_ph111_G5
zmp_ph111_G11
zmp_ph111_H1
zmp_ph111_B1a
zmp_ph111_B5
zmp_ph111_B8
zmp_ph111_B6
zmp_ph111_B7
zmp_ph111_B9
zmp_ph111_B10
zmp_ph114_G4
zmp_ph114_G11
zmp_ph114_H1
zmp_ph114_H2
zmp_ph114_A9
zmp_ph114_A11
zmp_ph114_B2
zmp_ph114_B4
zmp_ph114_D8
zmp_ph114_E2

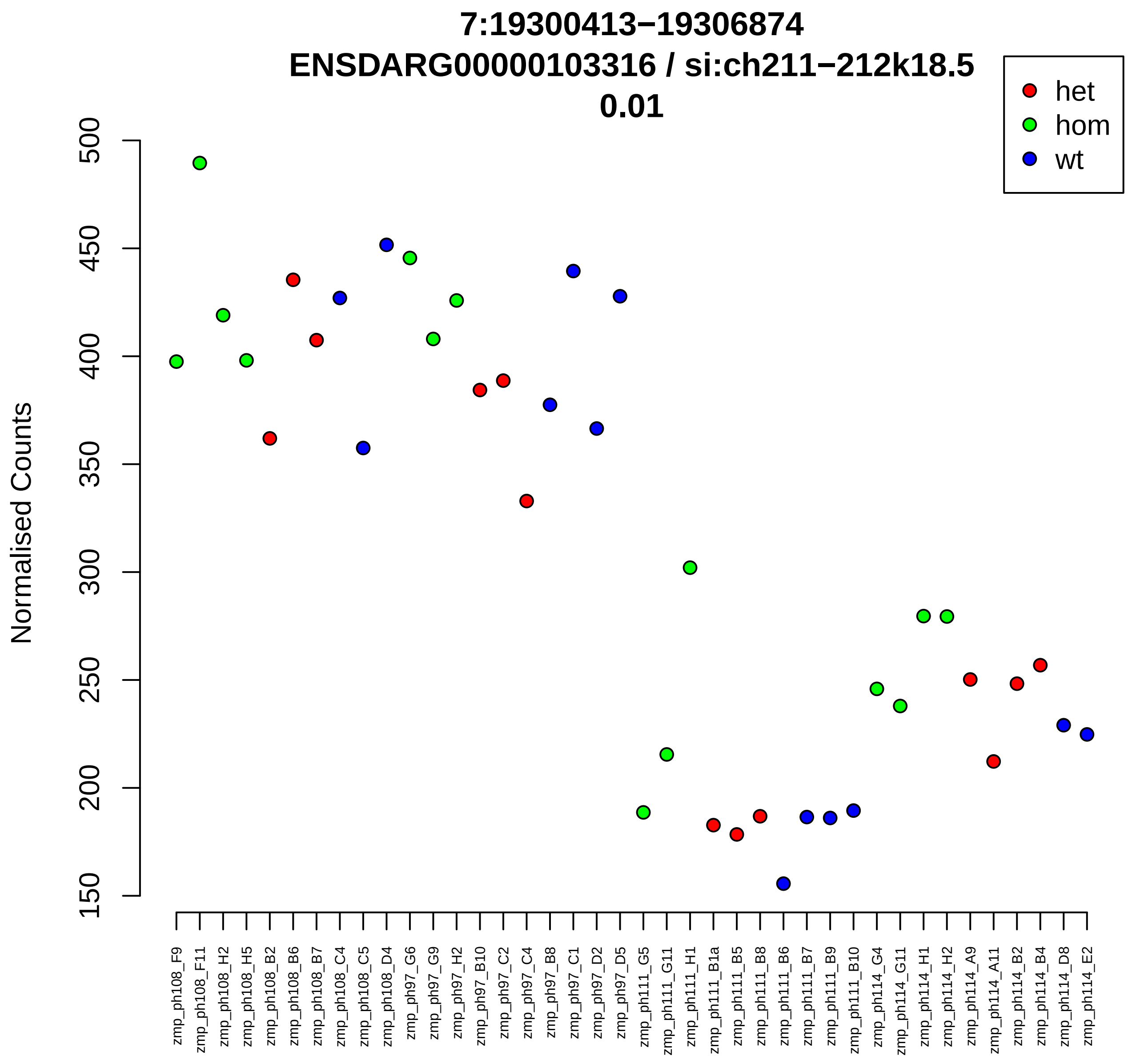

7:19300413−19306874
ENSDARG00000103316 / si:ch211−212k18.5
0.01
Normalised Counts
150
200
250
300
350
400
450
500
het
hom
wt
zmp_ph108_F9
zmp_ph108_F11
zmp_ph108_H2
zmp_ph108_H5
zmp_ph108_B2
zmp_ph108_B6
zmp_ph108_B7
zmp_ph108_C4
zmp_ph108_C5
zmp_ph108_D4
zmp_ph97_G6
zmp_ph97_G9
zmp_ph97_H2
zmp_ph97_B10
zmp_ph97_C2
zmp_ph97_C4
zmp_ph97_B8
zmp_ph97_C1
zmp_ph97_D2
zmp_ph97_D5
zmp_ph111_G5
zmp_ph111_G11
zmp_ph111_H1
zmp_ph111_B1a
zmp_ph111_B5
zmp_ph111_B8
zmp_ph111_B6
zmp_ph111_B7
zmp_ph111_B9
zmp_ph111_B10
zmp_ph114_G4
zmp_ph114_G11
zmp_ph114_H1
zmp_ph114_H2
zmp_ph114_A9
zmp_ph114_A11
zmp_ph114_B2
zmp_ph114_B4
zmp_ph114_D8
zmp_ph114_E2

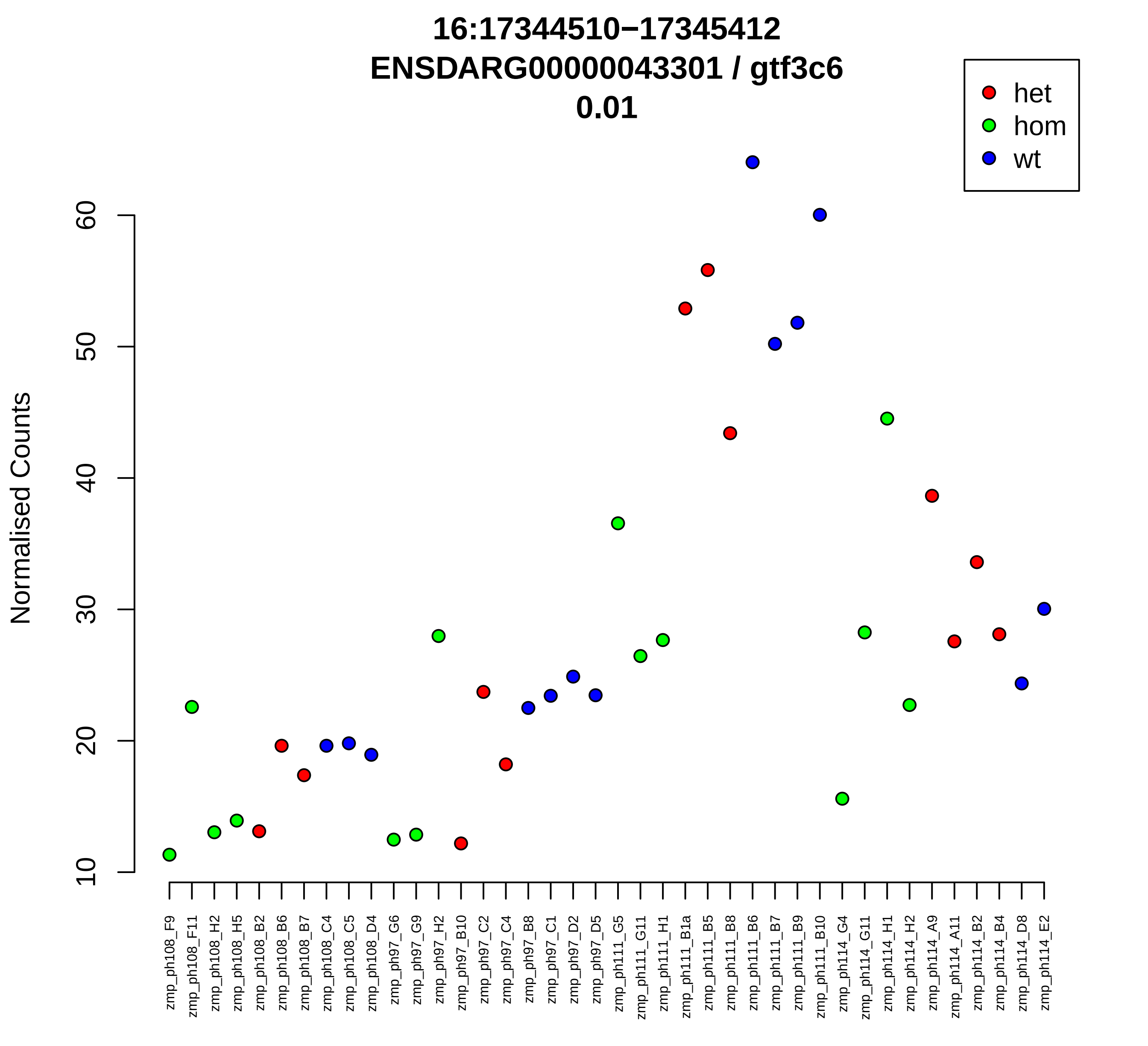

16:17344510−17345412
ENSDARG00000043301 / gtf3c6
0.01
het
hom
wt
Normalised Counts
10
20
30
40
50
60
zmp_ph108_F9
zmp_ph108_F11
zmp_ph108_H2
zmp_ph108_H5
zmp_ph108_B2
zmp_ph108_B6
zmp_ph108_B7
zmp_ph108_C4
zmp_ph108_C5
zmp_ph108_D4
zmp_ph97_G6
zmp_ph97_G9
zmp_ph97_H2
zmp_ph97_B10
zmp_ph97_C2
zmp_ph97_C4
zmp_ph97_B8
zmp_ph97_C1
zmp_ph97_D2
zmp_ph97_D5
zmp_ph111_G5
zmp_ph111_G11
zmp_ph111_H1
zmp_ph111_B1a
zmp_ph111_B5
zmp_ph111_B8
zmp_ph111_B6
zmp_ph111_B7
zmp_ph111_B9
zmp_ph111_B10
zmp_ph114_G4
zmp_ph114_G11
zmp_ph114_H1
zmp_ph114_H2
zmp_ph114_A9
zmp_ph114_A11
zmp_ph114_B2
zmp_ph114_B4
zmp_ph114_D8
zmp_ph114_E2

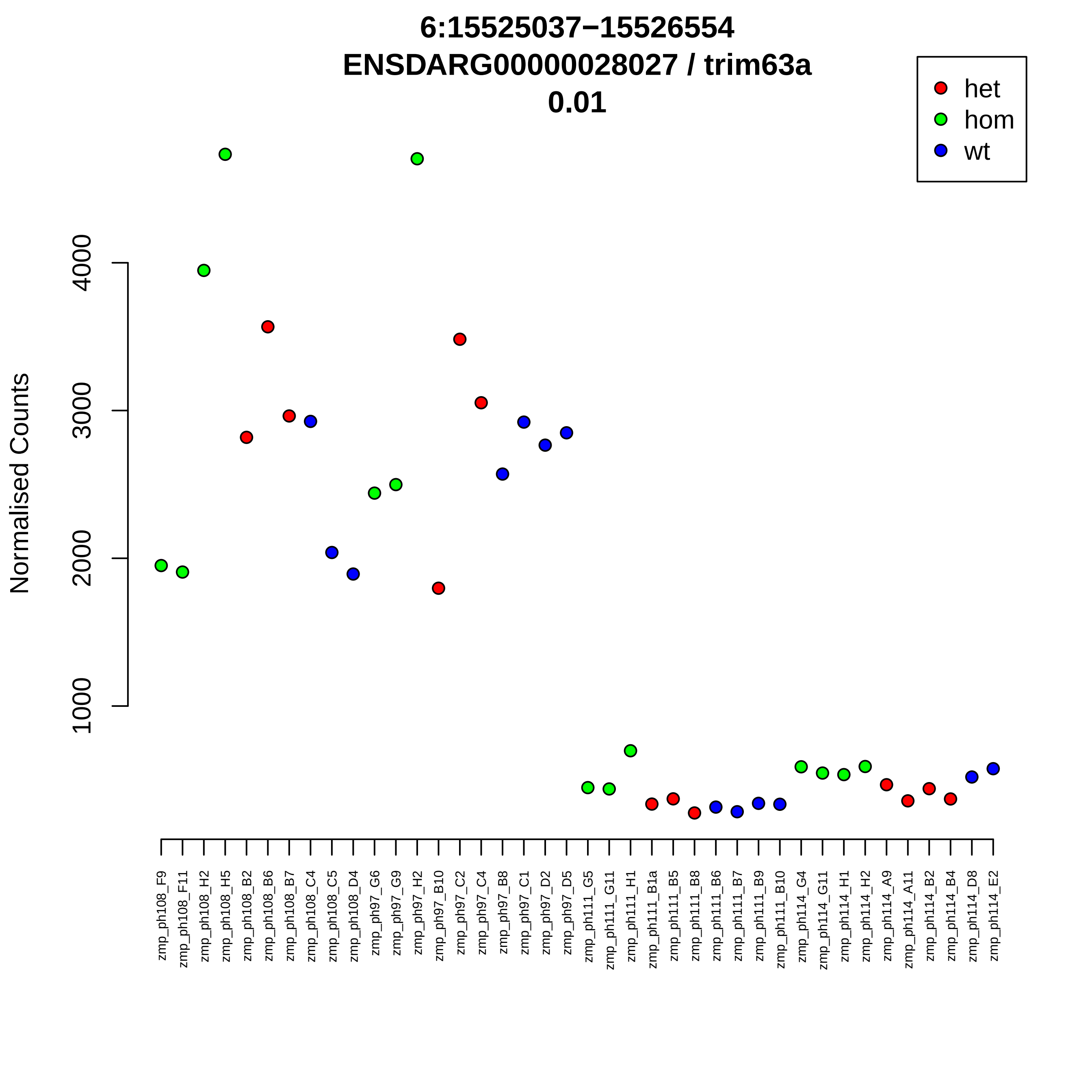

6:15525037−15526554
ENSDARG00000028027 / trim63a
0.01
het
hom
wt
Normalised Counts
1000
2000
3000
4000
zmp_ph108_F9
zmp_ph108_F11
zmp_ph108_H2
zmp_ph108_H5
zmp_ph108_B2
zmp_ph108_B6
zmp_ph108_B7
zmp_ph108_C4
zmp_ph108_C5
zmp_ph108_D4
zmp_ph97_G6
zmp_ph97_G9
zmp_ph97_H2
zmp_ph97_B10
zmp_ph97_C2
zmp_ph97_C4
zmp_ph97_B8
zmp_ph97_C1
zmp_ph97_D2
zmp_ph97_D5
zmp_ph111_G5
zmp_ph111_G11
zmp_ph111_H1
zmp_ph111_B1a
zmp_ph111_B5
zmp_ph111_B8
zmp_ph111_B6
zmp_ph111_B7
zmp_ph111_B9
zmp_ph111_B10
zmp_ph114_G4
zmp_ph114_G11
zmp_ph114_H1
zmp_ph114_H2
zmp_ph114_A9
zmp_ph114_A11
zmp_ph114_B2
zmp_ph114_B4
zmp_ph114_D8
zmp_ph114_E2

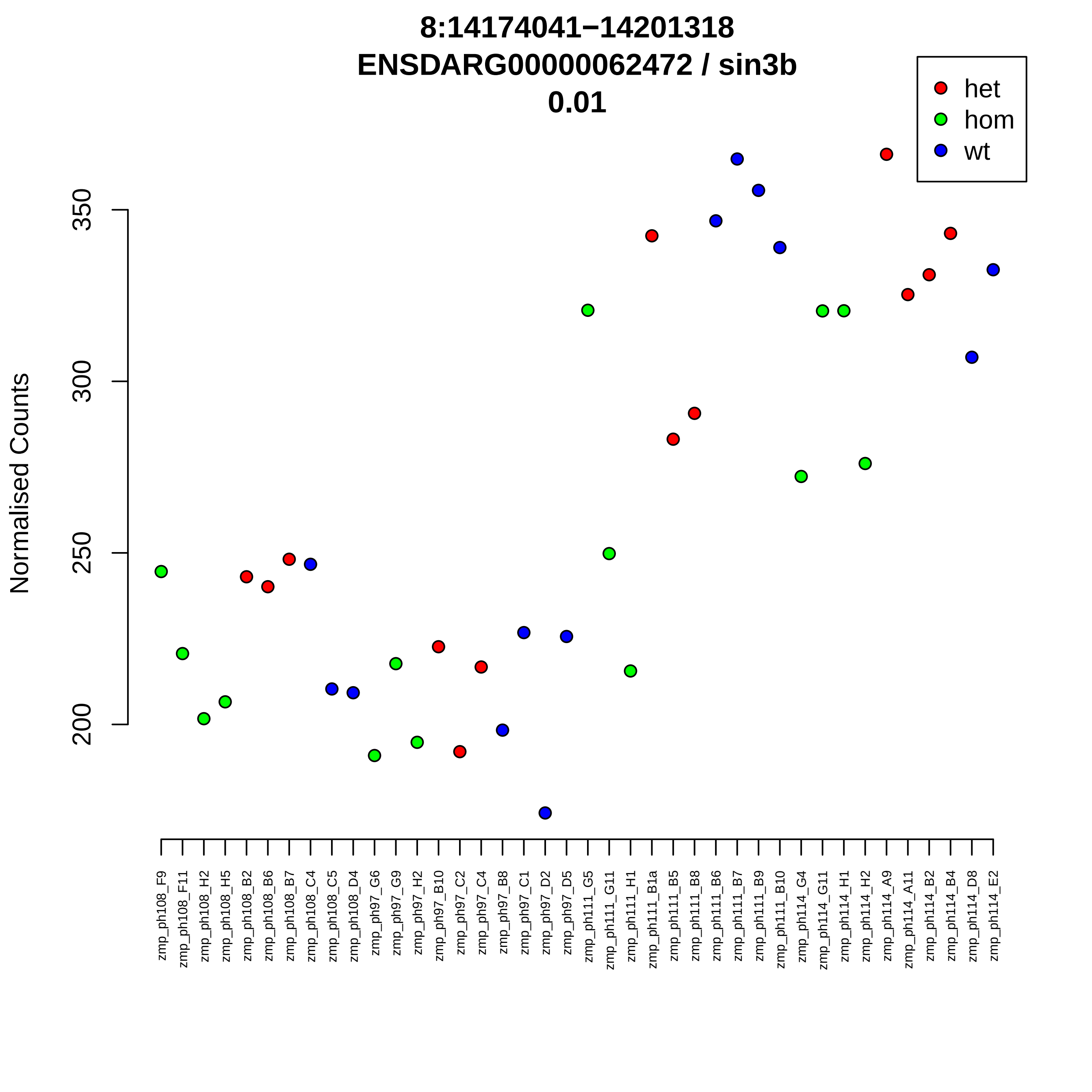
8:14174041−14201318
ENSDARG00000062472 / sin3b
0.01
het
hom
wt
Normalised Counts
200
250
300
350
zmp_ph108_F9
zmp_ph108_F11
zmp_ph108_H2
zmp_ph108_H5
zmp_ph108_B2
zmp_ph108_B6
zmp_ph108_B7
zmp_ph108_C4
zmp_ph108_C5
zmp_ph108_D4
zmp_ph97_G6
zmp_ph97_G9
zmp_ph97_H2
zmp_ph97_B10
zmp_ph97_C2
zmp_ph97_C4
zmp_ph97_B8
zmp_ph97_C1
zmp_ph97_D2
zmp_ph97_D5
zmp_ph111_G5
zmp_ph111_G11
zmp_ph111_H1
zmp_ph111_B1a
zmp_ph111_B5
zmp_ph111_B8
zmp_ph111_B6
zmp_ph111_B7
zmp_ph111_B9
zmp_ph111_B10
zmp_ph114_G4
zmp_ph114_G11
zmp_ph114_H1
zmp_ph114_H2
zmp_ph114_A9
zmp_ph114_A11
zmp_ph114_B2
zmp_ph114_B4
zmp_ph114_D8
zmp_ph114_E2

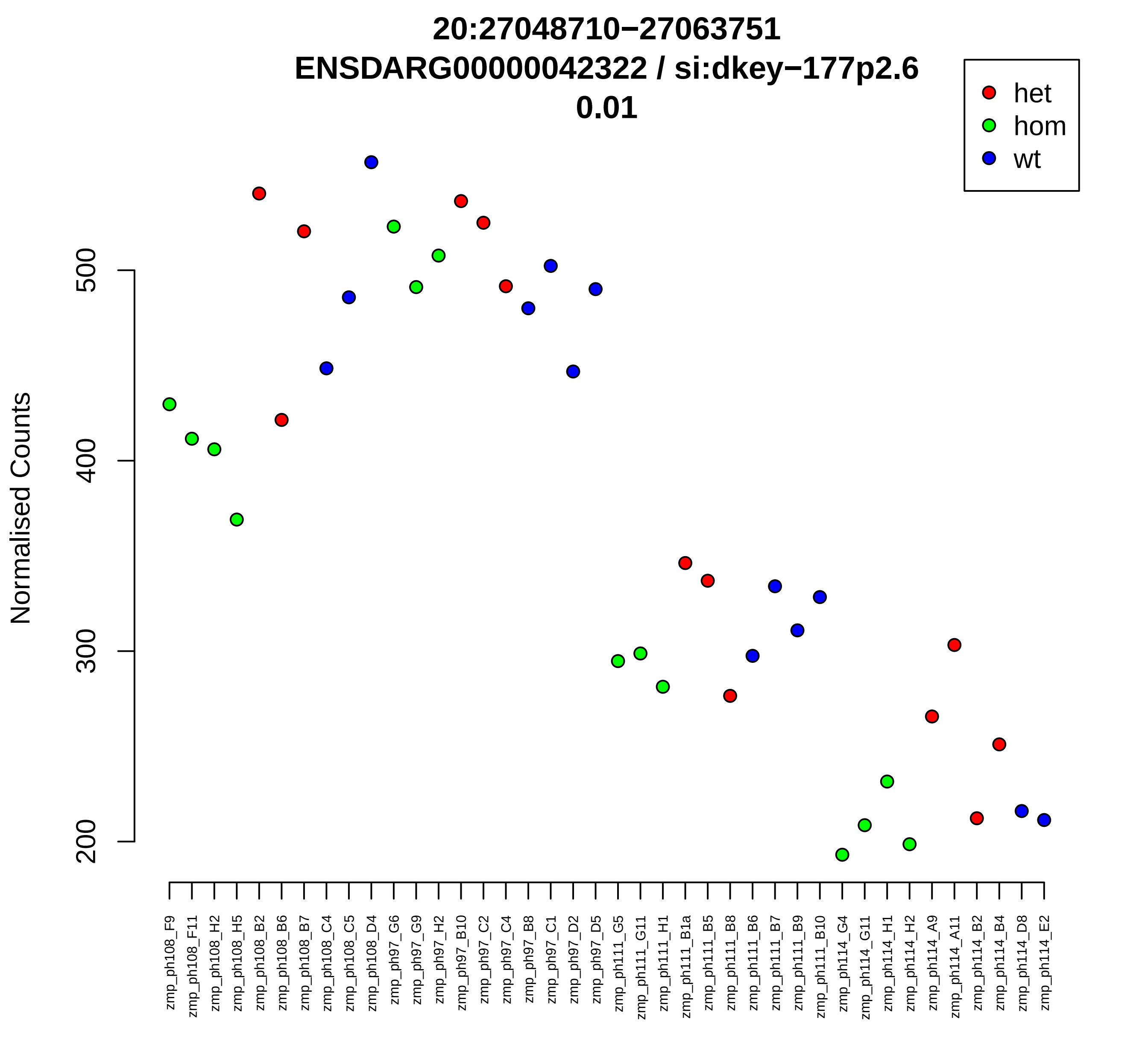

20:27048710−27063751
ENSDARG00000042322 / si:dkey−177p2.6
0.01
het
hom
wt
Normalised Counts
200
300
400
500
zmp_ph108_F9
zmp_ph108_F11
zmp_ph108_H2
zmp_ph108_H5
zmp_ph108_B2
zmp_ph108_B6
zmp_ph108_B7
zmp_ph108_C4
zmp_ph108_C5
zmp_ph108_D4
zmp_ph97_G6
zmp_ph97_G9
zmp_ph97_H2
zmp_ph97_B10
zmp_ph97_C2
zmp_ph97_C4
zmp_ph97_B8
zmp_ph97_C1
zmp_ph97_D2
zmp_ph97_D5
zmp_ph111_G5
zmp_ph111_G11
zmp_ph111_H1
zmp_ph111_B1a
zmp_ph111_B5
zmp_ph111_B8
zmp_ph111_B6
zmp_ph111_B7
zmp_ph111_B9
zmp_ph111_B10
zmp_ph114_G4
zmp_ph114_G11
zmp_ph114_H1
zmp_ph114_H2
zmp_ph114_A9
zmp_ph114_A11
zmp_ph114_B2
zmp_ph114_B4
zmp_ph114_D8
zmp_ph114_E2

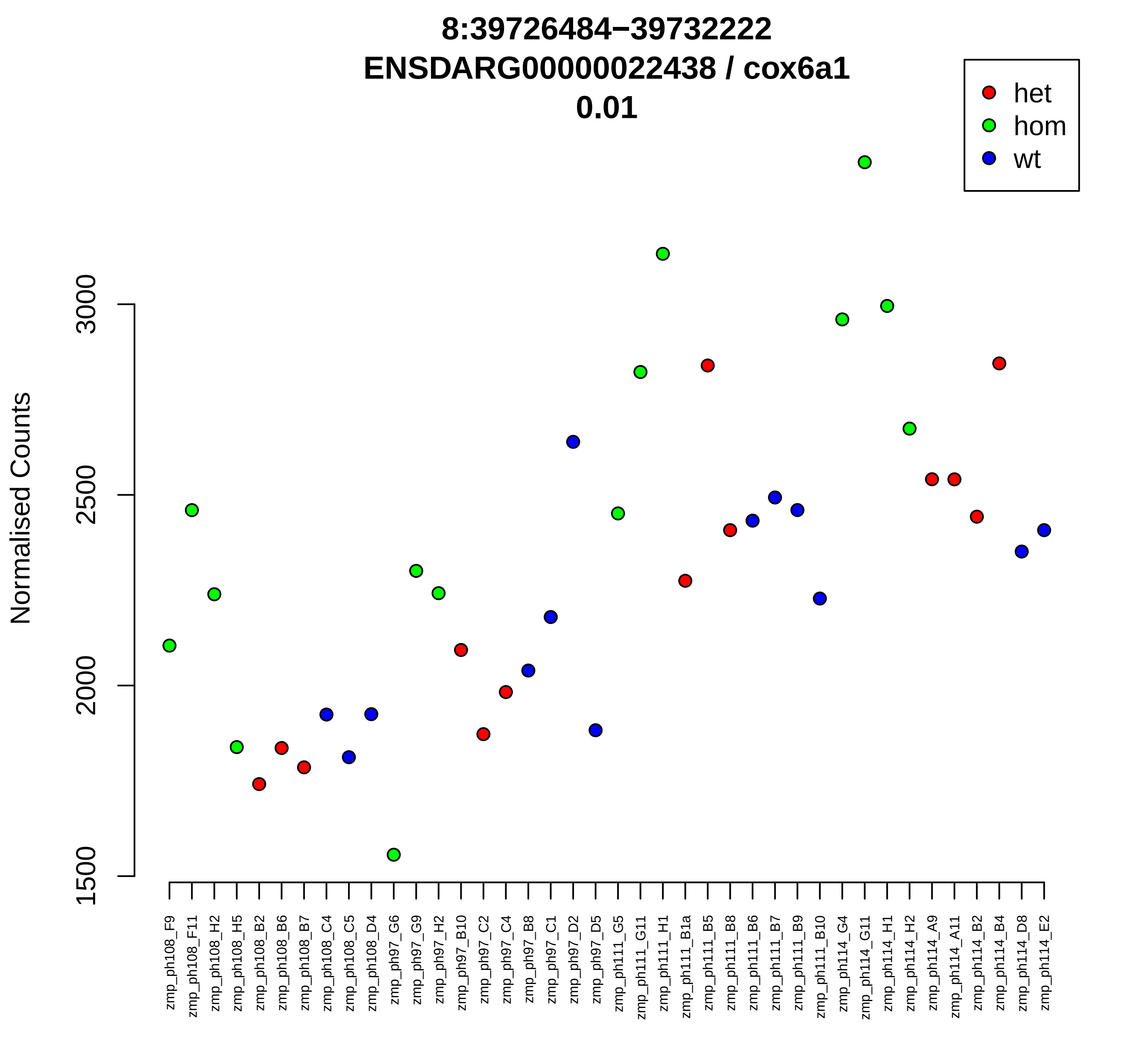
8:39726484−39732222
ENSDARG00000022438 / cox6a1
0.01
het
hom
wt
Normalised Counts
1500
2000
2500
3000
zmp_ph108_F9
zmp_ph108_F11
zmp_ph108_H2
zmp_ph108_H5
zmp_ph108_B2
zmp_ph108_B6
zmp_ph108_B7
zmp_ph108_C4
zmp_ph108_C5
zmp_ph108_D4
zmp_ph97_G6
zmp_ph97_G9
zmp_ph97_H2
zmp_ph97_B10
zmp_ph97_C2
zmp_ph97_C4
zmp_ph97_B8
zmp_ph97_C1
zmp_ph97_D2
zmp_ph97_D5
zmp_ph111_G5
zmp_ph111_G11
zmp_ph111_H1
zmp_ph111_B1a
zmp_ph111_B5
zmp_ph111_B8
zmp_ph111_B6
zmp_ph111_B7
zmp_ph111_B9
zmp_ph111_B10
zmp_ph114_G4
zmp_ph114_G11
zmp_ph114_H1
zmp_ph114_H2
zmp_ph114_A9
zmp_ph114_A11
zmp_ph114_B2
zmp_ph114_B4
zmp_ph114_D8
zmp_ph114_E2

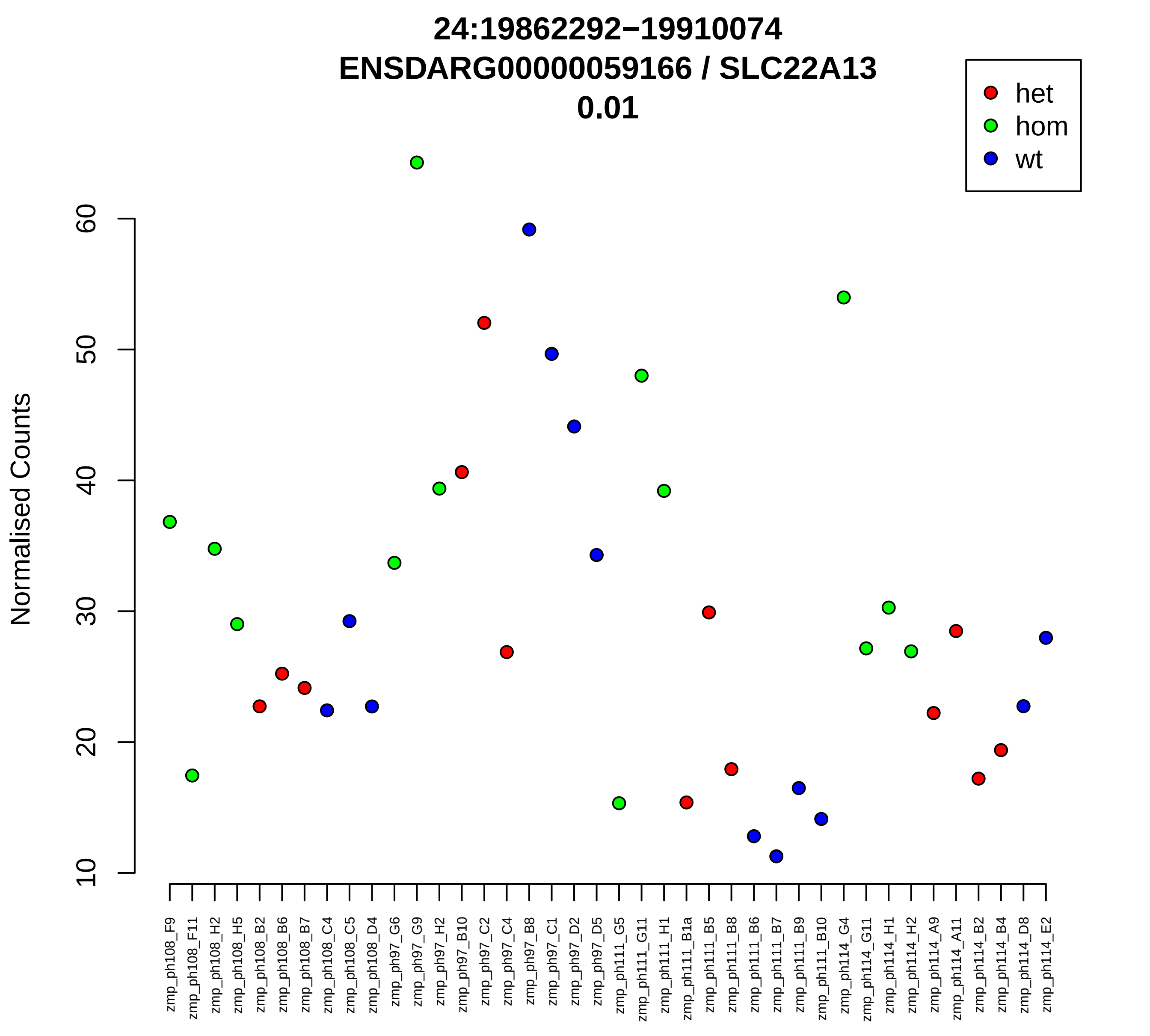
24:19862292−19910074
ENSDARG00000059166 / SLC22A13
0.01
het
hom
wt
Normalised Counts
10
20
30
40
50
60
zmp_ph108_F9
zmp_ph108_F11
zmp_ph108_H2
zmp_ph108_H5
zmp_ph108_B2
zmp_ph108_B6
zmp_ph108_B7
zmp_ph108_C4
zmp_ph108_C5
zmp_ph108_D4
zmp_ph97_G6
zmp_ph97_G9
zmp_ph97_H2
zmp_ph97_B10
zmp_ph97_C2
zmp_ph97_C4
zmp_ph97_B8
zmp_ph97_C1
zmp_ph97_D2
zmp_ph97_D5
zmp_ph111_G5
zmp_ph111_G11
zmp_ph111_H1
zmp_ph111_B1a
zmp_ph111_B5
zmp_ph111_B8
zmp_ph111_B6
zmp_ph111_B7
zmp_ph111_B9
zmp_ph111_B10
zmp_ph114_G4
zmp_ph114_G11
zmp_ph114_H1
zmp_ph114_H2
zmp_ph114_A9
zmp_ph114_A11
zmp_ph114_B2
zmp_ph114_B4
zmp_ph114_D8
zmp_ph114_E2

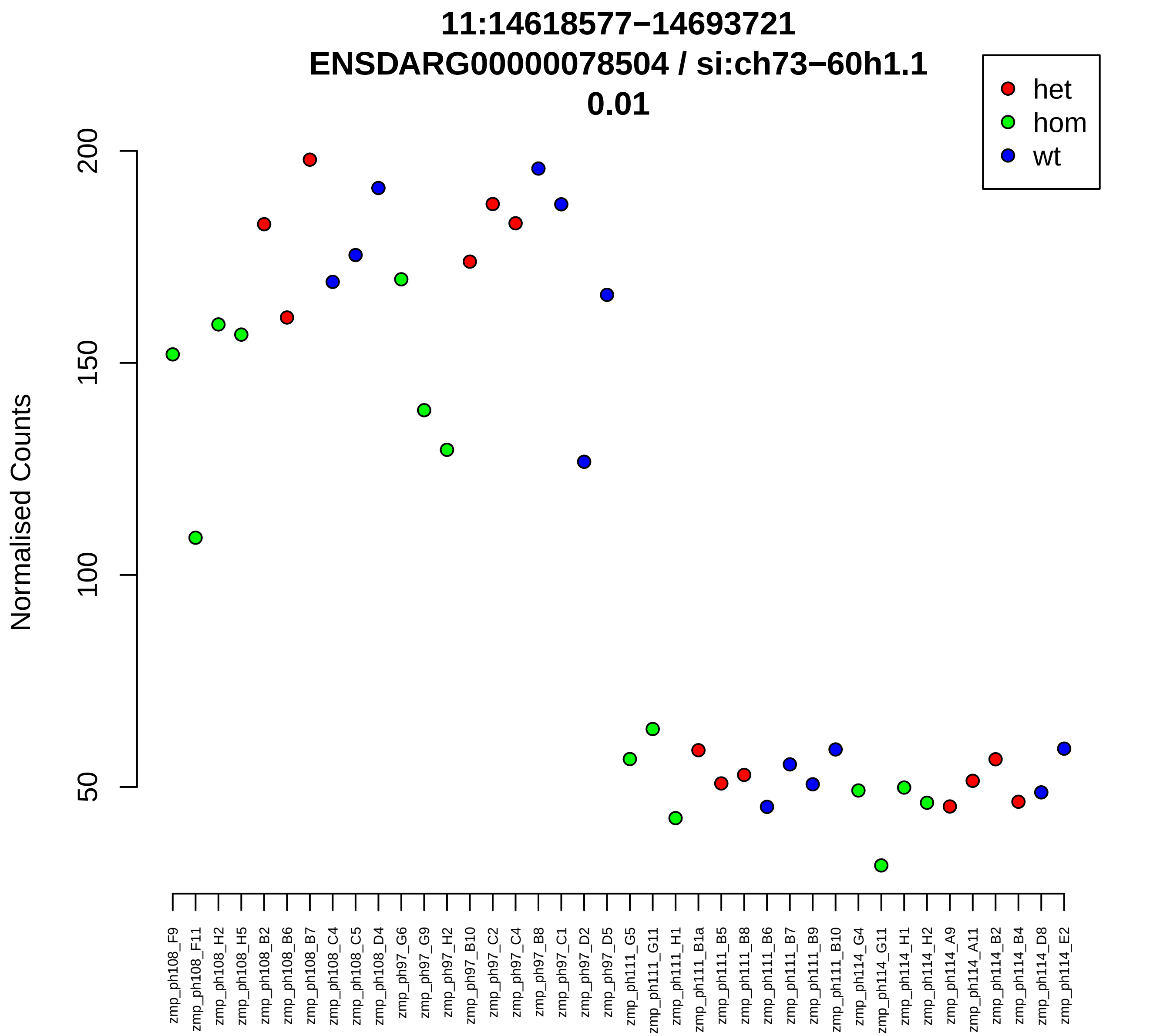
11:14618577−14693721
ENSDARG00000078504 / si:ch73−60h1.1
0.01
het
hom
wt
Normalised Counts
200
150
100
50
zmp_ph108_F9
zmp_ph108_F11
zmp_ph108_H2
zmp_ph108_H5
zmp_ph108_B2
zmp_ph108_B6
zmp_ph108_B7
zmp_ph108_C4
zmp_ph108_C5
zmp_ph108_D4
zmp_ph97_G6
zmp_ph97_G9
zmp_ph97_H2
zmp_ph97_B10
zmp_ph97_C2
zmp_ph97_C4
zmp_ph97_B8
zmp_ph97_C1
zmp_ph97_D2
zmp_ph97_D5
zmp_ph111_G5
zmp_ph111_G11
zmp_ph111_H1
zmp_ph111_B1a
zmp_ph111_B5
zmp_ph111_B8
zmp_ph111_B6
zmp_ph111_B7
zmp_ph111_B9
zmp_ph111_B10
zmp_ph114_G4
zmp_ph114_G11
zmp_ph114_H1
zmp_ph114_H2
zmp_ph114_A9
zmp_ph114_A11
zmp_ph114_B2
zmp_ph114_B4
zmp_ph114_D8
zmp_ph114_E2

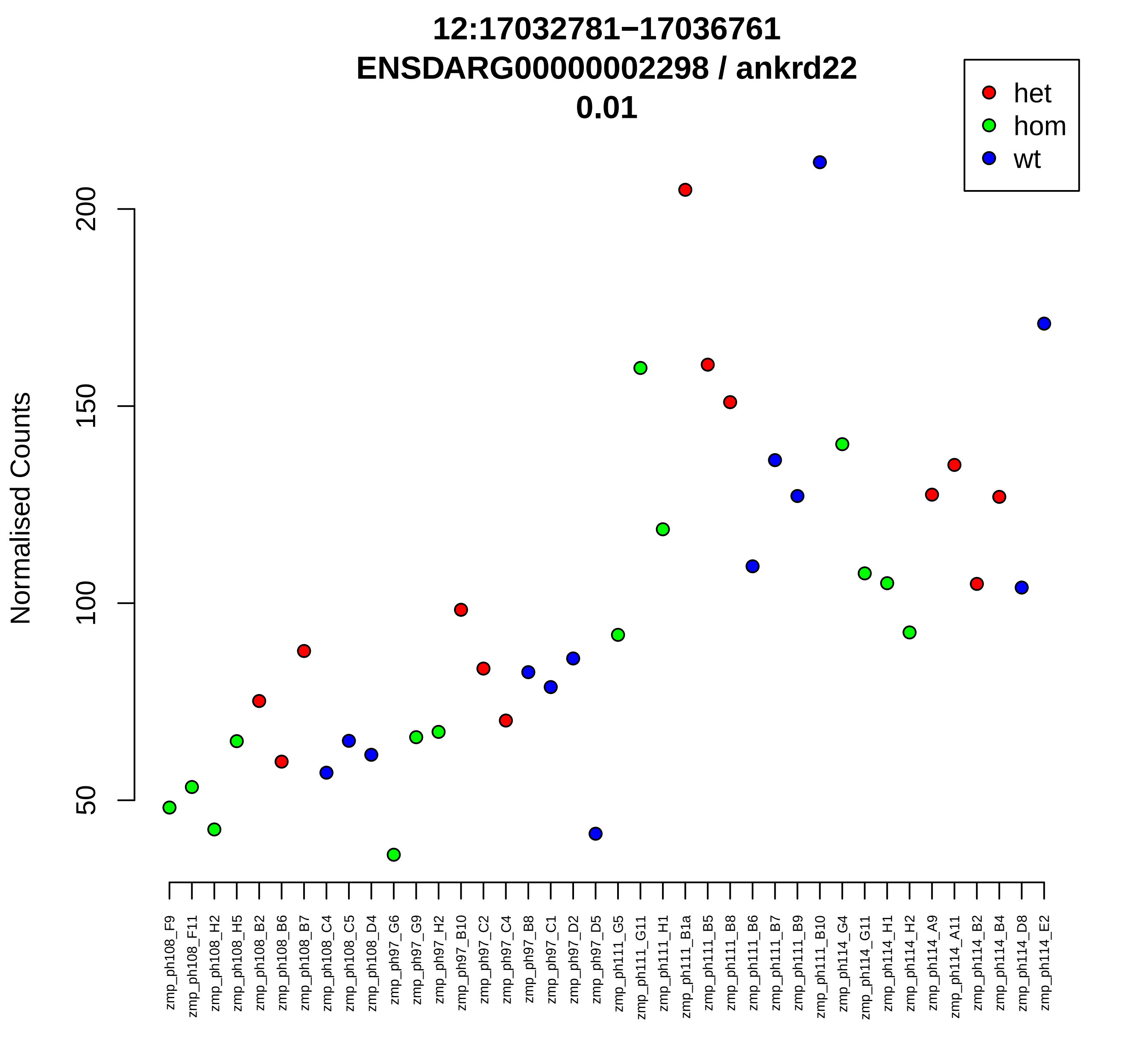
12:17032781−17036761
ENSDARG00000002298 / ankrd22
0.01
het
hom
wt
Normalised Counts
50
100
150
200
zmp_ph108_F9
zmp_ph108_F11
zmp_ph108_H2
zmp_ph108_H5
zmp_ph108_B2
zmp_ph108_B6
zmp_ph108_B7
zmp_ph108_C4
zmp_ph108_C5
zmp_ph108_D4
zmp_ph97_G6
zmp_ph97_G9
zmp_ph97_H2
zmp_ph97_B10
zmp_ph97_C2
zmp_ph97_C4
zmp_ph97_B8
zmp_ph97_C1
zmp_ph97_D2
zmp_ph97_D5
zmp_ph111_G5
zmp_ph111_G11
zmp_ph111_H1
zmp_ph111_B1a
zmp_ph111_B5
zmp_ph111_B8
zmp_ph111_B6
zmp_ph111_B7
zmp_ph111_B9
zmp_ph111_B10
zmp_ph114_G4
zmp_ph114_G11
zmp_ph114_H1
zmp_ph114_H2
zmp_ph114_A9
zmp_ph114_A11
zmp_ph114_B2
zmp_ph114_B4
zmp_ph114_D8
zmp_ph114_E2

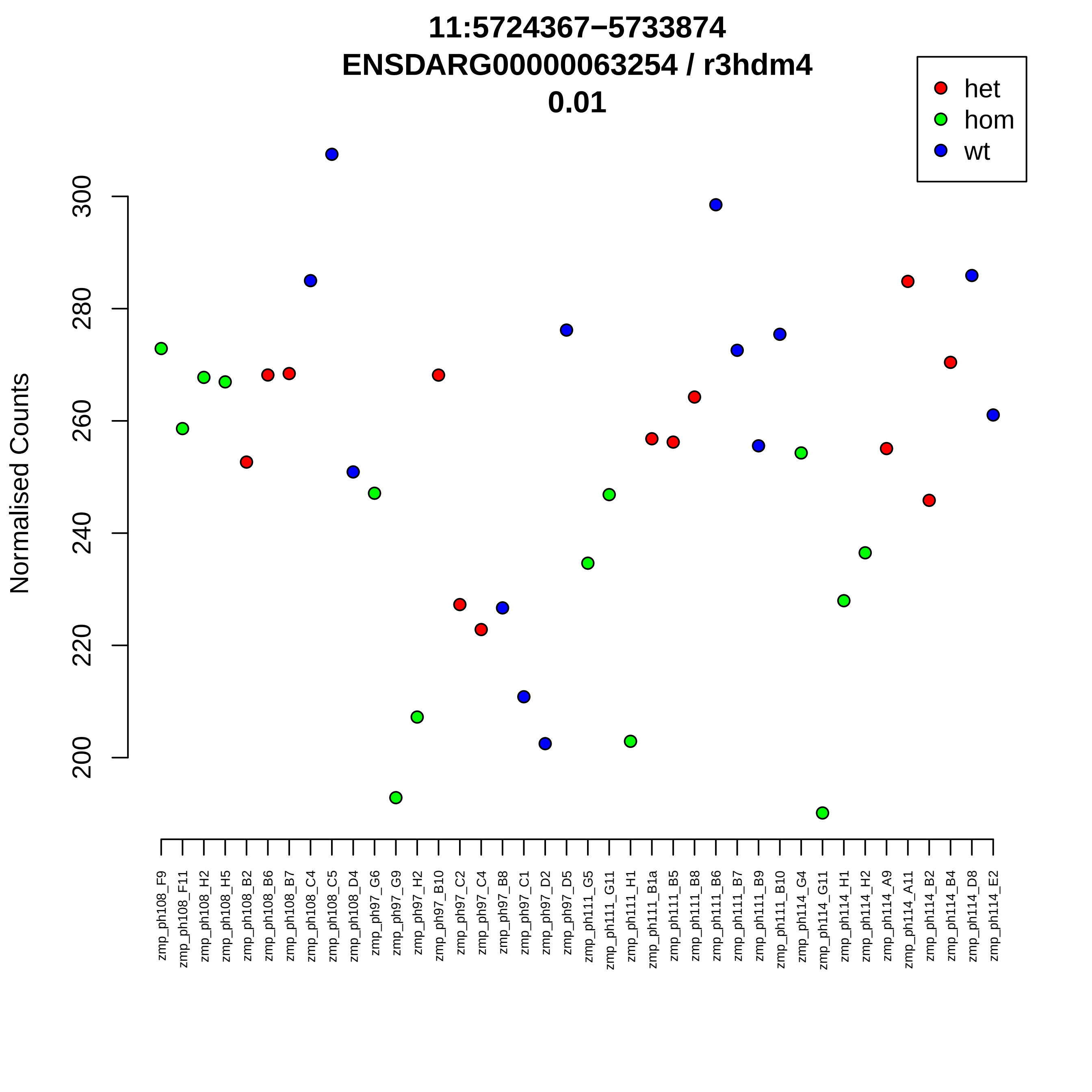
11:5724367−5733874
ENSDARG00000063254 / r3hdm4
0.01
het
hom
wt
Normalised Counts
200
220
240
260
280
300
zmp_ph108_F9
zmp_ph108_F11
zmp_ph108_H2
zmp_ph108_H5
zmp_ph108_B2
zmp_ph108_B6
zmp_ph108_B7
zmp_ph108_C4
zmp_ph108_C5
zmp_ph108_D4
zmp_ph97_G6
zmp_ph97_G9
zmp_ph97_H2
zmp_ph97_B10
zmp_ph97_C2
zmp_ph97_C4
zmp_ph97_B8
zmp_ph97_C1
zmp_ph97_D2
zmp_ph97_D5
zmp_ph111_G5
zmp_ph111_G11
zmp_ph111_H1
zmp_ph111_B1a
zmp_ph111_B5
zmp_ph111_B8
zmp_ph111_B6
zmp_ph111_B7
zmp_ph111_B9
zmp_ph111_B10
zmp_ph114_G4
zmp_ph114_G11
zmp_ph114_H1
zmp_ph114_H2
zmp_ph114_A9
zmp_ph114_A11
zmp_ph114_B2
zmp_ph114_B4
zmp_ph114_D8
zmp_ph114_E2

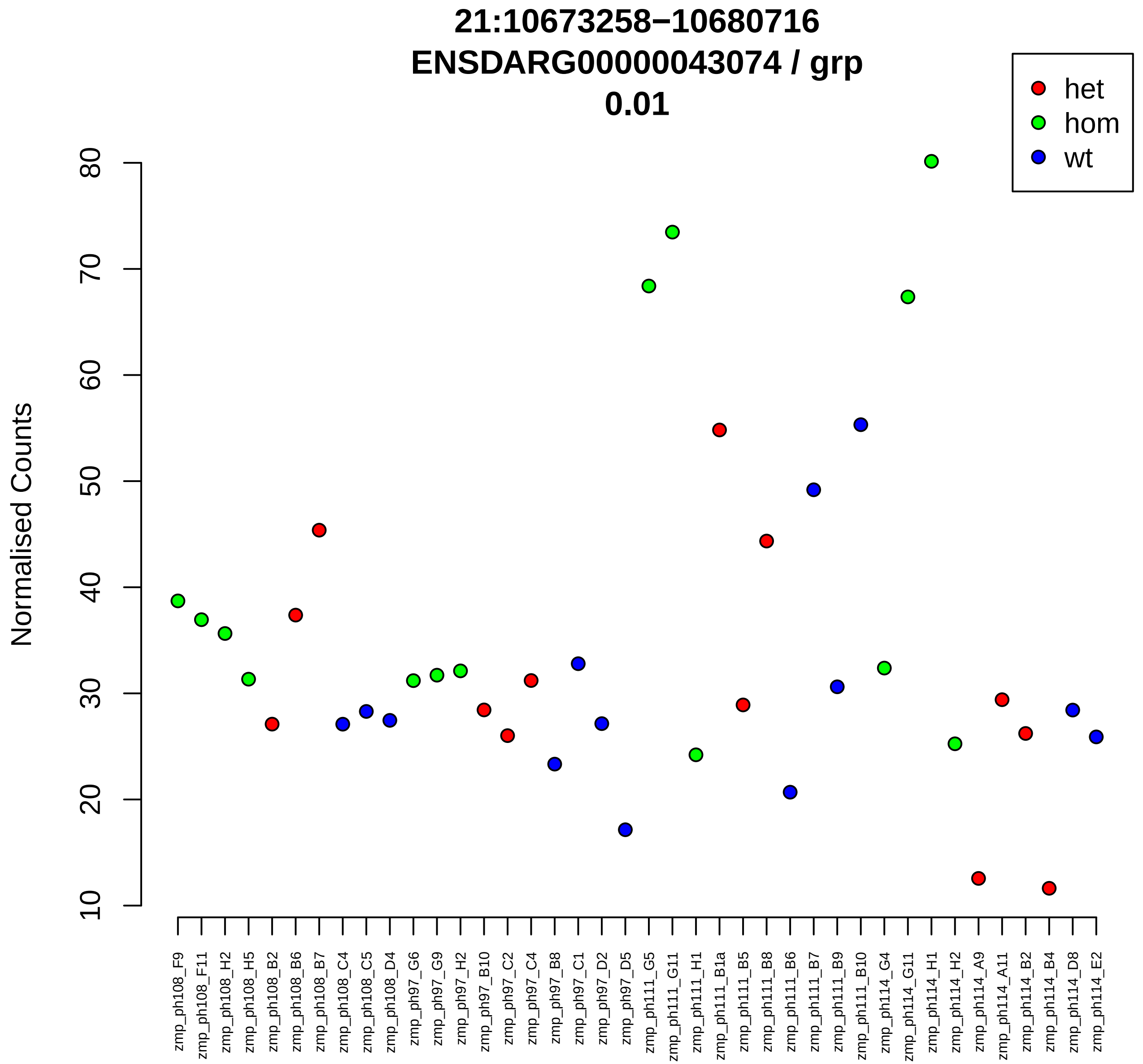

21:10673258−10680716
ENSDARG00000043074 / grp
0.01
het
hom
wt
Normalised Counts
10
20
30
40
50
60
70
80
zmp_ph108_F9
zmp_ph108_F11
zmp_ph108_H2
zmp_ph108_H5
zmp_ph108_B2
zmp_ph108_B6
zmp_ph108_B7
zmp_ph108_C4
zmp_ph108_C5
zmp_ph108_D4
zmp_ph97_G6
zmp_ph97_G9
zmp_ph97_H2
zmp_ph97_B10
zmp_ph97_C2
zmp_ph97_C4
zmp_ph97_B8
zmp_ph97_C1
zmp_ph97_D2
zmp_ph97_D5
zmp_ph111_G5
zmp_ph111_G11
zmp_ph111_H1
zmp_ph111_B1a
zmp_ph111_B5
zmp_ph111_B8
zmp_ph111_B6
zmp_ph111_B7
zmp_ph111_B9
zmp_ph111_B10
zmp_ph114_G4
zmp_ph114_G11
zmp_ph114_H1
zmp_ph114_H2
zmp_ph114_A9
zmp_ph114_A11
zmp_ph114_B2
zmp_ph114_B4
zmp_ph114_D8
zmp_ph114_E2

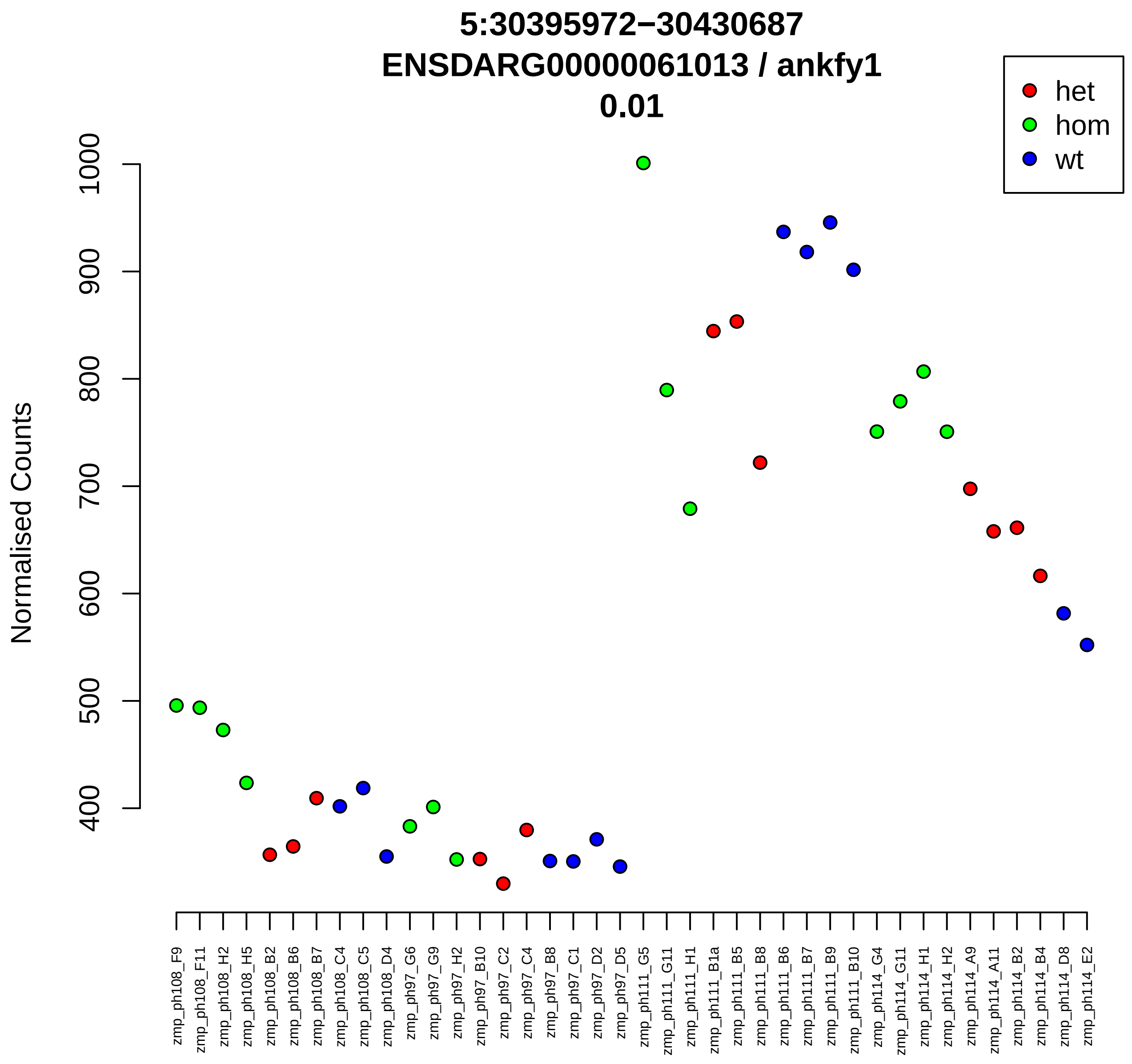

5:30395972−30430687
ENSDARG00000061013 / ankfy1
0.01
het
hom
wt
Normalised Counts
400
500
600
700
800
900
1000
zmp_ph108_F9
zmp_ph108_F11
zmp_ph108_H2
zmp_ph108_H5
zmp_ph108_B2
zmp_ph108_B6
zmp_ph108_B7
zmp_ph108_C4
zmp_ph108_C5
zmp_ph108_D4
zmp_ph97_G6
zmp_ph97_G9
zmp_ph97_H2
zmp_ph97_B10
zmp_ph97_C2
zmp_ph97_C4
zmp_ph97_B8
zmp_ph97_C1
zmp_ph97_D2
zmp_ph97_D5
zmp_ph111_G5
zmp_ph111_G11
zmp_ph111_H1
zmp_ph111_B1a
zmp_ph111_B5
zmp_ph111_B8
zmp_ph111_B6
zmp_ph111_B7
zmp_ph111_B9
zmp_ph111_B10
zmp_ph114_G4
zmp_ph114_G11
zmp_ph114_H1
zmp_ph114_H2
zmp_ph114_A9
zmp_ph114_A11
zmp_ph114_B2
zmp_ph114_B4
zmp_ph114_D8
zmp_ph114_E2

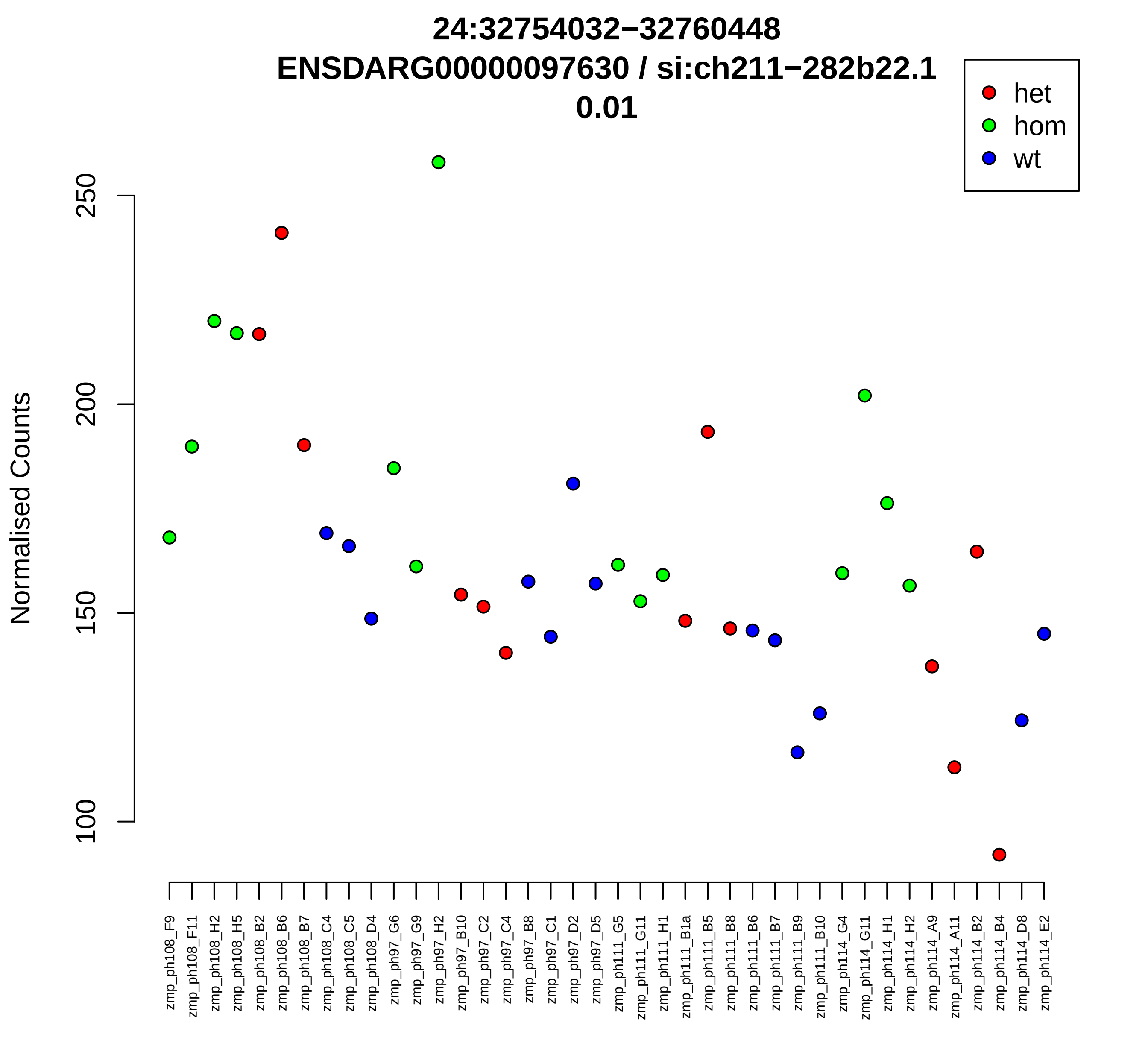
24:32754032−32760448
ENSDARG00000097630 / si:ch211−282b22.1
0.01
het
hom
wt
Normalised Counts
250
200
150
100
zmp_ph108_F9
zmp_ph108_F11
zmp_ph108_H2
zmp_ph108_H5
zmp_ph108_B2
zmp_ph108_B6
zmp_ph108_B7
zmp_ph108_C4
zmp_ph108_C5
zmp_ph108_D4
zmp_ph97_G6
zmp_ph97_G9
zmp_ph97_H2
zmp_ph97_B10
zmp_ph97_C2
zmp_ph97_C4
zmp_ph97_B8
zmp_ph97_C1
zmp_ph97_D2
zmp_ph97_D5
zmp_ph111_G5
zmp_ph111_G11
zmp_ph111_H1
zmp_ph111_B1a
zmp_ph111_B5
zmp_ph111_B8
zmp_ph111_B6
zmp_ph111_B7
zmp_ph111_B9
zmp_ph111_B10
zmp_ph114_G4
zmp_ph114_G11
zmp_ph114_H1
zmp_ph114_H2
zmp_ph114_A9
zmp_ph114_A11
zmp_ph114_B2
zmp_ph114_B4
zmp_ph114_D8
zmp_ph114_E2

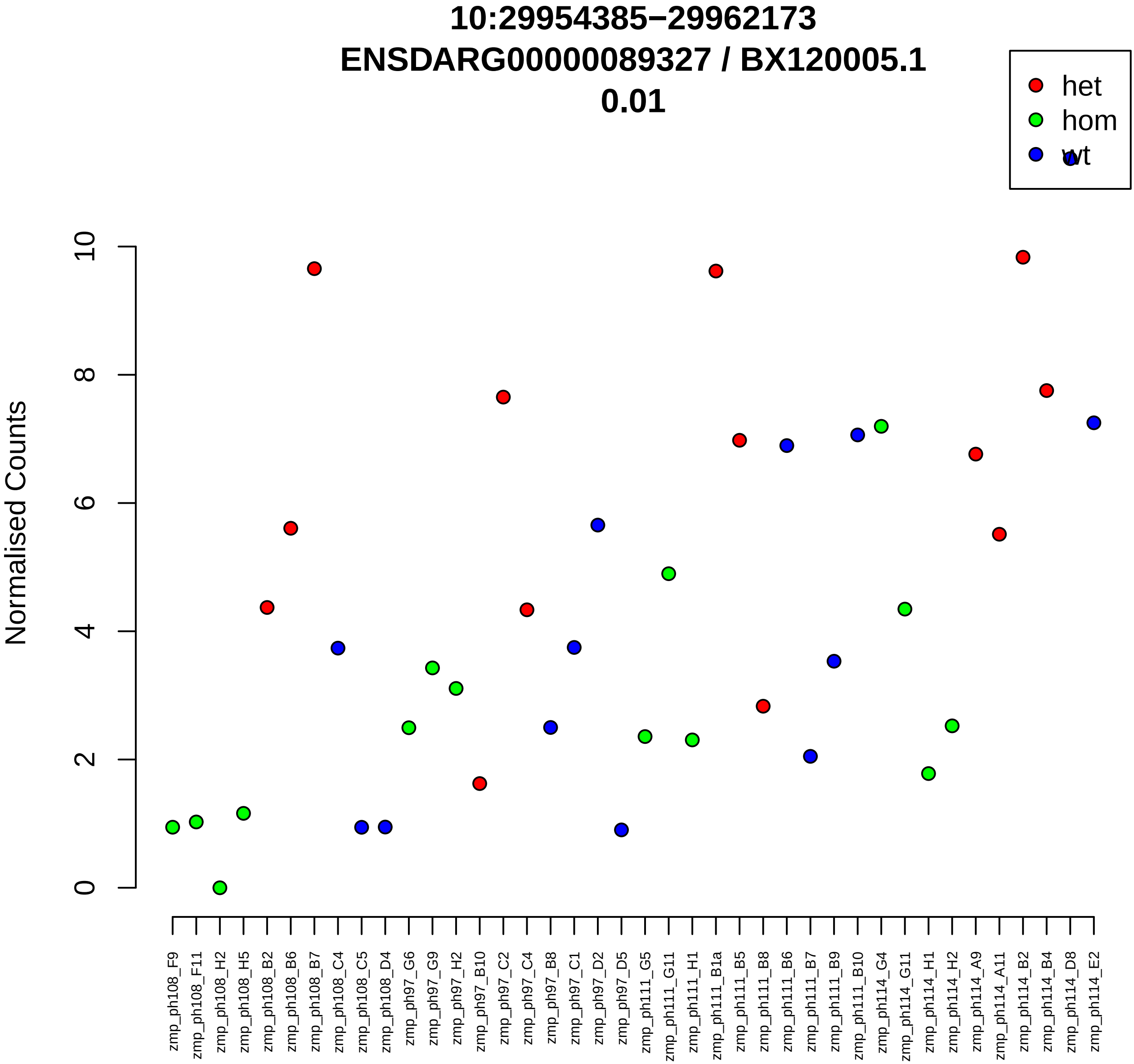

10:29954385−29962173
ENSDARG00000089327 / BX120005.1
0.01
het
hom
wt
Normalised Counts
0
2
4
6
8
10
zmp_ph108_F9
zmp_ph108_F11
zmp_ph108_H2
zmp_ph108_H5
zmp_ph108_B2
zmp_ph108_B6
zmp_ph108_B7
zmp_ph108_C4
zmp_ph108_C5
zmp_ph108_D4
zmp_ph97_G6
zmp_ph97_G9
zmp_ph97_H2
zmp_ph97_B10
zmp_ph97_C2
zmp_ph97_C4
zmp_ph97_B8
zmp_ph97_C1
zmp_ph97_D2
zmp_ph97_D5
zmp_ph111_G5
zmp_ph111_G11
zmp_ph111_H1
zmp_ph111_B1a
zmp_ph111_B5
zmp_ph111_B8
zmp_ph111_B6
zmp_ph111_B7
zmp_ph111_B9
zmp_ph111_B10
zmp_ph114_G4
zmp_ph114_G11
zmp_ph114_H1
zmp_ph114_H2
zmp_ph114_A9
zmp_ph114_A11
zmp_ph114_B2
zmp_ph114_B4
zmp_ph114_D8
zmp_ph114_E2

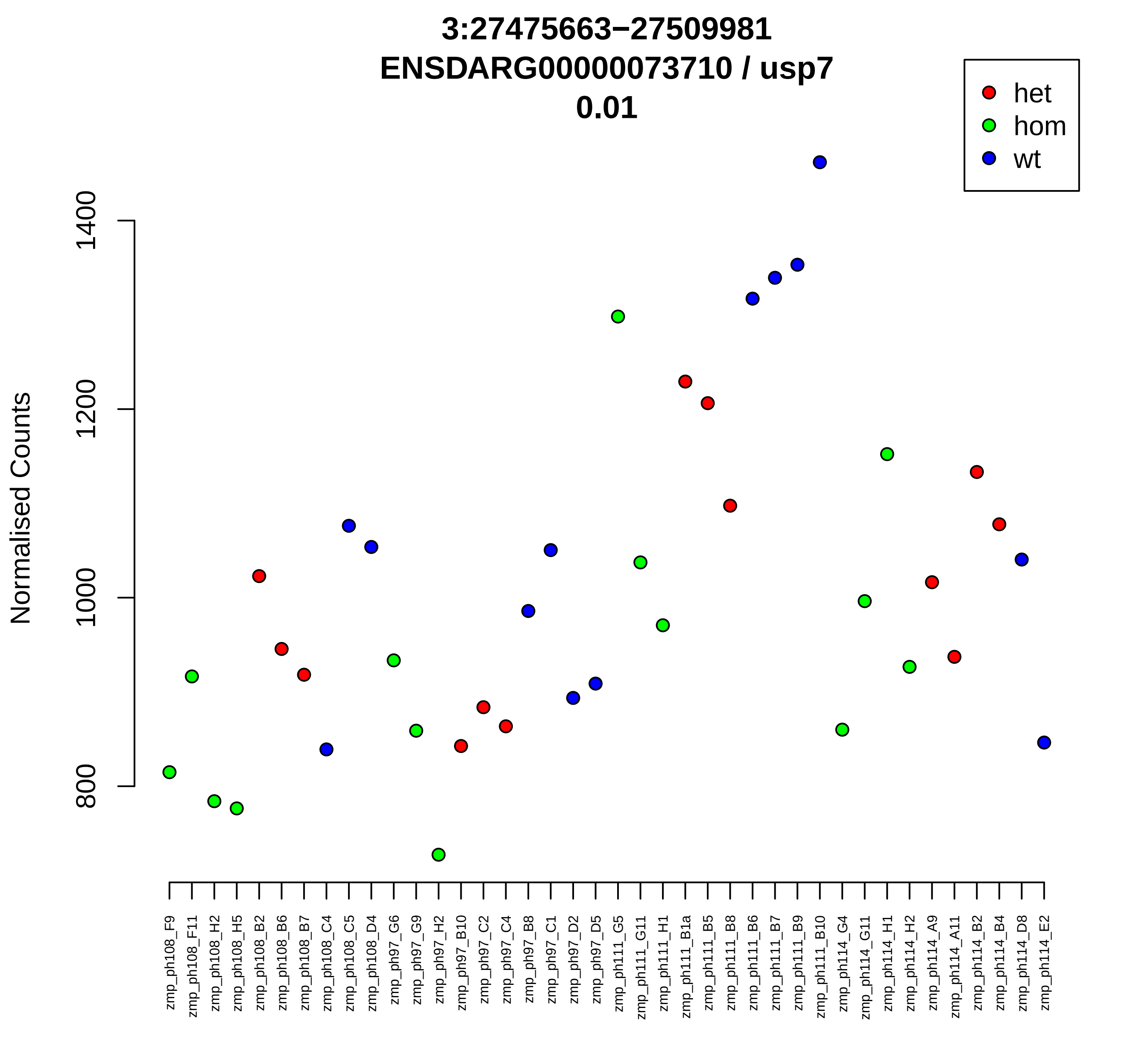
3:27475663−27509981
ENSDARG00000073710 / usp7
0.01
het
hom
wt
Normalised Counts
800
1000
1200
1400
zmp_ph108_F9
zmp_ph108_F11
zmp_ph108_H2
zmp_ph108_H5
zmp_ph108_B2
zmp_ph108_B6
zmp_ph108_B7
zmp_ph108_C4
zmp_ph108_C5
zmp_ph108_D4
zmp_ph97_G6
zmp_ph97_G9
zmp_ph97_H2
zmp_ph97_B10
zmp_ph97_C2
zmp_ph97_C4
zmp_ph97_B8
zmp_ph97_C1
zmp_ph97_D2
zmp_ph97_D5
zmp_ph111_G5
zmp_ph111_G11
zmp_ph111_H1
zmp_ph111_B1a
zmp_ph111_B5
zmp_ph111_B8
zmp_ph111_B6
zmp_ph111_B7
zmp_ph111_B9
zmp_ph111_B10
zmp_ph114_G4
zmp_ph114_G11
zmp_ph114_H1
zmp_ph114_H2
zmp_ph114_A9
zmp_ph114_A11
zmp_ph114_B2
zmp_ph114_B4
zmp_ph114_D8
zmp_ph114_E2

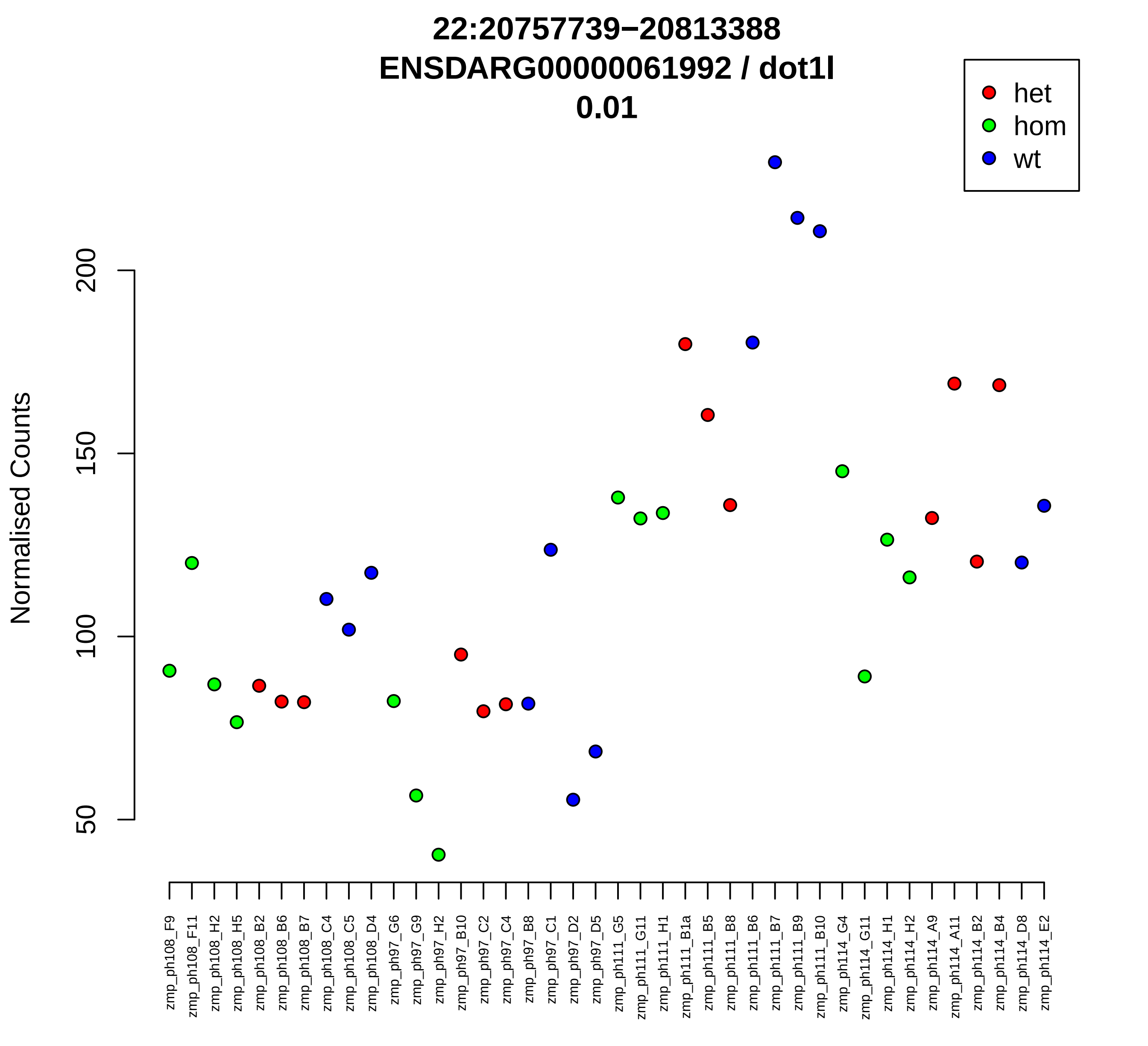

22:20757739−20813388
ENSDARG00000061992 / dot1l
0.01
het
hom
wt
Normalised Counts
50
100
150
200
zmp_ph108_F9
zmp_ph108_F11
zmp_ph108_H2
zmp_ph108_H5
zmp_ph108_B2
zmp_ph108_B6
zmp_ph108_B7
zmp_ph108_C4
zmp_ph108_C5
zmp_ph108_D4
zmp_ph97_G6
zmp_ph97_G9
zmp_ph97_H2
zmp_ph97_B10
zmp_ph97_C2
zmp_ph97_C4
zmp_ph97_B8
zmp_ph97_C1
zmp_ph97_D2
zmp_ph97_D5
zmp_ph111_G5
zmp_ph111_G11
zmp_ph111_H1
zmp_ph111_B1a
zmp_ph111_B5
zmp_ph111_B8
zmp_ph111_B6
zmp_ph111_B7
zmp_ph111_B9
zmp_ph111_B10
zmp_ph114_G4
zmp_ph114_G11
zmp_ph114_H1
zmp_ph114_H2
zmp_ph114_A9
zmp_ph114_A11
zmp_ph114_B2
zmp_ph114_B4
zmp_ph114_D8
zmp_ph114_E2

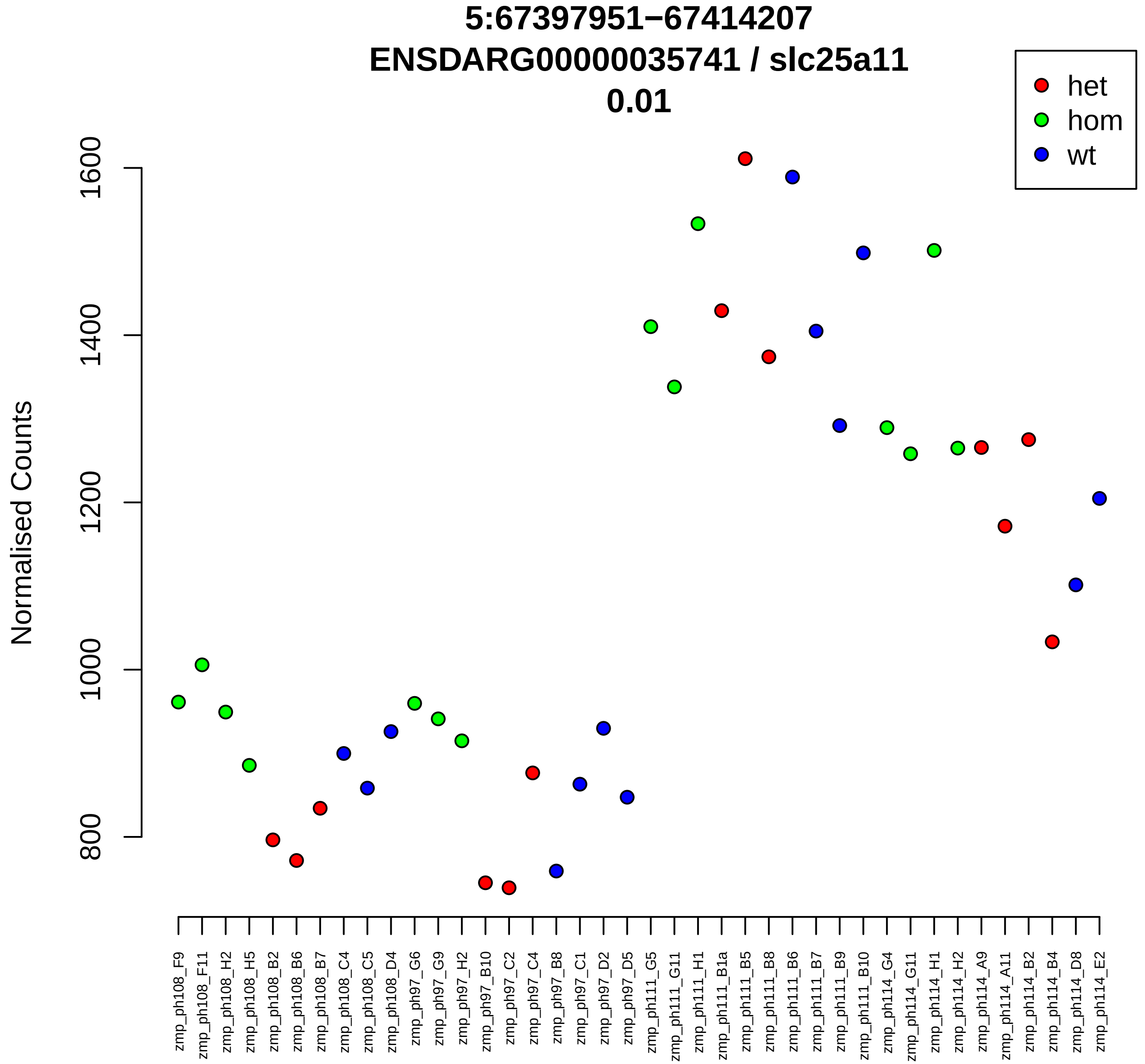
5:67397951−67414207
ENSDARG00000035741 / slc25a11
0.01
het
hom
wt
Normalised Counts
800
1000
1200
1400
1600
zmp_ph108_F9
zmp_ph108_F11
zmp_ph108_H2
zmp_ph108_H5
zmp_ph108_B2
zmp_ph108_B6
zmp_ph108_B7
zmp_ph108_C4
zmp_ph108_C5
zmp_ph108_D4
zmp_ph97_G6
zmp_ph97_G9
zmp_ph97_H2
zmp_ph97_B10
zmp_ph97_C2
zmp_ph97_C4
zmp_ph97_B8
zmp_ph97_C1
zmp_ph97_D2
zmp_ph97_D5
zmp_ph111_G5
zmp_ph111_G11
zmp_ph111_H1
zmp_ph111_B1a
zmp_ph111_B5
zmp_ph111_B8
zmp_ph111_B6
zmp_ph111_B7
zmp_ph111_B9
zmp_ph111_B10
zmp_ph114_G4
zmp_ph114_G11
zmp_ph114_H1
zmp_ph114_H2
zmp_ph114_A9
zmp_ph114_A11
zmp_ph114_B2
zmp_ph114_B4
zmp_ph114_D8
zmp_ph114_E2

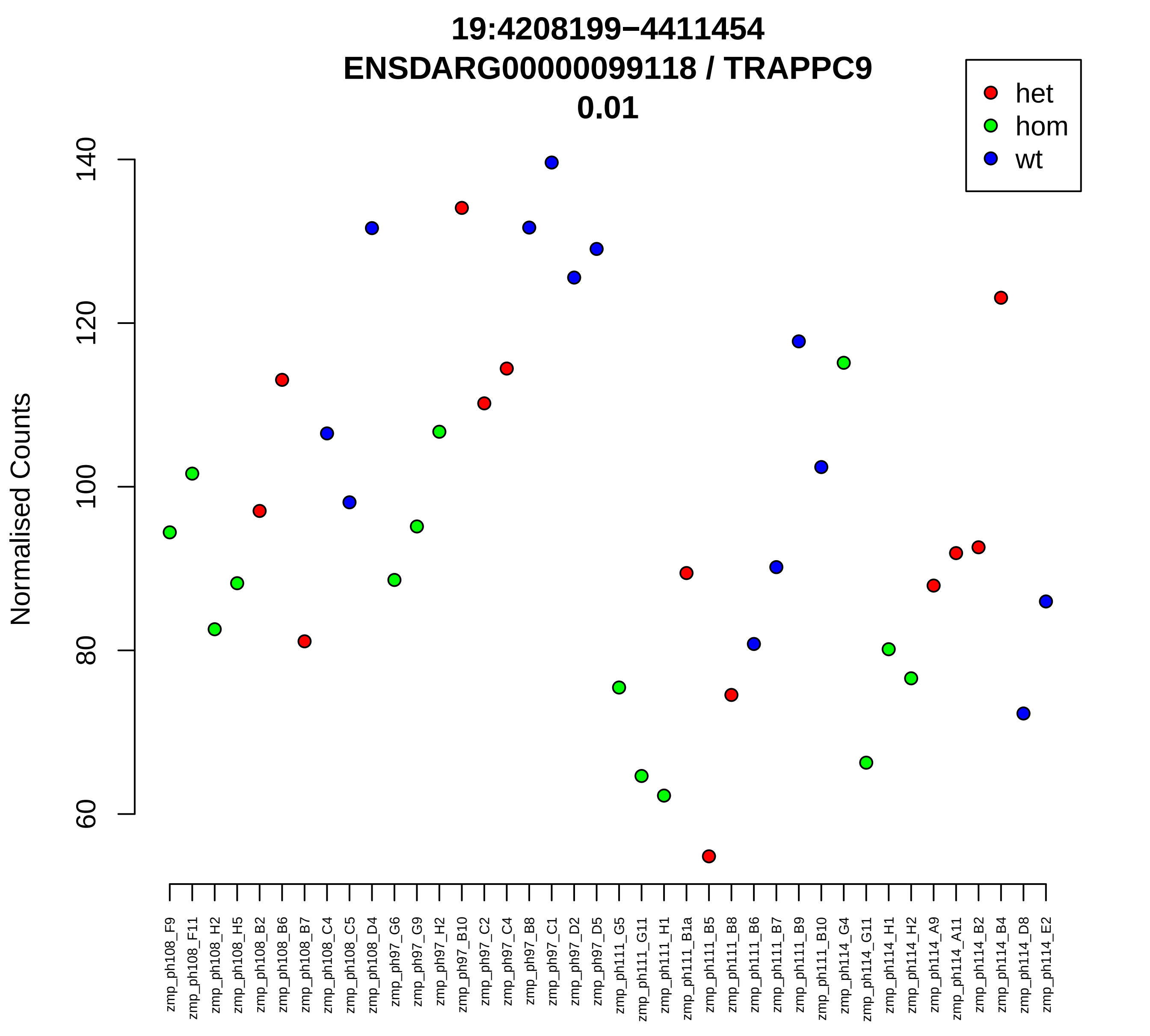

19:4208199−4411454
ENSDARG00000099118 / TRAPPC9
0.01
het
hom
wt
Normalised Counts
60
80
100
120
140
zmp_ph108_F9
zmp_ph108_F11
zmp_ph108_H2
zmp_ph108_H5
zmp_ph108_B2
zmp_ph108_B6
zmp_ph108_B7
zmp_ph108_C4
zmp_ph108_C5
zmp_ph108_D4
zmp_ph97_G6
zmp_ph97_G9
zmp_ph97_H2
zmp_ph97_B10
zmp_ph97_C2
zmp_ph97_C4
zmp_ph97_B8
zmp_ph97_C1
zmp_ph97_D2
zmp_ph97_D5
zmp_ph111_G5
zmp_ph111_G11
zmp_ph111_H1
zmp_ph111_B1a
zmp_ph111_B5
zmp_ph111_B8
zmp_ph111_B6
zmp_ph111_B7
zmp_ph111_B9
zmp_ph111_B10
zmp_ph114_G4
zmp_ph114_G11
zmp_ph114_H1
zmp_ph114_H2
zmp_ph114_A9
zmp_ph114_A11
zmp_ph114_B2
zmp_ph114_B4
zmp_ph114_D8
zmp_ph114_E2

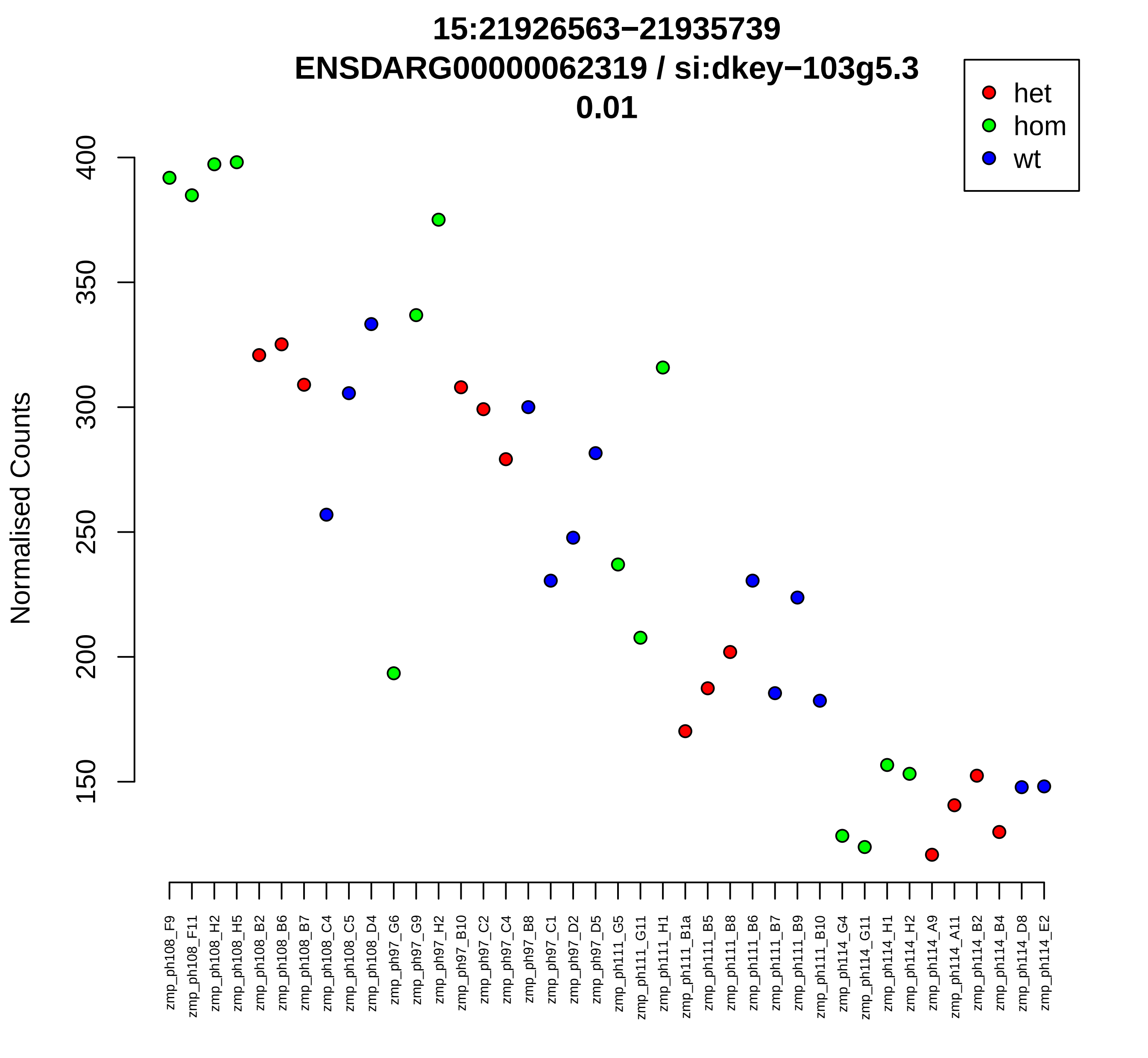

15:21926563−21935739
ENSDARG00000062319 / si:dkey−103g5.3
0.01
het
hom
wt
Normalised Counts
400
350
300
250
200
150
zmp_ph108_F9
zmp_ph108_F11
zmp_ph108_H2
zmp_ph108_H5
zmp_ph108_B2
zmp_ph108_B6
zmp_ph108_B7
zmp_ph108_C4
zmp_ph108_C5
zmp_ph108_D4
zmp_ph97_G6
zmp_ph97_G9
zmp_ph97_H2
zmp_ph97_B10
zmp_ph97_C2
zmp_ph97_C4
zmp_ph97_B8
zmp_ph97_C1
zmp_ph97_D2
zmp_ph97_D5
zmp_ph111_G5
zmp_ph111_G11
zmp_ph111_H1
zmp_ph111_B1a
zmp_ph111_B5
zmp_ph111_B8
zmp_ph111_B6
zmp_ph111_B7
zmp_ph111_B9
zmp_ph111_B10
zmp_ph114_G4
zmp_ph114_G11
zmp_ph114_H1
zmp_ph114_H2
zmp_ph114_A9
zmp_ph114_A11
zmp_ph114_B2
zmp_ph114_B4
zmp_ph114_D8
zmp_ph114_E2

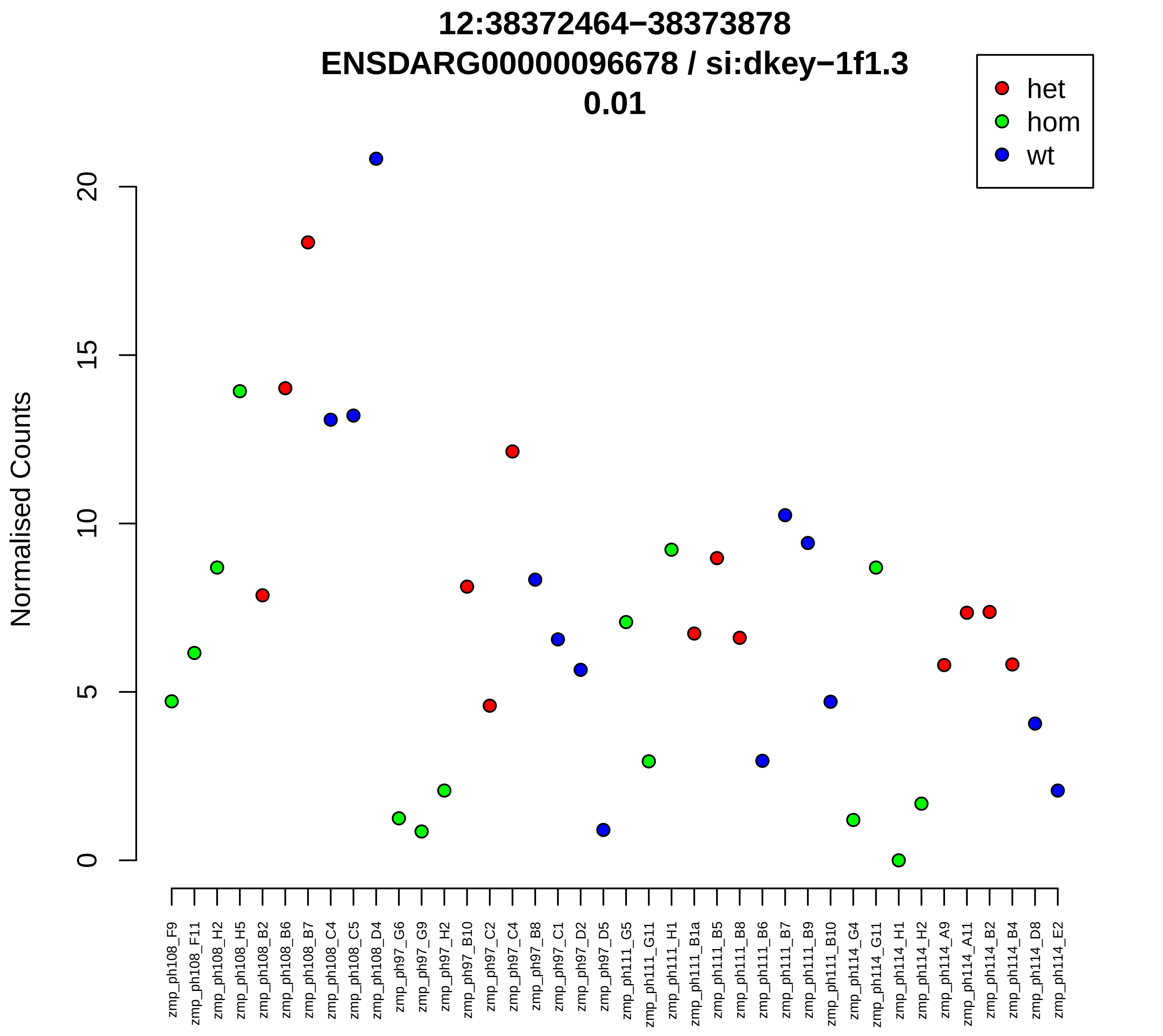
12:38372464−38373878
ENSDARG00000096678 / si:dkey−1f1.3
0.01
het
hom
wt
Normalised Counts
0
5
10
15
20
zmp_ph108_F9
zmp_ph108_F11
zmp_ph108_H2
zmp_ph108_H5
zmp_ph108_B2
zmp_ph108_B6
zmp_ph108_B7
zmp_ph108_C4
zmp_ph108_C5
zmp_ph108_D4
zmp_ph97_G6
zmp_ph97_G9
zmp_ph97_H2
zmp_ph97_B10
zmp_ph97_C2
zmp_ph97_C4
zmp_ph97_B8
zmp_ph97_C1
zmp_ph97_D2
zmp_ph97_D5
zmp_ph111_G5
zmp_ph111_G11
zmp_ph111_H1
zmp_ph111_B1a
zmp_ph111_B5
zmp_ph111_B8
zmp_ph111_B6
zmp_ph111_B7
zmp_ph111_B9
zmp_ph111_B10
zmp_ph114_G4
zmp_ph114_G11
zmp_ph114_H1
zmp_ph114_H2
zmp_ph114_A9
zmp_ph114_A11
zmp_ph114_B2
zmp_ph114_B4
zmp_ph114_D8
zmp_ph114_E2

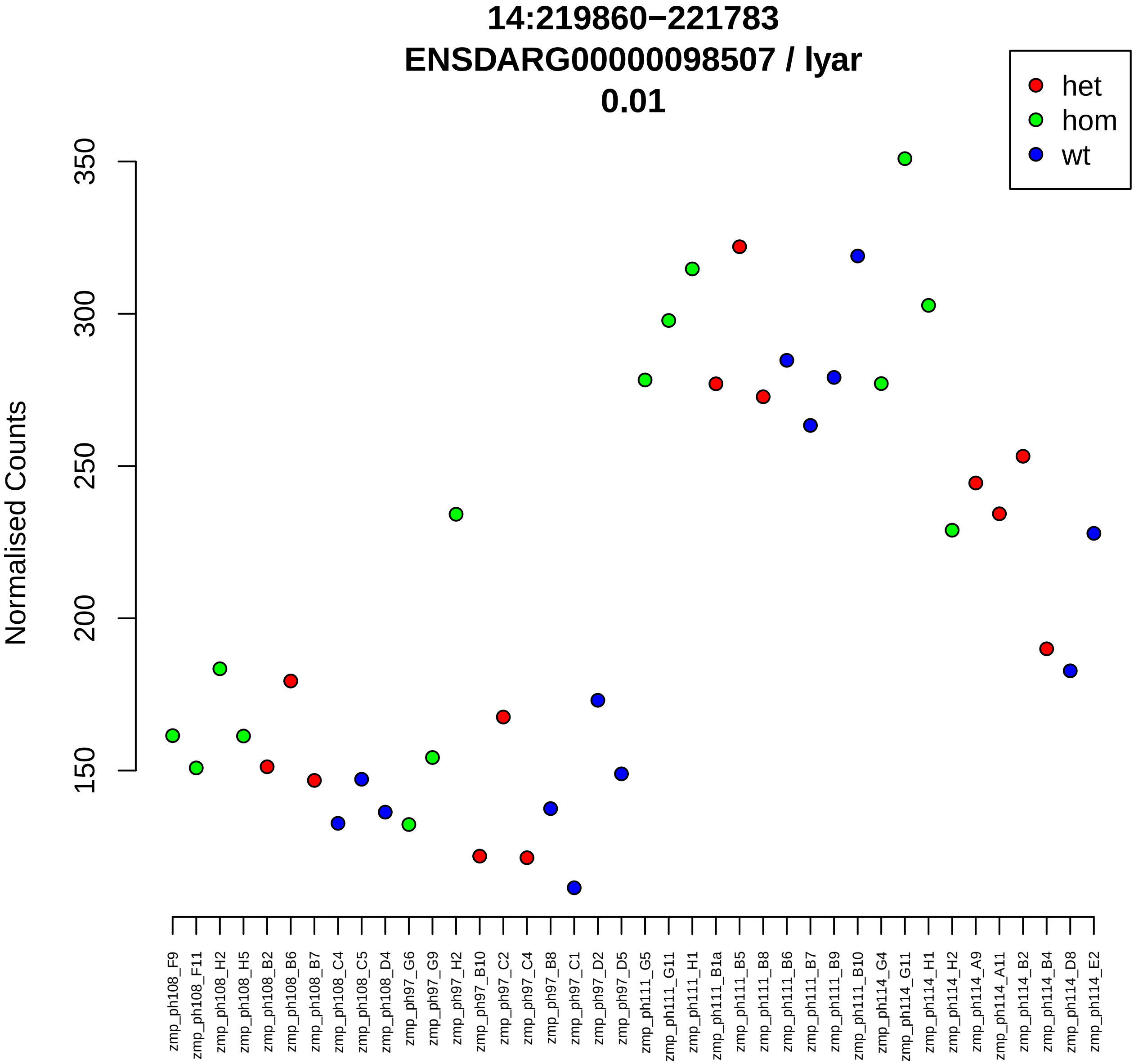
14:219860−221783
ENSDARG00000098507 / lyar
0.01
het
hom
wt
Normalised Counts
150
200
250
300
350
zmp_ph108_F9
zmp_ph108_F11
zmp_ph108_H2
zmp_ph108_H5
zmp_ph108_B2
zmp_ph108_B6
zmp_ph108_B7
zmp_ph108_C4
zmp_ph108_C5
zmp_ph108_D4
zmp_ph97_G6
zmp_ph97_G9
zmp_ph97_H2
zmp_ph97_B10
zmp_ph97_C2
zmp_ph97_C4
zmp_ph97_B8
zmp_ph97_C1
zmp_ph97_D2
zmp_ph97_D5
zmp_ph111_G5
zmp_ph111_G11
zmp_ph111_H1
zmp_ph111_B1a
zmp_ph111_B5
zmp_ph111_B8
zmp_ph111_B6
zmp_ph111_B7
zmp_ph111_B9
zmp_ph111_B10
zmp_ph114_G4
zmp_ph114_G11
zmp_ph114_H1
zmp_ph114_H2
zmp_ph114_A9
zmp_ph114_A11
zmp_ph114_B2
zmp_ph114_B4
zmp_ph114_D8
zmp_ph114_E2

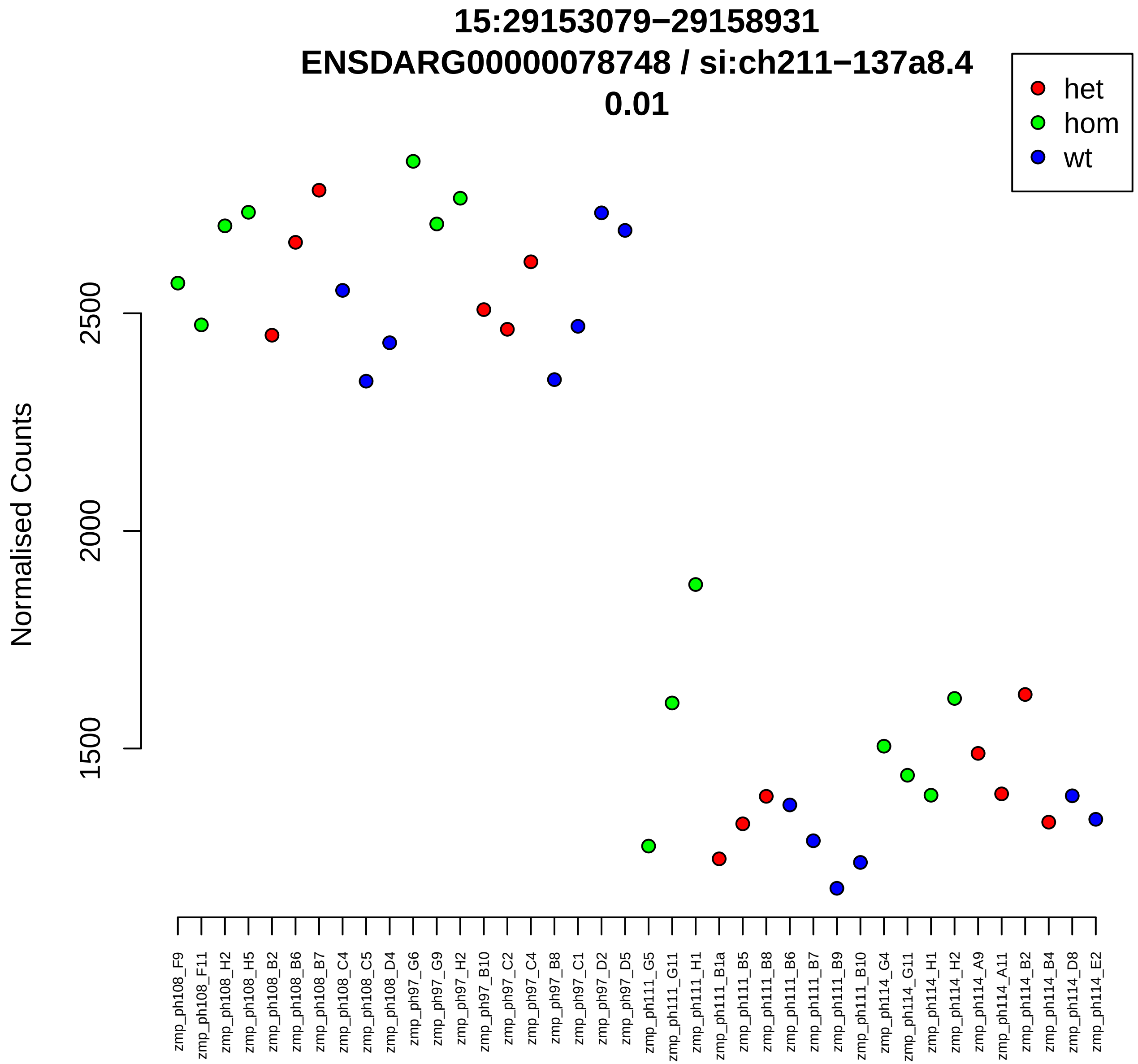

15:29153079−29158931
ENSDARG00000078748 / si:ch211−137a8.4
0.01
het
hom
wt
Normalised Counts
1500
2000
2500
zmp_ph108_F9
zmp_ph108_F11
zmp_ph108_H2
zmp_ph108_H5
zmp_ph108_B2
zmp_ph108_B6
zmp_ph108_B7
zmp_ph108_C4
zmp_ph108_C5
zmp_ph108_D4
zmp_ph97_G6
zmp_ph97_G9
zmp_ph97_H2
zmp_ph97_B10
zmp_ph97_C2
zmp_ph97_C4
zmp_ph97_B8
zmp_ph97_C1
zmp_ph97_D2
zmp_ph97_D5
zmp_ph111_G5
zmp_ph111_G11
zmp_ph111_H1
zmp_ph111_B1a
zmp_ph111_B5
zmp_ph111_B8
zmp_ph111_B6
zmp_ph111_B7
zmp_ph111_B9
zmp_ph111_B10
zmp_ph114_G4
zmp_ph114_G11
zmp_ph114_H1
zmp_ph114_H2
zmp_ph114_A9
zmp_ph114_A11
zmp_ph114_B2
zmp_ph114_B4
zmp_ph114_D8
zmp_ph114_E2

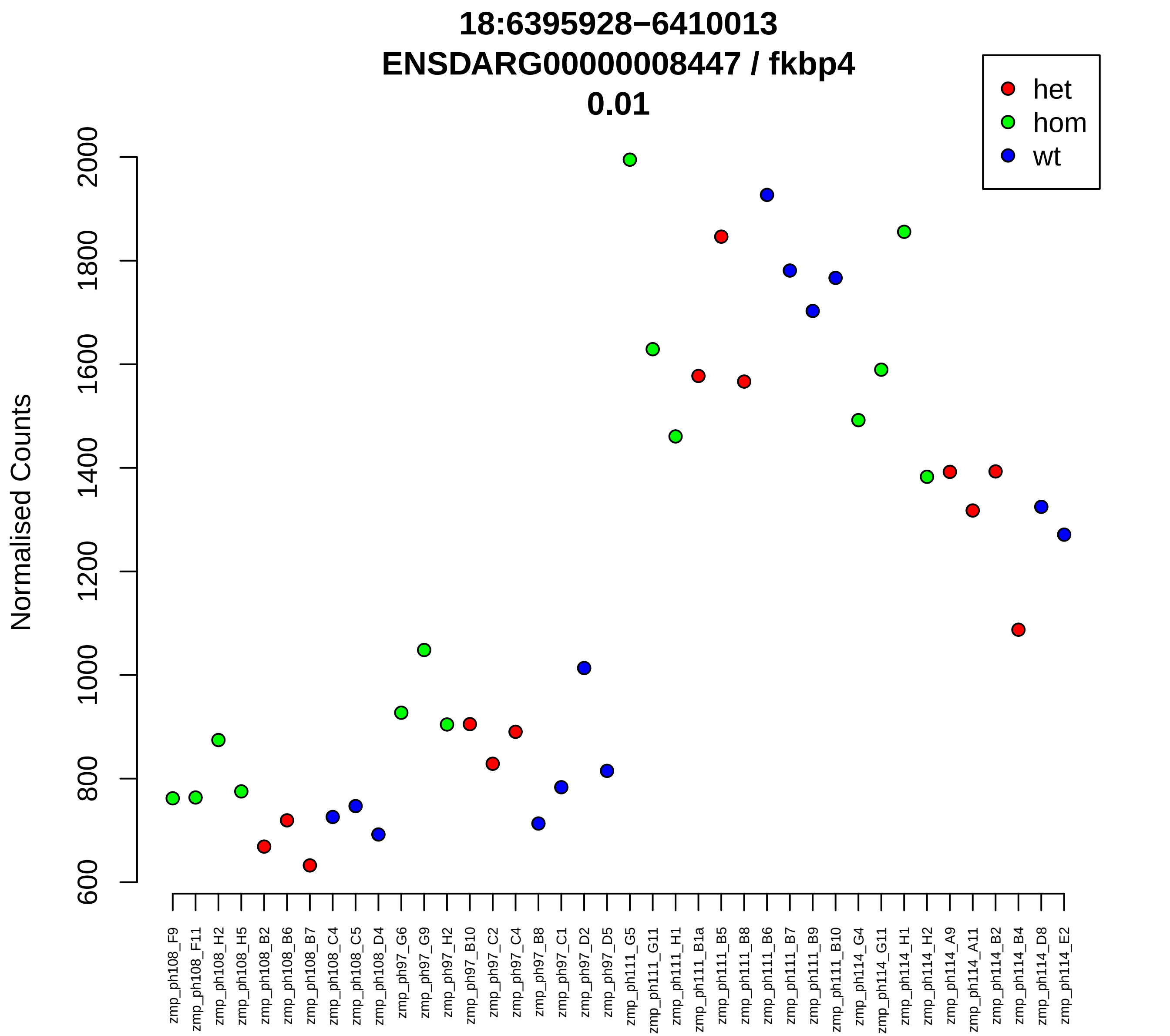
18:6395928−6410013
ENSDARG00000008447 / fkbp4
0.01
het
hom
wt
Normalised Counts
600
800
1000
1200
1400
1600
1800
2000
zmp_ph108_F9
zmp_ph108_F11
zmp_ph108_H2
zmp_ph108_H5
zmp_ph108_B2
zmp_ph108_B6
zmp_ph108_B7
zmp_ph108_C4
zmp_ph108_C5
zmp_ph108_D4
zmp_ph97_G6
zmp_ph97_G9
zmp_ph97_H2
zmp_ph97_B10
zmp_ph97_C2
zmp_ph97_C4
zmp_ph97_B8
zmp_ph97_C1
zmp_ph97_D2
zmp_ph97_D5
zmp_ph111_G5
zmp_ph111_G11
zmp_ph111_H1
zmp_ph111_B1a
zmp_ph111_B5
zmp_ph111_B8
zmp_ph111_B6
zmp_ph111_B7
zmp_ph111_B9
zmp_ph111_B10
zmp_ph114_G4
zmp_ph114_G11
zmp_ph114_H1
zmp_ph114_H2
zmp_ph114_A9
zmp_ph114_A11
zmp_ph114_B2
zmp_ph114_B4
zmp_ph114_D8
zmp_ph114_E2

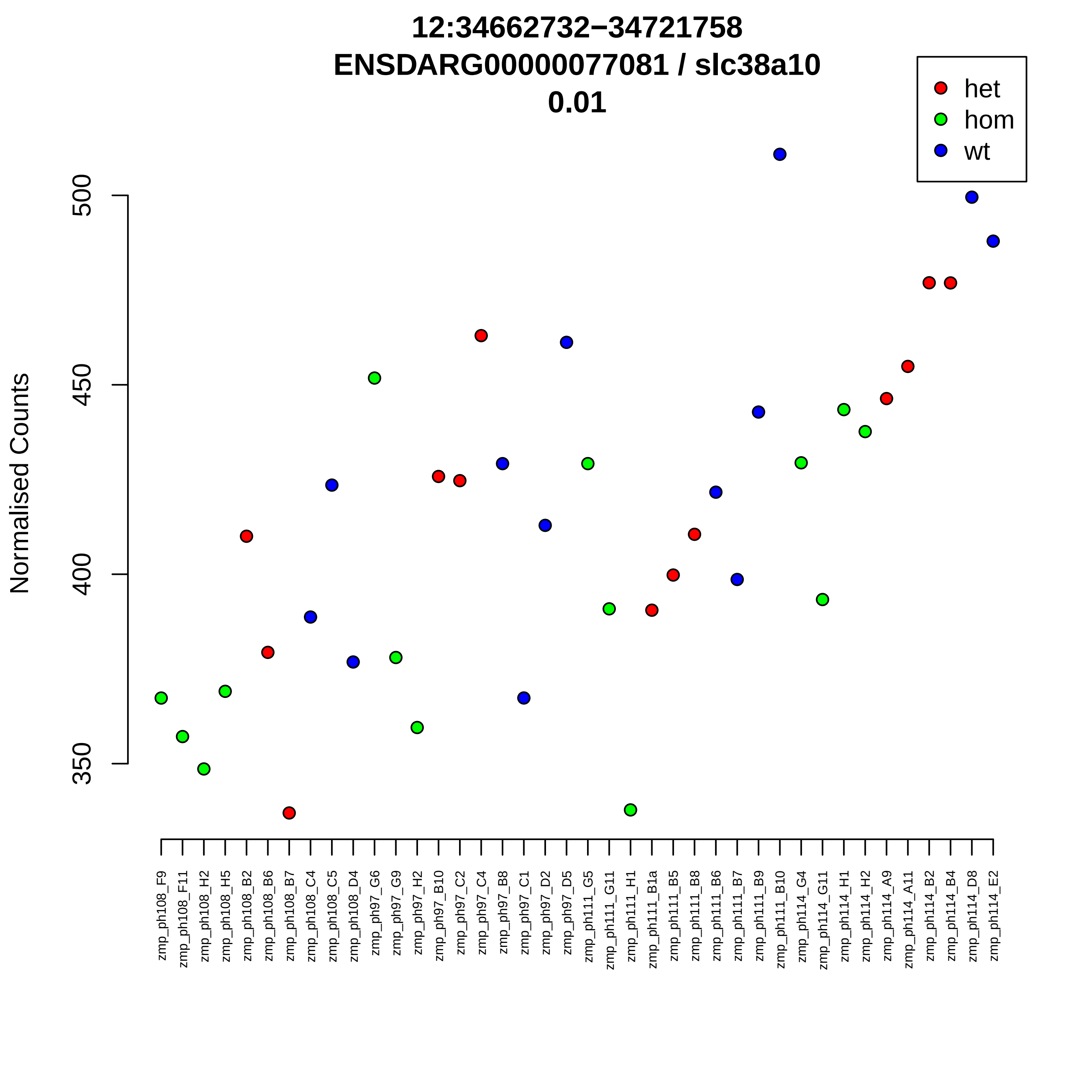
12:34662732−34721758
ENSDARG00000077081 / slc38a10
0.01
het
hom
wt
Normalised Counts
350
400
450
500
zmp_ph108_F9
zmp_ph108_F11
zmp_ph108_H2
zmp_ph108_H5
zmp_ph108_B2
zmp_ph108_B6
zmp_ph108_B7
zmp_ph108_C4
zmp_ph108_C5
zmp_ph108_D4
zmp_ph97_G6
zmp_ph97_G9
zmp_ph97_H2
zmp_ph97_B10
zmp_ph97_C2
zmp_ph97_C4
zmp_ph97_B8
zmp_ph97_C1
zmp_ph97_D2
zmp_ph97_D5
zmp_ph111_G5
zmp_ph111_G11
zmp_ph111_H1
zmp_ph111_B1a
zmp_ph111_B5
zmp_ph111_B8
zmp_ph111_B6
zmp_ph111_B7
zmp_ph111_B9
zmp_ph111_B10
zmp_ph114_G4
zmp_ph114_G11
zmp_ph114_H1
zmp_ph114_H2
zmp_ph114_A9
zmp_ph114_A11
zmp_ph114_B2
zmp_ph114_B4
zmp_ph114_D8
zmp_ph114_E2

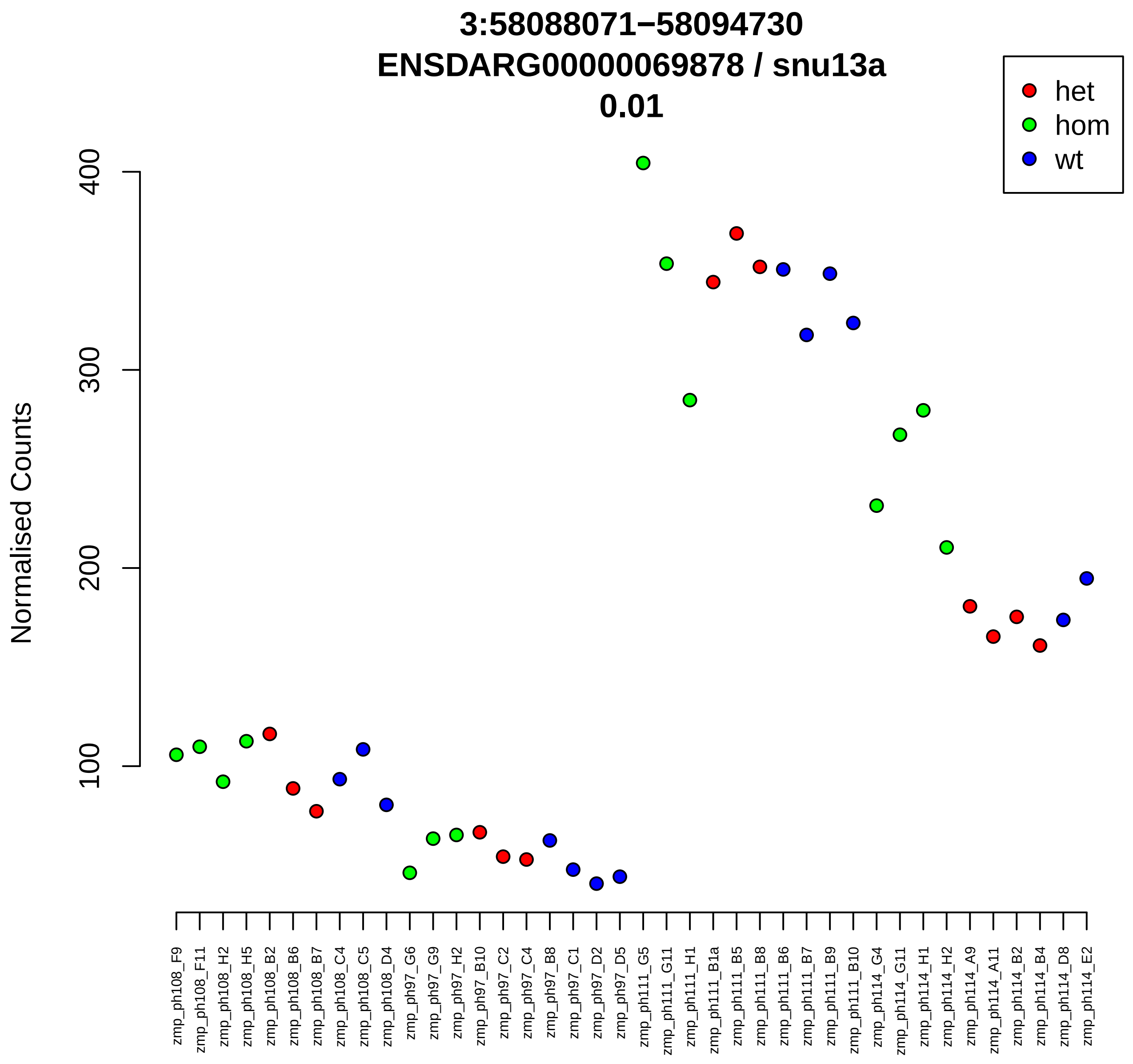

3:58088071−58094730
ENSDARG00000069878 / snu13a
0.01
het
hom
wt
Normalised Counts
100
200
300
400
zmp_ph108_F9
zmp_ph108_F11
zmp_ph108_H2
zmp_ph108_H5
zmp_ph108_B2
zmp_ph108_B6
zmp_ph108_B7
zmp_ph108_C4
zmp_ph108_C5
zmp_ph108_D4
zmp_ph97_G6
zmp_ph97_G9
zmp_ph97_H2
zmp_ph97_B10
zmp_ph97_C2
zmp_ph97_C4
zmp_ph97_B8
zmp_ph97_C1
zmp_ph97_D2
zmp_ph97_D5
zmp_ph111_G5
zmp_ph111_G11
zmp_ph111_H1
zmp_ph111_B1a
zmp_ph111_B5
zmp_ph111_B8
zmp_ph111_B6
zmp_ph111_B7
zmp_ph111_B9
zmp_ph111_B10
zmp_ph114_G4
zmp_ph114_G11
zmp_ph114_H1
zmp_ph114_H2
zmp_ph114_A9
zmp_ph114_A11
zmp_ph114_B2
zmp_ph114_B4
zmp_ph114_D8
zmp_ph114_E2

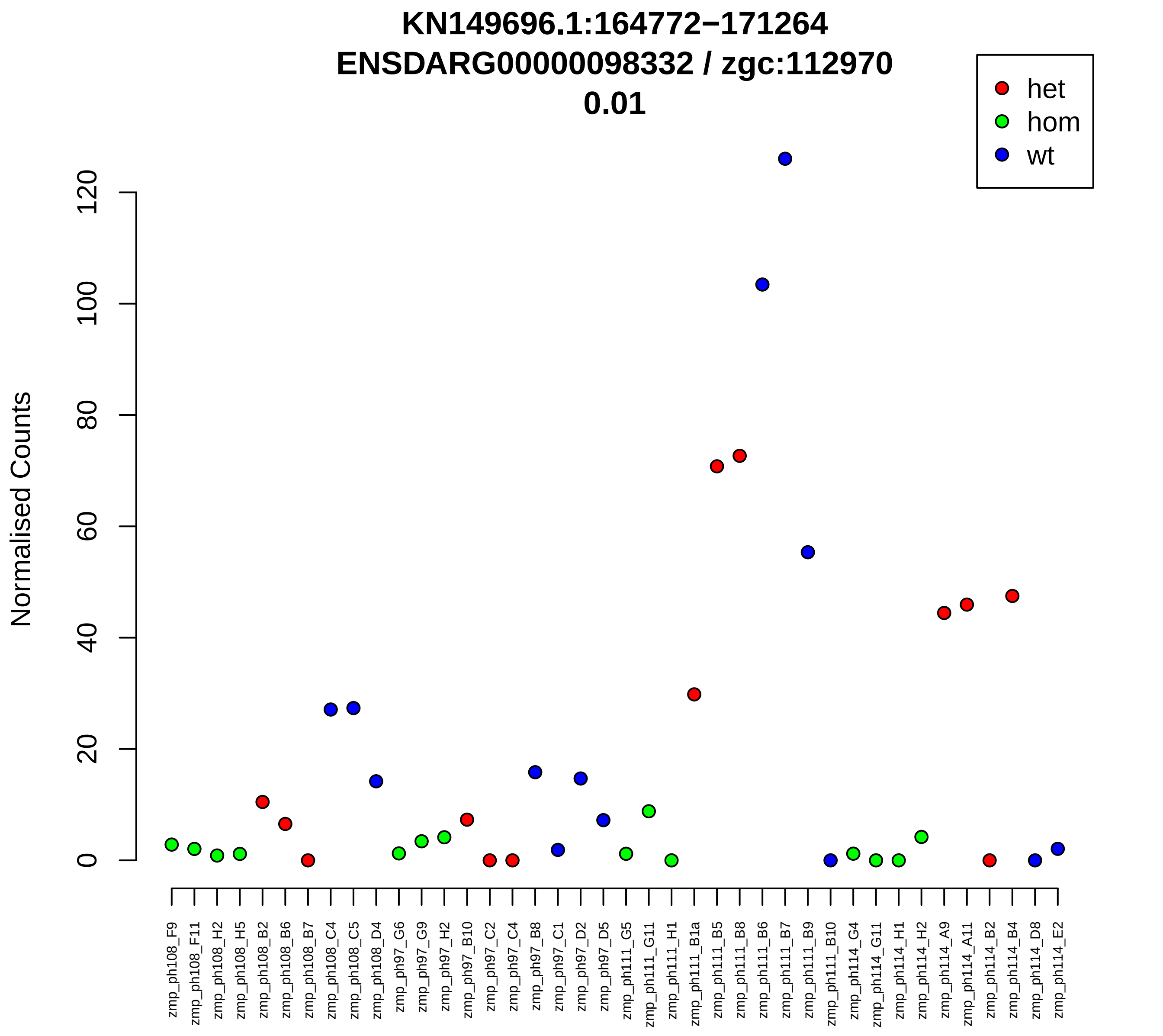
KN149696.1:164772−171264
ENSDARG00000098332 / zgc:112970
0.01
het
hom
wt
Normalised Counts
0
20
40
60
80
100
120
zmp_ph108_F9
zmp_ph108_F11
zmp_ph108_H2
zmp_ph108_H5
zmp_ph108_B2
zmp_ph108_B6
zmp_ph108_B7
zmp_ph108_C4
zmp_ph108_C5
zmp_ph108_D4
zmp_ph97_G6
zmp_ph97_G9
zmp_ph97_H2
zmp_ph97_B10
zmp_ph97_C2
zmp_ph97_C4
zmp_ph97_B8
zmp_ph97_C1
zmp_ph97_D2
zmp_ph97_D5
zmp_ph111_G5
zmp_ph111_G11
zmp_ph111_H1
zmp_ph111_B1a
zmp_ph111_B5
zmp_ph111_B8
zmp_ph111_B6
zmp_ph111_B7
zmp_ph111_B9
zmp_ph111_B10
zmp_ph114_G4
zmp_ph114_G11
zmp_ph114_H1
zmp_ph114_H2
zmp_ph114_A9
zmp_ph114_A11
zmp_ph114_B2
zmp_ph114_B4
zmp_ph114_D8
zmp_ph114_E2

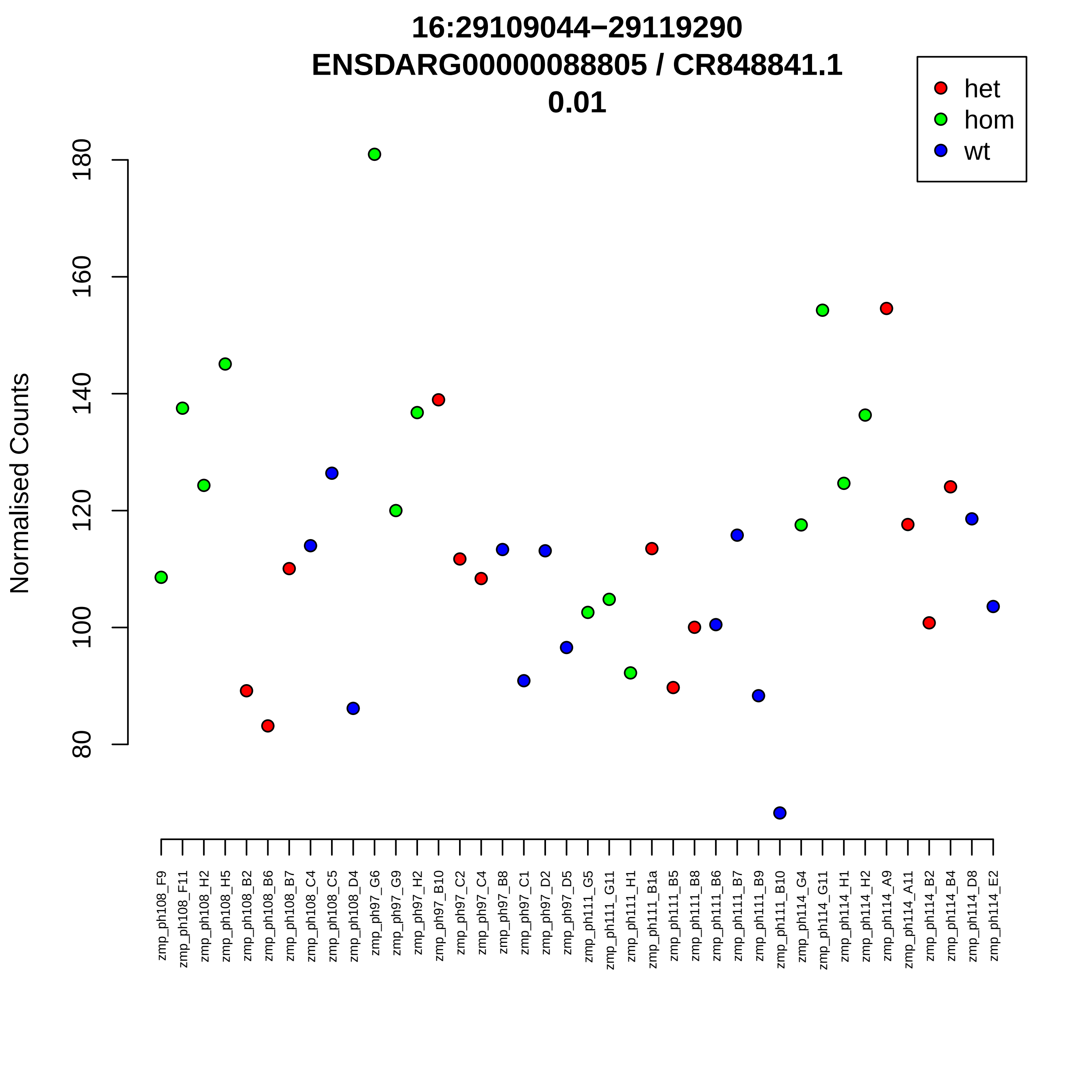
16:29109044−29119290
ENSDARG00000088805 / CR848841.1
0.01
het
hom
wt
Normalised Counts
80
100
120
140
160
180
zmp_ph108_F9
zmp_ph108_F11
zmp_ph108_H2
zmp_ph108_H5
zmp_ph108_B2
zmp_ph108_B6
zmp_ph108_B7
zmp_ph108_C4
zmp_ph108_C5
zmp_ph108_D4
zmp_ph97_G6
zmp_ph97_G9
zmp_ph97_H2
zmp_ph97_B10
zmp_ph97_C2
zmp_ph97_C4
zmp_ph97_B8
zmp_ph97_C1
zmp_ph97_D2
zmp_ph97_D5
zmp_ph111_G5
zmp_ph111_G11
zmp_ph111_H1
zmp_ph111_B1a
zmp_ph111_B5
zmp_ph111_B8
zmp_ph111_B6
zmp_ph111_B7
zmp_ph111_B9
zmp_ph111_B10
zmp_ph114_G4
zmp_ph114_G11
zmp_ph114_H1
zmp_ph114_H2
zmp_ph114_A9
zmp_ph114_A11
zmp_ph114_B2
zmp_ph114_B4
zmp_ph114_D8
zmp_ph114_E2

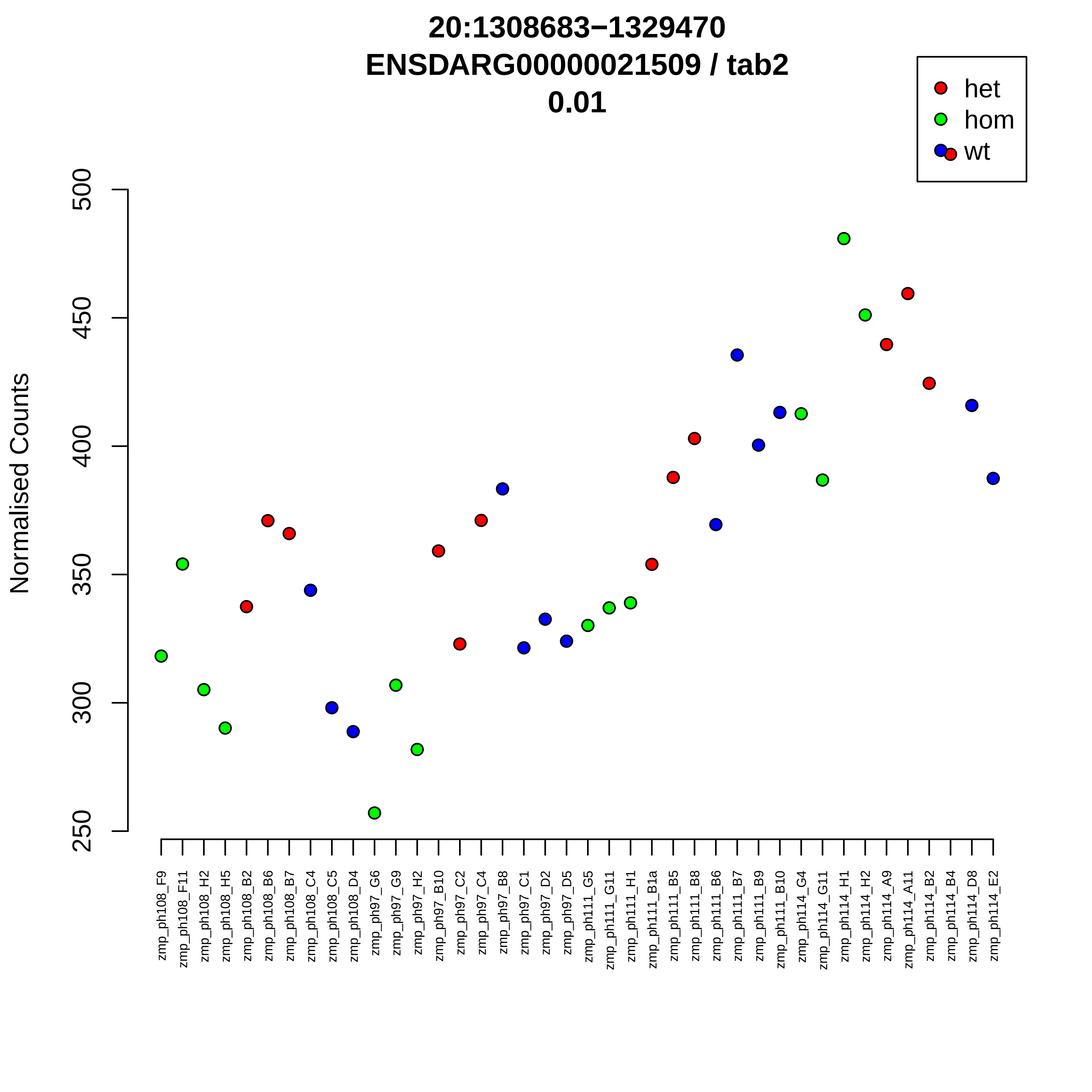

20:1308683−1329470
ENSDARG00000021509 / tab2
0.01
het
hom
wt
Normalised Counts
250
300
350
400
450
500
zmp_ph108_F9
zmp_ph108_F11
zmp_ph108_H2
zmp_ph108_H5
zmp_ph108_B2
zmp_ph108_B6
zmp_ph108_B7
zmp_ph108_C4
zmp_ph108_C5
zmp_ph108_D4
zmp_ph97_G6
zmp_ph97_G9
zmp_ph97_H2
zmp_ph97_B10
zmp_ph97_C2
zmp_ph97_C4
zmp_ph97_B8
zmp_ph97_C1
zmp_ph97_D2
zmp_ph97_D5
zmp_ph111_G5
zmp_ph111_G11
zmp_ph111_H1
zmp_ph111_B1a
zmp_ph111_B5
zmp_ph111_B8
zmp_ph111_B6
zmp_ph111_B7
zmp_ph111_B9
zmp_ph111_B10
zmp_ph114_G4
zmp_ph114_G11
zmp_ph114_H1
zmp_ph114_H2
zmp_ph114_A9
zmp_ph114_A11
zmp_ph114_B2
zmp_ph114_B4
zmp_ph114_D8
zmp_ph114_E2

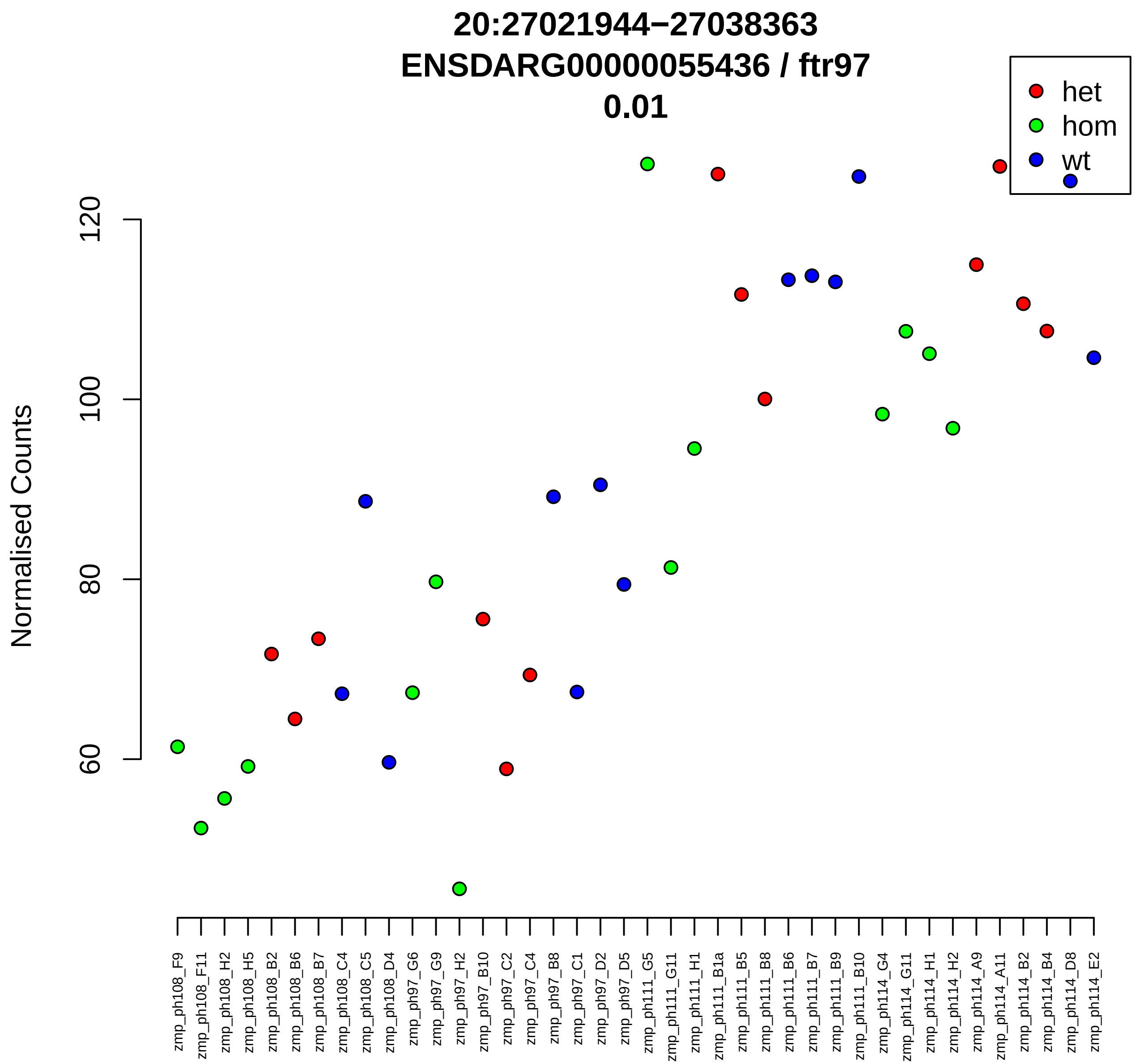
20:27021944−27038363
ENSDARG00000055436 / ftr97
0.01
het
hom
wt
Normalised Counts
60
80
100
120
zmp_ph108_F9
zmp_ph108_F11
zmp_ph108_H2
zmp_ph108_H5
zmp_ph108_B2
zmp_ph108_B6
zmp_ph108_B7
zmp_ph108_C4
zmp_ph108_C5
zmp_ph108_D4
zmp_ph97_G6
zmp_ph97_G9
zmp_ph97_H2
zmp_ph97_B10
zmp_ph97_C2
zmp_ph97_C4
zmp_ph97_B8
zmp_ph97_C1
zmp_ph97_D2
zmp_ph97_D5
zmp_ph111_G5
zmp_ph111_G11
zmp_ph111_H1
zmp_ph111_B1a
zmp_ph111_B5
zmp_ph111_B8
zmp_ph111_B6
zmp_ph111_B7
zmp_ph111_B9
zmp_ph111_B10
zmp_ph114_G4
zmp_ph114_G11
zmp_ph114_H1
zmp_ph114_H2
zmp_ph114_A9
zmp_ph114_A11
zmp_ph114_B2
zmp_ph114_B4
zmp_ph114_D8
zmp_ph114_E2

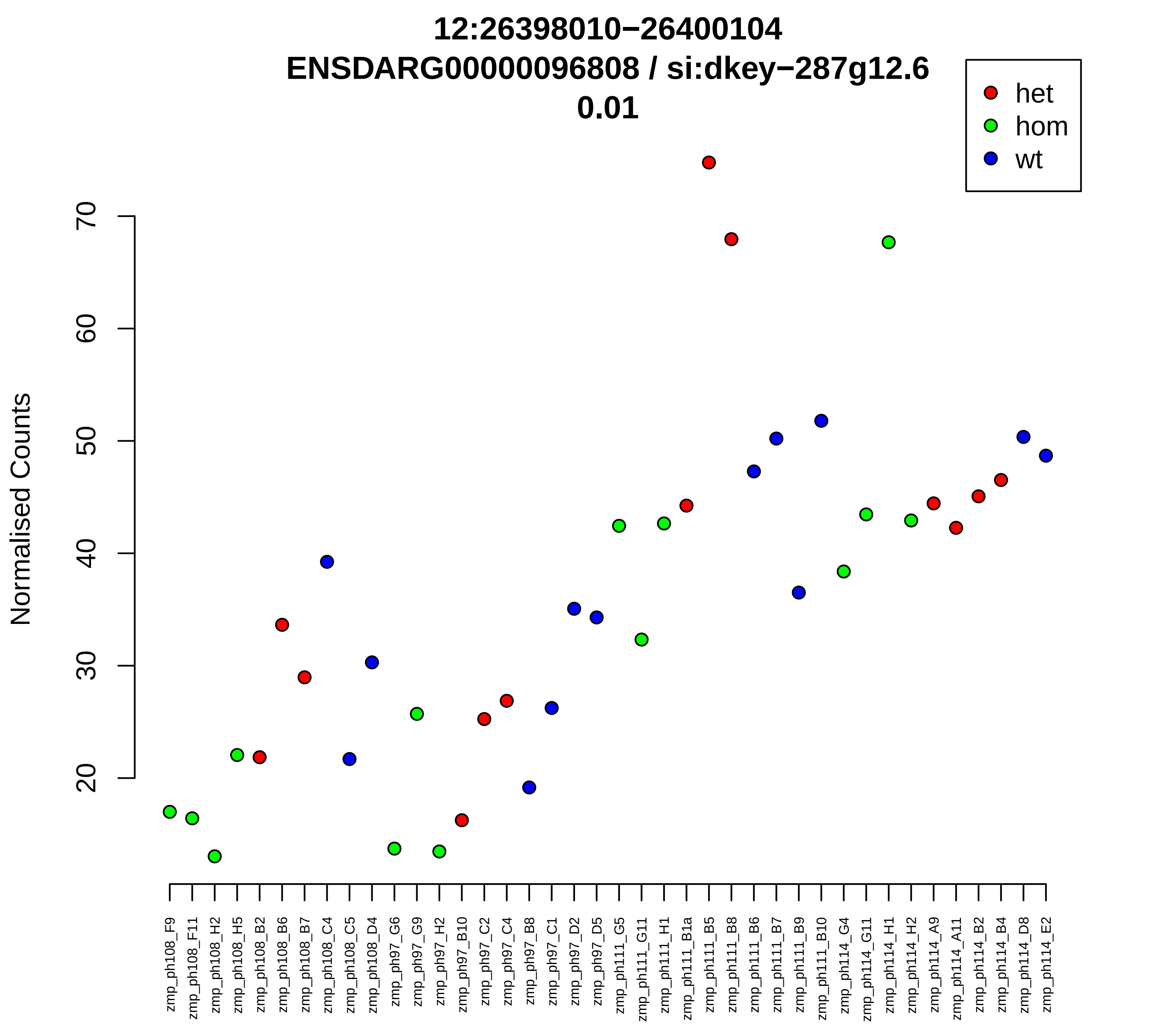
12:26398010−26400104
ENSDARG00000096808 / si:dkey−287g12.6
0.01
het
hom
wt
Normalised Counts
20
30
40
50
60
70
zmp_ph108_F9
zmp_ph108_F11
zmp_ph108_H2
zmp_ph108_H5
zmp_ph108_B2
zmp_ph108_B6
zmp_ph108_B7
zmp_ph108_C4
zmp_ph108_C5
zmp_ph108_D4
zmp_ph97_G6
zmp_ph97_G9
zmp_ph97_H2
zmp_ph97_B10
zmp_ph97_C2
zmp_ph97_C4
zmp_ph97_B8
zmp_ph97_C1
zmp_ph97_D2
zmp_ph97_D5
zmp_ph111_G5
zmp_ph111_G11
zmp_ph111_H1
zmp_ph111_B1a
zmp_ph111_B5
zmp_ph111_B8
zmp_ph111_B6
zmp_ph111_B7
zmp_ph111_B9
zmp_ph111_B10
zmp_ph114_G4
zmp_ph114_G11
zmp_ph114_H1
zmp_ph114_H2
zmp_ph114_A9
zmp_ph114_A11
zmp_ph114_B2
zmp_ph114_B4
zmp_ph114_D8
zmp_ph114_E2

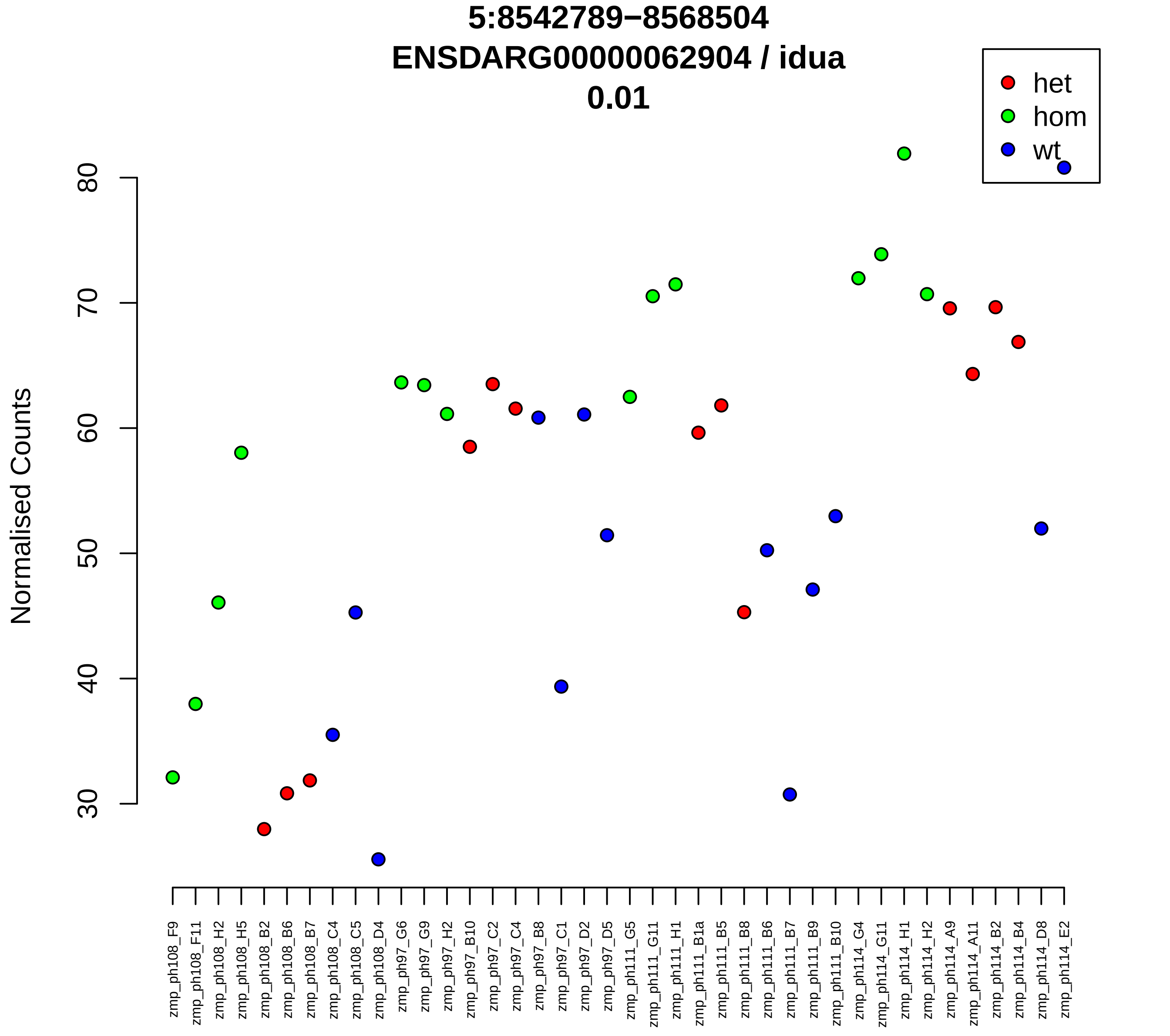
5:8542789−8568504
ENSDARG00000062904 / idua
0.01
het
hom
wt
Normalised Counts
30
40
50
60
70
80
zmp_ph108_F9
zmp_ph108_F11
zmp_ph108_H2
zmp_ph108_H5
zmp_ph108_B2
zmp_ph108_B6
zmp_ph108_B7
zmp_ph108_C4
zmp_ph108_C5
zmp_ph108_D4
zmp_ph97_G6
zmp_ph97_G9
zmp_ph97_H2
zmp_ph97_B10
zmp_ph97_C2
zmp_ph97_C4
zmp_ph97_B8
zmp_ph97_C1
zmp_ph97_D2
zmp_ph97_D5
zmp_ph111_G5
zmp_ph111_G11
zmp_ph111_H1
zmp_ph111_B1a
zmp_ph111_B5
zmp_ph111_B8
zmp_ph111_B6
zmp_ph111_B7
zmp_ph111_B9
zmp_ph111_B10
zmp_ph114_G4
zmp_ph114_G11
zmp_ph114_H1
zmp_ph114_H2
zmp_ph114_A9
zmp_ph114_A11
zmp_ph114_B2
zmp_ph114_B4
zmp_ph114_D8
zmp_ph114_E2

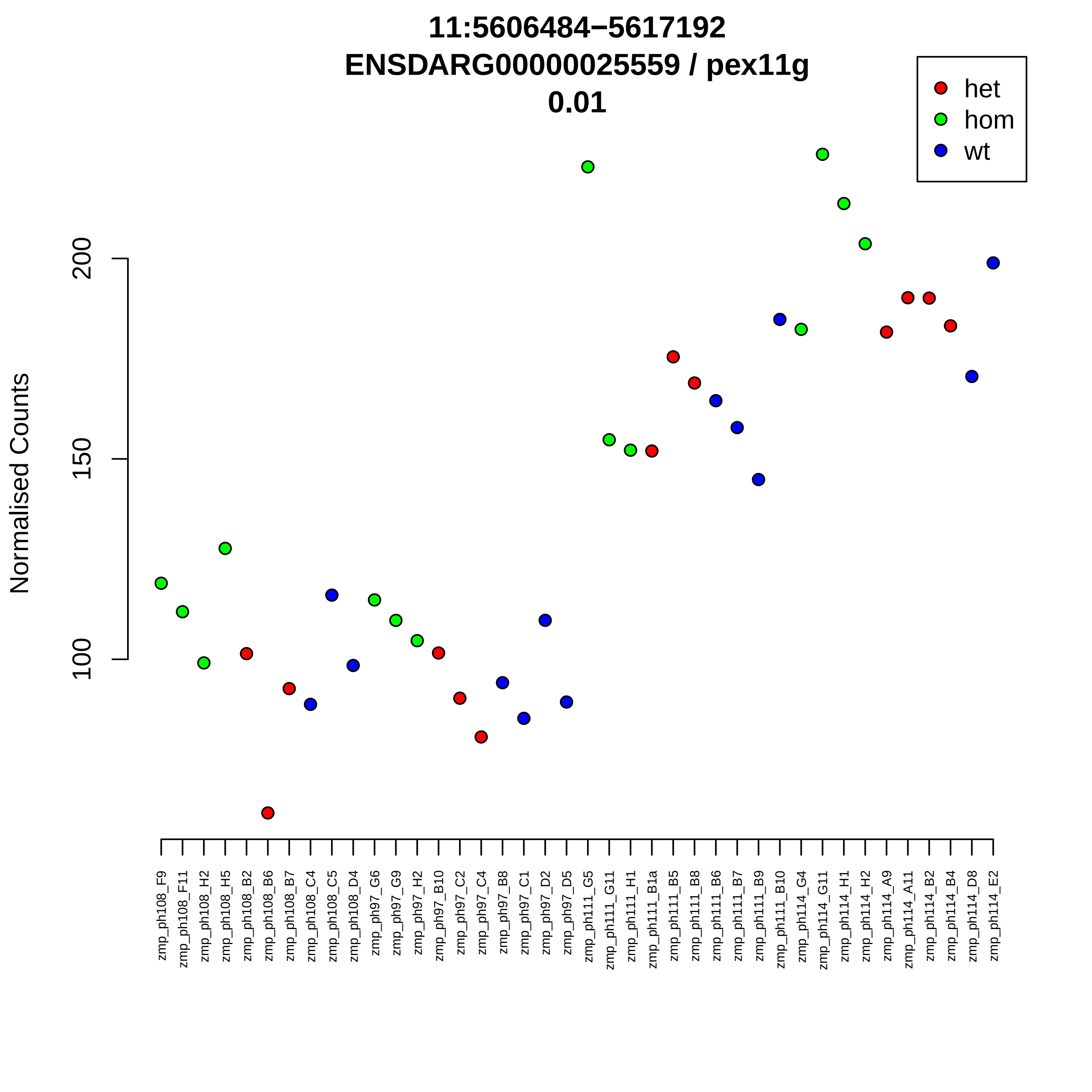

11:5606484−5617192
ENSDARG00000025559 / pex11g
0.01
het
hom
wt
Normalised Counts
100
150
200
zmp_ph108_F9
zmp_ph108_F11
zmp_ph108_H2
zmp_ph108_H5
zmp_ph108_B2
zmp_ph108_B6
zmp_ph108_B7
zmp_ph108_C4
zmp_ph108_C5
zmp_ph108_D4
zmp_ph97_G6
zmp_ph97_G9
zmp_ph97_H2
zmp_ph97_B10
zmp_ph97_C2
zmp_ph97_C4
zmp_ph97_B8
zmp_ph97_C1
zmp_ph97_D2
zmp_ph97_D5
zmp_ph111_G5
zmp_ph111_G11
zmp_ph111_H1
zmp_ph111_B1a
zmp_ph111_B5
zmp_ph111_B8
zmp_ph111_B6
zmp_ph111_B7
zmp_ph111_B9
zmp_ph111_B10
zmp_ph114_G4
zmp_ph114_G11
zmp_ph114_H1
zmp_ph114_H2
zmp_ph114_A9
zmp_ph114_A11
zmp_ph114_B2
zmp_ph114_B4
zmp_ph114_D8
zmp_ph114_E2

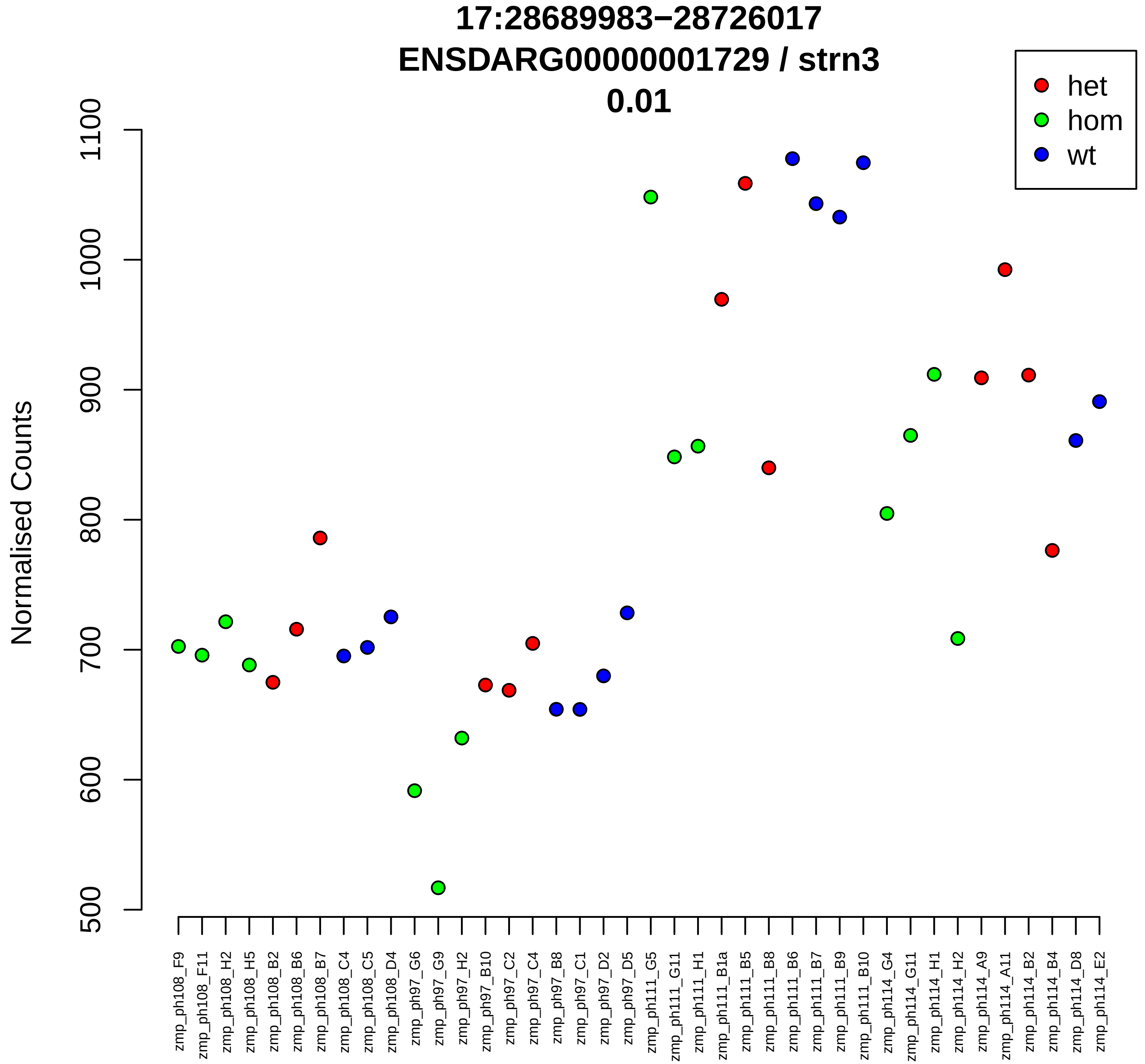
17:28689983−28726017
ENSDARG00000001729 / strn3
0.01
Normalised Counts
500
600
700
800
900
1000
1100
het
hom
wt
zmp_ph108_F9
zmp_ph108_F11
zmp_ph108_H2
zmp_ph108_H5
zmp_ph108_B2
zmp_ph108_B6
zmp_ph108_B7
zmp_ph108_C4
zmp_ph108_C5
zmp_ph108_D4
zmp_ph97_G6
zmp_ph97_G9
zmp_ph97_H2
zmp_ph97_B10
zmp_ph97_C2
zmp_ph97_C4
zmp_ph97_B8
zmp_ph97_C1
zmp_ph97_D2
zmp_ph97_D5
zmp_ph111_G5
zmp_ph111_G11
zmp_ph111_H1
zmp_ph111_B1a
zmp_ph111_B5
zmp_ph111_B8
zmp_ph111_B6
zmp_ph111_B7
zmp_ph111_B9
zmp_ph111_B10
zmp_ph114_G4
zmp_ph114_G11
zmp_ph114_H1
zmp_ph114_H2
zmp_ph114_A9
zmp_ph114_A11
zmp_ph114_B2
zmp_ph114_B4
zmp_ph114_D8
zmp_ph114_E2

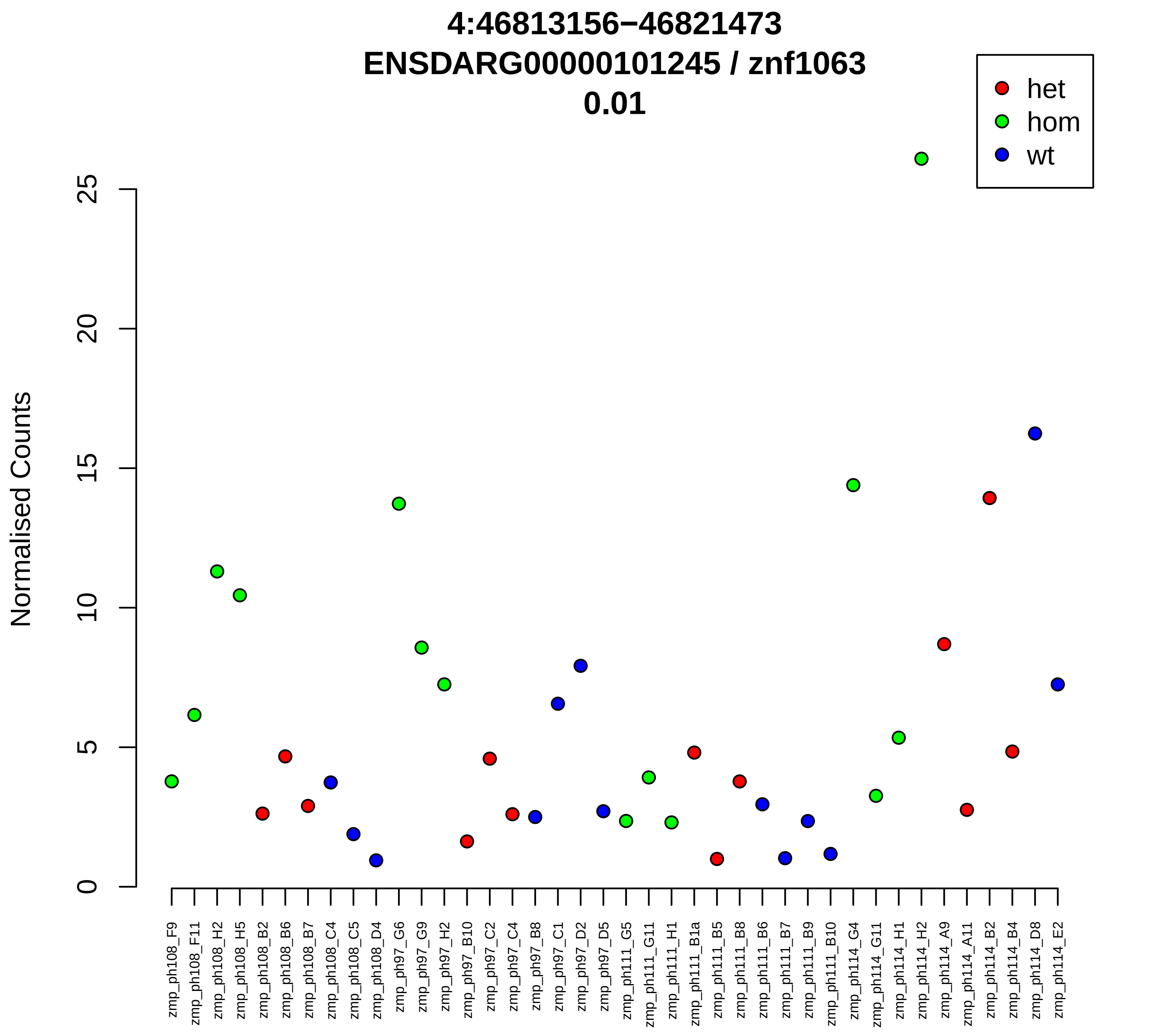
4:46813156−46821473
ENSDARG00000101245 / znf1063
0.01
het
hom
wt
Normalised Counts
0
5
10
15
20
25
zmp_ph108_F9
zmp_ph108_F11
zmp_ph108_H2
zmp_ph108_H5
zmp_ph108_B2
zmp_ph108_B6
zmp_ph108_B7
zmp_ph108_C4
zmp_ph108_C5
zmp_ph108_D4
zmp_ph97_G6
zmp_ph97_G9
zmp_ph97_H2
zmp_ph97_B10
zmp_ph97_C2
zmp_ph97_C4
zmp_ph97_B8
zmp_ph97_C1
zmp_ph97_D2
zmp_ph97_D5
zmp_ph111_G5
zmp_ph111_G11
zmp_ph111_H1
zmp_ph111_B1a
zmp_ph111_B5
zmp_ph111_B8
zmp_ph111_B6
zmp_ph111_B7
zmp_ph111_B9
zmp_ph111_B10
zmp_ph114_G4
zmp_ph114_G11
zmp_ph114_H1
zmp_ph114_H2
zmp_ph114_A9
zmp_ph114_A11
zmp_ph114_B2
zmp_ph114_B4
zmp_ph114_D8
zmp_ph114_E2

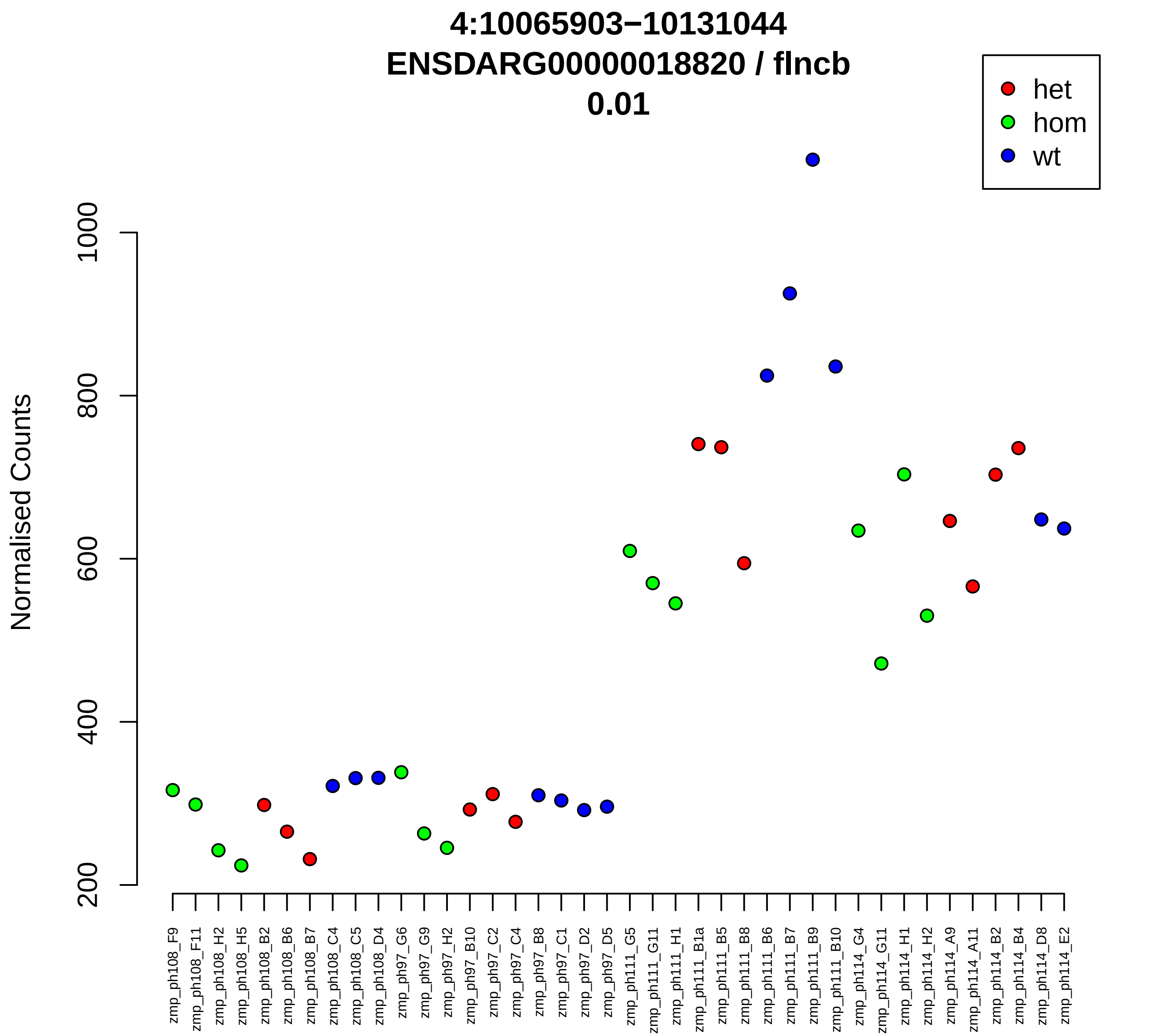
4:10065903−10131044
ENSDARG00000018820 / flncb
0.01
het
hom
wt
Normalised Counts
200
400
600
800
1000
zmp_ph108_F9
zmp_ph108_F11
zmp_ph108_H2
zmp_ph108_H5
zmp_ph108_B2
zmp_ph108_B6
zmp_ph108_B7
zmp_ph108_C4
zmp_ph108_C5
zmp_ph108_D4
zmp_ph97_G6
zmp_ph97_G9
zmp_ph97_H2
zmp_ph97_B10
zmp_ph97_C2
zmp_ph97_C4
zmp_ph97_B8
zmp_ph97_C1
zmp_ph97_D2
zmp_ph97_D5
zmp_ph111_G5
zmp_ph111_G11
zmp_ph111_H1
zmp_ph111_B1a
zmp_ph111_B5
zmp_ph111_B8
zmp_ph111_B6
zmp_ph111_B7
zmp_ph111_B9
zmp_ph111_B10
zmp_ph114_G4
zmp_ph114_G11
zmp_ph114_H1
zmp_ph114_H2
zmp_ph114_A9
zmp_ph114_A11
zmp_ph114_B2
zmp_ph114_B4
zmp_ph114_D8
zmp_ph114_E2

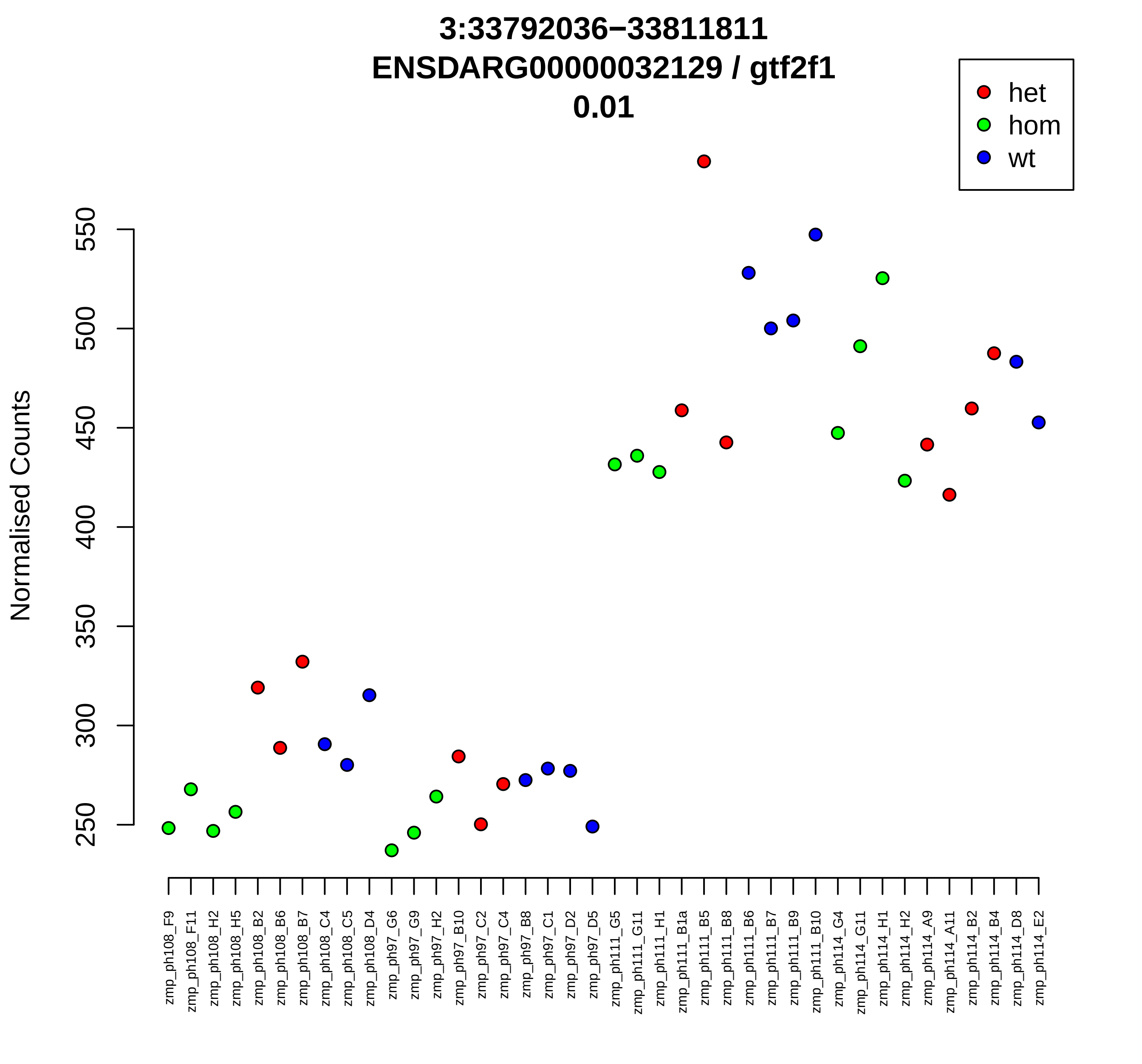

3:33792036−33811811
ENSDARG00000032129 / gtf2f1
0.01
het
hom
wt
Normalised Counts
250
300
350
400
450
500
550
zmp_ph108_F9
zmp_ph108_F11
zmp_ph108_H2
zmp_ph108_H5
zmp_ph108_B2
zmp_ph108_B6
zmp_ph108_B7
zmp_ph108_C4
zmp_ph108_C5
zmp_ph108_D4
zmp_ph97_G6
zmp_ph97_G9
zmp_ph97_H2
zmp_ph97_B10
zmp_ph97_C2
zmp_ph97_C4
zmp_ph97_B8
zmp_ph97_C1
zmp_ph97_D2
zmp_ph97_D5
zmp_ph111_G5
zmp_ph111_G11
zmp_ph111_H1
zmp_ph111_B1a
zmp_ph111_B5
zmp_ph111_B8
zmp_ph111_B6
zmp_ph111_B7
zmp_ph111_B9
zmp_ph111_B10
zmp_ph114_G4
zmp_ph114_G11
zmp_ph114_H1
zmp_ph114_H2
zmp_ph114_A9
zmp_ph114_A11
zmp_ph114_B2
zmp_ph114_B4
zmp_ph114_D8
zmp_ph114_E2

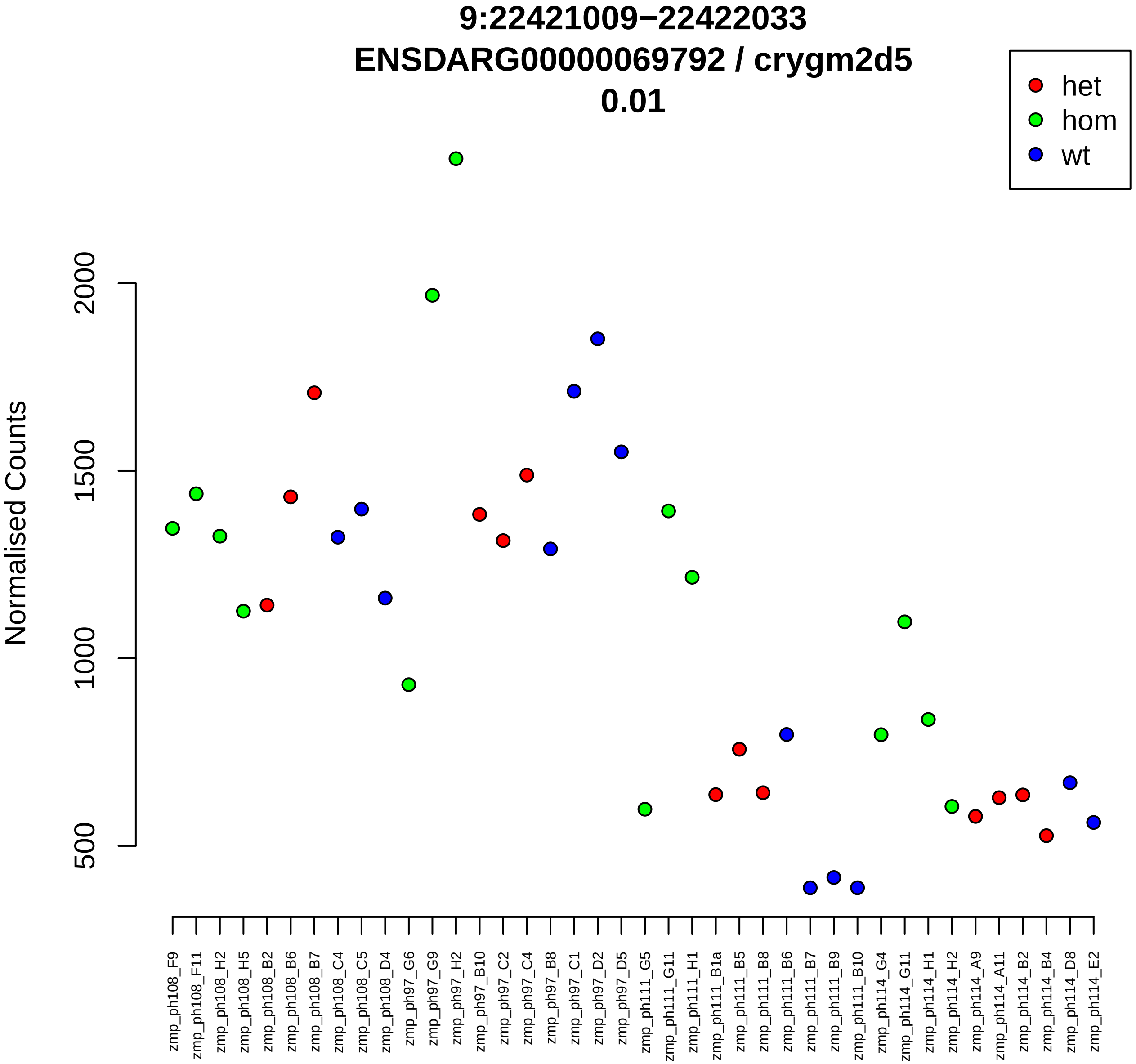
9:22421009−22422033
ENSDARG00000069792 / crygm2d5
0.01
het
hom
wt
Normalised Counts
500
1000
1500
2000
zmp_ph108_F9
zmp_ph108_F11
zmp_ph108_H2
zmp_ph108_H5
zmp_ph108_B2
zmp_ph108_B6
zmp_ph108_B7
zmp_ph108_C4
zmp_ph108_C5
zmp_ph108_D4
zmp_ph97_G6
zmp_ph97_G9
zmp_ph97_H2
zmp_ph97_B10
zmp_ph97_C2
zmp_ph97_C4
zmp_ph97_B8
zmp_ph97_C1
zmp_ph97_D2
zmp_ph97_D5
zmp_ph111_G5
zmp_ph111_G11
zmp_ph111_H1
zmp_ph111_B1a
zmp_ph111_B5
zmp_ph111_B8
zmp_ph111_B6
zmp_ph111_B7
zmp_ph111_B9
zmp_ph111_B10
zmp_ph114_G4
zmp_ph114_G11
zmp_ph114_H1
zmp_ph114_H2
zmp_ph114_A9
zmp_ph114_A11
zmp_ph114_B2
zmp_ph114_B4
zmp_ph114_D8
zmp_ph114_E2

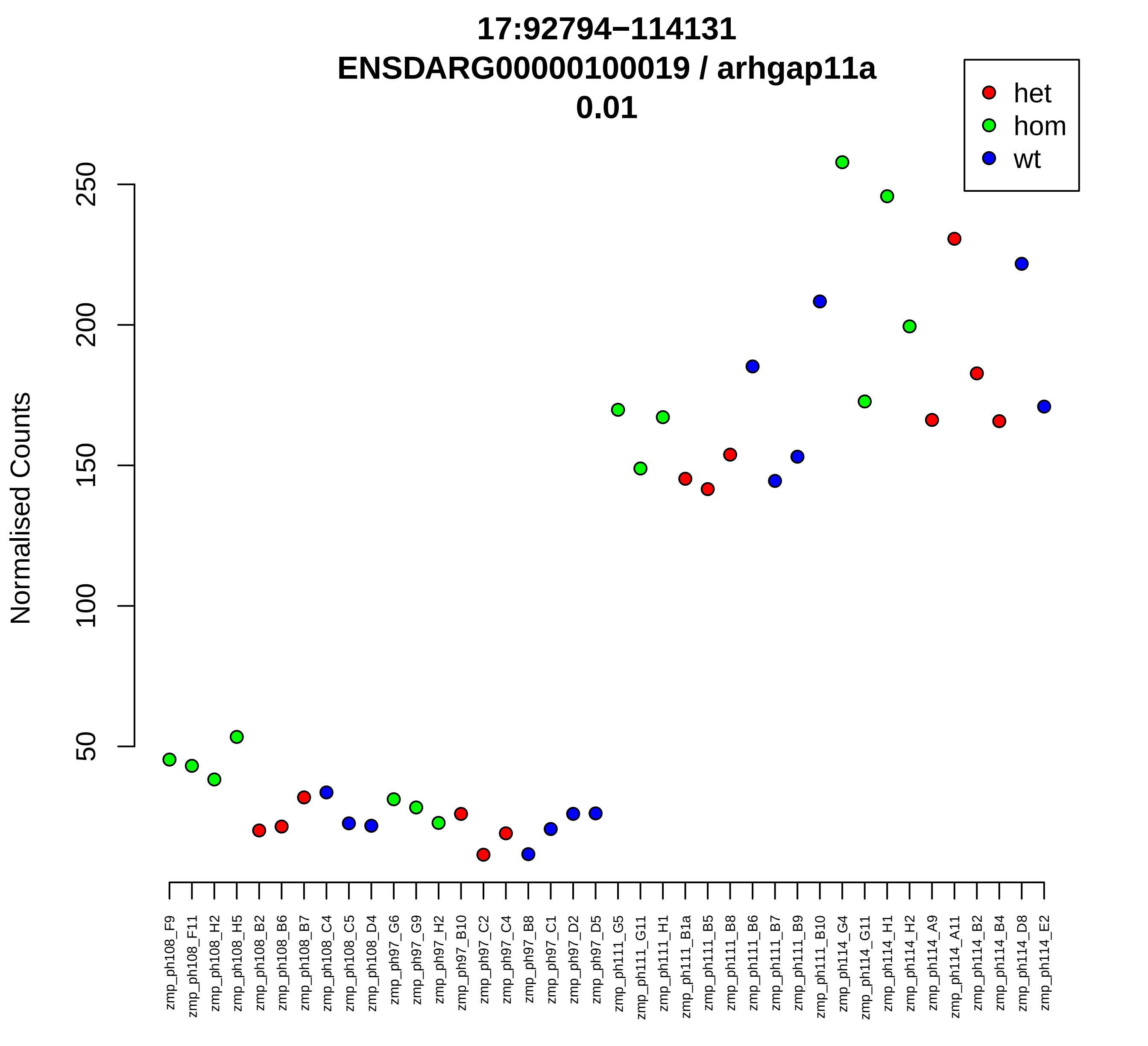

17:92794−114131
ENSDARG00000100019 / arhgap11a
0.01
het
hom
wt
Normalised Counts
50
100
150
200
250
zmp_ph108_F9
zmp_ph108_F11
zmp_ph108_H2
zmp_ph108_H5
zmp_ph108_B2
zmp_ph108_B6
zmp_ph108_B7
zmp_ph108_C4
zmp_ph108_C5
zmp_ph108_D4
zmp_ph97_G6
zmp_ph97_G9
zmp_ph97_H2
zmp_ph97_B10
zmp_ph97_C2
zmp_ph97_C4
zmp_ph97_B8
zmp_ph97_C1
zmp_ph97_D2
zmp_ph97_D5
zmp_ph111_G5
zmp_ph111_G11
zmp_ph111_H1
zmp_ph111_B1a
zmp_ph111_B5
zmp_ph111_B8
zmp_ph111_B6
zmp_ph111_B7
zmp_ph111_B9
zmp_ph111_B10
zmp_ph114_G4
zmp_ph114_G11
zmp_ph114_H1
zmp_ph114_H2
zmp_ph114_A9
zmp_ph114_A11
zmp_ph114_B2
zmp_ph114_B4
zmp_ph114_D8
zmp_ph114_E2

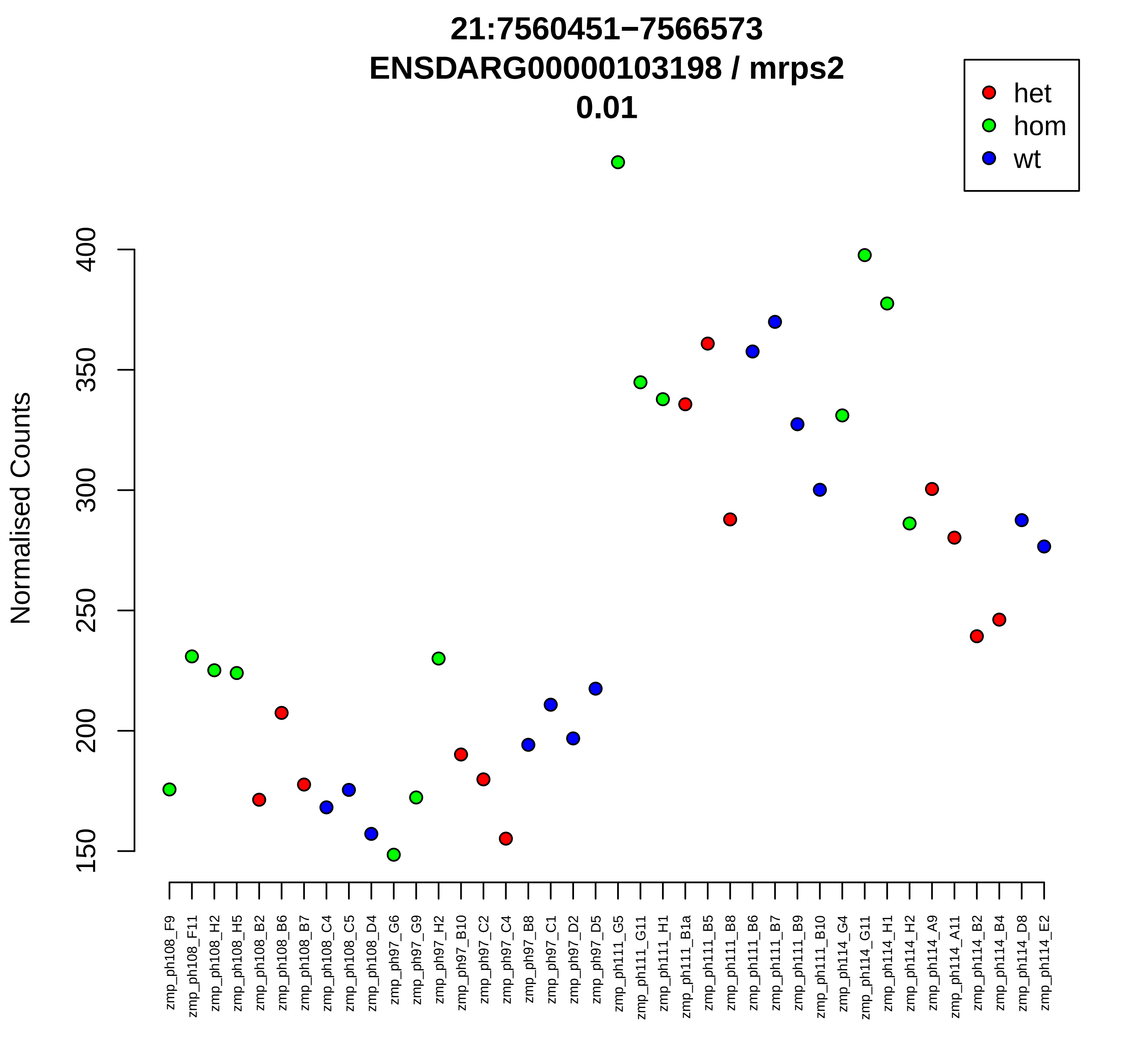
21:7560451−7566573
ENSDARG00000103198 / mrps2
0.01
het
hom
wt
Normalised Counts
150
200
250
300
350
400
zmp_ph108_F9
zmp_ph108_F11
zmp_ph108_H2
zmp_ph108_H5
zmp_ph108_B2
zmp_ph108_B6
zmp_ph108_B7
zmp_ph108_C4
zmp_ph108_C5
zmp_ph108_D4
zmp_ph97_G6
zmp_ph97_G9
zmp_ph97_H2
zmp_ph97_B10
zmp_ph97_C2
zmp_ph97_C4
zmp_ph97_B8
zmp_ph97_C1
zmp_ph97_D2
zmp_ph97_D5
zmp_ph111_G5
zmp_ph111_G11
zmp_ph111_H1
zmp_ph111_B1a
zmp_ph111_B5
zmp_ph111_B8
zmp_ph111_B6
zmp_ph111_B7
zmp_ph111_B9
zmp_ph111_B10
zmp_ph114_G4
zmp_ph114_G11
zmp_ph114_H1
zmp_ph114_H2
zmp_ph114_A9
zmp_ph114_A11
zmp_ph114_B2
zmp_ph114_B4
zmp_ph114_D8
zmp_ph114_E2

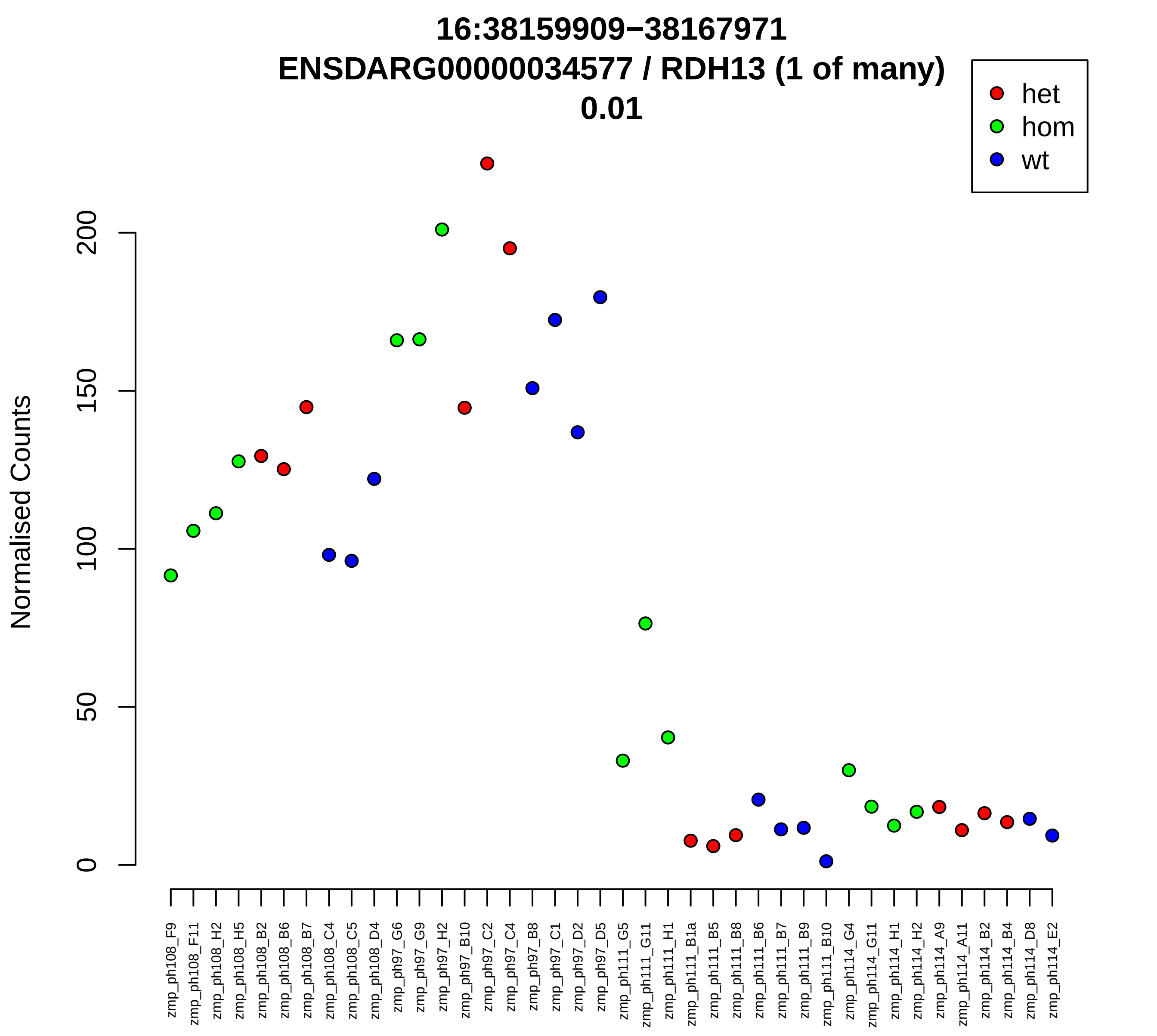

16:38159909−38167971
ENSDARG00000034577 / RDH13 (1 of many)
0.01
het
hom
wt
Normalised Counts
0
50
100
150
200
zmp_ph108_F9
zmp_ph108_F11
zmp_ph108_H2
zmp_ph108_H5
zmp_ph108_B2
zmp_ph108_B6
zmp_ph108_B7
zmp_ph108_C4
zmp_ph108_C5
zmp_ph108_D4
zmp_ph97_G6
zmp_ph97_G9
zmp_ph97_H2
zmp_ph97_B10
zmp_ph97_C2
zmp_ph97_C4
zmp_ph97_B8
zmp_ph97_C1
zmp_ph97_D2
zmp_ph97_D5
zmp_ph111_G5
zmp_ph111_G11
zmp_ph111_H1
zmp_ph111_B1a
zmp_ph111_B5
zmp_ph111_B8
zmp_ph111_B6
zmp_ph111_B7
zmp_ph111_B9
zmp_ph111_B10
zmp_ph114_G4
zmp_ph114_G11
zmp_ph114_H1
zmp_ph114_H2
zmp_ph114_A9
zmp_ph114_A11
zmp_ph114_B2
zmp_ph114_B4
zmp_ph114_D8
zmp_ph114_E2

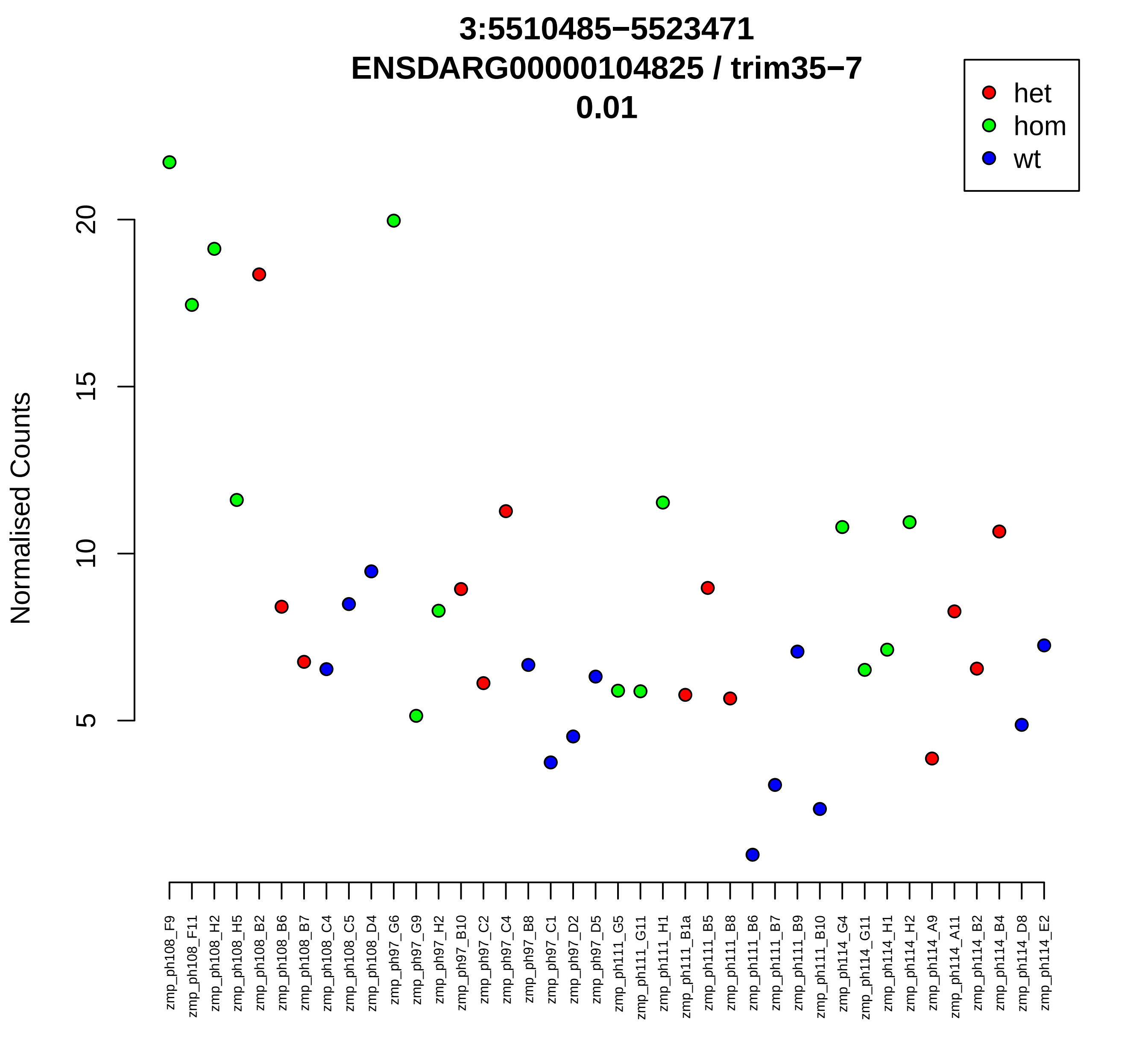

3:5510485−5523471
ENSDARG00000104825 / trim35−7
0.01
het
hom
wt
Normalised Counts
5
10
15
20
zmp_ph108_F9
zmp_ph108_F11
zmp_ph108_H2
zmp_ph108_H5
zmp_ph108_B2
zmp_ph108_B6
zmp_ph108_B7
zmp_ph108_C4
zmp_ph108_C5
zmp_ph108_D4
zmp_ph97_G6
zmp_ph97_G9
zmp_ph97_H2
zmp_ph97_B10
zmp_ph97_C2
zmp_ph97_C4
zmp_ph97_B8
zmp_ph97_C1
zmp_ph97_D2
zmp_ph97_D5
zmp_ph111_G5
zmp_ph111_G11
zmp_ph111_H1
zmp_ph111_B1a
zmp_ph111_B5
zmp_ph111_B8
zmp_ph111_B6
zmp_ph111_B7
zmp_ph111_B9
zmp_ph111_B10
zmp_ph114_G4
zmp_ph114_G11
zmp_ph114_H1
zmp_ph114_H2
zmp_ph114_A9
zmp_ph114_A11
zmp_ph114_B2
zmp_ph114_B4
zmp_ph114_D8
zmp_ph114_E2

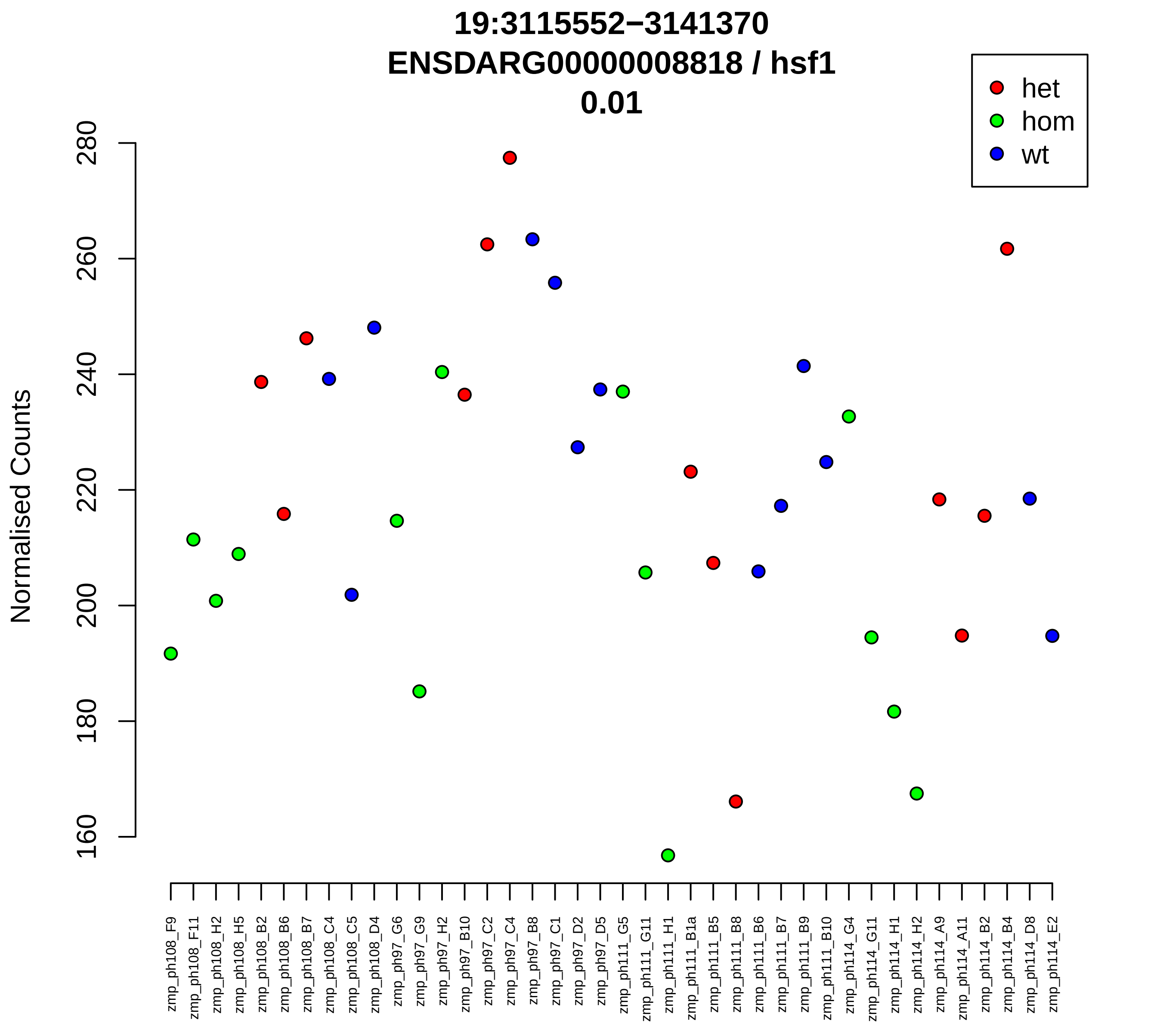

19:3115552−3141370
ENSDARG00000008818 / hsf1
0.01
het
hom
wt
Normalised Counts
160
180
200
220
240
260
280
zmp_ph108_F9
zmp_ph108_F11
zmp_ph108_H2
zmp_ph108_H5
zmp_ph108_B2
zmp_ph108_B6
zmp_ph108_B7
zmp_ph108_C4
zmp_ph108_C5
zmp_ph108_D4
zmp_ph97_G6
zmp_ph97_G9
zmp_ph97_H2
zmp_ph97_B10
zmp_ph97_C2
zmp_ph97_C4
zmp_ph97_B8
zmp_ph97_C1
zmp_ph97_D2
zmp_ph97_D5
zmp_ph111_G5
zmp_ph111_G11
zmp_ph111_H1
zmp_ph111_B1a
zmp_ph111_B5
zmp_ph111_B8
zmp_ph111_B6
zmp_ph111_B7
zmp_ph111_B9
zmp_ph111_B10
zmp_ph114_G4
zmp_ph114_G11
zmp_ph114_H1
zmp_ph114_H2
zmp_ph114_A9
zmp_ph114_A11
zmp_ph114_B2
zmp_ph114_B4
zmp_ph114_D8
zmp_ph114_E2

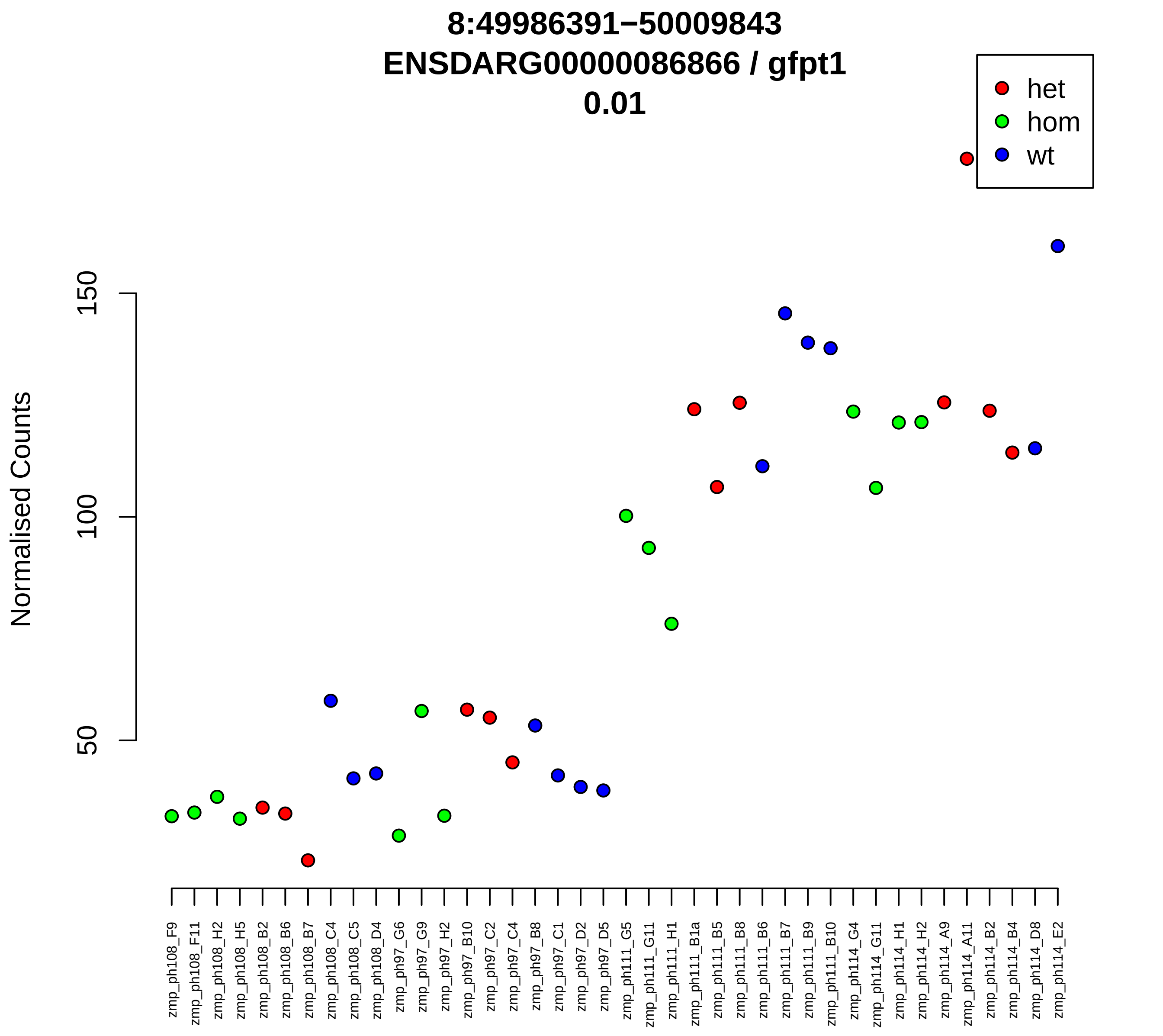
8:49986391−50009843
ENSDARG00000086866 / gfpt1
0.01
het
hom
wt
Normalised Counts
50
100
150
zmp_ph108_F9
zmp_ph108_F11
zmp_ph108_H2
zmp_ph108_H5
zmp_ph108_B2
zmp_ph108_B6
zmp_ph108_B7
zmp_ph108_C4
zmp_ph108_C5
zmp_ph108_D4
zmp_ph97_G6
zmp_ph97_G9
zmp_ph97_H2
zmp_ph97_B10
zmp_ph97_C2
zmp_ph97_C4
zmp_ph97_B8
zmp_ph97_C1
zmp_ph97_D2
zmp_ph97_D5
zmp_ph111_G5
zmp_ph111_G11
zmp_ph111_H1
zmp_ph111_B1a
zmp_ph111_B5
zmp_ph111_B8
zmp_ph111_B6
zmp_ph111_B7
zmp_ph111_B9
zmp_ph111_B10
zmp_ph114_G4
zmp_ph114_G11
zmp_ph114_H1
zmp_ph114_H2
zmp_ph114_A9
zmp_ph114_A11
zmp_ph114_B2
zmp_ph114_B4
zmp_ph114_D8
zmp_ph114_E2

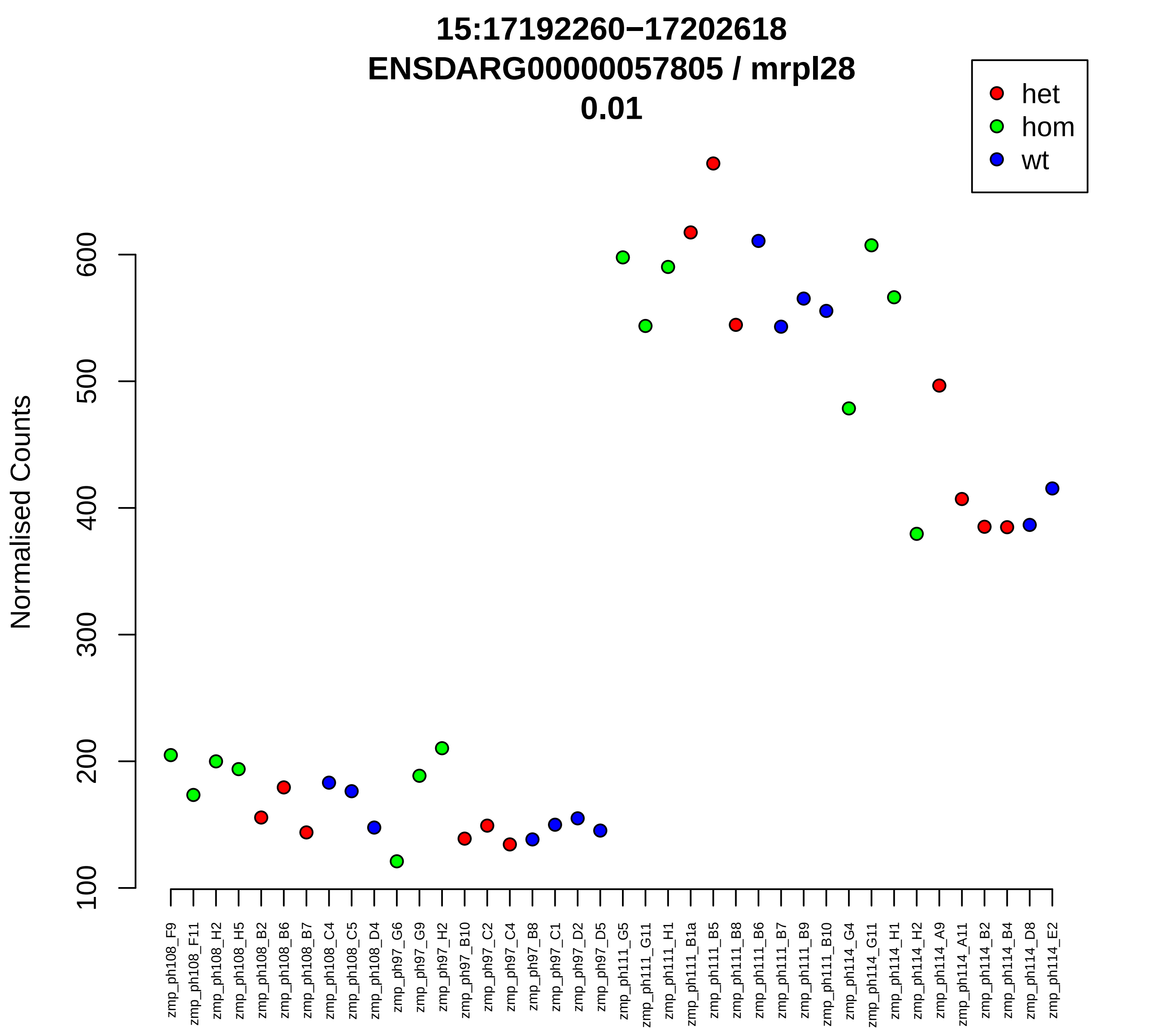

15:17192260−17202618
ENSDARG00000057805 / mrpl28
0.01
het
hom
wt
Normalised Counts
100
200
300
400
500
600
zmp_ph108_F9
zmp_ph108_F11
zmp_ph108_H2
zmp_ph108_H5
zmp_ph108_B2
zmp_ph108_B6
zmp_ph108_B7
zmp_ph108_C4
zmp_ph108_C5
zmp_ph108_D4
zmp_ph97_G6
zmp_ph97_G9
zmp_ph97_H2
zmp_ph97_B10
zmp_ph97_C2
zmp_ph97_C4
zmp_ph97_B8
zmp_ph97_C1
zmp_ph97_D2
zmp_ph97_D5
zmp_ph111_G5
zmp_ph111_G11
zmp_ph111_H1
zmp_ph111_B1a
zmp_ph111_B5
zmp_ph111_B8
zmp_ph111_B6
zmp_ph111_B7
zmp_ph111_B9
zmp_ph111_B10
zmp_ph114_G4
zmp_ph114_G11
zmp_ph114_H1
zmp_ph114_H2
zmp_ph114_A9
zmp_ph114_A11
zmp_ph114_B2
zmp_ph114_B4
zmp_ph114_D8
zmp_ph114_E2

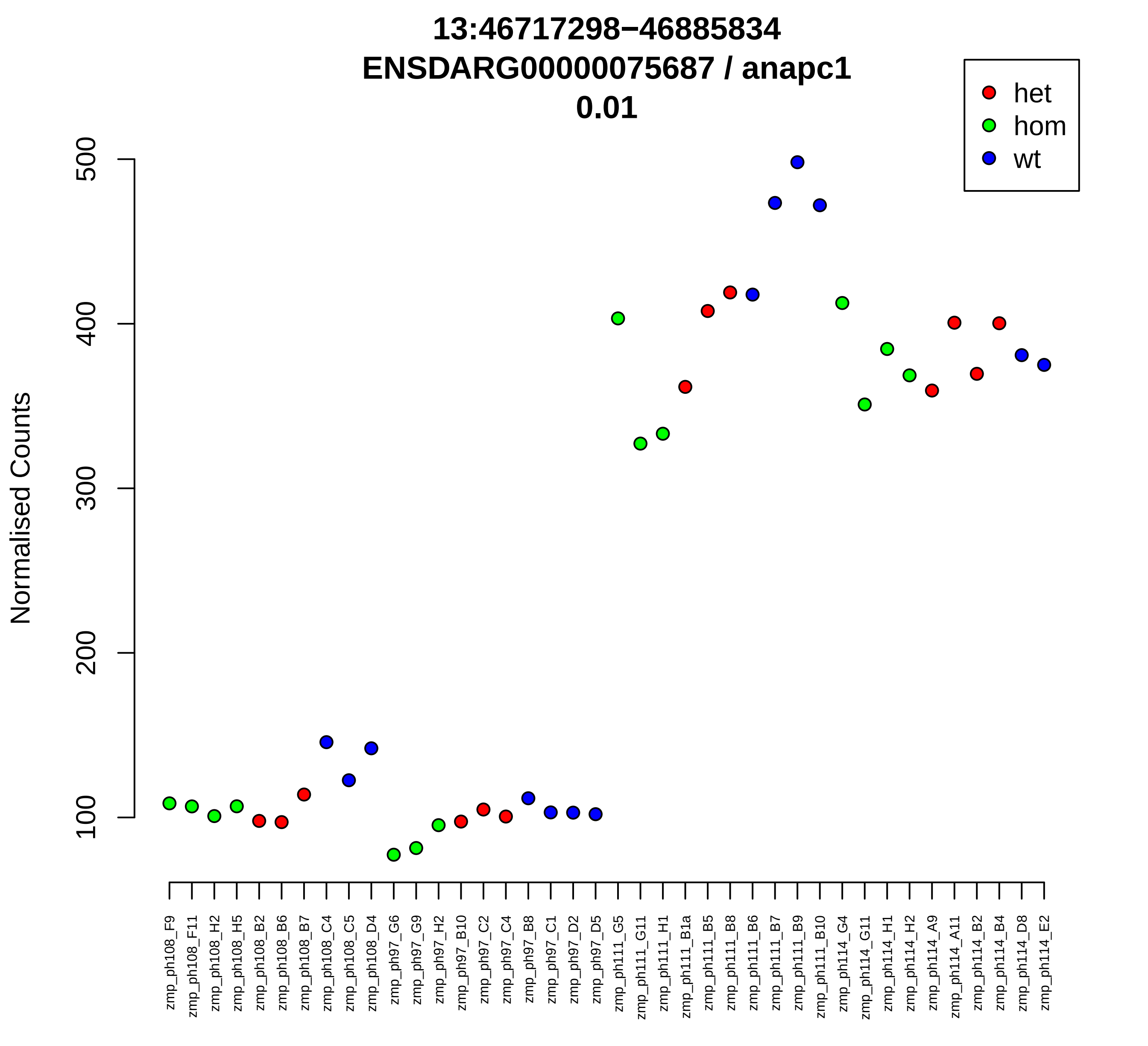

13:46717298−46885834
ENSDARG00000075687 / anapc1
0.01
het
hom
wt
Normalised Counts
100
200
300
400
500
zmp_ph108_F9
zmp_ph108_F11
zmp_ph108_H2
zmp_ph108_H5
zmp_ph108_B2
zmp_ph108_B6
zmp_ph108_B7
zmp_ph108_C4
zmp_ph108_C5
zmp_ph108_D4
zmp_ph97_G6
zmp_ph97_G9
zmp_ph97_H2
zmp_ph97_B10
zmp_ph97_C2
zmp_ph97_C4
zmp_ph97_B8
zmp_ph97_C1
zmp_ph97_D2
zmp_ph97_D5
zmp_ph111_G5
zmp_ph111_G11
zmp_ph111_H1
zmp_ph111_B1a
zmp_ph111_B5
zmp_ph111_B8
zmp_ph111_B6
zmp_ph111_B7
zmp_ph111_B9
zmp_ph111_B10
zmp_ph114_G4
zmp_ph114_G11
zmp_ph114_H1
zmp_ph114_H2
zmp_ph114_A9
zmp_ph114_A11
zmp_ph114_B2
zmp_ph114_B4
zmp_ph114_D8
zmp_ph114_E2

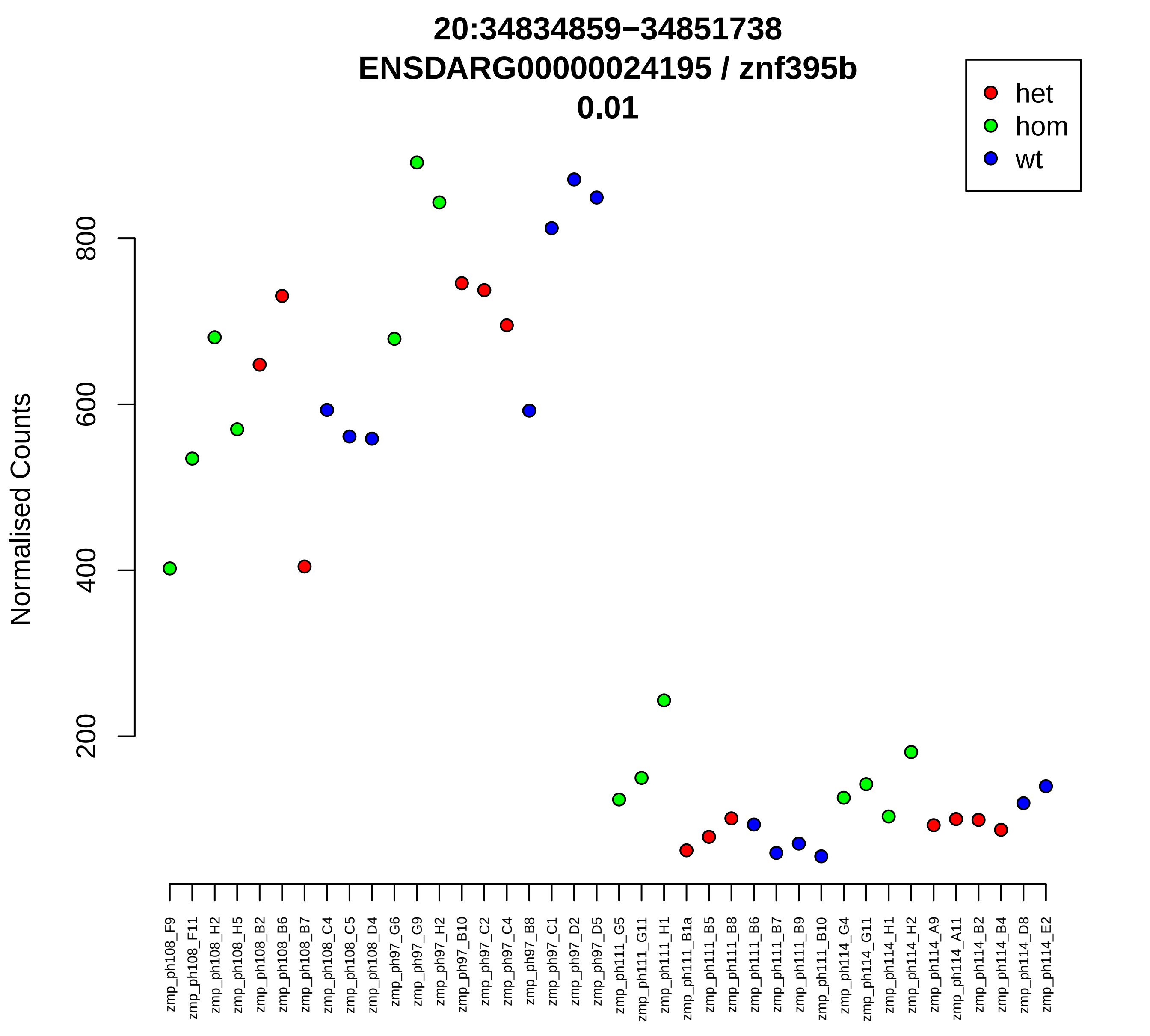
20:34834859−34851738
ENSDARG00000024195 / znf395b
0.01
het
hom
wt
Normalised Counts
200
400
600
800
zmp_ph108_F9
zmp_ph108_F11
zmp_ph108_H2
zmp_ph108_H5
zmp_ph108_B2
zmp_ph108_B6
zmp_ph108_B7
zmp_ph108_C4
zmp_ph108_C5
zmp_ph108_D4
zmp_ph97_G6
zmp_ph97_G9
zmp_ph97_H2
zmp_ph97_B10
zmp_ph97_C2
zmp_ph97_C4
zmp_ph97_B8
zmp_ph97_C1
zmp_ph97_D2
zmp_ph97_D5
zmp_ph111_G5
zmp_ph111_G11
zmp_ph111_H1
zmp_ph111_B1a
zmp_ph111_B5
zmp_ph111_B8
zmp_ph111_B6
zmp_ph111_B7
zmp_ph111_B9
zmp_ph111_B10
zmp_ph114_G4
zmp_ph114_G11
zmp_ph114_H1
zmp_ph114_H2
zmp_ph114_A9
zmp_ph114_A11
zmp_ph114_B2
zmp_ph114_B4
zmp_ph114_D8
zmp_ph114_E2

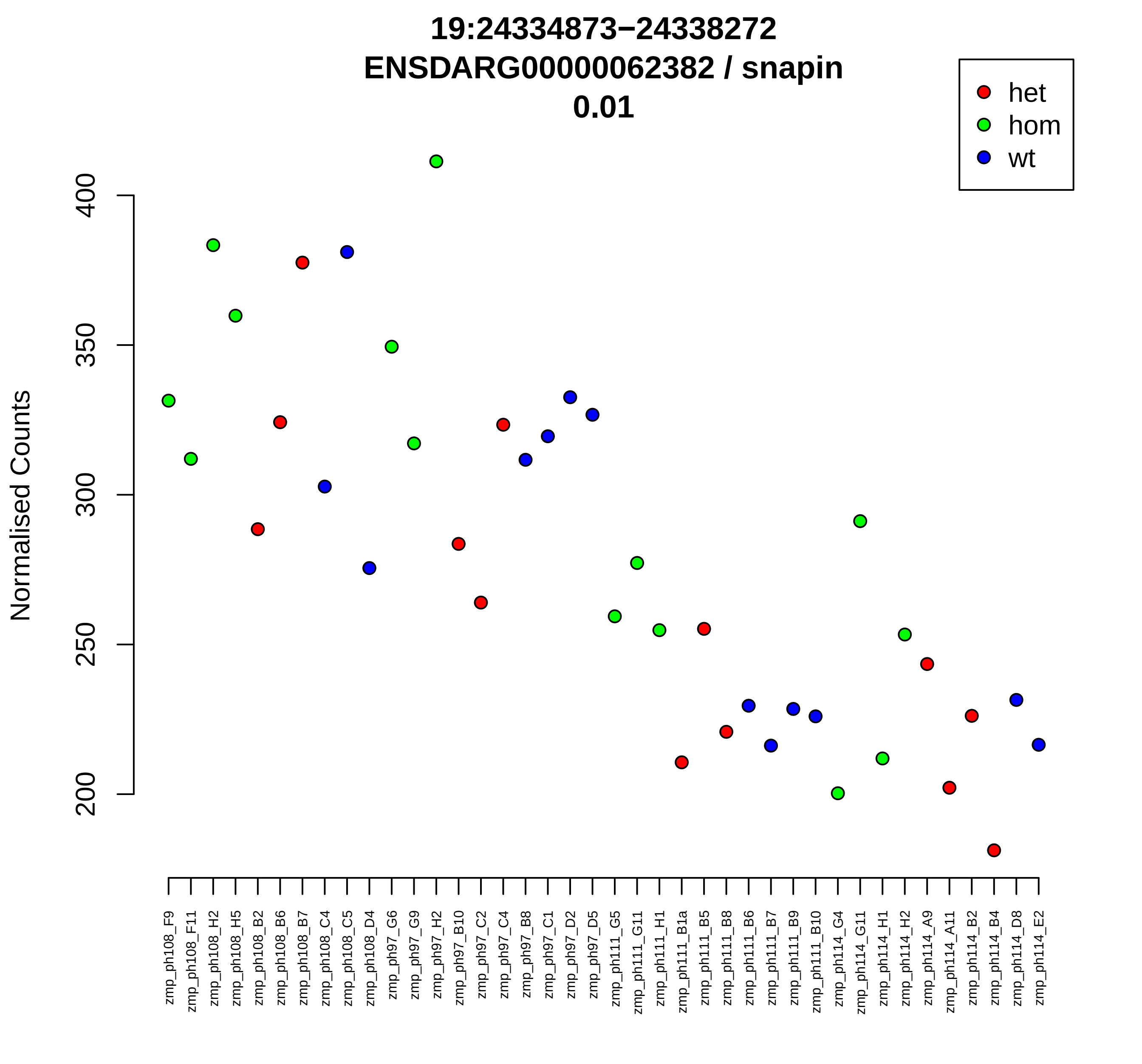

19:24334873−24338272
ENSDARG00000062382 / snapin
0.01
het
hom
wt
Normalised Counts
200
250
300
350
400
zmp_ph108_F9
zmp_ph108_F11
zmp_ph108_H2
zmp_ph108_H5
zmp_ph108_B2
zmp_ph108_B6
zmp_ph108_B7
zmp_ph108_C4
zmp_ph108_C5
zmp_ph108_D4
zmp_ph97_G6
zmp_ph97_G9
zmp_ph97_H2
zmp_ph97_B10
zmp_ph97_C2
zmp_ph97_C4
zmp_ph97_B8
zmp_ph97_C1
zmp_ph97_D2
zmp_ph97_D5
zmp_ph111_G5
zmp_ph111_G11
zmp_ph111_H1
zmp_ph111_B1a
zmp_ph111_B5
zmp_ph111_B8
zmp_ph111_B6
zmp_ph111_B7
zmp_ph111_B9
zmp_ph111_B10
zmp_ph114_G4
zmp_ph114_G11
zmp_ph114_H1
zmp_ph114_H2
zmp_ph114_A9
zmp_ph114_A11
zmp_ph114_B2
zmp_ph114_B4
zmp_ph114_D8
zmp_ph114_E2

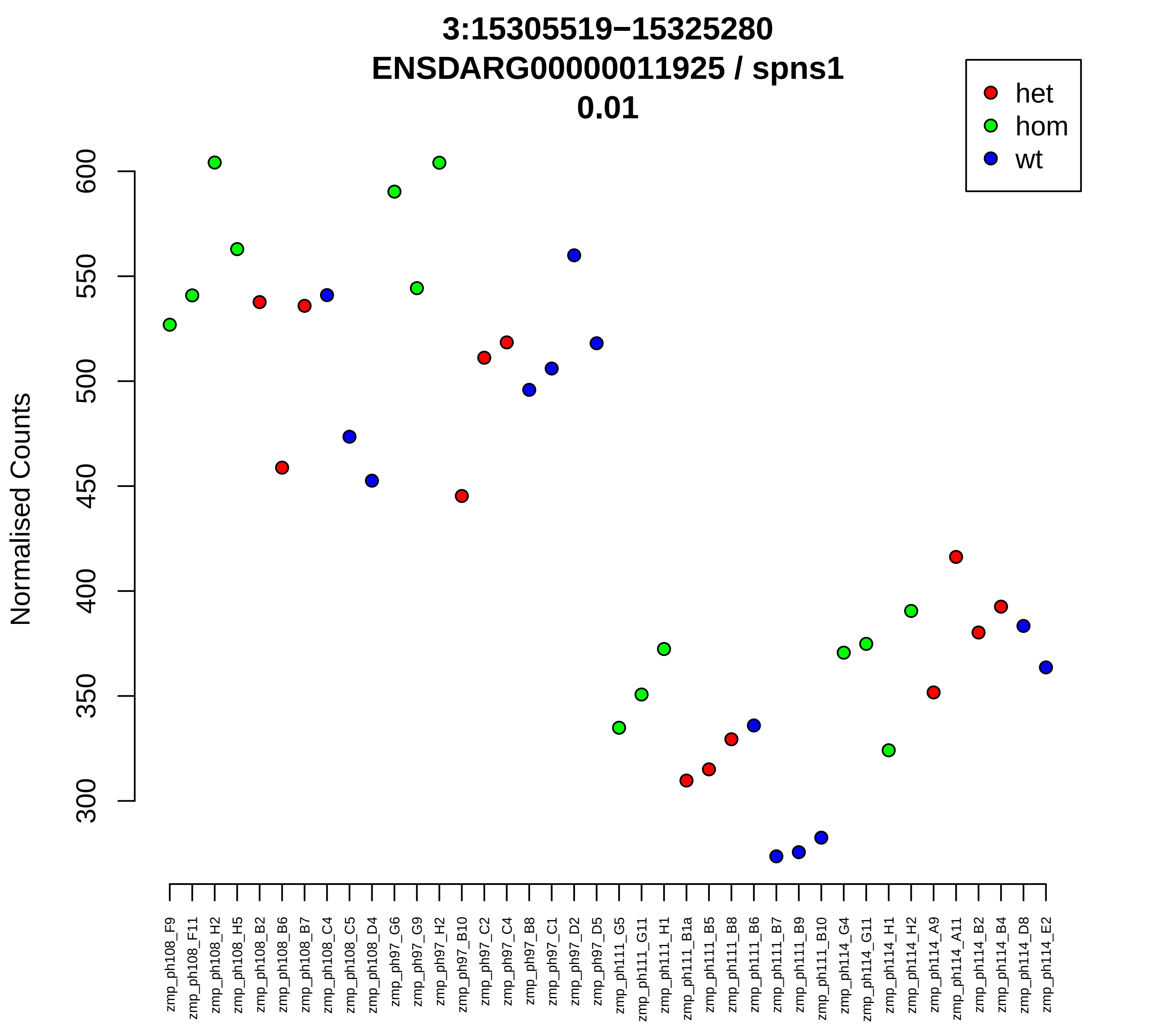
3:15305519−15325280
ENSDARG00000011925 / spns1
0.01
het
hom
wt
Normalised Counts
300
350
400
450
500
550
600
zmp_ph108_F9
zmp_ph108_F11
zmp_ph108_H2
zmp_ph108_H5
zmp_ph108_B2
zmp_ph108_B6
zmp_ph108_B7
zmp_ph108_C4
zmp_ph108_C5
zmp_ph108_D4
zmp_ph97_G6
zmp_ph97_G9
zmp_ph97_H2
zmp_ph97_B10
zmp_ph97_C2
zmp_ph97_C4
zmp_ph97_B8
zmp_ph97_C1
zmp_ph97_D2
zmp_ph97_D5
zmp_ph111_G5
zmp_ph111_G11
zmp_ph111_H1
zmp_ph111_B1a
zmp_ph111_B5
zmp_ph111_B8
zmp_ph111_B6
zmp_ph111_B7
zmp_ph111_B9
zmp_ph111_B10
zmp_ph114_G4
zmp_ph114_G11
zmp_ph114_H1
zmp_ph114_H2
zmp_ph114_A9
zmp_ph114_A11
zmp_ph114_B2
zmp_ph114_B4
zmp_ph114_D8
zmp_ph114_E2

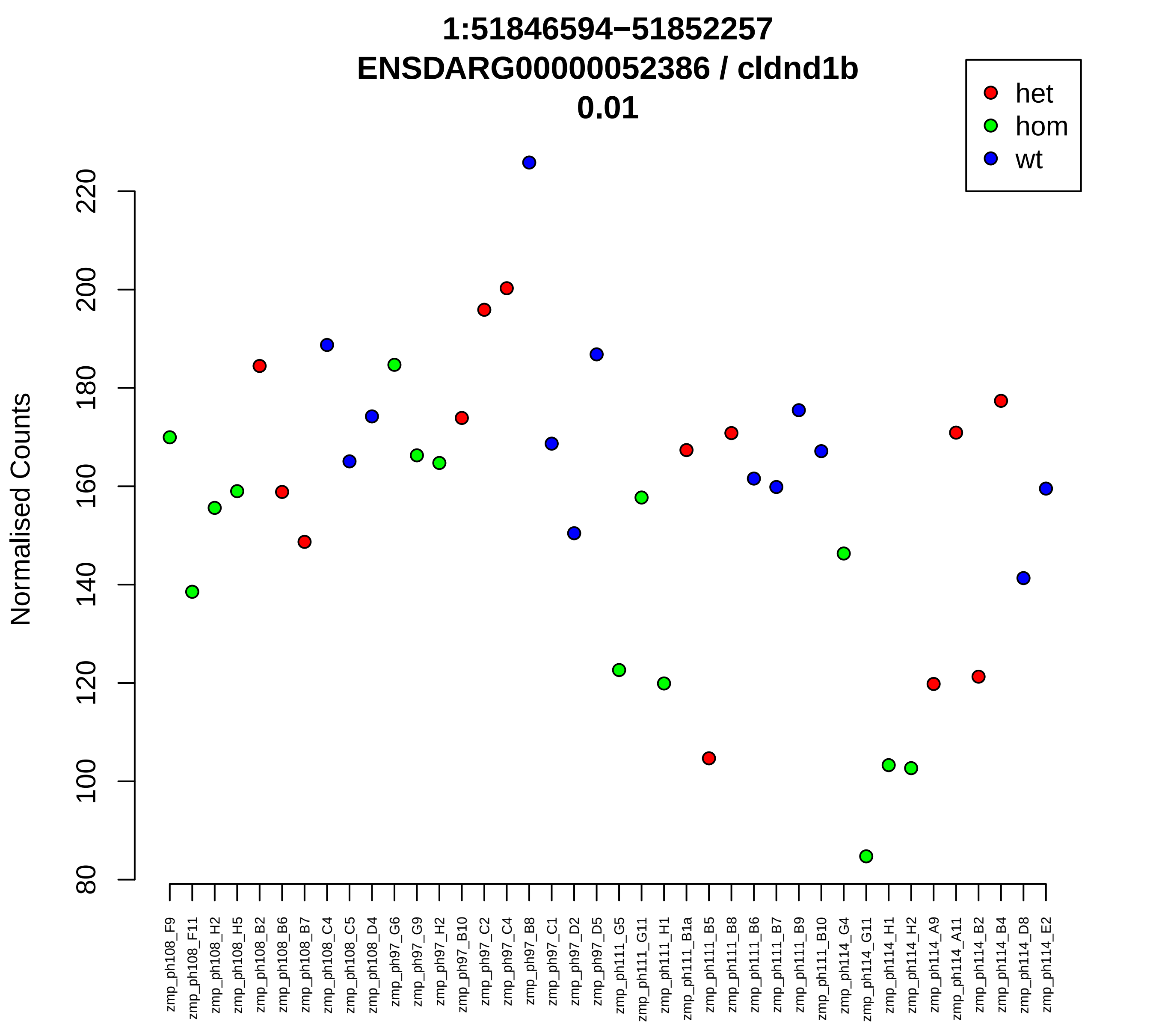

1:51846594−51852257
ENSDARG00000052386 / cldnd1b
0.01
het
hom
wt
Normalised Counts
80
100
120
140
160
180
200
220
zmp_ph108_F9
zmp_ph108_F11
zmp_ph108_H2
zmp_ph108_H5
zmp_ph108_B2
zmp_ph108_B6
zmp_ph108_B7
zmp_ph108_C4
zmp_ph108_C5
zmp_ph108_D4
zmp_ph97_G6
zmp_ph97_G9
zmp_ph97_H2
zmp_ph97_B10
zmp_ph97_C2
zmp_ph97_C4
zmp_ph97_B8
zmp_ph97_C1
zmp_ph97_D2
zmp_ph97_D5
zmp_ph111_G5
zmp_ph111_G11
zmp_ph111_H1
zmp_ph111_B1a
zmp_ph111_B5
zmp_ph111_B8
zmp_ph111_B6
zmp_ph111_B7
zmp_ph111_B9
zmp_ph111_B10
zmp_ph114_G4
zmp_ph114_G11
zmp_ph114_H1
zmp_ph114_H2
zmp_ph114_A9
zmp_ph114_A11
zmp_ph114_B2
zmp_ph114_B4
zmp_ph114_D8
zmp_ph114_E2

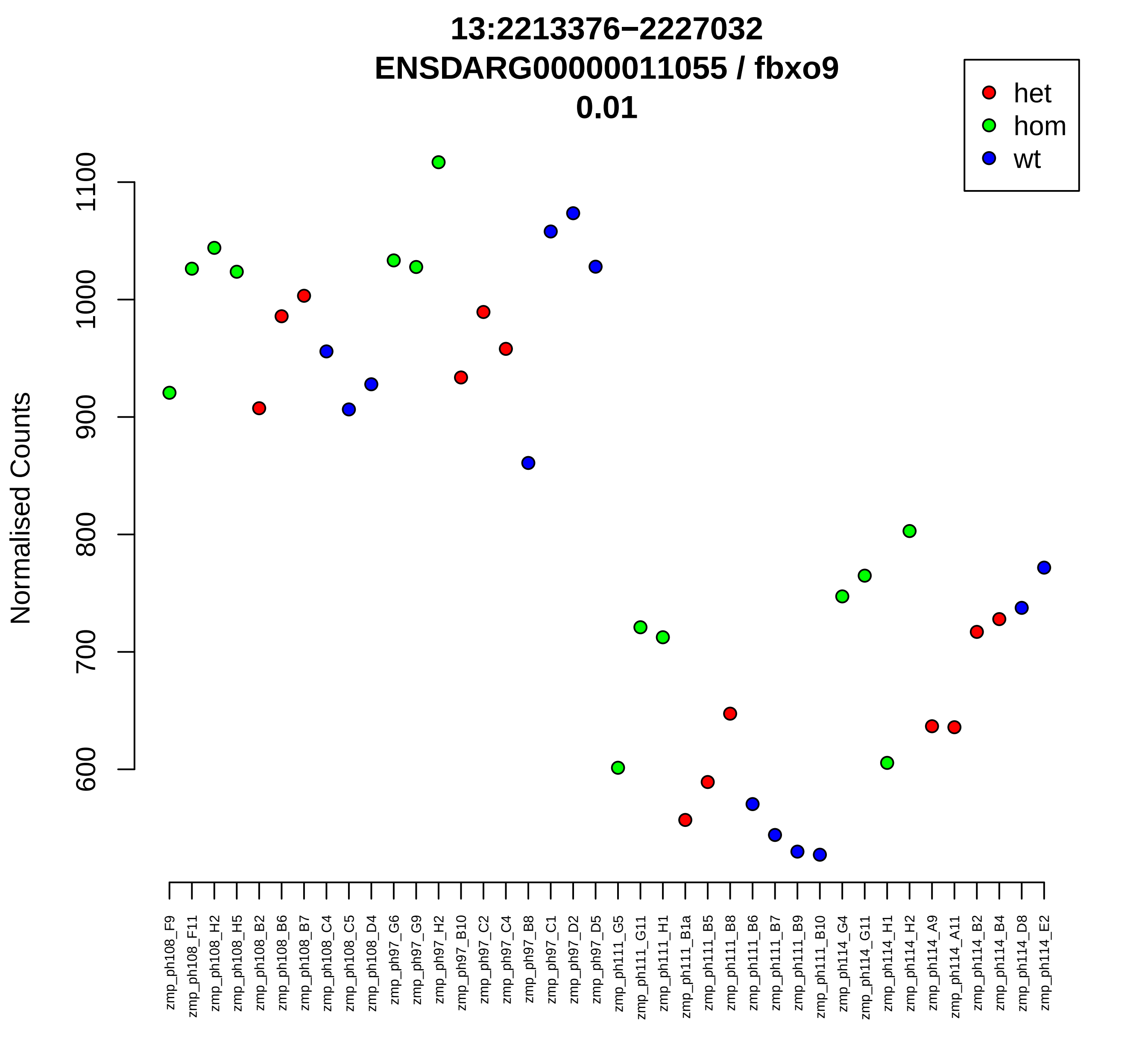
13:2213376−2227032
ENSDARG00000011055 / fbxo9
0.01
het
hom
wt
Normalised Counts
600
700
800
900
1000
1100
zmp_ph108_F9
zmp_ph108_F11
zmp_ph108_H2
zmp_ph108_H5
zmp_ph108_B2
zmp_ph108_B6
zmp_ph108_B7
zmp_ph108_C4
zmp_ph108_C5
zmp_ph108_D4
zmp_ph97_G6
zmp_ph97_G9
zmp_ph97_H2
zmp_ph97_B10
zmp_ph97_C2
zmp_ph97_C4
zmp_ph97_B8
zmp_ph97_C1
zmp_ph97_D2
zmp_ph97_D5
zmp_ph111_G5
zmp_ph111_G11
zmp_ph111_H1
zmp_ph111_B1a
zmp_ph111_B5
zmp_ph111_B8
zmp_ph111_B6
zmp_ph111_B7
zmp_ph111_B9
zmp_ph111_B10
zmp_ph114_G4
zmp_ph114_G11
zmp_ph114_H1
zmp_ph114_H2
zmp_ph114_A9
zmp_ph114_A11
zmp_ph114_B2
zmp_ph114_B4
zmp_ph114_D8
zmp_ph114_E2

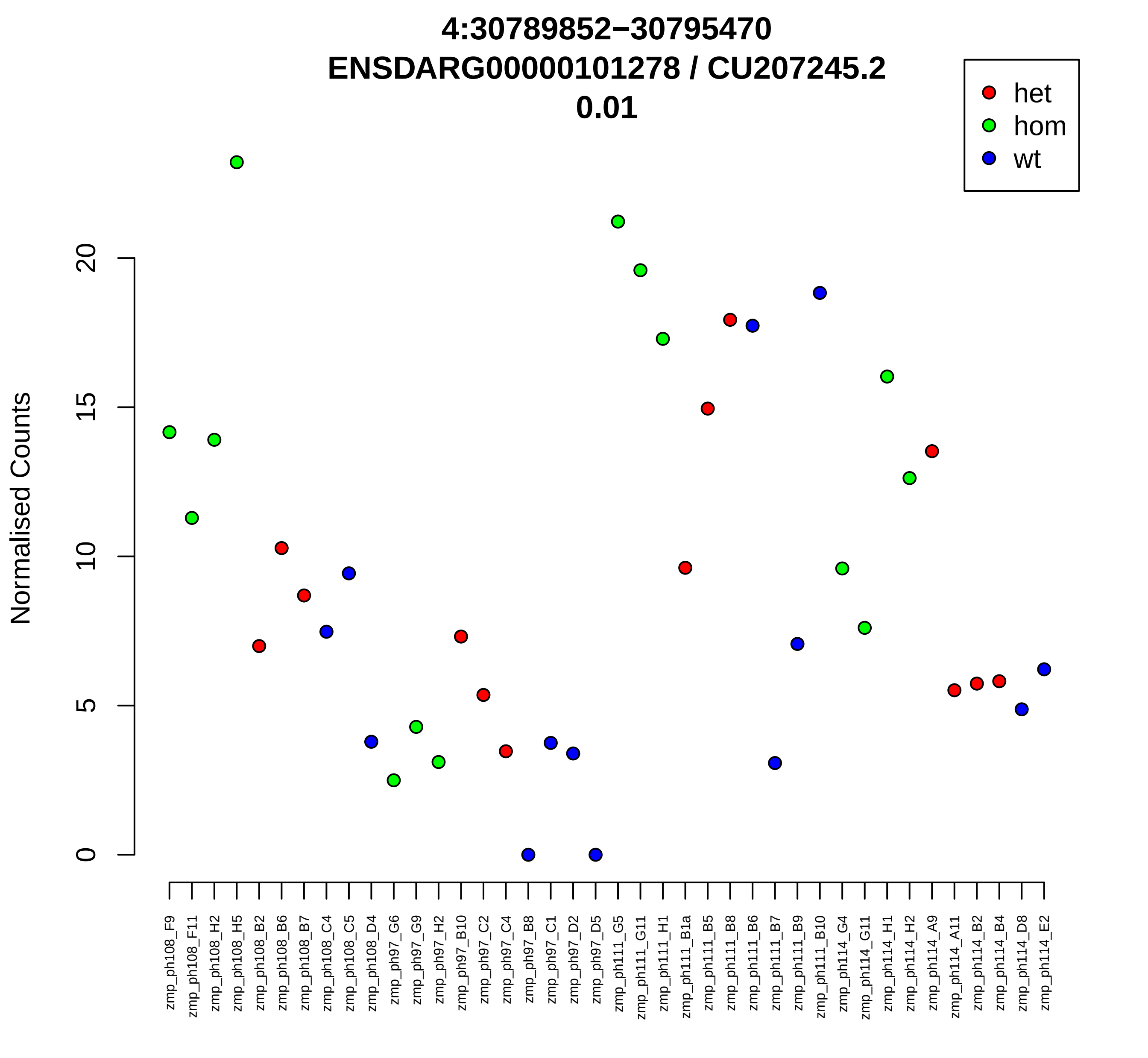
4:30789852−30795470
ENSDARG00000101278 / CU207245.2
0.01
het
hom
wt
Normalised Counts
0
5
10
15
20
zmp_ph108_F9
zmp_ph108_F11
zmp_ph108_H2
zmp_ph108_H5
zmp_ph108_B2
zmp_ph108_B6
zmp_ph108_B7
zmp_ph108_C4
zmp_ph108_C5
zmp_ph108_D4
zmp_ph97_G6
zmp_ph97_G9
zmp_ph97_H2
zmp_ph97_B10
zmp_ph97_C2
zmp_ph97_C4
zmp_ph97_B8
zmp_ph97_C1
zmp_ph97_D2
zmp_ph97_D5
zmp_ph111_G5
zmp_ph111_G11
zmp_ph111_H1
zmp_ph111_B1a
zmp_ph111_B5
zmp_ph111_B8
zmp_ph111_B6
zmp_ph111_B7
zmp_ph111_B9
zmp_ph111_B10
zmp_ph114_G4
zmp_ph114_G11
zmp_ph114_H1
zmp_ph114_H2
zmp_ph114_A9
zmp_ph114_A11
zmp_ph114_B2
zmp_ph114_B4
zmp_ph114_D8
zmp_ph114_E2

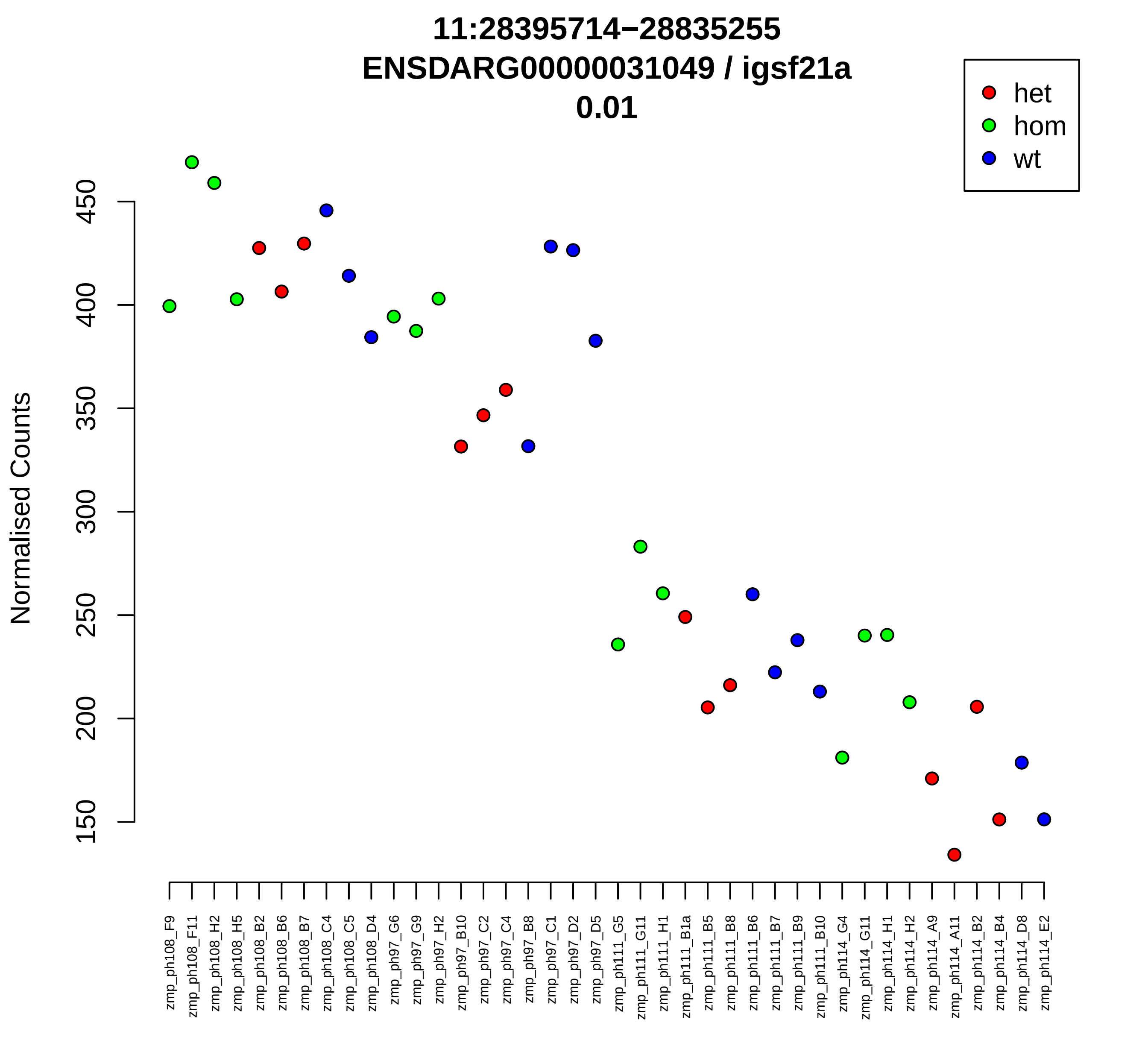
11:28395714−28835255
ENSDARG00000031049 / igsf21a
0.01
het
hom
wt
Normalised Counts
150
200
250
300
350
400
450
zmp_ph108_F9
zmp_ph108_F11
zmp_ph108_H2
zmp_ph108_H5
zmp_ph108_B2
zmp_ph108_B6
zmp_ph108_B7
zmp_ph108_C4
zmp_ph108_C5
zmp_ph108_D4
zmp_ph97_G6
zmp_ph97_G9
zmp_ph97_H2
zmp_ph97_B10
zmp_ph97_C2
zmp_ph97_C4
zmp_ph97_B8
zmp_ph97_C1
zmp_ph97_D2
zmp_ph97_D5
zmp_ph111_G5
zmp_ph111_G11
zmp_ph111_H1
zmp_ph111_B1a
zmp_ph111_B5
zmp_ph111_B8
zmp_ph111_B6
zmp_ph111_B7
zmp_ph111_B9
zmp_ph111_B10
zmp_ph114_G4
zmp_ph114_G11
zmp_ph114_H1
zmp_ph114_H2
zmp_ph114_A9
zmp_ph114_A11
zmp_ph114_B2
zmp_ph114_B4
zmp_ph114_D8
zmp_ph114_E2

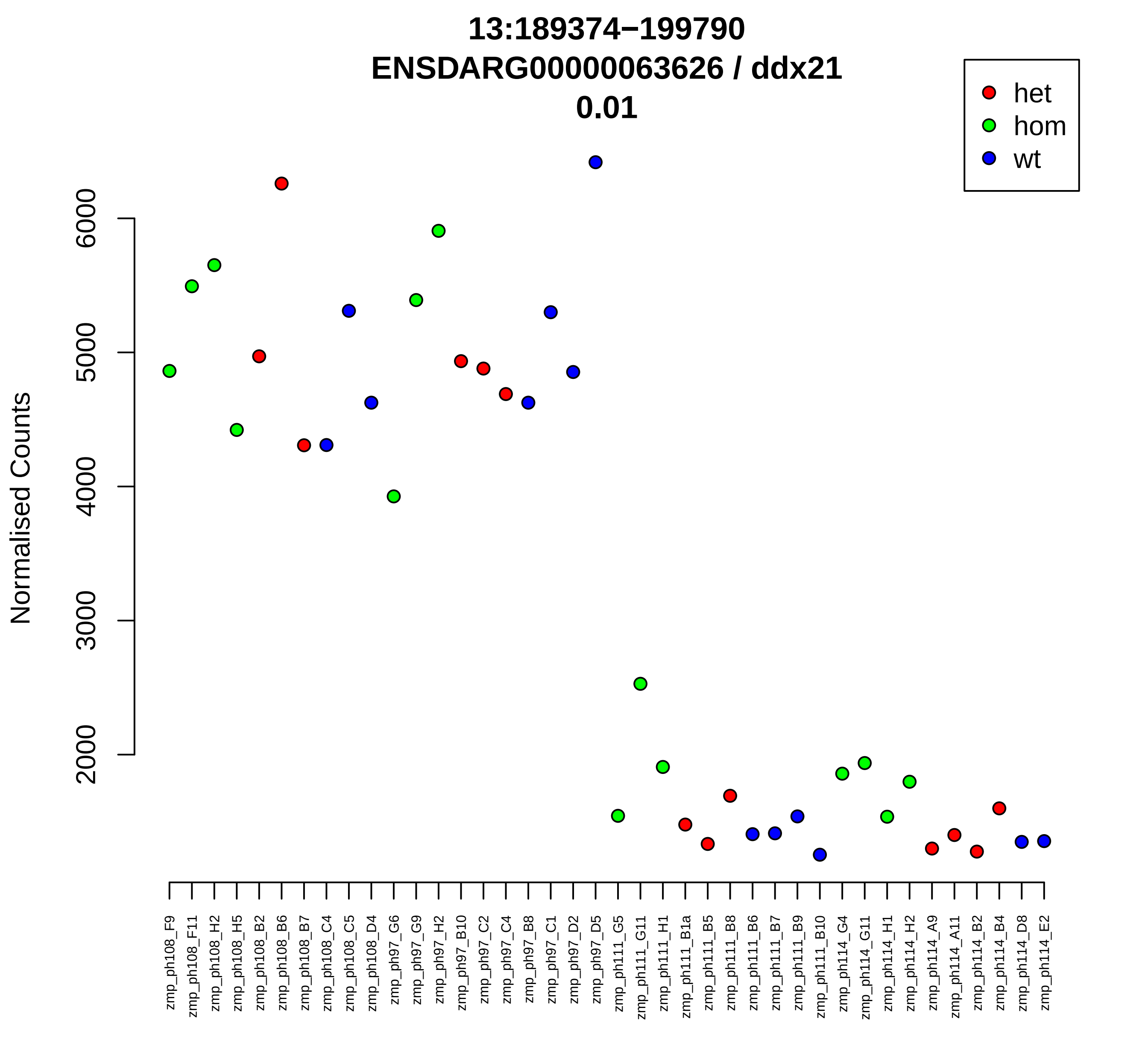

13:189374−199790
ENSDARG00000063626 / ddx21
0.01
het
hom
wt
Normalised Counts
2000
3000
4000
5000
6000
zmp_ph108_F9
zmp_ph108_F11
zmp_ph108_H2
zmp_ph108_H5
zmp_ph108_B2
zmp_ph108_B6
zmp_ph108_B7
zmp_ph108_C4
zmp_ph108_C5
zmp_ph108_D4
zmp_ph97_G6
zmp_ph97_G9
zmp_ph97_H2
zmp_ph97_B10
zmp_ph97_C2
zmp_ph97_C4
zmp_ph97_B8
zmp_ph97_C1
zmp_ph97_D2
zmp_ph97_D5
zmp_ph111_G5
zmp_ph111_G11
zmp_ph111_H1
zmp_ph111_B1a
zmp_ph111_B5
zmp_ph111_B8
zmp_ph111_B6
zmp_ph111_B7
zmp_ph111_B9
zmp_ph111_B10
zmp_ph114_G4
zmp_ph114_G11
zmp_ph114_H1
zmp_ph114_H2
zmp_ph114_A9
zmp_ph114_A11
zmp_ph114_B2
zmp_ph114_B4
zmp_ph114_D8
zmp_ph114_E2

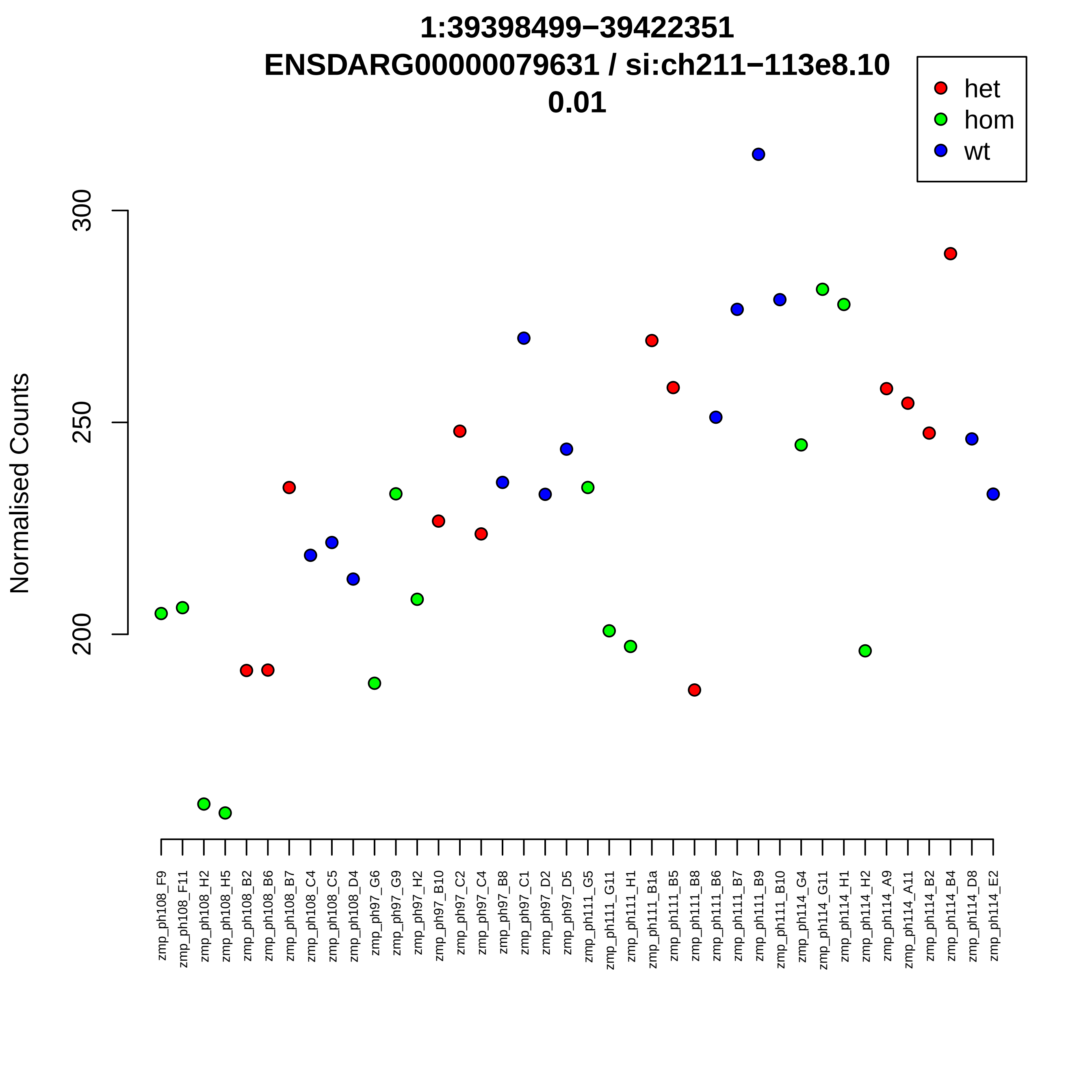

1:39398499−39422351
ENSDARG00000079631 / si:ch211−113e8.10
0.01
het
hom
wt
Normalised Counts
300
250
200
zmp_ph108_F9
zmp_ph108_F11
zmp_ph108_H2
zmp_ph108_H5
zmp_ph108_B2
zmp_ph108_B6
zmp_ph108_B7
zmp_ph108_C4
zmp_ph108_C5
zmp_ph108_D4
zmp_ph97_G6
zmp_ph97_G9
zmp_ph97_H2
zmp_ph97_B10
zmp_ph97_C2
zmp_ph97_C4
zmp_ph97_B8
zmp_ph97_C1
zmp_ph97_D2
zmp_ph97_D5
zmp_ph111_G5
zmp_ph111_G11
zmp_ph111_H1
zmp_ph111_B1a
zmp_ph111_B5
zmp_ph111_B8
zmp_ph111_B6
zmp_ph111_B7
zmp_ph111_B9
zmp_ph111_B10
zmp_ph114_G4
zmp_ph114_G11
zmp_ph114_H1
zmp_ph114_H2
zmp_ph114_A9
zmp_ph114_A11
zmp_ph114_B2
zmp_ph114_B4
zmp_ph114_D8
zmp_ph114_E2

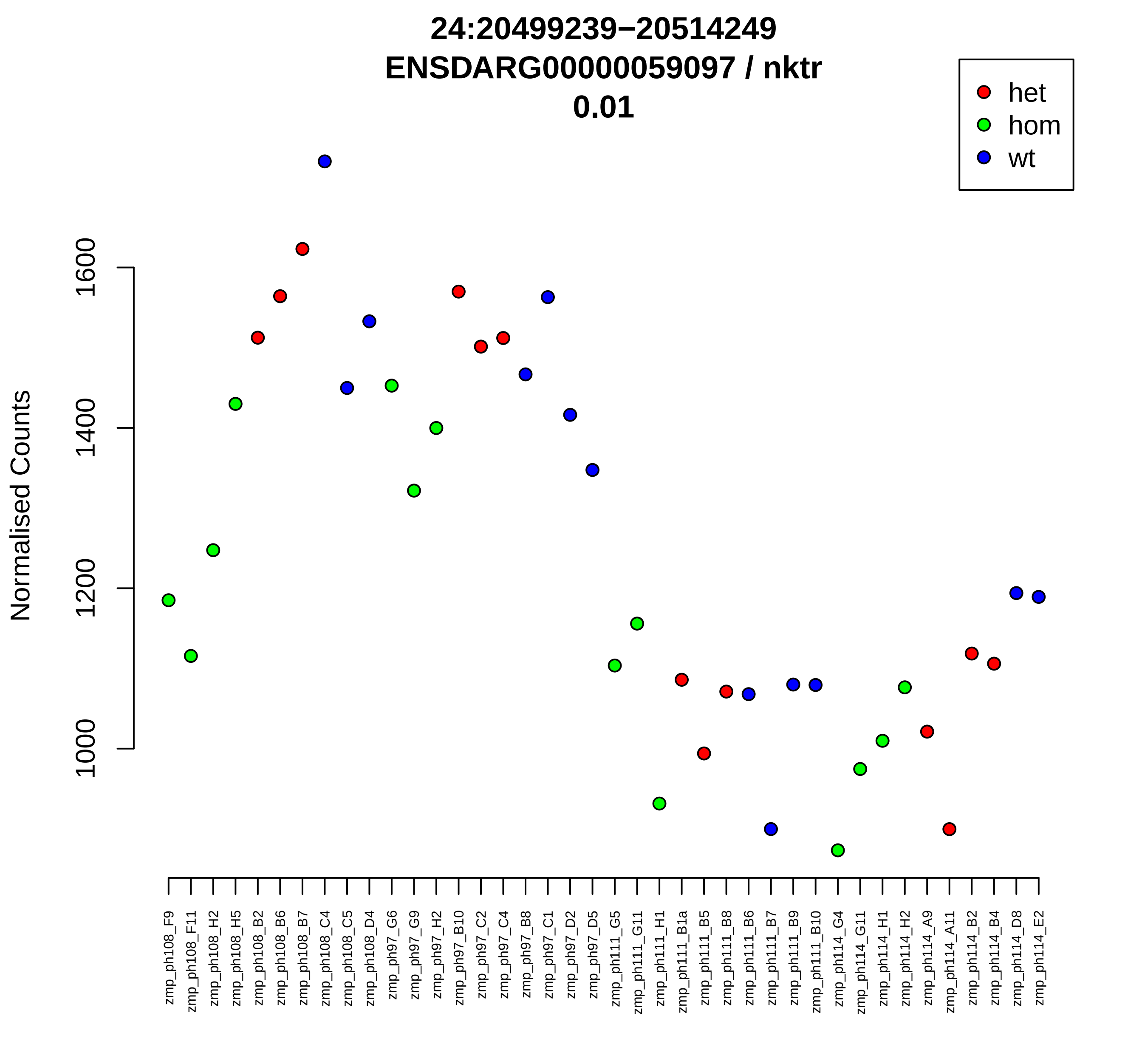
24:20499239−20514249
ENSDARG00000059097 / nktr
0.01
het
hom
wt
Normalised Counts
1000
1200
1400
1600
zmp_ph108_F9
zmp_ph108_F11
zmp_ph108_H2
zmp_ph108_H5
zmp_ph108_B2
zmp_ph108_B6
zmp_ph108_B7
zmp_ph108_C4
zmp_ph108_C5
zmp_ph108_D4
zmp_ph97_G6
zmp_ph97_G9
zmp_ph97_H2
zmp_ph97_B10
zmp_ph97_C2
zmp_ph97_C4
zmp_ph97_B8
zmp_ph97_C1
zmp_ph97_D2
zmp_ph97_D5
zmp_ph111_G5
zmp_ph111_G11
zmp_ph111_H1
zmp_ph111_B1a
zmp_ph111_B5
zmp_ph111_B8
zmp_ph111_B6
zmp_ph111_B7
zmp_ph111_B9
zmp_ph111_B10
zmp_ph114_G4
zmp_ph114_G11
zmp_ph114_H1
zmp_ph114_H2
zmp_ph114_A9
zmp_ph114_A11
zmp_ph114_B2
zmp_ph114_B4
zmp_ph114_D8
zmp_ph114_E2

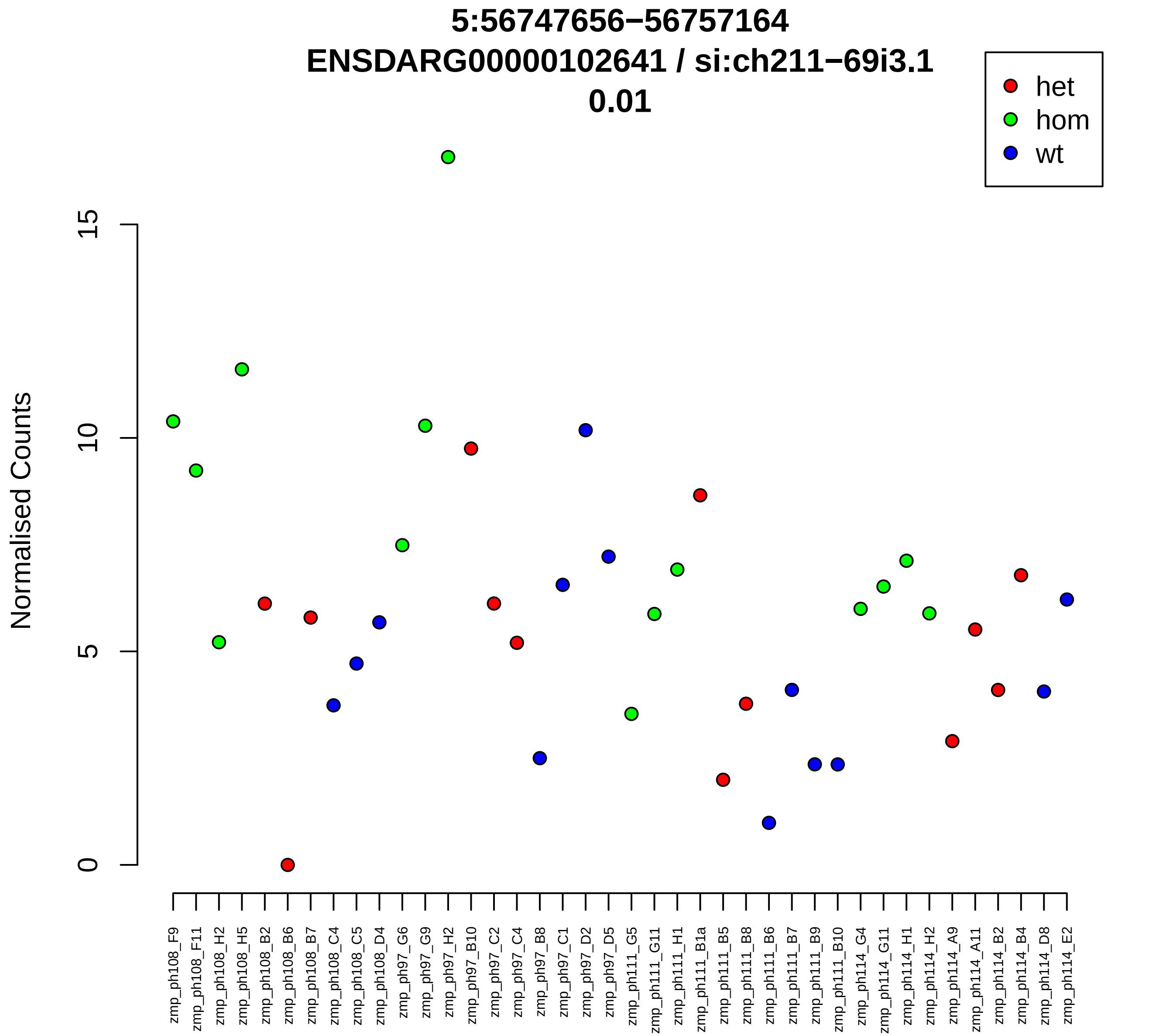

5:56747656−56757164
ENSDARG00000102641 / si:ch211−69i3.1
0.01
het
hom
wt
Normalised Counts
0
5
10
15
zmp_ph108_F9
zmp_ph108_F11
zmp_ph108_H2
zmp_ph108_H5
zmp_ph108_B2
zmp_ph108_B6
zmp_ph108_B7
zmp_ph108_C4
zmp_ph108_C5
zmp_ph108_D4
zmp_ph97_G6
zmp_ph97_G9
zmp_ph97_H2
zmp_ph97_B10
zmp_ph97_C2
zmp_ph97_C4
zmp_ph97_B8
zmp_ph97_C1
zmp_ph97_D2
zmp_ph97_D5
zmp_ph111_G5
zmp_ph111_G11
zmp_ph111_H1
zmp_ph111_B1a
zmp_ph111_B5
zmp_ph111_B8
zmp_ph111_B6
zmp_ph111_B7
zmp_ph111_B9
zmp_ph111_B10
zmp_ph114_G4
zmp_ph114_G11
zmp_ph114_H1
zmp_ph114_H2
zmp_ph114_A9
zmp_ph114_A11
zmp_ph114_B2
zmp_ph114_B4
zmp_ph114_D8
zmp_ph114_E2

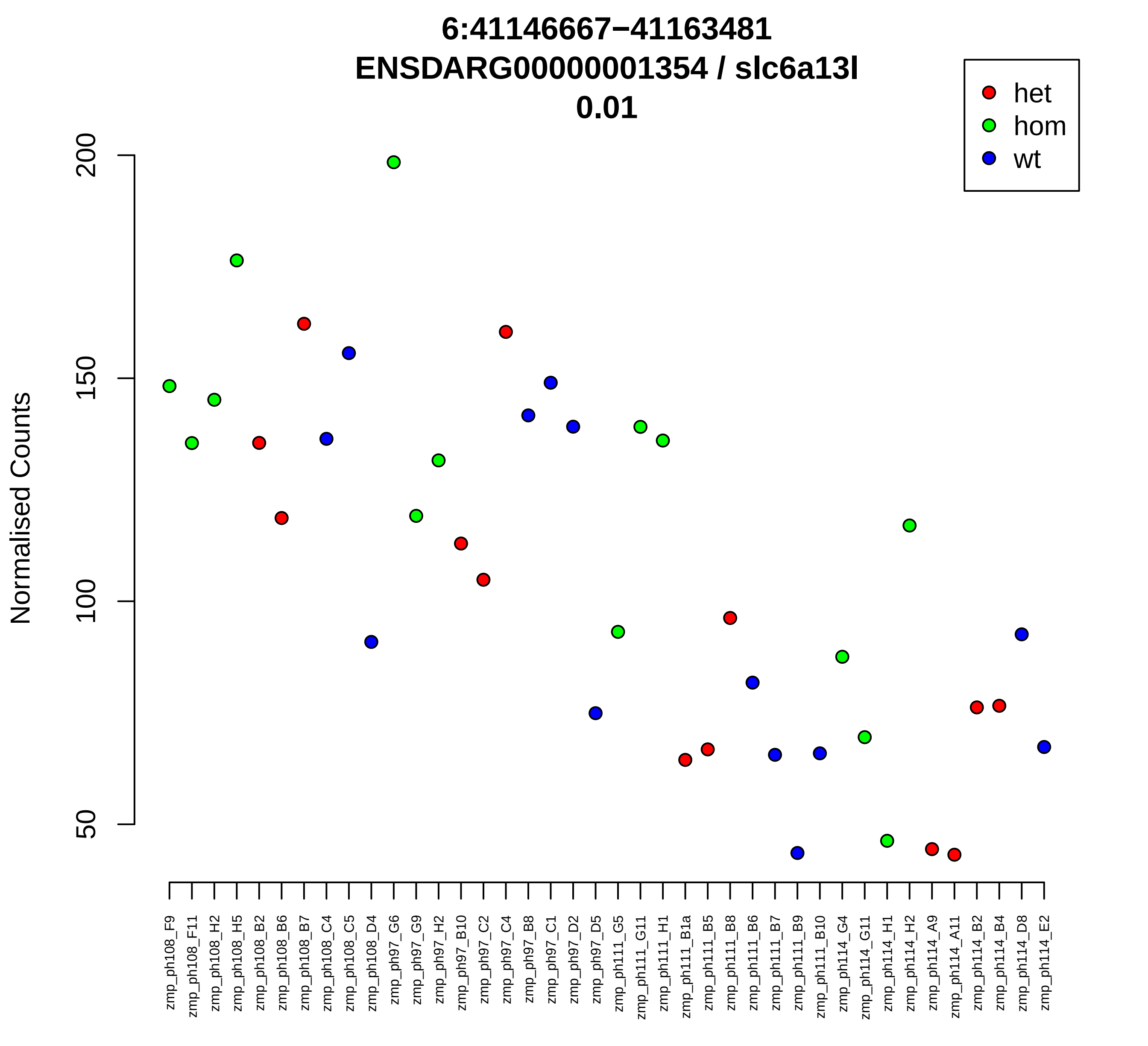

6:41146667−41163481
ENSDARG00000001354 / slc6a13l
0.01
het
hom
wt
Normalised Counts
200
150
100
50
zmp_ph108_F9
zmp_ph108_F11
zmp_ph108_H2
zmp_ph108_H5
zmp_ph108_B2
zmp_ph108_B6
zmp_ph108_B7
zmp_ph108_C4
zmp_ph108_C5
zmp_ph108_D4
zmp_ph97_G6
zmp_ph97_G9
zmp_ph97_H2
zmp_ph97_B10
zmp_ph97_C2
zmp_ph97_C4
zmp_ph97_B8
zmp_ph97_C1
zmp_ph97_D2
zmp_ph97_D5
zmp_ph111_G5
zmp_ph111_G11
zmp_ph111_H1
zmp_ph111_B1a
zmp_ph111_B5
zmp_ph111_B8
zmp_ph111_B6
zmp_ph111_B7
zmp_ph111_B9
zmp_ph111_B10
zmp_ph114_G4
zmp_ph114_G11
zmp_ph114_H1
zmp_ph114_H2
zmp_ph114_A9
zmp_ph114_A11
zmp_ph114_B2
zmp_ph114_B4
zmp_ph114_D8
zmp_ph114_E2

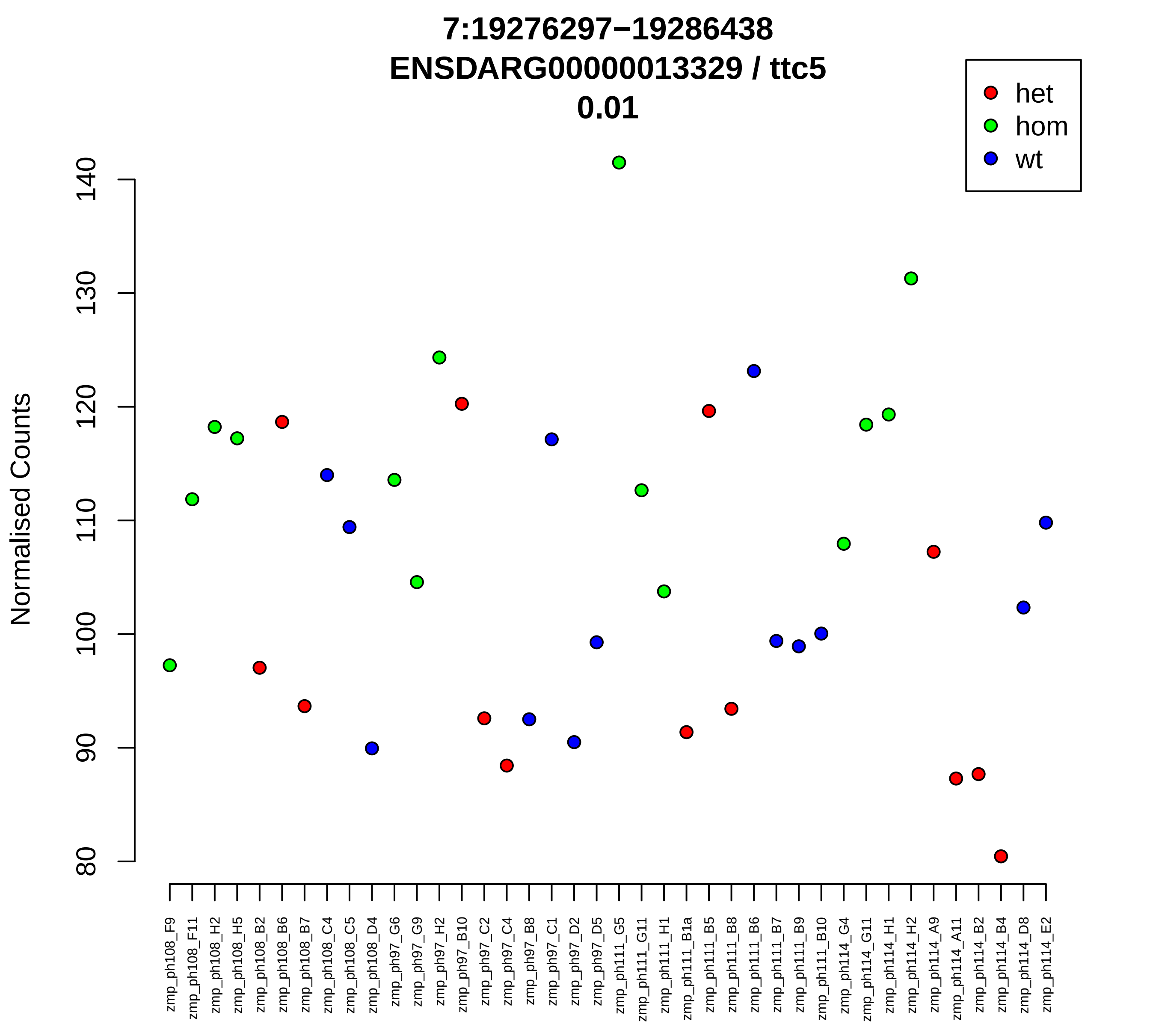
7:19276297−19286438
ENSDARG00000013329 / ttc5
0.01
het
hom
wt
Normalised Counts
80
90
100
110
120
130
140
zmp_ph108_F9
zmp_ph108_F11
zmp_ph108_H2
zmp_ph108_H5
zmp_ph108_B2
zmp_ph108_B6
zmp_ph108_B7
zmp_ph108_C4
zmp_ph108_C5
zmp_ph108_D4
zmp_ph97_G6
zmp_ph97_G9
zmp_ph97_H2
zmp_ph97_B10
zmp_ph97_C2
zmp_ph97_C4
zmp_ph97_B8
zmp_ph97_C1
zmp_ph97_D2
zmp_ph97_D5
zmp_ph111_G5
zmp_ph111_G11
zmp_ph111_H1
zmp_ph111_B1a
zmp_ph111_B5
zmp_ph111_B8
zmp_ph111_B6
zmp_ph111_B7
zmp_ph111_B9
zmp_ph111_B10
zmp_ph114_G4
zmp_ph114_G11
zmp_ph114_H1
zmp_ph114_H2
zmp_ph114_A9
zmp_ph114_A11
zmp_ph114_B2
zmp_ph114_B4
zmp_ph114_D8
zmp_ph114_E2

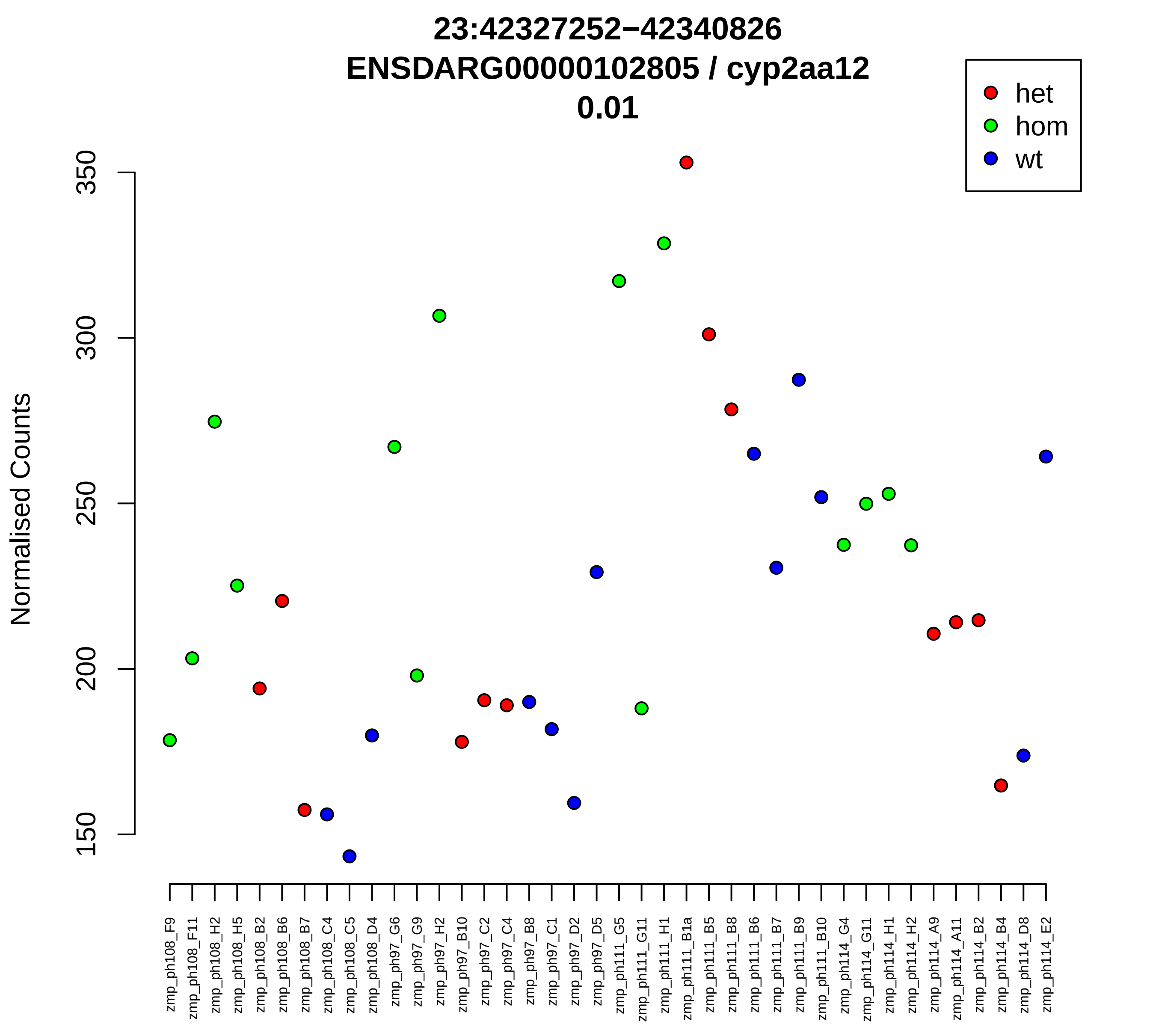
23:42327252−42340826
ENSDARG00000102805 / cyp2aa12
0.01
het
hom
wt
Normalised Counts
150
200
250
300
350
zmp_ph108_F9
zmp_ph108_F11
zmp_ph108_H2
zmp_ph108_H5
zmp_ph108_B2
zmp_ph108_B6
zmp_ph108_B7
zmp_ph108_C4
zmp_ph108_C5
zmp_ph108_D4
zmp_ph97_G6
zmp_ph97_G9
zmp_ph97_H2
zmp_ph97_B10
zmp_ph97_C2
zmp_ph97_C4
zmp_ph97_B8
zmp_ph97_C1
zmp_ph97_D2
zmp_ph97_D5
zmp_ph111_G5
zmp_ph111_G11
zmp_ph111_H1
zmp_ph111_B1a
zmp_ph111_B5
zmp_ph111_B8
zmp_ph111_B6
zmp_ph111_B7
zmp_ph111_B9
zmp_ph111_B10
zmp_ph114_G4
zmp_ph114_G11
zmp_ph114_H1
zmp_ph114_H2
zmp_ph114_A9
zmp_ph114_A11
zmp_ph114_B2
zmp_ph114_B4
zmp_ph114_D8
zmp_ph114_E2

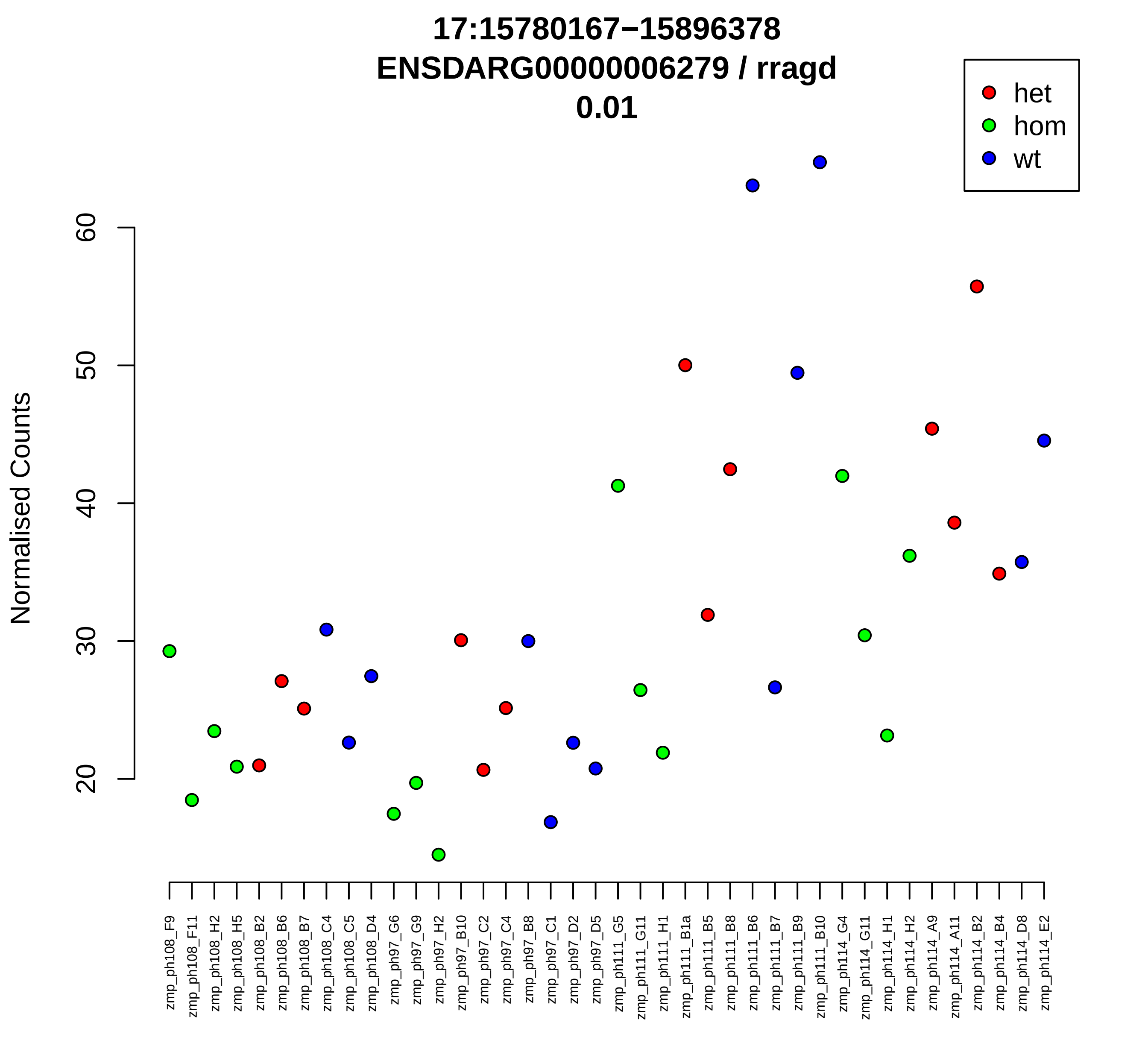

17:15780167−15896378
ENSDARG00000006279 / rragd
0.01
het
hom
wt
Normalised Counts
20
30
40
50
60
zmp_ph108_F9
zmp_ph108_F11
zmp_ph108_H2
zmp_ph108_H5
zmp_ph108_B2
zmp_ph108_B6
zmp_ph108_B7
zmp_ph108_C4
zmp_ph108_C5
zmp_ph108_D4
zmp_ph97_G6
zmp_ph97_G9
zmp_ph97_H2
zmp_ph97_B10
zmp_ph97_C2
zmp_ph97_C4
zmp_ph97_B8
zmp_ph97_C1
zmp_ph97_D2
zmp_ph97_D5
zmp_ph111_G5
zmp_ph111_G11
zmp_ph111_H1
zmp_ph111_B1a
zmp_ph111_B5
zmp_ph111_B8
zmp_ph111_B6
zmp_ph111_B7
zmp_ph111_B9
zmp_ph111_B10
zmp_ph114_G4
zmp_ph114_G11
zmp_ph114_H1
zmp_ph114_H2
zmp_ph114_A9
zmp_ph114_A11
zmp_ph114_B2
zmp_ph114_B4
zmp_ph114_D8
zmp_ph114_E2

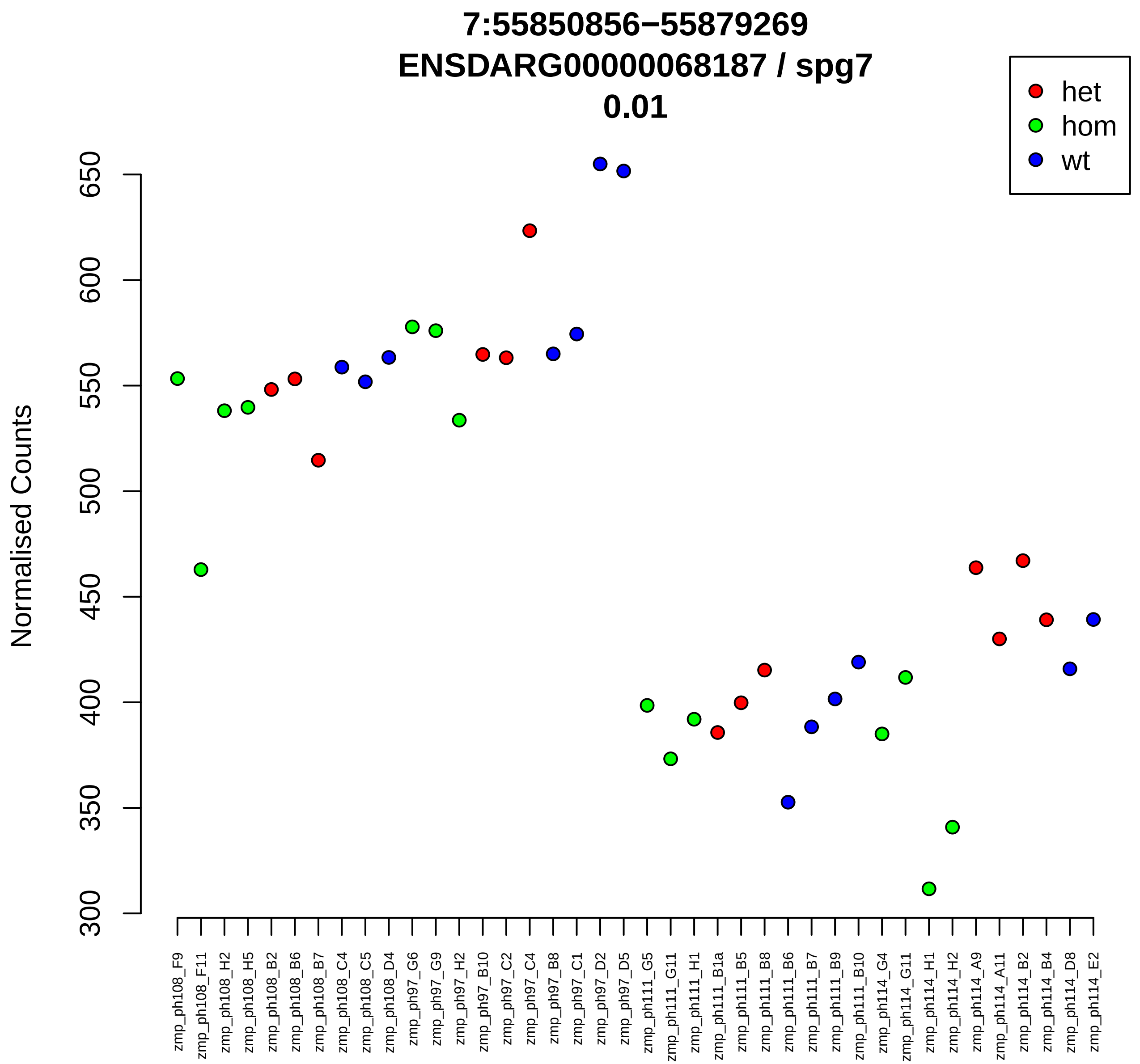
7:55850856−55879269
ENSDARG00000068187 / spg7
0.01
het
hom
wt
Normalised Counts
300
350
400
450
500
550
600
650
zmp_ph108_F9
zmp_ph108_F11
zmp_ph108_H2
zmp_ph108_H5
zmp_ph108_B2
zmp_ph108_B6
zmp_ph108_B7
zmp_ph108_C4
zmp_ph108_C5
zmp_ph108_D4
zmp_ph97_G6
zmp_ph97_G9
zmp_ph97_H2
zmp_ph97_B10
zmp_ph97_C2
zmp_ph97_C4
zmp_ph97_B8
zmp_ph97_C1
zmp_ph97_D2
zmp_ph97_D5
zmp_ph111_G5
zmp_ph111_G11
zmp_ph111_H1
zmp_ph111_B1a
zmp_ph111_B5
zmp_ph111_B8
zmp_ph111_B6
zmp_ph111_B7
zmp_ph111_B9
zmp_ph111_B10
zmp_ph114_G4
zmp_ph114_G11
zmp_ph114_H1
zmp_ph114_H2
zmp_ph114_A9
zmp_ph114_A11
zmp_ph114_B2
zmp_ph114_B4
zmp_ph114_D8
zmp_ph114_E2

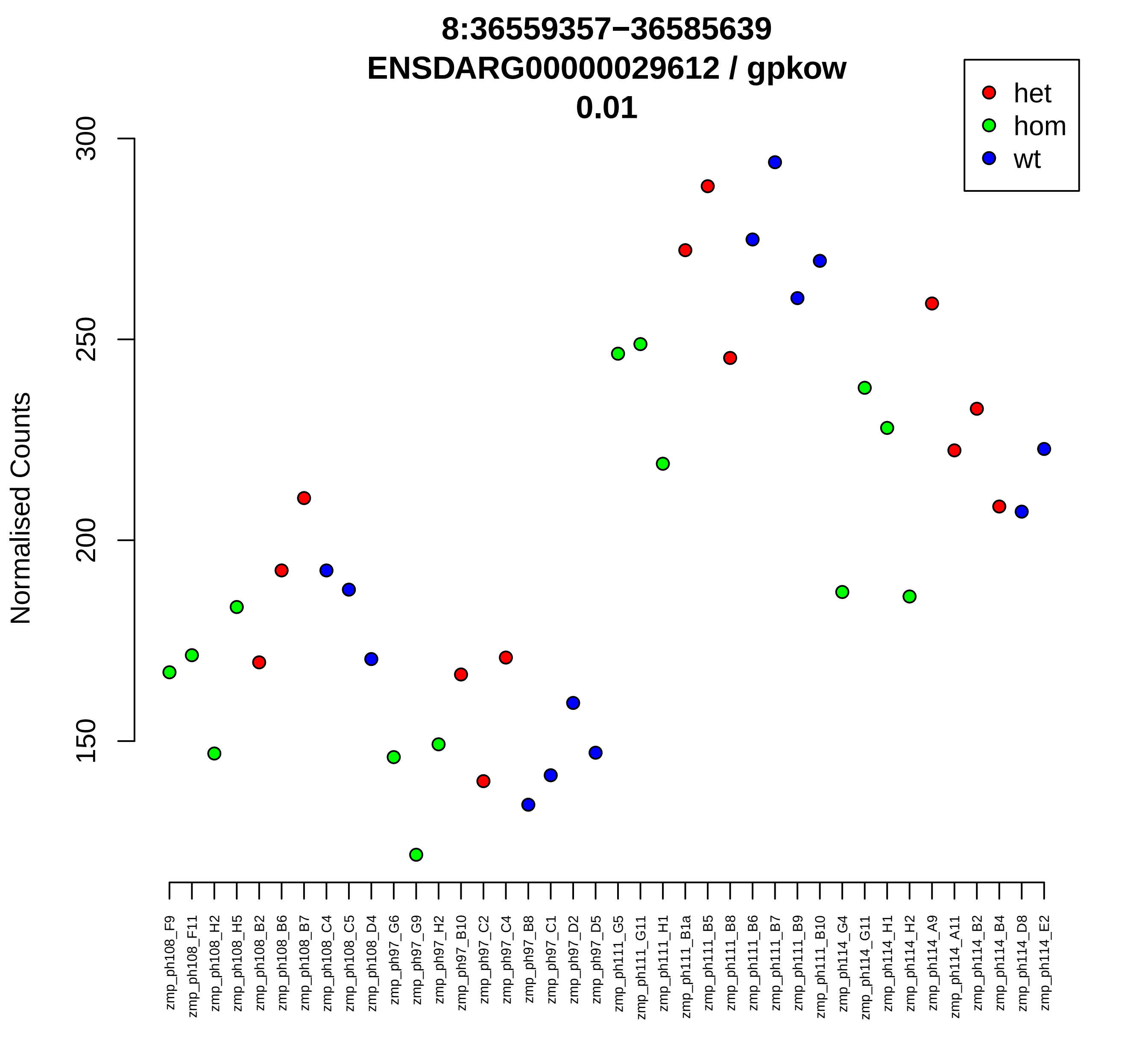
8:36559357−36585639
ENSDARG00000029612 / gpkow
0.01
het
hom
wt
Normalised Counts
150
200
250
300
zmp_ph108_F9
zmp_ph108_F11
zmp_ph108_H2
zmp_ph108_H5
zmp_ph108_B2
zmp_ph108_B6
zmp_ph108_B7
zmp_ph108_C4
zmp_ph108_C5
zmp_ph108_D4
zmp_ph97_G6
zmp_ph97_G9
zmp_ph97_H2
zmp_ph97_B10
zmp_ph97_C2
zmp_ph97_C4
zmp_ph97_B8
zmp_ph97_C1
zmp_ph97_D2
zmp_ph97_D5
zmp_ph111_G5
zmp_ph111_G11
zmp_ph111_H1
zmp_ph111_B1a
zmp_ph111_B5
zmp_ph111_B8
zmp_ph111_B6
zmp_ph111_B7
zmp_ph111_B9
zmp_ph111_B10
zmp_ph114_G4
zmp_ph114_G11
zmp_ph114_H1
zmp_ph114_H2
zmp_ph114_A9
zmp_ph114_A11
zmp_ph114_B2
zmp_ph114_B4
zmp_ph114_D8
zmp_ph114_E2

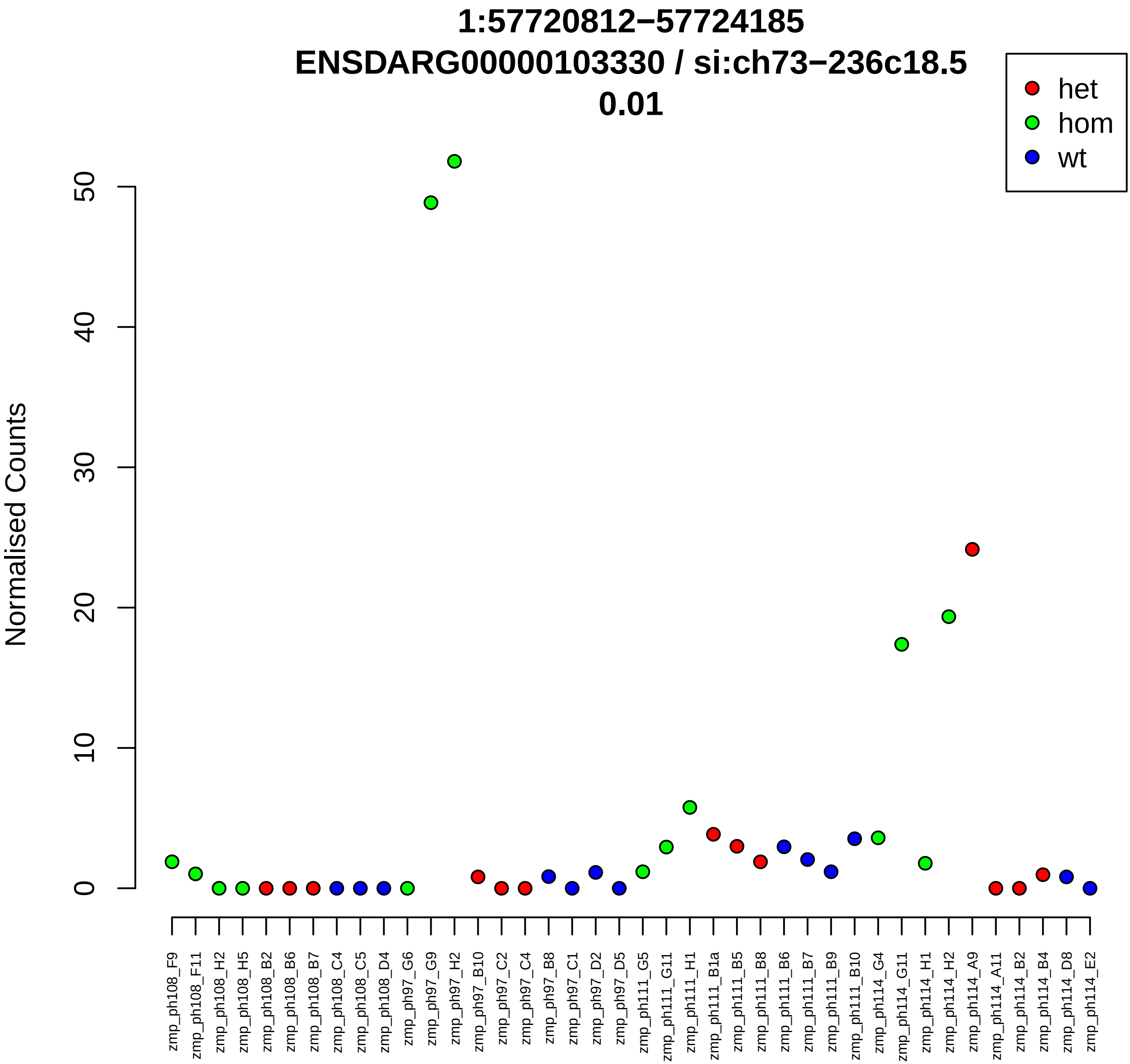
1:57720812−57724185
ENSDARG00000103330 / si:ch73−236c18.5
0.01
het
hom
wt
Normalised Counts
0
10
20
30
40
50
zmp_ph108_F9
zmp_ph108_F11
zmp_ph108_H2
zmp_ph108_H5
zmp_ph108_B2
zmp_ph108_B6
zmp_ph108_B7
zmp_ph108_C4
zmp_ph108_C5
zmp_ph108_D4
zmp_ph97_G6
zmp_ph97_G9
zmp_ph97_H2
zmp_ph97_B10
zmp_ph97_C2
zmp_ph97_C4
zmp_ph97_B8
zmp_ph97_C1
zmp_ph97_D2
zmp_ph97_D5
zmp_ph111_G5
zmp_ph111_G11
zmp_ph111_H1
zmp_ph111_B1a
zmp_ph111_B5
zmp_ph111_B8
zmp_ph111_B6
zmp_ph111_B7
zmp_ph111_B9
zmp_ph111_B10
zmp_ph114_G4
zmp_ph114_G11
zmp_ph114_H1
zmp_ph114_H2
zmp_ph114_A9
zmp_ph114_A11
zmp_ph114_B2
zmp_ph114_B4
zmp_ph114_D8
zmp_ph114_E2

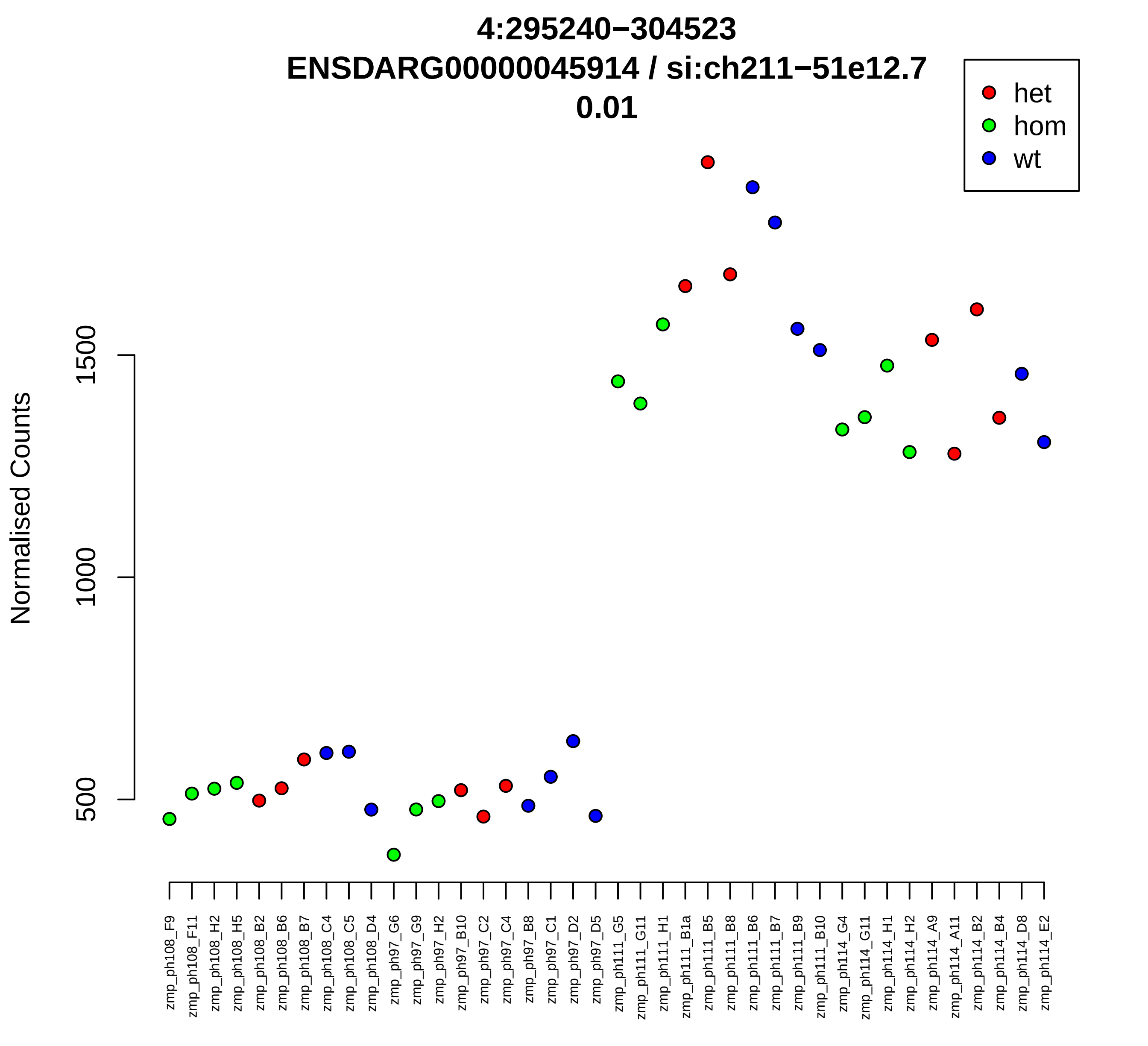
4:295240−304523
ENSDARG00000045914 / si:ch211−51e12.7
0.01
het
hom
wt
Normalised Counts
500
1000
1500
zmp_ph108_F9
zmp_ph108_F11
zmp_ph108_H2
zmp_ph108_H5
zmp_ph108_B2
zmp_ph108_B6
zmp_ph108_B7
zmp_ph108_C4
zmp_ph108_C5
zmp_ph108_D4
zmp_ph97_G6
zmp_ph97_G9
zmp_ph97_H2
zmp_ph97_B10
zmp_ph97_C2
zmp_ph97_C4
zmp_ph97_B8
zmp_ph97_C1
zmp_ph97_D2
zmp_ph97_D5
zmp_ph111_G5
zmp_ph111_G11
zmp_ph111_H1
zmp_ph111_B1a
zmp_ph111_B5
zmp_ph111_B8
zmp_ph111_B6
zmp_ph111_B7
zmp_ph111_B9
zmp_ph111_B10
zmp_ph114_G4
zmp_ph114_G11
zmp_ph114_H1
zmp_ph114_H2
zmp_ph114_A9
zmp_ph114_A11
zmp_ph114_B2
zmp_ph114_B4
zmp_ph114_D8
zmp_ph114_E2

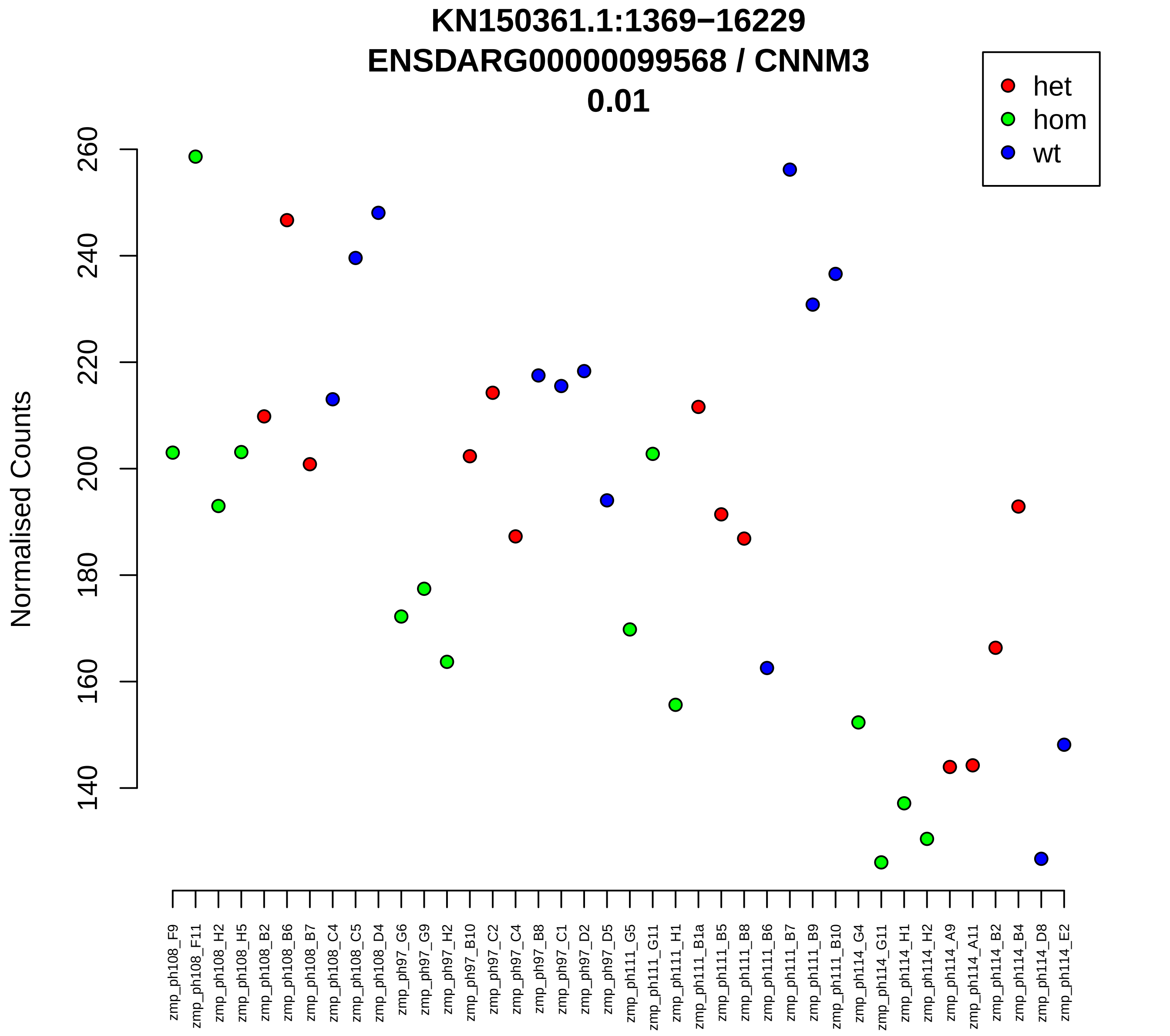
KN150361.1:1369−16229
ENSDARG00000099568 / CNNM3
0.01
het
hom
wt
Normalised Counts
140
160
180
200
220
240
260
zmp_ph108_F9
zmp_ph108_F11
zmp_ph108_H2
zmp_ph108_H5
zmp_ph108_B2
zmp_ph108_B6
zmp_ph108_B7
zmp_ph108_C4
zmp_ph108_C5
zmp_ph108_D4
zmp_ph97_G6
zmp_ph97_G9
zmp_ph97_H2
zmp_ph97_B10
zmp_ph97_C2
zmp_ph97_C4
zmp_ph97_B8
zmp_ph97_C1
zmp_ph97_D2
zmp_ph97_D5
zmp_ph111_G5
zmp_ph111_G11
zmp_ph111_H1
zmp_ph111_B1a
zmp_ph111_B5
zmp_ph111_B8
zmp_ph111_B6
zmp_ph111_B7
zmp_ph111_B9
zmp_ph111_B10
zmp_ph114_G4
zmp_ph114_G11
zmp_ph114_H1
zmp_ph114_H2
zmp_ph114_A9
zmp_ph114_A11
zmp_ph114_B2
zmp_ph114_B4
zmp_ph114_D8
zmp_ph114_E2

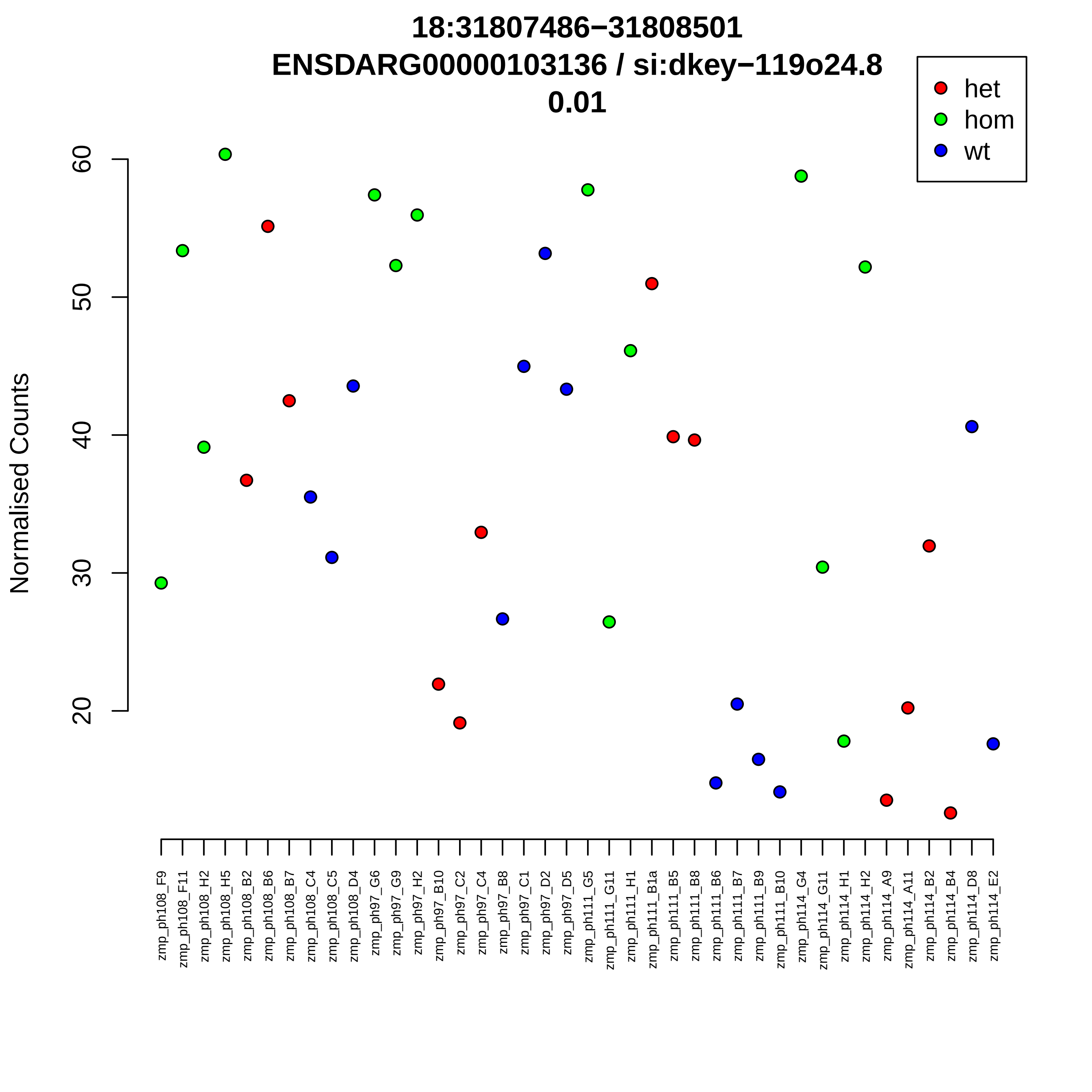

18:31807486−31808501
ENSDARG00000103136 / si:dkey−119o24.8
0.01
het
hom
wt
Normalised Counts
20
30
40
50
60
zmp_ph108_F9
zmp_ph108_F11
zmp_ph108_H2
zmp_ph108_H5
zmp_ph108_B2
zmp_ph108_B6
zmp_ph108_B7
zmp_ph108_C4
zmp_ph108_C5
zmp_ph108_D4
zmp_ph97_G6
zmp_ph97_G9
zmp_ph97_H2
zmp_ph97_B10
zmp_ph97_C2
zmp_ph97_C4
zmp_ph97_B8
zmp_ph97_C1
zmp_ph97_D2
zmp_ph97_D5
zmp_ph111_G5
zmp_ph111_G11
zmp_ph111_H1
zmp_ph111_B1a
zmp_ph111_B5
zmp_ph111_B8
zmp_ph111_B6
zmp_ph111_B7
zmp_ph111_B9
zmp_ph111_B10
zmp_ph114_G4
zmp_ph114_G11
zmp_ph114_H1
zmp_ph114_H2
zmp_ph114_A9
zmp_ph114_A11
zmp_ph114_B2
zmp_ph114_B4
zmp_ph114_D8
zmp_ph114_E2

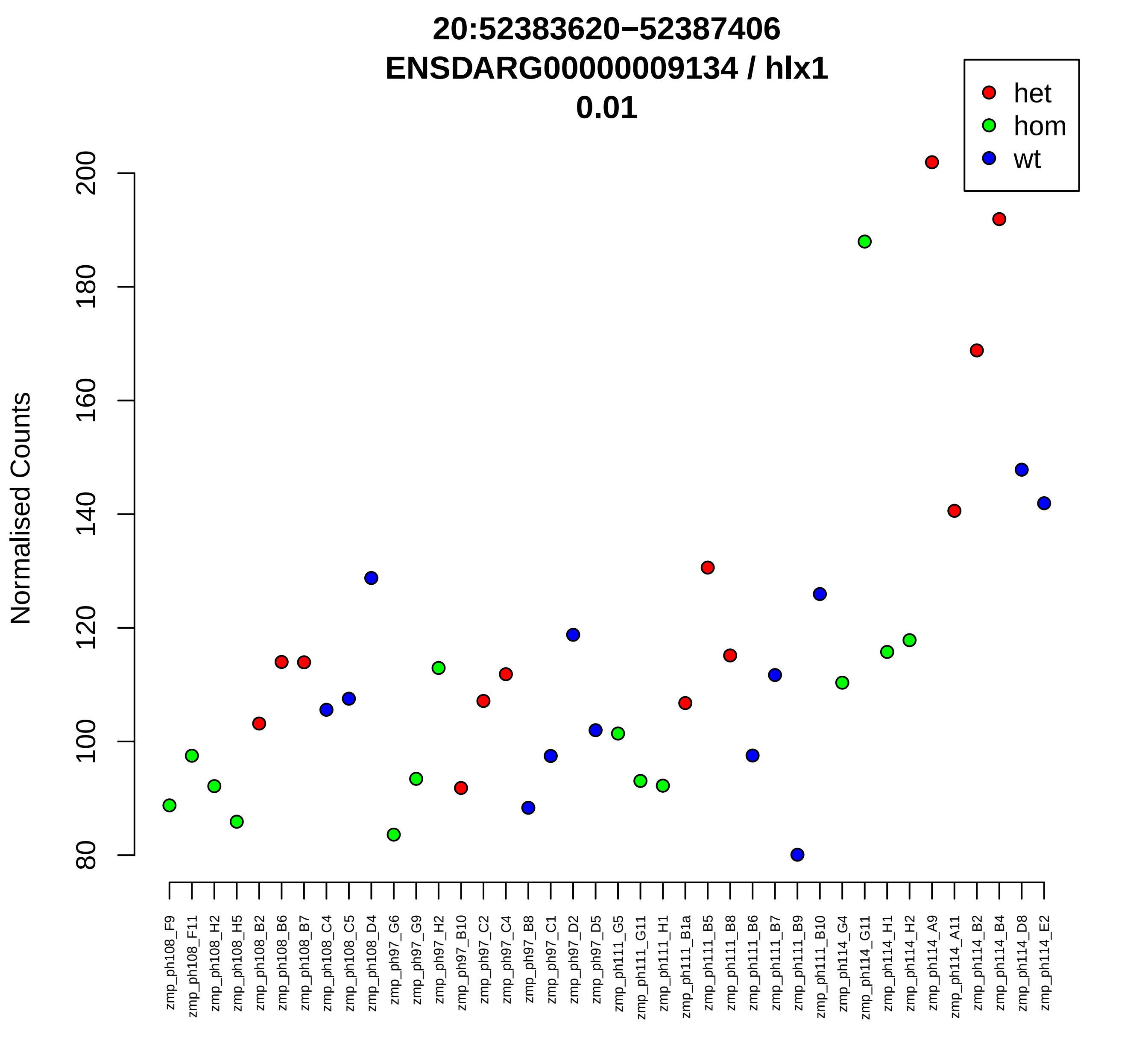
20:52383620−52387406
ENSDARG00000009134 / hlx1
0.01
het
hom
wt
Normalised Counts
80
100
120
140
160
180
200
zmp_ph108_F9
zmp_ph108_F11
zmp_ph108_H2
zmp_ph108_H5
zmp_ph108_B2
zmp_ph108_B6
zmp_ph108_B7
zmp_ph108_C4
zmp_ph108_C5
zmp_ph108_D4
zmp_ph97_G6
zmp_ph97_G9
zmp_ph97_H2
zmp_ph97_B10
zmp_ph97_C2
zmp_ph97_C4
zmp_ph97_B8
zmp_ph97_C1
zmp_ph97_D2
zmp_ph97_D5
zmp_ph111_G5
zmp_ph111_G11
zmp_ph111_H1
zmp_ph111_B1a
zmp_ph111_B5
zmp_ph111_B8
zmp_ph111_B6
zmp_ph111_B7
zmp_ph111_B9
zmp_ph111_B10
zmp_ph114_G4
zmp_ph114_G11
zmp_ph114_H1
zmp_ph114_H2
zmp_ph114_A9
zmp_ph114_A11
zmp_ph114_B2
zmp_ph114_B4
zmp_ph114_D8
zmp_ph114_E2

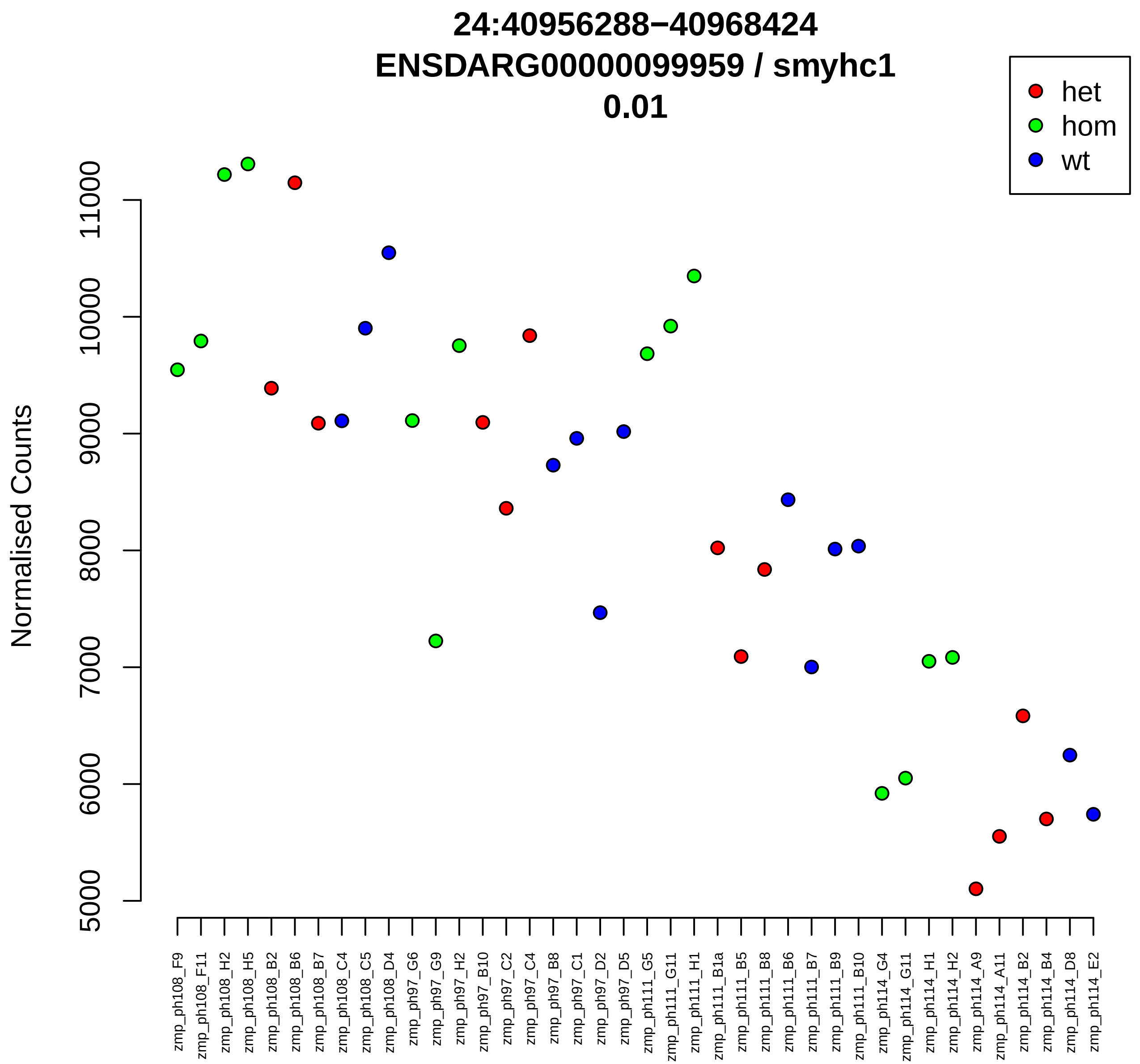

24:40956288−40968424
ENSDARG00000099959 / smyhc1
0.01
het
hom
wt
Normalised Counts
5000
6000
7000
8000
9000
10000
11000
zmp_ph108_F9
zmp_ph108_F11
zmp_ph108_H2
zmp_ph108_H5
zmp_ph108_B2
zmp_ph108_B6
zmp_ph108_B7
zmp_ph108_C4
zmp_ph108_C5
zmp_ph108_D4
zmp_ph97_G6
zmp_ph97_G9
zmp_ph97_H2
zmp_ph97_B10
zmp_ph97_C2
zmp_ph97_C4
zmp_ph97_B8
zmp_ph97_C1
zmp_ph97_D2
zmp_ph97_D5
zmp_ph111_G5
zmp_ph111_G11
zmp_ph111_H1
zmp_ph111_B1a
zmp_ph111_B5
zmp_ph111_B8
zmp_ph111_B6
zmp_ph111_B7
zmp_ph111_B9
zmp_ph111_B10
zmp_ph114_G4
zmp_ph114_G11
zmp_ph114_H1
zmp_ph114_H2
zmp_ph114_A9
zmp_ph114_A11
zmp_ph114_B2
zmp_ph114_B4
zmp_ph114_D8
zmp_ph114_E2

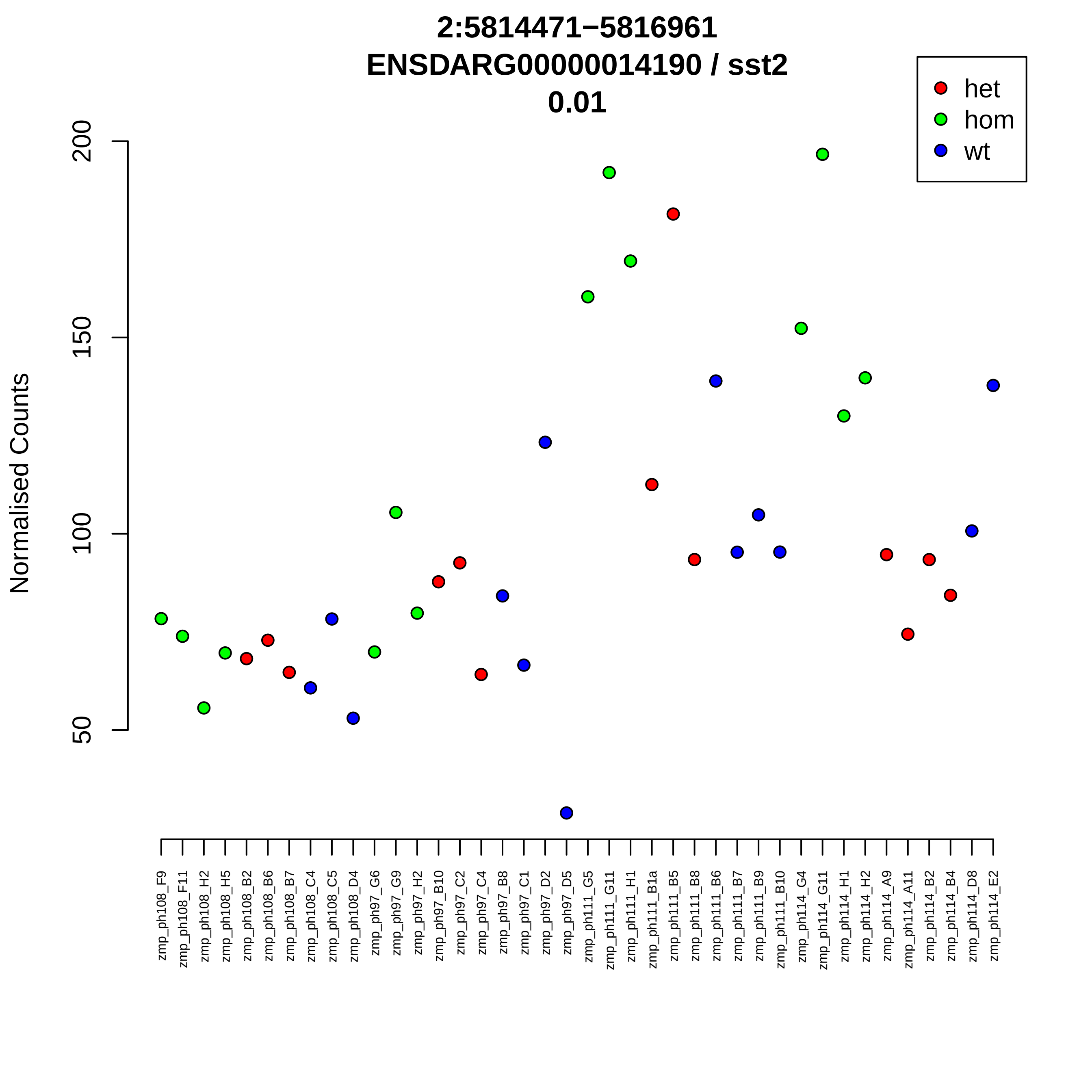
2:5814471−5816961
ENSDARG00000014190 / sst2
0.01
het
hom
wt
Normalised Counts
50
100
150
200
zmp_ph108_F9
zmp_ph108_F11
zmp_ph108_H2
zmp_ph108_H5
zmp_ph108_B2
zmp_ph108_B6
zmp_ph108_B7
zmp_ph108_C4
zmp_ph108_C5
zmp_ph108_D4
zmp_ph97_G6
zmp_ph97_G9
zmp_ph97_H2
zmp_ph97_B10
zmp_ph97_C2
zmp_ph97_C4
zmp_ph97_B8
zmp_ph97_C1
zmp_ph97_D2
zmp_ph97_D5
zmp_ph111_G5
zmp_ph111_G11
zmp_ph111_H1
zmp_ph111_B1a
zmp_ph111_B5
zmp_ph111_B8
zmp_ph111_B6
zmp_ph111_B7
zmp_ph111_B9
zmp_ph111_B10
zmp_ph114_G4
zmp_ph114_G11
zmp_ph114_H1
zmp_ph114_H2
zmp_ph114_A9
zmp_ph114_A11
zmp_ph114_B2
zmp_ph114_B4
zmp_ph114_D8
zmp_ph114_E2

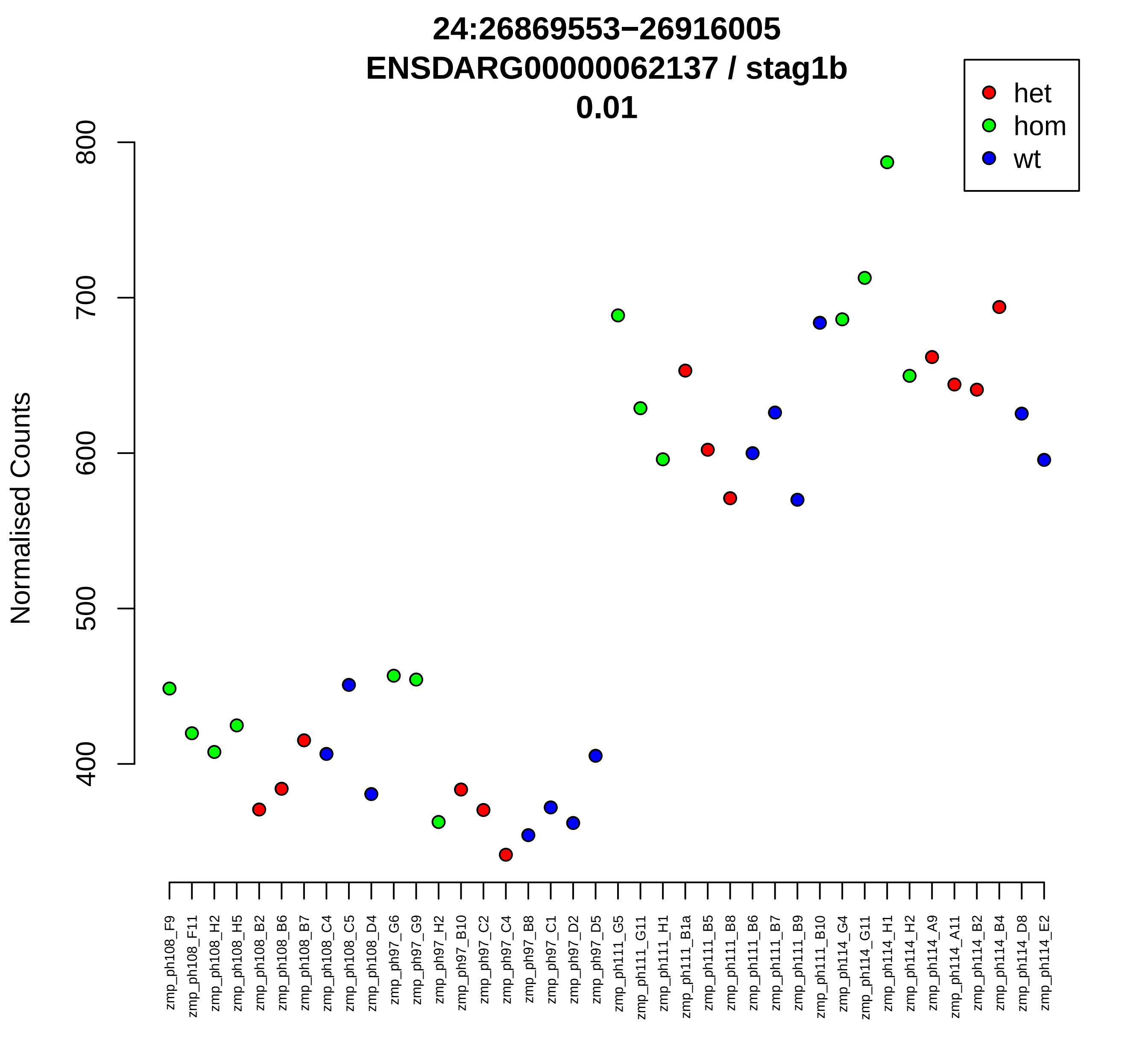

24:26869553−26916005
ENSDARG00000062137 / stag1b
0.01
het
hom
wt
Normalised Counts
400
500
600
700
800
zmp_ph108_F9
zmp_ph108_F11
zmp_ph108_H2
zmp_ph108_H5
zmp_ph108_B2
zmp_ph108_B6
zmp_ph108_B7
zmp_ph108_C4
zmp_ph108_C5
zmp_ph108_D4
zmp_ph97_G6
zmp_ph97_G9
zmp_ph97_H2
zmp_ph97_B10
zmp_ph97_C2
zmp_ph97_C4
zmp_ph97_B8
zmp_ph97_C1
zmp_ph97_D2
zmp_ph97_D5
zmp_ph111_G5
zmp_ph111_G11
zmp_ph111_H1
zmp_ph111_B1a
zmp_ph111_B5
zmp_ph111_B8
zmp_ph111_B6
zmp_ph111_B7
zmp_ph111_B9
zmp_ph111_B10
zmp_ph114_G4
zmp_ph114_G11
zmp_ph114_H1
zmp_ph114_H2
zmp_ph114_A9
zmp_ph114_A11
zmp_ph114_B2
zmp_ph114_B4
zmp_ph114_D8
zmp_ph114_E2

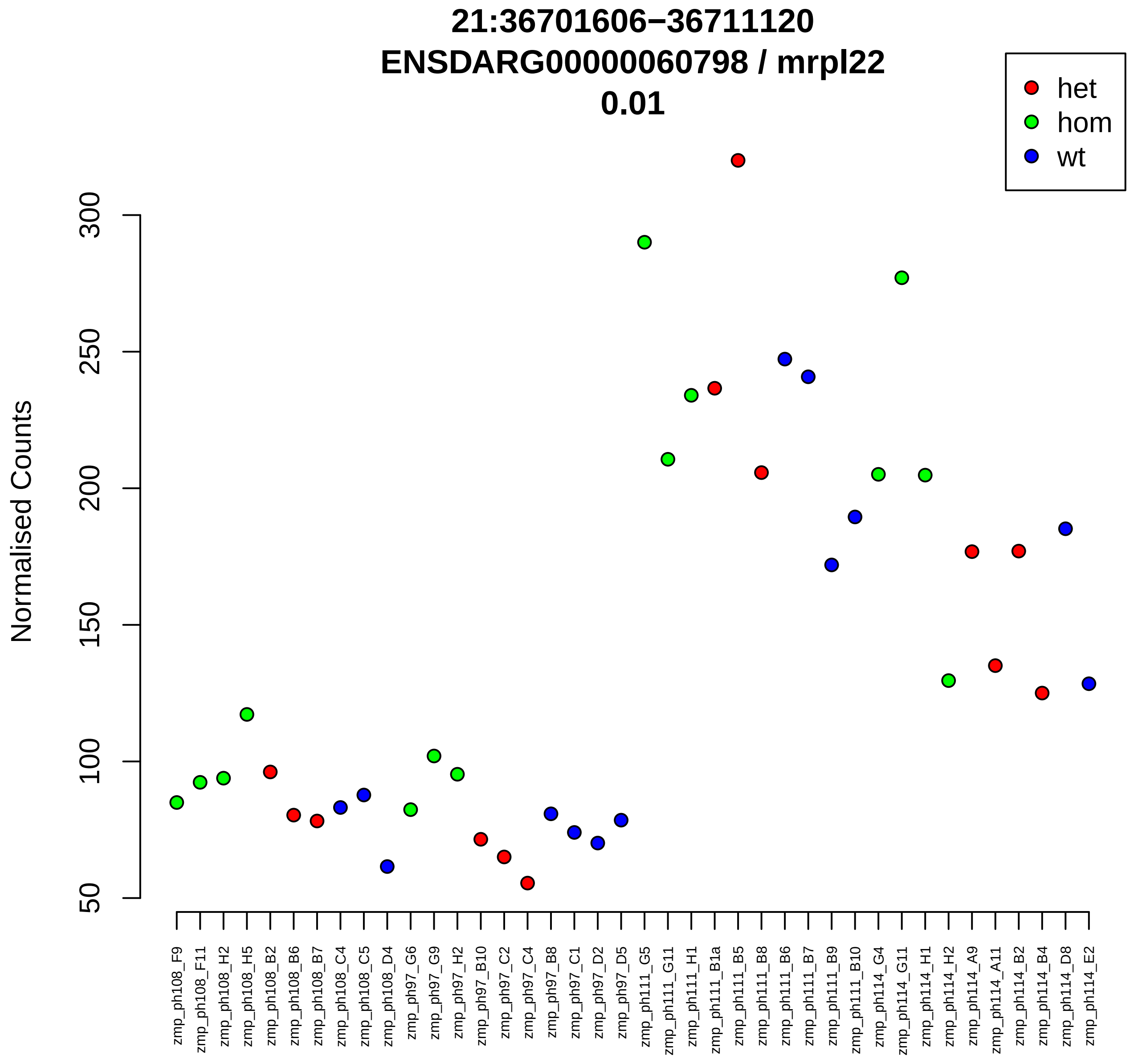

21:36701606−36711120
ENSDARG00000060798 / mrpl22
0.01
het
hom
wt
Normalised Counts
50
100
150
200
250
300
zmp_ph108_F9
zmp_ph108_F11
zmp_ph108_H2
zmp_ph108_H5
zmp_ph108_B2
zmp_ph108_B6
zmp_ph108_B7
zmp_ph108_C4
zmp_ph108_C5
zmp_ph108_D4
zmp_ph97_G6
zmp_ph97_G9
zmp_ph97_H2
zmp_ph97_B10
zmp_ph97_C2
zmp_ph97_C4
zmp_ph97_B8
zmp_ph97_C1
zmp_ph97_D2
zmp_ph97_D5
zmp_ph111_G5
zmp_ph111_G11
zmp_ph111_H1
zmp_ph111_B1a
zmp_ph111_B5
zmp_ph111_B8
zmp_ph111_B6
zmp_ph111_B7
zmp_ph111_B9
zmp_ph111_B10
zmp_ph114_G4
zmp_ph114_G11
zmp_ph114_H1
zmp_ph114_H2
zmp_ph114_A9
zmp_ph114_A11
zmp_ph114_B2
zmp_ph114_B4
zmp_ph114_D8
zmp_ph114_E2

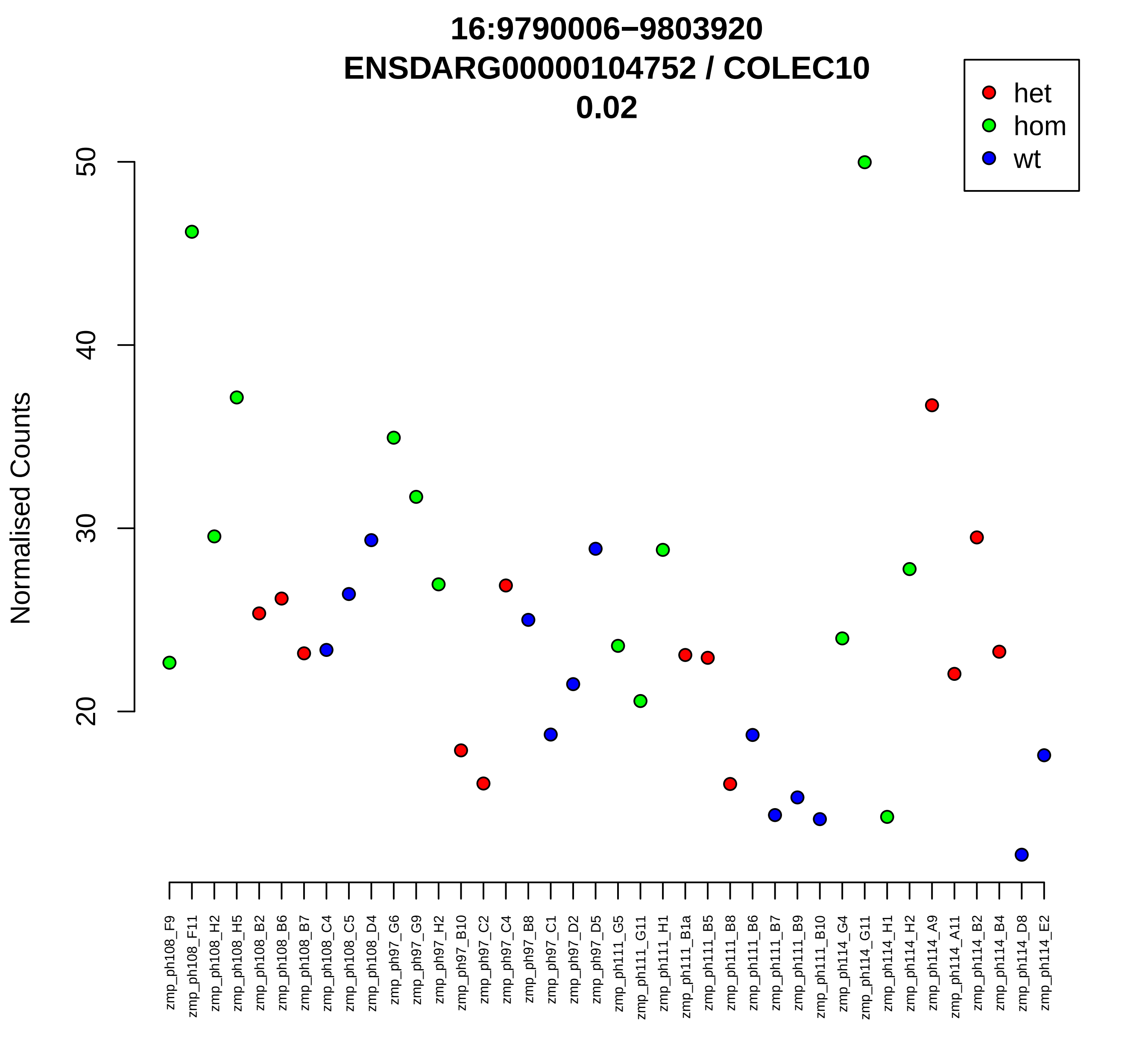

16:9790006−9803920
ENSDARG00000104752 / COLEC10
0.02
het
hom
wt
Normalised Counts
20
30
40
50
zmp_ph108_F9
zmp_ph108_F11
zmp_ph108_H2
zmp_ph108_H5
zmp_ph108_B2
zmp_ph108_B6
zmp_ph108_B7
zmp_ph108_C4
zmp_ph108_C5
zmp_ph108_D4
zmp_ph97_G6
zmp_ph97_G9
zmp_ph97_H2
zmp_ph97_B10
zmp_ph97_C2
zmp_ph97_C4
zmp_ph97_B8
zmp_ph97_C1
zmp_ph97_D2
zmp_ph97_D5
zmp_ph111_G5
zmp_ph111_G11
zmp_ph111_H1
zmp_ph111_B1a
zmp_ph111_B5
zmp_ph111_B8
zmp_ph111_B6
zmp_ph111_B7
zmp_ph111_B9
zmp_ph111_B10
zmp_ph114_G4
zmp_ph114_G11
zmp_ph114_H1
zmp_ph114_H2
zmp_ph114_A9
zmp_ph114_A11
zmp_ph114_B2
zmp_ph114_B4
zmp_ph114_D8
zmp_ph114_E2

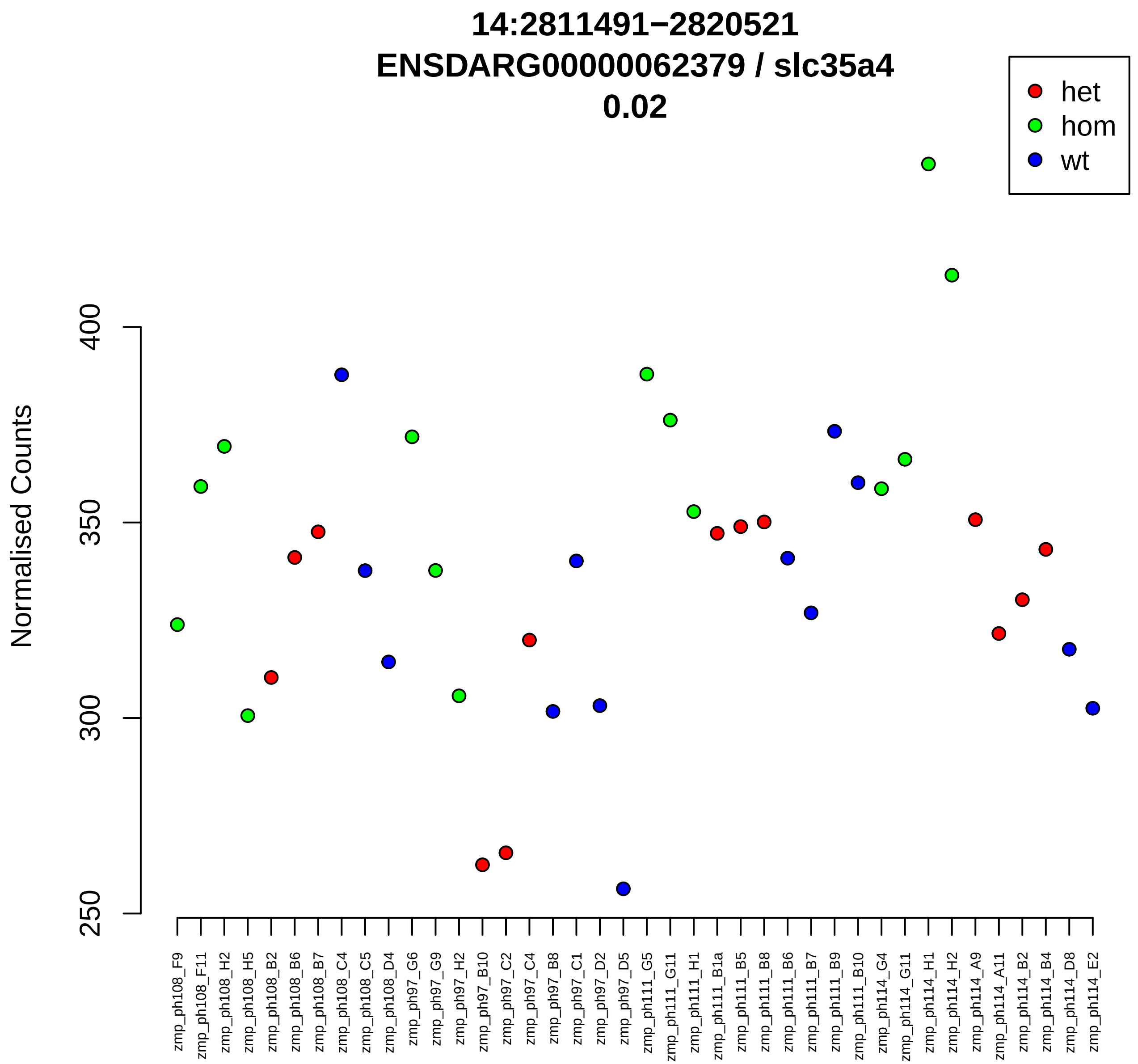

14:2811491−2820521
ENSDARG00000062379 / slc35a4
0.02
het
hom
wt
Normalised Counts
250
300
350
400
zmp_ph108_F9
zmp_ph108_F11
zmp_ph108_H2
zmp_ph108_H5
zmp_ph108_B2
zmp_ph108_B6
zmp_ph108_B7
zmp_ph108_C4
zmp_ph108_C5
zmp_ph108_D4
zmp_ph97_G6
zmp_ph97_G9
zmp_ph97_H2
zmp_ph97_B10
zmp_ph97_C2
zmp_ph97_C4
zmp_ph97_B8
zmp_ph97_C1
zmp_ph97_D2
zmp_ph97_D5
zmp_ph111_G5
zmp_ph111_G11
zmp_ph111_H1
zmp_ph111_B1a
zmp_ph111_B5
zmp_ph111_B8
zmp_ph111_B6
zmp_ph111_B7
zmp_ph111_B9
zmp_ph111_B10
zmp_ph114_G4
zmp_ph114_G11
zmp_ph114_H1
zmp_ph114_H2
zmp_ph114_A9
zmp_ph114_A11
zmp_ph114_B2
zmp_ph114_B4
zmp_ph114_D8
zmp_ph114_E2

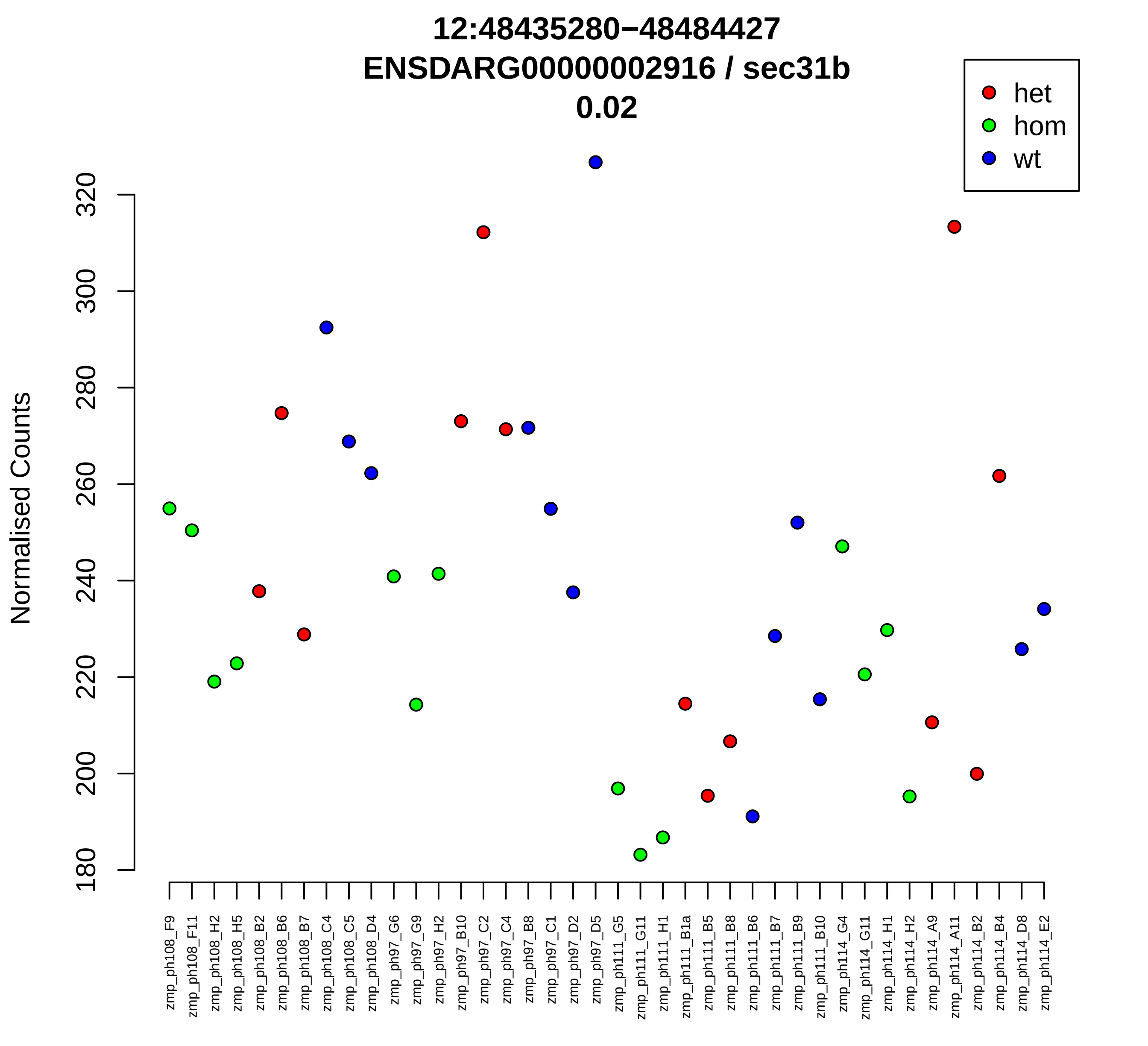
12:48435280−48484427
ENSDARG00000002916 / sec31b
0.02
het
hom
wt
Normalised Counts
180
200
220
240
260
280
300
320
zmp_ph108_F9
zmp_ph108_F11
zmp_ph108_H2
zmp_ph108_H5
zmp_ph108_B2
zmp_ph108_B6
zmp_ph108_B7
zmp_ph108_C4
zmp_ph108_C5
zmp_ph108_D4
zmp_ph97_G6
zmp_ph97_G9
zmp_ph97_H2
zmp_ph97_B10
zmp_ph97_C2
zmp_ph97_C4
zmp_ph97_B8
zmp_ph97_C1
zmp_ph97_D2
zmp_ph97_D5
zmp_ph111_G5
zmp_ph111_G11
zmp_ph111_H1
zmp_ph111_B1a
zmp_ph111_B5
zmp_ph111_B8
zmp_ph111_B6
zmp_ph111_B7
zmp_ph111_B9
zmp_ph111_B10
zmp_ph114_G4
zmp_ph114_G11
zmp_ph114_H1
zmp_ph114_H2
zmp_ph114_A9
zmp_ph114_A11
zmp_ph114_B2
zmp_ph114_B4
zmp_ph114_D8
zmp_ph114_E2

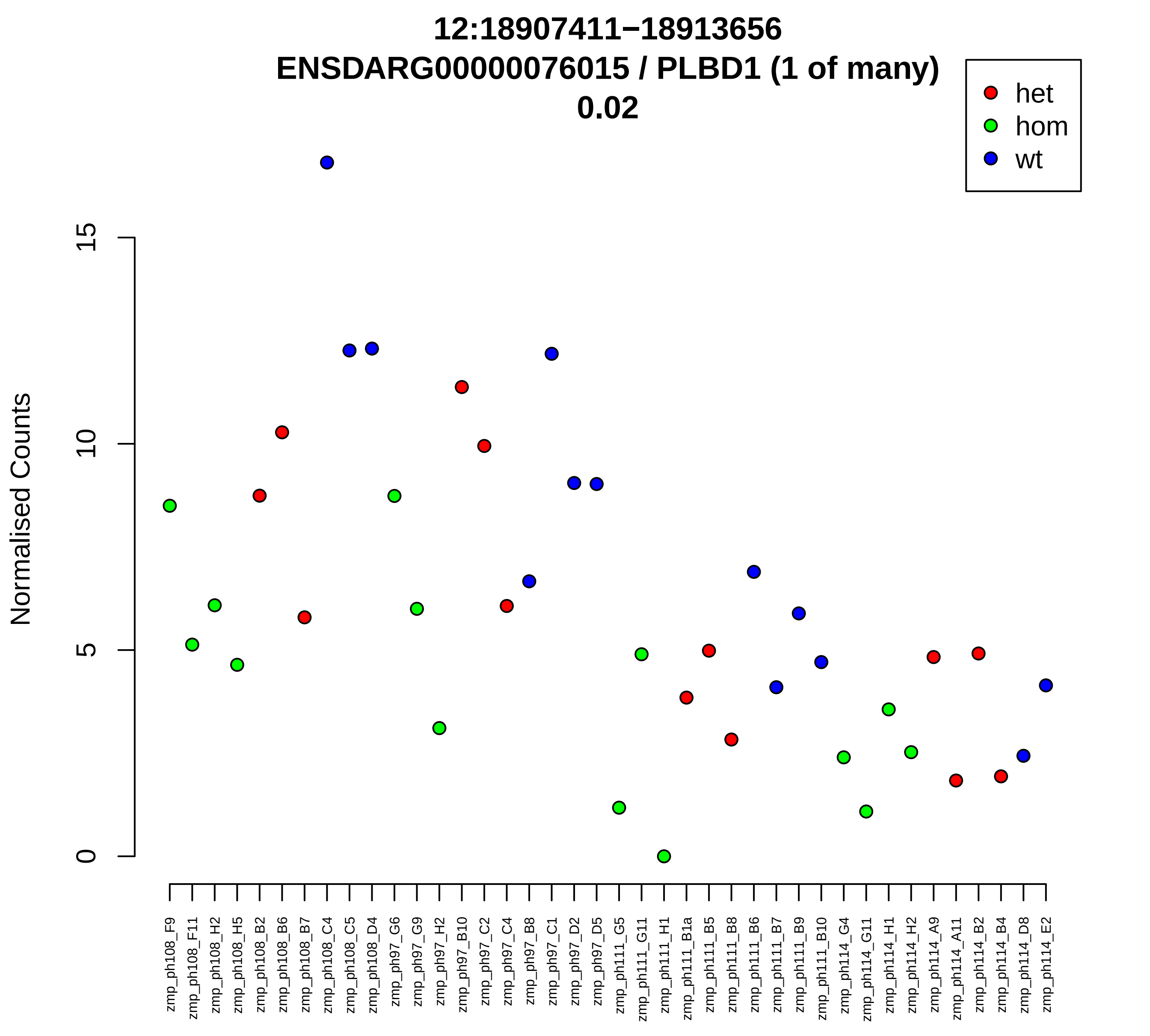

12:18907411−18913656
ENSDARG00000076015 / PLBD1 (1 of many)
0.02
het
hom
wt
Normalised Counts
0
5
10
15
zmp_ph108_F9
zmp_ph108_F11
zmp_ph108_H2
zmp_ph108_H5
zmp_ph108_B2
zmp_ph108_B6
zmp_ph108_B7
zmp_ph108_C4
zmp_ph108_C5
zmp_ph108_D4
zmp_ph97_G6
zmp_ph97_G9
zmp_ph97_H2
zmp_ph97_B10
zmp_ph97_C2
zmp_ph97_C4
zmp_ph97_B8
zmp_ph97_C1
zmp_ph97_D2
zmp_ph97_D5
zmp_ph111_G5
zmp_ph111_G11
zmp_ph111_H1
zmp_ph111_B1a
zmp_ph111_B5
zmp_ph111_B8
zmp_ph111_B6
zmp_ph111_B7
zmp_ph111_B9
zmp_ph111_B10
zmp_ph114_G4
zmp_ph114_G11
zmp_ph114_H1
zmp_ph114_H2
zmp_ph114_A9
zmp_ph114_A11
zmp_ph114_B2
zmp_ph114_B4
zmp_ph114_D8
zmp_ph114_E2

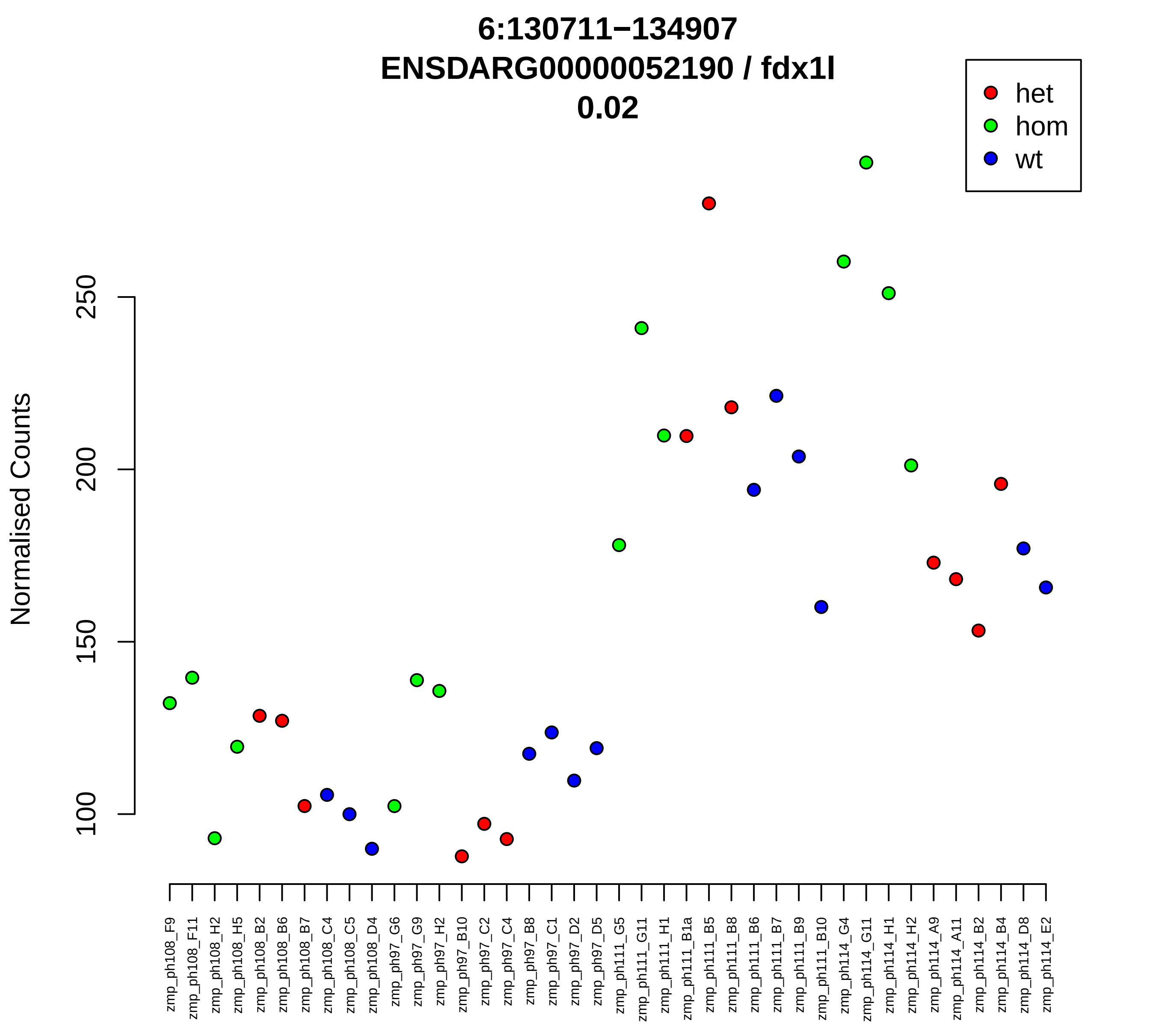

6:130711−134907
ENSDARG00000052190 / fdx1l
0.02
het
hom
wt
Normalised Counts
100
150
200
250
zmp_ph108_F9
zmp_ph108_F11
zmp_ph108_H2
zmp_ph108_H5
zmp_ph108_B2
zmp_ph108_B6
zmp_ph108_B7
zmp_ph108_C4
zmp_ph108_C5
zmp_ph108_D4
zmp_ph97_G6
zmp_ph97_G9
zmp_ph97_H2
zmp_ph97_B10
zmp_ph97_C2
zmp_ph97_C4
zmp_ph97_B8
zmp_ph97_C1
zmp_ph97_D2
zmp_ph97_D5
zmp_ph111_G5
zmp_ph111_G11
zmp_ph111_H1
zmp_ph111_B1a
zmp_ph111_B5
zmp_ph111_B8
zmp_ph111_B6
zmp_ph111_B7
zmp_ph111_B9
zmp_ph111_B10
zmp_ph114_G4
zmp_ph114_G11
zmp_ph114_H1
zmp_ph114_H2
zmp_ph114_A9
zmp_ph114_A11
zmp_ph114_B2
zmp_ph114_B4
zmp_ph114_D8
zmp_ph114_E2

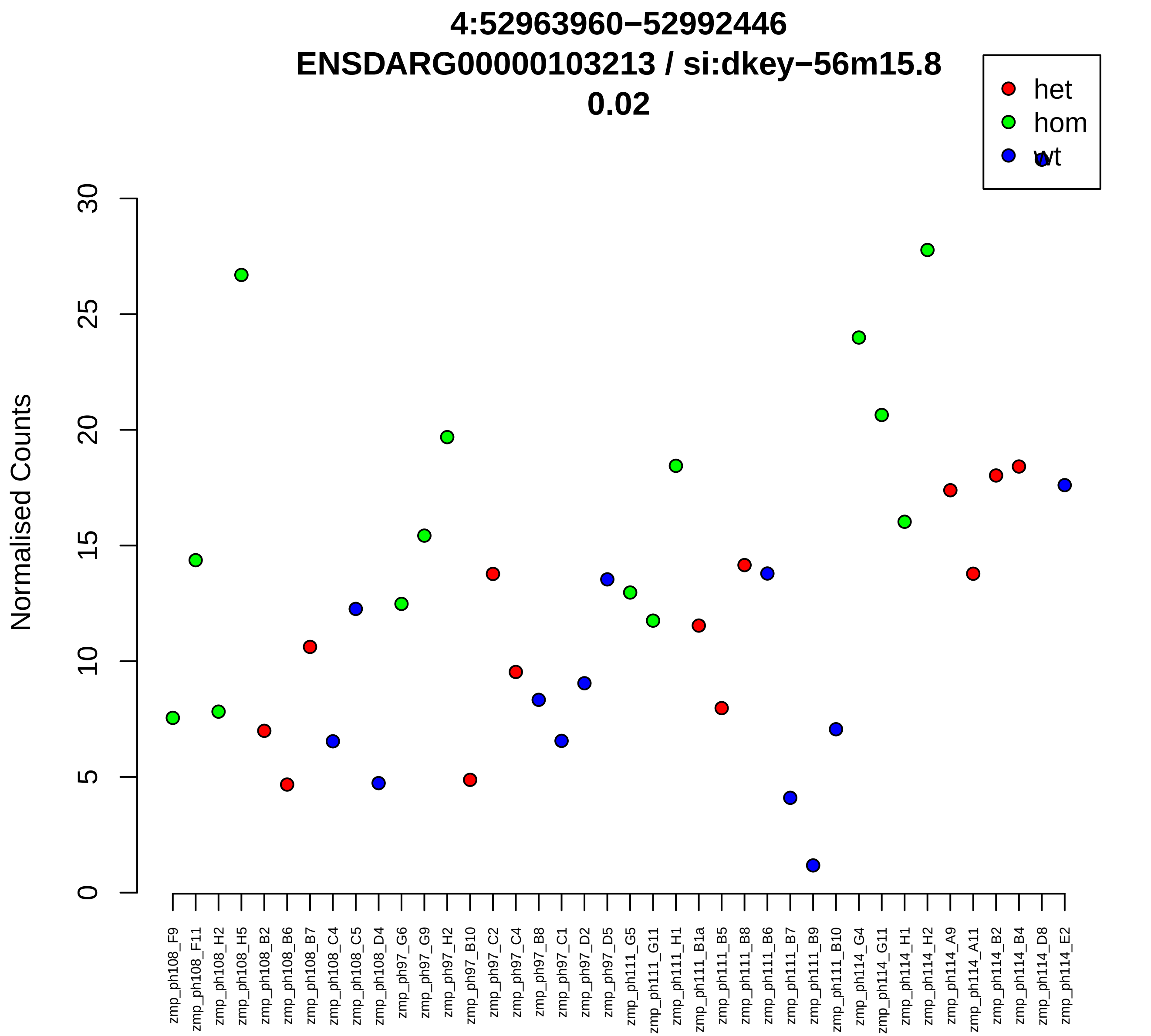

4:52963960−52992446
ENSDARG00000103213 / si:dkey−56m15.8
0.02
het
hom
wt
Normalised Counts
0
5
10
15
20
25
30
zmp_ph108_F9
zmp_ph108_F11
zmp_ph108_H2
zmp_ph108_H5
zmp_ph108_B2
zmp_ph108_B6
zmp_ph108_B7
zmp_ph108_C4
zmp_ph108_C5
zmp_ph108_D4
zmp_ph97_G6
zmp_ph97_G9
zmp_ph97_H2
zmp_ph97_B10
zmp_ph97_C2
zmp_ph97_C4
zmp_ph97_B8
zmp_ph97_C1
zmp_ph97_D2
zmp_ph97_D5
zmp_ph111_G5
zmp_ph111_G11
zmp_ph111_H1
zmp_ph111_B1a
zmp_ph111_B5
zmp_ph111_B8
zmp_ph111_B6
zmp_ph111_B7
zmp_ph111_B9
zmp_ph111_B10
zmp_ph114_G4
zmp_ph114_G11
zmp_ph114_H1
zmp_ph114_H2
zmp_ph114_A9
zmp_ph114_A11
zmp_ph114_B2
zmp_ph114_B4
zmp_ph114_D8
zmp_ph114_E2

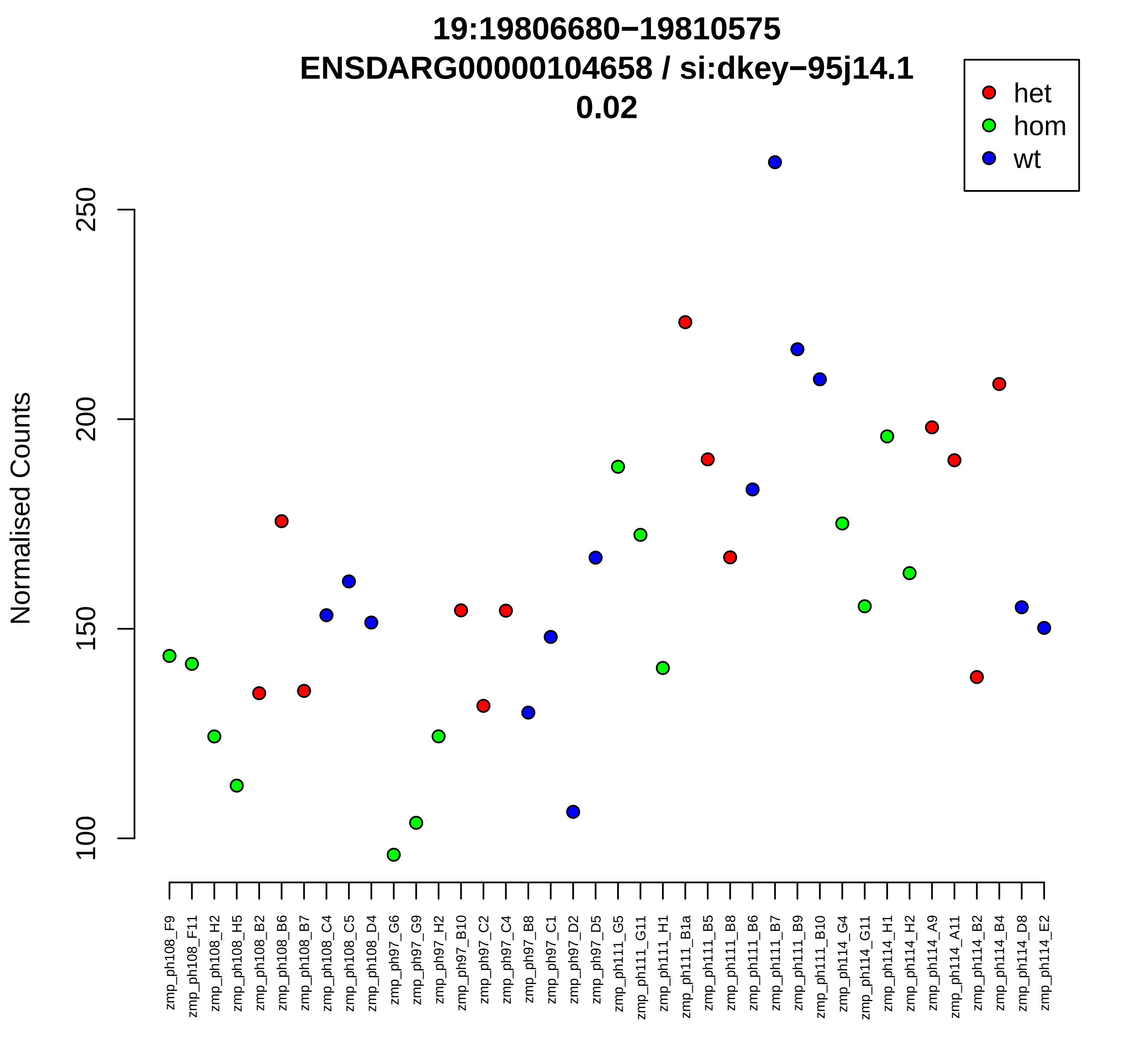

19:19806680−19810575
ENSDARG00000104658 / si:dkey−95j14.1
0.02
het
hom
wt
Normalised Counts
100
150
200
250
zmp_ph108_F9
zmp_ph108_F11
zmp_ph108_H2
zmp_ph108_H5
zmp_ph108_B2
zmp_ph108_B6
zmp_ph108_B7
zmp_ph108_C4
zmp_ph108_C5
zmp_ph108_D4
zmp_ph97_G6
zmp_ph97_G9
zmp_ph97_H2
zmp_ph97_B10
zmp_ph97_C2
zmp_ph97_C4
zmp_ph97_B8
zmp_ph97_C1
zmp_ph97_D2
zmp_ph97_D5
zmp_ph111_G5
zmp_ph111_G11
zmp_ph111_H1
zmp_ph111_B1a
zmp_ph111_B5
zmp_ph111_B8
zmp_ph111_B6
zmp_ph111_B7
zmp_ph111_B9
zmp_ph111_B10
zmp_ph114_G4
zmp_ph114_G11
zmp_ph114_H1
zmp_ph114_H2
zmp_ph114_A9
zmp_ph114_A11
zmp_ph114_B2
zmp_ph114_B4
zmp_ph114_D8
zmp_ph114_E2

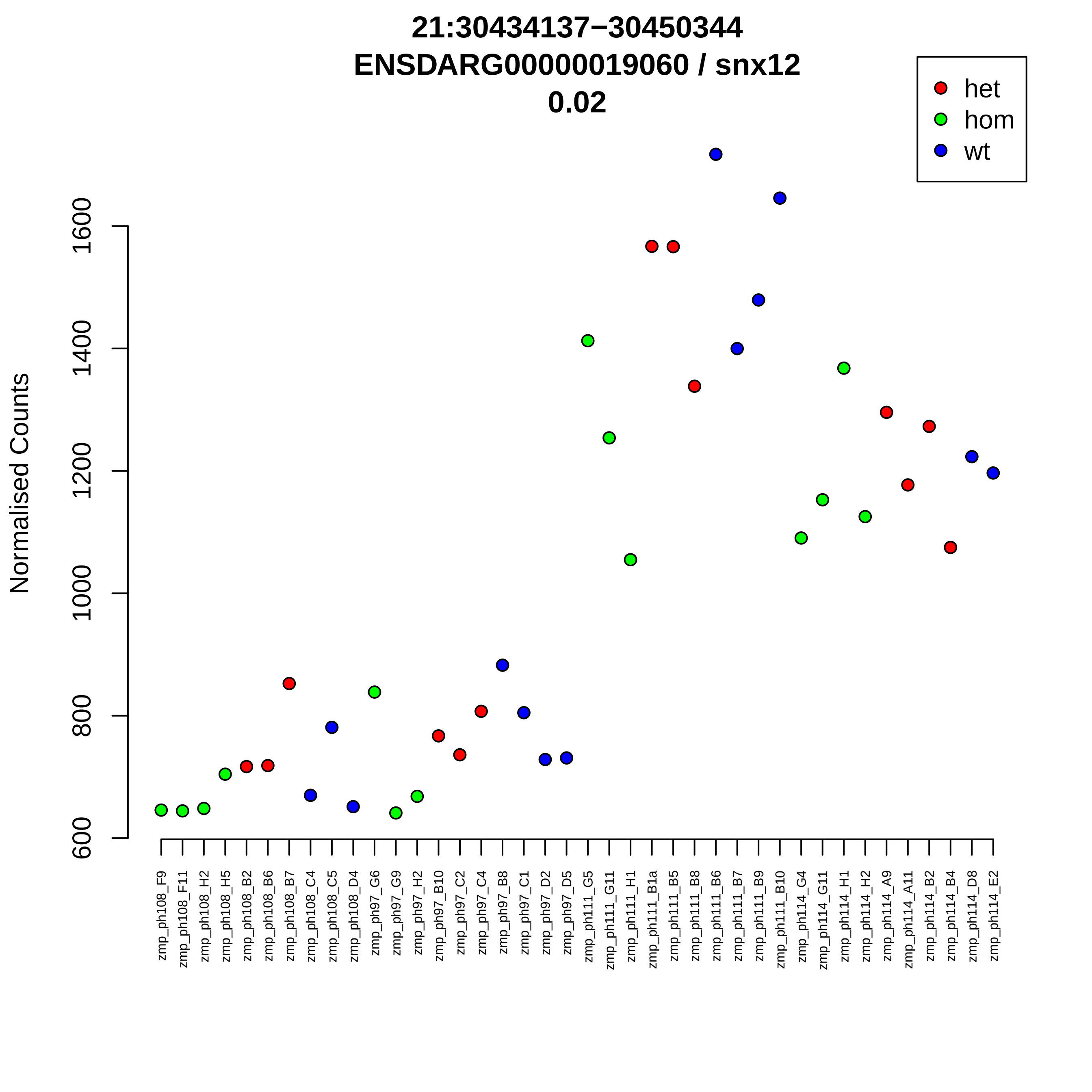

21:30434137−30450344
ENSDARG00000019060 / snx12
0.02
het
hom
wt
Normalised Counts
600
800
1000
1200
1400
1600
zmp_ph108_F9
zmp_ph108_F11
zmp_ph108_H2
zmp_ph108_H5
zmp_ph108_B2
zmp_ph108_B6
zmp_ph108_B7
zmp_ph108_C4
zmp_ph108_C5
zmp_ph108_D4
zmp_ph97_G6
zmp_ph97_G9
zmp_ph97_H2
zmp_ph97_B10
zmp_ph97_C2
zmp_ph97_C4
zmp_ph97_B8
zmp_ph97_C1
zmp_ph97_D2
zmp_ph97_D5
zmp_ph111_G5
zmp_ph111_G11
zmp_ph111_H1
zmp_ph111_B1a
zmp_ph111_B5
zmp_ph111_B8
zmp_ph111_B6
zmp_ph111_B7
zmp_ph111_B9
zmp_ph111_B10
zmp_ph114_G4
zmp_ph114_G11
zmp_ph114_H1
zmp_ph114_H2
zmp_ph114_A9
zmp_ph114_A11
zmp_ph114_B2
zmp_ph114_B4
zmp_ph114_D8
zmp_ph114_E2

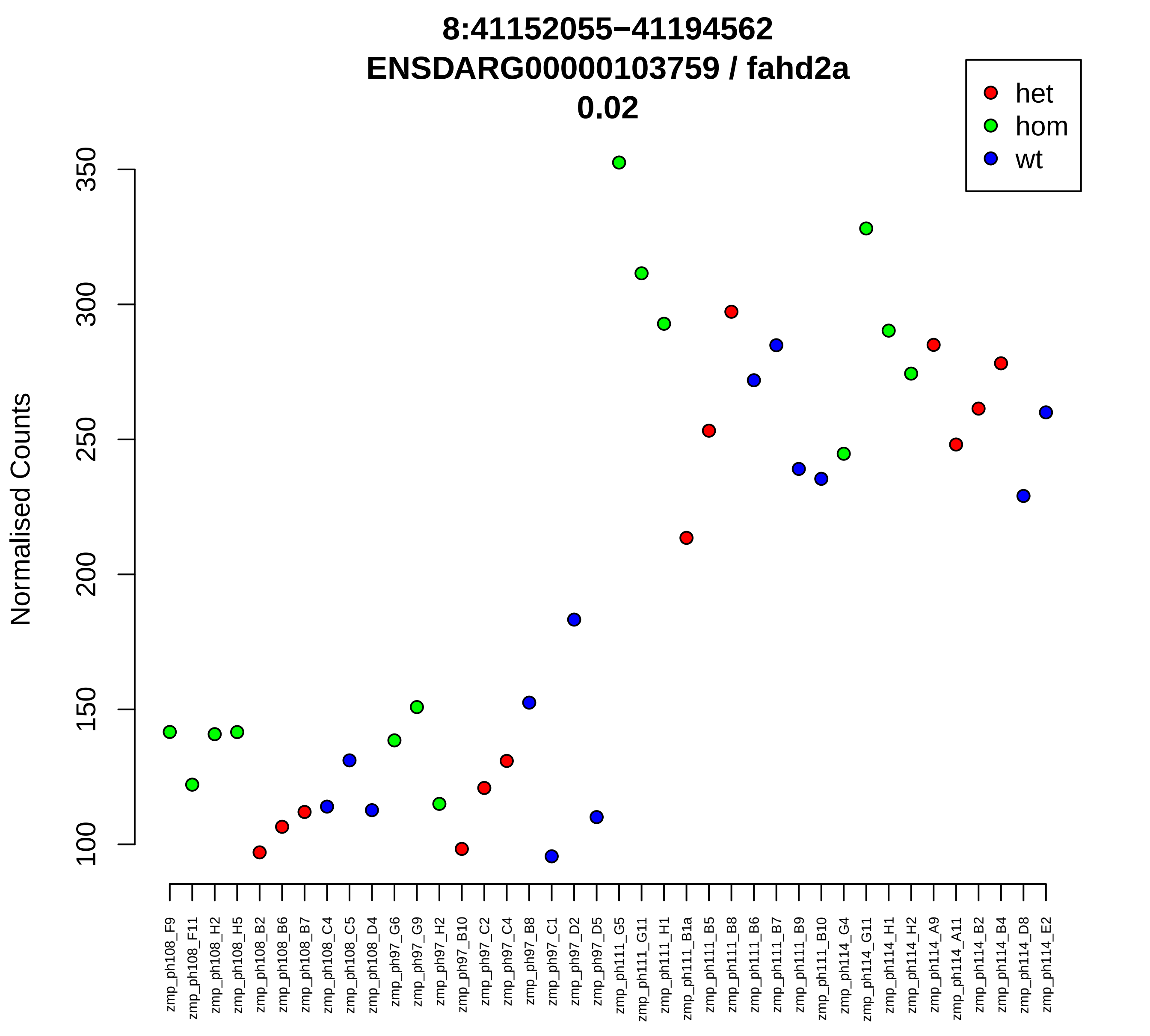
8:41152055−41194562
ENSDARG00000103759 / fahd2a
0.02
het
hom
wt
Normalised Counts
100
150
200
250
300
350
zmp_ph108_F9
zmp_ph108_F11
zmp_ph108_H2
zmp_ph108_H5
zmp_ph108_B2
zmp_ph108_B6
zmp_ph108_B7
zmp_ph108_C4
zmp_ph108_C5
zmp_ph108_D4
zmp_ph97_G6
zmp_ph97_G9
zmp_ph97_H2
zmp_ph97_B10
zmp_ph97_C2
zmp_ph97_C4
zmp_ph97_B8
zmp_ph97_C1
zmp_ph97_D2
zmp_ph97_D5
zmp_ph111_G5
zmp_ph111_G11
zmp_ph111_H1
zmp_ph111_B1a
zmp_ph111_B5
zmp_ph111_B8
zmp_ph111_B6
zmp_ph111_B7
zmp_ph111_B9
zmp_ph111_B10
zmp_ph114_G4
zmp_ph114_G11
zmp_ph114_H1
zmp_ph114_H2
zmp_ph114_A9
zmp_ph114_A11
zmp_ph114_B2
zmp_ph114_B4
zmp_ph114_D8
zmp_ph114_E2

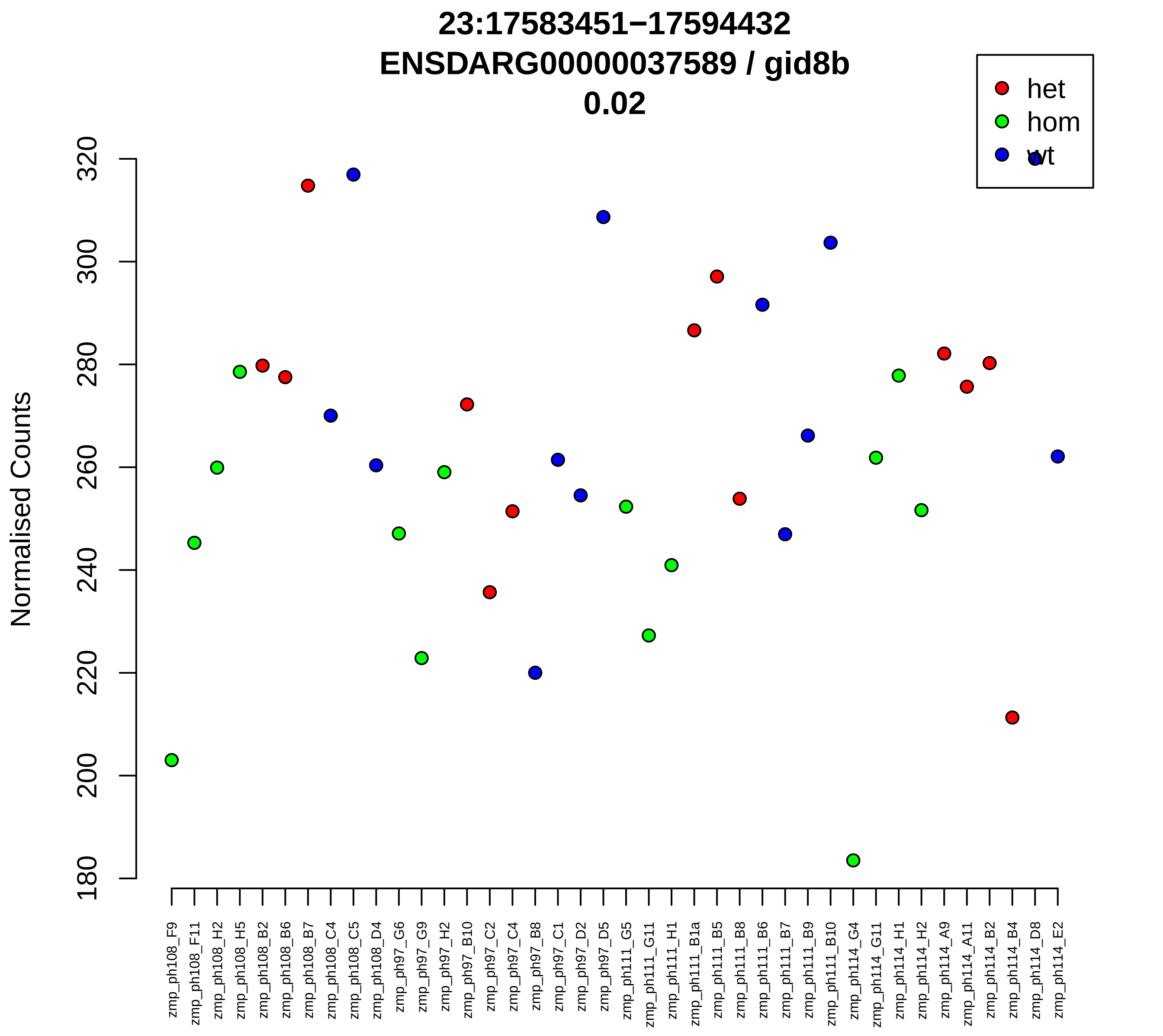

23:17583451−17594432
ENSDARG00000037589 / gid8b
0.02
het
hom
wt
Normalised Counts
180
200
220
240
260
280
300
320
zmp_ph108_F9
zmp_ph108_F11
zmp_ph108_H2
zmp_ph108_H5
zmp_ph108_B2
zmp_ph108_B6
zmp_ph108_B7
zmp_ph108_C4
zmp_ph108_C5
zmp_ph108_D4
zmp_ph97_G6
zmp_ph97_G9
zmp_ph97_H2
zmp_ph97_B10
zmp_ph97_C2
zmp_ph97_C4
zmp_ph97_B8
zmp_ph97_C1
zmp_ph97_D2
zmp_ph97_D5
zmp_ph111_G5
zmp_ph111_G11
zmp_ph111_H1
zmp_ph111_B1a
zmp_ph111_B5
zmp_ph111_B8
zmp_ph111_B6
zmp_ph111_B7
zmp_ph111_B9
zmp_ph111_B10
zmp_ph114_G4
zmp_ph114_G11
zmp_ph114_H1
zmp_ph114_H2
zmp_ph114_A9
zmp_ph114_A11
zmp_ph114_B2
zmp_ph114_B4
zmp_ph114_D8
zmp_ph114_E2

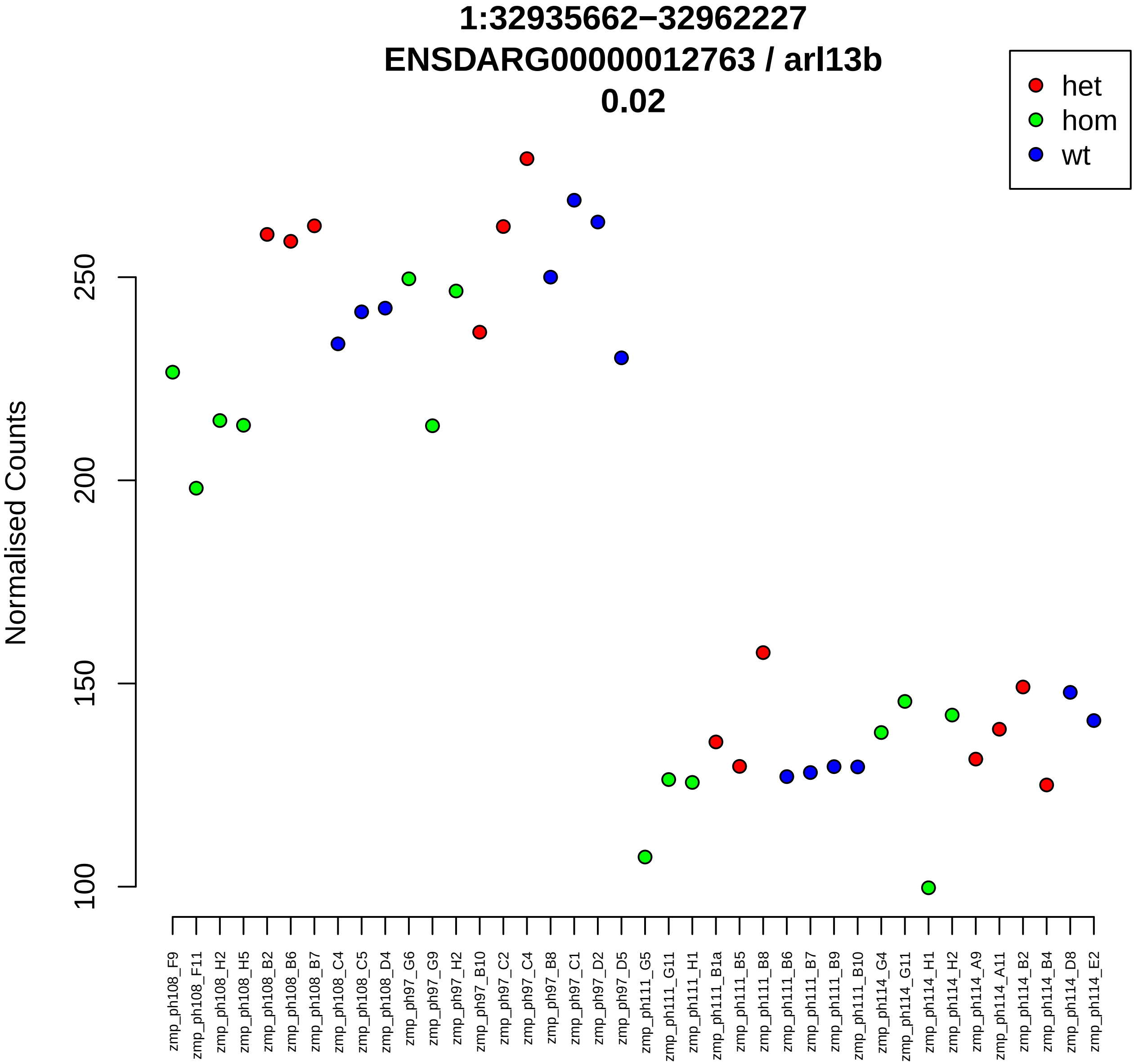

1:32935662−32962227
ENSDARG00000012763 / arl13b
0.02
het
hom
wt
Normalised Counts
100
150
200
250
zmp_ph108_F9
zmp_ph108_F11
zmp_ph108_H2
zmp_ph108_H5
zmp_ph108_B2
zmp_ph108_B6
zmp_ph108_B7
zmp_ph108_C4
zmp_ph108_C5
zmp_ph108_D4
zmp_ph97_G6
zmp_ph97_G9
zmp_ph97_H2
zmp_ph97_B10
zmp_ph97_C2
zmp_ph97_C4
zmp_ph97_B8
zmp_ph97_C1
zmp_ph97_D2
zmp_ph97_D5
zmp_ph111_G5
zmp_ph111_G11
zmp_ph111_H1
zmp_ph111_B1a
zmp_ph111_B5
zmp_ph111_B8
zmp_ph111_B6
zmp_ph111_B7
zmp_ph111_B9
zmp_ph111_B10
zmp_ph114_G4
zmp_ph114_G11
zmp_ph114_H1
zmp_ph114_H2
zmp_ph114_A9
zmp_ph114_A11
zmp_ph114_B2
zmp_ph114_B4
zmp_ph114_D8
zmp_ph114_E2

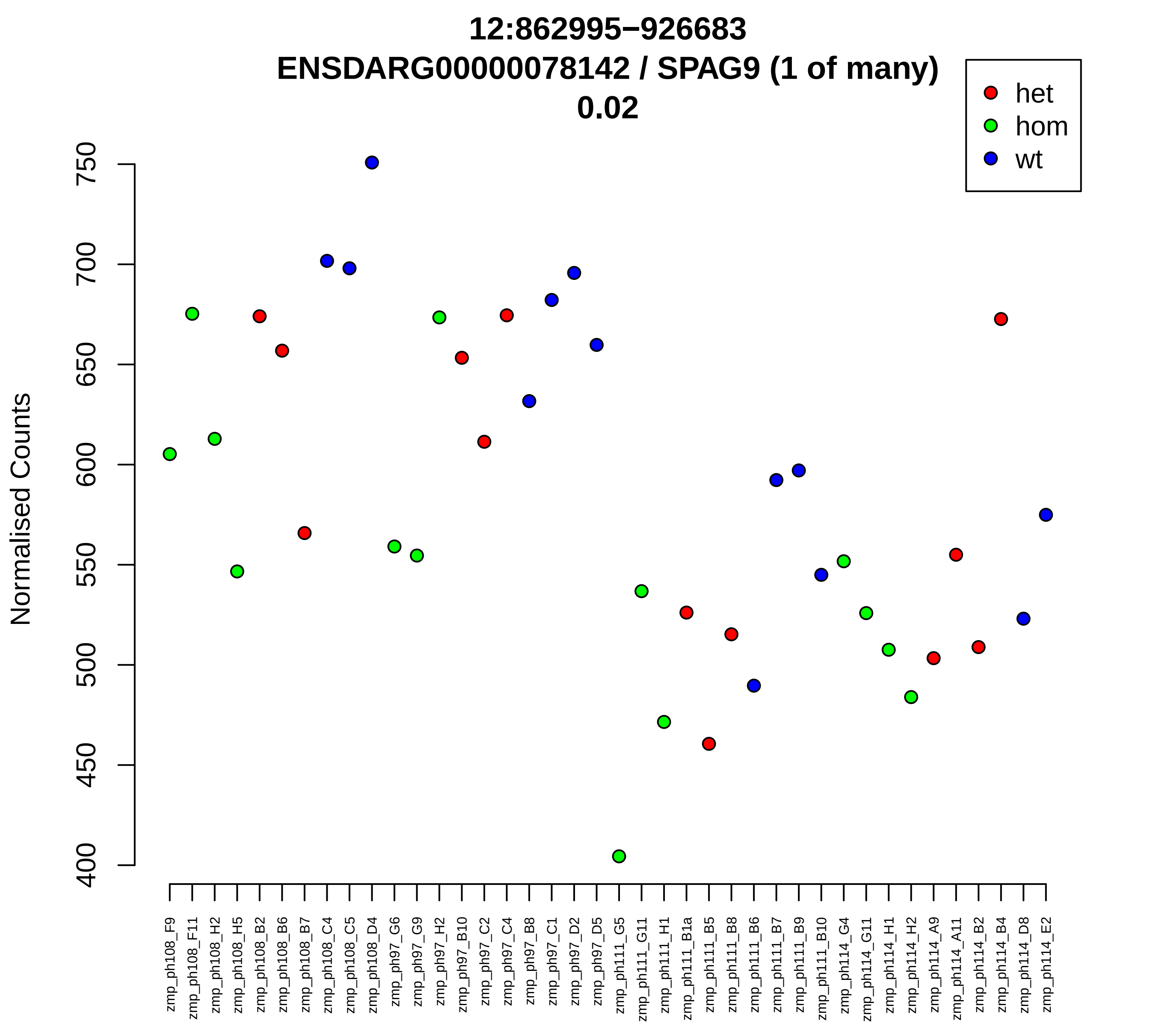
12:862995−926683
ENSDARG00000078142 / SPAG9 (1 of many)
0.02
het
hom
wt
Normalised Counts
400
450
500
550
600
650
700
750
zmp_ph108_F9
zmp_ph108_F11
zmp_ph108_H2
zmp_ph108_H5
zmp_ph108_B2
zmp_ph108_B6
zmp_ph108_B7
zmp_ph108_C4
zmp_ph108_C5
zmp_ph108_D4
zmp_ph97_G6
zmp_ph97_G9
zmp_ph97_H2
zmp_ph97_B10
zmp_ph97_C2
zmp_ph97_C4
zmp_ph97_B8
zmp_ph97_C1
zmp_ph97_D2
zmp_ph97_D5
zmp_ph111_G5
zmp_ph111_G11
zmp_ph111_H1
zmp_ph111_B1a
zmp_ph111_B5
zmp_ph111_B8
zmp_ph111_B6
zmp_ph111_B7
zmp_ph111_B9
zmp_ph111_B10
zmp_ph114_G4
zmp_ph114_G11
zmp_ph114_H1
zmp_ph114_H2
zmp_ph114_A9
zmp_ph114_A11
zmp_ph114_B2
zmp_ph114_B4
zmp_ph114_D8
zmp_ph114_E2

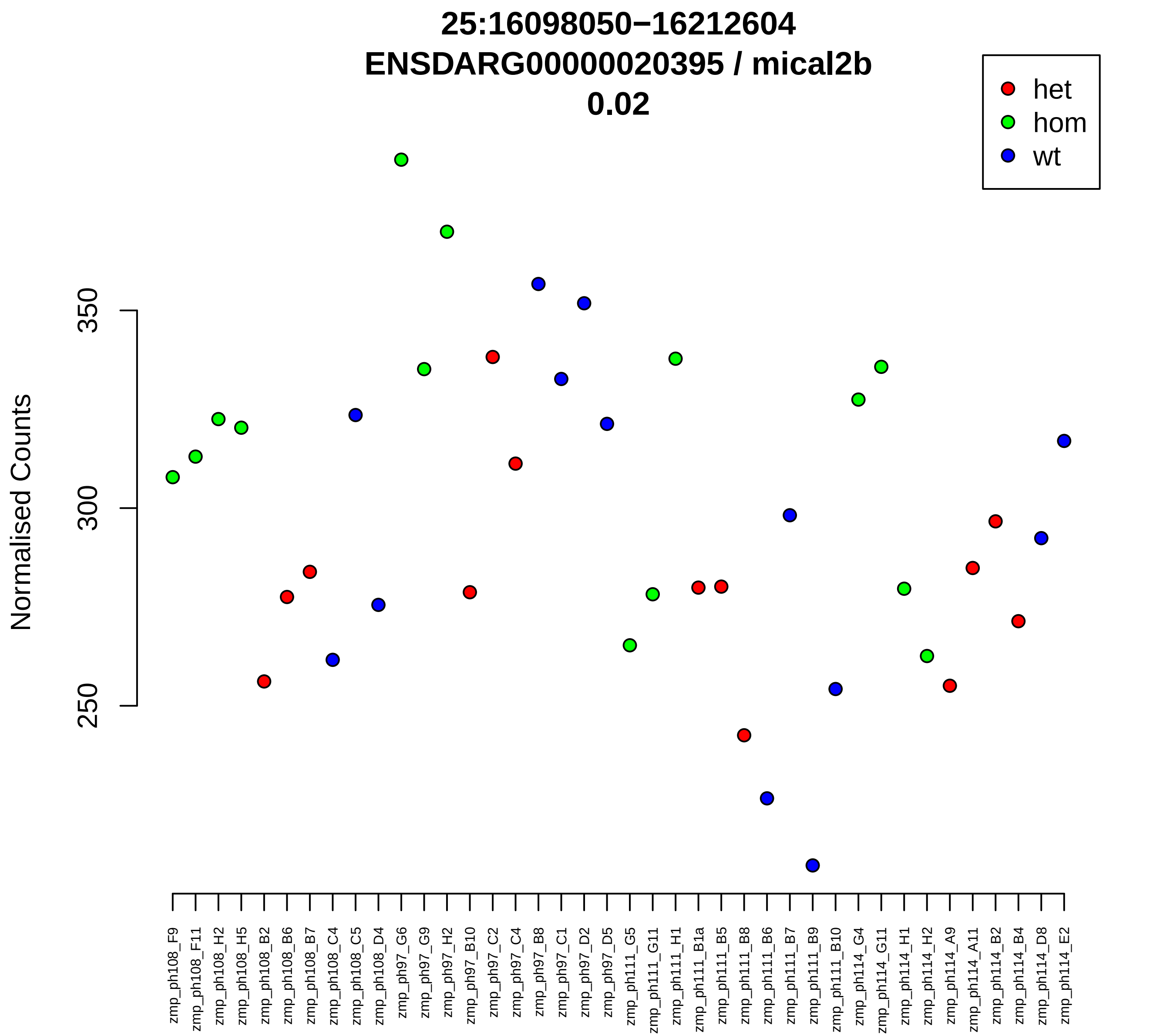

25:16098050−16212604
ENSDARG00000020395 / mical2b
0.02
het
hom
wt
Normalised Counts
250
300
350
zmp_ph108_F9
zmp_ph108_F11
zmp_ph108_H2
zmp_ph108_H5
zmp_ph108_B2
zmp_ph108_B6
zmp_ph108_B7
zmp_ph108_C4
zmp_ph108_C5
zmp_ph108_D4
zmp_ph97_G6
zmp_ph97_G9
zmp_ph97_H2
zmp_ph97_B10
zmp_ph97_C2
zmp_ph97_C4
zmp_ph97_B8
zmp_ph97_C1
zmp_ph97_D2
zmp_ph97_D5
zmp_ph111_G5
zmp_ph111_G11
zmp_ph111_H1
zmp_ph111_B1a
zmp_ph111_B5
zmp_ph111_B8
zmp_ph111_B6
zmp_ph111_B7
zmp_ph111_B9
zmp_ph111_B10
zmp_ph114_G4
zmp_ph114_G11
zmp_ph114_H1
zmp_ph114_H2
zmp_ph114_A9
zmp_ph114_A11
zmp_ph114_B2
zmp_ph114_B4
zmp_ph114_D8
zmp_ph114_E2

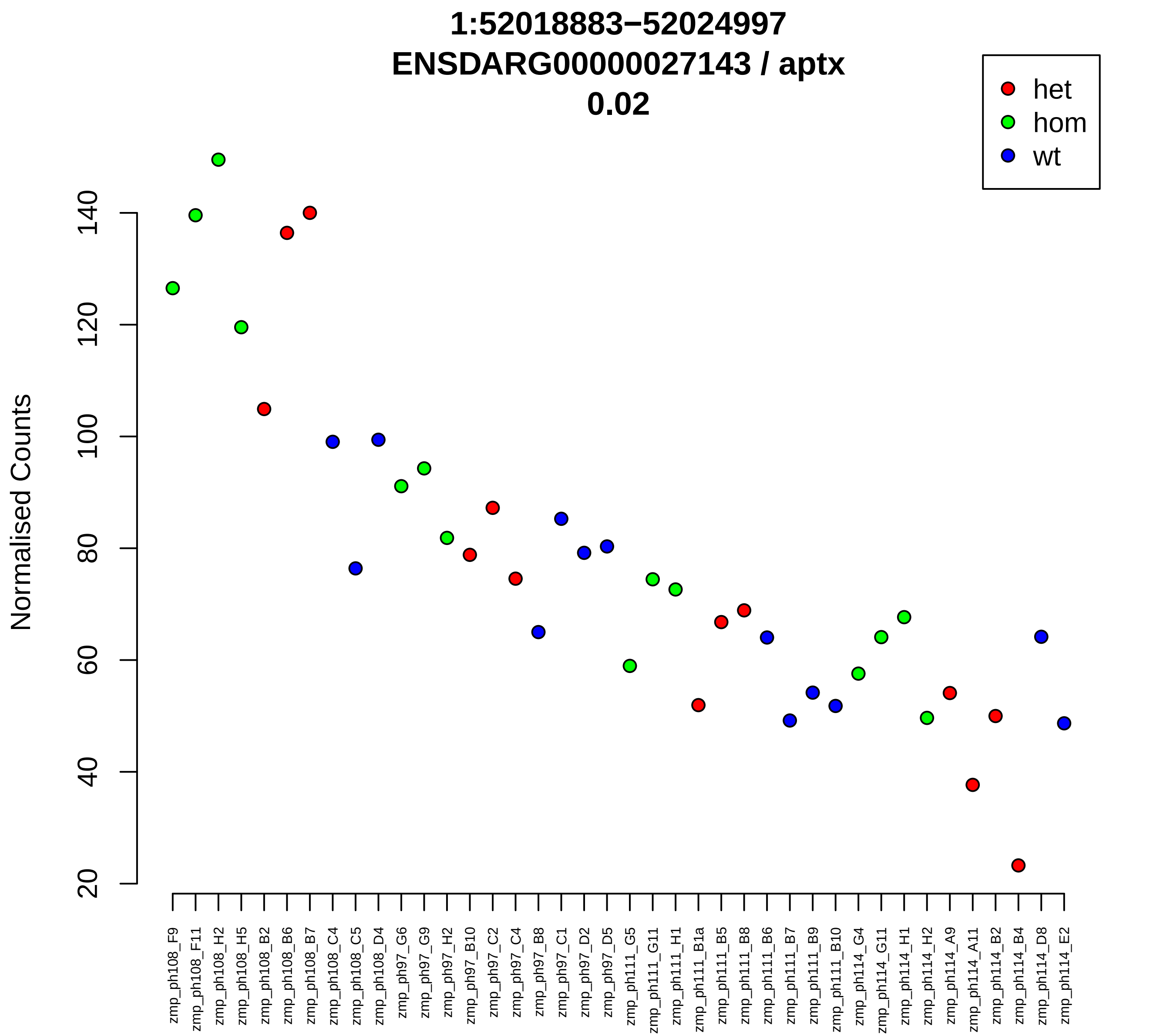

1:52018883−52024997
ENSDARG00000027143 / aptx
0.02
het
hom
wt
Normalised Counts
20
40
60
80
100
120
140
zmp_ph108_F9
zmp_ph108_F11
zmp_ph108_H2
zmp_ph108_H5
zmp_ph108_B2
zmp_ph108_B6
zmp_ph108_B7
zmp_ph108_C4
zmp_ph108_C5
zmp_ph108_D4
zmp_ph97_G6
zmp_ph97_G9
zmp_ph97_H2
zmp_ph97_B10
zmp_ph97_C2
zmp_ph97_C4
zmp_ph97_B8
zmp_ph97_C1
zmp_ph97_D2
zmp_ph97_D5
zmp_ph111_G5
zmp_ph111_G11
zmp_ph111_H1
zmp_ph111_B1a
zmp_ph111_B5
zmp_ph111_B8
zmp_ph111_B6
zmp_ph111_B7
zmp_ph111_B9
zmp_ph111_B10
zmp_ph114_G4
zmp_ph114_G11
zmp_ph114_H1
zmp_ph114_H2
zmp_ph114_A9
zmp_ph114_A11
zmp_ph114_B2
zmp_ph114_B4
zmp_ph114_D8
zmp_ph114_E2

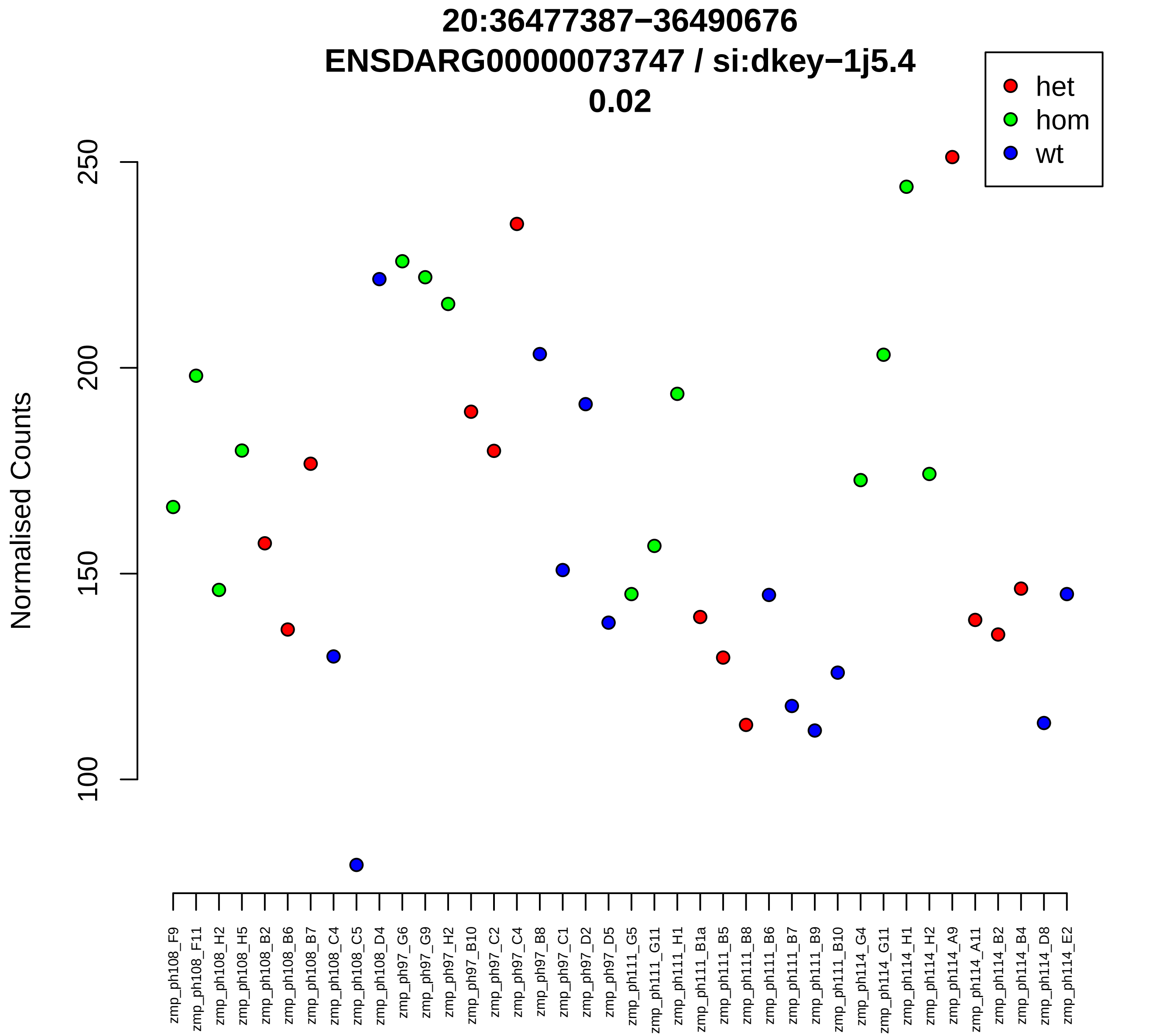

20:36477387−36490676
ENSDARG00000073747 / si:dkey−1j5.4
0.02
het
hom
wt
Normalised Counts
100
150
200
250
zmp_ph108_F9
zmp_ph108_F11
zmp_ph108_H2
zmp_ph108_H5
zmp_ph108_B2
zmp_ph108_B6
zmp_ph108_B7
zmp_ph108_C4
zmp_ph108_C5
zmp_ph108_D4
zmp_ph97_G6
zmp_ph97_G9
zmp_ph97_H2
zmp_ph97_B10
zmp_ph97_C2
zmp_ph97_C4
zmp_ph97_B8
zmp_ph97_C1
zmp_ph97_D2
zmp_ph97_D5
zmp_ph111_G5
zmp_ph111_G11
zmp_ph111_H1
zmp_ph111_B1a
zmp_ph111_B5
zmp_ph111_B8
zmp_ph111_B6
zmp_ph111_B7
zmp_ph111_B9
zmp_ph111_B10
zmp_ph114_G4
zmp_ph114_G11
zmp_ph114_H1
zmp_ph114_H2
zmp_ph114_A9
zmp_ph114_A11
zmp_ph114_B2
zmp_ph114_B4
zmp_ph114_D8
zmp_ph114_E2

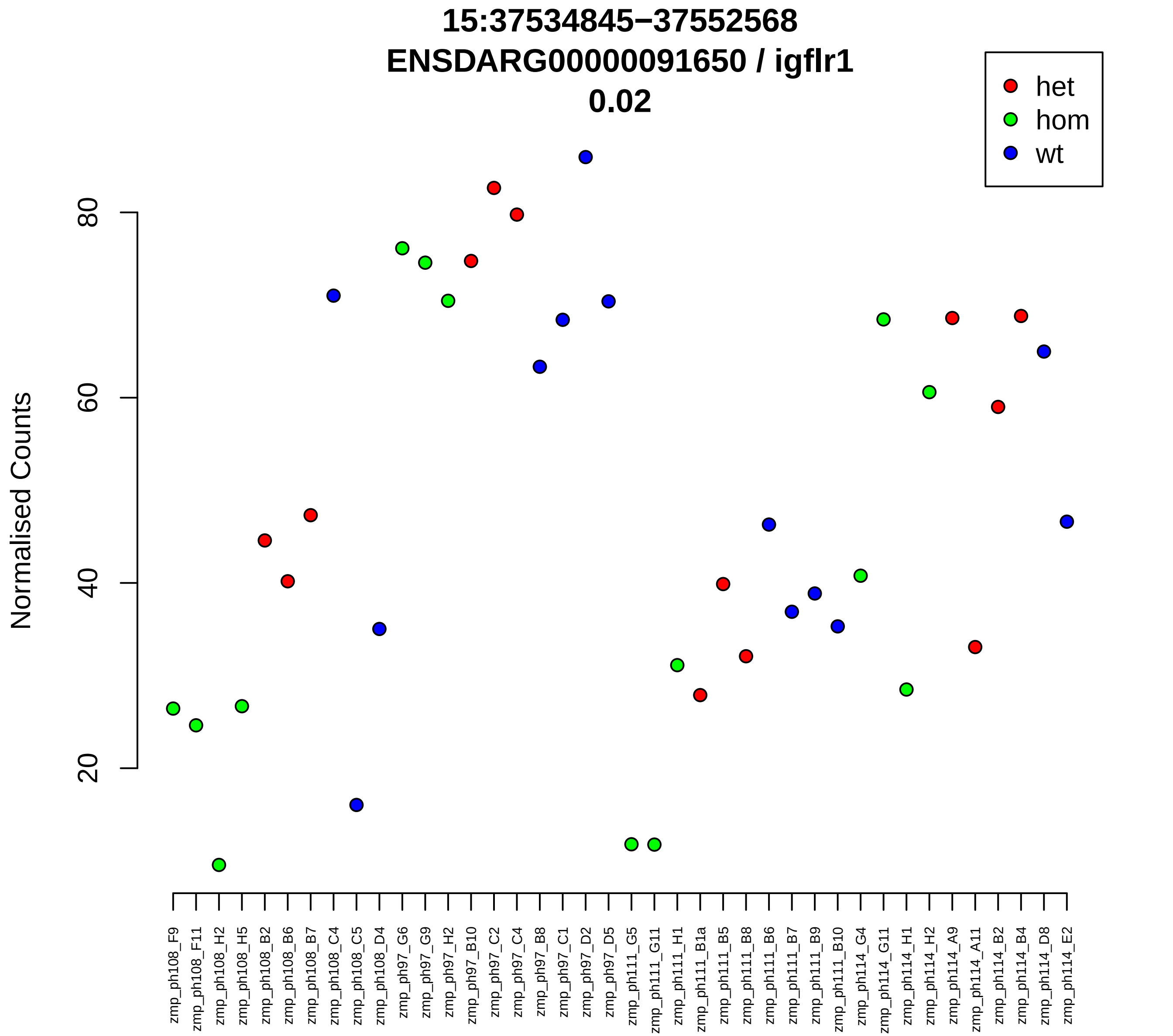
15:37534845−37552568
ENSDARG00000091650 / igflr1
0.02
het
hom
wt
Normalised Counts
20
40
60
80
zmp_ph108_F9
zmp_ph108_F11
zmp_ph108_H2
zmp_ph108_H5
zmp_ph108_B2
zmp_ph108_B6
zmp_ph108_B7
zmp_ph108_C4
zmp_ph108_C5
zmp_ph108_D4
zmp_ph97_G6
zmp_ph97_G9
zmp_ph97_H2
zmp_ph97_B10
zmp_ph97_C2
zmp_ph97_C4
zmp_ph97_B8
zmp_ph97_C1
zmp_ph97_D2
zmp_ph97_D5
zmp_ph111_G5
zmp_ph111_G11
zmp_ph111_H1
zmp_ph111_B1a
zmp_ph111_B5
zmp_ph111_B8
zmp_ph111_B6
zmp_ph111_B7
zmp_ph111_B9
zmp_ph111_B10
zmp_ph114_G4
zmp_ph114_G11
zmp_ph114_H1
zmp_ph114_H2
zmp_ph114_A9
zmp_ph114_A11
zmp_ph114_B2
zmp_ph114_B4
zmp_ph114_D8
zmp_ph114_E2

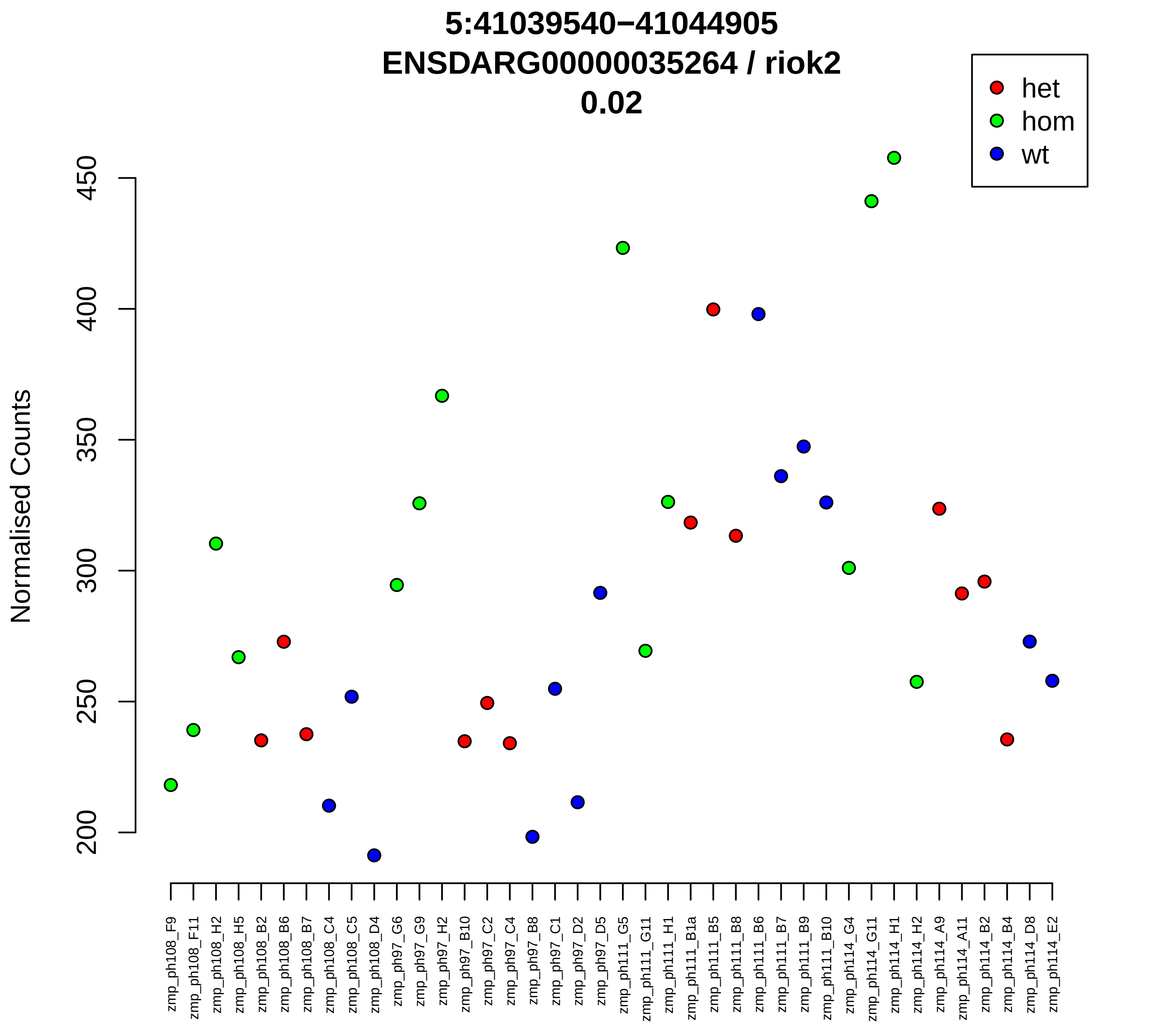

5:41039540−41044905
ENSDARG00000035264 / riok2
0.02
het
hom
wt
Normalised Counts
200
250
300
350
400
450
zmp_ph108_F9
zmp_ph108_F11
zmp_ph108_H2
zmp_ph108_H5
zmp_ph108_B2
zmp_ph108_B6
zmp_ph108_B7
zmp_ph108_C4
zmp_ph108_C5
zmp_ph108_D4
zmp_ph97_G6
zmp_ph97_G9
zmp_ph97_H2
zmp_ph97_B10
zmp_ph97_C2
zmp_ph97_C4
zmp_ph97_B8
zmp_ph97_C1
zmp_ph97_D2
zmp_ph97_D5
zmp_ph111_G5
zmp_ph111_G11
zmp_ph111_H1
zmp_ph111_B1a
zmp_ph111_B5
zmp_ph111_B8
zmp_ph111_B6
zmp_ph111_B7
zmp_ph111_B9
zmp_ph111_B10
zmp_ph114_G4
zmp_ph114_G11
zmp_ph114_H1
zmp_ph114_H2
zmp_ph114_A9
zmp_ph114_A11
zmp_ph114_B2
zmp_ph114_B4
zmp_ph114_D8
zmp_ph114_E2

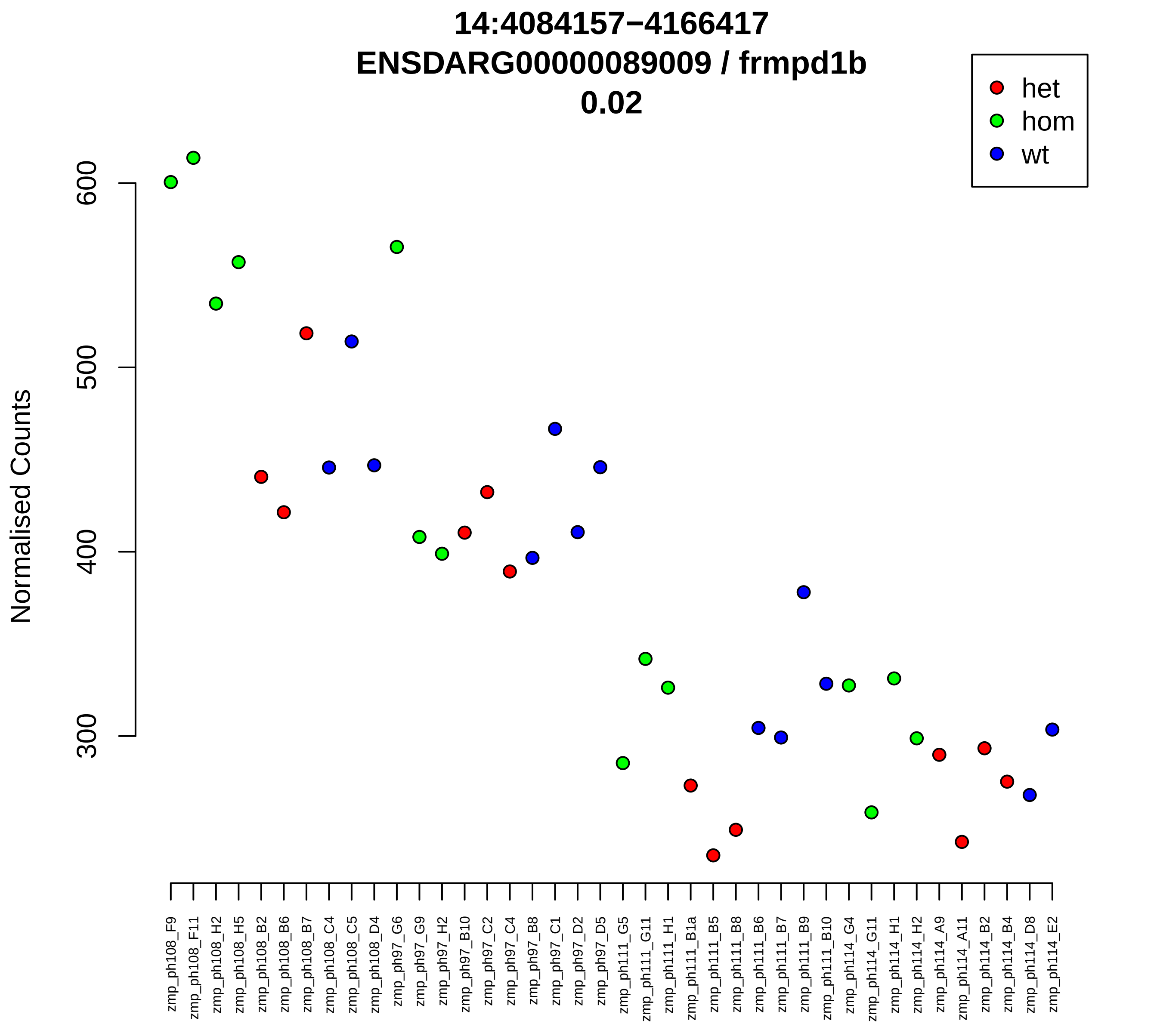

14:4084157−4166417
ENSDARG00000089009 / frmpd1b
0.02
het
hom
wt
Normalised Counts
600
500
400
300
zmp_ph108_F9
zmp_ph108_F11
zmp_ph108_H2
zmp_ph108_H5
zmp_ph108_B2
zmp_ph108_B6
zmp_ph108_B7
zmp_ph108_C4
zmp_ph108_C5
zmp_ph108_D4
zmp_ph97_G6
zmp_ph97_G9
zmp_ph97_H2
zmp_ph97_B10
zmp_ph97_C2
zmp_ph97_C4
zmp_ph97_B8
zmp_ph97_C1
zmp_ph97_D2
zmp_ph97_D5
zmp_ph111_G5
zmp_ph111_G11
zmp_ph111_H1
zmp_ph111_B1a
zmp_ph111_B5
zmp_ph111_B8
zmp_ph111_B6
zmp_ph111_B7
zmp_ph111_B9
zmp_ph111_B10
zmp_ph114_G4
zmp_ph114_G11
zmp_ph114_H1
zmp_ph114_H2
zmp_ph114_A9
zmp_ph114_A11
zmp_ph114_B2
zmp_ph114_B4
zmp_ph114_D8
zmp_ph114_E2

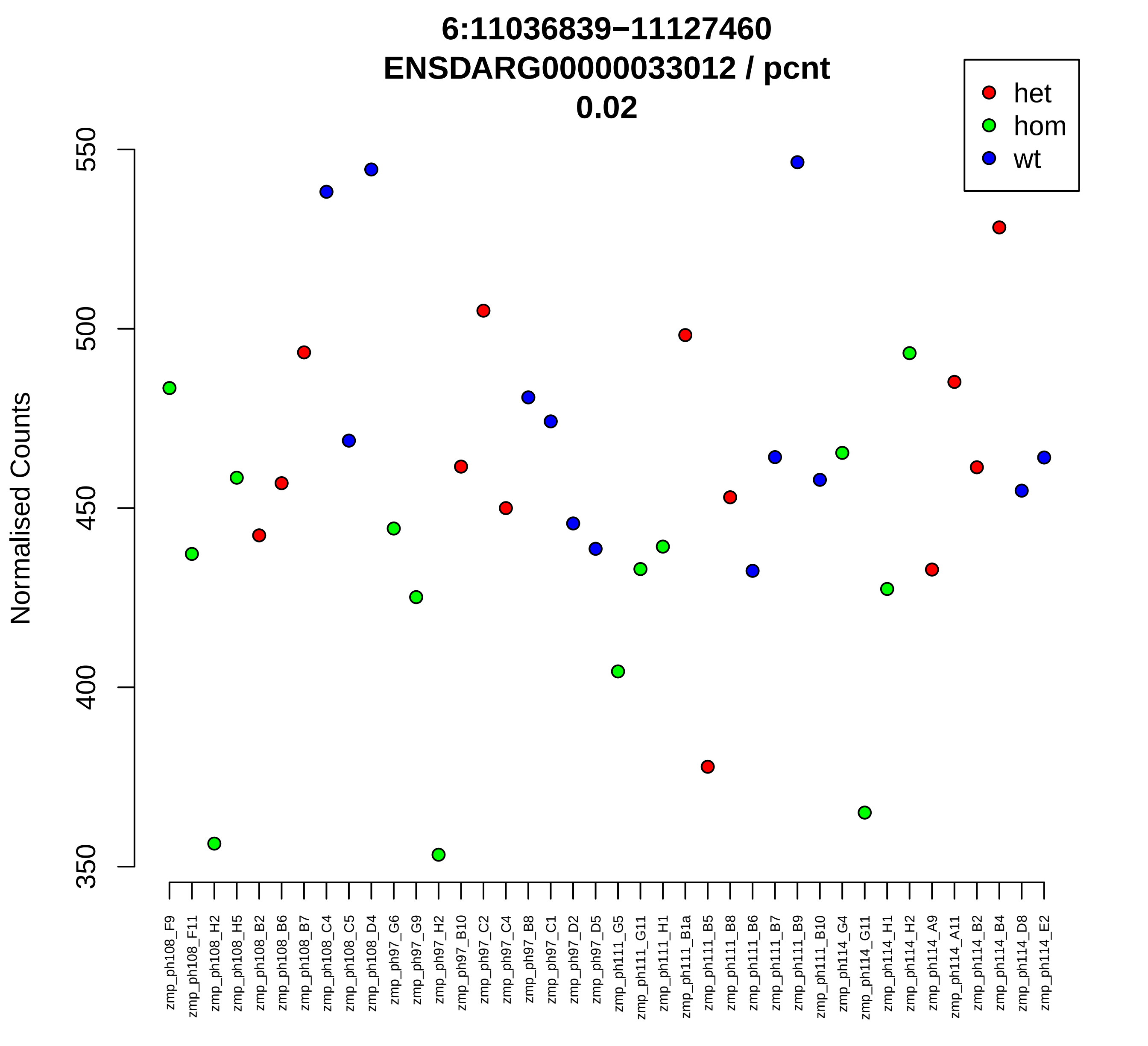
6:11036839−11127460
ENSDARG00000033012 / pcnt
0.02
het
hom
wt
Normalised Counts
550
500
450
400
350
zmp_ph108_F9
zmp_ph108_F11
zmp_ph108_H2
zmp_ph108_H5
zmp_ph108_B2
zmp_ph108_B6
zmp_ph108_B7
zmp_ph108_C4
zmp_ph108_C5
zmp_ph108_D4
zmp_ph97_G6
zmp_ph97_G9
zmp_ph97_H2
zmp_ph97_B10
zmp_ph97_C2
zmp_ph97_C4
zmp_ph97_B8
zmp_ph97_C1
zmp_ph97_D2
zmp_ph97_D5
zmp_ph111_G5
zmp_ph111_G11
zmp_ph111_H1
zmp_ph111_B1a
zmp_ph111_B5
zmp_ph111_B8
zmp_ph111_B6
zmp_ph111_B7
zmp_ph111_B9
zmp_ph111_B10
zmp_ph114_G4
zmp_ph114_G11
zmp_ph114_H1
zmp_ph114_H2
zmp_ph114_A9
zmp_ph114_A11
zmp_ph114_B2
zmp_ph114_B4
zmp_ph114_D8
zmp_ph114_E2

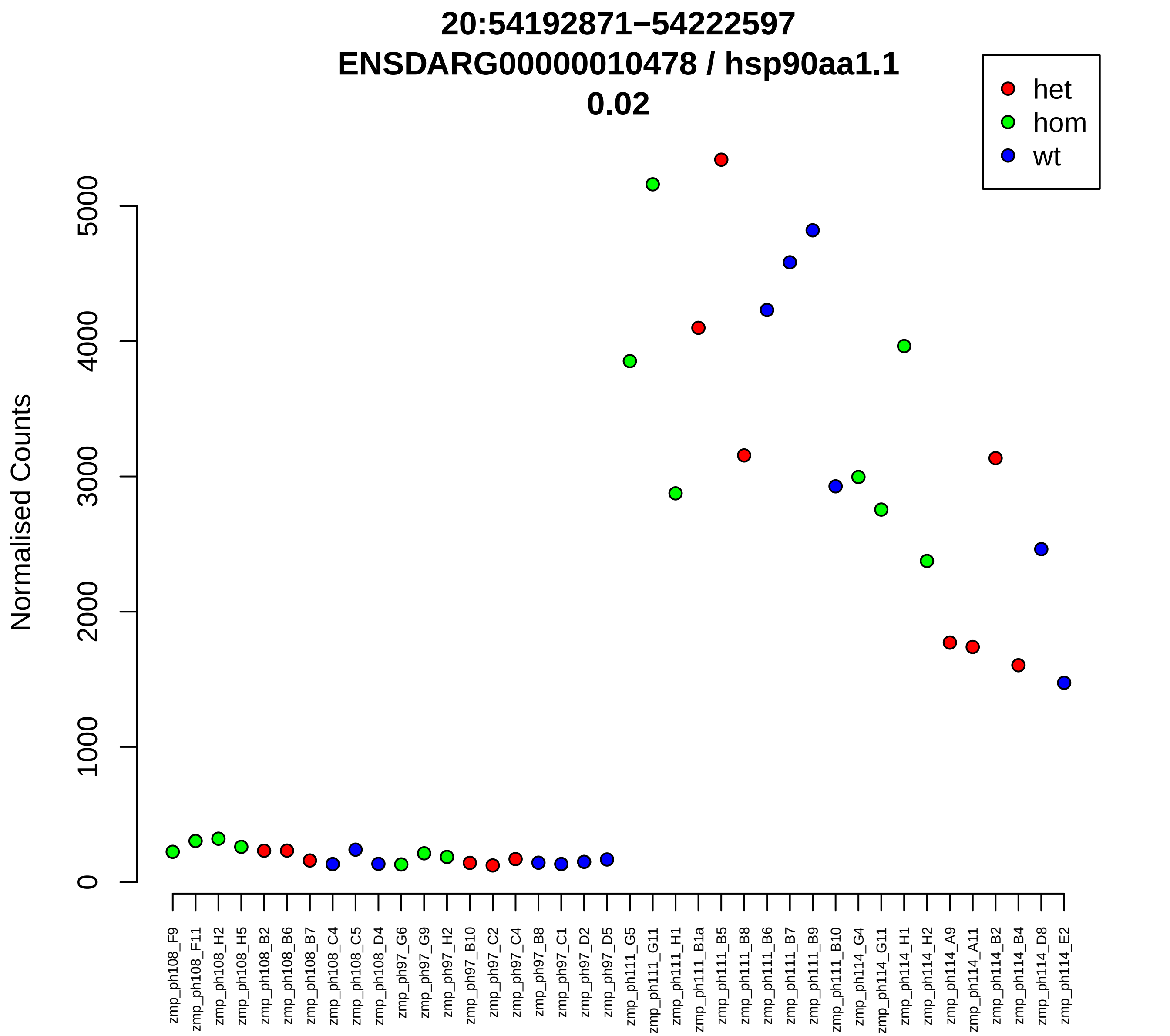
20:54192871−54222597
ENSDARG00000010478 / hsp90aa1.1
0.02
het
hom
wt
Normalised Counts
0
1000
2000
3000
4000
5000
zmp_ph108_F9
zmp_ph108_F11
zmp_ph108_H2
zmp_ph108_H5
zmp_ph108_B2
zmp_ph108_B6
zmp_ph108_B7
zmp_ph108_C4
zmp_ph108_C5
zmp_ph108_D4
zmp_ph97_G6
zmp_ph97_G9
zmp_ph97_H2
zmp_ph97_B10
zmp_ph97_C2
zmp_ph97_C4
zmp_ph97_B8
zmp_ph97_C1
zmp_ph97_D2
zmp_ph97_D5
zmp_ph111_G5
zmp_ph111_G11
zmp_ph111_H1
zmp_ph111_B1a
zmp_ph111_B5
zmp_ph111_B8
zmp_ph111_B6
zmp_ph111_B7
zmp_ph111_B9
zmp_ph111_B10
zmp_ph114_G4
zmp_ph114_G11
zmp_ph114_H1
zmp_ph114_H2
zmp_ph114_A9
zmp_ph114_A11
zmp_ph114_B2
zmp_ph114_B4
zmp_ph114_D8
zmp_ph114_E2

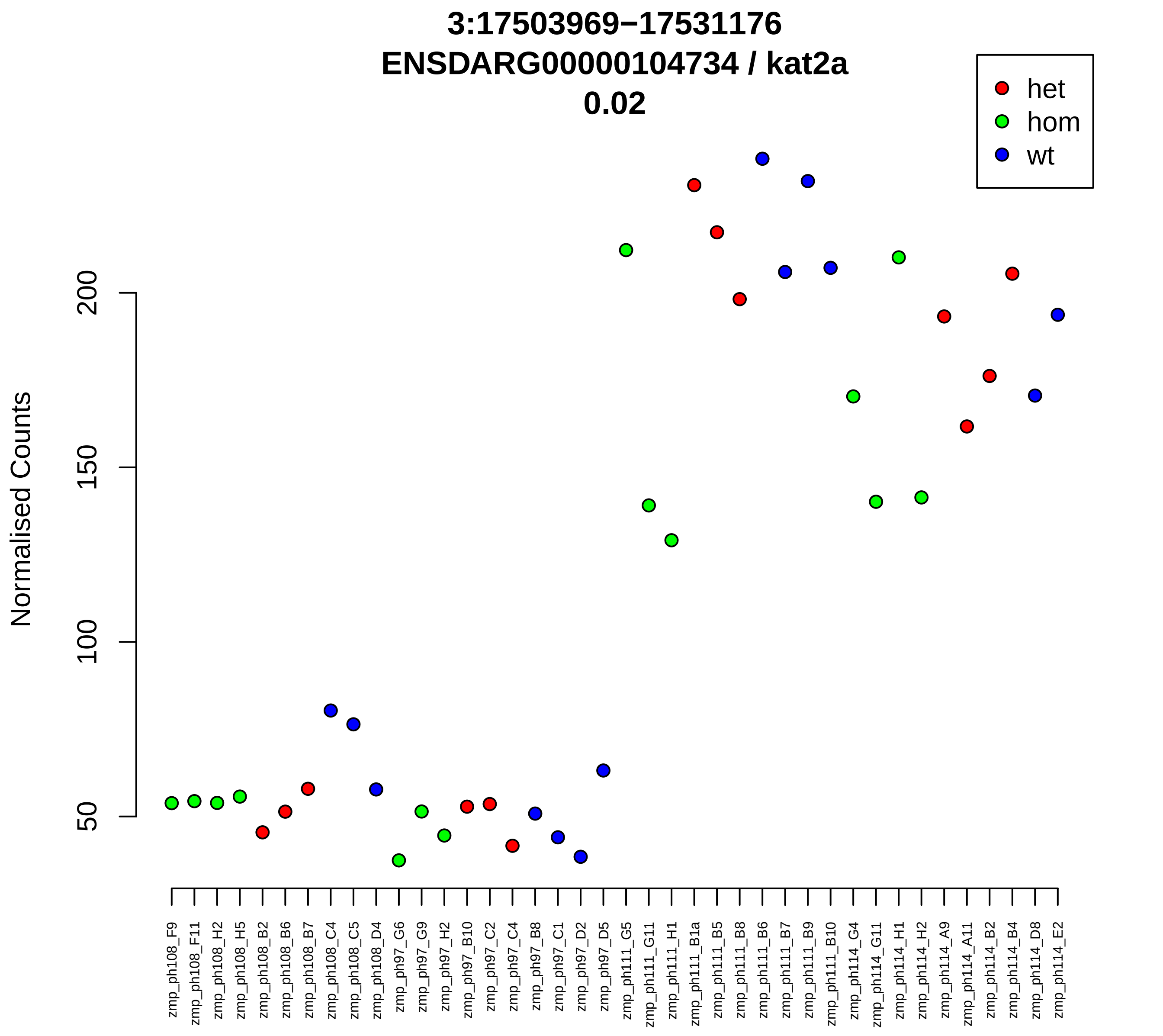

3:17503969−17531176
ENSDARG00000104734 / kat2a
0.02
het
hom
wt
Normalised Counts
50
100
150
200
zmp_ph108_F9
zmp_ph108_F11
zmp_ph108_H2
zmp_ph108_H5
zmp_ph108_B2
zmp_ph108_B6
zmp_ph108_B7
zmp_ph108_C4
zmp_ph108_C5
zmp_ph108_D4
zmp_ph97_G6
zmp_ph97_G9
zmp_ph97_H2
zmp_ph97_B10
zmp_ph97_C2
zmp_ph97_C4
zmp_ph97_B8
zmp_ph97_C1
zmp_ph97_D2
zmp_ph97_D5
zmp_ph111_G5
zmp_ph111_G11
zmp_ph111_H1
zmp_ph111_B1a
zmp_ph111_B5
zmp_ph111_B8
zmp_ph111_B6
zmp_ph111_B7
zmp_ph111_B9
zmp_ph111_B10
zmp_ph114_G4
zmp_ph114_G11
zmp_ph114_H1
zmp_ph114_H2
zmp_ph114_A9
zmp_ph114_A11
zmp_ph114_B2
zmp_ph114_B4
zmp_ph114_D8
zmp_ph114_E2

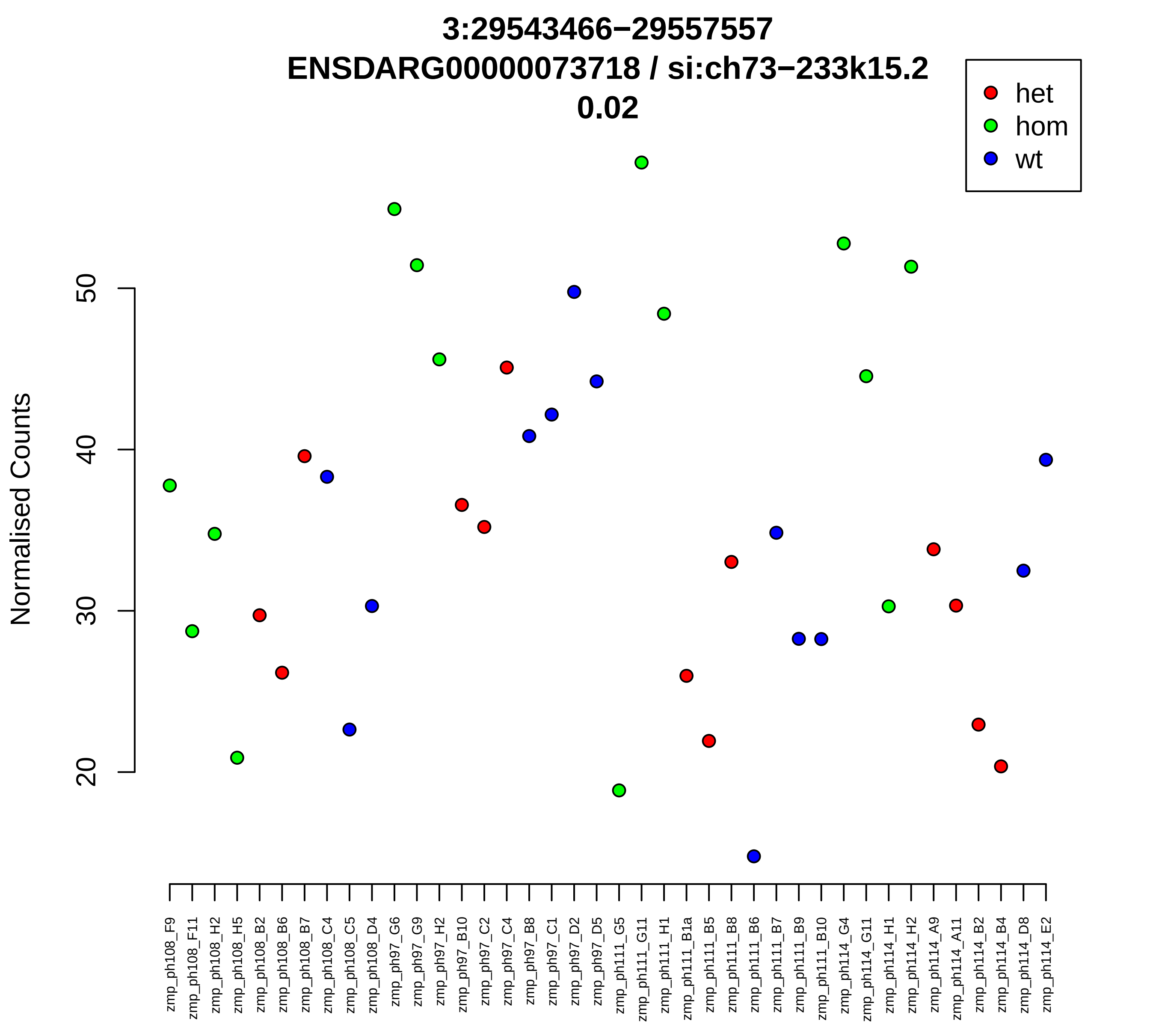

3:29543466−29557557
ENSDARG00000073718 / si:ch73−233k15.2
0.02
het
hom
wt
Normalised Counts
20
30
40
50
zmp_ph108_F9
zmp_ph108_F11
zmp_ph108_H2
zmp_ph108_H5
zmp_ph108_B2
zmp_ph108_B6
zmp_ph108_B7
zmp_ph108_C4
zmp_ph108_C5
zmp_ph108_D4
zmp_ph97_G6
zmp_ph97_G9
zmp_ph97_H2
zmp_ph97_B10
zmp_ph97_C2
zmp_ph97_C4
zmp_ph97_B8
zmp_ph97_C1
zmp_ph97_D2
zmp_ph97_D5
zmp_ph111_G5
zmp_ph111_G11
zmp_ph111_H1
zmp_ph111_B1a
zmp_ph111_B5
zmp_ph111_B8
zmp_ph111_B6
zmp_ph111_B7
zmp_ph111_B9
zmp_ph111_B10
zmp_ph114_G4
zmp_ph114_G11
zmp_ph114_H1
zmp_ph114_H2
zmp_ph114_A9
zmp_ph114_A11
zmp_ph114_B2
zmp_ph114_B4
zmp_ph114_D8
zmp_ph114_E2

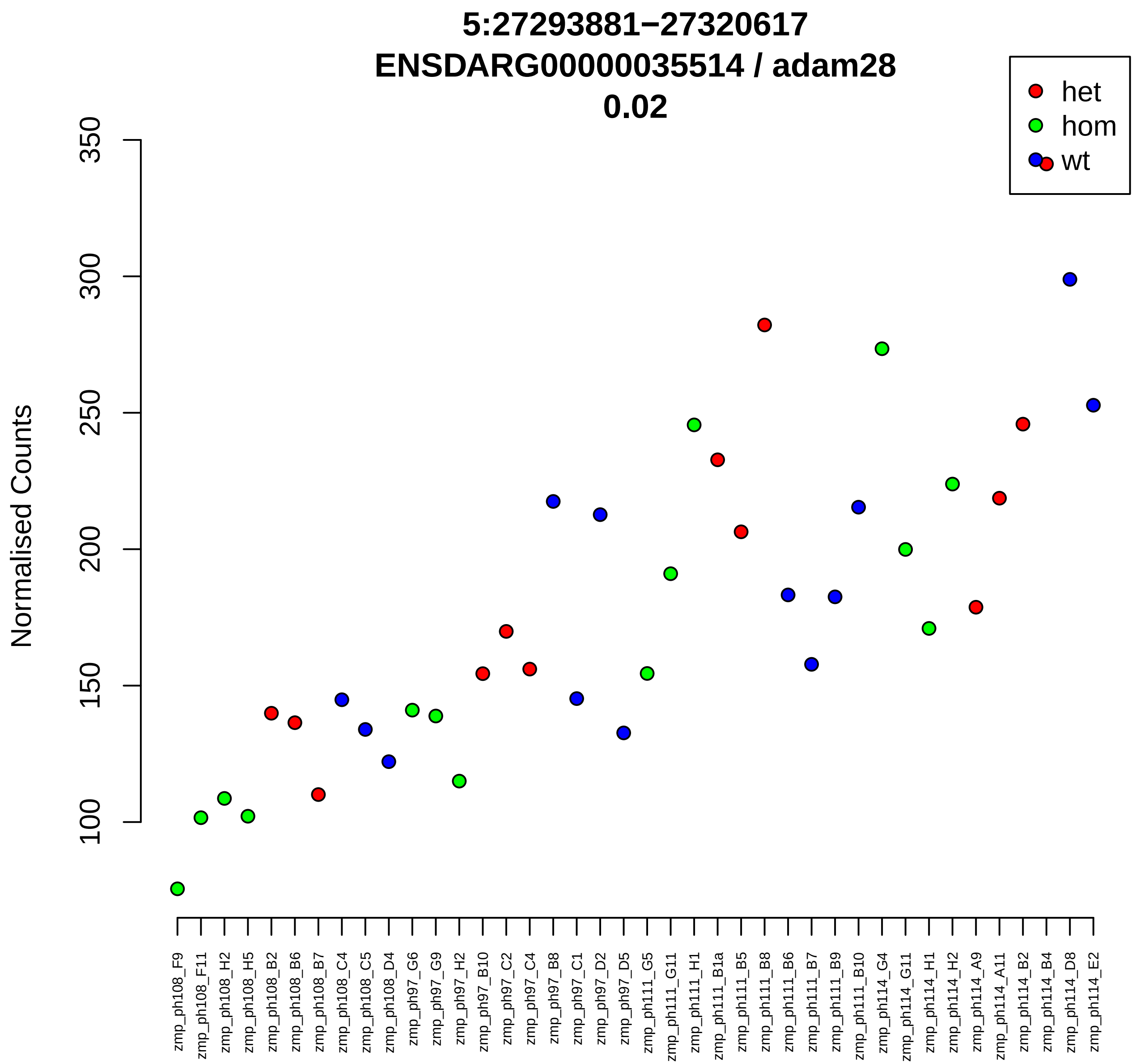

5:27293881−27320617
ENSDARG00000035514 / adam28
0.02
het
hom
wt
Normalised Counts
100
150
200
250
300
350
zmp_ph108_F9
zmp_ph108_F11
zmp_ph108_H2
zmp_ph108_H5
zmp_ph108_B2
zmp_ph108_B6
zmp_ph108_B7
zmp_ph108_C4
zmp_ph108_C5
zmp_ph108_D4
zmp_ph97_G6
zmp_ph97_G9
zmp_ph97_H2
zmp_ph97_B10
zmp_ph97_C2
zmp_ph97_C4
zmp_ph97_B8
zmp_ph97_C1
zmp_ph97_D2
zmp_ph97_D5
zmp_ph111_G5
zmp_ph111_G11
zmp_ph111_H1
zmp_ph111_B1a
zmp_ph111_B5
zmp_ph111_B8
zmp_ph111_B6
zmp_ph111_B7
zmp_ph111_B9
zmp_ph111_B10
zmp_ph114_G4
zmp_ph114_G11
zmp_ph114_H1
zmp_ph114_H2
zmp_ph114_A9
zmp_ph114_A11
zmp_ph114_B2
zmp_ph114_B4
zmp_ph114_D8
zmp_ph114_E2

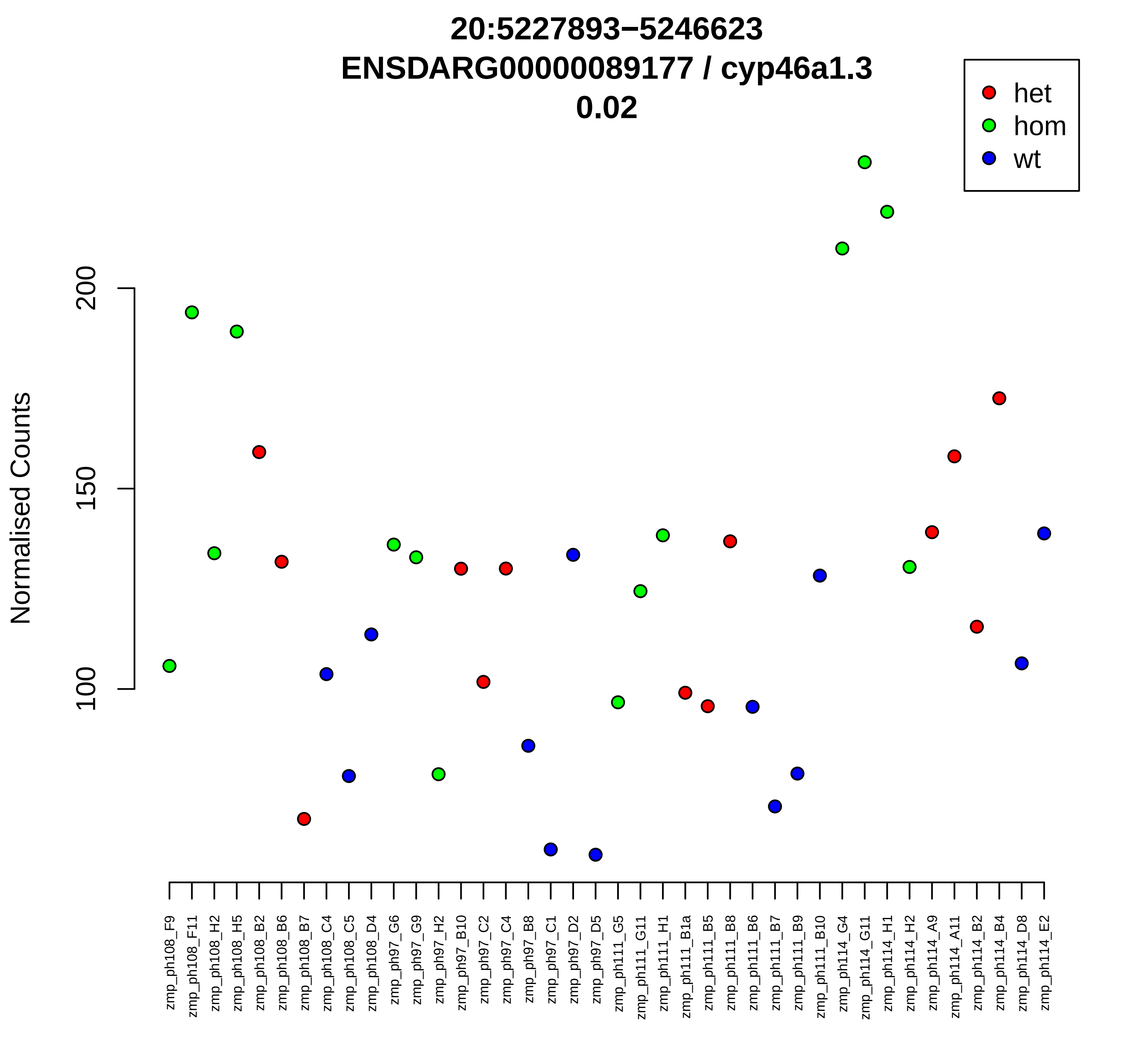
20:5227893−5246623
ENSDARG00000089177 / cyp46a1.3
0.02
het
hom
wt
Normalised Counts
100
150
200
zmp_ph108_F9
zmp_ph108_F11
zmp_ph108_H2
zmp_ph108_H5
zmp_ph108_B2
zmp_ph108_B6
zmp_ph108_B7
zmp_ph108_C4
zmp_ph108_C5
zmp_ph108_D4
zmp_ph97_G6
zmp_ph97_G9
zmp_ph97_H2
zmp_ph97_B10
zmp_ph97_C2
zmp_ph97_C4
zmp_ph97_B8
zmp_ph97_C1
zmp_ph97_D2
zmp_ph97_D5
zmp_ph111_G5
zmp_ph111_G11
zmp_ph111_H1
zmp_ph111_B1a
zmp_ph111_B5
zmp_ph111_B8
zmp_ph111_B6
zmp_ph111_B7
zmp_ph111_B9
zmp_ph111_B10
zmp_ph114_G4
zmp_ph114_G11
zmp_ph114_H1
zmp_ph114_H2
zmp_ph114_A9
zmp_ph114_A11
zmp_ph114_B2
zmp_ph114_B4
zmp_ph114_D8
zmp_ph114_E2

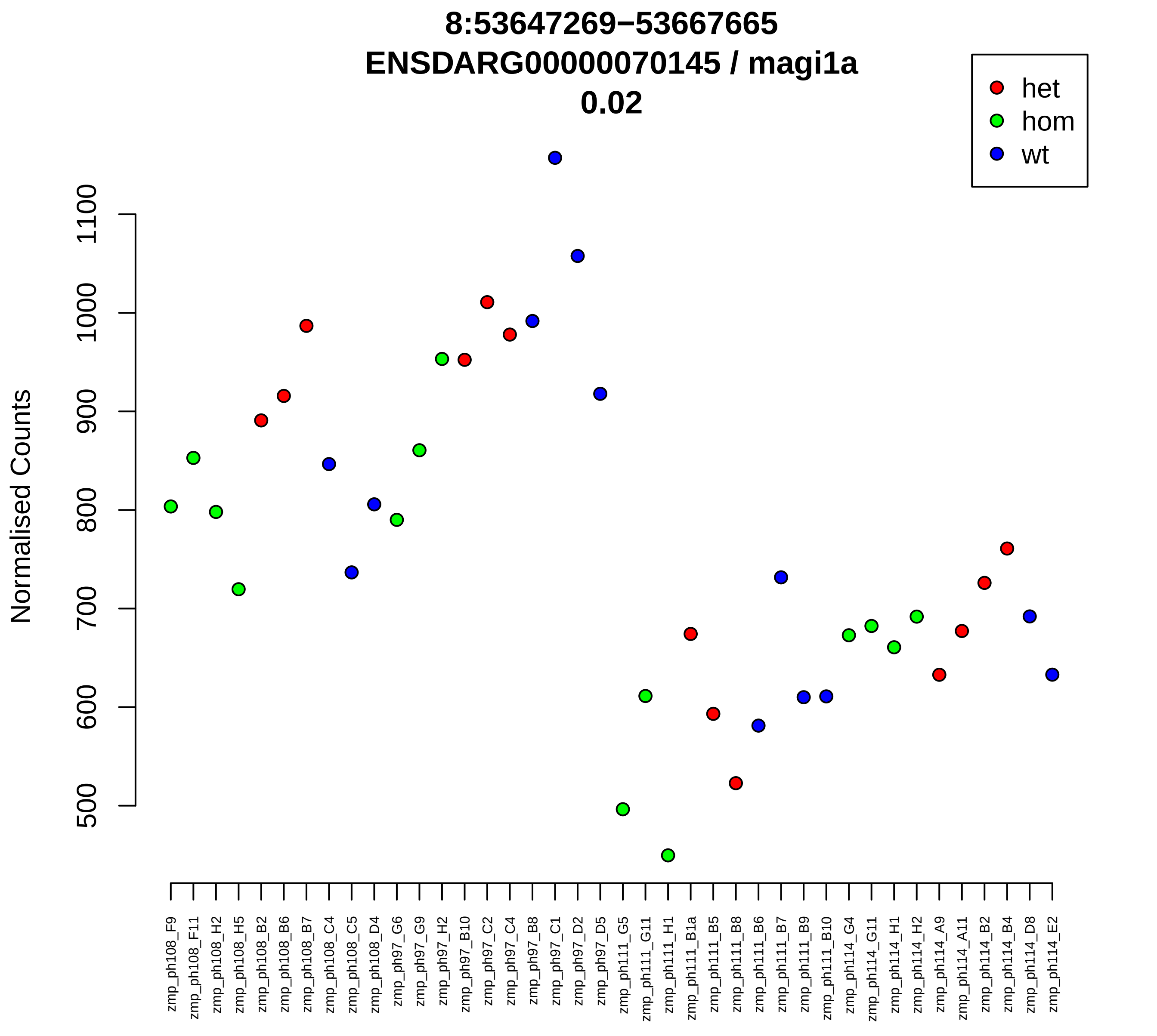

8:53647269−53667665
ENSDARG00000070145 / magi1a
0.02
het
hom
wt
Normalised Counts
500
600
700
800
900
1000
1100
zmp_ph108_F9
zmp_ph108_F11
zmp_ph108_H2
zmp_ph108_H5
zmp_ph108_B2
zmp_ph108_B6
zmp_ph108_B7
zmp_ph108_C4
zmp_ph108_C5
zmp_ph108_D4
zmp_ph97_G6
zmp_ph97_G9
zmp_ph97_H2
zmp_ph97_B10
zmp_ph97_C2
zmp_ph97_C4
zmp_ph97_B8
zmp_ph97_C1
zmp_ph97_D2
zmp_ph97_D5
zmp_ph111_G5
zmp_ph111_G11
zmp_ph111_H1
zmp_ph111_B1a
zmp_ph111_B5
zmp_ph111_B8
zmp_ph111_B6
zmp_ph111_B7
zmp_ph111_B9
zmp_ph111_B10
zmp_ph114_G4
zmp_ph114_G11
zmp_ph114_H1
zmp_ph114_H2
zmp_ph114_A9
zmp_ph114_A11
zmp_ph114_B2
zmp_ph114_B4
zmp_ph114_D8
zmp_ph114_E2

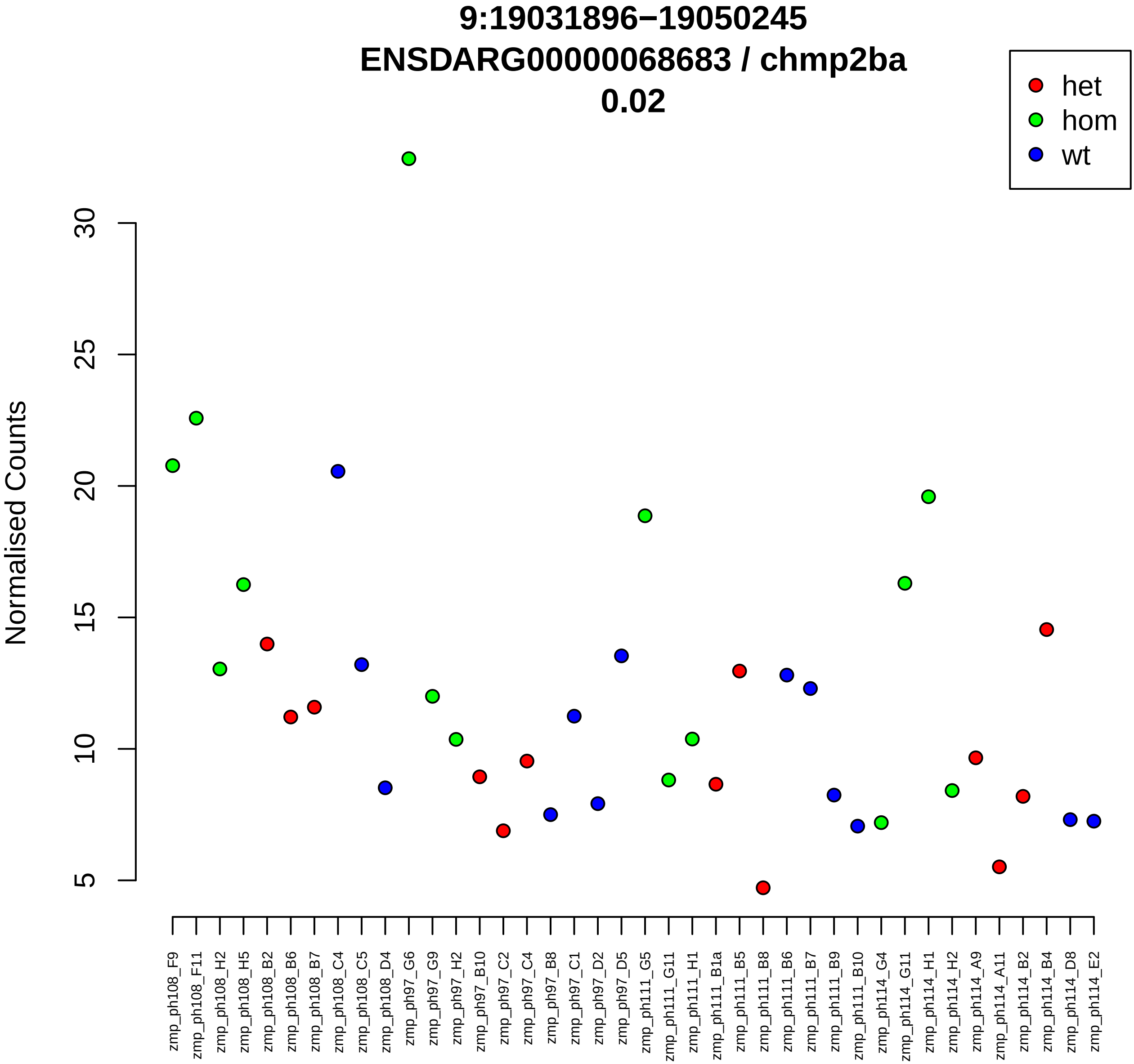

9:19031896−19050245
ENSDARG00000068683 / chmp2ba
0.02
het
hom
wt
Normalised Counts
5
10
15
20
25
30
zmp_ph108_F9
zmp_ph108_F11
zmp_ph108_H2
zmp_ph108_H5
zmp_ph108_B2
zmp_ph108_B6
zmp_ph108_B7
zmp_ph108_C4
zmp_ph108_C5
zmp_ph108_D4
zmp_ph97_G6
zmp_ph97_G9
zmp_ph97_H2
zmp_ph97_B10
zmp_ph97_C2
zmp_ph97_C4
zmp_ph97_B8
zmp_ph97_C1
zmp_ph97_D2
zmp_ph97_D5
zmp_ph111_G5
zmp_ph111_G11
zmp_ph111_H1
zmp_ph111_B1a
zmp_ph111_B5
zmp_ph111_B8
zmp_ph111_B6
zmp_ph111_B7
zmp_ph111_B9
zmp_ph111_B10
zmp_ph114_G4
zmp_ph114_G11
zmp_ph114_H1
zmp_ph114_H2
zmp_ph114_A9
zmp_ph114_A11
zmp_ph114_B2
zmp_ph114_B4
zmp_ph114_D8
zmp_ph114_E2

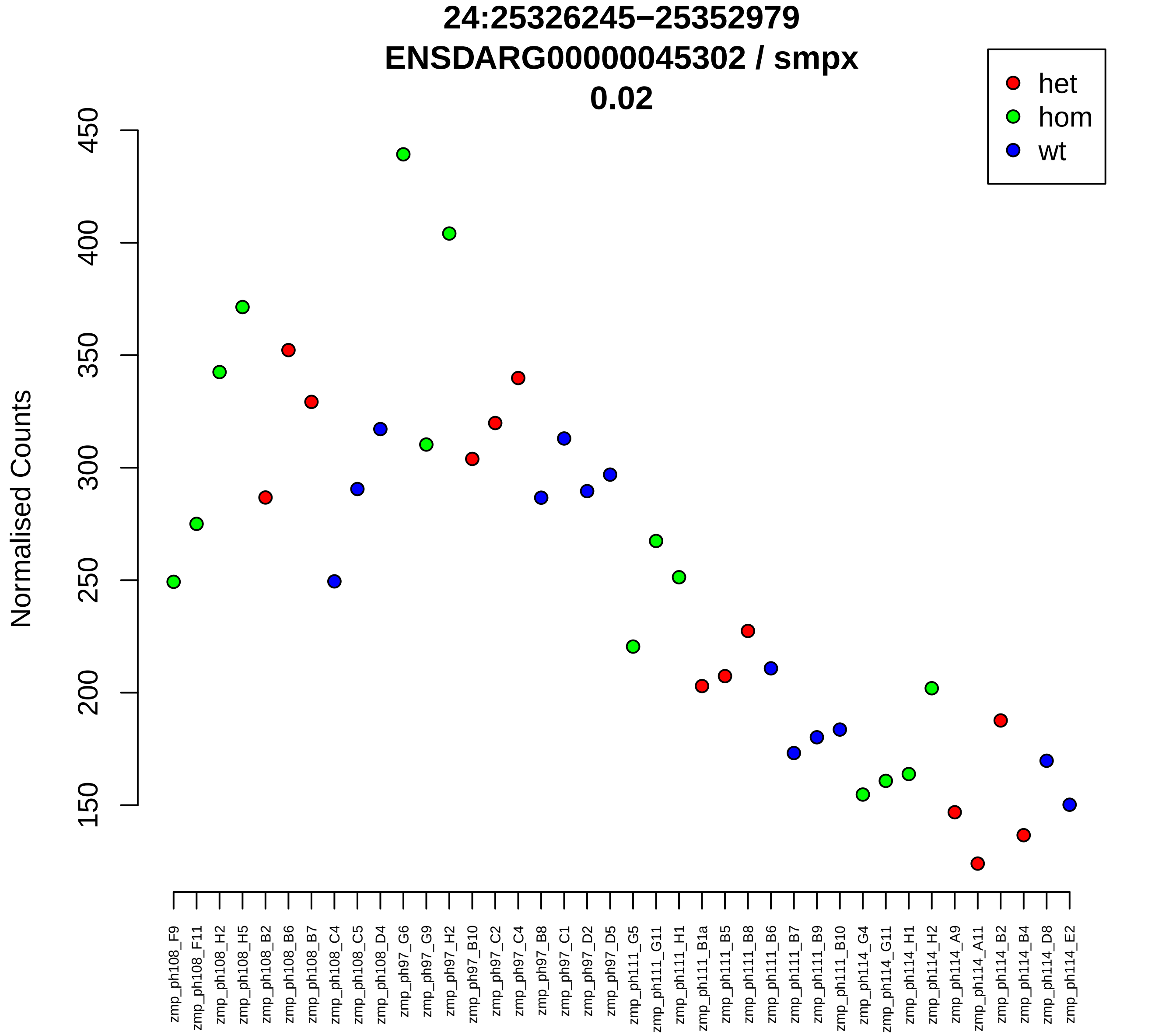

24:25326245−25352979
ENSDARG00000045302 / smpx
0.02
het
hom
wt
Normalised Counts
150
200
250
300
350
400
450
zmp_ph108_F9
zmp_ph108_F11
zmp_ph108_H2
zmp_ph108_H5
zmp_ph108_B2
zmp_ph108_B6
zmp_ph108_B7
zmp_ph108_C4
zmp_ph108_C5
zmp_ph108_D4
zmp_ph97_G6
zmp_ph97_G9
zmp_ph97_H2
zmp_ph97_B10
zmp_ph97_C2
zmp_ph97_C4
zmp_ph97_B8
zmp_ph97_C1
zmp_ph97_D2
zmp_ph97_D5
zmp_ph111_G5
zmp_ph111_G11
zmp_ph111_H1
zmp_ph111_B1a
zmp_ph111_B5
zmp_ph111_B8
zmp_ph111_B6
zmp_ph111_B7
zmp_ph111_B9
zmp_ph111_B10
zmp_ph114_G4
zmp_ph114_G11
zmp_ph114_H1
zmp_ph114_H2
zmp_ph114_A9
zmp_ph114_A11
zmp_ph114_B2
zmp_ph114_B4
zmp_ph114_D8
zmp_ph114_E2

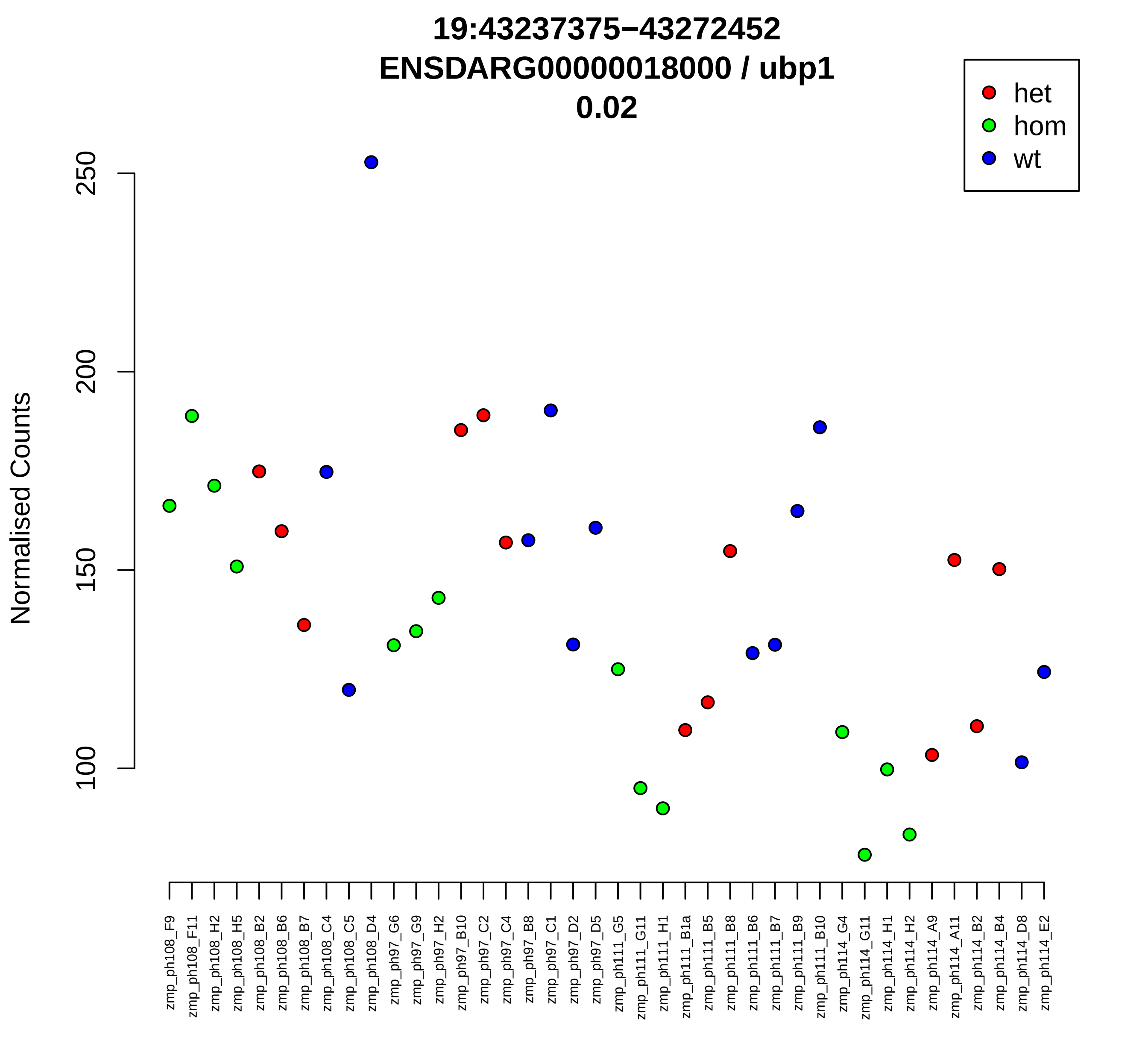

19:43237375−43272452
ENSDARG00000018000 / ubp1
0.02
het
hom
wt
Normalised Counts
250
200
150
100
zmp_ph108_F9
zmp_ph108_F11
zmp_ph108_H2
zmp_ph108_H5
zmp_ph108_B2
zmp_ph108_B6
zmp_ph108_B7
zmp_ph108_C4
zmp_ph108_C5
zmp_ph108_D4
zmp_ph97_G6
zmp_ph97_G9
zmp_ph97_H2
zmp_ph97_B10
zmp_ph97_C2
zmp_ph97_C4
zmp_ph97_B8
zmp_ph97_C1
zmp_ph97_D2
zmp_ph97_D5
zmp_ph111_G5
zmp_ph111_G11
zmp_ph111_H1
zmp_ph111_B1a
zmp_ph111_B5
zmp_ph111_B8
zmp_ph111_B6
zmp_ph111_B7
zmp_ph111_B9
zmp_ph111_B10
zmp_ph114_G4
zmp_ph114_G11
zmp_ph114_H1
zmp_ph114_H2
zmp_ph114_A9
zmp_ph114_A11
zmp_ph114_B2
zmp_ph114_B4
zmp_ph114_D8
zmp_ph114_E2

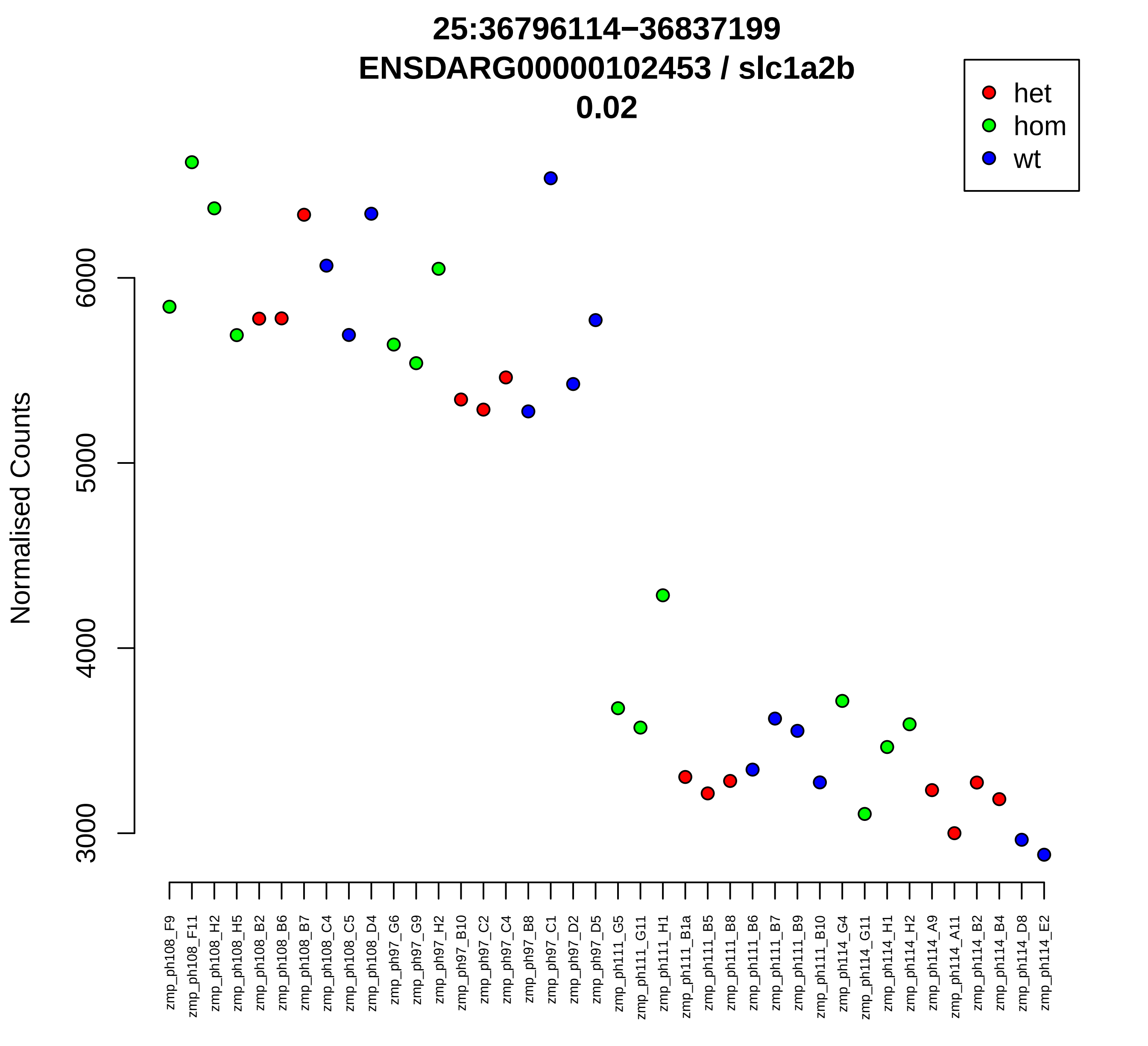

25:36796114−36837199
ENSDARG00000102453 / slc1a2b
0.02
het
hom
wt
Normalised Counts
3000
4000
5000
6000
zmp_ph108_F9
zmp_ph108_F11
zmp_ph108_H2
zmp_ph108_H5
zmp_ph108_B2
zmp_ph108_B6
zmp_ph108_B7
zmp_ph108_C4
zmp_ph108_C5
zmp_ph108_D4
zmp_ph97_G6
zmp_ph97_G9
zmp_ph97_H2
zmp_ph97_B10
zmp_ph97_C2
zmp_ph97_C4
zmp_ph97_B8
zmp_ph97_C1
zmp_ph97_D2
zmp_ph97_D5
zmp_ph111_G5
zmp_ph111_G11
zmp_ph111_H1
zmp_ph111_B1a
zmp_ph111_B5
zmp_ph111_B8
zmp_ph111_B6
zmp_ph111_B7
zmp_ph111_B9
zmp_ph111_B10
zmp_ph114_G4
zmp_ph114_G11
zmp_ph114_H1
zmp_ph114_H2
zmp_ph114_A9
zmp_ph114_A11
zmp_ph114_B2
zmp_ph114_B4
zmp_ph114_D8
zmp_ph114_E2

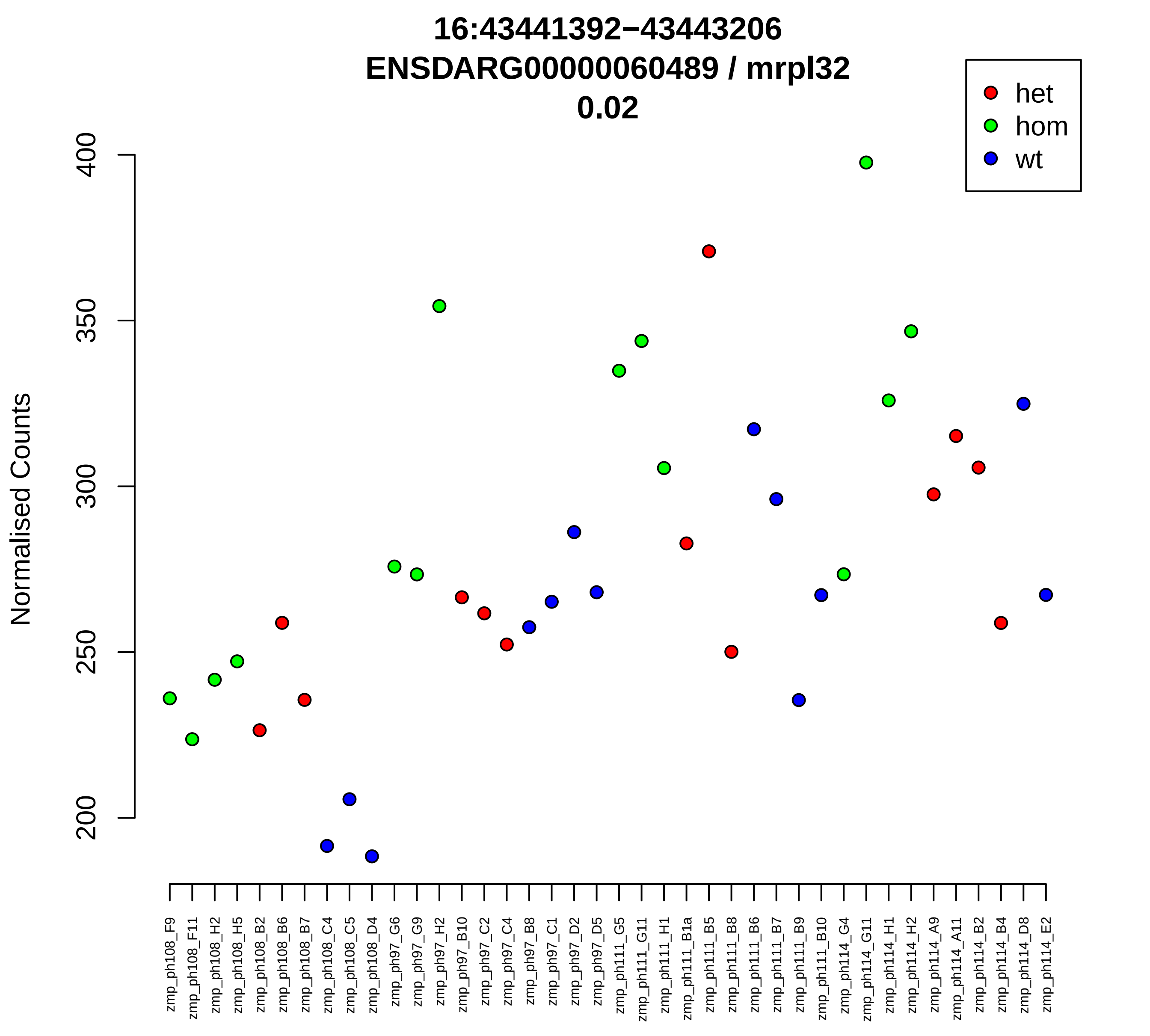
16:43441392−43443206
ENSDARG00000060489 / mrpl32
0.02
het
hom
wt
Normalised Counts
200
250
300
350
400
zmp_ph108_F9
zmp_ph108_F11
zmp_ph108_H2
zmp_ph108_H5
zmp_ph108_B2
zmp_ph108_B6
zmp_ph108_B7
zmp_ph108_C4
zmp_ph108_C5
zmp_ph108_D4
zmp_ph97_G6
zmp_ph97_G9
zmp_ph97_H2
zmp_ph97_B10
zmp_ph97_C2
zmp_ph97_C4
zmp_ph97_B8
zmp_ph97_C1
zmp_ph97_D2
zmp_ph97_D5
zmp_ph111_G5
zmp_ph111_G11
zmp_ph111_H1
zmp_ph111_B1a
zmp_ph111_B5
zmp_ph111_B8
zmp_ph111_B6
zmp_ph111_B7
zmp_ph111_B9
zmp_ph111_B10
zmp_ph114_G4
zmp_ph114_G11
zmp_ph114_H1
zmp_ph114_H2
zmp_ph114_A9
zmp_ph114_A11
zmp_ph114_B2
zmp_ph114_B4
zmp_ph114_D8
zmp_ph114_E2

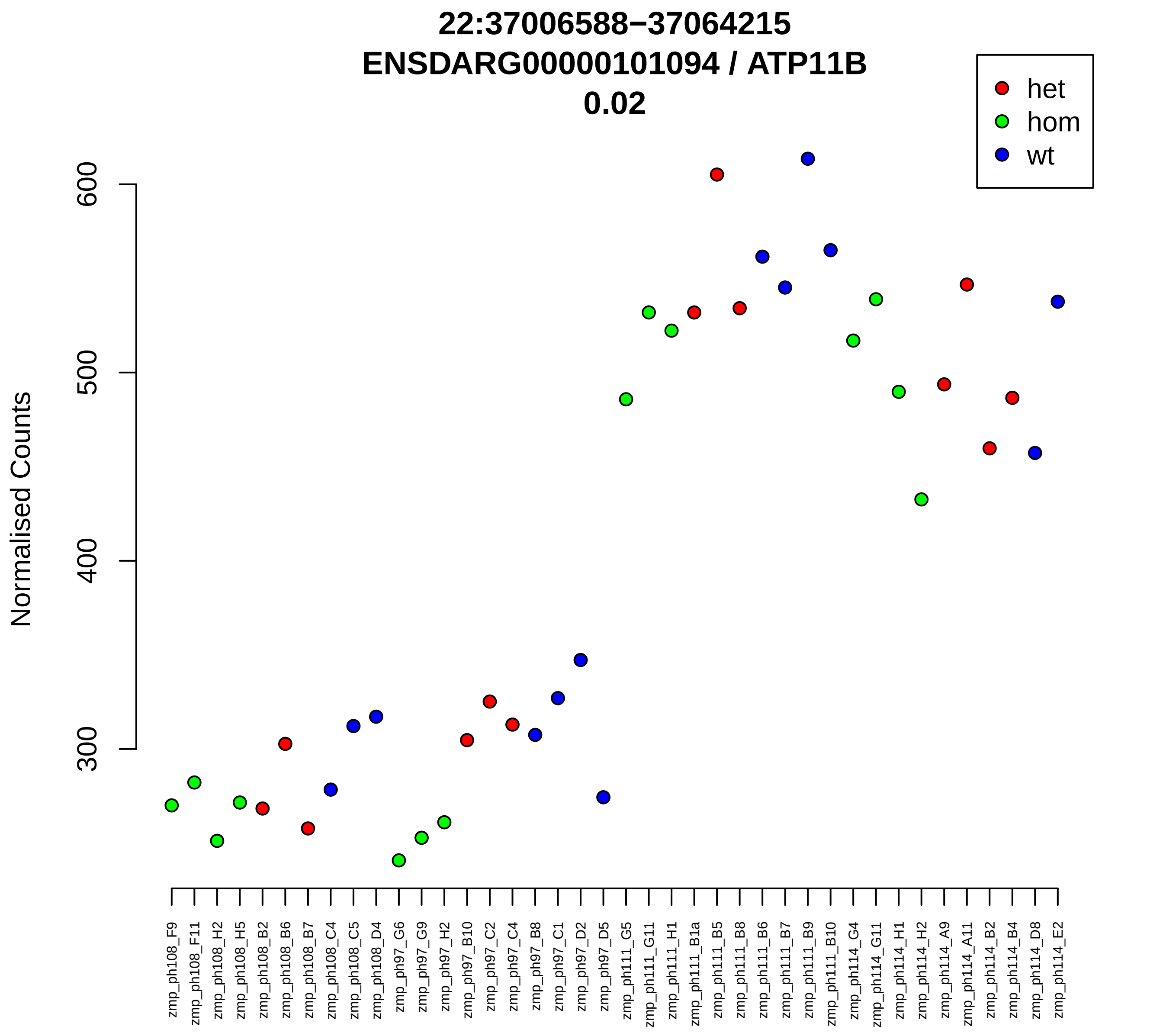

22:37006588−37064215
ENSDARG00000101094 / ATP11B
0.02
het
hom
wt
Normalised Counts
300
400
500
600
zmp_ph108_F9
zmp_ph108_F11
zmp_ph108_H2
zmp_ph108_H5
zmp_ph108_B2
zmp_ph108_B6
zmp_ph108_B7
zmp_ph108_C4
zmp_ph108_C5
zmp_ph108_D4
zmp_ph97_G6
zmp_ph97_G9
zmp_ph97_H2
zmp_ph97_B10
zmp_ph97_C2
zmp_ph97_C4
zmp_ph97_B8
zmp_ph97_C1
zmp_ph97_D2
zmp_ph97_D5
zmp_ph111_G5
zmp_ph111_G11
zmp_ph111_H1
zmp_ph111_B1a
zmp_ph111_B5
zmp_ph111_B8
zmp_ph111_B6
zmp_ph111_B7
zmp_ph111_B9
zmp_ph111_B10
zmp_ph114_G4
zmp_ph114_G11
zmp_ph114_H1
zmp_ph114_H2
zmp_ph114_A9
zmp_ph114_A11
zmp_ph114_B2
zmp_ph114_B4
zmp_ph114_D8
zmp_ph114_E2

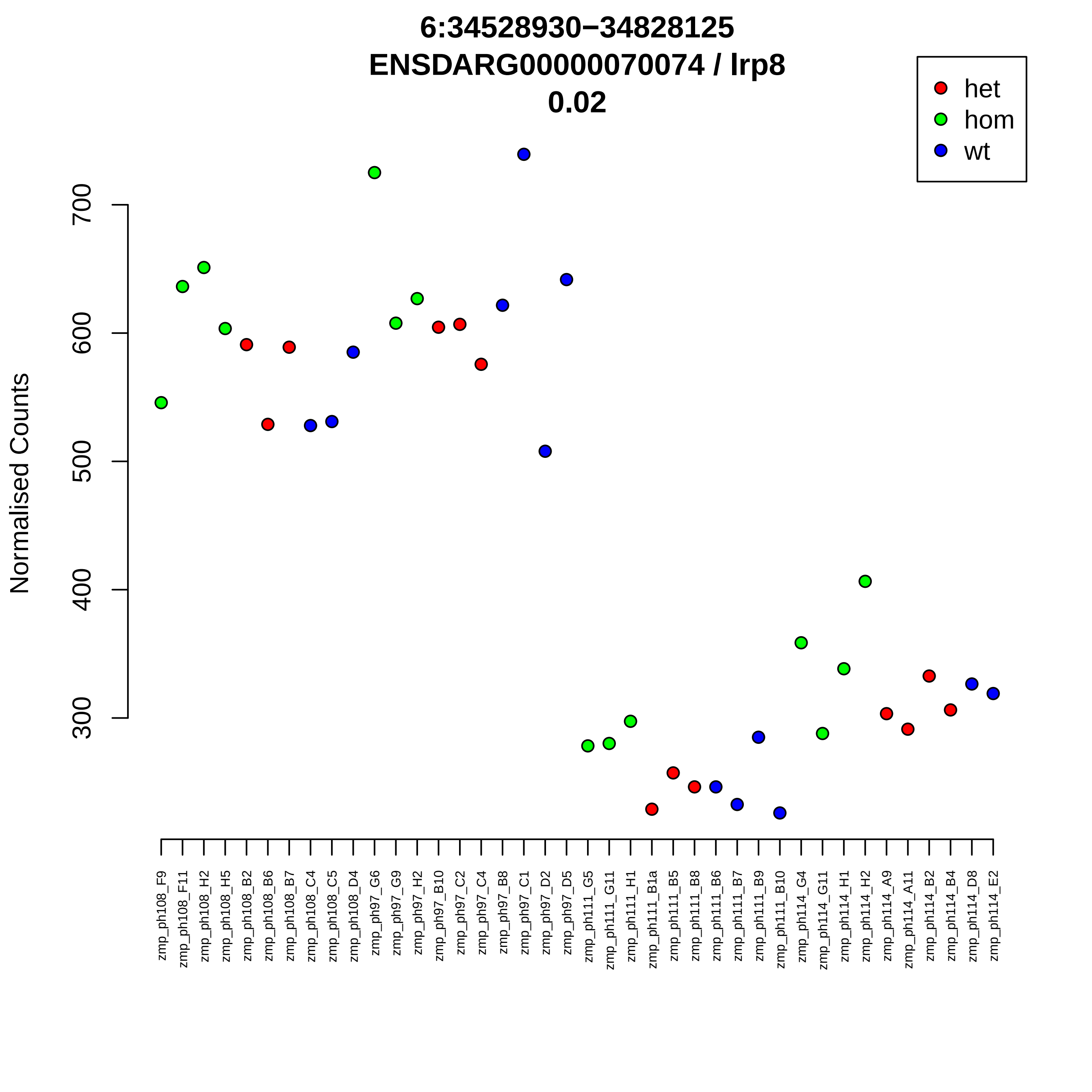
6:34528930−34828125
ENSDARG00000070074 / lrp8
0.02
het
hom
wt
Normalised Counts
300
400
500
600
700
zmp_ph108_F9
zmp_ph108_F11
zmp_ph108_H2
zmp_ph108_H5
zmp_ph108_B2
zmp_ph108_B6
zmp_ph108_B7
zmp_ph108_C4
zmp_ph108_C5
zmp_ph108_D4
zmp_ph97_G6
zmp_ph97_G9
zmp_ph97_H2
zmp_ph97_B10
zmp_ph97_C2
zmp_ph97_C4
zmp_ph97_B8
zmp_ph97_C1
zmp_ph97_D2
zmp_ph97_D5
zmp_ph111_G5
zmp_ph111_G11
zmp_ph111_H1
zmp_ph111_B1a
zmp_ph111_B5
zmp_ph111_B8
zmp_ph111_B6
zmp_ph111_B7
zmp_ph111_B9
zmp_ph111_B10
zmp_ph114_G4
zmp_ph114_G11
zmp_ph114_H1
zmp_ph114_H2
zmp_ph114_A9
zmp_ph114_A11
zmp_ph114_B2
zmp_ph114_B4
zmp_ph114_D8
zmp_ph114_E2

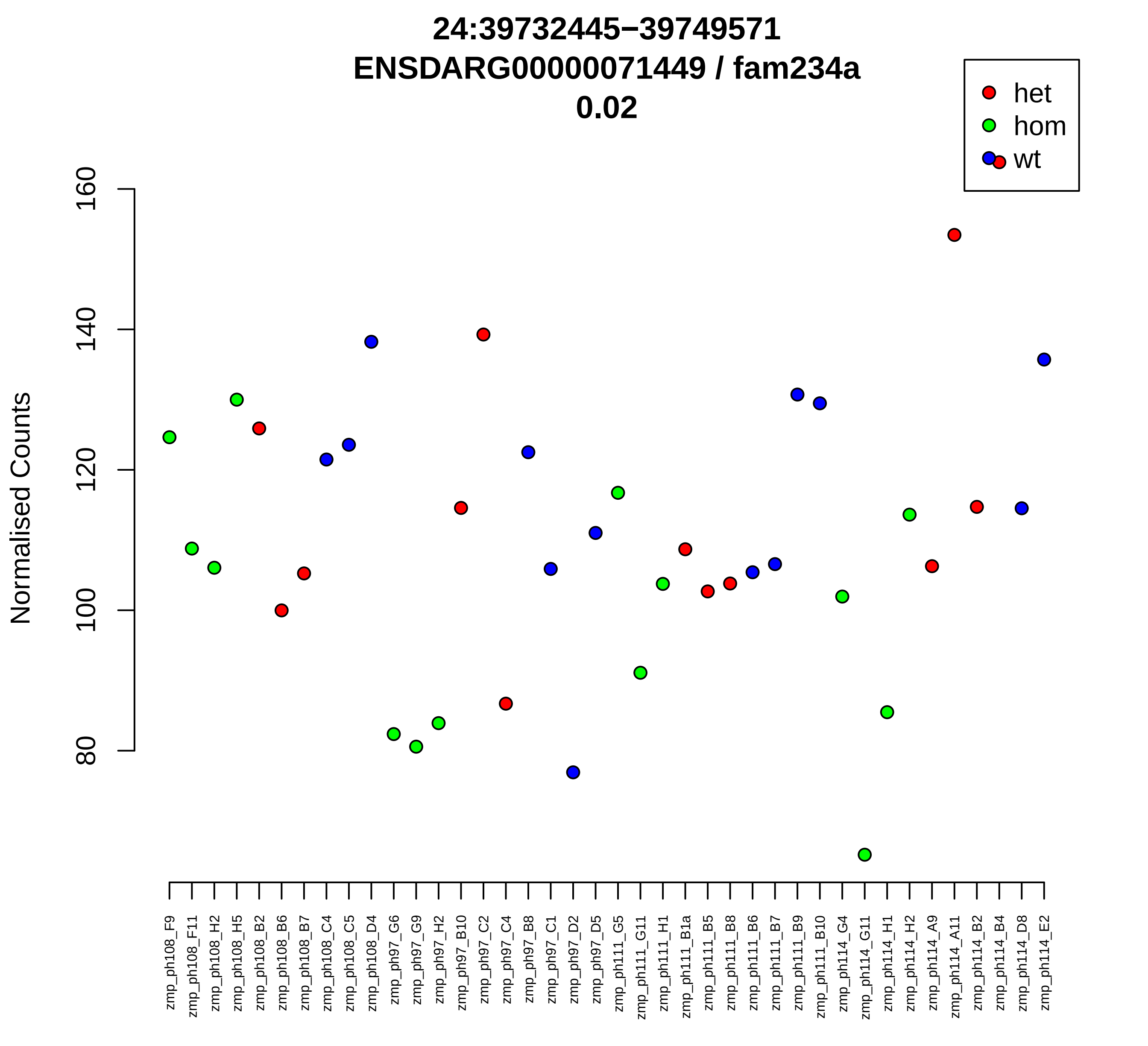
24:39732445−39749571
ENSDARG00000071449 / fam234a
0.02
het
hom
wt
Normalised Counts
80
100
120
140
160
zmp_ph108_F9
zmp_ph108_F11
zmp_ph108_H2
zmp_ph108_H5
zmp_ph108_B2
zmp_ph108_B6
zmp_ph108_B7
zmp_ph108_C4
zmp_ph108_C5
zmp_ph108_D4
zmp_ph97_G6
zmp_ph97_G9
zmp_ph97_H2
zmp_ph97_B10
zmp_ph97_C2
zmp_ph97_C4
zmp_ph97_B8
zmp_ph97_C1
zmp_ph97_D2
zmp_ph97_D5
zmp_ph111_G5
zmp_ph111_G11
zmp_ph111_H1
zmp_ph111_B1a
zmp_ph111_B5
zmp_ph111_B8
zmp_ph111_B6
zmp_ph111_B7
zmp_ph111_B9
zmp_ph111_B10
zmp_ph114_G4
zmp_ph114_G11
zmp_ph114_H1
zmp_ph114_H2
zmp_ph114_A9
zmp_ph114_A11
zmp_ph114_B2
zmp_ph114_B4
zmp_ph114_D8
zmp_ph114_E2

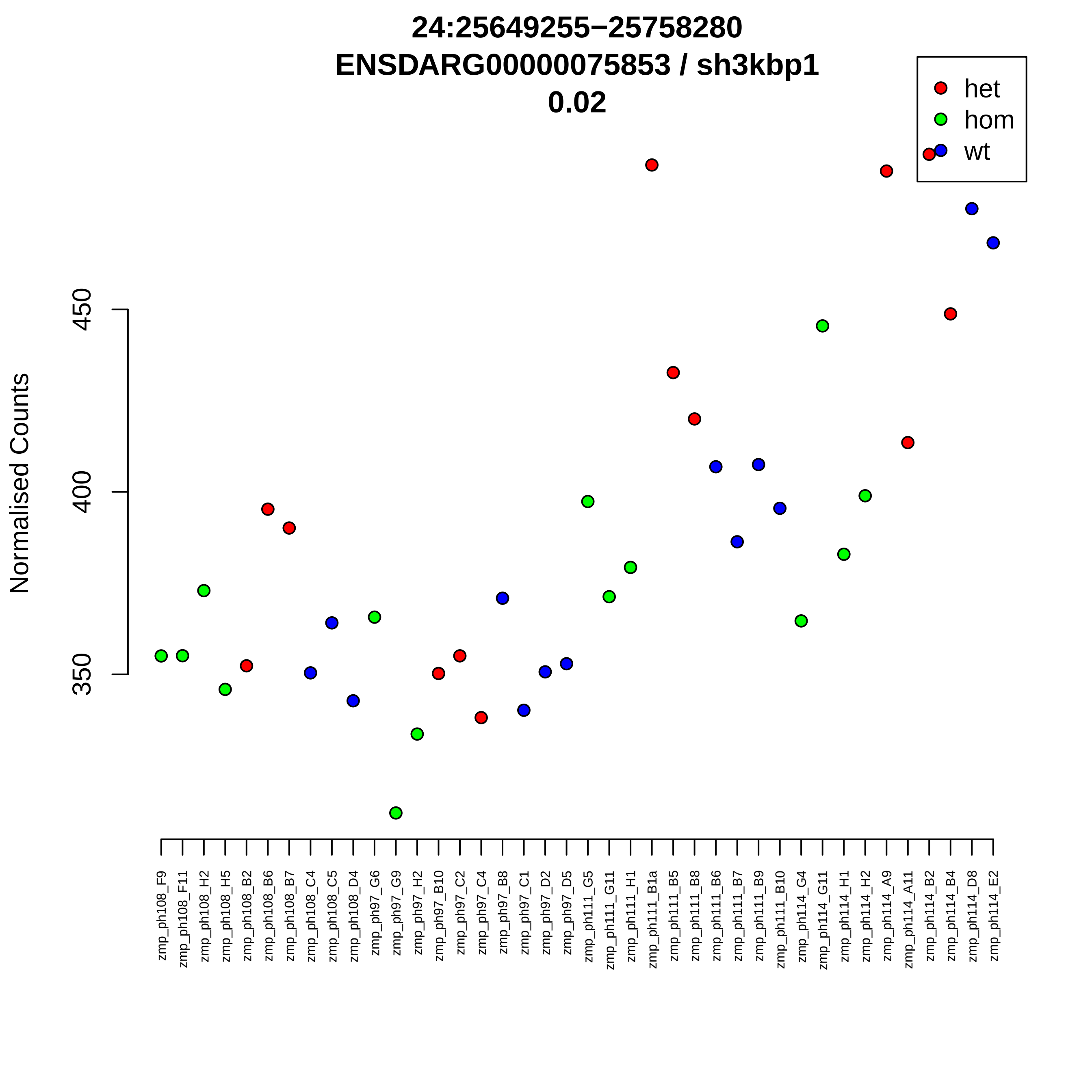
24:25649255−25758280
ENSDARG00000075853 / sh3kbp1
0.02
het
hom
wt
Normalised Counts
350
400
450
zmp_ph108_F9
zmp_ph108_F11
zmp_ph108_H2
zmp_ph108_H5
zmp_ph108_B2
zmp_ph108_B6
zmp_ph108_B7
zmp_ph108_C4
zmp_ph108_C5
zmp_ph108_D4
zmp_ph97_G6
zmp_ph97_G9
zmp_ph97_H2
zmp_ph97_B10
zmp_ph97_C2
zmp_ph97_C4
zmp_ph97_B8
zmp_ph97_C1
zmp_ph97_D2
zmp_ph97_D5
zmp_ph111_G5
zmp_ph111_G11
zmp_ph111_H1
zmp_ph111_B1a
zmp_ph111_B5
zmp_ph111_B8
zmp_ph111_B6
zmp_ph111_B7
zmp_ph111_B9
zmp_ph111_B10
zmp_ph114_G4
zmp_ph114_G11
zmp_ph114_H1
zmp_ph114_H2
zmp_ph114_A9
zmp_ph114_A11
zmp_ph114_B2
zmp_ph114_B4
zmp_ph114_D8
zmp_ph114_E2

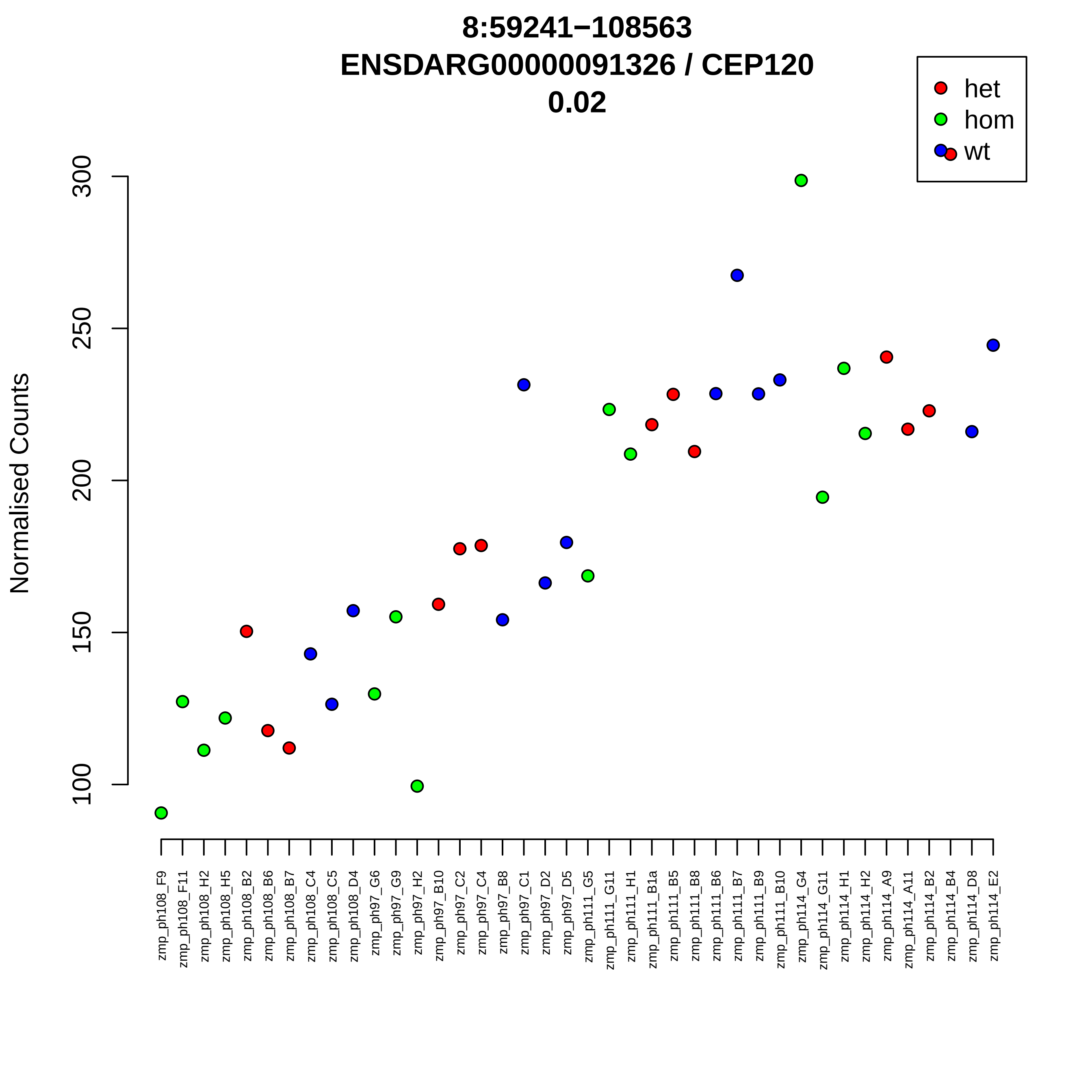

8:59241−108563
ENSDARG00000091326 / CEP120
0.02
het
hom
wt
Normalised Counts
100
150
200
250
300
zmp_ph108_F9
zmp_ph108_F11
zmp_ph108_H2
zmp_ph108_H5
zmp_ph108_B2
zmp_ph108_B6
zmp_ph108_B7
zmp_ph108_C4
zmp_ph108_C5
zmp_ph108_D4
zmp_ph97_G6
zmp_ph97_G9
zmp_ph97_H2
zmp_ph97_B10
zmp_ph97_C2
zmp_ph97_C4
zmp_ph97_B8
zmp_ph97_C1
zmp_ph97_D2
zmp_ph97_D5
zmp_ph111_G5
zmp_ph111_G11
zmp_ph111_H1
zmp_ph111_B1a
zmp_ph111_B5
zmp_ph111_B8
zmp_ph111_B6
zmp_ph111_B7
zmp_ph111_B9
zmp_ph111_B10
zmp_ph114_G4
zmp_ph114_G11
zmp_ph114_H1
zmp_ph114_H2
zmp_ph114_A9
zmp_ph114_A11
zmp_ph114_B2
zmp_ph114_B4
zmp_ph114_D8
zmp_ph114_E2

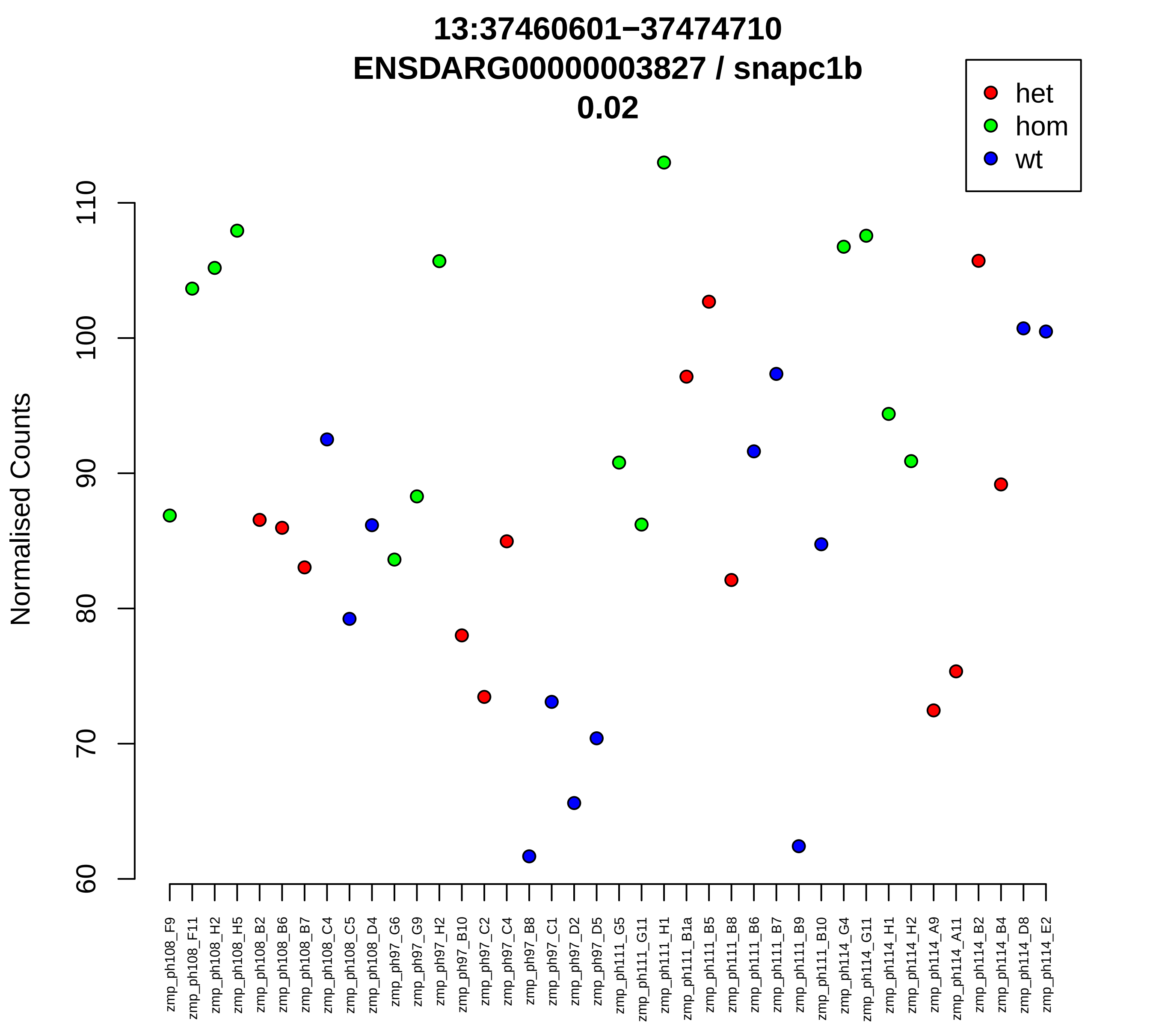
13:37460601−37474710
ENSDARG00000003827 / snapc1b
0.02
het
hom
wt
Normalised Counts
60
70
80
90
100
110
zmp_ph108_F9
zmp_ph108_F11
zmp_ph108_H2
zmp_ph108_H5
zmp_ph108_B2
zmp_ph108_B6
zmp_ph108_B7
zmp_ph108_C4
zmp_ph108_C5
zmp_ph108_D4
zmp_ph97_G6
zmp_ph97_G9
zmp_ph97_H2
zmp_ph97_B10
zmp_ph97_C2
zmp_ph97_C4
zmp_ph97_B8
zmp_ph97_C1
zmp_ph97_D2
zmp_ph97_D5
zmp_ph111_G5
zmp_ph111_G11
zmp_ph111_H1
zmp_ph111_B1a
zmp_ph111_B5
zmp_ph111_B8
zmp_ph111_B6
zmp_ph111_B7
zmp_ph111_B9
zmp_ph111_B10
zmp_ph114_G4
zmp_ph114_G11
zmp_ph114_H1
zmp_ph114_H2
zmp_ph114_A9
zmp_ph114_A11
zmp_ph114_B2
zmp_ph114_B4
zmp_ph114_D8
zmp_ph114_E2

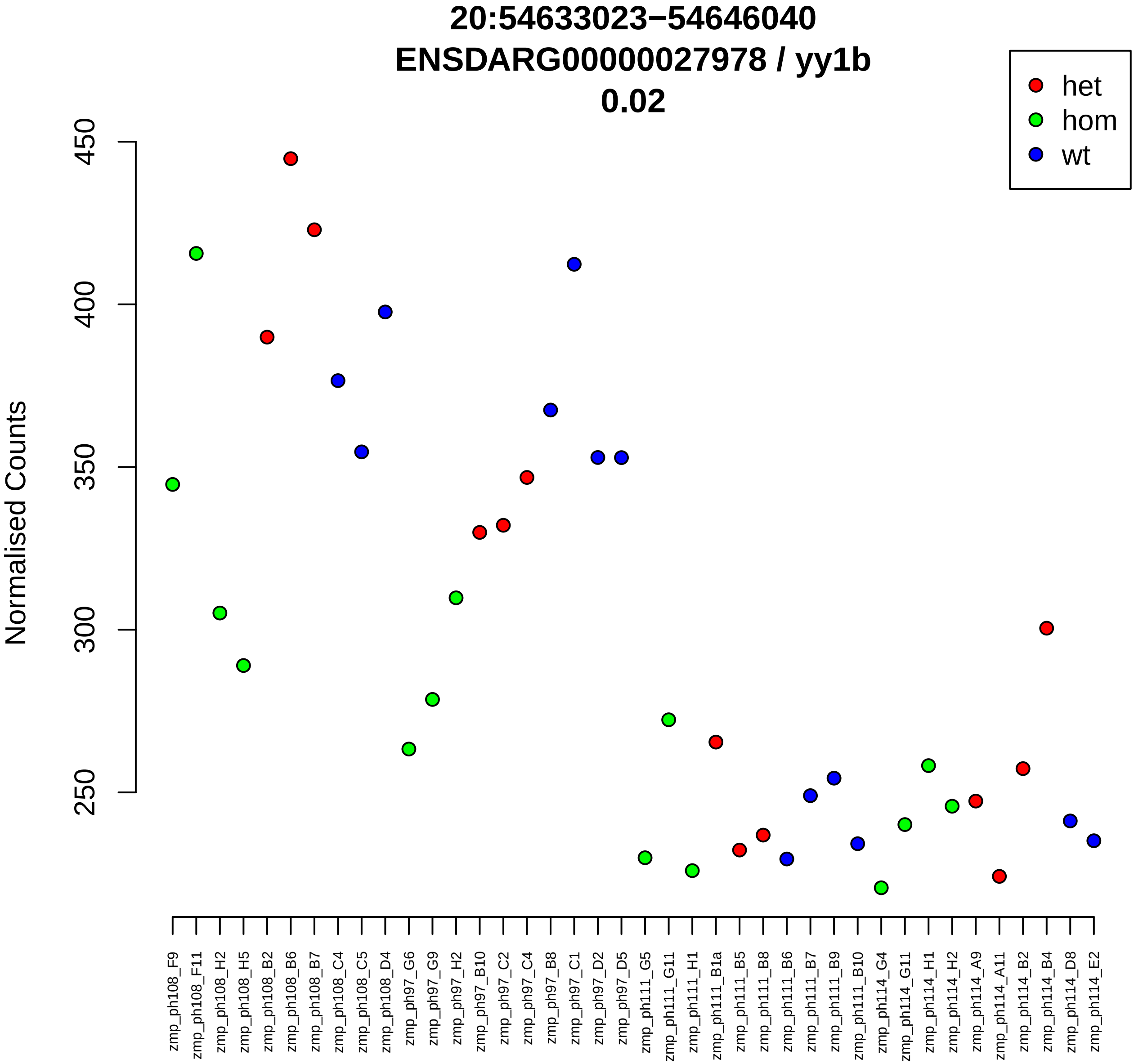

20:54633023−54646040
ENSDARG00000027978 / yy1b
0.02
het
hom
wt
Normalised Counts
250
300
350
400
450
zmp_ph108_F9
zmp_ph108_F11
zmp_ph108_H2
zmp_ph108_H5
zmp_ph108_B2
zmp_ph108_B6
zmp_ph108_B7
zmp_ph108_C4
zmp_ph108_C5
zmp_ph108_D4
zmp_ph97_G6
zmp_ph97_G9
zmp_ph97_H2
zmp_ph97_B10
zmp_ph97_C2
zmp_ph97_C4
zmp_ph97_B8
zmp_ph97_C1
zmp_ph97_D2
zmp_ph97_D5
zmp_ph111_G5
zmp_ph111_G11
zmp_ph111_H1
zmp_ph111_B1a
zmp_ph111_B5
zmp_ph111_B8
zmp_ph111_B6
zmp_ph111_B7
zmp_ph111_B9
zmp_ph111_B10
zmp_ph114_G4
zmp_ph114_G11
zmp_ph114_H1
zmp_ph114_H2
zmp_ph114_A9
zmp_ph114_A11
zmp_ph114_B2
zmp_ph114_B4
zmp_ph114_D8
zmp_ph114_E2

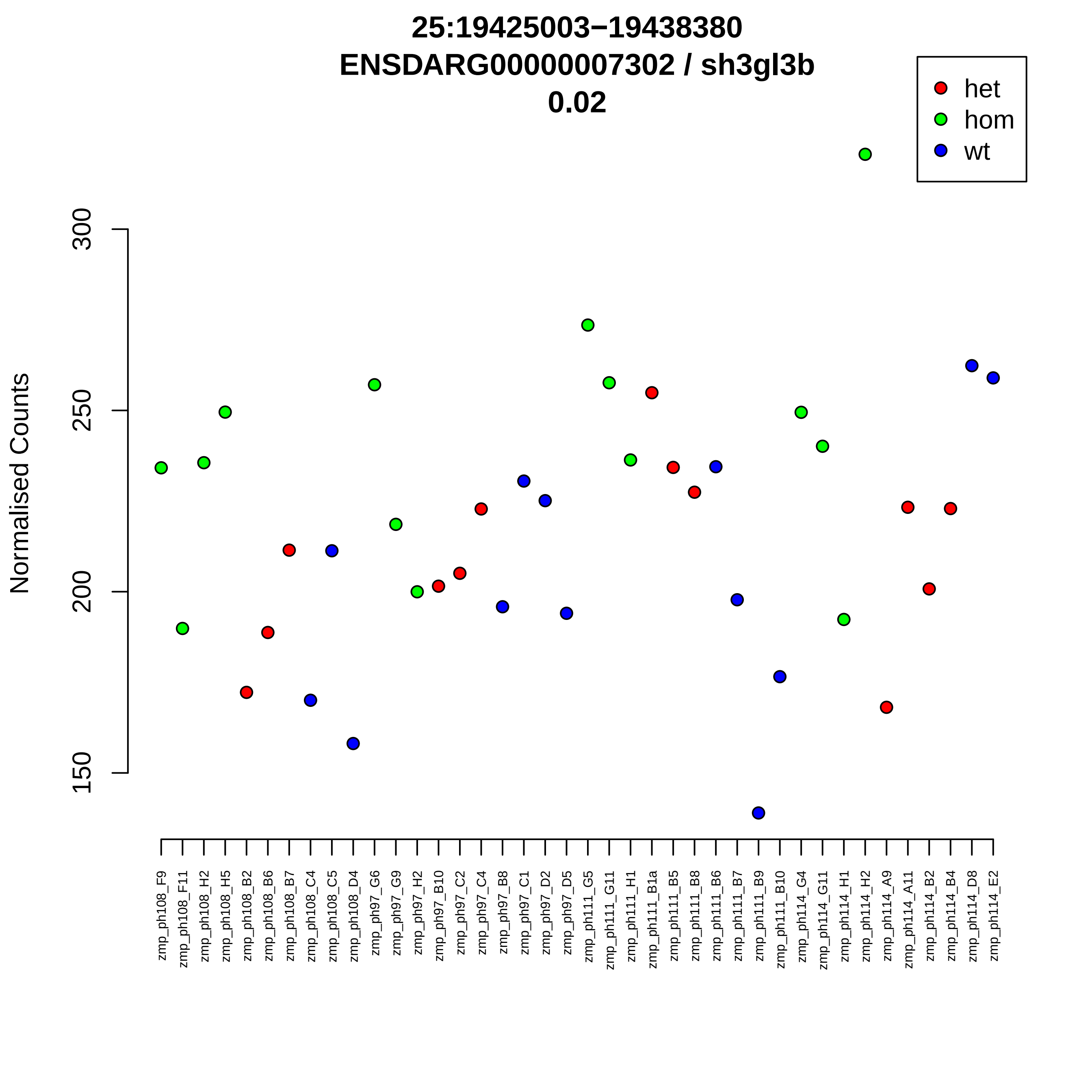
25:19425003−19438380
ENSDARG00000007302 / sh3gl3b
0.02
het
hom
wt
Normalised Counts
150
200
250
300
zmp_ph108_F9
zmp_ph108_F11
zmp_ph108_H2
zmp_ph108_H5
zmp_ph108_B2
zmp_ph108_B6
zmp_ph108_B7
zmp_ph108_C4
zmp_ph108_C5
zmp_ph108_D4
zmp_ph97_G6
zmp_ph97_G9
zmp_ph97_H2
zmp_ph97_B10
zmp_ph97_C2
zmp_ph97_C4
zmp_ph97_B8
zmp_ph97_C1
zmp_ph97_D2
zmp_ph97_D5
zmp_ph111_G5
zmp_ph111_G11
zmp_ph111_H1
zmp_ph111_B1a
zmp_ph111_B5
zmp_ph111_B8
zmp_ph111_B6
zmp_ph111_B7
zmp_ph111_B9
zmp_ph111_B10
zmp_ph114_G4
zmp_ph114_G11
zmp_ph114_H1
zmp_ph114_H2
zmp_ph114_A9
zmp_ph114_A11
zmp_ph114_B2
zmp_ph114_B4
zmp_ph114_D8
zmp_ph114_E2

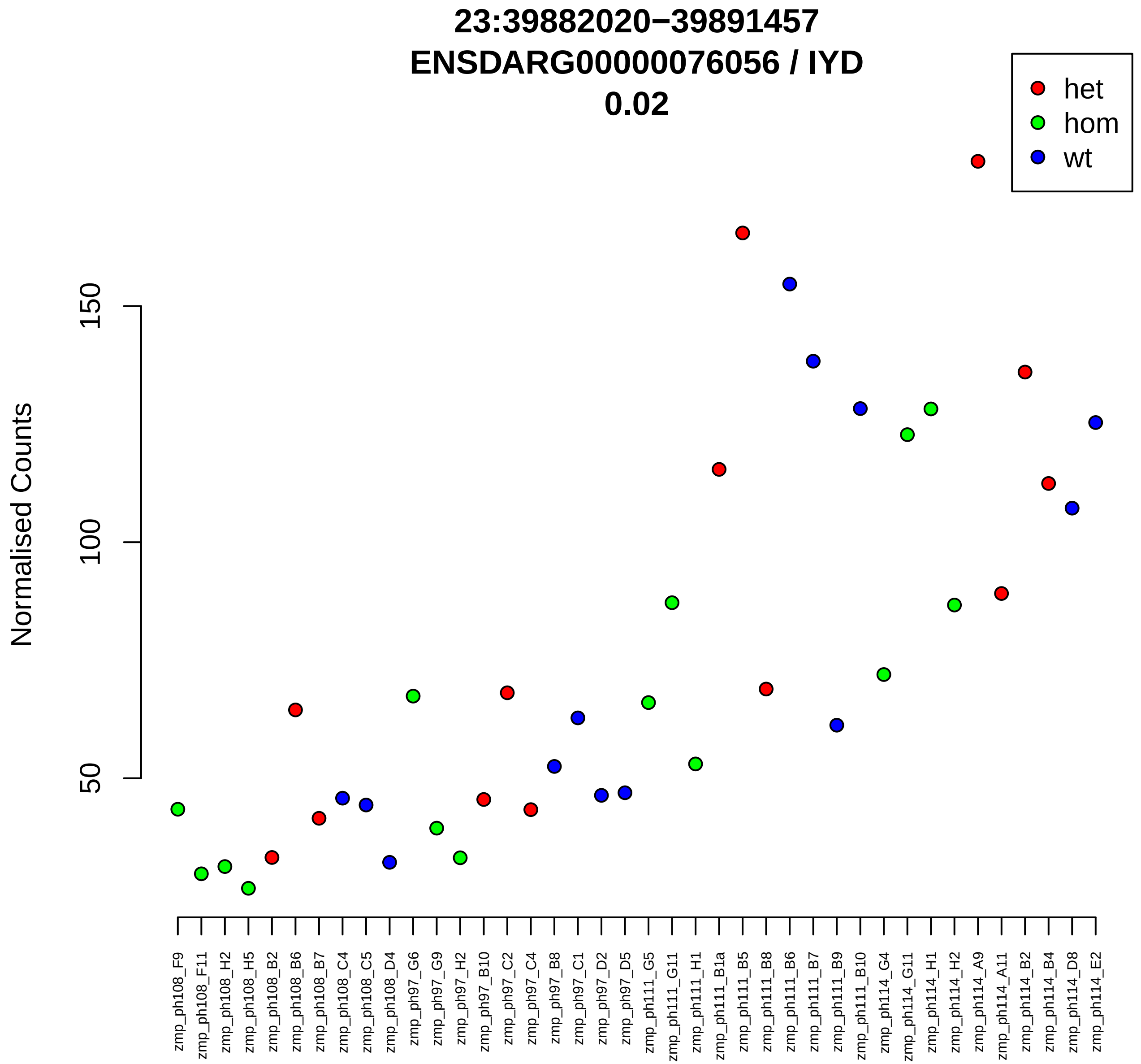
23:39882020−39891457
ENSDARG00000076056 / IYD
0.02
het
hom
wt
Normalised Counts
50
100
150
zmp_ph108_F9
zmp_ph108_F11
zmp_ph108_H2
zmp_ph108_H5
zmp_ph108_B2
zmp_ph108_B6
zmp_ph108_B7
zmp_ph108_C4
zmp_ph108_C5
zmp_ph108_D4
zmp_ph97_G6
zmp_ph97_G9
zmp_ph97_H2
zmp_ph97_B10
zmp_ph97_C2
zmp_ph97_C4
zmp_ph97_B8
zmp_ph97_C1
zmp_ph97_D2
zmp_ph97_D5
zmp_ph111_G5
zmp_ph111_G11
zmp_ph111_H1
zmp_ph111_B1a
zmp_ph111_B5
zmp_ph111_B8
zmp_ph111_B6
zmp_ph111_B7
zmp_ph111_B9
zmp_ph111_B10
zmp_ph114_G4
zmp_ph114_G11
zmp_ph114_H1
zmp_ph114_H2
zmp_ph114_A9
zmp_ph114_A11
zmp_ph114_B2
zmp_ph114_B4
zmp_ph114_D8
zmp_ph114_E2

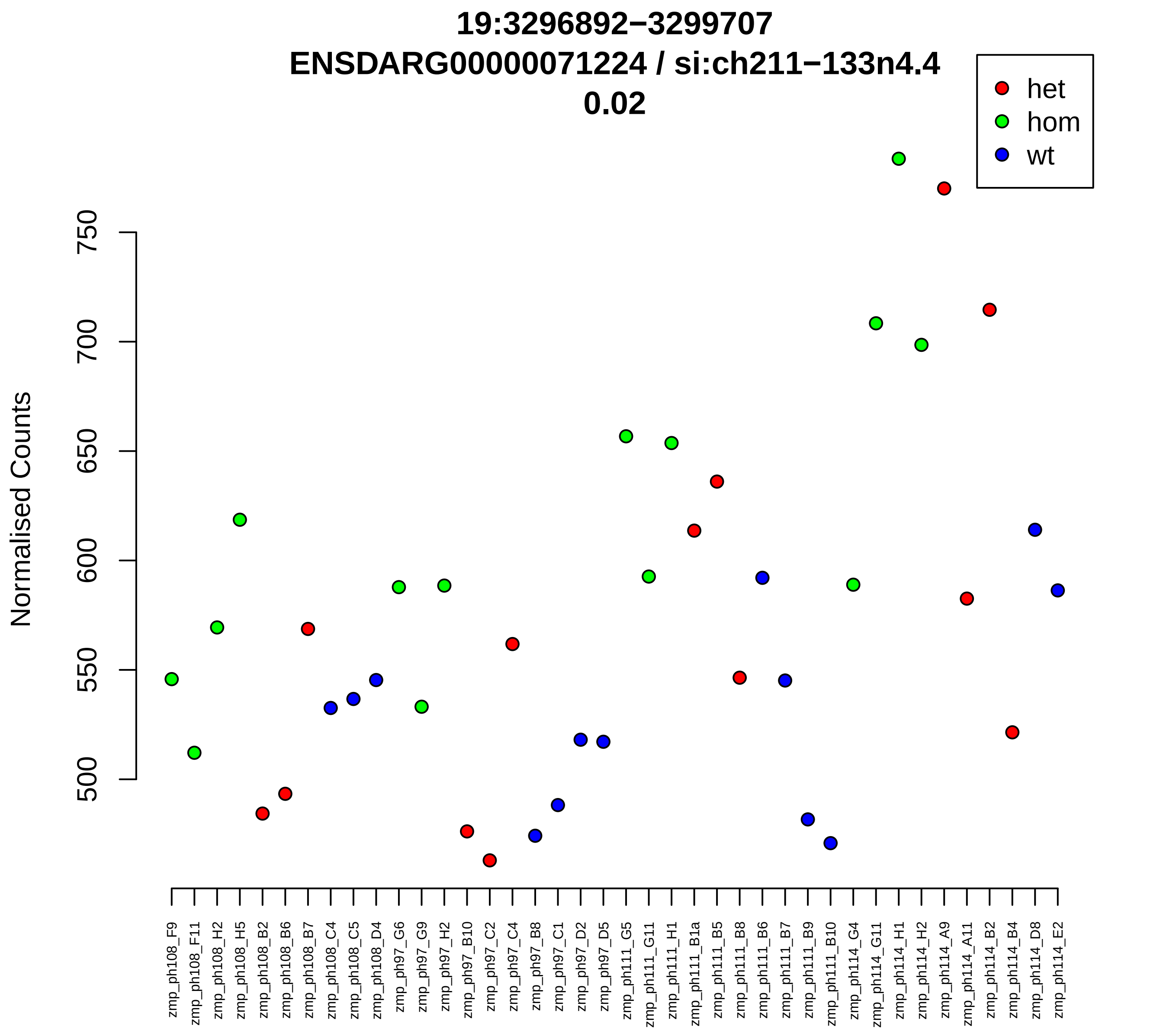
19:3296892−3299707
ENSDARG00000071224 / si:ch211−133n4.4
0.02
het
hom
wt
Normalised Counts
500
550
600
650
700
750
zmp_ph108_F9
zmp_ph108_F11
zmp_ph108_H2
zmp_ph108_H5
zmp_ph108_B2
zmp_ph108_B6
zmp_ph108_B7
zmp_ph108_C4
zmp_ph108_C5
zmp_ph108_D4
zmp_ph97_G6
zmp_ph97_G9
zmp_ph97_H2
zmp_ph97_B10
zmp_ph97_C2
zmp_ph97_C4
zmp_ph97_B8
zmp_ph97_C1
zmp_ph97_D2
zmp_ph97_D5
zmp_ph111_G5
zmp_ph111_G11
zmp_ph111_H1
zmp_ph111_B1a
zmp_ph111_B5
zmp_ph111_B8
zmp_ph111_B6
zmp_ph111_B7
zmp_ph111_B9
zmp_ph111_B10
zmp_ph114_G4
zmp_ph114_G11
zmp_ph114_H1
zmp_ph114_H2
zmp_ph114_A9
zmp_ph114_A11
zmp_ph114_B2
zmp_ph114_B4
zmp_ph114_D8
zmp_ph114_E2

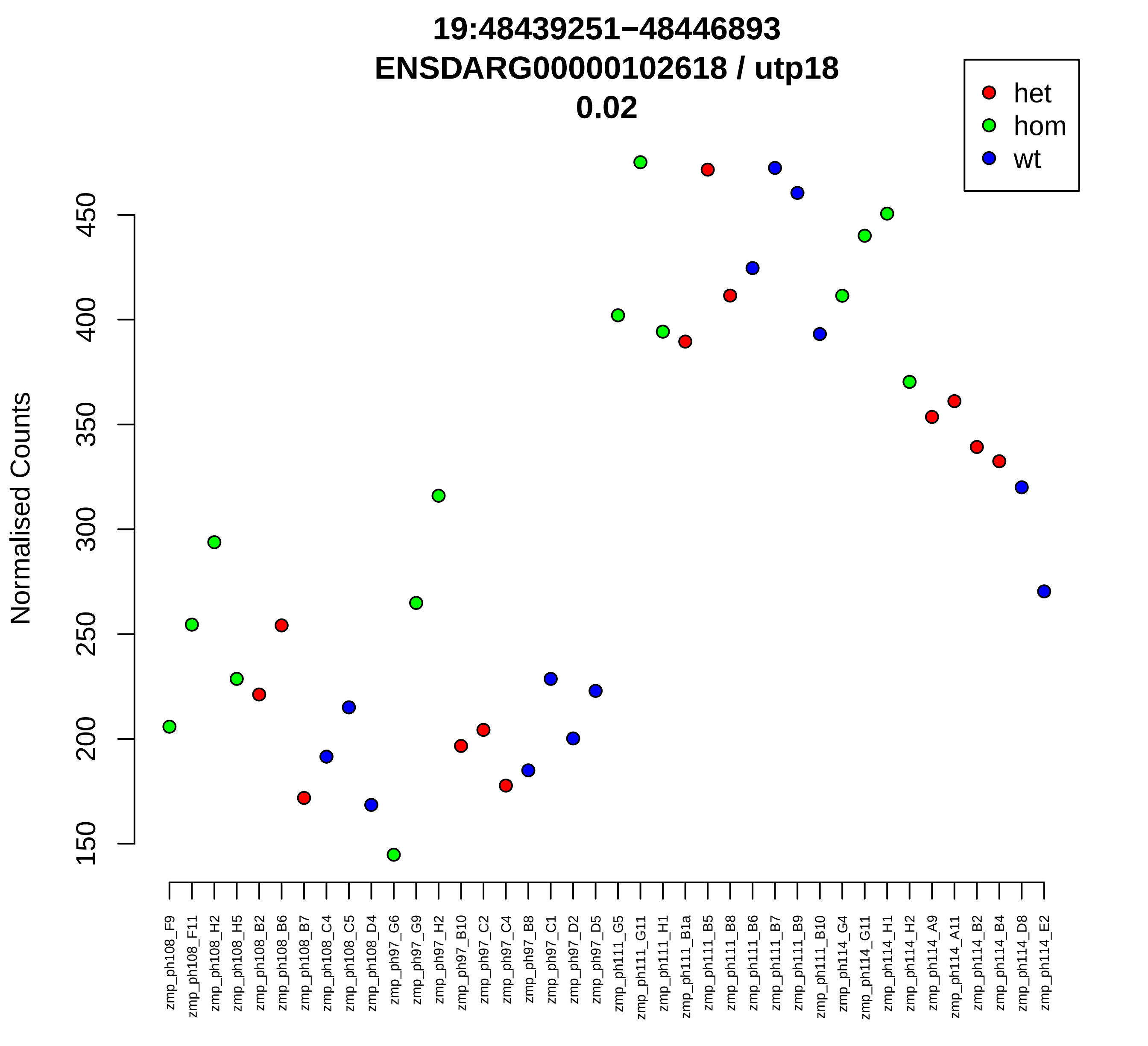

19:48439251−48446893
ENSDARG00000102618 / utp18
0.02
het
hom
wt
Normalised Counts
150
200
250
300
350
400
450
zmp_ph108_F9
zmp_ph108_F11
zmp_ph108_H2
zmp_ph108_H5
zmp_ph108_B2
zmp_ph108_B6
zmp_ph108_B7
zmp_ph108_C4
zmp_ph108_C5
zmp_ph108_D4
zmp_ph97_G6
zmp_ph97_G9
zmp_ph97_H2
zmp_ph97_B10
zmp_ph97_C2
zmp_ph97_C4
zmp_ph97_B8
zmp_ph97_C1
zmp_ph97_D2
zmp_ph97_D5
zmp_ph111_G5
zmp_ph111_G11
zmp_ph111_H1
zmp_ph111_B1a
zmp_ph111_B5
zmp_ph111_B8
zmp_ph111_B6
zmp_ph111_B7
zmp_ph111_B9
zmp_ph111_B10
zmp_ph114_G4
zmp_ph114_G11
zmp_ph114_H1
zmp_ph114_H2
zmp_ph114_A9
zmp_ph114_A11
zmp_ph114_B2
zmp_ph114_B4
zmp_ph114_D8
zmp_ph114_E2

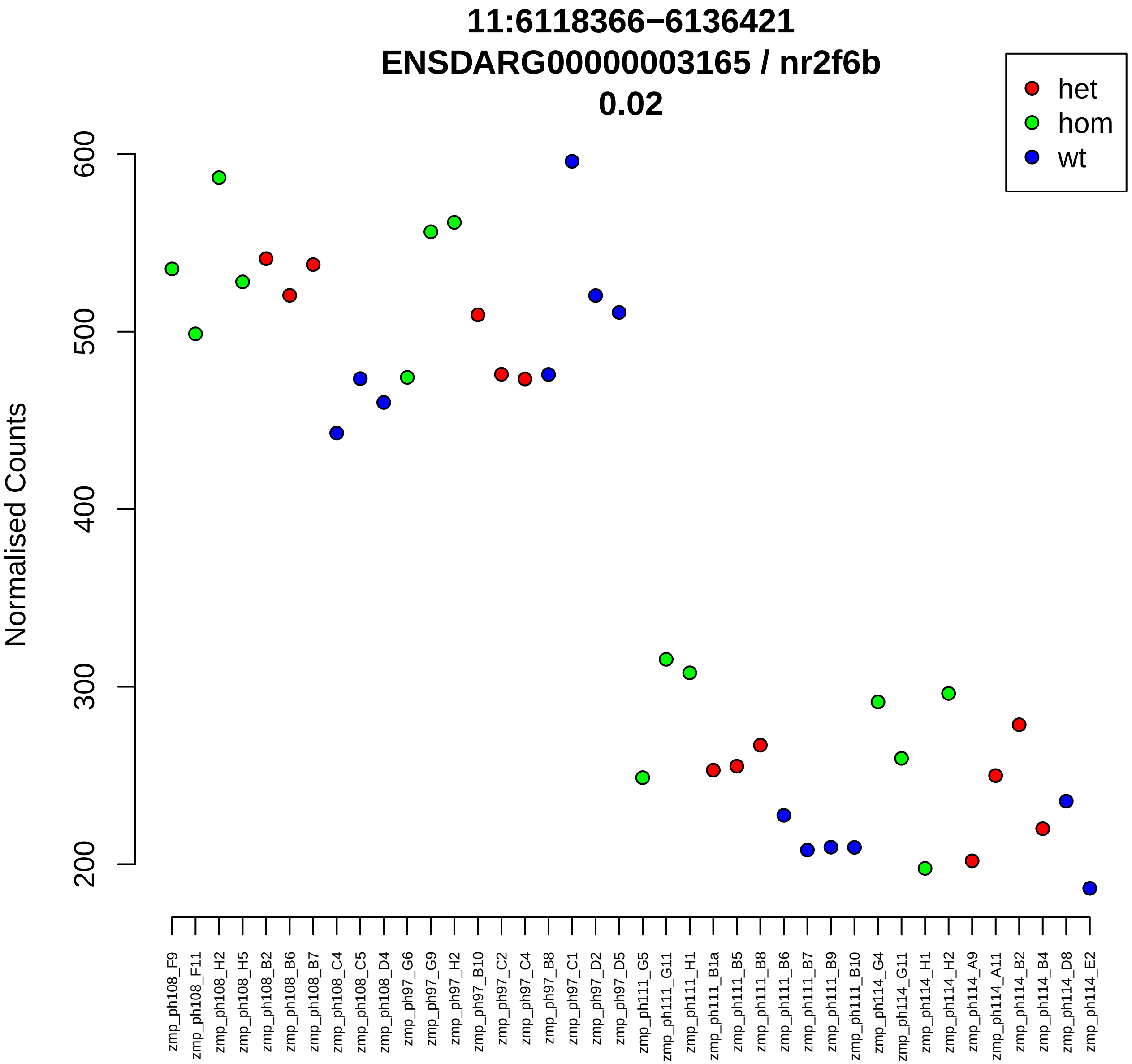

11:6118366−6136421
ENSDARG00000003165 / nr2f6b
0.02
het
hom
wt
Normalised Counts
600
500
400
300
200
zmp_ph108_F9
zmp_ph108_F11
zmp_ph108_H2
zmp_ph108_H5
zmp_ph108_B2
zmp_ph108_B6
zmp_ph108_B7
zmp_ph108_C4
zmp_ph108_C5
zmp_ph108_D4
zmp_ph97_G6
zmp_ph97_G9
zmp_ph97_H2
zmp_ph97_B10
zmp_ph97_C2
zmp_ph97_C4
zmp_ph97_B8
zmp_ph97_C1
zmp_ph97_D2
zmp_ph97_D5
zmp_ph111_G5
zmp_ph111_G11
zmp_ph111_H1
zmp_ph111_B1a
zmp_ph111_B5
zmp_ph111_B8
zmp_ph111_B6
zmp_ph111_B7
zmp_ph111_B9
zmp_ph111_B10
zmp_ph114_G4
zmp_ph114_G11
zmp_ph114_H1
zmp_ph114_H2
zmp_ph114_A9
zmp_ph114_A11
zmp_ph114_B2
zmp_ph114_B4
zmp_ph114_D8
zmp_ph114_E2

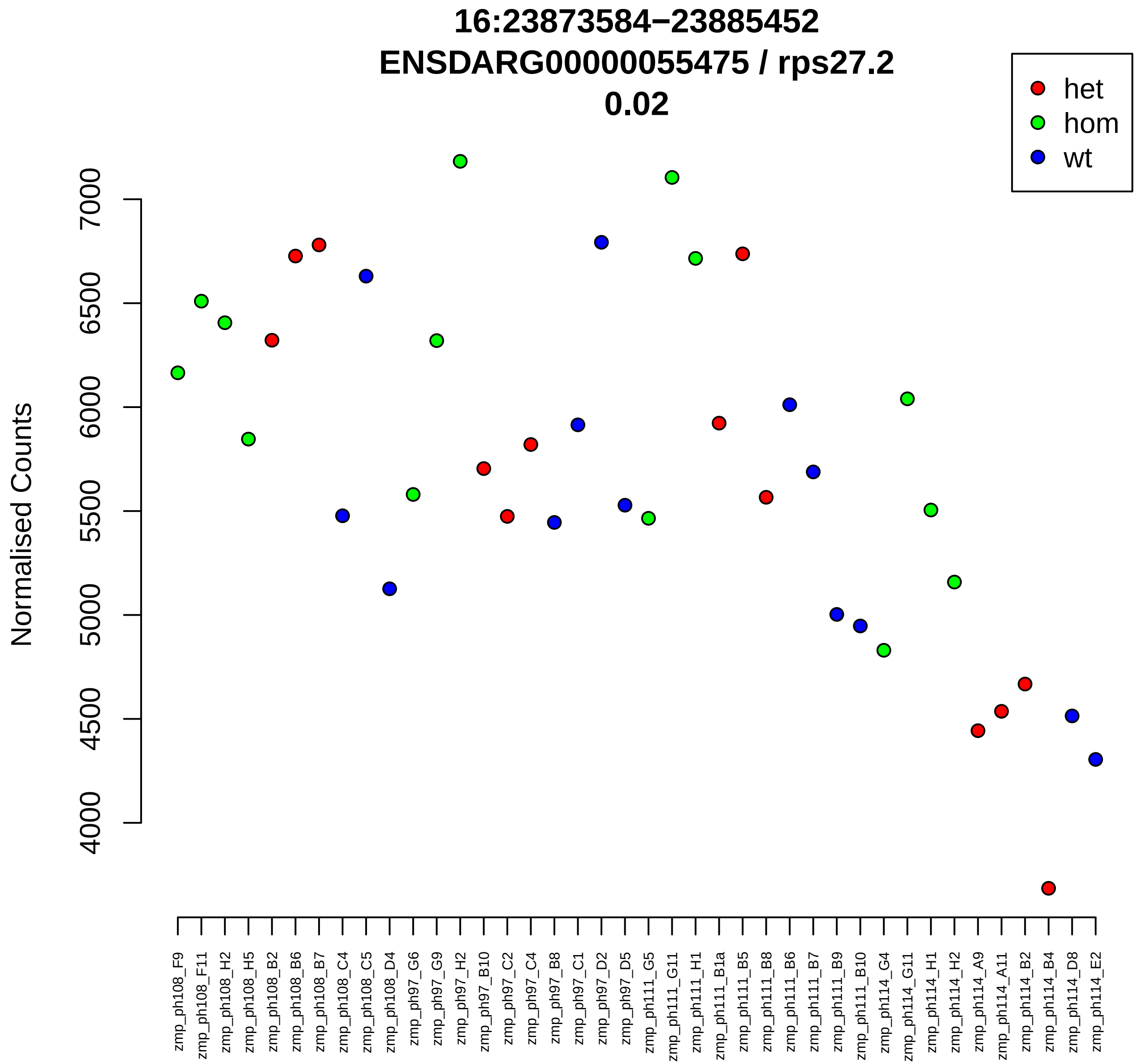

16:23873584−23885452
ENSDARG00000055475 / rps27.2
0.02
het
hom
wt
Normalised Counts
4000
4500
5000
5500
6000
6500
7000
zmp_ph108_F9
zmp_ph108_F11
zmp_ph108_H2
zmp_ph108_H5
zmp_ph108_B2
zmp_ph108_B6
zmp_ph108_B7
zmp_ph108_C4
zmp_ph108_C5
zmp_ph108_D4
zmp_ph97_G6
zmp_ph97_G9
zmp_ph97_H2
zmp_ph97_B10
zmp_ph97_C2
zmp_ph97_C4
zmp_ph97_B8
zmp_ph97_C1
zmp_ph97_D2
zmp_ph97_D5
zmp_ph111_G5
zmp_ph111_G11
zmp_ph111_H1
zmp_ph111_B1a
zmp_ph111_B5
zmp_ph111_B8
zmp_ph111_B6
zmp_ph111_B7
zmp_ph111_B9
zmp_ph111_B10
zmp_ph114_G4
zmp_ph114_G11
zmp_ph114_H1
zmp_ph114_H2
zmp_ph114_A9
zmp_ph114_A11
zmp_ph114_B2
zmp_ph114_B4
zmp_ph114_D8
zmp_ph114_E2

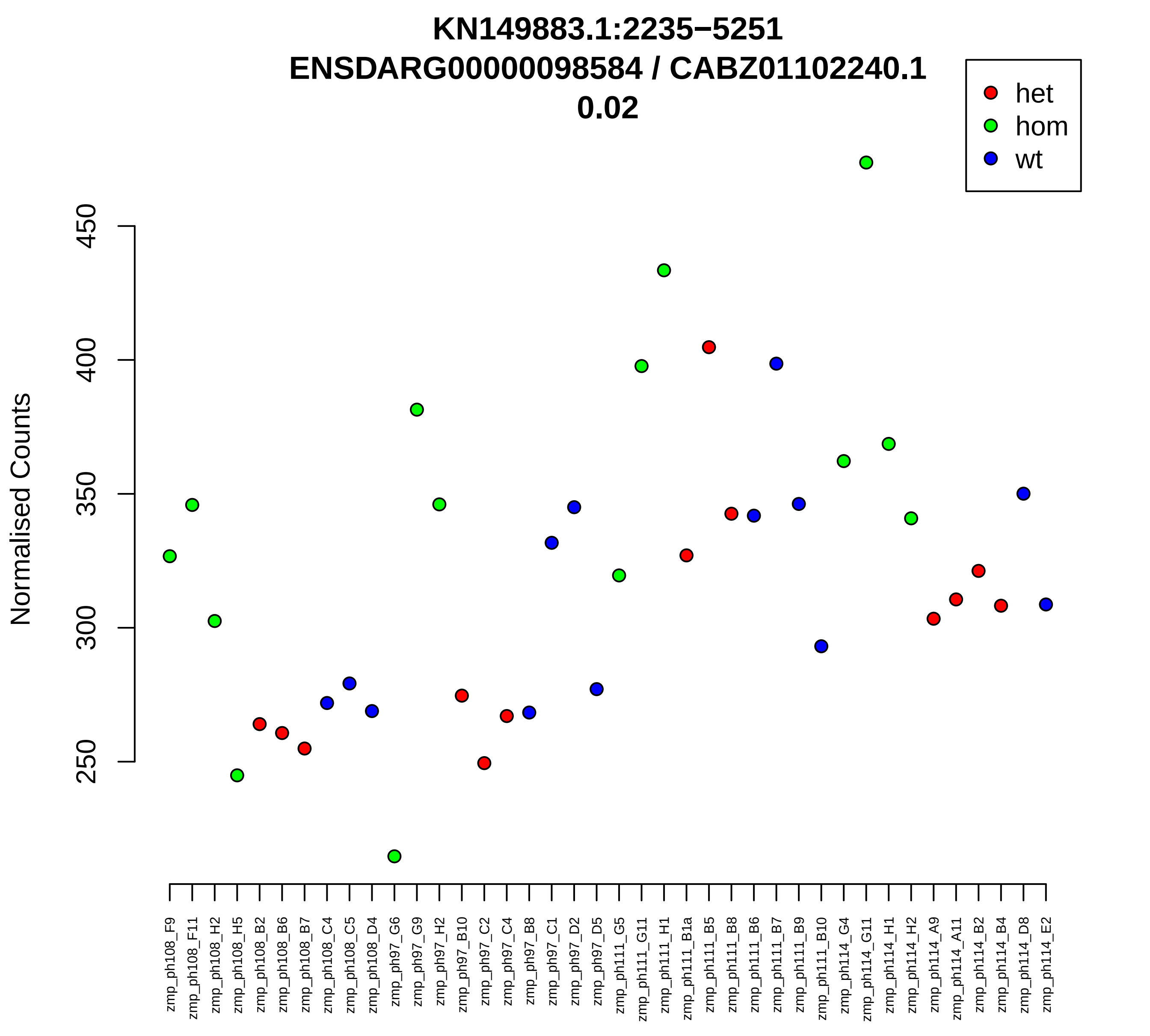
KN149883.1:2235−5251
ENSDARG00000098584 / CABZ01102240.1
0.02
het
hom
wt
Normalised Counts
250
300
350
400
450
zmp_ph108_F9
zmp_ph108_F11
zmp_ph108_H2
zmp_ph108_H5
zmp_ph108_B2
zmp_ph108_B6
zmp_ph108_B7
zmp_ph108_C4
zmp_ph108_C5
zmp_ph108_D4
zmp_ph97_G6
zmp_ph97_G9
zmp_ph97_H2
zmp_ph97_B10
zmp_ph97_C2
zmp_ph97_C4
zmp_ph97_B8
zmp_ph97_C1
zmp_ph97_D2
zmp_ph97_D5
zmp_ph111_G5
zmp_ph111_G11
zmp_ph111_H1
zmp_ph111_B1a
zmp_ph111_B5
zmp_ph111_B8
zmp_ph111_B6
zmp_ph111_B7
zmp_ph111_B9
zmp_ph111_B10
zmp_ph114_G4
zmp_ph114_G11
zmp_ph114_H1
zmp_ph114_H2
zmp_ph114_A9
zmp_ph114_A11
zmp_ph114_B2
zmp_ph114_B4
zmp_ph114_D8
zmp_ph114_E2

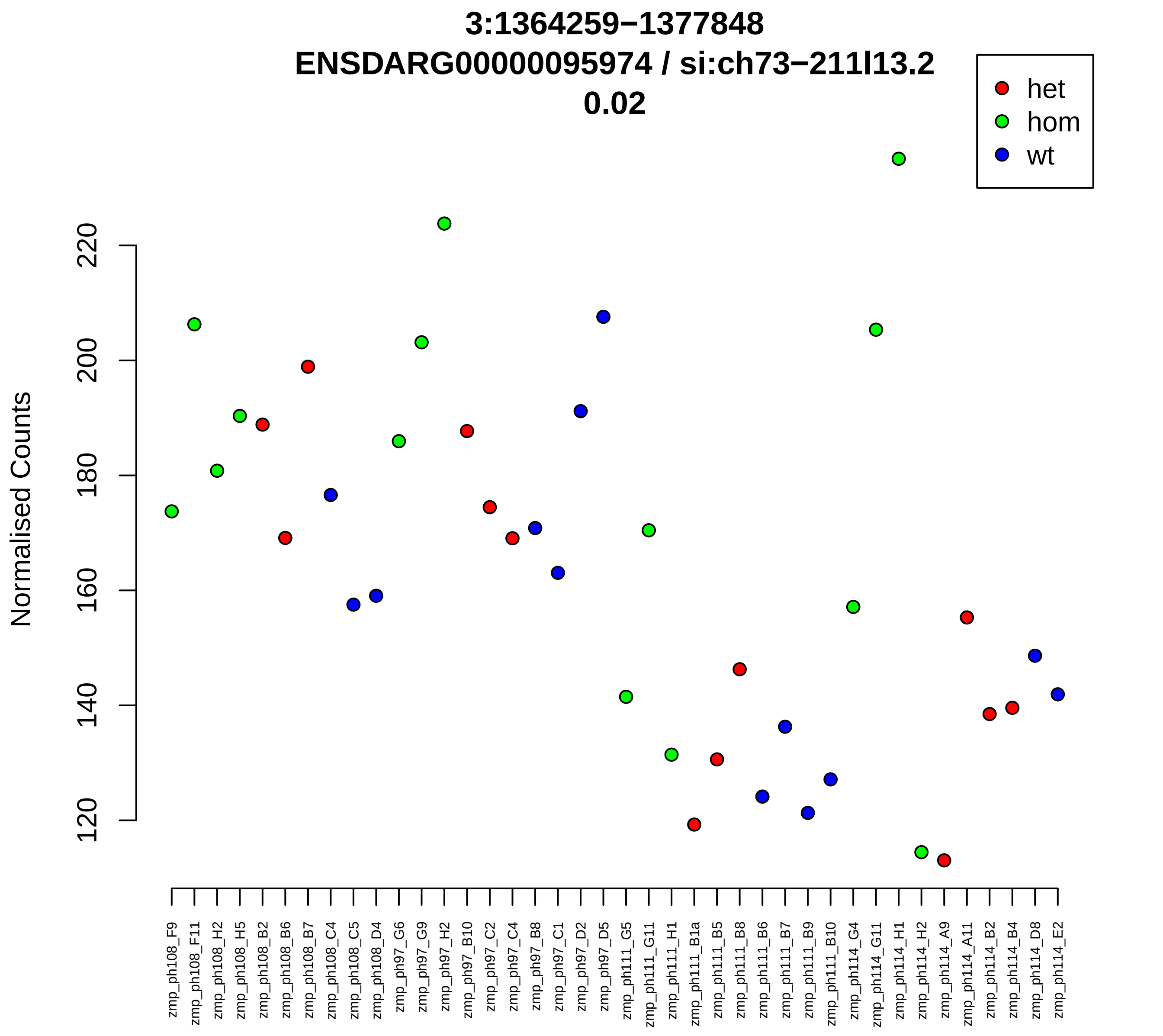

3:1364259−1377848
ENSDARG00000095974 / si:ch73−211l13.2
0.02
het
hom
wt
Normalised Counts
120
140
160
180
200
220
zmp_ph108_F9
zmp_ph108_F11
zmp_ph108_H2
zmp_ph108_H5
zmp_ph108_B2
zmp_ph108_B6
zmp_ph108_B7
zmp_ph108_C4
zmp_ph108_C5
zmp_ph108_D4
zmp_ph97_G6
zmp_ph97_G9
zmp_ph97_H2
zmp_ph97_B10
zmp_ph97_C2
zmp_ph97_C4
zmp_ph97_B8
zmp_ph97_C1
zmp_ph97_D2
zmp_ph97_D5
zmp_ph111_G5
zmp_ph111_G11
zmp_ph111_H1
zmp_ph111_B1a
zmp_ph111_B5
zmp_ph111_B8
zmp_ph111_B6
zmp_ph111_B7
zmp_ph111_B9
zmp_ph111_B10
zmp_ph114_G4
zmp_ph114_G11
zmp_ph114_H1
zmp_ph114_H2
zmp_ph114_A9
zmp_ph114_A11
zmp_ph114_B2
zmp_ph114_B4
zmp_ph114_D8
zmp_ph114_E2

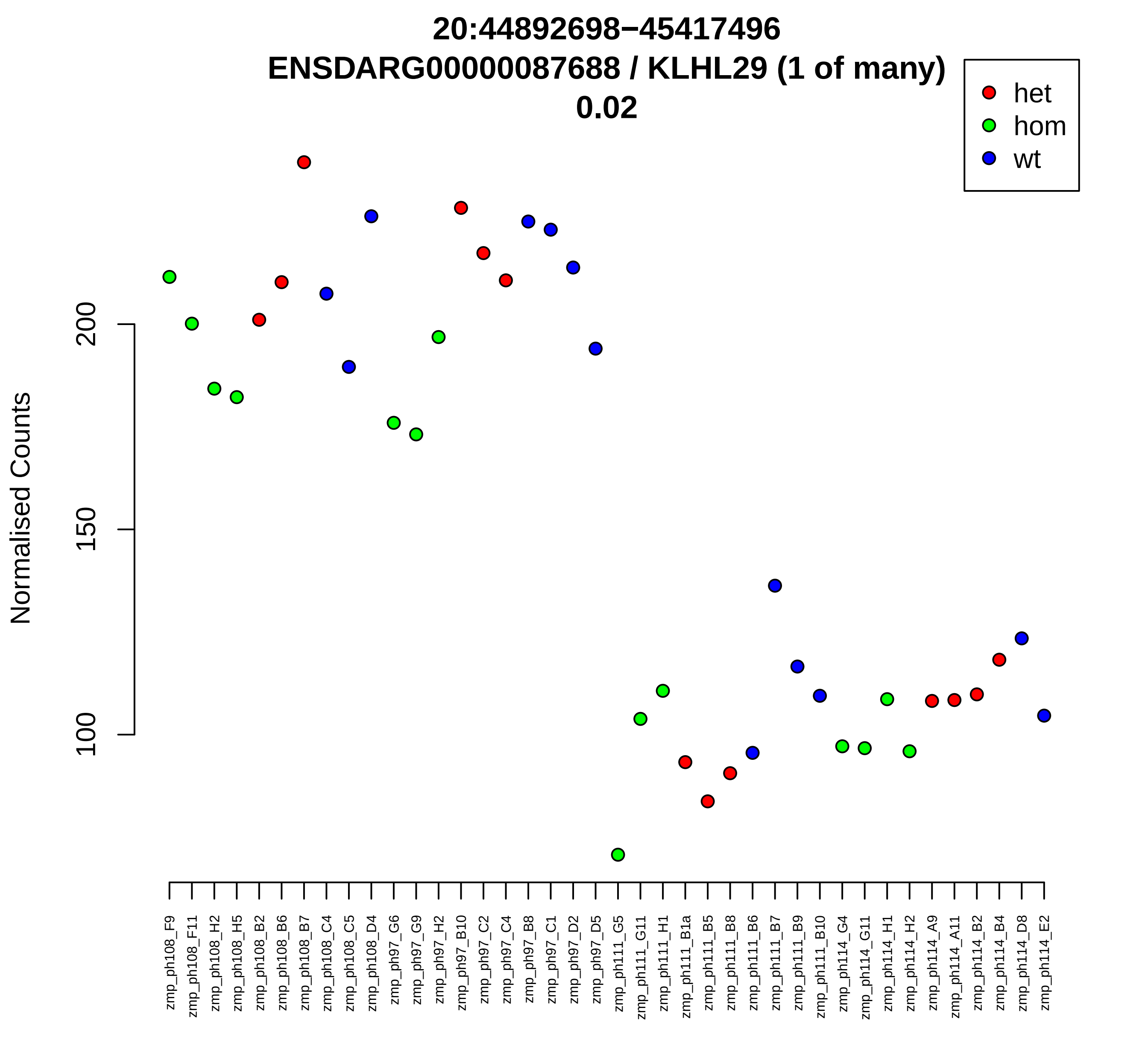
20:44892698−45417496
ENSDARG00000087688 / KLHL29 (1 of many)
0.02
het
hom
wt
Normalised Counts
100
150
200
zmp_ph108_F9
zmp_ph108_F11
zmp_ph108_H2
zmp_ph108_H5
zmp_ph108_B2
zmp_ph108_B6
zmp_ph108_B7
zmp_ph108_C4
zmp_ph108_C5
zmp_ph108_D4
zmp_ph97_G6
zmp_ph97_G9
zmp_ph97_H2
zmp_ph97_B10
zmp_ph97_C2
zmp_ph97_C4
zmp_ph97_B8
zmp_ph97_C1
zmp_ph97_D2
zmp_ph97_D5
zmp_ph111_G5
zmp_ph111_G11
zmp_ph111_H1
zmp_ph111_B1a
zmp_ph111_B5
zmp_ph111_B8
zmp_ph111_B6
zmp_ph111_B7
zmp_ph111_B9
zmp_ph111_B10
zmp_ph114_G4
zmp_ph114_G11
zmp_ph114_H1
zmp_ph114_H2
zmp_ph114_A9
zmp_ph114_A11
zmp_ph114_B2
zmp_ph114_B4
zmp_ph114_D8
zmp_ph114_E2

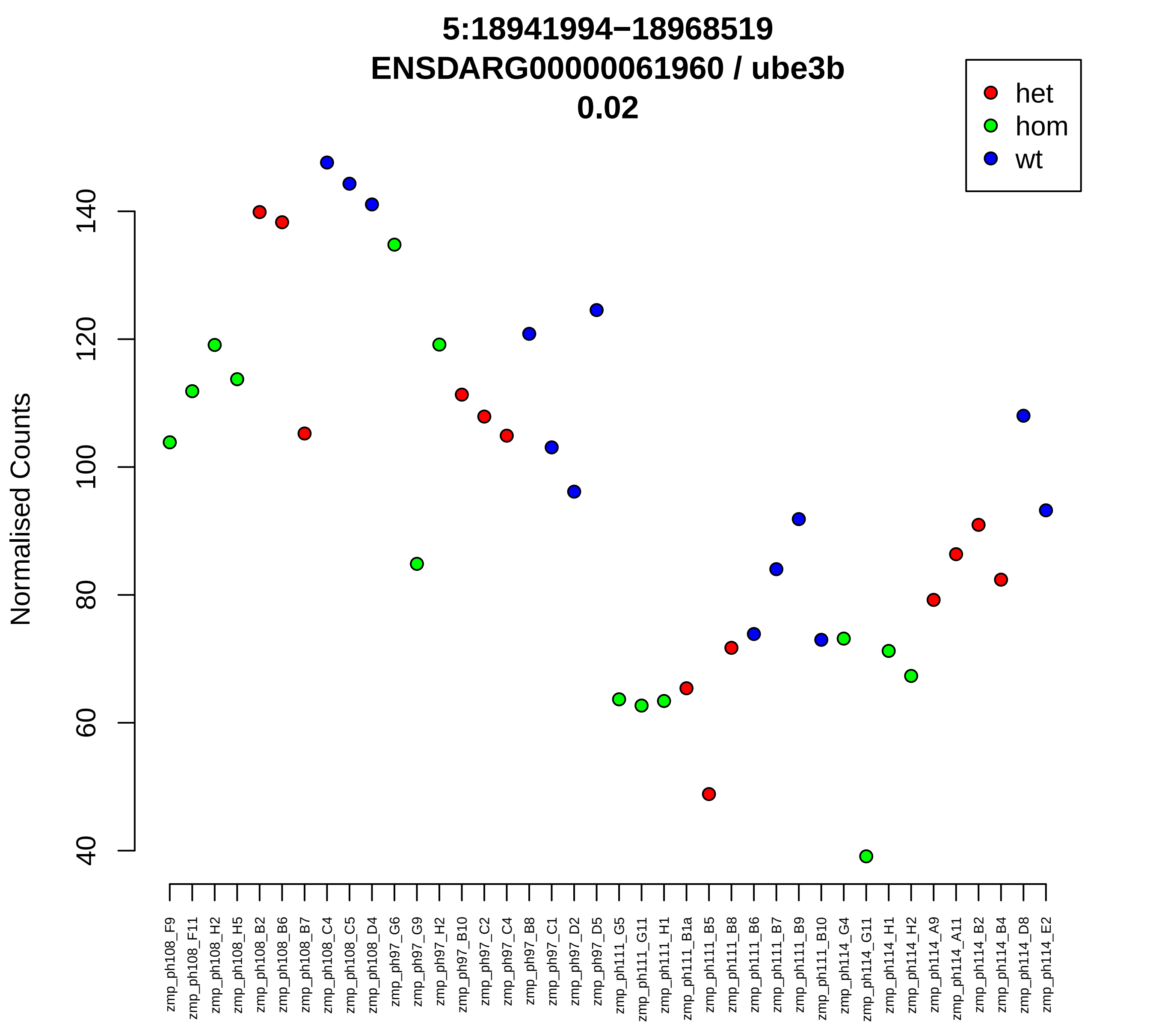

5:18941994−18968519
ENSDARG00000061960 / ube3b
0.02
het
hom
wt
Normalised Counts
40
60
80
100
120
140
zmp_ph108_F9
zmp_ph108_F11
zmp_ph108_H2
zmp_ph108_H5
zmp_ph108_B2
zmp_ph108_B6
zmp_ph108_B7
zmp_ph108_C4
zmp_ph108_C5
zmp_ph108_D4
zmp_ph97_G6
zmp_ph97_G9
zmp_ph97_H2
zmp_ph97_B10
zmp_ph97_C2
zmp_ph97_C4
zmp_ph97_B8
zmp_ph97_C1
zmp_ph97_D2
zmp_ph97_D5
zmp_ph111_G5
zmp_ph111_G11
zmp_ph111_H1
zmp_ph111_B1a
zmp_ph111_B5
zmp_ph111_B8
zmp_ph111_B6
zmp_ph111_B7
zmp_ph111_B9
zmp_ph111_B10
zmp_ph114_G4
zmp_ph114_G11
zmp_ph114_H1
zmp_ph114_H2
zmp_ph114_A9
zmp_ph114_A11
zmp_ph114_B2
zmp_ph114_B4
zmp_ph114_D8
zmp_ph114_E2

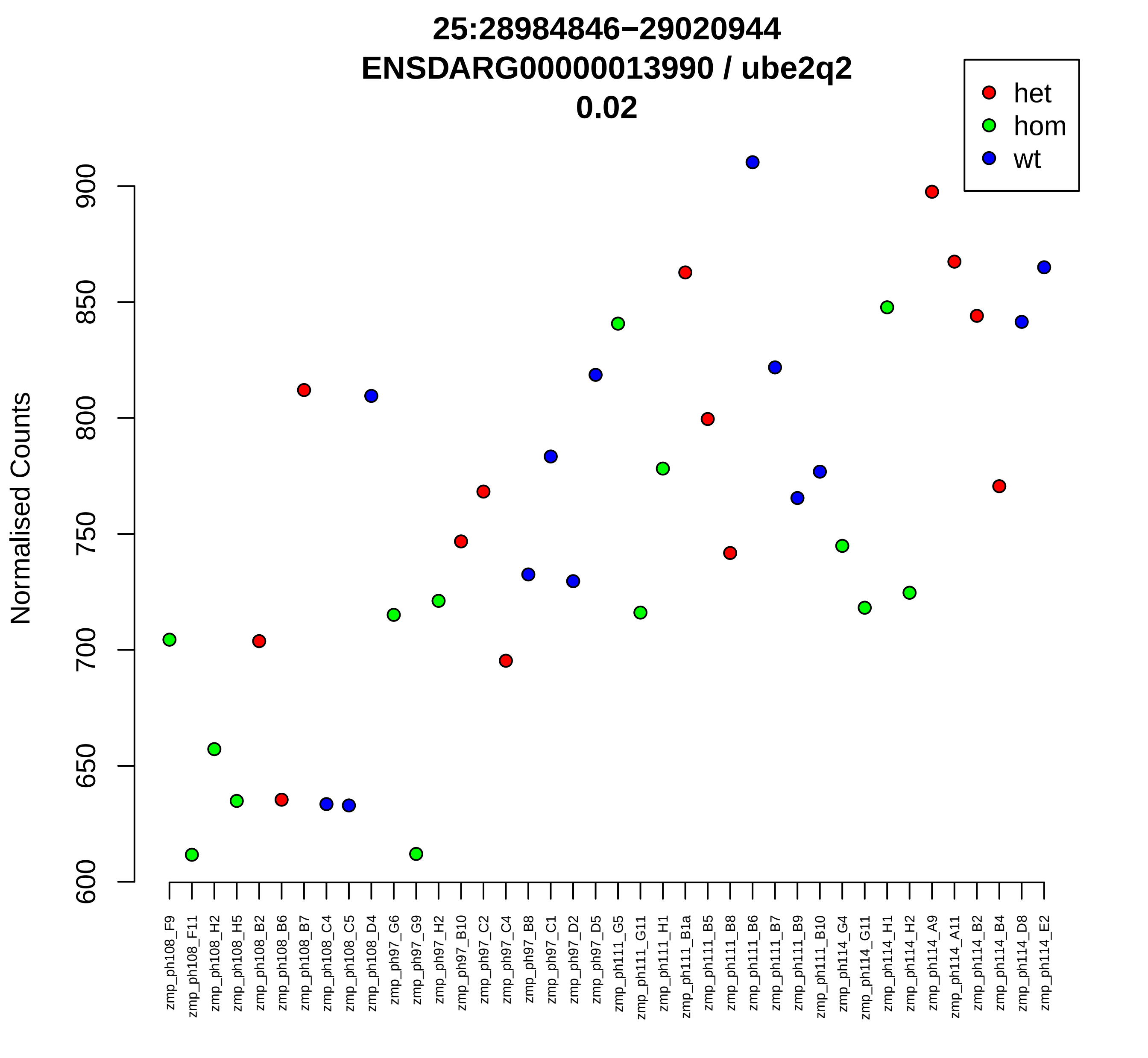

25:28984846−29020944
ENSDARG00000013990 / ube2q2
0.02
het
hom
wt
Normalised Counts
900
850
800
750
700
650
600
zmp_ph108_F9
zmp_ph108_F11
zmp_ph108_H2
zmp_ph108_H5
zmp_ph108_B2
zmp_ph108_B6
zmp_ph108_B7
zmp_ph108_C4
zmp_ph108_C5
zmp_ph108_D4
zmp_ph97_G6
zmp_ph97_G9
zmp_ph97_H2
zmp_ph97_B10
zmp_ph97_C2
zmp_ph97_C4
zmp_ph97_B8
zmp_ph97_C1
zmp_ph97_D2
zmp_ph97_D5
zmp_ph111_G5
zmp_ph111_G11
zmp_ph111_H1
zmp_ph111_B1a
zmp_ph111_B5
zmp_ph111_B8
zmp_ph111_B6
zmp_ph111_B7
zmp_ph111_B9
zmp_ph111_B10
zmp_ph114_G4
zmp_ph114_G11
zmp_ph114_H1
zmp_ph114_H2
zmp_ph114_A9
zmp_ph114_A11
zmp_ph114_B2
zmp_ph114_B4
zmp_ph114_D8
zmp_ph114_E2

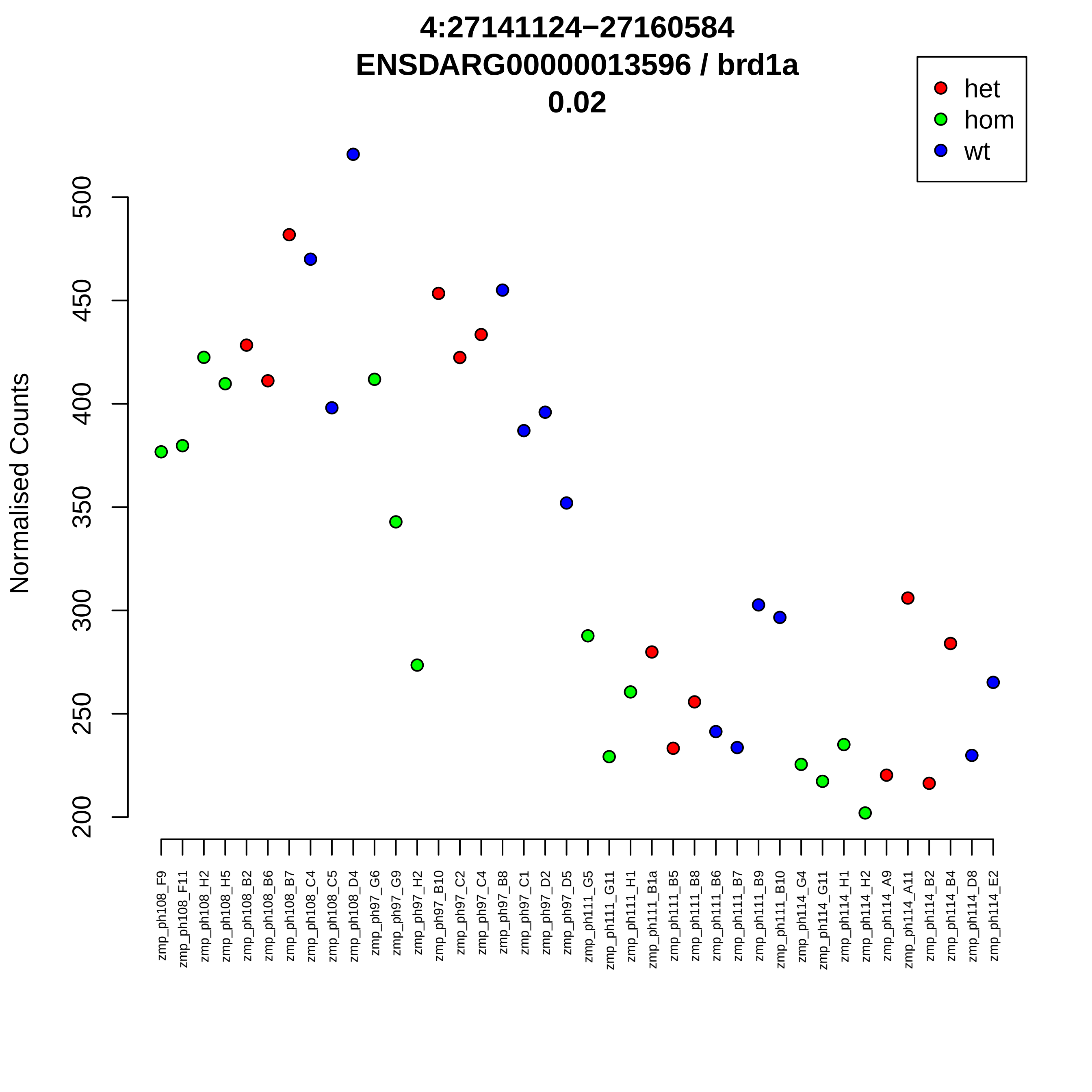

4:27141124−27160584
ENSDARG00000013596 / brd1a
0.02
het
hom
wt
Normalised Counts
200
250
300
350
400
450
500
zmp_ph108_F9
zmp_ph108_F11
zmp_ph108_H2
zmp_ph108_H5
zmp_ph108_B2
zmp_ph108_B6
zmp_ph108_B7
zmp_ph108_C4
zmp_ph108_C5
zmp_ph108_D4
zmp_ph97_G6
zmp_ph97_G9
zmp_ph97_H2
zmp_ph97_B10
zmp_ph97_C2
zmp_ph97_C4
zmp_ph97_B8
zmp_ph97_C1
zmp_ph97_D2
zmp_ph97_D5
zmp_ph111_G5
zmp_ph111_G11
zmp_ph111_H1
zmp_ph111_B1a
zmp_ph111_B5
zmp_ph111_B8
zmp_ph111_B6
zmp_ph111_B7
zmp_ph111_B9
zmp_ph111_B10
zmp_ph114_G4
zmp_ph114_G11
zmp_ph114_H1
zmp_ph114_H2
zmp_ph114_A9
zmp_ph114_A11
zmp_ph114_B2
zmp_ph114_B4
zmp_ph114_D8
zmp_ph114_E2

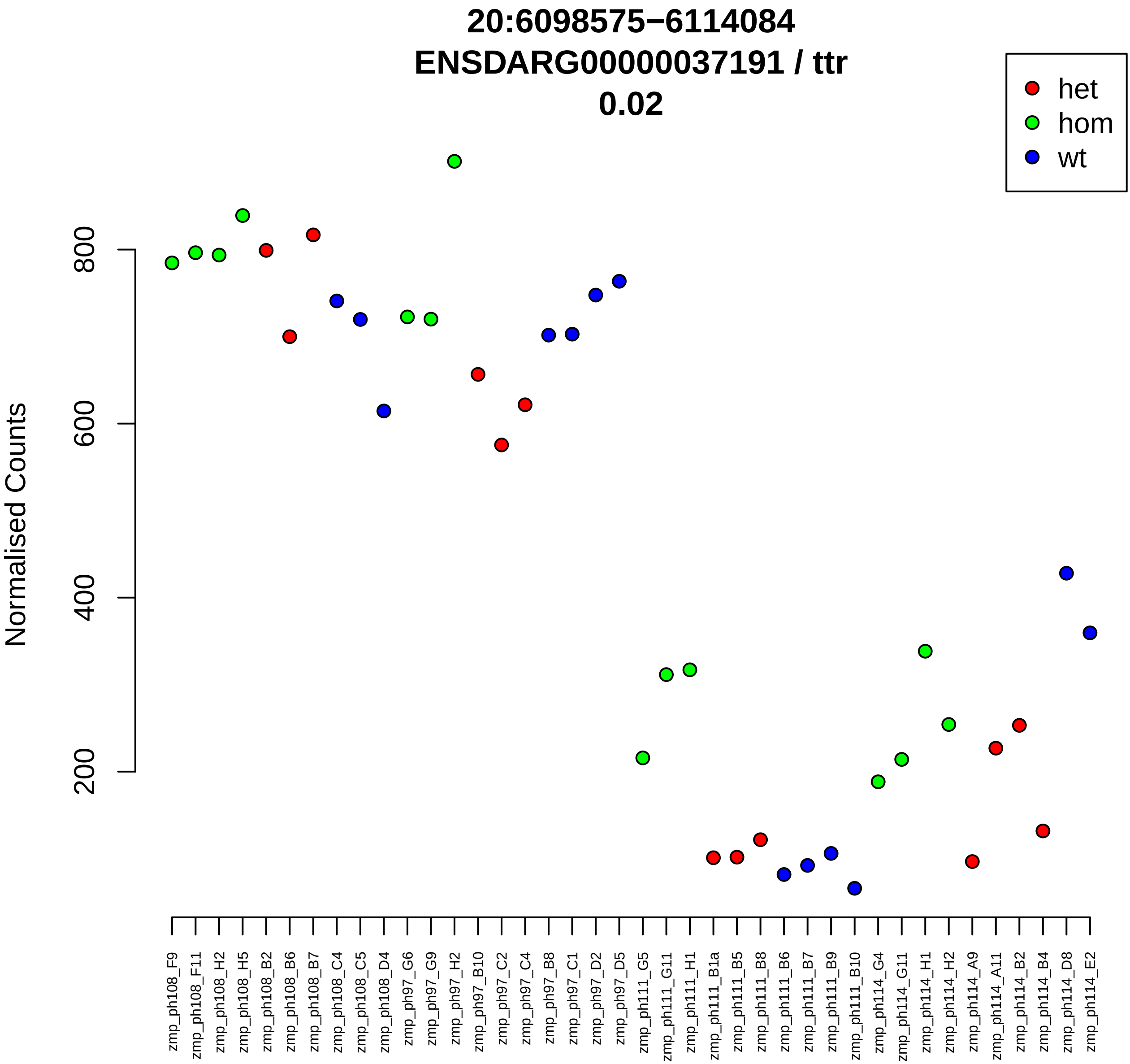

20:6098575−6114084
ENSDARG00000037191 / ttr
0.02
het
hom
wt
Normalised Counts
200
400
600
800
zmp_ph108_F9
zmp_ph108_F11
zmp_ph108_H2
zmp_ph108_H5
zmp_ph108_B2
zmp_ph108_B6
zmp_ph108_B7
zmp_ph108_C4
zmp_ph108_C5
zmp_ph108_D4
zmp_ph97_G6
zmp_ph97_G9
zmp_ph97_H2
zmp_ph97_B10
zmp_ph97_C2
zmp_ph97_C4
zmp_ph97_B8
zmp_ph97_C1
zmp_ph97_D2
zmp_ph97_D5
zmp_ph111_G5
zmp_ph111_G11
zmp_ph111_H1
zmp_ph111_B1a
zmp_ph111_B5
zmp_ph111_B8
zmp_ph111_B6
zmp_ph111_B7
zmp_ph111_B9
zmp_ph111_B10
zmp_ph114_G4
zmp_ph114_G11
zmp_ph114_H1
zmp_ph114_H2
zmp_ph114_A9
zmp_ph114_A11
zmp_ph114_B2
zmp_ph114_B4
zmp_ph114_D8
zmp_ph114_E2

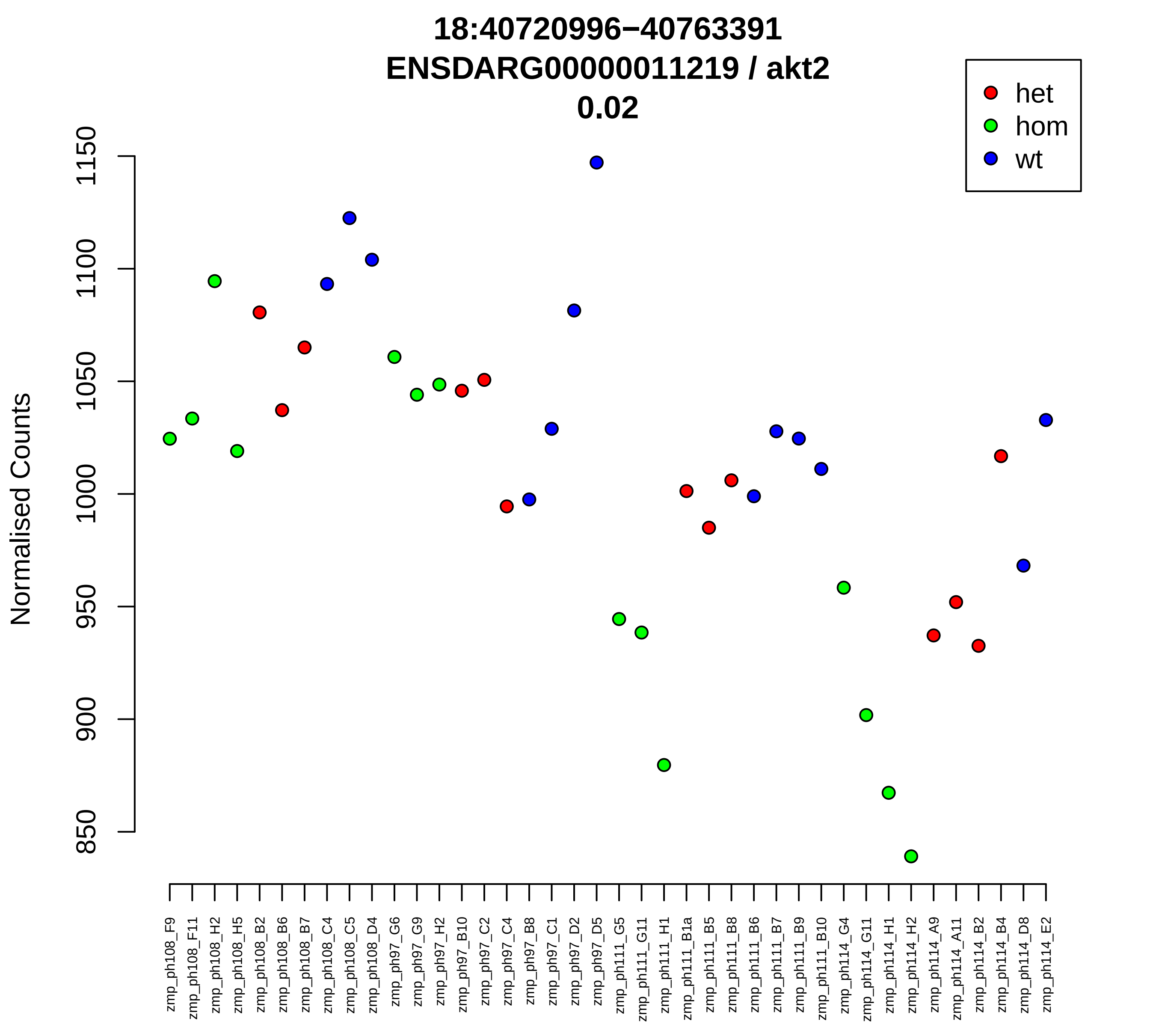

18:40720996−40763391
ENSDARG00000011219 / akt2
0.02
het
hom
wt
Normalised Counts
850
900
950
1000
1050
1100
1150
zmp_ph108_F9
zmp_ph108_F11
zmp_ph108_H2
zmp_ph108_H5
zmp_ph108_B2
zmp_ph108_B6
zmp_ph108_B7
zmp_ph108_C4
zmp_ph108_C5
zmp_ph108_D4
zmp_ph97_G6
zmp_ph97_G9
zmp_ph97_H2
zmp_ph97_B10
zmp_ph97_C2
zmp_ph97_C4
zmp_ph97_B8
zmp_ph97_C1
zmp_ph97_D2
zmp_ph97_D5
zmp_ph111_G5
zmp_ph111_G11
zmp_ph111_H1
zmp_ph111_B1a
zmp_ph111_B5
zmp_ph111_B8
zmp_ph111_B6
zmp_ph111_B7
zmp_ph111_B9
zmp_ph111_B10
zmp_ph114_G4
zmp_ph114_G11
zmp_ph114_H1
zmp_ph114_H2
zmp_ph114_A9
zmp_ph114_A11
zmp_ph114_B2
zmp_ph114_B4
zmp_ph114_D8
zmp_ph114_E2

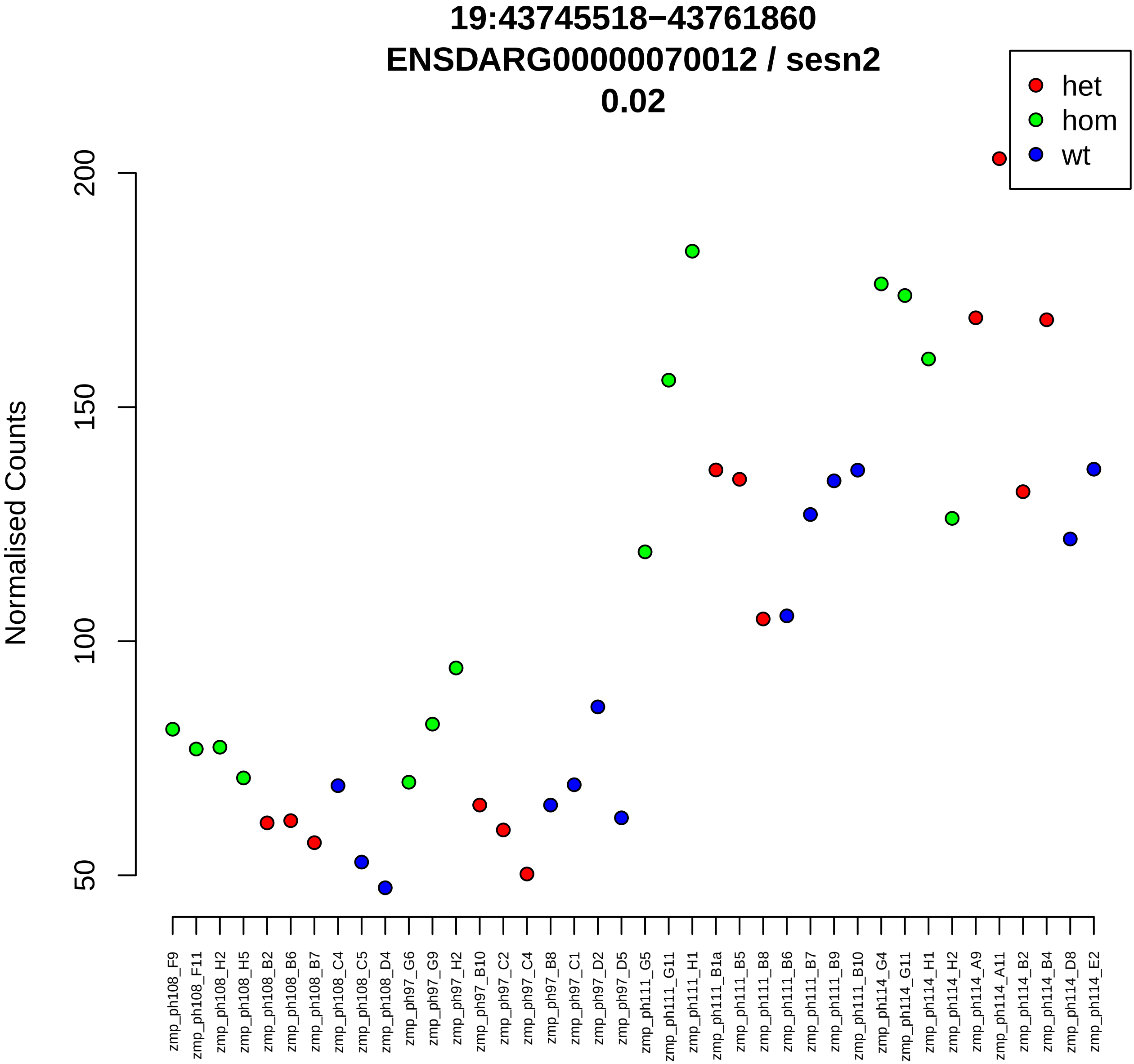

19:43745518−43761860
ENSDARG00000070012 / sesn2
0.02
het
hom
wt
Normalised Counts
50
100
150
200
zmp_ph108_F9
zmp_ph108_F11
zmp_ph108_H2
zmp_ph108_H5
zmp_ph108_B2
zmp_ph108_B6
zmp_ph108_B7
zmp_ph108_C4
zmp_ph108_C5
zmp_ph108_D4
zmp_ph97_G6
zmp_ph97_G9
zmp_ph97_H2
zmp_ph97_B10
zmp_ph97_C2
zmp_ph97_C4
zmp_ph97_B8
zmp_ph97_C1
zmp_ph97_D2
zmp_ph97_D5
zmp_ph111_G5
zmp_ph111_G11
zmp_ph111_H1
zmp_ph111_B1a
zmp_ph111_B5
zmp_ph111_B8
zmp_ph111_B6
zmp_ph111_B7
zmp_ph111_B9
zmp_ph111_B10
zmp_ph114_G4
zmp_ph114_G11
zmp_ph114_H1
zmp_ph114_H2
zmp_ph114_A9
zmp_ph114_A11
zmp_ph114_B2
zmp_ph114_B4
zmp_ph114_D8
zmp_ph114_E2

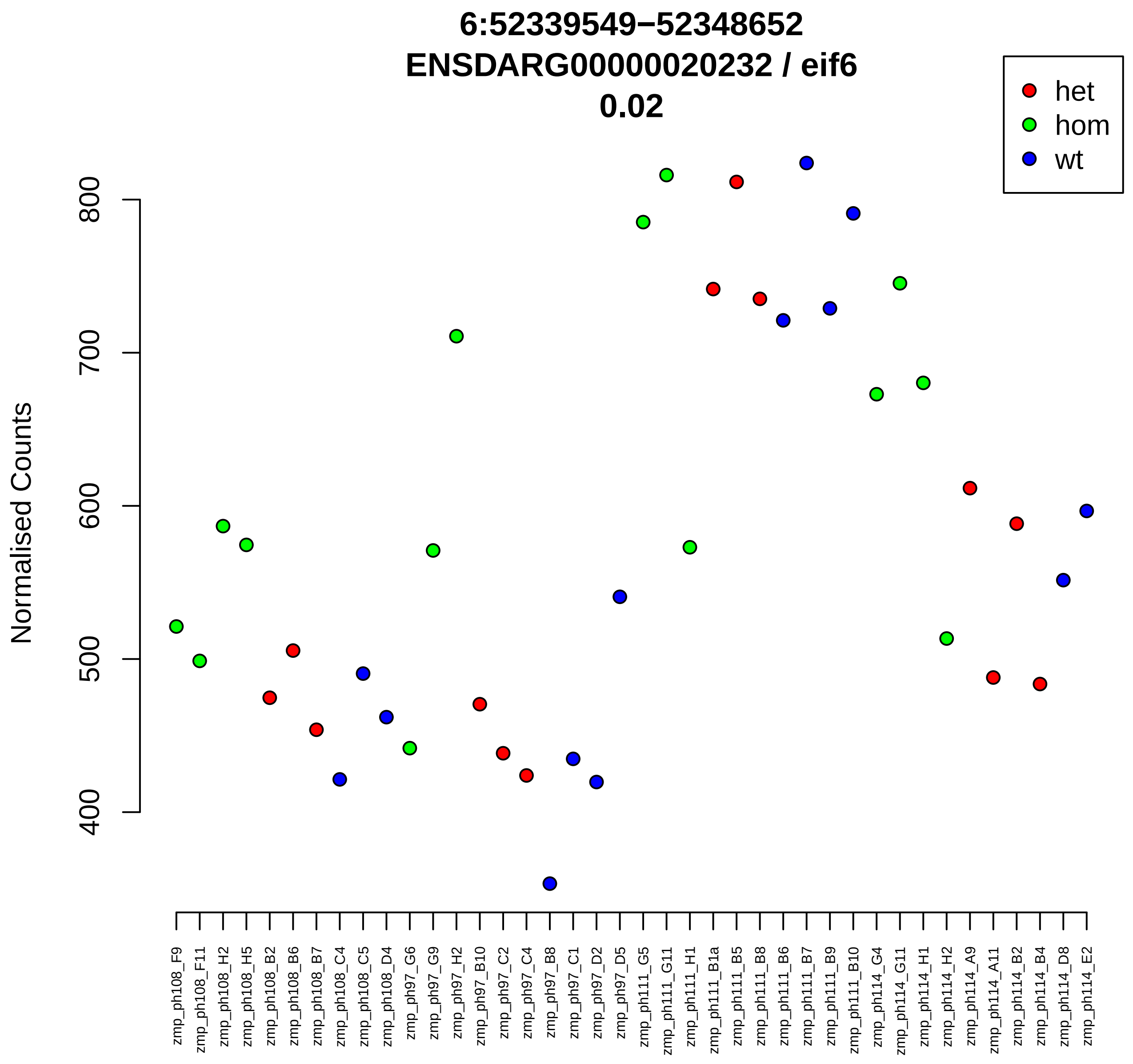
6:52339549−52348652
ENSDARG00000020232 / eif6
0.02
het
hom
wt
Normalised Counts
400
500
600
700
800
zmp_ph108_F9
zmp_ph108_F11
zmp_ph108_H2
zmp_ph108_H5
zmp_ph108_B2
zmp_ph108_B6
zmp_ph108_B7
zmp_ph108_C4
zmp_ph108_C5
zmp_ph108_D4
zmp_ph97_G6
zmp_ph97_G9
zmp_ph97_H2
zmp_ph97_B10
zmp_ph97_C2
zmp_ph97_C4
zmp_ph97_B8
zmp_ph97_C1
zmp_ph97_D2
zmp_ph97_D5
zmp_ph111_G5
zmp_ph111_G11
zmp_ph111_H1
zmp_ph111_B1a
zmp_ph111_B5
zmp_ph111_B8
zmp_ph111_B6
zmp_ph111_B7
zmp_ph111_B9
zmp_ph111_B10
zmp_ph114_G4
zmp_ph114_G11
zmp_ph114_H1
zmp_ph114_H2
zmp_ph114_A9
zmp_ph114_A11
zmp_ph114_B2
zmp_ph114_B4
zmp_ph114_D8
zmp_ph114_E2

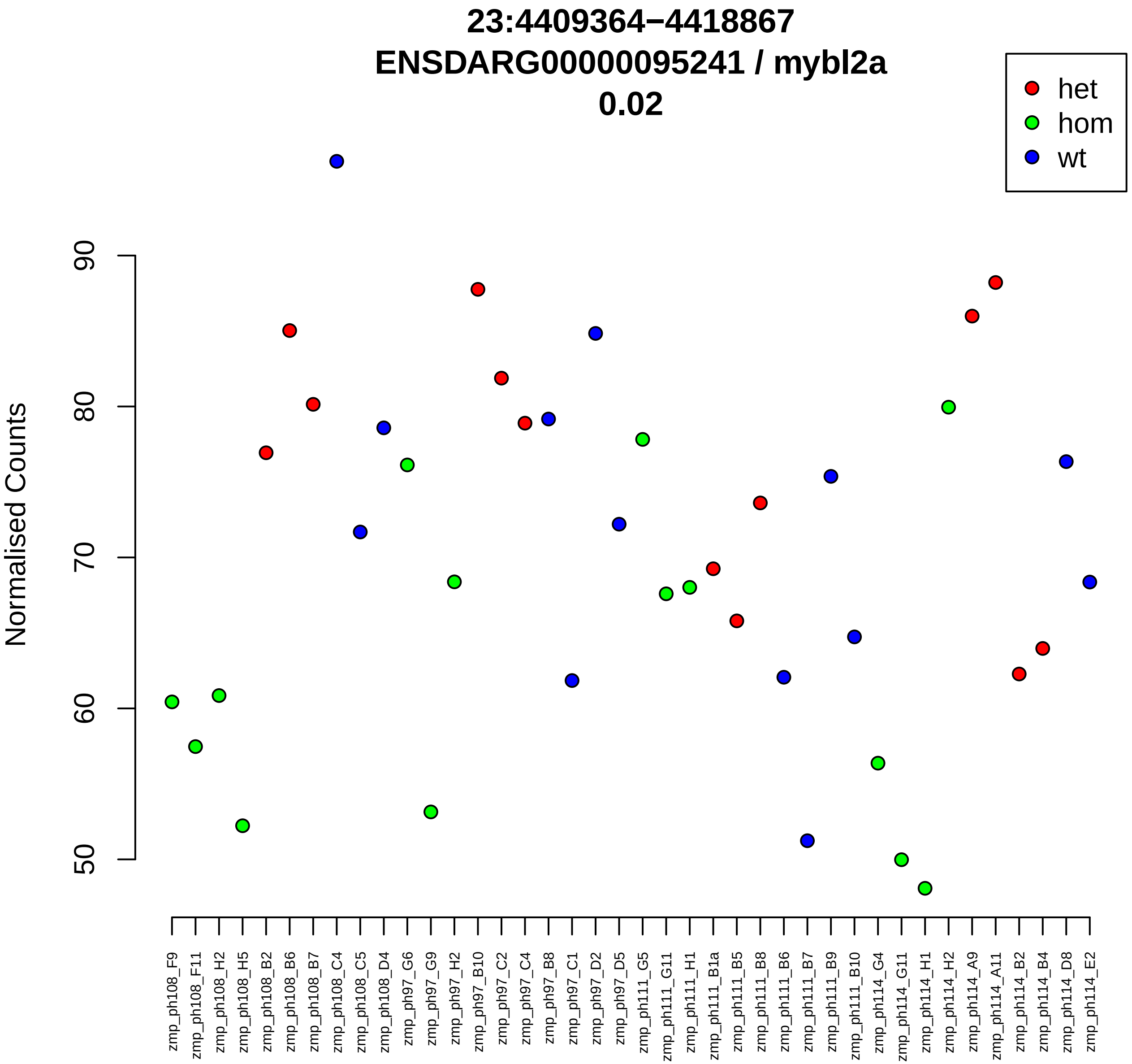

23:4409364−4418867
ENSDARG00000095241 / mybl2a
0.02
het
hom
wt
Normalised Counts
50
60
70
80
90
zmp_ph108_F9
zmp_ph108_F11
zmp_ph108_H2
zmp_ph108_H5
zmp_ph108_B2
zmp_ph108_B6
zmp_ph108_B7
zmp_ph108_C4
zmp_ph108_C5
zmp_ph108_D4
zmp_ph97_G6
zmp_ph97_G9
zmp_ph97_H2
zmp_ph97_B10
zmp_ph97_C2
zmp_ph97_C4
zmp_ph97_B8
zmp_ph97_C1
zmp_ph97_D2
zmp_ph97_D5
zmp_ph111_G5
zmp_ph111_G11
zmp_ph111_H1
zmp_ph111_B1a
zmp_ph111_B5
zmp_ph111_B8
zmp_ph111_B6
zmp_ph111_B7
zmp_ph111_B9
zmp_ph111_B10
zmp_ph114_G4
zmp_ph114_G11
zmp_ph114_H1
zmp_ph114_H2
zmp_ph114_A9
zmp_ph114_A11
zmp_ph114_B2
zmp_ph114_B4
zmp_ph114_D8
zmp_ph114_E2

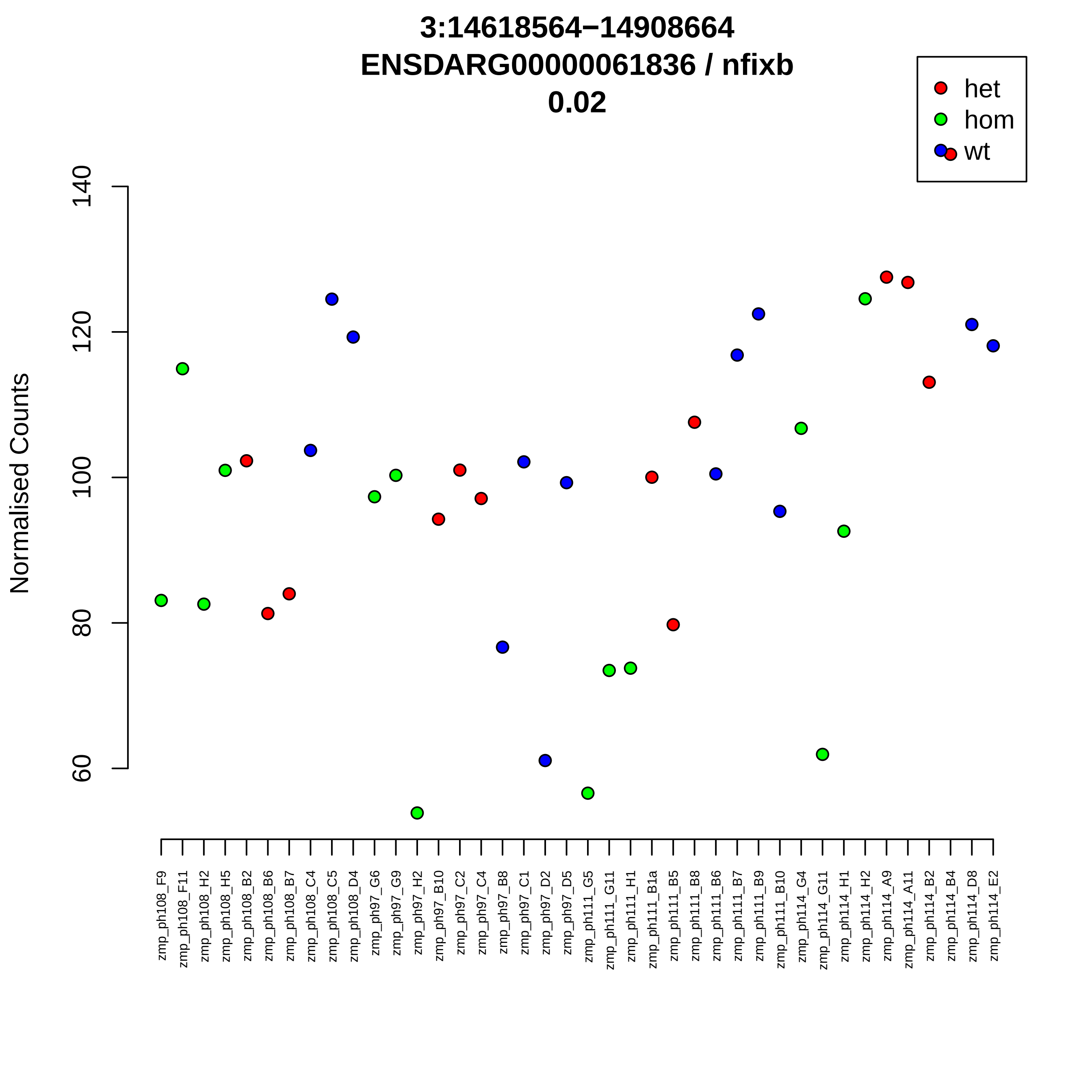

3:14618564−14908664
ENSDARG00000061836 / nfixb
0.02
het
hom
wt
Normalised Counts
140
120
100
80
60
zmp_ph108_F9
zmp_ph108_F11
zmp_ph108_H2
zmp_ph108_H5
zmp_ph108_B2
zmp_ph108_B6
zmp_ph108_B7
zmp_ph108_C4
zmp_ph108_C5
zmp_ph108_D4
zmp_ph97_G6
zmp_ph97_G9
zmp_ph97_H2
zmp_ph97_B10
zmp_ph97_C2
zmp_ph97_C4
zmp_ph97_B8
zmp_ph97_C1
zmp_ph97_D2
zmp_ph97_D5
zmp_ph111_G5
zmp_ph111_G11
zmp_ph111_H1
zmp_ph111_B1a
zmp_ph111_B5
zmp_ph111_B8
zmp_ph111_B6
zmp_ph111_B7
zmp_ph111_B9
zmp_ph111_B10
zmp_ph114_G4
zmp_ph114_G11
zmp_ph114_H1
zmp_ph114_H2
zmp_ph114_A9
zmp_ph114_A11
zmp_ph114_B2
zmp_ph114_B4
zmp_ph114_D8
zmp_ph114_E2

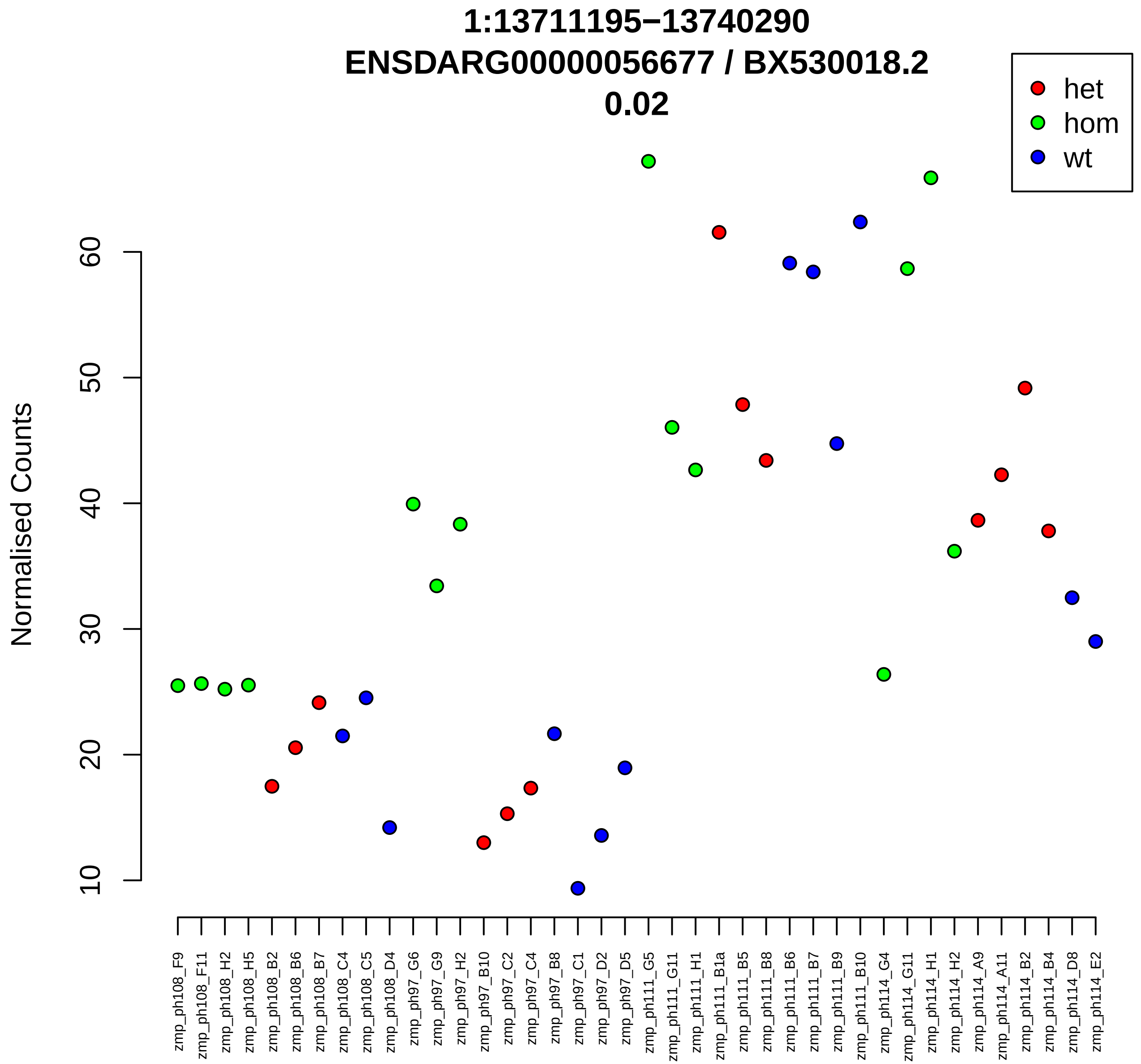
1:13711195−13740290
ENSDARG00000056677 / BX530018.2
0.02
het
hom
wt
Normalised Counts
10
20
30
40
50
60
zmp_ph108_F9
zmp_ph108_F11
zmp_ph108_H2
zmp_ph108_H5
zmp_ph108_B2
zmp_ph108_B6
zmp_ph108_B7
zmp_ph108_C4
zmp_ph108_C5
zmp_ph108_D4
zmp_ph97_G6
zmp_ph97_G9
zmp_ph97_H2
zmp_ph97_B10
zmp_ph97_C2
zmp_ph97_C4
zmp_ph97_B8
zmp_ph97_C1
zmp_ph97_D2
zmp_ph97_D5
zmp_ph111_G5
zmp_ph111_G11
zmp_ph111_H1
zmp_ph111_B1a
zmp_ph111_B5
zmp_ph111_B8
zmp_ph111_B6
zmp_ph111_B7
zmp_ph111_B9
zmp_ph111_B10
zmp_ph114_G4
zmp_ph114_G11
zmp_ph114_H1
zmp_ph114_H2
zmp_ph114_A9
zmp_ph114_A11
zmp_ph114_B2
zmp_ph114_B4
zmp_ph114_D8
zmp_ph114_E2

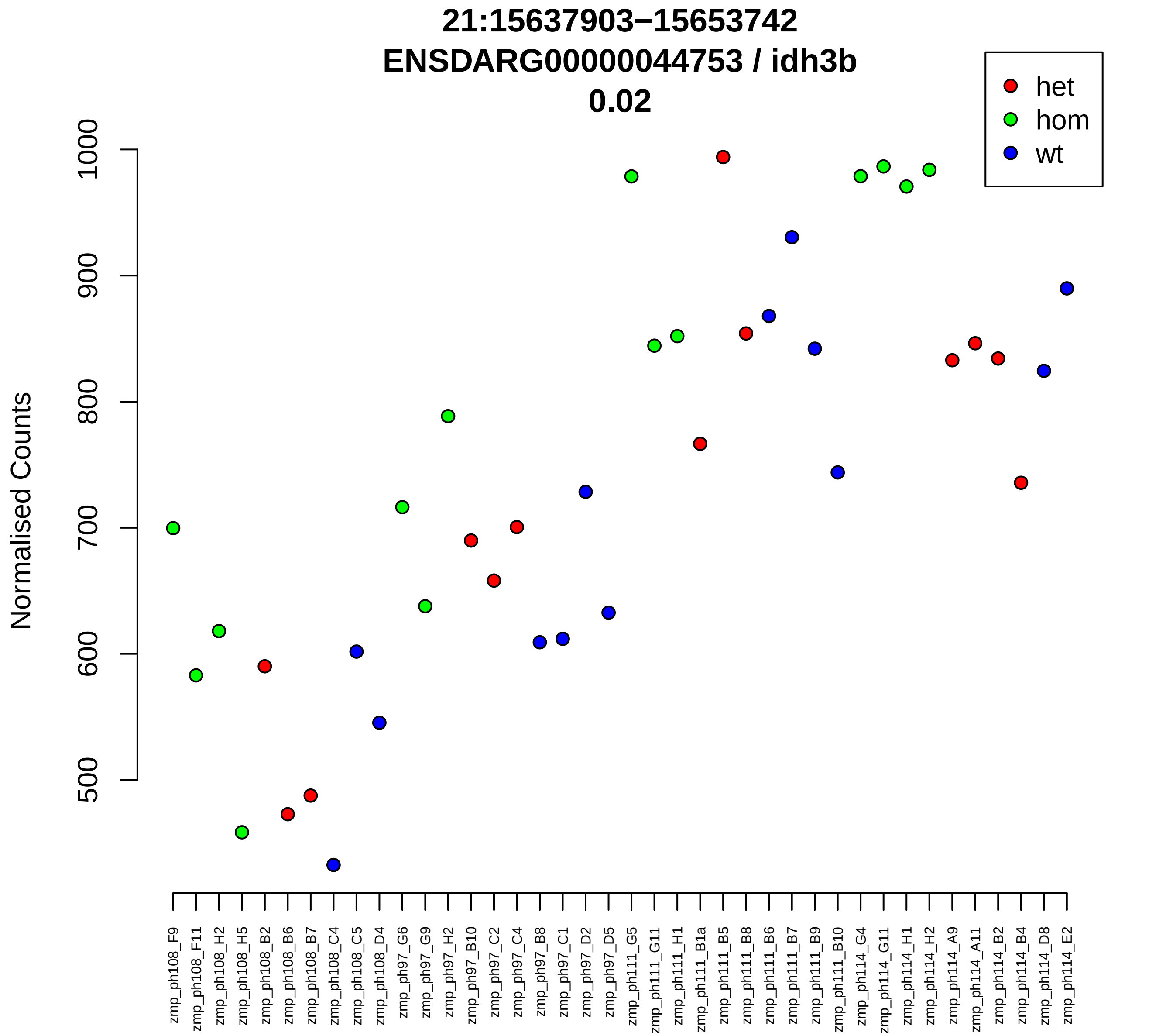

21:15637903−15653742
ENSDARG00000044753 / idh3b
0.02
het
hom
wt
Normalised Counts
500
600
700
800
900
1000
zmp_ph108_F9
zmp_ph108_F11
zmp_ph108_H2
zmp_ph108_H5
zmp_ph108_B2
zmp_ph108_B6
zmp_ph108_B7
zmp_ph108_C4
zmp_ph108_C5
zmp_ph108_D4
zmp_ph97_G6
zmp_ph97_G9
zmp_ph97_H2
zmp_ph97_B10
zmp_ph97_C2
zmp_ph97_C4
zmp_ph97_B8
zmp_ph97_C1
zmp_ph97_D2
zmp_ph97_D5
zmp_ph111_G5
zmp_ph111_G11
zmp_ph111_H1
zmp_ph111_B1a
zmp_ph111_B5
zmp_ph111_B8
zmp_ph111_B6
zmp_ph111_B7
zmp_ph111_B9
zmp_ph111_B10
zmp_ph114_G4
zmp_ph114_G11
zmp_ph114_H1
zmp_ph114_H2
zmp_ph114_A9
zmp_ph114_A11
zmp_ph114_B2
zmp_ph114_B4
zmp_ph114_D8
zmp_ph114_E2

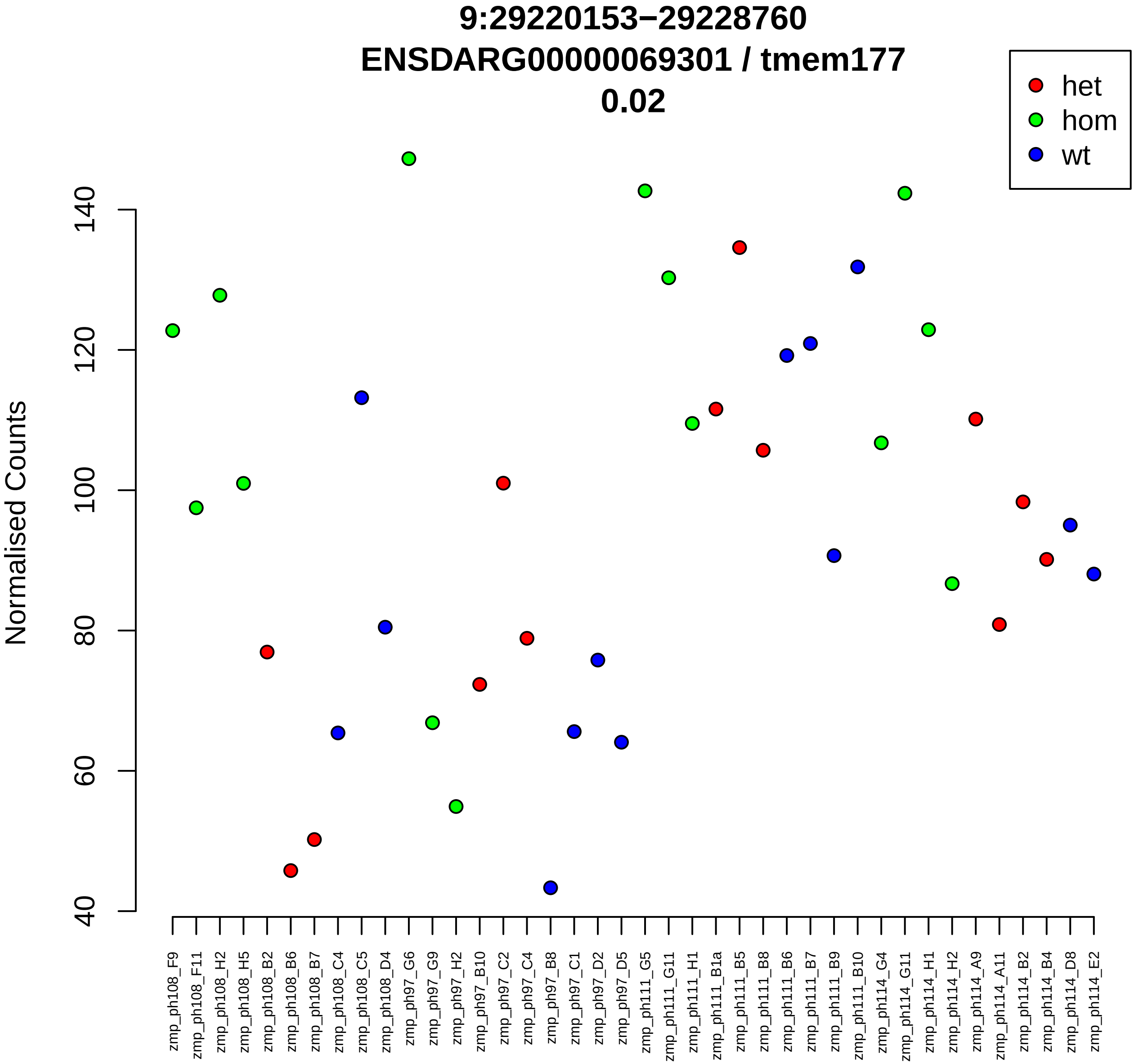

9:29220153−29228760
ENSDARG00000069301 / tmem177
0.02
het
hom
wt
Normalised Counts
40
60
80
100
120
140
zmp_ph108_F9
zmp_ph108_F11
zmp_ph108_H2
zmp_ph108_H5
zmp_ph108_B2
zmp_ph108_B6
zmp_ph108_B7
zmp_ph108_C4
zmp_ph108_C5
zmp_ph108_D4
zmp_ph97_G6
zmp_ph97_G9
zmp_ph97_H2
zmp_ph97_B10
zmp_ph97_C2
zmp_ph97_C4
zmp_ph97_B8
zmp_ph97_C1
zmp_ph97_D2
zmp_ph97_D5
zmp_ph111_G5
zmp_ph111_G11
zmp_ph111_H1
zmp_ph111_B1a
zmp_ph111_B5
zmp_ph111_B8
zmp_ph111_B6
zmp_ph111_B7
zmp_ph111_B9
zmp_ph111_B10
zmp_ph114_G4
zmp_ph114_G11
zmp_ph114_H1
zmp_ph114_H2
zmp_ph114_A9
zmp_ph114_A11
zmp_ph114_B2
zmp_ph114_B4
zmp_ph114_D8
zmp_ph114_E2

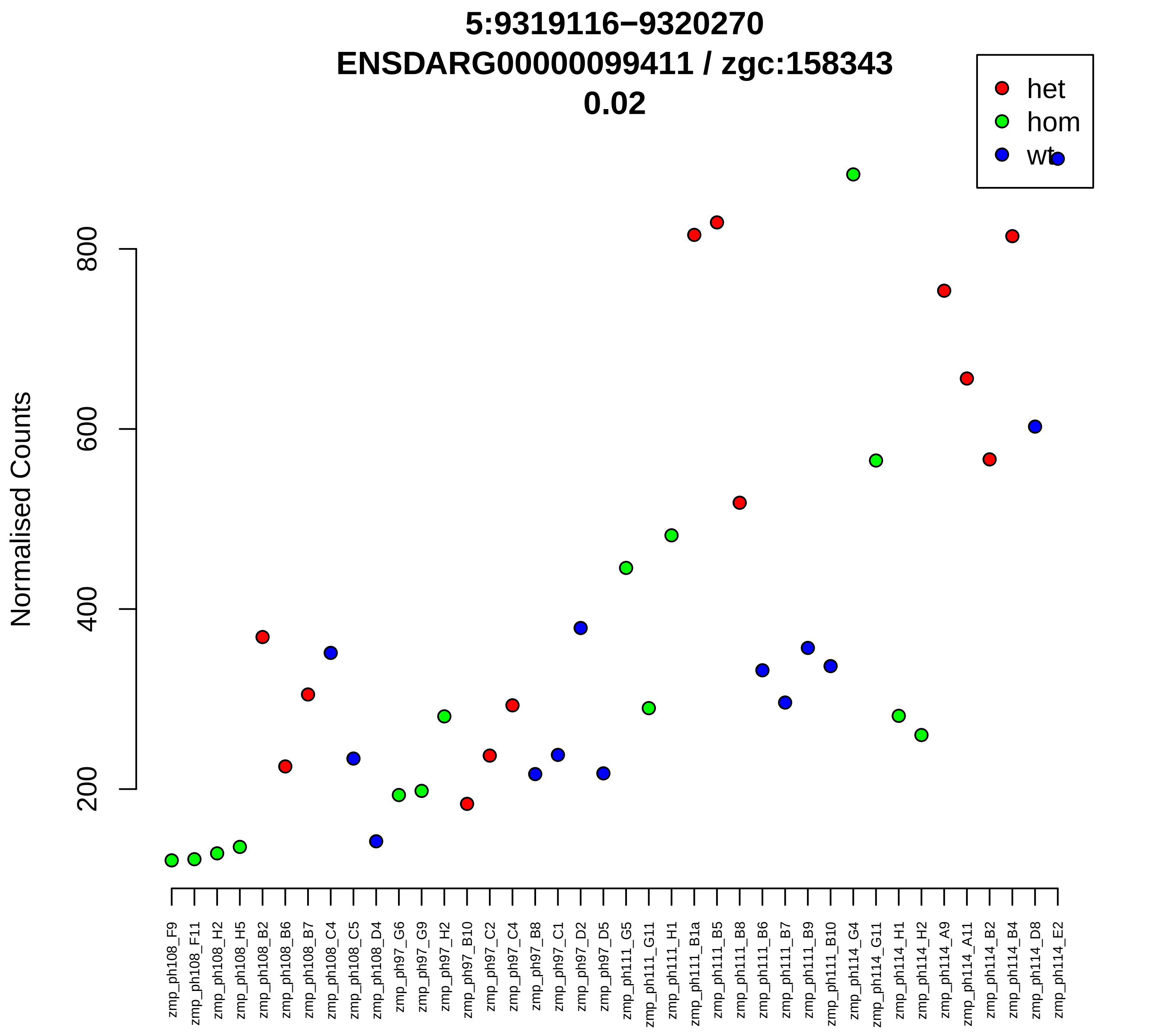

5:9319116−9320270
ENSDARG00000099411 / zgc:158343
0.02
het
hom
wt
Normalised Counts
200
400
600
800
zmp_ph108_F9
zmp_ph108_F11
zmp_ph108_H2
zmp_ph108_H5
zmp_ph108_B2
zmp_ph108_B6
zmp_ph108_B7
zmp_ph108_C4
zmp_ph108_C5
zmp_ph108_D4
zmp_ph97_G6
zmp_ph97_G9
zmp_ph97_H2
zmp_ph97_B10
zmp_ph97_C2
zmp_ph97_C4
zmp_ph97_B8
zmp_ph97_C1
zmp_ph97_D2
zmp_ph97_D5
zmp_ph111_G5
zmp_ph111_G11
zmp_ph111_H1
zmp_ph111_B1a
zmp_ph111_B5
zmp_ph111_B8
zmp_ph111_B6
zmp_ph111_B7
zmp_ph111_B9
zmp_ph111_B10
zmp_ph114_G4
zmp_ph114_G11
zmp_ph114_H1
zmp_ph114_H2
zmp_ph114_A9
zmp_ph114_A11
zmp_ph114_B2
zmp_ph114_B4
zmp_ph114_D8
zmp_ph114_E2

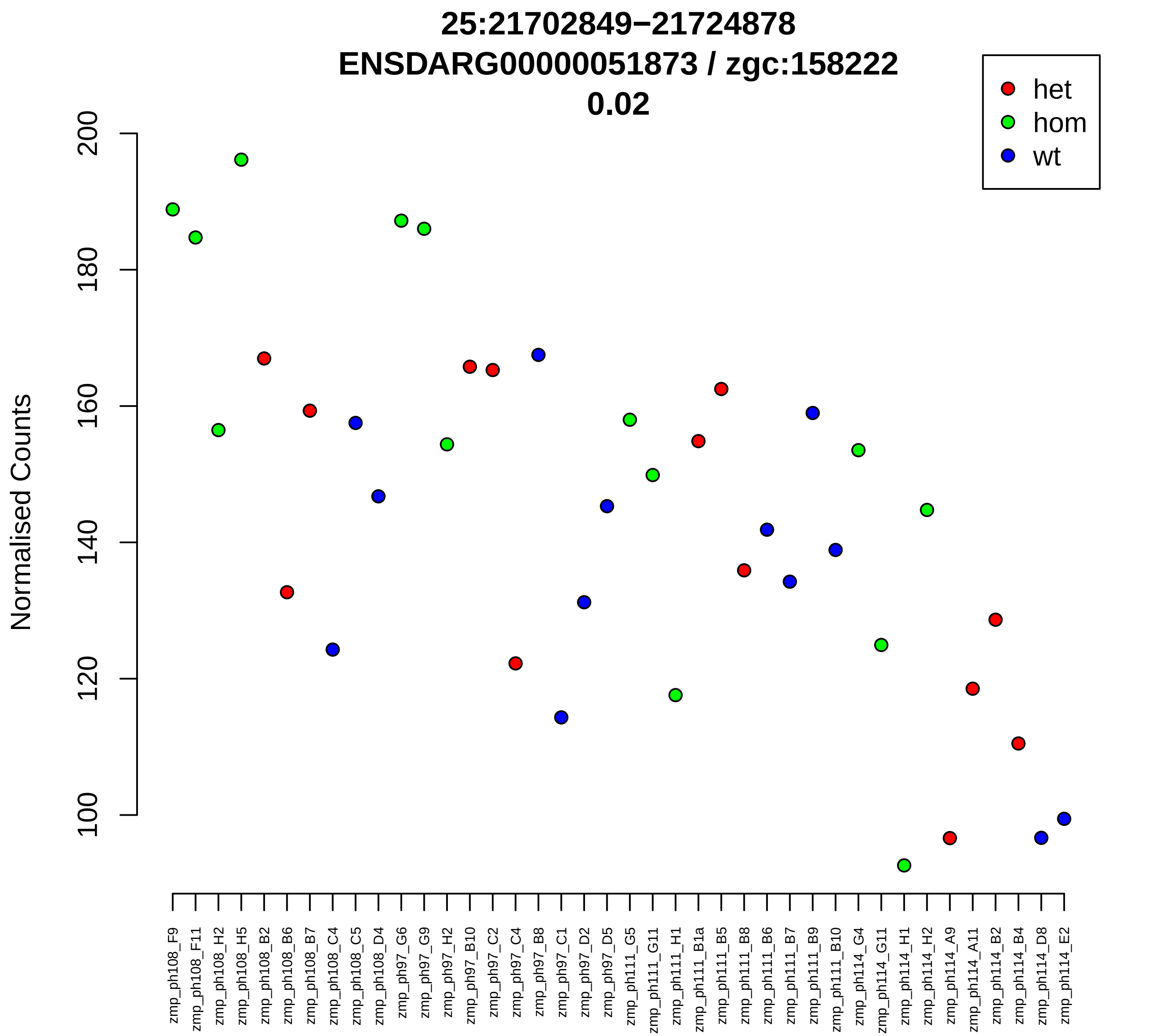

25:21702849−21724878
ENSDARG00000051873 / zgc:158222
0.02
Normalised Counts
200
180
160
140
120
100
het
hom
wt
zmp_ph108_F9
zmp_ph108_F11
zmp_ph108_H2
zmp_ph108_H5
zmp_ph108_B2
zmp_ph108_B6
zmp_ph108_B7
zmp_ph108_C4
zmp_ph108_C5
zmp_ph108_D4
zmp_ph97_G6
zmp_ph97_G9
zmp_ph97_H2
zmp_ph97_B10
zmp_ph97_C2
zmp_ph97_C4
zmp_ph97_B8
zmp_ph97_C1
zmp_ph97_D2
zmp_ph97_D5
zmp_ph111_G5
zmp_ph111_G11
zmp_ph111_H1
zmp_ph111_B1a
zmp_ph111_B5
zmp_ph111_B8
zmp_ph111_B6
zmp_ph111_B7
zmp_ph111_B9
zmp_ph111_B10
zmp_ph114_G4
zmp_ph114_G11
zmp_ph114_H1
zmp_ph114_H2
zmp_ph114_A9
zmp_ph114_A11
zmp_ph114_B2
zmp_ph114_B4
zmp_ph114_D8
zmp_ph114_E2

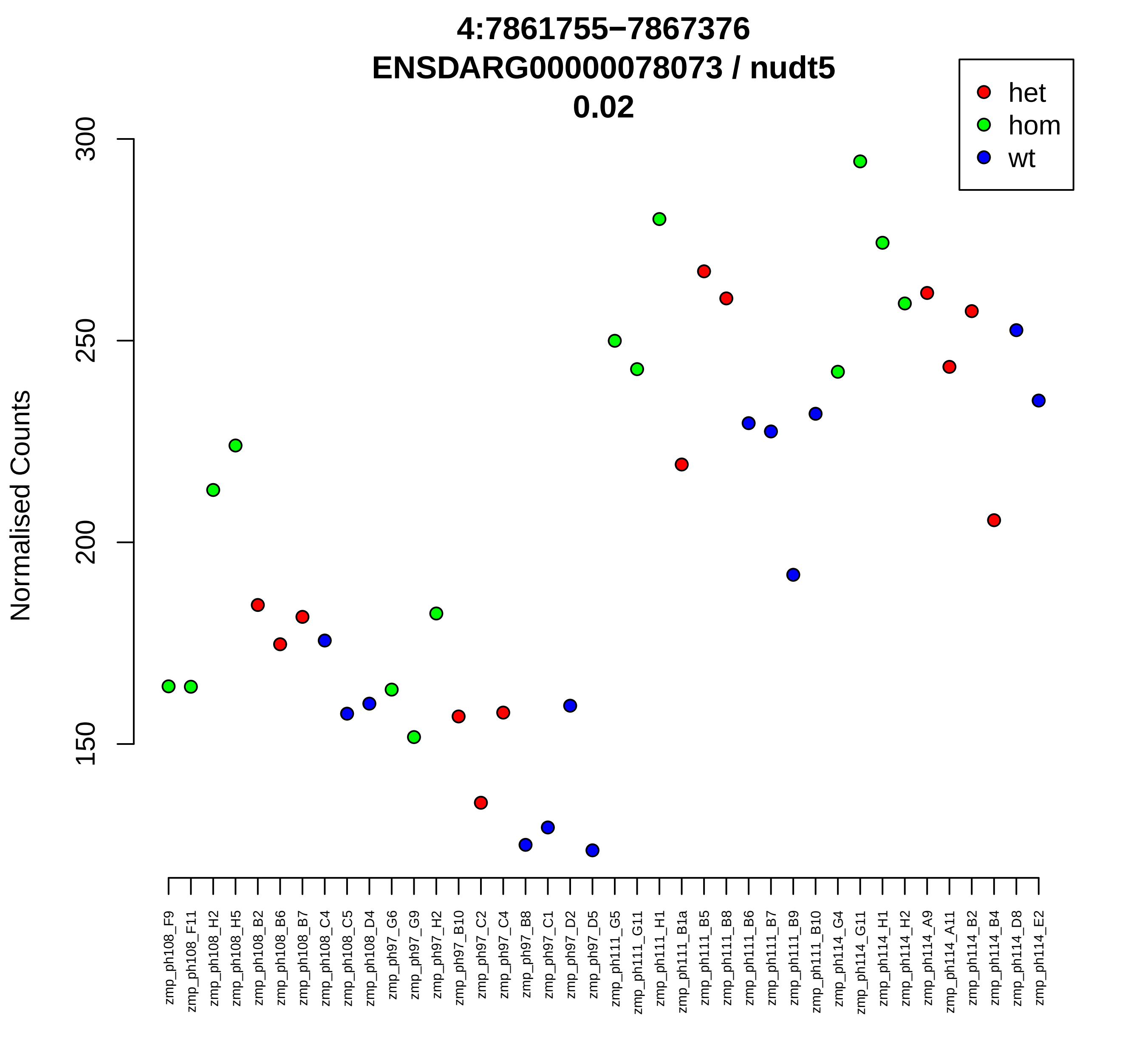
4:7861755−7867376
ENSDARG00000078073 / nudt5
0.02
het
hom
wt
Normalised Counts
150
200
250
300
zmp_ph108_F9
zmp_ph108_F11
zmp_ph108_H2
zmp_ph108_H5
zmp_ph108_B2
zmp_ph108_B6
zmp_ph108_B7
zmp_ph108_C4
zmp_ph108_C5
zmp_ph108_D4
zmp_ph97_G6
zmp_ph97_G9
zmp_ph97_H2
zmp_ph97_B10
zmp_ph97_C2
zmp_ph97_C4
zmp_ph97_B8
zmp_ph97_C1
zmp_ph97_D2
zmp_ph97_D5
zmp_ph111_G5
zmp_ph111_G11
zmp_ph111_H1
zmp_ph111_B1a
zmp_ph111_B5
zmp_ph111_B8
zmp_ph111_B6
zmp_ph111_B7
zmp_ph111_B9
zmp_ph111_B10
zmp_ph114_G4
zmp_ph114_G11
zmp_ph114_H1
zmp_ph114_H2
zmp_ph114_A9
zmp_ph114_A11
zmp_ph114_B2
zmp_ph114_B4
zmp_ph114_D8
zmp_ph114_E2

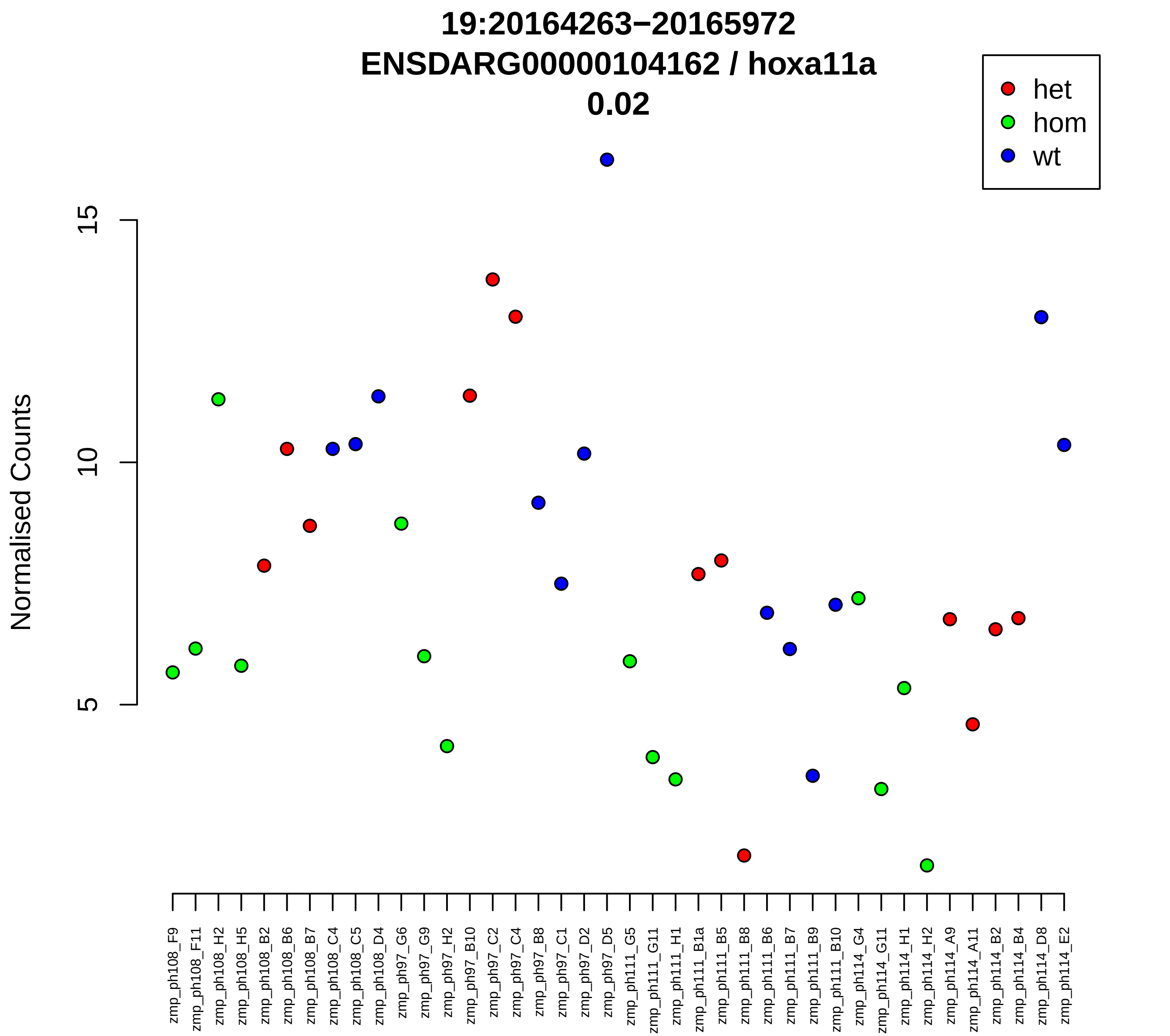

19:20164263−20165972
ENSDARG00000104162 / hoxa11a
0.02
het
hom
wt
Normalised Counts
5
10
15
zmp_ph108_F9
zmp_ph108_F11
zmp_ph108_H2
zmp_ph108_H5
zmp_ph108_B2
zmp_ph108_B6
zmp_ph108_B7
zmp_ph108_C4
zmp_ph108_C5
zmp_ph108_D4
zmp_ph97_G6
zmp_ph97_G9
zmp_ph97_H2
zmp_ph97_B10
zmp_ph97_C2
zmp_ph97_C4
zmp_ph97_B8
zmp_ph97_C1
zmp_ph97_D2
zmp_ph97_D5
zmp_ph111_G5
zmp_ph111_G11
zmp_ph111_H1
zmp_ph111_B1a
zmp_ph111_B5
zmp_ph111_B8
zmp_ph111_B6
zmp_ph111_B7
zmp_ph111_B9
zmp_ph111_B10
zmp_ph114_G4
zmp_ph114_G11
zmp_ph114_H1
zmp_ph114_H2
zmp_ph114_A9
zmp_ph114_A11
zmp_ph114_B2
zmp_ph114_B4
zmp_ph114_D8
zmp_ph114_E2

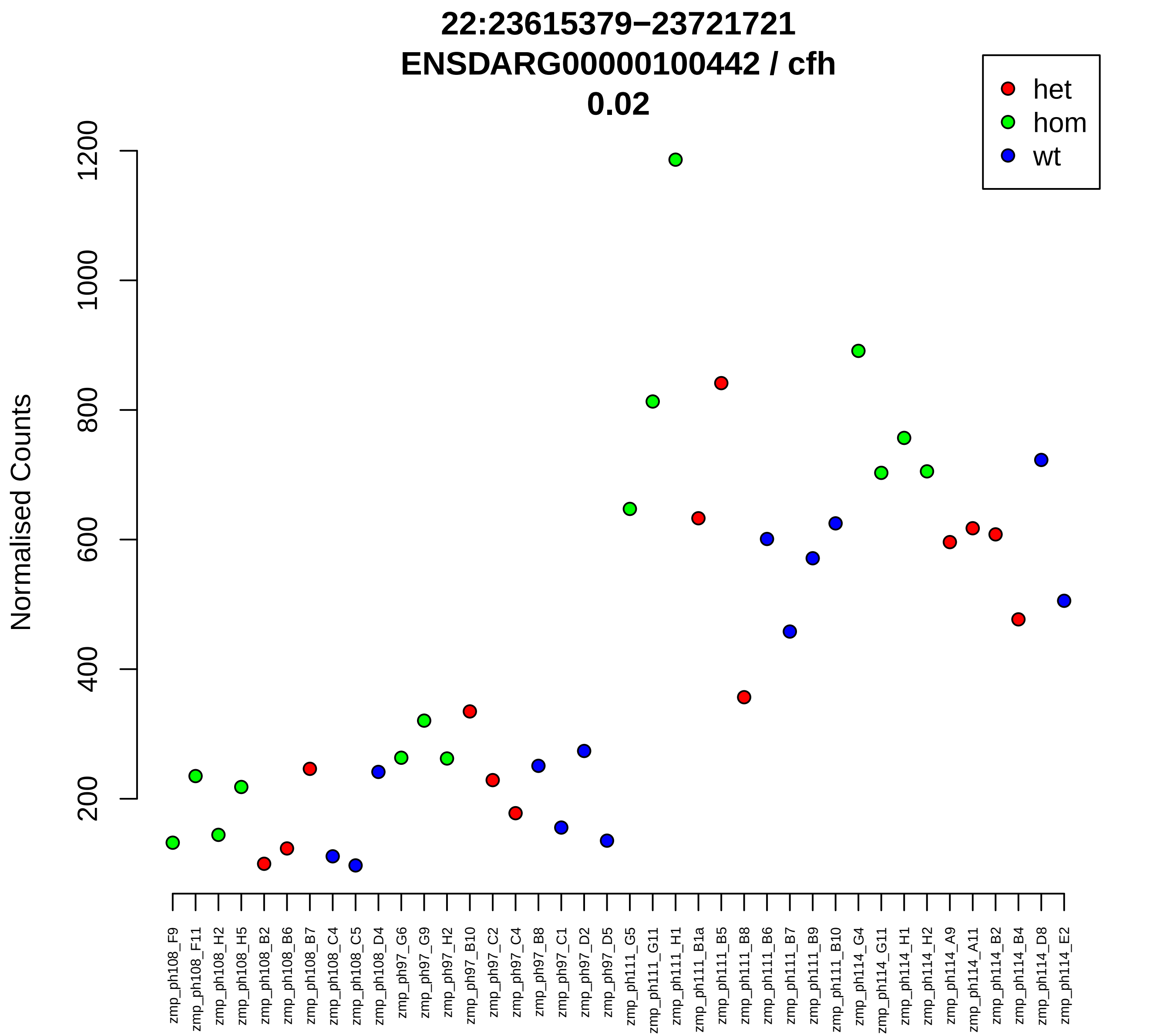

22:23615379−23721721
ENSDARG00000100442 / cfh
0.02
het
hom
wt
Normalised Counts
200
400
600
800
1000
1200
zmp_ph108_F9
zmp_ph108_F11
zmp_ph108_H2
zmp_ph108_H5
zmp_ph108_B2
zmp_ph108_B6
zmp_ph108_B7
zmp_ph108_C4
zmp_ph108_C5
zmp_ph108_D4
zmp_ph97_G6
zmp_ph97_G9
zmp_ph97_H2
zmp_ph97_B10
zmp_ph97_C2
zmp_ph97_C4
zmp_ph97_B8
zmp_ph97_C1
zmp_ph97_D2
zmp_ph97_D5
zmp_ph111_G5
zmp_ph111_G11
zmp_ph111_H1
zmp_ph111_B1a
zmp_ph111_B5
zmp_ph111_B8
zmp_ph111_B6
zmp_ph111_B7
zmp_ph111_B9
zmp_ph111_B10
zmp_ph114_G4
zmp_ph114_G11
zmp_ph114_H1
zmp_ph114_H2
zmp_ph114_A9
zmp_ph114_A11
zmp_ph114_B2
zmp_ph114_B4
zmp_ph114_D8
zmp_ph114_E2

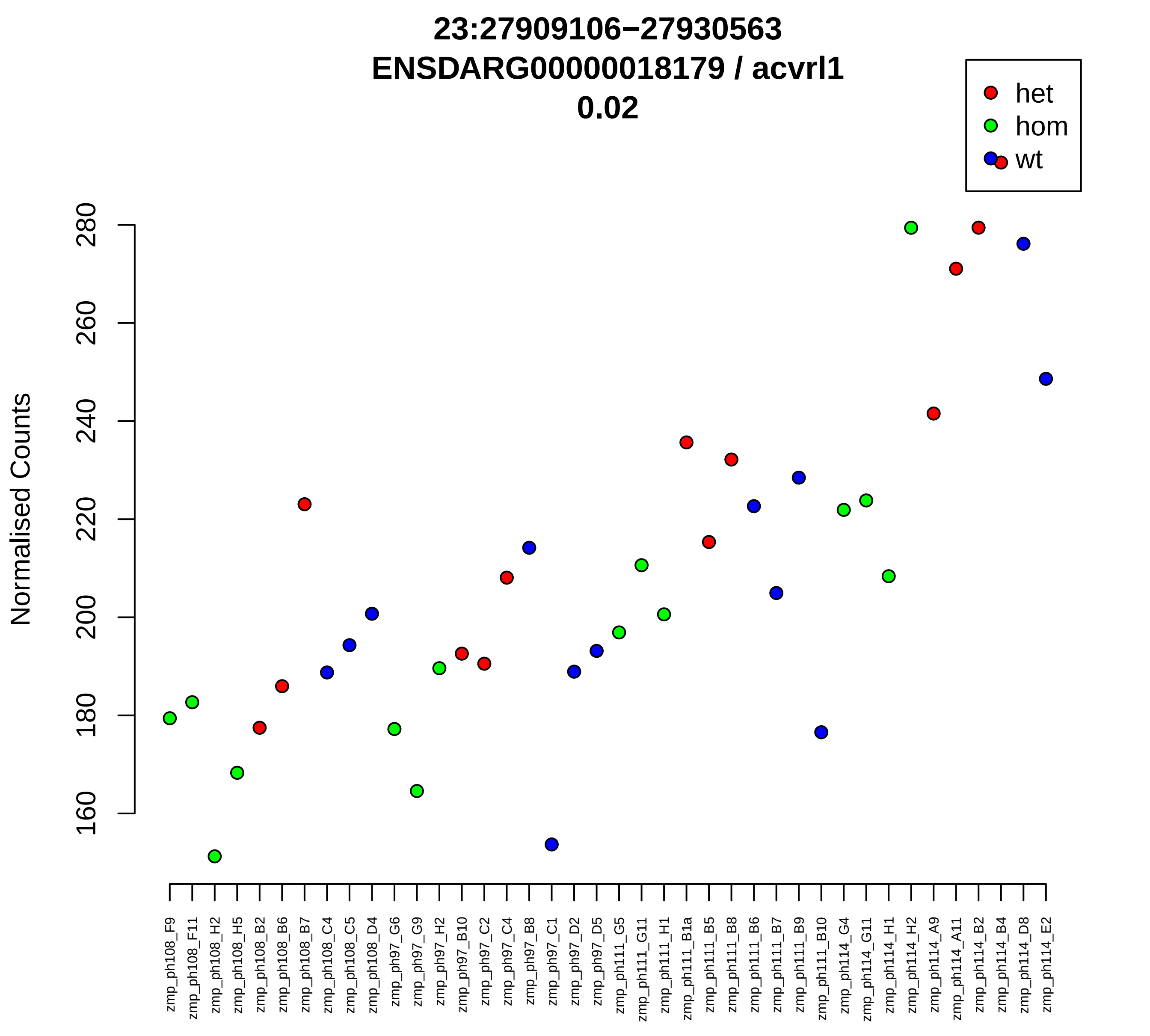

23:27909106−27930563
ENSDARG00000018179 / acvrl1
0.02
het
hom
wt
Normalised Counts
160
180
200
220
240
260
280
zmp_ph108_F9
zmp_ph108_F11
zmp_ph108_H2
zmp_ph108_H5
zmp_ph108_B2
zmp_ph108_B6
zmp_ph108_B7
zmp_ph108_C4
zmp_ph108_C5
zmp_ph108_D4
zmp_ph97_G6
zmp_ph97_G9
zmp_ph97_H2
zmp_ph97_B10
zmp_ph97_C2
zmp_ph97_C4
zmp_ph97_B8
zmp_ph97_C1
zmp_ph97_D2
zmp_ph97_D5
zmp_ph111_G5
zmp_ph111_G11
zmp_ph111_H1
zmp_ph111_B1a
zmp_ph111_B5
zmp_ph111_B8
zmp_ph111_B6
zmp_ph111_B7
zmp_ph111_B9
zmp_ph111_B10
zmp_ph114_G4
zmp_ph114_G11
zmp_ph114_H1
zmp_ph114_H2
zmp_ph114_A9
zmp_ph114_A11
zmp_ph114_B2
zmp_ph114_B4
zmp_ph114_D8
zmp_ph114_E2

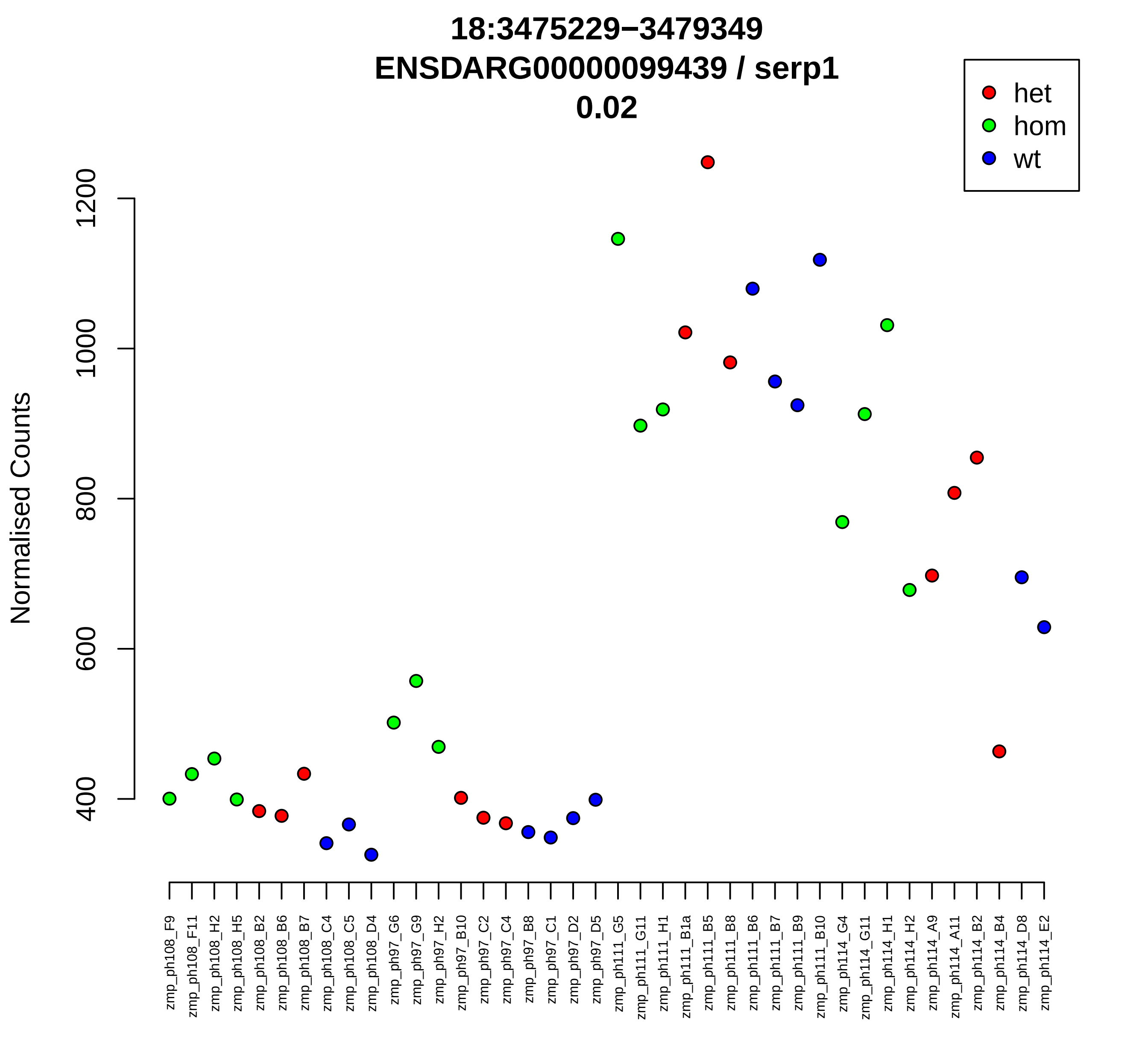
18:3475229−3479349
ENSDARG00000099439 / serp1
0.02
het
hom
wt
Normalised Counts
400
600
800
1000
1200
zmp_ph108_F9
zmp_ph108_F11
zmp_ph108_H2
zmp_ph108_H5
zmp_ph108_B2
zmp_ph108_B6
zmp_ph108_B7
zmp_ph108_C4
zmp_ph108_C5
zmp_ph108_D4
zmp_ph97_G6
zmp_ph97_G9
zmp_ph97_H2
zmp_ph97_B10
zmp_ph97_C2
zmp_ph97_C4
zmp_ph97_B8
zmp_ph97_C1
zmp_ph97_D2
zmp_ph97_D5
zmp_ph111_G5
zmp_ph111_G11
zmp_ph111_H1
zmp_ph111_B1a
zmp_ph111_B5
zmp_ph111_B8
zmp_ph111_B6
zmp_ph111_B7
zmp_ph111_B9
zmp_ph111_B10
zmp_ph114_G4
zmp_ph114_G11
zmp_ph114_H1
zmp_ph114_H2
zmp_ph114_A9
zmp_ph114_A11
zmp_ph114_B2
zmp_ph114_B4
zmp_ph114_D8
zmp_ph114_E2

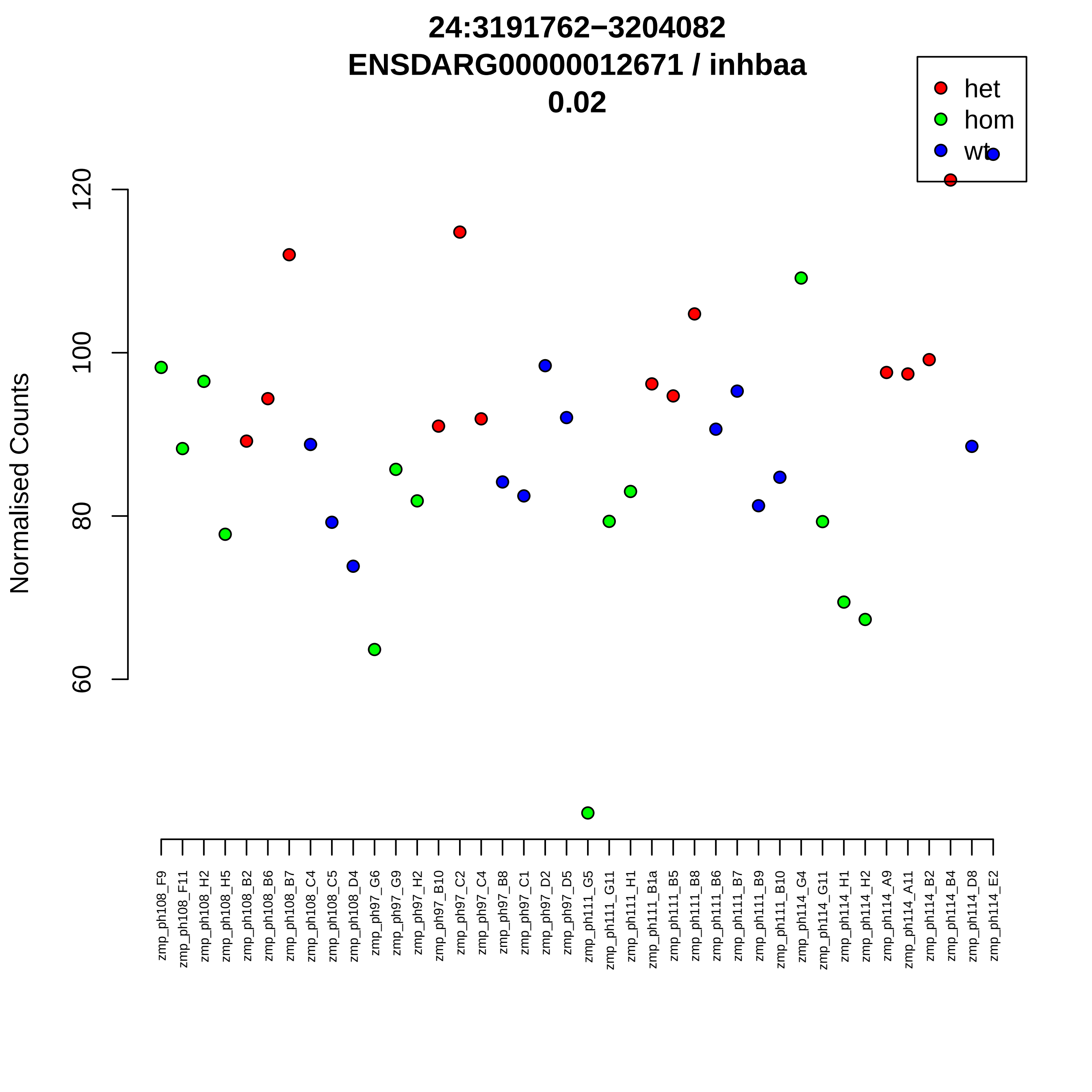

24:3191762−3204082
ENSDARG00000012671 / inhbaa
0.02
het
hom
wt
Normalised Counts
60
80
100
120
zmp_ph108_F9
zmp_ph108_F11
zmp_ph108_H2
zmp_ph108_H5
zmp_ph108_B2
zmp_ph108_B6
zmp_ph108_B7
zmp_ph108_C4
zmp_ph108_C5
zmp_ph108_D4
zmp_ph97_G6
zmp_ph97_G9
zmp_ph97_H2
zmp_ph97_B10
zmp_ph97_C2
zmp_ph97_C4
zmp_ph97_B8
zmp_ph97_C1
zmp_ph97_D2
zmp_ph97_D5
zmp_ph111_G5
zmp_ph111_G11
zmp_ph111_H1
zmp_ph111_B1a
zmp_ph111_B5
zmp_ph111_B8
zmp_ph111_B6
zmp_ph111_B7
zmp_ph111_B9
zmp_ph111_B10
zmp_ph114_G4
zmp_ph114_G11
zmp_ph114_H1
zmp_ph114_H2
zmp_ph114_A9
zmp_ph114_A11
zmp_ph114_B2
zmp_ph114_B4
zmp_ph114_D8
zmp_ph114_E2

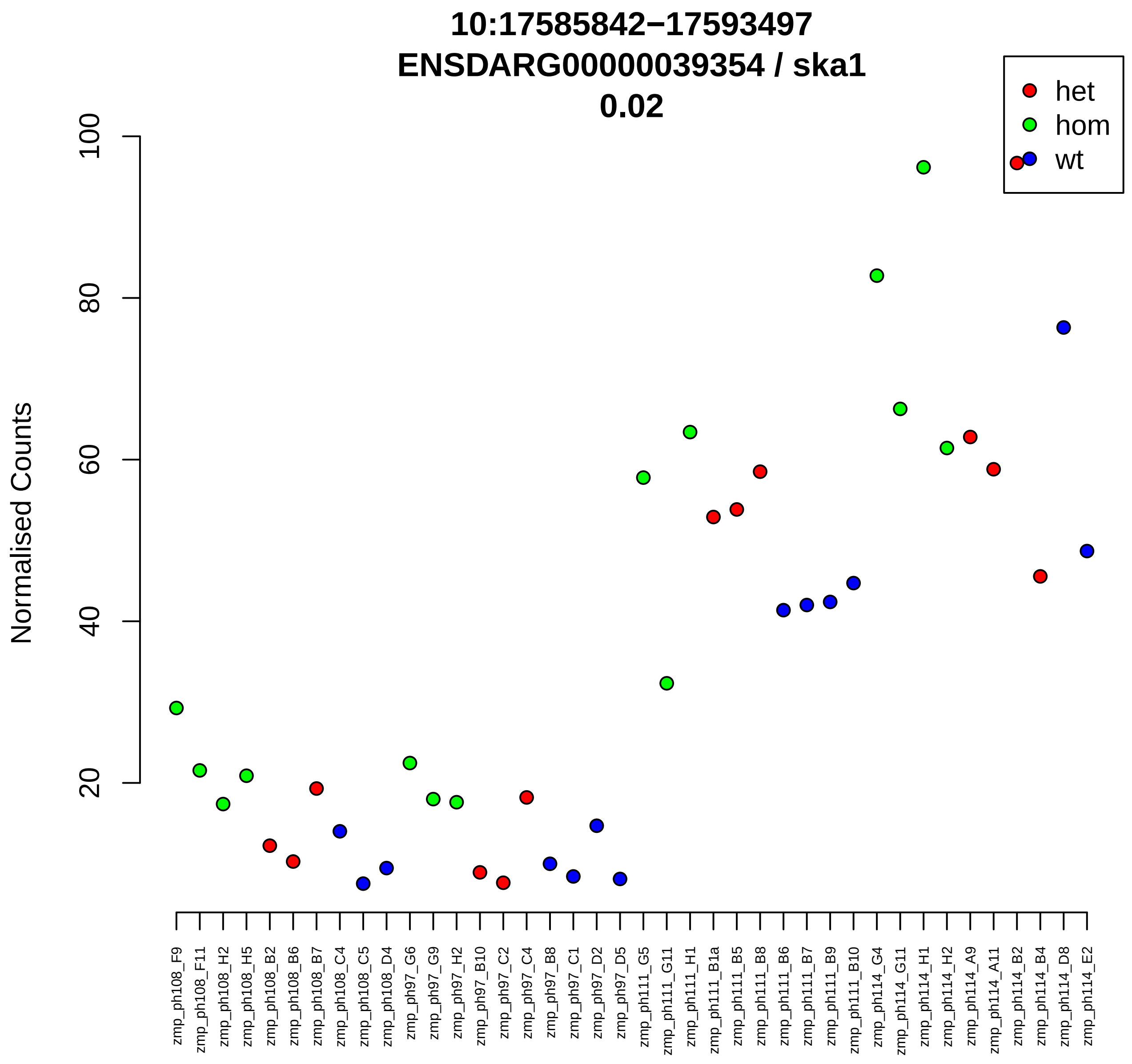

10:17585842−17593497
ENSDARG00000039354 / ska1
0.02
het
hom
wt
Normalised Counts
20
40
60
80
100
zmp_ph108_F9
zmp_ph108_F11
zmp_ph108_H2
zmp_ph108_H5
zmp_ph108_B2
zmp_ph108_B6
zmp_ph108_B7
zmp_ph108_C4
zmp_ph108_C5
zmp_ph108_D4
zmp_ph97_G6
zmp_ph97_G9
zmp_ph97_H2
zmp_ph97_B10
zmp_ph97_C2
zmp_ph97_C4
zmp_ph97_B8
zmp_ph97_C1
zmp_ph97_D2
zmp_ph97_D5
zmp_ph111_G5
zmp_ph111_G11
zmp_ph111_H1
zmp_ph111_B1a
zmp_ph111_B5
zmp_ph111_B8
zmp_ph111_B6
zmp_ph111_B7
zmp_ph111_B9
zmp_ph111_B10
zmp_ph114_G4
zmp_ph114_G11
zmp_ph114_H1
zmp_ph114_H2
zmp_ph114_A9
zmp_ph114_A11
zmp_ph114_B2
zmp_ph114_B4
zmp_ph114_D8
zmp_ph114_E2

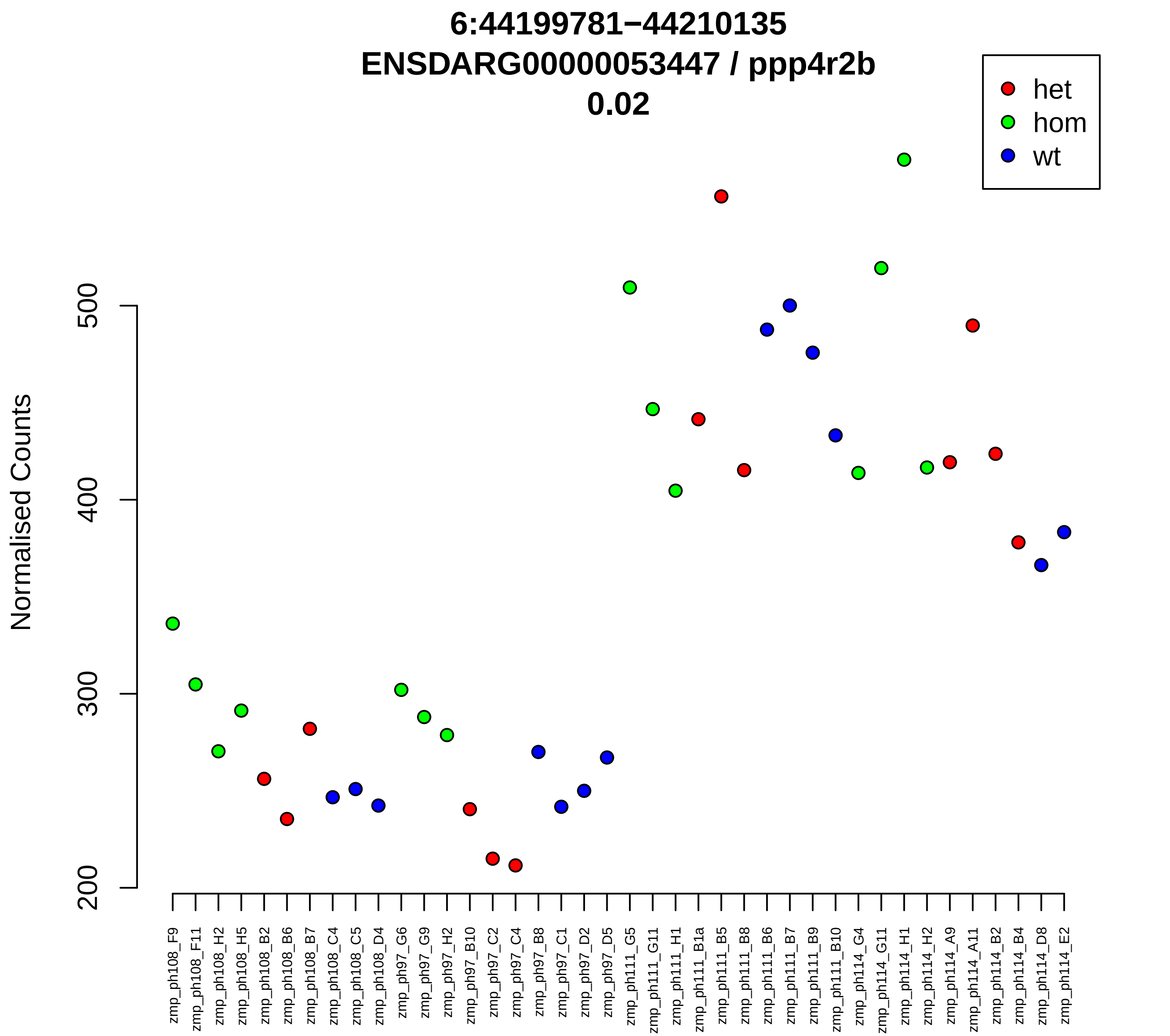

6:44199781−44210135
ENSDARG00000053447 / ppp4r2b
0.02
het
hom
wt
Normalised Counts
200
300
400
500
zmp_ph108_F9
zmp_ph108_F11
zmp_ph108_H2
zmp_ph108_H5
zmp_ph108_B2
zmp_ph108_B6
zmp_ph108_B7
zmp_ph108_C4
zmp_ph108_C5
zmp_ph108_D4
zmp_ph97_G6
zmp_ph97_G9
zmp_ph97_H2
zmp_ph97_B10
zmp_ph97_C2
zmp_ph97_C4
zmp_ph97_B8
zmp_ph97_C1
zmp_ph97_D2
zmp_ph97_D5
zmp_ph111_G5
zmp_ph111_G11
zmp_ph111_H1
zmp_ph111_B1a
zmp_ph111_B5
zmp_ph111_B8
zmp_ph111_B6
zmp_ph111_B7
zmp_ph111_B9
zmp_ph111_B10
zmp_ph114_G4
zmp_ph114_G11
zmp_ph114_H1
zmp_ph114_H2
zmp_ph114_A9
zmp_ph114_A11
zmp_ph114_B2
zmp_ph114_B4
zmp_ph114_D8
zmp_ph114_E2

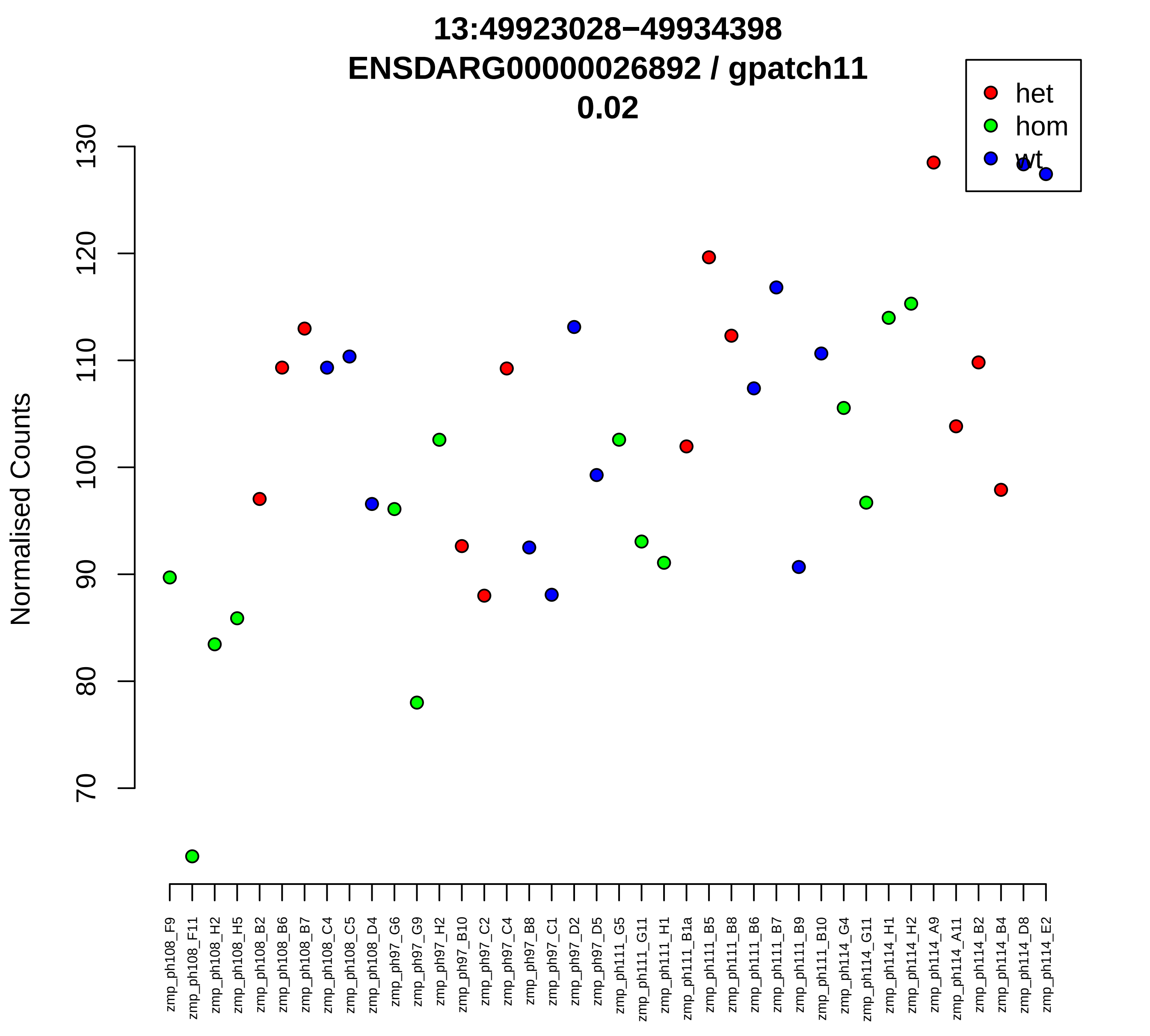

13:49923028−49934398
ENSDARG00000026892 / gpatch11
0.02
het
hom
wt
Normalised Counts
70
80
90
100
110
120
130
zmp_ph108_F9
zmp_ph108_F11
zmp_ph108_H2
zmp_ph108_H5
zmp_ph108_B2
zmp_ph108_B6
zmp_ph108_B7
zmp_ph108_C4
zmp_ph108_C5
zmp_ph108_D4
zmp_ph97_G6
zmp_ph97_G9
zmp_ph97_H2
zmp_ph97_B10
zmp_ph97_C2
zmp_ph97_C4
zmp_ph97_B8
zmp_ph97_C1
zmp_ph97_D2
zmp_ph97_D5
zmp_ph111_G5
zmp_ph111_G11
zmp_ph111_H1
zmp_ph111_B1a
zmp_ph111_B5
zmp_ph111_B8
zmp_ph111_B6
zmp_ph111_B7
zmp_ph111_B9
zmp_ph111_B10
zmp_ph114_G4
zmp_ph114_G11
zmp_ph114_H1
zmp_ph114_H2
zmp_ph114_A9
zmp_ph114_A11
zmp_ph114_B2
zmp_ph114_B4
zmp_ph114_D8
zmp_ph114_E2

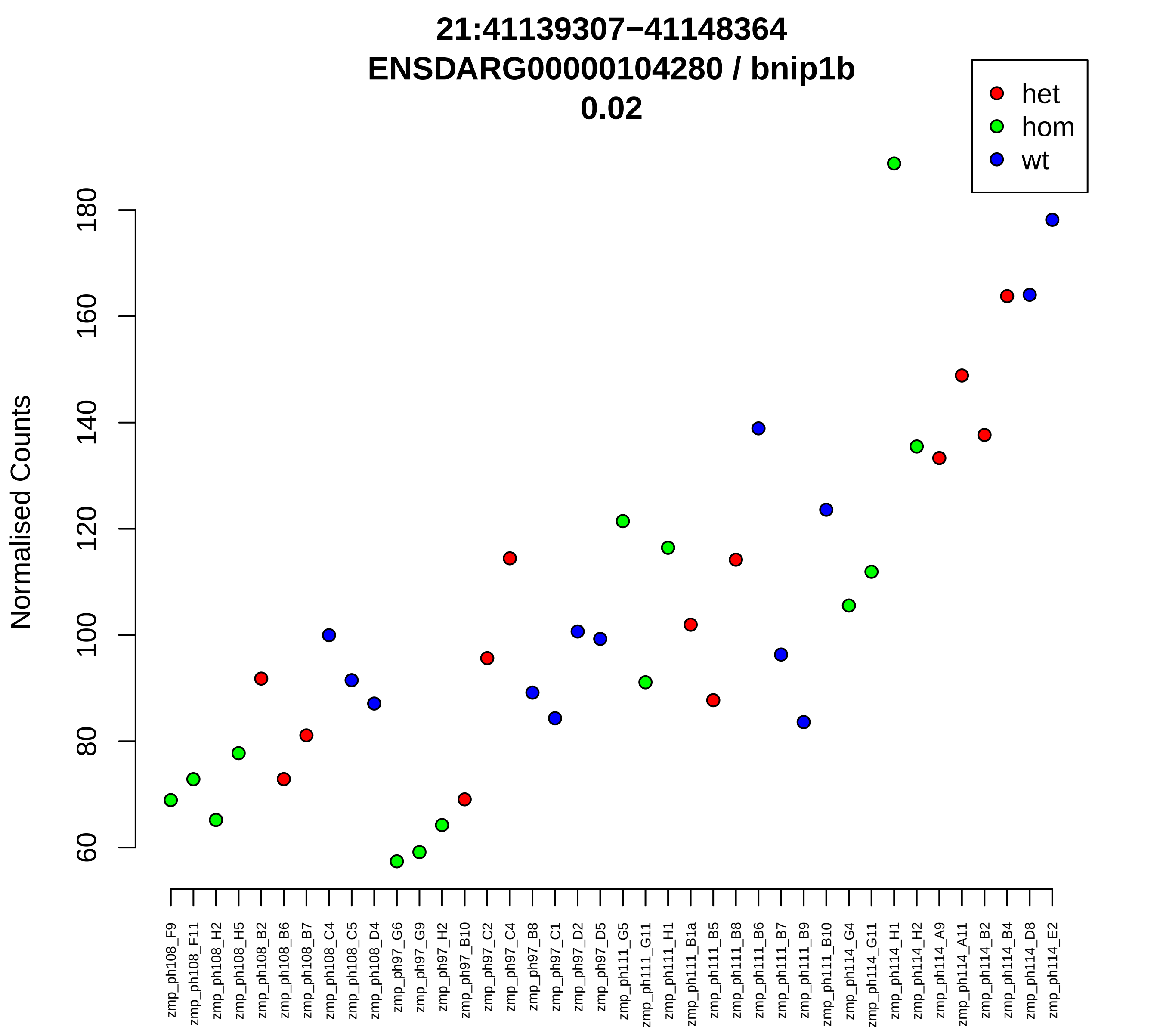
21:41139307−41148364
ENSDARG00000104280 / bnip1b
0.02
het
hom
wt
Normalised Counts
60
80
100
120
140
160
180
zmp_ph108_F9
zmp_ph108_F11
zmp_ph108_H2
zmp_ph108_H5
zmp_ph108_B2
zmp_ph108_B6
zmp_ph108_B7
zmp_ph108_C4
zmp_ph108_C5
zmp_ph108_D4
zmp_ph97_G6
zmp_ph97_G9
zmp_ph97_H2
zmp_ph97_B10
zmp_ph97_C2
zmp_ph97_C4
zmp_ph97_B8
zmp_ph97_C1
zmp_ph97_D2
zmp_ph97_D5
zmp_ph111_G5
zmp_ph111_G11
zmp_ph111_H1
zmp_ph111_B1a
zmp_ph111_B5
zmp_ph111_B8
zmp_ph111_B6
zmp_ph111_B7
zmp_ph111_B9
zmp_ph111_B10
zmp_ph114_G4
zmp_ph114_G11
zmp_ph114_H1
zmp_ph114_H2
zmp_ph114_A9
zmp_ph114_A11
zmp_ph114_B2
zmp_ph114_B4
zmp_ph114_D8
zmp_ph114_E2

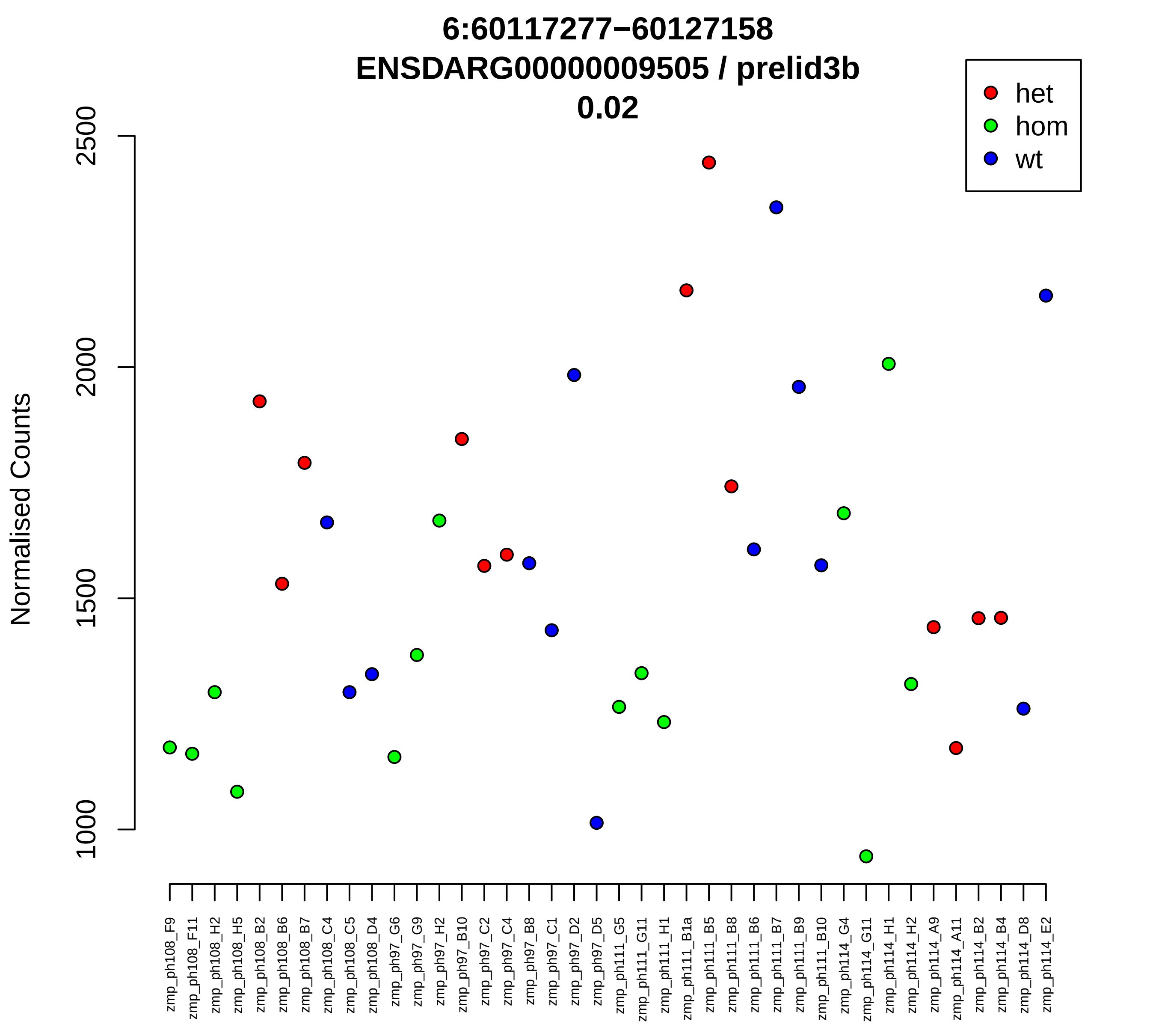

6:60117277−60127158
ENSDARG00000009505 / prelid3b
0.02
het
hom
wt
Normalised Counts
1000
1500
2000
2500
zmp_ph108_F9
zmp_ph108_F11
zmp_ph108_H2
zmp_ph108_H5
zmp_ph108_B2
zmp_ph108_B6
zmp_ph108_B7
zmp_ph108_C4
zmp_ph108_C5
zmp_ph108_D4
zmp_ph97_G6
zmp_ph97_G9
zmp_ph97_H2
zmp_ph97_B10
zmp_ph97_C2
zmp_ph97_C4
zmp_ph97_B8
zmp_ph97_C1
zmp_ph97_D2
zmp_ph97_D5
zmp_ph111_G5
zmp_ph111_G11
zmp_ph111_H1
zmp_ph111_B1a
zmp_ph111_B5
zmp_ph111_B8
zmp_ph111_B6
zmp_ph111_B7
zmp_ph111_B9
zmp_ph111_B10
zmp_ph114_G4
zmp_ph114_G11
zmp_ph114_H1
zmp_ph114_H2
zmp_ph114_A9
zmp_ph114_A11
zmp_ph114_B2
zmp_ph114_B4
zmp_ph114_D8
zmp_ph114_E2

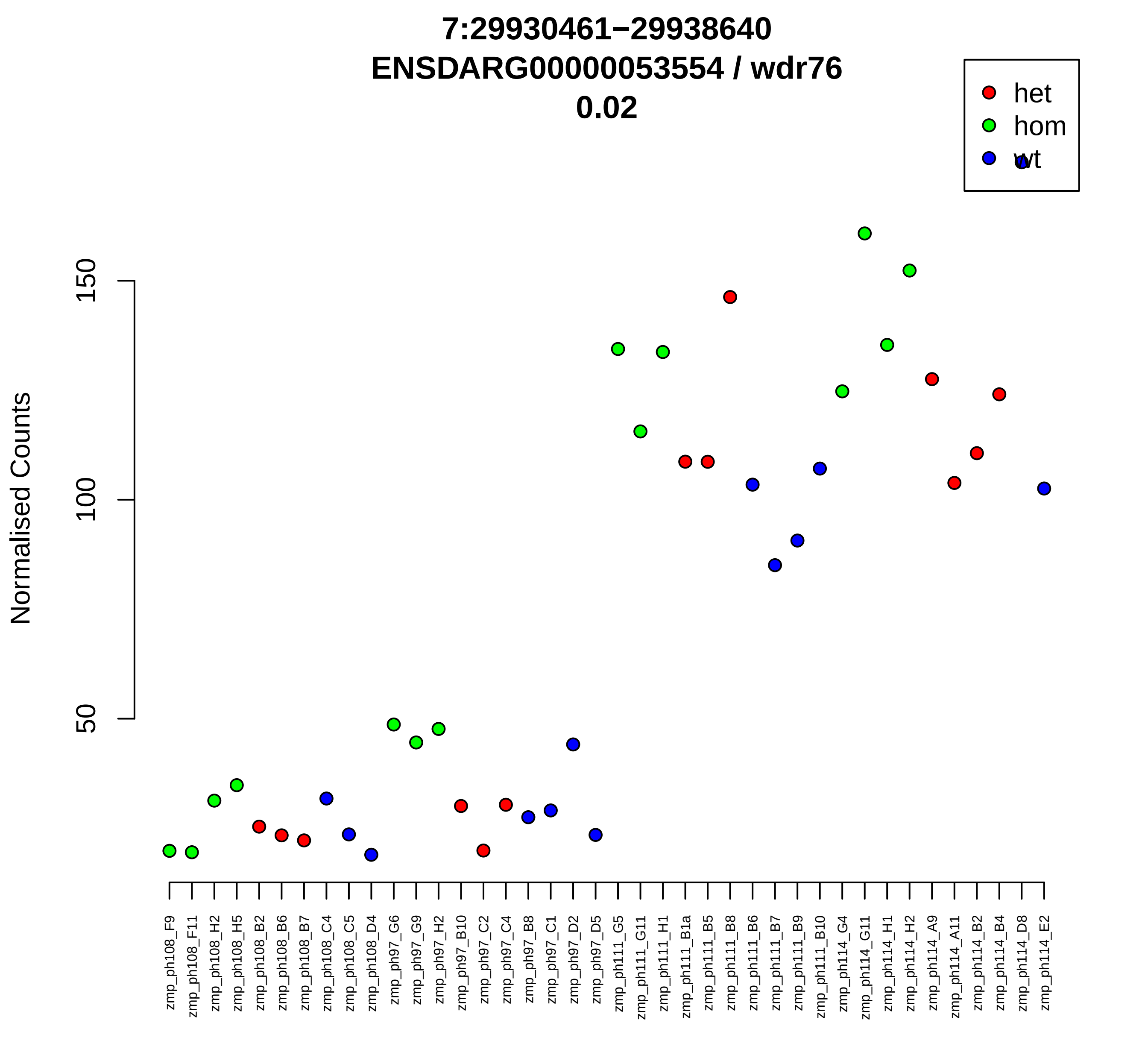

7:29930461−29938640
ENSDARG00000053554 / wdr76
0.02
het
hom
wt
Normalised Counts
50
100
150
zmp_ph108_F9
zmp_ph108_F11
zmp_ph108_H2
zmp_ph108_H5
zmp_ph108_B2
zmp_ph108_B6
zmp_ph108_B7
zmp_ph108_C4
zmp_ph108_C5
zmp_ph108_D4
zmp_ph97_G6
zmp_ph97_G9
zmp_ph97_H2
zmp_ph97_B10
zmp_ph97_C2
zmp_ph97_C4
zmp_ph97_B8
zmp_ph97_C1
zmp_ph97_D2
zmp_ph97_D5
zmp_ph111_G5
zmp_ph111_G11
zmp_ph111_H1
zmp_ph111_B1a
zmp_ph111_B5
zmp_ph111_B8
zmp_ph111_B6
zmp_ph111_B7
zmp_ph111_B9
zmp_ph111_B10
zmp_ph114_G4
zmp_ph114_G11
zmp_ph114_H1
zmp_ph114_H2
zmp_ph114_A9
zmp_ph114_A11
zmp_ph114_B2
zmp_ph114_B4
zmp_ph114_D8
zmp_ph114_E2

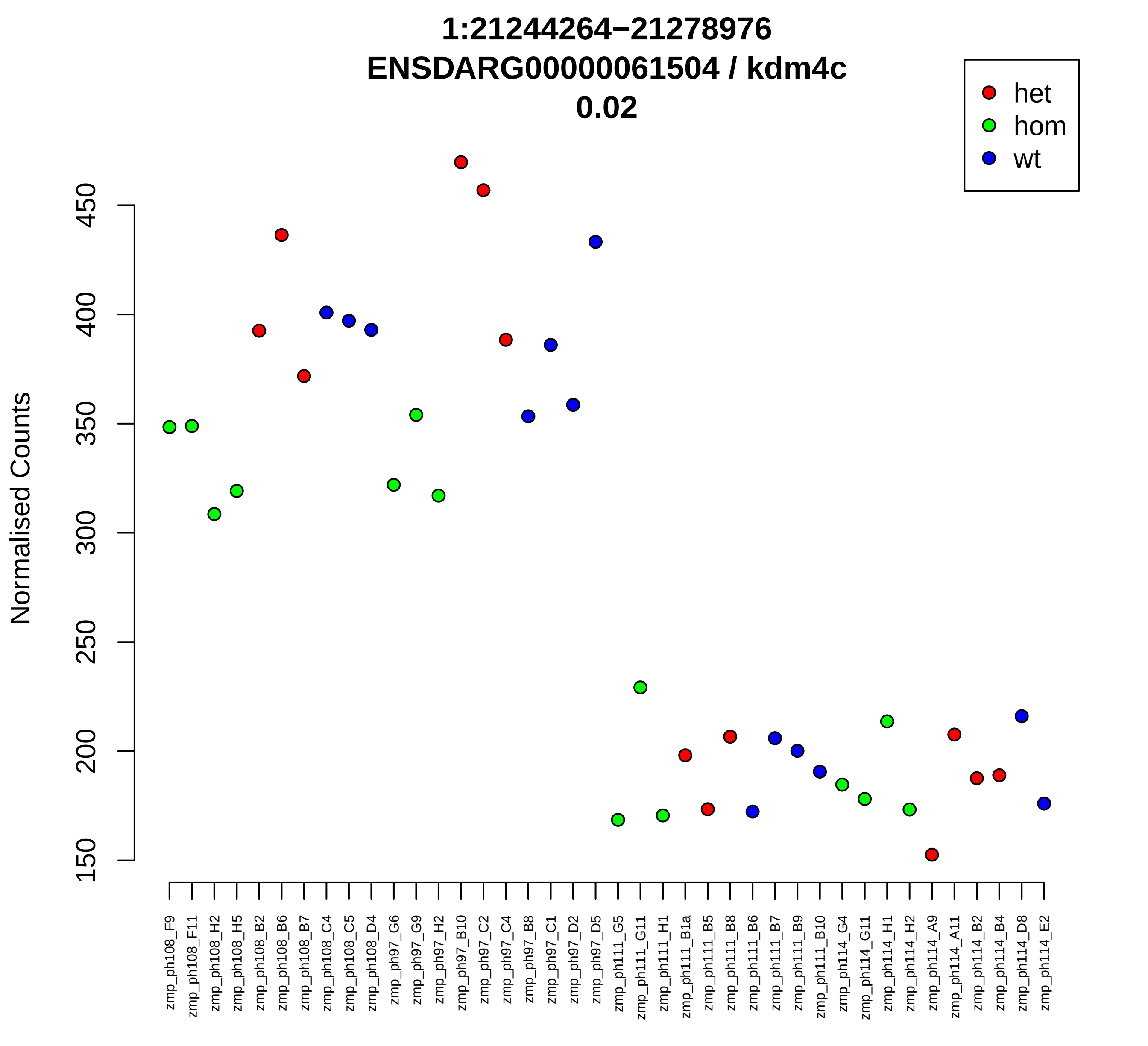
1:21244264−21278976
ENSDARG00000061504 / kdm4c
0.02
het
hom
wt
Normalised Counts
150
200
250
300
350
400
450
zmp_ph108_F9
zmp_ph108_F11
zmp_ph108_H2
zmp_ph108_H5
zmp_ph108_B2
zmp_ph108_B6
zmp_ph108_B7
zmp_ph108_C4
zmp_ph108_C5
zmp_ph108_D4
zmp_ph97_G6
zmp_ph97_G9
zmp_ph97_H2
zmp_ph97_B10
zmp_ph97_C2
zmp_ph97_C4
zmp_ph97_B8
zmp_ph97_C1
zmp_ph97_D2
zmp_ph97_D5
zmp_ph111_G5
zmp_ph111_G11
zmp_ph111_H1
zmp_ph111_B1a
zmp_ph111_B5
zmp_ph111_B8
zmp_ph111_B6
zmp_ph111_B7
zmp_ph111_B9
zmp_ph111_B10
zmp_ph114_G4
zmp_ph114_G11
zmp_ph114_H1
zmp_ph114_H2
zmp_ph114_A9
zmp_ph114_A11
zmp_ph114_B2
zmp_ph114_B4
zmp_ph114_D8
zmp_ph114_E2

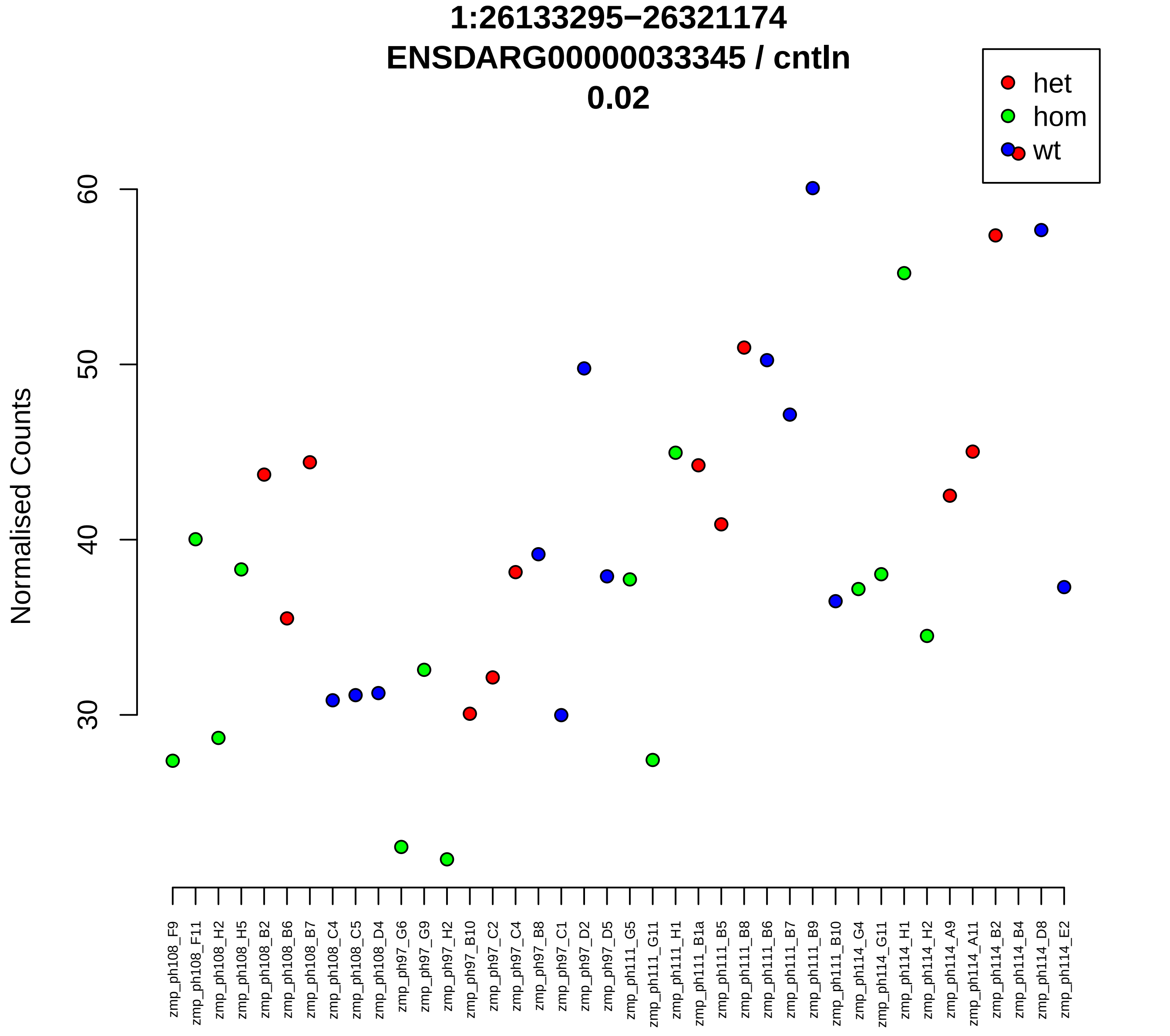

1:26133295−26321174
ENSDARG00000033345 / cntln
0.02
het
hom
wt
Normalised Counts
30
40
50
60
zmp_ph108_F9
zmp_ph108_F11
zmp_ph108_H2
zmp_ph108_H5
zmp_ph108_B2
zmp_ph108_B6
zmp_ph108_B7
zmp_ph108_C4
zmp_ph108_C5
zmp_ph108_D4
zmp_ph97_G6
zmp_ph97_G9
zmp_ph97_H2
zmp_ph97_B10
zmp_ph97_C2
zmp_ph97_C4
zmp_ph97_B8
zmp_ph97_C1
zmp_ph97_D2
zmp_ph97_D5
zmp_ph111_G5
zmp_ph111_G11
zmp_ph111_H1
zmp_ph111_B1a
zmp_ph111_B5
zmp_ph111_B8
zmp_ph111_B6
zmp_ph111_B7
zmp_ph111_B9
zmp_ph111_B10
zmp_ph114_G4
zmp_ph114_G11
zmp_ph114_H1
zmp_ph114_H2
zmp_ph114_A9
zmp_ph114_A11
zmp_ph114_B2
zmp_ph114_B4
zmp_ph114_D8
zmp_ph114_E2

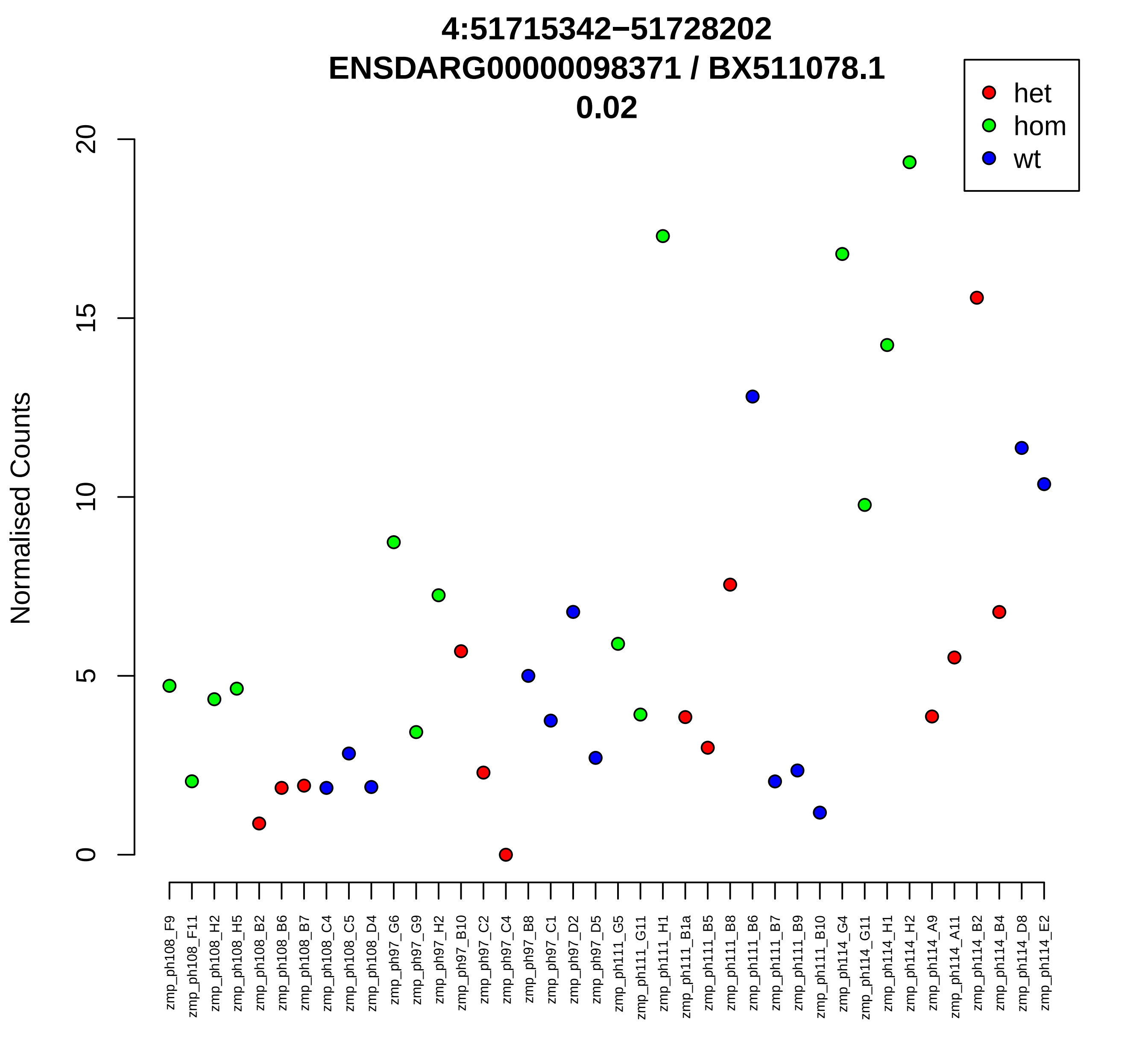
4:51715342−51728202
ENSDARG00000098371 / BX511078.1
0.02
het
hom
wt
Normalised Counts
0
5
10
15
20
zmp_ph108_F9
zmp_ph108_F11
zmp_ph108_H2
zmp_ph108_H5
zmp_ph108_B2
zmp_ph108_B6
zmp_ph108_B7
zmp_ph108_C4
zmp_ph108_C5
zmp_ph108_D4
zmp_ph97_G6
zmp_ph97_G9
zmp_ph97_H2
zmp_ph97_B10
zmp_ph97_C2
zmp_ph97_C4
zmp_ph97_B8
zmp_ph97_C1
zmp_ph97_D2
zmp_ph97_D5
zmp_ph111_G5
zmp_ph111_G11
zmp_ph111_H1
zmp_ph111_B1a
zmp_ph111_B5
zmp_ph111_B8
zmp_ph111_B6
zmp_ph111_B7
zmp_ph111_B9
zmp_ph111_B10
zmp_ph114_G4
zmp_ph114_G11
zmp_ph114_H1
zmp_ph114_H2
zmp_ph114_A9
zmp_ph114_A11
zmp_ph114_B2
zmp_ph114_B4
zmp_ph114_D8
zmp_ph114_E2

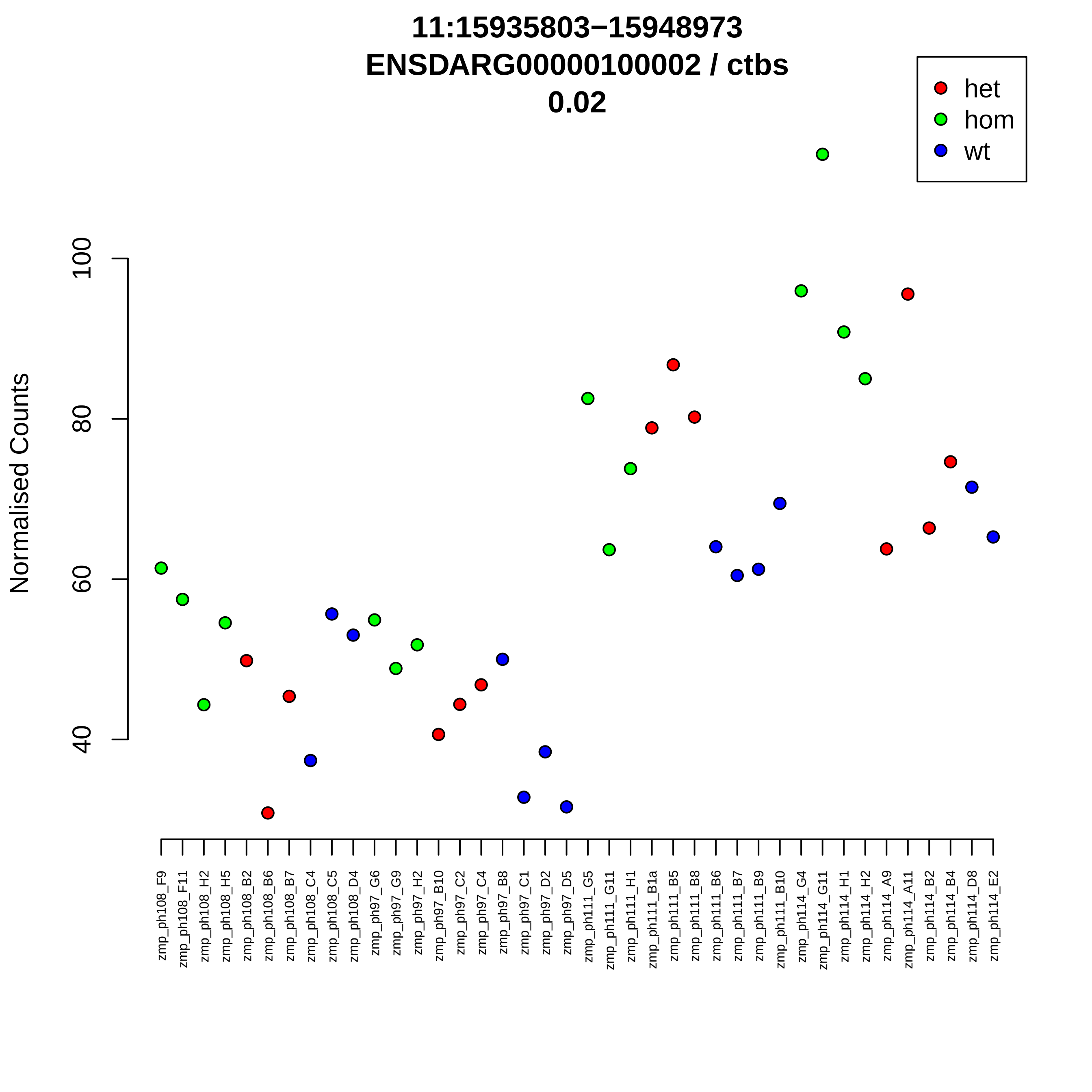

11:15935803−15948973
ENSDARG00000100002 / ctbs
0.02
het
hom
wt
Normalised Counts
40
60
80
100
zmp_ph108_F9
zmp_ph108_F11
zmp_ph108_H2
zmp_ph108_H5
zmp_ph108_B2
zmp_ph108_B6
zmp_ph108_B7
zmp_ph108_C4
zmp_ph108_C5
zmp_ph108_D4
zmp_ph97_G6
zmp_ph97_G9
zmp_ph97_H2
zmp_ph97_B10
zmp_ph97_C2
zmp_ph97_C4
zmp_ph97_B8
zmp_ph97_C1
zmp_ph97_D2
zmp_ph97_D5
zmp_ph111_G5
zmp_ph111_G11
zmp_ph111_H1
zmp_ph111_B1a
zmp_ph111_B5
zmp_ph111_B8
zmp_ph111_B6
zmp_ph111_B7
zmp_ph111_B9
zmp_ph111_B10
zmp_ph114_G4
zmp_ph114_G11
zmp_ph114_H1
zmp_ph114_H2
zmp_ph114_A9
zmp_ph114_A11
zmp_ph114_B2
zmp_ph114_B4
zmp_ph114_D8
zmp_ph114_E2

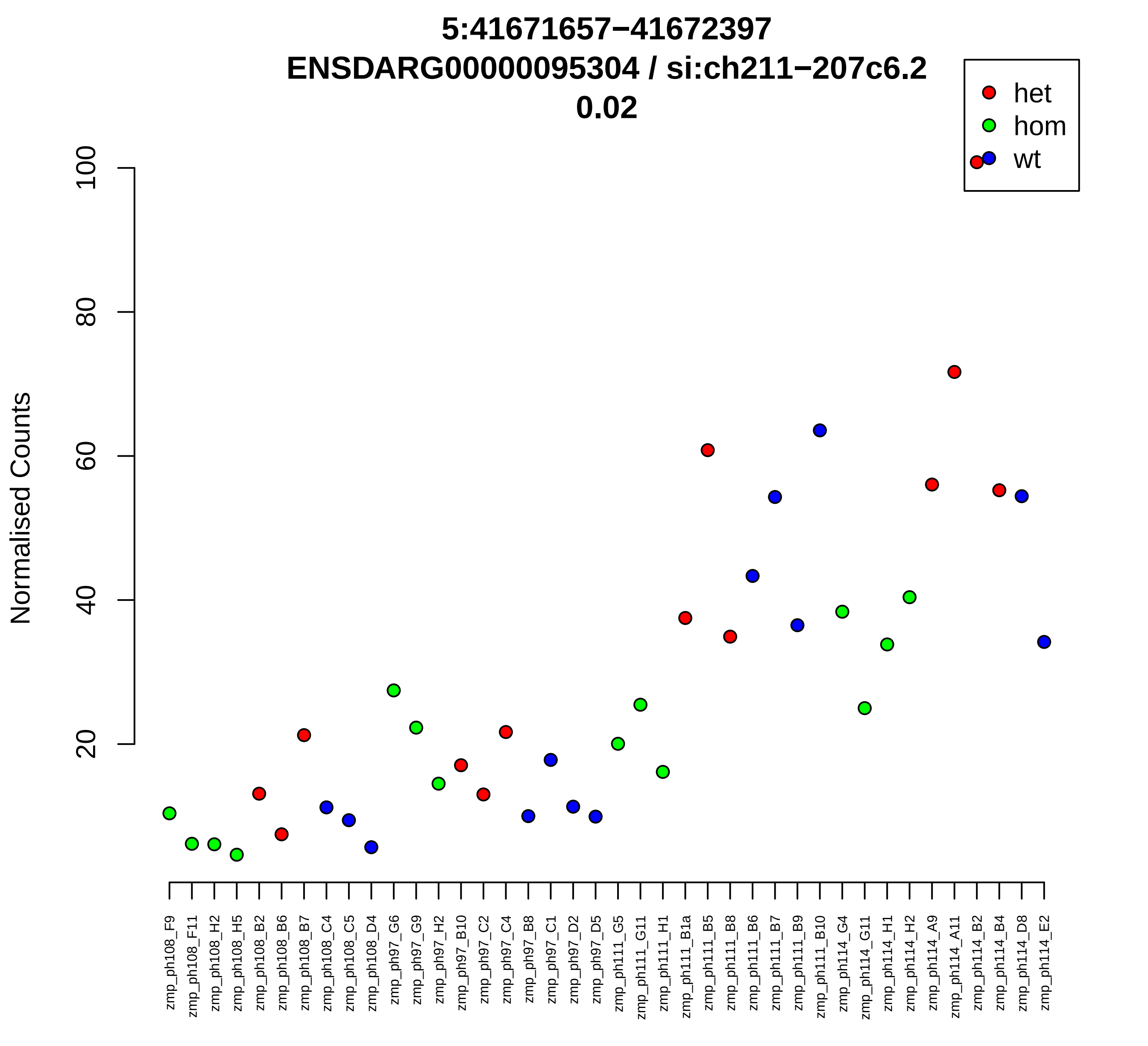
5:41671657−41672397
ENSDARG00000095304 / si:ch211−207c6.2
0.02
het
hom
wt
Normalised Counts
20
40
60
80
100
zmp_ph108_F9
zmp_ph108_F11
zmp_ph108_H2
zmp_ph108_H5
zmp_ph108_B2
zmp_ph108_B6
zmp_ph108_B7
zmp_ph108_C4
zmp_ph108_C5
zmp_ph108_D4
zmp_ph97_G6
zmp_ph97_G9
zmp_ph97_H2
zmp_ph97_B10
zmp_ph97_C2
zmp_ph97_C4
zmp_ph97_B8
zmp_ph97_C1
zmp_ph97_D2
zmp_ph97_D5
zmp_ph111_G5
zmp_ph111_G11
zmp_ph111_H1
zmp_ph111_B1a
zmp_ph111_B5
zmp_ph111_B8
zmp_ph111_B6
zmp_ph111_B7
zmp_ph111_B9
zmp_ph111_B10
zmp_ph114_G4
zmp_ph114_G11
zmp_ph114_H1
zmp_ph114_H2
zmp_ph114_A9
zmp_ph114_A11
zmp_ph114_B2
zmp_ph114_B4
zmp_ph114_D8
zmp_ph114_E2

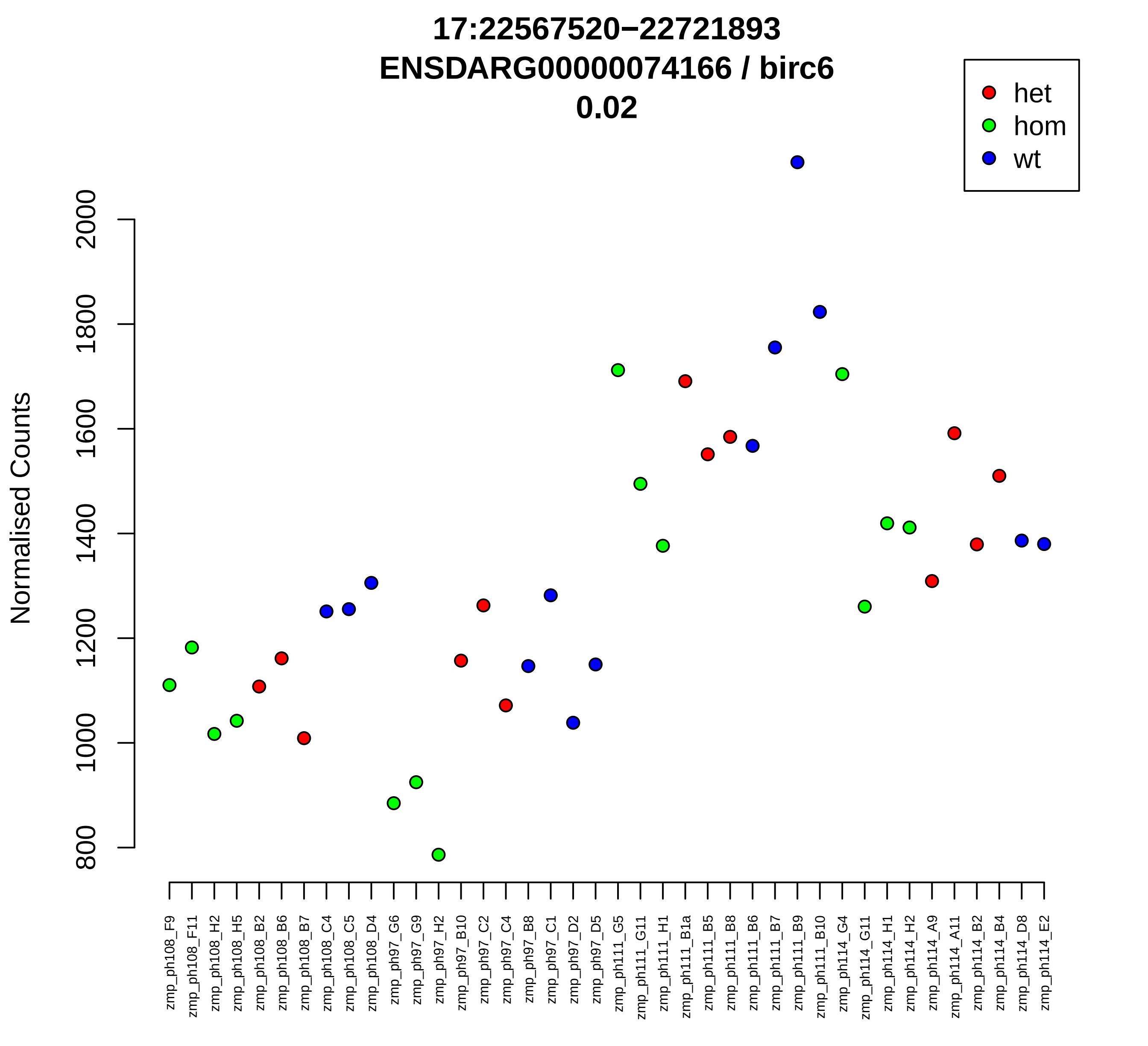

17:22567520−22721893
ENSDARG00000074166 / birc6
0.02
het
hom
wt
Normalised Counts
800
1000
1200
1400
1600
1800
2000
zmp_ph108_F9
zmp_ph108_F11
zmp_ph108_H2
zmp_ph108_H5
zmp_ph108_B2
zmp_ph108_B6
zmp_ph108_B7
zmp_ph108_C4
zmp_ph108_C5
zmp_ph108_D4
zmp_ph97_G6
zmp_ph97_G9
zmp_ph97_H2
zmp_ph97_B10
zmp_ph97_C2
zmp_ph97_C4
zmp_ph97_B8
zmp_ph97_C1
zmp_ph97_D2
zmp_ph97_D5
zmp_ph111_G5
zmp_ph111_G11
zmp_ph111_H1
zmp_ph111_B1a
zmp_ph111_B5
zmp_ph111_B8
zmp_ph111_B6
zmp_ph111_B7
zmp_ph111_B9
zmp_ph111_B10
zmp_ph114_G4
zmp_ph114_G11
zmp_ph114_H1
zmp_ph114_H2
zmp_ph114_A9
zmp_ph114_A11
zmp_ph114_B2
zmp_ph114_B4
zmp_ph114_D8
zmp_ph114_E2

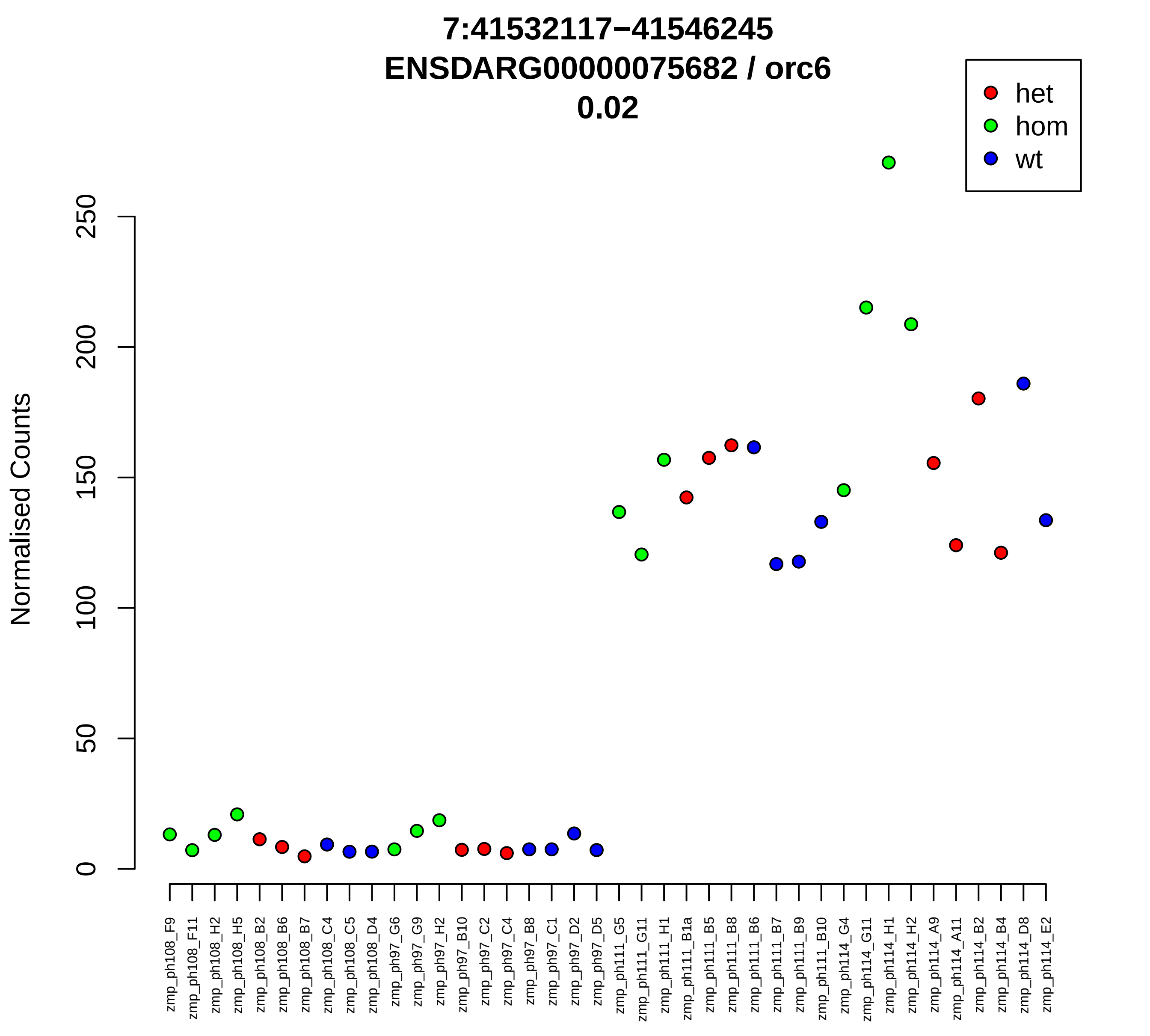

7:41532117−41546245
ENSDARG00000075682 / orc6
0.02
het
hom
wt
Normalised Counts
0
50
100
150
200
250
zmp_ph108_F9
zmp_ph108_F11
zmp_ph108_H2
zmp_ph108_H5
zmp_ph108_B2
zmp_ph108_B6
zmp_ph108_B7
zmp_ph108_C4
zmp_ph108_C5
zmp_ph108_D4
zmp_ph97_G6
zmp_ph97_G9
zmp_ph97_H2
zmp_ph97_B10
zmp_ph97_C2
zmp_ph97_C4
zmp_ph97_B8
zmp_ph97_C1
zmp_ph97_D2
zmp_ph97_D5
zmp_ph111_G5
zmp_ph111_G11
zmp_ph111_H1
zmp_ph111_B1a
zmp_ph111_B5
zmp_ph111_B8
zmp_ph111_B6
zmp_ph111_B7
zmp_ph111_B9
zmp_ph111_B10
zmp_ph114_G4
zmp_ph114_G11
zmp_ph114_H1
zmp_ph114_H2
zmp_ph114_A9
zmp_ph114_A11
zmp_ph114_B2
zmp_ph114_B4
zmp_ph114_D8
zmp_ph114_E2

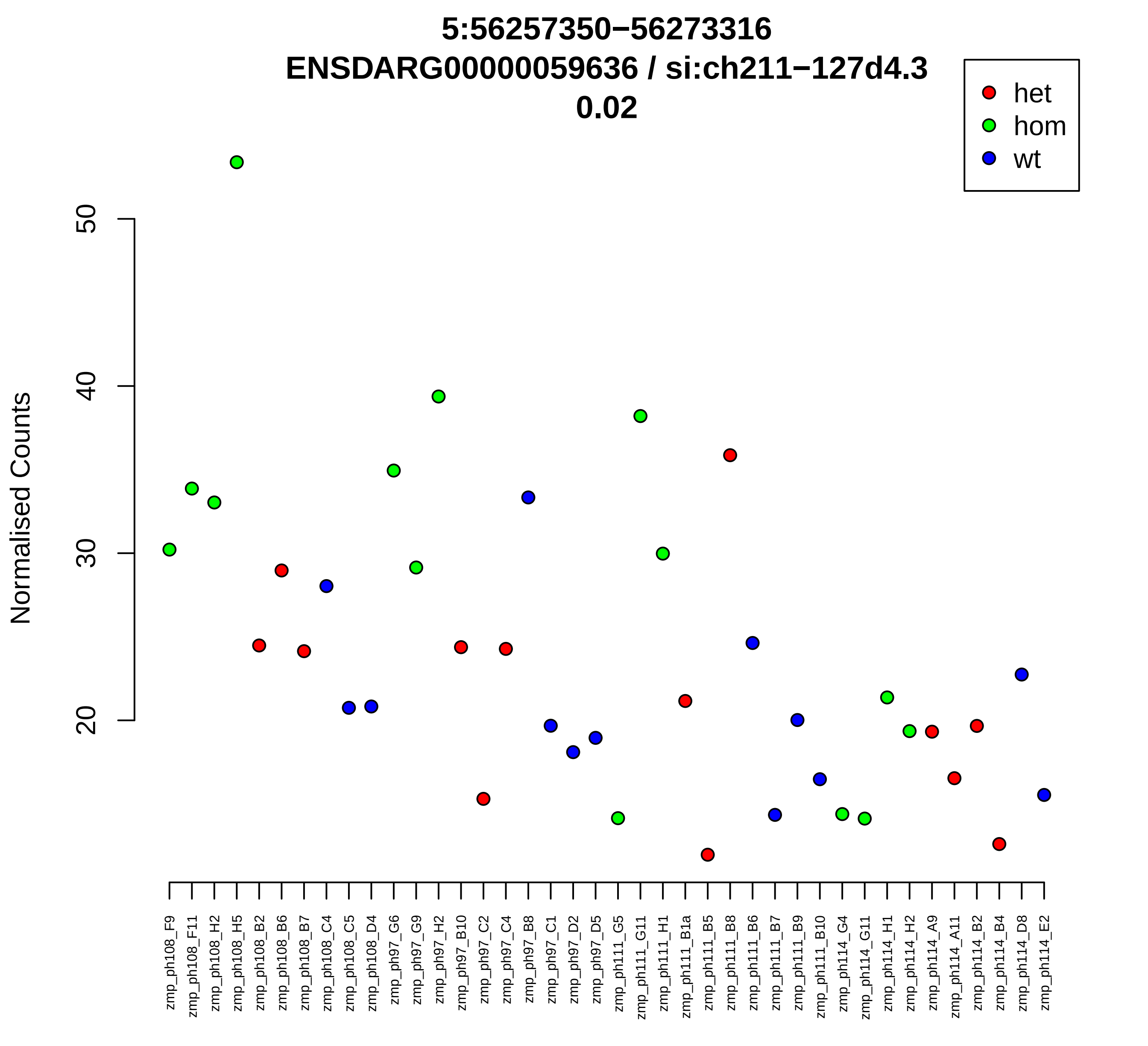

5:56257350−56273316
ENSDARG00000059636 / si:ch211−127d4.3
0.02
het
hom
wt
Normalised Counts
20
30
40
50
zmp_ph108_F9
zmp_ph108_F11
zmp_ph108_H2
zmp_ph108_H5
zmp_ph108_B2
zmp_ph108_B6
zmp_ph108_B7
zmp_ph108_C4
zmp_ph108_C5
zmp_ph108_D4
zmp_ph97_G6
zmp_ph97_G9
zmp_ph97_H2
zmp_ph97_B10
zmp_ph97_C2
zmp_ph97_C4
zmp_ph97_B8
zmp_ph97_C1
zmp_ph97_D2
zmp_ph97_D5
zmp_ph111_G5
zmp_ph111_G11
zmp_ph111_H1
zmp_ph111_B1a
zmp_ph111_B5
zmp_ph111_B8
zmp_ph111_B6
zmp_ph111_B7
zmp_ph111_B9
zmp_ph111_B10
zmp_ph114_G4
zmp_ph114_G11
zmp_ph114_H1
zmp_ph114_H2
zmp_ph114_A9
zmp_ph114_A11
zmp_ph114_B2
zmp_ph114_B4
zmp_ph114_D8
zmp_ph114_E2

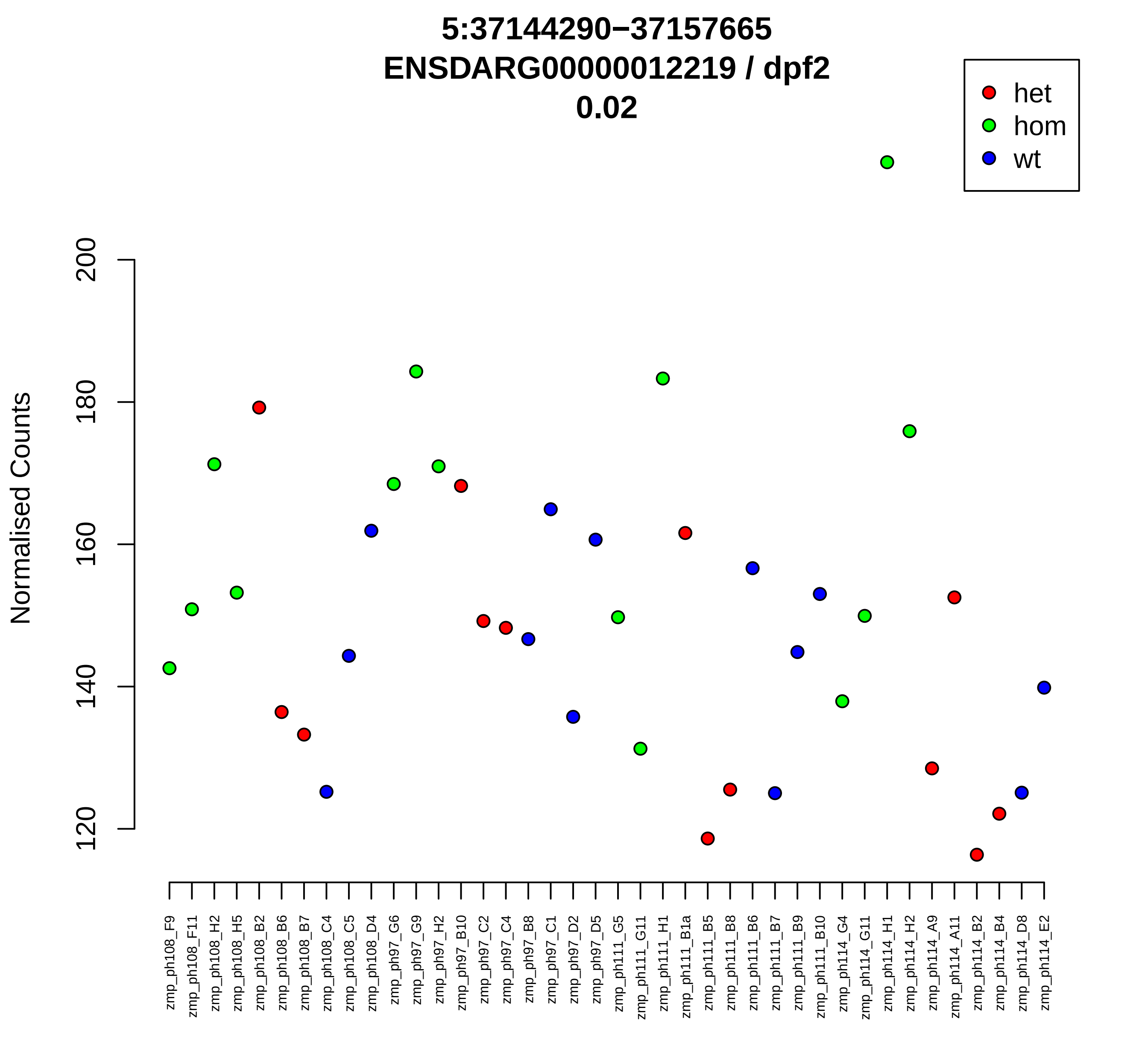

5:37144290−37157665
ENSDARG00000012219 / dpf2
0.02
het
hom
wt
Normalised Counts
120
140
160
180
200
zmp_ph108_F9
zmp_ph108_F11
zmp_ph108_H2
zmp_ph108_H5
zmp_ph108_B2
zmp_ph108_B6
zmp_ph108_B7
zmp_ph108_C4
zmp_ph108_C5
zmp_ph108_D4
zmp_ph97_G6
zmp_ph97_G9
zmp_ph97_H2
zmp_ph97_B10
zmp_ph97_C2
zmp_ph97_C4
zmp_ph97_B8
zmp_ph97_C1
zmp_ph97_D2
zmp_ph97_D5
zmp_ph111_G5
zmp_ph111_G11
zmp_ph111_H1
zmp_ph111_B1a
zmp_ph111_B5
zmp_ph111_B8
zmp_ph111_B6
zmp_ph111_B7
zmp_ph111_B9
zmp_ph111_B10
zmp_ph114_G4
zmp_ph114_G11
zmp_ph114_H1
zmp_ph114_H2
zmp_ph114_A9
zmp_ph114_A11
zmp_ph114_B2
zmp_ph114_B4
zmp_ph114_D8
zmp_ph114_E2

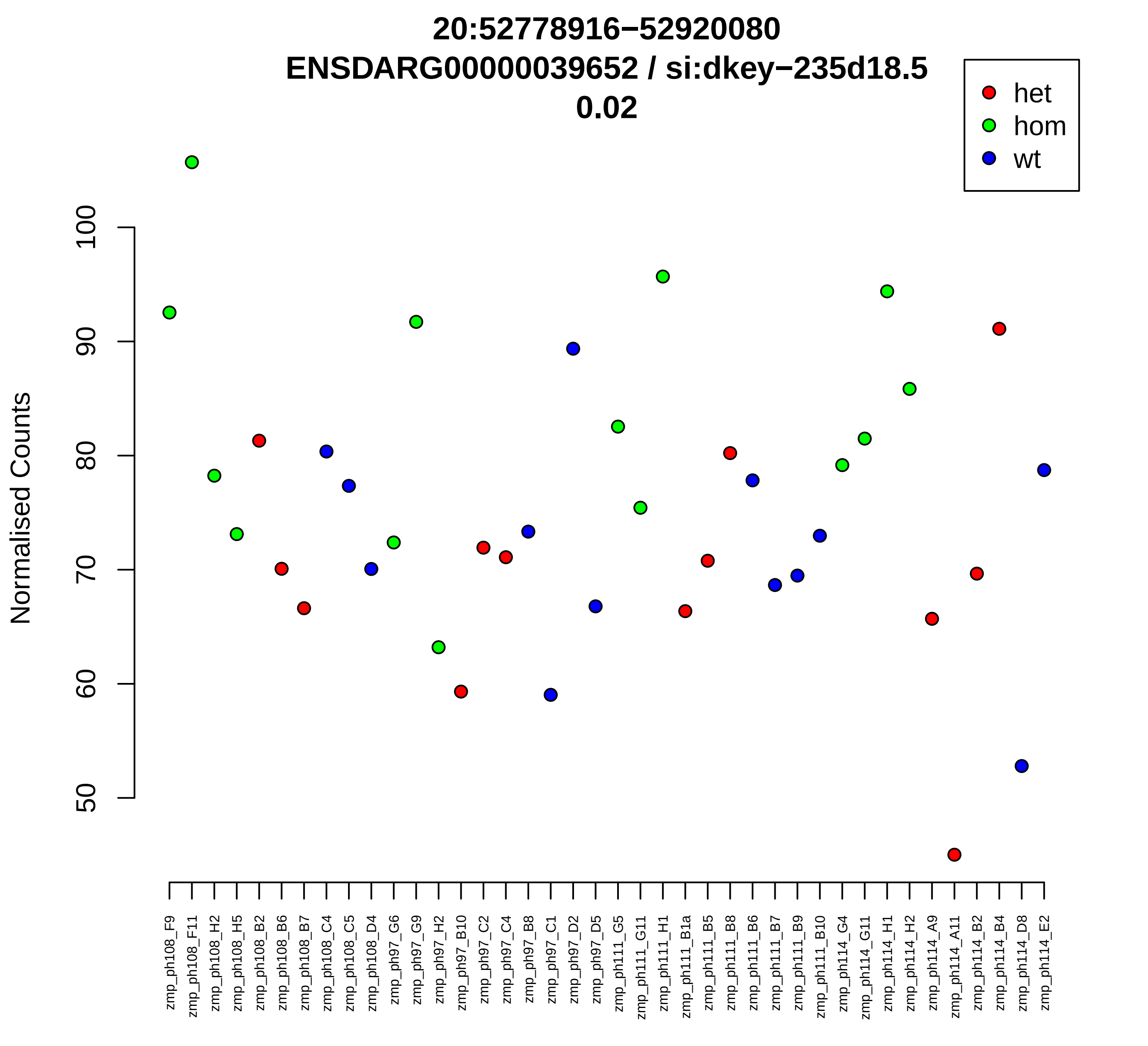
20:52778916−52920080
ENSDARG00000039652 / si:dkey−235d18.5
0.02
het
hom
wt
Normalised Counts
100
90
80
70
60
50
zmp_ph108_F9
zmp_ph108_F11
zmp_ph108_H2
zmp_ph108_H5
zmp_ph108_B2
zmp_ph108_B6
zmp_ph108_B7
zmp_ph108_C4
zmp_ph108_C5
zmp_ph108_D4
zmp_ph97_G6
zmp_ph97_G9
zmp_ph97_H2
zmp_ph97_B10
zmp_ph97_C2
zmp_ph97_C4
zmp_ph97_B8
zmp_ph97_C1
zmp_ph97_D2
zmp_ph97_D5
zmp_ph111_G5
zmp_ph111_G11
zmp_ph111_H1
zmp_ph111_B1a
zmp_ph111_B5
zmp_ph111_B8
zmp_ph111_B6
zmp_ph111_B7
zmp_ph111_B9
zmp_ph111_B10
zmp_ph114_G4
zmp_ph114_G11
zmp_ph114_H1
zmp_ph114_H2
zmp_ph114_A9
zmp_ph114_A11
zmp_ph114_B2
zmp_ph114_B4
zmp_ph114_D8
zmp_ph114_E2

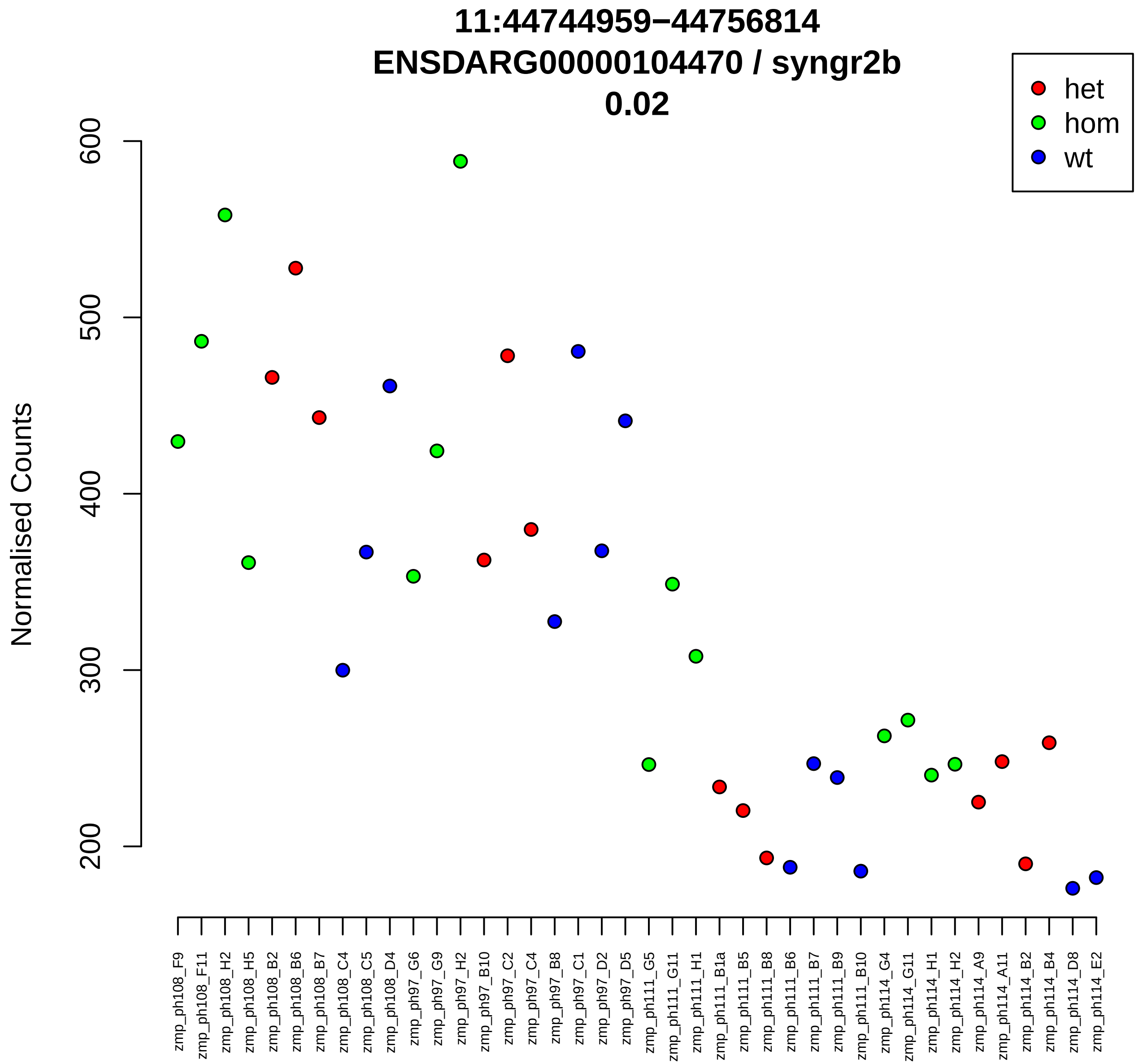

11:44744959−44756814
ENSDARG00000104470 / syngr2b
0.02
het
hom
wt
Normalised Counts
600
500
400
300
200
zmp_ph108_F9
zmp_ph108_F11
zmp_ph108_H2
zmp_ph108_H5
zmp_ph108_B2
zmp_ph108_B6
zmp_ph108_B7
zmp_ph108_C4
zmp_ph108_C5
zmp_ph108_D4
zmp_ph97_G6
zmp_ph97_G9
zmp_ph97_H2
zmp_ph97_B10
zmp_ph97_C2
zmp_ph97_C4
zmp_ph97_B8
zmp_ph97_C1
zmp_ph97_D2
zmp_ph97_D5
zmp_ph111_G5
zmp_ph111_G11
zmp_ph111_H1
zmp_ph111_B1a
zmp_ph111_B5
zmp_ph111_B8
zmp_ph111_B6
zmp_ph111_B7
zmp_ph111_B9
zmp_ph111_B10
zmp_ph114_G4
zmp_ph114_G11
zmp_ph114_H1
zmp_ph114_H2
zmp_ph114_A9
zmp_ph114_A11
zmp_ph114_B2
zmp_ph114_B4
zmp_ph114_D8
zmp_ph114_E2

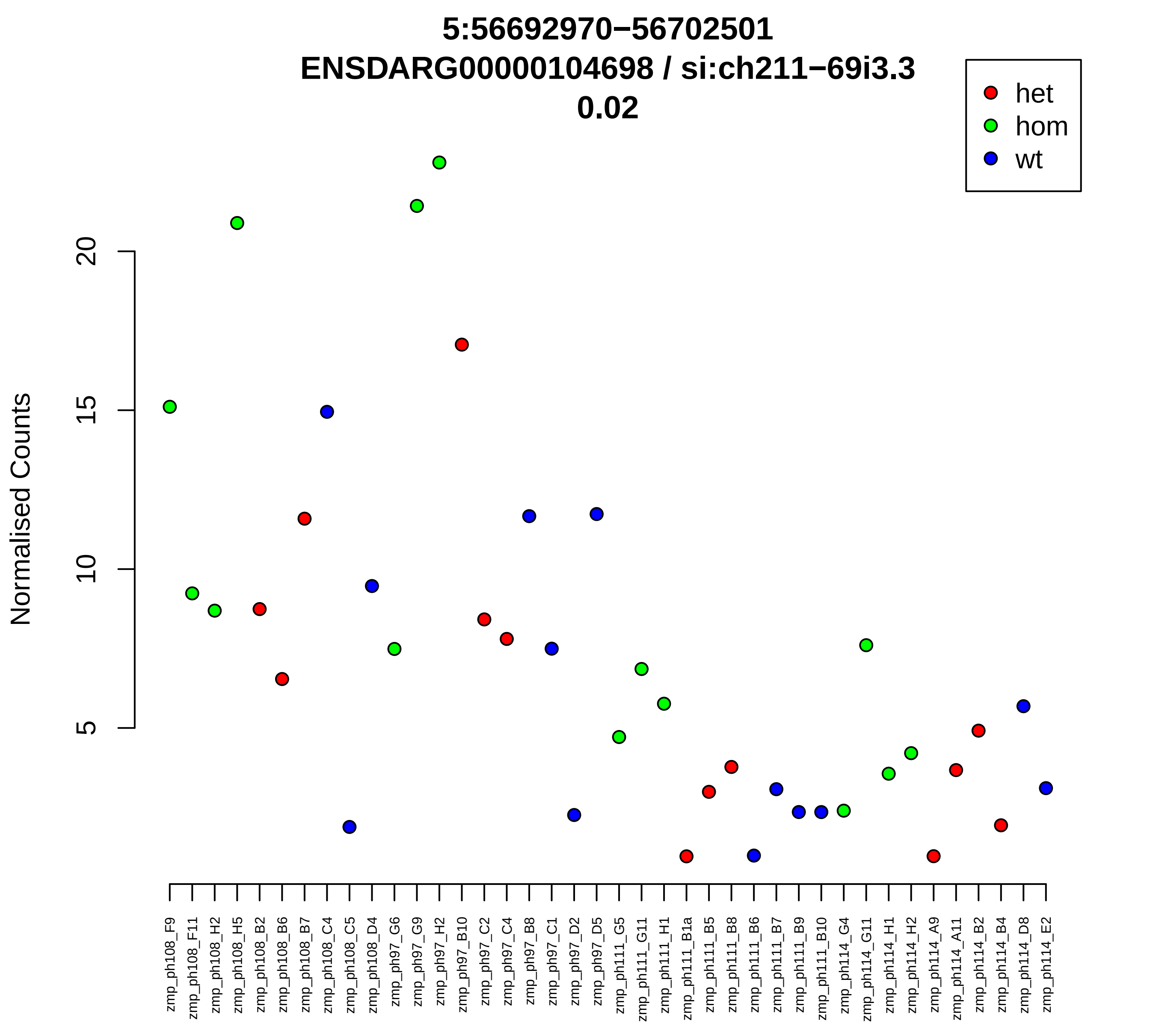

5:56692970−56702501
ENSDARG00000104698 / si:ch211−69i3.3
0.02
het
hom
wt
Normalised Counts
5
10
15
20
zmp_ph108_F9
zmp_ph108_F11
zmp_ph108_H2
zmp_ph108_H5
zmp_ph108_B2
zmp_ph108_B6
zmp_ph108_B7
zmp_ph108_C4
zmp_ph108_C5
zmp_ph108_D4
zmp_ph97_G6
zmp_ph97_G9
zmp_ph97_H2
zmp_ph97_B10
zmp_ph97_C2
zmp_ph97_C4
zmp_ph97_B8
zmp_ph97_C1
zmp_ph97_D2
zmp_ph97_D5
zmp_ph111_G5
zmp_ph111_G11
zmp_ph111_H1
zmp_ph111_B1a
zmp_ph111_B5
zmp_ph111_B8
zmp_ph111_B6
zmp_ph111_B7
zmp_ph111_B9
zmp_ph111_B10
zmp_ph114_G4
zmp_ph114_G11
zmp_ph114_H1
zmp_ph114_H2
zmp_ph114_A9
zmp_ph114_A11
zmp_ph114_B2
zmp_ph114_B4
zmp_ph114_D8
zmp_ph114_E2

3:20916575−20944785
ENSDARG00000006566 / nlk1
0.02

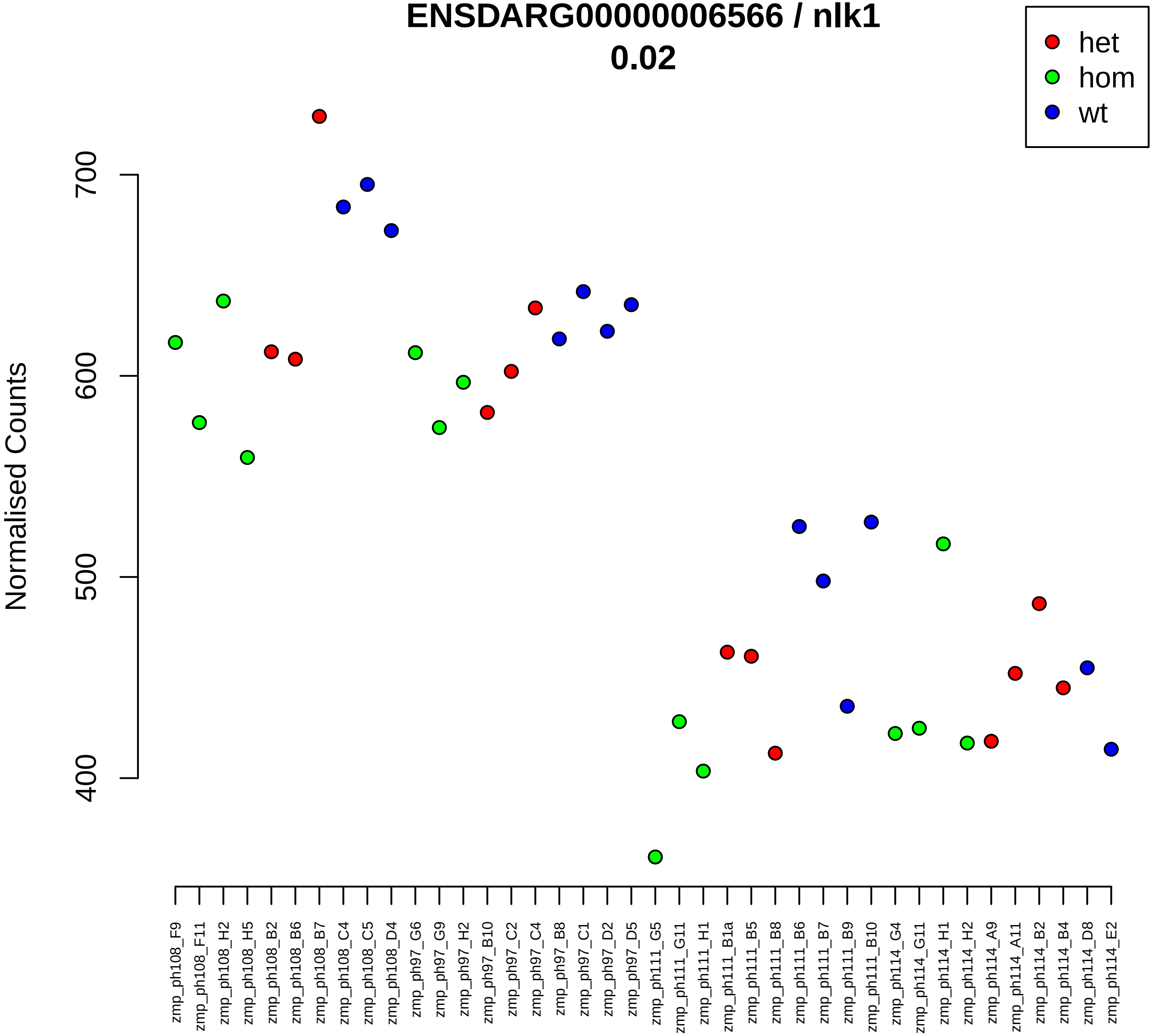

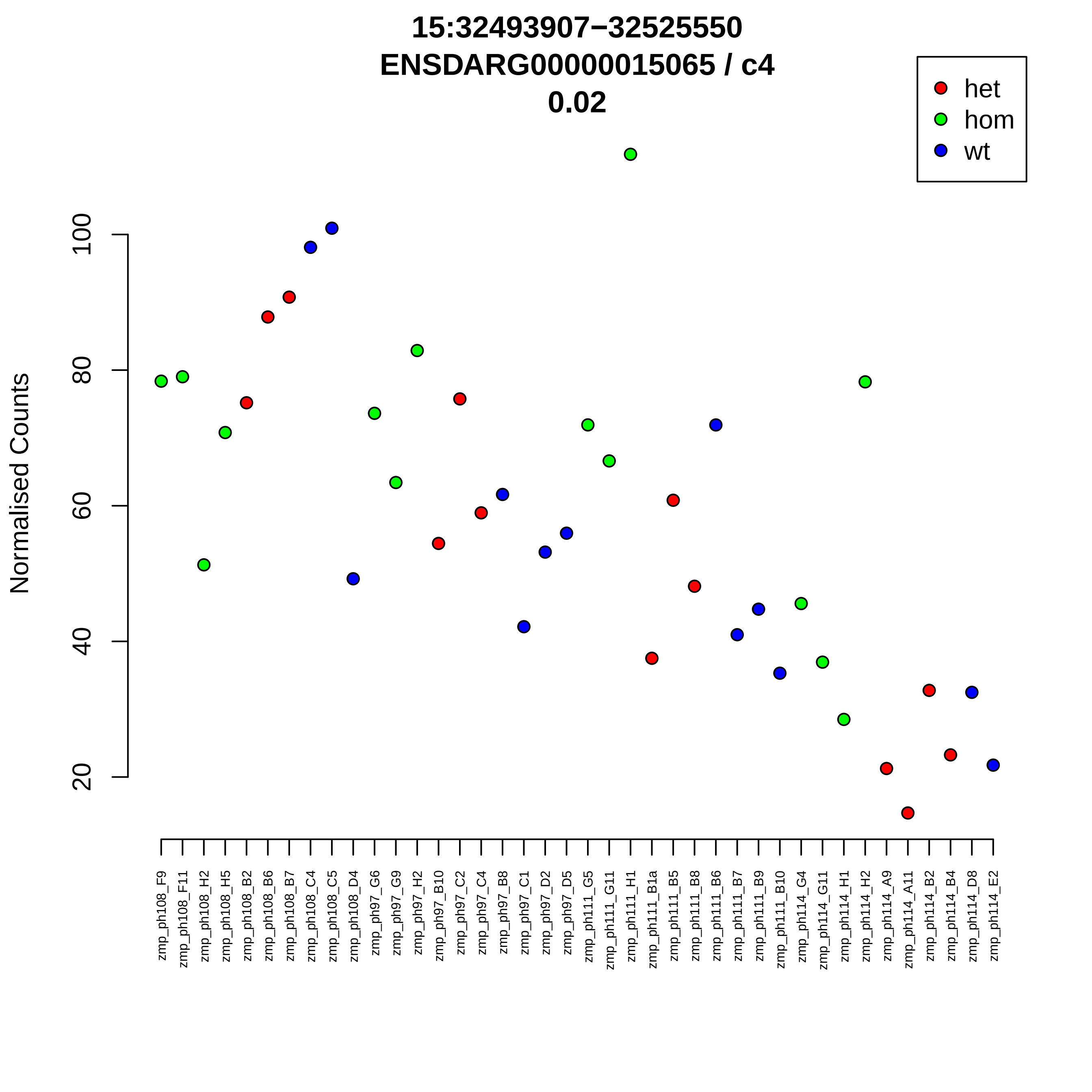
15:32493907−32525550
ENSDARG00000015065 / c4
0.02
het
hom
wt
Normalised Counts
20
40
60
80
100
zmp_ph108_F9
zmp_ph108_F11
zmp_ph108_H2
zmp_ph108_H5
zmp_ph108_B2
zmp_ph108_B6
zmp_ph108_B7
zmp_ph108_C4
zmp_ph108_C5
zmp_ph108_D4
zmp_ph97_G6
zmp_ph97_G9
zmp_ph97_H2
zmp_ph97_B10
zmp_ph97_C2
zmp_ph97_C4
zmp_ph97_B8
zmp_ph97_C1
zmp_ph97_D2
zmp_ph97_D5
zmp_ph111_G5
zmp_ph111_G11
zmp_ph111_H1
zmp_ph111_B1a
zmp_ph111_B5
zmp_ph111_B8
zmp_ph111_B6
zmp_ph111_B7
zmp_ph111_B9
zmp_ph111_B10
zmp_ph114_G4
zmp_ph114_G11
zmp_ph114_H1
zmp_ph114_H2
zmp_ph114_A9
zmp_ph114_A11
zmp_ph114_B2
zmp_ph114_B4
zmp_ph114_D8
zmp_ph114_E2

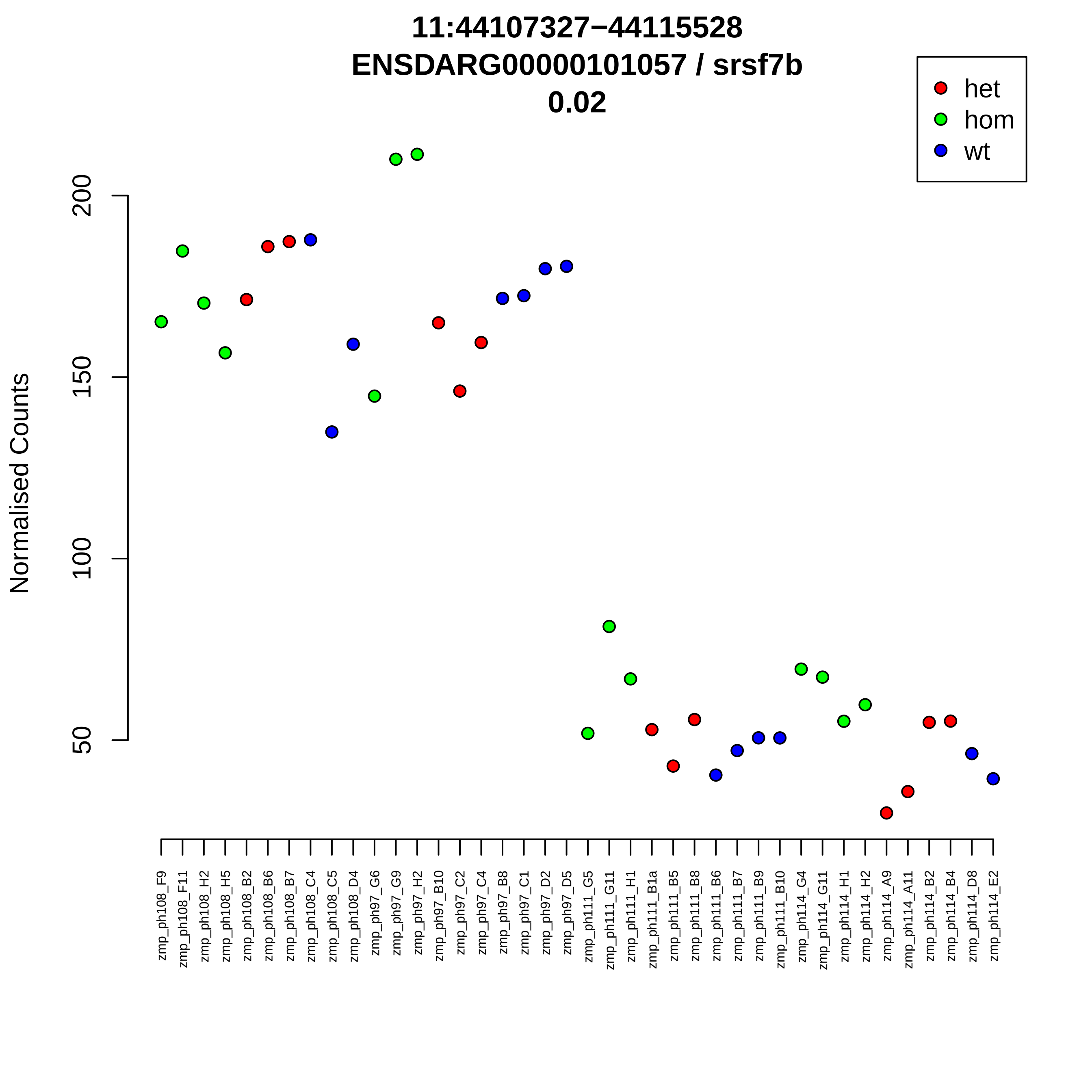
11:44107327−44115528
ENSDARG00000101057 / srsf7b
0.02
het
hom
wt
Normalised Counts
200
150
100
50
zmp_ph108_F9
zmp_ph108_F11
zmp_ph108_H2
zmp_ph108_H5
zmp_ph108_B2
zmp_ph108_B6
zmp_ph108_B7
zmp_ph108_C4
zmp_ph108_C5
zmp_ph108_D4
zmp_ph97_G6
zmp_ph97_G9
zmp_ph97_H2
zmp_ph97_B10
zmp_ph97_C2
zmp_ph97_C4
zmp_ph97_B8
zmp_ph97_C1
zmp_ph97_D2
zmp_ph97_D5
zmp_ph111_G5
zmp_ph111_G11
zmp_ph111_H1
zmp_ph111_B1a
zmp_ph111_B5
zmp_ph111_B8
zmp_ph111_B6
zmp_ph111_B7
zmp_ph111_B9
zmp_ph111_B10
zmp_ph114_G4
zmp_ph114_G11
zmp_ph114_H1
zmp_ph114_H2
zmp_ph114_A9
zmp_ph114_A11
zmp_ph114_B2
zmp_ph114_B4
zmp_ph114_D8
zmp_ph114_E2

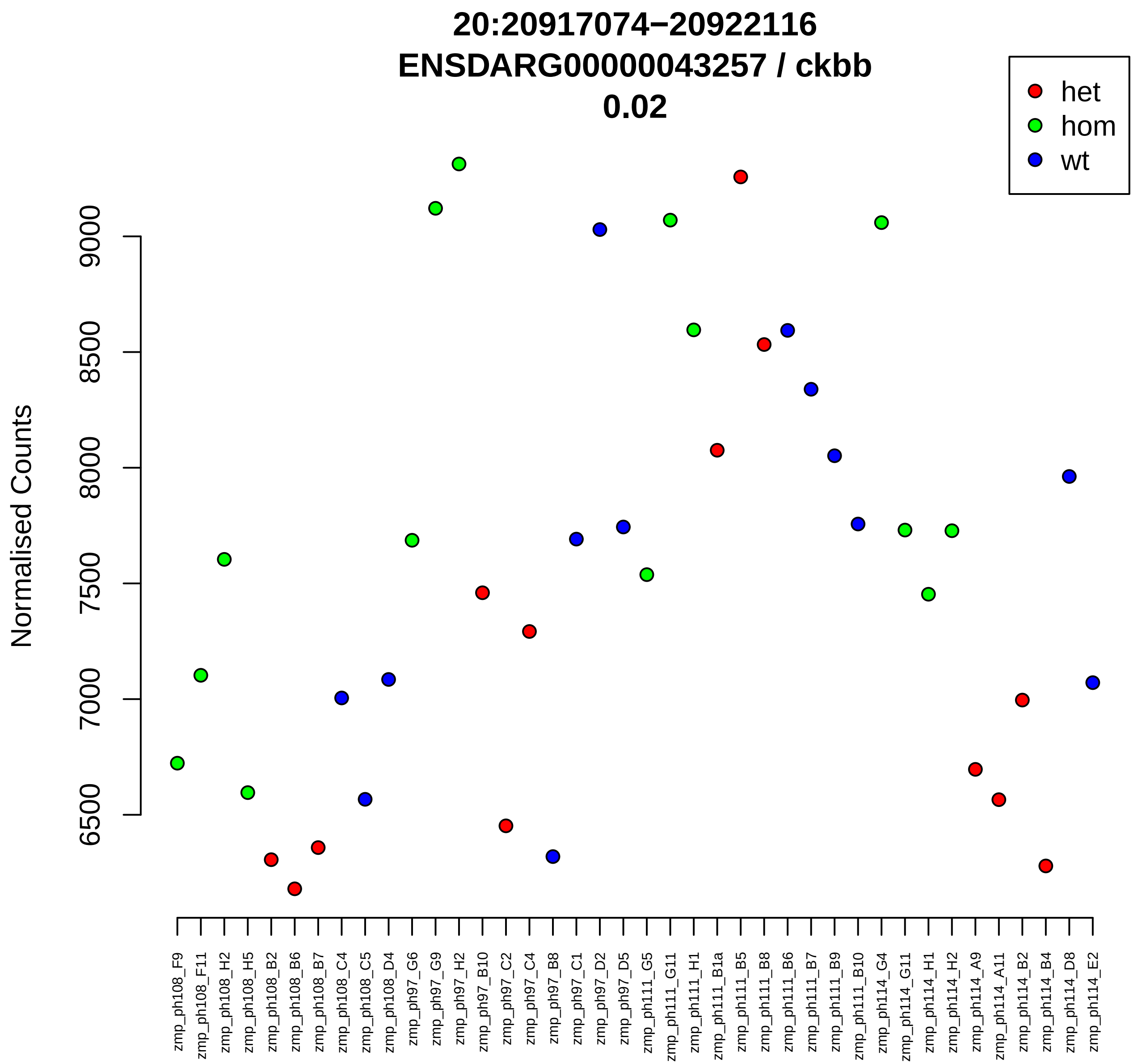
20:20917074−20922116
ENSDARG00000043257 / ckbb
0.02
het
hom
wt
Normalised Counts
6500
7000
7500
8000
8500
9000
zmp_ph108_F9
zmp_ph108_F11
zmp_ph108_H2
zmp_ph108_H5
zmp_ph108_B2
zmp_ph108_B6
zmp_ph108_B7
zmp_ph108_C4
zmp_ph108_C5
zmp_ph108_D4
zmp_ph97_G6
zmp_ph97_G9
zmp_ph97_H2
zmp_ph97_B10
zmp_ph97_C2
zmp_ph97_C4
zmp_ph97_B8
zmp_ph97_C1
zmp_ph97_D2
zmp_ph97_D5
zmp_ph111_G5
zmp_ph111_G11
zmp_ph111_H1
zmp_ph111_B1a
zmp_ph111_B5
zmp_ph111_B8
zmp_ph111_B6
zmp_ph111_B7
zmp_ph111_B9
zmp_ph111_B10
zmp_ph114_G4
zmp_ph114_G11
zmp_ph114_H1
zmp_ph114_H2
zmp_ph114_A9
zmp_ph114_A11
zmp_ph114_B2
zmp_ph114_B4
zmp_ph114_D8
zmp_ph114_E2

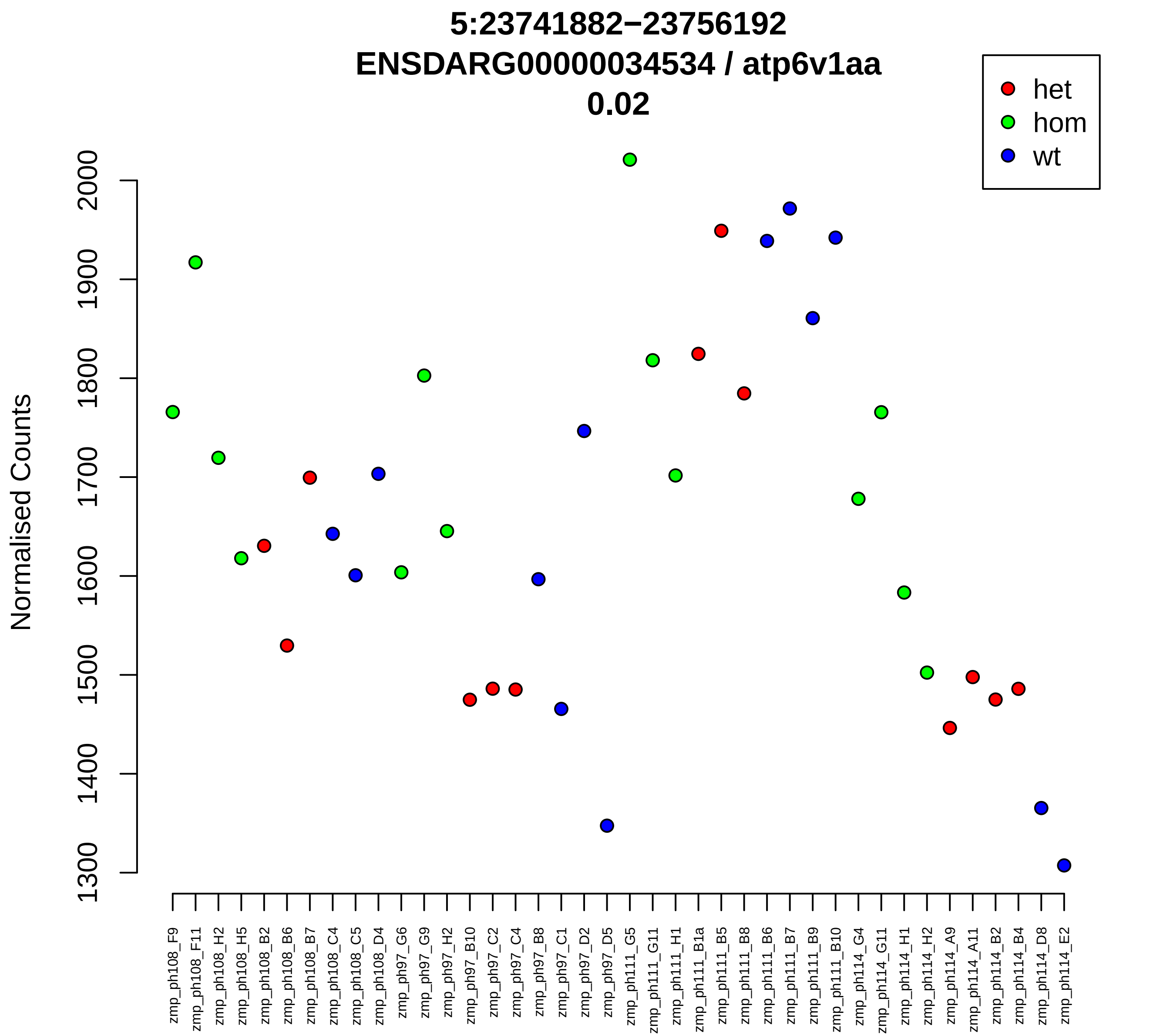

5:23741882−23756192
ENSDARG00000034534 / atp6v1aa
0.02
het
hom
wt
Normalised Counts
1300
1400
1500
1600
1700
1800
1900
2000
zmp_ph108_F9
zmp_ph108_F11
zmp_ph108_H2
zmp_ph108_H5
zmp_ph108_B2
zmp_ph108_B6
zmp_ph108_B7
zmp_ph108_C4
zmp_ph108_C5
zmp_ph108_D4
zmp_ph97_G6
zmp_ph97_G9
zmp_ph97_H2
zmp_ph97_B10
zmp_ph97_C2
zmp_ph97_C4
zmp_ph97_B8
zmp_ph97_C1
zmp_ph97_D2
zmp_ph97_D5
zmp_ph111_G5
zmp_ph111_G11
zmp_ph111_H1
zmp_ph111_B1a
zmp_ph111_B5
zmp_ph111_B8
zmp_ph111_B6
zmp_ph111_B7
zmp_ph111_B9
zmp_ph111_B10
zmp_ph114_G4
zmp_ph114_G11
zmp_ph114_H1
zmp_ph114_H2
zmp_ph114_A9
zmp_ph114_A11
zmp_ph114_B2
zmp_ph114_B4
zmp_ph114_D8
zmp_ph114_E2

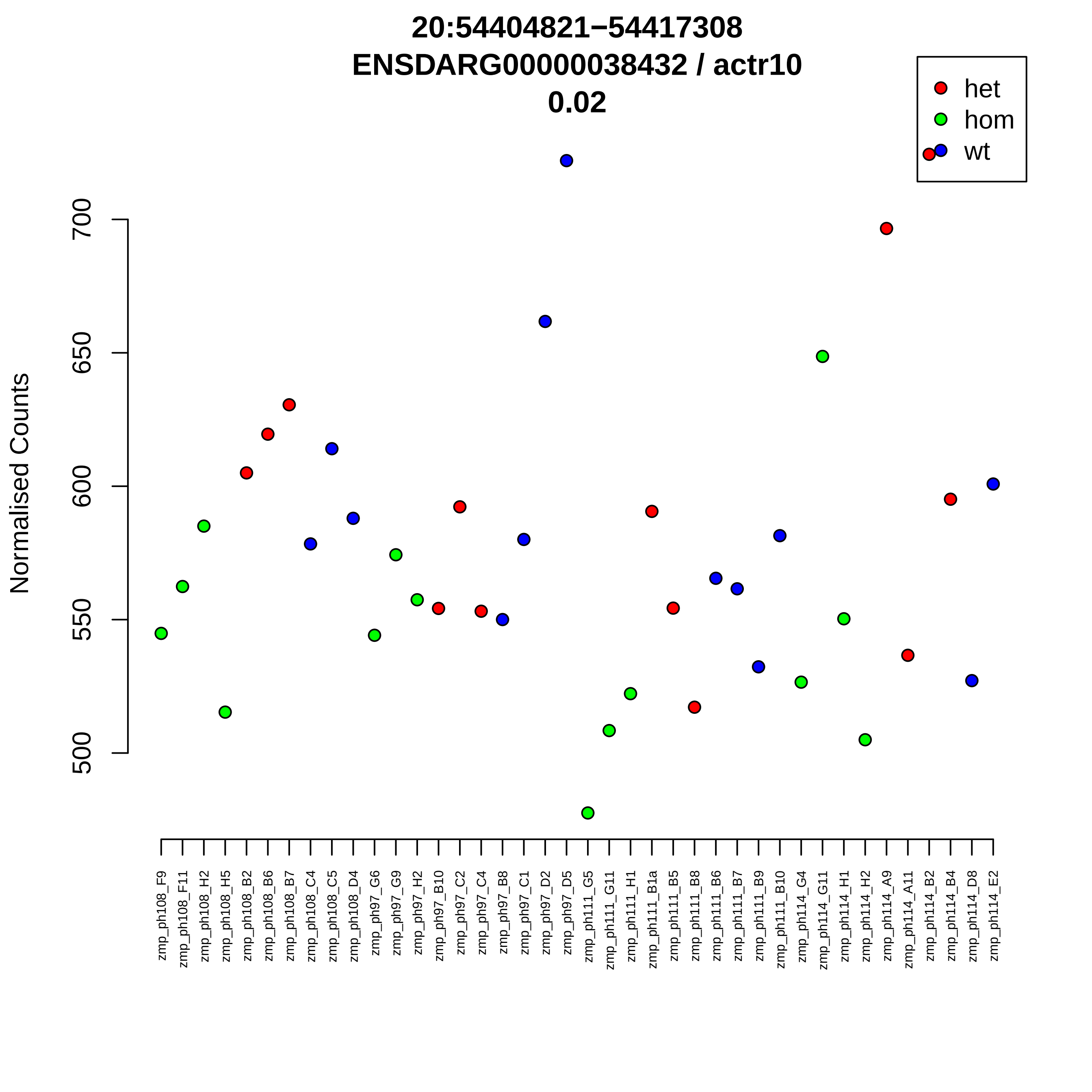

20:54404821−54417308
ENSDARG00000038432 / actr10
0.02
het
hom
wt
Normalised Counts
500
550
600
650
700
zmp_ph108_F9
zmp_ph108_F11
zmp_ph108_H2
zmp_ph108_H5
zmp_ph108_B2
zmp_ph108_B6
zmp_ph108_B7
zmp_ph108_C4
zmp_ph108_C5
zmp_ph108_D4
zmp_ph97_G6
zmp_ph97_G9
zmp_ph97_H2
zmp_ph97_B10
zmp_ph97_C2
zmp_ph97_C4
zmp_ph97_B8
zmp_ph97_C1
zmp_ph97_D2
zmp_ph97_D5
zmp_ph111_G5
zmp_ph111_G11
zmp_ph111_H1
zmp_ph111_B1a
zmp_ph111_B5
zmp_ph111_B8
zmp_ph111_B6
zmp_ph111_B7
zmp_ph111_B9
zmp_ph111_B10
zmp_ph114_G4
zmp_ph114_G11
zmp_ph114_H1
zmp_ph114_H2
zmp_ph114_A9
zmp_ph114_A11
zmp_ph114_B2
zmp_ph114_B4
zmp_ph114_D8
zmp_ph114_E2

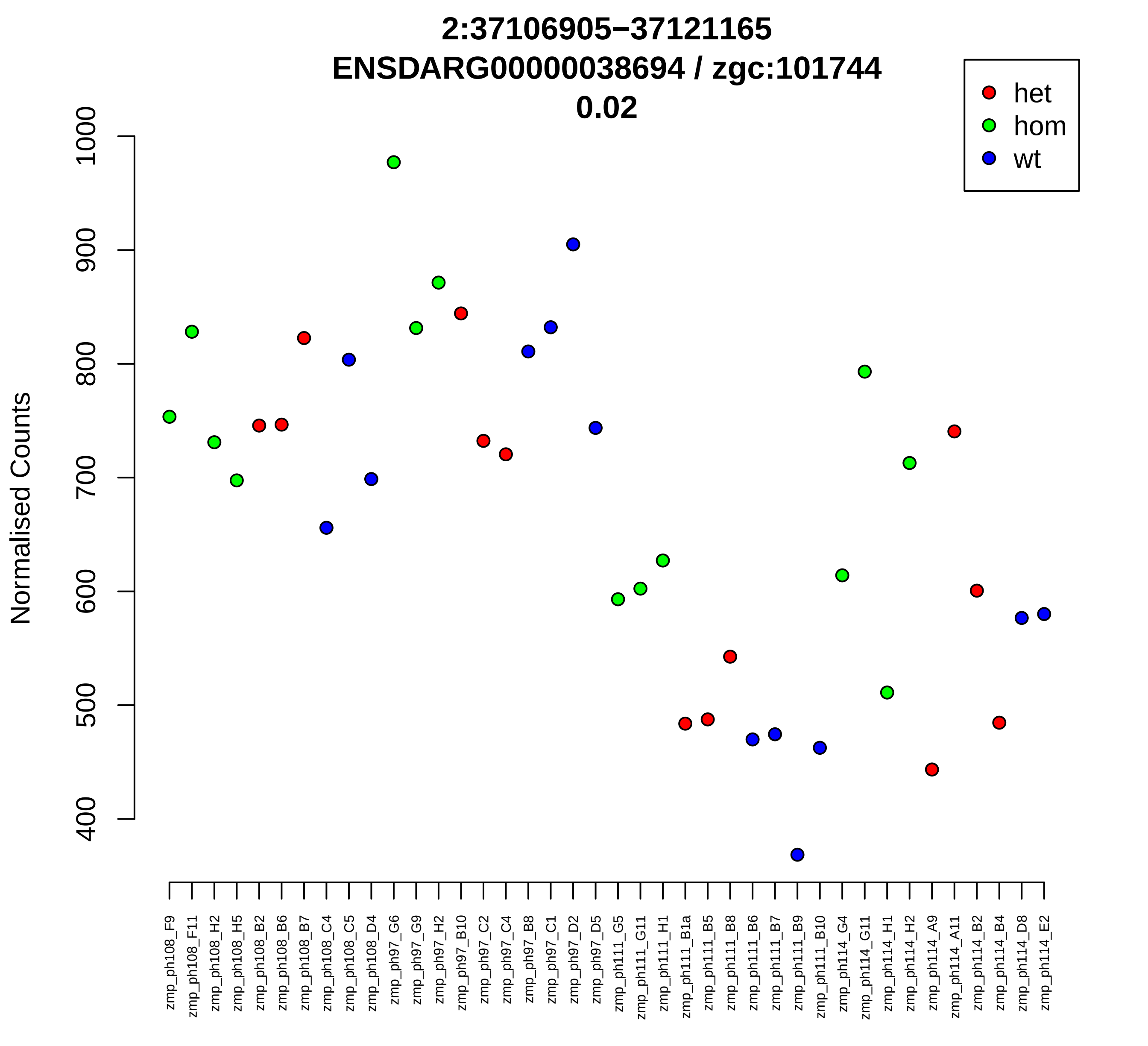
2:37106905−37121165
ENSDARG00000038694 / zgc:101744
0.02
het
hom
wt
Normalised Counts
1000
900
800
700
600
500
400
zmp_ph108_F9
zmp_ph108_F11
zmp_ph108_H2
zmp_ph108_H5
zmp_ph108_B2
zmp_ph108_B6
zmp_ph108_B7
zmp_ph108_C4
zmp_ph108_C5
zmp_ph108_D4
zmp_ph97_G6
zmp_ph97_G9
zmp_ph97_H2
zmp_ph97_B10
zmp_ph97_C2
zmp_ph97_C4
zmp_ph97_B8
zmp_ph97_C1
zmp_ph97_D2
zmp_ph97_D5
zmp_ph111_G5
zmp_ph111_G11
zmp_ph111_H1
zmp_ph111_B1a
zmp_ph111_B5
zmp_ph111_B8
zmp_ph111_B6
zmp_ph111_B7
zmp_ph111_B9
zmp_ph111_B10
zmp_ph114_G4
zmp_ph114_G11
zmp_ph114_H1
zmp_ph114_H2
zmp_ph114_A9
zmp_ph114_A11
zmp_ph114_B2
zmp_ph114_B4
zmp_ph114_D8
zmp_ph114_E2

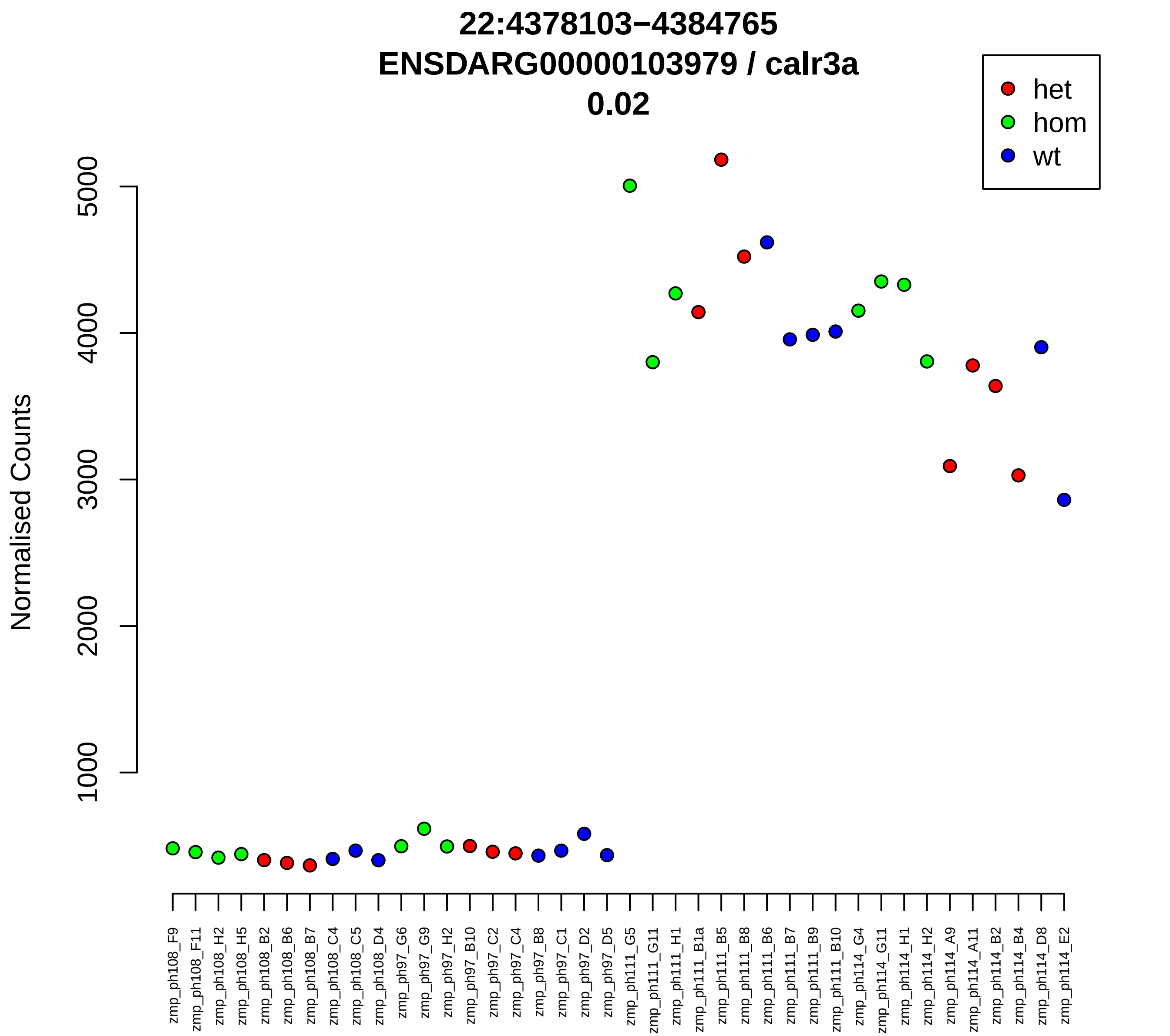
22:4378103−4384765
ENSDARG00000103979 / calr3a
0.02
het
hom
wt
Normalised Counts
1000
2000
3000
4000
5000
zmp_ph108_F9
zmp_ph108_F11
zmp_ph108_H2
zmp_ph108_H5
zmp_ph108_B2
zmp_ph108_B6
zmp_ph108_B7
zmp_ph108_C4
zmp_ph108_C5
zmp_ph108_D4
zmp_ph97_G6
zmp_ph97_G9
zmp_ph97_H2
zmp_ph97_B10
zmp_ph97_C2
zmp_ph97_C4
zmp_ph97_B8
zmp_ph97_C1
zmp_ph97_D2
zmp_ph97_D5
zmp_ph111_G5
zmp_ph111_G11
zmp_ph111_H1
zmp_ph111_B1a
zmp_ph111_B5
zmp_ph111_B8
zmp_ph111_B6
zmp_ph111_B7
zmp_ph111_B9
zmp_ph111_B10
zmp_ph114_G4
zmp_ph114_G11
zmp_ph114_H1
zmp_ph114_H2
zmp_ph114_A9
zmp_ph114_A11
zmp_ph114_B2
zmp_ph114_B4
zmp_ph114_D8
zmp_ph114_E2

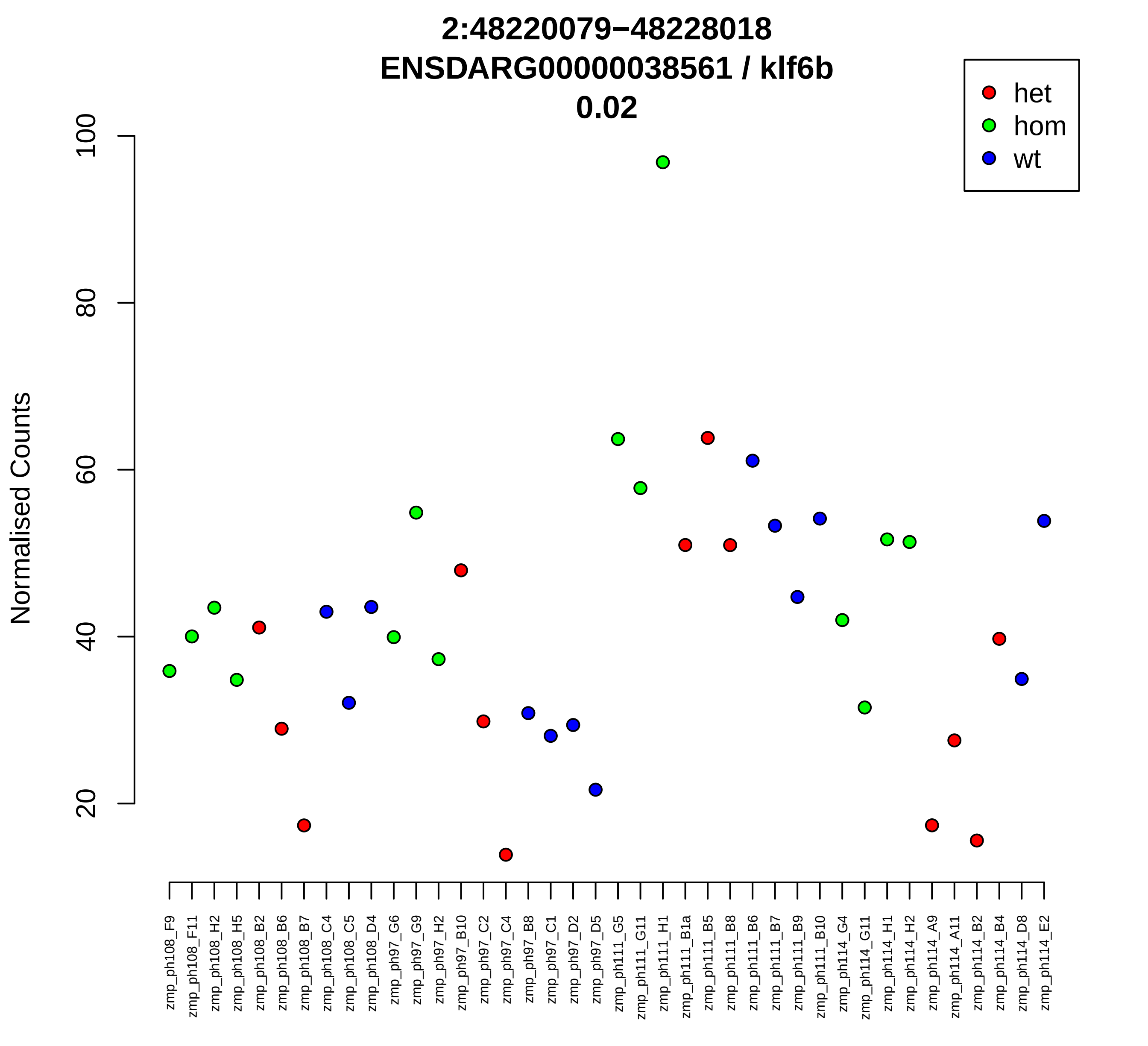
2:48220079−48228018
ENSDARG00000038561 / klf6b
0.02
het
hom
wt
Normalised Counts
100
80
60
40
20
zmp_ph108_F9
zmp_ph108_F11
zmp_ph108_H2
zmp_ph108_H5
zmp_ph108_B2
zmp_ph108_B6
zmp_ph108_B7
zmp_ph108_C4
zmp_ph108_C5
zmp_ph108_D4
zmp_ph97_G6
zmp_ph97_G9
zmp_ph97_H2
zmp_ph97_B10
zmp_ph97_C2
zmp_ph97_C4
zmp_ph97_B8
zmp_ph97_C1
zmp_ph97_D2
zmp_ph97_D5
zmp_ph111_G5
zmp_ph111_G11
zmp_ph111_H1
zmp_ph111_B1a
zmp_ph111_B5
zmp_ph111_B8
zmp_ph111_B6
zmp_ph111_B7
zmp_ph111_B9
zmp_ph111_B10
zmp_ph114_G4
zmp_ph114_G11
zmp_ph114_H1
zmp_ph114_H2
zmp_ph114_A9
zmp_ph114_A11
zmp_ph114_B2
zmp_ph114_B4
zmp_ph114_D8
zmp_ph114_E2

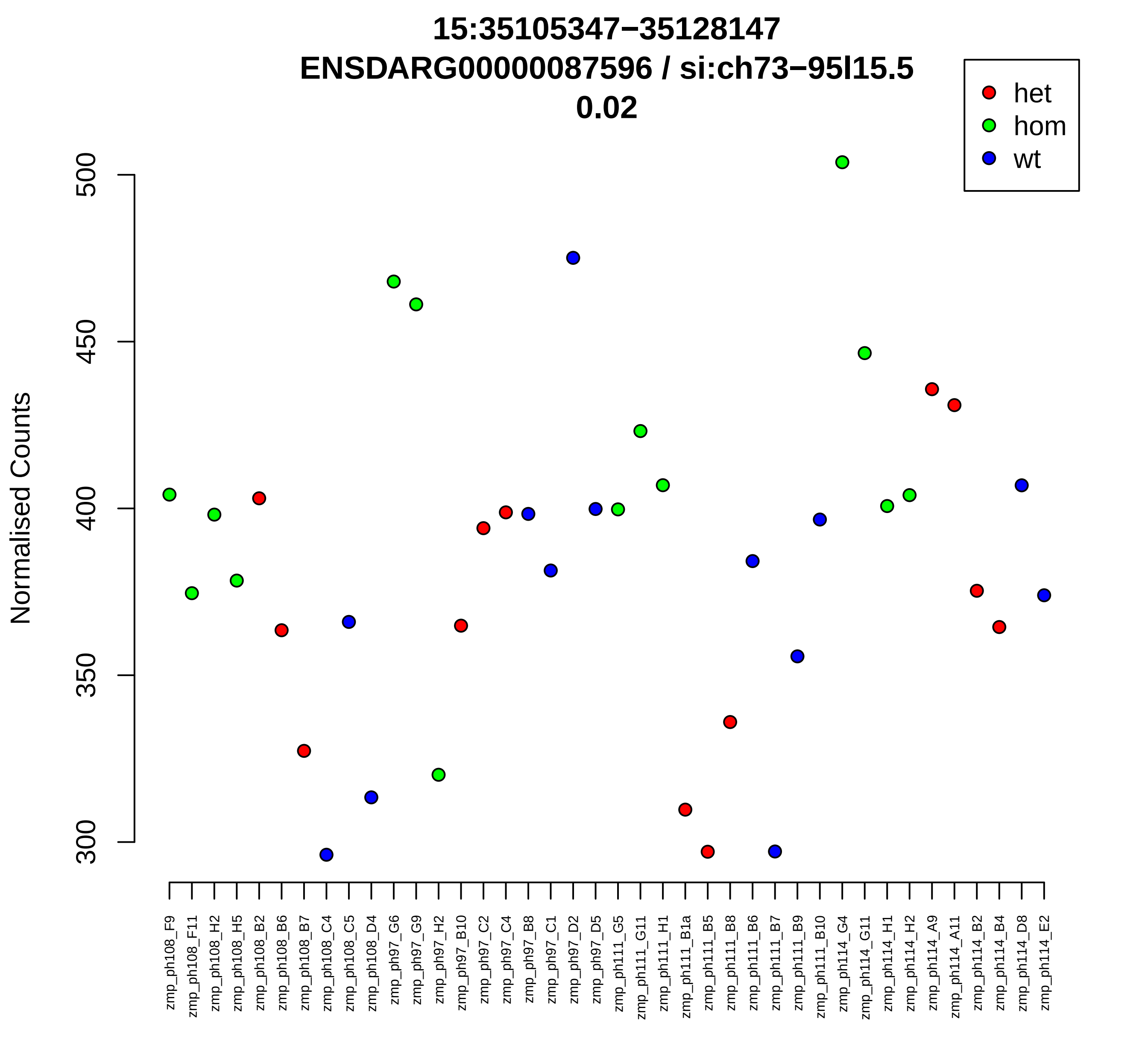

15:35105347−35128147
ENSDARG00000087596 / si:ch73−95l15.5
0.02
Normalised Counts
300
350
400
450
500
het
hom
wt
zmp_ph108_F9
zmp_ph108_F11
zmp_ph108_H2
zmp_ph108_H5
zmp_ph108_B2
zmp_ph108_B6
zmp_ph108_B7
zmp_ph108_C4
zmp_ph108_C5
zmp_ph108_D4
zmp_ph97_G6
zmp_ph97_G9
zmp_ph97_H2
zmp_ph97_B10
zmp_ph97_C2
zmp_ph97_C4
zmp_ph97_B8
zmp_ph97_C1
zmp_ph97_D2
zmp_ph97_D5
zmp_ph111_G5
zmp_ph111_G11
zmp_ph111_H1
zmp_ph111_B1a
zmp_ph111_B5
zmp_ph111_B8
zmp_ph111_B6
zmp_ph111_B7
zmp_ph111_B9
zmp_ph111_B10
zmp_ph114_G4
zmp_ph114_G11
zmp_ph114_H1
zmp_ph114_H2
zmp_ph114_A9
zmp_ph114_A11
zmp_ph114_B2
zmp_ph114_B4
zmp_ph114_D8
zmp_ph114_E2

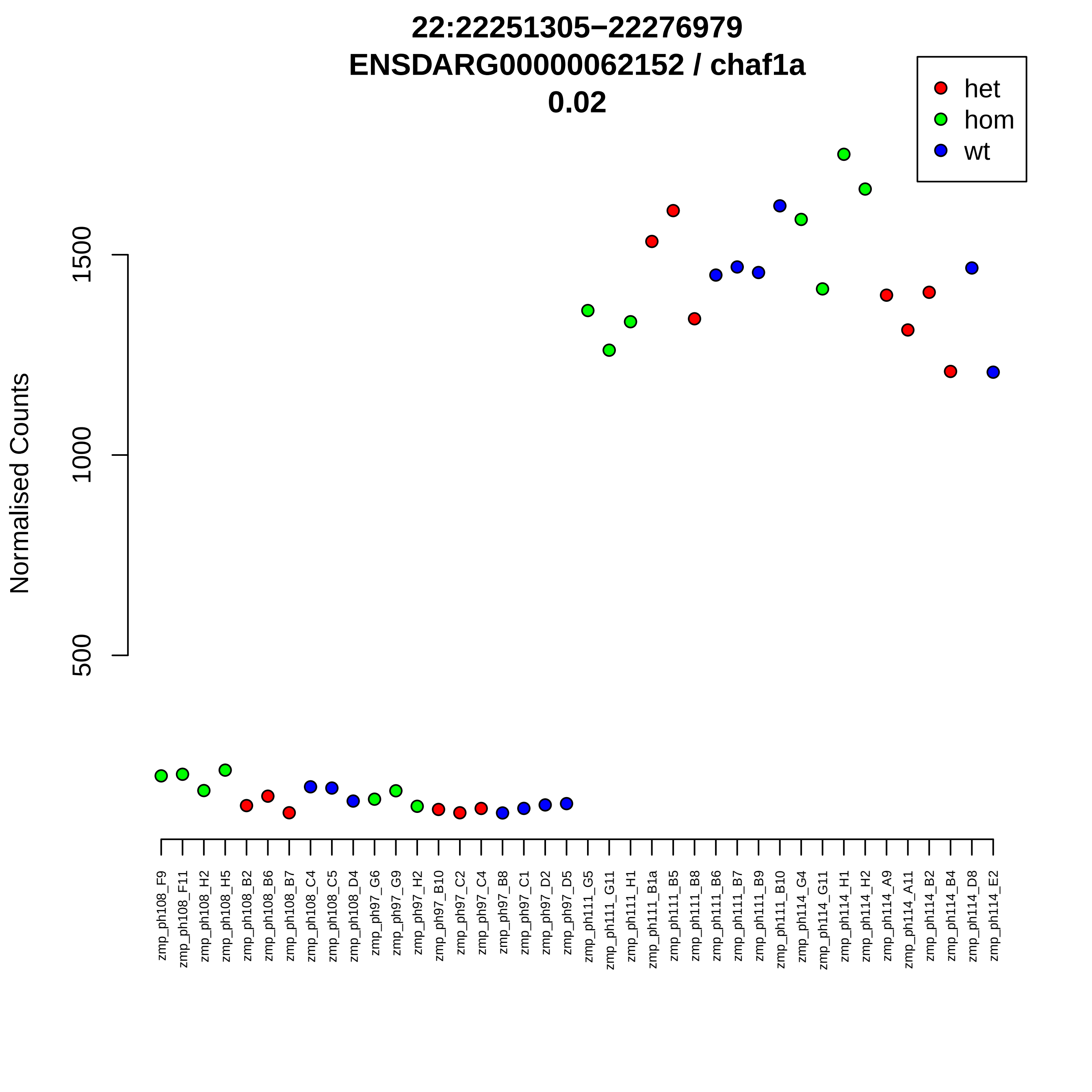

22:22251305−22276979
ENSDARG00000062152 / chaf1a
0.02
het
hom
wt
Normalised Counts
500
1000
1500
zmp_ph108_F9
zmp_ph108_F11
zmp_ph108_H2
zmp_ph108_H5
zmp_ph108_B2
zmp_ph108_B6
zmp_ph108_B7
zmp_ph108_C4
zmp_ph108_C5
zmp_ph108_D4
zmp_ph97_G6
zmp_ph97_G9
zmp_ph97_H2
zmp_ph97_B10
zmp_ph97_C2
zmp_ph97_C4
zmp_ph97_B8
zmp_ph97_C1
zmp_ph97_D2
zmp_ph97_D5
zmp_ph111_G5
zmp_ph111_G11
zmp_ph111_H1
zmp_ph111_B1a
zmp_ph111_B5
zmp_ph111_B8
zmp_ph111_B6
zmp_ph111_B7
zmp_ph111_B9
zmp_ph111_B10
zmp_ph114_G4
zmp_ph114_G11
zmp_ph114_H1
zmp_ph114_H2
zmp_ph114_A9
zmp_ph114_A11
zmp_ph114_B2
zmp_ph114_B4
zmp_ph114_D8
zmp_ph114_E2

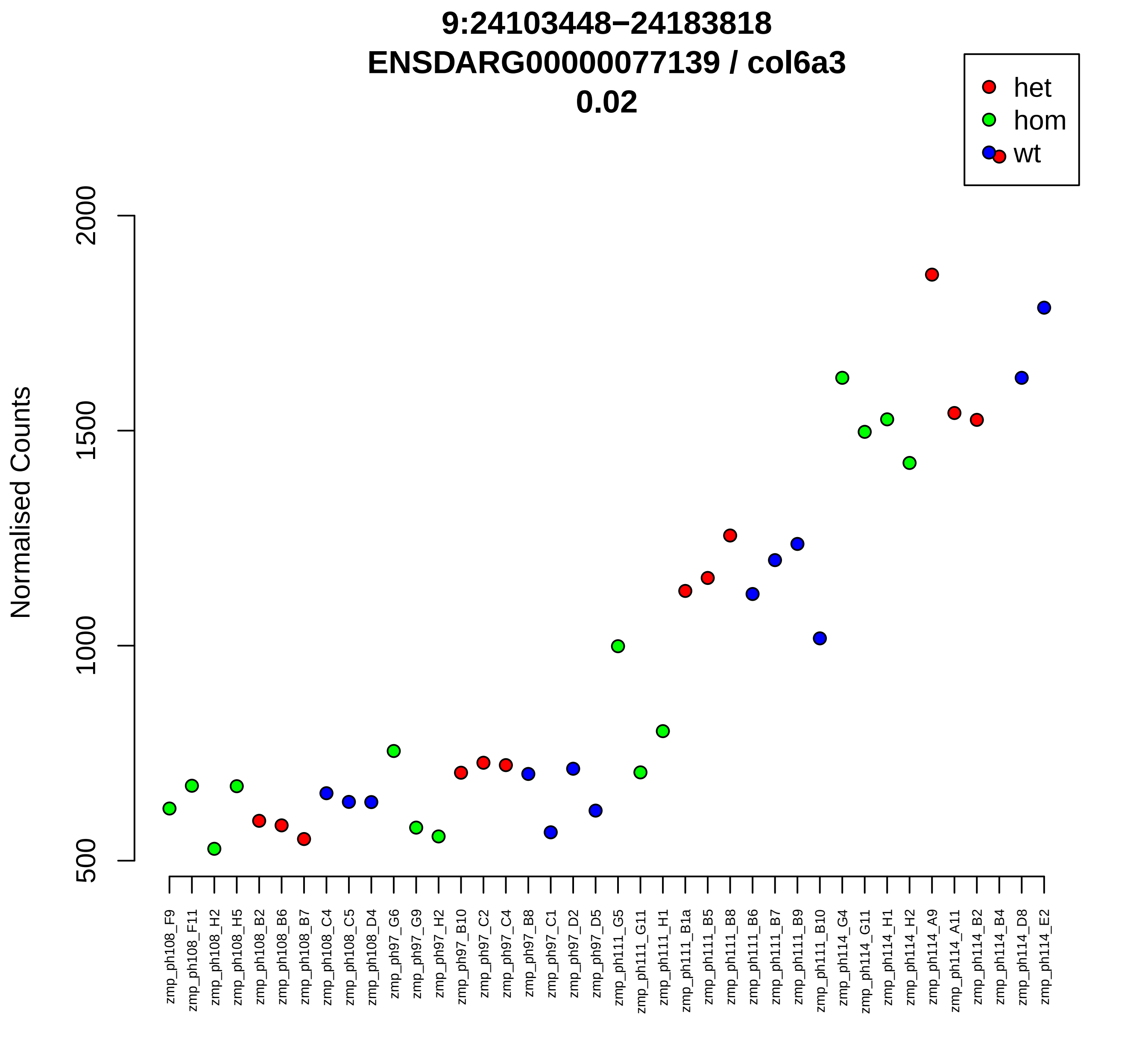

9:24103448−24183818
ENSDARG00000077139 / col6a3
0.02
het
hom
wt
Normalised Counts
500
1000
1500
2000
zmp_ph108_F9
zmp_ph108_F11
zmp_ph108_H2
zmp_ph108_H5
zmp_ph108_B2
zmp_ph108_B6
zmp_ph108_B7
zmp_ph108_C4
zmp_ph108_C5
zmp_ph108_D4
zmp_ph97_G6
zmp_ph97_G9
zmp_ph97_H2
zmp_ph97_B10
zmp_ph97_C2
zmp_ph97_C4
zmp_ph97_B8
zmp_ph97_C1
zmp_ph97_D2
zmp_ph97_D5
zmp_ph111_G5
zmp_ph111_G11
zmp_ph111_H1
zmp_ph111_B1a
zmp_ph111_B5
zmp_ph111_B8
zmp_ph111_B6
zmp_ph111_B7
zmp_ph111_B9
zmp_ph111_B10
zmp_ph114_G4
zmp_ph114_G11
zmp_ph114_H1
zmp_ph114_H2
zmp_ph114_A9
zmp_ph114_A11
zmp_ph114_B2
zmp_ph114_B4
zmp_ph114_D8
zmp_ph114_E2

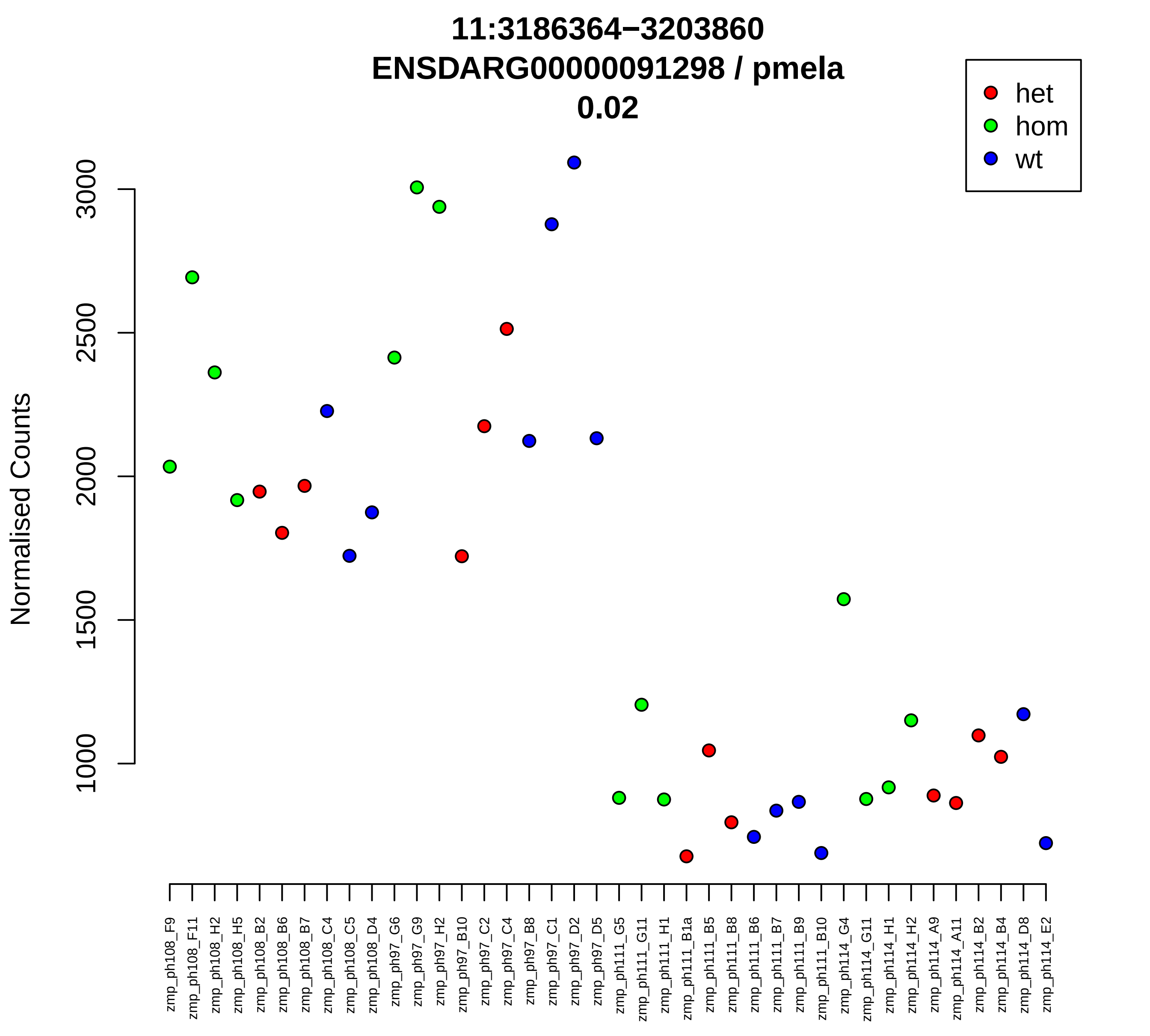
11:3186364−3203860
ENSDARG00000091298 / pmela
0.02
het
hom
wt
Normalised Counts
1000
1500
2000
2500
3000
zmp_ph108_F9
zmp_ph108_F11
zmp_ph108_H2
zmp_ph108_H5
zmp_ph108_B2
zmp_ph108_B6
zmp_ph108_B7
zmp_ph108_C4
zmp_ph108_C5
zmp_ph108_D4
zmp_ph97_G6
zmp_ph97_G9
zmp_ph97_H2
zmp_ph97_B10
zmp_ph97_C2
zmp_ph97_C4
zmp_ph97_B8
zmp_ph97_C1
zmp_ph97_D2
zmp_ph97_D5
zmp_ph111_G5
zmp_ph111_G11
zmp_ph111_H1
zmp_ph111_B1a
zmp_ph111_B5
zmp_ph111_B8
zmp_ph111_B6
zmp_ph111_B7
zmp_ph111_B9
zmp_ph111_B10
zmp_ph114_G4
zmp_ph114_G11
zmp_ph114_H1
zmp_ph114_H2
zmp_ph114_A9
zmp_ph114_A11
zmp_ph114_B2
zmp_ph114_B4
zmp_ph114_D8
zmp_ph114_E2

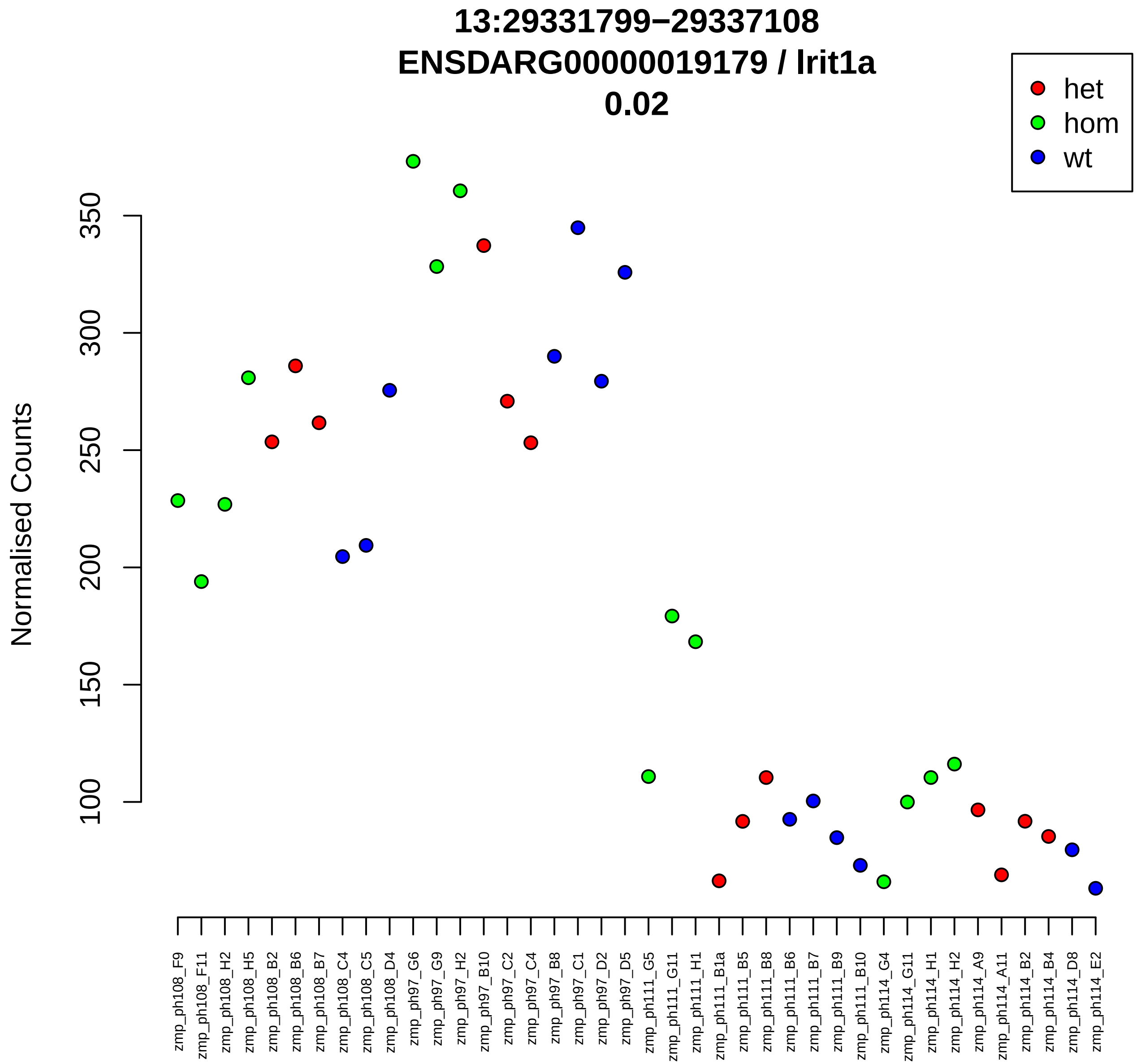

13:29331799−29337108
ENSDARG00000019179 / lrit1a
0.02
het
hom
wt
Normalised Counts
100
150
200
250
300
350
zmp_ph108_F9
zmp_ph108_F11
zmp_ph108_H2
zmp_ph108_H5
zmp_ph108_B2
zmp_ph108_B6
zmp_ph108_B7
zmp_ph108_C4
zmp_ph108_C5
zmp_ph108_D4
zmp_ph97_G6
zmp_ph97_G9
zmp_ph97_H2
zmp_ph97_B10
zmp_ph97_C2
zmp_ph97_C4
zmp_ph97_B8
zmp_ph97_C1
zmp_ph97_D2
zmp_ph97_D5
zmp_ph111_G5
zmp_ph111_G11
zmp_ph111_H1
zmp_ph111_B1a
zmp_ph111_B5
zmp_ph111_B8
zmp_ph111_B6
zmp_ph111_B7
zmp_ph111_B9
zmp_ph111_B10
zmp_ph114_G4
zmp_ph114_G11
zmp_ph114_H1
zmp_ph114_H2
zmp_ph114_A9
zmp_ph114_A11
zmp_ph114_B2
zmp_ph114_B4
zmp_ph114_D8
zmp_ph114_E2

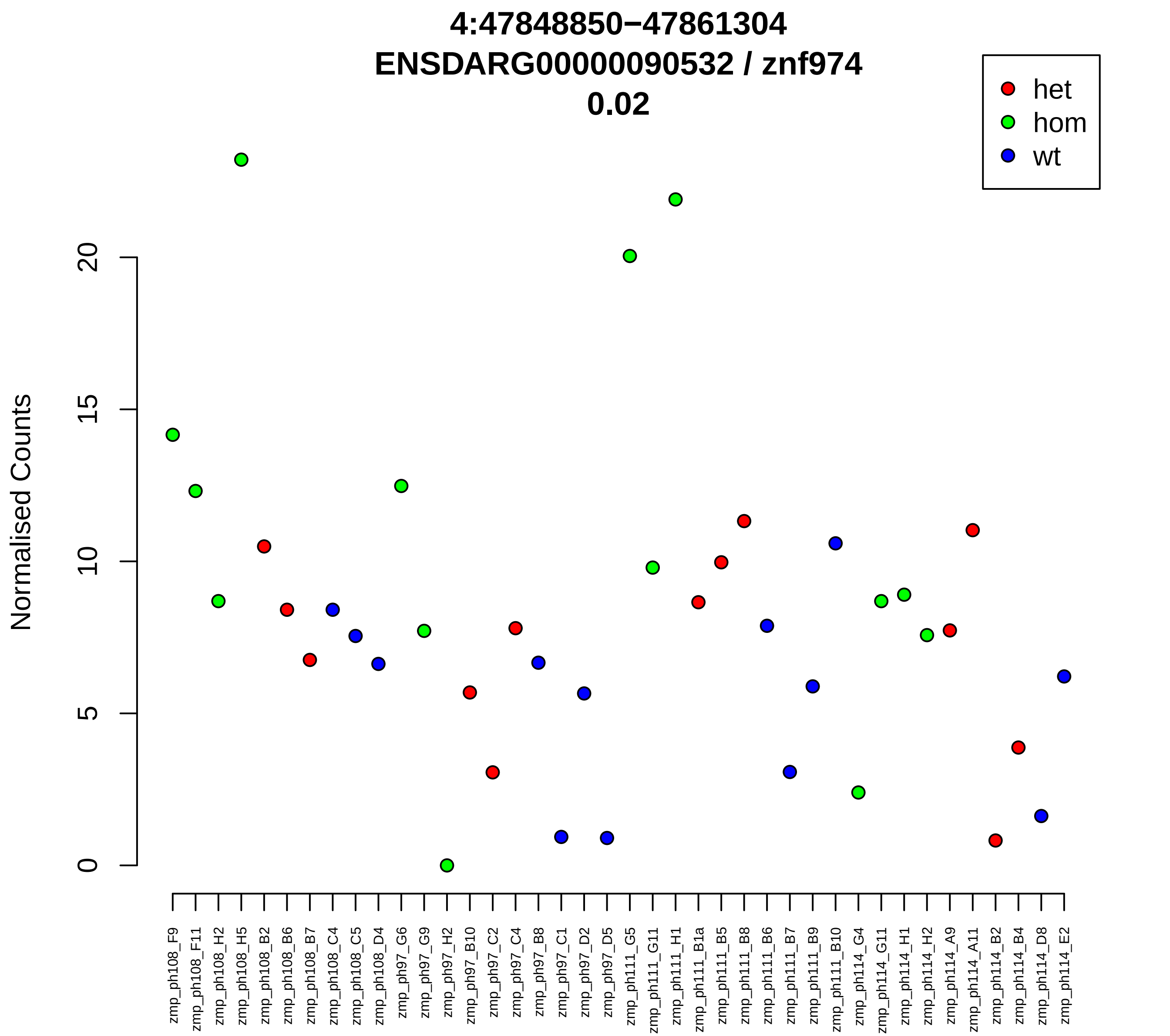

4:47848850−47861304
ENSDARG00000090532 / znf974
0.02
het
hom
wt
Normalised Counts
0
5
10
15
20
zmp_ph108_F9
zmp_ph108_F11
zmp_ph108_H2
zmp_ph108_H5
zmp_ph108_B2
zmp_ph108_B6
zmp_ph108_B7
zmp_ph108_C4
zmp_ph108_C5
zmp_ph108_D4
zmp_ph97_G6
zmp_ph97_G9
zmp_ph97_H2
zmp_ph97_B10
zmp_ph97_C2
zmp_ph97_C4
zmp_ph97_B8
zmp_ph97_C1
zmp_ph97_D2
zmp_ph97_D5
zmp_ph111_G5
zmp_ph111_G11
zmp_ph111_H1
zmp_ph111_B1a
zmp_ph111_B5
zmp_ph111_B8
zmp_ph111_B6
zmp_ph111_B7
zmp_ph111_B9
zmp_ph111_B10
zmp_ph114_G4
zmp_ph114_G11
zmp_ph114_H1
zmp_ph114_H2
zmp_ph114_A9
zmp_ph114_A11
zmp_ph114_B2
zmp_ph114_B4
zmp_ph114_D8
zmp_ph114_E2

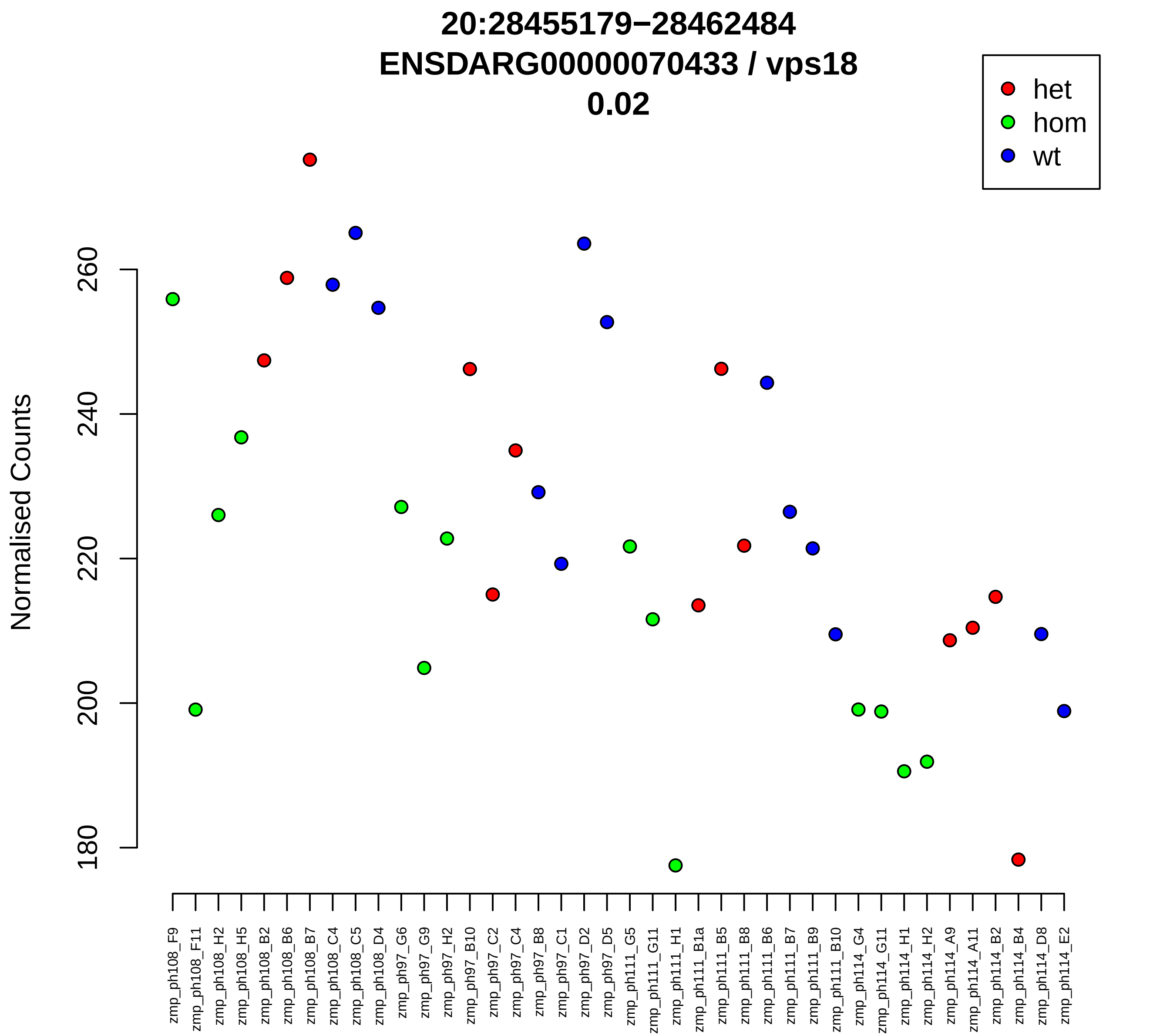
20:28455179−28462484
ENSDARG00000070433 / vps18
0.02
het
hom
wt
Normalised Counts
180
200
220
240
260
zmp_ph108_F9
zmp_ph108_F11
zmp_ph108_H2
zmp_ph108_H5
zmp_ph108_B2
zmp_ph108_B6
zmp_ph108_B7
zmp_ph108_C4
zmp_ph108_C5
zmp_ph108_D4
zmp_ph97_G6
zmp_ph97_G9
zmp_ph97_H2
zmp_ph97_B10
zmp_ph97_C2
zmp_ph97_C4
zmp_ph97_B8
zmp_ph97_C1
zmp_ph97_D2
zmp_ph97_D5
zmp_ph111_G5
zmp_ph111_G11
zmp_ph111_H1
zmp_ph111_B1a
zmp_ph111_B5
zmp_ph111_B8
zmp_ph111_B6
zmp_ph111_B7
zmp_ph111_B9
zmp_ph111_B10
zmp_ph114_G4
zmp_ph114_G11
zmp_ph114_H1
zmp_ph114_H2
zmp_ph114_A9
zmp_ph114_A11
zmp_ph114_B2
zmp_ph114_B4
zmp_ph114_D8
zmp_ph114_E2

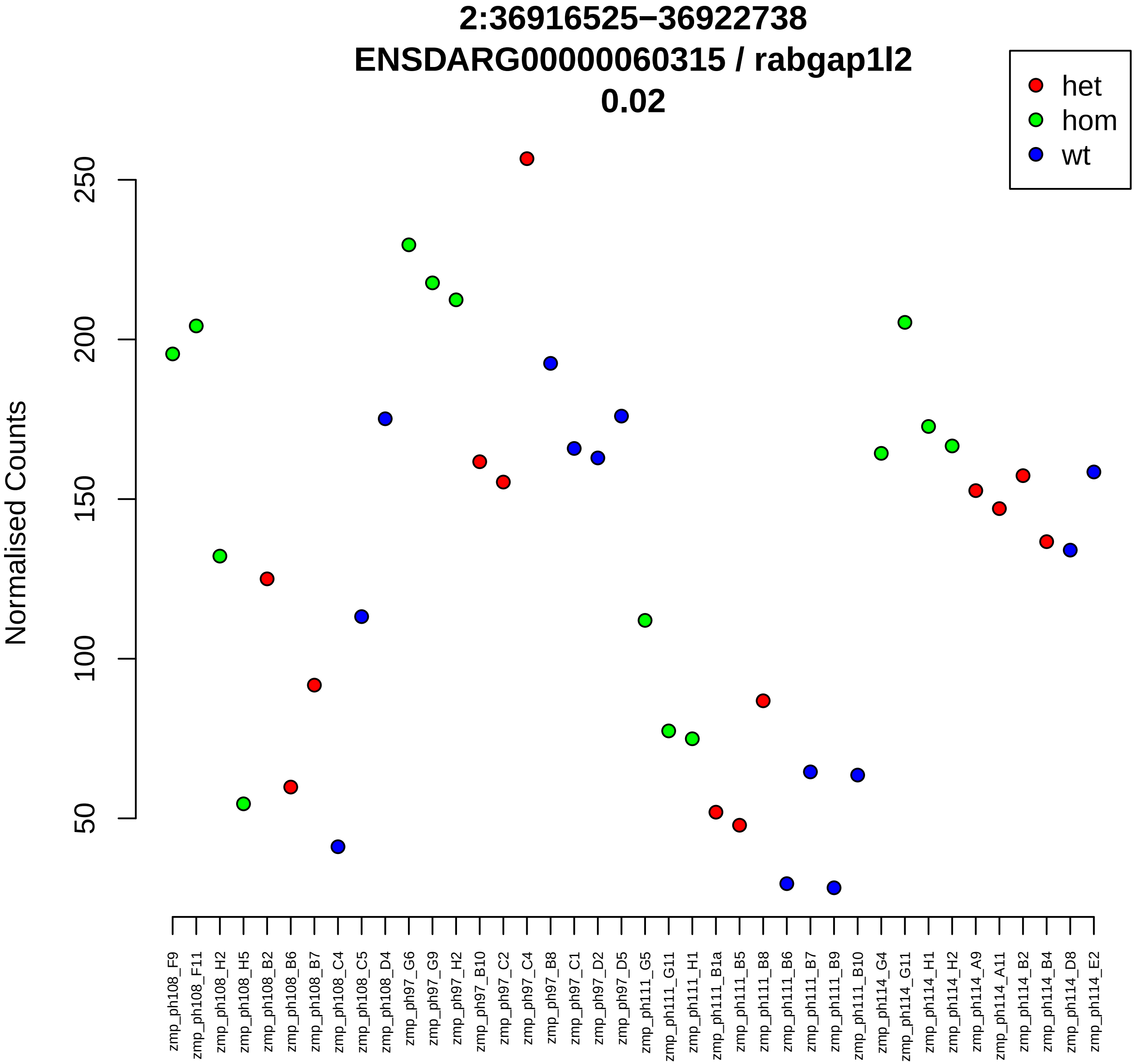

2:36916525−36922738
ENSDARG00000060315 / rabgap1l2
0.02
het
hom
wt
Normalised Counts
50
100
150
200
250
zmp_ph108_F9
zmp_ph108_F11
zmp_ph108_H2
zmp_ph108_H5
zmp_ph108_B2
zmp_ph108_B6
zmp_ph108_B7
zmp_ph108_C4
zmp_ph108_C5
zmp_ph108_D4
zmp_ph97_G6
zmp_ph97_G9
zmp_ph97_H2
zmp_ph97_B10
zmp_ph97_C2
zmp_ph97_C4
zmp_ph97_B8
zmp_ph97_C1
zmp_ph97_D2
zmp_ph97_D5
zmp_ph111_G5
zmp_ph111_G11
zmp_ph111_H1
zmp_ph111_B1a
zmp_ph111_B5
zmp_ph111_B8
zmp_ph111_B6
zmp_ph111_B7
zmp_ph111_B9
zmp_ph111_B10
zmp_ph114_G4
zmp_ph114_G11
zmp_ph114_H1
zmp_ph114_H2
zmp_ph114_A9
zmp_ph114_A11
zmp_ph114_B2
zmp_ph114_B4
zmp_ph114_D8
zmp_ph114_E2

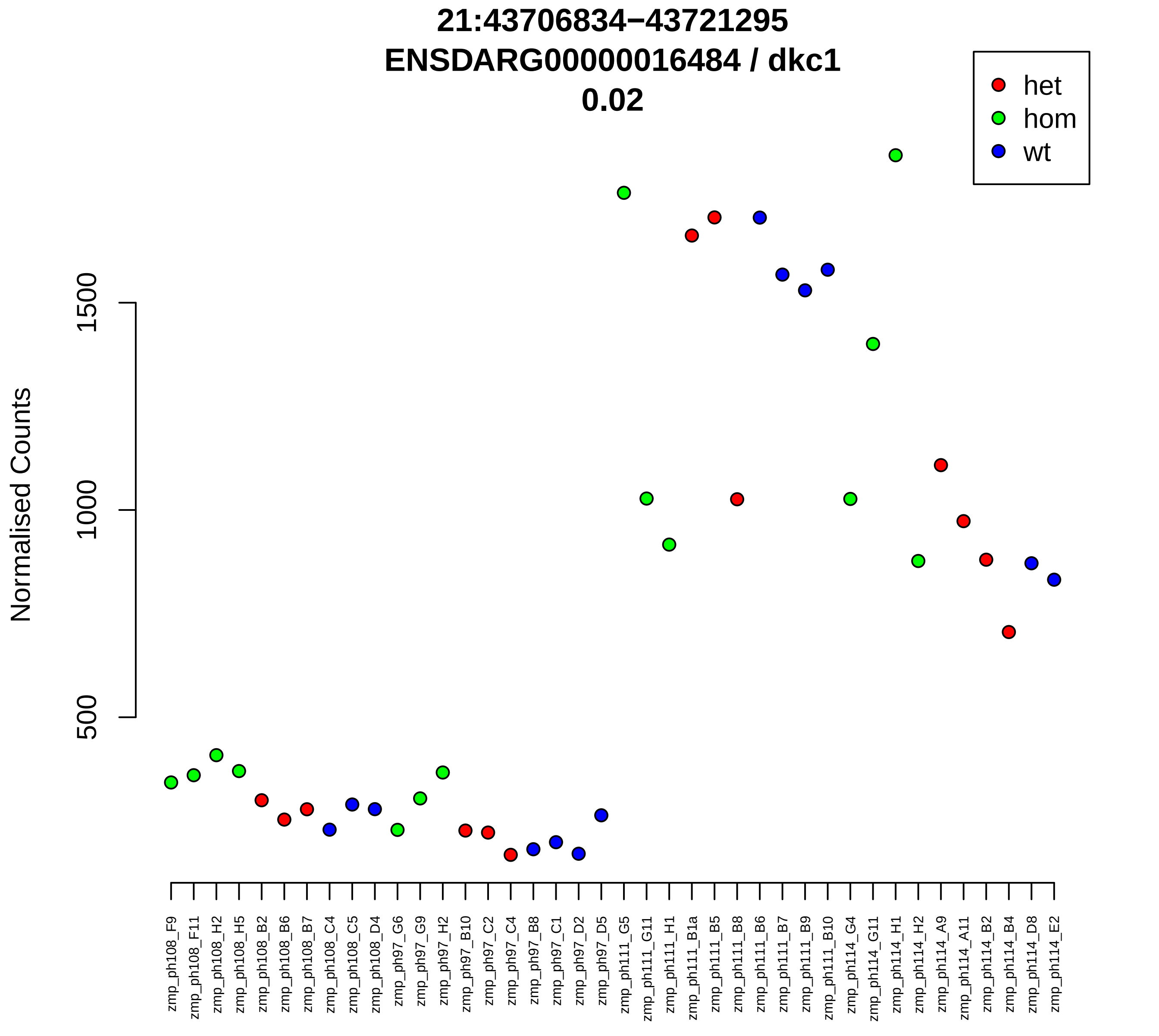

21:43706834−43721295
ENSDARG00000016484 / dkc1
0.02
het
hom
wt
Normalised Counts
500
1000
1500
zmp_ph108_F9
zmp_ph108_F11
zmp_ph108_H2
zmp_ph108_H5
zmp_ph108_B2
zmp_ph108_B6
zmp_ph108_B7
zmp_ph108_C4
zmp_ph108_C5
zmp_ph108_D4
zmp_ph97_G6
zmp_ph97_G9
zmp_ph97_H2
zmp_ph97_B10
zmp_ph97_C2
zmp_ph97_C4
zmp_ph97_B8
zmp_ph97_C1
zmp_ph97_D2
zmp_ph97_D5
zmp_ph111_G5
zmp_ph111_G11
zmp_ph111_H1
zmp_ph111_B1a
zmp_ph111_B5
zmp_ph111_B8
zmp_ph111_B6
zmp_ph111_B7
zmp_ph111_B9
zmp_ph111_B10
zmp_ph114_G4
zmp_ph114_G11
zmp_ph114_H1
zmp_ph114_H2
zmp_ph114_A9
zmp_ph114_A11
zmp_ph114_B2
zmp_ph114_B4
zmp_ph114_D8
zmp_ph114_E2

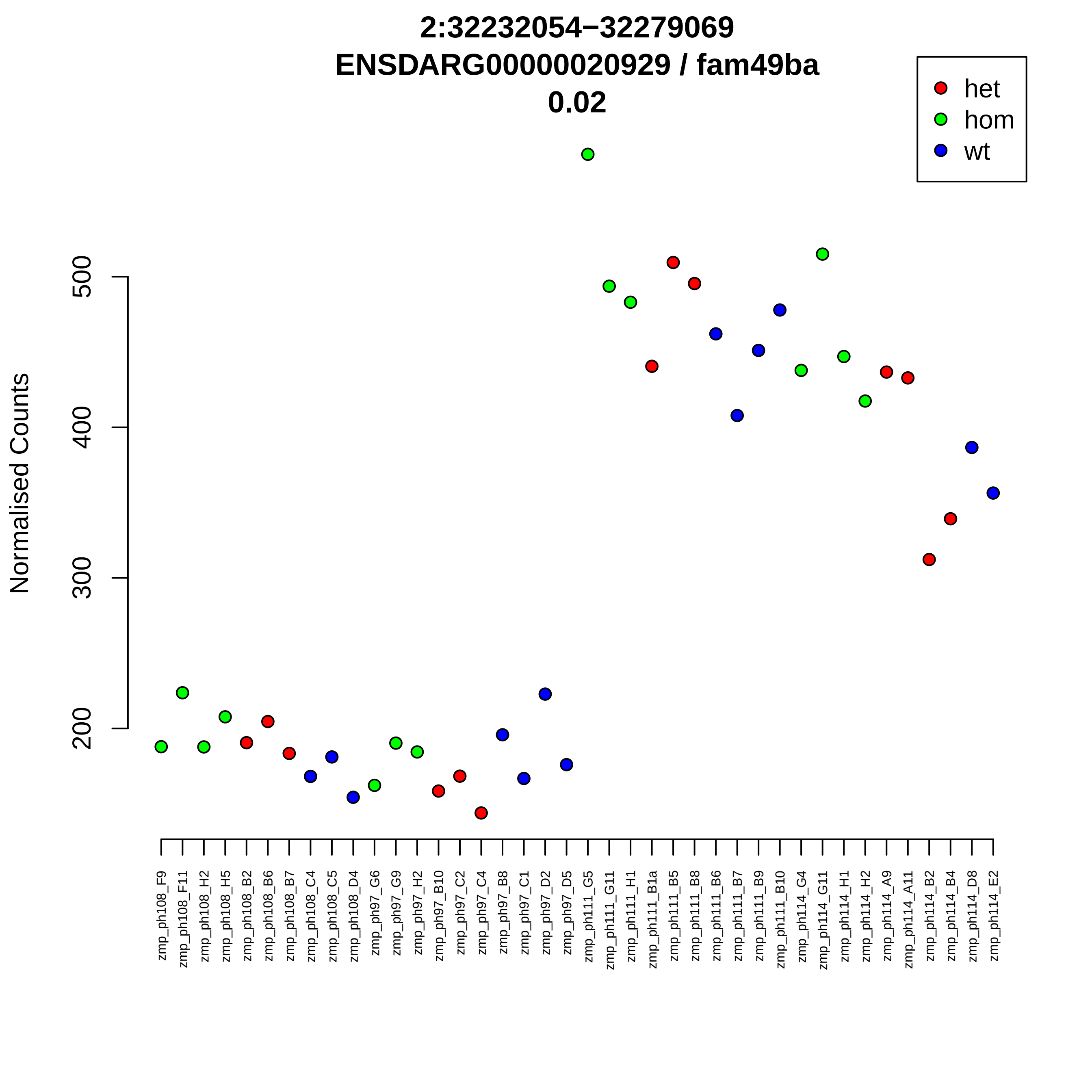

2:32232054−32279069
ENSDARG00000020929 / fam49ba
0.02
het
hom
wt
Normalised Counts
200
300
400
500
zmp_ph108_F9
zmp_ph108_F11
zmp_ph108_H2
zmp_ph108_H5
zmp_ph108_B2
zmp_ph108_B6
zmp_ph108_B7
zmp_ph108_C4
zmp_ph108_C5
zmp_ph108_D4
zmp_ph97_G6
zmp_ph97_G9
zmp_ph97_H2
zmp_ph97_B10
zmp_ph97_C2
zmp_ph97_C4
zmp_ph97_B8
zmp_ph97_C1
zmp_ph97_D2
zmp_ph97_D5
zmp_ph111_G5
zmp_ph111_G11
zmp_ph111_H1
zmp_ph111_B1a
zmp_ph111_B5
zmp_ph111_B8
zmp_ph111_B6
zmp_ph111_B7
zmp_ph111_B9
zmp_ph111_B10
zmp_ph114_G4
zmp_ph114_G11
zmp_ph114_H1
zmp_ph114_H2
zmp_ph114_A9
zmp_ph114_A11
zmp_ph114_B2
zmp_ph114_B4
zmp_ph114_D8
zmp_ph114_E2

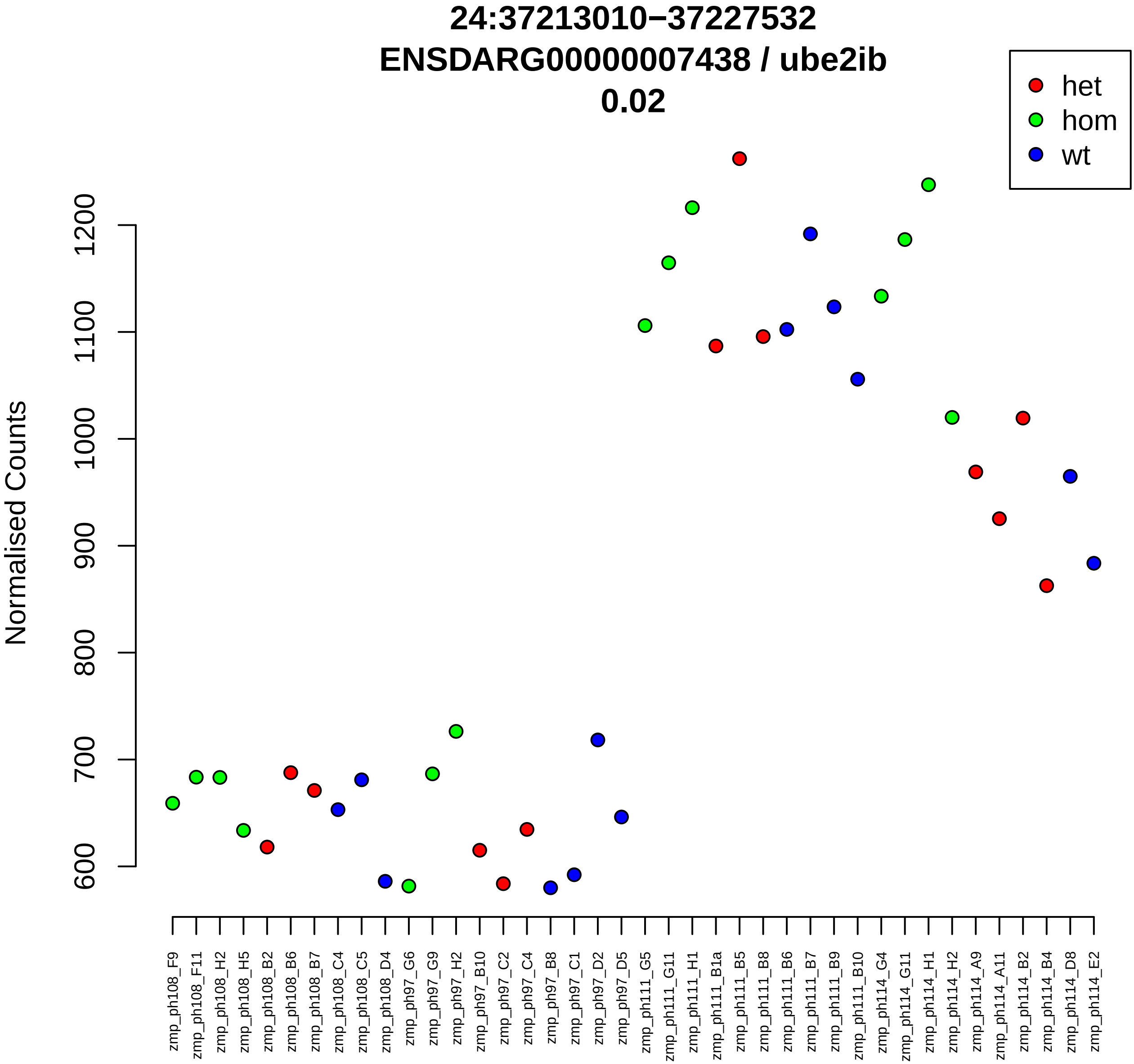

24:37213010−37227532
ENSDARG00000007438 / ube2ib
0.02
het
hom
wt
Normalised Counts
600
700
800
900
1000
1100
1200
zmp_ph108_F9
zmp_ph108_F11
zmp_ph108_H2
zmp_ph108_H5
zmp_ph108_B2
zmp_ph108_B6
zmp_ph108_B7
zmp_ph108_C4
zmp_ph108_C5
zmp_ph108_D4
zmp_ph97_G6
zmp_ph97_G9
zmp_ph97_H2
zmp_ph97_B10
zmp_ph97_C2
zmp_ph97_C4
zmp_ph97_B8
zmp_ph97_C1
zmp_ph97_D2
zmp_ph97_D5
zmp_ph111_G5
zmp_ph111_G11
zmp_ph111_H1
zmp_ph111_B1a
zmp_ph111_B5
zmp_ph111_B8
zmp_ph111_B6
zmp_ph111_B7
zmp_ph111_B9
zmp_ph111_B10
zmp_ph114_G4
zmp_ph114_G11
zmp_ph114_H1
zmp_ph114_H2
zmp_ph114_A9
zmp_ph114_A11
zmp_ph114_B2
zmp_ph114_B4
zmp_ph114_D8
zmp_ph114_E2

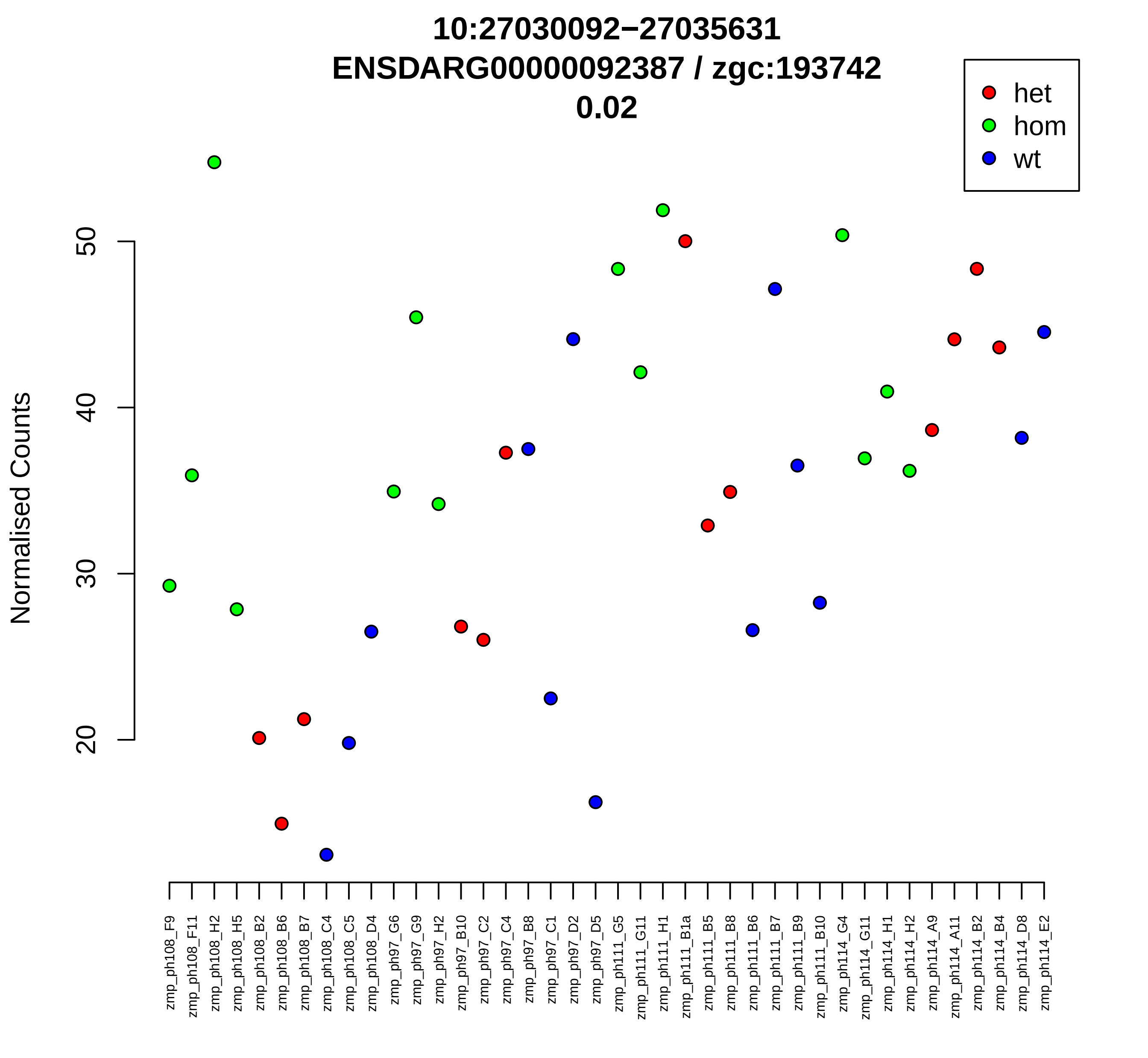
10:27030092−27035631
ENSDARG00000092387 / zgc:193742
0.02
het
hom
wt
Normalised Counts
20
30
40
50
zmp_ph108_F9
zmp_ph108_F11
zmp_ph108_H2
zmp_ph108_H5
zmp_ph108_B2
zmp_ph108_B6
zmp_ph108_B7
zmp_ph108_C4
zmp_ph108_C5
zmp_ph108_D4
zmp_ph97_G6
zmp_ph97_G9
zmp_ph97_H2
zmp_ph97_B10
zmp_ph97_C2
zmp_ph97_C4
zmp_ph97_B8
zmp_ph97_C1
zmp_ph97_D2
zmp_ph97_D5
zmp_ph111_G5
zmp_ph111_G11
zmp_ph111_H1
zmp_ph111_B1a
zmp_ph111_B5
zmp_ph111_B8
zmp_ph111_B6
zmp_ph111_B7
zmp_ph111_B9
zmp_ph111_B10
zmp_ph114_G4
zmp_ph114_G11
zmp_ph114_H1
zmp_ph114_H2
zmp_ph114_A9
zmp_ph114_A11
zmp_ph114_B2
zmp_ph114_B4
zmp_ph114_D8
zmp_ph114_E2

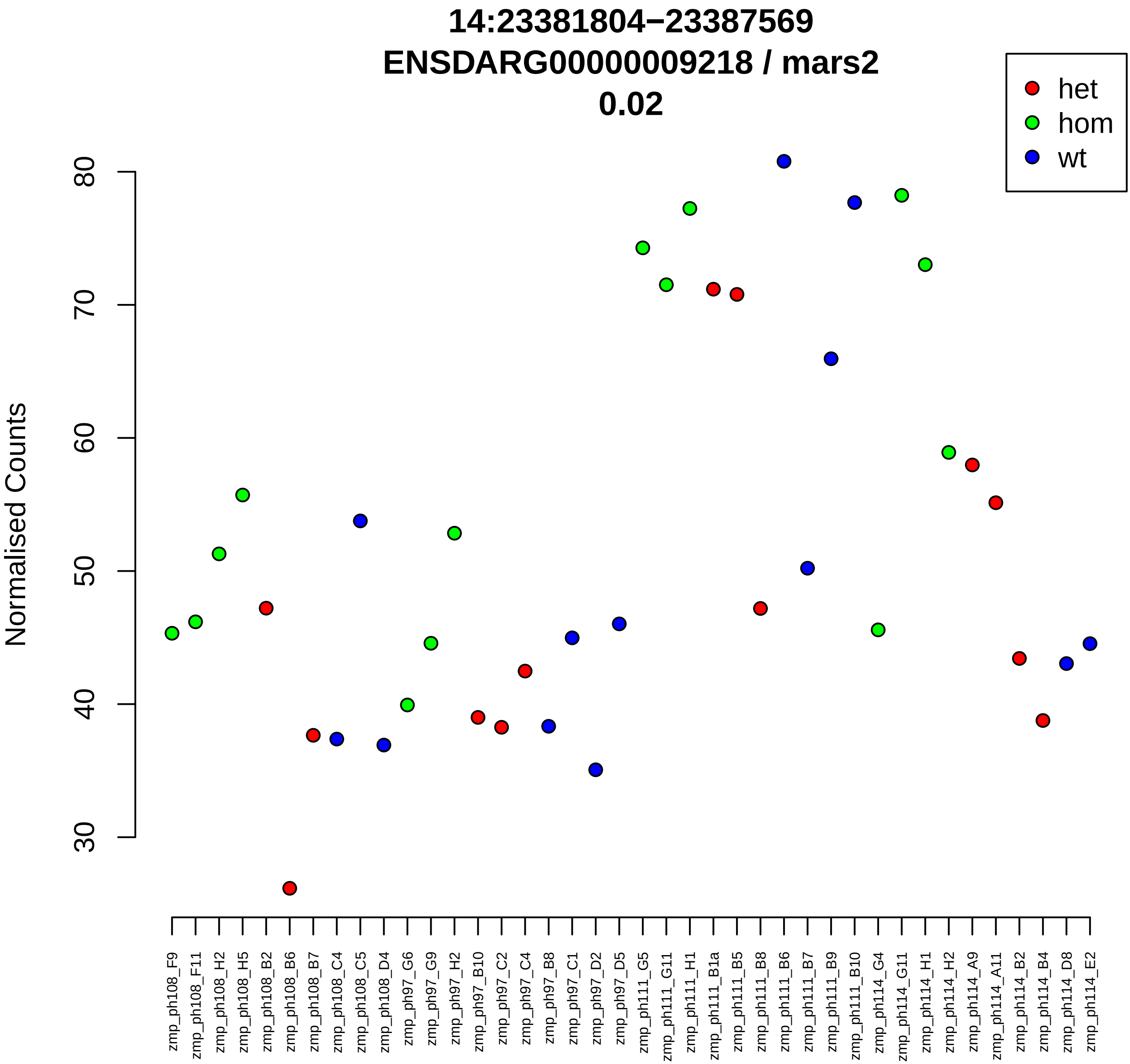
14:23381804−23387569
ENSDARG00000009218 / mars2
0.02
Normalised Counts
het
hom
wt
30
40
50
60
70
80
zmp_ph108_F9
zmp_ph108_F11
zmp_ph108_H2
zmp_ph108_H5
zmp_ph108_B2
zmp_ph108_B6
zmp_ph108_B7
zmp_ph108_C4
zmp_ph108_C5
zmp_ph108_D4
zmp_ph97_G6
zmp_ph97_G9
zmp_ph97_H2
zmp_ph97_B10
zmp_ph97_C2
zmp_ph97_C4
zmp_ph97_B8
zmp_ph97_C1
zmp_ph97_D2
zmp_ph97_D5
zmp_ph111_G5
zmp_ph111_G11
zmp_ph111_H1
zmp_ph111_B1a
zmp_ph111_B5
zmp_ph111_B8
zmp_ph111_B6
zmp_ph111_B7
zmp_ph111_B9
zmp_ph111_B10
zmp_ph114_G4
zmp_ph114_G11
zmp_ph114_H1
zmp_ph114_H2
zmp_ph114_A9
zmp_ph114_A11
zmp_ph114_B2
zmp_ph114_B4
zmp_ph114_D8
zmp_ph114_E2

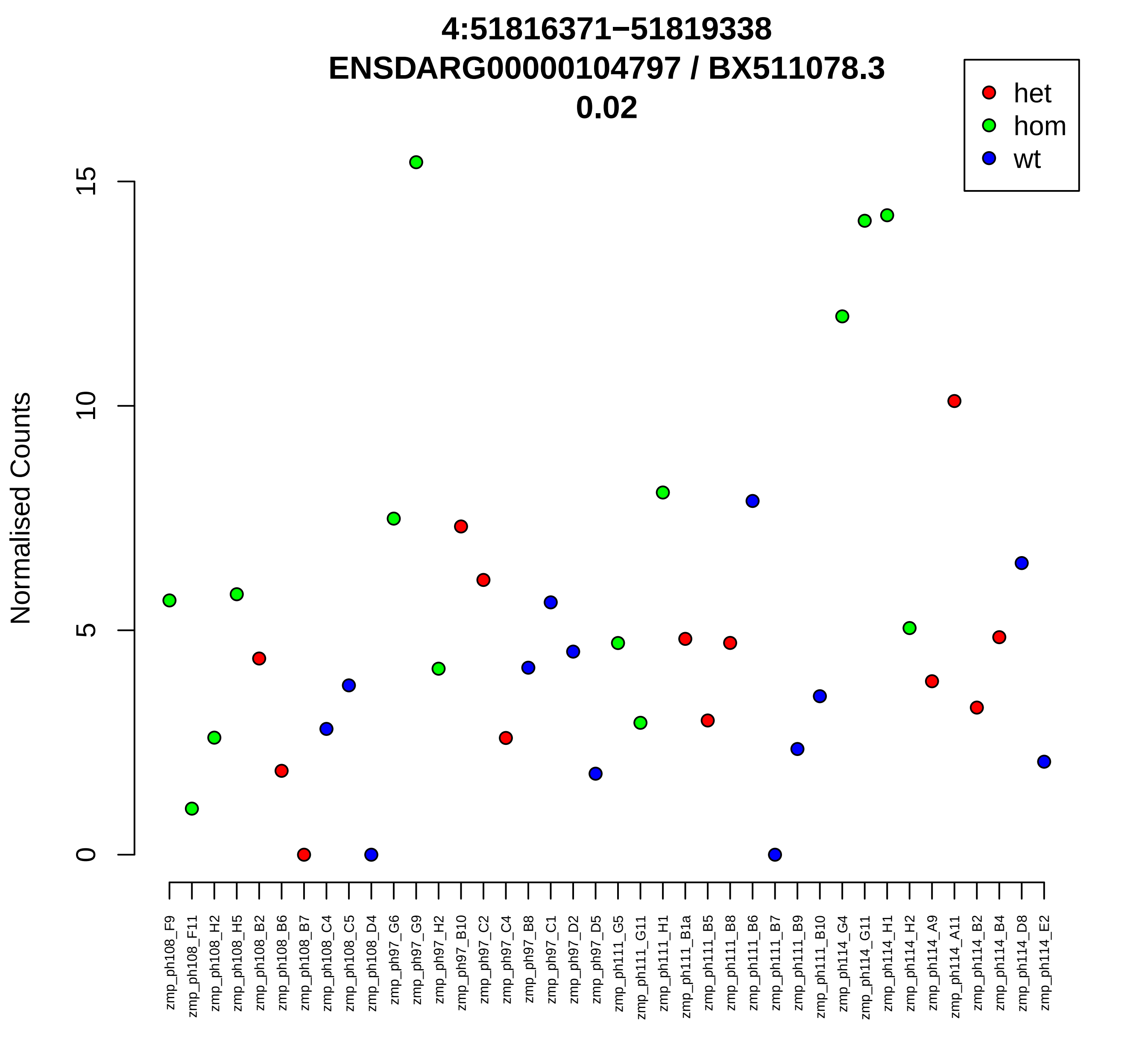

4:51816371−51819338
ENSDARG00000104797 / BX511078.3
0.02
het
hom
wt
Normalised Counts
0
5
10
15
zmp_ph108_F9
zmp_ph108_F11
zmp_ph108_H2
zmp_ph108_H5
zmp_ph108_B2
zmp_ph108_B6
zmp_ph108_B7
zmp_ph108_C4
zmp_ph108_C5
zmp_ph108_D4
zmp_ph97_G6
zmp_ph97_G9
zmp_ph97_H2
zmp_ph97_B10
zmp_ph97_C2
zmp_ph97_C4
zmp_ph97_B8
zmp_ph97_C1
zmp_ph97_D2
zmp_ph97_D5
zmp_ph111_G5
zmp_ph111_G11
zmp_ph111_H1
zmp_ph111_B1a
zmp_ph111_B5
zmp_ph111_B8
zmp_ph111_B6
zmp_ph111_B7
zmp_ph111_B9
zmp_ph111_B10
zmp_ph114_G4
zmp_ph114_G11
zmp_ph114_H1
zmp_ph114_H2
zmp_ph114_A9
zmp_ph114_A11
zmp_ph114_B2
zmp_ph114_B4
zmp_ph114_D8
zmp_ph114_E2

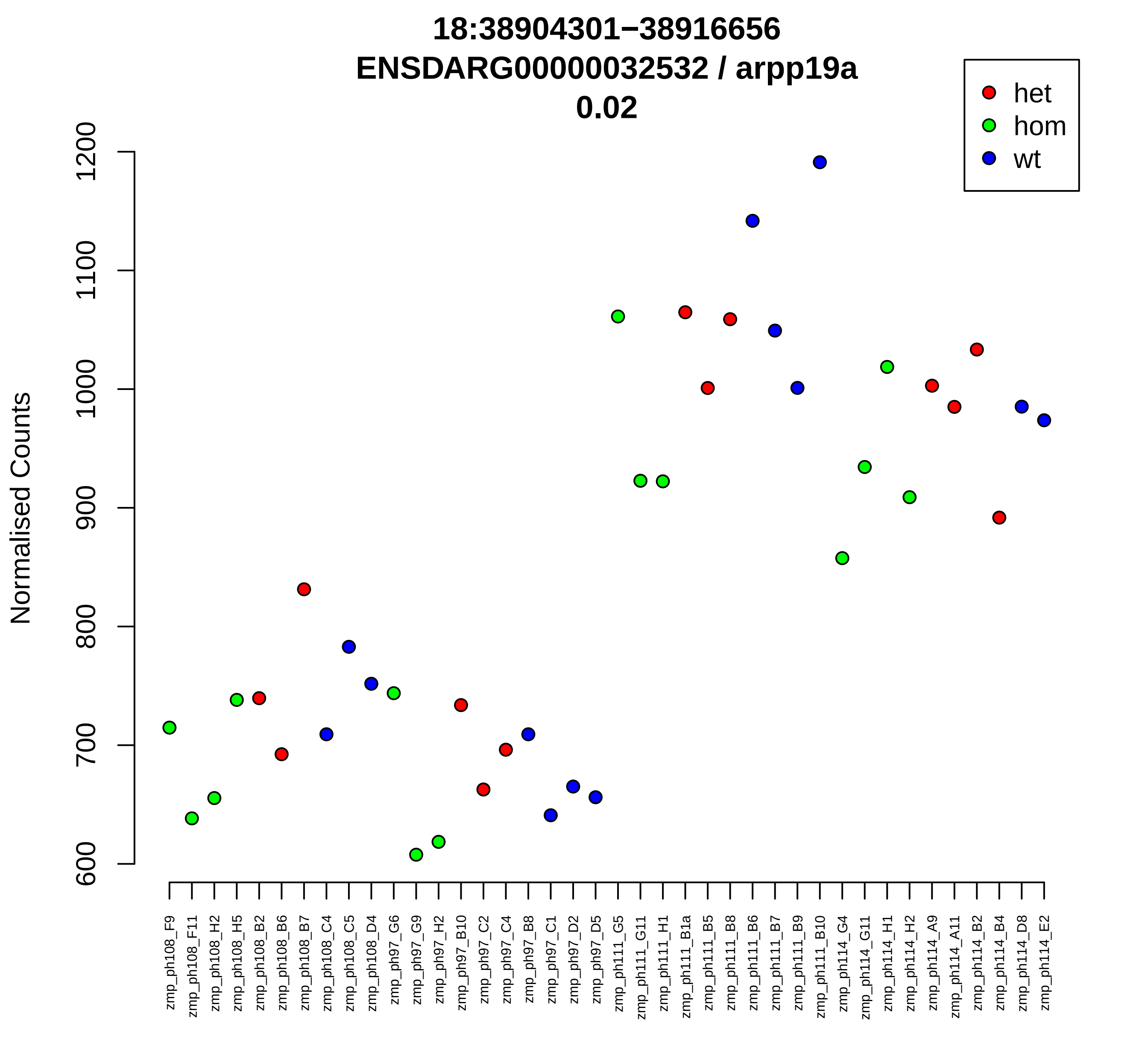

18:38904301−38916656
ENSDARG00000032532 / arpp19a
0.02
Normalised Counts
600
700
800
900
1000
1100
1200
het
hom
wt
zmp_ph108_F9
zmp_ph108_F11
zmp_ph108_H2
zmp_ph108_H5
zmp_ph108_B2
zmp_ph108_B6
zmp_ph108_B7
zmp_ph108_C4
zmp_ph108_C5
zmp_ph108_D4
zmp_ph97_G6
zmp_ph97_G9
zmp_ph97_H2
zmp_ph97_B10
zmp_ph97_C2
zmp_ph97_C4
zmp_ph97_B8
zmp_ph97_C1
zmp_ph97_D2
zmp_ph97_D5
zmp_ph111_G5
zmp_ph111_G11
zmp_ph111_H1
zmp_ph111_B1a
zmp_ph111_B5
zmp_ph111_B8
zmp_ph111_B6
zmp_ph111_B7
zmp_ph111_B9
zmp_ph111_B10
zmp_ph114_G4
zmp_ph114_G11
zmp_ph114_H1
zmp_ph114_H2
zmp_ph114_A9
zmp_ph114_A11
zmp_ph114_B2
zmp_ph114_B4
zmp_ph114_D8
zmp_ph114_E2

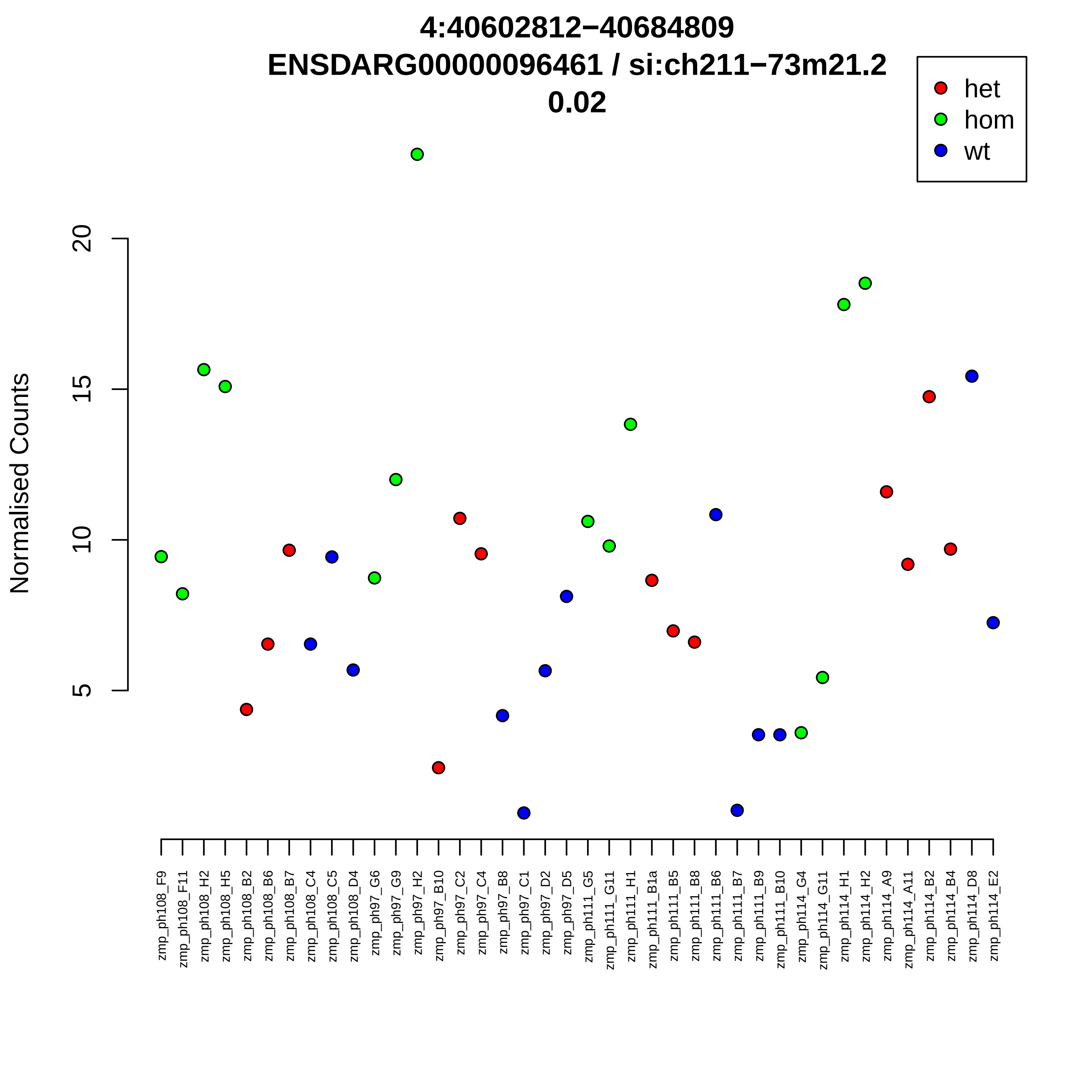

4:40602812−40684809
ENSDARG00000096461 / si:ch211−73m21.2
0.02
het
hom
wt
Normalised Counts
5
10
15
20
zmp_ph108_F9
zmp_ph108_F11
zmp_ph108_H2
zmp_ph108_H5
zmp_ph108_B2
zmp_ph108_B6
zmp_ph108_B7
zmp_ph108_C4
zmp_ph108_C5
zmp_ph108_D4
zmp_ph97_G6
zmp_ph97_G9
zmp_ph97_H2
zmp_ph97_B10
zmp_ph97_C2
zmp_ph97_C4
zmp_ph97_B8
zmp_ph97_C1
zmp_ph97_D2
zmp_ph97_D5
zmp_ph111_G5
zmp_ph111_G11
zmp_ph111_H1
zmp_ph111_B1a
zmp_ph111_B5
zmp_ph111_B8
zmp_ph111_B6
zmp_ph111_B7
zmp_ph111_B9
zmp_ph111_B10
zmp_ph114_G4
zmp_ph114_G11
zmp_ph114_H1
zmp_ph114_H2
zmp_ph114_A9
zmp_ph114_A11
zmp_ph114_B2
zmp_ph114_B4
zmp_ph114_D8
zmp_ph114_E2

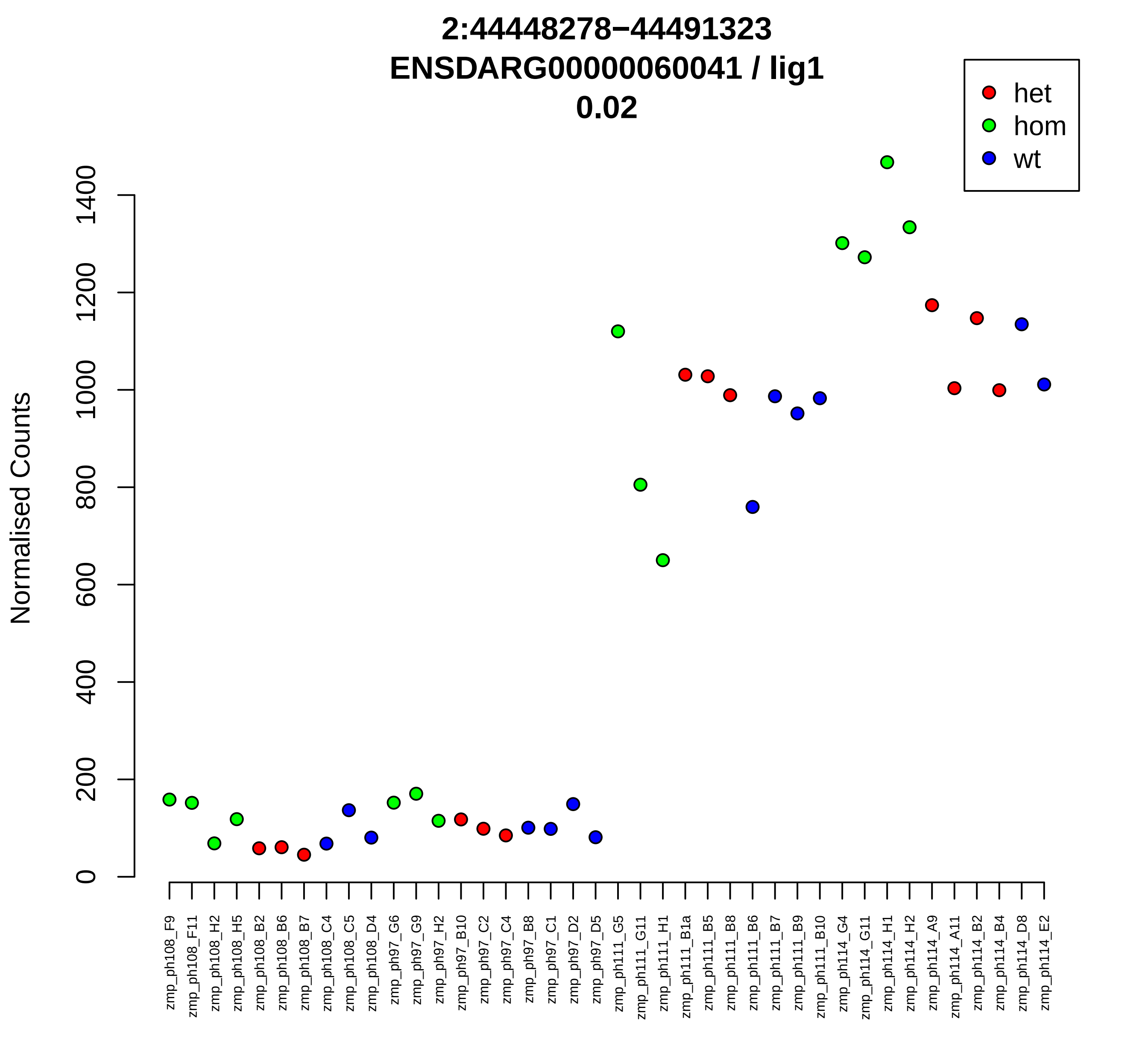
2:44448278−44491323
ENSDARG00000060041 / lig1
0.02
het
hom
wt
Normalised Counts
0
200
400
600
800
1000
1200
1400
zmp_ph108_F9
zmp_ph108_F11
zmp_ph108_H2
zmp_ph108_H5
zmp_ph108_B2
zmp_ph108_B6
zmp_ph108_B7
zmp_ph108_C4
zmp_ph108_C5
zmp_ph108_D4
zmp_ph97_G6
zmp_ph97_G9
zmp_ph97_H2
zmp_ph97_B10
zmp_ph97_C2
zmp_ph97_C4
zmp_ph97_B8
zmp_ph97_C1
zmp_ph97_D2
zmp_ph97_D5
zmp_ph111_G5
zmp_ph111_G11
zmp_ph111_H1
zmp_ph111_B1a
zmp_ph111_B5
zmp_ph111_B8
zmp_ph111_B6
zmp_ph111_B7
zmp_ph111_B9
zmp_ph111_B10
zmp_ph114_G4
zmp_ph114_G11
zmp_ph114_H1
zmp_ph114_H2
zmp_ph114_A9
zmp_ph114_A11
zmp_ph114_B2
zmp_ph114_B4
zmp_ph114_D8
zmp_ph114_E2

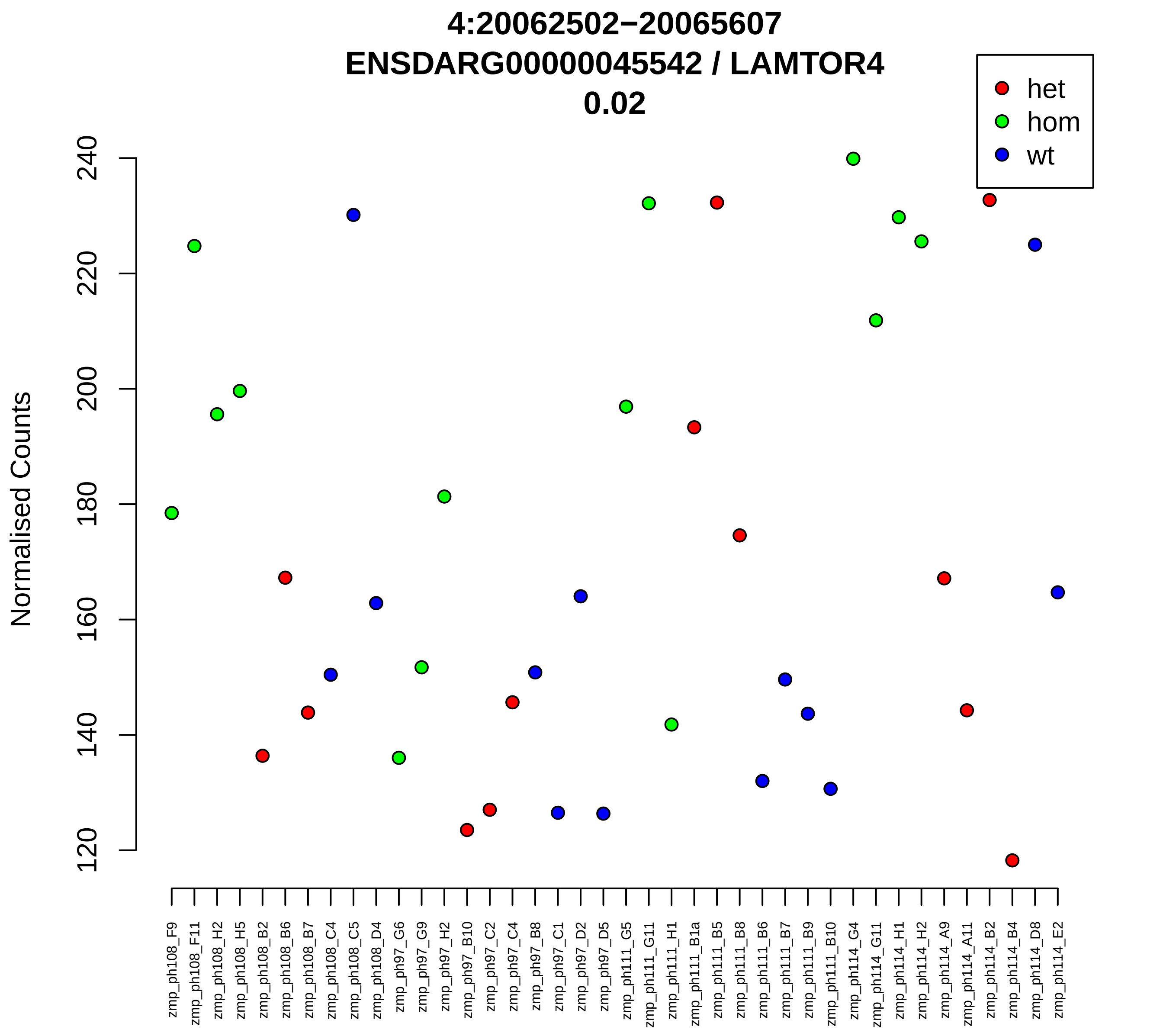
4:20062502−20065607
ENSDARG00000045542 / LAMTOR4
0.02
het
hom
wt
Normalised Counts
120
140
160
180
200
220
240
zmp_ph108_F9
zmp_ph108_F11
zmp_ph108_H2
zmp_ph108_H5
zmp_ph108_B2
zmp_ph108_B6
zmp_ph108_B7
zmp_ph108_C4
zmp_ph108_C5
zmp_ph108_D4
zmp_ph97_G6
zmp_ph97_G9
zmp_ph97_H2
zmp_ph97_B10
zmp_ph97_C2
zmp_ph97_C4
zmp_ph97_B8
zmp_ph97_C1
zmp_ph97_D2
zmp_ph97_D5
zmp_ph111_G5
zmp_ph111_G11
zmp_ph111_H1
zmp_ph111_B1a
zmp_ph111_B5
zmp_ph111_B8
zmp_ph111_B6
zmp_ph111_B7
zmp_ph111_B9
zmp_ph111_B10
zmp_ph114_G4
zmp_ph114_G11
zmp_ph114_H1
zmp_ph114_H2
zmp_ph114_A9
zmp_ph114_A11
zmp_ph114_B2
zmp_ph114_B4
zmp_ph114_D8
zmp_ph114_E2

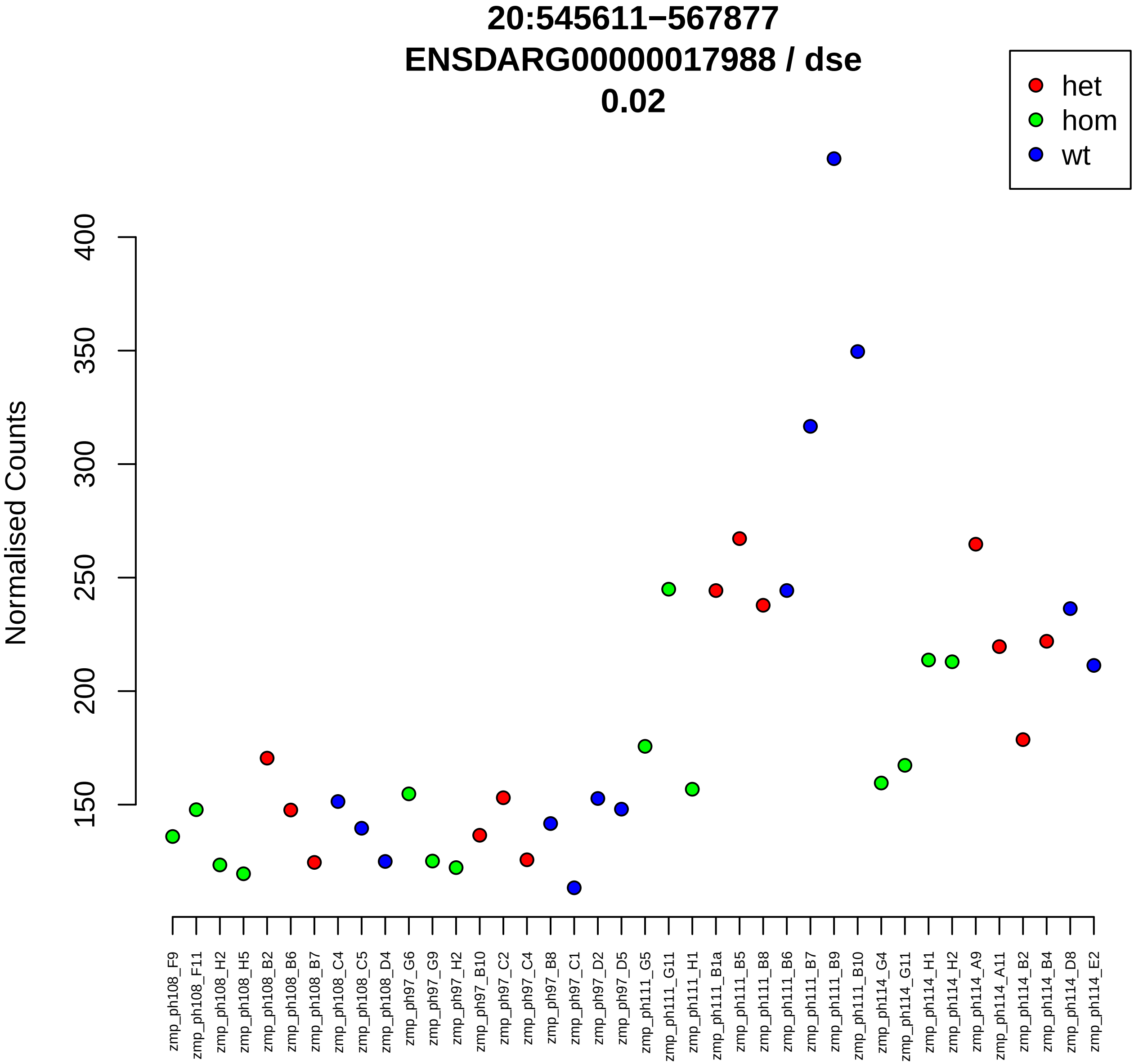

20:545611−567877
ENSDARG00000017988 / dse
0.02
het
hom
wt
Normalised Counts
150
200
250
300
350
400
zmp_ph108_F9
zmp_ph108_F11
zmp_ph108_H2
zmp_ph108_H5
zmp_ph108_B2
zmp_ph108_B6
zmp_ph108_B7
zmp_ph108_C4
zmp_ph108_C5
zmp_ph108_D4
zmp_ph97_G6
zmp_ph97_G9
zmp_ph97_H2
zmp_ph97_B10
zmp_ph97_C2
zmp_ph97_C4
zmp_ph97_B8
zmp_ph97_C1
zmp_ph97_D2
zmp_ph97_D5
zmp_ph111_G5
zmp_ph111_G11
zmp_ph111_H1
zmp_ph111_B1a
zmp_ph111_B5
zmp_ph111_B8
zmp_ph111_B6
zmp_ph111_B7
zmp_ph111_B9
zmp_ph111_B10
zmp_ph114_G4
zmp_ph114_G11
zmp_ph114_H1
zmp_ph114_H2
zmp_ph114_A9
zmp_ph114_A11
zmp_ph114_B2
zmp_ph114_B4
zmp_ph114_D8
zmp_ph114_E2

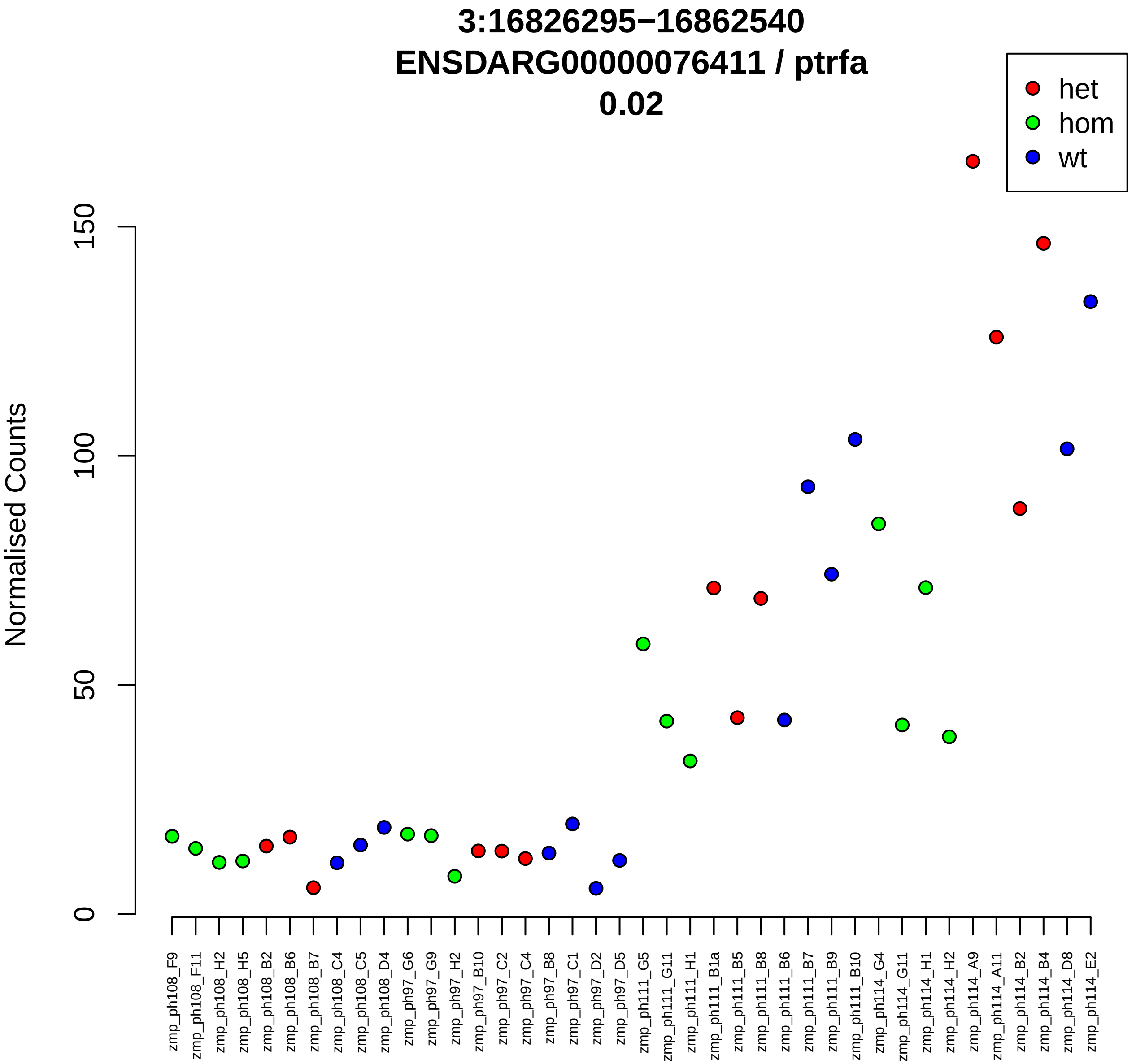

3:16826295−16862540
ENSDARG00000076411 / ptrfa
0.02
het
hom
wt
Normalised Counts
0
50
100
150
zmp_ph108_F9
zmp_ph108_F11
zmp_ph108_H2
zmp_ph108_H5
zmp_ph108_B2
zmp_ph108_B6
zmp_ph108_B7
zmp_ph108_C4
zmp_ph108_C5
zmp_ph108_D4
zmp_ph97_G6
zmp_ph97_G9
zmp_ph97_H2
zmp_ph97_B10
zmp_ph97_C2
zmp_ph97_C4
zmp_ph97_B8
zmp_ph97_C1
zmp_ph97_D2
zmp_ph97_D5
zmp_ph111_G5
zmp_ph111_G11
zmp_ph111_H1
zmp_ph111_B1a
zmp_ph111_B5
zmp_ph111_B8
zmp_ph111_B6
zmp_ph111_B7
zmp_ph111_B9
zmp_ph111_B10
zmp_ph114_G4
zmp_ph114_G11
zmp_ph114_H1
zmp_ph114_H2
zmp_ph114_A9
zmp_ph114_A11
zmp_ph114_B2
zmp_ph114_B4
zmp_ph114_D8
zmp_ph114_E2

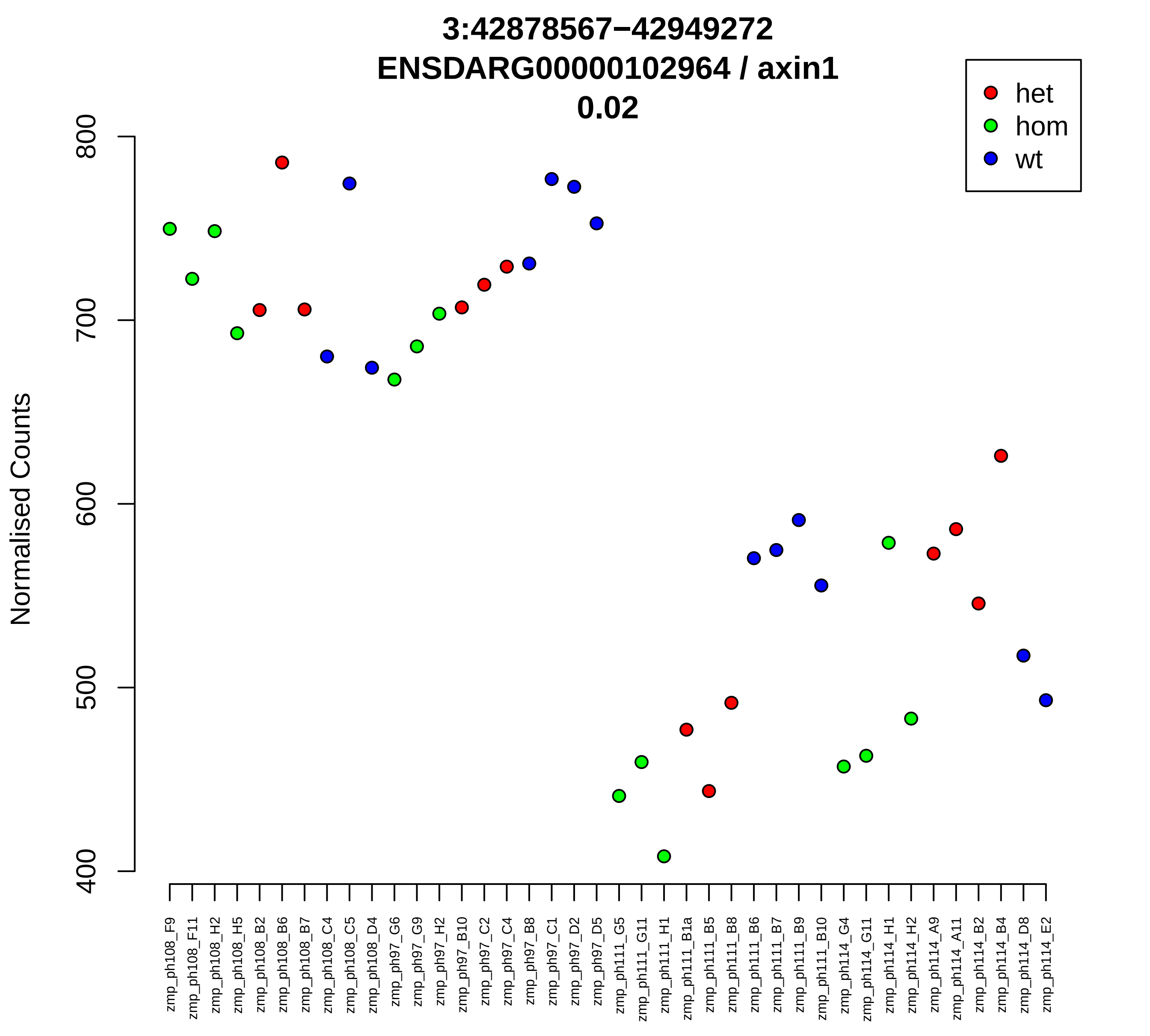
3:42878567−42949272
ENSDARG00000102964 / axin1
0.02
het
hom
wt
Normalised Counts
400
500
600
700
800
zmp_ph108_F9
zmp_ph108_F11
zmp_ph108_H2
zmp_ph108_H5
zmp_ph108_B2
zmp_ph108_B6
zmp_ph108_B7
zmp_ph108_C4
zmp_ph108_C5
zmp_ph108_D4
zmp_ph97_G6
zmp_ph97_G9
zmp_ph97_H2
zmp_ph97_B10
zmp_ph97_C2
zmp_ph97_C4
zmp_ph97_B8
zmp_ph97_C1
zmp_ph97_D2
zmp_ph97_D5
zmp_ph111_G5
zmp_ph111_G11
zmp_ph111_H1
zmp_ph111_B1a
zmp_ph111_B5
zmp_ph111_B8
zmp_ph111_B6
zmp_ph111_B7
zmp_ph111_B9
zmp_ph111_B10
zmp_ph114_G4
zmp_ph114_G11
zmp_ph114_H1
zmp_ph114_H2
zmp_ph114_A9
zmp_ph114_A11
zmp_ph114_B2
zmp_ph114_B4
zmp_ph114_D8
zmp_ph114_E2

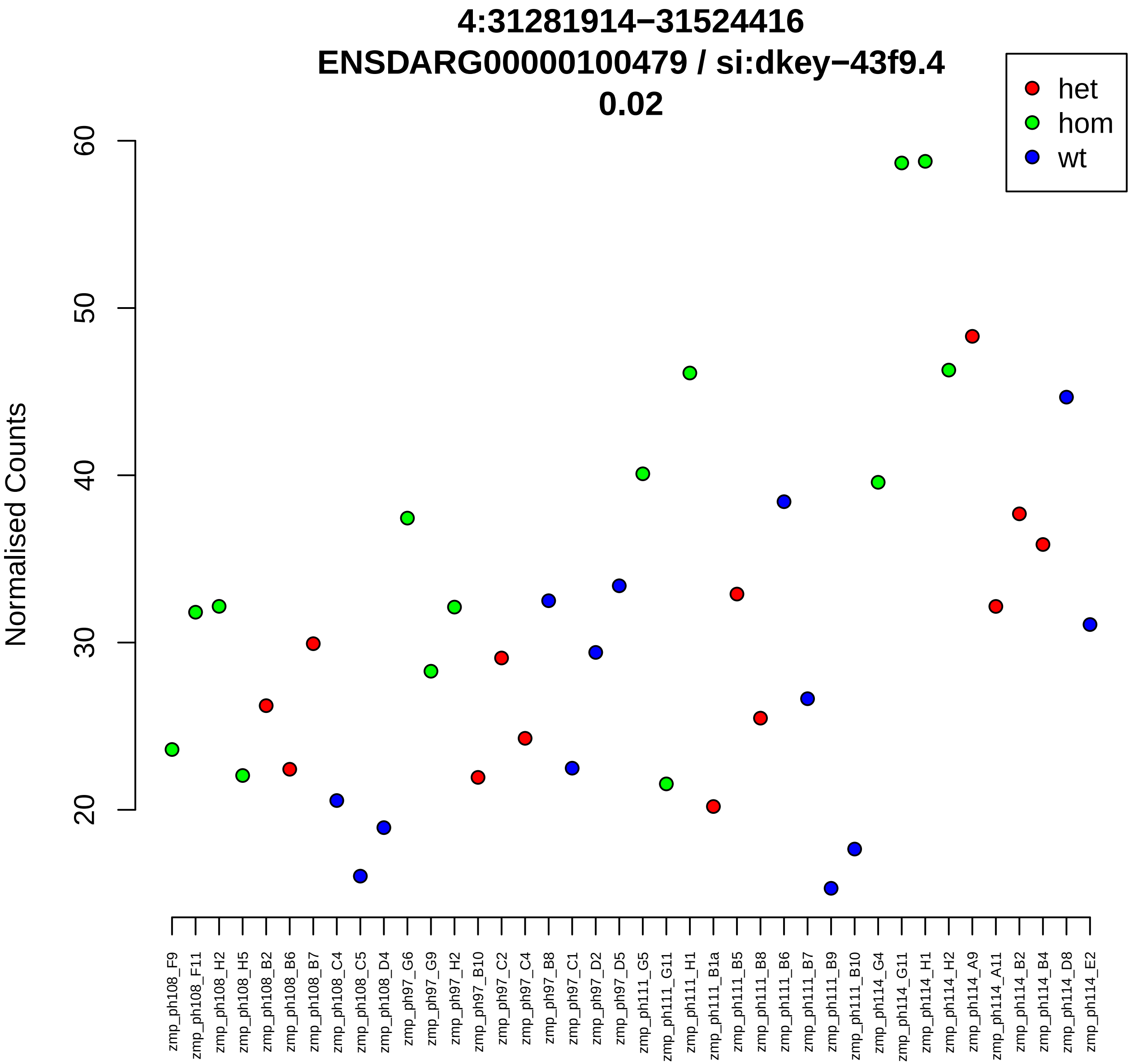
4:31281914−31524416
ENSDARG00000100479 / si:dkey−43f9.4
0.02
het
hom
wt
Normalised Counts
20
30
40
50
60
zmp_ph108_F9
zmp_ph108_F11
zmp_ph108_H2
zmp_ph108_H5
zmp_ph108_B2
zmp_ph108_B6
zmp_ph108_B7
zmp_ph108_C4
zmp_ph108_C5
zmp_ph108_D4
zmp_ph97_G6
zmp_ph97_G9
zmp_ph97_H2
zmp_ph97_B10
zmp_ph97_C2
zmp_ph97_C4
zmp_ph97_B8
zmp_ph97_C1
zmp_ph97_D2
zmp_ph97_D5
zmp_ph111_G5
zmp_ph111_G11
zmp_ph111_H1
zmp_ph111_B1a
zmp_ph111_B5
zmp_ph111_B8
zmp_ph111_B6
zmp_ph111_B7
zmp_ph111_B9
zmp_ph111_B10
zmp_ph114_G4
zmp_ph114_G11
zmp_ph114_H1
zmp_ph114_H2
zmp_ph114_A9
zmp_ph114_A11
zmp_ph114_B2
zmp_ph114_B4
zmp_ph114_D8
zmp_ph114_E2

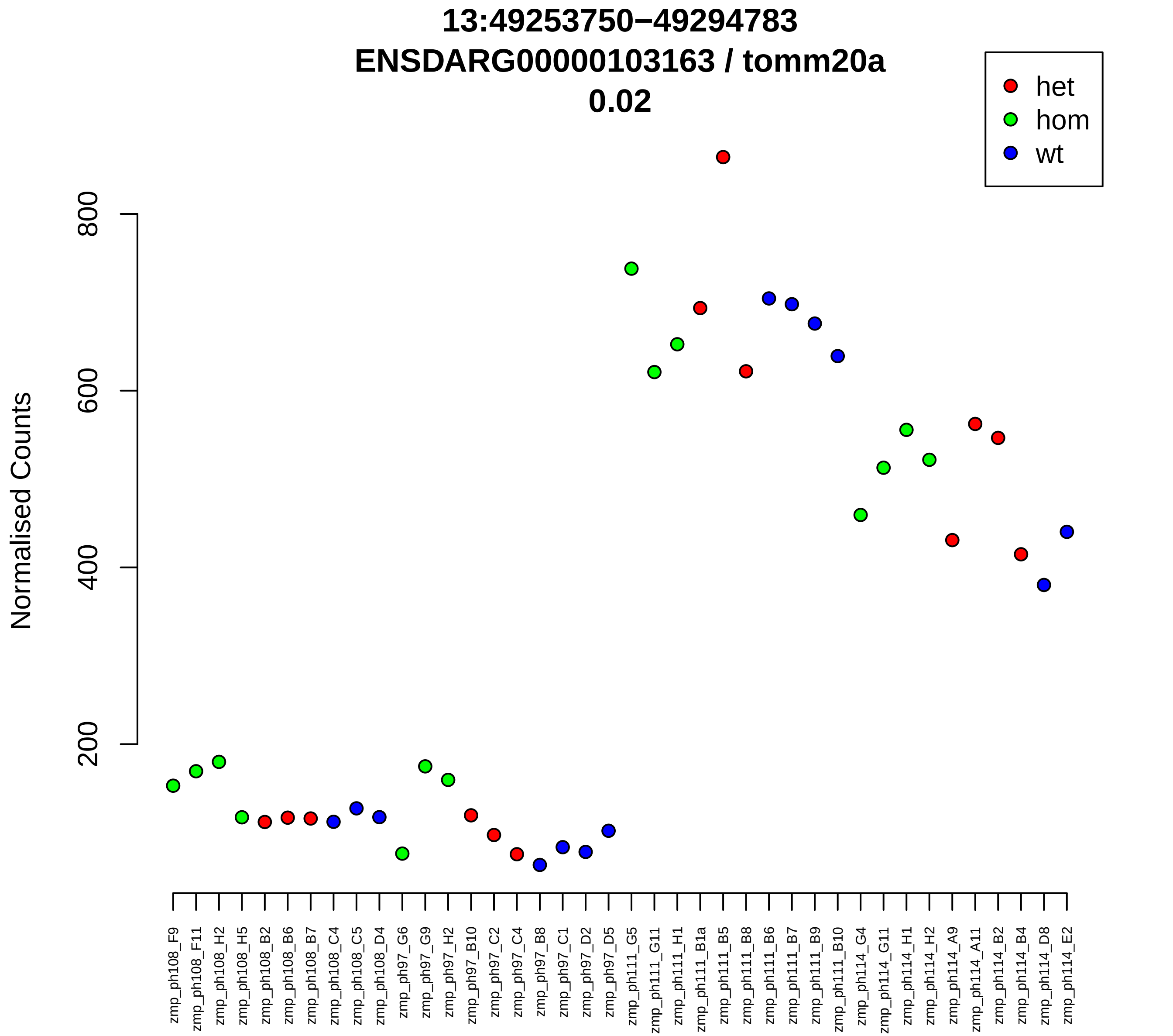
13:49253750−49294783
ENSDARG00000103163 / tomm20a
0.02
het
hom
wt
Normalised Counts
200
400
600
800
zmp_ph108_F9
zmp_ph108_F11
zmp_ph108_H2
zmp_ph108_H5
zmp_ph108_B2
zmp_ph108_B6
zmp_ph108_B7
zmp_ph108_C4
zmp_ph108_C5
zmp_ph108_D4
zmp_ph97_G6
zmp_ph97_G9
zmp_ph97_H2
zmp_ph97_B10
zmp_ph97_C2
zmp_ph97_C4
zmp_ph97_B8
zmp_ph97_C1
zmp_ph97_D2
zmp_ph97_D5
zmp_ph111_G5
zmp_ph111_G11
zmp_ph111_H1
zmp_ph111_B1a
zmp_ph111_B5
zmp_ph111_B8
zmp_ph111_B6
zmp_ph111_B7
zmp_ph111_B9
zmp_ph111_B10
zmp_ph114_G4
zmp_ph114_G11
zmp_ph114_H1
zmp_ph114_H2
zmp_ph114_A9
zmp_ph114_A11
zmp_ph114_B2
zmp_ph114_B4
zmp_ph114_D8
zmp_ph114_E2

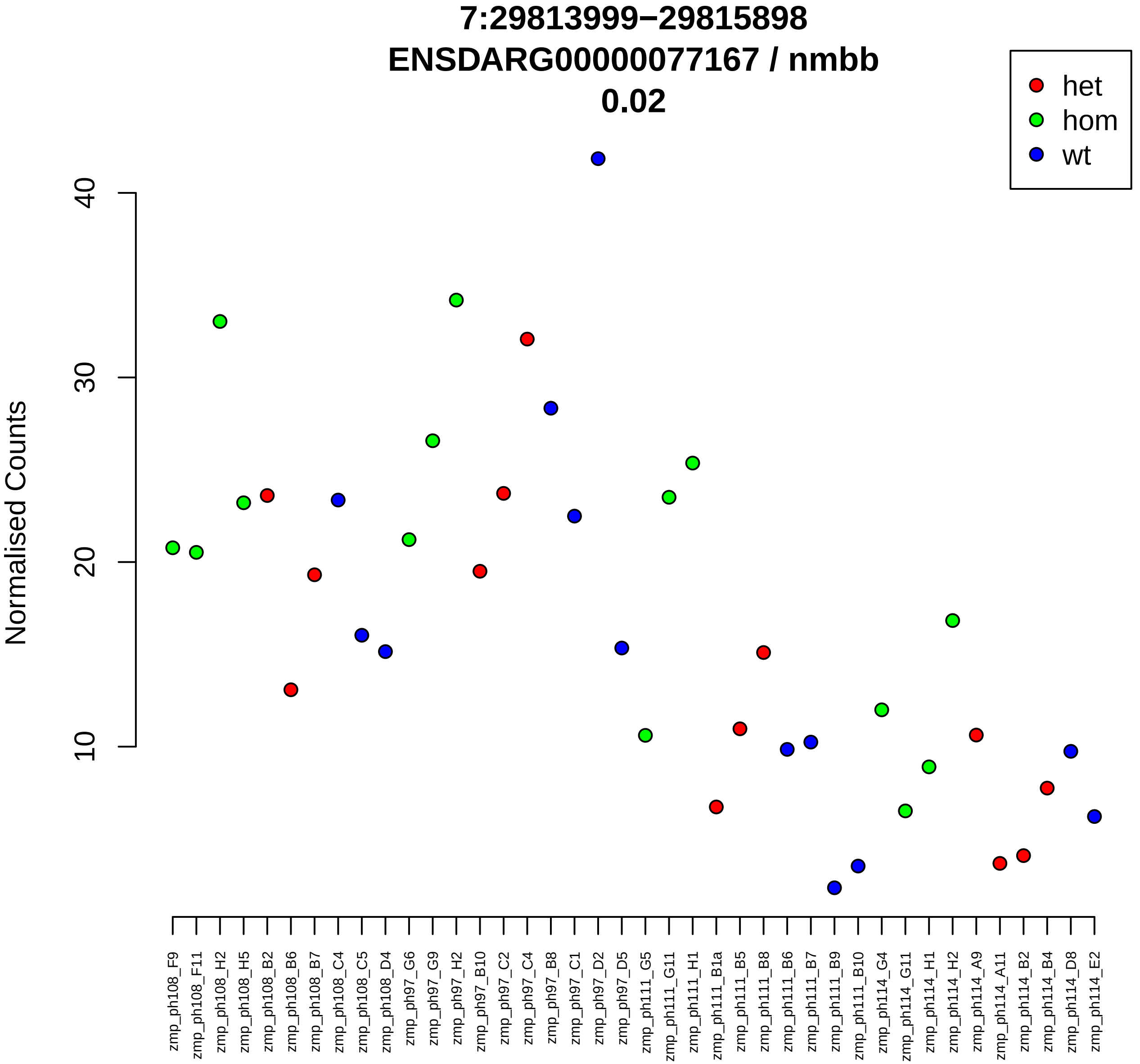
7:29813999−29815898
ENSDARG00000077167 / nmbb
0.02
het
hom
wt
Normalised Counts
10
20
30
40
zmp_ph108_F9
zmp_ph108_F11
zmp_ph108_H2
zmp_ph108_H5
zmp_ph108_B2
zmp_ph108_B6
zmp_ph108_B7
zmp_ph108_C4
zmp_ph108_C5
zmp_ph108_D4
zmp_ph97_G6
zmp_ph97_G9
zmp_ph97_H2
zmp_ph97_B10
zmp_ph97_C2
zmp_ph97_C4
zmp_ph97_B8
zmp_ph97_C1
zmp_ph97_D2
zmp_ph97_D5
zmp_ph111_G5
zmp_ph111_G11
zmp_ph111_H1
zmp_ph111_B1a
zmp_ph111_B5
zmp_ph111_B8
zmp_ph111_B6
zmp_ph111_B7
zmp_ph111_B9
zmp_ph111_B10
zmp_ph114_G4
zmp_ph114_G11
zmp_ph114_H1
zmp_ph114_H2
zmp_ph114_A9
zmp_ph114_A11
zmp_ph114_B2
zmp_ph114_B4
zmp_ph114_D8
zmp_ph114_E2

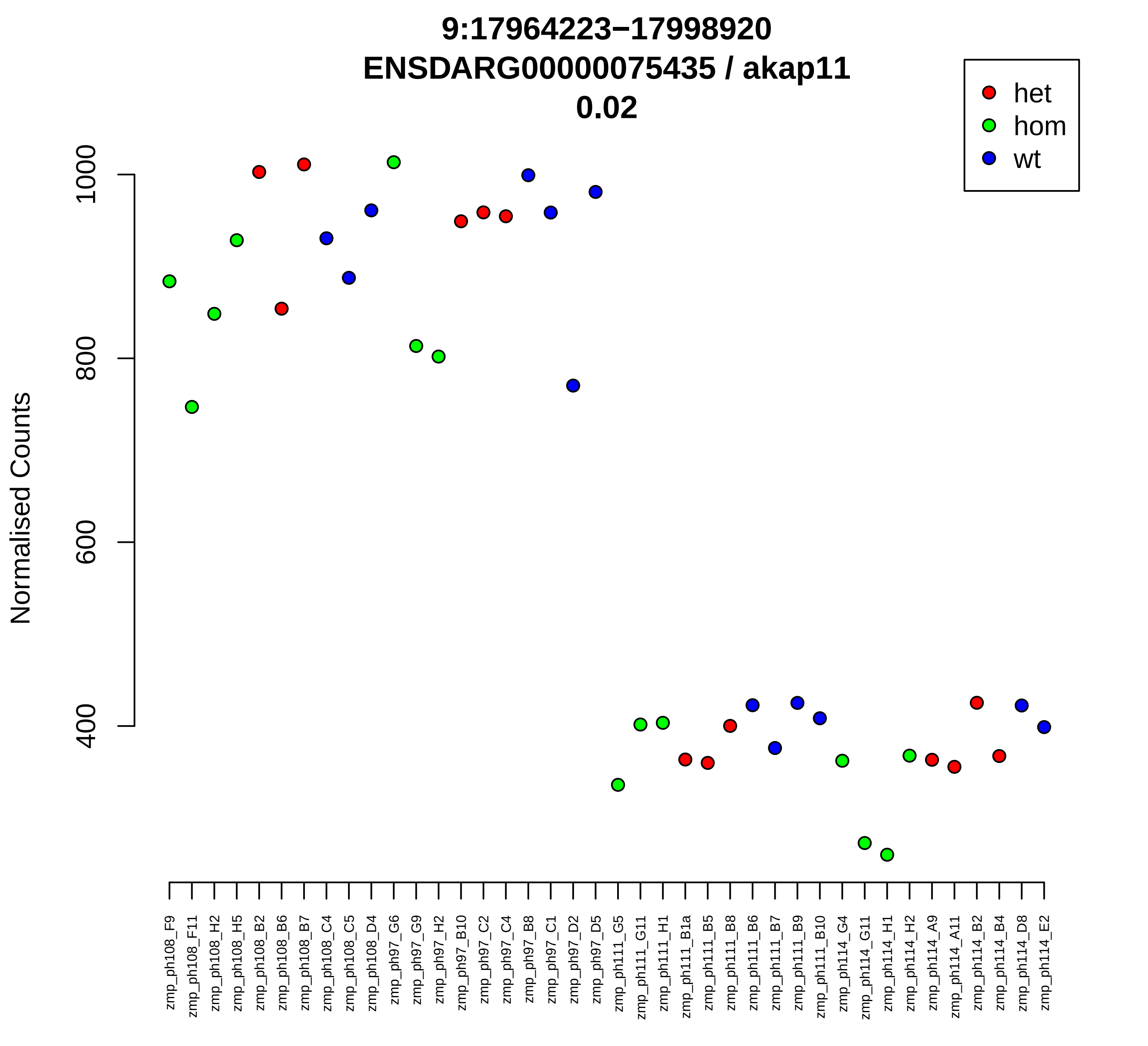

9:17964223−17998920
ENSDARG00000075435 / akap11
0.02
het
hom
wt
Normalised Counts
400
600
800
1000
zmp_ph108_F9
zmp_ph108_F11
zmp_ph108_H2
zmp_ph108_H5
zmp_ph108_B2
zmp_ph108_B6
zmp_ph108_B7
zmp_ph108_C4
zmp_ph108_C5
zmp_ph108_D4
zmp_ph97_G6
zmp_ph97_G9
zmp_ph97_H2
zmp_ph97_B10
zmp_ph97_C2
zmp_ph97_C4
zmp_ph97_B8
zmp_ph97_C1
zmp_ph97_D2
zmp_ph97_D5
zmp_ph111_G5
zmp_ph111_G11
zmp_ph111_H1
zmp_ph111_B1a
zmp_ph111_B5
zmp_ph111_B8
zmp_ph111_B6
zmp_ph111_B7
zmp_ph111_B9
zmp_ph111_B10
zmp_ph114_G4
zmp_ph114_G11
zmp_ph114_H1
zmp_ph114_H2
zmp_ph114_A9
zmp_ph114_A11
zmp_ph114_B2
zmp_ph114_B4
zmp_ph114_D8
zmp_ph114_E2

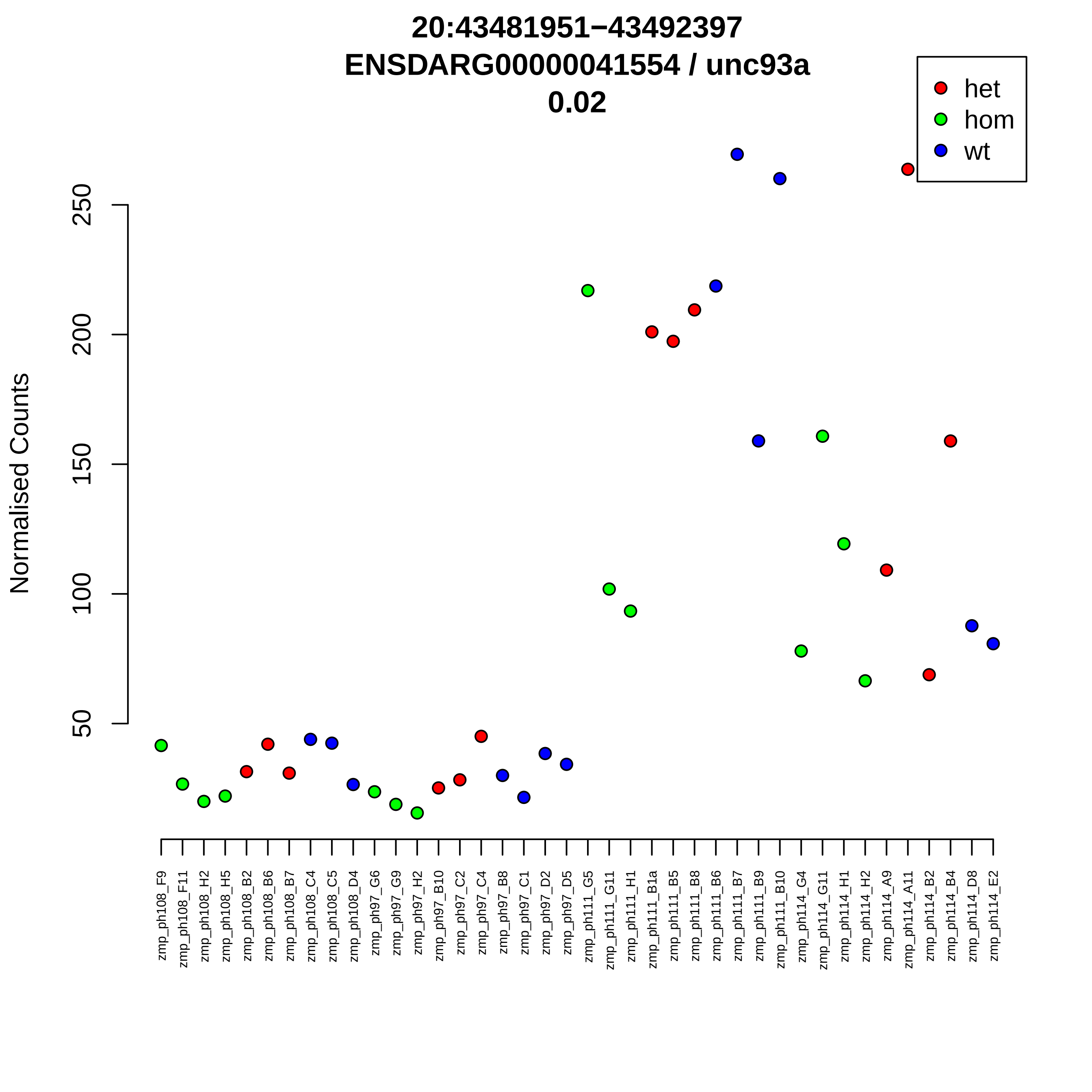

20:43481951−43492397
ENSDARG00000041554 / unc93a
0.02
het
hom
wt
Normalised Counts
50
100
150
200
250
zmp_ph108_F9
zmp_ph108_F11
zmp_ph108_H2
zmp_ph108_H5
zmp_ph108_B2
zmp_ph108_B6
zmp_ph108_B7
zmp_ph108_C4
zmp_ph108_C5
zmp_ph108_D4
zmp_ph97_G6
zmp_ph97_G9
zmp_ph97_H2
zmp_ph97_B10
zmp_ph97_C2
zmp_ph97_C4
zmp_ph97_B8
zmp_ph97_C1
zmp_ph97_D2
zmp_ph97_D5
zmp_ph111_G5
zmp_ph111_G11
zmp_ph111_H1
zmp_ph111_B1a
zmp_ph111_B5
zmp_ph111_B8
zmp_ph111_B6
zmp_ph111_B7
zmp_ph111_B9
zmp_ph111_B10
zmp_ph114_G4
zmp_ph114_G11
zmp_ph114_H1
zmp_ph114_H2
zmp_ph114_A9
zmp_ph114_A11
zmp_ph114_B2
zmp_ph114_B4
zmp_ph114_D8
zmp_ph114_E2

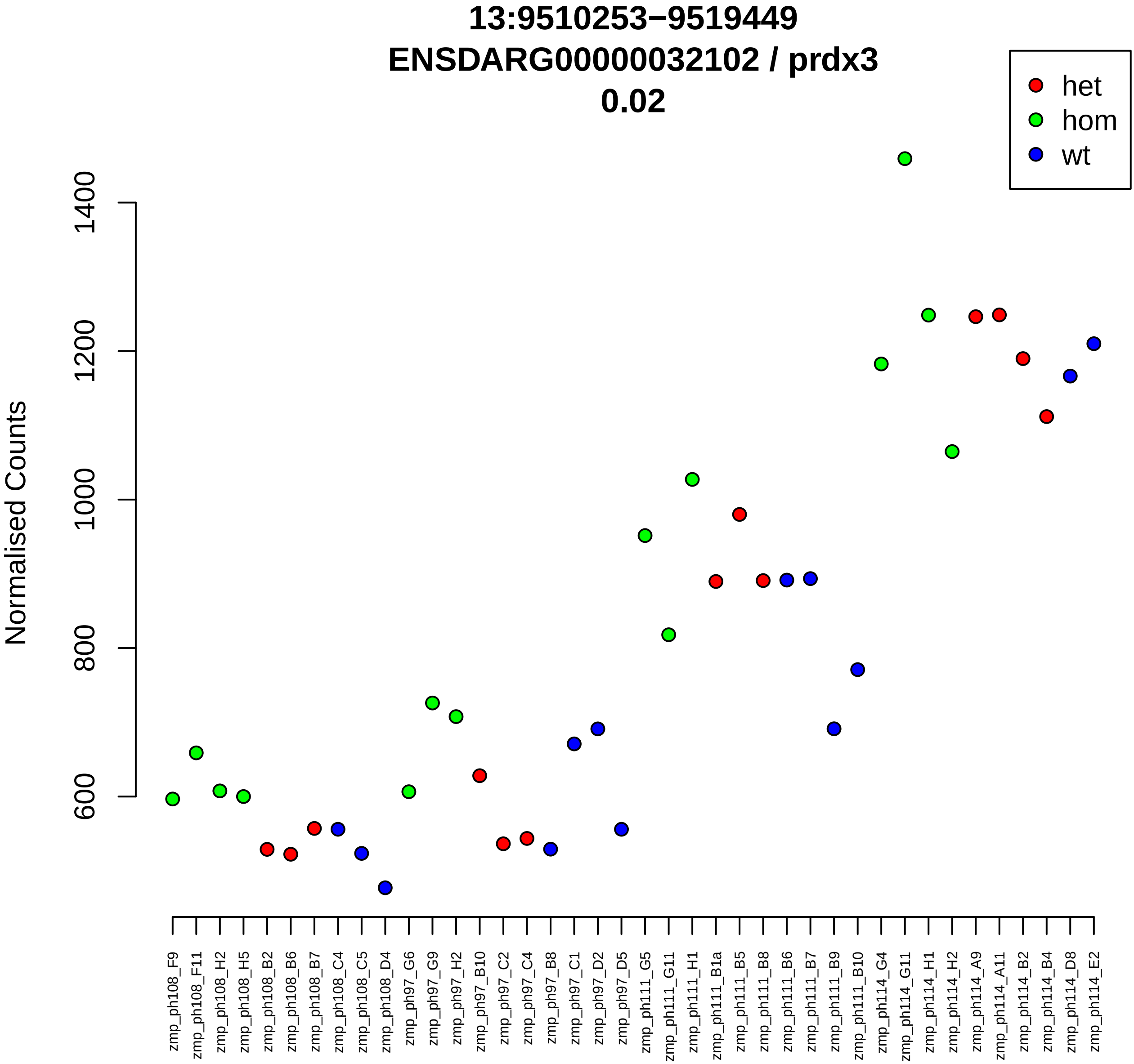
13:9510253−9519449
ENSDARG00000032102 / prdx3
0.02
het
hom
wt
Normalised Counts
600
800
1000
1200
1400
zmp_ph108_F9
zmp_ph108_F11
zmp_ph108_H2
zmp_ph108_H5
zmp_ph108_B2
zmp_ph108_B6
zmp_ph108_B7
zmp_ph108_C4
zmp_ph108_C5
zmp_ph108_D4
zmp_ph97_G6
zmp_ph97_G9
zmp_ph97_H2
zmp_ph97_B10
zmp_ph97_C2
zmp_ph97_C4
zmp_ph97_B8
zmp_ph97_C1
zmp_ph97_D2
zmp_ph97_D5
zmp_ph111_G5
zmp_ph111_G11
zmp_ph111_H1
zmp_ph111_B1a
zmp_ph111_B5
zmp_ph111_B8
zmp_ph111_B6
zmp_ph111_B7
zmp_ph111_B9
zmp_ph111_B10
zmp_ph114_G4
zmp_ph114_G11
zmp_ph114_H1
zmp_ph114_H2
zmp_ph114_A9
zmp_ph114_A11
zmp_ph114_B2
zmp_ph114_B4
zmp_ph114_D8
zmp_ph114_E2

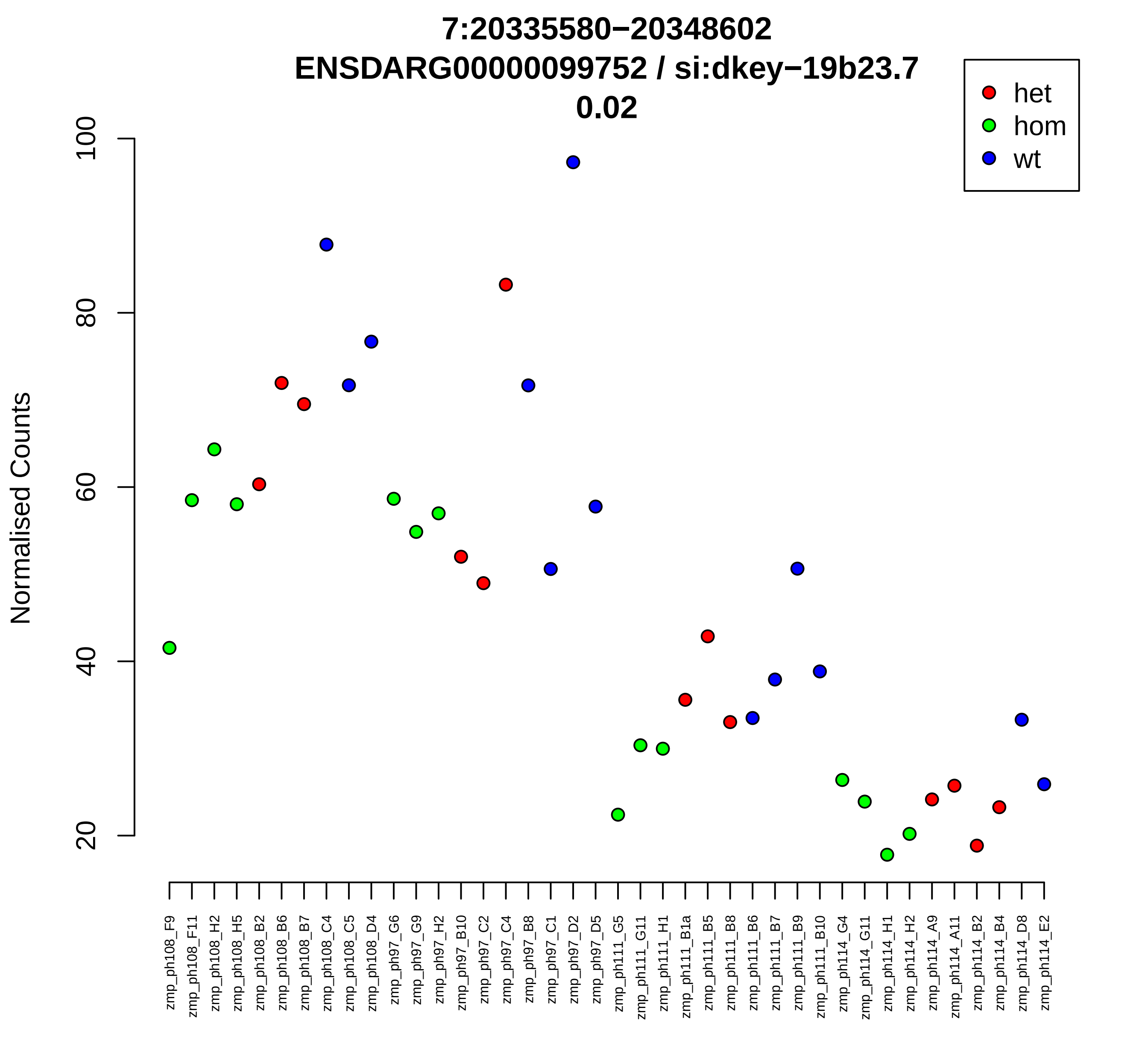
7:20335580−20348602
ENSDARG00000099752 / si:dkey−19b23.7
0.02
het
hom
wt
Normalised Counts
20
40
60
80
100
zmp_ph108_F9
zmp_ph108_F11
zmp_ph108_H2
zmp_ph108_H5
zmp_ph108_B2
zmp_ph108_B6
zmp_ph108_B7
zmp_ph108_C4
zmp_ph108_C5
zmp_ph108_D4
zmp_ph97_G6
zmp_ph97_G9
zmp_ph97_H2
zmp_ph97_B10
zmp_ph97_C2
zmp_ph97_C4
zmp_ph97_B8
zmp_ph97_C1
zmp_ph97_D2
zmp_ph97_D5
zmp_ph111_G5
zmp_ph111_G11
zmp_ph111_H1
zmp_ph111_B1a
zmp_ph111_B5
zmp_ph111_B8
zmp_ph111_B6
zmp_ph111_B7
zmp_ph111_B9
zmp_ph111_B10
zmp_ph114_G4
zmp_ph114_G11
zmp_ph114_H1
zmp_ph114_H2
zmp_ph114_A9
zmp_ph114_A11
zmp_ph114_B2
zmp_ph114_B4
zmp_ph114_D8
zmp_ph114_E2

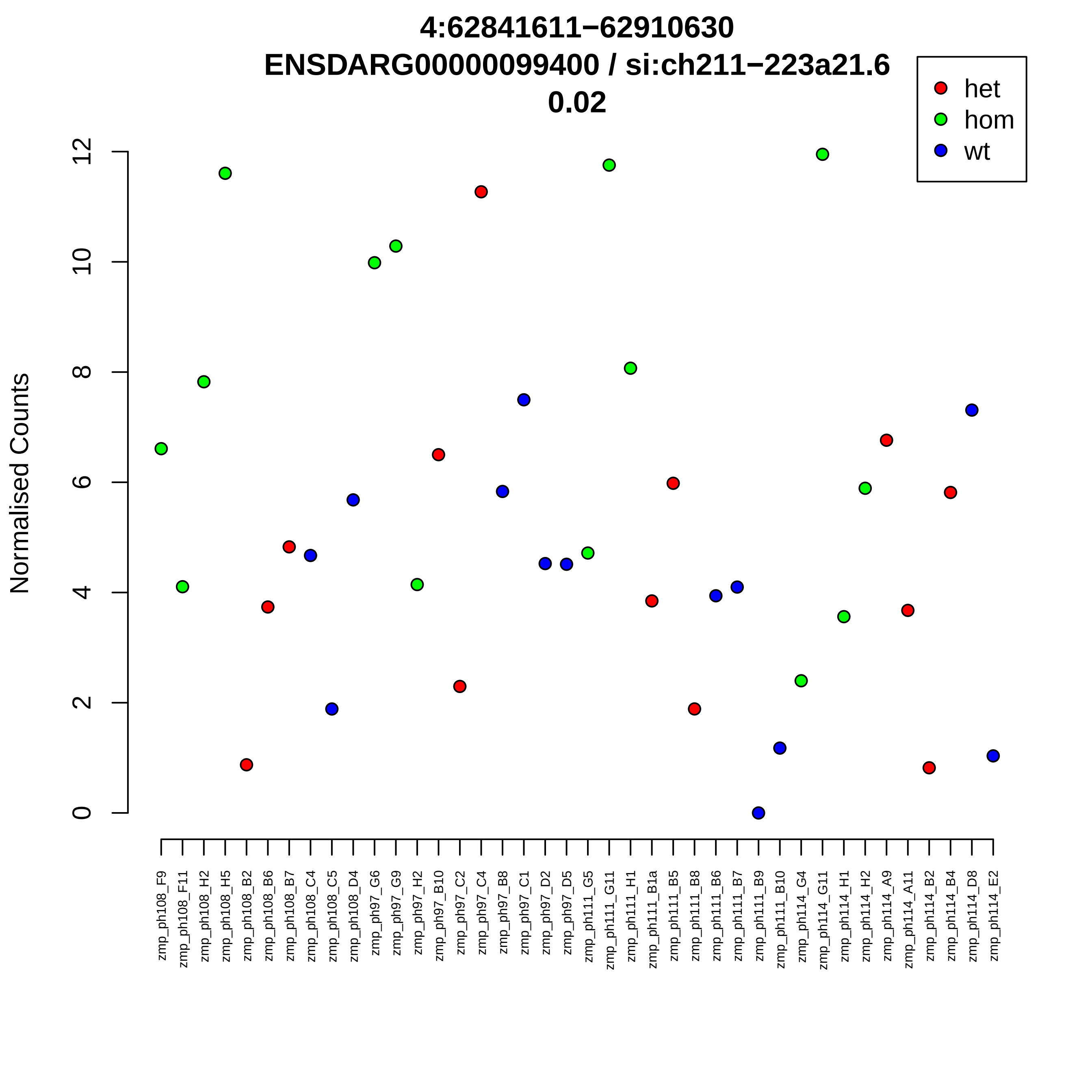
4:62841611−62910630
ENSDARG00000099400 / si:ch211−223a21.6
0.02
het
hom
wt
Normalised Counts
0
2
4
6
8
10
12
zmp_ph108_F9
zmp_ph108_F11
zmp_ph108_H2
zmp_ph108_H5
zmp_ph108_B2
zmp_ph108_B6
zmp_ph108_B7
zmp_ph108_C4
zmp_ph108_C5
zmp_ph108_D4
zmp_ph97_G6
zmp_ph97_G9
zmp_ph97_H2
zmp_ph97_B10
zmp_ph97_C2
zmp_ph97_C4
zmp_ph97_B8
zmp_ph97_C1
zmp_ph97_D2
zmp_ph97_D5
zmp_ph111_G5
zmp_ph111_G11
zmp_ph111_H1
zmp_ph111_B1a
zmp_ph111_B5
zmp_ph111_B8
zmp_ph111_B6
zmp_ph111_B7
zmp_ph111_B9
zmp_ph111_B10
zmp_ph114_G4
zmp_ph114_G11
zmp_ph114_H1
zmp_ph114_H2
zmp_ph114_A9
zmp_ph114_A11
zmp_ph114_B2
zmp_ph114_B4
zmp_ph114_D8
zmp_ph114_E2

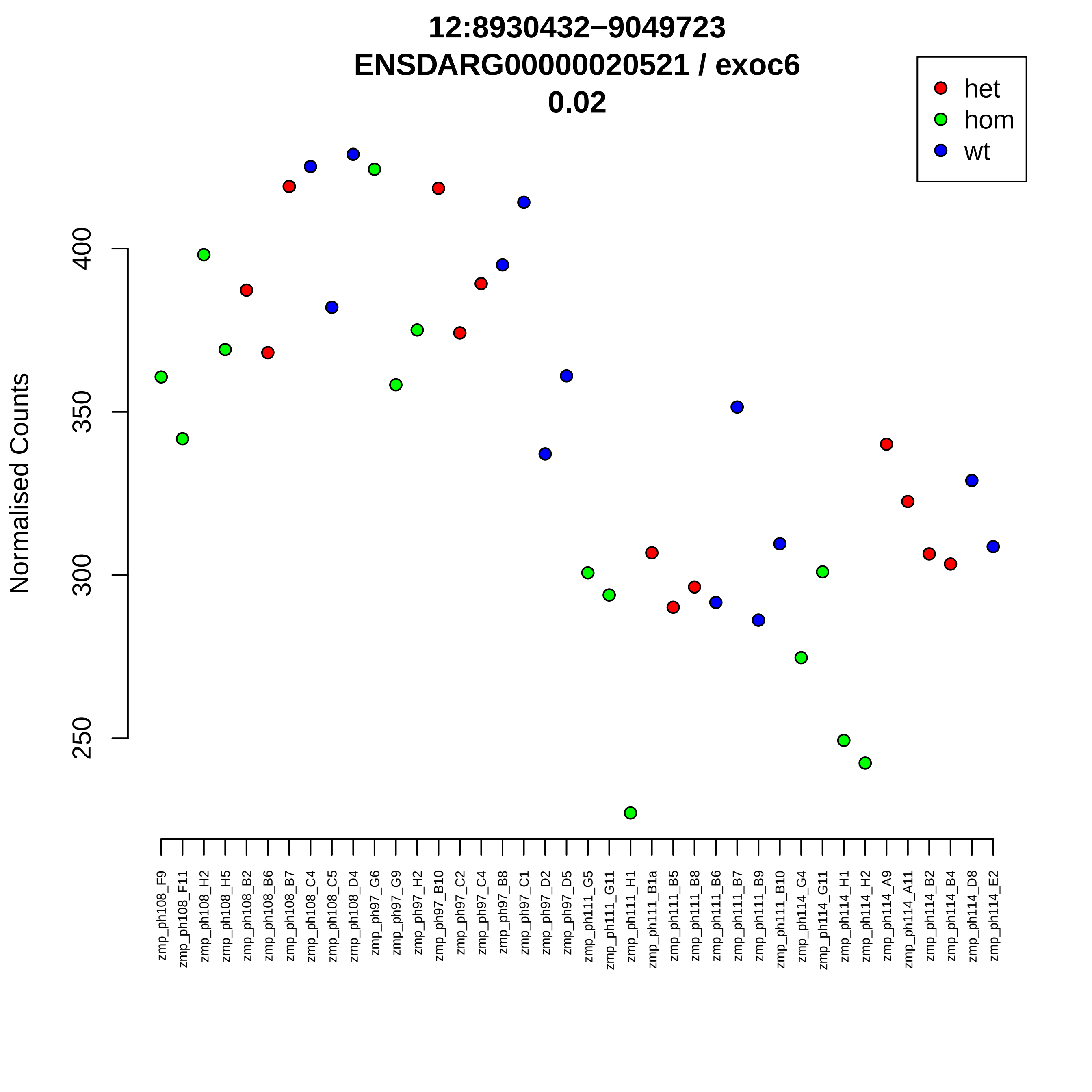
12:8930432−9049723
ENSDARG00000020521 / exoc6
0.02
het
hom
wt
Normalised Counts
250
300
350
400
zmp_ph108_F9
zmp_ph108_F11
zmp_ph108_H2
zmp_ph108_H5
zmp_ph108_B2
zmp_ph108_B6
zmp_ph108_B7
zmp_ph108_C4
zmp_ph108_C5
zmp_ph108_D4
zmp_ph97_G6
zmp_ph97_G9
zmp_ph97_H2
zmp_ph97_B10
zmp_ph97_C2
zmp_ph97_C4
zmp_ph97_B8
zmp_ph97_C1
zmp_ph97_D2
zmp_ph97_D5
zmp_ph111_G5
zmp_ph111_G11
zmp_ph111_H1
zmp_ph111_B1a
zmp_ph111_B5
zmp_ph111_B8
zmp_ph111_B6
zmp_ph111_B7
zmp_ph111_B9
zmp_ph111_B10
zmp_ph114_G4
zmp_ph114_G11
zmp_ph114_H1
zmp_ph114_H2
zmp_ph114_A9
zmp_ph114_A11
zmp_ph114_B2
zmp_ph114_B4
zmp_ph114_D8
zmp_ph114_E2

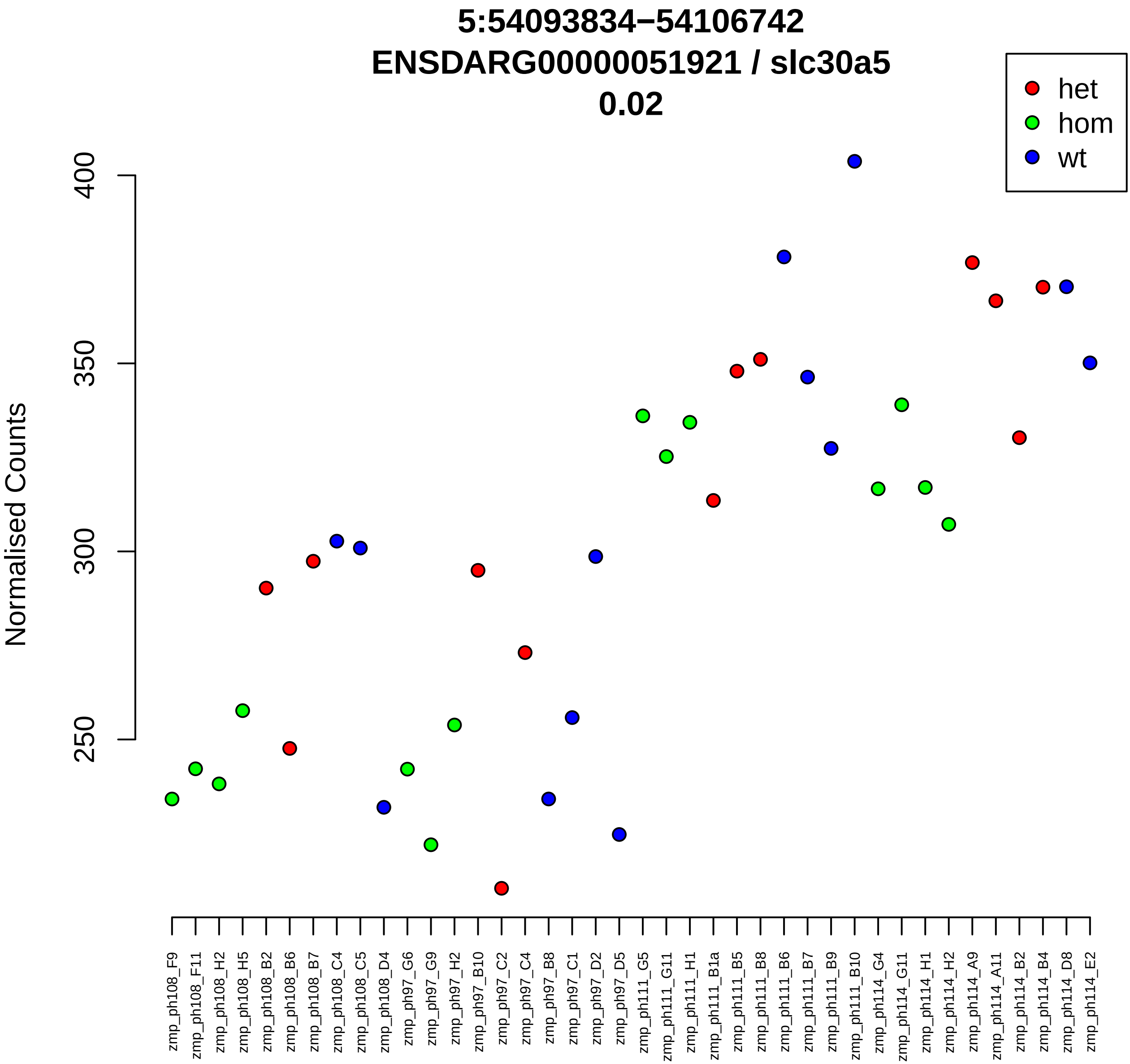

5:54093834−54106742
ENSDARG00000051921 / slc30a5
0.02
het
hom
wt
Normalised Counts
400
350
300
250
zmp_ph108_F9
zmp_ph108_F11
zmp_ph108_H2
zmp_ph108_H5
zmp_ph108_B2
zmp_ph108_B6
zmp_ph108_B7
zmp_ph108_C4
zmp_ph108_C5
zmp_ph108_D4
zmp_ph97_G6
zmp_ph97_G9
zmp_ph97_H2
zmp_ph97_B10
zmp_ph97_C2
zmp_ph97_C4
zmp_ph97_B8
zmp_ph97_C1
zmp_ph97_D2
zmp_ph97_D5
zmp_ph111_G5
zmp_ph111_G11
zmp_ph111_H1
zmp_ph111_B1a
zmp_ph111_B5
zmp_ph111_B8
zmp_ph111_B6
zmp_ph111_B7
zmp_ph111_B9
zmp_ph111_B10
zmp_ph114_G4
zmp_ph114_G11
zmp_ph114_H1
zmp_ph114_H2
zmp_ph114_A9
zmp_ph114_A11
zmp_ph114_B2
zmp_ph114_B4
zmp_ph114_D8
zmp_ph114_E2

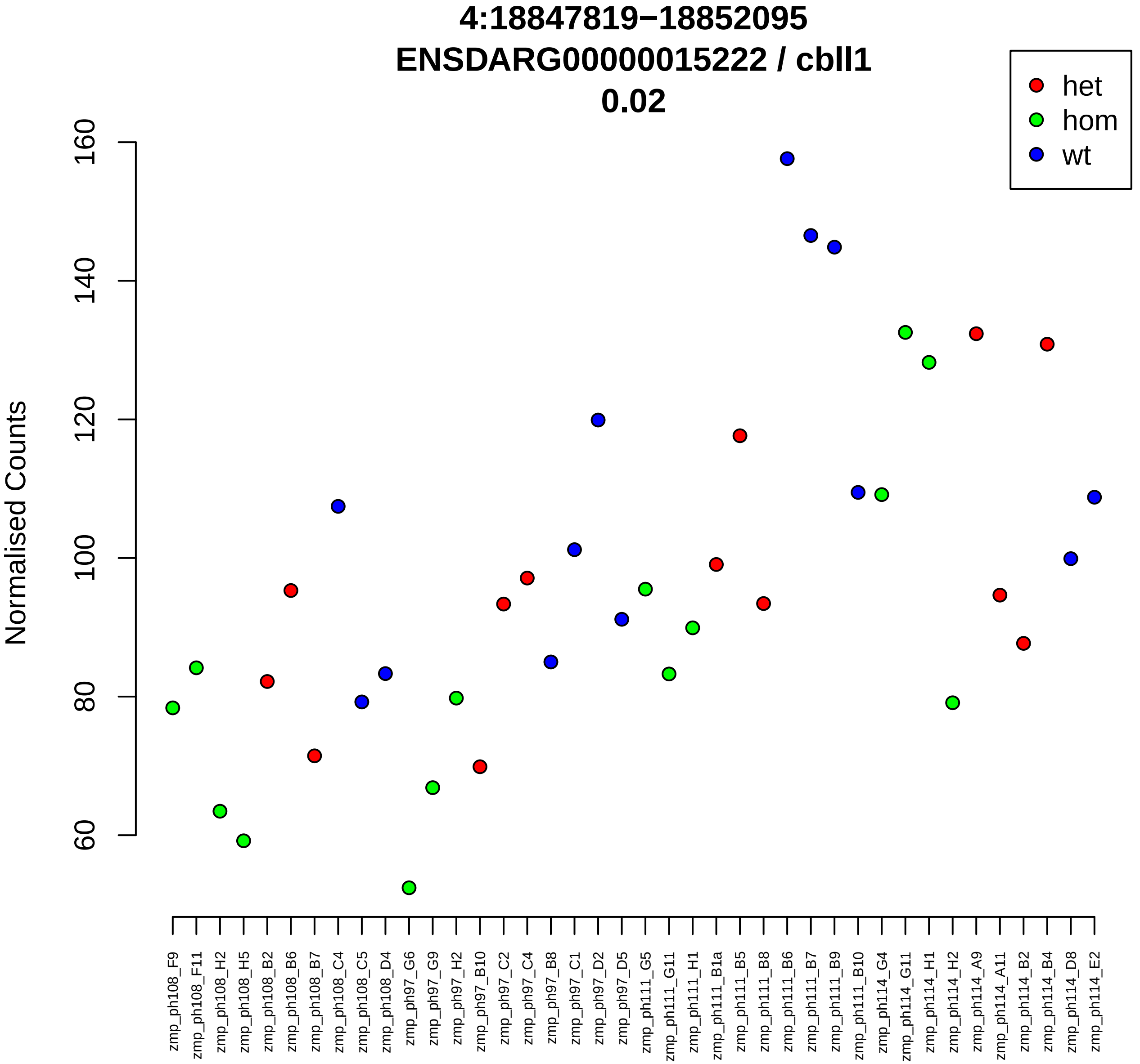

4:18847819−18852095
ENSDARG00000015222 / cbll1
0.02
het
hom
wt
Normalised Counts
60
80
100
120
140
160
zmp_ph108_F9
zmp_ph108_F11
zmp_ph108_H2
zmp_ph108_H5
zmp_ph108_B2
zmp_ph108_B6
zmp_ph108_B7
zmp_ph108_C4
zmp_ph108_C5
zmp_ph108_D4
zmp_ph97_G6
zmp_ph97_G9
zmp_ph97_H2
zmp_ph97_B10
zmp_ph97_C2
zmp_ph97_C4
zmp_ph97_B8
zmp_ph97_C1
zmp_ph97_D2
zmp_ph97_D5
zmp_ph111_G5
zmp_ph111_G11
zmp_ph111_H1
zmp_ph111_B1a
zmp_ph111_B5
zmp_ph111_B8
zmp_ph111_B6
zmp_ph111_B7
zmp_ph111_B9
zmp_ph111_B10
zmp_ph114_G4
zmp_ph114_G11
zmp_ph114_H1
zmp_ph114_H2
zmp_ph114_A9
zmp_ph114_A11
zmp_ph114_B2
zmp_ph114_B4
zmp_ph114_D8
zmp_ph114_E2

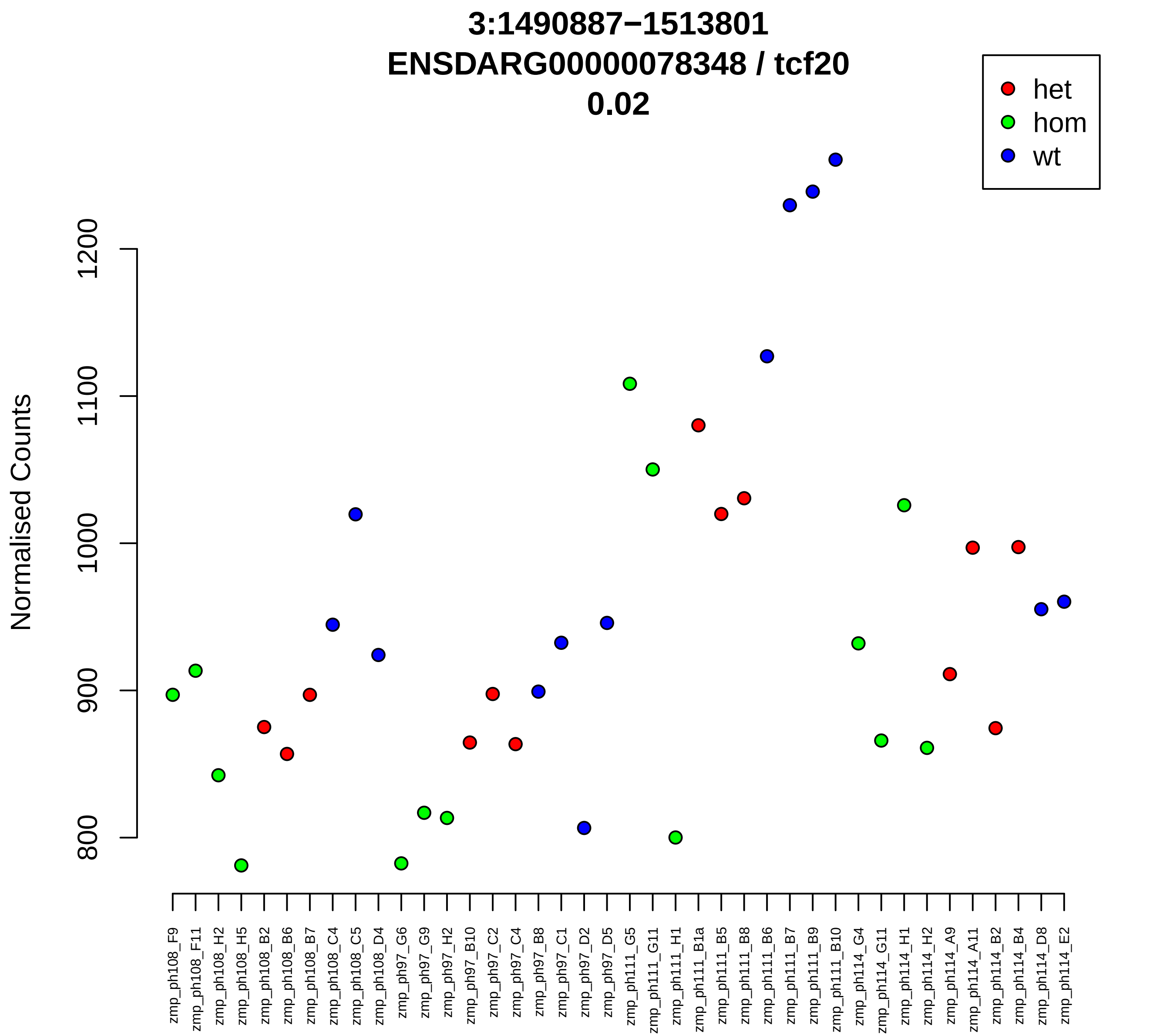

3:1490887−1513801
ENSDARG00000078348 / tcf20
0.02
het
hom
wt
Normalised Counts
800
900
1000
1100
1200
zmp_ph108_F9
zmp_ph108_F11
zmp_ph108_H2
zmp_ph108_H5
zmp_ph108_B2
zmp_ph108_B6
zmp_ph108_B7
zmp_ph108_C4
zmp_ph108_C5
zmp_ph108_D4
zmp_ph97_G6
zmp_ph97_G9
zmp_ph97_H2
zmp_ph97_B10
zmp_ph97_C2
zmp_ph97_C4
zmp_ph97_B8
zmp_ph97_C1
zmp_ph97_D2
zmp_ph97_D5
zmp_ph111_G5
zmp_ph111_G11
zmp_ph111_H1
zmp_ph111_B1a
zmp_ph111_B5
zmp_ph111_B8
zmp_ph111_B6
zmp_ph111_B7
zmp_ph111_B9
zmp_ph111_B10
zmp_ph114_G4
zmp_ph114_G11
zmp_ph114_H1
zmp_ph114_H2
zmp_ph114_A9
zmp_ph114_A11
zmp_ph114_B2
zmp_ph114_B4
zmp_ph114_D8
zmp_ph114_E2

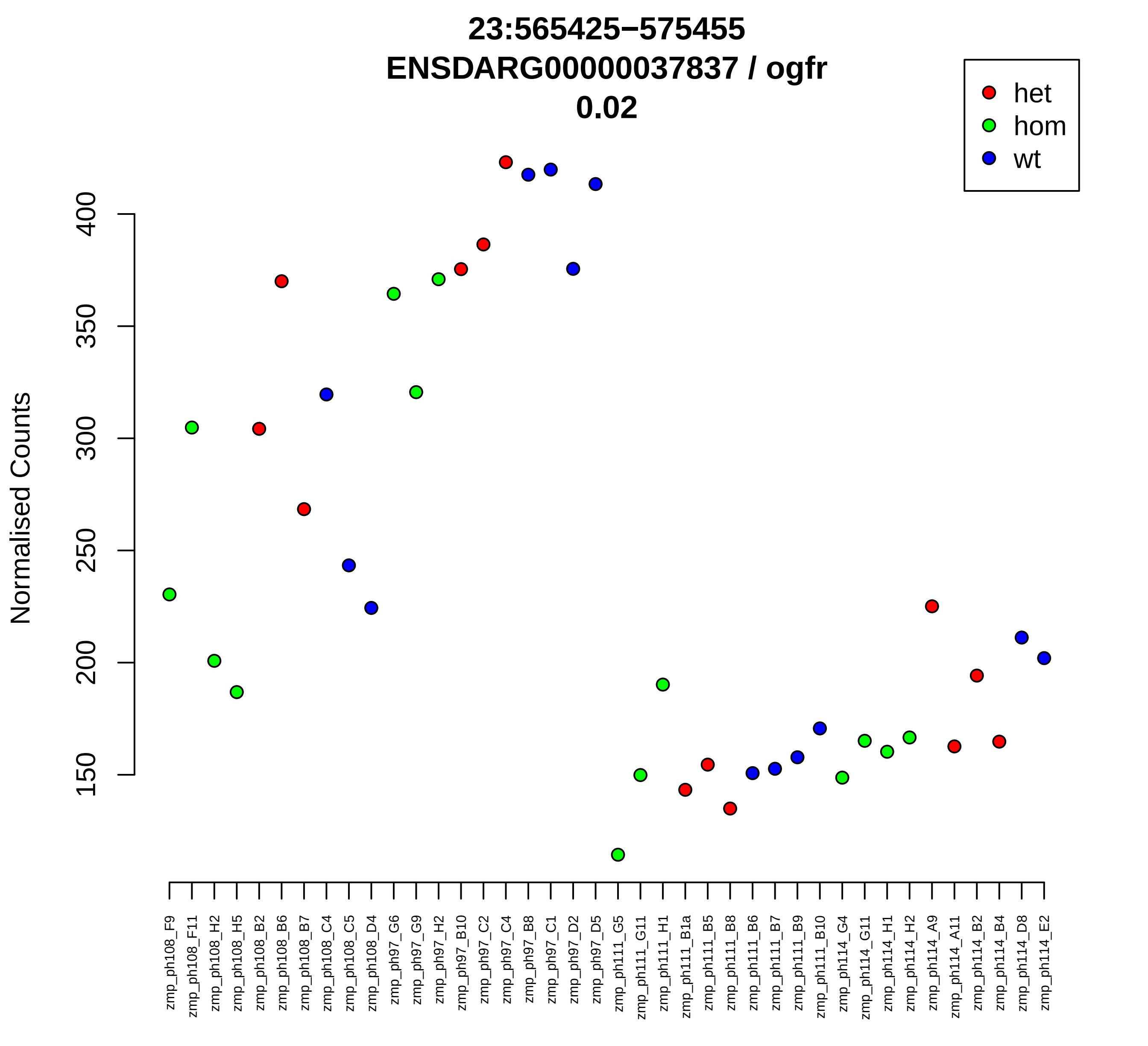

23:565425−575455
ENSDARG00000037837 / ogfr
0.02
het
hom
wt
Normalised Counts
150
200
250
300
350
400
zmp_ph108_F9
zmp_ph108_F11
zmp_ph108_H2
zmp_ph108_H5
zmp_ph108_B2
zmp_ph108_B6
zmp_ph108_B7
zmp_ph108_C4
zmp_ph108_C5
zmp_ph108_D4
zmp_ph97_G6
zmp_ph97_G9
zmp_ph97_H2
zmp_ph97_B10
zmp_ph97_C2
zmp_ph97_C4
zmp_ph97_B8
zmp_ph97_C1
zmp_ph97_D2
zmp_ph97_D5
zmp_ph111_G5
zmp_ph111_G11
zmp_ph111_H1
zmp_ph111_B1a
zmp_ph111_B5
zmp_ph111_B8
zmp_ph111_B6
zmp_ph111_B7
zmp_ph111_B9
zmp_ph111_B10
zmp_ph114_G4
zmp_ph114_G11
zmp_ph114_H1
zmp_ph114_H2
zmp_ph114_A9
zmp_ph114_A11
zmp_ph114_B2
zmp_ph114_B4
zmp_ph114_D8
zmp_ph114_E2

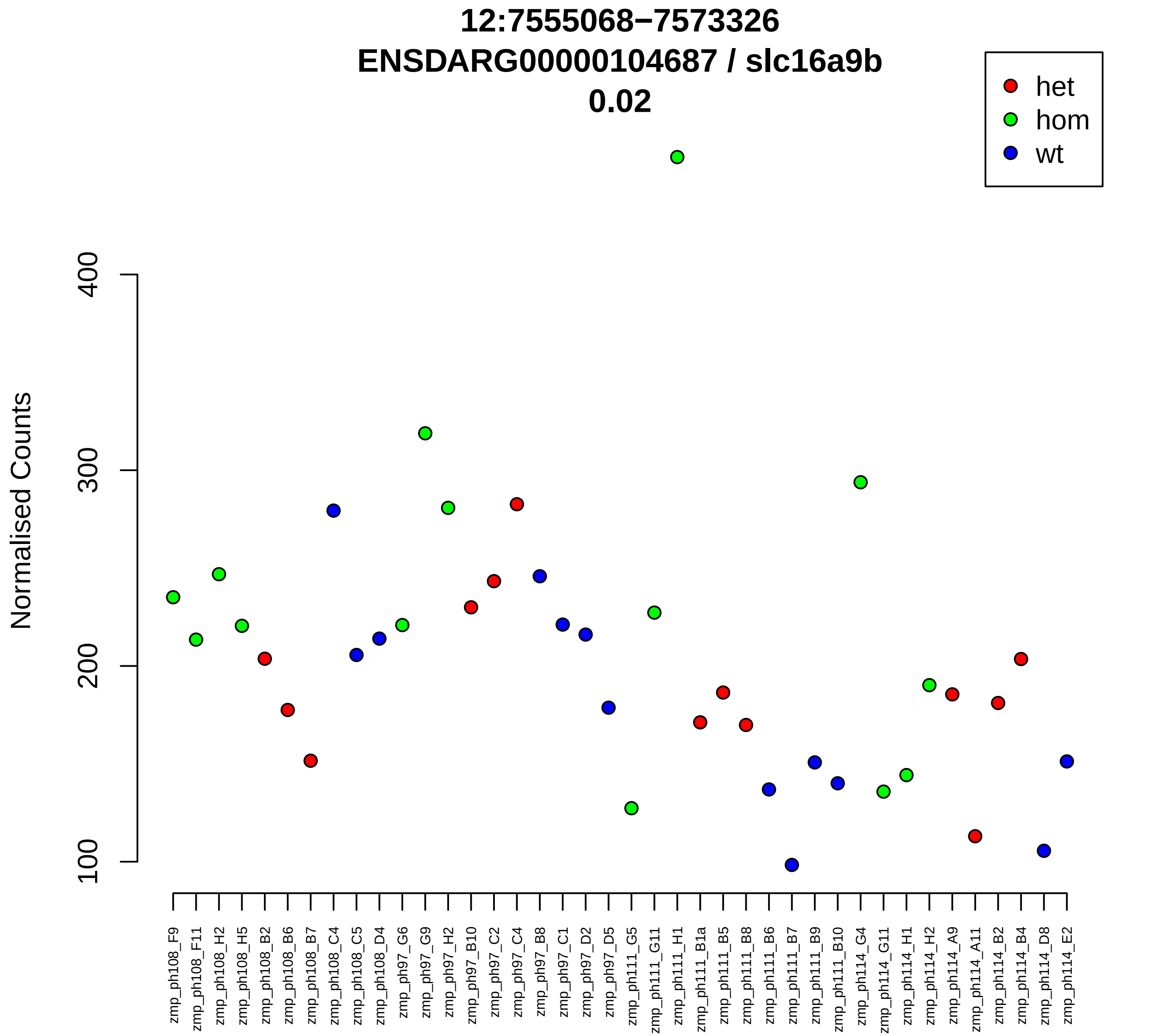
12:7555068−7573326
ENSDARG00000104687 / slc16a9b
0.02
het
hom
wt
Normalised Counts
100
200
300
400
zmp_ph108_F9
zmp_ph108_F11
zmp_ph108_H2
zmp_ph108_H5
zmp_ph108_B2
zmp_ph108_B6
zmp_ph108_B7
zmp_ph108_C4
zmp_ph108_C5
zmp_ph108_D4
zmp_ph97_G6
zmp_ph97_G9
zmp_ph97_H2
zmp_ph97_B10
zmp_ph97_C2
zmp_ph97_C4
zmp_ph97_B8
zmp_ph97_C1
zmp_ph97_D2
zmp_ph97_D5
zmp_ph111_G5
zmp_ph111_G11
zmp_ph111_H1
zmp_ph111_B1a
zmp_ph111_B5
zmp_ph111_B8
zmp_ph111_B6
zmp_ph111_B7
zmp_ph111_B9
zmp_ph111_B10
zmp_ph114_G4
zmp_ph114_G11
zmp_ph114_H1
zmp_ph114_H2
zmp_ph114_A9
zmp_ph114_A11
zmp_ph114_B2
zmp_ph114_B4
zmp_ph114_D8
zmp_ph114_E2

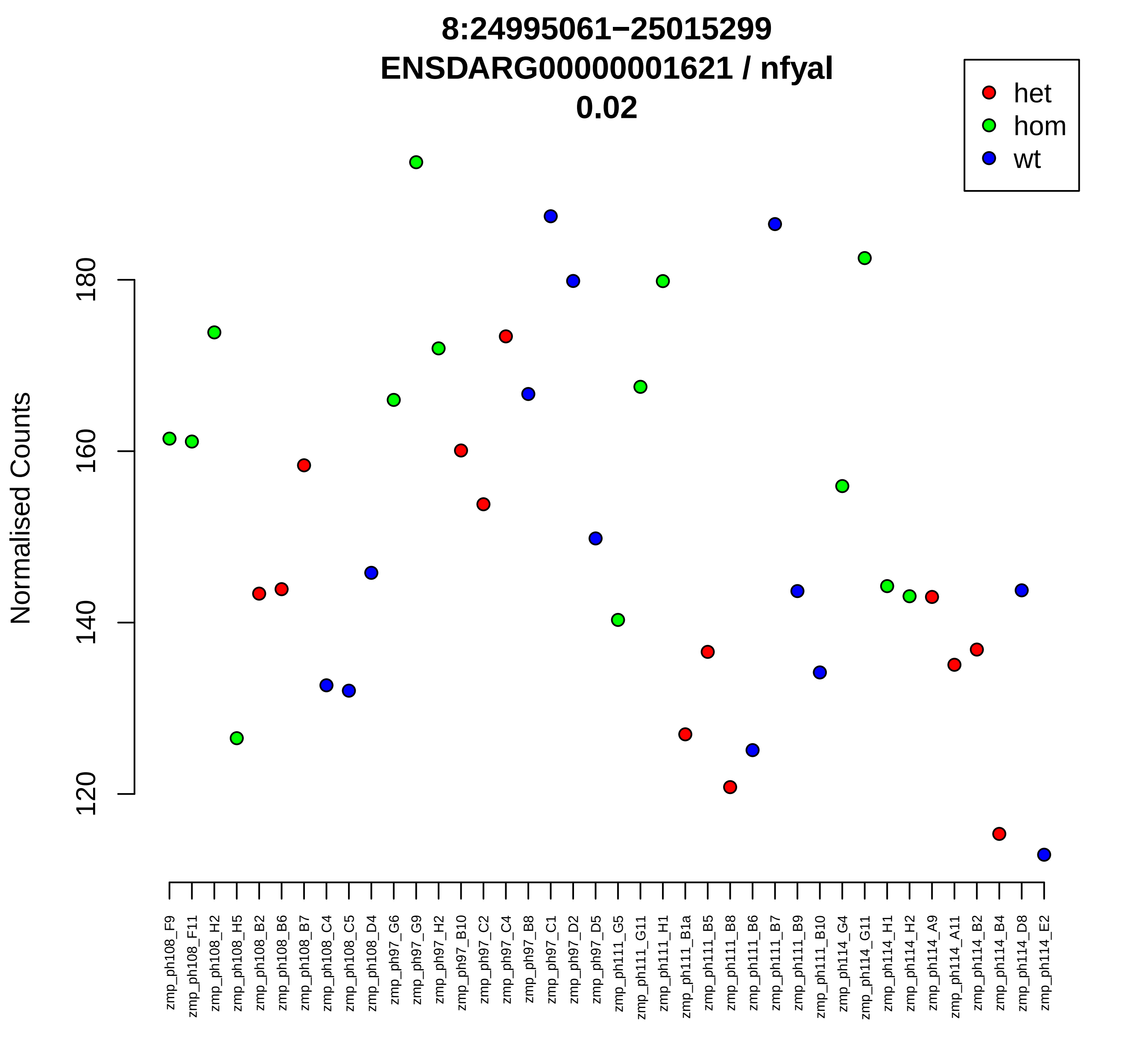
8:24995061−25015299
ENSDARG00000001621 / nfyal
0.02
het
hom
wt
Normalised Counts
120
140
160
180
zmp_ph108_F9
zmp_ph108_F11
zmp_ph108_H2
zmp_ph108_H5
zmp_ph108_B2
zmp_ph108_B6
zmp_ph108_B7
zmp_ph108_C4
zmp_ph108_C5
zmp_ph108_D4
zmp_ph97_G6
zmp_ph97_G9
zmp_ph97_H2
zmp_ph97_B10
zmp_ph97_C2
zmp_ph97_C4
zmp_ph97_B8
zmp_ph97_C1
zmp_ph97_D2
zmp_ph97_D5
zmp_ph111_G5
zmp_ph111_G11
zmp_ph111_H1
zmp_ph111_B1a
zmp_ph111_B5
zmp_ph111_B8
zmp_ph111_B6
zmp_ph111_B7
zmp_ph111_B9
zmp_ph111_B10
zmp_ph114_G4
zmp_ph114_G11
zmp_ph114_H1
zmp_ph114_H2
zmp_ph114_A9
zmp_ph114_A11
zmp_ph114_B2
zmp_ph114_B4
zmp_ph114_D8
zmp_ph114_E2

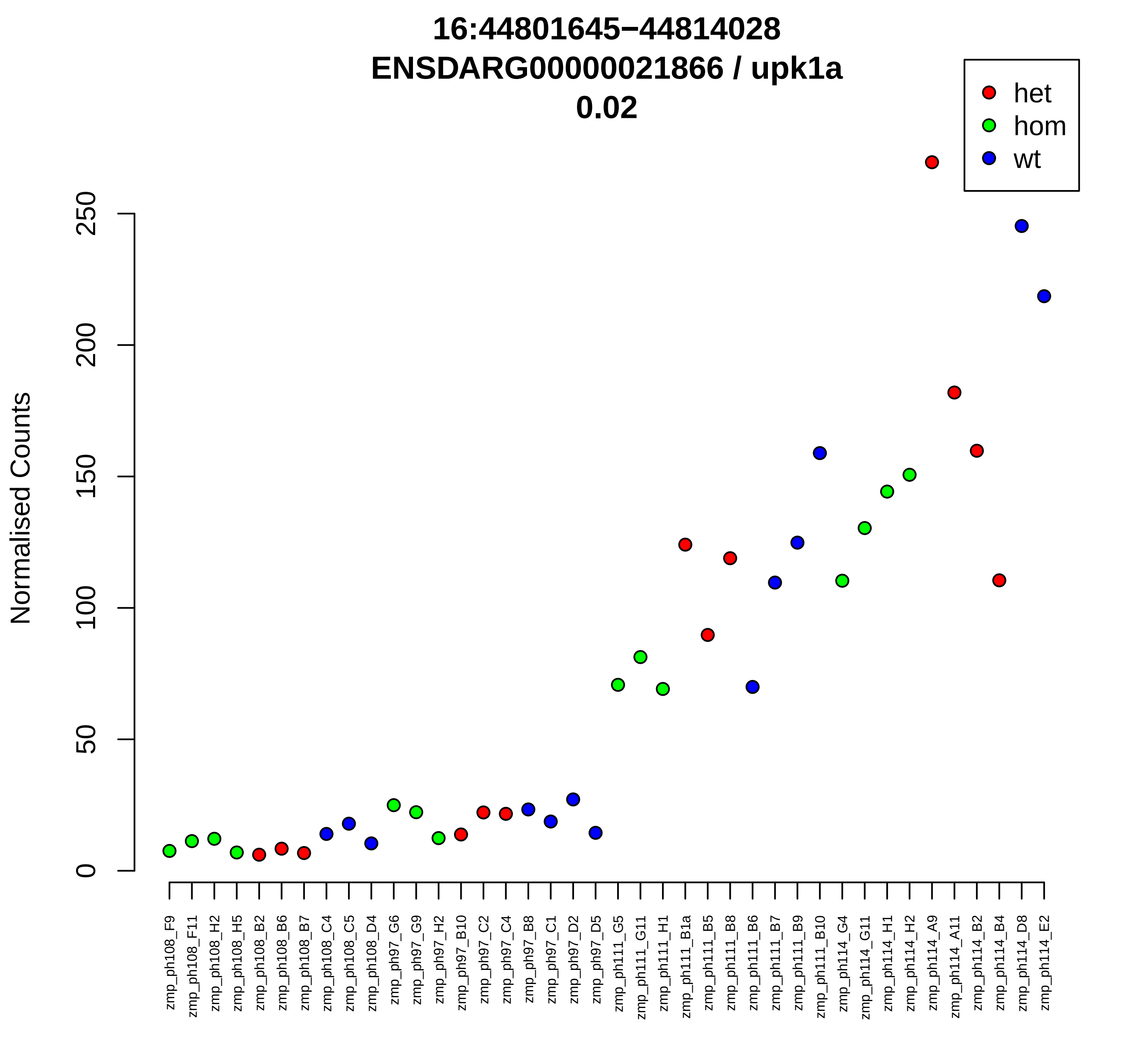

16:44801645−44814028
ENSDARG00000021866 / upk1a
0.02
het
hom
wt
Normalised Counts
0
50
100
150
200
250
zmp_ph108_F9
zmp_ph108_F11
zmp_ph108_H2
zmp_ph108_H5
zmp_ph108_B2
zmp_ph108_B6
zmp_ph108_B7
zmp_ph108_C4
zmp_ph108_C5
zmp_ph108_D4
zmp_ph97_G6
zmp_ph97_G9
zmp_ph97_H2
zmp_ph97_B10
zmp_ph97_C2
zmp_ph97_C4
zmp_ph97_B8
zmp_ph97_C1
zmp_ph97_D2
zmp_ph97_D5
zmp_ph111_G5
zmp_ph111_G11
zmp_ph111_H1
zmp_ph111_B1a
zmp_ph111_B5
zmp_ph111_B8
zmp_ph111_B6
zmp_ph111_B7
zmp_ph111_B9
zmp_ph111_B10
zmp_ph114_G4
zmp_ph114_G11
zmp_ph114_H1
zmp_ph114_H2
zmp_ph114_A9
zmp_ph114_A11
zmp_ph114_B2
zmp_ph114_B4
zmp_ph114_D8
zmp_ph114_E2

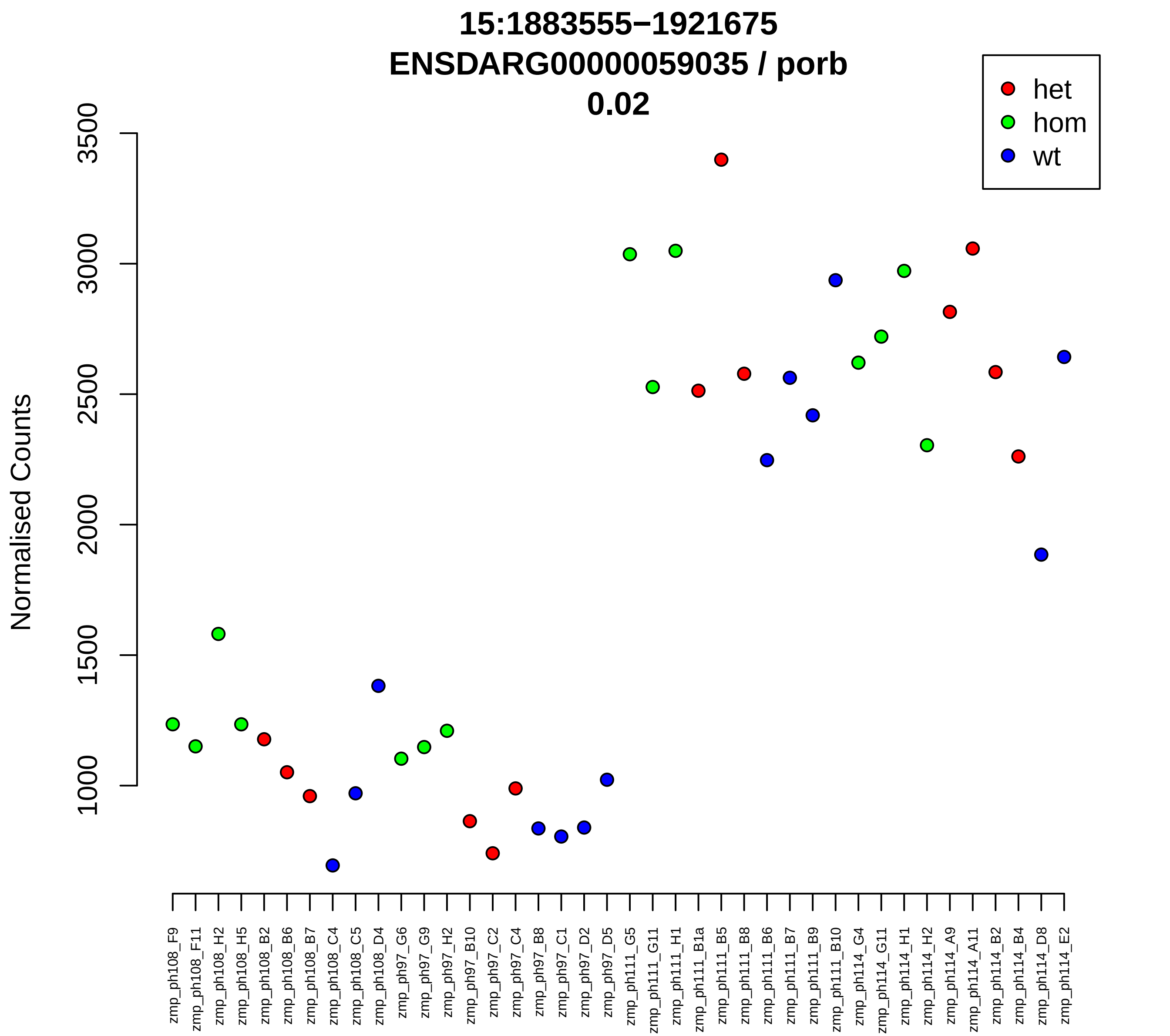

15:1883555−1921675
ENSDARG00000059035 / porb
0.02
het
hom
wt
Normalised Counts
1000
1500
2000
2500
3000
3500
zmp_ph108_F9
zmp_ph108_F11
zmp_ph108_H2
zmp_ph108_H5
zmp_ph108_B2
zmp_ph108_B6
zmp_ph108_B7
zmp_ph108_C4
zmp_ph108_C5
zmp_ph108_D4
zmp_ph97_G6
zmp_ph97_G9
zmp_ph97_H2
zmp_ph97_B10
zmp_ph97_C2
zmp_ph97_C4
zmp_ph97_B8
zmp_ph97_C1
zmp_ph97_D2
zmp_ph97_D5
zmp_ph111_G5
zmp_ph111_G11
zmp_ph111_H1
zmp_ph111_B1a
zmp_ph111_B5
zmp_ph111_B8
zmp_ph111_B6
zmp_ph111_B7
zmp_ph111_B9
zmp_ph111_B10
zmp_ph114_G4
zmp_ph114_G11
zmp_ph114_H1
zmp_ph114_H2
zmp_ph114_A9
zmp_ph114_A11
zmp_ph114_B2
zmp_ph114_B4
zmp_ph114_D8
zmp_ph114_E2

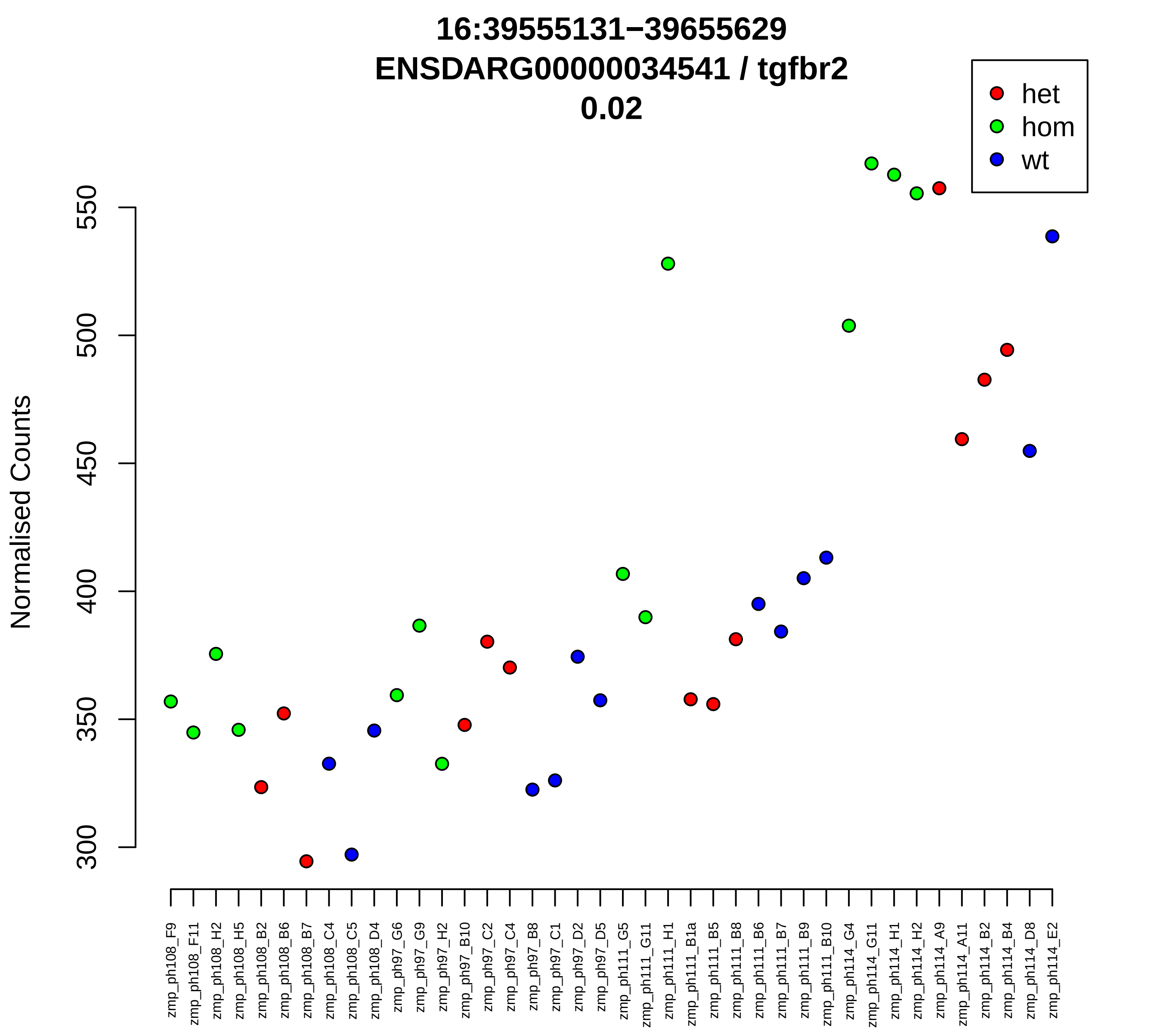

16:39555131−39655629
ENSDARG00000034541 / tgfbr2
0.02
het
hom
wt
Normalised Counts
300
350
400
450
500
550
zmp_ph108_F9
zmp_ph108_F11
zmp_ph108_H2
zmp_ph108_H5
zmp_ph108_B2
zmp_ph108_B6
zmp_ph108_B7
zmp_ph108_C4
zmp_ph108_C5
zmp_ph108_D4
zmp_ph97_G6
zmp_ph97_G9
zmp_ph97_H2
zmp_ph97_B10
zmp_ph97_C2
zmp_ph97_C4
zmp_ph97_B8
zmp_ph97_C1
zmp_ph97_D2
zmp_ph97_D5
zmp_ph111_G5
zmp_ph111_G11
zmp_ph111_H1
zmp_ph111_B1a
zmp_ph111_B5
zmp_ph111_B8
zmp_ph111_B6
zmp_ph111_B7
zmp_ph111_B9
zmp_ph111_B10
zmp_ph114_G4
zmp_ph114_G11
zmp_ph114_H1
zmp_ph114_H2
zmp_ph114_A9
zmp_ph114_A11
zmp_ph114_B2
zmp_ph114_B4
zmp_ph114_D8
zmp_ph114_E2

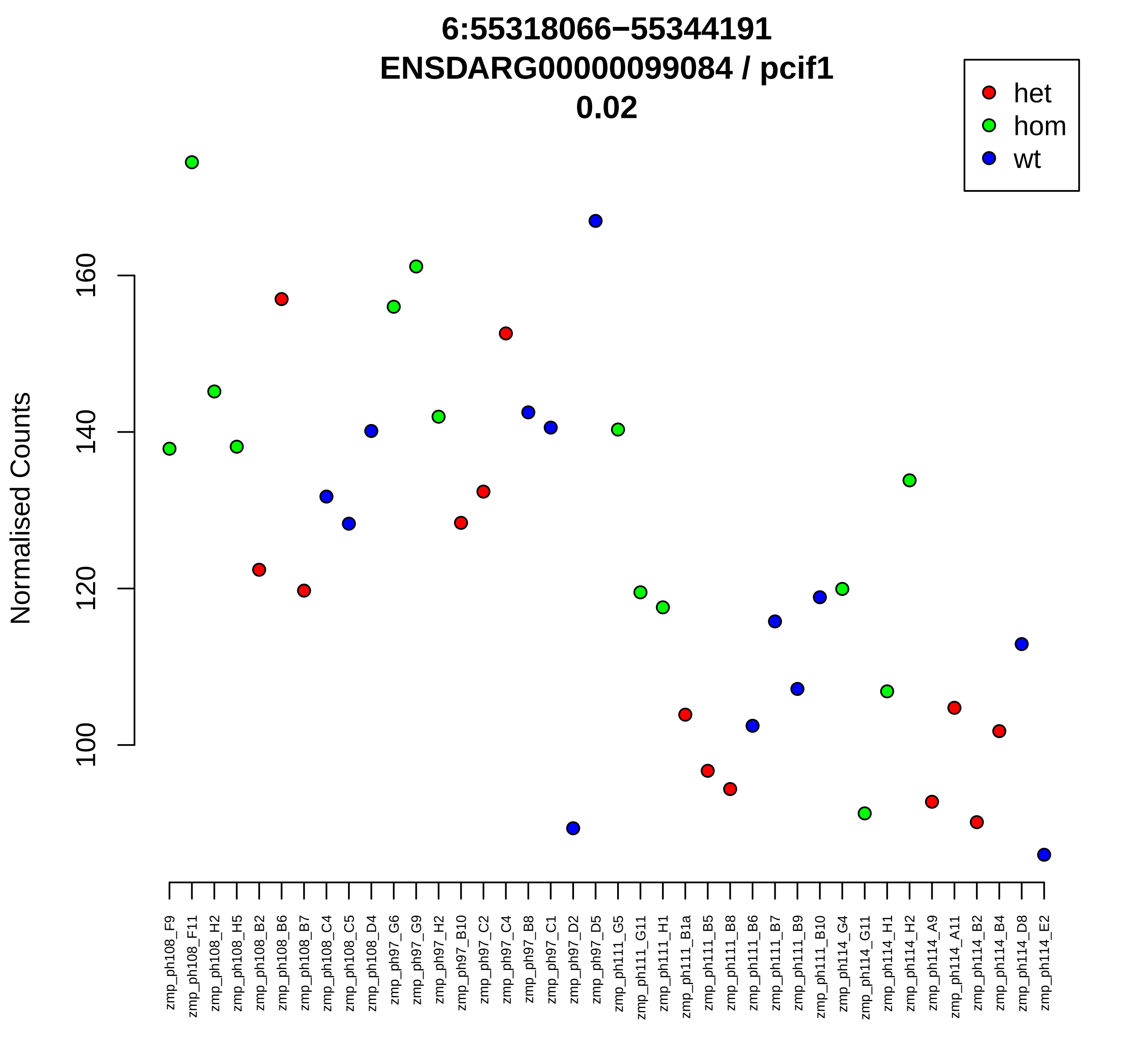
6:55318066−55344191
ENSDARG00000099084 / pcif1
0.02
het
hom
wt
Normalised Counts
100
120
140
160
zmp_ph108_F9
zmp_ph108_F11
zmp_ph108_H2
zmp_ph108_H5
zmp_ph108_B2
zmp_ph108_B6
zmp_ph108_B7
zmp_ph108_C4
zmp_ph108_C5
zmp_ph108_D4
zmp_ph97_G6
zmp_ph97_G9
zmp_ph97_H2
zmp_ph97_B10
zmp_ph97_C2
zmp_ph97_C4
zmp_ph97_B8
zmp_ph97_C1
zmp_ph97_D2
zmp_ph97_D5
zmp_ph111_G5
zmp_ph111_G11
zmp_ph111_H1
zmp_ph111_B1a
zmp_ph111_B5
zmp_ph111_B8
zmp_ph111_B6
zmp_ph111_B7
zmp_ph111_B9
zmp_ph111_B10
zmp_ph114_G4
zmp_ph114_G11
zmp_ph114_H1
zmp_ph114_H2
zmp_ph114_A9
zmp_ph114_A11
zmp_ph114_B2
zmp_ph114_B4
zmp_ph114_D8
zmp_ph114_E2

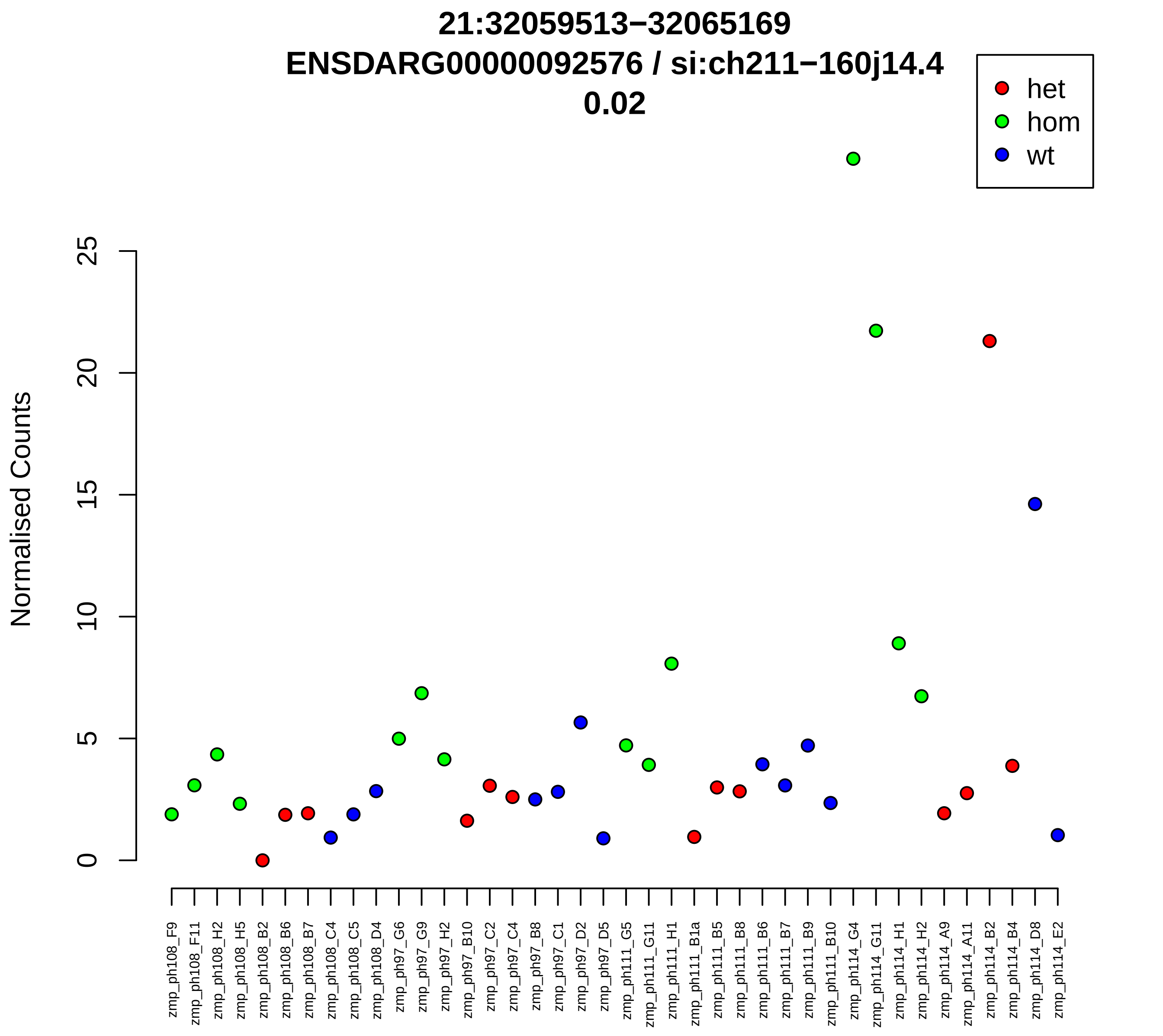

21:32059513−32065169
ENSDARG00000092576 / si:ch211−160j14.4
0.02
het
hom
wt
Normalised Counts
0
5
10
15
20
25
zmp_ph108_F9
zmp_ph108_F11
zmp_ph108_H2
zmp_ph108_H5
zmp_ph108_B2
zmp_ph108_B6
zmp_ph108_B7
zmp_ph108_C4
zmp_ph108_C5
zmp_ph108_D4
zmp_ph97_G6
zmp_ph97_G9
zmp_ph97_H2
zmp_ph97_B10
zmp_ph97_C2
zmp_ph97_C4
zmp_ph97_B8
zmp_ph97_C1
zmp_ph97_D2
zmp_ph97_D5
zmp_ph111_G5
zmp_ph111_G11
zmp_ph111_H1
zmp_ph111_B1a
zmp_ph111_B5
zmp_ph111_B8
zmp_ph111_B6
zmp_ph111_B7
zmp_ph111_B9
zmp_ph111_B10
zmp_ph114_G4
zmp_ph114_G11
zmp_ph114_H1
zmp_ph114_H2
zmp_ph114_A9
zmp_ph114_A11
zmp_ph114_B2
zmp_ph114_B4
zmp_ph114_D8
zmp_ph114_E2

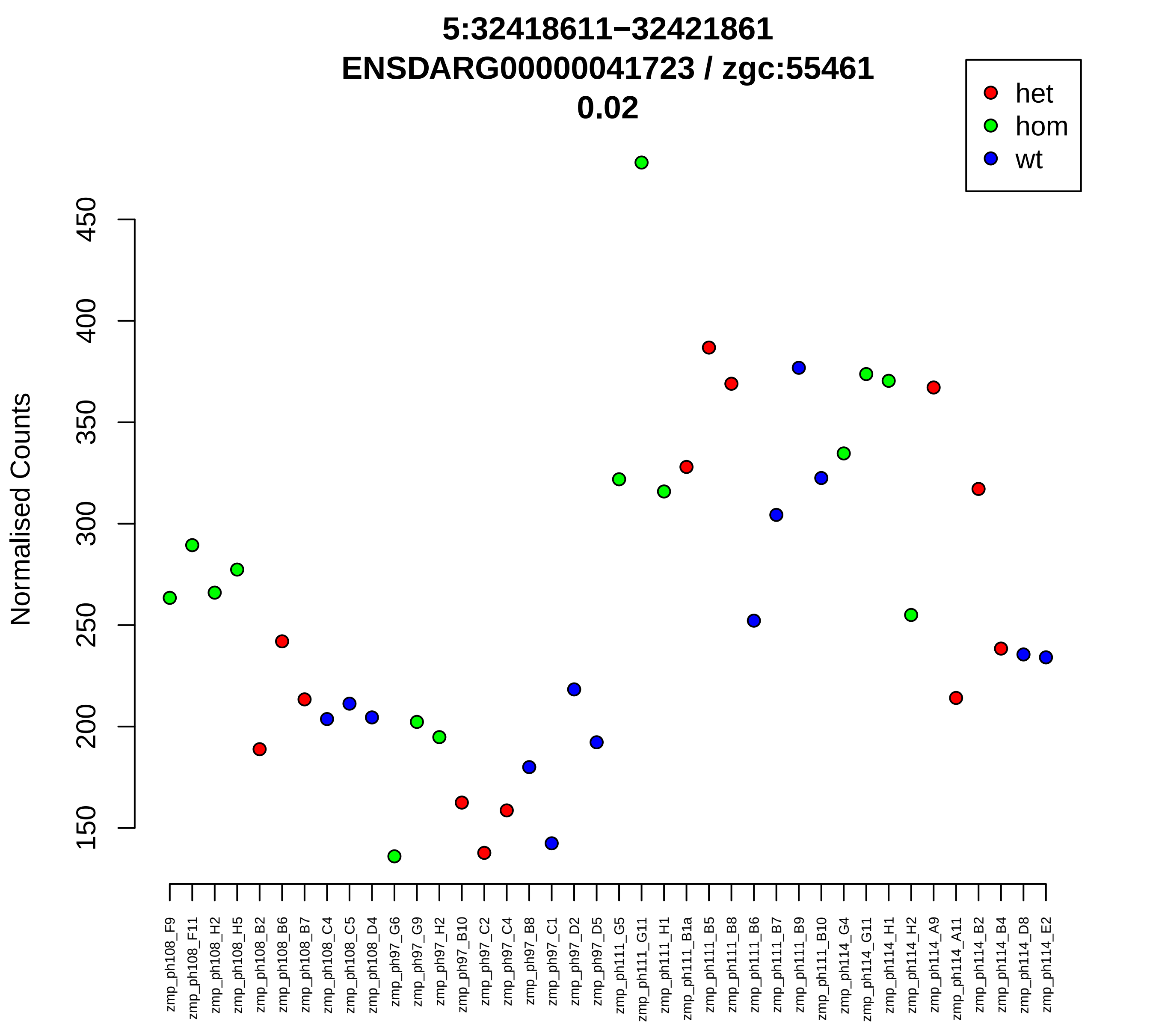

5:32418611−32421861
ENSDARG00000041723 / zgc:55461
0.02
het
hom
wt
Normalised Counts
150
200
250
300
350
400
450
zmp_ph108_F9
zmp_ph108_F11
zmp_ph108_H2
zmp_ph108_H5
zmp_ph108_B2
zmp_ph108_B6
zmp_ph108_B7
zmp_ph108_C4
zmp_ph108_C5
zmp_ph108_D4
zmp_ph97_G6
zmp_ph97_G9
zmp_ph97_H2
zmp_ph97_B10
zmp_ph97_C2
zmp_ph97_C4
zmp_ph97_B8
zmp_ph97_C1
zmp_ph97_D2
zmp_ph97_D5
zmp_ph111_G5
zmp_ph111_G11
zmp_ph111_H1
zmp_ph111_B1a
zmp_ph111_B5
zmp_ph111_B8
zmp_ph111_B6
zmp_ph111_B7
zmp_ph111_B9
zmp_ph111_B10
zmp_ph114_G4
zmp_ph114_G11
zmp_ph114_H1
zmp_ph114_H2
zmp_ph114_A9
zmp_ph114_A11
zmp_ph114_B2
zmp_ph114_B4
zmp_ph114_D8
zmp_ph114_E2

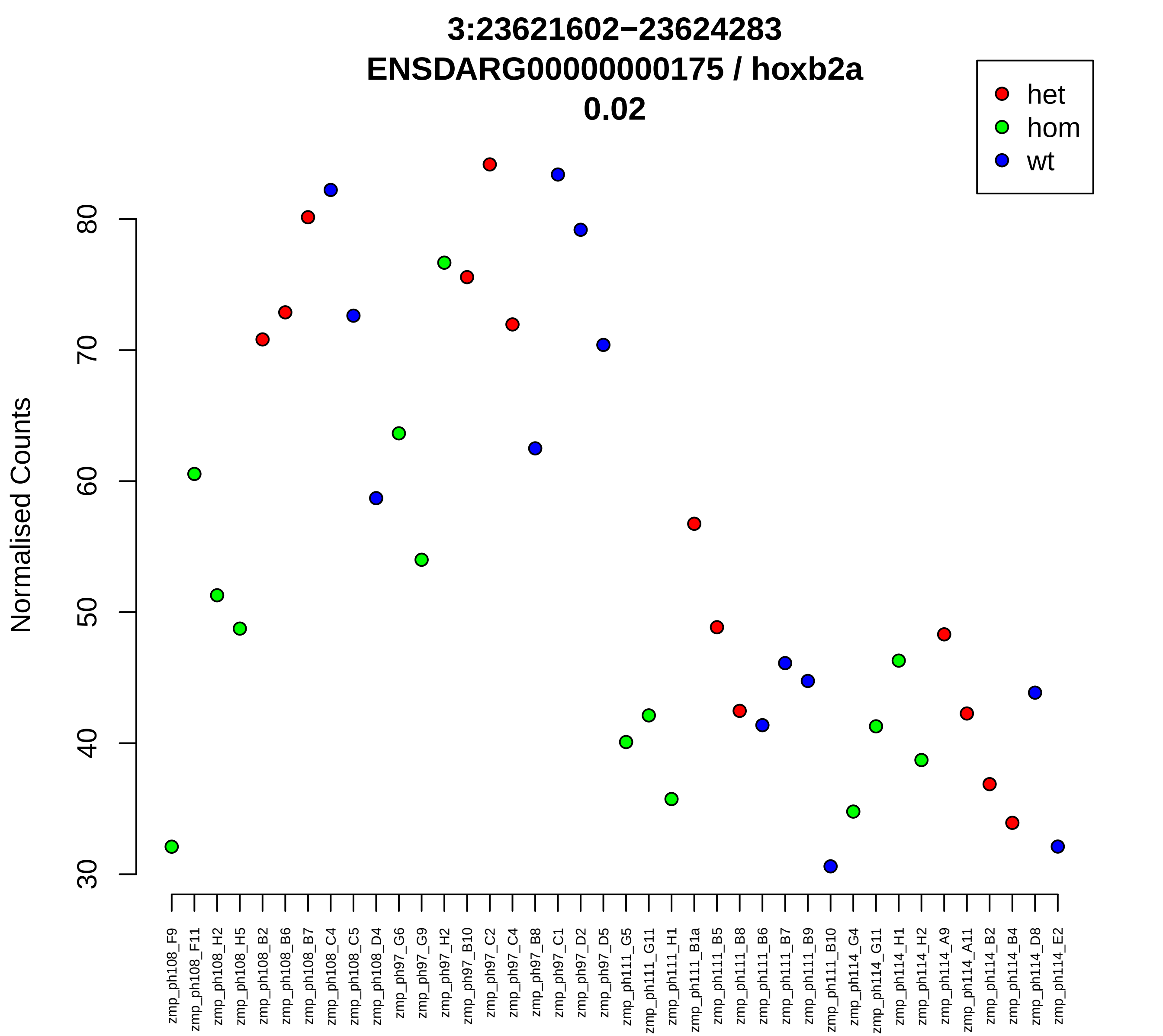

3:23621602−23624283
ENSDARG00000000175 / hoxb2a
0.02
het
hom
wt
Normalised Counts
30
40
50
60
70
80
zmp_ph108_F9
zmp_ph108_F11
zmp_ph108_H2
zmp_ph108_H5
zmp_ph108_B2
zmp_ph108_B6
zmp_ph108_B7
zmp_ph108_C4
zmp_ph108_C5
zmp_ph108_D4
zmp_ph97_G6
zmp_ph97_G9
zmp_ph97_H2
zmp_ph97_B10
zmp_ph97_C2
zmp_ph97_C4
zmp_ph97_B8
zmp_ph97_C1
zmp_ph97_D2
zmp_ph97_D5
zmp_ph111_G5
zmp_ph111_G11
zmp_ph111_H1
zmp_ph111_B1a
zmp_ph111_B5
zmp_ph111_B8
zmp_ph111_B6
zmp_ph111_B7
zmp_ph111_B9
zmp_ph111_B10
zmp_ph114_G4
zmp_ph114_G11
zmp_ph114_H1
zmp_ph114_H2
zmp_ph114_A9
zmp_ph114_A11
zmp_ph114_B2
zmp_ph114_B4
zmp_ph114_D8
zmp_ph114_E2

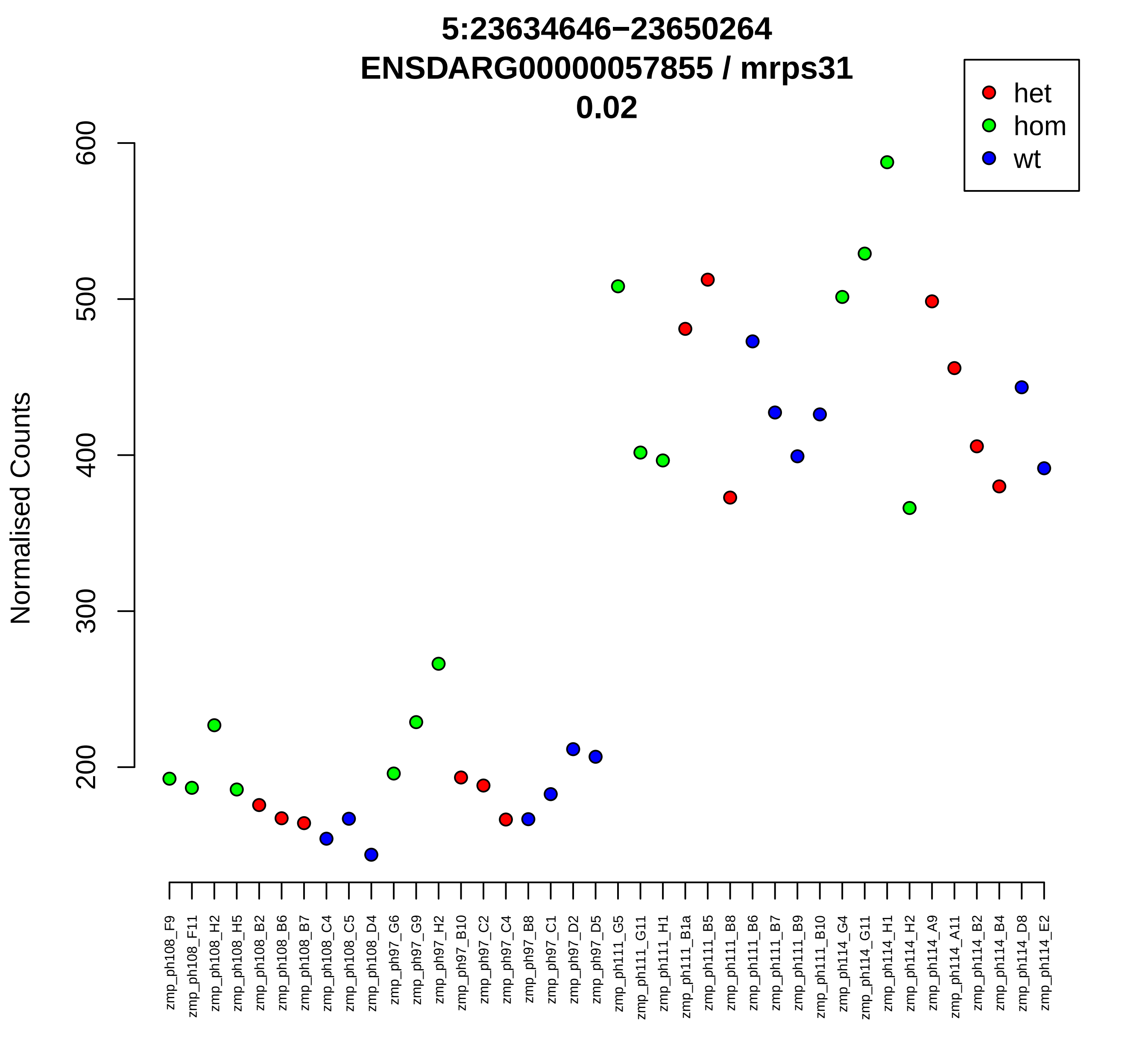
5:23634646−23650264
ENSDARG00000057855 / mrps31
0.02
het
hom
wt
Normalised Counts
200
300
400
500
600
zmp_ph108_F9
zmp_ph108_F11
zmp_ph108_H2
zmp_ph108_H5
zmp_ph108_B2
zmp_ph108_B6
zmp_ph108_B7
zmp_ph108_C4
zmp_ph108_C5
zmp_ph108_D4
zmp_ph97_G6
zmp_ph97_G9
zmp_ph97_H2
zmp_ph97_B10
zmp_ph97_C2
zmp_ph97_C4
zmp_ph97_B8
zmp_ph97_C1
zmp_ph97_D2
zmp_ph97_D5
zmp_ph111_G5
zmp_ph111_G11
zmp_ph111_H1
zmp_ph111_B1a
zmp_ph111_B5
zmp_ph111_B8
zmp_ph111_B6
zmp_ph111_B7
zmp_ph111_B9
zmp_ph111_B10
zmp_ph114_G4
zmp_ph114_G11
zmp_ph114_H1
zmp_ph114_H2
zmp_ph114_A9
zmp_ph114_A11
zmp_ph114_B2
zmp_ph114_B4
zmp_ph114_D8
zmp_ph114_E2

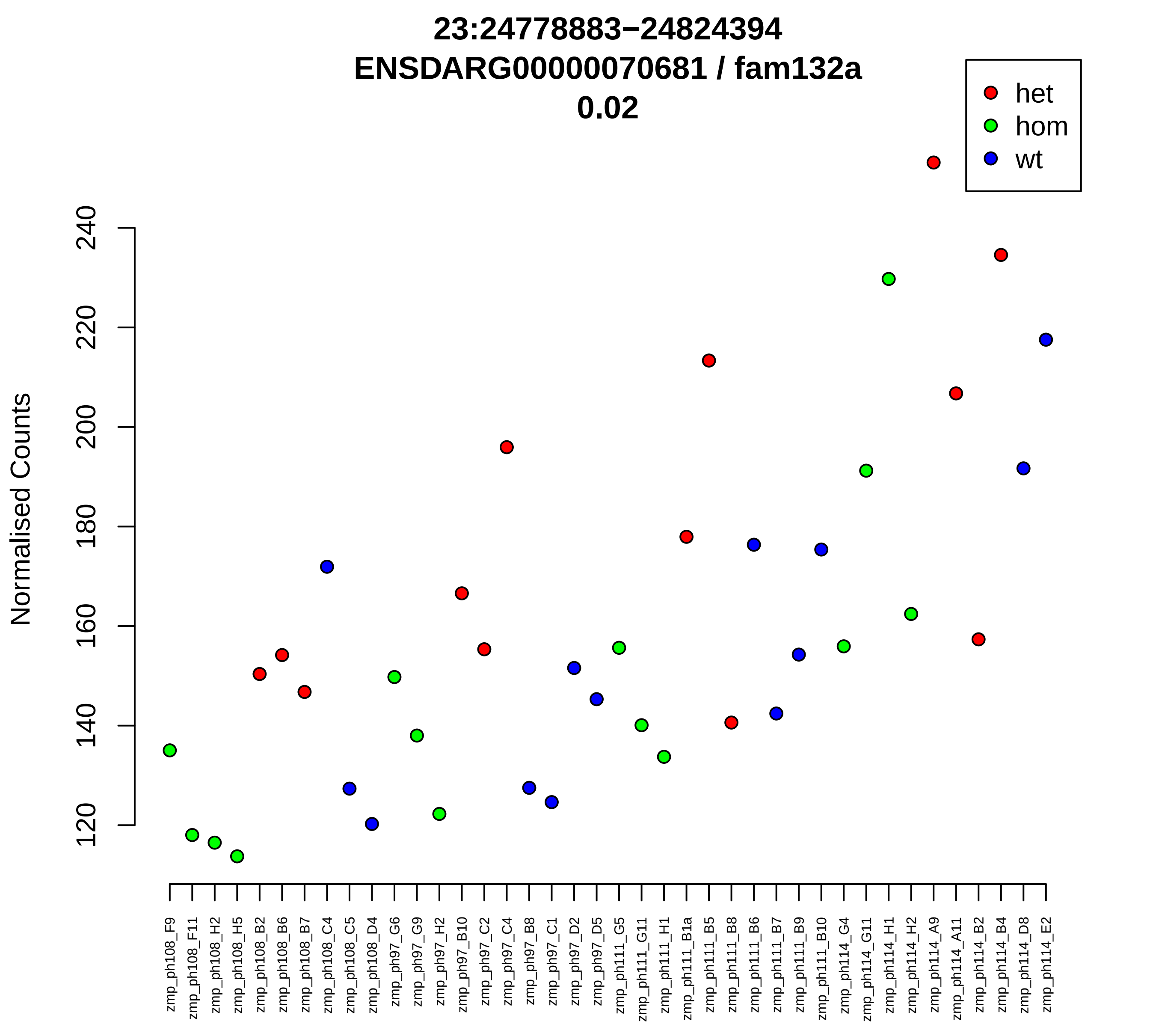

23:24778883−24824394
ENSDARG00000070681 / fam132a
0.02
het
hom
wt
Normalised Counts
120
140
160
180
200
220
240
zmp_ph108_F9
zmp_ph108_F11
zmp_ph108_H2
zmp_ph108_H5
zmp_ph108_B2
zmp_ph108_B6
zmp_ph108_B7
zmp_ph108_C4
zmp_ph108_C5
zmp_ph108_D4
zmp_ph97_G6
zmp_ph97_G9
zmp_ph97_H2
zmp_ph97_B10
zmp_ph97_C2
zmp_ph97_C4
zmp_ph97_B8
zmp_ph97_C1
zmp_ph97_D2
zmp_ph97_D5
zmp_ph111_G5
zmp_ph111_G11
zmp_ph111_H1
zmp_ph111_B1a
zmp_ph111_B5
zmp_ph111_B8
zmp_ph111_B6
zmp_ph111_B7
zmp_ph111_B9
zmp_ph111_B10
zmp_ph114_G4
zmp_ph114_G11
zmp_ph114_H1
zmp_ph114_H2
zmp_ph114_A9
zmp_ph114_A11
zmp_ph114_B2
zmp_ph114_B4
zmp_ph114_D8
zmp_ph114_E2

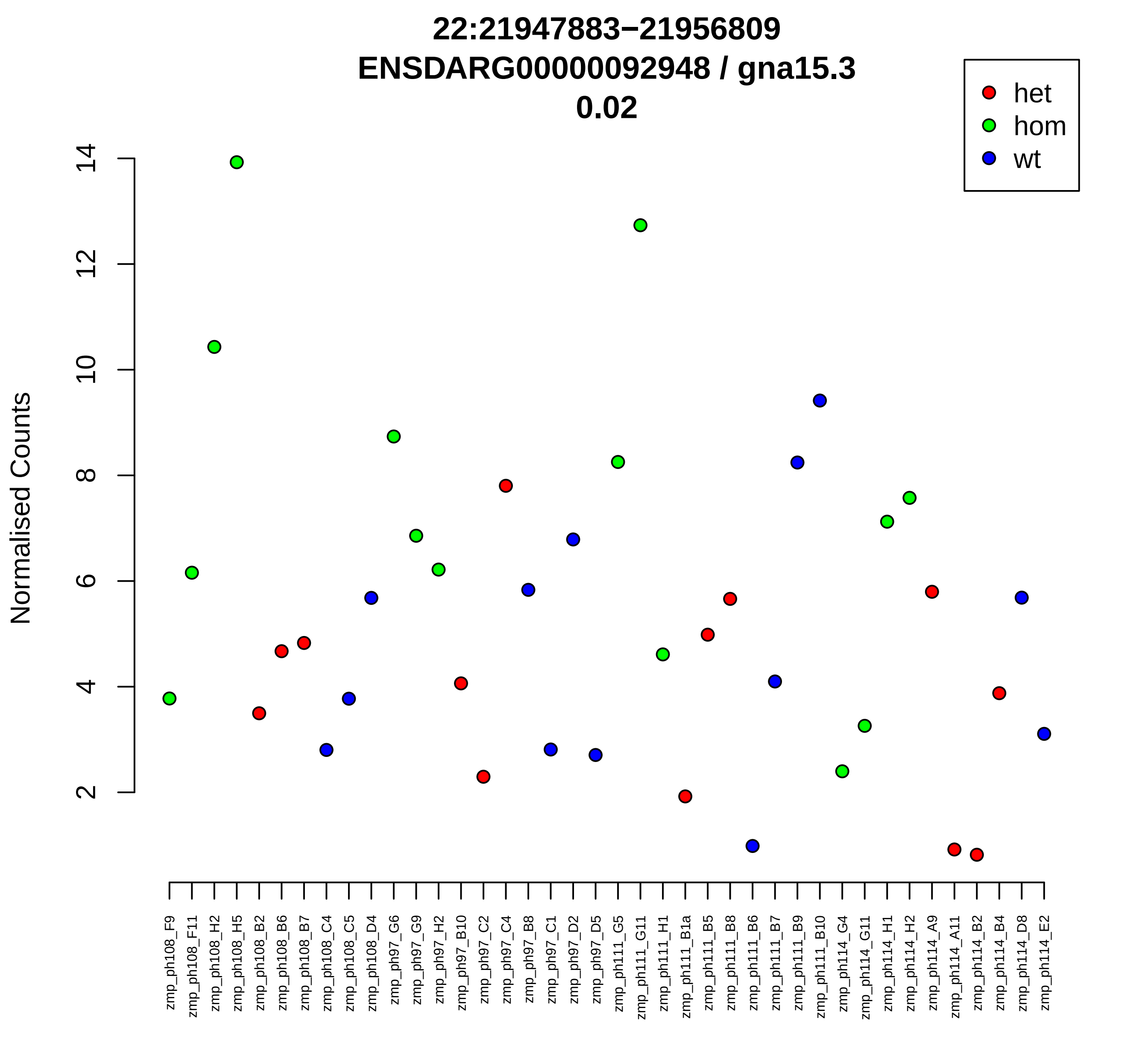

22:21947883−21956809
ENSDARG00000092948 / gna15.3
0.02
het
hom
wt
Normalised Counts
2
4
6
8
10
12
14
zmp_ph108_F9
zmp_ph108_F11
zmp_ph108_H2
zmp_ph108_H5
zmp_ph108_B2
zmp_ph108_B6
zmp_ph108_B7
zmp_ph108_C4
zmp_ph108_C5
zmp_ph108_D4
zmp_ph97_G6
zmp_ph97_G9
zmp_ph97_H2
zmp_ph97_B10
zmp_ph97_C2
zmp_ph97_C4
zmp_ph97_B8
zmp_ph97_C1
zmp_ph97_D2
zmp_ph97_D5
zmp_ph111_G5
zmp_ph111_G11
zmp_ph111_H1
zmp_ph111_B1a
zmp_ph111_B5
zmp_ph111_B8
zmp_ph111_B6
zmp_ph111_B7
zmp_ph111_B9
zmp_ph111_B10
zmp_ph114_G4
zmp_ph114_G11
zmp_ph114_H1
zmp_ph114_H2
zmp_ph114_A9
zmp_ph114_A11
zmp_ph114_B2
zmp_ph114_B4
zmp_ph114_D8
zmp_ph114_E2

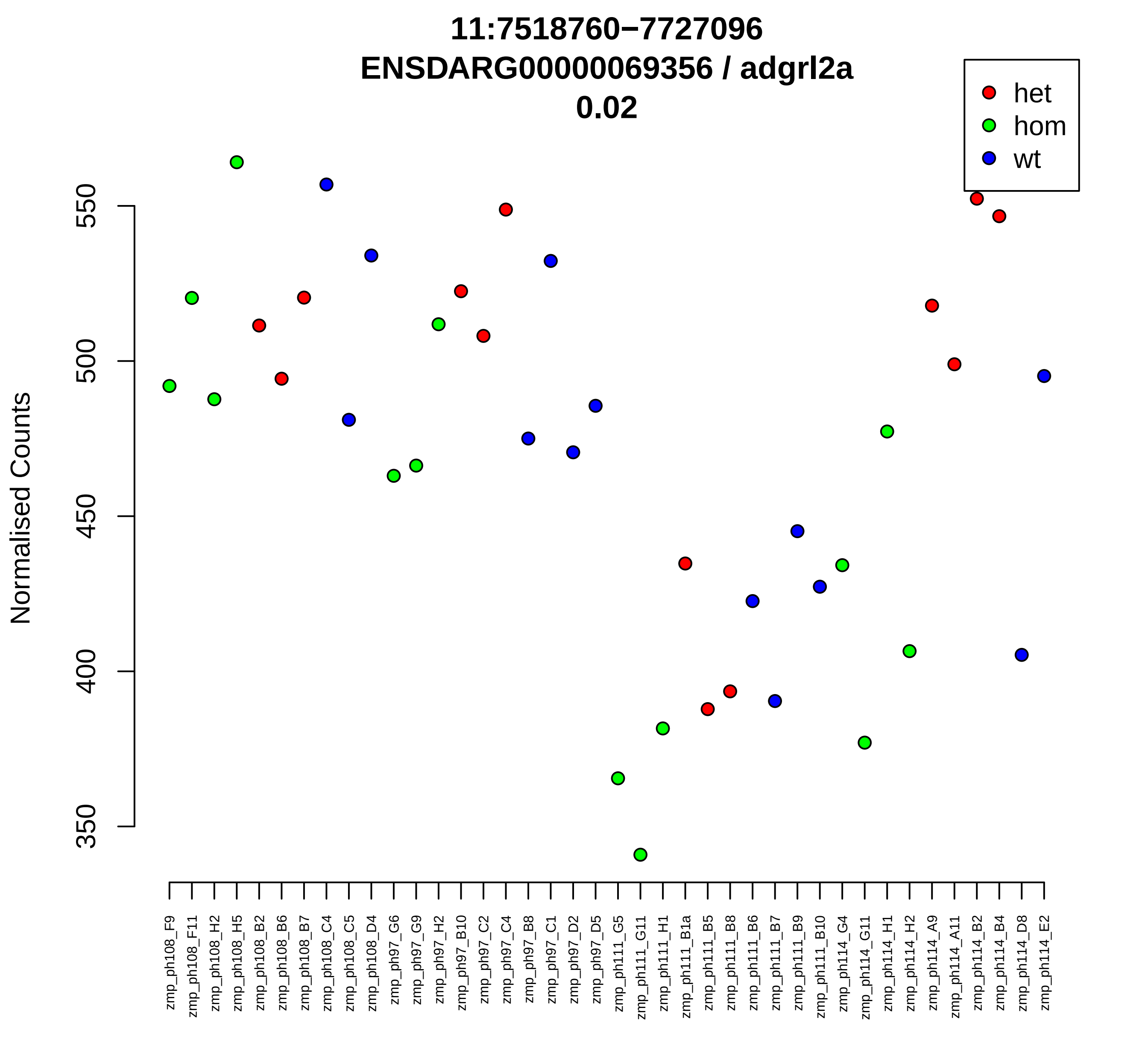
11:7518760−7727096
ENSDARG00000069356 / adgrl2a
0.02
het
hom
wt
Normalised Counts
350
400
450
500
550
zmp_ph108_F9
zmp_ph108_F11
zmp_ph108_H2
zmp_ph108_H5
zmp_ph108_B2
zmp_ph108_B6
zmp_ph108_B7
zmp_ph108_C4
zmp_ph108_C5
zmp_ph108_D4
zmp_ph97_G6
zmp_ph97_G9
zmp_ph97_H2
zmp_ph97_B10
zmp_ph97_C2
zmp_ph97_C4
zmp_ph97_B8
zmp_ph97_C1
zmp_ph97_D2
zmp_ph97_D5
zmp_ph111_G5
zmp_ph111_G11
zmp_ph111_H1
zmp_ph111_B1a
zmp_ph111_B5
zmp_ph111_B8
zmp_ph111_B6
zmp_ph111_B7
zmp_ph111_B9
zmp_ph111_B10
zmp_ph114_G4
zmp_ph114_G11
zmp_ph114_H1
zmp_ph114_H2
zmp_ph114_A9
zmp_ph114_A11
zmp_ph114_B2
zmp_ph114_B4
zmp_ph114_D8
zmp_ph114_E2

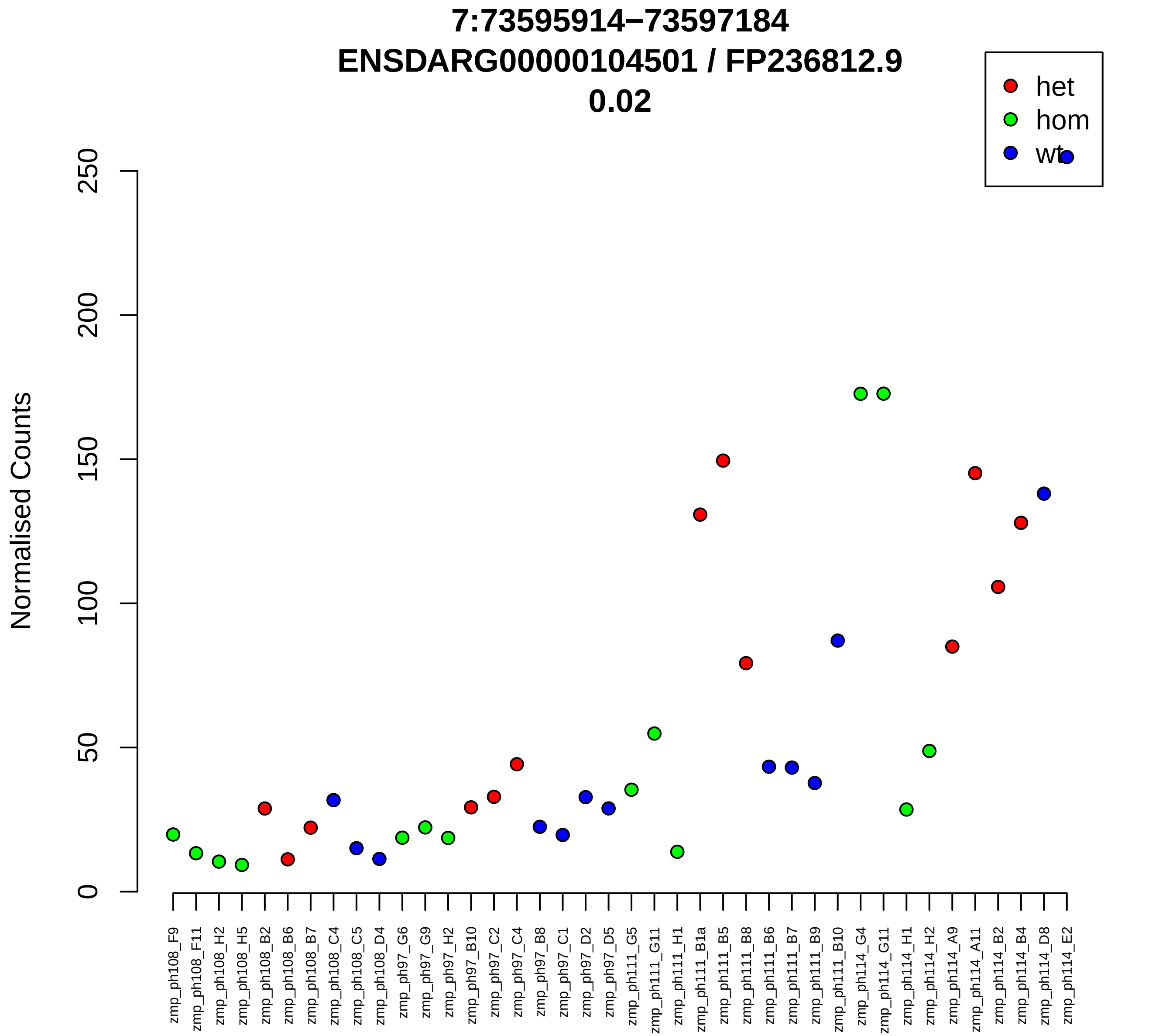

7:73595914−73597184
ENSDARG00000104501 / FP236812.9
0.02
het
hom
wt
Normalised Counts
0
50
100
150
200
250
zmp_ph108_F9
zmp_ph108_F11
zmp_ph108_H2
zmp_ph108_H5
zmp_ph108_B2
zmp_ph108_B6
zmp_ph108_B7
zmp_ph108_C4
zmp_ph108_C5
zmp_ph108_D4
zmp_ph97_G6
zmp_ph97_G9
zmp_ph97_H2
zmp_ph97_B10
zmp_ph97_C2
zmp_ph97_C4
zmp_ph97_B8
zmp_ph97_C1
zmp_ph97_D2
zmp_ph97_D5
zmp_ph111_G5
zmp_ph111_G11
zmp_ph111_H1
zmp_ph111_B1a
zmp_ph111_B5
zmp_ph111_B8
zmp_ph111_B6
zmp_ph111_B7
zmp_ph111_B9
zmp_ph111_B10
zmp_ph114_G4
zmp_ph114_G11
zmp_ph114_H1
zmp_ph114_H2
zmp_ph114_A9
zmp_ph114_A11
zmp_ph114_B2
zmp_ph114_B4
zmp_ph114_D8
zmp_ph114_E2

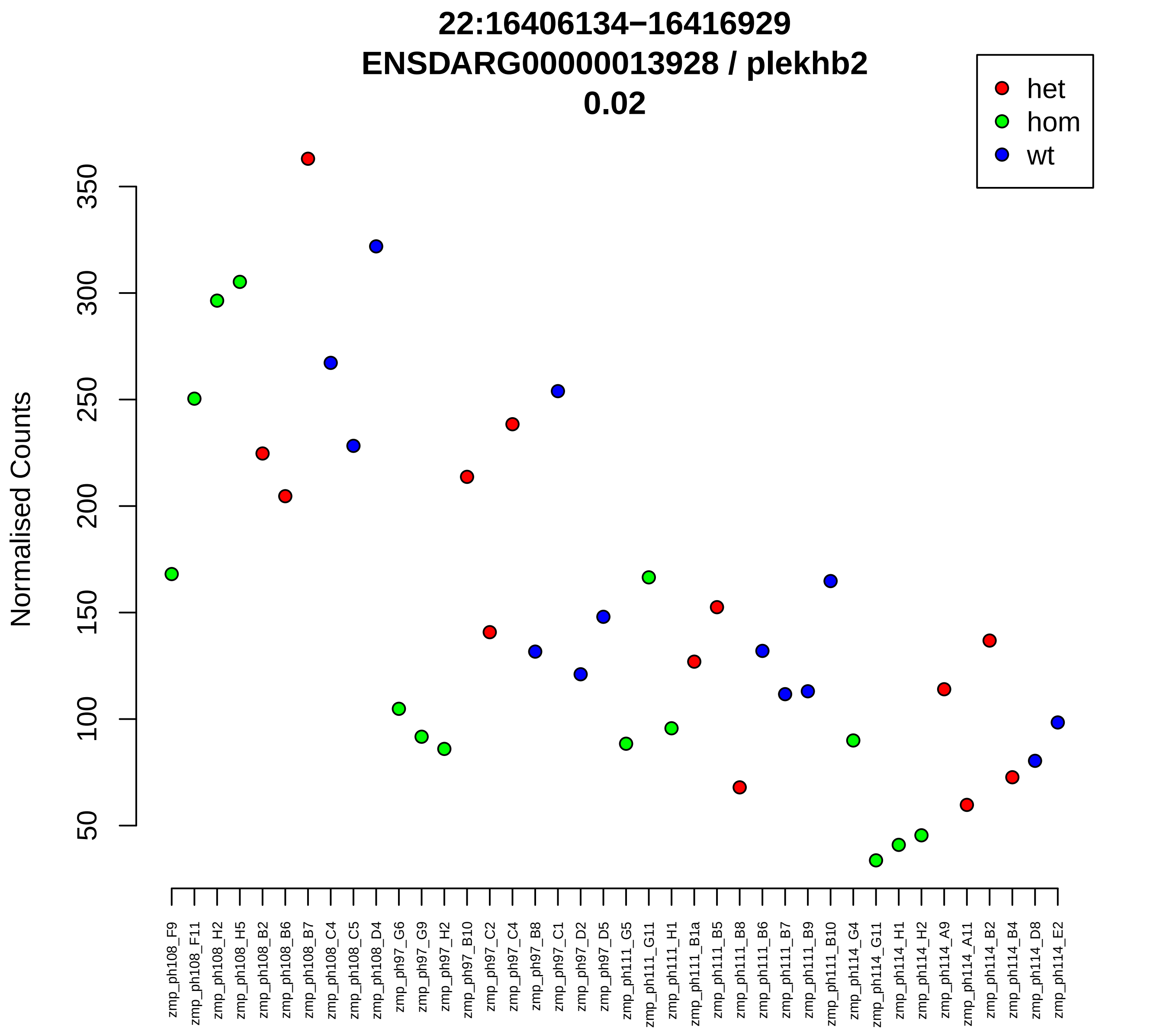
22:16406134−16416929
ENSDARG00000013928 / plekhb2
0.02
het
hom
wt
Normalised Counts
50
100
150
200
250
300
350
zmp_ph108_F9
zmp_ph108_F11
zmp_ph108_H2
zmp_ph108_H5
zmp_ph108_B2
zmp_ph108_B6
zmp_ph108_B7
zmp_ph108_C4
zmp_ph108_C5
zmp_ph108_D4
zmp_ph97_G6
zmp_ph97_G9
zmp_ph97_H2
zmp_ph97_B10
zmp_ph97_C2
zmp_ph97_C4
zmp_ph97_B8
zmp_ph97_C1
zmp_ph97_D2
zmp_ph97_D5
zmp_ph111_G5
zmp_ph111_G11
zmp_ph111_H1
zmp_ph111_B1a
zmp_ph111_B5
zmp_ph111_B8
zmp_ph111_B6
zmp_ph111_B7
zmp_ph111_B9
zmp_ph111_B10
zmp_ph114_G4
zmp_ph114_G11
zmp_ph114_H1
zmp_ph114_H2
zmp_ph114_A9
zmp_ph114_A11
zmp_ph114_B2
zmp_ph114_B4
zmp_ph114_D8
zmp_ph114_E2

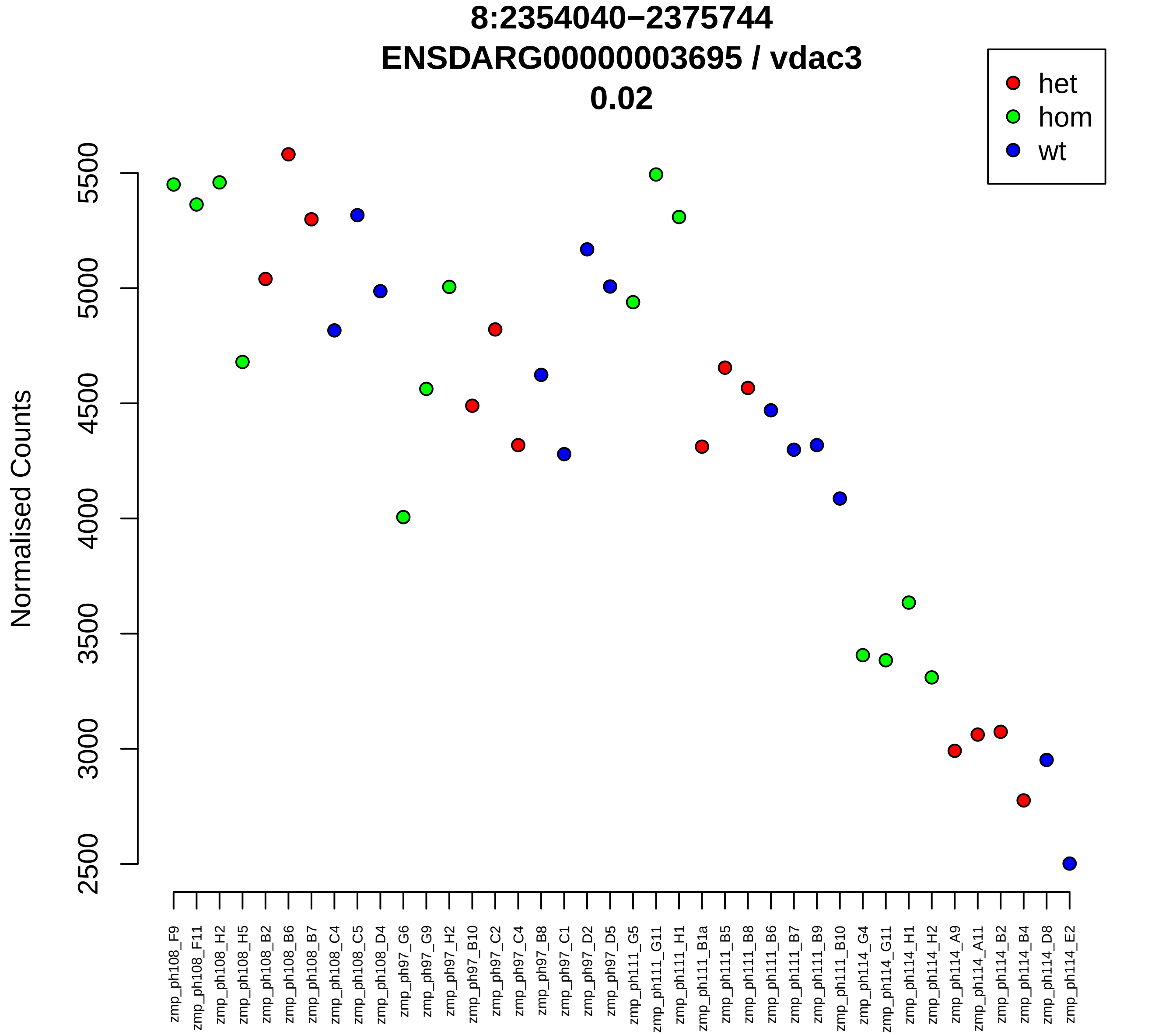

8:2354040−2375744
ENSDARG00000003695 / vdac3
0.02
het
hom
wt
Normalised Counts
2500
3000
3500
4000
4500
5000
5500
zmp_ph108_F9
zmp_ph108_F11
zmp_ph108_H2
zmp_ph108_H5
zmp_ph108_B2
zmp_ph108_B6
zmp_ph108_B7
zmp_ph108_C4
zmp_ph108_C5
zmp_ph108_D4
zmp_ph97_G6
zmp_ph97_G9
zmp_ph97_H2
zmp_ph97_B10
zmp_ph97_C2
zmp_ph97_C4
zmp_ph97_B8
zmp_ph97_C1
zmp_ph97_D2
zmp_ph97_D5
zmp_ph111_G5
zmp_ph111_G11
zmp_ph111_H1
zmp_ph111_B1a
zmp_ph111_B5
zmp_ph111_B8
zmp_ph111_B6
zmp_ph111_B7
zmp_ph111_B9
zmp_ph111_B10
zmp_ph114_G4
zmp_ph114_G11
zmp_ph114_H1
zmp_ph114_H2
zmp_ph114_A9
zmp_ph114_A11
zmp_ph114_B2
zmp_ph114_B4
zmp_ph114_D8
zmp_ph114_E2

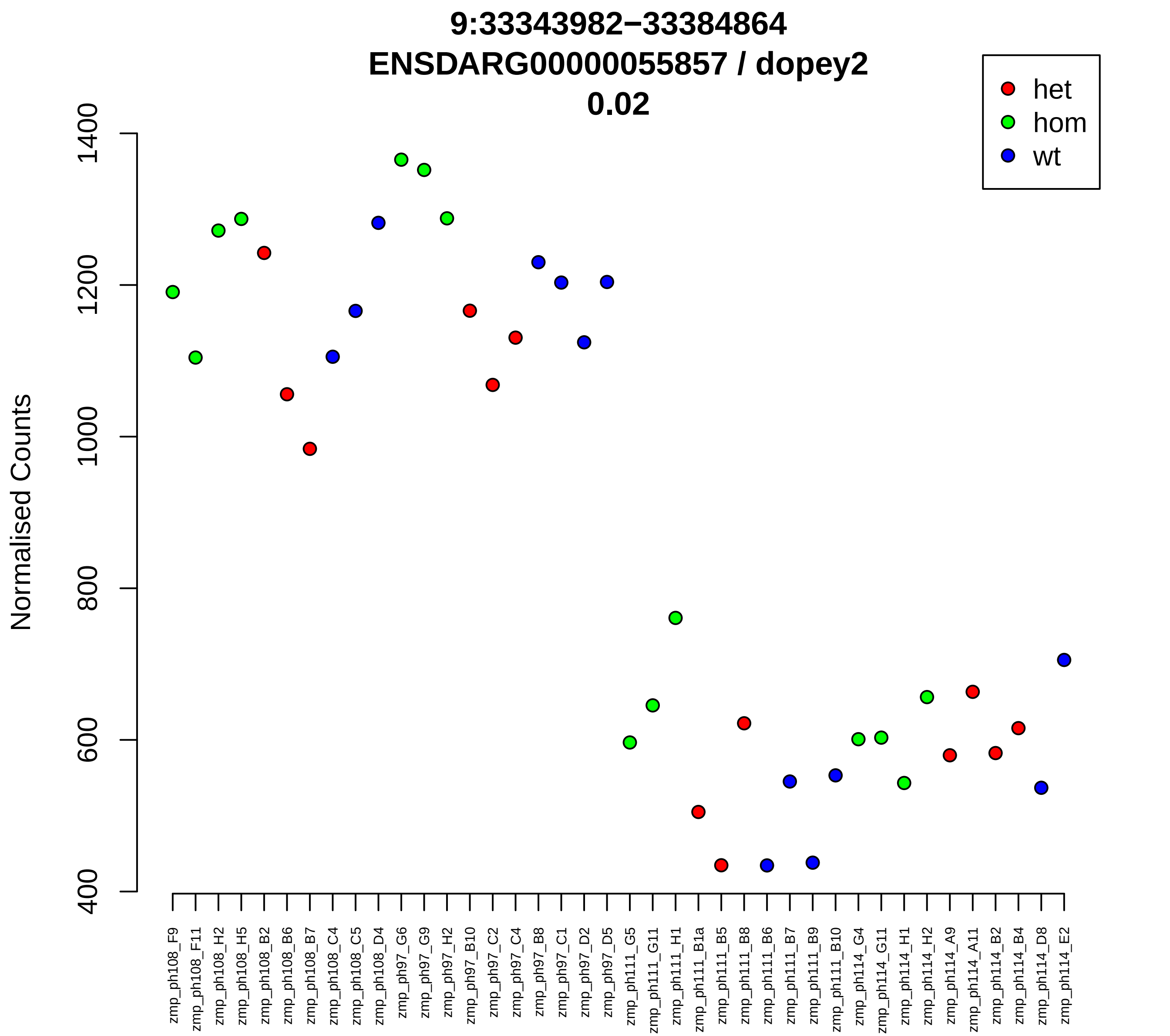
9:33343982−33384864
ENSDARG00000055857 / dopey2
0.02
het
hom
wt
Normalised Counts
400
600
800
1000
1200
1400
zmp_ph108_F9
zmp_ph108_F11
zmp_ph108_H2
zmp_ph108_H5
zmp_ph108_B2
zmp_ph108_B6
zmp_ph108_B7
zmp_ph108_C4
zmp_ph108_C5
zmp_ph108_D4
zmp_ph97_G6
zmp_ph97_G9
zmp_ph97_H2
zmp_ph97_B10
zmp_ph97_C2
zmp_ph97_C4
zmp_ph97_B8
zmp_ph97_C1
zmp_ph97_D2
zmp_ph97_D5
zmp_ph111_G5
zmp_ph111_G11
zmp_ph111_H1
zmp_ph111_B1a
zmp_ph111_B5
zmp_ph111_B8
zmp_ph111_B6
zmp_ph111_B7
zmp_ph111_B9
zmp_ph111_B10
zmp_ph114_G4
zmp_ph114_G11
zmp_ph114_H1
zmp_ph114_H2
zmp_ph114_A9
zmp_ph114_A11
zmp_ph114_B2
zmp_ph114_B4
zmp_ph114_D8
zmp_ph114_E2

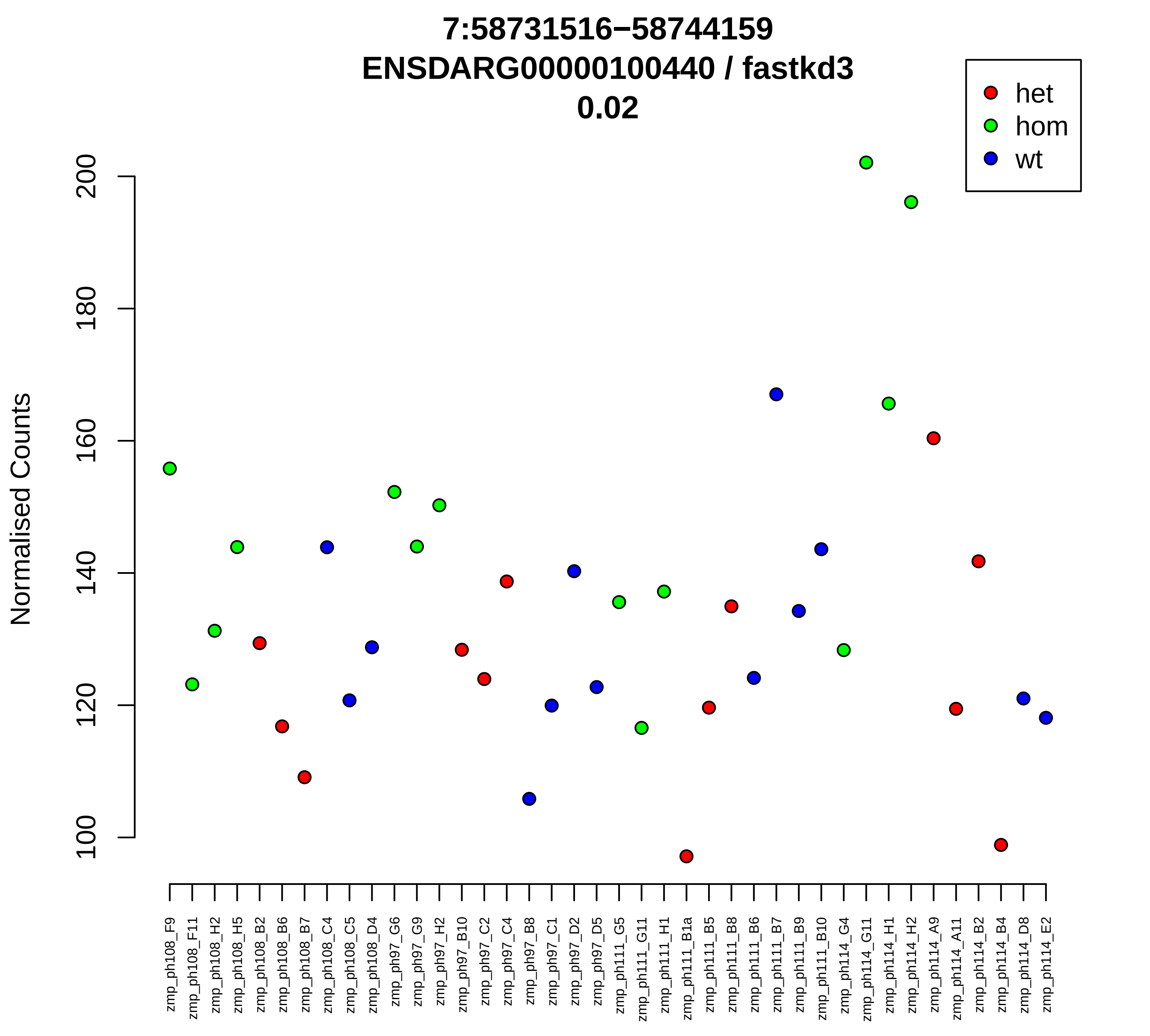

7:58731516−58744159
ENSDARG00000100440 / fastkd3
0.02
het
hom
wt
Normalised Counts
100
120
140
160
180
200
zmp_ph108_F9
zmp_ph108_F11
zmp_ph108_H2
zmp_ph108_H5
zmp_ph108_B2
zmp_ph108_B6
zmp_ph108_B7
zmp_ph108_C4
zmp_ph108_C5
zmp_ph108_D4
zmp_ph97_G6
zmp_ph97_G9
zmp_ph97_H2
zmp_ph97_B10
zmp_ph97_C2
zmp_ph97_C4
zmp_ph97_B8
zmp_ph97_C1
zmp_ph97_D2
zmp_ph97_D5
zmp_ph111_G5
zmp_ph111_G11
zmp_ph111_H1
zmp_ph111_B1a
zmp_ph111_B5
zmp_ph111_B8
zmp_ph111_B6
zmp_ph111_B7
zmp_ph111_B9
zmp_ph111_B10
zmp_ph114_G4
zmp_ph114_G11
zmp_ph114_H1
zmp_ph114_H2
zmp_ph114_A9
zmp_ph114_A11
zmp_ph114_B2
zmp_ph114_B4
zmp_ph114_D8
zmp_ph114_E2

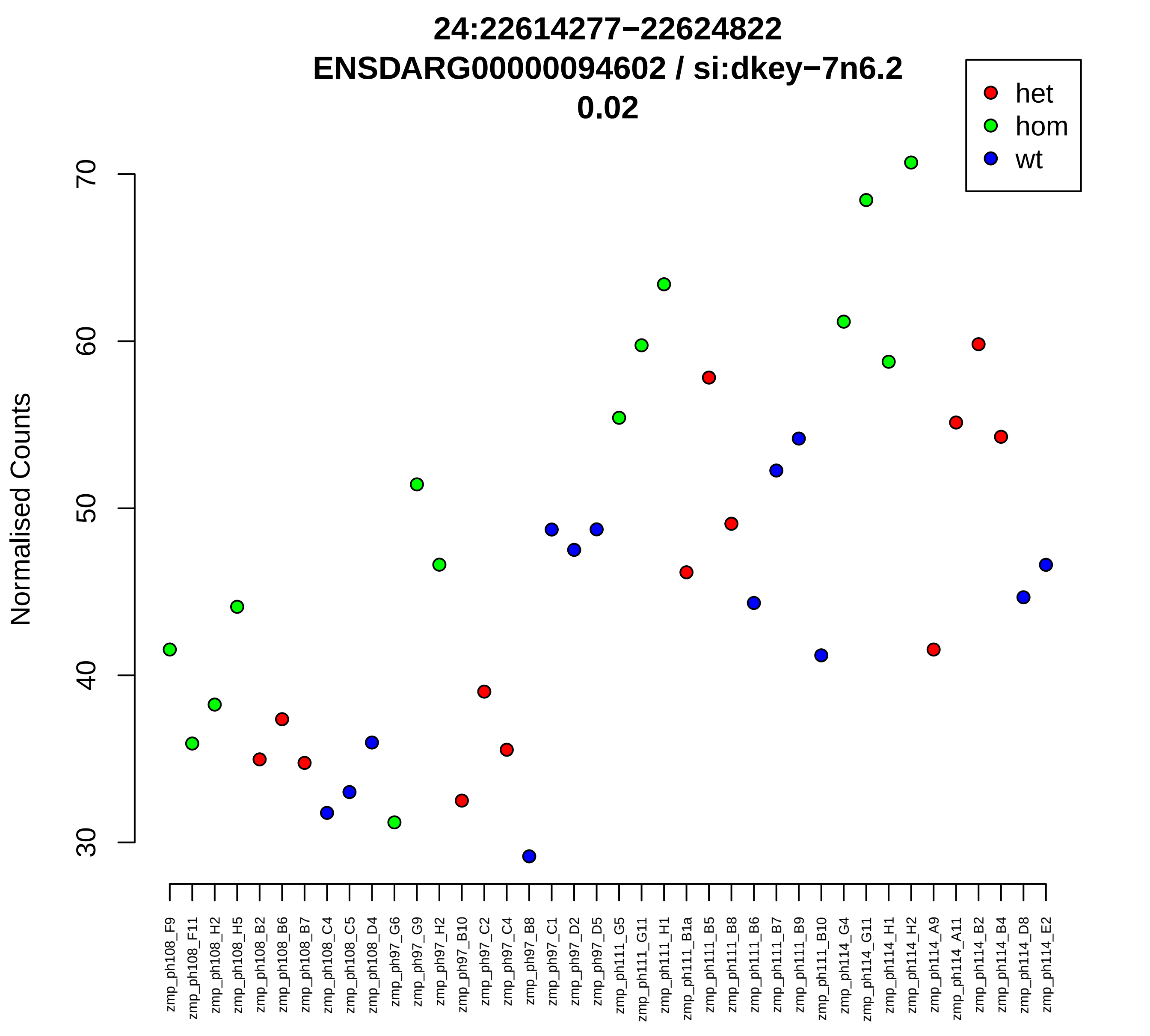

24:22614277−22624822
ENSDARG00000094602 / si:dkey−7n6.2
0.02
het
hom
wt
Normalised Counts
30
40
50
60
70
zmp_ph108_F9
zmp_ph108_F11
zmp_ph108_H2
zmp_ph108_H5
zmp_ph108_B2
zmp_ph108_B6
zmp_ph108_B7
zmp_ph108_C4
zmp_ph108_C5
zmp_ph108_D4
zmp_ph97_G6
zmp_ph97_G9
zmp_ph97_H2
zmp_ph97_B10
zmp_ph97_C2
zmp_ph97_C4
zmp_ph97_B8
zmp_ph97_C1
zmp_ph97_D2
zmp_ph97_D5
zmp_ph111_G5
zmp_ph111_G11
zmp_ph111_H1
zmp_ph111_B1a
zmp_ph111_B5
zmp_ph111_B8
zmp_ph111_B6
zmp_ph111_B7
zmp_ph111_B9
zmp_ph111_B10
zmp_ph114_G4
zmp_ph114_G11
zmp_ph114_H1
zmp_ph114_H2
zmp_ph114_A9
zmp_ph114_A11
zmp_ph114_B2
zmp_ph114_B4
zmp_ph114_D8
zmp_ph114_E2

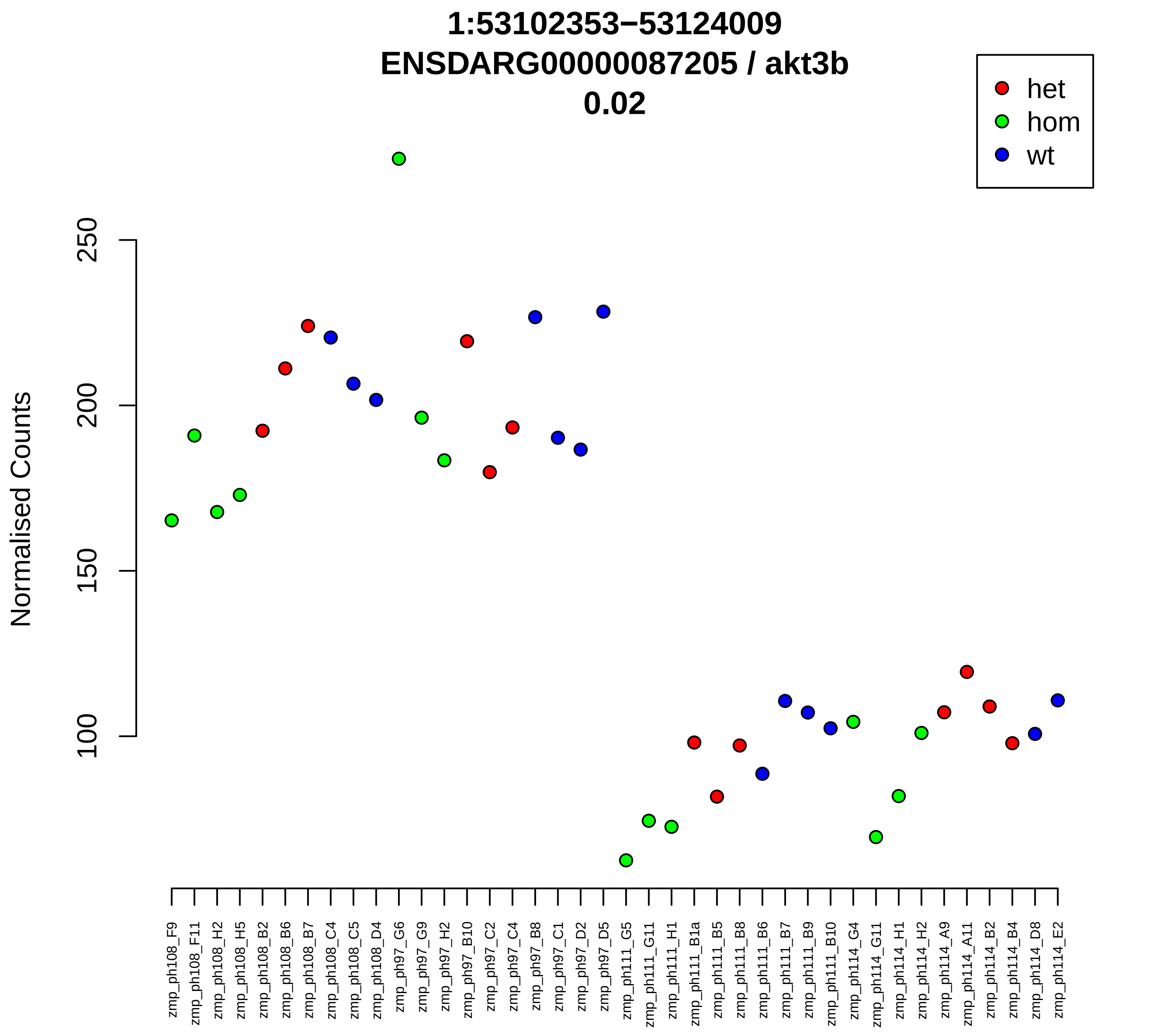
1:53102353−53124009
ENSDARG00000087205 / akt3b
0.02
het
hom
wt
Normalised Counts
100
150
200
250
zmp_ph108_F9
zmp_ph108_F11
zmp_ph108_H2
zmp_ph108_H5
zmp_ph108_B2
zmp_ph108_B6
zmp_ph108_B7
zmp_ph108_C4
zmp_ph108_C5
zmp_ph108_D4
zmp_ph97_G6
zmp_ph97_G9
zmp_ph97_H2
zmp_ph97_B10
zmp_ph97_C2
zmp_ph97_C4
zmp_ph97_B8
zmp_ph97_C1
zmp_ph97_D2
zmp_ph97_D5
zmp_ph111_G5
zmp_ph111_G11
zmp_ph111_H1
zmp_ph111_B1a
zmp_ph111_B5
zmp_ph111_B8
zmp_ph111_B6
zmp_ph111_B7
zmp_ph111_B9
zmp_ph111_B10
zmp_ph114_G4
zmp_ph114_G11
zmp_ph114_H1
zmp_ph114_H2
zmp_ph114_A9
zmp_ph114_A11
zmp_ph114_B2
zmp_ph114_B4
zmp_ph114_D8
zmp_ph114_E2

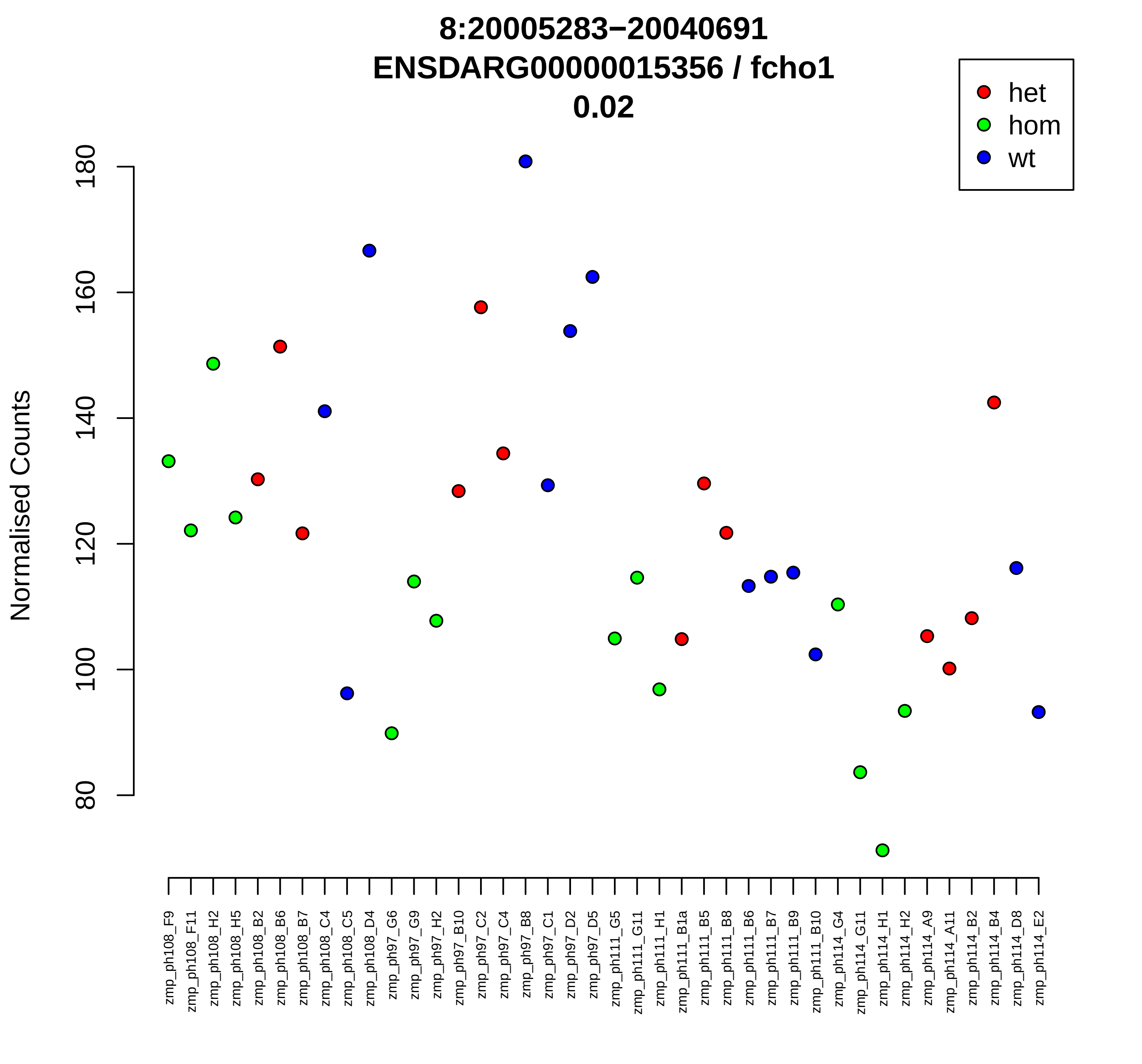
8:20005283−20040691
ENSDARG00000015356 / fcho1
0.02
het
hom
wt
Normalised Counts
180
160
140
120
100
80
zmp_ph108_F9
zmp_ph108_F11
zmp_ph108_H2
zmp_ph108_H5
zmp_ph108_B2
zmp_ph108_B6
zmp_ph108_B7
zmp_ph108_C4
zmp_ph108_C5
zmp_ph108_D4
zmp_ph97_G6
zmp_ph97_G9
zmp_ph97_H2
zmp_ph97_B10
zmp_ph97_C2
zmp_ph97_C4
zmp_ph97_B8
zmp_ph97_C1
zmp_ph97_D2
zmp_ph97_D5
zmp_ph111_G5
zmp_ph111_G11
zmp_ph111_H1
zmp_ph111_B1a
zmp_ph111_B5
zmp_ph111_B8
zmp_ph111_B6
zmp_ph111_B7
zmp_ph111_B9
zmp_ph111_B10
zmp_ph114_G4
zmp_ph114_G11
zmp_ph114_H1
zmp_ph114_H2
zmp_ph114_A9
zmp_ph114_A11
zmp_ph114_B2
zmp_ph114_B4
zmp_ph114_D8
zmp_ph114_E2

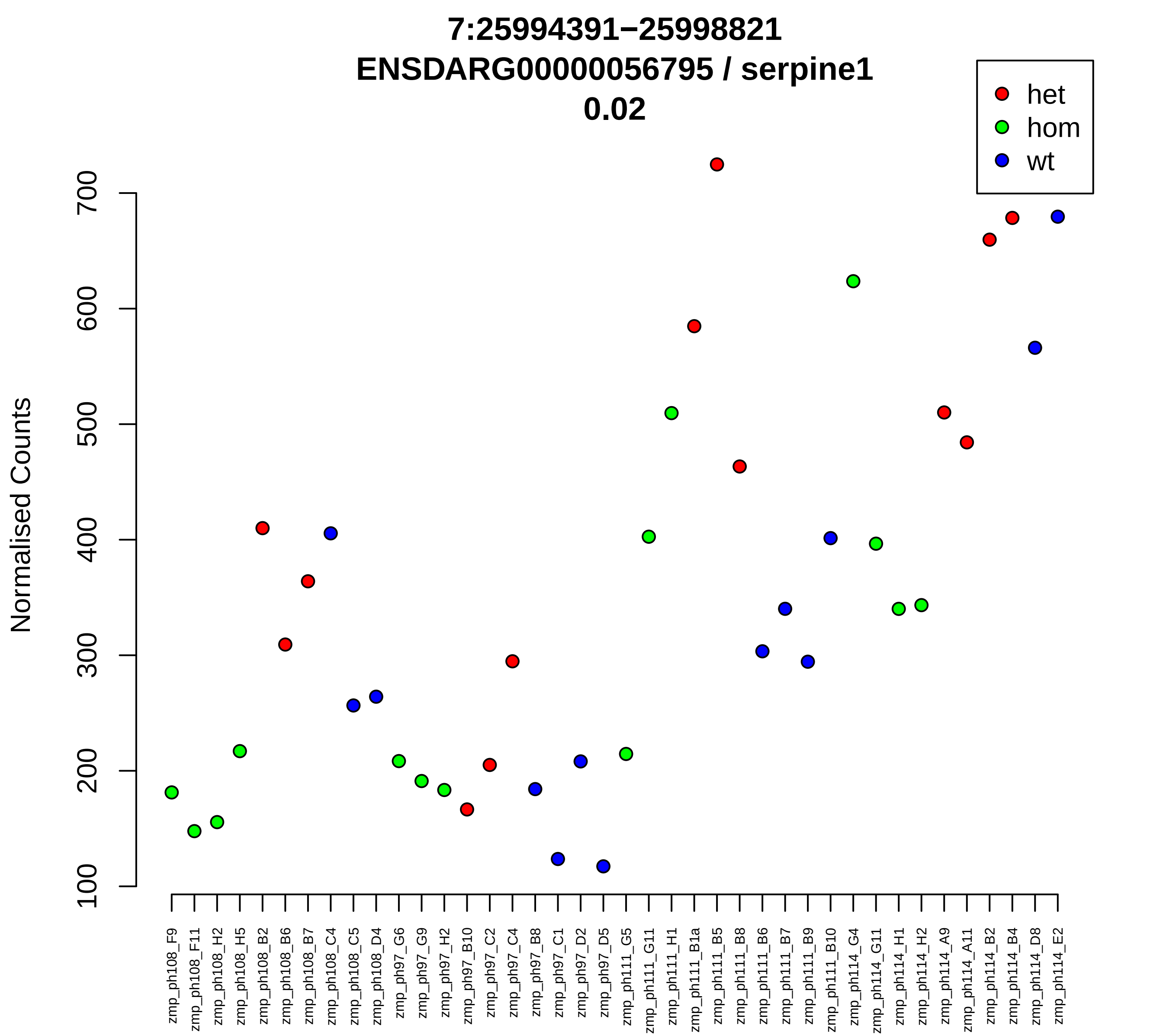
7:25994391−25998821
ENSDARG00000056795 / serpine1
0.02
het
hom
wt
Normalised Counts
100
200
300
400
500
600
700
zmp_ph108_F9
zmp_ph108_F11
zmp_ph108_H2
zmp_ph108_H5
zmp_ph108_B2
zmp_ph108_B6
zmp_ph108_B7
zmp_ph108_C4
zmp_ph108_C5
zmp_ph108_D4
zmp_ph97_G6
zmp_ph97_G9
zmp_ph97_H2
zmp_ph97_B10
zmp_ph97_C2
zmp_ph97_C4
zmp_ph97_B8
zmp_ph97_C1
zmp_ph97_D2
zmp_ph97_D5
zmp_ph111_G5
zmp_ph111_G11
zmp_ph111_H1
zmp_ph111_B1a
zmp_ph111_B5
zmp_ph111_B8
zmp_ph111_B6
zmp_ph111_B7
zmp_ph111_B9
zmp_ph111_B10
zmp_ph114_G4
zmp_ph114_G11
zmp_ph114_H1
zmp_ph114_H2
zmp_ph114_A9
zmp_ph114_A11
zmp_ph114_B2
zmp_ph114_B4
zmp_ph114_D8
zmp_ph114_E2

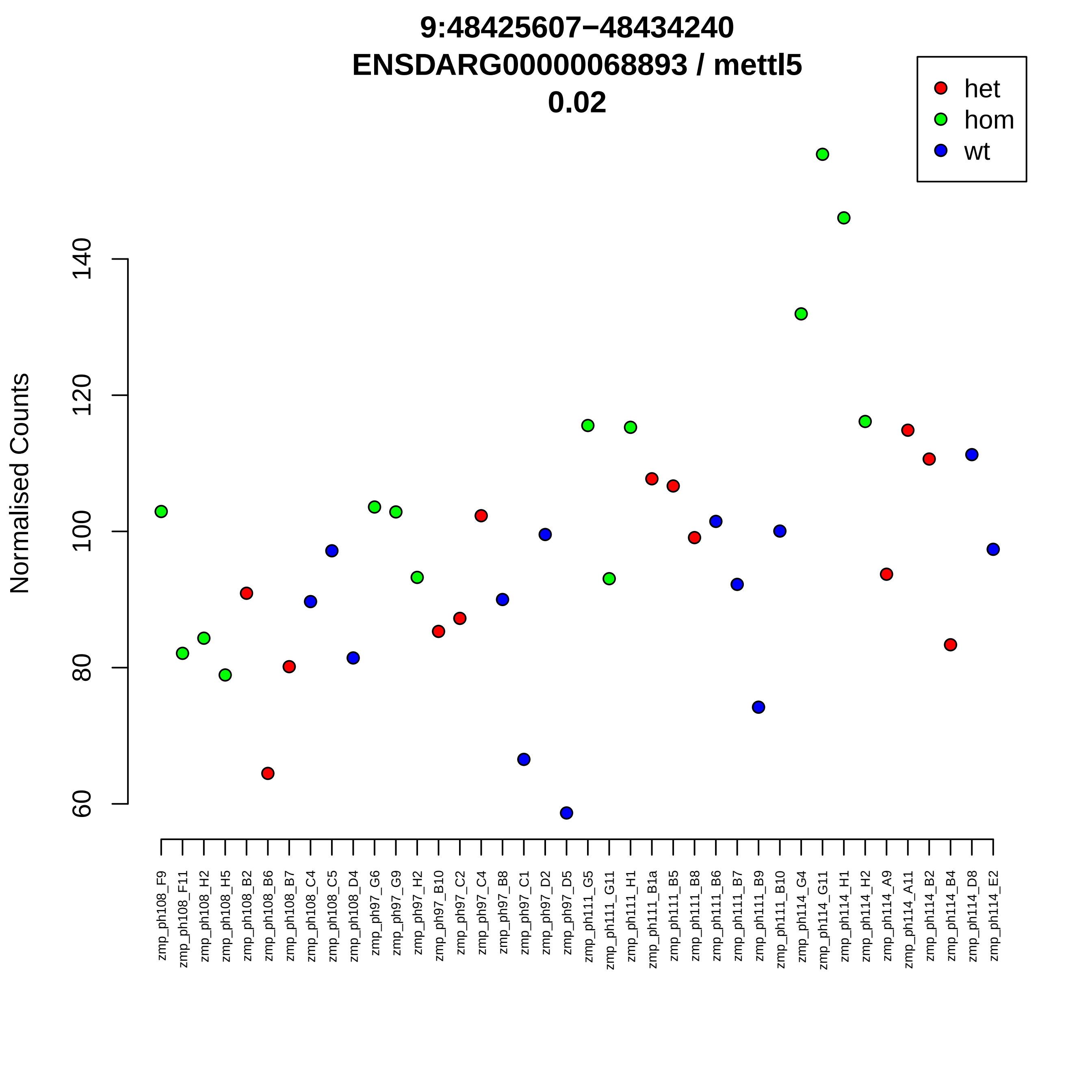

9:48425607−48434240
ENSDARG00000068893 / mettl5
0.02
het
hom
wt
Normalised Counts
60
80
100
120
140
zmp_ph108_F9
zmp_ph108_F11
zmp_ph108_H2
zmp_ph108_H5
zmp_ph108_B2
zmp_ph108_B6
zmp_ph108_B7
zmp_ph108_C4
zmp_ph108_C5
zmp_ph108_D4
zmp_ph97_G6
zmp_ph97_G9
zmp_ph97_H2
zmp_ph97_B10
zmp_ph97_C2
zmp_ph97_C4
zmp_ph97_B8
zmp_ph97_C1
zmp_ph97_D2
zmp_ph97_D5
zmp_ph111_G5
zmp_ph111_G11
zmp_ph111_H1
zmp_ph111_B1a
zmp_ph111_B5
zmp_ph111_B8
zmp_ph111_B6
zmp_ph111_B7
zmp_ph111_B9
zmp_ph111_B10
zmp_ph114_G4
zmp_ph114_G11
zmp_ph114_H1
zmp_ph114_H2
zmp_ph114_A9
zmp_ph114_A11
zmp_ph114_B2
zmp_ph114_B4
zmp_ph114_D8
zmp_ph114_E2

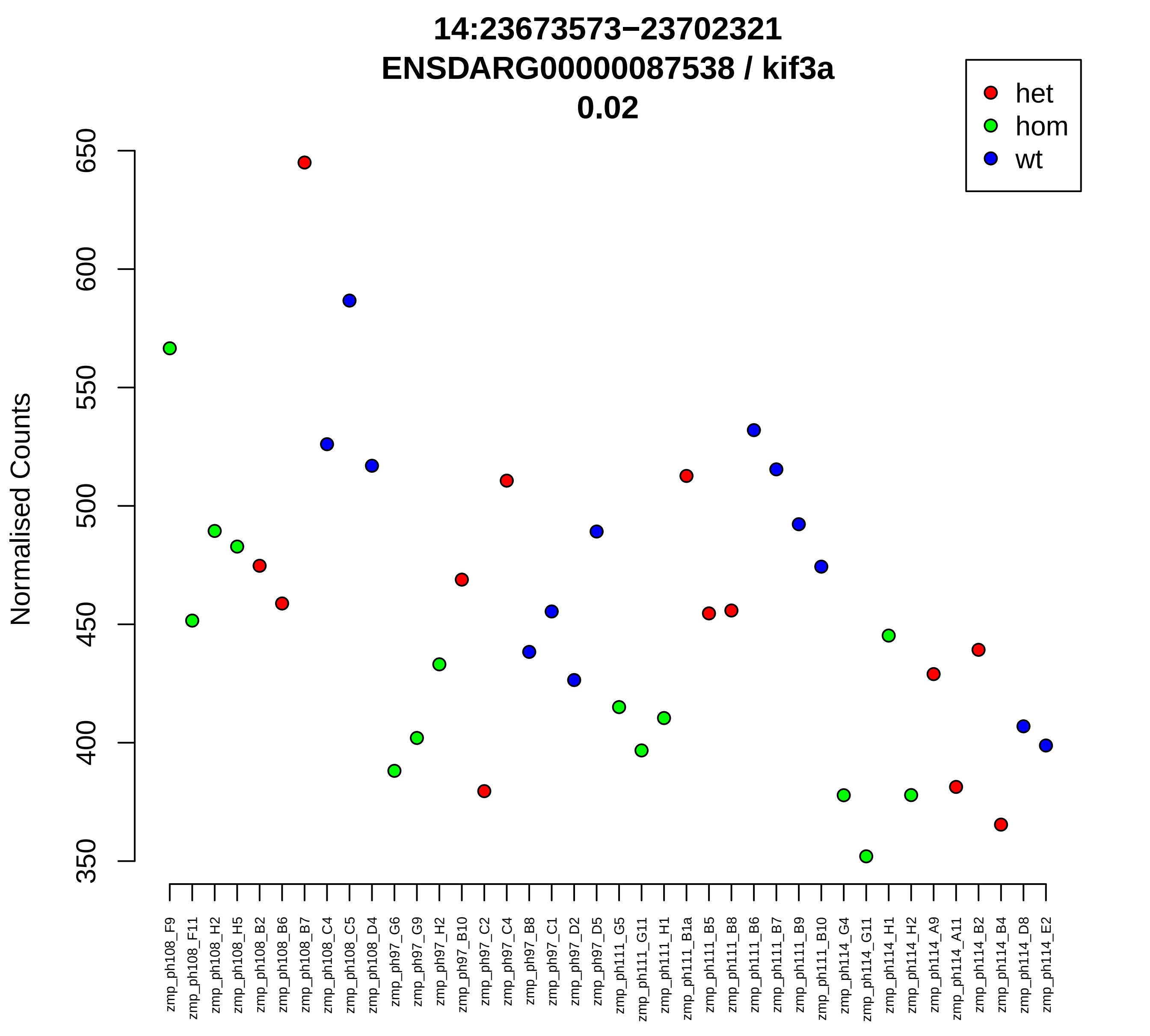

14:23673573−23702321
ENSDARG00000087538 / kif3a
0.02
het
hom
wt
Normalised Counts
350
400
450
500
550
600
650
zmp_ph108_F9
zmp_ph108_F11
zmp_ph108_H2
zmp_ph108_H5
zmp_ph108_B2
zmp_ph108_B6
zmp_ph108_B7
zmp_ph108_C4
zmp_ph108_C5
zmp_ph108_D4
zmp_ph97_G6
zmp_ph97_G9
zmp_ph97_H2
zmp_ph97_B10
zmp_ph97_C2
zmp_ph97_C4
zmp_ph97_B8
zmp_ph97_C1
zmp_ph97_D2
zmp_ph97_D5
zmp_ph111_G5
zmp_ph111_G11
zmp_ph111_H1
zmp_ph111_B1a
zmp_ph111_B5
zmp_ph111_B8
zmp_ph111_B6
zmp_ph111_B7
zmp_ph111_B9
zmp_ph111_B10
zmp_ph114_G4
zmp_ph114_G11
zmp_ph114_H1
zmp_ph114_H2
zmp_ph114_A9
zmp_ph114_A11
zmp_ph114_B2
zmp_ph114_B4
zmp_ph114_D8
zmp_ph114_E2

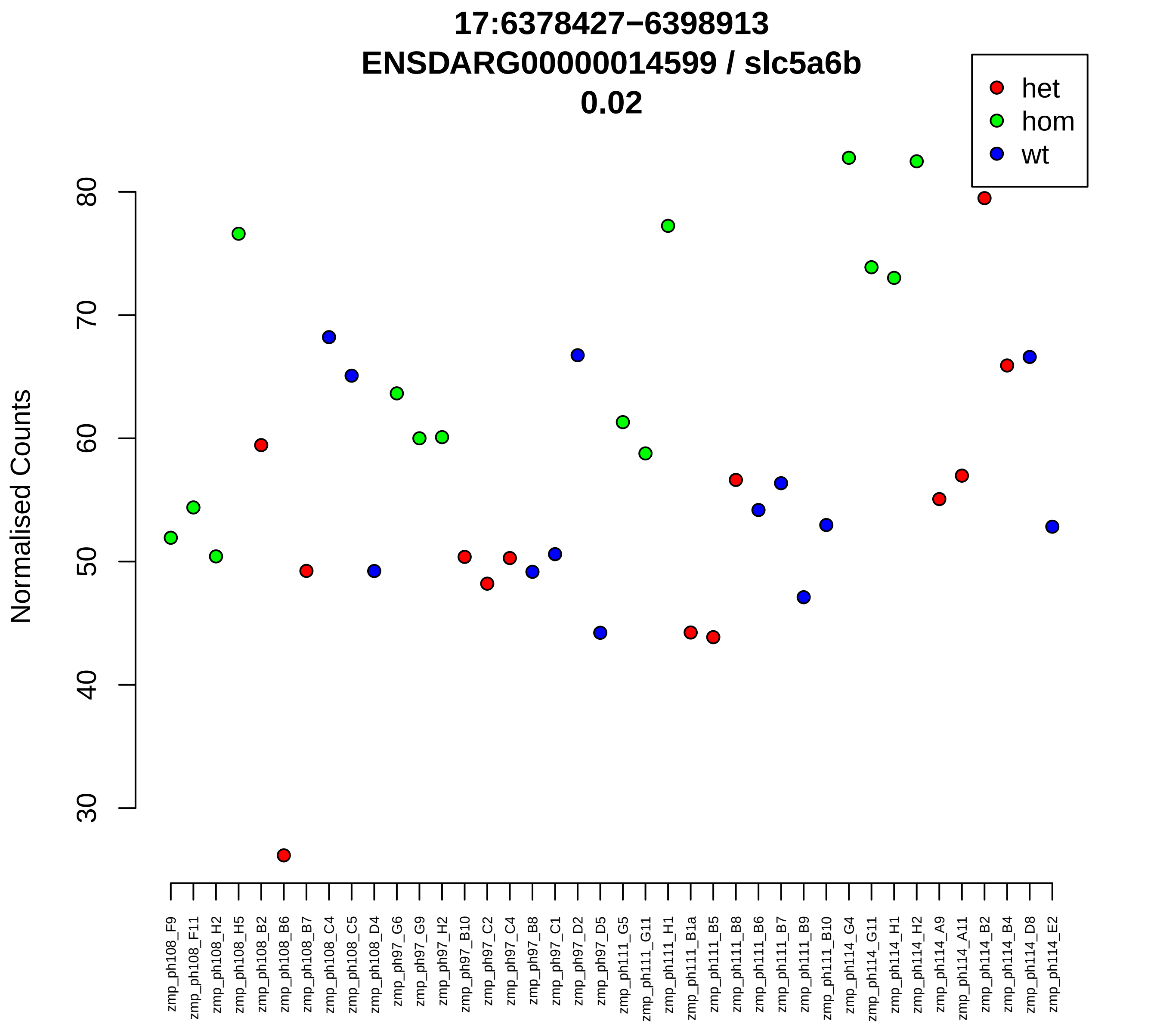
17:6378427−6398913
ENSDARG00000014599 / slc5a6b
0.02
het
hom
wt
Normalised Counts
30
40
50
60
70
80
zmp_ph108_F9
zmp_ph108_F11
zmp_ph108_H2
zmp_ph108_H5
zmp_ph108_B2
zmp_ph108_B6
zmp_ph108_B7
zmp_ph108_C4
zmp_ph108_C5
zmp_ph108_D4
zmp_ph97_G6
zmp_ph97_G9
zmp_ph97_H2
zmp_ph97_B10
zmp_ph97_C2
zmp_ph97_C4
zmp_ph97_B8
zmp_ph97_C1
zmp_ph97_D2
zmp_ph97_D5
zmp_ph111_G5
zmp_ph111_G11
zmp_ph111_H1
zmp_ph111_B1a
zmp_ph111_B5
zmp_ph111_B8
zmp_ph111_B6
zmp_ph111_B7
zmp_ph111_B9
zmp_ph111_B10
zmp_ph114_G4
zmp_ph114_G11
zmp_ph114_H1
zmp_ph114_H2
zmp_ph114_A9
zmp_ph114_A11
zmp_ph114_B2
zmp_ph114_B4
zmp_ph114_D8
zmp_ph114_E2

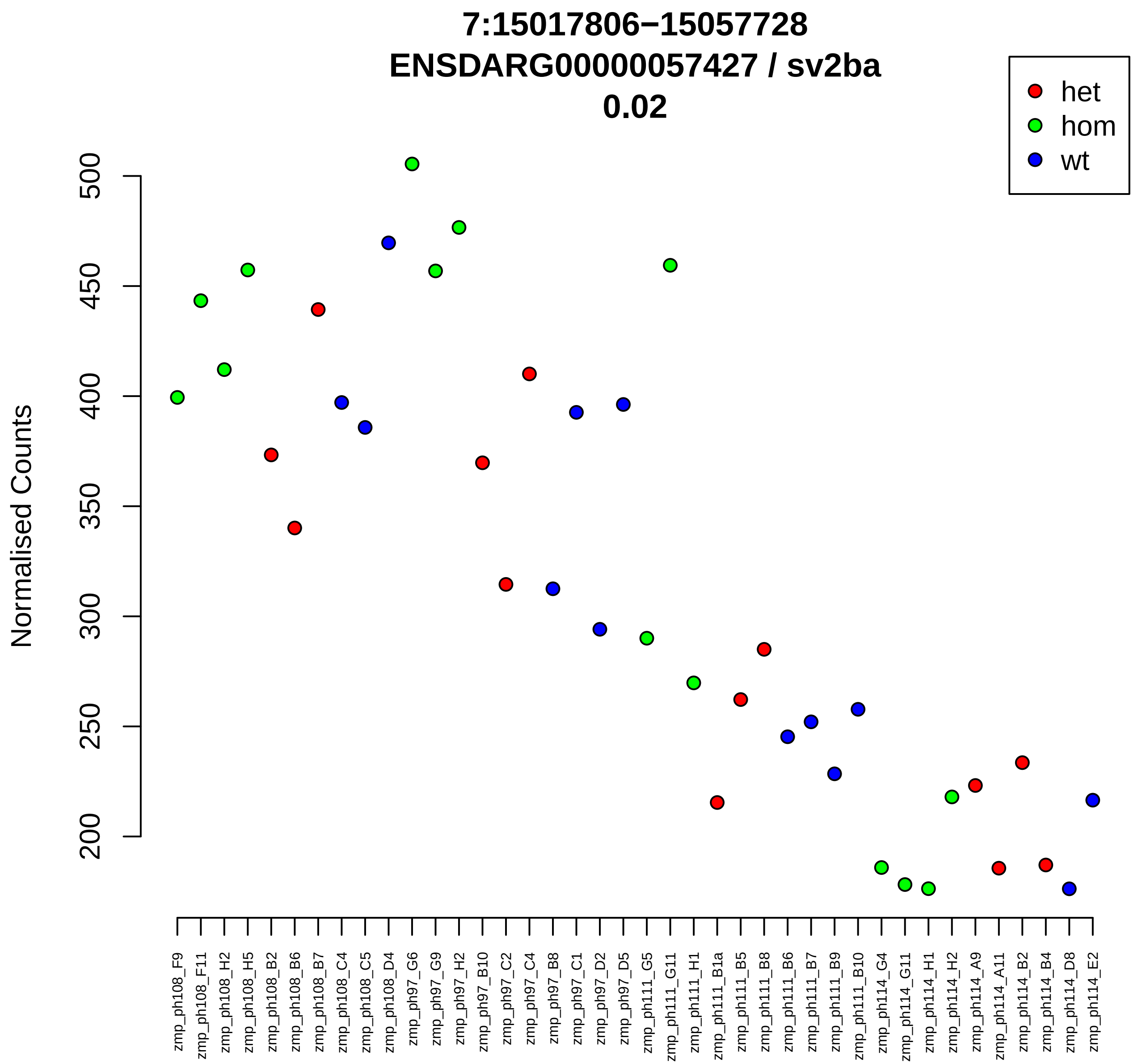

7:15017806−15057728
ENSDARG00000057427 / sv2ba
0.02
het
hom
wt
Normalised Counts
200
250
300
350
400
450
500
zmp_ph108_F9
zmp_ph108_F11
zmp_ph108_H2
zmp_ph108_H5
zmp_ph108_B2
zmp_ph108_B6
zmp_ph108_B7
zmp_ph108_C4
zmp_ph108_C5
zmp_ph108_D4
zmp_ph97_G6
zmp_ph97_G9
zmp_ph97_H2
zmp_ph97_B10
zmp_ph97_C2
zmp_ph97_C4
zmp_ph97_B8
zmp_ph97_C1
zmp_ph97_D2
zmp_ph97_D5
zmp_ph111_G5
zmp_ph111_G11
zmp_ph111_H1
zmp_ph111_B1a
zmp_ph111_B5
zmp_ph111_B8
zmp_ph111_B6
zmp_ph111_B7
zmp_ph111_B9
zmp_ph111_B10
zmp_ph114_G4
zmp_ph114_G11
zmp_ph114_H1
zmp_ph114_H2
zmp_ph114_A9
zmp_ph114_A11
zmp_ph114_B2
zmp_ph114_B4
zmp_ph114_D8
zmp_ph114_E2

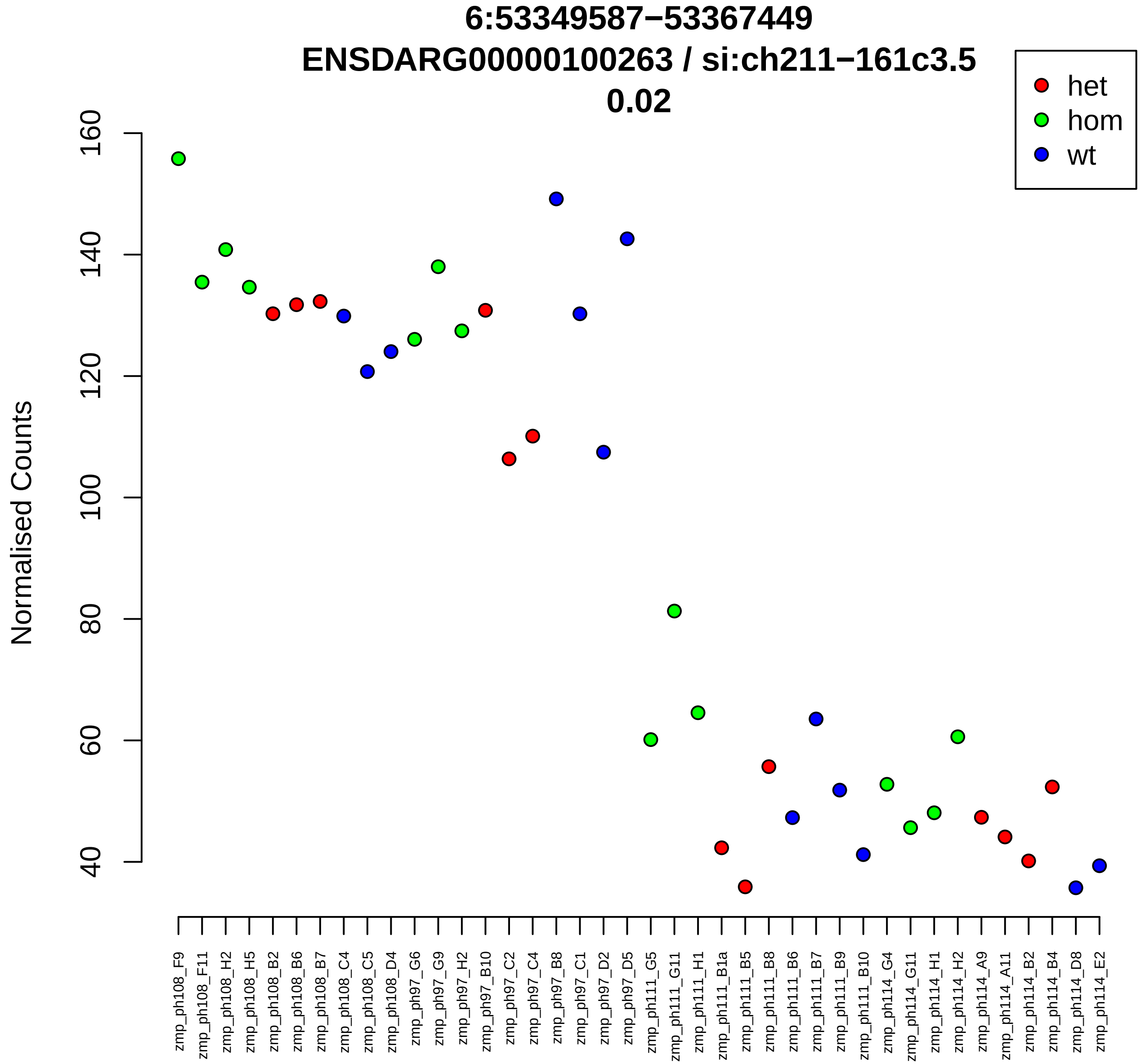

6:53349587−53367449
ENSDARG00000100263 / si:ch211−161c3.5
0.02
het
hom
wt
Normalised Counts
40
60
80
100
120
140
160
zmp_ph108_F9
zmp_ph108_F11
zmp_ph108_H2
zmp_ph108_H5
zmp_ph108_B2
zmp_ph108_B6
zmp_ph108_B7
zmp_ph108_C4
zmp_ph108_C5
zmp_ph108_D4
zmp_ph97_G6
zmp_ph97_G9
zmp_ph97_H2
zmp_ph97_B10
zmp_ph97_C2
zmp_ph97_C4
zmp_ph97_B8
zmp_ph97_C1
zmp_ph97_D2
zmp_ph97_D5
zmp_ph111_G5
zmp_ph111_G11
zmp_ph111_H1
zmp_ph111_B1a
zmp_ph111_B5
zmp_ph111_B8
zmp_ph111_B6
zmp_ph111_B7
zmp_ph111_B9
zmp_ph111_B10
zmp_ph114_G4
zmp_ph114_G11
zmp_ph114_H1
zmp_ph114_H2
zmp_ph114_A9
zmp_ph114_A11
zmp_ph114_B2
zmp_ph114_B4
zmp_ph114_D8
zmp_ph114_E2

10:3299525−3317901
ENSDARG00000021564 / VDAC3 (1 of many)
0.02

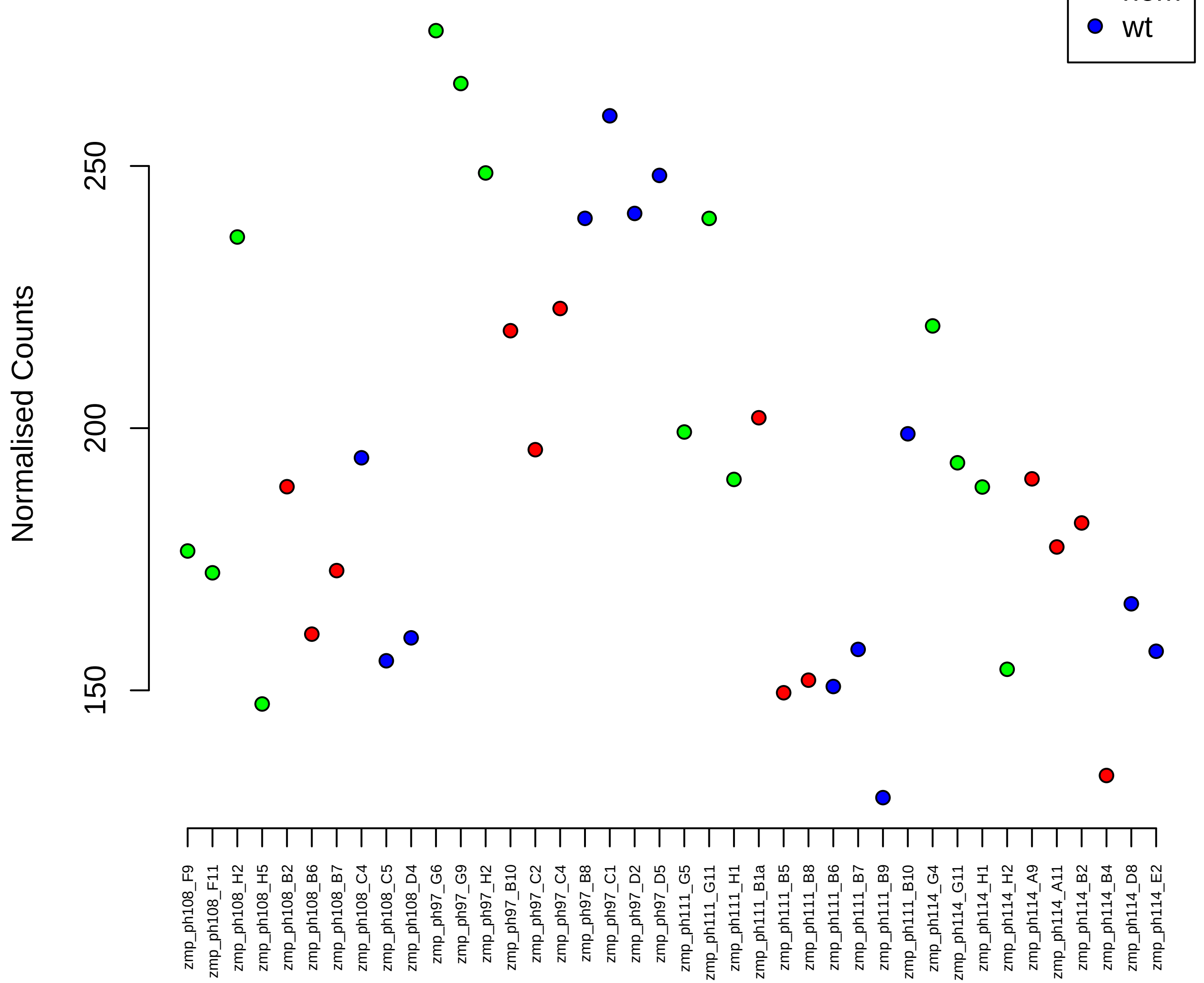

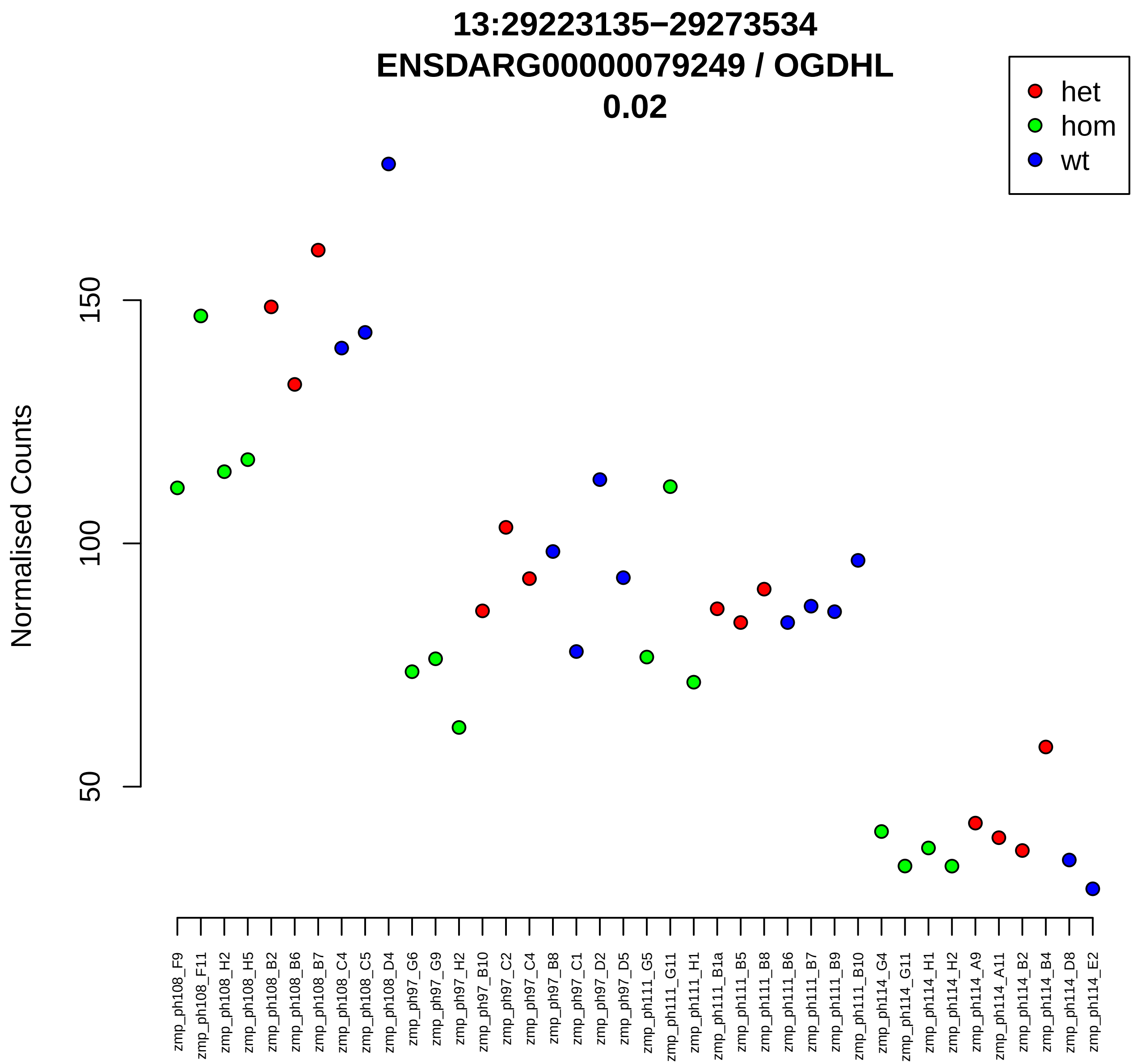
13:29223135−29273534
ENSDARG00000079249 / OGDHL
0.02
het
hom
wt
Normalised Counts
150
100
50
zmp_ph108_F9
zmp_ph108_F11
zmp_ph108_H2
zmp_ph108_H5
zmp_ph108_B2
zmp_ph108_B6
zmp_ph108_B7
zmp_ph108_C4
zmp_ph108_C5
zmp_ph108_D4
zmp_ph97_G6
zmp_ph97_G9
zmp_ph97_H2
zmp_ph97_B10
zmp_ph97_C2
zmp_ph97_C4
zmp_ph97_B8
zmp_ph97_C1
zmp_ph97_D2
zmp_ph97_D5
zmp_ph111_G5
zmp_ph111_G11
zmp_ph111_H1
zmp_ph111_B1a
zmp_ph111_B5
zmp_ph111_B8
zmp_ph111_B6
zmp_ph111_B7
zmp_ph111_B9
zmp_ph111_B10
zmp_ph114_G4
zmp_ph114_G11
zmp_ph114_H1
zmp_ph114_H2
zmp_ph114_A9
zmp_ph114_A11
zmp_ph114_B2
zmp_ph114_B4
zmp_ph114_D8
zmp_ph114_E2

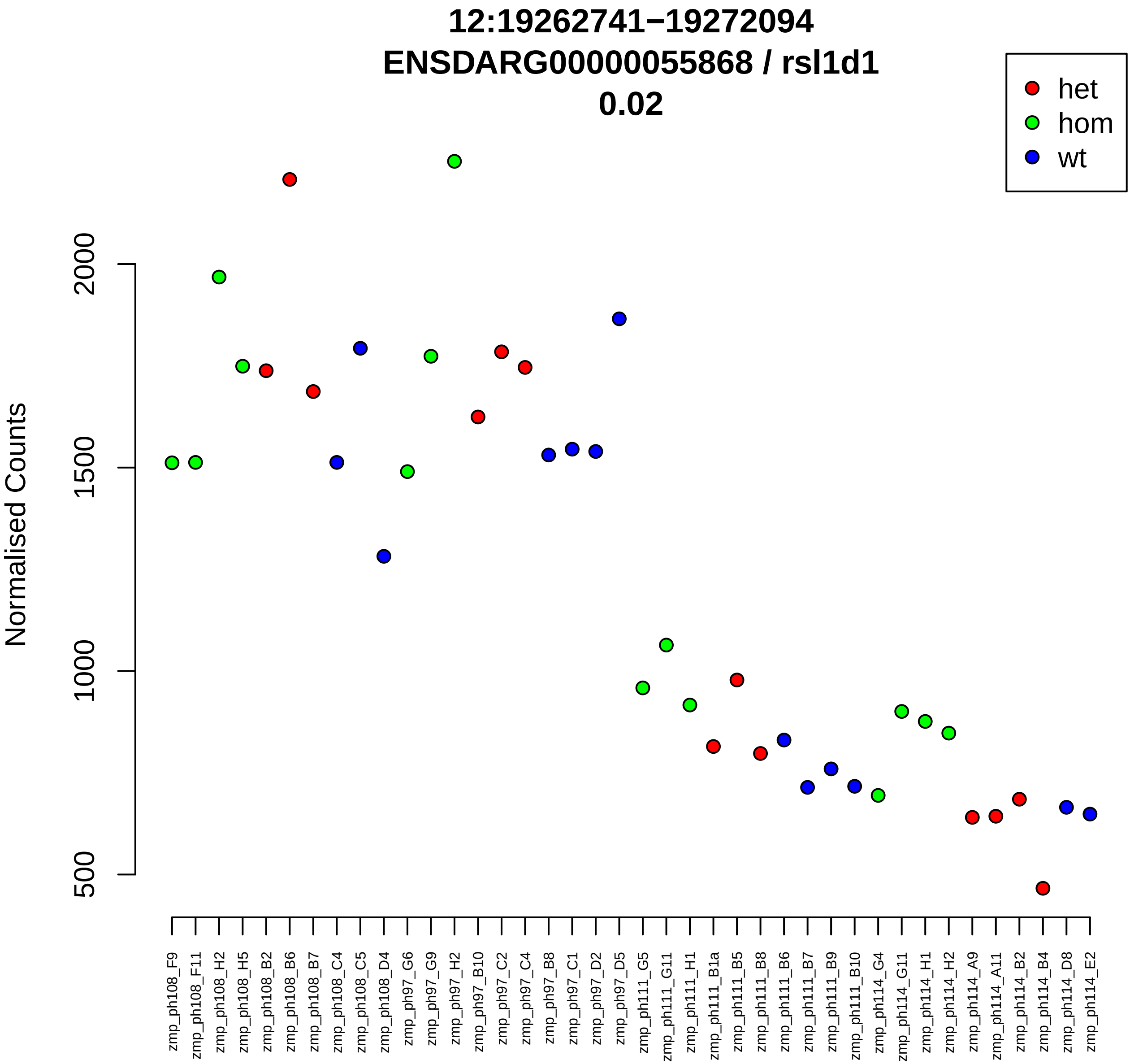
12:19262741−19272094
ENSDARG00000055868 / rsl1d1
0.02
het
hom
wt
Normalised Counts
500
1000
1500
2000
zmp_ph108_F9
zmp_ph108_F11
zmp_ph108_H2
zmp_ph108_H5
zmp_ph108_B2
zmp_ph108_B6
zmp_ph108_B7
zmp_ph108_C4
zmp_ph108_C5
zmp_ph108_D4
zmp_ph97_G6
zmp_ph97_G9
zmp_ph97_H2
zmp_ph97_B10
zmp_ph97_C2
zmp_ph97_C4
zmp_ph97_B8
zmp_ph97_C1
zmp_ph97_D2
zmp_ph97_D5
zmp_ph111_G5
zmp_ph111_G11
zmp_ph111_H1
zmp_ph111_B1a
zmp_ph111_B5
zmp_ph111_B8
zmp_ph111_B6
zmp_ph111_B7
zmp_ph111_B9
zmp_ph111_B10
zmp_ph114_G4
zmp_ph114_G11
zmp_ph114_H1
zmp_ph114_H2
zmp_ph114_A9
zmp_ph114_A11
zmp_ph114_B2
zmp_ph114_B4
zmp_ph114_D8
zmp_ph114_E2

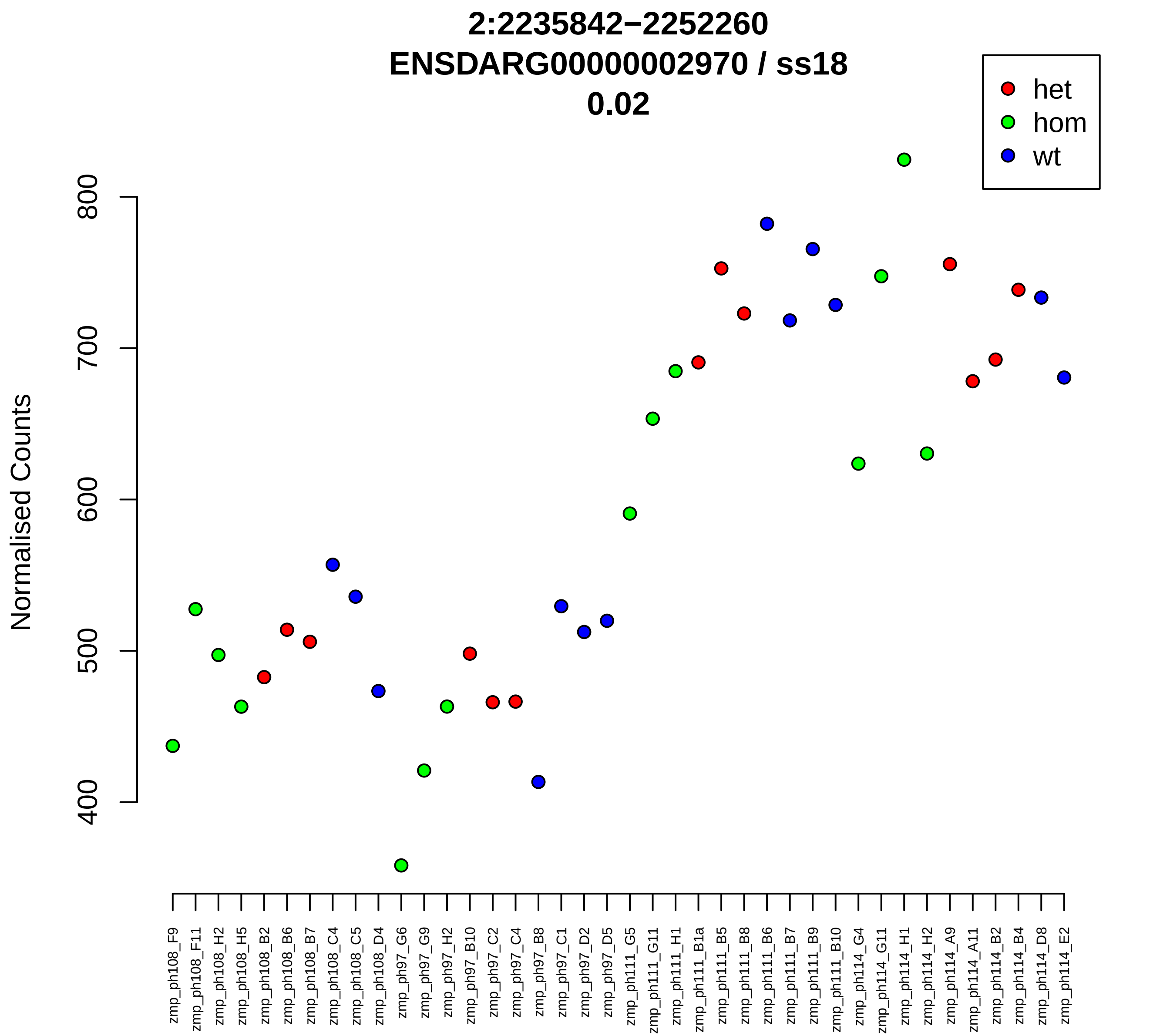

2:2235842−2252260
ENSDARG00000002970 / ss18
0.02
het
hom
wt
Normalised Counts
400
500
600
700
800
zmp_ph108_F9
zmp_ph108_F11
zmp_ph108_H2
zmp_ph108_H5
zmp_ph108_B2
zmp_ph108_B6
zmp_ph108_B7
zmp_ph108_C4
zmp_ph108_C5
zmp_ph108_D4
zmp_ph97_G6
zmp_ph97_G9
zmp_ph97_H2
zmp_ph97_B10
zmp_ph97_C2
zmp_ph97_C4
zmp_ph97_B8
zmp_ph97_C1
zmp_ph97_D2
zmp_ph97_D5
zmp_ph111_G5
zmp_ph111_G11
zmp_ph111_H1
zmp_ph111_B1a
zmp_ph111_B5
zmp_ph111_B8
zmp_ph111_B6
zmp_ph111_B7
zmp_ph111_B9
zmp_ph111_B10
zmp_ph114_G4
zmp_ph114_G11
zmp_ph114_H1
zmp_ph114_H2
zmp_ph114_A9
zmp_ph114_A11
zmp_ph114_B2
zmp_ph114_B4
zmp_ph114_D8
zmp_ph114_E2

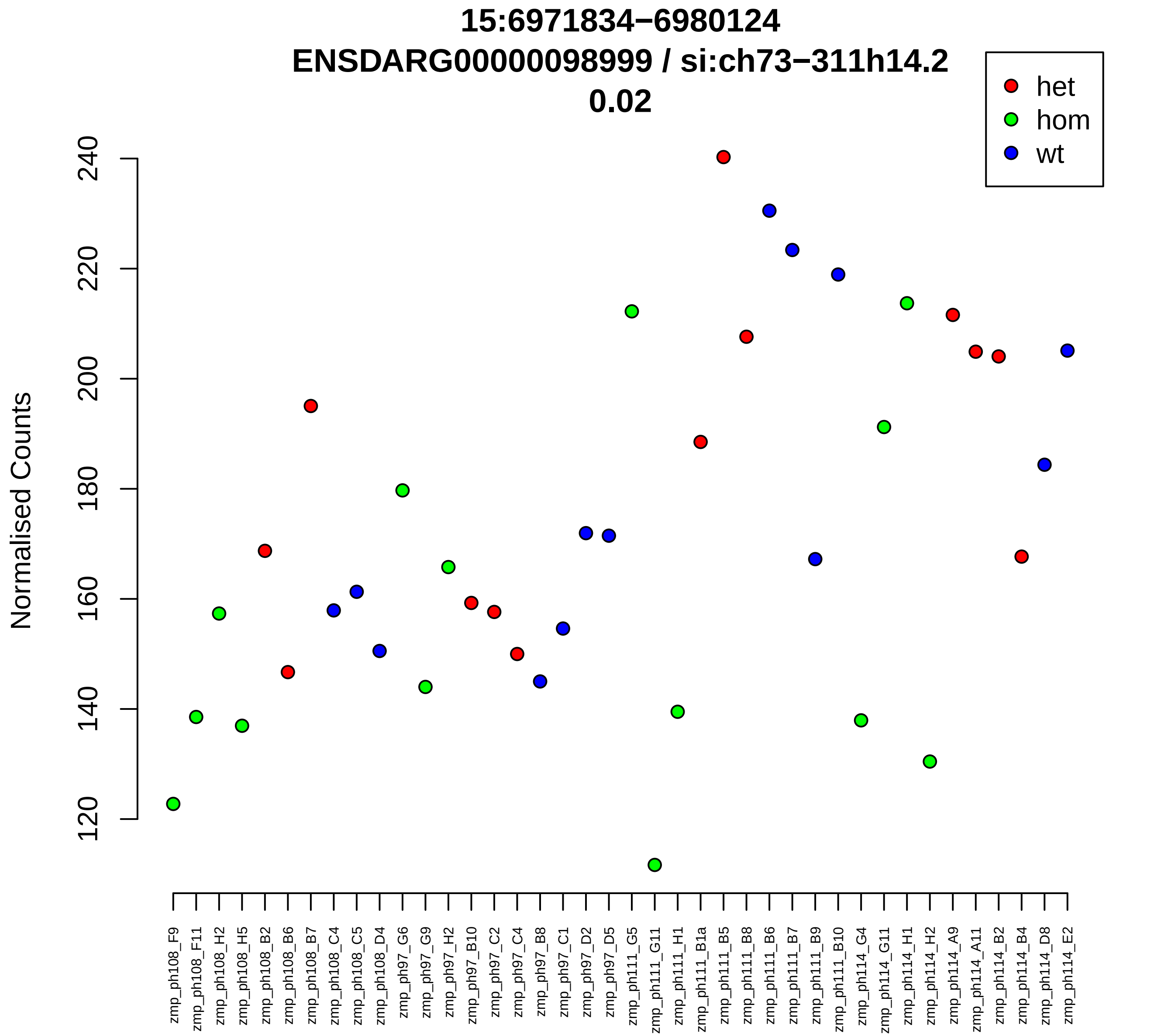
15:6971834−6980124
ENSDARG00000098999 / si:ch73−311h14.2
0.02
het
hom
wt
Normalised Counts
120
140
160
180
200
220
240
zmp_ph108_F9
zmp_ph108_F11
zmp_ph108_H2
zmp_ph108_H5
zmp_ph108_B2
zmp_ph108_B6
zmp_ph108_B7
zmp_ph108_C4
zmp_ph108_C5
zmp_ph108_D4
zmp_ph97_G6
zmp_ph97_G9
zmp_ph97_H2
zmp_ph97_B10
zmp_ph97_C2
zmp_ph97_C4
zmp_ph97_B8
zmp_ph97_C1
zmp_ph97_D2
zmp_ph97_D5
zmp_ph111_G5
zmp_ph111_G11
zmp_ph111_H1
zmp_ph111_B1a
zmp_ph111_B5
zmp_ph111_B8
zmp_ph111_B6
zmp_ph111_B7
zmp_ph111_B9
zmp_ph111_B10
zmp_ph114_G4
zmp_ph114_G11
zmp_ph114_H1
zmp_ph114_H2
zmp_ph114_A9
zmp_ph114_A11
zmp_ph114_B2
zmp_ph114_B4
zmp_ph114_D8
zmp_ph114_E2

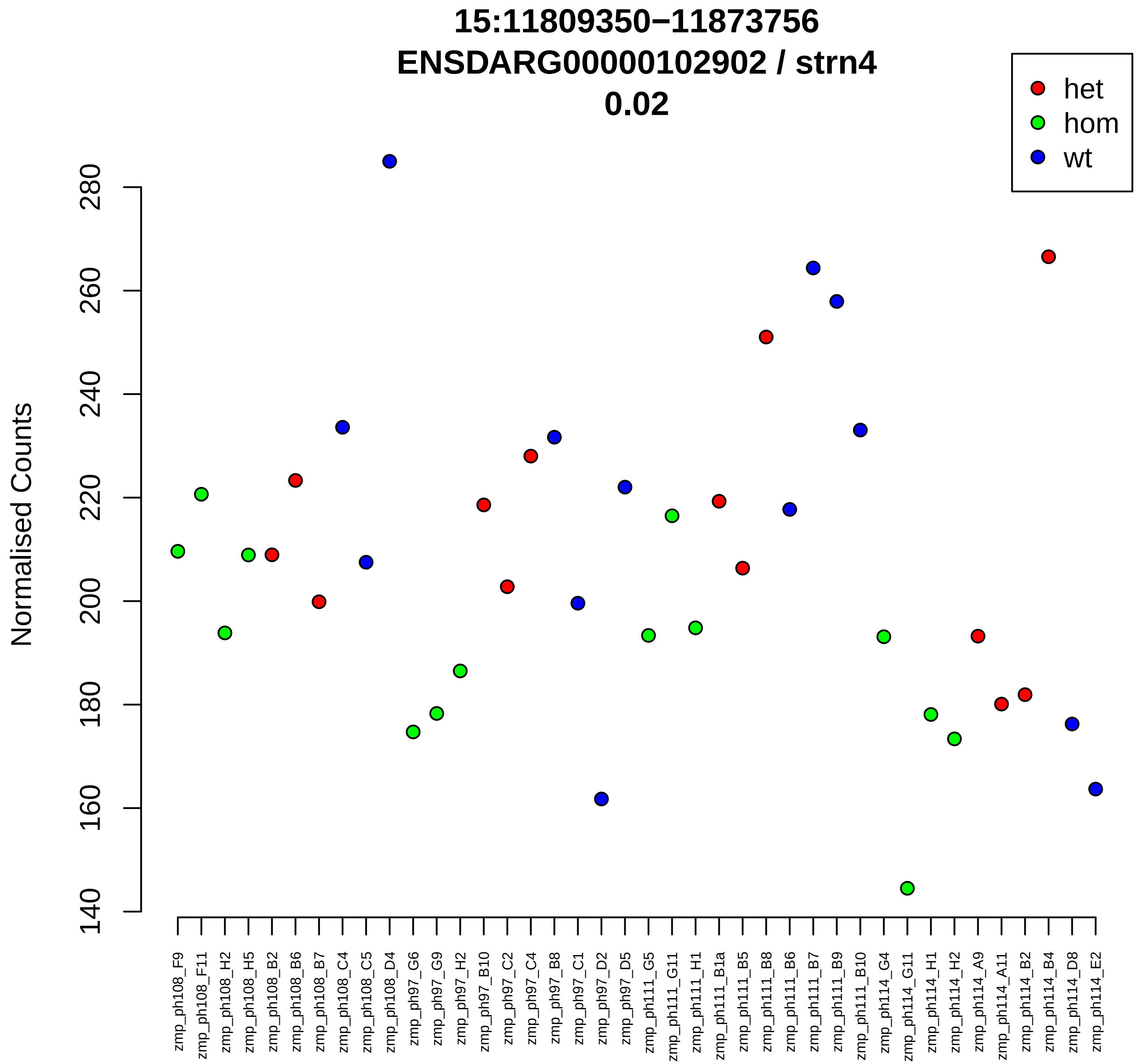
15:11809350−11873756
ENSDARG00000102902 / strn4
0.02
het
hom
wt
Normalised Counts
140
160
180
200
220
240
260
280
zmp_ph108_F9
zmp_ph108_F11
zmp_ph108_H2
zmp_ph108_H5
zmp_ph108_B2
zmp_ph108_B6
zmp_ph108_B7
zmp_ph108_C4
zmp_ph108_C5
zmp_ph108_D4
zmp_ph97_G6
zmp_ph97_G9
zmp_ph97_H2
zmp_ph97_B10
zmp_ph97_C2
zmp_ph97_C4
zmp_ph97_B8
zmp_ph97_C1
zmp_ph97_D2
zmp_ph97_D5
zmp_ph111_G5
zmp_ph111_G11
zmp_ph111_H1
zmp_ph111_B1a
zmp_ph111_B5
zmp_ph111_B8
zmp_ph111_B6
zmp_ph111_B7
zmp_ph111_B9
zmp_ph111_B10
zmp_ph114_G4
zmp_ph114_G11
zmp_ph114_H1
zmp_ph114_H2
zmp_ph114_A9
zmp_ph114_A11
zmp_ph114_B2
zmp_ph114_B4
zmp_ph114_D8
zmp_ph114_E2

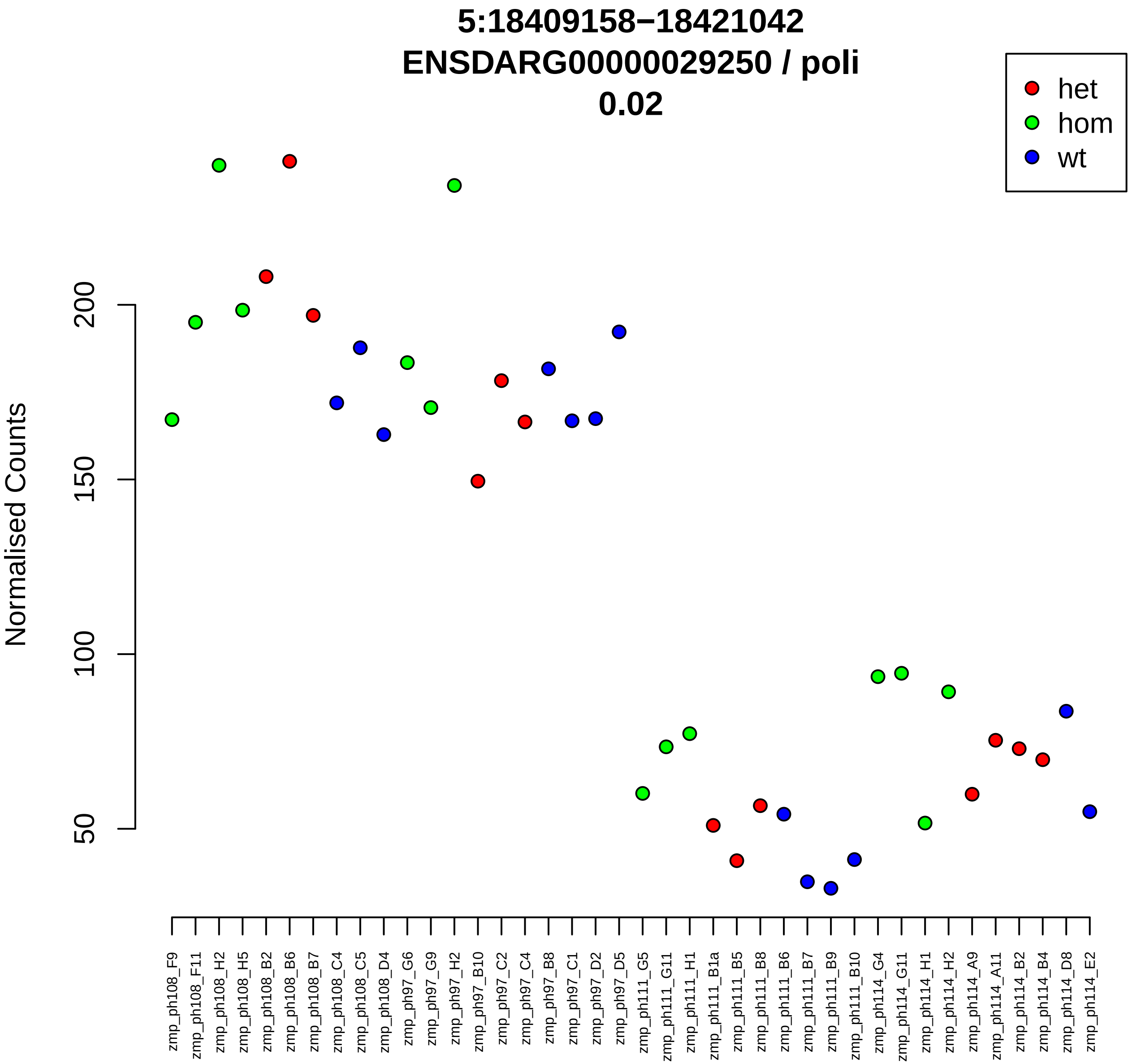

5:18409158−18421042
ENSDARG00000029250 / poli
0.02
het
hom
wt
Normalised Counts
50
100
150
200
zmp_ph108_F9
zmp_ph108_F11
zmp_ph108_H2
zmp_ph108_H5
zmp_ph108_B2
zmp_ph108_B6
zmp_ph108_B7
zmp_ph108_C4
zmp_ph108_C5
zmp_ph108_D4
zmp_ph97_G6
zmp_ph97_G9
zmp_ph97_H2
zmp_ph97_B10
zmp_ph97_C2
zmp_ph97_C4
zmp_ph97_B8
zmp_ph97_C1
zmp_ph97_D2
zmp_ph97_D5
zmp_ph111_G5
zmp_ph111_G11
zmp_ph111_H1
zmp_ph111_B1a
zmp_ph111_B5
zmp_ph111_B8
zmp_ph111_B6
zmp_ph111_B7
zmp_ph111_B9
zmp_ph111_B10
zmp_ph114_G4
zmp_ph114_G11
zmp_ph114_H1
zmp_ph114_H2
zmp_ph114_A9
zmp_ph114_A11
zmp_ph114_B2
zmp_ph114_B4
zmp_ph114_D8
zmp_ph114_E2

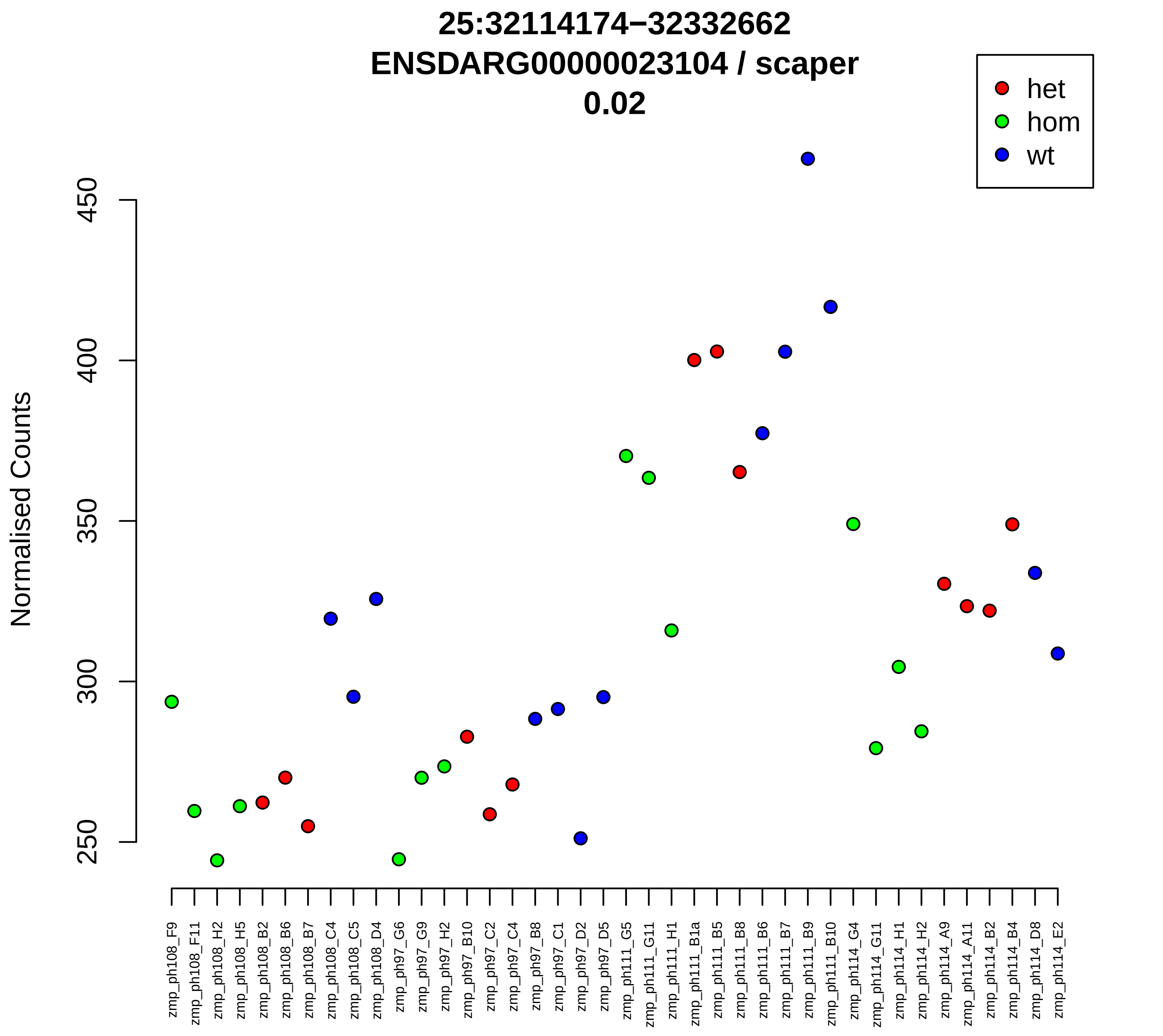

25:32114174−32332662
ENSDARG00000023104 / scaper
0.02
het
hom
wt
Normalised Counts
250
300
350
400
450
zmp_ph108_F9
zmp_ph108_F11
zmp_ph108_H2
zmp_ph108_H5
zmp_ph108_B2
zmp_ph108_B6
zmp_ph108_B7
zmp_ph108_C4
zmp_ph108_C5
zmp_ph108_D4
zmp_ph97_G6
zmp_ph97_G9
zmp_ph97_H2
zmp_ph97_B10
zmp_ph97_C2
zmp_ph97_C4
zmp_ph97_B8
zmp_ph97_C1
zmp_ph97_D2
zmp_ph97_D5
zmp_ph111_G5
zmp_ph111_G11
zmp_ph111_H1
zmp_ph111_B1a
zmp_ph111_B5
zmp_ph111_B8
zmp_ph111_B6
zmp_ph111_B7
zmp_ph111_B9
zmp_ph111_B10
zmp_ph114_G4
zmp_ph114_G11
zmp_ph114_H1
zmp_ph114_H2
zmp_ph114_A9
zmp_ph114_A11
zmp_ph114_B2
zmp_ph114_B4
zmp_ph114_D8
zmp_ph114_E2

6:54703198−54745934
ENSDARG00000052705 / pkp1b
0.02

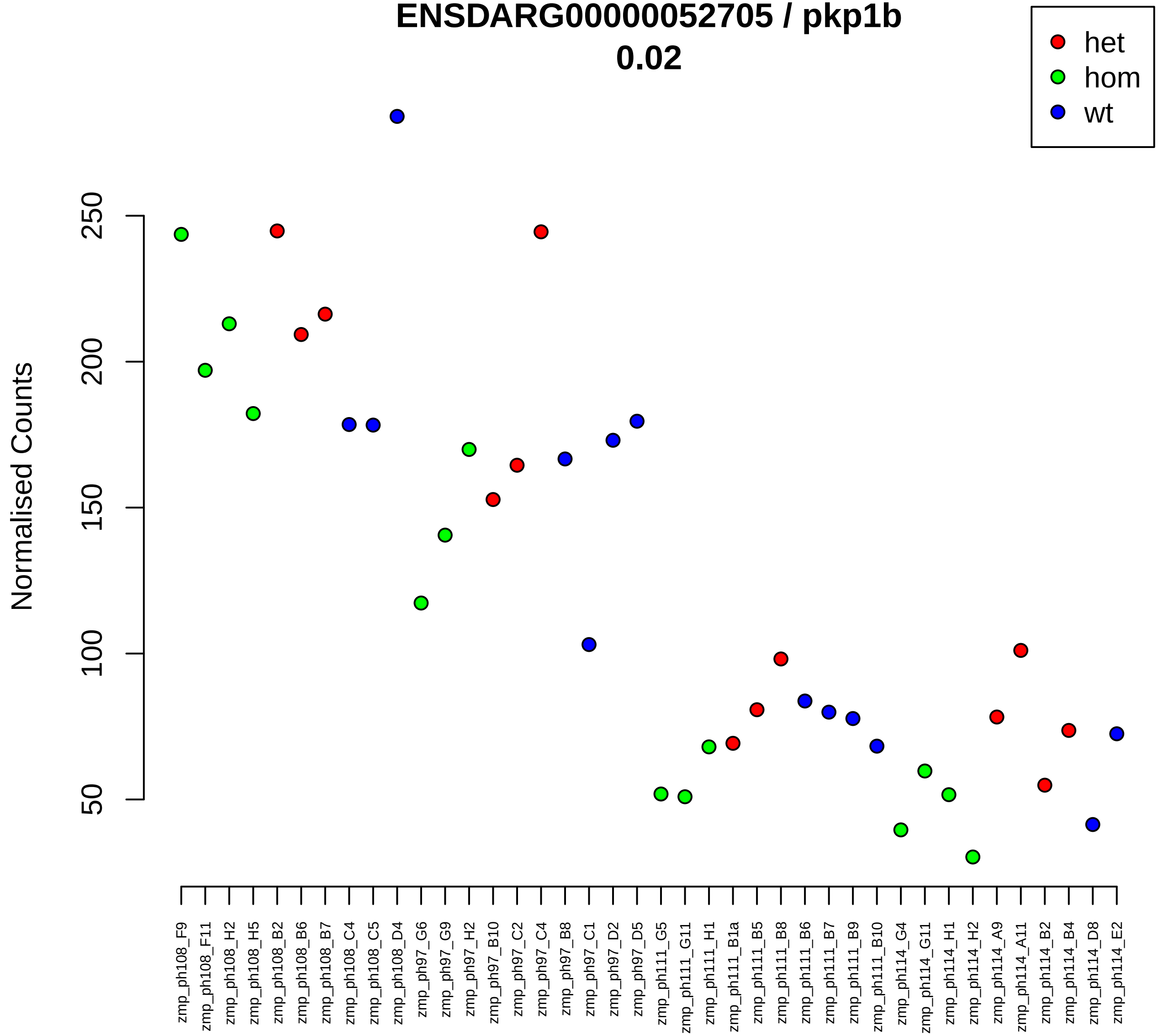

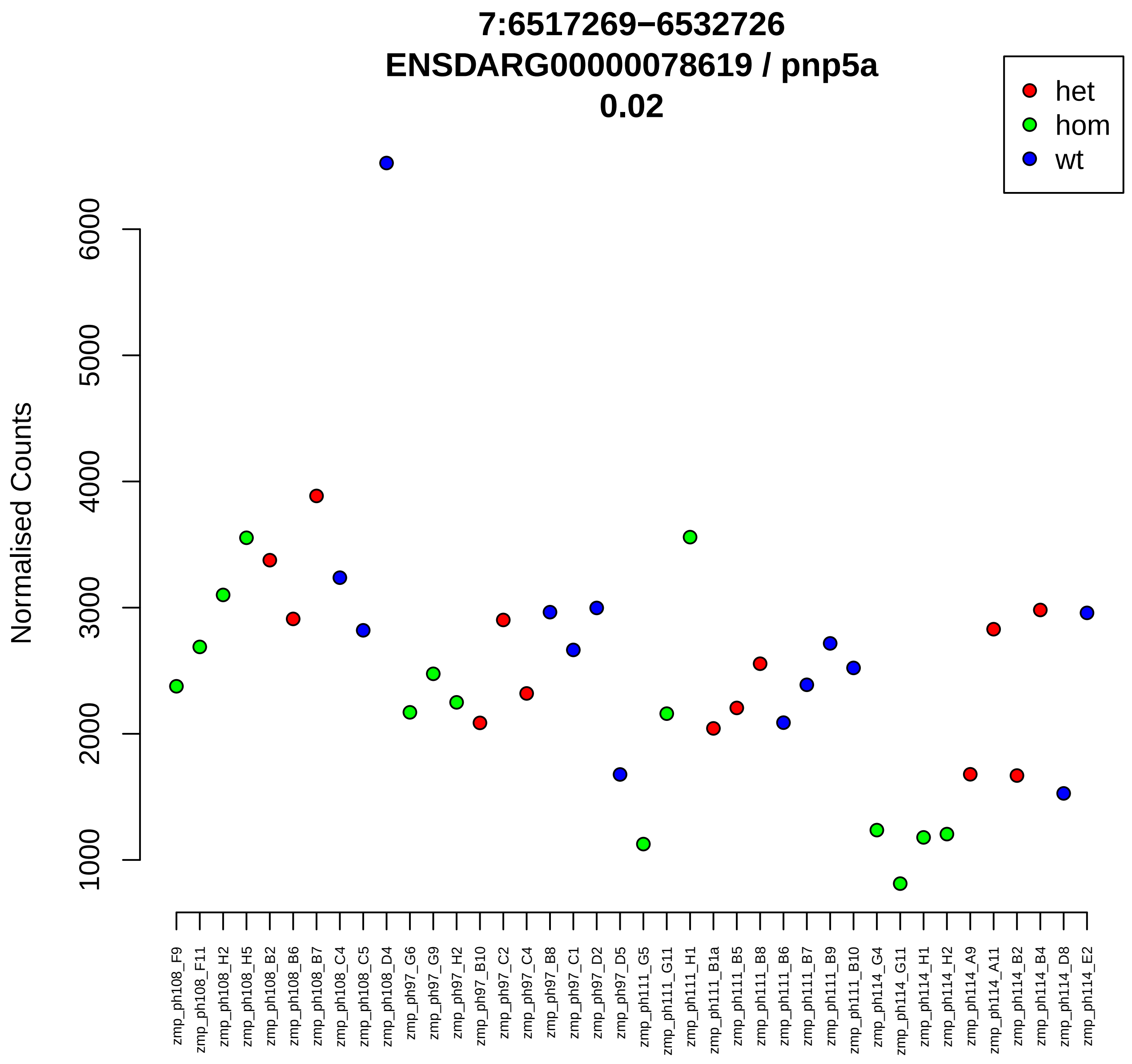

7:6517269−6532726
ENSDARG00000078619 / pnp5a
0.02
het
hom
wt
Normalised Counts
1000
2000
3000
4000
5000
6000
zmp_ph108_F9
zmp_ph108_F11
zmp_ph108_H2
zmp_ph108_H5
zmp_ph108_B2
zmp_ph108_B6
zmp_ph108_B7
zmp_ph108_C4
zmp_ph108_C5
zmp_ph108_D4
zmp_ph97_G6
zmp_ph97_G9
zmp_ph97_H2
zmp_ph97_B10
zmp_ph97_C2
zmp_ph97_C4
zmp_ph97_B8
zmp_ph97_C1
zmp_ph97_D2
zmp_ph97_D5
zmp_ph111_G5
zmp_ph111_G11
zmp_ph111_H1
zmp_ph111_B1a
zmp_ph111_B5
zmp_ph111_B8
zmp_ph111_B6
zmp_ph111_B7
zmp_ph111_B9
zmp_ph111_B10
zmp_ph114_G4
zmp_ph114_G11
zmp_ph114_H1
zmp_ph114_H2
zmp_ph114_A9
zmp_ph114_A11
zmp_ph114_B2
zmp_ph114_B4
zmp_ph114_D8
zmp_ph114_E2

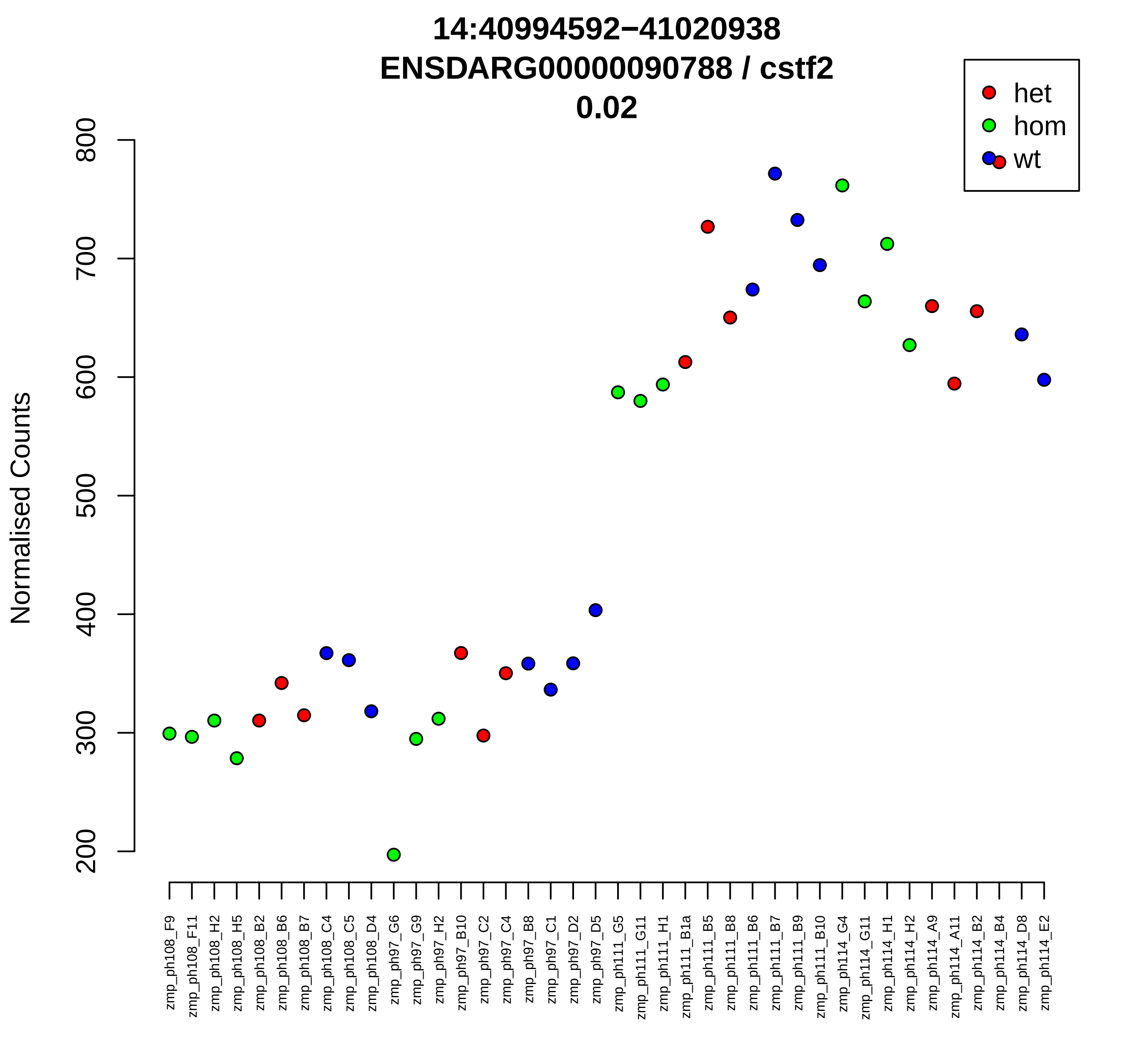

14:40994592−41020938
ENSDARG00000090788 / cstf2
0.02
het
hom
wt
Normalised Counts
200
300
400
500
600
700
800
zmp_ph108_F9
zmp_ph108_F11
zmp_ph108_H2
zmp_ph108_H5
zmp_ph108_B2
zmp_ph108_B6
zmp_ph108_B7
zmp_ph108_C4
zmp_ph108_C5
zmp_ph108_D4
zmp_ph97_G6
zmp_ph97_G9
zmp_ph97_H2
zmp_ph97_B10
zmp_ph97_C2
zmp_ph97_C4
zmp_ph97_B8
zmp_ph97_C1
zmp_ph97_D2
zmp_ph97_D5
zmp_ph111_G5
zmp_ph111_G11
zmp_ph111_H1
zmp_ph111_B1a
zmp_ph111_B5
zmp_ph111_B8
zmp_ph111_B6
zmp_ph111_B7
zmp_ph111_B9
zmp_ph111_B10
zmp_ph114_G4
zmp_ph114_G11
zmp_ph114_H1
zmp_ph114_H2
zmp_ph114_A9
zmp_ph114_A11
zmp_ph114_B2
zmp_ph114_B4
zmp_ph114_D8
zmp_ph114_E2

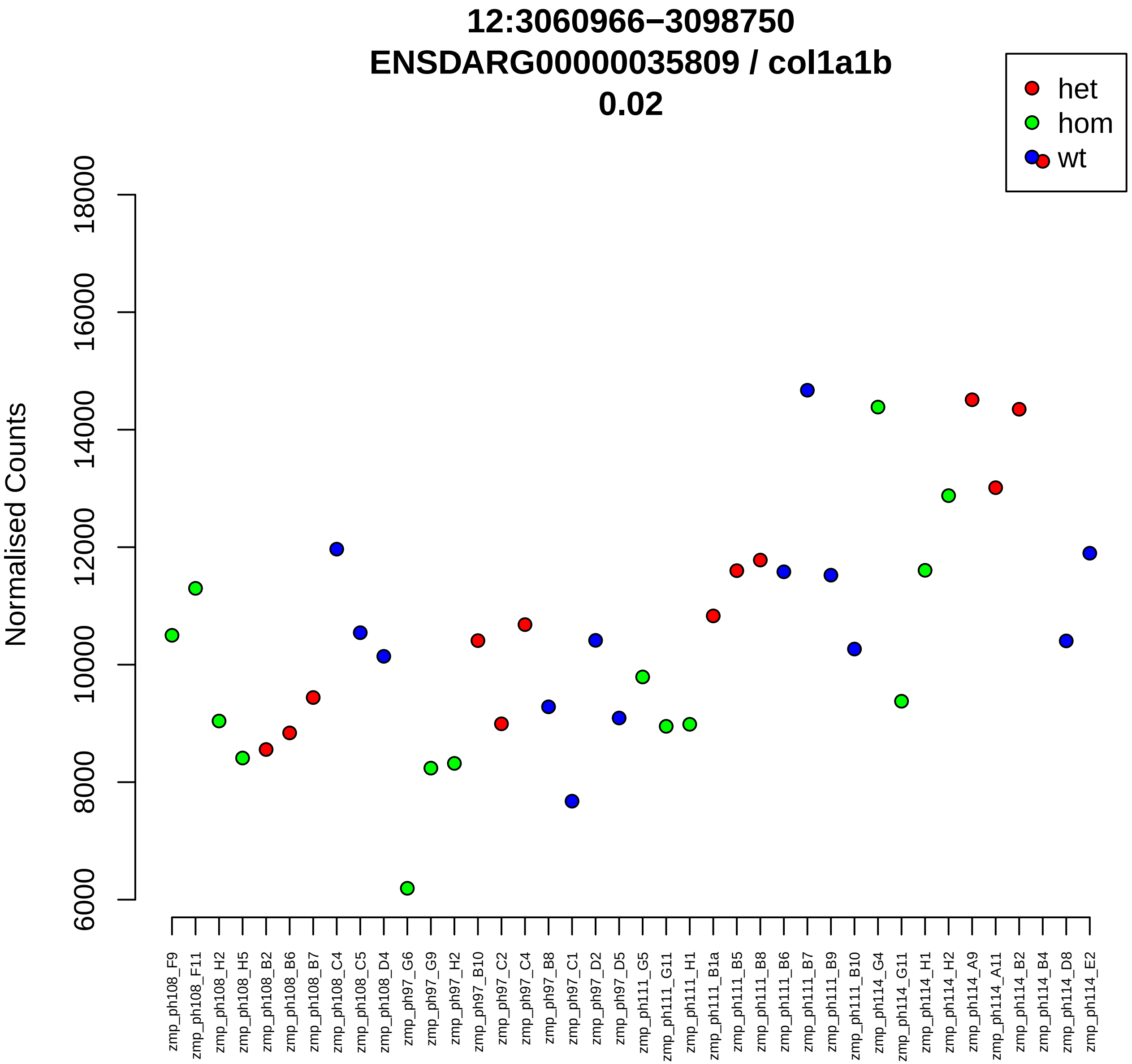

12:3060966−3098750
ENSDARG00000035809 / col1a1b
0.02
het
hom
wt
Normalised Counts
6000
8000
10000
12000
14000
16000
18000
zmp_ph108_F9
zmp_ph108_F11
zmp_ph108_H2
zmp_ph108_H5
zmp_ph108_B2
zmp_ph108_B6
zmp_ph108_B7
zmp_ph108_C4
zmp_ph108_C5
zmp_ph108_D4
zmp_ph97_G6
zmp_ph97_G9
zmp_ph97_H2
zmp_ph97_B10
zmp_ph97_C2
zmp_ph97_C4
zmp_ph97_B8
zmp_ph97_C1
zmp_ph97_D2
zmp_ph97_D5
zmp_ph111_G5
zmp_ph111_G11
zmp_ph111_H1
zmp_ph111_B1a
zmp_ph111_B5
zmp_ph111_B8
zmp_ph111_B6
zmp_ph111_B7
zmp_ph111_B9
zmp_ph111_B10
zmp_ph114_G4
zmp_ph114_G11
zmp_ph114_H1
zmp_ph114_H2
zmp_ph114_A9
zmp_ph114_A11
zmp_ph114_B2
zmp_ph114_B4
zmp_ph114_D8
zmp_ph114_E2

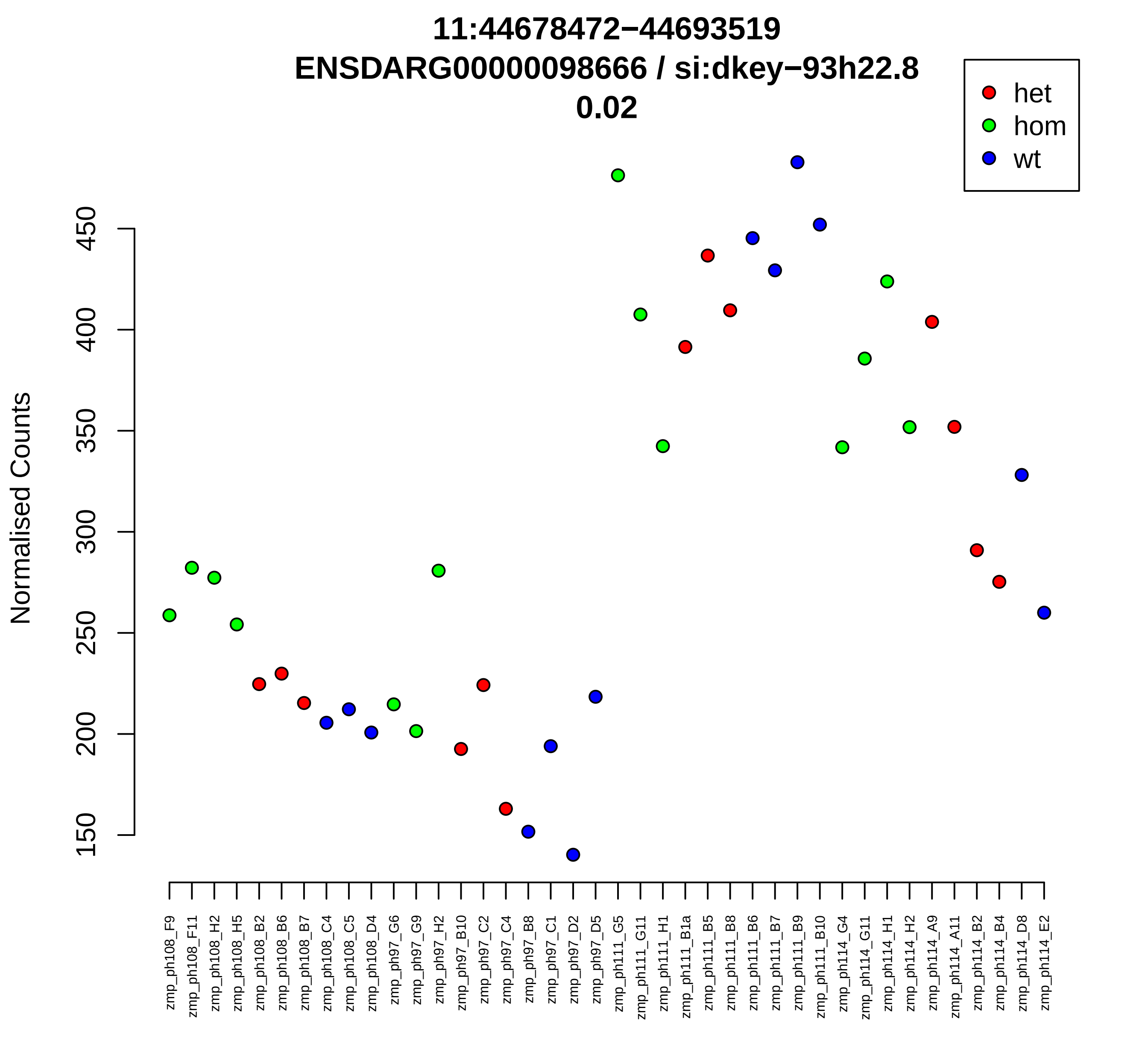
11:44678472−44693519
ENSDARG00000098666 / si:dkey−93h22.8
0.02
het
hom
wt
Normalised Counts
150
200
250
300
350
400
450
zmp_ph108_F9
zmp_ph108_F11
zmp_ph108_H2
zmp_ph108_H5
zmp_ph108_B2
zmp_ph108_B6
zmp_ph108_B7
zmp_ph108_C4
zmp_ph108_C5
zmp_ph108_D4
zmp_ph97_G6
zmp_ph97_G9
zmp_ph97_H2
zmp_ph97_B10
zmp_ph97_C2
zmp_ph97_C4
zmp_ph97_B8
zmp_ph97_C1
zmp_ph97_D2
zmp_ph97_D5
zmp_ph111_G5
zmp_ph111_G11
zmp_ph111_H1
zmp_ph111_B1a
zmp_ph111_B5
zmp_ph111_B8
zmp_ph111_B6
zmp_ph111_B7
zmp_ph111_B9
zmp_ph111_B10
zmp_ph114_G4
zmp_ph114_G11
zmp_ph114_H1
zmp_ph114_H2
zmp_ph114_A9
zmp_ph114_A11
zmp_ph114_B2
zmp_ph114_B4
zmp_ph114_D8
zmp_ph114_E2

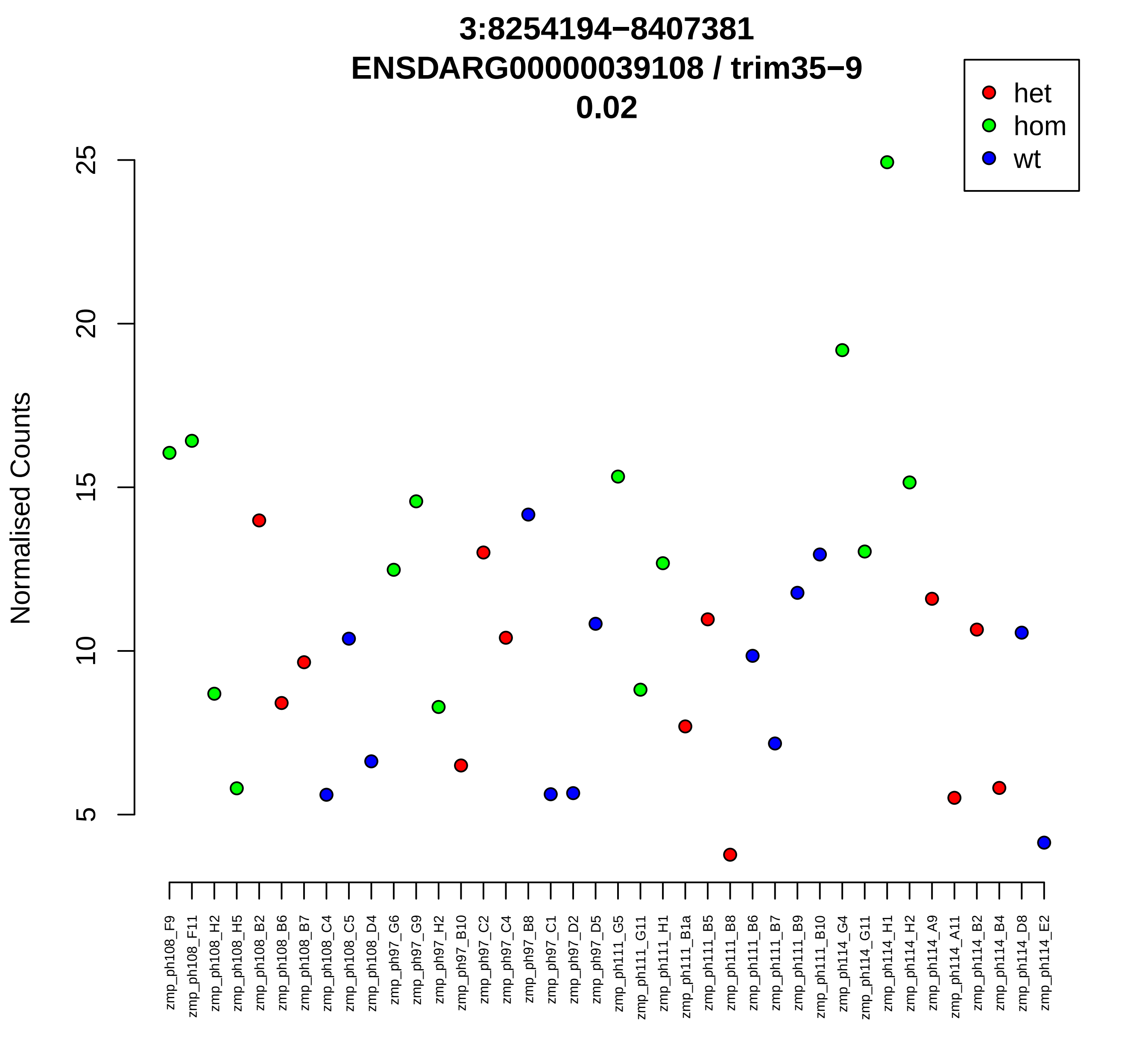

3:8254194−8407381
ENSDARG00000039108 / trim35−9
0.02
het
hom
wt
Normalised Counts
5
10
15
20
25
zmp_ph108_F9
zmp_ph108_F11
zmp_ph108_H2
zmp_ph108_H5
zmp_ph108_B2
zmp_ph108_B6
zmp_ph108_B7
zmp_ph108_C4
zmp_ph108_C5
zmp_ph108_D4
zmp_ph97_G6
zmp_ph97_G9
zmp_ph97_H2
zmp_ph97_B10
zmp_ph97_C2
zmp_ph97_C4
zmp_ph97_B8
zmp_ph97_C1
zmp_ph97_D2
zmp_ph97_D5
zmp_ph111_G5
zmp_ph111_G11
zmp_ph111_H1
zmp_ph111_B1a
zmp_ph111_B5
zmp_ph111_B8
zmp_ph111_B6
zmp_ph111_B7
zmp_ph111_B9
zmp_ph111_B10
zmp_ph114_G4
zmp_ph114_G11
zmp_ph114_H1
zmp_ph114_H2
zmp_ph114_A9
zmp_ph114_A11
zmp_ph114_B2
zmp_ph114_B4
zmp_ph114_D8
zmp_ph114_E2

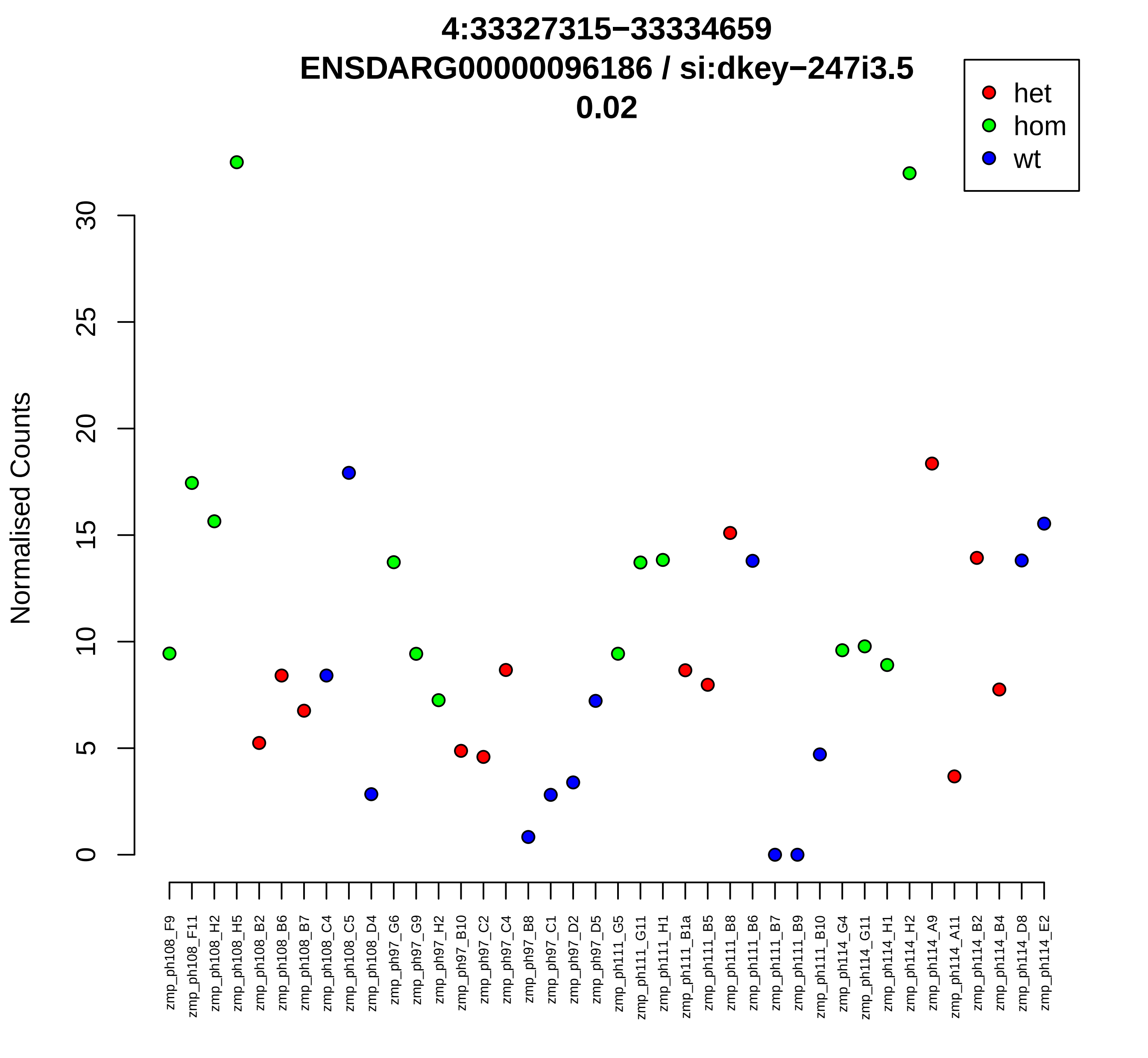

4:33327315−33334659
ENSDARG00000096186 / si:dkey−247i3.5
0.02
het
hom
wt
Normalised Counts
0
5
10
15
20
25
30
zmp_ph108_F9
zmp_ph108_F11
zmp_ph108_H2
zmp_ph108_H5
zmp_ph108_B2
zmp_ph108_B6
zmp_ph108_B7
zmp_ph108_C4
zmp_ph108_C5
zmp_ph108_D4
zmp_ph97_G6
zmp_ph97_G9
zmp_ph97_H2
zmp_ph97_B10
zmp_ph97_C2
zmp_ph97_C4
zmp_ph97_B8
zmp_ph97_C1
zmp_ph97_D2
zmp_ph97_D5
zmp_ph111_G5
zmp_ph111_G11
zmp_ph111_H1
zmp_ph111_B1a
zmp_ph111_B5
zmp_ph111_B8
zmp_ph111_B6
zmp_ph111_B7
zmp_ph111_B9
zmp_ph111_B10
zmp_ph114_G4
zmp_ph114_G11
zmp_ph114_H1
zmp_ph114_H2
zmp_ph114_A9
zmp_ph114_A11
zmp_ph114_B2
zmp_ph114_B4
zmp_ph114_D8
zmp_ph114_E2

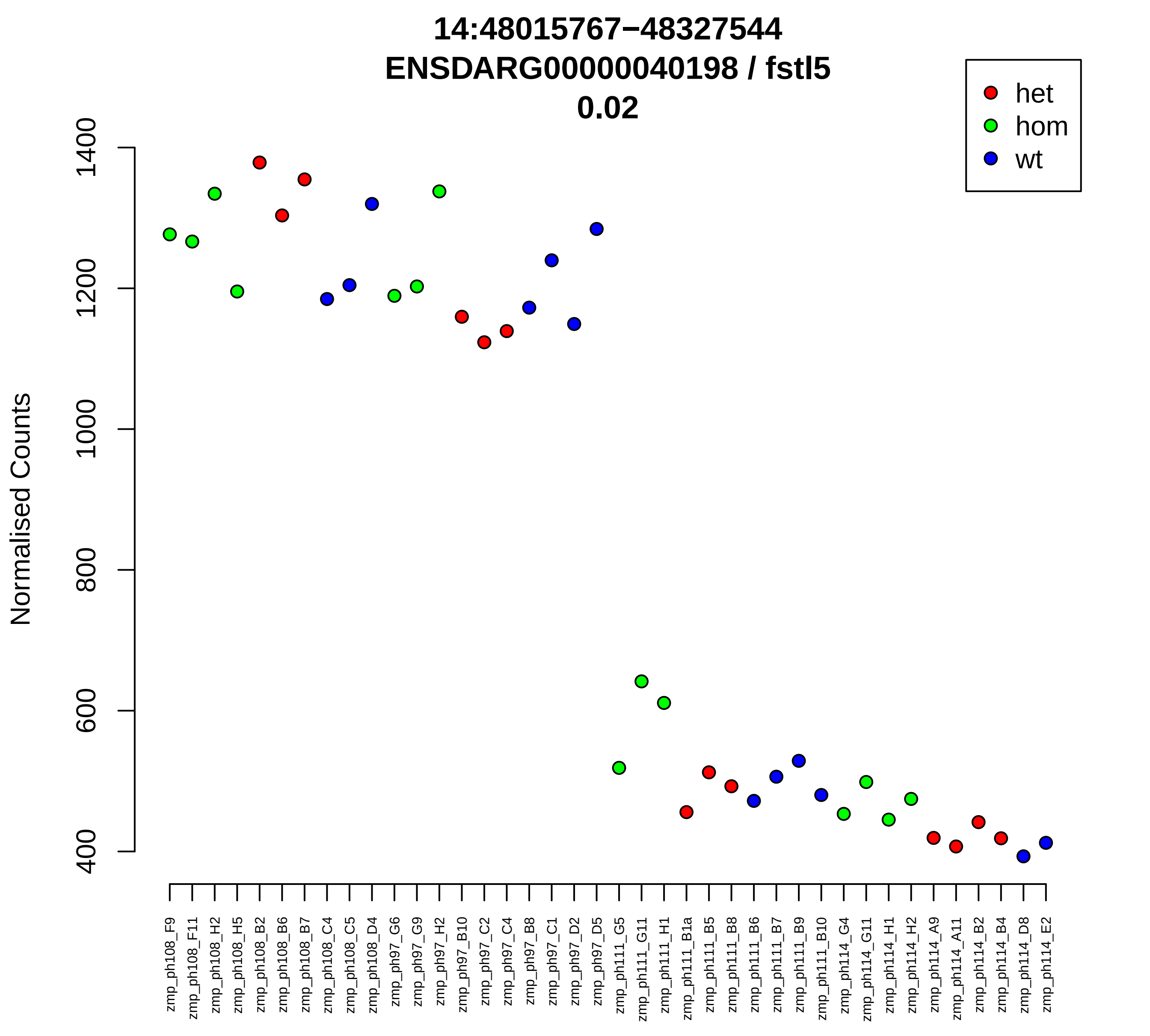

14:48015767−48327544
ENSDARG00000040198 / fstl5
0.02
het
hom
wt
Normalised Counts
400
600
800
1000
1200
1400
zmp_ph108_F9
zmp_ph108_F11
zmp_ph108_H2
zmp_ph108_H5
zmp_ph108_B2
zmp_ph108_B6
zmp_ph108_B7
zmp_ph108_C4
zmp_ph108_C5
zmp_ph108_D4
zmp_ph97_G6
zmp_ph97_G9
zmp_ph97_H2
zmp_ph97_B10
zmp_ph97_C2
zmp_ph97_C4
zmp_ph97_B8
zmp_ph97_C1
zmp_ph97_D2
zmp_ph97_D5
zmp_ph111_G5
zmp_ph111_G11
zmp_ph111_H1
zmp_ph111_B1a
zmp_ph111_B5
zmp_ph111_B8
zmp_ph111_B6
zmp_ph111_B7
zmp_ph111_B9
zmp_ph111_B10
zmp_ph114_G4
zmp_ph114_G11
zmp_ph114_H1
zmp_ph114_H2
zmp_ph114_A9
zmp_ph114_A11
zmp_ph114_B2
zmp_ph114_B4
zmp_ph114_D8
zmp_ph114_E2

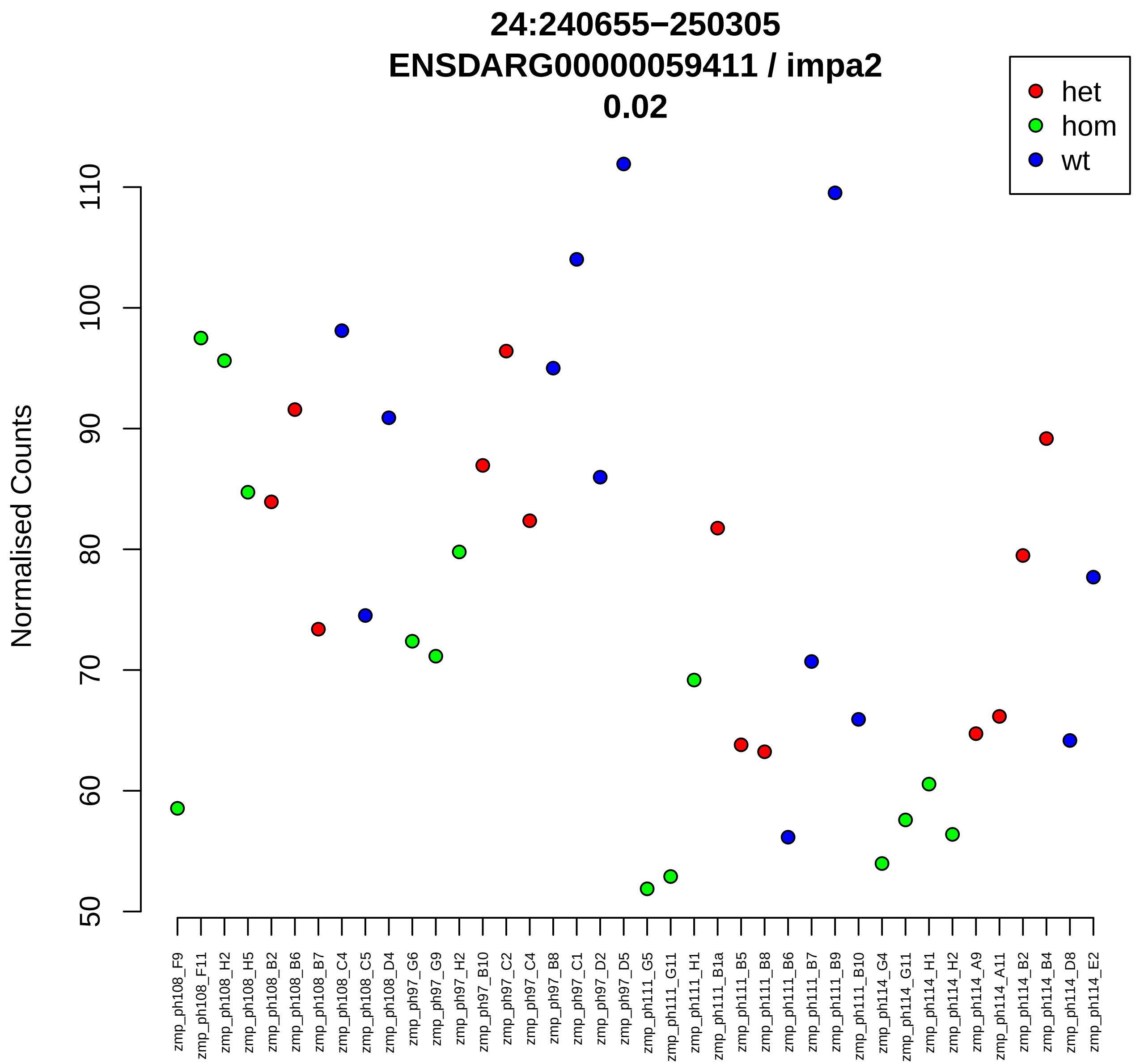

24:240655−250305
ENSDARG00000059411 / impa2
0.02
het
hom
wt
Normalised Counts
50
60
70
80
90
100
110
zmp_ph108_F9
zmp_ph108_F11
zmp_ph108_H2
zmp_ph108_H5
zmp_ph108_B2
zmp_ph108_B6
zmp_ph108_B7
zmp_ph108_C4
zmp_ph108_C5
zmp_ph108_D4
zmp_ph97_G6
zmp_ph97_G9
zmp_ph97_H2
zmp_ph97_B10
zmp_ph97_C2
zmp_ph97_C4
zmp_ph97_B8
zmp_ph97_C1
zmp_ph97_D2
zmp_ph97_D5
zmp_ph111_G5
zmp_ph111_G11
zmp_ph111_H1
zmp_ph111_B1a
zmp_ph111_B5
zmp_ph111_B8
zmp_ph111_B6
zmp_ph111_B7
zmp_ph111_B9
zmp_ph111_B10
zmp_ph114_G4
zmp_ph114_G11
zmp_ph114_H1
zmp_ph114_H2
zmp_ph114_A9
zmp_ph114_A11
zmp_ph114_B2
zmp_ph114_B4
zmp_ph114_D8
zmp_ph114_E2

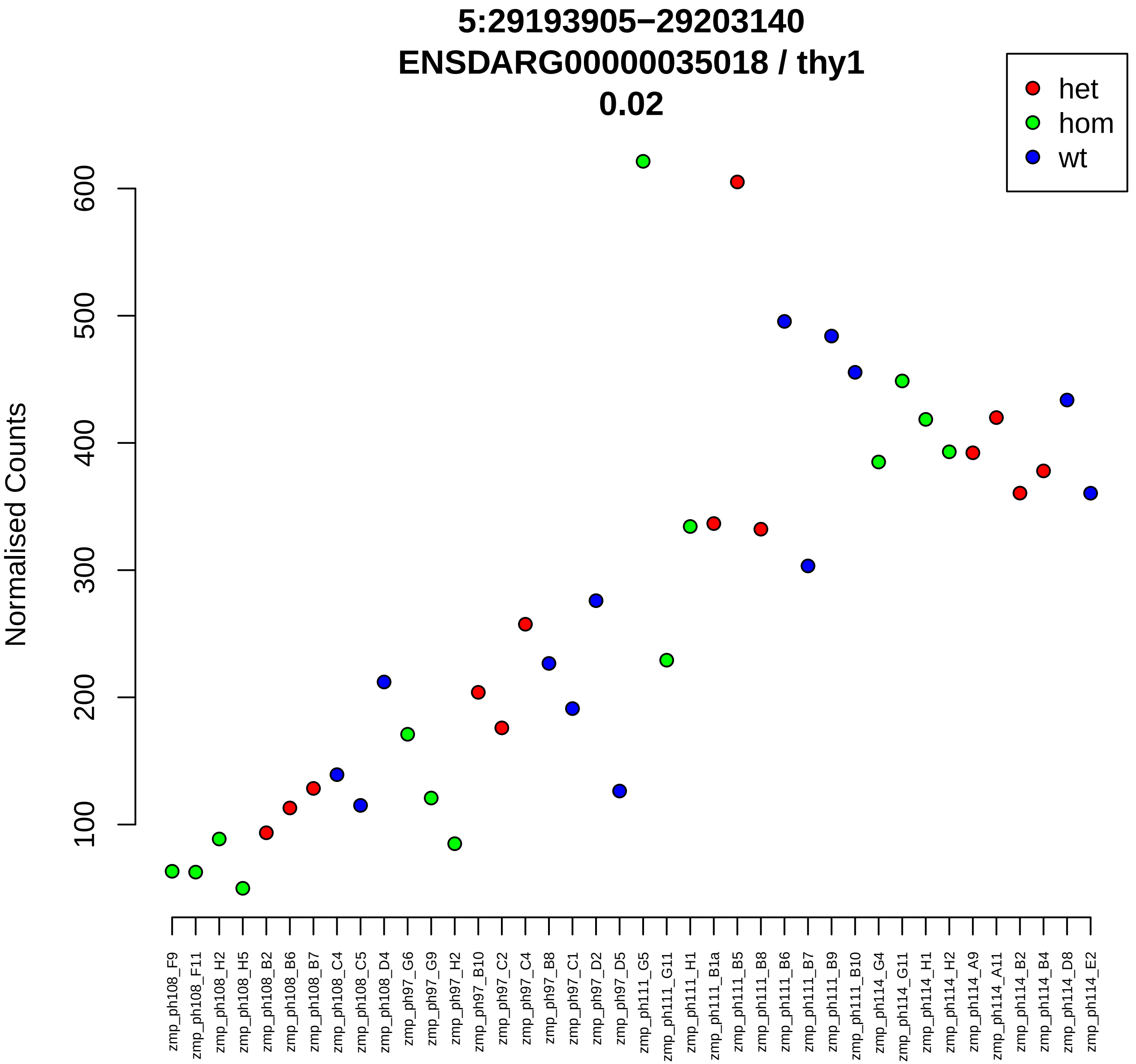
5:29193905−29203140
ENSDARG00000035018 / thy1
0.02
het
hom
wt
Normalised Counts
100
200
300
400
500
600
zmp_ph108_F9
zmp_ph108_F11
zmp_ph108_H2
zmp_ph108_H5
zmp_ph108_B2
zmp_ph108_B6
zmp_ph108_B7
zmp_ph108_C4
zmp_ph108_C5
zmp_ph108_D4
zmp_ph97_G6
zmp_ph97_G9
zmp_ph97_H2
zmp_ph97_B10
zmp_ph97_C2
zmp_ph97_C4
zmp_ph97_B8
zmp_ph97_C1
zmp_ph97_D2
zmp_ph97_D5
zmp_ph111_G5
zmp_ph111_G11
zmp_ph111_H1
zmp_ph111_B1a
zmp_ph111_B5
zmp_ph111_B8
zmp_ph111_B6
zmp_ph111_B7
zmp_ph111_B9
zmp_ph111_B10
zmp_ph114_G4
zmp_ph114_G11
zmp_ph114_H1
zmp_ph114_H2
zmp_ph114_A9
zmp_ph114_A11
zmp_ph114_B2
zmp_ph114_B4
zmp_ph114_D8
zmp_ph114_E2

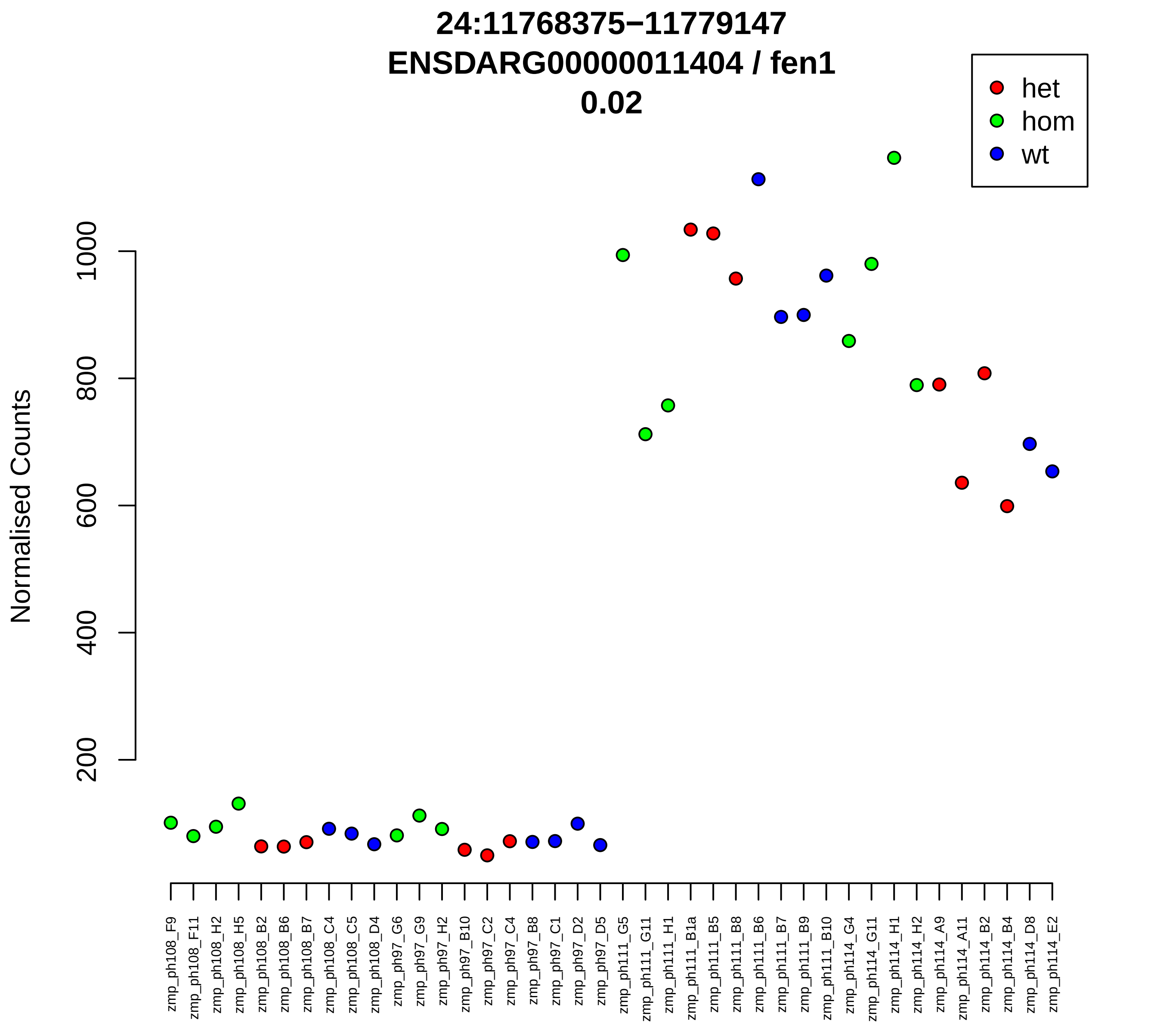

24:11768375−11779147
ENSDARG00000011404 / fen1
0.02
het
hom
wt
Normalised Counts
200
400
600
800
1000
zmp_ph108_F9
zmp_ph108_F11
zmp_ph108_H2
zmp_ph108_H5
zmp_ph108_B2
zmp_ph108_B6
zmp_ph108_B7
zmp_ph108_C4
zmp_ph108_C5
zmp_ph108_D4
zmp_ph97_G6
zmp_ph97_G9
zmp_ph97_H2
zmp_ph97_B10
zmp_ph97_C2
zmp_ph97_C4
zmp_ph97_B8
zmp_ph97_C1
zmp_ph97_D2
zmp_ph97_D5
zmp_ph111_G5
zmp_ph111_G11
zmp_ph111_H1
zmp_ph111_B1a
zmp_ph111_B5
zmp_ph111_B8
zmp_ph111_B6
zmp_ph111_B7
zmp_ph111_B9
zmp_ph111_B10
zmp_ph114_G4
zmp_ph114_G11
zmp_ph114_H1
zmp_ph114_H2
zmp_ph114_A9
zmp_ph114_A11
zmp_ph114_B2
zmp_ph114_B4
zmp_ph114_D8
zmp_ph114_E2

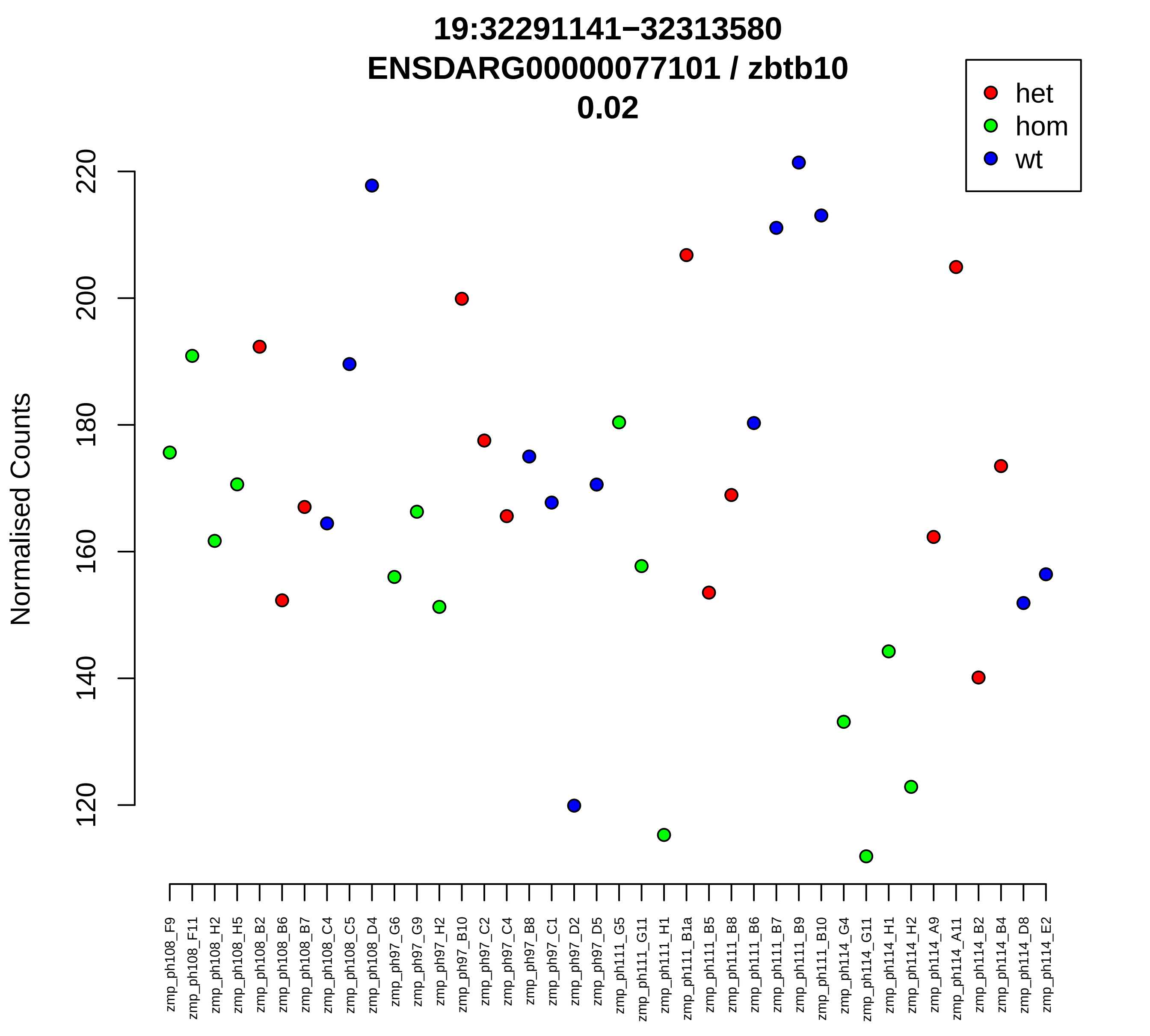
19:32291141−32313580
ENSDARG00000077101 / zbtb10
0.02
het
hom
wt
Normalised Counts
220
200
180
160
140
120
zmp_ph108_F9
zmp_ph108_F11
zmp_ph108_H2
zmp_ph108_H5
zmp_ph108_B2
zmp_ph108_B6
zmp_ph108_B7
zmp_ph108_C4
zmp_ph108_C5
zmp_ph108_D4
zmp_ph97_G6
zmp_ph97_G9
zmp_ph97_H2
zmp_ph97_B10
zmp_ph97_C2
zmp_ph97_C4
zmp_ph97_B8
zmp_ph97_C1
zmp_ph97_D2
zmp_ph97_D5
zmp_ph111_G5
zmp_ph111_G11
zmp_ph111_H1
zmp_ph111_B1a
zmp_ph111_B5
zmp_ph111_B8
zmp_ph111_B6
zmp_ph111_B7
zmp_ph111_B9
zmp_ph111_B10
zmp_ph114_G4
zmp_ph114_G11
zmp_ph114_H1
zmp_ph114_H2
zmp_ph114_A9
zmp_ph114_A11
zmp_ph114_B2
zmp_ph114_B4
zmp_ph114_D8
zmp_ph114_E2

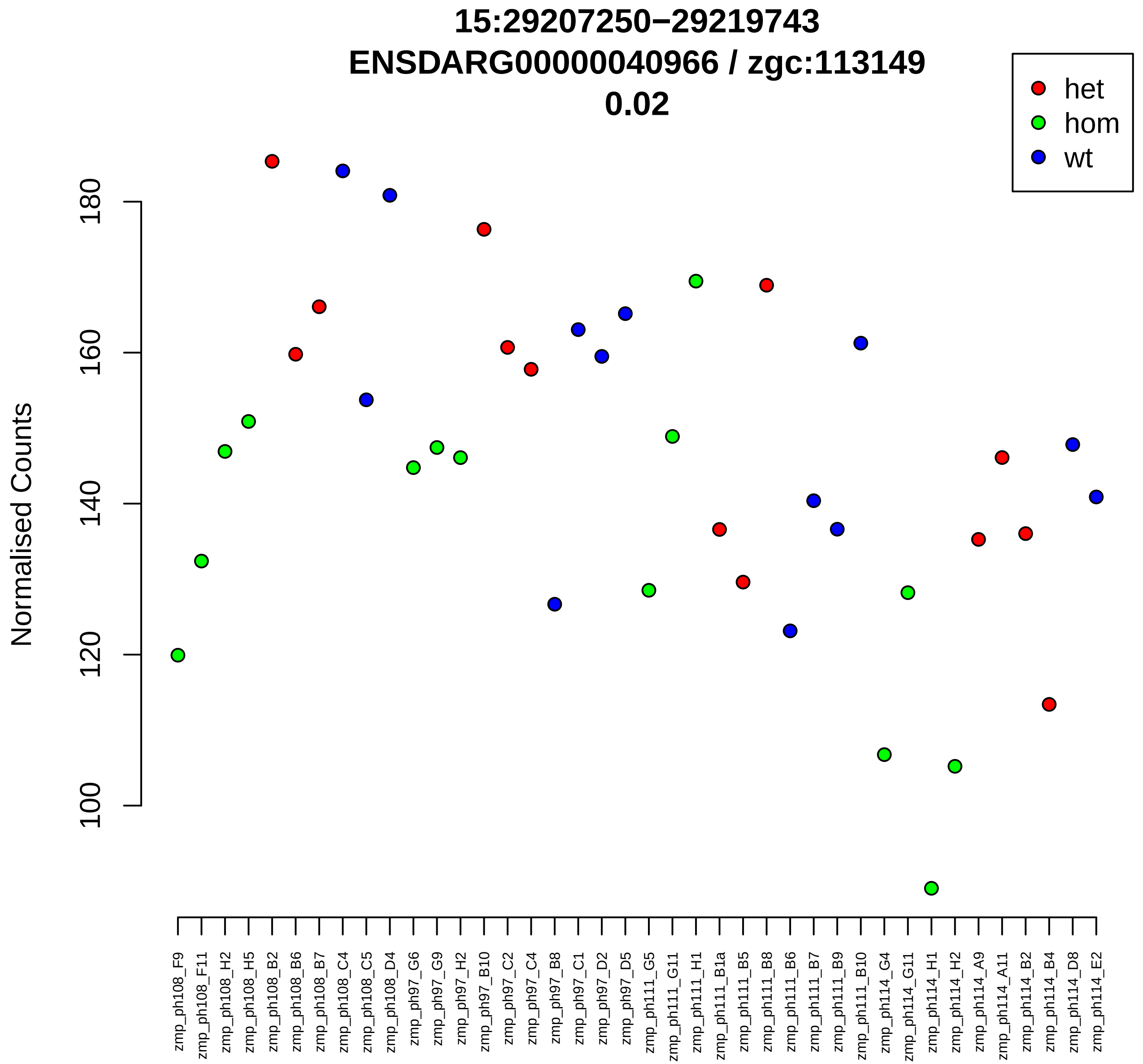
15:29207250−29219743
ENSDARG00000040966 / zgc:113149
0.02
het
hom
wt
Normalised Counts
100
120
140
160
180
zmp_ph108_F9
zmp_ph108_F11
zmp_ph108_H2
zmp_ph108_H5
zmp_ph108_B2
zmp_ph108_B6
zmp_ph108_B7
zmp_ph108_C4
zmp_ph108_C5
zmp_ph108_D4
zmp_ph97_G6
zmp_ph97_G9
zmp_ph97_H2
zmp_ph97_B10
zmp_ph97_C2
zmp_ph97_C4
zmp_ph97_B8
zmp_ph97_C1
zmp_ph97_D2
zmp_ph97_D5
zmp_ph111_G5
zmp_ph111_G11
zmp_ph111_H1
zmp_ph111_B1a
zmp_ph111_B5
zmp_ph111_B8
zmp_ph111_B6
zmp_ph111_B7
zmp_ph111_B9
zmp_ph111_B10
zmp_ph114_G4
zmp_ph114_G11
zmp_ph114_H1
zmp_ph114_H2
zmp_ph114_A9
zmp_ph114_A11
zmp_ph114_B2
zmp_ph114_B4
zmp_ph114_D8
zmp_ph114_E2

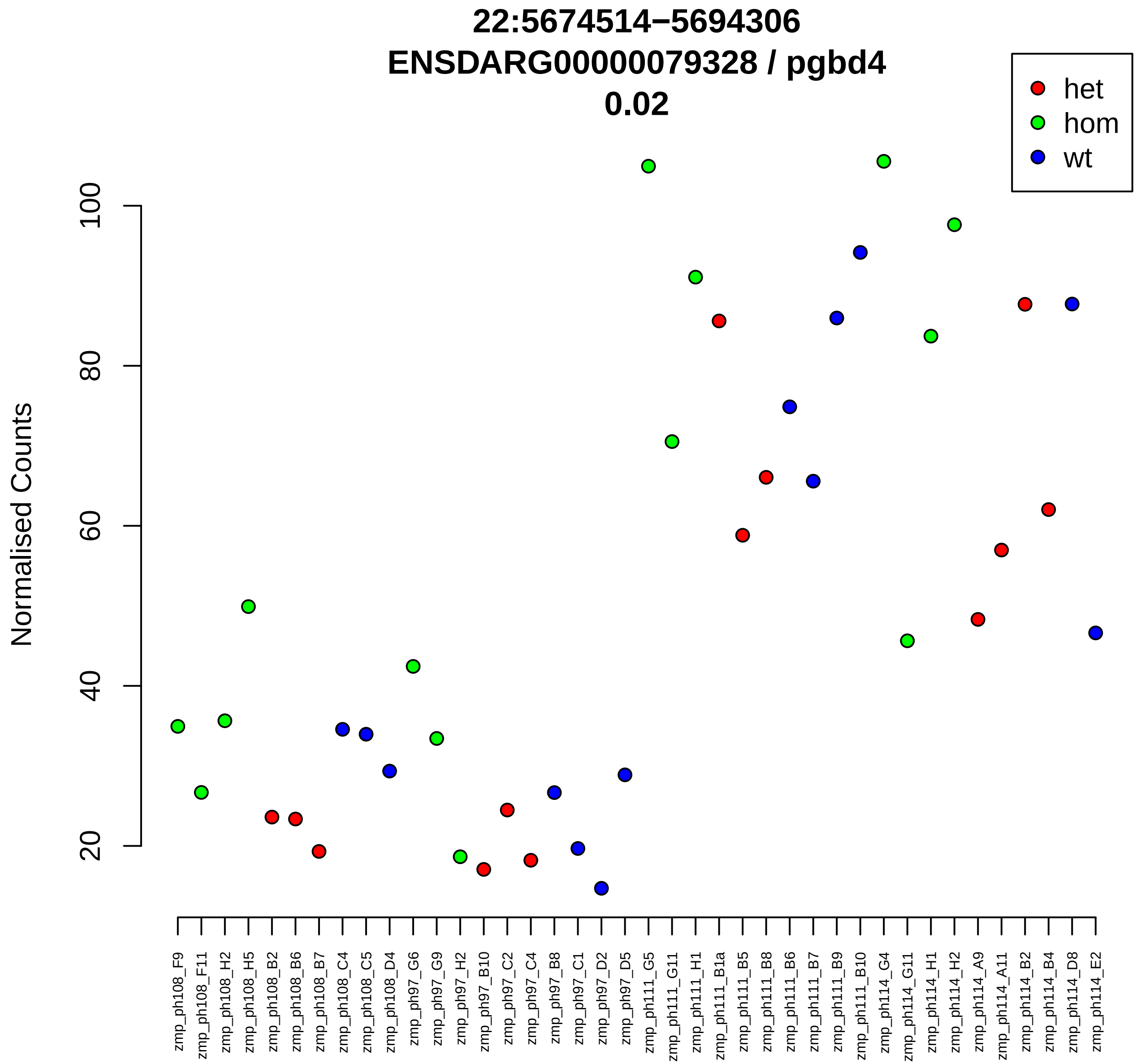
22:5674514−5694306
ENSDARG00000079328 / pgbd4
0.02
het
hom
wt
Normalised Counts
20
40
60
80
100
zmp_ph108_F9
zmp_ph108_F11
zmp_ph108_H2
zmp_ph108_H5
zmp_ph108_B2
zmp_ph108_B6
zmp_ph108_B7
zmp_ph108_C4
zmp_ph108_C5
zmp_ph108_D4
zmp_ph97_G6
zmp_ph97_G9
zmp_ph97_H2
zmp_ph97_B10
zmp_ph97_C2
zmp_ph97_C4
zmp_ph97_B8
zmp_ph97_C1
zmp_ph97_D2
zmp_ph97_D5
zmp_ph111_G5
zmp_ph111_G11
zmp_ph111_H1
zmp_ph111_B1a
zmp_ph111_B5
zmp_ph111_B8
zmp_ph111_B6
zmp_ph111_B7
zmp_ph111_B9
zmp_ph111_B10
zmp_ph114_G4
zmp_ph114_G11
zmp_ph114_H1
zmp_ph114_H2
zmp_ph114_A9
zmp_ph114_A11
zmp_ph114_B2
zmp_ph114_B4
zmp_ph114_D8
zmp_ph114_E2

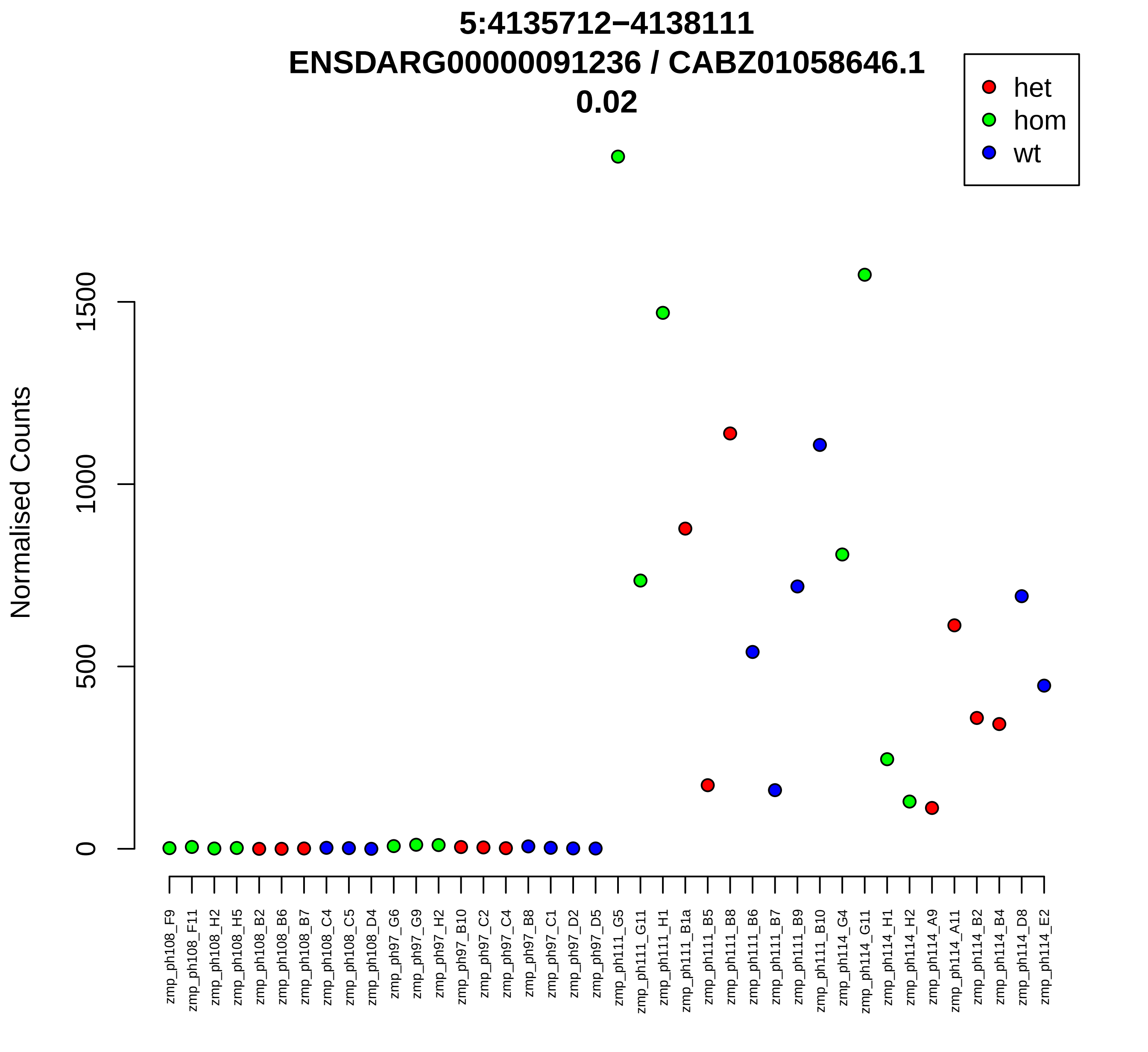
5:4135712−4138111
ENSDARG00000091236 / CABZ01058646.1
0.02
het
hom
wt
Normalised Counts
0
500
1000
1500
zmp_ph108_F9
zmp_ph108_F11
zmp_ph108_H2
zmp_ph108_H5
zmp_ph108_B2
zmp_ph108_B6
zmp_ph108_B7
zmp_ph108_C4
zmp_ph108_C5
zmp_ph108_D4
zmp_ph97_G6
zmp_ph97_G9
zmp_ph97_H2
zmp_ph97_B10
zmp_ph97_C2
zmp_ph97_C4
zmp_ph97_B8
zmp_ph97_C1
zmp_ph97_D2
zmp_ph97_D5
zmp_ph111_G5
zmp_ph111_G11
zmp_ph111_H1
zmp_ph111_B1a
zmp_ph111_B5
zmp_ph111_B8
zmp_ph111_B6
zmp_ph111_B7
zmp_ph111_B9
zmp_ph111_B10
zmp_ph114_G4
zmp_ph114_G11
zmp_ph114_H1
zmp_ph114_H2
zmp_ph114_A9
zmp_ph114_A11
zmp_ph114_B2
zmp_ph114_B4
zmp_ph114_D8
zmp_ph114_E2

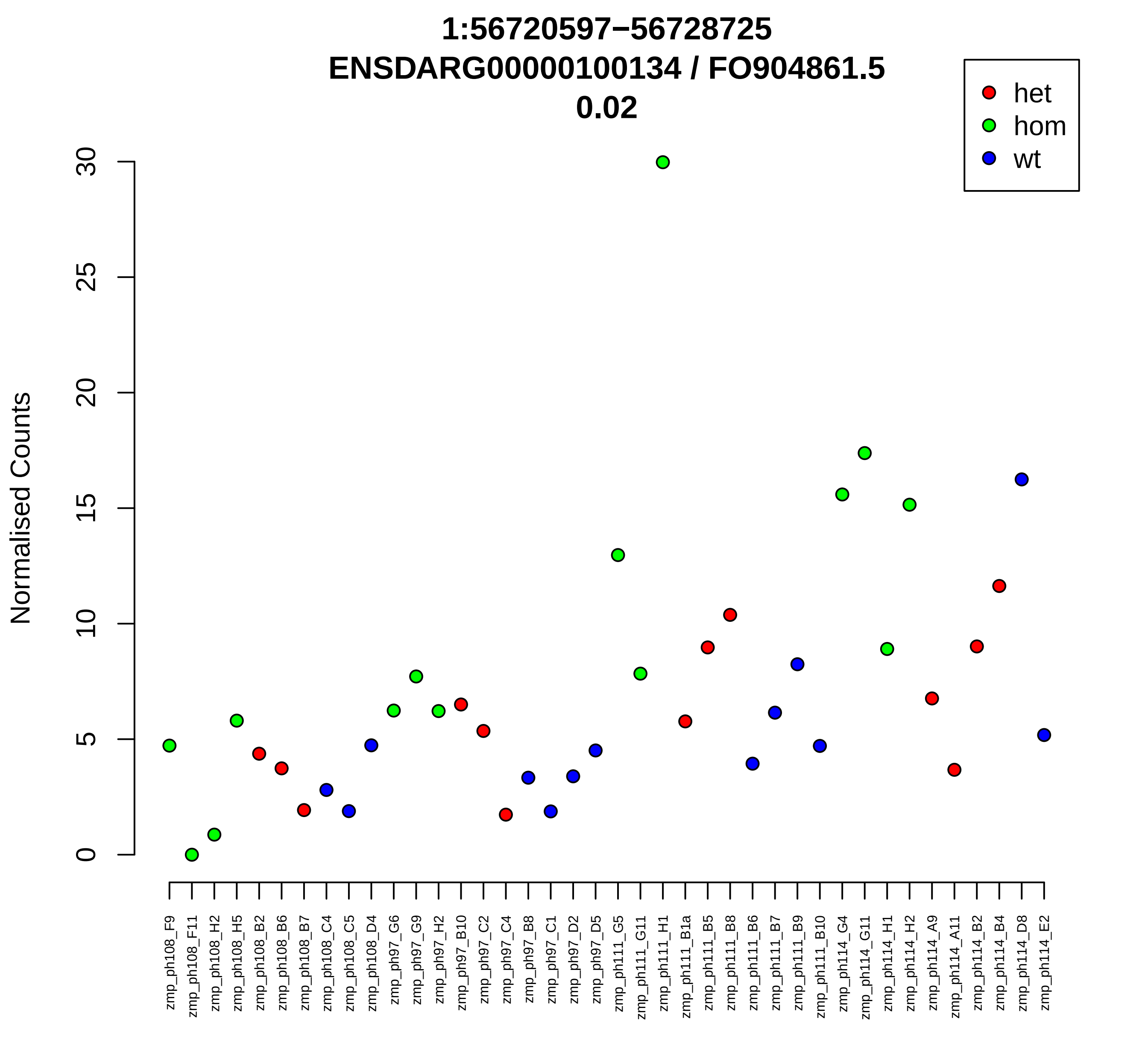

1:56720597−56728725
ENSDARG00000100134 / FO904861.5
0.02
het
hom
wt
Normalised Counts
0
5
10
15
20
25
30
zmp_ph108_F9
zmp_ph108_F11
zmp_ph108_H2
zmp_ph108_H5
zmp_ph108_B2
zmp_ph108_B6
zmp_ph108_B7
zmp_ph108_C4
zmp_ph108_C5
zmp_ph108_D4
zmp_ph97_G6
zmp_ph97_G9
zmp_ph97_H2
zmp_ph97_B10
zmp_ph97_C2
zmp_ph97_C4
zmp_ph97_B8
zmp_ph97_C1
zmp_ph97_D2
zmp_ph97_D5
zmp_ph111_G5
zmp_ph111_G11
zmp_ph111_H1
zmp_ph111_B1a
zmp_ph111_B5
zmp_ph111_B8
zmp_ph111_B6
zmp_ph111_B7
zmp_ph111_B9
zmp_ph111_B10
zmp_ph114_G4
zmp_ph114_G11
zmp_ph114_H1
zmp_ph114_H2
zmp_ph114_A9
zmp_ph114_A11
zmp_ph114_B2
zmp_ph114_B4
zmp_ph114_D8
zmp_ph114_E2

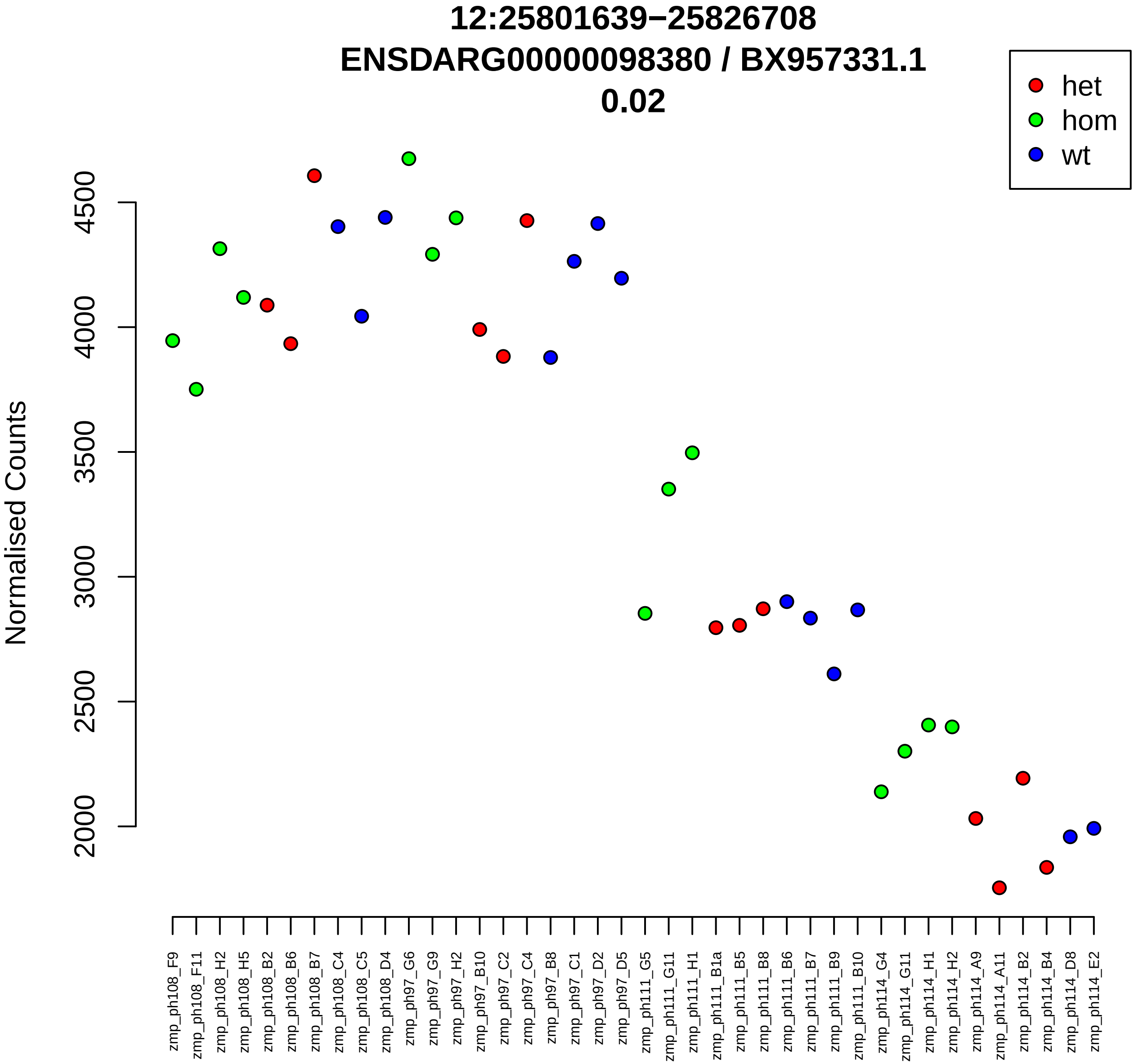
12:25801639−25826708
ENSDARG00000098380 / BX957331.1
0.02
het
hom
wt
Normalised Counts
2000
2500
3000
3500
4000
4500
zmp_ph108_F9
zmp_ph108_F11
zmp_ph108_H2
zmp_ph108_H5
zmp_ph108_B2
zmp_ph108_B6
zmp_ph108_B7
zmp_ph108_C4
zmp_ph108_C5
zmp_ph108_D4
zmp_ph97_G6
zmp_ph97_G9
zmp_ph97_H2
zmp_ph97_B10
zmp_ph97_C2
zmp_ph97_C4
zmp_ph97_B8
zmp_ph97_C1
zmp_ph97_D2
zmp_ph97_D5
zmp_ph111_G5
zmp_ph111_G11
zmp_ph111_H1
zmp_ph111_B1a
zmp_ph111_B5
zmp_ph111_B8
zmp_ph111_B6
zmp_ph111_B7
zmp_ph111_B9
zmp_ph111_B10
zmp_ph114_G4
zmp_ph114_G11
zmp_ph114_H1
zmp_ph114_H2
zmp_ph114_A9
zmp_ph114_A11
zmp_ph114_B2
zmp_ph114_B4
zmp_ph114_D8
zmp_ph114_E2

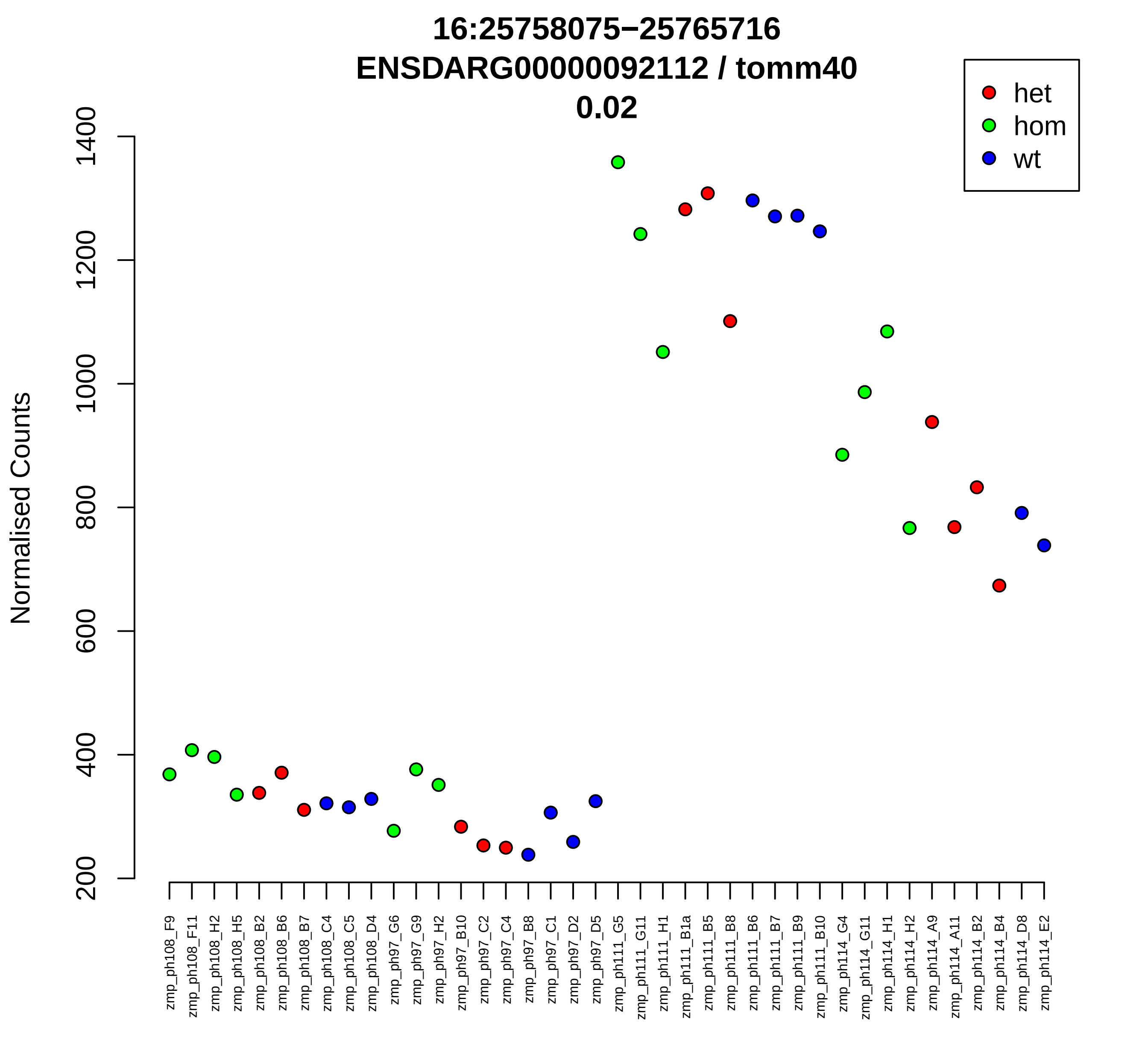

16:25758075−25765716
ENSDARG00000092112 / tomm40
0.02
het
hom
wt
Normalised Counts
200
400
600
800
1000
1200
1400
zmp_ph108_F9
zmp_ph108_F11
zmp_ph108_H2
zmp_ph108_H5
zmp_ph108_B2
zmp_ph108_B6
zmp_ph108_B7
zmp_ph108_C4
zmp_ph108_C5
zmp_ph108_D4
zmp_ph97_G6
zmp_ph97_G9
zmp_ph97_H2
zmp_ph97_B10
zmp_ph97_C2
zmp_ph97_C4
zmp_ph97_B8
zmp_ph97_C1
zmp_ph97_D2
zmp_ph97_D5
zmp_ph111_G5
zmp_ph111_G11
zmp_ph111_H1
zmp_ph111_B1a
zmp_ph111_B5
zmp_ph111_B8
zmp_ph111_B6
zmp_ph111_B7
zmp_ph111_B9
zmp_ph111_B10
zmp_ph114_G4
zmp_ph114_G11
zmp_ph114_H1
zmp_ph114_H2
zmp_ph114_A9
zmp_ph114_A11
zmp_ph114_B2
zmp_ph114_B4
zmp_ph114_D8
zmp_ph114_E2

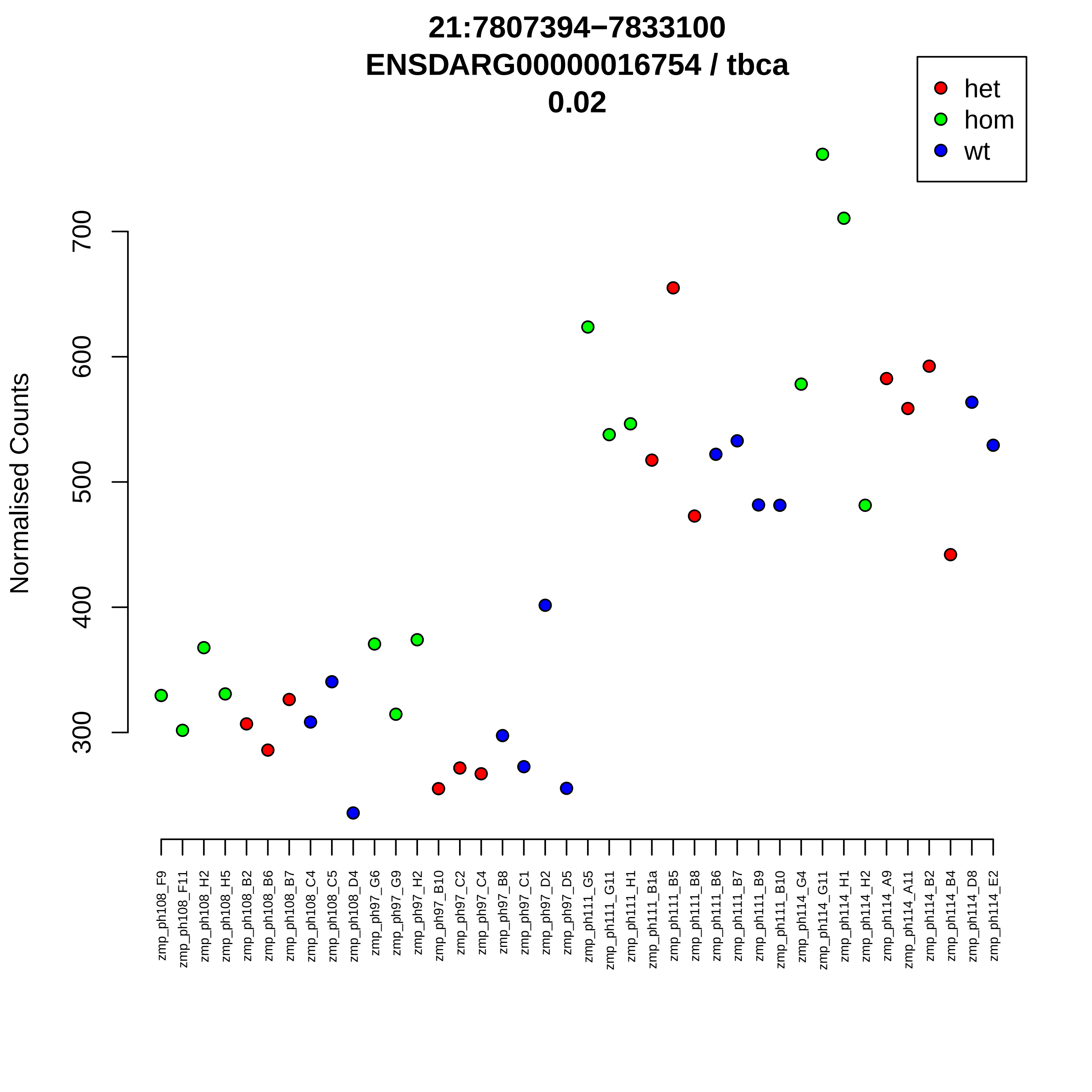

21:7807394−7833100
ENSDARG00000016754 / tbca
0.02
het
hom
wt
Normalised Counts
300
400
500
600
700
zmp_ph108_F9
zmp_ph108_F11
zmp_ph108_H2
zmp_ph108_H5
zmp_ph108_B2
zmp_ph108_B6
zmp_ph108_B7
zmp_ph108_C4
zmp_ph108_C5
zmp_ph108_D4
zmp_ph97_G6
zmp_ph97_G9
zmp_ph97_H2
zmp_ph97_B10
zmp_ph97_C2
zmp_ph97_C4
zmp_ph97_B8
zmp_ph97_C1
zmp_ph97_D2
zmp_ph97_D5
zmp_ph111_G5
zmp_ph111_G11
zmp_ph111_H1
zmp_ph111_B1a
zmp_ph111_B5
zmp_ph111_B8
zmp_ph111_B6
zmp_ph111_B7
zmp_ph111_B9
zmp_ph111_B10
zmp_ph114_G4
zmp_ph114_G11
zmp_ph114_H1
zmp_ph114_H2
zmp_ph114_A9
zmp_ph114_A11
zmp_ph114_B2
zmp_ph114_B4
zmp_ph114_D8
zmp_ph114_E2

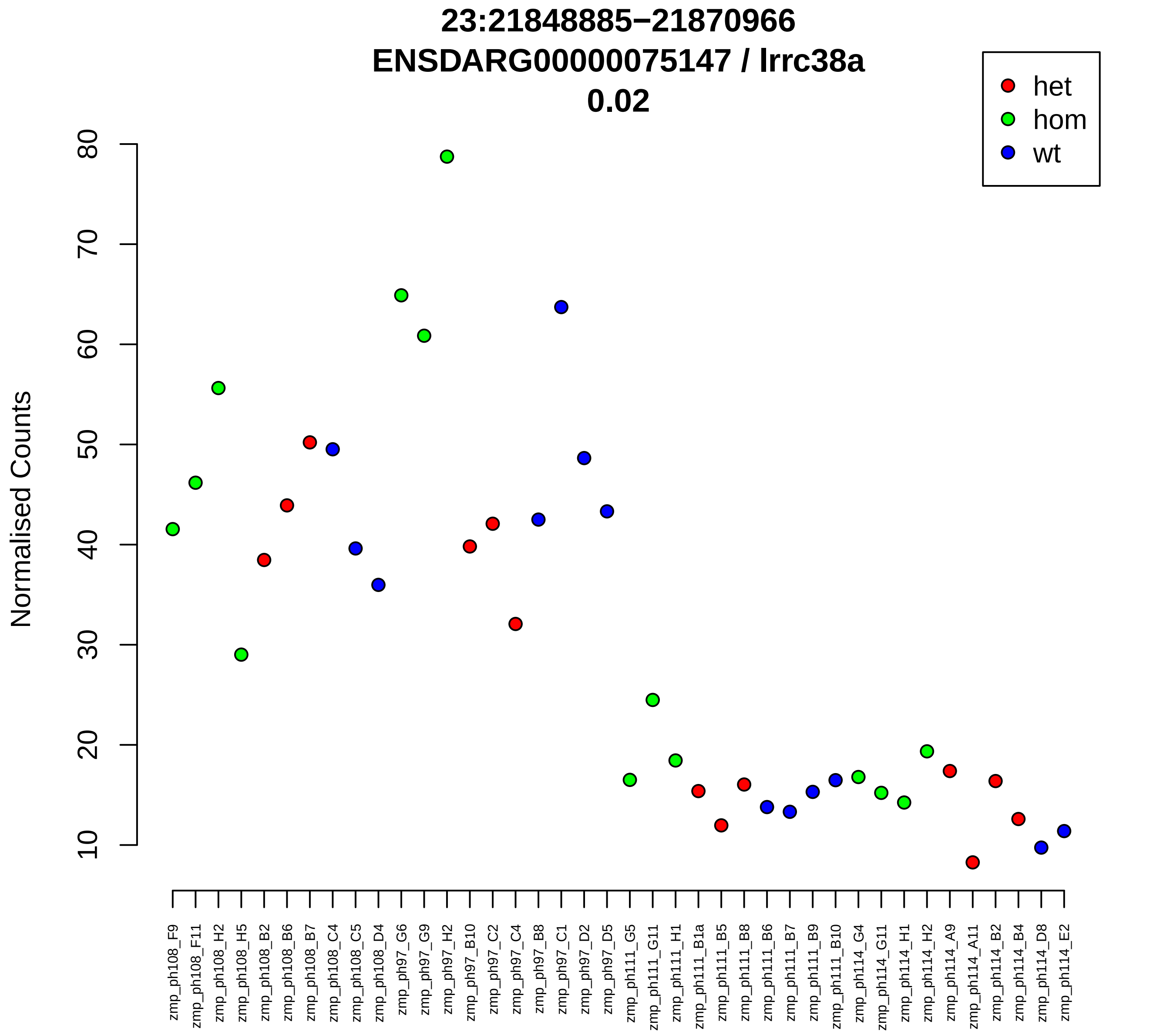
23:21848885−21870966
ENSDARG00000075147 / lrrc38a
0.02
het
hom
wt
Normalised Counts
10
20
30
40
50
60
70
80
zmp_ph108_F9
zmp_ph108_F11
zmp_ph108_H2
zmp_ph108_H5
zmp_ph108_B2
zmp_ph108_B6
zmp_ph108_B7
zmp_ph108_C4
zmp_ph108_C5
zmp_ph108_D4
zmp_ph97_G6
zmp_ph97_G9
zmp_ph97_H2
zmp_ph97_B10
zmp_ph97_C2
zmp_ph97_C4
zmp_ph97_B8
zmp_ph97_C1
zmp_ph97_D2
zmp_ph97_D5
zmp_ph111_G5
zmp_ph111_G11
zmp_ph111_H1
zmp_ph111_B1a
zmp_ph111_B5
zmp_ph111_B8
zmp_ph111_B6
zmp_ph111_B7
zmp_ph111_B9
zmp_ph111_B10
zmp_ph114_G4
zmp_ph114_G11
zmp_ph114_H1
zmp_ph114_H2
zmp_ph114_A9
zmp_ph114_A11
zmp_ph114_B2
zmp_ph114_B4
zmp_ph114_D8
zmp_ph114_E2

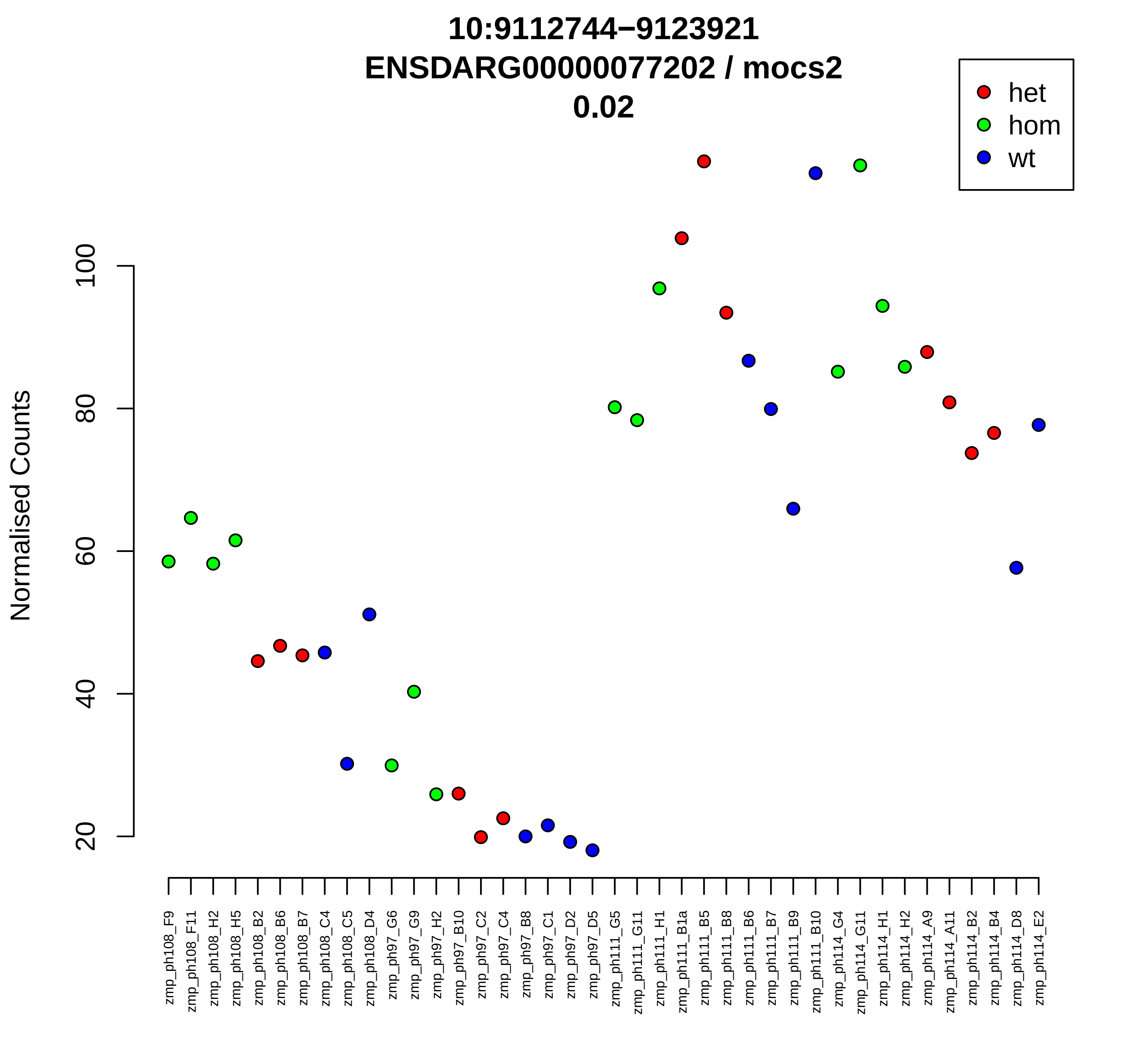
10:9112744−9123921
ENSDARG00000077202 / mocs2
0.02
het
hom
wt
Normalised Counts
20
40
60
80
100
zmp_ph108_F9
zmp_ph108_F11
zmp_ph108_H2
zmp_ph108_H5
zmp_ph108_B2
zmp_ph108_B6
zmp_ph108_B7
zmp_ph108_C4
zmp_ph108_C5
zmp_ph108_D4
zmp_ph97_G6
zmp_ph97_G9
zmp_ph97_H2
zmp_ph97_B10
zmp_ph97_C2
zmp_ph97_C4
zmp_ph97_B8
zmp_ph97_C1
zmp_ph97_D2
zmp_ph97_D5
zmp_ph111_G5
zmp_ph111_G11
zmp_ph111_H1
zmp_ph111_B1a
zmp_ph111_B5
zmp_ph111_B8
zmp_ph111_B6
zmp_ph111_B7
zmp_ph111_B9
zmp_ph111_B10
zmp_ph114_G4
zmp_ph114_G11
zmp_ph114_H1
zmp_ph114_H2
zmp_ph114_A9
zmp_ph114_A11
zmp_ph114_B2
zmp_ph114_B4
zmp_ph114_D8
zmp_ph114_E2

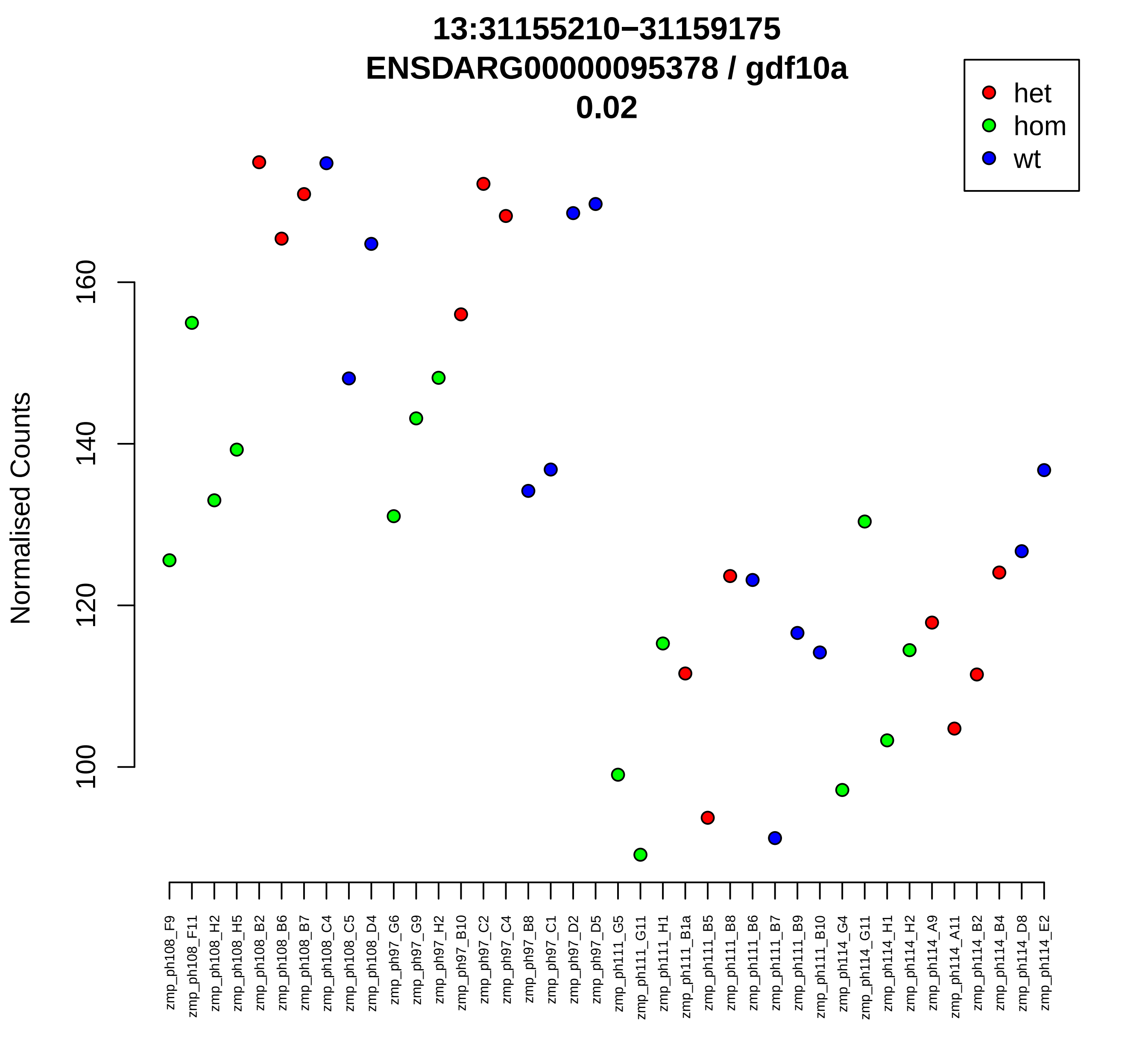

13:31155210−31159175
ENSDARG00000095378 / gdf10a
0.02
het
hom
wt
Normalised Counts
100
120
140
160
zmp_ph108_F9
zmp_ph108_F11
zmp_ph108_H2
zmp_ph108_H5
zmp_ph108_B2
zmp_ph108_B6
zmp_ph108_B7
zmp_ph108_C4
zmp_ph108_C5
zmp_ph108_D4
zmp_ph97_G6
zmp_ph97_G9
zmp_ph97_H2
zmp_ph97_B10
zmp_ph97_C2
zmp_ph97_C4
zmp_ph97_B8
zmp_ph97_C1
zmp_ph97_D2
zmp_ph97_D5
zmp_ph111_G5
zmp_ph111_G11
zmp_ph111_H1
zmp_ph111_B1a
zmp_ph111_B5
zmp_ph111_B8
zmp_ph111_B6
zmp_ph111_B7
zmp_ph111_B9
zmp_ph111_B10
zmp_ph114_G4
zmp_ph114_G11
zmp_ph114_H1
zmp_ph114_H2
zmp_ph114_A9
zmp_ph114_A11
zmp_ph114_B2
zmp_ph114_B4
zmp_ph114_D8
zmp_ph114_E2

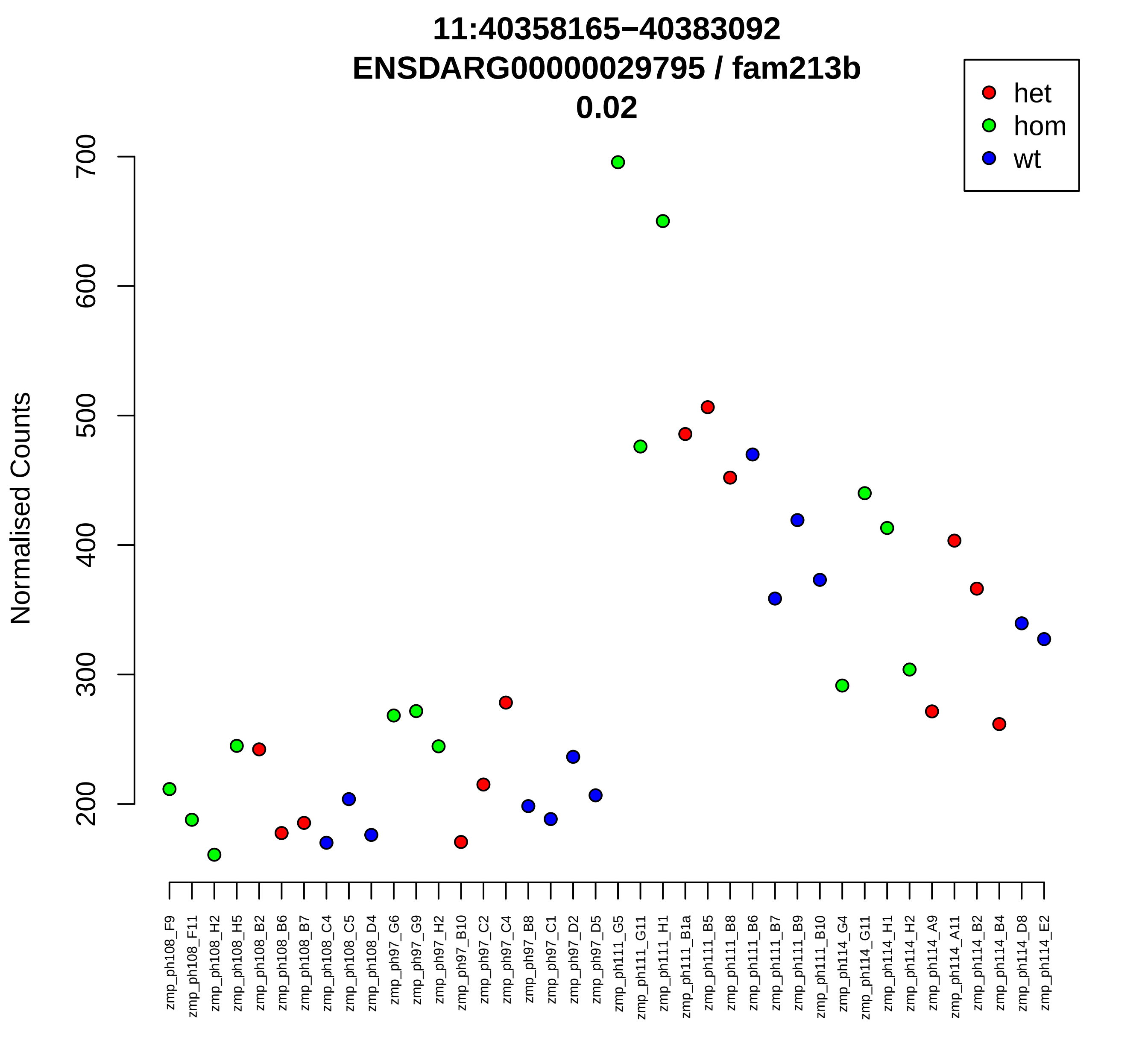
11:40358165−40383092
ENSDARG00000029795 / fam213b
0.02
het
hom
wt
Normalised Counts
200
300
400
500
600
700
zmp_ph108_F9
zmp_ph108_F11
zmp_ph108_H2
zmp_ph108_H5
zmp_ph108_B2
zmp_ph108_B6
zmp_ph108_B7
zmp_ph108_C4
zmp_ph108_C5
zmp_ph108_D4
zmp_ph97_G6
zmp_ph97_G9
zmp_ph97_H2
zmp_ph97_B10
zmp_ph97_C2
zmp_ph97_C4
zmp_ph97_B8
zmp_ph97_C1
zmp_ph97_D2
zmp_ph97_D5
zmp_ph111_G5
zmp_ph111_G11
zmp_ph111_H1
zmp_ph111_B1a
zmp_ph111_B5
zmp_ph111_B8
zmp_ph111_B6
zmp_ph111_B7
zmp_ph111_B9
zmp_ph111_B10
zmp_ph114_G4
zmp_ph114_G11
zmp_ph114_H1
zmp_ph114_H2
zmp_ph114_A9
zmp_ph114_A11
zmp_ph114_B2
zmp_ph114_B4
zmp_ph114_D8
zmp_ph114_E2

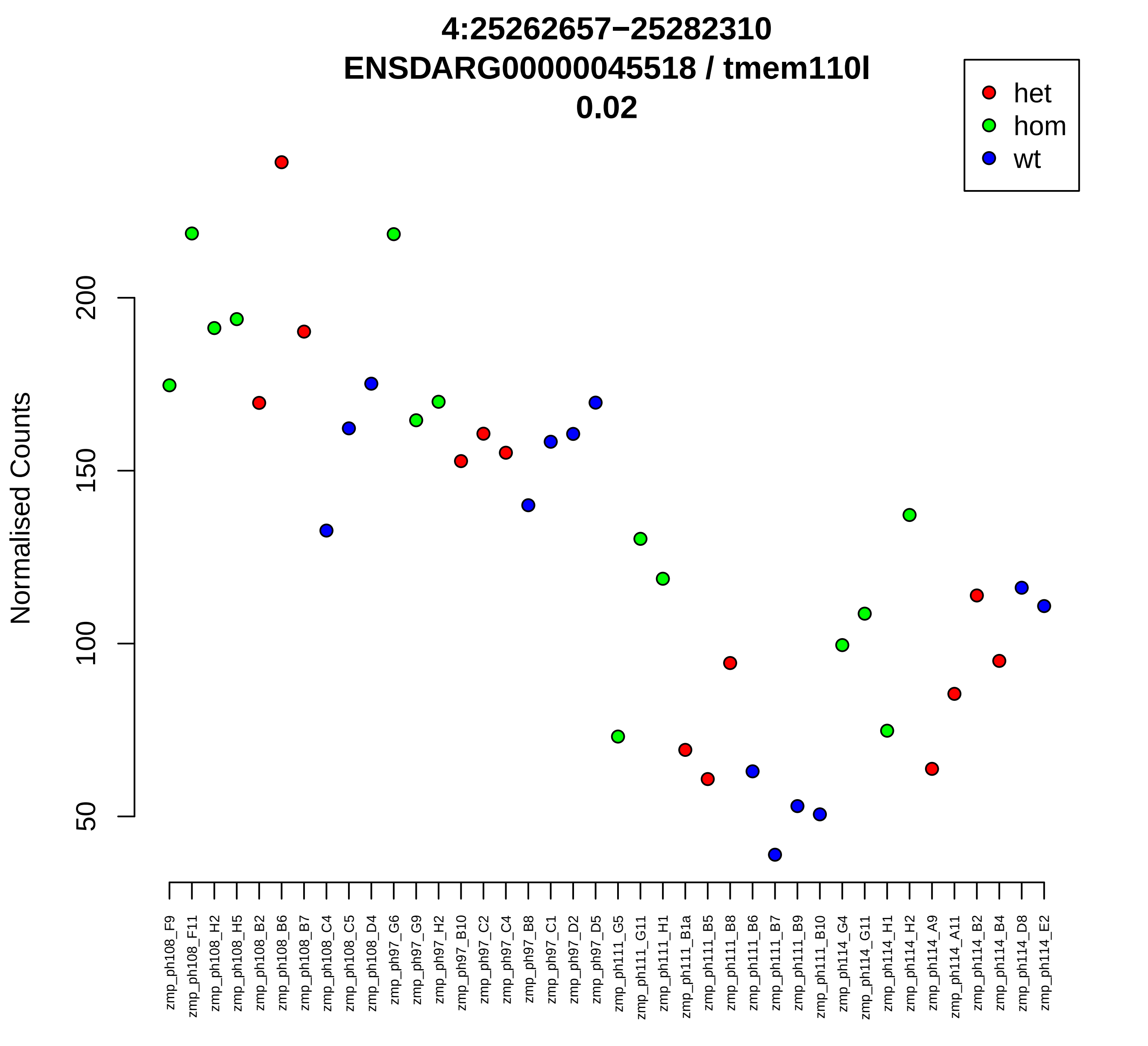

4:25262657−25282310
ENSDARG00000045518 / tmem110l
0.02
het
hom
wt
Normalised Counts
50
100
150
200
zmp_ph108_F9
zmp_ph108_F11
zmp_ph108_H2
zmp_ph108_H5
zmp_ph108_B2
zmp_ph108_B6
zmp_ph108_B7
zmp_ph108_C4
zmp_ph108_C5
zmp_ph108_D4
zmp_ph97_G6
zmp_ph97_G9
zmp_ph97_H2
zmp_ph97_B10
zmp_ph97_C2
zmp_ph97_C4
zmp_ph97_B8
zmp_ph97_C1
zmp_ph97_D2
zmp_ph97_D5
zmp_ph111_G5
zmp_ph111_G11
zmp_ph111_H1
zmp_ph111_B1a
zmp_ph111_B5
zmp_ph111_B8
zmp_ph111_B6
zmp_ph111_B7
zmp_ph111_B9
zmp_ph111_B10
zmp_ph114_G4
zmp_ph114_G11
zmp_ph114_H1
zmp_ph114_H2
zmp_ph114_A9
zmp_ph114_A11
zmp_ph114_B2
zmp_ph114_B4
zmp_ph114_D8
zmp_ph114_E2

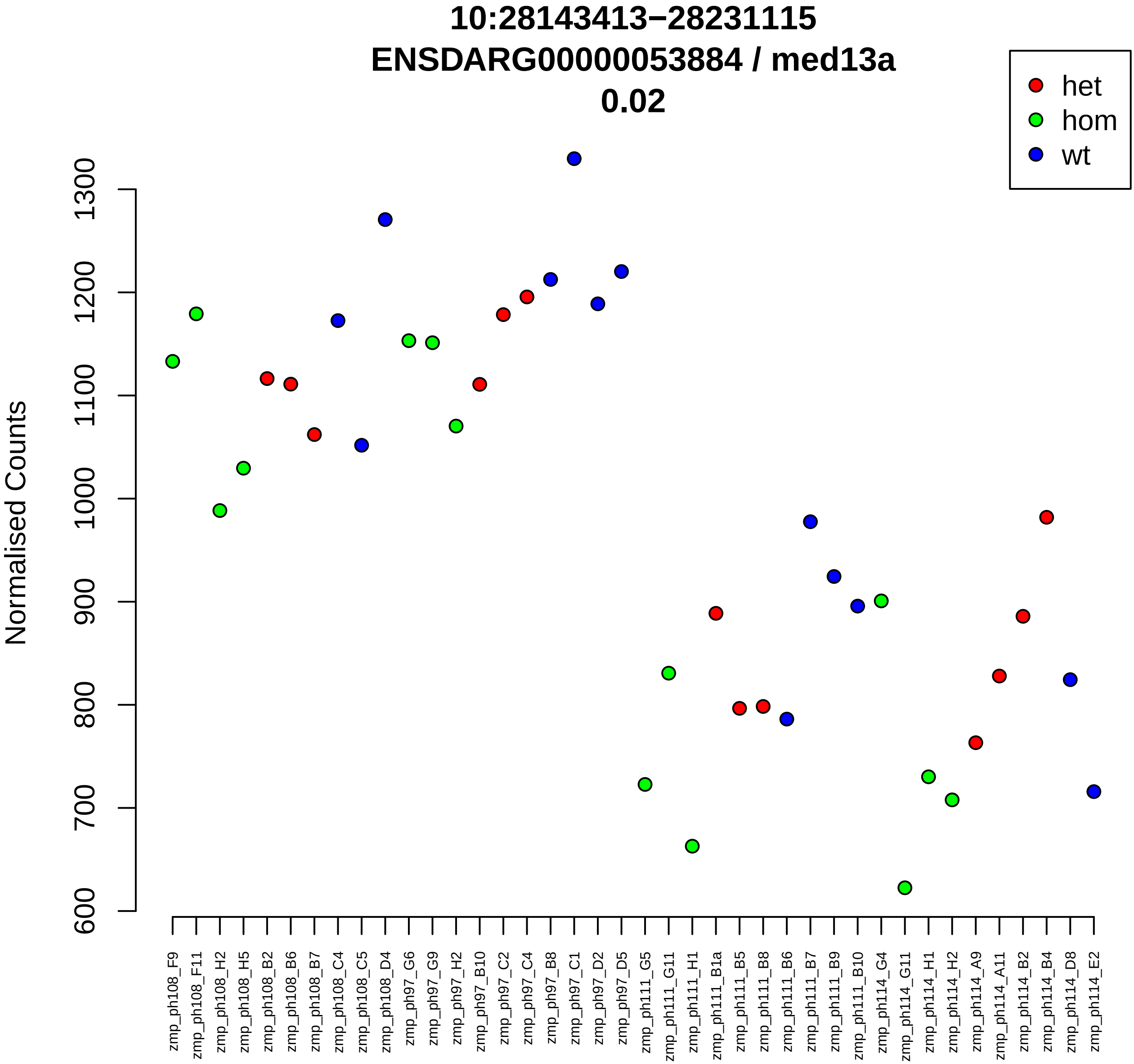

10:28143413−28231115
ENSDARG00000053884 / med13a
0.02
het
hom
wt
Normalised Counts
600
700
800
900
1000
1100
1200
1300
zmp_ph108_F9
zmp_ph108_F11
zmp_ph108_H2
zmp_ph108_H5
zmp_ph108_B2
zmp_ph108_B6
zmp_ph108_B7
zmp_ph108_C4
zmp_ph108_C5
zmp_ph108_D4
zmp_ph97_G6
zmp_ph97_G9
zmp_ph97_H2
zmp_ph97_B10
zmp_ph97_C2
zmp_ph97_C4
zmp_ph97_B8
zmp_ph97_C1
zmp_ph97_D2
zmp_ph97_D5
zmp_ph111_G5
zmp_ph111_G11
zmp_ph111_H1
zmp_ph111_B1a
zmp_ph111_B5
zmp_ph111_B8
zmp_ph111_B6
zmp_ph111_B7
zmp_ph111_B9
zmp_ph111_B10
zmp_ph114_G4
zmp_ph114_G11
zmp_ph114_H1
zmp_ph114_H2
zmp_ph114_A9
zmp_ph114_A11
zmp_ph114_B2
zmp_ph114_B4
zmp_ph114_D8
zmp_ph114_E2

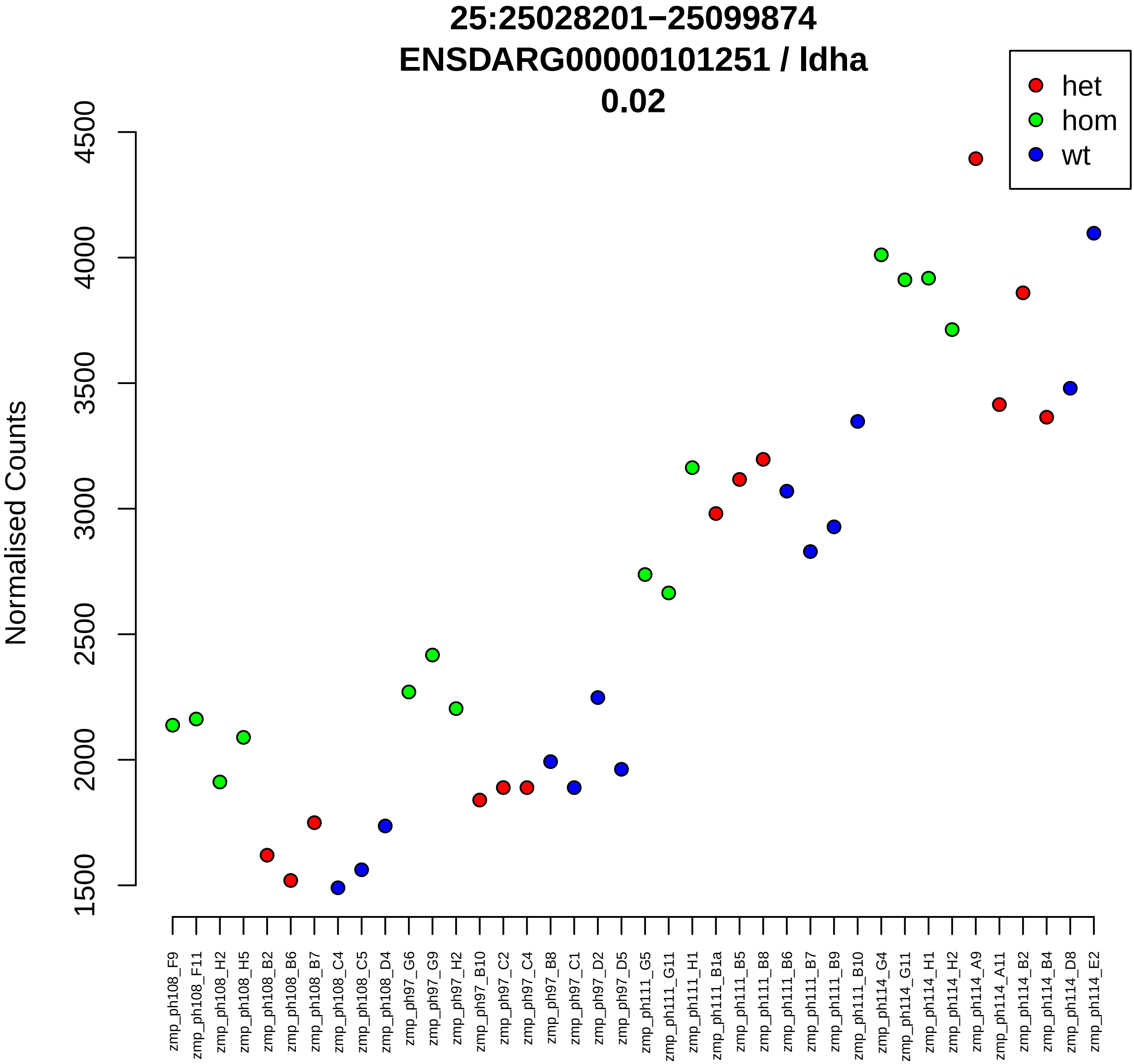
25:25028201−25099874
ENSDARG00000101251 / ldha
0.02
het
hom
wt
Normalised Counts
1500
2000
2500
3000
3500
4000
4500
zmp_ph108_F9
zmp_ph108_F11
zmp_ph108_H2
zmp_ph108_H5
zmp_ph108_B2
zmp_ph108_B6
zmp_ph108_B7
zmp_ph108_C4
zmp_ph108_C5
zmp_ph108_D4
zmp_ph97_G6
zmp_ph97_G9
zmp_ph97_H2
zmp_ph97_B10
zmp_ph97_C2
zmp_ph97_C4
zmp_ph97_B8
zmp_ph97_C1
zmp_ph97_D2
zmp_ph97_D5
zmp_ph111_G5
zmp_ph111_G11
zmp_ph111_H1
zmp_ph111_B1a
zmp_ph111_B5
zmp_ph111_B8
zmp_ph111_B6
zmp_ph111_B7
zmp_ph111_B9
zmp_ph111_B10
zmp_ph114_G4
zmp_ph114_G11
zmp_ph114_H1
zmp_ph114_H2
zmp_ph114_A9
zmp_ph114_A11
zmp_ph114_B2
zmp_ph114_B4
zmp_ph114_D8
zmp_ph114_E2

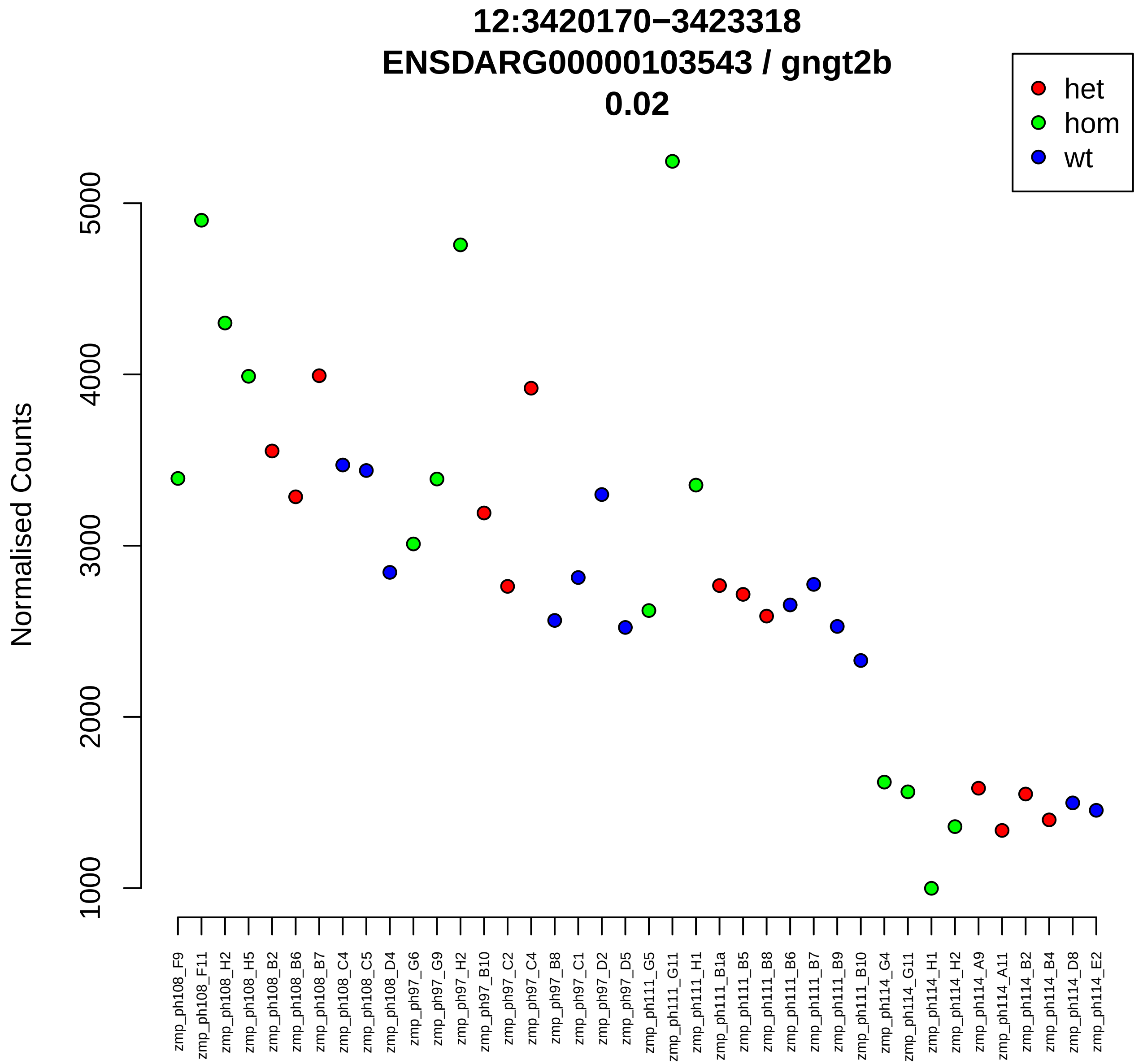

12:3420170−3423318
ENSDARG00000103543 / gngt2b
0.02
het
hom
wt
Normalised Counts
1000
2000
3000
4000
5000
zmp_ph108_F9
zmp_ph108_F11
zmp_ph108_H2
zmp_ph108_H5
zmp_ph108_B2
zmp_ph108_B6
zmp_ph108_B7
zmp_ph108_C4
zmp_ph108_C5
zmp_ph108_D4
zmp_ph97_G6
zmp_ph97_G9
zmp_ph97_H2
zmp_ph97_B10
zmp_ph97_C2
zmp_ph97_C4
zmp_ph97_B8
zmp_ph97_C1
zmp_ph97_D2
zmp_ph97_D5
zmp_ph111_G5
zmp_ph111_G11
zmp_ph111_H1
zmp_ph111_B1a
zmp_ph111_B5
zmp_ph111_B8
zmp_ph111_B6
zmp_ph111_B7
zmp_ph111_B9
zmp_ph111_B10
zmp_ph114_G4
zmp_ph114_G11
zmp_ph114_H1
zmp_ph114_H2
zmp_ph114_A9
zmp_ph114_A11
zmp_ph114_B2
zmp_ph114_B4
zmp_ph114_D8
zmp_ph114_E2

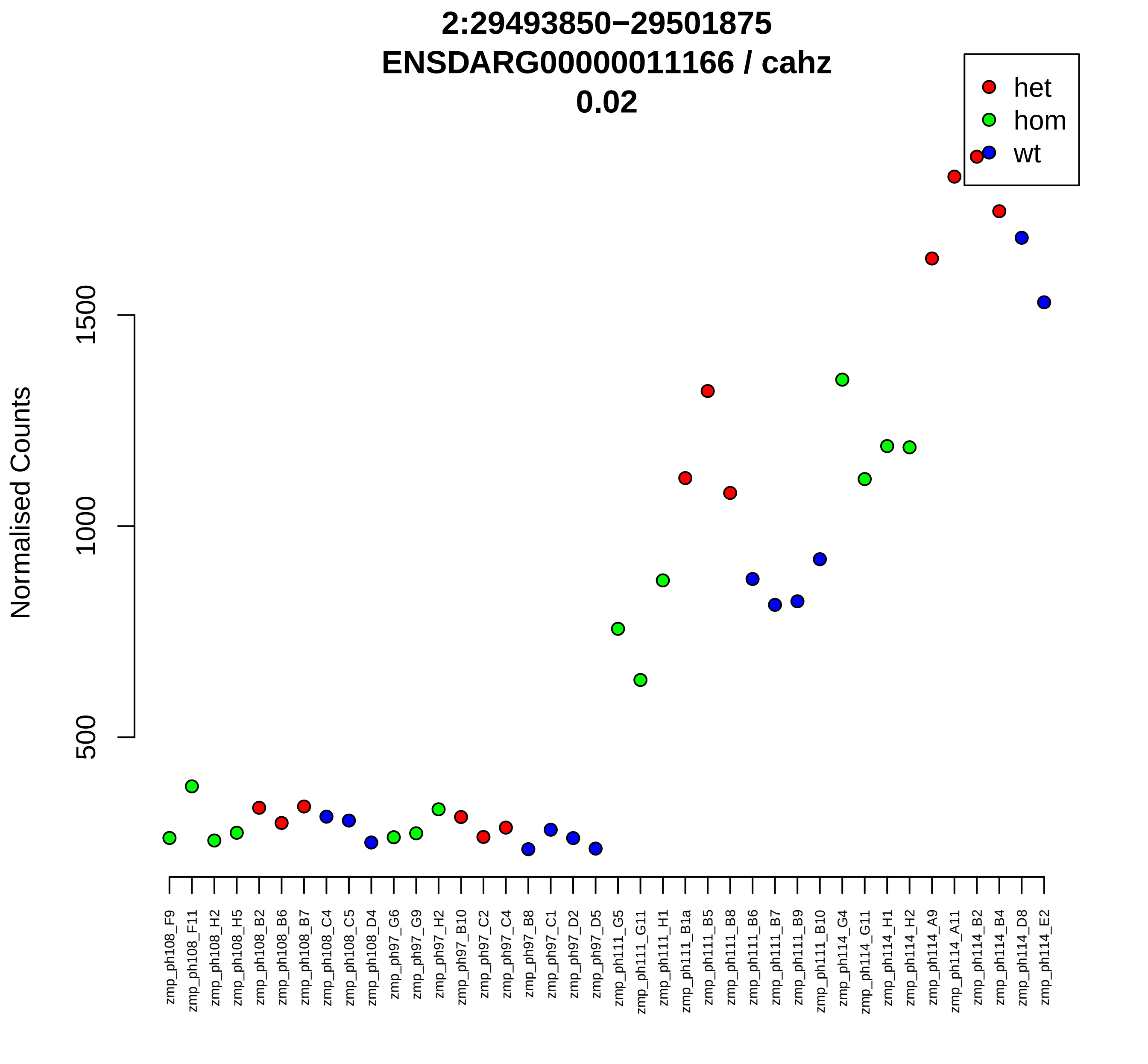
2:29493850−29501875
ENSDARG00000011166 / cahz
0.02
het
hom
wt
Normalised Counts
500
1000
1500
zmp_ph108_F9
zmp_ph108_F11
zmp_ph108_H2
zmp_ph108_H5
zmp_ph108_B2
zmp_ph108_B6
zmp_ph108_B7
zmp_ph108_C4
zmp_ph108_C5
zmp_ph108_D4
zmp_ph97_G6
zmp_ph97_G9
zmp_ph97_H2
zmp_ph97_B10
zmp_ph97_C2
zmp_ph97_C4
zmp_ph97_B8
zmp_ph97_C1
zmp_ph97_D2
zmp_ph97_D5
zmp_ph111_G5
zmp_ph111_G11
zmp_ph111_H1
zmp_ph111_B1a
zmp_ph111_B5
zmp_ph111_B8
zmp_ph111_B6
zmp_ph111_B7
zmp_ph111_B9
zmp_ph111_B10
zmp_ph114_G4
zmp_ph114_G11
zmp_ph114_H1
zmp_ph114_H2
zmp_ph114_A9
zmp_ph114_A11
zmp_ph114_B2
zmp_ph114_B4
zmp_ph114_D8
zmp_ph114_E2

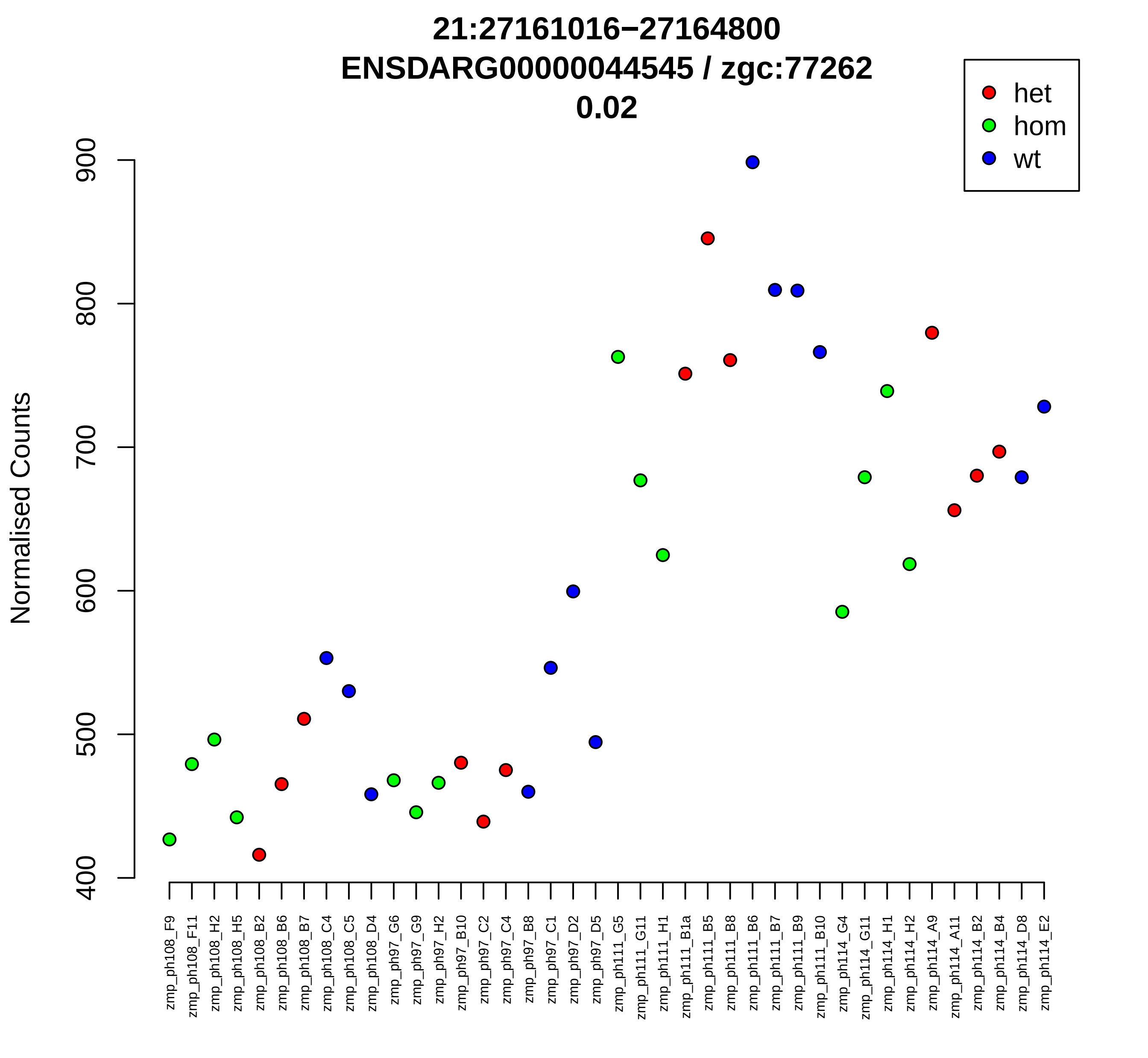

21:27161016−27164800
ENSDARG00000044545 / zgc:77262
0.02
het
hom
wt
Normalised Counts
400
500
600
700
800
900
zmp_ph108_F9
zmp_ph108_F11
zmp_ph108_H2
zmp_ph108_H5
zmp_ph108_B2
zmp_ph108_B6
zmp_ph108_B7
zmp_ph108_C4
zmp_ph108_C5
zmp_ph108_D4
zmp_ph97_G6
zmp_ph97_G9
zmp_ph97_H2
zmp_ph97_B10
zmp_ph97_C2
zmp_ph97_C4
zmp_ph97_B8
zmp_ph97_C1
zmp_ph97_D2
zmp_ph97_D5
zmp_ph111_G5
zmp_ph111_G11
zmp_ph111_H1
zmp_ph111_B1a
zmp_ph111_B5
zmp_ph111_B8
zmp_ph111_B6
zmp_ph111_B7
zmp_ph111_B9
zmp_ph111_B10
zmp_ph114_G4
zmp_ph114_G11
zmp_ph114_H1
zmp_ph114_H2
zmp_ph114_A9
zmp_ph114_A11
zmp_ph114_B2
zmp_ph114_B4
zmp_ph114_D8
zmp_ph114_E2

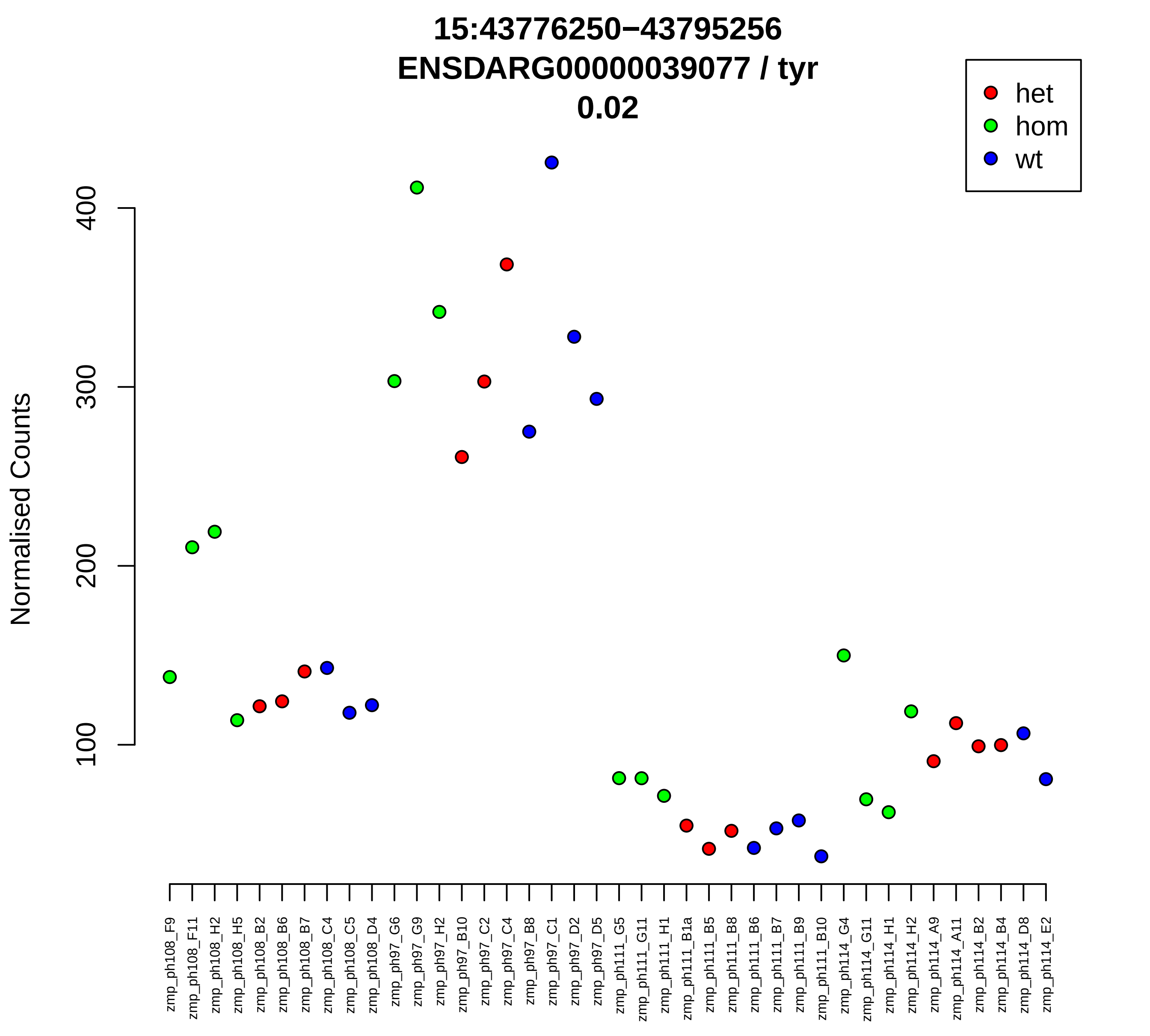
15:43776250−43795256
ENSDARG00000039077 / tyr
0.02
het
hom
wt
Normalised Counts
100
200
300
400
zmp_ph108_F9
zmp_ph108_F11
zmp_ph108_H2
zmp_ph108_H5
zmp_ph108_B2
zmp_ph108_B6
zmp_ph108_B7
zmp_ph108_C4
zmp_ph108_C5
zmp_ph108_D4
zmp_ph97_G6
zmp_ph97_G9
zmp_ph97_H2
zmp_ph97_B10
zmp_ph97_C2
zmp_ph97_C4
zmp_ph97_B8
zmp_ph97_C1
zmp_ph97_D2
zmp_ph97_D5
zmp_ph111_G5
zmp_ph111_G11
zmp_ph111_H1
zmp_ph111_B1a
zmp_ph111_B5
zmp_ph111_B8
zmp_ph111_B6
zmp_ph111_B7
zmp_ph111_B9
zmp_ph111_B10
zmp_ph114_G4
zmp_ph114_G11
zmp_ph114_H1
zmp_ph114_H2
zmp_ph114_A9
zmp_ph114_A11
zmp_ph114_B2
zmp_ph114_B4
zmp_ph114_D8
zmp_ph114_E2

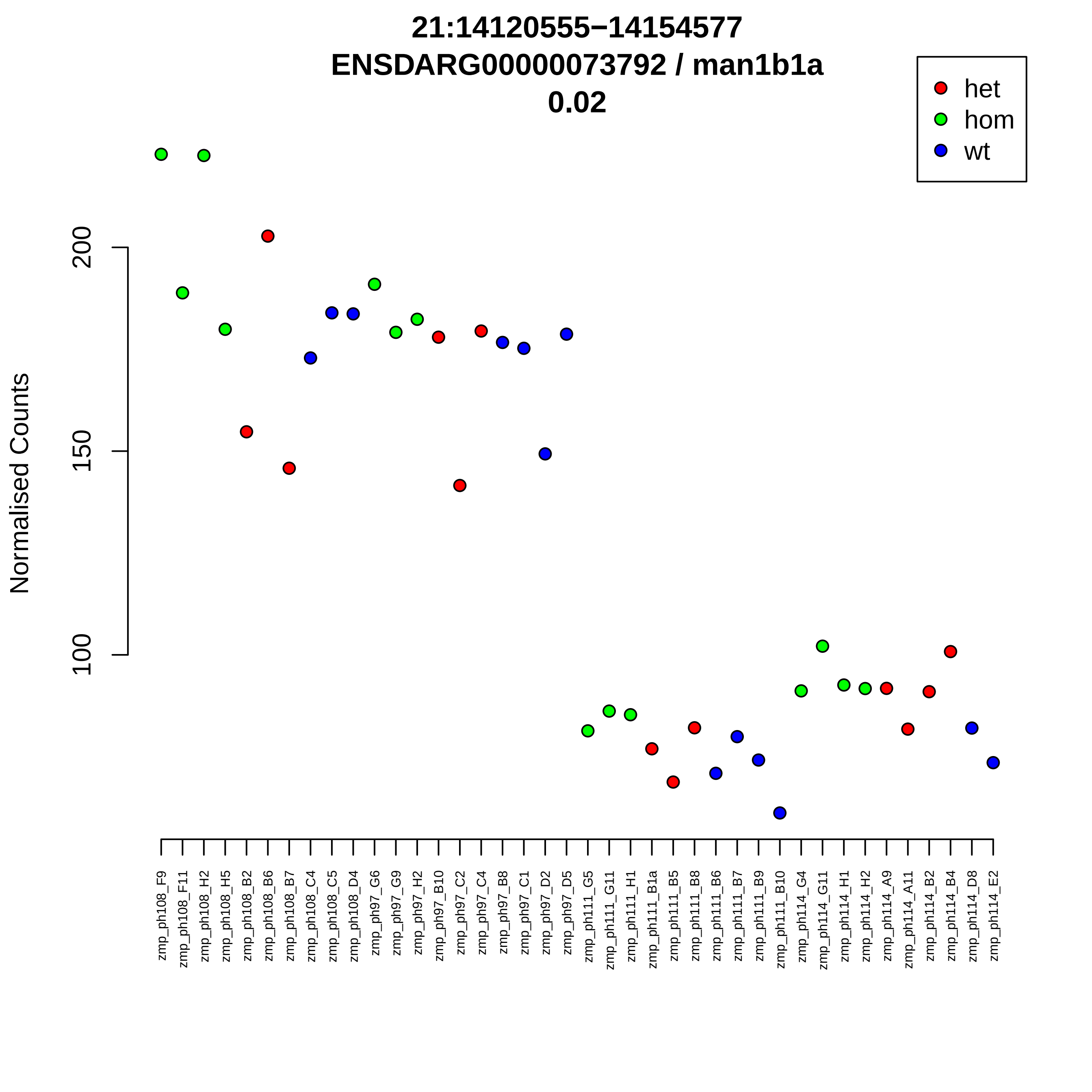
21:14120555−14154577
ENSDARG00000073792 / man1b1a
0.02
het
hom
wt
Normalised Counts
100
150
200
zmp_ph108_F9
zmp_ph108_F11
zmp_ph108_H2
zmp_ph108_H5
zmp_ph108_B2
zmp_ph108_B6
zmp_ph108_B7
zmp_ph108_C4
zmp_ph108_C5
zmp_ph108_D4
zmp_ph97_G6
zmp_ph97_G9
zmp_ph97_H2
zmp_ph97_B10
zmp_ph97_C2
zmp_ph97_C4
zmp_ph97_B8
zmp_ph97_C1
zmp_ph97_D2
zmp_ph97_D5
zmp_ph111_G5
zmp_ph111_G11
zmp_ph111_H1
zmp_ph111_B1a
zmp_ph111_B5
zmp_ph111_B8
zmp_ph111_B6
zmp_ph111_B7
zmp_ph111_B9
zmp_ph111_B10
zmp_ph114_G4
zmp_ph114_G11
zmp_ph114_H1
zmp_ph114_H2
zmp_ph114_A9
zmp_ph114_A11
zmp_ph114_B2
zmp_ph114_B4
zmp_ph114_D8
zmp_ph114_E2

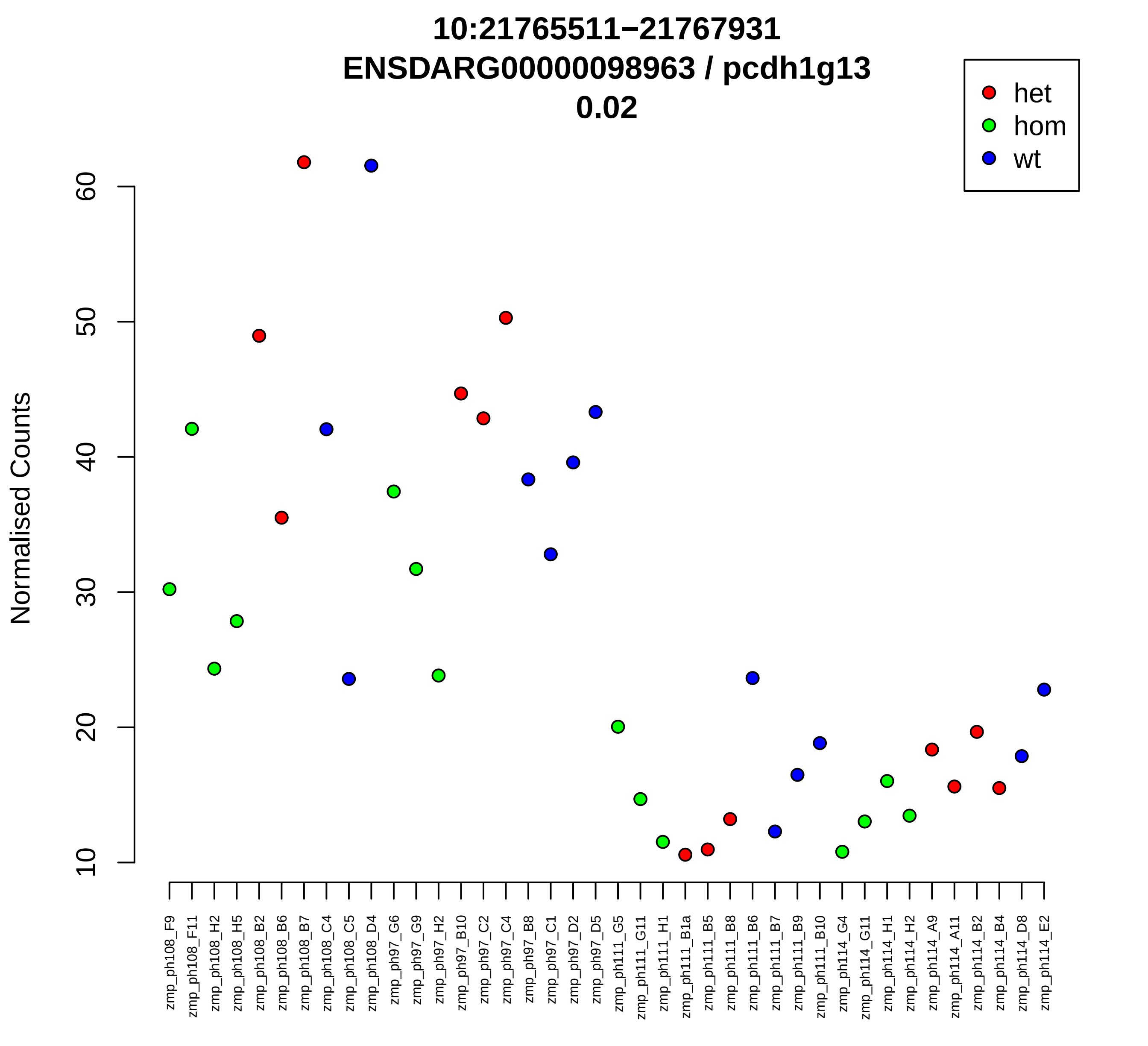

10:21765511−21767931
ENSDARG00000098963 / pcdh1g13
0.02
het
hom
wt
Normalised Counts
10
20
30
40
50
60
zmp_ph108_F9
zmp_ph108_F11
zmp_ph108_H2
zmp_ph108_H5
zmp_ph108_B2
zmp_ph108_B6
zmp_ph108_B7
zmp_ph108_C4
zmp_ph108_C5
zmp_ph108_D4
zmp_ph97_G6
zmp_ph97_G9
zmp_ph97_H2
zmp_ph97_B10
zmp_ph97_C2
zmp_ph97_C4
zmp_ph97_B8
zmp_ph97_C1
zmp_ph97_D2
zmp_ph97_D5
zmp_ph111_G5
zmp_ph111_G11
zmp_ph111_H1
zmp_ph111_B1a
zmp_ph111_B5
zmp_ph111_B8
zmp_ph111_B6
zmp_ph111_B7
zmp_ph111_B9
zmp_ph111_B10
zmp_ph114_G4
zmp_ph114_G11
zmp_ph114_H1
zmp_ph114_H2
zmp_ph114_A9
zmp_ph114_A11
zmp_ph114_B2
zmp_ph114_B4
zmp_ph114_D8
zmp_ph114_E2

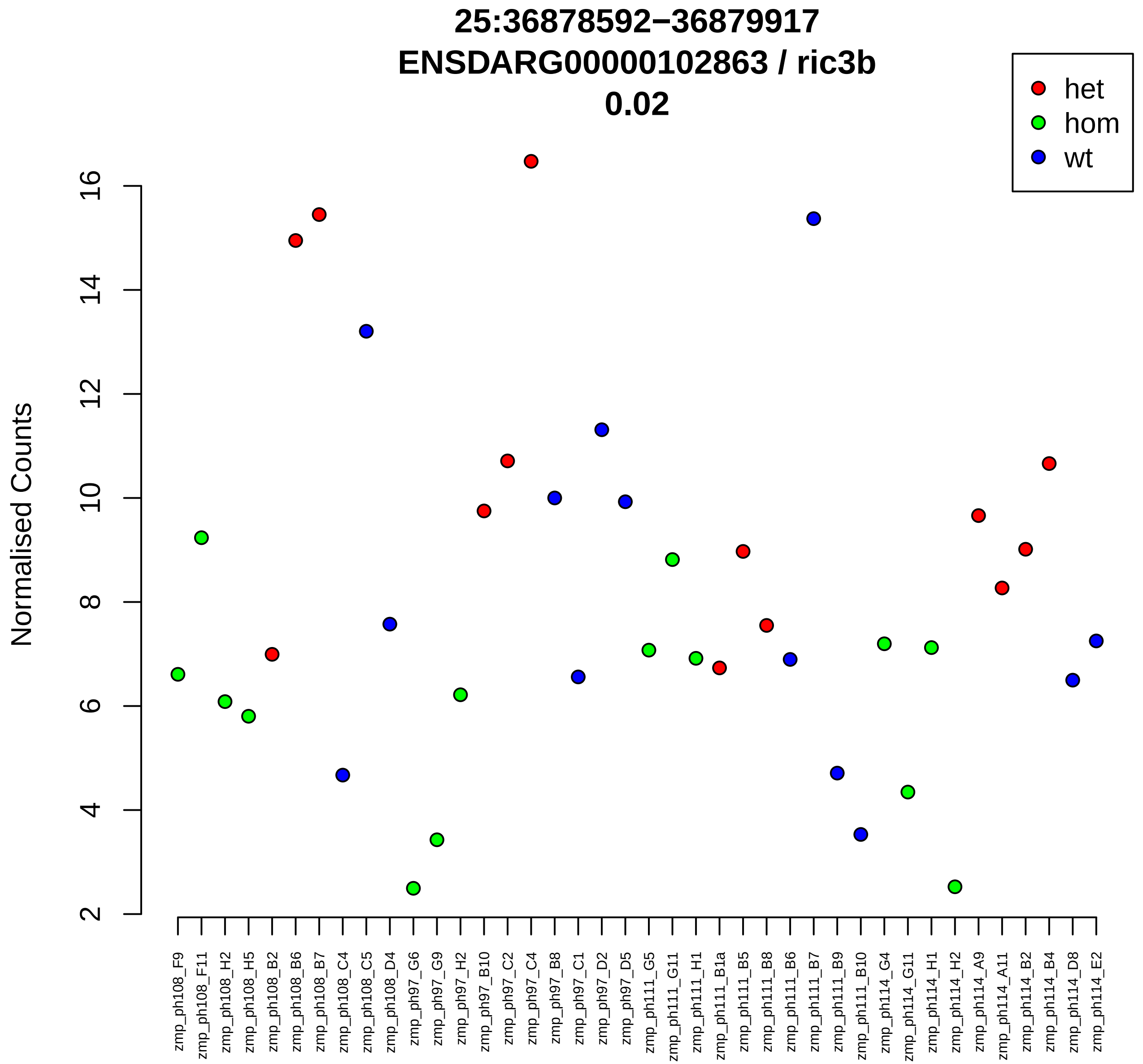

25:36878592−36879917
ENSDARG00000102863 / ric3b
0.02
het
hom
wt
Normalised Counts
2
4
6
8
10
12
14
16
zmp_ph108_F9
zmp_ph108_F11
zmp_ph108_H2
zmp_ph108_H5
zmp_ph108_B2
zmp_ph108_B6
zmp_ph108_B7
zmp_ph108_C4
zmp_ph108_C5
zmp_ph108_D4
zmp_ph97_G6
zmp_ph97_G9
zmp_ph97_H2
zmp_ph97_B10
zmp_ph97_C2
zmp_ph97_C4
zmp_ph97_B8
zmp_ph97_C1
zmp_ph97_D2
zmp_ph97_D5
zmp_ph111_G5
zmp_ph111_G11
zmp_ph111_H1
zmp_ph111_B1a
zmp_ph111_B5
zmp_ph111_B8
zmp_ph111_B6
zmp_ph111_B7
zmp_ph111_B9
zmp_ph111_B10
zmp_ph114_G4
zmp_ph114_G11
zmp_ph114_H1
zmp_ph114_H2
zmp_ph114_A9
zmp_ph114_A11
zmp_ph114_B2
zmp_ph114_B4
zmp_ph114_D8
zmp_ph114_E2

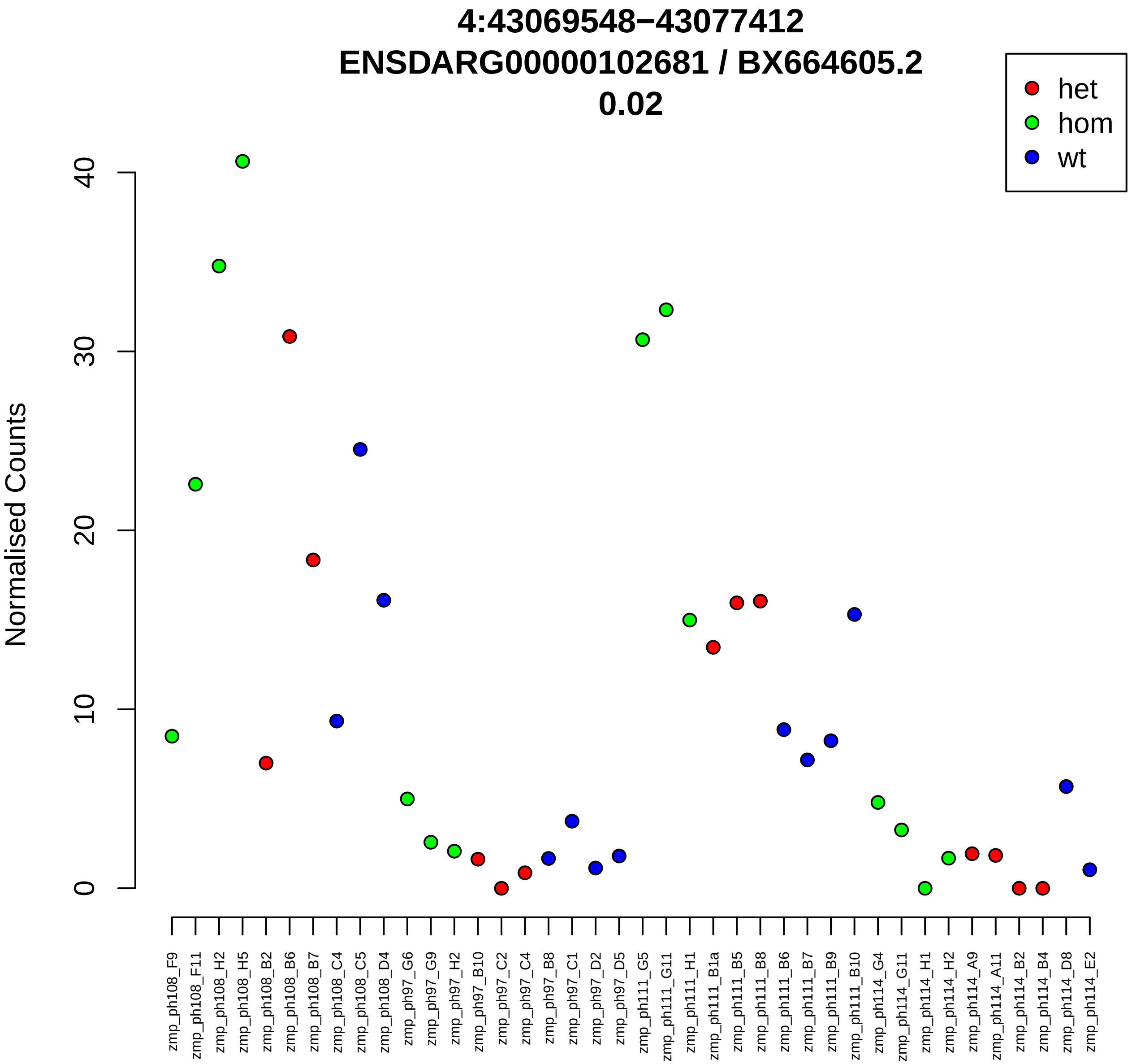

4:43069548−43077412
ENSDARG00000102681 / BX664605.2
0.02
het
hom
wt
Normalised Counts
0
10
20
30
40
zmp_ph108_F9
zmp_ph108_F11
zmp_ph108_H2
zmp_ph108_H5
zmp_ph108_B2
zmp_ph108_B6
zmp_ph108_B7
zmp_ph108_C4
zmp_ph108_C5
zmp_ph108_D4
zmp_ph97_G6
zmp_ph97_G9
zmp_ph97_H2
zmp_ph97_B10
zmp_ph97_C2
zmp_ph97_C4
zmp_ph97_B8
zmp_ph97_C1
zmp_ph97_D2
zmp_ph97_D5
zmp_ph111_G5
zmp_ph111_G11
zmp_ph111_H1
zmp_ph111_B1a
zmp_ph111_B5
zmp_ph111_B8
zmp_ph111_B6
zmp_ph111_B7
zmp_ph111_B9
zmp_ph111_B10
zmp_ph114_G4
zmp_ph114_G11
zmp_ph114_H1
zmp_ph114_H2
zmp_ph114_A9
zmp_ph114_A11
zmp_ph114_B2
zmp_ph114_B4
zmp_ph114_D8
zmp_ph114_E2

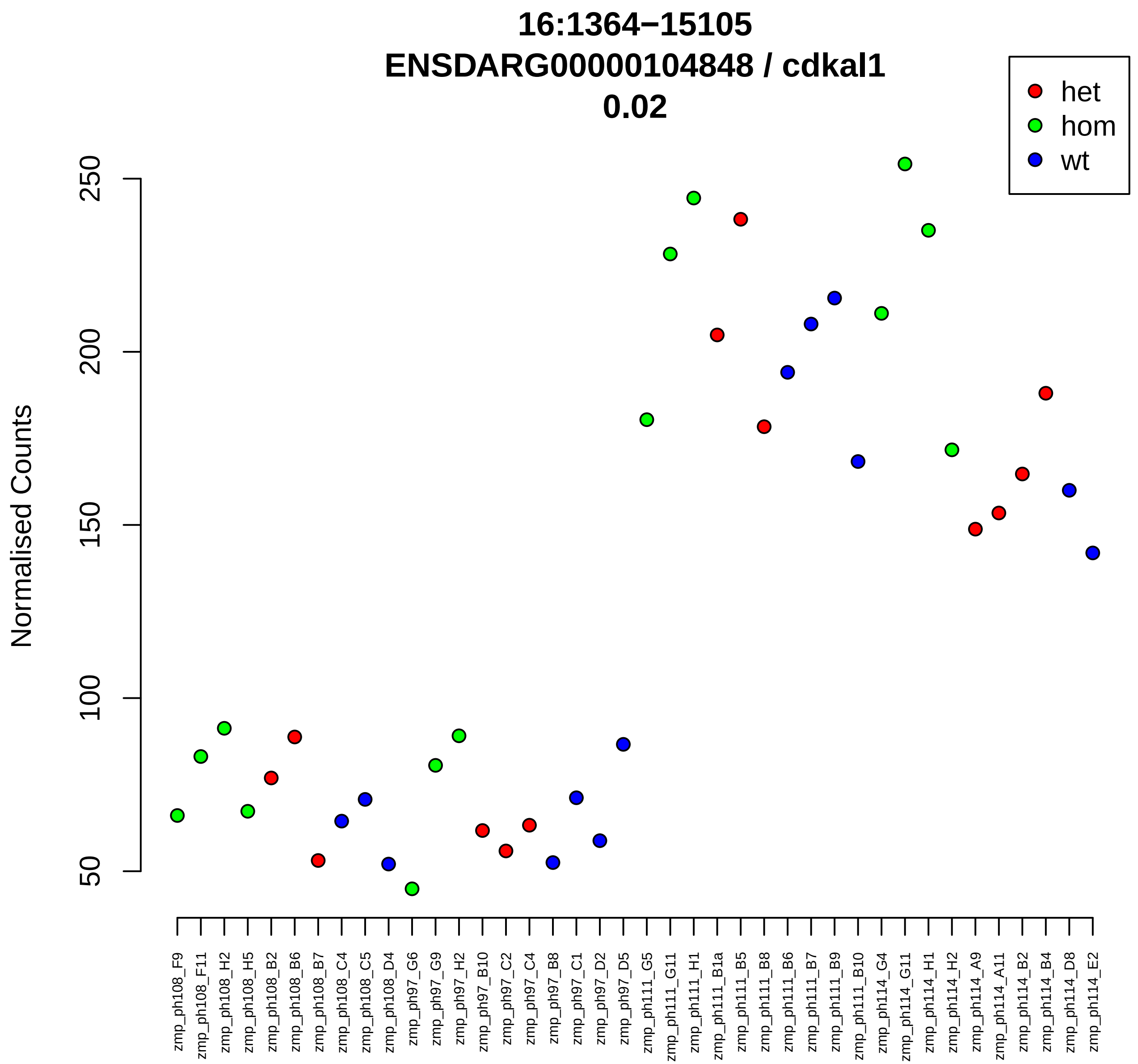
16:1364−15105
ENSDARG00000104848 / cdkal1
0.02
het
hom
wt
Normalised Counts
50
100
150
200
250
zmp_ph108_F9
zmp_ph108_F11
zmp_ph108_H2
zmp_ph108_H5
zmp_ph108_B2
zmp_ph108_B6
zmp_ph108_B7
zmp_ph108_C4
zmp_ph108_C5
zmp_ph108_D4
zmp_ph97_G6
zmp_ph97_G9
zmp_ph97_H2
zmp_ph97_B10
zmp_ph97_C2
zmp_ph97_C4
zmp_ph97_B8
zmp_ph97_C1
zmp_ph97_D2
zmp_ph97_D5
zmp_ph111_G5
zmp_ph111_G11
zmp_ph111_H1
zmp_ph111_B1a
zmp_ph111_B5
zmp_ph111_B8
zmp_ph111_B6
zmp_ph111_B7
zmp_ph111_B9
zmp_ph111_B10
zmp_ph114_G4
zmp_ph114_G11
zmp_ph114_H1
zmp_ph114_H2
zmp_ph114_A9
zmp_ph114_A11
zmp_ph114_B2
zmp_ph114_B4
zmp_ph114_D8
zmp_ph114_E2

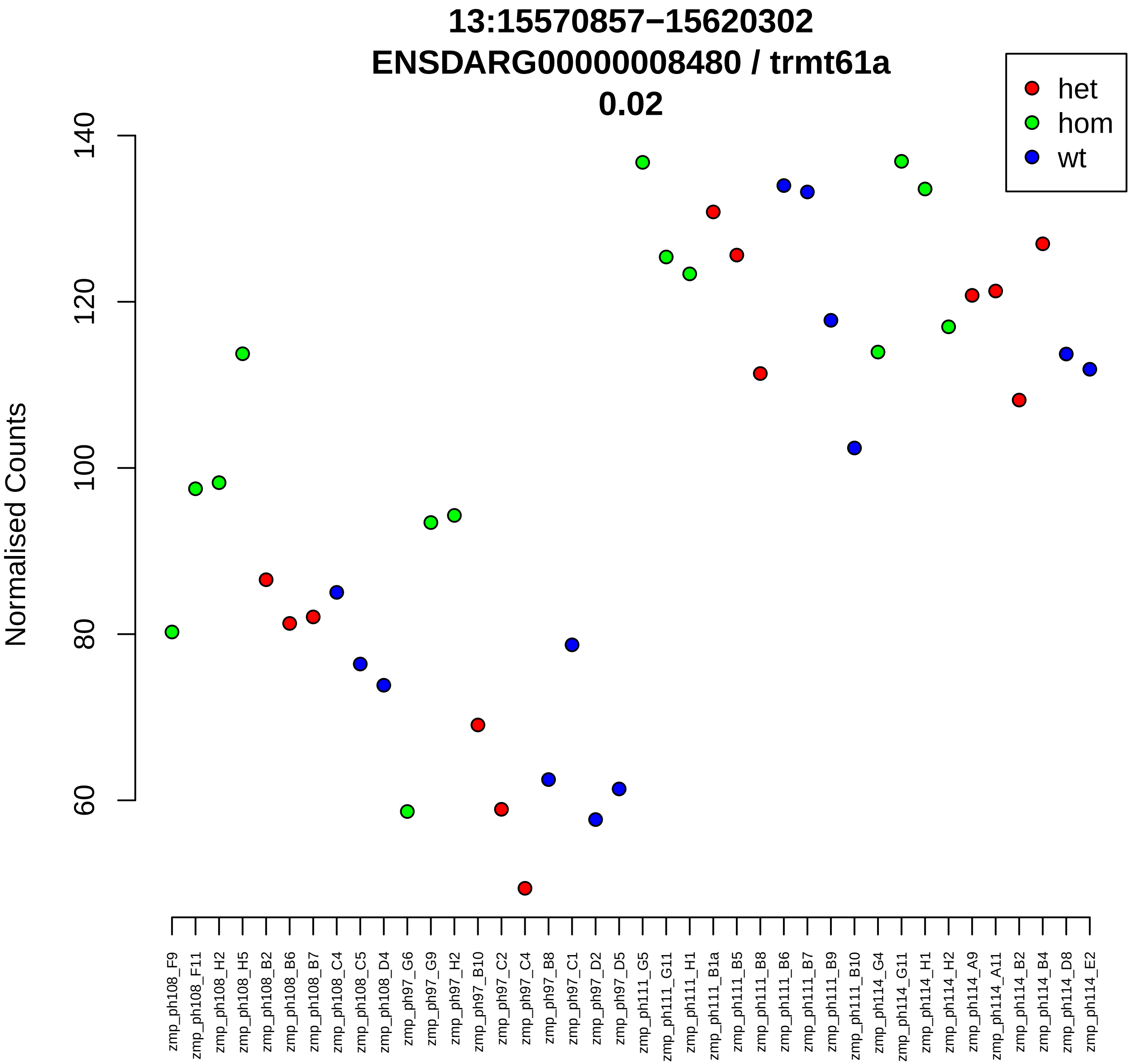
13:15570857−15620302
ENSDARG00000008480 / trmt61a
0.02
het
hom
wt
Normalised Counts
140
120
100
80
60
zmp_ph108_F9
zmp_ph108_F11
zmp_ph108_H2
zmp_ph108_H5
zmp_ph108_B2
zmp_ph108_B6
zmp_ph108_B7
zmp_ph108_C4
zmp_ph108_C5
zmp_ph108_D4
zmp_ph97_G6
zmp_ph97_G9
zmp_ph97_H2
zmp_ph97_B10
zmp_ph97_C2
zmp_ph97_C4
zmp_ph97_B8
zmp_ph97_C1
zmp_ph97_D2
zmp_ph97_D5
zmp_ph111_G5
zmp_ph111_G11
zmp_ph111_H1
zmp_ph111_B1a
zmp_ph111_B5
zmp_ph111_B8
zmp_ph111_B6
zmp_ph111_B7
zmp_ph111_B9
zmp_ph111_B10
zmp_ph114_G4
zmp_ph114_G11
zmp_ph114_H1
zmp_ph114_H2
zmp_ph114_A9
zmp_ph114_A11
zmp_ph114_B2
zmp_ph114_B4
zmp_ph114_D8
zmp_ph114_E2

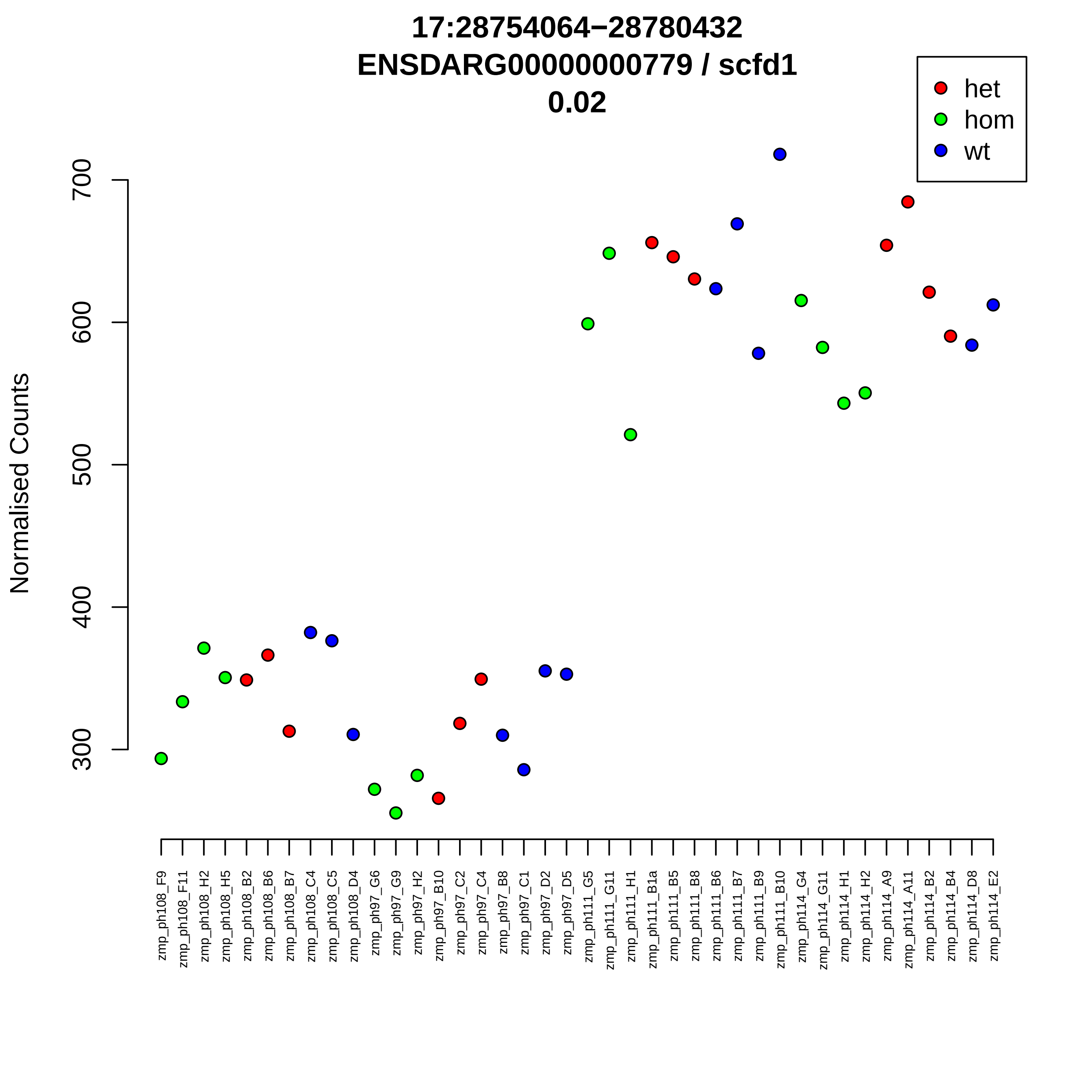
17:28754064−28780432
ENSDARG00000000779 / scfd1
0.02
het
hom
wt
Normalised Counts
300
400
500
600
700
zmp_ph108_F9
zmp_ph108_F11
zmp_ph108_H2
zmp_ph108_H5
zmp_ph108_B2
zmp_ph108_B6
zmp_ph108_B7
zmp_ph108_C4
zmp_ph108_C5
zmp_ph108_D4
zmp_ph97_G6
zmp_ph97_G9
zmp_ph97_H2
zmp_ph97_B10
zmp_ph97_C2
zmp_ph97_C4
zmp_ph97_B8
zmp_ph97_C1
zmp_ph97_D2
zmp_ph97_D5
zmp_ph111_G5
zmp_ph111_G11
zmp_ph111_H1
zmp_ph111_B1a
zmp_ph111_B5
zmp_ph111_B8
zmp_ph111_B6
zmp_ph111_B7
zmp_ph111_B9
zmp_ph111_B10
zmp_ph114_G4
zmp_ph114_G11
zmp_ph114_H1
zmp_ph114_H2
zmp_ph114_A9
zmp_ph114_A11
zmp_ph114_B2
zmp_ph114_B4
zmp_ph114_D8
zmp_ph114_E2

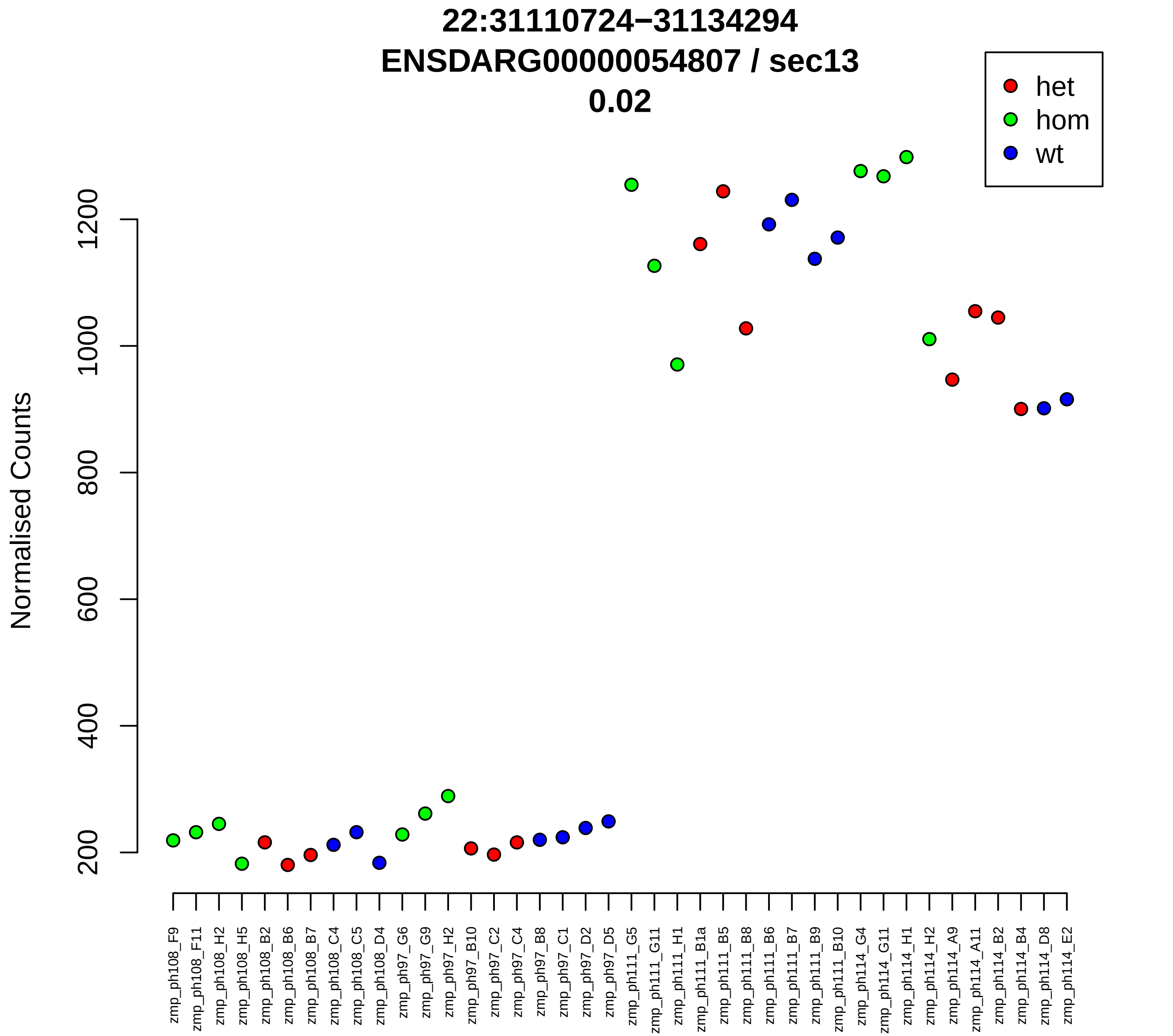
22:31110724−31134294
ENSDARG00000054807 / sec13
0.02
het
hom
wt
Normalised Counts
200
400
600
800
1000
1200
zmp_ph108_F9
zmp_ph108_F11
zmp_ph108_H2
zmp_ph108_H5
zmp_ph108_B2
zmp_ph108_B6
zmp_ph108_B7
zmp_ph108_C4
zmp_ph108_C5
zmp_ph108_D4
zmp_ph97_G6
zmp_ph97_G9
zmp_ph97_H2
zmp_ph97_B10
zmp_ph97_C2
zmp_ph97_C4
zmp_ph97_B8
zmp_ph97_C1
zmp_ph97_D2
zmp_ph97_D5
zmp_ph111_G5
zmp_ph111_G11
zmp_ph111_H1
zmp_ph111_B1a
zmp_ph111_B5
zmp_ph111_B8
zmp_ph111_B6
zmp_ph111_B7
zmp_ph111_B9
zmp_ph111_B10
zmp_ph114_G4
zmp_ph114_G11
zmp_ph114_H1
zmp_ph114_H2
zmp_ph114_A9
zmp_ph114_A11
zmp_ph114_B2
zmp_ph114_B4
zmp_ph114_D8
zmp_ph114_E2

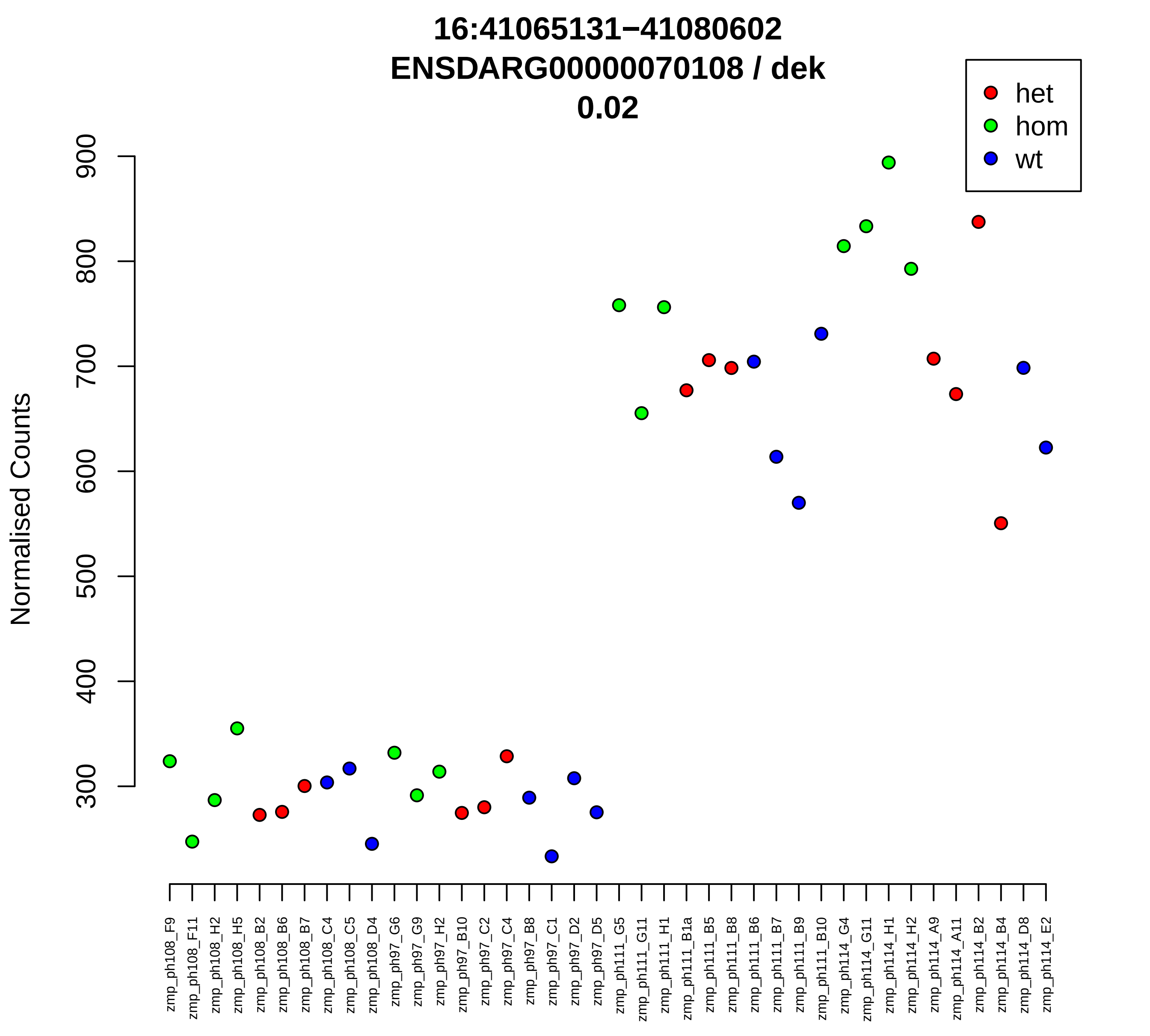

16:41065131−41080602
ENSDARG00000070108 / dek
0.02
het
hom
wt
Normalised Counts
300
400
500
600
700
800
900
zmp_ph108_F9
zmp_ph108_F11
zmp_ph108_H2
zmp_ph108_H5
zmp_ph108_B2
zmp_ph108_B6
zmp_ph108_B7
zmp_ph108_C4
zmp_ph108_C5
zmp_ph108_D4
zmp_ph97_G6
zmp_ph97_G9
zmp_ph97_H2
zmp_ph97_B10
zmp_ph97_C2
zmp_ph97_C4
zmp_ph97_B8
zmp_ph97_C1
zmp_ph97_D2
zmp_ph97_D5
zmp_ph111_G5
zmp_ph111_G11
zmp_ph111_H1
zmp_ph111_B1a
zmp_ph111_B5
zmp_ph111_B8
zmp_ph111_B6
zmp_ph111_B7
zmp_ph111_B9
zmp_ph111_B10
zmp_ph114_G4
zmp_ph114_G11
zmp_ph114_H1
zmp_ph114_H2
zmp_ph114_A9
zmp_ph114_A11
zmp_ph114_B2
zmp_ph114_B4
zmp_ph114_D8
zmp_ph114_E2

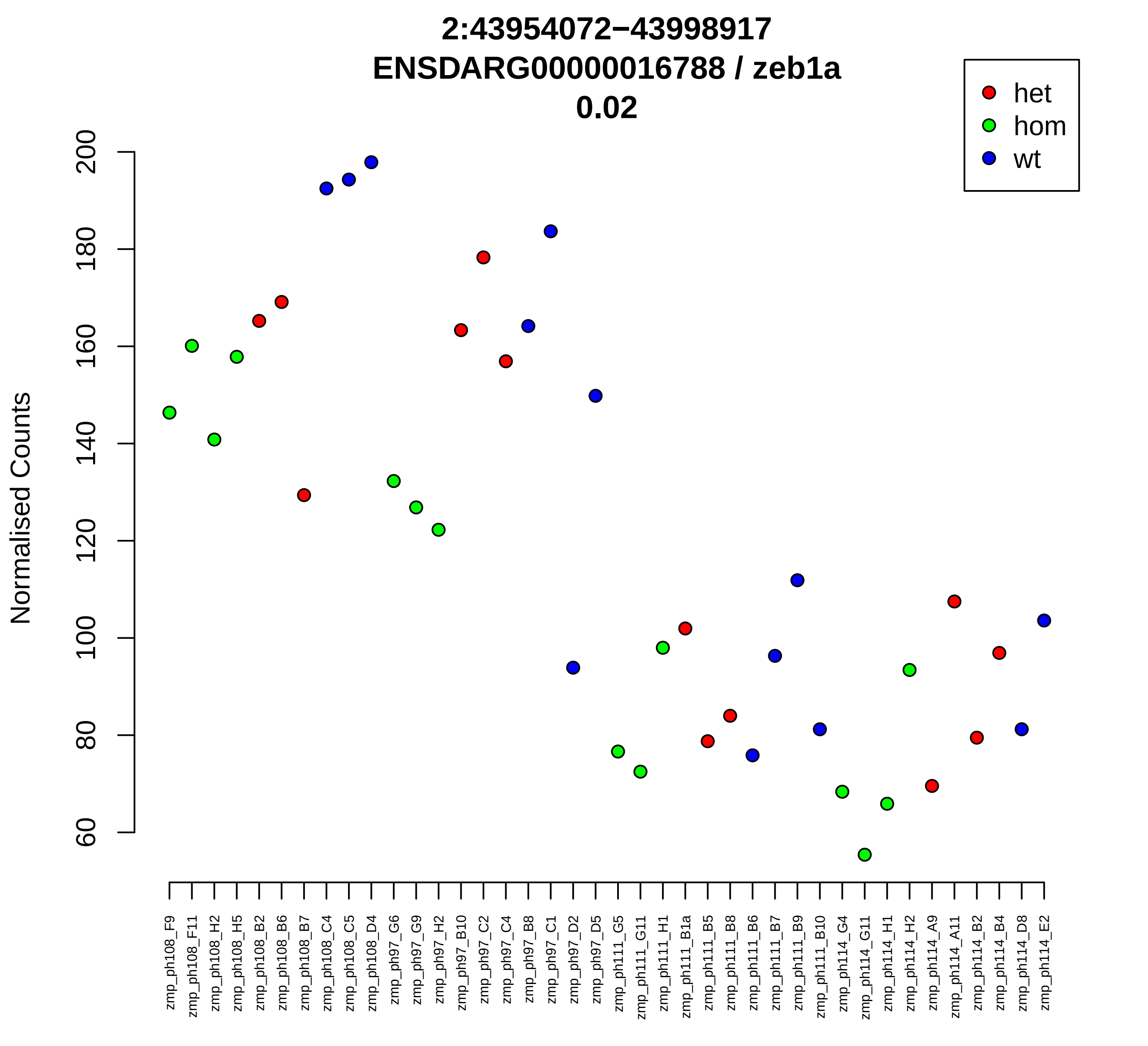

2:43954072−43998917
ENSDARG00000016788 / zeb1a
0.02
het
hom
wt
Normalised Counts
60
80
100
120
140
160
180
200
zmp_ph108_F9
zmp_ph108_F11
zmp_ph108_H2
zmp_ph108_H5
zmp_ph108_B2
zmp_ph108_B6
zmp_ph108_B7
zmp_ph108_C4
zmp_ph108_C5
zmp_ph108_D4
zmp_ph97_G6
zmp_ph97_G9
zmp_ph97_H2
zmp_ph97_B10
zmp_ph97_C2
zmp_ph97_C4
zmp_ph97_B8
zmp_ph97_C1
zmp_ph97_D2
zmp_ph97_D5
zmp_ph111_G5
zmp_ph111_G11
zmp_ph111_H1
zmp_ph111_B1a
zmp_ph111_B5
zmp_ph111_B8
zmp_ph111_B6
zmp_ph111_B7
zmp_ph111_B9
zmp_ph111_B10
zmp_ph114_G4
zmp_ph114_G11
zmp_ph114_H1
zmp_ph114_H2
zmp_ph114_A9
zmp_ph114_A11
zmp_ph114_B2
zmp_ph114_B4
zmp_ph114_D8
zmp_ph114_E2

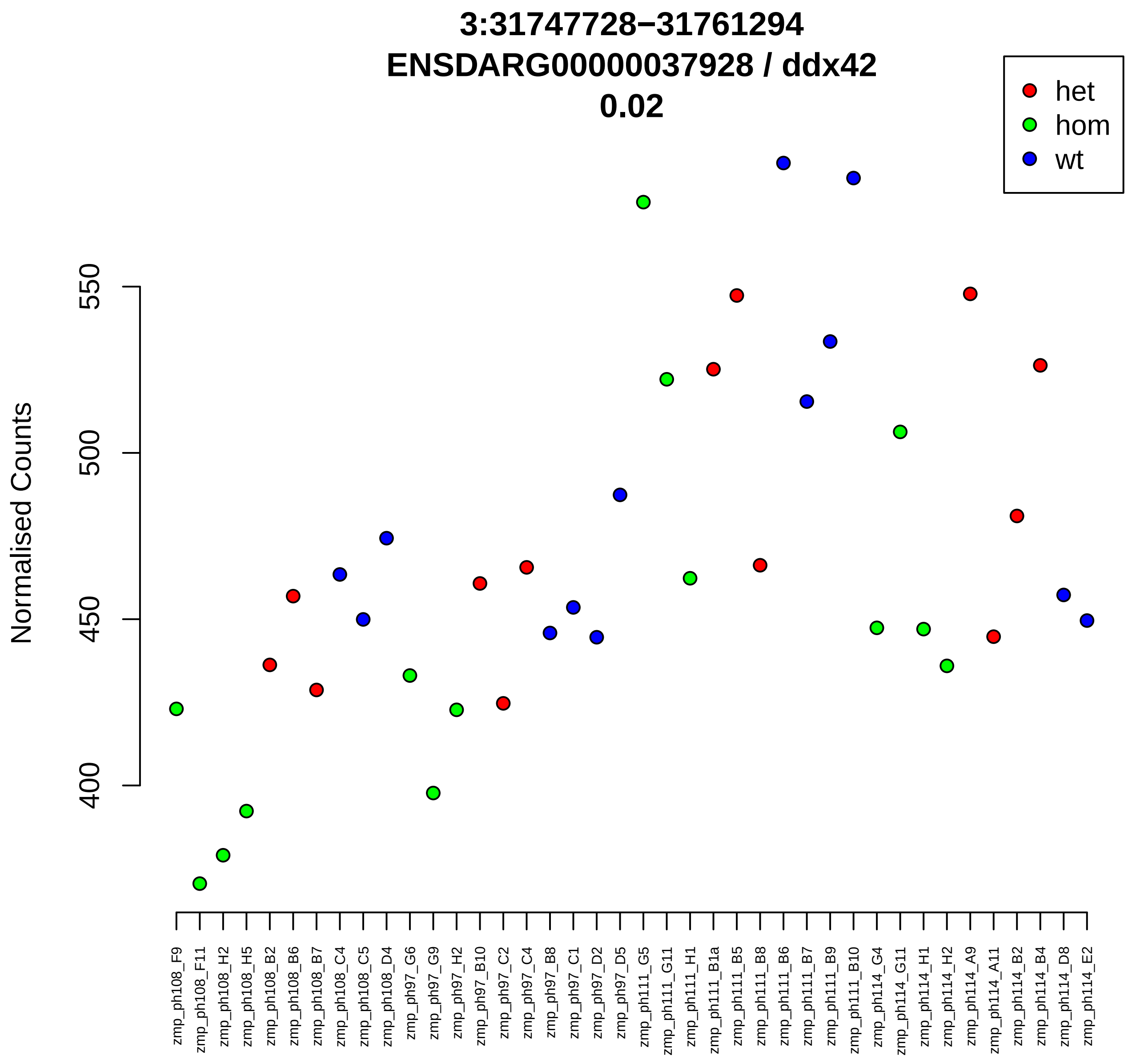
3:31747728−31761294
ENSDARG00000037928 / ddx42
0.02
het
hom
wt
Normalised Counts
400
450
500
550
zmp_ph108_F9
zmp_ph108_F11
zmp_ph108_H2
zmp_ph108_H5
zmp_ph108_B2
zmp_ph108_B6
zmp_ph108_B7
zmp_ph108_C4
zmp_ph108_C5
zmp_ph108_D4
zmp_ph97_G6
zmp_ph97_G9
zmp_ph97_H2
zmp_ph97_B10
zmp_ph97_C2
zmp_ph97_C4
zmp_ph97_B8
zmp_ph97_C1
zmp_ph97_D2
zmp_ph97_D5
zmp_ph111_G5
zmp_ph111_G11
zmp_ph111_H1
zmp_ph111_B1a
zmp_ph111_B5
zmp_ph111_B8
zmp_ph111_B6
zmp_ph111_B7
zmp_ph111_B9
zmp_ph111_B10
zmp_ph114_G4
zmp_ph114_G11
zmp_ph114_H1
zmp_ph114_H2
zmp_ph114_A9
zmp_ph114_A11
zmp_ph114_B2
zmp_ph114_B4
zmp_ph114_D8
zmp_ph114_E2

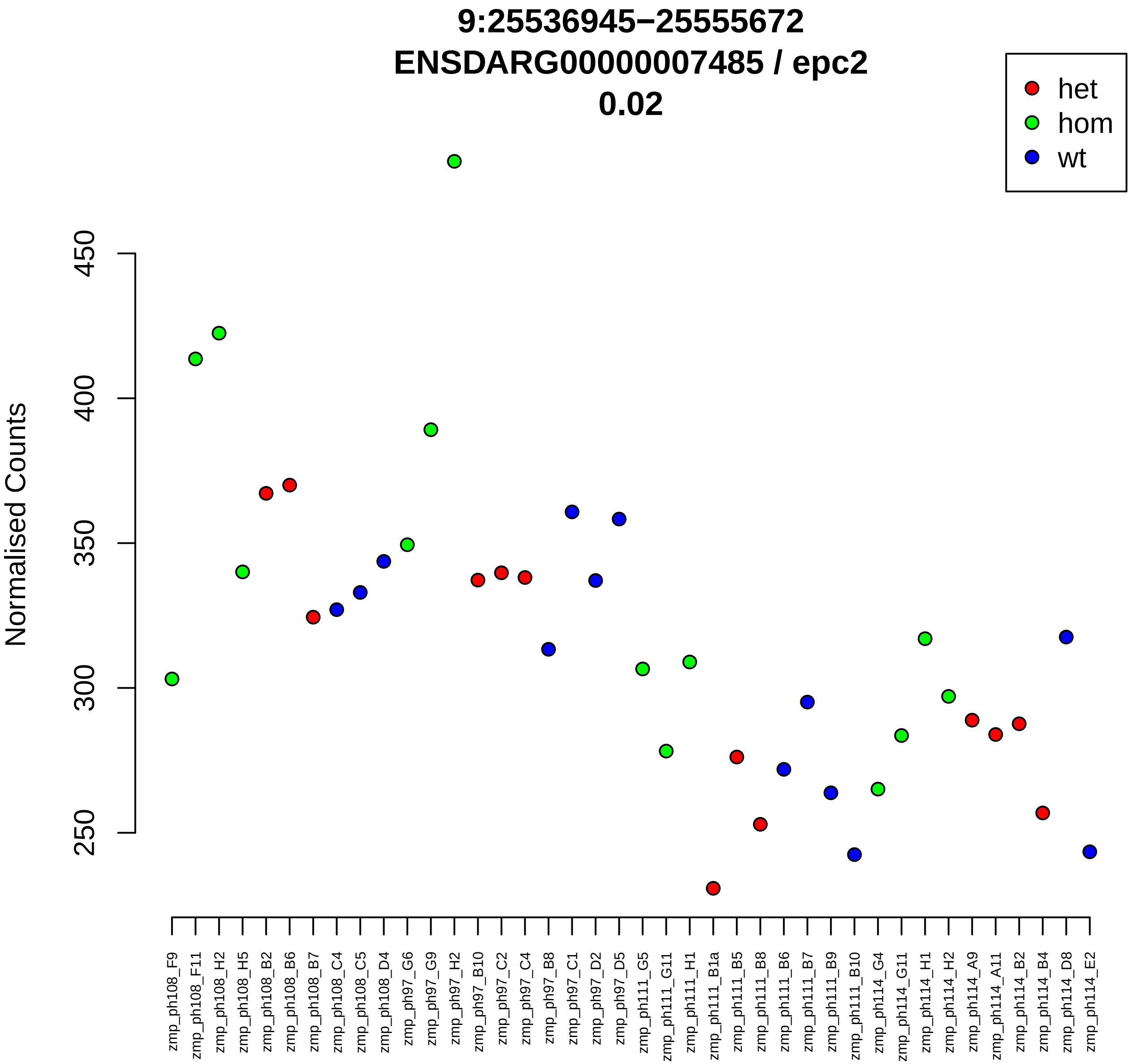
9:25536945−25555672
ENSDARG00000007485 / epc2
0.02
het
hom
wt
Normalised Counts
450
400
350
300
250
zmp_ph108_F9
zmp_ph108_F11
zmp_ph108_H2
zmp_ph108_H5
zmp_ph108_B2
zmp_ph108_B6
zmp_ph108_B7
zmp_ph108_C4
zmp_ph108_C5
zmp_ph108_D4
zmp_ph97_G6
zmp_ph97_G9
zmp_ph97_H2
zmp_ph97_B10
zmp_ph97_C2
zmp_ph97_C4
zmp_ph97_B8
zmp_ph97_C1
zmp_ph97_D2
zmp_ph97_D5
zmp_ph111_G5
zmp_ph111_G11
zmp_ph111_H1
zmp_ph111_B1a
zmp_ph111_B5
zmp_ph111_B8
zmp_ph111_B6
zmp_ph111_B7
zmp_ph111_B9
zmp_ph111_B10
zmp_ph114_G4
zmp_ph114_G11
zmp_ph114_H1
zmp_ph114_H2
zmp_ph114_A9
zmp_ph114_A11
zmp_ph114_B2
zmp_ph114_B4
zmp_ph114_D8
zmp_ph114_E2

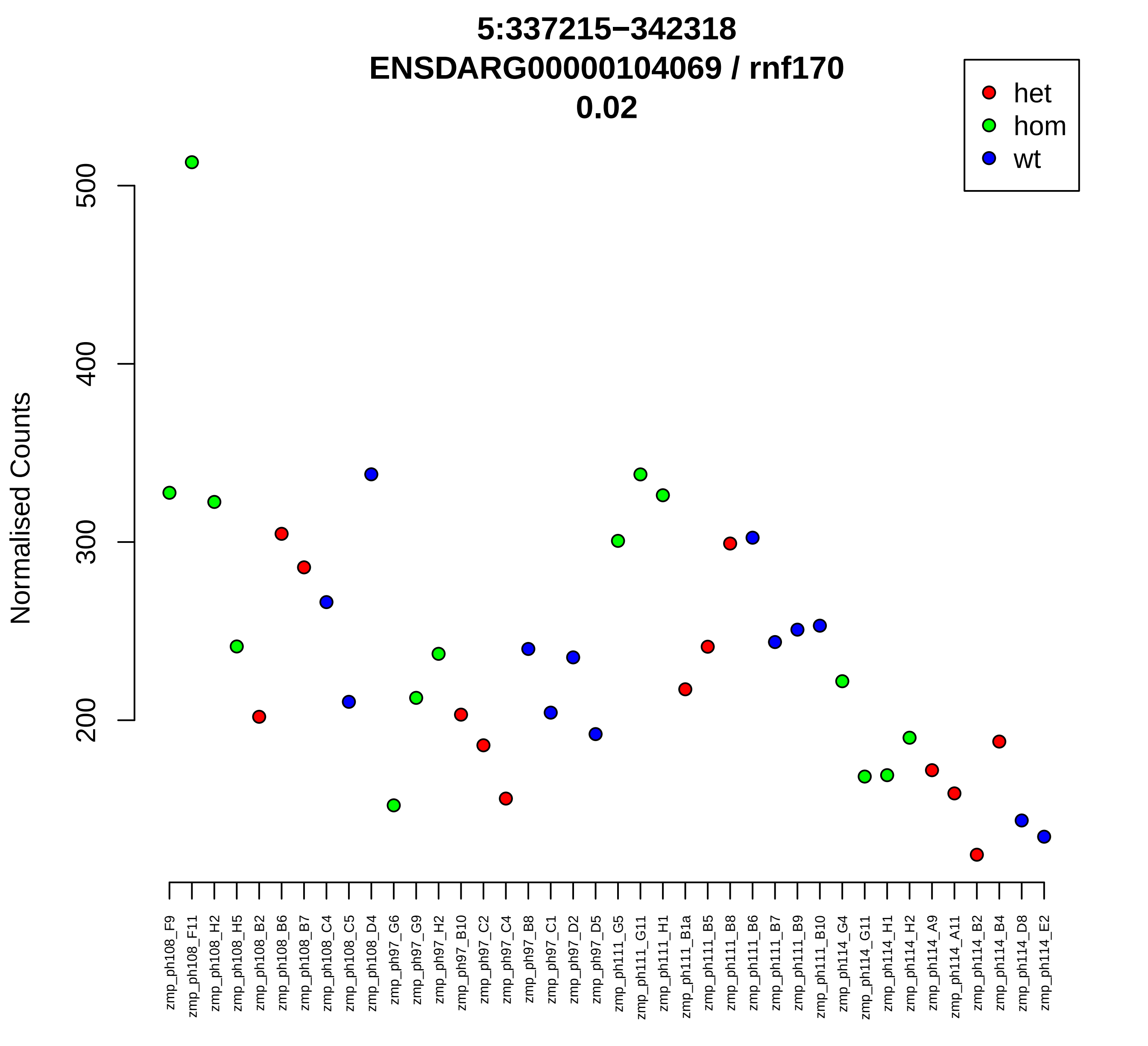

5:337215−342318
ENSDARG00000104069 / rnf170
0.02
het
hom
wt
Normalised Counts
500
400
300
200
zmp_ph108_F9
zmp_ph108_F11
zmp_ph108_H2
zmp_ph108_H5
zmp_ph108_B2
zmp_ph108_B6
zmp_ph108_B7
zmp_ph108_C4
zmp_ph108_C5
zmp_ph108_D4
zmp_ph97_G6
zmp_ph97_G9
zmp_ph97_H2
zmp_ph97_B10
zmp_ph97_C2
zmp_ph97_C4
zmp_ph97_B8
zmp_ph97_C1
zmp_ph97_D2
zmp_ph97_D5
zmp_ph111_G5
zmp_ph111_G11
zmp_ph111_H1
zmp_ph111_B1a
zmp_ph111_B5
zmp_ph111_B8
zmp_ph111_B6
zmp_ph111_B7
zmp_ph111_B9
zmp_ph111_B10
zmp_ph114_G4
zmp_ph114_G11
zmp_ph114_H1
zmp_ph114_H2
zmp_ph114_A9
zmp_ph114_A11
zmp_ph114_B2
zmp_ph114_B4
zmp_ph114_D8
zmp_ph114_E2

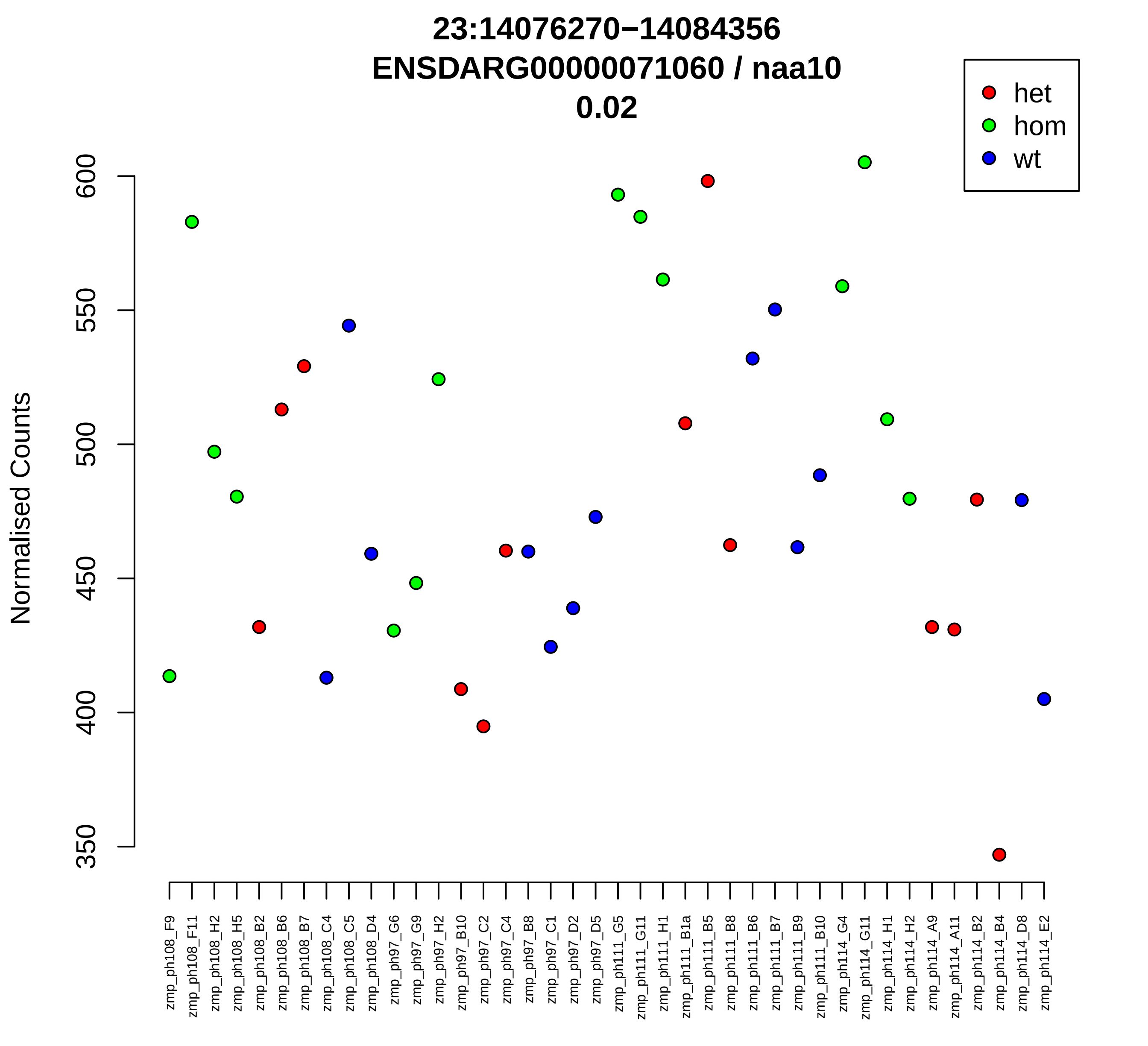
23:14076270−14084356
ENSDARG00000071060 / naa10
0.02
het
hom
wt
Normalised Counts
350
400
450
500
550
600
zmp_ph108_F9
zmp_ph108_F11
zmp_ph108_H2
zmp_ph108_H5
zmp_ph108_B2
zmp_ph108_B6
zmp_ph108_B7
zmp_ph108_C4
zmp_ph108_C5
zmp_ph108_D4
zmp_ph97_G6
zmp_ph97_G9
zmp_ph97_H2
zmp_ph97_B10
zmp_ph97_C2
zmp_ph97_C4
zmp_ph97_B8
zmp_ph97_C1
zmp_ph97_D2
zmp_ph97_D5
zmp_ph111_G5
zmp_ph111_G11
zmp_ph111_H1
zmp_ph111_B1a
zmp_ph111_B5
zmp_ph111_B8
zmp_ph111_B6
zmp_ph111_B7
zmp_ph111_B9
zmp_ph111_B10
zmp_ph114_G4
zmp_ph114_G11
zmp_ph114_H1
zmp_ph114_H2
zmp_ph114_A9
zmp_ph114_A11
zmp_ph114_B2
zmp_ph114_B4
zmp_ph114_D8
zmp_ph114_E2

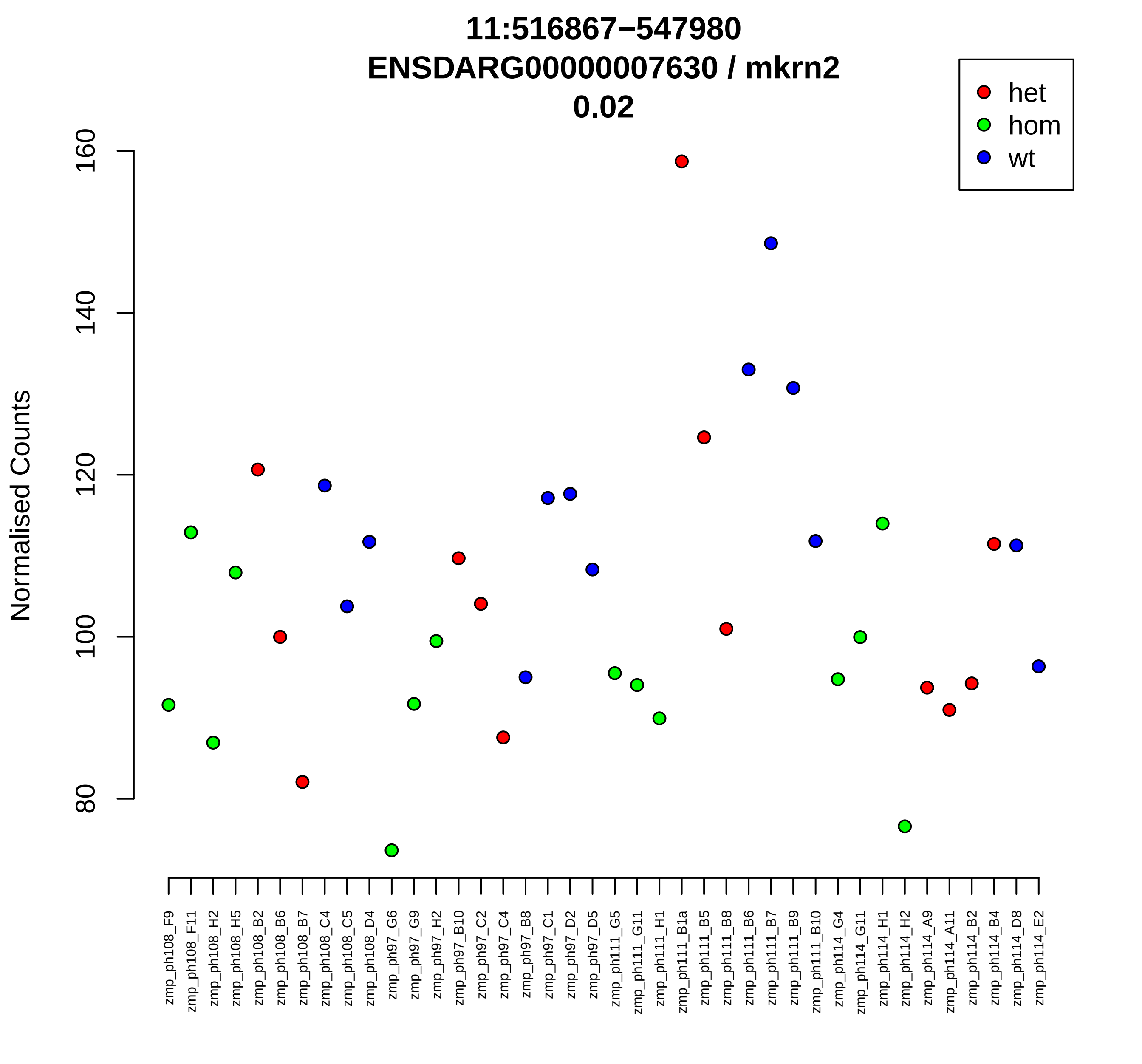

11:516867−547980
ENSDARG00000007630 / mkrn2
0.02
het
hom
wt
Normalised Counts
80
100
120
140
160
zmp_ph108_F9
zmp_ph108_F11
zmp_ph108_H2
zmp_ph108_H5
zmp_ph108_B2
zmp_ph108_B6
zmp_ph108_B7
zmp_ph108_C4
zmp_ph108_C5
zmp_ph108_D4
zmp_ph97_G6
zmp_ph97_G9
zmp_ph97_H2
zmp_ph97_B10
zmp_ph97_C2
zmp_ph97_C4
zmp_ph97_B8
zmp_ph97_C1
zmp_ph97_D2
zmp_ph97_D5
zmp_ph111_G5
zmp_ph111_G11
zmp_ph111_H1
zmp_ph111_B1a
zmp_ph111_B5
zmp_ph111_B8
zmp_ph111_B6
zmp_ph111_B7
zmp_ph111_B9
zmp_ph111_B10
zmp_ph114_G4
zmp_ph114_G11
zmp_ph114_H1
zmp_ph114_H2
zmp_ph114_A9
zmp_ph114_A11
zmp_ph114_B2
zmp_ph114_B4
zmp_ph114_D8
zmp_ph114_E2

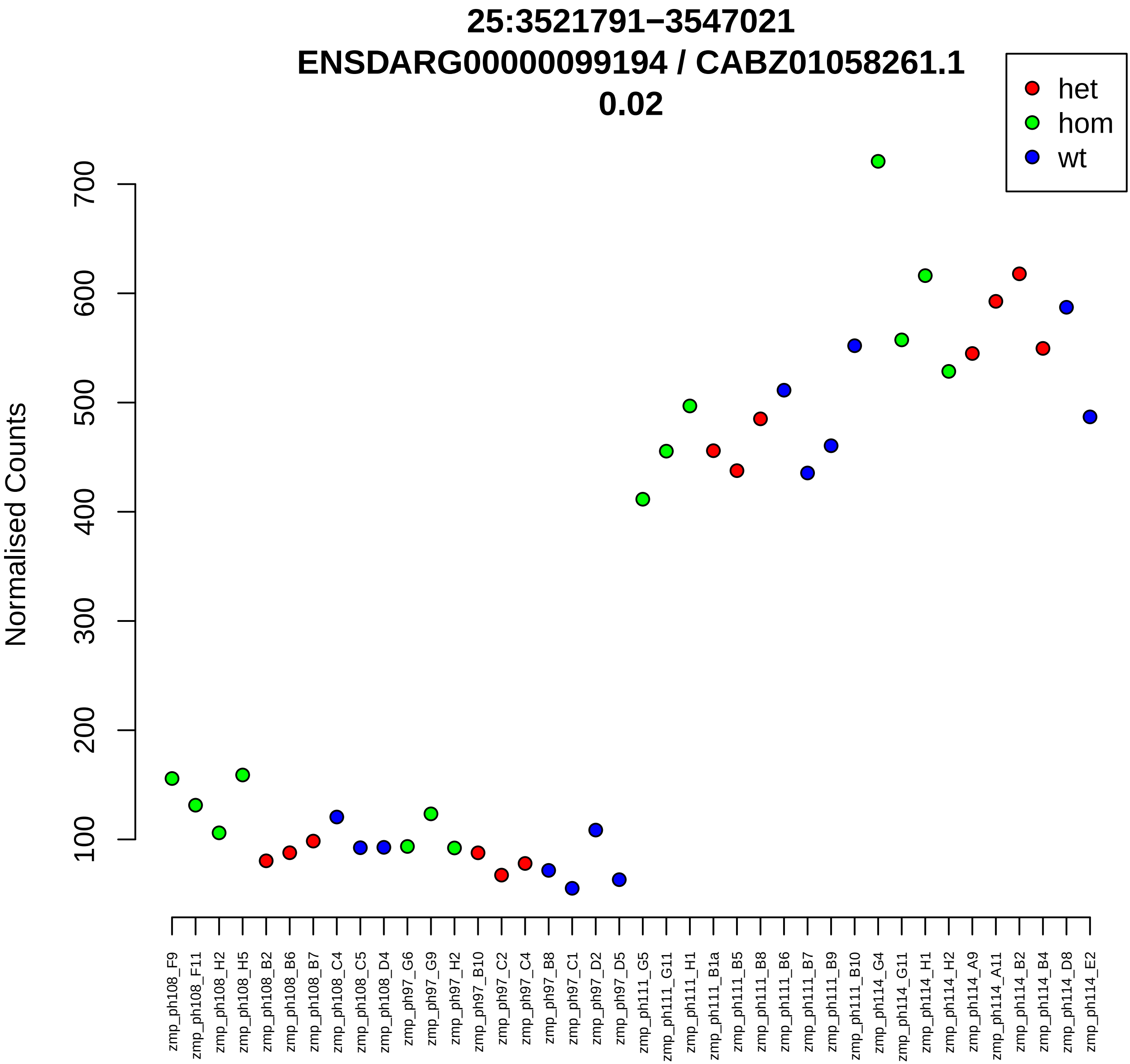
25:3521791−3547021
ENSDARG00000099194 / CABZ01058261.1
0.02
het
hom
wt
Normalised Counts
100
200
300
400
500
600
700
zmp_ph108_F9
zmp_ph108_F11
zmp_ph108_H2
zmp_ph108_H5
zmp_ph108_B2
zmp_ph108_B6
zmp_ph108_B7
zmp_ph108_C4
zmp_ph108_C5
zmp_ph108_D4
zmp_ph97_G6
zmp_ph97_G9
zmp_ph97_H2
zmp_ph97_B10
zmp_ph97_C2
zmp_ph97_C4
zmp_ph97_B8
zmp_ph97_C1
zmp_ph97_D2
zmp_ph97_D5
zmp_ph111_G5
zmp_ph111_G11
zmp_ph111_H1
zmp_ph111_B1a
zmp_ph111_B5
zmp_ph111_B8
zmp_ph111_B6
zmp_ph111_B7
zmp_ph111_B9
zmp_ph111_B10
zmp_ph114_G4
zmp_ph114_G11
zmp_ph114_H1
zmp_ph114_H2
zmp_ph114_A9
zmp_ph114_A11
zmp_ph114_B2
zmp_ph114_B4
zmp_ph114_D8
zmp_ph114_E2

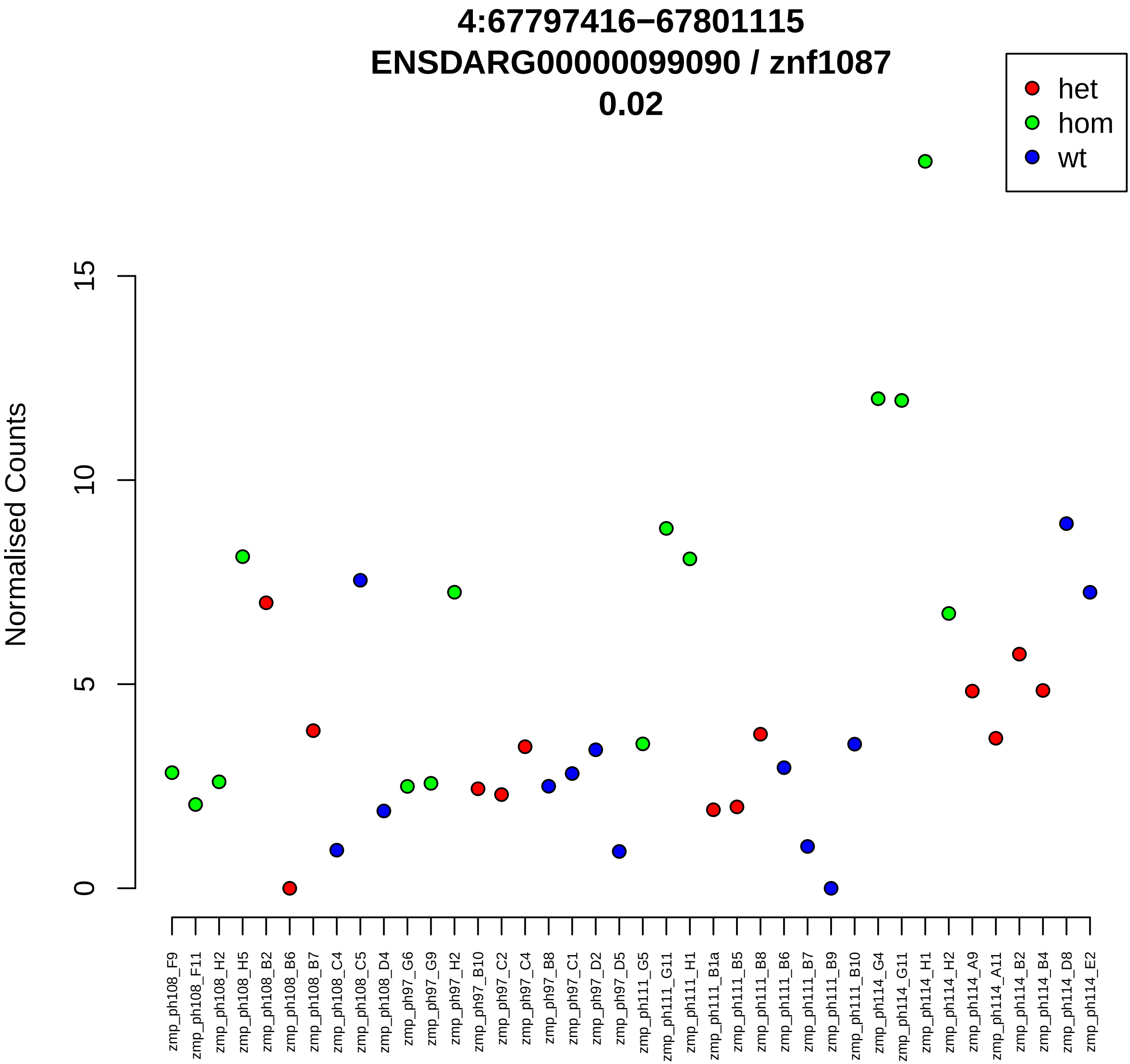
4:67797416−67801115
ENSDARG00000099090 / znf1087
0.02
het
hom
wt
Normalised Counts
0
5
10
15
zmp_ph108_F9
zmp_ph108_F11
zmp_ph108_H2
zmp_ph108_H5
zmp_ph108_B2
zmp_ph108_B6
zmp_ph108_B7
zmp_ph108_C4
zmp_ph108_C5
zmp_ph108_D4
zmp_ph97_G6
zmp_ph97_G9
zmp_ph97_H2
zmp_ph97_B10
zmp_ph97_C2
zmp_ph97_C4
zmp_ph97_B8
zmp_ph97_C1
zmp_ph97_D2
zmp_ph97_D5
zmp_ph111_G5
zmp_ph111_G11
zmp_ph111_H1
zmp_ph111_B1a
zmp_ph111_B5
zmp_ph111_B8
zmp_ph111_B6
zmp_ph111_B7
zmp_ph111_B9
zmp_ph111_B10
zmp_ph114_G4
zmp_ph114_G11
zmp_ph114_H1
zmp_ph114_H2
zmp_ph114_A9
zmp_ph114_A11
zmp_ph114_B2
zmp_ph114_B4
zmp_ph114_D8
zmp_ph114_E2

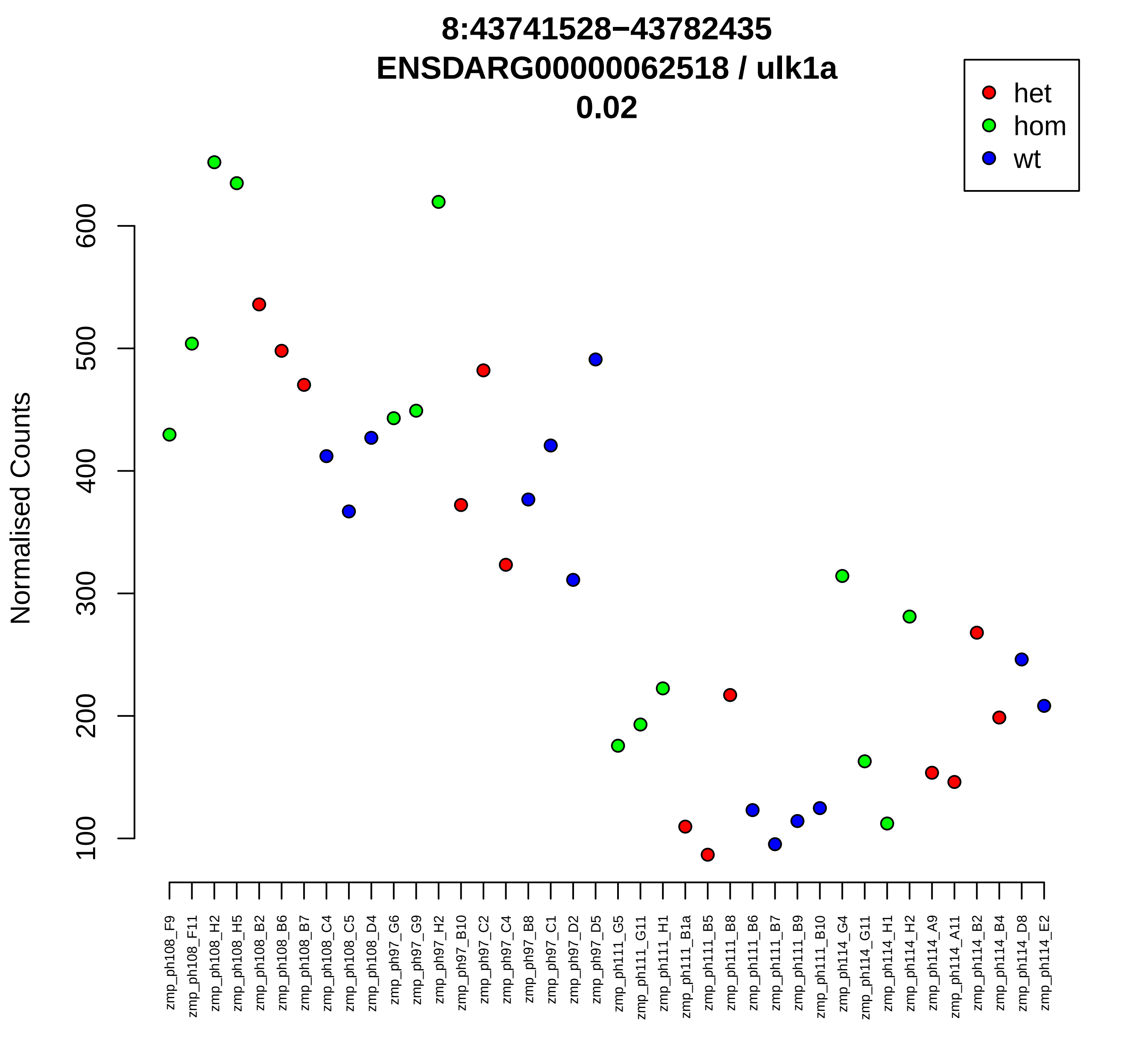

8:43741528−43782435
ENSDARG00000062518 / ulk1a
0.02
het
hom
wt
Normalised Counts
100
200
300
400
500
600
zmp_ph108_F9
zmp_ph108_F11
zmp_ph108_H2
zmp_ph108_H5
zmp_ph108_B2
zmp_ph108_B6
zmp_ph108_B7
zmp_ph108_C4
zmp_ph108_C5
zmp_ph108_D4
zmp_ph97_G6
zmp_ph97_G9
zmp_ph97_H2
zmp_ph97_B10
zmp_ph97_C2
zmp_ph97_C4
zmp_ph97_B8
zmp_ph97_C1
zmp_ph97_D2
zmp_ph97_D5
zmp_ph111_G5
zmp_ph111_G11
zmp_ph111_H1
zmp_ph111_B1a
zmp_ph111_B5
zmp_ph111_B8
zmp_ph111_B6
zmp_ph111_B7
zmp_ph111_B9
zmp_ph111_B10
zmp_ph114_G4
zmp_ph114_G11
zmp_ph114_H1
zmp_ph114_H2
zmp_ph114_A9
zmp_ph114_A11
zmp_ph114_B2
zmp_ph114_B4
zmp_ph114_D8
zmp_ph114_E2

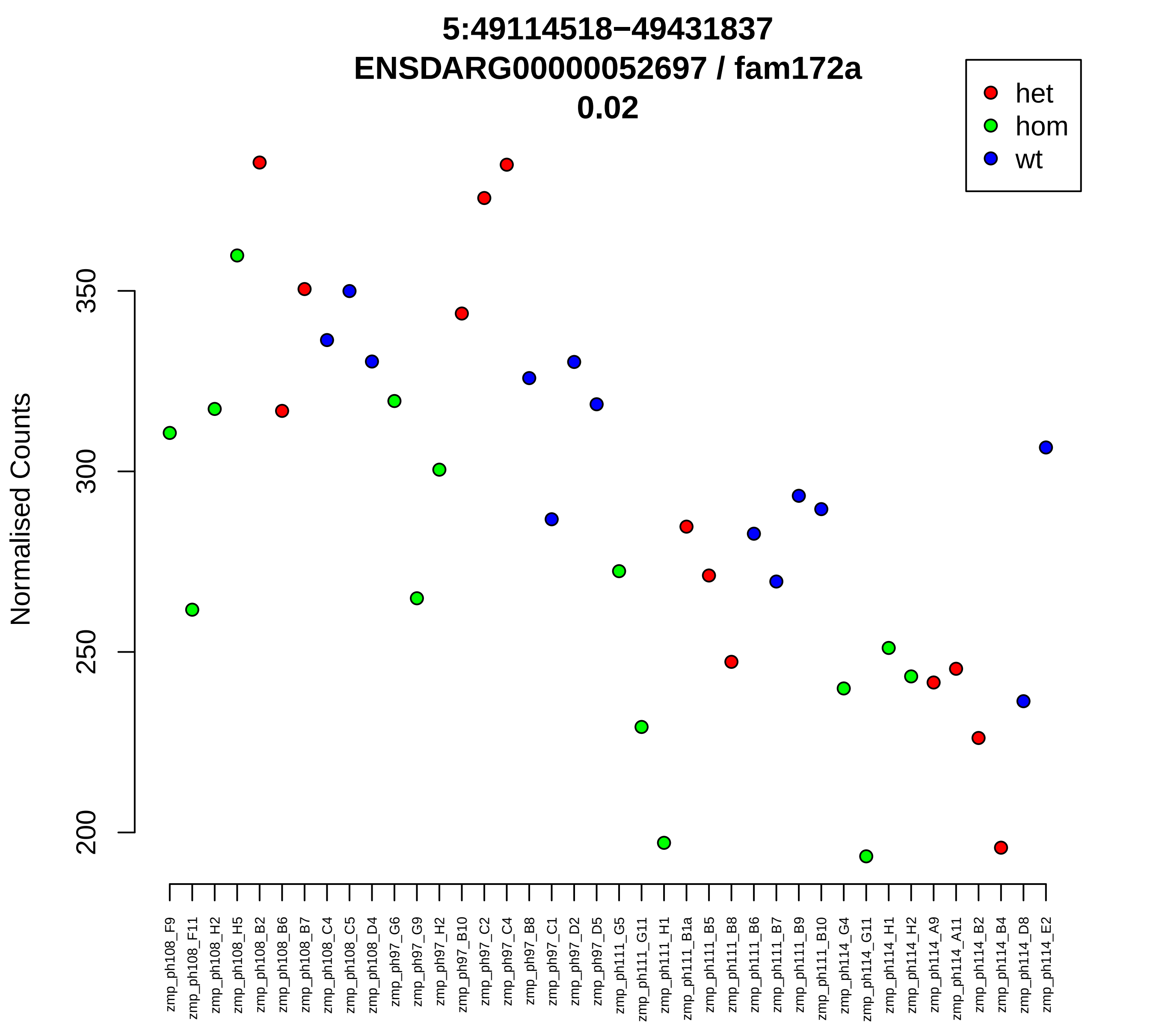
5:49114518−49431837
ENSDARG00000052697 / fam172a
0.02
het
hom
wt
Normalised Counts
200
250
300
350
zmp_ph108_F9
zmp_ph108_F11
zmp_ph108_H2
zmp_ph108_H5
zmp_ph108_B2
zmp_ph108_B6
zmp_ph108_B7
zmp_ph108_C4
zmp_ph108_C5
zmp_ph108_D4
zmp_ph97_G6
zmp_ph97_G9
zmp_ph97_H2
zmp_ph97_B10
zmp_ph97_C2
zmp_ph97_C4
zmp_ph97_B8
zmp_ph97_C1
zmp_ph97_D2
zmp_ph97_D5
zmp_ph111_G5
zmp_ph111_G11
zmp_ph111_H1
zmp_ph111_B1a
zmp_ph111_B5
zmp_ph111_B8
zmp_ph111_B6
zmp_ph111_B7
zmp_ph111_B9
zmp_ph111_B10
zmp_ph114_G4
zmp_ph114_G11
zmp_ph114_H1
zmp_ph114_H2
zmp_ph114_A9
zmp_ph114_A11
zmp_ph114_B2
zmp_ph114_B4
zmp_ph114_D8
zmp_ph114_E2

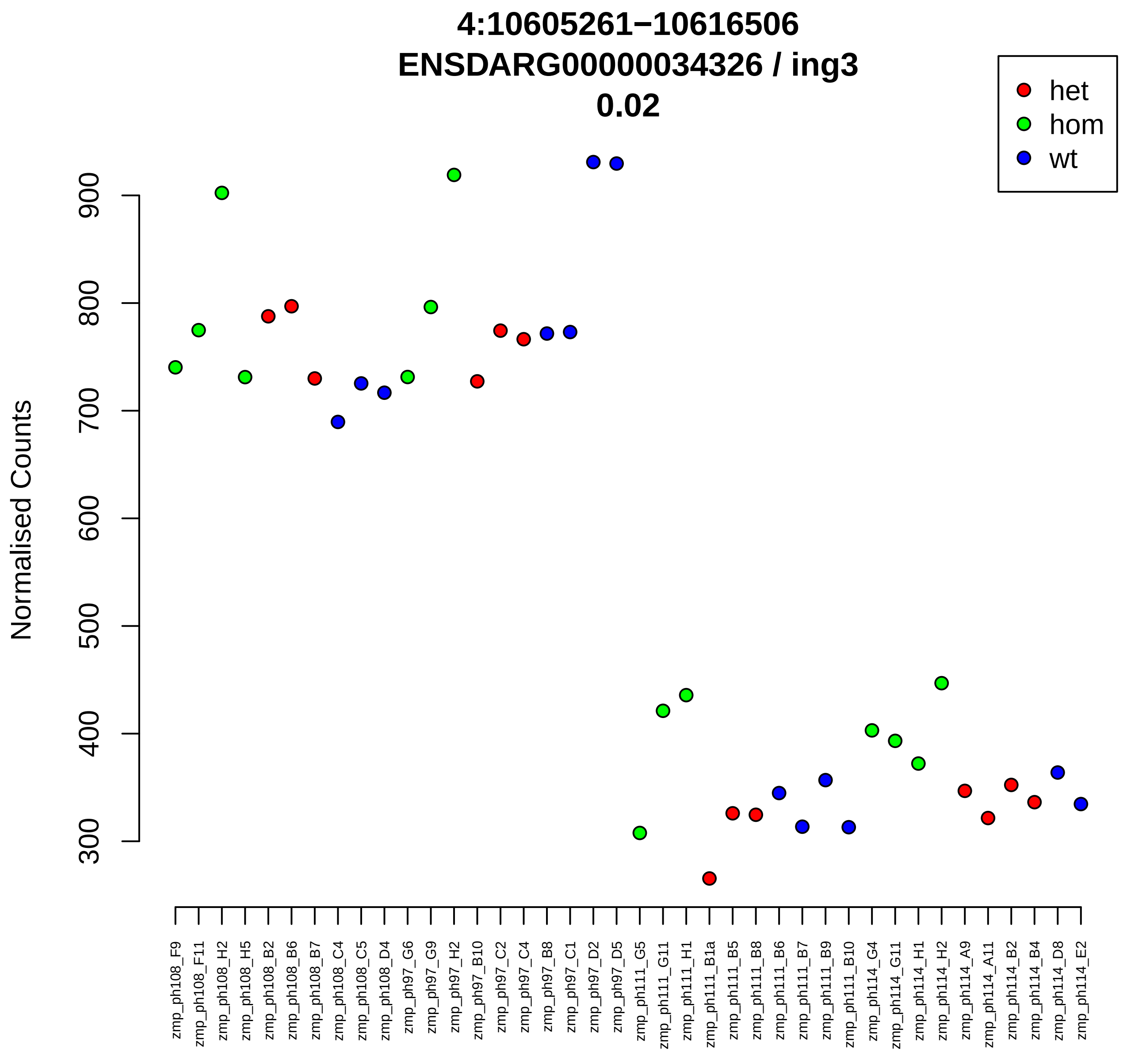

4:10605261−10616506
ENSDARG00000034326 / ing3
0.02
het
hom
wt
Normalised Counts
300
400
500
600
700
800
900
zmp_ph108_F9
zmp_ph108_F11
zmp_ph108_H2
zmp_ph108_H5
zmp_ph108_B2
zmp_ph108_B6
zmp_ph108_B7
zmp_ph108_C4
zmp_ph108_C5
zmp_ph108_D4
zmp_ph97_G6
zmp_ph97_G9
zmp_ph97_H2
zmp_ph97_B10
zmp_ph97_C2
zmp_ph97_C4
zmp_ph97_B8
zmp_ph97_C1
zmp_ph97_D2
zmp_ph97_D5
zmp_ph111_G5
zmp_ph111_G11
zmp_ph111_H1
zmp_ph111_B1a
zmp_ph111_B5
zmp_ph111_B8
zmp_ph111_B6
zmp_ph111_B7
zmp_ph111_B9
zmp_ph111_B10
zmp_ph114_G4
zmp_ph114_G11
zmp_ph114_H1
zmp_ph114_H2
zmp_ph114_A9
zmp_ph114_A11
zmp_ph114_B2
zmp_ph114_B4
zmp_ph114_D8
zmp_ph114_E2

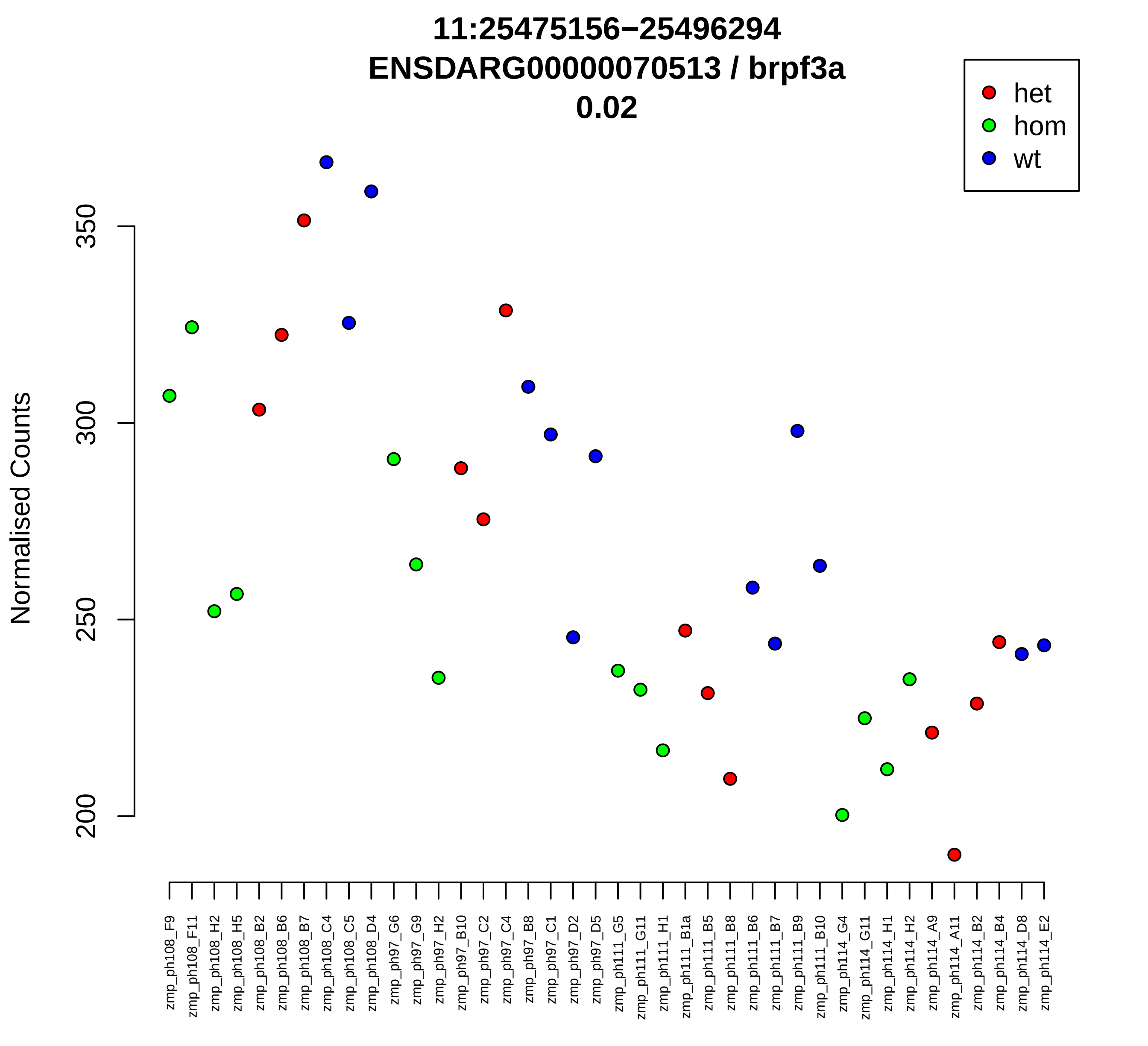

11:25475156−25496294
ENSDARG00000070513 / brpf3a
0.02
het
hom
wt
Normalised Counts
200
250
300
350
zmp_ph108_F9
zmp_ph108_F11
zmp_ph108_H2
zmp_ph108_H5
zmp_ph108_B2
zmp_ph108_B6
zmp_ph108_B7
zmp_ph108_C4
zmp_ph108_C5
zmp_ph108_D4
zmp_ph97_G6
zmp_ph97_G9
zmp_ph97_H2
zmp_ph97_B10
zmp_ph97_C2
zmp_ph97_C4
zmp_ph97_B8
zmp_ph97_C1
zmp_ph97_D2
zmp_ph97_D5
zmp_ph111_G5
zmp_ph111_G11
zmp_ph111_H1
zmp_ph111_B1a
zmp_ph111_B5
zmp_ph111_B8
zmp_ph111_B6
zmp_ph111_B7
zmp_ph111_B9
zmp_ph111_B10
zmp_ph114_G4
zmp_ph114_G11
zmp_ph114_H1
zmp_ph114_H2
zmp_ph114_A9
zmp_ph114_A11
zmp_ph114_B2
zmp_ph114_B4
zmp_ph114_D8
zmp_ph114_E2

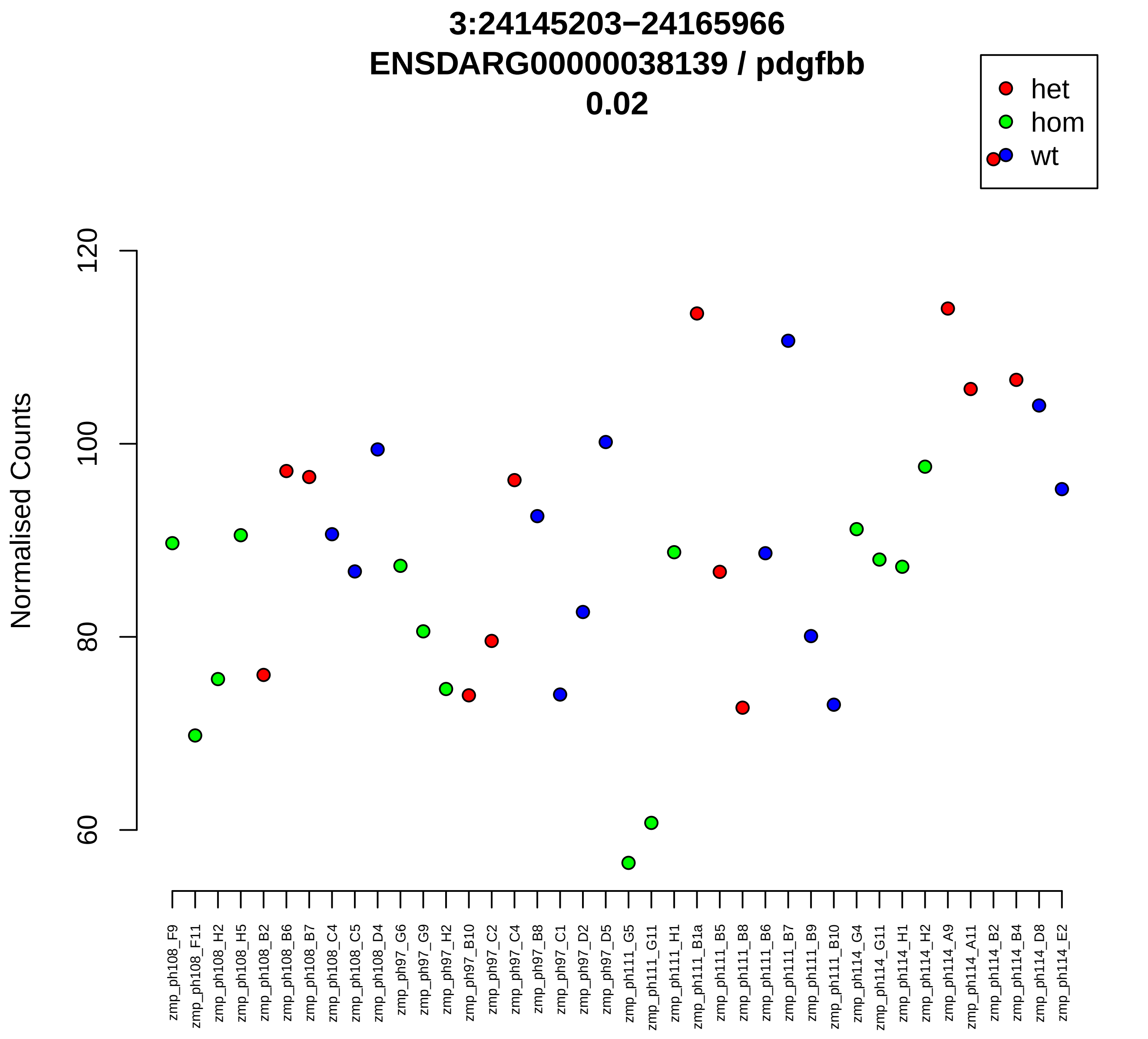
3:24145203−24165966
ENSDARG00000038139 / pdgfbb
0.02
het
hom
wt
Normalised Counts
60
80
100
120
zmp_ph108_F9
zmp_ph108_F11
zmp_ph108_H2
zmp_ph108_H5
zmp_ph108_B2
zmp_ph108_B6
zmp_ph108_B7
zmp_ph108_C4
zmp_ph108_C5
zmp_ph108_D4
zmp_ph97_G6
zmp_ph97_G9
zmp_ph97_H2
zmp_ph97_B10
zmp_ph97_C2
zmp_ph97_C4
zmp_ph97_B8
zmp_ph97_C1
zmp_ph97_D2
zmp_ph97_D5
zmp_ph111_G5
zmp_ph111_G11
zmp_ph111_H1
zmp_ph111_B1a
zmp_ph111_B5
zmp_ph111_B8
zmp_ph111_B6
zmp_ph111_B7
zmp_ph111_B9
zmp_ph111_B10
zmp_ph114_G4
zmp_ph114_G11
zmp_ph114_H1
zmp_ph114_H2
zmp_ph114_A9
zmp_ph114_A11
zmp_ph114_B2
zmp_ph114_B4
zmp_ph114_D8
zmp_ph114_E2

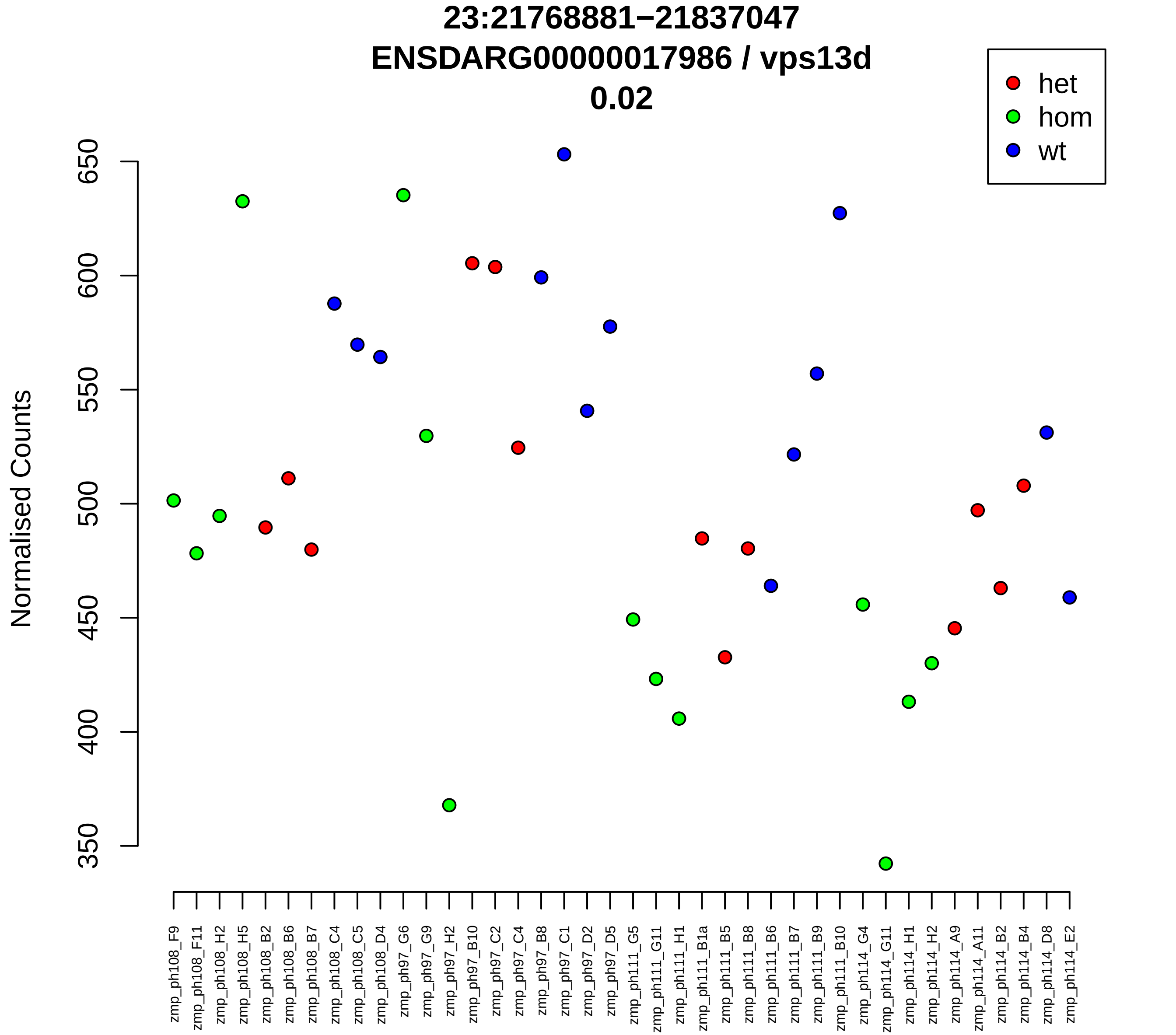
23:21768881−21837047
ENSDARG00000017986 / vps13d
0.02
Normalised Counts
350
400
450
500
550
600
650
het
hom
wt
zmp_ph108_F9
zmp_ph108_F11
zmp_ph108_H2
zmp_ph108_H5
zmp_ph108_B2
zmp_ph108_B6
zmp_ph108_B7
zmp_ph108_C4
zmp_ph108_C5
zmp_ph108_D4
zmp_ph97_G6
zmp_ph97_G9
zmp_ph97_H2
zmp_ph97_B10
zmp_ph97_C2
zmp_ph97_C4
zmp_ph97_B8
zmp_ph97_C1
zmp_ph97_D2
zmp_ph97_D5
zmp_ph111_G5
zmp_ph111_G11
zmp_ph111_H1
zmp_ph111_B1a
zmp_ph111_B5
zmp_ph111_B8
zmp_ph111_B6
zmp_ph111_B7
zmp_ph111_B9
zmp_ph111_B10
zmp_ph114_G4
zmp_ph114_G11
zmp_ph114_H1
zmp_ph114_H2
zmp_ph114_A9
zmp_ph114_A11
zmp_ph114_B2
zmp_ph114_B4
zmp_ph114_D8
zmp_ph114_E2

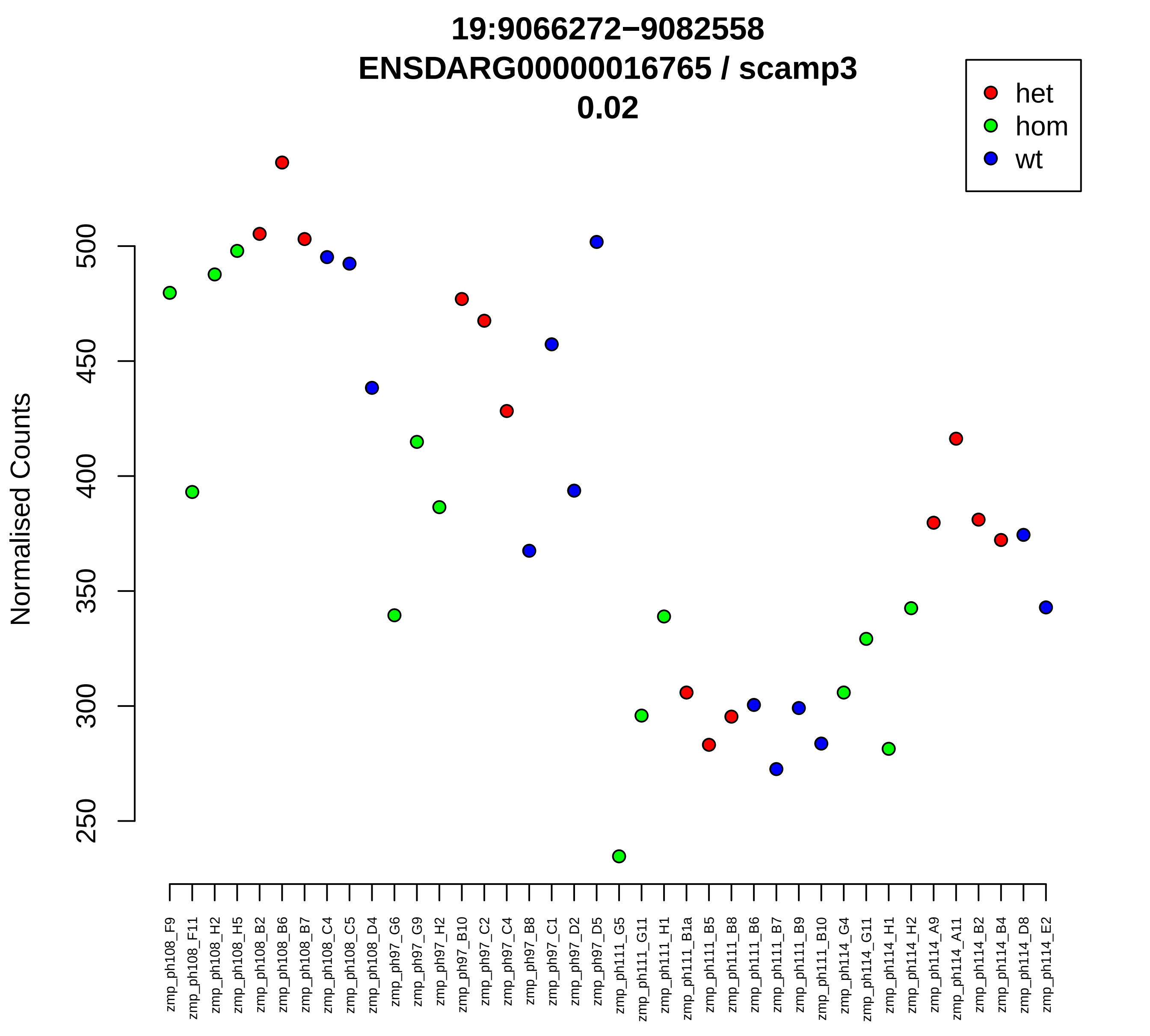
19:9066272−9082558
ENSDARG00000016765 / scamp3
0.02
het
hom
wt
Normalised Counts
250
300
350
400
450
500
zmp_ph108_F9
zmp_ph108_F11
zmp_ph108_H2
zmp_ph108_H5
zmp_ph108_B2
zmp_ph108_B6
zmp_ph108_B7
zmp_ph108_C4
zmp_ph108_C5
zmp_ph108_D4
zmp_ph97_G6
zmp_ph97_G9
zmp_ph97_H2
zmp_ph97_B10
zmp_ph97_C2
zmp_ph97_C4
zmp_ph97_B8
zmp_ph97_C1
zmp_ph97_D2
zmp_ph97_D5
zmp_ph111_G5
zmp_ph111_G11
zmp_ph111_H1
zmp_ph111_B1a
zmp_ph111_B5
zmp_ph111_B8
zmp_ph111_B6
zmp_ph111_B7
zmp_ph111_B9
zmp_ph111_B10
zmp_ph114_G4
zmp_ph114_G11
zmp_ph114_H1
zmp_ph114_H2
zmp_ph114_A9
zmp_ph114_A11
zmp_ph114_B2
zmp_ph114_B4
zmp_ph114_D8
zmp_ph114_E2

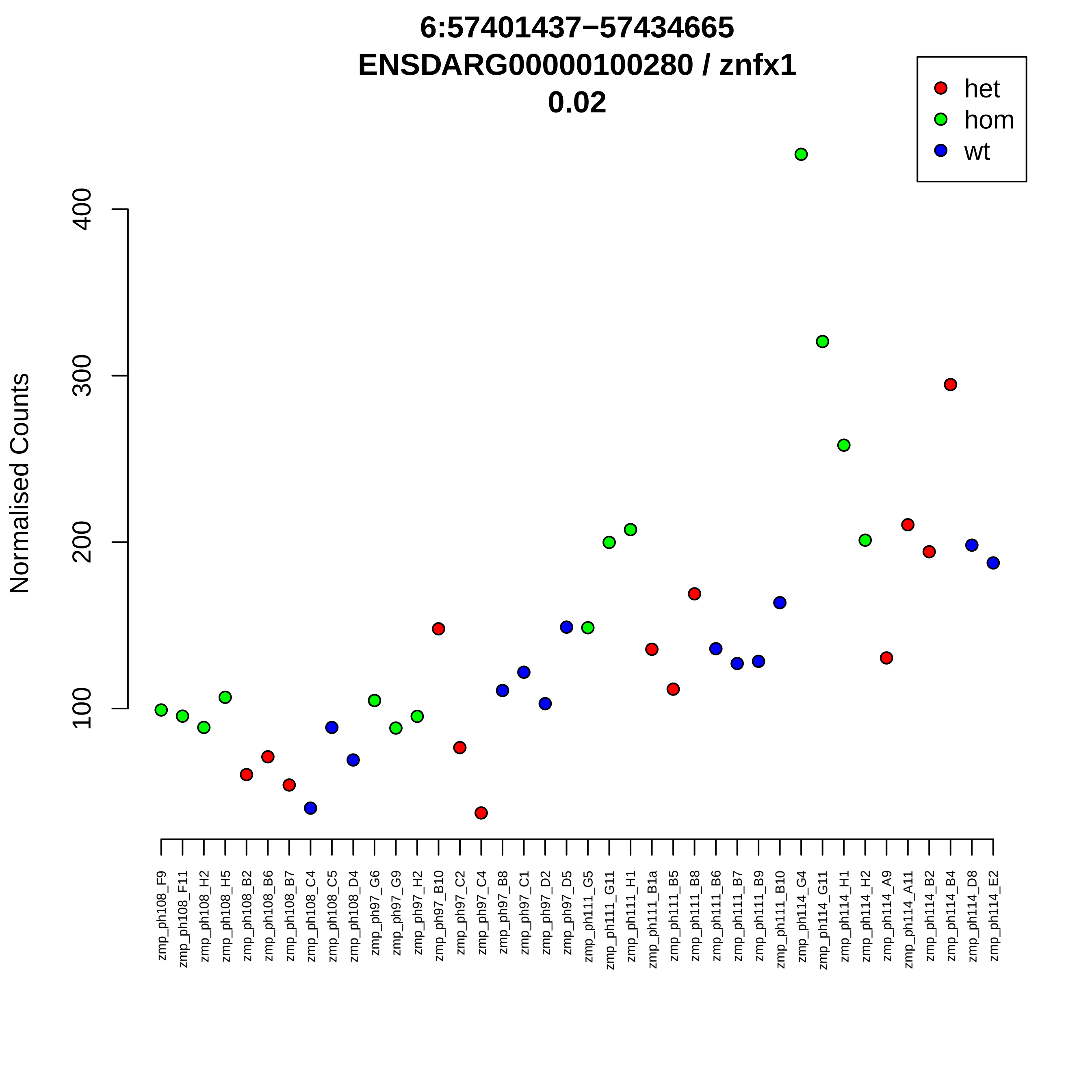
6:57401437−57434665
ENSDARG00000100280 / znfx1
0.02
het
hom
wt
Normalised Counts
100
200
300
400
zmp_ph108_F9
zmp_ph108_F11
zmp_ph108_H2
zmp_ph108_H5
zmp_ph108_B2
zmp_ph108_B6
zmp_ph108_B7
zmp_ph108_C4
zmp_ph108_C5
zmp_ph108_D4
zmp_ph97_G6
zmp_ph97_G9
zmp_ph97_H2
zmp_ph97_B10
zmp_ph97_C2
zmp_ph97_C4
zmp_ph97_B8
zmp_ph97_C1
zmp_ph97_D2
zmp_ph97_D5
zmp_ph111_G5
zmp_ph111_G11
zmp_ph111_H1
zmp_ph111_B1a
zmp_ph111_B5
zmp_ph111_B8
zmp_ph111_B6
zmp_ph111_B7
zmp_ph111_B9
zmp_ph111_B10
zmp_ph114_G4
zmp_ph114_G11
zmp_ph114_H1
zmp_ph114_H2
zmp_ph114_A9
zmp_ph114_A11
zmp_ph114_B2
zmp_ph114_B4
zmp_ph114_D8
zmp_ph114_E2

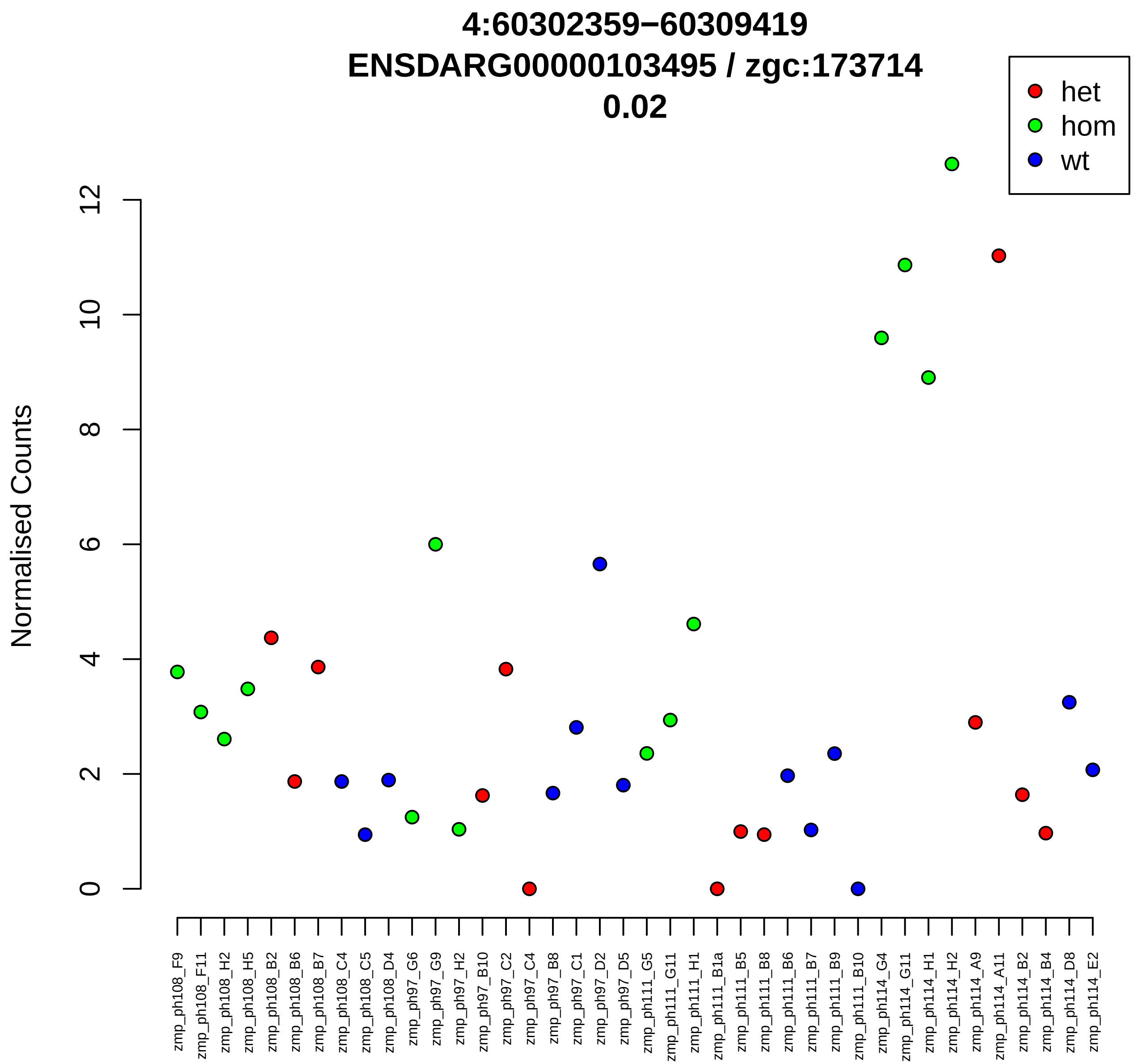
4:60302359−60309419
ENSDARG00000103495 / zgc:173714
0.02
het
hom
wt
Normalised Counts
0
2
4
6
8
10
12
zmp_ph108_F9
zmp_ph108_F11
zmp_ph108_H2
zmp_ph108_H5
zmp_ph108_B2
zmp_ph108_B6
zmp_ph108_B7
zmp_ph108_C4
zmp_ph108_C5
zmp_ph108_D4
zmp_ph97_G6
zmp_ph97_G9
zmp_ph97_H2
zmp_ph97_B10
zmp_ph97_C2
zmp_ph97_C4
zmp_ph97_B8
zmp_ph97_C1
zmp_ph97_D2
zmp_ph97_D5
zmp_ph111_G5
zmp_ph111_G11
zmp_ph111_H1
zmp_ph111_B1a
zmp_ph111_B5
zmp_ph111_B8
zmp_ph111_B6
zmp_ph111_B7
zmp_ph111_B9
zmp_ph111_B10
zmp_ph114_G4
zmp_ph114_G11
zmp_ph114_H1
zmp_ph114_H2
zmp_ph114_A9
zmp_ph114_A11
zmp_ph114_B2
zmp_ph114_B4
zmp_ph114_D8
zmp_ph114_E2

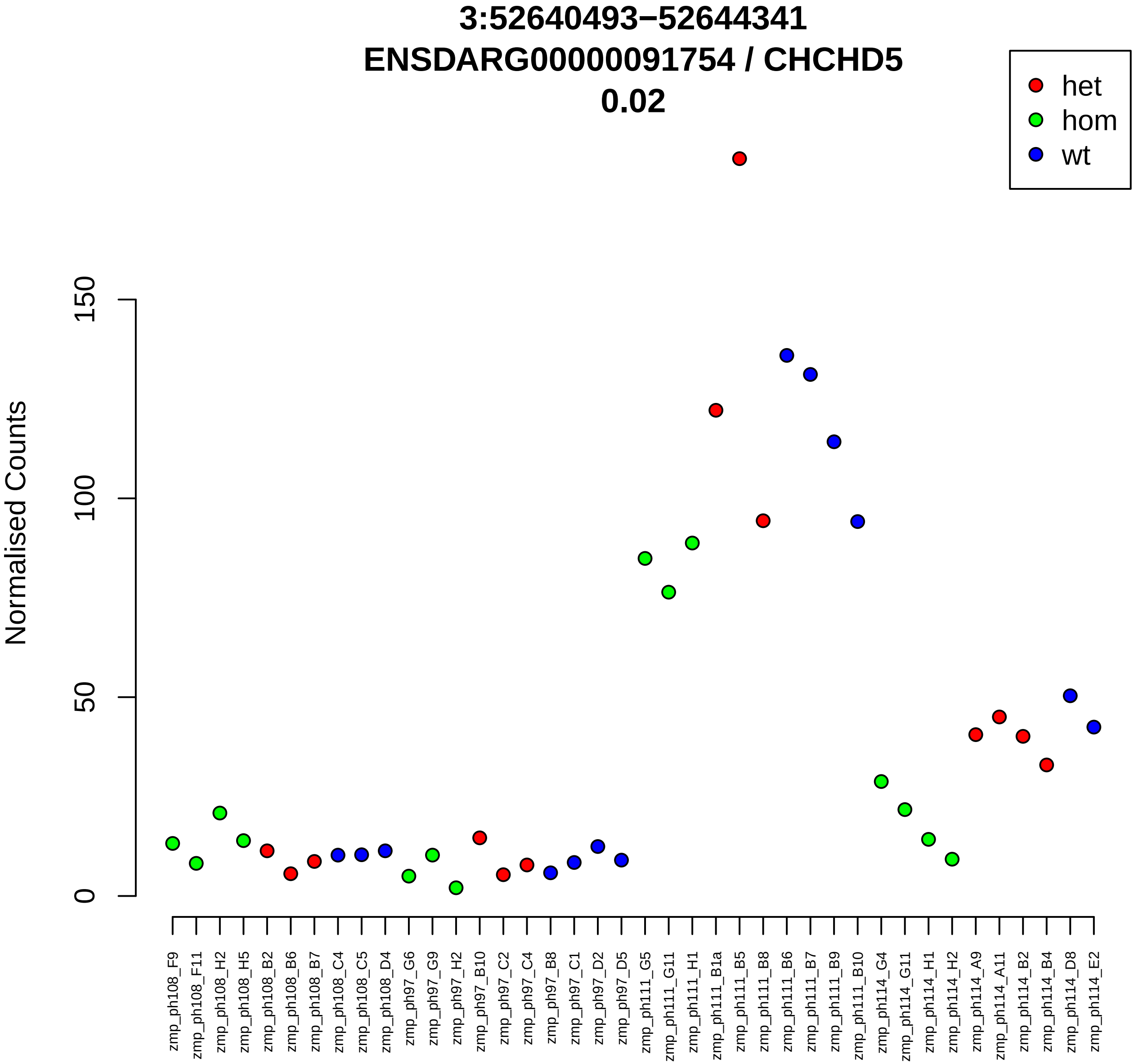

3:52640493−52644341
ENSDARG00000091754 / CHCHD5
0.02
het
hom
wt
Normalised Counts
0
50
100
150
zmp_ph108_F9
zmp_ph108_F11
zmp_ph108_H2
zmp_ph108_H5
zmp_ph108_B2
zmp_ph108_B6
zmp_ph108_B7
zmp_ph108_C4
zmp_ph108_C5
zmp_ph108_D4
zmp_ph97_G6
zmp_ph97_G9
zmp_ph97_H2
zmp_ph97_B10
zmp_ph97_C2
zmp_ph97_C4
zmp_ph97_B8
zmp_ph97_C1
zmp_ph97_D2
zmp_ph97_D5
zmp_ph111_G5
zmp_ph111_G11
zmp_ph111_H1
zmp_ph111_B1a
zmp_ph111_B5
zmp_ph111_B8
zmp_ph111_B6
zmp_ph111_B7
zmp_ph111_B9
zmp_ph111_B10
zmp_ph114_G4
zmp_ph114_G11
zmp_ph114_H1
zmp_ph114_H2
zmp_ph114_A9
zmp_ph114_A11
zmp_ph114_B2
zmp_ph114_B4
zmp_ph114_D8
zmp_ph114_E2

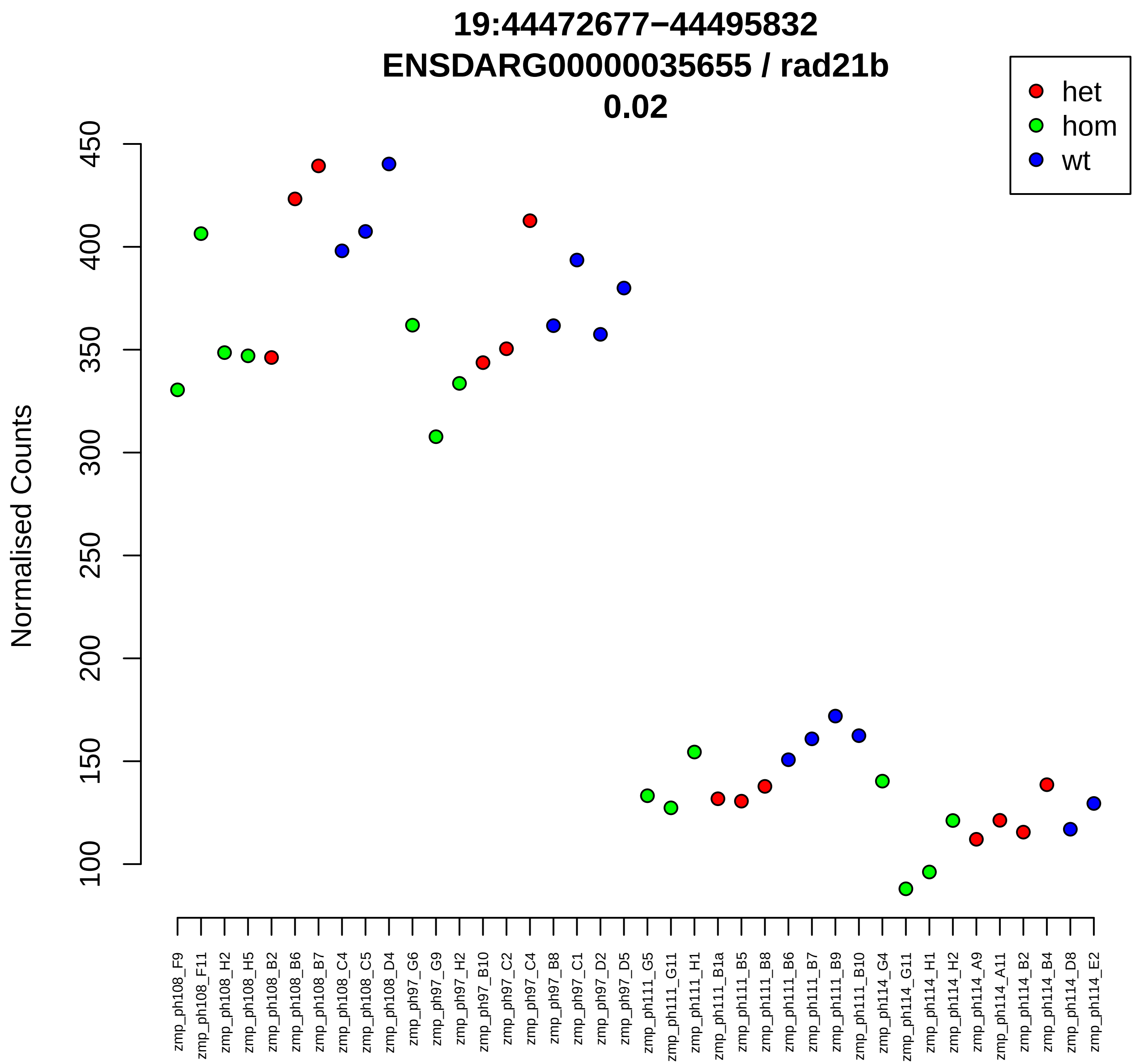

19:44472677−44495832
ENSDARG00000035655 / rad21b
0.02
Normalised Counts
100
150
200
250
300
350
400
450
het
hom
wt
zmp_ph108_F9
zmp_ph108_F11
zmp_ph108_H2
zmp_ph108_H5
zmp_ph108_B2
zmp_ph108_B6
zmp_ph108_B7
zmp_ph108_C4
zmp_ph108_C5
zmp_ph108_D4
zmp_ph97_G6
zmp_ph97_G9
zmp_ph97_H2
zmp_ph97_B10
zmp_ph97_C2
zmp_ph97_C4
zmp_ph97_B8
zmp_ph97_C1
zmp_ph97_D2
zmp_ph97_D5
zmp_ph111_G5
zmp_ph111_G11
zmp_ph111_H1
zmp_ph111_B1a
zmp_ph111_B5
zmp_ph111_B8
zmp_ph111_B6
zmp_ph111_B7
zmp_ph111_B9
zmp_ph111_B10
zmp_ph114_G4
zmp_ph114_G11
zmp_ph114_H1
zmp_ph114_H2
zmp_ph114_A9
zmp_ph114_A11
zmp_ph114_B2
zmp_ph114_B4
zmp_ph114_D8
zmp_ph114_E2

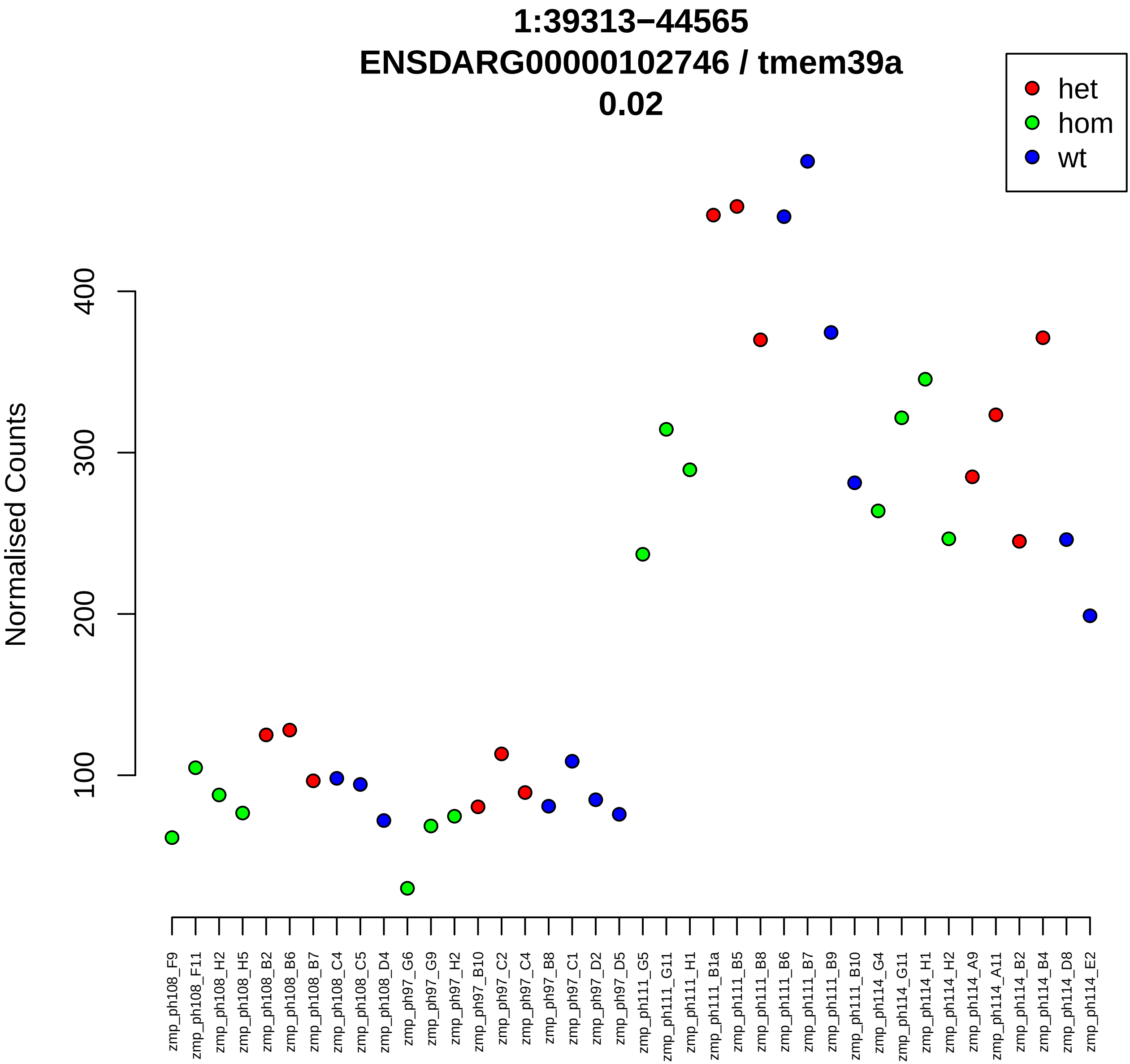
1:39313−44565
ENSDARG00000102746 / tmem39a
0.02
het
hom
wt
Normalised Counts
100
200
300
400
zmp_ph108_F9
zmp_ph108_F11
zmp_ph108_H2
zmp_ph108_H5
zmp_ph108_B2
zmp_ph108_B6
zmp_ph108_B7
zmp_ph108_C4
zmp_ph108_C5
zmp_ph108_D4
zmp_ph97_G6
zmp_ph97_G9
zmp_ph97_H2
zmp_ph97_B10
zmp_ph97_C2
zmp_ph97_C4
zmp_ph97_B8
zmp_ph97_C1
zmp_ph97_D2
zmp_ph97_D5
zmp_ph111_G5
zmp_ph111_G11
zmp_ph111_H1
zmp_ph111_B1a
zmp_ph111_B5
zmp_ph111_B8
zmp_ph111_B6
zmp_ph111_B7
zmp_ph111_B9
zmp_ph111_B10
zmp_ph114_G4
zmp_ph114_G11
zmp_ph114_H1
zmp_ph114_H2
zmp_ph114_A9
zmp_ph114_A11
zmp_ph114_B2
zmp_ph114_B4
zmp_ph114_D8
zmp_ph114_E2

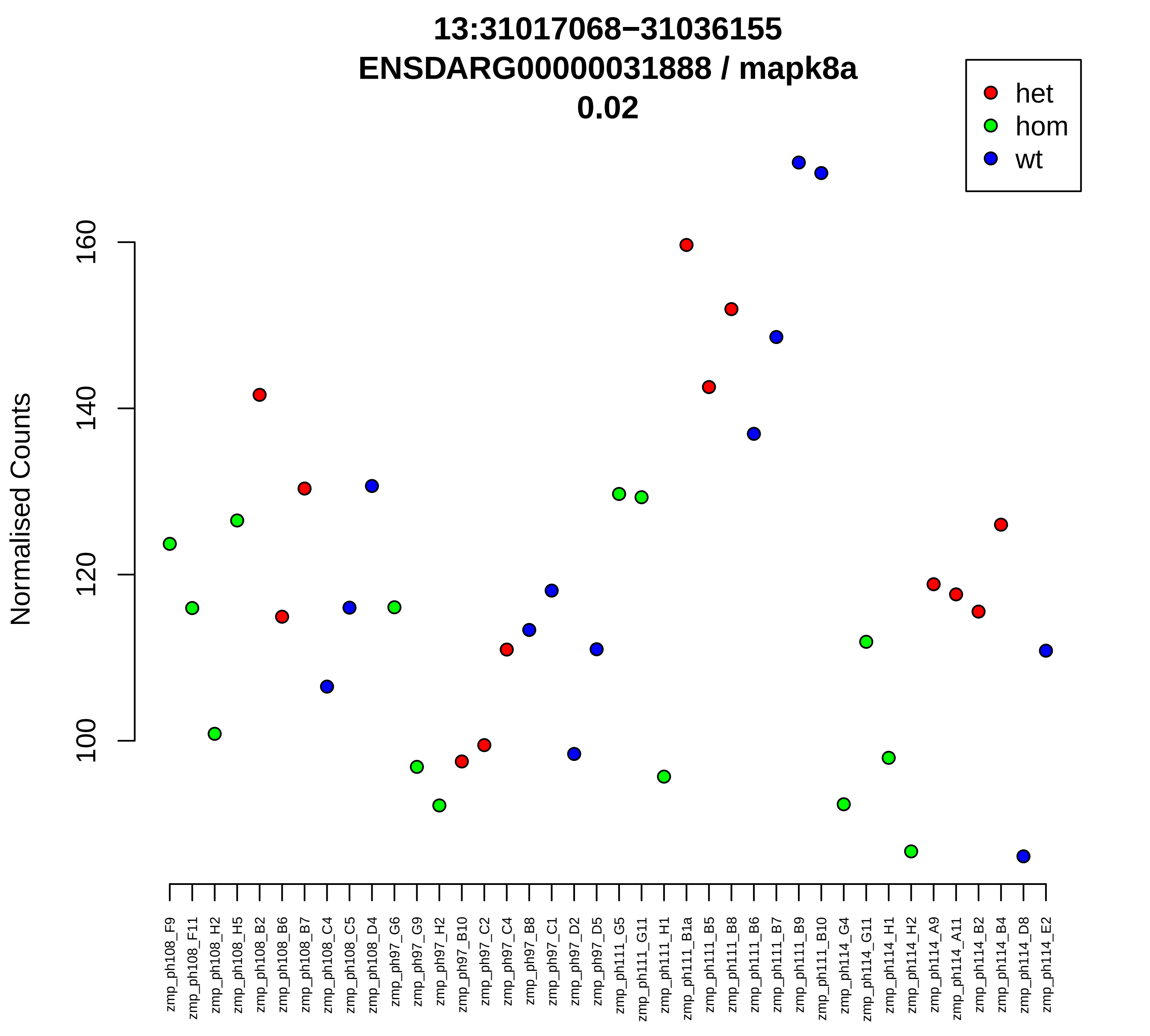
13:31017068−31036155
ENSDARG00000031888 / mapk8a
0.02
het
hom
wt
Normalised Counts
100
120
140
160
zmp_ph108_F9
zmp_ph108_F11
zmp_ph108_H2
zmp_ph108_H5
zmp_ph108_B2
zmp_ph108_B6
zmp_ph108_B7
zmp_ph108_C4
zmp_ph108_C5
zmp_ph108_D4
zmp_ph97_G6
zmp_ph97_G9
zmp_ph97_H2
zmp_ph97_B10
zmp_ph97_C2
zmp_ph97_C4
zmp_ph97_B8
zmp_ph97_C1
zmp_ph97_D2
zmp_ph97_D5
zmp_ph111_G5
zmp_ph111_G11
zmp_ph111_H1
zmp_ph111_B1a
zmp_ph111_B5
zmp_ph111_B8
zmp_ph111_B6
zmp_ph111_B7
zmp_ph111_B9
zmp_ph111_B10
zmp_ph114_G4
zmp_ph114_G11
zmp_ph114_H1
zmp_ph114_H2
zmp_ph114_A9
zmp_ph114_A11
zmp_ph114_B2
zmp_ph114_B4
zmp_ph114_D8
zmp_ph114_E2

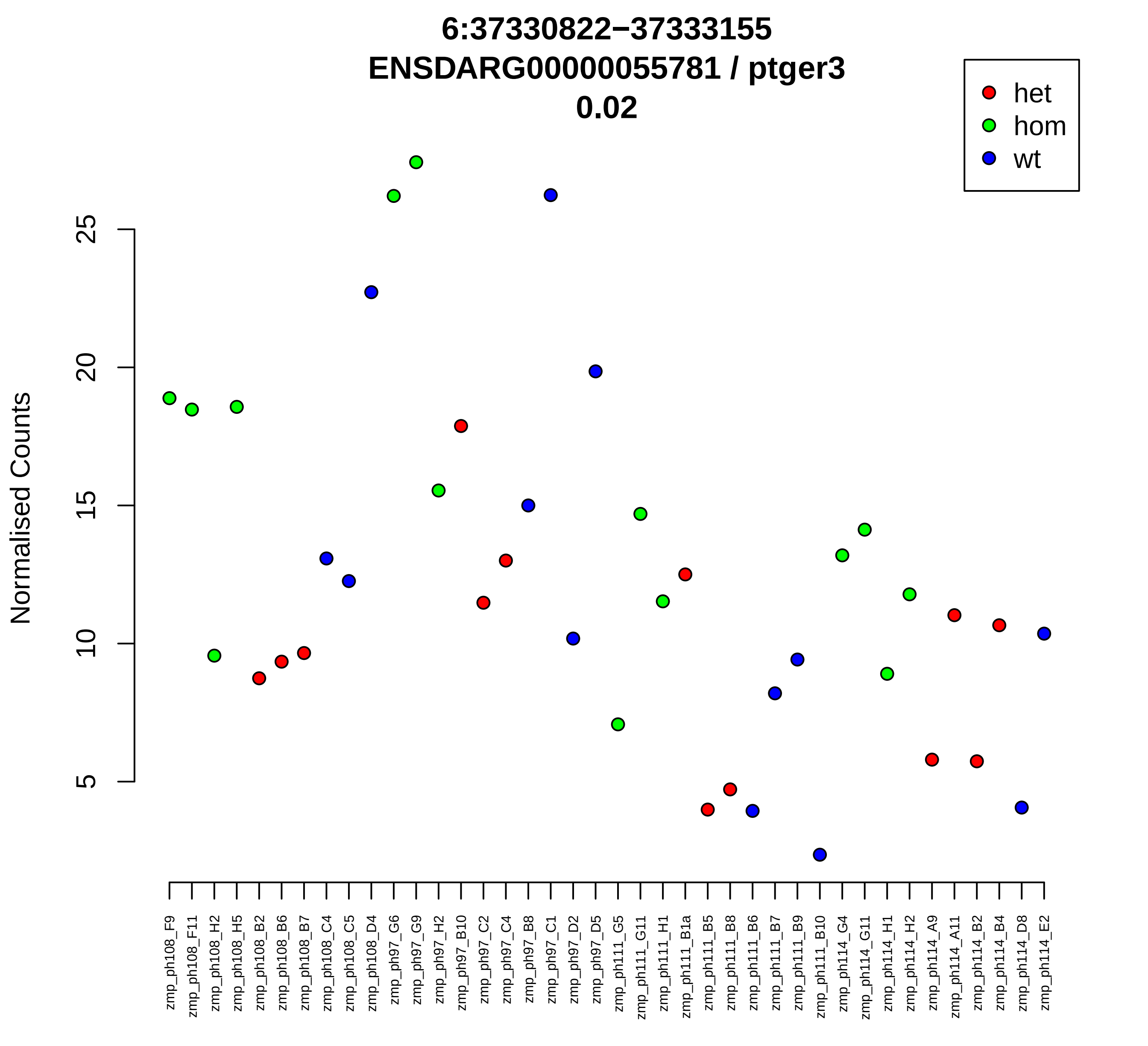

6:37330822−37333155
ENSDARG00000055781 / ptger3
0.02
het
hom
wt
Normalised Counts
5
10
15
20
25
zmp_ph108_F9
zmp_ph108_F11
zmp_ph108_H2
zmp_ph108_H5
zmp_ph108_B2
zmp_ph108_B6
zmp_ph108_B7
zmp_ph108_C4
zmp_ph108_C5
zmp_ph108_D4
zmp_ph97_G6
zmp_ph97_G9
zmp_ph97_H2
zmp_ph97_B10
zmp_ph97_C2
zmp_ph97_C4
zmp_ph97_B8
zmp_ph97_C1
zmp_ph97_D2
zmp_ph97_D5
zmp_ph111_G5
zmp_ph111_G11
zmp_ph111_H1
zmp_ph111_B1a
zmp_ph111_B5
zmp_ph111_B8
zmp_ph111_B6
zmp_ph111_B7
zmp_ph111_B9
zmp_ph111_B10
zmp_ph114_G4
zmp_ph114_G11
zmp_ph114_H1
zmp_ph114_H2
zmp_ph114_A9
zmp_ph114_A11
zmp_ph114_B2
zmp_ph114_B4
zmp_ph114_D8
zmp_ph114_E2

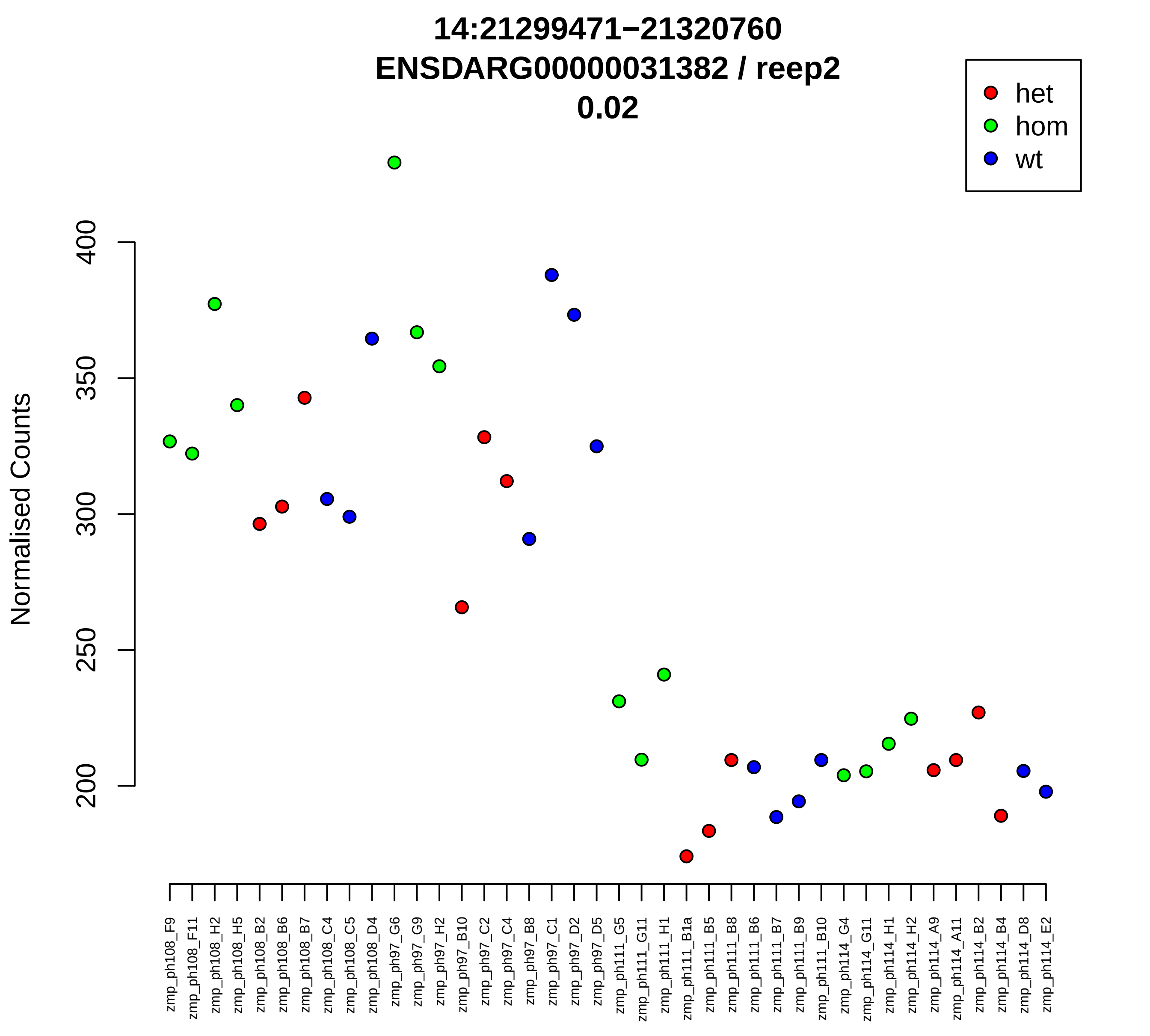

14:21299471−21320760
ENSDARG00000031382 / reep2
0.02
het
hom
wt
Normalised Counts
200
250
300
350
400
zmp_ph108_F9
zmp_ph108_F11
zmp_ph108_H2
zmp_ph108_H5
zmp_ph108_B2
zmp_ph108_B6
zmp_ph108_B7
zmp_ph108_C4
zmp_ph108_C5
zmp_ph108_D4
zmp_ph97_G6
zmp_ph97_G9
zmp_ph97_H2
zmp_ph97_B10
zmp_ph97_C2
zmp_ph97_C4
zmp_ph97_B8
zmp_ph97_C1
zmp_ph97_D2
zmp_ph97_D5
zmp_ph111_G5
zmp_ph111_G11
zmp_ph111_H1
zmp_ph111_B1a
zmp_ph111_B5
zmp_ph111_B8
zmp_ph111_B6
zmp_ph111_B7
zmp_ph111_B9
zmp_ph111_B10
zmp_ph114_G4
zmp_ph114_G11
zmp_ph114_H1
zmp_ph114_H2
zmp_ph114_A9
zmp_ph114_A11
zmp_ph114_B2
zmp_ph114_B4
zmp_ph114_D8
zmp_ph114_E2

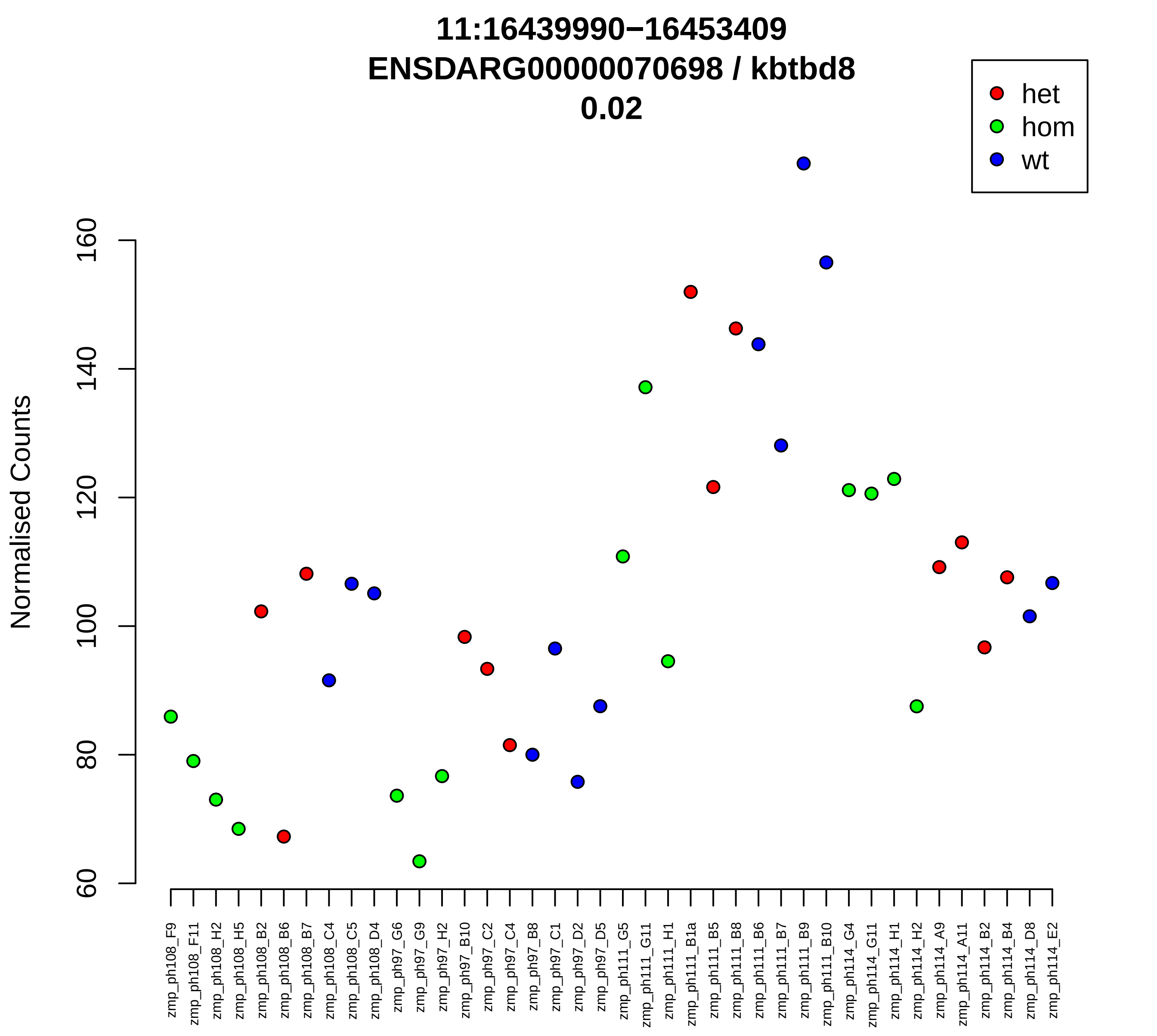

11:16439990−16453409
ENSDARG00000070698 / kbtbd8
0.02
het
hom
wt
Normalised Counts
60
80
100
120
140
160
zmp_ph108_F9
zmp_ph108_F11
zmp_ph108_H2
zmp_ph108_H5
zmp_ph108_B2
zmp_ph108_B6
zmp_ph108_B7
zmp_ph108_C4
zmp_ph108_C5
zmp_ph108_D4
zmp_ph97_G6
zmp_ph97_G9
zmp_ph97_H2
zmp_ph97_B10
zmp_ph97_C2
zmp_ph97_C4
zmp_ph97_B8
zmp_ph97_C1
zmp_ph97_D2
zmp_ph97_D5
zmp_ph111_G5
zmp_ph111_G11
zmp_ph111_H1
zmp_ph111_B1a
zmp_ph111_B5
zmp_ph111_B8
zmp_ph111_B6
zmp_ph111_B7
zmp_ph111_B9
zmp_ph111_B10
zmp_ph114_G4
zmp_ph114_G11
zmp_ph114_H1
zmp_ph114_H2
zmp_ph114_A9
zmp_ph114_A11
zmp_ph114_B2
zmp_ph114_B4
zmp_ph114_D8
zmp_ph114_E2

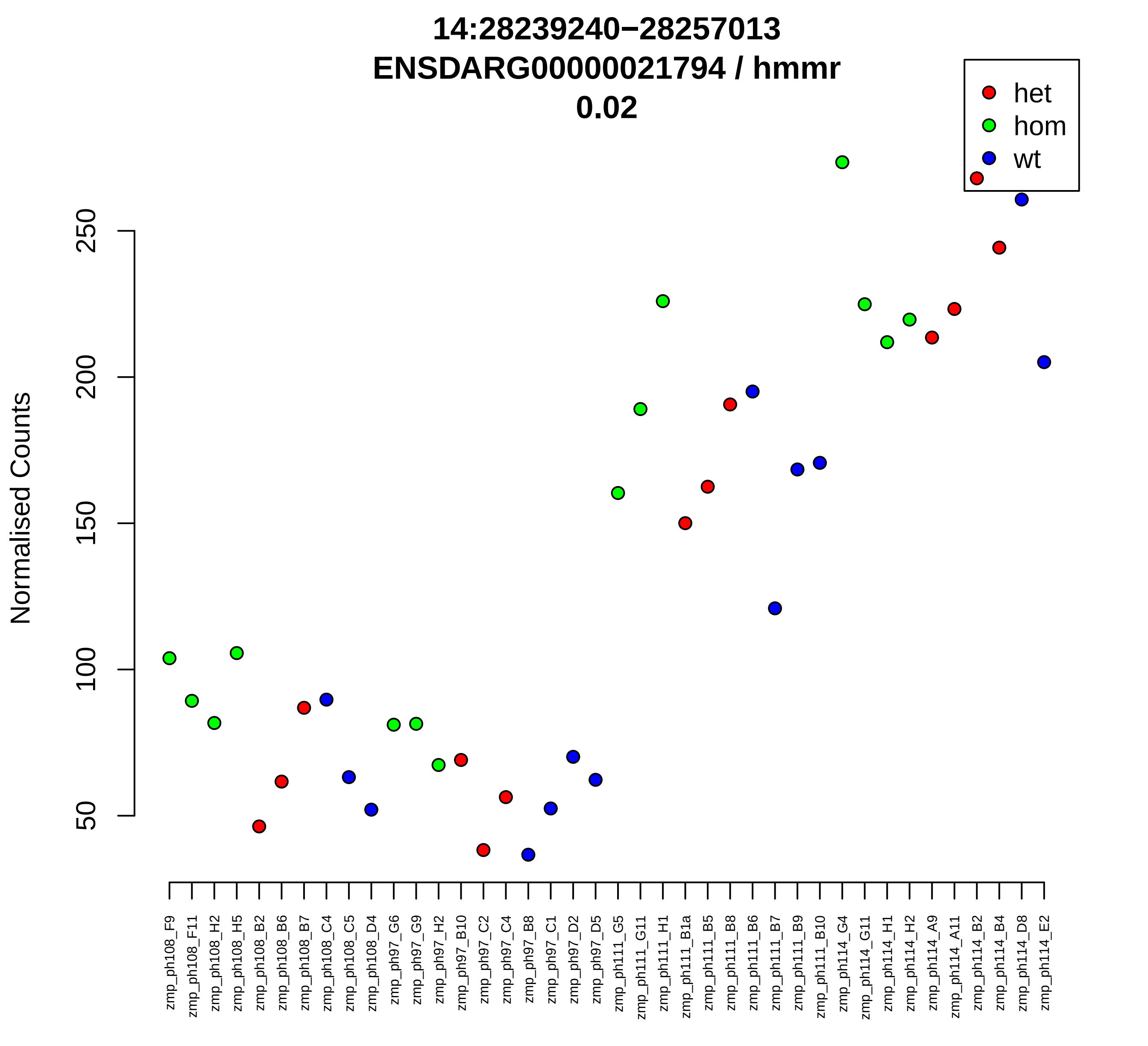
14:28239240−28257013
ENSDARG00000021794 / hmmr
0.02
het
hom
wt
Normalised Counts
50
100
150
200
250
zmp_ph108_F9
zmp_ph108_F11
zmp_ph108_H2
zmp_ph108_H5
zmp_ph108_B2
zmp_ph108_B6
zmp_ph108_B7
zmp_ph108_C4
zmp_ph108_C5
zmp_ph108_D4
zmp_ph97_G6
zmp_ph97_G9
zmp_ph97_H2
zmp_ph97_B10
zmp_ph97_C2
zmp_ph97_C4
zmp_ph97_B8
zmp_ph97_C1
zmp_ph97_D2
zmp_ph97_D5
zmp_ph111_G5
zmp_ph111_G11
zmp_ph111_H1
zmp_ph111_B1a
zmp_ph111_B5
zmp_ph111_B8
zmp_ph111_B6
zmp_ph111_B7
zmp_ph111_B9
zmp_ph111_B10
zmp_ph114_G4
zmp_ph114_G11
zmp_ph114_H1
zmp_ph114_H2
zmp_ph114_A9
zmp_ph114_A11
zmp_ph114_B2
zmp_ph114_B4
zmp_ph114_D8
zmp_ph114_E2

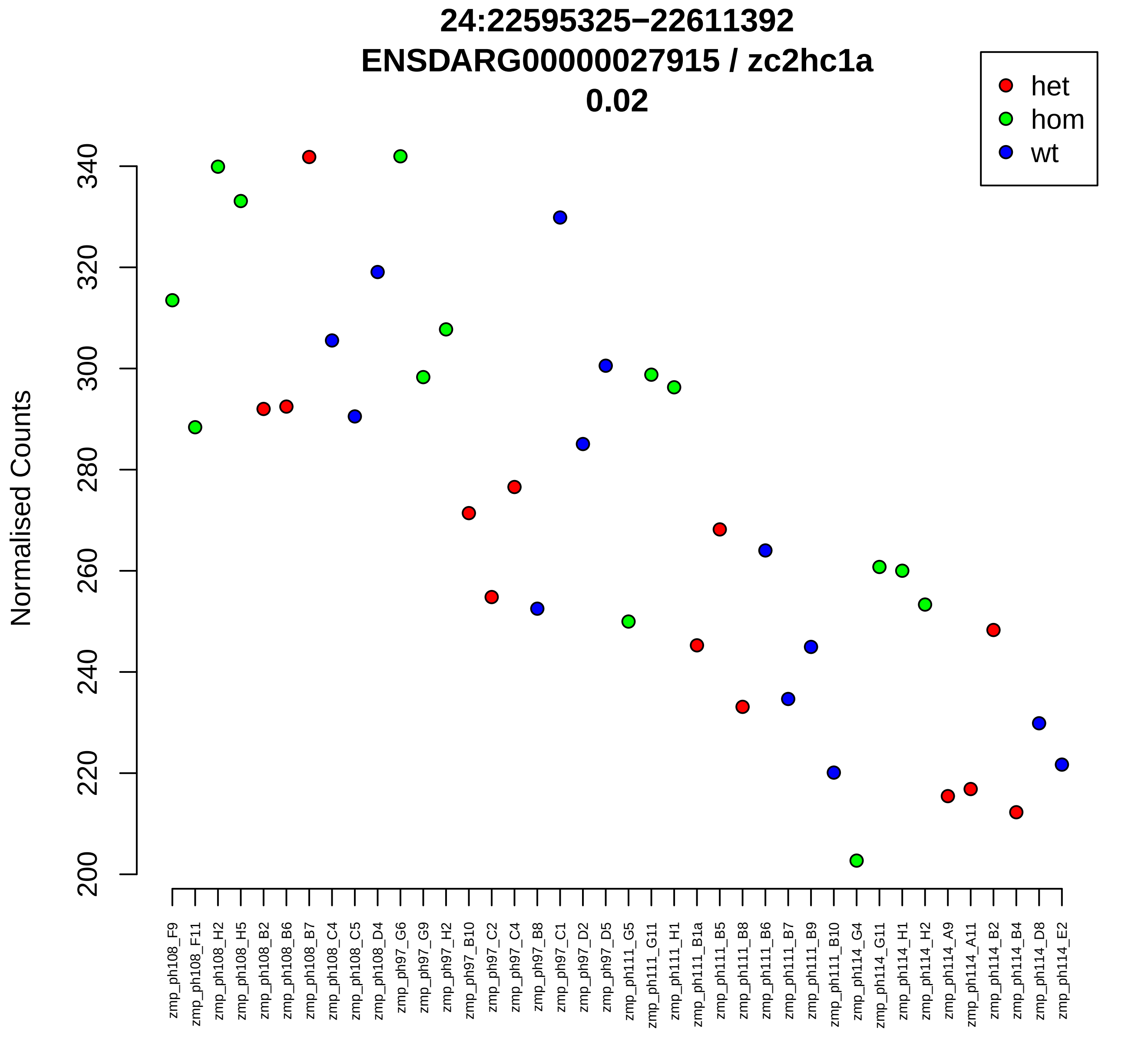

24:22595325−22611392
ENSDARG00000027915 / zc2hc1a
0.02
het
hom
wt
Normalised Counts
200
220
240
260
280
300
320
340
zmp_ph108_F9
zmp_ph108_F11
zmp_ph108_H2
zmp_ph108_H5
zmp_ph108_B2
zmp_ph108_B6
zmp_ph108_B7
zmp_ph108_C4
zmp_ph108_C5
zmp_ph108_D4
zmp_ph97_G6
zmp_ph97_G9
zmp_ph97_H2
zmp_ph97_B10
zmp_ph97_C2
zmp_ph97_C4
zmp_ph97_B8
zmp_ph97_C1
zmp_ph97_D2
zmp_ph97_D5
zmp_ph111_G5
zmp_ph111_G11
zmp_ph111_H1
zmp_ph111_B1a
zmp_ph111_B5
zmp_ph111_B8
zmp_ph111_B6
zmp_ph111_B7
zmp_ph111_B9
zmp_ph111_B10
zmp_ph114_G4
zmp_ph114_G11
zmp_ph114_H1
zmp_ph114_H2
zmp_ph114_A9
zmp_ph114_A11
zmp_ph114_B2
zmp_ph114_B4
zmp_ph114_D8
zmp_ph114_E2

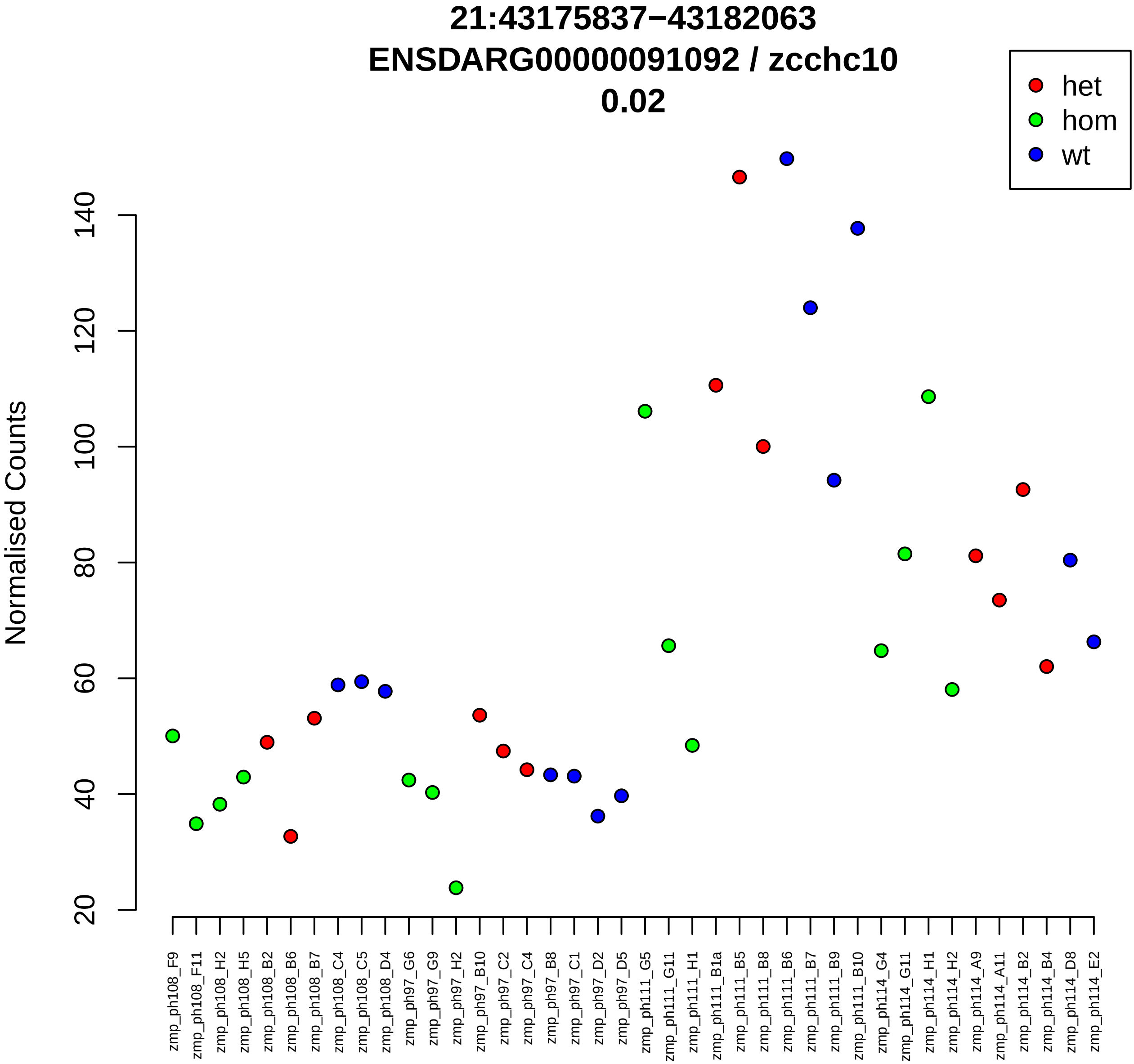

21:43175837−43182063
ENSDARG00000091092 / zcchc10
0.02
het
hom
wt
Normalised Counts
20
40
60
80
100
120
140
zmp_ph108_F9
zmp_ph108_F11
zmp_ph108_H2
zmp_ph108_H5
zmp_ph108_B2
zmp_ph108_B6
zmp_ph108_B7
zmp_ph108_C4
zmp_ph108_C5
zmp_ph108_D4
zmp_ph97_G6
zmp_ph97_G9
zmp_ph97_H2
zmp_ph97_B10
zmp_ph97_C2
zmp_ph97_C4
zmp_ph97_B8
zmp_ph97_C1
zmp_ph97_D2
zmp_ph97_D5
zmp_ph111_G5
zmp_ph111_G11
zmp_ph111_H1
zmp_ph111_B1a
zmp_ph111_B5
zmp_ph111_B8
zmp_ph111_B6
zmp_ph111_B7
zmp_ph111_B9
zmp_ph111_B10
zmp_ph114_G4
zmp_ph114_G11
zmp_ph114_H1
zmp_ph114_H2
zmp_ph114_A9
zmp_ph114_A11
zmp_ph114_B2
zmp_ph114_B4
zmp_ph114_D8
zmp_ph114_E2

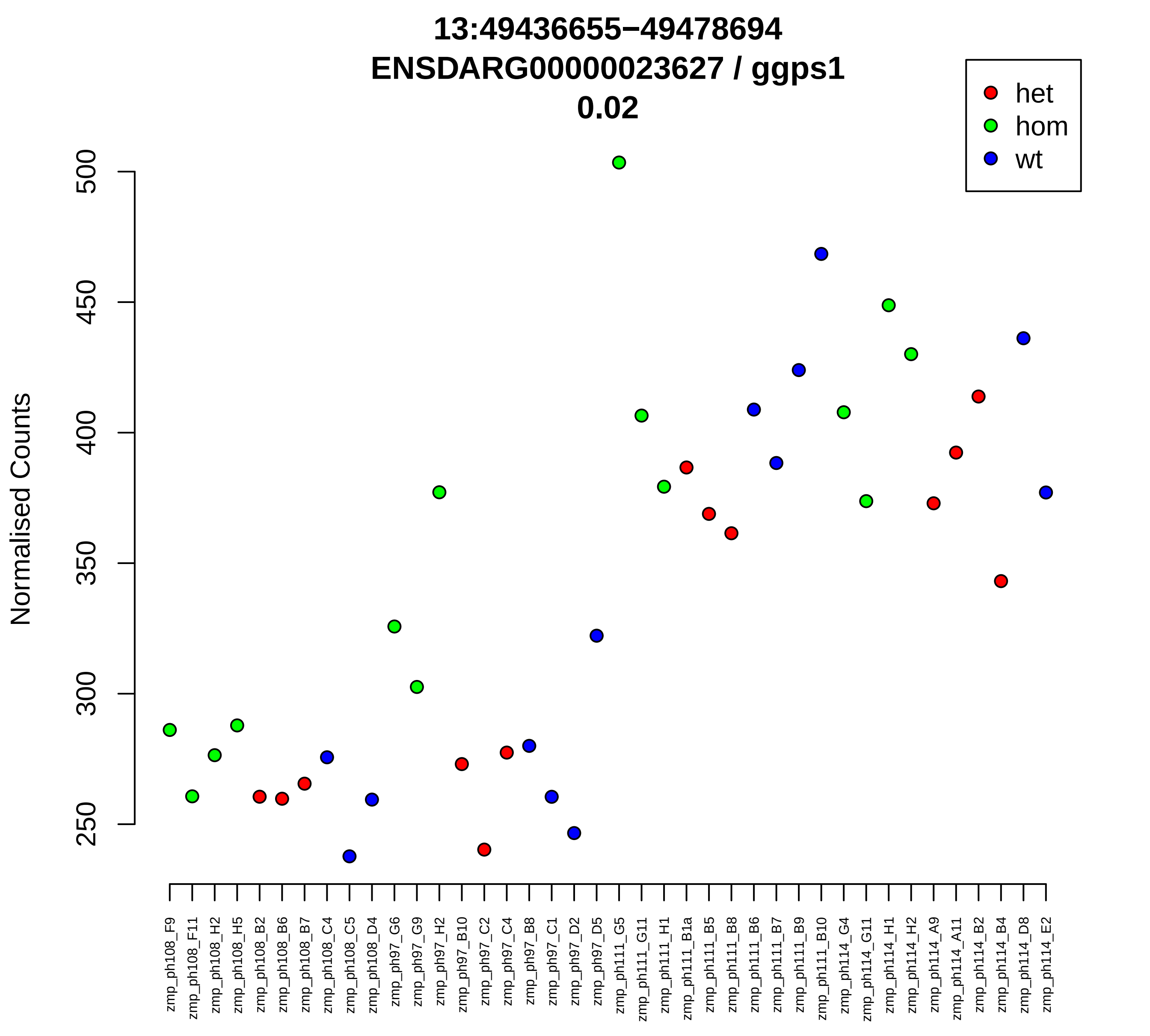

13:49436655−49478694
ENSDARG00000023627 / ggps1
0.02
het
hom
wt
Normalised Counts
250
300
350
400
450
500
zmp_ph108_F9
zmp_ph108_F11
zmp_ph108_H2
zmp_ph108_H5
zmp_ph108_B2
zmp_ph108_B6
zmp_ph108_B7
zmp_ph108_C4
zmp_ph108_C5
zmp_ph108_D4
zmp_ph97_G6
zmp_ph97_G9
zmp_ph97_H2
zmp_ph97_B10
zmp_ph97_C2
zmp_ph97_C4
zmp_ph97_B8
zmp_ph97_C1
zmp_ph97_D2
zmp_ph97_D5
zmp_ph111_G5
zmp_ph111_G11
zmp_ph111_H1
zmp_ph111_B1a
zmp_ph111_B5
zmp_ph111_B8
zmp_ph111_B6
zmp_ph111_B7
zmp_ph111_B9
zmp_ph111_B10
zmp_ph114_G4
zmp_ph114_G11
zmp_ph114_H1
zmp_ph114_H2
zmp_ph114_A9
zmp_ph114_A11
zmp_ph114_B2
zmp_ph114_B4
zmp_ph114_D8
zmp_ph114_E2

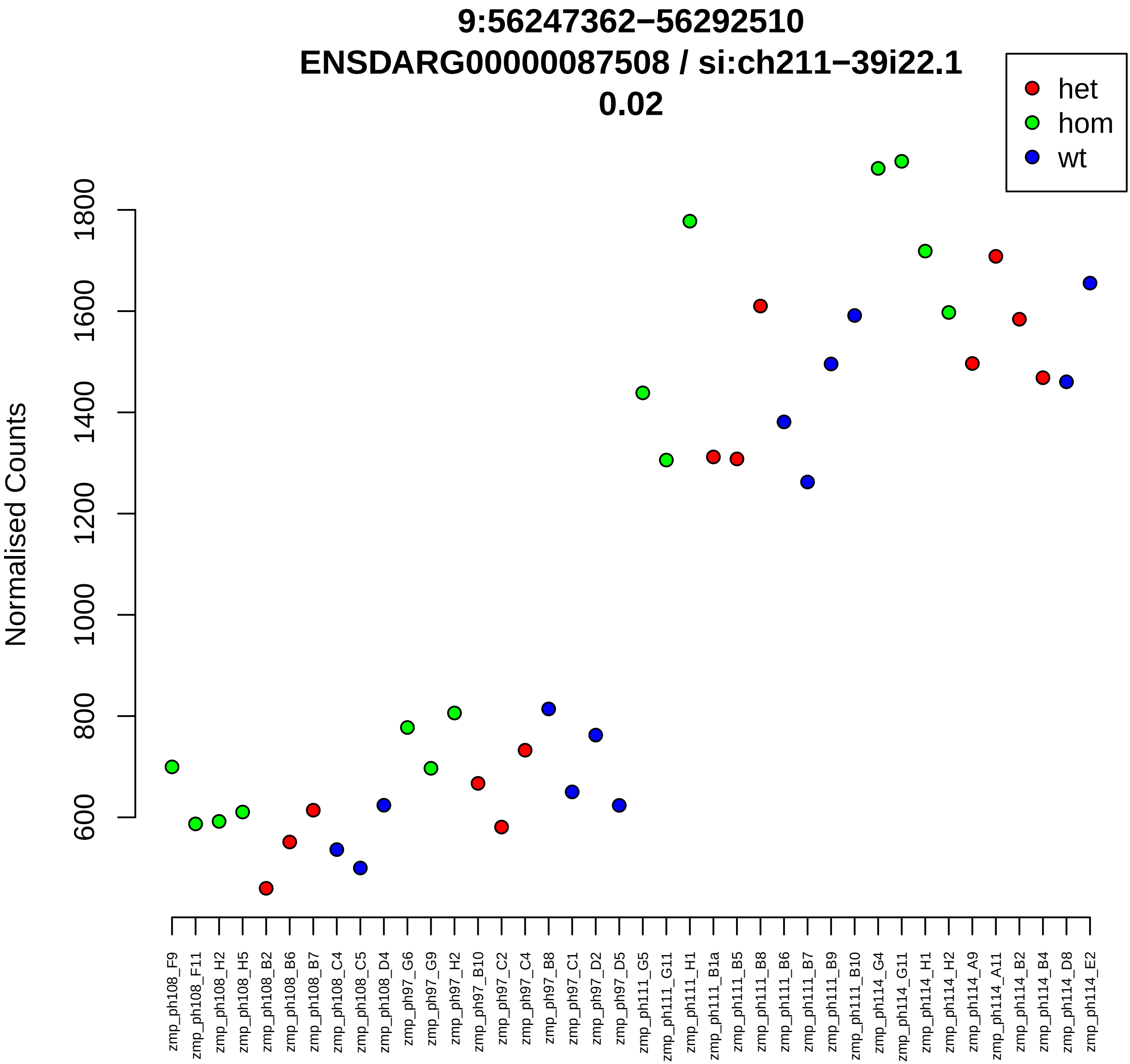
9:56247362−56292510
ENSDARG00000087508 / si:ch211−39i22.1
0.02
het
hom
wt
Normalised Counts
600
800
1000
1200
1400
1600
1800
zmp_ph108_F9
zmp_ph108_F11
zmp_ph108_H2
zmp_ph108_H5
zmp_ph108_B2
zmp_ph108_B6
zmp_ph108_B7
zmp_ph108_C4
zmp_ph108_C5
zmp_ph108_D4
zmp_ph97_G6
zmp_ph97_G9
zmp_ph97_H2
zmp_ph97_B10
zmp_ph97_C2
zmp_ph97_C4
zmp_ph97_B8
zmp_ph97_C1
zmp_ph97_D2
zmp_ph97_D5
zmp_ph111_G5
zmp_ph111_G11
zmp_ph111_H1
zmp_ph111_B1a
zmp_ph111_B5
zmp_ph111_B8
zmp_ph111_B6
zmp_ph111_B7
zmp_ph111_B9
zmp_ph111_B10
zmp_ph114_G4
zmp_ph114_G11
zmp_ph114_H1
zmp_ph114_H2
zmp_ph114_A9
zmp_ph114_A11
zmp_ph114_B2
zmp_ph114_B4
zmp_ph114_D8
zmp_ph114_E2

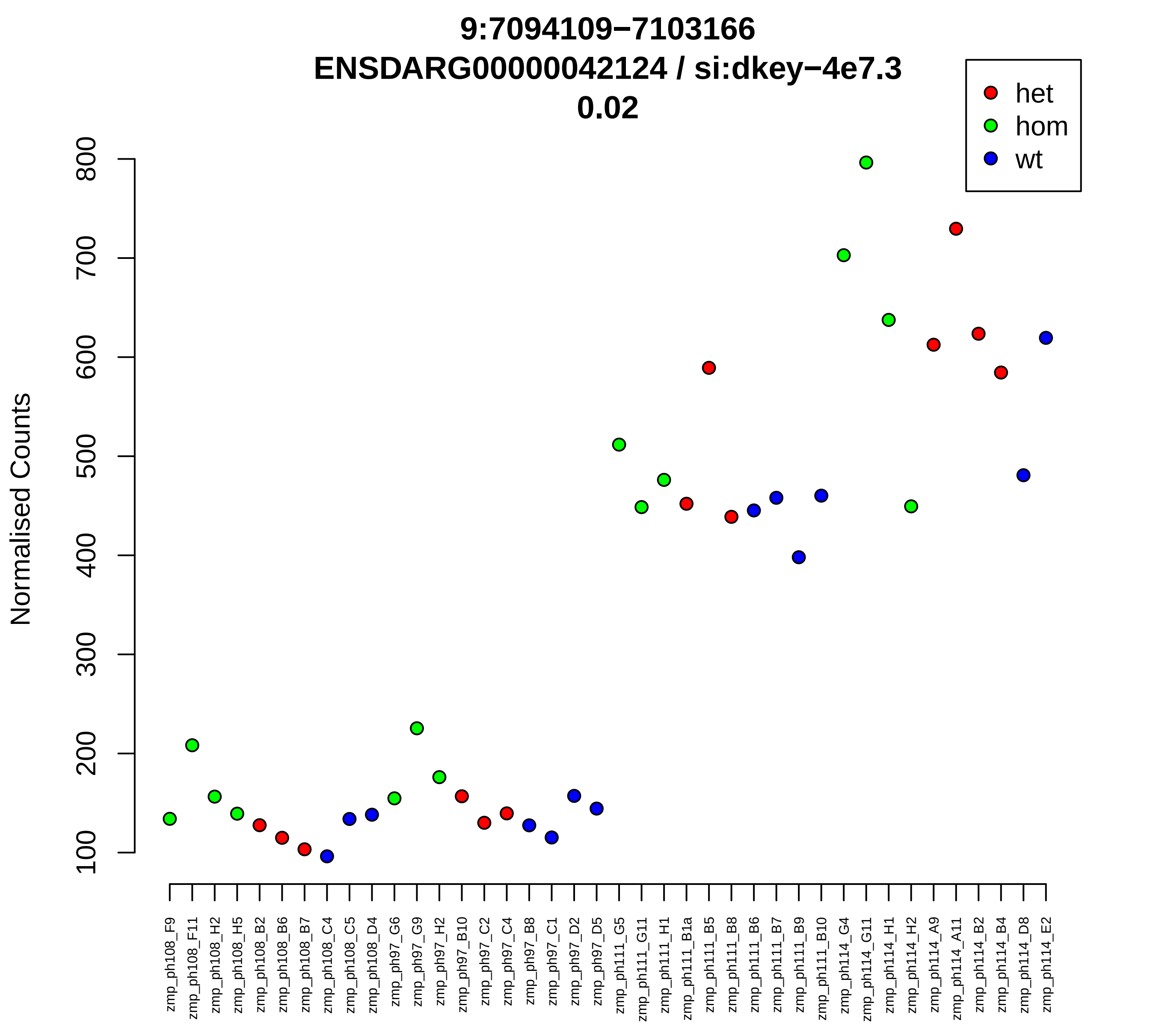

9:7094109−7103166
ENSDARG00000042124 / si:dkey−4e7.3
0.02
het
hom
wt
Normalised Counts
100
200
300
400
500
600
700
800
zmp_ph108_F9
zmp_ph108_F11
zmp_ph108_H2
zmp_ph108_H5
zmp_ph108_B2
zmp_ph108_B6
zmp_ph108_B7
zmp_ph108_C4
zmp_ph108_C5
zmp_ph108_D4
zmp_ph97_G6
zmp_ph97_G9
zmp_ph97_H2
zmp_ph97_B10
zmp_ph97_C2
zmp_ph97_C4
zmp_ph97_B8
zmp_ph97_C1
zmp_ph97_D2
zmp_ph97_D5
zmp_ph111_G5
zmp_ph111_G11
zmp_ph111_H1
zmp_ph111_B1a
zmp_ph111_B5
zmp_ph111_B8
zmp_ph111_B6
zmp_ph111_B7
zmp_ph111_B9
zmp_ph111_B10
zmp_ph114_G4
zmp_ph114_G11
zmp_ph114_H1
zmp_ph114_H2
zmp_ph114_A9
zmp_ph114_A11
zmp_ph114_B2
zmp_ph114_B4
zmp_ph114_D8
zmp_ph114_E2

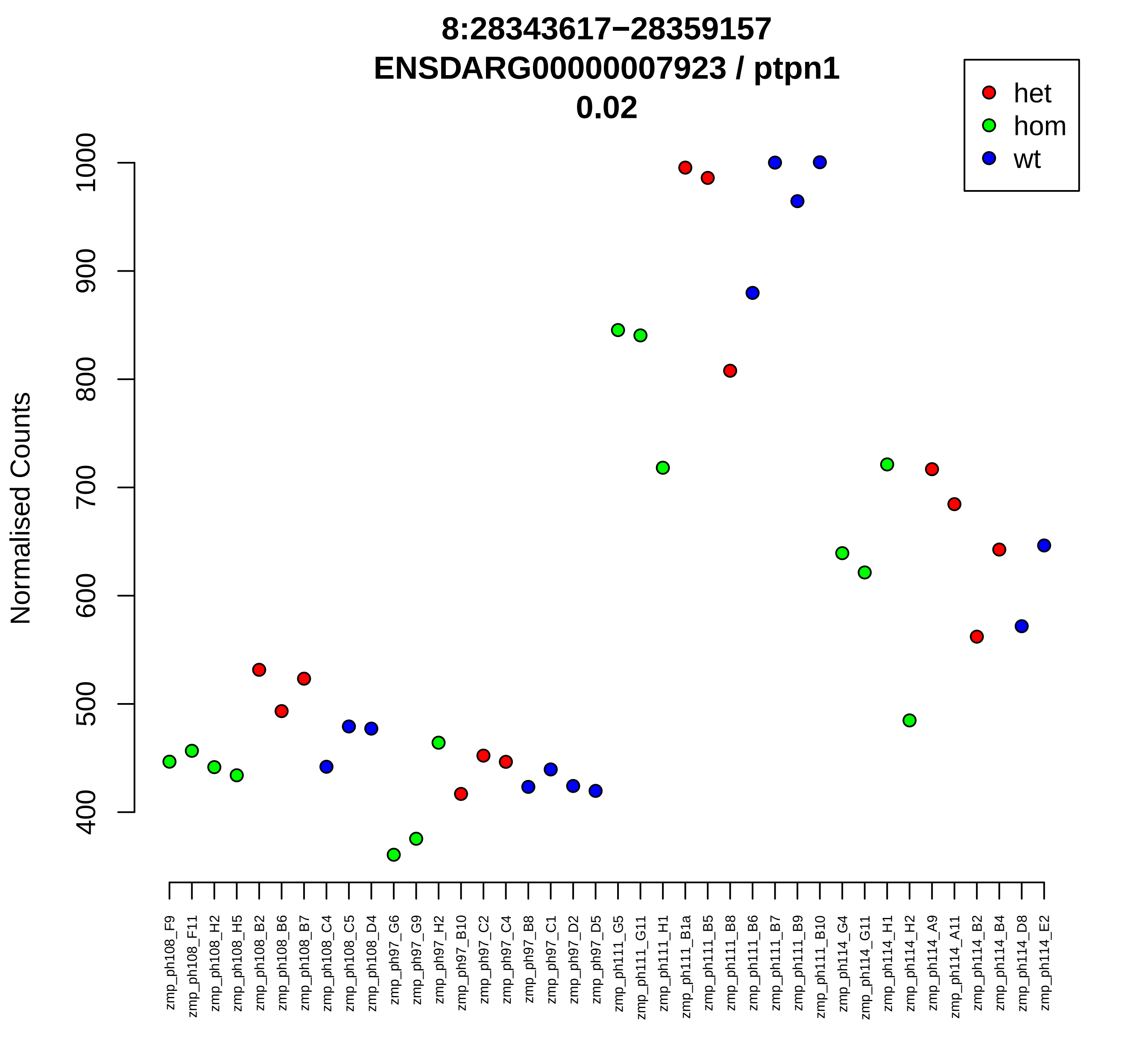

8:28343617−28359157
ENSDARG00000007923 / ptpn1
0.02
het
hom
wt
Normalised Counts
1000
900
800
700
600
500
400
zmp_ph108_F9
zmp_ph108_F11
zmp_ph108_H2
zmp_ph108_H5
zmp_ph108_B2
zmp_ph108_B6
zmp_ph108_B7
zmp_ph108_C4
zmp_ph108_C5
zmp_ph108_D4
zmp_ph97_G6
zmp_ph97_G9
zmp_ph97_H2
zmp_ph97_B10
zmp_ph97_C2
zmp_ph97_C4
zmp_ph97_B8
zmp_ph97_C1
zmp_ph97_D2
zmp_ph97_D5
zmp_ph111_G5
zmp_ph111_G11
zmp_ph111_H1
zmp_ph111_B1a
zmp_ph111_B5
zmp_ph111_B8
zmp_ph111_B6
zmp_ph111_B7
zmp_ph111_B9
zmp_ph111_B10
zmp_ph114_G4
zmp_ph114_G11
zmp_ph114_H1
zmp_ph114_H2
zmp_ph114_A9
zmp_ph114_A11
zmp_ph114_B2
zmp_ph114_B4
zmp_ph114_D8
zmp_ph114_E2

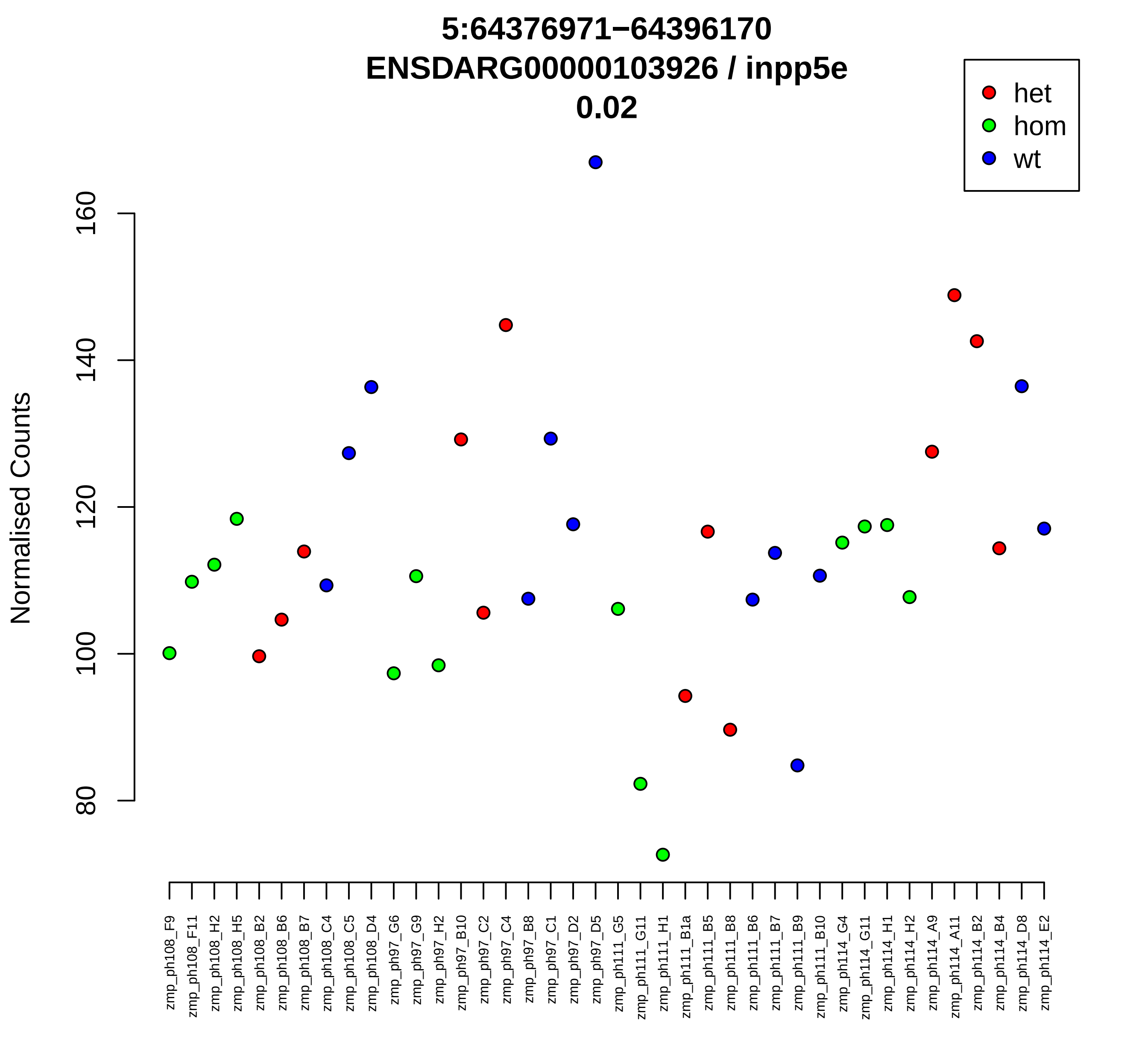

5:64376971−64396170
ENSDARG00000103926 / inpp5e
0.02
het
hom
wt
Normalised Counts
80
100
120
140
160
zmp_ph108_F9
zmp_ph108_F11
zmp_ph108_H2
zmp_ph108_H5
zmp_ph108_B2
zmp_ph108_B6
zmp_ph108_B7
zmp_ph108_C4
zmp_ph108_C5
zmp_ph108_D4
zmp_ph97_G6
zmp_ph97_G9
zmp_ph97_H2
zmp_ph97_B10
zmp_ph97_C2
zmp_ph97_C4
zmp_ph97_B8
zmp_ph97_C1
zmp_ph97_D2
zmp_ph97_D5
zmp_ph111_G5
zmp_ph111_G11
zmp_ph111_H1
zmp_ph111_B1a
zmp_ph111_B5
zmp_ph111_B8
zmp_ph111_B6
zmp_ph111_B7
zmp_ph111_B9
zmp_ph111_B10
zmp_ph114_G4
zmp_ph114_G11
zmp_ph114_H1
zmp_ph114_H2
zmp_ph114_A9
zmp_ph114_A11
zmp_ph114_B2
zmp_ph114_B4
zmp_ph114_D8
zmp_ph114_E2

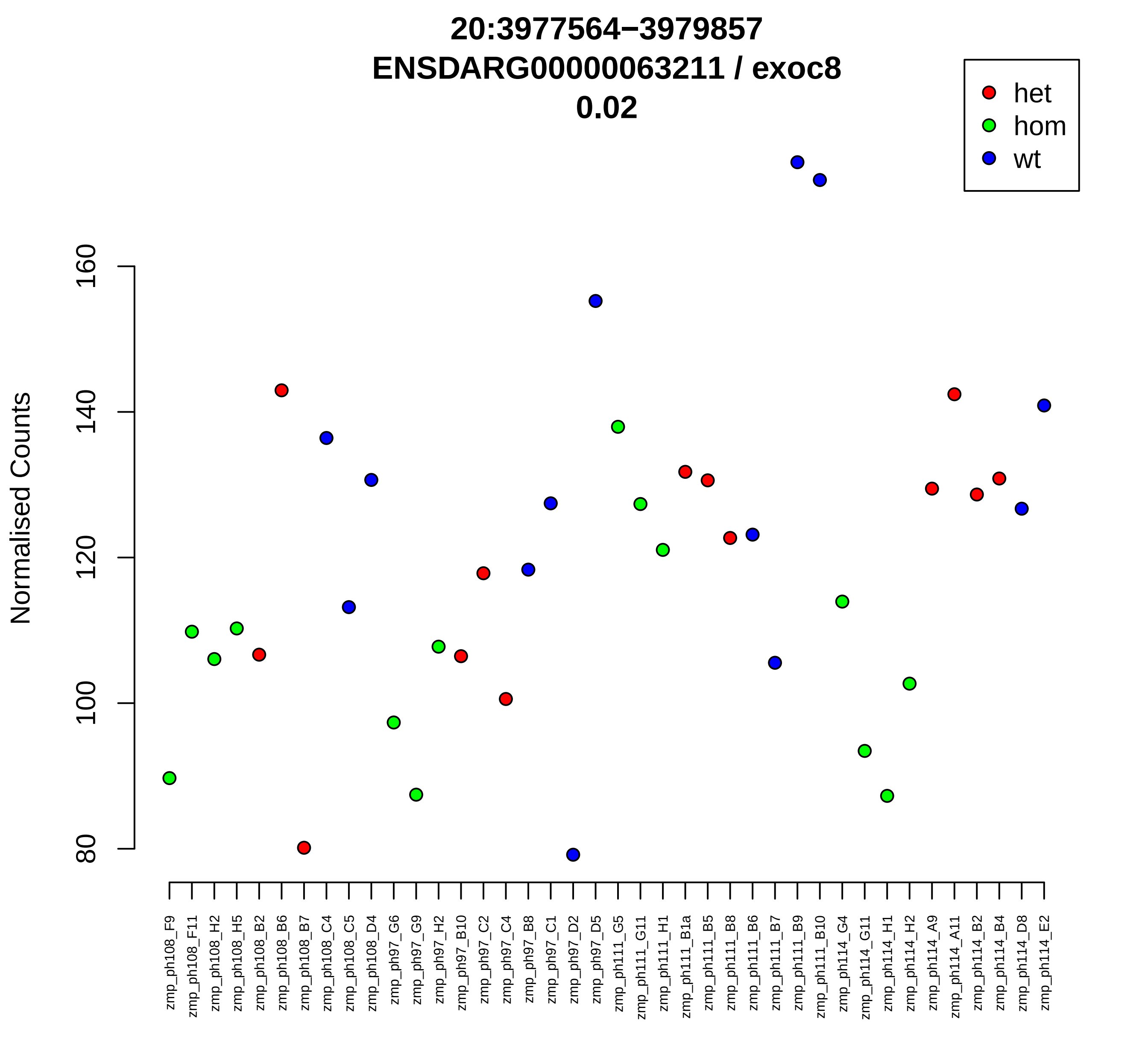
20:3977564−3979857
ENSDARG00000063211 / exoc8
0.02
het
hom
wt
Normalised Counts
80
100
120
140
160
zmp_ph108_F9
zmp_ph108_F11
zmp_ph108_H2
zmp_ph108_H5
zmp_ph108_B2
zmp_ph108_B6
zmp_ph108_B7
zmp_ph108_C4
zmp_ph108_C5
zmp_ph108_D4
zmp_ph97_G6
zmp_ph97_G9
zmp_ph97_H2
zmp_ph97_B10
zmp_ph97_C2
zmp_ph97_C4
zmp_ph97_B8
zmp_ph97_C1
zmp_ph97_D2
zmp_ph97_D5
zmp_ph111_G5
zmp_ph111_G11
zmp_ph111_H1
zmp_ph111_B1a
zmp_ph111_B5
zmp_ph111_B8
zmp_ph111_B6
zmp_ph111_B7
zmp_ph111_B9
zmp_ph111_B10
zmp_ph114_G4
zmp_ph114_G11
zmp_ph114_H1
zmp_ph114_H2
zmp_ph114_A9
zmp_ph114_A11
zmp_ph114_B2
zmp_ph114_B4
zmp_ph114_D8
zmp_ph114_E2

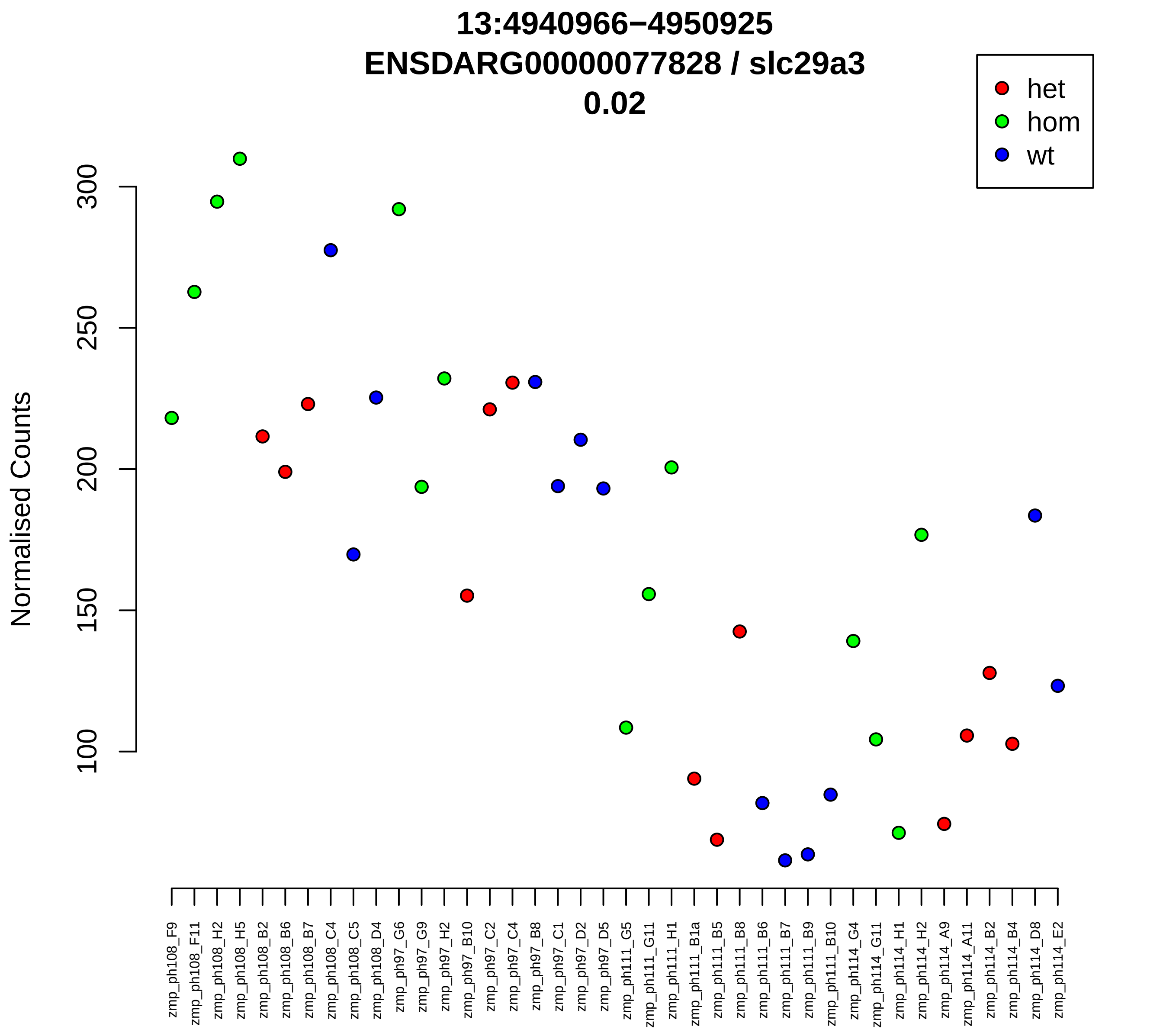

13:4940966−4950925
ENSDARG00000077828 / slc29a3
0.02
het
hom
wt
Normalised Counts
100
150
200
250
300
zmp_ph108_F9
zmp_ph108_F11
zmp_ph108_H2
zmp_ph108_H5
zmp_ph108_B2
zmp_ph108_B6
zmp_ph108_B7
zmp_ph108_C4
zmp_ph108_C5
zmp_ph108_D4
zmp_ph97_G6
zmp_ph97_G9
zmp_ph97_H2
zmp_ph97_B10
zmp_ph97_C2
zmp_ph97_C4
zmp_ph97_B8
zmp_ph97_C1
zmp_ph97_D2
zmp_ph97_D5
zmp_ph111_G5
zmp_ph111_G11
zmp_ph111_H1
zmp_ph111_B1a
zmp_ph111_B5
zmp_ph111_B8
zmp_ph111_B6
zmp_ph111_B7
zmp_ph111_B9
zmp_ph111_B10
zmp_ph114_G4
zmp_ph114_G11
zmp_ph114_H1
zmp_ph114_H2
zmp_ph114_A9
zmp_ph114_A11
zmp_ph114_B2
zmp_ph114_B4
zmp_ph114_D8
zmp_ph114_E2

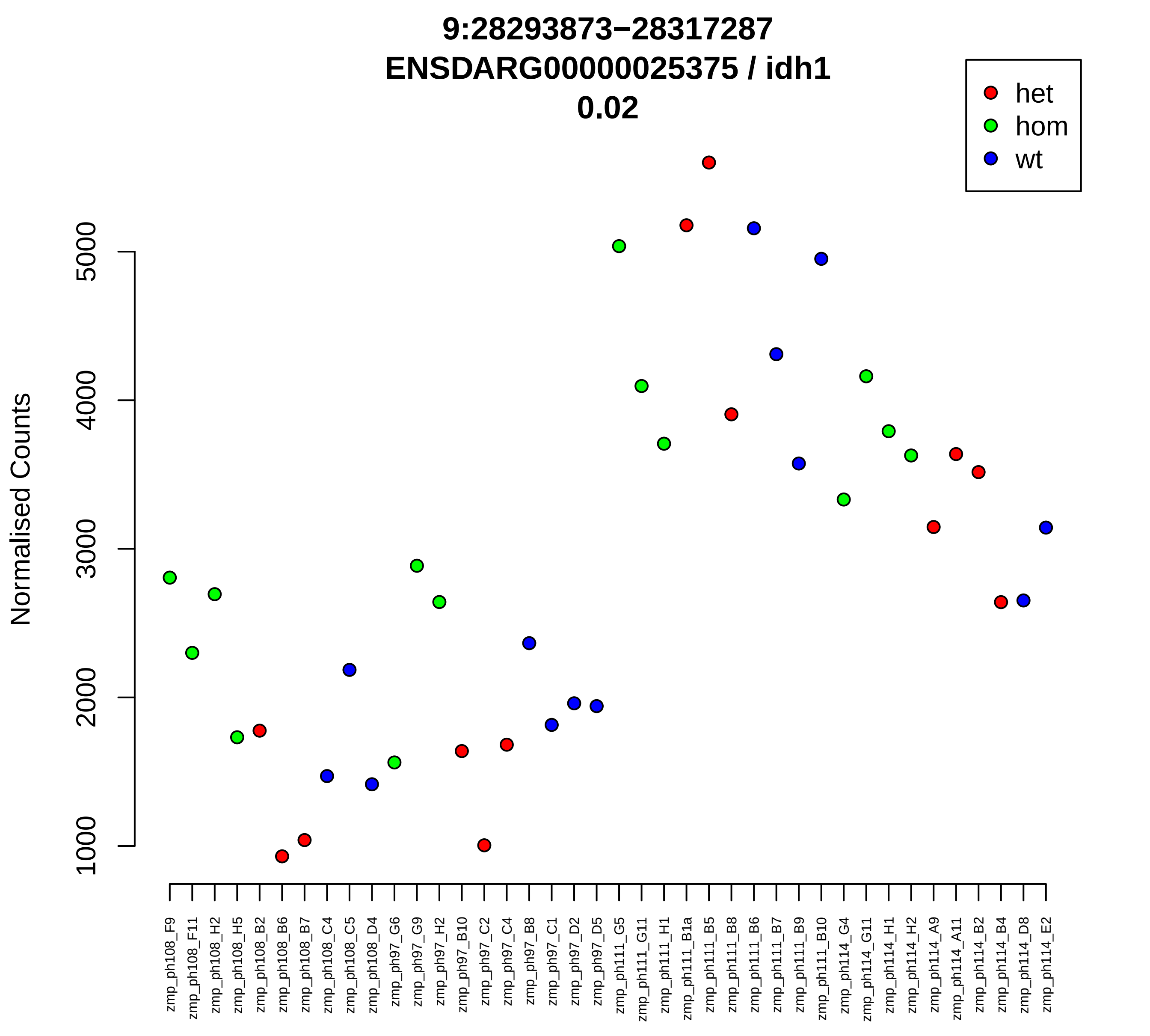

9:28293873−28317287
ENSDARG00000025375 / idh1
0.02
het
hom
wt
Normalised Counts
1000
2000
3000
4000
5000
zmp_ph108_F9
zmp_ph108_F11
zmp_ph108_H2
zmp_ph108_H5
zmp_ph108_B2
zmp_ph108_B6
zmp_ph108_B7
zmp_ph108_C4
zmp_ph108_C5
zmp_ph108_D4
zmp_ph97_G6
zmp_ph97_G9
zmp_ph97_H2
zmp_ph97_B10
zmp_ph97_C2
zmp_ph97_C4
zmp_ph97_B8
zmp_ph97_C1
zmp_ph97_D2
zmp_ph97_D5
zmp_ph111_G5
zmp_ph111_G11
zmp_ph111_H1
zmp_ph111_B1a
zmp_ph111_B5
zmp_ph111_B8
zmp_ph111_B6
zmp_ph111_B7
zmp_ph111_B9
zmp_ph111_B10
zmp_ph114_G4
zmp_ph114_G11
zmp_ph114_H1
zmp_ph114_H2
zmp_ph114_A9
zmp_ph114_A11
zmp_ph114_B2
zmp_ph114_B4
zmp_ph114_D8
zmp_ph114_E2

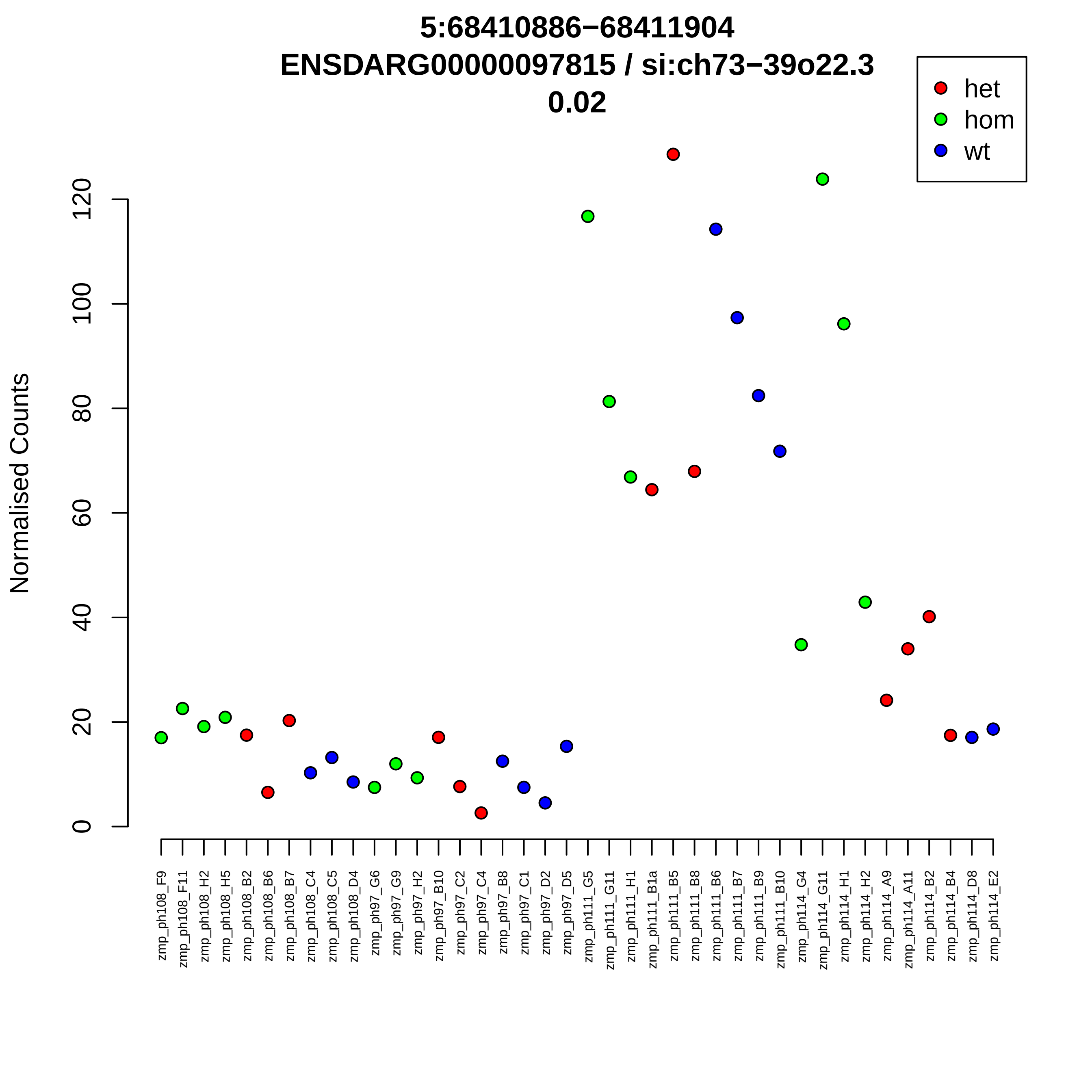

5:68410886−68411904
ENSDARG00000097815 / si:ch73−39o22.3
0.02
het
hom
wt
Normalised Counts
0
20
40
60
80
100
120
zmp_ph108_F9
zmp_ph108_F11
zmp_ph108_H2
zmp_ph108_H5
zmp_ph108_B2
zmp_ph108_B6
zmp_ph108_B7
zmp_ph108_C4
zmp_ph108_C5
zmp_ph108_D4
zmp_ph97_G6
zmp_ph97_G9
zmp_ph97_H2
zmp_ph97_B10
zmp_ph97_C2
zmp_ph97_C4
zmp_ph97_B8
zmp_ph97_C1
zmp_ph97_D2
zmp_ph97_D5
zmp_ph111_G5
zmp_ph111_G11
zmp_ph111_H1
zmp_ph111_B1a
zmp_ph111_B5
zmp_ph111_B8
zmp_ph111_B6
zmp_ph111_B7
zmp_ph111_B9
zmp_ph111_B10
zmp_ph114_G4
zmp_ph114_G11
zmp_ph114_H1
zmp_ph114_H2
zmp_ph114_A9
zmp_ph114_A11
zmp_ph114_B2
zmp_ph114_B4
zmp_ph114_D8
zmp_ph114_E2

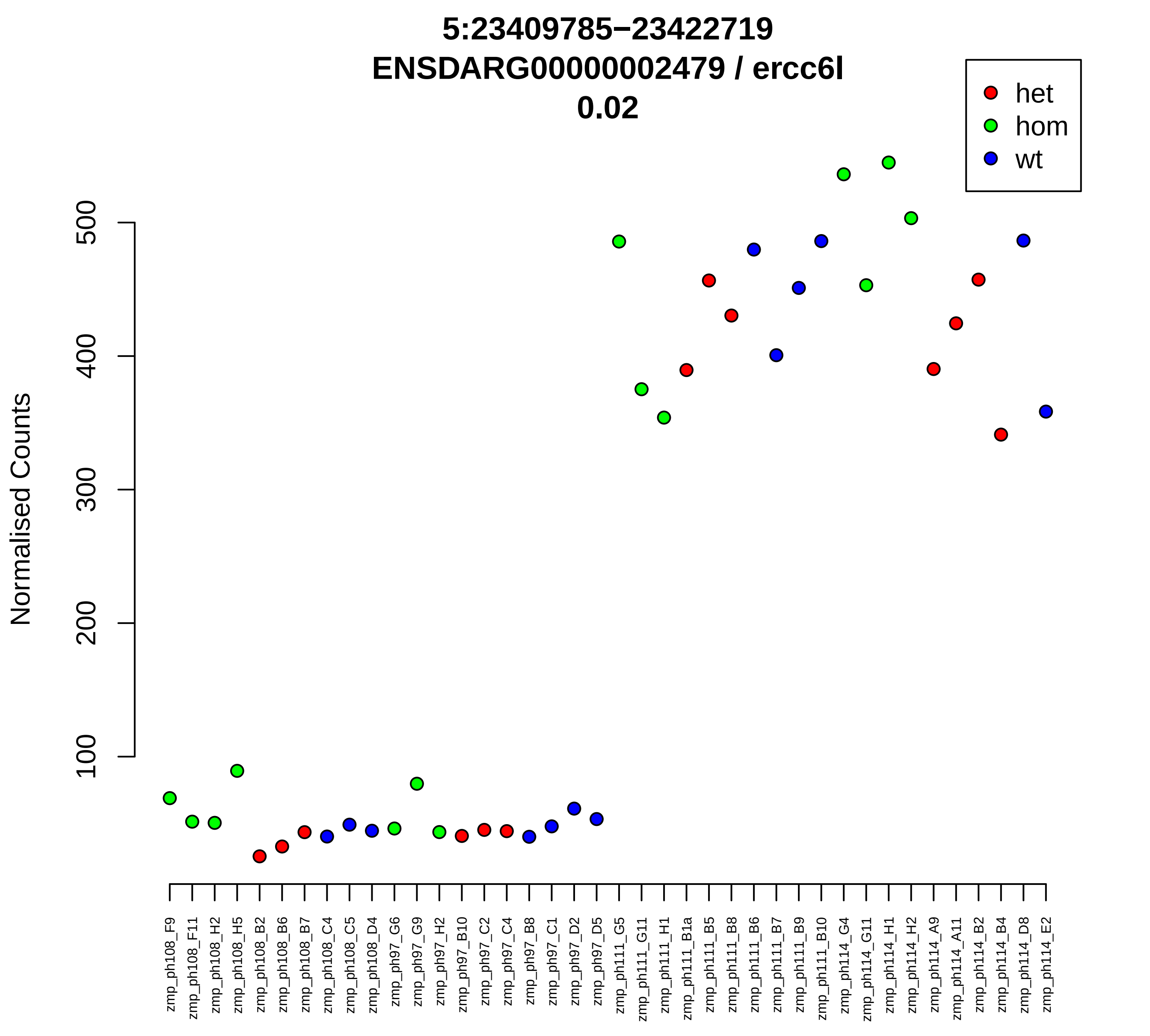
5:23409785−23422719
ENSDARG00000002479 / ercc6l
0.02
het
hom
wt
Normalised Counts
100
200
300
400
500
zmp_ph108_F9
zmp_ph108_F11
zmp_ph108_H2
zmp_ph108_H5
zmp_ph108_B2
zmp_ph108_B6
zmp_ph108_B7
zmp_ph108_C4
zmp_ph108_C5
zmp_ph108_D4
zmp_ph97_G6
zmp_ph97_G9
zmp_ph97_H2
zmp_ph97_B10
zmp_ph97_C2
zmp_ph97_C4
zmp_ph97_B8
zmp_ph97_C1
zmp_ph97_D2
zmp_ph97_D5
zmp_ph111_G5
zmp_ph111_G11
zmp_ph111_H1
zmp_ph111_B1a
zmp_ph111_B5
zmp_ph111_B8
zmp_ph111_B6
zmp_ph111_B7
zmp_ph111_B9
zmp_ph111_B10
zmp_ph114_G4
zmp_ph114_G11
zmp_ph114_H1
zmp_ph114_H2
zmp_ph114_A9
zmp_ph114_A11
zmp_ph114_B2
zmp_ph114_B4
zmp_ph114_D8
zmp_ph114_E2

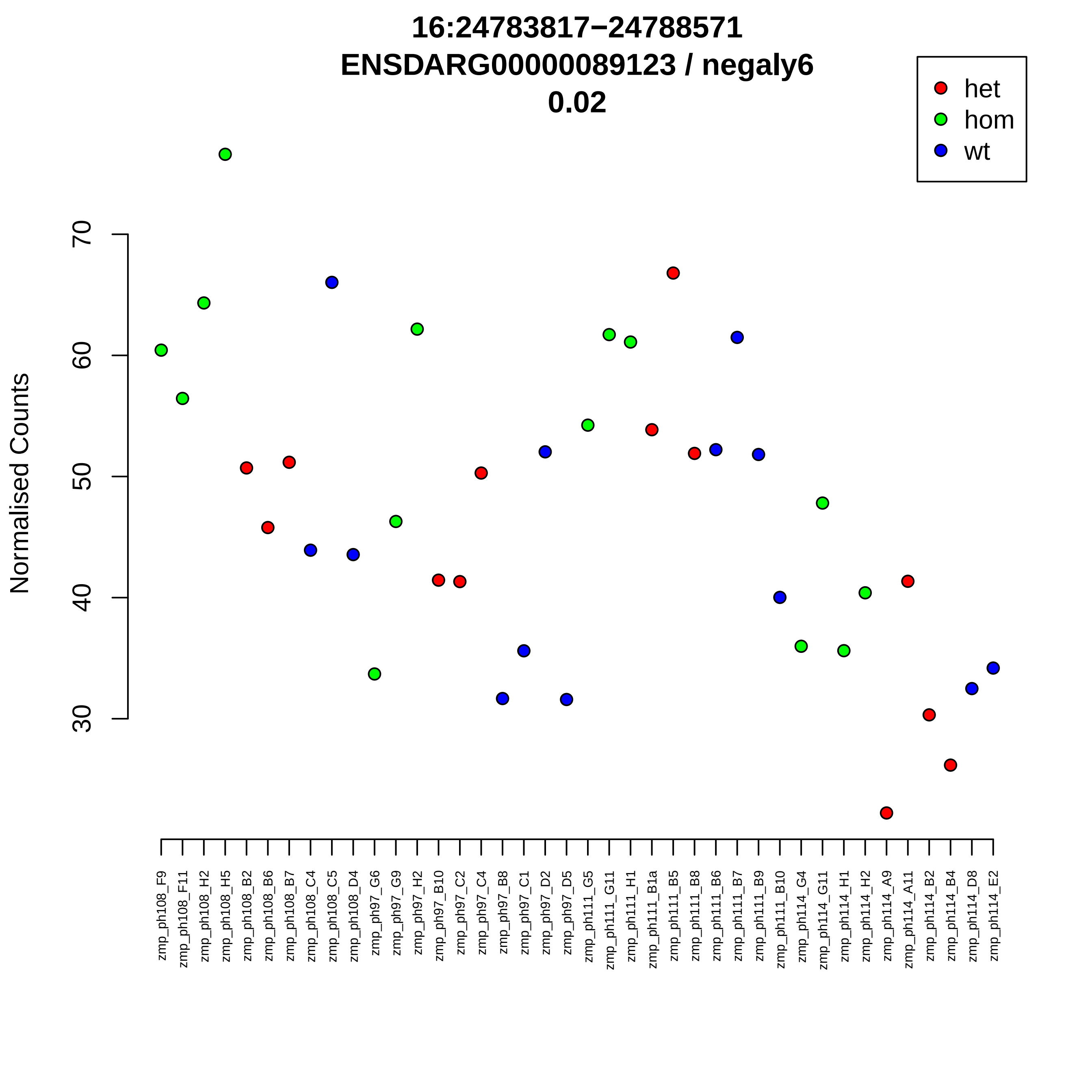
16:24783817−24788571
ENSDARG00000089123 / negaly6
0.02
het
hom
wt
Normalised Counts
30
40
50
60
70
zmp_ph108_F9
zmp_ph108_F11
zmp_ph108_H2
zmp_ph108_H5
zmp_ph108_B2
zmp_ph108_B6
zmp_ph108_B7
zmp_ph108_C4
zmp_ph108_C5
zmp_ph108_D4
zmp_ph97_G6
zmp_ph97_G9
zmp_ph97_H2
zmp_ph97_B10
zmp_ph97_C2
zmp_ph97_C4
zmp_ph97_B8
zmp_ph97_C1
zmp_ph97_D2
zmp_ph97_D5
zmp_ph111_G5
zmp_ph111_G11
zmp_ph111_H1
zmp_ph111_B1a
zmp_ph111_B5
zmp_ph111_B8
zmp_ph111_B6
zmp_ph111_B7
zmp_ph111_B9
zmp_ph111_B10
zmp_ph114_G4
zmp_ph114_G11
zmp_ph114_H1
zmp_ph114_H2
zmp_ph114_A9
zmp_ph114_A11
zmp_ph114_B2
zmp_ph114_B4
zmp_ph114_D8
zmp_ph114_E2

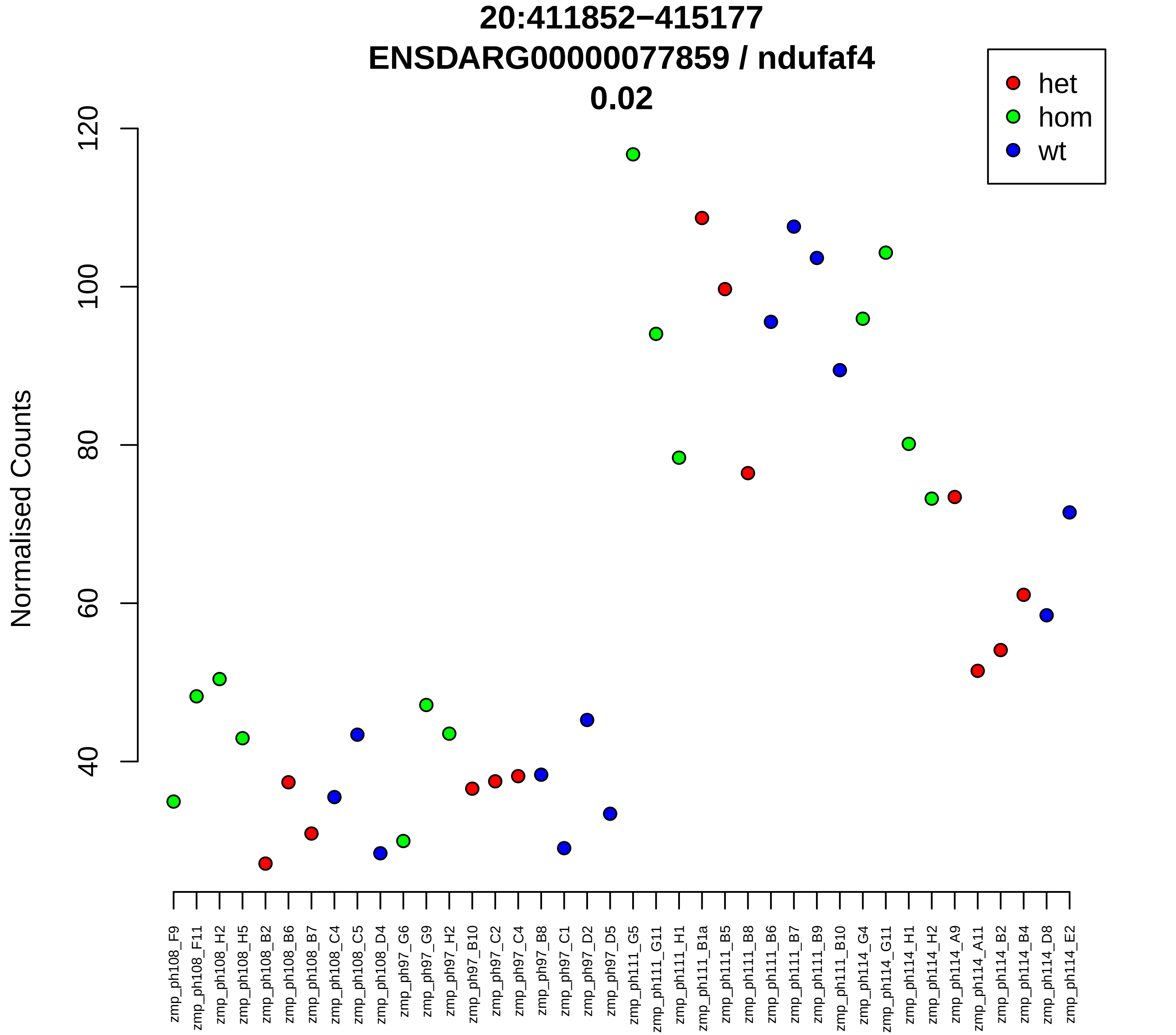

20:411852−415177
ENSDARG00000077859 / ndufaf4
0.02
het
hom
wt
Normalised Counts
120
100
80
60
40
zmp_ph108_F9
zmp_ph108_F11
zmp_ph108_H2
zmp_ph108_H5
zmp_ph108_B2
zmp_ph108_B6
zmp_ph108_B7
zmp_ph108_C4
zmp_ph108_C5
zmp_ph108_D4
zmp_ph97_G6
zmp_ph97_G9
zmp_ph97_H2
zmp_ph97_B10
zmp_ph97_C2
zmp_ph97_C4
zmp_ph97_B8
zmp_ph97_C1
zmp_ph97_D2
zmp_ph97_D5
zmp_ph111_G5
zmp_ph111_G11
zmp_ph111_H1
zmp_ph111_B1a
zmp_ph111_B5
zmp_ph111_B8
zmp_ph111_B6
zmp_ph111_B7
zmp_ph111_B9
zmp_ph111_B10
zmp_ph114_G4
zmp_ph114_G11
zmp_ph114_H1
zmp_ph114_H2
zmp_ph114_A9
zmp_ph114_A11
zmp_ph114_B2
zmp_ph114_B4
zmp_ph114_D8
zmp_ph114_E2

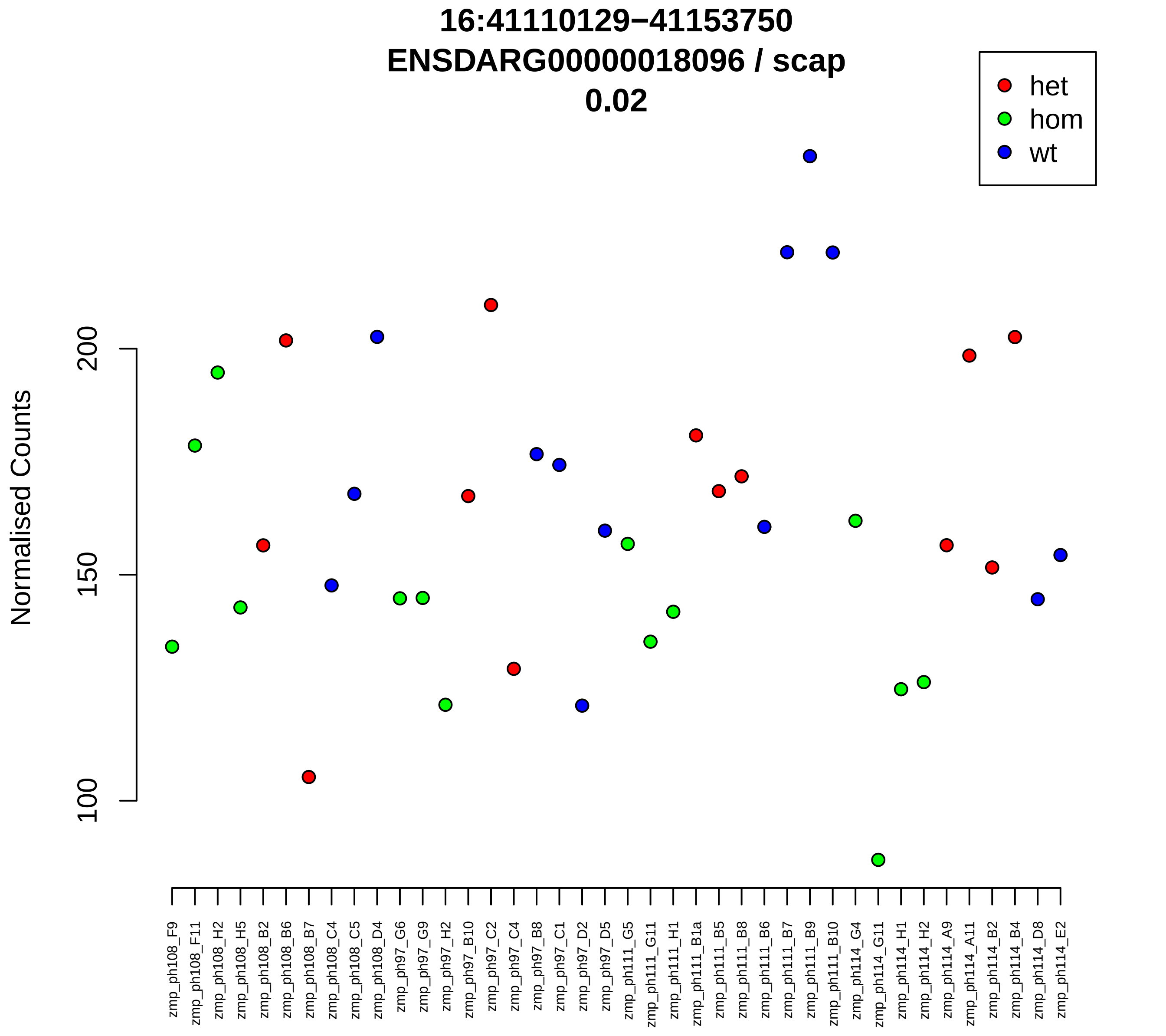
16:41110129−41153750
ENSDARG00000018096 / scap
0.02
het
hom
wt
Normalised Counts
100
150
200
zmp_ph108_F9
zmp_ph108_F11
zmp_ph108_H2
zmp_ph108_H5
zmp_ph108_B2
zmp_ph108_B6
zmp_ph108_B7
zmp_ph108_C4
zmp_ph108_C5
zmp_ph108_D4
zmp_ph97_G6
zmp_ph97_G9
zmp_ph97_H2
zmp_ph97_B10
zmp_ph97_C2
zmp_ph97_C4
zmp_ph97_B8
zmp_ph97_C1
zmp_ph97_D2
zmp_ph97_D5
zmp_ph111_G5
zmp_ph111_G11
zmp_ph111_H1
zmp_ph111_B1a
zmp_ph111_B5
zmp_ph111_B8
zmp_ph111_B6
zmp_ph111_B7
zmp_ph111_B9
zmp_ph111_B10
zmp_ph114_G4
zmp_ph114_G11
zmp_ph114_H1
zmp_ph114_H2
zmp_ph114_A9
zmp_ph114_A11
zmp_ph114_B2
zmp_ph114_B4
zmp_ph114_D8
zmp_ph114_E2

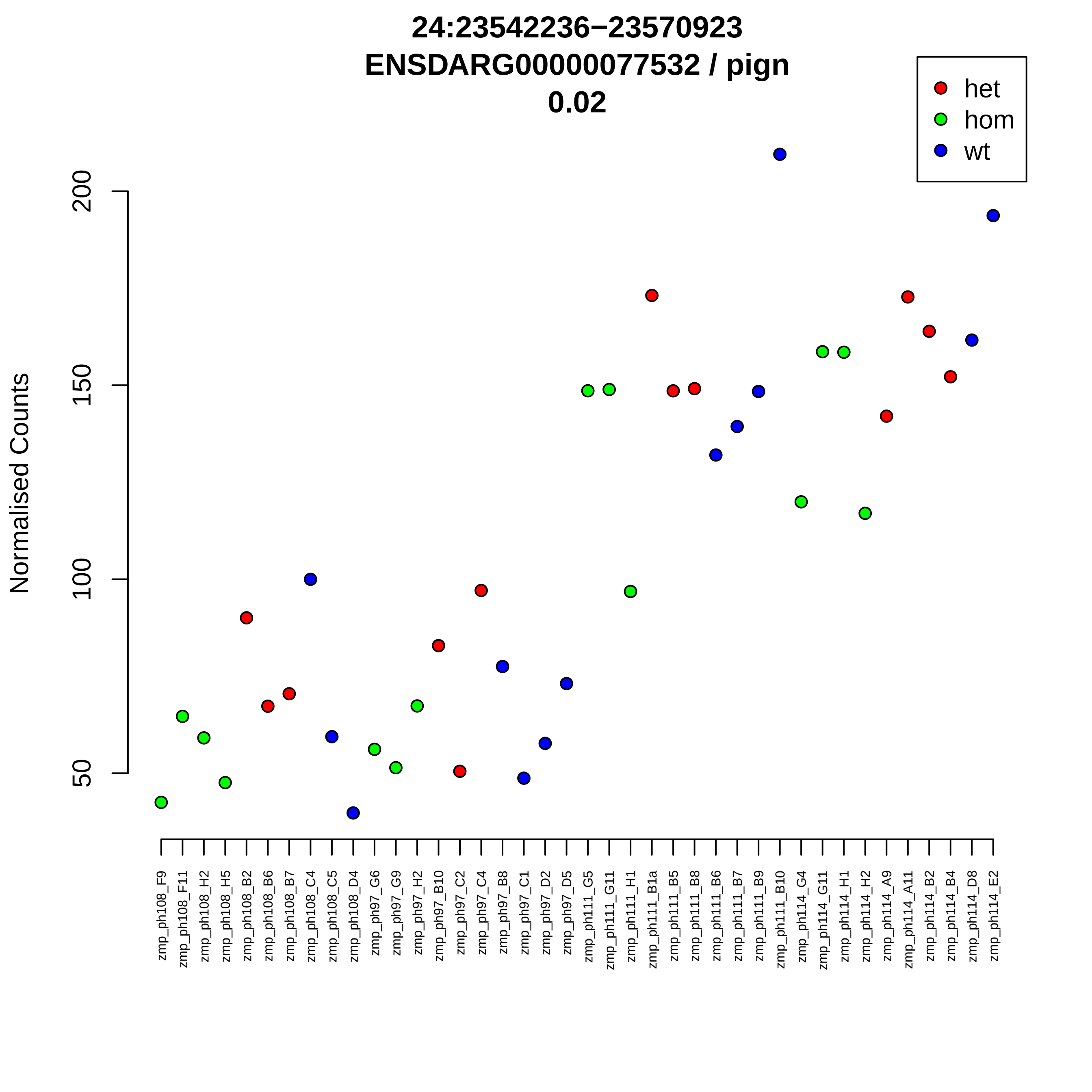
24:23542236−23570923
ENSDARG00000077532 / pign
0.02
het
hom
wt
Normalised Counts
50
100
150
200
zmp_ph108_F9
zmp_ph108_F11
zmp_ph108_H2
zmp_ph108_H5
zmp_ph108_B2
zmp_ph108_B6
zmp_ph108_B7
zmp_ph108_C4
zmp_ph108_C5
zmp_ph108_D4
zmp_ph97_G6
zmp_ph97_G9
zmp_ph97_H2
zmp_ph97_B10
zmp_ph97_C2
zmp_ph97_C4
zmp_ph97_B8
zmp_ph97_C1
zmp_ph97_D2
zmp_ph97_D5
zmp_ph111_G5
zmp_ph111_G11
zmp_ph111_H1
zmp_ph111_B1a
zmp_ph111_B5
zmp_ph111_B8
zmp_ph111_B6
zmp_ph111_B7
zmp_ph111_B9
zmp_ph111_B10
zmp_ph114_G4
zmp_ph114_G11
zmp_ph114_H1
zmp_ph114_H2
zmp_ph114_A9
zmp_ph114_A11
zmp_ph114_B2
zmp_ph114_B4
zmp_ph114_D8
zmp_ph114_E2

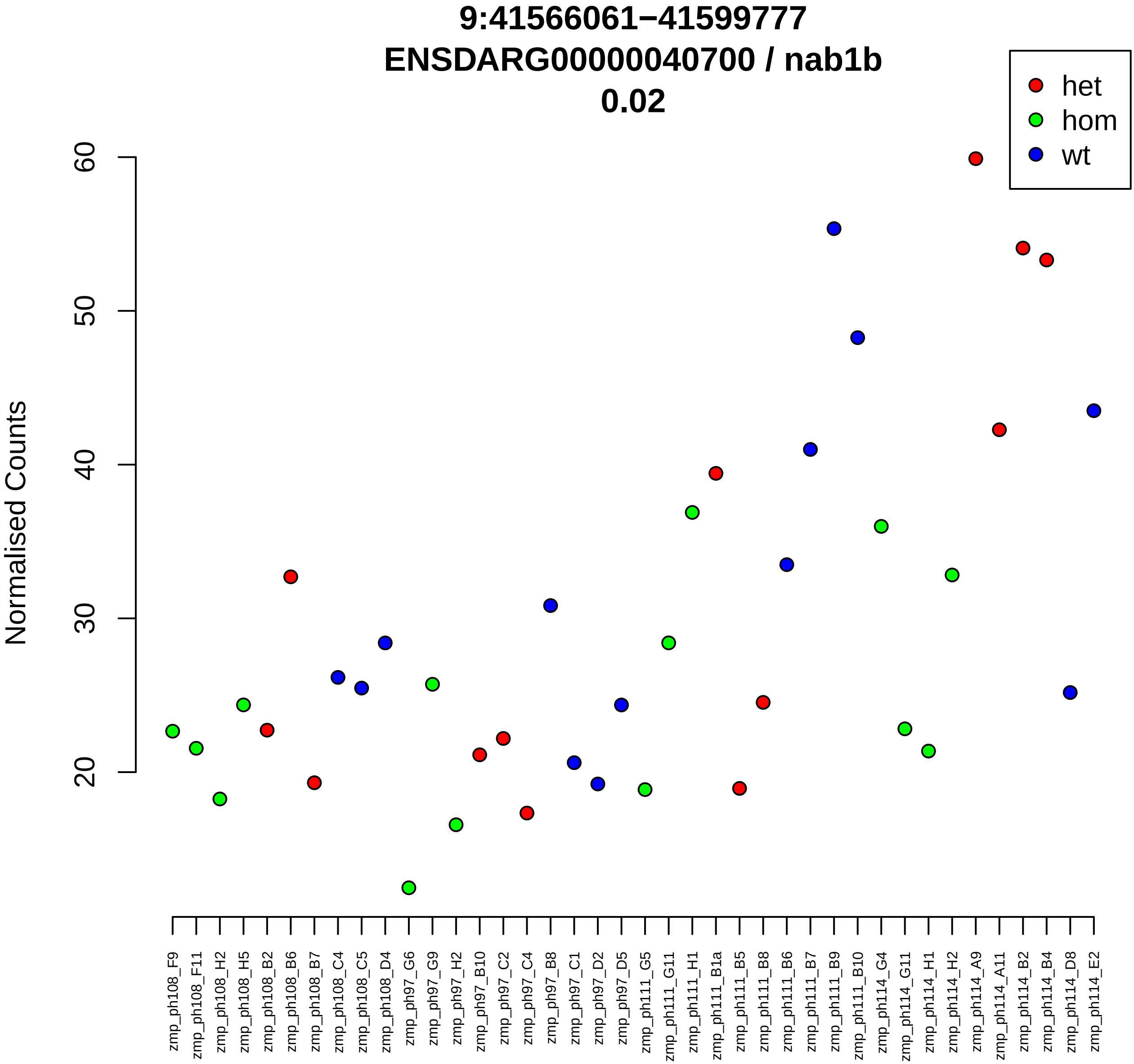
9:41566061−41599777
ENSDARG00000040700 / nab1b
0.02
het
hom
wt
Normalised Counts
20
30
40
50
60
zmp_ph108_F9
zmp_ph108_F11
zmp_ph108_H2
zmp_ph108_H5
zmp_ph108_B2
zmp_ph108_B6
zmp_ph108_B7
zmp_ph108_C4
zmp_ph108_C5
zmp_ph108_D4
zmp_ph97_G6
zmp_ph97_G9
zmp_ph97_H2
zmp_ph97_B10
zmp_ph97_C2
zmp_ph97_C4
zmp_ph97_B8
zmp_ph97_C1
zmp_ph97_D2
zmp_ph97_D5
zmp_ph111_G5
zmp_ph111_G11
zmp_ph111_H1
zmp_ph111_B1a
zmp_ph111_B5
zmp_ph111_B8
zmp_ph111_B6
zmp_ph111_B7
zmp_ph111_B9
zmp_ph111_B10
zmp_ph114_G4
zmp_ph114_G11
zmp_ph114_H1
zmp_ph114_H2
zmp_ph114_A9
zmp_ph114_A11
zmp_ph114_B2
zmp_ph114_B4
zmp_ph114_D8
zmp_ph114_E2

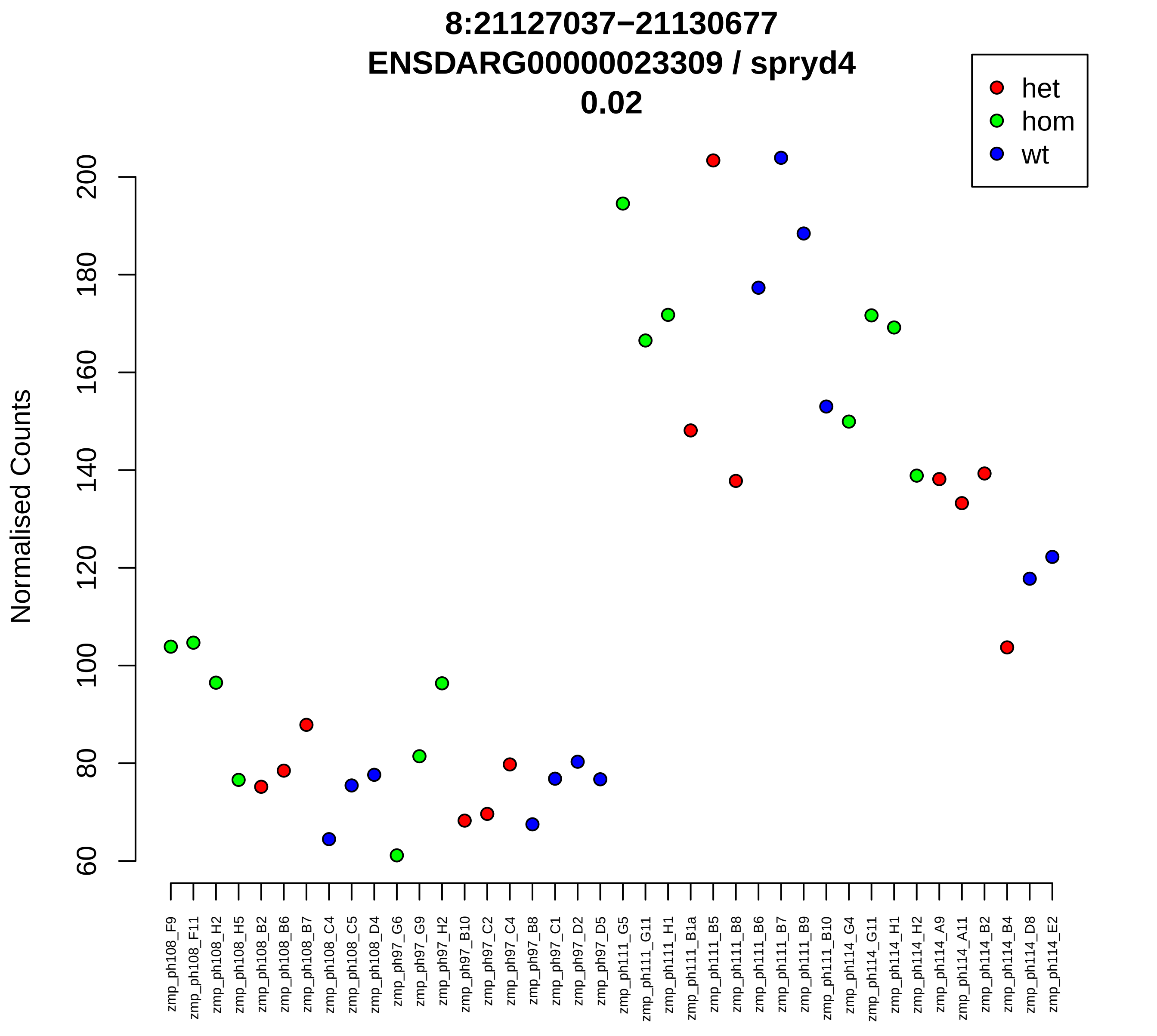

8:21127037−21130677
ENSDARG00000023309 / spryd4
0.02
het
hom
wt
Normalised Counts
60
80
100
120
140
160
180
200
zmp_ph108_F9
zmp_ph108_F11
zmp_ph108_H2
zmp_ph108_H5
zmp_ph108_B2
zmp_ph108_B6
zmp_ph108_B7
zmp_ph108_C4
zmp_ph108_C5
zmp_ph108_D4
zmp_ph97_G6
zmp_ph97_G9
zmp_ph97_H2
zmp_ph97_B10
zmp_ph97_C2
zmp_ph97_C4
zmp_ph97_B8
zmp_ph97_C1
zmp_ph97_D2
zmp_ph97_D5
zmp_ph111_G5
zmp_ph111_G11
zmp_ph111_H1
zmp_ph111_B1a
zmp_ph111_B5
zmp_ph111_B8
zmp_ph111_B6
zmp_ph111_B7
zmp_ph111_B9
zmp_ph111_B10
zmp_ph114_G4
zmp_ph114_G11
zmp_ph114_H1
zmp_ph114_H2
zmp_ph114_A9
zmp_ph114_A11
zmp_ph114_B2
zmp_ph114_B4
zmp_ph114_D8
zmp_ph114_E2

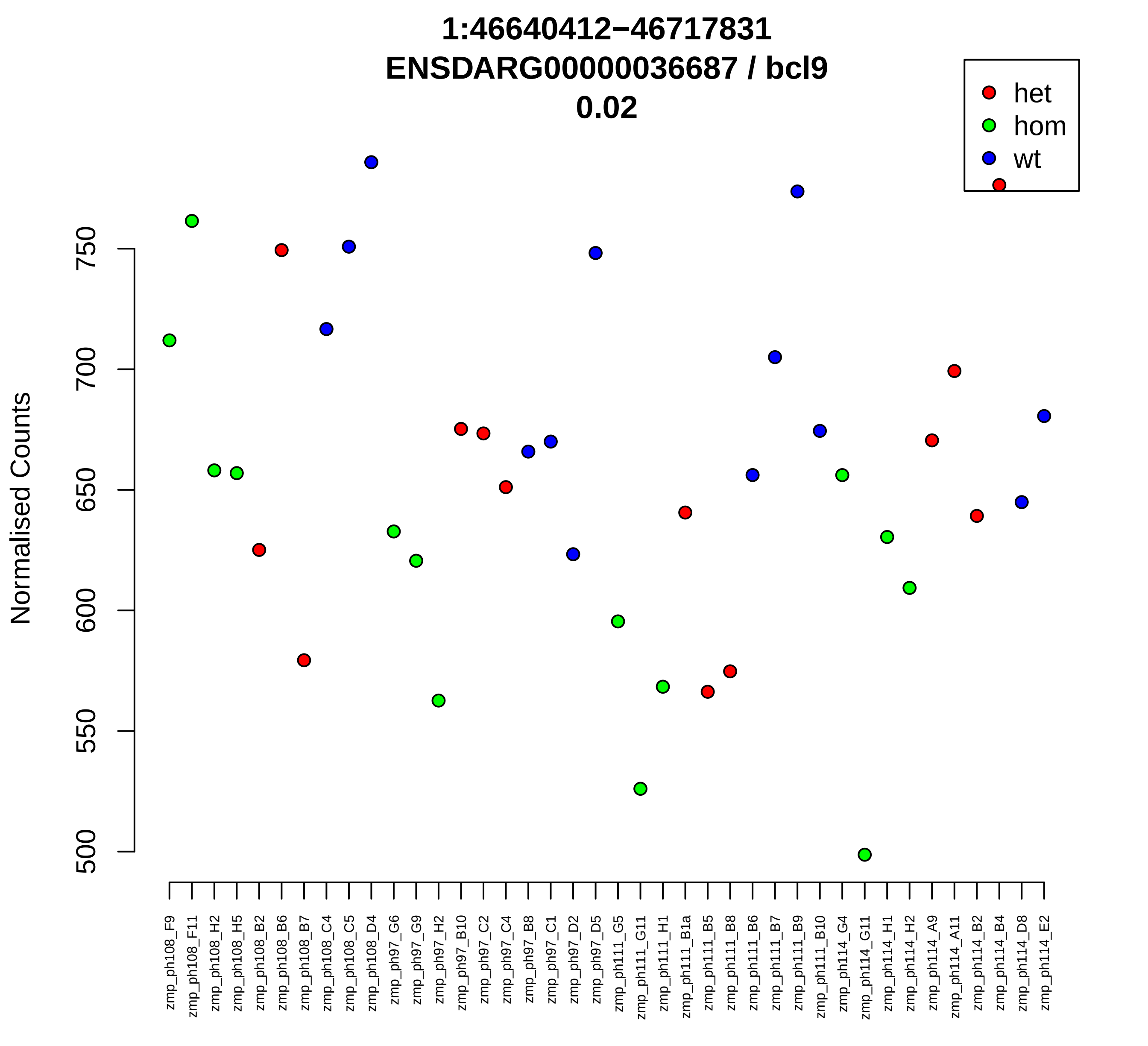
1:46640412−46717831
ENSDARG00000036687 / bcl9
0.02
het
hom
wt
Normalised Counts
500
550
600
650
700
750
zmp_ph108_F9
zmp_ph108_F11
zmp_ph108_H2
zmp_ph108_H5
zmp_ph108_B2
zmp_ph108_B6
zmp_ph108_B7
zmp_ph108_C4
zmp_ph108_C5
zmp_ph108_D4
zmp_ph97_G6
zmp_ph97_G9
zmp_ph97_H2
zmp_ph97_B10
zmp_ph97_C2
zmp_ph97_C4
zmp_ph97_B8
zmp_ph97_C1
zmp_ph97_D2
zmp_ph97_D5
zmp_ph111_G5
zmp_ph111_G11
zmp_ph111_H1
zmp_ph111_B1a
zmp_ph111_B5
zmp_ph111_B8
zmp_ph111_B6
zmp_ph111_B7
zmp_ph111_B9
zmp_ph111_B10
zmp_ph114_G4
zmp_ph114_G11
zmp_ph114_H1
zmp_ph114_H2
zmp_ph114_A9
zmp_ph114_A11
zmp_ph114_B2
zmp_ph114_B4
zmp_ph114_D8
zmp_ph114_E2

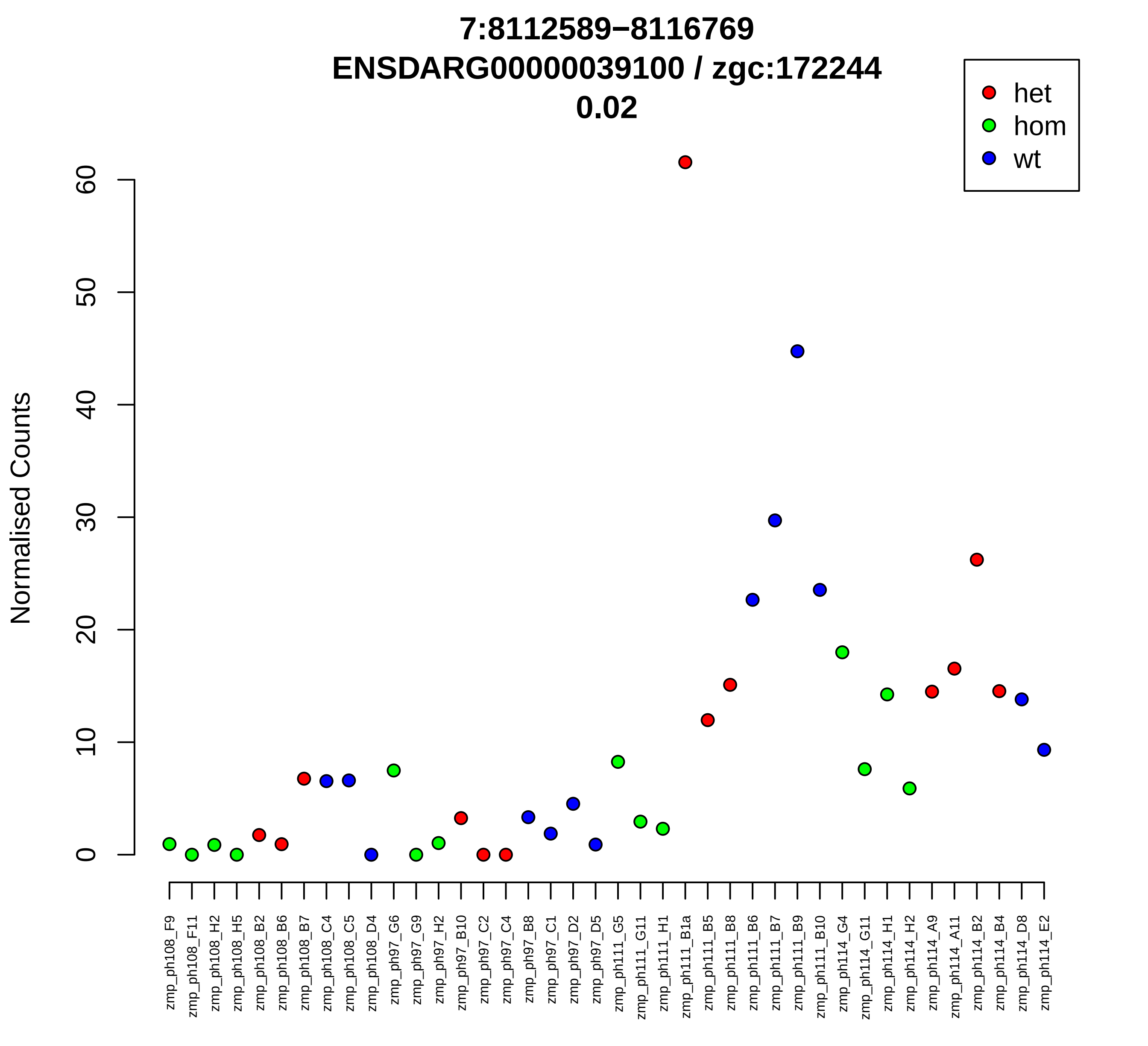

7:8112589−8116769
ENSDARG00000039100 / zgc:172244
0.02
het
hom
wt
Normalised Counts
0
10
20
30
40
50
60
zmp_ph108_F9
zmp_ph108_F11
zmp_ph108_H2
zmp_ph108_H5
zmp_ph108_B2
zmp_ph108_B6
zmp_ph108_B7
zmp_ph108_C4
zmp_ph108_C5
zmp_ph108_D4
zmp_ph97_G6
zmp_ph97_G9
zmp_ph97_H2
zmp_ph97_B10
zmp_ph97_C2
zmp_ph97_C4
zmp_ph97_B8
zmp_ph97_C1
zmp_ph97_D2
zmp_ph97_D5
zmp_ph111_G5
zmp_ph111_G11
zmp_ph111_H1
zmp_ph111_B1a
zmp_ph111_B5
zmp_ph111_B8
zmp_ph111_B6
zmp_ph111_B7
zmp_ph111_B9
zmp_ph111_B10
zmp_ph114_G4
zmp_ph114_G11
zmp_ph114_H1
zmp_ph114_H2
zmp_ph114_A9
zmp_ph114_A11
zmp_ph114_B2
zmp_ph114_B4
zmp_ph114_D8
zmp_ph114_E2

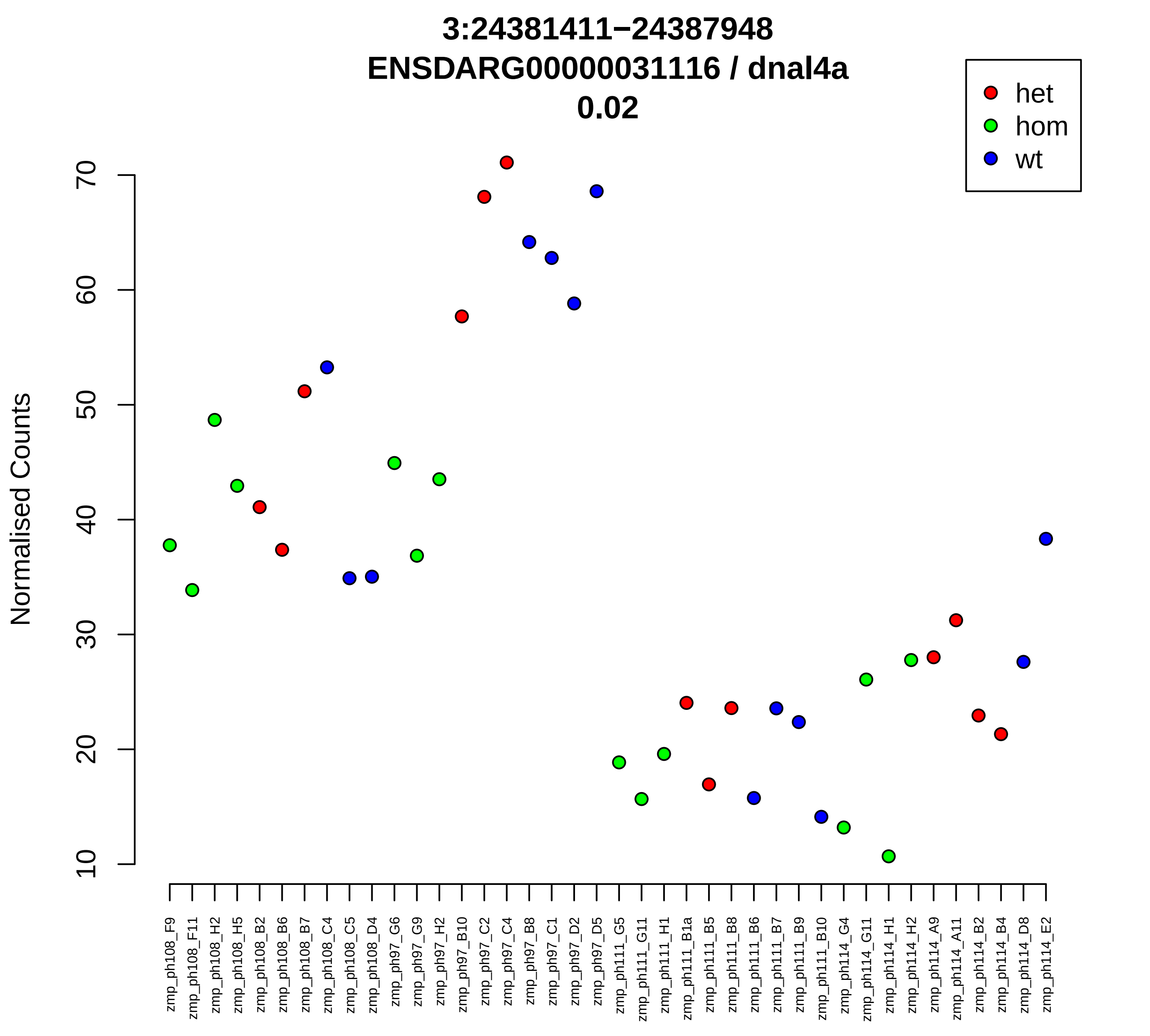

3:24381411−24387948
ENSDARG00000031116 / dnal4a
0.02
het
hom
wt
Normalised Counts
10
20
30
40
50
60
70
zmp_ph108_F9
zmp_ph108_F11
zmp_ph108_H2
zmp_ph108_H5
zmp_ph108_B2
zmp_ph108_B6
zmp_ph108_B7
zmp_ph108_C4
zmp_ph108_C5
zmp_ph108_D4
zmp_ph97_G6
zmp_ph97_G9
zmp_ph97_H2
zmp_ph97_B10
zmp_ph97_C2
zmp_ph97_C4
zmp_ph97_B8
zmp_ph97_C1
zmp_ph97_D2
zmp_ph97_D5
zmp_ph111_G5
zmp_ph111_G11
zmp_ph111_H1
zmp_ph111_B1a
zmp_ph111_B5
zmp_ph111_B8
zmp_ph111_B6
zmp_ph111_B7
zmp_ph111_B9
zmp_ph111_B10
zmp_ph114_G4
zmp_ph114_G11
zmp_ph114_H1
zmp_ph114_H2
zmp_ph114_A9
zmp_ph114_A11
zmp_ph114_B2
zmp_ph114_B4
zmp_ph114_D8
zmp_ph114_E2

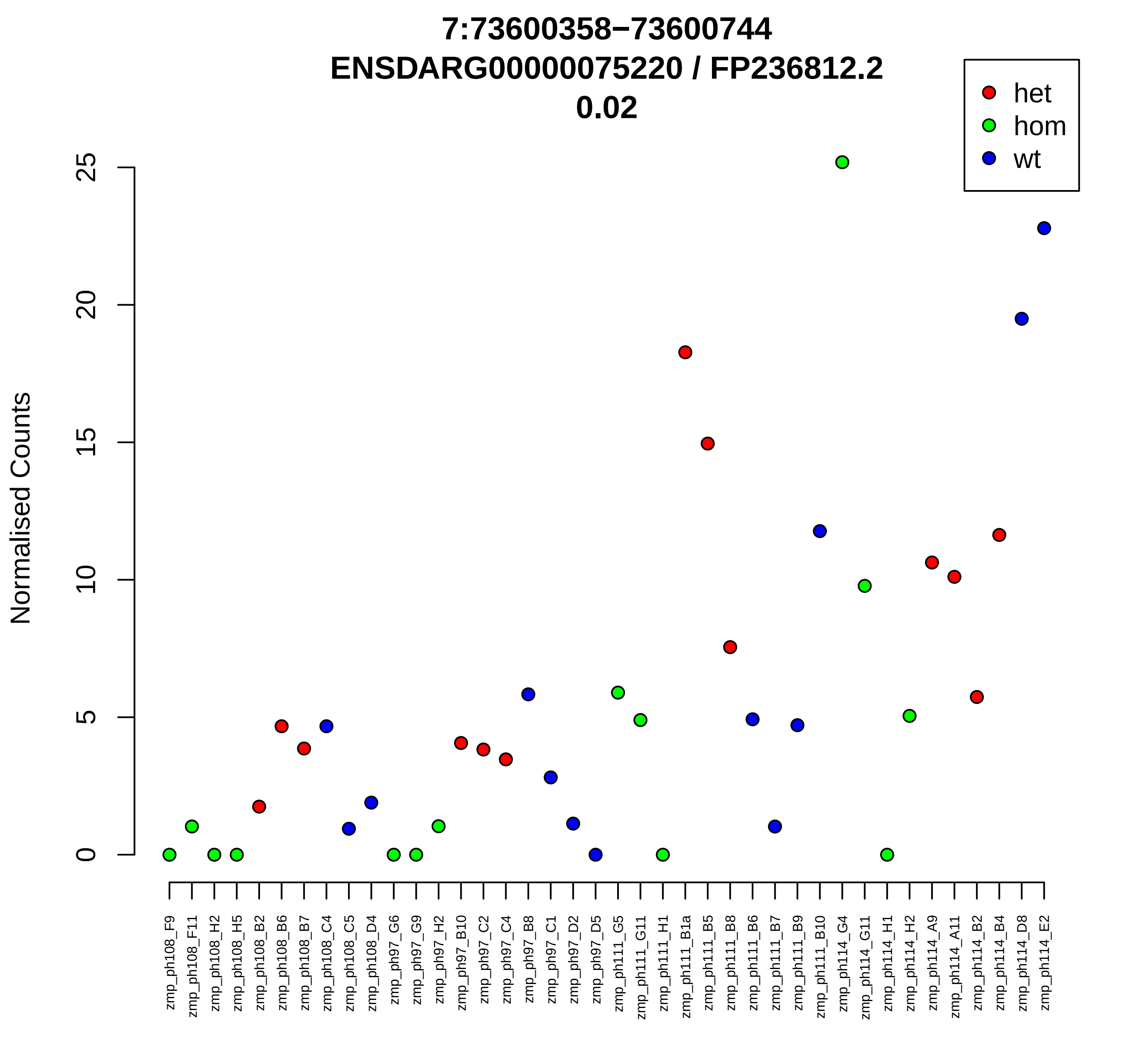
7:73600358−73600744
ENSDARG00000075220 / FP236812.2
0.02
het
hom
wt
Normalised Counts
0
5
10
15
20
25
zmp_ph108_F9
zmp_ph108_F11
zmp_ph108_H2
zmp_ph108_H5
zmp_ph108_B2
zmp_ph108_B6
zmp_ph108_B7
zmp_ph108_C4
zmp_ph108_C5
zmp_ph108_D4
zmp_ph97_G6
zmp_ph97_G9
zmp_ph97_H2
zmp_ph97_B10
zmp_ph97_C2
zmp_ph97_C4
zmp_ph97_B8
zmp_ph97_C1
zmp_ph97_D2
zmp_ph97_D5
zmp_ph111_G5
zmp_ph111_G11
zmp_ph111_H1
zmp_ph111_B1a
zmp_ph111_B5
zmp_ph111_B8
zmp_ph111_B6
zmp_ph111_B7
zmp_ph111_B9
zmp_ph111_B10
zmp_ph114_G4
zmp_ph114_G11
zmp_ph114_H1
zmp_ph114_H2
zmp_ph114_A9
zmp_ph114_A11
zmp_ph114_B2
zmp_ph114_B4
zmp_ph114_D8
zmp_ph114_E2

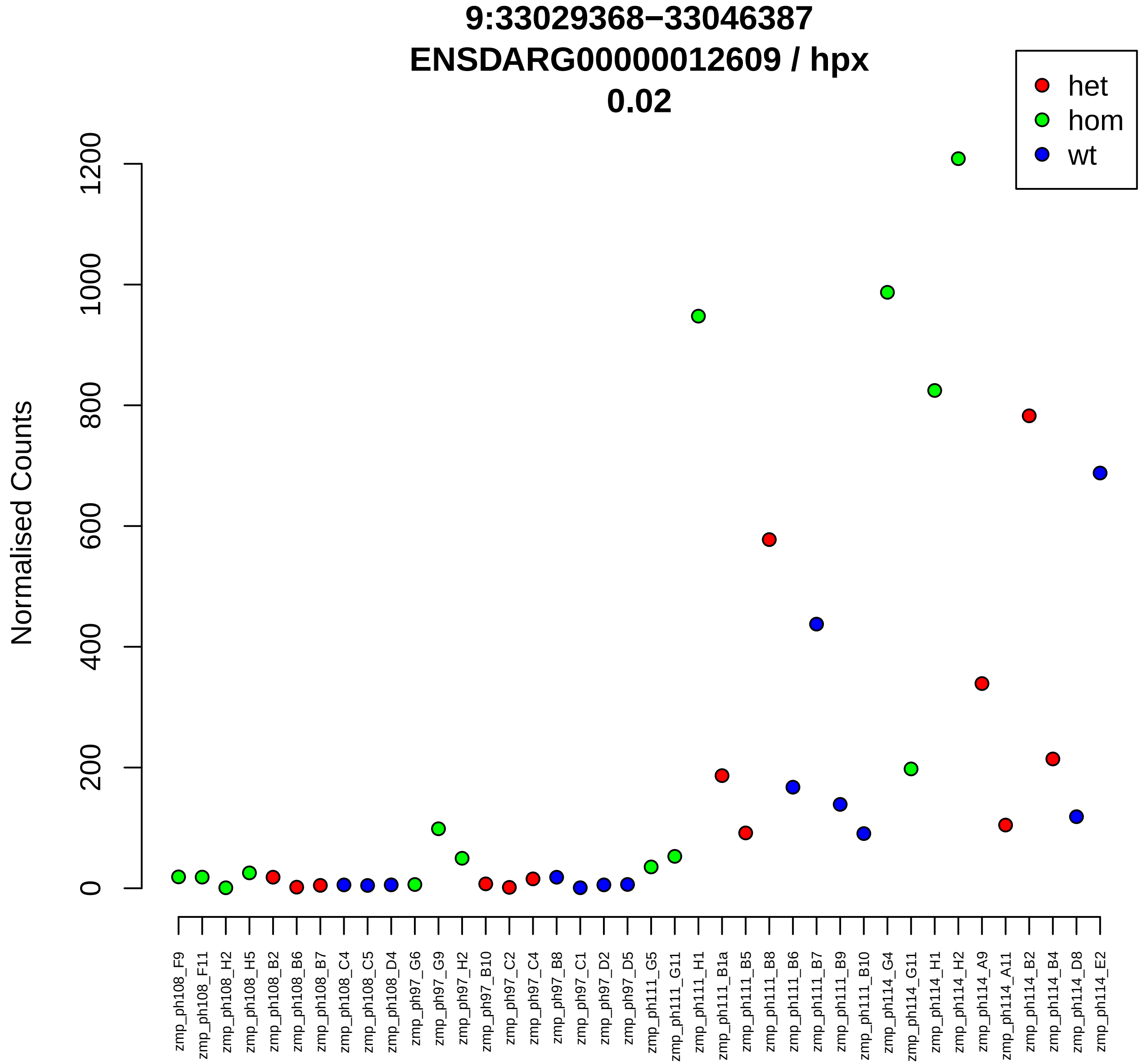

9:33029368−33046387
ENSDARG00000012609 / hpx
0.02
het
hom
wt
Normalised Counts
0
200
400
600
800
1000
1200
zmp_ph108_F9
zmp_ph108_F11
zmp_ph108_H2
zmp_ph108_H5
zmp_ph108_B2
zmp_ph108_B6
zmp_ph108_B7
zmp_ph108_C4
zmp_ph108_C5
zmp_ph108_D4
zmp_ph97_G6
zmp_ph97_G9
zmp_ph97_H2
zmp_ph97_B10
zmp_ph97_C2
zmp_ph97_C4
zmp_ph97_B8
zmp_ph97_C1
zmp_ph97_D2
zmp_ph97_D5
zmp_ph111_G5
zmp_ph111_G11
zmp_ph111_H1
zmp_ph111_B1a
zmp_ph111_B5
zmp_ph111_B8
zmp_ph111_B6
zmp_ph111_B7
zmp_ph111_B9
zmp_ph111_B10
zmp_ph114_G4
zmp_ph114_G11
zmp_ph114_H1
zmp_ph114_H2
zmp_ph114_A9
zmp_ph114_A11
zmp_ph114_B2
zmp_ph114_B4
zmp_ph114_D8
zmp_ph114_E2

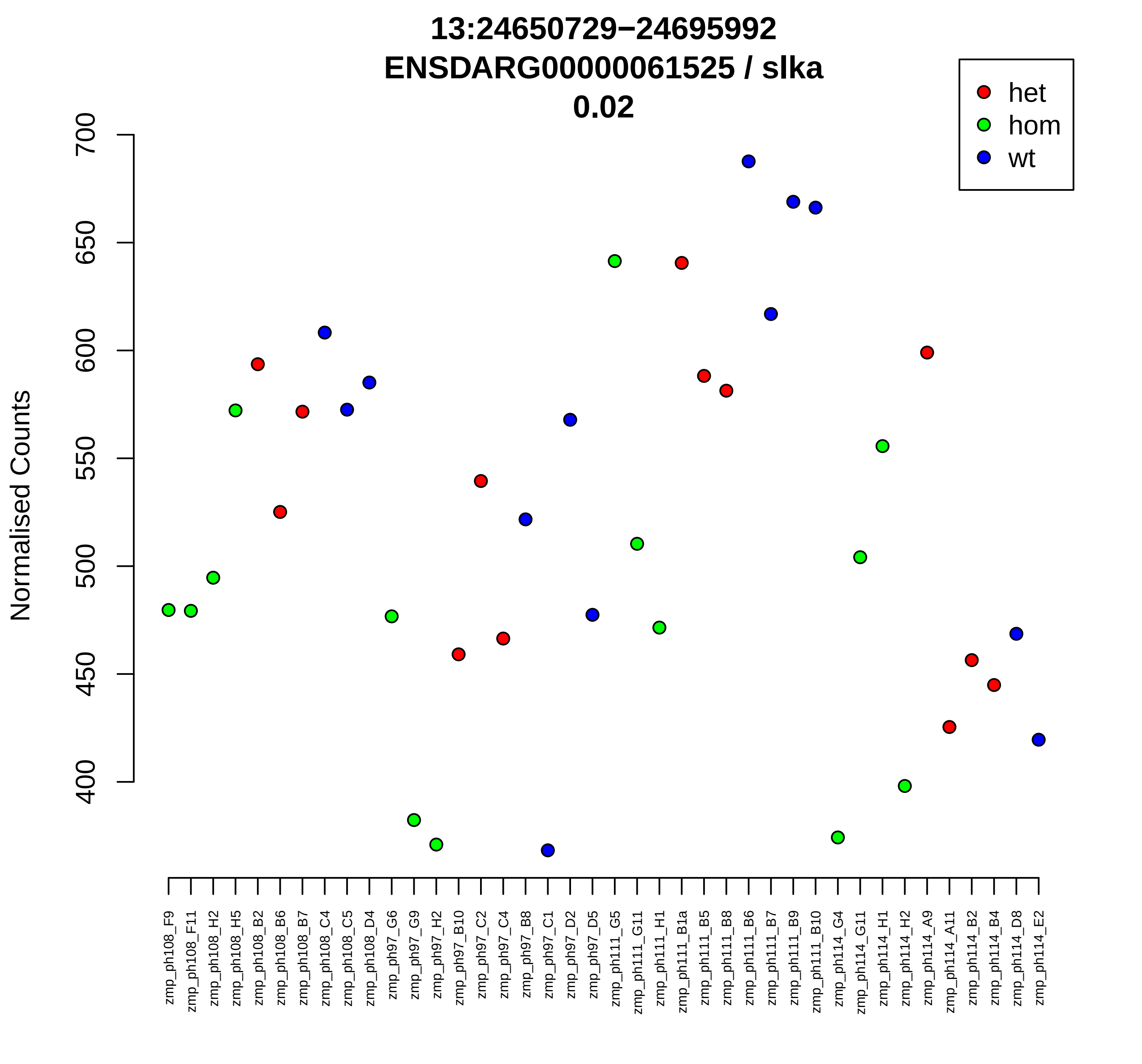

13:24650729−24695992
ENSDARG00000061525 / slka
0.02
het
hom
wt
Normalised Counts
400
450
500
550
600
650
700
zmp_ph108_F9
zmp_ph108_F11
zmp_ph108_H2
zmp_ph108_H5
zmp_ph108_B2
zmp_ph108_B6
zmp_ph108_B7
zmp_ph108_C4
zmp_ph108_C5
zmp_ph108_D4
zmp_ph97_G6
zmp_ph97_G9
zmp_ph97_H2
zmp_ph97_B10
zmp_ph97_C2
zmp_ph97_C4
zmp_ph97_B8
zmp_ph97_C1
zmp_ph97_D2
zmp_ph97_D5
zmp_ph111_G5
zmp_ph111_G11
zmp_ph111_H1
zmp_ph111_B1a
zmp_ph111_B5
zmp_ph111_B8
zmp_ph111_B6
zmp_ph111_B7
zmp_ph111_B9
zmp_ph111_B10
zmp_ph114_G4
zmp_ph114_G11
zmp_ph114_H1
zmp_ph114_H2
zmp_ph114_A9
zmp_ph114_A11
zmp_ph114_B2
zmp_ph114_B4
zmp_ph114_D8
zmp_ph114_E2

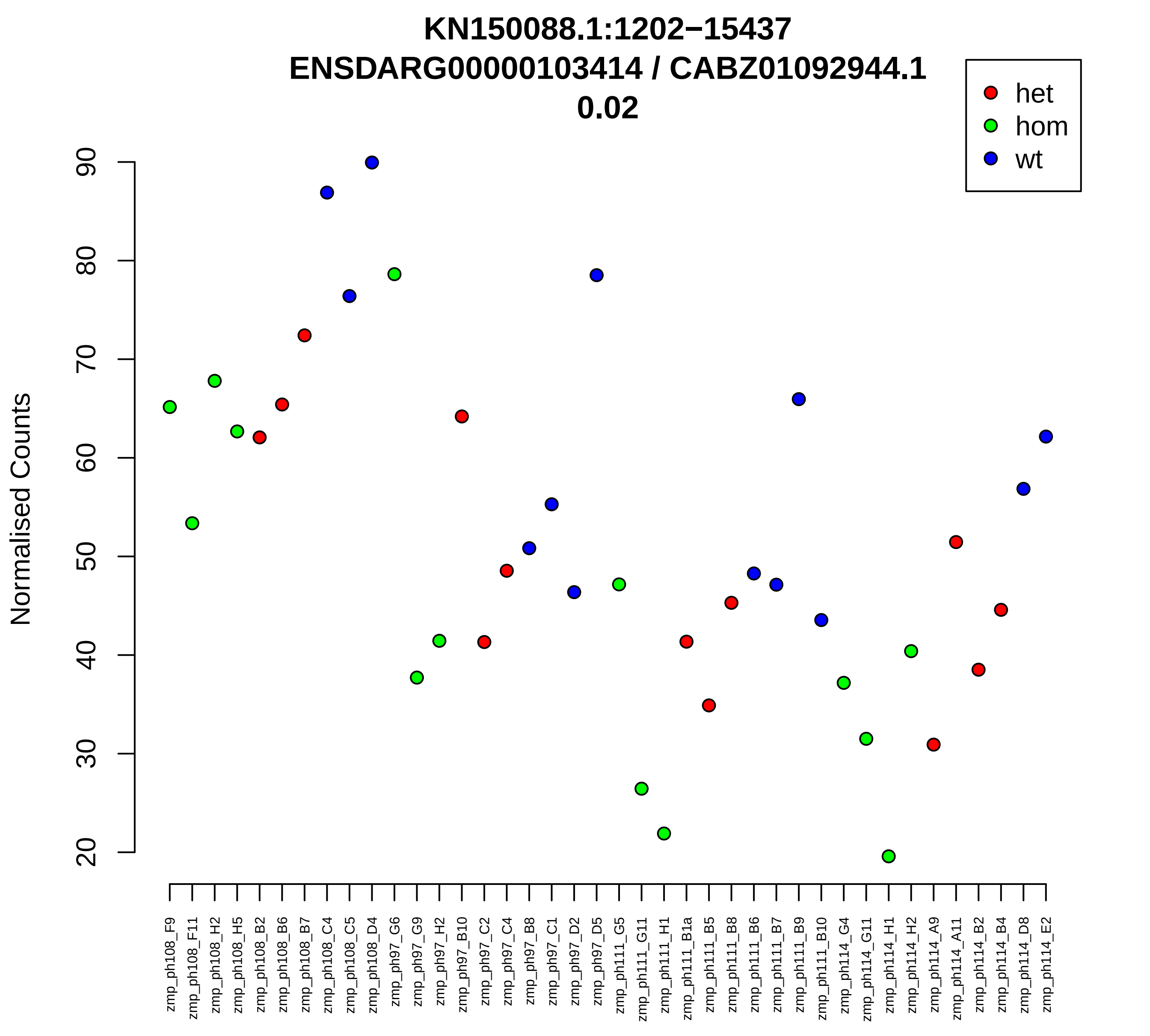
KN150088.1:1202−15437
ENSDARG00000103414 / CABZ01092944.1
0.02
het
hom
wt
Normalised Counts
20
30
40
50
60
70
80
90
zmp_ph108_F9
zmp_ph108_F11
zmp_ph108_H2
zmp_ph108_H5
zmp_ph108_B2
zmp_ph108_B6
zmp_ph108_B7
zmp_ph108_C4
zmp_ph108_C5
zmp_ph108_D4
zmp_ph97_G6
zmp_ph97_G9
zmp_ph97_H2
zmp_ph97_B10
zmp_ph97_C2
zmp_ph97_C4
zmp_ph97_B8
zmp_ph97_C1
zmp_ph97_D2
zmp_ph97_D5
zmp_ph111_G5
zmp_ph111_G11
zmp_ph111_H1
zmp_ph111_B1a
zmp_ph111_B5
zmp_ph111_B8
zmp_ph111_B6
zmp_ph111_B7
zmp_ph111_B9
zmp_ph111_B10
zmp_ph114_G4
zmp_ph114_G11
zmp_ph114_H1
zmp_ph114_H2
zmp_ph114_A9
zmp_ph114_A11
zmp_ph114_B2
zmp_ph114_B4
zmp_ph114_D8
zmp_ph114_E2

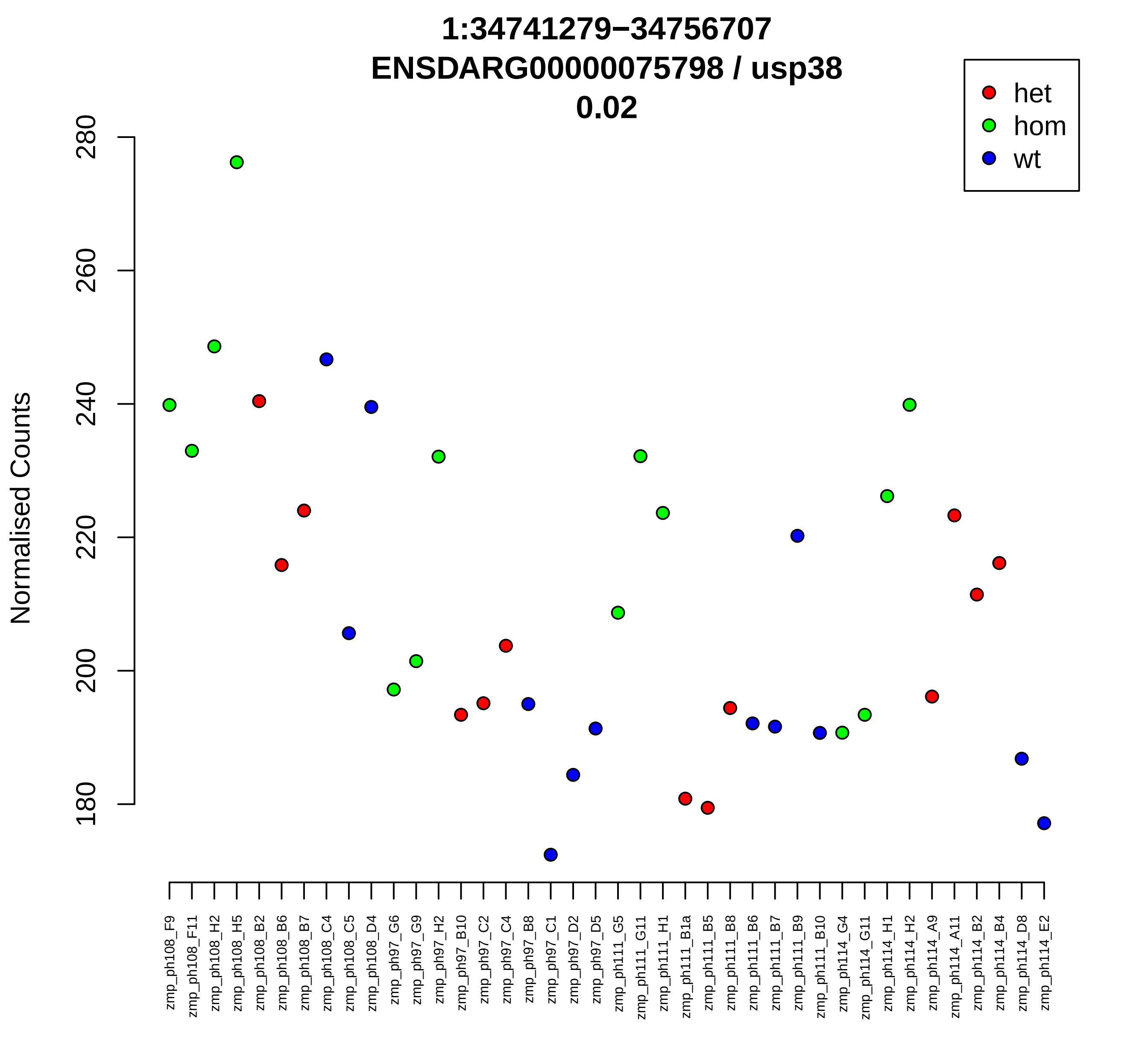

1:34741279−34756707
ENSDARG00000075798 / usp38
0.02
het
hom
wt
Normalised Counts
180
200
220
240
260
280
zmp_ph108_F9
zmp_ph108_F11
zmp_ph108_H2
zmp_ph108_H5
zmp_ph108_B2
zmp_ph108_B6
zmp_ph108_B7
zmp_ph108_C4
zmp_ph108_C5
zmp_ph108_D4
zmp_ph97_G6
zmp_ph97_G9
zmp_ph97_H2
zmp_ph97_B10
zmp_ph97_C2
zmp_ph97_C4
zmp_ph97_B8
zmp_ph97_C1
zmp_ph97_D2
zmp_ph97_D5
zmp_ph111_G5
zmp_ph111_G11
zmp_ph111_H1
zmp_ph111_B1a
zmp_ph111_B5
zmp_ph111_B8
zmp_ph111_B6
zmp_ph111_B7
zmp_ph111_B9
zmp_ph111_B10
zmp_ph114_G4
zmp_ph114_G11
zmp_ph114_H1
zmp_ph114_H2
zmp_ph114_A9
zmp_ph114_A11
zmp_ph114_B2
zmp_ph114_B4
zmp_ph114_D8
zmp_ph114_E2

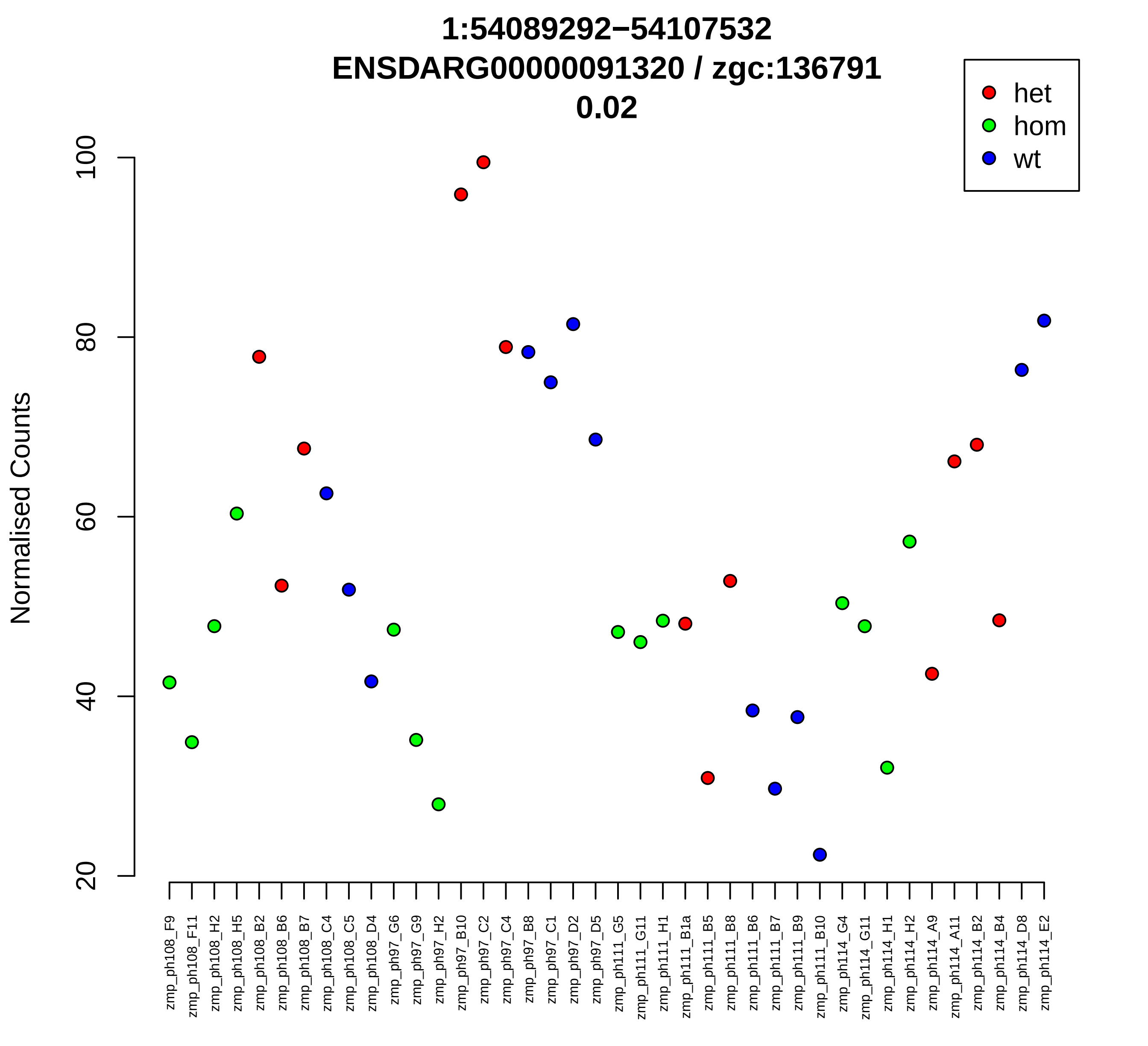

1:54089292−54107532
ENSDARG00000091320 / zgc:136791
0.02
het
hom
wt
Normalised Counts
20
40
60
80
100
zmp_ph108_F9
zmp_ph108_F11
zmp_ph108_H2
zmp_ph108_H5
zmp_ph108_B2
zmp_ph108_B6
zmp_ph108_B7
zmp_ph108_C4
zmp_ph108_C5
zmp_ph108_D4
zmp_ph97_G6
zmp_ph97_G9
zmp_ph97_H2
zmp_ph97_B10
zmp_ph97_C2
zmp_ph97_C4
zmp_ph97_B8
zmp_ph97_C1
zmp_ph97_D2
zmp_ph97_D5
zmp_ph111_G5
zmp_ph111_G11
zmp_ph111_H1
zmp_ph111_B1a
zmp_ph111_B5
zmp_ph111_B8
zmp_ph111_B6
zmp_ph111_B7
zmp_ph111_B9
zmp_ph111_B10
zmp_ph114_G4
zmp_ph114_G11
zmp_ph114_H1
zmp_ph114_H2
zmp_ph114_A9
zmp_ph114_A11
zmp_ph114_B2
zmp_ph114_B4
zmp_ph114_D8
zmp_ph114_E2

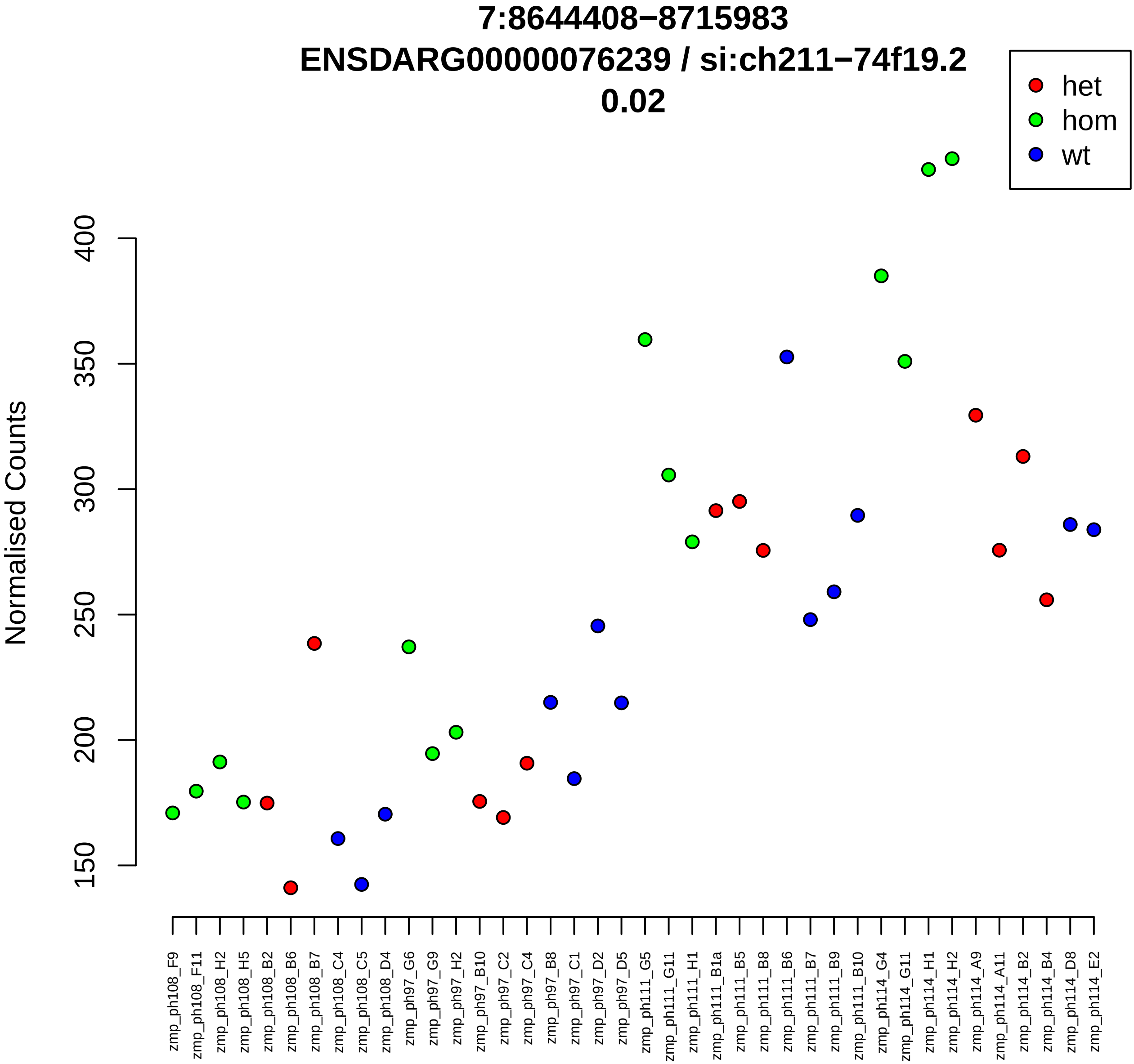
7:8644408−8715983
ENSDARG00000076239 / si:ch211−74f19.2
0.02
het
hom
wt
Normalised Counts
150
200
250
300
350
400
zmp_ph108_F9
zmp_ph108_F11
zmp_ph108_H2
zmp_ph108_H5
zmp_ph108_B2
zmp_ph108_B6
zmp_ph108_B7
zmp_ph108_C4
zmp_ph108_C5
zmp_ph108_D4
zmp_ph97_G6
zmp_ph97_G9
zmp_ph97_H2
zmp_ph97_B10
zmp_ph97_C2
zmp_ph97_C4
zmp_ph97_B8
zmp_ph97_C1
zmp_ph97_D2
zmp_ph97_D5
zmp_ph111_G5
zmp_ph111_G11
zmp_ph111_H1
zmp_ph111_B1a
zmp_ph111_B5
zmp_ph111_B8
zmp_ph111_B6
zmp_ph111_B7
zmp_ph111_B9
zmp_ph111_B10
zmp_ph114_G4
zmp_ph114_G11
zmp_ph114_H1
zmp_ph114_H2
zmp_ph114_A9
zmp_ph114_A11
zmp_ph114_B2
zmp_ph114_B4
zmp_ph114_D8
zmp_ph114_E2

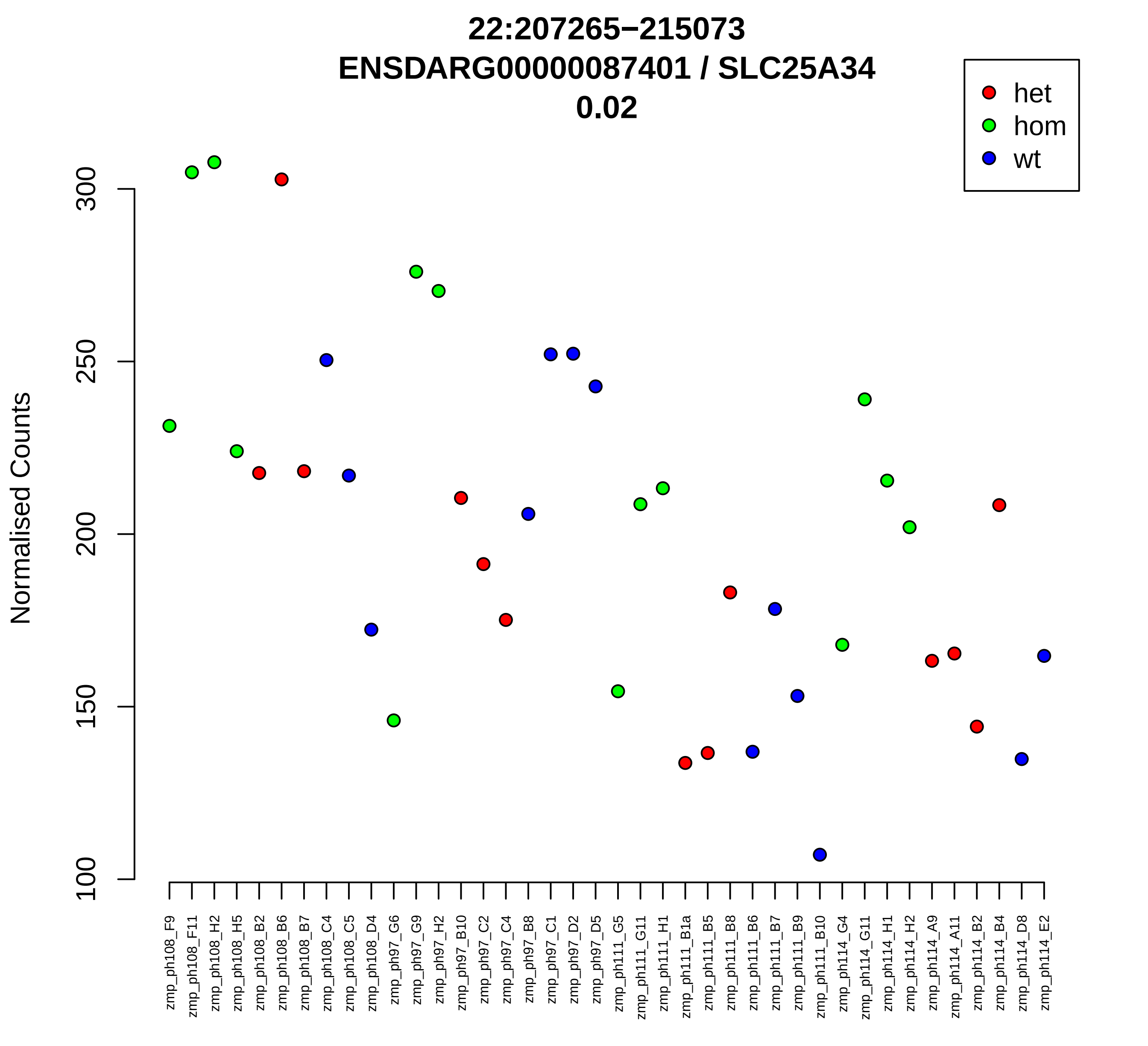

22:207265−215073
ENSDARG00000087401 / SLC25A34
0.02
het
hom
wt
Normalised Counts
100
150
200
250
300
zmp_ph108_F9
zmp_ph108_F11
zmp_ph108_H2
zmp_ph108_H5
zmp_ph108_B2
zmp_ph108_B6
zmp_ph108_B7
zmp_ph108_C4
zmp_ph108_C5
zmp_ph108_D4
zmp_ph97_G6
zmp_ph97_G9
zmp_ph97_H2
zmp_ph97_B10
zmp_ph97_C2
zmp_ph97_C4
zmp_ph97_B8
zmp_ph97_C1
zmp_ph97_D2
zmp_ph97_D5
zmp_ph111_G5
zmp_ph111_G11
zmp_ph111_H1
zmp_ph111_B1a
zmp_ph111_B5
zmp_ph111_B8
zmp_ph111_B6
zmp_ph111_B7
zmp_ph111_B9
zmp_ph111_B10
zmp_ph114_G4
zmp_ph114_G11
zmp_ph114_H1
zmp_ph114_H2
zmp_ph114_A9
zmp_ph114_A11
zmp_ph114_B2
zmp_ph114_B4
zmp_ph114_D8
zmp_ph114_E2

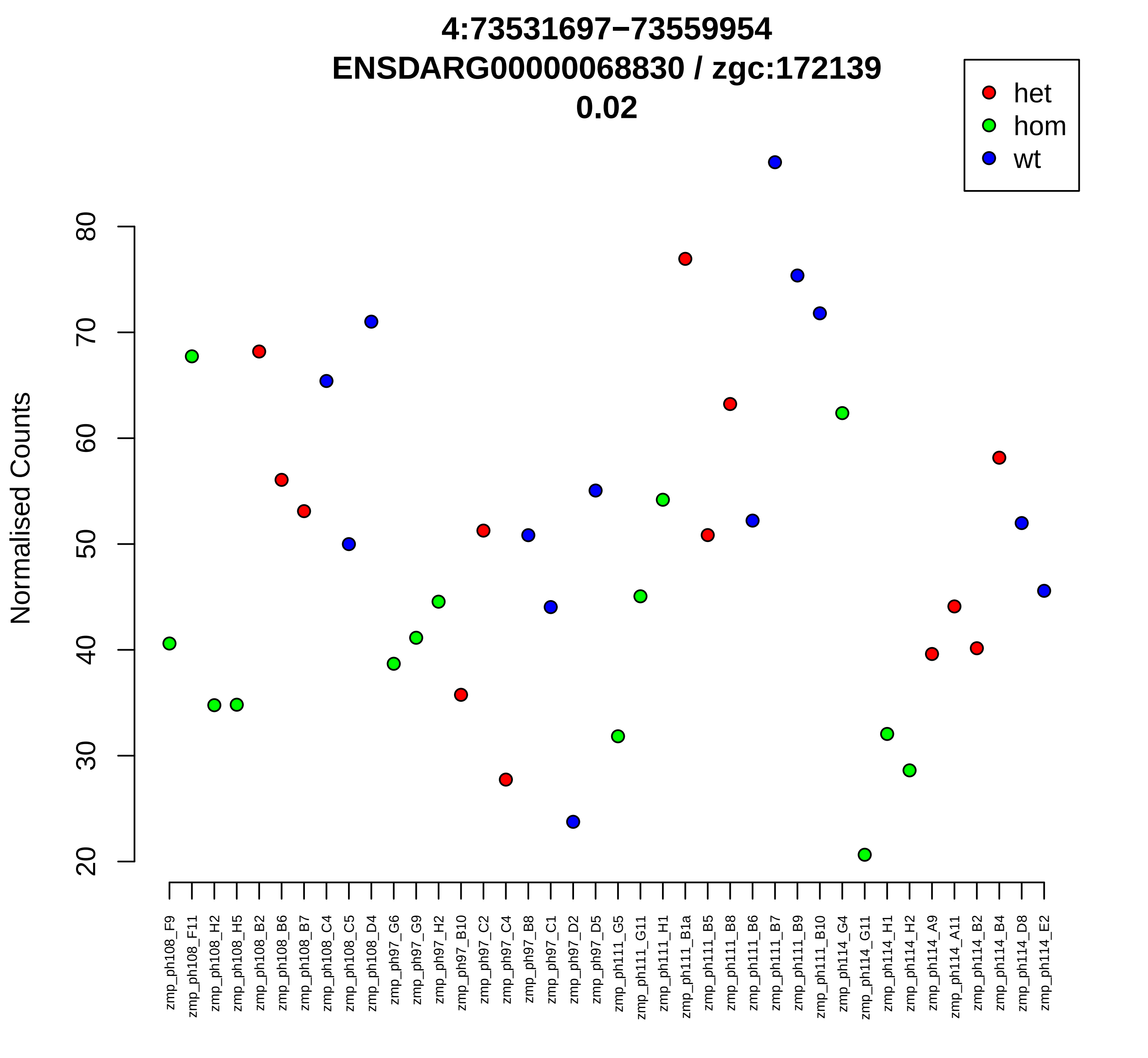
4:73531697−73559954
ENSDARG00000068830 / zgc:172139
0.02
het
hom
wt
Normalised Counts
20
30
40
50
60
70
80
zmp_ph108_F9
zmp_ph108_F11
zmp_ph108_H2
zmp_ph108_H5
zmp_ph108_B2
zmp_ph108_B6
zmp_ph108_B7
zmp_ph108_C4
zmp_ph108_C5
zmp_ph108_D4
zmp_ph97_G6
zmp_ph97_G9
zmp_ph97_H2
zmp_ph97_B10
zmp_ph97_C2
zmp_ph97_C4
zmp_ph97_B8
zmp_ph97_C1
zmp_ph97_D2
zmp_ph97_D5
zmp_ph111_G5
zmp_ph111_G11
zmp_ph111_H1
zmp_ph111_B1a
zmp_ph111_B5
zmp_ph111_B8
zmp_ph111_B6
zmp_ph111_B7
zmp_ph111_B9
zmp_ph111_B10
zmp_ph114_G4
zmp_ph114_G11
zmp_ph114_H1
zmp_ph114_H2
zmp_ph114_A9
zmp_ph114_A11
zmp_ph114_B2
zmp_ph114_B4
zmp_ph114_D8
zmp_ph114_E2

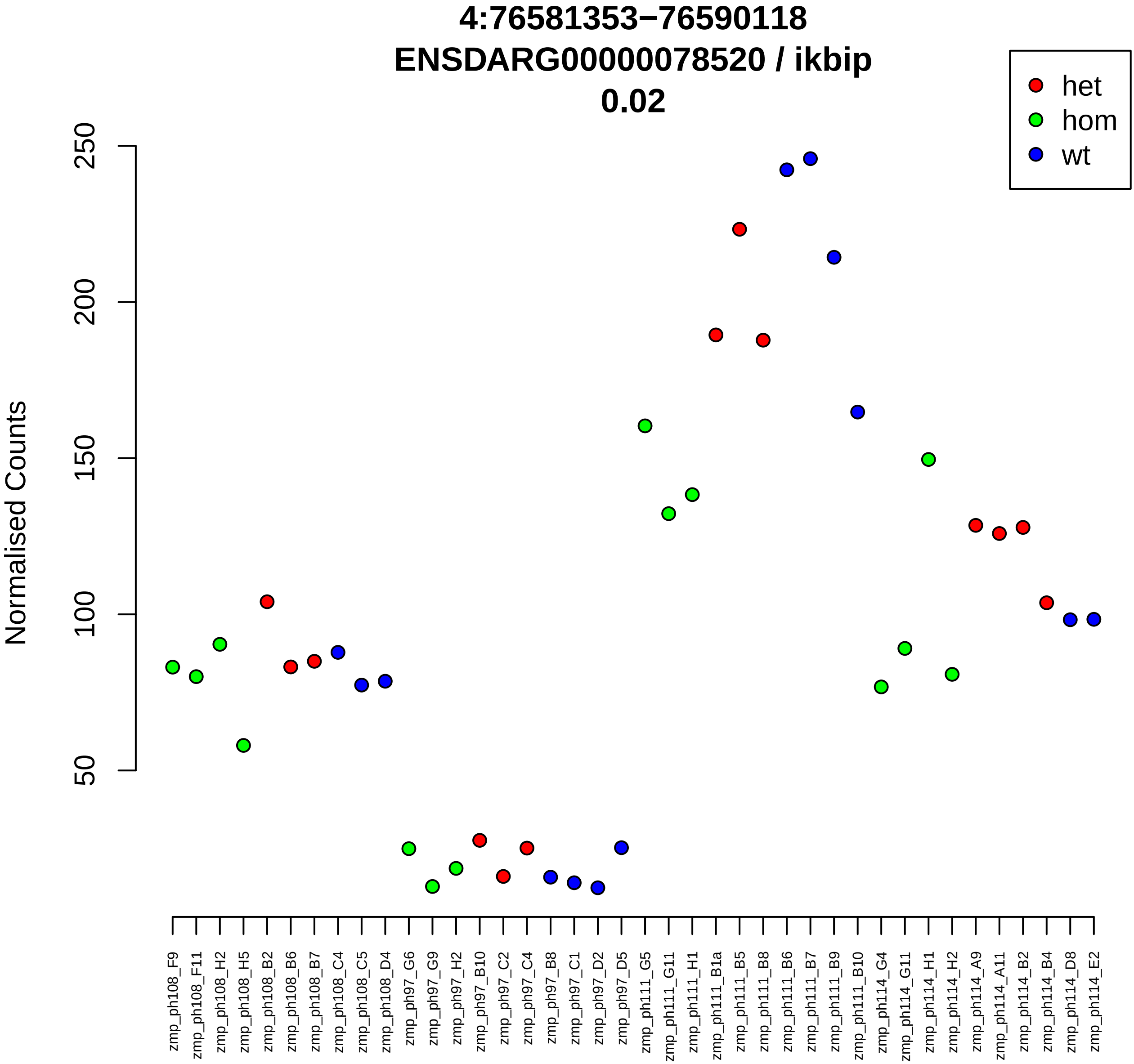

4:76581353−76590118
ENSDARG00000078520 / ikbip
0.02
het
hom
wt
Normalised Counts
50
100
150
200
250
zmp_ph108_F9
zmp_ph108_F11
zmp_ph108_H2
zmp_ph108_H5
zmp_ph108_B2
zmp_ph108_B6
zmp_ph108_B7
zmp_ph108_C4
zmp_ph108_C5
zmp_ph108_D4
zmp_ph97_G6
zmp_ph97_G9
zmp_ph97_H2
zmp_ph97_B10
zmp_ph97_C2
zmp_ph97_C4
zmp_ph97_B8
zmp_ph97_C1
zmp_ph97_D2
zmp_ph97_D5
zmp_ph111_G5
zmp_ph111_G11
zmp_ph111_H1
zmp_ph111_B1a
zmp_ph111_B5
zmp_ph111_B8
zmp_ph111_B6
zmp_ph111_B7
zmp_ph111_B9
zmp_ph111_B10
zmp_ph114_G4
zmp_ph114_G11
zmp_ph114_H1
zmp_ph114_H2
zmp_ph114_A9
zmp_ph114_A11
zmp_ph114_B2
zmp_ph114_B4
zmp_ph114_D8
zmp_ph114_E2

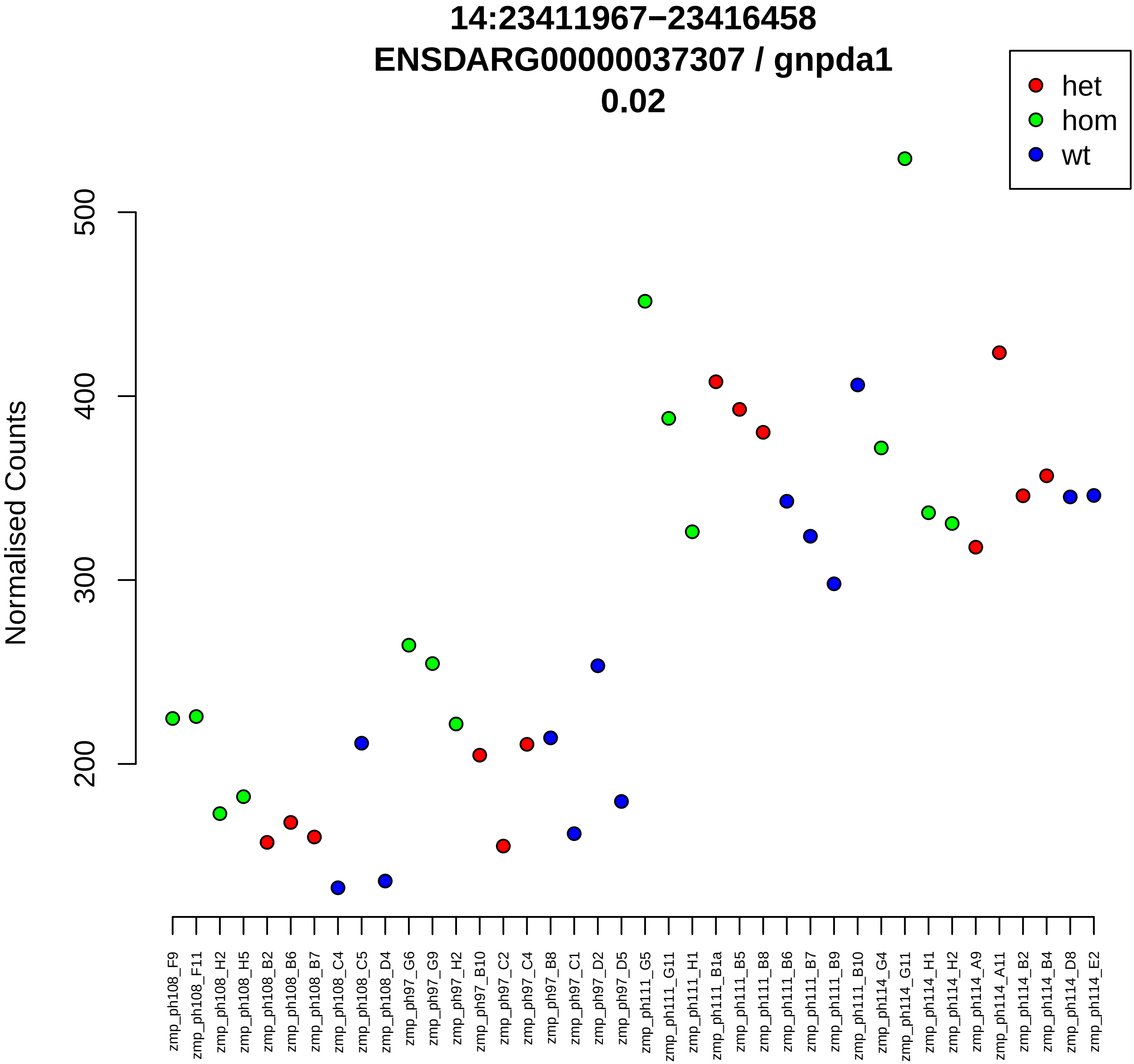
14:23411967−23416458
ENSDARG00000037307 / gnpda1
0.02
het
hom
wt
Normalised Counts
200
300
400
500
zmp_ph108_F9
zmp_ph108_F11
zmp_ph108_H2
zmp_ph108_H5
zmp_ph108_B2
zmp_ph108_B6
zmp_ph108_B7
zmp_ph108_C4
zmp_ph108_C5
zmp_ph108_D4
zmp_ph97_G6
zmp_ph97_G9
zmp_ph97_H2
zmp_ph97_B10
zmp_ph97_C2
zmp_ph97_C4
zmp_ph97_B8
zmp_ph97_C1
zmp_ph97_D2
zmp_ph97_D5
zmp_ph111_G5
zmp_ph111_G11
zmp_ph111_H1
zmp_ph111_B1a
zmp_ph111_B5
zmp_ph111_B8
zmp_ph111_B6
zmp_ph111_B7
zmp_ph111_B9
zmp_ph111_B10
zmp_ph114_G4
zmp_ph114_G11
zmp_ph114_H1
zmp_ph114_H2
zmp_ph114_A9
zmp_ph114_A11
zmp_ph114_B2
zmp_ph114_B4
zmp_ph114_D8
zmp_ph114_E2

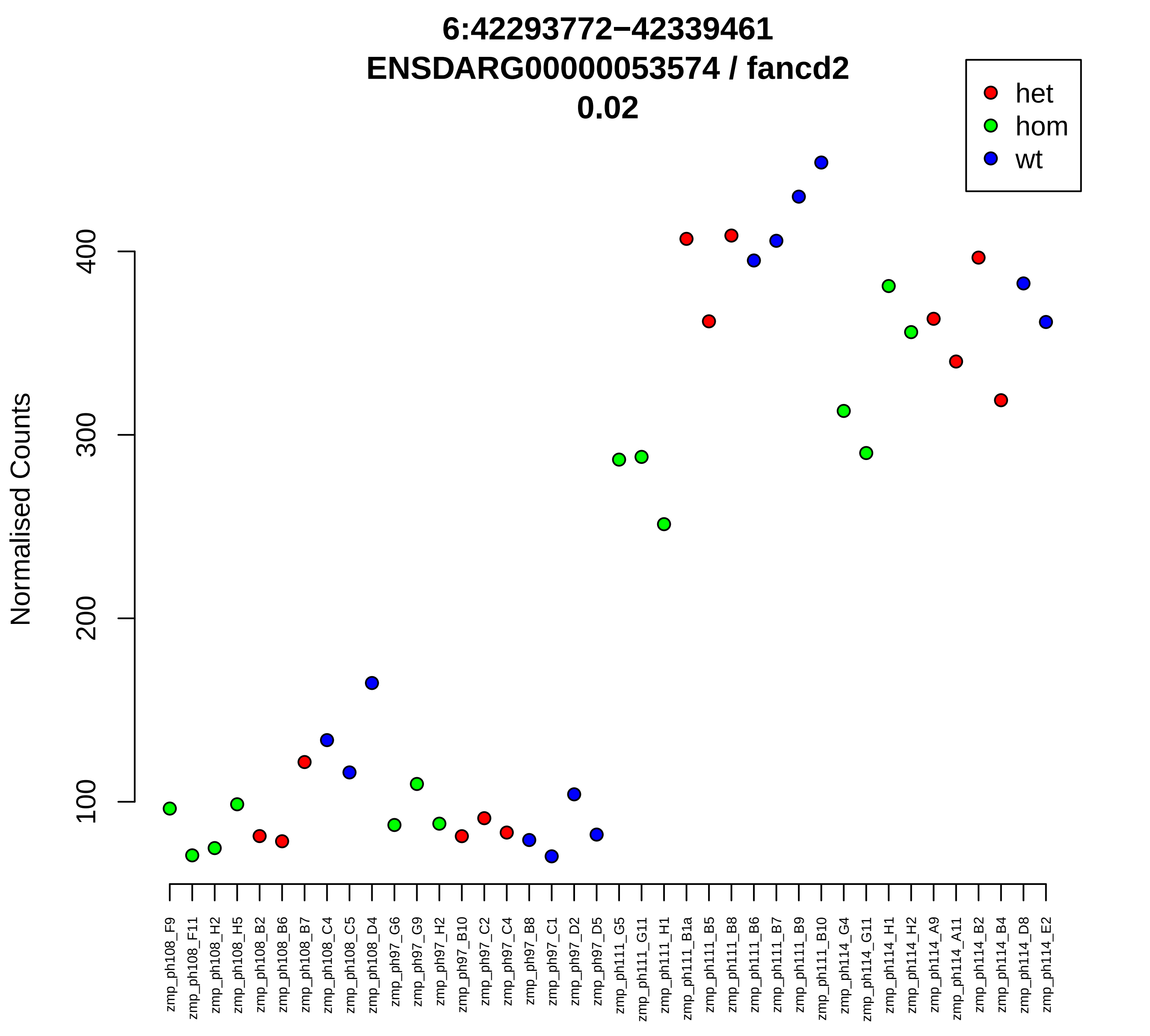
6:42293772−42339461
ENSDARG00000053574 / fancd2
0.02
het
hom
wt
Normalised Counts
100
200
300
400
zmp_ph108_F9
zmp_ph108_F11
zmp_ph108_H2
zmp_ph108_H5
zmp_ph108_B2
zmp_ph108_B6
zmp_ph108_B7
zmp_ph108_C4
zmp_ph108_C5
zmp_ph108_D4
zmp_ph97_G6
zmp_ph97_G9
zmp_ph97_H2
zmp_ph97_B10
zmp_ph97_C2
zmp_ph97_C4
zmp_ph97_B8
zmp_ph97_C1
zmp_ph97_D2
zmp_ph97_D5
zmp_ph111_G5
zmp_ph111_G11
zmp_ph111_H1
zmp_ph111_B1a
zmp_ph111_B5
zmp_ph111_B8
zmp_ph111_B6
zmp_ph111_B7
zmp_ph111_B9
zmp_ph111_B10
zmp_ph114_G4
zmp_ph114_G11
zmp_ph114_H1
zmp_ph114_H2
zmp_ph114_A9
zmp_ph114_A11
zmp_ph114_B2
zmp_ph114_B4
zmp_ph114_D8
zmp_ph114_E2

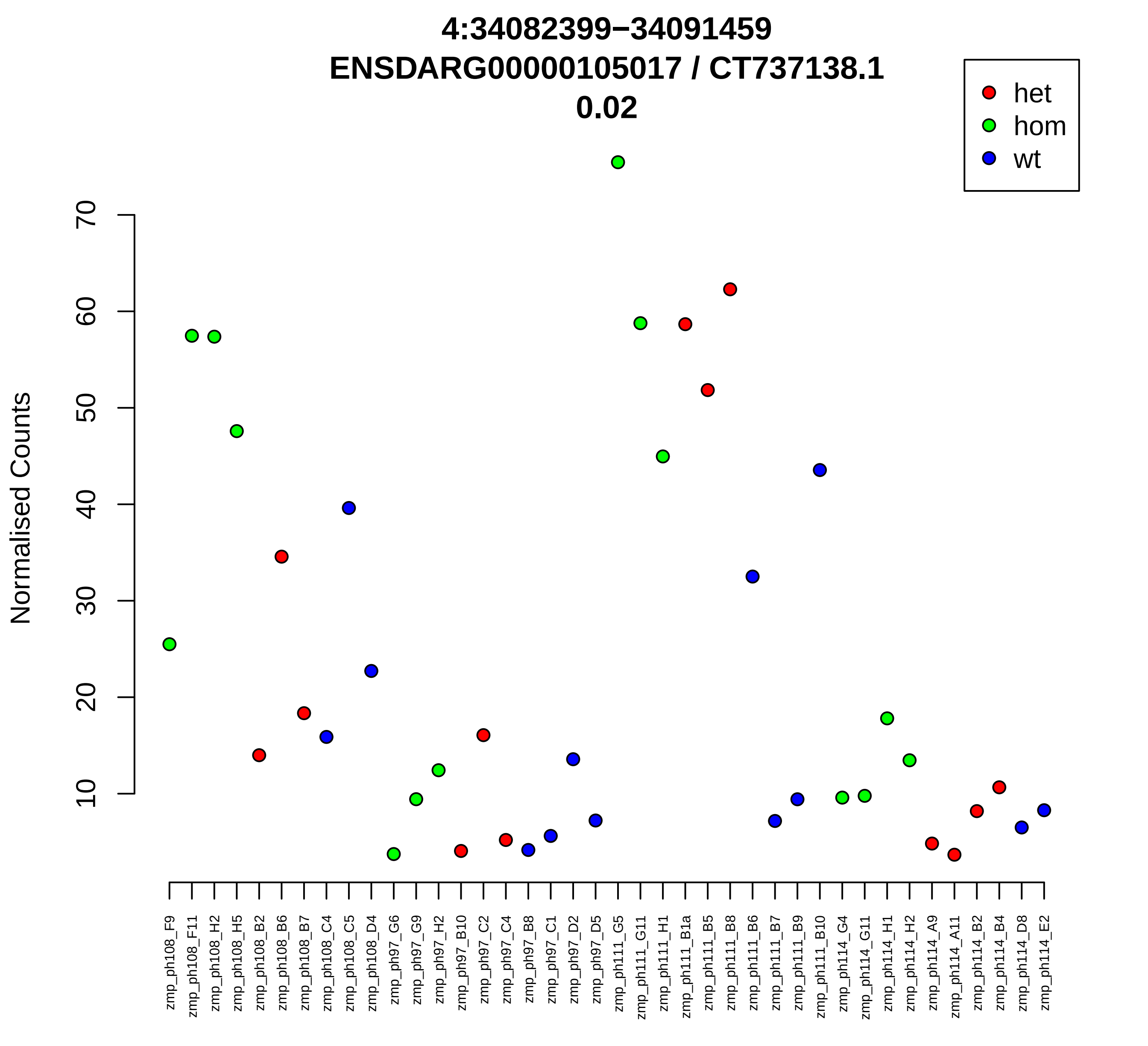

4:34082399−34091459
ENSDARG00000105017 / CT737138.1
0.02
het
hom
wt
Normalised Counts
10
20
30
40
50
60
70
zmp_ph108_F9
zmp_ph108_F11
zmp_ph108_H2
zmp_ph108_H5
zmp_ph108_B2
zmp_ph108_B6
zmp_ph108_B7
zmp_ph108_C4
zmp_ph108_C5
zmp_ph108_D4
zmp_ph97_G6
zmp_ph97_G9
zmp_ph97_H2
zmp_ph97_B10
zmp_ph97_C2
zmp_ph97_C4
zmp_ph97_B8
zmp_ph97_C1
zmp_ph97_D2
zmp_ph97_D5
zmp_ph111_G5
zmp_ph111_G11
zmp_ph111_H1
zmp_ph111_B1a
zmp_ph111_B5
zmp_ph111_B8
zmp_ph111_B6
zmp_ph111_B7
zmp_ph111_B9
zmp_ph111_B10
zmp_ph114_G4
zmp_ph114_G11
zmp_ph114_H1
zmp_ph114_H2
zmp_ph114_A9
zmp_ph114_A11
zmp_ph114_B2
zmp_ph114_B4
zmp_ph114_D8
zmp_ph114_E2

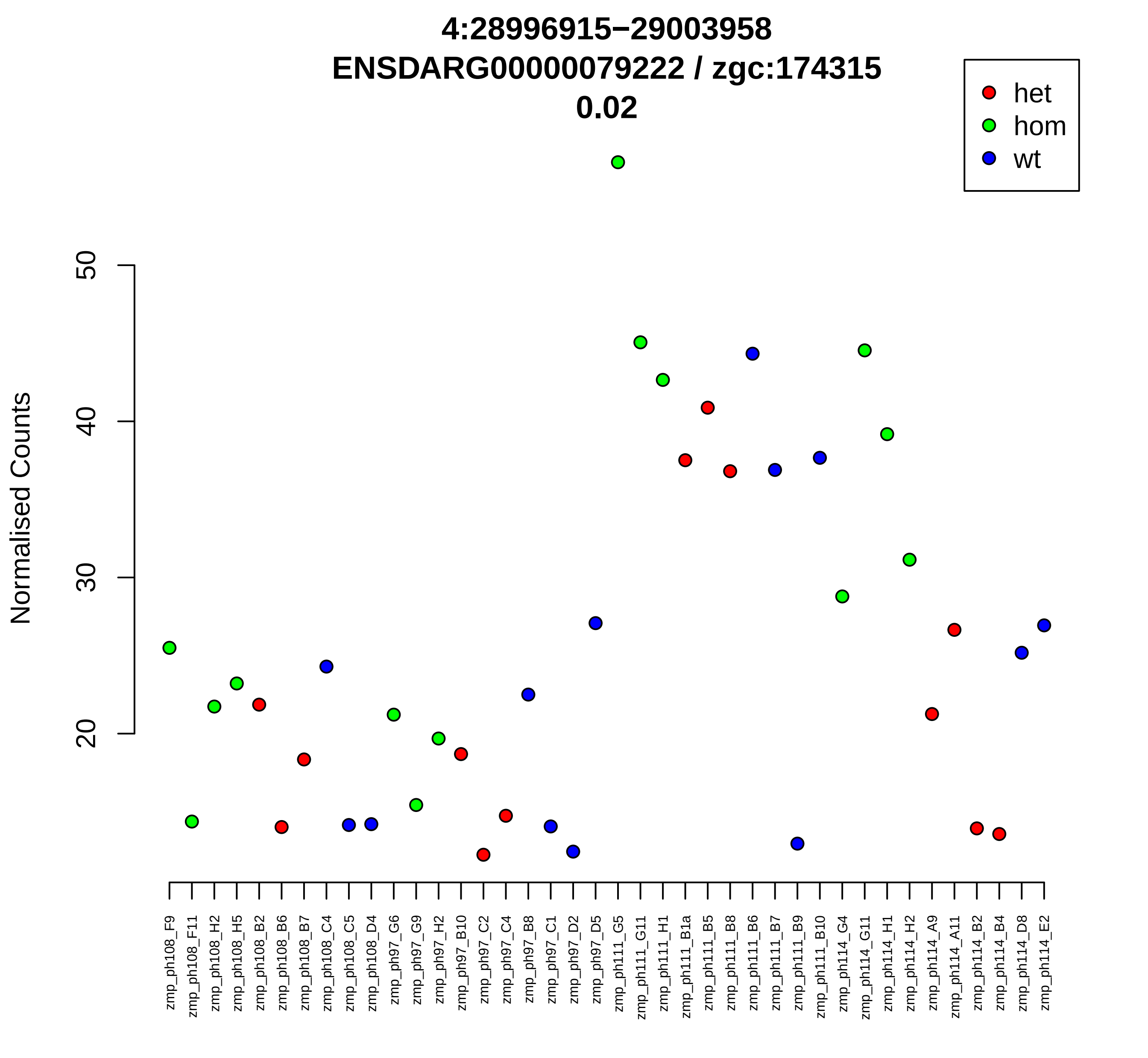
4:28996915−29003958
ENSDARG00000079222 / zgc:174315
0.02
het
hom
wt
Normalised Counts
20
30
40
50
zmp_ph108_F9
zmp_ph108_F11
zmp_ph108_H2
zmp_ph108_H5
zmp_ph108_B2
zmp_ph108_B6
zmp_ph108_B7
zmp_ph108_C4
zmp_ph108_C5
zmp_ph108_D4
zmp_ph97_G6
zmp_ph97_G9
zmp_ph97_H2
zmp_ph97_B10
zmp_ph97_C2
zmp_ph97_C4
zmp_ph97_B8
zmp_ph97_C1
zmp_ph97_D2
zmp_ph97_D5
zmp_ph111_G5
zmp_ph111_G11
zmp_ph111_H1
zmp_ph111_B1a
zmp_ph111_B5
zmp_ph111_B8
zmp_ph111_B6
zmp_ph111_B7
zmp_ph111_B9
zmp_ph111_B10
zmp_ph114_G4
zmp_ph114_G11
zmp_ph114_H1
zmp_ph114_H2
zmp_ph114_A9
zmp_ph114_A11
zmp_ph114_B2
zmp_ph114_B4
zmp_ph114_D8
zmp_ph114_E2

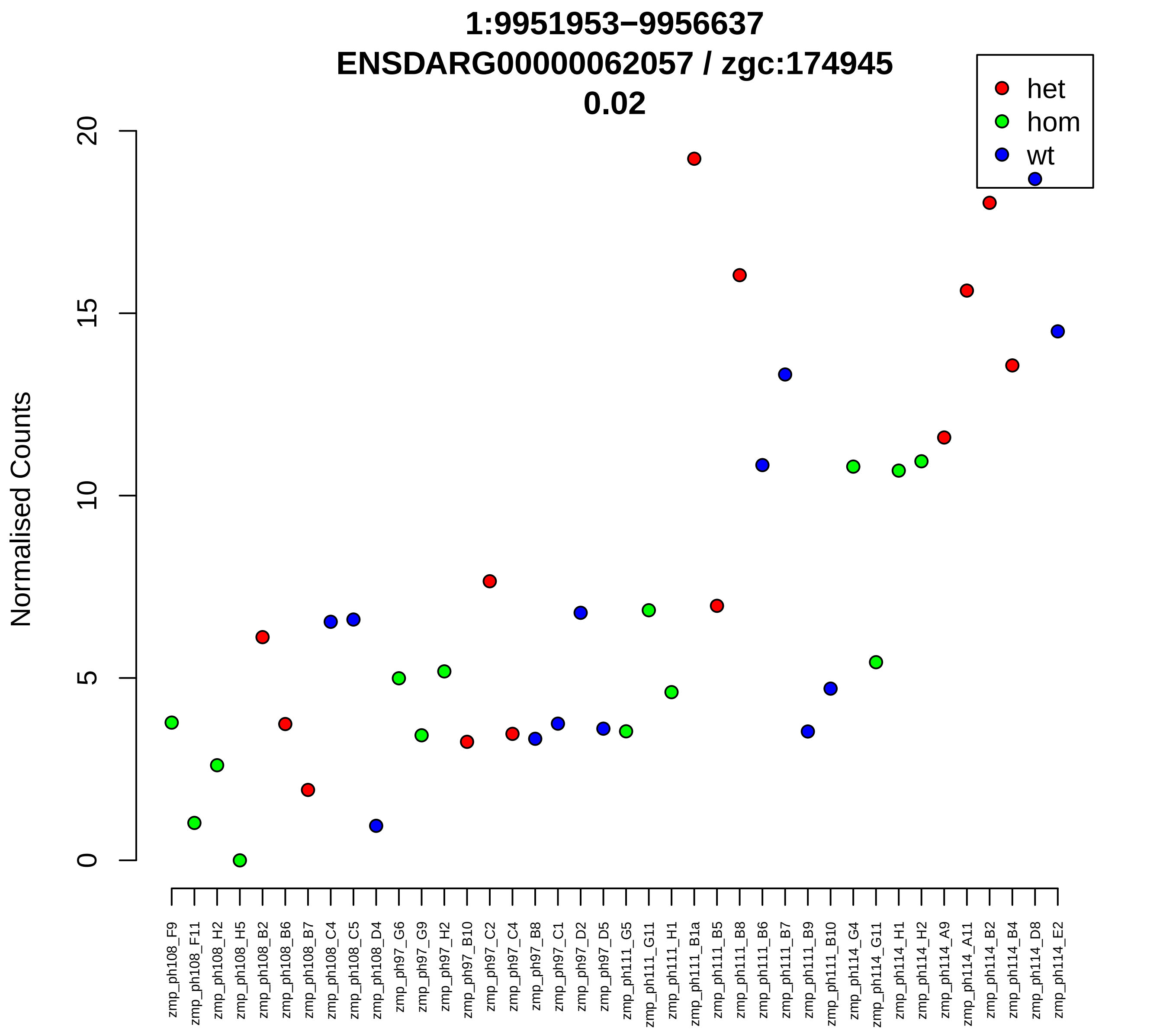

1:9951953−9956637
ENSDARG00000062057 / zgc:174945
0.02
het
hom
wt
Normalised Counts
0
5
10
15
20
zmp_ph108_F9
zmp_ph108_F11
zmp_ph108_H2
zmp_ph108_H5
zmp_ph108_B2
zmp_ph108_B6
zmp_ph108_B7
zmp_ph108_C4
zmp_ph108_C5
zmp_ph108_D4
zmp_ph97_G6
zmp_ph97_G9
zmp_ph97_H2
zmp_ph97_B10
zmp_ph97_C2
zmp_ph97_C4
zmp_ph97_B8
zmp_ph97_C1
zmp_ph97_D2
zmp_ph97_D5
zmp_ph111_G5
zmp_ph111_G11
zmp_ph111_H1
zmp_ph111_B1a
zmp_ph111_B5
zmp_ph111_B8
zmp_ph111_B6
zmp_ph111_B7
zmp_ph111_B9
zmp_ph111_B10
zmp_ph114_G4
zmp_ph114_G11
zmp_ph114_H1
zmp_ph114_H2
zmp_ph114_A9
zmp_ph114_A11
zmp_ph114_B2
zmp_ph114_B4
zmp_ph114_D8
zmp_ph114_E2

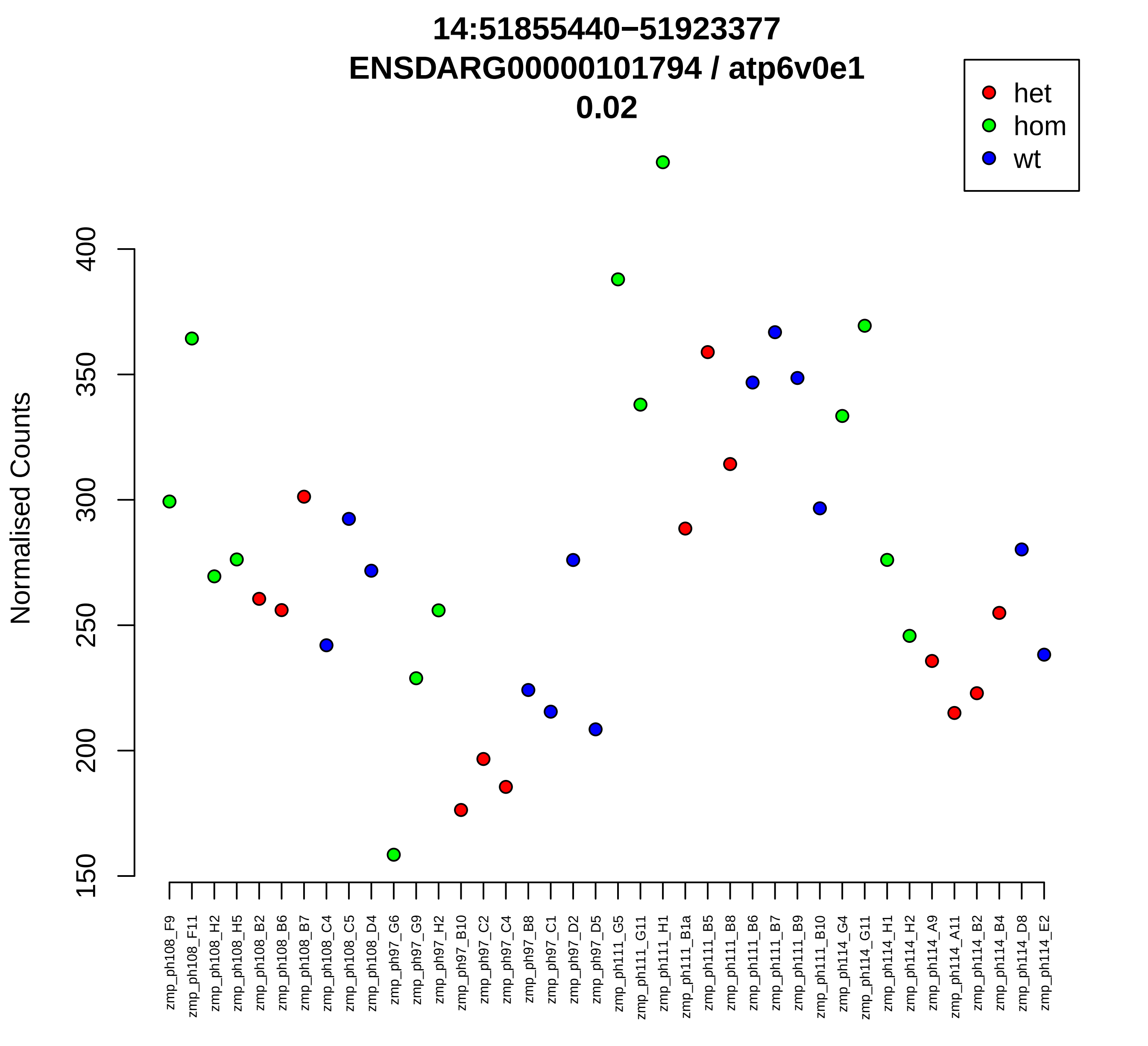
14:51855440−51923377
ENSDARG00000101794 / atp6v0e1
0.02
het
hom
wt
Normalised Counts
150
200
250
300
350
400
zmp_ph108_F9
zmp_ph108_F11
zmp_ph108_H2
zmp_ph108_H5
zmp_ph108_B2
zmp_ph108_B6
zmp_ph108_B7
zmp_ph108_C4
zmp_ph108_C5
zmp_ph108_D4
zmp_ph97_G6
zmp_ph97_G9
zmp_ph97_H2
zmp_ph97_B10
zmp_ph97_C2
zmp_ph97_C4
zmp_ph97_B8
zmp_ph97_C1
zmp_ph97_D2
zmp_ph97_D5
zmp_ph111_G5
zmp_ph111_G11
zmp_ph111_H1
zmp_ph111_B1a
zmp_ph111_B5
zmp_ph111_B8
zmp_ph111_B6
zmp_ph111_B7
zmp_ph111_B9
zmp_ph111_B10
zmp_ph114_G4
zmp_ph114_G11
zmp_ph114_H1
zmp_ph114_H2
zmp_ph114_A9
zmp_ph114_A11
zmp_ph114_B2
zmp_ph114_B4
zmp_ph114_D8
zmp_ph114_E2

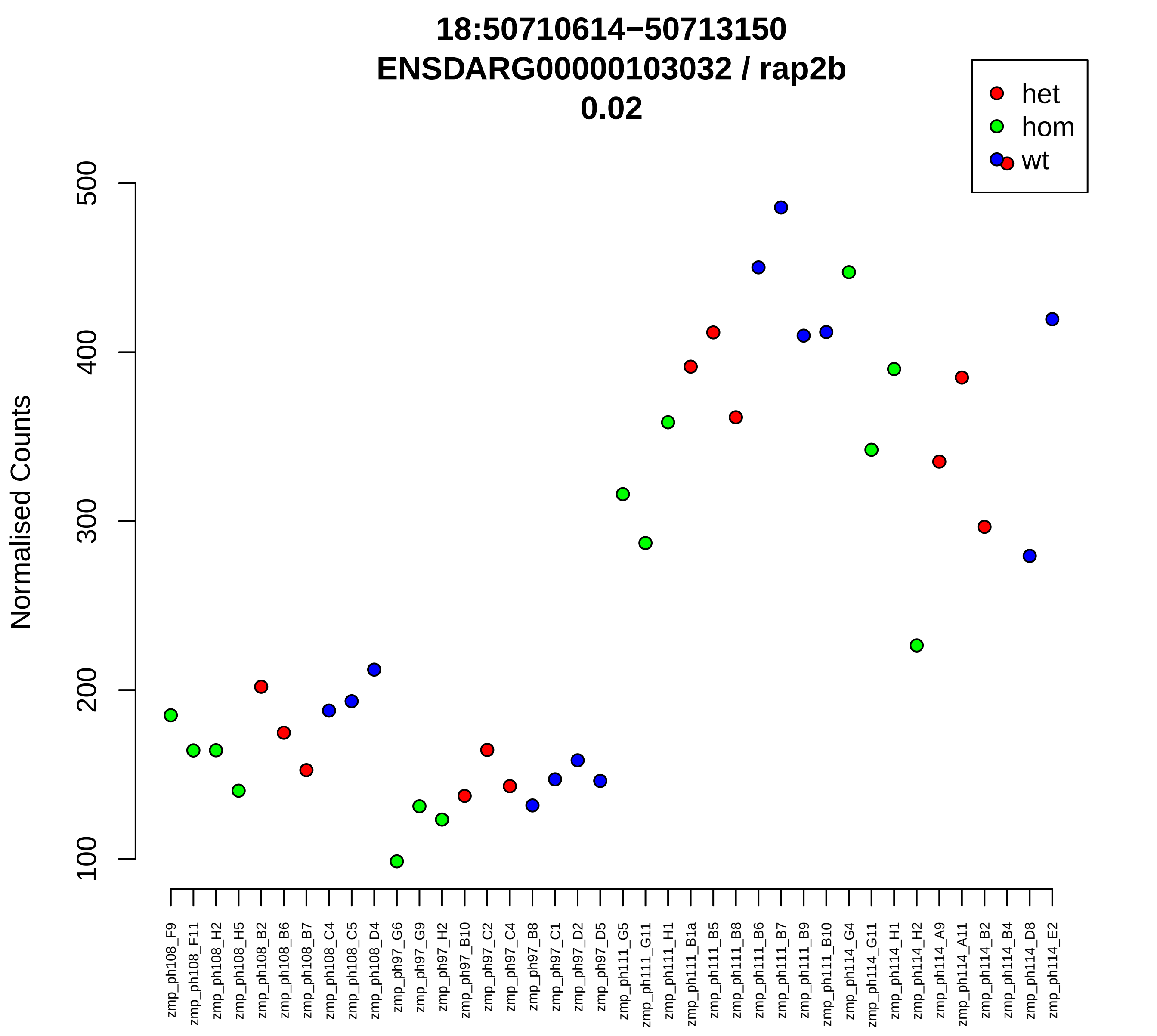

18:50710614−50713150
ENSDARG00000103032 / rap2b
0.02
het
hom
wt
Normalised Counts
100
200
300
400
500
zmp_ph108_F9
zmp_ph108_F11
zmp_ph108_H2
zmp_ph108_H5
zmp_ph108_B2
zmp_ph108_B6
zmp_ph108_B7
zmp_ph108_C4
zmp_ph108_C5
zmp_ph108_D4
zmp_ph97_G6
zmp_ph97_G9
zmp_ph97_H2
zmp_ph97_B10
zmp_ph97_C2
zmp_ph97_C4
zmp_ph97_B8
zmp_ph97_C1
zmp_ph97_D2
zmp_ph97_D5
zmp_ph111_G5
zmp_ph111_G11
zmp_ph111_H1
zmp_ph111_B1a
zmp_ph111_B5
zmp_ph111_B8
zmp_ph111_B6
zmp_ph111_B7
zmp_ph111_B9
zmp_ph111_B10
zmp_ph114_G4
zmp_ph114_G11
zmp_ph114_H1
zmp_ph114_H2
zmp_ph114_A9
zmp_ph114_A11
zmp_ph114_B2
zmp_ph114_B4
zmp_ph114_D8
zmp_ph114_E2

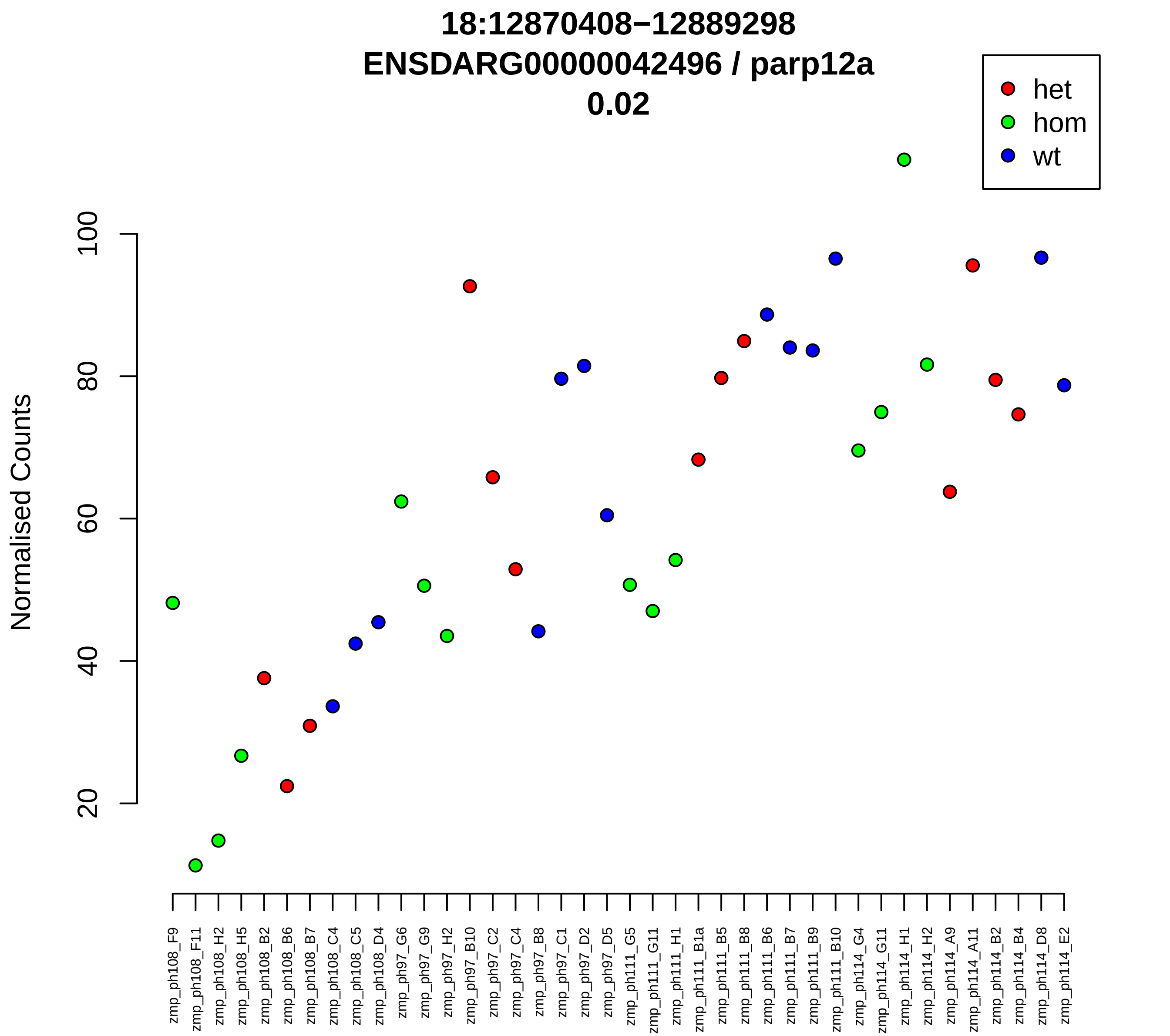

18:12870408−12889298
ENSDARG00000042496 / parp12a
0.02
het
hom
wt
Normalised Counts
20
40
60
80
100
zmp_ph108_F9
zmp_ph108_F11
zmp_ph108_H2
zmp_ph108_H5
zmp_ph108_B2
zmp_ph108_B6
zmp_ph108_B7
zmp_ph108_C4
zmp_ph108_C5
zmp_ph108_D4
zmp_ph97_G6
zmp_ph97_G9
zmp_ph97_H2
zmp_ph97_B10
zmp_ph97_C2
zmp_ph97_C4
zmp_ph97_B8
zmp_ph97_C1
zmp_ph97_D2
zmp_ph97_D5
zmp_ph111_G5
zmp_ph111_G11
zmp_ph111_H1
zmp_ph111_B1a
zmp_ph111_B5
zmp_ph111_B8
zmp_ph111_B6
zmp_ph111_B7
zmp_ph111_B9
zmp_ph111_B10
zmp_ph114_G4
zmp_ph114_G11
zmp_ph114_H1
zmp_ph114_H2
zmp_ph114_A9
zmp_ph114_A11
zmp_ph114_B2
zmp_ph114_B4
zmp_ph114_D8
zmp_ph114_E2

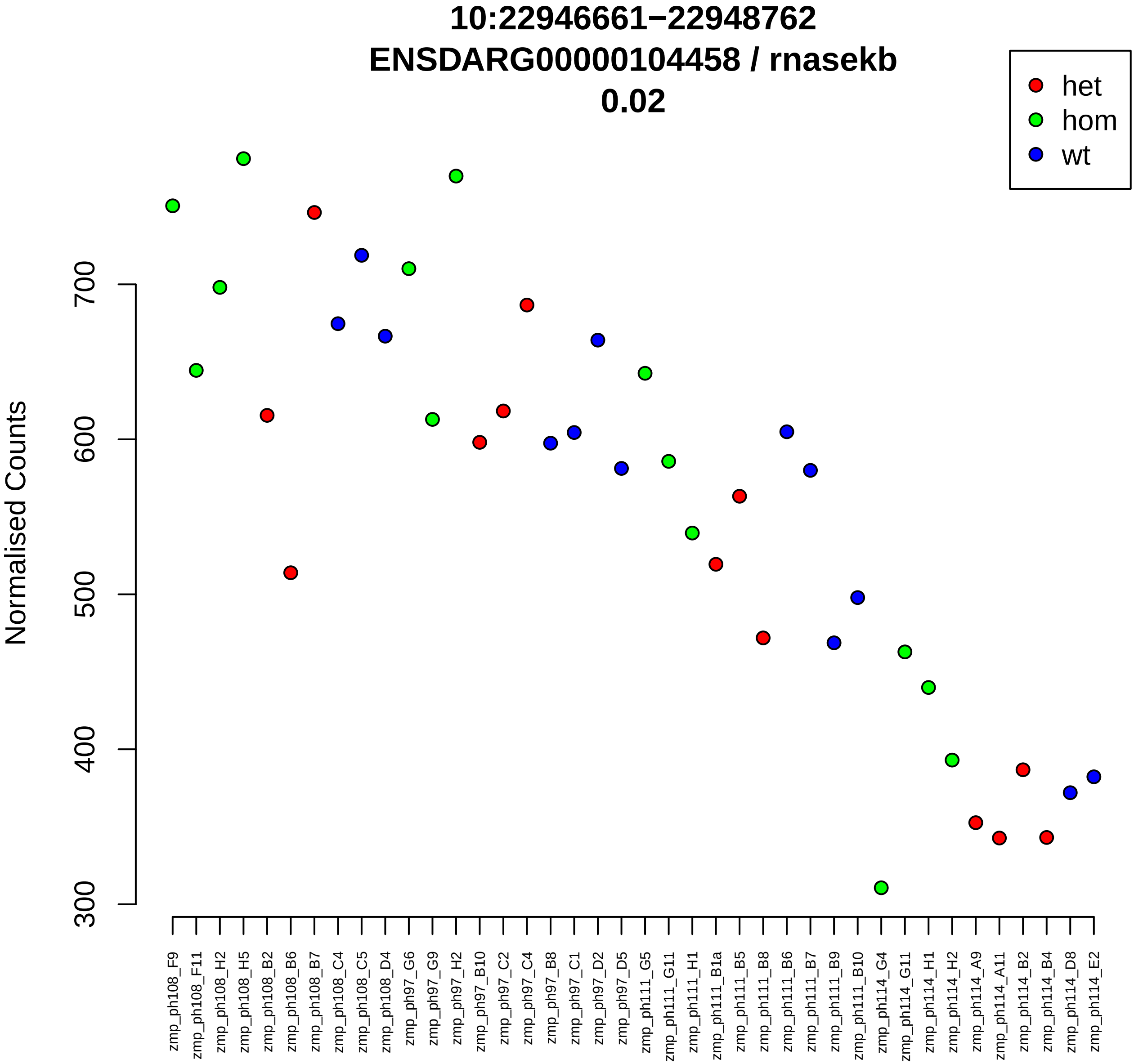

10:22946661−22948762
ENSDARG00000104458 / rnasekb
0.02
het
hom
wt
Normalised Counts
300
400
500
600
700
zmp_ph108_F9
zmp_ph108_F11
zmp_ph108_H2
zmp_ph108_H5
zmp_ph108_B2
zmp_ph108_B6
zmp_ph108_B7
zmp_ph108_C4
zmp_ph108_C5
zmp_ph108_D4
zmp_ph97_G6
zmp_ph97_G9
zmp_ph97_H2
zmp_ph97_B10
zmp_ph97_C2
zmp_ph97_C4
zmp_ph97_B8
zmp_ph97_C1
zmp_ph97_D2
zmp_ph97_D5
zmp_ph111_G5
zmp_ph111_G11
zmp_ph111_H1
zmp_ph111_B1a
zmp_ph111_B5
zmp_ph111_B8
zmp_ph111_B6
zmp_ph111_B7
zmp_ph111_B9
zmp_ph111_B10
zmp_ph114_G4
zmp_ph114_G11
zmp_ph114_H1
zmp_ph114_H2
zmp_ph114_A9
zmp_ph114_A11
zmp_ph114_B2
zmp_ph114_B4
zmp_ph114_D8
zmp_ph114_E2

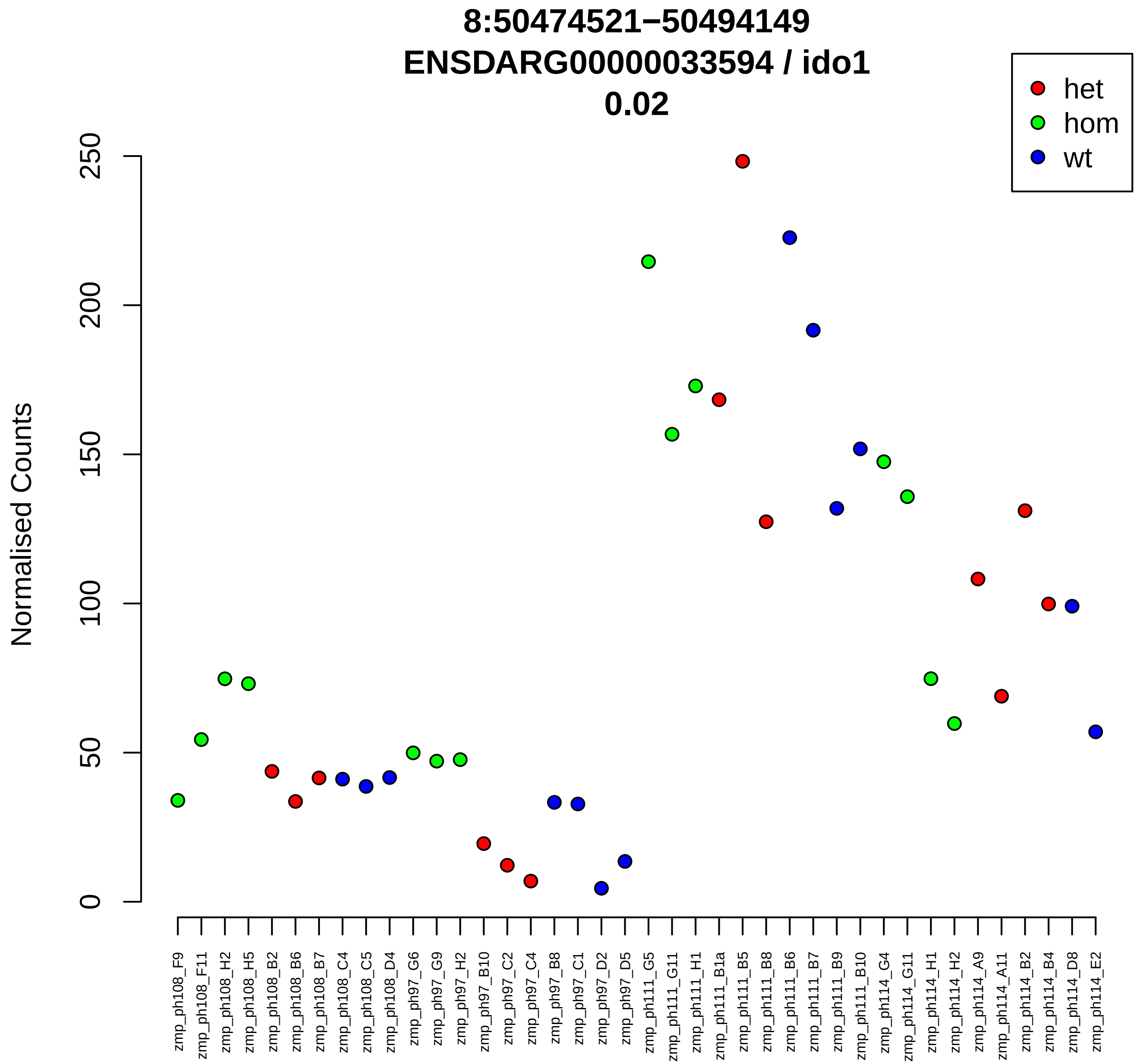

8:50474521−50494149
ENSDARG00000033594 / ido1
0.02
het
hom
wt
Normalised Counts
0
50
100
150
200
250
zmp_ph108_F9
zmp_ph108_F11
zmp_ph108_H2
zmp_ph108_H5
zmp_ph108_B2
zmp_ph108_B6
zmp_ph108_B7
zmp_ph108_C4
zmp_ph108_C5
zmp_ph108_D4
zmp_ph97_G6
zmp_ph97_G9
zmp_ph97_H2
zmp_ph97_B10
zmp_ph97_C2
zmp_ph97_C4
zmp_ph97_B8
zmp_ph97_C1
zmp_ph97_D2
zmp_ph97_D5
zmp_ph111_G5
zmp_ph111_G11
zmp_ph111_H1
zmp_ph111_B1a
zmp_ph111_B5
zmp_ph111_B8
zmp_ph111_B6
zmp_ph111_B7
zmp_ph111_B9
zmp_ph111_B10
zmp_ph114_G4
zmp_ph114_G11
zmp_ph114_H1
zmp_ph114_H2
zmp_ph114_A9
zmp_ph114_A11
zmp_ph114_B2
zmp_ph114_B4
zmp_ph114_D8
zmp_ph114_E2

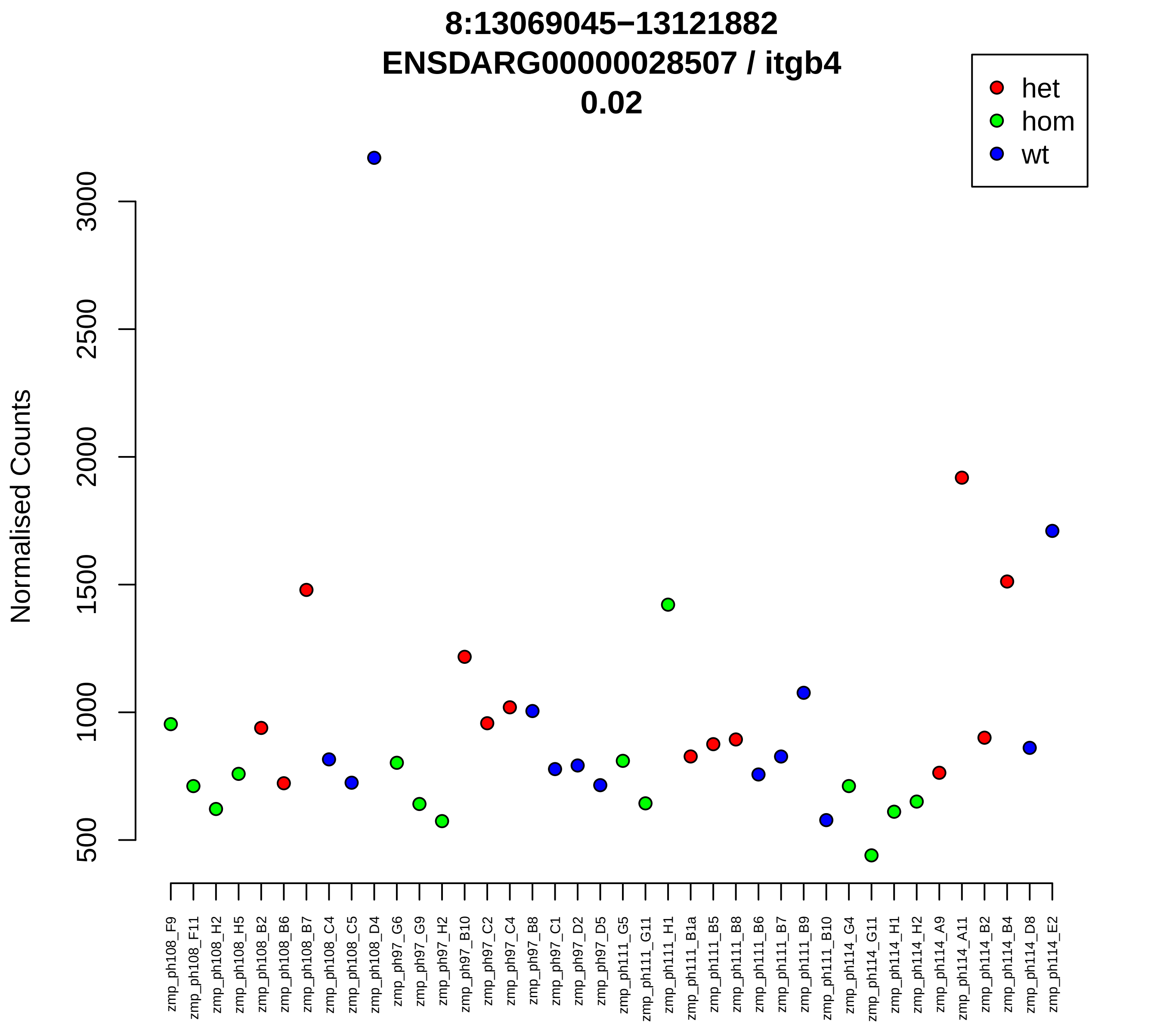

8:13069045−13121882
ENSDARG00000028507 / itgb4
0.02
het
hom
wt
Normalised Counts
500
1000
1500
2000
2500
3000
zmp_ph108_F9
zmp_ph108_F11
zmp_ph108_H2
zmp_ph108_H5
zmp_ph108_B2
zmp_ph108_B6
zmp_ph108_B7
zmp_ph108_C4
zmp_ph108_C5
zmp_ph108_D4
zmp_ph97_G6
zmp_ph97_G9
zmp_ph97_H2
zmp_ph97_B10
zmp_ph97_C2
zmp_ph97_C4
zmp_ph97_B8
zmp_ph97_C1
zmp_ph97_D2
zmp_ph97_D5
zmp_ph111_G5
zmp_ph111_G11
zmp_ph111_H1
zmp_ph111_B1a
zmp_ph111_B5
zmp_ph111_B8
zmp_ph111_B6
zmp_ph111_B7
zmp_ph111_B9
zmp_ph111_B10
zmp_ph114_G4
zmp_ph114_G11
zmp_ph114_H1
zmp_ph114_H2
zmp_ph114_A9
zmp_ph114_A11
zmp_ph114_B2
zmp_ph114_B4
zmp_ph114_D8
zmp_ph114_E2

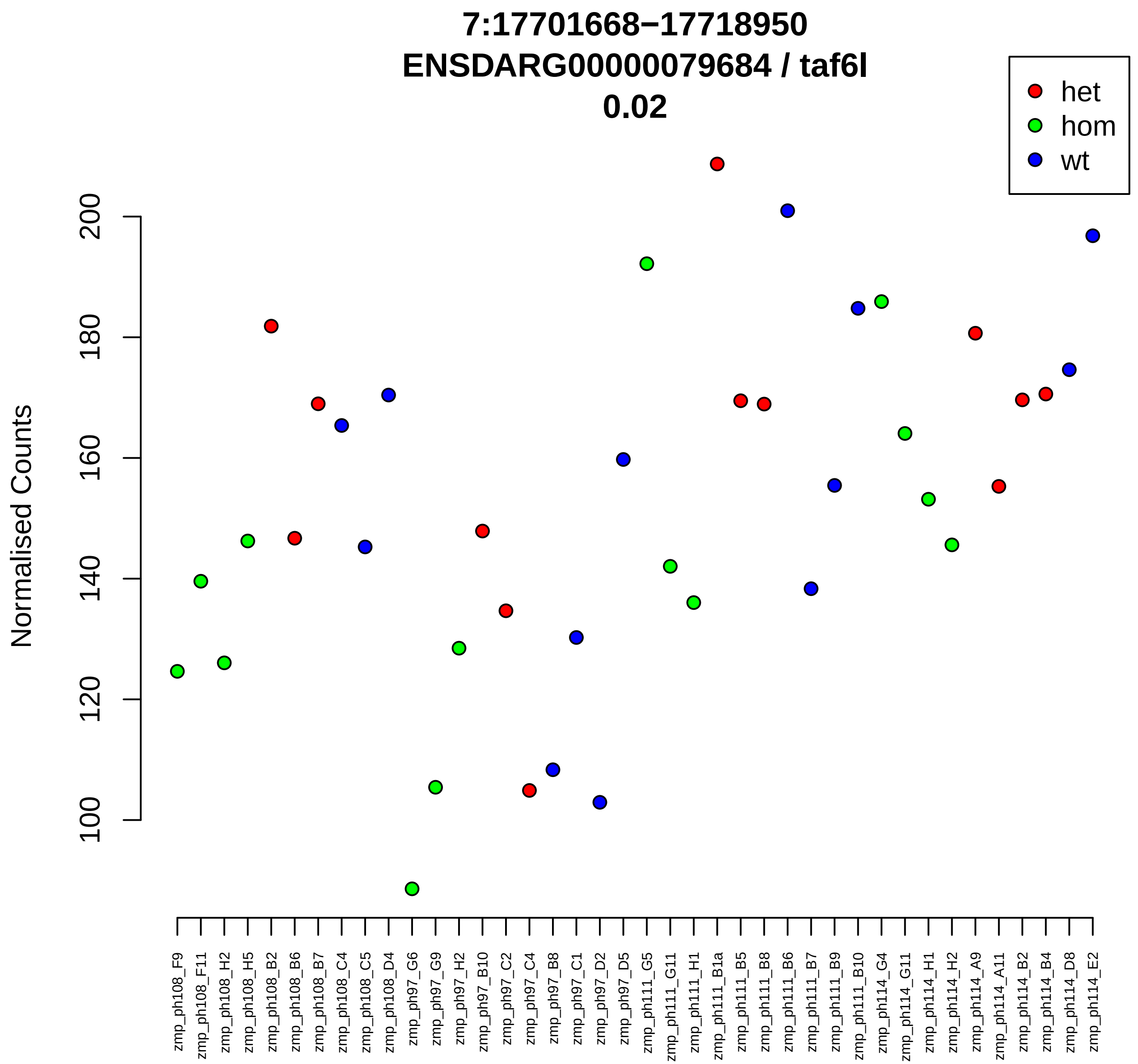

7:17701668−17718950
ENSDARG00000079684 / taf6l
0.02
het
hom
wt
Normalised Counts
100
120
140
160
180
200
zmp_ph108_F9
zmp_ph108_F11
zmp_ph108_H2
zmp_ph108_H5
zmp_ph108_B2
zmp_ph108_B6
zmp_ph108_B7
zmp_ph108_C4
zmp_ph108_C5
zmp_ph108_D4
zmp_ph97_G6
zmp_ph97_G9
zmp_ph97_H2
zmp_ph97_B10
zmp_ph97_C2
zmp_ph97_C4
zmp_ph97_B8
zmp_ph97_C1
zmp_ph97_D2
zmp_ph97_D5
zmp_ph111_G5
zmp_ph111_G11
zmp_ph111_H1
zmp_ph111_B1a
zmp_ph111_B5
zmp_ph111_B8
zmp_ph111_B6
zmp_ph111_B7
zmp_ph111_B9
zmp_ph111_B10
zmp_ph114_G4
zmp_ph114_G11
zmp_ph114_H1
zmp_ph114_H2
zmp_ph114_A9
zmp_ph114_A11
zmp_ph114_B2
zmp_ph114_B4
zmp_ph114_D8
zmp_ph114_E2

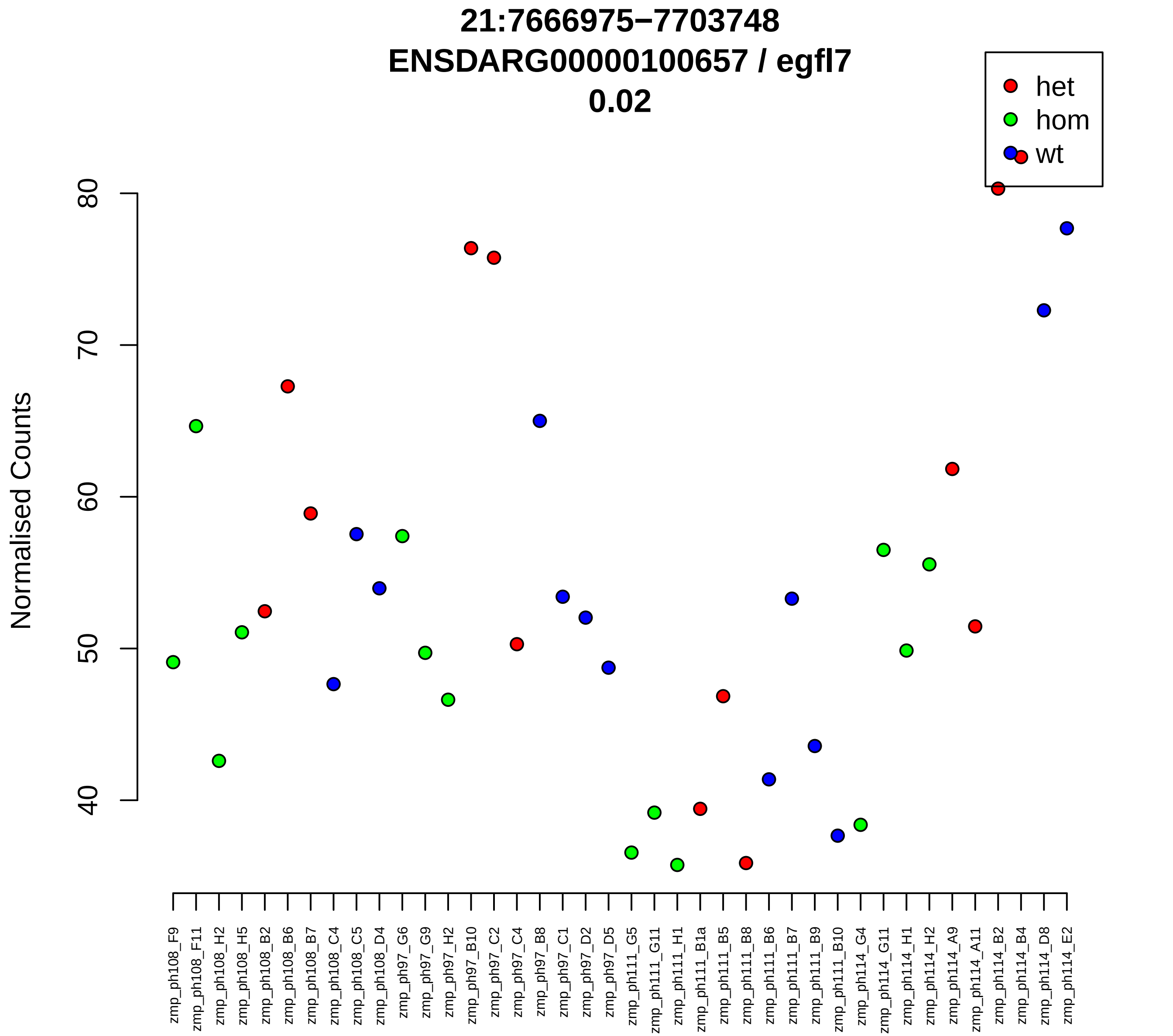

21:7666975−7703748
ENSDARG00000100657 / egfl7
0.02
het
hom
wt
Normalised Counts
40
50
60
70
80
zmp_ph108_F9
zmp_ph108_F11
zmp_ph108_H2
zmp_ph108_H5
zmp_ph108_B2
zmp_ph108_B6
zmp_ph108_B7
zmp_ph108_C4
zmp_ph108_C5
zmp_ph108_D4
zmp_ph97_G6
zmp_ph97_G9
zmp_ph97_H2
zmp_ph97_B10
zmp_ph97_C2
zmp_ph97_C4
zmp_ph97_B8
zmp_ph97_C1
zmp_ph97_D2
zmp_ph97_D5
zmp_ph111_G5
zmp_ph111_G11
zmp_ph111_H1
zmp_ph111_B1a
zmp_ph111_B5
zmp_ph111_B8
zmp_ph111_B6
zmp_ph111_B7
zmp_ph111_B9
zmp_ph111_B10
zmp_ph114_G4
zmp_ph114_G11
zmp_ph114_H1
zmp_ph114_H2
zmp_ph114_A9
zmp_ph114_A11
zmp_ph114_B2
zmp_ph114_B4
zmp_ph114_D8
zmp_ph114_E2

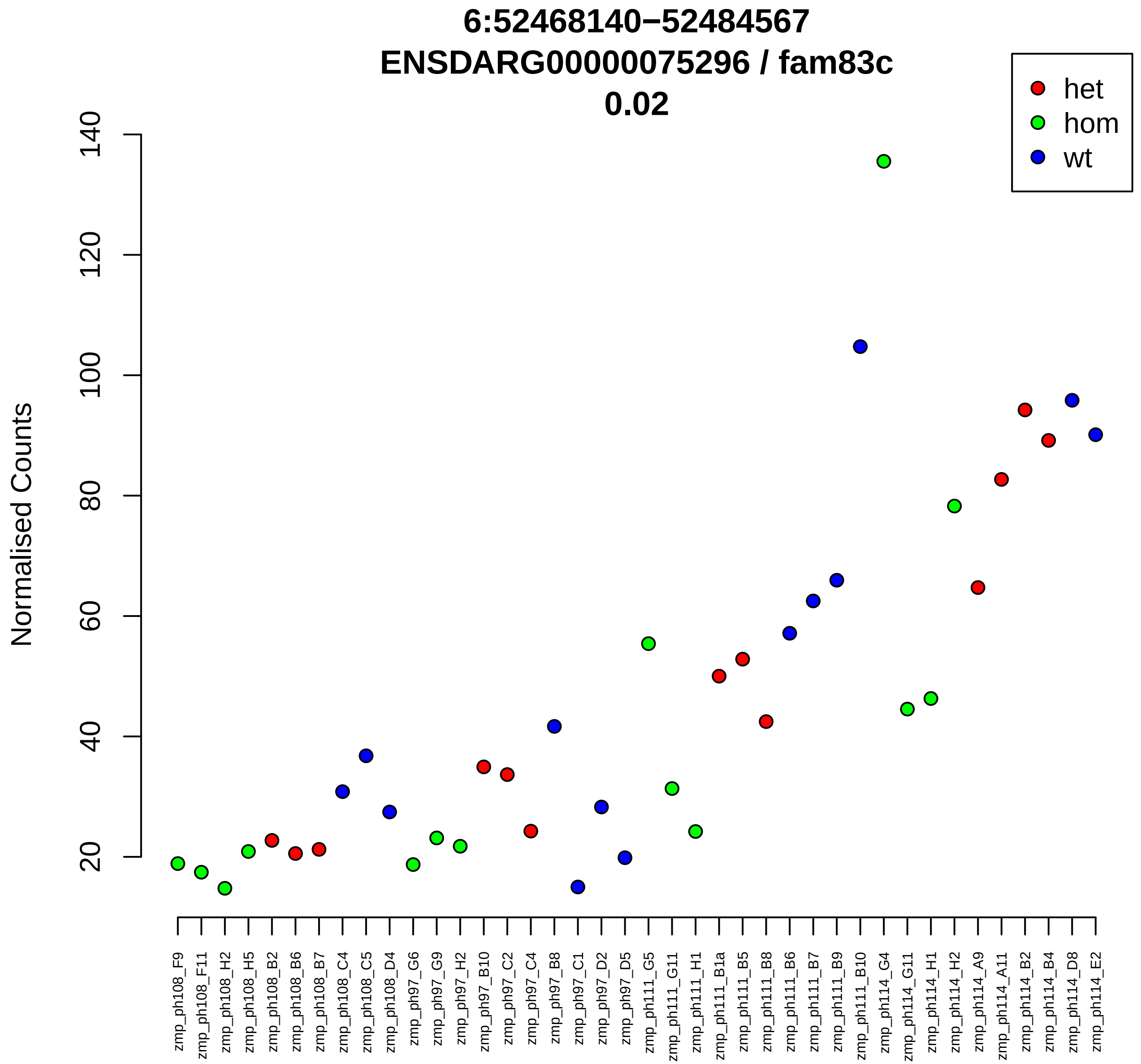

6:52468140−52484567
ENSDARG00000075296 / fam83c
0.02
het
hom
wt
Normalised Counts
20
40
60
80
100
120
140
zmp_ph108_F9
zmp_ph108_F11
zmp_ph108_H2
zmp_ph108_H5
zmp_ph108_B2
zmp_ph108_B6
zmp_ph108_B7
zmp_ph108_C4
zmp_ph108_C5
zmp_ph108_D4
zmp_ph97_G6
zmp_ph97_G9
zmp_ph97_H2
zmp_ph97_B10
zmp_ph97_C2
zmp_ph97_C4
zmp_ph97_B8
zmp_ph97_C1
zmp_ph97_D2
zmp_ph97_D5
zmp_ph111_G5
zmp_ph111_G11
zmp_ph111_H1
zmp_ph111_B1a
zmp_ph111_B5
zmp_ph111_B8
zmp_ph111_B6
zmp_ph111_B7
zmp_ph111_B9
zmp_ph111_B10
zmp_ph114_G4
zmp_ph114_G11
zmp_ph114_H1
zmp_ph114_H2
zmp_ph114_A9
zmp_ph114_A11
zmp_ph114_B2
zmp_ph114_B4
zmp_ph114_D8
zmp_ph114_E2

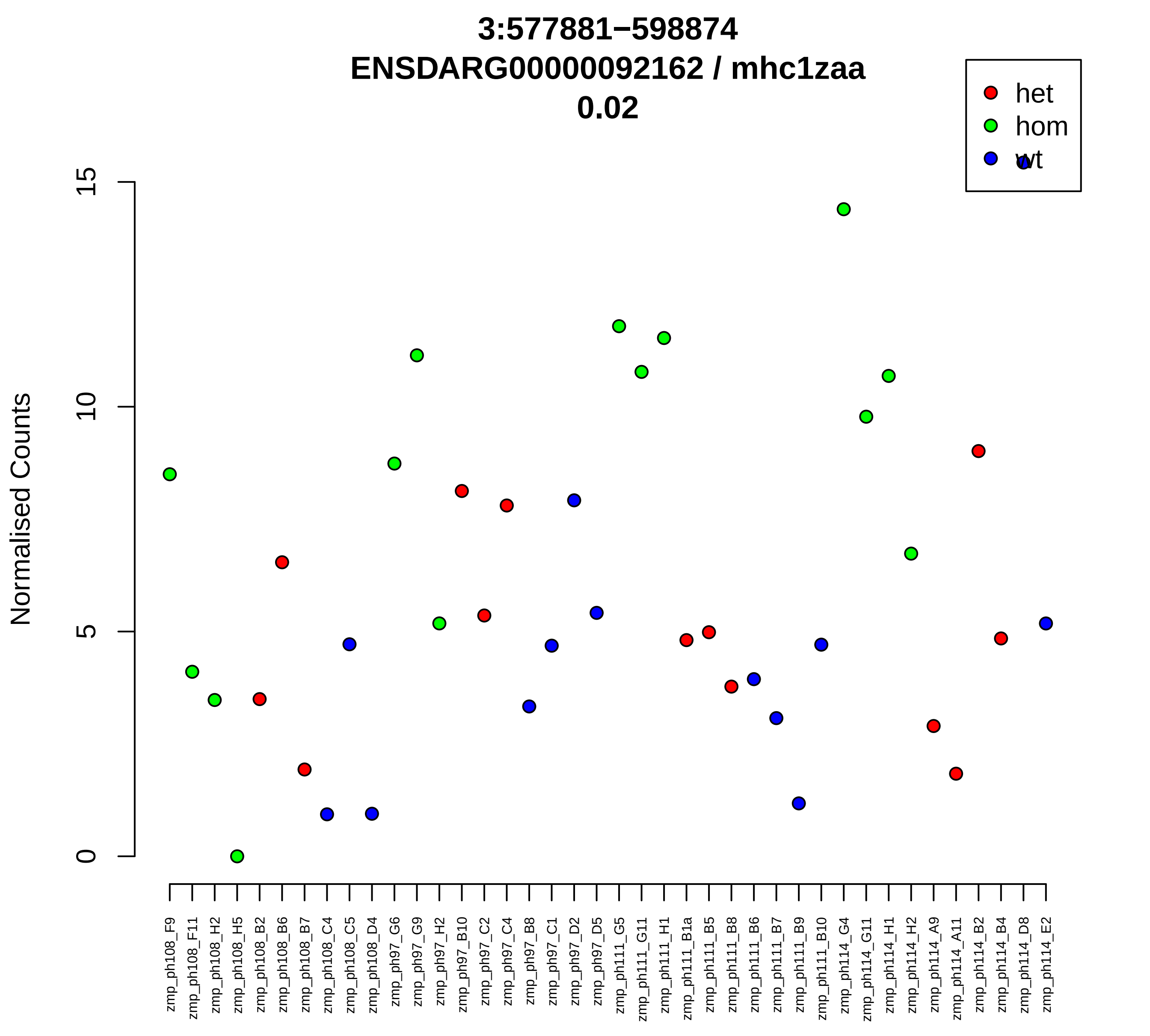
3:577881−598874
ENSDARG00000092162 / mhc1zaa
0.02
het
hom
wt
Normalised Counts
0
5
10
15
zmp_ph108_F9
zmp_ph108_F11
zmp_ph108_H2
zmp_ph108_H5
zmp_ph108_B2
zmp_ph108_B6
zmp_ph108_B7
zmp_ph108_C4
zmp_ph108_C5
zmp_ph108_D4
zmp_ph97_G6
zmp_ph97_G9
zmp_ph97_H2
zmp_ph97_B10
zmp_ph97_C2
zmp_ph97_C4
zmp_ph97_B8
zmp_ph97_C1
zmp_ph97_D2
zmp_ph97_D5
zmp_ph111_G5
zmp_ph111_G11
zmp_ph111_H1
zmp_ph111_B1a
zmp_ph111_B5
zmp_ph111_B8
zmp_ph111_B6
zmp_ph111_B7
zmp_ph111_B9
zmp_ph111_B10
zmp_ph114_G4
zmp_ph114_G11
zmp_ph114_H1
zmp_ph114_H2
zmp_ph114_A9
zmp_ph114_A11
zmp_ph114_B2
zmp_ph114_B4
zmp_ph114_D8
zmp_ph114_E2

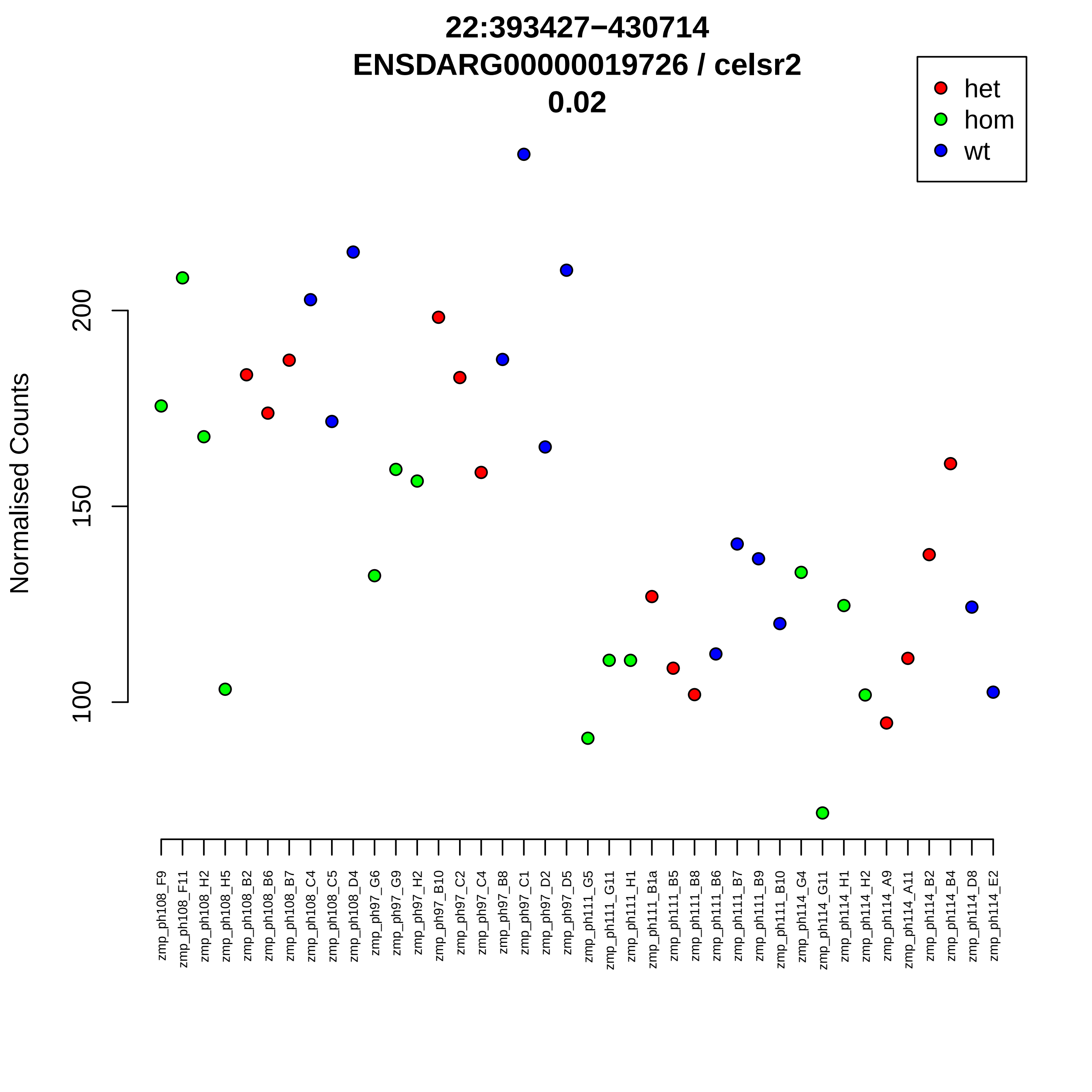
22:393427−430714
ENSDARG00000019726 / celsr2
0.02
het
hom
wt
Normalised Counts
100
150
200
zmp_ph108_F9
zmp_ph108_F11
zmp_ph108_H2
zmp_ph108_H5
zmp_ph108_B2
zmp_ph108_B6
zmp_ph108_B7
zmp_ph108_C4
zmp_ph108_C5
zmp_ph108_D4
zmp_ph97_G6
zmp_ph97_G9
zmp_ph97_H2
zmp_ph97_B10
zmp_ph97_C2
zmp_ph97_C4
zmp_ph97_B8
zmp_ph97_C1
zmp_ph97_D2
zmp_ph97_D5
zmp_ph111_G5
zmp_ph111_G11
zmp_ph111_H1
zmp_ph111_B1a
zmp_ph111_B5
zmp_ph111_B8
zmp_ph111_B6
zmp_ph111_B7
zmp_ph111_B9
zmp_ph111_B10
zmp_ph114_G4
zmp_ph114_G11
zmp_ph114_H1
zmp_ph114_H2
zmp_ph114_A9
zmp_ph114_A11
zmp_ph114_B2
zmp_ph114_B4
zmp_ph114_D8
zmp_ph114_E2

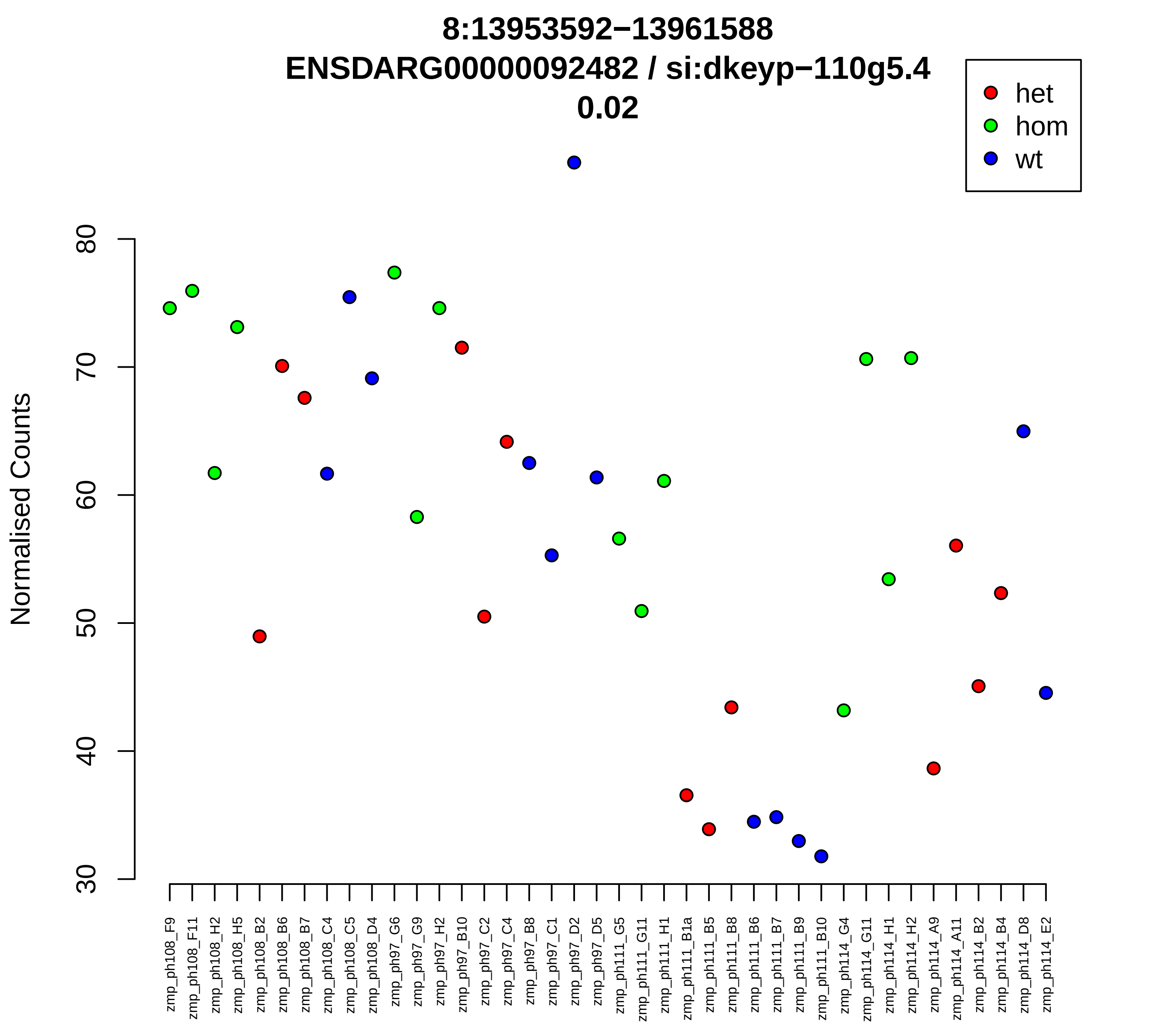

8:13953592−13961588
ENSDARG00000092482 / si:dkeyp−110g5.4
0.02
het
hom
wt
Normalised Counts
30
40
50
60
70
80
zmp_ph108_F9
zmp_ph108_F11
zmp_ph108_H2
zmp_ph108_H5
zmp_ph108_B2
zmp_ph108_B6
zmp_ph108_B7
zmp_ph108_C4
zmp_ph108_C5
zmp_ph108_D4
zmp_ph97_G6
zmp_ph97_G9
zmp_ph97_H2
zmp_ph97_B10
zmp_ph97_C2
zmp_ph97_C4
zmp_ph97_B8
zmp_ph97_C1
zmp_ph97_D2
zmp_ph97_D5
zmp_ph111_G5
zmp_ph111_G11
zmp_ph111_H1
zmp_ph111_B1a
zmp_ph111_B5
zmp_ph111_B8
zmp_ph111_B6
zmp_ph111_B7
zmp_ph111_B9
zmp_ph111_B10
zmp_ph114_G4
zmp_ph114_G11
zmp_ph114_H1
zmp_ph114_H2
zmp_ph114_A9
zmp_ph114_A11
zmp_ph114_B2
zmp_ph114_B4
zmp_ph114_D8
zmp_ph114_E2

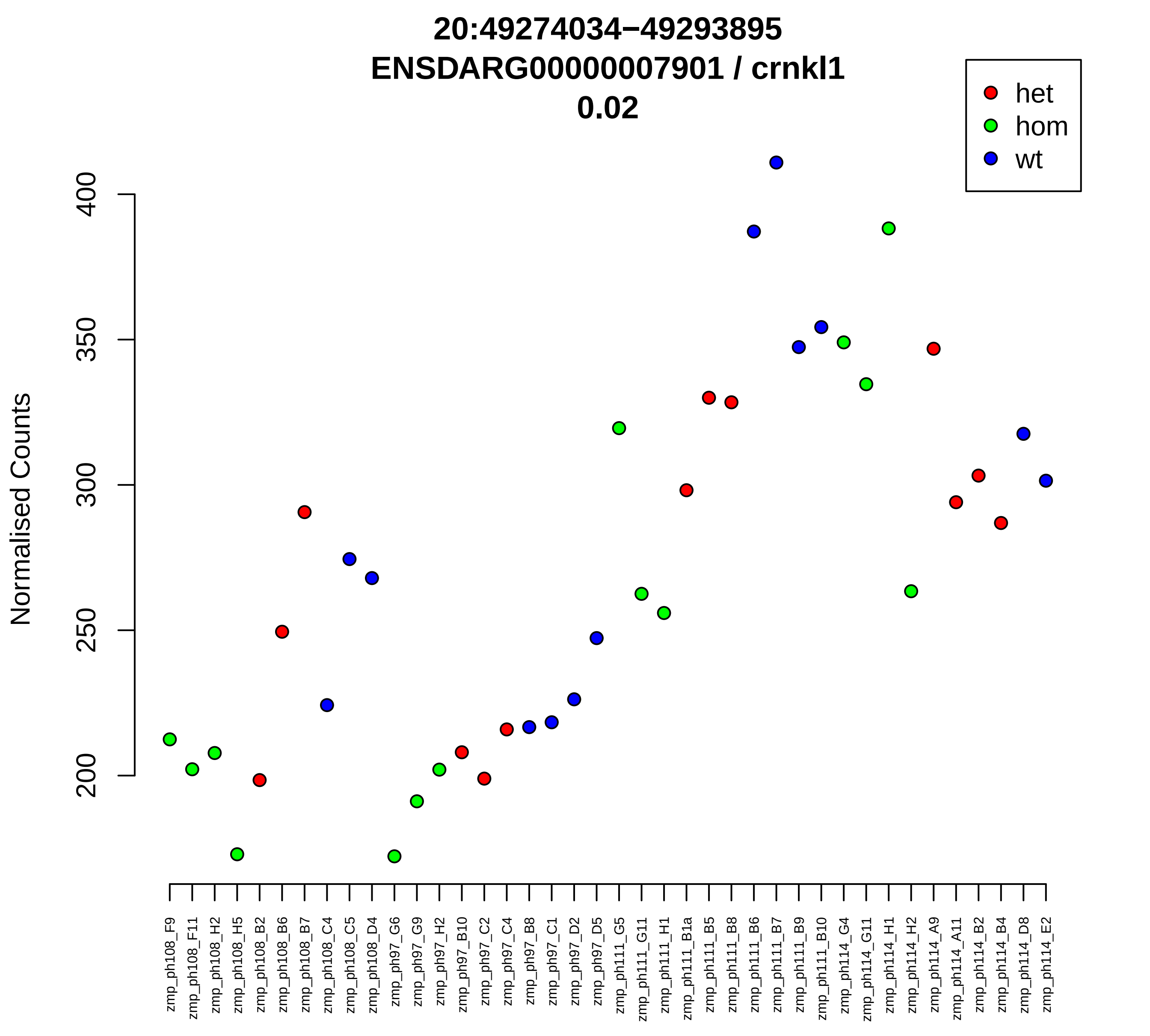

20:49274034−49293895
ENSDARG00000007901 / crnkl1
0.02
het
hom
wt
Normalised Counts
200
250
300
350
400
zmp_ph108_F9
zmp_ph108_F11
zmp_ph108_H2
zmp_ph108_H5
zmp_ph108_B2
zmp_ph108_B6
zmp_ph108_B7
zmp_ph108_C4
zmp_ph108_C5
zmp_ph108_D4
zmp_ph97_G6
zmp_ph97_G9
zmp_ph97_H2
zmp_ph97_B10
zmp_ph97_C2
zmp_ph97_C4
zmp_ph97_B8
zmp_ph97_C1
zmp_ph97_D2
zmp_ph97_D5
zmp_ph111_G5
zmp_ph111_G11
zmp_ph111_H1
zmp_ph111_B1a
zmp_ph111_B5
zmp_ph111_B8
zmp_ph111_B6
zmp_ph111_B7
zmp_ph111_B9
zmp_ph111_B10
zmp_ph114_G4
zmp_ph114_G11
zmp_ph114_H1
zmp_ph114_H2
zmp_ph114_A9
zmp_ph114_A11
zmp_ph114_B2
zmp_ph114_B4
zmp_ph114_D8
zmp_ph114_E2

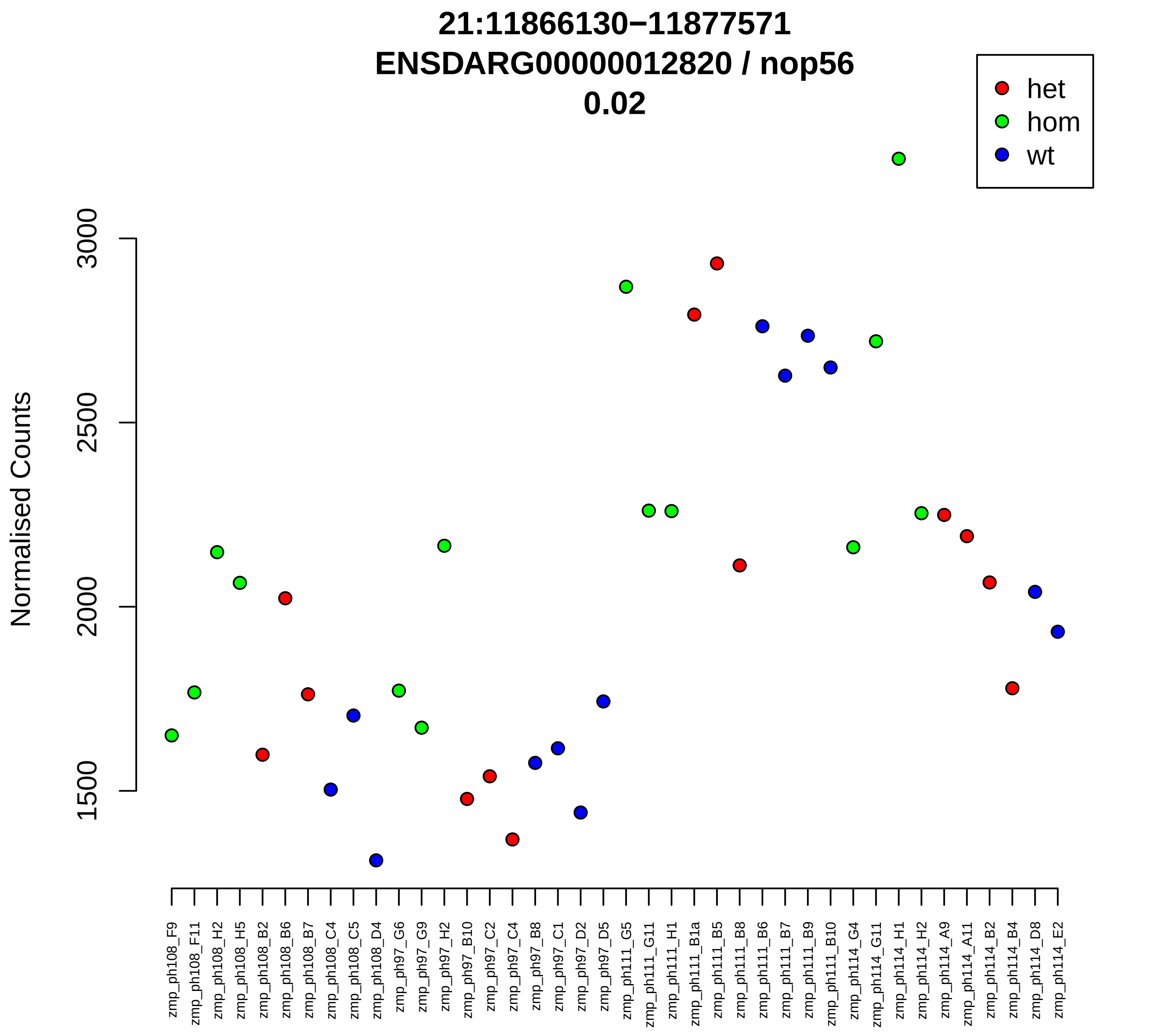

21:11866130−11877571
ENSDARG00000012820 / nop56
0.02
het
hom
wt
Normalised Counts
1500
2000
2500
3000
zmp_ph108_F9
zmp_ph108_F11
zmp_ph108_H2
zmp_ph108_H5
zmp_ph108_B2
zmp_ph108_B6
zmp_ph108_B7
zmp_ph108_C4
zmp_ph108_C5
zmp_ph108_D4
zmp_ph97_G6
zmp_ph97_G9
zmp_ph97_H2
zmp_ph97_B10
zmp_ph97_C2
zmp_ph97_C4
zmp_ph97_B8
zmp_ph97_C1
zmp_ph97_D2
zmp_ph97_D5
zmp_ph111_G5
zmp_ph111_G11
zmp_ph111_H1
zmp_ph111_B1a
zmp_ph111_B5
zmp_ph111_B8
zmp_ph111_B6
zmp_ph111_B7
zmp_ph111_B9
zmp_ph111_B10
zmp_ph114_G4
zmp_ph114_G11
zmp_ph114_H1
zmp_ph114_H2
zmp_ph114_A9
zmp_ph114_A11
zmp_ph114_B2
zmp_ph114_B4
zmp_ph114_D8
zmp_ph114_E2

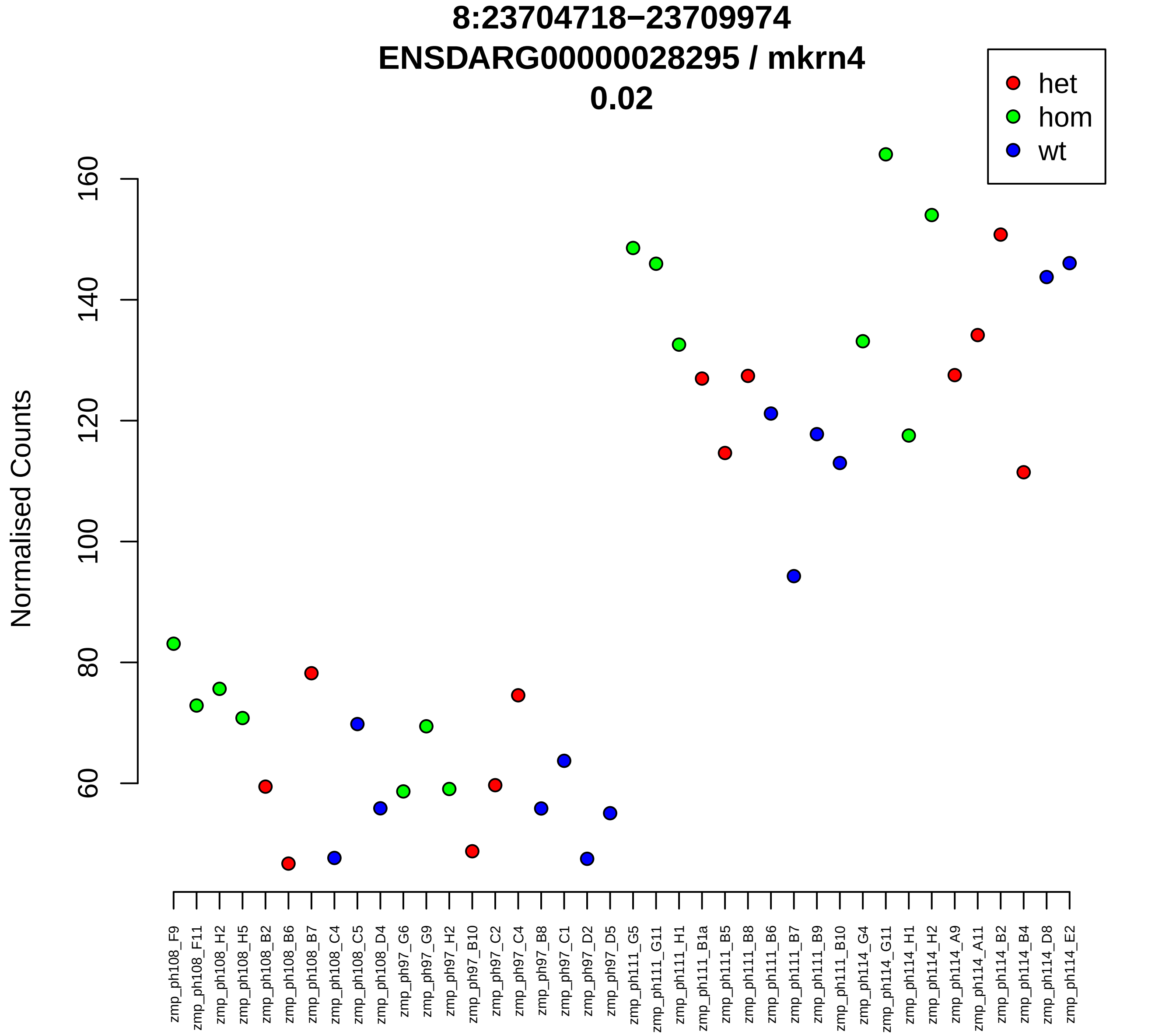

8:23704718−23709974
ENSDARG00000028295 / mkrn4
0.02
het
hom
wt
Normalised Counts
60
80
100
120
140
160
zmp_ph108_F9
zmp_ph108_F11
zmp_ph108_H2
zmp_ph108_H5
zmp_ph108_B2
zmp_ph108_B6
zmp_ph108_B7
zmp_ph108_C4
zmp_ph108_C5
zmp_ph108_D4
zmp_ph97_G6
zmp_ph97_G9
zmp_ph97_H2
zmp_ph97_B10
zmp_ph97_C2
zmp_ph97_C4
zmp_ph97_B8
zmp_ph97_C1
zmp_ph97_D2
zmp_ph97_D5
zmp_ph111_G5
zmp_ph111_G11
zmp_ph111_H1
zmp_ph111_B1a
zmp_ph111_B5
zmp_ph111_B8
zmp_ph111_B6
zmp_ph111_B7
zmp_ph111_B9
zmp_ph111_B10
zmp_ph114_G4
zmp_ph114_G11
zmp_ph114_H1
zmp_ph114_H2
zmp_ph114_A9
zmp_ph114_A11
zmp_ph114_B2
zmp_ph114_B4
zmp_ph114_D8
zmp_ph114_E2

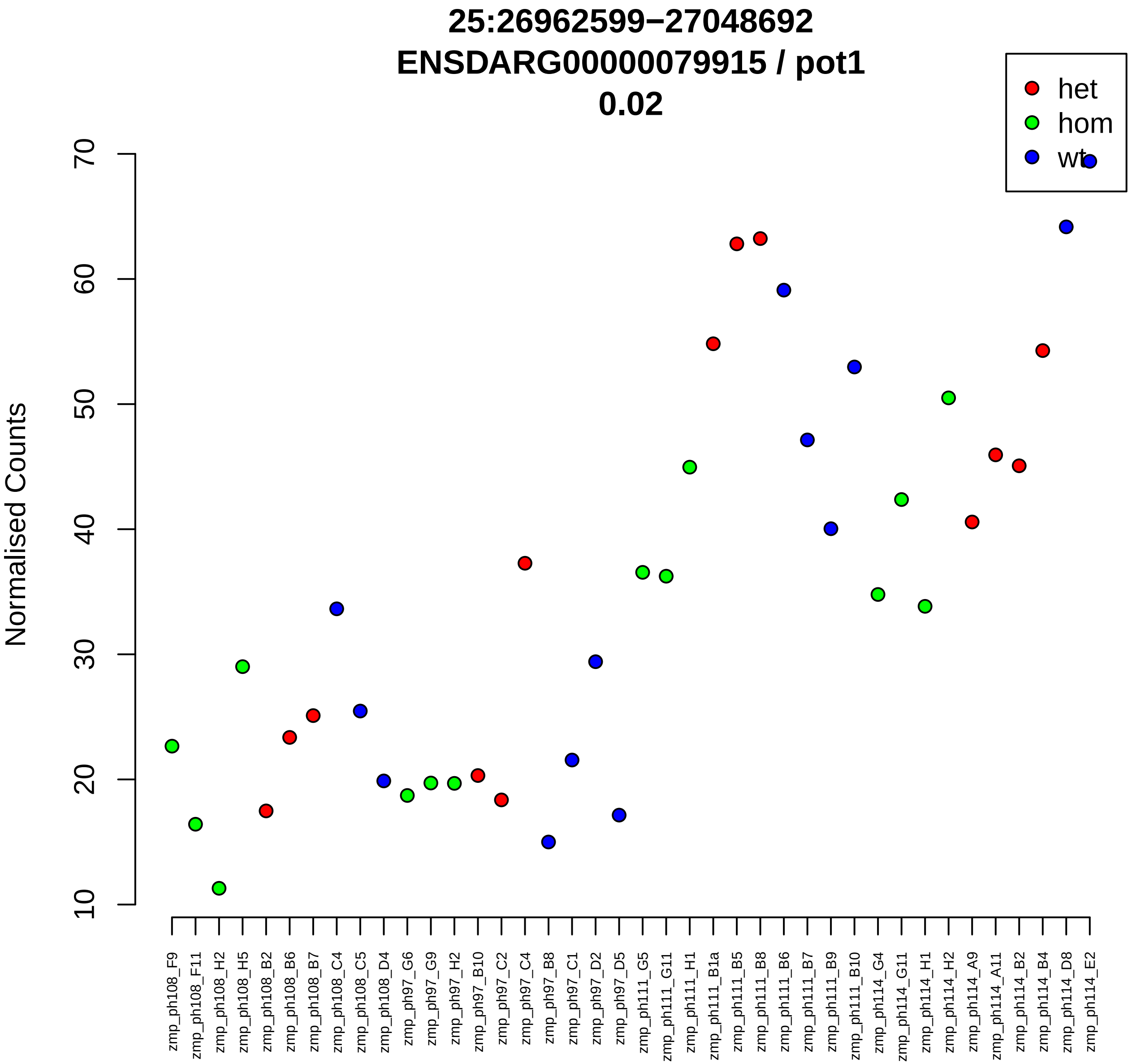

25:26962599−27048692
ENSDARG00000079915 / pot1
0.02
het
hom
wt
Normalised Counts
10
20
30
40
50
60
70
zmp_ph108_F9
zmp_ph108_F11
zmp_ph108_H2
zmp_ph108_H5
zmp_ph108_B2
zmp_ph108_B6
zmp_ph108_B7
zmp_ph108_C4
zmp_ph108_C5
zmp_ph108_D4
zmp_ph97_G6
zmp_ph97_G9
zmp_ph97_H2
zmp_ph97_B10
zmp_ph97_C2
zmp_ph97_C4
zmp_ph97_B8
zmp_ph97_C1
zmp_ph97_D2
zmp_ph97_D5
zmp_ph111_G5
zmp_ph111_G11
zmp_ph111_H1
zmp_ph111_B1a
zmp_ph111_B5
zmp_ph111_B8
zmp_ph111_B6
zmp_ph111_B7
zmp_ph111_B9
zmp_ph111_B10
zmp_ph114_G4
zmp_ph114_G11
zmp_ph114_H1
zmp_ph114_H2
zmp_ph114_A9
zmp_ph114_A11
zmp_ph114_B2
zmp_ph114_B4
zmp_ph114_D8
zmp_ph114_E2

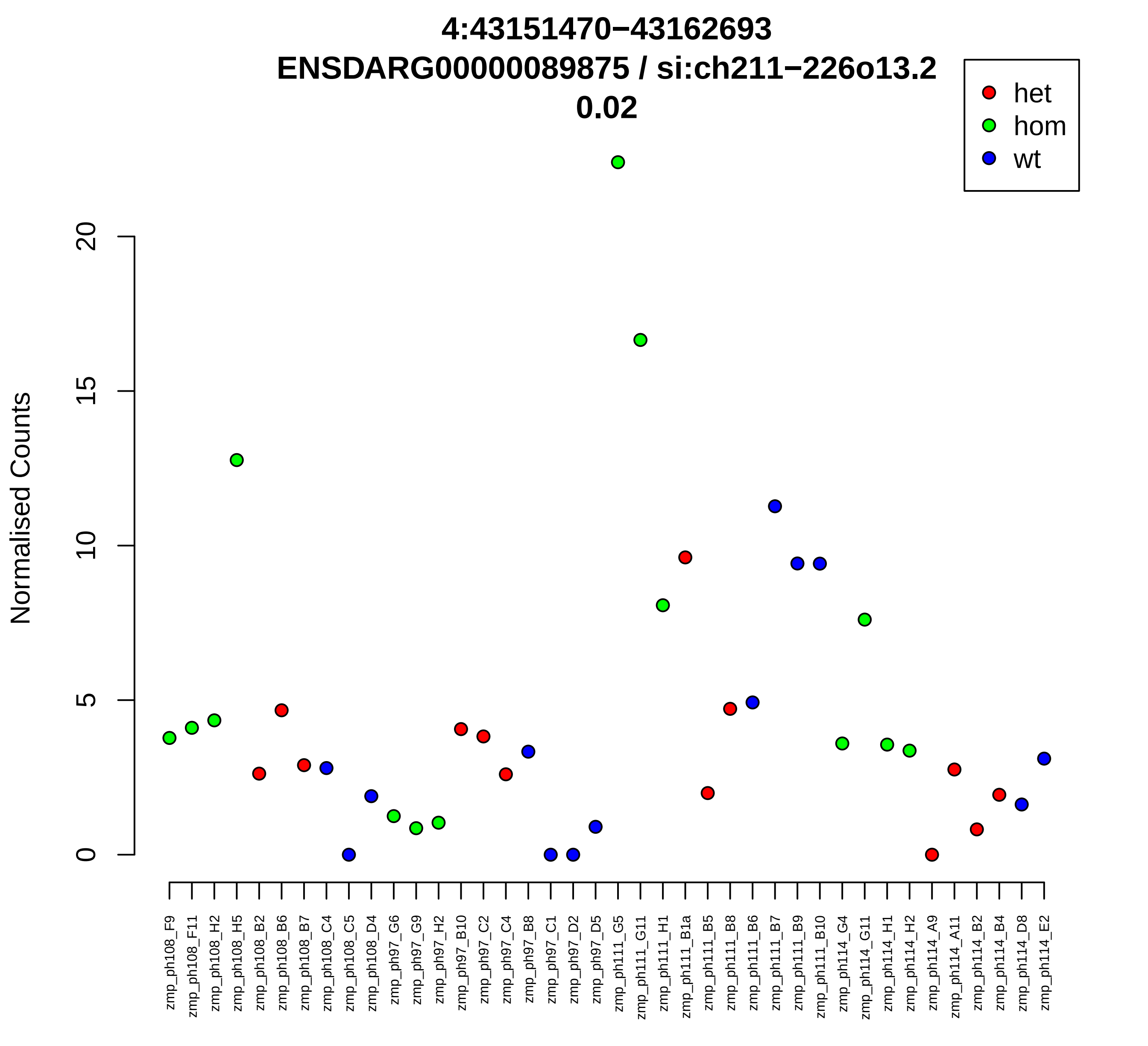
4:43151470−43162693
ENSDARG00000089875 / si:ch211−226o13.2
0.02
het
hom
wt
Normalised Counts
0
5
10
15
20
zmp_ph108_F9
zmp_ph108_F11
zmp_ph108_H2
zmp_ph108_H5
zmp_ph108_B2
zmp_ph108_B6
zmp_ph108_B7
zmp_ph108_C4
zmp_ph108_C5
zmp_ph108_D4
zmp_ph97_G6
zmp_ph97_G9
zmp_ph97_H2
zmp_ph97_B10
zmp_ph97_C2
zmp_ph97_C4
zmp_ph97_B8
zmp_ph97_C1
zmp_ph97_D2
zmp_ph97_D5
zmp_ph111_G5
zmp_ph111_G11
zmp_ph111_H1
zmp_ph111_B1a
zmp_ph111_B5
zmp_ph111_B8
zmp_ph111_B6
zmp_ph111_B7
zmp_ph111_B9
zmp_ph111_B10
zmp_ph114_G4
zmp_ph114_G11
zmp_ph114_H1
zmp_ph114_H2
zmp_ph114_A9
zmp_ph114_A11
zmp_ph114_B2
zmp_ph114_B4
zmp_ph114_D8
zmp_ph114_E2

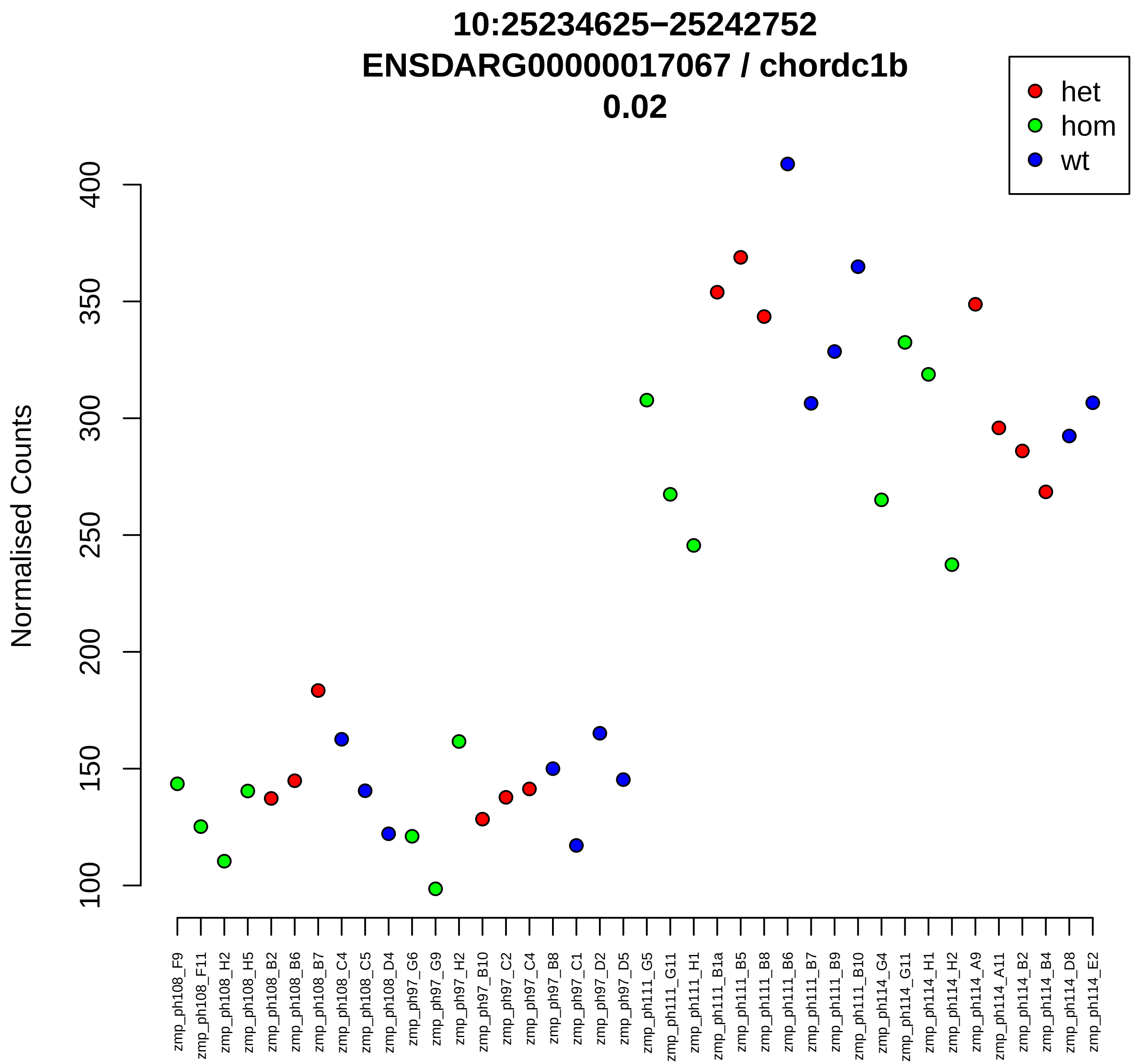
10:25234625−25242752
ENSDARG00000017067 / chordc1b
0.02
het
hom
wt
Normalised Counts
100
150
200
250
300
350
400
zmp_ph108_F9
zmp_ph108_F11
zmp_ph108_H2
zmp_ph108_H5
zmp_ph108_B2
zmp_ph108_B6
zmp_ph108_B7
zmp_ph108_C4
zmp_ph108_C5
zmp_ph108_D4
zmp_ph97_G6
zmp_ph97_G9
zmp_ph97_H2
zmp_ph97_B10
zmp_ph97_C2
zmp_ph97_C4
zmp_ph97_B8
zmp_ph97_C1
zmp_ph97_D2
zmp_ph97_D5
zmp_ph111_G5
zmp_ph111_G11
zmp_ph111_H1
zmp_ph111_B1a
zmp_ph111_B5
zmp_ph111_B8
zmp_ph111_B6
zmp_ph111_B7
zmp_ph111_B9
zmp_ph111_B10
zmp_ph114_G4
zmp_ph114_G11
zmp_ph114_H1
zmp_ph114_H2
zmp_ph114_A9
zmp_ph114_A11
zmp_ph114_B2
zmp_ph114_B4
zmp_ph114_D8
zmp_ph114_E2

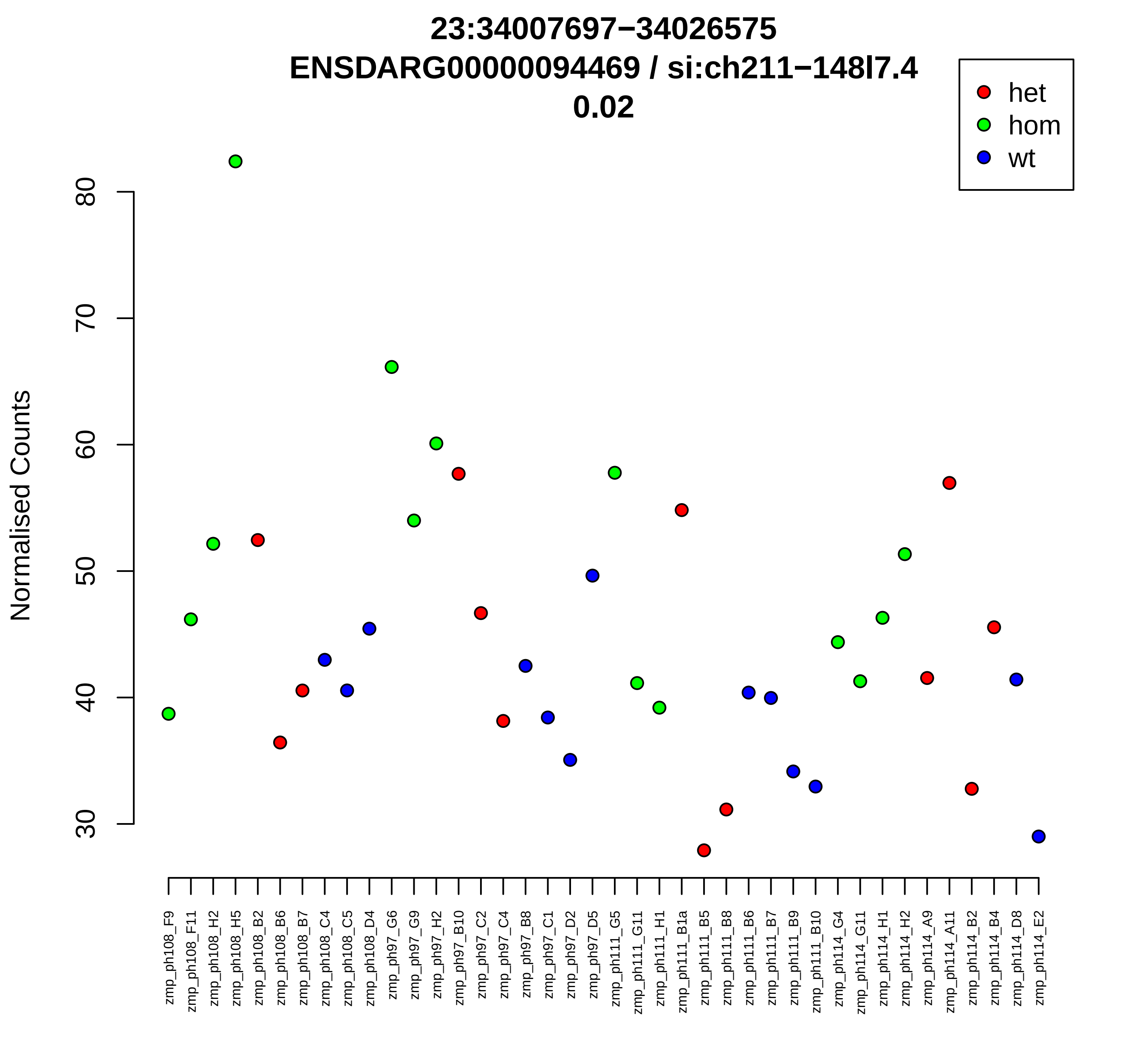
23:34007697−34026575
ENSDARG00000094469 / si:ch211−148l7.4
0.02
het
hom
wt
Normalised Counts
30
40
50
60
70
80
zmp_ph108_F9
zmp_ph108_F11
zmp_ph108_H2
zmp_ph108_H5
zmp_ph108_B2
zmp_ph108_B6
zmp_ph108_B7
zmp_ph108_C4
zmp_ph108_C5
zmp_ph108_D4
zmp_ph97_G6
zmp_ph97_G9
zmp_ph97_H2
zmp_ph97_B10
zmp_ph97_C2
zmp_ph97_C4
zmp_ph97_B8
zmp_ph97_C1
zmp_ph97_D2
zmp_ph97_D5
zmp_ph111_G5
zmp_ph111_G11
zmp_ph111_H1
zmp_ph111_B1a
zmp_ph111_B5
zmp_ph111_B8
zmp_ph111_B6
zmp_ph111_B7
zmp_ph111_B9
zmp_ph111_B10
zmp_ph114_G4
zmp_ph114_G11
zmp_ph114_H1
zmp_ph114_H2
zmp_ph114_A9
zmp_ph114_A11
zmp_ph114_B2
zmp_ph114_B4
zmp_ph114_D8
zmp_ph114_E2

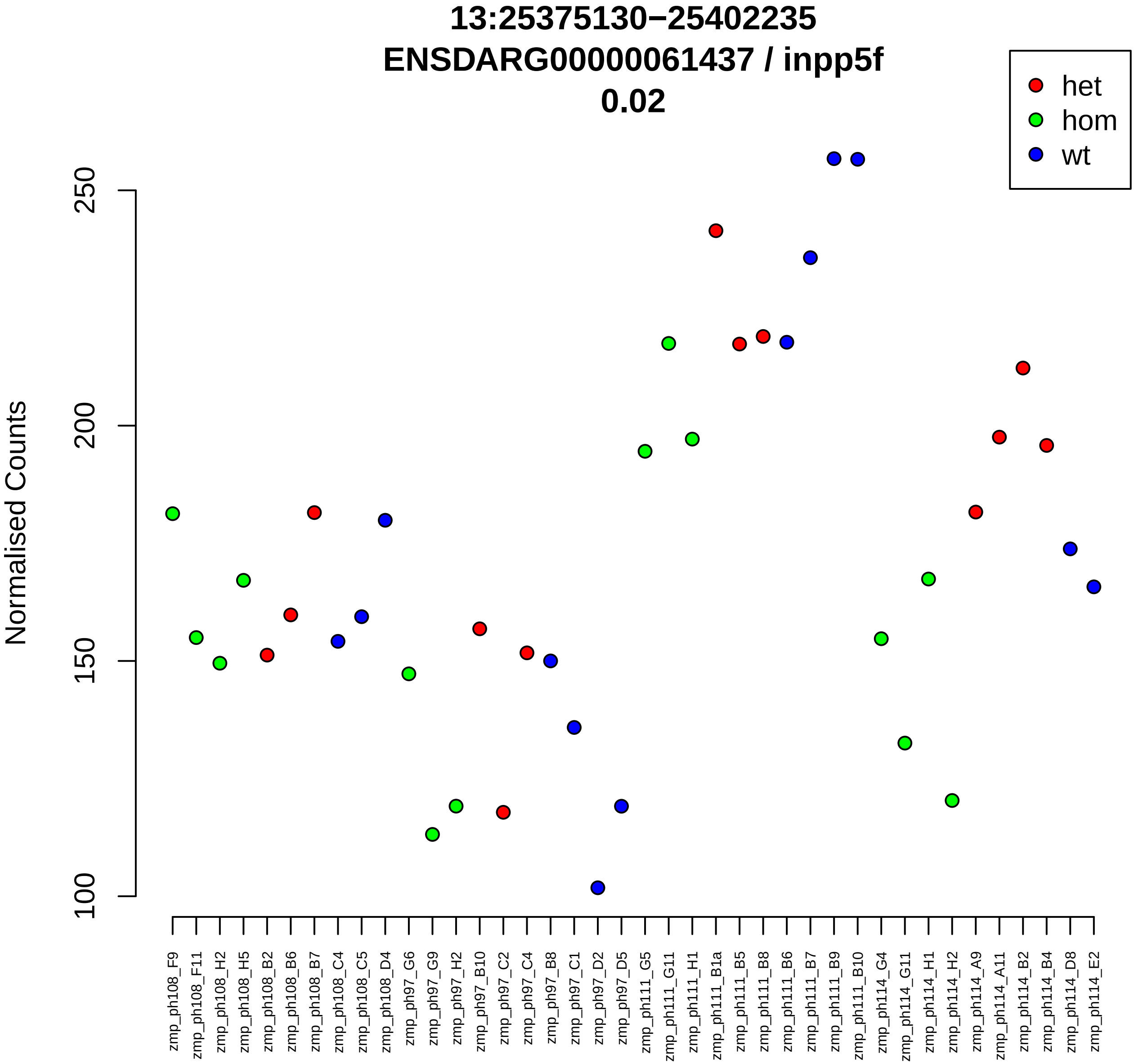

13:25375130−25402235
ENSDARG00000061437 / inpp5f
0.02
het
hom
wt
Normalised Counts
100
150
200
250
zmp_ph108_F9
zmp_ph108_F11
zmp_ph108_H2
zmp_ph108_H5
zmp_ph108_B2
zmp_ph108_B6
zmp_ph108_B7
zmp_ph108_C4
zmp_ph108_C5
zmp_ph108_D4
zmp_ph97_G6
zmp_ph97_G9
zmp_ph97_H2
zmp_ph97_B10
zmp_ph97_C2
zmp_ph97_C4
zmp_ph97_B8
zmp_ph97_C1
zmp_ph97_D2
zmp_ph97_D5
zmp_ph111_G5
zmp_ph111_G11
zmp_ph111_H1
zmp_ph111_B1a
zmp_ph111_B5
zmp_ph111_B8
zmp_ph111_B6
zmp_ph111_B7
zmp_ph111_B9
zmp_ph111_B10
zmp_ph114_G4
zmp_ph114_G11
zmp_ph114_H1
zmp_ph114_H2
zmp_ph114_A9
zmp_ph114_A11
zmp_ph114_B2
zmp_ph114_B4
zmp_ph114_D8
zmp_ph114_E2

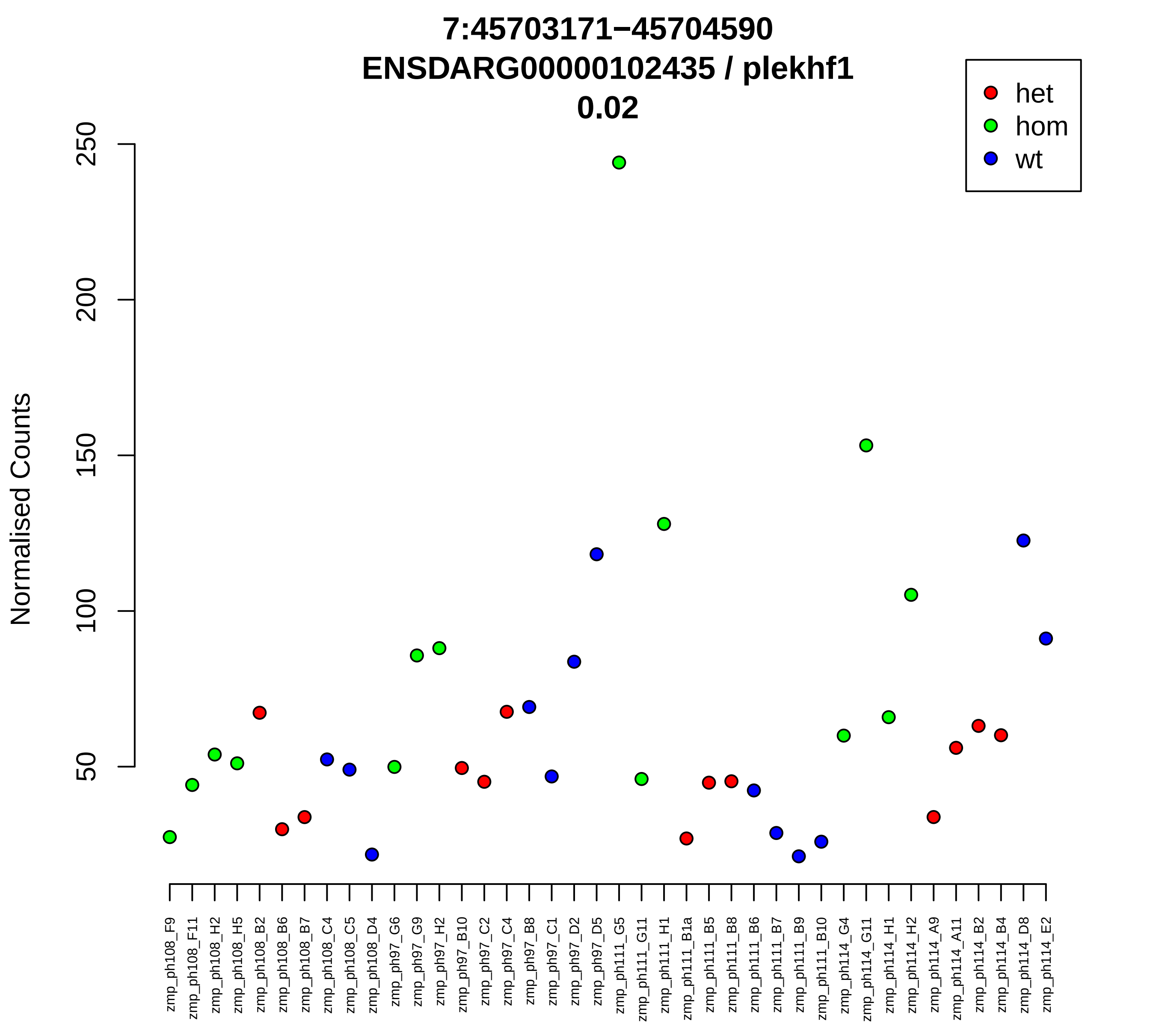

7:45703171−45704590
ENSDARG00000102435 / plekhf1
0.02
het
hom
wt
Normalised Counts
50
100
150
200
250
zmp_ph108_F9
zmp_ph108_F11
zmp_ph108_H2
zmp_ph108_H5
zmp_ph108_B2
zmp_ph108_B6
zmp_ph108_B7
zmp_ph108_C4
zmp_ph108_C5
zmp_ph108_D4
zmp_ph97_G6
zmp_ph97_G9
zmp_ph97_H2
zmp_ph97_B10
zmp_ph97_C2
zmp_ph97_C4
zmp_ph97_B8
zmp_ph97_C1
zmp_ph97_D2
zmp_ph97_D5
zmp_ph111_G5
zmp_ph111_G11
zmp_ph111_H1
zmp_ph111_B1a
zmp_ph111_B5
zmp_ph111_B8
zmp_ph111_B6
zmp_ph111_B7
zmp_ph111_B9
zmp_ph111_B10
zmp_ph114_G4
zmp_ph114_G11
zmp_ph114_H1
zmp_ph114_H2
zmp_ph114_A9
zmp_ph114_A11
zmp_ph114_B2
zmp_ph114_B4
zmp_ph114_D8
zmp_ph114_E2

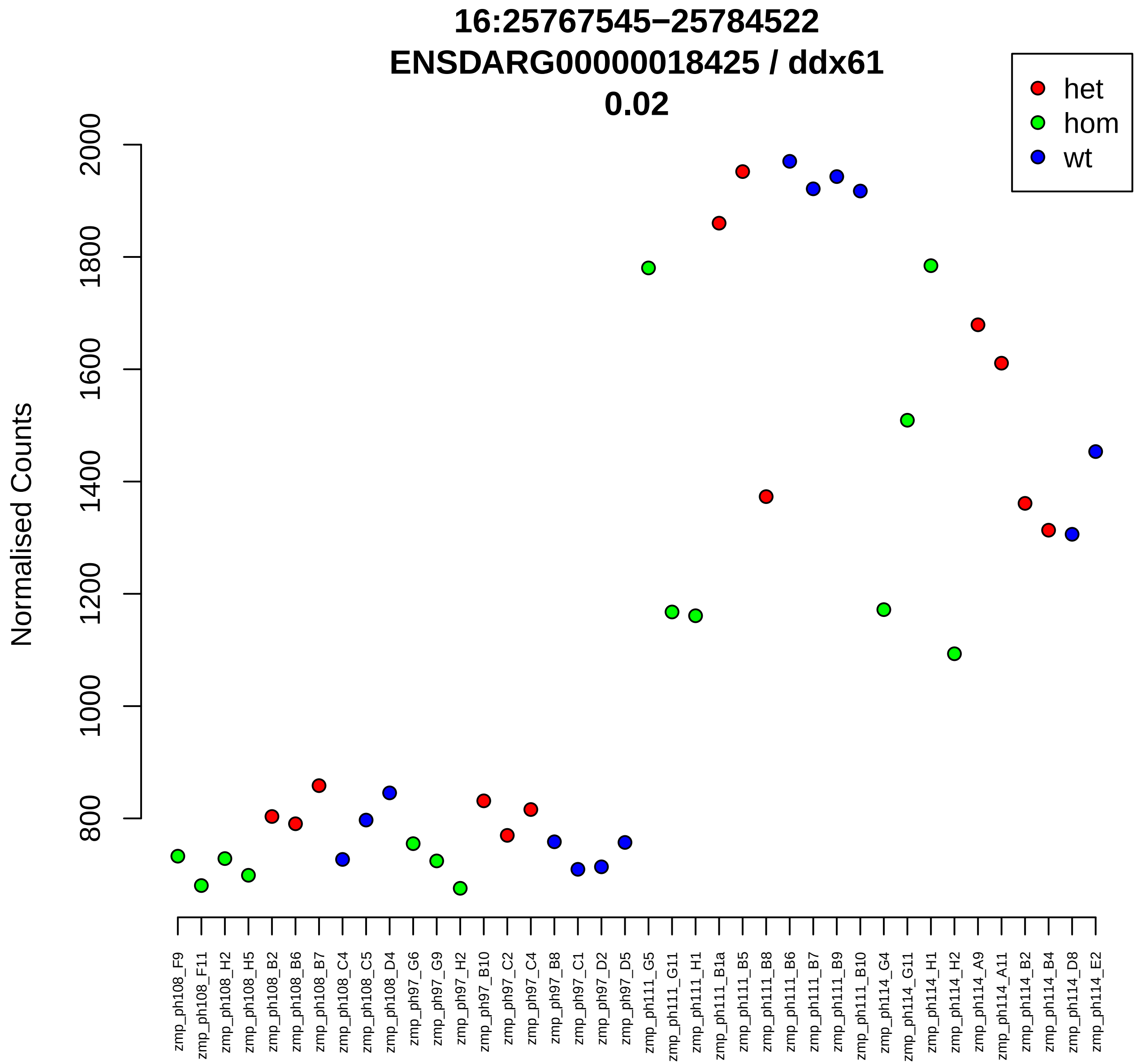
16:25767545−25784522
ENSDARG00000018425 / ddx61
0.02
het
hom
wt
Normalised Counts
800
1000
1200
1400
1600
1800
2000
zmp_ph108_F9
zmp_ph108_F11
zmp_ph108_H2
zmp_ph108_H5
zmp_ph108_B2
zmp_ph108_B6
zmp_ph108_B7
zmp_ph108_C4
zmp_ph108_C5
zmp_ph108_D4
zmp_ph97_G6
zmp_ph97_G9
zmp_ph97_H2
zmp_ph97_B10
zmp_ph97_C2
zmp_ph97_C4
zmp_ph97_B8
zmp_ph97_C1
zmp_ph97_D2
zmp_ph97_D5
zmp_ph111_G5
zmp_ph111_G11
zmp_ph111_H1
zmp_ph111_B1a
zmp_ph111_B5
zmp_ph111_B8
zmp_ph111_B6
zmp_ph111_B7
zmp_ph111_B9
zmp_ph111_B10
zmp_ph114_G4
zmp_ph114_G11
zmp_ph114_H1
zmp_ph114_H2
zmp_ph114_A9
zmp_ph114_A11
zmp_ph114_B2
zmp_ph114_B4
zmp_ph114_D8
zmp_ph114_E2

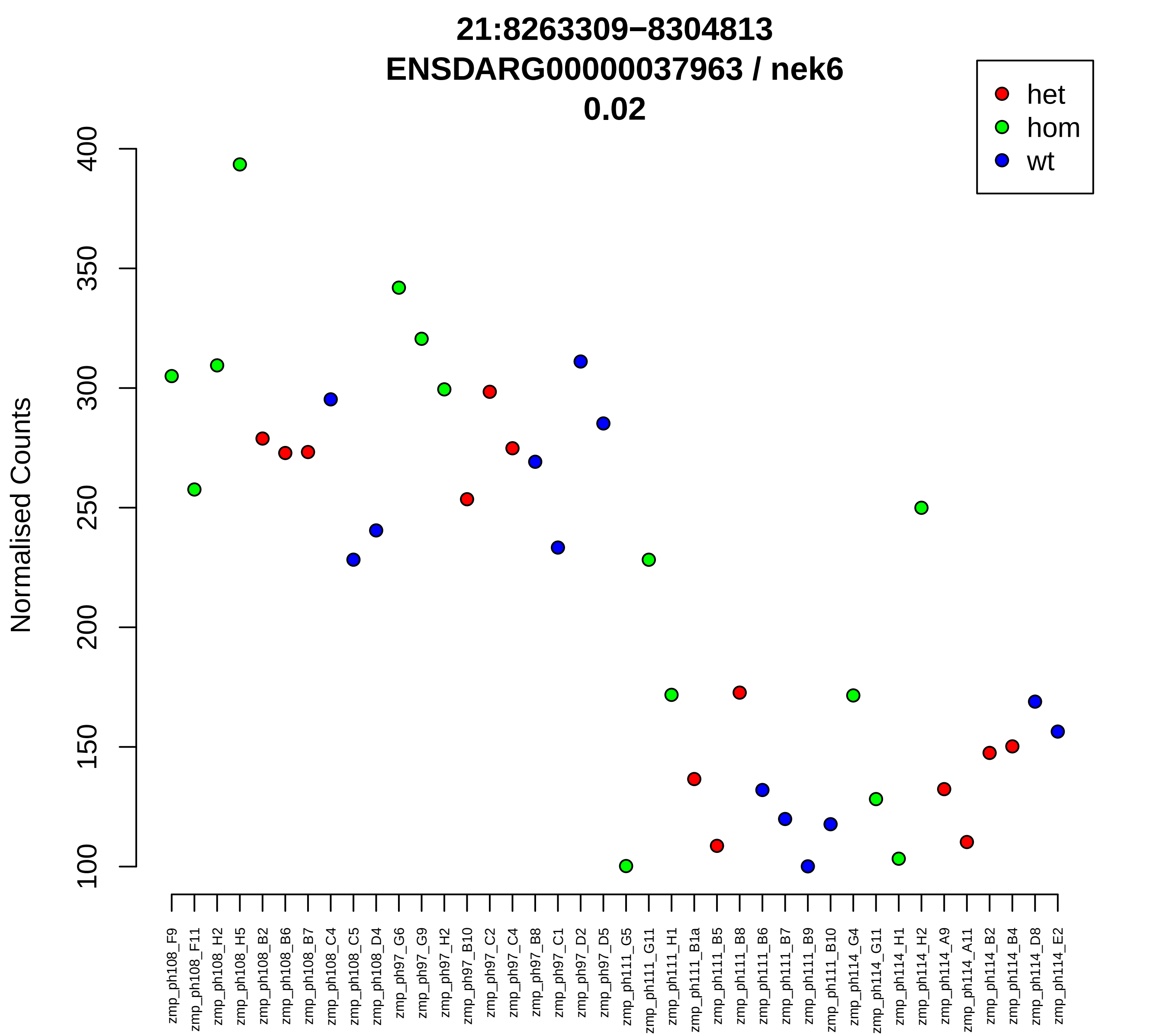
21:8263309−8304813
ENSDARG00000037963 / nek6
0.02
Normalised Counts
100
150
200
250
300
350
400
het
hom
wt
zmp_ph108_F9
zmp_ph108_F11
zmp_ph108_H2
zmp_ph108_H5
zmp_ph108_B2
zmp_ph108_B6
zmp_ph108_B7
zmp_ph108_C4
zmp_ph108_C5
zmp_ph108_D4
zmp_ph97_G6
zmp_ph97_G9
zmp_ph97_H2
zmp_ph97_B10
zmp_ph97_C2
zmp_ph97_C4
zmp_ph97_B8
zmp_ph97_C1
zmp_ph97_D2
zmp_ph97_D5
zmp_ph111_G5
zmp_ph111_G11
zmp_ph111_H1
zmp_ph111_B1a
zmp_ph111_B5
zmp_ph111_B8
zmp_ph111_B6
zmp_ph111_B7
zmp_ph111_B9
zmp_ph111_B10
zmp_ph114_G4
zmp_ph114_G11
zmp_ph114_H1
zmp_ph114_H2
zmp_ph114_A9
zmp_ph114_A11
zmp_ph114_B2
zmp_ph114_B4
zmp_ph114_D8
zmp_ph114_E2

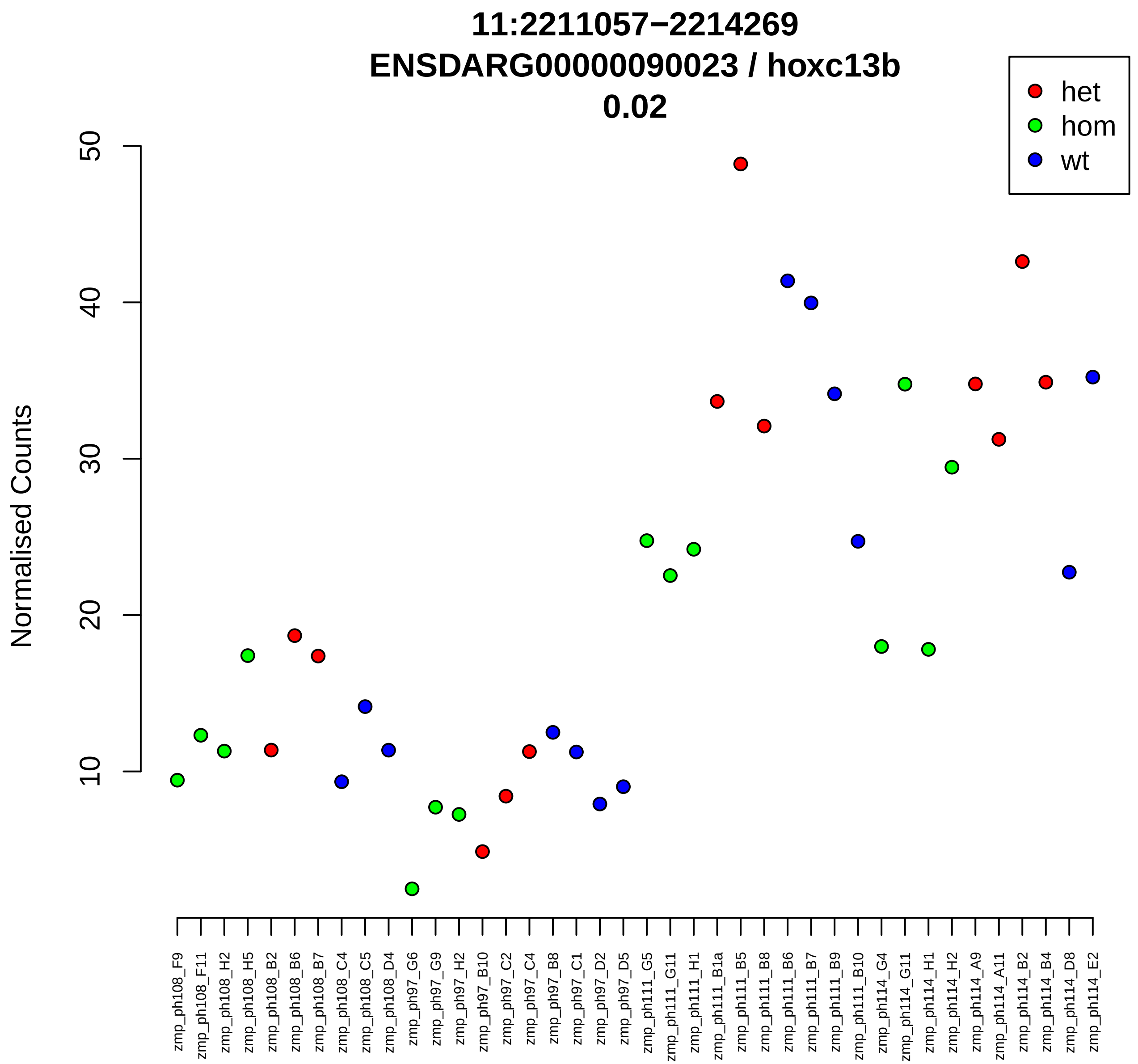
11:2211057−2214269
ENSDARG00000090023 / hoxc13b
0.02
het
hom
wt
Normalised Counts
10
20
30
40
50
zmp_ph108_F9
zmp_ph108_F11
zmp_ph108_H2
zmp_ph108_H5
zmp_ph108_B2
zmp_ph108_B6
zmp_ph108_B7
zmp_ph108_C4
zmp_ph108_C5
zmp_ph108_D4
zmp_ph97_G6
zmp_ph97_G9
zmp_ph97_H2
zmp_ph97_B10
zmp_ph97_C2
zmp_ph97_C4
zmp_ph97_B8
zmp_ph97_C1
zmp_ph97_D2
zmp_ph97_D5
zmp_ph111_G5
zmp_ph111_G11
zmp_ph111_H1
zmp_ph111_B1a
zmp_ph111_B5
zmp_ph111_B8
zmp_ph111_B6
zmp_ph111_B7
zmp_ph111_B9
zmp_ph111_B10
zmp_ph114_G4
zmp_ph114_G11
zmp_ph114_H1
zmp_ph114_H2
zmp_ph114_A9
zmp_ph114_A11
zmp_ph114_B2
zmp_ph114_B4
zmp_ph114_D8
zmp_ph114_E2

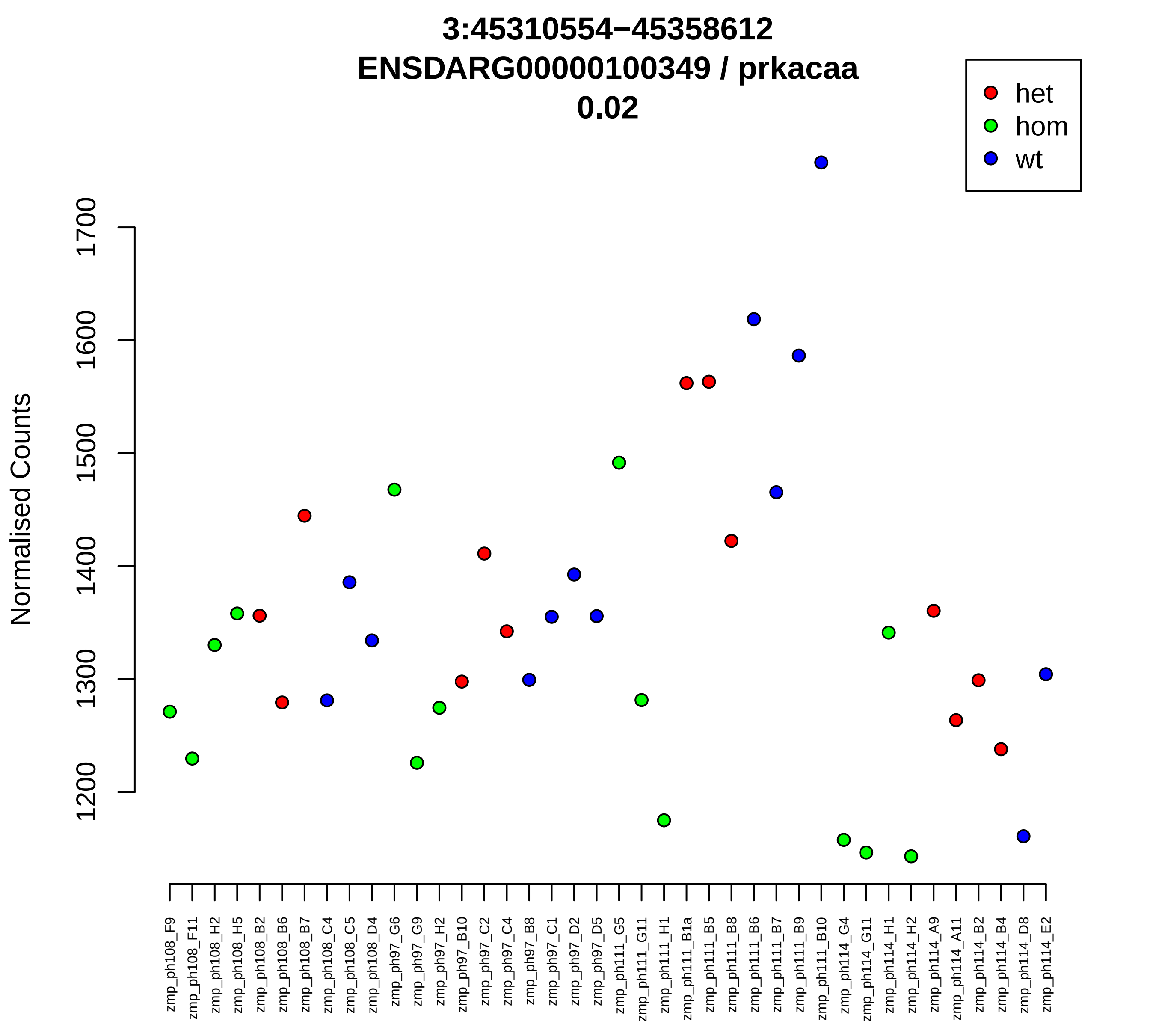

3:45310554−45358612
ENSDARG00000100349 / prkacaa
0.02
het
hom
wt
Normalised Counts
1200
1300
1400
1500
1600
1700
zmp_ph108_F9
zmp_ph108_F11
zmp_ph108_H2
zmp_ph108_H5
zmp_ph108_B2
zmp_ph108_B6
zmp_ph108_B7
zmp_ph108_C4
zmp_ph108_C5
zmp_ph108_D4
zmp_ph97_G6
zmp_ph97_G9
zmp_ph97_H2
zmp_ph97_B10
zmp_ph97_C2
zmp_ph97_C4
zmp_ph97_B8
zmp_ph97_C1
zmp_ph97_D2
zmp_ph97_D5
zmp_ph111_G5
zmp_ph111_G11
zmp_ph111_H1
zmp_ph111_B1a
zmp_ph111_B5
zmp_ph111_B8
zmp_ph111_B6
zmp_ph111_B7
zmp_ph111_B9
zmp_ph111_B10
zmp_ph114_G4
zmp_ph114_G11
zmp_ph114_H1
zmp_ph114_H2
zmp_ph114_A9
zmp_ph114_A11
zmp_ph114_B2
zmp_ph114_B4
zmp_ph114_D8
zmp_ph114_E2

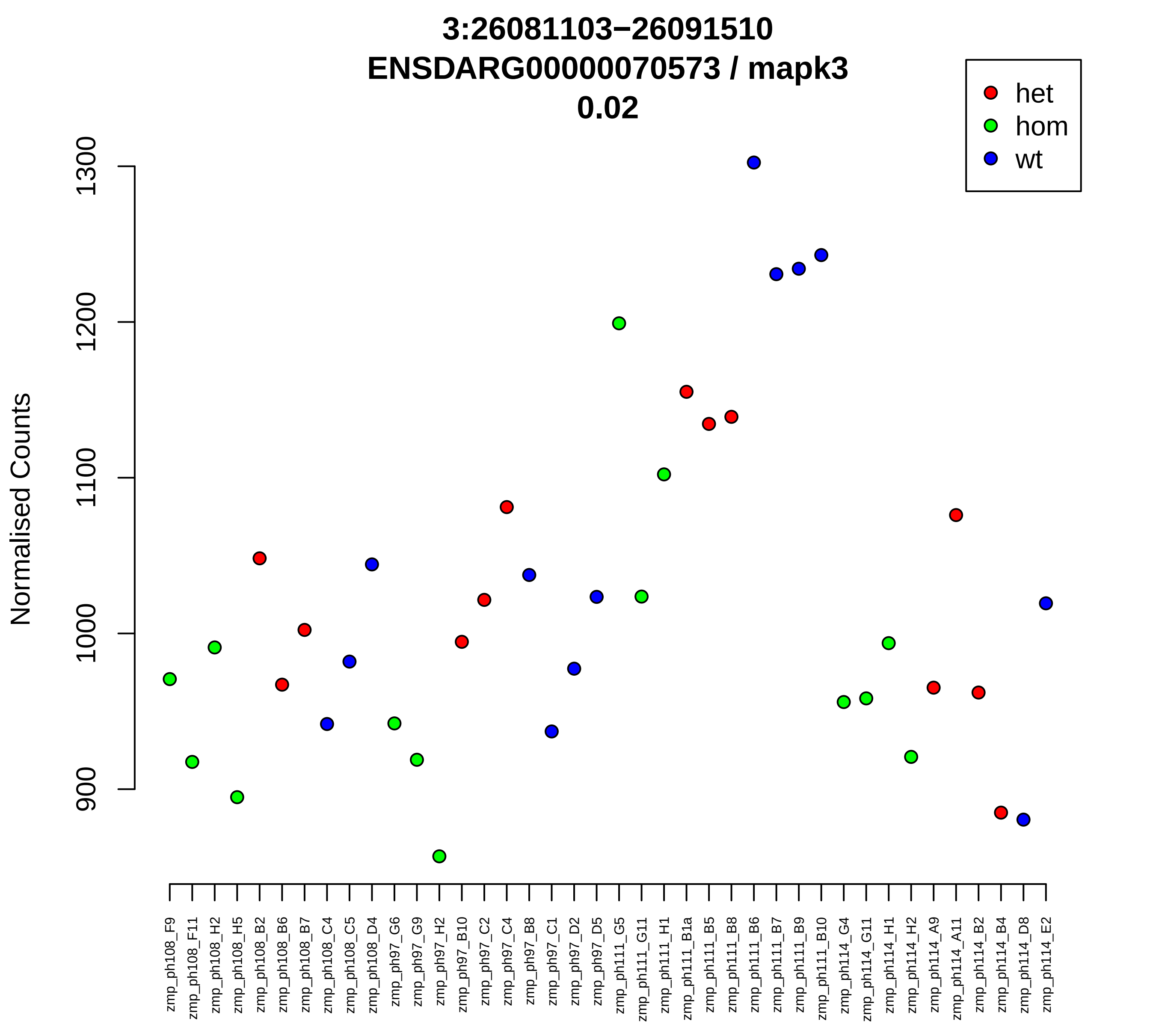
3:26081103−26091510
ENSDARG00000070573 / mapk3
0.02
het
hom
wt
Normalised Counts
900
1000
1100
1200
1300
zmp_ph108_F9
zmp_ph108_F11
zmp_ph108_H2
zmp_ph108_H5
zmp_ph108_B2
zmp_ph108_B6
zmp_ph108_B7
zmp_ph108_C4
zmp_ph108_C5
zmp_ph108_D4
zmp_ph97_G6
zmp_ph97_G9
zmp_ph97_H2
zmp_ph97_B10
zmp_ph97_C2
zmp_ph97_C4
zmp_ph97_B8
zmp_ph97_C1
zmp_ph97_D2
zmp_ph97_D5
zmp_ph111_G5
zmp_ph111_G11
zmp_ph111_H1
zmp_ph111_B1a
zmp_ph111_B5
zmp_ph111_B8
zmp_ph111_B6
zmp_ph111_B7
zmp_ph111_B9
zmp_ph111_B10
zmp_ph114_G4
zmp_ph114_G11
zmp_ph114_H1
zmp_ph114_H2
zmp_ph114_A9
zmp_ph114_A11
zmp_ph114_B2
zmp_ph114_B4
zmp_ph114_D8
zmp_ph114_E2

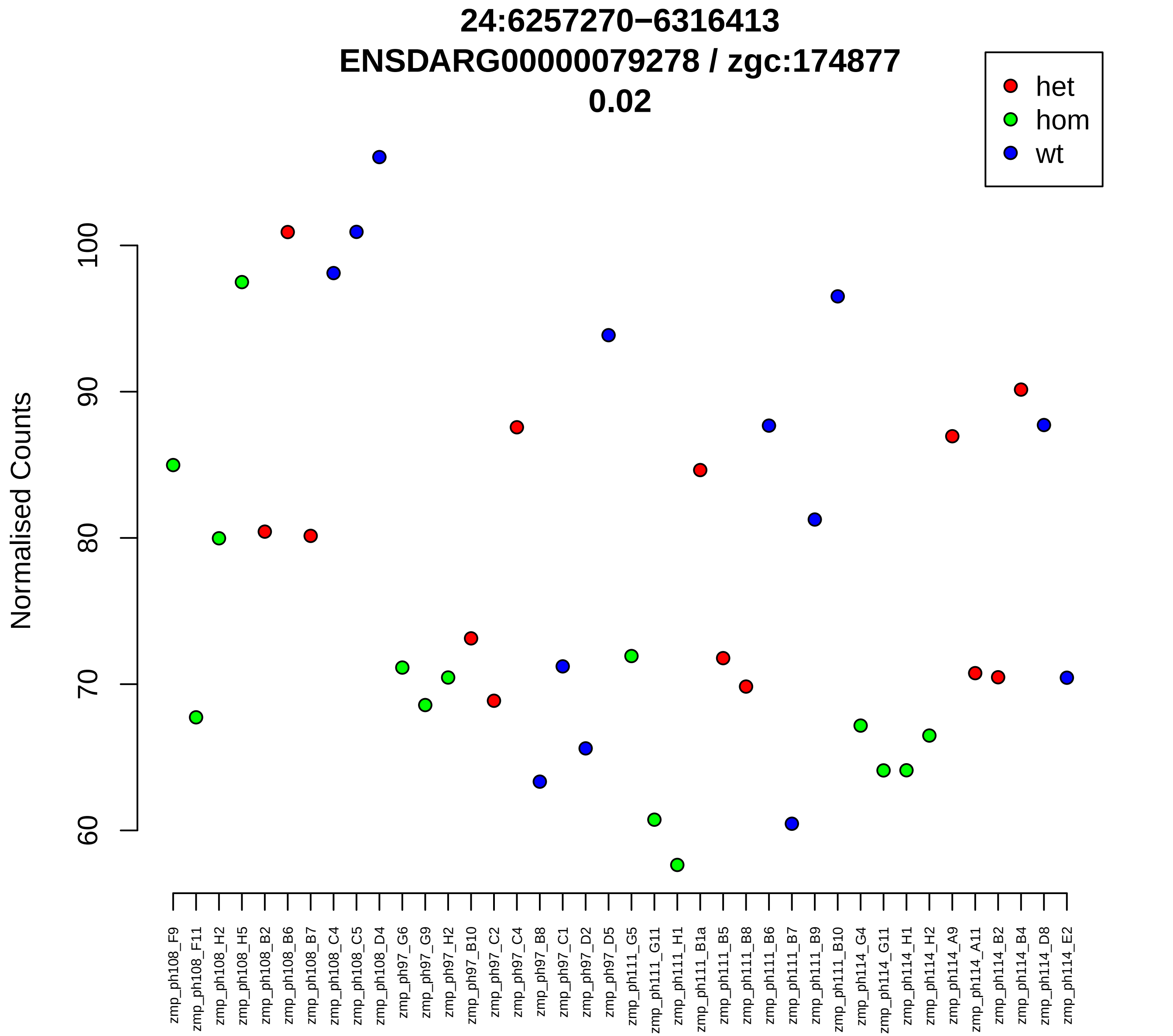

24:6257270−6316413
ENSDARG00000079278 / zgc:174877
0.02
het
hom
wt
Normalised Counts
60
70
80
90
100
zmp_ph108_F9
zmp_ph108_F11
zmp_ph108_H2
zmp_ph108_H5
zmp_ph108_B2
zmp_ph108_B6
zmp_ph108_B7
zmp_ph108_C4
zmp_ph108_C5
zmp_ph108_D4
zmp_ph97_G6
zmp_ph97_G9
zmp_ph97_H2
zmp_ph97_B10
zmp_ph97_C2
zmp_ph97_C4
zmp_ph97_B8
zmp_ph97_C1
zmp_ph97_D2
zmp_ph97_D5
zmp_ph111_G5
zmp_ph111_G11
zmp_ph111_H1
zmp_ph111_B1a
zmp_ph111_B5
zmp_ph111_B8
zmp_ph111_B6
zmp_ph111_B7
zmp_ph111_B9
zmp_ph111_B10
zmp_ph114_G4
zmp_ph114_G11
zmp_ph114_H1
zmp_ph114_H2
zmp_ph114_A9
zmp_ph114_A11
zmp_ph114_B2
zmp_ph114_B4
zmp_ph114_D8
zmp_ph114_E2

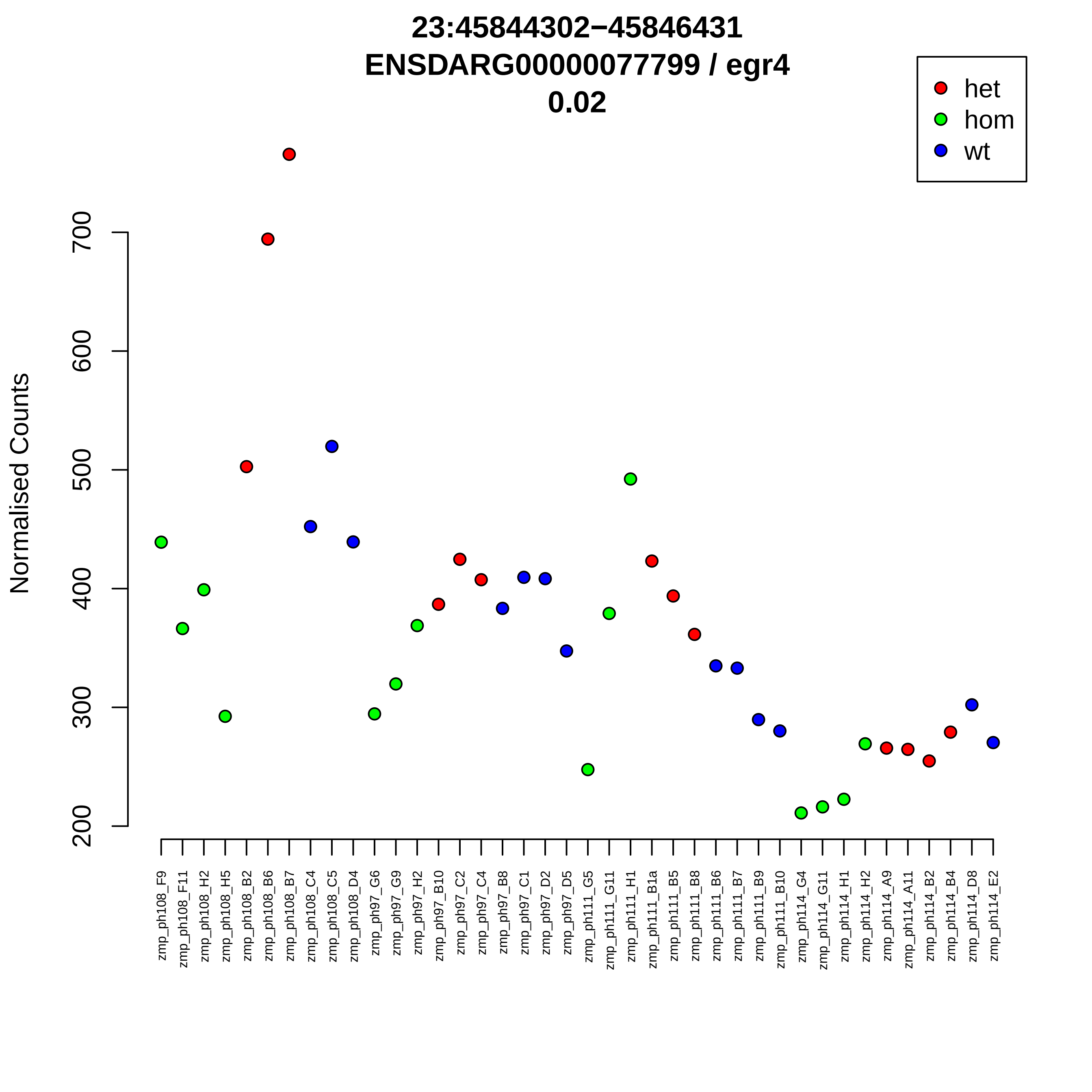
23:45844302−45846431
ENSDARG00000077799 / egr4
0.02
het
hom
wt
Normalised Counts
200
300
400
500
600
700
zmp_ph108_F9
zmp_ph108_F11
zmp_ph108_H2
zmp_ph108_H5
zmp_ph108_B2
zmp_ph108_B6
zmp_ph108_B7
zmp_ph108_C4
zmp_ph108_C5
zmp_ph108_D4
zmp_ph97_G6
zmp_ph97_G9
zmp_ph97_H2
zmp_ph97_B10
zmp_ph97_C2
zmp_ph97_C4
zmp_ph97_B8
zmp_ph97_C1
zmp_ph97_D2
zmp_ph97_D5
zmp_ph111_G5
zmp_ph111_G11
zmp_ph111_H1
zmp_ph111_B1a
zmp_ph111_B5
zmp_ph111_B8
zmp_ph111_B6
zmp_ph111_B7
zmp_ph111_B9
zmp_ph111_B10
zmp_ph114_G4
zmp_ph114_G11
zmp_ph114_H1
zmp_ph114_H2
zmp_ph114_A9
zmp_ph114_A11
zmp_ph114_B2
zmp_ph114_B4
zmp_ph114_D8
zmp_ph114_E2

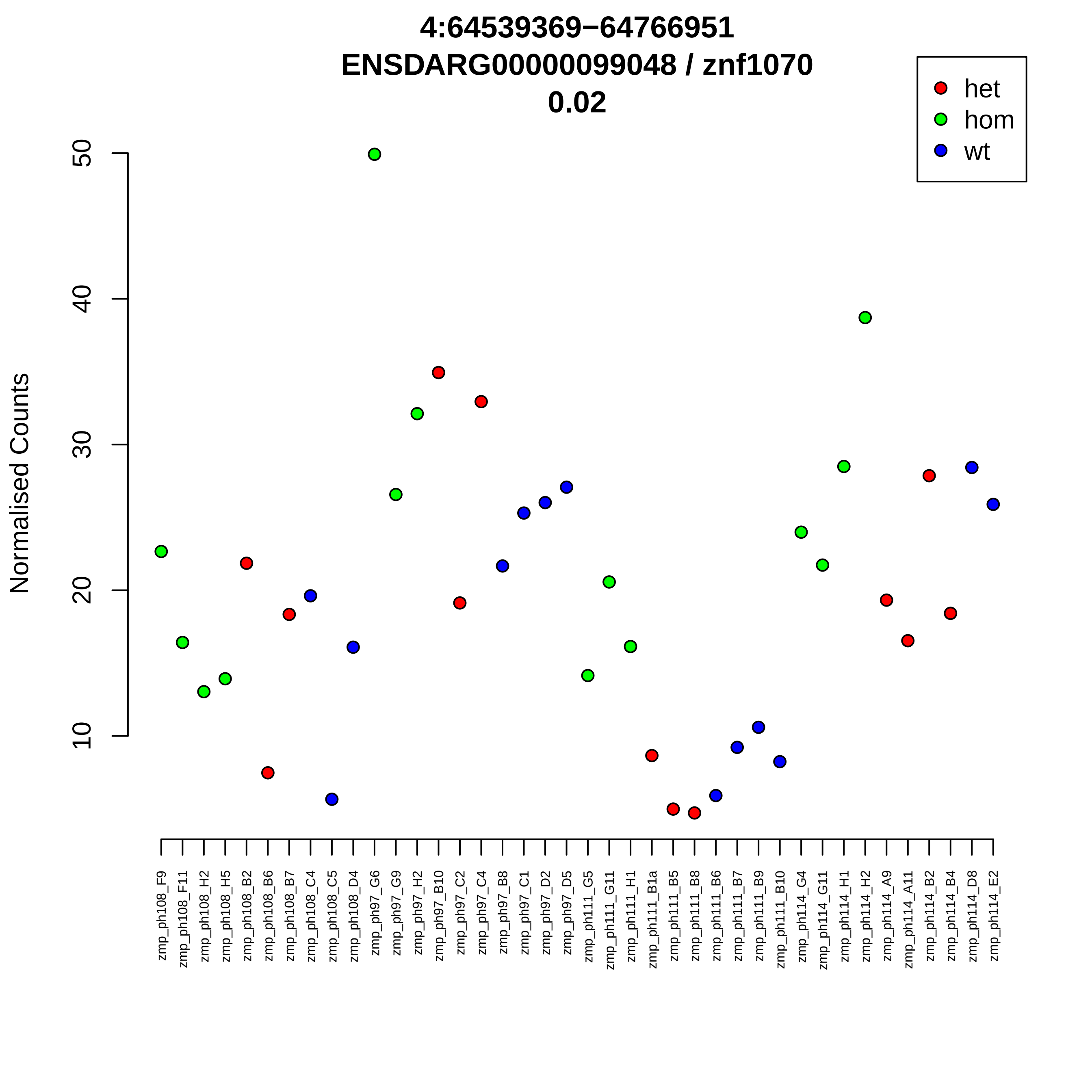
4:64539369−64766951
ENSDARG00000099048 / znf1070
0.02
het
hom
wt
Normalised Counts
10
20
30
40
50
zmp_ph108_F9
zmp_ph108_F11
zmp_ph108_H2
zmp_ph108_H5
zmp_ph108_B2
zmp_ph108_B6
zmp_ph108_B7
zmp_ph108_C4
zmp_ph108_C5
zmp_ph108_D4
zmp_ph97_G6
zmp_ph97_G9
zmp_ph97_H2
zmp_ph97_B10
zmp_ph97_C2
zmp_ph97_C4
zmp_ph97_B8
zmp_ph97_C1
zmp_ph97_D2
zmp_ph97_D5
zmp_ph111_G5
zmp_ph111_G11
zmp_ph111_H1
zmp_ph111_B1a
zmp_ph111_B5
zmp_ph111_B8
zmp_ph111_B6
zmp_ph111_B7
zmp_ph111_B9
zmp_ph111_B10
zmp_ph114_G4
zmp_ph114_G11
zmp_ph114_H1
zmp_ph114_H2
zmp_ph114_A9
zmp_ph114_A11
zmp_ph114_B2
zmp_ph114_B4
zmp_ph114_D8
zmp_ph114_E2

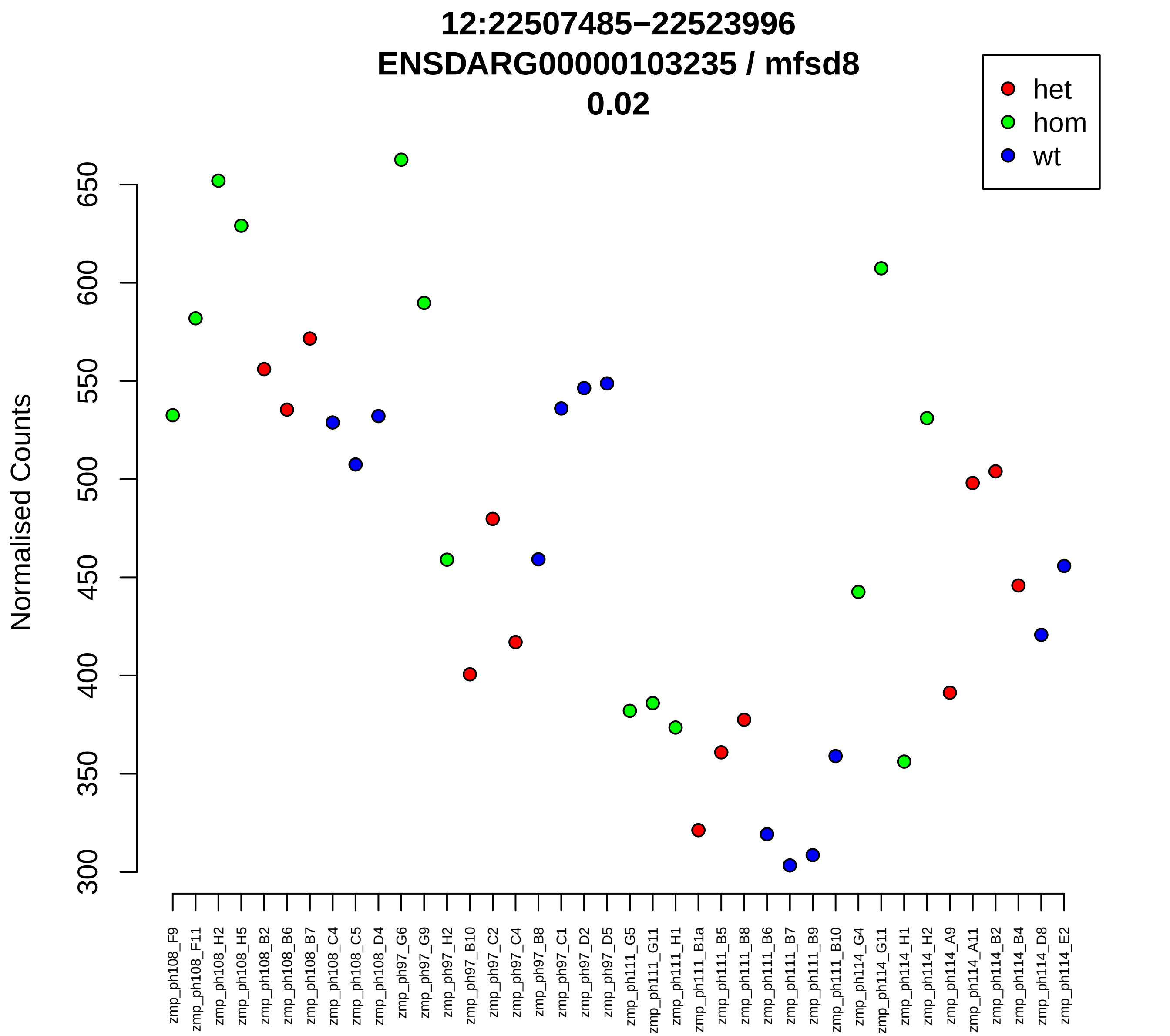

12:22507485−22523996
ENSDARG00000103235 / mfsd8
0.02
het
hom
wt
Normalised Counts
300
350
400
450
500
550
600
650
zmp_ph108_F9
zmp_ph108_F11
zmp_ph108_H2
zmp_ph108_H5
zmp_ph108_B2
zmp_ph108_B6
zmp_ph108_B7
zmp_ph108_C4
zmp_ph108_C5
zmp_ph108_D4
zmp_ph97_G6
zmp_ph97_G9
zmp_ph97_H2
zmp_ph97_B10
zmp_ph97_C2
zmp_ph97_C4
zmp_ph97_B8
zmp_ph97_C1
zmp_ph97_D2
zmp_ph97_D5
zmp_ph111_G5
zmp_ph111_G11
zmp_ph111_H1
zmp_ph111_B1a
zmp_ph111_B5
zmp_ph111_B8
zmp_ph111_B6
zmp_ph111_B7
zmp_ph111_B9
zmp_ph111_B10
zmp_ph114_G4
zmp_ph114_G11
zmp_ph114_H1
zmp_ph114_H2
zmp_ph114_A9
zmp_ph114_A11
zmp_ph114_B2
zmp_ph114_B4
zmp_ph114_D8
zmp_ph114_E2

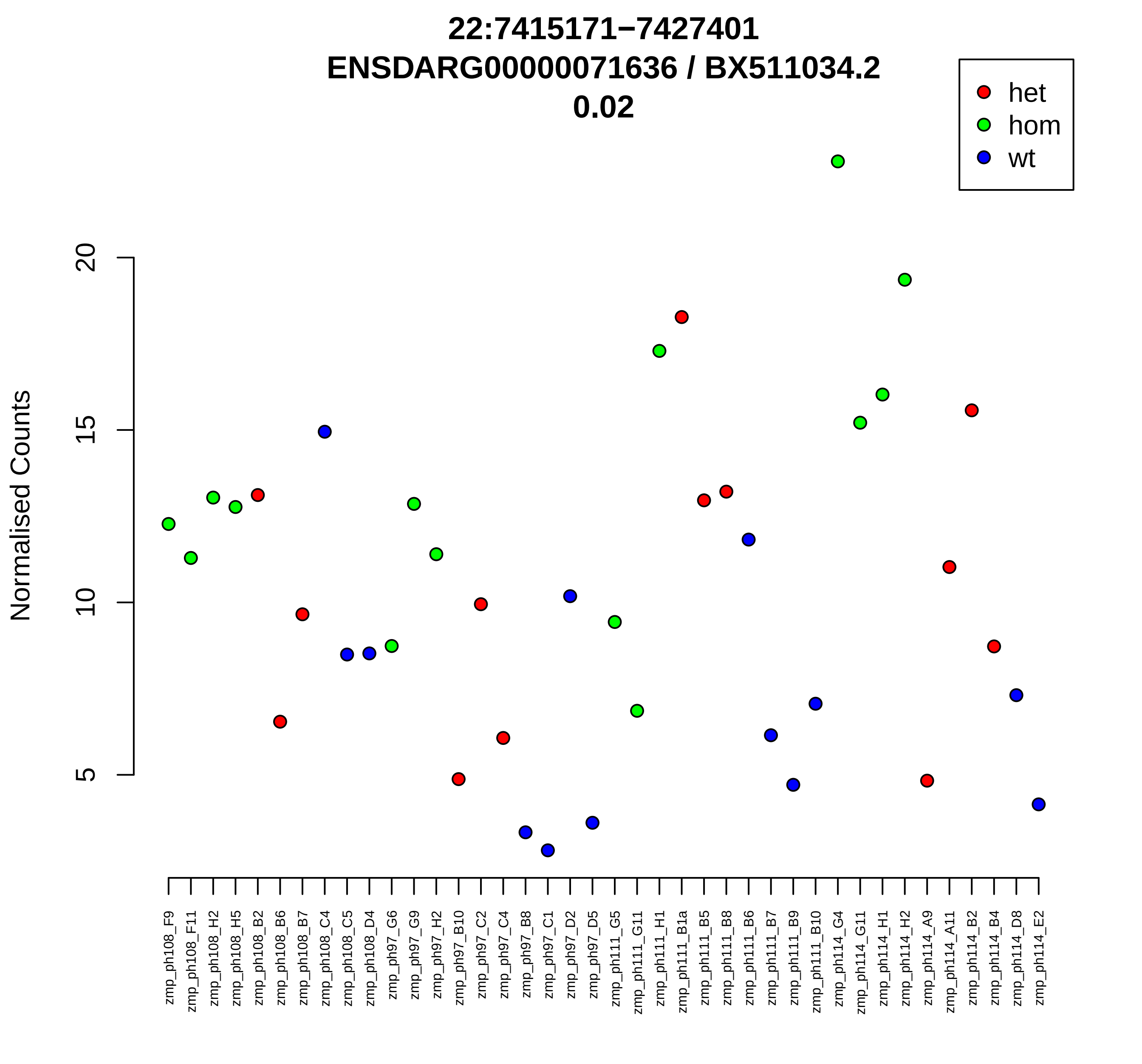
22:7415171−7427401
ENSDARG00000071636 / BX511034.2
0.02
het
hom
wt
Normalised Counts
5
10
15
20
zmp_ph108_F9
zmp_ph108_F11
zmp_ph108_H2
zmp_ph108_H5
zmp_ph108_B2
zmp_ph108_B6
zmp_ph108_B7
zmp_ph108_C4
zmp_ph108_C5
zmp_ph108_D4
zmp_ph97_G6
zmp_ph97_G9
zmp_ph97_H2
zmp_ph97_B10
zmp_ph97_C2
zmp_ph97_C4
zmp_ph97_B8
zmp_ph97_C1
zmp_ph97_D2
zmp_ph97_D5
zmp_ph111_G5
zmp_ph111_G11
zmp_ph111_H1
zmp_ph111_B1a
zmp_ph111_B5
zmp_ph111_B8
zmp_ph111_B6
zmp_ph111_B7
zmp_ph111_B9
zmp_ph111_B10
zmp_ph114_G4
zmp_ph114_G11
zmp_ph114_H1
zmp_ph114_H2
zmp_ph114_A9
zmp_ph114_A11
zmp_ph114_B2
zmp_ph114_B4
zmp_ph114_D8
zmp_ph114_E2

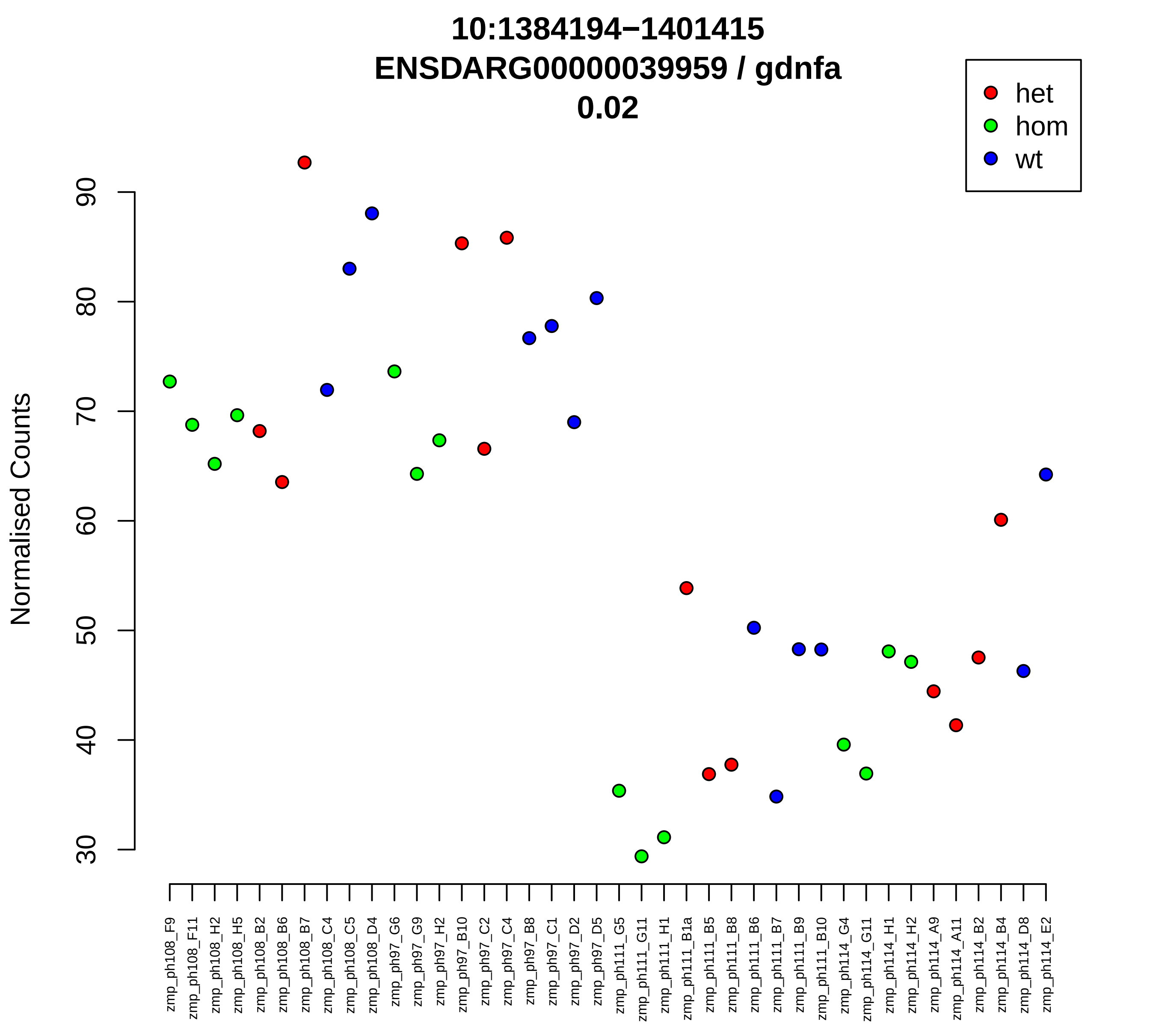

10:1384194−1401415
ENSDARG00000039959 / gdnfa
0.02
het
hom
wt
Normalised Counts
30
40
50
60
70
80
90
zmp_ph108_F9
zmp_ph108_F11
zmp_ph108_H2
zmp_ph108_H5
zmp_ph108_B2
zmp_ph108_B6
zmp_ph108_B7
zmp_ph108_C4
zmp_ph108_C5
zmp_ph108_D4
zmp_ph97_G6
zmp_ph97_G9
zmp_ph97_H2
zmp_ph97_B10
zmp_ph97_C2
zmp_ph97_C4
zmp_ph97_B8
zmp_ph97_C1
zmp_ph97_D2
zmp_ph97_D5
zmp_ph111_G5
zmp_ph111_G11
zmp_ph111_H1
zmp_ph111_B1a
zmp_ph111_B5
zmp_ph111_B8
zmp_ph111_B6
zmp_ph111_B7
zmp_ph111_B9
zmp_ph111_B10
zmp_ph114_G4
zmp_ph114_G11
zmp_ph114_H1
zmp_ph114_H2
zmp_ph114_A9
zmp_ph114_A11
zmp_ph114_B2
zmp_ph114_B4
zmp_ph114_D8
zmp_ph114_E2

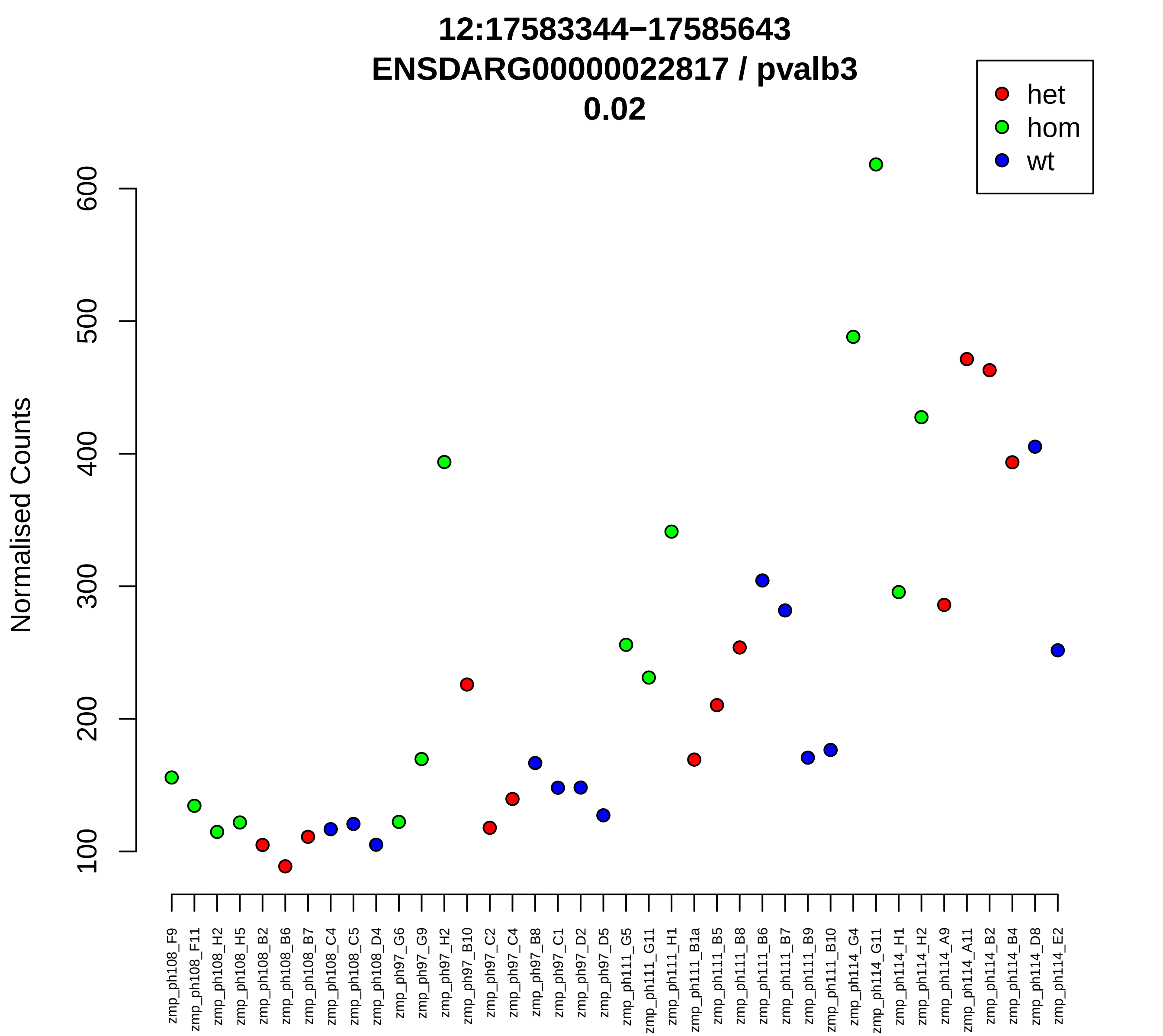
12:17583344−17585643
ENSDARG00000022817 / pvalb3
0.02
Normalised Counts
het
hom
wt
100
200
300
400
500
600
zmp_ph108_F9
zmp_ph108_F11
zmp_ph108_H2
zmp_ph108_H5
zmp_ph108_B2
zmp_ph108_B6
zmp_ph108_B7
zmp_ph108_C4
zmp_ph108_C5
zmp_ph108_D4
zmp_ph97_G6
zmp_ph97_G9
zmp_ph97_H2
zmp_ph97_B10
zmp_ph97_C2
zmp_ph97_C4
zmp_ph97_B8
zmp_ph97_C1
zmp_ph97_D2
zmp_ph97_D5
zmp_ph111_G5
zmp_ph111_G11
zmp_ph111_H1
zmp_ph111_B1a
zmp_ph111_B5
zmp_ph111_B8
zmp_ph111_B6
zmp_ph111_B7
zmp_ph111_B9
zmp_ph111_B10
zmp_ph114_G4
zmp_ph114_G11
zmp_ph114_H1
zmp_ph114_H2
zmp_ph114_A9
zmp_ph114_A11
zmp_ph114_B2
zmp_ph114_B4
zmp_ph114_D8
zmp_ph114_E2

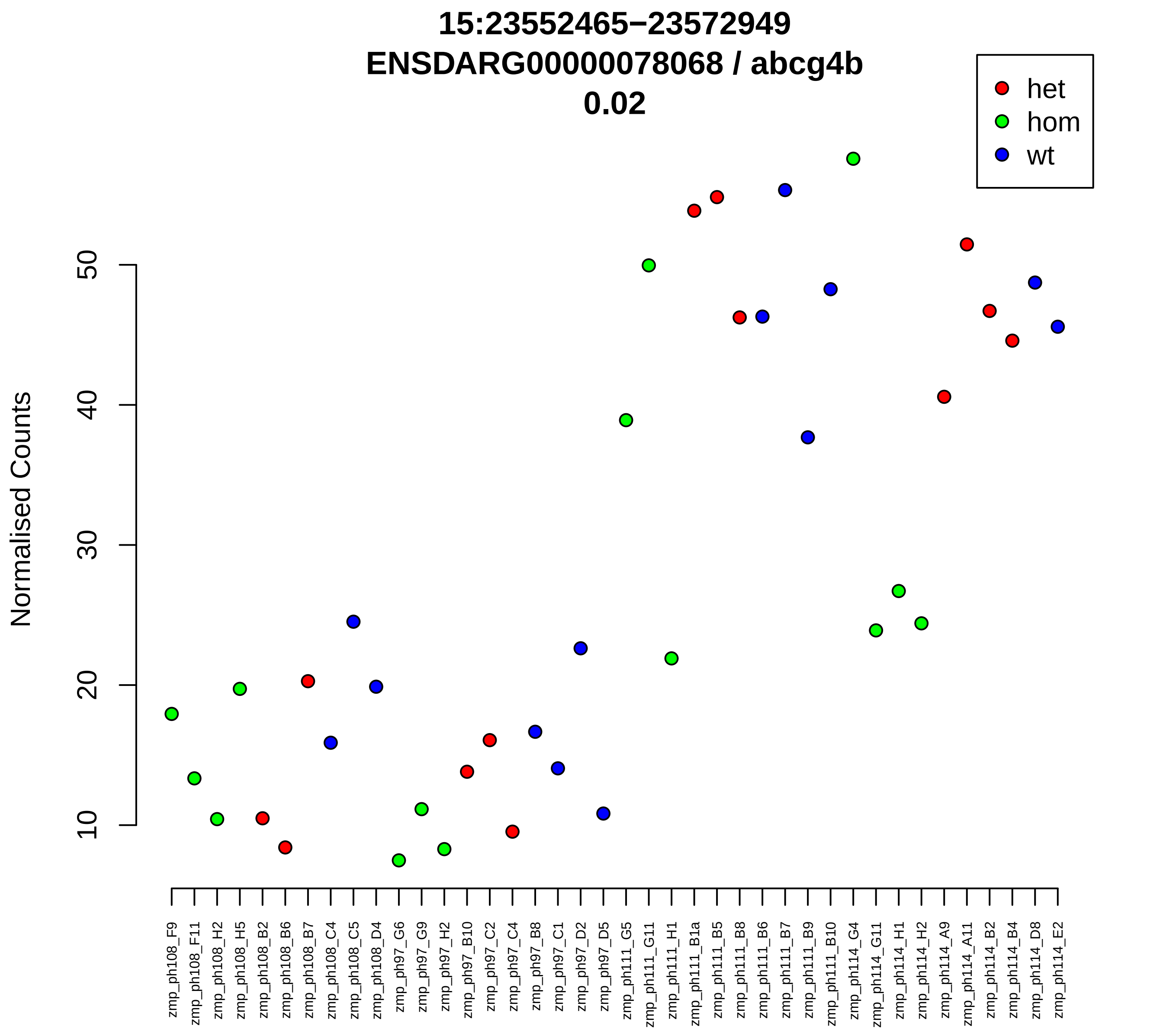

15:23552465−23572949
ENSDARG00000078068 / abcg4b
0.02
het
hom
wt
Normalised Counts
10
20
30
40
50
zmp_ph108_F9
zmp_ph108_F11
zmp_ph108_H2
zmp_ph108_H5
zmp_ph108_B2
zmp_ph108_B6
zmp_ph108_B7
zmp_ph108_C4
zmp_ph108_C5
zmp_ph108_D4
zmp_ph97_G6
zmp_ph97_G9
zmp_ph97_H2
zmp_ph97_B10
zmp_ph97_C2
zmp_ph97_C4
zmp_ph97_B8
zmp_ph97_C1
zmp_ph97_D2
zmp_ph97_D5
zmp_ph111_G5
zmp_ph111_G11
zmp_ph111_H1
zmp_ph111_B1a
zmp_ph111_B5
zmp_ph111_B8
zmp_ph111_B6
zmp_ph111_B7
zmp_ph111_B9
zmp_ph111_B10
zmp_ph114_G4
zmp_ph114_G11
zmp_ph114_H1
zmp_ph114_H2
zmp_ph114_A9
zmp_ph114_A11
zmp_ph114_B2
zmp_ph114_B4
zmp_ph114_D8
zmp_ph114_E2

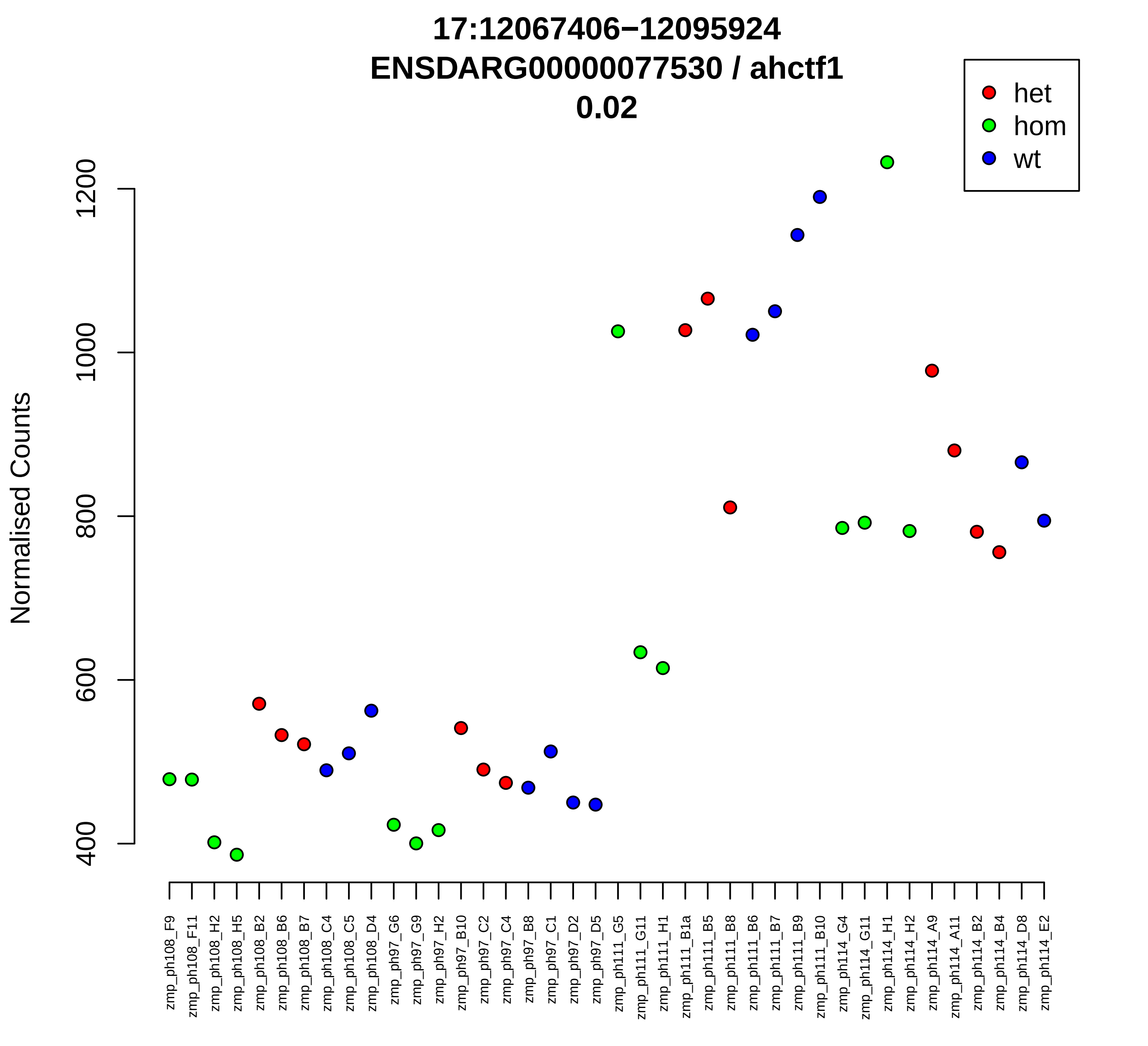
17:12067406−12095924
ENSDARG00000077530 / ahctf1
0.02
het
hom
wt
Normalised Counts
400
600
800
1000
1200
zmp_ph108_F9
zmp_ph108_F11
zmp_ph108_H2
zmp_ph108_H5
zmp_ph108_B2
zmp_ph108_B6
zmp_ph108_B7
zmp_ph108_C4
zmp_ph108_C5
zmp_ph108_D4
zmp_ph97_G6
zmp_ph97_G9
zmp_ph97_H2
zmp_ph97_B10
zmp_ph97_C2
zmp_ph97_C4
zmp_ph97_B8
zmp_ph97_C1
zmp_ph97_D2
zmp_ph97_D5
zmp_ph111_G5
zmp_ph111_G11
zmp_ph111_H1
zmp_ph111_B1a
zmp_ph111_B5
zmp_ph111_B8
zmp_ph111_B6
zmp_ph111_B7
zmp_ph111_B9
zmp_ph111_B10
zmp_ph114_G4
zmp_ph114_G11
zmp_ph114_H1
zmp_ph114_H2
zmp_ph114_A9
zmp_ph114_A11
zmp_ph114_B2
zmp_ph114_B4
zmp_ph114_D8
zmp_ph114_E2

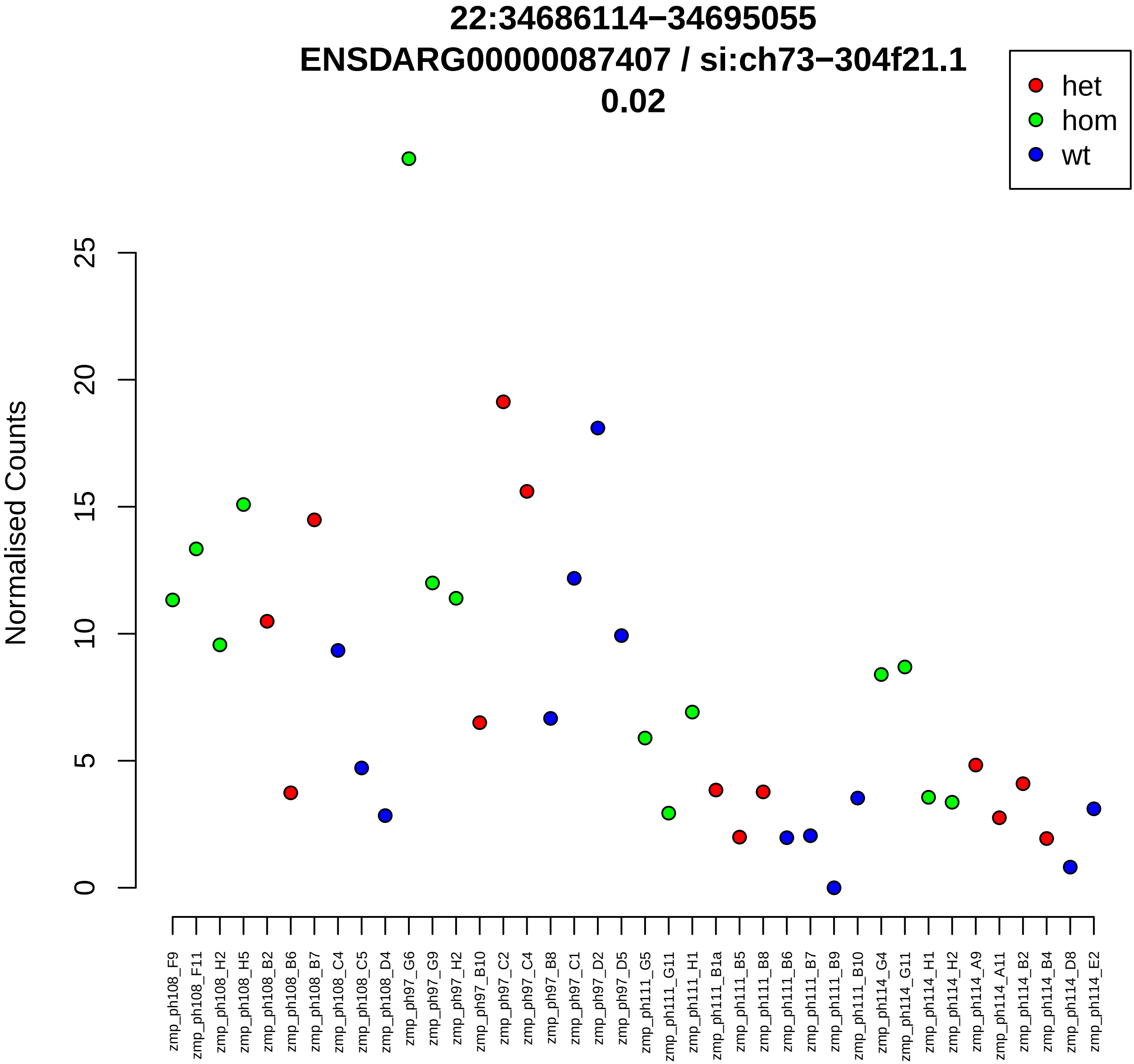

22:34686114−34695055
ENSDARG00000087407 / si:ch73−304f21.1
0.02
het
hom
wt
Normalised Counts
0
5
10
15
20
25
zmp_ph108_F9
zmp_ph108_F11
zmp_ph108_H2
zmp_ph108_H5
zmp_ph108_B2
zmp_ph108_B6
zmp_ph108_B7
zmp_ph108_C4
zmp_ph108_C5
zmp_ph108_D4
zmp_ph97_G6
zmp_ph97_G9
zmp_ph97_H2
zmp_ph97_B10
zmp_ph97_C2
zmp_ph97_C4
zmp_ph97_B8
zmp_ph97_C1
zmp_ph97_D2
zmp_ph97_D5
zmp_ph111_G5
zmp_ph111_G11
zmp_ph111_H1
zmp_ph111_B1a
zmp_ph111_B5
zmp_ph111_B8
zmp_ph111_B6
zmp_ph111_B7
zmp_ph111_B9
zmp_ph111_B10
zmp_ph114_G4
zmp_ph114_G11
zmp_ph114_H1
zmp_ph114_H2
zmp_ph114_A9
zmp_ph114_A11
zmp_ph114_B2
zmp_ph114_B4
zmp_ph114_D8
zmp_ph114_E2

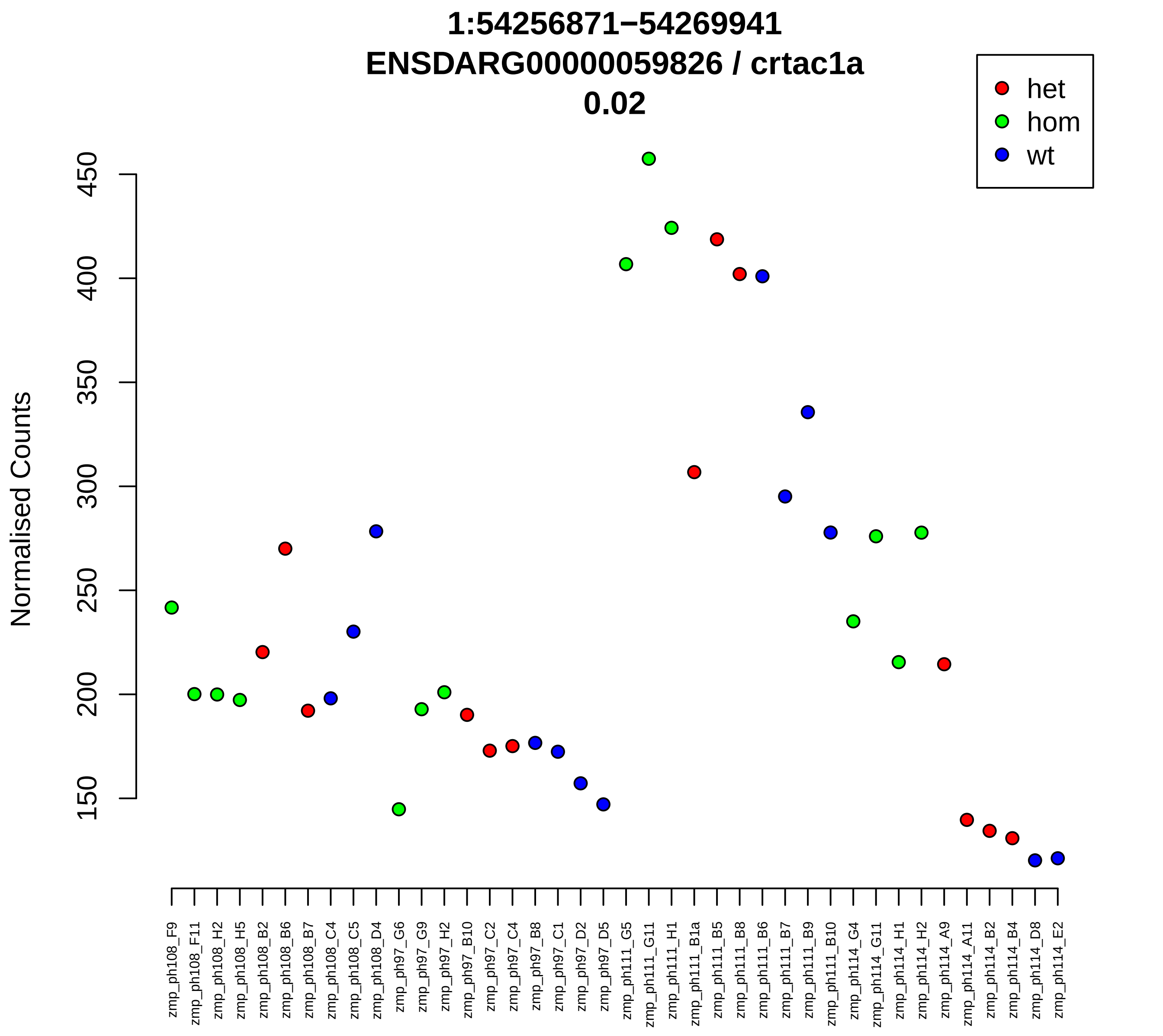

1:54256871−54269941
ENSDARG00000059826 / crtac1a
0.02
het
hom
wt
Normalised Counts
150
200
250
300
350
400
450
zmp_ph108_F9
zmp_ph108_F11
zmp_ph108_H2
zmp_ph108_H5
zmp_ph108_B2
zmp_ph108_B6
zmp_ph108_B7
zmp_ph108_C4
zmp_ph108_C5
zmp_ph108_D4
zmp_ph97_G6
zmp_ph97_G9
zmp_ph97_H2
zmp_ph97_B10
zmp_ph97_C2
zmp_ph97_C4
zmp_ph97_B8
zmp_ph97_C1
zmp_ph97_D2
zmp_ph97_D5
zmp_ph111_G5
zmp_ph111_G11
zmp_ph111_H1
zmp_ph111_B1a
zmp_ph111_B5
zmp_ph111_B8
zmp_ph111_B6
zmp_ph111_B7
zmp_ph111_B9
zmp_ph111_B10
zmp_ph114_G4
zmp_ph114_G11
zmp_ph114_H1
zmp_ph114_H2
zmp_ph114_A9
zmp_ph114_A11
zmp_ph114_B2
zmp_ph114_B4
zmp_ph114_D8
zmp_ph114_E2

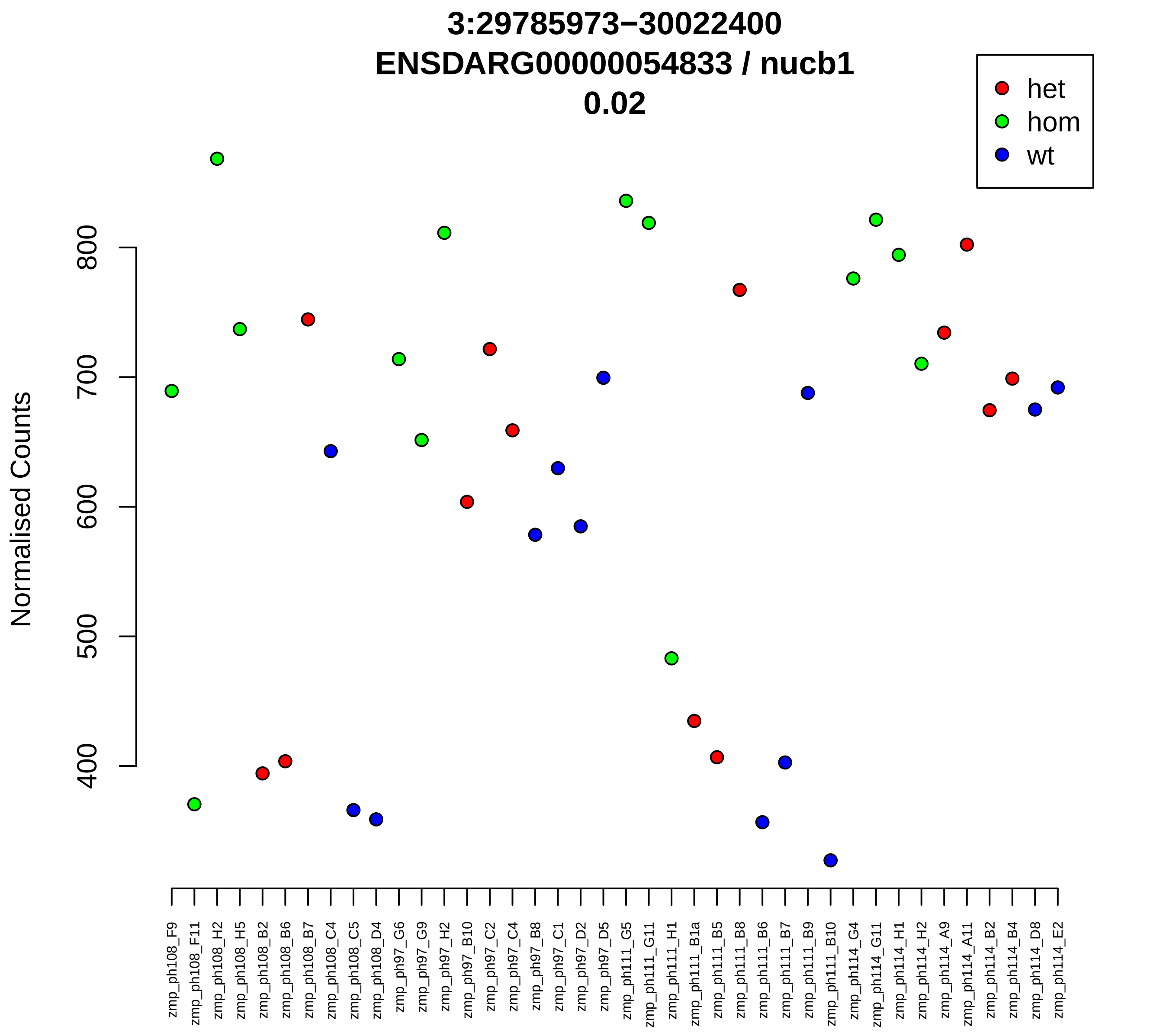
3:29785973−30022400
ENSDARG00000054833 / nucb1
0.02
het
hom
wt
Normalised Counts
400
500
600
700
800
zmp_ph108_F9
zmp_ph108_F11
zmp_ph108_H2
zmp_ph108_H5
zmp_ph108_B2
zmp_ph108_B6
zmp_ph108_B7
zmp_ph108_C4
zmp_ph108_C5
zmp_ph108_D4
zmp_ph97_G6
zmp_ph97_G9
zmp_ph97_H2
zmp_ph97_B10
zmp_ph97_C2
zmp_ph97_C4
zmp_ph97_B8
zmp_ph97_C1
zmp_ph97_D2
zmp_ph97_D5
zmp_ph111_G5
zmp_ph111_G11
zmp_ph111_H1
zmp_ph111_B1a
zmp_ph111_B5
zmp_ph111_B8
zmp_ph111_B6
zmp_ph111_B7
zmp_ph111_B9
zmp_ph111_B10
zmp_ph114_G4
zmp_ph114_G11
zmp_ph114_H1
zmp_ph114_H2
zmp_ph114_A9
zmp_ph114_A11
zmp_ph114_B2
zmp_ph114_B4
zmp_ph114_D8
zmp_ph114_E2

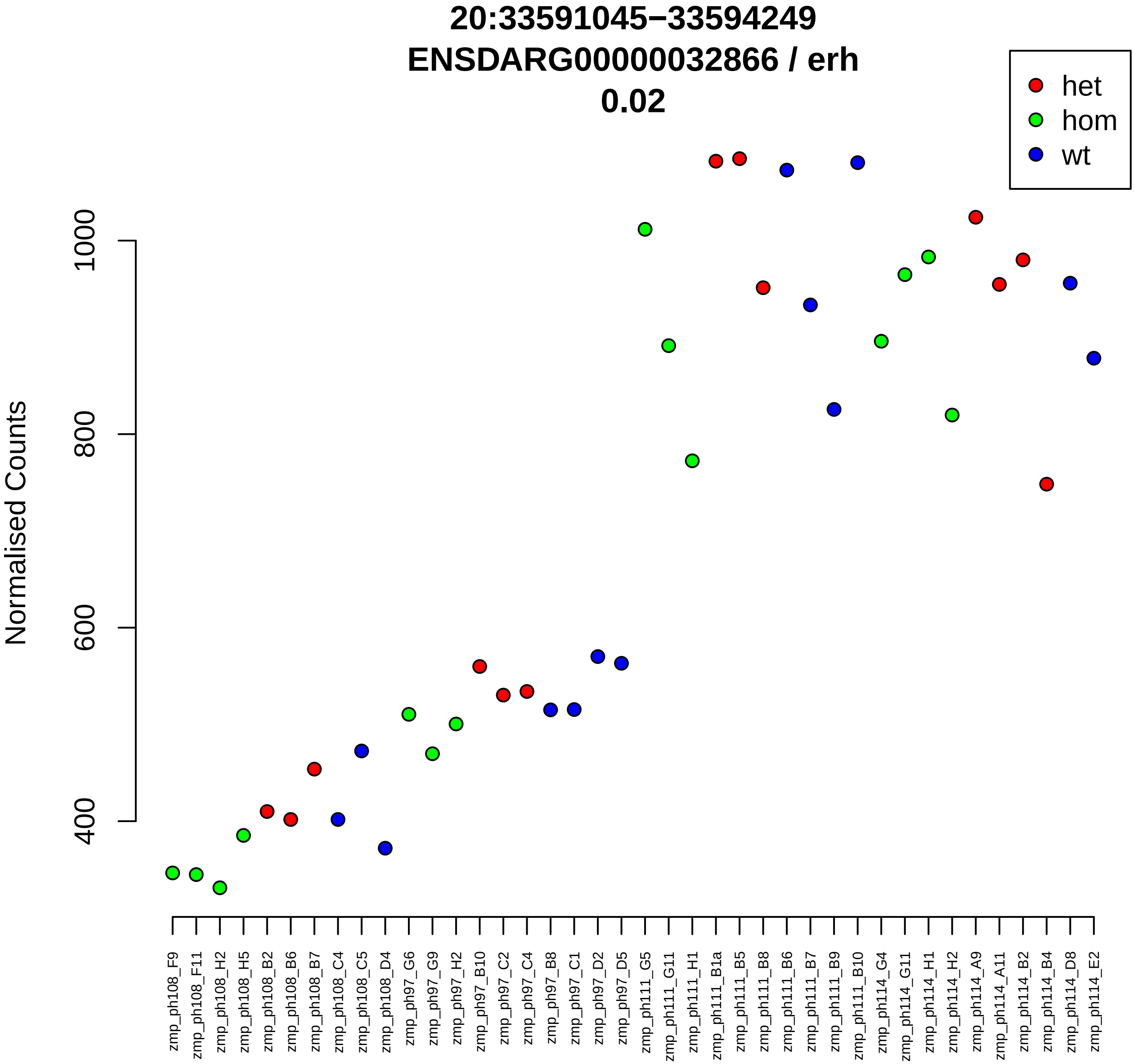

20:33591045−33594249
ENSDARG00000032866 / erh
0.02
het
hom
wt
Normalised Counts
400
600
800
1000
zmp_ph108_F9
zmp_ph108_F11
zmp_ph108_H2
zmp_ph108_H5
zmp_ph108_B2
zmp_ph108_B6
zmp_ph108_B7
zmp_ph108_C4
zmp_ph108_C5
zmp_ph108_D4
zmp_ph97_G6
zmp_ph97_G9
zmp_ph97_H2
zmp_ph97_B10
zmp_ph97_C2
zmp_ph97_C4
zmp_ph97_B8
zmp_ph97_C1
zmp_ph97_D2
zmp_ph97_D5
zmp_ph111_G5
zmp_ph111_G11
zmp_ph111_H1
zmp_ph111_B1a
zmp_ph111_B5
zmp_ph111_B8
zmp_ph111_B6
zmp_ph111_B7
zmp_ph111_B9
zmp_ph111_B10
zmp_ph114_G4
zmp_ph114_G11
zmp_ph114_H1
zmp_ph114_H2
zmp_ph114_A9
zmp_ph114_A11
zmp_ph114_B2
zmp_ph114_B4
zmp_ph114_D8
zmp_ph114_E2

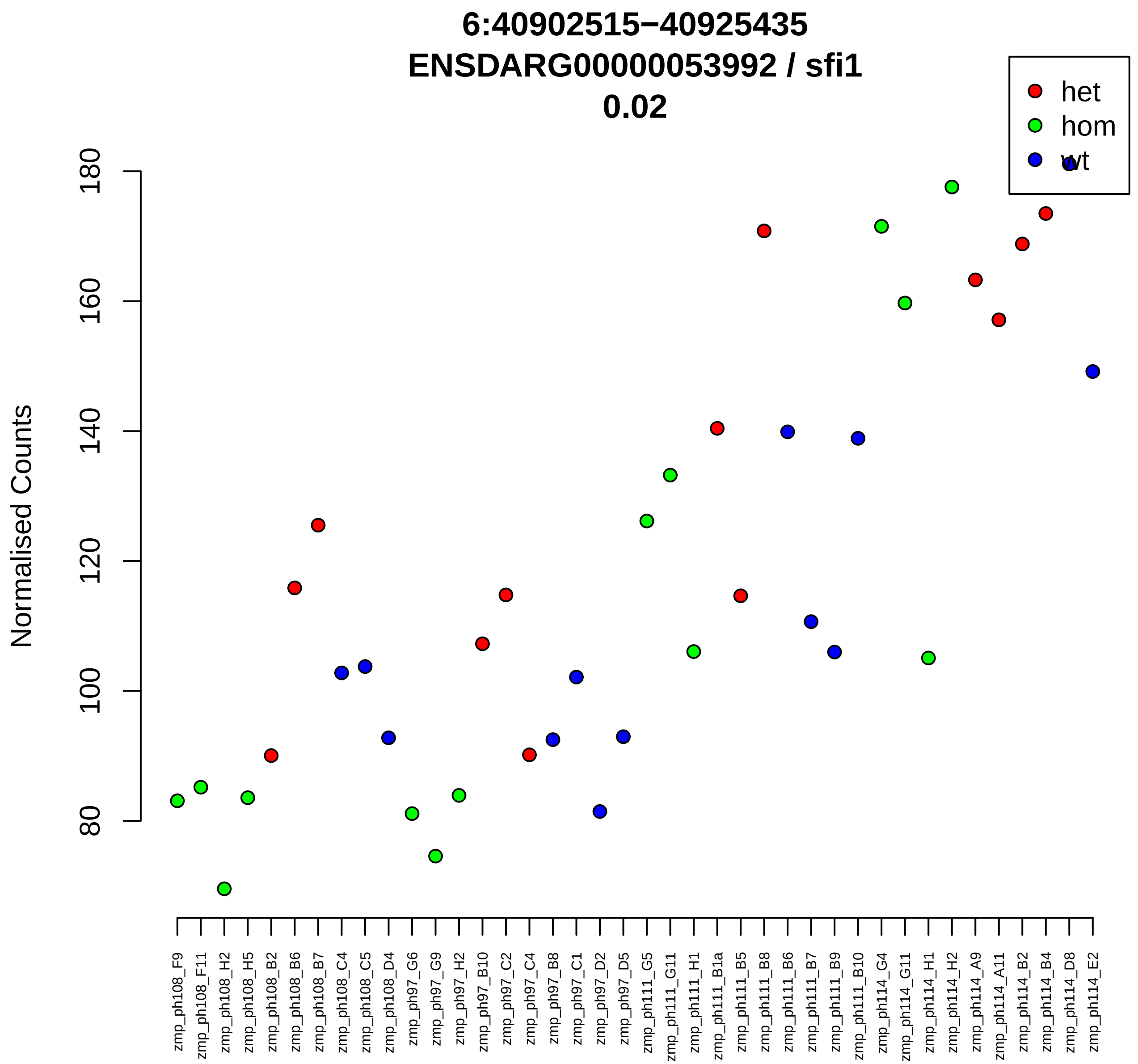
6:40902515−40925435
ENSDARG00000053992 / sfi1
0.02
het
hom
wt
Normalised Counts
80
100
120
140
160
180
zmp_ph108_F9
zmp_ph108_F11
zmp_ph108_H2
zmp_ph108_H5
zmp_ph108_B2
zmp_ph108_B6
zmp_ph108_B7
zmp_ph108_C4
zmp_ph108_C5
zmp_ph108_D4
zmp_ph97_G6
zmp_ph97_G9
zmp_ph97_H2
zmp_ph97_B10
zmp_ph97_C2
zmp_ph97_C4
zmp_ph97_B8
zmp_ph97_C1
zmp_ph97_D2
zmp_ph97_D5
zmp_ph111_G5
zmp_ph111_G11
zmp_ph111_H1
zmp_ph111_B1a
zmp_ph111_B5
zmp_ph111_B8
zmp_ph111_B6
zmp_ph111_B7
zmp_ph111_B9
zmp_ph111_B10
zmp_ph114_G4
zmp_ph114_G11
zmp_ph114_H1
zmp_ph114_H2
zmp_ph114_A9
zmp_ph114_A11
zmp_ph114_B2
zmp_ph114_B4
zmp_ph114_D8
zmp_ph114_E2

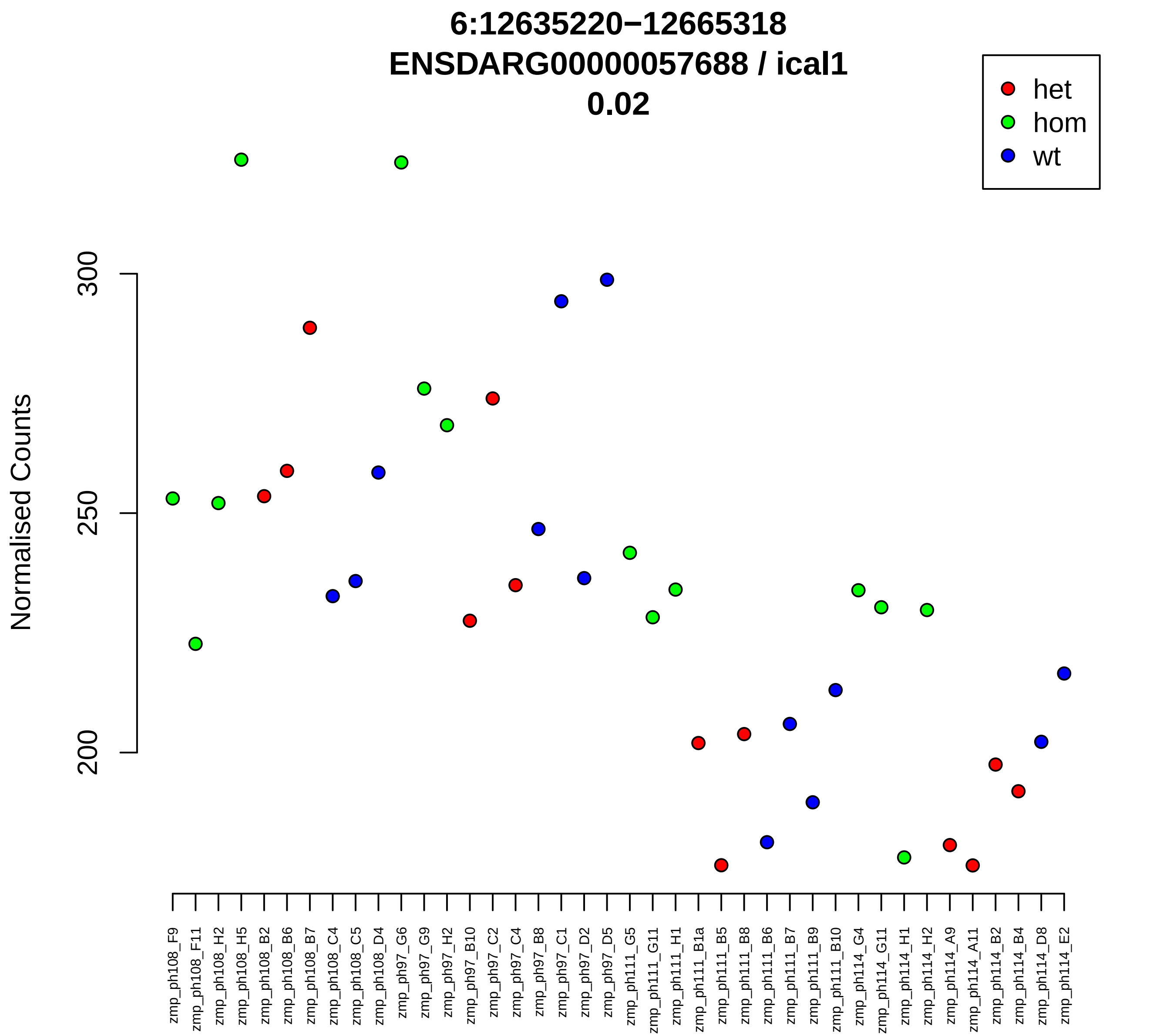
6:12635220−12665318
ENSDARG00000057688 / ical1
0.02
het
hom
wt
Normalised Counts
200
250
300
zmp_ph108_F9
zmp_ph108_F11
zmp_ph108_H2
zmp_ph108_H5
zmp_ph108_B2
zmp_ph108_B6
zmp_ph108_B7
zmp_ph108_C4
zmp_ph108_C5
zmp_ph108_D4
zmp_ph97_G6
zmp_ph97_G9
zmp_ph97_H2
zmp_ph97_B10
zmp_ph97_C2
zmp_ph97_C4
zmp_ph97_B8
zmp_ph97_C1
zmp_ph97_D2
zmp_ph97_D5
zmp_ph111_G5
zmp_ph111_G11
zmp_ph111_H1
zmp_ph111_B1a
zmp_ph111_B5
zmp_ph111_B8
zmp_ph111_B6
zmp_ph111_B7
zmp_ph111_B9
zmp_ph111_B10
zmp_ph114_G4
zmp_ph114_G11
zmp_ph114_H1
zmp_ph114_H2
zmp_ph114_A9
zmp_ph114_A11
zmp_ph114_B2
zmp_ph114_B4
zmp_ph114_D8
zmp_ph114_E2

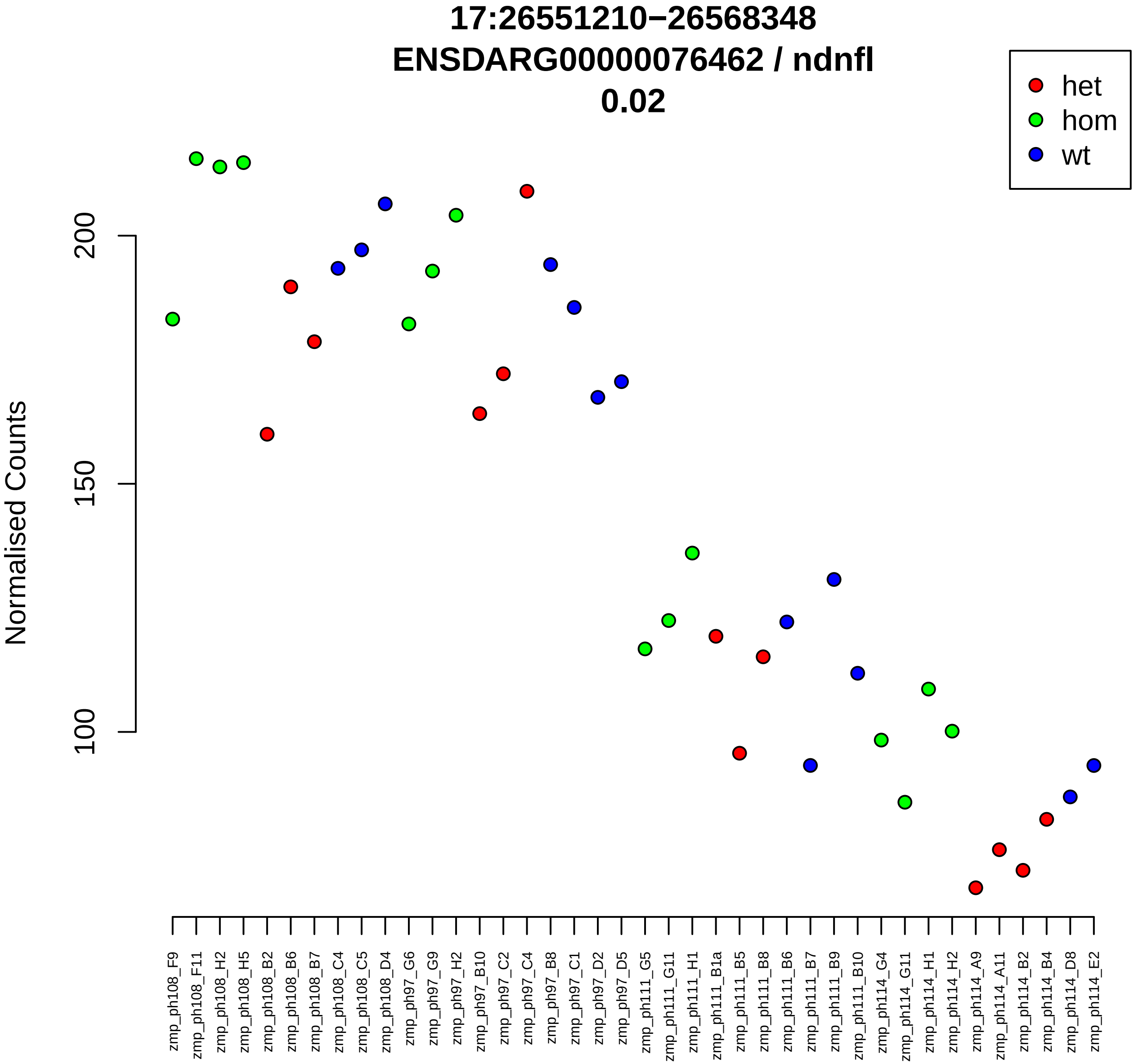
17:26551210−26568348
ENSDARG00000076462 / ndnfl
0.02
het
hom
wt
Normalised Counts
200
150
100
zmp_ph108_F9
zmp_ph108_F11
zmp_ph108_H2
zmp_ph108_H5
zmp_ph108_B2
zmp_ph108_B6
zmp_ph108_B7
zmp_ph108_C4
zmp_ph108_C5
zmp_ph108_D4
zmp_ph97_G6
zmp_ph97_G9
zmp_ph97_H2
zmp_ph97_B10
zmp_ph97_C2
zmp_ph97_C4
zmp_ph97_B8
zmp_ph97_C1
zmp_ph97_D2
zmp_ph97_D5
zmp_ph111_G5
zmp_ph111_G11
zmp_ph111_H1
zmp_ph111_B1a
zmp_ph111_B5
zmp_ph111_B8
zmp_ph111_B6
zmp_ph111_B7
zmp_ph111_B9
zmp_ph111_B10
zmp_ph114_G4
zmp_ph114_G11
zmp_ph114_H1
zmp_ph114_H2
zmp_ph114_A9
zmp_ph114_A11
zmp_ph114_B2
zmp_ph114_B4
zmp_ph114_D8
zmp_ph114_E2

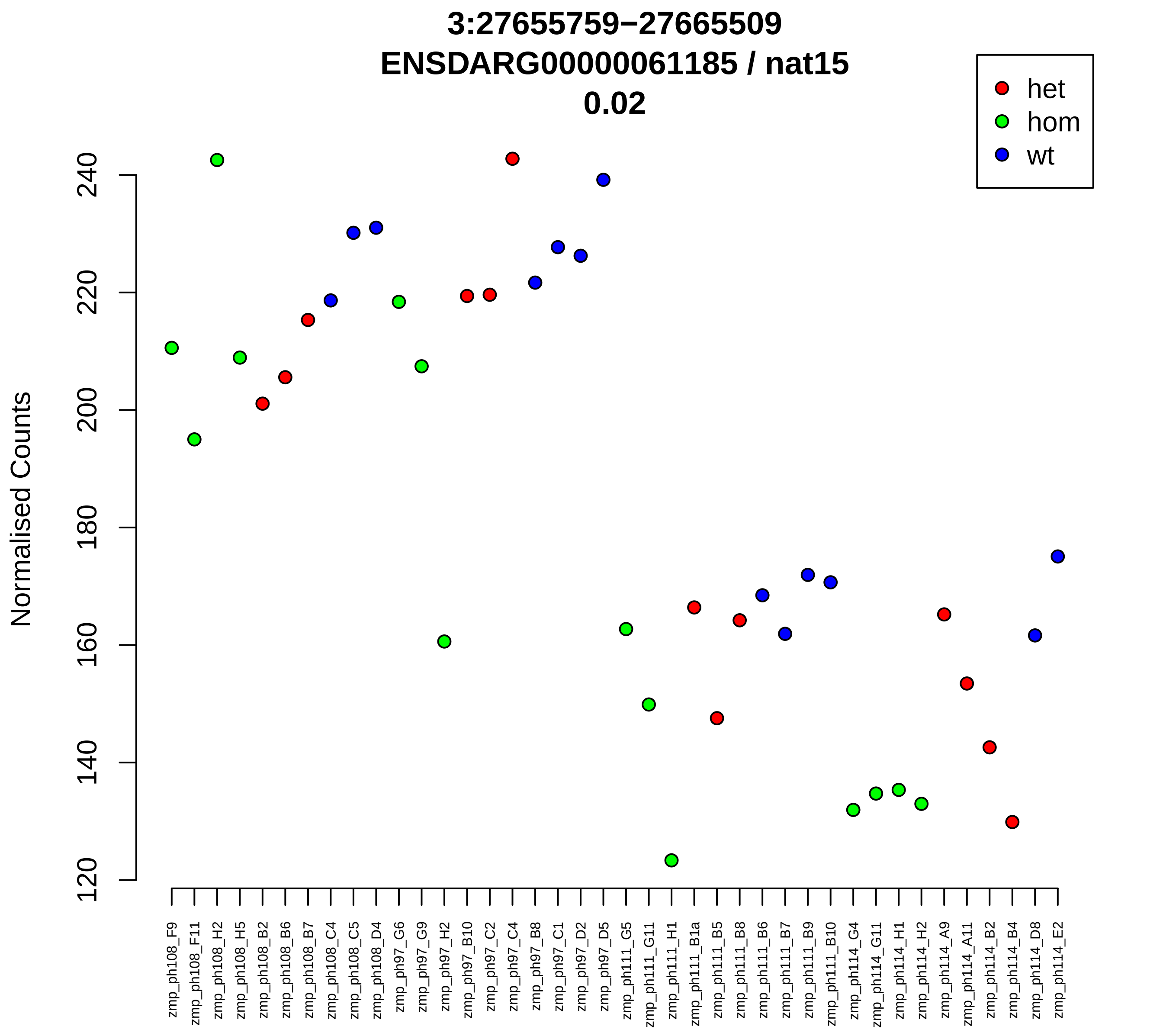
3:27655759−27665509
ENSDARG00000061185 / nat15
0.02
het
hom
wt
Normalised Counts
120
140
160
180
200
220
240
zmp_ph108_F9
zmp_ph108_F11
zmp_ph108_H2
zmp_ph108_H5
zmp_ph108_B2
zmp_ph108_B6
zmp_ph108_B7
zmp_ph108_C4
zmp_ph108_C5
zmp_ph108_D4
zmp_ph97_G6
zmp_ph97_G9
zmp_ph97_H2
zmp_ph97_B10
zmp_ph97_C2
zmp_ph97_C4
zmp_ph97_B8
zmp_ph97_C1
zmp_ph97_D2
zmp_ph97_D5
zmp_ph111_G5
zmp_ph111_G11
zmp_ph111_H1
zmp_ph111_B1a
zmp_ph111_B5
zmp_ph111_B8
zmp_ph111_B6
zmp_ph111_B7
zmp_ph111_B9
zmp_ph111_B10
zmp_ph114_G4
zmp_ph114_G11
zmp_ph114_H1
zmp_ph114_H2
zmp_ph114_A9
zmp_ph114_A11
zmp_ph114_B2
zmp_ph114_B4
zmp_ph114_D8
zmp_ph114_E2

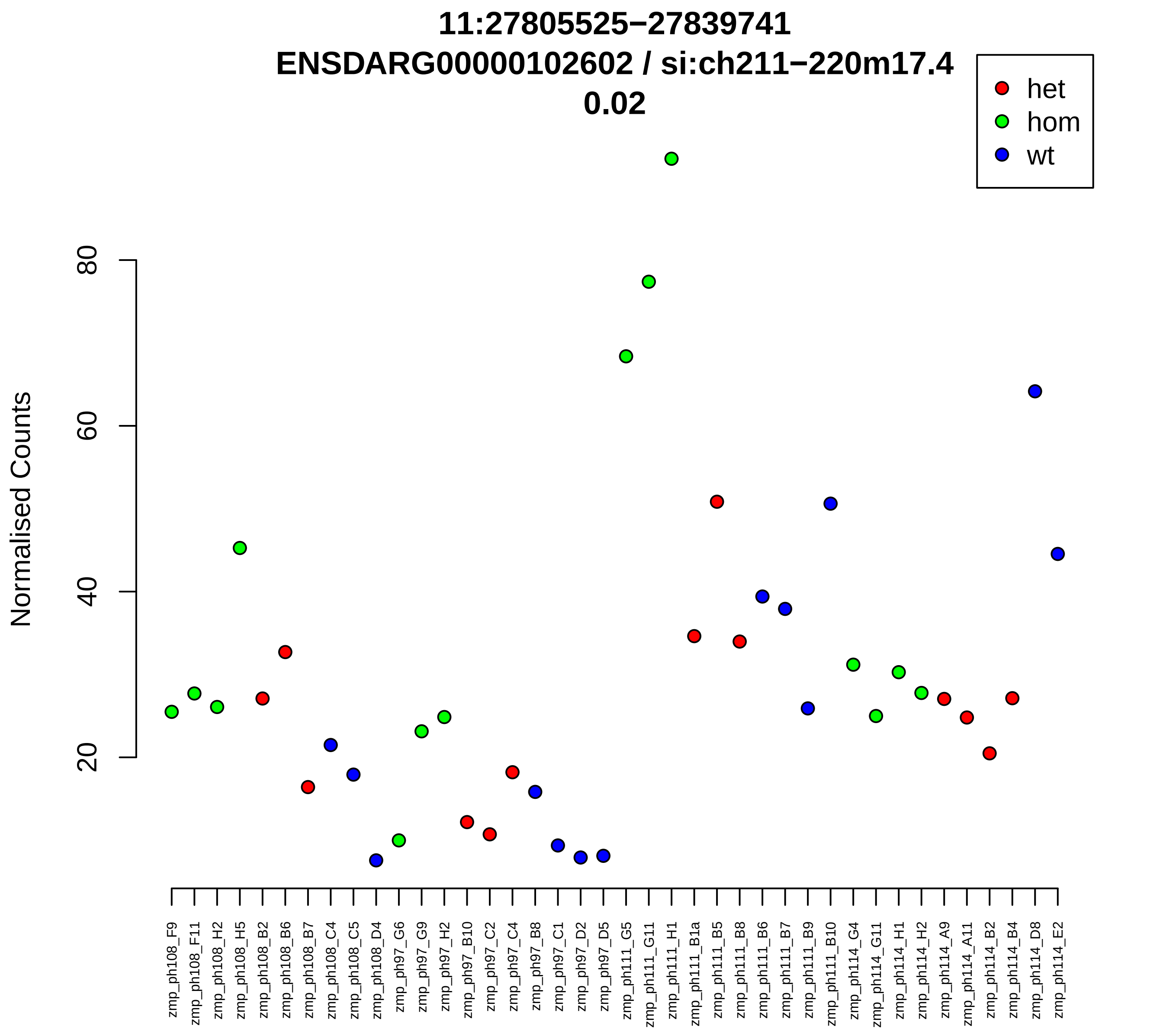
11:27805525−27839741
ENSDARG00000102602 / si:ch211−220m17.4
0.02
het
hom
wt
Normalised Counts
20
40
60
80
zmp_ph108_F9
zmp_ph108_F11
zmp_ph108_H2
zmp_ph108_H5
zmp_ph108_B2
zmp_ph108_B6
zmp_ph108_B7
zmp_ph108_C4
zmp_ph108_C5
zmp_ph108_D4
zmp_ph97_G6
zmp_ph97_G9
zmp_ph97_H2
zmp_ph97_B10
zmp_ph97_C2
zmp_ph97_C4
zmp_ph97_B8
zmp_ph97_C1
zmp_ph97_D2
zmp_ph97_D5
zmp_ph111_G5
zmp_ph111_G11
zmp_ph111_H1
zmp_ph111_B1a
zmp_ph111_B5
zmp_ph111_B8
zmp_ph111_B6
zmp_ph111_B7
zmp_ph111_B9
zmp_ph111_B10
zmp_ph114_G4
zmp_ph114_G11
zmp_ph114_H1
zmp_ph114_H2
zmp_ph114_A9
zmp_ph114_A11
zmp_ph114_B2
zmp_ph114_B4
zmp_ph114_D8
zmp_ph114_E2

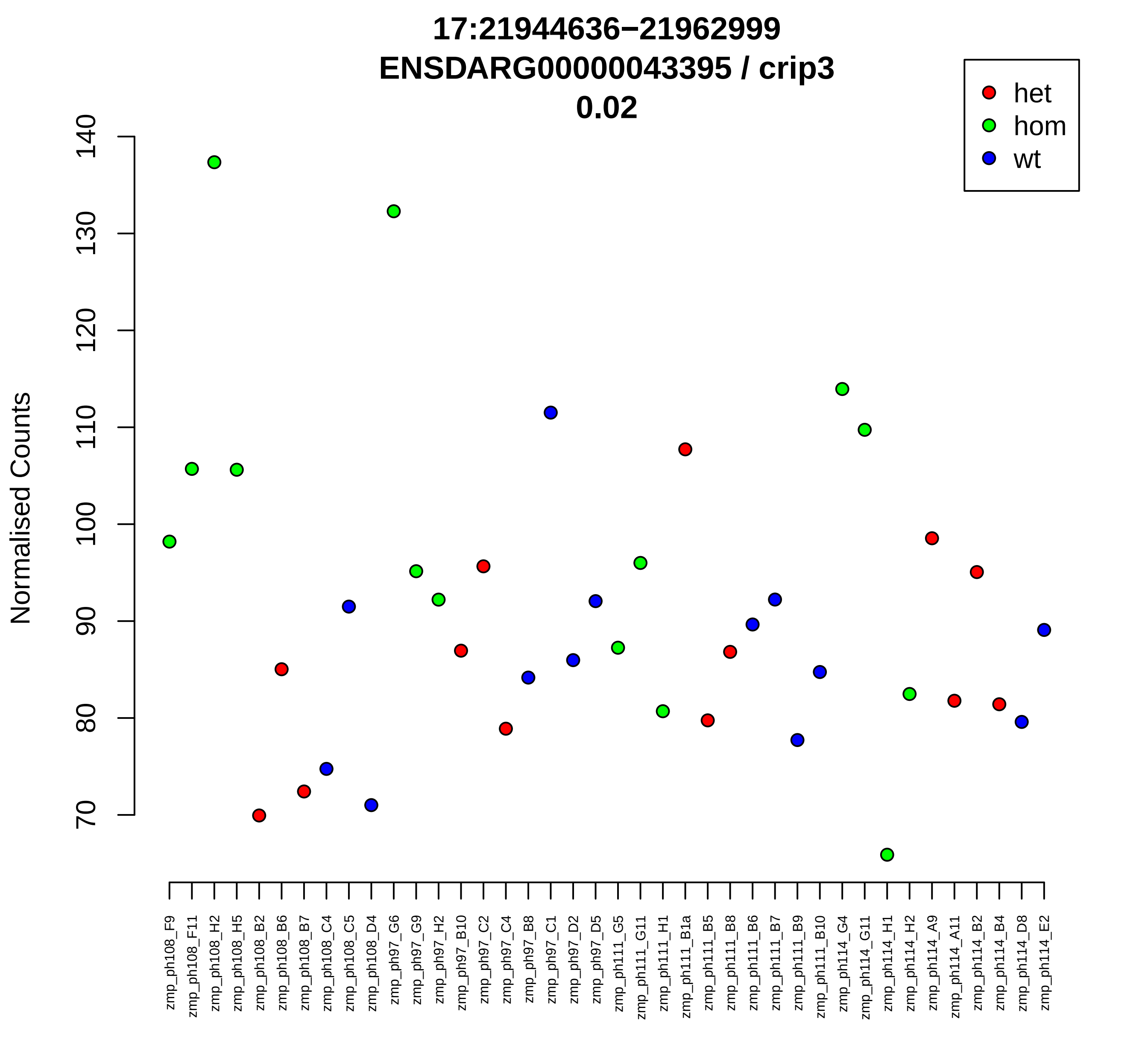

17:21944636−21962999
ENSDARG00000043395 / crip3
0.02
het
hom
wt
Normalised Counts
70
80
90
100
110
120
130
140
zmp_ph108_F9
zmp_ph108_F11
zmp_ph108_H2
zmp_ph108_H5
zmp_ph108_B2
zmp_ph108_B6
zmp_ph108_B7
zmp_ph108_C4
zmp_ph108_C5
zmp_ph108_D4
zmp_ph97_G6
zmp_ph97_G9
zmp_ph97_H2
zmp_ph97_B10
zmp_ph97_C2
zmp_ph97_C4
zmp_ph97_B8
zmp_ph97_C1
zmp_ph97_D2
zmp_ph97_D5
zmp_ph111_G5
zmp_ph111_G11
zmp_ph111_H1
zmp_ph111_B1a
zmp_ph111_B5
zmp_ph111_B8
zmp_ph111_B6
zmp_ph111_B7
zmp_ph111_B9
zmp_ph111_B10
zmp_ph114_G4
zmp_ph114_G11
zmp_ph114_H1
zmp_ph114_H2
zmp_ph114_A9
zmp_ph114_A11
zmp_ph114_B2
zmp_ph114_B4
zmp_ph114_D8
zmp_ph114_E2

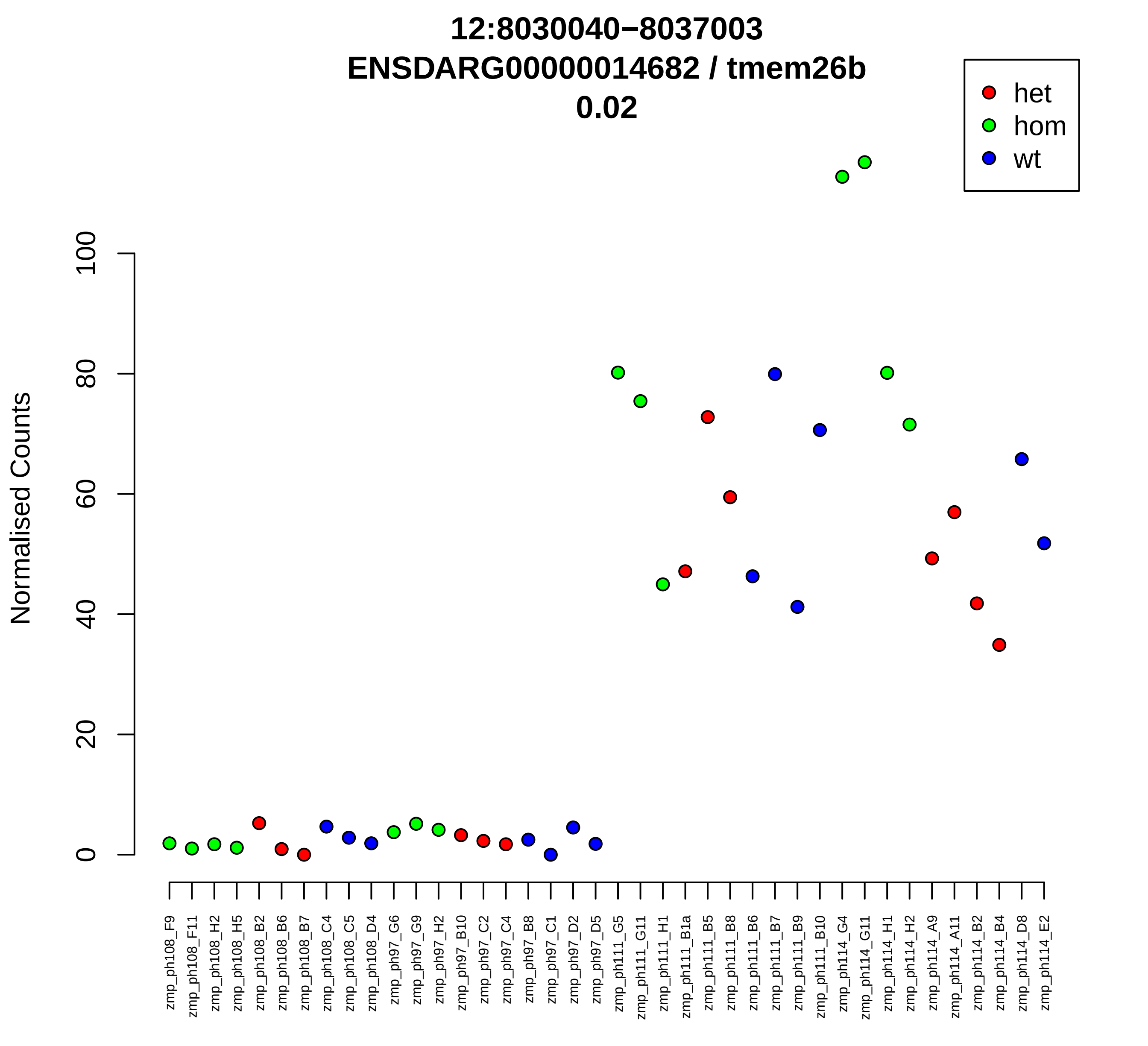

12:8030040−8037003
ENSDARG00000014682 / tmem26b
0.02
het
hom
wt
Normalised Counts
0
20
40
60
80
100
zmp_ph108_F9
zmp_ph108_F11
zmp_ph108_H2
zmp_ph108_H5
zmp_ph108_B2
zmp_ph108_B6
zmp_ph108_B7
zmp_ph108_C4
zmp_ph108_C5
zmp_ph108_D4
zmp_ph97_G6
zmp_ph97_G9
zmp_ph97_H2
zmp_ph97_B10
zmp_ph97_C2
zmp_ph97_C4
zmp_ph97_B8
zmp_ph97_C1
zmp_ph97_D2
zmp_ph97_D5
zmp_ph111_G5
zmp_ph111_G11
zmp_ph111_H1
zmp_ph111_B1a
zmp_ph111_B5
zmp_ph111_B8
zmp_ph111_B6
zmp_ph111_B7
zmp_ph111_B9
zmp_ph111_B10
zmp_ph114_G4
zmp_ph114_G11
zmp_ph114_H1
zmp_ph114_H2
zmp_ph114_A9
zmp_ph114_A11
zmp_ph114_B2
zmp_ph114_B4
zmp_ph114_D8
zmp_ph114_E2

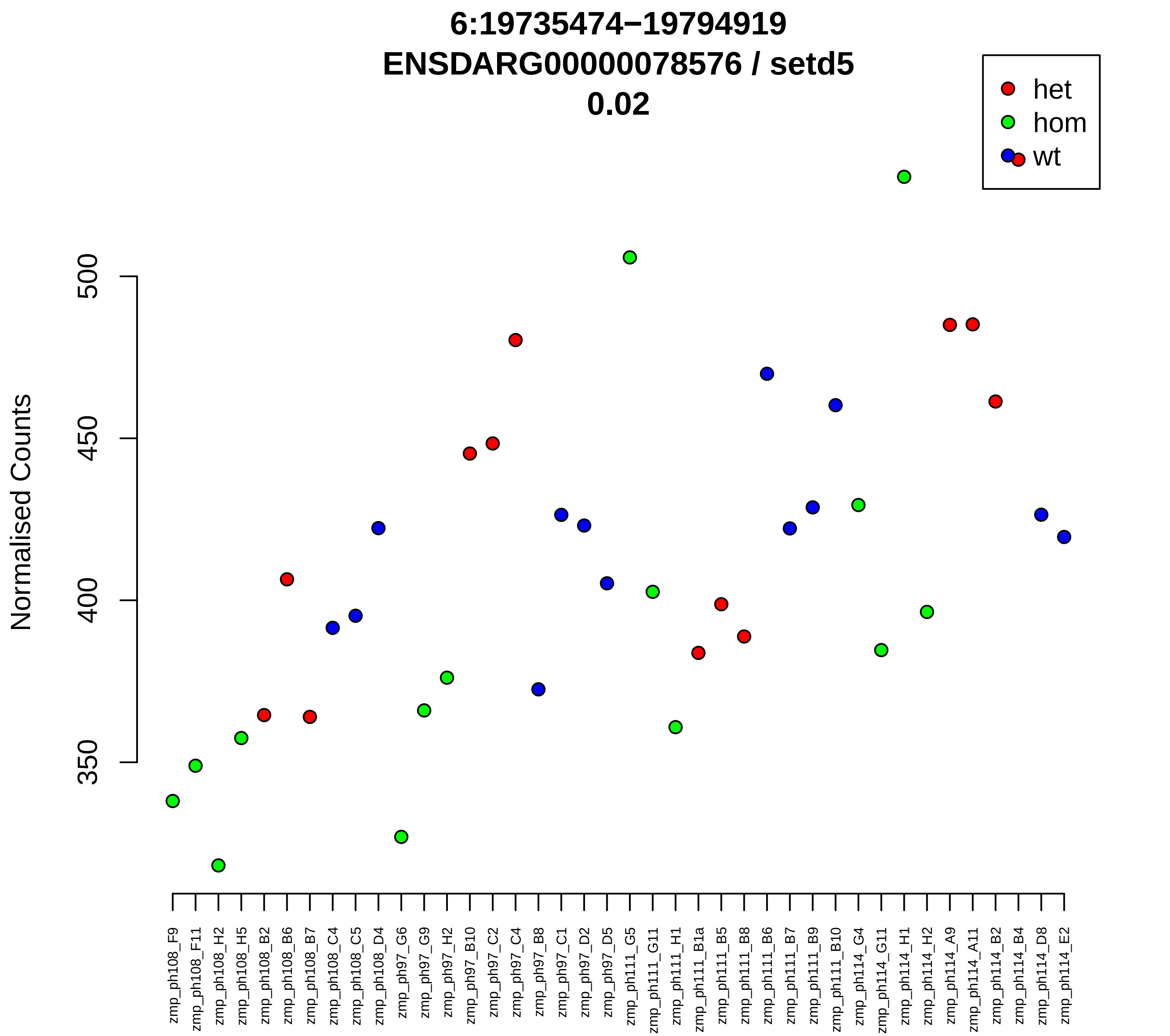

6:19735474−19794919
ENSDARG00000078576 / setd5
0.02
het
hom
wt
Normalised Counts
350
400
450
500
zmp_ph108_F9
zmp_ph108_F11
zmp_ph108_H2
zmp_ph108_H5
zmp_ph108_B2
zmp_ph108_B6
zmp_ph108_B7
zmp_ph108_C4
zmp_ph108_C5
zmp_ph108_D4
zmp_ph97_G6
zmp_ph97_G9
zmp_ph97_H2
zmp_ph97_B10
zmp_ph97_C2
zmp_ph97_C4
zmp_ph97_B8
zmp_ph97_C1
zmp_ph97_D2
zmp_ph97_D5
zmp_ph111_G5
zmp_ph111_G11
zmp_ph111_H1
zmp_ph111_B1a
zmp_ph111_B5
zmp_ph111_B8
zmp_ph111_B6
zmp_ph111_B7
zmp_ph111_B9
zmp_ph111_B10
zmp_ph114_G4
zmp_ph114_G11
zmp_ph114_H1
zmp_ph114_H2
zmp_ph114_A9
zmp_ph114_A11
zmp_ph114_B2
zmp_ph114_B4
zmp_ph114_D8
zmp_ph114_E2

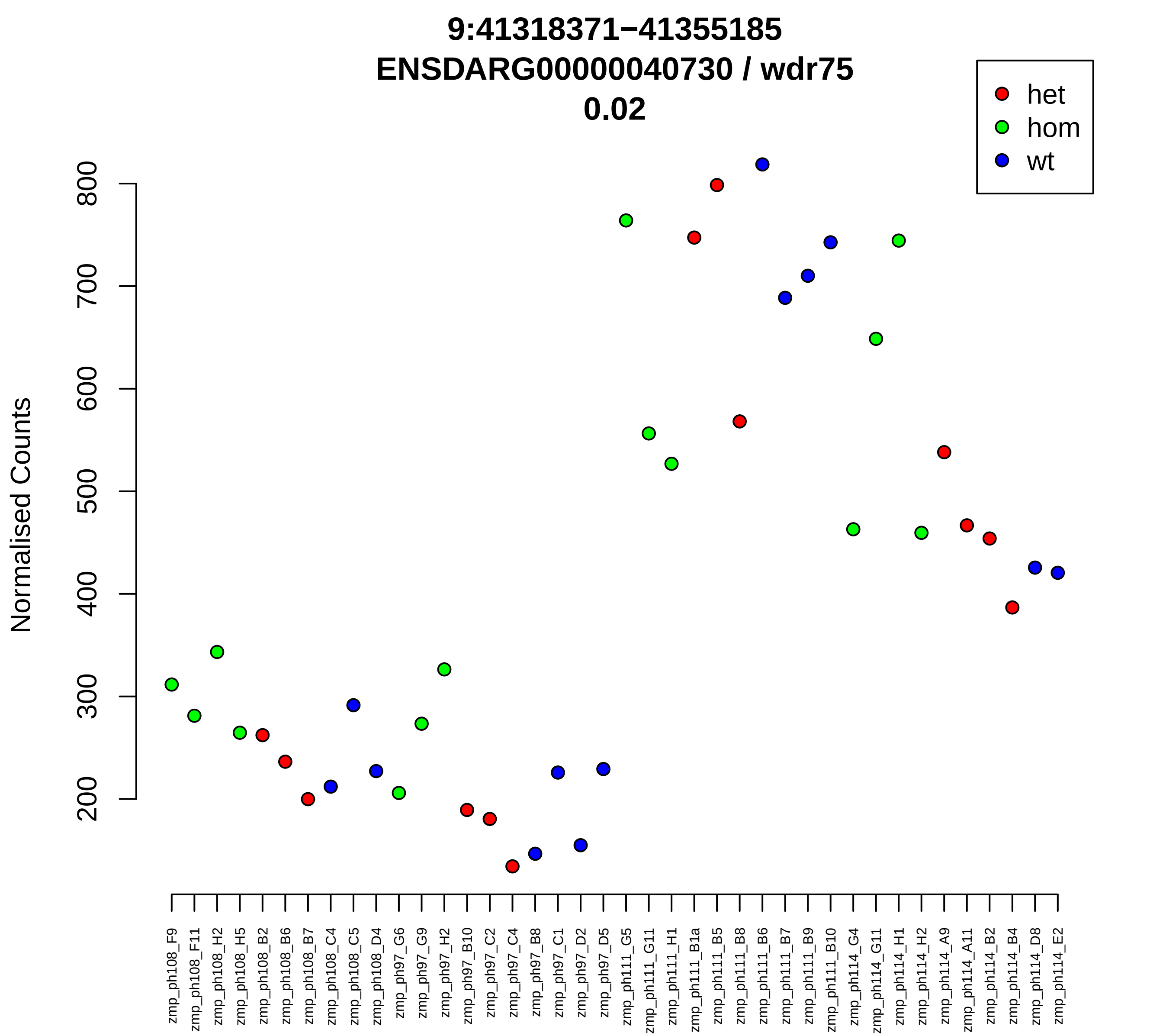
9:41318371−41355185
ENSDARG00000040730 / wdr75
0.02
het
hom
wt
Normalised Counts
200
300
400
500
600
700
800
zmp_ph108_F9
zmp_ph108_F11
zmp_ph108_H2
zmp_ph108_H5
zmp_ph108_B2
zmp_ph108_B6
zmp_ph108_B7
zmp_ph108_C4
zmp_ph108_C5
zmp_ph108_D4
zmp_ph97_G6
zmp_ph97_G9
zmp_ph97_H2
zmp_ph97_B10
zmp_ph97_C2
zmp_ph97_C4
zmp_ph97_B8
zmp_ph97_C1
zmp_ph97_D2
zmp_ph97_D5
zmp_ph111_G5
zmp_ph111_G11
zmp_ph111_H1
zmp_ph111_B1a
zmp_ph111_B5
zmp_ph111_B8
zmp_ph111_B6
zmp_ph111_B7
zmp_ph111_B9
zmp_ph111_B10
zmp_ph114_G4
zmp_ph114_G11
zmp_ph114_H1
zmp_ph114_H2
zmp_ph114_A9
zmp_ph114_A11
zmp_ph114_B2
zmp_ph114_B4
zmp_ph114_D8
zmp_ph114_E2

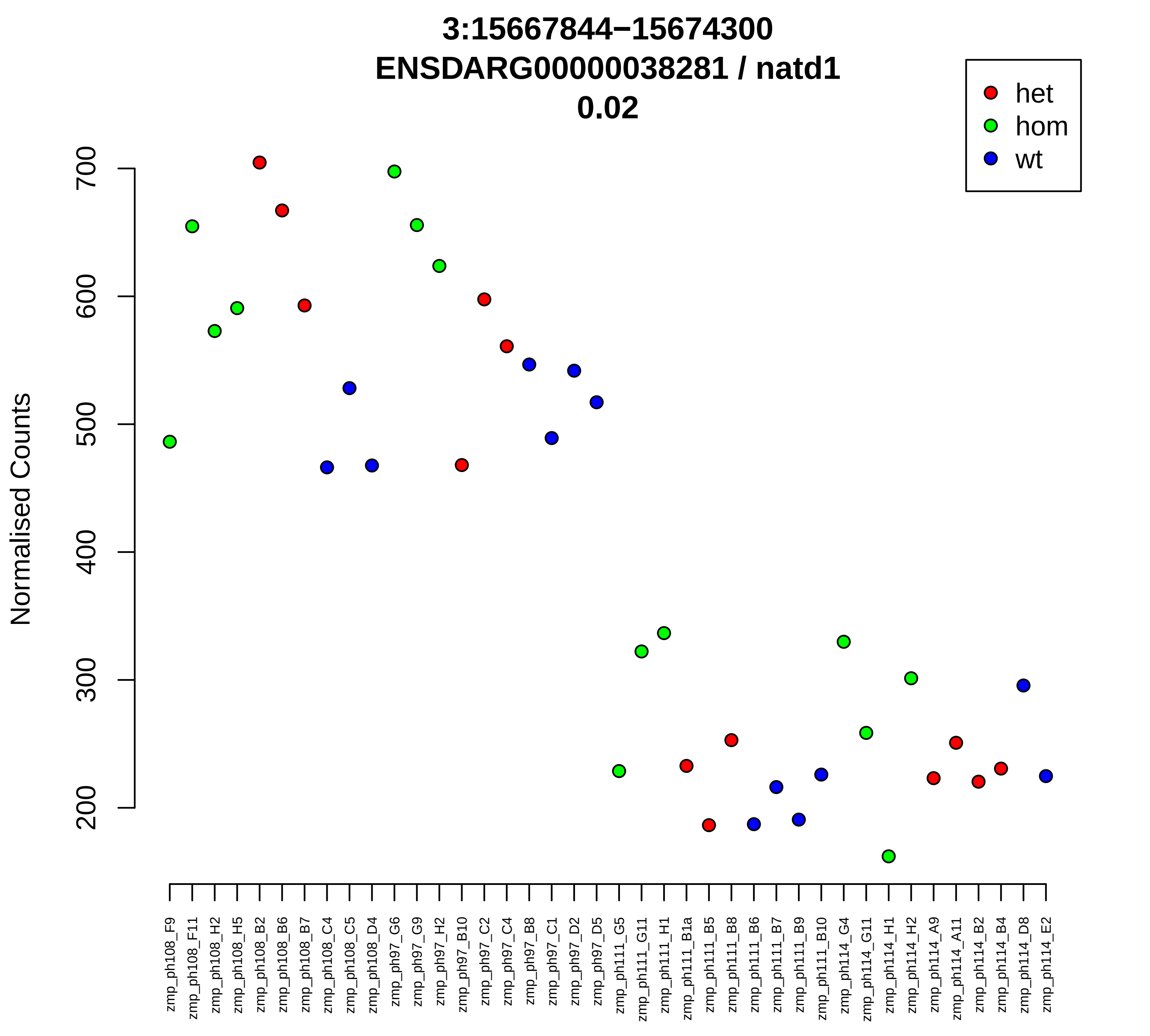

3:15667844−15674300
ENSDARG00000038281 / natd1
0.02
het
hom
wt
Normalised Counts
200
300
400
500
600
700
zmp_ph108_F9
zmp_ph108_F11
zmp_ph108_H2
zmp_ph108_H5
zmp_ph108_B2
zmp_ph108_B6
zmp_ph108_B7
zmp_ph108_C4
zmp_ph108_C5
zmp_ph108_D4
zmp_ph97_G6
zmp_ph97_G9
zmp_ph97_H2
zmp_ph97_B10
zmp_ph97_C2
zmp_ph97_C4
zmp_ph97_B8
zmp_ph97_C1
zmp_ph97_D2
zmp_ph97_D5
zmp_ph111_G5
zmp_ph111_G11
zmp_ph111_H1
zmp_ph111_B1a
zmp_ph111_B5
zmp_ph111_B8
zmp_ph111_B6
zmp_ph111_B7
zmp_ph111_B9
zmp_ph111_B10
zmp_ph114_G4
zmp_ph114_G11
zmp_ph114_H1
zmp_ph114_H2
zmp_ph114_A9
zmp_ph114_A11
zmp_ph114_B2
zmp_ph114_B4
zmp_ph114_D8
zmp_ph114_E2

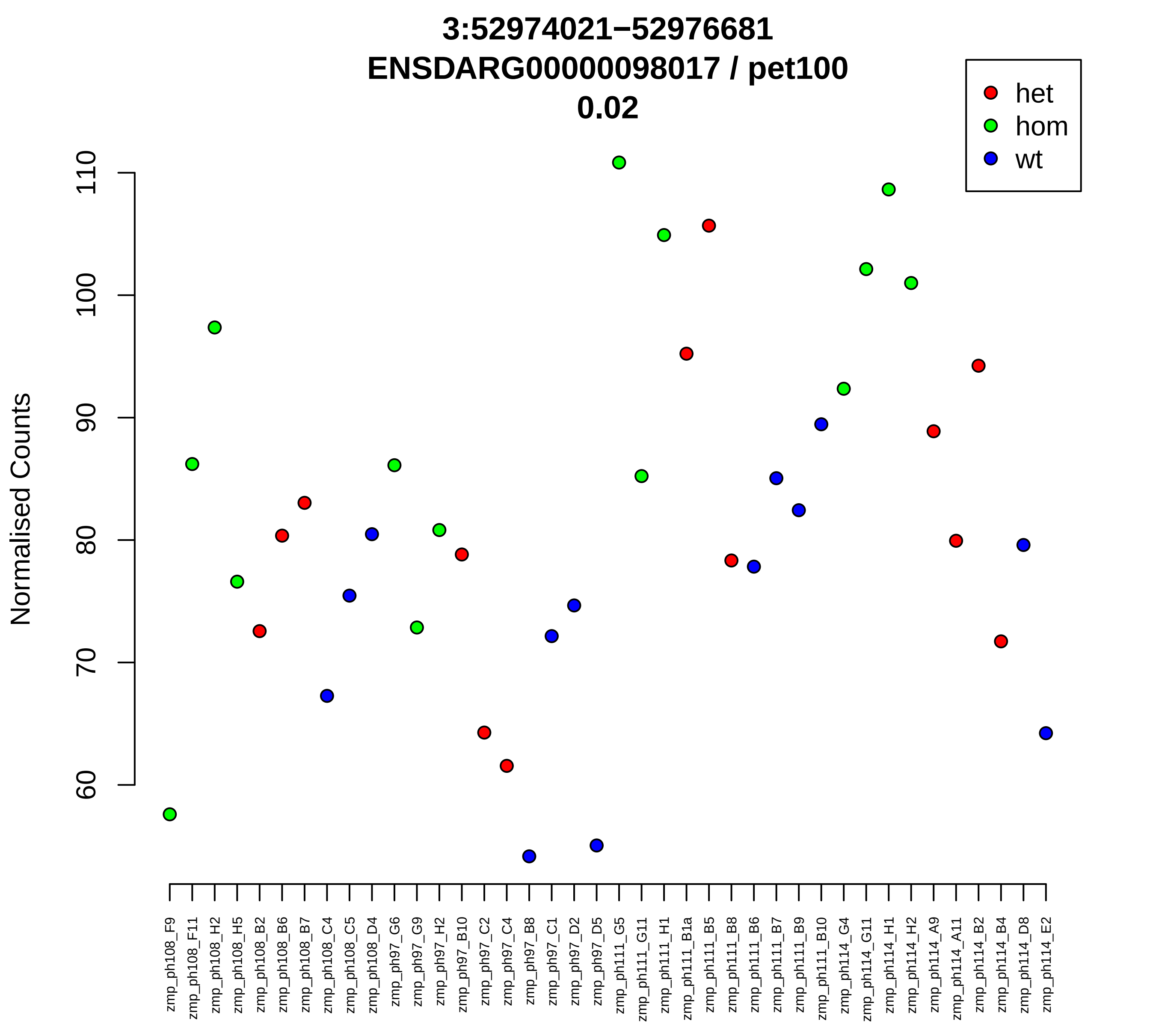
3:52974021−52976681
ENSDARG00000098017 / pet100
0.02
het
hom
wt
Normalised Counts
60
70
80
90
100
110
zmp_ph108_F9
zmp_ph108_F11
zmp_ph108_H2
zmp_ph108_H5
zmp_ph108_B2
zmp_ph108_B6
zmp_ph108_B7
zmp_ph108_C4
zmp_ph108_C5
zmp_ph108_D4
zmp_ph97_G6
zmp_ph97_G9
zmp_ph97_H2
zmp_ph97_B10
zmp_ph97_C2
zmp_ph97_C4
zmp_ph97_B8
zmp_ph97_C1
zmp_ph97_D2
zmp_ph97_D5
zmp_ph111_G5
zmp_ph111_G11
zmp_ph111_H1
zmp_ph111_B1a
zmp_ph111_B5
zmp_ph111_B8
zmp_ph111_B6
zmp_ph111_B7
zmp_ph111_B9
zmp_ph111_B10
zmp_ph114_G4
zmp_ph114_G11
zmp_ph114_H1
zmp_ph114_H2
zmp_ph114_A9
zmp_ph114_A11
zmp_ph114_B2
zmp_ph114_B4
zmp_ph114_D8
zmp_ph114_E2

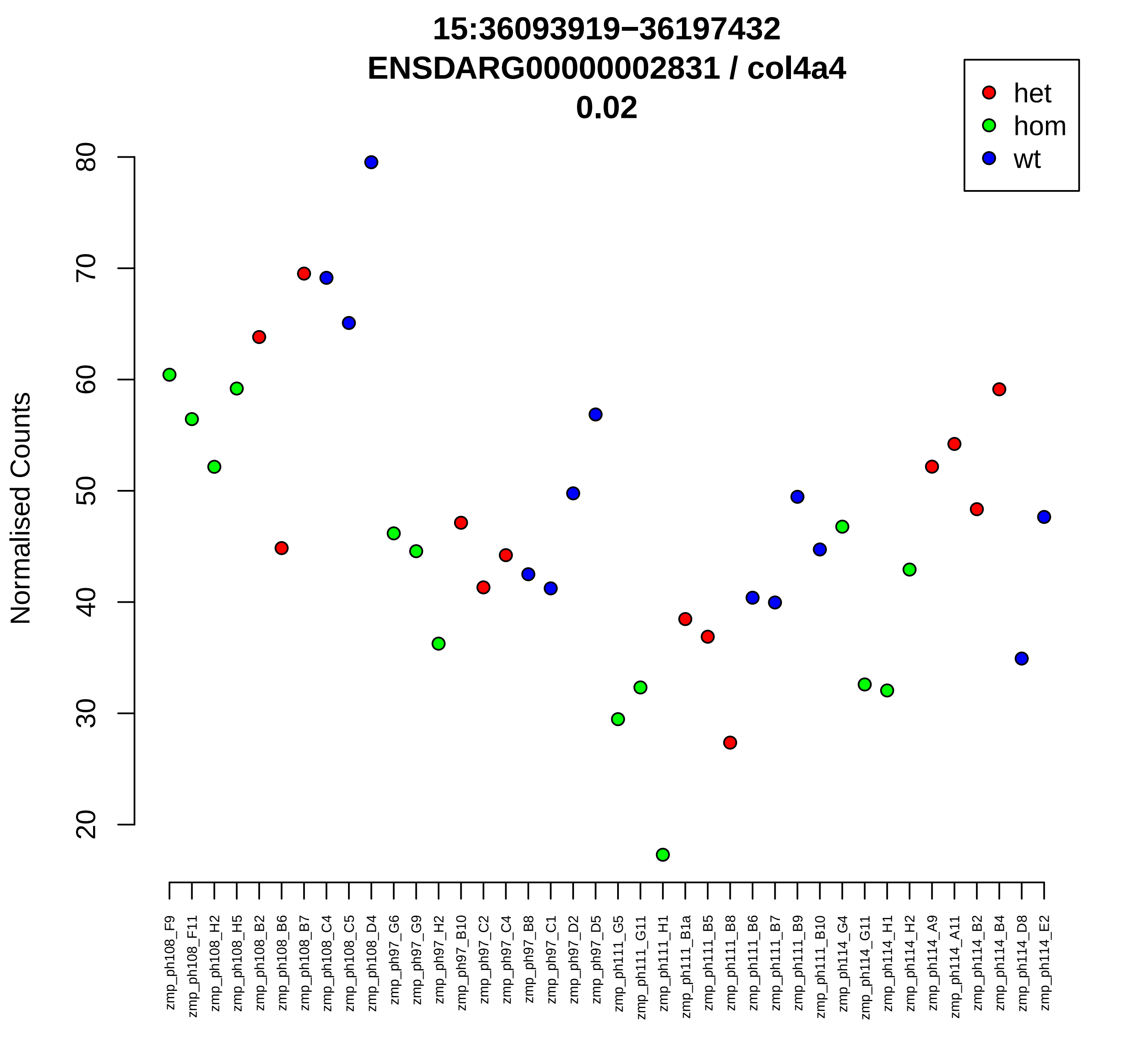
15:36093919−36197432
ENSDARG00000002831 / col4a4
0.02
het
hom
wt
Normalised Counts
20
30
40
50
60
70
80
zmp_ph108_F9
zmp_ph108_F11
zmp_ph108_H2
zmp_ph108_H5
zmp_ph108_B2
zmp_ph108_B6
zmp_ph108_B7
zmp_ph108_C4
zmp_ph108_C5
zmp_ph108_D4
zmp_ph97_G6
zmp_ph97_G9
zmp_ph97_H2
zmp_ph97_B10
zmp_ph97_C2
zmp_ph97_C4
zmp_ph97_B8
zmp_ph97_C1
zmp_ph97_D2
zmp_ph97_D5
zmp_ph111_G5
zmp_ph111_G11
zmp_ph111_H1
zmp_ph111_B1a
zmp_ph111_B5
zmp_ph111_B8
zmp_ph111_B6
zmp_ph111_B7
zmp_ph111_B9
zmp_ph111_B10
zmp_ph114_G4
zmp_ph114_G11
zmp_ph114_H1
zmp_ph114_H2
zmp_ph114_A9
zmp_ph114_A11
zmp_ph114_B2
zmp_ph114_B4
zmp_ph114_D8
zmp_ph114_E2

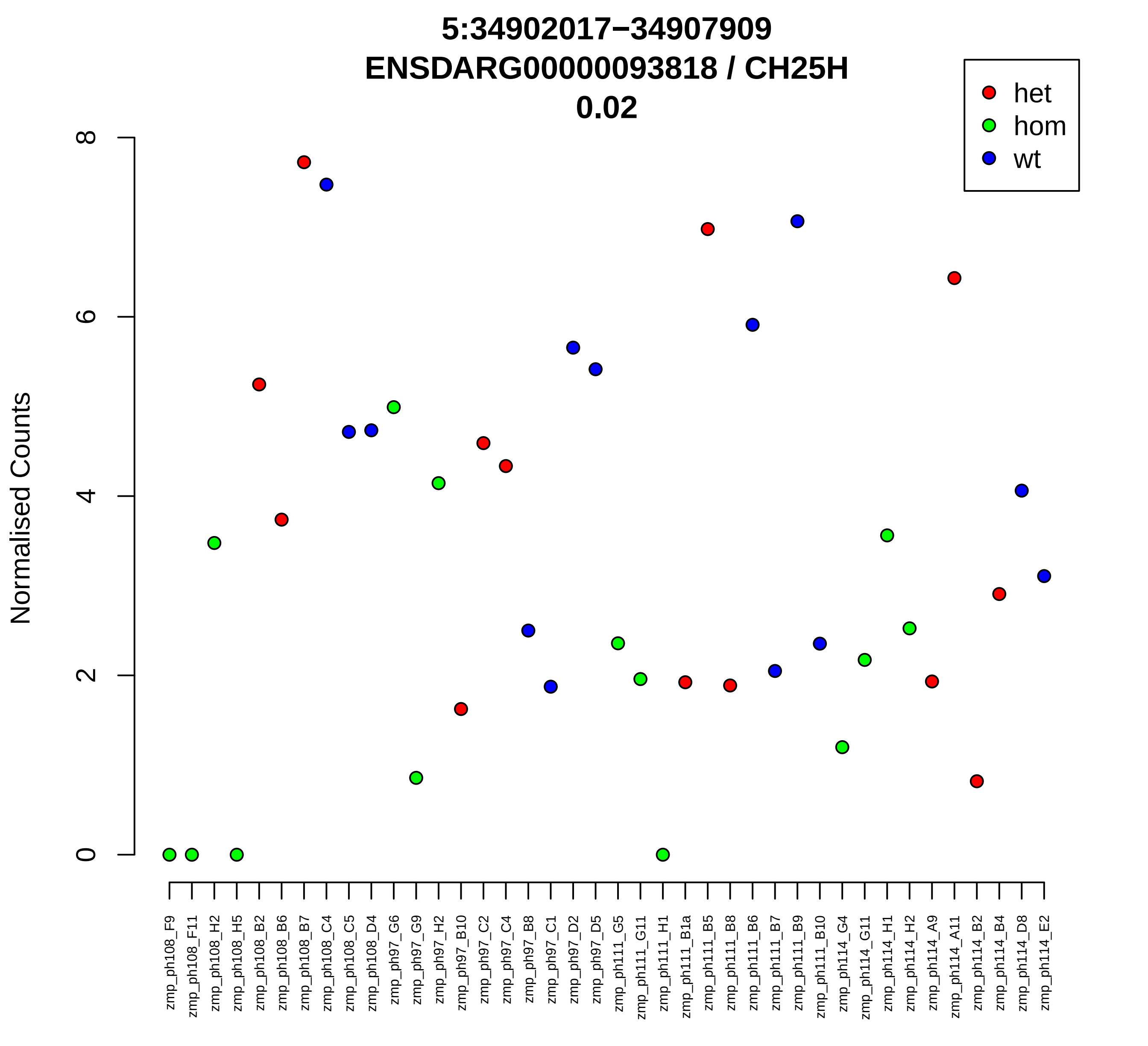
5:34902017−34907909
ENSDARG00000093818 / CH25H
0.02
het
hom
wt
Normalised Counts
0
2
4
6
8
zmp_ph108_F9
zmp_ph108_F11
zmp_ph108_H2
zmp_ph108_H5
zmp_ph108_B2
zmp_ph108_B6
zmp_ph108_B7
zmp_ph108_C4
zmp_ph108_C5
zmp_ph108_D4
zmp_ph97_G6
zmp_ph97_G9
zmp_ph97_H2
zmp_ph97_B10
zmp_ph97_C2
zmp_ph97_C4
zmp_ph97_B8
zmp_ph97_C1
zmp_ph97_D2
zmp_ph97_D5
zmp_ph111_G5
zmp_ph111_G11
zmp_ph111_H1
zmp_ph111_B1a
zmp_ph111_B5
zmp_ph111_B8
zmp_ph111_B6
zmp_ph111_B7
zmp_ph111_B9
zmp_ph111_B10
zmp_ph114_G4
zmp_ph114_G11
zmp_ph114_H1
zmp_ph114_H2
zmp_ph114_A9
zmp_ph114_A11
zmp_ph114_B2
zmp_ph114_B4
zmp_ph114_D8
zmp_ph114_E2

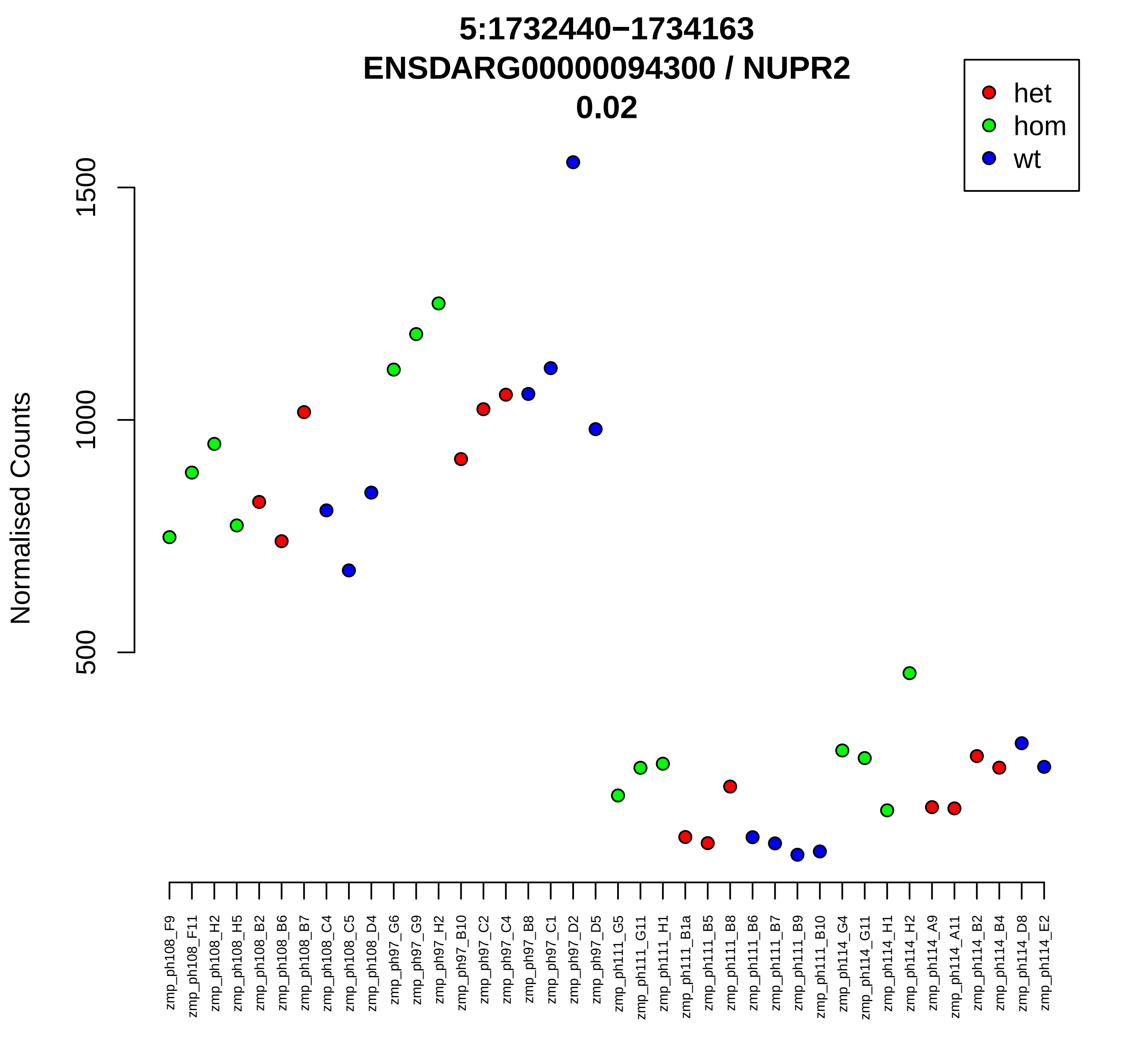
5:1732440−1734163
ENSDARG00000094300 / NUPR2
0.02
het
hom
wt
Normalised Counts
500
1000
1500
zmp_ph108_F9
zmp_ph108_F11
zmp_ph108_H2
zmp_ph108_H5
zmp_ph108_B2
zmp_ph108_B6
zmp_ph108_B7
zmp_ph108_C4
zmp_ph108_C5
zmp_ph108_D4
zmp_ph97_G6
zmp_ph97_G9
zmp_ph97_H2
zmp_ph97_B10
zmp_ph97_C2
zmp_ph97_C4
zmp_ph97_B8
zmp_ph97_C1
zmp_ph97_D2
zmp_ph97_D5
zmp_ph111_G5
zmp_ph111_G11
zmp_ph111_H1
zmp_ph111_B1a
zmp_ph111_B5
zmp_ph111_B8
zmp_ph111_B6
zmp_ph111_B7
zmp_ph111_B9
zmp_ph111_B10
zmp_ph114_G4
zmp_ph114_G11
zmp_ph114_H1
zmp_ph114_H2
zmp_ph114_A9
zmp_ph114_A11
zmp_ph114_B2
zmp_ph114_B4
zmp_ph114_D8
zmp_ph114_E2

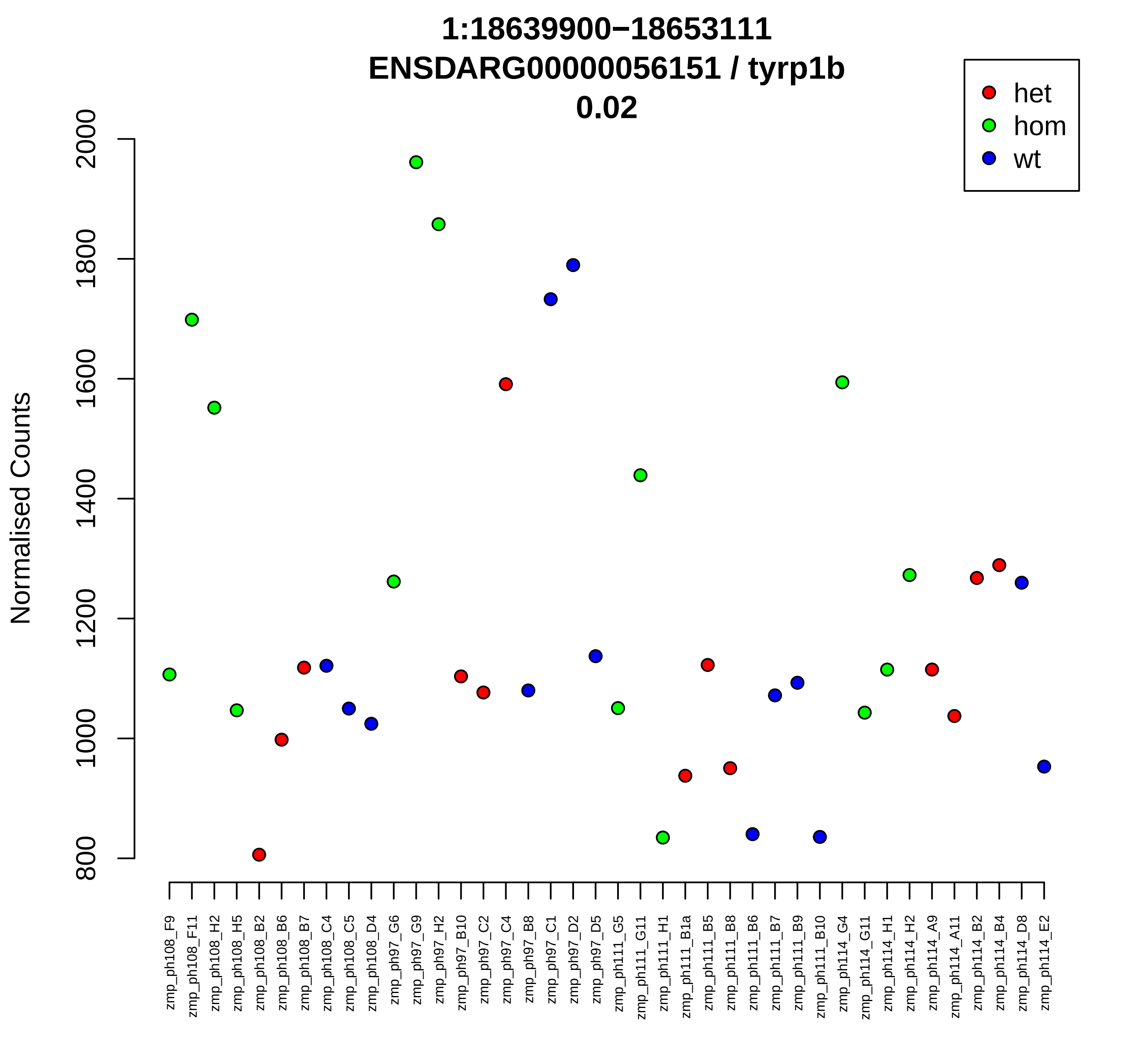
1:18639900−18653111
ENSDARG00000056151 / tyrp1b
0.02
het
hom
wt
Normalised Counts
800
1000
1200
1400
1600
1800
2000
zmp_ph108_F9
zmp_ph108_F11
zmp_ph108_H2
zmp_ph108_H5
zmp_ph108_B2
zmp_ph108_B6
zmp_ph108_B7
zmp_ph108_C4
zmp_ph108_C5
zmp_ph108_D4
zmp_ph97_G6
zmp_ph97_G9
zmp_ph97_H2
zmp_ph97_B10
zmp_ph97_C2
zmp_ph97_C4
zmp_ph97_B8
zmp_ph97_C1
zmp_ph97_D2
zmp_ph97_D5
zmp_ph111_G5
zmp_ph111_G11
zmp_ph111_H1
zmp_ph111_B1a
zmp_ph111_B5
zmp_ph111_B8
zmp_ph111_B6
zmp_ph111_B7
zmp_ph111_B9
zmp_ph111_B10
zmp_ph114_G4
zmp_ph114_G11
zmp_ph114_H1
zmp_ph114_H2
zmp_ph114_A9
zmp_ph114_A11
zmp_ph114_B2
zmp_ph114_B4
zmp_ph114_D8
zmp_ph114_E2

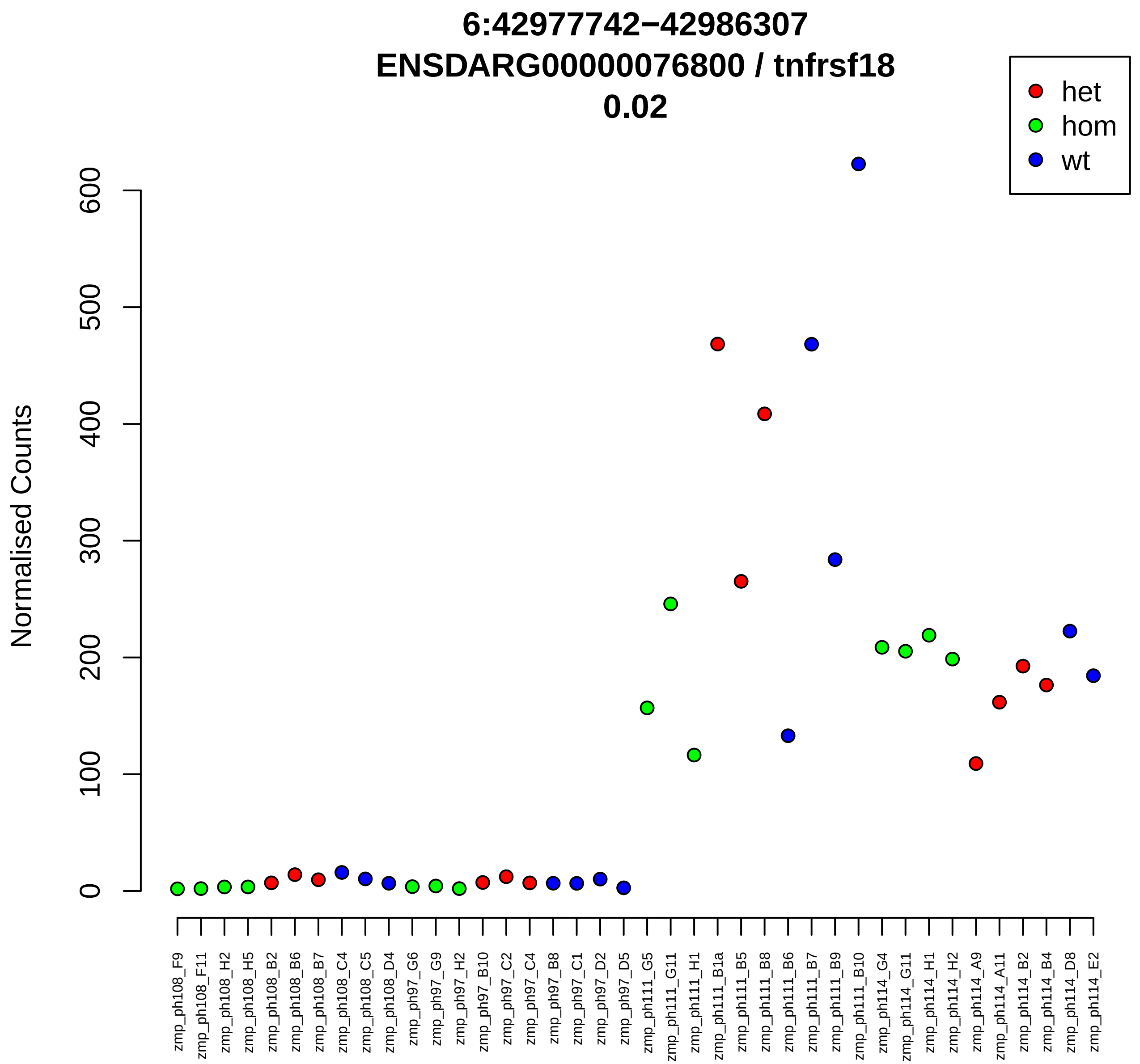

6:42977742−42986307
ENSDARG00000076800 / tnfrsf18
0.02
het
hom
wt
Normalised Counts
0
100
200
300
400
500
600
zmp_ph108_F9
zmp_ph108_F11
zmp_ph108_H2
zmp_ph108_H5
zmp_ph108_B2
zmp_ph108_B6
zmp_ph108_B7
zmp_ph108_C4
zmp_ph108_C5
zmp_ph108_D4
zmp_ph97_G6
zmp_ph97_G9
zmp_ph97_H2
zmp_ph97_B10
zmp_ph97_C2
zmp_ph97_C4
zmp_ph97_B8
zmp_ph97_C1
zmp_ph97_D2
zmp_ph97_D5
zmp_ph111_G5
zmp_ph111_G11
zmp_ph111_H1
zmp_ph111_B1a
zmp_ph111_B5
zmp_ph111_B8
zmp_ph111_B6
zmp_ph111_B7
zmp_ph111_B9
zmp_ph111_B10
zmp_ph114_G4
zmp_ph114_G11
zmp_ph114_H1
zmp_ph114_H2
zmp_ph114_A9
zmp_ph114_A11
zmp_ph114_B2
zmp_ph114_B4
zmp_ph114_D8
zmp_ph114_E2

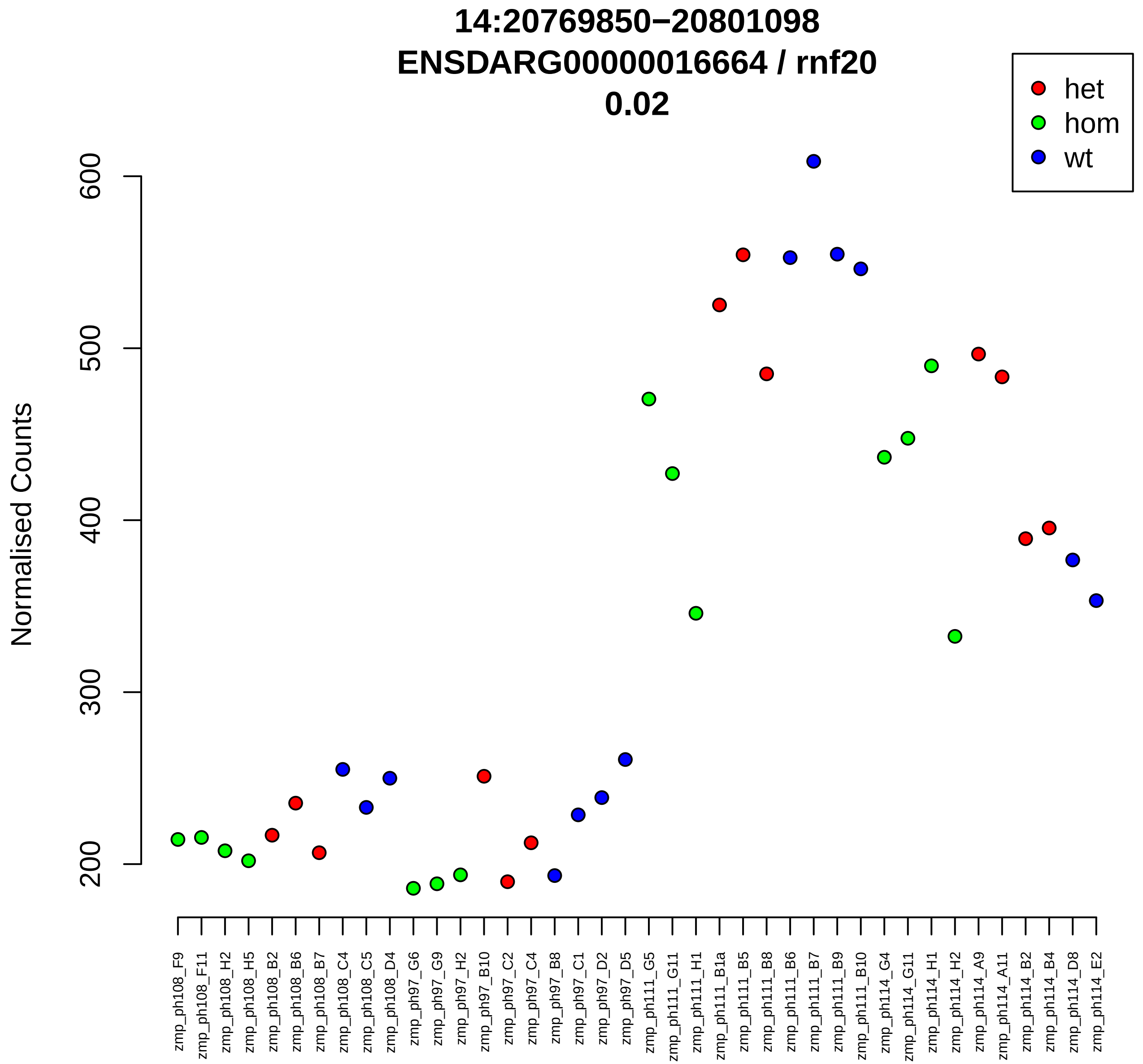

14:20769850−20801098
ENSDARG00000016664 / rnf20
0.02
het
hom
wt
Normalised Counts
200
300
400
500
600
zmp_ph108_F9
zmp_ph108_F11
zmp_ph108_H2
zmp_ph108_H5
zmp_ph108_B2
zmp_ph108_B6
zmp_ph108_B7
zmp_ph108_C4
zmp_ph108_C5
zmp_ph108_D4
zmp_ph97_G6
zmp_ph97_G9
zmp_ph97_H2
zmp_ph97_B10
zmp_ph97_C2
zmp_ph97_C4
zmp_ph97_B8
zmp_ph97_C1
zmp_ph97_D2
zmp_ph97_D5
zmp_ph111_G5
zmp_ph111_G11
zmp_ph111_H1
zmp_ph111_B1a
zmp_ph111_B5
zmp_ph111_B8
zmp_ph111_B6
zmp_ph111_B7
zmp_ph111_B9
zmp_ph111_B10
zmp_ph114_G4
zmp_ph114_G11
zmp_ph114_H1
zmp_ph114_H2
zmp_ph114_A9
zmp_ph114_A11
zmp_ph114_B2
zmp_ph114_B4
zmp_ph114_D8
zmp_ph114_E2

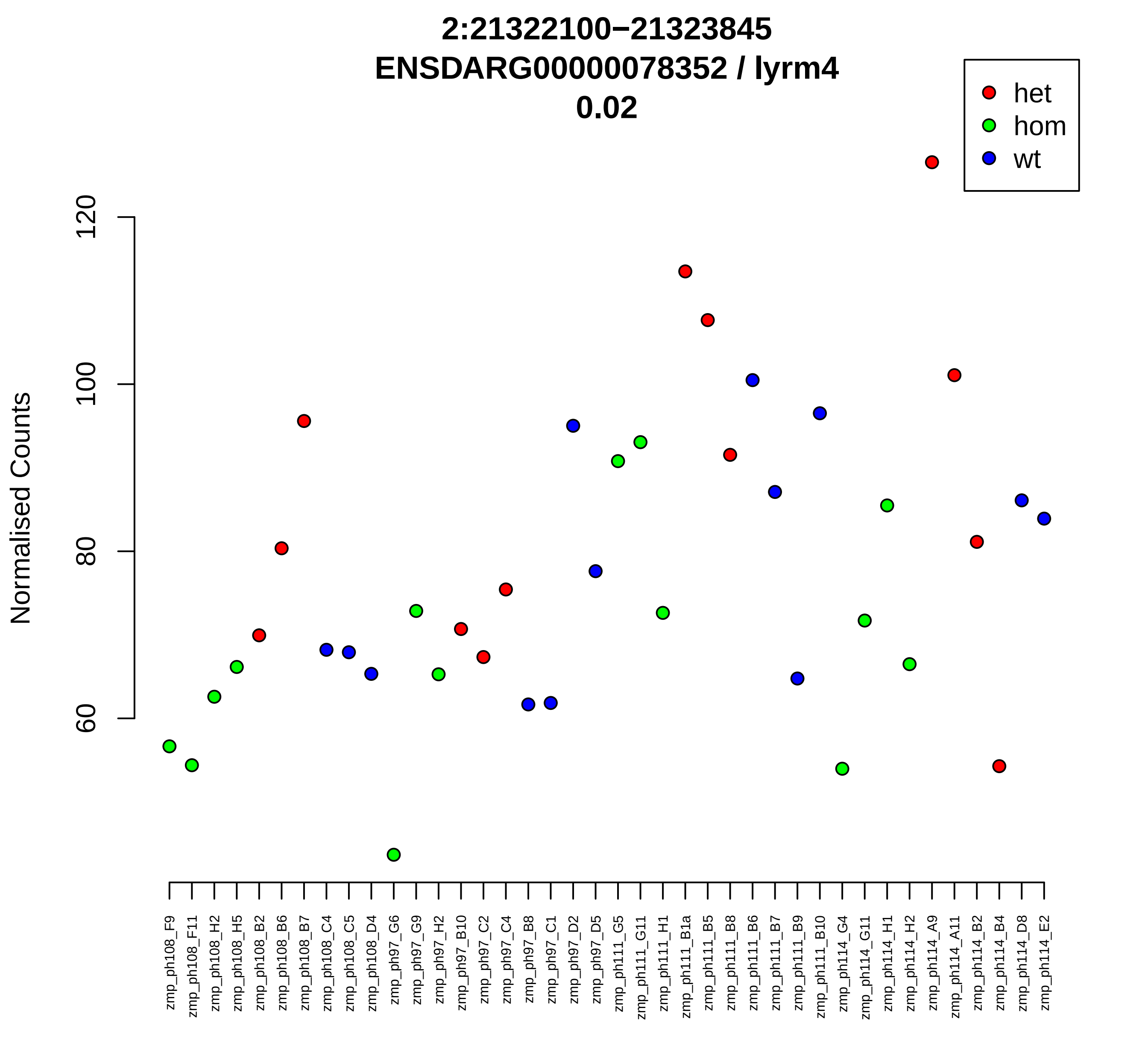
2:21322100−21323845
ENSDARG00000078352 / lyrm4
0.02
het
hom
wt
Normalised Counts
60
80
100
120
zmp_ph108_F9
zmp_ph108_F11
zmp_ph108_H2
zmp_ph108_H5
zmp_ph108_B2
zmp_ph108_B6
zmp_ph108_B7
zmp_ph108_C4
zmp_ph108_C5
zmp_ph108_D4
zmp_ph97_G6
zmp_ph97_G9
zmp_ph97_H2
zmp_ph97_B10
zmp_ph97_C2
zmp_ph97_C4
zmp_ph97_B8
zmp_ph97_C1
zmp_ph97_D2
zmp_ph97_D5
zmp_ph111_G5
zmp_ph111_G11
zmp_ph111_H1
zmp_ph111_B1a
zmp_ph111_B5
zmp_ph111_B8
zmp_ph111_B6
zmp_ph111_B7
zmp_ph111_B9
zmp_ph111_B10
zmp_ph114_G4
zmp_ph114_G11
zmp_ph114_H1
zmp_ph114_H2
zmp_ph114_A9
zmp_ph114_A11
zmp_ph114_B2
zmp_ph114_B4
zmp_ph114_D8
zmp_ph114_E2

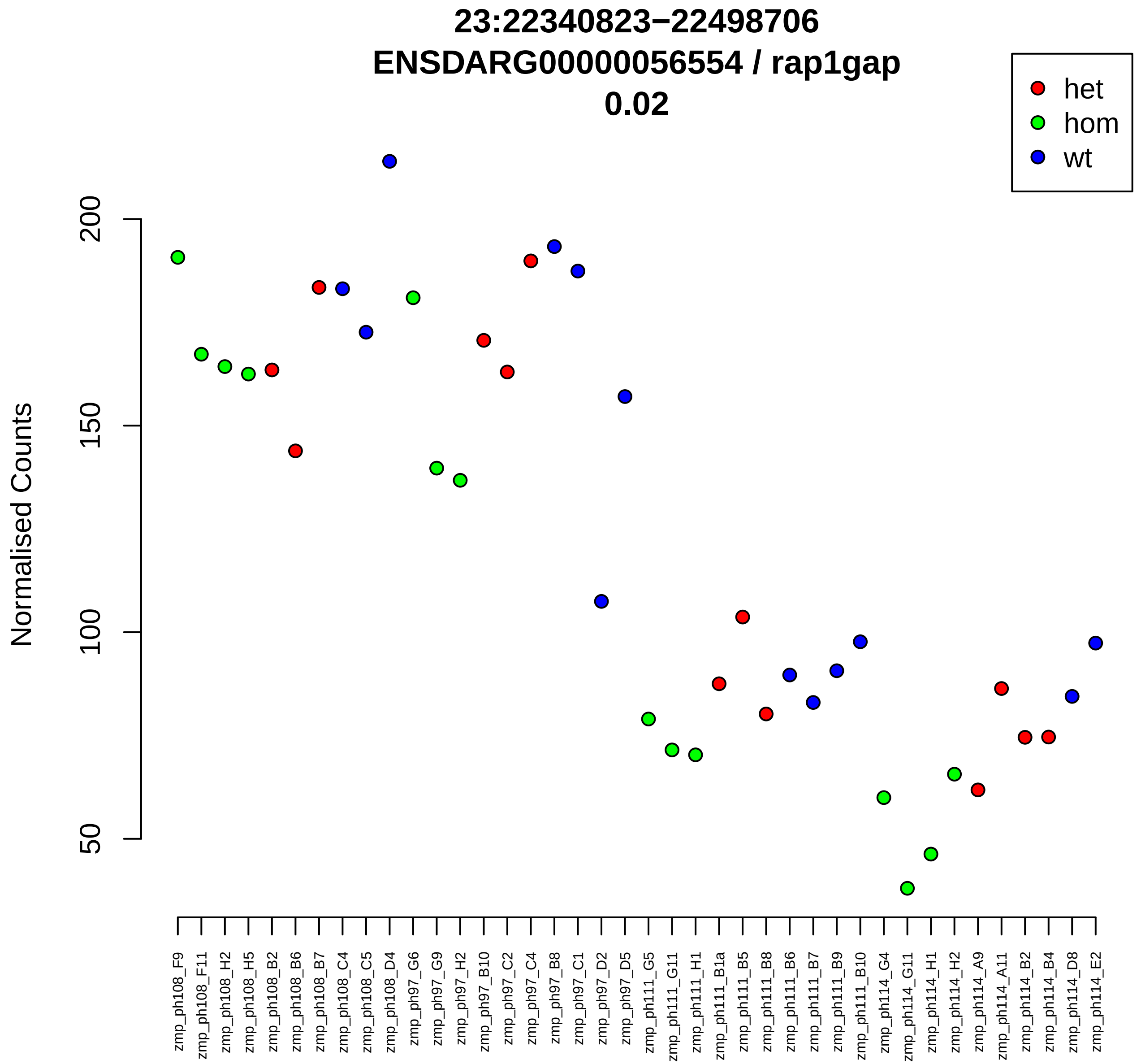

23:22340823−22498706
ENSDARG00000056554 / rap1gap
0.02
het
hom
wt
Normalised Counts
50
100
150
200
zmp_ph108_F9
zmp_ph108_F11
zmp_ph108_H2
zmp_ph108_H5
zmp_ph108_B2
zmp_ph108_B6
zmp_ph108_B7
zmp_ph108_C4
zmp_ph108_C5
zmp_ph108_D4
zmp_ph97_G6
zmp_ph97_G9
zmp_ph97_H2
zmp_ph97_B10
zmp_ph97_C2
zmp_ph97_C4
zmp_ph97_B8
zmp_ph97_C1
zmp_ph97_D2
zmp_ph97_D5
zmp_ph111_G5
zmp_ph111_G11
zmp_ph111_H1
zmp_ph111_B1a
zmp_ph111_B5
zmp_ph111_B8
zmp_ph111_B6
zmp_ph111_B7
zmp_ph111_B9
zmp_ph111_B10
zmp_ph114_G4
zmp_ph114_G11
zmp_ph114_H1
zmp_ph114_H2
zmp_ph114_A9
zmp_ph114_A11
zmp_ph114_B2
zmp_ph114_B4
zmp_ph114_D8
zmp_ph114_E2

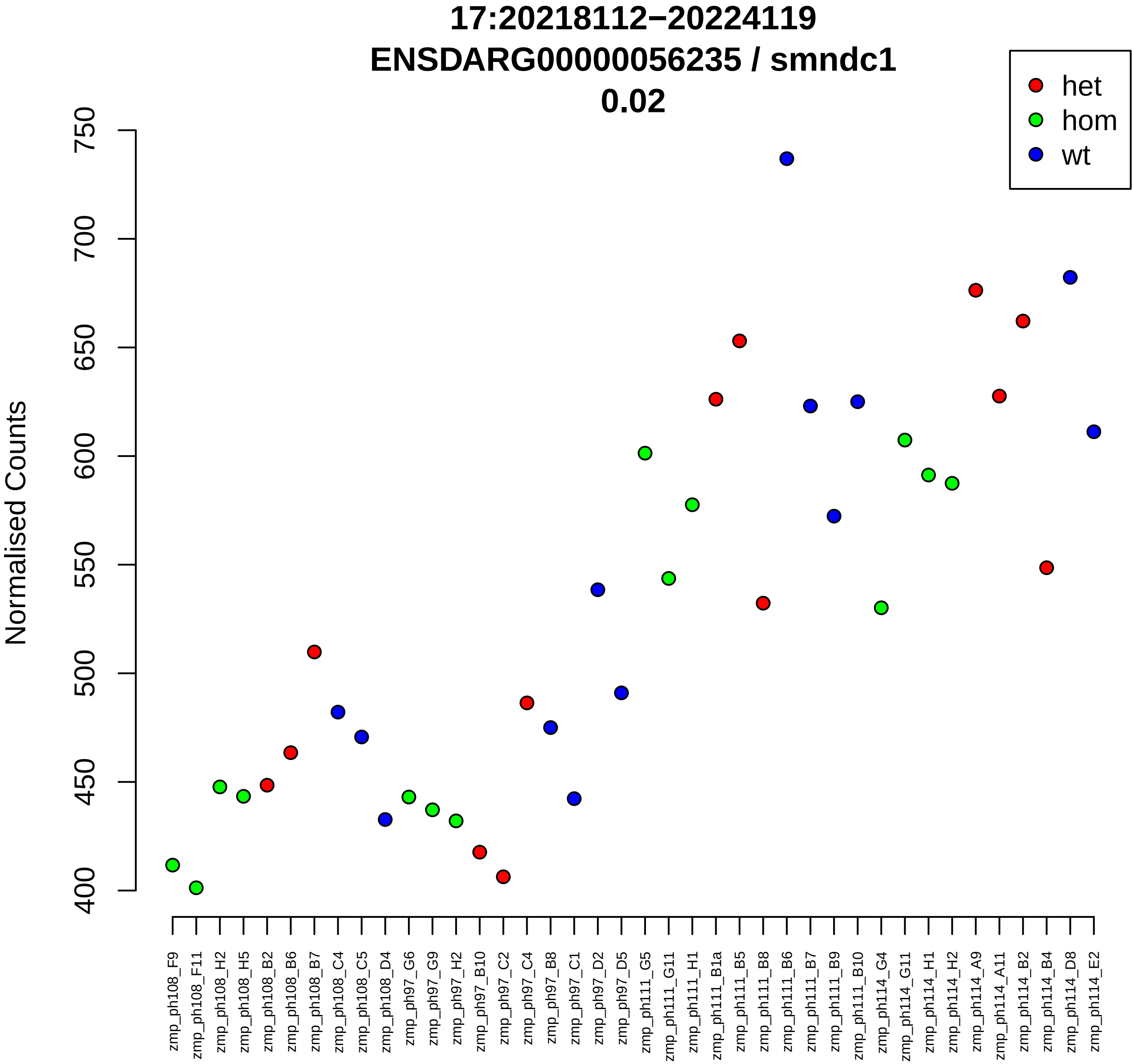

17:20218112−20224119
ENSDARG00000056235 / smndc1
0.02
het
hom
wt
Normalised Counts
400
450
500
550
600
650
700
750
zmp_ph108_F9
zmp_ph108_F11
zmp_ph108_H2
zmp_ph108_H5
zmp_ph108_B2
zmp_ph108_B6
zmp_ph108_B7
zmp_ph108_C4
zmp_ph108_C5
zmp_ph108_D4
zmp_ph97_G6
zmp_ph97_G9
zmp_ph97_H2
zmp_ph97_B10
zmp_ph97_C2
zmp_ph97_C4
zmp_ph97_B8
zmp_ph97_C1
zmp_ph97_D2
zmp_ph97_D5
zmp_ph111_G5
zmp_ph111_G11
zmp_ph111_H1
zmp_ph111_B1a
zmp_ph111_B5
zmp_ph111_B8
zmp_ph111_B6
zmp_ph111_B7
zmp_ph111_B9
zmp_ph111_B10
zmp_ph114_G4
zmp_ph114_G11
zmp_ph114_H1
zmp_ph114_H2
zmp_ph114_A9
zmp_ph114_A11
zmp_ph114_B2
zmp_ph114_B4
zmp_ph114_D8
zmp_ph114_E2

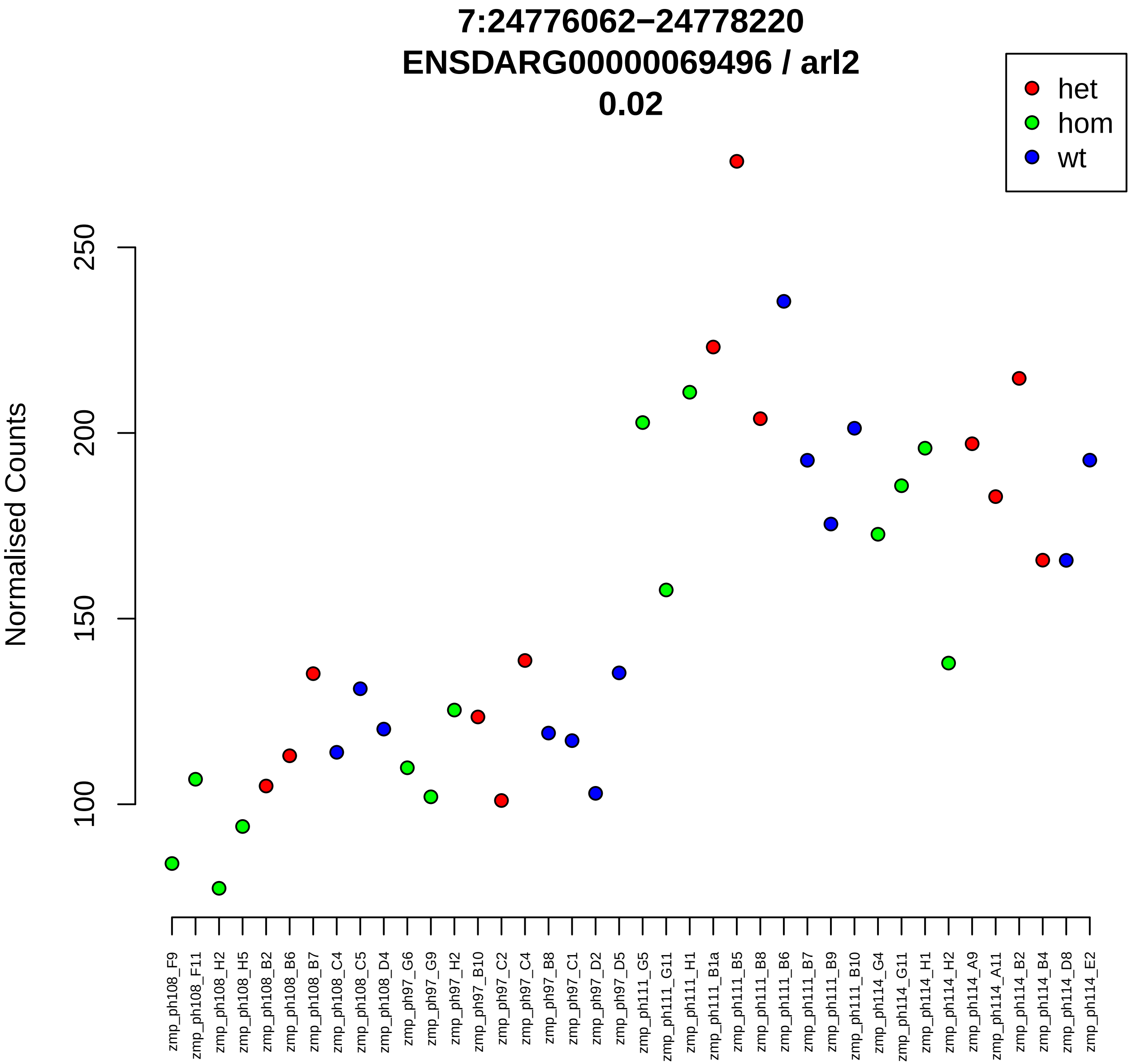
7:24776062−24778220
ENSDARG00000069496 / arl2
0.02
het
hom
wt
Normalised Counts
100
150
200
250
zmp_ph108_F9
zmp_ph108_F11
zmp_ph108_H2
zmp_ph108_H5
zmp_ph108_B2
zmp_ph108_B6
zmp_ph108_B7
zmp_ph108_C4
zmp_ph108_C5
zmp_ph108_D4
zmp_ph97_G6
zmp_ph97_G9
zmp_ph97_H2
zmp_ph97_B10
zmp_ph97_C2
zmp_ph97_C4
zmp_ph97_B8
zmp_ph97_C1
zmp_ph97_D2
zmp_ph97_D5
zmp_ph111_G5
zmp_ph111_G11
zmp_ph111_H1
zmp_ph111_B1a
zmp_ph111_B5
zmp_ph111_B8
zmp_ph111_B6
zmp_ph111_B7
zmp_ph111_B9
zmp_ph111_B10
zmp_ph114_G4
zmp_ph114_G11
zmp_ph114_H1
zmp_ph114_H2
zmp_ph114_A9
zmp_ph114_A11
zmp_ph114_B2
zmp_ph114_B4
zmp_ph114_D8
zmp_ph114_E2

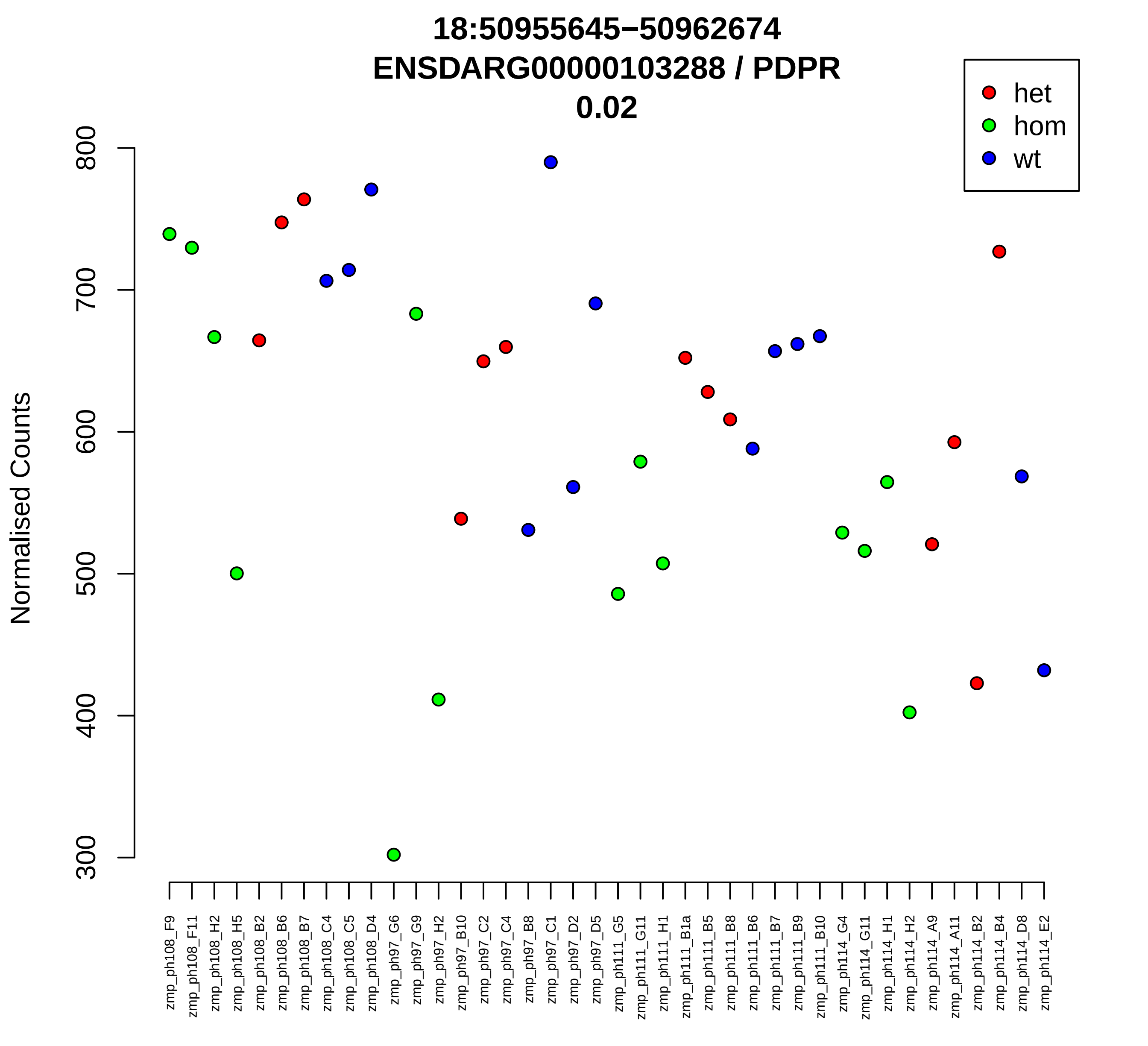

18:50955645−50962674
ENSDARG00000103288 / PDPR
0.02
het
hom
wt
Normalised Counts
300
400
500
600
700
800
zmp_ph108_F9
zmp_ph108_F11
zmp_ph108_H2
zmp_ph108_H5
zmp_ph108_B2
zmp_ph108_B6
zmp_ph108_B7
zmp_ph108_C4
zmp_ph108_C5
zmp_ph108_D4
zmp_ph97_G6
zmp_ph97_G9
zmp_ph97_H2
zmp_ph97_B10
zmp_ph97_C2
zmp_ph97_C4
zmp_ph97_B8
zmp_ph97_C1
zmp_ph97_D2
zmp_ph97_D5
zmp_ph111_G5
zmp_ph111_G11
zmp_ph111_H1
zmp_ph111_B1a
zmp_ph111_B5
zmp_ph111_B8
zmp_ph111_B6
zmp_ph111_B7
zmp_ph111_B9
zmp_ph111_B10
zmp_ph114_G4
zmp_ph114_G11
zmp_ph114_H1
zmp_ph114_H2
zmp_ph114_A9
zmp_ph114_A11
zmp_ph114_B2
zmp_ph114_B4
zmp_ph114_D8
zmp_ph114_E2

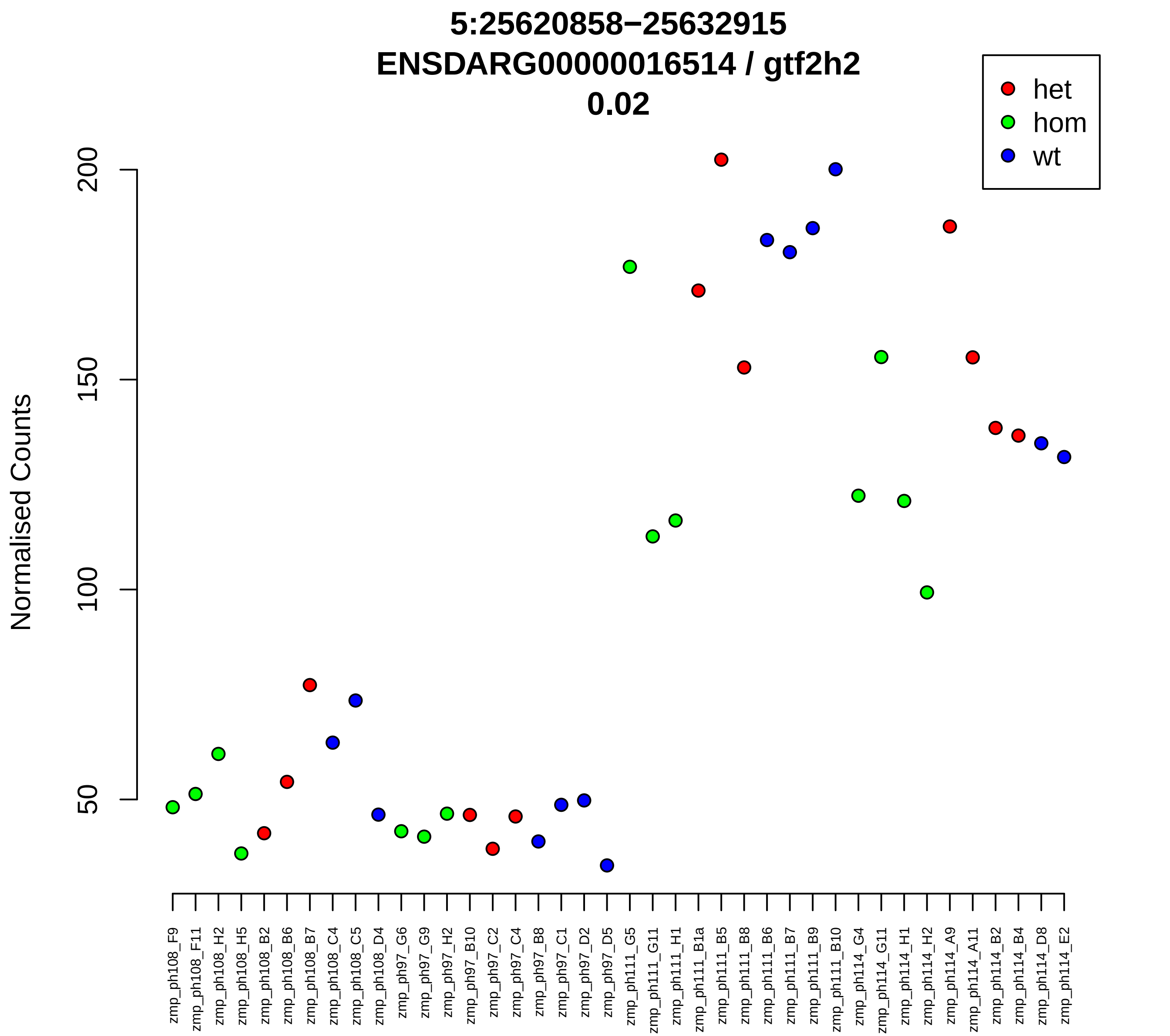
5:25620858−25632915
ENSDARG00000016514 / gtf2h2
0.02
het
hom
wt
Normalised Counts
200
150
100
50
zmp_ph108_F9
zmp_ph108_F11
zmp_ph108_H2
zmp_ph108_H5
zmp_ph108_B2
zmp_ph108_B6
zmp_ph108_B7
zmp_ph108_C4
zmp_ph108_C5
zmp_ph108_D4
zmp_ph97_G6
zmp_ph97_G9
zmp_ph97_H2
zmp_ph97_B10
zmp_ph97_C2
zmp_ph97_C4
zmp_ph97_B8
zmp_ph97_C1
zmp_ph97_D2
zmp_ph97_D5
zmp_ph111_G5
zmp_ph111_G11
zmp_ph111_H1
zmp_ph111_B1a
zmp_ph111_B5
zmp_ph111_B8
zmp_ph111_B6
zmp_ph111_B7
zmp_ph111_B9
zmp_ph111_B10
zmp_ph114_G4
zmp_ph114_G11
zmp_ph114_H1
zmp_ph114_H2
zmp_ph114_A9
zmp_ph114_A11
zmp_ph114_B2
zmp_ph114_B4
zmp_ph114_D8
zmp_ph114_E2

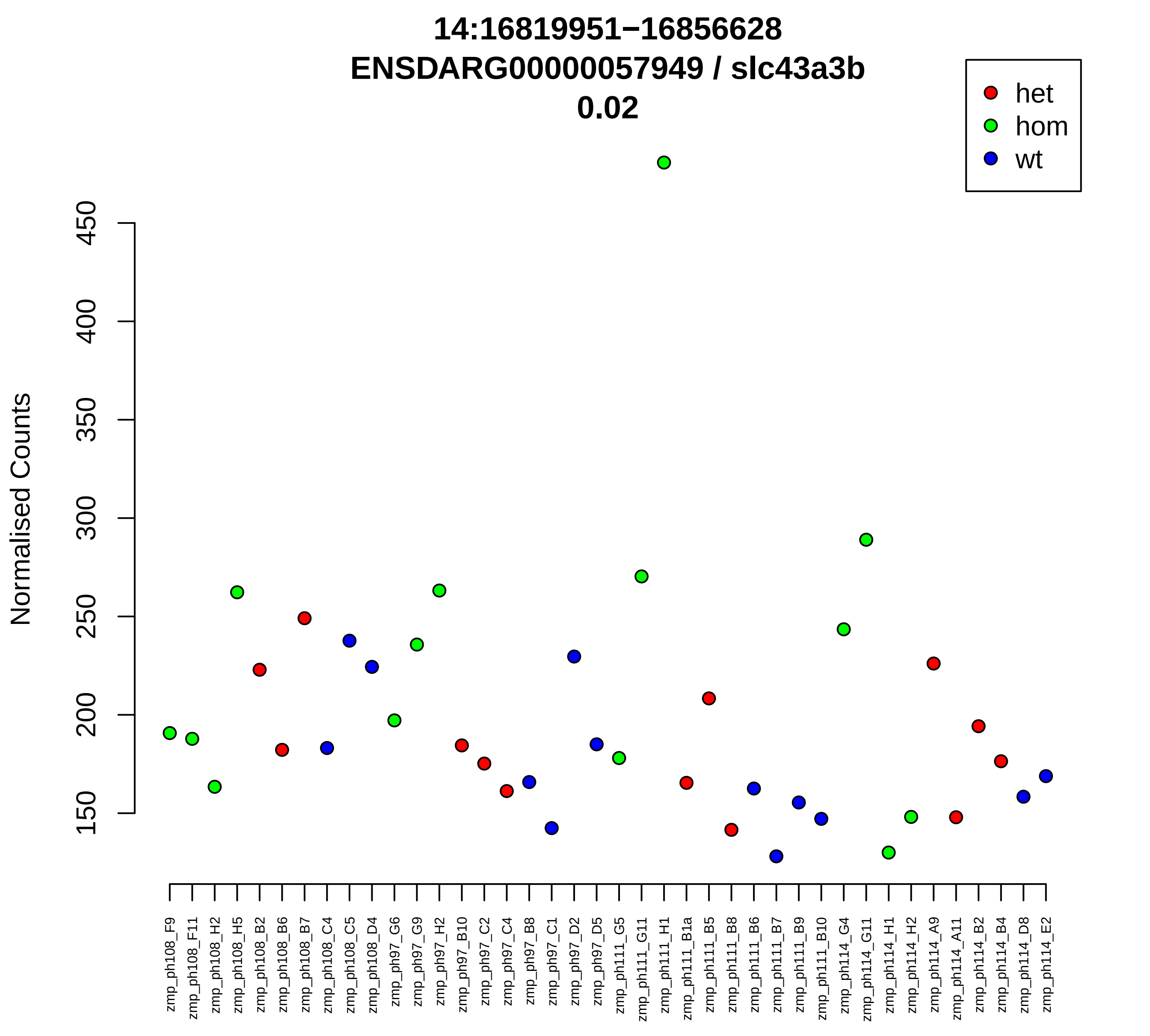
14:16819951−16856628
ENSDARG00000057949 / slc43a3b
0.02
het
hom
wt
Normalised Counts
150
200
250
300
350
400
450
zmp_ph108_F9
zmp_ph108_F11
zmp_ph108_H2
zmp_ph108_H5
zmp_ph108_B2
zmp_ph108_B6
zmp_ph108_B7
zmp_ph108_C4
zmp_ph108_C5
zmp_ph108_D4
zmp_ph97_G6
zmp_ph97_G9
zmp_ph97_H2
zmp_ph97_B10
zmp_ph97_C2
zmp_ph97_C4
zmp_ph97_B8
zmp_ph97_C1
zmp_ph97_D2
zmp_ph97_D5
zmp_ph111_G5
zmp_ph111_G11
zmp_ph111_H1
zmp_ph111_B1a
zmp_ph111_B5
zmp_ph111_B8
zmp_ph111_B6
zmp_ph111_B7
zmp_ph111_B9
zmp_ph111_B10
zmp_ph114_G4
zmp_ph114_G11
zmp_ph114_H1
zmp_ph114_H2
zmp_ph114_A9
zmp_ph114_A11
zmp_ph114_B2
zmp_ph114_B4
zmp_ph114_D8
zmp_ph114_E2

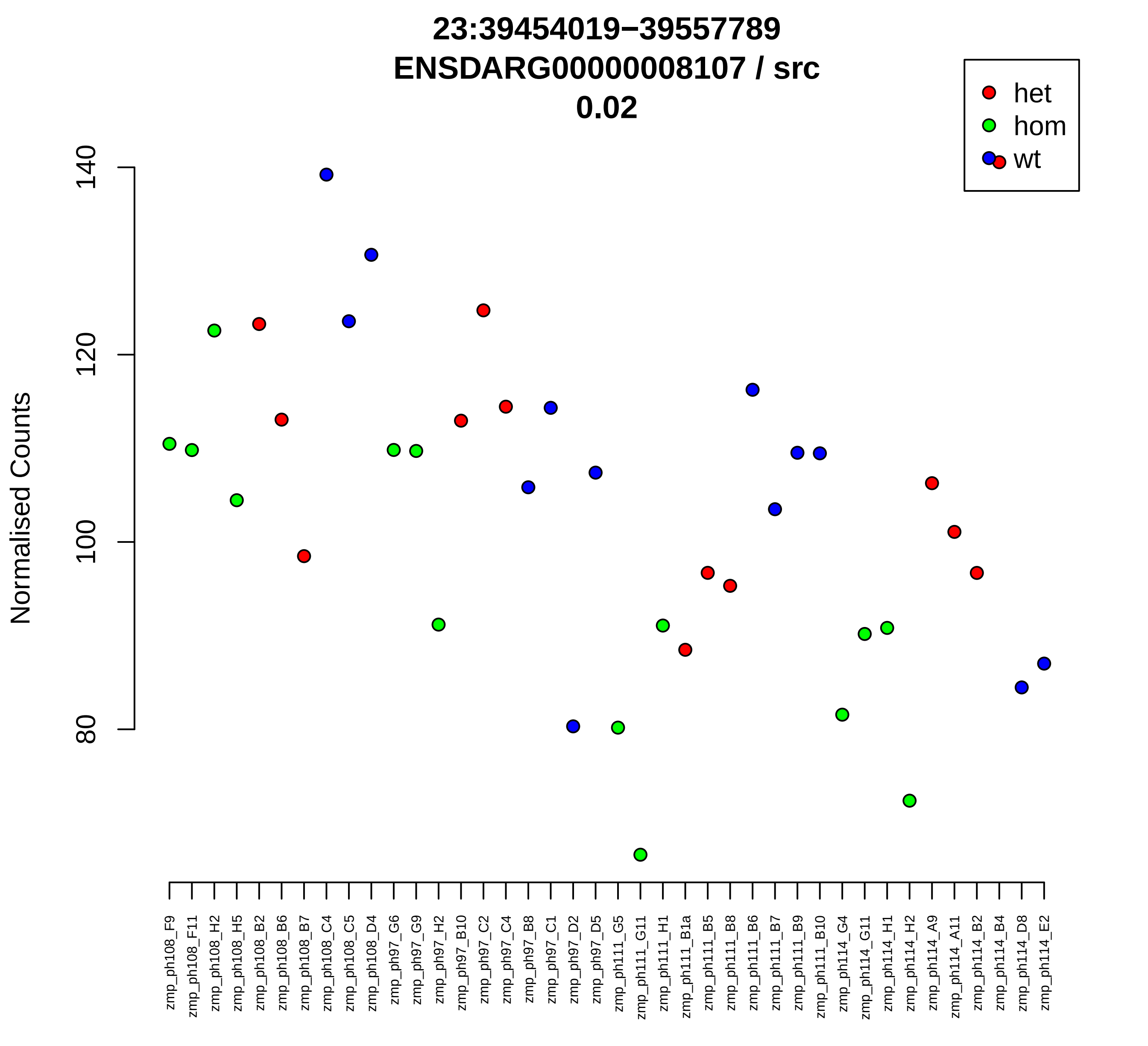

23:39454019−39557789
ENSDARG00000008107 / src
0.02
het
hom
wt
Normalised Counts
140
120
100
80
zmp_ph108_F9
zmp_ph108_F11
zmp_ph108_H2
zmp_ph108_H5
zmp_ph108_B2
zmp_ph108_B6
zmp_ph108_B7
zmp_ph108_C4
zmp_ph108_C5
zmp_ph108_D4
zmp_ph97_G6
zmp_ph97_G9
zmp_ph97_H2
zmp_ph97_B10
zmp_ph97_C2
zmp_ph97_C4
zmp_ph97_B8
zmp_ph97_C1
zmp_ph97_D2
zmp_ph97_D5
zmp_ph111_G5
zmp_ph111_G11
zmp_ph111_H1
zmp_ph111_B1a
zmp_ph111_B5
zmp_ph111_B8
zmp_ph111_B6
zmp_ph111_B7
zmp_ph111_B9
zmp_ph111_B10
zmp_ph114_G4
zmp_ph114_G11
zmp_ph114_H1
zmp_ph114_H2
zmp_ph114_A9
zmp_ph114_A11
zmp_ph114_B2
zmp_ph114_B4
zmp_ph114_D8
zmp_ph114_E2

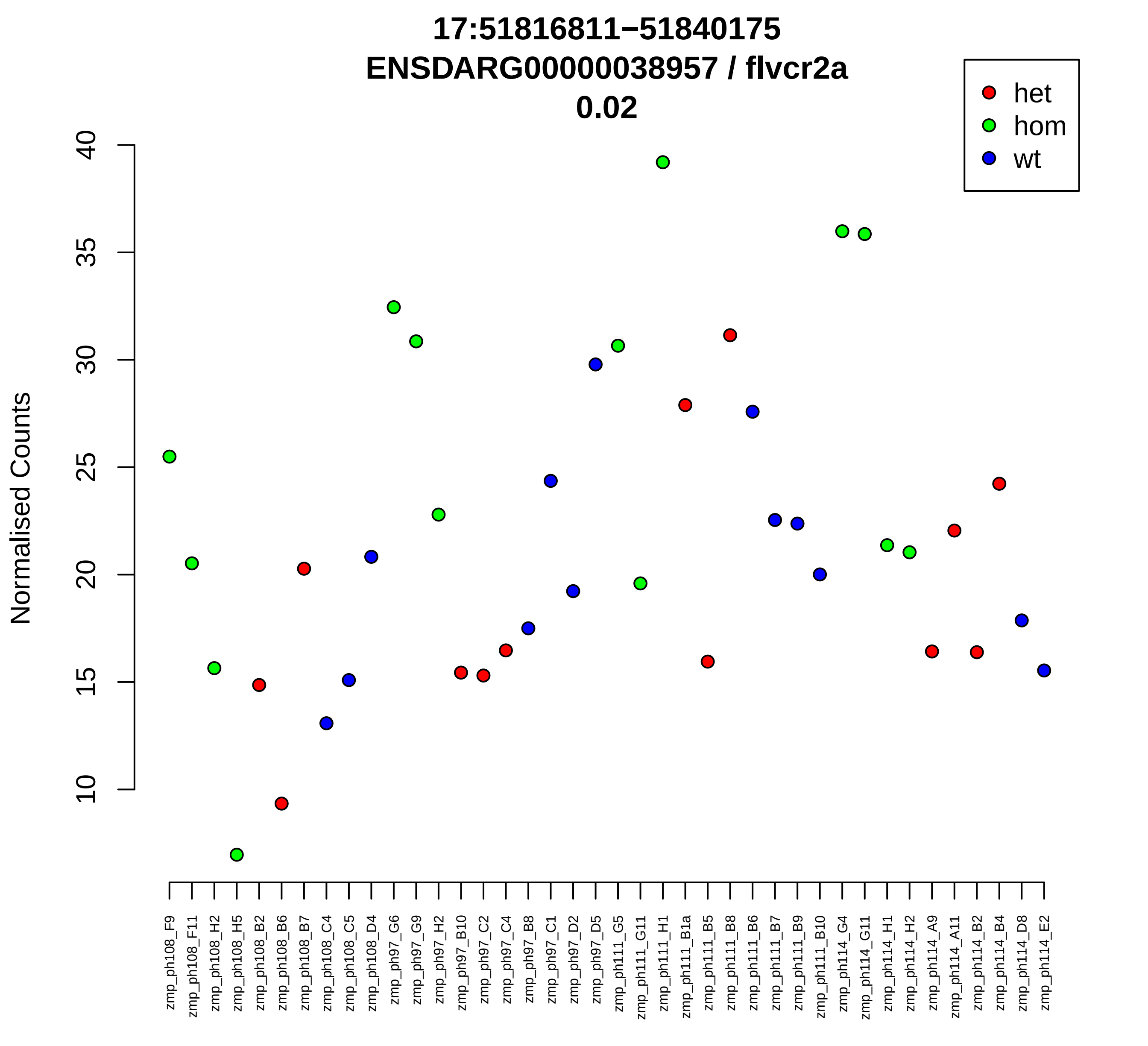

17:51816811−51840175
ENSDARG00000038957 / flvcr2a
0.02
het
hom
wt
Normalised Counts
10
15
20
25
30
35
40
zmp_ph108_F9
zmp_ph108_F11
zmp_ph108_H2
zmp_ph108_H5
zmp_ph108_B2
zmp_ph108_B6
zmp_ph108_B7
zmp_ph108_C4
zmp_ph108_C5
zmp_ph108_D4
zmp_ph97_G6
zmp_ph97_G9
zmp_ph97_H2
zmp_ph97_B10
zmp_ph97_C2
zmp_ph97_C4
zmp_ph97_B8
zmp_ph97_C1
zmp_ph97_D2
zmp_ph97_D5
zmp_ph111_G5
zmp_ph111_G11
zmp_ph111_H1
zmp_ph111_B1a
zmp_ph111_B5
zmp_ph111_B8
zmp_ph111_B6
zmp_ph111_B7
zmp_ph111_B9
zmp_ph111_B10
zmp_ph114_G4
zmp_ph114_G11
zmp_ph114_H1
zmp_ph114_H2
zmp_ph114_A9
zmp_ph114_A11
zmp_ph114_B2
zmp_ph114_B4
zmp_ph114_D8
zmp_ph114_E2

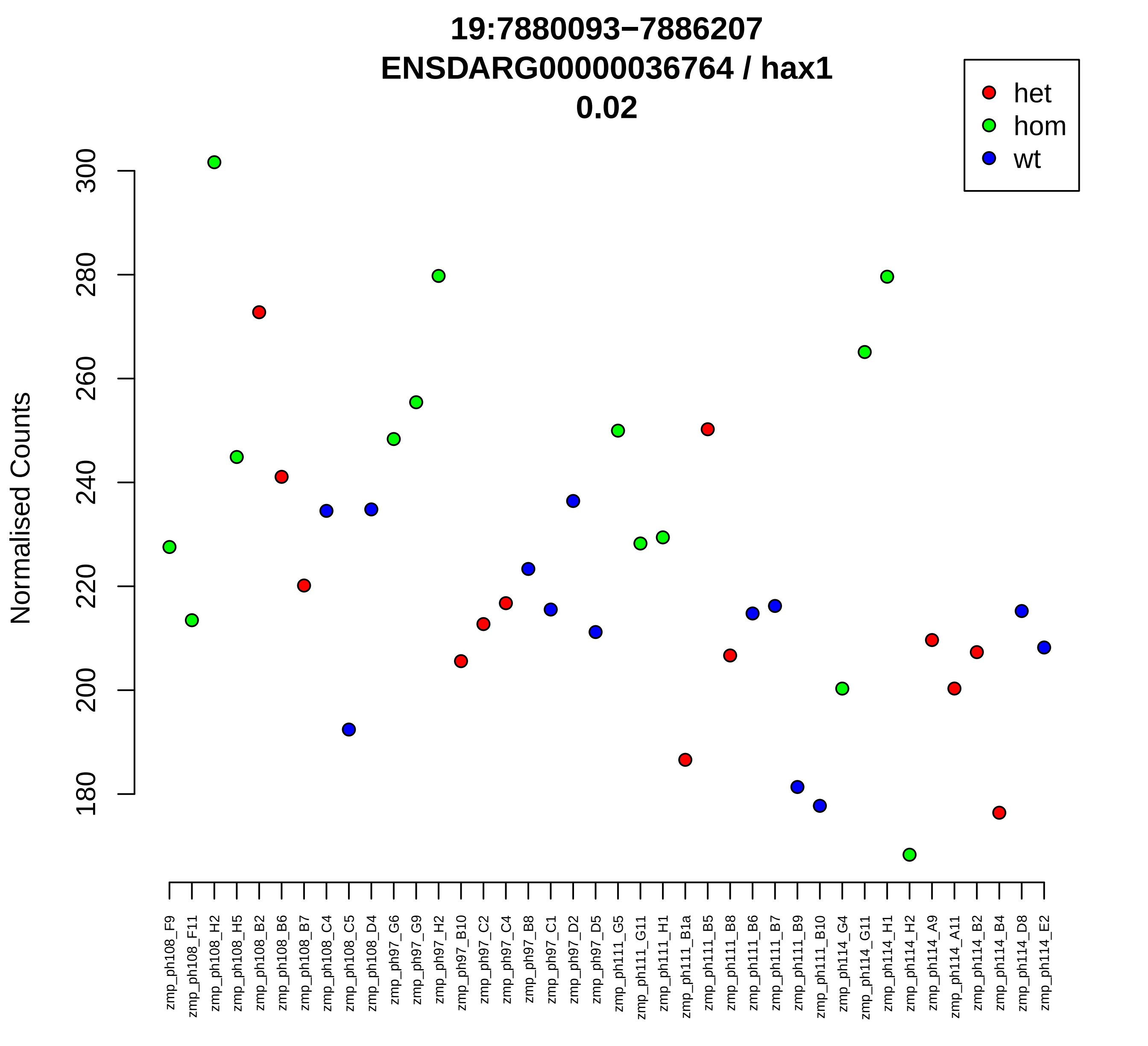
19:7880093−7886207
ENSDARG00000036764 / hax1
0.02
het
hom
wt
Normalised Counts
180
200
220
240
260
280
300
zmp_ph108_F9
zmp_ph108_F11
zmp_ph108_H2
zmp_ph108_H5
zmp_ph108_B2
zmp_ph108_B6
zmp_ph108_B7
zmp_ph108_C4
zmp_ph108_C5
zmp_ph108_D4
zmp_ph97_G6
zmp_ph97_G9
zmp_ph97_H2
zmp_ph97_B10
zmp_ph97_C2
zmp_ph97_C4
zmp_ph97_B8
zmp_ph97_C1
zmp_ph97_D2
zmp_ph97_D5
zmp_ph111_G5
zmp_ph111_G11
zmp_ph111_H1
zmp_ph111_B1a
zmp_ph111_B5
zmp_ph111_B8
zmp_ph111_B6
zmp_ph111_B7
zmp_ph111_B9
zmp_ph111_B10
zmp_ph114_G4
zmp_ph114_G11
zmp_ph114_H1
zmp_ph114_H2
zmp_ph114_A9
zmp_ph114_A11
zmp_ph114_B2
zmp_ph114_B4
zmp_ph114_D8
zmp_ph114_E2

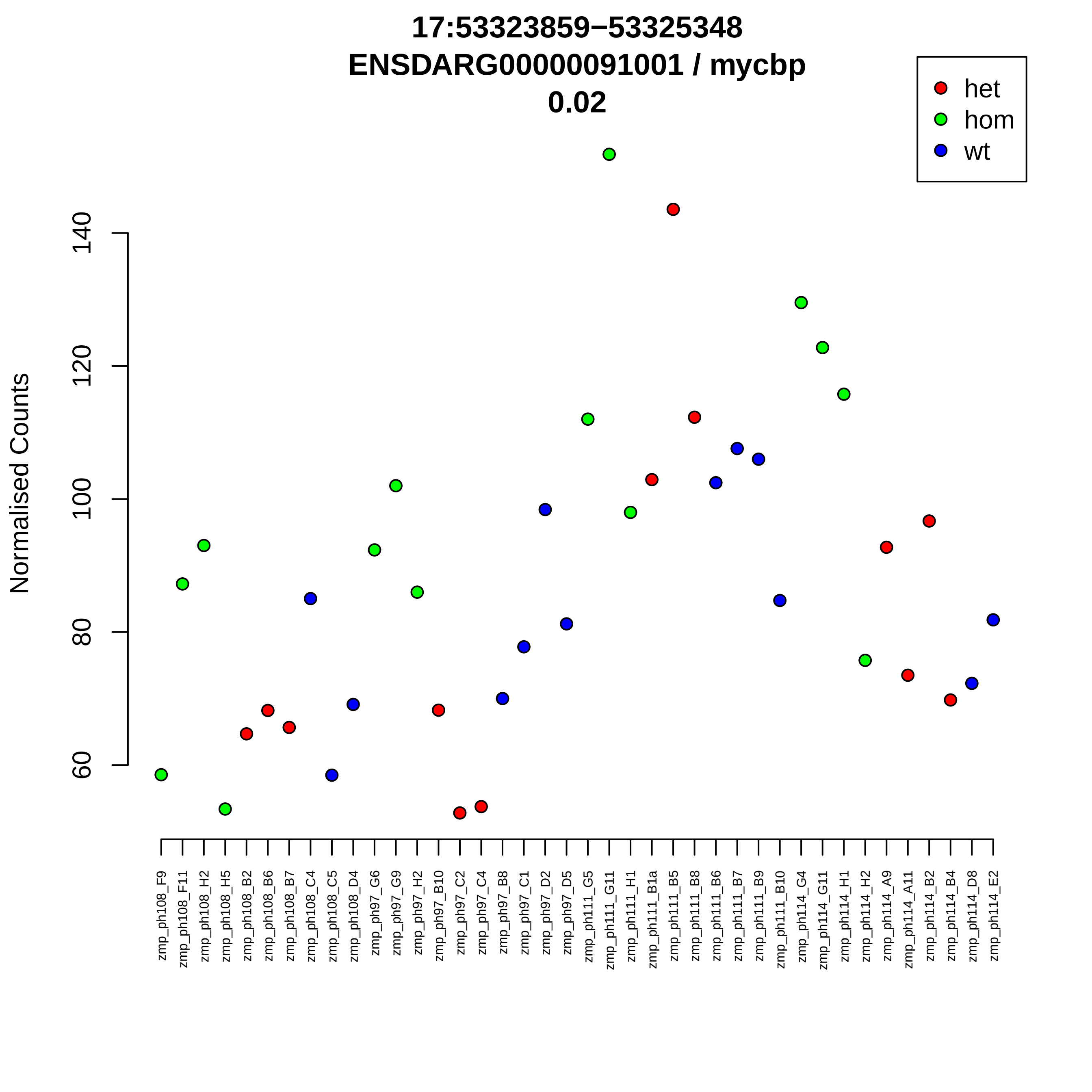

17:53323859−53325348
ENSDARG00000091001 / mycbp
0.02
het
hom
wt
Normalised Counts
60
80
100
120
140
zmp_ph108_F9
zmp_ph108_F11
zmp_ph108_H2
zmp_ph108_H5
zmp_ph108_B2
zmp_ph108_B6
zmp_ph108_B7
zmp_ph108_C4
zmp_ph108_C5
zmp_ph108_D4
zmp_ph97_G6
zmp_ph97_G9
zmp_ph97_H2
zmp_ph97_B10
zmp_ph97_C2
zmp_ph97_C4
zmp_ph97_B8
zmp_ph97_C1
zmp_ph97_D2
zmp_ph97_D5
zmp_ph111_G5
zmp_ph111_G11
zmp_ph111_H1
zmp_ph111_B1a
zmp_ph111_B5
zmp_ph111_B8
zmp_ph111_B6
zmp_ph111_B7
zmp_ph111_B9
zmp_ph111_B10
zmp_ph114_G4
zmp_ph114_G11
zmp_ph114_H1
zmp_ph114_H2
zmp_ph114_A9
zmp_ph114_A11
zmp_ph114_B2
zmp_ph114_B4
zmp_ph114_D8
zmp_ph114_E2

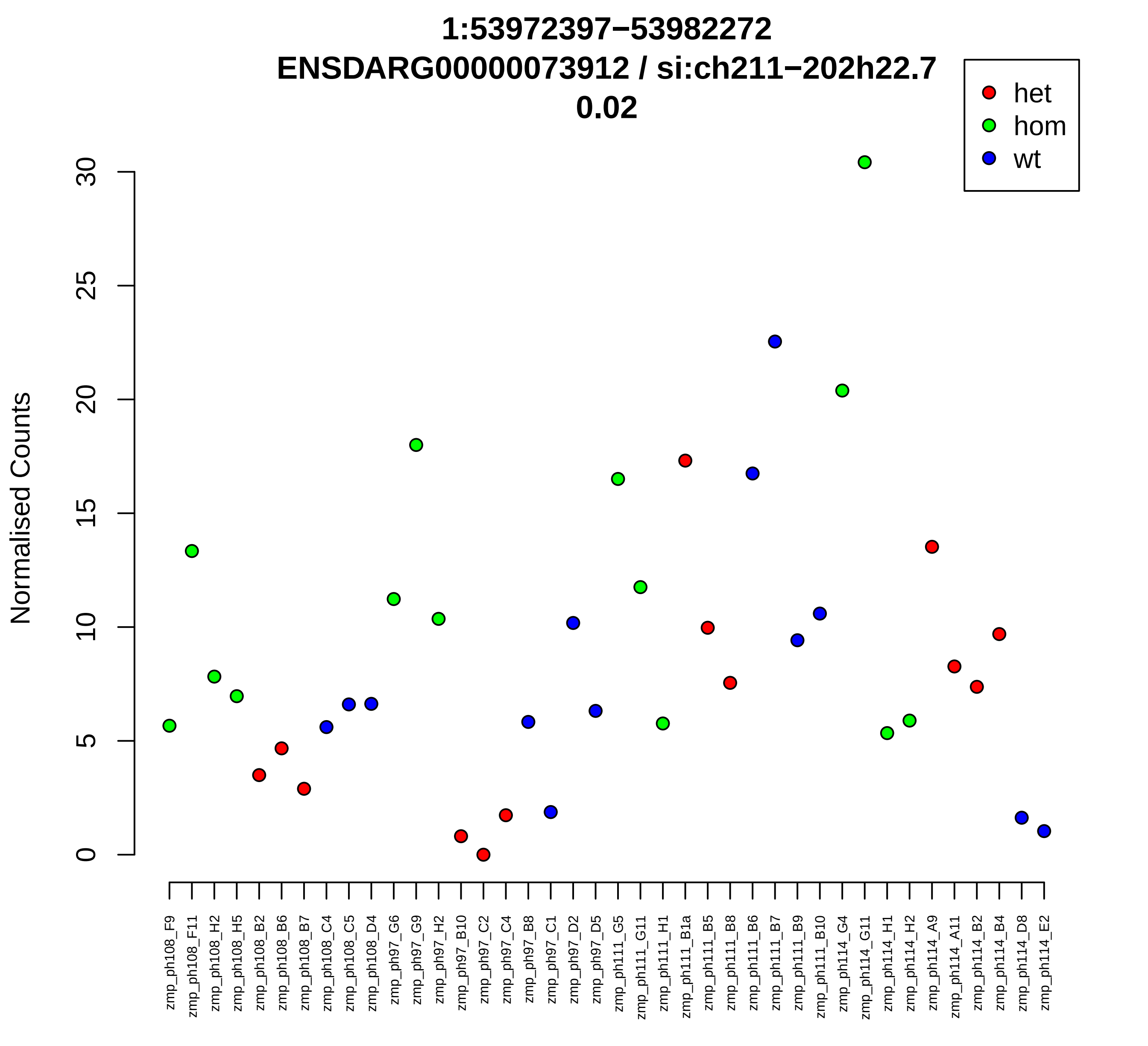

1:53972397−53982272
ENSDARG00000073912 / si:ch211−202h22.7
0.02
het
hom
wt
Normalised Counts
0
5
10
15
20
25
30
zmp_ph108_F9
zmp_ph108_F11
zmp_ph108_H2
zmp_ph108_H5
zmp_ph108_B2
zmp_ph108_B6
zmp_ph108_B7
zmp_ph108_C4
zmp_ph108_C5
zmp_ph108_D4
zmp_ph97_G6
zmp_ph97_G9
zmp_ph97_H2
zmp_ph97_B10
zmp_ph97_C2
zmp_ph97_C4
zmp_ph97_B8
zmp_ph97_C1
zmp_ph97_D2
zmp_ph97_D5
zmp_ph111_G5
zmp_ph111_G11
zmp_ph111_H1
zmp_ph111_B1a
zmp_ph111_B5
zmp_ph111_B8
zmp_ph111_B6
zmp_ph111_B7
zmp_ph111_B9
zmp_ph111_B10
zmp_ph114_G4
zmp_ph114_G11
zmp_ph114_H1
zmp_ph114_H2
zmp_ph114_A9
zmp_ph114_A11
zmp_ph114_B2
zmp_ph114_B4
zmp_ph114_D8
zmp_ph114_E2

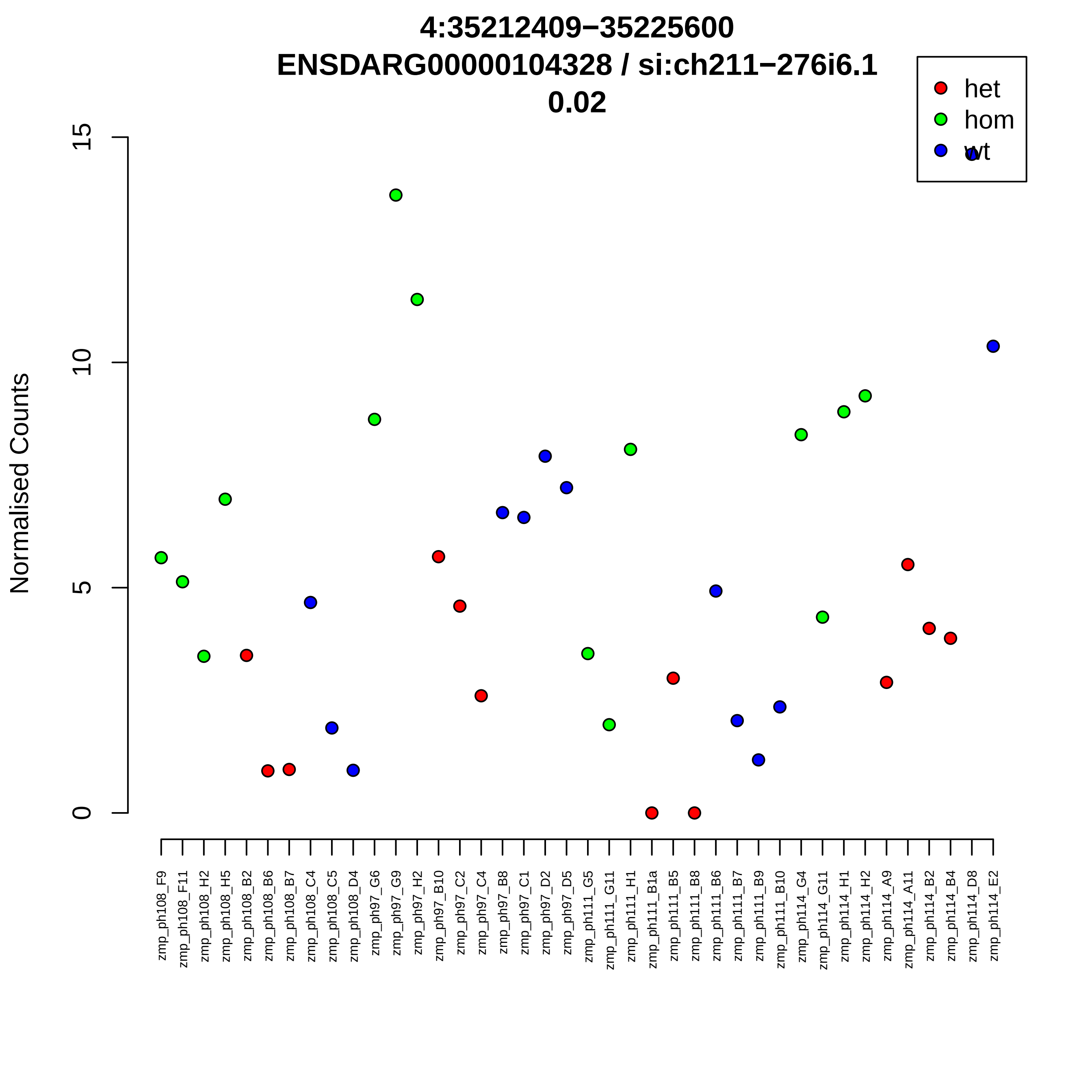
4:35212409−35225600
ENSDARG00000104328 / si:ch211−276i6.1
0.02
het
hom
wt
Normalised Counts
0
5
10
15
zmp_ph108_F9
zmp_ph108_F11
zmp_ph108_H2
zmp_ph108_H5
zmp_ph108_B2
zmp_ph108_B6
zmp_ph108_B7
zmp_ph108_C4
zmp_ph108_C5
zmp_ph108_D4
zmp_ph97_G6
zmp_ph97_G9
zmp_ph97_H2
zmp_ph97_B10
zmp_ph97_C2
zmp_ph97_C4
zmp_ph97_B8
zmp_ph97_C1
zmp_ph97_D2
zmp_ph97_D5
zmp_ph111_G5
zmp_ph111_G11
zmp_ph111_H1
zmp_ph111_B1a
zmp_ph111_B5
zmp_ph111_B8
zmp_ph111_B6
zmp_ph111_B7
zmp_ph111_B9
zmp_ph111_B10
zmp_ph114_G4
zmp_ph114_G11
zmp_ph114_H1
zmp_ph114_H2
zmp_ph114_A9
zmp_ph114_A11
zmp_ph114_B2
zmp_ph114_B4
zmp_ph114_D8
zmp_ph114_E2

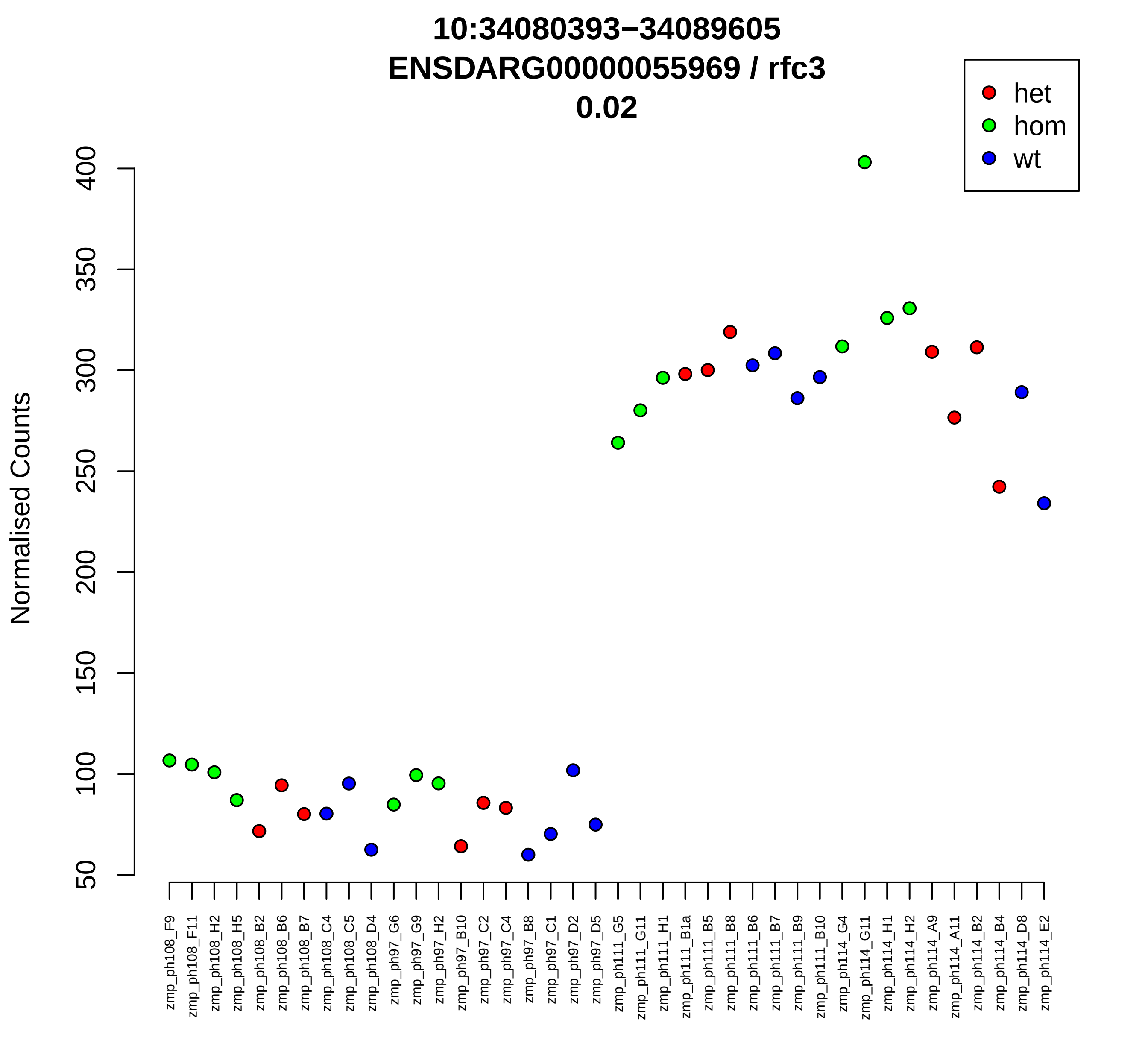
10:34080393−34089605
ENSDARG00000055969 / rfc3
0.02
het
hom
wt
Normalised Counts
400
350
300
250
200
150
100
50
zmp_ph108_F9
zmp_ph108_F11
zmp_ph108_H2
zmp_ph108_H5
zmp_ph108_B2
zmp_ph108_B6
zmp_ph108_B7
zmp_ph108_C4
zmp_ph108_C5
zmp_ph108_D4
zmp_ph97_G6
zmp_ph97_G9
zmp_ph97_H2
zmp_ph97_B10
zmp_ph97_C2
zmp_ph97_C4
zmp_ph97_B8
zmp_ph97_C1
zmp_ph97_D2
zmp_ph97_D5
zmp_ph111_G5
zmp_ph111_G11
zmp_ph111_H1
zmp_ph111_B1a
zmp_ph111_B5
zmp_ph111_B8
zmp_ph111_B6
zmp_ph111_B7
zmp_ph111_B9
zmp_ph111_B10
zmp_ph114_G4
zmp_ph114_G11
zmp_ph114_H1
zmp_ph114_H2
zmp_ph114_A9
zmp_ph114_A11
zmp_ph114_B2
zmp_ph114_B4
zmp_ph114_D8
zmp_ph114_E2

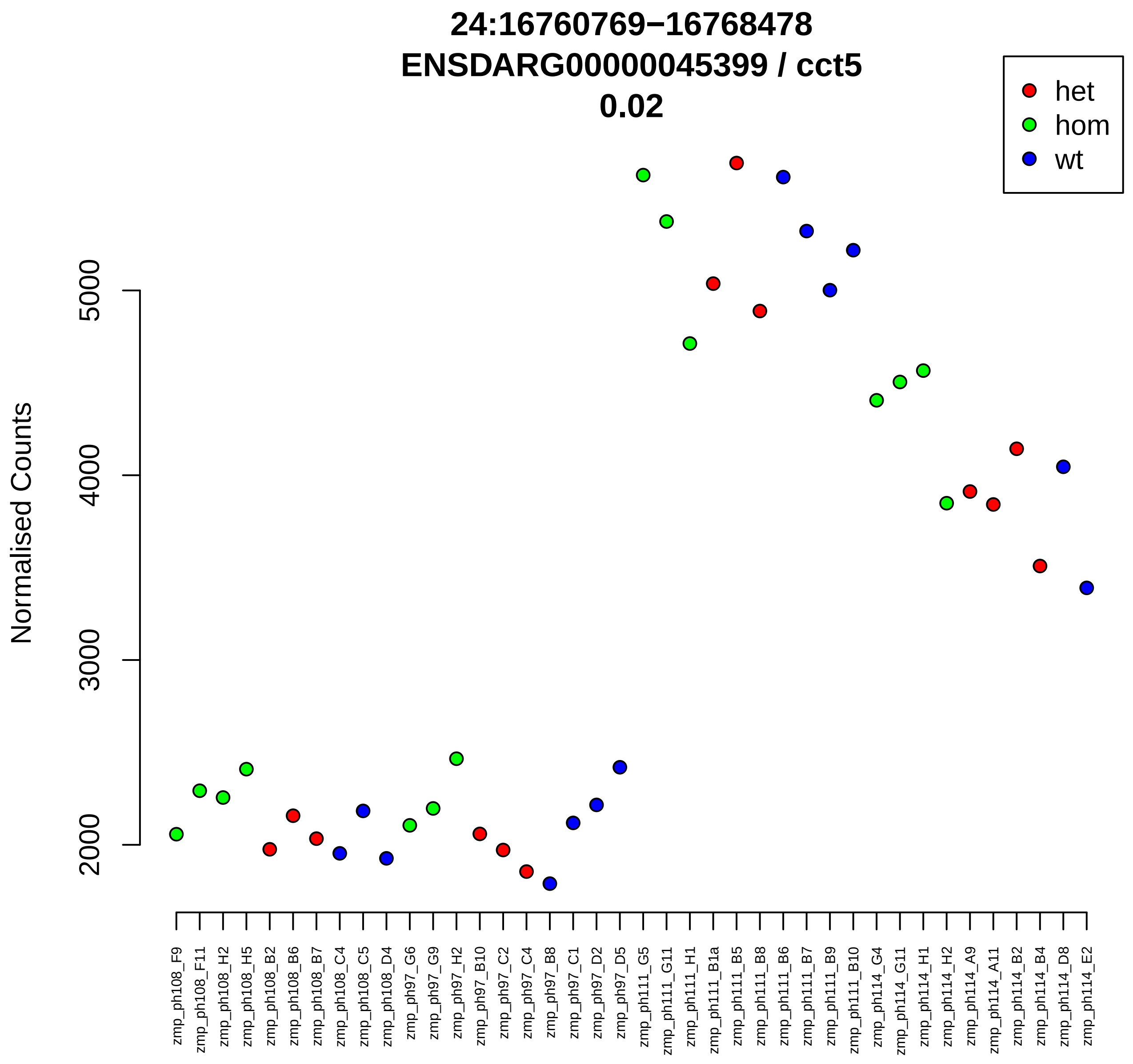

24:16760769−16768478
ENSDARG00000045399 / cct5
0.02
het
hom
wt
Normalised Counts
2000
3000
4000
5000
zmp_ph108_F9
zmp_ph108_F11
zmp_ph108_H2
zmp_ph108_H5
zmp_ph108_B2
zmp_ph108_B6
zmp_ph108_B7
zmp_ph108_C4
zmp_ph108_C5
zmp_ph108_D4
zmp_ph97_G6
zmp_ph97_G9
zmp_ph97_H2
zmp_ph97_B10
zmp_ph97_C2
zmp_ph97_C4
zmp_ph97_B8
zmp_ph97_C1
zmp_ph97_D2
zmp_ph97_D5
zmp_ph111_G5
zmp_ph111_G11
zmp_ph111_H1
zmp_ph111_B1a
zmp_ph111_B5
zmp_ph111_B8
zmp_ph111_B6
zmp_ph111_B7
zmp_ph111_B9
zmp_ph111_B10
zmp_ph114_G4
zmp_ph114_G11
zmp_ph114_H1
zmp_ph114_H2
zmp_ph114_A9
zmp_ph114_A11
zmp_ph114_B2
zmp_ph114_B4
zmp_ph114_D8
zmp_ph114_E2

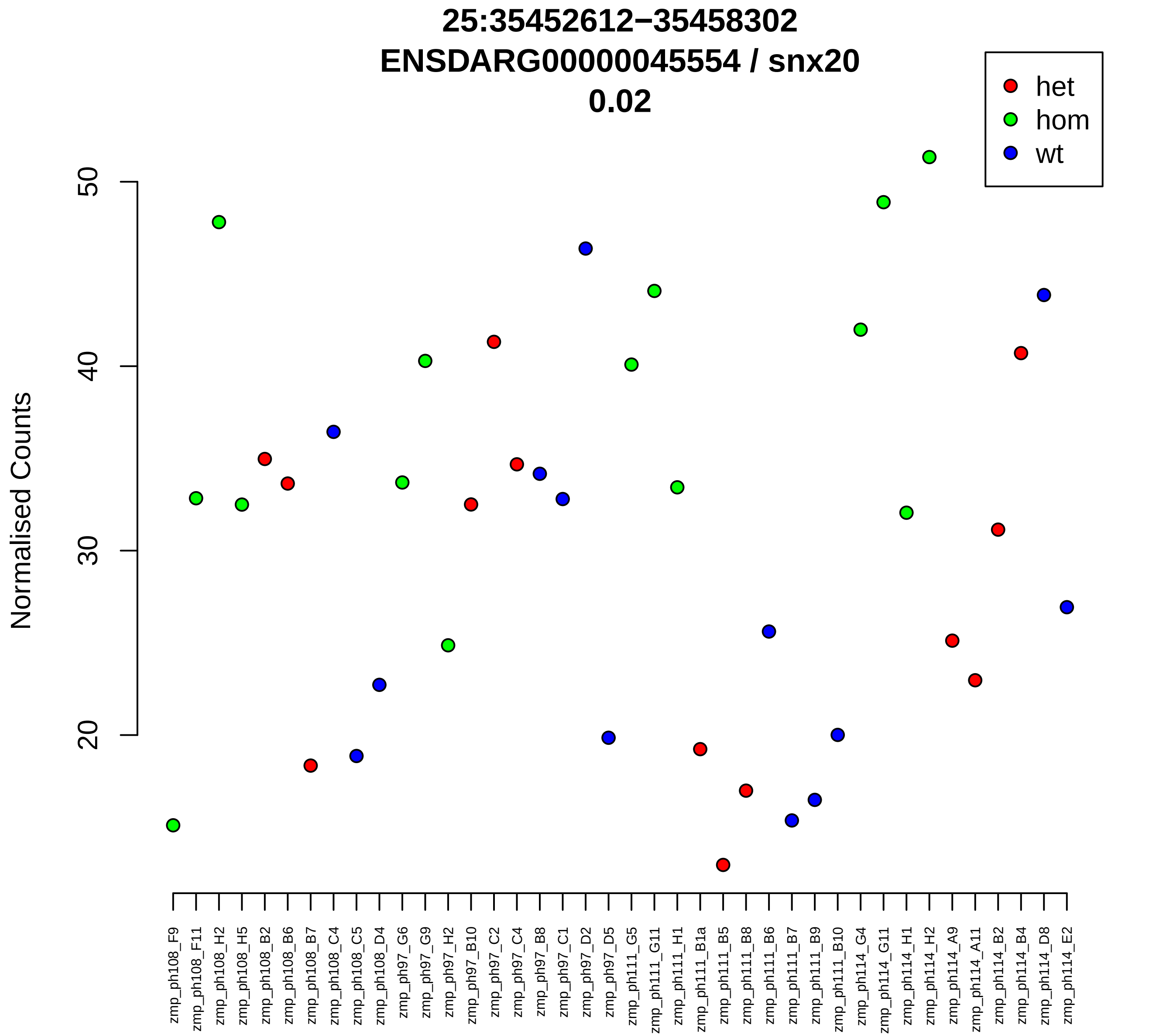
25:35452612−35458302
ENSDARG00000045554 / snx20
0.02
het
hom
wt
Normalised Counts
20
30
40
50
zmp_ph108_F9
zmp_ph108_F11
zmp_ph108_H2
zmp_ph108_H5
zmp_ph108_B2
zmp_ph108_B6
zmp_ph108_B7
zmp_ph108_C4
zmp_ph108_C5
zmp_ph108_D4
zmp_ph97_G6
zmp_ph97_G9
zmp_ph97_H2
zmp_ph97_B10
zmp_ph97_C2
zmp_ph97_C4
zmp_ph97_B8
zmp_ph97_C1
zmp_ph97_D2
zmp_ph97_D5
zmp_ph111_G5
zmp_ph111_G11
zmp_ph111_H1
zmp_ph111_B1a
zmp_ph111_B5
zmp_ph111_B8
zmp_ph111_B6
zmp_ph111_B7
zmp_ph111_B9
zmp_ph111_B10
zmp_ph114_G4
zmp_ph114_G11
zmp_ph114_H1
zmp_ph114_H2
zmp_ph114_A9
zmp_ph114_A11
zmp_ph114_B2
zmp_ph114_B4
zmp_ph114_D8
zmp_ph114_E2

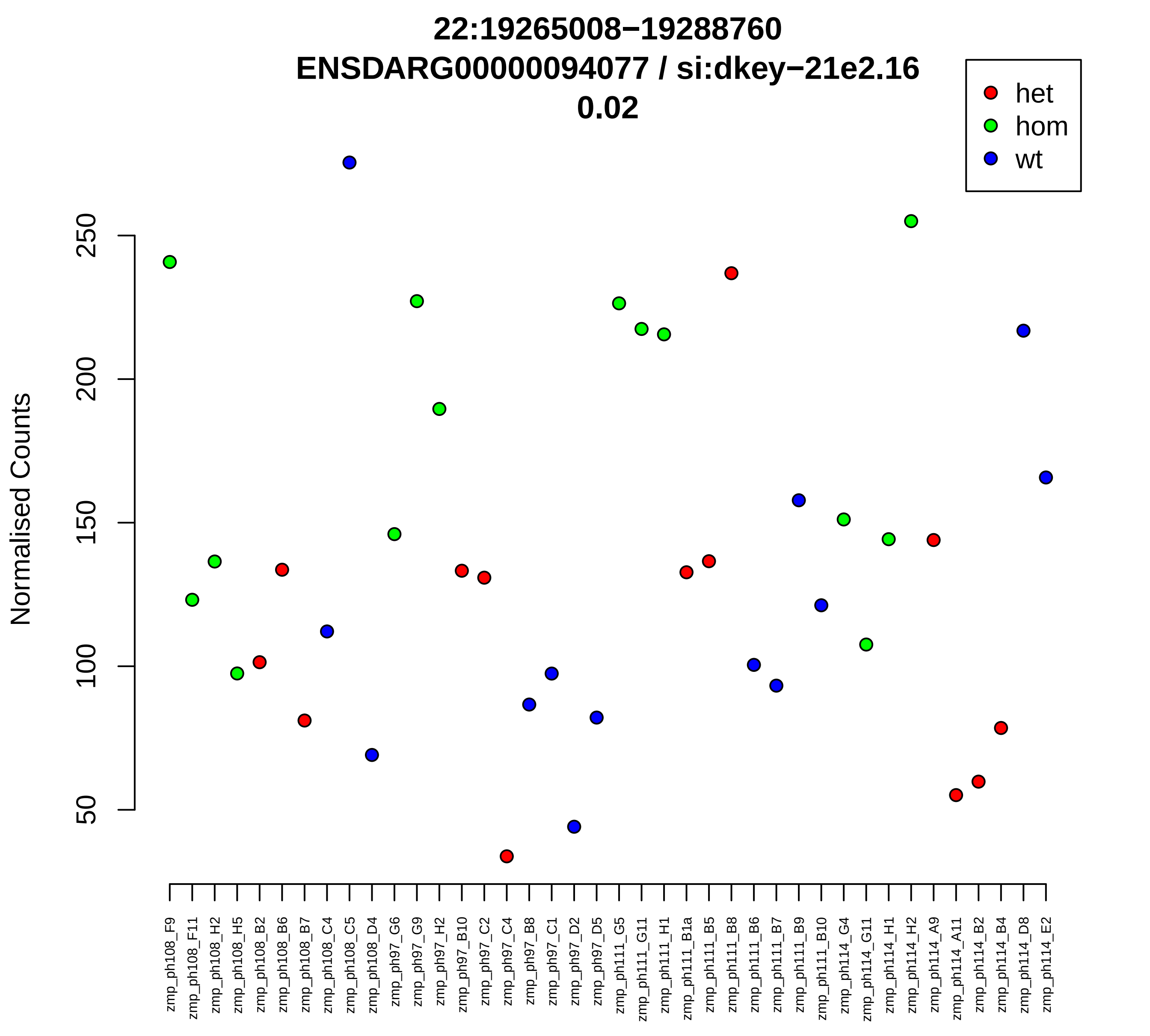
22:19265008−19288760
ENSDARG00000094077 / si:dkey−21e2.16
0.02
het
hom
wt
Normalised Counts
50
100
150
200
250
zmp_ph108_F9
zmp_ph108_F11
zmp_ph108_H2
zmp_ph108_H5
zmp_ph108_B2
zmp_ph108_B6
zmp_ph108_B7
zmp_ph108_C4
zmp_ph108_C5
zmp_ph108_D4
zmp_ph97_G6
zmp_ph97_G9
zmp_ph97_H2
zmp_ph97_B10
zmp_ph97_C2
zmp_ph97_C4
zmp_ph97_B8
zmp_ph97_C1
zmp_ph97_D2
zmp_ph97_D5
zmp_ph111_G5
zmp_ph111_G11
zmp_ph111_H1
zmp_ph111_B1a
zmp_ph111_B5
zmp_ph111_B8
zmp_ph111_B6
zmp_ph111_B7
zmp_ph111_B9
zmp_ph111_B10
zmp_ph114_G4
zmp_ph114_G11
zmp_ph114_H1
zmp_ph114_H2
zmp_ph114_A9
zmp_ph114_A11
zmp_ph114_B2
zmp_ph114_B4
zmp_ph114_D8
zmp_ph114_E2

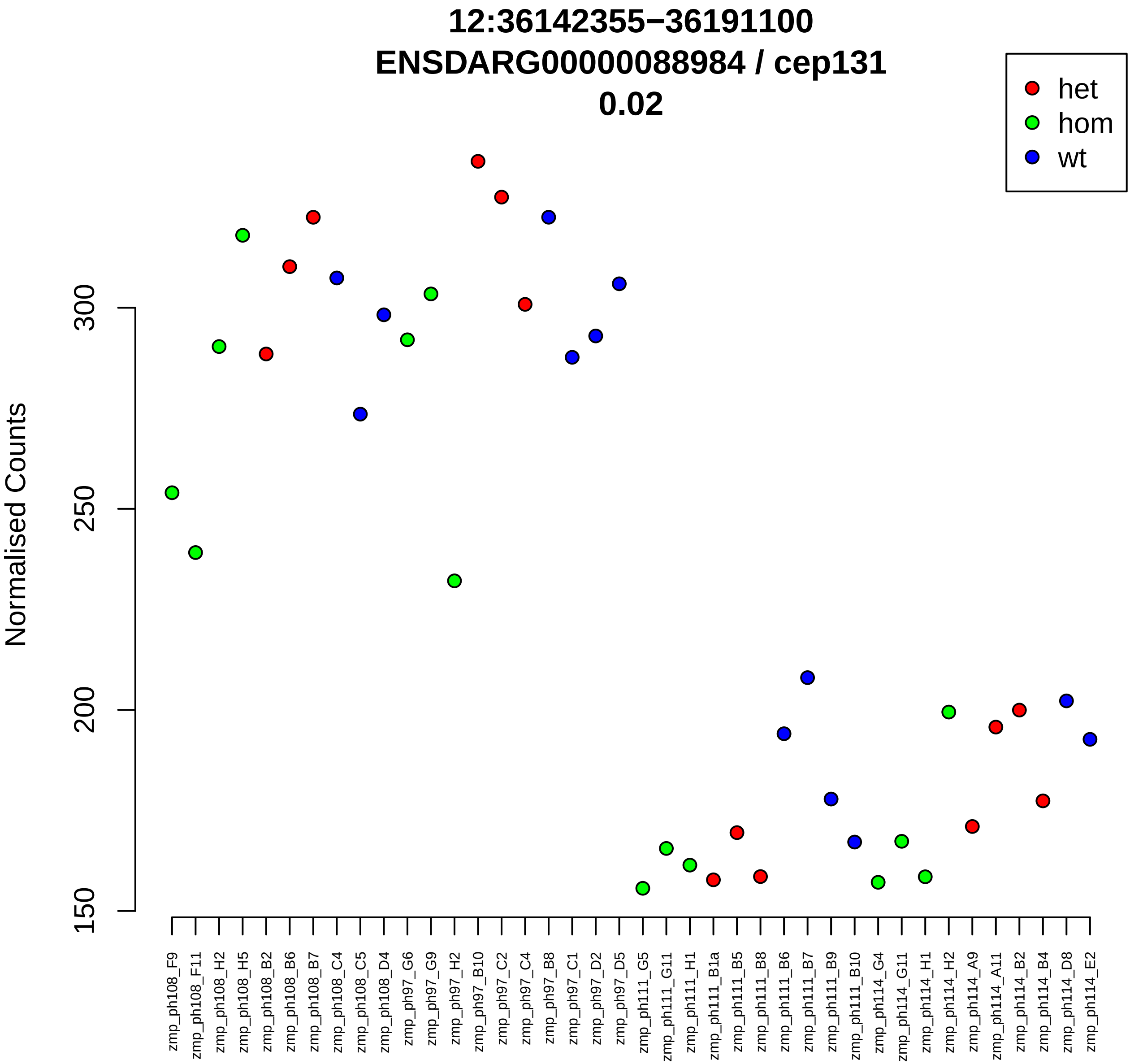
12:36142355−36191100
ENSDARG00000088984 / cep131
0.02
het
hom
wt
Normalised Counts
150
200
250
300
zmp_ph108_F9
zmp_ph108_F11
zmp_ph108_H2
zmp_ph108_H5
zmp_ph108_B2
zmp_ph108_B6
zmp_ph108_B7
zmp_ph108_C4
zmp_ph108_C5
zmp_ph108_D4
zmp_ph97_G6
zmp_ph97_G9
zmp_ph97_H2
zmp_ph97_B10
zmp_ph97_C2
zmp_ph97_C4
zmp_ph97_B8
zmp_ph97_C1
zmp_ph97_D2
zmp_ph97_D5
zmp_ph111_G5
zmp_ph111_G11
zmp_ph111_H1
zmp_ph111_B1a
zmp_ph111_B5
zmp_ph111_B8
zmp_ph111_B6
zmp_ph111_B7
zmp_ph111_B9
zmp_ph111_B10
zmp_ph114_G4
zmp_ph114_G11
zmp_ph114_H1
zmp_ph114_H2
zmp_ph114_A9
zmp_ph114_A11
zmp_ph114_B2
zmp_ph114_B4
zmp_ph114_D8
zmp_ph114_E2

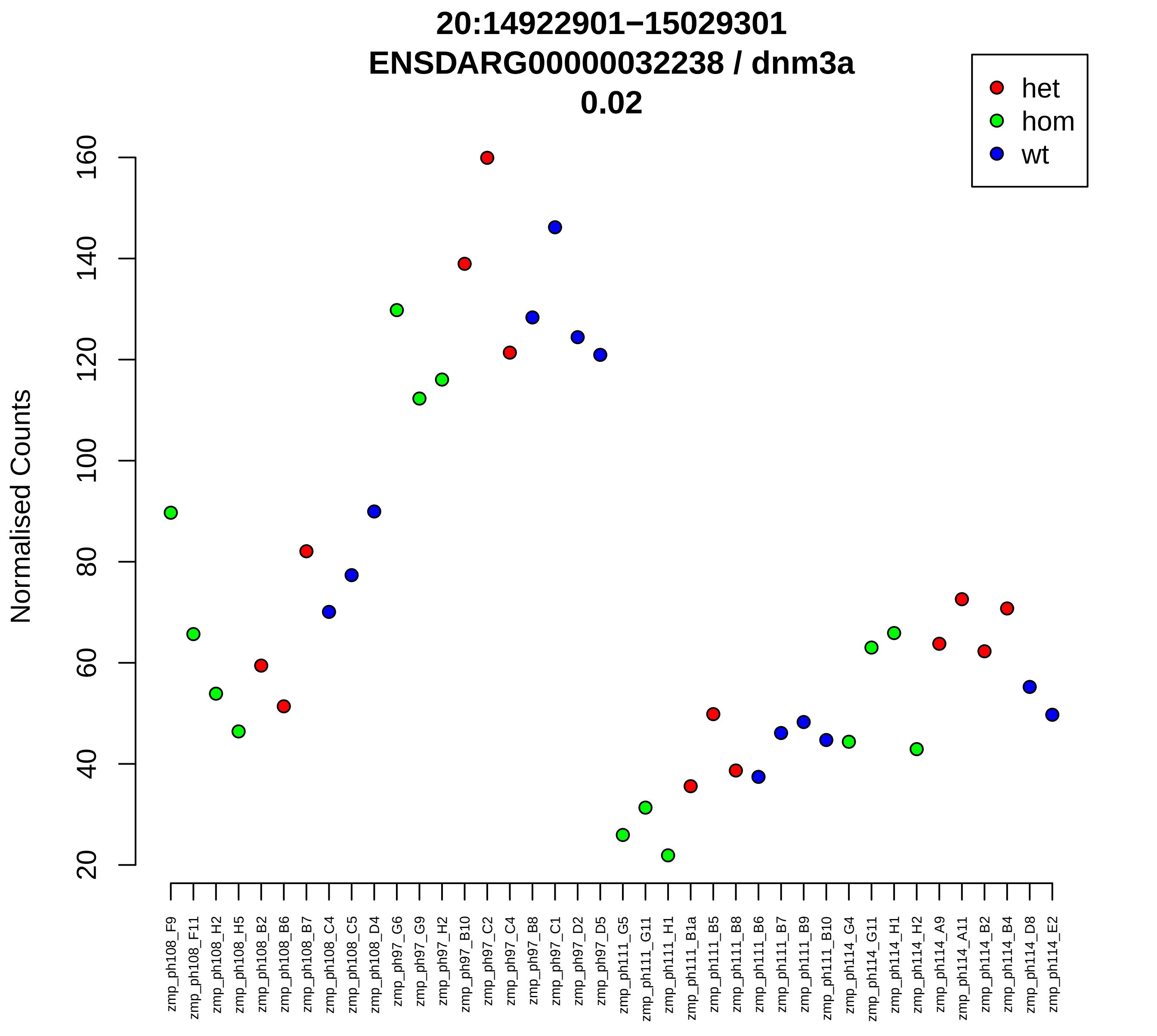
20:14922901−15029301
ENSDARG00000032238 / dnm3a
0.02
het
hom
wt
Normalised Counts
20
40
60
80
100
120
140
160
zmp_ph108_F9
zmp_ph108_F11
zmp_ph108_H2
zmp_ph108_H5
zmp_ph108_B2
zmp_ph108_B6
zmp_ph108_B7
zmp_ph108_C4
zmp_ph108_C5
zmp_ph108_D4
zmp_ph97_G6
zmp_ph97_G9
zmp_ph97_H2
zmp_ph97_B10
zmp_ph97_C2
zmp_ph97_C4
zmp_ph97_B8
zmp_ph97_C1
zmp_ph97_D2
zmp_ph97_D5
zmp_ph111_G5
zmp_ph111_G11
zmp_ph111_H1
zmp_ph111_B1a
zmp_ph111_B5
zmp_ph111_B8
zmp_ph111_B6
zmp_ph111_B7
zmp_ph111_B9
zmp_ph111_B10
zmp_ph114_G4
zmp_ph114_G11
zmp_ph114_H1
zmp_ph114_H2
zmp_ph114_A9
zmp_ph114_A11
zmp_ph114_B2
zmp_ph114_B4
zmp_ph114_D8
zmp_ph114_E2

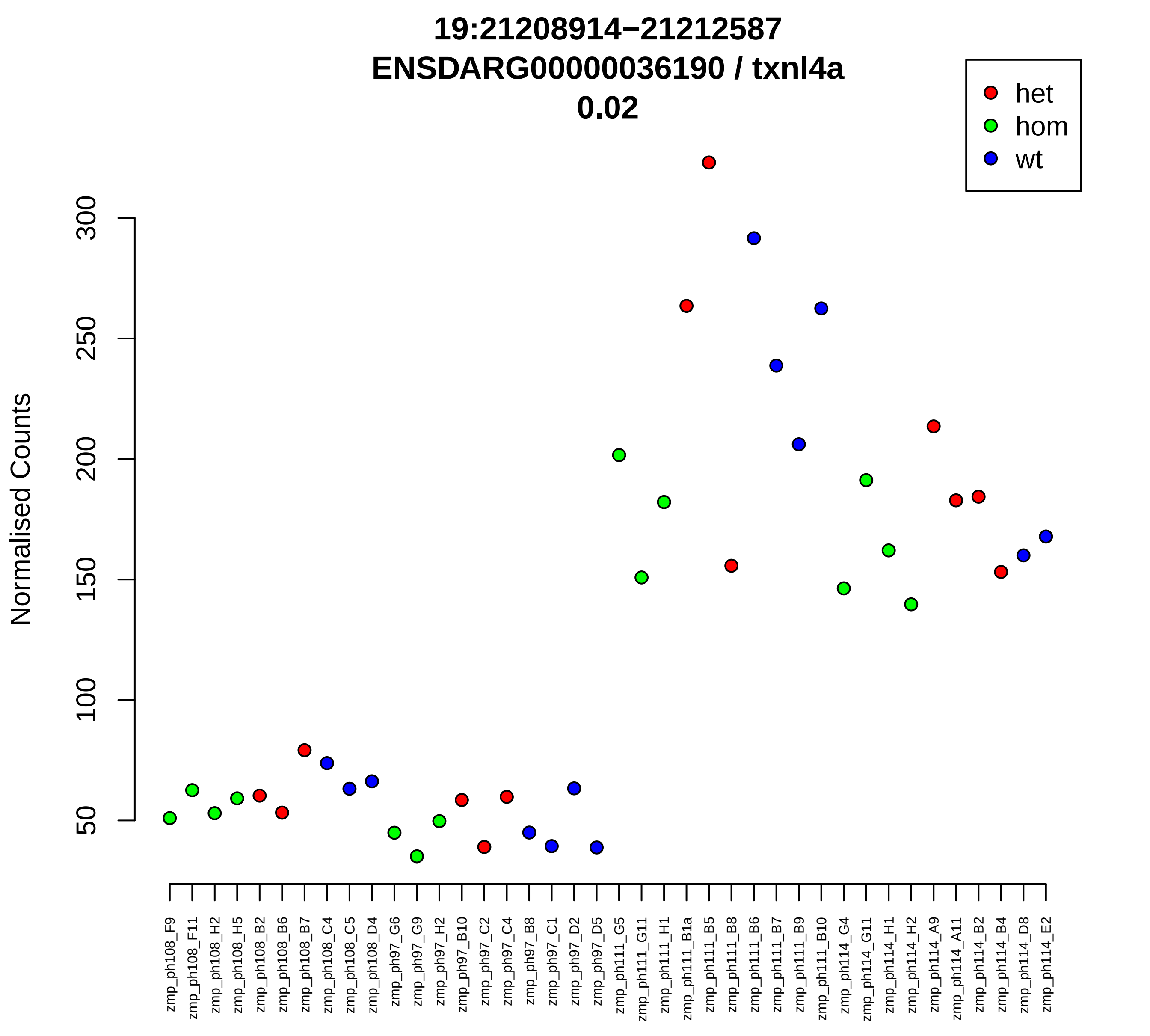

19:21208914−21212587
ENSDARG00000036190 / txnl4a
0.02
het
hom
wt
Normalised Counts
50
100
150
200
250
300
zmp_ph108_F9
zmp_ph108_F11
zmp_ph108_H2
zmp_ph108_H5
zmp_ph108_B2
zmp_ph108_B6
zmp_ph108_B7
zmp_ph108_C4
zmp_ph108_C5
zmp_ph108_D4
zmp_ph97_G6
zmp_ph97_G9
zmp_ph97_H2
zmp_ph97_B10
zmp_ph97_C2
zmp_ph97_C4
zmp_ph97_B8
zmp_ph97_C1
zmp_ph97_D2
zmp_ph97_D5
zmp_ph111_G5
zmp_ph111_G11
zmp_ph111_H1
zmp_ph111_B1a
zmp_ph111_B5
zmp_ph111_B8
zmp_ph111_B6
zmp_ph111_B7
zmp_ph111_B9
zmp_ph111_B10
zmp_ph114_G4
zmp_ph114_G11
zmp_ph114_H1
zmp_ph114_H2
zmp_ph114_A9
zmp_ph114_A11
zmp_ph114_B2
zmp_ph114_B4
zmp_ph114_D8
zmp_ph114_E2

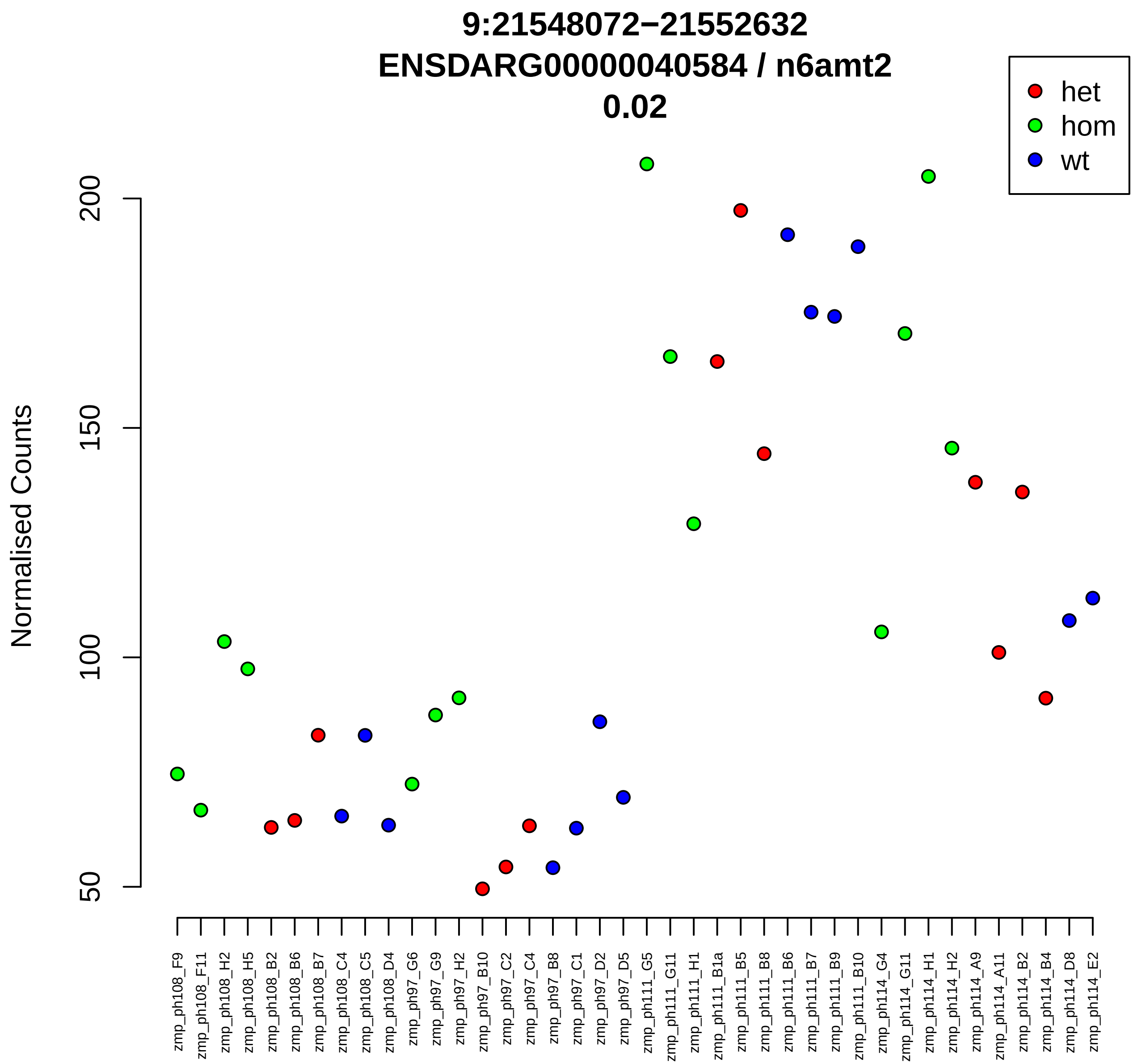
9:21548072−21552632
ENSDARG00000040584 / n6amt2
0.02
het
hom
wt
Normalised Counts
50
100
150
200
zmp_ph108_F9
zmp_ph108_F11
zmp_ph108_H2
zmp_ph108_H5
zmp_ph108_B2
zmp_ph108_B6
zmp_ph108_B7
zmp_ph108_C4
zmp_ph108_C5
zmp_ph108_D4
zmp_ph97_G6
zmp_ph97_G9
zmp_ph97_H2
zmp_ph97_B10
zmp_ph97_C2
zmp_ph97_C4
zmp_ph97_B8
zmp_ph97_C1
zmp_ph97_D2
zmp_ph97_D5
zmp_ph111_G5
zmp_ph111_G11
zmp_ph111_H1
zmp_ph111_B1a
zmp_ph111_B5
zmp_ph111_B8
zmp_ph111_B6
zmp_ph111_B7
zmp_ph111_B9
zmp_ph111_B10
zmp_ph114_G4
zmp_ph114_G11
zmp_ph114_H1
zmp_ph114_H2
zmp_ph114_A9
zmp_ph114_A11
zmp_ph114_B2
zmp_ph114_B4
zmp_ph114_D8
zmp_ph114_E2

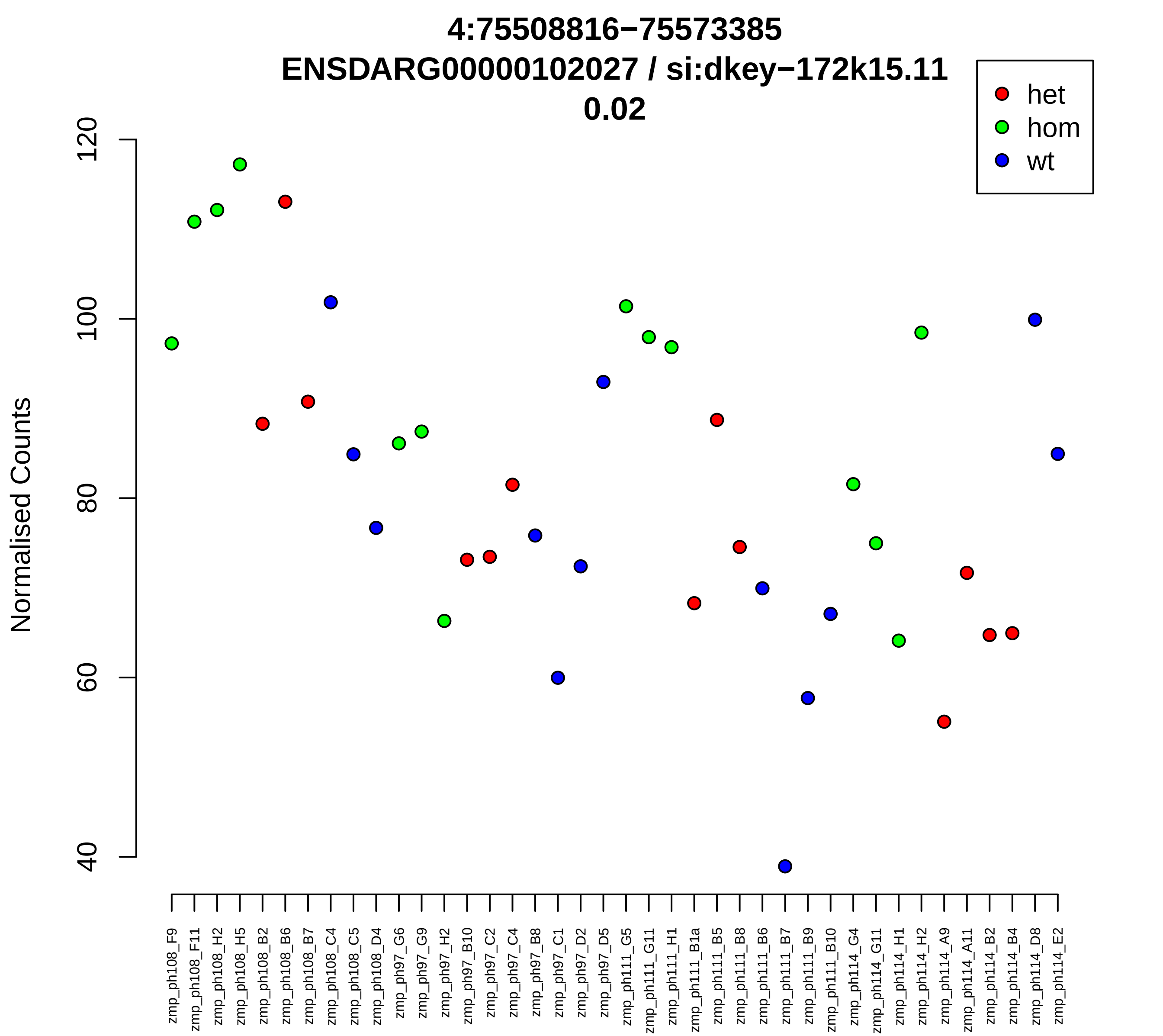
4:75508816−75573385
ENSDARG00000102027 / si:dkey−172k15.11
0.02
het
hom
wt
Normalised Counts
40
60
80
100
120
zmp_ph108_F9
zmp_ph108_F11
zmp_ph108_H2
zmp_ph108_H5
zmp_ph108_B2
zmp_ph108_B6
zmp_ph108_B7
zmp_ph108_C4
zmp_ph108_C5
zmp_ph108_D4
zmp_ph97_G6
zmp_ph97_G9
zmp_ph97_H2
zmp_ph97_B10
zmp_ph97_C2
zmp_ph97_C4
zmp_ph97_B8
zmp_ph97_C1
zmp_ph97_D2
zmp_ph97_D5
zmp_ph111_G5
zmp_ph111_G11
zmp_ph111_H1
zmp_ph111_B1a
zmp_ph111_B5
zmp_ph111_B8
zmp_ph111_B6
zmp_ph111_B7
zmp_ph111_B9
zmp_ph111_B10
zmp_ph114_G4
zmp_ph114_G11
zmp_ph114_H1
zmp_ph114_H2
zmp_ph114_A9
zmp_ph114_A11
zmp_ph114_B2
zmp_ph114_B4
zmp_ph114_D8
zmp_ph114_E2

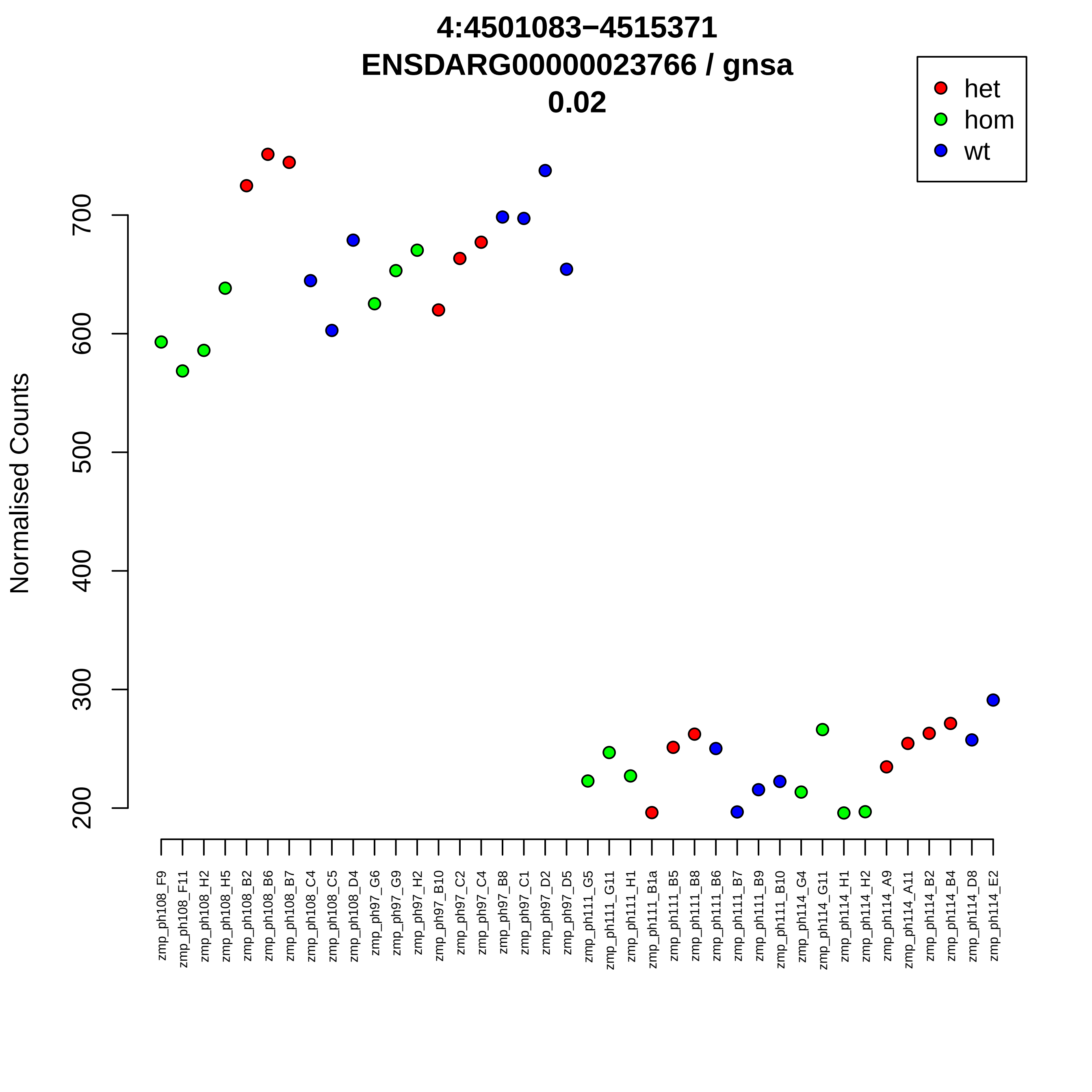
4:4501083−4515371
ENSDARG00000023766 / gnsa
0.02
het
hom
wt
Normalised Counts
200
300
400
500
600
700
zmp_ph108_F9
zmp_ph108_F11
zmp_ph108_H2
zmp_ph108_H5
zmp_ph108_B2
zmp_ph108_B6
zmp_ph108_B7
zmp_ph108_C4
zmp_ph108_C5
zmp_ph108_D4
zmp_ph97_G6
zmp_ph97_G9
zmp_ph97_H2
zmp_ph97_B10
zmp_ph97_C2
zmp_ph97_C4
zmp_ph97_B8
zmp_ph97_C1
zmp_ph97_D2
zmp_ph97_D5
zmp_ph111_G5
zmp_ph111_G11
zmp_ph111_H1
zmp_ph111_B1a
zmp_ph111_B5
zmp_ph111_B8
zmp_ph111_B6
zmp_ph111_B7
zmp_ph111_B9
zmp_ph111_B10
zmp_ph114_G4
zmp_ph114_G11
zmp_ph114_H1
zmp_ph114_H2
zmp_ph114_A9
zmp_ph114_A11
zmp_ph114_B2
zmp_ph114_B4
zmp_ph114_D8
zmp_ph114_E2

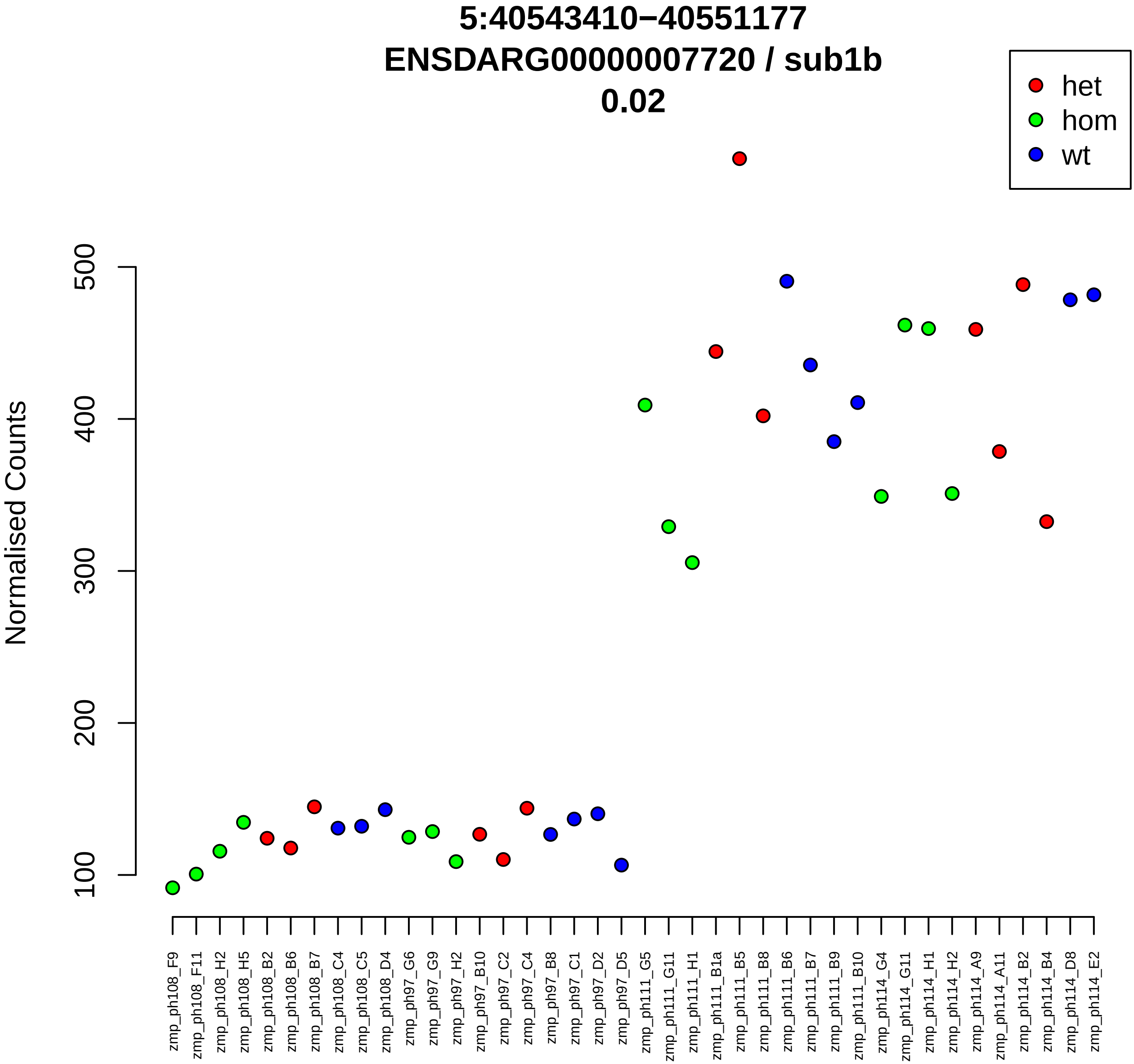
5:40543410−40551177
ENSDARG00000007720 / sub1b
0.02
het
hom
wt
Normalised Counts
100
200
300
400
500
zmp_ph108_F9
zmp_ph108_F11
zmp_ph108_H2
zmp_ph108_H5
zmp_ph108_B2
zmp_ph108_B6
zmp_ph108_B7
zmp_ph108_C4
zmp_ph108_C5
zmp_ph108_D4
zmp_ph97_G6
zmp_ph97_G9
zmp_ph97_H2
zmp_ph97_B10
zmp_ph97_C2
zmp_ph97_C4
zmp_ph97_B8
zmp_ph97_C1
zmp_ph97_D2
zmp_ph97_D5
zmp_ph111_G5
zmp_ph111_G11
zmp_ph111_H1
zmp_ph111_B1a
zmp_ph111_B5
zmp_ph111_B8
zmp_ph111_B6
zmp_ph111_B7
zmp_ph111_B9
zmp_ph111_B10
zmp_ph114_G4
zmp_ph114_G11
zmp_ph114_H1
zmp_ph114_H2
zmp_ph114_A9
zmp_ph114_A11
zmp_ph114_B2
zmp_ph114_B4
zmp_ph114_D8
zmp_ph114_E2

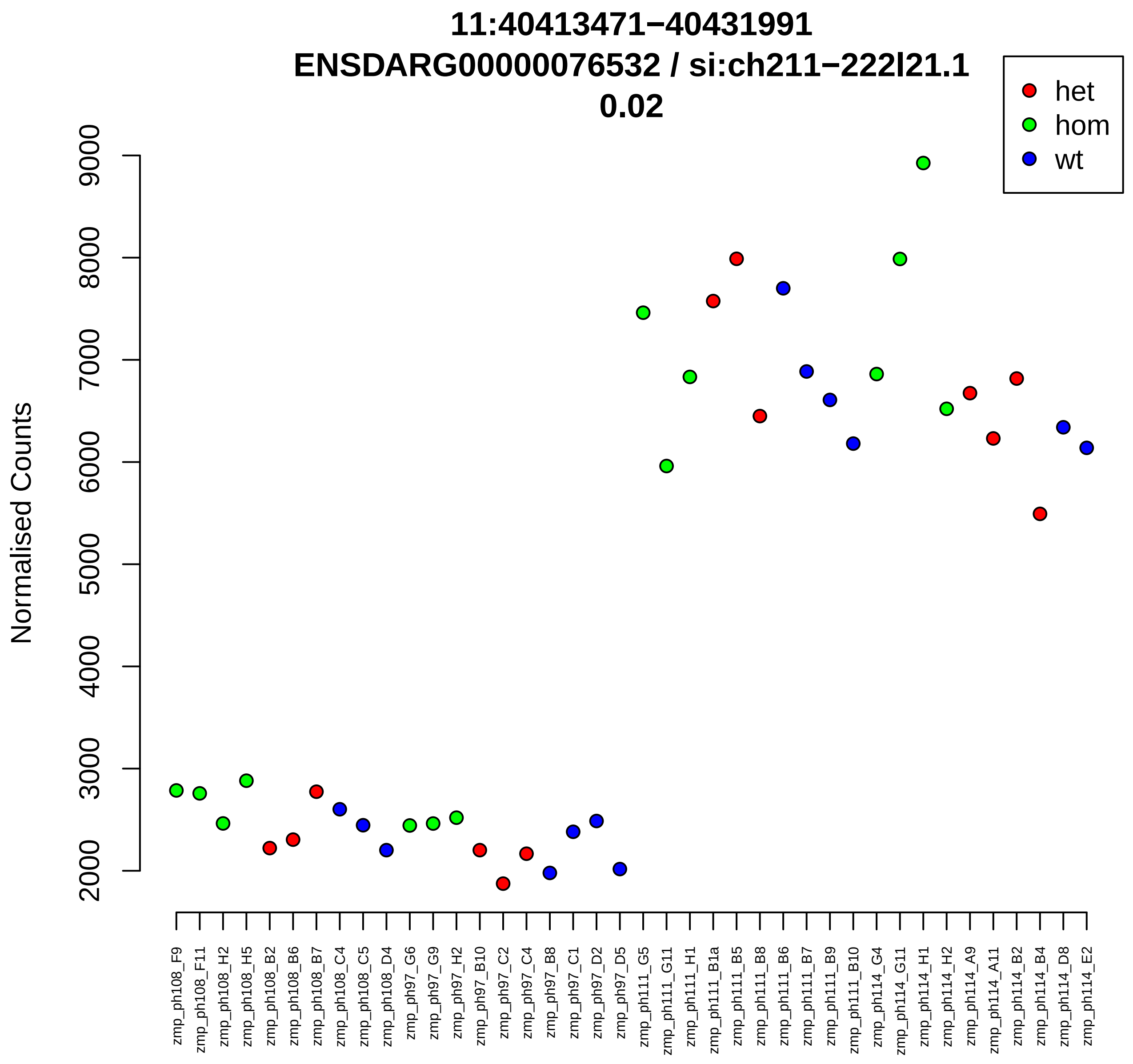
11:40413471−40431991
ENSDARG00000076532 / si:ch211−222l21.1
0.02
Normalised Counts
2000
3000
4000
5000
6000
7000
8000
9000
het
hom
wt
zmp_ph108_F9
zmp_ph108_F11
zmp_ph108_H2
zmp_ph108_H5
zmp_ph108_B2
zmp_ph108_B6
zmp_ph108_B7
zmp_ph108_C4
zmp_ph108_C5
zmp_ph108_D4
zmp_ph97_G6
zmp_ph97_G9
zmp_ph97_H2
zmp_ph97_B10
zmp_ph97_C2
zmp_ph97_C4
zmp_ph97_B8
zmp_ph97_C1
zmp_ph97_D2
zmp_ph97_D5
zmp_ph111_G5
zmp_ph111_G11
zmp_ph111_H1
zmp_ph111_B1a
zmp_ph111_B5
zmp_ph111_B8
zmp_ph111_B6
zmp_ph111_B7
zmp_ph111_B9
zmp_ph111_B10
zmp_ph114_G4
zmp_ph114_G11
zmp_ph114_H1
zmp_ph114_H2
zmp_ph114_A9
zmp_ph114_A11
zmp_ph114_B2
zmp_ph114_B4
zmp_ph114_D8
zmp_ph114_E2

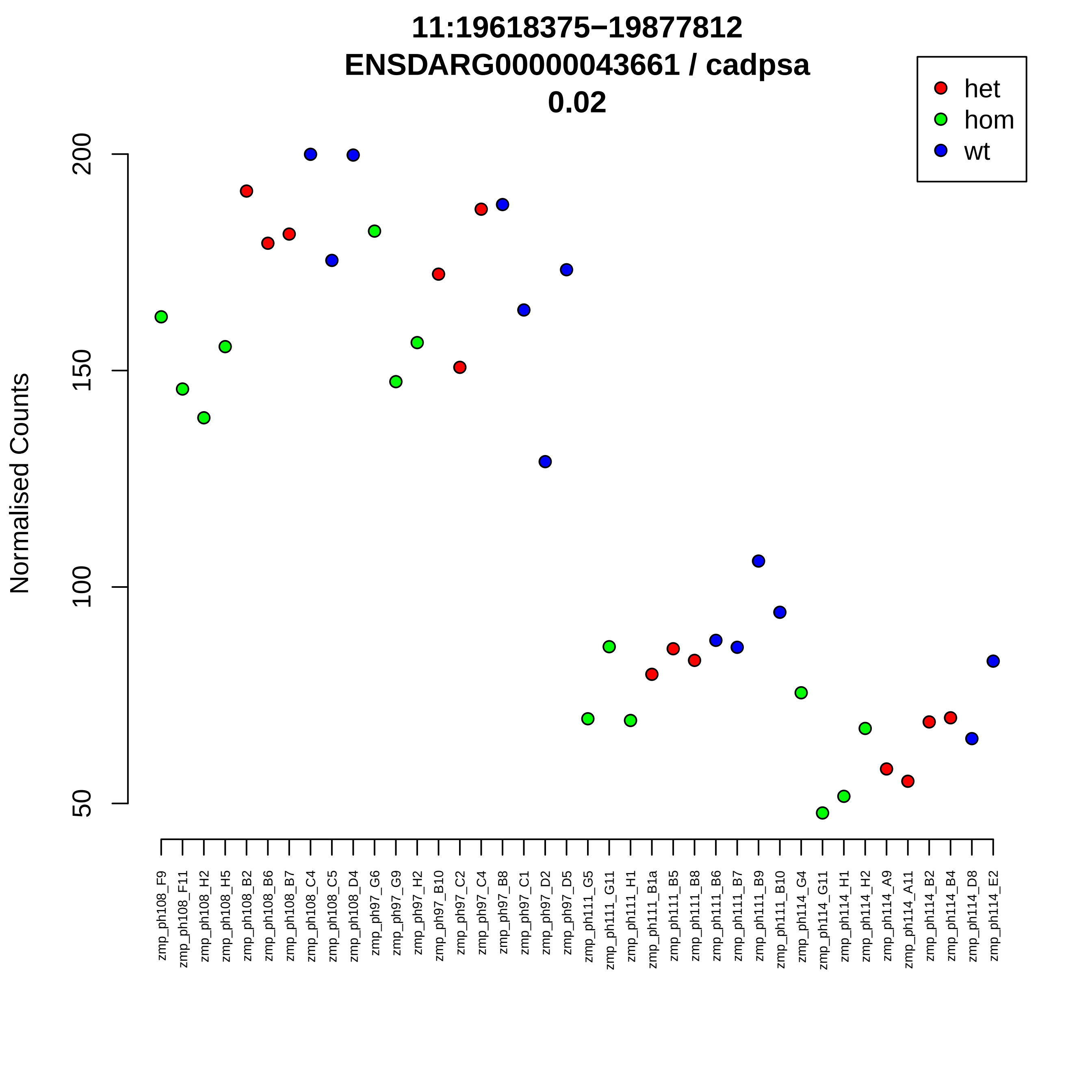

11:19618375−19877812
ENSDARG00000043661 / cadpsa
0.02
het
hom
wt
Normalised Counts
200
150
100
50
zmp_ph108_F9
zmp_ph108_F11
zmp_ph108_H2
zmp_ph108_H5
zmp_ph108_B2
zmp_ph108_B6
zmp_ph108_B7
zmp_ph108_C4
zmp_ph108_C5
zmp_ph108_D4
zmp_ph97_G6
zmp_ph97_G9
zmp_ph97_H2
zmp_ph97_B10
zmp_ph97_C2
zmp_ph97_C4
zmp_ph97_B8
zmp_ph97_C1
zmp_ph97_D2
zmp_ph97_D5
zmp_ph111_G5
zmp_ph111_G11
zmp_ph111_H1
zmp_ph111_B1a
zmp_ph111_B5
zmp_ph111_B8
zmp_ph111_B6
zmp_ph111_B7
zmp_ph111_B9
zmp_ph111_B10
zmp_ph114_G4
zmp_ph114_G11
zmp_ph114_H1
zmp_ph114_H2
zmp_ph114_A9
zmp_ph114_A11
zmp_ph114_B2
zmp_ph114_B4
zmp_ph114_D8
zmp_ph114_E2

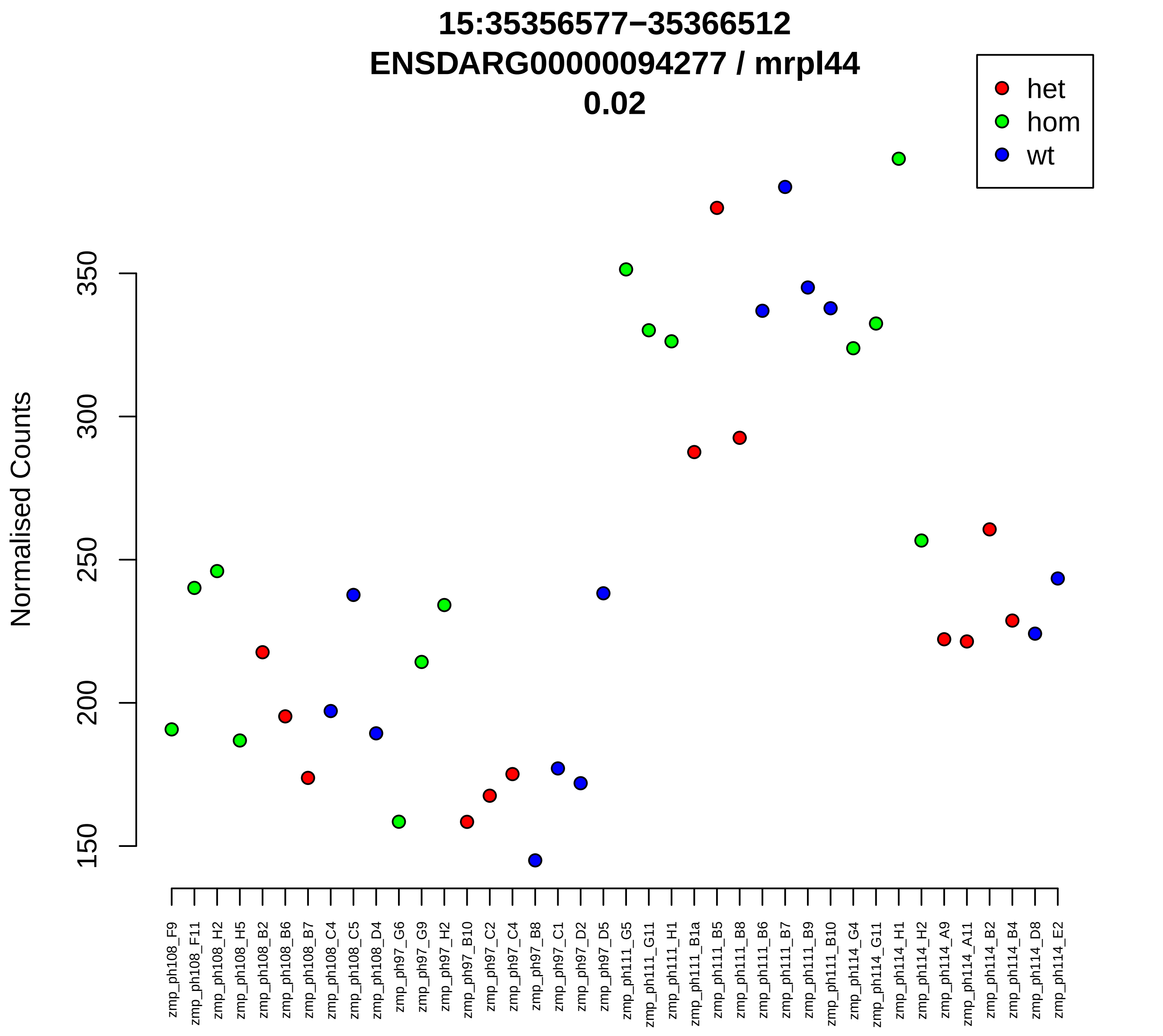
15:35356577−35366512
ENSDARG00000094277 / mrpl44
0.02
het
hom
wt
Normalised Counts
150
200
250
300
350
zmp_ph108_F9
zmp_ph108_F11
zmp_ph108_H2
zmp_ph108_H5
zmp_ph108_B2
zmp_ph108_B6
zmp_ph108_B7
zmp_ph108_C4
zmp_ph108_C5
zmp_ph108_D4
zmp_ph97_G6
zmp_ph97_G9
zmp_ph97_H2
zmp_ph97_B10
zmp_ph97_C2
zmp_ph97_C4
zmp_ph97_B8
zmp_ph97_C1
zmp_ph97_D2
zmp_ph97_D5
zmp_ph111_G5
zmp_ph111_G11
zmp_ph111_H1
zmp_ph111_B1a
zmp_ph111_B5
zmp_ph111_B8
zmp_ph111_B6
zmp_ph111_B7
zmp_ph111_B9
zmp_ph111_B10
zmp_ph114_G4
zmp_ph114_G11
zmp_ph114_H1
zmp_ph114_H2
zmp_ph114_A9
zmp_ph114_A11
zmp_ph114_B2
zmp_ph114_B4
zmp_ph114_D8
zmp_ph114_E2

24:33895310−33915493
ENSDARG00000056672 / abcb8
0.02

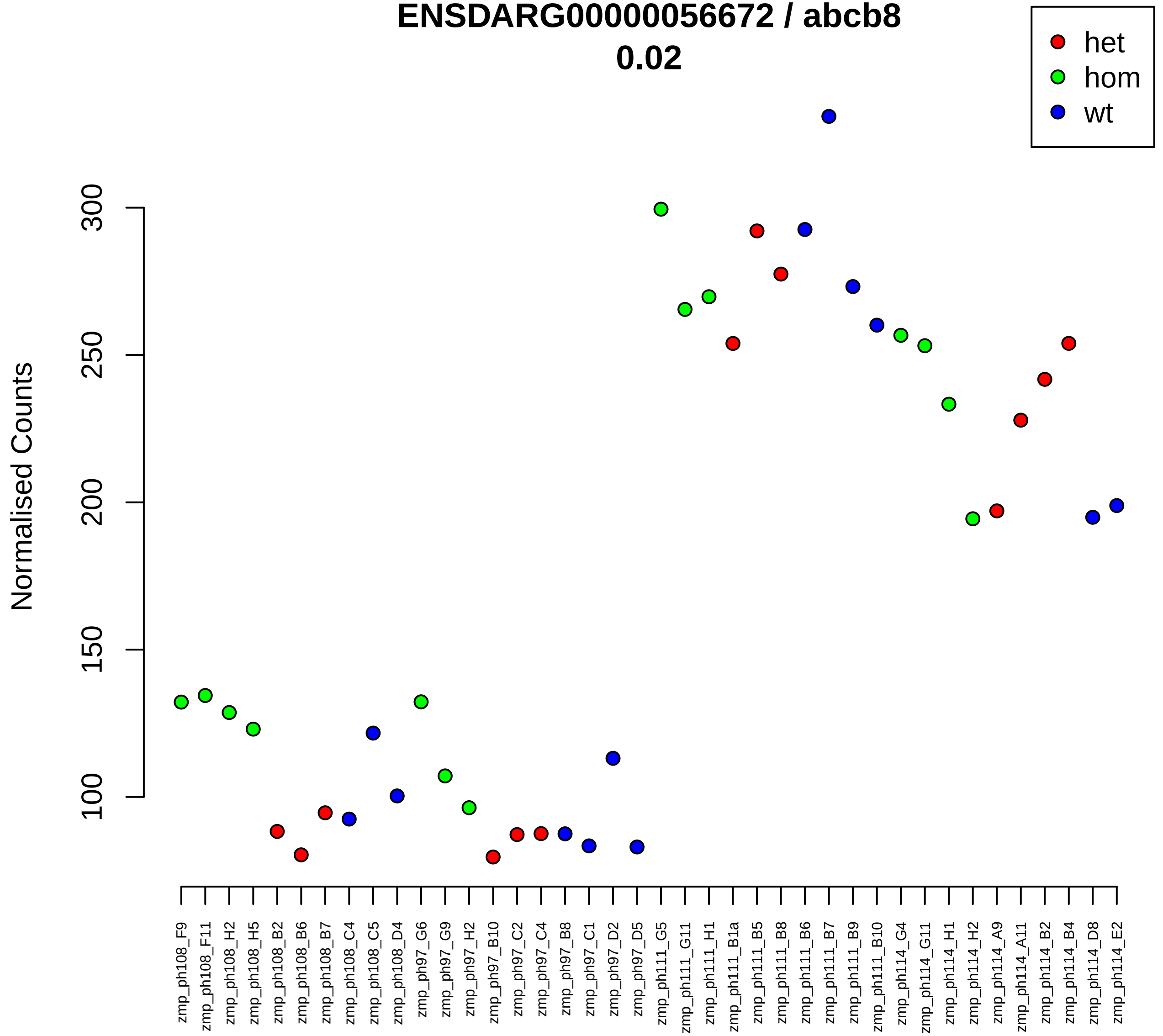

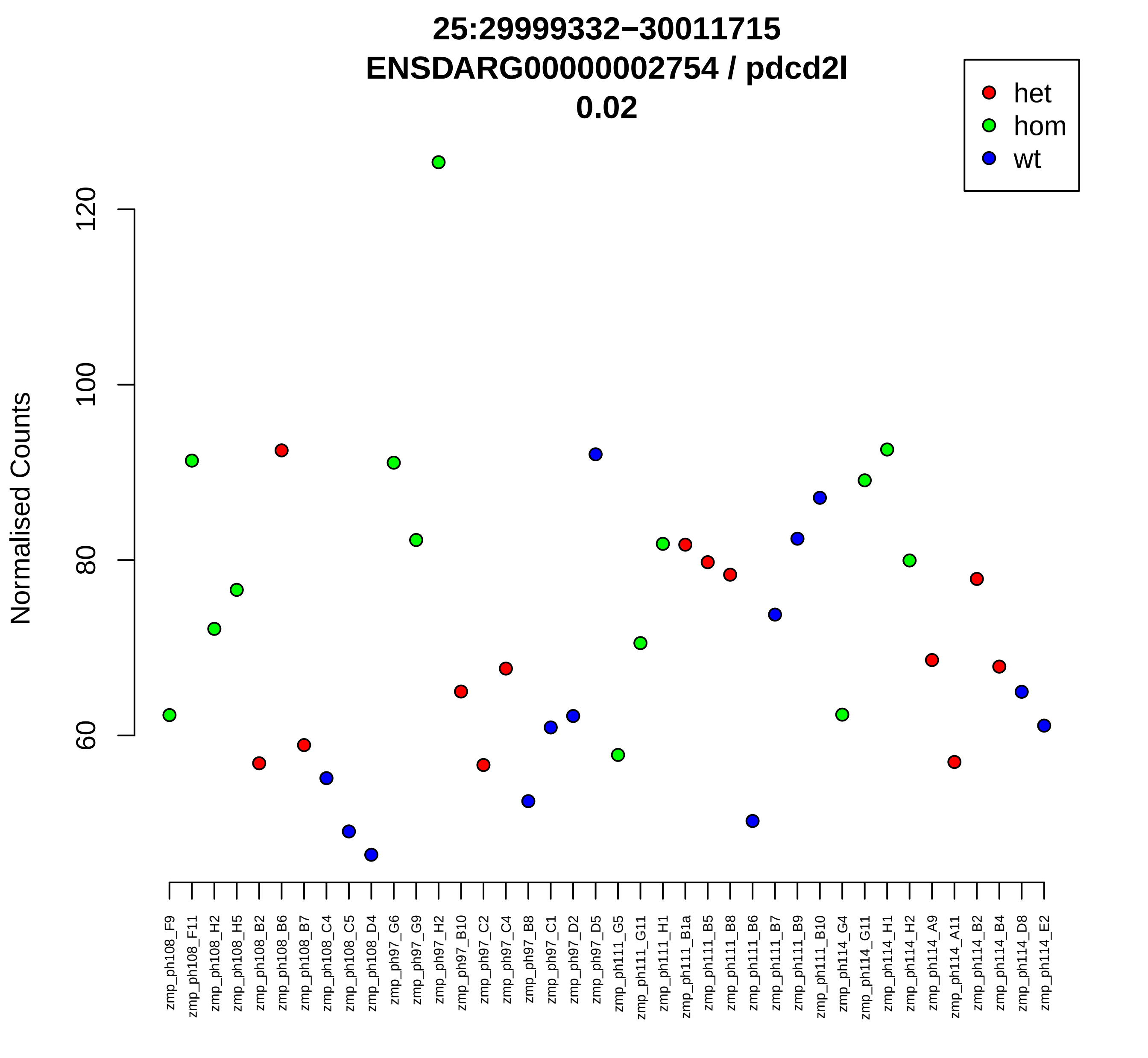
25:29999332−30011715
ENSDARG00000002754 / pdcd2l
0.02
het
hom
wt
Normalised Counts
60
80
100
120
zmp_ph108_F9
zmp_ph108_F11
zmp_ph108_H2
zmp_ph108_H5
zmp_ph108_B2
zmp_ph108_B6
zmp_ph108_B7
zmp_ph108_C4
zmp_ph108_C5
zmp_ph108_D4
zmp_ph97_G6
zmp_ph97_G9
zmp_ph97_H2
zmp_ph97_B10
zmp_ph97_C2
zmp_ph97_C4
zmp_ph97_B8
zmp_ph97_C1
zmp_ph97_D2
zmp_ph97_D5
zmp_ph111_G5
zmp_ph111_G11
zmp_ph111_H1
zmp_ph111_B1a
zmp_ph111_B5
zmp_ph111_B8
zmp_ph111_B6
zmp_ph111_B7
zmp_ph111_B9
zmp_ph111_B10
zmp_ph114_G4
zmp_ph114_G11
zmp_ph114_H1
zmp_ph114_H2
zmp_ph114_A9
zmp_ph114_A11
zmp_ph114_B2
zmp_ph114_B4
zmp_ph114_D8
zmp_ph114_E2

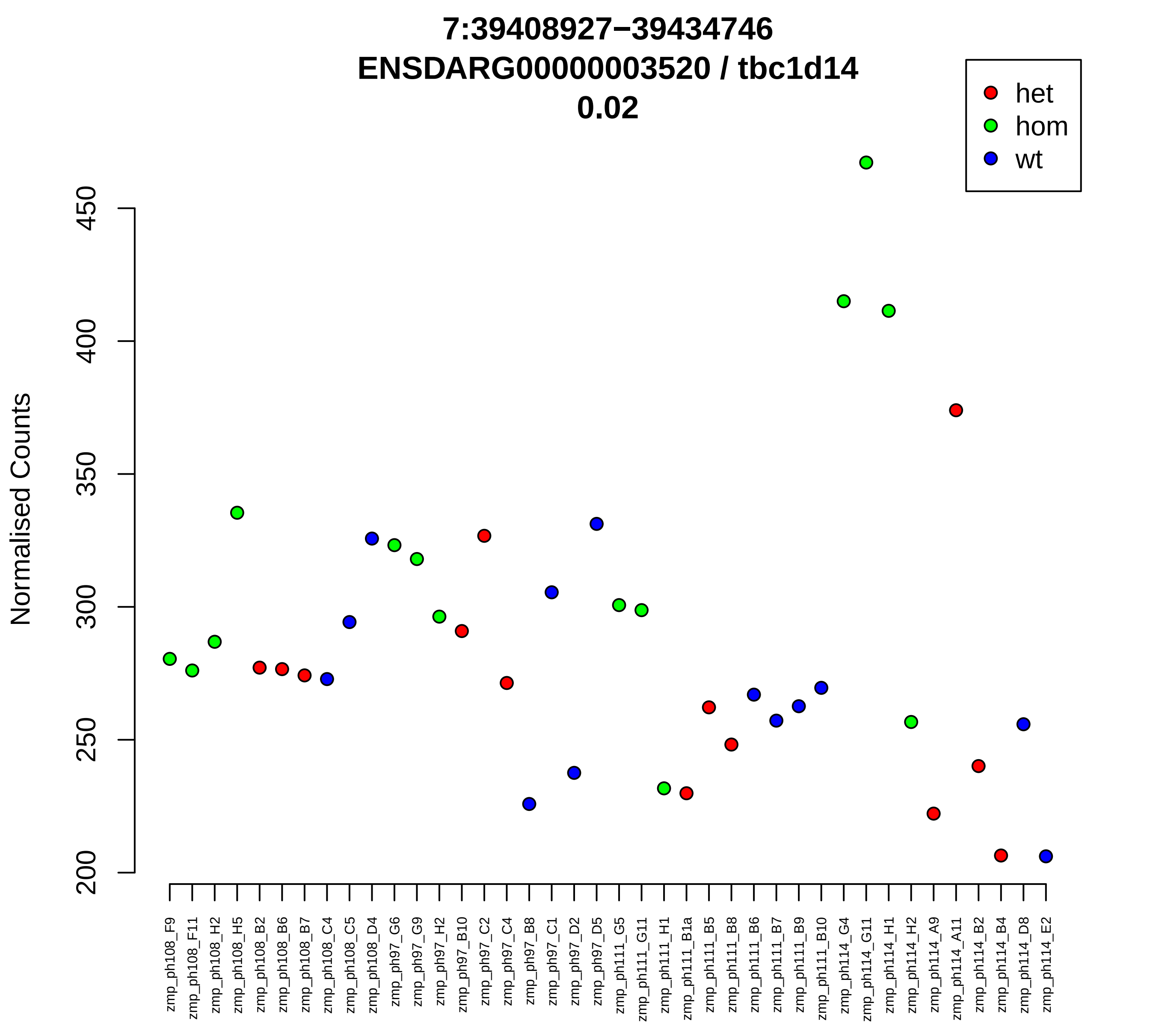
7:39408927−39434746
ENSDARG00000003520 / tbc1d14
0.02
het
hom
wt
Normalised Counts
200
250
300
350
400
450
zmp_ph108_F9
zmp_ph108_F11
zmp_ph108_H2
zmp_ph108_H5
zmp_ph108_B2
zmp_ph108_B6
zmp_ph108_B7
zmp_ph108_C4
zmp_ph108_C5
zmp_ph108_D4
zmp_ph97_G6
zmp_ph97_G9
zmp_ph97_H2
zmp_ph97_B10
zmp_ph97_C2
zmp_ph97_C4
zmp_ph97_B8
zmp_ph97_C1
zmp_ph97_D2
zmp_ph97_D5
zmp_ph111_G5
zmp_ph111_G11
zmp_ph111_H1
zmp_ph111_B1a
zmp_ph111_B5
zmp_ph111_B8
zmp_ph111_B6
zmp_ph111_B7
zmp_ph111_B9
zmp_ph111_B10
zmp_ph114_G4
zmp_ph114_G11
zmp_ph114_H1
zmp_ph114_H2
zmp_ph114_A9
zmp_ph114_A11
zmp_ph114_B2
zmp_ph114_B4
zmp_ph114_D8
zmp_ph114_E2

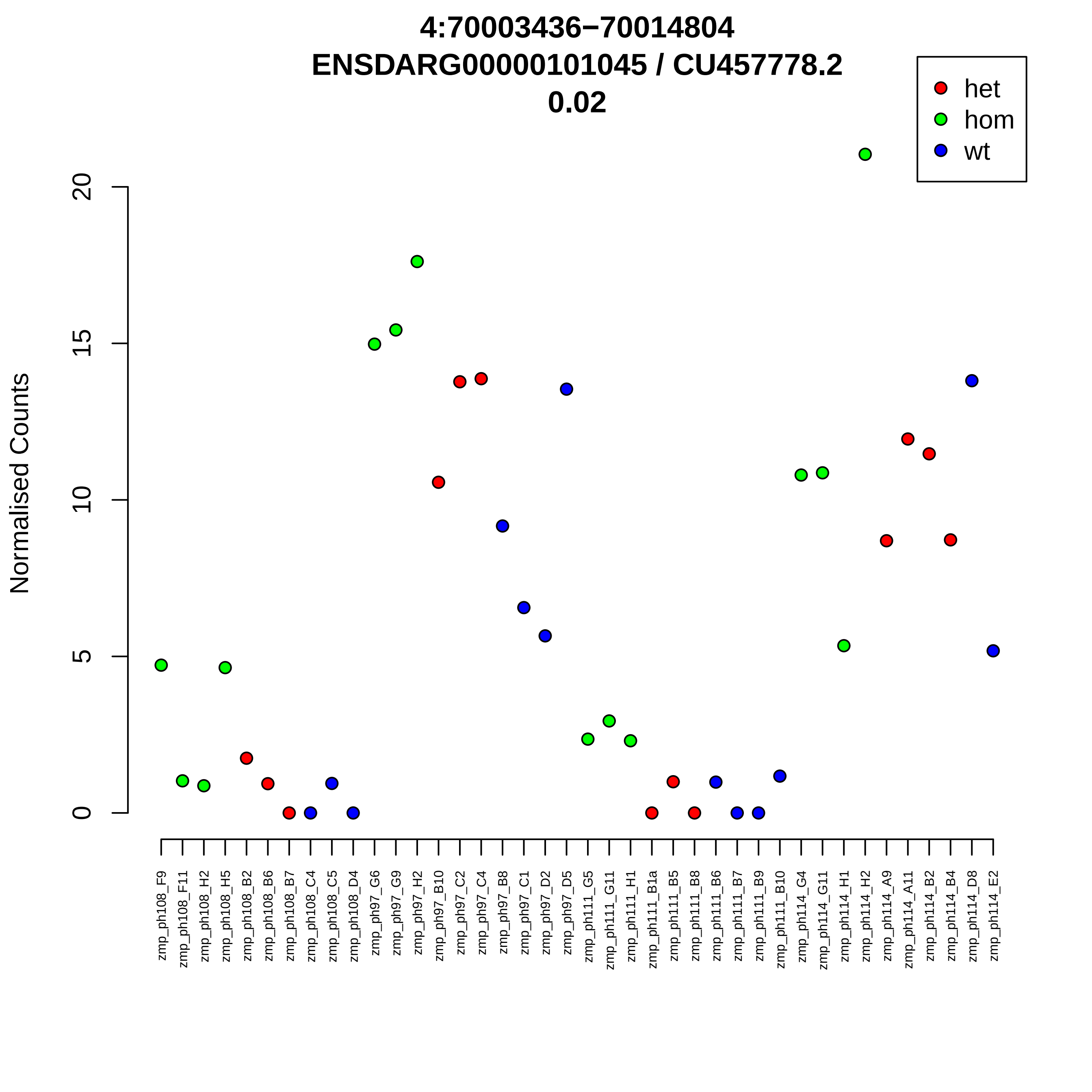
4:70003436−70014804
ENSDARG00000101045 / CU457778.2
0.02
het
hom
wt
Normalised Counts
0
5
10
15
20
zmp_ph108_F9
zmp_ph108_F11
zmp_ph108_H2
zmp_ph108_H5
zmp_ph108_B2
zmp_ph108_B6
zmp_ph108_B7
zmp_ph108_C4
zmp_ph108_C5
zmp_ph108_D4
zmp_ph97_G6
zmp_ph97_G9
zmp_ph97_H2
zmp_ph97_B10
zmp_ph97_C2
zmp_ph97_C4
zmp_ph97_B8
zmp_ph97_C1
zmp_ph97_D2
zmp_ph97_D5
zmp_ph111_G5
zmp_ph111_G11
zmp_ph111_H1
zmp_ph111_B1a
zmp_ph111_B5
zmp_ph111_B8
zmp_ph111_B6
zmp_ph111_B7
zmp_ph111_B9
zmp_ph111_B10
zmp_ph114_G4
zmp_ph114_G11
zmp_ph114_H1
zmp_ph114_H2
zmp_ph114_A9
zmp_ph114_A11
zmp_ph114_B2
zmp_ph114_B4
zmp_ph114_D8
zmp_ph114_E2

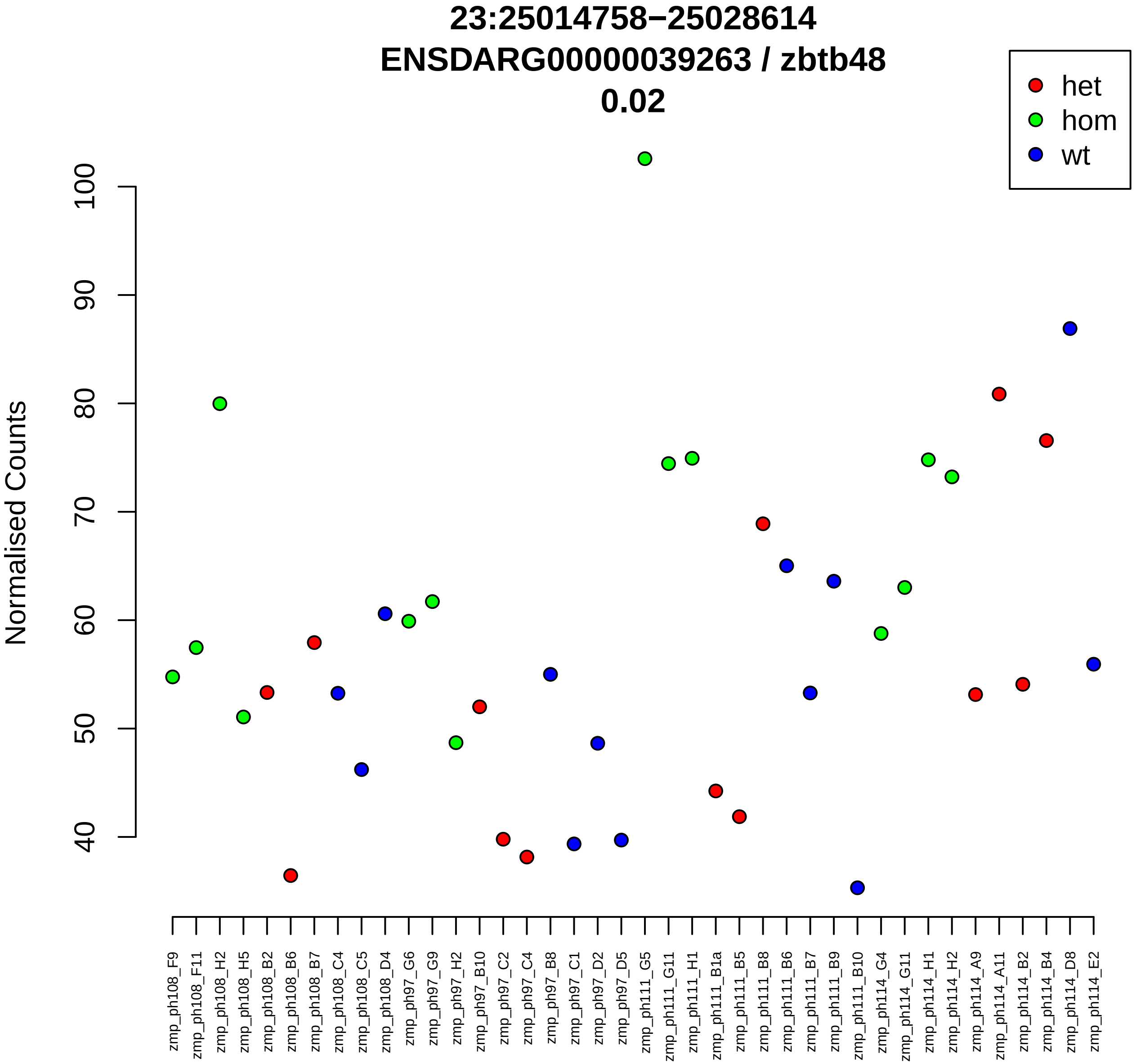
23:25014758−25028614
ENSDARG00000039263 / zbtb48
0.02
het
hom
wt
Normalised Counts
100
90
80
70
60
50
40
zmp_ph108_F9
zmp_ph108_F11
zmp_ph108_H2
zmp_ph108_H5
zmp_ph108_B2
zmp_ph108_B6
zmp_ph108_B7
zmp_ph108_C4
zmp_ph108_C5
zmp_ph108_D4
zmp_ph97_G6
zmp_ph97_G9
zmp_ph97_H2
zmp_ph97_B10
zmp_ph97_C2
zmp_ph97_C4
zmp_ph97_B8
zmp_ph97_C1
zmp_ph97_D2
zmp_ph97_D5
zmp_ph111_G5
zmp_ph111_G11
zmp_ph111_H1
zmp_ph111_B1a
zmp_ph111_B5
zmp_ph111_B8
zmp_ph111_B6
zmp_ph111_B7
zmp_ph111_B9
zmp_ph111_B10
zmp_ph114_G4
zmp_ph114_G11
zmp_ph114_H1
zmp_ph114_H2
zmp_ph114_A9
zmp_ph114_A11
zmp_ph114_B2
zmp_ph114_B4
zmp_ph114_D8
zmp_ph114_E2

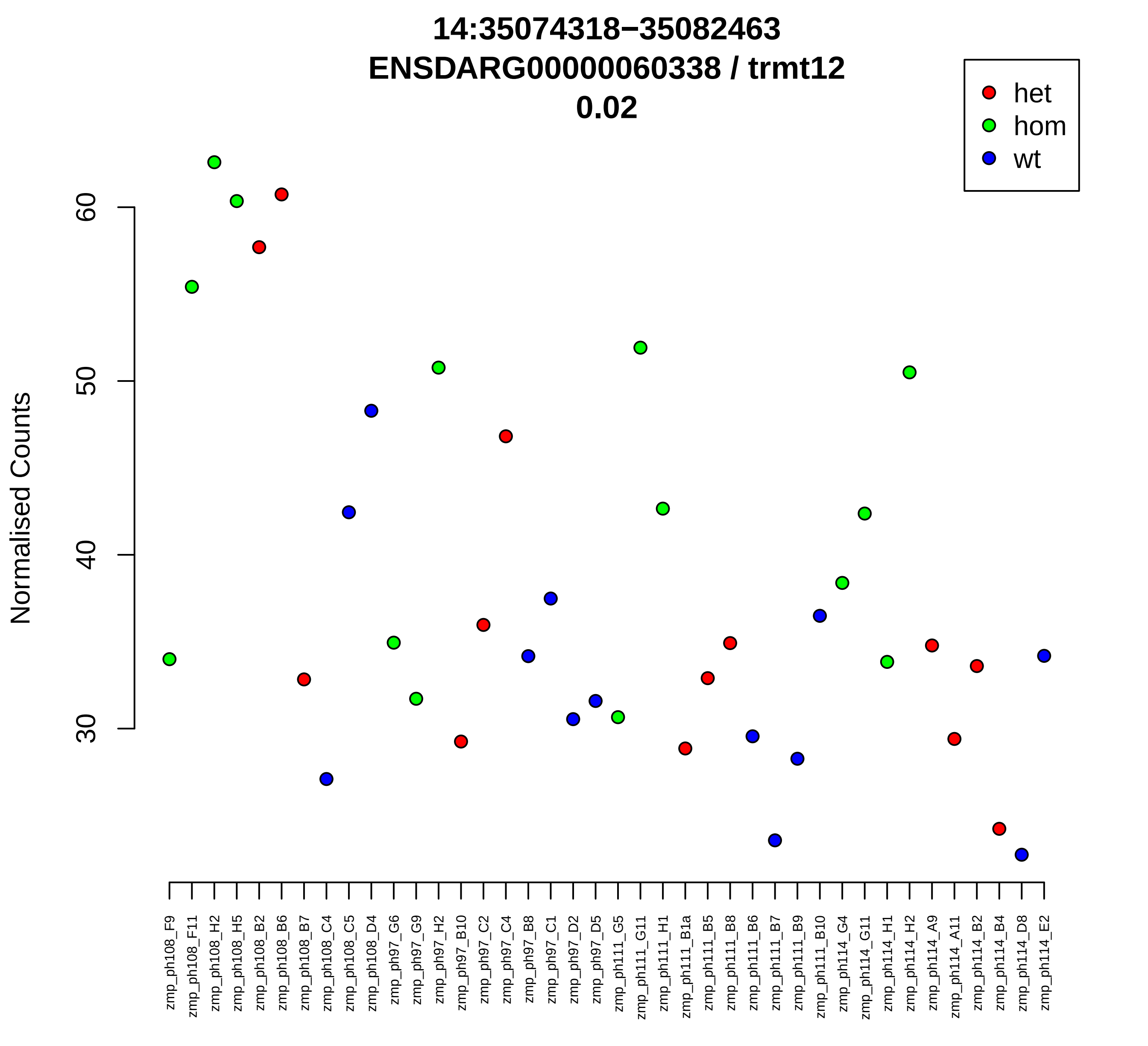
14:35074318−35082463
ENSDARG00000060338 / trmt12
0.02
het
hom
wt
Normalised Counts
30
40
50
60
zmp_ph108_F9
zmp_ph108_F11
zmp_ph108_H2
zmp_ph108_H5
zmp_ph108_B2
zmp_ph108_B6
zmp_ph108_B7
zmp_ph108_C4
zmp_ph108_C5
zmp_ph108_D4
zmp_ph97_G6
zmp_ph97_G9
zmp_ph97_H2
zmp_ph97_B10
zmp_ph97_C2
zmp_ph97_C4
zmp_ph97_B8
zmp_ph97_C1
zmp_ph97_D2
zmp_ph97_D5
zmp_ph111_G5
zmp_ph111_G11
zmp_ph111_H1
zmp_ph111_B1a
zmp_ph111_B5
zmp_ph111_B8
zmp_ph111_B6
zmp_ph111_B7
zmp_ph111_B9
zmp_ph111_B10
zmp_ph114_G4
zmp_ph114_G11
zmp_ph114_H1
zmp_ph114_H2
zmp_ph114_A9
zmp_ph114_A11
zmp_ph114_B2
zmp_ph114_B4
zmp_ph114_D8
zmp_ph114_E2

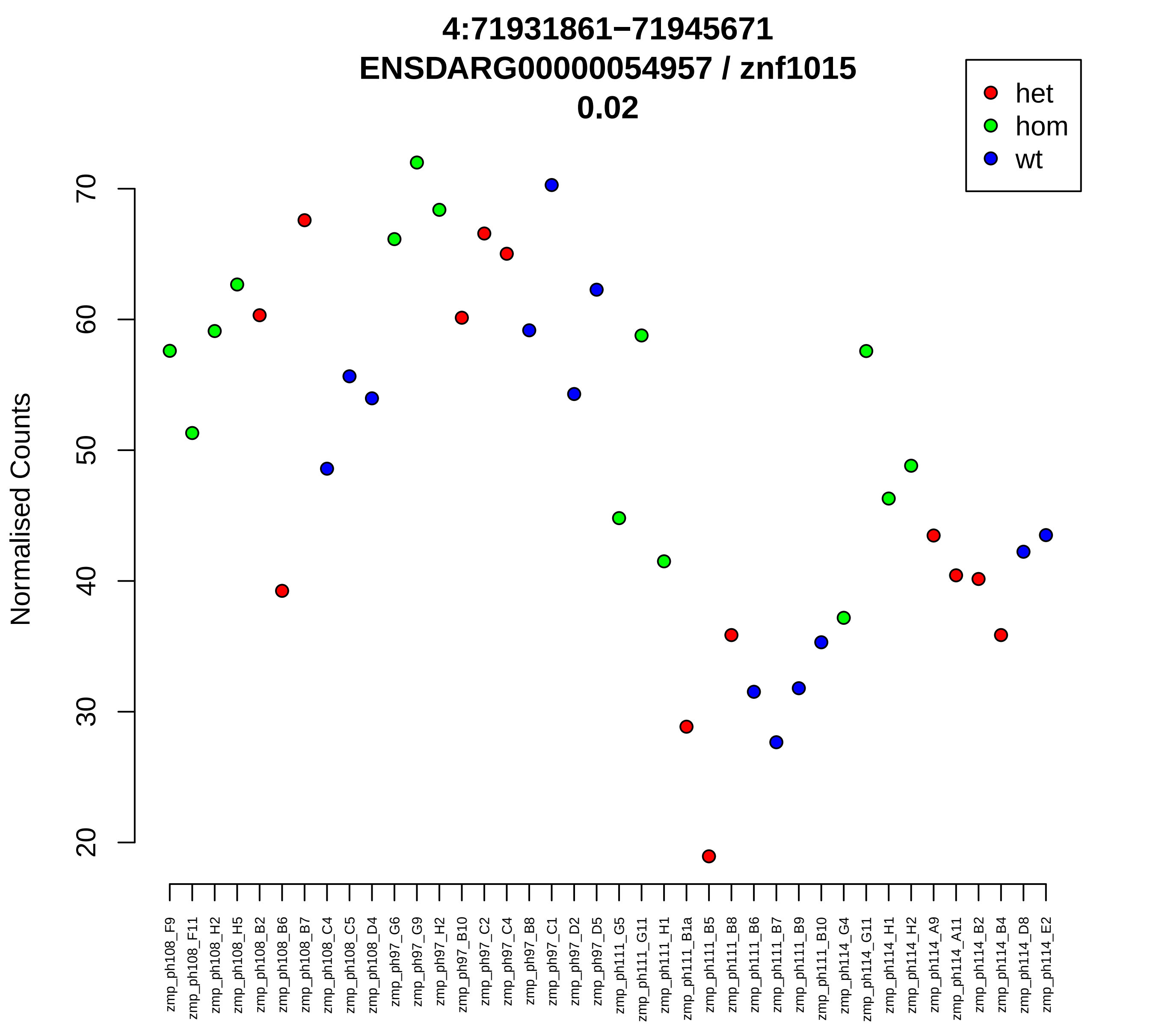
4:71931861−71945671
ENSDARG00000054957 / znf1015
0.02
het
hom
wt
Normalised Counts
20
30
40
50
60
70
zmp_ph108_F9
zmp_ph108_F11
zmp_ph108_H2
zmp_ph108_H5
zmp_ph108_B2
zmp_ph108_B6
zmp_ph108_B7
zmp_ph108_C4
zmp_ph108_C5
zmp_ph108_D4
zmp_ph97_G6
zmp_ph97_G9
zmp_ph97_H2
zmp_ph97_B10
zmp_ph97_C2
zmp_ph97_C4
zmp_ph97_B8
zmp_ph97_C1
zmp_ph97_D2
zmp_ph97_D5
zmp_ph111_G5
zmp_ph111_G11
zmp_ph111_H1
zmp_ph111_B1a
zmp_ph111_B5
zmp_ph111_B8
zmp_ph111_B6
zmp_ph111_B7
zmp_ph111_B9
zmp_ph111_B10
zmp_ph114_G4
zmp_ph114_G11
zmp_ph114_H1
zmp_ph114_H2
zmp_ph114_A9
zmp_ph114_A11
zmp_ph114_B2
zmp_ph114_B4
zmp_ph114_D8
zmp_ph114_E2

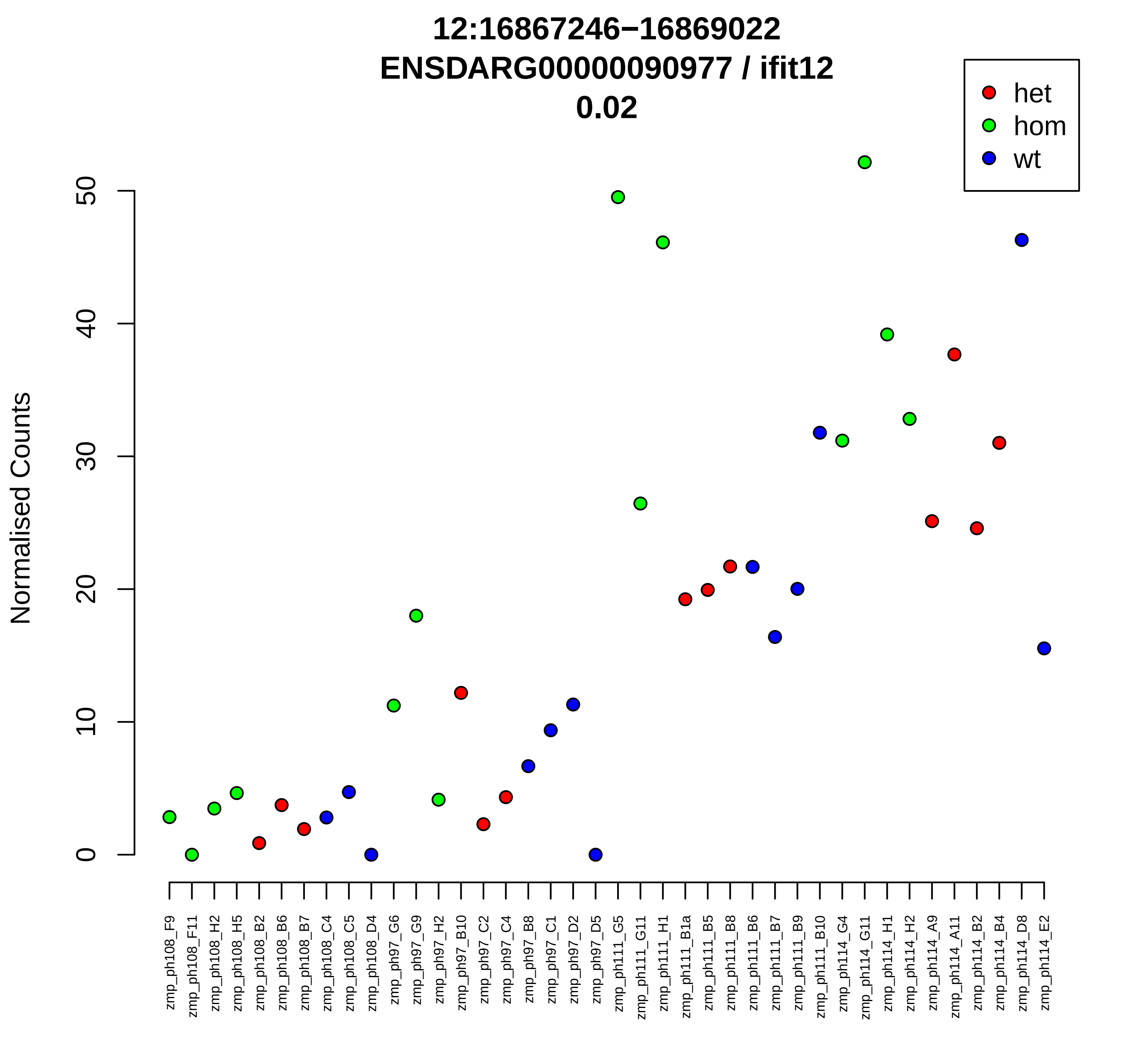

12:16867246−16869022
ENSDARG00000090977 / ifit12
0.02
het
hom
wt
Normalised Counts
0
10
20
30
40
50
zmp_ph108_F9
zmp_ph108_F11
zmp_ph108_H2
zmp_ph108_H5
zmp_ph108_B2
zmp_ph108_B6
zmp_ph108_B7
zmp_ph108_C4
zmp_ph108_C5
zmp_ph108_D4
zmp_ph97_G6
zmp_ph97_G9
zmp_ph97_H2
zmp_ph97_B10
zmp_ph97_C2
zmp_ph97_C4
zmp_ph97_B8
zmp_ph97_C1
zmp_ph97_D2
zmp_ph97_D5
zmp_ph111_G5
zmp_ph111_G11
zmp_ph111_H1
zmp_ph111_B1a
zmp_ph111_B5
zmp_ph111_B8
zmp_ph111_B6
zmp_ph111_B7
zmp_ph111_B9
zmp_ph111_B10
zmp_ph114_G4
zmp_ph114_G11
zmp_ph114_H1
zmp_ph114_H2
zmp_ph114_A9
zmp_ph114_A11
zmp_ph114_B2
zmp_ph114_B4
zmp_ph114_D8
zmp_ph114_E2

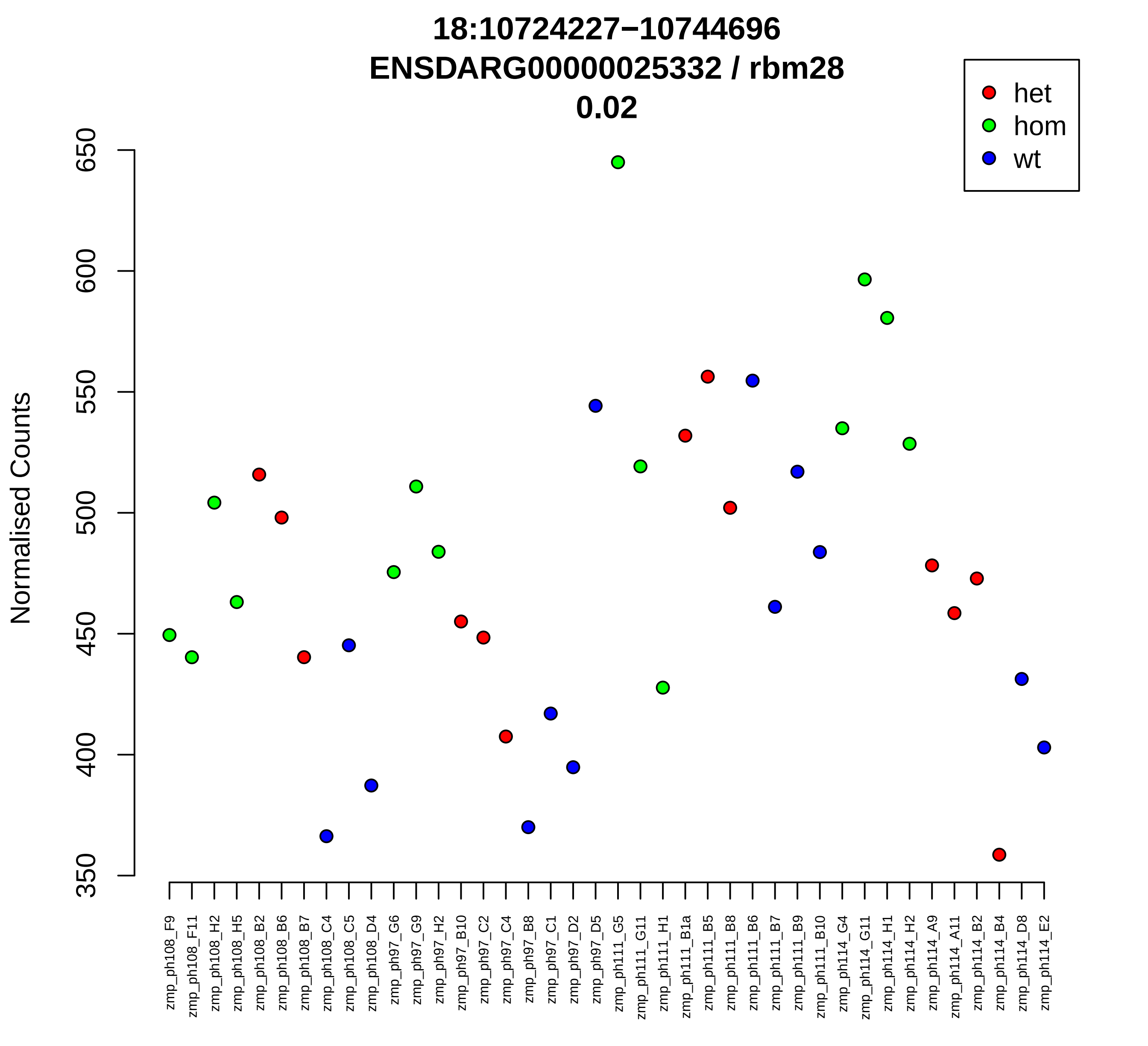

18:10724227−10744696
ENSDARG00000025332 / rbm28
0.02
het
hom
wt
Normalised Counts
350
400
450
500
550
600
650
zmp_ph108_F9
zmp_ph108_F11
zmp_ph108_H2
zmp_ph108_H5
zmp_ph108_B2
zmp_ph108_B6
zmp_ph108_B7
zmp_ph108_C4
zmp_ph108_C5
zmp_ph108_D4
zmp_ph97_G6
zmp_ph97_G9
zmp_ph97_H2
zmp_ph97_B10
zmp_ph97_C2
zmp_ph97_C4
zmp_ph97_B8
zmp_ph97_C1
zmp_ph97_D2
zmp_ph97_D5
zmp_ph111_G5
zmp_ph111_G11
zmp_ph111_H1
zmp_ph111_B1a
zmp_ph111_B5
zmp_ph111_B8
zmp_ph111_B6
zmp_ph111_B7
zmp_ph111_B9
zmp_ph111_B10
zmp_ph114_G4
zmp_ph114_G11
zmp_ph114_H1
zmp_ph114_H2
zmp_ph114_A9
zmp_ph114_A11
zmp_ph114_B2
zmp_ph114_B4
zmp_ph114_D8
zmp_ph114_E2

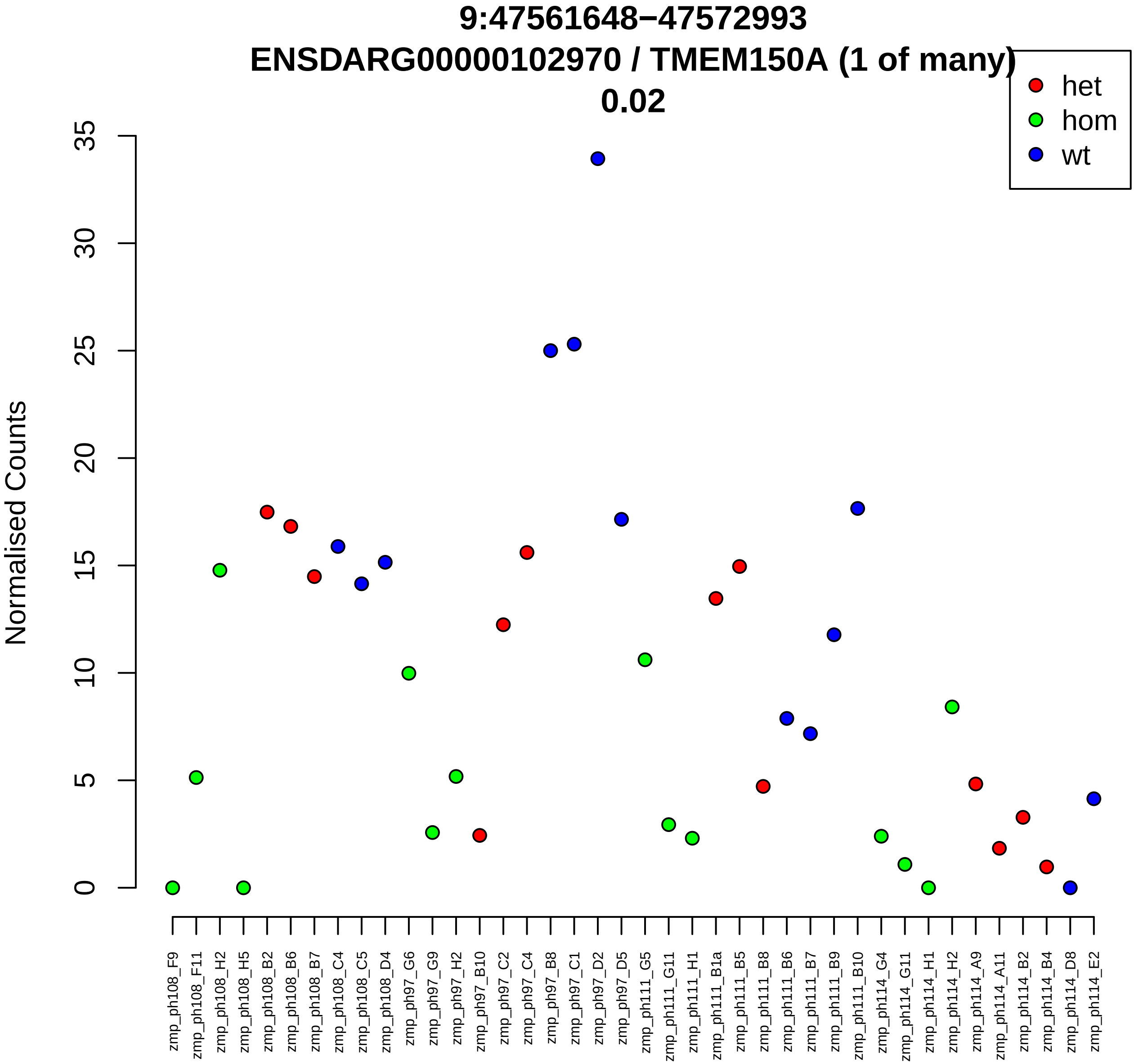

9:47561648−47572993
ENSDARG00000102970 / TMEM150A (1 of many)
0.02
het
hom
wt
Normalised Counts
0
5
10
15
20
25
30
35
zmp_ph108_F9
zmp_ph108_F11
zmp_ph108_H2
zmp_ph108_H5
zmp_ph108_B2
zmp_ph108_B6
zmp_ph108_B7
zmp_ph108_C4
zmp_ph108_C5
zmp_ph108_D4
zmp_ph97_G6
zmp_ph97_G9
zmp_ph97_H2
zmp_ph97_B10
zmp_ph97_C2
zmp_ph97_C4
zmp_ph97_B8
zmp_ph97_C1
zmp_ph97_D2
zmp_ph97_D5
zmp_ph111_G5
zmp_ph111_G11
zmp_ph111_H1
zmp_ph111_B1a
zmp_ph111_B5
zmp_ph111_B8
zmp_ph111_B6
zmp_ph111_B7
zmp_ph111_B9
zmp_ph111_B10
zmp_ph114_G4
zmp_ph114_G11
zmp_ph114_H1
zmp_ph114_H2
zmp_ph114_A9
zmp_ph114_A11
zmp_ph114_B2
zmp_ph114_B4
zmp_ph114_D8
zmp_ph114_E2

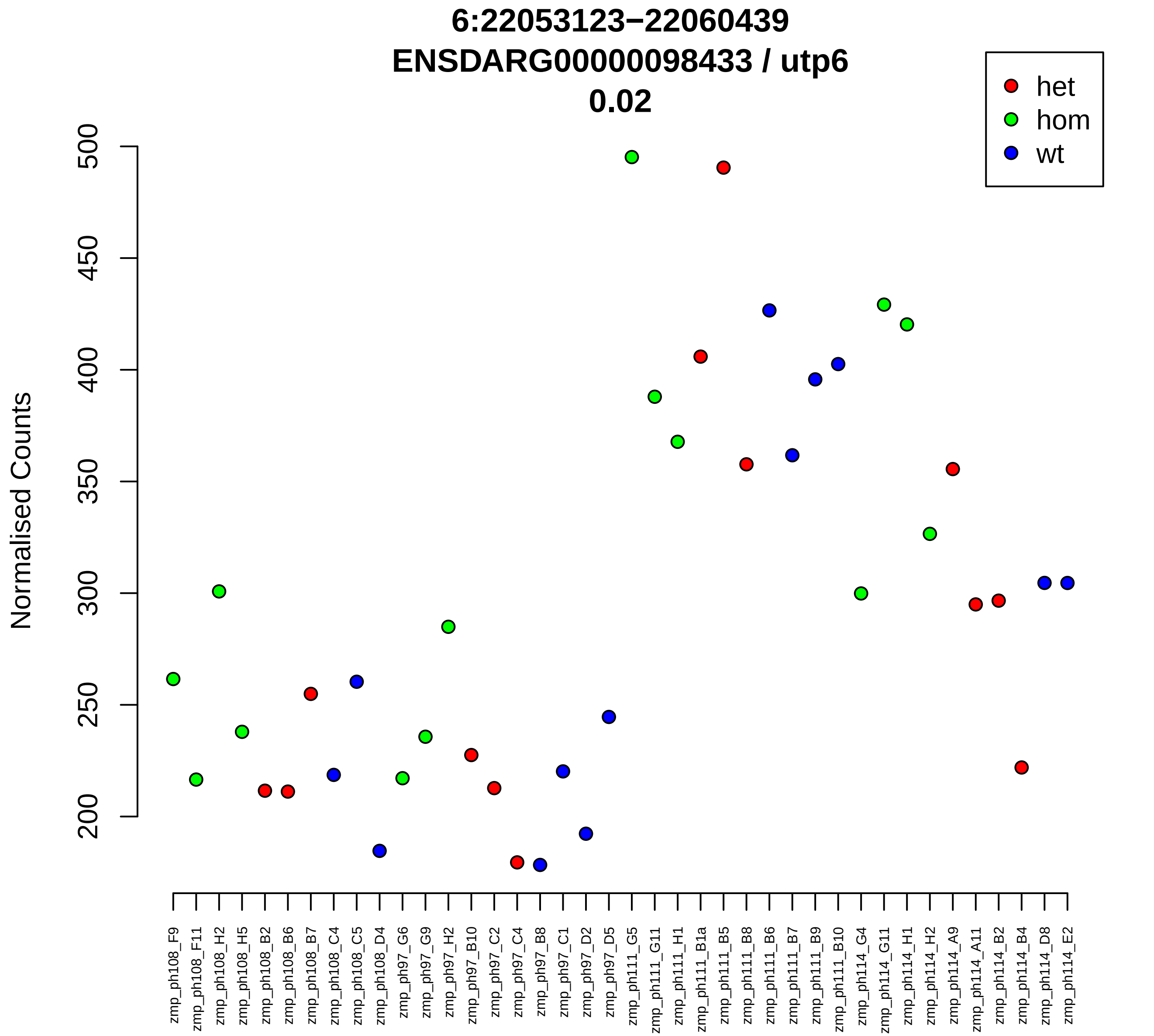
6:22053123−22060439
ENSDARG00000098433 / utp6
0.02
het
hom
wt
Normalised Counts
200
250
300
350
400
450
500
zmp_ph108_F9
zmp_ph108_F11
zmp_ph108_H2
zmp_ph108_H5
zmp_ph108_B2
zmp_ph108_B6
zmp_ph108_B7
zmp_ph108_C4
zmp_ph108_C5
zmp_ph108_D4
zmp_ph97_G6
zmp_ph97_G9
zmp_ph97_H2
zmp_ph97_B10
zmp_ph97_C2
zmp_ph97_C4
zmp_ph97_B8
zmp_ph97_C1
zmp_ph97_D2
zmp_ph97_D5
zmp_ph111_G5
zmp_ph111_G11
zmp_ph111_H1
zmp_ph111_B1a
zmp_ph111_B5
zmp_ph111_B8
zmp_ph111_B6
zmp_ph111_B7
zmp_ph111_B9
zmp_ph111_B10
zmp_ph114_G4
zmp_ph114_G11
zmp_ph114_H1
zmp_ph114_H2
zmp_ph114_A9
zmp_ph114_A11
zmp_ph114_B2
zmp_ph114_B4
zmp_ph114_D8
zmp_ph114_E2

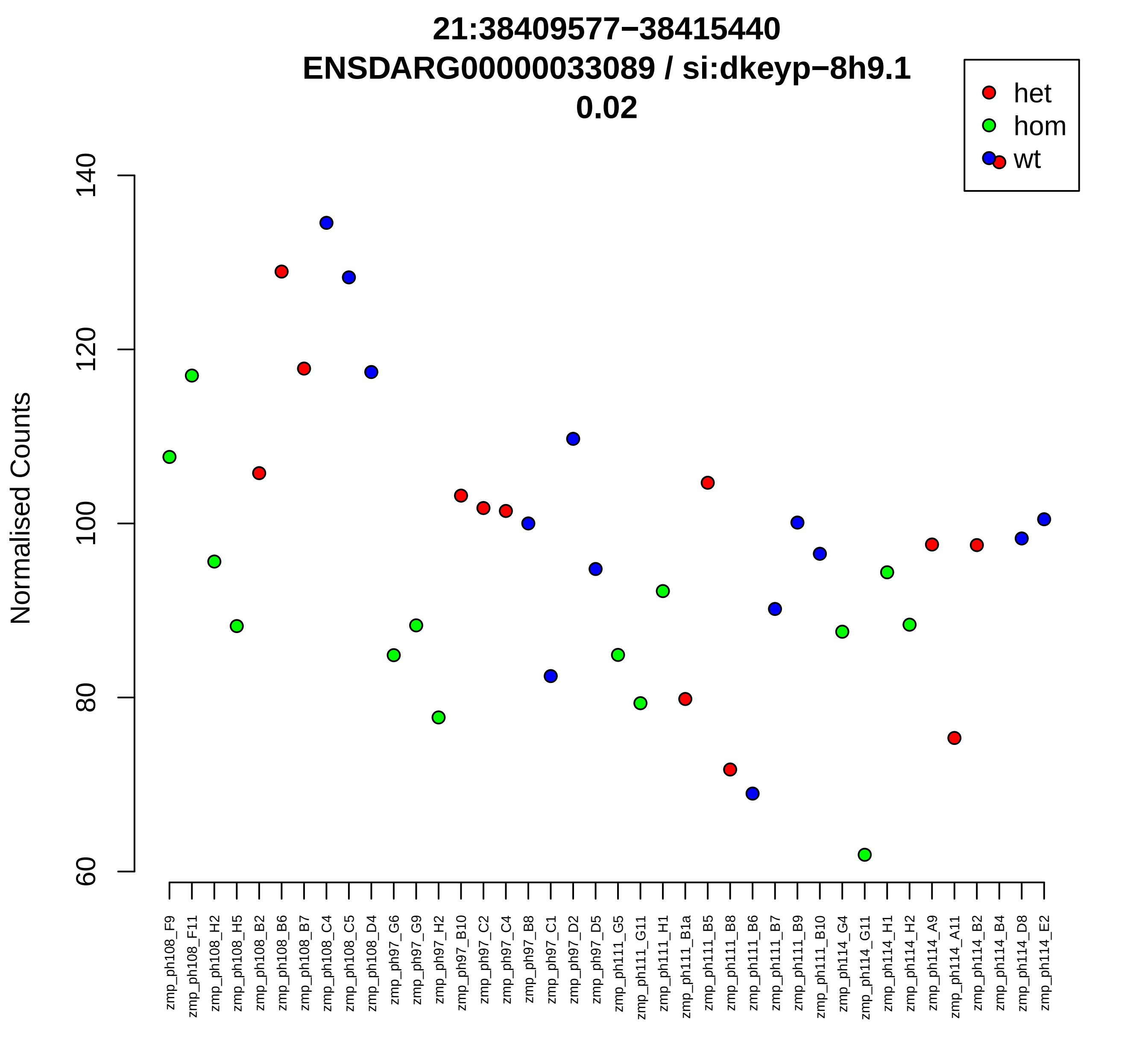
21:38409577−38415440
ENSDARG00000033089 / si:dkeyp−8h9.1
0.02
het
hom
wt
Normalised Counts
140
120
100
80
60
zmp_ph108_F9
zmp_ph108_F11
zmp_ph108_H2
zmp_ph108_H5
zmp_ph108_B2
zmp_ph108_B6
zmp_ph108_B7
zmp_ph108_C4
zmp_ph108_C5
zmp_ph108_D4
zmp_ph97_G6
zmp_ph97_G9
zmp_ph97_H2
zmp_ph97_B10
zmp_ph97_C2
zmp_ph97_C4
zmp_ph97_B8
zmp_ph97_C1
zmp_ph97_D2
zmp_ph97_D5
zmp_ph111_G5
zmp_ph111_G11
zmp_ph111_H1
zmp_ph111_B1a
zmp_ph111_B5
zmp_ph111_B8
zmp_ph111_B6
zmp_ph111_B7
zmp_ph111_B9
zmp_ph111_B10
zmp_ph114_G4
zmp_ph114_G11
zmp_ph114_H1
zmp_ph114_H2
zmp_ph114_A9
zmp_ph114_A11
zmp_ph114_B2
zmp_ph114_B4
zmp_ph114_D8
zmp_ph114_E2

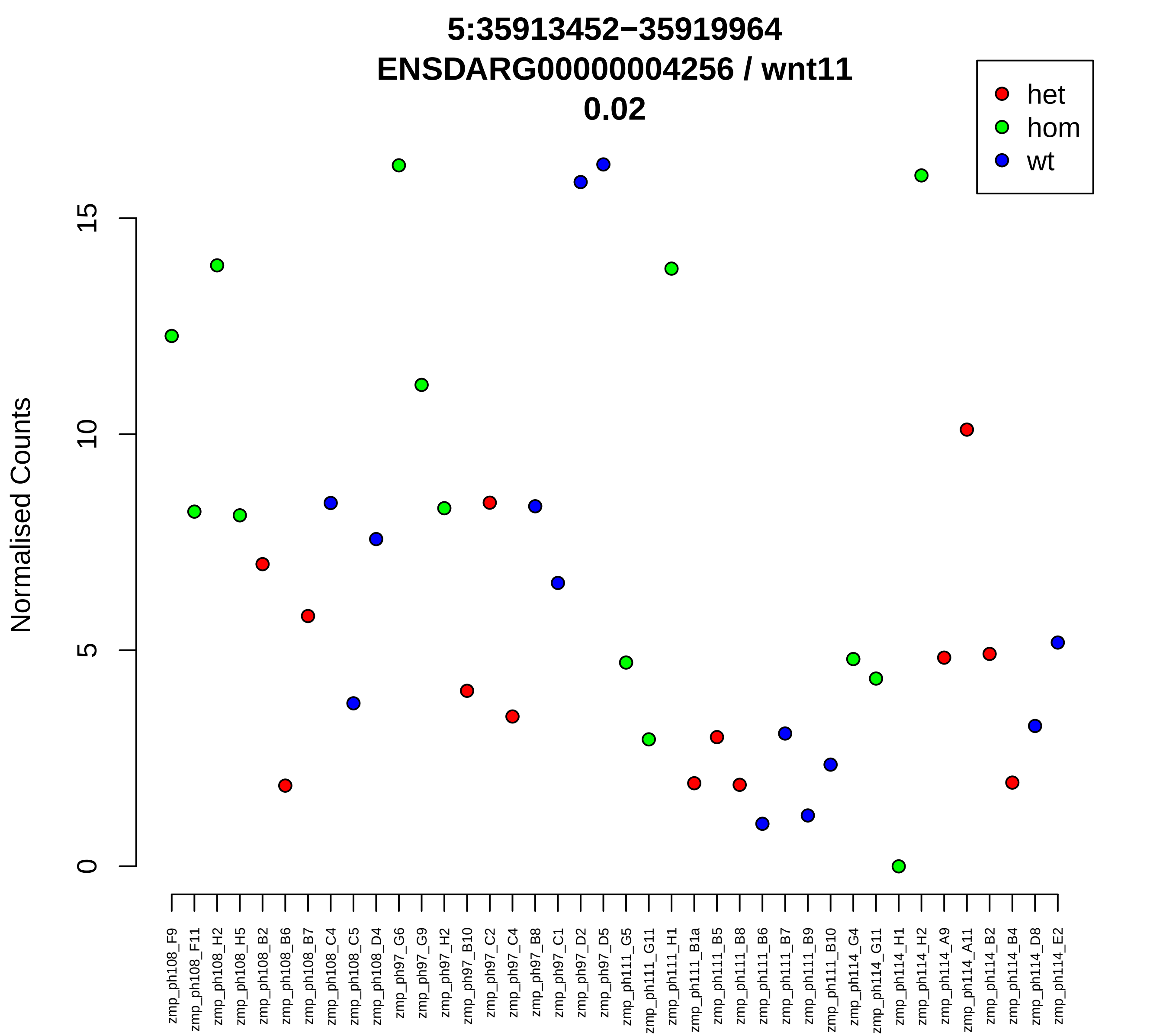

5:35913452−35919964
ENSDARG00000004256 / wnt11
0.02
het
hom
wt
Normalised Counts
0
5
10
15
zmp_ph108_F9
zmp_ph108_F11
zmp_ph108_H2
zmp_ph108_H5
zmp_ph108_B2
zmp_ph108_B6
zmp_ph108_B7
zmp_ph108_C4
zmp_ph108_C5
zmp_ph108_D4
zmp_ph97_G6
zmp_ph97_G9
zmp_ph97_H2
zmp_ph97_B10
zmp_ph97_C2
zmp_ph97_C4
zmp_ph97_B8
zmp_ph97_C1
zmp_ph97_D2
zmp_ph97_D5
zmp_ph111_G5
zmp_ph111_G11
zmp_ph111_H1
zmp_ph111_B1a
zmp_ph111_B5
zmp_ph111_B8
zmp_ph111_B6
zmp_ph111_B7
zmp_ph111_B9
zmp_ph111_B10
zmp_ph114_G4
zmp_ph114_G11
zmp_ph114_H1
zmp_ph114_H2
zmp_ph114_A9
zmp_ph114_A11
zmp_ph114_B2
zmp_ph114_B4
zmp_ph114_D8
zmp_ph114_E2

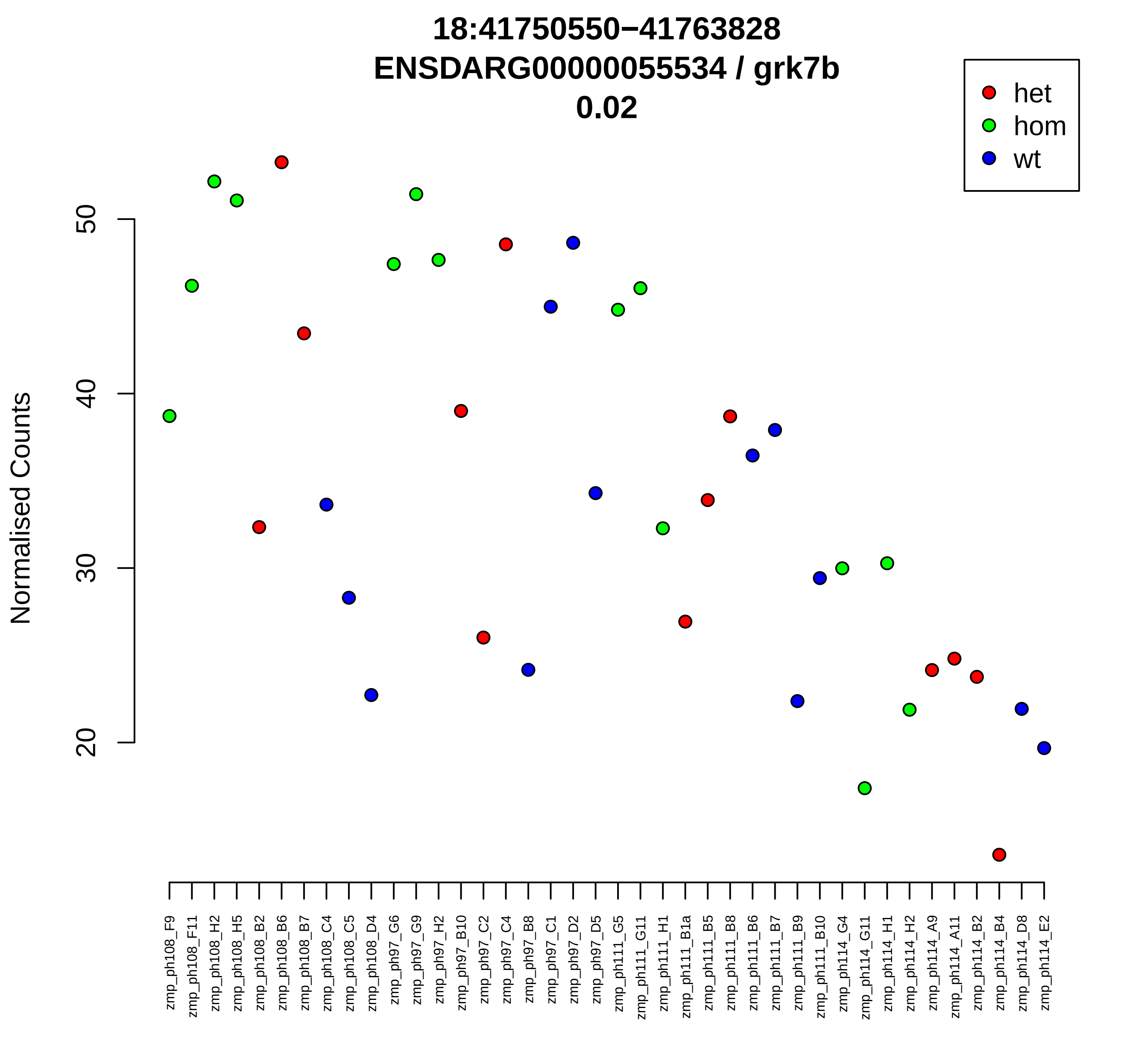
18:41750550−41763828
ENSDARG00000055534 / grk7b
0.02
het
hom
wt
Normalised Counts
20
30
40
50
zmp_ph108_F9
zmp_ph108_F11
zmp_ph108_H2
zmp_ph108_H5
zmp_ph108_B2
zmp_ph108_B6
zmp_ph108_B7
zmp_ph108_C4
zmp_ph108_C5
zmp_ph108_D4
zmp_ph97_G6
zmp_ph97_G9
zmp_ph97_H2
zmp_ph97_B10
zmp_ph97_C2
zmp_ph97_C4
zmp_ph97_B8
zmp_ph97_C1
zmp_ph97_D2
zmp_ph97_D5
zmp_ph111_G5
zmp_ph111_G11
zmp_ph111_H1
zmp_ph111_B1a
zmp_ph111_B5
zmp_ph111_B8
zmp_ph111_B6
zmp_ph111_B7
zmp_ph111_B9
zmp_ph111_B10
zmp_ph114_G4
zmp_ph114_G11
zmp_ph114_H1
zmp_ph114_H2
zmp_ph114_A9
zmp_ph114_A11
zmp_ph114_B2
zmp_ph114_B4
zmp_ph114_D8
zmp_ph114_E2

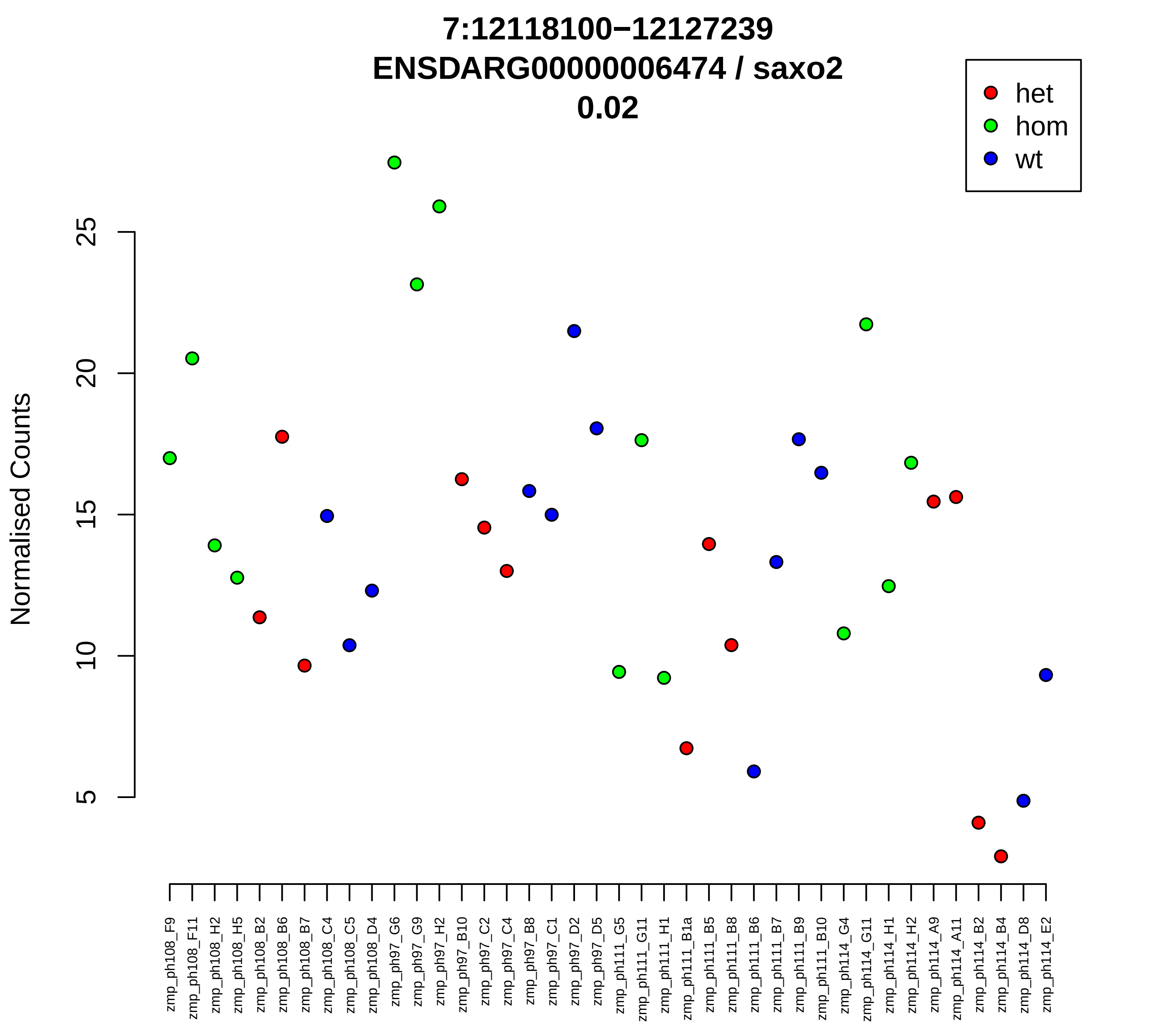
7:12118100−12127239
ENSDARG00000006474 / saxo2
0.02
het
hom
wt
Normalised Counts
5
10
15
20
25
zmp_ph108_F9
zmp_ph108_F11
zmp_ph108_H2
zmp_ph108_H5
zmp_ph108_B2
zmp_ph108_B6
zmp_ph108_B7
zmp_ph108_C4
zmp_ph108_C5
zmp_ph108_D4
zmp_ph97_G6
zmp_ph97_G9
zmp_ph97_H2
zmp_ph97_B10
zmp_ph97_C2
zmp_ph97_C4
zmp_ph97_B8
zmp_ph97_C1
zmp_ph97_D2
zmp_ph97_D5
zmp_ph111_G5
zmp_ph111_G11
zmp_ph111_H1
zmp_ph111_B1a
zmp_ph111_B5
zmp_ph111_B8
zmp_ph111_B6
zmp_ph111_B7
zmp_ph111_B9
zmp_ph111_B10
zmp_ph114_G4
zmp_ph114_G11
zmp_ph114_H1
zmp_ph114_H2
zmp_ph114_A9
zmp_ph114_A11
zmp_ph114_B2
zmp_ph114_B4
zmp_ph114_D8
zmp_ph114_E2

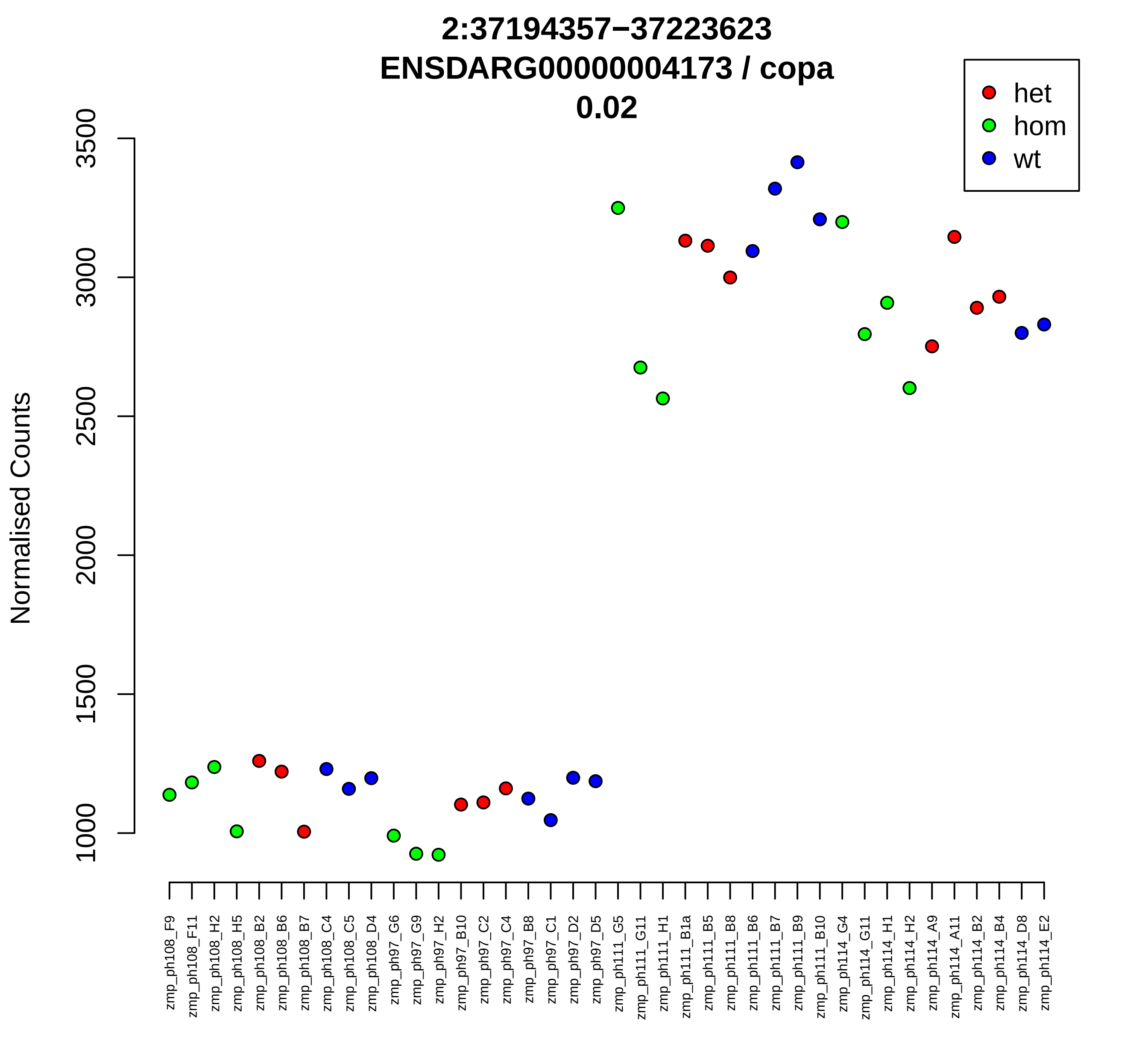

2:37194357−37223623
ENSDARG00000004173 / copa
0.02
Normalised Counts
1000
1500
2000
2500
3000
3500
het
hom
wt
zmp_ph108_F9
zmp_ph108_F11
zmp_ph108_H2
zmp_ph108_H5
zmp_ph108_B2
zmp_ph108_B6
zmp_ph108_B7
zmp_ph108_C4
zmp_ph108_C5
zmp_ph108_D4
zmp_ph97_G6
zmp_ph97_G9
zmp_ph97_H2
zmp_ph97_B10
zmp_ph97_C2
zmp_ph97_C4
zmp_ph97_B8
zmp_ph97_C1
zmp_ph97_D2
zmp_ph97_D5
zmp_ph111_G5
zmp_ph111_G11
zmp_ph111_H1
zmp_ph111_B1a
zmp_ph111_B5
zmp_ph111_B8
zmp_ph111_B6
zmp_ph111_B7
zmp_ph111_B9
zmp_ph111_B10
zmp_ph114_G4
zmp_ph114_G11
zmp_ph114_H1
zmp_ph114_H2
zmp_ph114_A9
zmp_ph114_A11
zmp_ph114_B2
zmp_ph114_B4
zmp_ph114_D8
zmp_ph114_E2

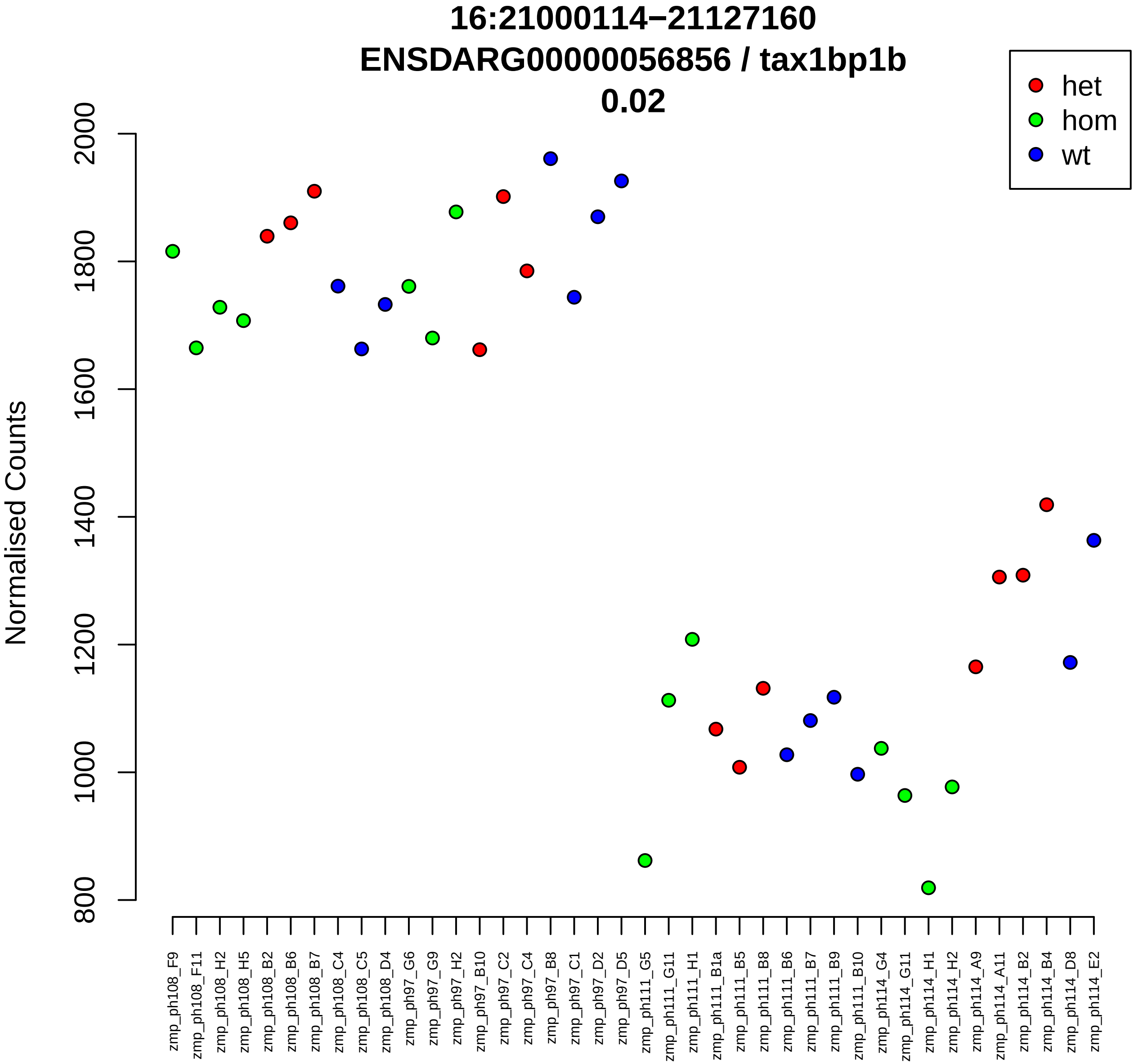

16:21000114−21127160
ENSDARG00000056856 / tax1bp1b
0.02
het
hom
wt
Normalised Counts
800
1000
1200
1400
1600
1800
2000
zmp_ph108_F9
zmp_ph108_F11
zmp_ph108_H2
zmp_ph108_H5
zmp_ph108_B2
zmp_ph108_B6
zmp_ph108_B7
zmp_ph108_C4
zmp_ph108_C5
zmp_ph108_D4
zmp_ph97_G6
zmp_ph97_G9
zmp_ph97_H2
zmp_ph97_B10
zmp_ph97_C2
zmp_ph97_C4
zmp_ph97_B8
zmp_ph97_C1
zmp_ph97_D2
zmp_ph97_D5
zmp_ph111_G5
zmp_ph111_G11
zmp_ph111_H1
zmp_ph111_B1a
zmp_ph111_B5
zmp_ph111_B8
zmp_ph111_B6
zmp_ph111_B7
zmp_ph111_B9
zmp_ph111_B10
zmp_ph114_G4
zmp_ph114_G11
zmp_ph114_H1
zmp_ph114_H2
zmp_ph114_A9
zmp_ph114_A11
zmp_ph114_B2
zmp_ph114_B4
zmp_ph114_D8
zmp_ph114_E2

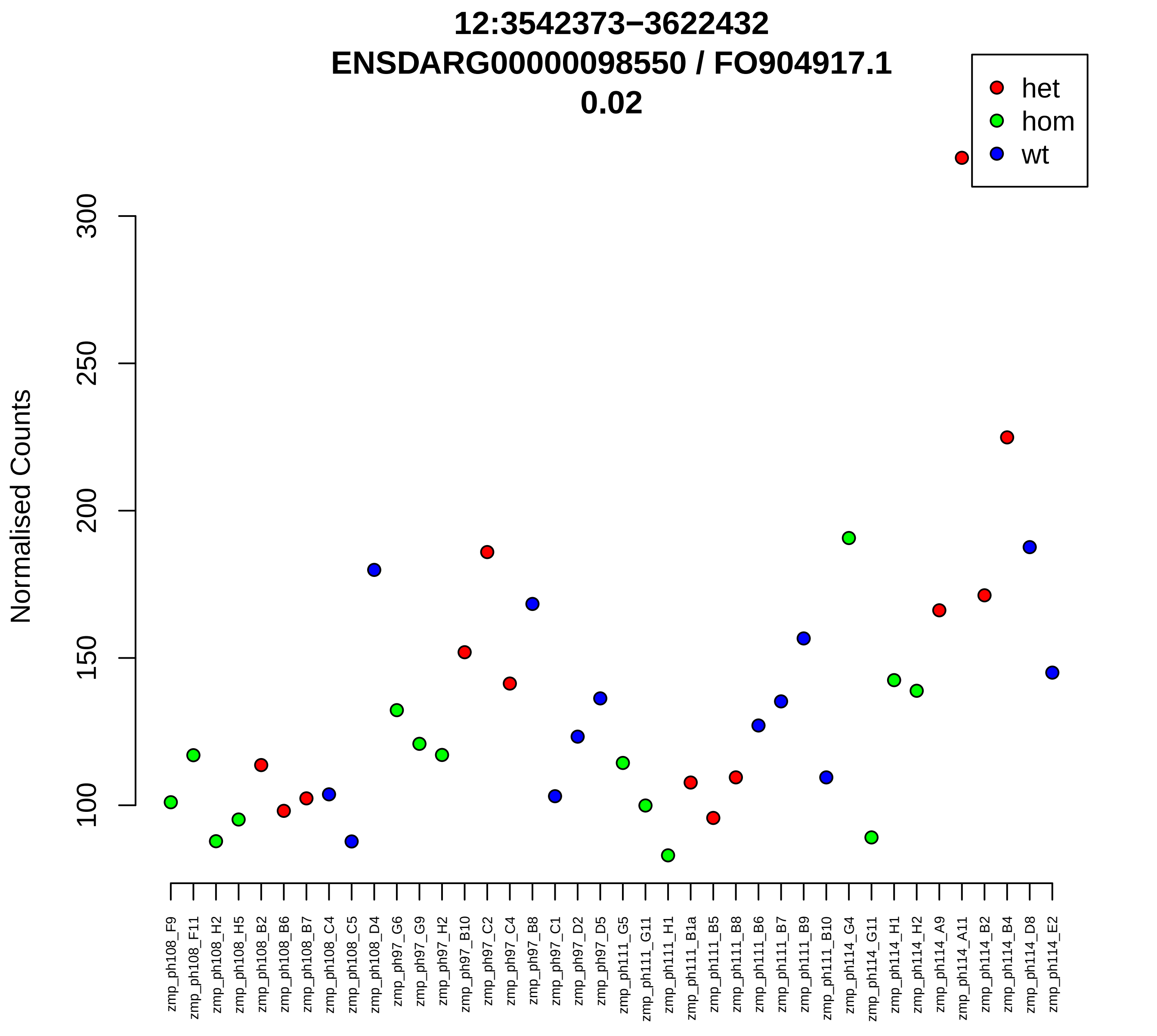

12:3542373−3622432
ENSDARG00000098550 / FO904917.1
0.02
het
hom
wt
Normalised Counts
100
150
200
250
300
zmp_ph108_F9
zmp_ph108_F11
zmp_ph108_H2
zmp_ph108_H5
zmp_ph108_B2
zmp_ph108_B6
zmp_ph108_B7
zmp_ph108_C4
zmp_ph108_C5
zmp_ph108_D4
zmp_ph97_G6
zmp_ph97_G9
zmp_ph97_H2
zmp_ph97_B10
zmp_ph97_C2
zmp_ph97_C4
zmp_ph97_B8
zmp_ph97_C1
zmp_ph97_D2
zmp_ph97_D5
zmp_ph111_G5
zmp_ph111_G11
zmp_ph111_H1
zmp_ph111_B1a
zmp_ph111_B5
zmp_ph111_B8
zmp_ph111_B6
zmp_ph111_B7
zmp_ph111_B9
zmp_ph111_B10
zmp_ph114_G4
zmp_ph114_G11
zmp_ph114_H1
zmp_ph114_H2
zmp_ph114_A9
zmp_ph114_A11
zmp_ph114_B2
zmp_ph114_B4
zmp_ph114_D8
zmp_ph114_E2

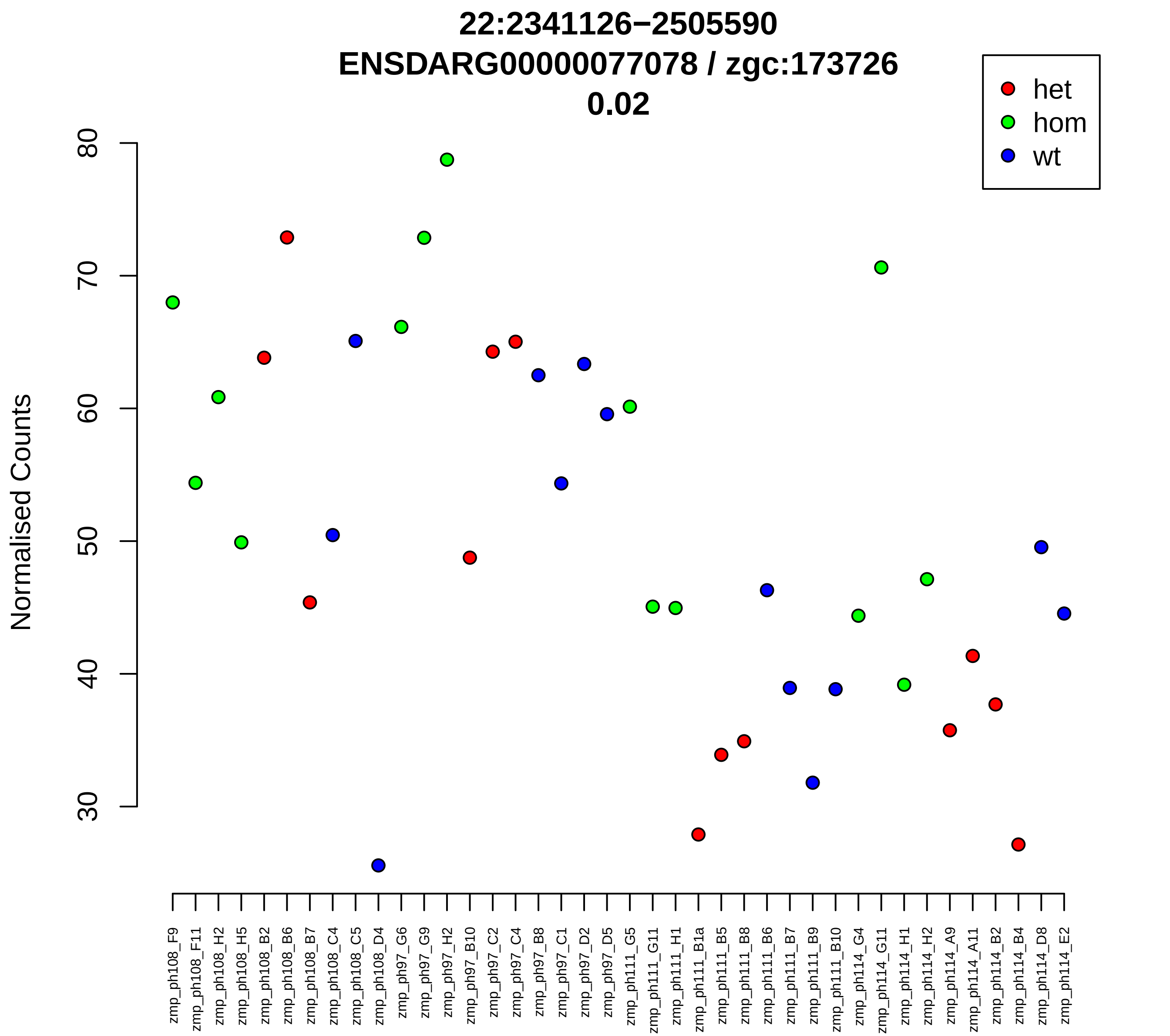
22:2341126−2505590
ENSDARG00000077078 / zgc:173726
0.02
het
hom
wt
Normalised Counts
30
40
50
60
70
80
zmp_ph108_F9
zmp_ph108_F11
zmp_ph108_H2
zmp_ph108_H5
zmp_ph108_B2
zmp_ph108_B6
zmp_ph108_B7
zmp_ph108_C4
zmp_ph108_C5
zmp_ph108_D4
zmp_ph97_G6
zmp_ph97_G9
zmp_ph97_H2
zmp_ph97_B10
zmp_ph97_C2
zmp_ph97_C4
zmp_ph97_B8
zmp_ph97_C1
zmp_ph97_D2
zmp_ph97_D5
zmp_ph111_G5
zmp_ph111_G11
zmp_ph111_H1
zmp_ph111_B1a
zmp_ph111_B5
zmp_ph111_B8
zmp_ph111_B6
zmp_ph111_B7
zmp_ph111_B9
zmp_ph111_B10
zmp_ph114_G4
zmp_ph114_G11
zmp_ph114_H1
zmp_ph114_H2
zmp_ph114_A9
zmp_ph114_A11
zmp_ph114_B2
zmp_ph114_B4
zmp_ph114_D8
zmp_ph114_E2

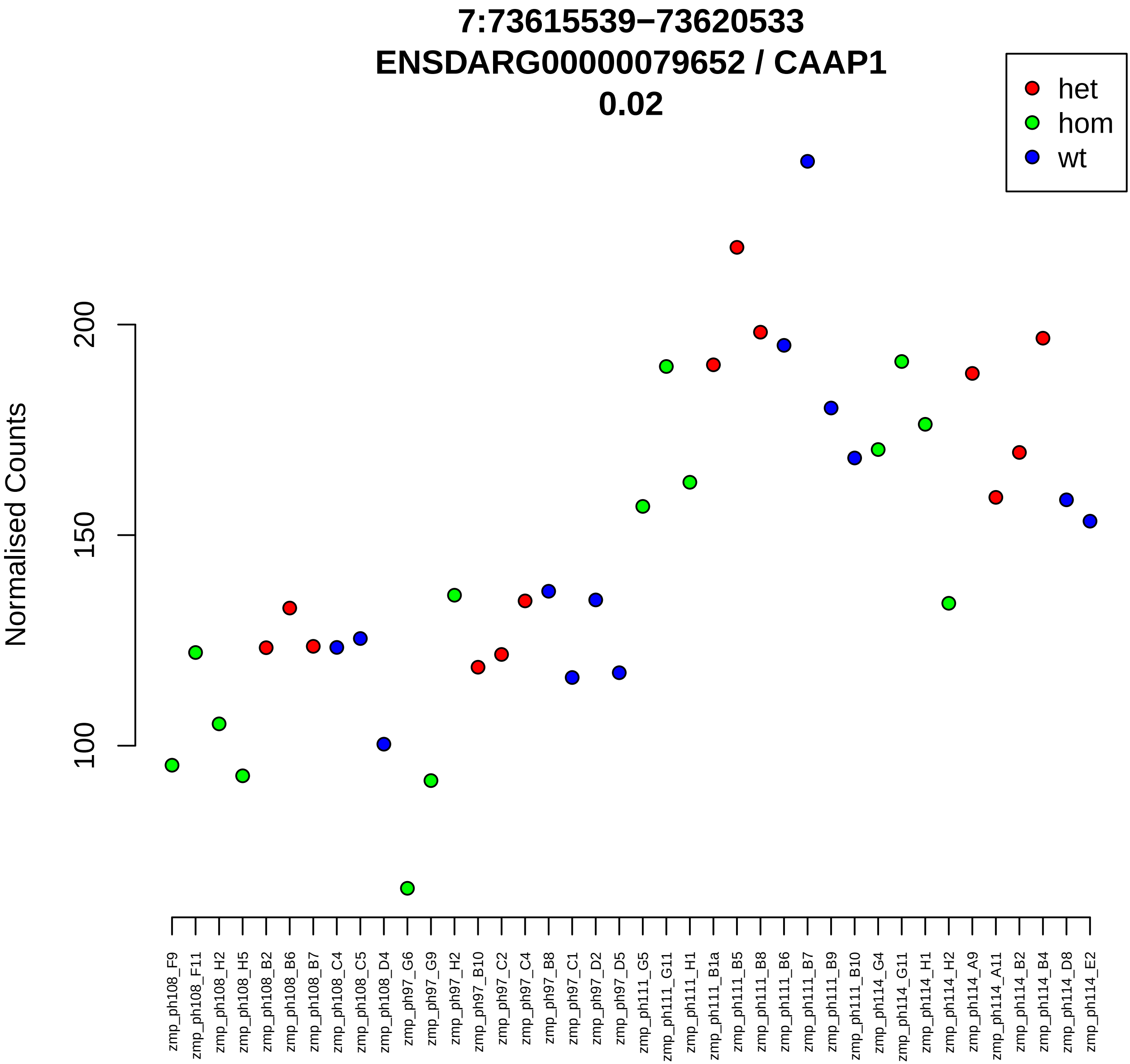

7:73615539−73620533
ENSDARG00000079652 / CAAP1
0.02
het
hom
wt
Normalised Counts
100
150
200
zmp_ph108_F9
zmp_ph108_F11
zmp_ph108_H2
zmp_ph108_H5
zmp_ph108_B2
zmp_ph108_B6
zmp_ph108_B7
zmp_ph108_C4
zmp_ph108_C5
zmp_ph108_D4
zmp_ph97_G6
zmp_ph97_G9
zmp_ph97_H2
zmp_ph97_B10
zmp_ph97_C2
zmp_ph97_C4
zmp_ph97_B8
zmp_ph97_C1
zmp_ph97_D2
zmp_ph97_D5
zmp_ph111_G5
zmp_ph111_G11
zmp_ph111_H1
zmp_ph111_B1a
zmp_ph111_B5
zmp_ph111_B8
zmp_ph111_B6
zmp_ph111_B7
zmp_ph111_B9
zmp_ph111_B10
zmp_ph114_G4
zmp_ph114_G11
zmp_ph114_H1
zmp_ph114_H2
zmp_ph114_A9
zmp_ph114_A11
zmp_ph114_B2
zmp_ph114_B4
zmp_ph114_D8
zmp_ph114_E2

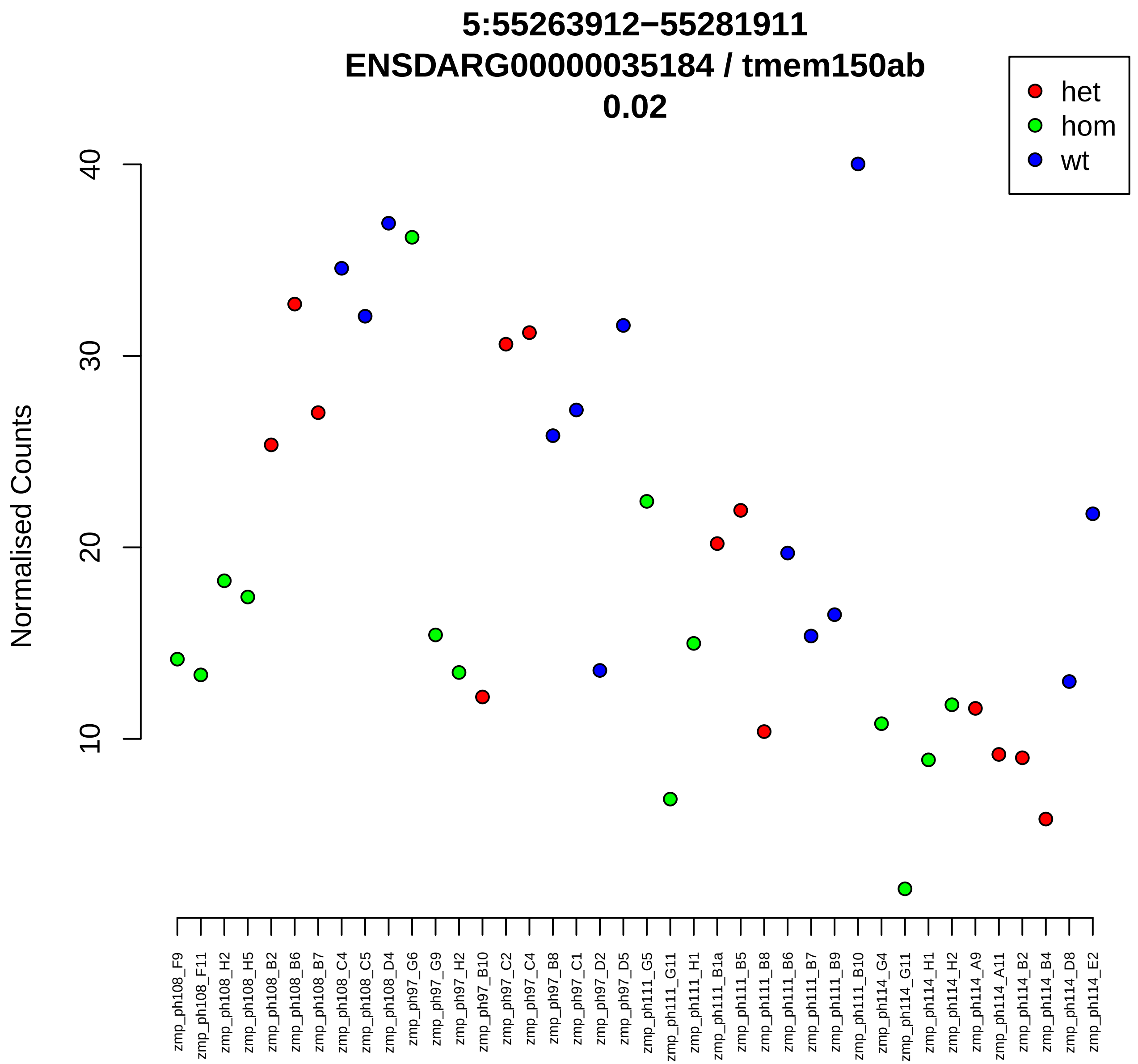
5:55263912−55281911
ENSDARG00000035184 / tmem150ab
0.02
het
hom
wt
Normalised Counts
10
20
30
40
zmp_ph108_F9
zmp_ph108_F11
zmp_ph108_H2
zmp_ph108_H5
zmp_ph108_B2
zmp_ph108_B6
zmp_ph108_B7
zmp_ph108_C4
zmp_ph108_C5
zmp_ph108_D4
zmp_ph97_G6
zmp_ph97_G9
zmp_ph97_H2
zmp_ph97_B10
zmp_ph97_C2
zmp_ph97_C4
zmp_ph97_B8
zmp_ph97_C1
zmp_ph97_D2
zmp_ph97_D5
zmp_ph111_G5
zmp_ph111_G11
zmp_ph111_H1
zmp_ph111_B1a
zmp_ph111_B5
zmp_ph111_B8
zmp_ph111_B6
zmp_ph111_B7
zmp_ph111_B9
zmp_ph111_B10
zmp_ph114_G4
zmp_ph114_G11
zmp_ph114_H1
zmp_ph114_H2
zmp_ph114_A9
zmp_ph114_A11
zmp_ph114_B2
zmp_ph114_B4
zmp_ph114_D8
zmp_ph114_E2

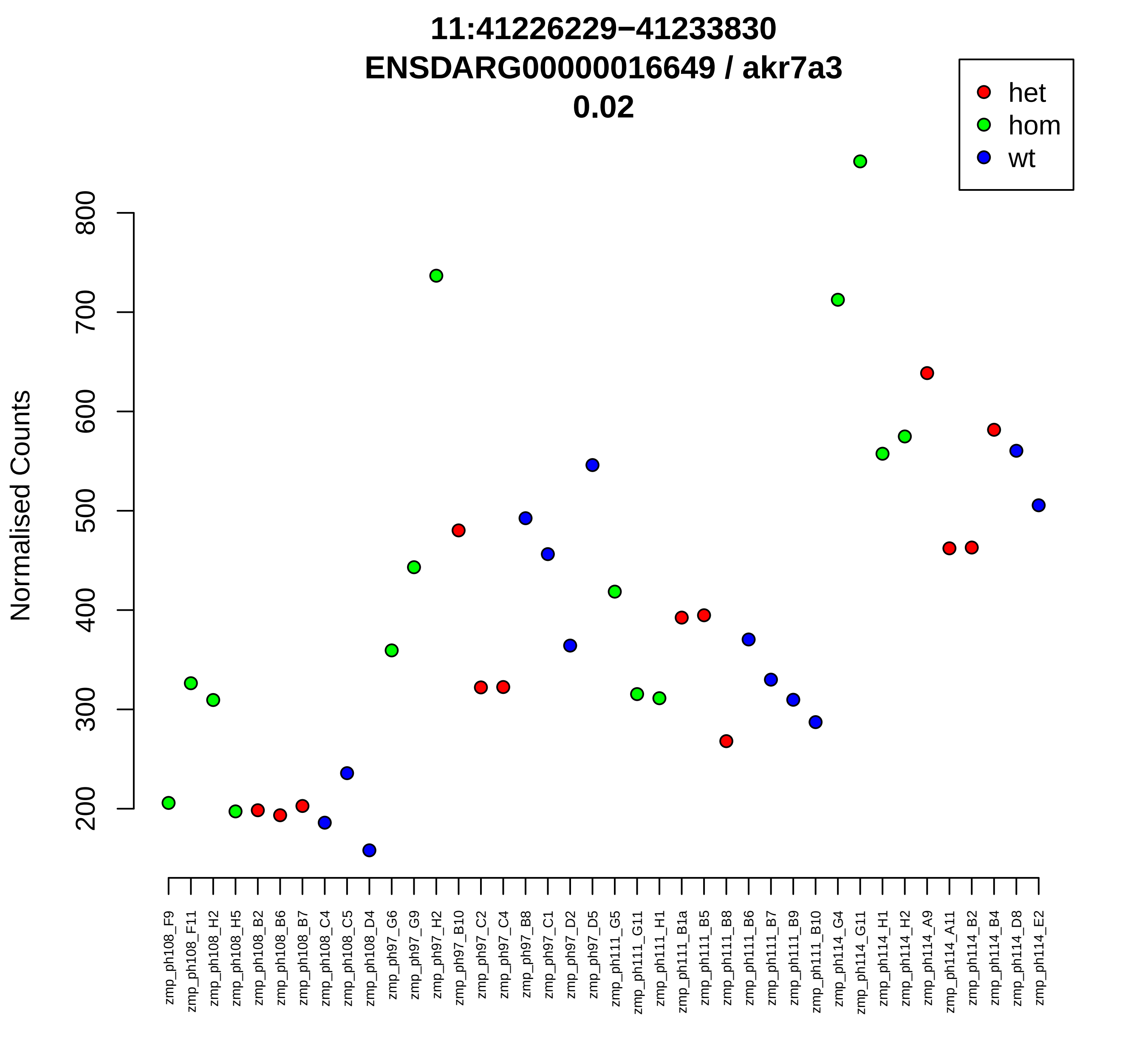
11:41226229−41233830
ENSDARG00000016649 / akr7a3
0.02
het
hom
wt
Normalised Counts
200
300
400
500
600
700
800
zmp_ph108_F9
zmp_ph108_F11
zmp_ph108_H2
zmp_ph108_H5
zmp_ph108_B2
zmp_ph108_B6
zmp_ph108_B7
zmp_ph108_C4
zmp_ph108_C5
zmp_ph108_D4
zmp_ph97_G6
zmp_ph97_G9
zmp_ph97_H2
zmp_ph97_B10
zmp_ph97_C2
zmp_ph97_C4
zmp_ph97_B8
zmp_ph97_C1
zmp_ph97_D2
zmp_ph97_D5
zmp_ph111_G5
zmp_ph111_G11
zmp_ph111_H1
zmp_ph111_B1a
zmp_ph111_B5
zmp_ph111_B8
zmp_ph111_B6
zmp_ph111_B7
zmp_ph111_B9
zmp_ph111_B10
zmp_ph114_G4
zmp_ph114_G11
zmp_ph114_H1
zmp_ph114_H2
zmp_ph114_A9
zmp_ph114_A11
zmp_ph114_B2
zmp_ph114_B4
zmp_ph114_D8
zmp_ph114_E2

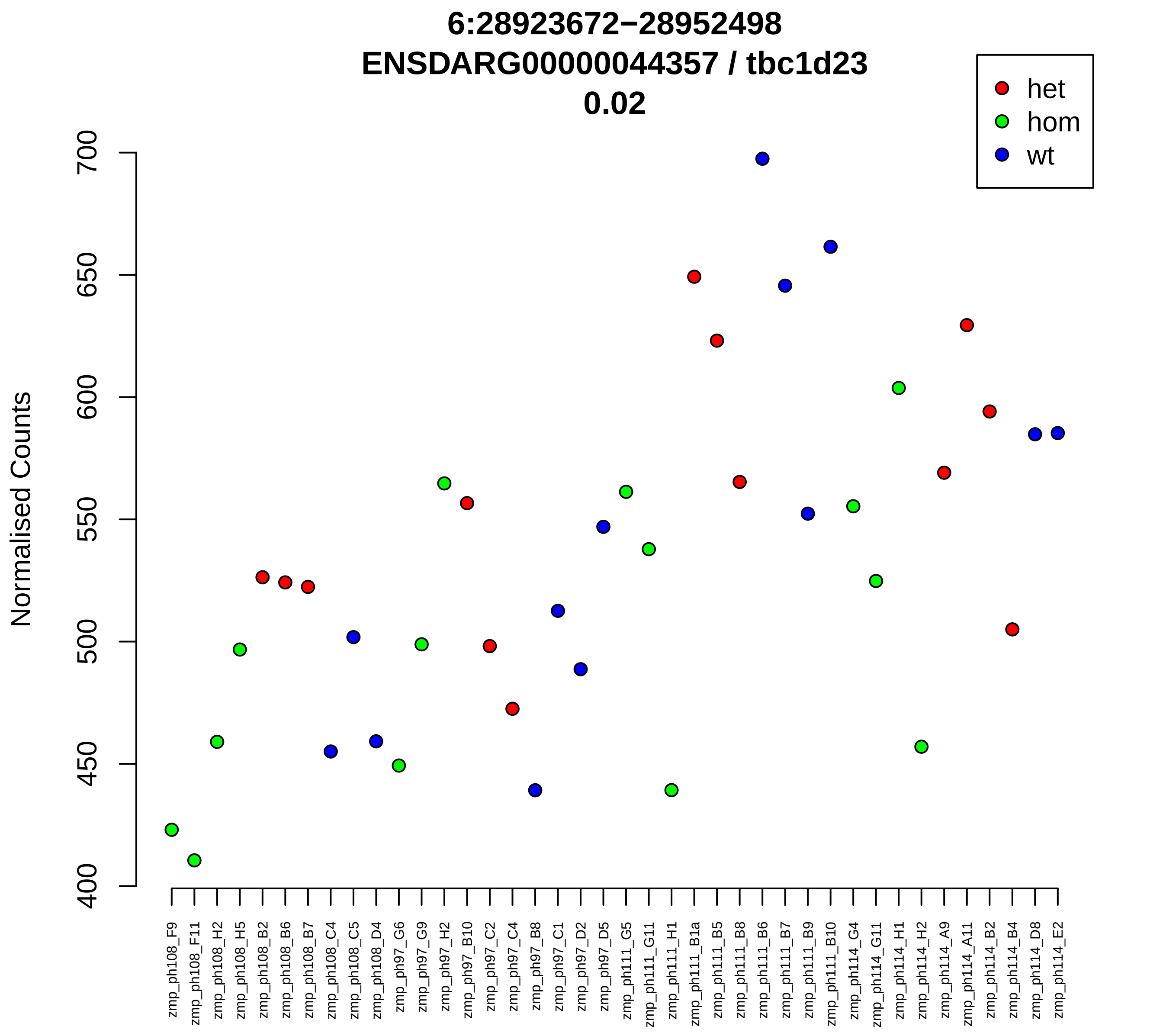
6:28923672−28952498
ENSDARG00000044357 / tbc1d23
0.02
het
hom
wt
Normalised Counts
400
450
500
550
600
650
700
zmp_ph108_F9
zmp_ph108_F11
zmp_ph108_H2
zmp_ph108_H5
zmp_ph108_B2
zmp_ph108_B6
zmp_ph108_B7
zmp_ph108_C4
zmp_ph108_C5
zmp_ph108_D4
zmp_ph97_G6
zmp_ph97_G9
zmp_ph97_H2
zmp_ph97_B10
zmp_ph97_C2
zmp_ph97_C4
zmp_ph97_B8
zmp_ph97_C1
zmp_ph97_D2
zmp_ph97_D5
zmp_ph111_G5
zmp_ph111_G11
zmp_ph111_H1
zmp_ph111_B1a
zmp_ph111_B5
zmp_ph111_B8
zmp_ph111_B6
zmp_ph111_B7
zmp_ph111_B9
zmp_ph111_B10
zmp_ph114_G4
zmp_ph114_G11
zmp_ph114_H1
zmp_ph114_H2
zmp_ph114_A9
zmp_ph114_A11
zmp_ph114_B2
zmp_ph114_B4
zmp_ph114_D8
zmp_ph114_E2

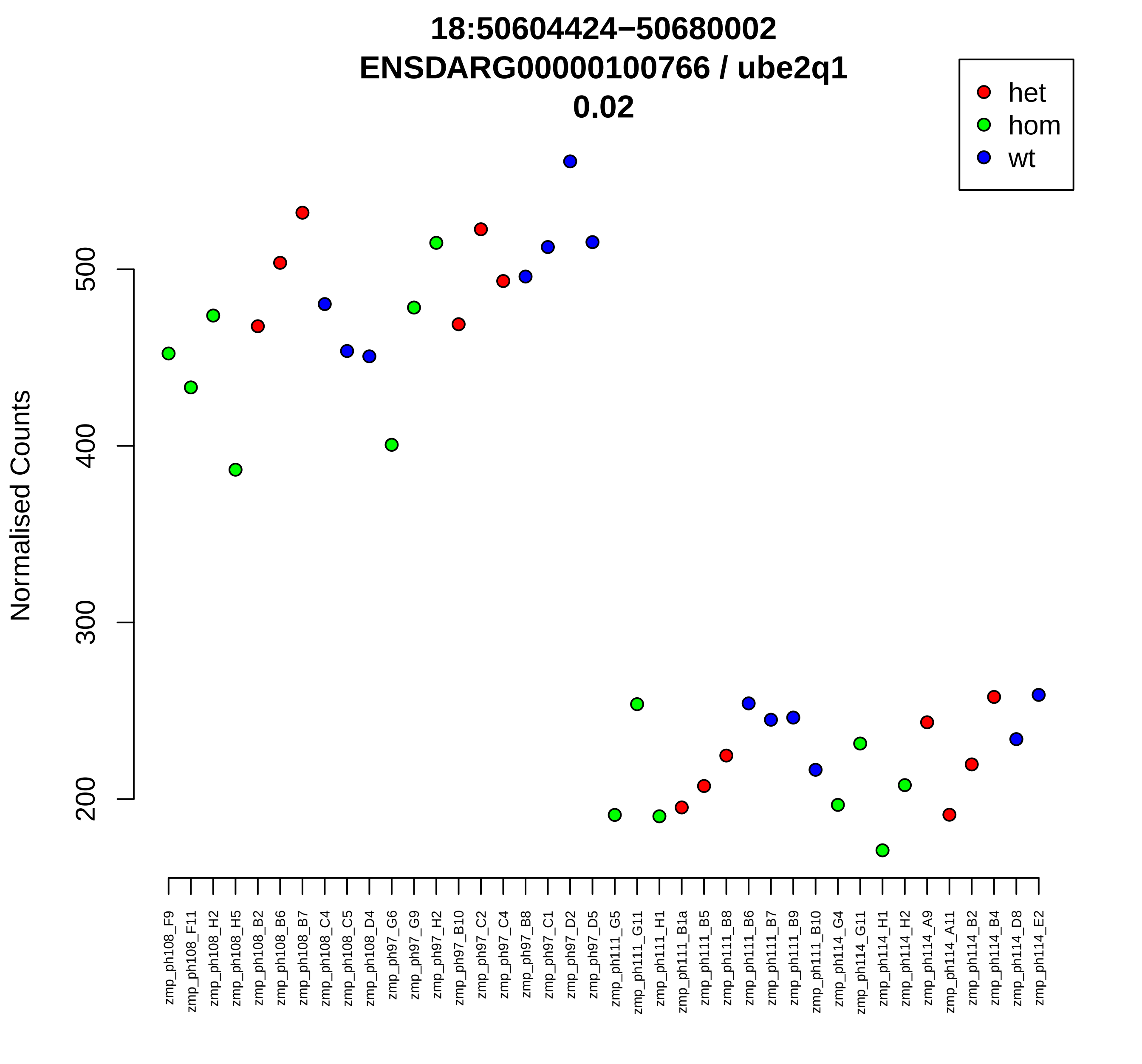

18:50604424−50680002
ENSDARG00000100766 / ube2q1
0.02
het
hom
wt
Normalised Counts
200
300
400
500
zmp_ph108_F9
zmp_ph108_F11
zmp_ph108_H2
zmp_ph108_H5
zmp_ph108_B2
zmp_ph108_B6
zmp_ph108_B7
zmp_ph108_C4
zmp_ph108_C5
zmp_ph108_D4
zmp_ph97_G6
zmp_ph97_G9
zmp_ph97_H2
zmp_ph97_B10
zmp_ph97_C2
zmp_ph97_C4
zmp_ph97_B8
zmp_ph97_C1
zmp_ph97_D2
zmp_ph97_D5
zmp_ph111_G5
zmp_ph111_G11
zmp_ph111_H1
zmp_ph111_B1a
zmp_ph111_B5
zmp_ph111_B8
zmp_ph111_B6
zmp_ph111_B7
zmp_ph111_B9
zmp_ph111_B10
zmp_ph114_G4
zmp_ph114_G11
zmp_ph114_H1
zmp_ph114_H2
zmp_ph114_A9
zmp_ph114_A11
zmp_ph114_B2
zmp_ph114_B4
zmp_ph114_D8
zmp_ph114_E2

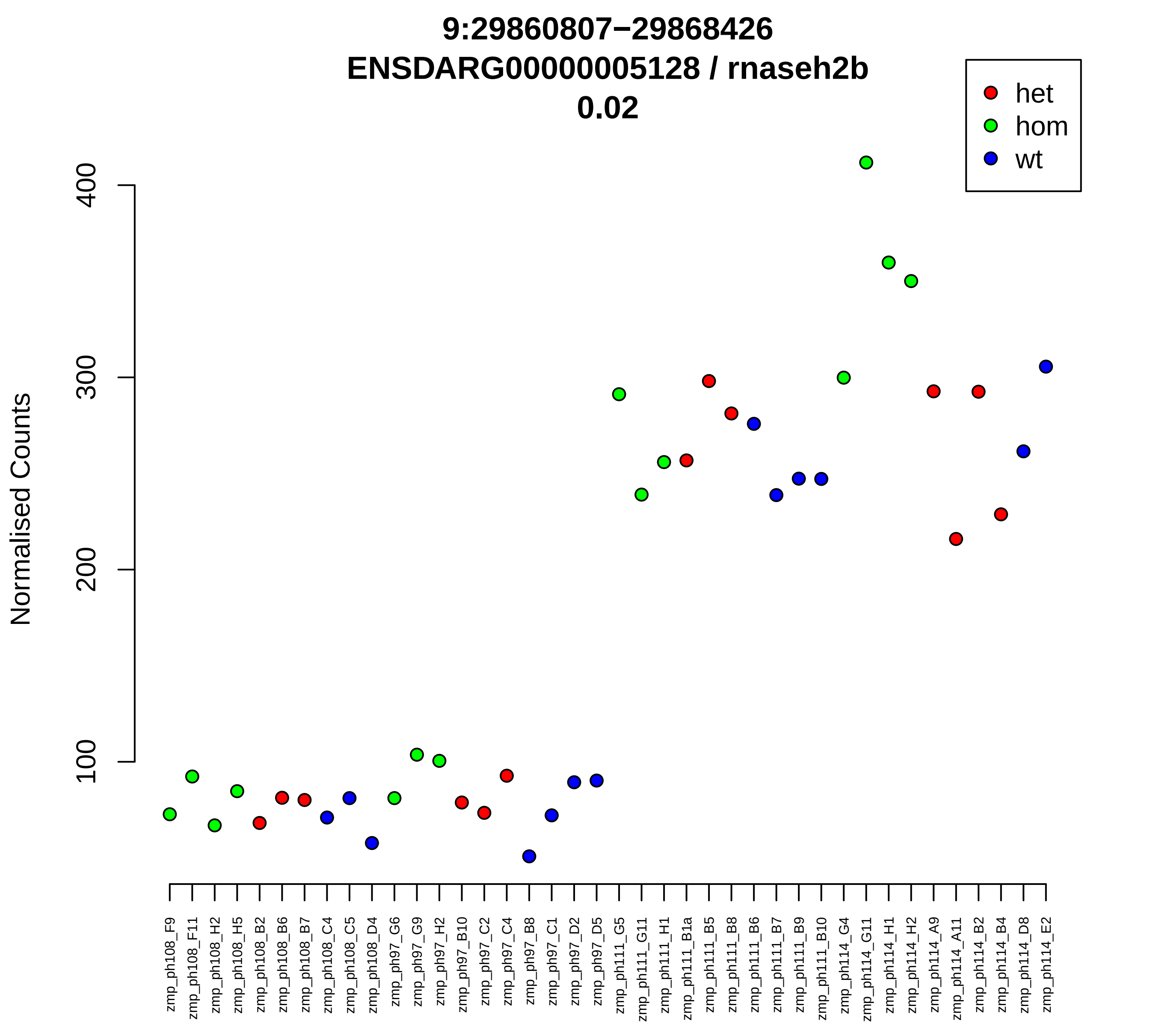
9:29860807−29868426
ENSDARG00000005128 / rnaseh2b
0.02
het
hom
wt
Normalised Counts
100
200
300
400
zmp_ph108_F9
zmp_ph108_F11
zmp_ph108_H2
zmp_ph108_H5
zmp_ph108_B2
zmp_ph108_B6
zmp_ph108_B7
zmp_ph108_C4
zmp_ph108_C5
zmp_ph108_D4
zmp_ph97_G6
zmp_ph97_G9
zmp_ph97_H2
zmp_ph97_B10
zmp_ph97_C2
zmp_ph97_C4
zmp_ph97_B8
zmp_ph97_C1
zmp_ph97_D2
zmp_ph97_D5
zmp_ph111_G5
zmp_ph111_G11
zmp_ph111_H1
zmp_ph111_B1a
zmp_ph111_B5
zmp_ph111_B8
zmp_ph111_B6
zmp_ph111_B7
zmp_ph111_B9
zmp_ph111_B10
zmp_ph114_G4
zmp_ph114_G11
zmp_ph114_H1
zmp_ph114_H2
zmp_ph114_A9
zmp_ph114_A11
zmp_ph114_B2
zmp_ph114_B4
zmp_ph114_D8
zmp_ph114_E2

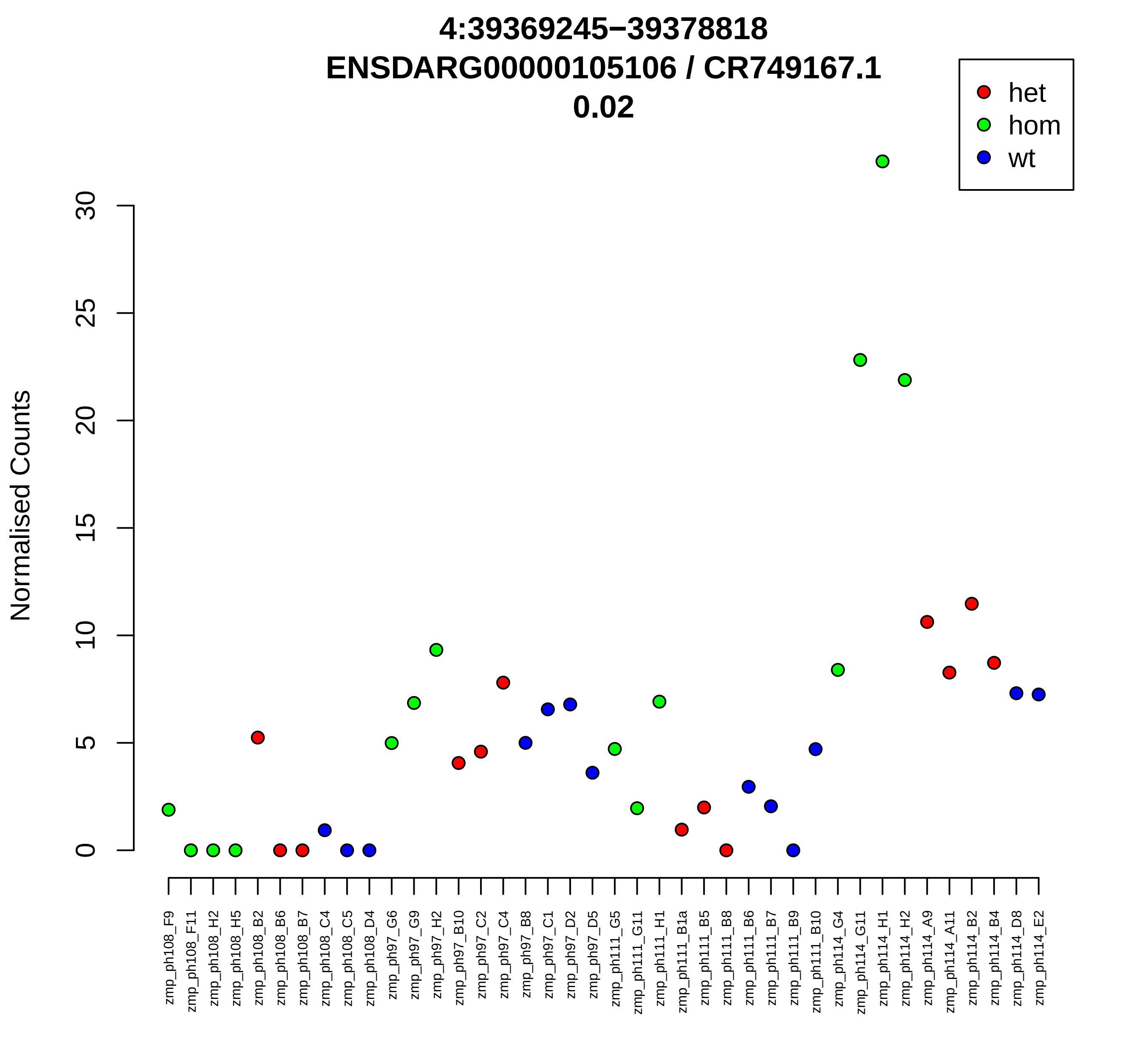

4:39369245−39378818
ENSDARG00000105106 / CR749167.1
0.02
het
hom
wt
Normalised Counts
0
5
10
15
20
25
30
zmp_ph108_F9
zmp_ph108_F11
zmp_ph108_H2
zmp_ph108_H5
zmp_ph108_B2
zmp_ph108_B6
zmp_ph108_B7
zmp_ph108_C4
zmp_ph108_C5
zmp_ph108_D4
zmp_ph97_G6
zmp_ph97_G9
zmp_ph97_H2
zmp_ph97_B10
zmp_ph97_C2
zmp_ph97_C4
zmp_ph97_B8
zmp_ph97_C1
zmp_ph97_D2
zmp_ph97_D5
zmp_ph111_G5
zmp_ph111_G11
zmp_ph111_H1
zmp_ph111_B1a
zmp_ph111_B5
zmp_ph111_B8
zmp_ph111_B6
zmp_ph111_B7
zmp_ph111_B9
zmp_ph111_B10
zmp_ph114_G4
zmp_ph114_G11
zmp_ph114_H1
zmp_ph114_H2
zmp_ph114_A9
zmp_ph114_A11
zmp_ph114_B2
zmp_ph114_B4
zmp_ph114_D8
zmp_ph114_E2

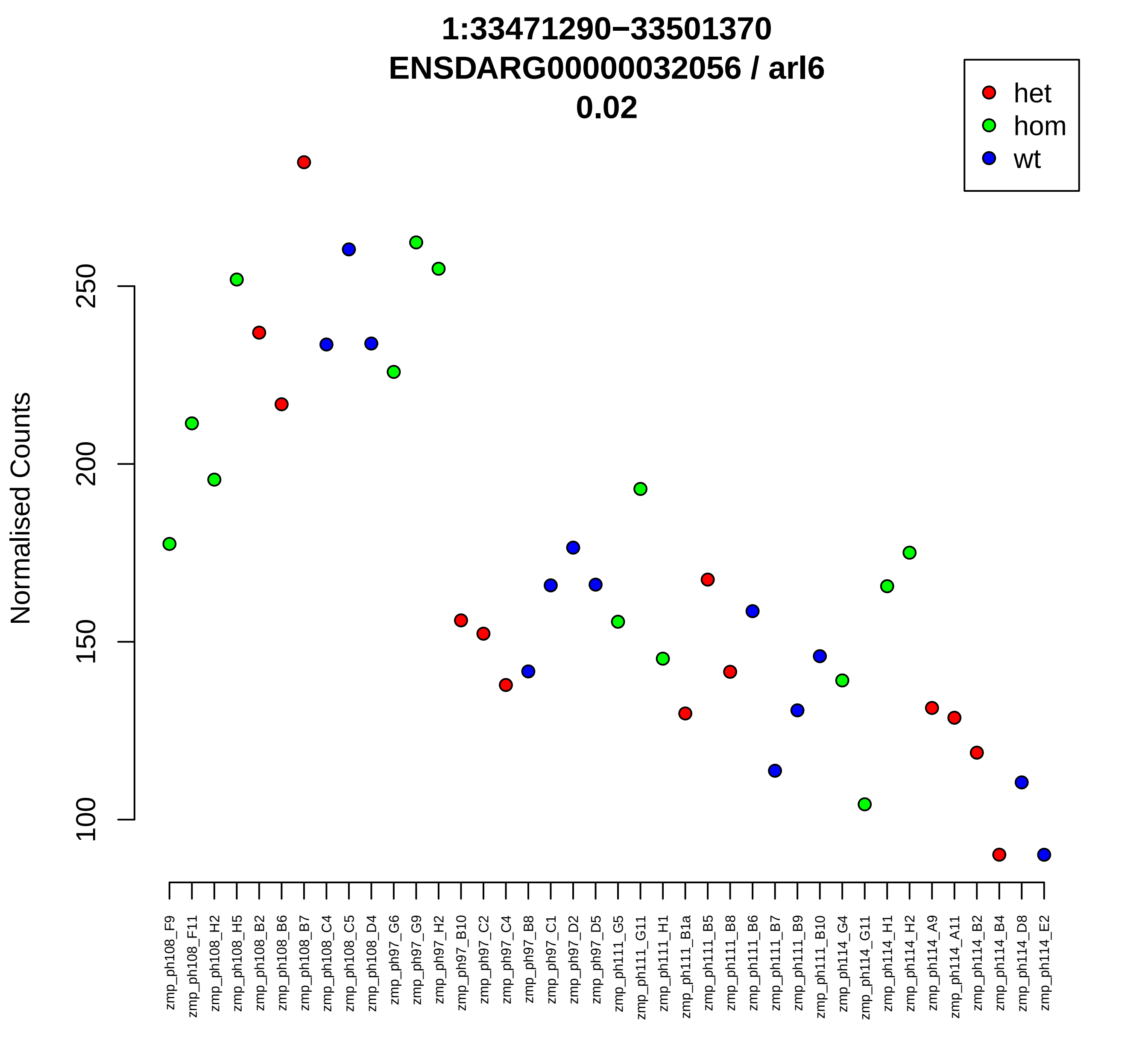

1:33471290−33501370
ENSDARG00000032056 / arl6
0.02
het
hom
wt
Normalised Counts
100
150
200
250
zmp_ph108_F9
zmp_ph108_F11
zmp_ph108_H2
zmp_ph108_H5
zmp_ph108_B2
zmp_ph108_B6
zmp_ph108_B7
zmp_ph108_C4
zmp_ph108_C5
zmp_ph108_D4
zmp_ph97_G6
zmp_ph97_G9
zmp_ph97_H2
zmp_ph97_B10
zmp_ph97_C2
zmp_ph97_C4
zmp_ph97_B8
zmp_ph97_C1
zmp_ph97_D2
zmp_ph97_D5
zmp_ph111_G5
zmp_ph111_G11
zmp_ph111_H1
zmp_ph111_B1a
zmp_ph111_B5
zmp_ph111_B8
zmp_ph111_B6
zmp_ph111_B7
zmp_ph111_B9
zmp_ph111_B10
zmp_ph114_G4
zmp_ph114_G11
zmp_ph114_H1
zmp_ph114_H2
zmp_ph114_A9
zmp_ph114_A11
zmp_ph114_B2
zmp_ph114_B4
zmp_ph114_D8
zmp_ph114_E2

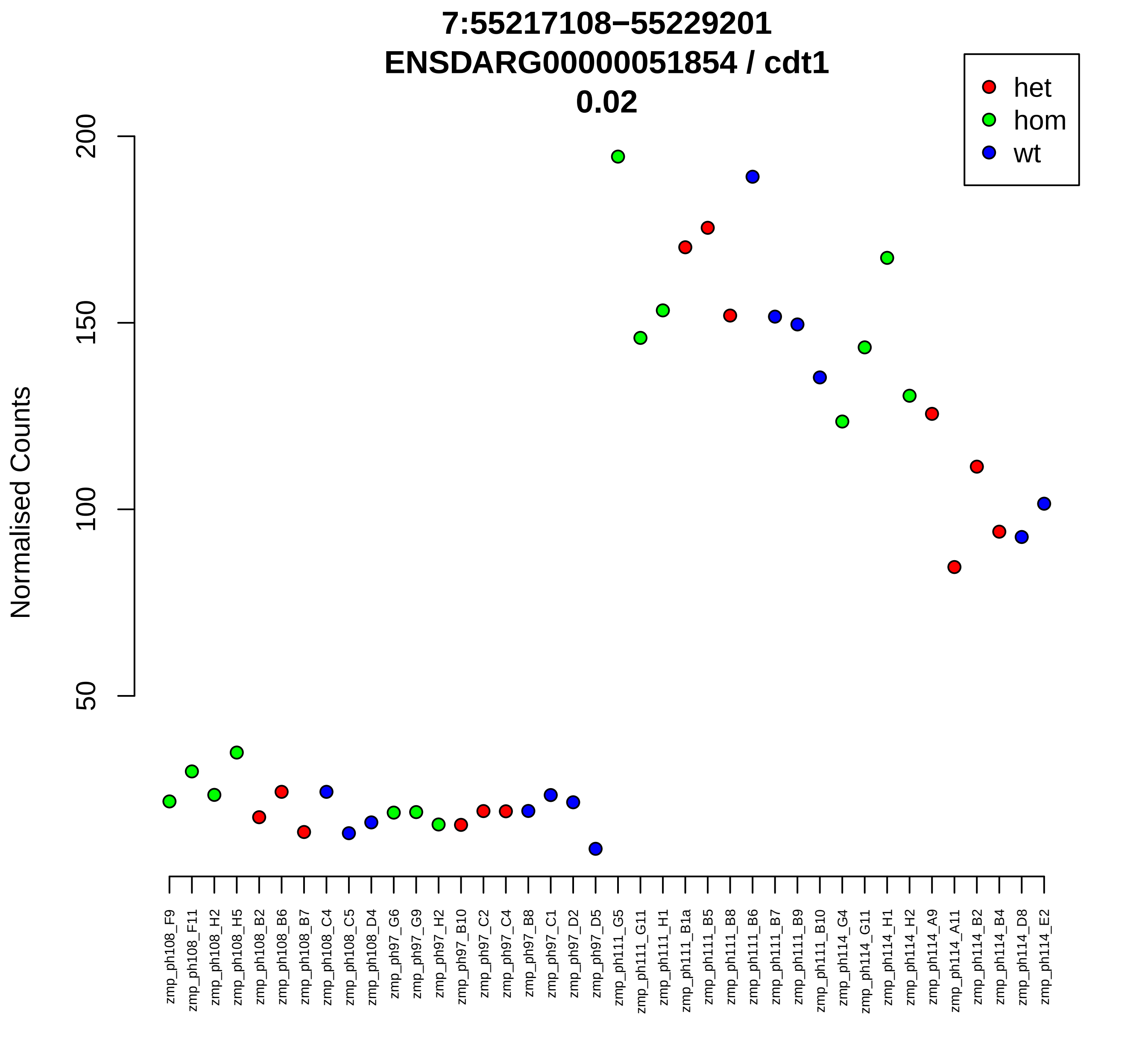

7:55217108−55229201
ENSDARG00000051854 / cdt1
0.02
het
hom
wt
Normalised Counts
50
100
150
200
zmp_ph108_F9
zmp_ph108_F11
zmp_ph108_H2
zmp_ph108_H5
zmp_ph108_B2
zmp_ph108_B6
zmp_ph108_B7
zmp_ph108_C4
zmp_ph108_C5
zmp_ph108_D4
zmp_ph97_G6
zmp_ph97_G9
zmp_ph97_H2
zmp_ph97_B10
zmp_ph97_C2
zmp_ph97_C4
zmp_ph97_B8
zmp_ph97_C1
zmp_ph97_D2
zmp_ph97_D5
zmp_ph111_G5
zmp_ph111_G11
zmp_ph111_H1
zmp_ph111_B1a
zmp_ph111_B5
zmp_ph111_B8
zmp_ph111_B6
zmp_ph111_B7
zmp_ph111_B9
zmp_ph111_B10
zmp_ph114_G4
zmp_ph114_G11
zmp_ph114_H1
zmp_ph114_H2
zmp_ph114_A9
zmp_ph114_A11
zmp_ph114_B2
zmp_ph114_B4
zmp_ph114_D8
zmp_ph114_E2

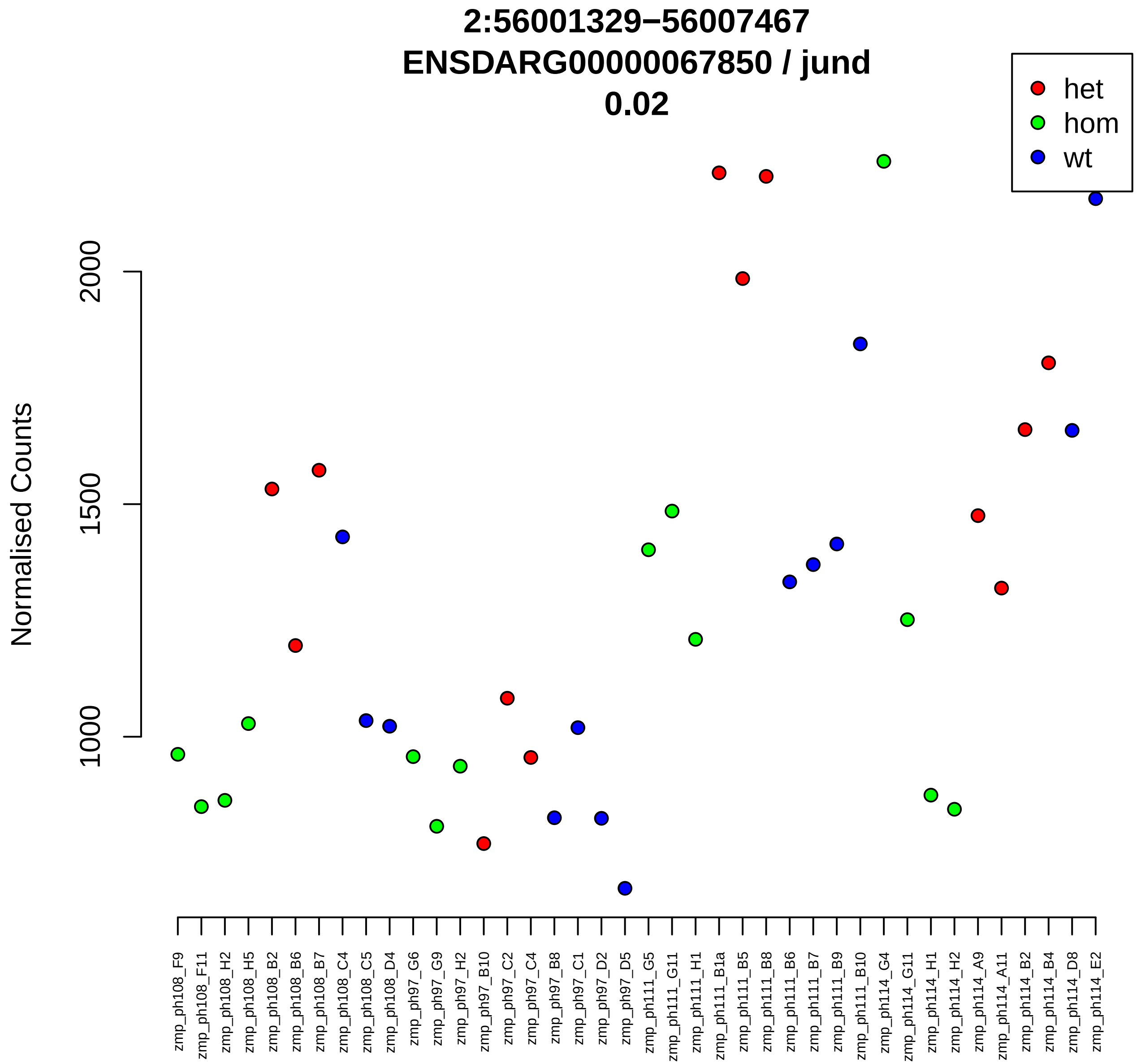

2:56001329−56007467
ENSDARG00000067850 / jund
0.02
het
hom
wt
Normalised Counts
1000
1500
2000
zmp_ph108_F9
zmp_ph108_F11
zmp_ph108_H2
zmp_ph108_H5
zmp_ph108_B2
zmp_ph108_B6
zmp_ph108_B7
zmp_ph108_C4
zmp_ph108_C5
zmp_ph108_D4
zmp_ph97_G6
zmp_ph97_G9
zmp_ph97_H2
zmp_ph97_B10
zmp_ph97_C2
zmp_ph97_C4
zmp_ph97_B8
zmp_ph97_C1
zmp_ph97_D2
zmp_ph97_D5
zmp_ph111_G5
zmp_ph111_G11
zmp_ph111_H1
zmp_ph111_B1a
zmp_ph111_B5
zmp_ph111_B8
zmp_ph111_B6
zmp_ph111_B7
zmp_ph111_B9
zmp_ph111_B10
zmp_ph114_G4
zmp_ph114_G11
zmp_ph114_H1
zmp_ph114_H2
zmp_ph114_A9
zmp_ph114_A11
zmp_ph114_B2
zmp_ph114_B4
zmp_ph114_D8
zmp_ph114_E2

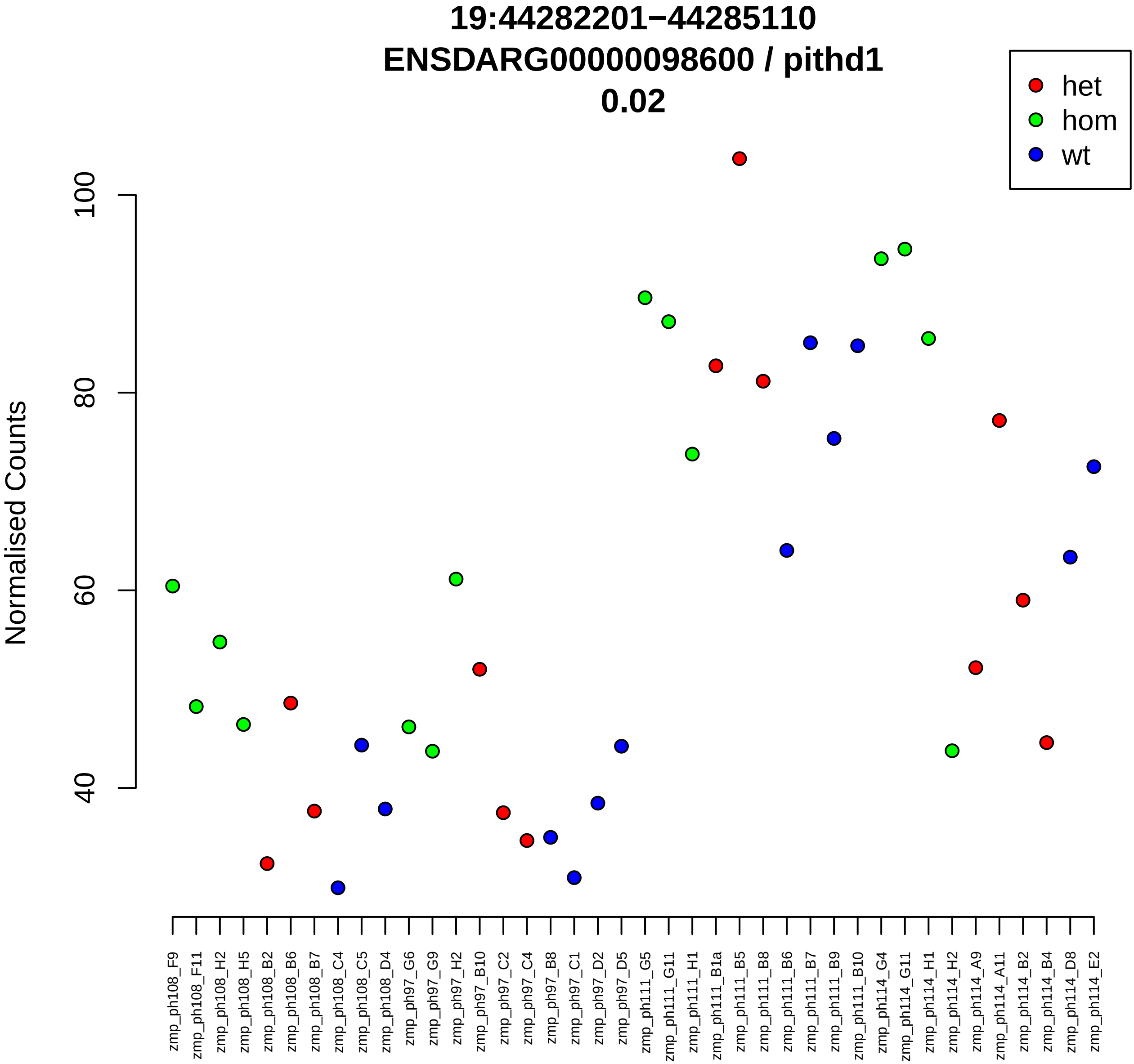

19:44282201−44285110
ENSDARG00000098600 / pithd1
0.02
het
hom
wt
Normalised Counts
100
80
60
40
zmp_ph108_F9
zmp_ph108_F11
zmp_ph108_H2
zmp_ph108_H5
zmp_ph108_B2
zmp_ph108_B6
zmp_ph108_B7
zmp_ph108_C4
zmp_ph108_C5
zmp_ph108_D4
zmp_ph97_G6
zmp_ph97_G9
zmp_ph97_H2
zmp_ph97_B10
zmp_ph97_C2
zmp_ph97_C4
zmp_ph97_B8
zmp_ph97_C1
zmp_ph97_D2
zmp_ph97_D5
zmp_ph111_G5
zmp_ph111_G11
zmp_ph111_H1
zmp_ph111_B1a
zmp_ph111_B5
zmp_ph111_B8
zmp_ph111_B6
zmp_ph111_B7
zmp_ph111_B9
zmp_ph111_B10
zmp_ph114_G4
zmp_ph114_G11
zmp_ph114_H1
zmp_ph114_H2
zmp_ph114_A9
zmp_ph114_A11
zmp_ph114_B2
zmp_ph114_B4
zmp_ph114_D8
zmp_ph114_E2

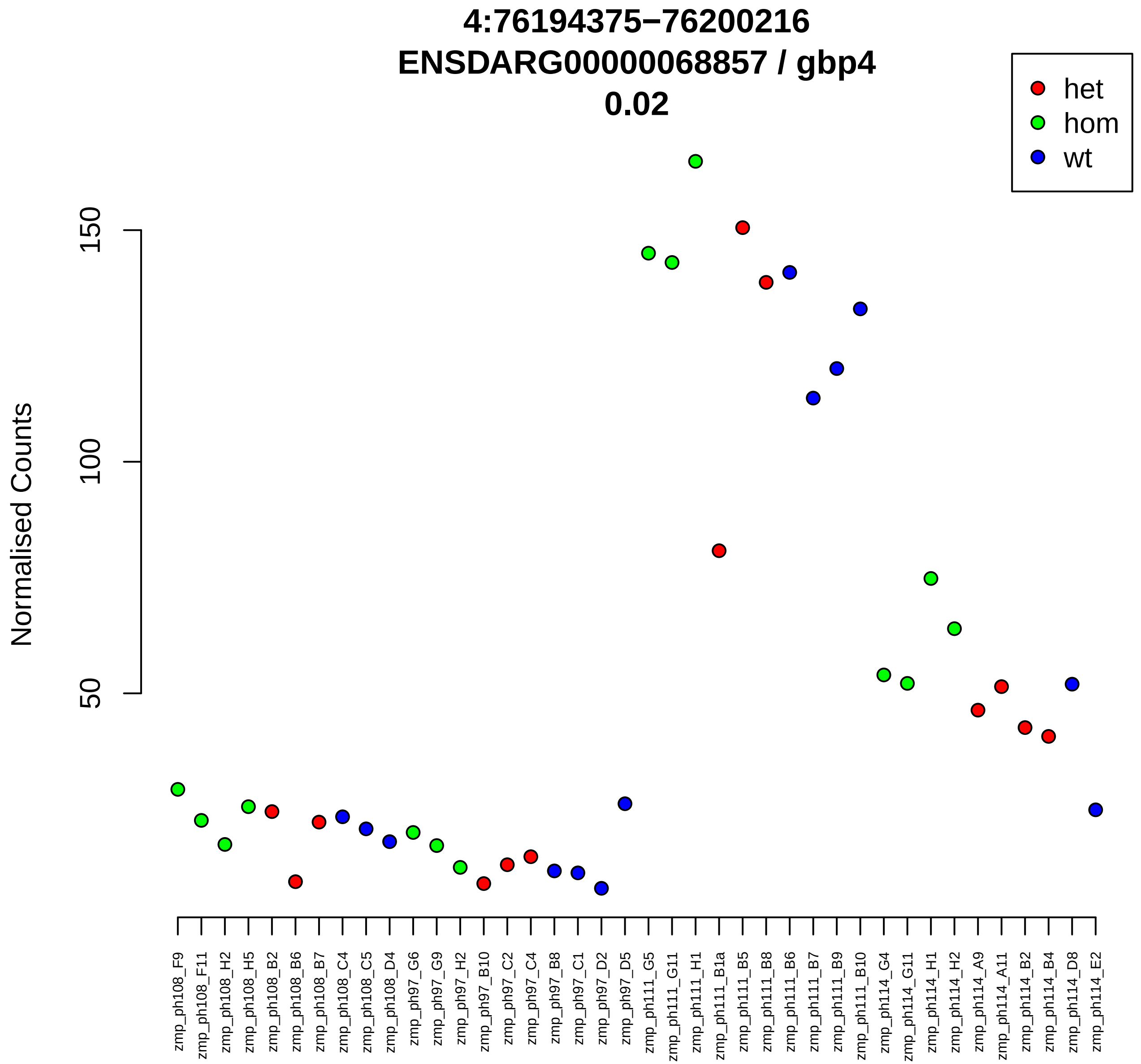
4:76194375−76200216
ENSDARG00000068857 / gbp4
0.02
het
hom
wt
Normalised Counts
150
100
50
zmp_ph108_F9
zmp_ph108_F11
zmp_ph108_H2
zmp_ph108_H5
zmp_ph108_B2
zmp_ph108_B6
zmp_ph108_B7
zmp_ph108_C4
zmp_ph108_C5
zmp_ph108_D4
zmp_ph97_G6
zmp_ph97_G9
zmp_ph97_H2
zmp_ph97_B10
zmp_ph97_C2
zmp_ph97_C4
zmp_ph97_B8
zmp_ph97_C1
zmp_ph97_D2
zmp_ph97_D5
zmp_ph111_G5
zmp_ph111_G11
zmp_ph111_H1
zmp_ph111_B1a
zmp_ph111_B5
zmp_ph111_B8
zmp_ph111_B6
zmp_ph111_B7
zmp_ph111_B9
zmp_ph111_B10
zmp_ph114_G4
zmp_ph114_G11
zmp_ph114_H1
zmp_ph114_H2
zmp_ph114_A9
zmp_ph114_A11
zmp_ph114_B2
zmp_ph114_B4
zmp_ph114_D8
zmp_ph114_E2

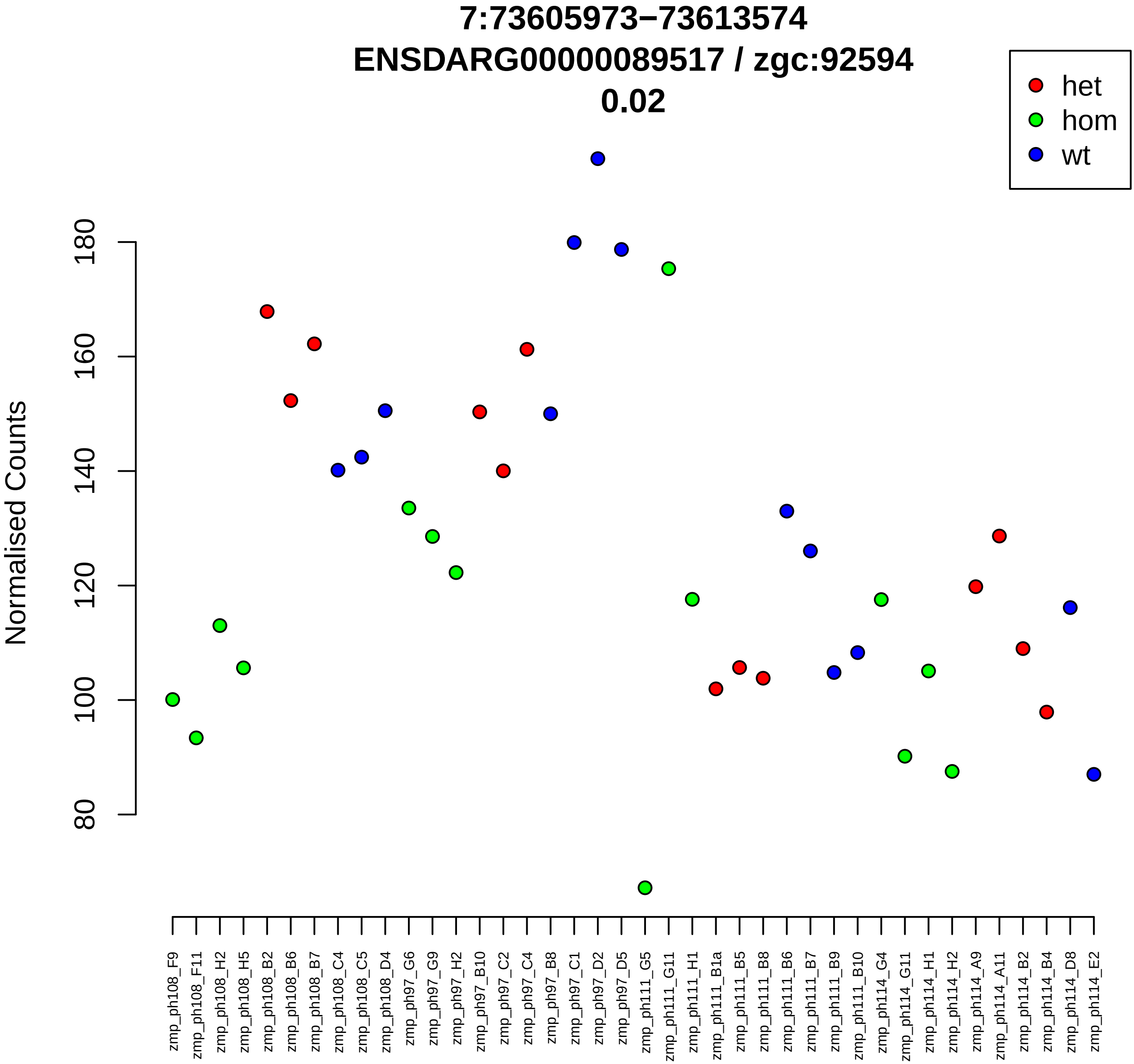
7:73605973−73613574
ENSDARG00000089517 / zgc:92594
0.02
het
hom
wt
Normalised Counts
80
100
120
140
160
180
zmp_ph108_F9
zmp_ph108_F11
zmp_ph108_H2
zmp_ph108_H5
zmp_ph108_B2
zmp_ph108_B6
zmp_ph108_B7
zmp_ph108_C4
zmp_ph108_C5
zmp_ph108_D4
zmp_ph97_G6
zmp_ph97_G9
zmp_ph97_H2
zmp_ph97_B10
zmp_ph97_C2
zmp_ph97_C4
zmp_ph97_B8
zmp_ph97_C1
zmp_ph97_D2
zmp_ph97_D5
zmp_ph111_G5
zmp_ph111_G11
zmp_ph111_H1
zmp_ph111_B1a
zmp_ph111_B5
zmp_ph111_B8
zmp_ph111_B6
zmp_ph111_B7
zmp_ph111_B9
zmp_ph111_B10
zmp_ph114_G4
zmp_ph114_G11
zmp_ph114_H1
zmp_ph114_H2
zmp_ph114_A9
zmp_ph114_A11
zmp_ph114_B2
zmp_ph114_B4
zmp_ph114_D8
zmp_ph114_E2

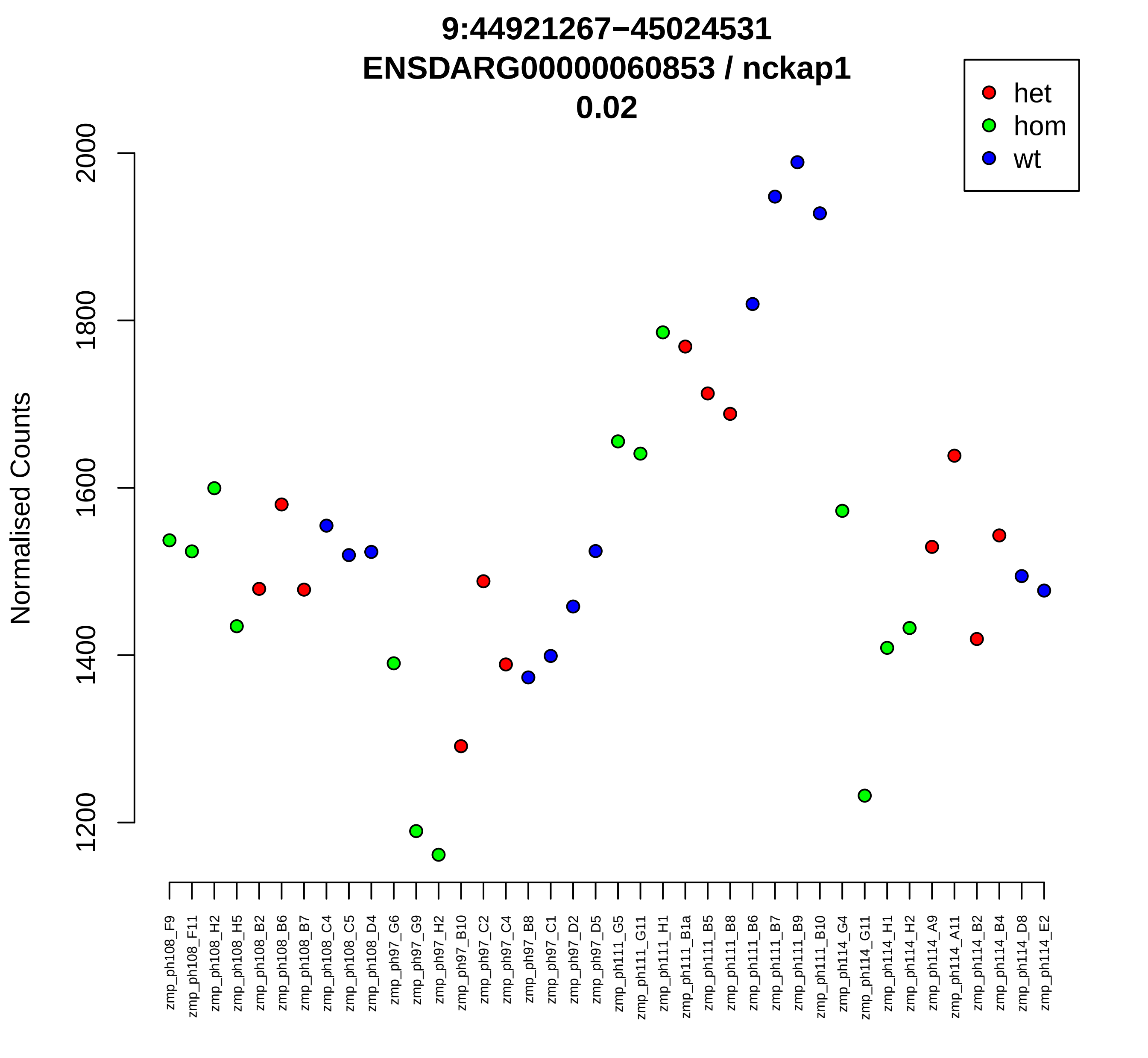

9:44921267−45024531
ENSDARG00000060853 / nckap1
0.02
het
hom
wt
Normalised Counts
1200
1400
1600
1800
2000
zmp_ph108_F9
zmp_ph108_F11
zmp_ph108_H2
zmp_ph108_H5
zmp_ph108_B2
zmp_ph108_B6
zmp_ph108_B7
zmp_ph108_C4
zmp_ph108_C5
zmp_ph108_D4
zmp_ph97_G6
zmp_ph97_G9
zmp_ph97_H2
zmp_ph97_B10
zmp_ph97_C2
zmp_ph97_C4
zmp_ph97_B8
zmp_ph97_C1
zmp_ph97_D2
zmp_ph97_D5
zmp_ph111_G5
zmp_ph111_G11
zmp_ph111_H1
zmp_ph111_B1a
zmp_ph111_B5
zmp_ph111_B8
zmp_ph111_B6
zmp_ph111_B7
zmp_ph111_B9
zmp_ph111_B10
zmp_ph114_G4
zmp_ph114_G11
zmp_ph114_H1
zmp_ph114_H2
zmp_ph114_A9
zmp_ph114_A11
zmp_ph114_B2
zmp_ph114_B4
zmp_ph114_D8
zmp_ph114_E2

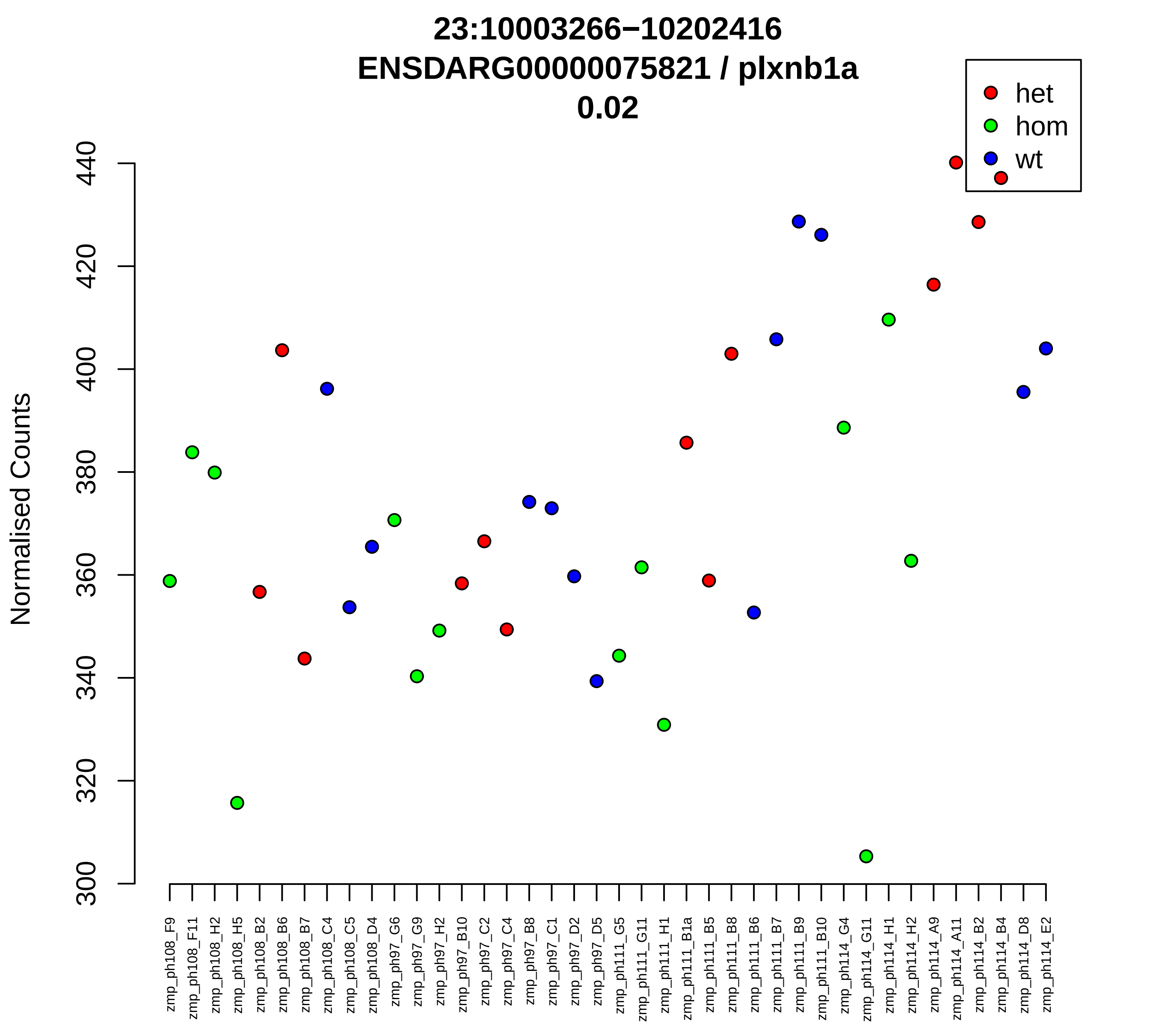

23:10003266−10202416
ENSDARG00000075821 / plxnb1a
0.02
het
hom
wt
Normalised Counts
300
320
340
360
380
400
420
440
zmp_ph108_F9
zmp_ph108_F11
zmp_ph108_H2
zmp_ph108_H5
zmp_ph108_B2
zmp_ph108_B6
zmp_ph108_B7
zmp_ph108_C4
zmp_ph108_C5
zmp_ph108_D4
zmp_ph97_G6
zmp_ph97_G9
zmp_ph97_H2
zmp_ph97_B10
zmp_ph97_C2
zmp_ph97_C4
zmp_ph97_B8
zmp_ph97_C1
zmp_ph97_D2
zmp_ph97_D5
zmp_ph111_G5
zmp_ph111_G11
zmp_ph111_H1
zmp_ph111_B1a
zmp_ph111_B5
zmp_ph111_B8
zmp_ph111_B6
zmp_ph111_B7
zmp_ph111_B9
zmp_ph111_B10
zmp_ph114_G4
zmp_ph114_G11
zmp_ph114_H1
zmp_ph114_H2
zmp_ph114_A9
zmp_ph114_A11
zmp_ph114_B2
zmp_ph114_B4
zmp_ph114_D8
zmp_ph114_E2

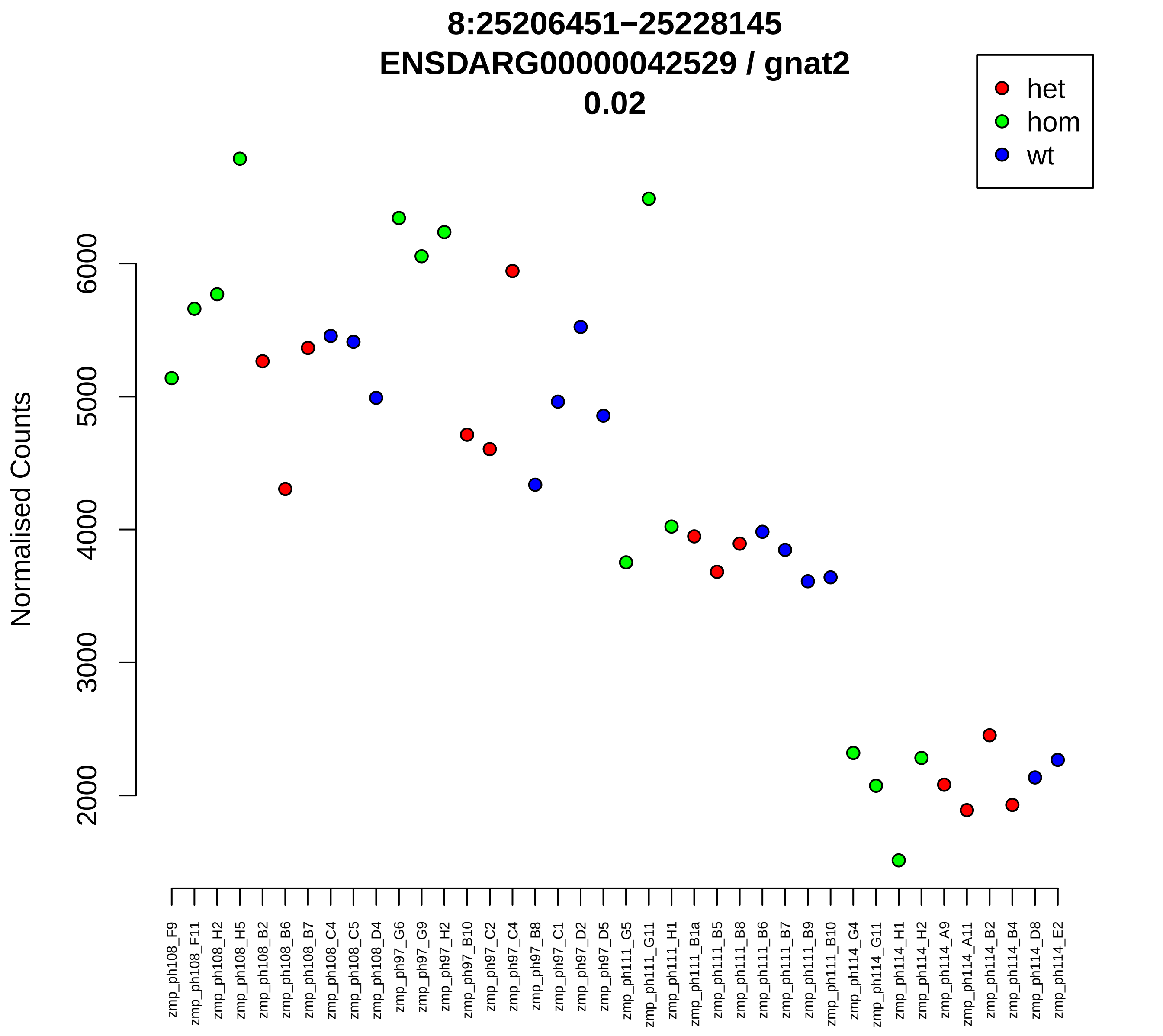

8:25206451−25228145
ENSDARG00000042529 / gnat2
0.02
het
hom
wt
Normalised Counts
2000
3000
4000
5000
6000
zmp_ph108_F9
zmp_ph108_F11
zmp_ph108_H2
zmp_ph108_H5
zmp_ph108_B2
zmp_ph108_B6
zmp_ph108_B7
zmp_ph108_C4
zmp_ph108_C5
zmp_ph108_D4
zmp_ph97_G6
zmp_ph97_G9
zmp_ph97_H2
zmp_ph97_B10
zmp_ph97_C2
zmp_ph97_C4
zmp_ph97_B8
zmp_ph97_C1
zmp_ph97_D2
zmp_ph97_D5
zmp_ph111_G5
zmp_ph111_G11
zmp_ph111_H1
zmp_ph111_B1a
zmp_ph111_B5
zmp_ph111_B8
zmp_ph111_B6
zmp_ph111_B7
zmp_ph111_B9
zmp_ph111_B10
zmp_ph114_G4
zmp_ph114_G11
zmp_ph114_H1
zmp_ph114_H2
zmp_ph114_A9
zmp_ph114_A11
zmp_ph114_B2
zmp_ph114_B4
zmp_ph114_D8
zmp_ph114_E2

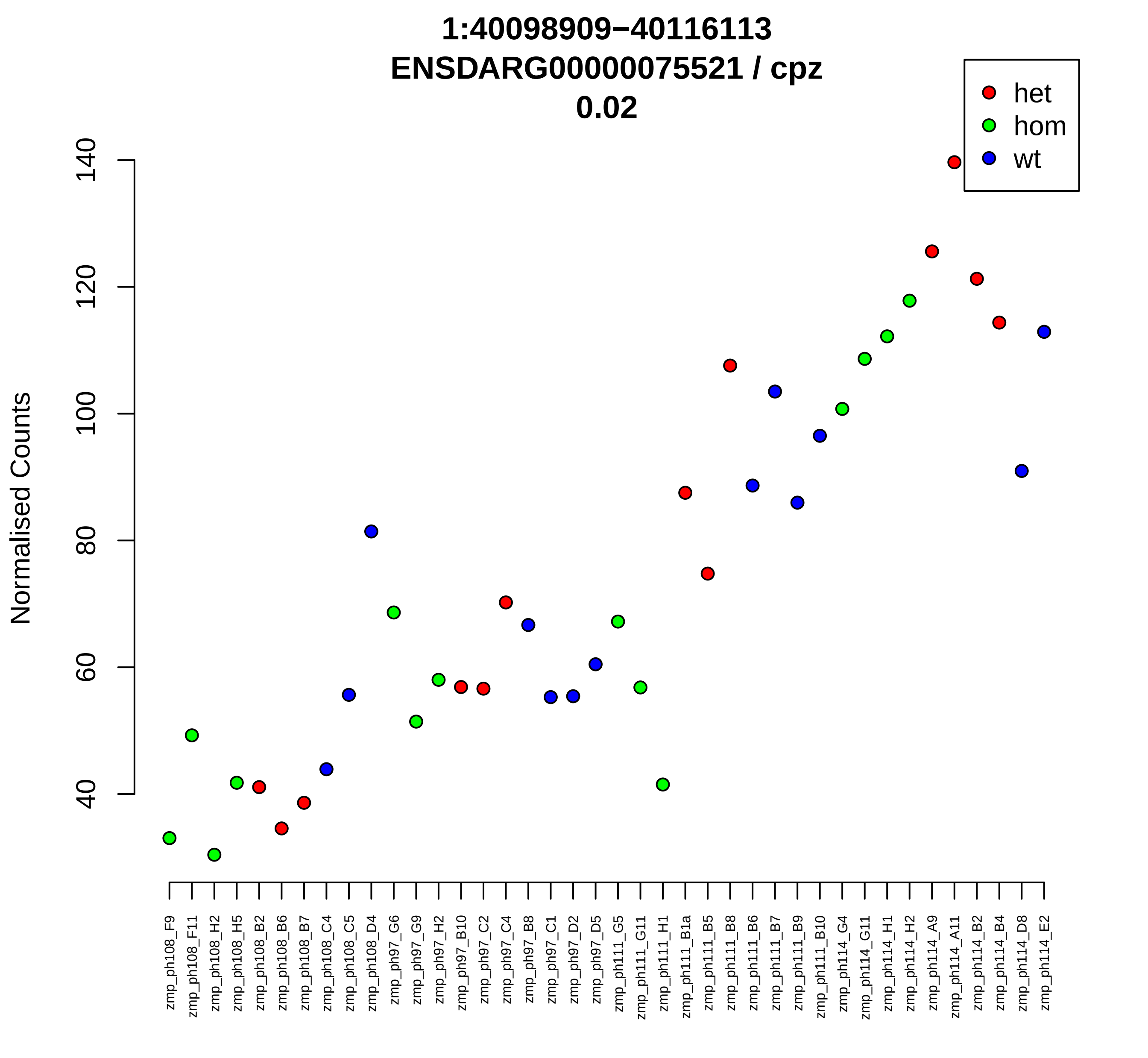
1:40098909−40116113
ENSDARG00000075521 / cpz
0.02
het
hom
wt
Normalised Counts
40
60
80
100
120
140
zmp_ph108_F9
zmp_ph108_F11
zmp_ph108_H2
zmp_ph108_H5
zmp_ph108_B2
zmp_ph108_B6
zmp_ph108_B7
zmp_ph108_C4
zmp_ph108_C5
zmp_ph108_D4
zmp_ph97_G6
zmp_ph97_G9
zmp_ph97_H2
zmp_ph97_B10
zmp_ph97_C2
zmp_ph97_C4
zmp_ph97_B8
zmp_ph97_C1
zmp_ph97_D2
zmp_ph97_D5
zmp_ph111_G5
zmp_ph111_G11
zmp_ph111_H1
zmp_ph111_B1a
zmp_ph111_B5
zmp_ph111_B8
zmp_ph111_B6
zmp_ph111_B7
zmp_ph111_B9
zmp_ph111_B10
zmp_ph114_G4
zmp_ph114_G11
zmp_ph114_H1
zmp_ph114_H2
zmp_ph114_A9
zmp_ph114_A11
zmp_ph114_B2
zmp_ph114_B4
zmp_ph114_D8
zmp_ph114_E2

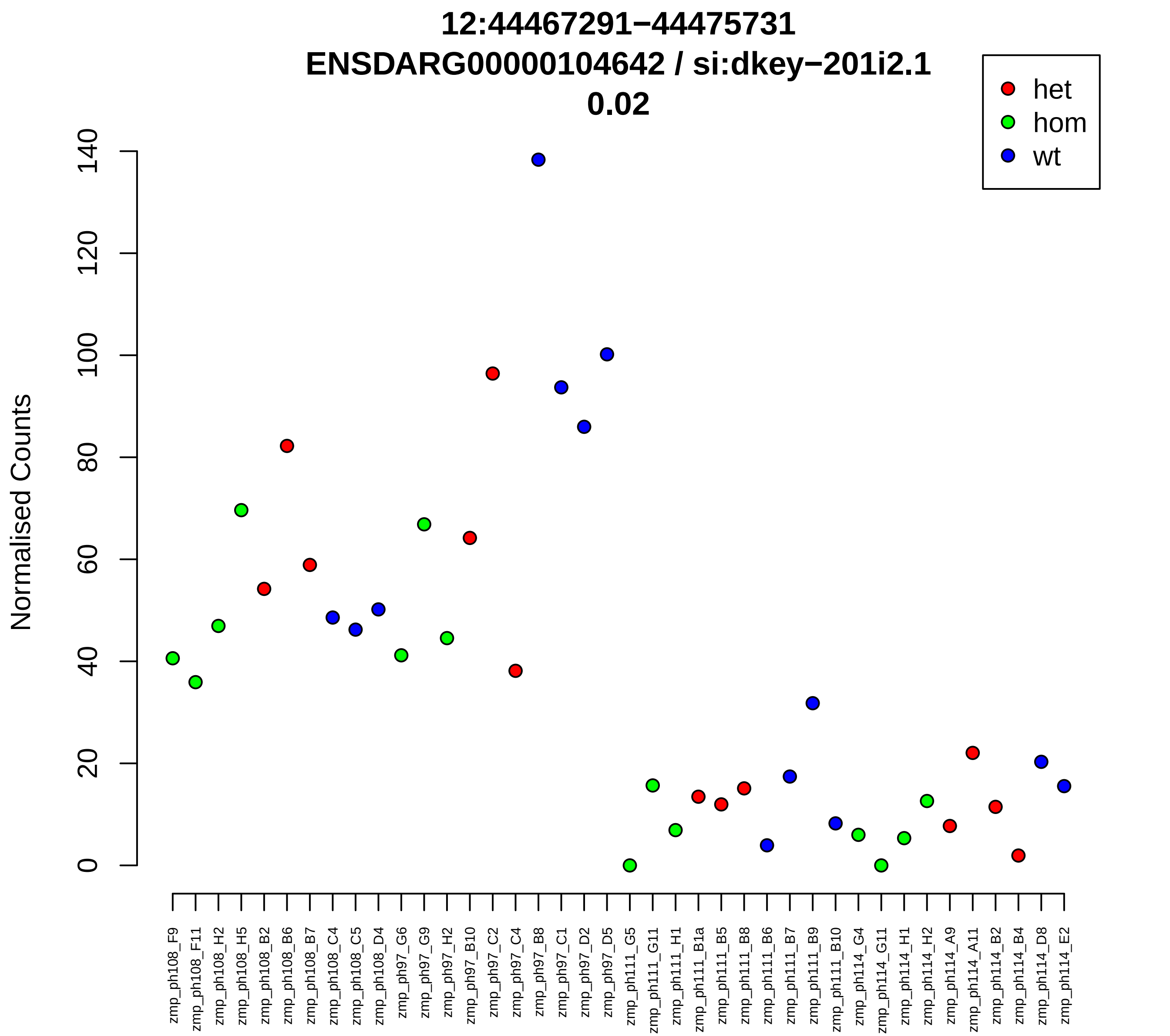
12:44467291−44475731
ENSDARG00000104642 / si:dkey−201i2.1
0.02
het
hom
wt
Normalised Counts
0
20
40
60
80
100
120
140
zmp_ph108_F9
zmp_ph108_F11
zmp_ph108_H2
zmp_ph108_H5
zmp_ph108_B2
zmp_ph108_B6
zmp_ph108_B7
zmp_ph108_C4
zmp_ph108_C5
zmp_ph108_D4
zmp_ph97_G6
zmp_ph97_G9
zmp_ph97_H2
zmp_ph97_B10
zmp_ph97_C2
zmp_ph97_C4
zmp_ph97_B8
zmp_ph97_C1
zmp_ph97_D2
zmp_ph97_D5
zmp_ph111_G5
zmp_ph111_G11
zmp_ph111_H1
zmp_ph111_B1a
zmp_ph111_B5
zmp_ph111_B8
zmp_ph111_B6
zmp_ph111_B7
zmp_ph111_B9
zmp_ph111_B10
zmp_ph114_G4
zmp_ph114_G11
zmp_ph114_H1
zmp_ph114_H2
zmp_ph114_A9
zmp_ph114_A11
zmp_ph114_B2
zmp_ph114_B4
zmp_ph114_D8
zmp_ph114_E2

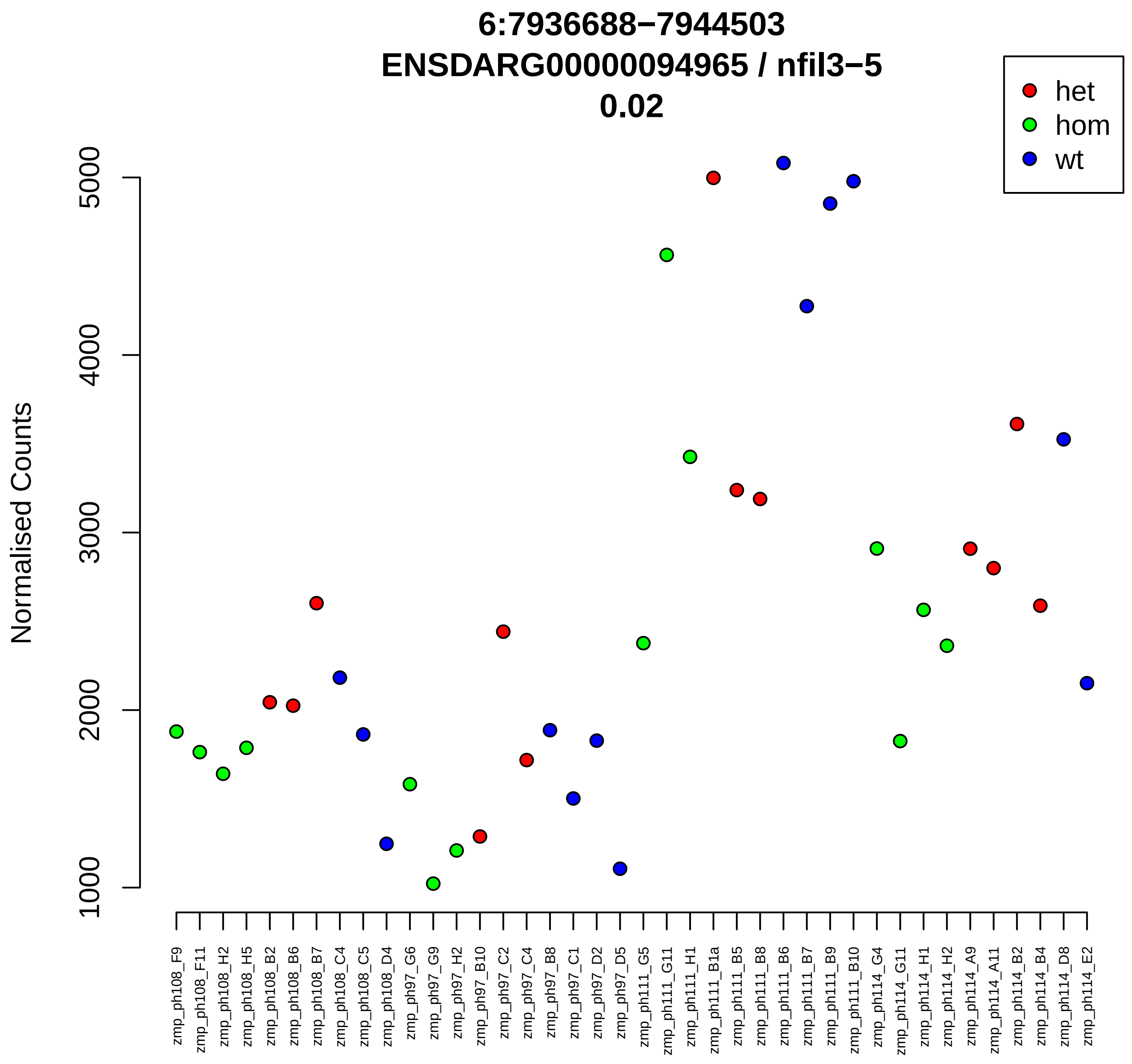

6:7936688−7944503
ENSDARG00000094965 / nfil3−5
0.02
het
hom
wt
Normalised Counts
1000
2000
3000
4000
5000
zmp_ph108_F9
zmp_ph108_F11
zmp_ph108_H2
zmp_ph108_H5
zmp_ph108_B2
zmp_ph108_B6
zmp_ph108_B7
zmp_ph108_C4
zmp_ph108_C5
zmp_ph108_D4
zmp_ph97_G6
zmp_ph97_G9
zmp_ph97_H2
zmp_ph97_B10
zmp_ph97_C2
zmp_ph97_C4
zmp_ph97_B8
zmp_ph97_C1
zmp_ph97_D2
zmp_ph97_D5
zmp_ph111_G5
zmp_ph111_G11
zmp_ph111_H1
zmp_ph111_B1a
zmp_ph111_B5
zmp_ph111_B8
zmp_ph111_B6
zmp_ph111_B7
zmp_ph111_B9
zmp_ph111_B10
zmp_ph114_G4
zmp_ph114_G11
zmp_ph114_H1
zmp_ph114_H2
zmp_ph114_A9
zmp_ph114_A11
zmp_ph114_B2
zmp_ph114_B4
zmp_ph114_D8
zmp_ph114_E2

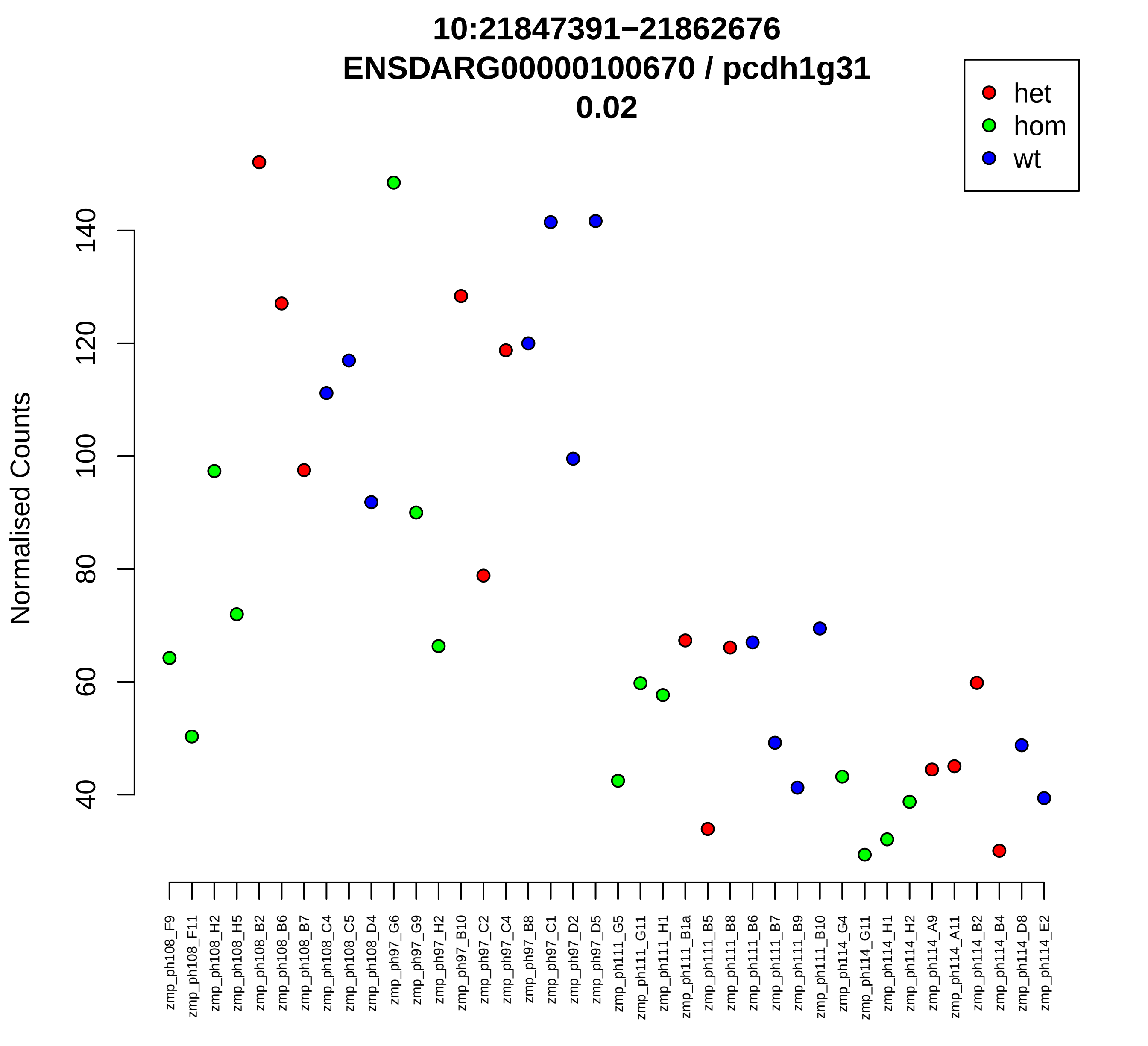

10:21847391−21862676
ENSDARG00000100670 / pcdh1g31
0.02
het
hom
wt
Normalised Counts
40
60
80
100
120
140
zmp_ph108_F9
zmp_ph108_F11
zmp_ph108_H2
zmp_ph108_H5
zmp_ph108_B2
zmp_ph108_B6
zmp_ph108_B7
zmp_ph108_C4
zmp_ph108_C5
zmp_ph108_D4
zmp_ph97_G6
zmp_ph97_G9
zmp_ph97_H2
zmp_ph97_B10
zmp_ph97_C2
zmp_ph97_C4
zmp_ph97_B8
zmp_ph97_C1
zmp_ph97_D2
zmp_ph97_D5
zmp_ph111_G5
zmp_ph111_G11
zmp_ph111_H1
zmp_ph111_B1a
zmp_ph111_B5
zmp_ph111_B8
zmp_ph111_B6
zmp_ph111_B7
zmp_ph111_B9
zmp_ph111_B10
zmp_ph114_G4
zmp_ph114_G11
zmp_ph114_H1
zmp_ph114_H2
zmp_ph114_A9
zmp_ph114_A11
zmp_ph114_B2
zmp_ph114_B4
zmp_ph114_D8
zmp_ph114_E2

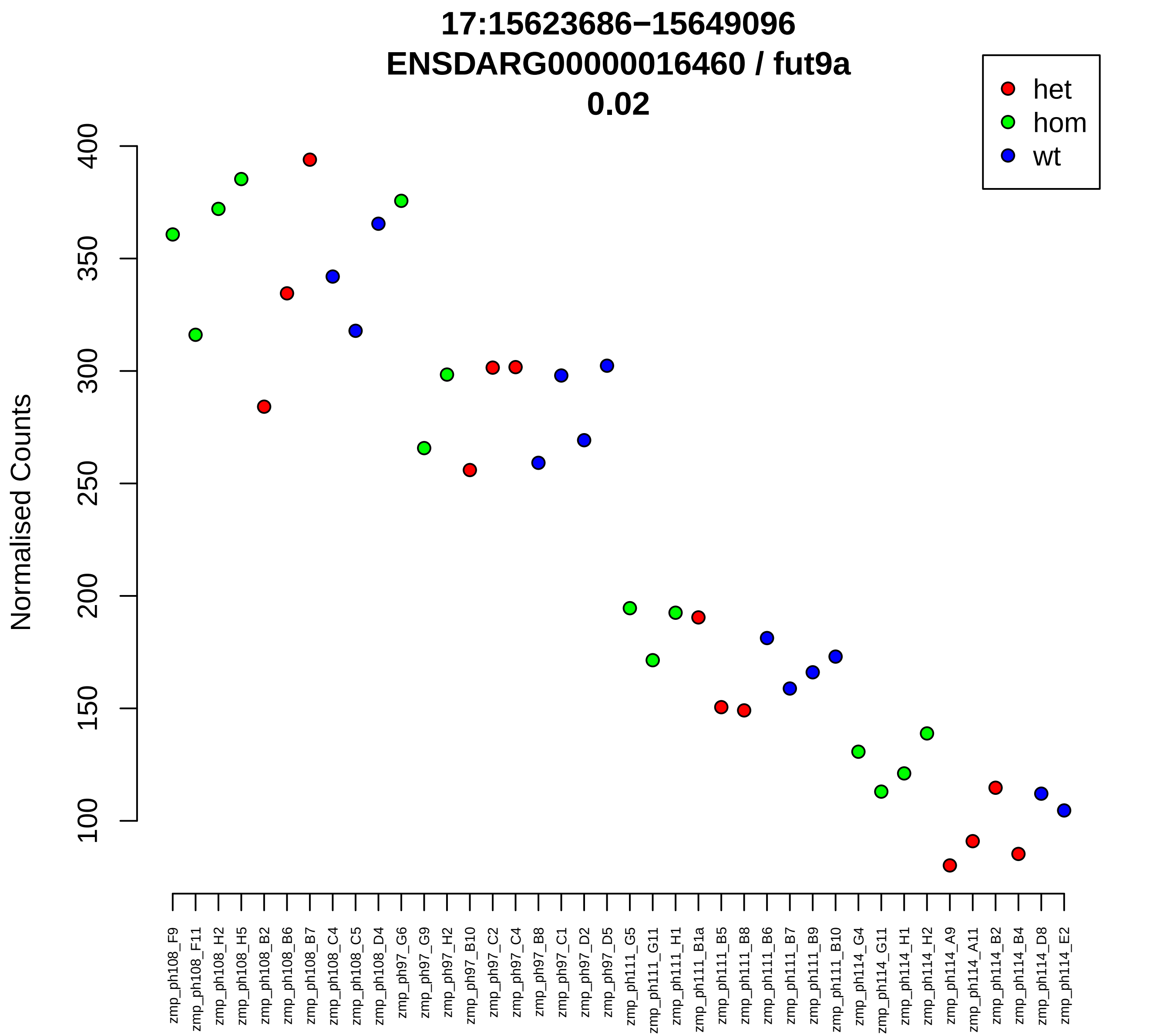

17:15623686−15649096
ENSDARG00000016460 / fut9a
0.02
het
hom
wt
Normalised Counts
100
150
200
250
300
350
400
zmp_ph108_F9
zmp_ph108_F11
zmp_ph108_H2
zmp_ph108_H5
zmp_ph108_B2
zmp_ph108_B6
zmp_ph108_B7
zmp_ph108_C4
zmp_ph108_C5
zmp_ph108_D4
zmp_ph97_G6
zmp_ph97_G9
zmp_ph97_H2
zmp_ph97_B10
zmp_ph97_C2
zmp_ph97_C4
zmp_ph97_B8
zmp_ph97_C1
zmp_ph97_D2
zmp_ph97_D5
zmp_ph111_G5
zmp_ph111_G11
zmp_ph111_H1
zmp_ph111_B1a
zmp_ph111_B5
zmp_ph111_B8
zmp_ph111_B6
zmp_ph111_B7
zmp_ph111_B9
zmp_ph111_B10
zmp_ph114_G4
zmp_ph114_G11
zmp_ph114_H1
zmp_ph114_H2
zmp_ph114_A9
zmp_ph114_A11
zmp_ph114_B2
zmp_ph114_B4
zmp_ph114_D8
zmp_ph114_E2

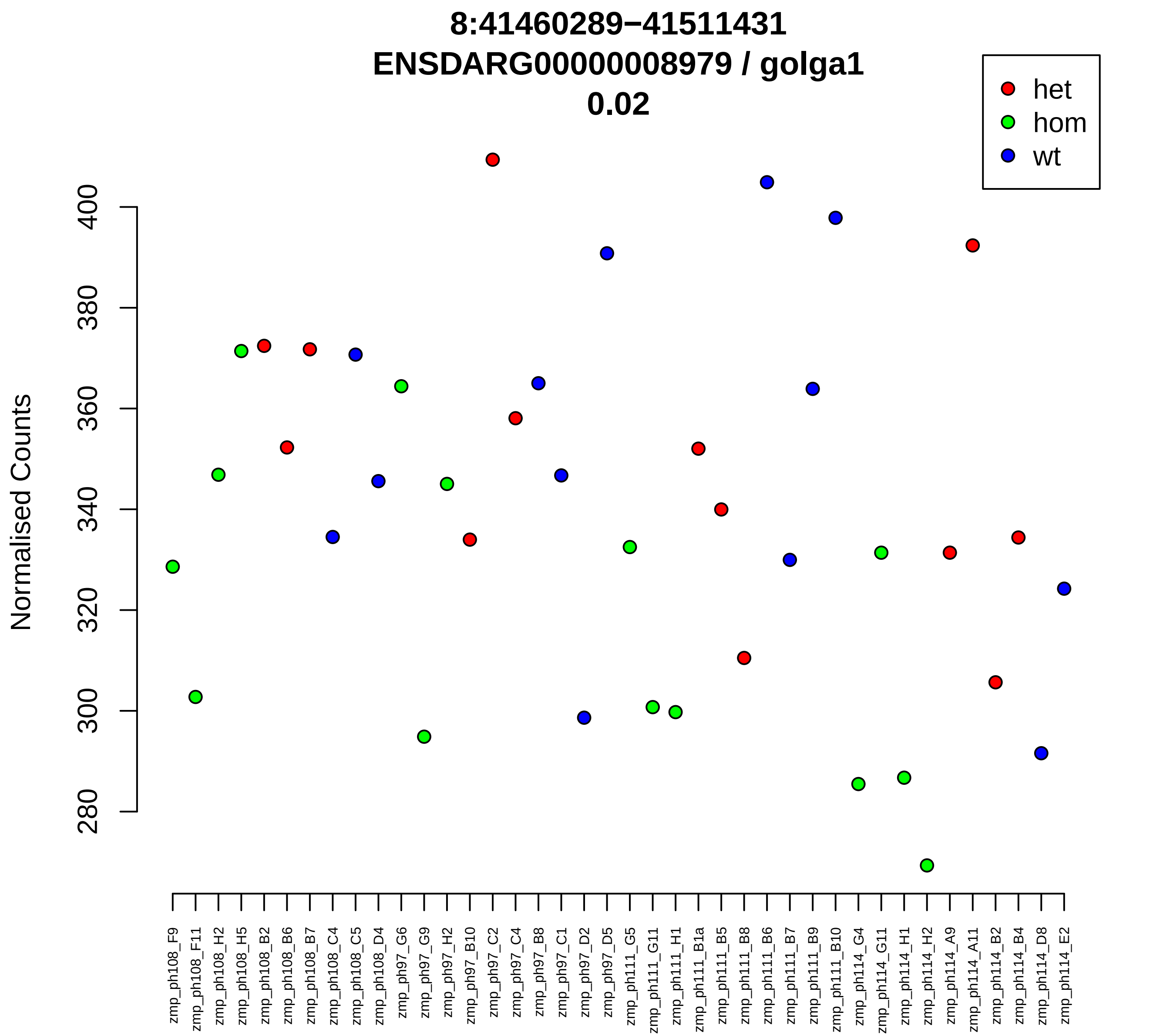
8:41460289−41511431
ENSDARG00000008979 / golga1
0.02
het
hom
wt
Normalised Counts
280
300
320
340
360
380
400
zmp_ph108_F9
zmp_ph108_F11
zmp_ph108_H2
zmp_ph108_H5
zmp_ph108_B2
zmp_ph108_B6
zmp_ph108_B7
zmp_ph108_C4
zmp_ph108_C5
zmp_ph108_D4
zmp_ph97_G6
zmp_ph97_G9
zmp_ph97_H2
zmp_ph97_B10
zmp_ph97_C2
zmp_ph97_C4
zmp_ph97_B8
zmp_ph97_C1
zmp_ph97_D2
zmp_ph97_D5
zmp_ph111_G5
zmp_ph111_G11
zmp_ph111_H1
zmp_ph111_B1a
zmp_ph111_B5
zmp_ph111_B8
zmp_ph111_B6
zmp_ph111_B7
zmp_ph111_B9
zmp_ph111_B10
zmp_ph114_G4
zmp_ph114_G11
zmp_ph114_H1
zmp_ph114_H2
zmp_ph114_A9
zmp_ph114_A11
zmp_ph114_B2
zmp_ph114_B4
zmp_ph114_D8
zmp_ph114_E2

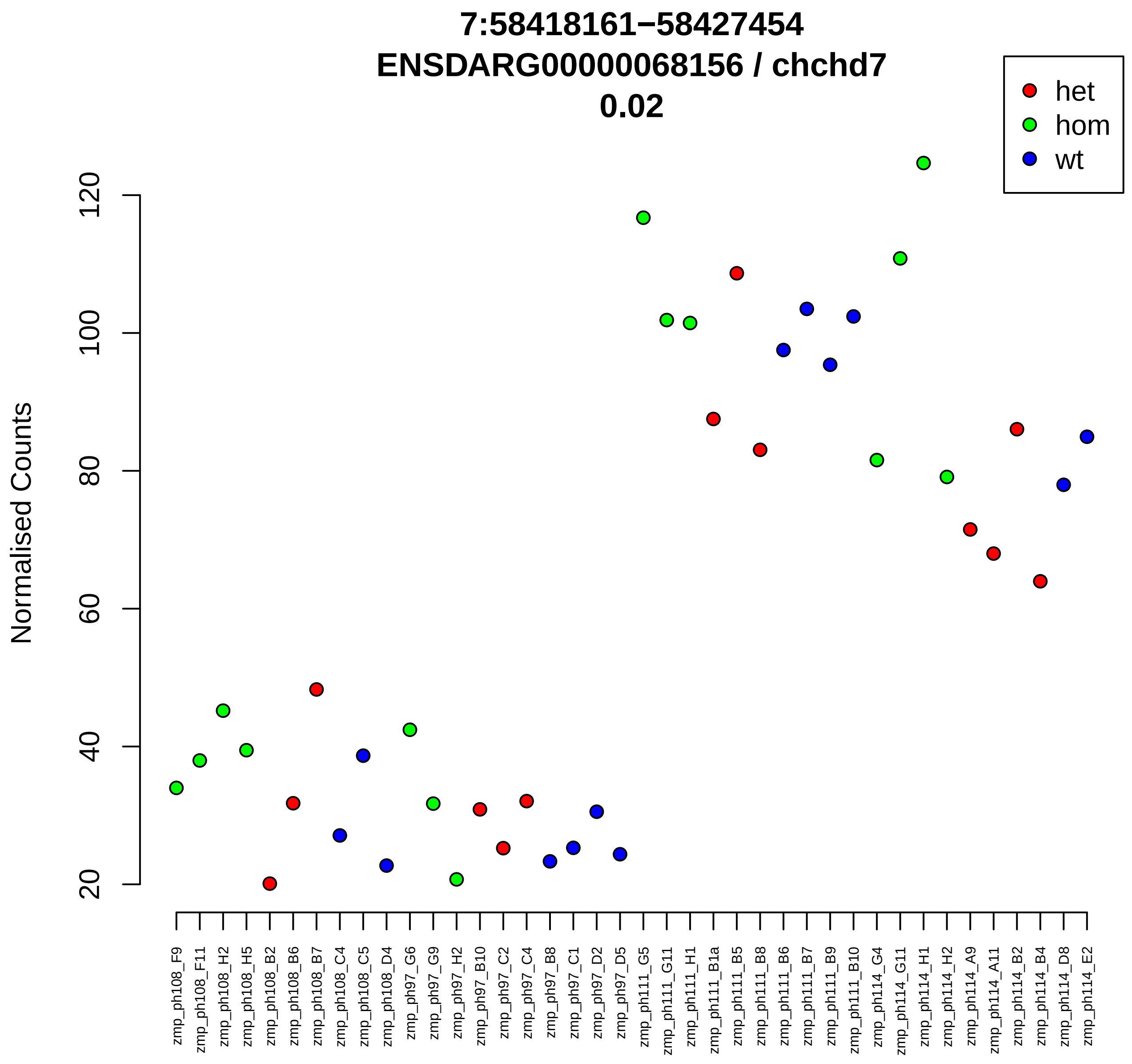

7:58418161−58427454
ENSDARG00000068156 / chchd7
0.02
het
hom
wt
Normalised Counts
20
40
60
80
100
120
zmp_ph108_F9
zmp_ph108_F11
zmp_ph108_H2
zmp_ph108_H5
zmp_ph108_B2
zmp_ph108_B6
zmp_ph108_B7
zmp_ph108_C4
zmp_ph108_C5
zmp_ph108_D4
zmp_ph97_G6
zmp_ph97_G9
zmp_ph97_H2
zmp_ph97_B10
zmp_ph97_C2
zmp_ph97_C4
zmp_ph97_B8
zmp_ph97_C1
zmp_ph97_D2
zmp_ph97_D5
zmp_ph111_G5
zmp_ph111_G11
zmp_ph111_H1
zmp_ph111_B1a
zmp_ph111_B5
zmp_ph111_B8
zmp_ph111_B6
zmp_ph111_B7
zmp_ph111_B9
zmp_ph111_B10
zmp_ph114_G4
zmp_ph114_G11
zmp_ph114_H1
zmp_ph114_H2
zmp_ph114_A9
zmp_ph114_A11
zmp_ph114_B2
zmp_ph114_B4
zmp_ph114_D8
zmp_ph114_E2

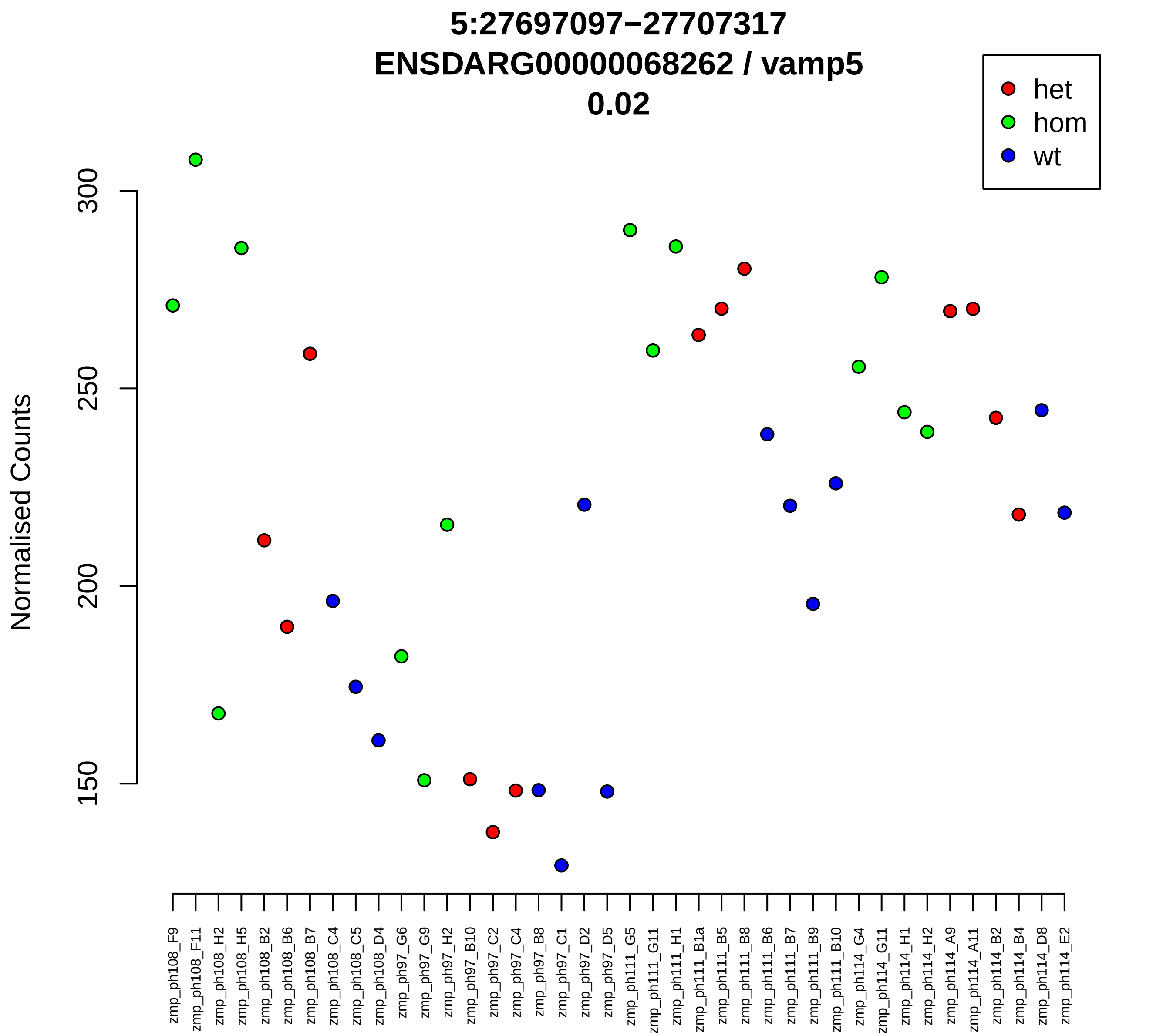
5:27697097−27707317
ENSDARG00000068262 / vamp5
0.02
het
hom
wt
Normalised Counts
300
250
200
150
zmp_ph108_F9
zmp_ph108_F11
zmp_ph108_H2
zmp_ph108_H5
zmp_ph108_B2
zmp_ph108_B6
zmp_ph108_B7
zmp_ph108_C4
zmp_ph108_C5
zmp_ph108_D4
zmp_ph97_G6
zmp_ph97_G9
zmp_ph97_H2
zmp_ph97_B10
zmp_ph97_C2
zmp_ph97_C4
zmp_ph97_B8
zmp_ph97_C1
zmp_ph97_D2
zmp_ph97_D5
zmp_ph111_G5
zmp_ph111_G11
zmp_ph111_H1
zmp_ph111_B1a
zmp_ph111_B5
zmp_ph111_B8
zmp_ph111_B6
zmp_ph111_B7
zmp_ph111_B9
zmp_ph111_B10
zmp_ph114_G4
zmp_ph114_G11
zmp_ph114_H1
zmp_ph114_H2
zmp_ph114_A9
zmp_ph114_A11
zmp_ph114_B2
zmp_ph114_B4
zmp_ph114_D8
zmp_ph114_E2

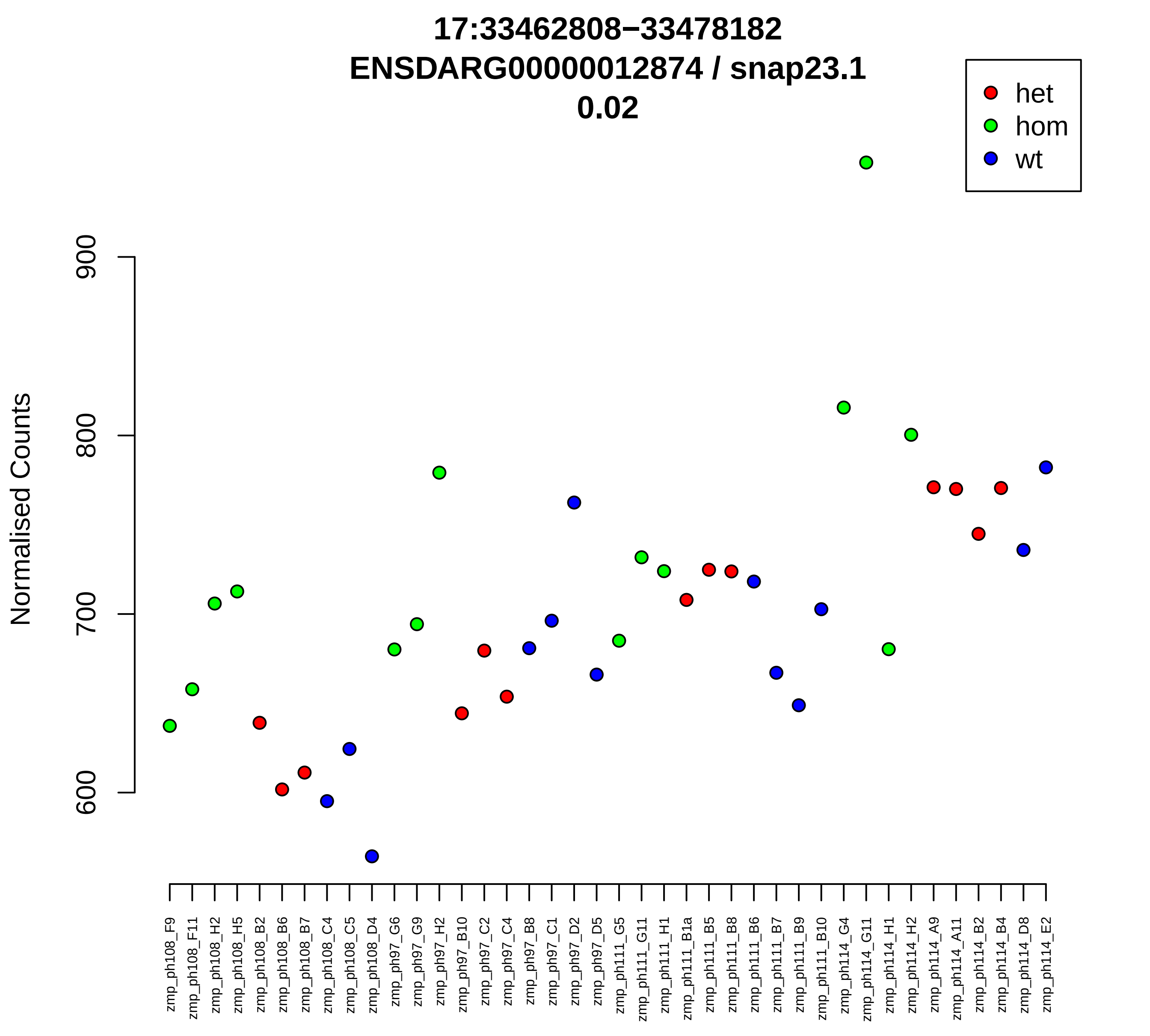
17:33462808−33478182
ENSDARG00000012874 / snap23.1
0.02
het
hom
wt
Normalised Counts
600
700
800
900
zmp_ph108_F9
zmp_ph108_F11
zmp_ph108_H2
zmp_ph108_H5
zmp_ph108_B2
zmp_ph108_B6
zmp_ph108_B7
zmp_ph108_C4
zmp_ph108_C5
zmp_ph108_D4
zmp_ph97_G6
zmp_ph97_G9
zmp_ph97_H2
zmp_ph97_B10
zmp_ph97_C2
zmp_ph97_C4
zmp_ph97_B8
zmp_ph97_C1
zmp_ph97_D2
zmp_ph97_D5
zmp_ph111_G5
zmp_ph111_G11
zmp_ph111_H1
zmp_ph111_B1a
zmp_ph111_B5
zmp_ph111_B8
zmp_ph111_B6
zmp_ph111_B7
zmp_ph111_B9
zmp_ph111_B10
zmp_ph114_G4
zmp_ph114_G11
zmp_ph114_H1
zmp_ph114_H2
zmp_ph114_A9
zmp_ph114_A11
zmp_ph114_B2
zmp_ph114_B4
zmp_ph114_D8
zmp_ph114_E2

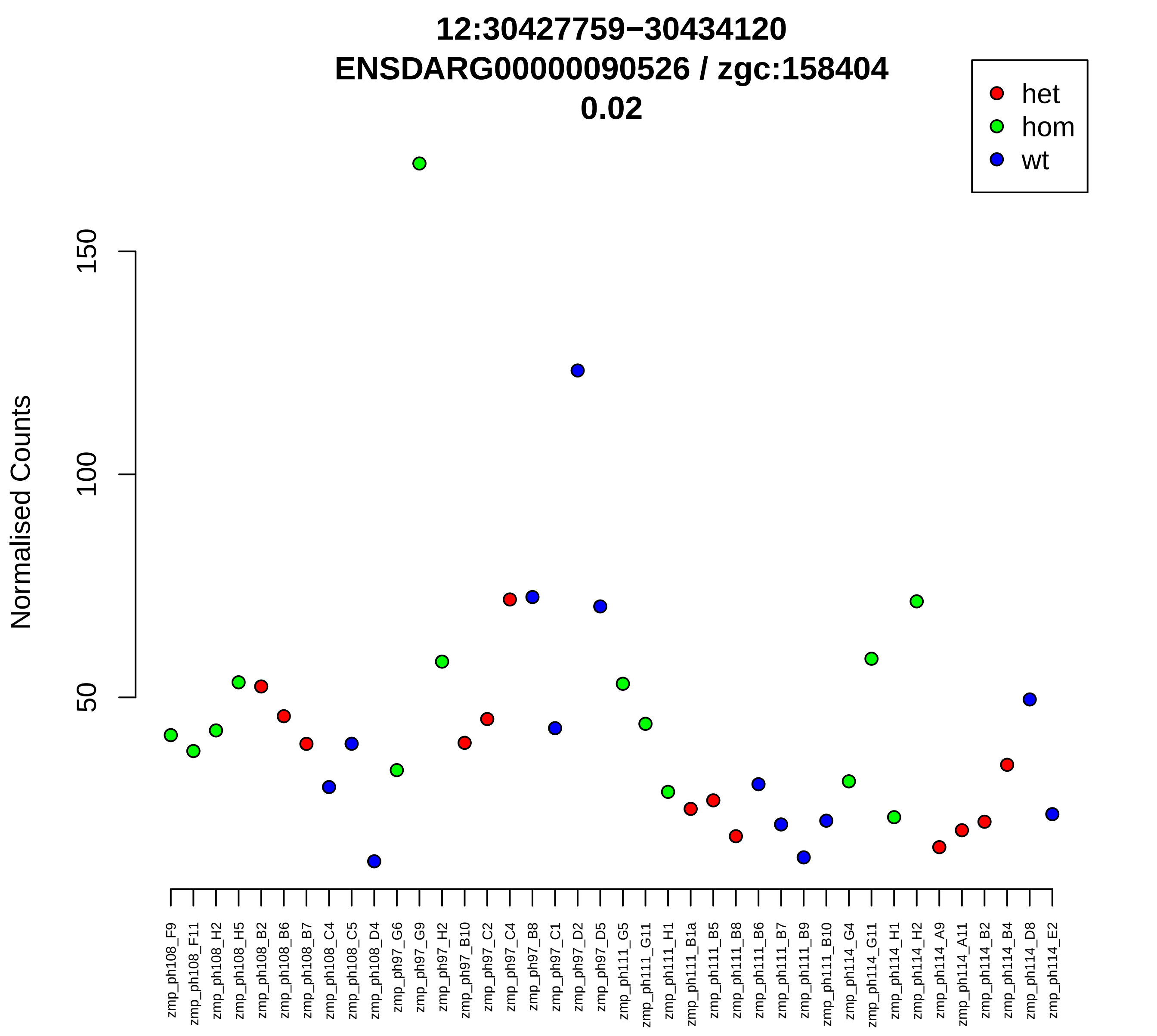

12:30427759−30434120
ENSDARG00000090526 / zgc:158404
0.02
het
hom
wt
Normalised Counts
50
100
150
zmp_ph108_F9
zmp_ph108_F11
zmp_ph108_H2
zmp_ph108_H5
zmp_ph108_B2
zmp_ph108_B6
zmp_ph108_B7
zmp_ph108_C4
zmp_ph108_C5
zmp_ph108_D4
zmp_ph97_G6
zmp_ph97_G9
zmp_ph97_H2
zmp_ph97_B10
zmp_ph97_C2
zmp_ph97_C4
zmp_ph97_B8
zmp_ph97_C1
zmp_ph97_D2
zmp_ph97_D5
zmp_ph111_G5
zmp_ph111_G11
zmp_ph111_H1
zmp_ph111_B1a
zmp_ph111_B5
zmp_ph111_B8
zmp_ph111_B6
zmp_ph111_B7
zmp_ph111_B9
zmp_ph111_B10
zmp_ph114_G4
zmp_ph114_G11
zmp_ph114_H1
zmp_ph114_H2
zmp_ph114_A9
zmp_ph114_A11
zmp_ph114_B2
zmp_ph114_B4
zmp_ph114_D8
zmp_ph114_E2

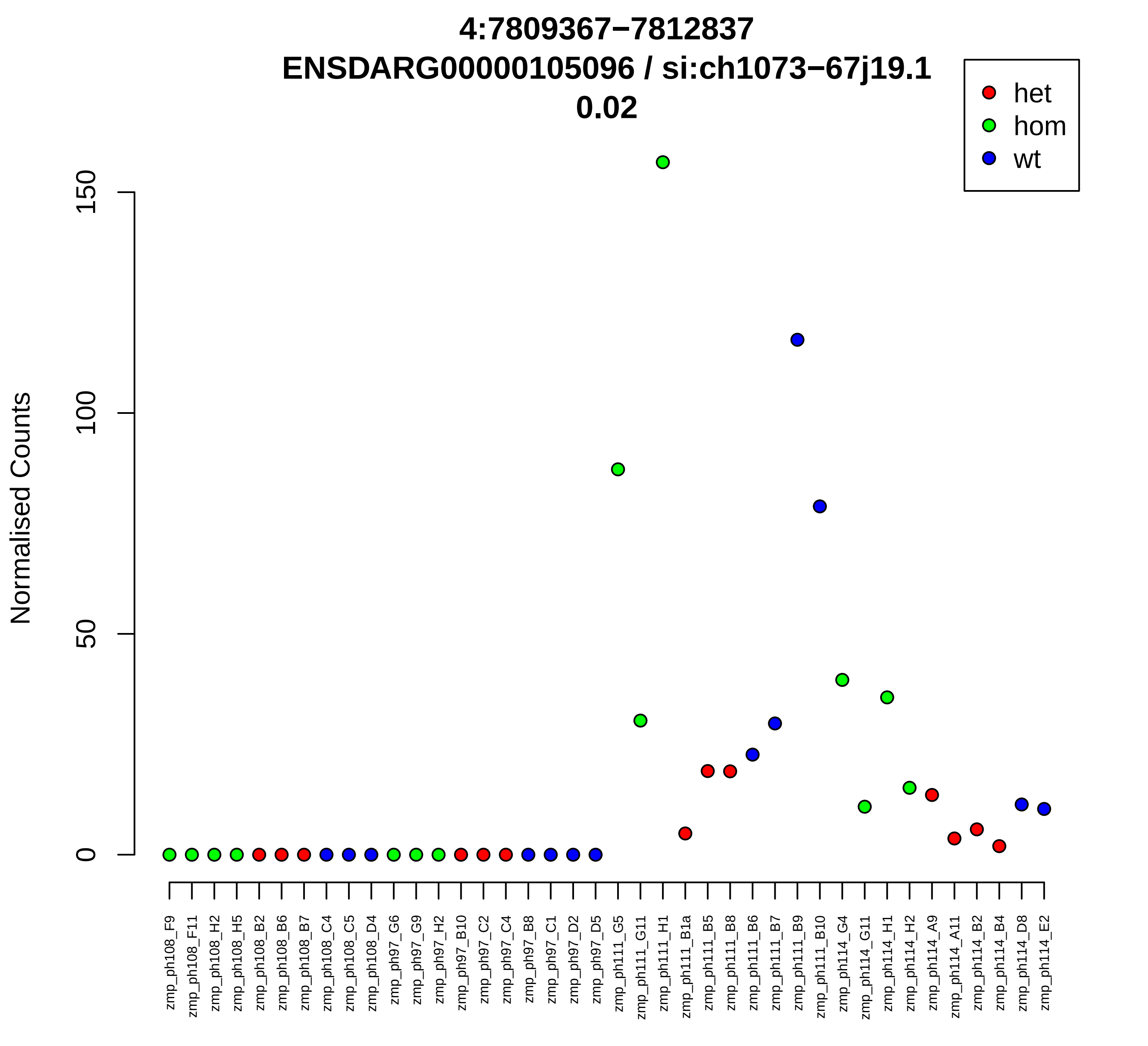
4:7809367−7812837
ENSDARG00000105096 / si:ch1073−67j19.1
0.02
het
hom
wt
Normalised Counts
0
50
100
150
zmp_ph108_F9
zmp_ph108_F11
zmp_ph108_H2
zmp_ph108_H5
zmp_ph108_B2
zmp_ph108_B6
zmp_ph108_B7
zmp_ph108_C4
zmp_ph108_C5
zmp_ph108_D4
zmp_ph97_G6
zmp_ph97_G9
zmp_ph97_H2
zmp_ph97_B10
zmp_ph97_C2
zmp_ph97_C4
zmp_ph97_B8
zmp_ph97_C1
zmp_ph97_D2
zmp_ph97_D5
zmp_ph111_G5
zmp_ph111_G11
zmp_ph111_H1
zmp_ph111_B1a
zmp_ph111_B5
zmp_ph111_B8
zmp_ph111_B6
zmp_ph111_B7
zmp_ph111_B9
zmp_ph111_B10
zmp_ph114_G4
zmp_ph114_G11
zmp_ph114_H1
zmp_ph114_H2
zmp_ph114_A9
zmp_ph114_A11
zmp_ph114_B2
zmp_ph114_B4
zmp_ph114_D8
zmp_ph114_E2

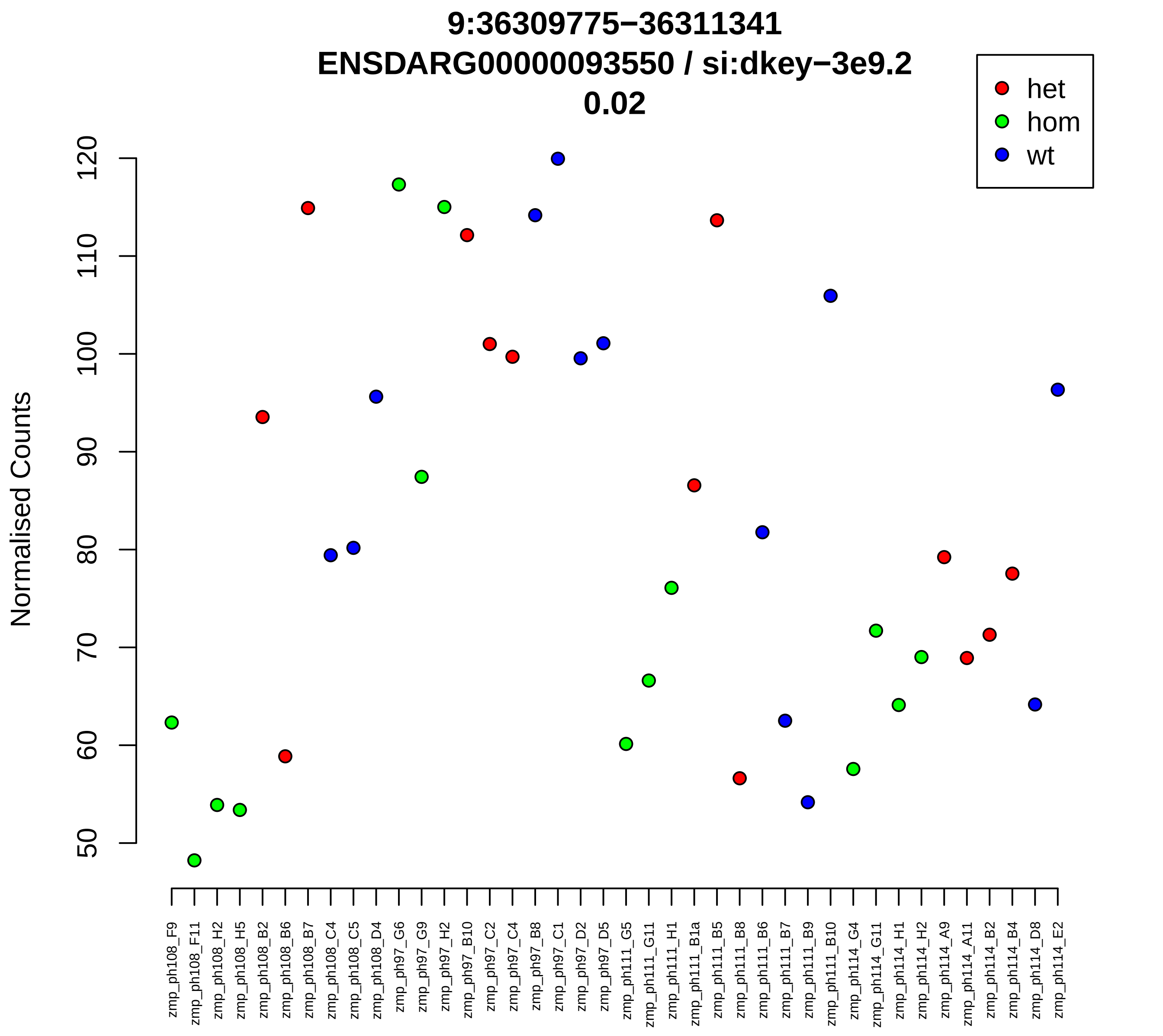

9:36309775−36311341
ENSDARG00000093550 / si:dkey−3e9.2
0.02
het
hom
wt
Normalised Counts
50
60
70
80
90
100
110
120
zmp_ph108_F9
zmp_ph108_F11
zmp_ph108_H2
zmp_ph108_H5
zmp_ph108_B2
zmp_ph108_B6
zmp_ph108_B7
zmp_ph108_C4
zmp_ph108_C5
zmp_ph108_D4
zmp_ph97_G6
zmp_ph97_G9
zmp_ph97_H2
zmp_ph97_B10
zmp_ph97_C2
zmp_ph97_C4
zmp_ph97_B8
zmp_ph97_C1
zmp_ph97_D2
zmp_ph97_D5
zmp_ph111_G5
zmp_ph111_G11
zmp_ph111_H1
zmp_ph111_B1a
zmp_ph111_B5
zmp_ph111_B8
zmp_ph111_B6
zmp_ph111_B7
zmp_ph111_B9
zmp_ph111_B10
zmp_ph114_G4
zmp_ph114_G11
zmp_ph114_H1
zmp_ph114_H2
zmp_ph114_A9
zmp_ph114_A11
zmp_ph114_B2
zmp_ph114_B4
zmp_ph114_D8
zmp_ph114_E2

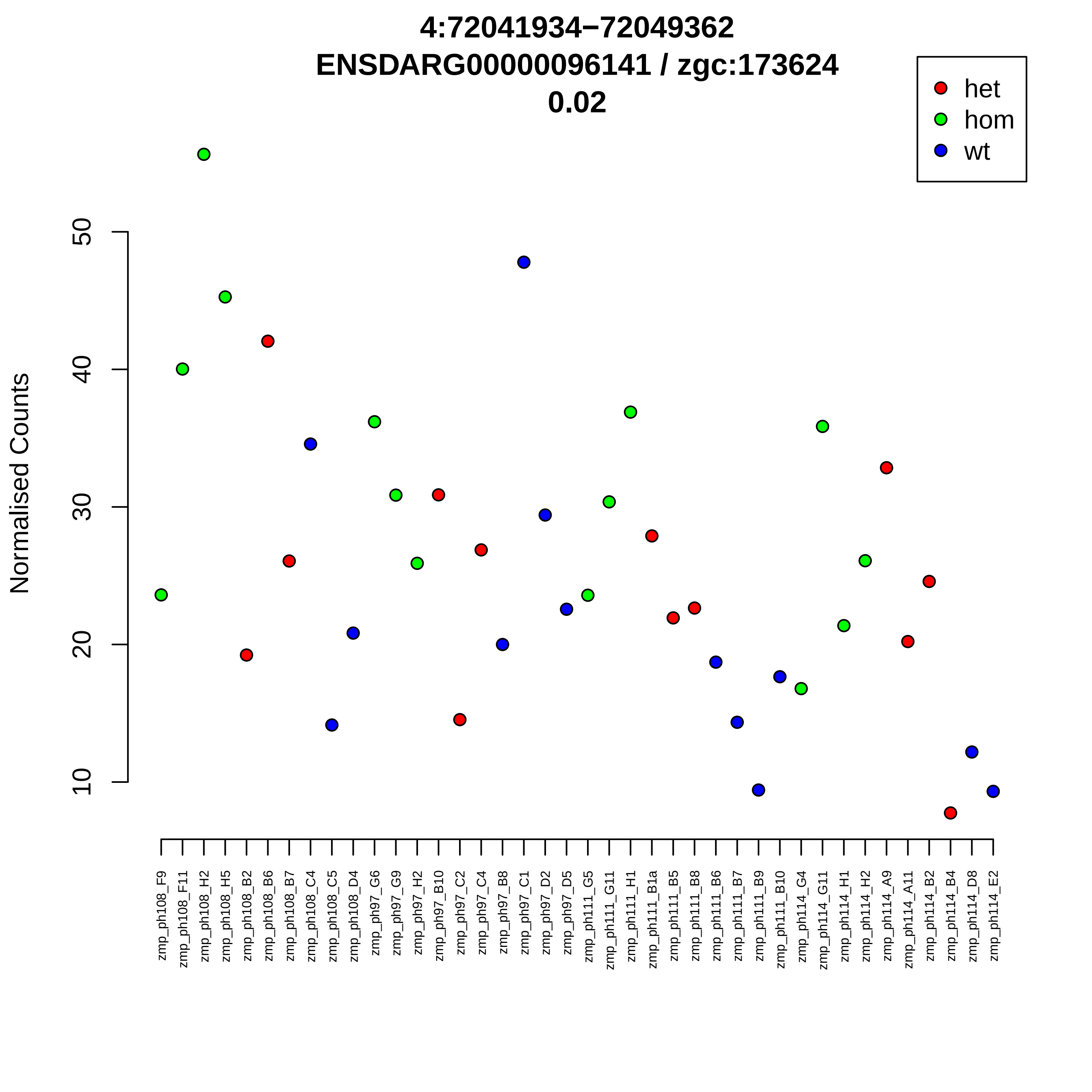

4:72041934−72049362
ENSDARG00000096141 / zgc:173624
0.02
het
hom
wt
Normalised Counts
10
20
30
40
50
zmp_ph108_F9
zmp_ph108_F11
zmp_ph108_H2
zmp_ph108_H5
zmp_ph108_B2
zmp_ph108_B6
zmp_ph108_B7
zmp_ph108_C4
zmp_ph108_C5
zmp_ph108_D4
zmp_ph97_G6
zmp_ph97_G9
zmp_ph97_H2
zmp_ph97_B10
zmp_ph97_C2
zmp_ph97_C4
zmp_ph97_B8
zmp_ph97_C1
zmp_ph97_D2
zmp_ph97_D5
zmp_ph111_G5
zmp_ph111_G11
zmp_ph111_H1
zmp_ph111_B1a
zmp_ph111_B5
zmp_ph111_B8
zmp_ph111_B6
zmp_ph111_B7
zmp_ph111_B9
zmp_ph111_B10
zmp_ph114_G4
zmp_ph114_G11
zmp_ph114_H1
zmp_ph114_H2
zmp_ph114_A9
zmp_ph114_A11
zmp_ph114_B2
zmp_ph114_B4
zmp_ph114_D8
zmp_ph114_E2

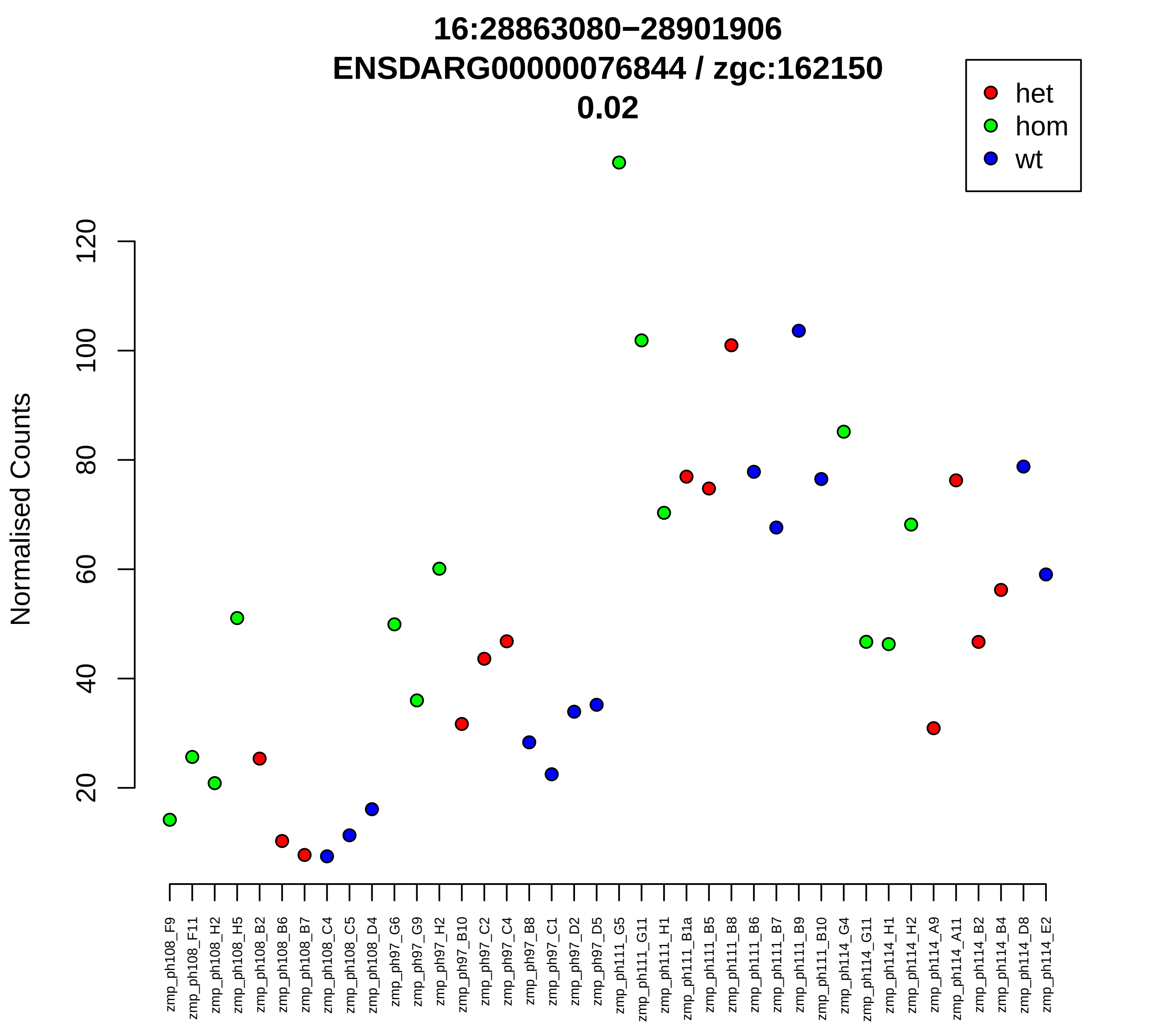
16:28863080−28901906
ENSDARG00000076844 / zgc:162150
0.02
het
hom
wt
Normalised Counts
20
40
60
80
100
120
zmp_ph108_F9
zmp_ph108_F11
zmp_ph108_H2
zmp_ph108_H5
zmp_ph108_B2
zmp_ph108_B6
zmp_ph108_B7
zmp_ph108_C4
zmp_ph108_C5
zmp_ph108_D4
zmp_ph97_G6
zmp_ph97_G9
zmp_ph97_H2
zmp_ph97_B10
zmp_ph97_C2
zmp_ph97_C4
zmp_ph97_B8
zmp_ph97_C1
zmp_ph97_D2
zmp_ph97_D5
zmp_ph111_G5
zmp_ph111_G11
zmp_ph111_H1
zmp_ph111_B1a
zmp_ph111_B5
zmp_ph111_B8
zmp_ph111_B6
zmp_ph111_B7
zmp_ph111_B9
zmp_ph111_B10
zmp_ph114_G4
zmp_ph114_G11
zmp_ph114_H1
zmp_ph114_H2
zmp_ph114_A9
zmp_ph114_A11
zmp_ph114_B2
zmp_ph114_B4
zmp_ph114_D8
zmp_ph114_E2

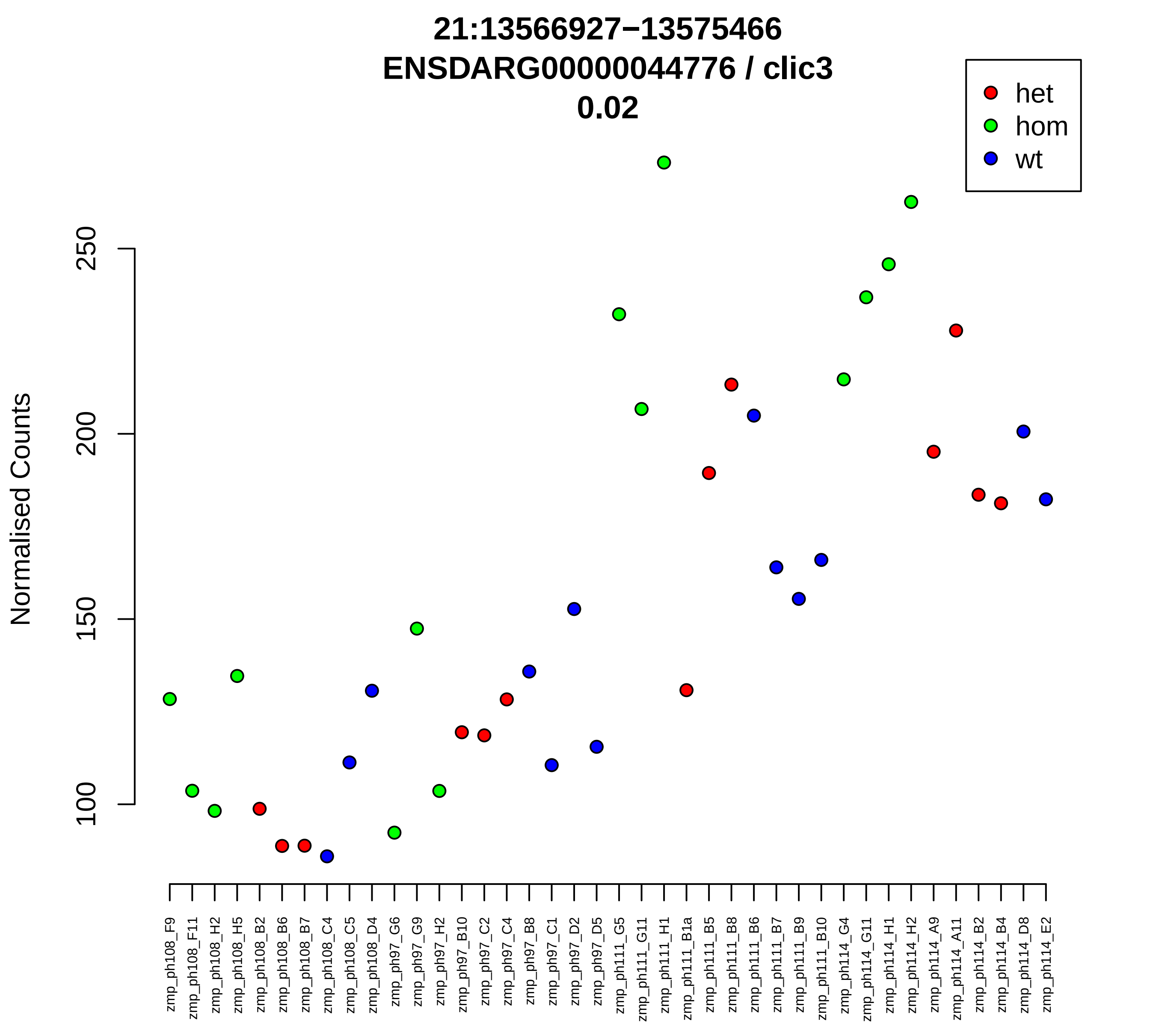

21:13566927−13575466
ENSDARG00000044776 / clic3
0.02
het
hom
wt
Normalised Counts
100
150
200
250
zmp_ph108_F9
zmp_ph108_F11
zmp_ph108_H2
zmp_ph108_H5
zmp_ph108_B2
zmp_ph108_B6
zmp_ph108_B7
zmp_ph108_C4
zmp_ph108_C5
zmp_ph108_D4
zmp_ph97_G6
zmp_ph97_G9
zmp_ph97_H2
zmp_ph97_B10
zmp_ph97_C2
zmp_ph97_C4
zmp_ph97_B8
zmp_ph97_C1
zmp_ph97_D2
zmp_ph97_D5
zmp_ph111_G5
zmp_ph111_G11
zmp_ph111_H1
zmp_ph111_B1a
zmp_ph111_B5
zmp_ph111_B8
zmp_ph111_B6
zmp_ph111_B7
zmp_ph111_B9
zmp_ph111_B10
zmp_ph114_G4
zmp_ph114_G11
zmp_ph114_H1
zmp_ph114_H2
zmp_ph114_A9
zmp_ph114_A11
zmp_ph114_B2
zmp_ph114_B4
zmp_ph114_D8
zmp_ph114_E2

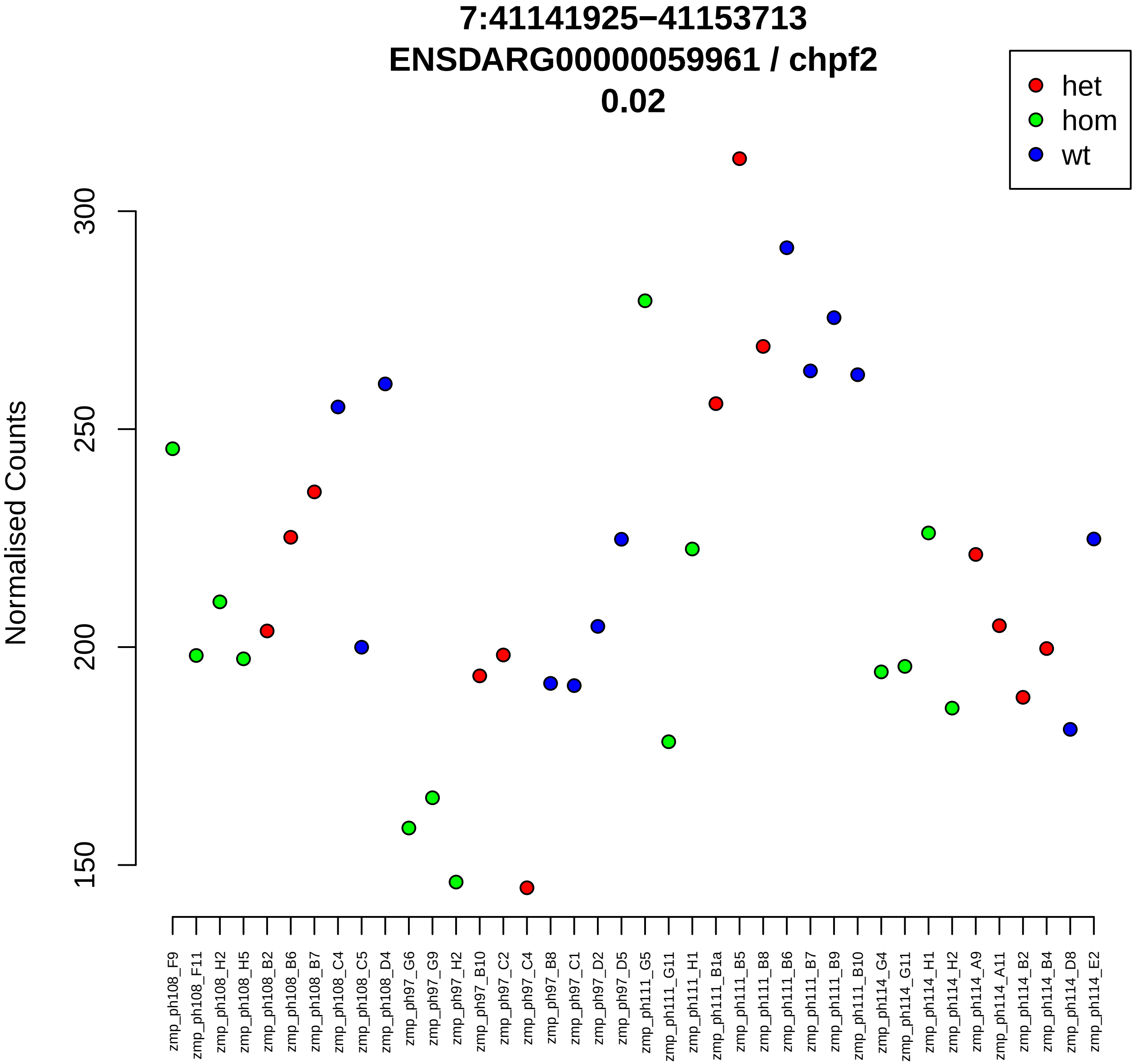
7:41141925−41153713
ENSDARG00000059961 / chpf2
0.02
het
hom
wt
Normalised Counts
150
200
250
300
zmp_ph108_F9
zmp_ph108_F11
zmp_ph108_H2
zmp_ph108_H5
zmp_ph108_B2
zmp_ph108_B6
zmp_ph108_B7
zmp_ph108_C4
zmp_ph108_C5
zmp_ph108_D4
zmp_ph97_G6
zmp_ph97_G9
zmp_ph97_H2
zmp_ph97_B10
zmp_ph97_C2
zmp_ph97_C4
zmp_ph97_B8
zmp_ph97_C1
zmp_ph97_D2
zmp_ph97_D5
zmp_ph111_G5
zmp_ph111_G11
zmp_ph111_H1
zmp_ph111_B1a
zmp_ph111_B5
zmp_ph111_B8
zmp_ph111_B6
zmp_ph111_B7
zmp_ph111_B9
zmp_ph111_B10
zmp_ph114_G4
zmp_ph114_G11
zmp_ph114_H1
zmp_ph114_H2
zmp_ph114_A9
zmp_ph114_A11
zmp_ph114_B2
zmp_ph114_B4
zmp_ph114_D8
zmp_ph114_E2

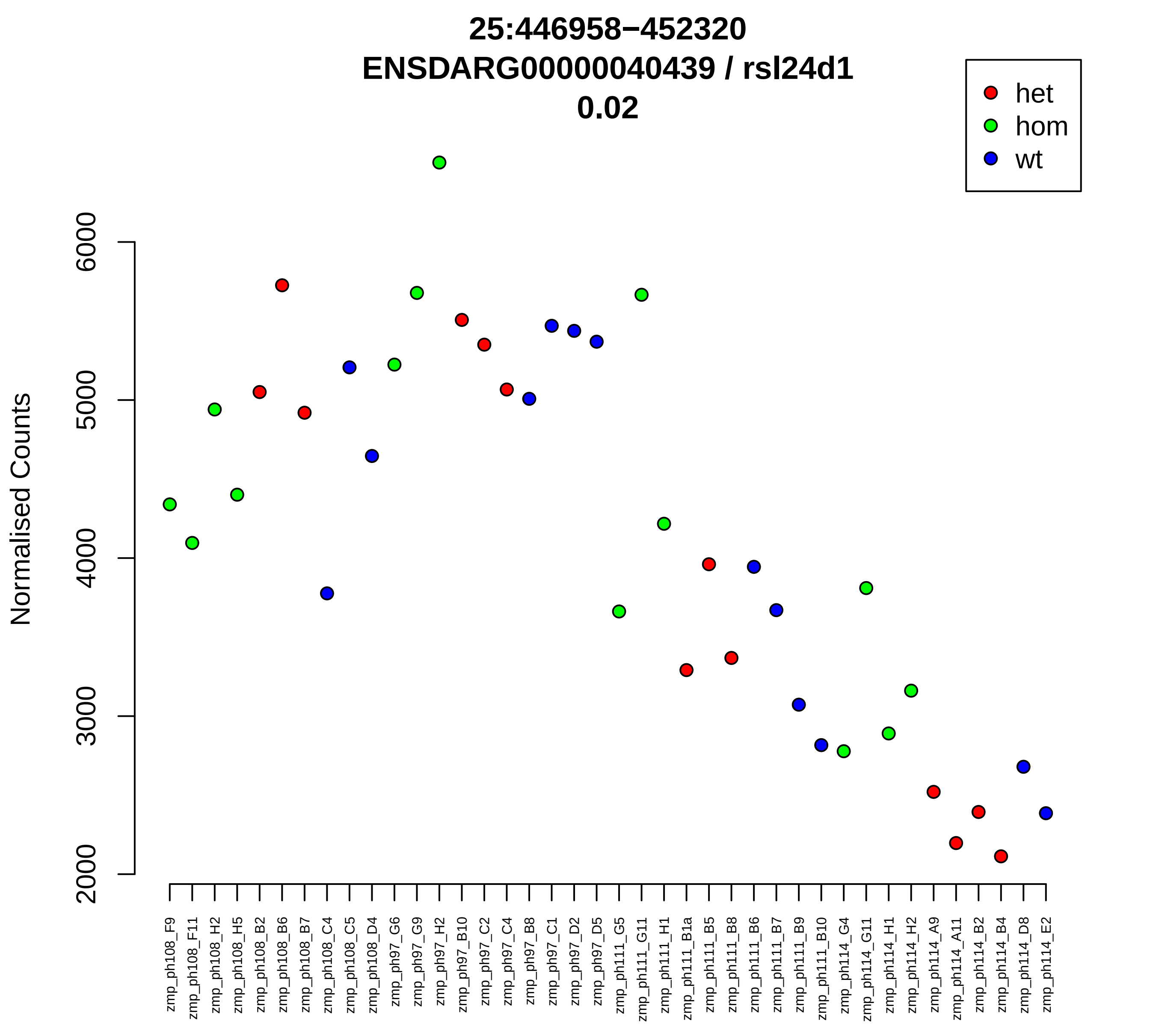
25:446958−452320
ENSDARG00000040439 / rsl24d1
0.02
het
hom
wt
Normalised Counts
2000
3000
4000
5000
6000
zmp_ph108_F9
zmp_ph108_F11
zmp_ph108_H2
zmp_ph108_H5
zmp_ph108_B2
zmp_ph108_B6
zmp_ph108_B7
zmp_ph108_C4
zmp_ph108_C5
zmp_ph108_D4
zmp_ph97_G6
zmp_ph97_G9
zmp_ph97_H2
zmp_ph97_B10
zmp_ph97_C2
zmp_ph97_C4
zmp_ph97_B8
zmp_ph97_C1
zmp_ph97_D2
zmp_ph97_D5
zmp_ph111_G5
zmp_ph111_G11
zmp_ph111_H1
zmp_ph111_B1a
zmp_ph111_B5
zmp_ph111_B8
zmp_ph111_B6
zmp_ph111_B7
zmp_ph111_B9
zmp_ph111_B10
zmp_ph114_G4
zmp_ph114_G11
zmp_ph114_H1
zmp_ph114_H2
zmp_ph114_A9
zmp_ph114_A11
zmp_ph114_B2
zmp_ph114_B4
zmp_ph114_D8
zmp_ph114_E2

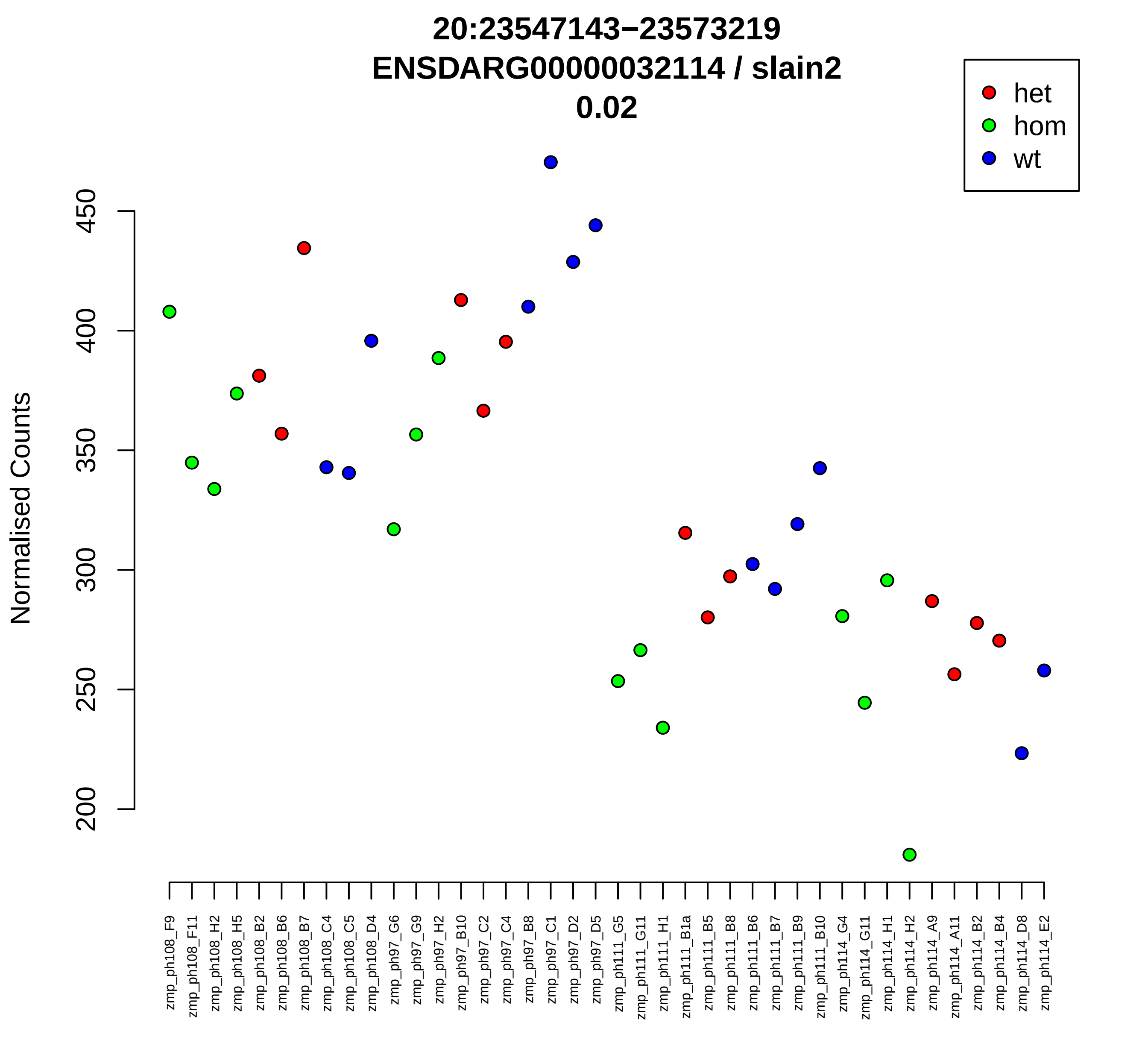

20:23547143−23573219
ENSDARG00000032114 / slain2
0.02
het
hom
wt
Normalised Counts
200
250
300
350
400
450
zmp_ph108_F9
zmp_ph108_F11
zmp_ph108_H2
zmp_ph108_H5
zmp_ph108_B2
zmp_ph108_B6
zmp_ph108_B7
zmp_ph108_C4
zmp_ph108_C5
zmp_ph108_D4
zmp_ph97_G6
zmp_ph97_G9
zmp_ph97_H2
zmp_ph97_B10
zmp_ph97_C2
zmp_ph97_C4
zmp_ph97_B8
zmp_ph97_C1
zmp_ph97_D2
zmp_ph97_D5
zmp_ph111_G5
zmp_ph111_G11
zmp_ph111_H1
zmp_ph111_B1a
zmp_ph111_B5
zmp_ph111_B8
zmp_ph111_B6
zmp_ph111_B7
zmp_ph111_B9
zmp_ph111_B10
zmp_ph114_G4
zmp_ph114_G11
zmp_ph114_H1
zmp_ph114_H2
zmp_ph114_A9
zmp_ph114_A11
zmp_ph114_B2
zmp_ph114_B4
zmp_ph114_D8
zmp_ph114_E2

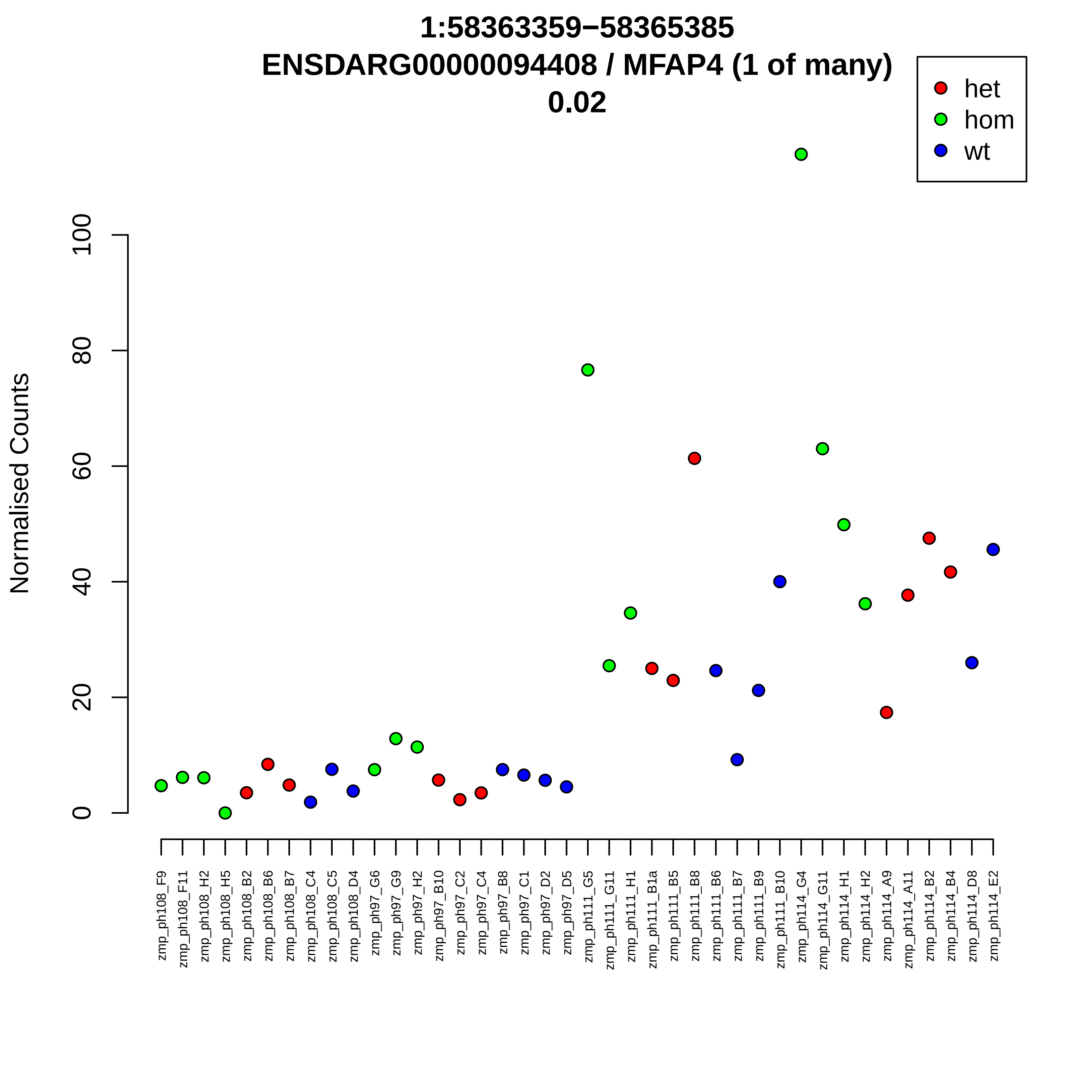

1:58363359−58365385
ENSDARG00000094408 / MFAP4 (1 of many)
0.02
het
hom
wt
Normalised Counts
0
20
40
60
80
100
zmp_ph108_F9
zmp_ph108_F11
zmp_ph108_H2
zmp_ph108_H5
zmp_ph108_B2
zmp_ph108_B6
zmp_ph108_B7
zmp_ph108_C4
zmp_ph108_C5
zmp_ph108_D4
zmp_ph97_G6
zmp_ph97_G9
zmp_ph97_H2
zmp_ph97_B10
zmp_ph97_C2
zmp_ph97_C4
zmp_ph97_B8
zmp_ph97_C1
zmp_ph97_D2
zmp_ph97_D5
zmp_ph111_G5
zmp_ph111_G11
zmp_ph111_H1
zmp_ph111_B1a
zmp_ph111_B5
zmp_ph111_B8
zmp_ph111_B6
zmp_ph111_B7
zmp_ph111_B9
zmp_ph111_B10
zmp_ph114_G4
zmp_ph114_G11
zmp_ph114_H1
zmp_ph114_H2
zmp_ph114_A9
zmp_ph114_A11
zmp_ph114_B2
zmp_ph114_B4
zmp_ph114_D8
zmp_ph114_E2

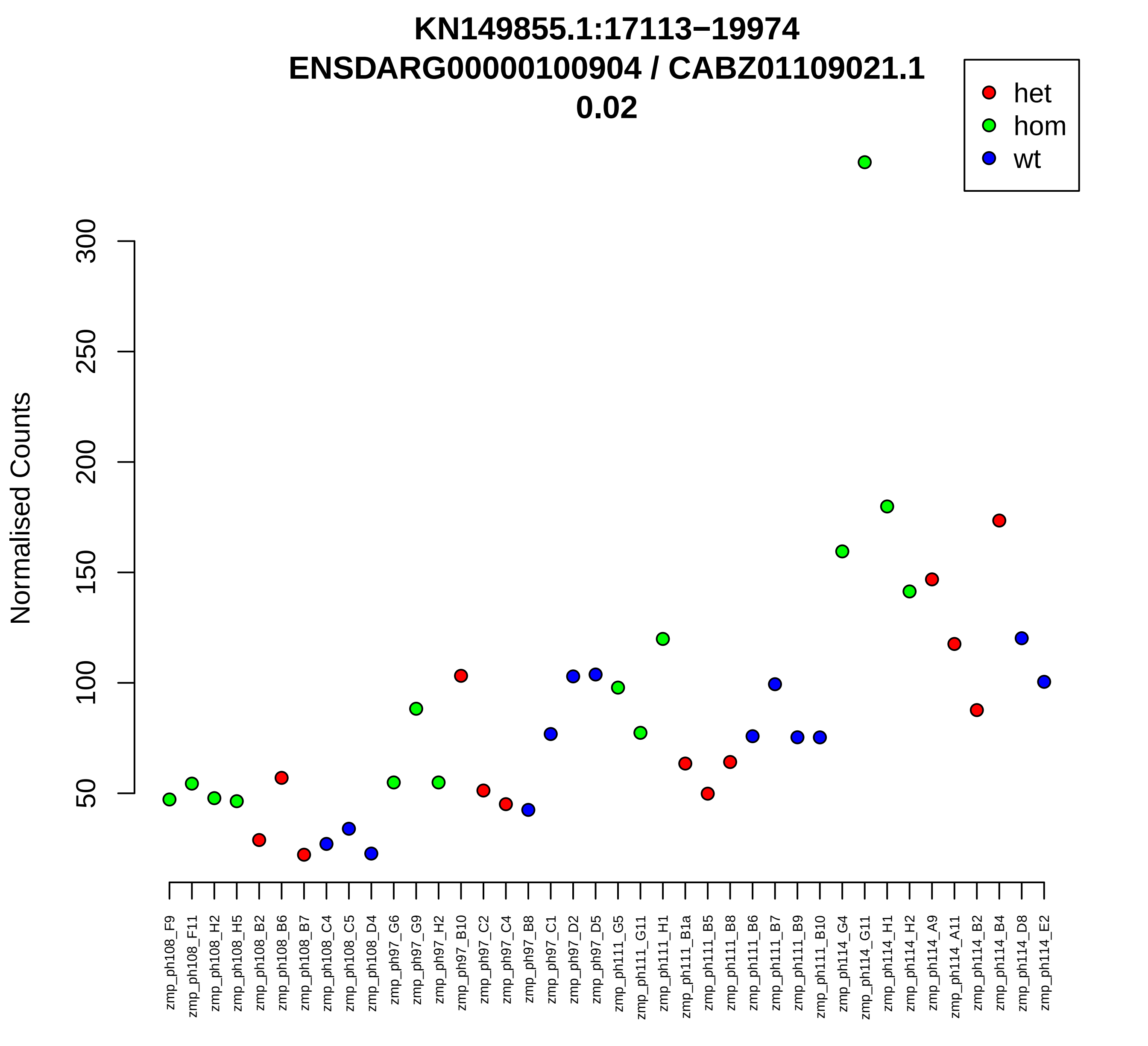
KN149855.1:17113−19974
ENSDARG00000100904 / CABZ01109021.1
0.02
het
hom
wt
Normalised Counts
50
100
150
200
250
300
zmp_ph108_F9
zmp_ph108_F11
zmp_ph108_H2
zmp_ph108_H5
zmp_ph108_B2
zmp_ph108_B6
zmp_ph108_B7
zmp_ph108_C4
zmp_ph108_C5
zmp_ph108_D4
zmp_ph97_G6
zmp_ph97_G9
zmp_ph97_H2
zmp_ph97_B10
zmp_ph97_C2
zmp_ph97_C4
zmp_ph97_B8
zmp_ph97_C1
zmp_ph97_D2
zmp_ph97_D5
zmp_ph111_G5
zmp_ph111_G11
zmp_ph111_H1
zmp_ph111_B1a
zmp_ph111_B5
zmp_ph111_B8
zmp_ph111_B6
zmp_ph111_B7
zmp_ph111_B9
zmp_ph111_B10
zmp_ph114_G4
zmp_ph114_G11
zmp_ph114_H1
zmp_ph114_H2
zmp_ph114_A9
zmp_ph114_A11
zmp_ph114_B2
zmp_ph114_B4
zmp_ph114_D8
zmp_ph114_E2

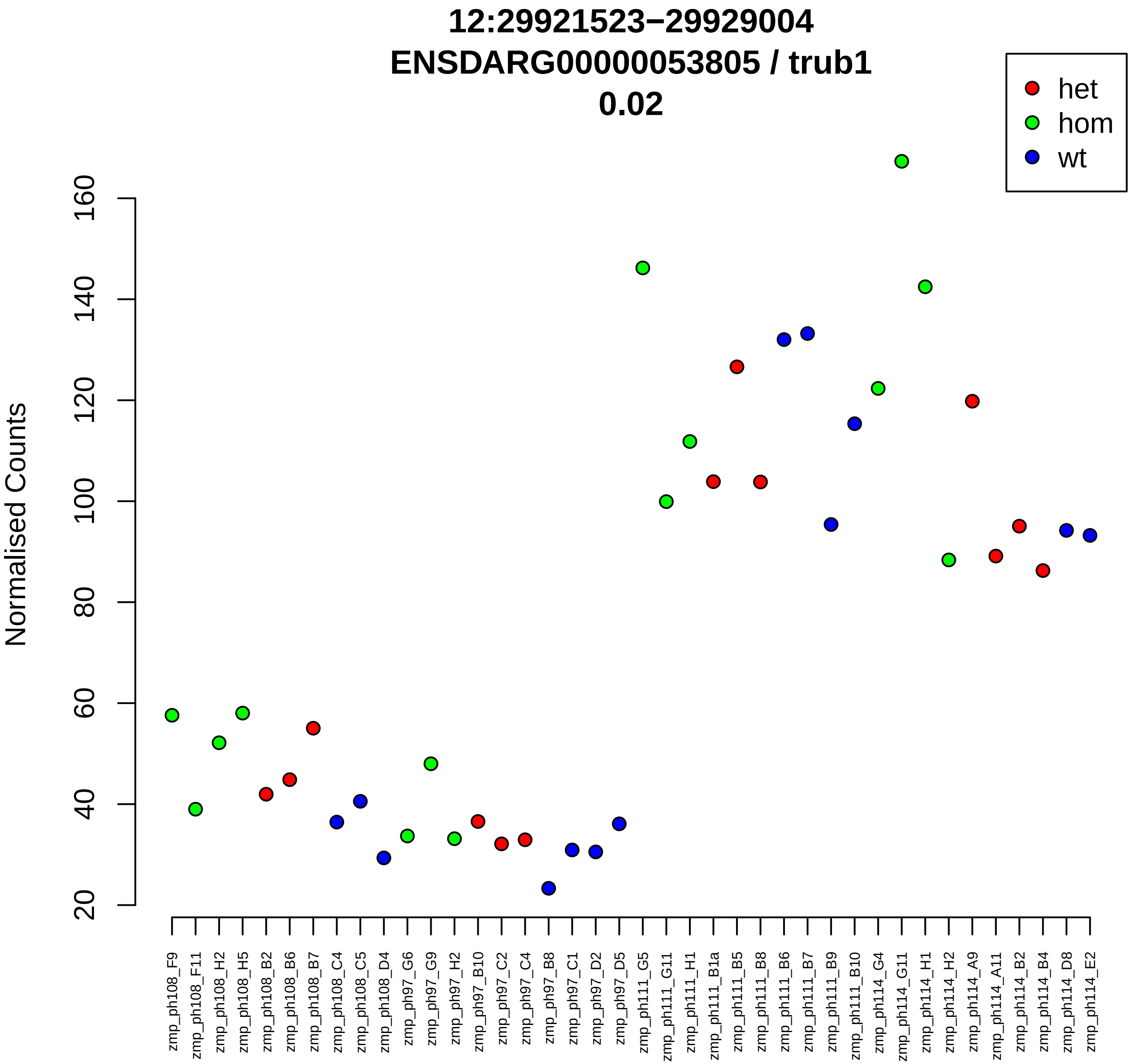

12:29921523−29929004
ENSDARG00000053805 / trub1
0.02
het
hom
wt
Normalised Counts
20
40
60
80
100
120
140
160
zmp_ph108_F9
zmp_ph108_F11
zmp_ph108_H2
zmp_ph108_H5
zmp_ph108_B2
zmp_ph108_B6
zmp_ph108_B7
zmp_ph108_C4
zmp_ph108_C5
zmp_ph108_D4
zmp_ph97_G6
zmp_ph97_G9
zmp_ph97_H2
zmp_ph97_B10
zmp_ph97_C2
zmp_ph97_C4
zmp_ph97_B8
zmp_ph97_C1
zmp_ph97_D2
zmp_ph97_D5
zmp_ph111_G5
zmp_ph111_G11
zmp_ph111_H1
zmp_ph111_B1a
zmp_ph111_B5
zmp_ph111_B8
zmp_ph111_B6
zmp_ph111_B7
zmp_ph111_B9
zmp_ph111_B10
zmp_ph114_G4
zmp_ph114_G11
zmp_ph114_H1
zmp_ph114_H2
zmp_ph114_A9
zmp_ph114_A11
zmp_ph114_B2
zmp_ph114_B4
zmp_ph114_D8
zmp_ph114_E2

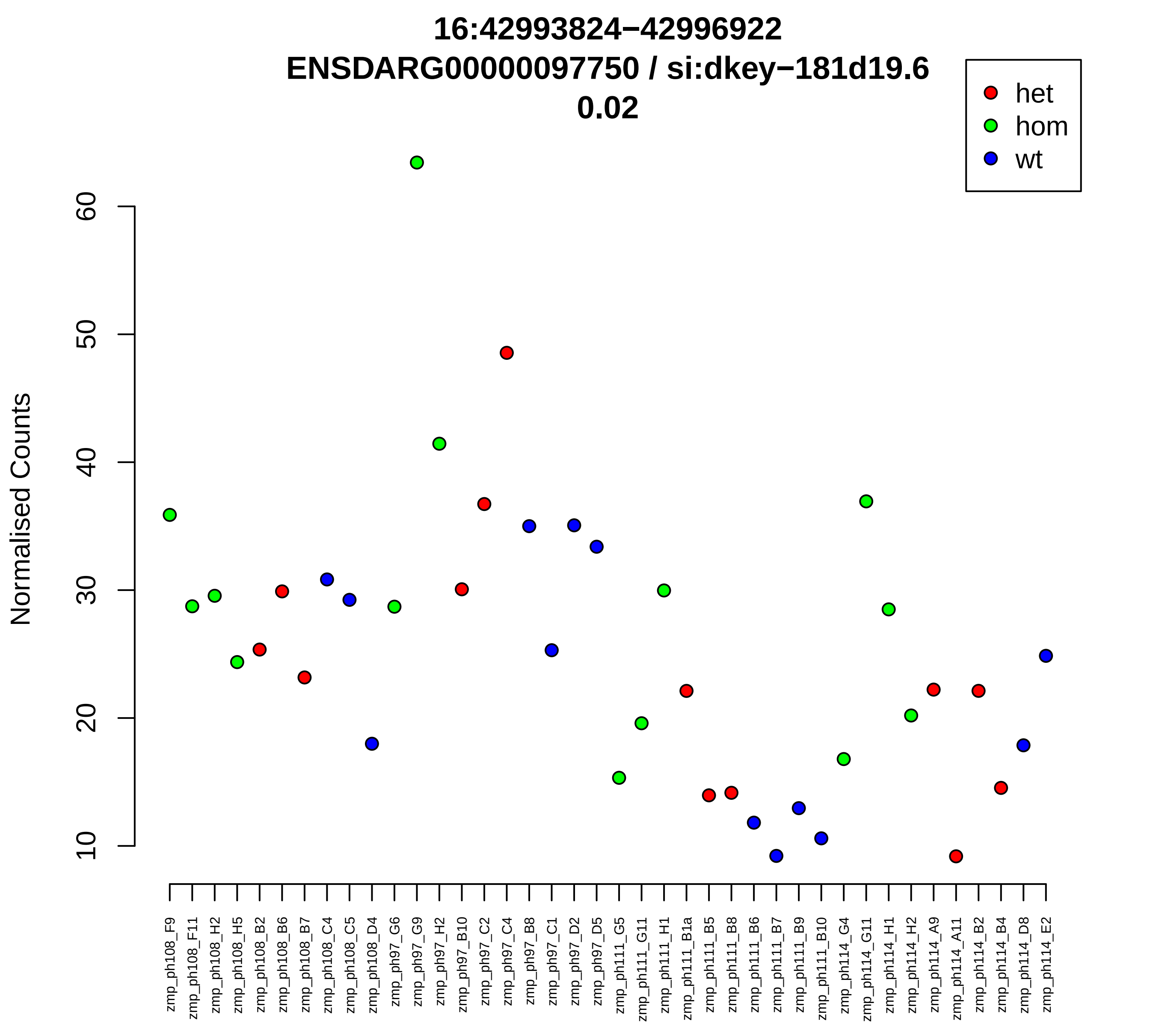
16:42993824−42996922
ENSDARG00000097750 / si:dkey−181d19.6
0.02
het
hom
wt
Normalised Counts
10
20
30
40
50
60
zmp_ph108_F9
zmp_ph108_F11
zmp_ph108_H2
zmp_ph108_H5
zmp_ph108_B2
zmp_ph108_B6
zmp_ph108_B7
zmp_ph108_C4
zmp_ph108_C5
zmp_ph108_D4
zmp_ph97_G6
zmp_ph97_G9
zmp_ph97_H2
zmp_ph97_B10
zmp_ph97_C2
zmp_ph97_C4
zmp_ph97_B8
zmp_ph97_C1
zmp_ph97_D2
zmp_ph97_D5
zmp_ph111_G5
zmp_ph111_G11
zmp_ph111_H1
zmp_ph111_B1a
zmp_ph111_B5
zmp_ph111_B8
zmp_ph111_B6
zmp_ph111_B7
zmp_ph111_B9
zmp_ph111_B10
zmp_ph114_G4
zmp_ph114_G11
zmp_ph114_H1
zmp_ph114_H2
zmp_ph114_A9
zmp_ph114_A11
zmp_ph114_B2
zmp_ph114_B4
zmp_ph114_D8
zmp_ph114_E2

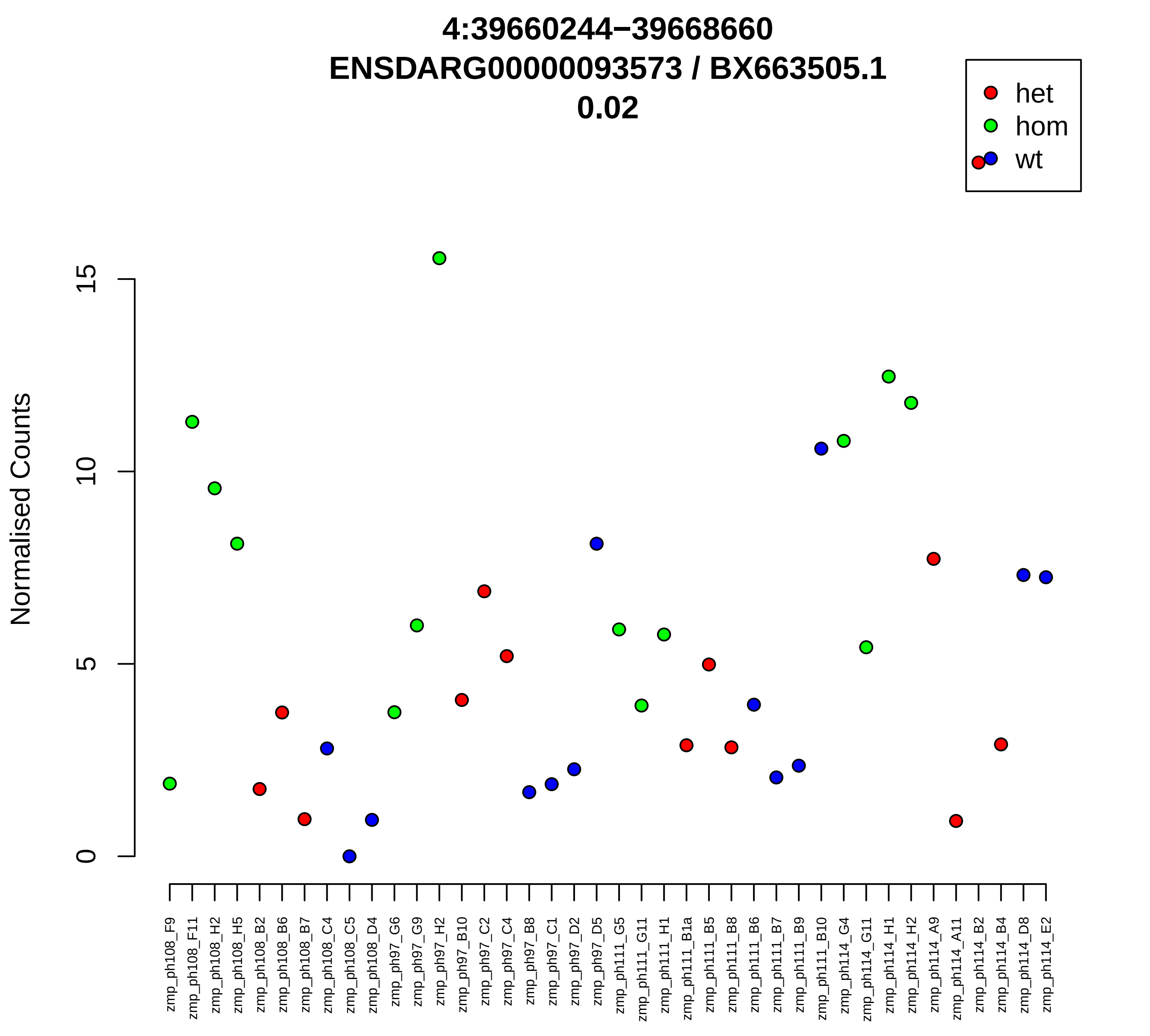
4:39660244−39668660
ENSDARG00000093573 / BX663505.1
0.02
het
hom
wt
Normalised Counts
0
5
10
15
zmp_ph108_F9
zmp_ph108_F11
zmp_ph108_H2
zmp_ph108_H5
zmp_ph108_B2
zmp_ph108_B6
zmp_ph108_B7
zmp_ph108_C4
zmp_ph108_C5
zmp_ph108_D4
zmp_ph97_G6
zmp_ph97_G9
zmp_ph97_H2
zmp_ph97_B10
zmp_ph97_C2
zmp_ph97_C4
zmp_ph97_B8
zmp_ph97_C1
zmp_ph97_D2
zmp_ph97_D5
zmp_ph111_G5
zmp_ph111_G11
zmp_ph111_H1
zmp_ph111_B1a
zmp_ph111_B5
zmp_ph111_B8
zmp_ph111_B6
zmp_ph111_B7
zmp_ph111_B9
zmp_ph111_B10
zmp_ph114_G4
zmp_ph114_G11
zmp_ph114_H1
zmp_ph114_H2
zmp_ph114_A9
zmp_ph114_A11
zmp_ph114_B2
zmp_ph114_B4
zmp_ph114_D8
zmp_ph114_E2

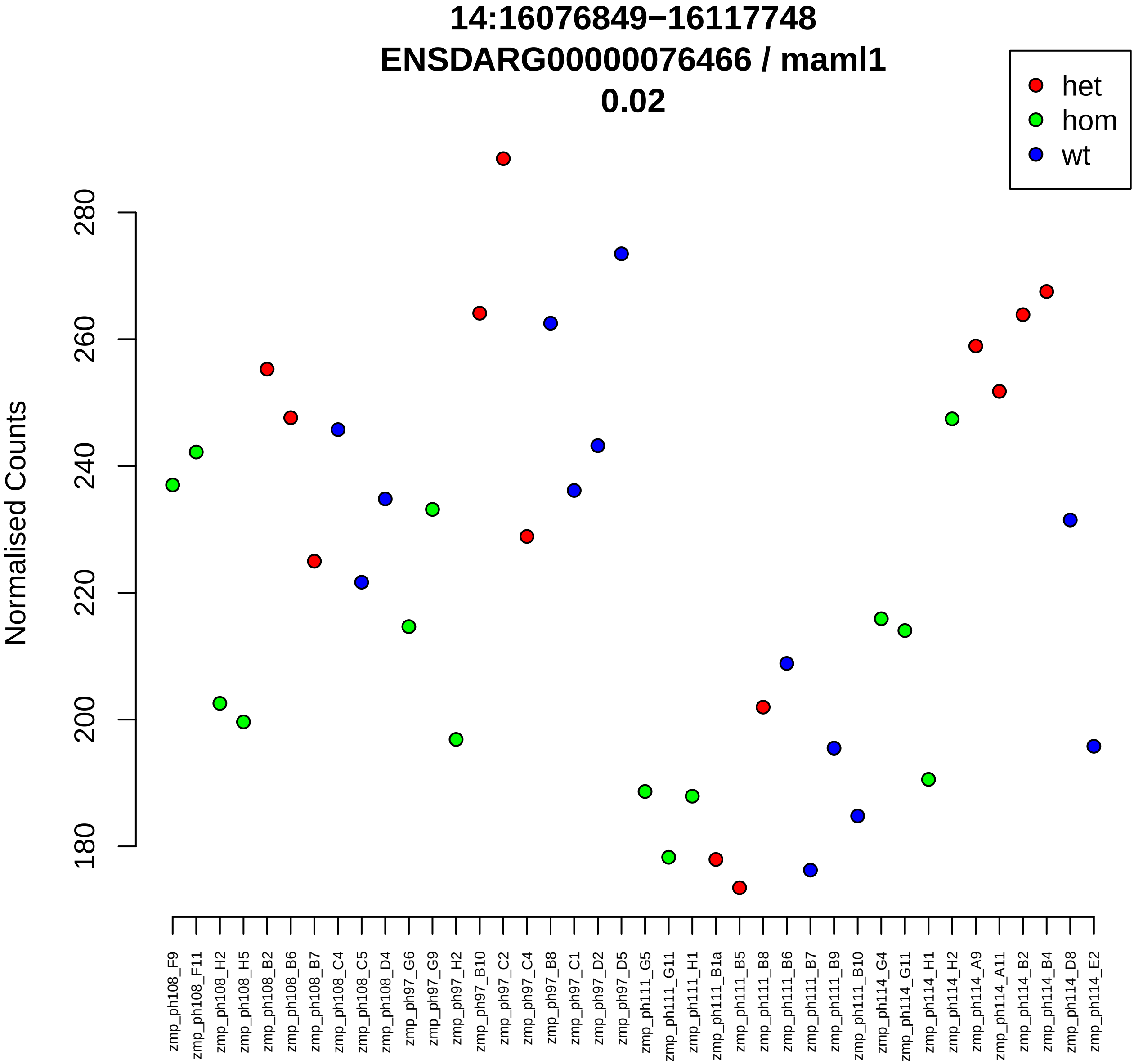

14:16076849−16117748
ENSDARG00000076466 / maml1
0.02
het
hom
wt
Normalised Counts
180
200
220
240
260
280
zmp_ph108_F9
zmp_ph108_F11
zmp_ph108_H2
zmp_ph108_H5
zmp_ph108_B2
zmp_ph108_B6
zmp_ph108_B7
zmp_ph108_C4
zmp_ph108_C5
zmp_ph108_D4
zmp_ph97_G6
zmp_ph97_G9
zmp_ph97_H2
zmp_ph97_B10
zmp_ph97_C2
zmp_ph97_C4
zmp_ph97_B8
zmp_ph97_C1
zmp_ph97_D2
zmp_ph97_D5
zmp_ph111_G5
zmp_ph111_G11
zmp_ph111_H1
zmp_ph111_B1a
zmp_ph111_B5
zmp_ph111_B8
zmp_ph111_B6
zmp_ph111_B7
zmp_ph111_B9
zmp_ph111_B10
zmp_ph114_G4
zmp_ph114_G11
zmp_ph114_H1
zmp_ph114_H2
zmp_ph114_A9
zmp_ph114_A11
zmp_ph114_B2
zmp_ph114_B4
zmp_ph114_D8
zmp_ph114_E2

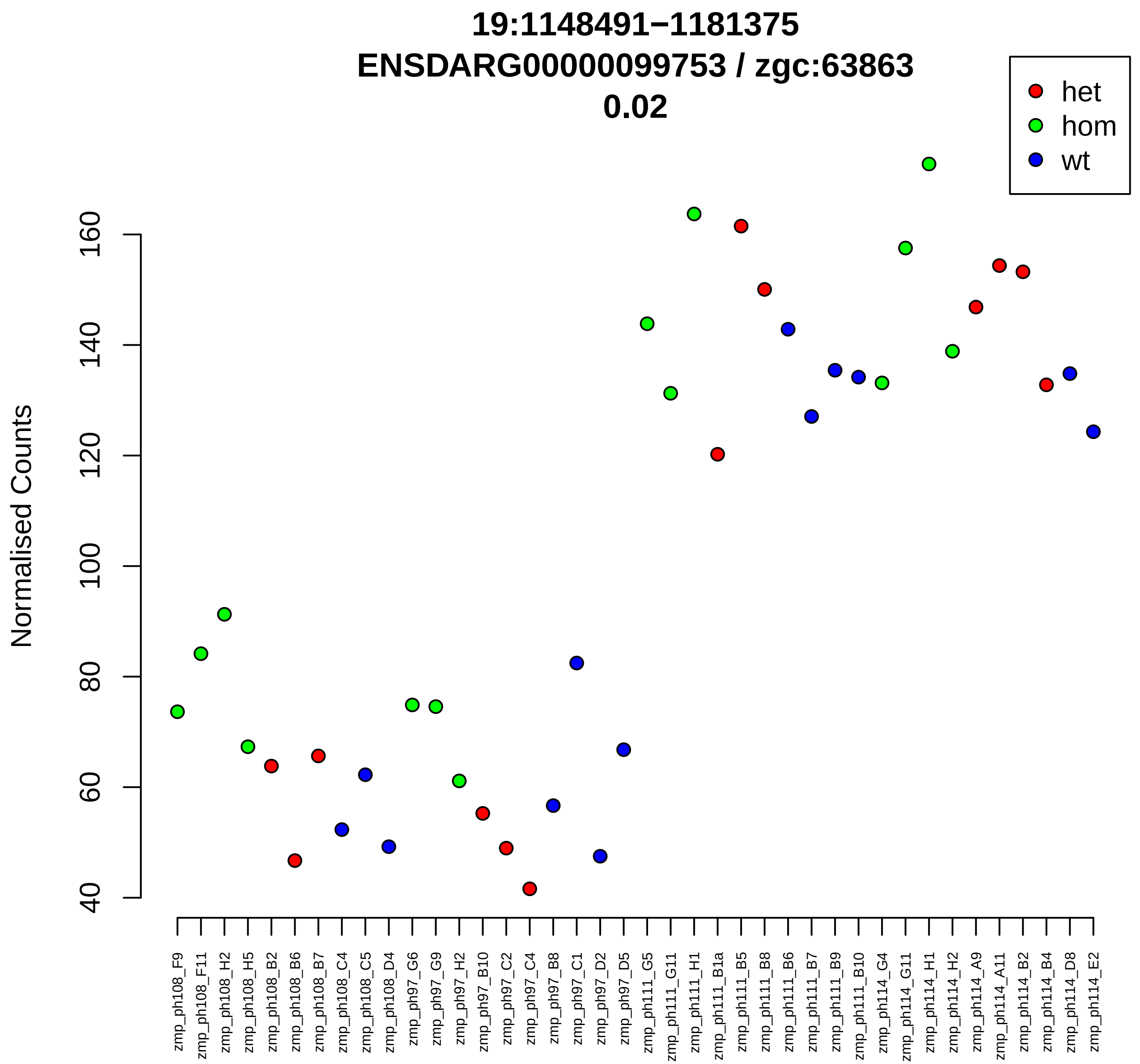

19:1148491−1181375
ENSDARG00000099753 / zgc:63863
0.02
het
hom
wt
Normalised Counts
40
60
80
100
120
140
160
zmp_ph108_F9
zmp_ph108_F11
zmp_ph108_H2
zmp_ph108_H5
zmp_ph108_B2
zmp_ph108_B6
zmp_ph108_B7
zmp_ph108_C4
zmp_ph108_C5
zmp_ph108_D4
zmp_ph97_G6
zmp_ph97_G9
zmp_ph97_H2
zmp_ph97_B10
zmp_ph97_C2
zmp_ph97_C4
zmp_ph97_B8
zmp_ph97_C1
zmp_ph97_D2
zmp_ph97_D5
zmp_ph111_G5
zmp_ph111_G11
zmp_ph111_H1
zmp_ph111_B1a
zmp_ph111_B5
zmp_ph111_B8
zmp_ph111_B6
zmp_ph111_B7
zmp_ph111_B9
zmp_ph111_B10
zmp_ph114_G4
zmp_ph114_G11
zmp_ph114_H1
zmp_ph114_H2
zmp_ph114_A9
zmp_ph114_A11
zmp_ph114_B2
zmp_ph114_B4
zmp_ph114_D8
zmp_ph114_E2

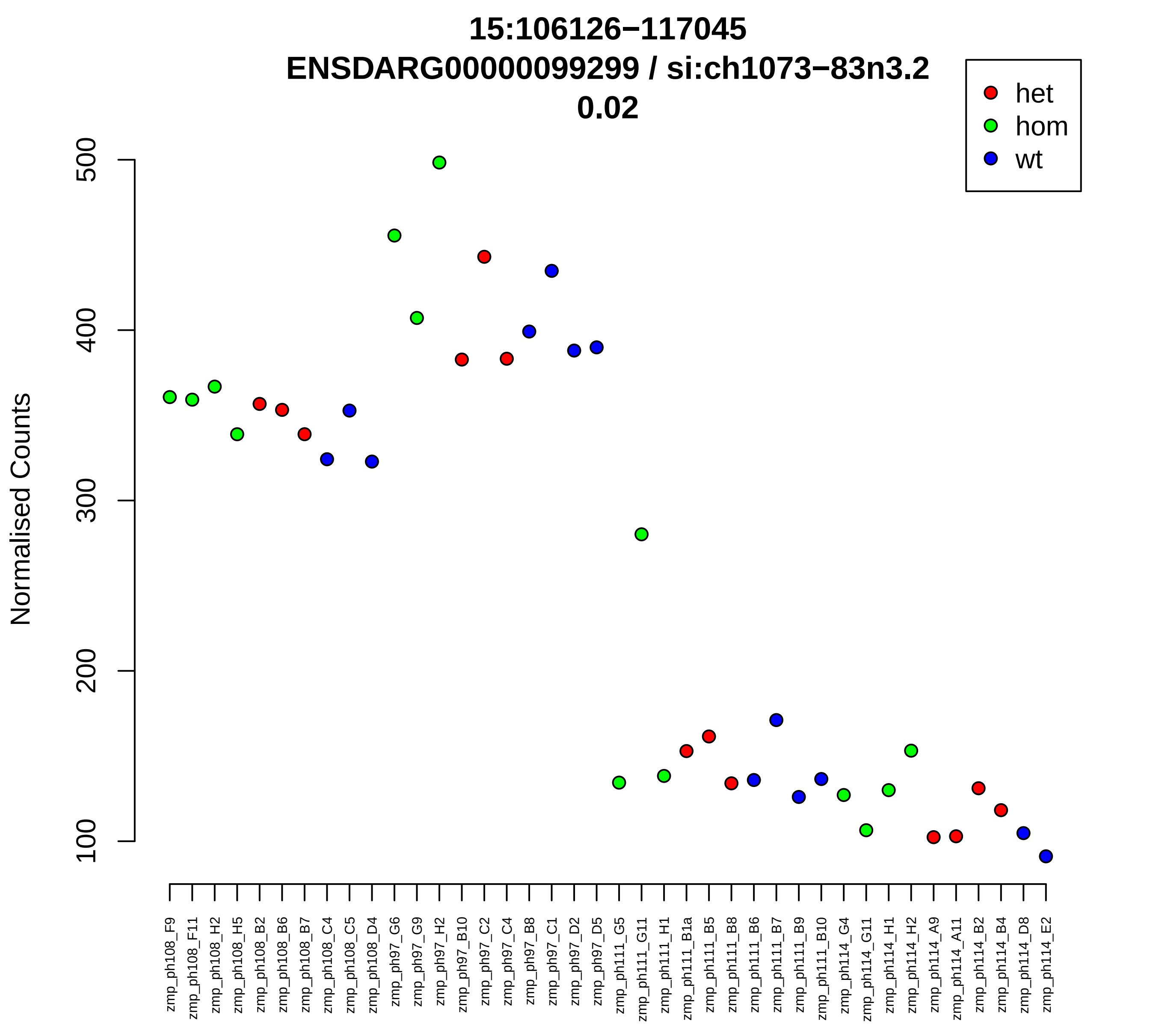

15:106126−117045
ENSDARG00000099299 / si:ch1073−83n3.2
0.02
het
hom
wt
Normalised Counts
100
200
300
400
500
zmp_ph108_F9
zmp_ph108_F11
zmp_ph108_H2
zmp_ph108_H5
zmp_ph108_B2
zmp_ph108_B6
zmp_ph108_B7
zmp_ph108_C4
zmp_ph108_C5
zmp_ph108_D4
zmp_ph97_G6
zmp_ph97_G9
zmp_ph97_H2
zmp_ph97_B10
zmp_ph97_C2
zmp_ph97_C4
zmp_ph97_B8
zmp_ph97_C1
zmp_ph97_D2
zmp_ph97_D5
zmp_ph111_G5
zmp_ph111_G11
zmp_ph111_H1
zmp_ph111_B1a
zmp_ph111_B5
zmp_ph111_B8
zmp_ph111_B6
zmp_ph111_B7
zmp_ph111_B9
zmp_ph111_B10
zmp_ph114_G4
zmp_ph114_G11
zmp_ph114_H1
zmp_ph114_H2
zmp_ph114_A9
zmp_ph114_A11
zmp_ph114_B2
zmp_ph114_B4
zmp_ph114_D8
zmp_ph114_E2

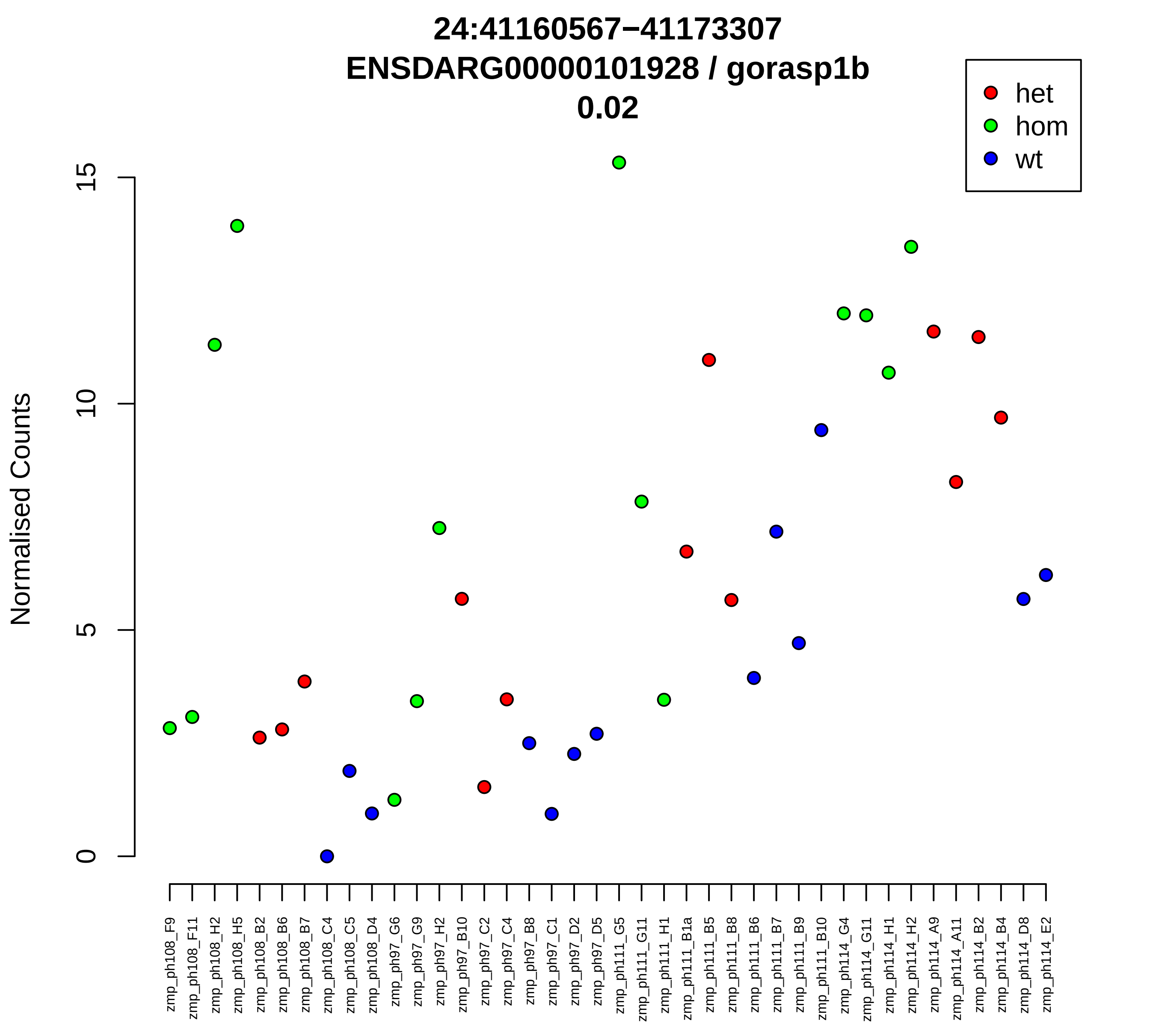

24:41160567−41173307
ENSDARG00000101928 / gorasp1b
0.02
het
hom
wt
Normalised Counts
0
5
10
15
zmp_ph108_F9
zmp_ph108_F11
zmp_ph108_H2
zmp_ph108_H5
zmp_ph108_B2
zmp_ph108_B6
zmp_ph108_B7
zmp_ph108_C4
zmp_ph108_C5
zmp_ph108_D4
zmp_ph97_G6
zmp_ph97_G9
zmp_ph97_H2
zmp_ph97_B10
zmp_ph97_C2
zmp_ph97_C4
zmp_ph97_B8
zmp_ph97_C1
zmp_ph97_D2
zmp_ph97_D5
zmp_ph111_G5
zmp_ph111_G11
zmp_ph111_H1
zmp_ph111_B1a
zmp_ph111_B5
zmp_ph111_B8
zmp_ph111_B6
zmp_ph111_B7
zmp_ph111_B9
zmp_ph111_B10
zmp_ph114_G4
zmp_ph114_G11
zmp_ph114_H1
zmp_ph114_H2
zmp_ph114_A9
zmp_ph114_A11
zmp_ph114_B2
zmp_ph114_B4
zmp_ph114_D8
zmp_ph114_E2

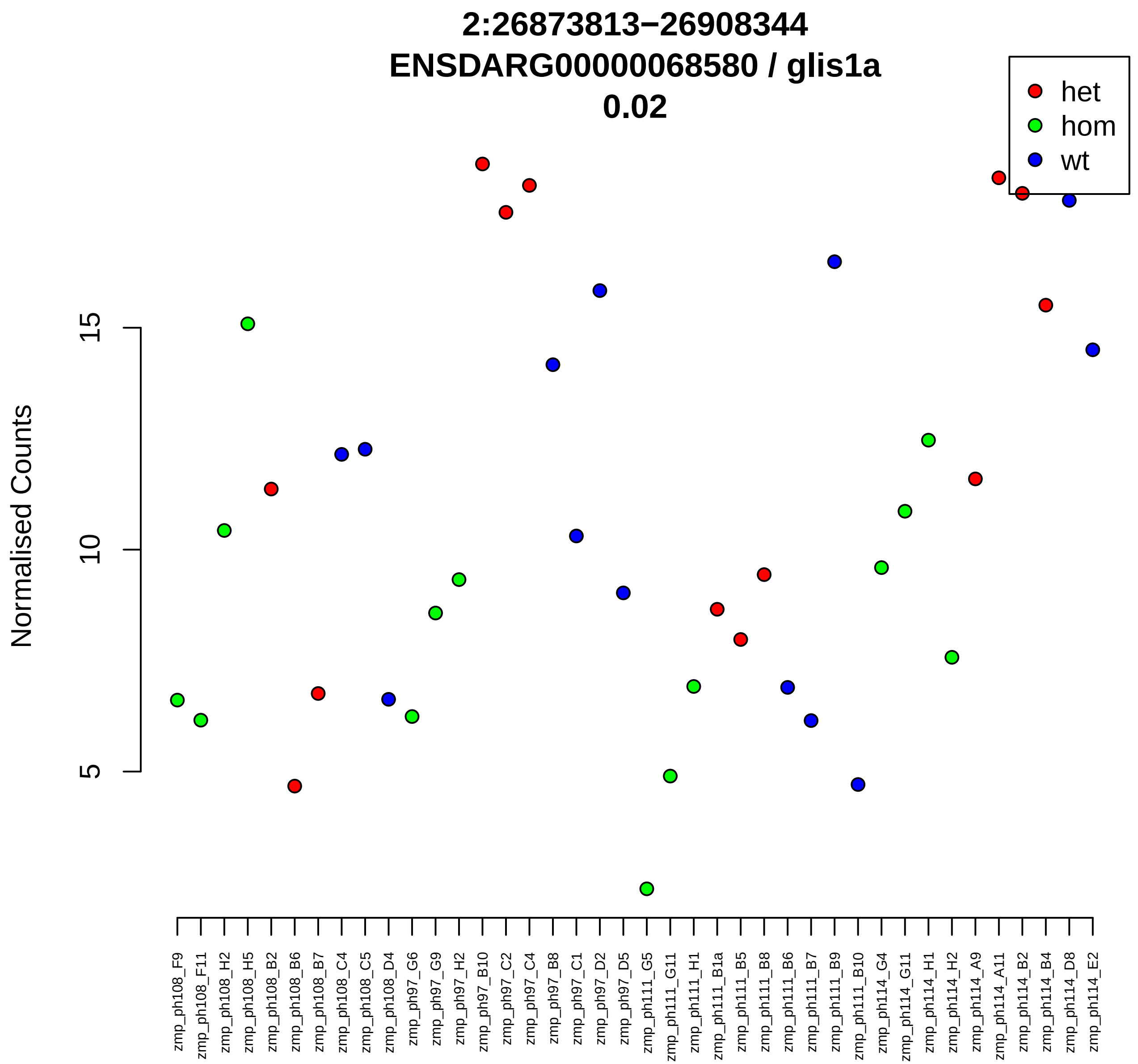
2:26873813−26908344
ENSDARG00000068580 / glis1a
0.02
het
hom
wt
Normalised Counts
5
10
15
zmp_ph108_F9
zmp_ph108_F11
zmp_ph108_H2
zmp_ph108_H5
zmp_ph108_B2
zmp_ph108_B6
zmp_ph108_B7
zmp_ph108_C4
zmp_ph108_C5
zmp_ph108_D4
zmp_ph97_G6
zmp_ph97_G9
zmp_ph97_H2
zmp_ph97_B10
zmp_ph97_C2
zmp_ph97_C4
zmp_ph97_B8
zmp_ph97_C1
zmp_ph97_D2
zmp_ph97_D5
zmp_ph111_G5
zmp_ph111_G11
zmp_ph111_H1
zmp_ph111_B1a
zmp_ph111_B5
zmp_ph111_B8
zmp_ph111_B6
zmp_ph111_B7
zmp_ph111_B9
zmp_ph111_B10
zmp_ph114_G4
zmp_ph114_G11
zmp_ph114_H1
zmp_ph114_H2
zmp_ph114_A9
zmp_ph114_A11
zmp_ph114_B2
zmp_ph114_B4
zmp_ph114_D8
zmp_ph114_E2

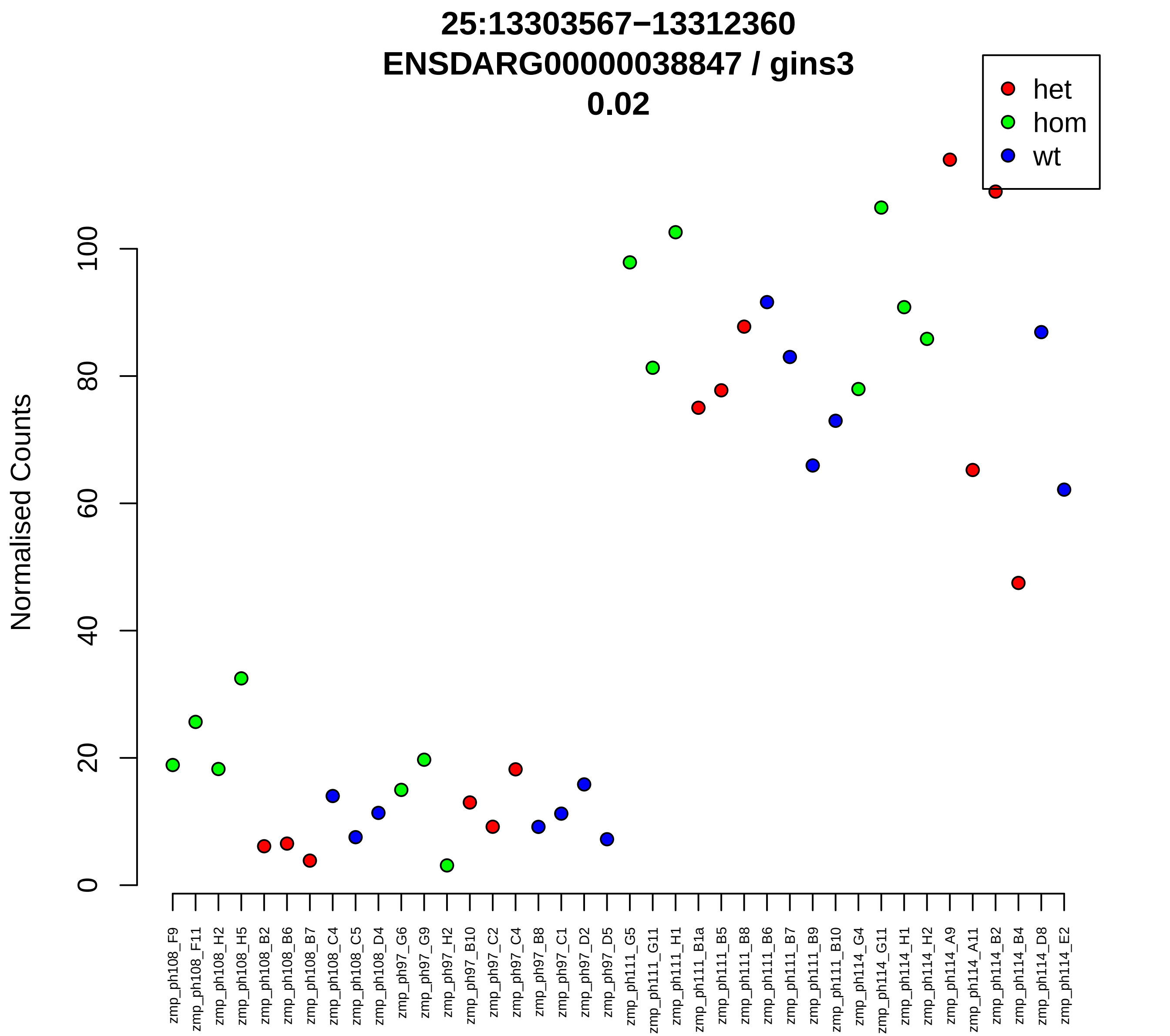

25:13303567−13312360
ENSDARG00000038847 / gins3
0.02
het
hom
wt
Normalised Counts
0
20
40
60
80
100
zmp_ph108_F9
zmp_ph108_F11
zmp_ph108_H2
zmp_ph108_H5
zmp_ph108_B2
zmp_ph108_B6
zmp_ph108_B7
zmp_ph108_C4
zmp_ph108_C5
zmp_ph108_D4
zmp_ph97_G6
zmp_ph97_G9
zmp_ph97_H2
zmp_ph97_B10
zmp_ph97_C2
zmp_ph97_C4
zmp_ph97_B8
zmp_ph97_C1
zmp_ph97_D2
zmp_ph97_D5
zmp_ph111_G5
zmp_ph111_G11
zmp_ph111_H1
zmp_ph111_B1a
zmp_ph111_B5
zmp_ph111_B8
zmp_ph111_B6
zmp_ph111_B7
zmp_ph111_B9
zmp_ph111_B10
zmp_ph114_G4
zmp_ph114_G11
zmp_ph114_H1
zmp_ph114_H2
zmp_ph114_A9
zmp_ph114_A11
zmp_ph114_B2
zmp_ph114_B4
zmp_ph114_D8
zmp_ph114_E2

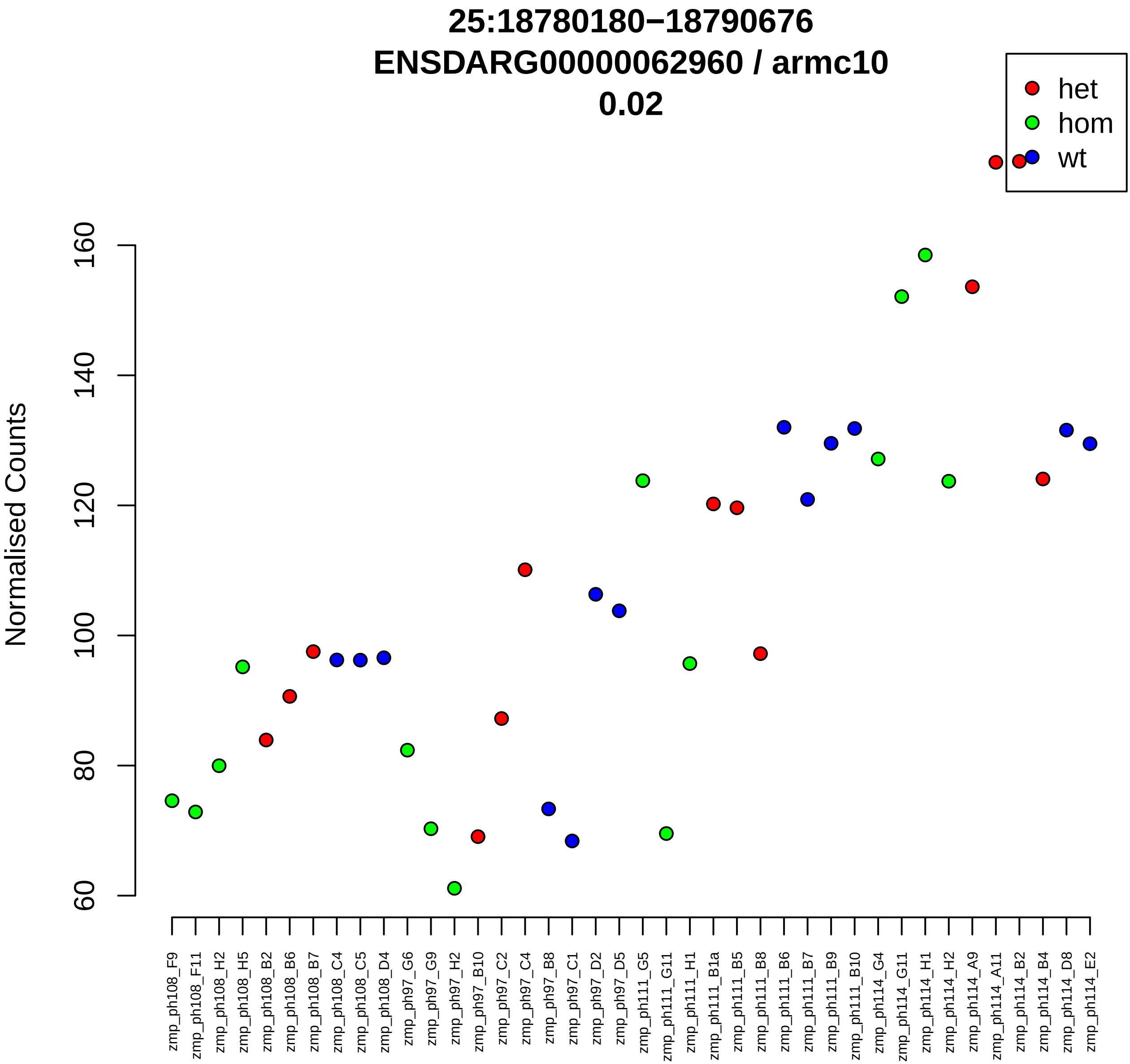

25:18780180−18790676
ENSDARG00000062960 / armc10
0.02
het
hom
wt
Normalised Counts
60
80
100
120
140
160
zmp_ph108_F9
zmp_ph108_F11
zmp_ph108_H2
zmp_ph108_H5
zmp_ph108_B2
zmp_ph108_B6
zmp_ph108_B7
zmp_ph108_C4
zmp_ph108_C5
zmp_ph108_D4
zmp_ph97_G6
zmp_ph97_G9
zmp_ph97_H2
zmp_ph97_B10
zmp_ph97_C2
zmp_ph97_C4
zmp_ph97_B8
zmp_ph97_C1
zmp_ph97_D2
zmp_ph97_D5
zmp_ph111_G5
zmp_ph111_G11
zmp_ph111_H1
zmp_ph111_B1a
zmp_ph111_B5
zmp_ph111_B8
zmp_ph111_B6
zmp_ph111_B7
zmp_ph111_B9
zmp_ph111_B10
zmp_ph114_G4
zmp_ph114_G11
zmp_ph114_H1
zmp_ph114_H2
zmp_ph114_A9
zmp_ph114_A11
zmp_ph114_B2
zmp_ph114_B4
zmp_ph114_D8
zmp_ph114_E2

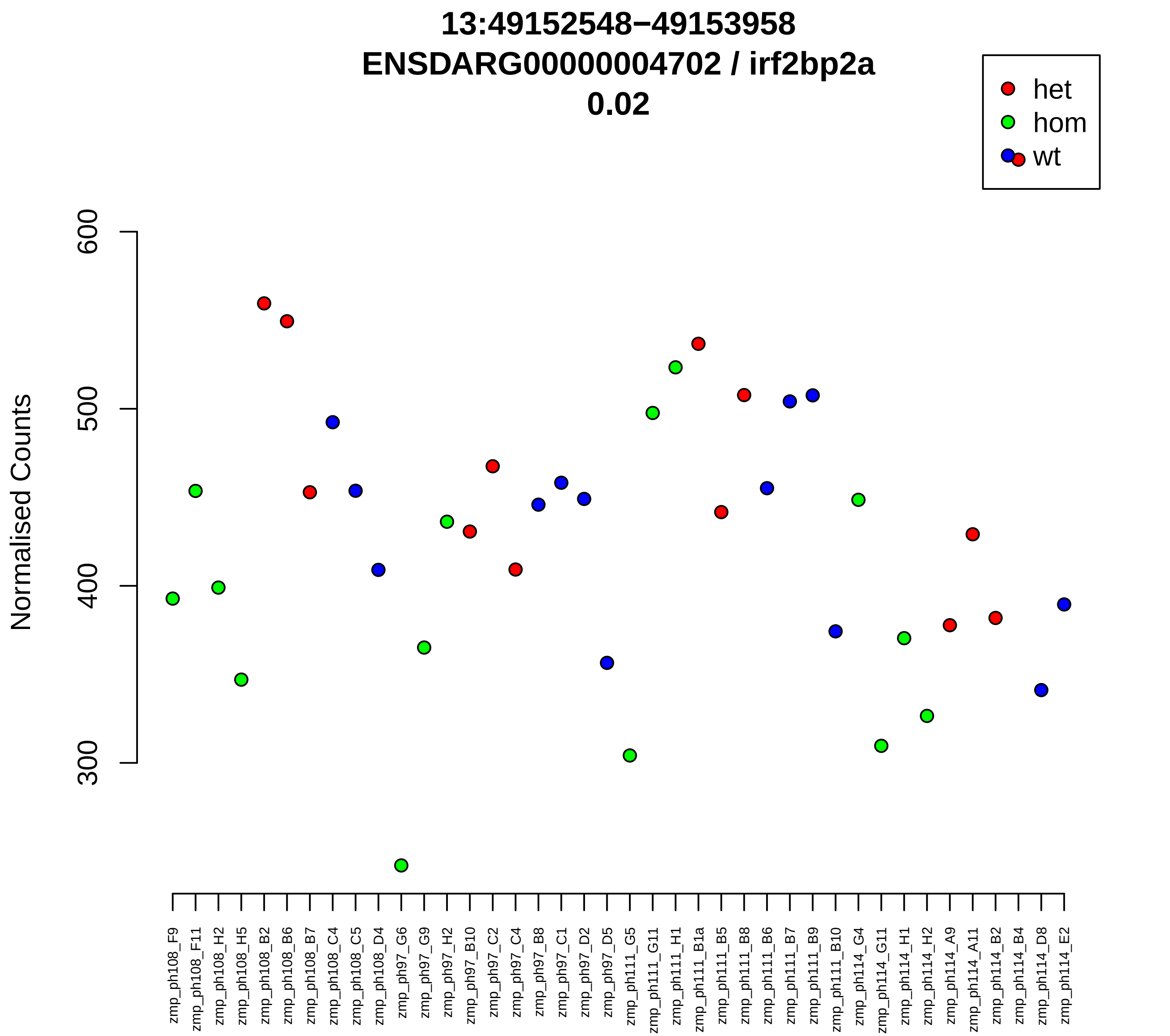

13:49152548−49153958
ENSDARG00000004702 / irf2bp2a
0.02
het
hom
wt
Normalised Counts
600
500
400
300
zmp_ph108_F9
zmp_ph108_F11
zmp_ph108_H2
zmp_ph108_H5
zmp_ph108_B2
zmp_ph108_B6
zmp_ph108_B7
zmp_ph108_C4
zmp_ph108_C5
zmp_ph108_D4
zmp_ph97_G6
zmp_ph97_G9
zmp_ph97_H2
zmp_ph97_B10
zmp_ph97_C2
zmp_ph97_C4
zmp_ph97_B8
zmp_ph97_C1
zmp_ph97_D2
zmp_ph97_D5
zmp_ph111_G5
zmp_ph111_G11
zmp_ph111_H1
zmp_ph111_B1a
zmp_ph111_B5
zmp_ph111_B8
zmp_ph111_B6
zmp_ph111_B7
zmp_ph111_B9
zmp_ph111_B10
zmp_ph114_G4
zmp_ph114_G11
zmp_ph114_H1
zmp_ph114_H2
zmp_ph114_A9
zmp_ph114_A11
zmp_ph114_B2
zmp_ph114_B4
zmp_ph114_D8
zmp_ph114_E2

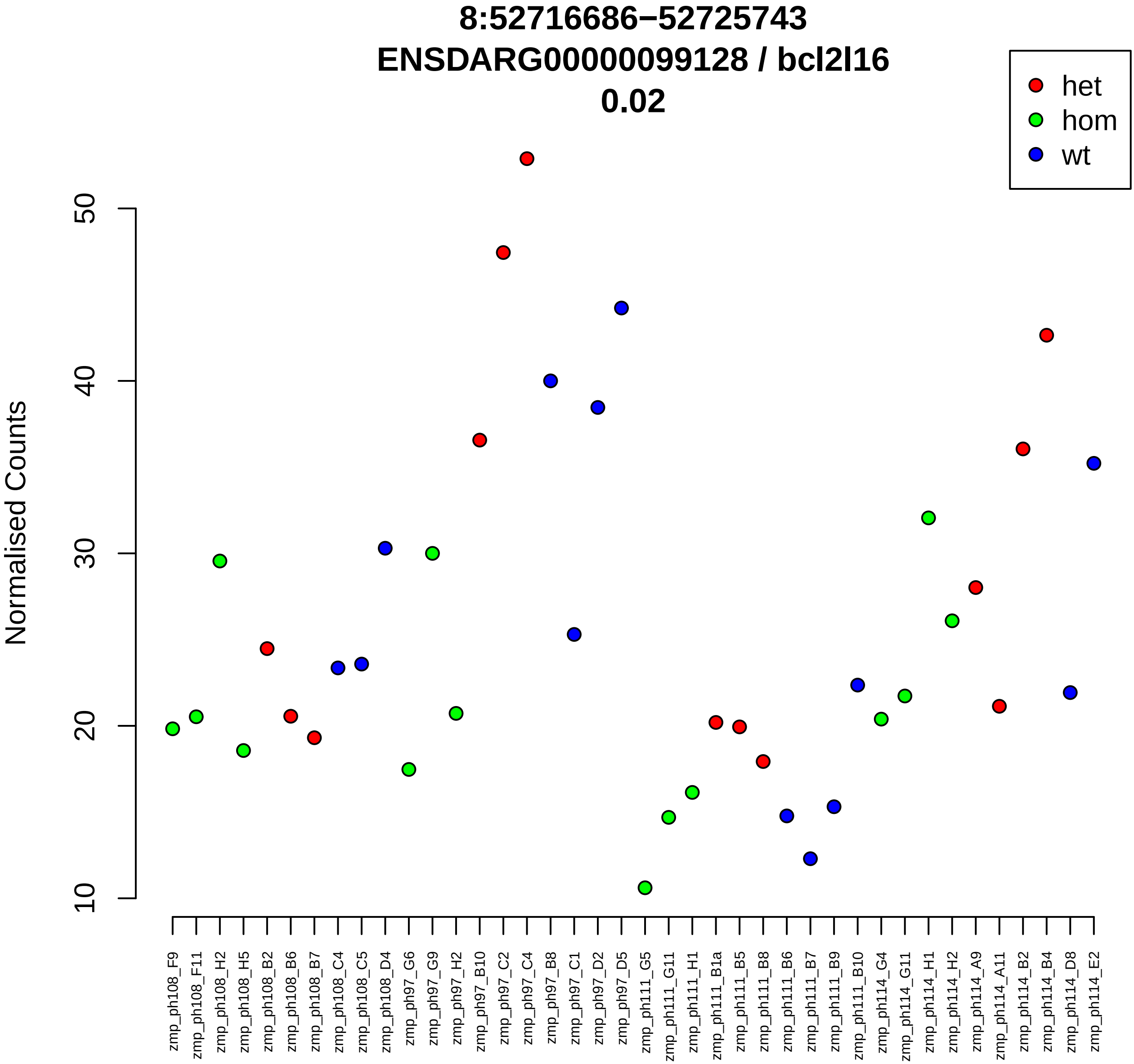

8:52716686−52725743
ENSDARG00000099128 / bcl2l16
0.02
het
hom
wt
Normalised Counts
10
20
30
40
50
zmp_ph108_F9
zmp_ph108_F11
zmp_ph108_H2
zmp_ph108_H5
zmp_ph108_B2
zmp_ph108_B6
zmp_ph108_B7
zmp_ph108_C4
zmp_ph108_C5
zmp_ph108_D4
zmp_ph97_G6
zmp_ph97_G9
zmp_ph97_H2
zmp_ph97_B10
zmp_ph97_C2
zmp_ph97_C4
zmp_ph97_B8
zmp_ph97_C1
zmp_ph97_D2
zmp_ph97_D5
zmp_ph111_G5
zmp_ph111_G11
zmp_ph111_H1
zmp_ph111_B1a
zmp_ph111_B5
zmp_ph111_B8
zmp_ph111_B6
zmp_ph111_B7
zmp_ph111_B9
zmp_ph111_B10
zmp_ph114_G4
zmp_ph114_G11
zmp_ph114_H1
zmp_ph114_H2
zmp_ph114_A9
zmp_ph114_A11
zmp_ph114_B2
zmp_ph114_B4
zmp_ph114_D8
zmp_ph114_E2

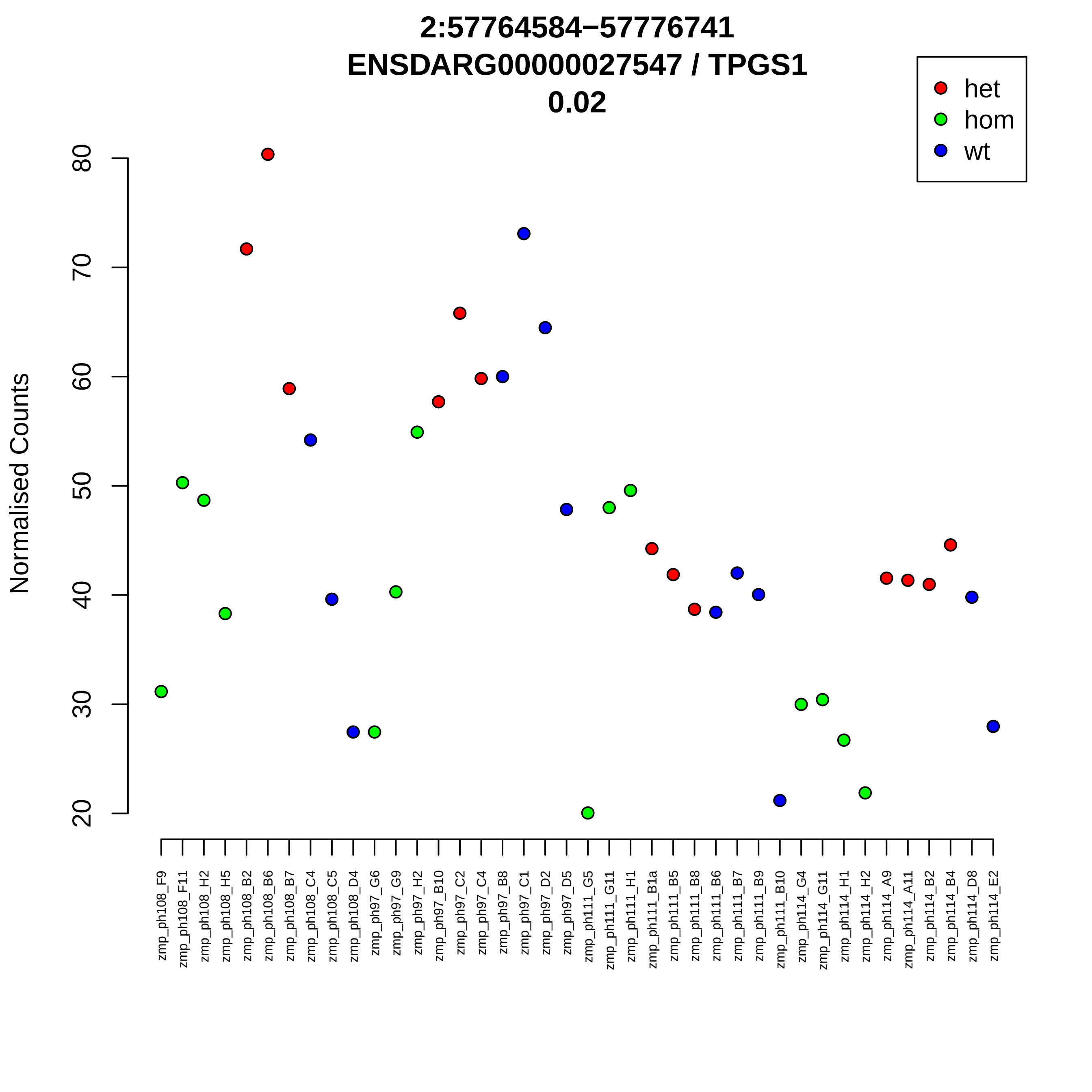

2:57764584−57776741
ENSDARG00000027547 / TPGS1
0.02
het
hom
wt
Normalised Counts
20
30
40
50
60
70
80
zmp_ph108_F9
zmp_ph108_F11
zmp_ph108_H2
zmp_ph108_H5
zmp_ph108_B2
zmp_ph108_B6
zmp_ph108_B7
zmp_ph108_C4
zmp_ph108_C5
zmp_ph108_D4
zmp_ph97_G6
zmp_ph97_G9
zmp_ph97_H2
zmp_ph97_B10
zmp_ph97_C2
zmp_ph97_C4
zmp_ph97_B8
zmp_ph97_C1
zmp_ph97_D2
zmp_ph97_D5
zmp_ph111_G5
zmp_ph111_G11
zmp_ph111_H1
zmp_ph111_B1a
zmp_ph111_B5
zmp_ph111_B8
zmp_ph111_B6
zmp_ph111_B7
zmp_ph111_B9
zmp_ph111_B10
zmp_ph114_G4
zmp_ph114_G11
zmp_ph114_H1
zmp_ph114_H2
zmp_ph114_A9
zmp_ph114_A11
zmp_ph114_B2
zmp_ph114_B4
zmp_ph114_D8
zmp_ph114_E2

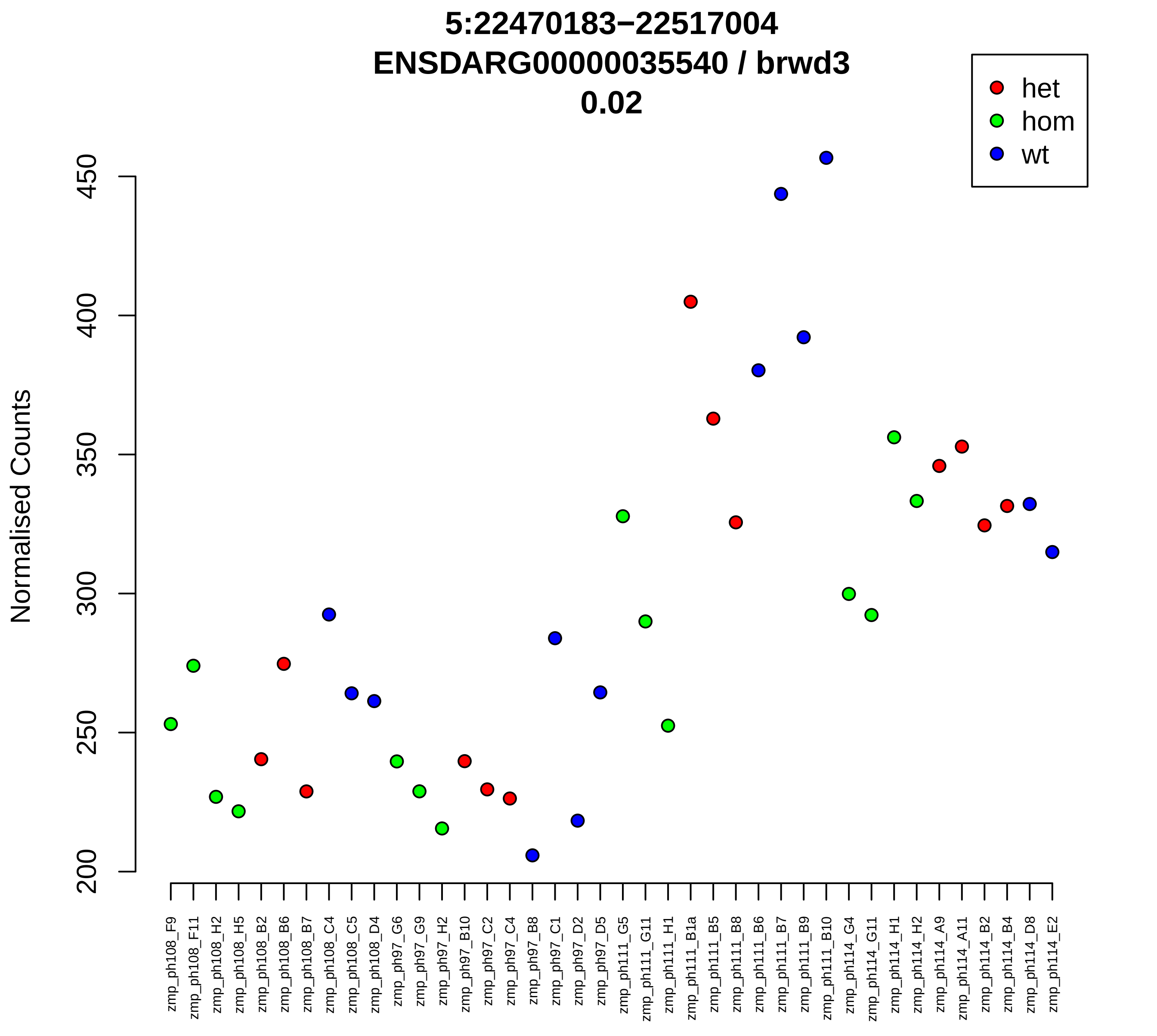

5:22470183−22517004
ENSDARG00000035540 / brwd3
0.02
het
hom
wt
Normalised Counts
200
250
300
350
400
450
zmp_ph108_F9
zmp_ph108_F11
zmp_ph108_H2
zmp_ph108_H5
zmp_ph108_B2
zmp_ph108_B6
zmp_ph108_B7
zmp_ph108_C4
zmp_ph108_C5
zmp_ph108_D4
zmp_ph97_G6
zmp_ph97_G9
zmp_ph97_H2
zmp_ph97_B10
zmp_ph97_C2
zmp_ph97_C4
zmp_ph97_B8
zmp_ph97_C1
zmp_ph97_D2
zmp_ph97_D5
zmp_ph111_G5
zmp_ph111_G11
zmp_ph111_H1
zmp_ph111_B1a
zmp_ph111_B5
zmp_ph111_B8
zmp_ph111_B6
zmp_ph111_B7
zmp_ph111_B9
zmp_ph111_B10
zmp_ph114_G4
zmp_ph114_G11
zmp_ph114_H1
zmp_ph114_H2
zmp_ph114_A9
zmp_ph114_A11
zmp_ph114_B2
zmp_ph114_B4
zmp_ph114_D8
zmp_ph114_E2

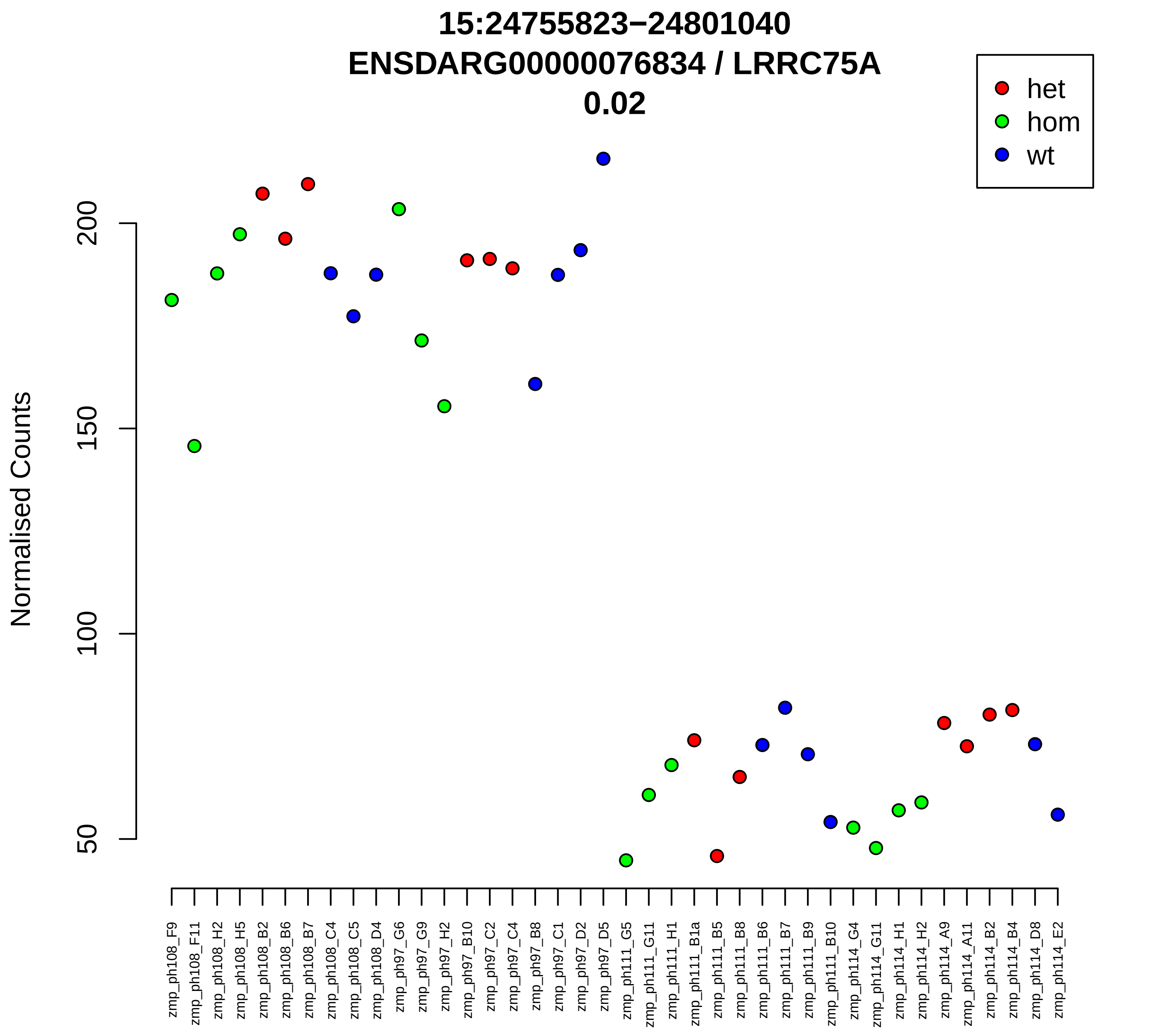

15:24755823−24801040
ENSDARG00000076834 / LRRC75A
0.02
het
hom
wt
Normalised Counts
50
100
150
200
zmp_ph108_F9
zmp_ph108_F11
zmp_ph108_H2
zmp_ph108_H5
zmp_ph108_B2
zmp_ph108_B6
zmp_ph108_B7
zmp_ph108_C4
zmp_ph108_C5
zmp_ph108_D4
zmp_ph97_G6
zmp_ph97_G9
zmp_ph97_H2
zmp_ph97_B10
zmp_ph97_C2
zmp_ph97_C4
zmp_ph97_B8
zmp_ph97_C1
zmp_ph97_D2
zmp_ph97_D5
zmp_ph111_G5
zmp_ph111_G11
zmp_ph111_H1
zmp_ph111_B1a
zmp_ph111_B5
zmp_ph111_B8
zmp_ph111_B6
zmp_ph111_B7
zmp_ph111_B9
zmp_ph111_B10
zmp_ph114_G4
zmp_ph114_G11
zmp_ph114_H1
zmp_ph114_H2
zmp_ph114_A9
zmp_ph114_A11
zmp_ph114_B2
zmp_ph114_B4
zmp_ph114_D8
zmp_ph114_E2

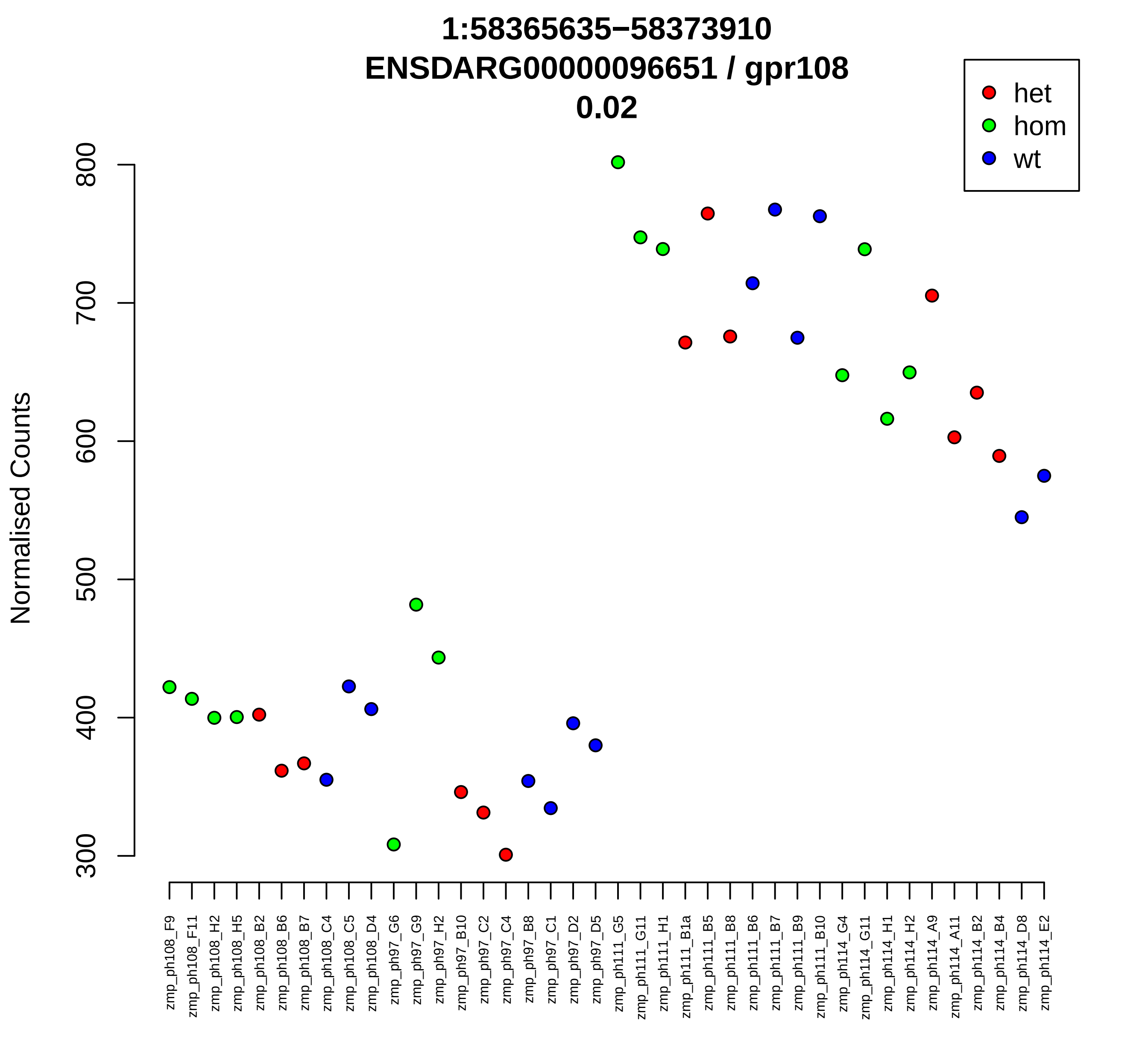

1:58365635−58373910
ENSDARG00000096651 / gpr108
0.02
het
hom
wt
Normalised Counts
300
400
500
600
700
800
zmp_ph108_F9
zmp_ph108_F11
zmp_ph108_H2
zmp_ph108_H5
zmp_ph108_B2
zmp_ph108_B6
zmp_ph108_B7
zmp_ph108_C4
zmp_ph108_C5
zmp_ph108_D4
zmp_ph97_G6
zmp_ph97_G9
zmp_ph97_H2
zmp_ph97_B10
zmp_ph97_C2
zmp_ph97_C4
zmp_ph97_B8
zmp_ph97_C1
zmp_ph97_D2
zmp_ph97_D5
zmp_ph111_G5
zmp_ph111_G11
zmp_ph111_H1
zmp_ph111_B1a
zmp_ph111_B5
zmp_ph111_B8
zmp_ph111_B6
zmp_ph111_B7
zmp_ph111_B9
zmp_ph111_B10
zmp_ph114_G4
zmp_ph114_G11
zmp_ph114_H1
zmp_ph114_H2
zmp_ph114_A9
zmp_ph114_A11
zmp_ph114_B2
zmp_ph114_B4
zmp_ph114_D8
zmp_ph114_E2

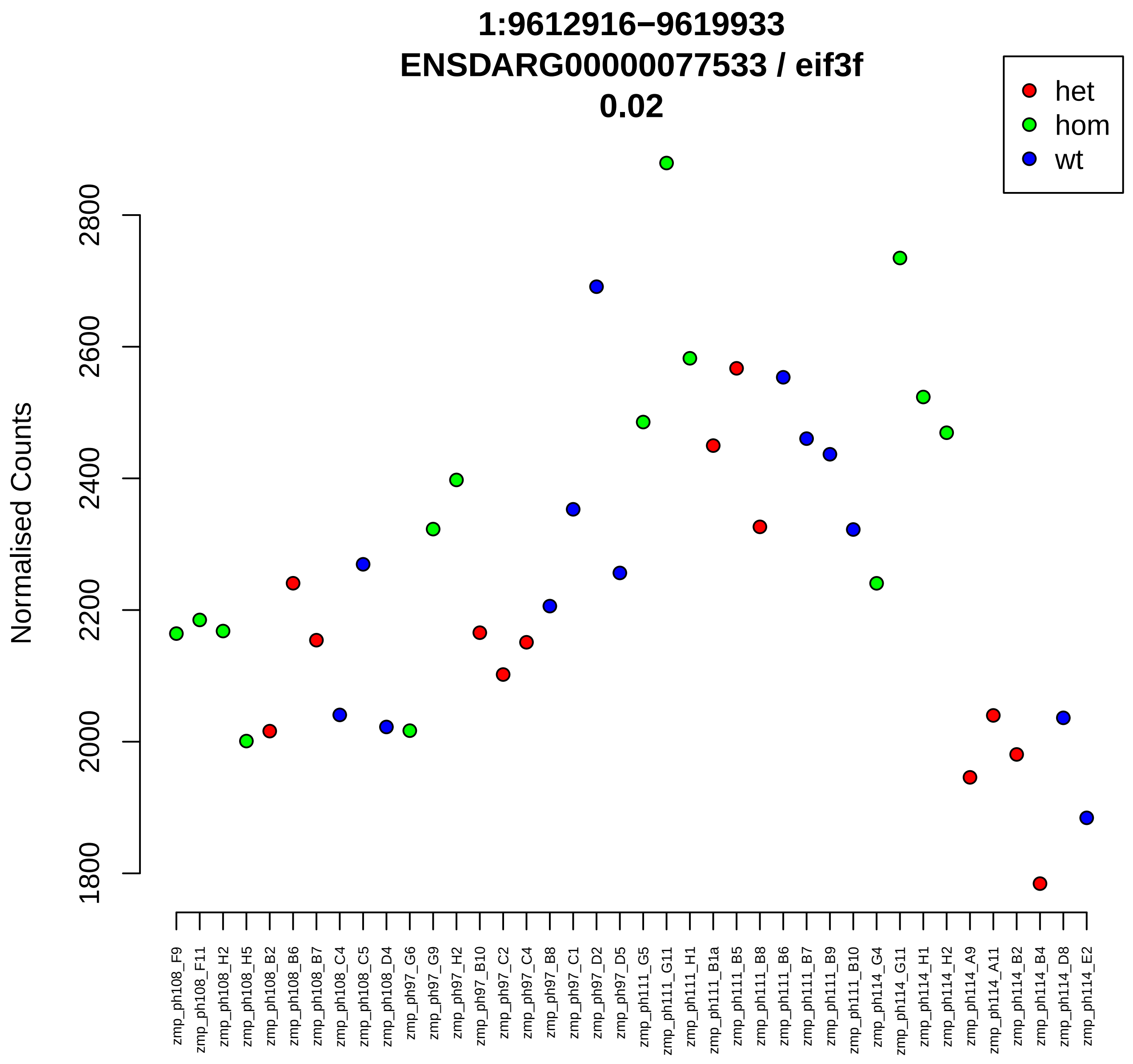
1:9612916−9619933
ENSDARG00000077533 / eif3f
0.02
het
hom
wt
Normalised Counts
1800
2000
2200
2400
2600
2800
zmp_ph108_F9
zmp_ph108_F11
zmp_ph108_H2
zmp_ph108_H5
zmp_ph108_B2
zmp_ph108_B6
zmp_ph108_B7
zmp_ph108_C4
zmp_ph108_C5
zmp_ph108_D4
zmp_ph97_G6
zmp_ph97_G9
zmp_ph97_H2
zmp_ph97_B10
zmp_ph97_C2
zmp_ph97_C4
zmp_ph97_B8
zmp_ph97_C1
zmp_ph97_D2
zmp_ph97_D5
zmp_ph111_G5
zmp_ph111_G11
zmp_ph111_H1
zmp_ph111_B1a
zmp_ph111_B5
zmp_ph111_B8
zmp_ph111_B6
zmp_ph111_B7
zmp_ph111_B9
zmp_ph111_B10
zmp_ph114_G4
zmp_ph114_G11
zmp_ph114_H1
zmp_ph114_H2
zmp_ph114_A9
zmp_ph114_A11
zmp_ph114_B2
zmp_ph114_B4
zmp_ph114_D8
zmp_ph114_E2

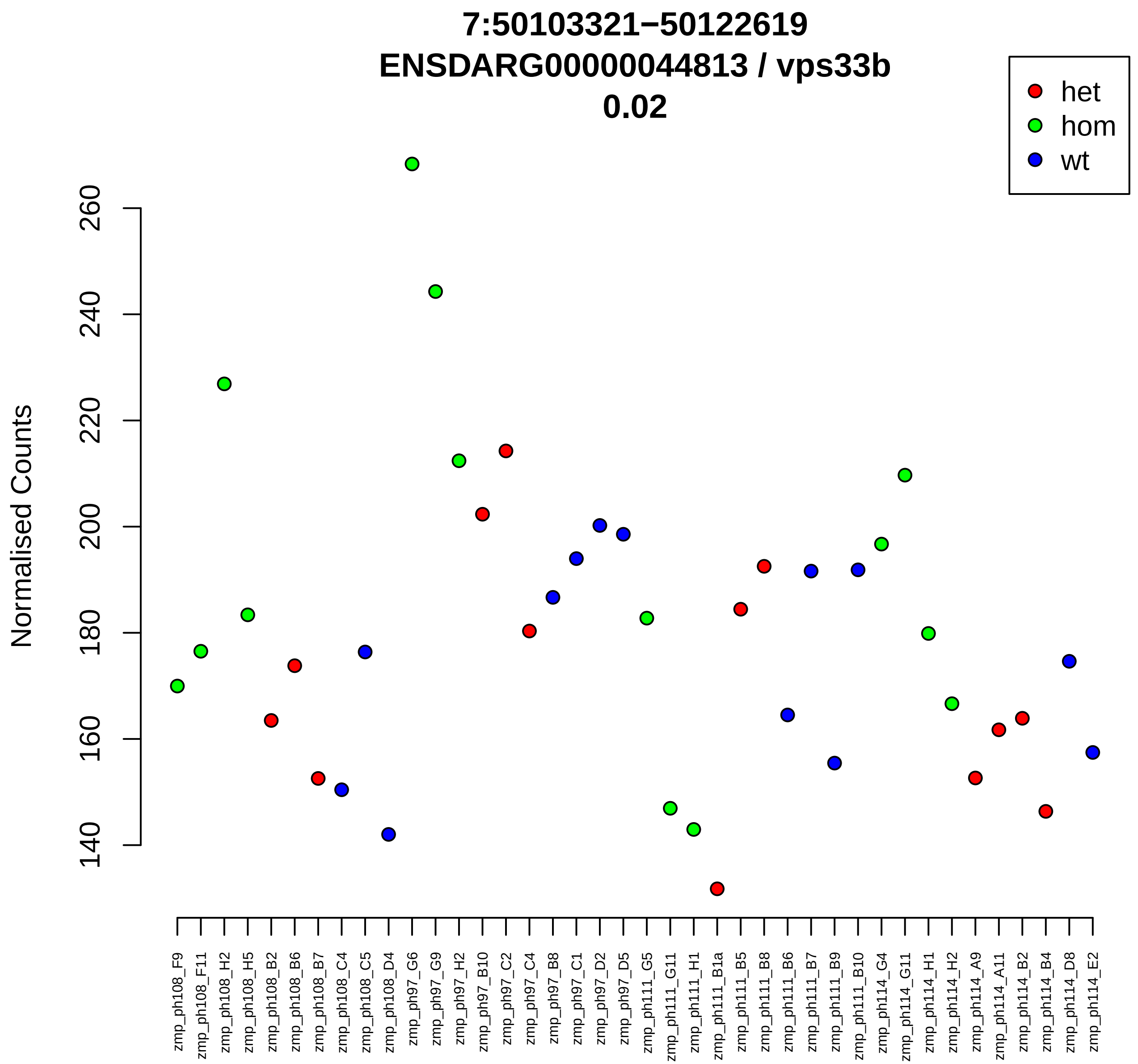

7:50103321−50122619
ENSDARG00000044813 / vps33b
0.02
het
hom
wt
Normalised Counts
140
160
180
200
220
240
260
zmp_ph108_F9
zmp_ph108_F11
zmp_ph108_H2
zmp_ph108_H5
zmp_ph108_B2
zmp_ph108_B6
zmp_ph108_B7
zmp_ph108_C4
zmp_ph108_C5
zmp_ph108_D4
zmp_ph97_G6
zmp_ph97_G9
zmp_ph97_H2
zmp_ph97_B10
zmp_ph97_C2
zmp_ph97_C4
zmp_ph97_B8
zmp_ph97_C1
zmp_ph97_D2
zmp_ph97_D5
zmp_ph111_G5
zmp_ph111_G11
zmp_ph111_H1
zmp_ph111_B1a
zmp_ph111_B5
zmp_ph111_B8
zmp_ph111_B6
zmp_ph111_B7
zmp_ph111_B9
zmp_ph111_B10
zmp_ph114_G4
zmp_ph114_G11
zmp_ph114_H1
zmp_ph114_H2
zmp_ph114_A9
zmp_ph114_A11
zmp_ph114_B2
zmp_ph114_B4
zmp_ph114_D8
zmp_ph114_E2

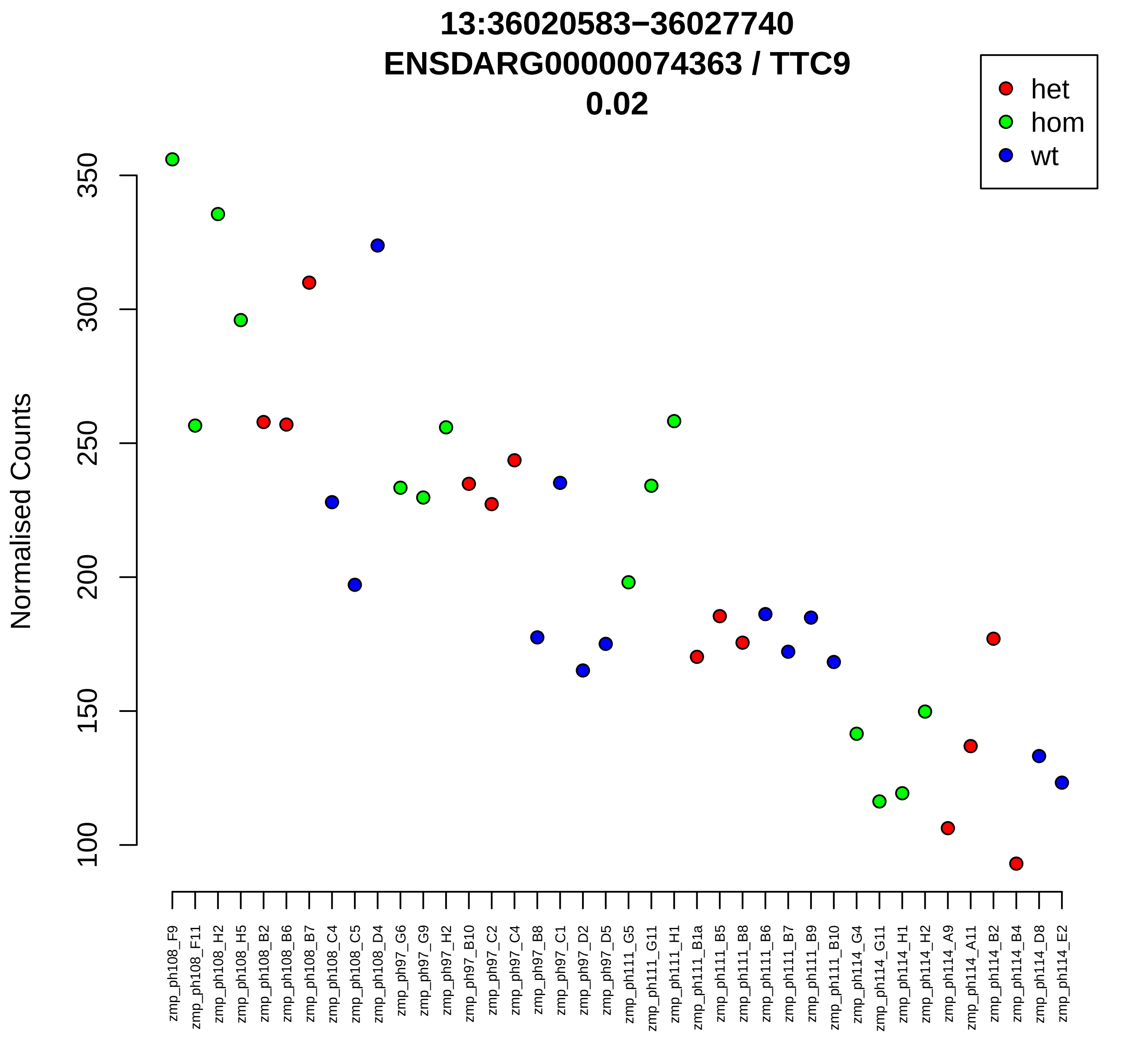

13:36020583−36027740
ENSDARG00000074363 / TTC9
0.02
het
hom
wt
Normalised Counts
100
150
200
250
300
350
zmp_ph108_F9
zmp_ph108_F11
zmp_ph108_H2
zmp_ph108_H5
zmp_ph108_B2
zmp_ph108_B6
zmp_ph108_B7
zmp_ph108_C4
zmp_ph108_C5
zmp_ph108_D4
zmp_ph97_G6
zmp_ph97_G9
zmp_ph97_H2
zmp_ph97_B10
zmp_ph97_C2
zmp_ph97_C4
zmp_ph97_B8
zmp_ph97_C1
zmp_ph97_D2
zmp_ph97_D5
zmp_ph111_G5
zmp_ph111_G11
zmp_ph111_H1
zmp_ph111_B1a
zmp_ph111_B5
zmp_ph111_B8
zmp_ph111_B6
zmp_ph111_B7
zmp_ph111_B9
zmp_ph111_B10
zmp_ph114_G4
zmp_ph114_G11
zmp_ph114_H1
zmp_ph114_H2
zmp_ph114_A9
zmp_ph114_A11
zmp_ph114_B2
zmp_ph114_B4
zmp_ph114_D8
zmp_ph114_E2

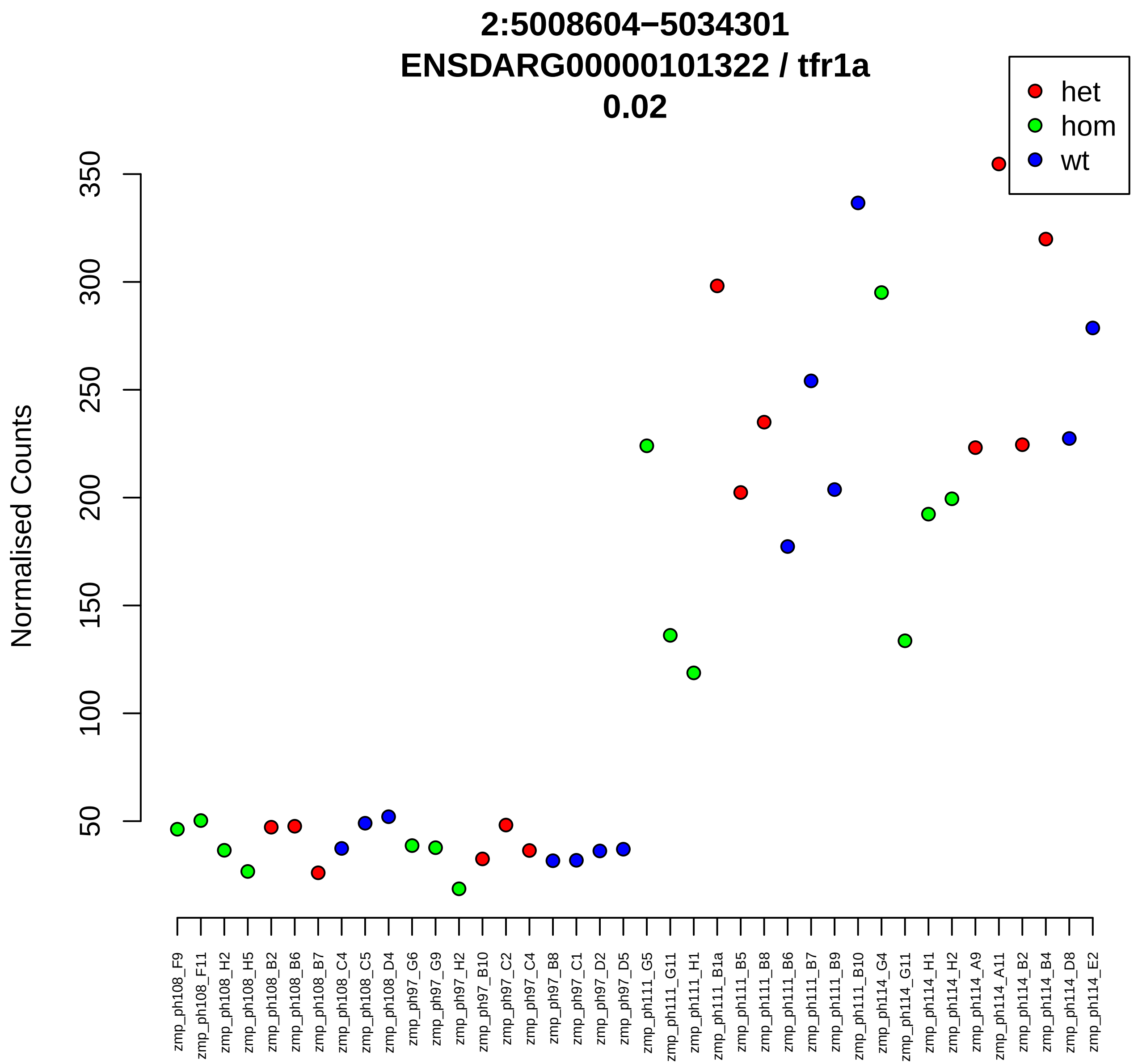
2:5008604−5034301
ENSDARG00000101322 / tfr1a
0.02
het
hom
wt
Normalised Counts
50
100
150
200
250
300
350
zmp_ph108_F9
zmp_ph108_F11
zmp_ph108_H2
zmp_ph108_H5
zmp_ph108_B2
zmp_ph108_B6
zmp_ph108_B7
zmp_ph108_C4
zmp_ph108_C5
zmp_ph108_D4
zmp_ph97_G6
zmp_ph97_G9
zmp_ph97_H2
zmp_ph97_B10
zmp_ph97_C2
zmp_ph97_C4
zmp_ph97_B8
zmp_ph97_C1
zmp_ph97_D2
zmp_ph97_D5
zmp_ph111_G5
zmp_ph111_G11
zmp_ph111_H1
zmp_ph111_B1a
zmp_ph111_B5
zmp_ph111_B8
zmp_ph111_B6
zmp_ph111_B7
zmp_ph111_B9
zmp_ph111_B10
zmp_ph114_G4
zmp_ph114_G11
zmp_ph114_H1
zmp_ph114_H2
zmp_ph114_A9
zmp_ph114_A11
zmp_ph114_B2
zmp_ph114_B4
zmp_ph114_D8
zmp_ph114_E2

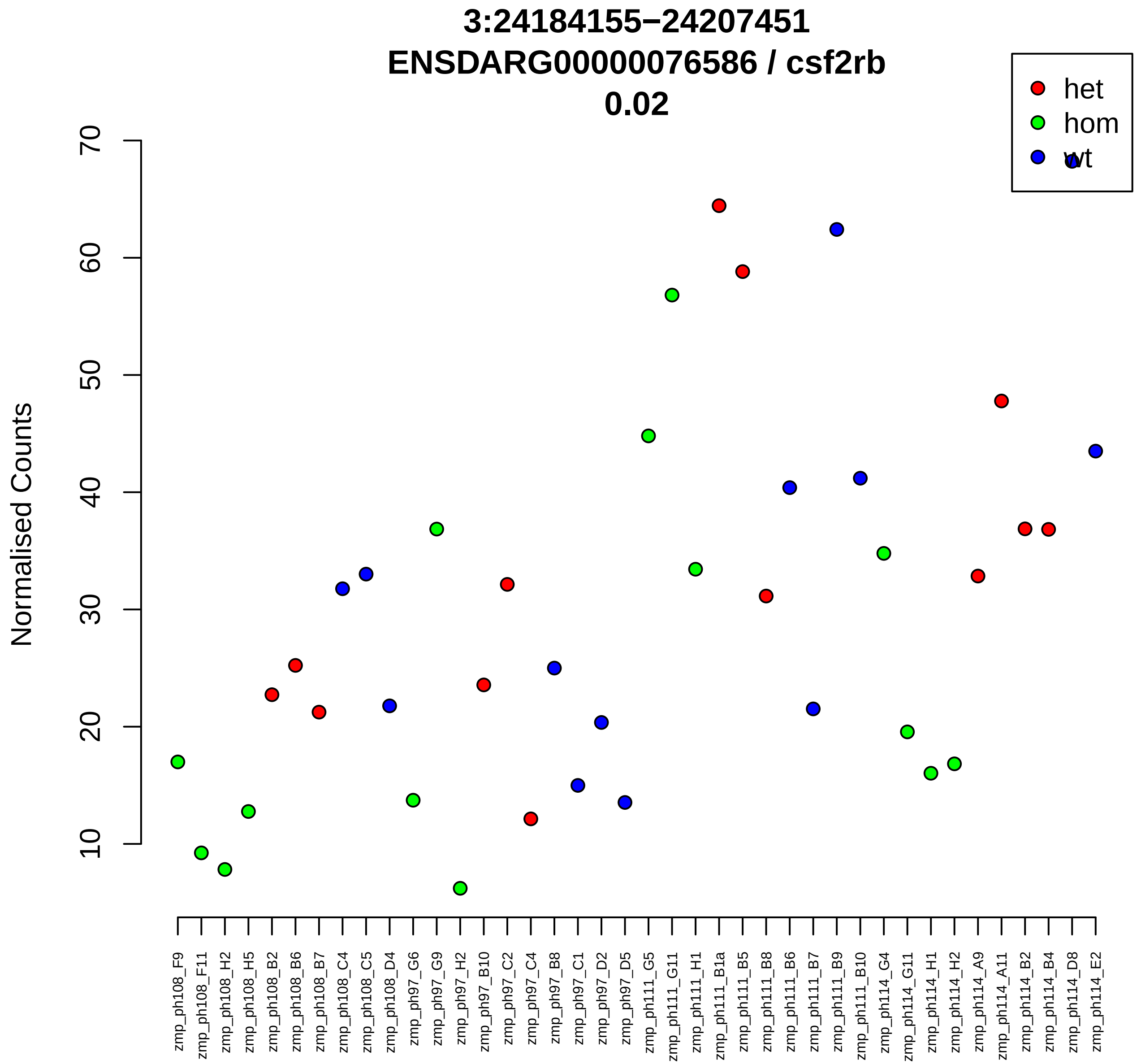
3:24184155−24207451
ENSDARG00000076586 / csf2rb
0.02
het
hom
wt
Normalised Counts
10
20
30
40
50
60
70
zmp_ph108_F9
zmp_ph108_F11
zmp_ph108_H2
zmp_ph108_H5
zmp_ph108_B2
zmp_ph108_B6
zmp_ph108_B7
zmp_ph108_C4
zmp_ph108_C5
zmp_ph108_D4
zmp_ph97_G6
zmp_ph97_G9
zmp_ph97_H2
zmp_ph97_B10
zmp_ph97_C2
zmp_ph97_C4
zmp_ph97_B8
zmp_ph97_C1
zmp_ph97_D2
zmp_ph97_D5
zmp_ph111_G5
zmp_ph111_G11
zmp_ph111_H1
zmp_ph111_B1a
zmp_ph111_B5
zmp_ph111_B8
zmp_ph111_B6
zmp_ph111_B7
zmp_ph111_B9
zmp_ph111_B10
zmp_ph114_G4
zmp_ph114_G11
zmp_ph114_H1
zmp_ph114_H2
zmp_ph114_A9
zmp_ph114_A11
zmp_ph114_B2
zmp_ph114_B4
zmp_ph114_D8
zmp_ph114_E2

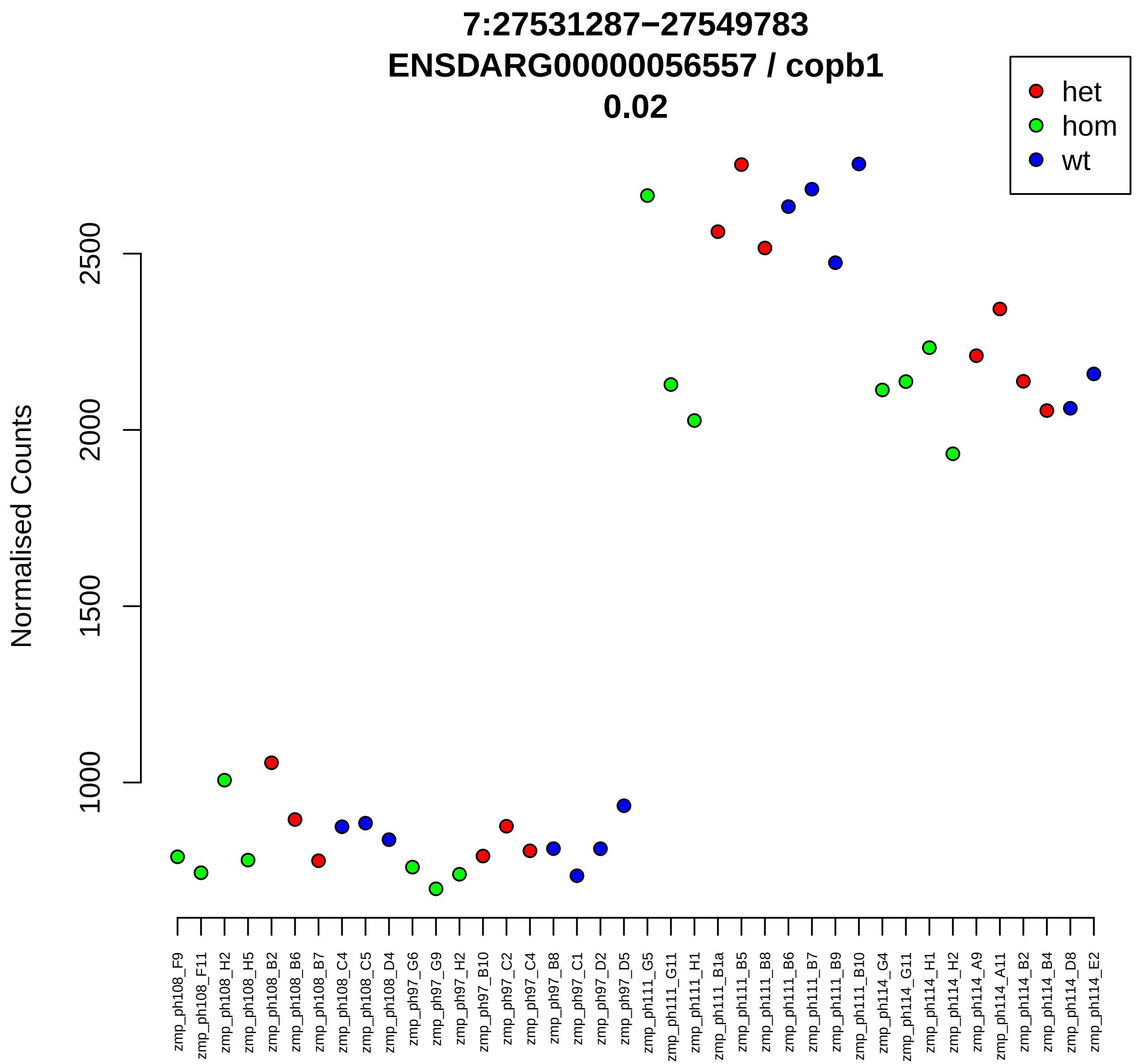

7:27531287−27549783
ENSDARG00000056557 / copb1
0.02
het
hom
wt
Normalised Counts
1000
1500
2000
2500
zmp_ph108_F9
zmp_ph108_F11
zmp_ph108_H2
zmp_ph108_H5
zmp_ph108_B2
zmp_ph108_B6
zmp_ph108_B7
zmp_ph108_C4
zmp_ph108_C5
zmp_ph108_D4
zmp_ph97_G6
zmp_ph97_G9
zmp_ph97_H2
zmp_ph97_B10
zmp_ph97_C2
zmp_ph97_C4
zmp_ph97_B8
zmp_ph97_C1
zmp_ph97_D2
zmp_ph97_D5
zmp_ph111_G5
zmp_ph111_G11
zmp_ph111_H1
zmp_ph111_B1a
zmp_ph111_B5
zmp_ph111_B8
zmp_ph111_B6
zmp_ph111_B7
zmp_ph111_B9
zmp_ph111_B10
zmp_ph114_G4
zmp_ph114_G11
zmp_ph114_H1
zmp_ph114_H2
zmp_ph114_A9
zmp_ph114_A11
zmp_ph114_B2
zmp_ph114_B4
zmp_ph114_D8
zmp_ph114_E2

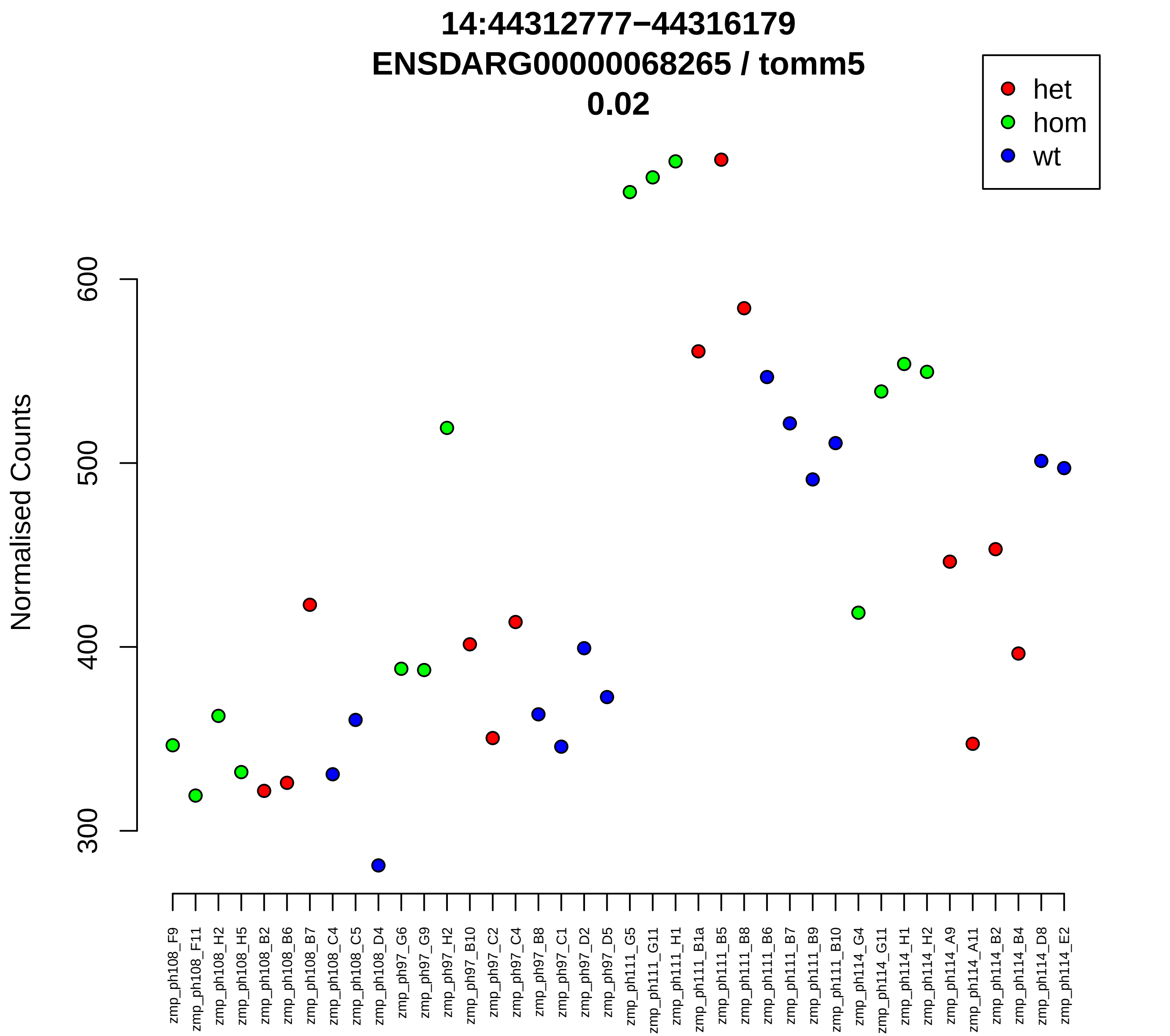
14:44312777−44316179
ENSDARG00000068265 / tomm5
0.02
het
hom
wt
Normalised Counts
300
400
500
600
zmp_ph108_F9
zmp_ph108_F11
zmp_ph108_H2
zmp_ph108_H5
zmp_ph108_B2
zmp_ph108_B6
zmp_ph108_B7
zmp_ph108_C4
zmp_ph108_C5
zmp_ph108_D4
zmp_ph97_G6
zmp_ph97_G9
zmp_ph97_H2
zmp_ph97_B10
zmp_ph97_C2
zmp_ph97_C4
zmp_ph97_B8
zmp_ph97_C1
zmp_ph97_D2
zmp_ph97_D5
zmp_ph111_G5
zmp_ph111_G11
zmp_ph111_H1
zmp_ph111_B1a
zmp_ph111_B5
zmp_ph111_B8
zmp_ph111_B6
zmp_ph111_B7
zmp_ph111_B9
zmp_ph111_B10
zmp_ph114_G4
zmp_ph114_G11
zmp_ph114_H1
zmp_ph114_H2
zmp_ph114_A9
zmp_ph114_A11
zmp_ph114_B2
zmp_ph114_B4
zmp_ph114_D8
zmp_ph114_E2

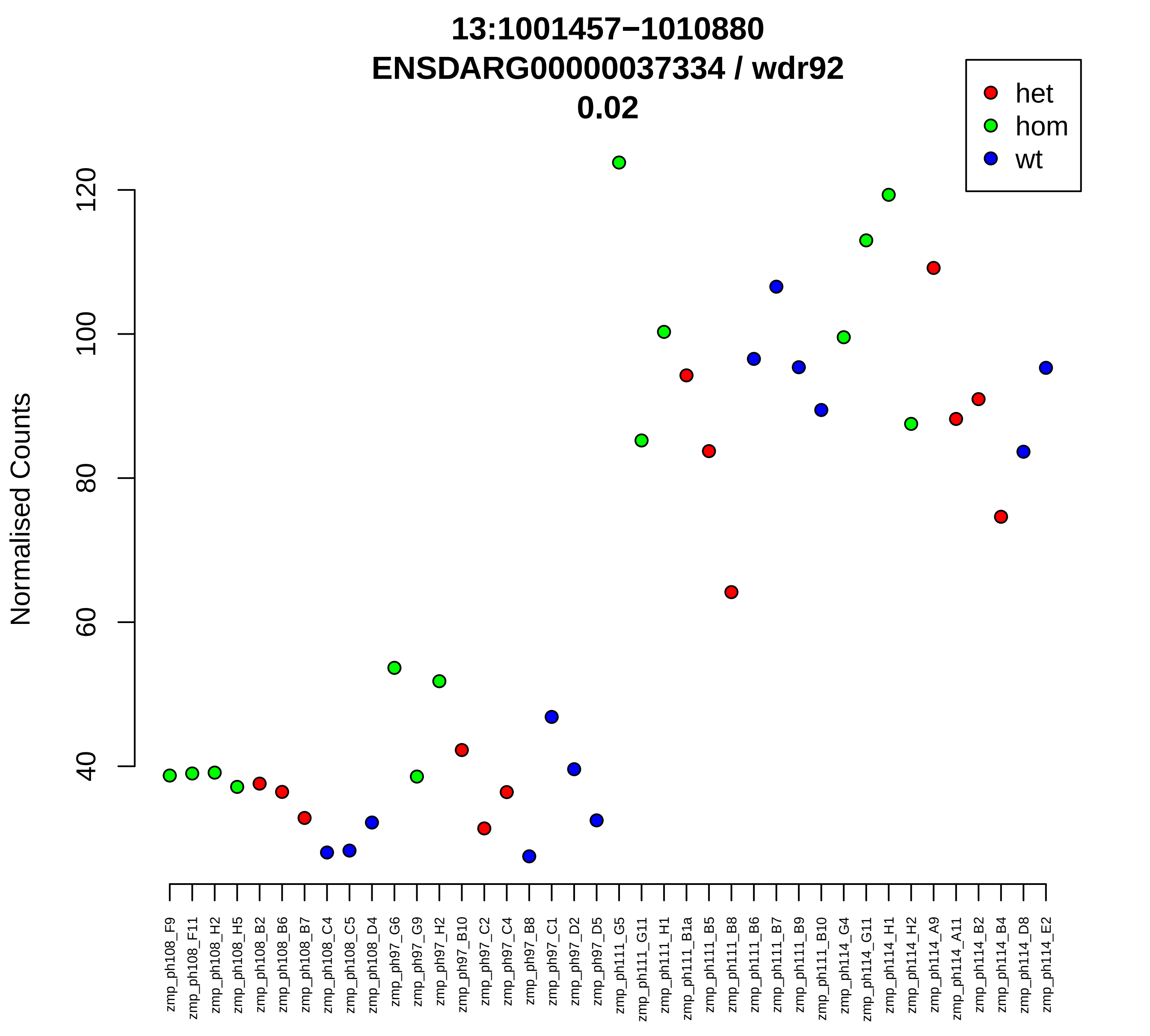
13:1001457−1010880
ENSDARG00000037334 / wdr92
0.02
het
hom
wt
Normalised Counts
40
60
80
100
120
zmp_ph108_F9
zmp_ph108_F11
zmp_ph108_H2
zmp_ph108_H5
zmp_ph108_B2
zmp_ph108_B6
zmp_ph108_B7
zmp_ph108_C4
zmp_ph108_C5
zmp_ph108_D4
zmp_ph97_G6
zmp_ph97_G9
zmp_ph97_H2
zmp_ph97_B10
zmp_ph97_C2
zmp_ph97_C4
zmp_ph97_B8
zmp_ph97_C1
zmp_ph97_D2
zmp_ph97_D5
zmp_ph111_G5
zmp_ph111_G11
zmp_ph111_H1
zmp_ph111_B1a
zmp_ph111_B5
zmp_ph111_B8
zmp_ph111_B6
zmp_ph111_B7
zmp_ph111_B9
zmp_ph111_B10
zmp_ph114_G4
zmp_ph114_G11
zmp_ph114_H1
zmp_ph114_H2
zmp_ph114_A9
zmp_ph114_A11
zmp_ph114_B2
zmp_ph114_B4
zmp_ph114_D8
zmp_ph114_E2

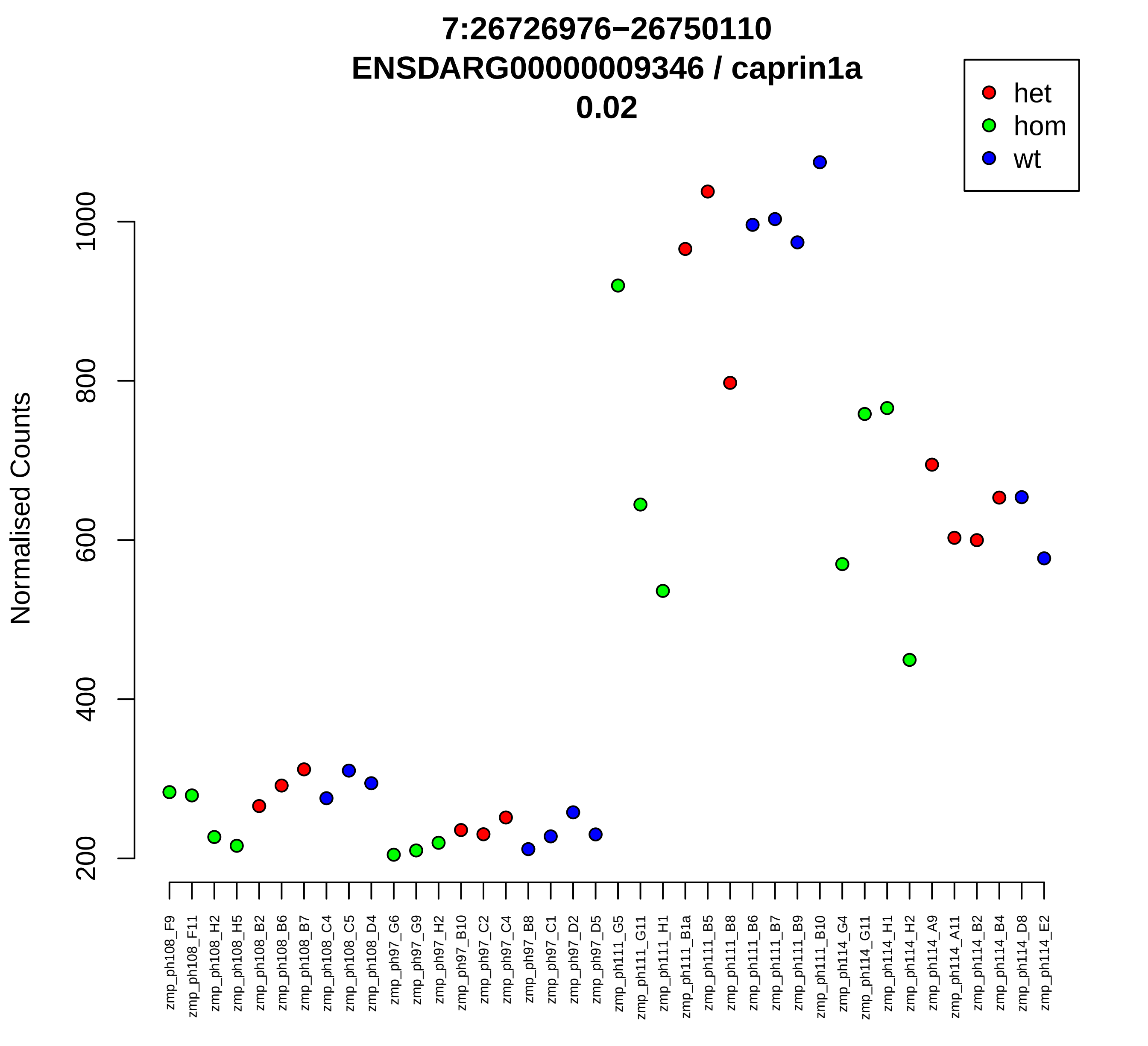

7:26726976−26750110
ENSDARG00000009346 / caprin1a
0.02
het
hom
wt
Normalised Counts
200
400
600
800
1000
zmp_ph108_F9
zmp_ph108_F11
zmp_ph108_H2
zmp_ph108_H5
zmp_ph108_B2
zmp_ph108_B6
zmp_ph108_B7
zmp_ph108_C4
zmp_ph108_C5
zmp_ph108_D4
zmp_ph97_G6
zmp_ph97_G9
zmp_ph97_H2
zmp_ph97_B10
zmp_ph97_C2
zmp_ph97_C4
zmp_ph97_B8
zmp_ph97_C1
zmp_ph97_D2
zmp_ph97_D5
zmp_ph111_G5
zmp_ph111_G11
zmp_ph111_H1
zmp_ph111_B1a
zmp_ph111_B5
zmp_ph111_B8
zmp_ph111_B6
zmp_ph111_B7
zmp_ph111_B9
zmp_ph111_B10
zmp_ph114_G4
zmp_ph114_G11
zmp_ph114_H1
zmp_ph114_H2
zmp_ph114_A9
zmp_ph114_A11
zmp_ph114_B2
zmp_ph114_B4
zmp_ph114_D8
zmp_ph114_E2

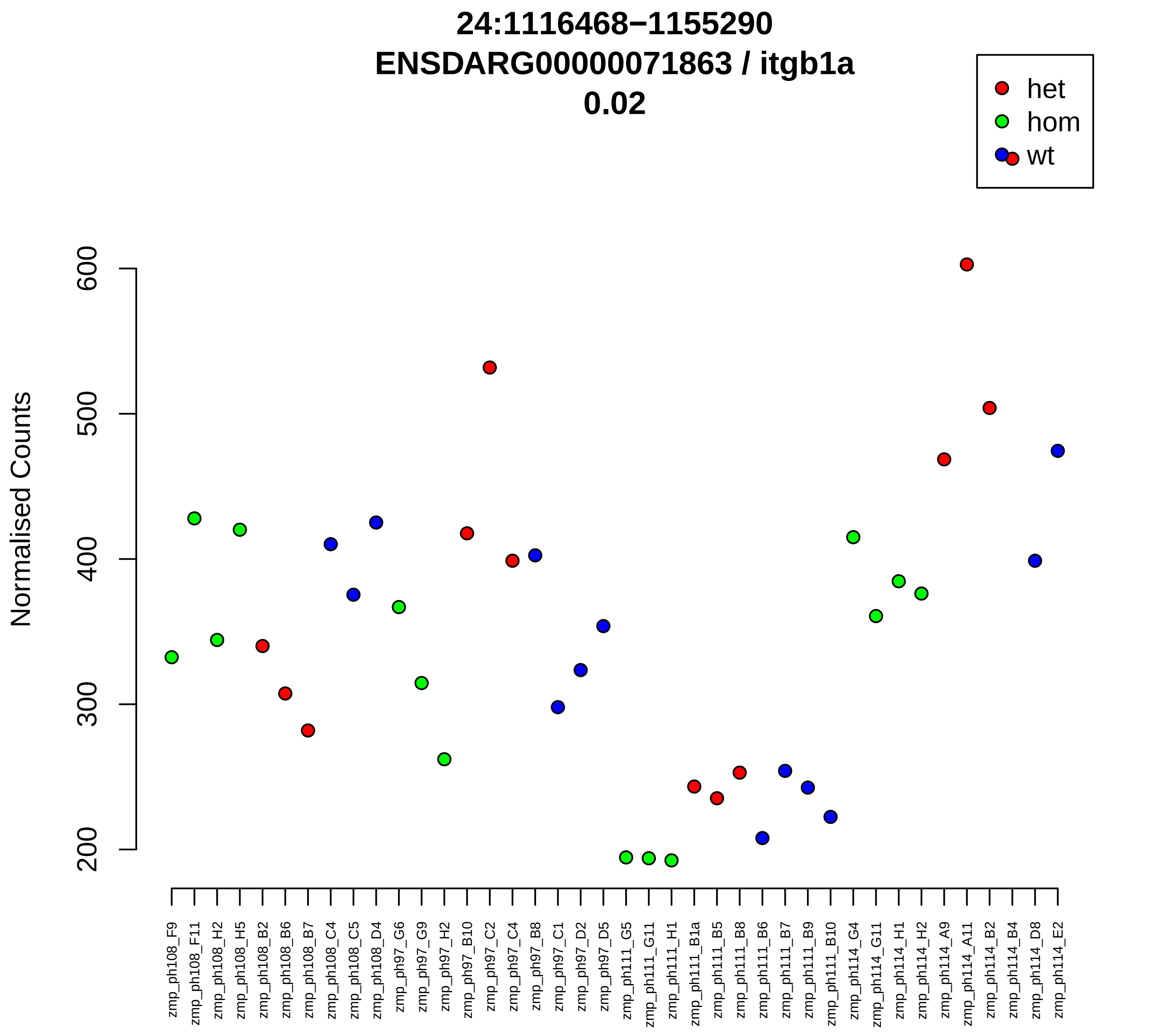

24:1116468−1155290
ENSDARG00000071863 / itgb1a
0.02
het
hom
wt
Normalised Counts
200
300
400
500
600
zmp_ph108_F9
zmp_ph108_F11
zmp_ph108_H2
zmp_ph108_H5
zmp_ph108_B2
zmp_ph108_B6
zmp_ph108_B7
zmp_ph108_C4
zmp_ph108_C5
zmp_ph108_D4
zmp_ph97_G6
zmp_ph97_G9
zmp_ph97_H2
zmp_ph97_B10
zmp_ph97_C2
zmp_ph97_C4
zmp_ph97_B8
zmp_ph97_C1
zmp_ph97_D2
zmp_ph97_D5
zmp_ph111_G5
zmp_ph111_G11
zmp_ph111_H1
zmp_ph111_B1a
zmp_ph111_B5
zmp_ph111_B8
zmp_ph111_B6
zmp_ph111_B7
zmp_ph111_B9
zmp_ph111_B10
zmp_ph114_G4
zmp_ph114_G11
zmp_ph114_H1
zmp_ph114_H2
zmp_ph114_A9
zmp_ph114_A11
zmp_ph114_B2
zmp_ph114_B4
zmp_ph114_D8
zmp_ph114_E2

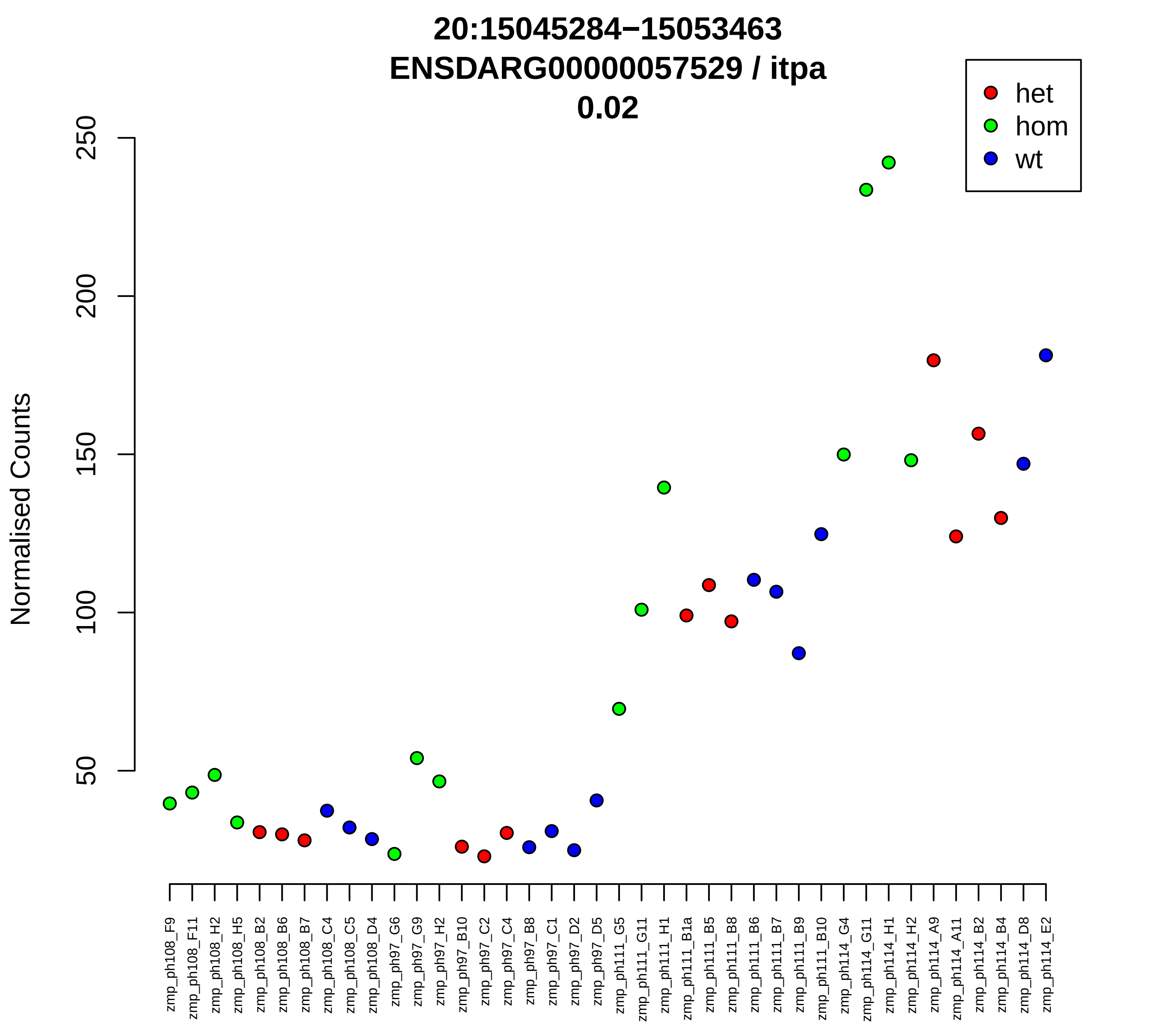
20:15045284−15053463
ENSDARG00000057529 / itpa
0.02
het
hom
wt
Normalised Counts
50
100
150
200
250
zmp_ph108_F9
zmp_ph108_F11
zmp_ph108_H2
zmp_ph108_H5
zmp_ph108_B2
zmp_ph108_B6
zmp_ph108_B7
zmp_ph108_C4
zmp_ph108_C5
zmp_ph108_D4
zmp_ph97_G6
zmp_ph97_G9
zmp_ph97_H2
zmp_ph97_B10
zmp_ph97_C2
zmp_ph97_C4
zmp_ph97_B8
zmp_ph97_C1
zmp_ph97_D2
zmp_ph97_D5
zmp_ph111_G5
zmp_ph111_G11
zmp_ph111_H1
zmp_ph111_B1a
zmp_ph111_B5
zmp_ph111_B8
zmp_ph111_B6
zmp_ph111_B7
zmp_ph111_B9
zmp_ph111_B10
zmp_ph114_G4
zmp_ph114_G11
zmp_ph114_H1
zmp_ph114_H2
zmp_ph114_A9
zmp_ph114_A11
zmp_ph114_B2
zmp_ph114_B4
zmp_ph114_D8
zmp_ph114_E2

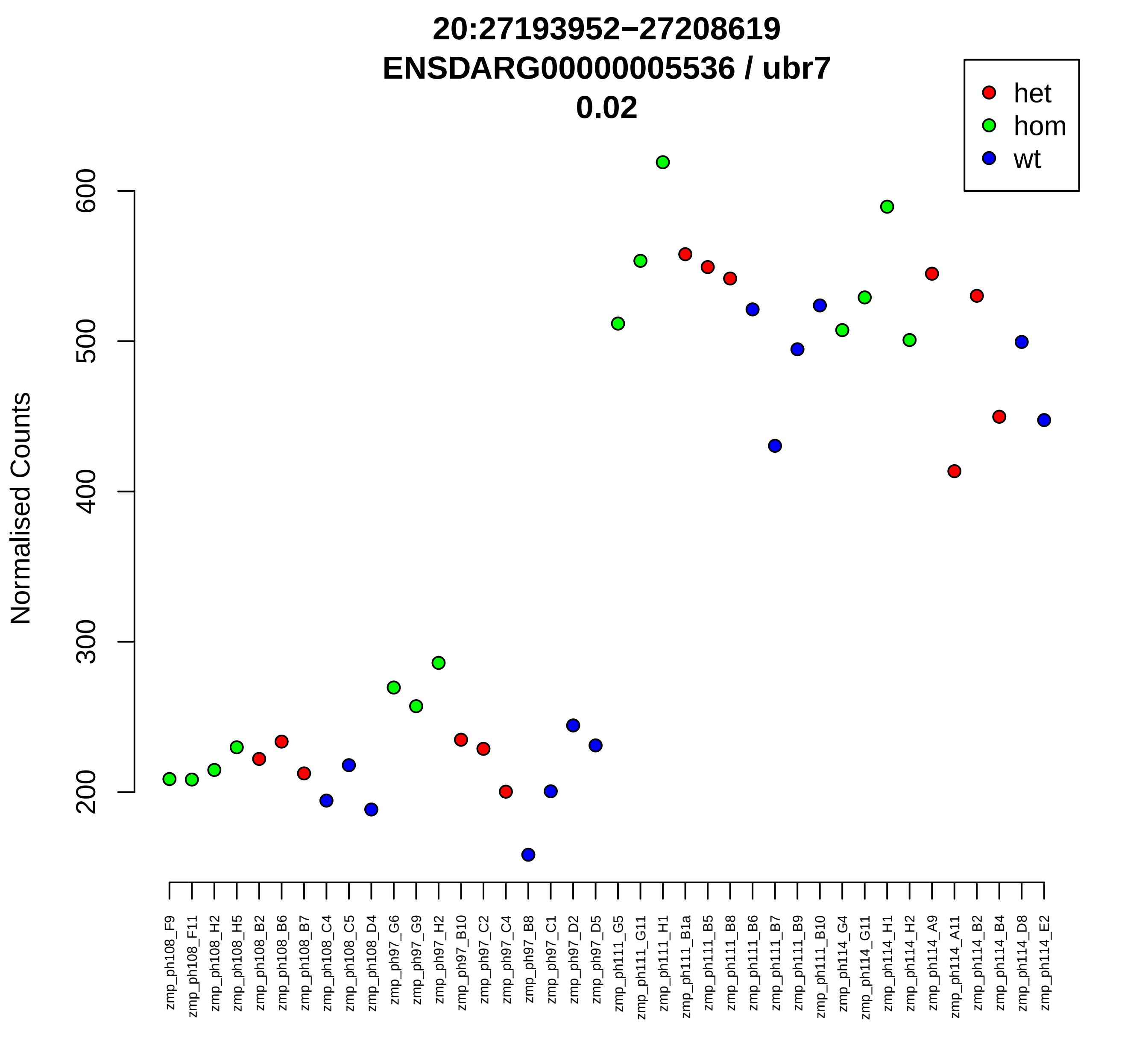

20:27193952−27208619
ENSDARG00000005536 / ubr7
0.02
het
hom
wt
Normalised Counts
200
300
400
500
600
zmp_ph108_F9
zmp_ph108_F11
zmp_ph108_H2
zmp_ph108_H5
zmp_ph108_B2
zmp_ph108_B6
zmp_ph108_B7
zmp_ph108_C4
zmp_ph108_C5
zmp_ph108_D4
zmp_ph97_G6
zmp_ph97_G9
zmp_ph97_H2
zmp_ph97_B10
zmp_ph97_C2
zmp_ph97_C4
zmp_ph97_B8
zmp_ph97_C1
zmp_ph97_D2
zmp_ph97_D5
zmp_ph111_G5
zmp_ph111_G11
zmp_ph111_H1
zmp_ph111_B1a
zmp_ph111_B5
zmp_ph111_B8
zmp_ph111_B6
zmp_ph111_B7
zmp_ph111_B9
zmp_ph111_B10
zmp_ph114_G4
zmp_ph114_G11
zmp_ph114_H1
zmp_ph114_H2
zmp_ph114_A9
zmp_ph114_A11
zmp_ph114_B2
zmp_ph114_B4
zmp_ph114_D8
zmp_ph114_E2

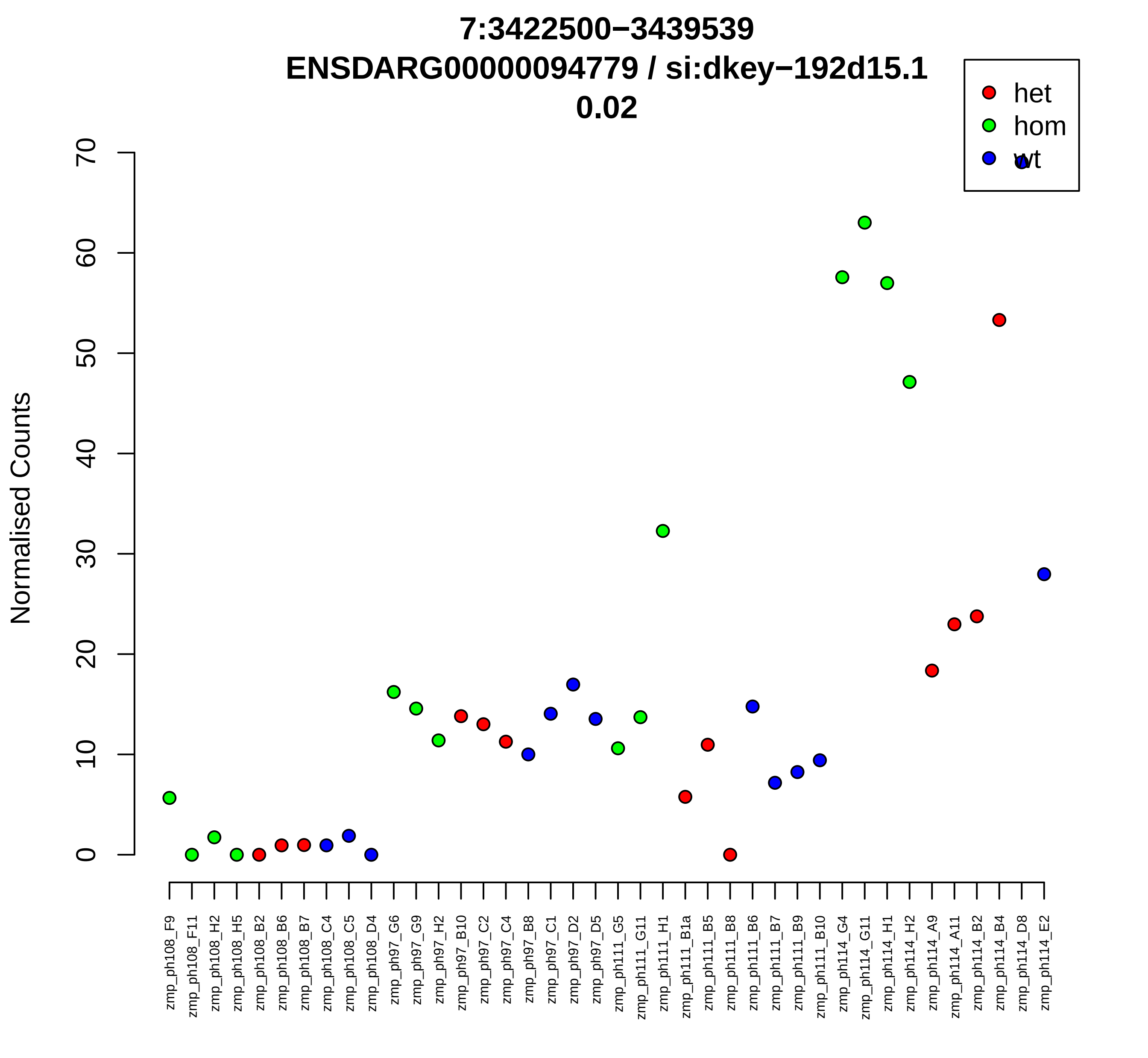
7:3422500−3439539
ENSDARG00000094779 / si:dkey−192d15.1
0.02
het
hom
wt
Normalised Counts
0
10
20
30
40
50
60
70
zmp_ph108_F9
zmp_ph108_F11
zmp_ph108_H2
zmp_ph108_H5
zmp_ph108_B2
zmp_ph108_B6
zmp_ph108_B7
zmp_ph108_C4
zmp_ph108_C5
zmp_ph108_D4
zmp_ph97_G6
zmp_ph97_G9
zmp_ph97_H2
zmp_ph97_B10
zmp_ph97_C2
zmp_ph97_C4
zmp_ph97_B8
zmp_ph97_C1
zmp_ph97_D2
zmp_ph97_D5
zmp_ph111_G5
zmp_ph111_G11
zmp_ph111_H1
zmp_ph111_B1a
zmp_ph111_B5
zmp_ph111_B8
zmp_ph111_B6
zmp_ph111_B7
zmp_ph111_B9
zmp_ph111_B10
zmp_ph114_G4
zmp_ph114_G11
zmp_ph114_H1
zmp_ph114_H2
zmp_ph114_A9
zmp_ph114_A11
zmp_ph114_B2
zmp_ph114_B4
zmp_ph114_D8
zmp_ph114_E2

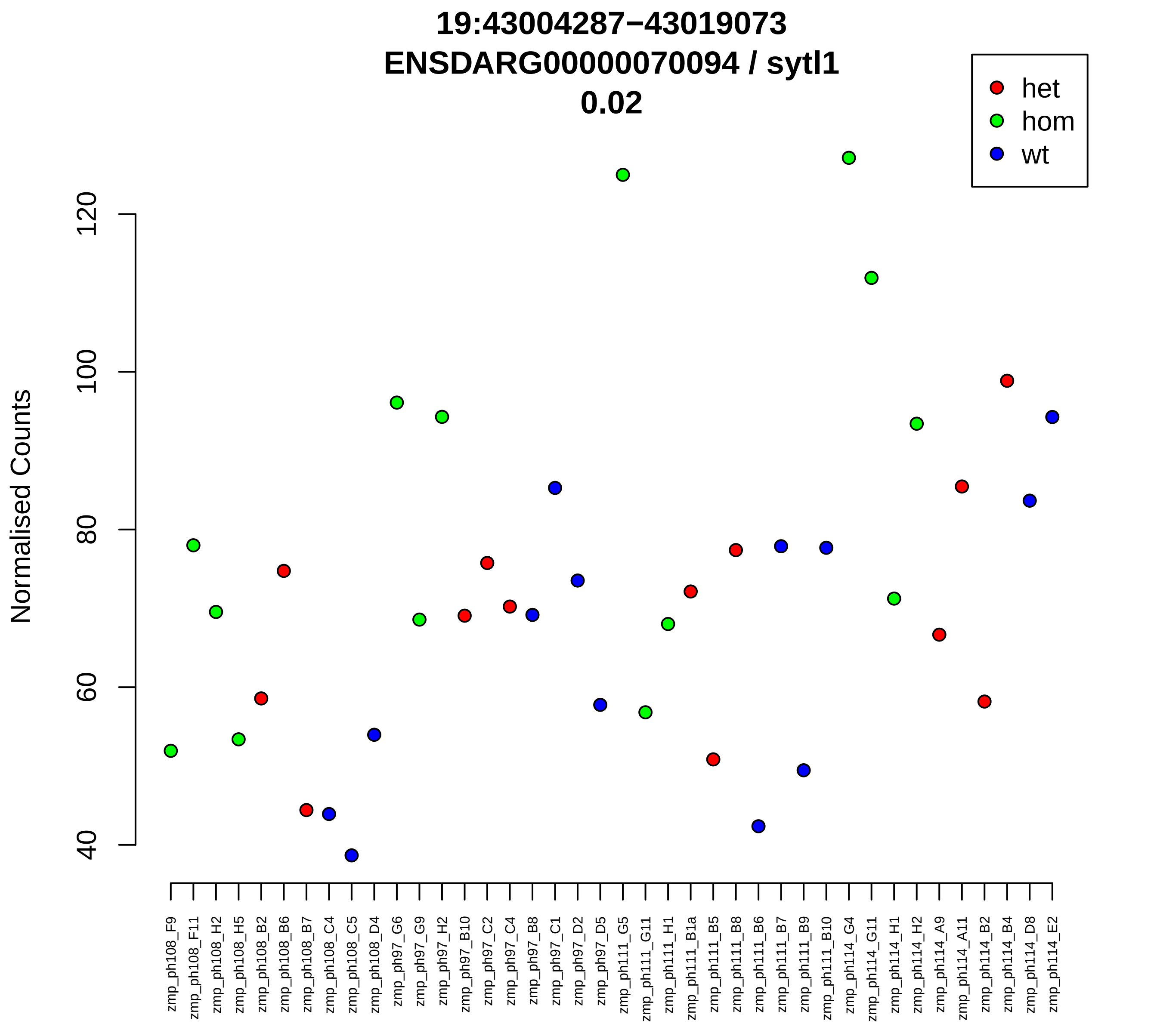

19:43004287−43019073
ENSDARG00000070094 / sytl1
0.02
het
hom
wt
Normalised Counts
40
60
80
100
120
zmp_ph108_F9
zmp_ph108_F11
zmp_ph108_H2
zmp_ph108_H5
zmp_ph108_B2
zmp_ph108_B6
zmp_ph108_B7
zmp_ph108_C4
zmp_ph108_C5
zmp_ph108_D4
zmp_ph97_G6
zmp_ph97_G9
zmp_ph97_H2
zmp_ph97_B10
zmp_ph97_C2
zmp_ph97_C4
zmp_ph97_B8
zmp_ph97_C1
zmp_ph97_D2
zmp_ph97_D5
zmp_ph111_G5
zmp_ph111_G11
zmp_ph111_H1
zmp_ph111_B1a
zmp_ph111_B5
zmp_ph111_B8
zmp_ph111_B6
zmp_ph111_B7
zmp_ph111_B9
zmp_ph111_B10
zmp_ph114_G4
zmp_ph114_G11
zmp_ph114_H1
zmp_ph114_H2
zmp_ph114_A9
zmp_ph114_A11
zmp_ph114_B2
zmp_ph114_B4
zmp_ph114_D8
zmp_ph114_E2

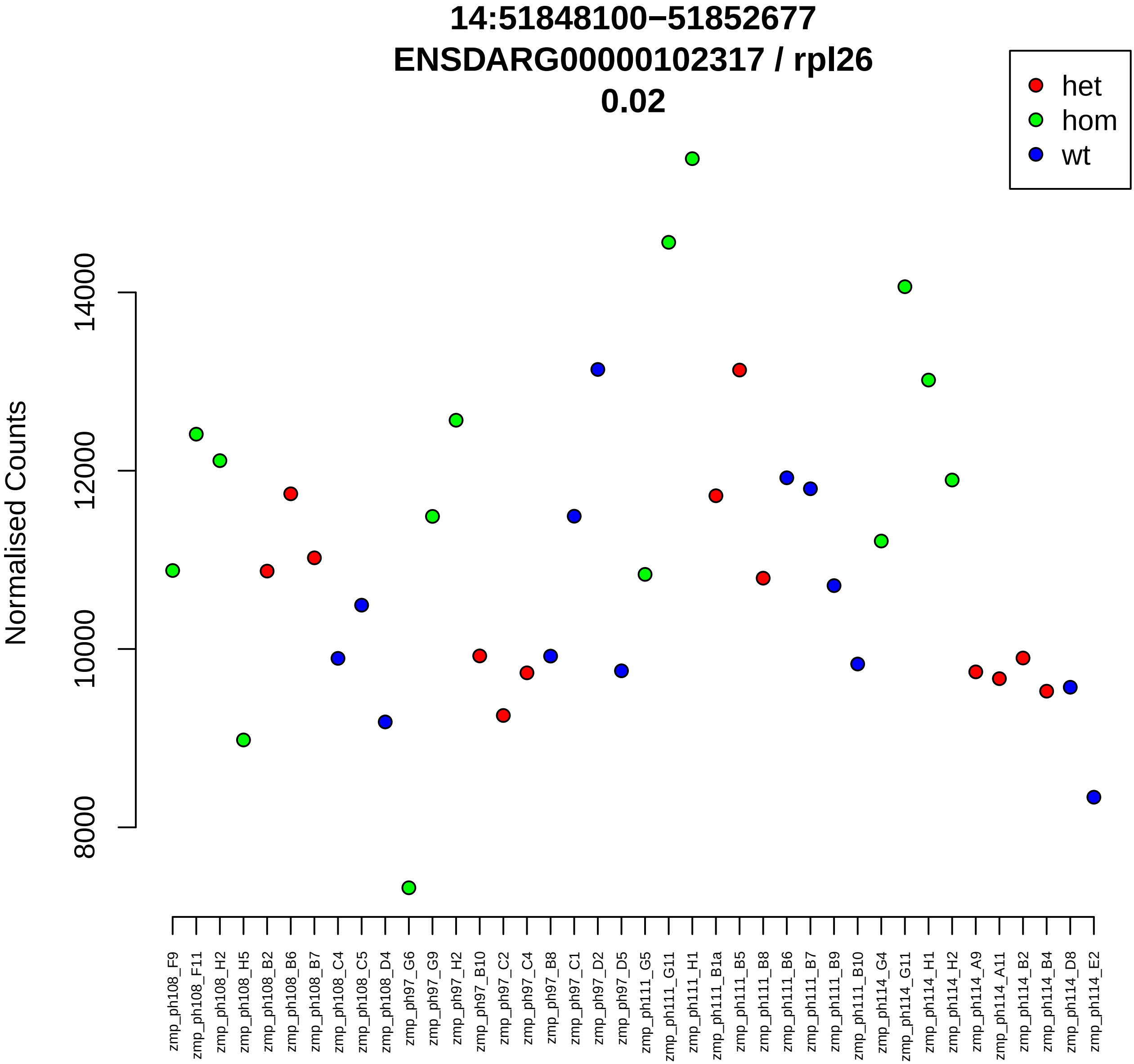

14:51848100−51852677
ENSDARG00000102317 / rpl26
0.02
het
hom
wt
Normalised Counts
8000
10000
12000
14000
zmp_ph108_F9
zmp_ph108_F11
zmp_ph108_H2
zmp_ph108_H5
zmp_ph108_B2
zmp_ph108_B6
zmp_ph108_B7
zmp_ph108_C4
zmp_ph108_C5
zmp_ph108_D4
zmp_ph97_G6
zmp_ph97_G9
zmp_ph97_H2
zmp_ph97_B10
zmp_ph97_C2
zmp_ph97_C4
zmp_ph97_B8
zmp_ph97_C1
zmp_ph97_D2
zmp_ph97_D5
zmp_ph111_G5
zmp_ph111_G11
zmp_ph111_H1
zmp_ph111_B1a
zmp_ph111_B5
zmp_ph111_B8
zmp_ph111_B6
zmp_ph111_B7
zmp_ph111_B9
zmp_ph111_B10
zmp_ph114_G4
zmp_ph114_G11
zmp_ph114_H1
zmp_ph114_H2
zmp_ph114_A9
zmp_ph114_A11
zmp_ph114_B2
zmp_ph114_B4
zmp_ph114_D8
zmp_ph114_E2

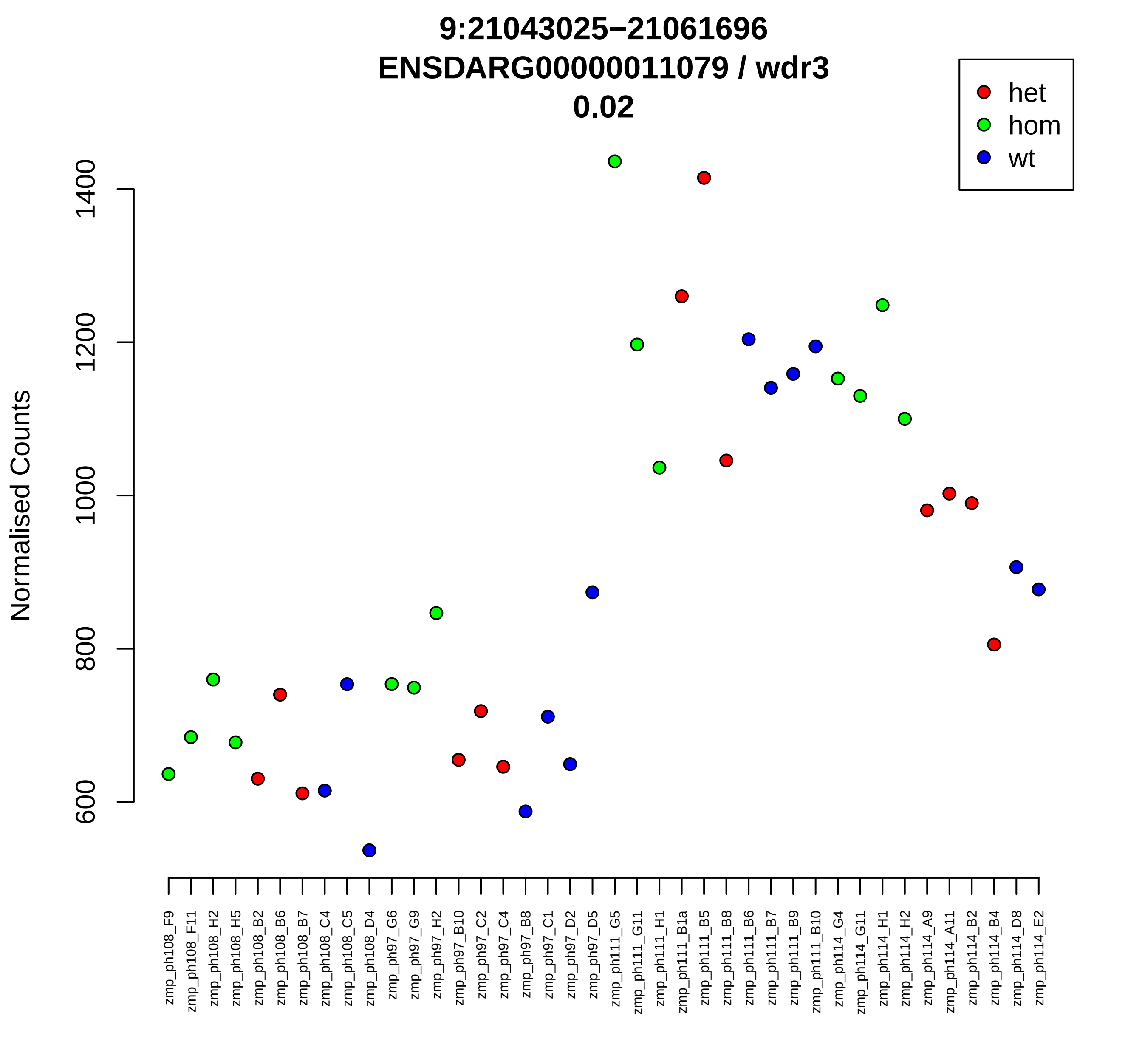
9:21043025−21061696
ENSDARG00000011079 / wdr3
0.02
het
hom
wt
Normalised Counts
600
800
1000
1200
1400
zmp_ph108_F9
zmp_ph108_F11
zmp_ph108_H2
zmp_ph108_H5
zmp_ph108_B2
zmp_ph108_B6
zmp_ph108_B7
zmp_ph108_C4
zmp_ph108_C5
zmp_ph108_D4
zmp_ph97_G6
zmp_ph97_G9
zmp_ph97_H2
zmp_ph97_B10
zmp_ph97_C2
zmp_ph97_C4
zmp_ph97_B8
zmp_ph97_C1
zmp_ph97_D2
zmp_ph97_D5
zmp_ph111_G5
zmp_ph111_G11
zmp_ph111_H1
zmp_ph111_B1a
zmp_ph111_B5
zmp_ph111_B8
zmp_ph111_B6
zmp_ph111_B7
zmp_ph111_B9
zmp_ph111_B10
zmp_ph114_G4
zmp_ph114_G11
zmp_ph114_H1
zmp_ph114_H2
zmp_ph114_A9
zmp_ph114_A11
zmp_ph114_B2
zmp_ph114_B4
zmp_ph114_D8
zmp_ph114_E2

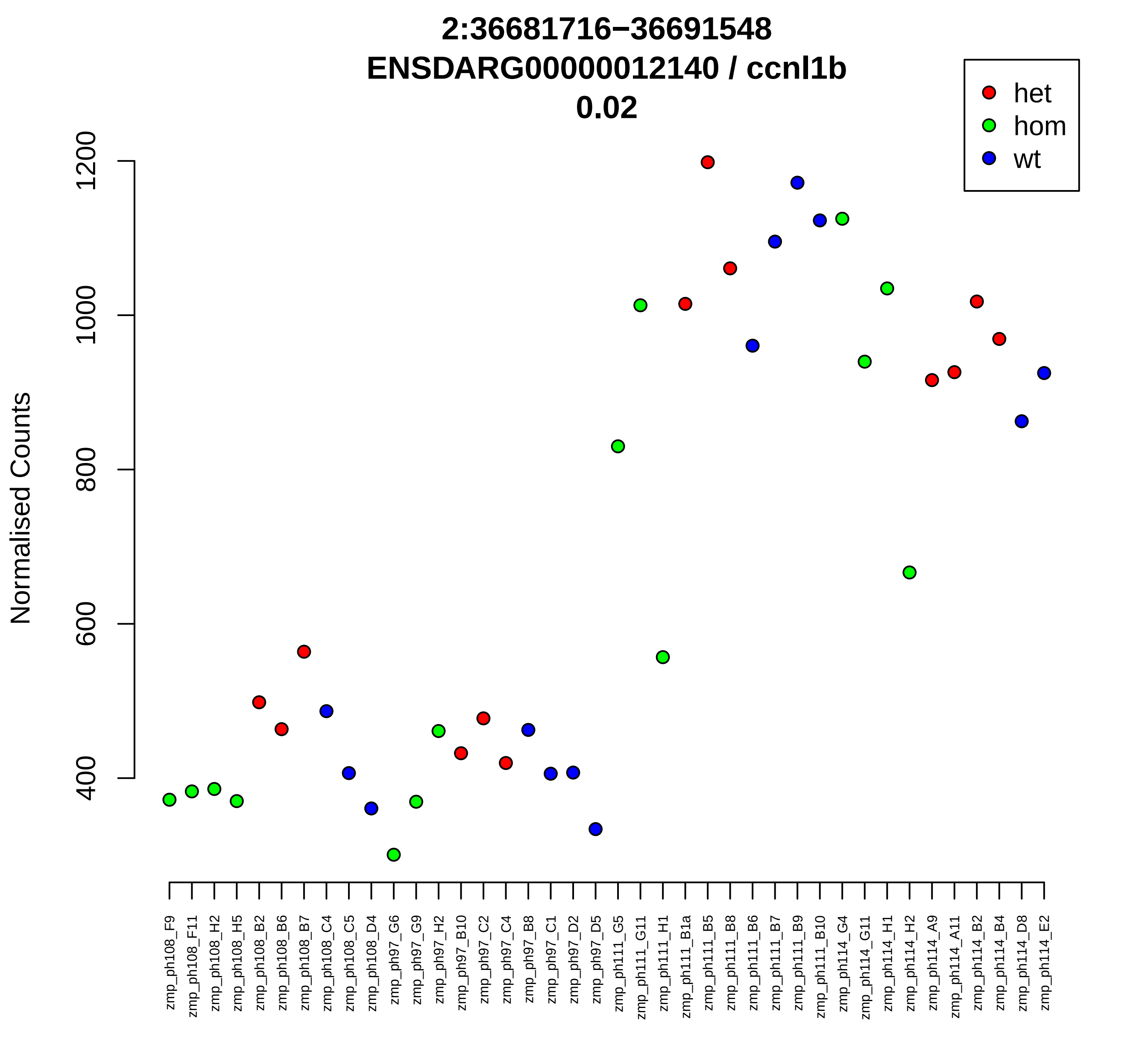

2:36681716−36691548
ENSDARG00000012140 / ccnl1b
0.02
het
hom
wt
Normalised Counts
400
600
800
1000
1200
zmp_ph108_F9
zmp_ph108_F11
zmp_ph108_H2
zmp_ph108_H5
zmp_ph108_B2
zmp_ph108_B6
zmp_ph108_B7
zmp_ph108_C4
zmp_ph108_C5
zmp_ph108_D4
zmp_ph97_G6
zmp_ph97_G9
zmp_ph97_H2
zmp_ph97_B10
zmp_ph97_C2
zmp_ph97_C4
zmp_ph97_B8
zmp_ph97_C1
zmp_ph97_D2
zmp_ph97_D5
zmp_ph111_G5
zmp_ph111_G11
zmp_ph111_H1
zmp_ph111_B1a
zmp_ph111_B5
zmp_ph111_B8
zmp_ph111_B6
zmp_ph111_B7
zmp_ph111_B9
zmp_ph111_B10
zmp_ph114_G4
zmp_ph114_G11
zmp_ph114_H1
zmp_ph114_H2
zmp_ph114_A9
zmp_ph114_A11
zmp_ph114_B2
zmp_ph114_B4
zmp_ph114_D8
zmp_ph114_E2

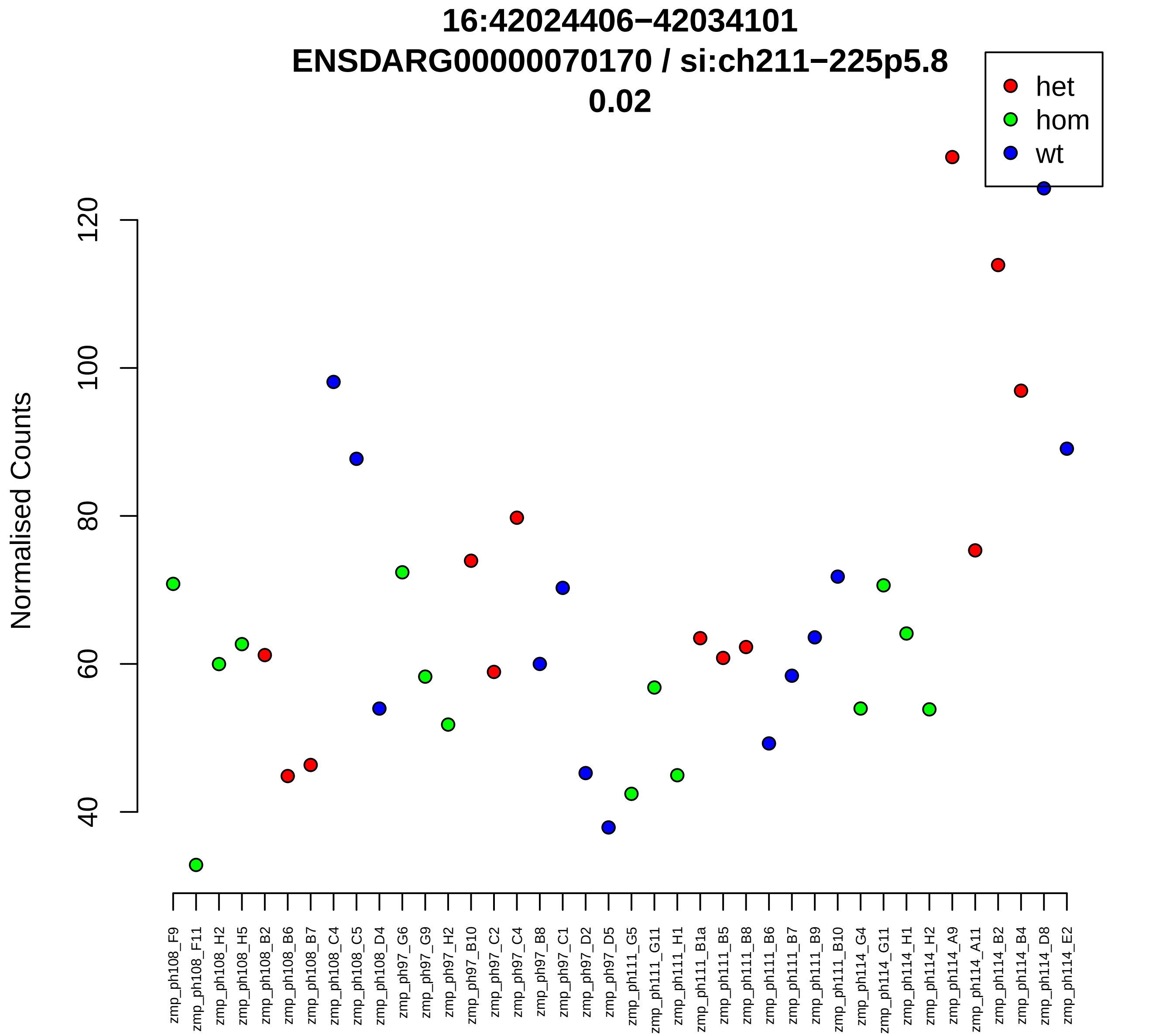
16:42024406−42034101
ENSDARG00000070170 / si:ch211−225p5.8
0.02
het
hom
wt
Normalised Counts
40
60
80
100
120
zmp_ph108_F9
zmp_ph108_F11
zmp_ph108_H2
zmp_ph108_H5
zmp_ph108_B2
zmp_ph108_B6
zmp_ph108_B7
zmp_ph108_C4
zmp_ph108_C5
zmp_ph108_D4
zmp_ph97_G6
zmp_ph97_G9
zmp_ph97_H2
zmp_ph97_B10
zmp_ph97_C2
zmp_ph97_C4
zmp_ph97_B8
zmp_ph97_C1
zmp_ph97_D2
zmp_ph97_D5
zmp_ph111_G5
zmp_ph111_G11
zmp_ph111_H1
zmp_ph111_B1a
zmp_ph111_B5
zmp_ph111_B8
zmp_ph111_B6
zmp_ph111_B7
zmp_ph111_B9
zmp_ph111_B10
zmp_ph114_G4
zmp_ph114_G11
zmp_ph114_H1
zmp_ph114_H2
zmp_ph114_A9
zmp_ph114_A11
zmp_ph114_B2
zmp_ph114_B4
zmp_ph114_D8
zmp_ph114_E2

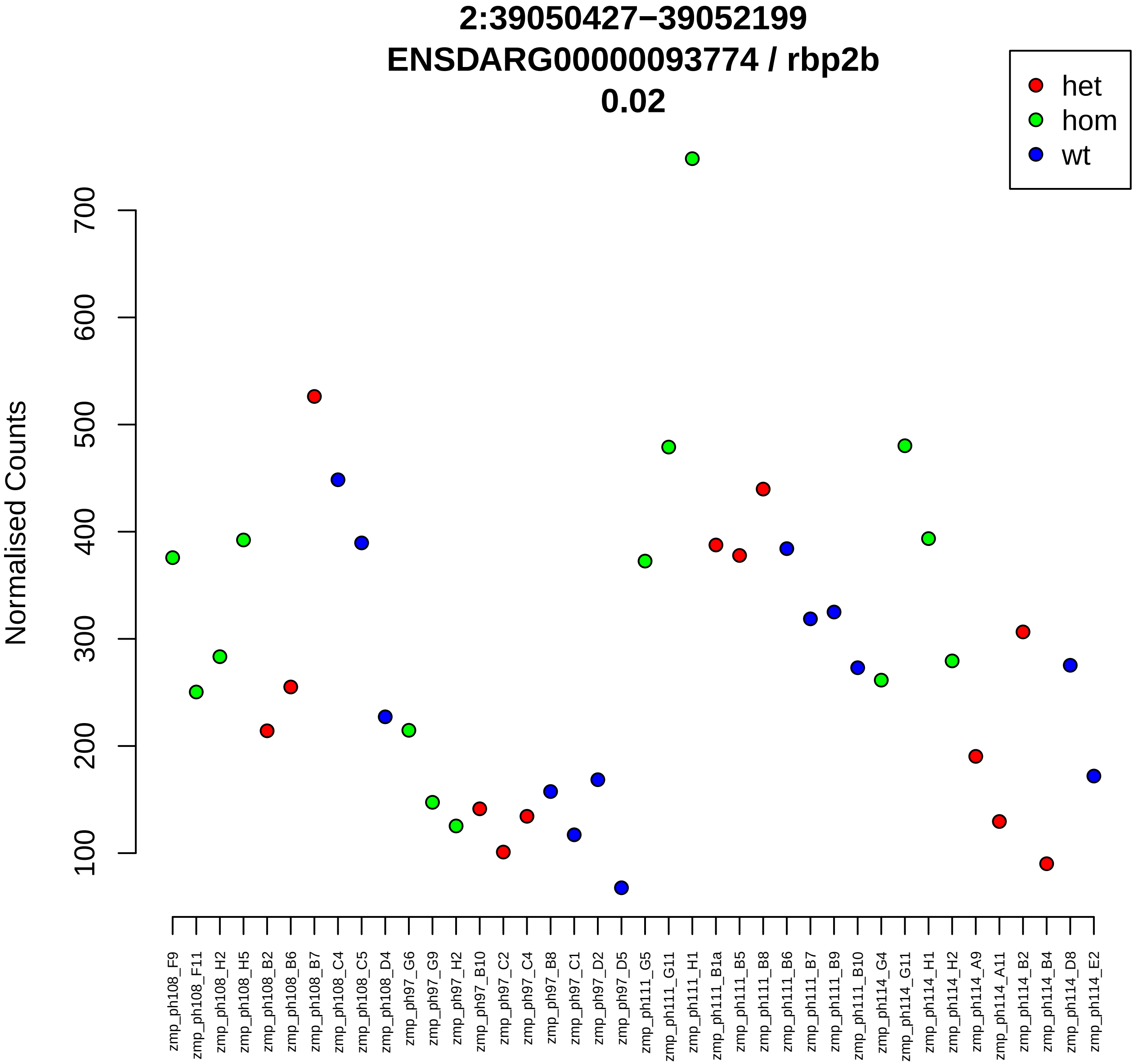

2:39050427−39052199
ENSDARG00000093774 / rbp2b
0.02
het
hom
wt
Normalised Counts
100
200
300
400
500
600
700
zmp_ph108_F9
zmp_ph108_F11
zmp_ph108_H2
zmp_ph108_H5
zmp_ph108_B2
zmp_ph108_B6
zmp_ph108_B7
zmp_ph108_C4
zmp_ph108_C5
zmp_ph108_D4
zmp_ph97_G6
zmp_ph97_G9
zmp_ph97_H2
zmp_ph97_B10
zmp_ph97_C2
zmp_ph97_C4
zmp_ph97_B8
zmp_ph97_C1
zmp_ph97_D2
zmp_ph97_D5
zmp_ph111_G5
zmp_ph111_G11
zmp_ph111_H1
zmp_ph111_B1a
zmp_ph111_B5
zmp_ph111_B8
zmp_ph111_B6
zmp_ph111_B7
zmp_ph111_B9
zmp_ph111_B10
zmp_ph114_G4
zmp_ph114_G11
zmp_ph114_H1
zmp_ph114_H2
zmp_ph114_A9
zmp_ph114_A11
zmp_ph114_B2
zmp_ph114_B4
zmp_ph114_D8
zmp_ph114_E2

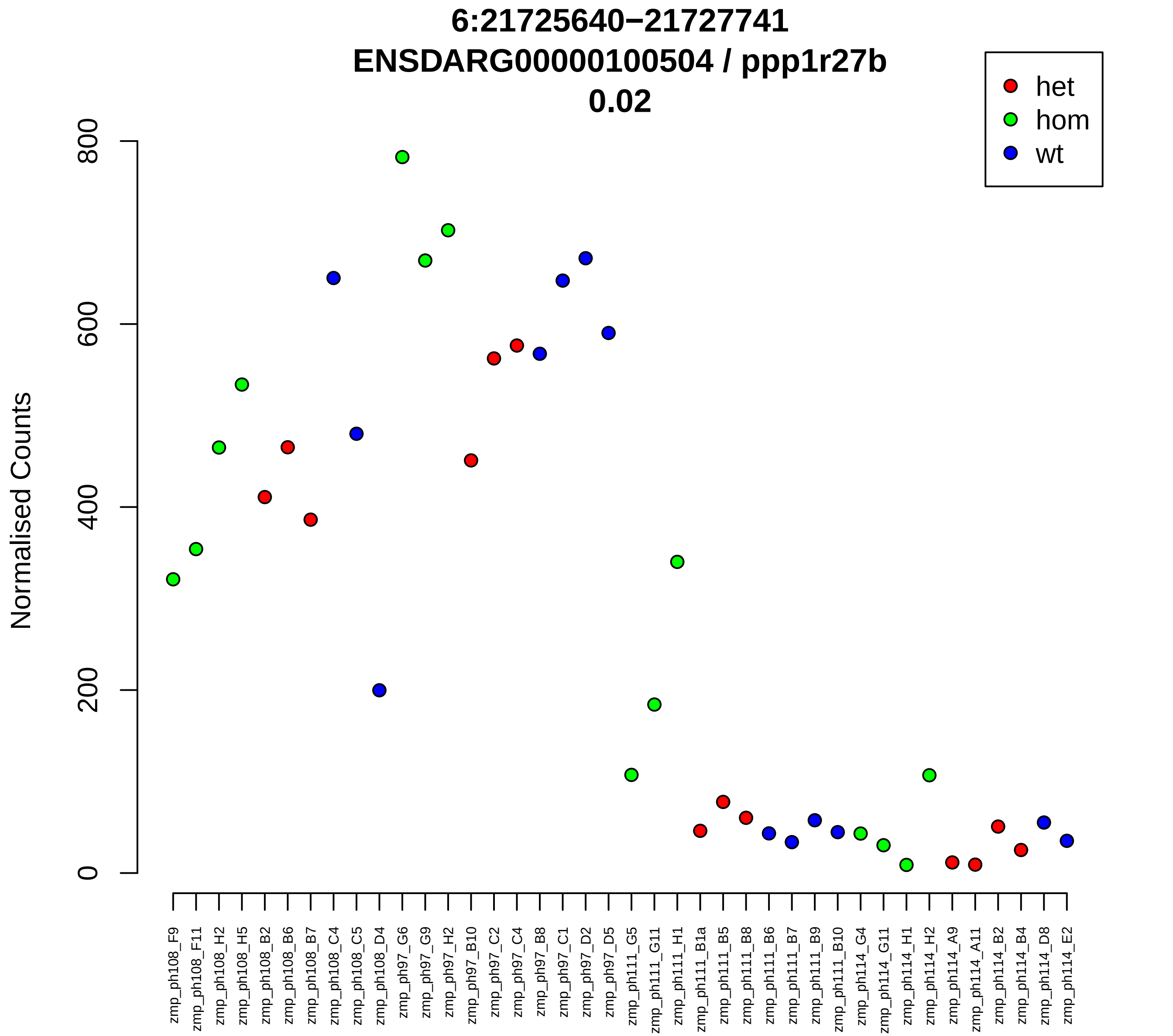
6:21725640−21727741
ENSDARG00000100504 / ppp1r27b
0.02
het
hom
wt
Normalised Counts
0
200
400
600
800
zmp_ph108_F9
zmp_ph108_F11
zmp_ph108_H2
zmp_ph108_H5
zmp_ph108_B2
zmp_ph108_B6
zmp_ph108_B7
zmp_ph108_C4
zmp_ph108_C5
zmp_ph108_D4
zmp_ph97_G6
zmp_ph97_G9
zmp_ph97_H2
zmp_ph97_B10
zmp_ph97_C2
zmp_ph97_C4
zmp_ph97_B8
zmp_ph97_C1
zmp_ph97_D2
zmp_ph97_D5
zmp_ph111_G5
zmp_ph111_G11
zmp_ph111_H1
zmp_ph111_B1a
zmp_ph111_B5
zmp_ph111_B8
zmp_ph111_B6
zmp_ph111_B7
zmp_ph111_B9
zmp_ph111_B10
zmp_ph114_G4
zmp_ph114_G11
zmp_ph114_H1
zmp_ph114_H2
zmp_ph114_A9
zmp_ph114_A11
zmp_ph114_B2
zmp_ph114_B4
zmp_ph114_D8
zmp_ph114_E2

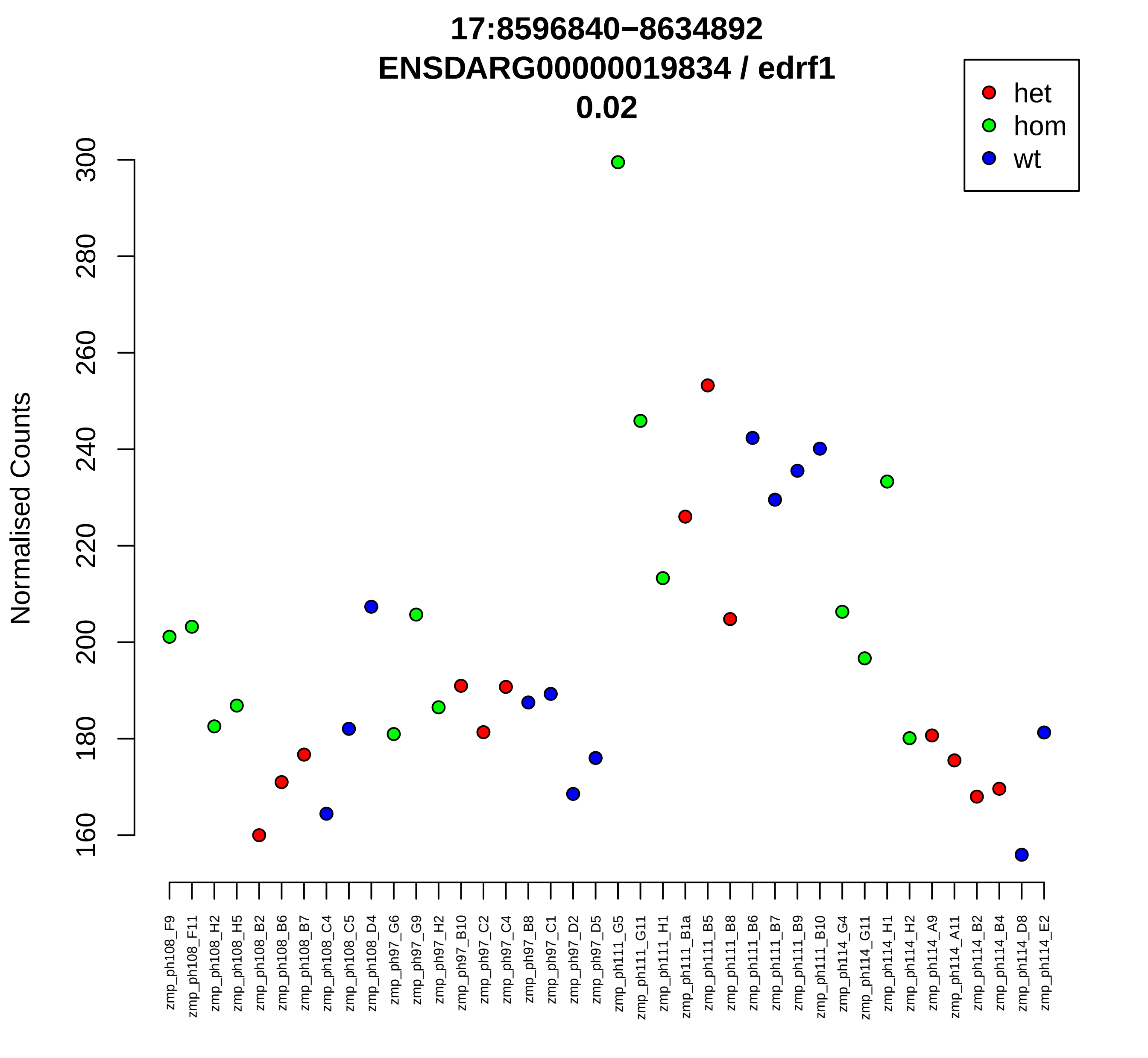
17:8596840−8634892
ENSDARG00000019834 / edrf1
0.02
het
hom
wt
Normalised Counts
160
180
200
220
240
260
280
300
zmp_ph108_F9
zmp_ph108_F11
zmp_ph108_H2
zmp_ph108_H5
zmp_ph108_B2
zmp_ph108_B6
zmp_ph108_B7
zmp_ph108_C4
zmp_ph108_C5
zmp_ph108_D4
zmp_ph97_G6
zmp_ph97_G9
zmp_ph97_H2
zmp_ph97_B10
zmp_ph97_C2
zmp_ph97_C4
zmp_ph97_B8
zmp_ph97_C1
zmp_ph97_D2
zmp_ph97_D5
zmp_ph111_G5
zmp_ph111_G11
zmp_ph111_H1
zmp_ph111_B1a
zmp_ph111_B5
zmp_ph111_B8
zmp_ph111_B6
zmp_ph111_B7
zmp_ph111_B9
zmp_ph111_B10
zmp_ph114_G4
zmp_ph114_G11
zmp_ph114_H1
zmp_ph114_H2
zmp_ph114_A9
zmp_ph114_A11
zmp_ph114_B2
zmp_ph114_B4
zmp_ph114_D8
zmp_ph114_E2

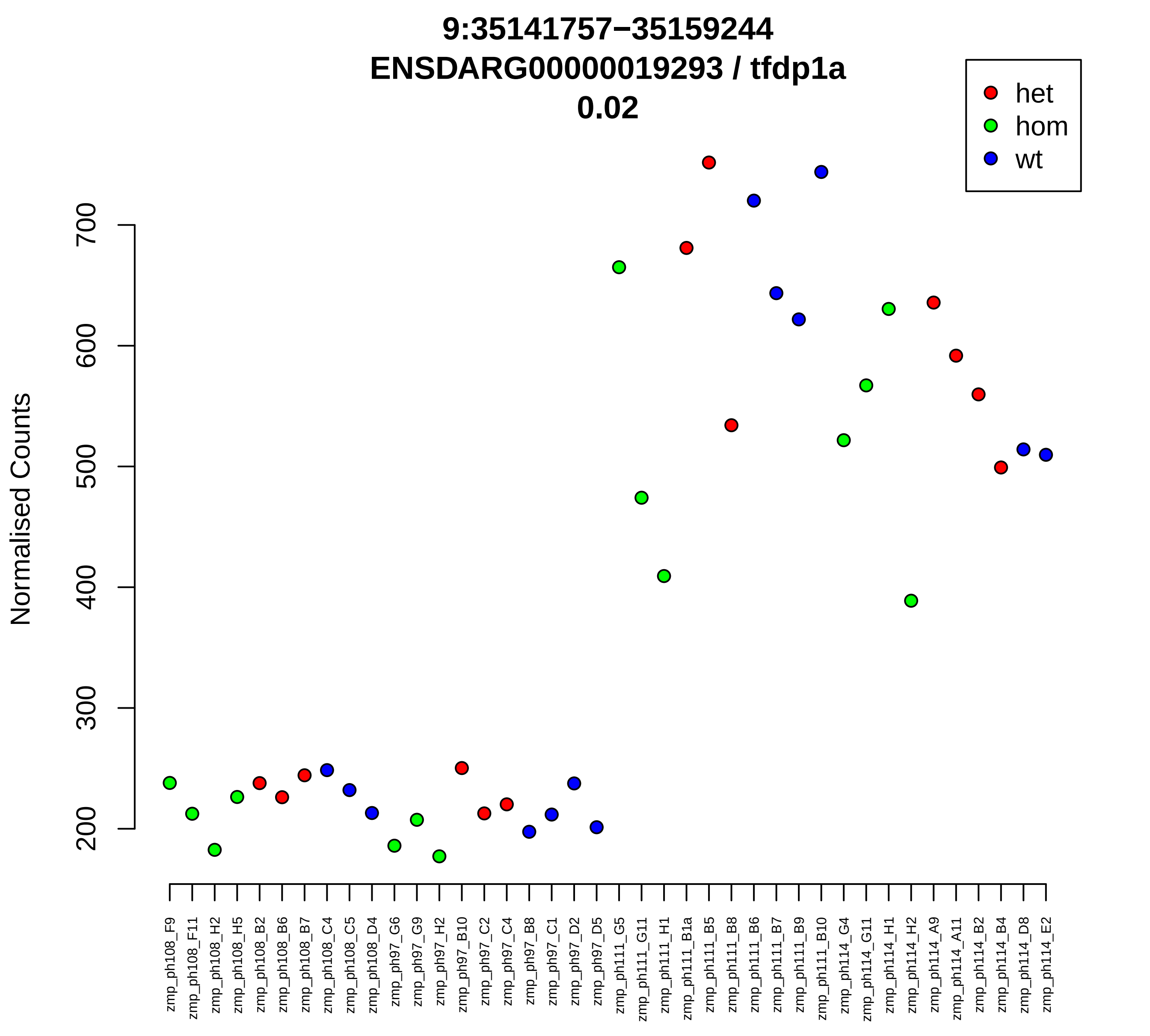

9:35141757−35159244
ENSDARG00000019293 / tfdp1a
0.02
het
hom
wt
Normalised Counts
200
300
400
500
600
700
zmp_ph108_F9
zmp_ph108_F11
zmp_ph108_H2
zmp_ph108_H5
zmp_ph108_B2
zmp_ph108_B6
zmp_ph108_B7
zmp_ph108_C4
zmp_ph108_C5
zmp_ph108_D4
zmp_ph97_G6
zmp_ph97_G9
zmp_ph97_H2
zmp_ph97_B10
zmp_ph97_C2
zmp_ph97_C4
zmp_ph97_B8
zmp_ph97_C1
zmp_ph97_D2
zmp_ph97_D5
zmp_ph111_G5
zmp_ph111_G11
zmp_ph111_H1
zmp_ph111_B1a
zmp_ph111_B5
zmp_ph111_B8
zmp_ph111_B6
zmp_ph111_B7
zmp_ph111_B9
zmp_ph111_B10
zmp_ph114_G4
zmp_ph114_G11
zmp_ph114_H1
zmp_ph114_H2
zmp_ph114_A9
zmp_ph114_A11
zmp_ph114_B2
zmp_ph114_B4
zmp_ph114_D8
zmp_ph114_E2

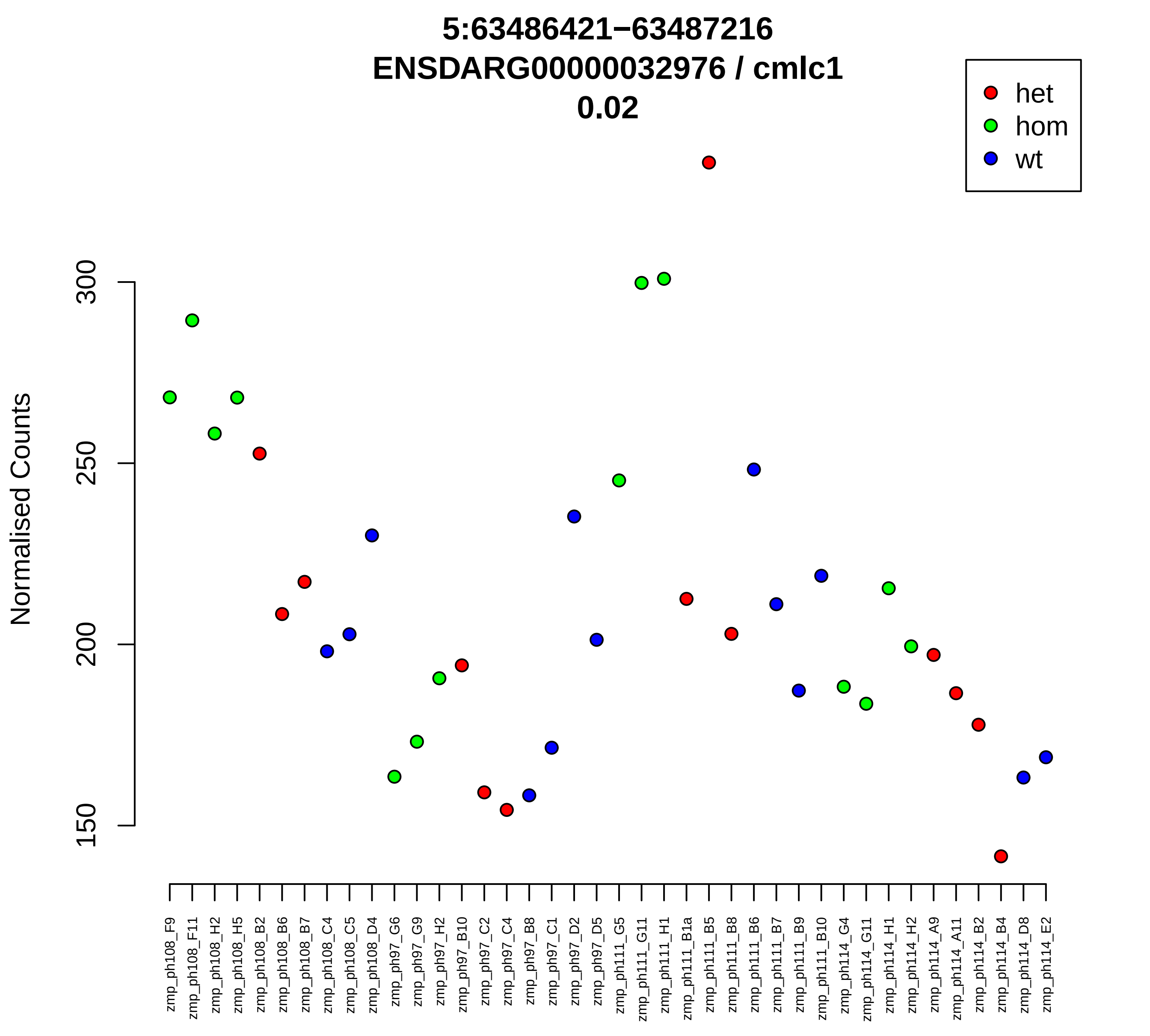
5:63486421−63487216
ENSDARG00000032976 / cmlc1
0.02
het
hom
wt
Normalised Counts
150
200
250
300
zmp_ph108_F9
zmp_ph108_F11
zmp_ph108_H2
zmp_ph108_H5
zmp_ph108_B2
zmp_ph108_B6
zmp_ph108_B7
zmp_ph108_C4
zmp_ph108_C5
zmp_ph108_D4
zmp_ph97_G6
zmp_ph97_G9
zmp_ph97_H2
zmp_ph97_B10
zmp_ph97_C2
zmp_ph97_C4
zmp_ph97_B8
zmp_ph97_C1
zmp_ph97_D2
zmp_ph97_D5
zmp_ph111_G5
zmp_ph111_G11
zmp_ph111_H1
zmp_ph111_B1a
zmp_ph111_B5
zmp_ph111_B8
zmp_ph111_B6
zmp_ph111_B7
zmp_ph111_B9
zmp_ph111_B10
zmp_ph114_G4
zmp_ph114_G11
zmp_ph114_H1
zmp_ph114_H2
zmp_ph114_A9
zmp_ph114_A11
zmp_ph114_B2
zmp_ph114_B4
zmp_ph114_D8
zmp_ph114_E2

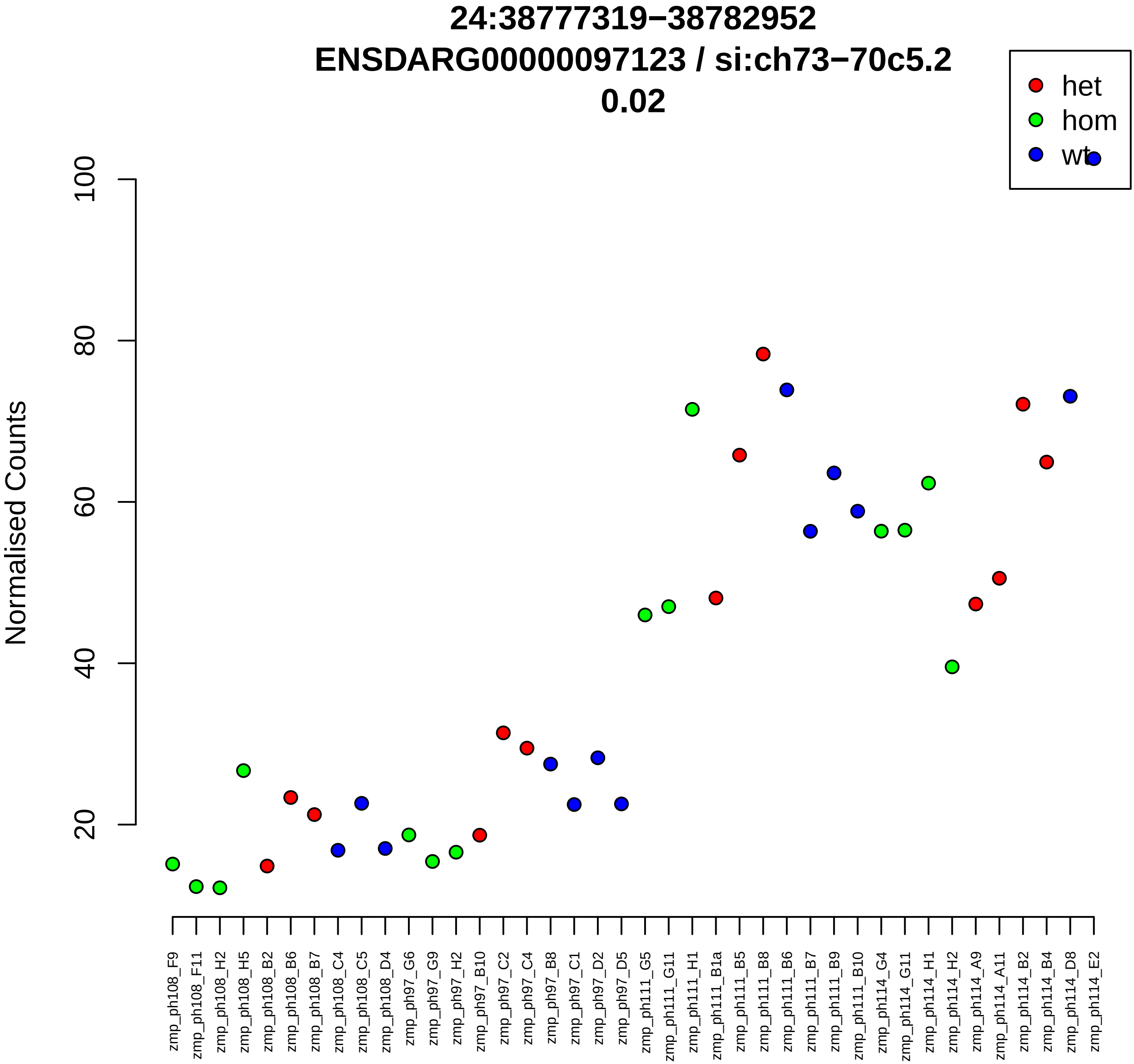

24:38777319−38782952
ENSDARG00000097123 / si:ch73−70c5.2
0.02
het
hom
wt
Normalised Counts
20
40
60
80
100
zmp_ph108_F9
zmp_ph108_F11
zmp_ph108_H2
zmp_ph108_H5
zmp_ph108_B2
zmp_ph108_B6
zmp_ph108_B7
zmp_ph108_C4
zmp_ph108_C5
zmp_ph108_D4
zmp_ph97_G6
zmp_ph97_G9
zmp_ph97_H2
zmp_ph97_B10
zmp_ph97_C2
zmp_ph97_C4
zmp_ph97_B8
zmp_ph97_C1
zmp_ph97_D2
zmp_ph97_D5
zmp_ph111_G5
zmp_ph111_G11
zmp_ph111_H1
zmp_ph111_B1a
zmp_ph111_B5
zmp_ph111_B8
zmp_ph111_B6
zmp_ph111_B7
zmp_ph111_B9
zmp_ph111_B10
zmp_ph114_G4
zmp_ph114_G11
zmp_ph114_H1
zmp_ph114_H2
zmp_ph114_A9
zmp_ph114_A11
zmp_ph114_B2
zmp_ph114_B4
zmp_ph114_D8
zmp_ph114_E2

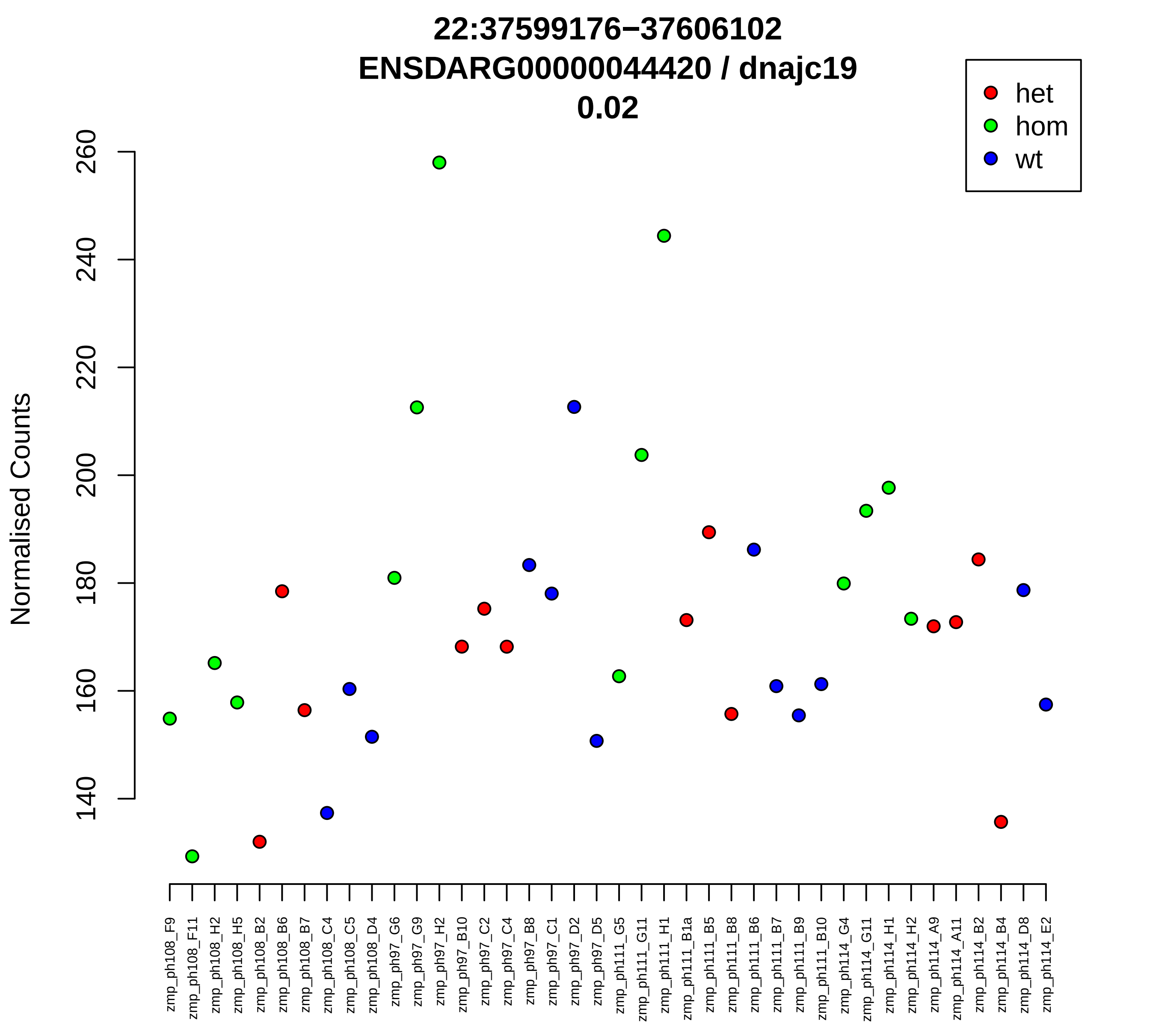
22:37599176−37606102
ENSDARG00000044420 / dnajc19
0.02
het
hom
wt
Normalised Counts
140
160
180
200
220
240
260
zmp_ph108_F9
zmp_ph108_F11
zmp_ph108_H2
zmp_ph108_H5
zmp_ph108_B2
zmp_ph108_B6
zmp_ph108_B7
zmp_ph108_C4
zmp_ph108_C5
zmp_ph108_D4
zmp_ph97_G6
zmp_ph97_G9
zmp_ph97_H2
zmp_ph97_B10
zmp_ph97_C2
zmp_ph97_C4
zmp_ph97_B8
zmp_ph97_C1
zmp_ph97_D2
zmp_ph97_D5
zmp_ph111_G5
zmp_ph111_G11
zmp_ph111_H1
zmp_ph111_B1a
zmp_ph111_B5
zmp_ph111_B8
zmp_ph111_B6
zmp_ph111_B7
zmp_ph111_B9
zmp_ph111_B10
zmp_ph114_G4
zmp_ph114_G11
zmp_ph114_H1
zmp_ph114_H2
zmp_ph114_A9
zmp_ph114_A11
zmp_ph114_B2
zmp_ph114_B4
zmp_ph114_D8
zmp_ph114_E2

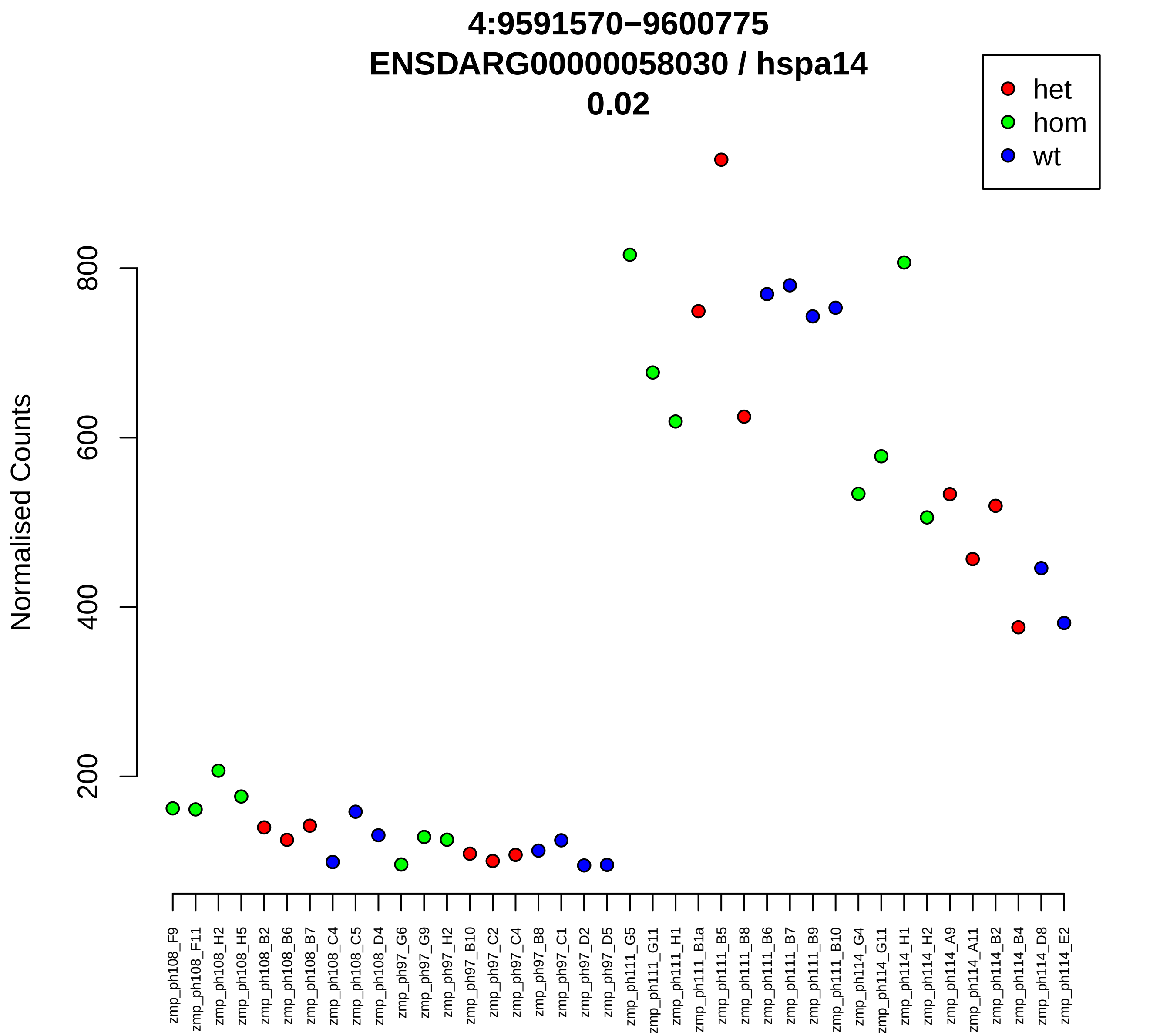

4:9591570−9600775
ENSDARG00000058030 / hspa14
0.02
het
hom
wt
Normalised Counts
200
400
600
800
zmp_ph108_F9
zmp_ph108_F11
zmp_ph108_H2
zmp_ph108_H5
zmp_ph108_B2
zmp_ph108_B6
zmp_ph108_B7
zmp_ph108_C4
zmp_ph108_C5
zmp_ph108_D4
zmp_ph97_G6
zmp_ph97_G9
zmp_ph97_H2
zmp_ph97_B10
zmp_ph97_C2
zmp_ph97_C4
zmp_ph97_B8
zmp_ph97_C1
zmp_ph97_D2
zmp_ph97_D5
zmp_ph111_G5
zmp_ph111_G11
zmp_ph111_H1
zmp_ph111_B1a
zmp_ph111_B5
zmp_ph111_B8
zmp_ph111_B6
zmp_ph111_B7
zmp_ph111_B9
zmp_ph111_B10
zmp_ph114_G4
zmp_ph114_G11
zmp_ph114_H1
zmp_ph114_H2
zmp_ph114_A9
zmp_ph114_A11
zmp_ph114_B2
zmp_ph114_B4
zmp_ph114_D8
zmp_ph114_E2

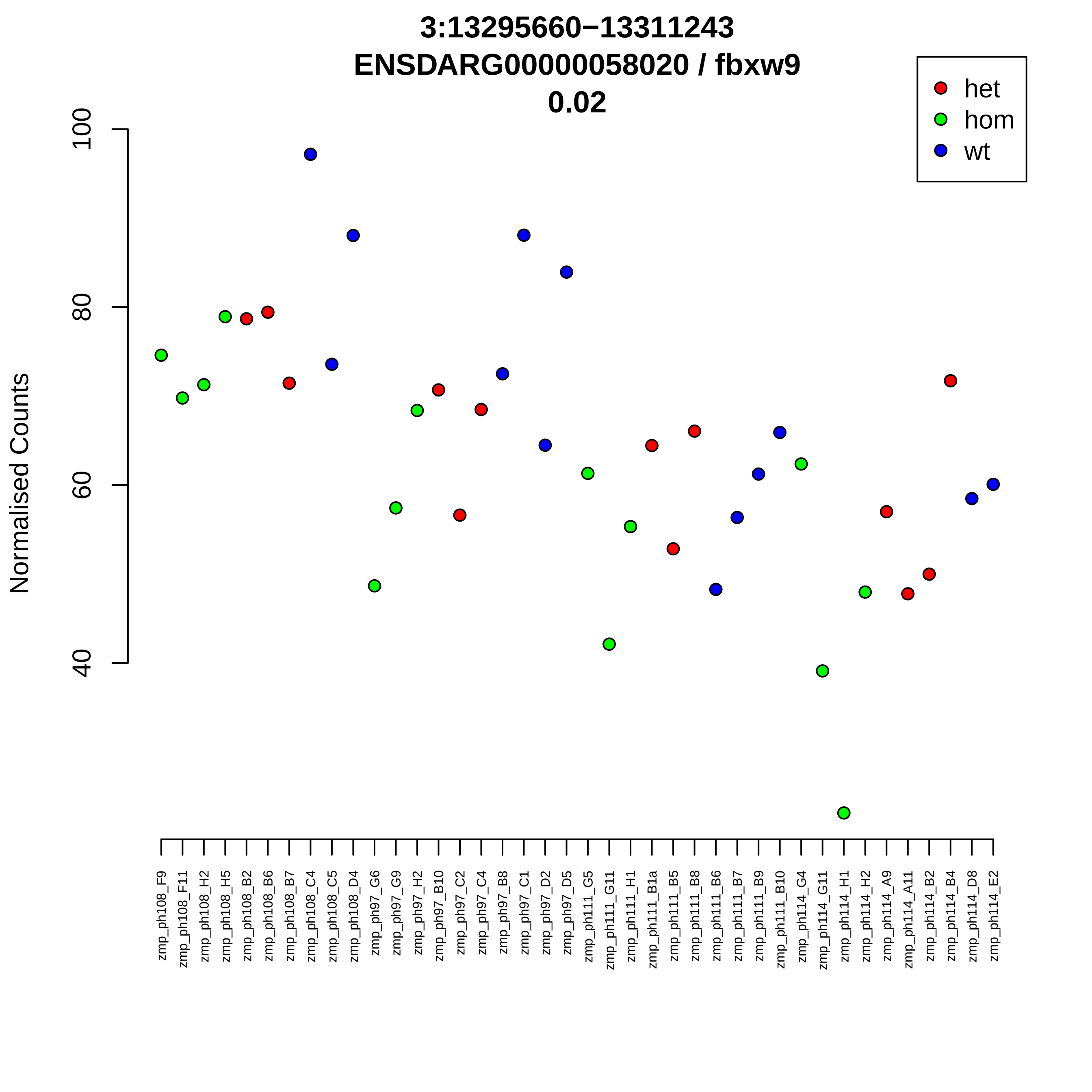
3:13295660−13311243
ENSDARG00000058020 / fbxw9
0.02
het
hom
wt
Normalised Counts
100
80
60
40
zmp_ph108_F9
zmp_ph108_F11
zmp_ph108_H2
zmp_ph108_H5
zmp_ph108_B2
zmp_ph108_B6
zmp_ph108_B7
zmp_ph108_C4
zmp_ph108_C5
zmp_ph108_D4
zmp_ph97_G6
zmp_ph97_G9
zmp_ph97_H2
zmp_ph97_B10
zmp_ph97_C2
zmp_ph97_C4
zmp_ph97_B8
zmp_ph97_C1
zmp_ph97_D2
zmp_ph97_D5
zmp_ph111_G5
zmp_ph111_G11
zmp_ph111_H1
zmp_ph111_B1a
zmp_ph111_B5
zmp_ph111_B8
zmp_ph111_B6
zmp_ph111_B7
zmp_ph111_B9
zmp_ph111_B10
zmp_ph114_G4
zmp_ph114_G11
zmp_ph114_H1
zmp_ph114_H2
zmp_ph114_A9
zmp_ph114_A11
zmp_ph114_B2
zmp_ph114_B4
zmp_ph114_D8
zmp_ph114_E2

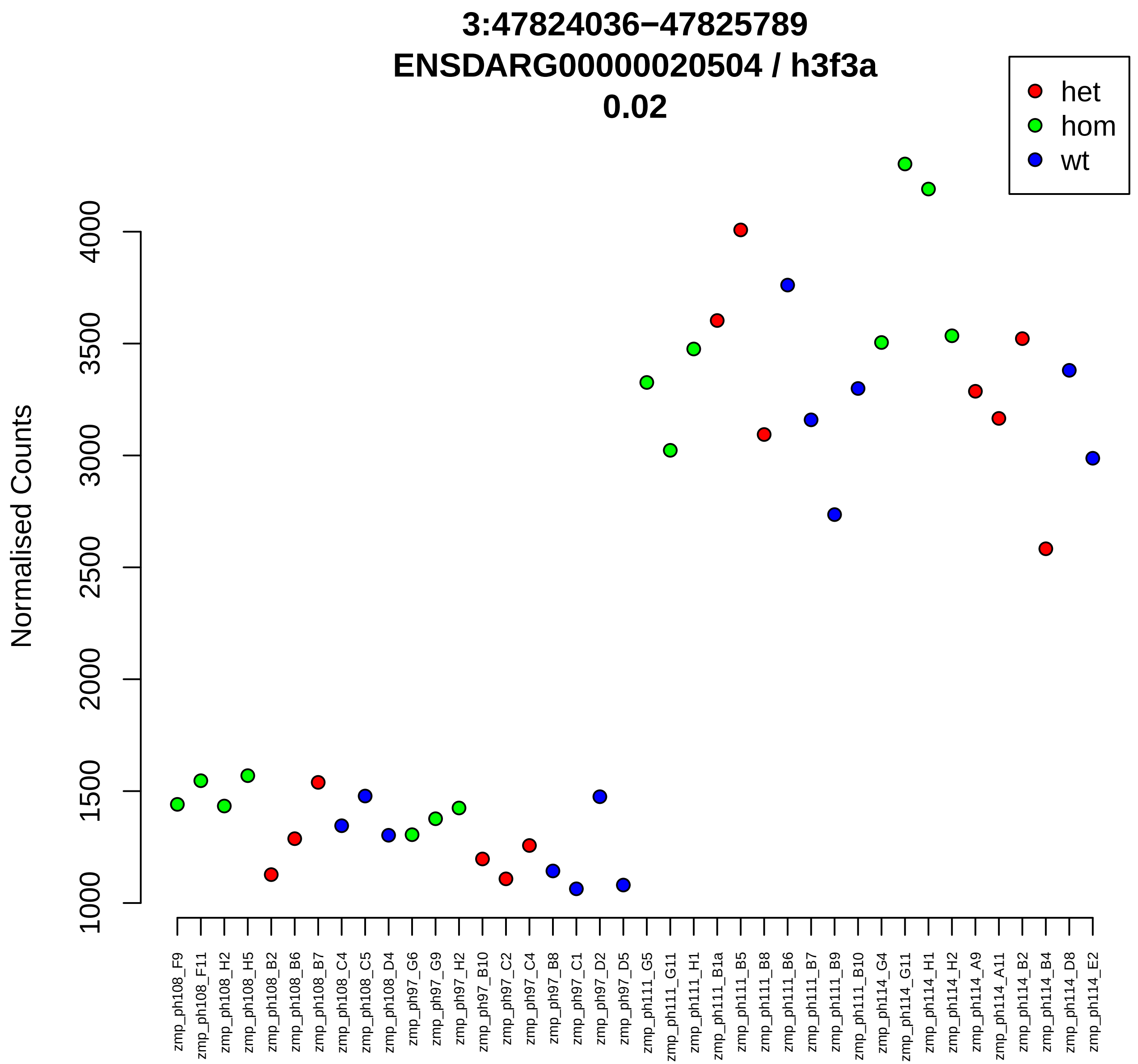
3:47824036−47825789
ENSDARG00000020504 / h3f3a
0.02
het
hom
wt
Normalised Counts
1000
1500
2000
2500
3000
3500
4000
zmp_ph108_F9
zmp_ph108_F11
zmp_ph108_H2
zmp_ph108_H5
zmp_ph108_B2
zmp_ph108_B6
zmp_ph108_B7
zmp_ph108_C4
zmp_ph108_C5
zmp_ph108_D4
zmp_ph97_G6
zmp_ph97_G9
zmp_ph97_H2
zmp_ph97_B10
zmp_ph97_C2
zmp_ph97_C4
zmp_ph97_B8
zmp_ph97_C1
zmp_ph97_D2
zmp_ph97_D5
zmp_ph111_G5
zmp_ph111_G11
zmp_ph111_H1
zmp_ph111_B1a
zmp_ph111_B5
zmp_ph111_B8
zmp_ph111_B6
zmp_ph111_B7
zmp_ph111_B9
zmp_ph111_B10
zmp_ph114_G4
zmp_ph114_G11
zmp_ph114_H1
zmp_ph114_H2
zmp_ph114_A9
zmp_ph114_A11
zmp_ph114_B2
zmp_ph114_B4
zmp_ph114_D8
zmp_ph114_E2

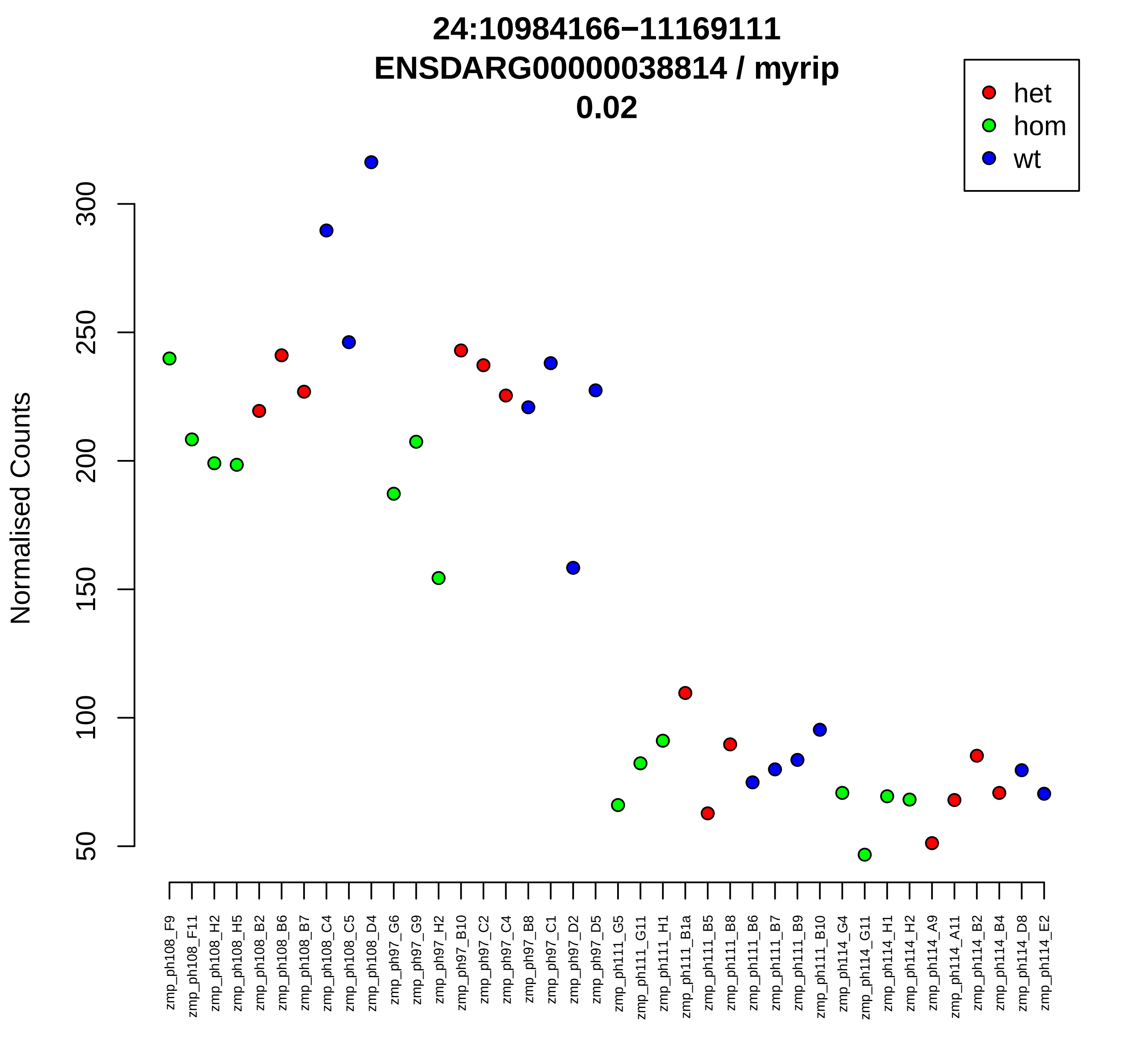

24:10984166−11169111
ENSDARG00000038814 / myrip
0.02
het
hom
wt
Normalised Counts
50
100
150
200
250
300
zmp_ph108_F9
zmp_ph108_F11
zmp_ph108_H2
zmp_ph108_H5
zmp_ph108_B2
zmp_ph108_B6
zmp_ph108_B7
zmp_ph108_C4
zmp_ph108_C5
zmp_ph108_D4
zmp_ph97_G6
zmp_ph97_G9
zmp_ph97_H2
zmp_ph97_B10
zmp_ph97_C2
zmp_ph97_C4
zmp_ph97_B8
zmp_ph97_C1
zmp_ph97_D2
zmp_ph97_D5
zmp_ph111_G5
zmp_ph111_G11
zmp_ph111_H1
zmp_ph111_B1a
zmp_ph111_B5
zmp_ph111_B8
zmp_ph111_B6
zmp_ph111_B7
zmp_ph111_B9
zmp_ph111_B10
zmp_ph114_G4
zmp_ph114_G11
zmp_ph114_H1
zmp_ph114_H2
zmp_ph114_A9
zmp_ph114_A11
zmp_ph114_B2
zmp_ph114_B4
zmp_ph114_D8
zmp_ph114_E2

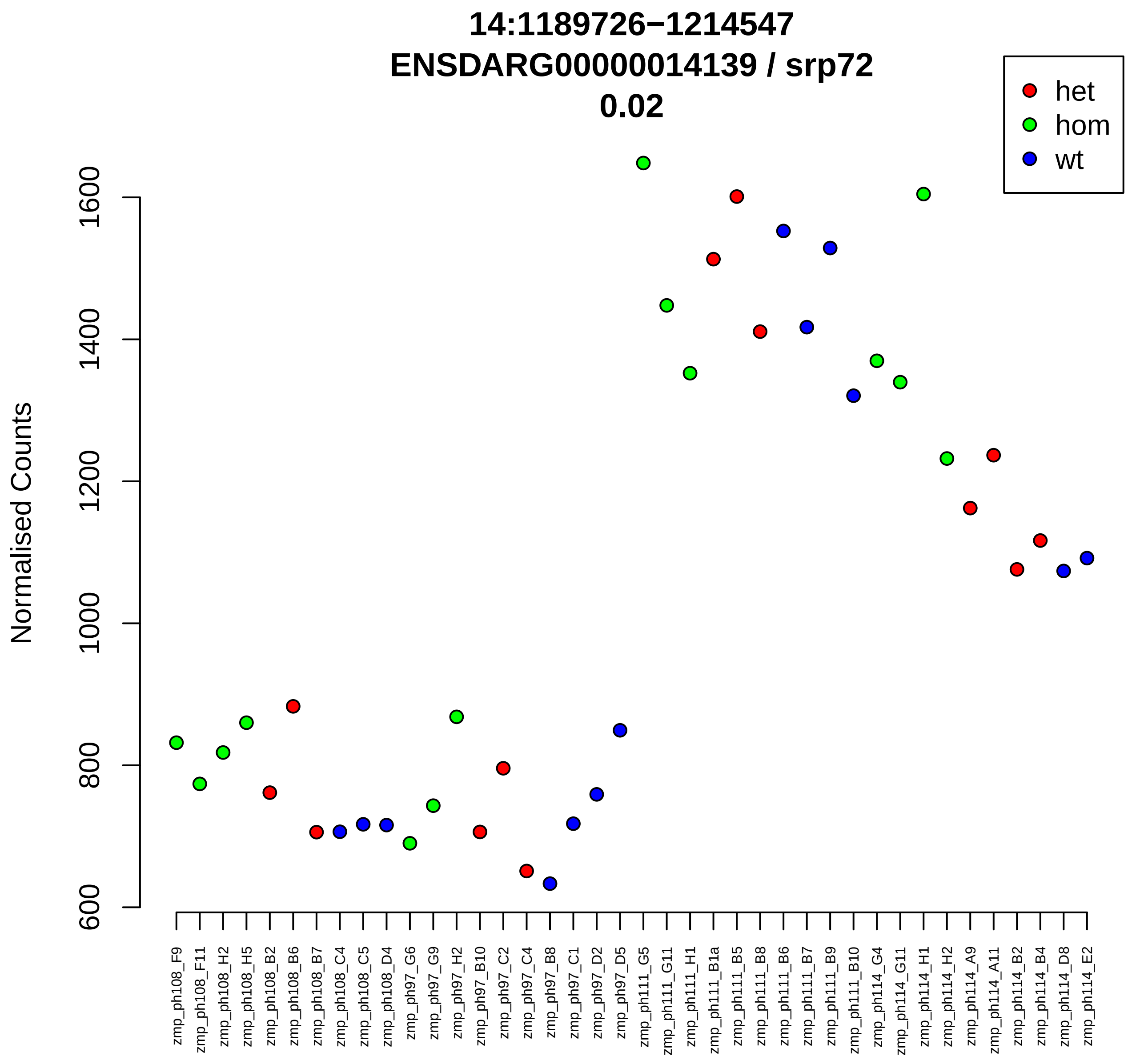

14:1189726−1214547
ENSDARG00000014139 / srp72
0.02
het
hom
wt
Normalised Counts
600
800
1000
1200
1400
1600
zmp_ph108_F9
zmp_ph108_F11
zmp_ph108_H2
zmp_ph108_H5
zmp_ph108_B2
zmp_ph108_B6
zmp_ph108_B7
zmp_ph108_C4
zmp_ph108_C5
zmp_ph108_D4
zmp_ph97_G6
zmp_ph97_G9
zmp_ph97_H2
zmp_ph97_B10
zmp_ph97_C2
zmp_ph97_C4
zmp_ph97_B8
zmp_ph97_C1
zmp_ph97_D2
zmp_ph97_D5
zmp_ph111_G5
zmp_ph111_G11
zmp_ph111_H1
zmp_ph111_B1a
zmp_ph111_B5
zmp_ph111_B8
zmp_ph111_B6
zmp_ph111_B7
zmp_ph111_B9
zmp_ph111_B10
zmp_ph114_G4
zmp_ph114_G11
zmp_ph114_H1
zmp_ph114_H2
zmp_ph114_A9
zmp_ph114_A11
zmp_ph114_B2
zmp_ph114_B4
zmp_ph114_D8
zmp_ph114_E2

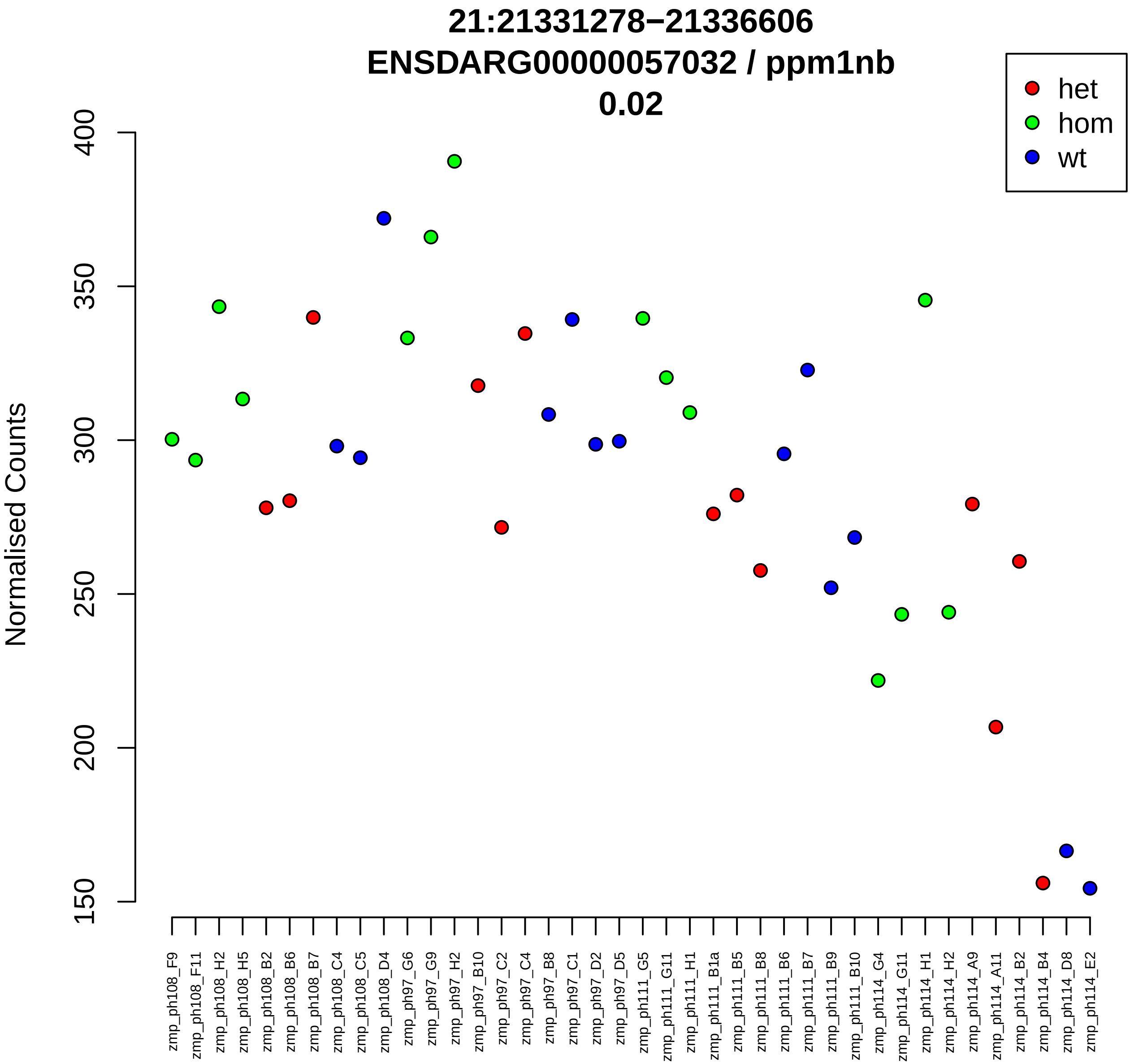
21:21331278−21336606
ENSDARG00000057032 / ppm1nb
0.02
het
hom
wt
Normalised Counts
400
350
300
250
200
150
zmp_ph108_F9
zmp_ph108_F11
zmp_ph108_H2
zmp_ph108_H5
zmp_ph108_B2
zmp_ph108_B6
zmp_ph108_B7
zmp_ph108_C4
zmp_ph108_C5
zmp_ph108_D4
zmp_ph97_G6
zmp_ph97_G9
zmp_ph97_H2
zmp_ph97_B10
zmp_ph97_C2
zmp_ph97_C4
zmp_ph97_B8
zmp_ph97_C1
zmp_ph97_D2
zmp_ph97_D5
zmp_ph111_G5
zmp_ph111_G11
zmp_ph111_H1
zmp_ph111_B1a
zmp_ph111_B5
zmp_ph111_B8
zmp_ph111_B6
zmp_ph111_B7
zmp_ph111_B9
zmp_ph111_B10
zmp_ph114_G4
zmp_ph114_G11
zmp_ph114_H1
zmp_ph114_H2
zmp_ph114_A9
zmp_ph114_A11
zmp_ph114_B2
zmp_ph114_B4
zmp_ph114_D8
zmp_ph114_E2

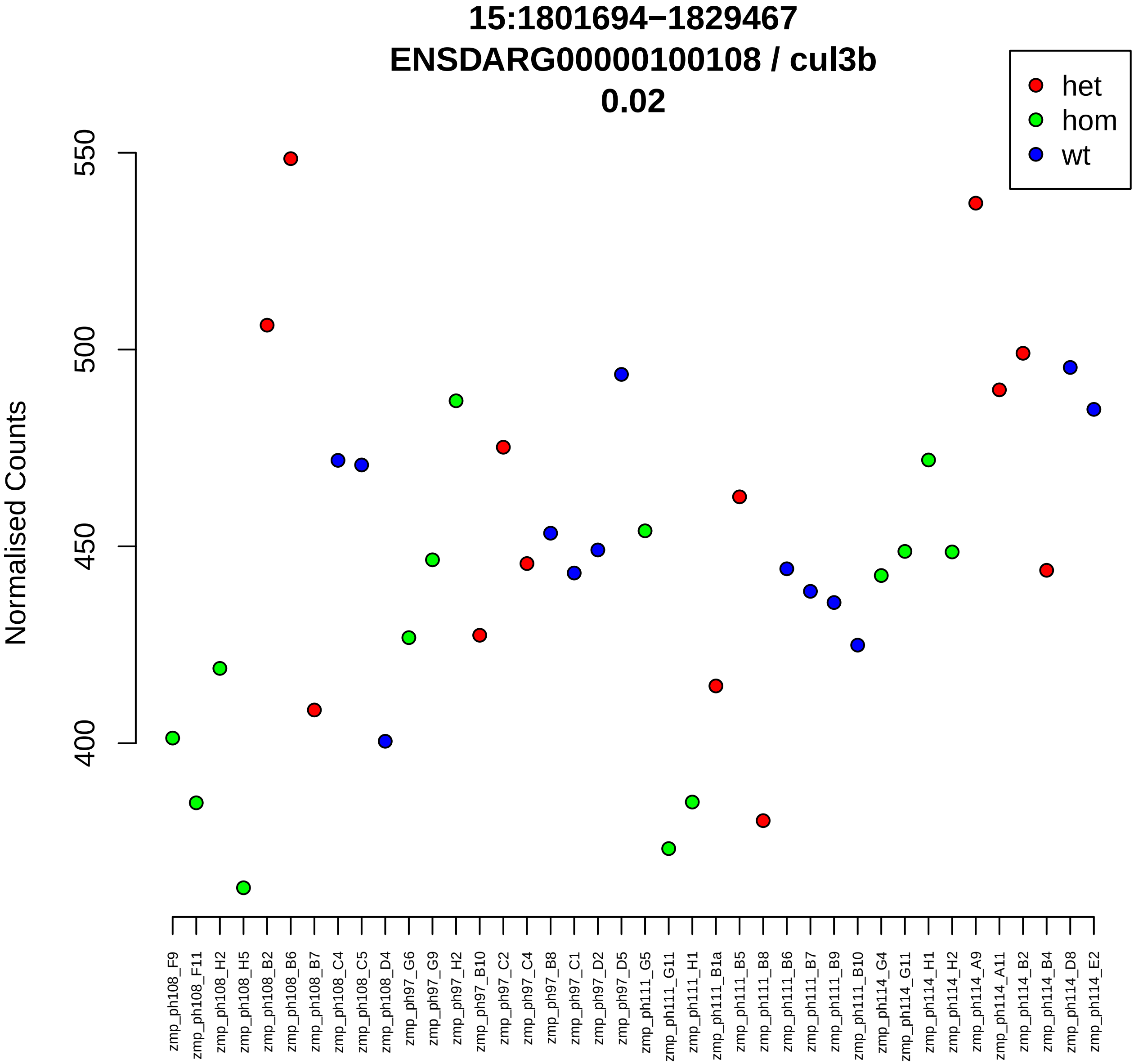
15:1801694−1829467
ENSDARG00000100108 / cul3b
0.02
het
hom
wt
Normalised Counts
550
500
450
400
zmp_ph108_F9
zmp_ph108_F11
zmp_ph108_H2
zmp_ph108_H5
zmp_ph108_B2
zmp_ph108_B6
zmp_ph108_B7
zmp_ph108_C4
zmp_ph108_C5
zmp_ph108_D4
zmp_ph97_G6
zmp_ph97_G9
zmp_ph97_H2
zmp_ph97_B10
zmp_ph97_C2
zmp_ph97_C4
zmp_ph97_B8
zmp_ph97_C1
zmp_ph97_D2
zmp_ph97_D5
zmp_ph111_G5
zmp_ph111_G11
zmp_ph111_H1
zmp_ph111_B1a
zmp_ph111_B5
zmp_ph111_B8
zmp_ph111_B6
zmp_ph111_B7
zmp_ph111_B9
zmp_ph111_B10
zmp_ph114_G4
zmp_ph114_G11
zmp_ph114_H1
zmp_ph114_H2
zmp_ph114_A9
zmp_ph114_A11
zmp_ph114_B2
zmp_ph114_B4
zmp_ph114_D8
zmp_ph114_E2

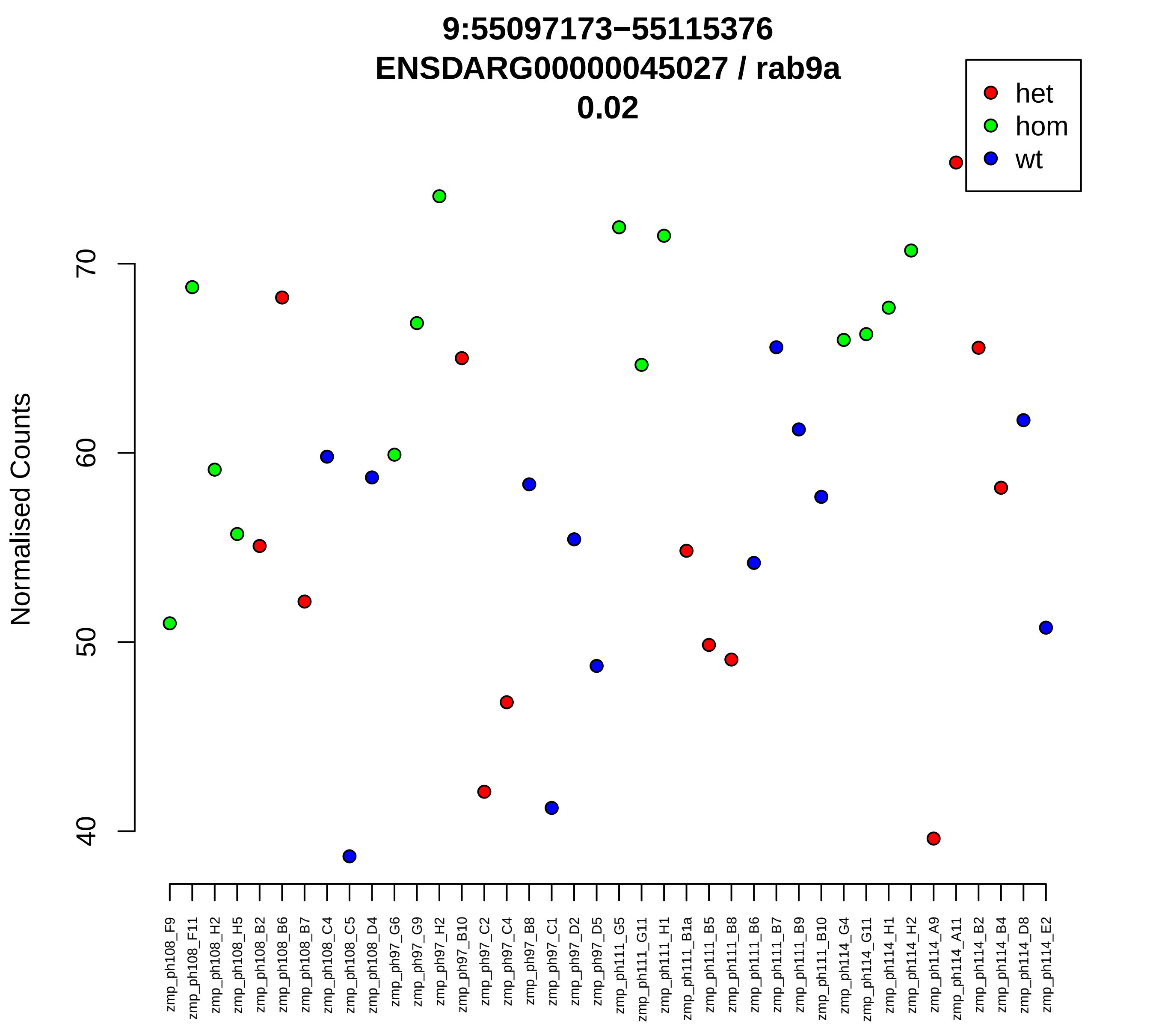
9:55097173−55115376
ENSDARG00000045027 / rab9a
0.02
het
hom
wt
Normalised Counts
40
50
60
70
zmp_ph108_F9
zmp_ph108_F11
zmp_ph108_H2
zmp_ph108_H5
zmp_ph108_B2
zmp_ph108_B6
zmp_ph108_B7
zmp_ph108_C4
zmp_ph108_C5
zmp_ph108_D4
zmp_ph97_G6
zmp_ph97_G9
zmp_ph97_H2
zmp_ph97_B10
zmp_ph97_C2
zmp_ph97_C4
zmp_ph97_B8
zmp_ph97_C1
zmp_ph97_D2
zmp_ph97_D5
zmp_ph111_G5
zmp_ph111_G11
zmp_ph111_H1
zmp_ph111_B1a
zmp_ph111_B5
zmp_ph111_B8
zmp_ph111_B6
zmp_ph111_B7
zmp_ph111_B9
zmp_ph111_B10
zmp_ph114_G4
zmp_ph114_G11
zmp_ph114_H1
zmp_ph114_H2
zmp_ph114_A9
zmp_ph114_A11
zmp_ph114_B2
zmp_ph114_B4
zmp_ph114_D8
zmp_ph114_E2

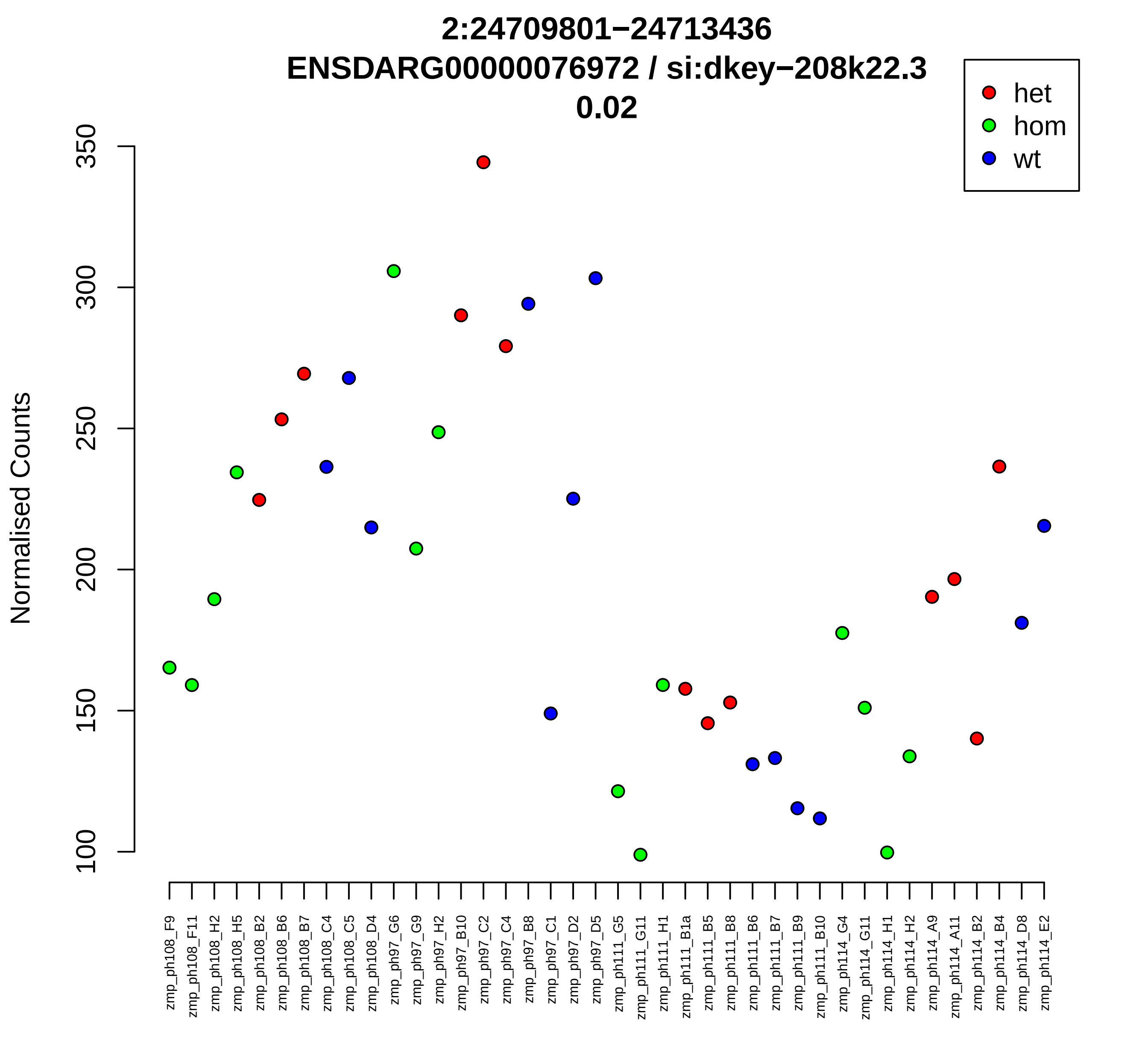

2:24709801−24713436
ENSDARG00000076972 / si:dkey−208k22.3
0.02
het
hom
wt
Normalised Counts
100
150
200
250
300
350
zmp_ph108_F9
zmp_ph108_F11
zmp_ph108_H2
zmp_ph108_H5
zmp_ph108_B2
zmp_ph108_B6
zmp_ph108_B7
zmp_ph108_C4
zmp_ph108_C5
zmp_ph108_D4
zmp_ph97_G6
zmp_ph97_G9
zmp_ph97_H2
zmp_ph97_B10
zmp_ph97_C2
zmp_ph97_C4
zmp_ph97_B8
zmp_ph97_C1
zmp_ph97_D2
zmp_ph97_D5
zmp_ph111_G5
zmp_ph111_G11
zmp_ph111_H1
zmp_ph111_B1a
zmp_ph111_B5
zmp_ph111_B8
zmp_ph111_B6
zmp_ph111_B7
zmp_ph111_B9
zmp_ph111_B10
zmp_ph114_G4
zmp_ph114_G11
zmp_ph114_H1
zmp_ph114_H2
zmp_ph114_A9
zmp_ph114_A11
zmp_ph114_B2
zmp_ph114_B4
zmp_ph114_D8
zmp_ph114_E2

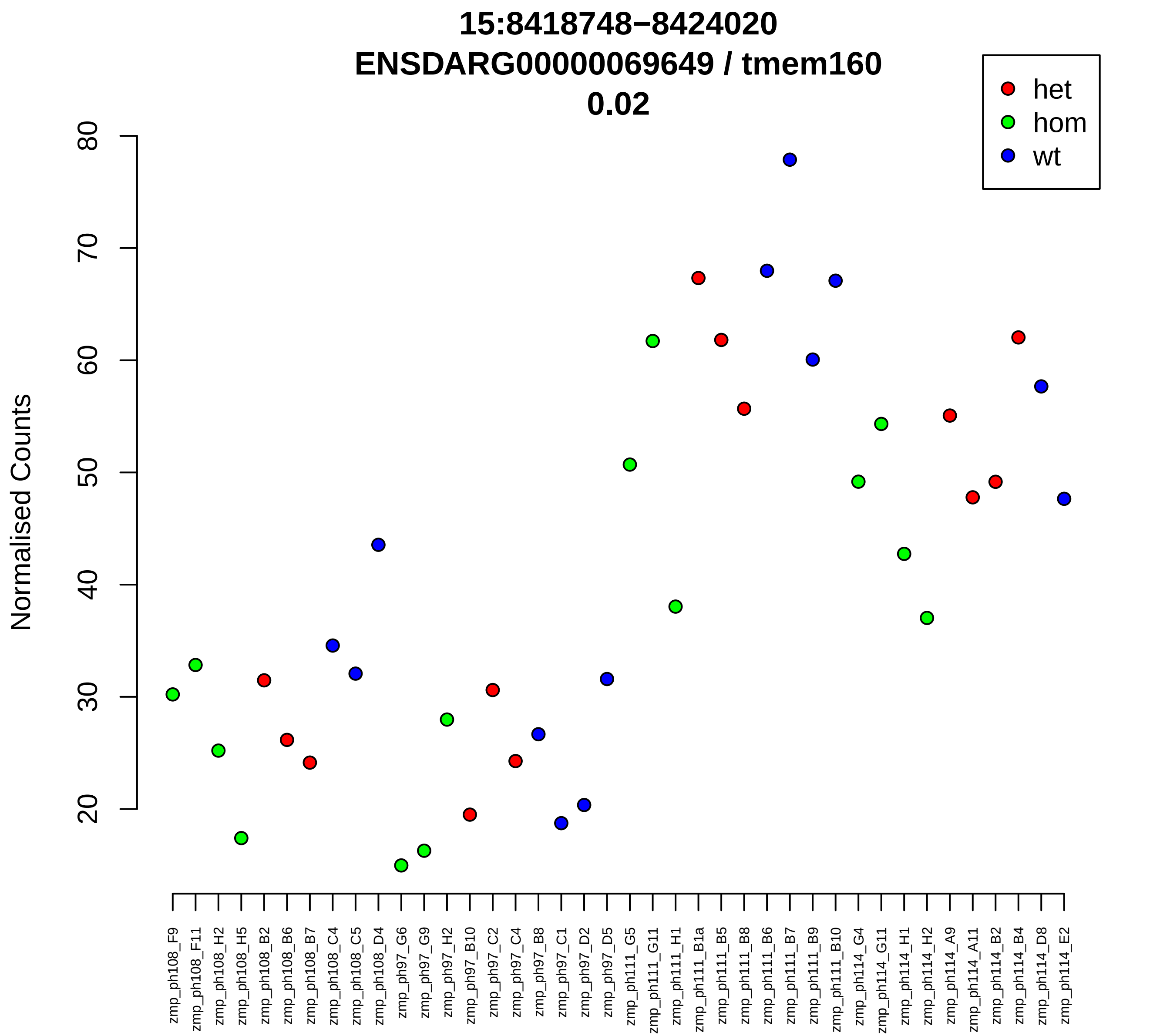
15:8418748−8424020
ENSDARG00000069649 / tmem160
0.02
Normalised Counts
het
hom
wt
20
30
40
50
60
70
80
zmp_ph108_F9
zmp_ph108_F11
zmp_ph108_H2
zmp_ph108_H5
zmp_ph108_B2
zmp_ph108_B6
zmp_ph108_B7
zmp_ph108_C4
zmp_ph108_C5
zmp_ph108_D4
zmp_ph97_G6
zmp_ph97_G9
zmp_ph97_H2
zmp_ph97_B10
zmp_ph97_C2
zmp_ph97_C4
zmp_ph97_B8
zmp_ph97_C1
zmp_ph97_D2
zmp_ph97_D5
zmp_ph111_G5
zmp_ph111_G11
zmp_ph111_H1
zmp_ph111_B1a
zmp_ph111_B5
zmp_ph111_B8
zmp_ph111_B6
zmp_ph111_B7
zmp_ph111_B9
zmp_ph111_B10
zmp_ph114_G4
zmp_ph114_G11
zmp_ph114_H1
zmp_ph114_H2
zmp_ph114_A9
zmp_ph114_A11
zmp_ph114_B2
zmp_ph114_B4
zmp_ph114_D8
zmp_ph114_E2

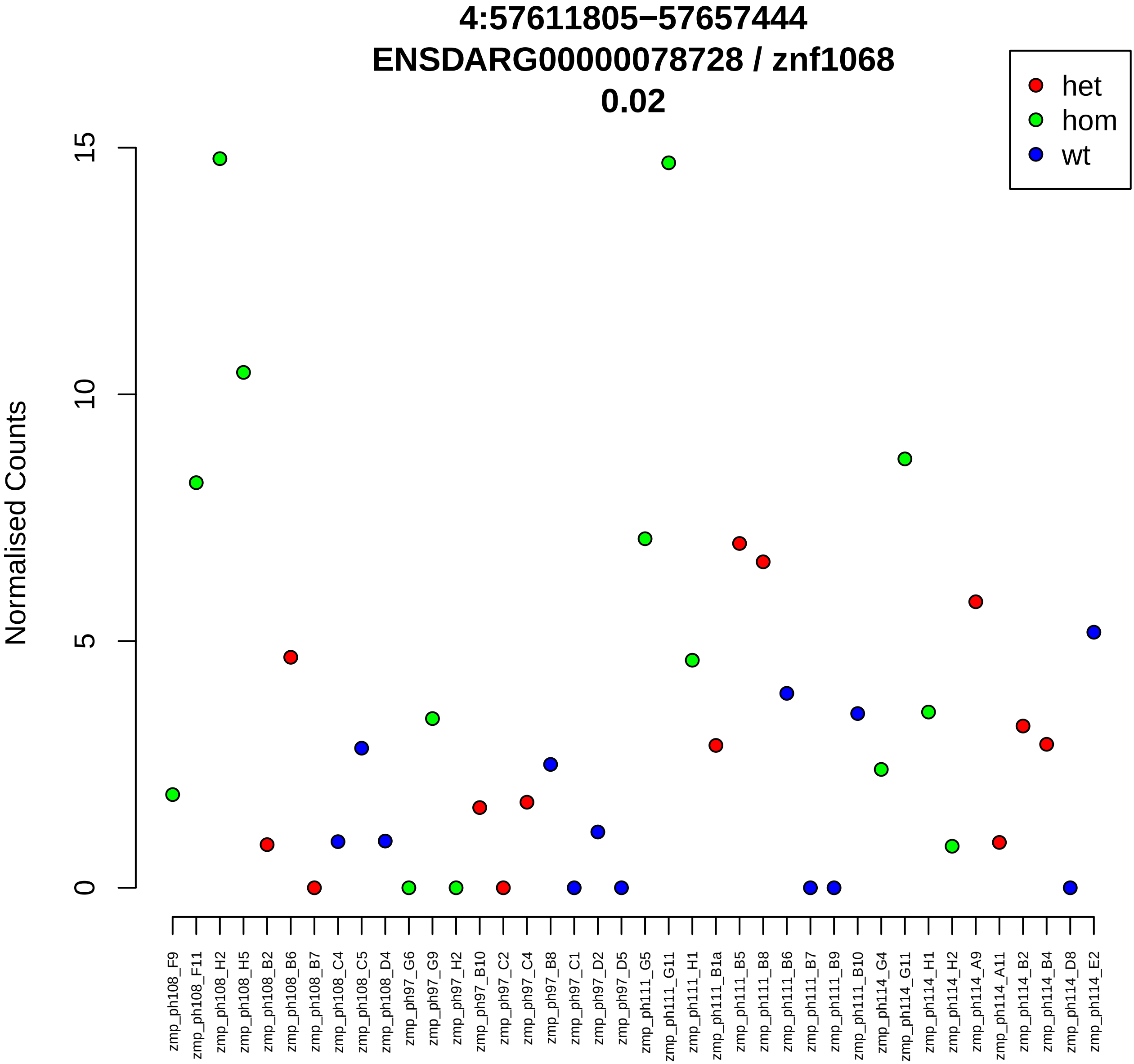
4:57611805−57657444
ENSDARG00000078728 / znf1068
0.02
het
hom
wt
Normalised Counts
0
5
10
15
zmp_ph108_F9
zmp_ph108_F11
zmp_ph108_H2
zmp_ph108_H5
zmp_ph108_B2
zmp_ph108_B6
zmp_ph108_B7
zmp_ph108_C4
zmp_ph108_C5
zmp_ph108_D4
zmp_ph97_G6
zmp_ph97_G9
zmp_ph97_H2
zmp_ph97_B10
zmp_ph97_C2
zmp_ph97_C4
zmp_ph97_B8
zmp_ph97_C1
zmp_ph97_D2
zmp_ph97_D5
zmp_ph111_G5
zmp_ph111_G11
zmp_ph111_H1
zmp_ph111_B1a
zmp_ph111_B5
zmp_ph111_B8
zmp_ph111_B6
zmp_ph111_B7
zmp_ph111_B9
zmp_ph111_B10
zmp_ph114_G4
zmp_ph114_G11
zmp_ph114_H1
zmp_ph114_H2
zmp_ph114_A9
zmp_ph114_A11
zmp_ph114_B2
zmp_ph114_B4
zmp_ph114_D8
zmp_ph114_E2

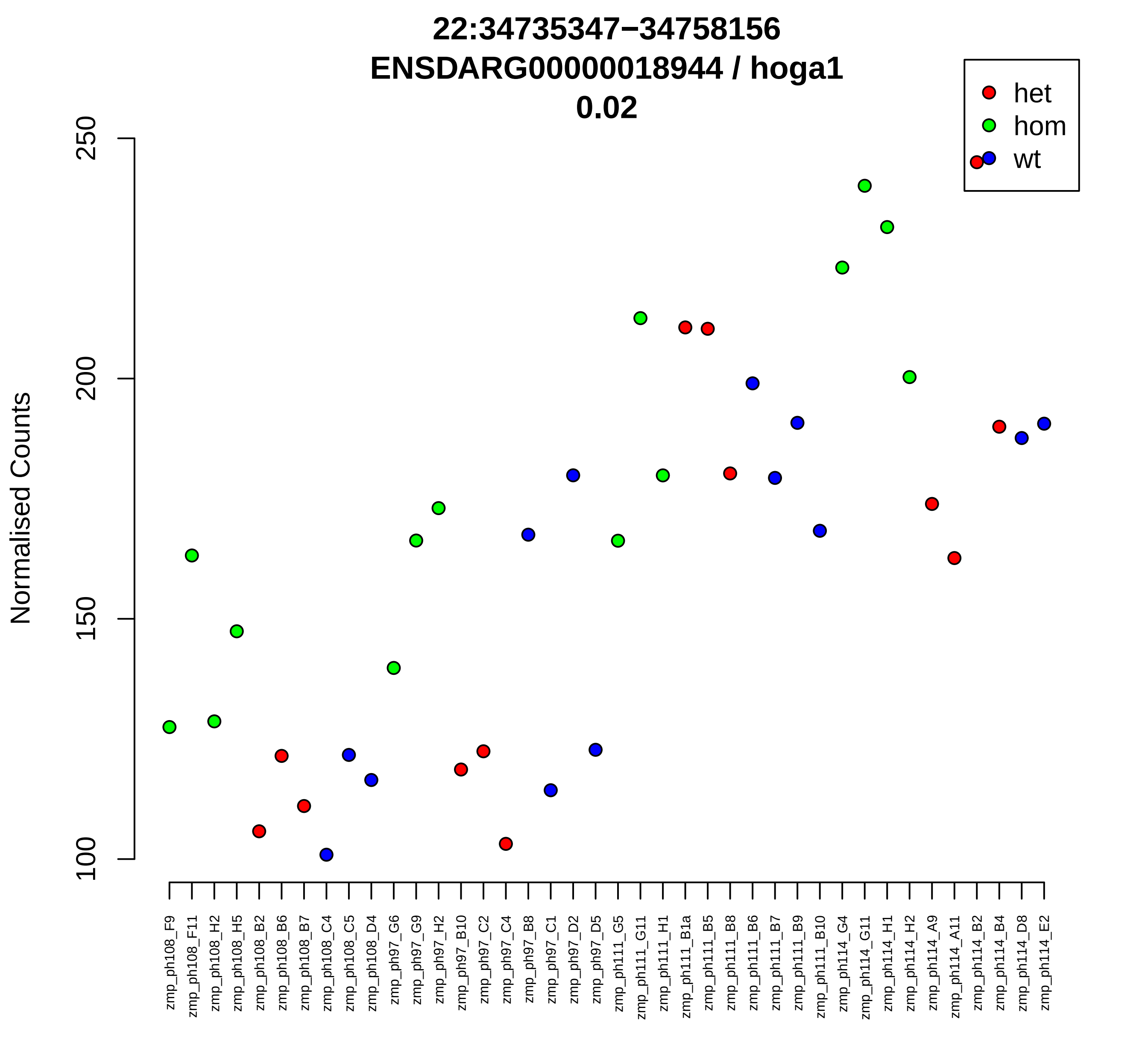
22:34735347−34758156
ENSDARG00000018944 / hoga1
0.02
Normalised Counts
250
200
150
100
het
hom
wt
zmp_ph108_F9
zmp_ph108_F11
zmp_ph108_H2
zmp_ph108_H5
zmp_ph108_B2
zmp_ph108_B6
zmp_ph108_B7
zmp_ph108_C4
zmp_ph108_C5
zmp_ph108_D4
zmp_ph97_G6
zmp_ph97_G9
zmp_ph97_H2
zmp_ph97_B10
zmp_ph97_C2
zmp_ph97_C4
zmp_ph97_B8
zmp_ph97_C1
zmp_ph97_D2
zmp_ph97_D5
zmp_ph111_G5
zmp_ph111_G11
zmp_ph111_H1
zmp_ph111_B1a
zmp_ph111_B5
zmp_ph111_B8
zmp_ph111_B6
zmp_ph111_B7
zmp_ph111_B9
zmp_ph111_B10
zmp_ph114_G4
zmp_ph114_G11
zmp_ph114_H1
zmp_ph114_H2
zmp_ph114_A9
zmp_ph114_A11
zmp_ph114_B2
zmp_ph114_B4
zmp_ph114_D8
zmp_ph114_E2

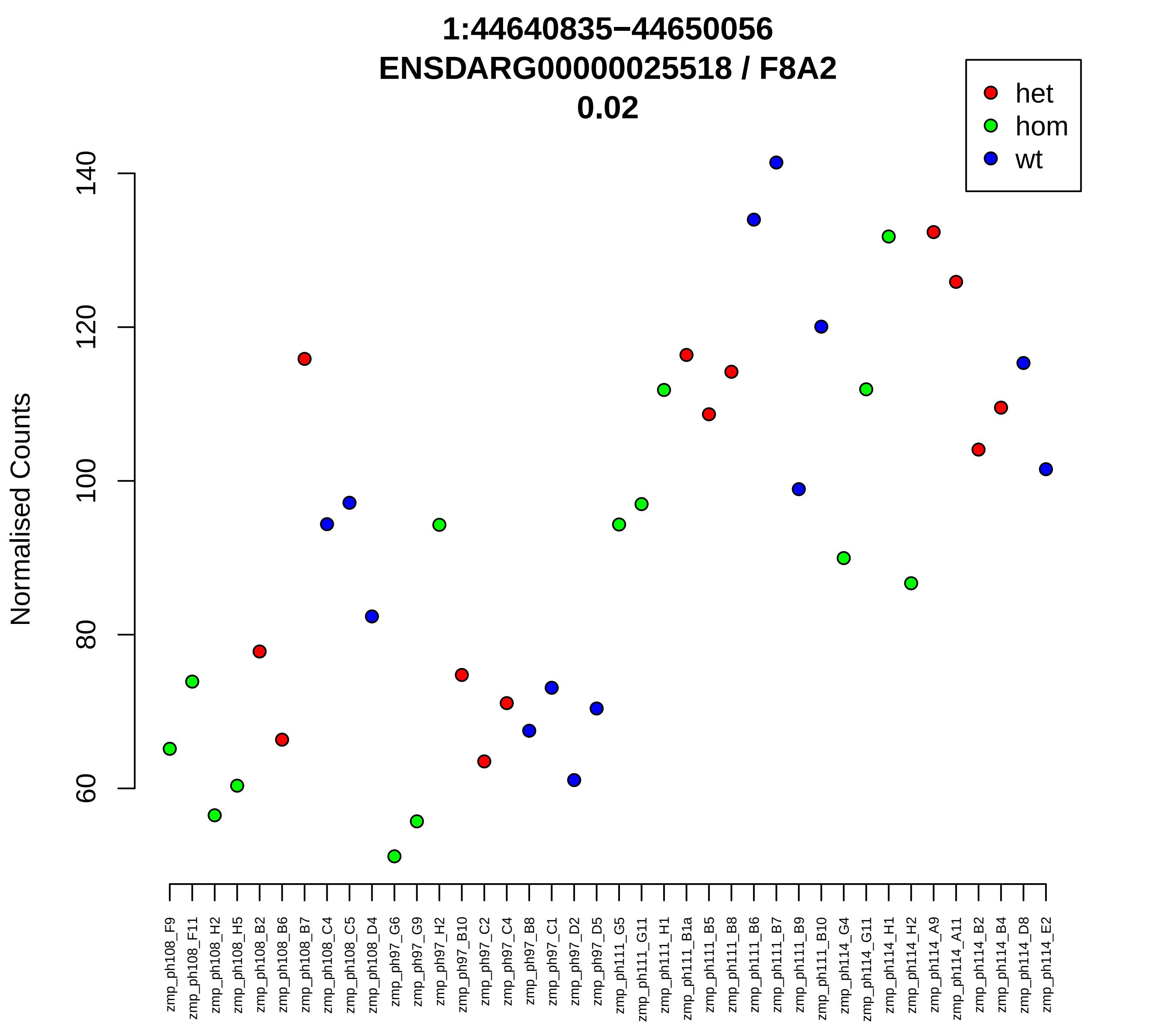
1:44640835−44650056
ENSDARG00000025518 / F8A2
0.02
het
hom
wt
Normalised Counts
60
80
100
120
140
zmp_ph108_F9
zmp_ph108_F11
zmp_ph108_H2
zmp_ph108_H5
zmp_ph108_B2
zmp_ph108_B6
zmp_ph108_B7
zmp_ph108_C4
zmp_ph108_C5
zmp_ph108_D4
zmp_ph97_G6
zmp_ph97_G9
zmp_ph97_H2
zmp_ph97_B10
zmp_ph97_C2
zmp_ph97_C4
zmp_ph97_B8
zmp_ph97_C1
zmp_ph97_D2
zmp_ph97_D5
zmp_ph111_G5
zmp_ph111_G11
zmp_ph111_H1
zmp_ph111_B1a
zmp_ph111_B5
zmp_ph111_B8
zmp_ph111_B6
zmp_ph111_B7
zmp_ph111_B9
zmp_ph111_B10
zmp_ph114_G4
zmp_ph114_G11
zmp_ph114_H1
zmp_ph114_H2
zmp_ph114_A9
zmp_ph114_A11
zmp_ph114_B2
zmp_ph114_B4
zmp_ph114_D8
zmp_ph114_E2

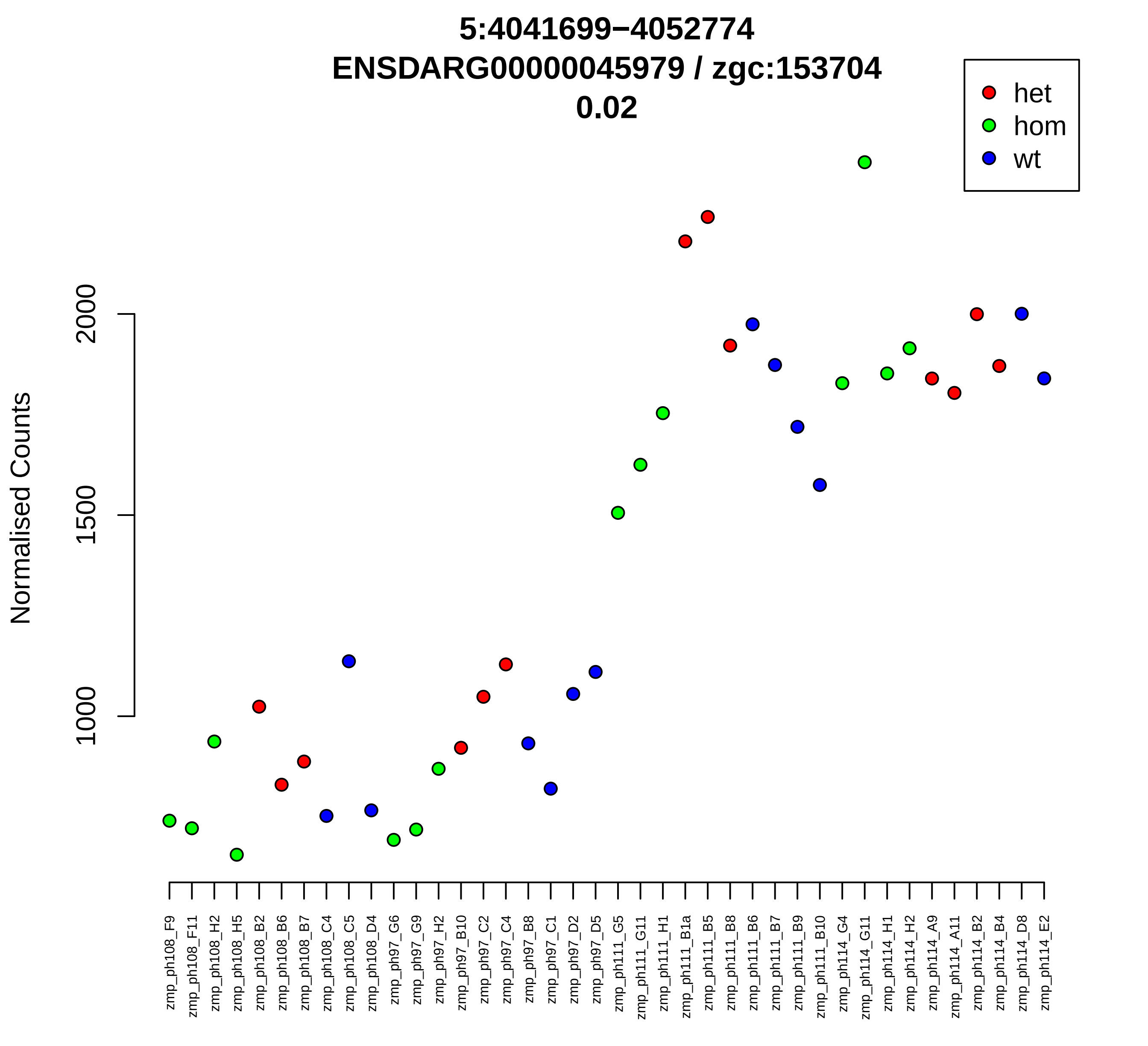
5:4041699−4052774
ENSDARG00000045979 / zgc:153704
0.02
het
hom
wt
Normalised Counts
1000
1500
2000
zmp_ph108_F9
zmp_ph108_F11
zmp_ph108_H2
zmp_ph108_H5
zmp_ph108_B2
zmp_ph108_B6
zmp_ph108_B7
zmp_ph108_C4
zmp_ph108_C5
zmp_ph108_D4
zmp_ph97_G6
zmp_ph97_G9
zmp_ph97_H2
zmp_ph97_B10
zmp_ph97_C2
zmp_ph97_C4
zmp_ph97_B8
zmp_ph97_C1
zmp_ph97_D2
zmp_ph97_D5
zmp_ph111_G5
zmp_ph111_G11
zmp_ph111_H1
zmp_ph111_B1a
zmp_ph111_B5
zmp_ph111_B8
zmp_ph111_B6
zmp_ph111_B7
zmp_ph111_B9
zmp_ph111_B10
zmp_ph114_G4
zmp_ph114_G11
zmp_ph114_H1
zmp_ph114_H2
zmp_ph114_A9
zmp_ph114_A11
zmp_ph114_B2
zmp_ph114_B4
zmp_ph114_D8
zmp_ph114_E2

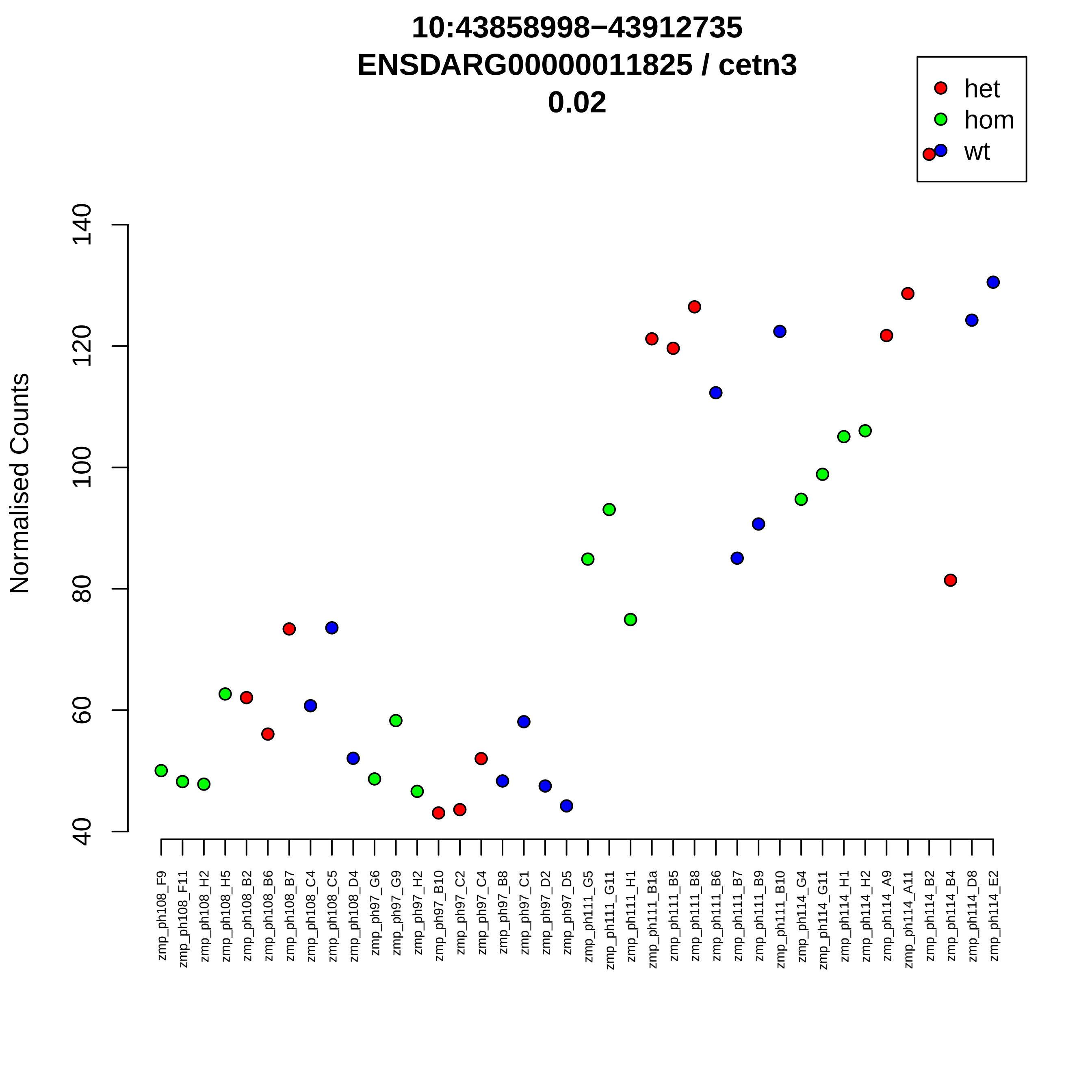

10:43858998−43912735
ENSDARG00000011825 / cetn3
0.02
het
hom
wt
Normalised Counts
40
60
80
100
120
140
zmp_ph108_F9
zmp_ph108_F11
zmp_ph108_H2
zmp_ph108_H5
zmp_ph108_B2
zmp_ph108_B6
zmp_ph108_B7
zmp_ph108_C4
zmp_ph108_C5
zmp_ph108_D4
zmp_ph97_G6
zmp_ph97_G9
zmp_ph97_H2
zmp_ph97_B10
zmp_ph97_C2
zmp_ph97_C4
zmp_ph97_B8
zmp_ph97_C1
zmp_ph97_D2
zmp_ph97_D5
zmp_ph111_G5
zmp_ph111_G11
zmp_ph111_H1
zmp_ph111_B1a
zmp_ph111_B5
zmp_ph111_B8
zmp_ph111_B6
zmp_ph111_B7
zmp_ph111_B9
zmp_ph111_B10
zmp_ph114_G4
zmp_ph114_G11
zmp_ph114_H1
zmp_ph114_H2
zmp_ph114_A9
zmp_ph114_A11
zmp_ph114_B2
zmp_ph114_B4
zmp_ph114_D8
zmp_ph114_E2

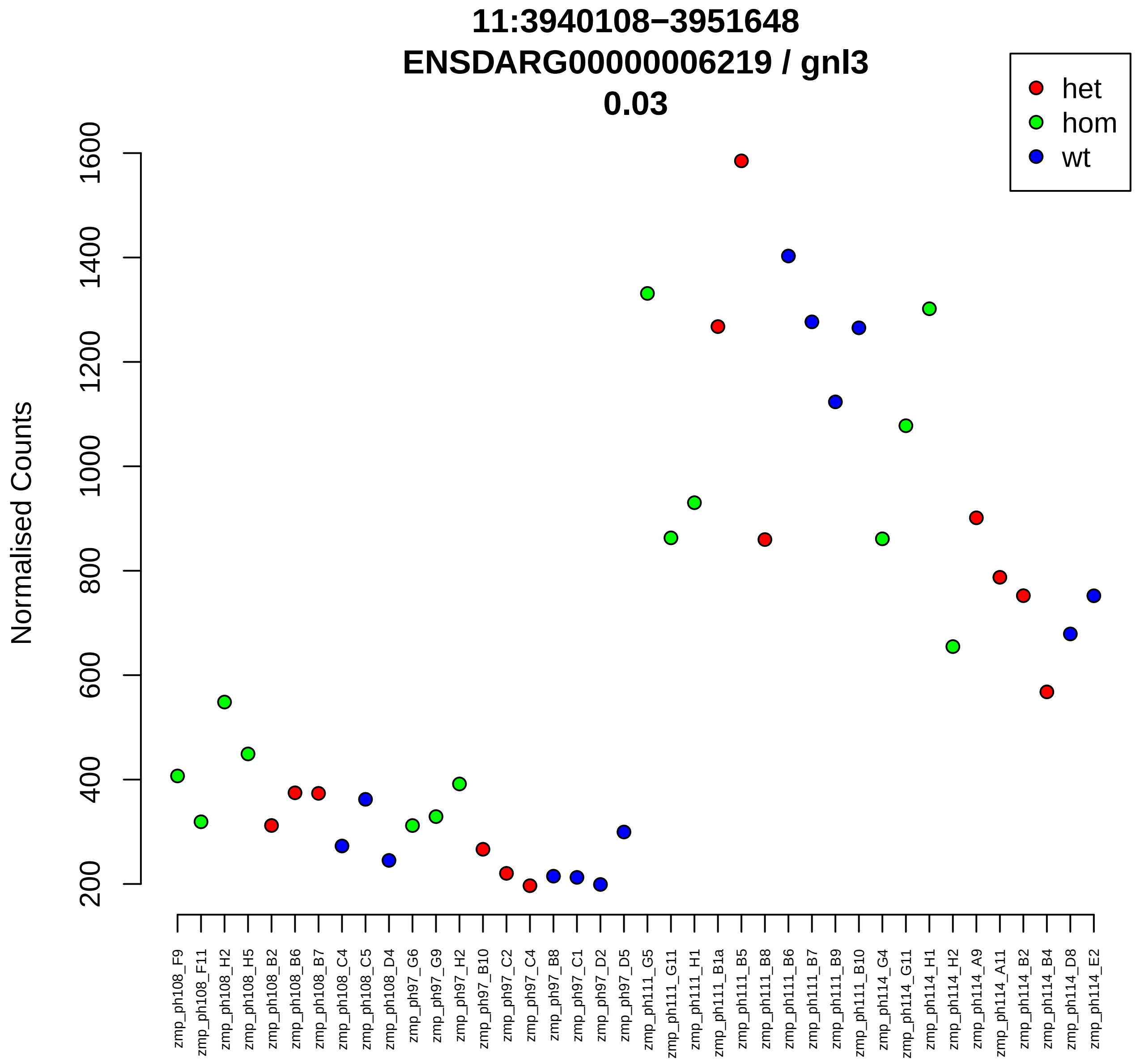

11:3940108−3951648
ENSDARG00000006219 / gnl3
0.03
het
hom
wt
Normalised Counts
200
400
600
800
1000
1200
1400
1600
zmp_ph108_F9
zmp_ph108_F11
zmp_ph108_H2
zmp_ph108_H5
zmp_ph108_B2
zmp_ph108_B6
zmp_ph108_B7
zmp_ph108_C4
zmp_ph108_C5
zmp_ph108_D4
zmp_ph97_G6
zmp_ph97_G9
zmp_ph97_H2
zmp_ph97_B10
zmp_ph97_C2
zmp_ph97_C4
zmp_ph97_B8
zmp_ph97_C1
zmp_ph97_D2
zmp_ph97_D5
zmp_ph111_G5
zmp_ph111_G11
zmp_ph111_H1
zmp_ph111_B1a
zmp_ph111_B5
zmp_ph111_B8
zmp_ph111_B6
zmp_ph111_B7
zmp_ph111_B9
zmp_ph111_B10
zmp_ph114_G4
zmp_ph114_G11
zmp_ph114_H1
zmp_ph114_H2
zmp_ph114_A9
zmp_ph114_A11
zmp_ph114_B2
zmp_ph114_B4
zmp_ph114_D8
zmp_ph114_E2

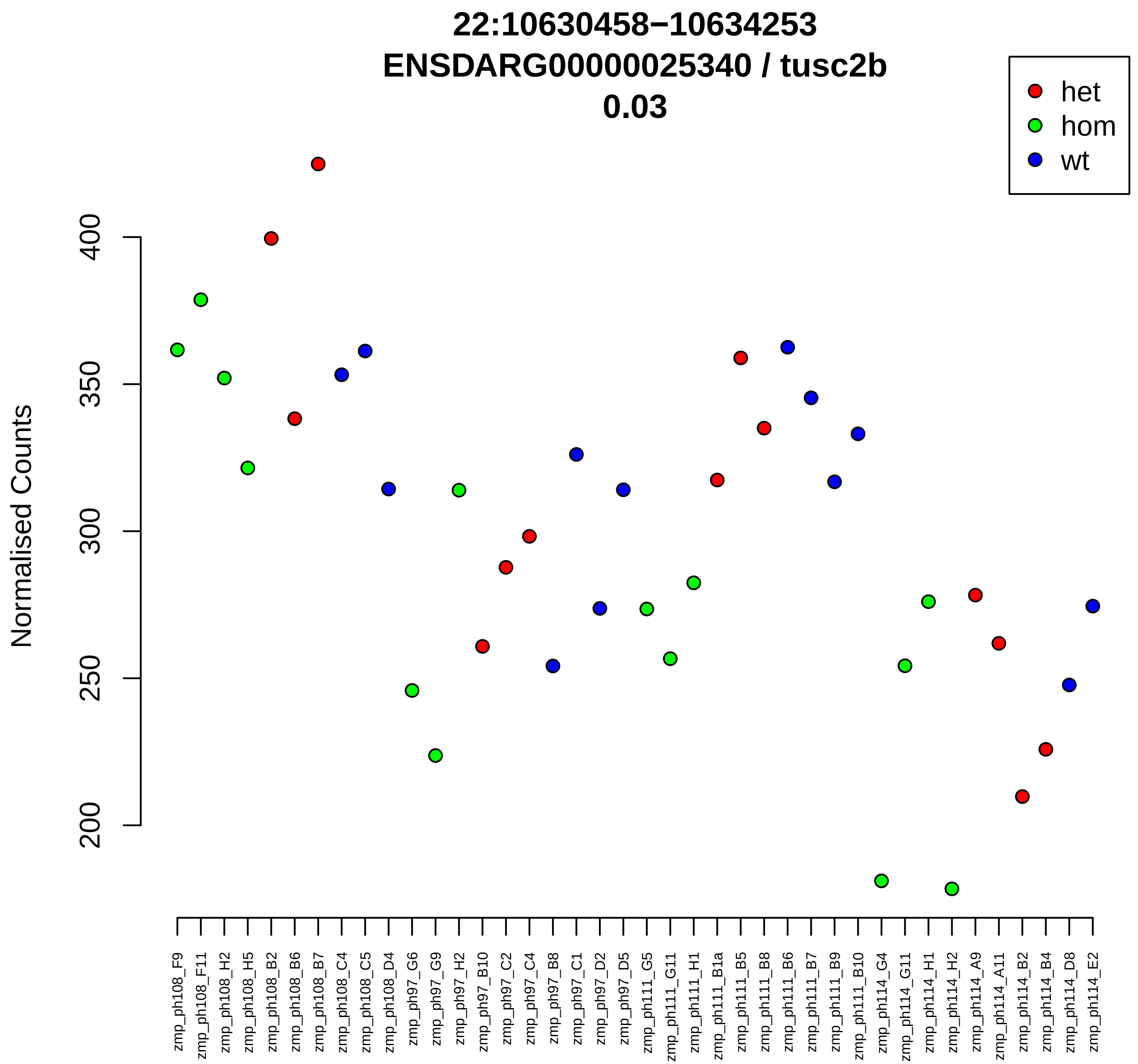
22:10630458−10634253
ENSDARG00000025340 / tusc2b
0.03
het
hom
wt
Normalised Counts
400
350
300
250
200
zmp_ph108_F9
zmp_ph108_F11
zmp_ph108_H2
zmp_ph108_H5
zmp_ph108_B2
zmp_ph108_B6
zmp_ph108_B7
zmp_ph108_C4
zmp_ph108_C5
zmp_ph108_D4
zmp_ph97_G6
zmp_ph97_G9
zmp_ph97_H2
zmp_ph97_B10
zmp_ph97_C2
zmp_ph97_C4
zmp_ph97_B8
zmp_ph97_C1
zmp_ph97_D2
zmp_ph97_D5
zmp_ph111_G5
zmp_ph111_G11
zmp_ph111_H1
zmp_ph111_B1a
zmp_ph111_B5
zmp_ph111_B8
zmp_ph111_B6
zmp_ph111_B7
zmp_ph111_B9
zmp_ph111_B10
zmp_ph114_G4
zmp_ph114_G11
zmp_ph114_H1
zmp_ph114_H2
zmp_ph114_A9
zmp_ph114_A11
zmp_ph114_B2
zmp_ph114_B4
zmp_ph114_D8
zmp_ph114_E2

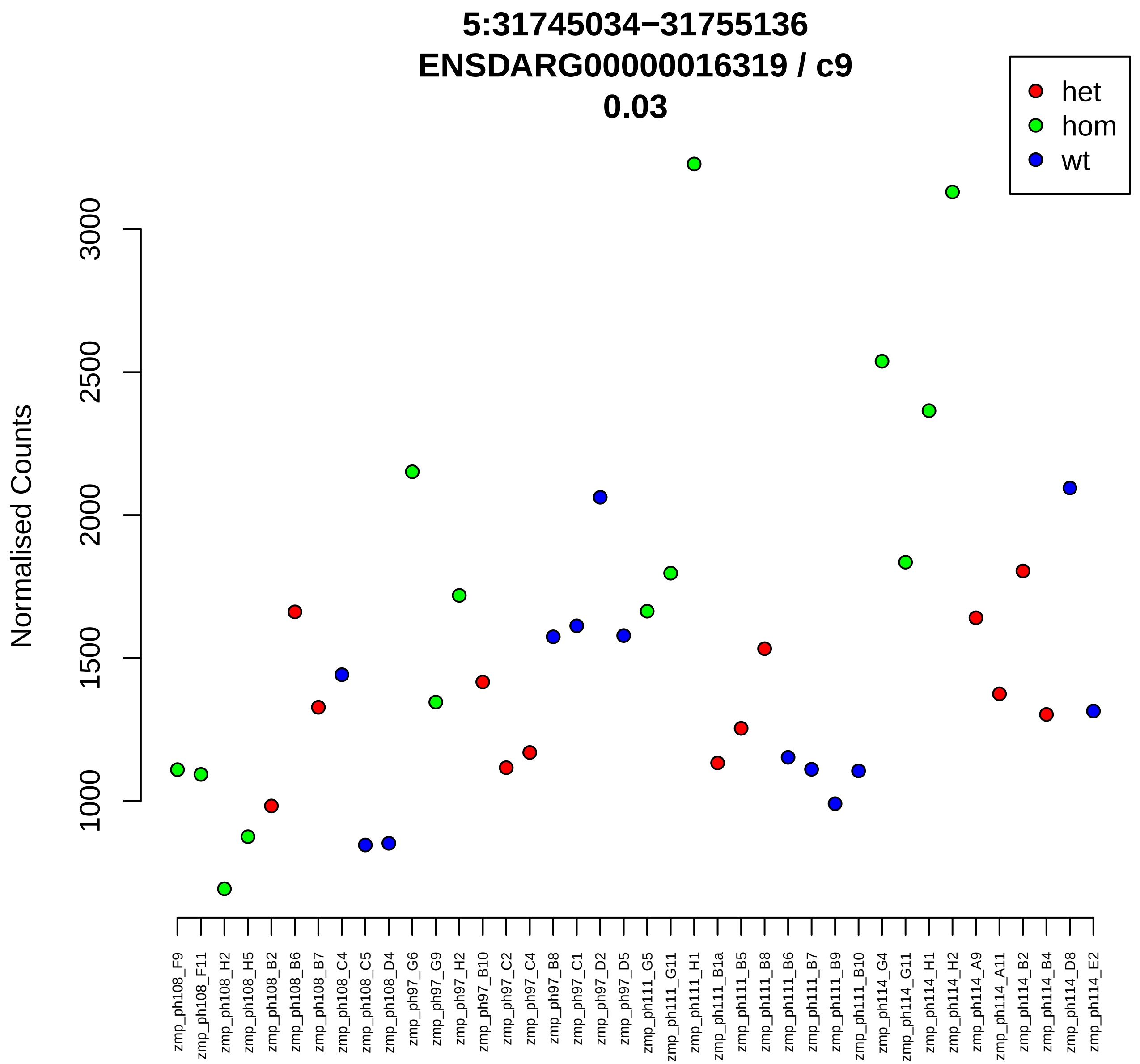

5:31745034−31755136
ENSDARG00000016319 / c9
0.03
het
hom
wt
Normalised Counts
1000
1500
2000
2500
3000
zmp_ph108_F9
zmp_ph108_F11
zmp_ph108_H2
zmp_ph108_H5
zmp_ph108_B2
zmp_ph108_B6
zmp_ph108_B7
zmp_ph108_C4
zmp_ph108_C5
zmp_ph108_D4
zmp_ph97_G6
zmp_ph97_G9
zmp_ph97_H2
zmp_ph97_B10
zmp_ph97_C2
zmp_ph97_C4
zmp_ph97_B8
zmp_ph97_C1
zmp_ph97_D2
zmp_ph97_D5
zmp_ph111_G5
zmp_ph111_G11
zmp_ph111_H1
zmp_ph111_B1a
zmp_ph111_B5
zmp_ph111_B8
zmp_ph111_B6
zmp_ph111_B7
zmp_ph111_B9
zmp_ph111_B10
zmp_ph114_G4
zmp_ph114_G11
zmp_ph114_H1
zmp_ph114_H2
zmp_ph114_A9
zmp_ph114_A11
zmp_ph114_B2
zmp_ph114_B4
zmp_ph114_D8
zmp_ph114_E2

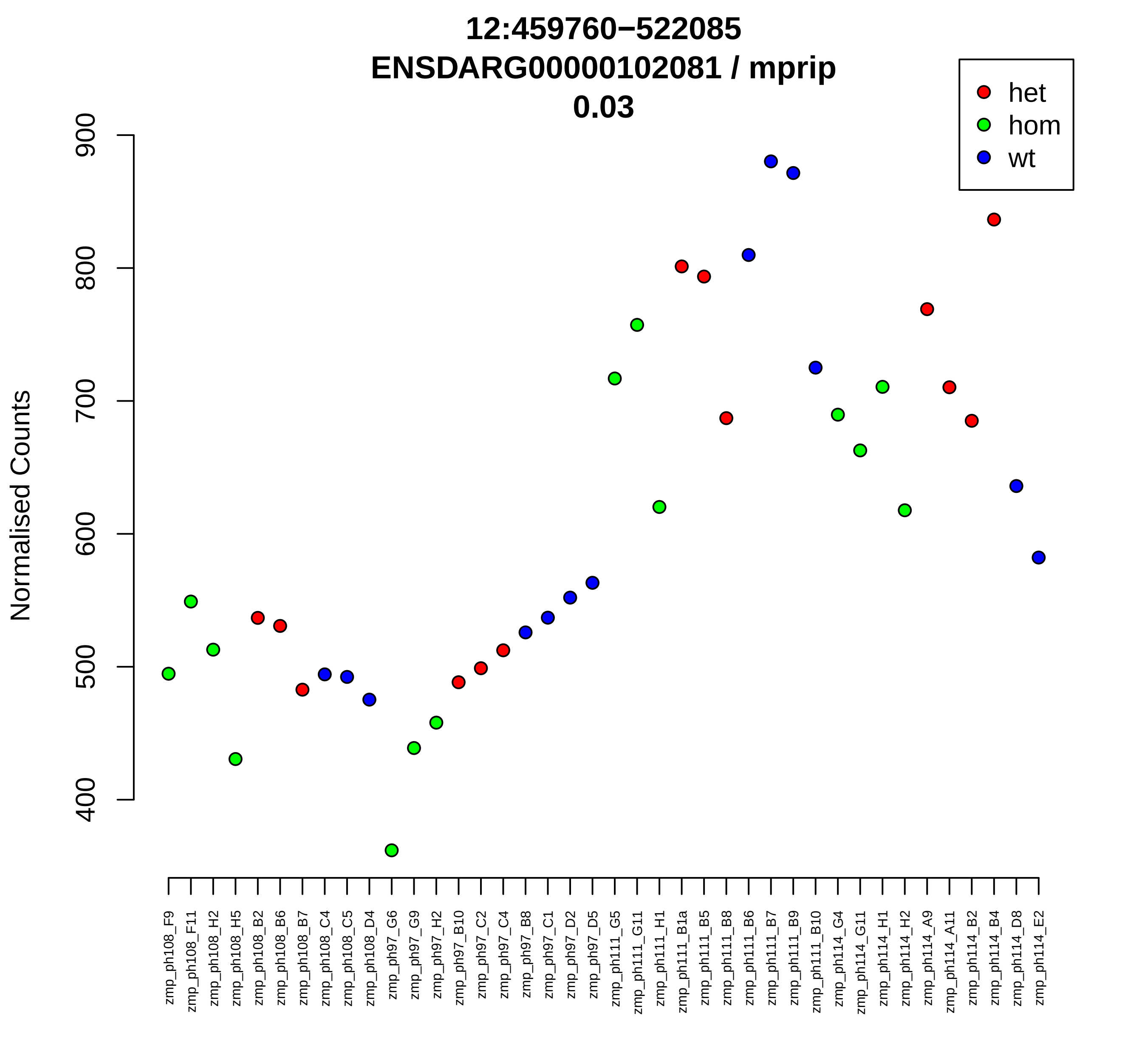

12:459760−522085
ENSDARG00000102081 / mprip
0.03
het
hom
wt
Normalised Counts
400
500
600
700
800
900
zmp_ph108_F9
zmp_ph108_F11
zmp_ph108_H2
zmp_ph108_H5
zmp_ph108_B2
zmp_ph108_B6
zmp_ph108_B7
zmp_ph108_C4
zmp_ph108_C5
zmp_ph108_D4
zmp_ph97_G6
zmp_ph97_G9
zmp_ph97_H2
zmp_ph97_B10
zmp_ph97_C2
zmp_ph97_C4
zmp_ph97_B8
zmp_ph97_C1
zmp_ph97_D2
zmp_ph97_D5
zmp_ph111_G5
zmp_ph111_G11
zmp_ph111_H1
zmp_ph111_B1a
zmp_ph111_B5
zmp_ph111_B8
zmp_ph111_B6
zmp_ph111_B7
zmp_ph111_B9
zmp_ph111_B10
zmp_ph114_G4
zmp_ph114_G11
zmp_ph114_H1
zmp_ph114_H2
zmp_ph114_A9
zmp_ph114_A11
zmp_ph114_B2
zmp_ph114_B4
zmp_ph114_D8
zmp_ph114_E2

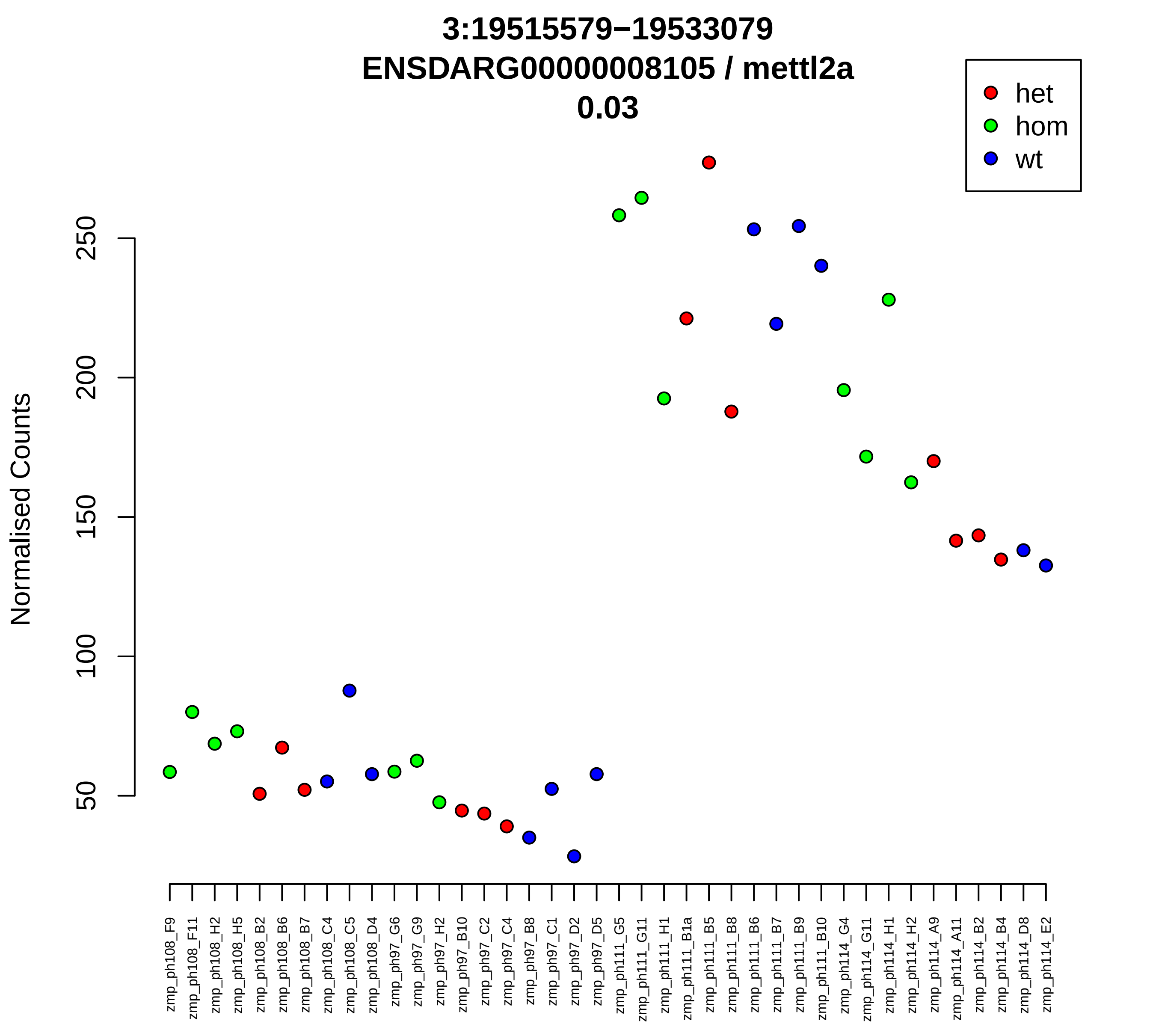

3:19515579−19533079
ENSDARG00000008105 / mettl2a
0.03
het
hom
wt
Normalised Counts
50
100
150
200
250
zmp_ph108_F9
zmp_ph108_F11
zmp_ph108_H2
zmp_ph108_H5
zmp_ph108_B2
zmp_ph108_B6
zmp_ph108_B7
zmp_ph108_C4
zmp_ph108_C5
zmp_ph108_D4
zmp_ph97_G6
zmp_ph97_G9
zmp_ph97_H2
zmp_ph97_B10
zmp_ph97_C2
zmp_ph97_C4
zmp_ph97_B8
zmp_ph97_C1
zmp_ph97_D2
zmp_ph97_D5
zmp_ph111_G5
zmp_ph111_G11
zmp_ph111_H1
zmp_ph111_B1a
zmp_ph111_B5
zmp_ph111_B8
zmp_ph111_B6
zmp_ph111_B7
zmp_ph111_B9
zmp_ph111_B10
zmp_ph114_G4
zmp_ph114_G11
zmp_ph114_H1
zmp_ph114_H2
zmp_ph114_A9
zmp_ph114_A11
zmp_ph114_B2
zmp_ph114_B4
zmp_ph114_D8
zmp_ph114_E2

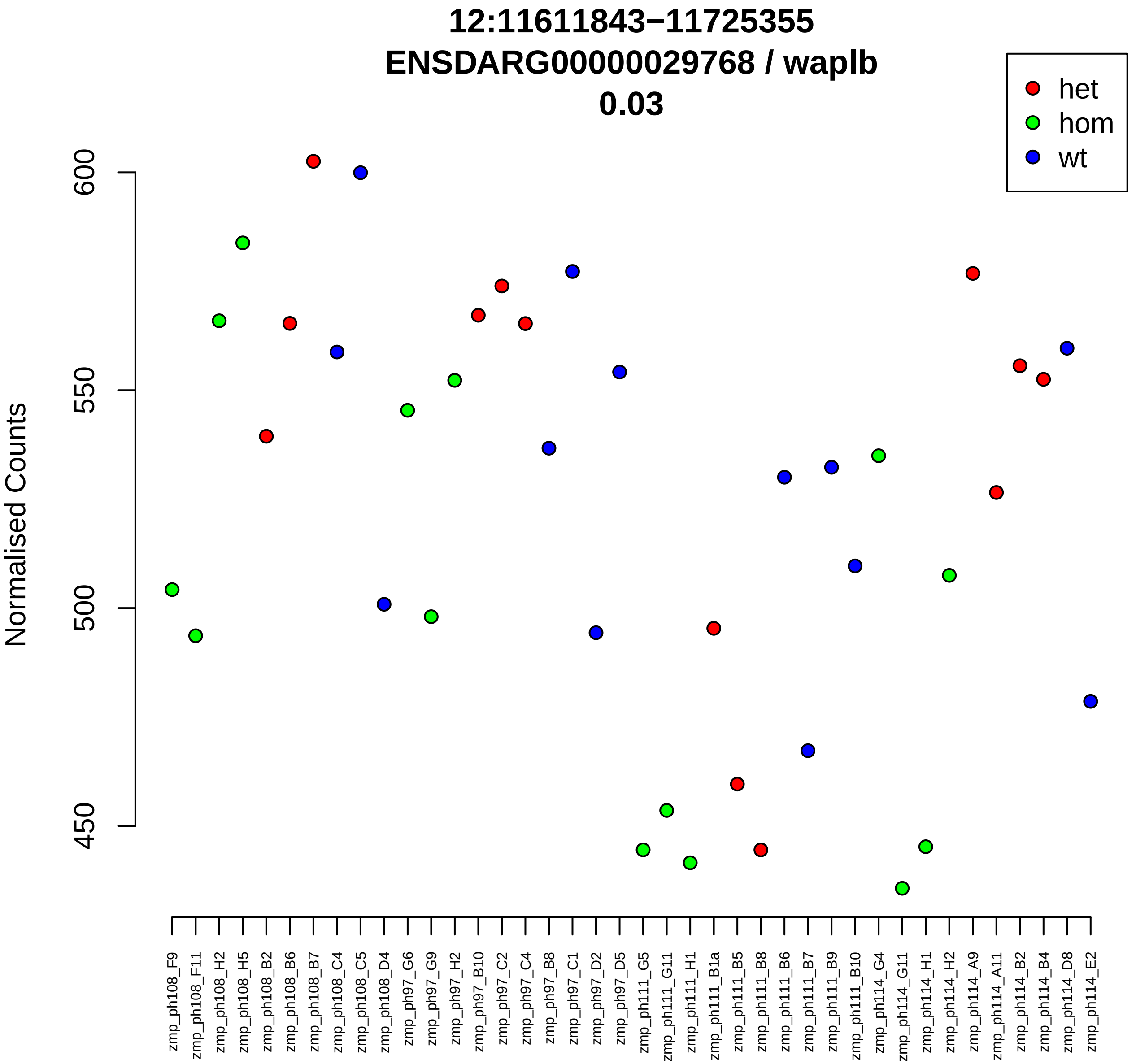

12:11611843−11725355
ENSDARG00000029768 / waplb
0.03
het
hom
wt
Normalised Counts
600
550
500
450
zmp_ph108_F9
zmp_ph108_F11
zmp_ph108_H2
zmp_ph108_H5
zmp_ph108_B2
zmp_ph108_B6
zmp_ph108_B7
zmp_ph108_C4
zmp_ph108_C5
zmp_ph108_D4
zmp_ph97_G6
zmp_ph97_G9
zmp_ph97_H2
zmp_ph97_B10
zmp_ph97_C2
zmp_ph97_C4
zmp_ph97_B8
zmp_ph97_C1
zmp_ph97_D2
zmp_ph97_D5
zmp_ph111_G5
zmp_ph111_G11
zmp_ph111_H1
zmp_ph111_B1a
zmp_ph111_B5
zmp_ph111_B8
zmp_ph111_B6
zmp_ph111_B7
zmp_ph111_B9
zmp_ph111_B10
zmp_ph114_G4
zmp_ph114_G11
zmp_ph114_H1
zmp_ph114_H2
zmp_ph114_A9
zmp_ph114_A11
zmp_ph114_B2
zmp_ph114_B4
zmp_ph114_D8
zmp_ph114_E2

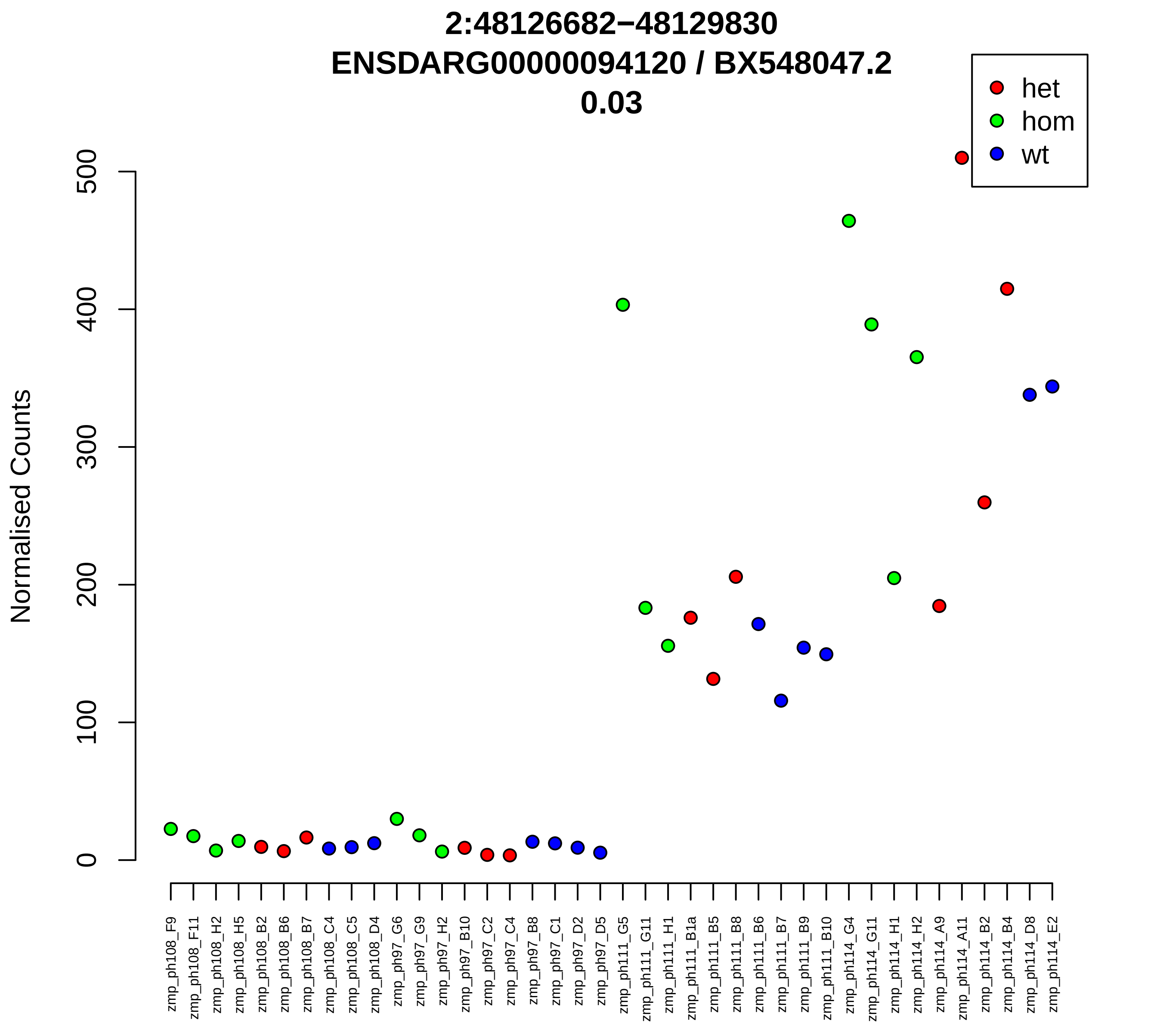

2:48126682−48129830
ENSDARG00000094120 / BX548047.2
0.03
het
hom
wt
Normalised Counts
0
100
200
300
400
500
zmp_ph108_F9
zmp_ph108_F11
zmp_ph108_H2
zmp_ph108_H5
zmp_ph108_B2
zmp_ph108_B6
zmp_ph108_B7
zmp_ph108_C4
zmp_ph108_C5
zmp_ph108_D4
zmp_ph97_G6
zmp_ph97_G9
zmp_ph97_H2
zmp_ph97_B10
zmp_ph97_C2
zmp_ph97_C4
zmp_ph97_B8
zmp_ph97_C1
zmp_ph97_D2
zmp_ph97_D5
zmp_ph111_G5
zmp_ph111_G11
zmp_ph111_H1
zmp_ph111_B1a
zmp_ph111_B5
zmp_ph111_B8
zmp_ph111_B6
zmp_ph111_B7
zmp_ph111_B9
zmp_ph111_B10
zmp_ph114_G4
zmp_ph114_G11
zmp_ph114_H1
zmp_ph114_H2
zmp_ph114_A9
zmp_ph114_A11
zmp_ph114_B2
zmp_ph114_B4
zmp_ph114_D8
zmp_ph114_E2

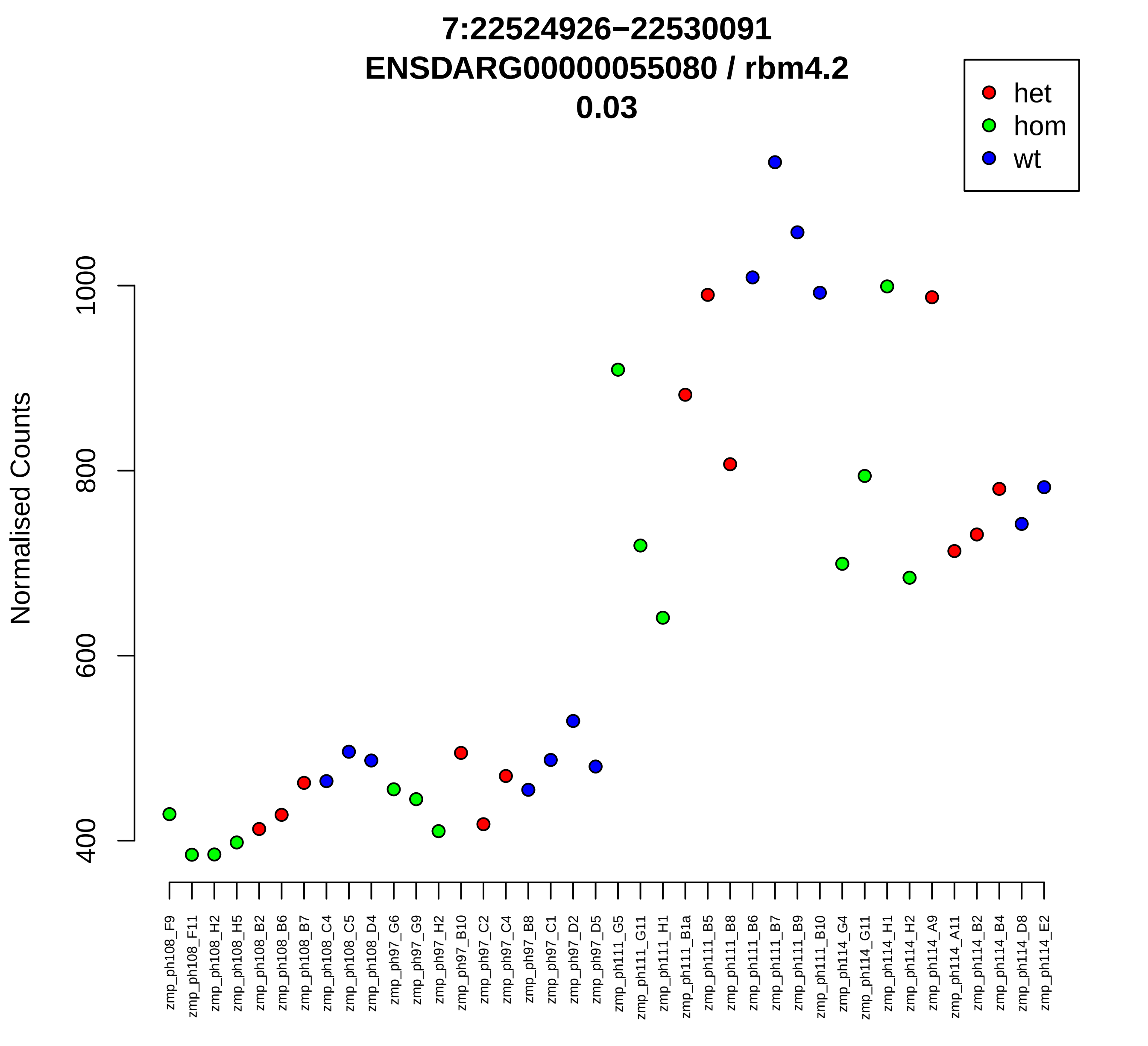

7:22524926−22530091
ENSDARG00000055080 / rbm4.2
0.03
het
hom
wt
Normalised Counts
400
600
800
1000
zmp_ph108_F9
zmp_ph108_F11
zmp_ph108_H2
zmp_ph108_H5
zmp_ph108_B2
zmp_ph108_B6
zmp_ph108_B7
zmp_ph108_C4
zmp_ph108_C5
zmp_ph108_D4
zmp_ph97_G6
zmp_ph97_G9
zmp_ph97_H2
zmp_ph97_B10
zmp_ph97_C2
zmp_ph97_C4
zmp_ph97_B8
zmp_ph97_C1
zmp_ph97_D2
zmp_ph97_D5
zmp_ph111_G5
zmp_ph111_G11
zmp_ph111_H1
zmp_ph111_B1a
zmp_ph111_B5
zmp_ph111_B8
zmp_ph111_B6
zmp_ph111_B7
zmp_ph111_B9
zmp_ph111_B10
zmp_ph114_G4
zmp_ph114_G11
zmp_ph114_H1
zmp_ph114_H2
zmp_ph114_A9
zmp_ph114_A11
zmp_ph114_B2
zmp_ph114_B4
zmp_ph114_D8
zmp_ph114_E2

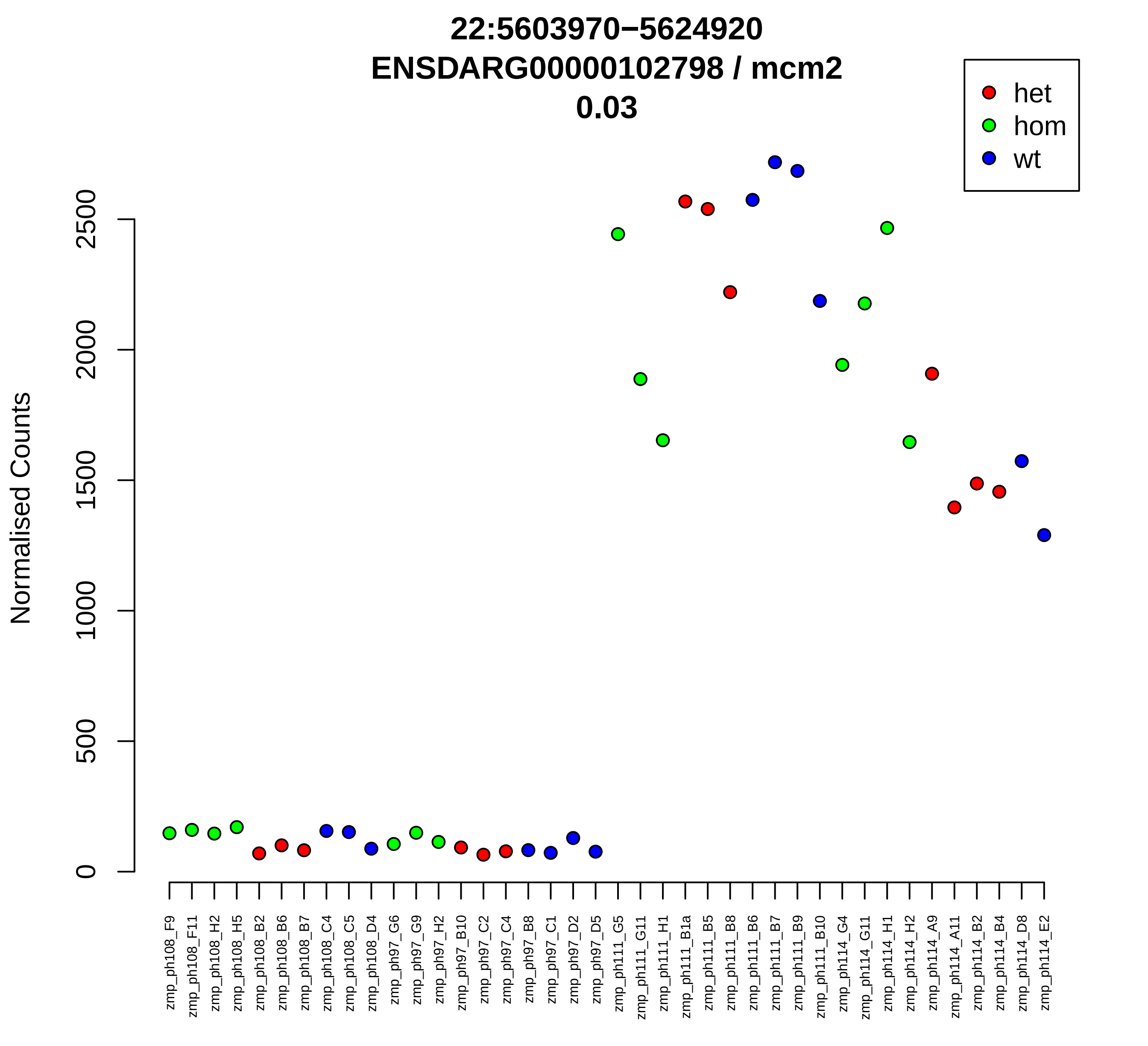
22:5603970−5624920
ENSDARG00000102798 / mcm2
0.03
het
hom
wt
Normalised Counts
0
500
1000
1500
2000
2500
zmp_ph108_F9
zmp_ph108_F11
zmp_ph108_H2
zmp_ph108_H5
zmp_ph108_B2
zmp_ph108_B6
zmp_ph108_B7
zmp_ph108_C4
zmp_ph108_C5
zmp_ph108_D4
zmp_ph97_G6
zmp_ph97_G9
zmp_ph97_H2
zmp_ph97_B10
zmp_ph97_C2
zmp_ph97_C4
zmp_ph97_B8
zmp_ph97_C1
zmp_ph97_D2
zmp_ph97_D5
zmp_ph111_G5
zmp_ph111_G11
zmp_ph111_H1
zmp_ph111_B1a
zmp_ph111_B5
zmp_ph111_B8
zmp_ph111_B6
zmp_ph111_B7
zmp_ph111_B9
zmp_ph111_B10
zmp_ph114_G4
zmp_ph114_G11
zmp_ph114_H1
zmp_ph114_H2
zmp_ph114_A9
zmp_ph114_A11
zmp_ph114_B2
zmp_ph114_B4
zmp_ph114_D8
zmp_ph114_E2

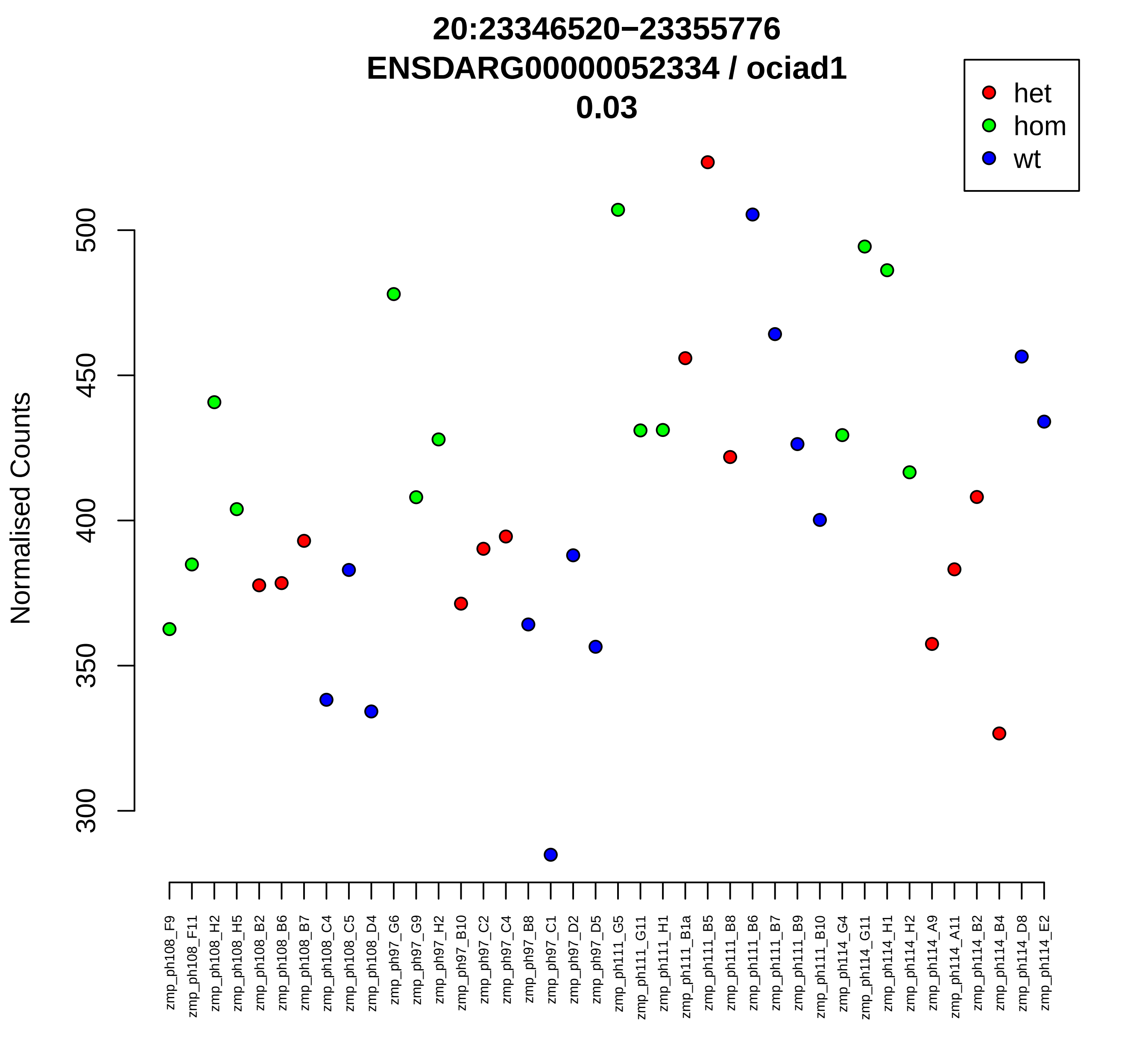

20:23346520−23355776
ENSDARG00000052334 / ociad1
0.03
het
hom
wt
Normalised Counts
300
350
400
450
500
zmp_ph108_F9
zmp_ph108_F11
zmp_ph108_H2
zmp_ph108_H5
zmp_ph108_B2
zmp_ph108_B6
zmp_ph108_B7
zmp_ph108_C4
zmp_ph108_C5
zmp_ph108_D4
zmp_ph97_G6
zmp_ph97_G9
zmp_ph97_H2
zmp_ph97_B10
zmp_ph97_C2
zmp_ph97_C4
zmp_ph97_B8
zmp_ph97_C1
zmp_ph97_D2
zmp_ph97_D5
zmp_ph111_G5
zmp_ph111_G11
zmp_ph111_H1
zmp_ph111_B1a
zmp_ph111_B5
zmp_ph111_B8
zmp_ph111_B6
zmp_ph111_B7
zmp_ph111_B9
zmp_ph111_B10
zmp_ph114_G4
zmp_ph114_G11
zmp_ph114_H1
zmp_ph114_H2
zmp_ph114_A9
zmp_ph114_A11
zmp_ph114_B2
zmp_ph114_B4
zmp_ph114_D8
zmp_ph114_E2

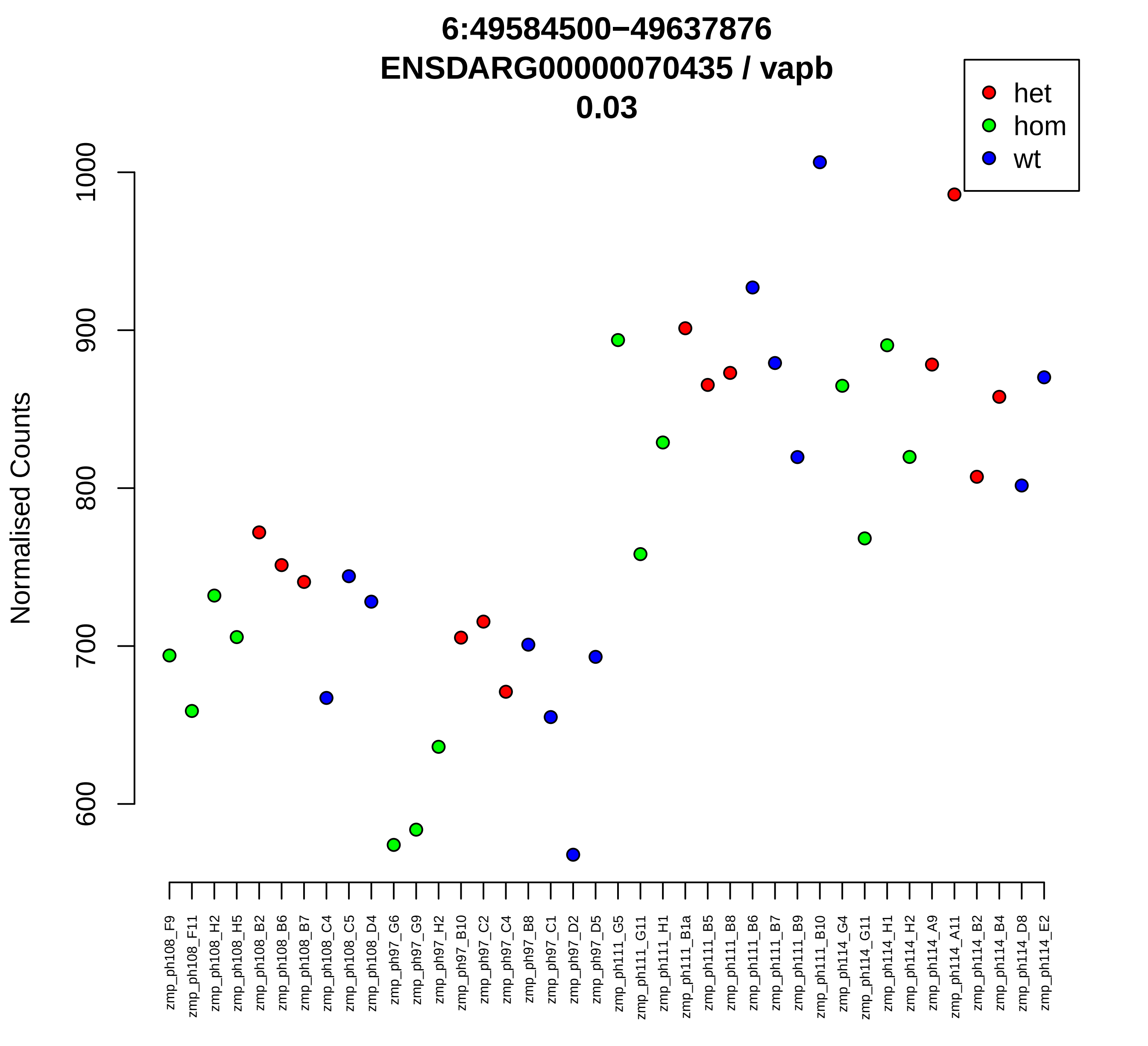
6:49584500−49637876
ENSDARG00000070435 / vapb
0.03
het
hom
wt
Normalised Counts
600
700
800
900
1000
zmp_ph108_F9
zmp_ph108_F11
zmp_ph108_H2
zmp_ph108_H5
zmp_ph108_B2
zmp_ph108_B6
zmp_ph108_B7
zmp_ph108_C4
zmp_ph108_C5
zmp_ph108_D4
zmp_ph97_G6
zmp_ph97_G9
zmp_ph97_H2
zmp_ph97_B10
zmp_ph97_C2
zmp_ph97_C4
zmp_ph97_B8
zmp_ph97_C1
zmp_ph97_D2
zmp_ph97_D5
zmp_ph111_G5
zmp_ph111_G11
zmp_ph111_H1
zmp_ph111_B1a
zmp_ph111_B5
zmp_ph111_B8
zmp_ph111_B6
zmp_ph111_B7
zmp_ph111_B9
zmp_ph111_B10
zmp_ph114_G4
zmp_ph114_G11
zmp_ph114_H1
zmp_ph114_H2
zmp_ph114_A9
zmp_ph114_A11
zmp_ph114_B2
zmp_ph114_B4
zmp_ph114_D8
zmp_ph114_E2

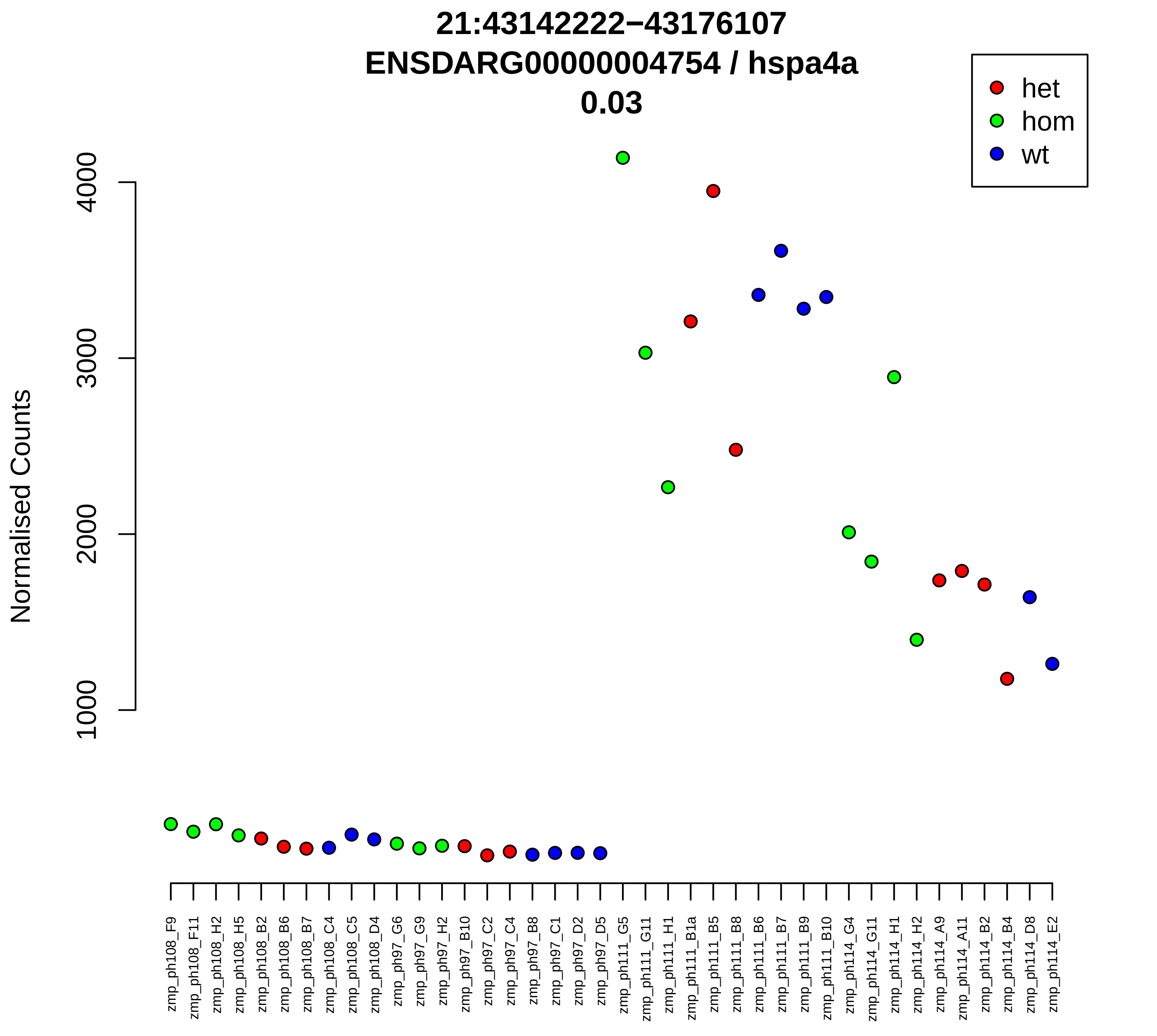
21:43142222−43176107
ENSDARG00000004754 / hspa4a
0.03
het
hom
wt
Normalised Counts
1000
2000
3000
4000
zmp_ph108_F9
zmp_ph108_F11
zmp_ph108_H2
zmp_ph108_H5
zmp_ph108_B2
zmp_ph108_B6
zmp_ph108_B7
zmp_ph108_C4
zmp_ph108_C5
zmp_ph108_D4
zmp_ph97_G6
zmp_ph97_G9
zmp_ph97_H2
zmp_ph97_B10
zmp_ph97_C2
zmp_ph97_C4
zmp_ph97_B8
zmp_ph97_C1
zmp_ph97_D2
zmp_ph97_D5
zmp_ph111_G5
zmp_ph111_G11
zmp_ph111_H1
zmp_ph111_B1a
zmp_ph111_B5
zmp_ph111_B8
zmp_ph111_B6
zmp_ph111_B7
zmp_ph111_B9
zmp_ph111_B10
zmp_ph114_G4
zmp_ph114_G11
zmp_ph114_H1
zmp_ph114_H2
zmp_ph114_A9
zmp_ph114_A11
zmp_ph114_B2
zmp_ph114_B4
zmp_ph114_D8
zmp_ph114_E2

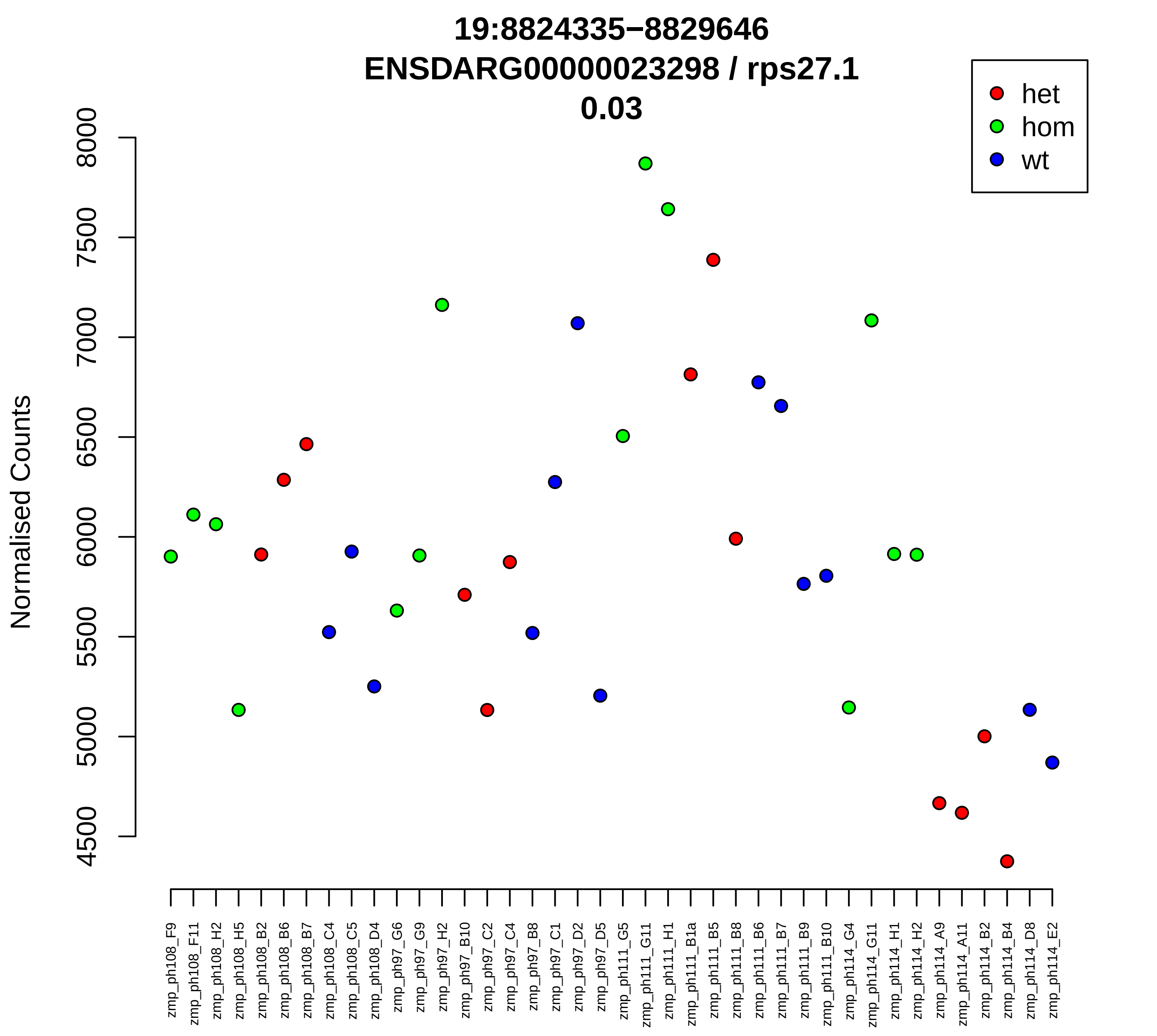
19:8824335−8829646
ENSDARG00000023298 / rps27.1
0.03
het
hom
wt
Normalised Counts
4500
5000
5500
6000
6500
7000
7500
8000
zmp_ph108_F9
zmp_ph108_F11
zmp_ph108_H2
zmp_ph108_H5
zmp_ph108_B2
zmp_ph108_B6
zmp_ph108_B7
zmp_ph108_C4
zmp_ph108_C5
zmp_ph108_D4
zmp_ph97_G6
zmp_ph97_G9
zmp_ph97_H2
zmp_ph97_B10
zmp_ph97_C2
zmp_ph97_C4
zmp_ph97_B8
zmp_ph97_C1
zmp_ph97_D2
zmp_ph97_D5
zmp_ph111_G5
zmp_ph111_G11
zmp_ph111_H1
zmp_ph111_B1a
zmp_ph111_B5
zmp_ph111_B8
zmp_ph111_B6
zmp_ph111_B7
zmp_ph111_B9
zmp_ph111_B10
zmp_ph114_G4
zmp_ph114_G11
zmp_ph114_H1
zmp_ph114_H2
zmp_ph114_A9
zmp_ph114_A11
zmp_ph114_B2
zmp_ph114_B4
zmp_ph114_D8
zmp_ph114_E2

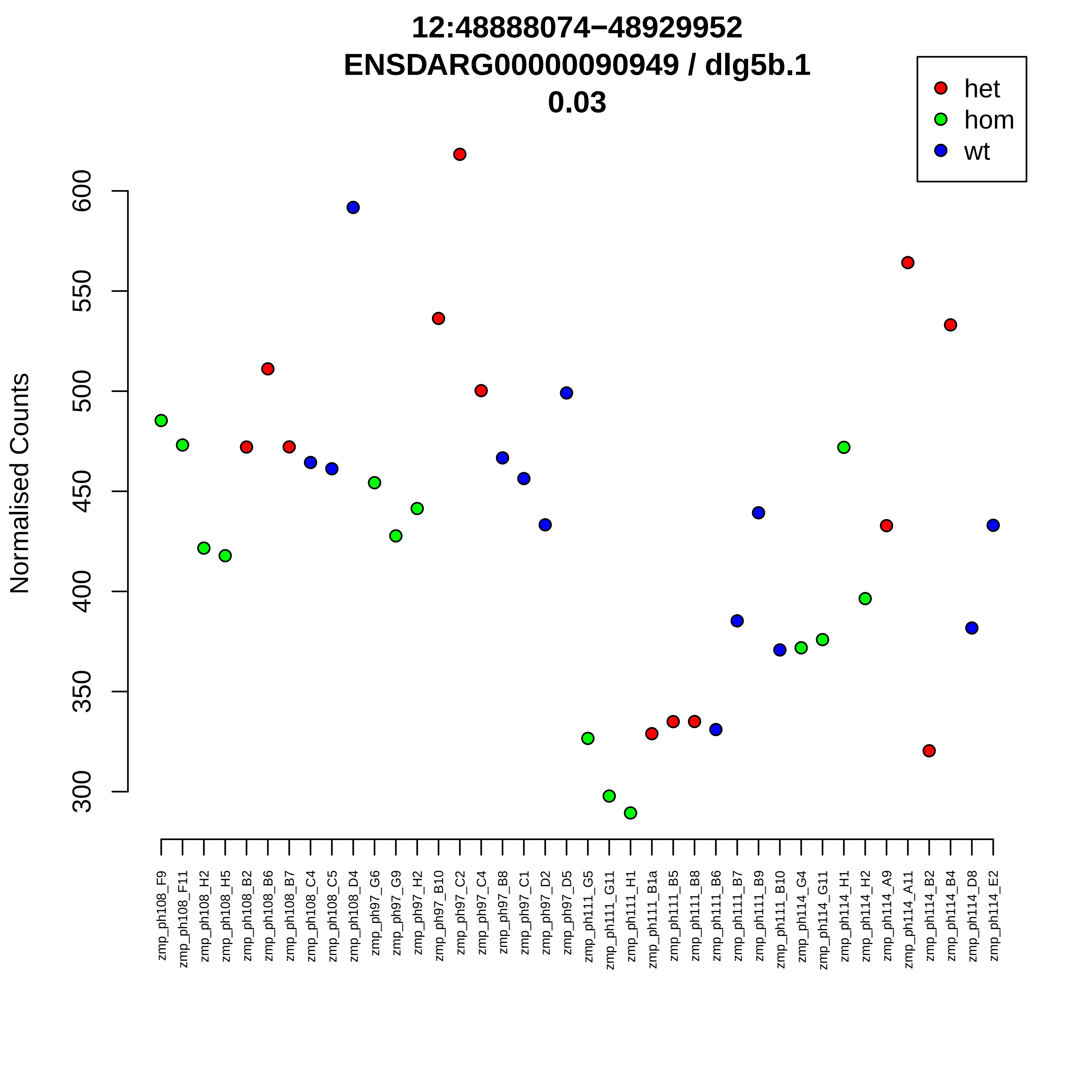

12:48888074−48929952
ENSDARG00000090949 / dlg5b.1
0.03
het
hom
wt
Normalised Counts
300
350
400
450
500
550
600
zmp_ph108_F9
zmp_ph108_F11
zmp_ph108_H2
zmp_ph108_H5
zmp_ph108_B2
zmp_ph108_B6
zmp_ph108_B7
zmp_ph108_C4
zmp_ph108_C5
zmp_ph108_D4
zmp_ph97_G6
zmp_ph97_G9
zmp_ph97_H2
zmp_ph97_B10
zmp_ph97_C2
zmp_ph97_C4
zmp_ph97_B8
zmp_ph97_C1
zmp_ph97_D2
zmp_ph97_D5
zmp_ph111_G5
zmp_ph111_G11
zmp_ph111_H1
zmp_ph111_B1a
zmp_ph111_B5
zmp_ph111_B8
zmp_ph111_B6
zmp_ph111_B7
zmp_ph111_B9
zmp_ph111_B10
zmp_ph114_G4
zmp_ph114_G11
zmp_ph114_H1
zmp_ph114_H2
zmp_ph114_A9
zmp_ph114_A11
zmp_ph114_B2
zmp_ph114_B4
zmp_ph114_D8
zmp_ph114_E2

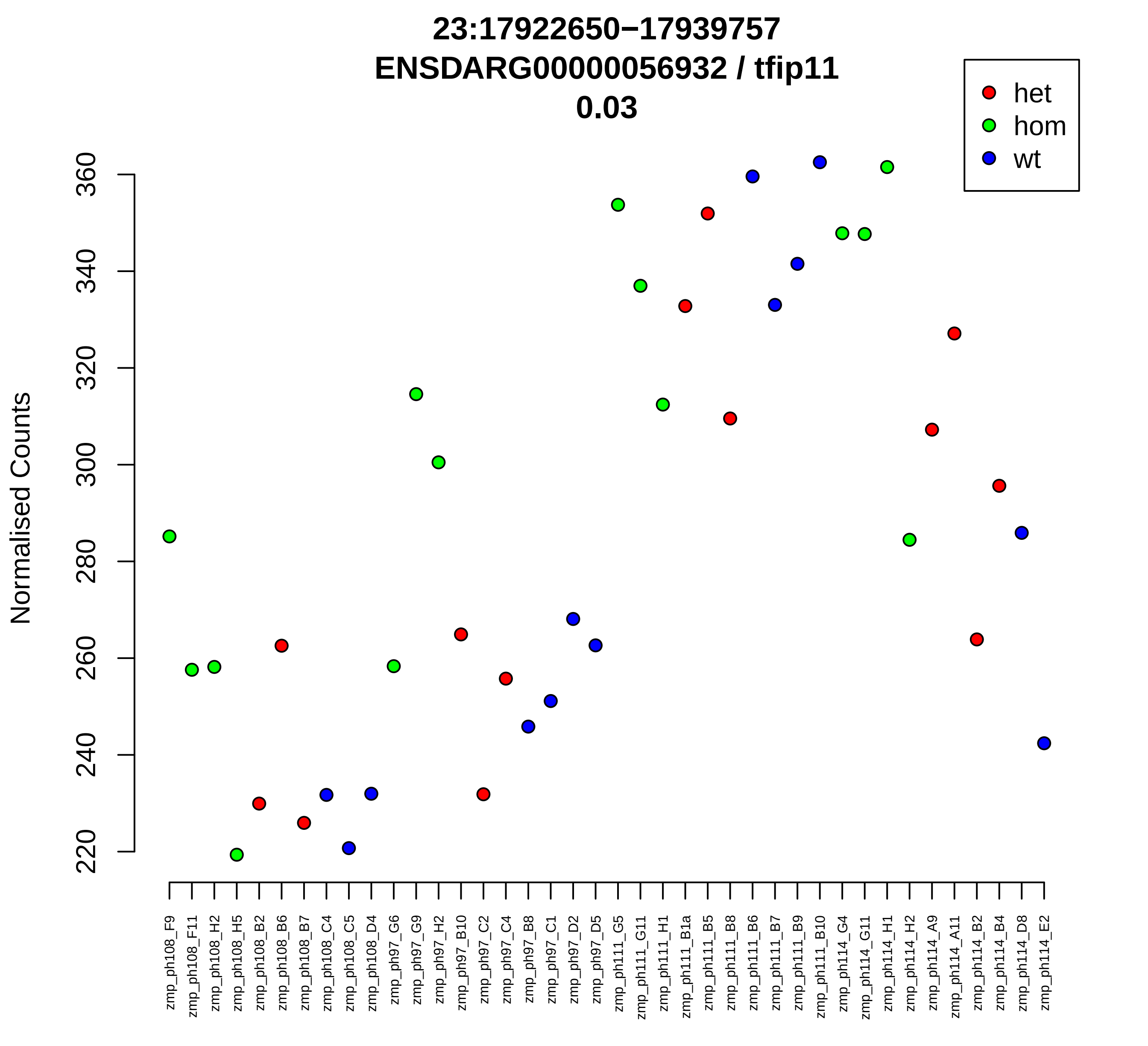

23:17922650−17939757
ENSDARG00000056932 / tfip11
0.03
het
hom
wt
Normalised Counts
220
240
260
280
300
320
340
360
zmp_ph108_F9
zmp_ph108_F11
zmp_ph108_H2
zmp_ph108_H5
zmp_ph108_B2
zmp_ph108_B6
zmp_ph108_B7
zmp_ph108_C4
zmp_ph108_C5
zmp_ph108_D4
zmp_ph97_G6
zmp_ph97_G9
zmp_ph97_H2
zmp_ph97_B10
zmp_ph97_C2
zmp_ph97_C4
zmp_ph97_B8
zmp_ph97_C1
zmp_ph97_D2
zmp_ph97_D5
zmp_ph111_G5
zmp_ph111_G11
zmp_ph111_H1
zmp_ph111_B1a
zmp_ph111_B5
zmp_ph111_B8
zmp_ph111_B6
zmp_ph111_B7
zmp_ph111_B9
zmp_ph111_B10
zmp_ph114_G4
zmp_ph114_G11
zmp_ph114_H1
zmp_ph114_H2
zmp_ph114_A9
zmp_ph114_A11
zmp_ph114_B2
zmp_ph114_B4
zmp_ph114_D8
zmp_ph114_E2

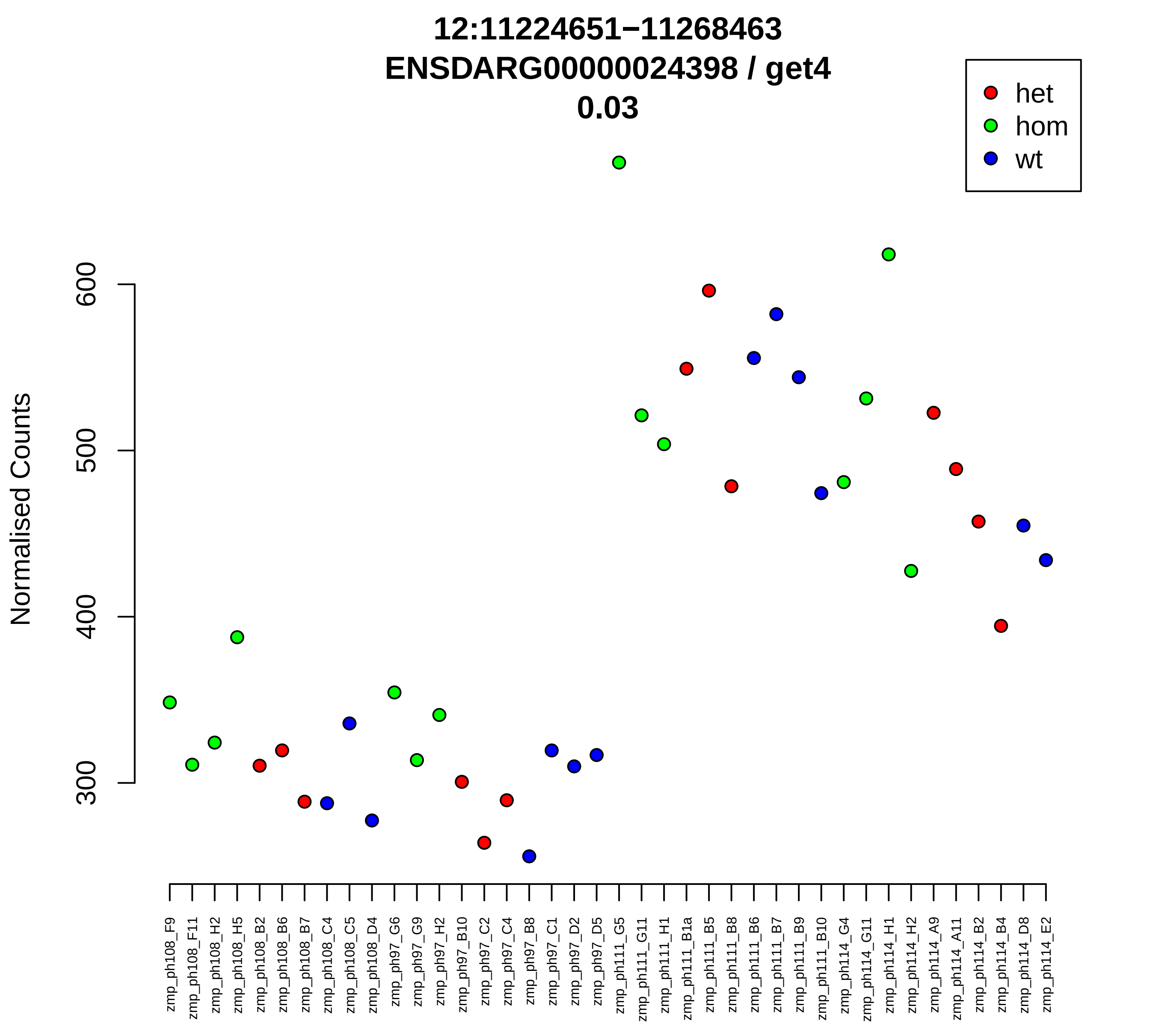

12:11224651−11268463
ENSDARG00000024398 / get4
0.03
het
hom
wt
Normalised Counts
300
400
500
600
zmp_ph108_F9
zmp_ph108_F11
zmp_ph108_H2
zmp_ph108_H5
zmp_ph108_B2
zmp_ph108_B6
zmp_ph108_B7
zmp_ph108_C4
zmp_ph108_C5
zmp_ph108_D4
zmp_ph97_G6
zmp_ph97_G9
zmp_ph97_H2
zmp_ph97_B10
zmp_ph97_C2
zmp_ph97_C4
zmp_ph97_B8
zmp_ph97_C1
zmp_ph97_D2
zmp_ph97_D5
zmp_ph111_G5
zmp_ph111_G11
zmp_ph111_H1
zmp_ph111_B1a
zmp_ph111_B5
zmp_ph111_B8
zmp_ph111_B6
zmp_ph111_B7
zmp_ph111_B9
zmp_ph111_B10
zmp_ph114_G4
zmp_ph114_G11
zmp_ph114_H1
zmp_ph114_H2
zmp_ph114_A9
zmp_ph114_A11
zmp_ph114_B2
zmp_ph114_B4
zmp_ph114_D8
zmp_ph114_E2

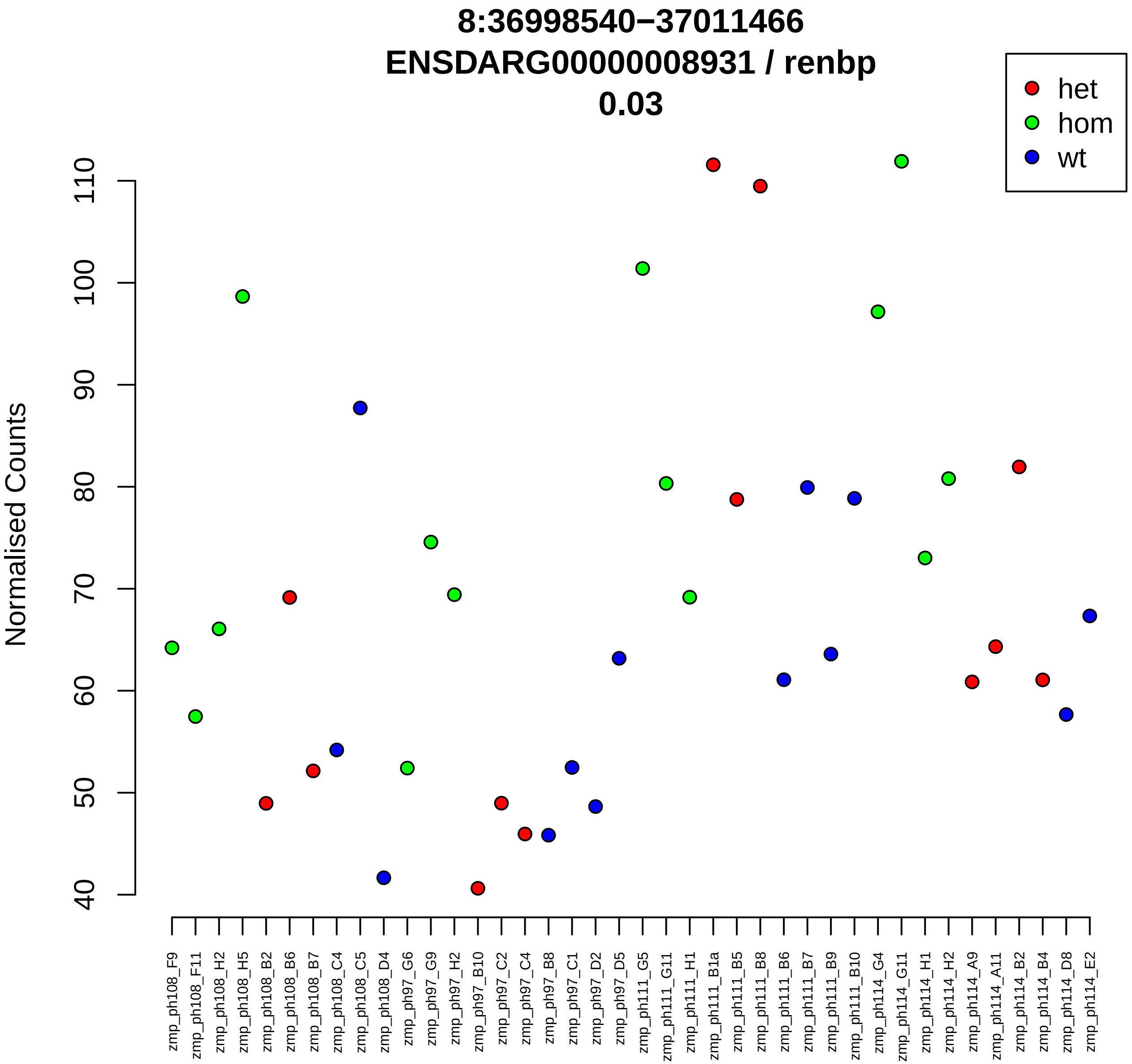
8:36998540−37011466
ENSDARG00000008931 / renbp
0.03
het
hom
wt
Normalised Counts
40
50
60
70
80
90
100
110
zmp_ph108_F9
zmp_ph108_F11
zmp_ph108_H2
zmp_ph108_H5
zmp_ph108_B2
zmp_ph108_B6
zmp_ph108_B7
zmp_ph108_C4
zmp_ph108_C5
zmp_ph108_D4
zmp_ph97_G6
zmp_ph97_G9
zmp_ph97_H2
zmp_ph97_B10
zmp_ph97_C2
zmp_ph97_C4
zmp_ph97_B8
zmp_ph97_C1
zmp_ph97_D2
zmp_ph97_D5
zmp_ph111_G5
zmp_ph111_G11
zmp_ph111_H1
zmp_ph111_B1a
zmp_ph111_B5
zmp_ph111_B8
zmp_ph111_B6
zmp_ph111_B7
zmp_ph111_B9
zmp_ph111_B10
zmp_ph114_G4
zmp_ph114_G11
zmp_ph114_H1
zmp_ph114_H2
zmp_ph114_A9
zmp_ph114_A11
zmp_ph114_B2
zmp_ph114_B4
zmp_ph114_D8
zmp_ph114_E2

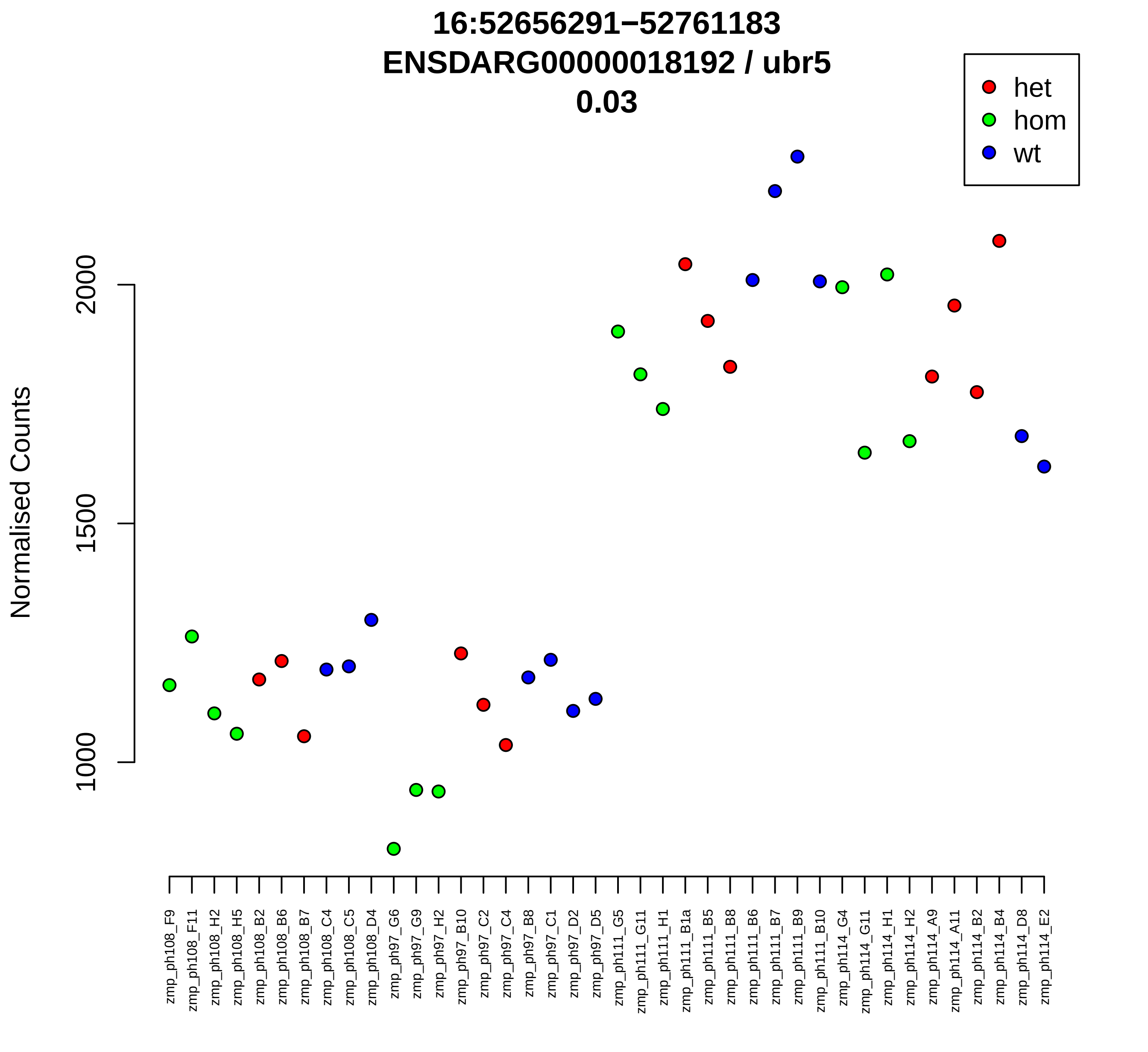

16:52656291−52761183
ENSDARG00000018192 / ubr5
0.03
het
hom
wt
Normalised Counts
1000
1500
2000
zmp_ph108_F9
zmp_ph108_F11
zmp_ph108_H2
zmp_ph108_H5
zmp_ph108_B2
zmp_ph108_B6
zmp_ph108_B7
zmp_ph108_C4
zmp_ph108_C5
zmp_ph108_D4
zmp_ph97_G6
zmp_ph97_G9
zmp_ph97_H2
zmp_ph97_B10
zmp_ph97_C2
zmp_ph97_C4
zmp_ph97_B8
zmp_ph97_C1
zmp_ph97_D2
zmp_ph97_D5
zmp_ph111_G5
zmp_ph111_G11
zmp_ph111_H1
zmp_ph111_B1a
zmp_ph111_B5
zmp_ph111_B8
zmp_ph111_B6
zmp_ph111_B7
zmp_ph111_B9
zmp_ph111_B10
zmp_ph114_G4
zmp_ph114_G11
zmp_ph114_H1
zmp_ph114_H2
zmp_ph114_A9
zmp_ph114_A11
zmp_ph114_B2
zmp_ph114_B4
zmp_ph114_D8
zmp_ph114_E2

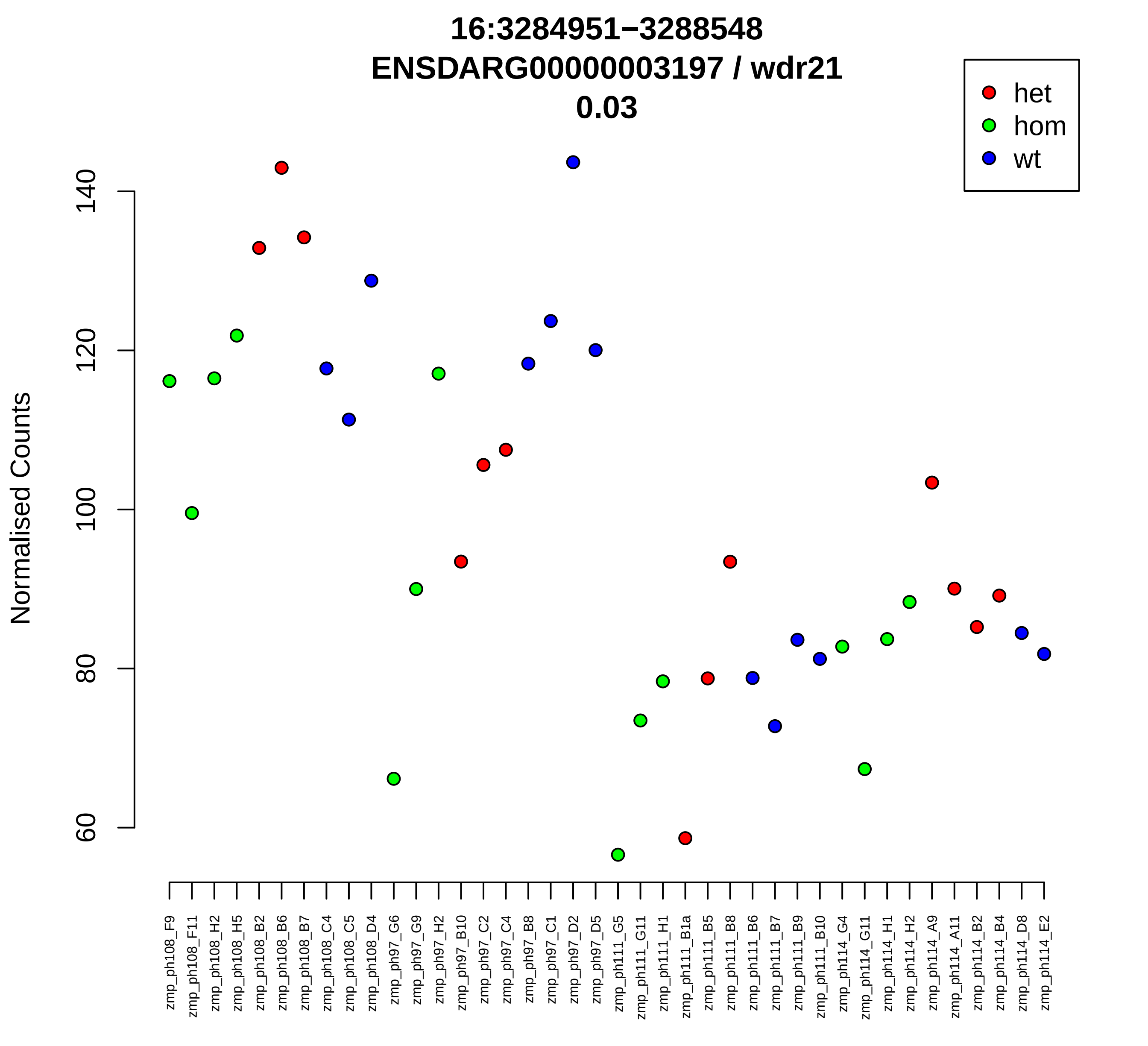

16:3284951−3288548
ENSDARG00000003197 / wdr21
0.03
het
hom
wt
Normalised Counts
60
80
100
120
140
zmp_ph108_F9
zmp_ph108_F11
zmp_ph108_H2
zmp_ph108_H5
zmp_ph108_B2
zmp_ph108_B6
zmp_ph108_B7
zmp_ph108_C4
zmp_ph108_C5
zmp_ph108_D4
zmp_ph97_G6
zmp_ph97_G9
zmp_ph97_H2
zmp_ph97_B10
zmp_ph97_C2
zmp_ph97_C4
zmp_ph97_B8
zmp_ph97_C1
zmp_ph97_D2
zmp_ph97_D5
zmp_ph111_G5
zmp_ph111_G11
zmp_ph111_H1
zmp_ph111_B1a
zmp_ph111_B5
zmp_ph111_B8
zmp_ph111_B6
zmp_ph111_B7
zmp_ph111_B9
zmp_ph111_B10
zmp_ph114_G4
zmp_ph114_G11
zmp_ph114_H1
zmp_ph114_H2
zmp_ph114_A9
zmp_ph114_A11
zmp_ph114_B2
zmp_ph114_B4
zmp_ph114_D8
zmp_ph114_E2

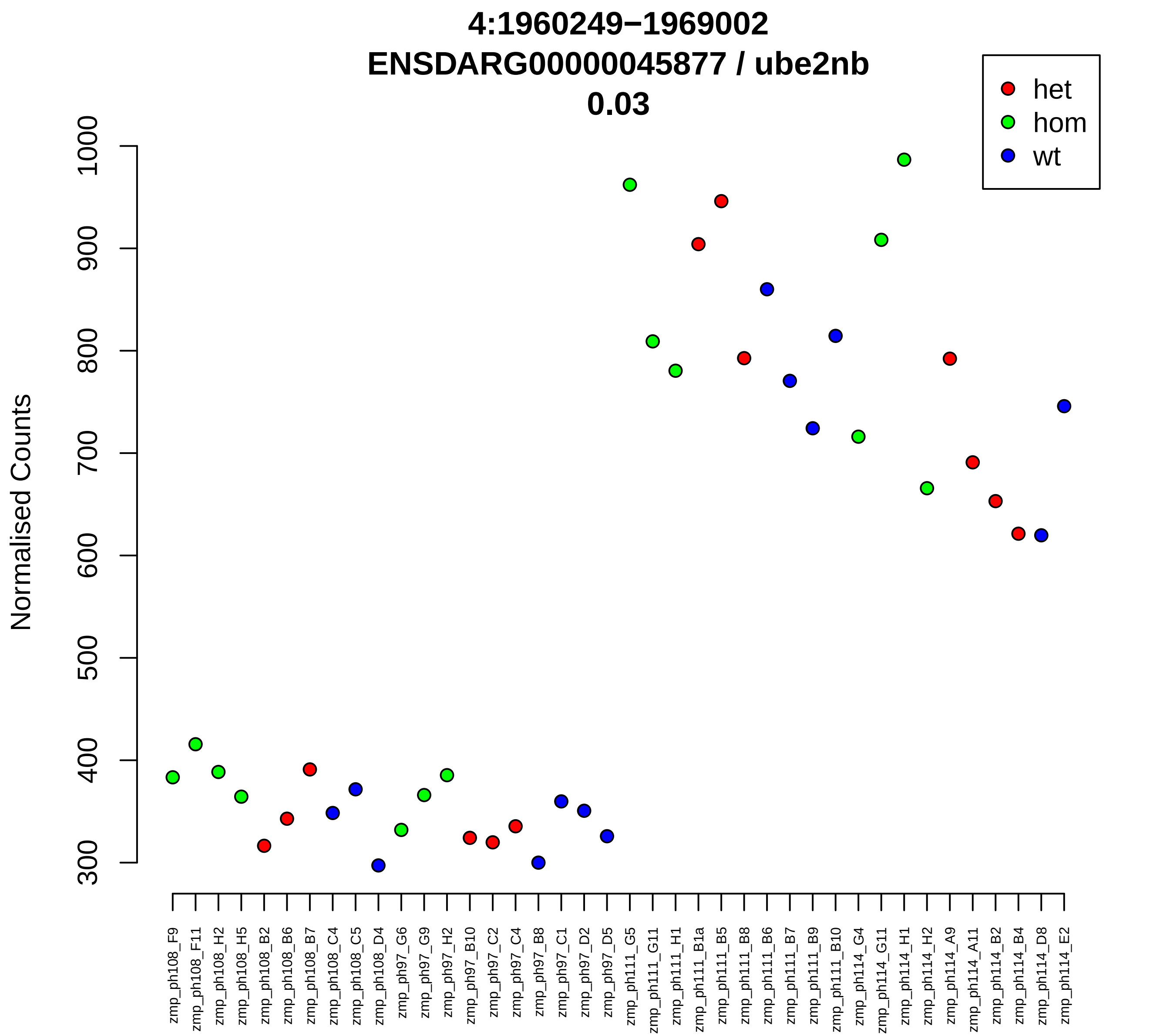
4:1960249−1969002
ENSDARG00000045877 / ube2nb
0.03
het
hom
wt
Normalised Counts
300
400
500
600
700
800
900
1000
zmp_ph108_F9
zmp_ph108_F11
zmp_ph108_H2
zmp_ph108_H5
zmp_ph108_B2
zmp_ph108_B6
zmp_ph108_B7
zmp_ph108_C4
zmp_ph108_C5
zmp_ph108_D4
zmp_ph97_G6
zmp_ph97_G9
zmp_ph97_H2
zmp_ph97_B10
zmp_ph97_C2
zmp_ph97_C4
zmp_ph97_B8
zmp_ph97_C1
zmp_ph97_D2
zmp_ph97_D5
zmp_ph111_G5
zmp_ph111_G11
zmp_ph111_H1
zmp_ph111_B1a
zmp_ph111_B5
zmp_ph111_B8
zmp_ph111_B6
zmp_ph111_B7
zmp_ph111_B9
zmp_ph111_B10
zmp_ph114_G4
zmp_ph114_G11
zmp_ph114_H1
zmp_ph114_H2
zmp_ph114_A9
zmp_ph114_A11
zmp_ph114_B2
zmp_ph114_B4
zmp_ph114_D8
zmp_ph114_E2

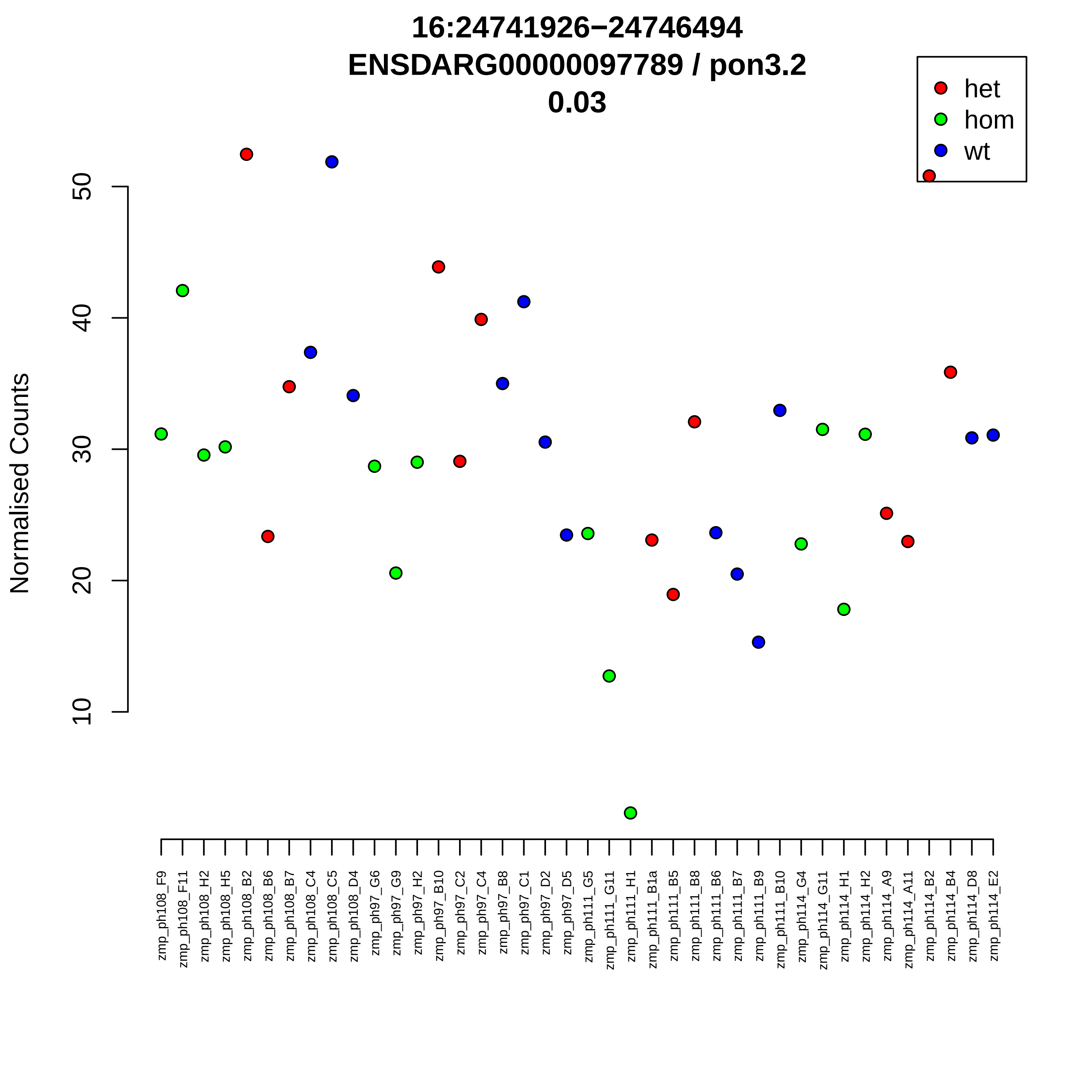

16:24741926−24746494
ENSDARG00000097789 / pon3.2
0.03
het
hom
wt
Normalised Counts
10
20
30
40
50
zmp_ph108_F9
zmp_ph108_F11
zmp_ph108_H2
zmp_ph108_H5
zmp_ph108_B2
zmp_ph108_B6
zmp_ph108_B7
zmp_ph108_C4
zmp_ph108_C5
zmp_ph108_D4
zmp_ph97_G6
zmp_ph97_G9
zmp_ph97_H2
zmp_ph97_B10
zmp_ph97_C2
zmp_ph97_C4
zmp_ph97_B8
zmp_ph97_C1
zmp_ph97_D2
zmp_ph97_D5
zmp_ph111_G5
zmp_ph111_G11
zmp_ph111_H1
zmp_ph111_B1a
zmp_ph111_B5
zmp_ph111_B8
zmp_ph111_B6
zmp_ph111_B7
zmp_ph111_B9
zmp_ph111_B10
zmp_ph114_G4
zmp_ph114_G11
zmp_ph114_H1
zmp_ph114_H2
zmp_ph114_A9
zmp_ph114_A11
zmp_ph114_B2
zmp_ph114_B4
zmp_ph114_D8
zmp_ph114_E2

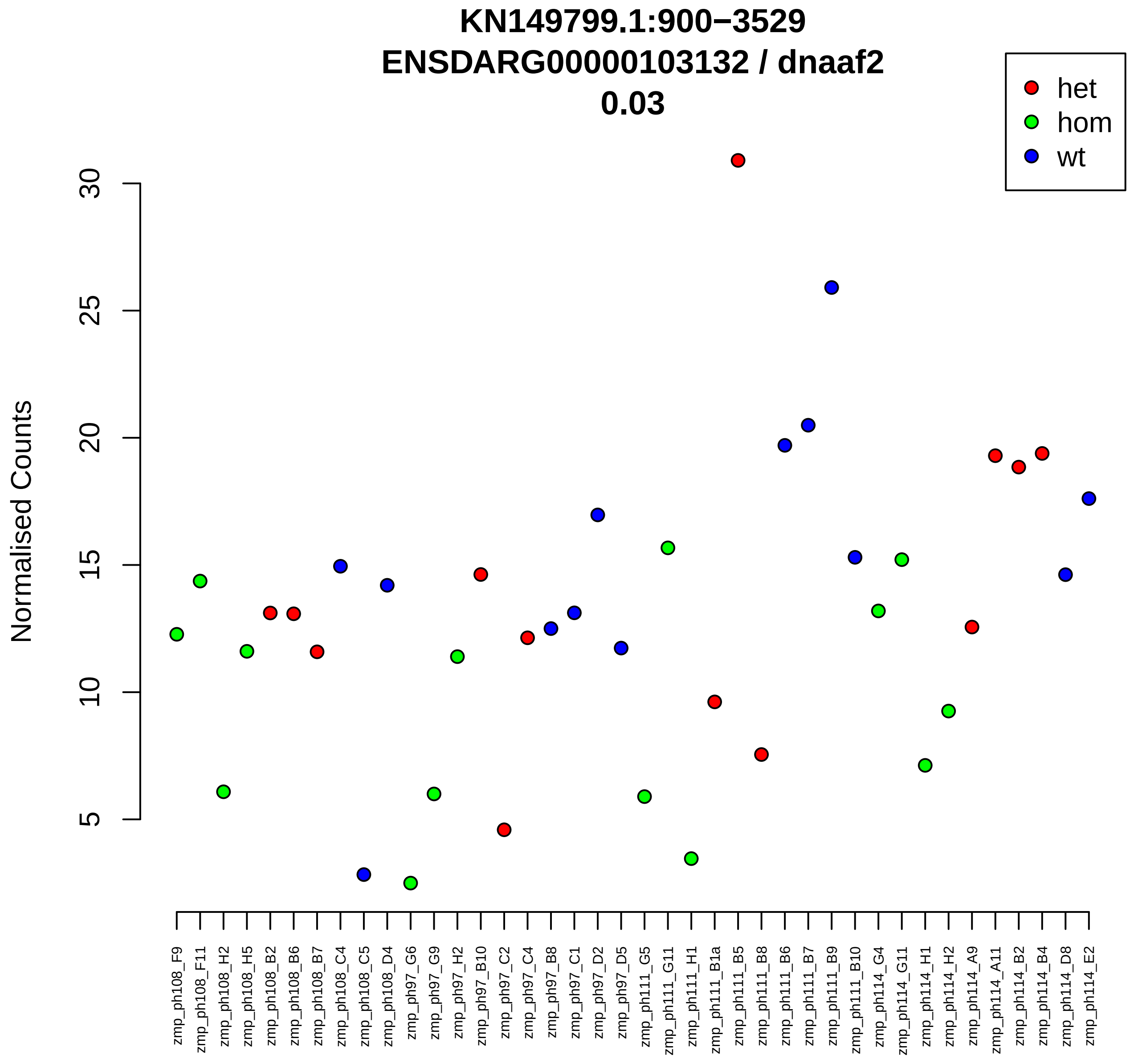

KN149799.1:900−3529
ENSDARG00000103132 / dnaaf2
0.03
het
hom
wt
Normalised Counts
5
10
15
20
25
30
zmp_ph108_F9
zmp_ph108_F11
zmp_ph108_H2
zmp_ph108_H5
zmp_ph108_B2
zmp_ph108_B6
zmp_ph108_B7
zmp_ph108_C4
zmp_ph108_C5
zmp_ph108_D4
zmp_ph97_G6
zmp_ph97_G9
zmp_ph97_H2
zmp_ph97_B10
zmp_ph97_C2
zmp_ph97_C4
zmp_ph97_B8
zmp_ph97_C1
zmp_ph97_D2
zmp_ph97_D5
zmp_ph111_G5
zmp_ph111_G11
zmp_ph111_H1
zmp_ph111_B1a
zmp_ph111_B5
zmp_ph111_B8
zmp_ph111_B6
zmp_ph111_B7
zmp_ph111_B9
zmp_ph111_B10
zmp_ph114_G4
zmp_ph114_G11
zmp_ph114_H1
zmp_ph114_H2
zmp_ph114_A9
zmp_ph114_A11
zmp_ph114_B2
zmp_ph114_B4
zmp_ph114_D8
zmp_ph114_E2

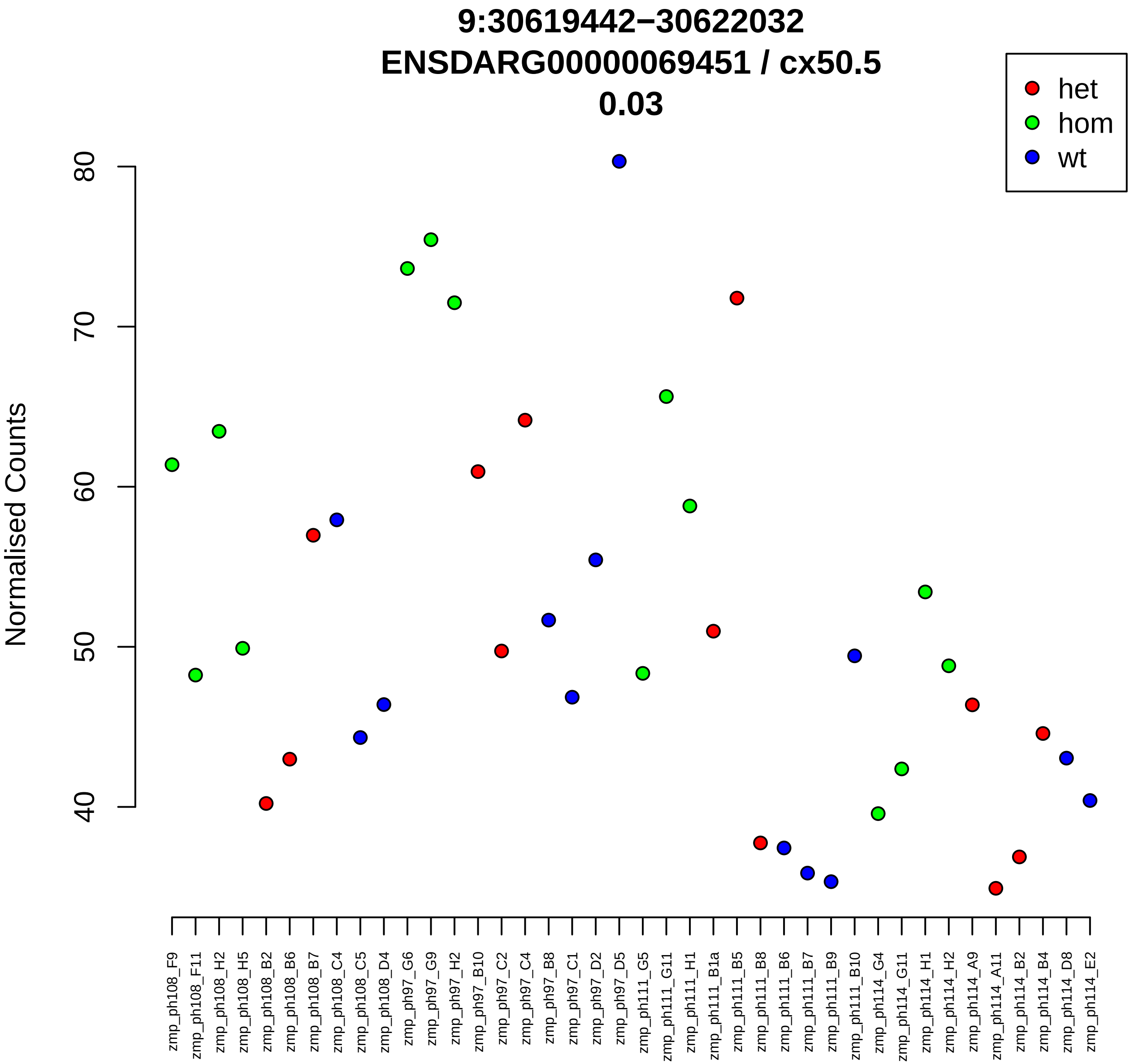

9:30619442−30622032
ENSDARG00000069451 / cx50.5
0.03
het
hom
wt
Normalised Counts
40
50
60
70
80
zmp_ph108_F9
zmp_ph108_F11
zmp_ph108_H2
zmp_ph108_H5
zmp_ph108_B2
zmp_ph108_B6
zmp_ph108_B7
zmp_ph108_C4
zmp_ph108_C5
zmp_ph108_D4
zmp_ph97_G6
zmp_ph97_G9
zmp_ph97_H2
zmp_ph97_B10
zmp_ph97_C2
zmp_ph97_C4
zmp_ph97_B8
zmp_ph97_C1
zmp_ph97_D2
zmp_ph97_D5
zmp_ph111_G5
zmp_ph111_G11
zmp_ph111_H1
zmp_ph111_B1a
zmp_ph111_B5
zmp_ph111_B8
zmp_ph111_B6
zmp_ph111_B7
zmp_ph111_B9
zmp_ph111_B10
zmp_ph114_G4
zmp_ph114_G11
zmp_ph114_H1
zmp_ph114_H2
zmp_ph114_A9
zmp_ph114_A11
zmp_ph114_B2
zmp_ph114_B4
zmp_ph114_D8
zmp_ph114_E2

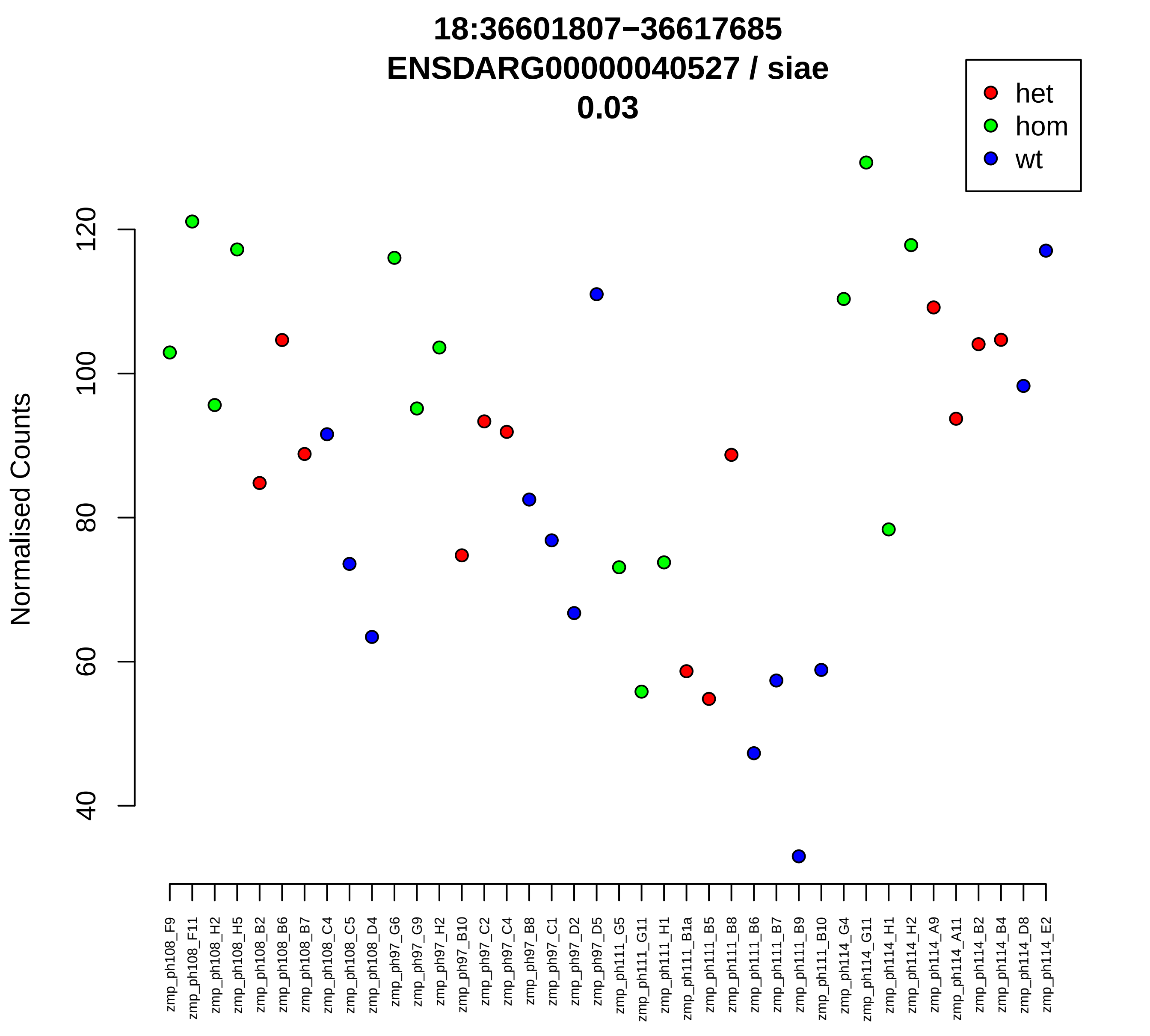

18:36601807−36617685
ENSDARG00000040527 / siae
0.03
het
hom
wt
Normalised Counts
40
60
80
100
120
zmp_ph108_F9
zmp_ph108_F11
zmp_ph108_H2
zmp_ph108_H5
zmp_ph108_B2
zmp_ph108_B6
zmp_ph108_B7
zmp_ph108_C4
zmp_ph108_C5
zmp_ph108_D4
zmp_ph97_G6
zmp_ph97_G9
zmp_ph97_H2
zmp_ph97_B10
zmp_ph97_C2
zmp_ph97_C4
zmp_ph97_B8
zmp_ph97_C1
zmp_ph97_D2
zmp_ph97_D5
zmp_ph111_G5
zmp_ph111_G11
zmp_ph111_H1
zmp_ph111_B1a
zmp_ph111_B5
zmp_ph111_B8
zmp_ph111_B6
zmp_ph111_B7
zmp_ph111_B9
zmp_ph111_B10
zmp_ph114_G4
zmp_ph114_G11
zmp_ph114_H1
zmp_ph114_H2
zmp_ph114_A9
zmp_ph114_A11
zmp_ph114_B2
zmp_ph114_B4
zmp_ph114_D8
zmp_ph114_E2

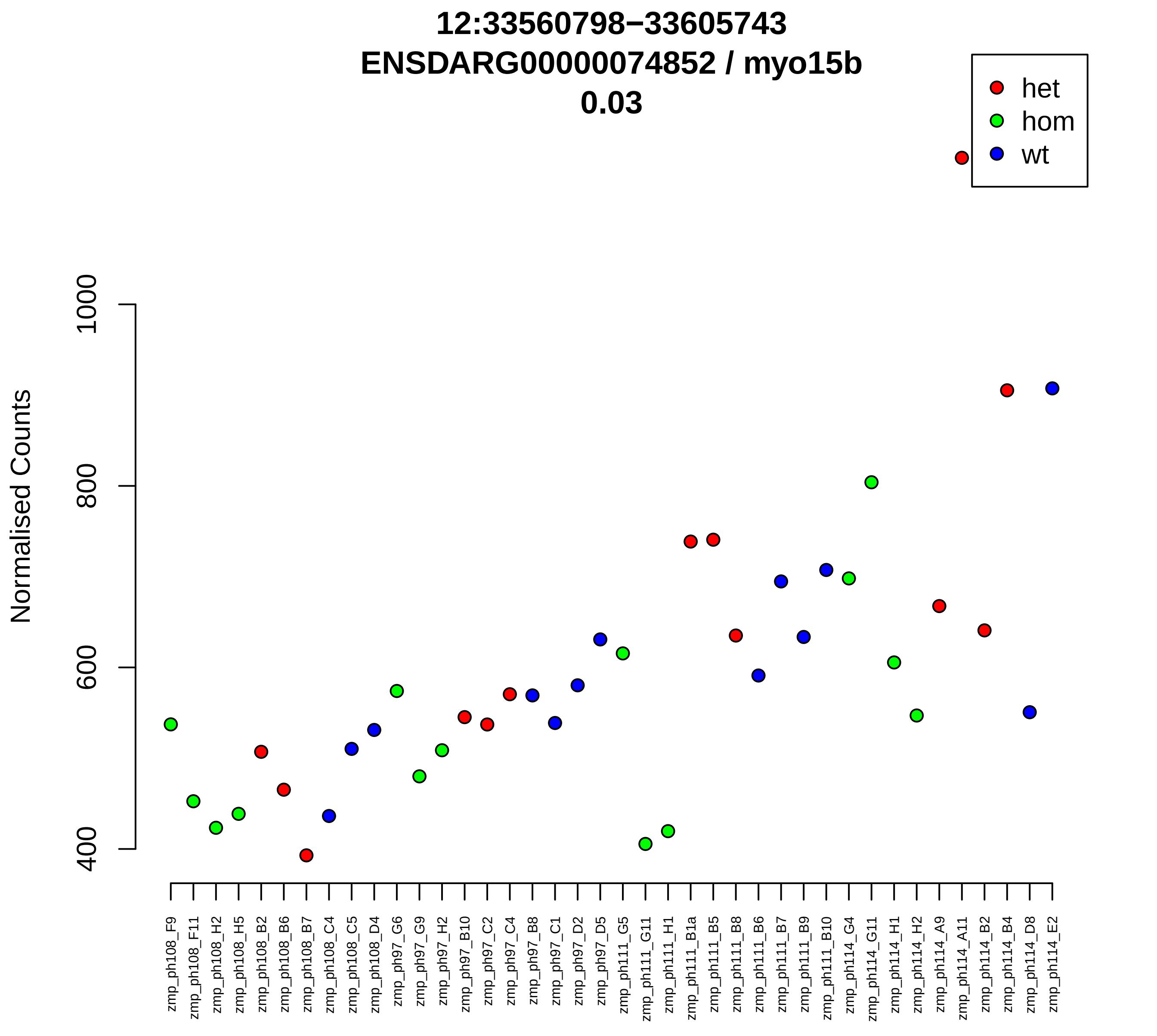
12:33560798−33605743
ENSDARG00000074852 / myo15b
0.03
het
hom
wt
Normalised Counts
400
600
800
1000
zmp_ph108_F9
zmp_ph108_F11
zmp_ph108_H2
zmp_ph108_H5
zmp_ph108_B2
zmp_ph108_B6
zmp_ph108_B7
zmp_ph108_C4
zmp_ph108_C5
zmp_ph108_D4
zmp_ph97_G6
zmp_ph97_G9
zmp_ph97_H2
zmp_ph97_B10
zmp_ph97_C2
zmp_ph97_C4
zmp_ph97_B8
zmp_ph97_C1
zmp_ph97_D2
zmp_ph97_D5
zmp_ph111_G5
zmp_ph111_G11
zmp_ph111_H1
zmp_ph111_B1a
zmp_ph111_B5
zmp_ph111_B8
zmp_ph111_B6
zmp_ph111_B7
zmp_ph111_B9
zmp_ph111_B10
zmp_ph114_G4
zmp_ph114_G11
zmp_ph114_H1
zmp_ph114_H2
zmp_ph114_A9
zmp_ph114_A11
zmp_ph114_B2
zmp_ph114_B4
zmp_ph114_D8
zmp_ph114_E2

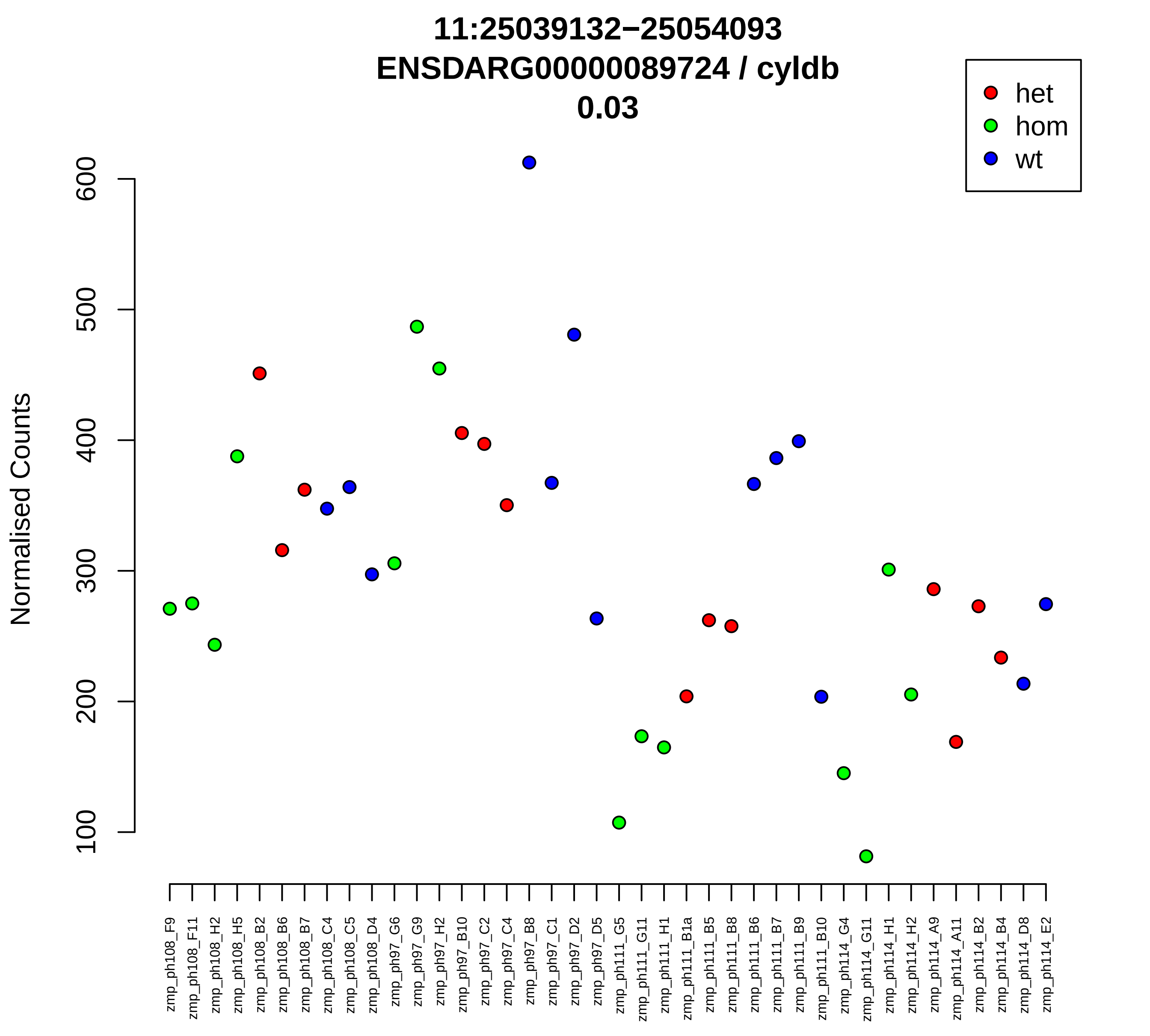
11:25039132−25054093
ENSDARG00000089724 / cyldb
0.03
het
hom
wt
Normalised Counts
100
200
300
400
500
600
zmp_ph108_F9
zmp_ph108_F11
zmp_ph108_H2
zmp_ph108_H5
zmp_ph108_B2
zmp_ph108_B6
zmp_ph108_B7
zmp_ph108_C4
zmp_ph108_C5
zmp_ph108_D4
zmp_ph97_G6
zmp_ph97_G9
zmp_ph97_H2
zmp_ph97_B10
zmp_ph97_C2
zmp_ph97_C4
zmp_ph97_B8
zmp_ph97_C1
zmp_ph97_D2
zmp_ph97_D5
zmp_ph111_G5
zmp_ph111_G11
zmp_ph111_H1
zmp_ph111_B1a
zmp_ph111_B5
zmp_ph111_B8
zmp_ph111_B6
zmp_ph111_B7
zmp_ph111_B9
zmp_ph111_B10
zmp_ph114_G4
zmp_ph114_G11
zmp_ph114_H1
zmp_ph114_H2
zmp_ph114_A9
zmp_ph114_A11
zmp_ph114_B2
zmp_ph114_B4
zmp_ph114_D8
zmp_ph114_E2

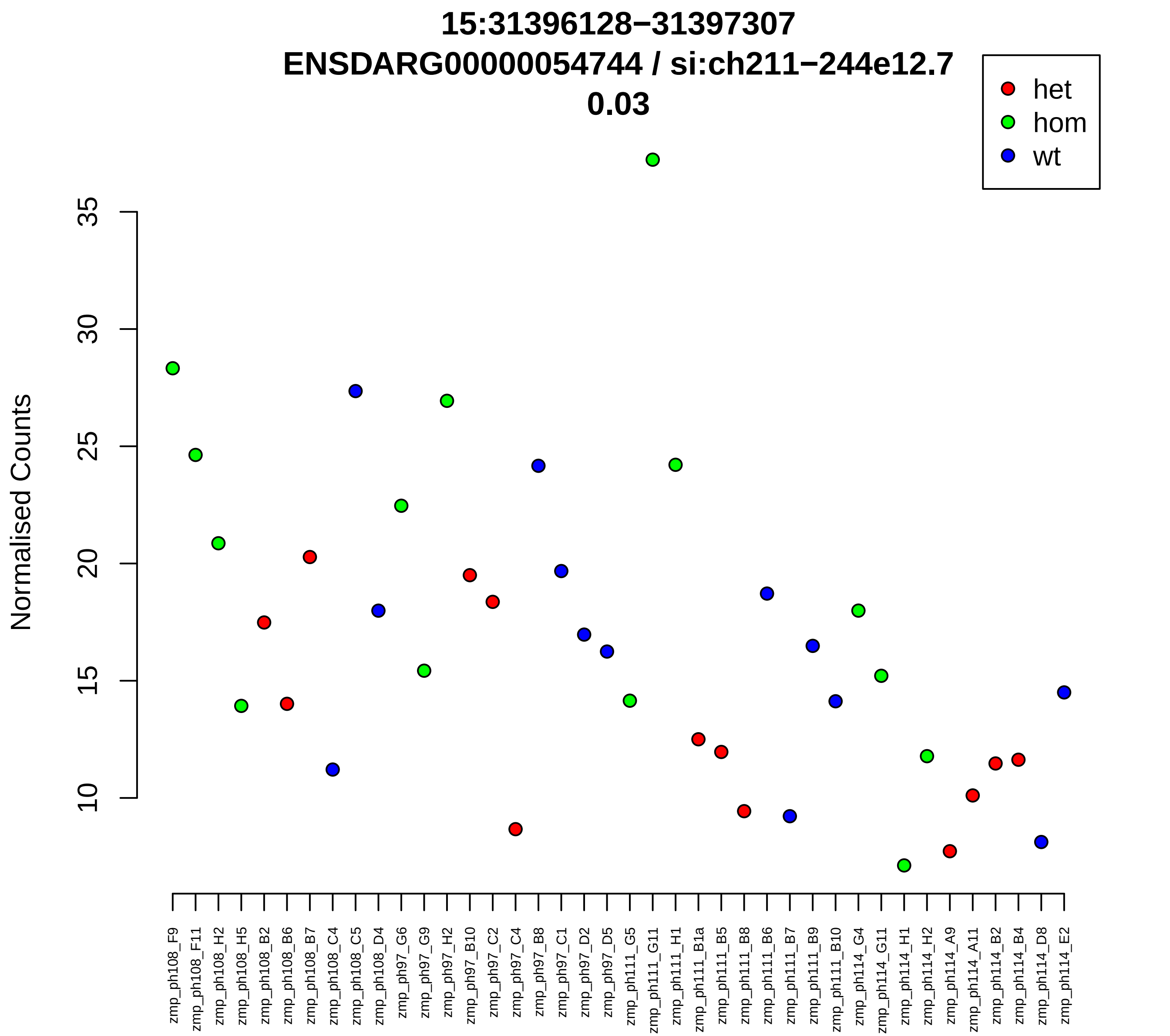

15:31396128−31397307
ENSDARG00000054744 / si:ch211−244e12.7
0.03
het
hom
wt
Normalised Counts
10
15
20
25
30
35
zmp_ph108_F9
zmp_ph108_F11
zmp_ph108_H2
zmp_ph108_H5
zmp_ph108_B2
zmp_ph108_B6
zmp_ph108_B7
zmp_ph108_C4
zmp_ph108_C5
zmp_ph108_D4
zmp_ph97_G6
zmp_ph97_G9
zmp_ph97_H2
zmp_ph97_B10
zmp_ph97_C2
zmp_ph97_C4
zmp_ph97_B8
zmp_ph97_C1
zmp_ph97_D2
zmp_ph97_D5
zmp_ph111_G5
zmp_ph111_G11
zmp_ph111_H1
zmp_ph111_B1a
zmp_ph111_B5
zmp_ph111_B8
zmp_ph111_B6
zmp_ph111_B7
zmp_ph111_B9
zmp_ph111_B10
zmp_ph114_G4
zmp_ph114_G11
zmp_ph114_H1
zmp_ph114_H2
zmp_ph114_A9
zmp_ph114_A11
zmp_ph114_B2
zmp_ph114_B4
zmp_ph114_D8
zmp_ph114_E2

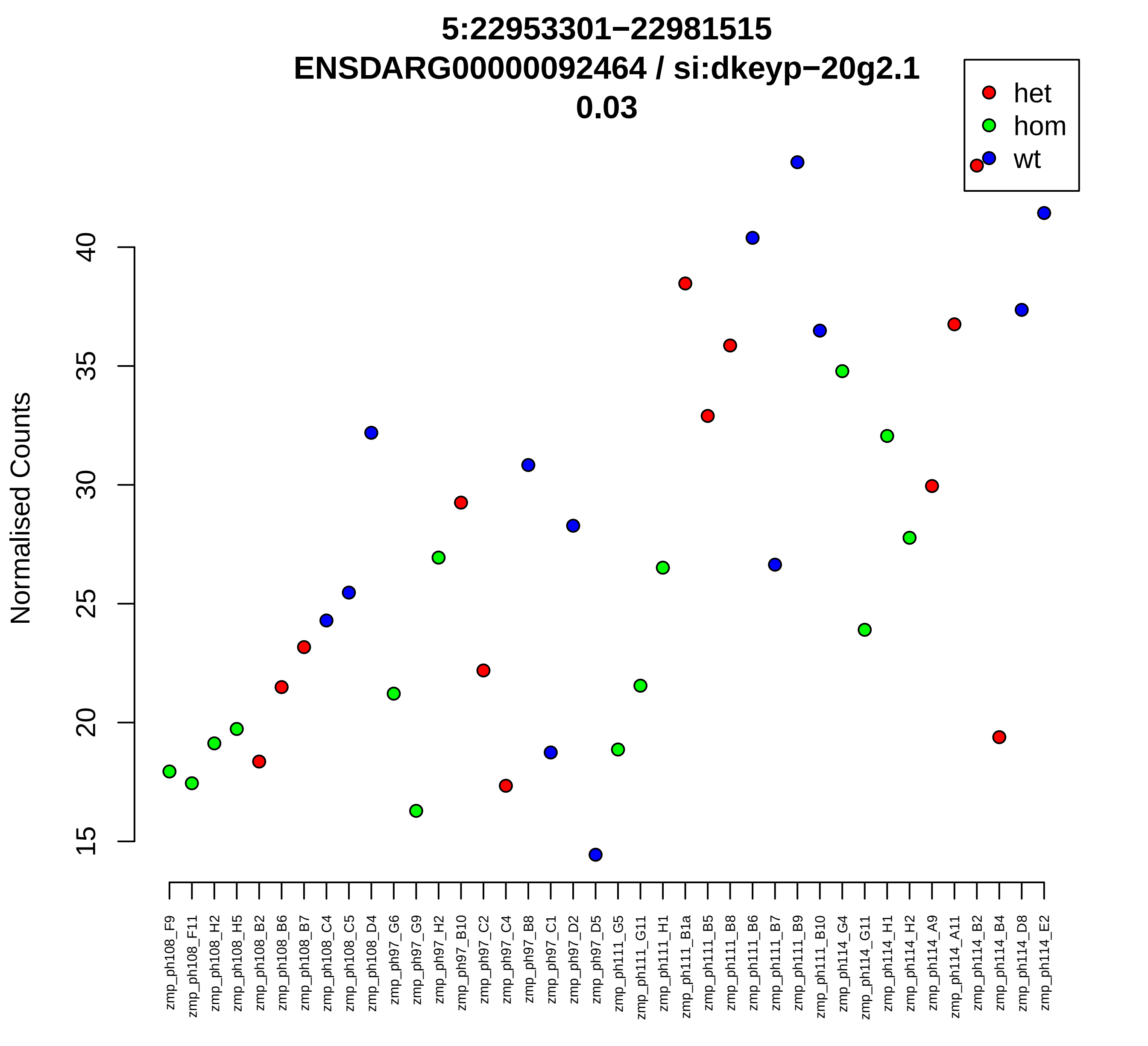
5:22953301−22981515
ENSDARG00000092464 / si:dkeyp−20g2.1
0.03
het
hom
wt
Normalised Counts
15
20
25
30
35
40
zmp_ph108_F9
zmp_ph108_F11
zmp_ph108_H2
zmp_ph108_H5
zmp_ph108_B2
zmp_ph108_B6
zmp_ph108_B7
zmp_ph108_C4
zmp_ph108_C5
zmp_ph108_D4
zmp_ph97_G6
zmp_ph97_G9
zmp_ph97_H2
zmp_ph97_B10
zmp_ph97_C2
zmp_ph97_C4
zmp_ph97_B8
zmp_ph97_C1
zmp_ph97_D2
zmp_ph97_D5
zmp_ph111_G5
zmp_ph111_G11
zmp_ph111_H1
zmp_ph111_B1a
zmp_ph111_B5
zmp_ph111_B8
zmp_ph111_B6
zmp_ph111_B7
zmp_ph111_B9
zmp_ph111_B10
zmp_ph114_G4
zmp_ph114_G11
zmp_ph114_H1
zmp_ph114_H2
zmp_ph114_A9
zmp_ph114_A11
zmp_ph114_B2
zmp_ph114_B4
zmp_ph114_D8
zmp_ph114_E2

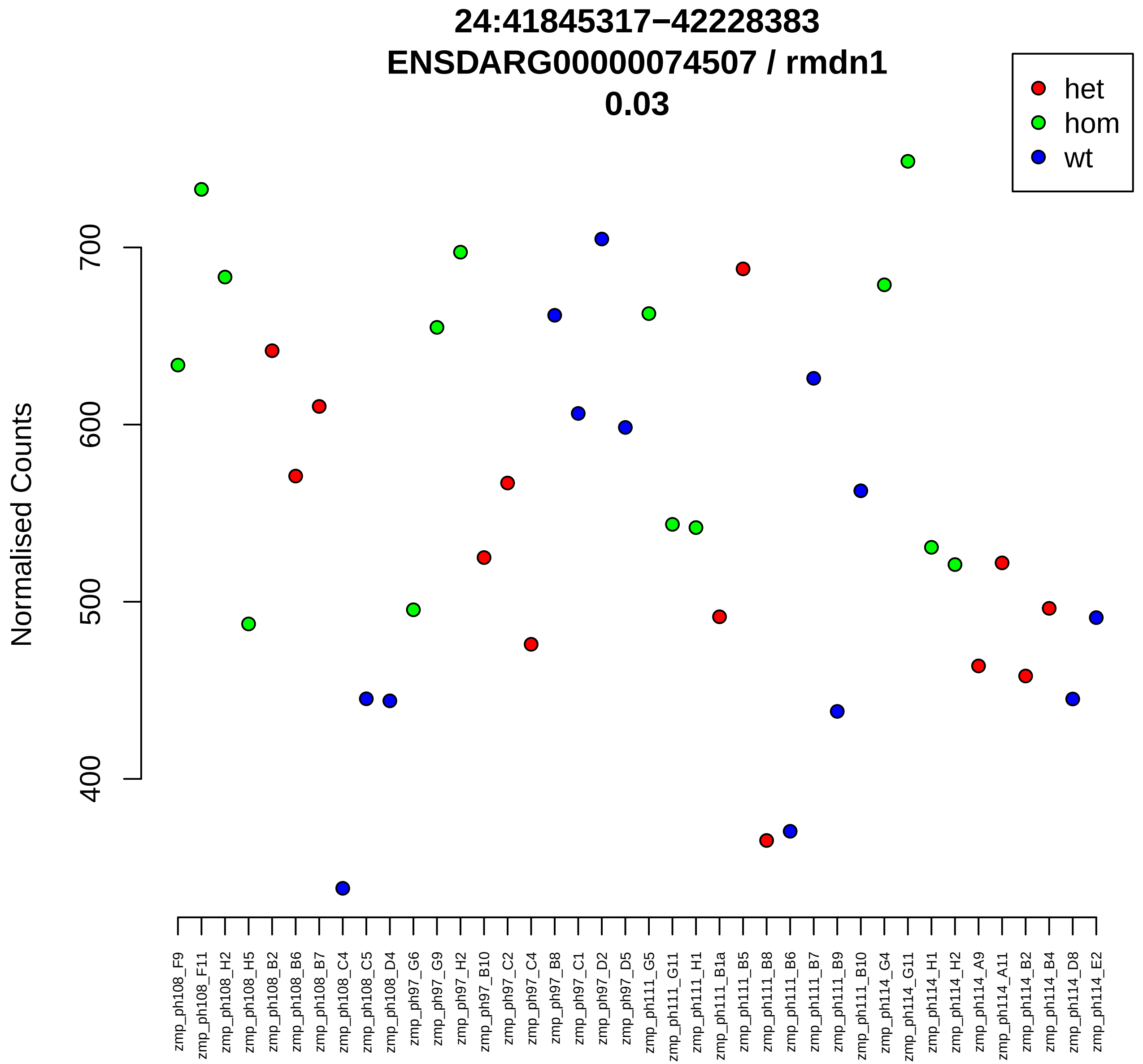
24:41845317−42228383
ENSDARG00000074507 / rmdn1
0.03
het
hom
wt
Normalised Counts
400
500
600
700
zmp_ph108_F9
zmp_ph108_F11
zmp_ph108_H2
zmp_ph108_H5
zmp_ph108_B2
zmp_ph108_B6
zmp_ph108_B7
zmp_ph108_C4
zmp_ph108_C5
zmp_ph108_D4
zmp_ph97_G6
zmp_ph97_G9
zmp_ph97_H2
zmp_ph97_B10
zmp_ph97_C2
zmp_ph97_C4
zmp_ph97_B8
zmp_ph97_C1
zmp_ph97_D2
zmp_ph97_D5
zmp_ph111_G5
zmp_ph111_G11
zmp_ph111_H1
zmp_ph111_B1a
zmp_ph111_B5
zmp_ph111_B8
zmp_ph111_B6
zmp_ph111_B7
zmp_ph111_B9
zmp_ph111_B10
zmp_ph114_G4
zmp_ph114_G11
zmp_ph114_H1
zmp_ph114_H2
zmp_ph114_A9
zmp_ph114_A11
zmp_ph114_B2
zmp_ph114_B4
zmp_ph114_D8
zmp_ph114_E2

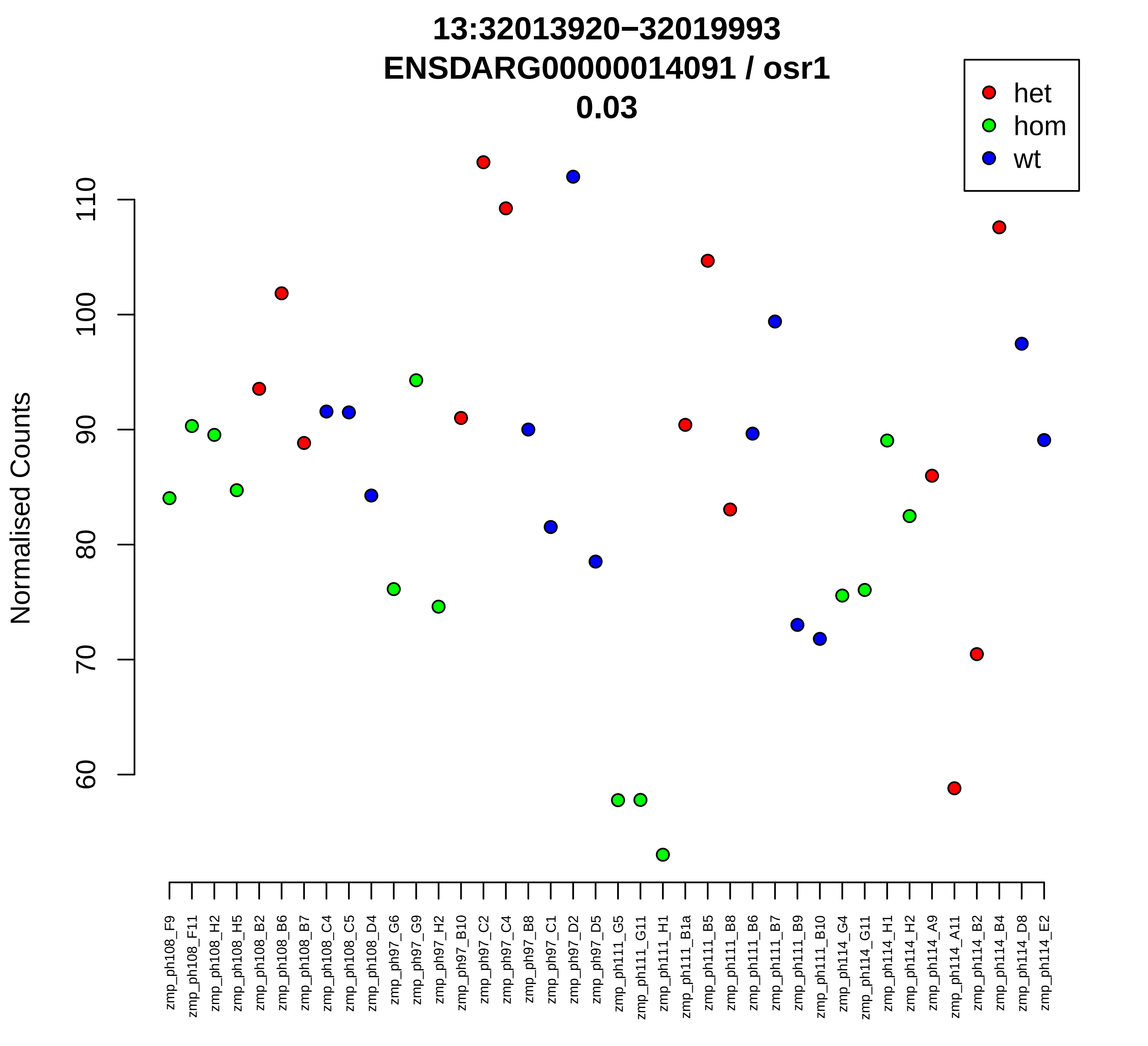
13:32013920−32019993
ENSDARG00000014091 / osr1
0.03
het
hom
wt
Normalised Counts
60
70
80
90
100
110
zmp_ph108_F9
zmp_ph108_F11
zmp_ph108_H2
zmp_ph108_H5
zmp_ph108_B2
zmp_ph108_B6
zmp_ph108_B7
zmp_ph108_C4
zmp_ph108_C5
zmp_ph108_D4
zmp_ph97_G6
zmp_ph97_G9
zmp_ph97_H2
zmp_ph97_B10
zmp_ph97_C2
zmp_ph97_C4
zmp_ph97_B8
zmp_ph97_C1
zmp_ph97_D2
zmp_ph97_D5
zmp_ph111_G5
zmp_ph111_G11
zmp_ph111_H1
zmp_ph111_B1a
zmp_ph111_B5
zmp_ph111_B8
zmp_ph111_B6
zmp_ph111_B7
zmp_ph111_B9
zmp_ph111_B10
zmp_ph114_G4
zmp_ph114_G11
zmp_ph114_H1
zmp_ph114_H2
zmp_ph114_A9
zmp_ph114_A11
zmp_ph114_B2
zmp_ph114_B4
zmp_ph114_D8
zmp_ph114_E2

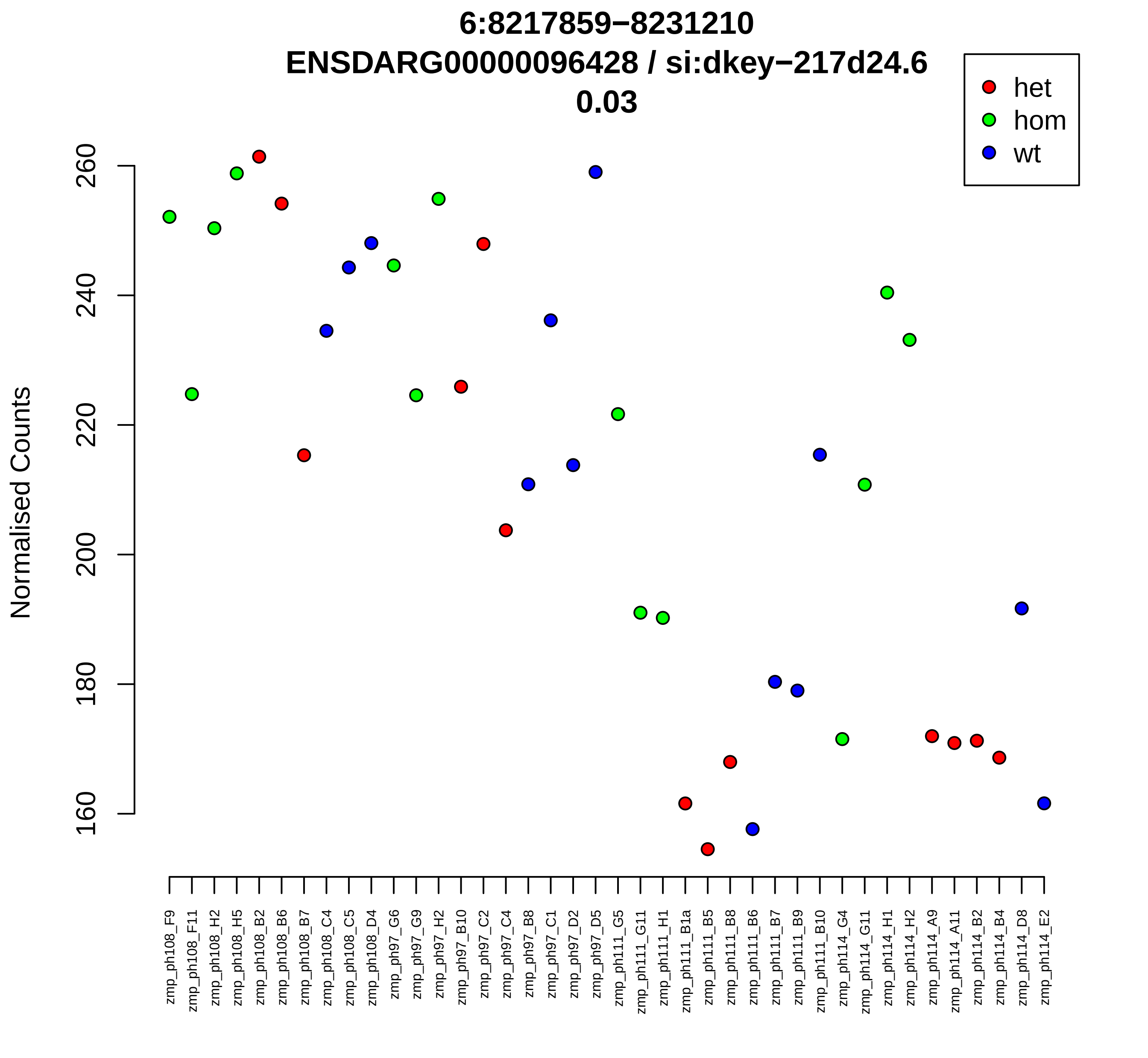

6:8217859−8231210
ENSDARG00000096428 / si:dkey−217d24.6
0.03
het
hom
wt
Normalised Counts
160
180
200
220
240
260
zmp_ph108_F9
zmp_ph108_F11
zmp_ph108_H2
zmp_ph108_H5
zmp_ph108_B2
zmp_ph108_B6
zmp_ph108_B7
zmp_ph108_C4
zmp_ph108_C5
zmp_ph108_D4
zmp_ph97_G6
zmp_ph97_G9
zmp_ph97_H2
zmp_ph97_B10
zmp_ph97_C2
zmp_ph97_C4
zmp_ph97_B8
zmp_ph97_C1
zmp_ph97_D2
zmp_ph97_D5
zmp_ph111_G5
zmp_ph111_G11
zmp_ph111_H1
zmp_ph111_B1a
zmp_ph111_B5
zmp_ph111_B8
zmp_ph111_B6
zmp_ph111_B7
zmp_ph111_B9
zmp_ph111_B10
zmp_ph114_G4
zmp_ph114_G11
zmp_ph114_H1
zmp_ph114_H2
zmp_ph114_A9
zmp_ph114_A11
zmp_ph114_B2
zmp_ph114_B4
zmp_ph114_D8
zmp_ph114_E2

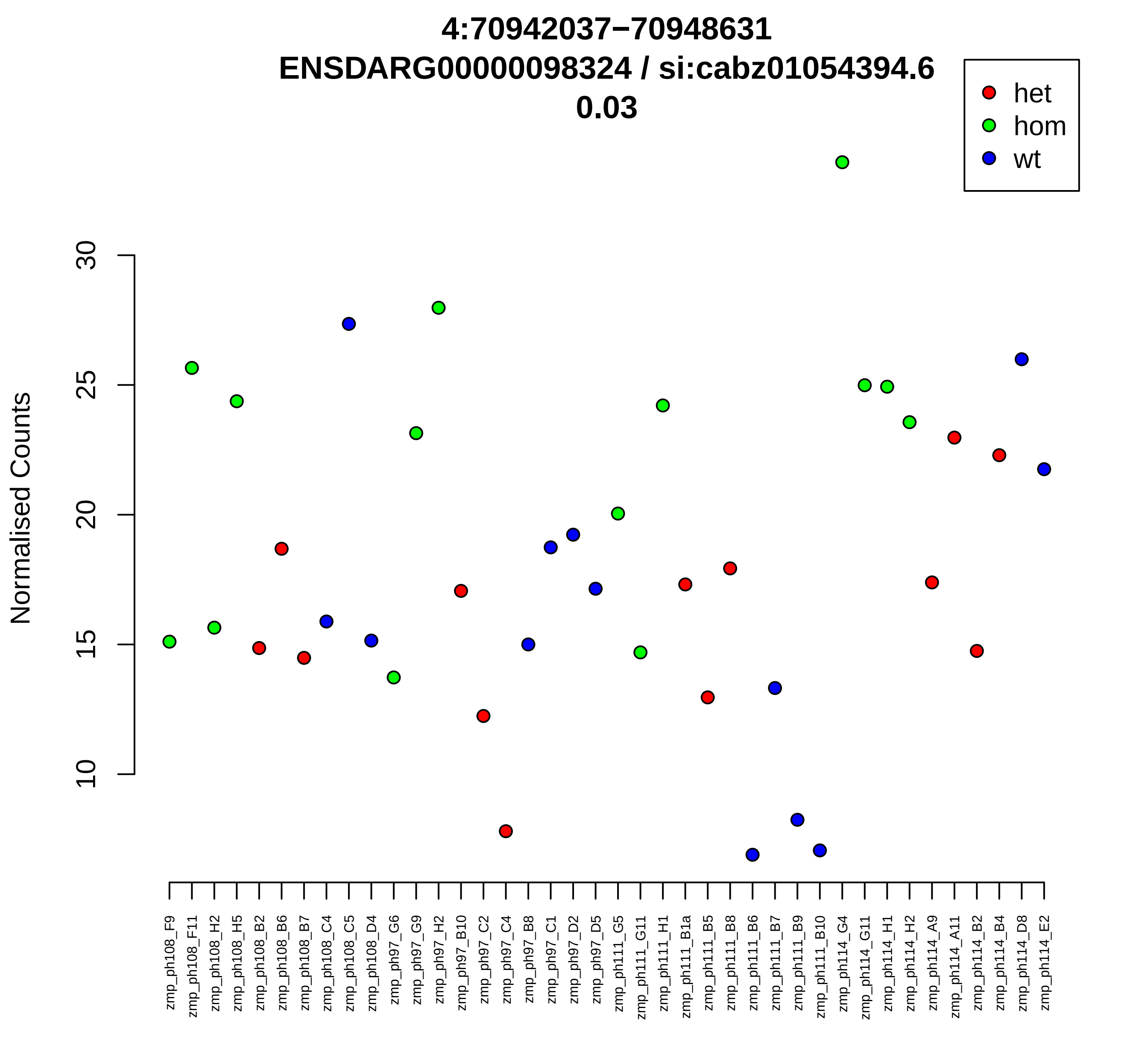
4:70942037−70948631
ENSDARG00000098324 / si:cabz01054394.6
0.03
het
hom
wt
Normalised Counts
10
15
20
25
30
zmp_ph108_F9
zmp_ph108_F11
zmp_ph108_H2
zmp_ph108_H5
zmp_ph108_B2
zmp_ph108_B6
zmp_ph108_B7
zmp_ph108_C4
zmp_ph108_C5
zmp_ph108_D4
zmp_ph97_G6
zmp_ph97_G9
zmp_ph97_H2
zmp_ph97_B10
zmp_ph97_C2
zmp_ph97_C4
zmp_ph97_B8
zmp_ph97_C1
zmp_ph97_D2
zmp_ph97_D5
zmp_ph111_G5
zmp_ph111_G11
zmp_ph111_H1
zmp_ph111_B1a
zmp_ph111_B5
zmp_ph111_B8
zmp_ph111_B6
zmp_ph111_B7
zmp_ph111_B9
zmp_ph111_B10
zmp_ph114_G4
zmp_ph114_G11
zmp_ph114_H1
zmp_ph114_H2
zmp_ph114_A9
zmp_ph114_A11
zmp_ph114_B2
zmp_ph114_B4
zmp_ph114_D8
zmp_ph114_E2

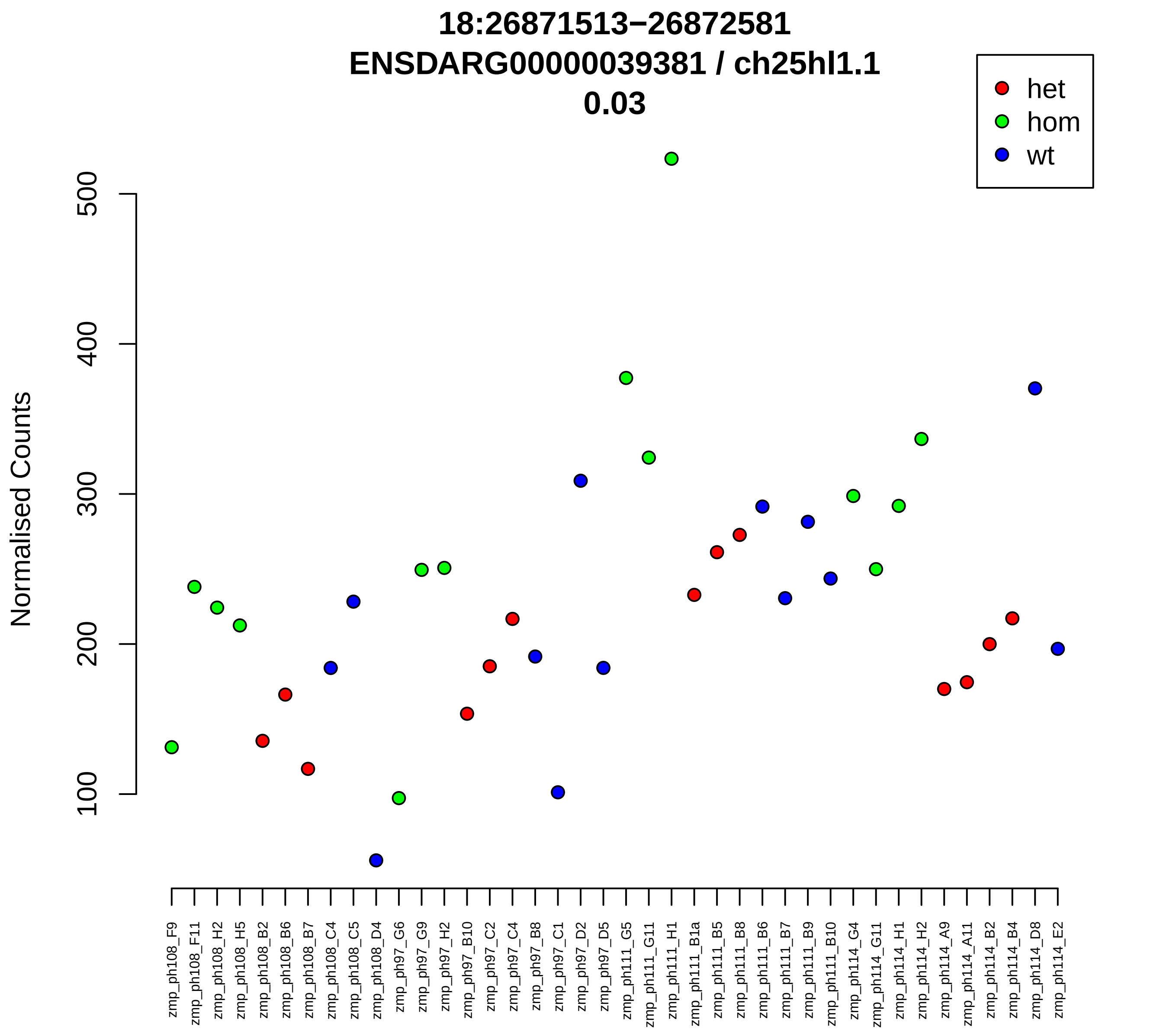

18:26871513−26872581
ENSDARG00000039381 / ch25hl1.1
0.03
het
hom
wt
Normalised Counts
100
200
300
400
500
zmp_ph108_F9
zmp_ph108_F11
zmp_ph108_H2
zmp_ph108_H5
zmp_ph108_B2
zmp_ph108_B6
zmp_ph108_B7
zmp_ph108_C4
zmp_ph108_C5
zmp_ph108_D4
zmp_ph97_G6
zmp_ph97_G9
zmp_ph97_H2
zmp_ph97_B10
zmp_ph97_C2
zmp_ph97_C4
zmp_ph97_B8
zmp_ph97_C1
zmp_ph97_D2
zmp_ph97_D5
zmp_ph111_G5
zmp_ph111_G11
zmp_ph111_H1
zmp_ph111_B1a
zmp_ph111_B5
zmp_ph111_B8
zmp_ph111_B6
zmp_ph111_B7
zmp_ph111_B9
zmp_ph111_B10
zmp_ph114_G4
zmp_ph114_G11
zmp_ph114_H1
zmp_ph114_H2
zmp_ph114_A9
zmp_ph114_A11
zmp_ph114_B2
zmp_ph114_B4
zmp_ph114_D8
zmp_ph114_E2

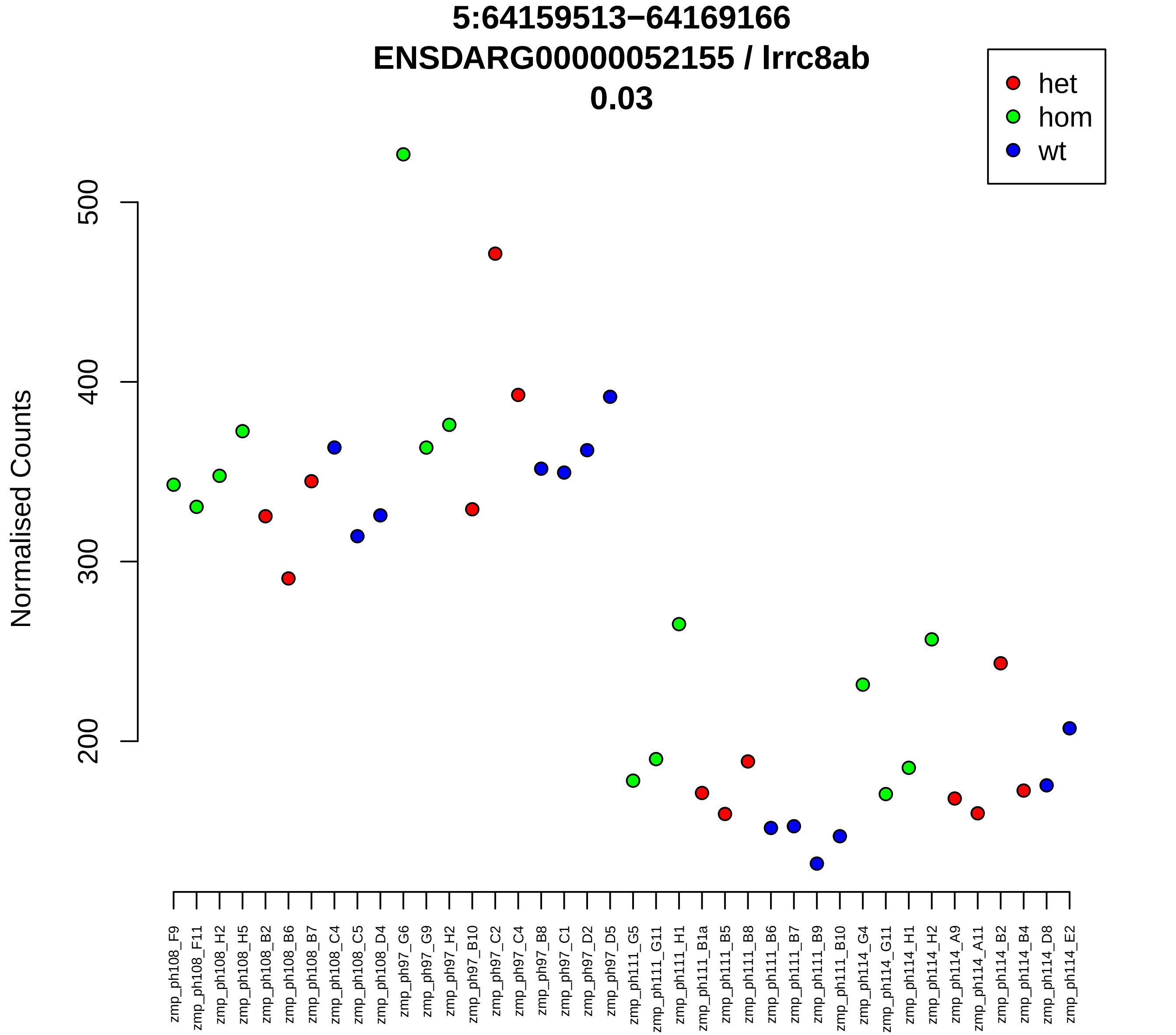
5:64159513−64169166
ENSDARG00000052155 / lrrc8ab
0.03
het
hom
wt
Normalised Counts
200
300
400
500
zmp_ph108_F9
zmp_ph108_F11
zmp_ph108_H2
zmp_ph108_H5
zmp_ph108_B2
zmp_ph108_B6
zmp_ph108_B7
zmp_ph108_C4
zmp_ph108_C5
zmp_ph108_D4
zmp_ph97_G6
zmp_ph97_G9
zmp_ph97_H2
zmp_ph97_B10
zmp_ph97_C2
zmp_ph97_C4
zmp_ph97_B8
zmp_ph97_C1
zmp_ph97_D2
zmp_ph97_D5
zmp_ph111_G5
zmp_ph111_G11
zmp_ph111_H1
zmp_ph111_B1a
zmp_ph111_B5
zmp_ph111_B8
zmp_ph111_B6
zmp_ph111_B7
zmp_ph111_B9
zmp_ph111_B10
zmp_ph114_G4
zmp_ph114_G11
zmp_ph114_H1
zmp_ph114_H2
zmp_ph114_A9
zmp_ph114_A11
zmp_ph114_B2
zmp_ph114_B4
zmp_ph114_D8
zmp_ph114_E2

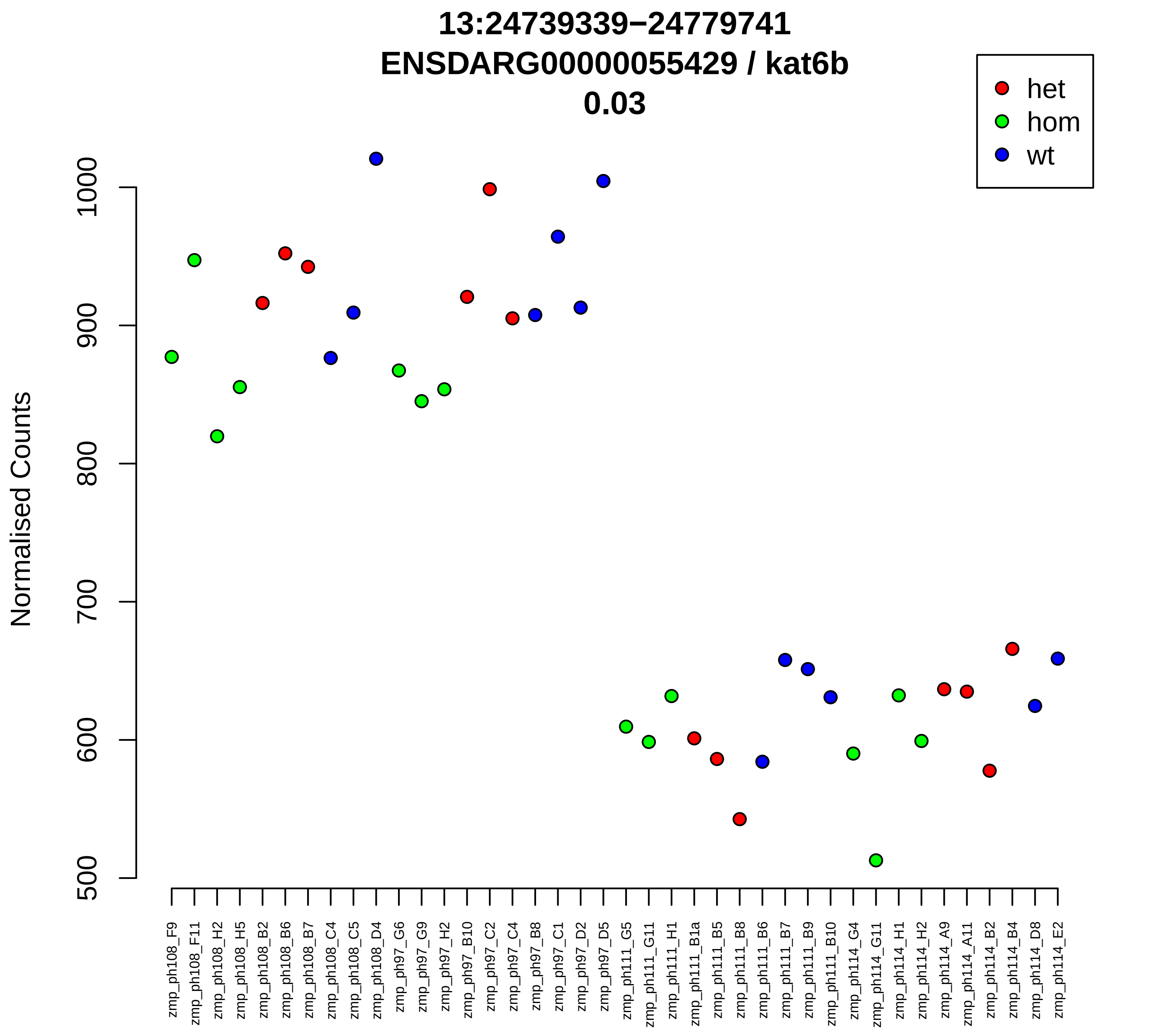

13:24739339−24779741
ENSDARG00000055429 / kat6b
0.03
het
hom
wt
Normalised Counts
500
600
700
800
900
1000
zmp_ph108_F9
zmp_ph108_F11
zmp_ph108_H2
zmp_ph108_H5
zmp_ph108_B2
zmp_ph108_B6
zmp_ph108_B7
zmp_ph108_C4
zmp_ph108_C5
zmp_ph108_D4
zmp_ph97_G6
zmp_ph97_G9
zmp_ph97_H2
zmp_ph97_B10
zmp_ph97_C2
zmp_ph97_C4
zmp_ph97_B8
zmp_ph97_C1
zmp_ph97_D2
zmp_ph97_D5
zmp_ph111_G5
zmp_ph111_G11
zmp_ph111_H1
zmp_ph111_B1a
zmp_ph111_B5
zmp_ph111_B8
zmp_ph111_B6
zmp_ph111_B7
zmp_ph111_B9
zmp_ph111_B10
zmp_ph114_G4
zmp_ph114_G11
zmp_ph114_H1
zmp_ph114_H2
zmp_ph114_A9
zmp_ph114_A11
zmp_ph114_B2
zmp_ph114_B4
zmp_ph114_D8
zmp_ph114_E2

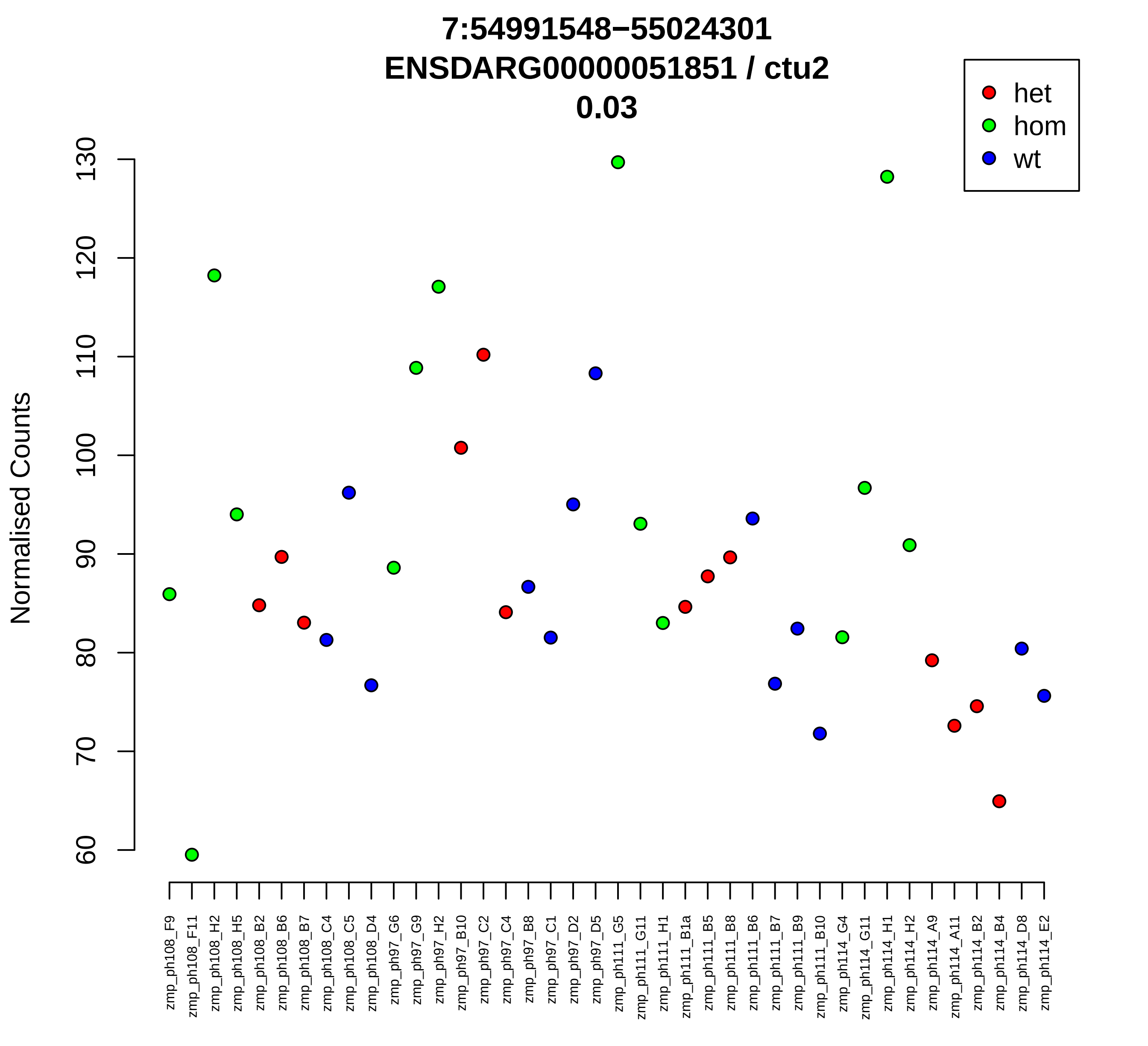

7:54991548−55024301
ENSDARG00000051851 / ctu2
0.03
het
hom
wt
Normalised Counts
60
70
80
90
100
110
120
130
zmp_ph108_F9
zmp_ph108_F11
zmp_ph108_H2
zmp_ph108_H5
zmp_ph108_B2
zmp_ph108_B6
zmp_ph108_B7
zmp_ph108_C4
zmp_ph108_C5
zmp_ph108_D4
zmp_ph97_G6
zmp_ph97_G9
zmp_ph97_H2
zmp_ph97_B10
zmp_ph97_C2
zmp_ph97_C4
zmp_ph97_B8
zmp_ph97_C1
zmp_ph97_D2
zmp_ph97_D5
zmp_ph111_G5
zmp_ph111_G11
zmp_ph111_H1
zmp_ph111_B1a
zmp_ph111_B5
zmp_ph111_B8
zmp_ph111_B6
zmp_ph111_B7
zmp_ph111_B9
zmp_ph111_B10
zmp_ph114_G4
zmp_ph114_G11
zmp_ph114_H1
zmp_ph114_H2
zmp_ph114_A9
zmp_ph114_A11
zmp_ph114_B2
zmp_ph114_B4
zmp_ph114_D8
zmp_ph114_E2

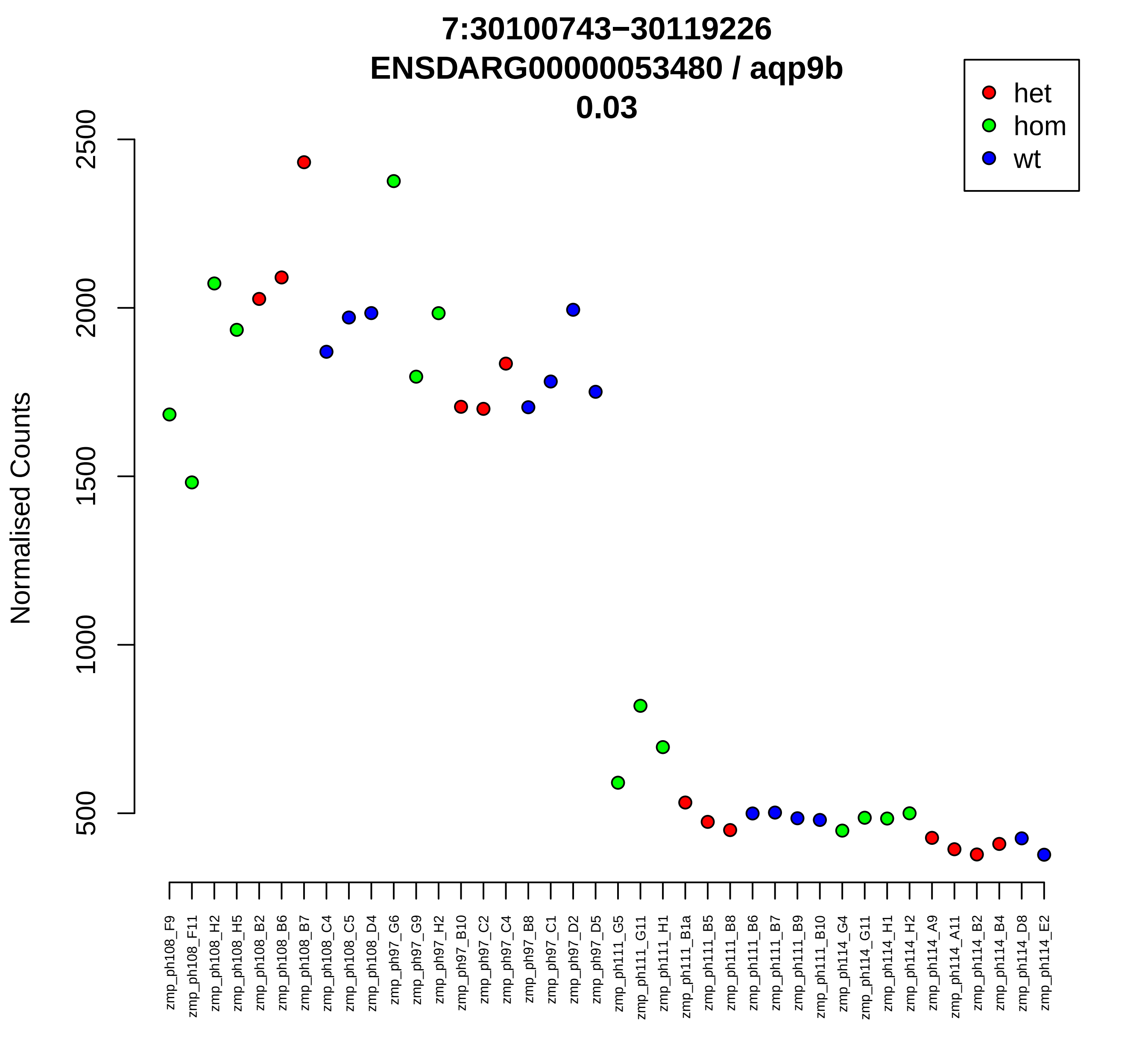
7:30100743−30119226
ENSDARG00000053480 / aqp9b
0.03
het
hom
wt
Normalised Counts
500
1000
1500
2000
2500
zmp_ph108_F9
zmp_ph108_F11
zmp_ph108_H2
zmp_ph108_H5
zmp_ph108_B2
zmp_ph108_B6
zmp_ph108_B7
zmp_ph108_C4
zmp_ph108_C5
zmp_ph108_D4
zmp_ph97_G6
zmp_ph97_G9
zmp_ph97_H2
zmp_ph97_B10
zmp_ph97_C2
zmp_ph97_C4
zmp_ph97_B8
zmp_ph97_C1
zmp_ph97_D2
zmp_ph97_D5
zmp_ph111_G5
zmp_ph111_G11
zmp_ph111_H1
zmp_ph111_B1a
zmp_ph111_B5
zmp_ph111_B8
zmp_ph111_B6
zmp_ph111_B7
zmp_ph111_B9
zmp_ph111_B10
zmp_ph114_G4
zmp_ph114_G11
zmp_ph114_H1
zmp_ph114_H2
zmp_ph114_A9
zmp_ph114_A11
zmp_ph114_B2
zmp_ph114_B4
zmp_ph114_D8
zmp_ph114_E2

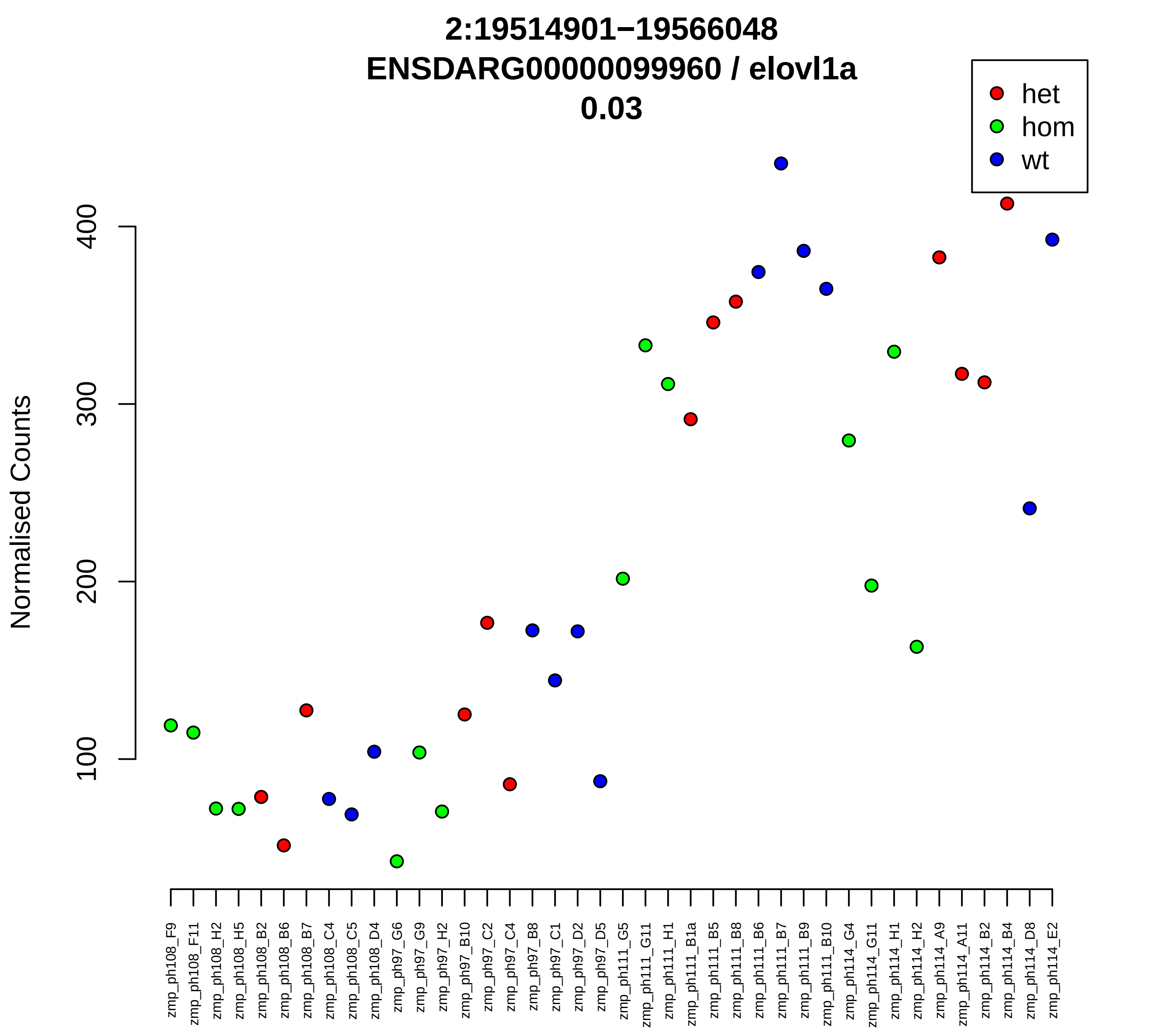

2:19514901−19566048
ENSDARG00000099960 / elovl1a
0.03
het
hom
wt
Normalised Counts
100
200
300
400
zmp_ph108_F9
zmp_ph108_F11
zmp_ph108_H2
zmp_ph108_H5
zmp_ph108_B2
zmp_ph108_B6
zmp_ph108_B7
zmp_ph108_C4
zmp_ph108_C5
zmp_ph108_D4
zmp_ph97_G6
zmp_ph97_G9
zmp_ph97_H2
zmp_ph97_B10
zmp_ph97_C2
zmp_ph97_C4
zmp_ph97_B8
zmp_ph97_C1
zmp_ph97_D2
zmp_ph97_D5
zmp_ph111_G5
zmp_ph111_G11
zmp_ph111_H1
zmp_ph111_B1a
zmp_ph111_B5
zmp_ph111_B8
zmp_ph111_B6
zmp_ph111_B7
zmp_ph111_B9
zmp_ph111_B10
zmp_ph114_G4
zmp_ph114_G11
zmp_ph114_H1
zmp_ph114_H2
zmp_ph114_A9
zmp_ph114_A11
zmp_ph114_B2
zmp_ph114_B4
zmp_ph114_D8
zmp_ph114_E2

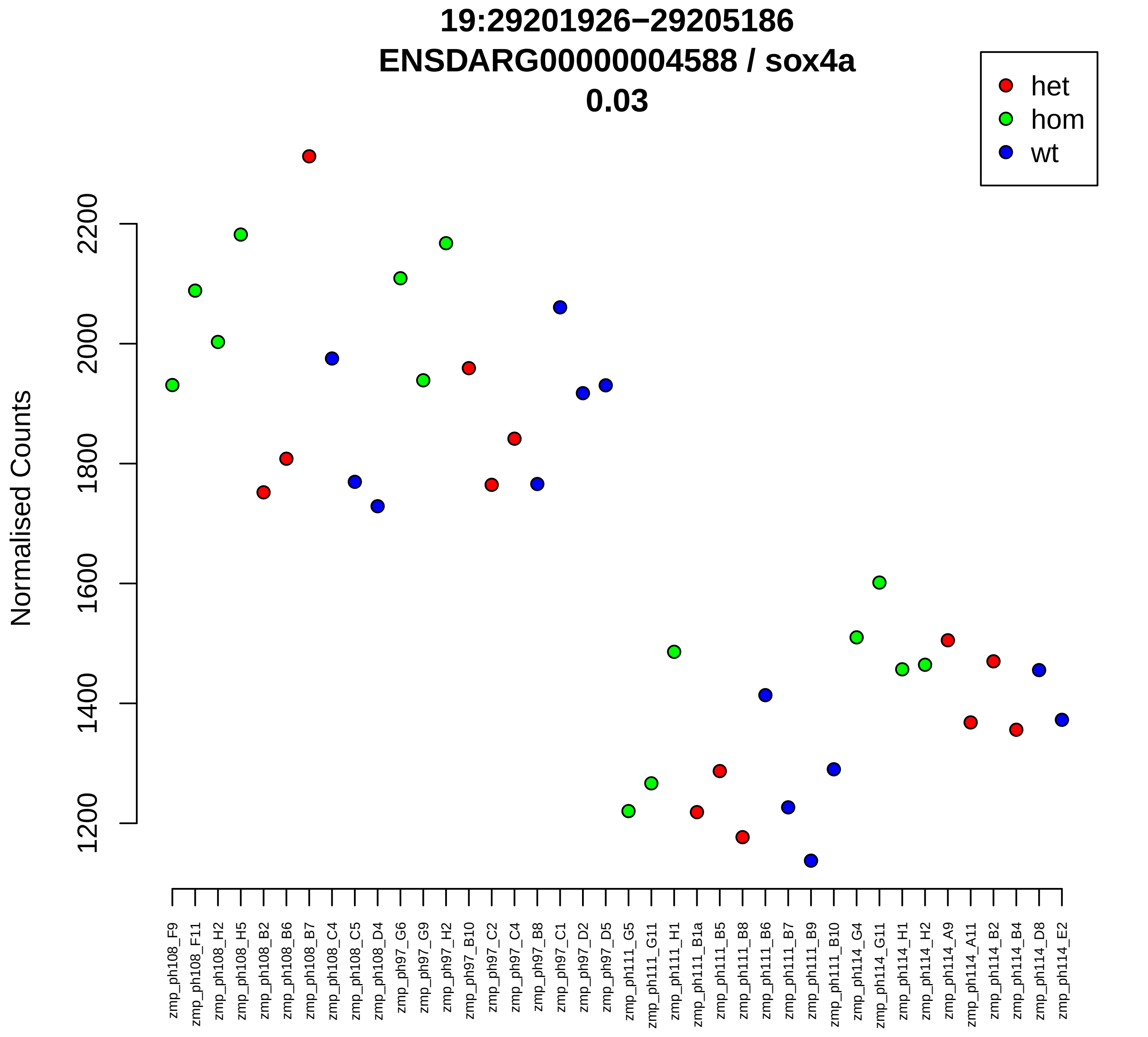

19:29201926−29205186
ENSDARG00000004588 / sox4a
0.03
het
hom
wt
Normalised Counts
1200
1400
1600
1800
2000
2200
zmp_ph108_F9
zmp_ph108_F11
zmp_ph108_H2
zmp_ph108_H5
zmp_ph108_B2
zmp_ph108_B6
zmp_ph108_B7
zmp_ph108_C4
zmp_ph108_C5
zmp_ph108_D4
zmp_ph97_G6
zmp_ph97_G9
zmp_ph97_H2
zmp_ph97_B10
zmp_ph97_C2
zmp_ph97_C4
zmp_ph97_B8
zmp_ph97_C1
zmp_ph97_D2
zmp_ph97_D5
zmp_ph111_G5
zmp_ph111_G11
zmp_ph111_H1
zmp_ph111_B1a
zmp_ph111_B5
zmp_ph111_B8
zmp_ph111_B6
zmp_ph111_B7
zmp_ph111_B9
zmp_ph111_B10
zmp_ph114_G4
zmp_ph114_G11
zmp_ph114_H1
zmp_ph114_H2
zmp_ph114_A9
zmp_ph114_A11
zmp_ph114_B2
zmp_ph114_B4
zmp_ph114_D8
zmp_ph114_E2

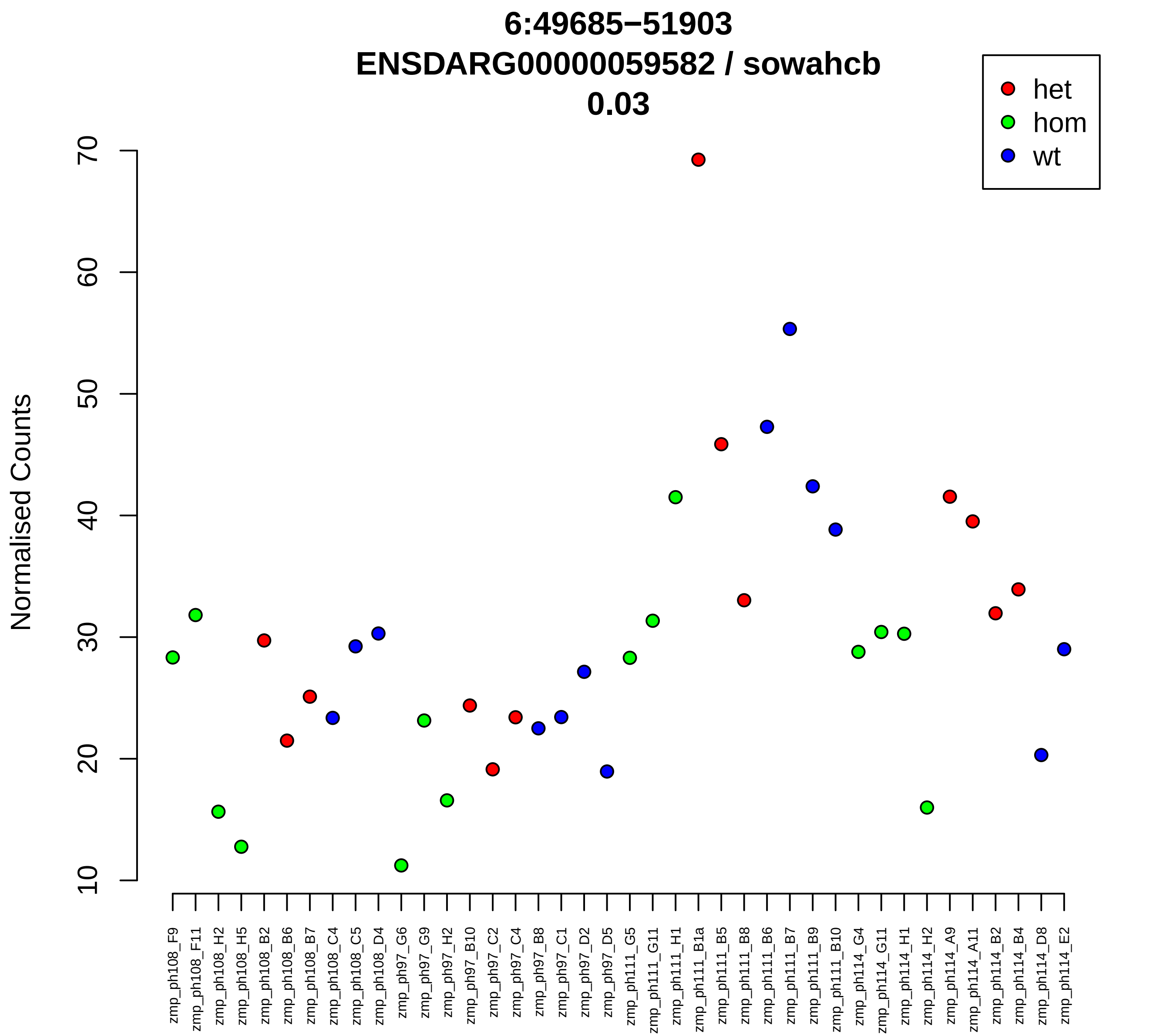
6:49685−51903
ENSDARG00000059582 / sowahcb
0.03
het
hom
wt
Normalised Counts
10
20
30
40
50
60
70
zmp_ph108_F9
zmp_ph108_F11
zmp_ph108_H2
zmp_ph108_H5
zmp_ph108_B2
zmp_ph108_B6
zmp_ph108_B7
zmp_ph108_C4
zmp_ph108_C5
zmp_ph108_D4
zmp_ph97_G6
zmp_ph97_G9
zmp_ph97_H2
zmp_ph97_B10
zmp_ph97_C2
zmp_ph97_C4
zmp_ph97_B8
zmp_ph97_C1
zmp_ph97_D2
zmp_ph97_D5
zmp_ph111_G5
zmp_ph111_G11
zmp_ph111_H1
zmp_ph111_B1a
zmp_ph111_B5
zmp_ph111_B8
zmp_ph111_B6
zmp_ph111_B7
zmp_ph111_B9
zmp_ph111_B10
zmp_ph114_G4
zmp_ph114_G11
zmp_ph114_H1
zmp_ph114_H2
zmp_ph114_A9
zmp_ph114_A11
zmp_ph114_B2
zmp_ph114_B4
zmp_ph114_D8
zmp_ph114_E2

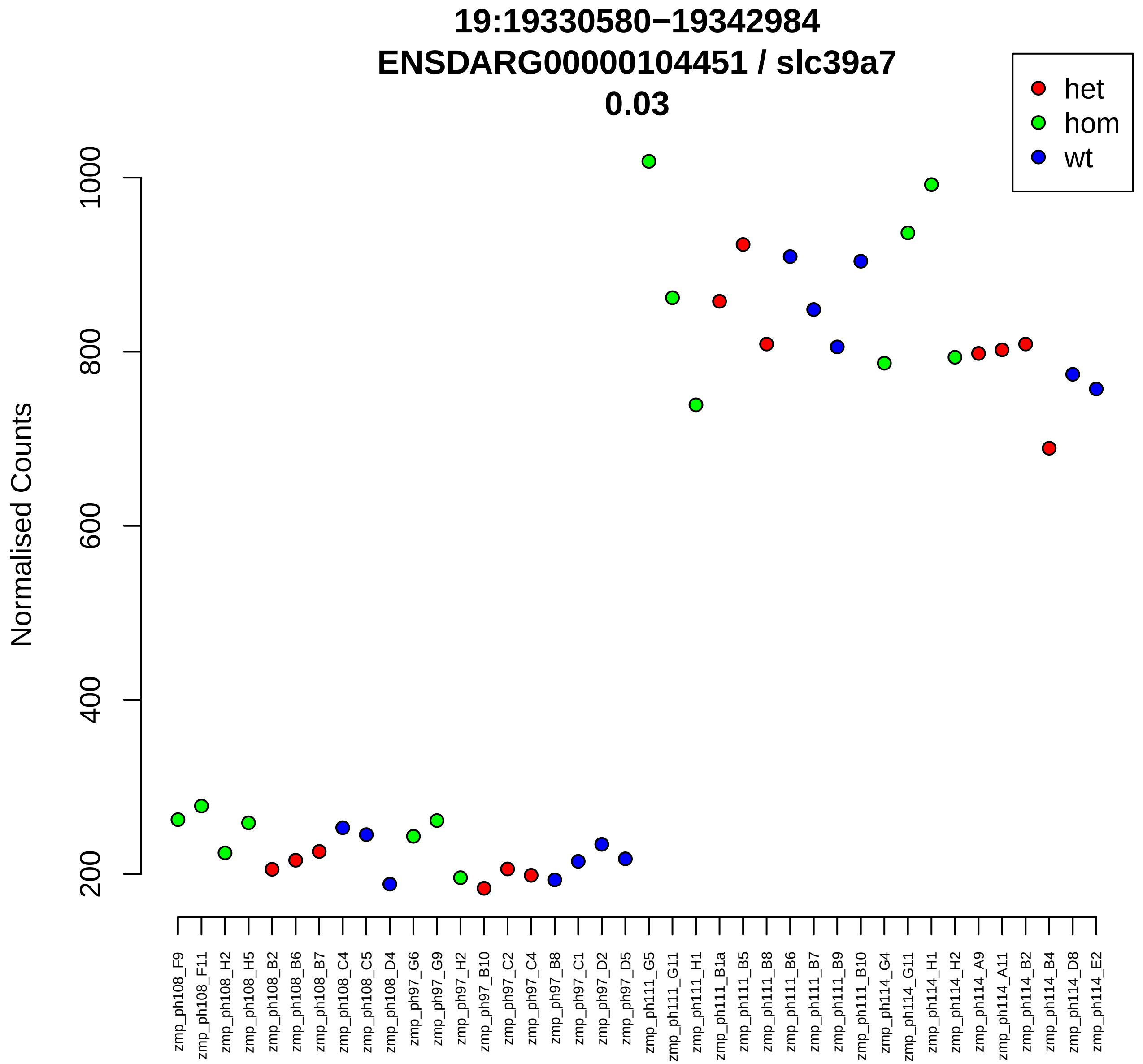

19:19330580−19342984
ENSDARG00000104451 / slc39a7
0.03
het
hom
wt
Normalised Counts
200
400
600
800
1000
zmp_ph108_F9
zmp_ph108_F11
zmp_ph108_H2
zmp_ph108_H5
zmp_ph108_B2
zmp_ph108_B6
zmp_ph108_B7
zmp_ph108_C4
zmp_ph108_C5
zmp_ph108_D4
zmp_ph97_G6
zmp_ph97_G9
zmp_ph97_H2
zmp_ph97_B10
zmp_ph97_C2
zmp_ph97_C4
zmp_ph97_B8
zmp_ph97_C1
zmp_ph97_D2
zmp_ph97_D5
zmp_ph111_G5
zmp_ph111_G11
zmp_ph111_H1
zmp_ph111_B1a
zmp_ph111_B5
zmp_ph111_B8
zmp_ph111_B6
zmp_ph111_B7
zmp_ph111_B9
zmp_ph111_B10
zmp_ph114_G4
zmp_ph114_G11
zmp_ph114_H1
zmp_ph114_H2
zmp_ph114_A9
zmp_ph114_A11
zmp_ph114_B2
zmp_ph114_B4
zmp_ph114_D8
zmp_ph114_E2

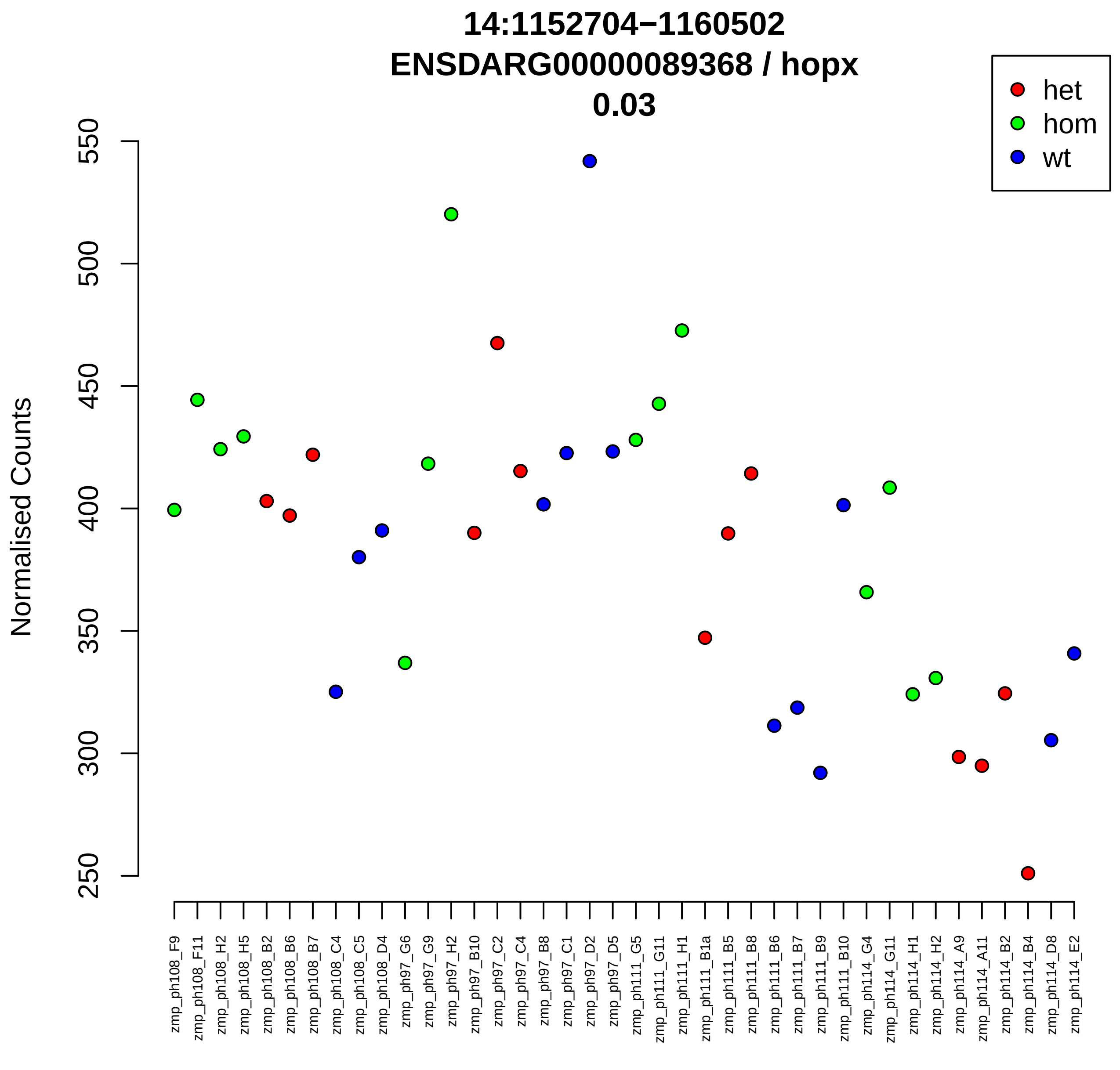

14:1152704−1160502
ENSDARG00000089368 / hopx
0.03
het
hom
wt
Normalised Counts
250
300
350
400
450
500
550
zmp_ph108_F9
zmp_ph108_F11
zmp_ph108_H2
zmp_ph108_H5
zmp_ph108_B2
zmp_ph108_B6
zmp_ph108_B7
zmp_ph108_C4
zmp_ph108_C5
zmp_ph108_D4
zmp_ph97_G6
zmp_ph97_G9
zmp_ph97_H2
zmp_ph97_B10
zmp_ph97_C2
zmp_ph97_C4
zmp_ph97_B8
zmp_ph97_C1
zmp_ph97_D2
zmp_ph97_D5
zmp_ph111_G5
zmp_ph111_G11
zmp_ph111_H1
zmp_ph111_B1a
zmp_ph111_B5
zmp_ph111_B8
zmp_ph111_B6
zmp_ph111_B7
zmp_ph111_B9
zmp_ph111_B10
zmp_ph114_G4
zmp_ph114_G11
zmp_ph114_H1
zmp_ph114_H2
zmp_ph114_A9
zmp_ph114_A11
zmp_ph114_B2
zmp_ph114_B4
zmp_ph114_D8
zmp_ph114_E2

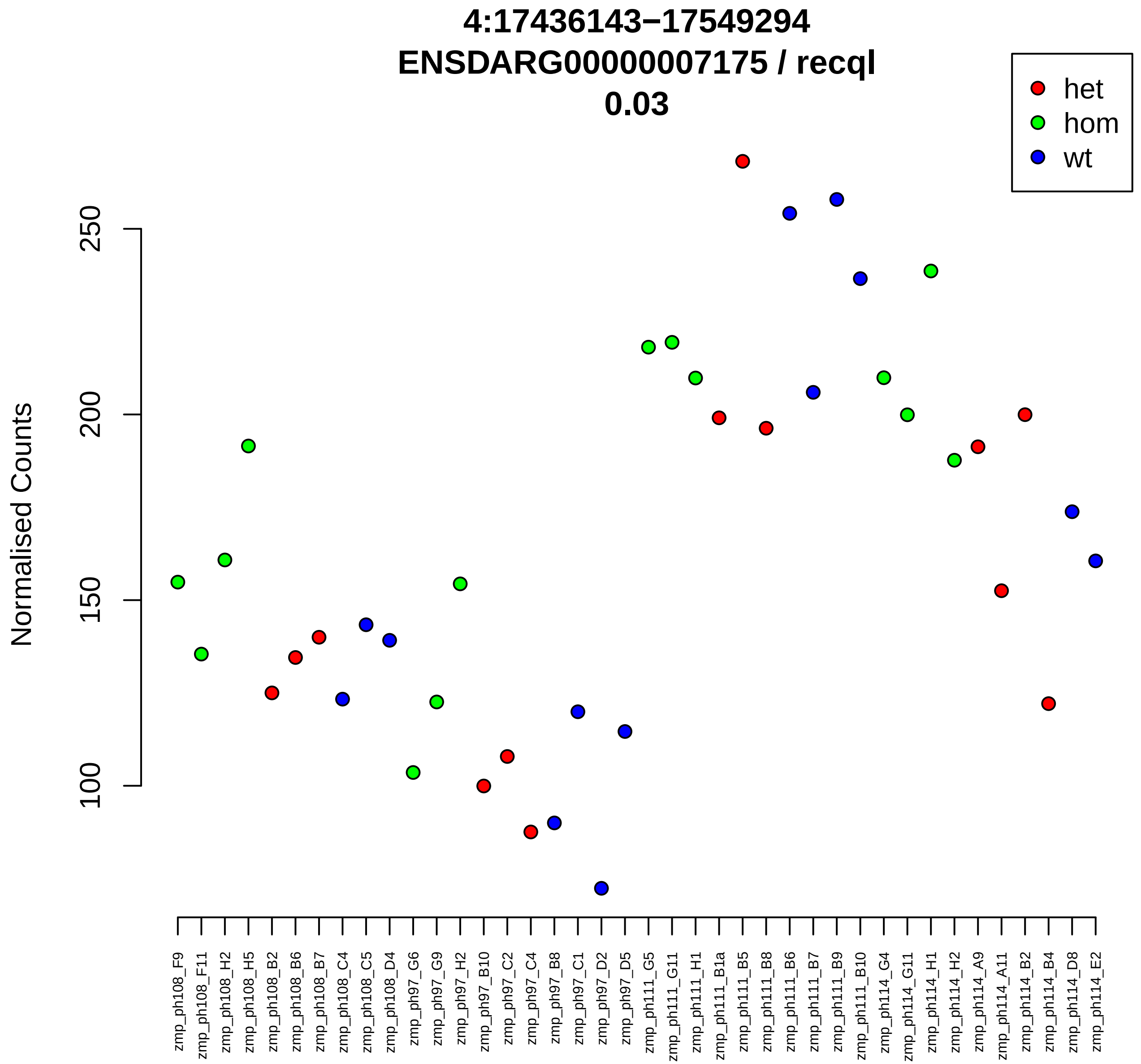

4:17436143−17549294
ENSDARG00000007175 / recql
0.03
het
hom
wt
Normalised Counts
100
150
200
250
zmp_ph108_F9
zmp_ph108_F11
zmp_ph108_H2
zmp_ph108_H5
zmp_ph108_B2
zmp_ph108_B6
zmp_ph108_B7
zmp_ph108_C4
zmp_ph108_C5
zmp_ph108_D4
zmp_ph97_G6
zmp_ph97_G9
zmp_ph97_H2
zmp_ph97_B10
zmp_ph97_C2
zmp_ph97_C4
zmp_ph97_B8
zmp_ph97_C1
zmp_ph97_D2
zmp_ph97_D5
zmp_ph111_G5
zmp_ph111_G11
zmp_ph111_H1
zmp_ph111_B1a
zmp_ph111_B5
zmp_ph111_B8
zmp_ph111_B6
zmp_ph111_B7
zmp_ph111_B9
zmp_ph111_B10
zmp_ph114_G4
zmp_ph114_G11
zmp_ph114_H1
zmp_ph114_H2
zmp_ph114_A9
zmp_ph114_A11
zmp_ph114_B2
zmp_ph114_B4
zmp_ph114_D8
zmp_ph114_E2

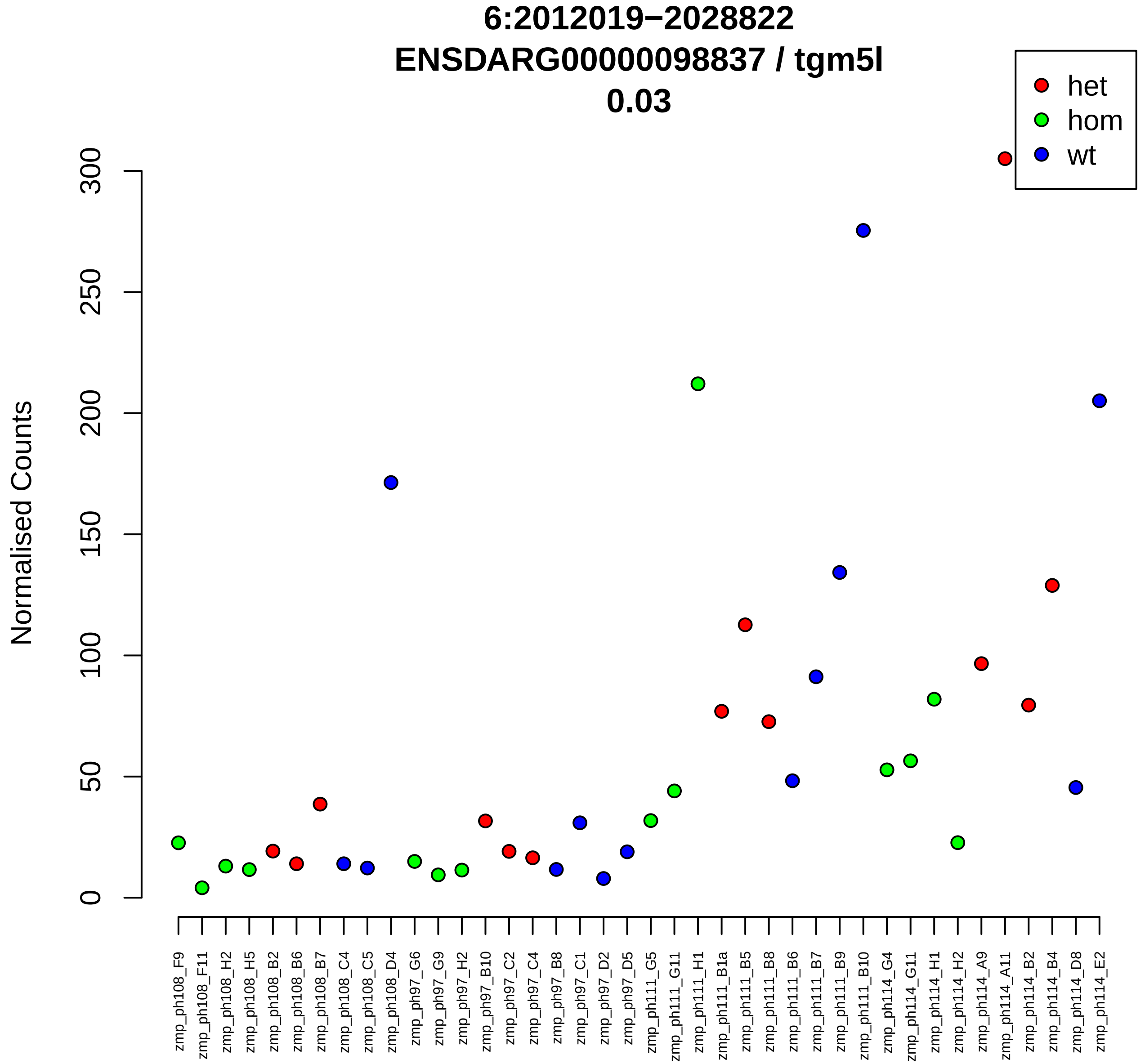
6:2012019−2028822
ENSDARG00000098837 / tgm5l
0.03
het
hom
wt
Normalised Counts
0
50
100
150
200
250
300
zmp_ph108_F9
zmp_ph108_F11
zmp_ph108_H2
zmp_ph108_H5
zmp_ph108_B2
zmp_ph108_B6
zmp_ph108_B7
zmp_ph108_C4
zmp_ph108_C5
zmp_ph108_D4
zmp_ph97_G6
zmp_ph97_G9
zmp_ph97_H2
zmp_ph97_B10
zmp_ph97_C2
zmp_ph97_C4
zmp_ph97_B8
zmp_ph97_C1
zmp_ph97_D2
zmp_ph97_D5
zmp_ph111_G5
zmp_ph111_G11
zmp_ph111_H1
zmp_ph111_B1a
zmp_ph111_B5
zmp_ph111_B8
zmp_ph111_B6
zmp_ph111_B7
zmp_ph111_B9
zmp_ph111_B10
zmp_ph114_G4
zmp_ph114_G11
zmp_ph114_H1
zmp_ph114_H2
zmp_ph114_A9
zmp_ph114_A11
zmp_ph114_B2
zmp_ph114_B4
zmp_ph114_D8
zmp_ph114_E2

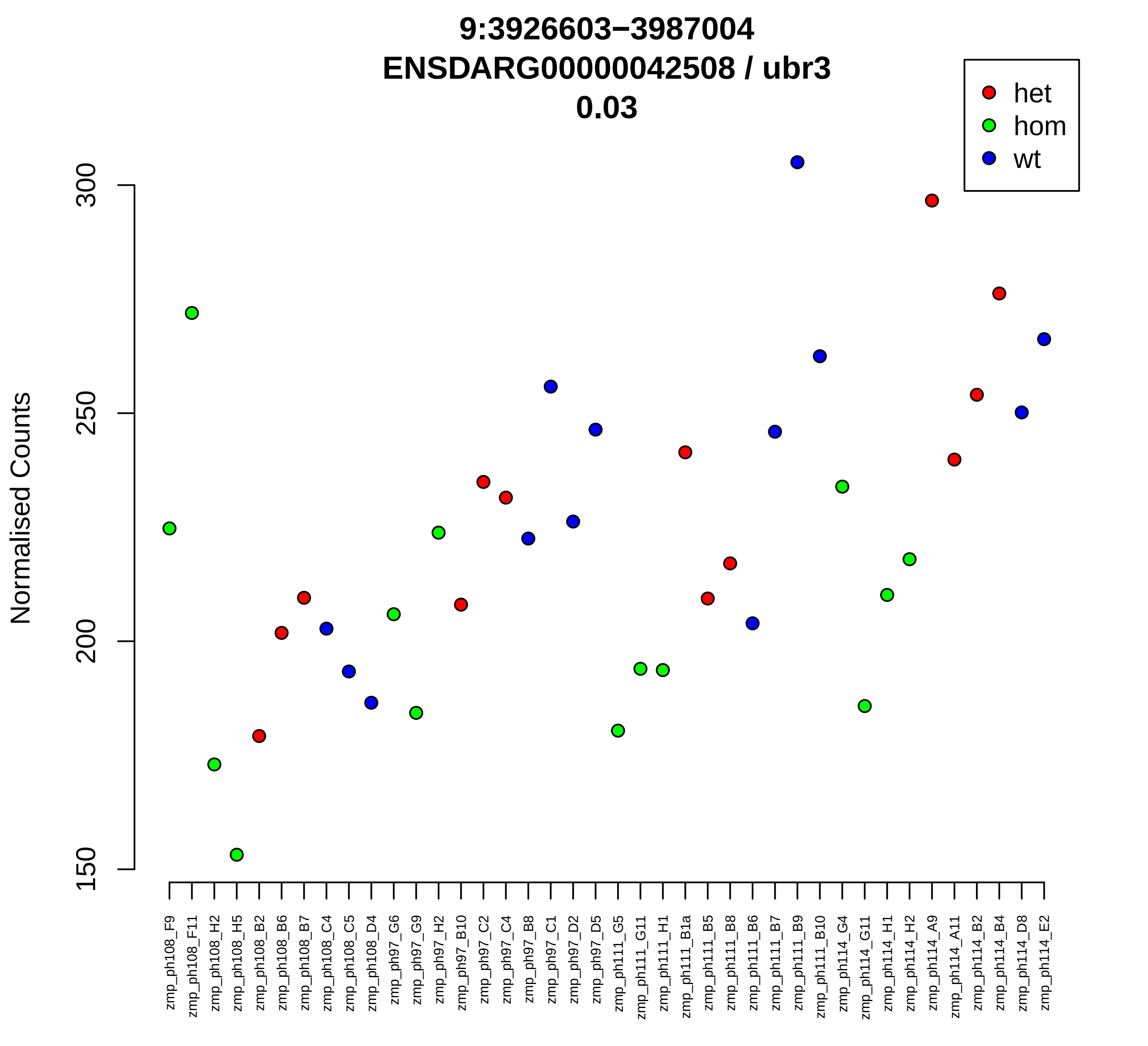
9:3926603−3987004
ENSDARG00000042508 / ubr3
0.03
het
hom
wt
Normalised Counts
150
200
250
300
zmp_ph108_F9
zmp_ph108_F11
zmp_ph108_H2
zmp_ph108_H5
zmp_ph108_B2
zmp_ph108_B6
zmp_ph108_B7
zmp_ph108_C4
zmp_ph108_C5
zmp_ph108_D4
zmp_ph97_G6
zmp_ph97_G9
zmp_ph97_H2
zmp_ph97_B10
zmp_ph97_C2
zmp_ph97_C4
zmp_ph97_B8
zmp_ph97_C1
zmp_ph97_D2
zmp_ph97_D5
zmp_ph111_G5
zmp_ph111_G11
zmp_ph111_H1
zmp_ph111_B1a
zmp_ph111_B5
zmp_ph111_B8
zmp_ph111_B6
zmp_ph111_B7
zmp_ph111_B9
zmp_ph111_B10
zmp_ph114_G4
zmp_ph114_G11
zmp_ph114_H1
zmp_ph114_H2
zmp_ph114_A9
zmp_ph114_A11
zmp_ph114_B2
zmp_ph114_B4
zmp_ph114_D8
zmp_ph114_E2

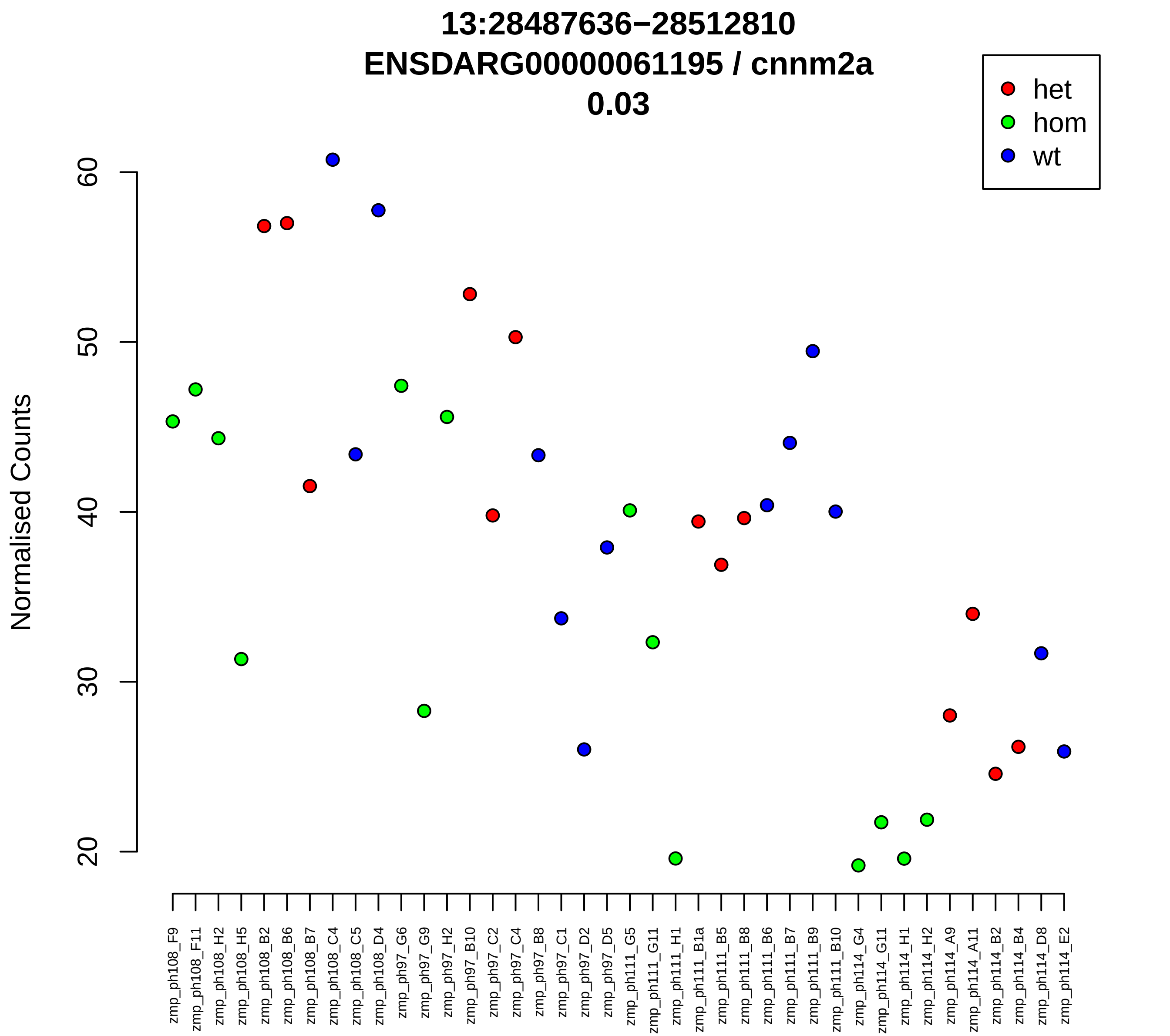

13:28487636−28512810
ENSDARG00000061195 / cnnm2a
0.03
het
hom
wt
Normalised Counts
20
30
40
50
60
zmp_ph108_F9
zmp_ph108_F11
zmp_ph108_H2
zmp_ph108_H5
zmp_ph108_B2
zmp_ph108_B6
zmp_ph108_B7
zmp_ph108_C4
zmp_ph108_C5
zmp_ph108_D4
zmp_ph97_G6
zmp_ph97_G9
zmp_ph97_H2
zmp_ph97_B10
zmp_ph97_C2
zmp_ph97_C4
zmp_ph97_B8
zmp_ph97_C1
zmp_ph97_D2
zmp_ph97_D5
zmp_ph111_G5
zmp_ph111_G11
zmp_ph111_H1
zmp_ph111_B1a
zmp_ph111_B5
zmp_ph111_B8
zmp_ph111_B6
zmp_ph111_B7
zmp_ph111_B9
zmp_ph111_B10
zmp_ph114_G4
zmp_ph114_G11
zmp_ph114_H1
zmp_ph114_H2
zmp_ph114_A9
zmp_ph114_A11
zmp_ph114_B2
zmp_ph114_B4
zmp_ph114_D8
zmp_ph114_E2

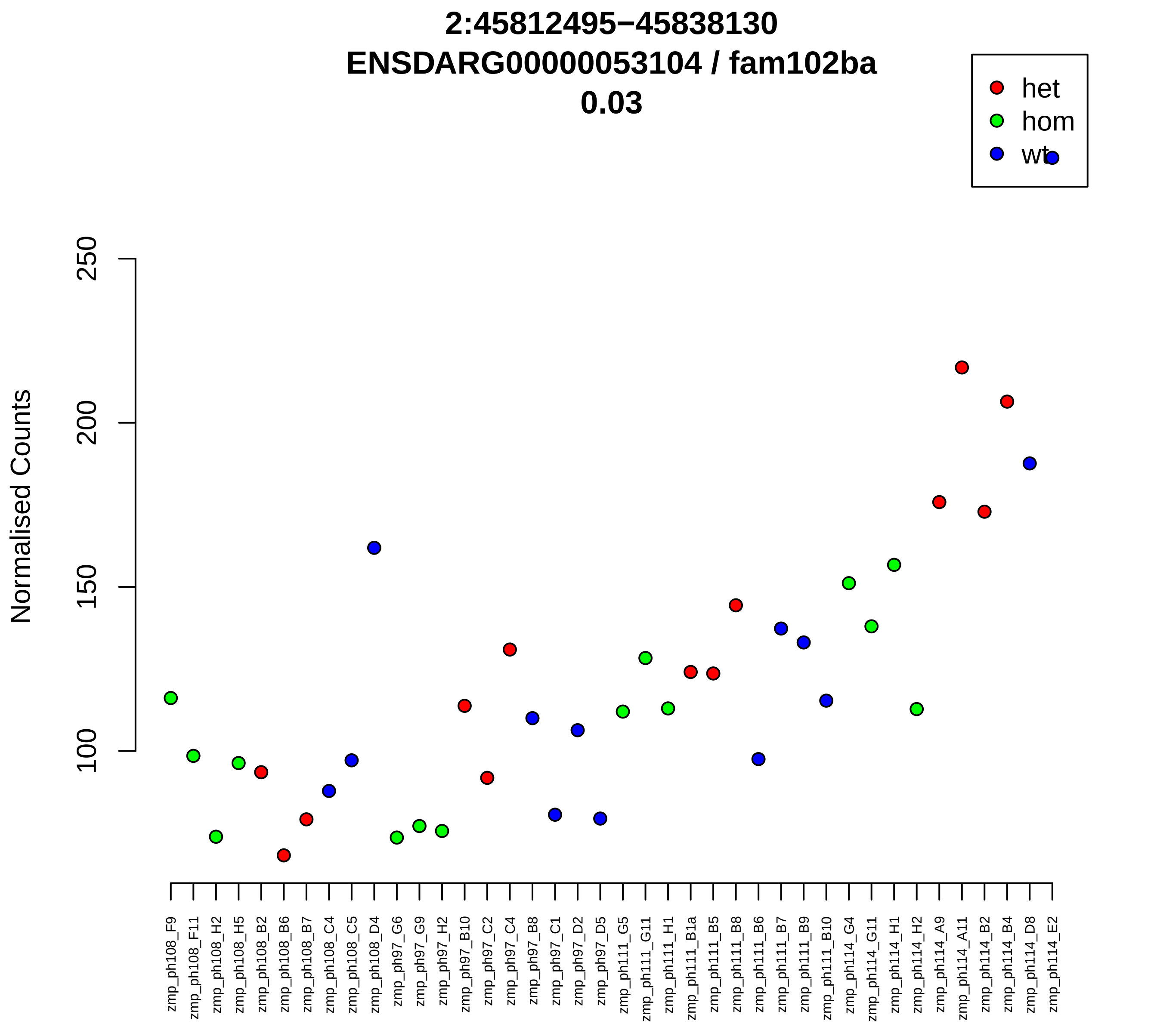
2:45812495−45838130
ENSDARG00000053104 / fam102ba
0.03
het
hom
wt
Normalised Counts
100
150
200
250
zmp_ph108_F9
zmp_ph108_F11
zmp_ph108_H2
zmp_ph108_H5
zmp_ph108_B2
zmp_ph108_B6
zmp_ph108_B7
zmp_ph108_C4
zmp_ph108_C5
zmp_ph108_D4
zmp_ph97_G6
zmp_ph97_G9
zmp_ph97_H2
zmp_ph97_B10
zmp_ph97_C2
zmp_ph97_C4
zmp_ph97_B8
zmp_ph97_C1
zmp_ph97_D2
zmp_ph97_D5
zmp_ph111_G5
zmp_ph111_G11
zmp_ph111_H1
zmp_ph111_B1a
zmp_ph111_B5
zmp_ph111_B8
zmp_ph111_B6
zmp_ph111_B7
zmp_ph111_B9
zmp_ph111_B10
zmp_ph114_G4
zmp_ph114_G11
zmp_ph114_H1
zmp_ph114_H2
zmp_ph114_A9
zmp_ph114_A11
zmp_ph114_B2
zmp_ph114_B4
zmp_ph114_D8
zmp_ph114_E2

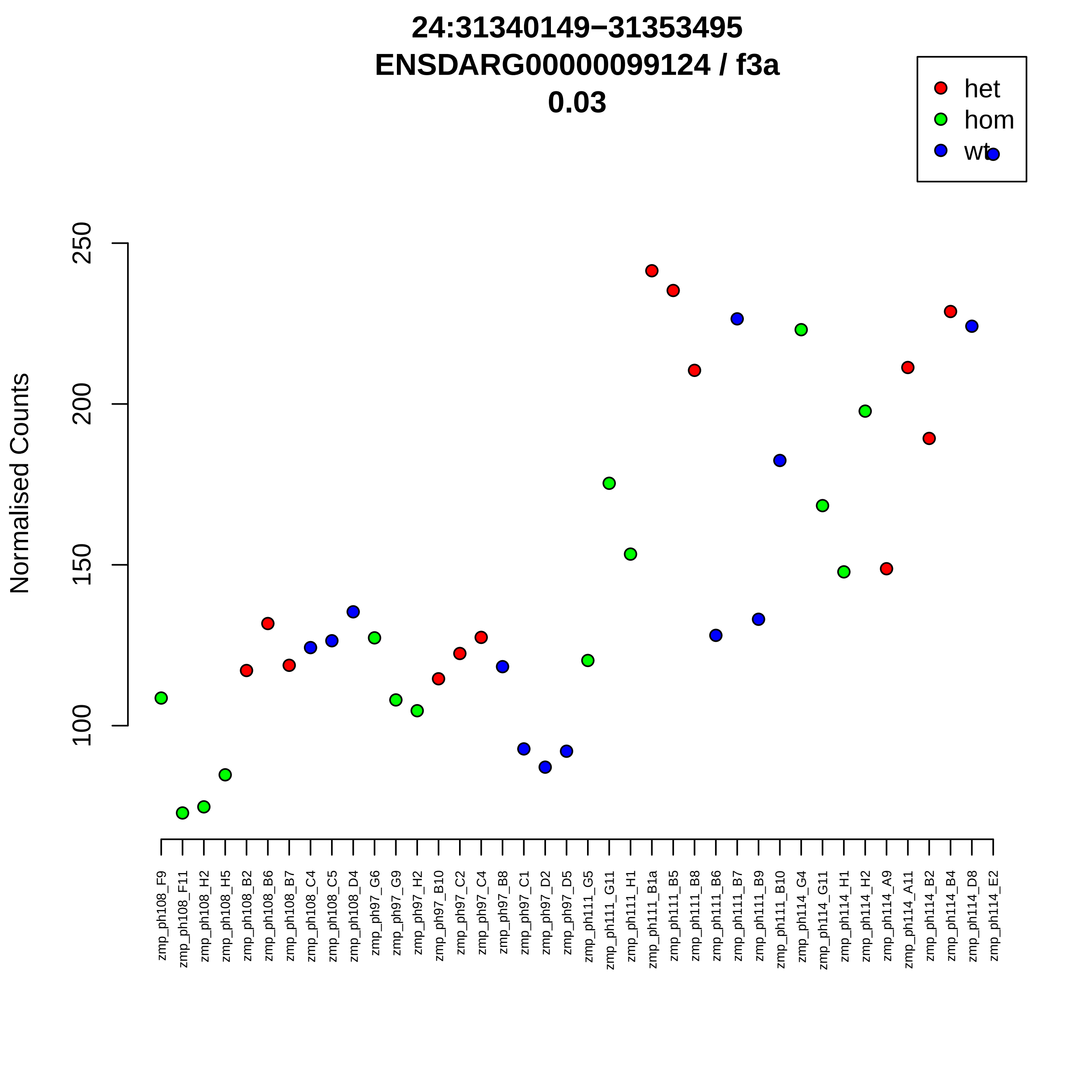
24:31340149−31353495
ENSDARG00000099124 / f3a
0.03
het
hom
wt
Normalised Counts
100
150
200
250
zmp_ph108_F9
zmp_ph108_F11
zmp_ph108_H2
zmp_ph108_H5
zmp_ph108_B2
zmp_ph108_B6
zmp_ph108_B7
zmp_ph108_C4
zmp_ph108_C5
zmp_ph108_D4
zmp_ph97_G6
zmp_ph97_G9
zmp_ph97_H2
zmp_ph97_B10
zmp_ph97_C2
zmp_ph97_C4
zmp_ph97_B8
zmp_ph97_C1
zmp_ph97_D2
zmp_ph97_D5
zmp_ph111_G5
zmp_ph111_G11
zmp_ph111_H1
zmp_ph111_B1a
zmp_ph111_B5
zmp_ph111_B8
zmp_ph111_B6
zmp_ph111_B7
zmp_ph111_B9
zmp_ph111_B10
zmp_ph114_G4
zmp_ph114_G11
zmp_ph114_H1
zmp_ph114_H2
zmp_ph114_A9
zmp_ph114_A11
zmp_ph114_B2
zmp_ph114_B4
zmp_ph114_D8
zmp_ph114_E2

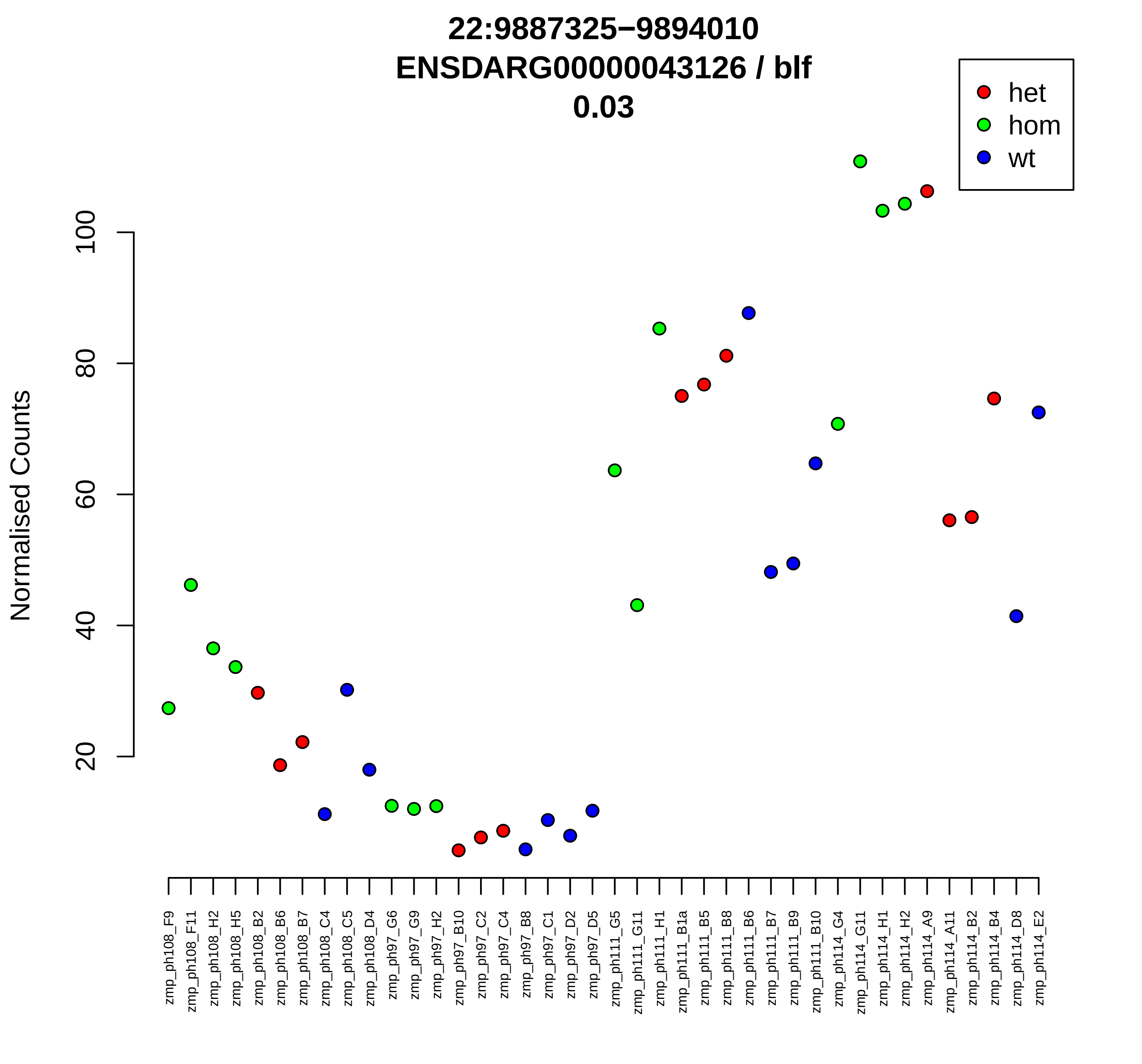
22:9887325−9894010
ENSDARG00000043126 / blf
0.03
het
hom
wt
Normalised Counts
20
40
60
80
100
zmp_ph108_F9
zmp_ph108_F11
zmp_ph108_H2
zmp_ph108_H5
zmp_ph108_B2
zmp_ph108_B6
zmp_ph108_B7
zmp_ph108_C4
zmp_ph108_C5
zmp_ph108_D4
zmp_ph97_G6
zmp_ph97_G9
zmp_ph97_H2
zmp_ph97_B10
zmp_ph97_C2
zmp_ph97_C4
zmp_ph97_B8
zmp_ph97_C1
zmp_ph97_D2
zmp_ph97_D5
zmp_ph111_G5
zmp_ph111_G11
zmp_ph111_H1
zmp_ph111_B1a
zmp_ph111_B5
zmp_ph111_B8
zmp_ph111_B6
zmp_ph111_B7
zmp_ph111_B9
zmp_ph111_B10
zmp_ph114_G4
zmp_ph114_G11
zmp_ph114_H1
zmp_ph114_H2
zmp_ph114_A9
zmp_ph114_A11
zmp_ph114_B2
zmp_ph114_B4
zmp_ph114_D8
zmp_ph114_E2

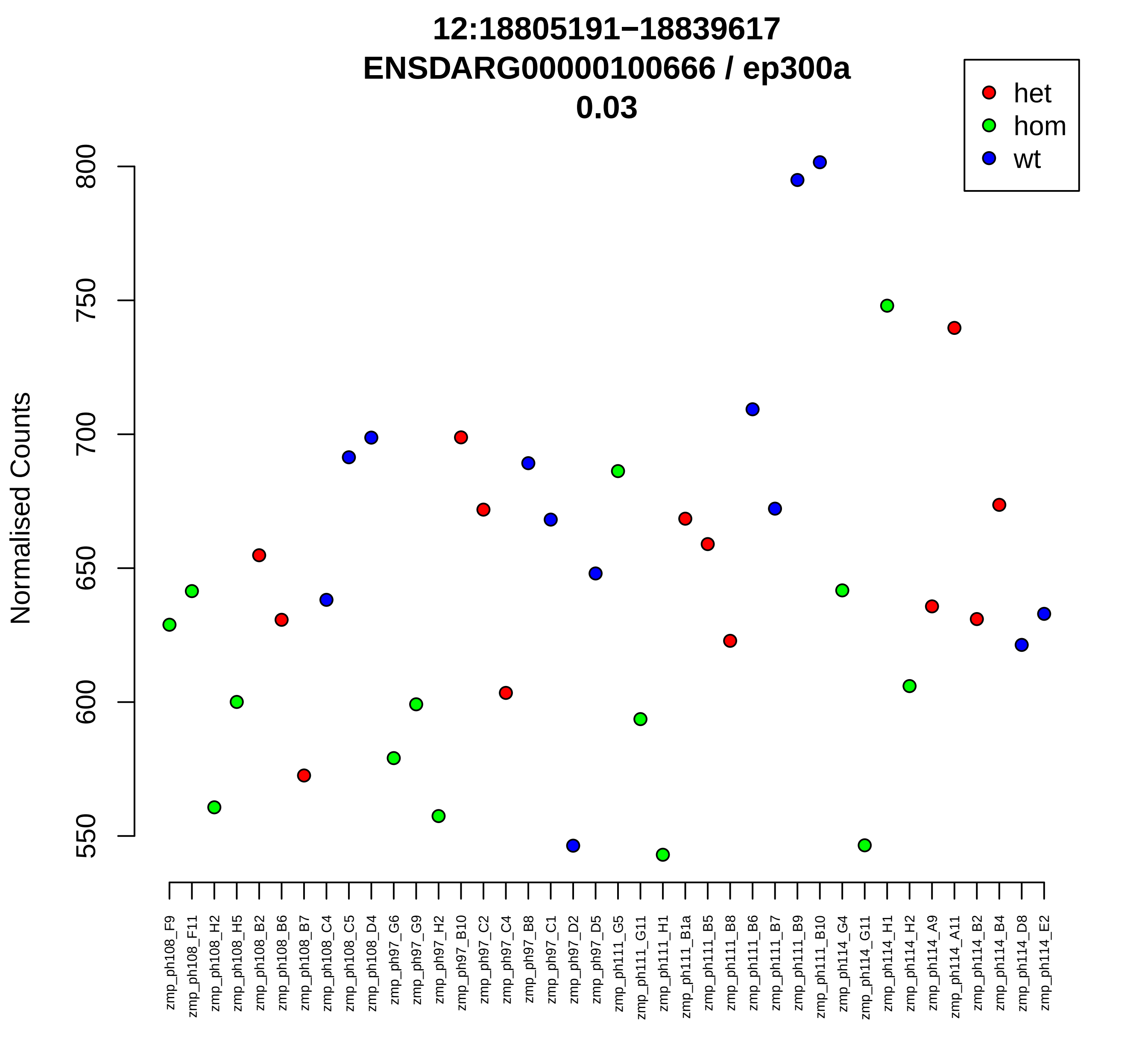

12:18805191−18839617
ENSDARG00000100666 / ep300a
0.03
het
hom
wt
Normalised Counts
550
600
650
700
750
800
zmp_ph108_F9
zmp_ph108_F11
zmp_ph108_H2
zmp_ph108_H5
zmp_ph108_B2
zmp_ph108_B6
zmp_ph108_B7
zmp_ph108_C4
zmp_ph108_C5
zmp_ph108_D4
zmp_ph97_G6
zmp_ph97_G9
zmp_ph97_H2
zmp_ph97_B10
zmp_ph97_C2
zmp_ph97_C4
zmp_ph97_B8
zmp_ph97_C1
zmp_ph97_D2
zmp_ph97_D5
zmp_ph111_G5
zmp_ph111_G11
zmp_ph111_H1
zmp_ph111_B1a
zmp_ph111_B5
zmp_ph111_B8
zmp_ph111_B6
zmp_ph111_B7
zmp_ph111_B9
zmp_ph111_B10
zmp_ph114_G4
zmp_ph114_G11
zmp_ph114_H1
zmp_ph114_H2
zmp_ph114_A9
zmp_ph114_A11
zmp_ph114_B2
zmp_ph114_B4
zmp_ph114_D8
zmp_ph114_E2

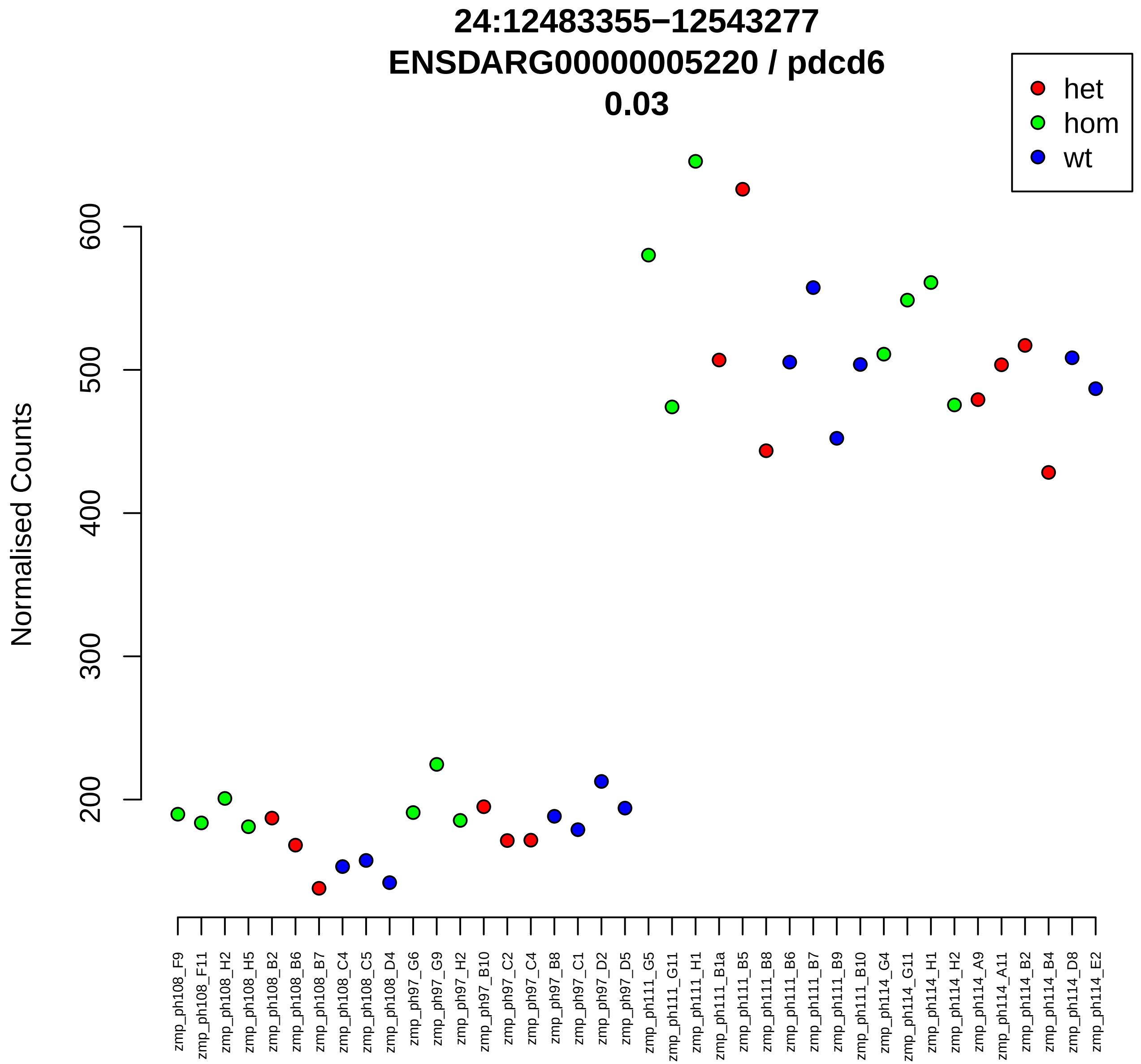
24:12483355−12543277
ENSDARG00000005220 / pdcd6
0.03
het
hom
wt
Normalised Counts
200
300
400
500
600
zmp_ph108_F9
zmp_ph108_F11
zmp_ph108_H2
zmp_ph108_H5
zmp_ph108_B2
zmp_ph108_B6
zmp_ph108_B7
zmp_ph108_C4
zmp_ph108_C5
zmp_ph108_D4
zmp_ph97_G6
zmp_ph97_G9
zmp_ph97_H2
zmp_ph97_B10
zmp_ph97_C2
zmp_ph97_C4
zmp_ph97_B8
zmp_ph97_C1
zmp_ph97_D2
zmp_ph97_D5
zmp_ph111_G5
zmp_ph111_G11
zmp_ph111_H1
zmp_ph111_B1a
zmp_ph111_B5
zmp_ph111_B8
zmp_ph111_B6
zmp_ph111_B7
zmp_ph111_B9
zmp_ph111_B10
zmp_ph114_G4
zmp_ph114_G11
zmp_ph114_H1
zmp_ph114_H2
zmp_ph114_A9
zmp_ph114_A11
zmp_ph114_B2
zmp_ph114_B4
zmp_ph114_D8
zmp_ph114_E2

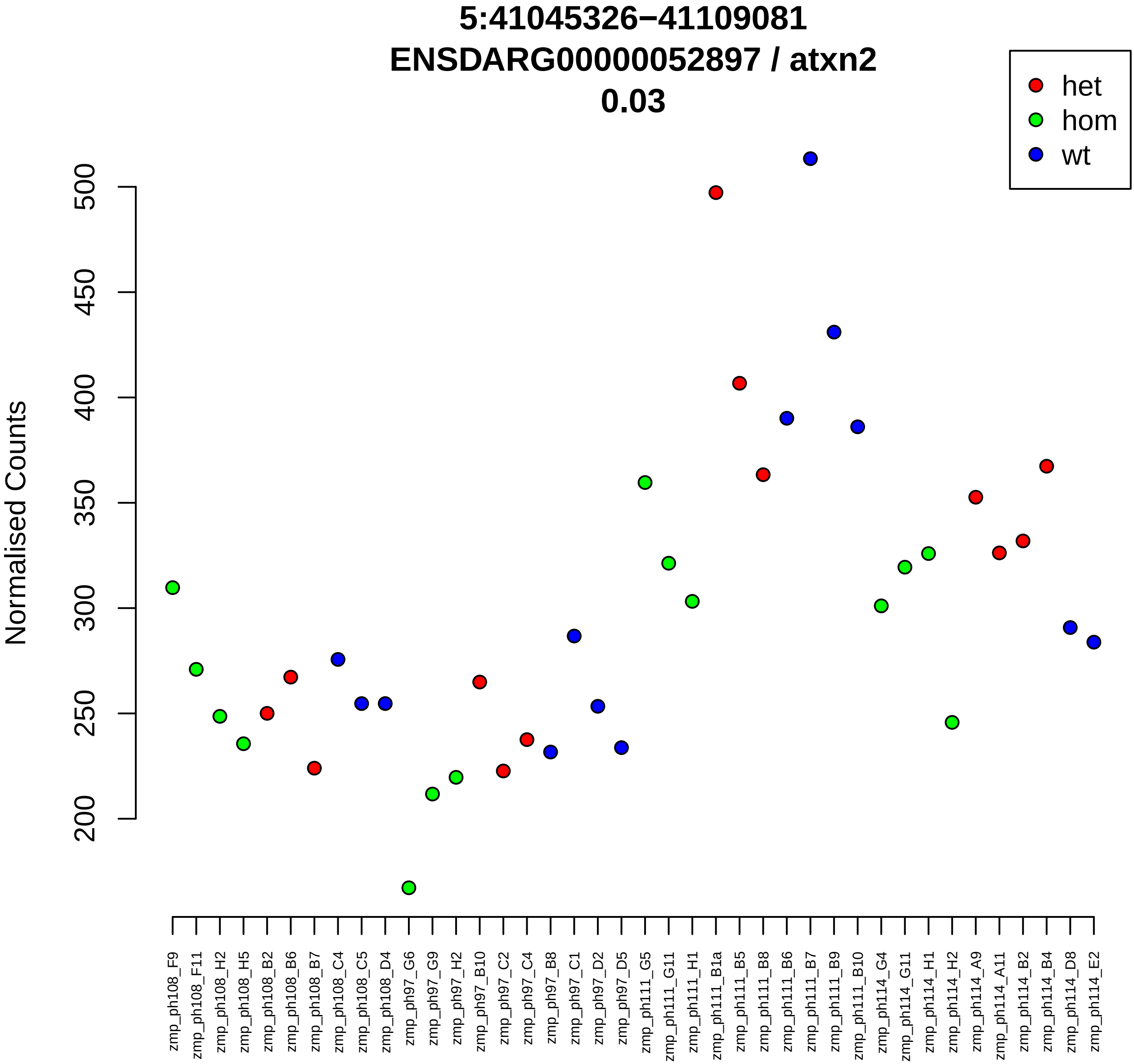
5:41045326−41109081
ENSDARG00000052897 / atxn2
0.03
het
hom
wt
Normalised Counts
200
250
300
350
400
450
500
zmp_ph108_F9
zmp_ph108_F11
zmp_ph108_H2
zmp_ph108_H5
zmp_ph108_B2
zmp_ph108_B6
zmp_ph108_B7
zmp_ph108_C4
zmp_ph108_C5
zmp_ph108_D4
zmp_ph97_G6
zmp_ph97_G9
zmp_ph97_H2
zmp_ph97_B10
zmp_ph97_C2
zmp_ph97_C4
zmp_ph97_B8
zmp_ph97_C1
zmp_ph97_D2
zmp_ph97_D5
zmp_ph111_G5
zmp_ph111_G11
zmp_ph111_H1
zmp_ph111_B1a
zmp_ph111_B5
zmp_ph111_B8
zmp_ph111_B6
zmp_ph111_B7
zmp_ph111_B9
zmp_ph111_B10
zmp_ph114_G4
zmp_ph114_G11
zmp_ph114_H1
zmp_ph114_H2
zmp_ph114_A9
zmp_ph114_A11
zmp_ph114_B2
zmp_ph114_B4
zmp_ph114_D8
zmp_ph114_E2

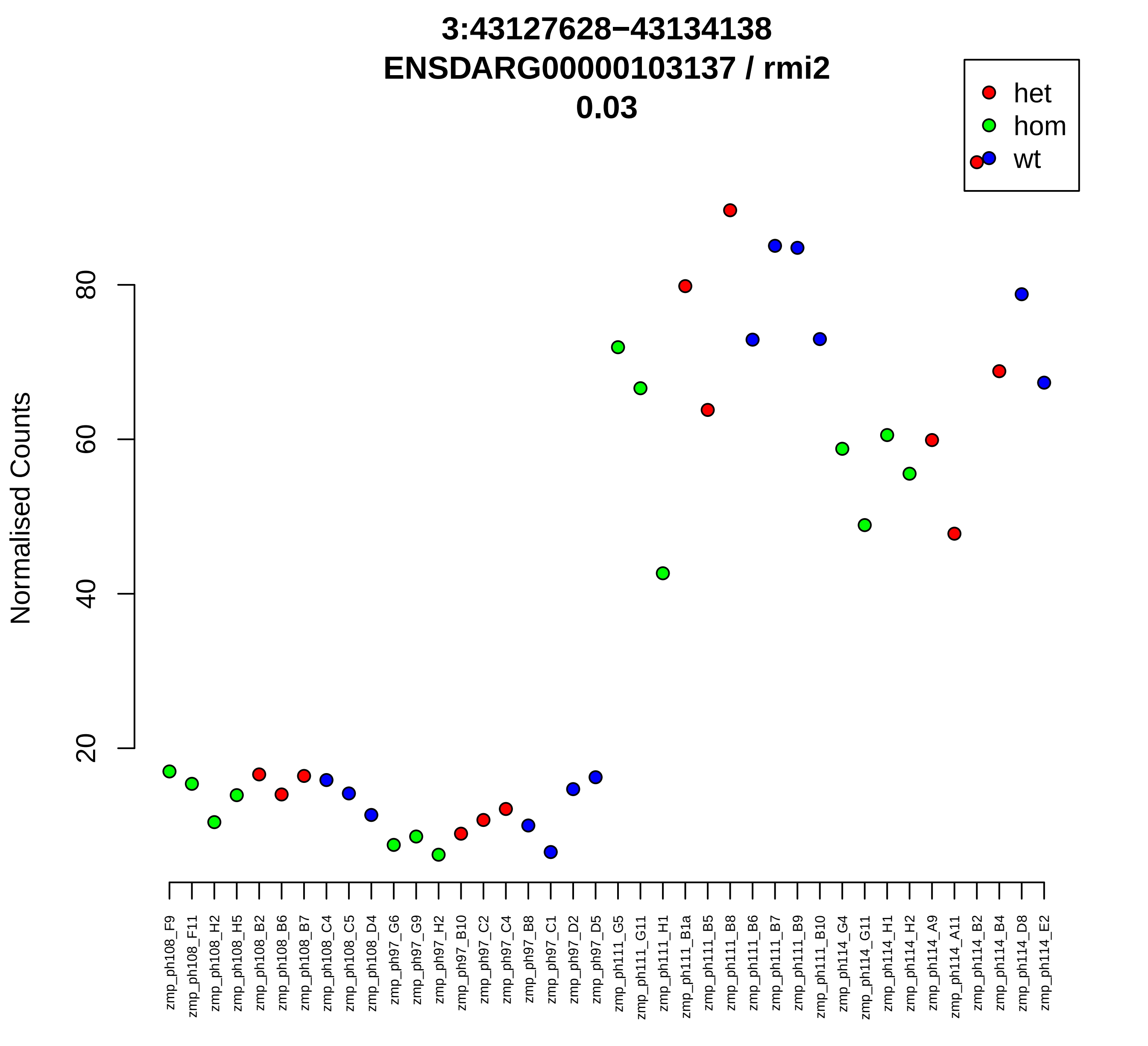

3:43127628−43134138
ENSDARG00000103137 / rmi2
0.03
het
hom
wt
Normalised Counts
20
40
60
80
zmp_ph108_F9
zmp_ph108_F11
zmp_ph108_H2
zmp_ph108_H5
zmp_ph108_B2
zmp_ph108_B6
zmp_ph108_B7
zmp_ph108_C4
zmp_ph108_C5
zmp_ph108_D4
zmp_ph97_G6
zmp_ph97_G9
zmp_ph97_H2
zmp_ph97_B10
zmp_ph97_C2
zmp_ph97_C4
zmp_ph97_B8
zmp_ph97_C1
zmp_ph97_D2
zmp_ph97_D5
zmp_ph111_G5
zmp_ph111_G11
zmp_ph111_H1
zmp_ph111_B1a
zmp_ph111_B5
zmp_ph111_B8
zmp_ph111_B6
zmp_ph111_B7
zmp_ph111_B9
zmp_ph111_B10
zmp_ph114_G4
zmp_ph114_G11
zmp_ph114_H1
zmp_ph114_H2
zmp_ph114_A9
zmp_ph114_A11
zmp_ph114_B2
zmp_ph114_B4
zmp_ph114_D8
zmp_ph114_E2

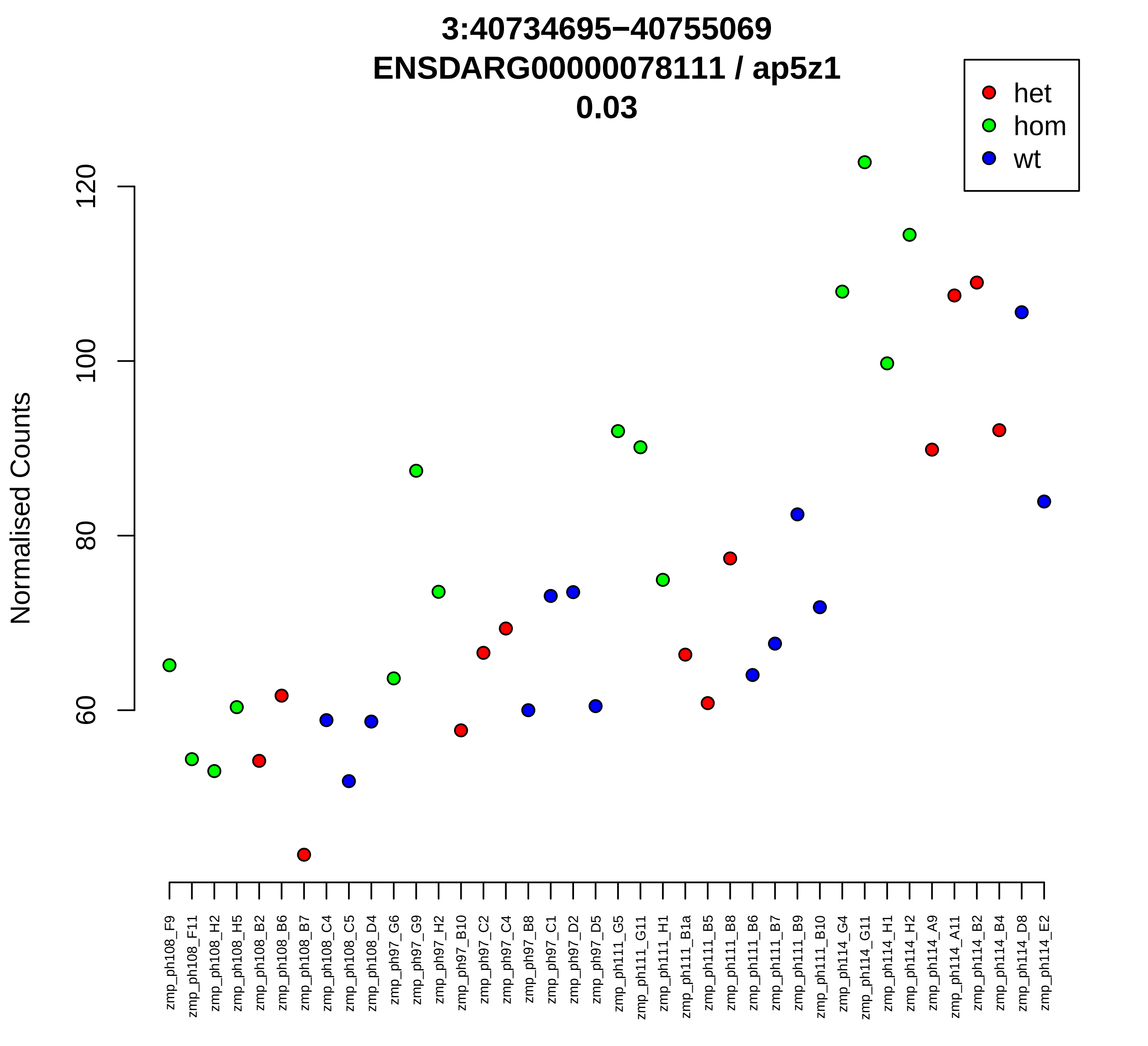

3:40734695−40755069
ENSDARG00000078111 / ap5z1
0.03
het
hom
wt
Normalised Counts
60
80
100
120
zmp_ph108_F9
zmp_ph108_F11
zmp_ph108_H2
zmp_ph108_H5
zmp_ph108_B2
zmp_ph108_B6
zmp_ph108_B7
zmp_ph108_C4
zmp_ph108_C5
zmp_ph108_D4
zmp_ph97_G6
zmp_ph97_G9
zmp_ph97_H2
zmp_ph97_B10
zmp_ph97_C2
zmp_ph97_C4
zmp_ph97_B8
zmp_ph97_C1
zmp_ph97_D2
zmp_ph97_D5
zmp_ph111_G5
zmp_ph111_G11
zmp_ph111_H1
zmp_ph111_B1a
zmp_ph111_B5
zmp_ph111_B8
zmp_ph111_B6
zmp_ph111_B7
zmp_ph111_B9
zmp_ph111_B10
zmp_ph114_G4
zmp_ph114_G11
zmp_ph114_H1
zmp_ph114_H2
zmp_ph114_A9
zmp_ph114_A11
zmp_ph114_B2
zmp_ph114_B4
zmp_ph114_D8
zmp_ph114_E2

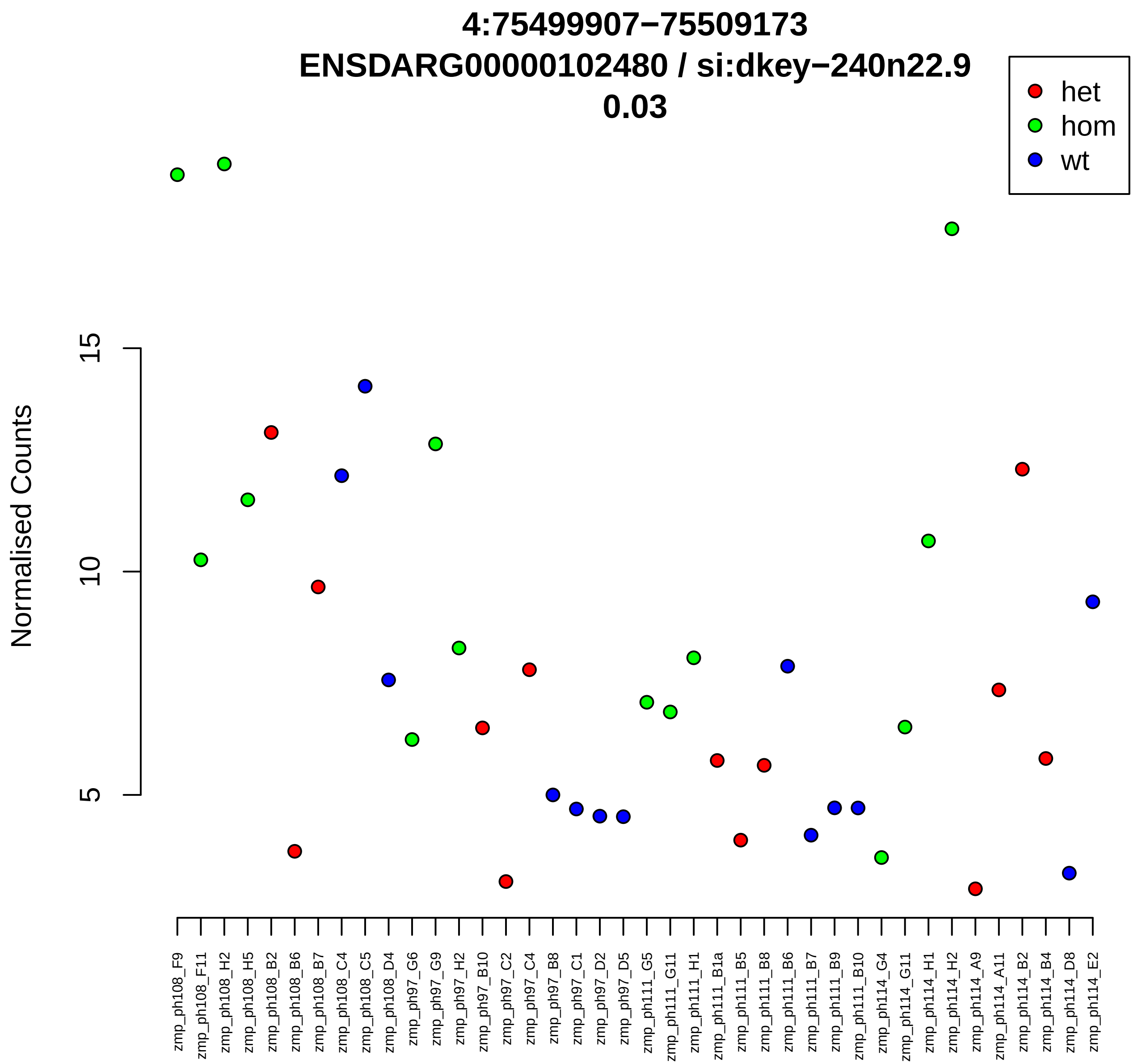

4:75499907−75509173
ENSDARG00000102480 / si:dkey−240n22.9
0.03
het
hom
wt
Normalised Counts
5
10
15
zmp_ph108_F9
zmp_ph108_F11
zmp_ph108_H2
zmp_ph108_H5
zmp_ph108_B2
zmp_ph108_B6
zmp_ph108_B7
zmp_ph108_C4
zmp_ph108_C5
zmp_ph108_D4
zmp_ph97_G6
zmp_ph97_G9
zmp_ph97_H2
zmp_ph97_B10
zmp_ph97_C2
zmp_ph97_C4
zmp_ph97_B8
zmp_ph97_C1
zmp_ph97_D2
zmp_ph97_D5
zmp_ph111_G5
zmp_ph111_G11
zmp_ph111_H1
zmp_ph111_B1a
zmp_ph111_B5
zmp_ph111_B8
zmp_ph111_B6
zmp_ph111_B7
zmp_ph111_B9
zmp_ph111_B10
zmp_ph114_G4
zmp_ph114_G11
zmp_ph114_H1
zmp_ph114_H2
zmp_ph114_A9
zmp_ph114_A11
zmp_ph114_B2
zmp_ph114_B4
zmp_ph114_D8
zmp_ph114_E2

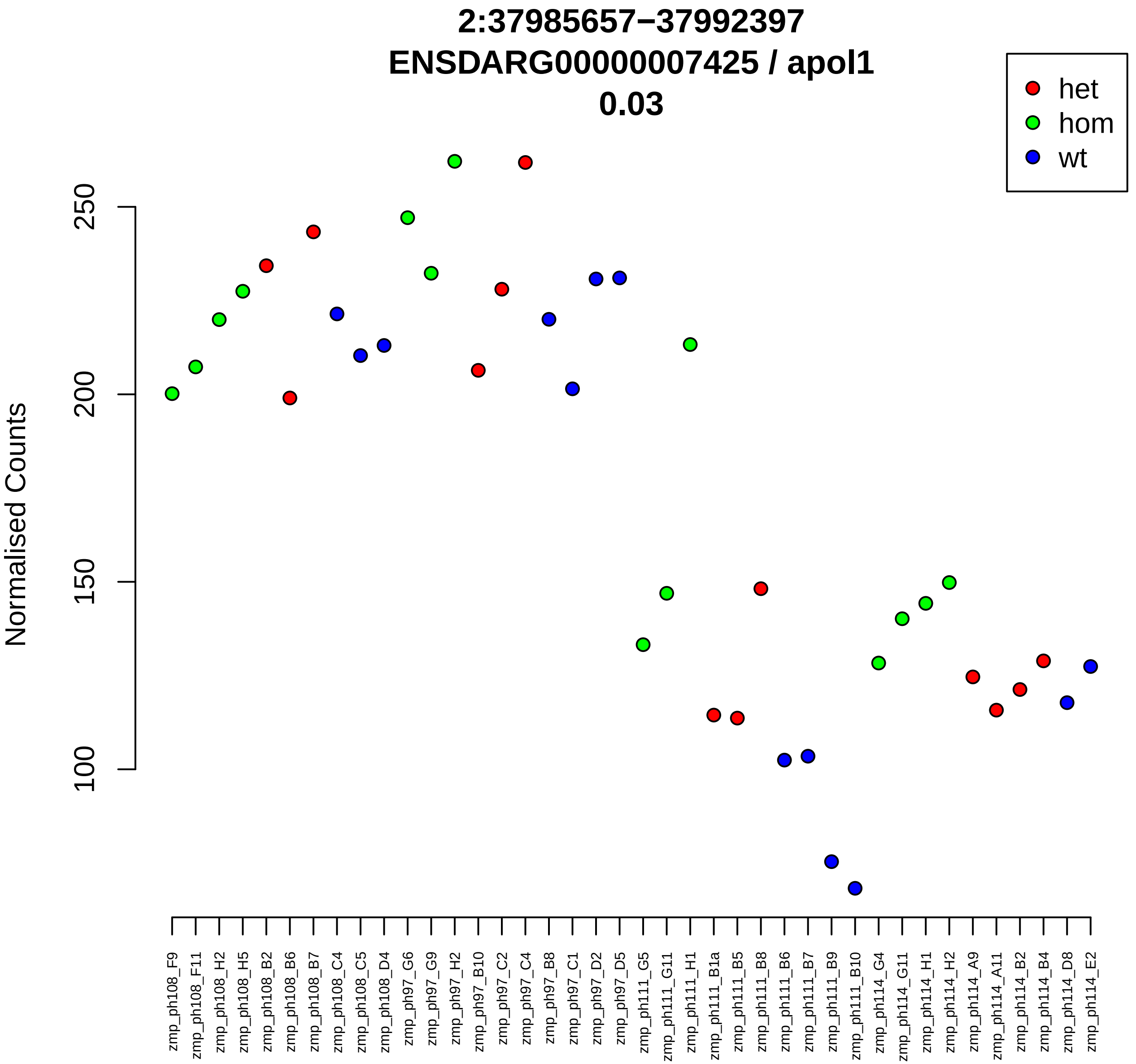

2:37985657−37992397
ENSDARG00000007425 / apol1
0.03
het
hom
wt
Normalised Counts
100
150
200
250
zmp_ph108_F9
zmp_ph108_F11
zmp_ph108_H2
zmp_ph108_H5
zmp_ph108_B2
zmp_ph108_B6
zmp_ph108_B7
zmp_ph108_C4
zmp_ph108_C5
zmp_ph108_D4
zmp_ph97_G6
zmp_ph97_G9
zmp_ph97_H2
zmp_ph97_B10
zmp_ph97_C2
zmp_ph97_C4
zmp_ph97_B8
zmp_ph97_C1
zmp_ph97_D2
zmp_ph97_D5
zmp_ph111_G5
zmp_ph111_G11
zmp_ph111_H1
zmp_ph111_B1a
zmp_ph111_B5
zmp_ph111_B8
zmp_ph111_B6
zmp_ph111_B7
zmp_ph111_B9
zmp_ph111_B10
zmp_ph114_G4
zmp_ph114_G11
zmp_ph114_H1
zmp_ph114_H2
zmp_ph114_A9
zmp_ph114_A11
zmp_ph114_B2
zmp_ph114_B4
zmp_ph114_D8
zmp_ph114_E2

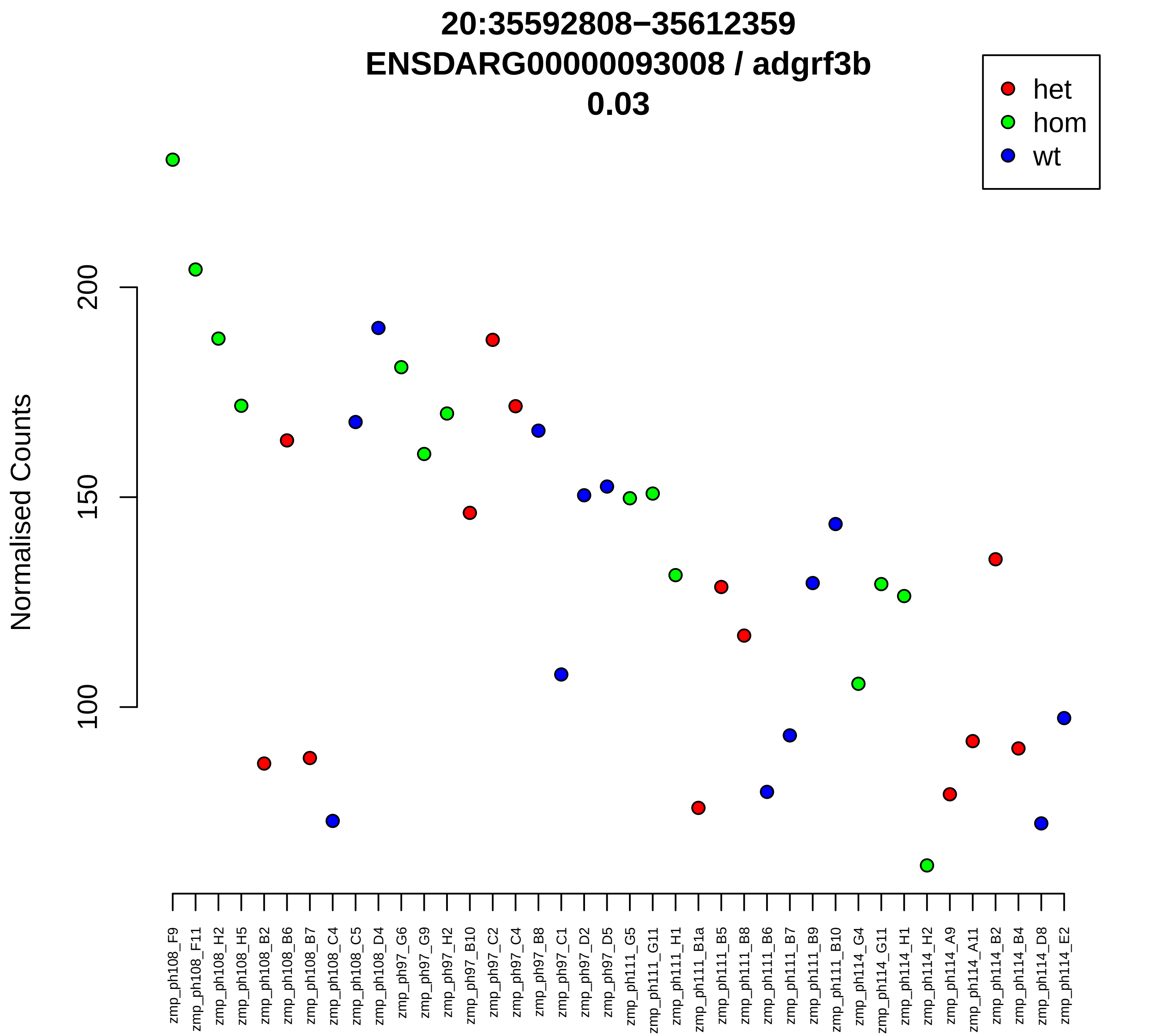

20:35592808−35612359
ENSDARG00000093008 / adgrf3b
0.03
het
hom
wt
Normalised Counts
100
150
200
zmp_ph108_F9
zmp_ph108_F11
zmp_ph108_H2
zmp_ph108_H5
zmp_ph108_B2
zmp_ph108_B6
zmp_ph108_B7
zmp_ph108_C4
zmp_ph108_C5
zmp_ph108_D4
zmp_ph97_G6
zmp_ph97_G9
zmp_ph97_H2
zmp_ph97_B10
zmp_ph97_C2
zmp_ph97_C4
zmp_ph97_B8
zmp_ph97_C1
zmp_ph97_D2
zmp_ph97_D5
zmp_ph111_G5
zmp_ph111_G11
zmp_ph111_H1
zmp_ph111_B1a
zmp_ph111_B5
zmp_ph111_B8
zmp_ph111_B6
zmp_ph111_B7
zmp_ph111_B9
zmp_ph111_B10
zmp_ph114_G4
zmp_ph114_G11
zmp_ph114_H1
zmp_ph114_H2
zmp_ph114_A9
zmp_ph114_A11
zmp_ph114_B2
zmp_ph114_B4
zmp_ph114_D8
zmp_ph114_E2

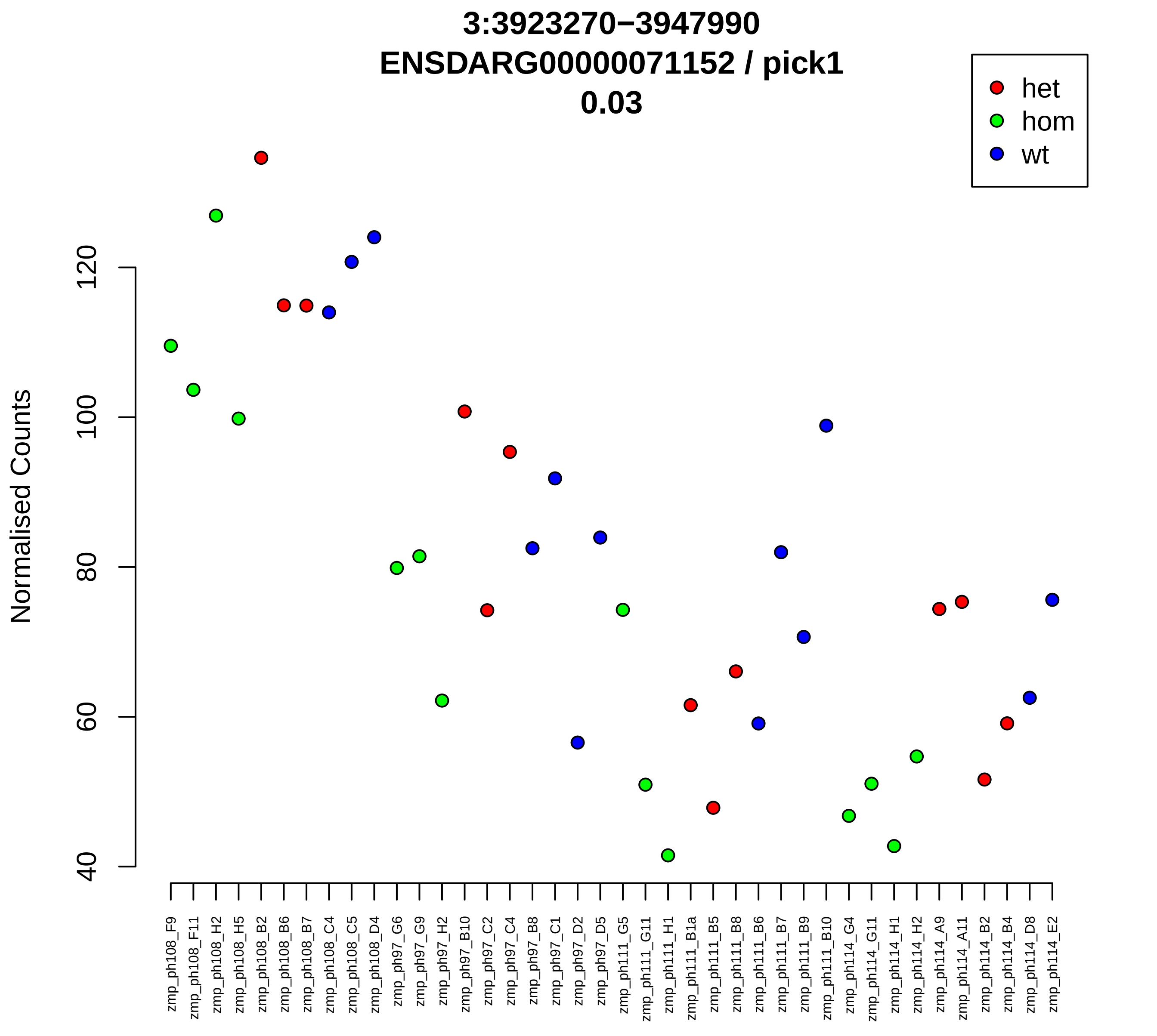
3:3923270−3947990
ENSDARG00000071152 / pick1
0.03
het
hom
wt
Normalised Counts
40
60
80
100
120
zmp_ph108_F9
zmp_ph108_F11
zmp_ph108_H2
zmp_ph108_H5
zmp_ph108_B2
zmp_ph108_B6
zmp_ph108_B7
zmp_ph108_C4
zmp_ph108_C5
zmp_ph108_D4
zmp_ph97_G6
zmp_ph97_G9
zmp_ph97_H2
zmp_ph97_B10
zmp_ph97_C2
zmp_ph97_C4
zmp_ph97_B8
zmp_ph97_C1
zmp_ph97_D2
zmp_ph97_D5
zmp_ph111_G5
zmp_ph111_G11
zmp_ph111_H1
zmp_ph111_B1a
zmp_ph111_B5
zmp_ph111_B8
zmp_ph111_B6
zmp_ph111_B7
zmp_ph111_B9
zmp_ph111_B10
zmp_ph114_G4
zmp_ph114_G11
zmp_ph114_H1
zmp_ph114_H2
zmp_ph114_A9
zmp_ph114_A11
zmp_ph114_B2
zmp_ph114_B4
zmp_ph114_D8
zmp_ph114_E2

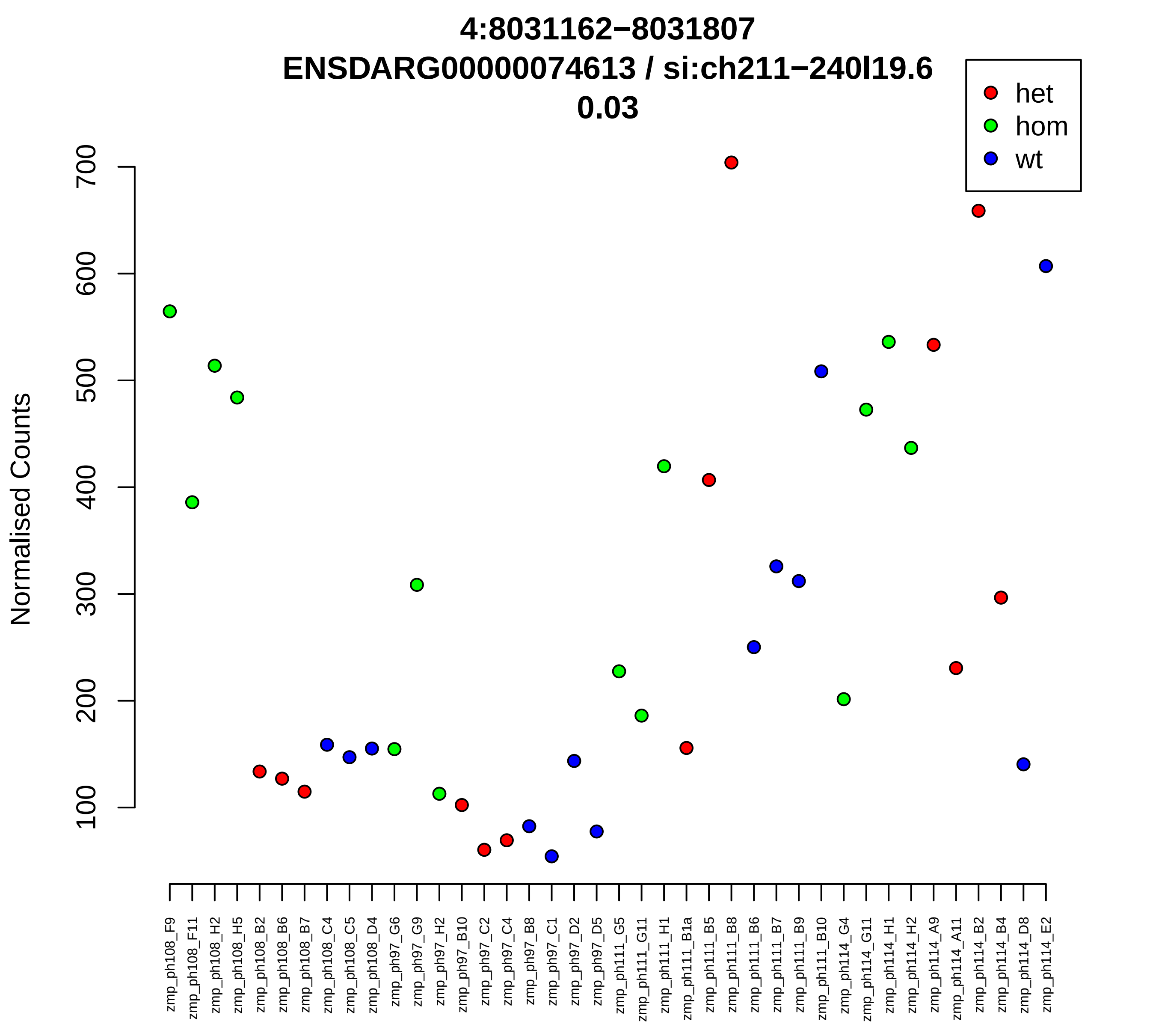
4:8031162−8031807
ENSDARG00000074613 / si:ch211−240l19.6
0.03
het
hom
wt
Normalised Counts
100
200
300
400
500
600
700
zmp_ph108_F9
zmp_ph108_F11
zmp_ph108_H2
zmp_ph108_H5
zmp_ph108_B2
zmp_ph108_B6
zmp_ph108_B7
zmp_ph108_C4
zmp_ph108_C5
zmp_ph108_D4
zmp_ph97_G6
zmp_ph97_G9
zmp_ph97_H2
zmp_ph97_B10
zmp_ph97_C2
zmp_ph97_C4
zmp_ph97_B8
zmp_ph97_C1
zmp_ph97_D2
zmp_ph97_D5
zmp_ph111_G5
zmp_ph111_G11
zmp_ph111_H1
zmp_ph111_B1a
zmp_ph111_B5
zmp_ph111_B8
zmp_ph111_B6
zmp_ph111_B7
zmp_ph111_B9
zmp_ph111_B10
zmp_ph114_G4
zmp_ph114_G11
zmp_ph114_H1
zmp_ph114_H2
zmp_ph114_A9
zmp_ph114_A11
zmp_ph114_B2
zmp_ph114_B4
zmp_ph114_D8
zmp_ph114_E2

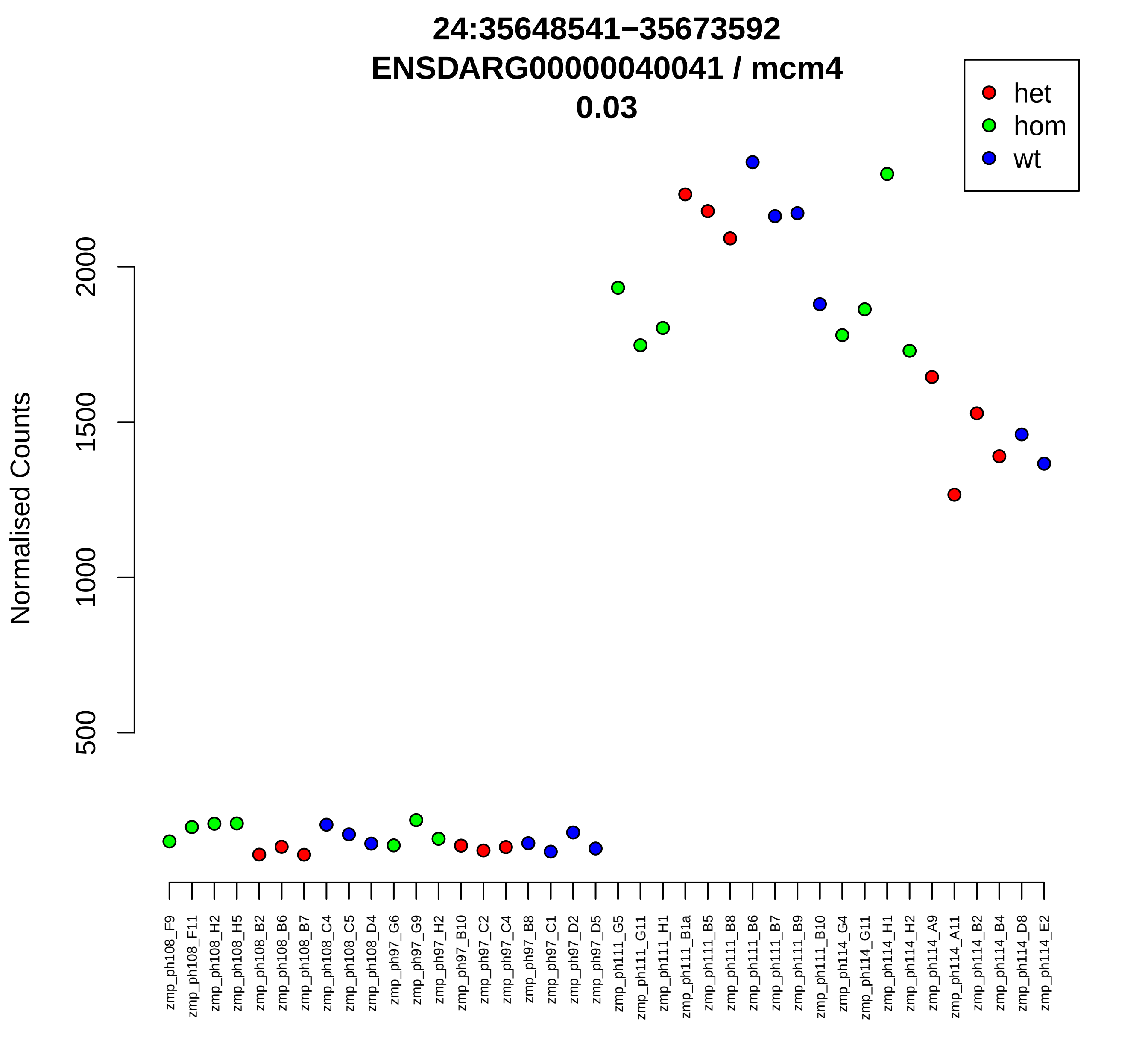

24:35648541−35673592
ENSDARG00000040041 / mcm4
0.03
het
hom
wt
Normalised Counts
500
1000
1500
2000
zmp_ph108_F9
zmp_ph108_F11
zmp_ph108_H2
zmp_ph108_H5
zmp_ph108_B2
zmp_ph108_B6
zmp_ph108_B7
zmp_ph108_C4
zmp_ph108_C5
zmp_ph108_D4
zmp_ph97_G6
zmp_ph97_G9
zmp_ph97_H2
zmp_ph97_B10
zmp_ph97_C2
zmp_ph97_C4
zmp_ph97_B8
zmp_ph97_C1
zmp_ph97_D2
zmp_ph97_D5
zmp_ph111_G5
zmp_ph111_G11
zmp_ph111_H1
zmp_ph111_B1a
zmp_ph111_B5
zmp_ph111_B8
zmp_ph111_B6
zmp_ph111_B7
zmp_ph111_B9
zmp_ph111_B10
zmp_ph114_G4
zmp_ph114_G11
zmp_ph114_H1
zmp_ph114_H2
zmp_ph114_A9
zmp_ph114_A11
zmp_ph114_B2
zmp_ph114_B4
zmp_ph114_D8
zmp_ph114_E2

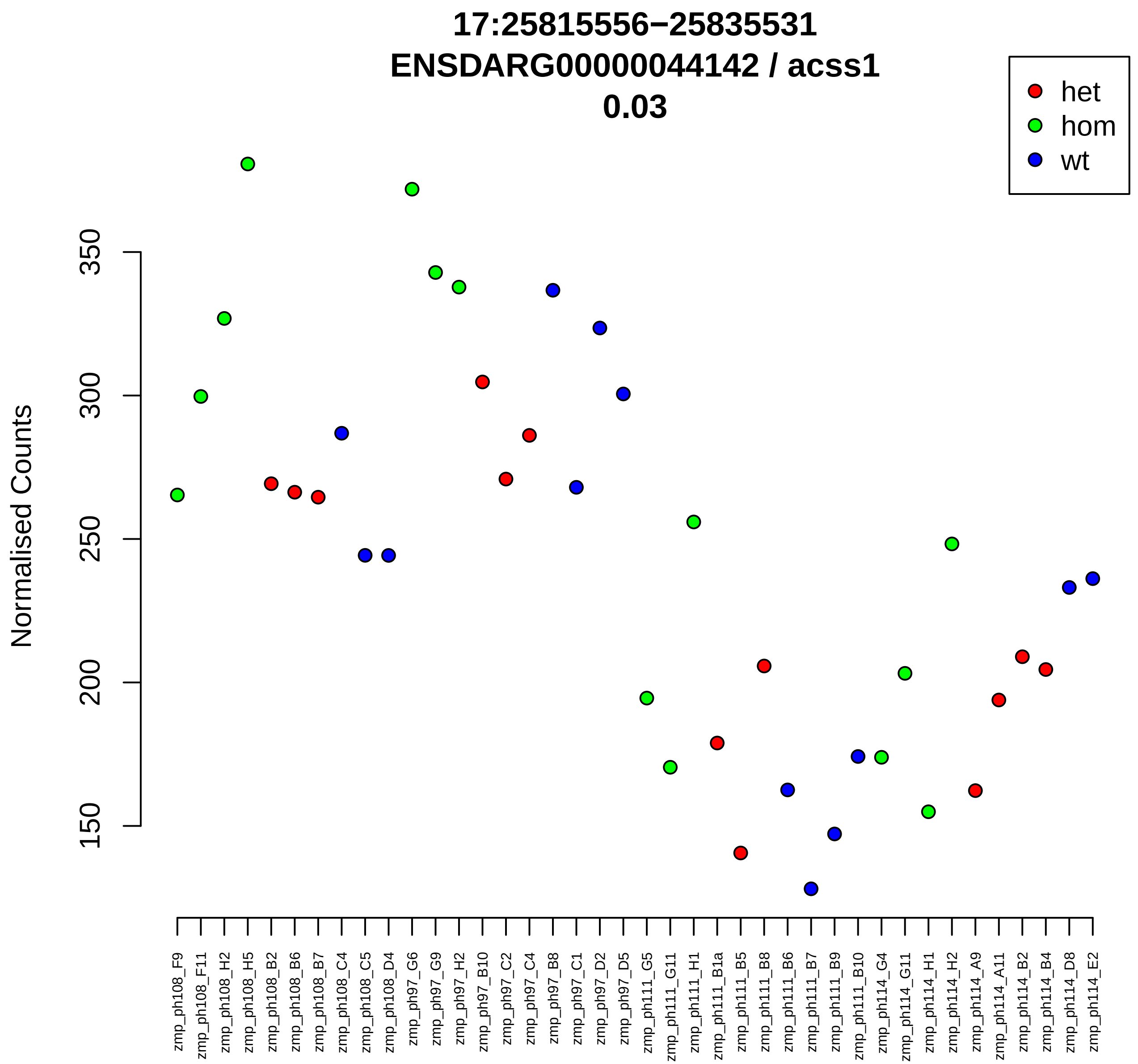

17:25815556−25835531
ENSDARG00000044142 / acss1
0.03
het
hom
wt
Normalised Counts
150
200
250
300
350
zmp_ph108_F9
zmp_ph108_F11
zmp_ph108_H2
zmp_ph108_H5
zmp_ph108_B2
zmp_ph108_B6
zmp_ph108_B7
zmp_ph108_C4
zmp_ph108_C5
zmp_ph108_D4
zmp_ph97_G6
zmp_ph97_G9
zmp_ph97_H2
zmp_ph97_B10
zmp_ph97_C2
zmp_ph97_C4
zmp_ph97_B8
zmp_ph97_C1
zmp_ph97_D2
zmp_ph97_D5
zmp_ph111_G5
zmp_ph111_G11
zmp_ph111_H1
zmp_ph111_B1a
zmp_ph111_B5
zmp_ph111_B8
zmp_ph111_B6
zmp_ph111_B7
zmp_ph111_B9
zmp_ph111_B10
zmp_ph114_G4
zmp_ph114_G11
zmp_ph114_H1
zmp_ph114_H2
zmp_ph114_A9
zmp_ph114_A11
zmp_ph114_B2
zmp_ph114_B4
zmp_ph114_D8
zmp_ph114_E2

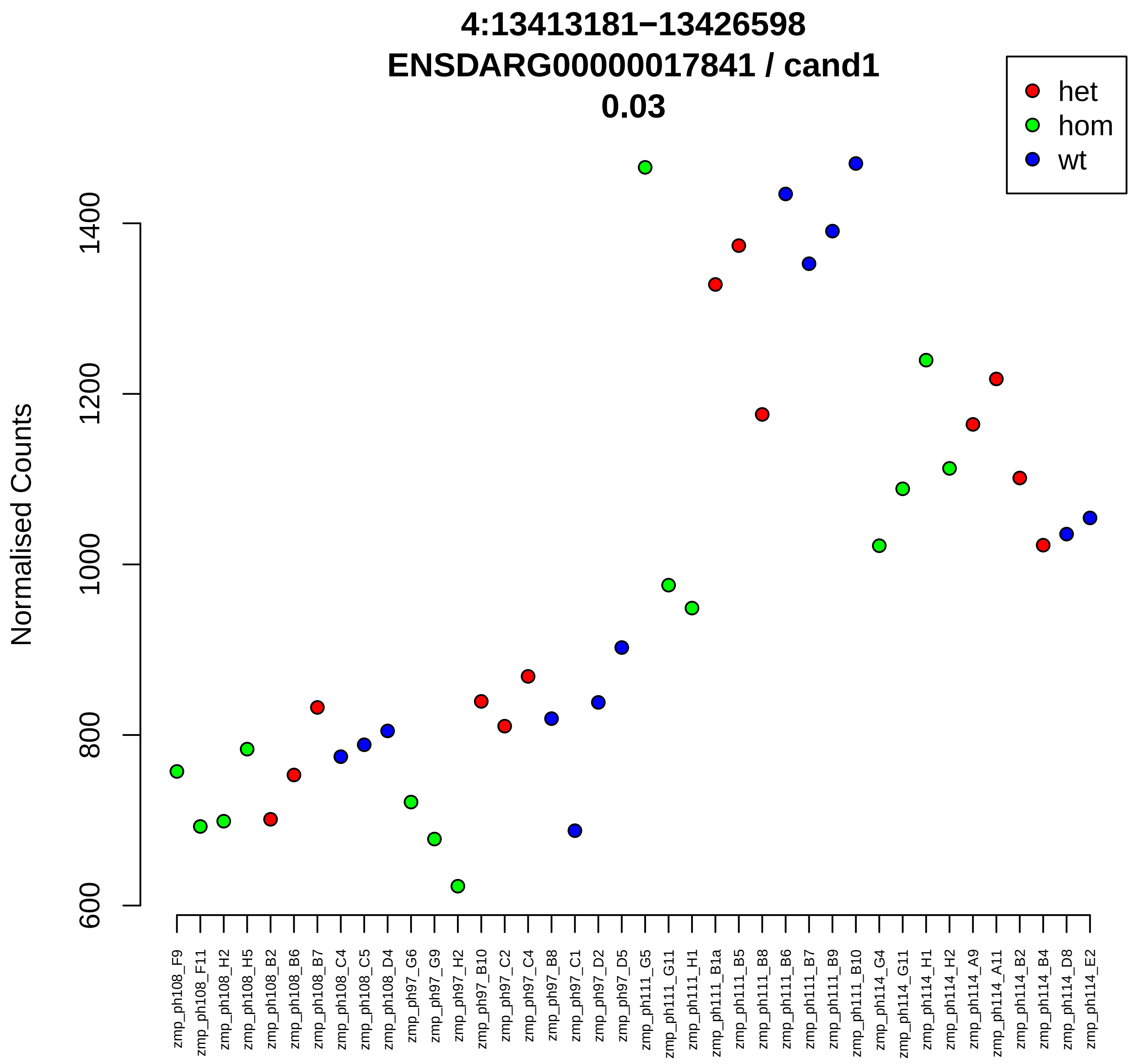
4:13413181−13426598
ENSDARG00000017841 / cand1
0.03
het
hom
wt
Normalised Counts
600
800
1000
1200
1400
zmp_ph108_F9
zmp_ph108_F11
zmp_ph108_H2
zmp_ph108_H5
zmp_ph108_B2
zmp_ph108_B6
zmp_ph108_B7
zmp_ph108_C4
zmp_ph108_C5
zmp_ph108_D4
zmp_ph97_G6
zmp_ph97_G9
zmp_ph97_H2
zmp_ph97_B10
zmp_ph97_C2
zmp_ph97_C4
zmp_ph97_B8
zmp_ph97_C1
zmp_ph97_D2
zmp_ph97_D5
zmp_ph111_G5
zmp_ph111_G11
zmp_ph111_H1
zmp_ph111_B1a
zmp_ph111_B5
zmp_ph111_B8
zmp_ph111_B6
zmp_ph111_B7
zmp_ph111_B9
zmp_ph111_B10
zmp_ph114_G4
zmp_ph114_G11
zmp_ph114_H1
zmp_ph114_H2
zmp_ph114_A9
zmp_ph114_A11
zmp_ph114_B2
zmp_ph114_B4
zmp_ph114_D8
zmp_ph114_E2

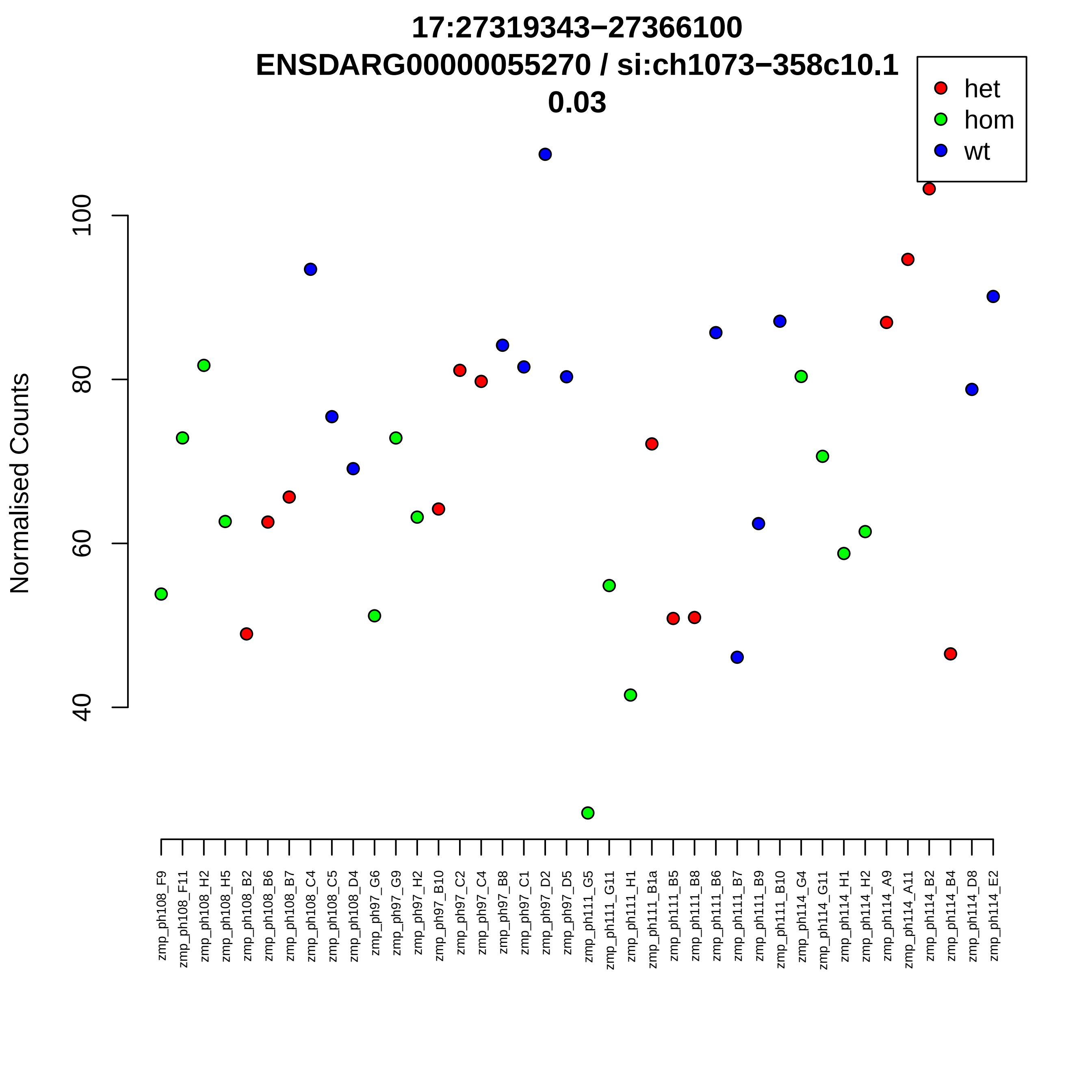
17:27319343−27366100
ENSDARG00000055270 / si:ch1073−358c10.1
0.03
het
hom
wt
Normalised Counts
40
60
80
100
zmp_ph108_F9
zmp_ph108_F11
zmp_ph108_H2
zmp_ph108_H5
zmp_ph108_B2
zmp_ph108_B6
zmp_ph108_B7
zmp_ph108_C4
zmp_ph108_C5
zmp_ph108_D4
zmp_ph97_G6
zmp_ph97_G9
zmp_ph97_H2
zmp_ph97_B10
zmp_ph97_C2
zmp_ph97_C4
zmp_ph97_B8
zmp_ph97_C1
zmp_ph97_D2
zmp_ph97_D5
zmp_ph111_G5
zmp_ph111_G11
zmp_ph111_H1
zmp_ph111_B1a
zmp_ph111_B5
zmp_ph111_B8
zmp_ph111_B6
zmp_ph111_B7
zmp_ph111_B9
zmp_ph111_B10
zmp_ph114_G4
zmp_ph114_G11
zmp_ph114_H1
zmp_ph114_H2
zmp_ph114_A9
zmp_ph114_A11
zmp_ph114_B2
zmp_ph114_B4
zmp_ph114_D8
zmp_ph114_E2

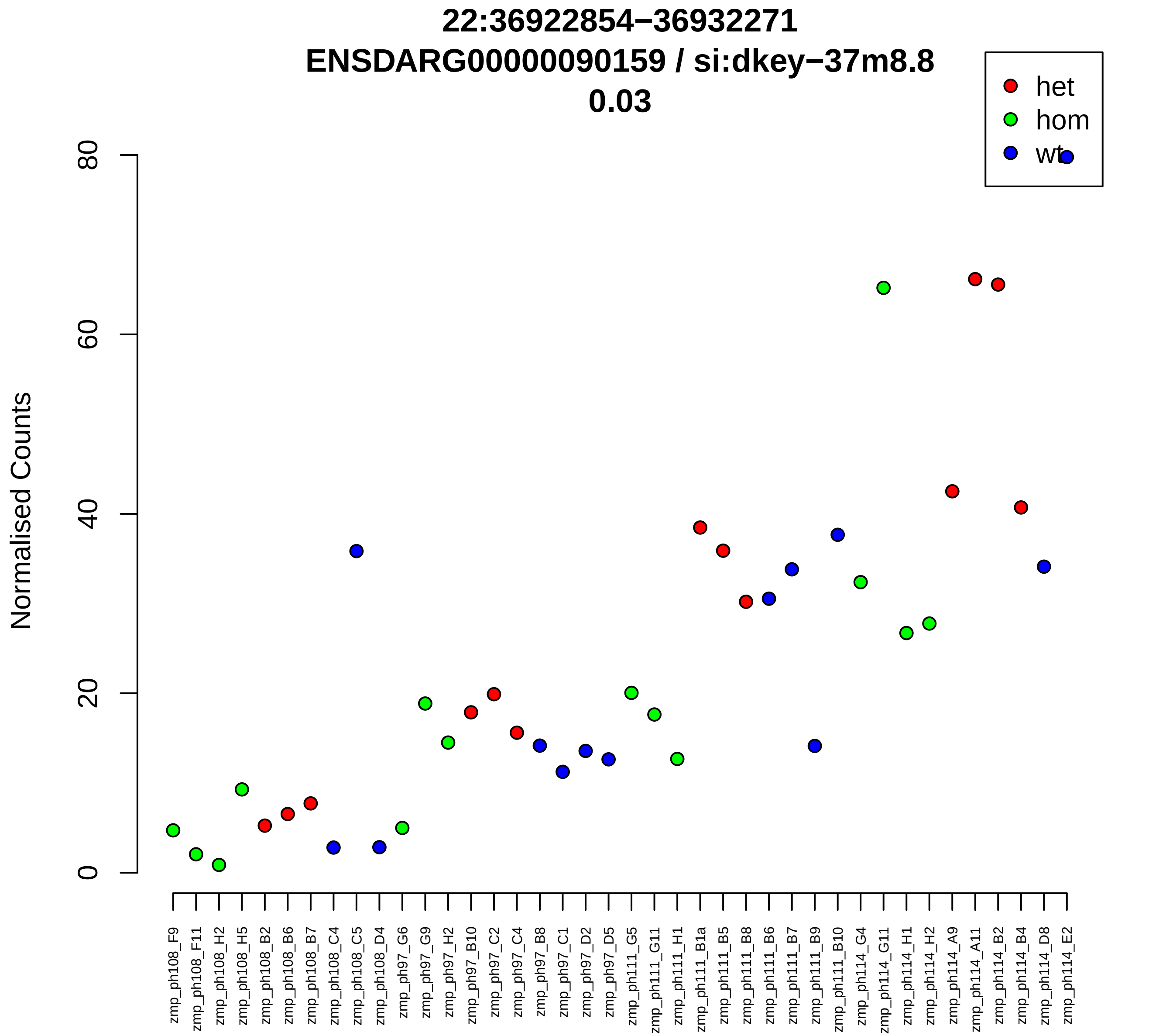

22:36922854−36932271
ENSDARG00000090159 / si:dkey−37m8.8
0.03
het
hom
wt
Normalised Counts
0
20
40
60
80
zmp_ph108_F9
zmp_ph108_F11
zmp_ph108_H2
zmp_ph108_H5
zmp_ph108_B2
zmp_ph108_B6
zmp_ph108_B7
zmp_ph108_C4
zmp_ph108_C5
zmp_ph108_D4
zmp_ph97_G6
zmp_ph97_G9
zmp_ph97_H2
zmp_ph97_B10
zmp_ph97_C2
zmp_ph97_C4
zmp_ph97_B8
zmp_ph97_C1
zmp_ph97_D2
zmp_ph97_D5
zmp_ph111_G5
zmp_ph111_G11
zmp_ph111_H1
zmp_ph111_B1a
zmp_ph111_B5
zmp_ph111_B8
zmp_ph111_B6
zmp_ph111_B7
zmp_ph111_B9
zmp_ph111_B10
zmp_ph114_G4
zmp_ph114_G11
zmp_ph114_H1
zmp_ph114_H2
zmp_ph114_A9
zmp_ph114_A11
zmp_ph114_B2
zmp_ph114_B4
zmp_ph114_D8
zmp_ph114_E2

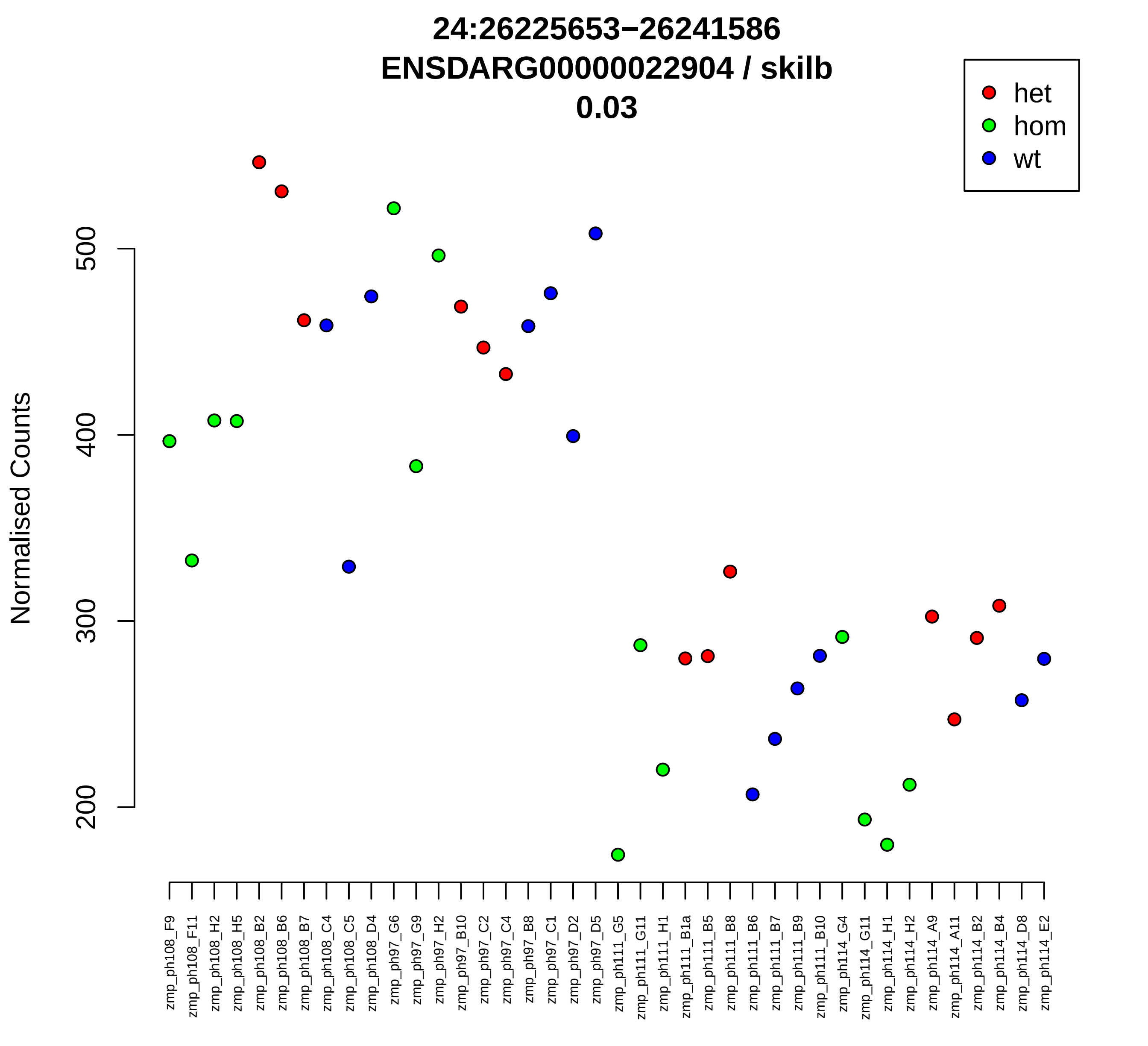
24:26225653−26241586
ENSDARG00000022904 / skilb
0.03
het
hom
wt
Normalised Counts
200
300
400
500
zmp_ph108_F9
zmp_ph108_F11
zmp_ph108_H2
zmp_ph108_H5
zmp_ph108_B2
zmp_ph108_B6
zmp_ph108_B7
zmp_ph108_C4
zmp_ph108_C5
zmp_ph108_D4
zmp_ph97_G6
zmp_ph97_G9
zmp_ph97_H2
zmp_ph97_B10
zmp_ph97_C2
zmp_ph97_C4
zmp_ph97_B8
zmp_ph97_C1
zmp_ph97_D2
zmp_ph97_D5
zmp_ph111_G5
zmp_ph111_G11
zmp_ph111_H1
zmp_ph111_B1a
zmp_ph111_B5
zmp_ph111_B8
zmp_ph111_B6
zmp_ph111_B7
zmp_ph111_B9
zmp_ph111_B10
zmp_ph114_G4
zmp_ph114_G11
zmp_ph114_H1
zmp_ph114_H2
zmp_ph114_A9
zmp_ph114_A11
zmp_ph114_B2
zmp_ph114_B4
zmp_ph114_D8
zmp_ph114_E2

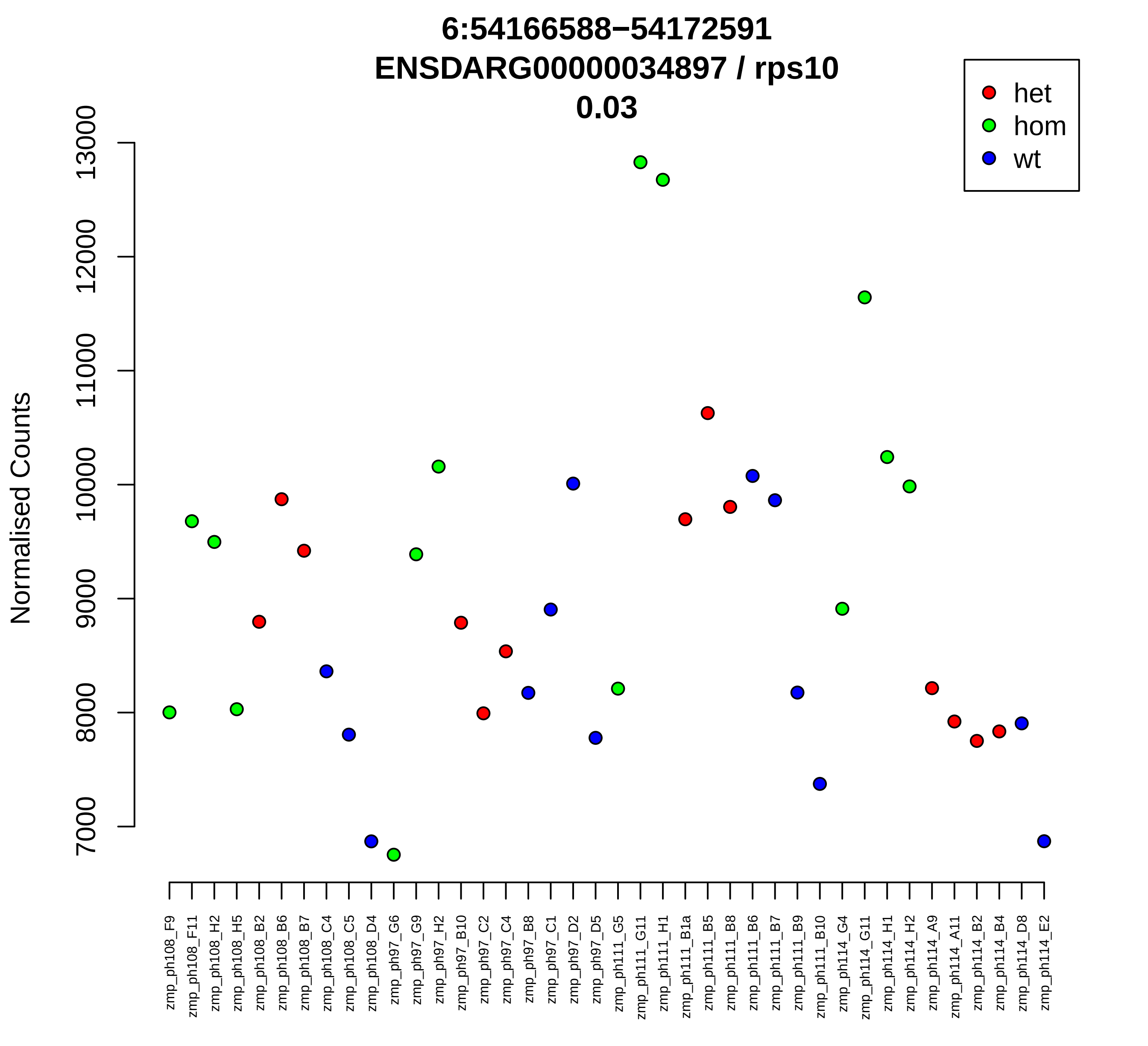
6:54166588−54172591
ENSDARG00000034897 / rps10
0.03
het
hom
wt
Normalised Counts
7000
8000
9000
10000
11000
12000
13000
zmp_ph108_F9
zmp_ph108_F11
zmp_ph108_H2
zmp_ph108_H5
zmp_ph108_B2
zmp_ph108_B6
zmp_ph108_B7
zmp_ph108_C4
zmp_ph108_C5
zmp_ph108_D4
zmp_ph97_G6
zmp_ph97_G9
zmp_ph97_H2
zmp_ph97_B10
zmp_ph97_C2
zmp_ph97_C4
zmp_ph97_B8
zmp_ph97_C1
zmp_ph97_D2
zmp_ph97_D5
zmp_ph111_G5
zmp_ph111_G11
zmp_ph111_H1
zmp_ph111_B1a
zmp_ph111_B5
zmp_ph111_B8
zmp_ph111_B6
zmp_ph111_B7
zmp_ph111_B9
zmp_ph111_B10
zmp_ph114_G4
zmp_ph114_G11
zmp_ph114_H1
zmp_ph114_H2
zmp_ph114_A9
zmp_ph114_A11
zmp_ph114_B2
zmp_ph114_B4
zmp_ph114_D8
zmp_ph114_E2

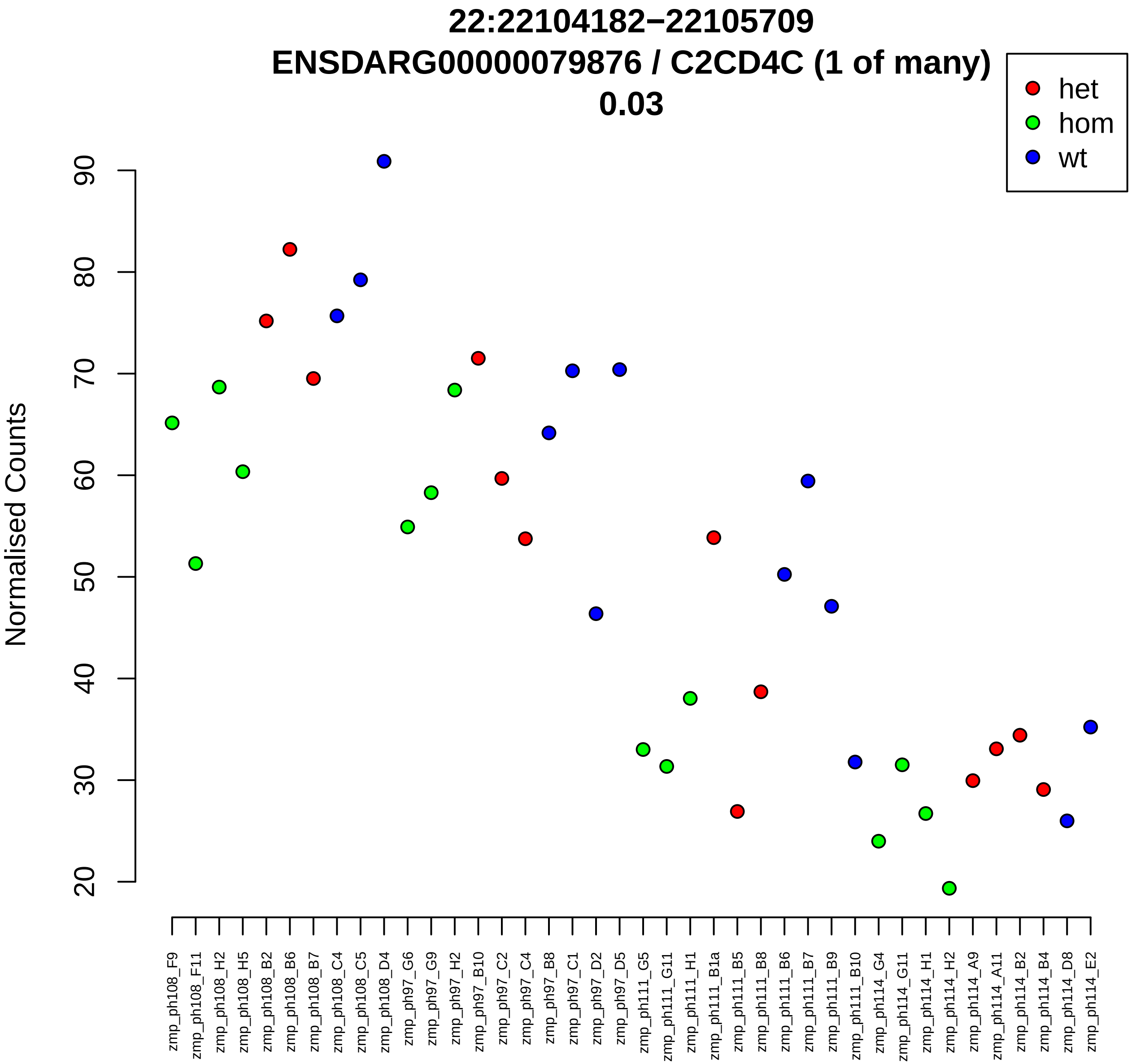

22:22104182−22105709
ENSDARG00000079876 / C2CD4C (1 of many)
0.03
het
hom
wt
Normalised Counts
20
30
40
50
60
70
80
90
zmp_ph108_F9
zmp_ph108_F11
zmp_ph108_H2
zmp_ph108_H5
zmp_ph108_B2
zmp_ph108_B6
zmp_ph108_B7
zmp_ph108_C4
zmp_ph108_C5
zmp_ph108_D4
zmp_ph97_G6
zmp_ph97_G9
zmp_ph97_H2
zmp_ph97_B10
zmp_ph97_C2
zmp_ph97_C4
zmp_ph97_B8
zmp_ph97_C1
zmp_ph97_D2
zmp_ph97_D5
zmp_ph111_G5
zmp_ph111_G11
zmp_ph111_H1
zmp_ph111_B1a
zmp_ph111_B5
zmp_ph111_B8
zmp_ph111_B6
zmp_ph111_B7
zmp_ph111_B9
zmp_ph111_B10
zmp_ph114_G4
zmp_ph114_G11
zmp_ph114_H1
zmp_ph114_H2
zmp_ph114_A9
zmp_ph114_A11
zmp_ph114_B2
zmp_ph114_B4
zmp_ph114_D8
zmp_ph114_E2

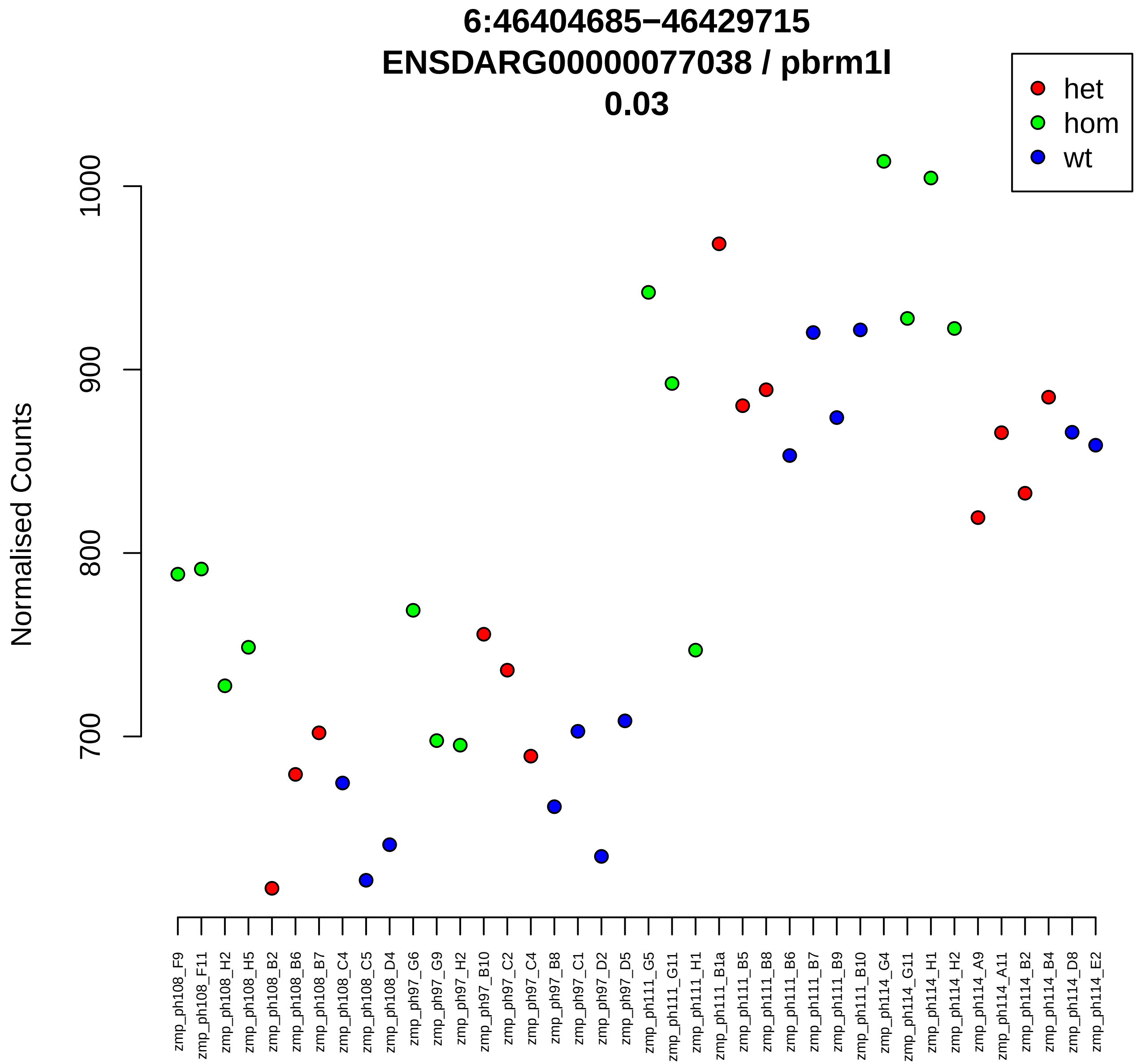
6:46404685−46429715
ENSDARG00000077038 / pbrm1l
0.03
het
hom
wt
Normalised Counts
1000
900
800
700
zmp_ph108_F9
zmp_ph108_F11
zmp_ph108_H2
zmp_ph108_H5
zmp_ph108_B2
zmp_ph108_B6
zmp_ph108_B7
zmp_ph108_C4
zmp_ph108_C5
zmp_ph108_D4
zmp_ph97_G6
zmp_ph97_G9
zmp_ph97_H2
zmp_ph97_B10
zmp_ph97_C2
zmp_ph97_C4
zmp_ph97_B8
zmp_ph97_C1
zmp_ph97_D2
zmp_ph97_D5
zmp_ph111_G5
zmp_ph111_G11
zmp_ph111_H1
zmp_ph111_B1a
zmp_ph111_B5
zmp_ph111_B8
zmp_ph111_B6
zmp_ph111_B7
zmp_ph111_B9
zmp_ph111_B10
zmp_ph114_G4
zmp_ph114_G11
zmp_ph114_H1
zmp_ph114_H2
zmp_ph114_A9
zmp_ph114_A11
zmp_ph114_B2
zmp_ph114_B4
zmp_ph114_D8
zmp_ph114_E2

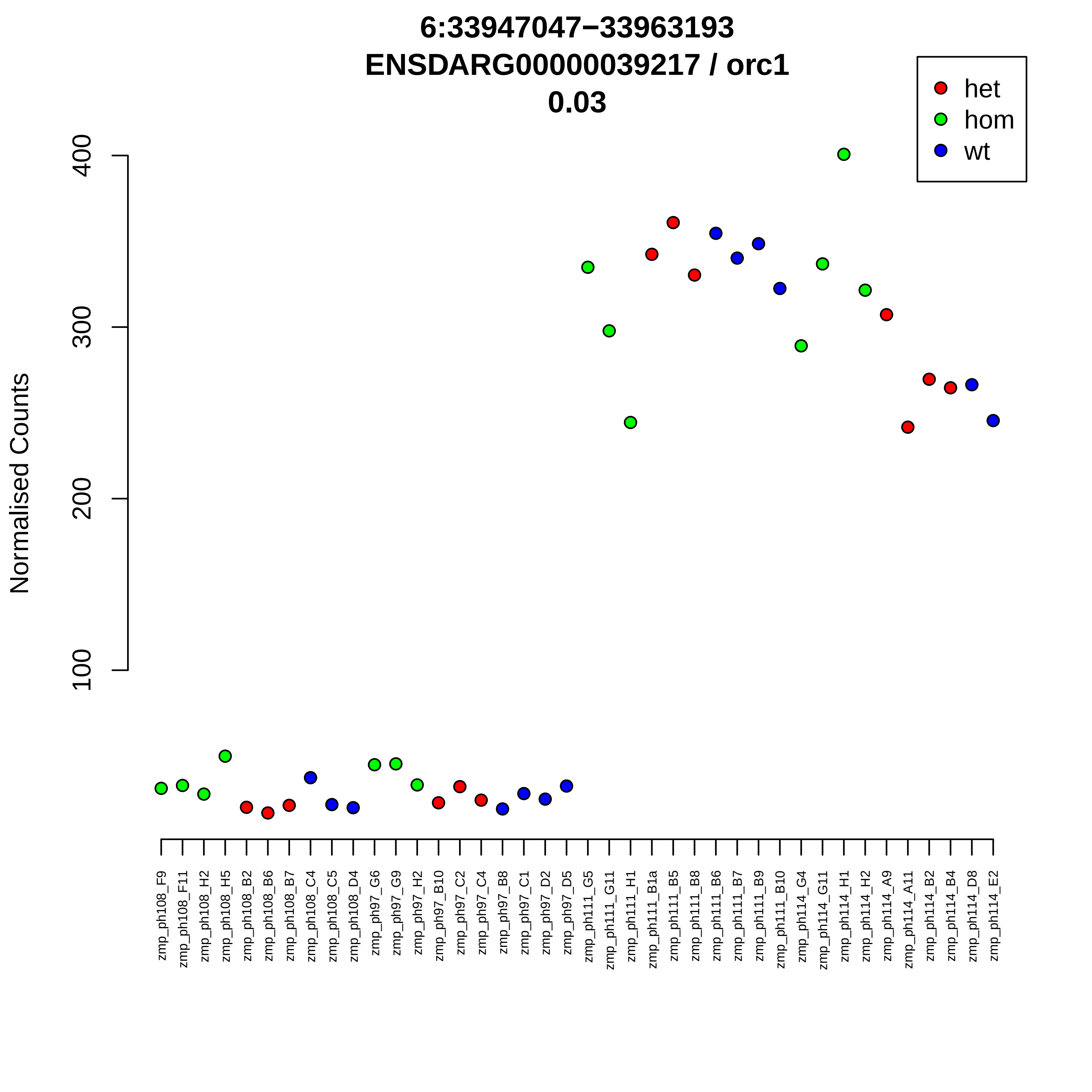

6:33947047−33963193
ENSDARG00000039217 / orc1
0.03
het
hom
wt
Normalised Counts
100
200
300
400
zmp_ph108_F9
zmp_ph108_F11
zmp_ph108_H2
zmp_ph108_H5
zmp_ph108_B2
zmp_ph108_B6
zmp_ph108_B7
zmp_ph108_C4
zmp_ph108_C5
zmp_ph108_D4
zmp_ph97_G6
zmp_ph97_G9
zmp_ph97_H2
zmp_ph97_B10
zmp_ph97_C2
zmp_ph97_C4
zmp_ph97_B8
zmp_ph97_C1
zmp_ph97_D2
zmp_ph97_D5
zmp_ph111_G5
zmp_ph111_G11
zmp_ph111_H1
zmp_ph111_B1a
zmp_ph111_B5
zmp_ph111_B8
zmp_ph111_B6
zmp_ph111_B7
zmp_ph111_B9
zmp_ph111_B10
zmp_ph114_G4
zmp_ph114_G11
zmp_ph114_H1
zmp_ph114_H2
zmp_ph114_A9
zmp_ph114_A11
zmp_ph114_B2
zmp_ph114_B4
zmp_ph114_D8
zmp_ph114_E2

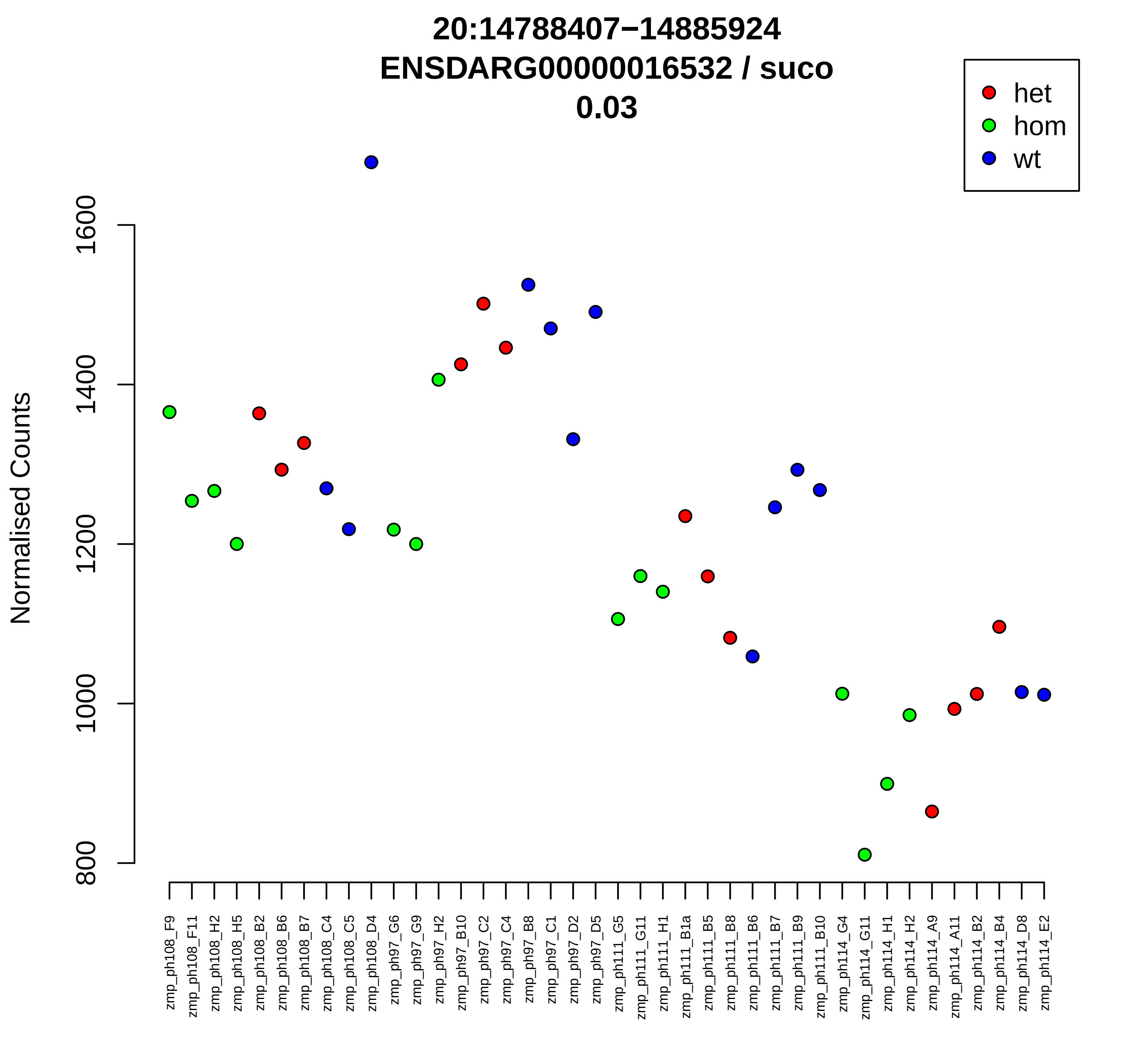

20:14788407−14885924
ENSDARG00000016532 / suco
0.03
het
hom
wt
Normalised Counts
800
1000
1200
1400
1600
zmp_ph108_F9
zmp_ph108_F11
zmp_ph108_H2
zmp_ph108_H5
zmp_ph108_B2
zmp_ph108_B6
zmp_ph108_B7
zmp_ph108_C4
zmp_ph108_C5
zmp_ph108_D4
zmp_ph97_G6
zmp_ph97_G9
zmp_ph97_H2
zmp_ph97_B10
zmp_ph97_C2
zmp_ph97_C4
zmp_ph97_B8
zmp_ph97_C1
zmp_ph97_D2
zmp_ph97_D5
zmp_ph111_G5
zmp_ph111_G11
zmp_ph111_H1
zmp_ph111_B1a
zmp_ph111_B5
zmp_ph111_B8
zmp_ph111_B6
zmp_ph111_B7
zmp_ph111_B9
zmp_ph111_B10
zmp_ph114_G4
zmp_ph114_G11
zmp_ph114_H1
zmp_ph114_H2
zmp_ph114_A9
zmp_ph114_A11
zmp_ph114_B2
zmp_ph114_B4
zmp_ph114_D8
zmp_ph114_E2

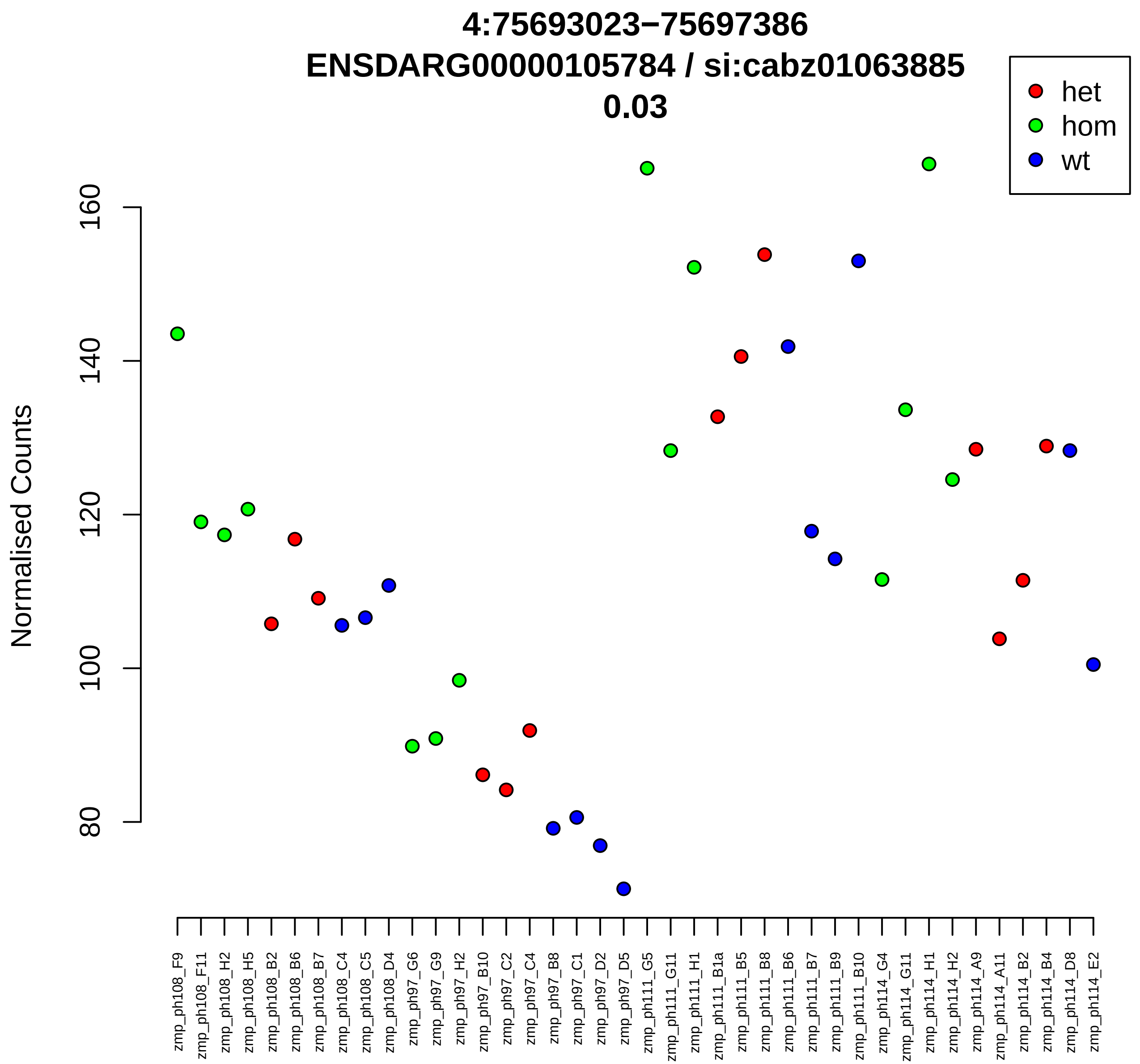
4:75693023−75697386
ENSDARG00000105784 / si:cabz01063885
0.03
het
hom
wt
Normalised Counts
80
100
120
140
160
zmp_ph108_F9
zmp_ph108_F11
zmp_ph108_H2
zmp_ph108_H5
zmp_ph108_B2
zmp_ph108_B6
zmp_ph108_B7
zmp_ph108_C4
zmp_ph108_C5
zmp_ph108_D4
zmp_ph97_G6
zmp_ph97_G9
zmp_ph97_H2
zmp_ph97_B10
zmp_ph97_C2
zmp_ph97_C4
zmp_ph97_B8
zmp_ph97_C1
zmp_ph97_D2
zmp_ph97_D5
zmp_ph111_G5
zmp_ph111_G11
zmp_ph111_H1
zmp_ph111_B1a
zmp_ph111_B5
zmp_ph111_B8
zmp_ph111_B6
zmp_ph111_B7
zmp_ph111_B9
zmp_ph111_B10
zmp_ph114_G4
zmp_ph114_G11
zmp_ph114_H1
zmp_ph114_H2
zmp_ph114_A9
zmp_ph114_A11
zmp_ph114_B2
zmp_ph114_B4
zmp_ph114_D8
zmp_ph114_E2

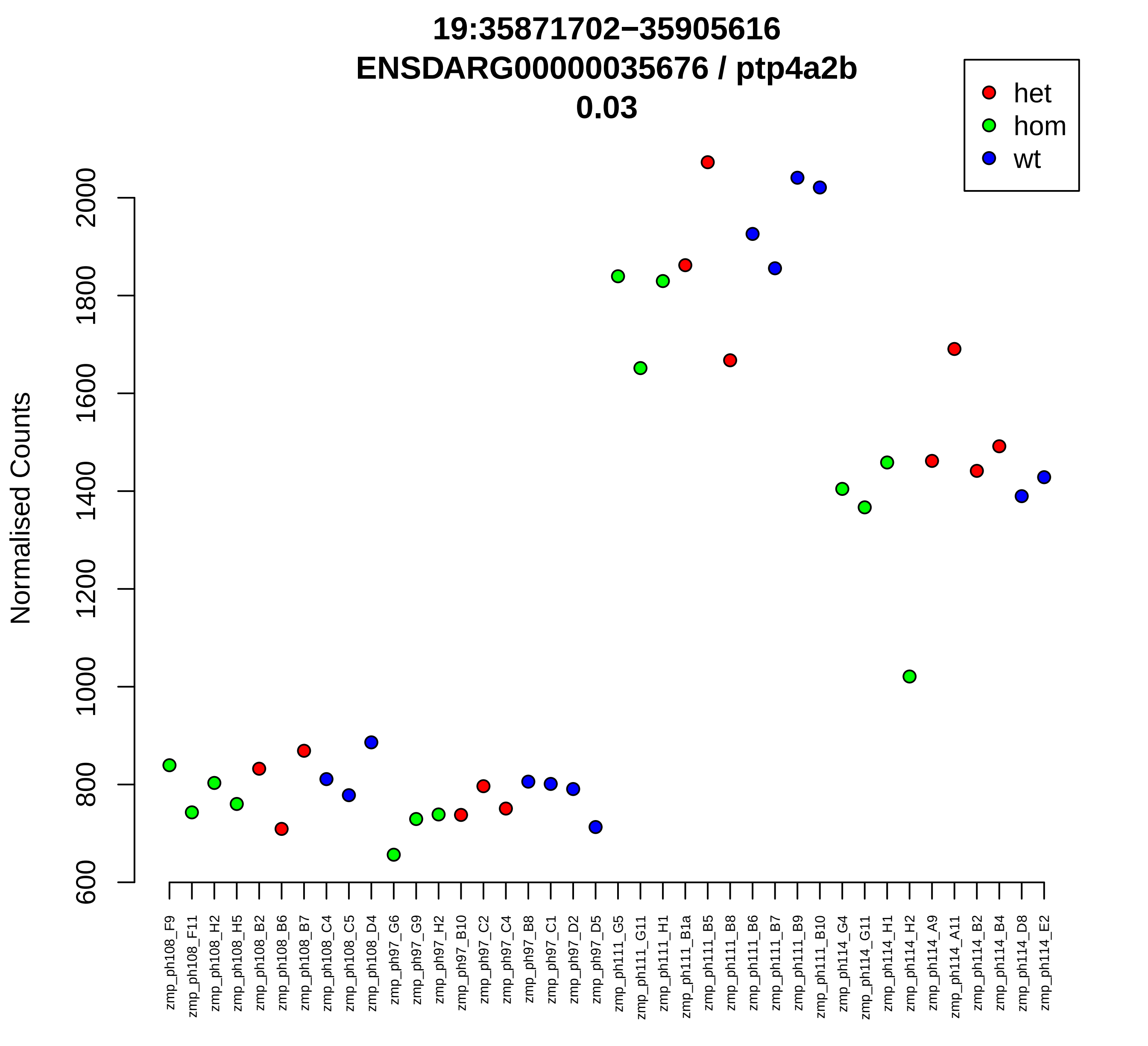
19:35871702−35905616
ENSDARG00000035676 / ptp4a2b
0.03
het
hom
wt
Normalised Counts
600
800
1000
1200
1400
1600
1800
2000
zmp_ph108_F9
zmp_ph108_F11
zmp_ph108_H2
zmp_ph108_H5
zmp_ph108_B2
zmp_ph108_B6
zmp_ph108_B7
zmp_ph108_C4
zmp_ph108_C5
zmp_ph108_D4
zmp_ph97_G6
zmp_ph97_G9
zmp_ph97_H2
zmp_ph97_B10
zmp_ph97_C2
zmp_ph97_C4
zmp_ph97_B8
zmp_ph97_C1
zmp_ph97_D2
zmp_ph97_D5
zmp_ph111_G5
zmp_ph111_G11
zmp_ph111_H1
zmp_ph111_B1a
zmp_ph111_B5
zmp_ph111_B8
zmp_ph111_B6
zmp_ph111_B7
zmp_ph111_B9
zmp_ph111_B10
zmp_ph114_G4
zmp_ph114_G11
zmp_ph114_H1
zmp_ph114_H2
zmp_ph114_A9
zmp_ph114_A11
zmp_ph114_B2
zmp_ph114_B4
zmp_ph114_D8
zmp_ph114_E2

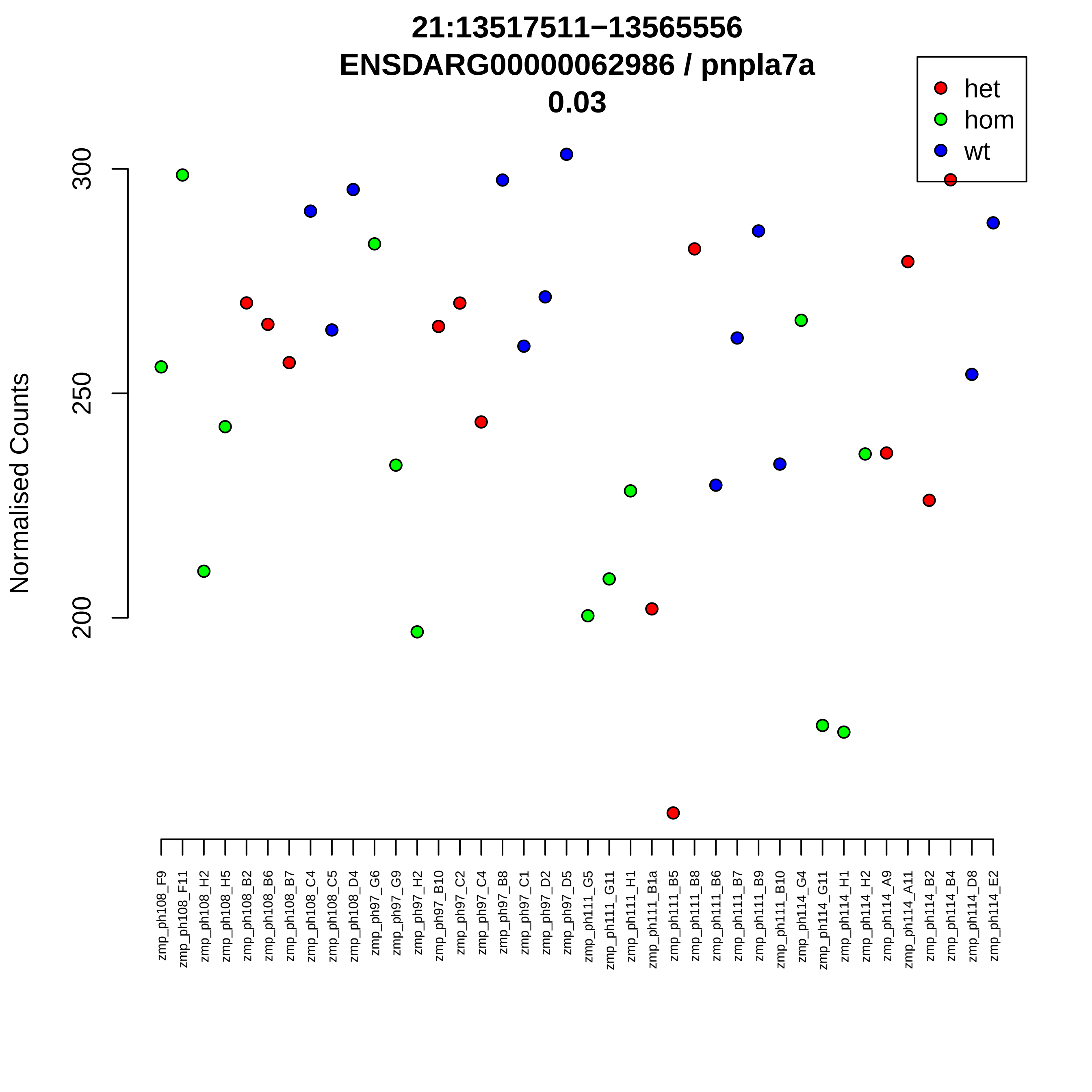
21:13517511−13565556
ENSDARG00000062986 / pnpla7a
0.03
het
hom
wt
Normalised Counts
300
250
200
zmp_ph108_F9
zmp_ph108_F11
zmp_ph108_H2
zmp_ph108_H5
zmp_ph108_B2
zmp_ph108_B6
zmp_ph108_B7
zmp_ph108_C4
zmp_ph108_C5
zmp_ph108_D4
zmp_ph97_G6
zmp_ph97_G9
zmp_ph97_H2
zmp_ph97_B10
zmp_ph97_C2
zmp_ph97_C4
zmp_ph97_B8
zmp_ph97_C1
zmp_ph97_D2
zmp_ph97_D5
zmp_ph111_G5
zmp_ph111_G11
zmp_ph111_H1
zmp_ph111_B1a
zmp_ph111_B5
zmp_ph111_B8
zmp_ph111_B6
zmp_ph111_B7
zmp_ph111_B9
zmp_ph111_B10
zmp_ph114_G4
zmp_ph114_G11
zmp_ph114_H1
zmp_ph114_H2
zmp_ph114_A9
zmp_ph114_A11
zmp_ph114_B2
zmp_ph114_B4
zmp_ph114_D8
zmp_ph114_E2

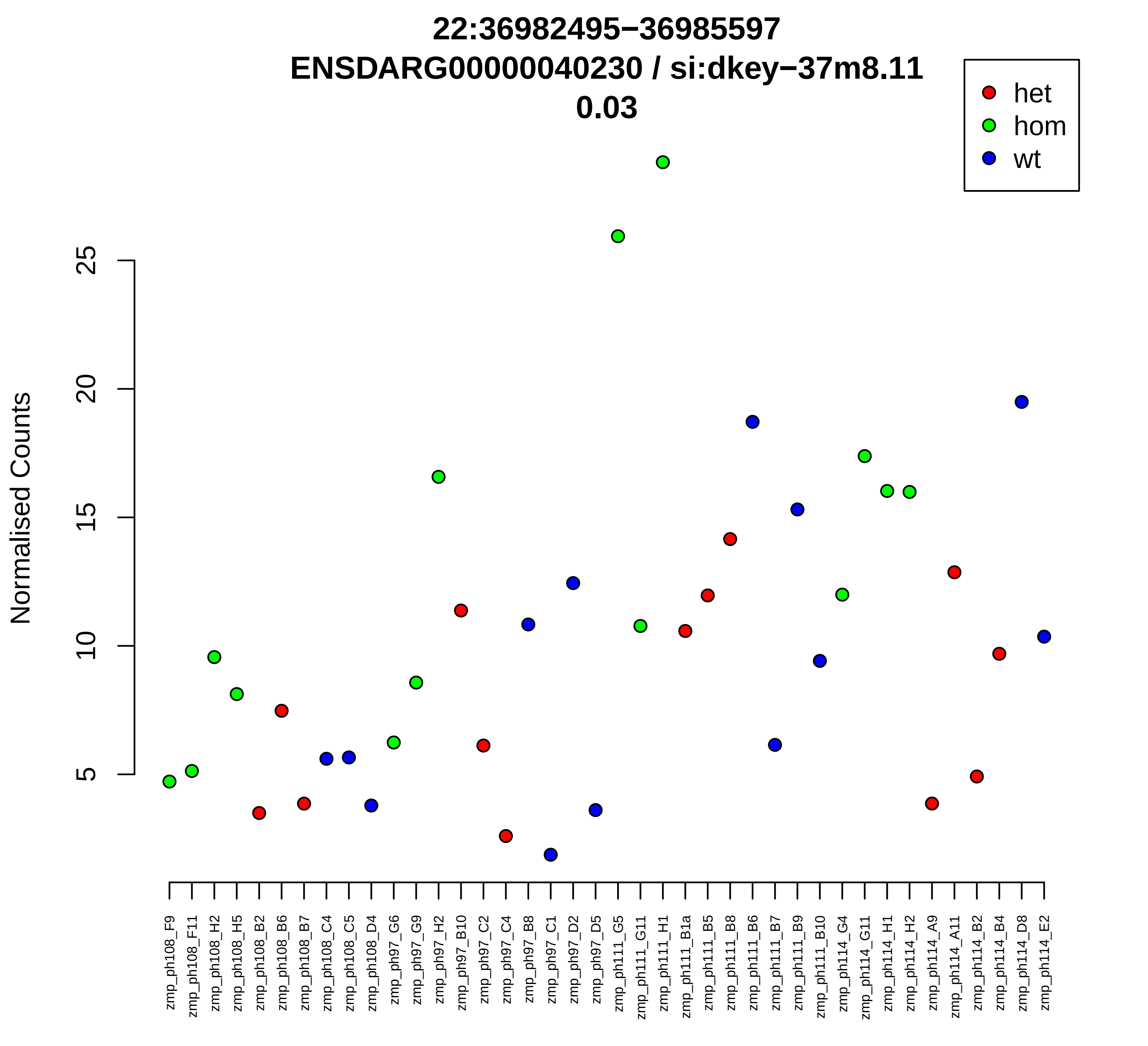
22:36982495−36985597
ENSDARG00000040230 / si:dkey−37m8.11
0.03
het
hom
wt
Normalised Counts
5
10
15
20
25
zmp_ph108_F9
zmp_ph108_F11
zmp_ph108_H2
zmp_ph108_H5
zmp_ph108_B2
zmp_ph108_B6
zmp_ph108_B7
zmp_ph108_C4
zmp_ph108_C5
zmp_ph108_D4
zmp_ph97_G6
zmp_ph97_G9
zmp_ph97_H2
zmp_ph97_B10
zmp_ph97_C2
zmp_ph97_C4
zmp_ph97_B8
zmp_ph97_C1
zmp_ph97_D2
zmp_ph97_D5
zmp_ph111_G5
zmp_ph111_G11
zmp_ph111_H1
zmp_ph111_B1a
zmp_ph111_B5
zmp_ph111_B8
zmp_ph111_B6
zmp_ph111_B7
zmp_ph111_B9
zmp_ph111_B10
zmp_ph114_G4
zmp_ph114_G11
zmp_ph114_H1
zmp_ph114_H2
zmp_ph114_A9
zmp_ph114_A11
zmp_ph114_B2
zmp_ph114_B4
zmp_ph114_D8
zmp_ph114_E2

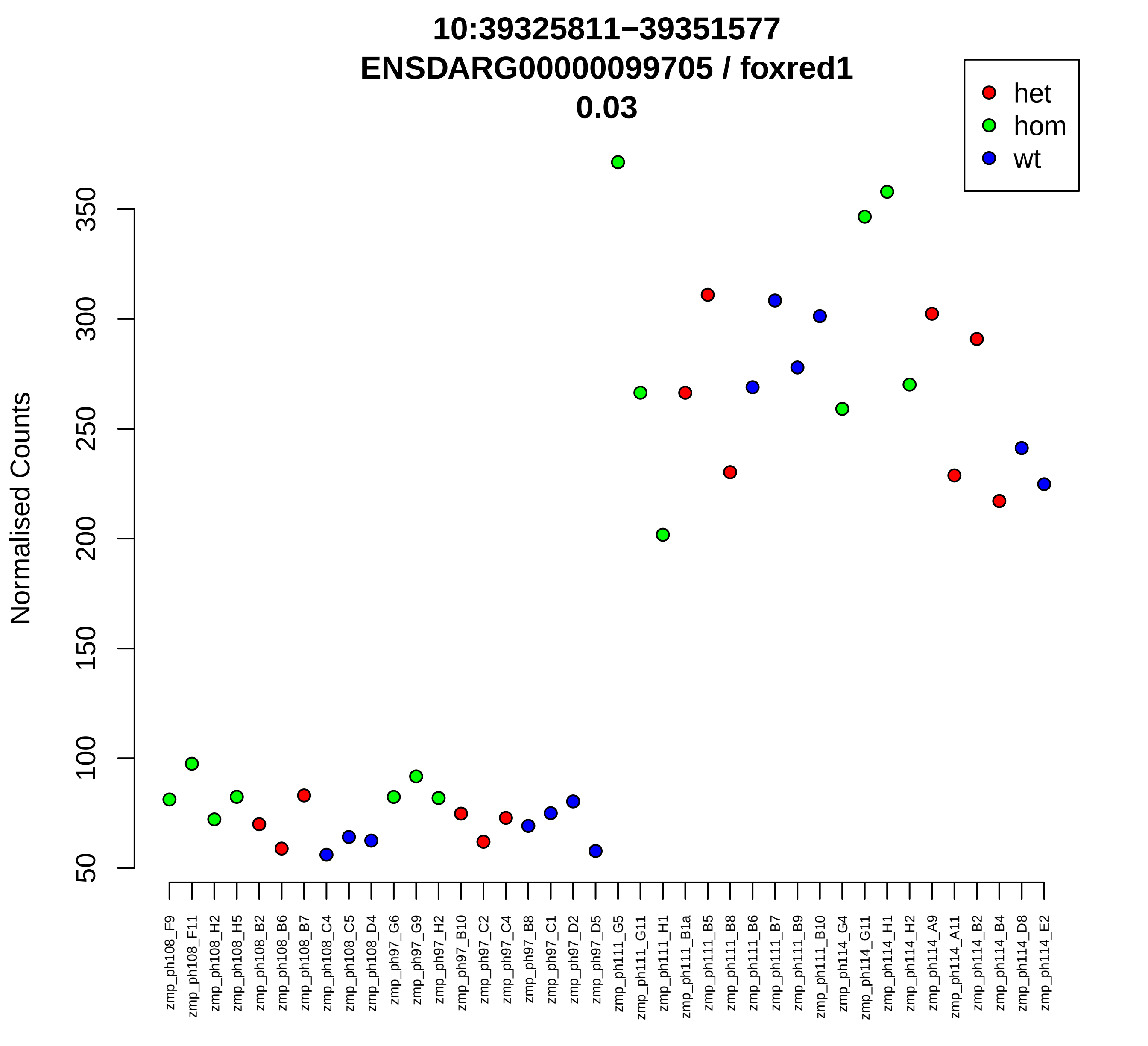

10:39325811−39351577
ENSDARG00000099705 / foxred1
0.03
het
hom
wt
Normalised Counts
50
100
150
200
250
300
350
zmp_ph108_F9
zmp_ph108_F11
zmp_ph108_H2
zmp_ph108_H5
zmp_ph108_B2
zmp_ph108_B6
zmp_ph108_B7
zmp_ph108_C4
zmp_ph108_C5
zmp_ph108_D4
zmp_ph97_G6
zmp_ph97_G9
zmp_ph97_H2
zmp_ph97_B10
zmp_ph97_C2
zmp_ph97_C4
zmp_ph97_B8
zmp_ph97_C1
zmp_ph97_D2
zmp_ph97_D5
zmp_ph111_G5
zmp_ph111_G11
zmp_ph111_H1
zmp_ph111_B1a
zmp_ph111_B5
zmp_ph111_B8
zmp_ph111_B6
zmp_ph111_B7
zmp_ph111_B9
zmp_ph111_B10
zmp_ph114_G4
zmp_ph114_G11
zmp_ph114_H1
zmp_ph114_H2
zmp_ph114_A9
zmp_ph114_A11
zmp_ph114_B2
zmp_ph114_B4
zmp_ph114_D8
zmp_ph114_E2

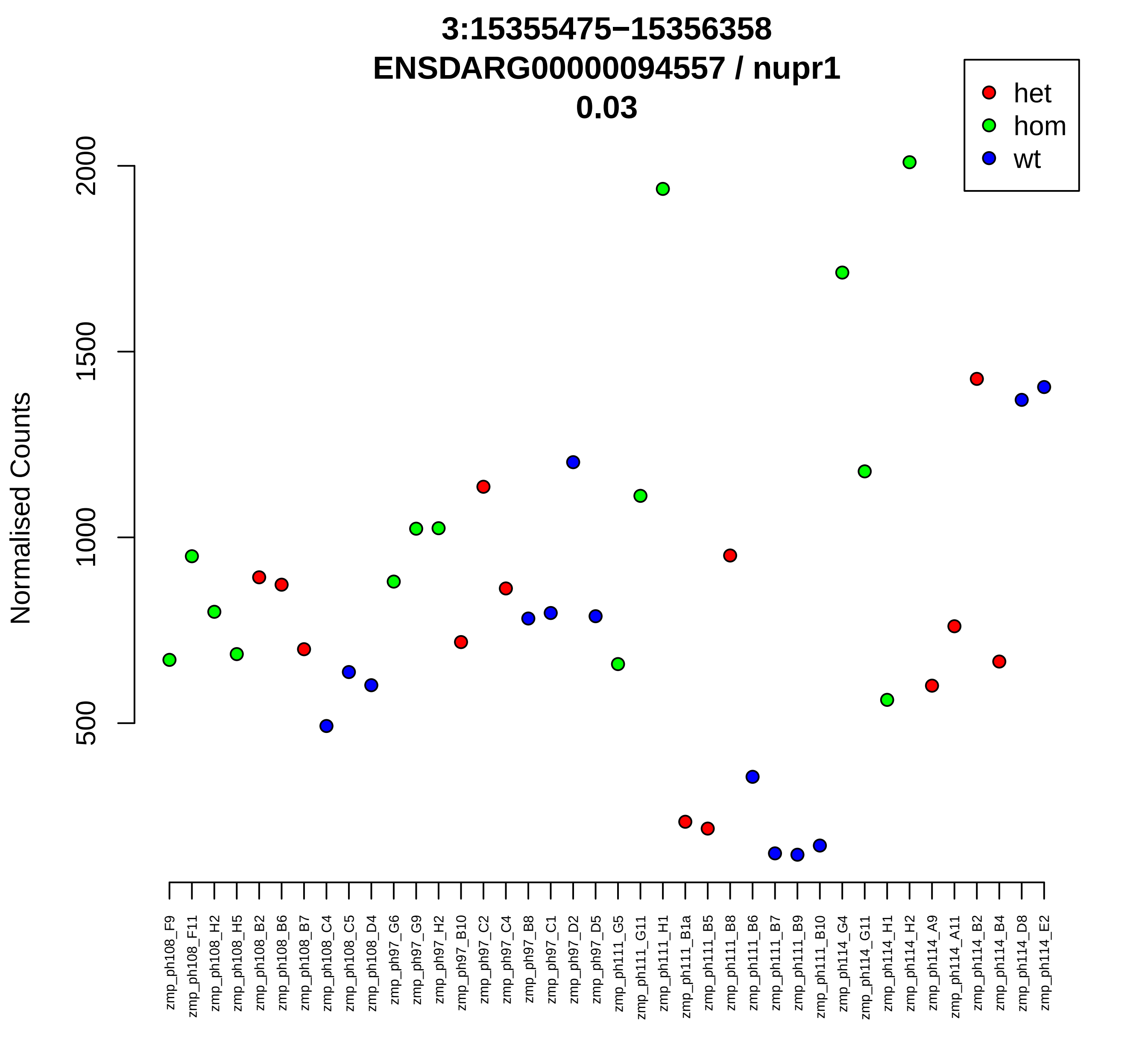

3:15355475−15356358
ENSDARG00000094557 / nupr1
0.03
het
hom
wt
Normalised Counts
500
1000
1500
2000
zmp_ph108_F9
zmp_ph108_F11
zmp_ph108_H2
zmp_ph108_H5
zmp_ph108_B2
zmp_ph108_B6
zmp_ph108_B7
zmp_ph108_C4
zmp_ph108_C5
zmp_ph108_D4
zmp_ph97_G6
zmp_ph97_G9
zmp_ph97_H2
zmp_ph97_B10
zmp_ph97_C2
zmp_ph97_C4
zmp_ph97_B8
zmp_ph97_C1
zmp_ph97_D2
zmp_ph97_D5
zmp_ph111_G5
zmp_ph111_G11
zmp_ph111_H1
zmp_ph111_B1a
zmp_ph111_B5
zmp_ph111_B8
zmp_ph111_B6
zmp_ph111_B7
zmp_ph111_B9
zmp_ph111_B10
zmp_ph114_G4
zmp_ph114_G11
zmp_ph114_H1
zmp_ph114_H2
zmp_ph114_A9
zmp_ph114_A11
zmp_ph114_B2
zmp_ph114_B4
zmp_ph114_D8
zmp_ph114_E2

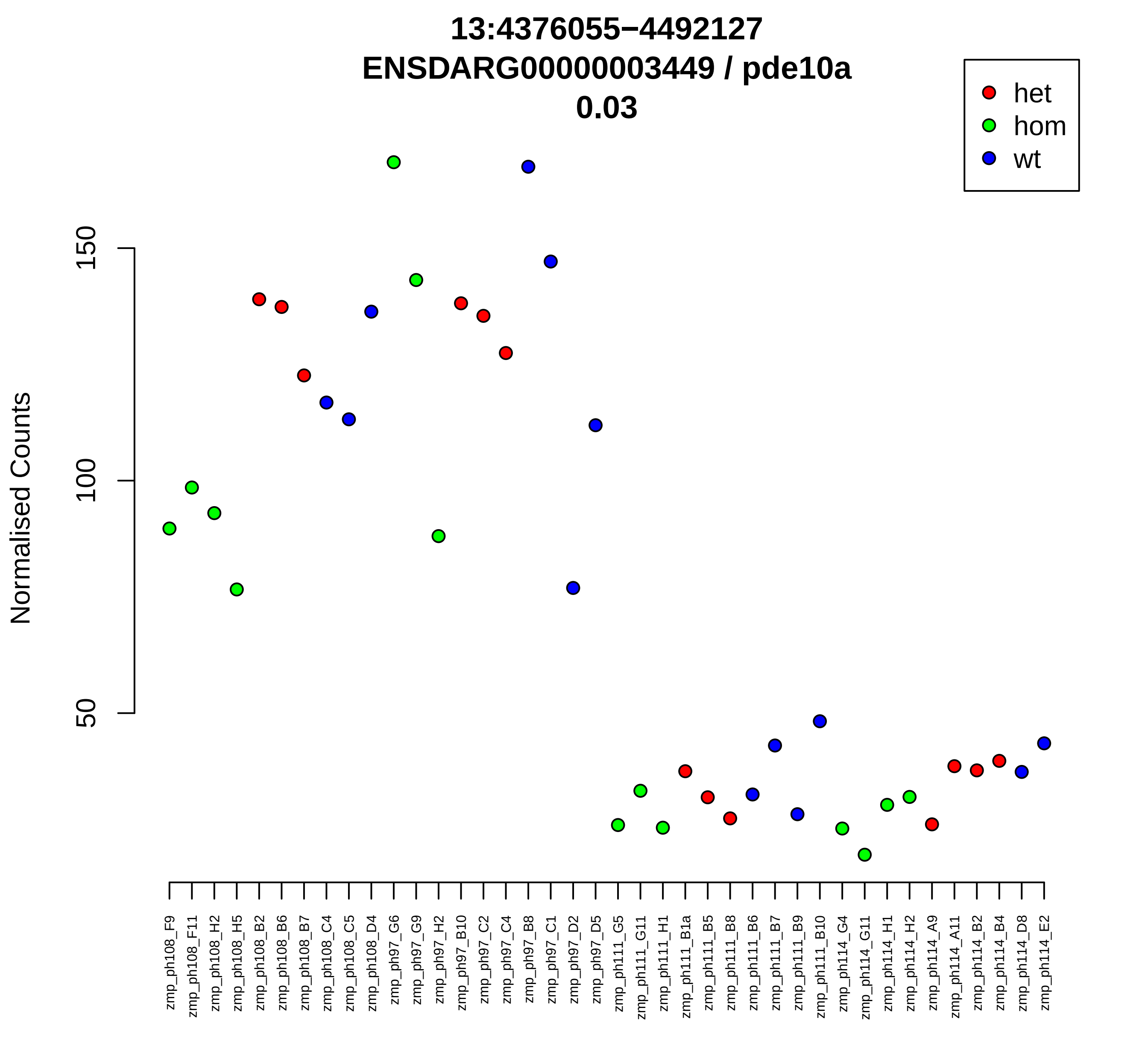
13:4376055−4492127
ENSDARG00000003449 / pde10a
0.03
het
hom
wt
Normalised Counts
50
100
150
zmp_ph108_F9
zmp_ph108_F11
zmp_ph108_H2
zmp_ph108_H5
zmp_ph108_B2
zmp_ph108_B6
zmp_ph108_B7
zmp_ph108_C4
zmp_ph108_C5
zmp_ph108_D4
zmp_ph97_G6
zmp_ph97_G9
zmp_ph97_H2
zmp_ph97_B10
zmp_ph97_C2
zmp_ph97_C4
zmp_ph97_B8
zmp_ph97_C1
zmp_ph97_D2
zmp_ph97_D5
zmp_ph111_G5
zmp_ph111_G11
zmp_ph111_H1
zmp_ph111_B1a
zmp_ph111_B5
zmp_ph111_B8
zmp_ph111_B6
zmp_ph111_B7
zmp_ph111_B9
zmp_ph111_B10
zmp_ph114_G4
zmp_ph114_G11
zmp_ph114_H1
zmp_ph114_H2
zmp_ph114_A9
zmp_ph114_A11
zmp_ph114_B2
zmp_ph114_B4
zmp_ph114_D8
zmp_ph114_E2

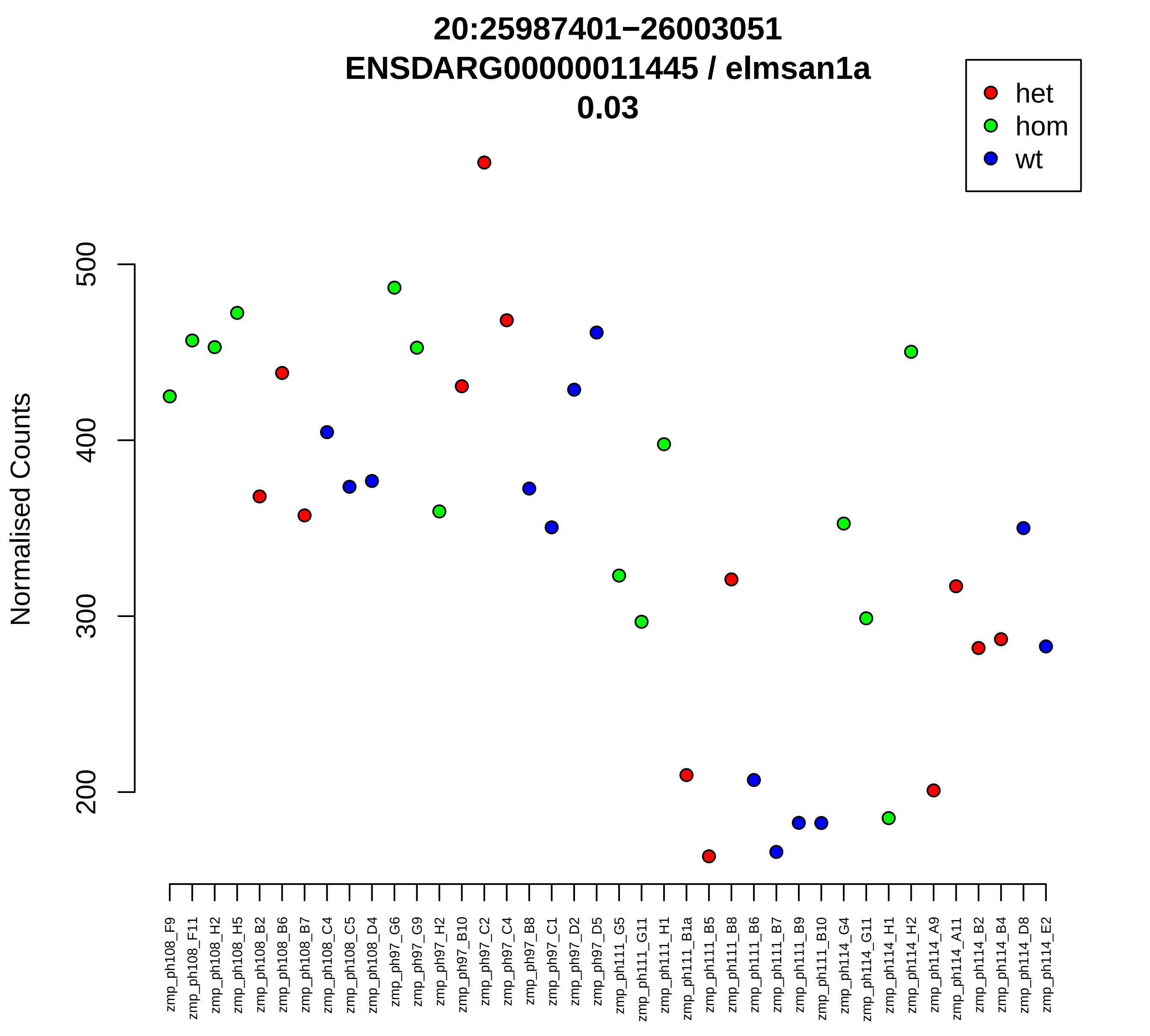
20:25987401−26003051
ENSDARG00000011445 / elmsan1a
0.03
het
hom
wt
Normalised Counts
200
300
400
500
zmp_ph108_F9
zmp_ph108_F11
zmp_ph108_H2
zmp_ph108_H5
zmp_ph108_B2
zmp_ph108_B6
zmp_ph108_B7
zmp_ph108_C4
zmp_ph108_C5
zmp_ph108_D4
zmp_ph97_G6
zmp_ph97_G9
zmp_ph97_H2
zmp_ph97_B10
zmp_ph97_C2
zmp_ph97_C4
zmp_ph97_B8
zmp_ph97_C1
zmp_ph97_D2
zmp_ph97_D5
zmp_ph111_G5
zmp_ph111_G11
zmp_ph111_H1
zmp_ph111_B1a
zmp_ph111_B5
zmp_ph111_B8
zmp_ph111_B6
zmp_ph111_B7
zmp_ph111_B9
zmp_ph111_B10
zmp_ph114_G4
zmp_ph114_G11
zmp_ph114_H1
zmp_ph114_H2
zmp_ph114_A9
zmp_ph114_A11
zmp_ph114_B2
zmp_ph114_B4
zmp_ph114_D8
zmp_ph114_E2

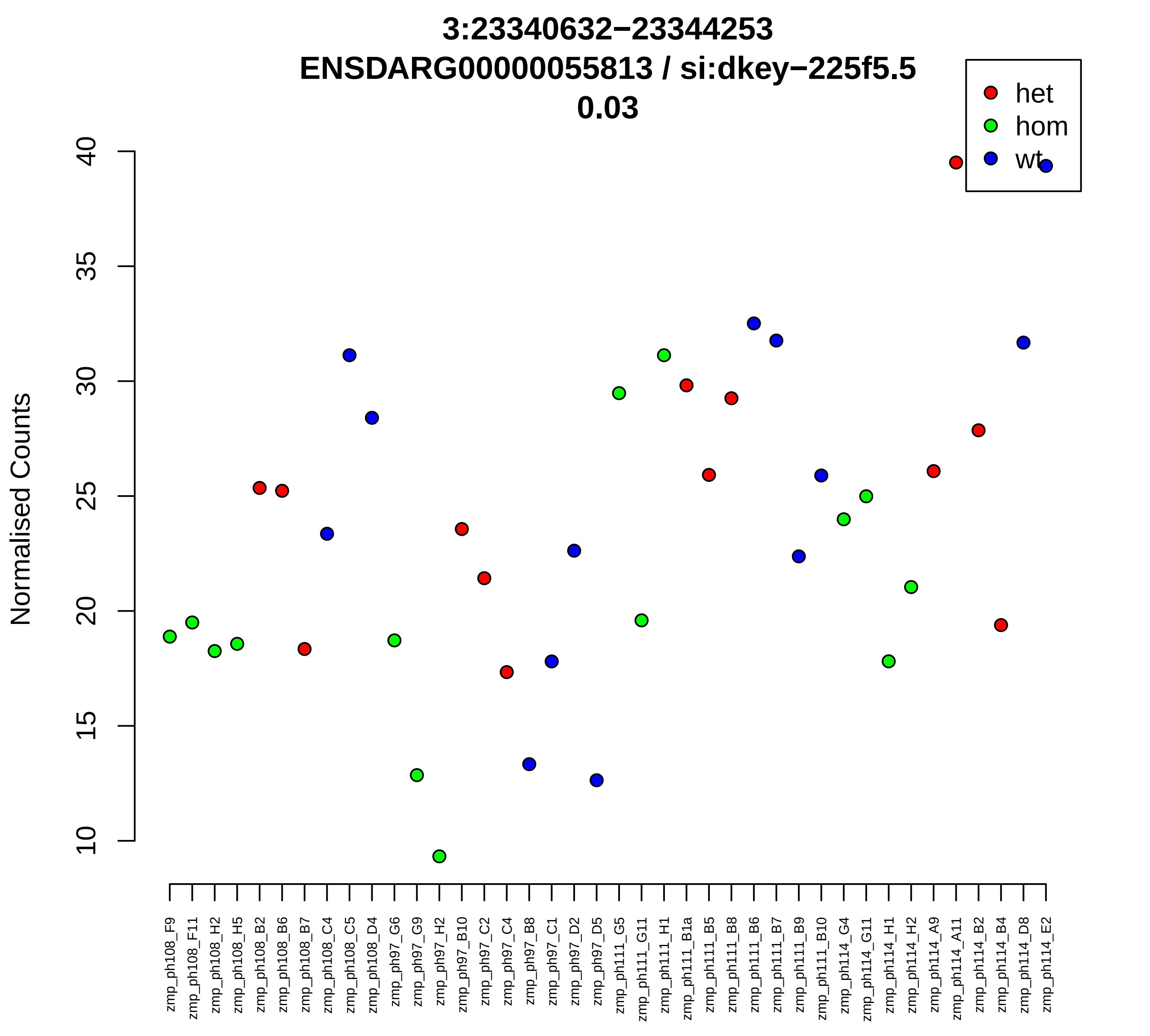

3:23340632−23344253
ENSDARG00000055813 / si:dkey−225f5.5
0.03
het
hom
wt
Normalised Counts
10
15
20
25
30
35
40
zmp_ph108_F9
zmp_ph108_F11
zmp_ph108_H2
zmp_ph108_H5
zmp_ph108_B2
zmp_ph108_B6
zmp_ph108_B7
zmp_ph108_C4
zmp_ph108_C5
zmp_ph108_D4
zmp_ph97_G6
zmp_ph97_G9
zmp_ph97_H2
zmp_ph97_B10
zmp_ph97_C2
zmp_ph97_C4
zmp_ph97_B8
zmp_ph97_C1
zmp_ph97_D2
zmp_ph97_D5
zmp_ph111_G5
zmp_ph111_G11
zmp_ph111_H1
zmp_ph111_B1a
zmp_ph111_B5
zmp_ph111_B8
zmp_ph111_B6
zmp_ph111_B7
zmp_ph111_B9
zmp_ph111_B10
zmp_ph114_G4
zmp_ph114_G11
zmp_ph114_H1
zmp_ph114_H2
zmp_ph114_A9
zmp_ph114_A11
zmp_ph114_B2
zmp_ph114_B4
zmp_ph114_D8
zmp_ph114_E2

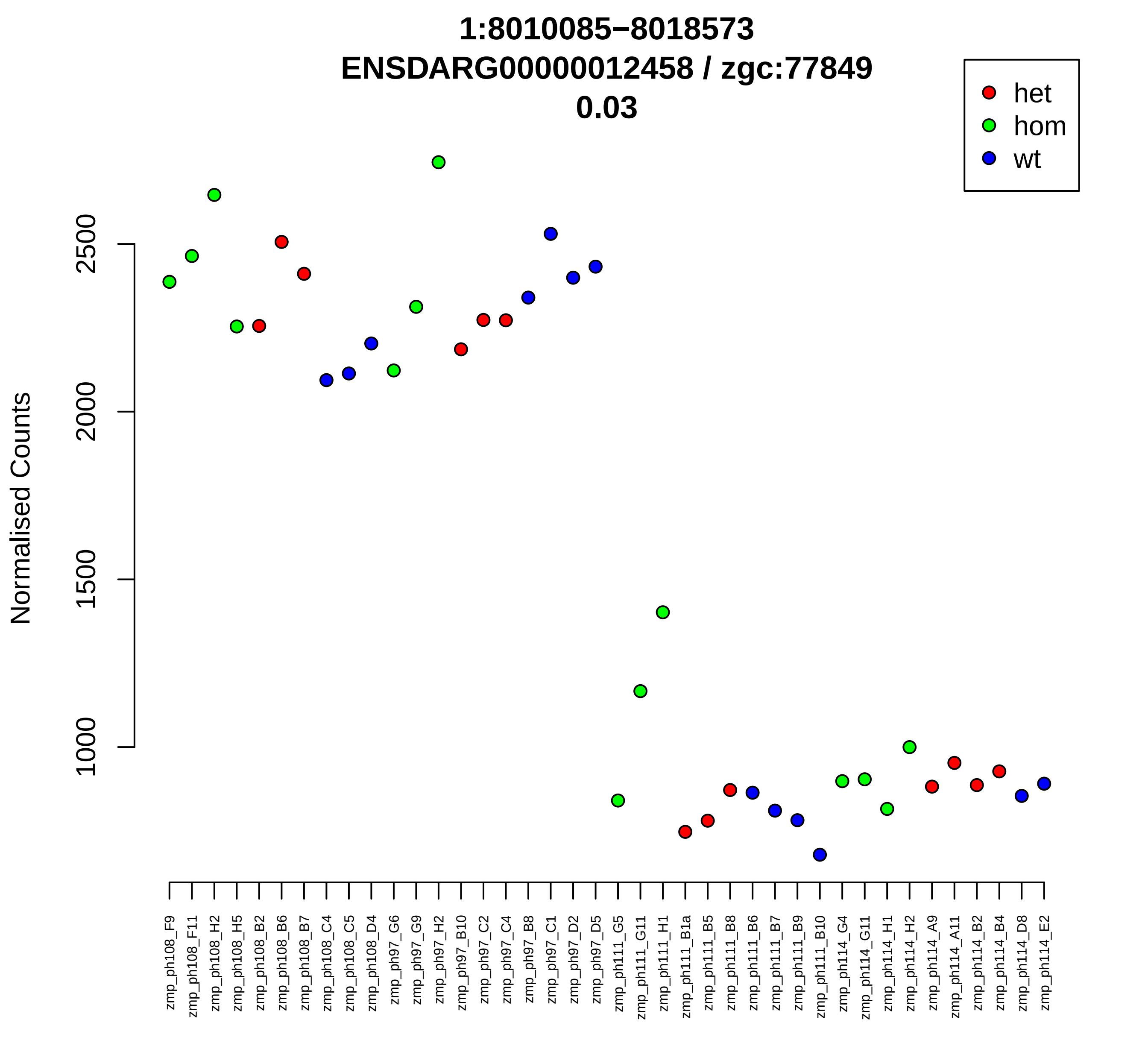

1:8010085−8018573
ENSDARG00000012458 / zgc:77849
0.03
het
hom
wt
Normalised Counts
1000
1500
2000
2500
zmp_ph108_F9
zmp_ph108_F11
zmp_ph108_H2
zmp_ph108_H5
zmp_ph108_B2
zmp_ph108_B6
zmp_ph108_B7
zmp_ph108_C4
zmp_ph108_C5
zmp_ph108_D4
zmp_ph97_G6
zmp_ph97_G9
zmp_ph97_H2
zmp_ph97_B10
zmp_ph97_C2
zmp_ph97_C4
zmp_ph97_B8
zmp_ph97_C1
zmp_ph97_D2
zmp_ph97_D5
zmp_ph111_G5
zmp_ph111_G11
zmp_ph111_H1
zmp_ph111_B1a
zmp_ph111_B5
zmp_ph111_B8
zmp_ph111_B6
zmp_ph111_B7
zmp_ph111_B9
zmp_ph111_B10
zmp_ph114_G4
zmp_ph114_G11
zmp_ph114_H1
zmp_ph114_H2
zmp_ph114_A9
zmp_ph114_A11
zmp_ph114_B2
zmp_ph114_B4
zmp_ph114_D8
zmp_ph114_E2

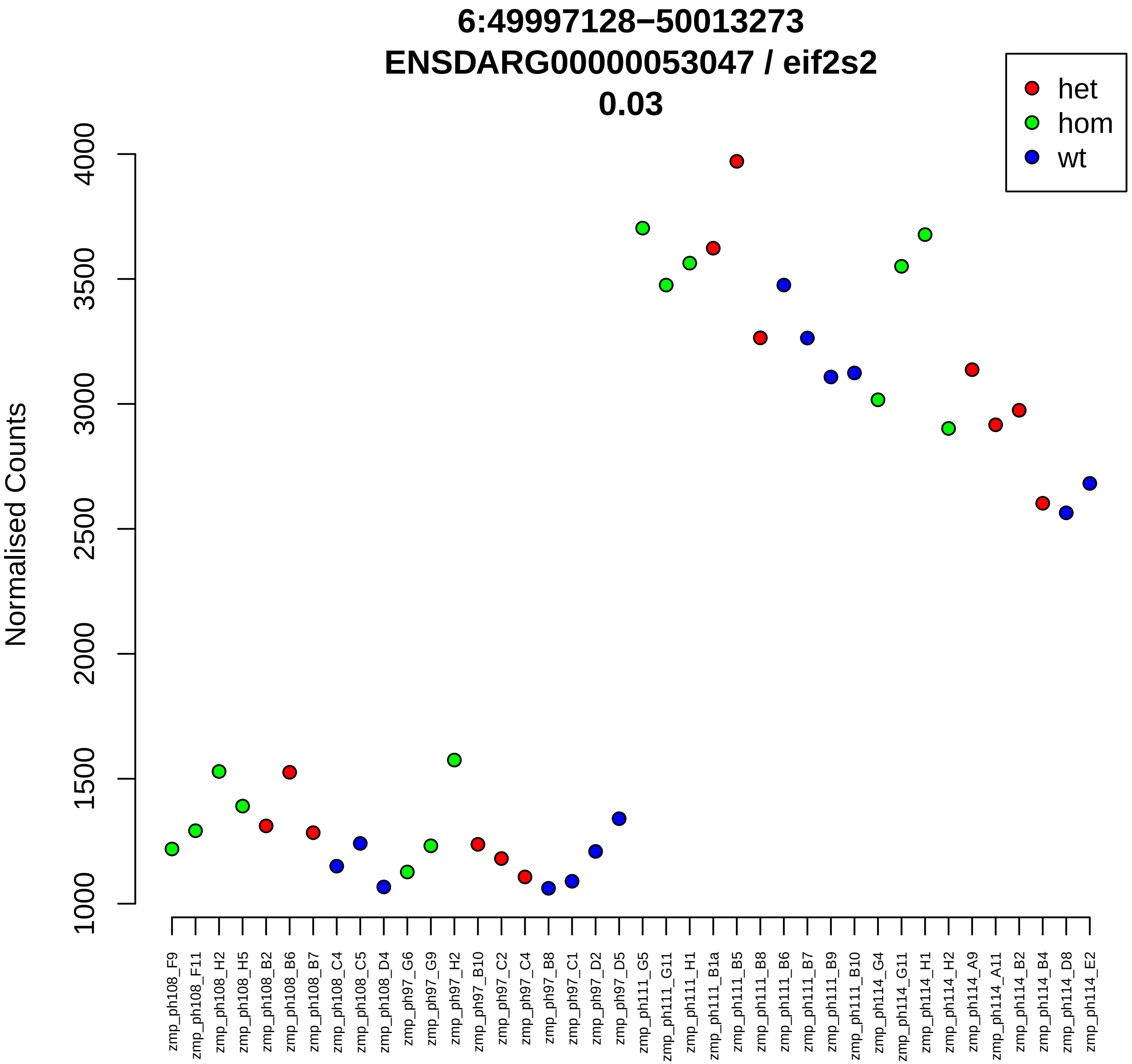
6:49997128−50013273
ENSDARG00000053047 / eif2s2
0.03
het
hom
wt
Normalised Counts
1000
1500
2000
2500
3000
3500
4000
zmp_ph108_F9
zmp_ph108_F11
zmp_ph108_H2
zmp_ph108_H5
zmp_ph108_B2
zmp_ph108_B6
zmp_ph108_B7
zmp_ph108_C4
zmp_ph108_C5
zmp_ph108_D4
zmp_ph97_G6
zmp_ph97_G9
zmp_ph97_H2
zmp_ph97_B10
zmp_ph97_C2
zmp_ph97_C4
zmp_ph97_B8
zmp_ph97_C1
zmp_ph97_D2
zmp_ph97_D5
zmp_ph111_G5
zmp_ph111_G11
zmp_ph111_H1
zmp_ph111_B1a
zmp_ph111_B5
zmp_ph111_B8
zmp_ph111_B6
zmp_ph111_B7
zmp_ph111_B9
zmp_ph111_B10
zmp_ph114_G4
zmp_ph114_G11
zmp_ph114_H1
zmp_ph114_H2
zmp_ph114_A9
zmp_ph114_A11
zmp_ph114_B2
zmp_ph114_B4
zmp_ph114_D8
zmp_ph114_E2

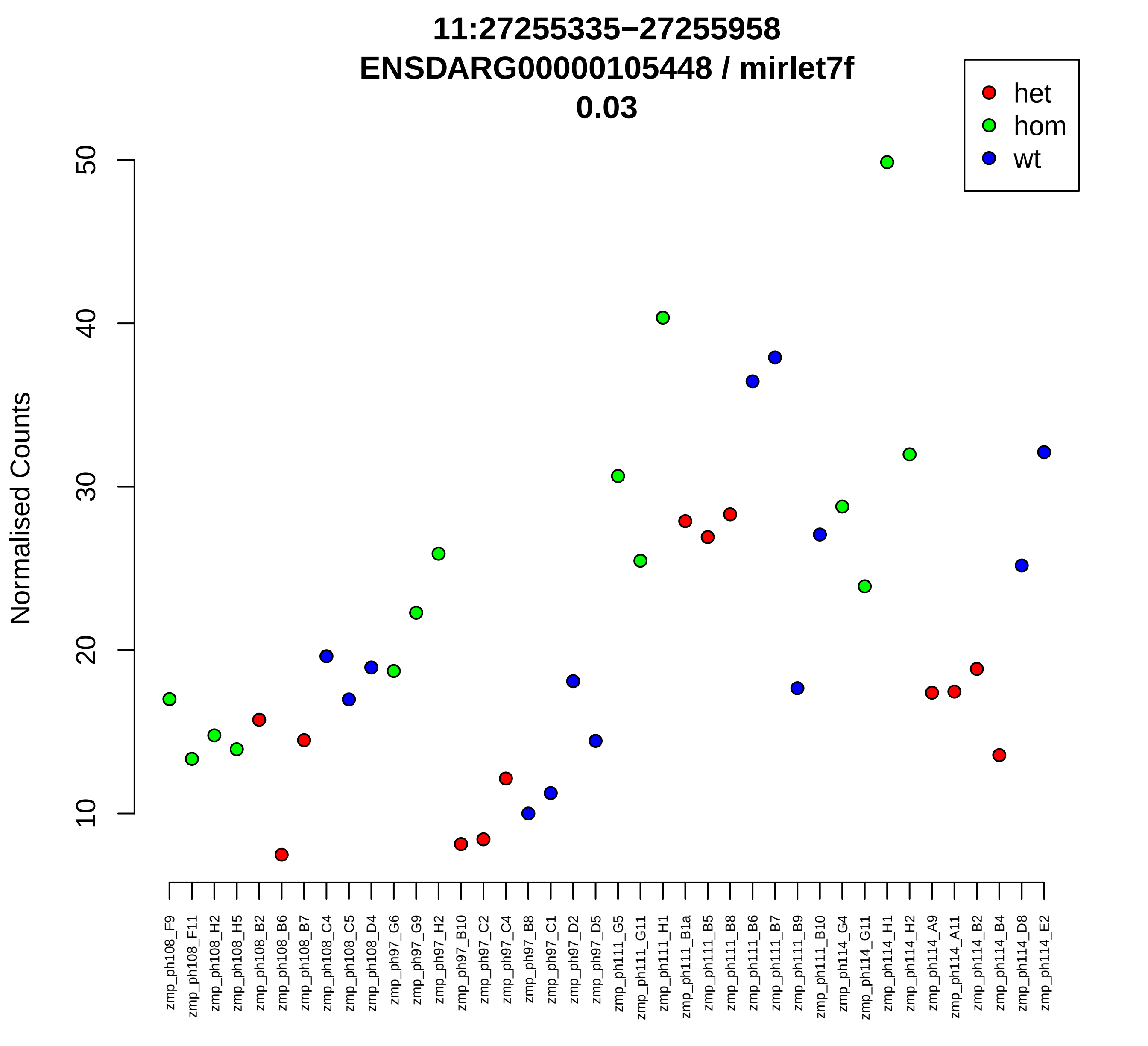
11:27255335−27255958
ENSDARG00000105448 / mirlet7f
0.03
het
hom
wt
Normalised Counts
10
20
30
40
50
zmp_ph108_F9
zmp_ph108_F11
zmp_ph108_H2
zmp_ph108_H5
zmp_ph108_B2
zmp_ph108_B6
zmp_ph108_B7
zmp_ph108_C4
zmp_ph108_C5
zmp_ph108_D4
zmp_ph97_G6
zmp_ph97_G9
zmp_ph97_H2
zmp_ph97_B10
zmp_ph97_C2
zmp_ph97_C4
zmp_ph97_B8
zmp_ph97_C1
zmp_ph97_D2
zmp_ph97_D5
zmp_ph111_G5
zmp_ph111_G11
zmp_ph111_H1
zmp_ph111_B1a
zmp_ph111_B5
zmp_ph111_B8
zmp_ph111_B6
zmp_ph111_B7
zmp_ph111_B9
zmp_ph111_B10
zmp_ph114_G4
zmp_ph114_G11
zmp_ph114_H1
zmp_ph114_H2
zmp_ph114_A9
zmp_ph114_A11
zmp_ph114_B2
zmp_ph114_B4
zmp_ph114_D8
zmp_ph114_E2

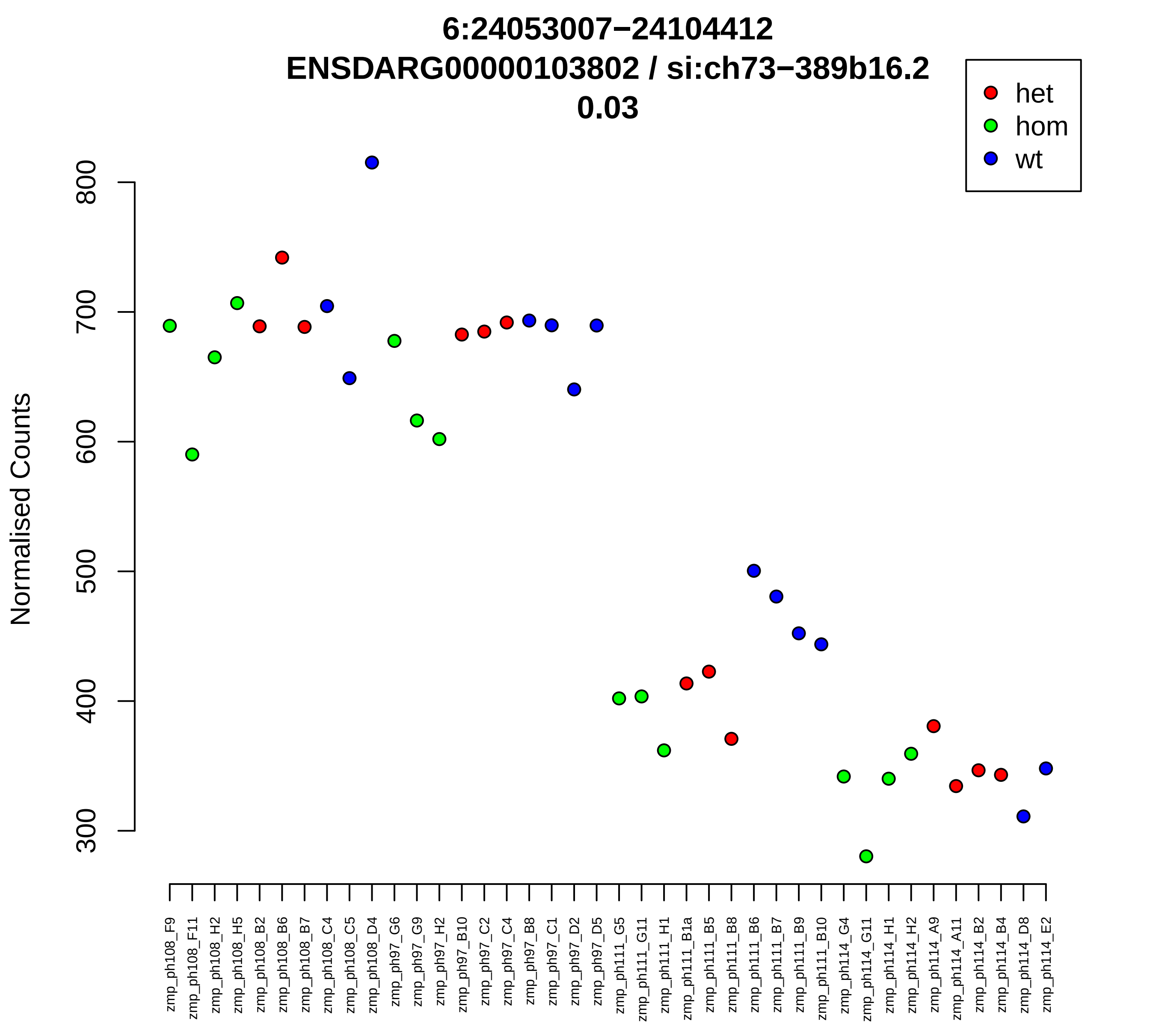
6:24053007−24104412
ENSDARG00000103802 / si:ch73−389b16.2
0.03
het
hom
wt
Normalised Counts
300
400
500
600
700
800
zmp_ph108_F9
zmp_ph108_F11
zmp_ph108_H2
zmp_ph108_H5
zmp_ph108_B2
zmp_ph108_B6
zmp_ph108_B7
zmp_ph108_C4
zmp_ph108_C5
zmp_ph108_D4
zmp_ph97_G6
zmp_ph97_G9
zmp_ph97_H2
zmp_ph97_B10
zmp_ph97_C2
zmp_ph97_C4
zmp_ph97_B8
zmp_ph97_C1
zmp_ph97_D2
zmp_ph97_D5
zmp_ph111_G5
zmp_ph111_G11
zmp_ph111_H1
zmp_ph111_B1a
zmp_ph111_B5
zmp_ph111_B8
zmp_ph111_B6
zmp_ph111_B7
zmp_ph111_B9
zmp_ph111_B10
zmp_ph114_G4
zmp_ph114_G11
zmp_ph114_H1
zmp_ph114_H2
zmp_ph114_A9
zmp_ph114_A11
zmp_ph114_B2
zmp_ph114_B4
zmp_ph114_D8
zmp_ph114_E2

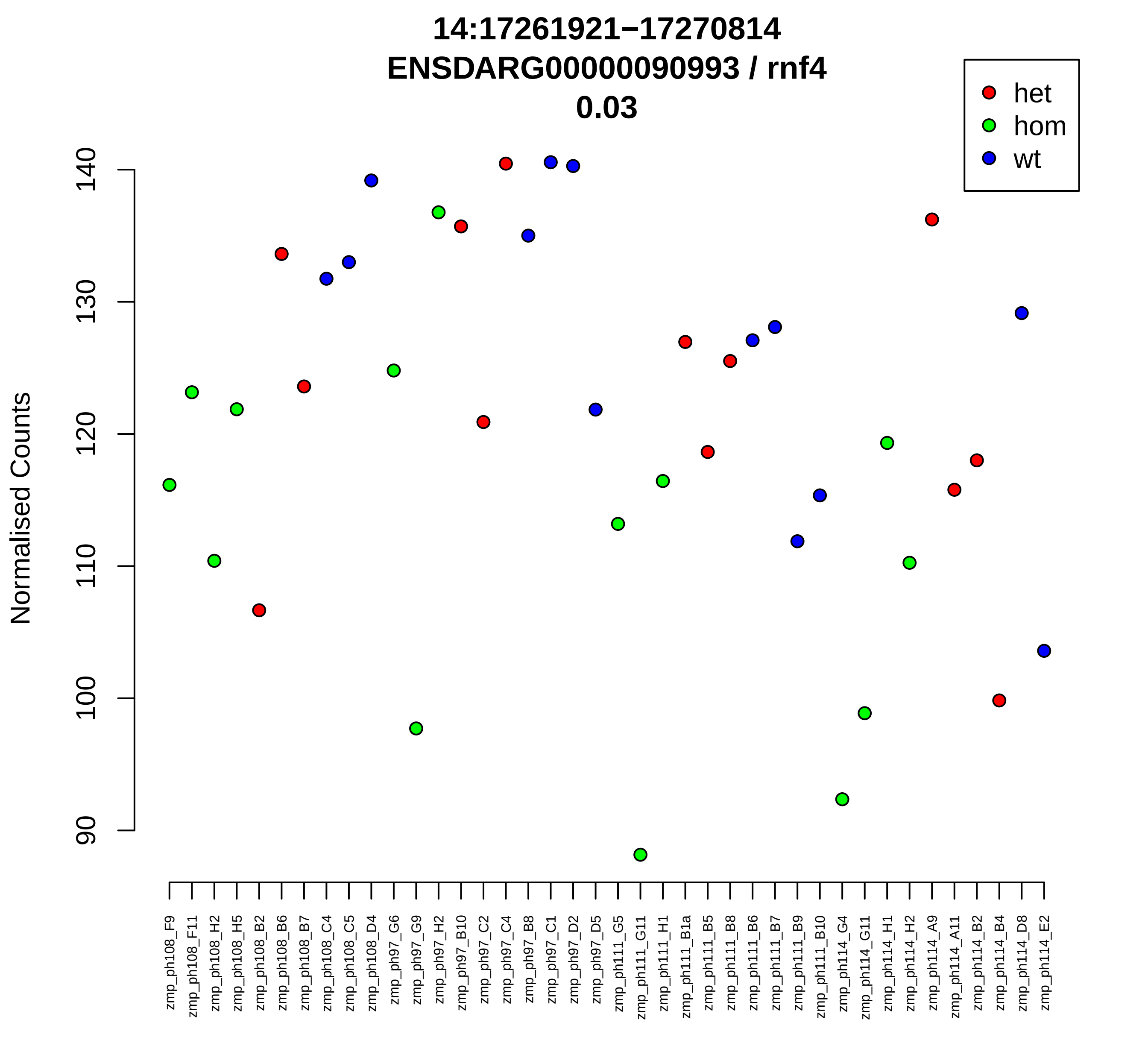

14:17261921−17270814
ENSDARG00000090993 / rnf4
0.03
het
hom
wt
Normalised Counts
90
100
110
120
130
140
zmp_ph108_F9
zmp_ph108_F11
zmp_ph108_H2
zmp_ph108_H5
zmp_ph108_B2
zmp_ph108_B6
zmp_ph108_B7
zmp_ph108_C4
zmp_ph108_C5
zmp_ph108_D4
zmp_ph97_G6
zmp_ph97_G9
zmp_ph97_H2
zmp_ph97_B10
zmp_ph97_C2
zmp_ph97_C4
zmp_ph97_B8
zmp_ph97_C1
zmp_ph97_D2
zmp_ph97_D5
zmp_ph111_G5
zmp_ph111_G11
zmp_ph111_H1
zmp_ph111_B1a
zmp_ph111_B5
zmp_ph111_B8
zmp_ph111_B6
zmp_ph111_B7
zmp_ph111_B9
zmp_ph111_B10
zmp_ph114_G4
zmp_ph114_G11
zmp_ph114_H1
zmp_ph114_H2
zmp_ph114_A9
zmp_ph114_A11
zmp_ph114_B2
zmp_ph114_B4
zmp_ph114_D8
zmp_ph114_E2

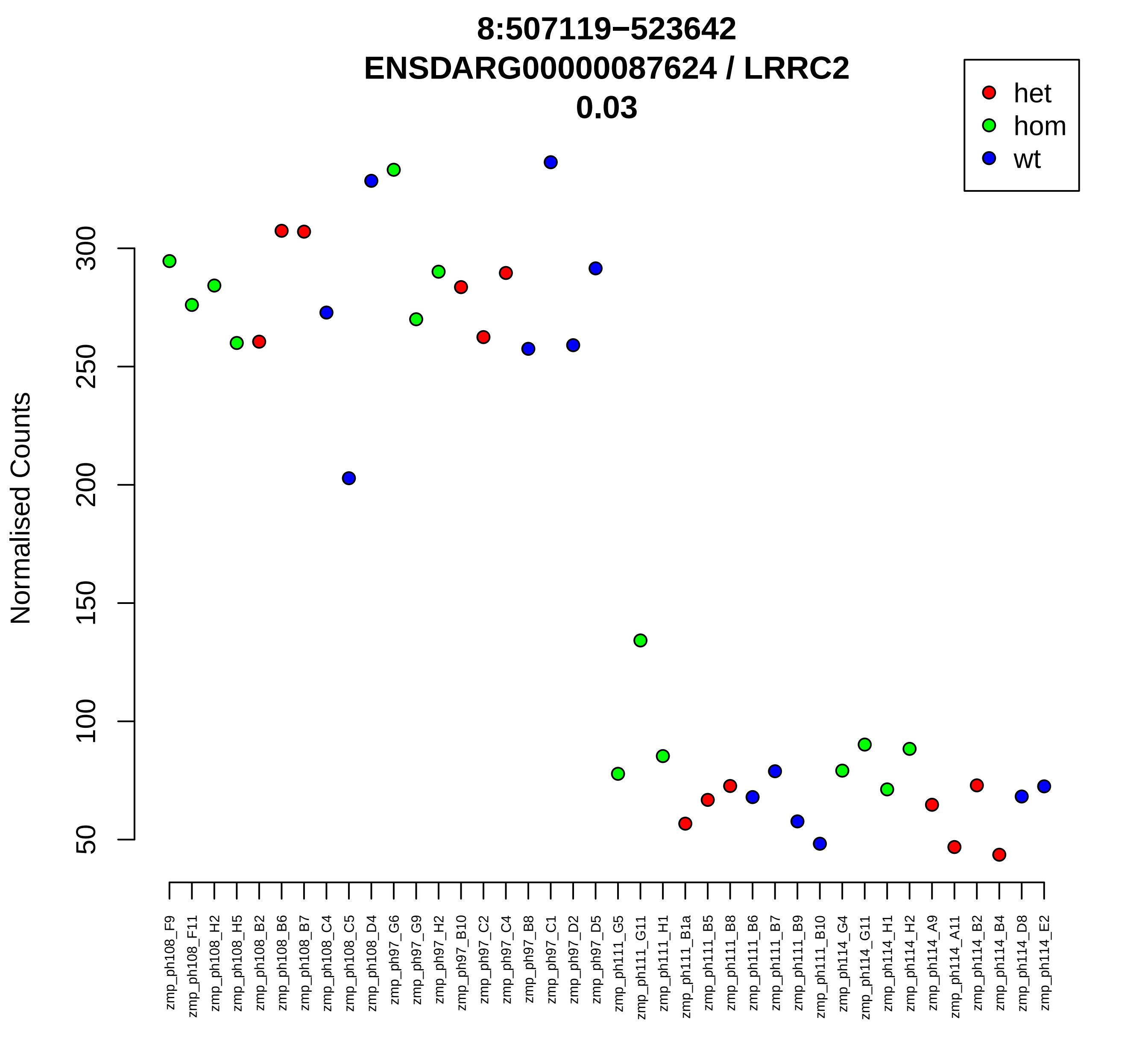
8:507119−523642
ENSDARG00000087624 / LRRC2
0.03
het
hom
wt
Normalised Counts
50
100
150
200
250
300
zmp_ph108_F9
zmp_ph108_F11
zmp_ph108_H2
zmp_ph108_H5
zmp_ph108_B2
zmp_ph108_B6
zmp_ph108_B7
zmp_ph108_C4
zmp_ph108_C5
zmp_ph108_D4
zmp_ph97_G6
zmp_ph97_G9
zmp_ph97_H2
zmp_ph97_B10
zmp_ph97_C2
zmp_ph97_C4
zmp_ph97_B8
zmp_ph97_C1
zmp_ph97_D2
zmp_ph97_D5
zmp_ph111_G5
zmp_ph111_G11
zmp_ph111_H1
zmp_ph111_B1a
zmp_ph111_B5
zmp_ph111_B8
zmp_ph111_B6
zmp_ph111_B7
zmp_ph111_B9
zmp_ph111_B10
zmp_ph114_G4
zmp_ph114_G11
zmp_ph114_H1
zmp_ph114_H2
zmp_ph114_A9
zmp_ph114_A11
zmp_ph114_B2
zmp_ph114_B4
zmp_ph114_D8
zmp_ph114_E2

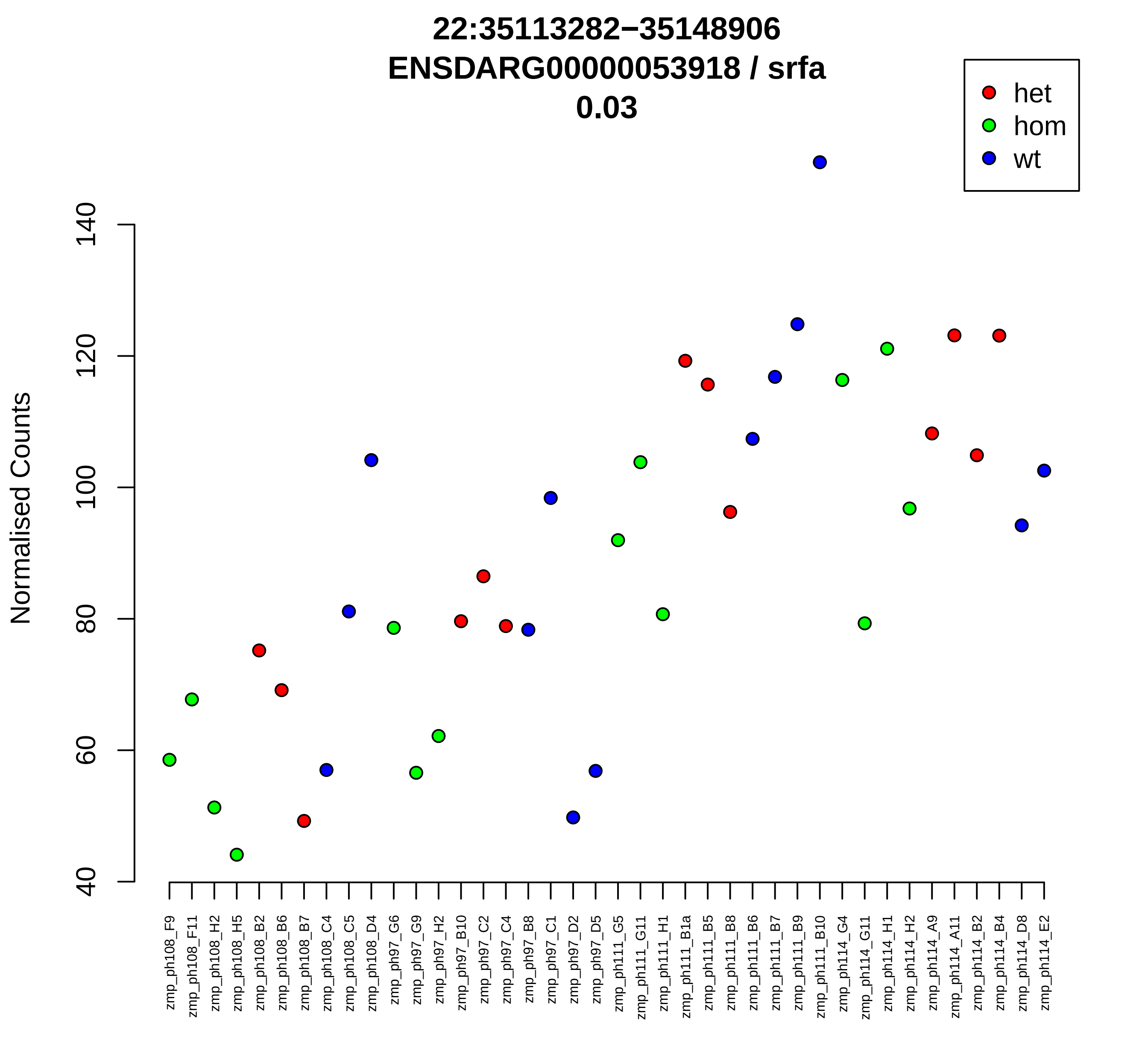

22:35113282−35148906
ENSDARG00000053918 / srfa
0.03
het
hom
wt
Normalised Counts
40
60
80
100
120
140
zmp_ph108_F9
zmp_ph108_F11
zmp_ph108_H2
zmp_ph108_H5
zmp_ph108_B2
zmp_ph108_B6
zmp_ph108_B7
zmp_ph108_C4
zmp_ph108_C5
zmp_ph108_D4
zmp_ph97_G6
zmp_ph97_G9
zmp_ph97_H2
zmp_ph97_B10
zmp_ph97_C2
zmp_ph97_C4
zmp_ph97_B8
zmp_ph97_C1
zmp_ph97_D2
zmp_ph97_D5
zmp_ph111_G5
zmp_ph111_G11
zmp_ph111_H1
zmp_ph111_B1a
zmp_ph111_B5
zmp_ph111_B8
zmp_ph111_B6
zmp_ph111_B7
zmp_ph111_B9
zmp_ph111_B10
zmp_ph114_G4
zmp_ph114_G11
zmp_ph114_H1
zmp_ph114_H2
zmp_ph114_A9
zmp_ph114_A11
zmp_ph114_B2
zmp_ph114_B4
zmp_ph114_D8
zmp_ph114_E2

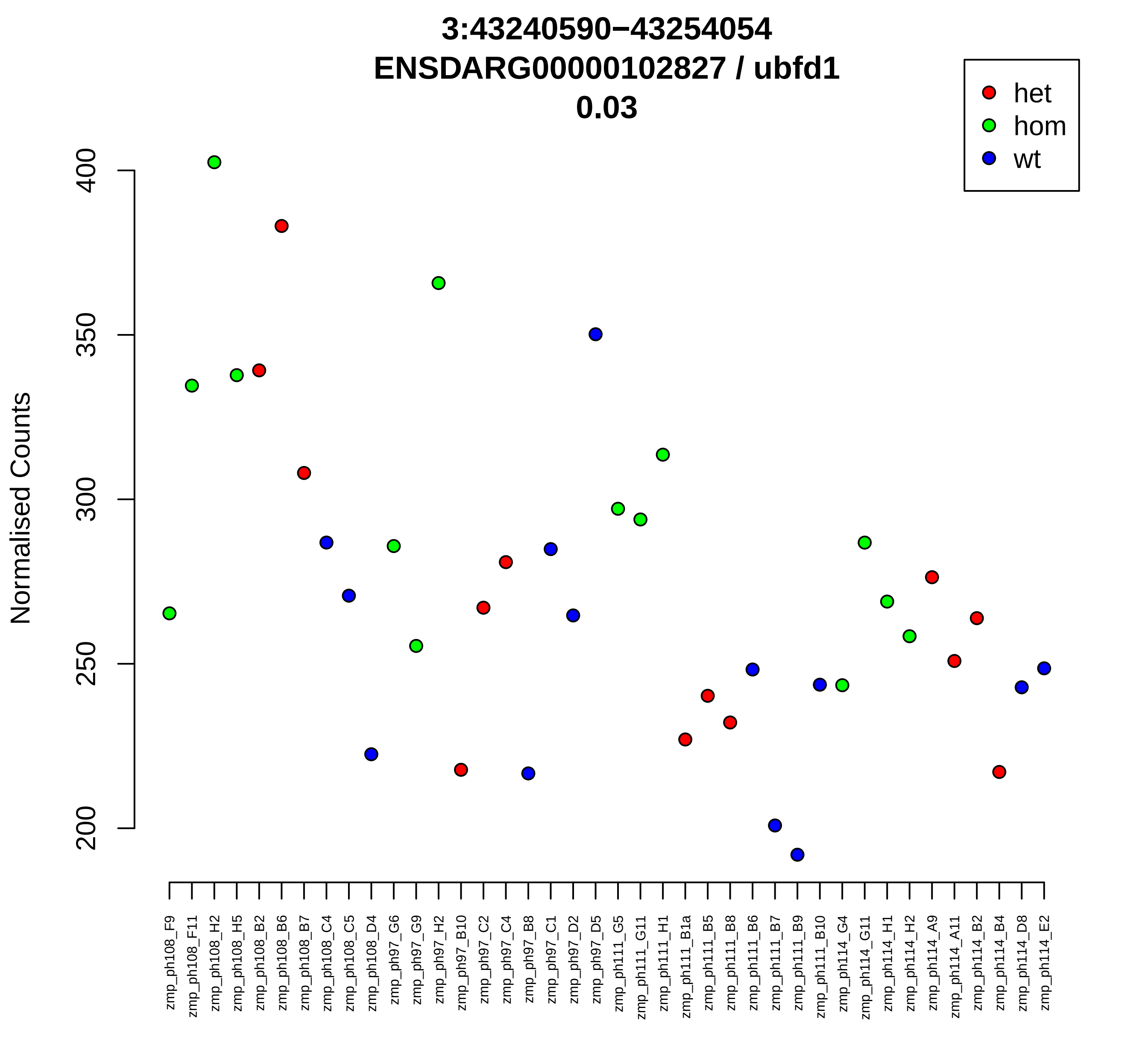

3:43240590−43254054
ENSDARG00000102827 / ubfd1
0.03
het
hom
wt
Normalised Counts
200
250
300
350
400
zmp_ph108_F9
zmp_ph108_F11
zmp_ph108_H2
zmp_ph108_H5
zmp_ph108_B2
zmp_ph108_B6
zmp_ph108_B7
zmp_ph108_C4
zmp_ph108_C5
zmp_ph108_D4
zmp_ph97_G6
zmp_ph97_G9
zmp_ph97_H2
zmp_ph97_B10
zmp_ph97_C2
zmp_ph97_C4
zmp_ph97_B8
zmp_ph97_C1
zmp_ph97_D2
zmp_ph97_D5
zmp_ph111_G5
zmp_ph111_G11
zmp_ph111_H1
zmp_ph111_B1a
zmp_ph111_B5
zmp_ph111_B8
zmp_ph111_B6
zmp_ph111_B7
zmp_ph111_B9
zmp_ph111_B10
zmp_ph114_G4
zmp_ph114_G11
zmp_ph114_H1
zmp_ph114_H2
zmp_ph114_A9
zmp_ph114_A11
zmp_ph114_B2
zmp_ph114_B4
zmp_ph114_D8
zmp_ph114_E2

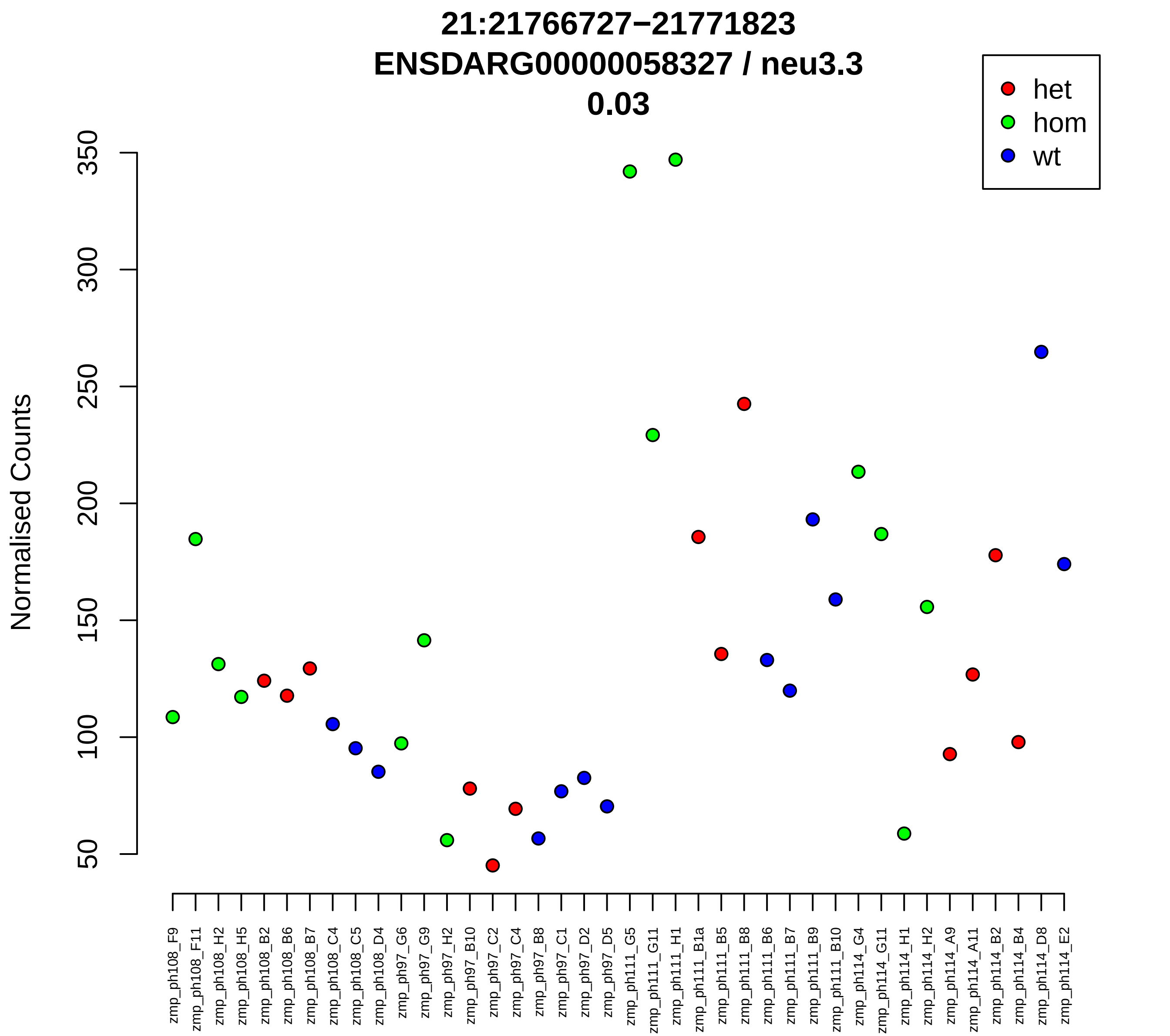

21:21766727−21771823
ENSDARG00000058327 / neu3.3
0.03
het
hom
wt
Normalised Counts
50
100
150
200
250
300
350
zmp_ph108_F9
zmp_ph108_F11
zmp_ph108_H2
zmp_ph108_H5
zmp_ph108_B2
zmp_ph108_B6
zmp_ph108_B7
zmp_ph108_C4
zmp_ph108_C5
zmp_ph108_D4
zmp_ph97_G6
zmp_ph97_G9
zmp_ph97_H2
zmp_ph97_B10
zmp_ph97_C2
zmp_ph97_C4
zmp_ph97_B8
zmp_ph97_C1
zmp_ph97_D2
zmp_ph97_D5
zmp_ph111_G5
zmp_ph111_G11
zmp_ph111_H1
zmp_ph111_B1a
zmp_ph111_B5
zmp_ph111_B8
zmp_ph111_B6
zmp_ph111_B7
zmp_ph111_B9
zmp_ph111_B10
zmp_ph114_G4
zmp_ph114_G11
zmp_ph114_H1
zmp_ph114_H2
zmp_ph114_A9
zmp_ph114_A11
zmp_ph114_B2
zmp_ph114_B4
zmp_ph114_D8
zmp_ph114_E2

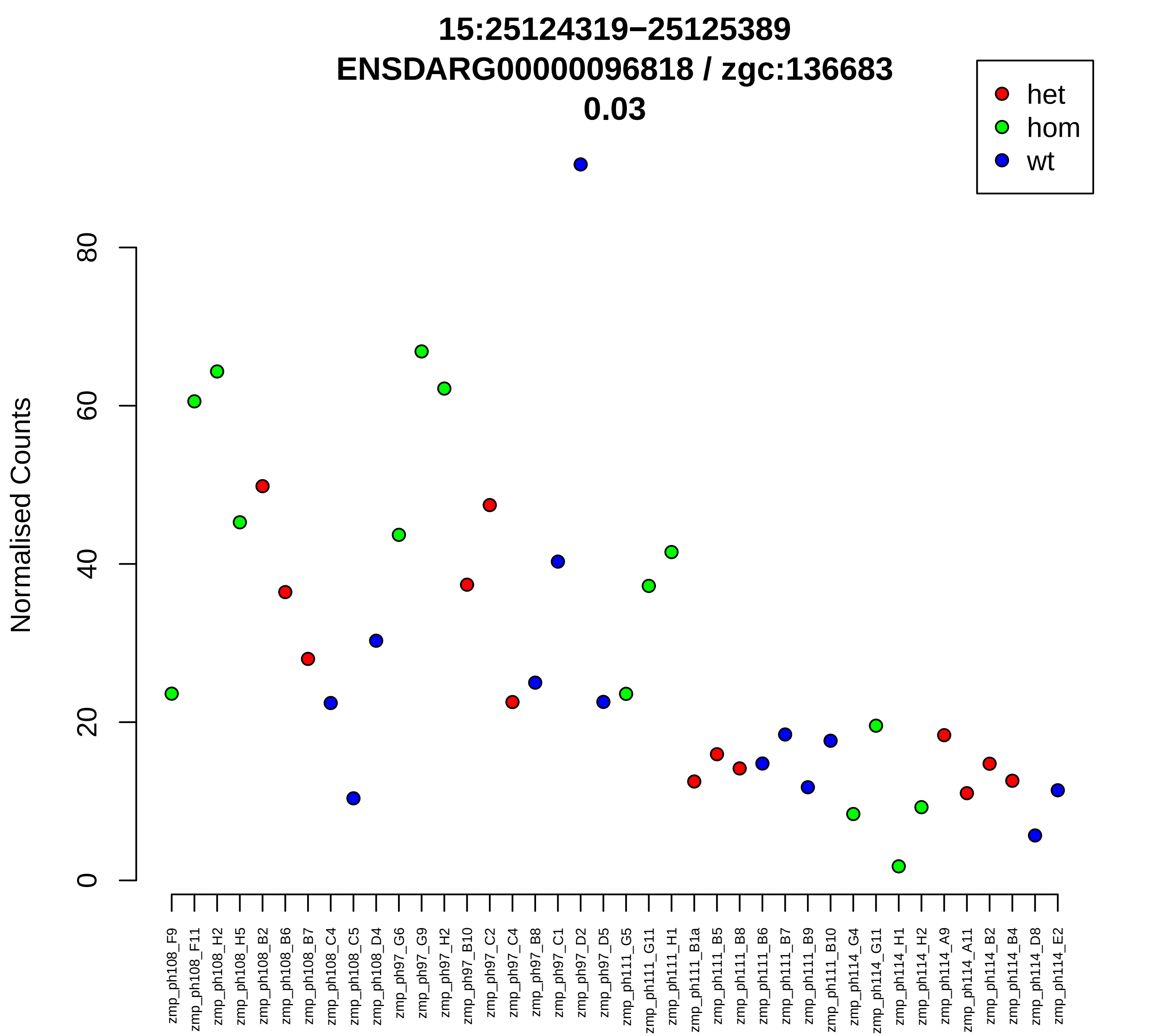
15:25124319−25125389
ENSDARG00000096818 / zgc:136683
0.03
het
hom
wt
Normalised Counts
0
20
40
60
80
zmp_ph108_F9
zmp_ph108_F11
zmp_ph108_H2
zmp_ph108_H5
zmp_ph108_B2
zmp_ph108_B6
zmp_ph108_B7
zmp_ph108_C4
zmp_ph108_C5
zmp_ph108_D4
zmp_ph97_G6
zmp_ph97_G9
zmp_ph97_H2
zmp_ph97_B10
zmp_ph97_C2
zmp_ph97_C4
zmp_ph97_B8
zmp_ph97_C1
zmp_ph97_D2
zmp_ph97_D5
zmp_ph111_G5
zmp_ph111_G11
zmp_ph111_H1
zmp_ph111_B1a
zmp_ph111_B5
zmp_ph111_B8
zmp_ph111_B6
zmp_ph111_B7
zmp_ph111_B9
zmp_ph111_B10
zmp_ph114_G4
zmp_ph114_G11
zmp_ph114_H1
zmp_ph114_H2
zmp_ph114_A9
zmp_ph114_A11
zmp_ph114_B2
zmp_ph114_B4
zmp_ph114_D8
zmp_ph114_E2

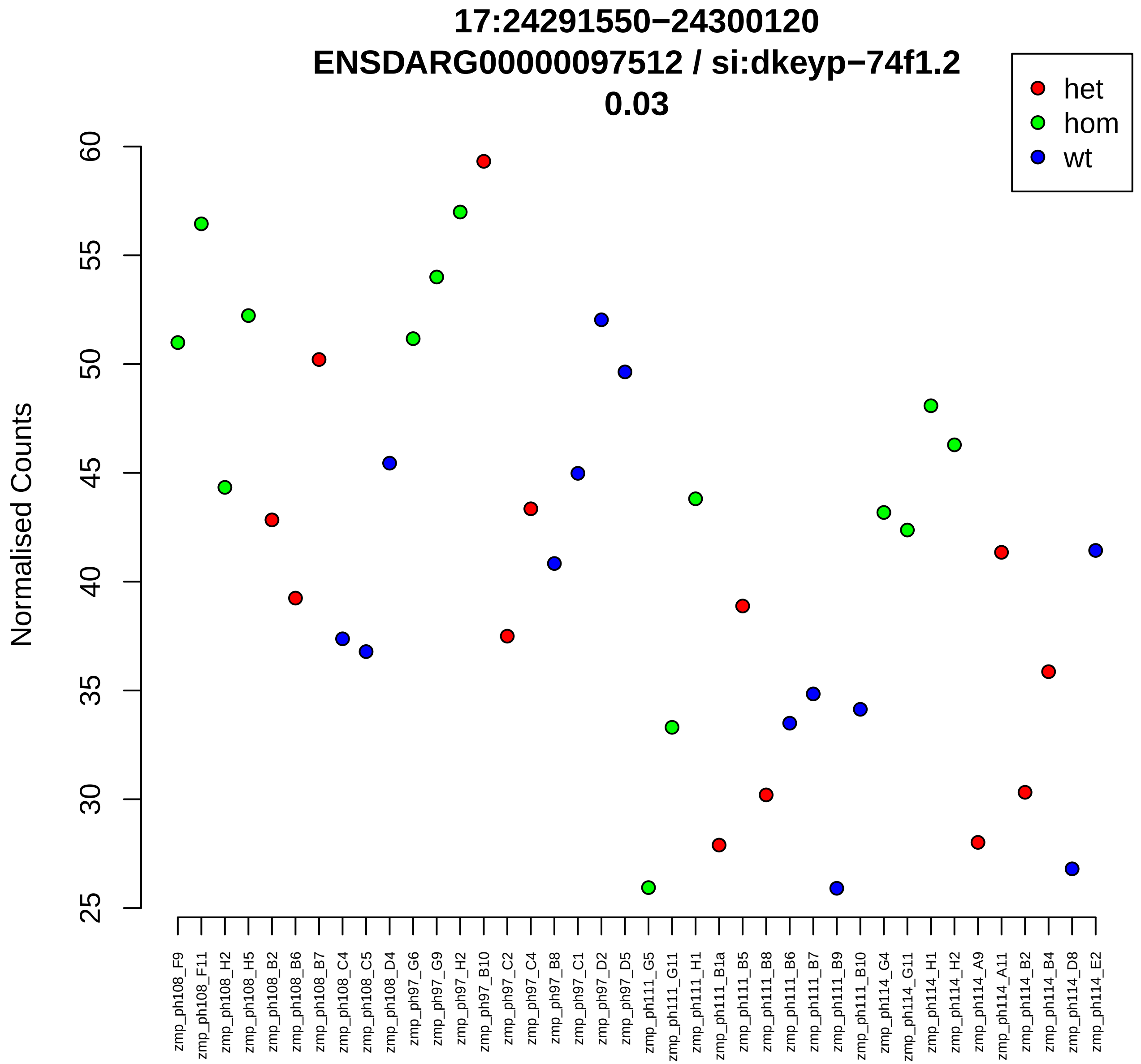

17:24291550−24300120
ENSDARG00000097512 / si:dkeyp−74f1.2
0.03
het
hom
wt
Normalised Counts
25
30
35
40
45
50
55
60
zmp_ph108_F9
zmp_ph108_F11
zmp_ph108_H2
zmp_ph108_H5
zmp_ph108_B2
zmp_ph108_B6
zmp_ph108_B7
zmp_ph108_C4
zmp_ph108_C5
zmp_ph108_D4
zmp_ph97_G6
zmp_ph97_G9
zmp_ph97_H2
zmp_ph97_B10
zmp_ph97_C2
zmp_ph97_C4
zmp_ph97_B8
zmp_ph97_C1
zmp_ph97_D2
zmp_ph97_D5
zmp_ph111_G5
zmp_ph111_G11
zmp_ph111_H1
zmp_ph111_B1a
zmp_ph111_B5
zmp_ph111_B8
zmp_ph111_B6
zmp_ph111_B7
zmp_ph111_B9
zmp_ph111_B10
zmp_ph114_G4
zmp_ph114_G11
zmp_ph114_H1
zmp_ph114_H2
zmp_ph114_A9
zmp_ph114_A11
zmp_ph114_B2
zmp_ph114_B4
zmp_ph114_D8
zmp_ph114_E2

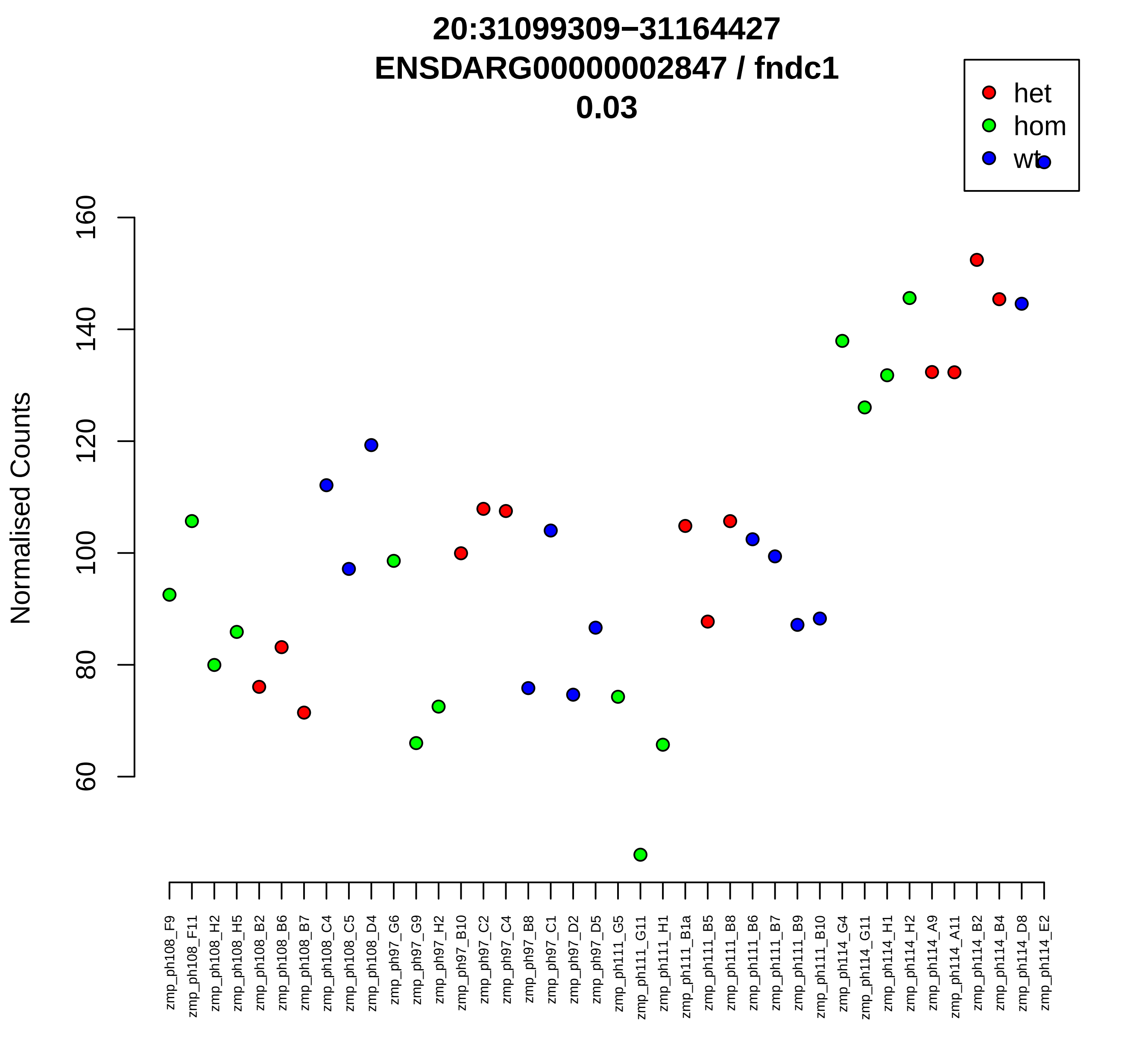

20:31099309−31164427
ENSDARG00000002847 / fndc1
0.03
het
hom
wt
Normalised Counts
60
80
100
120
140
160
zmp_ph108_F9
zmp_ph108_F11
zmp_ph108_H2
zmp_ph108_H5
zmp_ph108_B2
zmp_ph108_B6
zmp_ph108_B7
zmp_ph108_C4
zmp_ph108_C5
zmp_ph108_D4
zmp_ph97_G6
zmp_ph97_G9
zmp_ph97_H2
zmp_ph97_B10
zmp_ph97_C2
zmp_ph97_C4
zmp_ph97_B8
zmp_ph97_C1
zmp_ph97_D2
zmp_ph97_D5
zmp_ph111_G5
zmp_ph111_G11
zmp_ph111_H1
zmp_ph111_B1a
zmp_ph111_B5
zmp_ph111_B8
zmp_ph111_B6
zmp_ph111_B7
zmp_ph111_B9
zmp_ph111_B10
zmp_ph114_G4
zmp_ph114_G11
zmp_ph114_H1
zmp_ph114_H2
zmp_ph114_A9
zmp_ph114_A11
zmp_ph114_B2
zmp_ph114_B4
zmp_ph114_D8
zmp_ph114_E2

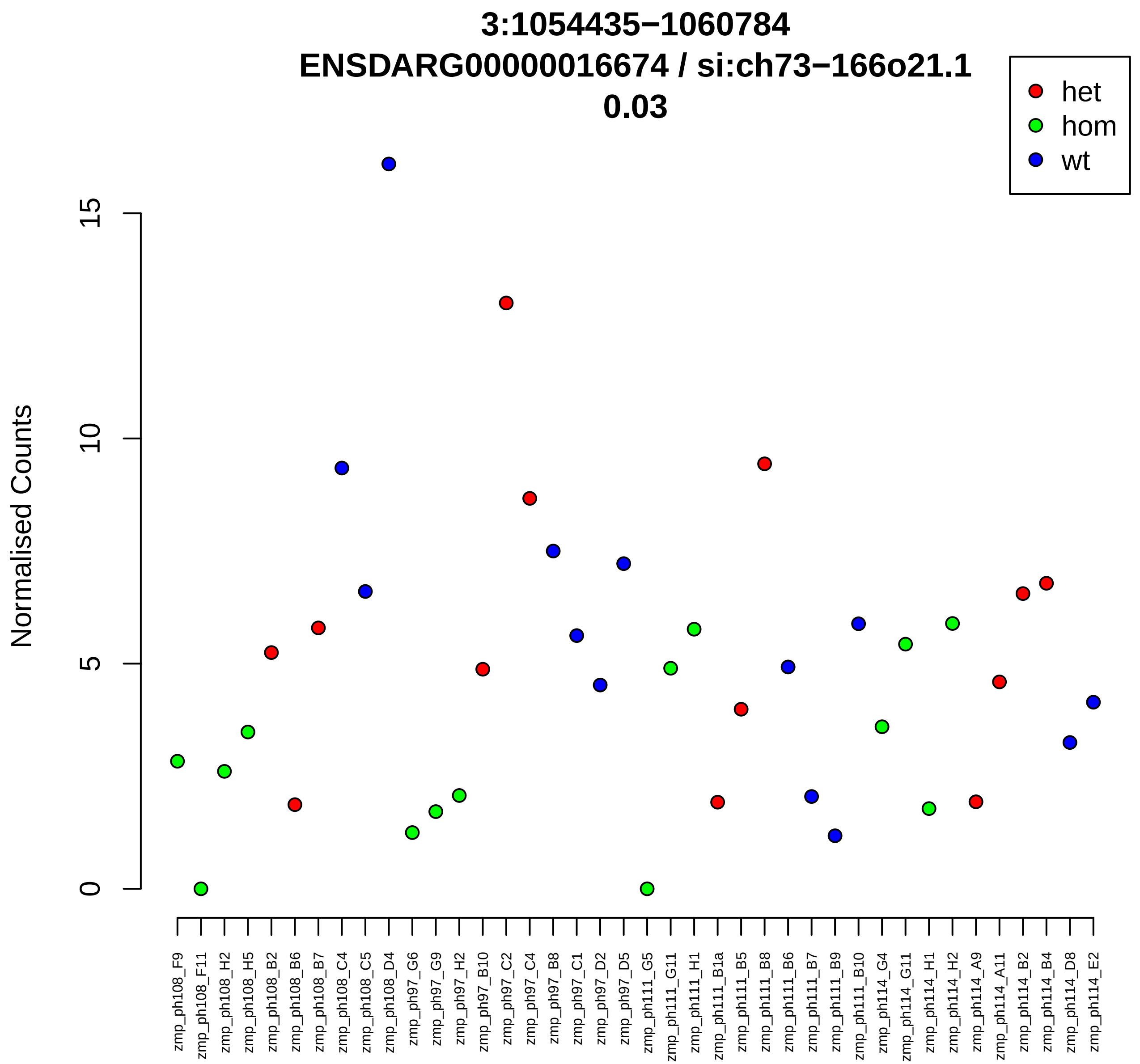
3:1054435−1060784
ENSDARG00000016674 / si:ch73−166o21.1
0.03
het
hom
wt
Normalised Counts
0
5
10
15
zmp_ph108_F9
zmp_ph108_F11
zmp_ph108_H2
zmp_ph108_H5
zmp_ph108_B2
zmp_ph108_B6
zmp_ph108_B7
zmp_ph108_C4
zmp_ph108_C5
zmp_ph108_D4
zmp_ph97_G6
zmp_ph97_G9
zmp_ph97_H2
zmp_ph97_B10
zmp_ph97_C2
zmp_ph97_C4
zmp_ph97_B8
zmp_ph97_C1
zmp_ph97_D2
zmp_ph97_D5
zmp_ph111_G5
zmp_ph111_G11
zmp_ph111_H1
zmp_ph111_B1a
zmp_ph111_B5
zmp_ph111_B8
zmp_ph111_B6
zmp_ph111_B7
zmp_ph111_B9
zmp_ph111_B10
zmp_ph114_G4
zmp_ph114_G11
zmp_ph114_H1
zmp_ph114_H2
zmp_ph114_A9
zmp_ph114_A11
zmp_ph114_B2
zmp_ph114_B4
zmp_ph114_D8
zmp_ph114_E2

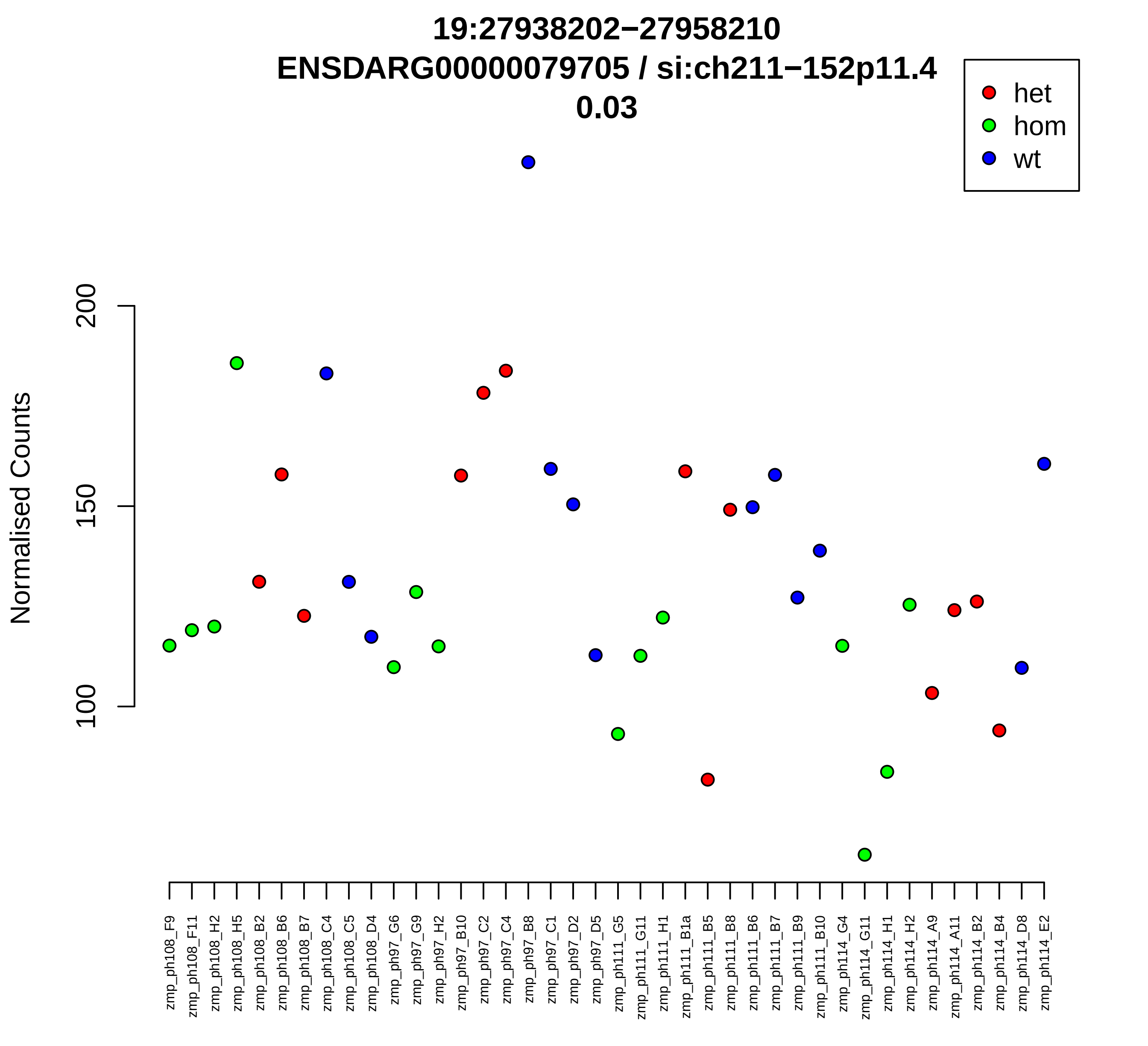

19:27938202−27958210
ENSDARG00000079705 / si:ch211−152p11.4
0.03
het
hom
wt
Normalised Counts
100
150
200
zmp_ph108_F9
zmp_ph108_F11
zmp_ph108_H2
zmp_ph108_H5
zmp_ph108_B2
zmp_ph108_B6
zmp_ph108_B7
zmp_ph108_C4
zmp_ph108_C5
zmp_ph108_D4
zmp_ph97_G6
zmp_ph97_G9
zmp_ph97_H2
zmp_ph97_B10
zmp_ph97_C2
zmp_ph97_C4
zmp_ph97_B8
zmp_ph97_C1
zmp_ph97_D2
zmp_ph97_D5
zmp_ph111_G5
zmp_ph111_G11
zmp_ph111_H1
zmp_ph111_B1a
zmp_ph111_B5
zmp_ph111_B8
zmp_ph111_B6
zmp_ph111_B7
zmp_ph111_B9
zmp_ph111_B10
zmp_ph114_G4
zmp_ph114_G11
zmp_ph114_H1
zmp_ph114_H2
zmp_ph114_A9
zmp_ph114_A11
zmp_ph114_B2
zmp_ph114_B4
zmp_ph114_D8
zmp_ph114_E2

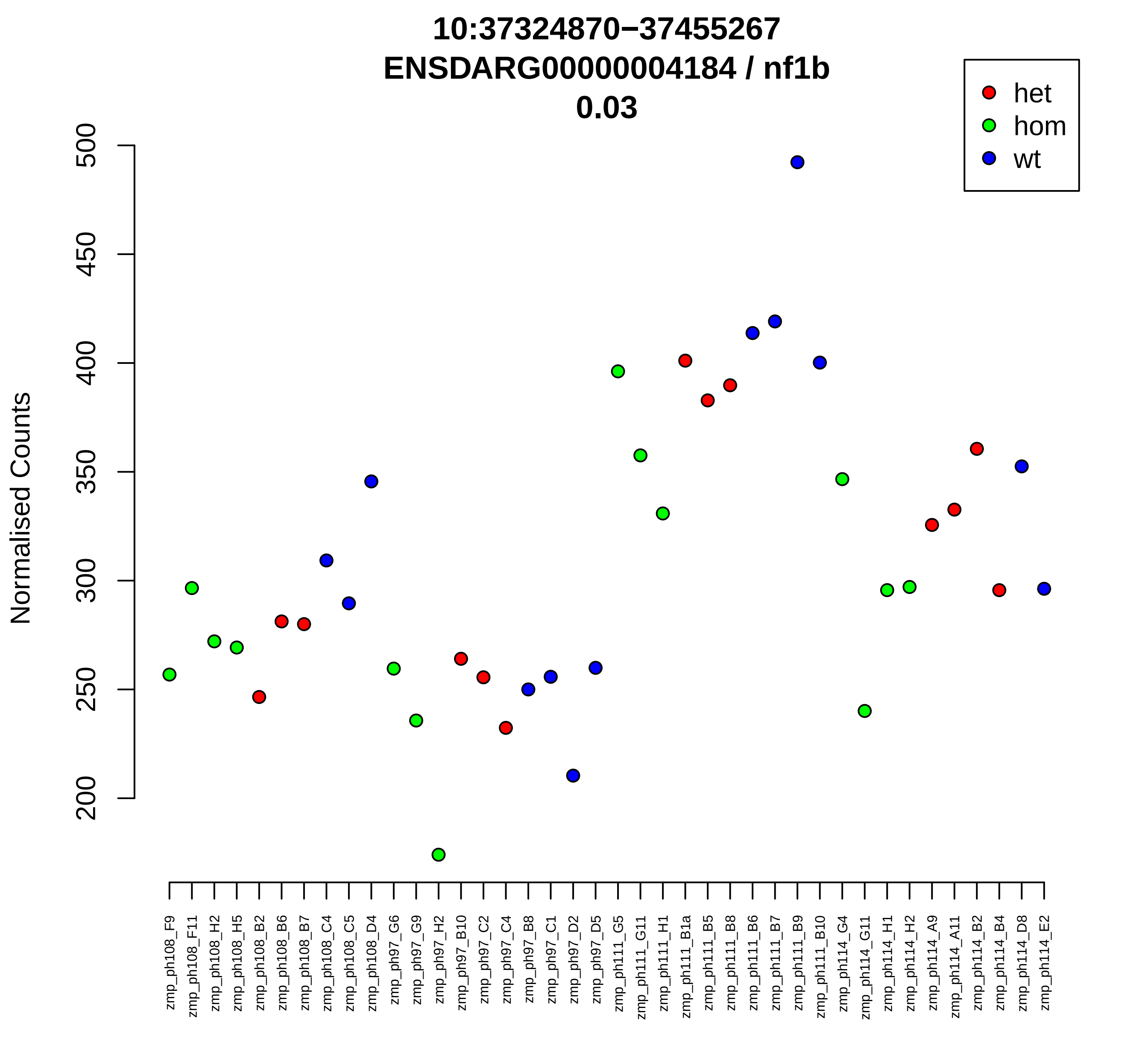

10:37324870−37455267
ENSDARG00000004184 / nf1b
0.03
het
hom
wt
Normalised Counts
200
250
300
350
400
450
500
zmp_ph108_F9
zmp_ph108_F11
zmp_ph108_H2
zmp_ph108_H5
zmp_ph108_B2
zmp_ph108_B6
zmp_ph108_B7
zmp_ph108_C4
zmp_ph108_C5
zmp_ph108_D4
zmp_ph97_G6
zmp_ph97_G9
zmp_ph97_H2
zmp_ph97_B10
zmp_ph97_C2
zmp_ph97_C4
zmp_ph97_B8
zmp_ph97_C1
zmp_ph97_D2
zmp_ph97_D5
zmp_ph111_G5
zmp_ph111_G11
zmp_ph111_H1
zmp_ph111_B1a
zmp_ph111_B5
zmp_ph111_B8
zmp_ph111_B6
zmp_ph111_B7
zmp_ph111_B9
zmp_ph111_B10
zmp_ph114_G4
zmp_ph114_G11
zmp_ph114_H1
zmp_ph114_H2
zmp_ph114_A9
zmp_ph114_A11
zmp_ph114_B2
zmp_ph114_B4
zmp_ph114_D8
zmp_ph114_E2

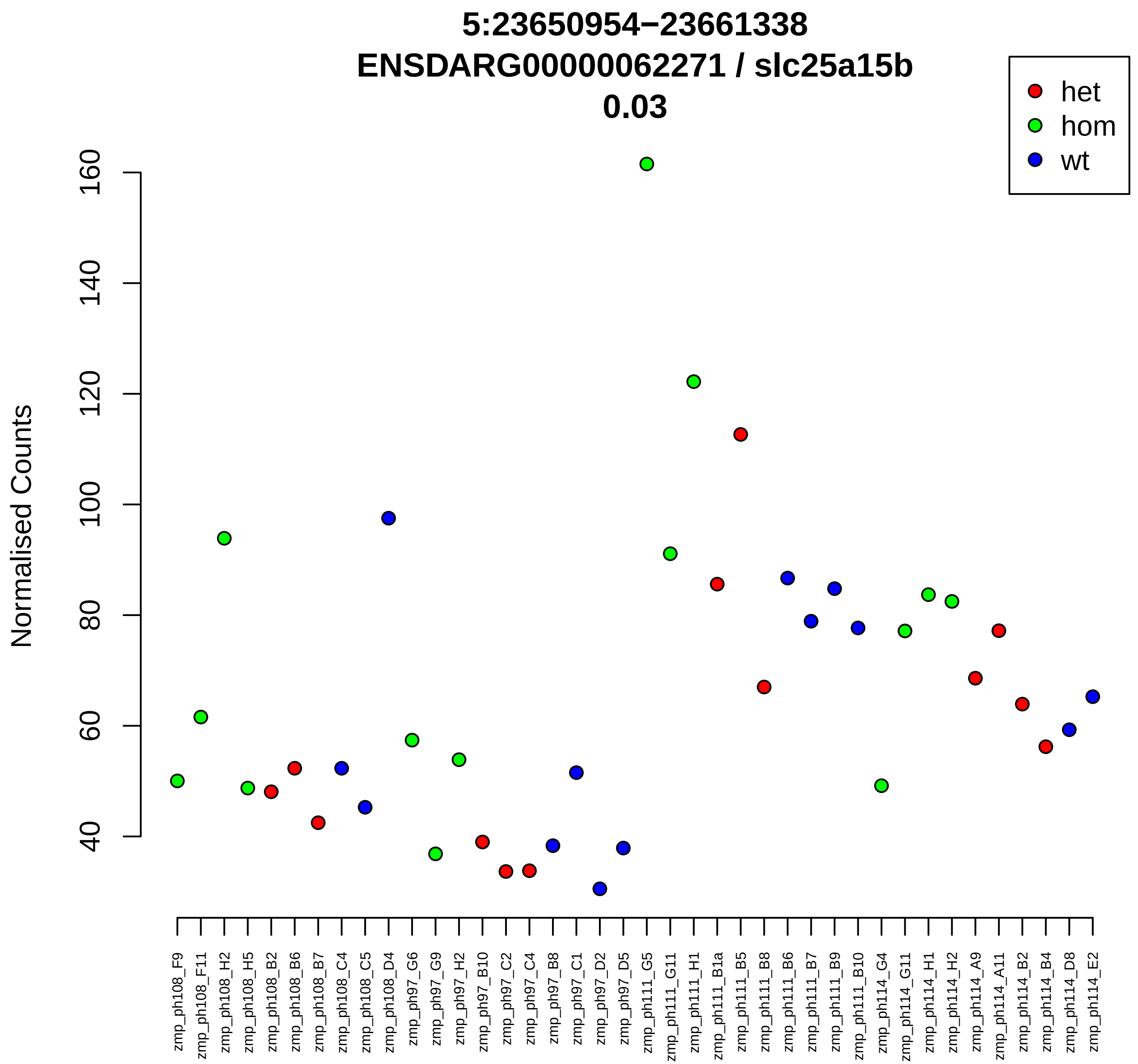

5:23650954−23661338
ENSDARG00000062271 / slc25a15b
0.03
het
hom
wt
Normalised Counts
40
60
80
100
120
140
160
zmp_ph108_F9
zmp_ph108_F11
zmp_ph108_H2
zmp_ph108_H5
zmp_ph108_B2
zmp_ph108_B6
zmp_ph108_B7
zmp_ph108_C4
zmp_ph108_C5
zmp_ph108_D4
zmp_ph97_G6
zmp_ph97_G9
zmp_ph97_H2
zmp_ph97_B10
zmp_ph97_C2
zmp_ph97_C4
zmp_ph97_B8
zmp_ph97_C1
zmp_ph97_D2
zmp_ph97_D5
zmp_ph111_G5
zmp_ph111_G11
zmp_ph111_H1
zmp_ph111_B1a
zmp_ph111_B5
zmp_ph111_B8
zmp_ph111_B6
zmp_ph111_B7
zmp_ph111_B9
zmp_ph111_B10
zmp_ph114_G4
zmp_ph114_G11
zmp_ph114_H1
zmp_ph114_H2
zmp_ph114_A9
zmp_ph114_A11
zmp_ph114_B2
zmp_ph114_B4
zmp_ph114_D8
zmp_ph114_E2

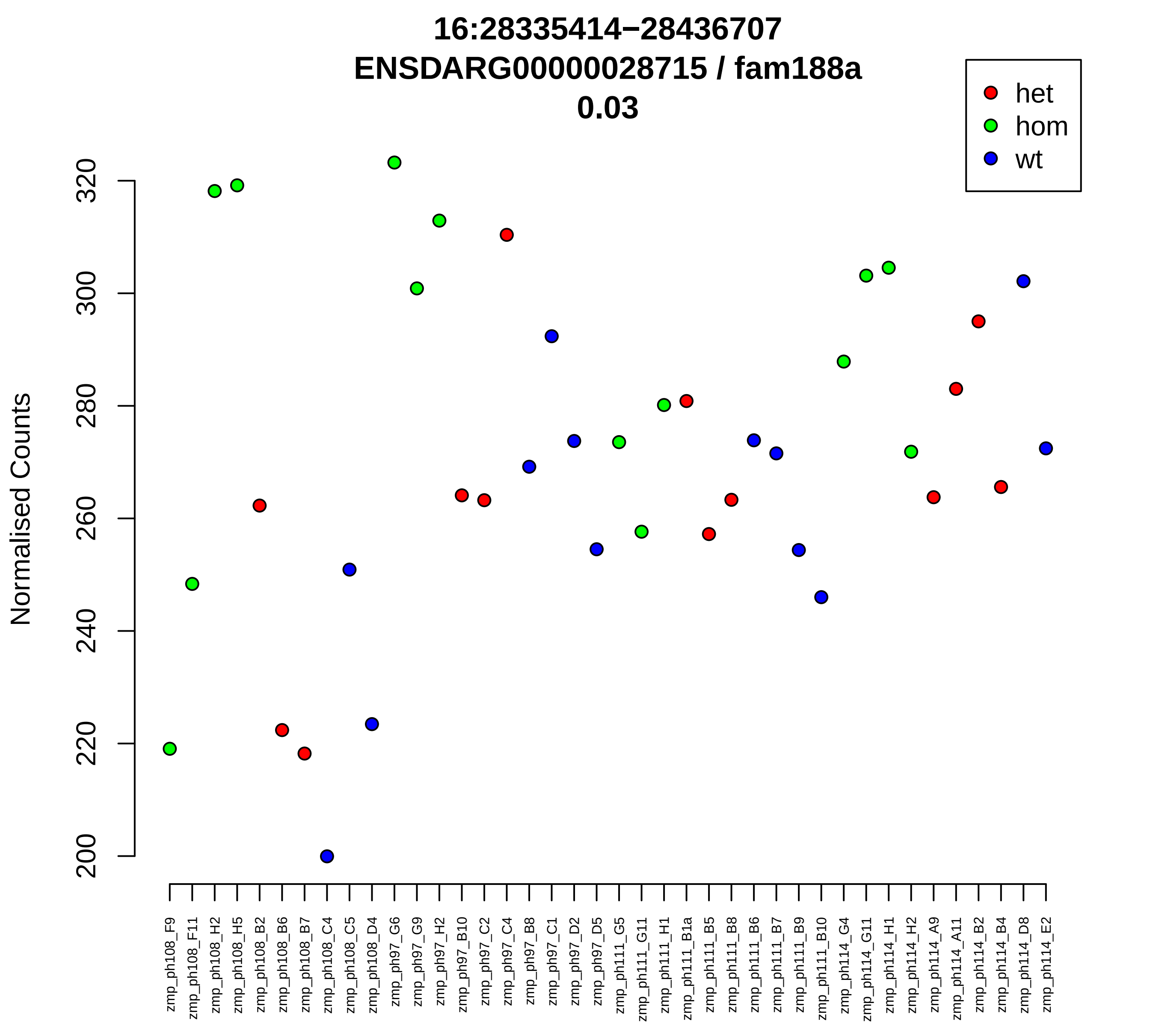
16:28335414−28436707
ENSDARG00000028715 / fam188a
0.03
het
hom
wt
Normalised Counts
200
220
240
260
280
300
320
zmp_ph108_F9
zmp_ph108_F11
zmp_ph108_H2
zmp_ph108_H5
zmp_ph108_B2
zmp_ph108_B6
zmp_ph108_B7
zmp_ph108_C4
zmp_ph108_C5
zmp_ph108_D4
zmp_ph97_G6
zmp_ph97_G9
zmp_ph97_H2
zmp_ph97_B10
zmp_ph97_C2
zmp_ph97_C4
zmp_ph97_B8
zmp_ph97_C1
zmp_ph97_D2
zmp_ph97_D5
zmp_ph111_G5
zmp_ph111_G11
zmp_ph111_H1
zmp_ph111_B1a
zmp_ph111_B5
zmp_ph111_B8
zmp_ph111_B6
zmp_ph111_B7
zmp_ph111_B9
zmp_ph111_B10
zmp_ph114_G4
zmp_ph114_G11
zmp_ph114_H1
zmp_ph114_H2
zmp_ph114_A9
zmp_ph114_A11
zmp_ph114_B2
zmp_ph114_B4
zmp_ph114_D8
zmp_ph114_E2

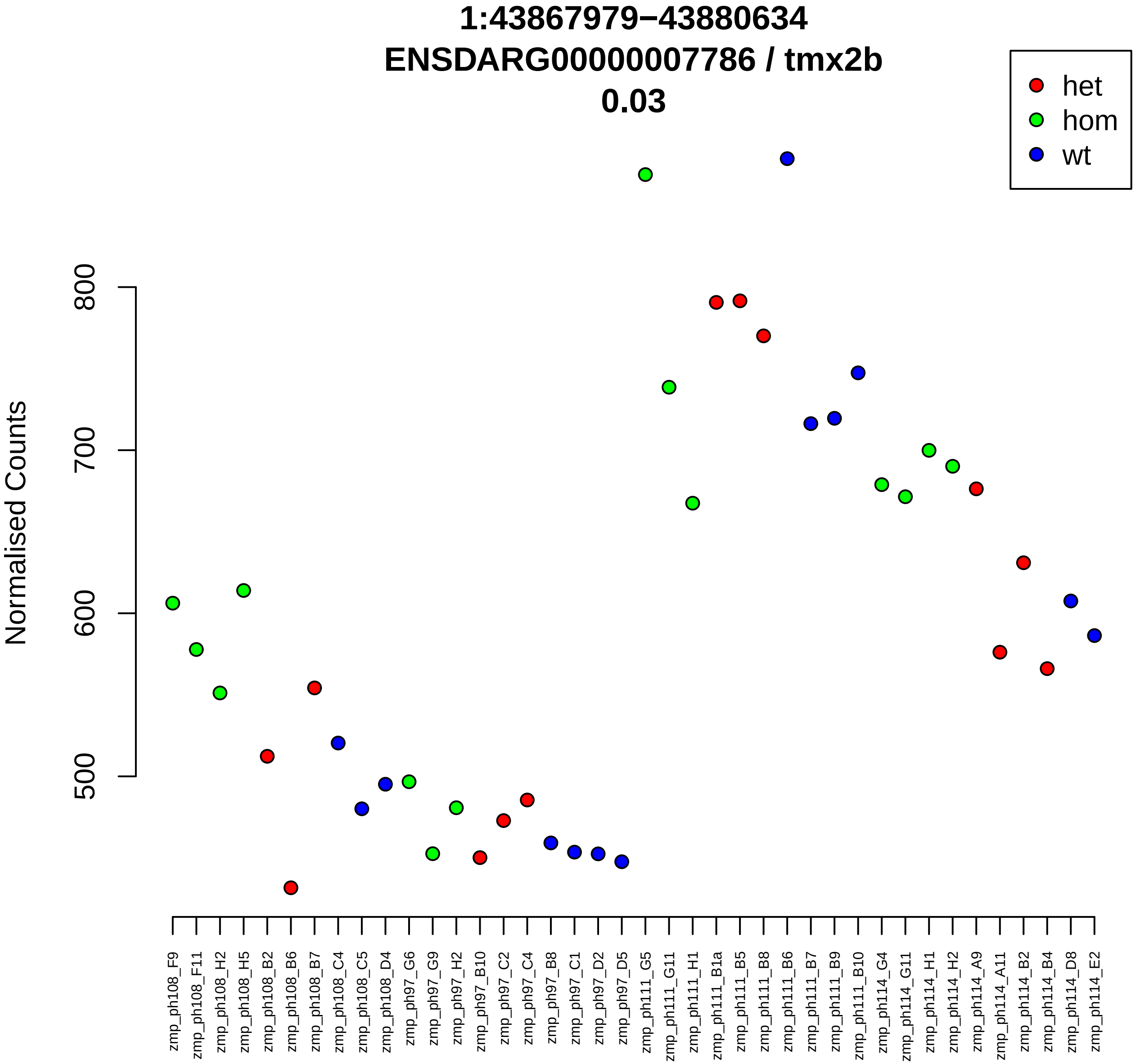

1:43867979−43880634
ENSDARG00000007786 / tmx2b
0.03
het
hom
wt
Normalised Counts
500
600
700
800
zmp_ph108_F9
zmp_ph108_F11
zmp_ph108_H2
zmp_ph108_H5
zmp_ph108_B2
zmp_ph108_B6
zmp_ph108_B7
zmp_ph108_C4
zmp_ph108_C5
zmp_ph108_D4
zmp_ph97_G6
zmp_ph97_G9
zmp_ph97_H2
zmp_ph97_B10
zmp_ph97_C2
zmp_ph97_C4
zmp_ph97_B8
zmp_ph97_C1
zmp_ph97_D2
zmp_ph97_D5
zmp_ph111_G5
zmp_ph111_G11
zmp_ph111_H1
zmp_ph111_B1a
zmp_ph111_B5
zmp_ph111_B8
zmp_ph111_B6
zmp_ph111_B7
zmp_ph111_B9
zmp_ph111_B10
zmp_ph114_G4
zmp_ph114_G11
zmp_ph114_H1
zmp_ph114_H2
zmp_ph114_A9
zmp_ph114_A11
zmp_ph114_B2
zmp_ph114_B4
zmp_ph114_D8
zmp_ph114_E2

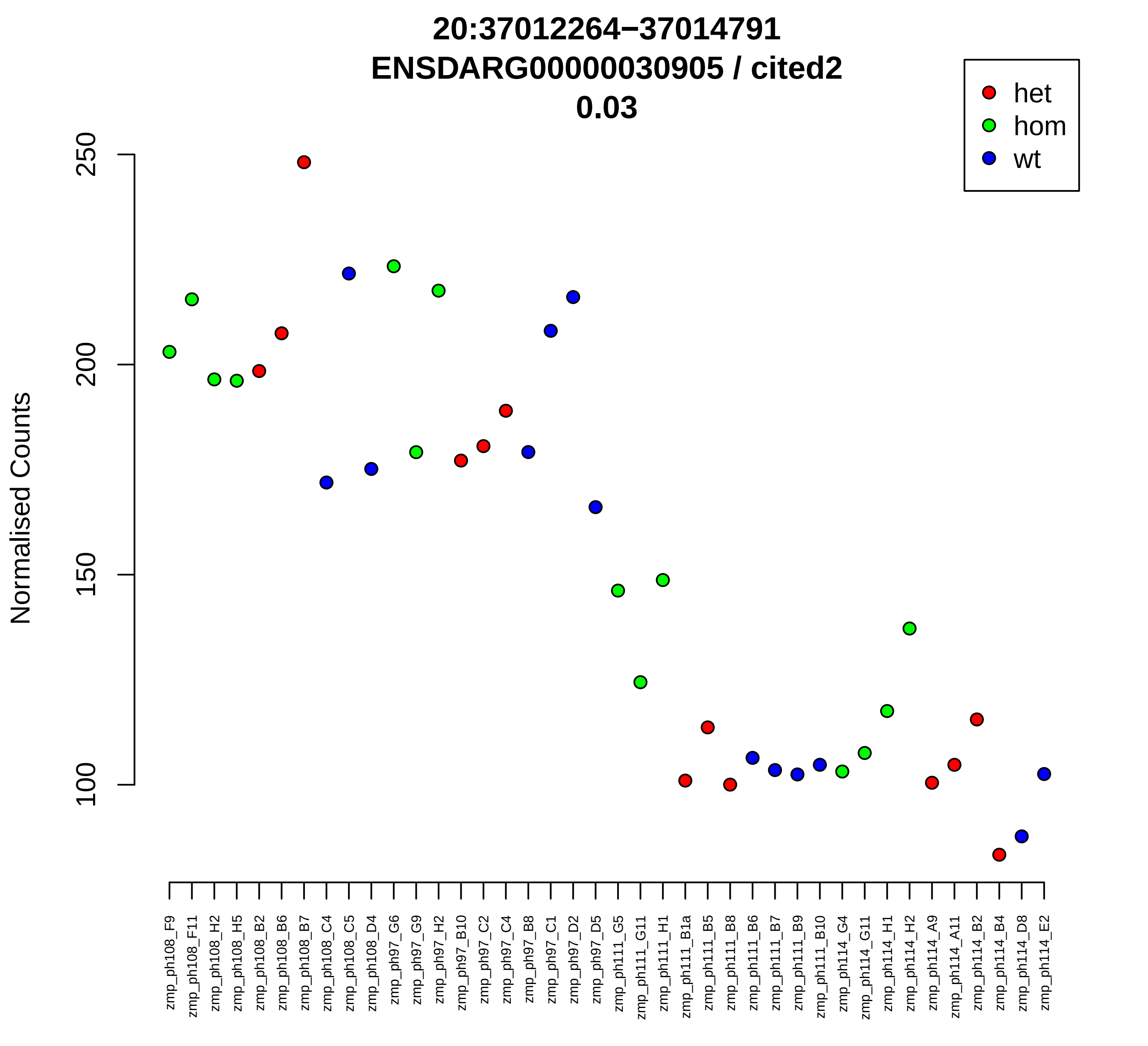

20:37012264−37014791
ENSDARG00000030905 / cited2
0.03
het
hom
wt
Normalised Counts
250
200
150
100
zmp_ph108_F9
zmp_ph108_F11
zmp_ph108_H2
zmp_ph108_H5
zmp_ph108_B2
zmp_ph108_B6
zmp_ph108_B7
zmp_ph108_C4
zmp_ph108_C5
zmp_ph108_D4
zmp_ph97_G6
zmp_ph97_G9
zmp_ph97_H2
zmp_ph97_B10
zmp_ph97_C2
zmp_ph97_C4
zmp_ph97_B8
zmp_ph97_C1
zmp_ph97_D2
zmp_ph97_D5
zmp_ph111_G5
zmp_ph111_G11
zmp_ph111_H1
zmp_ph111_B1a
zmp_ph111_B5
zmp_ph111_B8
zmp_ph111_B6
zmp_ph111_B7
zmp_ph111_B9
zmp_ph111_B10
zmp_ph114_G4
zmp_ph114_G11
zmp_ph114_H1
zmp_ph114_H2
zmp_ph114_A9
zmp_ph114_A11
zmp_ph114_B2
zmp_ph114_B4
zmp_ph114_D8
zmp_ph114_E2

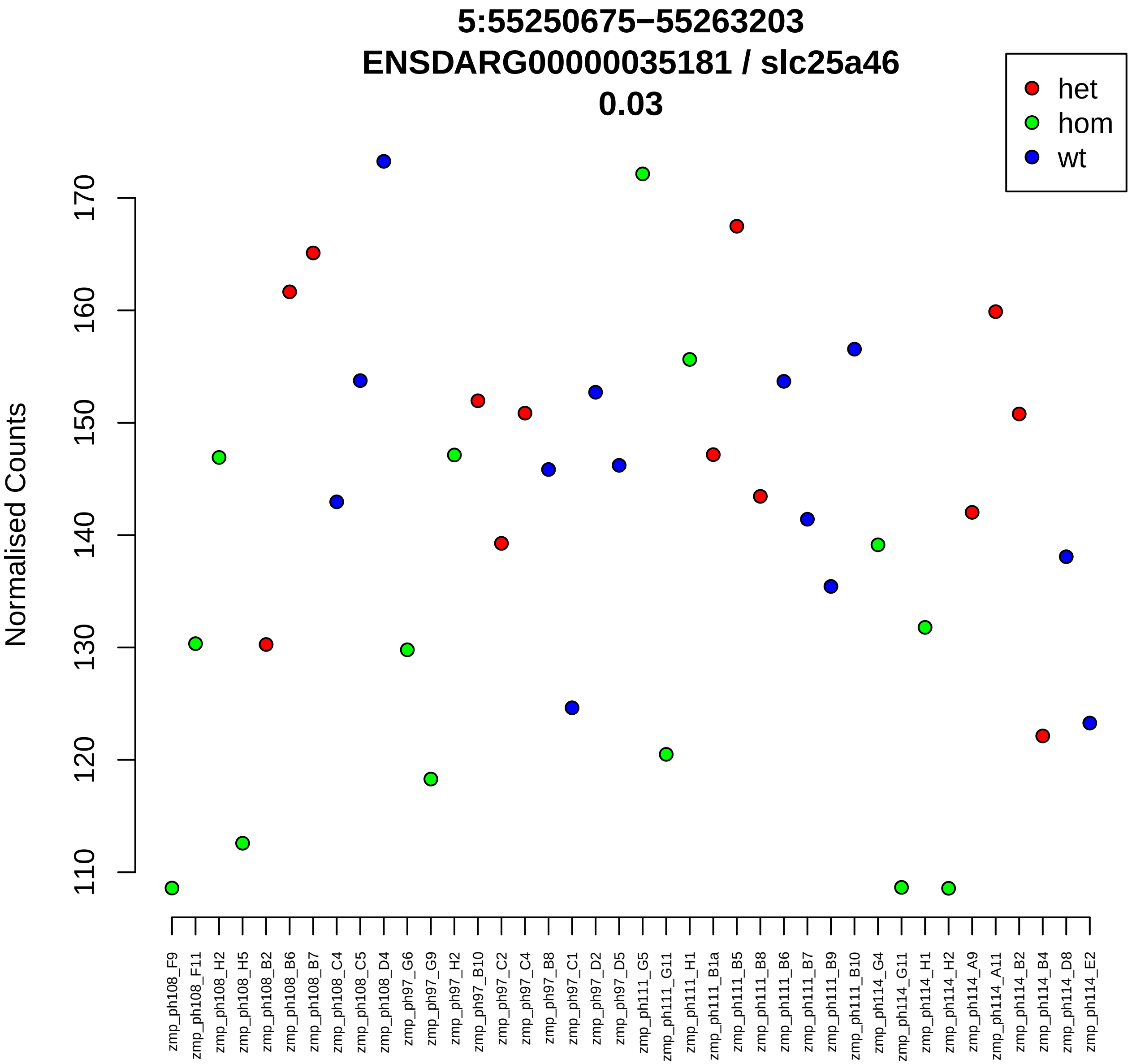
5:55250675−55263203
ENSDARG00000035181 / slc25a46
0.03
het
hom
wt
Normalised Counts
110
120
130
140
150
160
170
zmp_ph108_F9
zmp_ph108_F11
zmp_ph108_H2
zmp_ph108_H5
zmp_ph108_B2
zmp_ph108_B6
zmp_ph108_B7
zmp_ph108_C4
zmp_ph108_C5
zmp_ph108_D4
zmp_ph97_G6
zmp_ph97_G9
zmp_ph97_H2
zmp_ph97_B10
zmp_ph97_C2
zmp_ph97_C4
zmp_ph97_B8
zmp_ph97_C1
zmp_ph97_D2
zmp_ph97_D5
zmp_ph111_G5
zmp_ph111_G11
zmp_ph111_H1
zmp_ph111_B1a
zmp_ph111_B5
zmp_ph111_B8
zmp_ph111_B6
zmp_ph111_B7
zmp_ph111_B9
zmp_ph111_B10
zmp_ph114_G4
zmp_ph114_G11
zmp_ph114_H1
zmp_ph114_H2
zmp_ph114_A9
zmp_ph114_A11
zmp_ph114_B2
zmp_ph114_B4
zmp_ph114_D8
zmp_ph114_E2

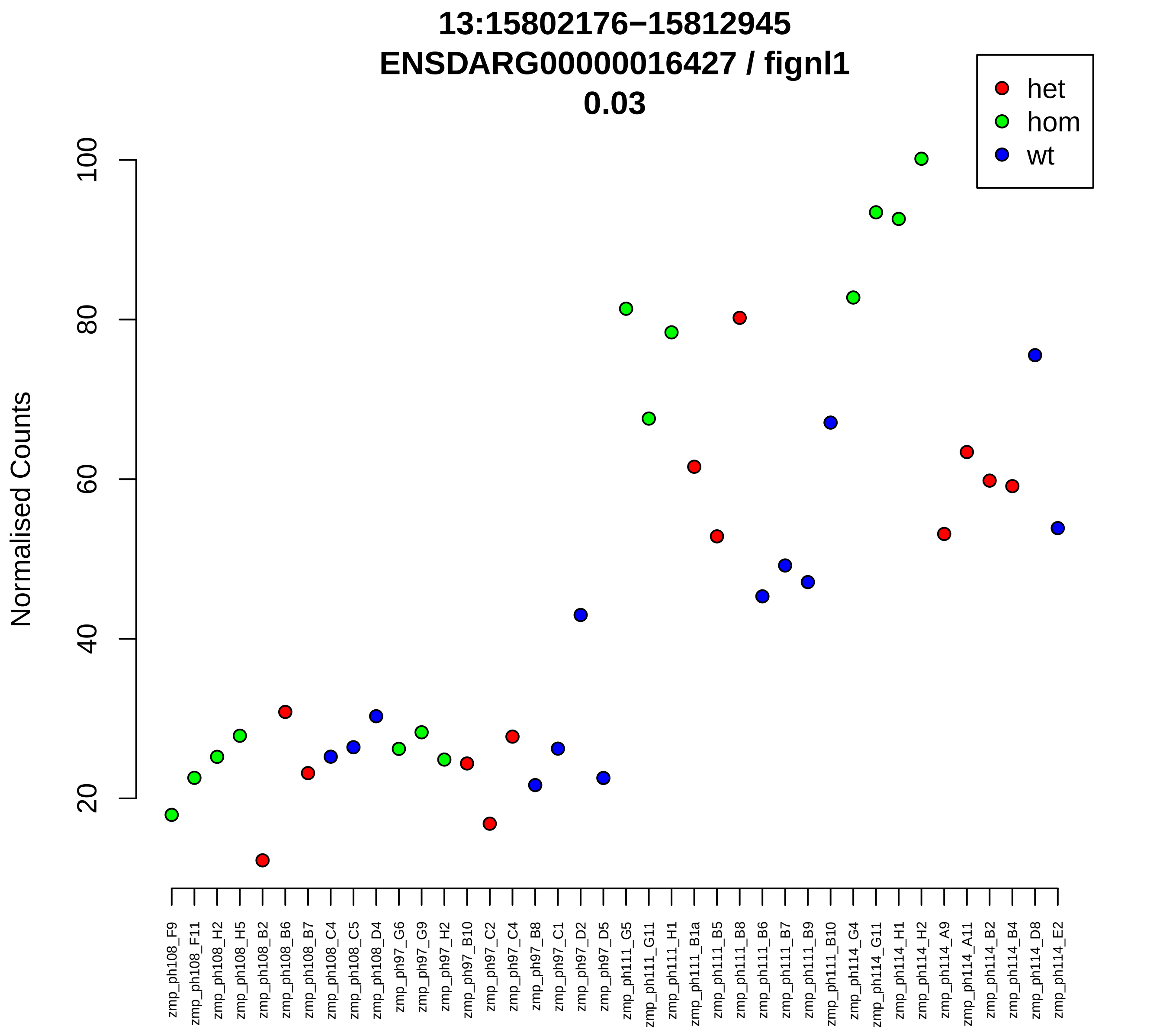

13:15802176−15812945
ENSDARG00000016427 / fignl1
0.03
het
hom
wt
Normalised Counts
20
40
60
80
100
zmp_ph108_F9
zmp_ph108_F11
zmp_ph108_H2
zmp_ph108_H5
zmp_ph108_B2
zmp_ph108_B6
zmp_ph108_B7
zmp_ph108_C4
zmp_ph108_C5
zmp_ph108_D4
zmp_ph97_G6
zmp_ph97_G9
zmp_ph97_H2
zmp_ph97_B10
zmp_ph97_C2
zmp_ph97_C4
zmp_ph97_B8
zmp_ph97_C1
zmp_ph97_D2
zmp_ph97_D5
zmp_ph111_G5
zmp_ph111_G11
zmp_ph111_H1
zmp_ph111_B1a
zmp_ph111_B5
zmp_ph111_B8
zmp_ph111_B6
zmp_ph111_B7
zmp_ph111_B9
zmp_ph111_B10
zmp_ph114_G4
zmp_ph114_G11
zmp_ph114_H1
zmp_ph114_H2
zmp_ph114_A9
zmp_ph114_A11
zmp_ph114_B2
zmp_ph114_B4
zmp_ph114_D8
zmp_ph114_E2

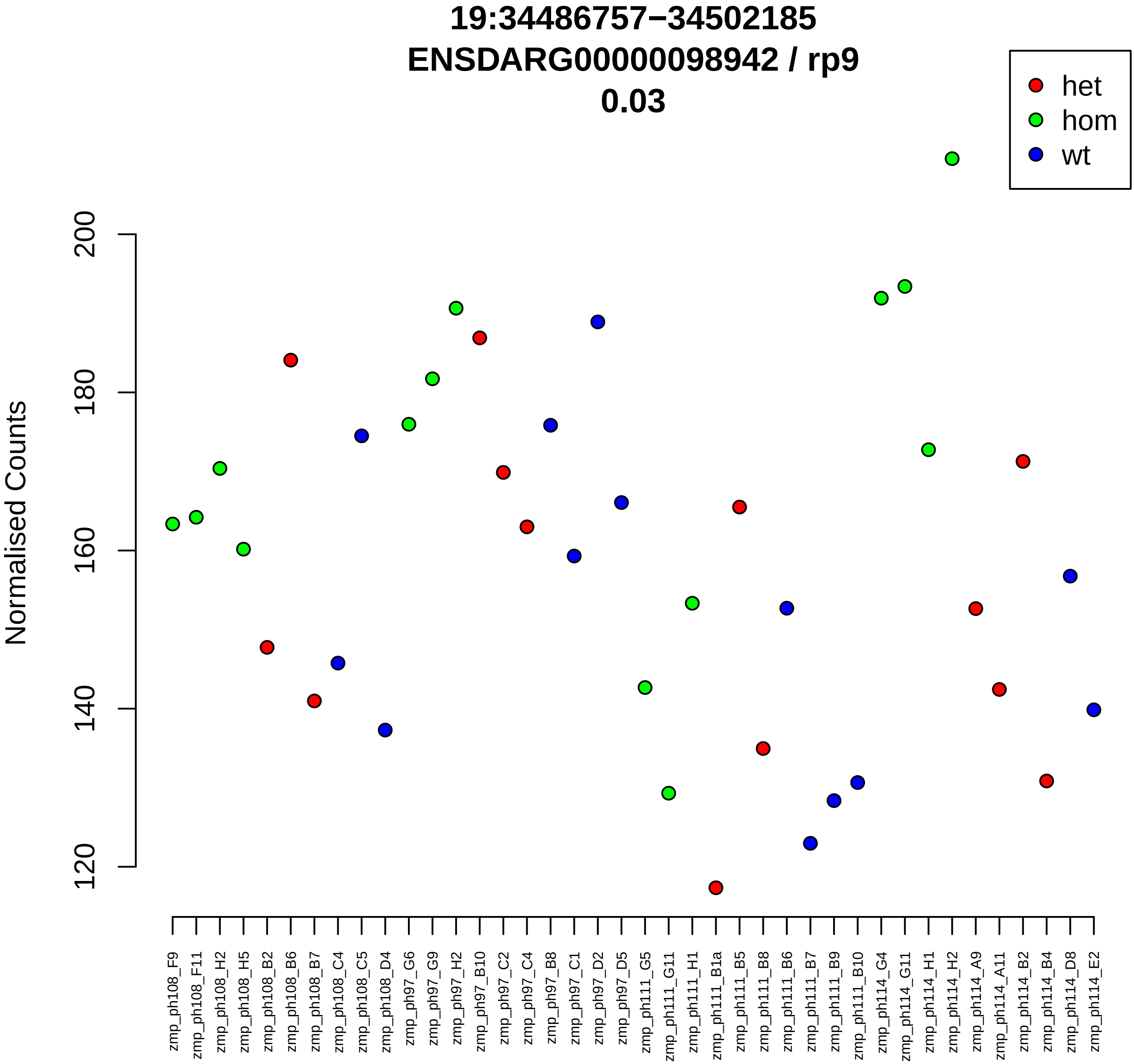
19:34486757−34502185
ENSDARG00000098942 / rp9
0.03
het
hom
wt
Normalised Counts
120
140
160
180
200
zmp_ph108_F9
zmp_ph108_F11
zmp_ph108_H2
zmp_ph108_H5
zmp_ph108_B2
zmp_ph108_B6
zmp_ph108_B7
zmp_ph108_C4
zmp_ph108_C5
zmp_ph108_D4
zmp_ph97_G6
zmp_ph97_G9
zmp_ph97_H2
zmp_ph97_B10
zmp_ph97_C2
zmp_ph97_C4
zmp_ph97_B8
zmp_ph97_C1
zmp_ph97_D2
zmp_ph97_D5
zmp_ph111_G5
zmp_ph111_G11
zmp_ph111_H1
zmp_ph111_B1a
zmp_ph111_B5
zmp_ph111_B8
zmp_ph111_B6
zmp_ph111_B7
zmp_ph111_B9
zmp_ph111_B10
zmp_ph114_G4
zmp_ph114_G11
zmp_ph114_H1
zmp_ph114_H2
zmp_ph114_A9
zmp_ph114_A11
zmp_ph114_B2
zmp_ph114_B4
zmp_ph114_D8
zmp_ph114_E2

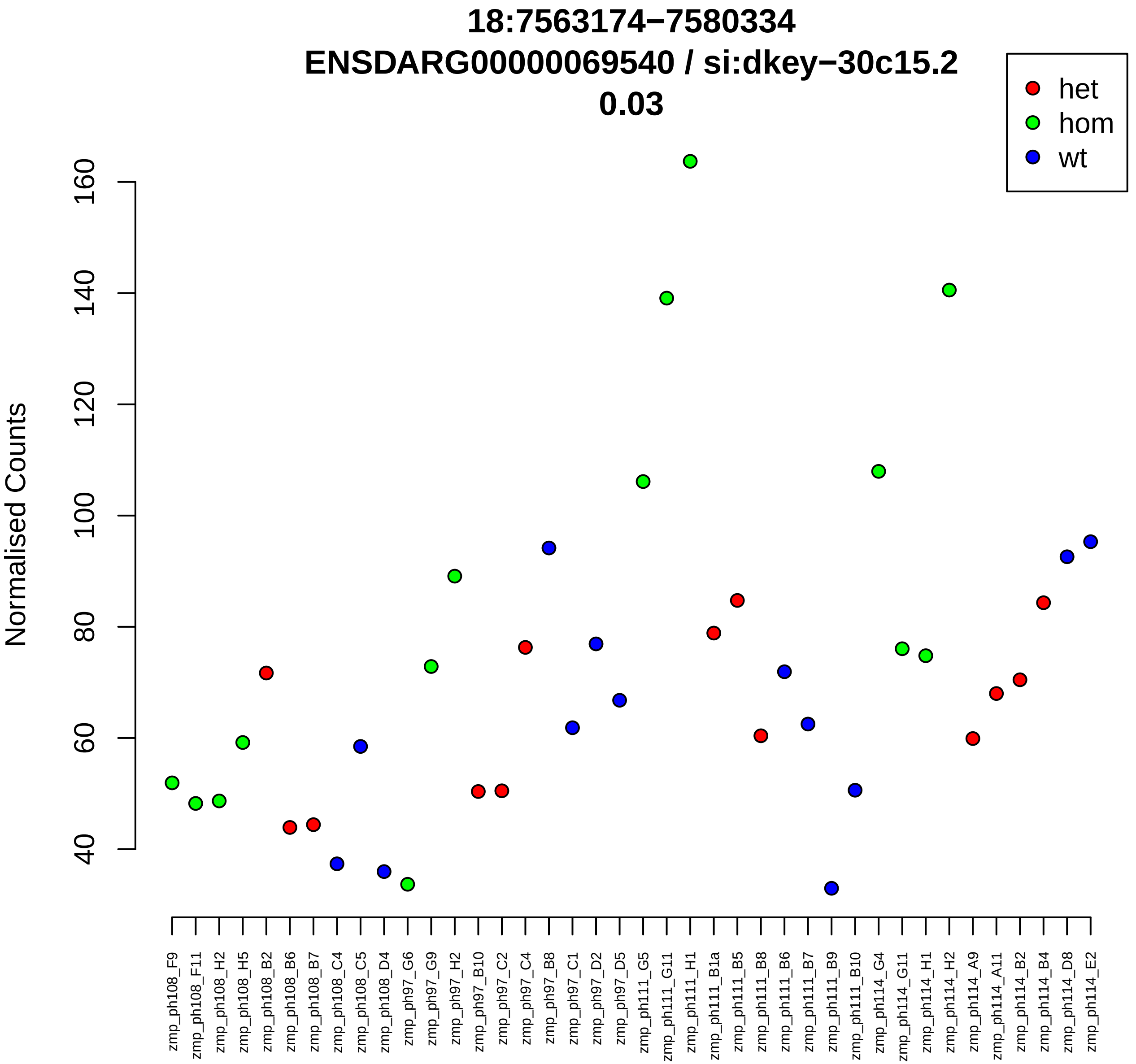

18:7563174−7580334
ENSDARG00000069540 / si:dkey−30c15.2
0.03
het
hom
wt
Normalised Counts
40
60
80
100
120
140
160
zmp_ph108_F9
zmp_ph108_F11
zmp_ph108_H2
zmp_ph108_H5
zmp_ph108_B2
zmp_ph108_B6
zmp_ph108_B7
zmp_ph108_C4
zmp_ph108_C5
zmp_ph108_D4
zmp_ph97_G6
zmp_ph97_G9
zmp_ph97_H2
zmp_ph97_B10
zmp_ph97_C2
zmp_ph97_C4
zmp_ph97_B8
zmp_ph97_C1
zmp_ph97_D2
zmp_ph97_D5
zmp_ph111_G5
zmp_ph111_G11
zmp_ph111_H1
zmp_ph111_B1a
zmp_ph111_B5
zmp_ph111_B8
zmp_ph111_B6
zmp_ph111_B7
zmp_ph111_B9
zmp_ph111_B10
zmp_ph114_G4
zmp_ph114_G11
zmp_ph114_H1
zmp_ph114_H2
zmp_ph114_A9
zmp_ph114_A11
zmp_ph114_B2
zmp_ph114_B4
zmp_ph114_D8
zmp_ph114_E2

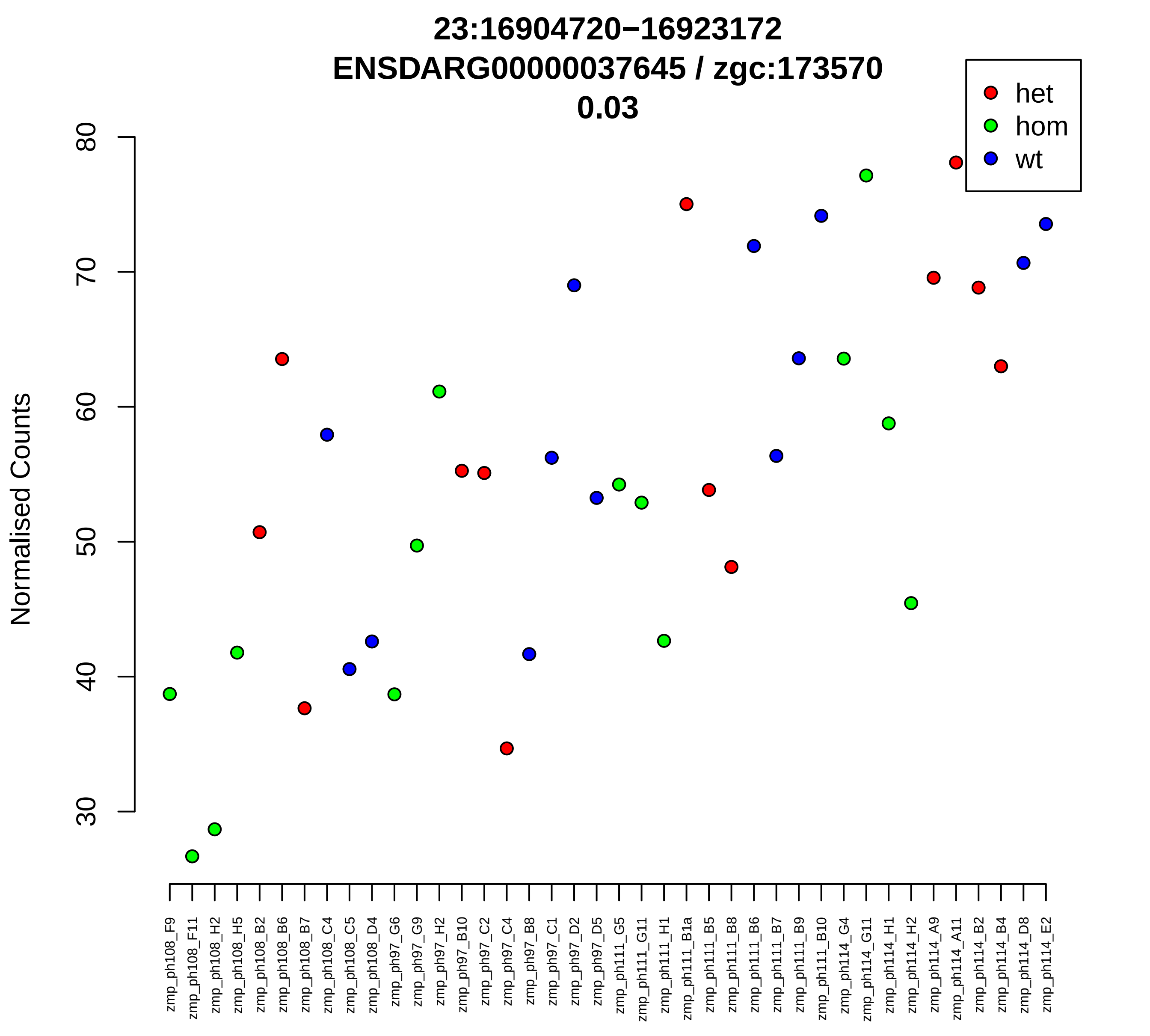

23:16904720−16923172
ENSDARG00000037645 / zgc:173570
0.03
Normalised Counts
het
hom
wt
30
40
50
60
70
80
zmp_ph108_F9
zmp_ph108_F11
zmp_ph108_H2
zmp_ph108_H5
zmp_ph108_B2
zmp_ph108_B6
zmp_ph108_B7
zmp_ph108_C4
zmp_ph108_C5
zmp_ph108_D4
zmp_ph97_G6
zmp_ph97_G9
zmp_ph97_H2
zmp_ph97_B10
zmp_ph97_C2
zmp_ph97_C4
zmp_ph97_B8
zmp_ph97_C1
zmp_ph97_D2
zmp_ph97_D5
zmp_ph111_G5
zmp_ph111_G11
zmp_ph111_H1
zmp_ph111_B1a
zmp_ph111_B5
zmp_ph111_B8
zmp_ph111_B6
zmp_ph111_B7
zmp_ph111_B9
zmp_ph111_B10
zmp_ph114_G4
zmp_ph114_G11
zmp_ph114_H1
zmp_ph114_H2
zmp_ph114_A9
zmp_ph114_A11
zmp_ph114_B2
zmp_ph114_B4
zmp_ph114_D8
zmp_ph114_E2

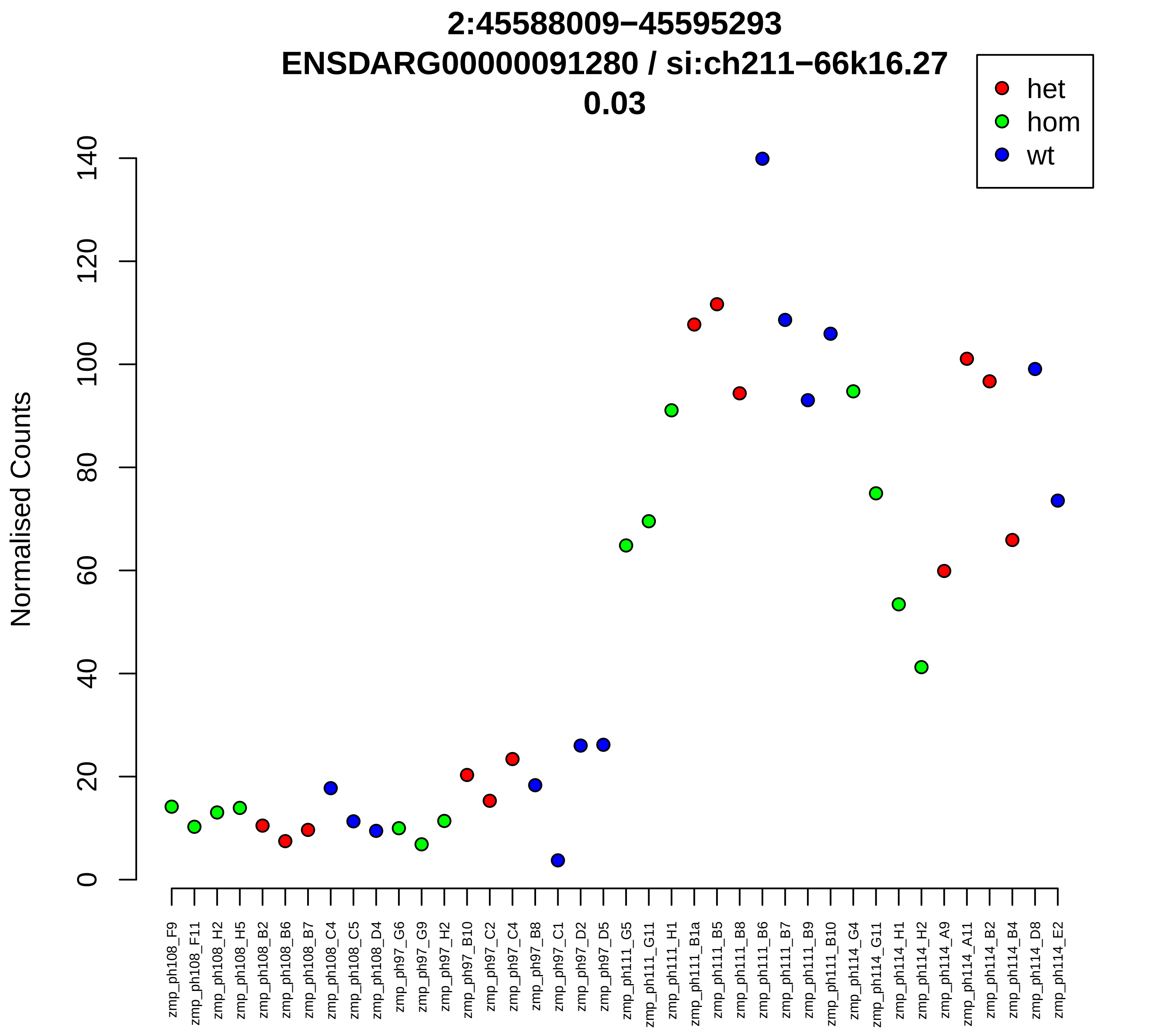

2:45588009−45595293
ENSDARG00000091280 / si:ch211−66k16.27
0.03
het
hom
wt
Normalised Counts
0
20
40
60
80
100
120
140
zmp_ph108_F9
zmp_ph108_F11
zmp_ph108_H2
zmp_ph108_H5
zmp_ph108_B2
zmp_ph108_B6
zmp_ph108_B7
zmp_ph108_C4
zmp_ph108_C5
zmp_ph108_D4
zmp_ph97_G6
zmp_ph97_G9
zmp_ph97_H2
zmp_ph97_B10
zmp_ph97_C2
zmp_ph97_C4
zmp_ph97_B8
zmp_ph97_C1
zmp_ph97_D2
zmp_ph97_D5
zmp_ph111_G5
zmp_ph111_G11
zmp_ph111_H1
zmp_ph111_B1a
zmp_ph111_B5
zmp_ph111_B8
zmp_ph111_B6
zmp_ph111_B7
zmp_ph111_B9
zmp_ph111_B10
zmp_ph114_G4
zmp_ph114_G11
zmp_ph114_H1
zmp_ph114_H2
zmp_ph114_A9
zmp_ph114_A11
zmp_ph114_B2
zmp_ph114_B4
zmp_ph114_D8
zmp_ph114_E2

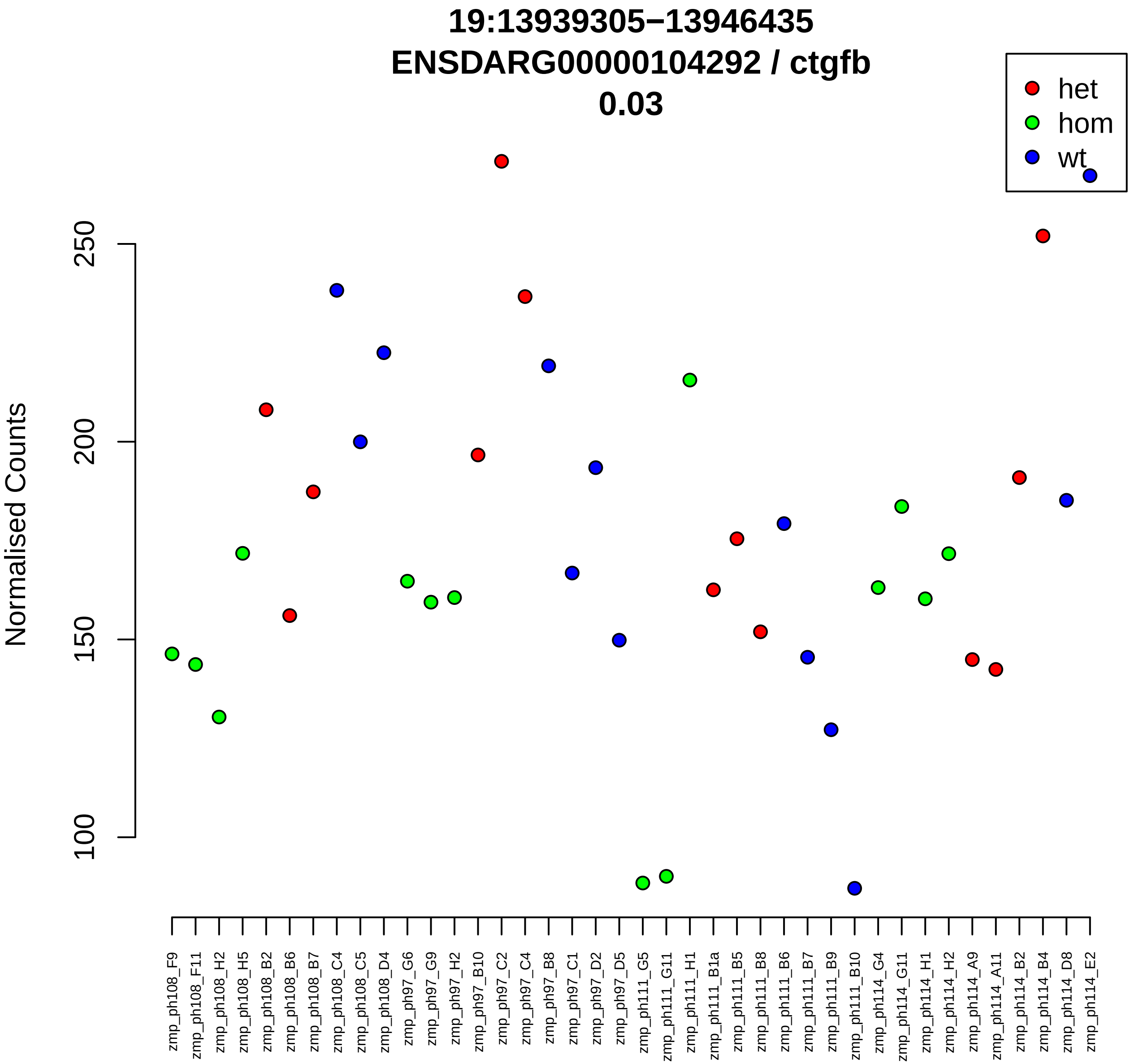
19:13939305−13946435
ENSDARG00000104292 / ctgfb
0.03
het
hom
wt
Normalised Counts
100
150
200
250
zmp_ph108_F9
zmp_ph108_F11
zmp_ph108_H2
zmp_ph108_H5
zmp_ph108_B2
zmp_ph108_B6
zmp_ph108_B7
zmp_ph108_C4
zmp_ph108_C5
zmp_ph108_D4
zmp_ph97_G6
zmp_ph97_G9
zmp_ph97_H2
zmp_ph97_B10
zmp_ph97_C2
zmp_ph97_C4
zmp_ph97_B8
zmp_ph97_C1
zmp_ph97_D2
zmp_ph97_D5
zmp_ph111_G5
zmp_ph111_G11
zmp_ph111_H1
zmp_ph111_B1a
zmp_ph111_B5
zmp_ph111_B8
zmp_ph111_B6
zmp_ph111_B7
zmp_ph111_B9
zmp_ph111_B10
zmp_ph114_G4
zmp_ph114_G11
zmp_ph114_H1
zmp_ph114_H2
zmp_ph114_A9
zmp_ph114_A11
zmp_ph114_B2
zmp_ph114_B4
zmp_ph114_D8
zmp_ph114_E2

5:64175502−64180136
ENSDARG00000035129 / zgc:101858
0.03

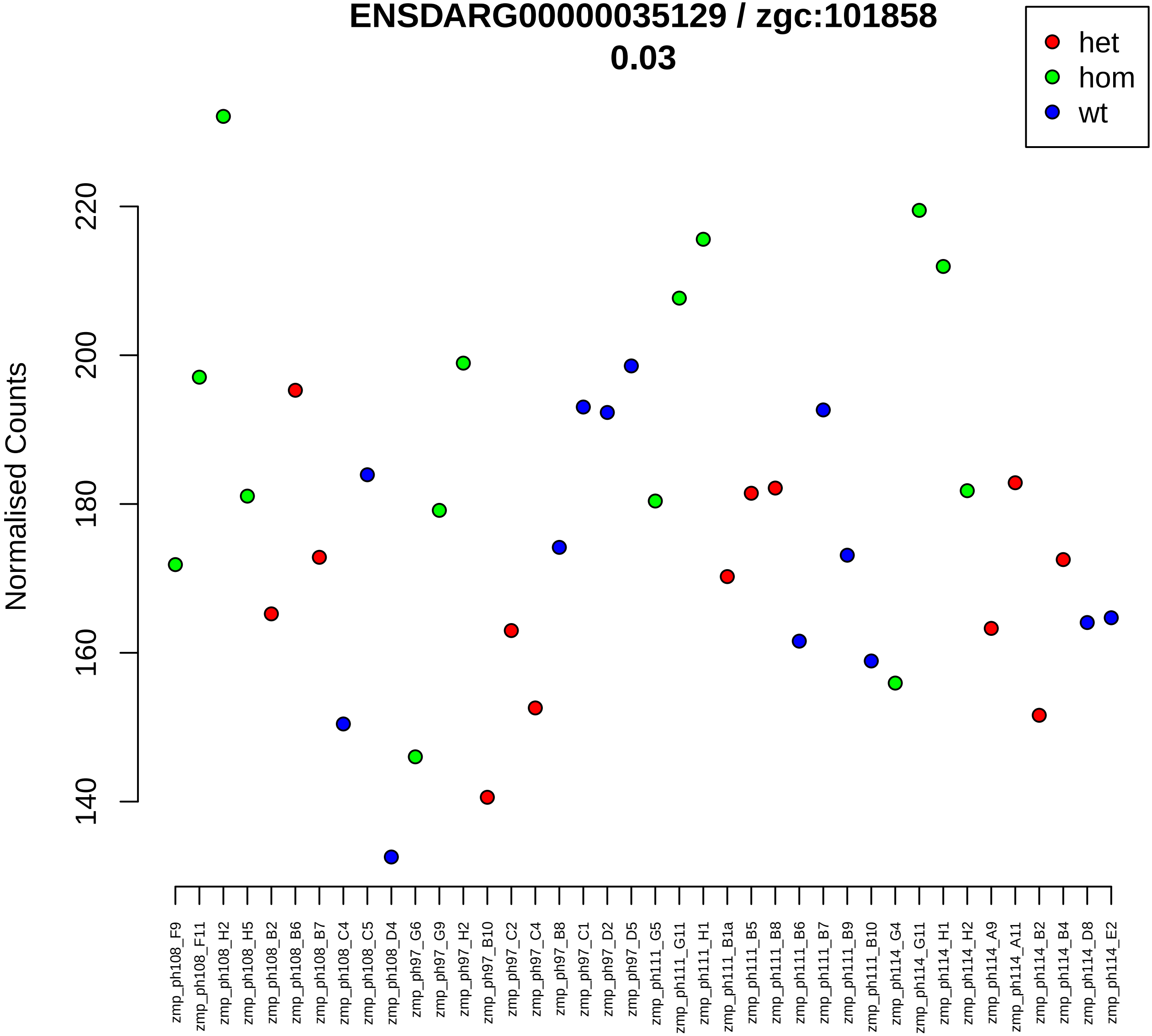

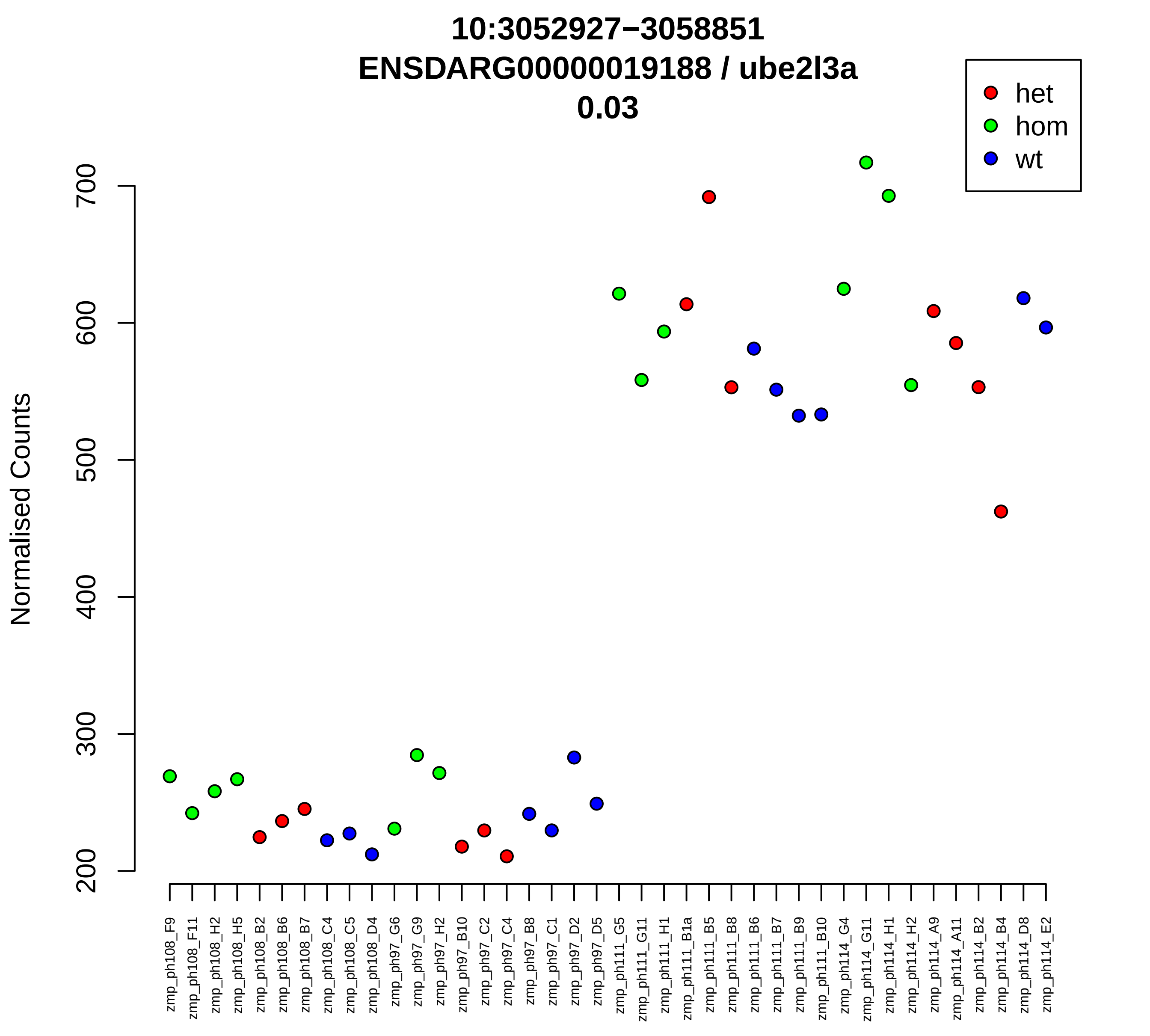
10:3052927−3058851
ENSDARG00000019188 / ube2l3a
0.03
het
hom
wt
Normalised Counts
200
300
400
500
600
700
zmp_ph108_F9
zmp_ph108_F11
zmp_ph108_H2
zmp_ph108_H5
zmp_ph108_B2
zmp_ph108_B6
zmp_ph108_B7
zmp_ph108_C4
zmp_ph108_C5
zmp_ph108_D4
zmp_ph97_G6
zmp_ph97_G9
zmp_ph97_H2
zmp_ph97_B10
zmp_ph97_C2
zmp_ph97_C4
zmp_ph97_B8
zmp_ph97_C1
zmp_ph97_D2
zmp_ph97_D5
zmp_ph111_G5
zmp_ph111_G11
zmp_ph111_H1
zmp_ph111_B1a
zmp_ph111_B5
zmp_ph111_B8
zmp_ph111_B6
zmp_ph111_B7
zmp_ph111_B9
zmp_ph111_B10
zmp_ph114_G4
zmp_ph114_G11
zmp_ph114_H1
zmp_ph114_H2
zmp_ph114_A9
zmp_ph114_A11
zmp_ph114_B2
zmp_ph114_B4
zmp_ph114_D8
zmp_ph114_E2

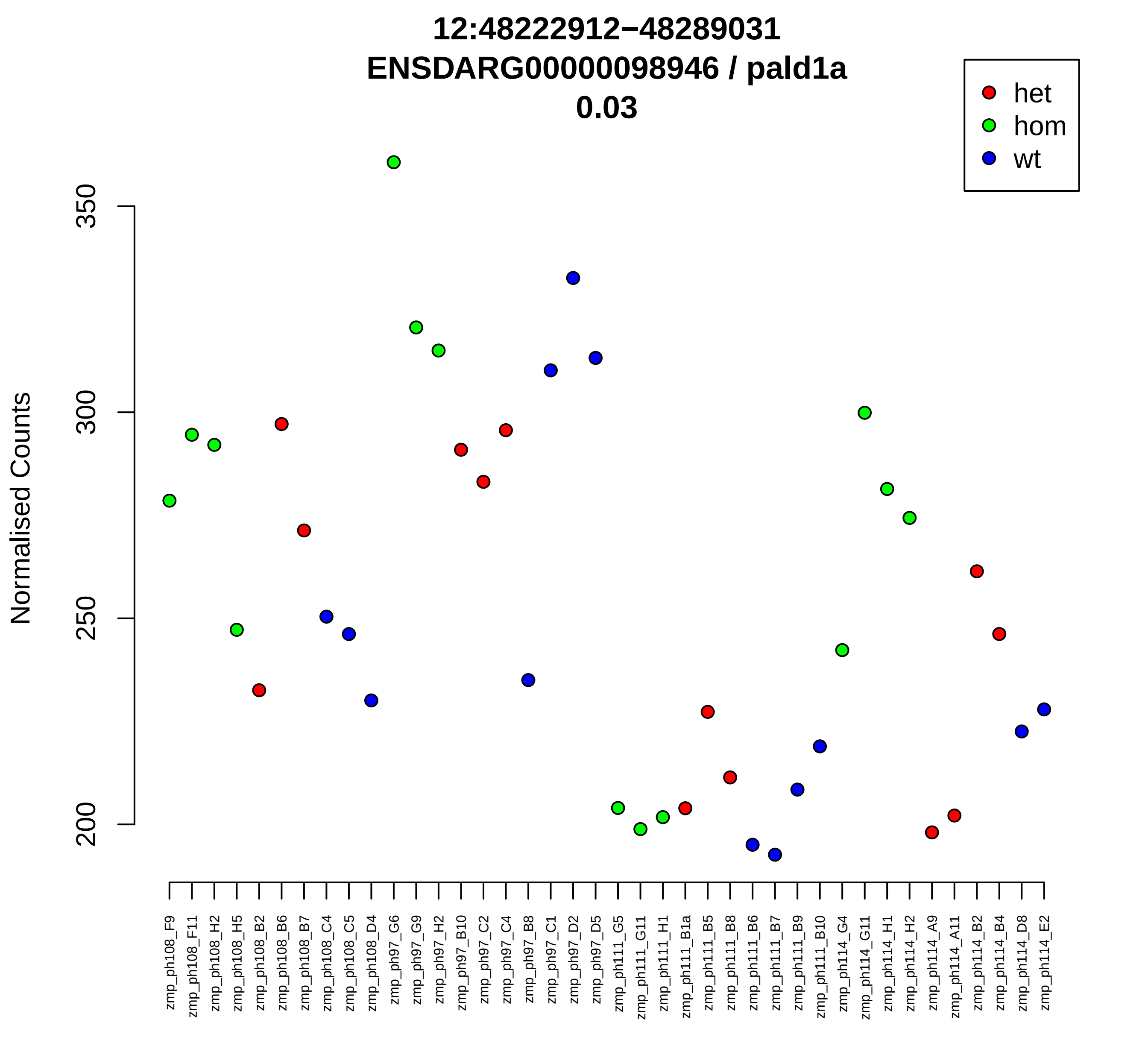

12:48222912−48289031
ENSDARG00000098946 / pald1a
0.03
het
hom
wt
Normalised Counts
350
300
250
200
zmp_ph108_F9
zmp_ph108_F11
zmp_ph108_H2
zmp_ph108_H5
zmp_ph108_B2
zmp_ph108_B6
zmp_ph108_B7
zmp_ph108_C4
zmp_ph108_C5
zmp_ph108_D4
zmp_ph97_G6
zmp_ph97_G9
zmp_ph97_H2
zmp_ph97_B10
zmp_ph97_C2
zmp_ph97_C4
zmp_ph97_B8
zmp_ph97_C1
zmp_ph97_D2
zmp_ph97_D5
zmp_ph111_G5
zmp_ph111_G11
zmp_ph111_H1
zmp_ph111_B1a
zmp_ph111_B5
zmp_ph111_B8
zmp_ph111_B6
zmp_ph111_B7
zmp_ph111_B9
zmp_ph111_B10
zmp_ph114_G4
zmp_ph114_G11
zmp_ph114_H1
zmp_ph114_H2
zmp_ph114_A9
zmp_ph114_A11
zmp_ph114_B2
zmp_ph114_B4
zmp_ph114_D8
zmp_ph114_E2

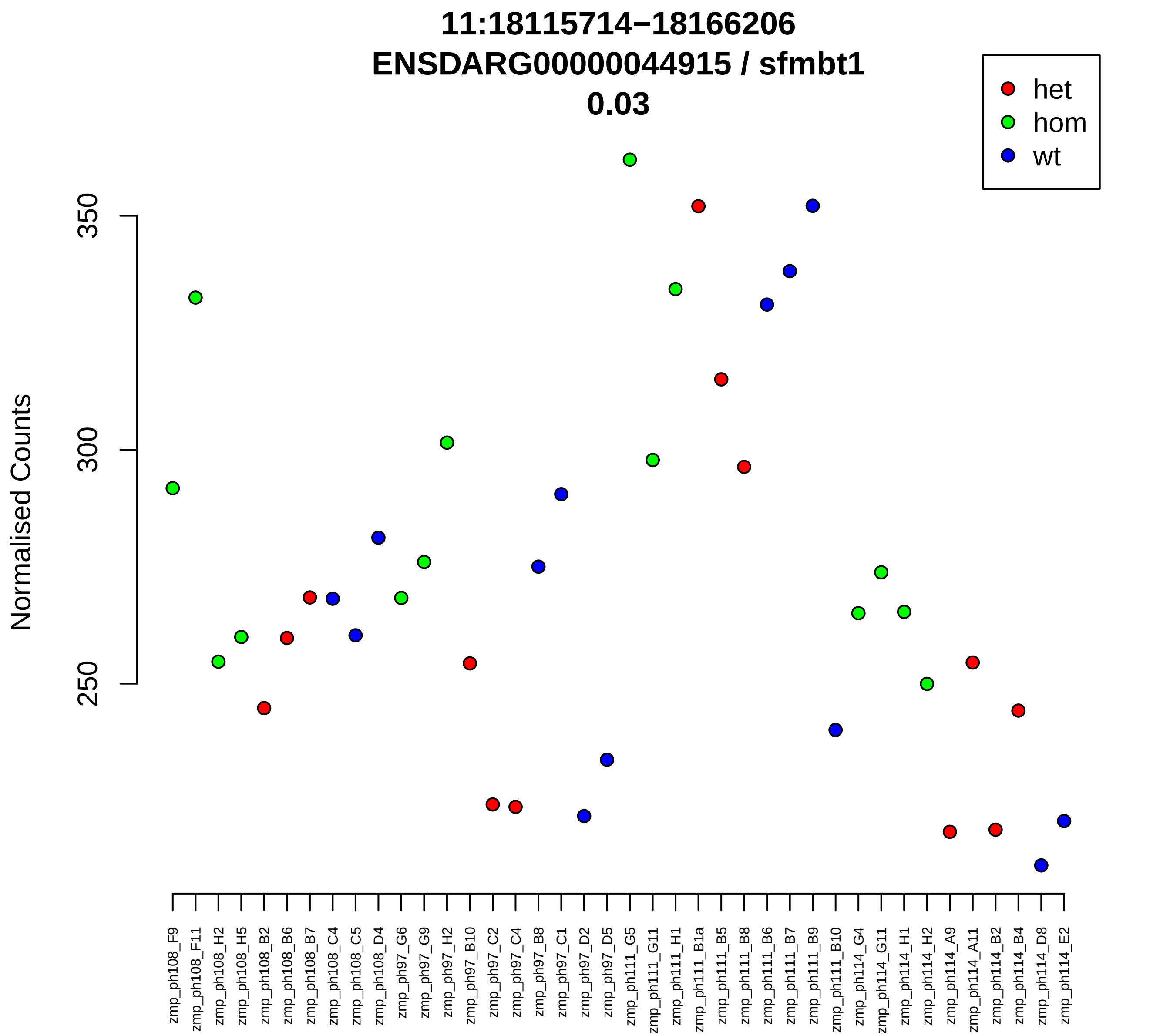
11:18115714−18166206
ENSDARG00000044915 / sfmbt1
0.03
het
hom
wt
Normalised Counts
250
300
350
zmp_ph108_F9
zmp_ph108_F11
zmp_ph108_H2
zmp_ph108_H5
zmp_ph108_B2
zmp_ph108_B6
zmp_ph108_B7
zmp_ph108_C4
zmp_ph108_C5
zmp_ph108_D4
zmp_ph97_G6
zmp_ph97_G9
zmp_ph97_H2
zmp_ph97_B10
zmp_ph97_C2
zmp_ph97_C4
zmp_ph97_B8
zmp_ph97_C1
zmp_ph97_D2
zmp_ph97_D5
zmp_ph111_G5
zmp_ph111_G11
zmp_ph111_H1
zmp_ph111_B1a
zmp_ph111_B5
zmp_ph111_B8
zmp_ph111_B6
zmp_ph111_B7
zmp_ph111_B9
zmp_ph111_B10
zmp_ph114_G4
zmp_ph114_G11
zmp_ph114_H1
zmp_ph114_H2
zmp_ph114_A9
zmp_ph114_A11
zmp_ph114_B2
zmp_ph114_B4
zmp_ph114_D8
zmp_ph114_E2

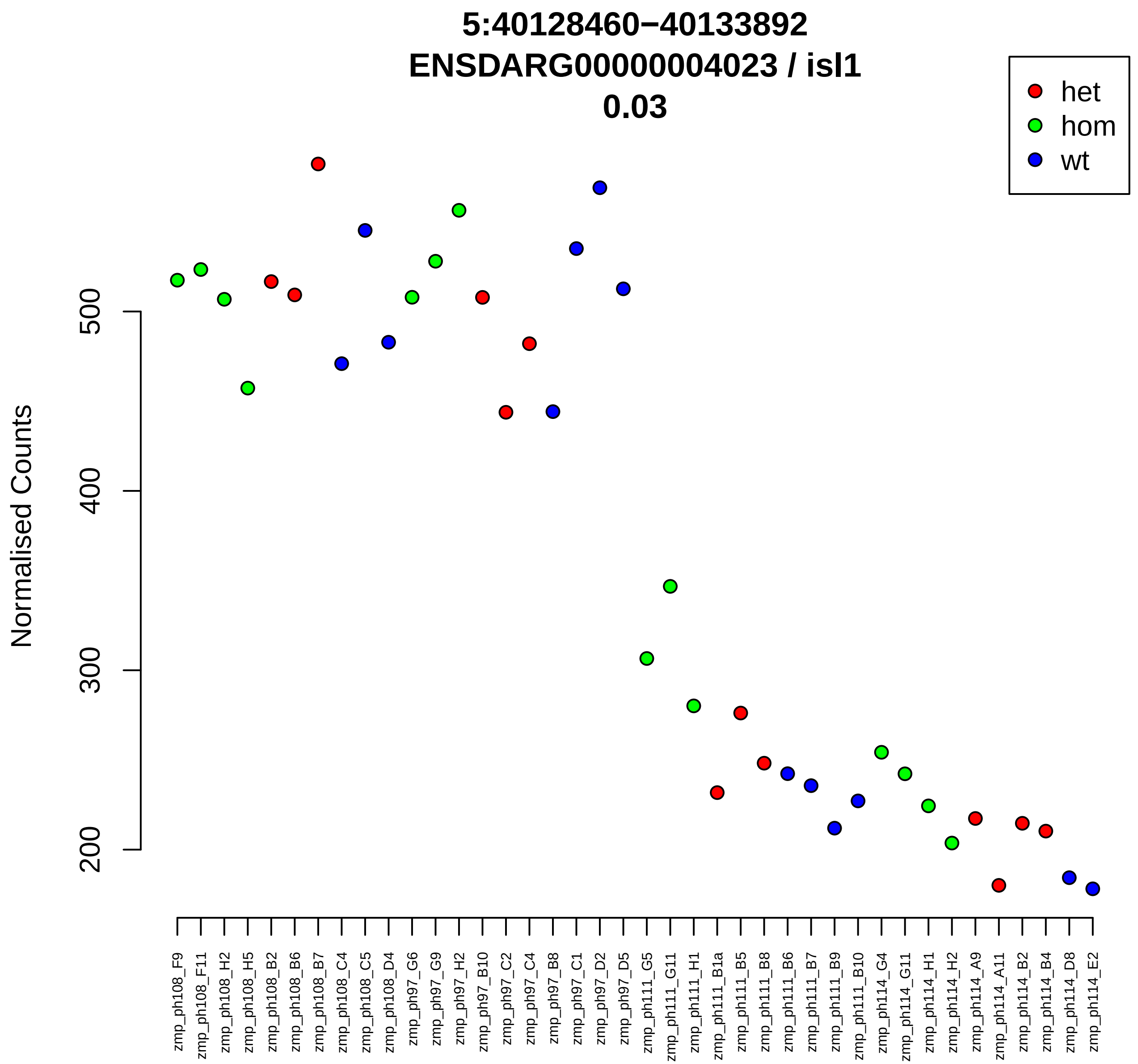

5:40128460−40133892
ENSDARG00000004023 / isl1
0.03
het
hom
wt
Normalised Counts
200
300
400
500
zmp_ph108_F9
zmp_ph108_F11
zmp_ph108_H2
zmp_ph108_H5
zmp_ph108_B2
zmp_ph108_B6
zmp_ph108_B7
zmp_ph108_C4
zmp_ph108_C5
zmp_ph108_D4
zmp_ph97_G6
zmp_ph97_G9
zmp_ph97_H2
zmp_ph97_B10
zmp_ph97_C2
zmp_ph97_C4
zmp_ph97_B8
zmp_ph97_C1
zmp_ph97_D2
zmp_ph97_D5
zmp_ph111_G5
zmp_ph111_G11
zmp_ph111_H1
zmp_ph111_B1a
zmp_ph111_B5
zmp_ph111_B8
zmp_ph111_B6
zmp_ph111_B7
zmp_ph111_B9
zmp_ph111_B10
zmp_ph114_G4
zmp_ph114_G11
zmp_ph114_H1
zmp_ph114_H2
zmp_ph114_A9
zmp_ph114_A11
zmp_ph114_B2
zmp_ph114_B4
zmp_ph114_D8
zmp_ph114_E2

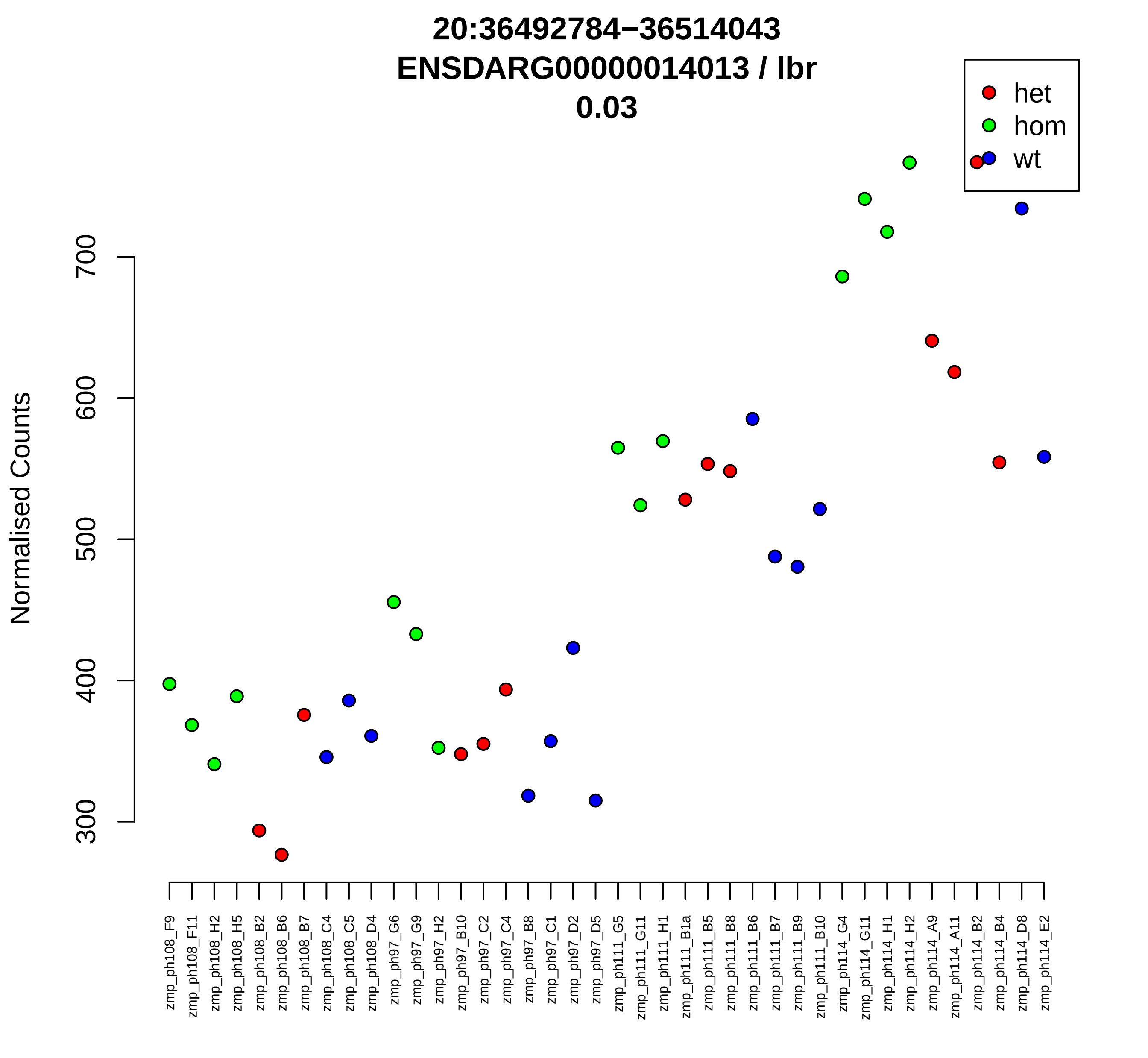

20:36492784−36514043
ENSDARG00000014013 / lbr
0.03
het
hom
wt
Normalised Counts
300
400
500
600
700
zmp_ph108_F9
zmp_ph108_F11
zmp_ph108_H2
zmp_ph108_H5
zmp_ph108_B2
zmp_ph108_B6
zmp_ph108_B7
zmp_ph108_C4
zmp_ph108_C5
zmp_ph108_D4
zmp_ph97_G6
zmp_ph97_G9
zmp_ph97_H2
zmp_ph97_B10
zmp_ph97_C2
zmp_ph97_C4
zmp_ph97_B8
zmp_ph97_C1
zmp_ph97_D2
zmp_ph97_D5
zmp_ph111_G5
zmp_ph111_G11
zmp_ph111_H1
zmp_ph111_B1a
zmp_ph111_B5
zmp_ph111_B8
zmp_ph111_B6
zmp_ph111_B7
zmp_ph111_B9
zmp_ph111_B10
zmp_ph114_G4
zmp_ph114_G11
zmp_ph114_H1
zmp_ph114_H2
zmp_ph114_A9
zmp_ph114_A11
zmp_ph114_B2
zmp_ph114_B4
zmp_ph114_D8
zmp_ph114_E2

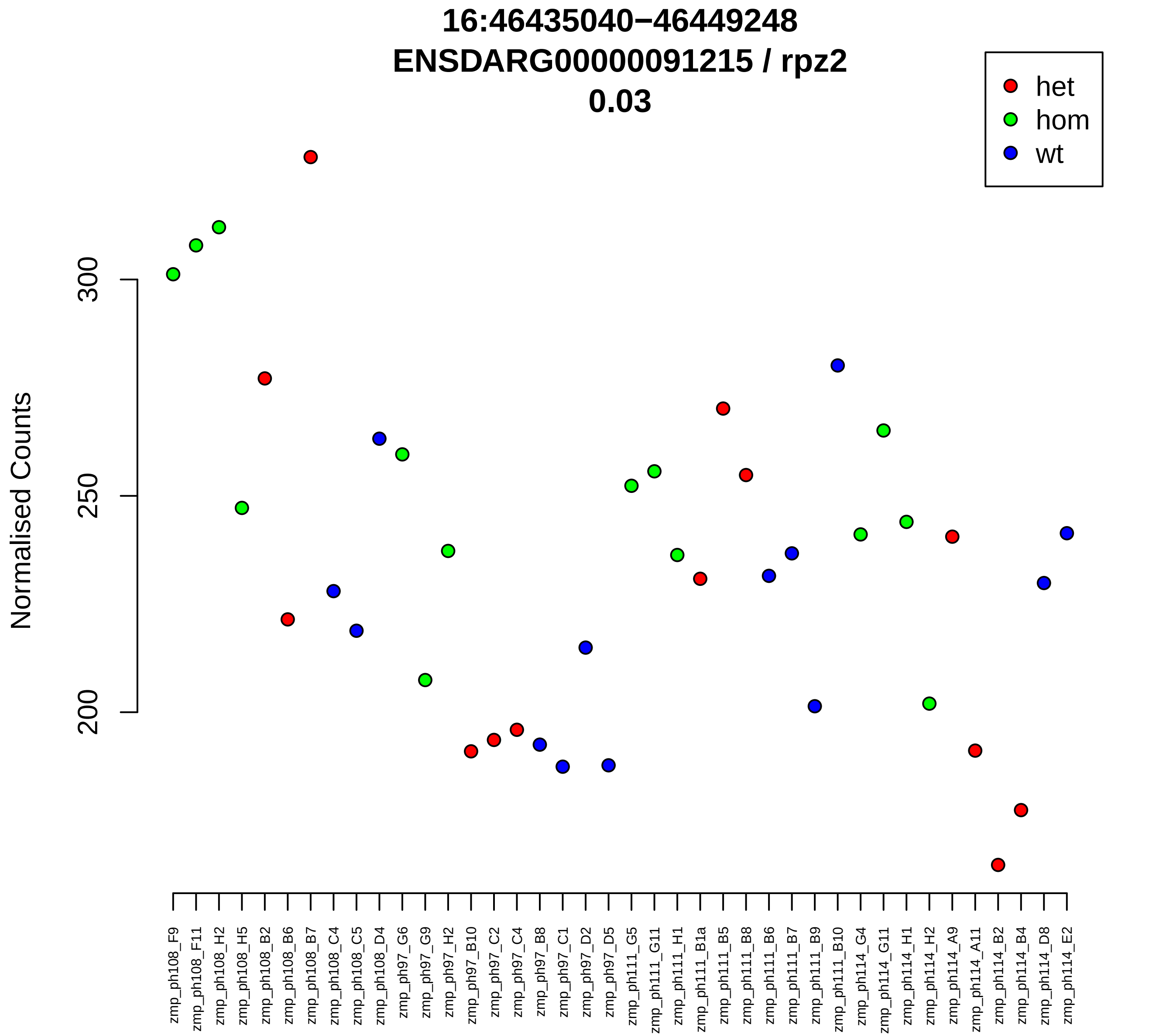
16:46435040−46449248
ENSDARG00000091215 / rpz2
0.03
het
hom
wt
Normalised Counts
300
250
200
zmp_ph108_F9
zmp_ph108_F11
zmp_ph108_H2
zmp_ph108_H5
zmp_ph108_B2
zmp_ph108_B6
zmp_ph108_B7
zmp_ph108_C4
zmp_ph108_C5
zmp_ph108_D4
zmp_ph97_G6
zmp_ph97_G9
zmp_ph97_H2
zmp_ph97_B10
zmp_ph97_C2
zmp_ph97_C4
zmp_ph97_B8
zmp_ph97_C1
zmp_ph97_D2
zmp_ph97_D5
zmp_ph111_G5
zmp_ph111_G11
zmp_ph111_H1
zmp_ph111_B1a
zmp_ph111_B5
zmp_ph111_B8
zmp_ph111_B6
zmp_ph111_B7
zmp_ph111_B9
zmp_ph111_B10
zmp_ph114_G4
zmp_ph114_G11
zmp_ph114_H1
zmp_ph114_H2
zmp_ph114_A9
zmp_ph114_A11
zmp_ph114_B2
zmp_ph114_B4
zmp_ph114_D8
zmp_ph114_E2

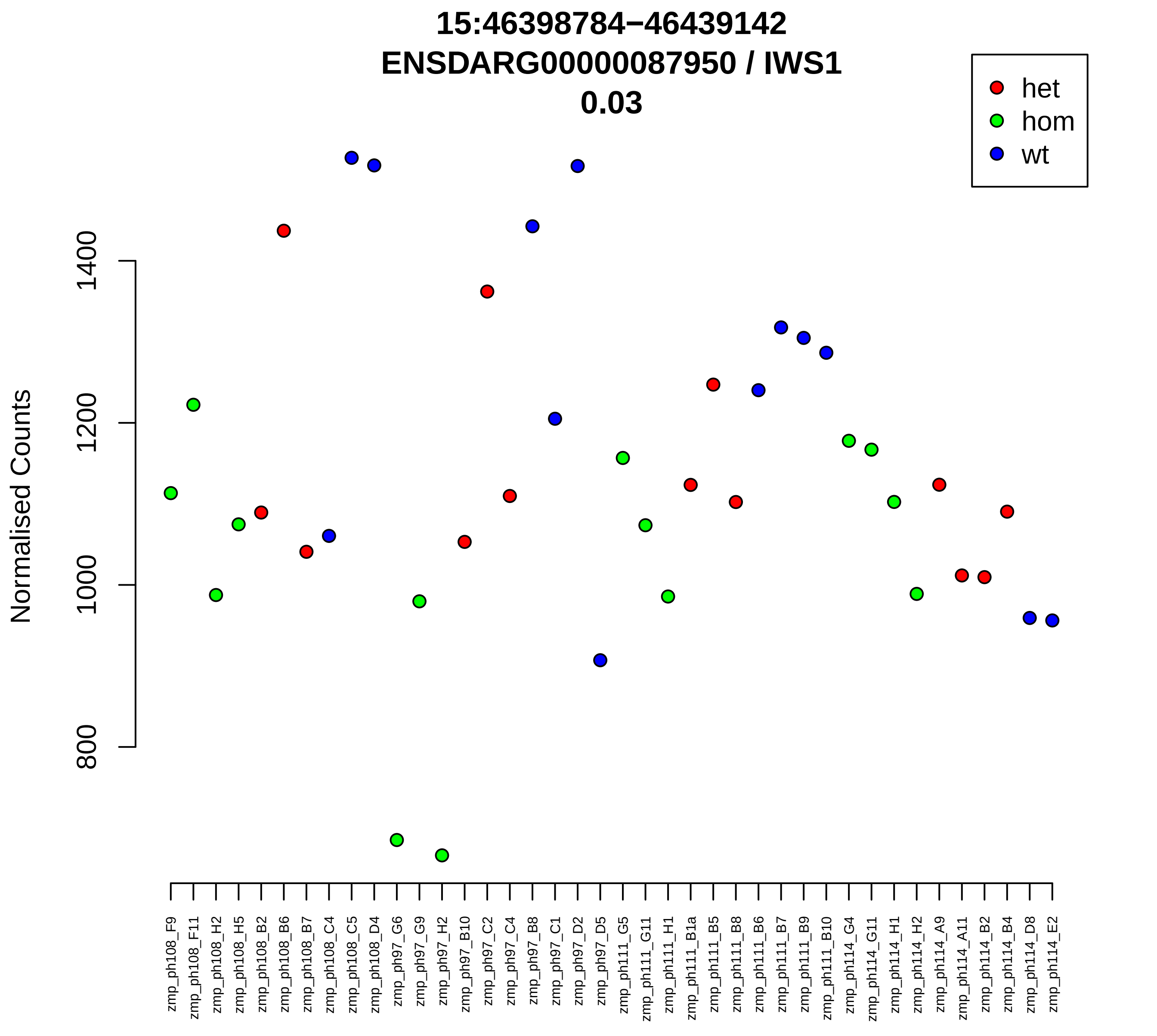
15:46398784−46439142
ENSDARG00000087950 / IWS1
0.03
het
hom
wt
Normalised Counts
800
1000
1200
1400
zmp_ph108_F9
zmp_ph108_F11
zmp_ph108_H2
zmp_ph108_H5
zmp_ph108_B2
zmp_ph108_B6
zmp_ph108_B7
zmp_ph108_C4
zmp_ph108_C5
zmp_ph108_D4
zmp_ph97_G6
zmp_ph97_G9
zmp_ph97_H2
zmp_ph97_B10
zmp_ph97_C2
zmp_ph97_C4
zmp_ph97_B8
zmp_ph97_C1
zmp_ph97_D2
zmp_ph97_D5
zmp_ph111_G5
zmp_ph111_G11
zmp_ph111_H1
zmp_ph111_B1a
zmp_ph111_B5
zmp_ph111_B8
zmp_ph111_B6
zmp_ph111_B7
zmp_ph111_B9
zmp_ph111_B10
zmp_ph114_G4
zmp_ph114_G11
zmp_ph114_H1
zmp_ph114_H2
zmp_ph114_A9
zmp_ph114_A11
zmp_ph114_B2
zmp_ph114_B4
zmp_ph114_D8
zmp_ph114_E2

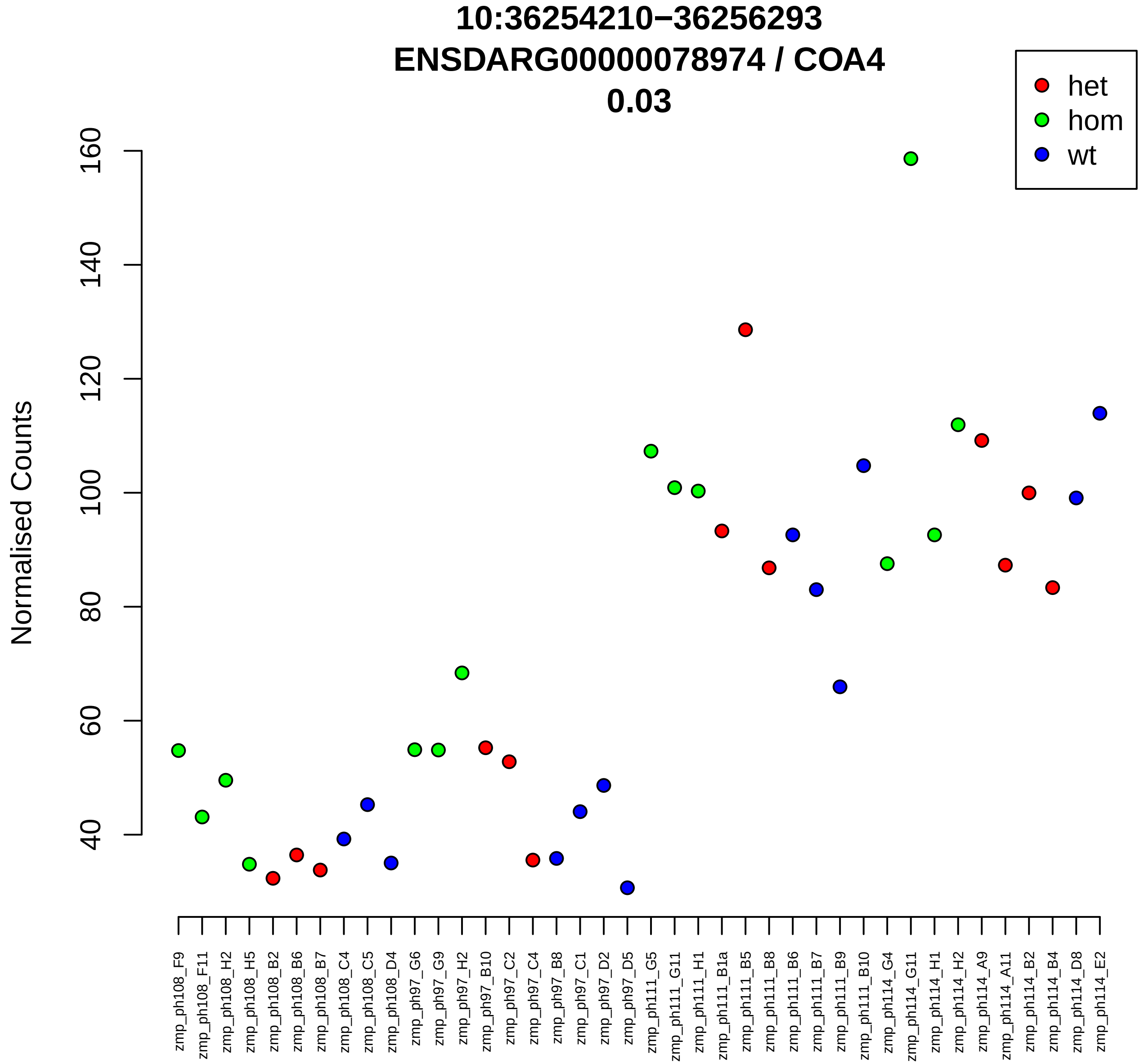

10:36254210−36256293
ENSDARG00000078974 / COA4
0.03
het
hom
wt
Normalised Counts
40
60
80
100
120
140
160
zmp_ph108_F9
zmp_ph108_F11
zmp_ph108_H2
zmp_ph108_H5
zmp_ph108_B2
zmp_ph108_B6
zmp_ph108_B7
zmp_ph108_C4
zmp_ph108_C5
zmp_ph108_D4
zmp_ph97_G6
zmp_ph97_G9
zmp_ph97_H2
zmp_ph97_B10
zmp_ph97_C2
zmp_ph97_C4
zmp_ph97_B8
zmp_ph97_C1
zmp_ph97_D2
zmp_ph97_D5
zmp_ph111_G5
zmp_ph111_G11
zmp_ph111_H1
zmp_ph111_B1a
zmp_ph111_B5
zmp_ph111_B8
zmp_ph111_B6
zmp_ph111_B7
zmp_ph111_B9
zmp_ph111_B10
zmp_ph114_G4
zmp_ph114_G11
zmp_ph114_H1
zmp_ph114_H2
zmp_ph114_A9
zmp_ph114_A11
zmp_ph114_B2
zmp_ph114_B4
zmp_ph114_D8
zmp_ph114_E2

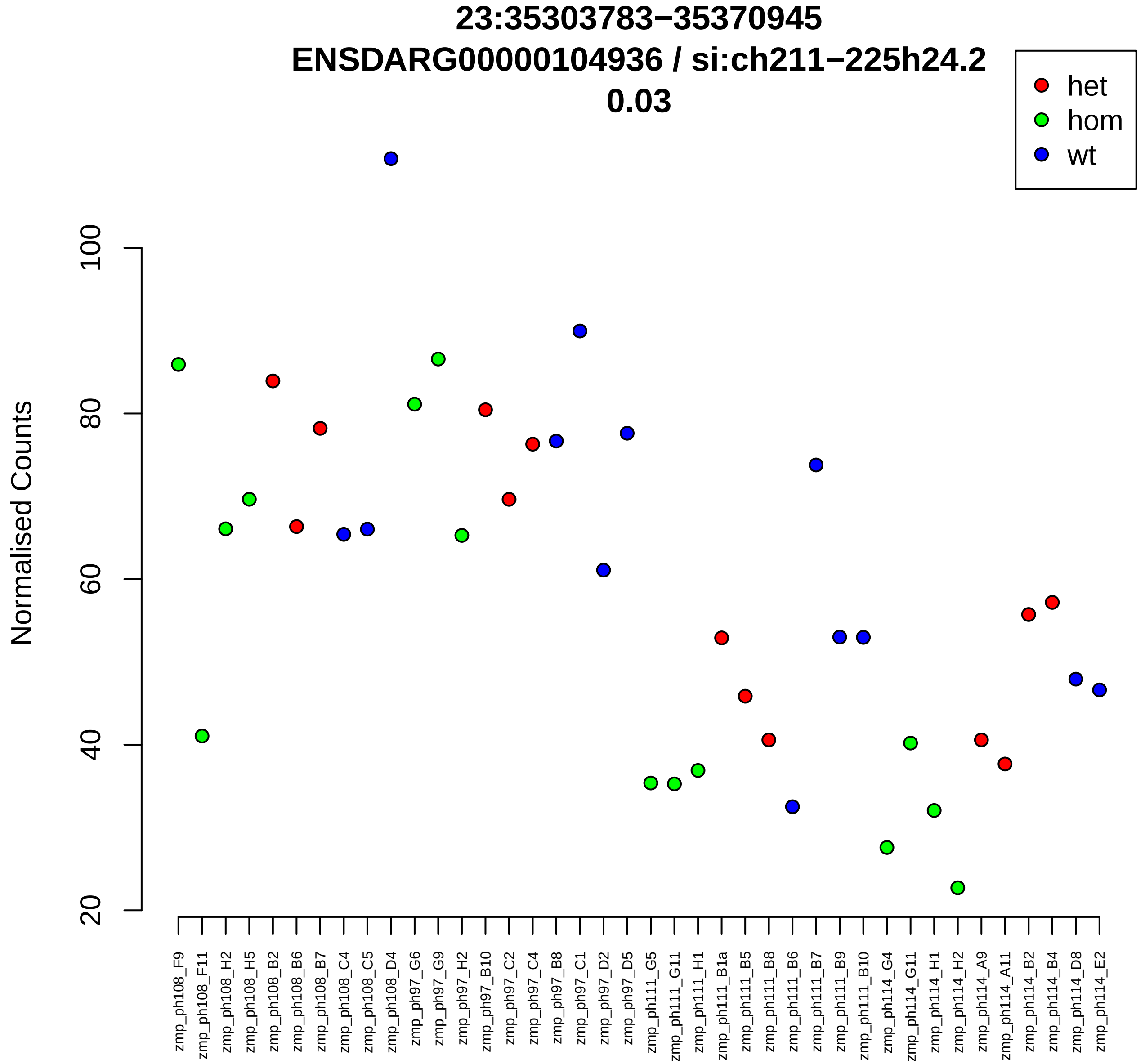
23:35303783−35370945
ENSDARG00000104936 / si:ch211−225h24.2
0.03
het
hom
wt
Normalised Counts
20
40
60
80
100
zmp_ph108_F9
zmp_ph108_F11
zmp_ph108_H2
zmp_ph108_H5
zmp_ph108_B2
zmp_ph108_B6
zmp_ph108_B7
zmp_ph108_C4
zmp_ph108_C5
zmp_ph108_D4
zmp_ph97_G6
zmp_ph97_G9
zmp_ph97_H2
zmp_ph97_B10
zmp_ph97_C2
zmp_ph97_C4
zmp_ph97_B8
zmp_ph97_C1
zmp_ph97_D2
zmp_ph97_D5
zmp_ph111_G5
zmp_ph111_G11
zmp_ph111_H1
zmp_ph111_B1a
zmp_ph111_B5
zmp_ph111_B8
zmp_ph111_B6
zmp_ph111_B7
zmp_ph111_B9
zmp_ph111_B10
zmp_ph114_G4
zmp_ph114_G11
zmp_ph114_H1
zmp_ph114_H2
zmp_ph114_A9
zmp_ph114_A11
zmp_ph114_B2
zmp_ph114_B4
zmp_ph114_D8
zmp_ph114_E2

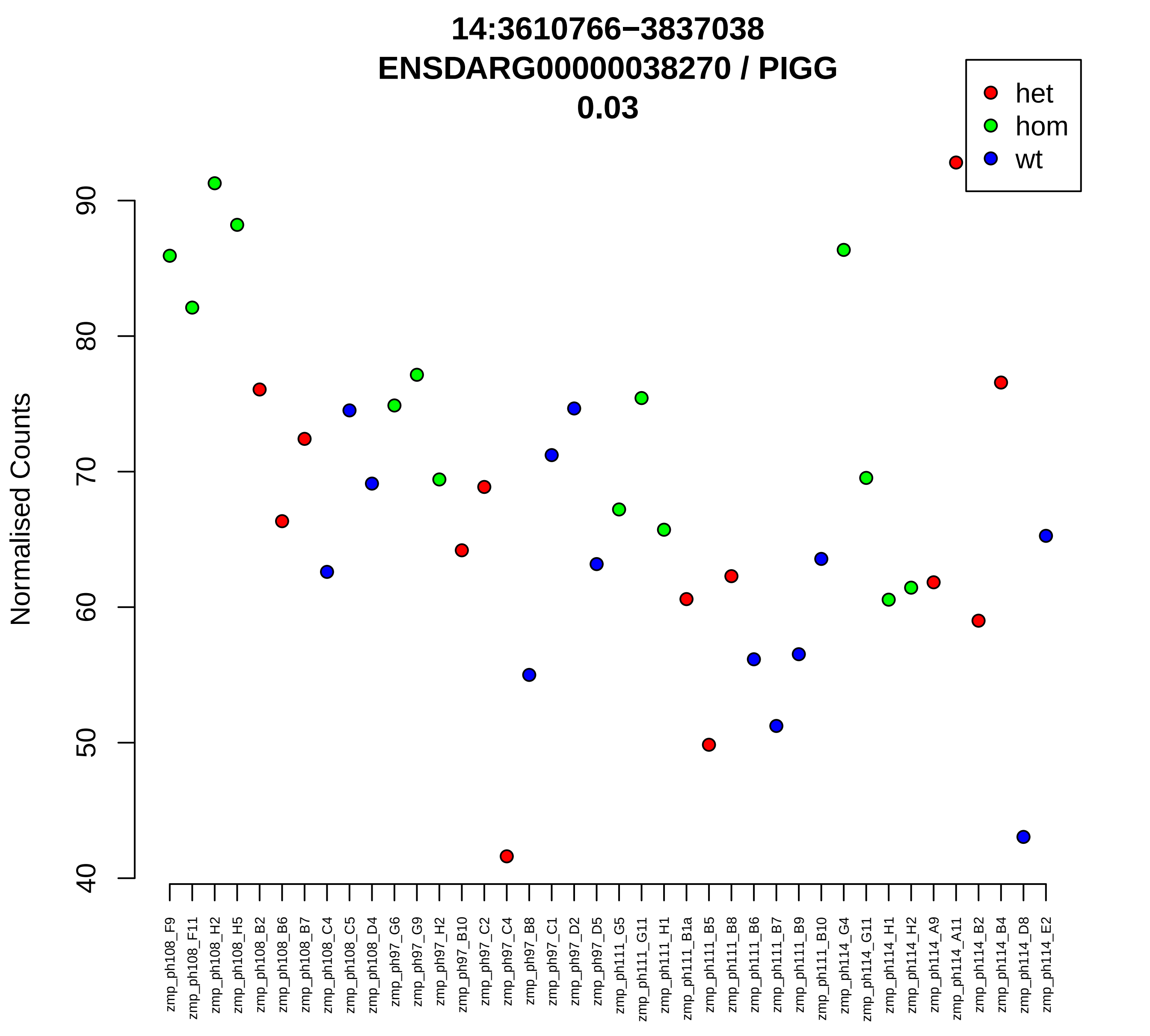
14:3610766−3837038
ENSDARG00000038270 / PIGG
0.03
het
hom
wt
Normalised Counts
40
50
60
70
80
90
zmp_ph108_F9
zmp_ph108_F11
zmp_ph108_H2
zmp_ph108_H5
zmp_ph108_B2
zmp_ph108_B6
zmp_ph108_B7
zmp_ph108_C4
zmp_ph108_C5
zmp_ph108_D4
zmp_ph97_G6
zmp_ph97_G9
zmp_ph97_H2
zmp_ph97_B10
zmp_ph97_C2
zmp_ph97_C4
zmp_ph97_B8
zmp_ph97_C1
zmp_ph97_D2
zmp_ph97_D5
zmp_ph111_G5
zmp_ph111_G11
zmp_ph111_H1
zmp_ph111_B1a
zmp_ph111_B5
zmp_ph111_B8
zmp_ph111_B6
zmp_ph111_B7
zmp_ph111_B9
zmp_ph111_B10
zmp_ph114_G4
zmp_ph114_G11
zmp_ph114_H1
zmp_ph114_H2
zmp_ph114_A9
zmp_ph114_A11
zmp_ph114_B2
zmp_ph114_B4
zmp_ph114_D8
zmp_ph114_E2

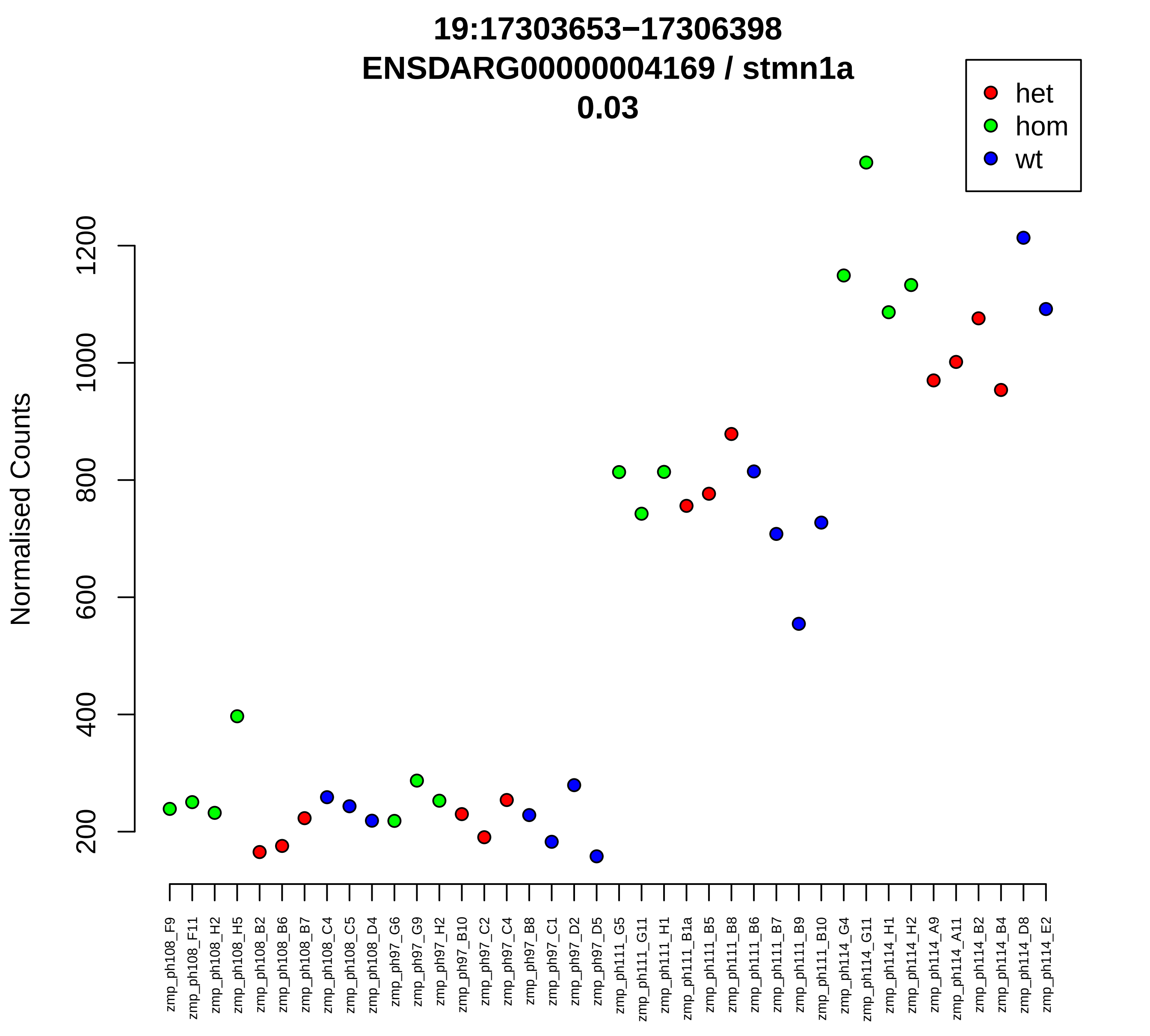

19:17303653−17306398
ENSDARG00000004169 / stmn1a
0.03
het
hom
wt
Normalised Counts
200
400
600
800
1000
1200
zmp_ph108_F9
zmp_ph108_F11
zmp_ph108_H2
zmp_ph108_H5
zmp_ph108_B2
zmp_ph108_B6
zmp_ph108_B7
zmp_ph108_C4
zmp_ph108_C5
zmp_ph108_D4
zmp_ph97_G6
zmp_ph97_G9
zmp_ph97_H2
zmp_ph97_B10
zmp_ph97_C2
zmp_ph97_C4
zmp_ph97_B8
zmp_ph97_C1
zmp_ph97_D2
zmp_ph97_D5
zmp_ph111_G5
zmp_ph111_G11
zmp_ph111_H1
zmp_ph111_B1a
zmp_ph111_B5
zmp_ph111_B8
zmp_ph111_B6
zmp_ph111_B7
zmp_ph111_B9
zmp_ph111_B10
zmp_ph114_G4
zmp_ph114_G11
zmp_ph114_H1
zmp_ph114_H2
zmp_ph114_A9
zmp_ph114_A11
zmp_ph114_B2
zmp_ph114_B4
zmp_ph114_D8
zmp_ph114_E2

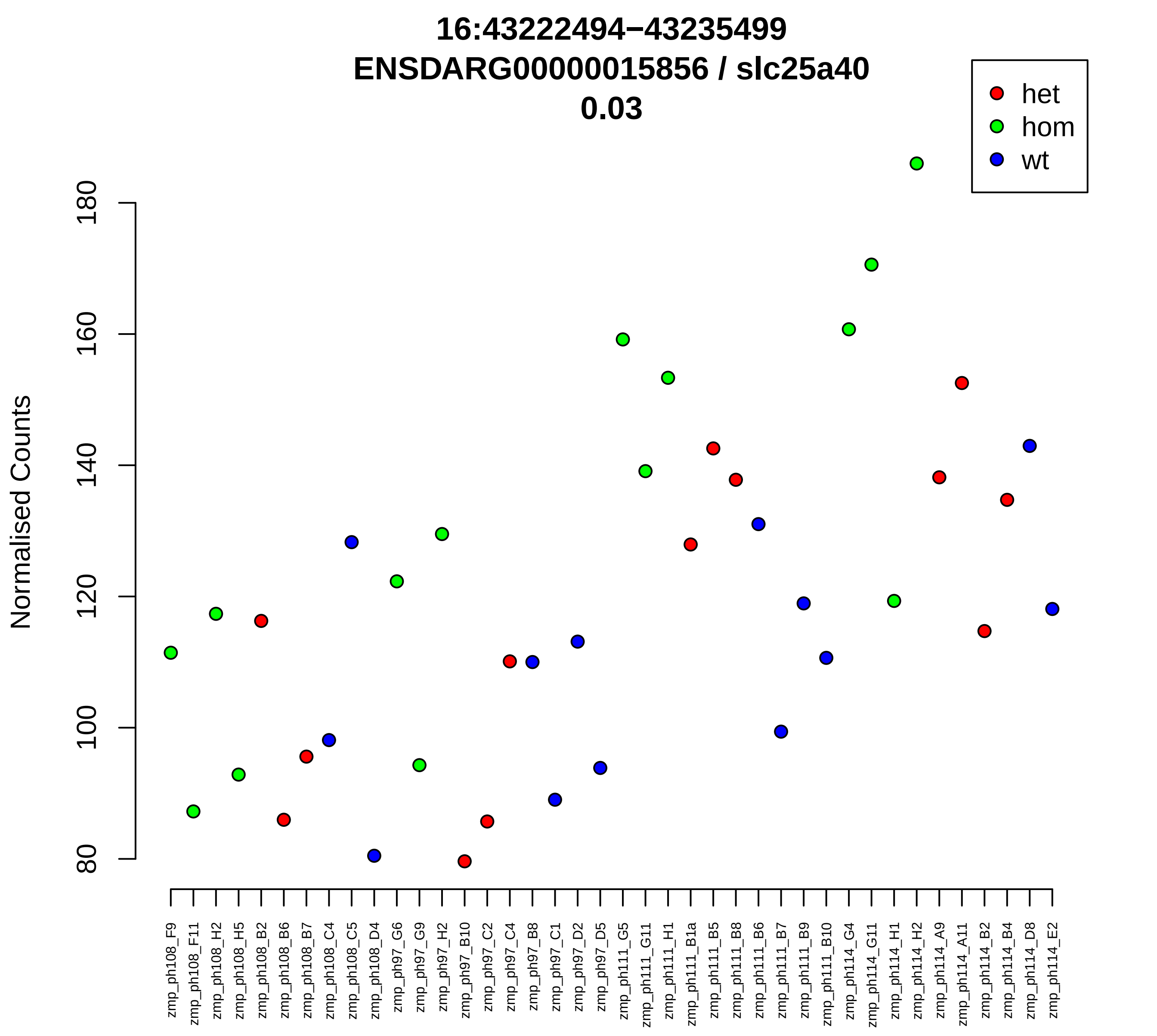

16:43222494−43235499
ENSDARG00000015856 / slc25a40
0.03
het
hom
wt
Normalised Counts
80
100
120
140
160
180
zmp_ph108_F9
zmp_ph108_F11
zmp_ph108_H2
zmp_ph108_H5
zmp_ph108_B2
zmp_ph108_B6
zmp_ph108_B7
zmp_ph108_C4
zmp_ph108_C5
zmp_ph108_D4
zmp_ph97_G6
zmp_ph97_G9
zmp_ph97_H2
zmp_ph97_B10
zmp_ph97_C2
zmp_ph97_C4
zmp_ph97_B8
zmp_ph97_C1
zmp_ph97_D2
zmp_ph97_D5
zmp_ph111_G5
zmp_ph111_G11
zmp_ph111_H1
zmp_ph111_B1a
zmp_ph111_B5
zmp_ph111_B8
zmp_ph111_B6
zmp_ph111_B7
zmp_ph111_B9
zmp_ph111_B10
zmp_ph114_G4
zmp_ph114_G11
zmp_ph114_H1
zmp_ph114_H2
zmp_ph114_A9
zmp_ph114_A11
zmp_ph114_B2
zmp_ph114_B4
zmp_ph114_D8
zmp_ph114_E2

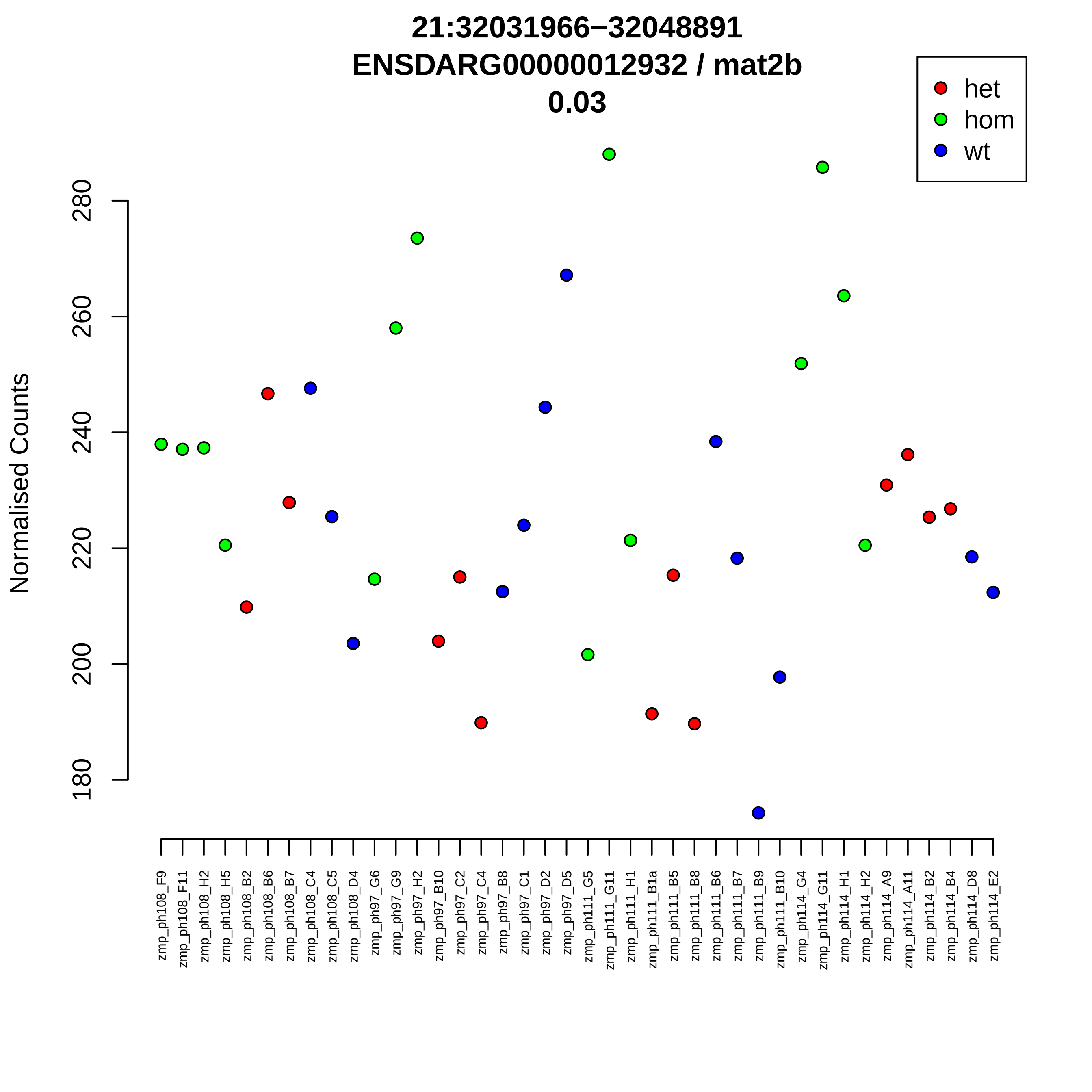

21:32031966−32048891
ENSDARG00000012932 / mat2b
0.03
het
hom
wt
Normalised Counts
180
200
220
240
260
280
zmp_ph108_F9
zmp_ph108_F11
zmp_ph108_H2
zmp_ph108_H5
zmp_ph108_B2
zmp_ph108_B6
zmp_ph108_B7
zmp_ph108_C4
zmp_ph108_C5
zmp_ph108_D4
zmp_ph97_G6
zmp_ph97_G9
zmp_ph97_H2
zmp_ph97_B10
zmp_ph97_C2
zmp_ph97_C4
zmp_ph97_B8
zmp_ph97_C1
zmp_ph97_D2
zmp_ph97_D5
zmp_ph111_G5
zmp_ph111_G11
zmp_ph111_H1
zmp_ph111_B1a
zmp_ph111_B5
zmp_ph111_B8
zmp_ph111_B6
zmp_ph111_B7
zmp_ph111_B9
zmp_ph111_B10
zmp_ph114_G4
zmp_ph114_G11
zmp_ph114_H1
zmp_ph114_H2
zmp_ph114_A9
zmp_ph114_A11
zmp_ph114_B2
zmp_ph114_B4
zmp_ph114_D8
zmp_ph114_E2

21:2334042−2335082
ENSDARG00000104488 / si:ch73−299h12.9
0.03

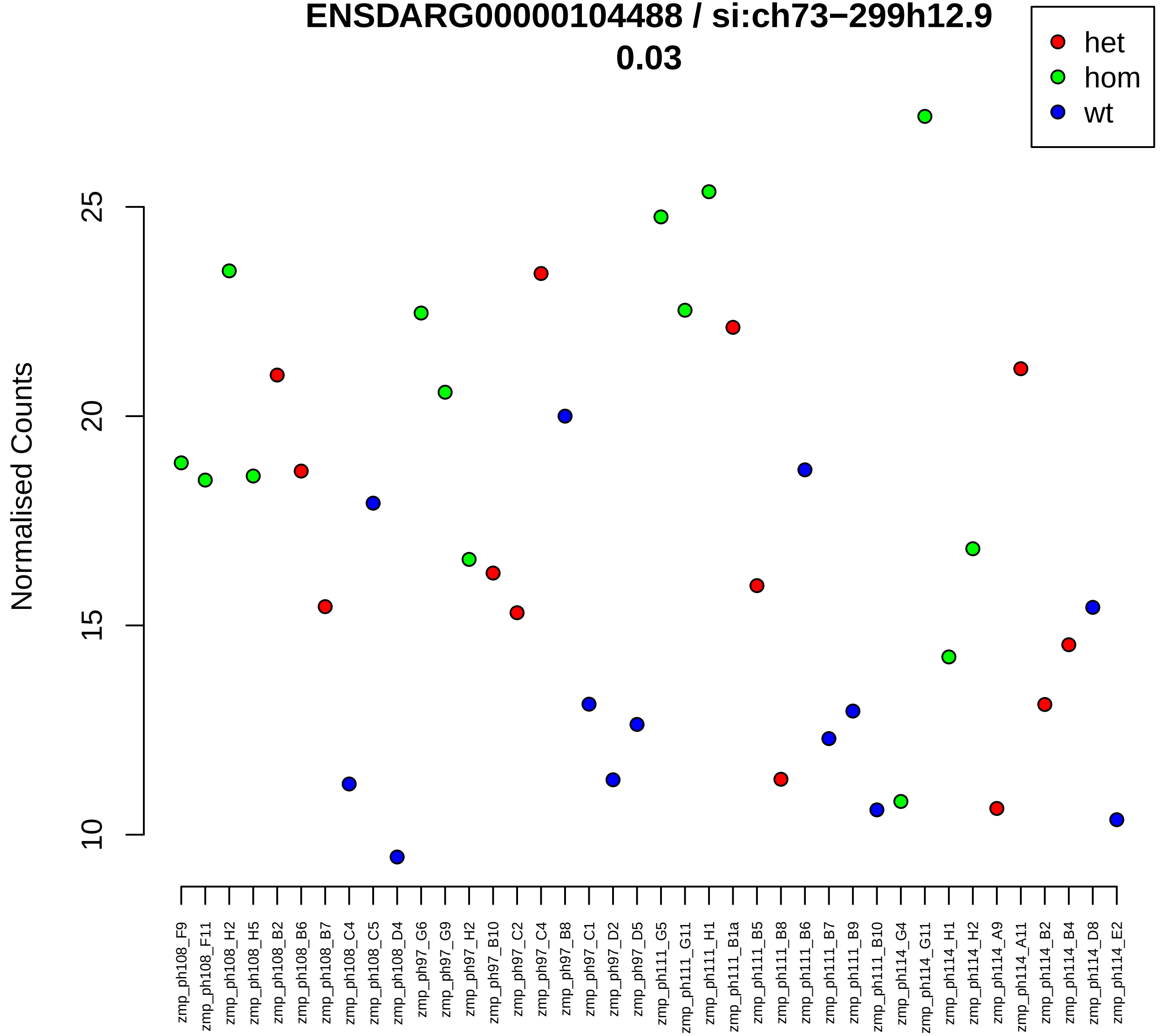

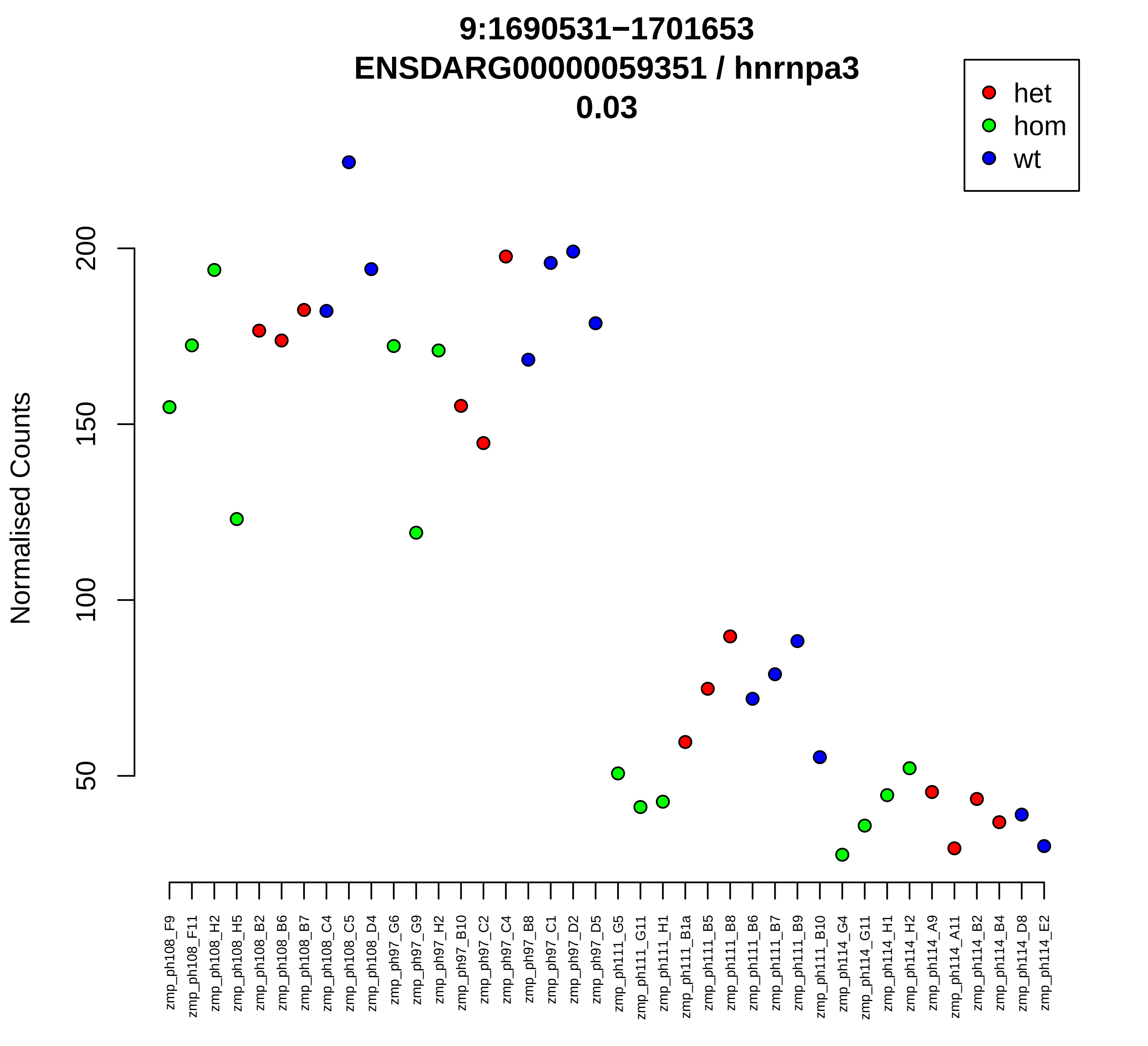

9:1690531−1701653
ENSDARG00000059351 / hnrnpa3
0.03
het
hom
wt
Normalised Counts
50
100
150
200
zmp_ph108_F9
zmp_ph108_F11
zmp_ph108_H2
zmp_ph108_H5
zmp_ph108_B2
zmp_ph108_B6
zmp_ph108_B7
zmp_ph108_C4
zmp_ph108_C5
zmp_ph108_D4
zmp_ph97_G6
zmp_ph97_G9
zmp_ph97_H2
zmp_ph97_B10
zmp_ph97_C2
zmp_ph97_C4
zmp_ph97_B8
zmp_ph97_C1
zmp_ph97_D2
zmp_ph97_D5
zmp_ph111_G5
zmp_ph111_G11
zmp_ph111_H1
zmp_ph111_B1a
zmp_ph111_B5
zmp_ph111_B8
zmp_ph111_B6
zmp_ph111_B7
zmp_ph111_B9
zmp_ph111_B10
zmp_ph114_G4
zmp_ph114_G11
zmp_ph114_H1
zmp_ph114_H2
zmp_ph114_A9
zmp_ph114_A11
zmp_ph114_B2
zmp_ph114_B4
zmp_ph114_D8
zmp_ph114_E2

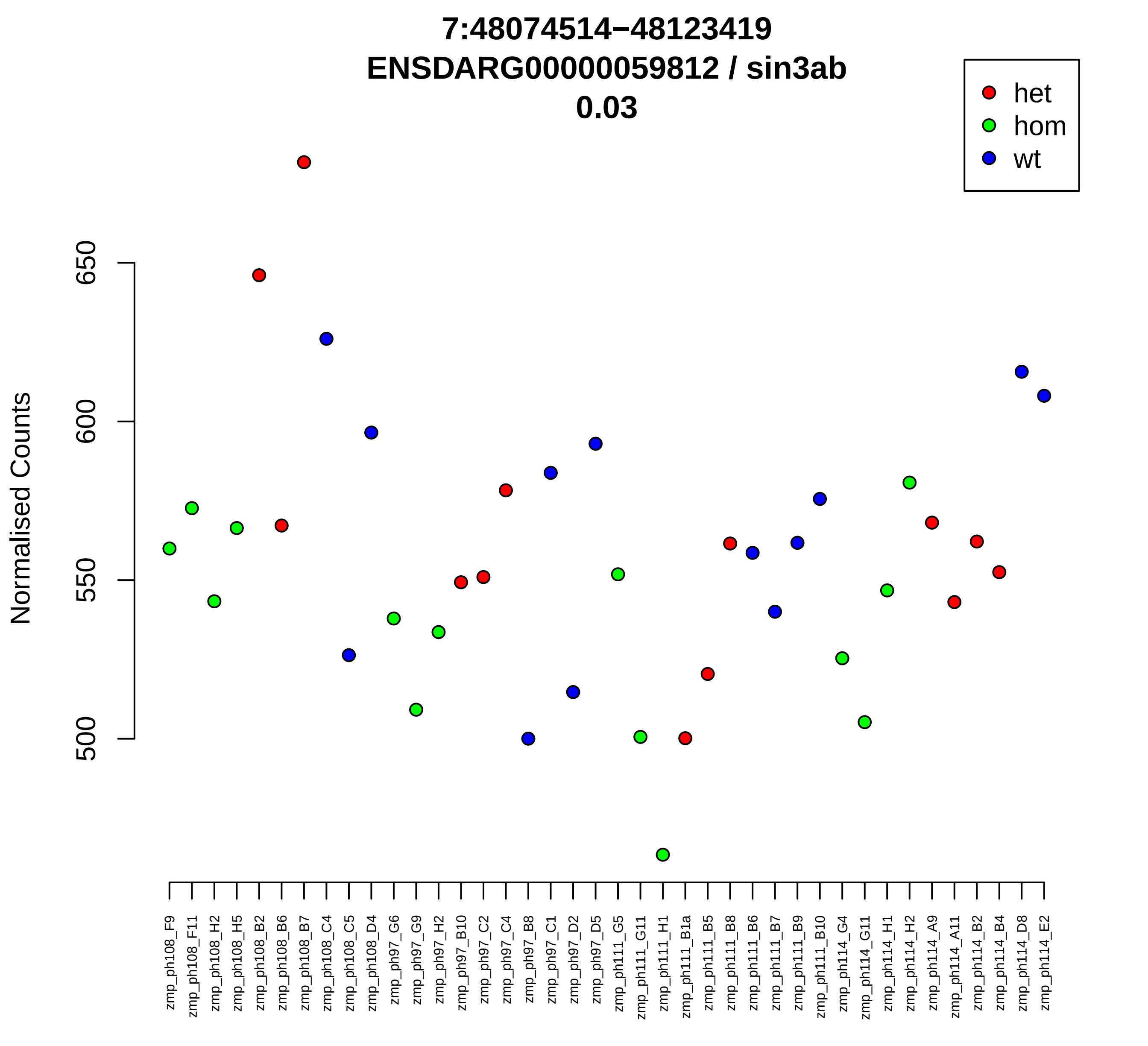

7:48074514−48123419
ENSDARG00000059812 / sin3ab
0.03
het
hom
wt
Normalised Counts
500
550
600
650
zmp_ph108_F9
zmp_ph108_F11
zmp_ph108_H2
zmp_ph108_H5
zmp_ph108_B2
zmp_ph108_B6
zmp_ph108_B7
zmp_ph108_C4
zmp_ph108_C5
zmp_ph108_D4
zmp_ph97_G6
zmp_ph97_G9
zmp_ph97_H2
zmp_ph97_B10
zmp_ph97_C2
zmp_ph97_C4
zmp_ph97_B8
zmp_ph97_C1
zmp_ph97_D2
zmp_ph97_D5
zmp_ph111_G5
zmp_ph111_G11
zmp_ph111_H1
zmp_ph111_B1a
zmp_ph111_B5
zmp_ph111_B8
zmp_ph111_B6
zmp_ph111_B7
zmp_ph111_B9
zmp_ph111_B10
zmp_ph114_G4
zmp_ph114_G11
zmp_ph114_H1
zmp_ph114_H2
zmp_ph114_A9
zmp_ph114_A11
zmp_ph114_B2
zmp_ph114_B4
zmp_ph114_D8
zmp_ph114_E2

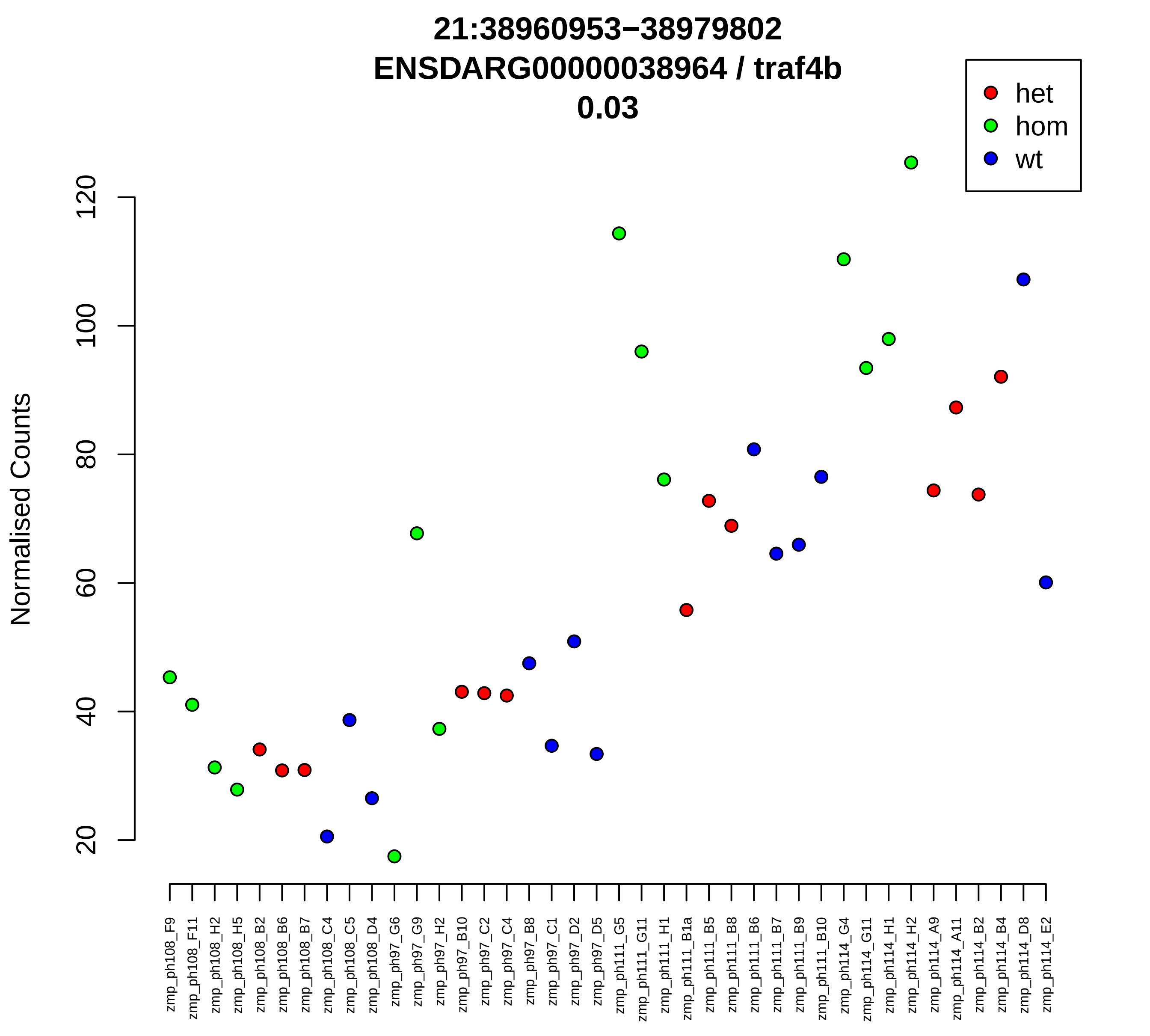

21:38960953−38979802
ENSDARG00000038964 / traf4b
0.03
het
hom
wt
Normalised Counts
20
40
60
80
100
120
zmp_ph108_F9
zmp_ph108_F11
zmp_ph108_H2
zmp_ph108_H5
zmp_ph108_B2
zmp_ph108_B6
zmp_ph108_B7
zmp_ph108_C4
zmp_ph108_C5
zmp_ph108_D4
zmp_ph97_G6
zmp_ph97_G9
zmp_ph97_H2
zmp_ph97_B10
zmp_ph97_C2
zmp_ph97_C4
zmp_ph97_B8
zmp_ph97_C1
zmp_ph97_D2
zmp_ph97_D5
zmp_ph111_G5
zmp_ph111_G11
zmp_ph111_H1
zmp_ph111_B1a
zmp_ph111_B5
zmp_ph111_B8
zmp_ph111_B6
zmp_ph111_B7
zmp_ph111_B9
zmp_ph111_B10
zmp_ph114_G4
zmp_ph114_G11
zmp_ph114_H1
zmp_ph114_H2
zmp_ph114_A9
zmp_ph114_A11
zmp_ph114_B2
zmp_ph114_B4
zmp_ph114_D8
zmp_ph114_E2

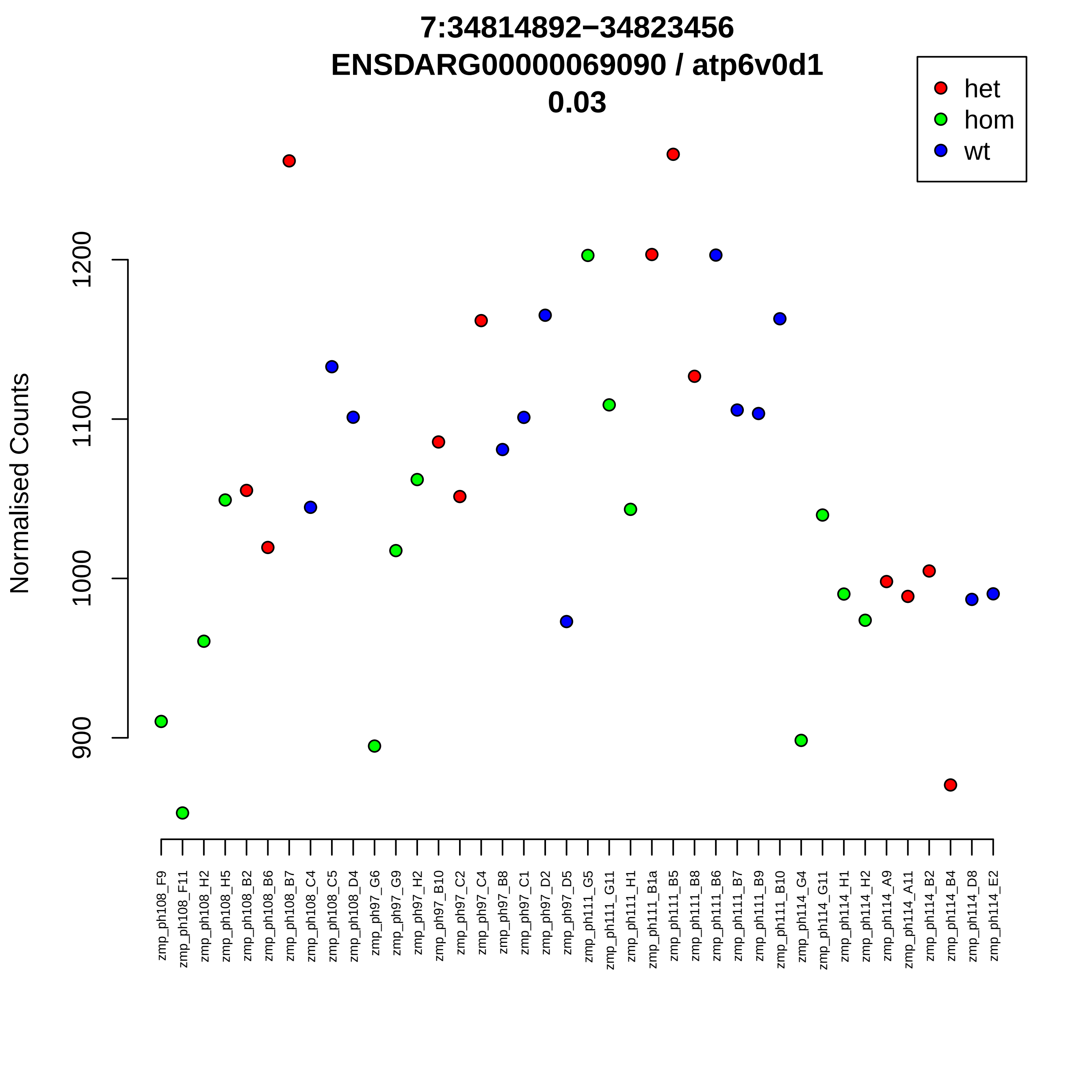

7:34814892−34823456
ENSDARG00000069090 / atp6v0d1
0.03
het
hom
wt
Normalised Counts
900
1000
1100
1200
zmp_ph108_F9
zmp_ph108_F11
zmp_ph108_H2
zmp_ph108_H5
zmp_ph108_B2
zmp_ph108_B6
zmp_ph108_B7
zmp_ph108_C4
zmp_ph108_C5
zmp_ph108_D4
zmp_ph97_G6
zmp_ph97_G9
zmp_ph97_H2
zmp_ph97_B10
zmp_ph97_C2
zmp_ph97_C4
zmp_ph97_B8
zmp_ph97_C1
zmp_ph97_D2
zmp_ph97_D5
zmp_ph111_G5
zmp_ph111_G11
zmp_ph111_H1
zmp_ph111_B1a
zmp_ph111_B5
zmp_ph111_B8
zmp_ph111_B6
zmp_ph111_B7
zmp_ph111_B9
zmp_ph111_B10
zmp_ph114_G4
zmp_ph114_G11
zmp_ph114_H1
zmp_ph114_H2
zmp_ph114_A9
zmp_ph114_A11
zmp_ph114_B2
zmp_ph114_B4
zmp_ph114_D8
zmp_ph114_E2

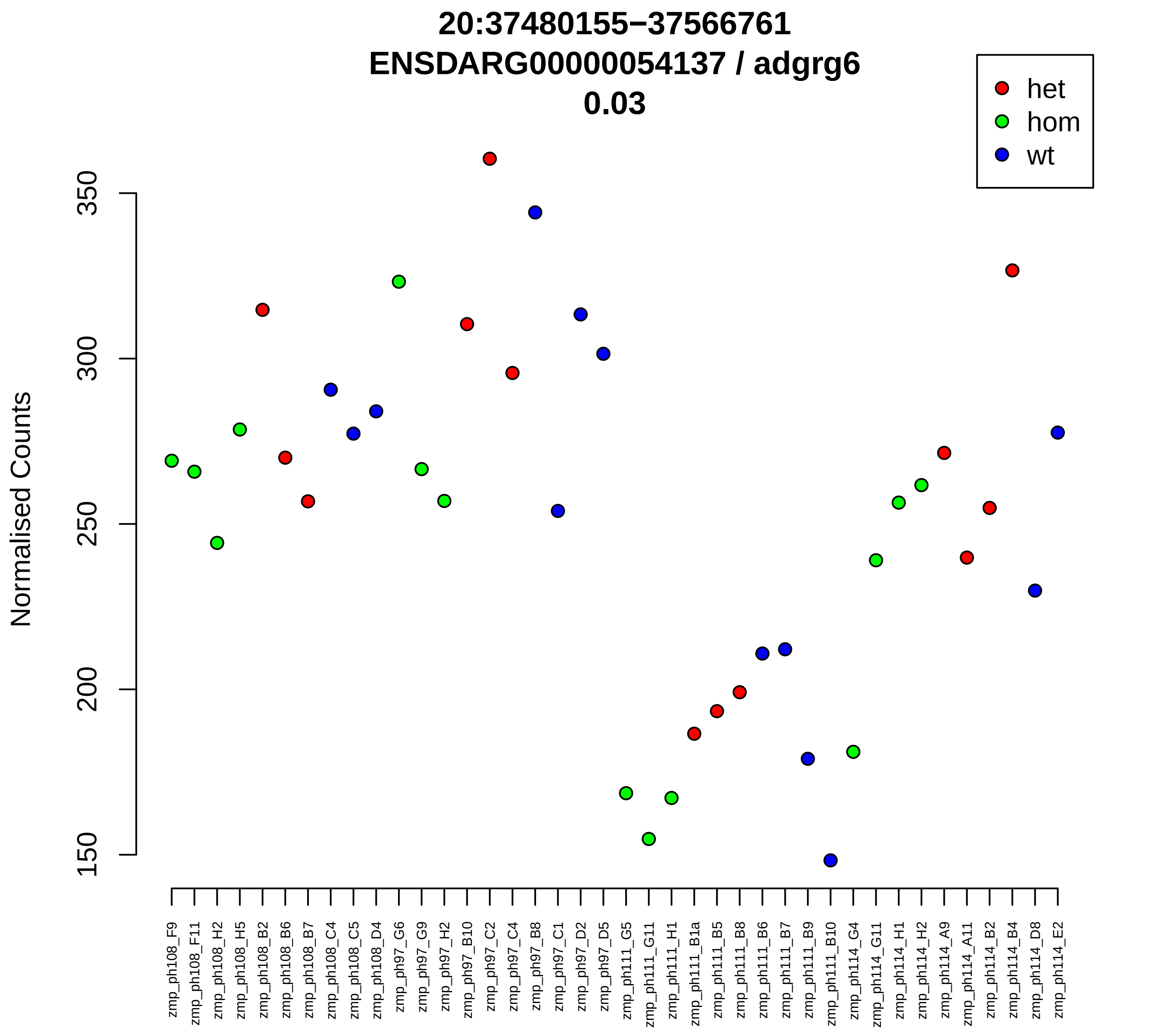

20:37480155−37566761
ENSDARG00000054137 / adgrg6
0.03
het
hom
wt
Normalised Counts
150
200
250
300
350
zmp_ph108_F9
zmp_ph108_F11
zmp_ph108_H2
zmp_ph108_H5
zmp_ph108_B2
zmp_ph108_B6
zmp_ph108_B7
zmp_ph108_C4
zmp_ph108_C5
zmp_ph108_D4
zmp_ph97_G6
zmp_ph97_G9
zmp_ph97_H2
zmp_ph97_B10
zmp_ph97_C2
zmp_ph97_C4
zmp_ph97_B8
zmp_ph97_C1
zmp_ph97_D2
zmp_ph97_D5
zmp_ph111_G5
zmp_ph111_G11
zmp_ph111_H1
zmp_ph111_B1a
zmp_ph111_B5
zmp_ph111_B8
zmp_ph111_B6
zmp_ph111_B7
zmp_ph111_B9
zmp_ph111_B10
zmp_ph114_G4
zmp_ph114_G11
zmp_ph114_H1
zmp_ph114_H2
zmp_ph114_A9
zmp_ph114_A11
zmp_ph114_B2
zmp_ph114_B4
zmp_ph114_D8
zmp_ph114_E2

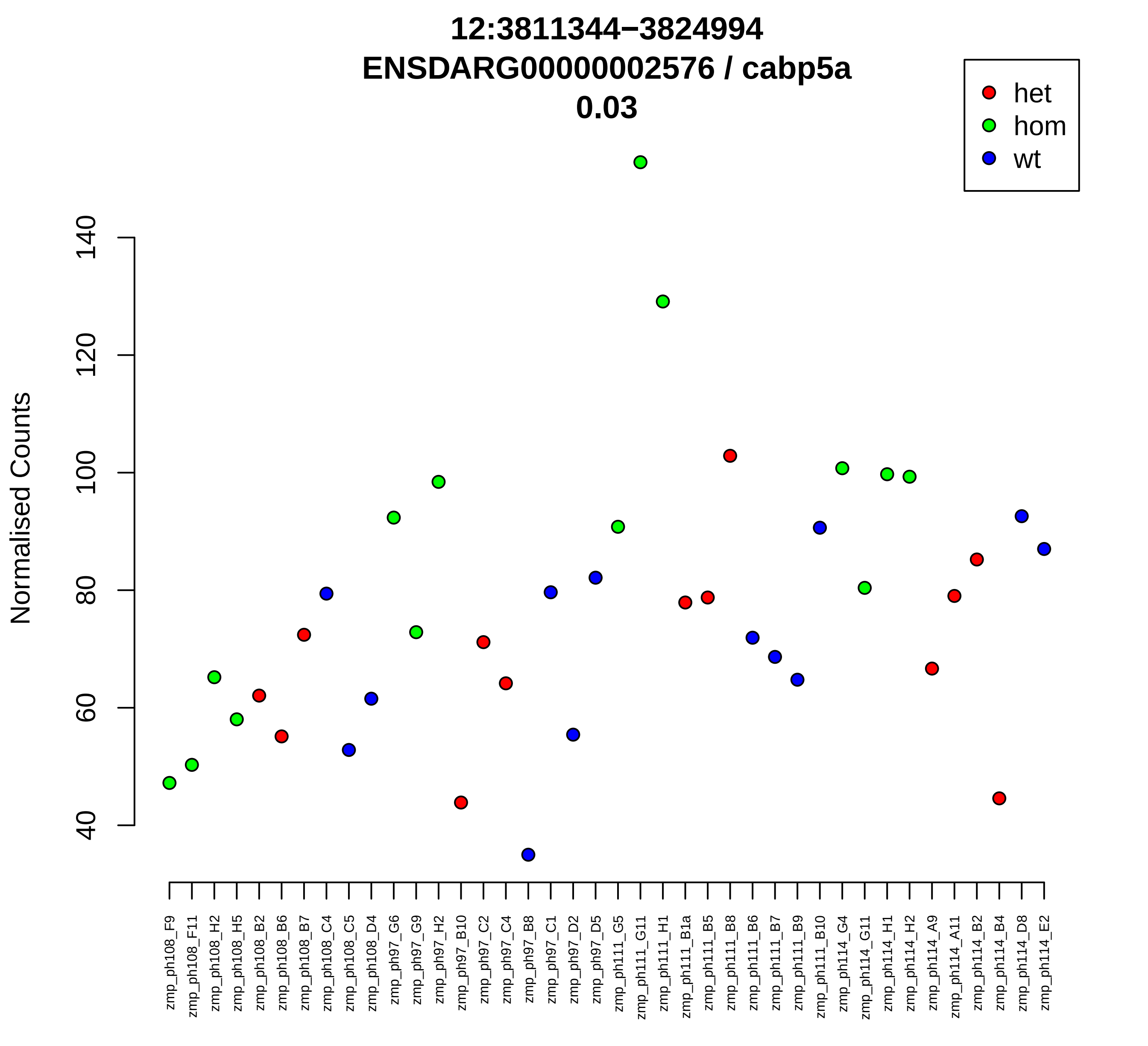
12:3811344−3824994
ENSDARG00000002576 / cabp5a
0.03
het
hom
wt
Normalised Counts
40
60
80
100
120
140
zmp_ph108_F9
zmp_ph108_F11
zmp_ph108_H2
zmp_ph108_H5
zmp_ph108_B2
zmp_ph108_B6
zmp_ph108_B7
zmp_ph108_C4
zmp_ph108_C5
zmp_ph108_D4
zmp_ph97_G6
zmp_ph97_G9
zmp_ph97_H2
zmp_ph97_B10
zmp_ph97_C2
zmp_ph97_C4
zmp_ph97_B8
zmp_ph97_C1
zmp_ph97_D2
zmp_ph97_D5
zmp_ph111_G5
zmp_ph111_G11
zmp_ph111_H1
zmp_ph111_B1a
zmp_ph111_B5
zmp_ph111_B8
zmp_ph111_B6
zmp_ph111_B7
zmp_ph111_B9
zmp_ph111_B10
zmp_ph114_G4
zmp_ph114_G11
zmp_ph114_H1
zmp_ph114_H2
zmp_ph114_A9
zmp_ph114_A11
zmp_ph114_B2
zmp_ph114_B4
zmp_ph114_D8
zmp_ph114_E2

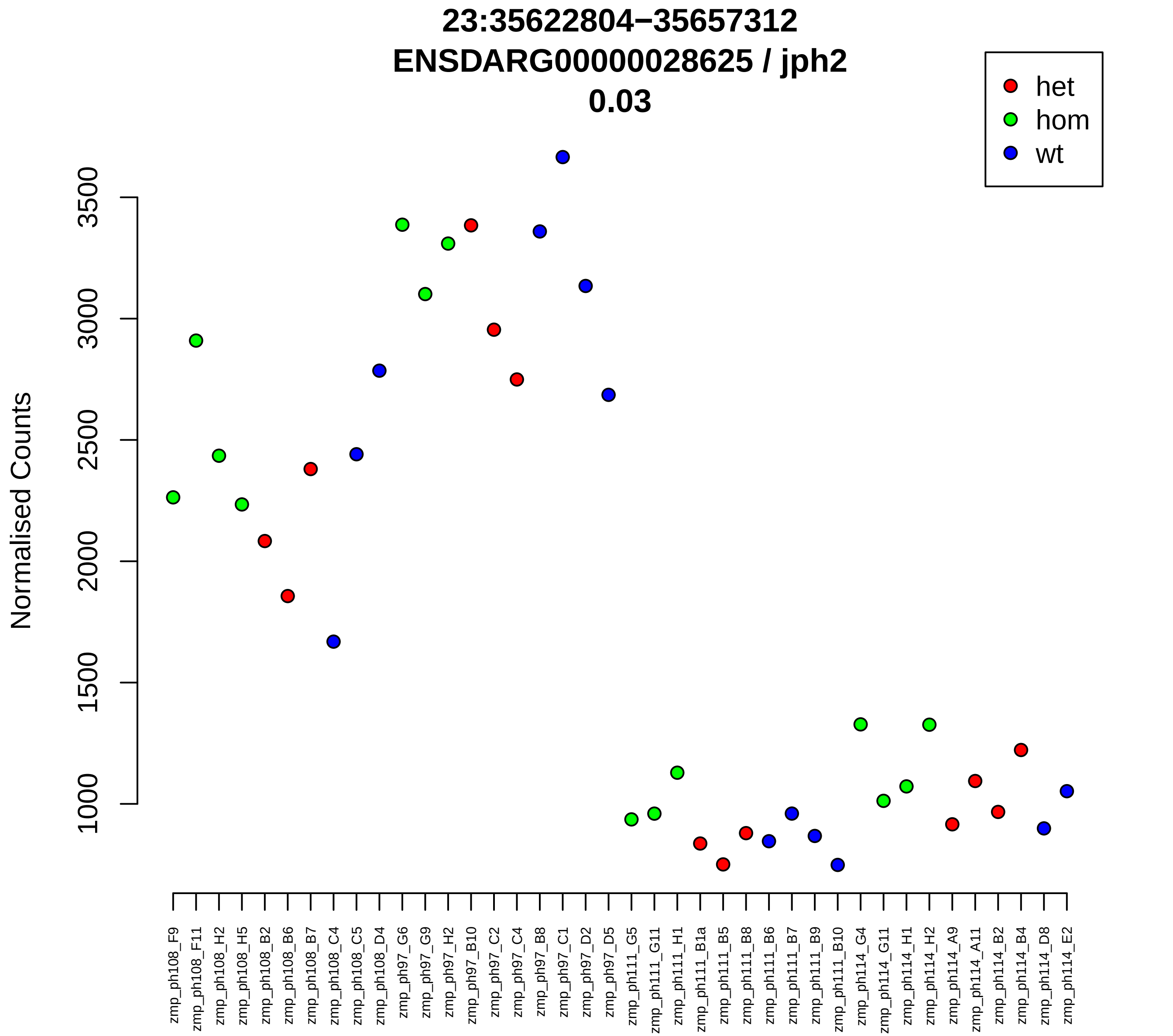

23:35622804−35657312
ENSDARG00000028625 / jph2
0.03
het
hom
wt
Normalised Counts
1000
1500
2000
2500
3000
3500
zmp_ph108_F9
zmp_ph108_F11
zmp_ph108_H2
zmp_ph108_H5
zmp_ph108_B2
zmp_ph108_B6
zmp_ph108_B7
zmp_ph108_C4
zmp_ph108_C5
zmp_ph108_D4
zmp_ph97_G6
zmp_ph97_G9
zmp_ph97_H2
zmp_ph97_B10
zmp_ph97_C2
zmp_ph97_C4
zmp_ph97_B8
zmp_ph97_C1
zmp_ph97_D2
zmp_ph97_D5
zmp_ph111_G5
zmp_ph111_G11
zmp_ph111_H1
zmp_ph111_B1a
zmp_ph111_B5
zmp_ph111_B8
zmp_ph111_B6
zmp_ph111_B7
zmp_ph111_B9
zmp_ph111_B10
zmp_ph114_G4
zmp_ph114_G11
zmp_ph114_H1
zmp_ph114_H2
zmp_ph114_A9
zmp_ph114_A11
zmp_ph114_B2
zmp_ph114_B4
zmp_ph114_D8
zmp_ph114_E2

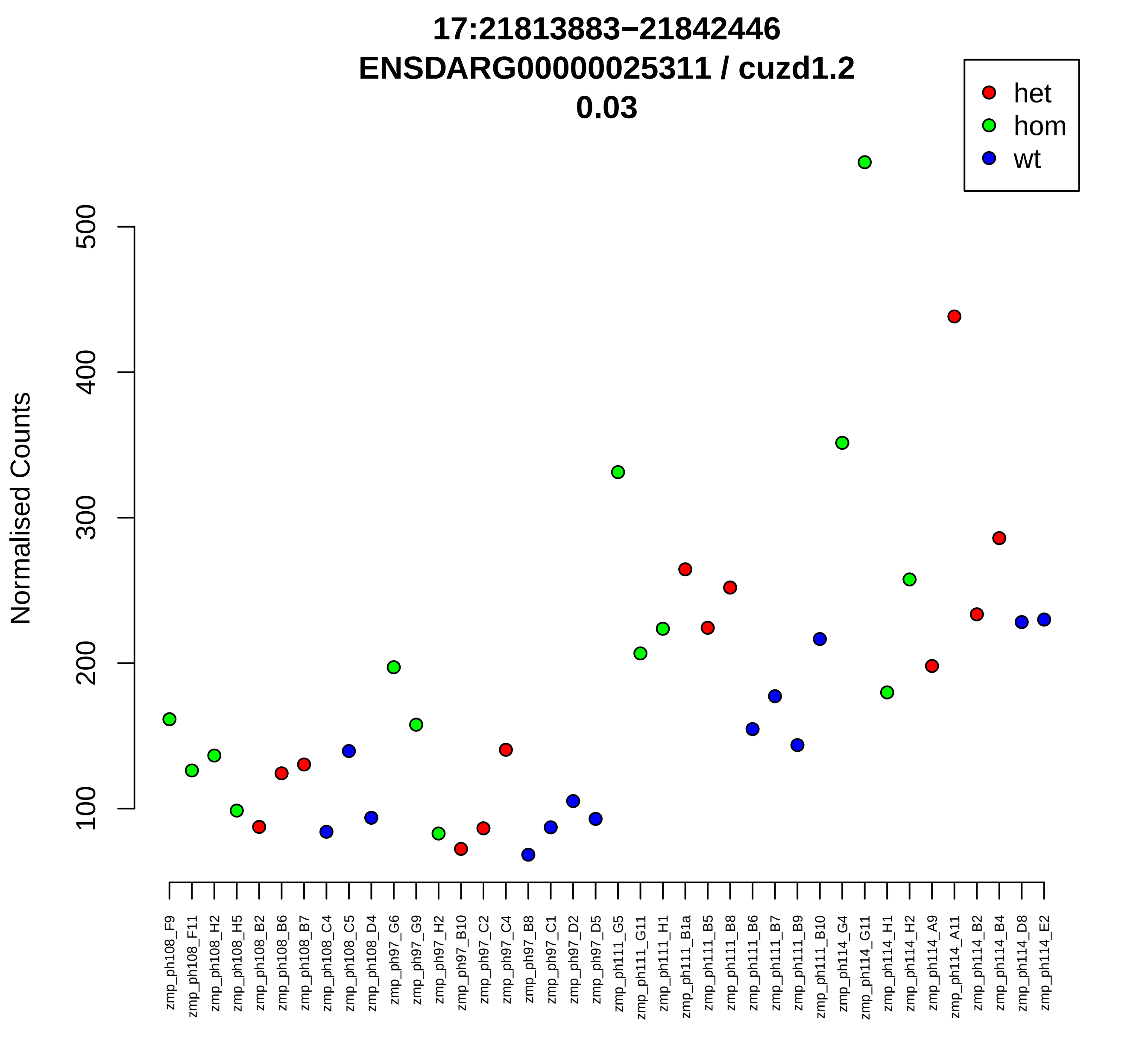
17:21813883−21842446
ENSDARG00000025311 / cuzd1.2
0.03
het
hom
wt
Normalised Counts
100
200
300
400
500
zmp_ph108_F9
zmp_ph108_F11
zmp_ph108_H2
zmp_ph108_H5
zmp_ph108_B2
zmp_ph108_B6
zmp_ph108_B7
zmp_ph108_C4
zmp_ph108_C5
zmp_ph108_D4
zmp_ph97_G6
zmp_ph97_G9
zmp_ph97_H2
zmp_ph97_B10
zmp_ph97_C2
zmp_ph97_C4
zmp_ph97_B8
zmp_ph97_C1
zmp_ph97_D2
zmp_ph97_D5
zmp_ph111_G5
zmp_ph111_G11
zmp_ph111_H1
zmp_ph111_B1a
zmp_ph111_B5
zmp_ph111_B8
zmp_ph111_B6
zmp_ph111_B7
zmp_ph111_B9
zmp_ph111_B10
zmp_ph114_G4
zmp_ph114_G11
zmp_ph114_H1
zmp_ph114_H2
zmp_ph114_A9
zmp_ph114_A11
zmp_ph114_B2
zmp_ph114_B4
zmp_ph114_D8
zmp_ph114_E2

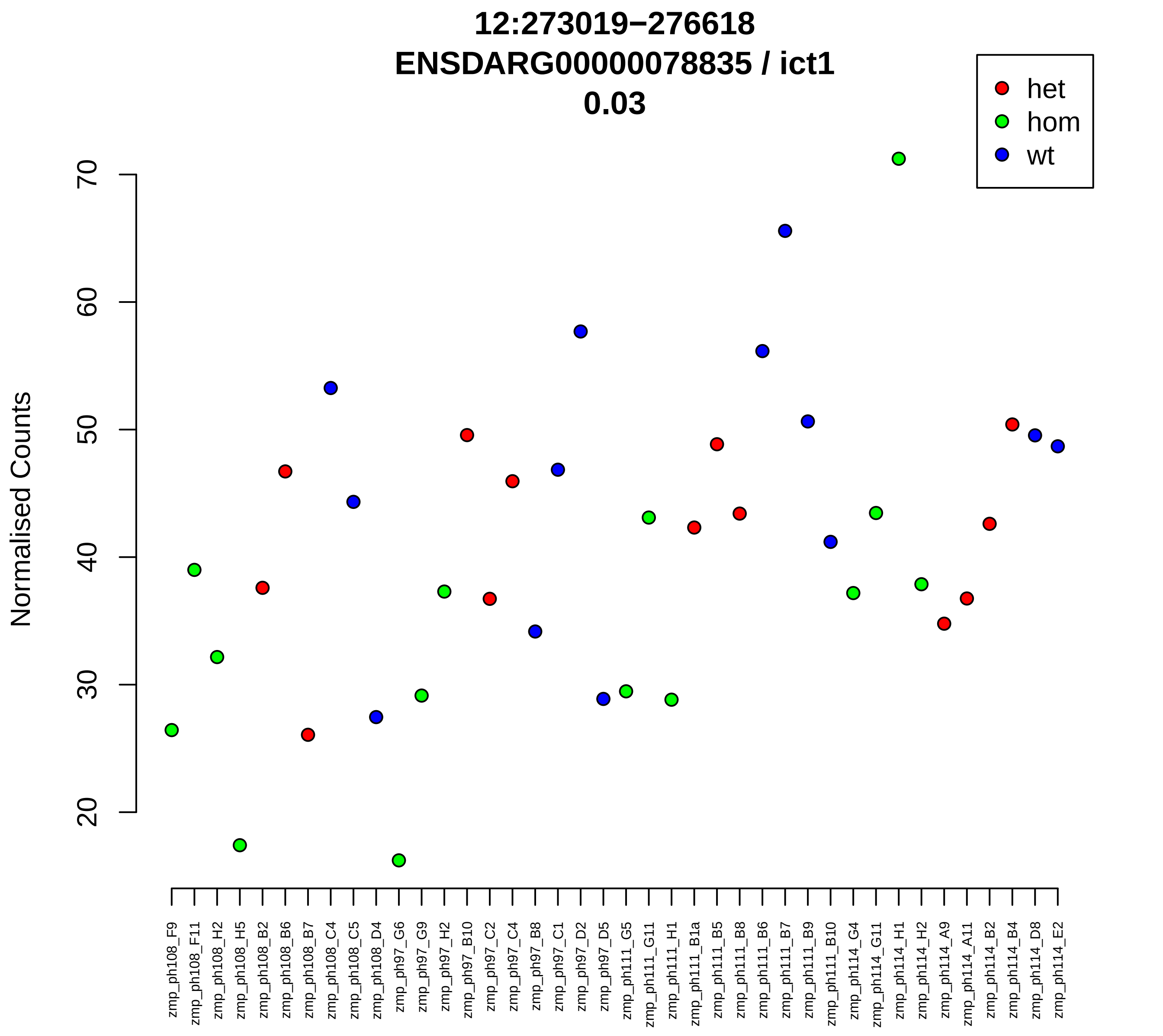
12:273019−276618
ENSDARG00000078835 / ict1
0.03
het
hom
wt
Normalised Counts
20
30
40
50
60
70
zmp_ph108_F9
zmp_ph108_F11
zmp_ph108_H2
zmp_ph108_H5
zmp_ph108_B2
zmp_ph108_B6
zmp_ph108_B7
zmp_ph108_C4
zmp_ph108_C5
zmp_ph108_D4
zmp_ph97_G6
zmp_ph97_G9
zmp_ph97_H2
zmp_ph97_B10
zmp_ph97_C2
zmp_ph97_C4
zmp_ph97_B8
zmp_ph97_C1
zmp_ph97_D2
zmp_ph97_D5
zmp_ph111_G5
zmp_ph111_G11
zmp_ph111_H1
zmp_ph111_B1a
zmp_ph111_B5
zmp_ph111_B8
zmp_ph111_B6
zmp_ph111_B7
zmp_ph111_B9
zmp_ph111_B10
zmp_ph114_G4
zmp_ph114_G11
zmp_ph114_H1
zmp_ph114_H2
zmp_ph114_A9
zmp_ph114_A11
zmp_ph114_B2
zmp_ph114_B4
zmp_ph114_D8
zmp_ph114_E2

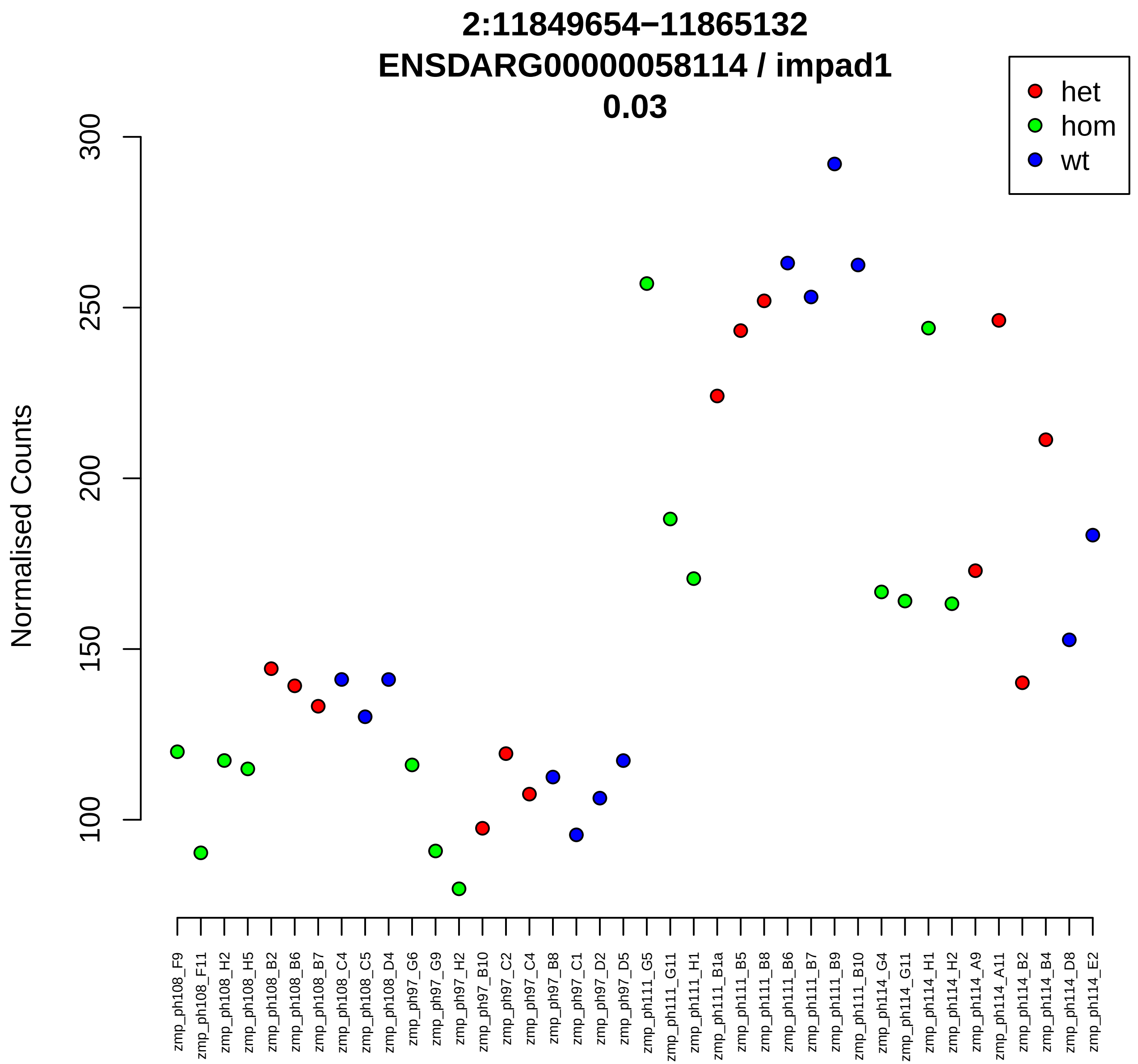

2:11849654−11865132
ENSDARG00000058114 / impad1
0.03
het
hom
wt
Normalised Counts
100
150
200
250
300
zmp_ph108_F9
zmp_ph108_F11
zmp_ph108_H2
zmp_ph108_H5
zmp_ph108_B2
zmp_ph108_B6
zmp_ph108_B7
zmp_ph108_C4
zmp_ph108_C5
zmp_ph108_D4
zmp_ph97_G6
zmp_ph97_G9
zmp_ph97_H2
zmp_ph97_B10
zmp_ph97_C2
zmp_ph97_C4
zmp_ph97_B8
zmp_ph97_C1
zmp_ph97_D2
zmp_ph97_D5
zmp_ph111_G5
zmp_ph111_G11
zmp_ph111_H1
zmp_ph111_B1a
zmp_ph111_B5
zmp_ph111_B8
zmp_ph111_B6
zmp_ph111_B7
zmp_ph111_B9
zmp_ph111_B10
zmp_ph114_G4
zmp_ph114_G11
zmp_ph114_H1
zmp_ph114_H2
zmp_ph114_A9
zmp_ph114_A11
zmp_ph114_B2
zmp_ph114_B4
zmp_ph114_D8
zmp_ph114_E2

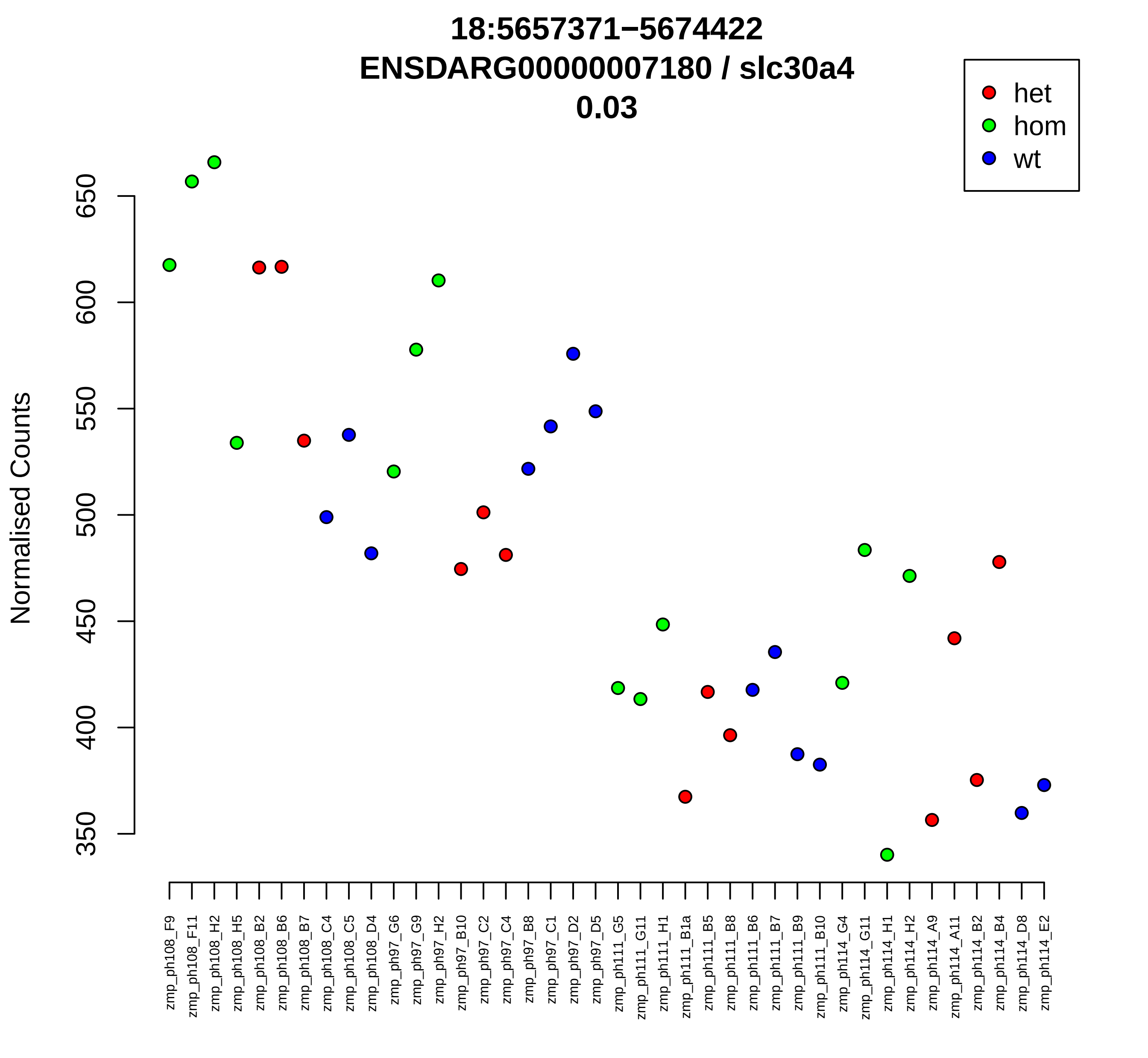

18:5657371−5674422
ENSDARG00000007180 / slc30a4
0.03
het
hom
wt
Normalised Counts
350
400
450
500
550
600
650
zmp_ph108_F9
zmp_ph108_F11
zmp_ph108_H2
zmp_ph108_H5
zmp_ph108_B2
zmp_ph108_B6
zmp_ph108_B7
zmp_ph108_C4
zmp_ph108_C5
zmp_ph108_D4
zmp_ph97_G6
zmp_ph97_G9
zmp_ph97_H2
zmp_ph97_B10
zmp_ph97_C2
zmp_ph97_C4
zmp_ph97_B8
zmp_ph97_C1
zmp_ph97_D2
zmp_ph97_D5
zmp_ph111_G5
zmp_ph111_G11
zmp_ph111_H1
zmp_ph111_B1a
zmp_ph111_B5
zmp_ph111_B8
zmp_ph111_B6
zmp_ph111_B7
zmp_ph111_B9
zmp_ph111_B10
zmp_ph114_G4
zmp_ph114_G11
zmp_ph114_H1
zmp_ph114_H2
zmp_ph114_A9
zmp_ph114_A11
zmp_ph114_B2
zmp_ph114_B4
zmp_ph114_D8
zmp_ph114_E2

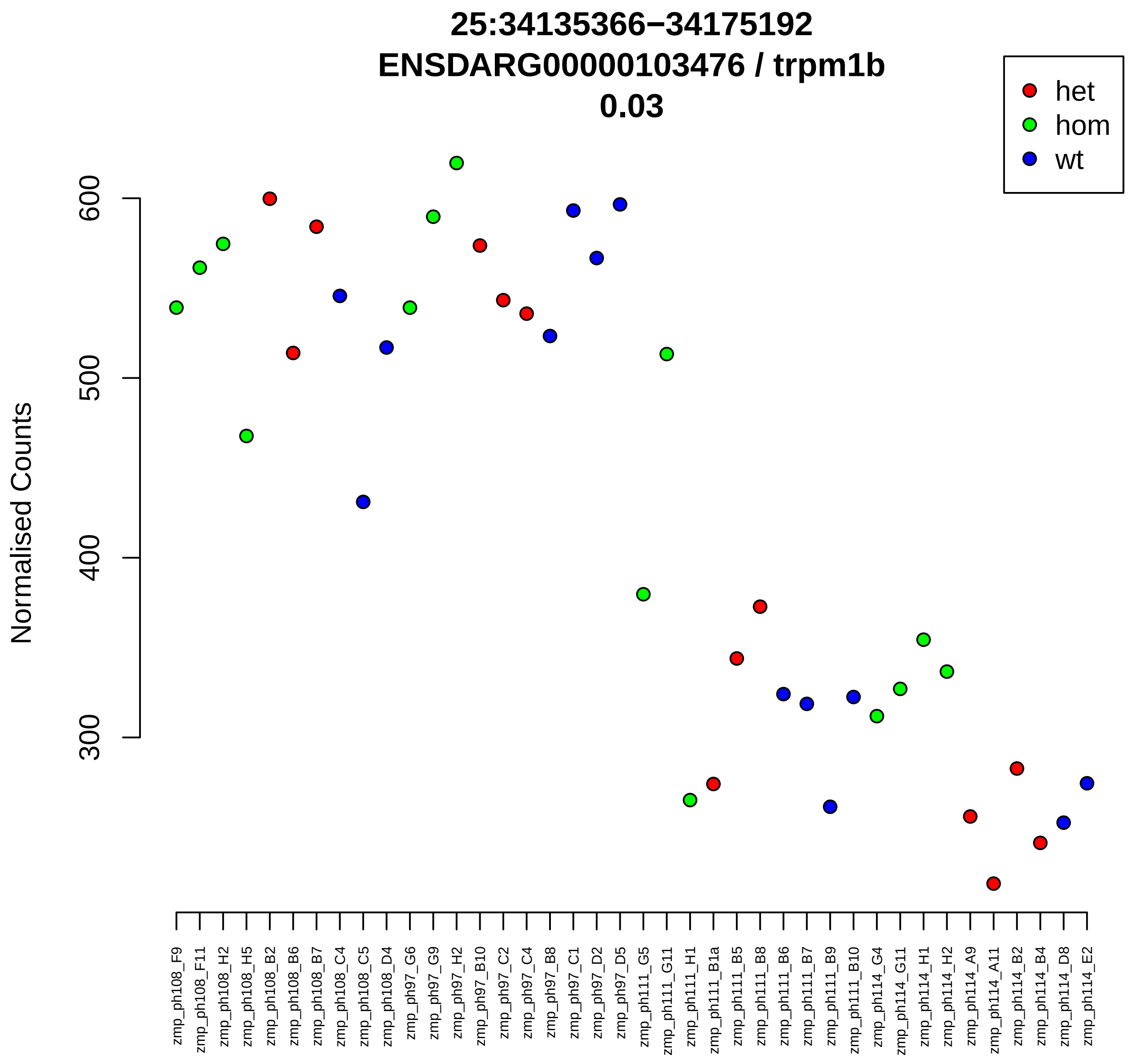
25:34135366−34175192
ENSDARG00000103476 / trpm1b
0.03
het
hom
wt
Normalised Counts
600
500
400
300
zmp_ph108_F9
zmp_ph108_F11
zmp_ph108_H2
zmp_ph108_H5
zmp_ph108_B2
zmp_ph108_B6
zmp_ph108_B7
zmp_ph108_C4
zmp_ph108_C5
zmp_ph108_D4
zmp_ph97_G6
zmp_ph97_G9
zmp_ph97_H2
zmp_ph97_B10
zmp_ph97_C2
zmp_ph97_C4
zmp_ph97_B8
zmp_ph97_C1
zmp_ph97_D2
zmp_ph97_D5
zmp_ph111_G5
zmp_ph111_G11
zmp_ph111_H1
zmp_ph111_B1a
zmp_ph111_B5
zmp_ph111_B8
zmp_ph111_B6
zmp_ph111_B7
zmp_ph111_B9
zmp_ph111_B10
zmp_ph114_G4
zmp_ph114_G11
zmp_ph114_H1
zmp_ph114_H2
zmp_ph114_A9
zmp_ph114_A11
zmp_ph114_B2
zmp_ph114_B4
zmp_ph114_D8
zmp_ph114_E2

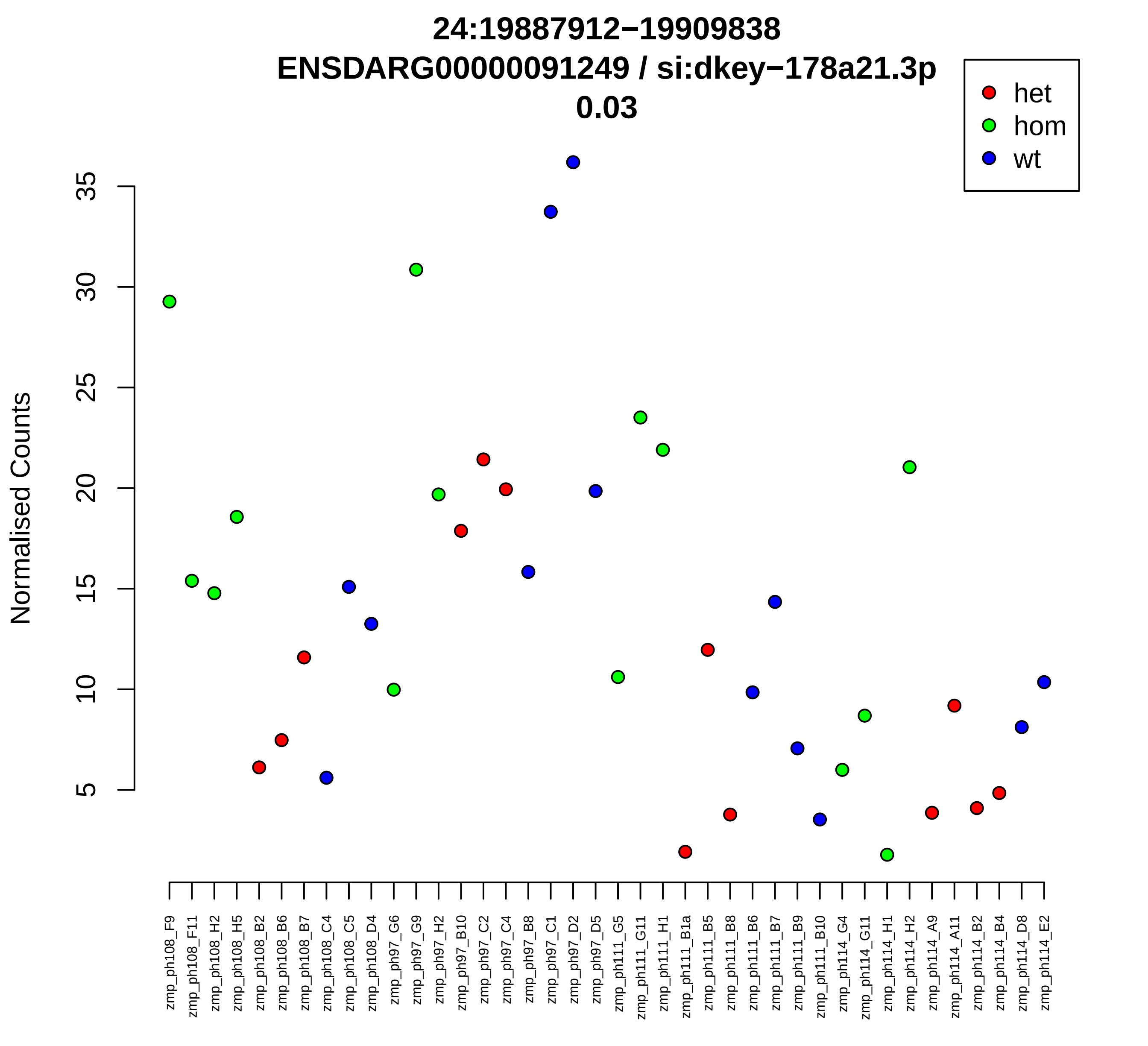

24:19887912−19909838
ENSDARG00000091249 / si:dkey−178a21.3p
0.03
het
hom
wt
Normalised Counts
5
10
15
20
25
30
35
zmp_ph108_F9
zmp_ph108_F11
zmp_ph108_H2
zmp_ph108_H5
zmp_ph108_B2
zmp_ph108_B6
zmp_ph108_B7
zmp_ph108_C4
zmp_ph108_C5
zmp_ph108_D4
zmp_ph97_G6
zmp_ph97_G9
zmp_ph97_H2
zmp_ph97_B10
zmp_ph97_C2
zmp_ph97_C4
zmp_ph97_B8
zmp_ph97_C1
zmp_ph97_D2
zmp_ph97_D5
zmp_ph111_G5
zmp_ph111_G11
zmp_ph111_H1
zmp_ph111_B1a
zmp_ph111_B5
zmp_ph111_B8
zmp_ph111_B6
zmp_ph111_B7
zmp_ph111_B9
zmp_ph111_B10
zmp_ph114_G4
zmp_ph114_G11
zmp_ph114_H1
zmp_ph114_H2
zmp_ph114_A9
zmp_ph114_A11
zmp_ph114_B2
zmp_ph114_B4
zmp_ph114_D8
zmp_ph114_E2

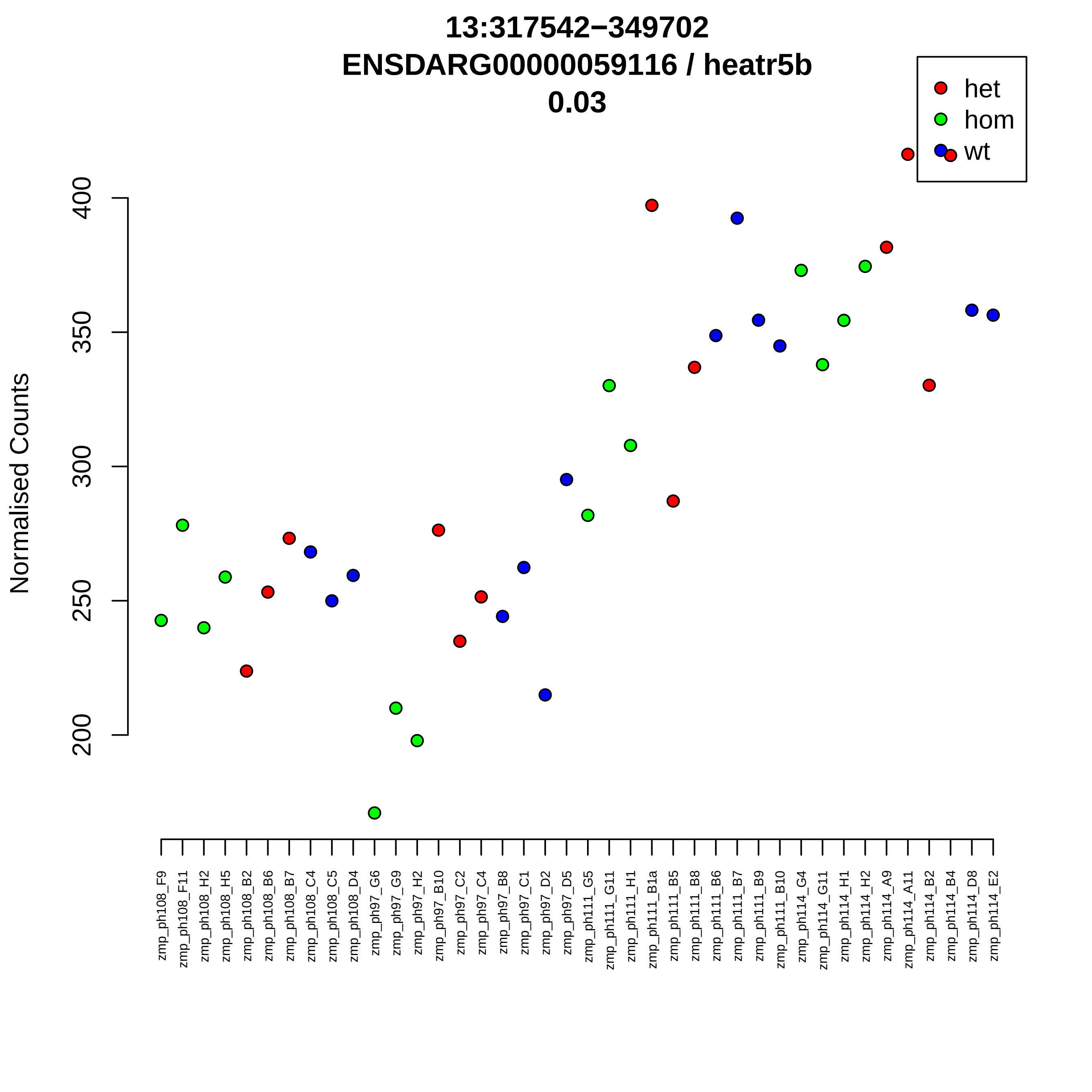

13:317542−349702
ENSDARG00000059116 / heatr5b
0.03
het
hom
wt
Normalised Counts
200
250
300
350
400
zmp_ph108_F9
zmp_ph108_F11
zmp_ph108_H2
zmp_ph108_H5
zmp_ph108_B2
zmp_ph108_B6
zmp_ph108_B7
zmp_ph108_C4
zmp_ph108_C5
zmp_ph108_D4
zmp_ph97_G6
zmp_ph97_G9
zmp_ph97_H2
zmp_ph97_B10
zmp_ph97_C2
zmp_ph97_C4
zmp_ph97_B8
zmp_ph97_C1
zmp_ph97_D2
zmp_ph97_D5
zmp_ph111_G5
zmp_ph111_G11
zmp_ph111_H1
zmp_ph111_B1a
zmp_ph111_B5
zmp_ph111_B8
zmp_ph111_B6
zmp_ph111_B7
zmp_ph111_B9
zmp_ph111_B10
zmp_ph114_G4
zmp_ph114_G11
zmp_ph114_H1
zmp_ph114_H2
zmp_ph114_A9
zmp_ph114_A11
zmp_ph114_B2
zmp_ph114_B4
zmp_ph114_D8
zmp_ph114_E2

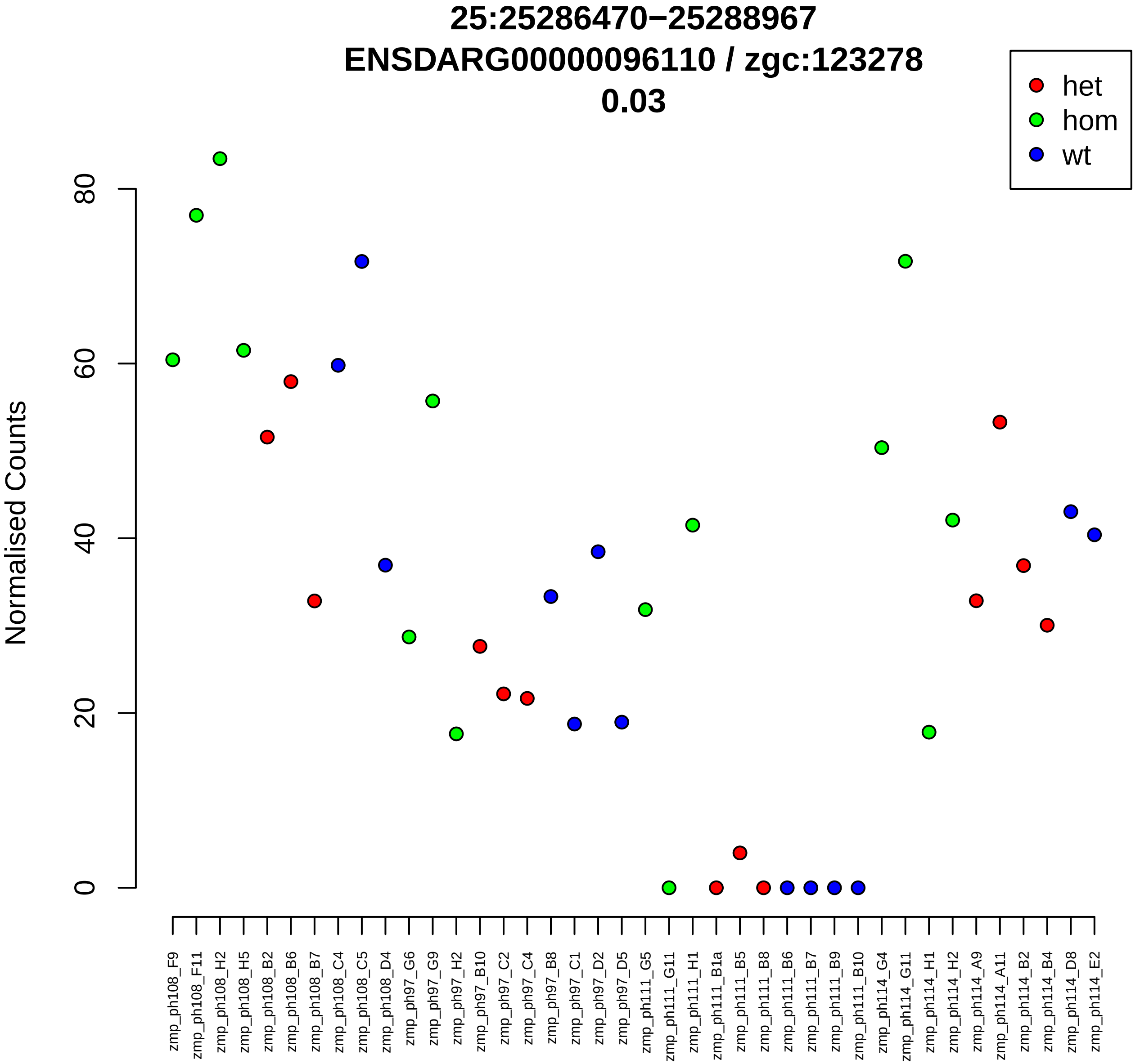
25:25286470−25288967
ENSDARG00000096110 / zgc:123278
0.03
het
hom
wt
Normalised Counts
0
20
40
60
80
zmp_ph108_F9
zmp_ph108_F11
zmp_ph108_H2
zmp_ph108_H5
zmp_ph108_B2
zmp_ph108_B6
zmp_ph108_B7
zmp_ph108_C4
zmp_ph108_C5
zmp_ph108_D4
zmp_ph97_G6
zmp_ph97_G9
zmp_ph97_H2
zmp_ph97_B10
zmp_ph97_C2
zmp_ph97_C4
zmp_ph97_B8
zmp_ph97_C1
zmp_ph97_D2
zmp_ph97_D5
zmp_ph111_G5
zmp_ph111_G11
zmp_ph111_H1
zmp_ph111_B1a
zmp_ph111_B5
zmp_ph111_B8
zmp_ph111_B6
zmp_ph111_B7
zmp_ph111_B9
zmp_ph111_B10
zmp_ph114_G4
zmp_ph114_G11
zmp_ph114_H1
zmp_ph114_H2
zmp_ph114_A9
zmp_ph114_A11
zmp_ph114_B2
zmp_ph114_B4
zmp_ph114_D8
zmp_ph114_E2

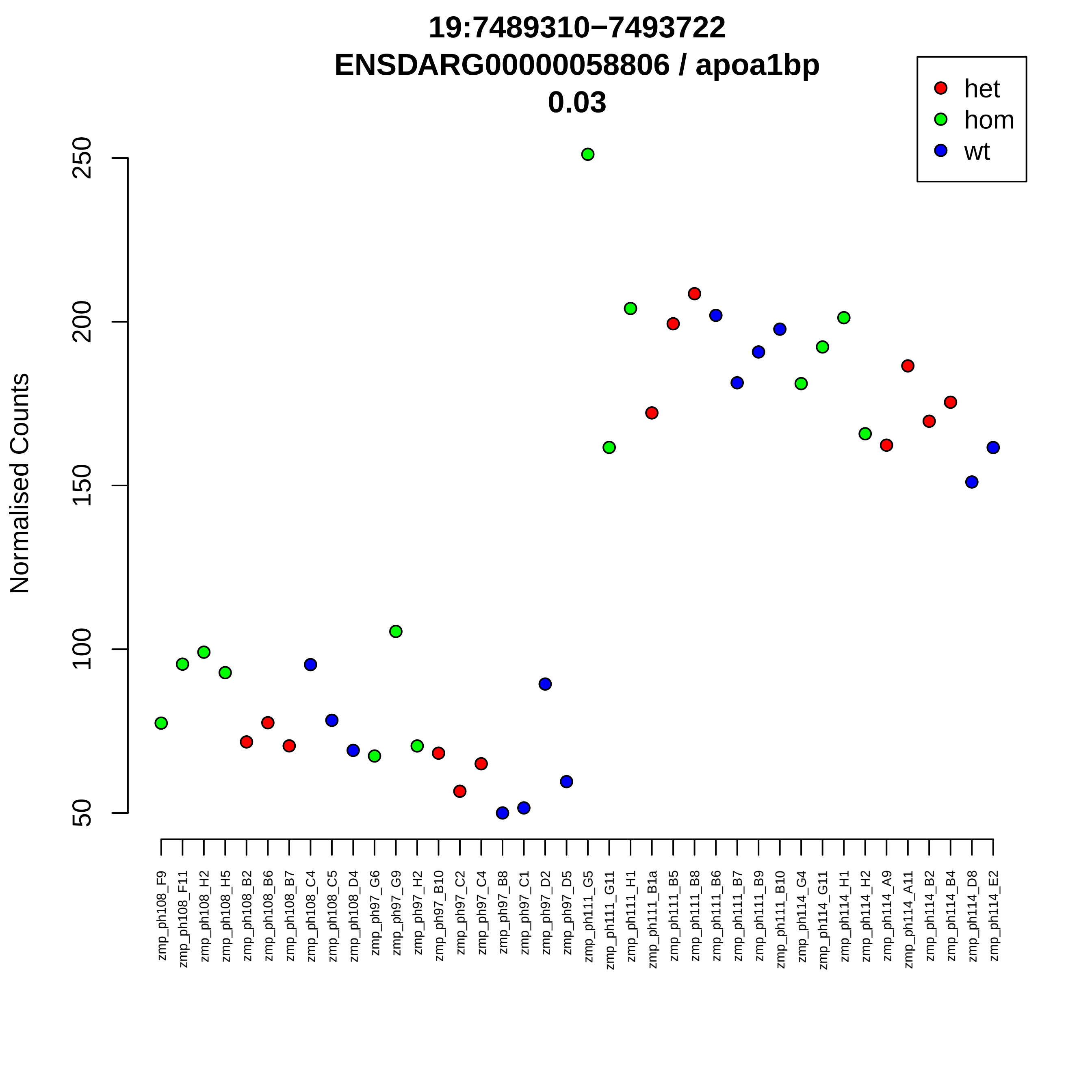
19:7489310−7493722
ENSDARG00000058806 / apoa1bp
0.03
het
hom
wt
Normalised Counts
50
100
150
200
250
zmp_ph108_F9
zmp_ph108_F11
zmp_ph108_H2
zmp_ph108_H5
zmp_ph108_B2
zmp_ph108_B6
zmp_ph108_B7
zmp_ph108_C4
zmp_ph108_C5
zmp_ph108_D4
zmp_ph97_G6
zmp_ph97_G9
zmp_ph97_H2
zmp_ph97_B10
zmp_ph97_C2
zmp_ph97_C4
zmp_ph97_B8
zmp_ph97_C1
zmp_ph97_D2
zmp_ph97_D5
zmp_ph111_G5
zmp_ph111_G11
zmp_ph111_H1
zmp_ph111_B1a
zmp_ph111_B5
zmp_ph111_B8
zmp_ph111_B6
zmp_ph111_B7
zmp_ph111_B9
zmp_ph111_B10
zmp_ph114_G4
zmp_ph114_G11
zmp_ph114_H1
zmp_ph114_H2
zmp_ph114_A9
zmp_ph114_A11
zmp_ph114_B2
zmp_ph114_B4
zmp_ph114_D8
zmp_ph114_E2

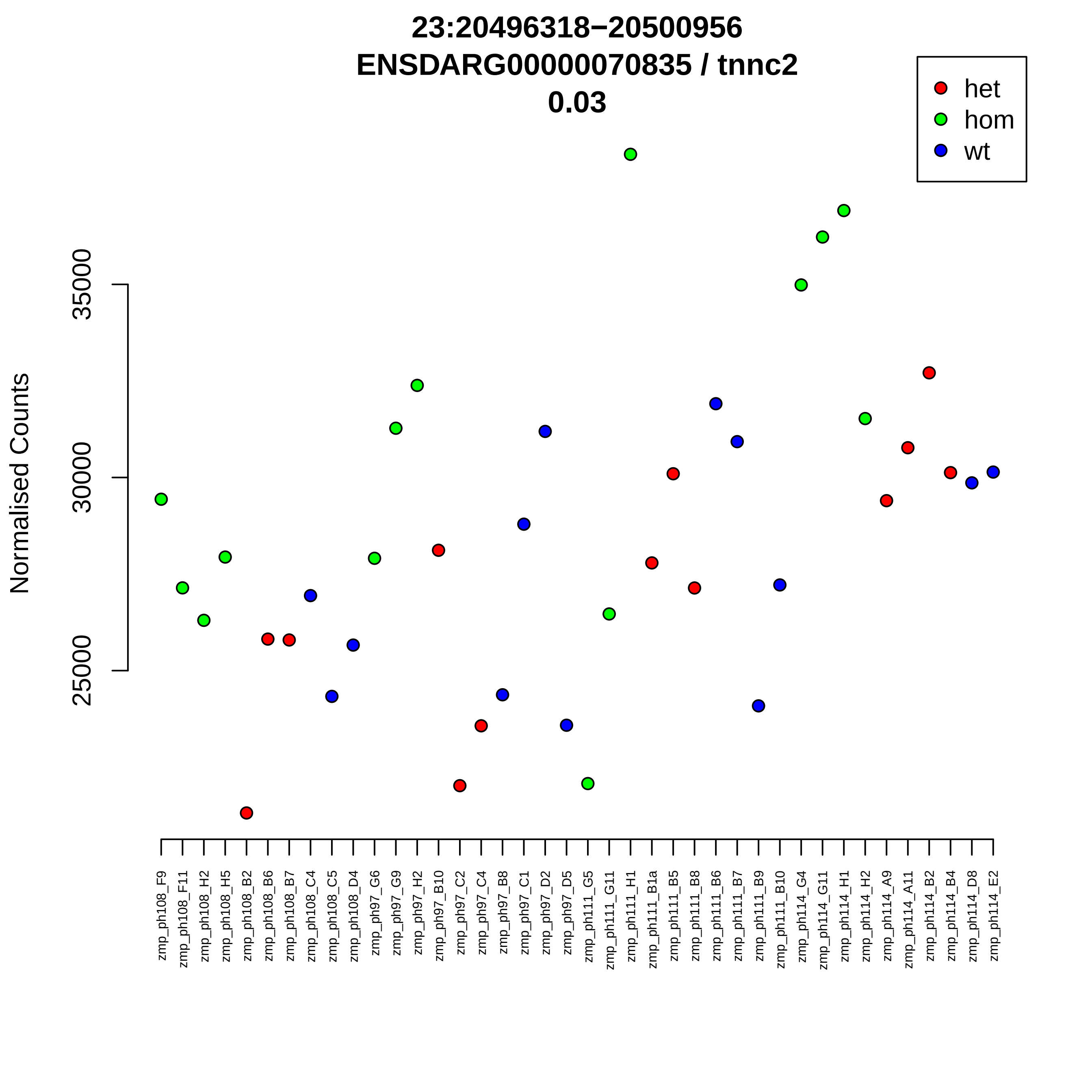

23:20496318−20500956
ENSDARG00000070835 / tnnc2
0.03
het
hom
wt
Normalised Counts
25000
30000
35000
zmp_ph108_F9
zmp_ph108_F11
zmp_ph108_H2
zmp_ph108_H5
zmp_ph108_B2
zmp_ph108_B6
zmp_ph108_B7
zmp_ph108_C4
zmp_ph108_C5
zmp_ph108_D4
zmp_ph97_G6
zmp_ph97_G9
zmp_ph97_H2
zmp_ph97_B10
zmp_ph97_C2
zmp_ph97_C4
zmp_ph97_B8
zmp_ph97_C1
zmp_ph97_D2
zmp_ph97_D5
zmp_ph111_G5
zmp_ph111_G11
zmp_ph111_H1
zmp_ph111_B1a
zmp_ph111_B5
zmp_ph111_B8
zmp_ph111_B6
zmp_ph111_B7
zmp_ph111_B9
zmp_ph111_B10
zmp_ph114_G4
zmp_ph114_G11
zmp_ph114_H1
zmp_ph114_H2
zmp_ph114_A9
zmp_ph114_A11
zmp_ph114_B2
zmp_ph114_B4
zmp_ph114_D8
zmp_ph114_E2

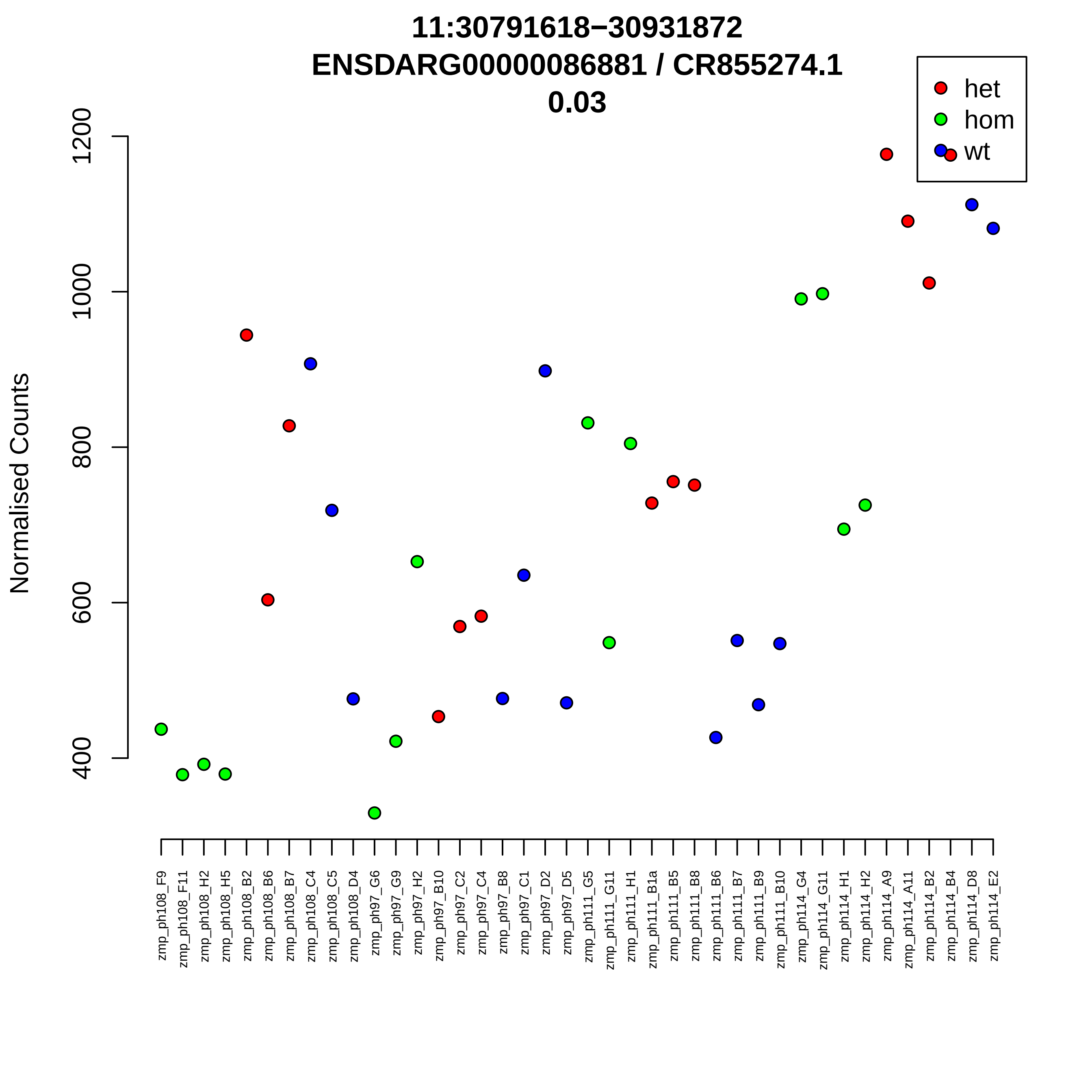
11:30791618−30931872
ENSDARG00000086881 / CR855274.1
0.03
het
hom
wt
Normalised Counts
400
600
800
1000
1200
zmp_ph108_F9
zmp_ph108_F11
zmp_ph108_H2
zmp_ph108_H5
zmp_ph108_B2
zmp_ph108_B6
zmp_ph108_B7
zmp_ph108_C4
zmp_ph108_C5
zmp_ph108_D4
zmp_ph97_G6
zmp_ph97_G9
zmp_ph97_H2
zmp_ph97_B10
zmp_ph97_C2
zmp_ph97_C4
zmp_ph97_B8
zmp_ph97_C1
zmp_ph97_D2
zmp_ph97_D5
zmp_ph111_G5
zmp_ph111_G11
zmp_ph111_H1
zmp_ph111_B1a
zmp_ph111_B5
zmp_ph111_B8
zmp_ph111_B6
zmp_ph111_B7
zmp_ph111_B9
zmp_ph111_B10
zmp_ph114_G4
zmp_ph114_G11
zmp_ph114_H1
zmp_ph114_H2
zmp_ph114_A9
zmp_ph114_A11
zmp_ph114_B2
zmp_ph114_B4
zmp_ph114_D8
zmp_ph114_E2

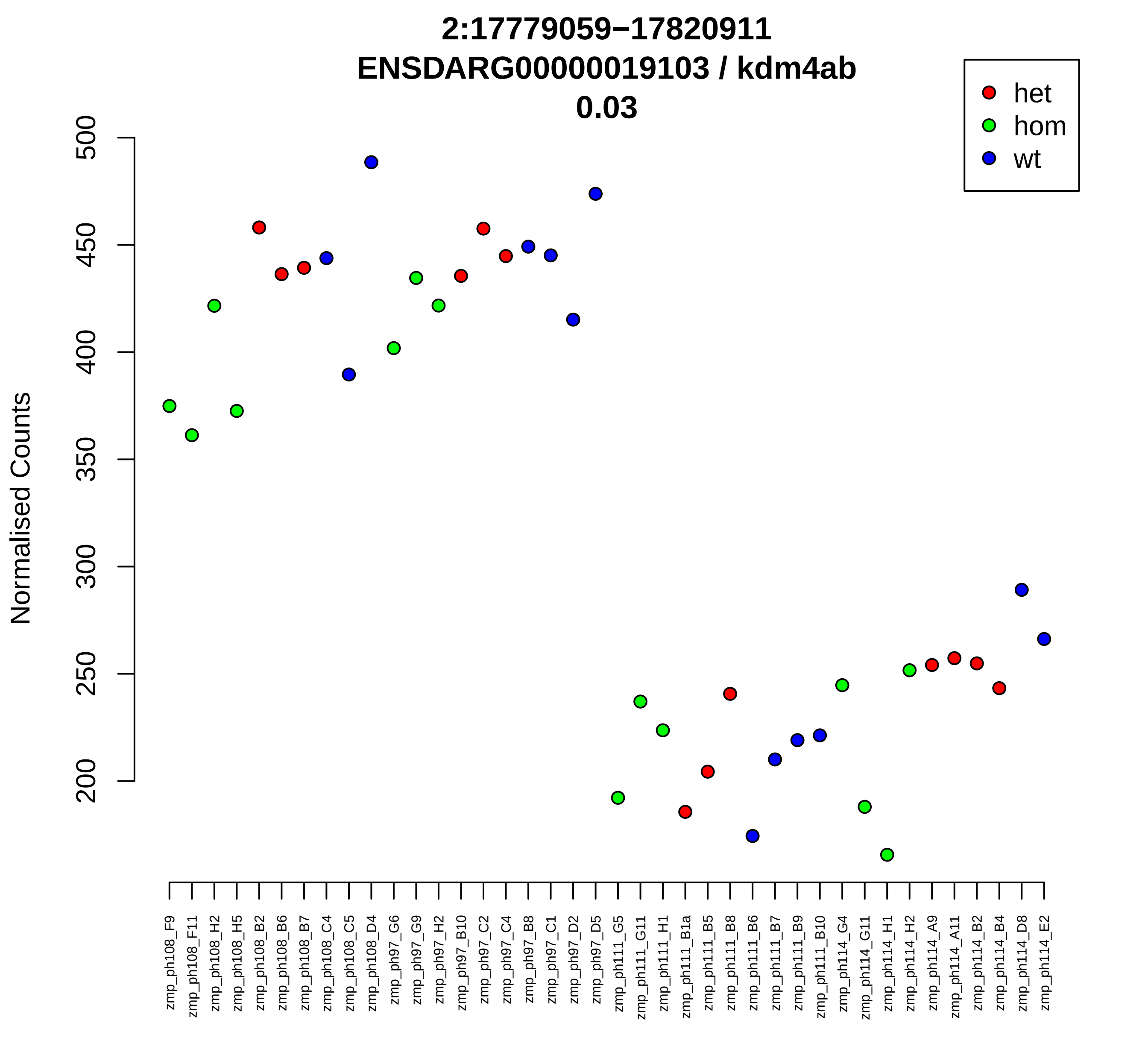
2:17779059−17820911
ENSDARG00000019103 / kdm4ab
0.03
Normalised Counts
500
450
400
350
300
250
200
het
hom
wt
zmp_ph108_F9
zmp_ph108_F11
zmp_ph108_H2
zmp_ph108_H5
zmp_ph108_B2
zmp_ph108_B6
zmp_ph108_B7
zmp_ph108_C4
zmp_ph108_C5
zmp_ph108_D4
zmp_ph97_G6
zmp_ph97_G9
zmp_ph97_H2
zmp_ph97_B10
zmp_ph97_C2
zmp_ph97_C4
zmp_ph97_B8
zmp_ph97_C1
zmp_ph97_D2
zmp_ph97_D5
zmp_ph111_G5
zmp_ph111_G11
zmp_ph111_H1
zmp_ph111_B1a
zmp_ph111_B5
zmp_ph111_B8
zmp_ph111_B6
zmp_ph111_B7
zmp_ph111_B9
zmp_ph111_B10
zmp_ph114_G4
zmp_ph114_G11
zmp_ph114_H1
zmp_ph114_H2
zmp_ph114_A9
zmp_ph114_A11
zmp_ph114_B2
zmp_ph114_B4
zmp_ph114_D8
zmp_ph114_E2

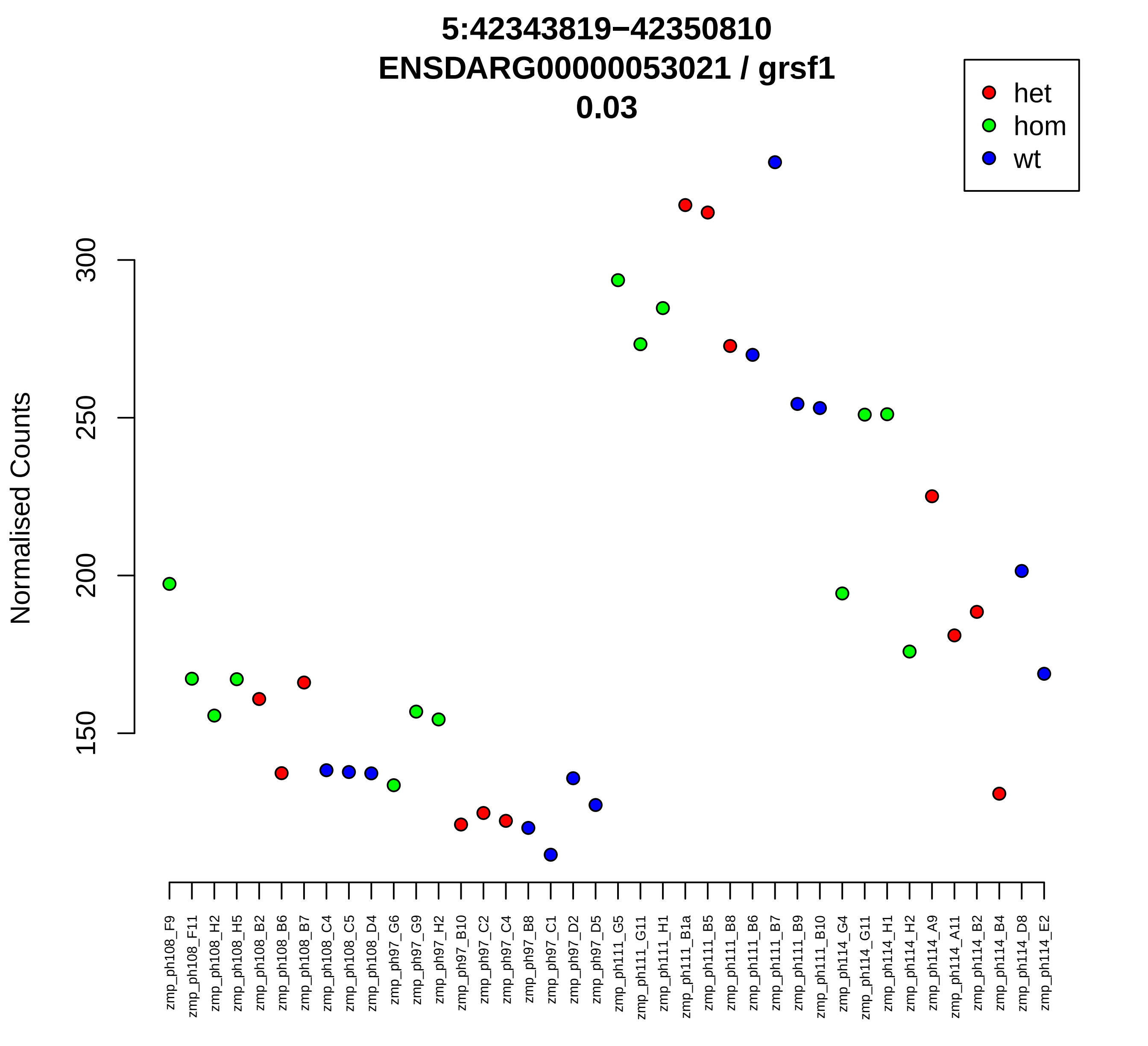

5:42343819−42350810
ENSDARG00000053021 / grsf1
0.03
het
hom
wt
Normalised Counts
150
200
250
300
zmp_ph108_F9
zmp_ph108_F11
zmp_ph108_H2
zmp_ph108_H5
zmp_ph108_B2
zmp_ph108_B6
zmp_ph108_B7
zmp_ph108_C4
zmp_ph108_C5
zmp_ph108_D4
zmp_ph97_G6
zmp_ph97_G9
zmp_ph97_H2
zmp_ph97_B10
zmp_ph97_C2
zmp_ph97_C4
zmp_ph97_B8
zmp_ph97_C1
zmp_ph97_D2
zmp_ph97_D5
zmp_ph111_G5
zmp_ph111_G11
zmp_ph111_H1
zmp_ph111_B1a
zmp_ph111_B5
zmp_ph111_B8
zmp_ph111_B6
zmp_ph111_B7
zmp_ph111_B9
zmp_ph111_B10
zmp_ph114_G4
zmp_ph114_G11
zmp_ph114_H1
zmp_ph114_H2
zmp_ph114_A9
zmp_ph114_A11
zmp_ph114_B2
zmp_ph114_B4
zmp_ph114_D8
zmp_ph114_E2

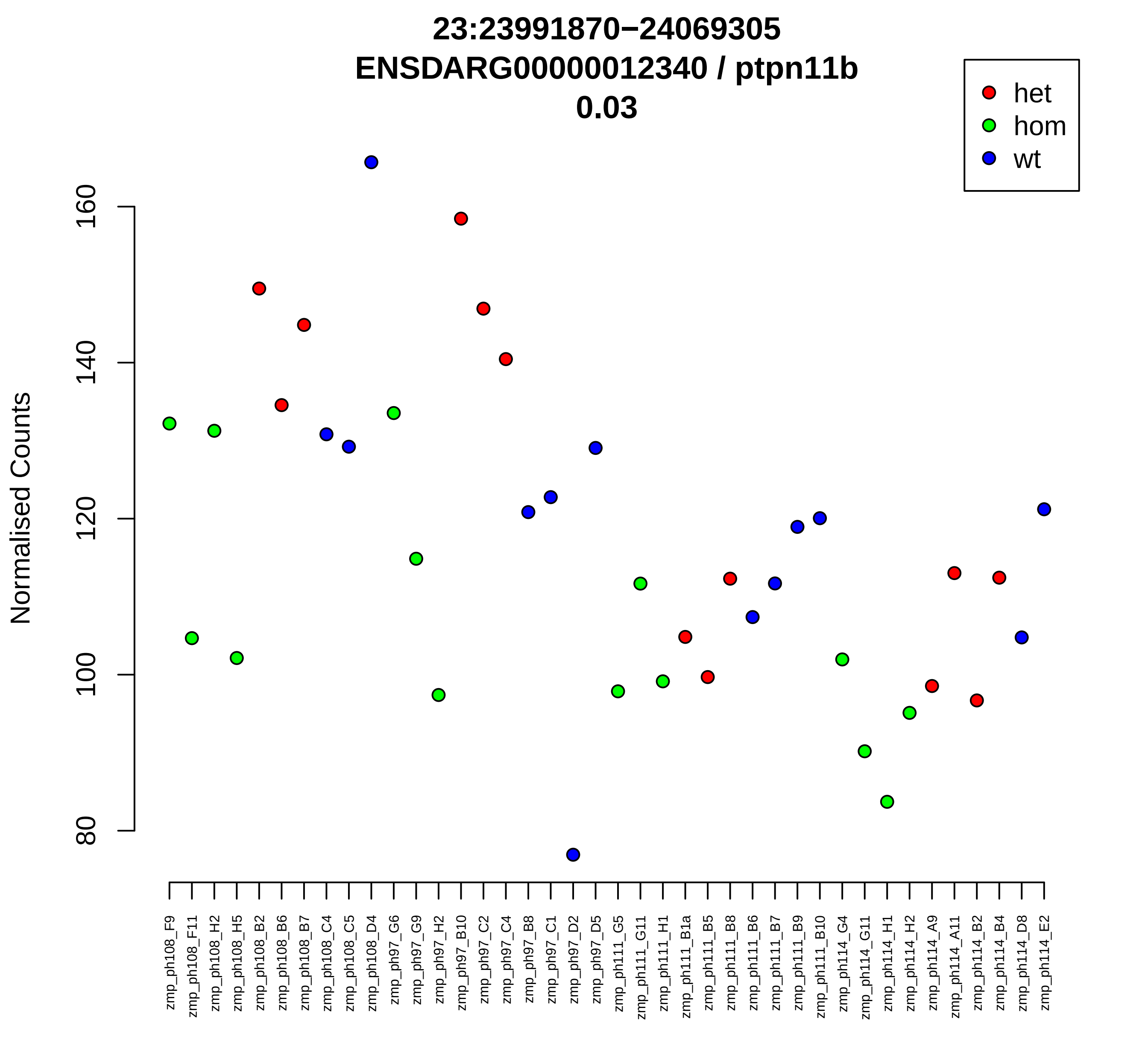
23:23991870−24069305
ENSDARG00000012340 / ptpn11b
0.03
het
hom
wt
Normalised Counts
80
100
120
140
160
zmp_ph108_F9
zmp_ph108_F11
zmp_ph108_H2
zmp_ph108_H5
zmp_ph108_B2
zmp_ph108_B6
zmp_ph108_B7
zmp_ph108_C4
zmp_ph108_C5
zmp_ph108_D4
zmp_ph97_G6
zmp_ph97_G9
zmp_ph97_H2
zmp_ph97_B10
zmp_ph97_C2
zmp_ph97_C4
zmp_ph97_B8
zmp_ph97_C1
zmp_ph97_D2
zmp_ph97_D5
zmp_ph111_G5
zmp_ph111_G11
zmp_ph111_H1
zmp_ph111_B1a
zmp_ph111_B5
zmp_ph111_B8
zmp_ph111_B6
zmp_ph111_B7
zmp_ph111_B9
zmp_ph111_B10
zmp_ph114_G4
zmp_ph114_G11
zmp_ph114_H1
zmp_ph114_H2
zmp_ph114_A9
zmp_ph114_A11
zmp_ph114_B2
zmp_ph114_B4
zmp_ph114_D8
zmp_ph114_E2

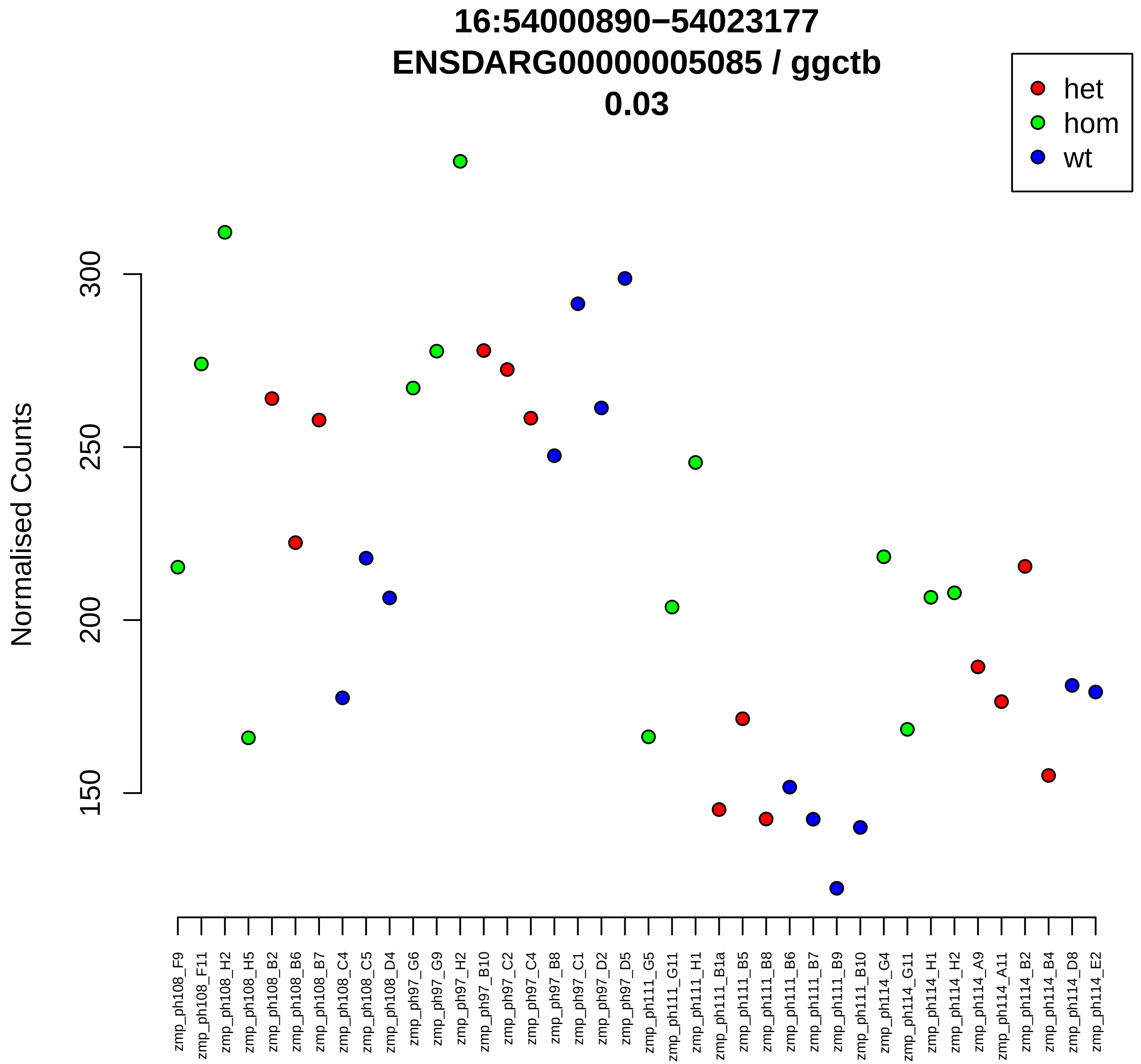

16:54000890−54023177
ENSDARG00000005085 / ggctb
0.03
het
hom
wt
Normalised Counts
150
200
250
300
zmp_ph108_F9
zmp_ph108_F11
zmp_ph108_H2
zmp_ph108_H5
zmp_ph108_B2
zmp_ph108_B6
zmp_ph108_B7
zmp_ph108_C4
zmp_ph108_C5
zmp_ph108_D4
zmp_ph97_G6
zmp_ph97_G9
zmp_ph97_H2
zmp_ph97_B10
zmp_ph97_C2
zmp_ph97_C4
zmp_ph97_B8
zmp_ph97_C1
zmp_ph97_D2
zmp_ph97_D5
zmp_ph111_G5
zmp_ph111_G11
zmp_ph111_H1
zmp_ph111_B1a
zmp_ph111_B5
zmp_ph111_B8
zmp_ph111_B6
zmp_ph111_B7
zmp_ph111_B9
zmp_ph111_B10
zmp_ph114_G4
zmp_ph114_G11
zmp_ph114_H1
zmp_ph114_H2
zmp_ph114_A9
zmp_ph114_A11
zmp_ph114_B2
zmp_ph114_B4
zmp_ph114_D8
zmp_ph114_E2

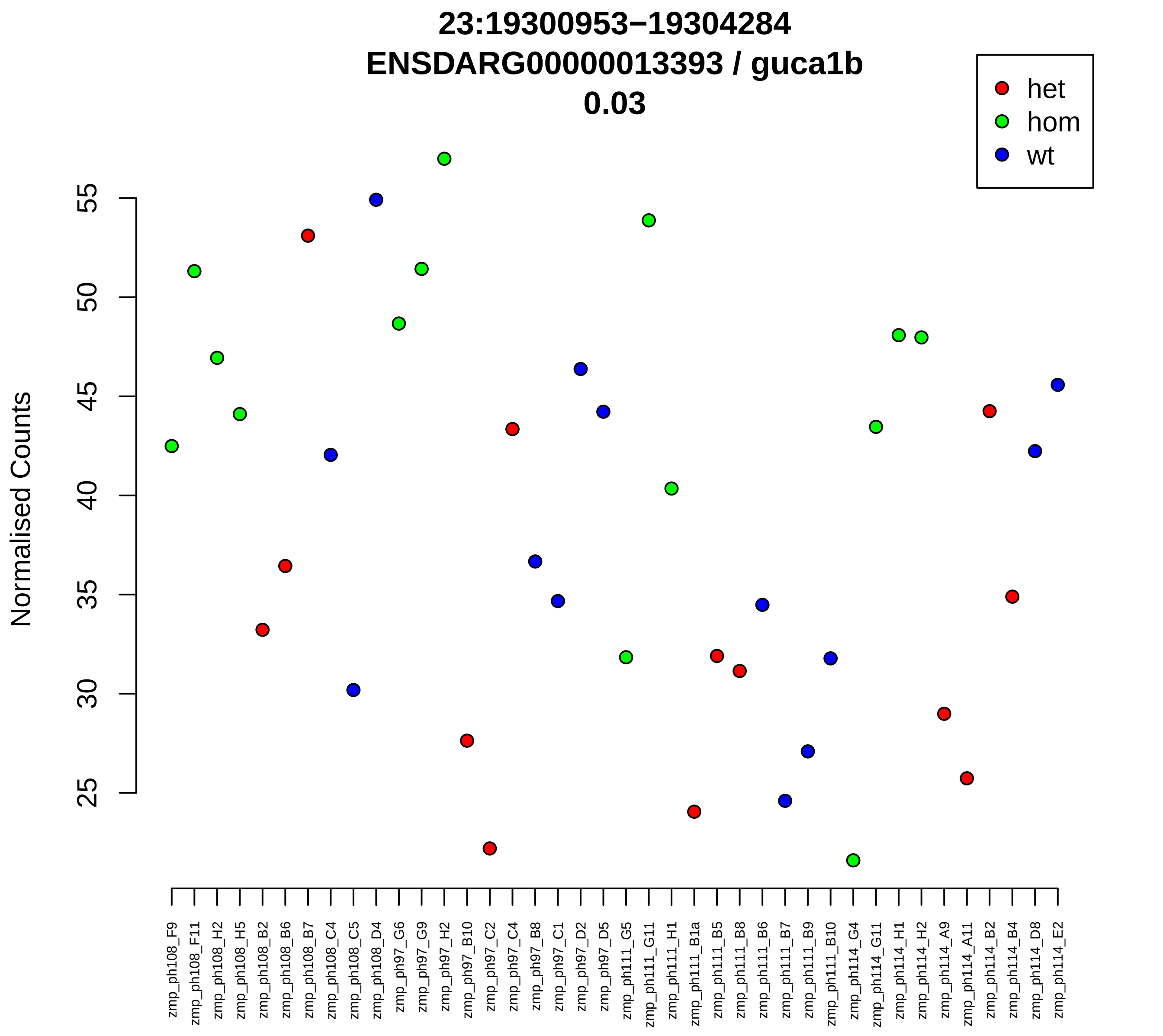
23:19300953−19304284
ENSDARG00000013393 / guca1b
0.03
het
hom
wt
Normalised Counts
25
30
35
40
45
50
55
zmp_ph108_F9
zmp_ph108_F11
zmp_ph108_H2
zmp_ph108_H5
zmp_ph108_B2
zmp_ph108_B6
zmp_ph108_B7
zmp_ph108_C4
zmp_ph108_C5
zmp_ph108_D4
zmp_ph97_G6
zmp_ph97_G9
zmp_ph97_H2
zmp_ph97_B10
zmp_ph97_C2
zmp_ph97_C4
zmp_ph97_B8
zmp_ph97_C1
zmp_ph97_D2
zmp_ph97_D5
zmp_ph111_G5
zmp_ph111_G11
zmp_ph111_H1
zmp_ph111_B1a
zmp_ph111_B5
zmp_ph111_B8
zmp_ph111_B6
zmp_ph111_B7
zmp_ph111_B9
zmp_ph111_B10
zmp_ph114_G4
zmp_ph114_G11
zmp_ph114_H1
zmp_ph114_H2
zmp_ph114_A9
zmp_ph114_A11
zmp_ph114_B2
zmp_ph114_B4
zmp_ph114_D8
zmp_ph114_E2

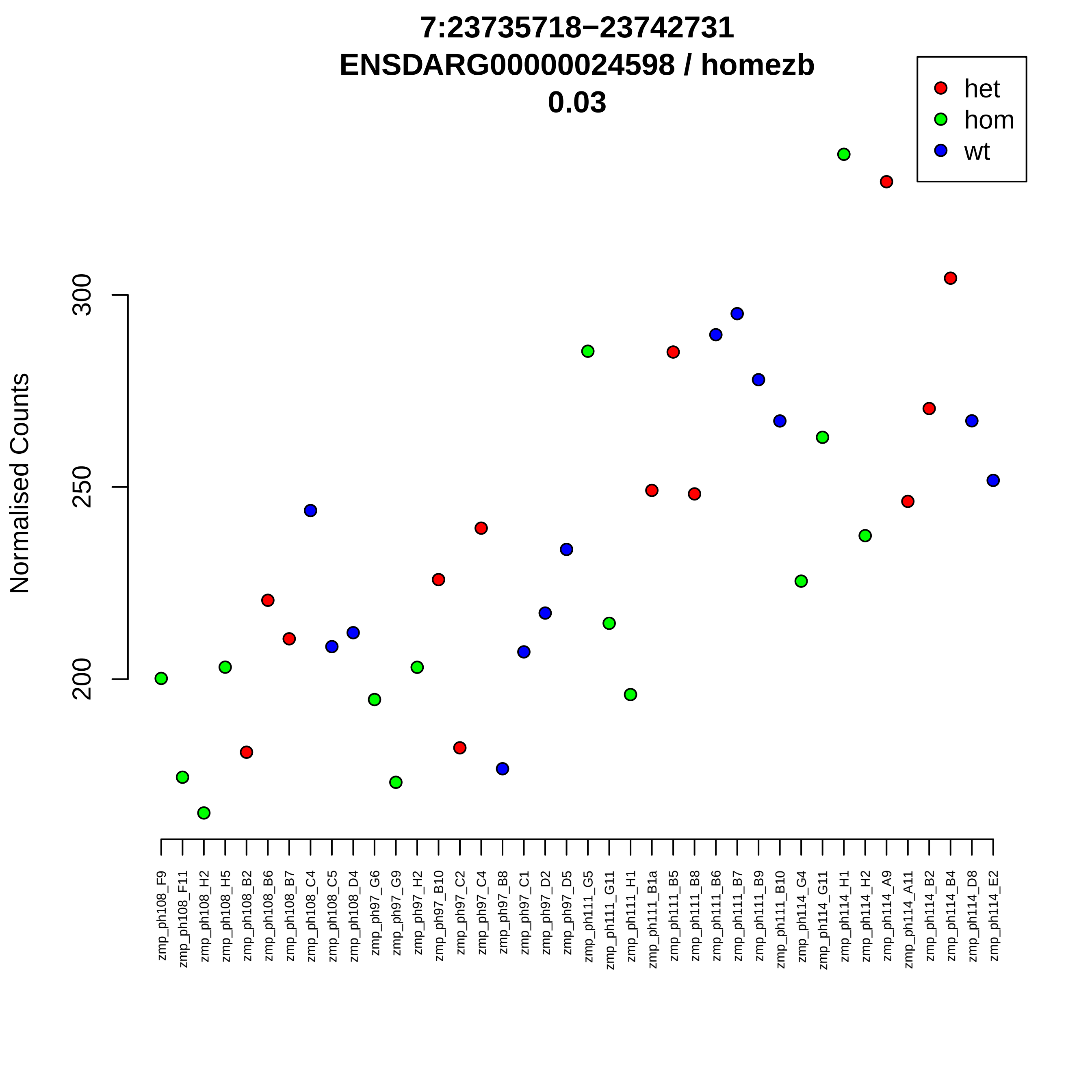
7:23735718−23742731
ENSDARG00000024598 / homezb
0.03
het
hom
wt
Normalised Counts
200
250
300
zmp_ph108_F9
zmp_ph108_F11
zmp_ph108_H2
zmp_ph108_H5
zmp_ph108_B2
zmp_ph108_B6
zmp_ph108_B7
zmp_ph108_C4
zmp_ph108_C5
zmp_ph108_D4
zmp_ph97_G6
zmp_ph97_G9
zmp_ph97_H2
zmp_ph97_B10
zmp_ph97_C2
zmp_ph97_C4
zmp_ph97_B8
zmp_ph97_C1
zmp_ph97_D2
zmp_ph97_D5
zmp_ph111_G5
zmp_ph111_G11
zmp_ph111_H1
zmp_ph111_B1a
zmp_ph111_B5
zmp_ph111_B8
zmp_ph111_B6
zmp_ph111_B7
zmp_ph111_B9
zmp_ph111_B10
zmp_ph114_G4
zmp_ph114_G11
zmp_ph114_H1
zmp_ph114_H2
zmp_ph114_A9
zmp_ph114_A11
zmp_ph114_B2
zmp_ph114_B4
zmp_ph114_D8
zmp_ph114_E2

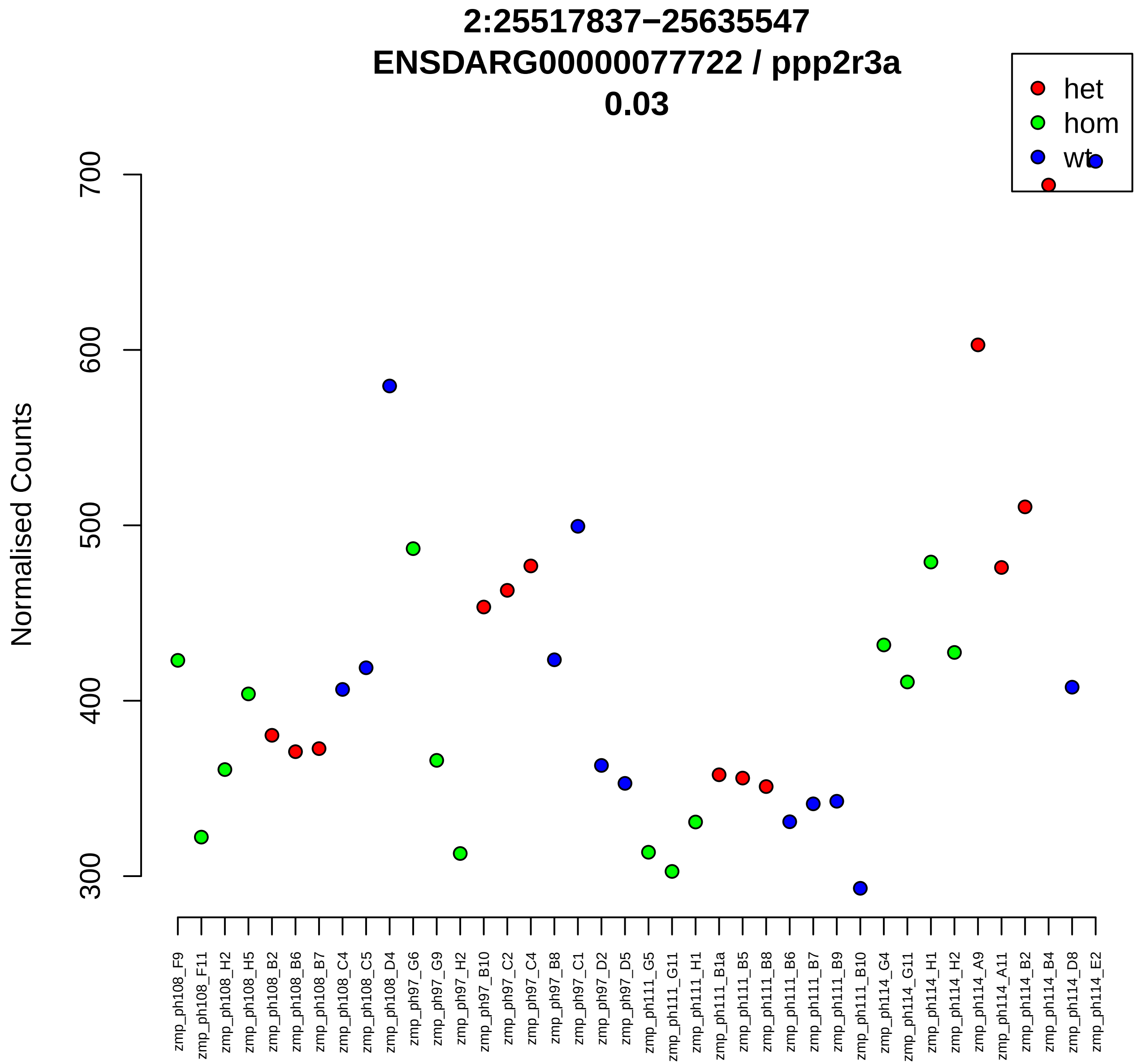
2:25517837−25635547
ENSDARG00000077722 / ppp2r3a
0.03
het
hom
wt
Normalised Counts
700
600
500
400
300
zmp_ph108_F9
zmp_ph108_F11
zmp_ph108_H2
zmp_ph108_H5
zmp_ph108_B2
zmp_ph108_B6
zmp_ph108_B7
zmp_ph108_C4
zmp_ph108_C5
zmp_ph108_D4
zmp_ph97_G6
zmp_ph97_G9
zmp_ph97_H2
zmp_ph97_B10
zmp_ph97_C2
zmp_ph97_C4
zmp_ph97_B8
zmp_ph97_C1
zmp_ph97_D2
zmp_ph97_D5
zmp_ph111_G5
zmp_ph111_G11
zmp_ph111_H1
zmp_ph111_B1a
zmp_ph111_B5
zmp_ph111_B8
zmp_ph111_B6
zmp_ph111_B7
zmp_ph111_B9
zmp_ph111_B10
zmp_ph114_G4
zmp_ph114_G11
zmp_ph114_H1
zmp_ph114_H2
zmp_ph114_A9
zmp_ph114_A11
zmp_ph114_B2
zmp_ph114_B4
zmp_ph114_D8
zmp_ph114_E2

1:249897−262959
ENSDARG00000058803 / grk1a
0.03

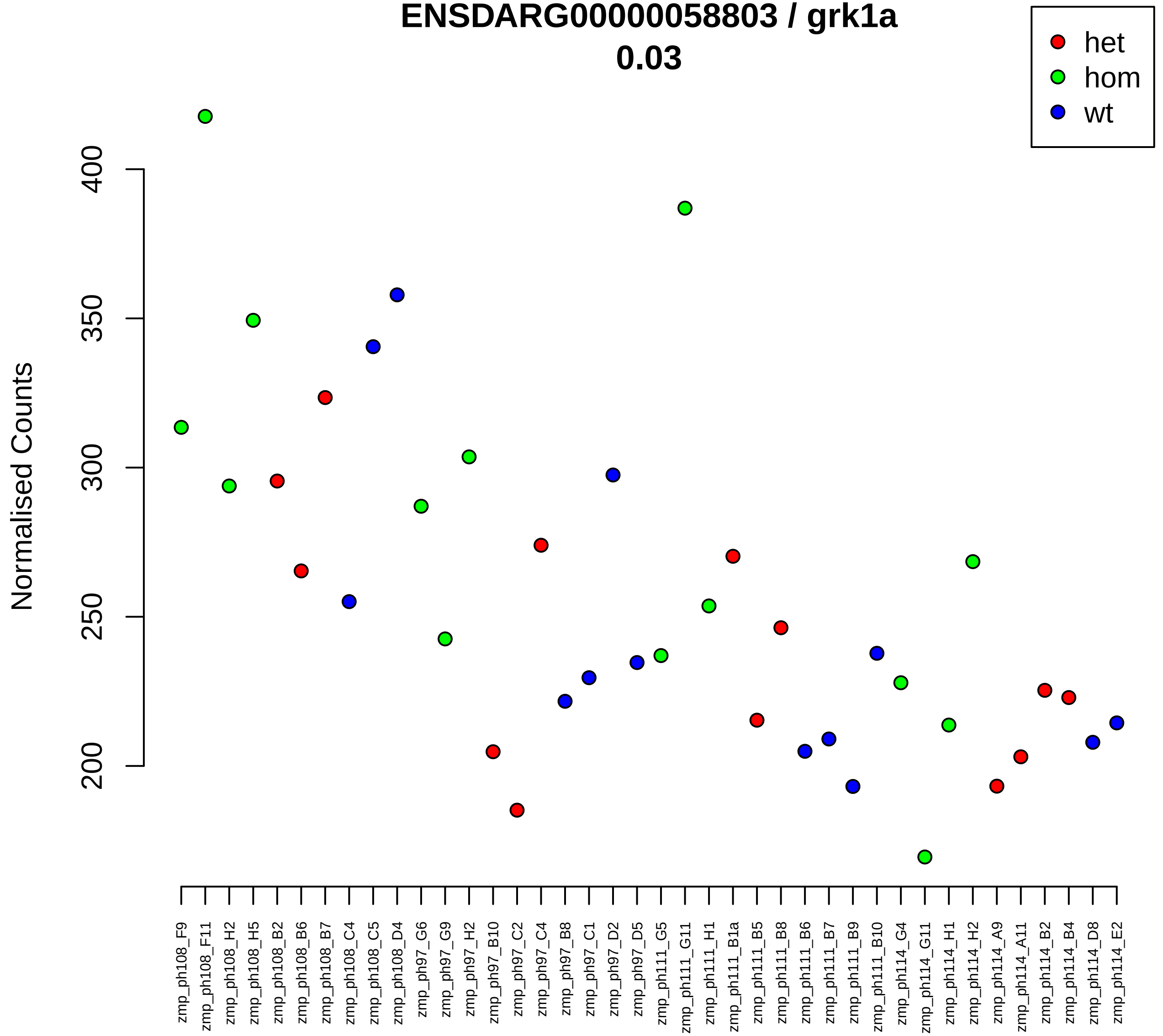

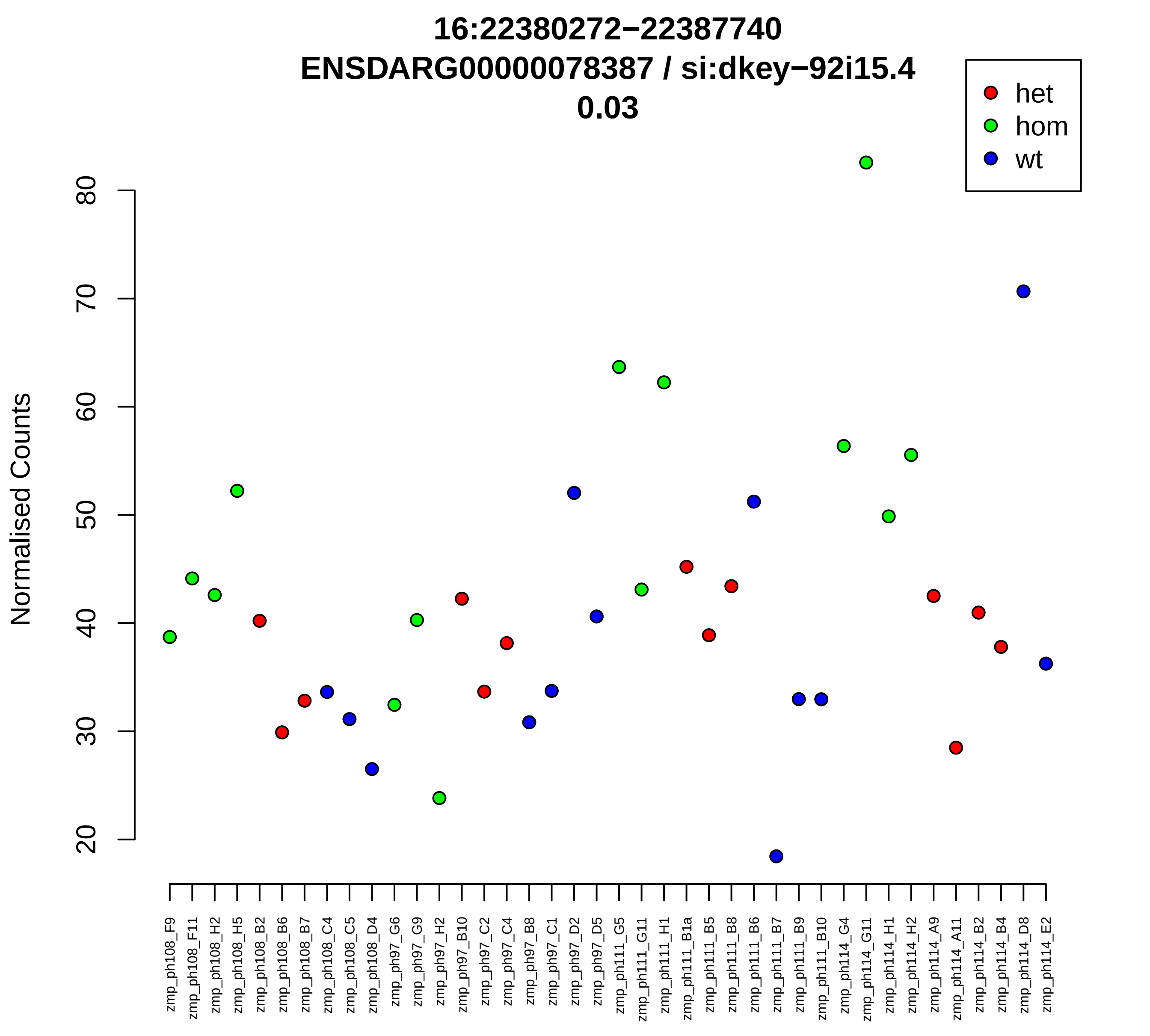
16:22380272−22387740
ENSDARG00000078387 / si:dkey−92i15.4
0.03
het
hom
wt
Normalised Counts
20
30
40
50
60
70
80
zmp_ph108_F9
zmp_ph108_F11
zmp_ph108_H2
zmp_ph108_H5
zmp_ph108_B2
zmp_ph108_B6
zmp_ph108_B7
zmp_ph108_C4
zmp_ph108_C5
zmp_ph108_D4
zmp_ph97_G6
zmp_ph97_G9
zmp_ph97_H2
zmp_ph97_B10
zmp_ph97_C2
zmp_ph97_C4
zmp_ph97_B8
zmp_ph97_C1
zmp_ph97_D2
zmp_ph97_D5
zmp_ph111_G5
zmp_ph111_G11
zmp_ph111_H1
zmp_ph111_B1a
zmp_ph111_B5
zmp_ph111_B8
zmp_ph111_B6
zmp_ph111_B7
zmp_ph111_B9
zmp_ph111_B10
zmp_ph114_G4
zmp_ph114_G11
zmp_ph114_H1
zmp_ph114_H2
zmp_ph114_A9
zmp_ph114_A11
zmp_ph114_B2
zmp_ph114_B4
zmp_ph114_D8
zmp_ph114_E2

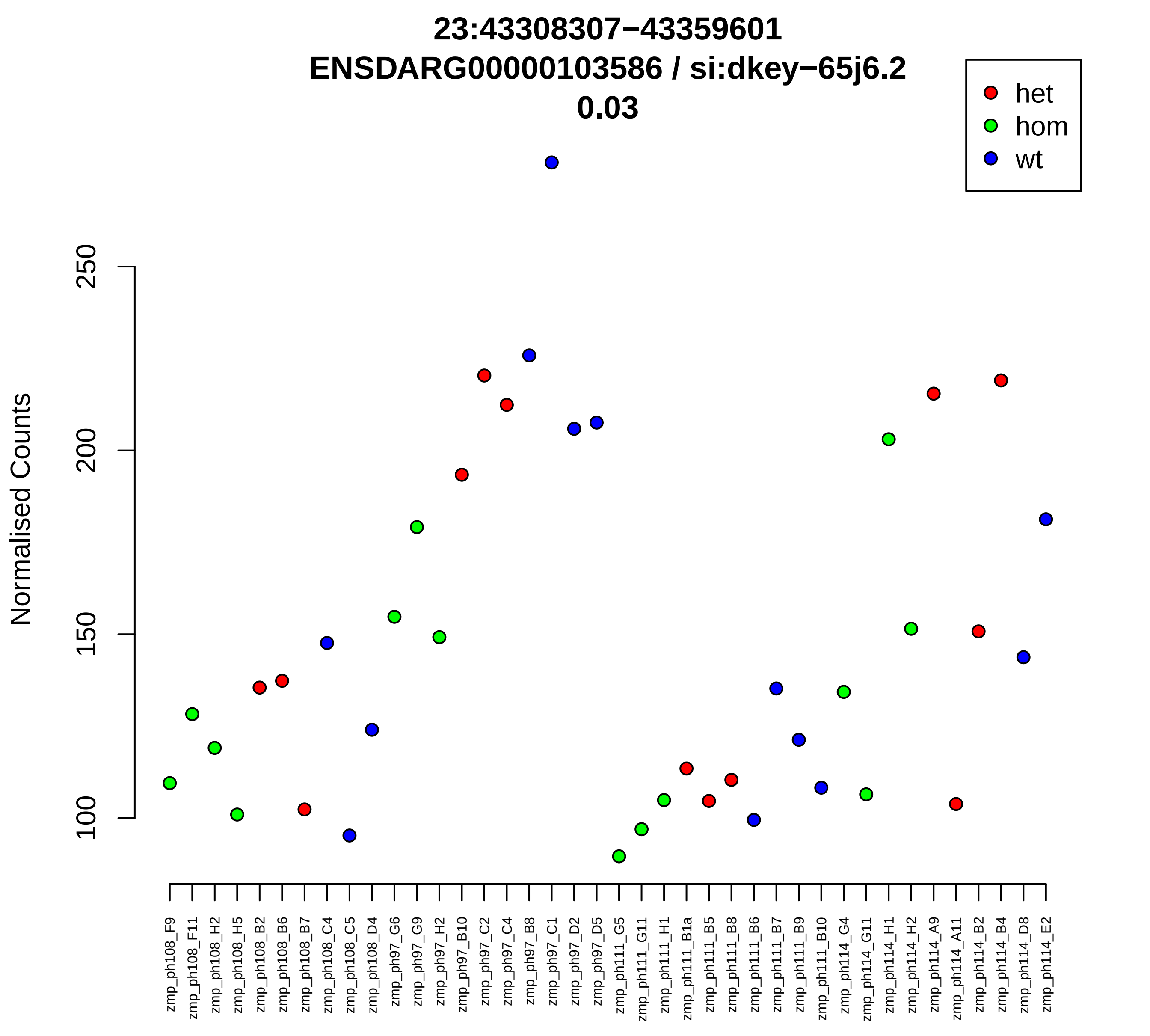
23:43308307−43359601
ENSDARG00000103586 / si:dkey−65j6.2
0.03
het
hom
wt
Normalised Counts
100
150
200
250
zmp_ph108_F9
zmp_ph108_F11
zmp_ph108_H2
zmp_ph108_H5
zmp_ph108_B2
zmp_ph108_B6
zmp_ph108_B7
zmp_ph108_C4
zmp_ph108_C5
zmp_ph108_D4
zmp_ph97_G6
zmp_ph97_G9
zmp_ph97_H2
zmp_ph97_B10
zmp_ph97_C2
zmp_ph97_C4
zmp_ph97_B8
zmp_ph97_C1
zmp_ph97_D2
zmp_ph97_D5
zmp_ph111_G5
zmp_ph111_G11
zmp_ph111_H1
zmp_ph111_B1a
zmp_ph111_B5
zmp_ph111_B8
zmp_ph111_B6
zmp_ph111_B7
zmp_ph111_B9
zmp_ph111_B10
zmp_ph114_G4
zmp_ph114_G11
zmp_ph114_H1
zmp_ph114_H2
zmp_ph114_A9
zmp_ph114_A11
zmp_ph114_B2
zmp_ph114_B4
zmp_ph114_D8
zmp_ph114_E2

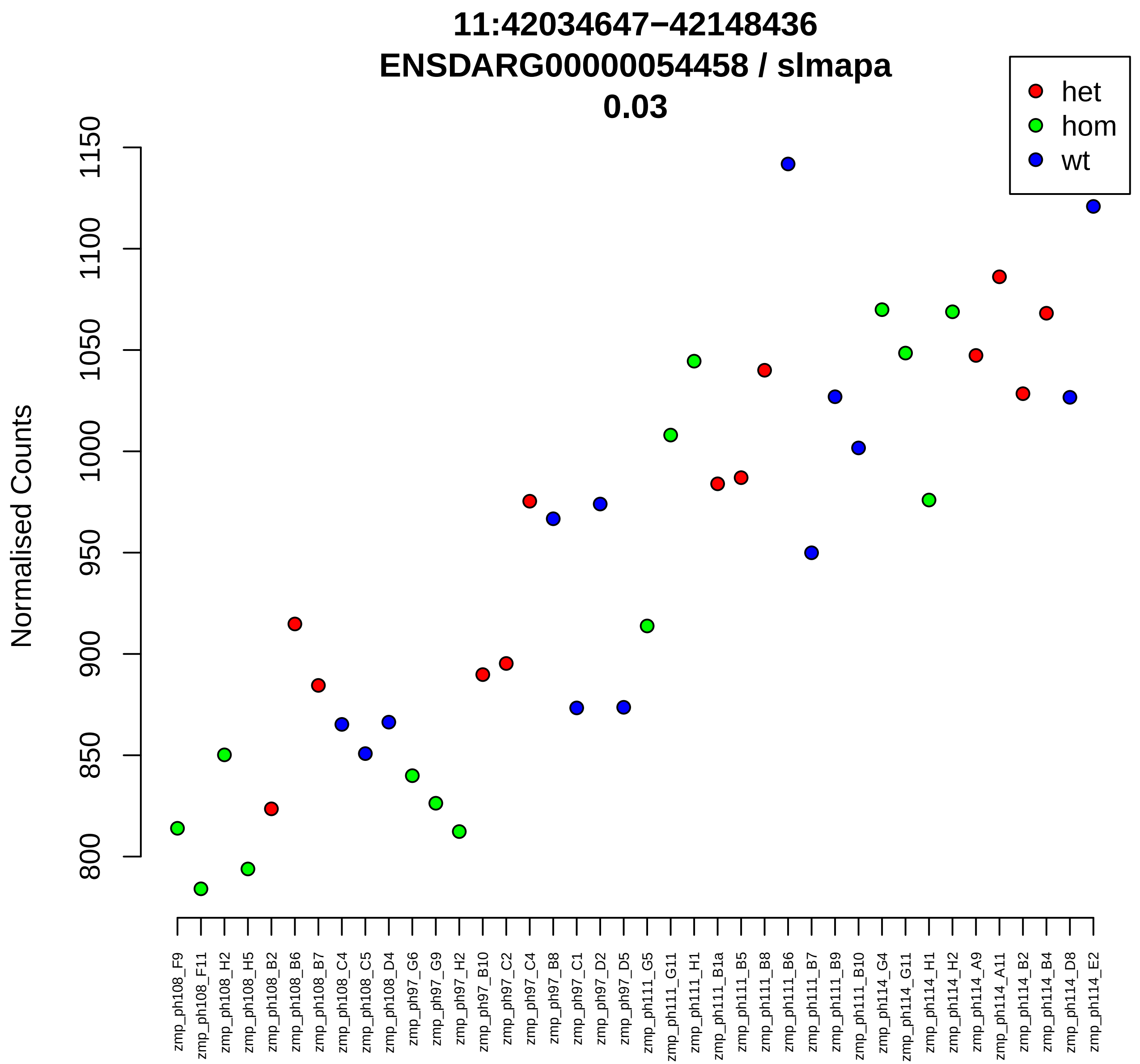
11:42034647−42148436
ENSDARG00000054458 / slmapa
0.03
het
hom
wt
Normalised Counts
800
850
900
950
1000
1050
1100
1150
zmp_ph108_F9
zmp_ph108_F11
zmp_ph108_H2
zmp_ph108_H5
zmp_ph108_B2
zmp_ph108_B6
zmp_ph108_B7
zmp_ph108_C4
zmp_ph108_C5
zmp_ph108_D4
zmp_ph97_G6
zmp_ph97_G9
zmp_ph97_H2
zmp_ph97_B10
zmp_ph97_C2
zmp_ph97_C4
zmp_ph97_B8
zmp_ph97_C1
zmp_ph97_D2
zmp_ph97_D5
zmp_ph111_G5
zmp_ph111_G11
zmp_ph111_H1
zmp_ph111_B1a
zmp_ph111_B5
zmp_ph111_B8
zmp_ph111_B6
zmp_ph111_B7
zmp_ph111_B9
zmp_ph111_B10
zmp_ph114_G4
zmp_ph114_G11
zmp_ph114_H1
zmp_ph114_H2
zmp_ph114_A9
zmp_ph114_A11
zmp_ph114_B2
zmp_ph114_B4
zmp_ph114_D8
zmp_ph114_E2

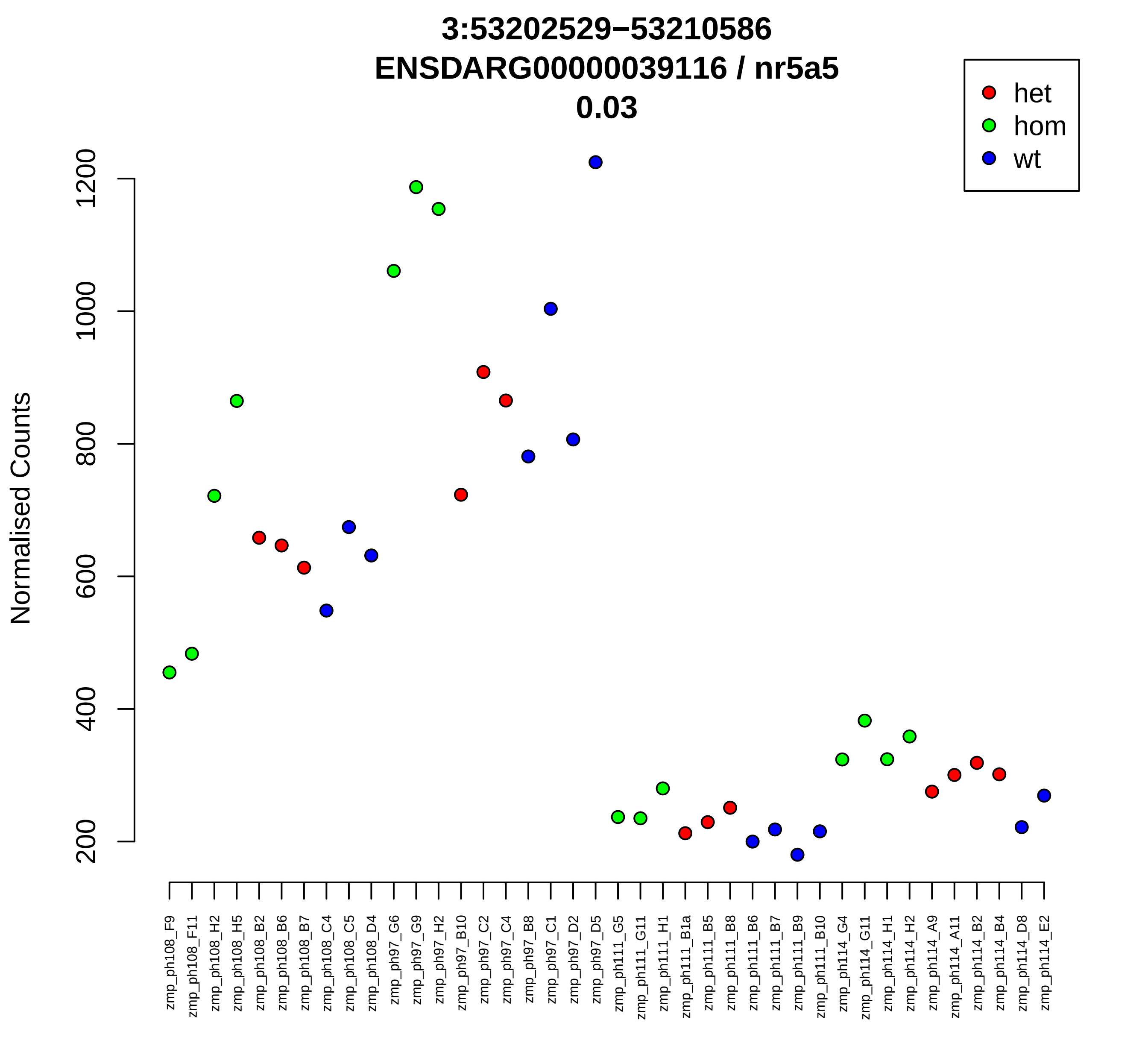

3:53202529−53210586
ENSDARG00000039116 / nr5a5
0.03
het
hom
wt
Normalised Counts
200
400
600
800
1000
1200
zmp_ph108_F9
zmp_ph108_F11
zmp_ph108_H2
zmp_ph108_H5
zmp_ph108_B2
zmp_ph108_B6
zmp_ph108_B7
zmp_ph108_C4
zmp_ph108_C5
zmp_ph108_D4
zmp_ph97_G6
zmp_ph97_G9
zmp_ph97_H2
zmp_ph97_B10
zmp_ph97_C2
zmp_ph97_C4
zmp_ph97_B8
zmp_ph97_C1
zmp_ph97_D2
zmp_ph97_D5
zmp_ph111_G5
zmp_ph111_G11
zmp_ph111_H1
zmp_ph111_B1a
zmp_ph111_B5
zmp_ph111_B8
zmp_ph111_B6
zmp_ph111_B7
zmp_ph111_B9
zmp_ph111_B10
zmp_ph114_G4
zmp_ph114_G11
zmp_ph114_H1
zmp_ph114_H2
zmp_ph114_A9
zmp_ph114_A11
zmp_ph114_B2
zmp_ph114_B4
zmp_ph114_D8
zmp_ph114_E2

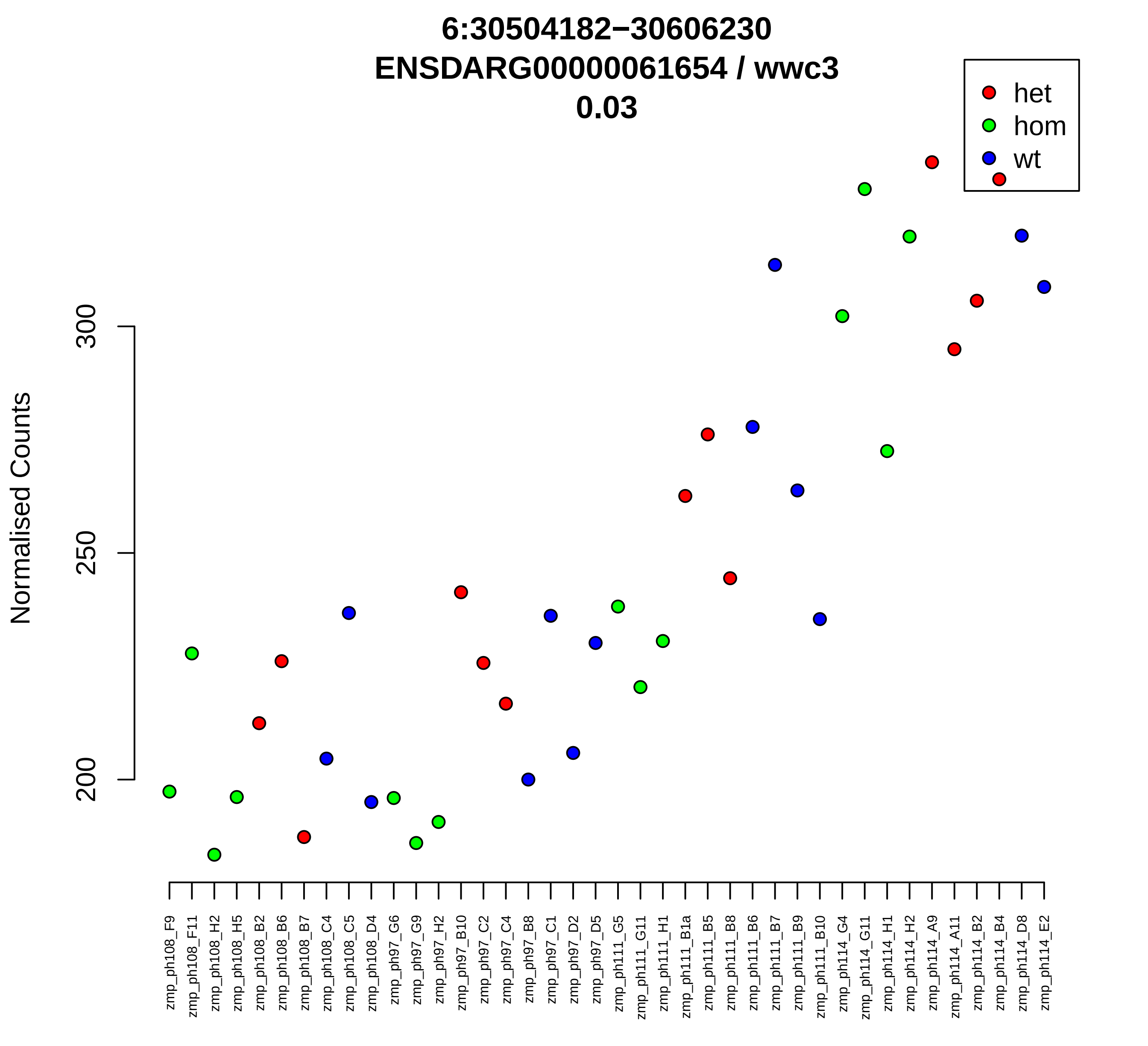

6:30504182−30606230
ENSDARG00000061654 / wwc3
0.03
het
hom
wt
Normalised Counts
200
250
300
zmp_ph108_F9
zmp_ph108_F11
zmp_ph108_H2
zmp_ph108_H5
zmp_ph108_B2
zmp_ph108_B6
zmp_ph108_B7
zmp_ph108_C4
zmp_ph108_C5
zmp_ph108_D4
zmp_ph97_G6
zmp_ph97_G9
zmp_ph97_H2
zmp_ph97_B10
zmp_ph97_C2
zmp_ph97_C4
zmp_ph97_B8
zmp_ph97_C1
zmp_ph97_D2
zmp_ph97_D5
zmp_ph111_G5
zmp_ph111_G11
zmp_ph111_H1
zmp_ph111_B1a
zmp_ph111_B5
zmp_ph111_B8
zmp_ph111_B6
zmp_ph111_B7
zmp_ph111_B9
zmp_ph111_B10
zmp_ph114_G4
zmp_ph114_G11
zmp_ph114_H1
zmp_ph114_H2
zmp_ph114_A9
zmp_ph114_A11
zmp_ph114_B2
zmp_ph114_B4
zmp_ph114_D8
zmp_ph114_E2

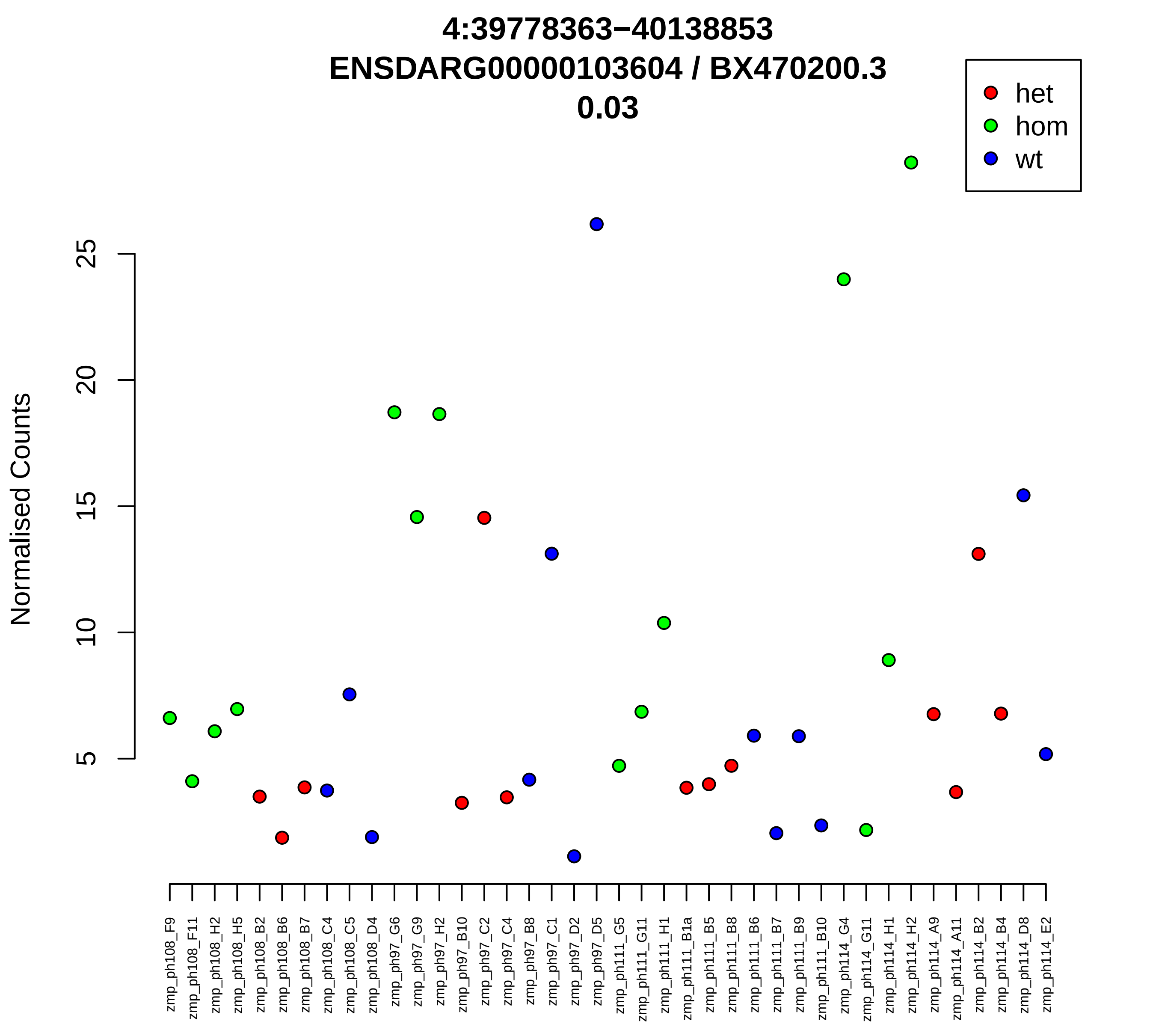
4:39778363−40138853
ENSDARG00000103604 / BX470200.3
0.03
het
hom
wt
Normalised Counts
5
10
15
20
25
zmp_ph108_F9
zmp_ph108_F11
zmp_ph108_H2
zmp_ph108_H5
zmp_ph108_B2
zmp_ph108_B6
zmp_ph108_B7
zmp_ph108_C4
zmp_ph108_C5
zmp_ph108_D4
zmp_ph97_G6
zmp_ph97_G9
zmp_ph97_H2
zmp_ph97_B10
zmp_ph97_C2
zmp_ph97_C4
zmp_ph97_B8
zmp_ph97_C1
zmp_ph97_D2
zmp_ph97_D5
zmp_ph111_G5
zmp_ph111_G11
zmp_ph111_H1
zmp_ph111_B1a
zmp_ph111_B5
zmp_ph111_B8
zmp_ph111_B6
zmp_ph111_B7
zmp_ph111_B9
zmp_ph111_B10
zmp_ph114_G4
zmp_ph114_G11
zmp_ph114_H1
zmp_ph114_H2
zmp_ph114_A9
zmp_ph114_A11
zmp_ph114_B2
zmp_ph114_B4
zmp_ph114_D8
zmp_ph114_E2

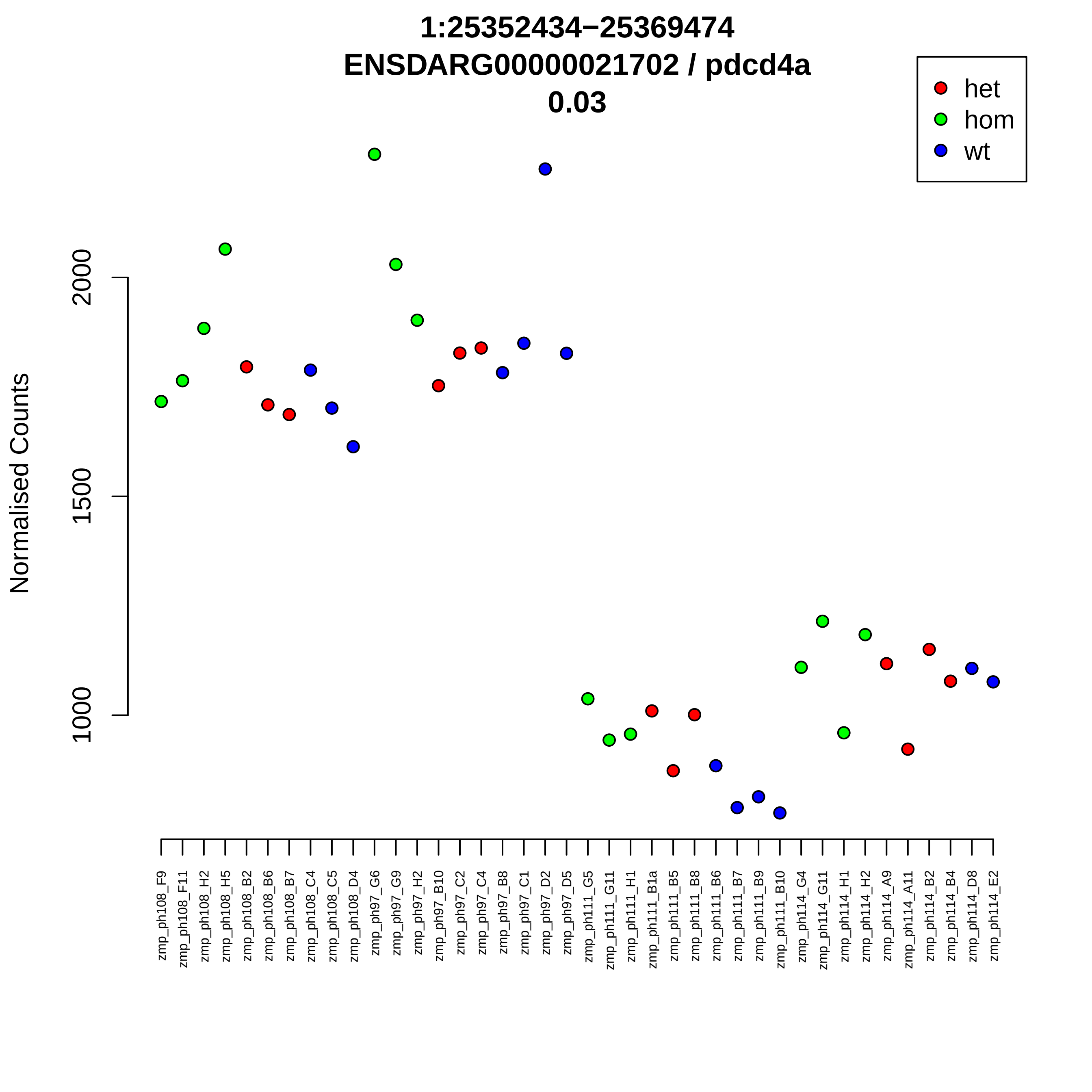
1:25352434−25369474
ENSDARG00000021702 / pdcd4a
0.03
het
hom
wt
Normalised Counts
1000
1500
2000
zmp_ph108_F9
zmp_ph108_F11
zmp_ph108_H2
zmp_ph108_H5
zmp_ph108_B2
zmp_ph108_B6
zmp_ph108_B7
zmp_ph108_C4
zmp_ph108_C5
zmp_ph108_D4
zmp_ph97_G6
zmp_ph97_G9
zmp_ph97_H2
zmp_ph97_B10
zmp_ph97_C2
zmp_ph97_C4
zmp_ph97_B8
zmp_ph97_C1
zmp_ph97_D2
zmp_ph97_D5
zmp_ph111_G5
zmp_ph111_G11
zmp_ph111_H1
zmp_ph111_B1a
zmp_ph111_B5
zmp_ph111_B8
zmp_ph111_B6
zmp_ph111_B7
zmp_ph111_B9
zmp_ph111_B10
zmp_ph114_G4
zmp_ph114_G11
zmp_ph114_H1
zmp_ph114_H2
zmp_ph114_A9
zmp_ph114_A11
zmp_ph114_B2
zmp_ph114_B4
zmp_ph114_D8
zmp_ph114_E2

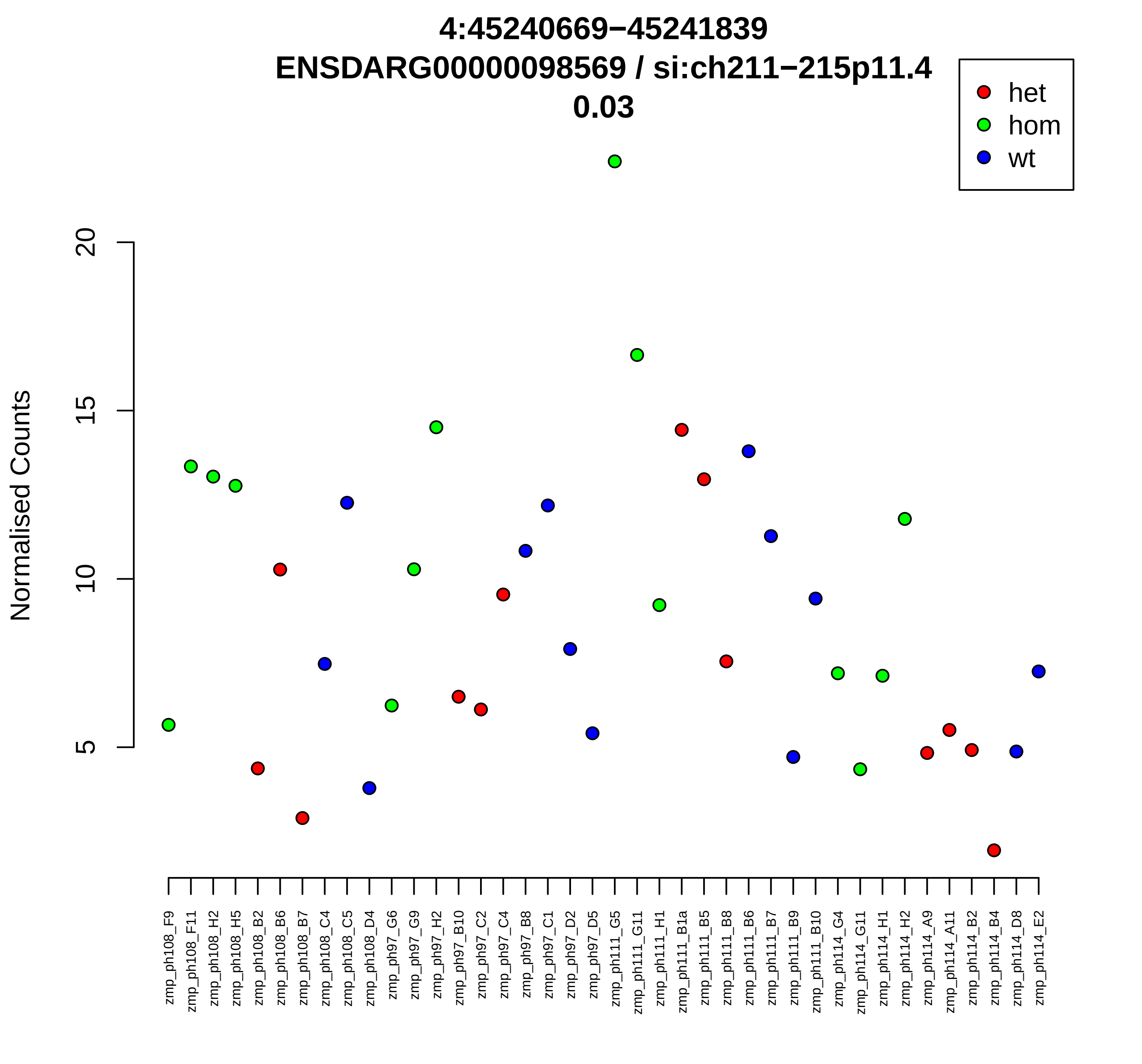
4:45240669−45241839
ENSDARG00000098569 / si:ch211−215p11.4
0.03
het
hom
wt
Normalised Counts
5
10
15
20
zmp_ph108_F9
zmp_ph108_F11
zmp_ph108_H2
zmp_ph108_H5
zmp_ph108_B2
zmp_ph108_B6
zmp_ph108_B7
zmp_ph108_C4
zmp_ph108_C5
zmp_ph108_D4
zmp_ph97_G6
zmp_ph97_G9
zmp_ph97_H2
zmp_ph97_B10
zmp_ph97_C2
zmp_ph97_C4
zmp_ph97_B8
zmp_ph97_C1
zmp_ph97_D2
zmp_ph97_D5
zmp_ph111_G5
zmp_ph111_G11
zmp_ph111_H1
zmp_ph111_B1a
zmp_ph111_B5
zmp_ph111_B8
zmp_ph111_B6
zmp_ph111_B7
zmp_ph111_B9
zmp_ph111_B10
zmp_ph114_G4
zmp_ph114_G11
zmp_ph114_H1
zmp_ph114_H2
zmp_ph114_A9
zmp_ph114_A11
zmp_ph114_B2
zmp_ph114_B4
zmp_ph114_D8
zmp_ph114_E2

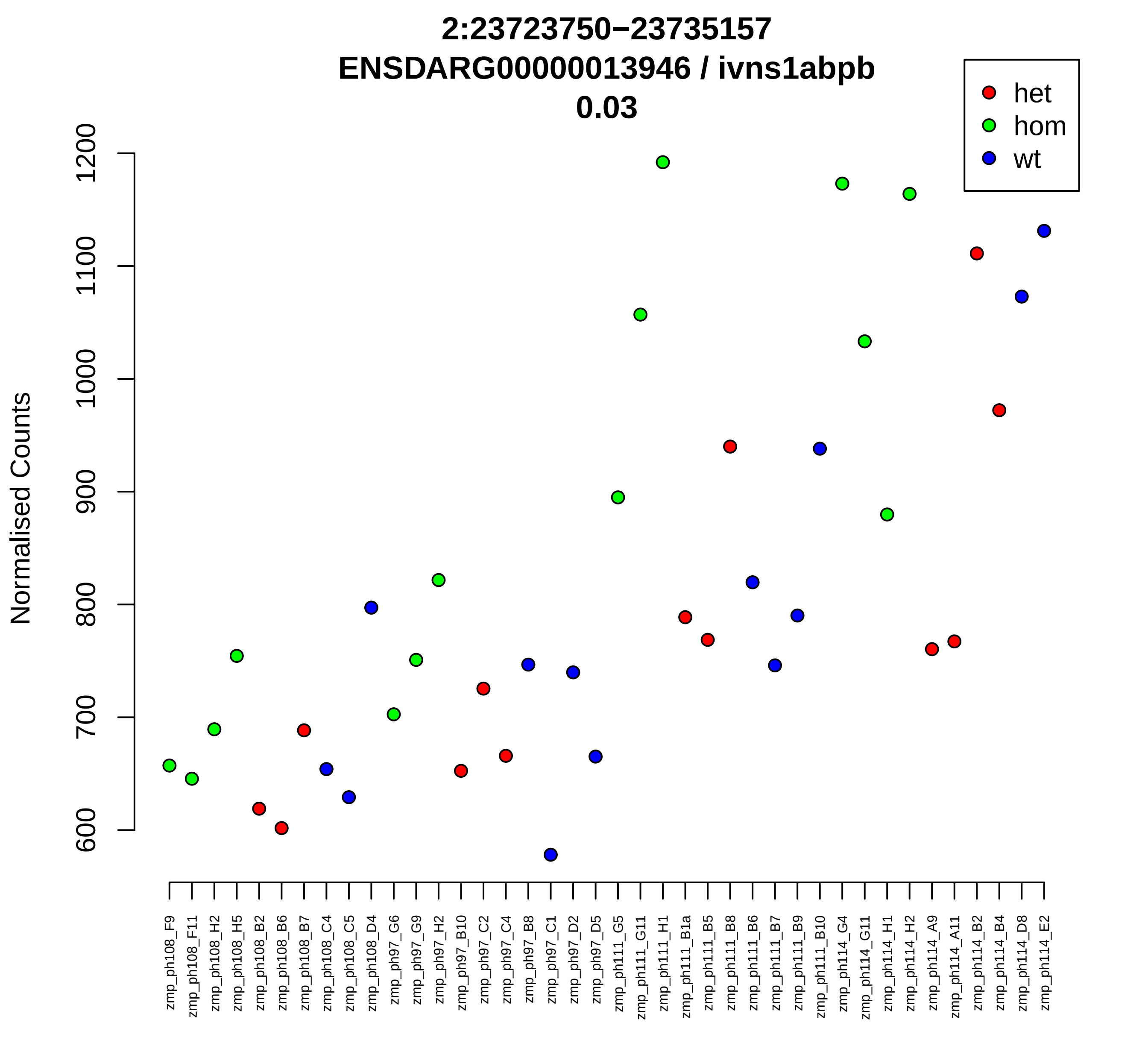

2:23723750−23735157
ENSDARG00000013946 / ivns1abpb
0.03
het
hom
wt
Normalised Counts
600
700
800
900
1000
1100
1200
zmp_ph108_F9
zmp_ph108_F11
zmp_ph108_H2
zmp_ph108_H5
zmp_ph108_B2
zmp_ph108_B6
zmp_ph108_B7
zmp_ph108_C4
zmp_ph108_C5
zmp_ph108_D4
zmp_ph97_G6
zmp_ph97_G9
zmp_ph97_H2
zmp_ph97_B10
zmp_ph97_C2
zmp_ph97_C4
zmp_ph97_B8
zmp_ph97_C1
zmp_ph97_D2
zmp_ph97_D5
zmp_ph111_G5
zmp_ph111_G11
zmp_ph111_H1
zmp_ph111_B1a
zmp_ph111_B5
zmp_ph111_B8
zmp_ph111_B6
zmp_ph111_B7
zmp_ph111_B9
zmp_ph111_B10
zmp_ph114_G4
zmp_ph114_G11
zmp_ph114_H1
zmp_ph114_H2
zmp_ph114_A9
zmp_ph114_A11
zmp_ph114_B2
zmp_ph114_B4
zmp_ph114_D8
zmp_ph114_E2

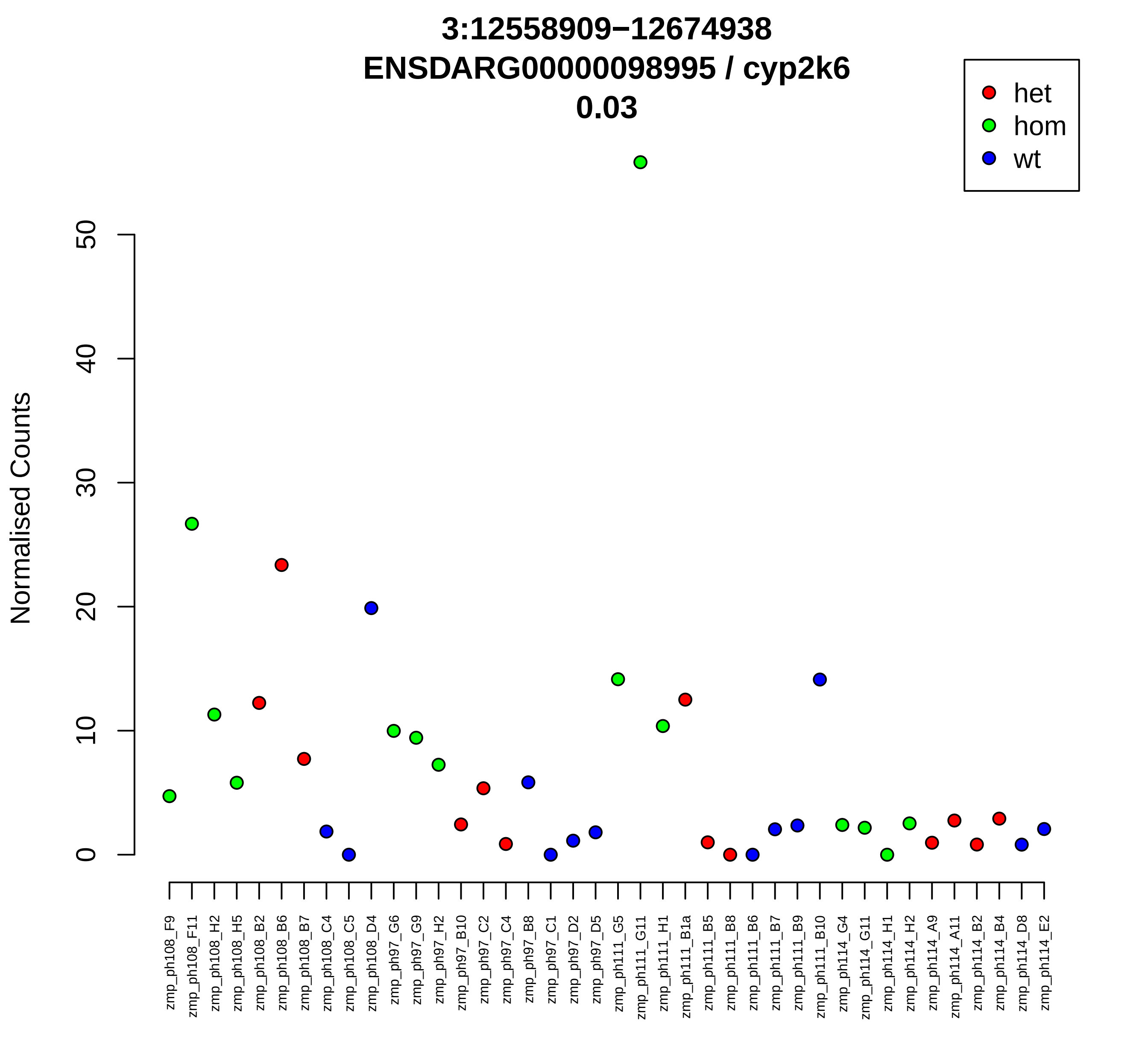

3:12558909−12674938
ENSDARG00000098995 / cyp2k6
0.03
het
hom
wt
Normalised Counts
0
10
20
30
40
50
zmp_ph108_F9
zmp_ph108_F11
zmp_ph108_H2
zmp_ph108_H5
zmp_ph108_B2
zmp_ph108_B6
zmp_ph108_B7
zmp_ph108_C4
zmp_ph108_C5
zmp_ph108_D4
zmp_ph97_G6
zmp_ph97_G9
zmp_ph97_H2
zmp_ph97_B10
zmp_ph97_C2
zmp_ph97_C4
zmp_ph97_B8
zmp_ph97_C1
zmp_ph97_D2
zmp_ph97_D5
zmp_ph111_G5
zmp_ph111_G11
zmp_ph111_H1
zmp_ph111_B1a
zmp_ph111_B5
zmp_ph111_B8
zmp_ph111_B6
zmp_ph111_B7
zmp_ph111_B9
zmp_ph111_B10
zmp_ph114_G4
zmp_ph114_G11
zmp_ph114_H1
zmp_ph114_H2
zmp_ph114_A9
zmp_ph114_A11
zmp_ph114_B2
zmp_ph114_B4
zmp_ph114_D8
zmp_ph114_E2

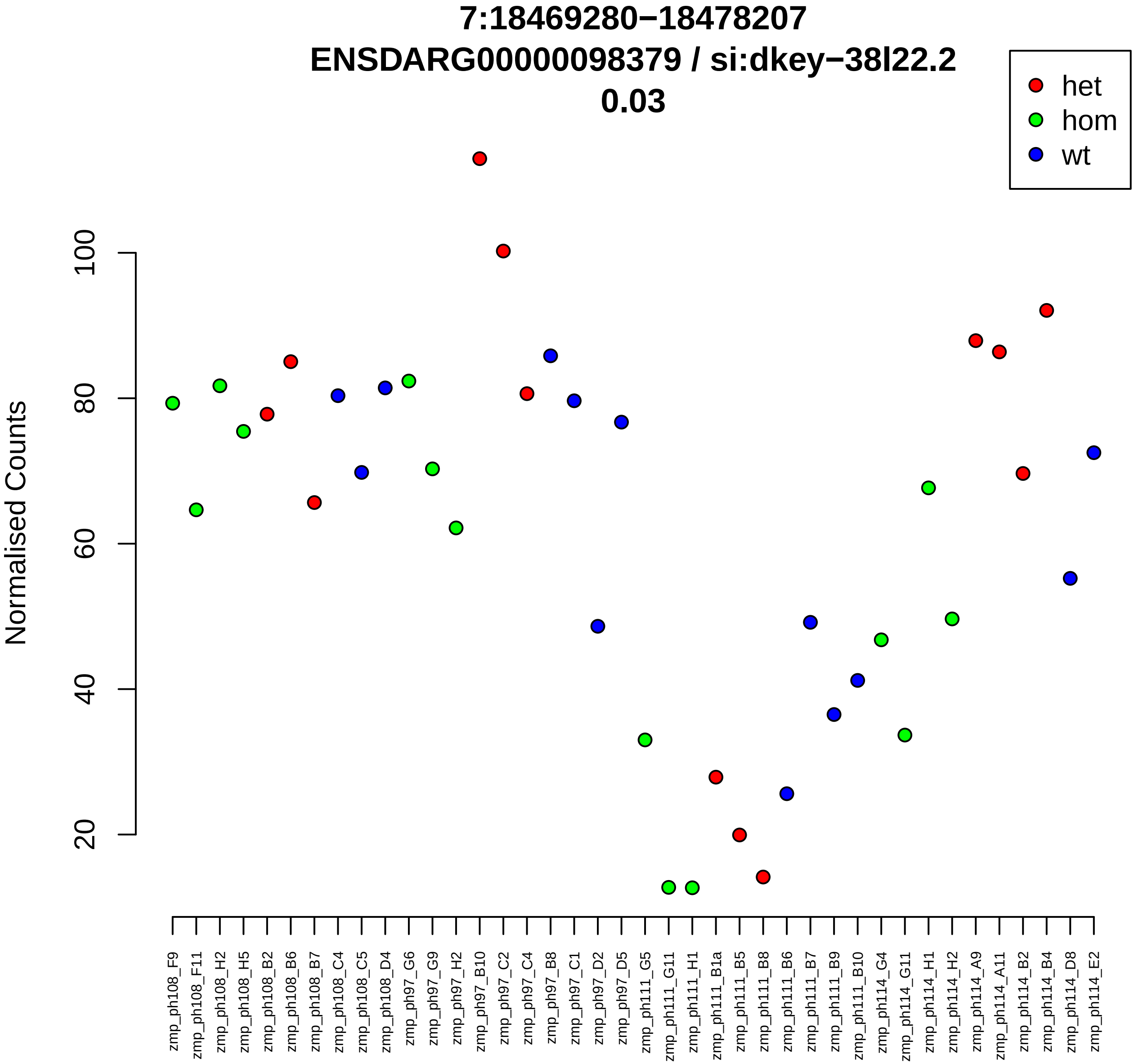

7:18469280−18478207
ENSDARG00000098379 / si:dkey−38l22.2
0.03
het
hom
wt
Normalised Counts
20
40
60
80
100
zmp_ph108_F9
zmp_ph108_F11
zmp_ph108_H2
zmp_ph108_H5
zmp_ph108_B2
zmp_ph108_B6
zmp_ph108_B7
zmp_ph108_C4
zmp_ph108_C5
zmp_ph108_D4
zmp_ph97_G6
zmp_ph97_G9
zmp_ph97_H2
zmp_ph97_B10
zmp_ph97_C2
zmp_ph97_C4
zmp_ph97_B8
zmp_ph97_C1
zmp_ph97_D2
zmp_ph97_D5
zmp_ph111_G5
zmp_ph111_G11
zmp_ph111_H1
zmp_ph111_B1a
zmp_ph111_B5
zmp_ph111_B8
zmp_ph111_B6
zmp_ph111_B7
zmp_ph111_B9
zmp_ph111_B10
zmp_ph114_G4
zmp_ph114_G11
zmp_ph114_H1
zmp_ph114_H2
zmp_ph114_A9
zmp_ph114_A11
zmp_ph114_B2
zmp_ph114_B4
zmp_ph114_D8
zmp_ph114_E2

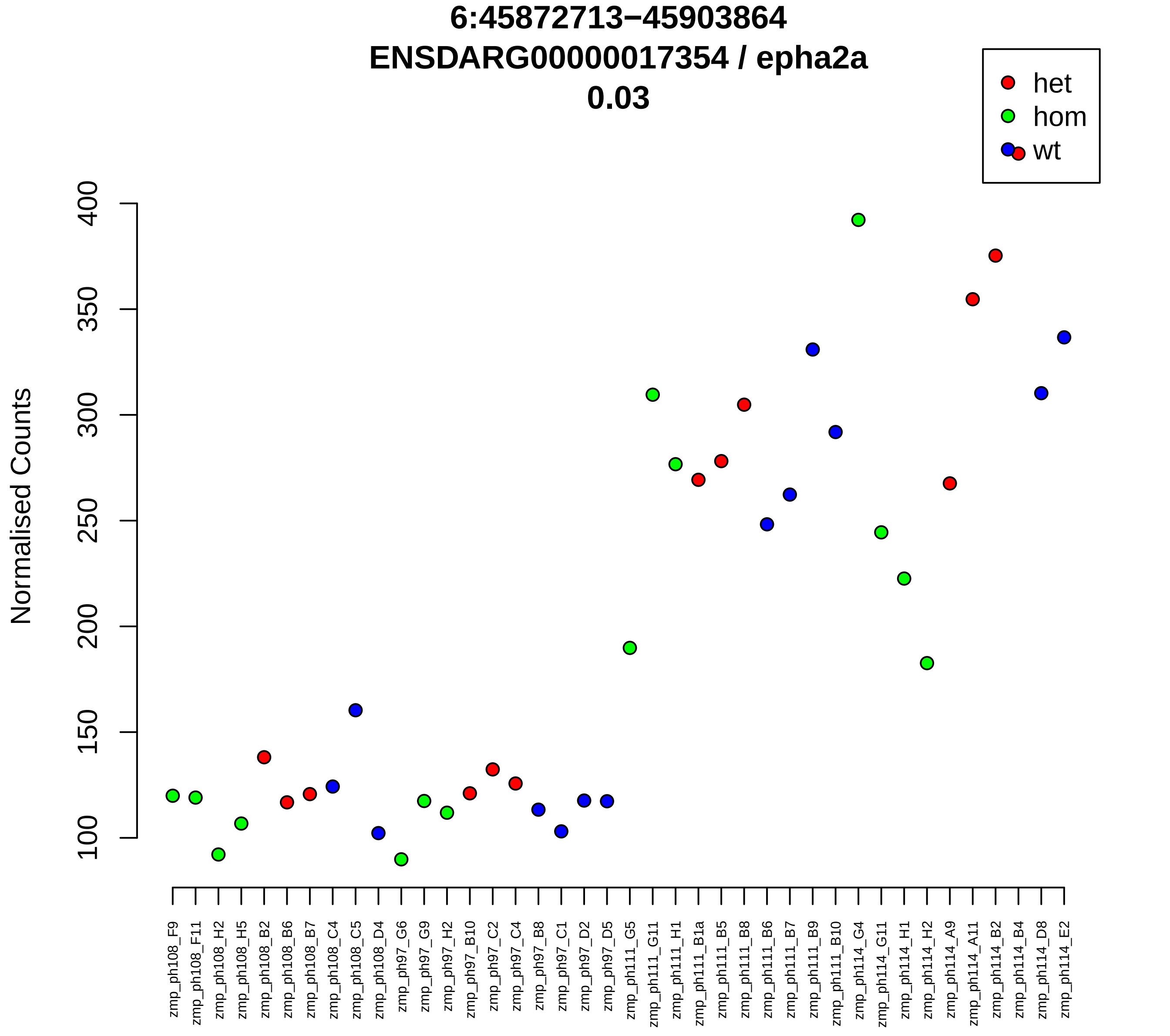

6:45872713−45903864
ENSDARG00000017354 / epha2a
0.03
het
hom
wt
Normalised Counts
100
150
200
250
300
350
400
zmp_ph108_F9
zmp_ph108_F11
zmp_ph108_H2
zmp_ph108_H5
zmp_ph108_B2
zmp_ph108_B6
zmp_ph108_B7
zmp_ph108_C4
zmp_ph108_C5
zmp_ph108_D4
zmp_ph97_G6
zmp_ph97_G9
zmp_ph97_H2
zmp_ph97_B10
zmp_ph97_C2
zmp_ph97_C4
zmp_ph97_B8
zmp_ph97_C1
zmp_ph97_D2
zmp_ph97_D5
zmp_ph111_G5
zmp_ph111_G11
zmp_ph111_H1
zmp_ph111_B1a
zmp_ph111_B5
zmp_ph111_B8
zmp_ph111_B6
zmp_ph111_B7
zmp_ph111_B9
zmp_ph111_B10
zmp_ph114_G4
zmp_ph114_G11
zmp_ph114_H1
zmp_ph114_H2
zmp_ph114_A9
zmp_ph114_A11
zmp_ph114_B2
zmp_ph114_B4
zmp_ph114_D8
zmp_ph114_E2

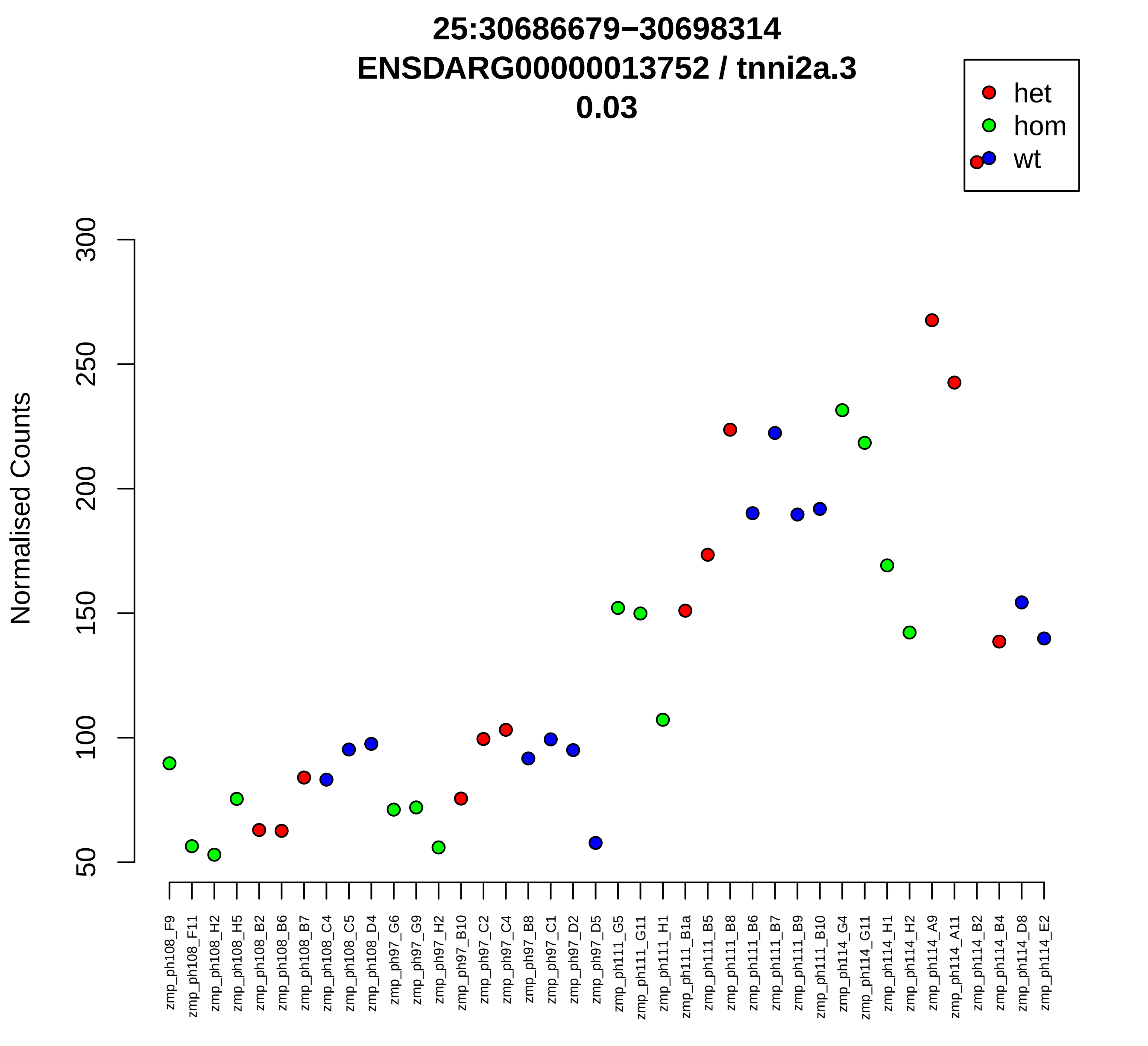
25:30686679−30698314
ENSDARG00000013752 / tnni2a.3
0.03
het
hom
wt
Normalised Counts
50
100
150
200
250
300
zmp_ph108_F9
zmp_ph108_F11
zmp_ph108_H2
zmp_ph108_H5
zmp_ph108_B2
zmp_ph108_B6
zmp_ph108_B7
zmp_ph108_C4
zmp_ph108_C5
zmp_ph108_D4
zmp_ph97_G6
zmp_ph97_G9
zmp_ph97_H2
zmp_ph97_B10
zmp_ph97_C2
zmp_ph97_C4
zmp_ph97_B8
zmp_ph97_C1
zmp_ph97_D2
zmp_ph97_D5
zmp_ph111_G5
zmp_ph111_G11
zmp_ph111_H1
zmp_ph111_B1a
zmp_ph111_B5
zmp_ph111_B8
zmp_ph111_B6
zmp_ph111_B7
zmp_ph111_B9
zmp_ph111_B10
zmp_ph114_G4
zmp_ph114_G11
zmp_ph114_H1
zmp_ph114_H2
zmp_ph114_A9
zmp_ph114_A11
zmp_ph114_B2
zmp_ph114_B4
zmp_ph114_D8
zmp_ph114_E2

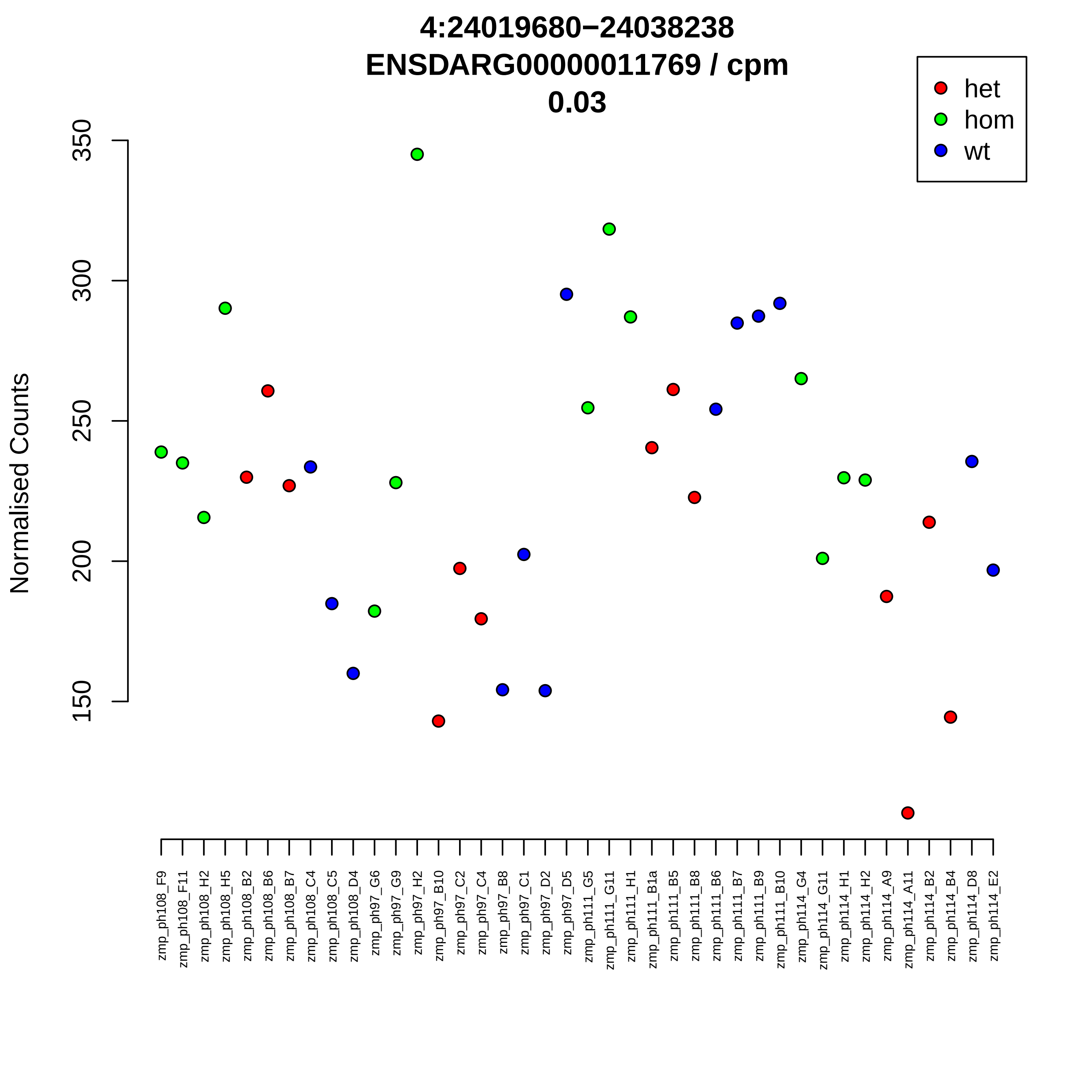

4:24019680−24038238
ENSDARG00000011769 / cpm
0.03
het
hom
wt
Normalised Counts
150
200
250
300
350
zmp_ph108_F9
zmp_ph108_F11
zmp_ph108_H2
zmp_ph108_H5
zmp_ph108_B2
zmp_ph108_B6
zmp_ph108_B7
zmp_ph108_C4
zmp_ph108_C5
zmp_ph108_D4
zmp_ph97_G6
zmp_ph97_G9
zmp_ph97_H2
zmp_ph97_B10
zmp_ph97_C2
zmp_ph97_C4
zmp_ph97_B8
zmp_ph97_C1
zmp_ph97_D2
zmp_ph97_D5
zmp_ph111_G5
zmp_ph111_G11
zmp_ph111_H1
zmp_ph111_B1a
zmp_ph111_B5
zmp_ph111_B8
zmp_ph111_B6
zmp_ph111_B7
zmp_ph111_B9
zmp_ph111_B10
zmp_ph114_G4
zmp_ph114_G11
zmp_ph114_H1
zmp_ph114_H2
zmp_ph114_A9
zmp_ph114_A11
zmp_ph114_B2
zmp_ph114_B4
zmp_ph114_D8
zmp_ph114_E2

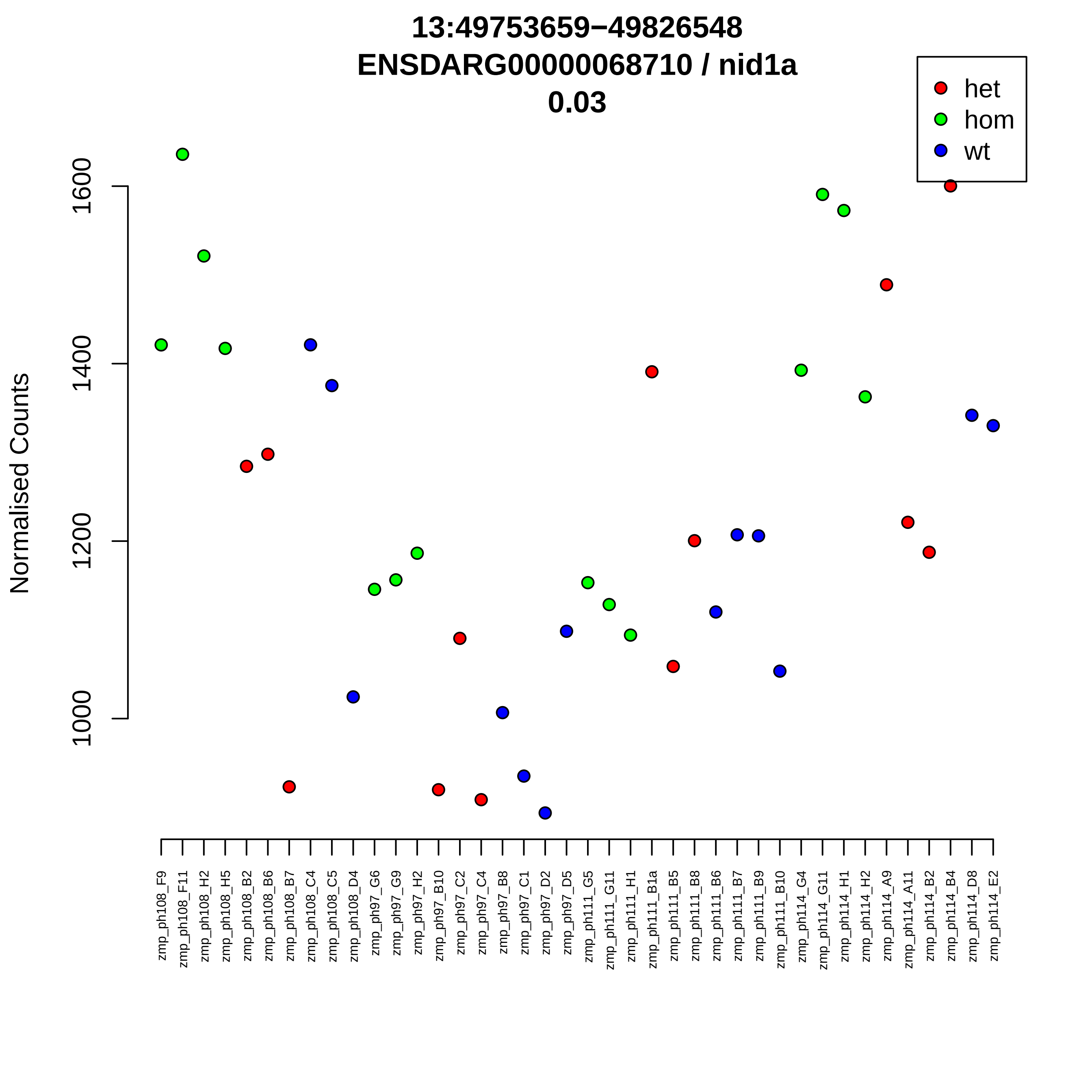
13:49753659−49826548
ENSDARG00000068710 / nid1a
0.03
het
hom
wt
Normalised Counts
1000
1200
1400
1600
zmp_ph108_F9
zmp_ph108_F11
zmp_ph108_H2
zmp_ph108_H5
zmp_ph108_B2
zmp_ph108_B6
zmp_ph108_B7
zmp_ph108_C4
zmp_ph108_C5
zmp_ph108_D4
zmp_ph97_G6
zmp_ph97_G9
zmp_ph97_H2
zmp_ph97_B10
zmp_ph97_C2
zmp_ph97_C4
zmp_ph97_B8
zmp_ph97_C1
zmp_ph97_D2
zmp_ph97_D5
zmp_ph111_G5
zmp_ph111_G11
zmp_ph111_H1
zmp_ph111_B1a
zmp_ph111_B5
zmp_ph111_B8
zmp_ph111_B6
zmp_ph111_B7
zmp_ph111_B9
zmp_ph111_B10
zmp_ph114_G4
zmp_ph114_G11
zmp_ph114_H1
zmp_ph114_H2
zmp_ph114_A9
zmp_ph114_A11
zmp_ph114_B2
zmp_ph114_B4
zmp_ph114_D8
zmp_ph114_E2

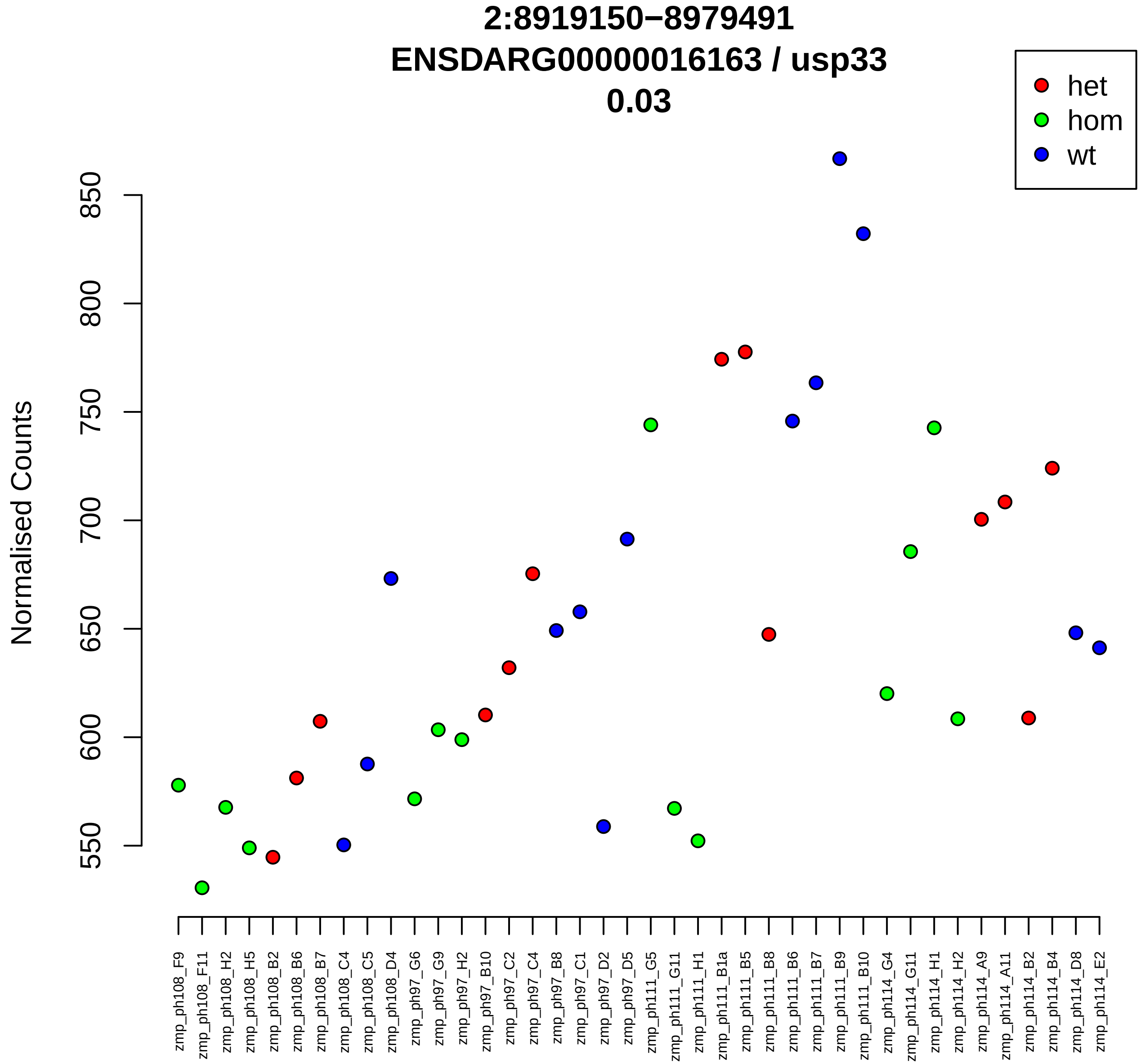
2:8919150−8979491
ENSDARG00000016163 / usp33
0.03
het
hom
wt
Normalised Counts
550
600
650
700
750
800
850
zmp_ph108_F9
zmp_ph108_F11
zmp_ph108_H2
zmp_ph108_H5
zmp_ph108_B2
zmp_ph108_B6
zmp_ph108_B7
zmp_ph108_C4
zmp_ph108_C5
zmp_ph108_D4
zmp_ph97_G6
zmp_ph97_G9
zmp_ph97_H2
zmp_ph97_B10
zmp_ph97_C2
zmp_ph97_C4
zmp_ph97_B8
zmp_ph97_C1
zmp_ph97_D2
zmp_ph97_D5
zmp_ph111_G5
zmp_ph111_G11
zmp_ph111_H1
zmp_ph111_B1a
zmp_ph111_B5
zmp_ph111_B8
zmp_ph111_B6
zmp_ph111_B7
zmp_ph111_B9
zmp_ph111_B10
zmp_ph114_G4
zmp_ph114_G11
zmp_ph114_H1
zmp_ph114_H2
zmp_ph114_A9
zmp_ph114_A11
zmp_ph114_B2
zmp_ph114_B4
zmp_ph114_D8
zmp_ph114_E2

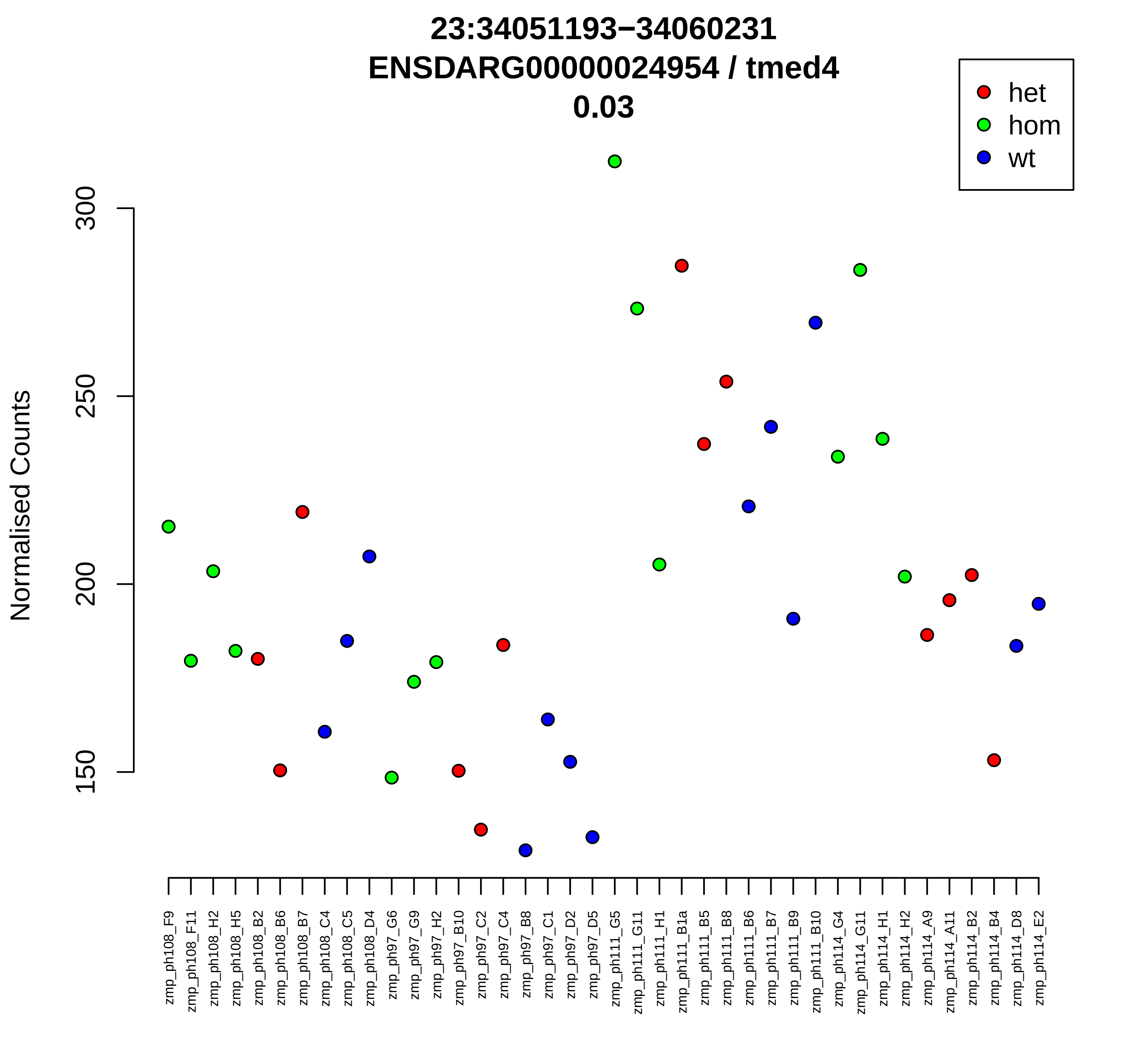

23:34051193−34060231
ENSDARG00000024954 / tmed4
0.03
het
hom
wt
Normalised Counts
300
250
200
150
zmp_ph108_F9
zmp_ph108_F11
zmp_ph108_H2
zmp_ph108_H5
zmp_ph108_B2
zmp_ph108_B6
zmp_ph108_B7
zmp_ph108_C4
zmp_ph108_C5
zmp_ph108_D4
zmp_ph97_G6
zmp_ph97_G9
zmp_ph97_H2
zmp_ph97_B10
zmp_ph97_C2
zmp_ph97_C4
zmp_ph97_B8
zmp_ph97_C1
zmp_ph97_D2
zmp_ph97_D5
zmp_ph111_G5
zmp_ph111_G11
zmp_ph111_H1
zmp_ph111_B1a
zmp_ph111_B5
zmp_ph111_B8
zmp_ph111_B6
zmp_ph111_B7
zmp_ph111_B9
zmp_ph111_B10
zmp_ph114_G4
zmp_ph114_G11
zmp_ph114_H1
zmp_ph114_H2
zmp_ph114_A9
zmp_ph114_A11
zmp_ph114_B2
zmp_ph114_B4
zmp_ph114_D8
zmp_ph114_E2

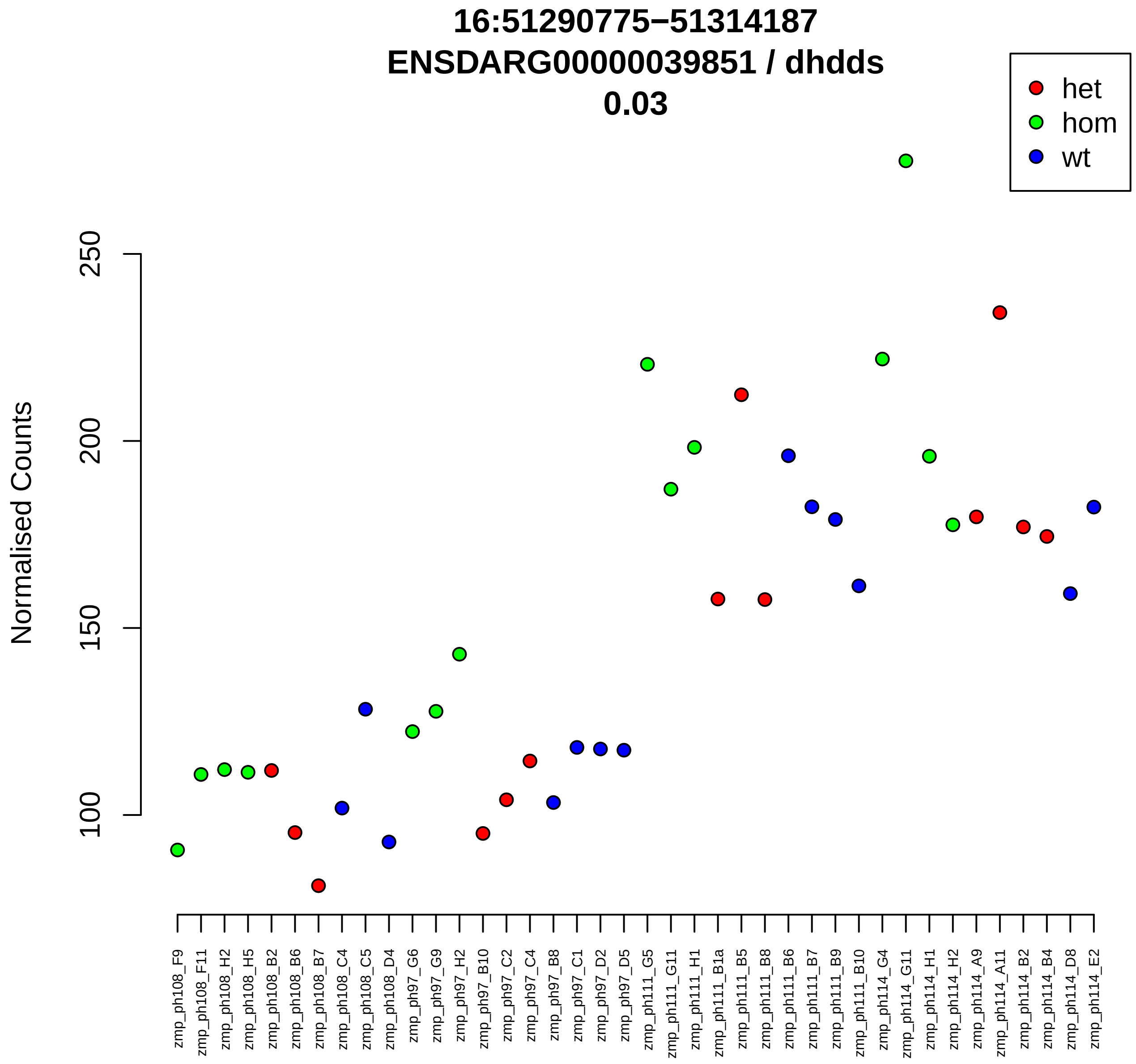
16:51290775−51314187
ENSDARG00000039851 / dhdds
0.03
het
hom
wt
Normalised Counts
100
150
200
250
zmp_ph108_F9
zmp_ph108_F11
zmp_ph108_H2
zmp_ph108_H5
zmp_ph108_B2
zmp_ph108_B6
zmp_ph108_B7
zmp_ph108_C4
zmp_ph108_C5
zmp_ph108_D4
zmp_ph97_G6
zmp_ph97_G9
zmp_ph97_H2
zmp_ph97_B10
zmp_ph97_C2
zmp_ph97_C4
zmp_ph97_B8
zmp_ph97_C1
zmp_ph97_D2
zmp_ph97_D5
zmp_ph111_G5
zmp_ph111_G11
zmp_ph111_H1
zmp_ph111_B1a
zmp_ph111_B5
zmp_ph111_B8
zmp_ph111_B6
zmp_ph111_B7
zmp_ph111_B9
zmp_ph111_B10
zmp_ph114_G4
zmp_ph114_G11
zmp_ph114_H1
zmp_ph114_H2
zmp_ph114_A9
zmp_ph114_A11
zmp_ph114_B2
zmp_ph114_B4
zmp_ph114_D8
zmp_ph114_E2

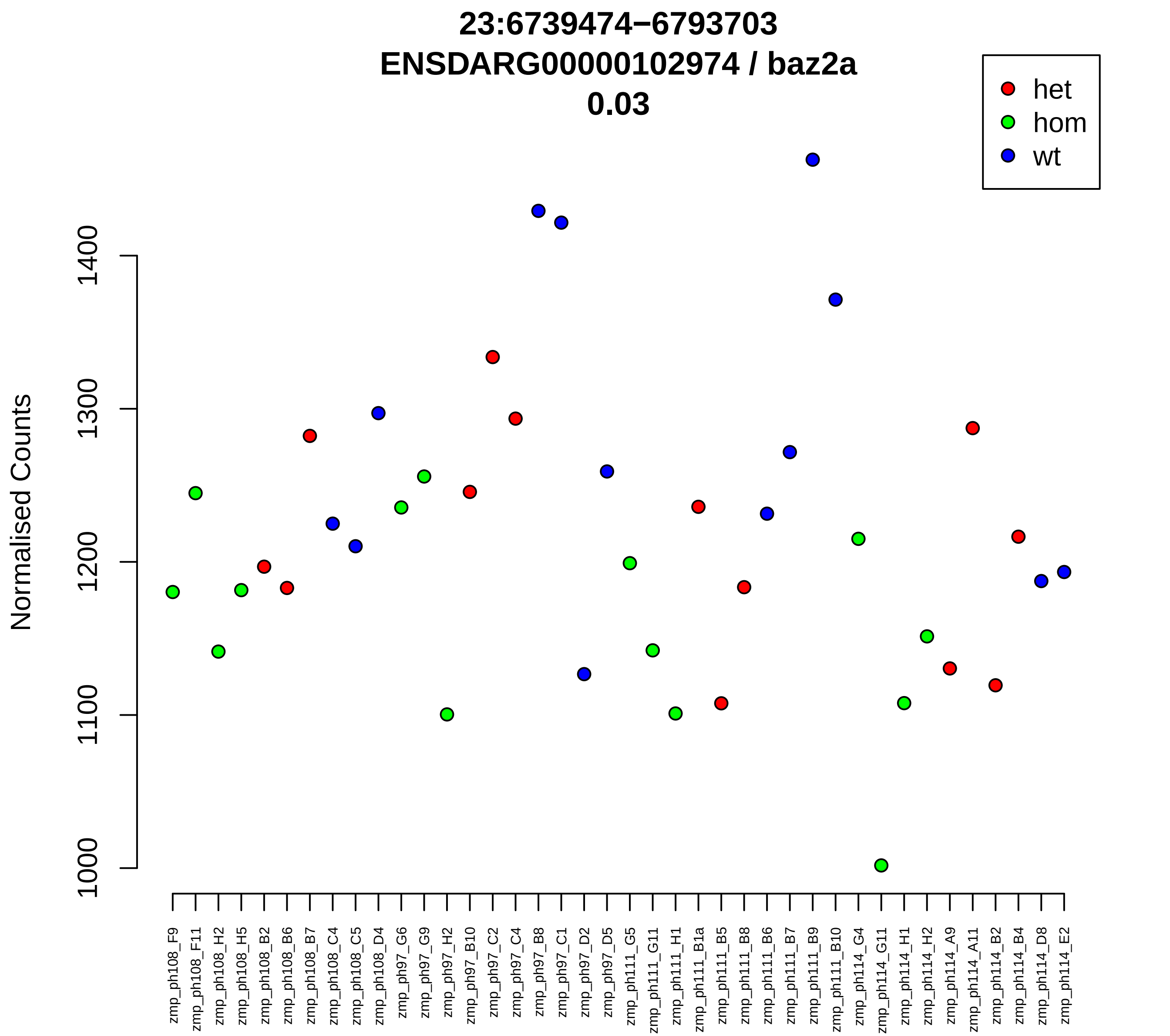
23:6739474−6793703
ENSDARG00000102974 / baz2a
0.03
het
hom
wt
Normalised Counts
1000
1100
1200
1300
1400
zmp_ph108_F9
zmp_ph108_F11
zmp_ph108_H2
zmp_ph108_H5
zmp_ph108_B2
zmp_ph108_B6
zmp_ph108_B7
zmp_ph108_C4
zmp_ph108_C5
zmp_ph108_D4
zmp_ph97_G6
zmp_ph97_G9
zmp_ph97_H2
zmp_ph97_B10
zmp_ph97_C2
zmp_ph97_C4
zmp_ph97_B8
zmp_ph97_C1
zmp_ph97_D2
zmp_ph97_D5
zmp_ph111_G5
zmp_ph111_G11
zmp_ph111_H1
zmp_ph111_B1a
zmp_ph111_B5
zmp_ph111_B8
zmp_ph111_B6
zmp_ph111_B7
zmp_ph111_B9
zmp_ph111_B10
zmp_ph114_G4
zmp_ph114_G11
zmp_ph114_H1
zmp_ph114_H2
zmp_ph114_A9
zmp_ph114_A11
zmp_ph114_B2
zmp_ph114_B4
zmp_ph114_D8
zmp_ph114_E2

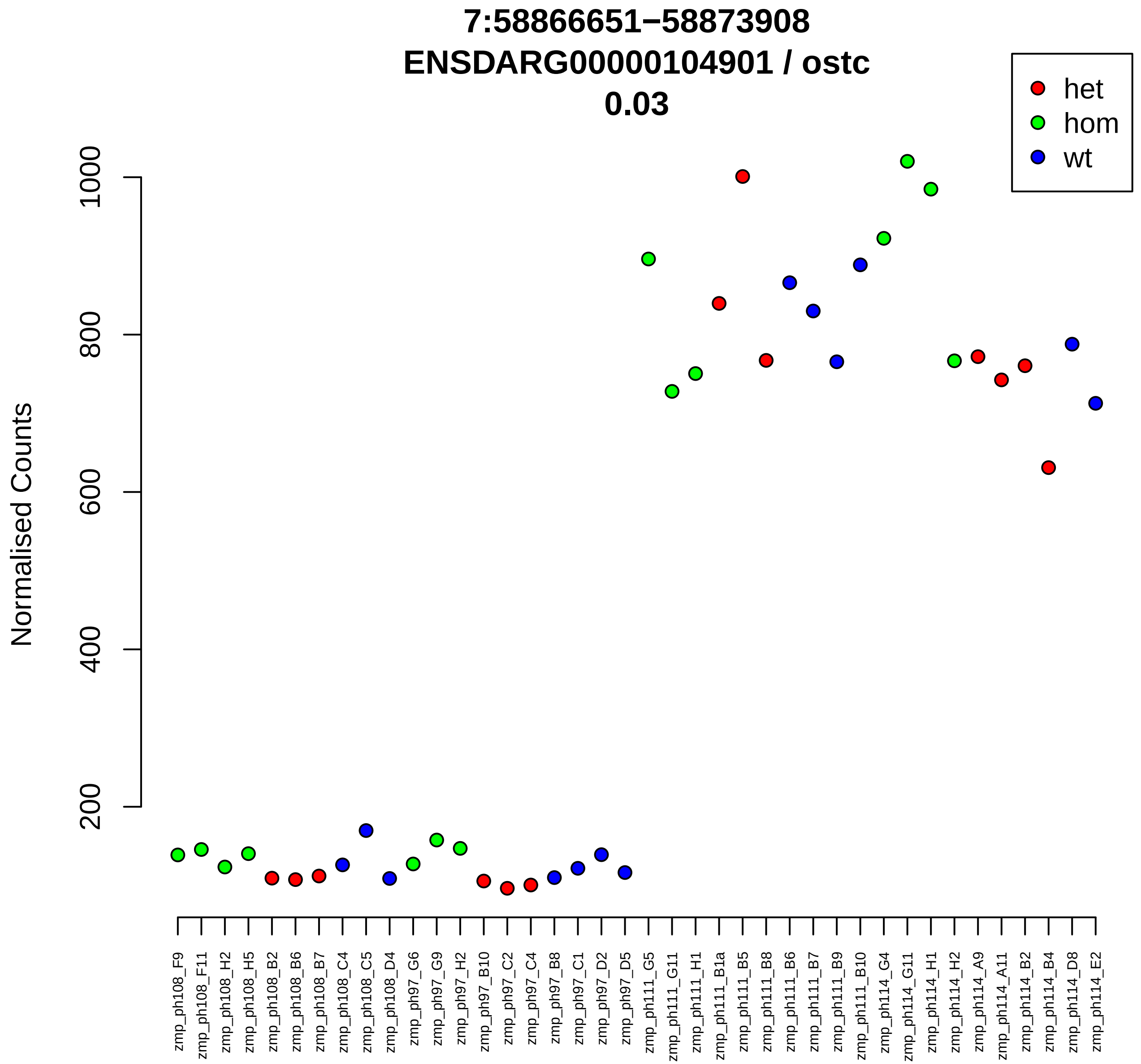
7:58866651−58873908
ENSDARG00000104901 / ostc
0.03
het
hom
wt
Normalised Counts
200
400
600
800
1000
zmp_ph108_F9
zmp_ph108_F11
zmp_ph108_H2
zmp_ph108_H5
zmp_ph108_B2
zmp_ph108_B6
zmp_ph108_B7
zmp_ph108_C4
zmp_ph108_C5
zmp_ph108_D4
zmp_ph97_G6
zmp_ph97_G9
zmp_ph97_H2
zmp_ph97_B10
zmp_ph97_C2
zmp_ph97_C4
zmp_ph97_B8
zmp_ph97_C1
zmp_ph97_D2
zmp_ph97_D5
zmp_ph111_G5
zmp_ph111_G11
zmp_ph111_H1
zmp_ph111_B1a
zmp_ph111_B5
zmp_ph111_B8
zmp_ph111_B6
zmp_ph111_B7
zmp_ph111_B9
zmp_ph111_B10
zmp_ph114_G4
zmp_ph114_G11
zmp_ph114_H1
zmp_ph114_H2
zmp_ph114_A9
zmp_ph114_A11
zmp_ph114_B2
zmp_ph114_B4
zmp_ph114_D8
zmp_ph114_E2

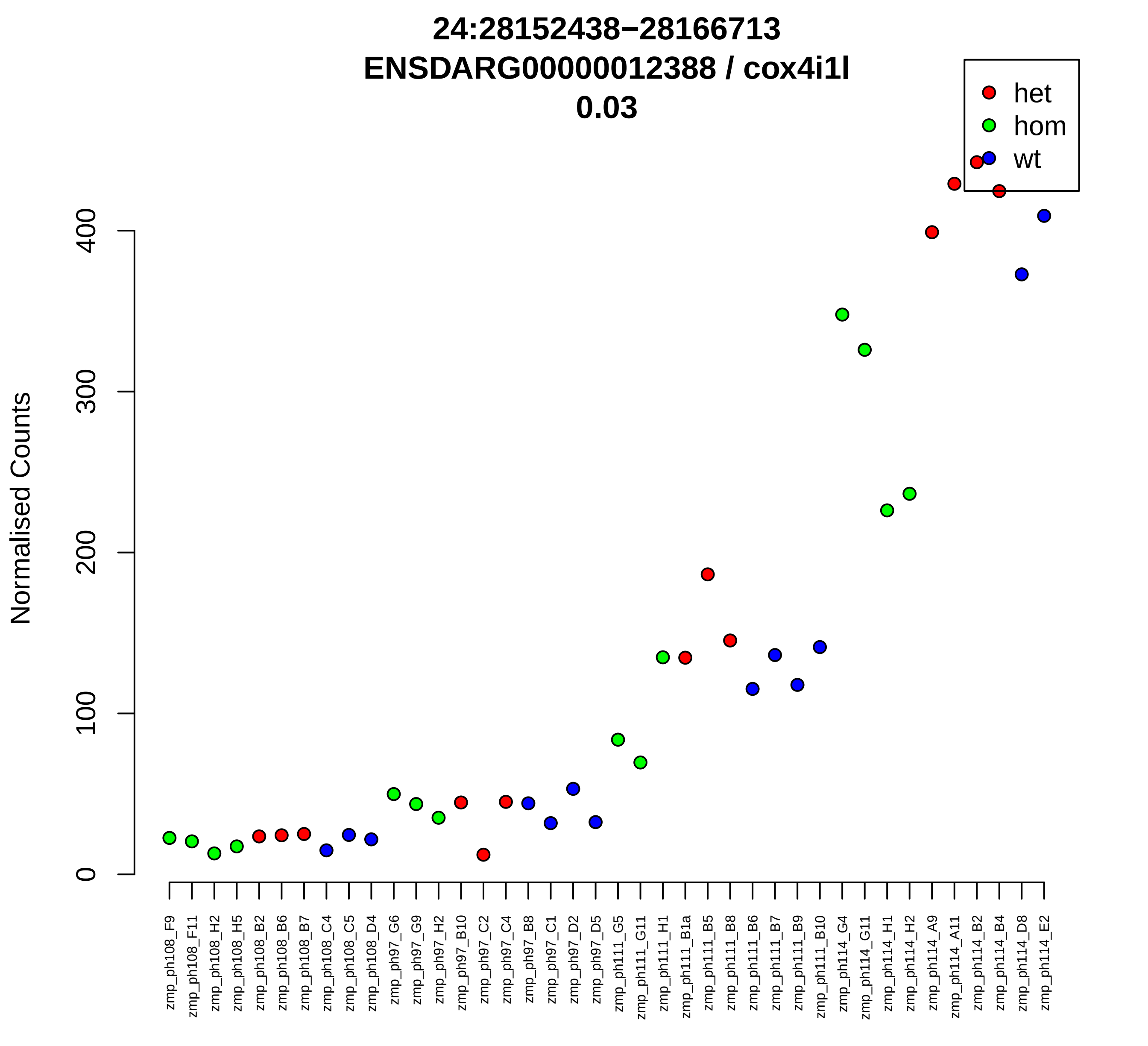

24:28152438−28166713
ENSDARG00000012388 / cox4i1l
0.03
het
hom
wt
Normalised Counts
0
100
200
300
400
zmp_ph108_F9
zmp_ph108_F11
zmp_ph108_H2
zmp_ph108_H5
zmp_ph108_B2
zmp_ph108_B6
zmp_ph108_B7
zmp_ph108_C4
zmp_ph108_C5
zmp_ph108_D4
zmp_ph97_G6
zmp_ph97_G9
zmp_ph97_H2
zmp_ph97_B10
zmp_ph97_C2
zmp_ph97_C4
zmp_ph97_B8
zmp_ph97_C1
zmp_ph97_D2
zmp_ph97_D5
zmp_ph111_G5
zmp_ph111_G11
zmp_ph111_H1
zmp_ph111_B1a
zmp_ph111_B5
zmp_ph111_B8
zmp_ph111_B6
zmp_ph111_B7
zmp_ph111_B9
zmp_ph111_B10
zmp_ph114_G4
zmp_ph114_G11
zmp_ph114_H1
zmp_ph114_H2
zmp_ph114_A9
zmp_ph114_A11
zmp_ph114_B2
zmp_ph114_B4
zmp_ph114_D8
zmp_ph114_E2

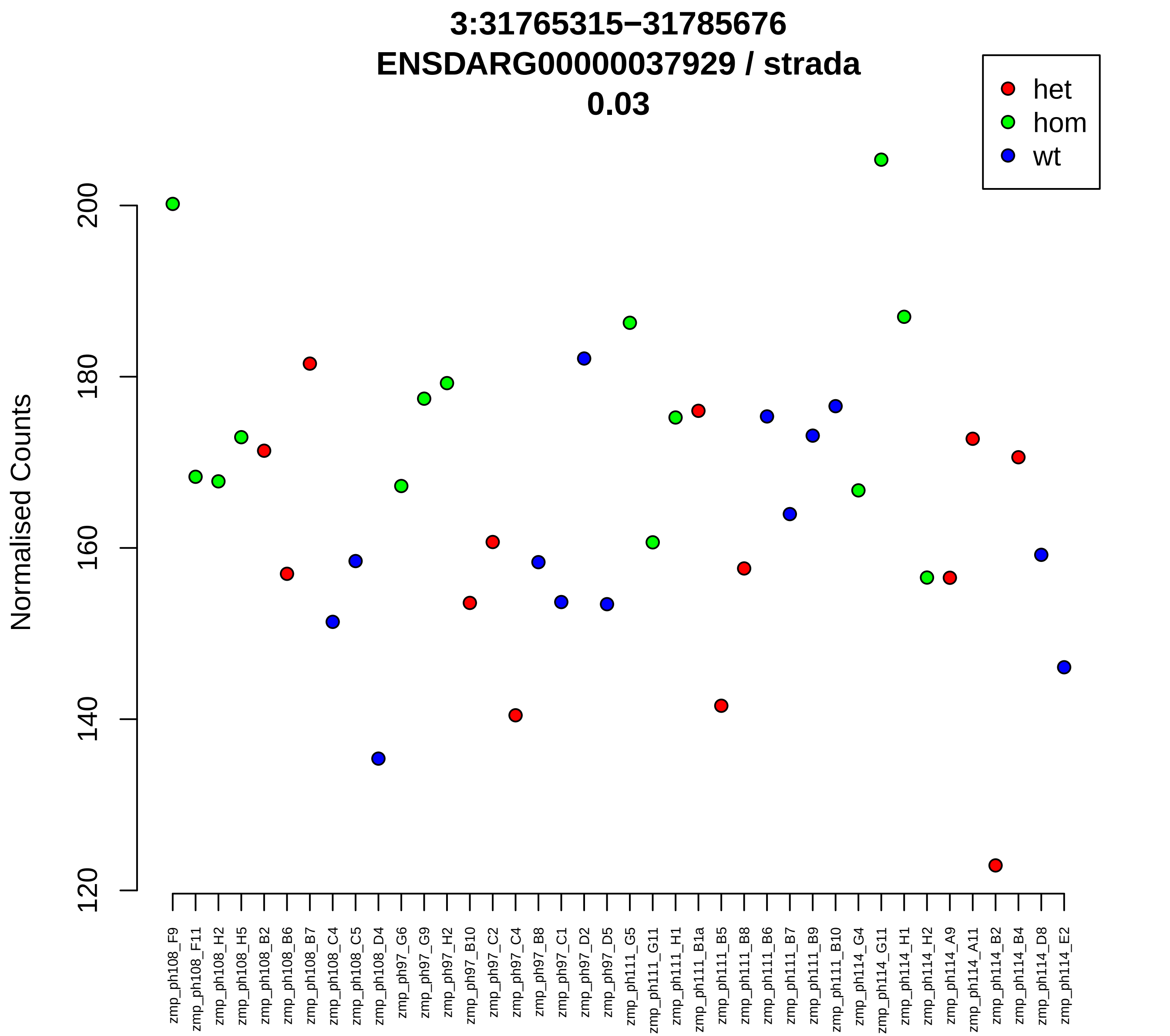
3:31765315−31785676
ENSDARG00000037929 / strada
0.03
het
hom
wt
Normalised Counts
120
140
160
180
200
zmp_ph108_F9
zmp_ph108_F11
zmp_ph108_H2
zmp_ph108_H5
zmp_ph108_B2
zmp_ph108_B6
zmp_ph108_B7
zmp_ph108_C4
zmp_ph108_C5
zmp_ph108_D4
zmp_ph97_G6
zmp_ph97_G9
zmp_ph97_H2
zmp_ph97_B10
zmp_ph97_C2
zmp_ph97_C4
zmp_ph97_B8
zmp_ph97_C1
zmp_ph97_D2
zmp_ph97_D5
zmp_ph111_G5
zmp_ph111_G11
zmp_ph111_H1
zmp_ph111_B1a
zmp_ph111_B5
zmp_ph111_B8
zmp_ph111_B6
zmp_ph111_B7
zmp_ph111_B9
zmp_ph111_B10
zmp_ph114_G4
zmp_ph114_G11
zmp_ph114_H1
zmp_ph114_H2
zmp_ph114_A9
zmp_ph114_A11
zmp_ph114_B2
zmp_ph114_B4
zmp_ph114_D8
zmp_ph114_E2

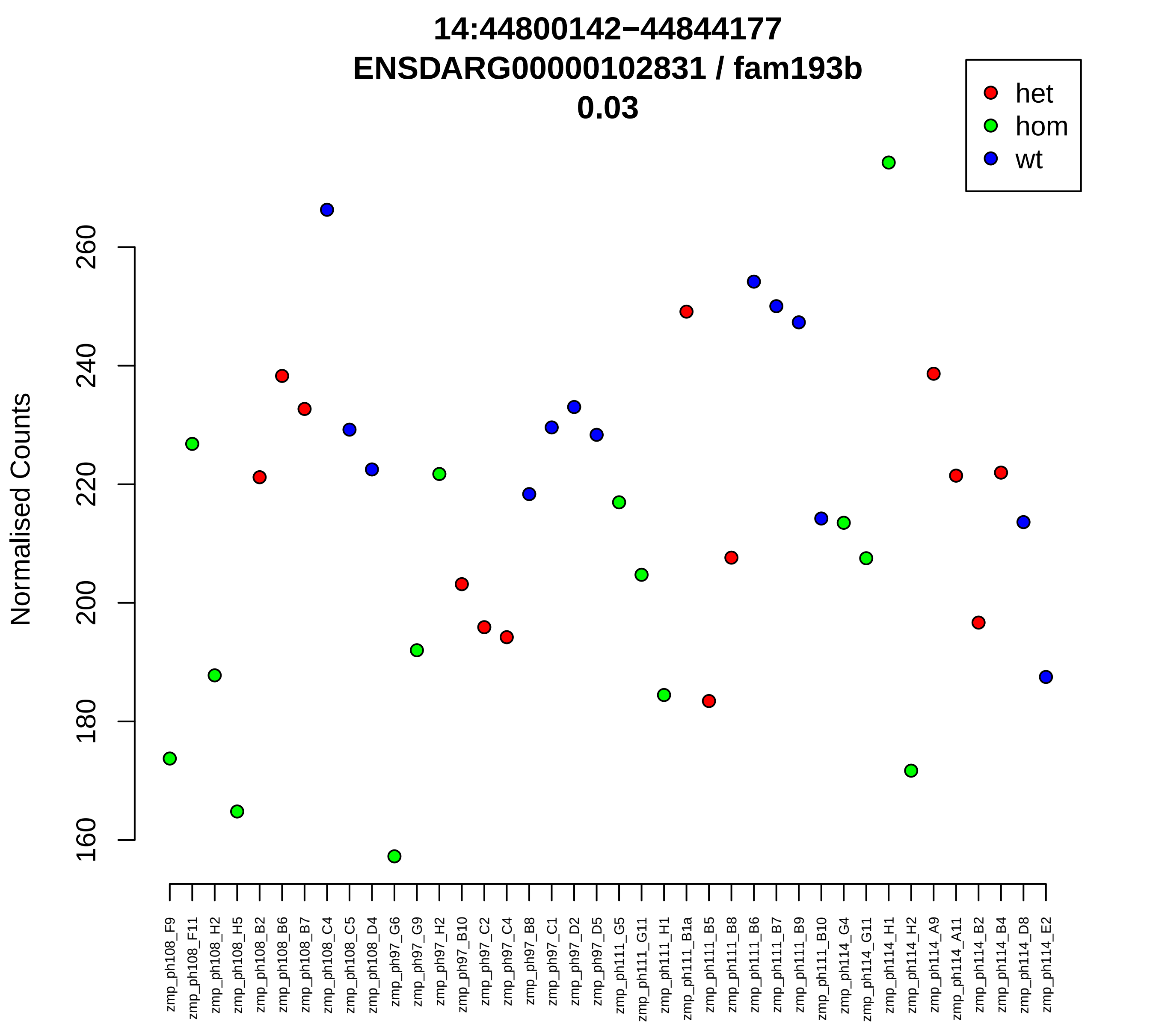
14:44800142−44844177
ENSDARG00000102831 / fam193b
0.03
het
hom
wt
Normalised Counts
160
180
200
220
240
260
zmp_ph108_F9
zmp_ph108_F11
zmp_ph108_H2
zmp_ph108_H5
zmp_ph108_B2
zmp_ph108_B6
zmp_ph108_B7
zmp_ph108_C4
zmp_ph108_C5
zmp_ph108_D4
zmp_ph97_G6
zmp_ph97_G9
zmp_ph97_H2
zmp_ph97_B10
zmp_ph97_C2
zmp_ph97_C4
zmp_ph97_B8
zmp_ph97_C1
zmp_ph97_D2
zmp_ph97_D5
zmp_ph111_G5
zmp_ph111_G11
zmp_ph111_H1
zmp_ph111_B1a
zmp_ph111_B5
zmp_ph111_B8
zmp_ph111_B6
zmp_ph111_B7
zmp_ph111_B9
zmp_ph111_B10
zmp_ph114_G4
zmp_ph114_G11
zmp_ph114_H1
zmp_ph114_H2
zmp_ph114_A9
zmp_ph114_A11
zmp_ph114_B2
zmp_ph114_B4
zmp_ph114_D8
zmp_ph114_E2

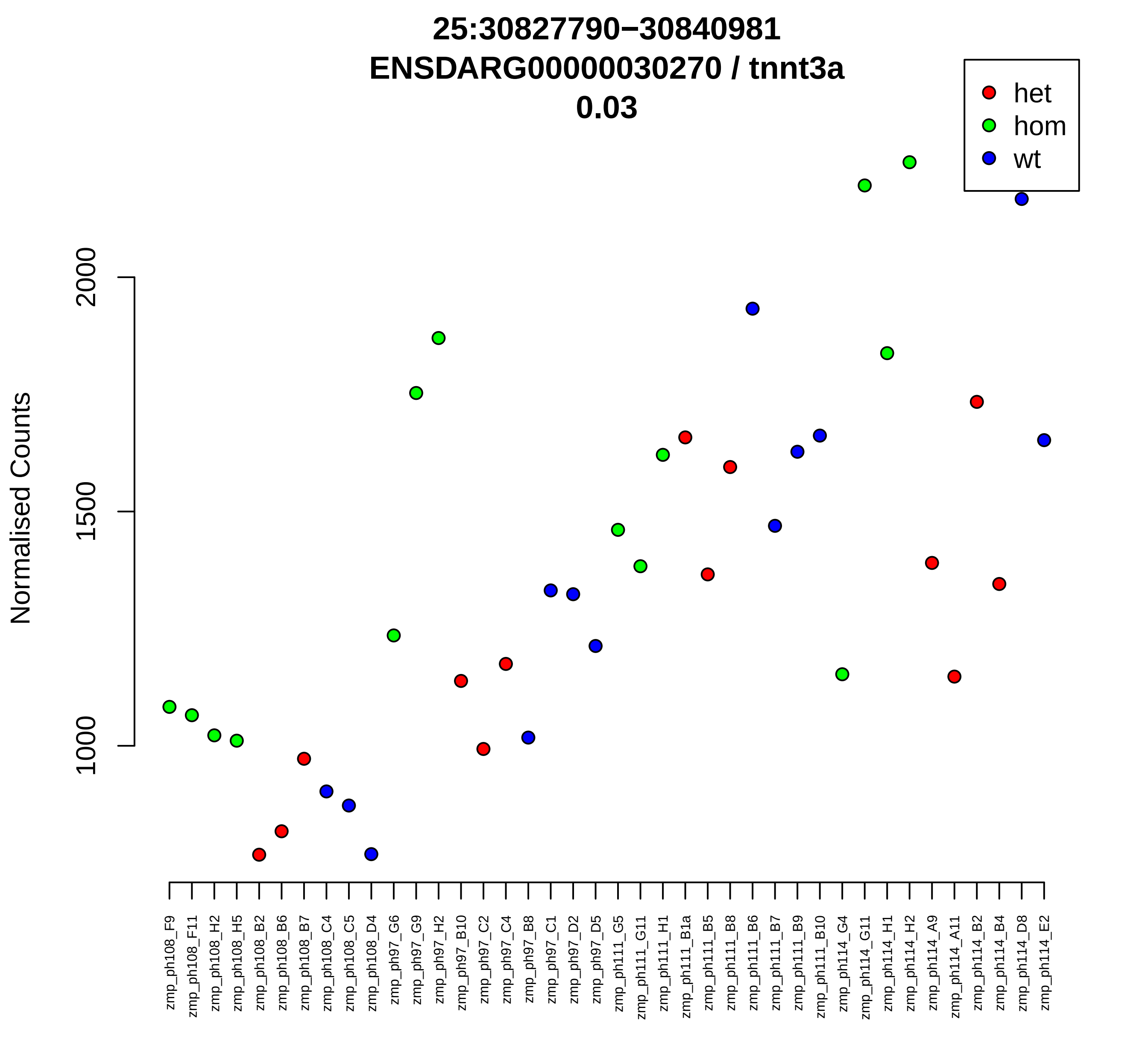

25:30827790−30840981
ENSDARG00000030270 / tnnt3a
0.03
het
hom
wt
Normalised Counts
1000
1500
2000
zmp_ph108_F9
zmp_ph108_F11
zmp_ph108_H2
zmp_ph108_H5
zmp_ph108_B2
zmp_ph108_B6
zmp_ph108_B7
zmp_ph108_C4
zmp_ph108_C5
zmp_ph108_D4
zmp_ph97_G6
zmp_ph97_G9
zmp_ph97_H2
zmp_ph97_B10
zmp_ph97_C2
zmp_ph97_C4
zmp_ph97_B8
zmp_ph97_C1
zmp_ph97_D2
zmp_ph97_D5
zmp_ph111_G5
zmp_ph111_G11
zmp_ph111_H1
zmp_ph111_B1a
zmp_ph111_B5
zmp_ph111_B8
zmp_ph111_B6
zmp_ph111_B7
zmp_ph111_B9
zmp_ph111_B10
zmp_ph114_G4
zmp_ph114_G11
zmp_ph114_H1
zmp_ph114_H2
zmp_ph114_A9
zmp_ph114_A11
zmp_ph114_B2
zmp_ph114_B4
zmp_ph114_D8
zmp_ph114_E2

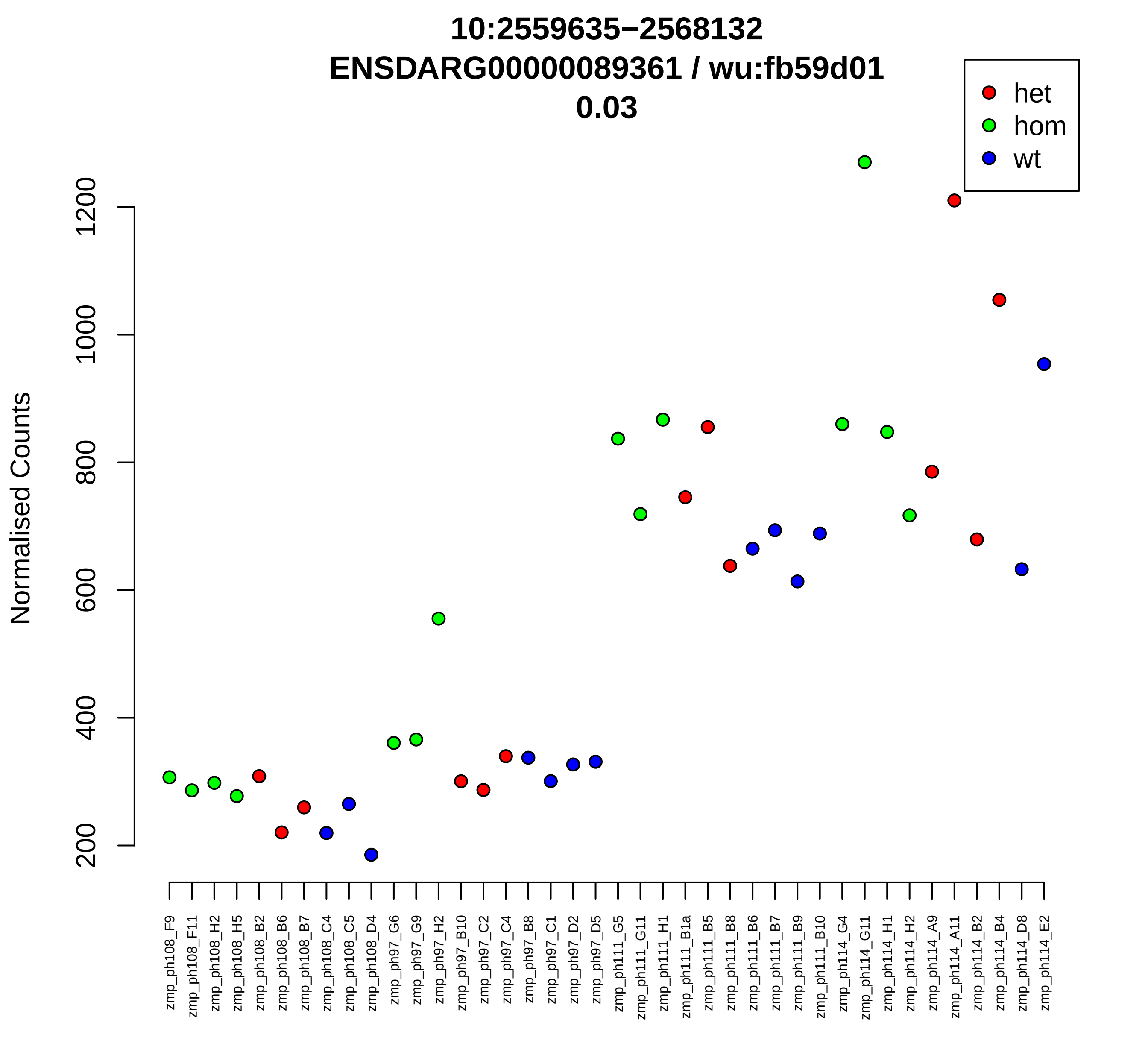
10:2559635−2568132
ENSDARG00000089361 / wu:fb59d01
0.03
het
hom
wt
Normalised Counts
200
400
600
800
1000
1200
zmp_ph108_F9
zmp_ph108_F11
zmp_ph108_H2
zmp_ph108_H5
zmp_ph108_B2
zmp_ph108_B6
zmp_ph108_B7
zmp_ph108_C4
zmp_ph108_C5
zmp_ph108_D4
zmp_ph97_G6
zmp_ph97_G9
zmp_ph97_H2
zmp_ph97_B10
zmp_ph97_C2
zmp_ph97_C4
zmp_ph97_B8
zmp_ph97_C1
zmp_ph97_D2
zmp_ph97_D5
zmp_ph111_G5
zmp_ph111_G11
zmp_ph111_H1
zmp_ph111_B1a
zmp_ph111_B5
zmp_ph111_B8
zmp_ph111_B6
zmp_ph111_B7
zmp_ph111_B9
zmp_ph111_B10
zmp_ph114_G4
zmp_ph114_G11
zmp_ph114_H1
zmp_ph114_H2
zmp_ph114_A9
zmp_ph114_A11
zmp_ph114_B2
zmp_ph114_B4
zmp_ph114_D8
zmp_ph114_E2

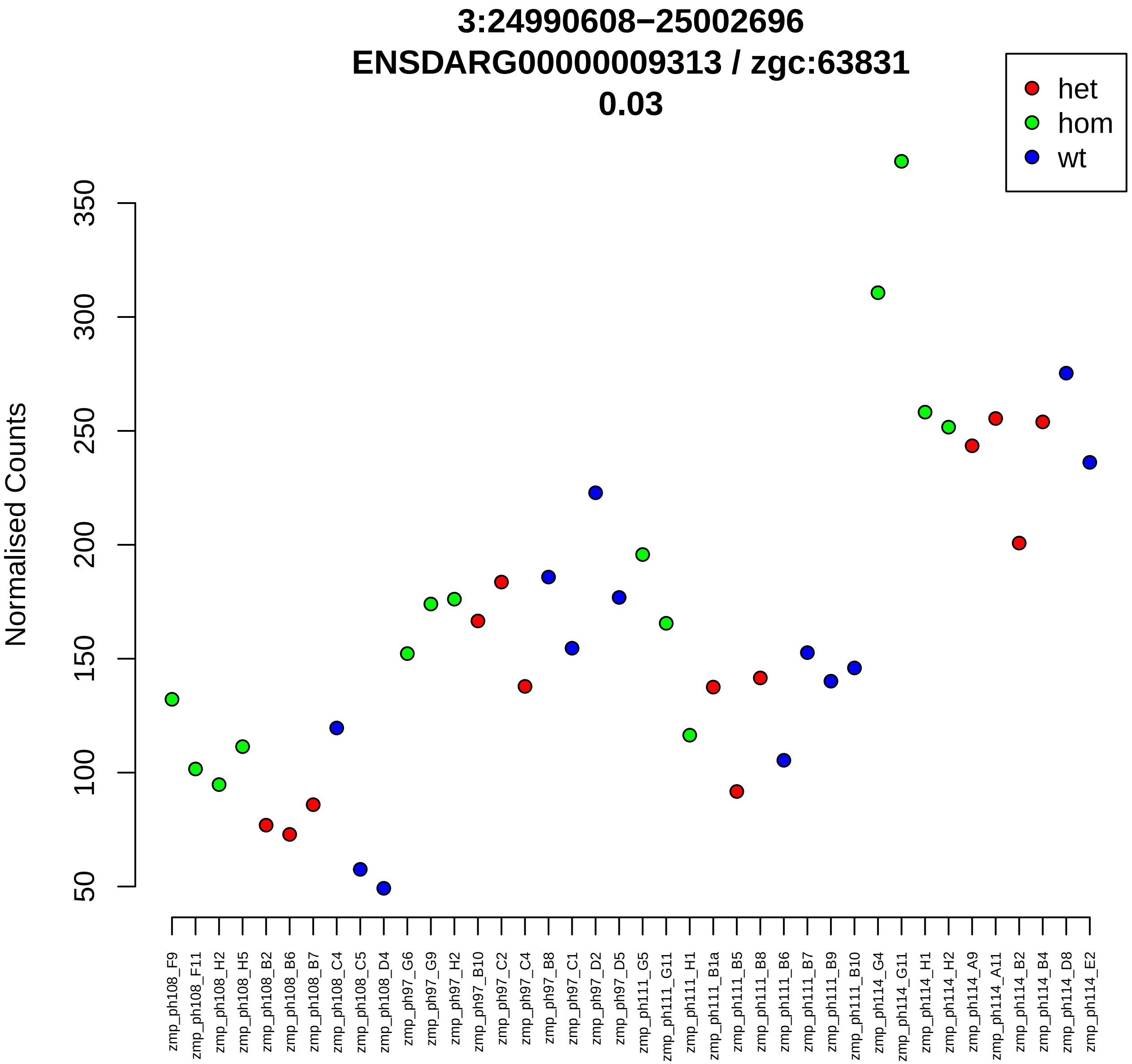
3:24990608−25002696
ENSDARG00000009313 / zgc:63831
0.03
het
hom
wt
Normalised Counts
50
100
150
200
250
300
350
zmp_ph108_F9
zmp_ph108_F11
zmp_ph108_H2
zmp_ph108_H5
zmp_ph108_B2
zmp_ph108_B6
zmp_ph108_B7
zmp_ph108_C4
zmp_ph108_C5
zmp_ph108_D4
zmp_ph97_G6
zmp_ph97_G9
zmp_ph97_H2
zmp_ph97_B10
zmp_ph97_C2
zmp_ph97_C4
zmp_ph97_B8
zmp_ph97_C1
zmp_ph97_D2
zmp_ph97_D5
zmp_ph111_G5
zmp_ph111_G11
zmp_ph111_H1
zmp_ph111_B1a
zmp_ph111_B5
zmp_ph111_B8
zmp_ph111_B6
zmp_ph111_B7
zmp_ph111_B9
zmp_ph111_B10
zmp_ph114_G4
zmp_ph114_G11
zmp_ph114_H1
zmp_ph114_H2
zmp_ph114_A9
zmp_ph114_A11
zmp_ph114_B2
zmp_ph114_B4
zmp_ph114_D8
zmp_ph114_E2

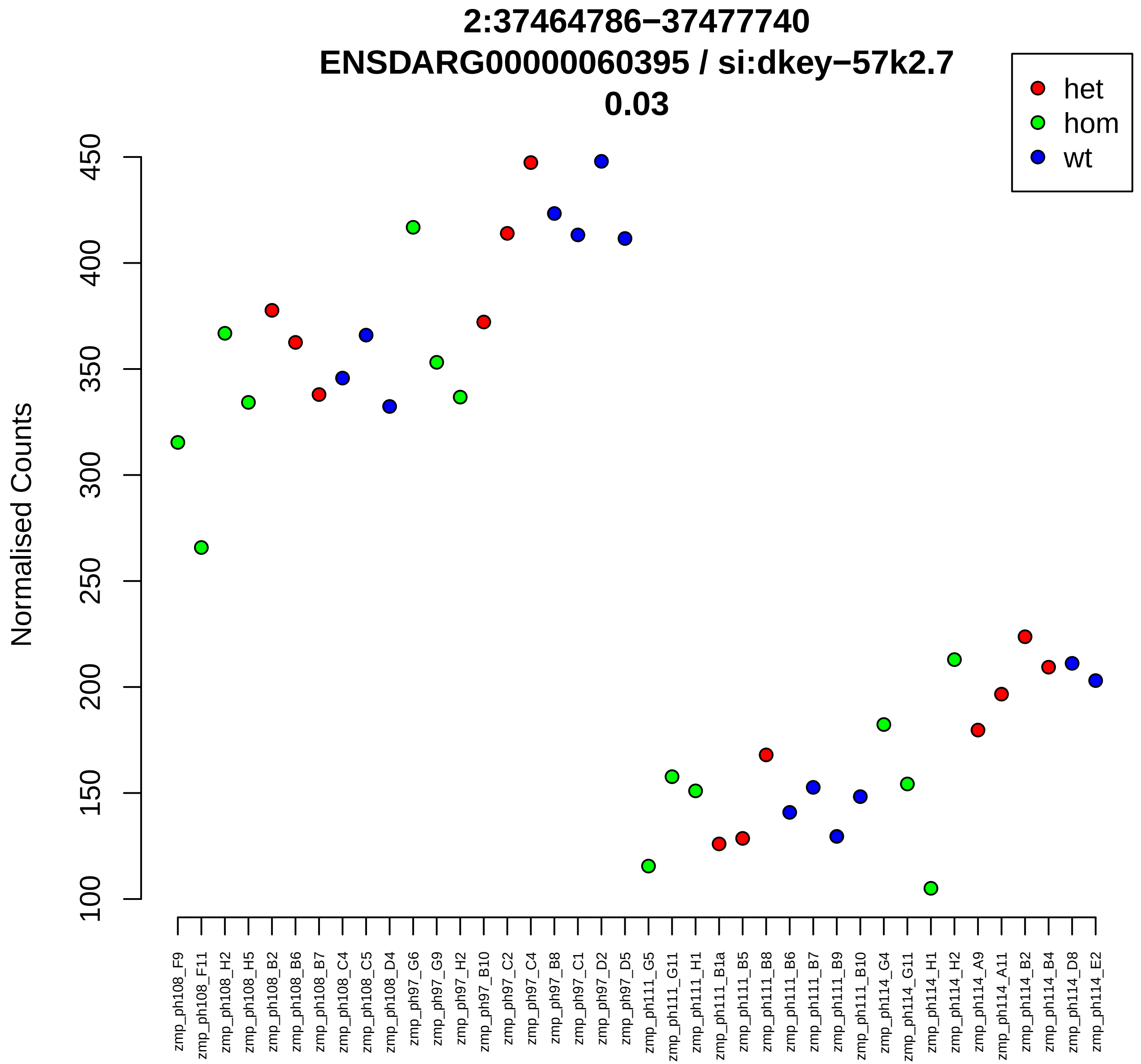

2:37464786−37477740
ENSDARG00000060395 / si:dkey−57k2.7
0.03
het
hom
wt
Normalised Counts
100
150
200
250
300
350
400
450
zmp_ph108_F9
zmp_ph108_F11
zmp_ph108_H2
zmp_ph108_H5
zmp_ph108_B2
zmp_ph108_B6
zmp_ph108_B7
zmp_ph108_C4
zmp_ph108_C5
zmp_ph108_D4
zmp_ph97_G6
zmp_ph97_G9
zmp_ph97_H2
zmp_ph97_B10
zmp_ph97_C2
zmp_ph97_C4
zmp_ph97_B8
zmp_ph97_C1
zmp_ph97_D2
zmp_ph97_D5
zmp_ph111_G5
zmp_ph111_G11
zmp_ph111_H1
zmp_ph111_B1a
zmp_ph111_B5
zmp_ph111_B8
zmp_ph111_B6
zmp_ph111_B7
zmp_ph111_B9
zmp_ph111_B10
zmp_ph114_G4
zmp_ph114_G11
zmp_ph114_H1
zmp_ph114_H2
zmp_ph114_A9
zmp_ph114_A11
zmp_ph114_B2
zmp_ph114_B4
zmp_ph114_D8
zmp_ph114_E2

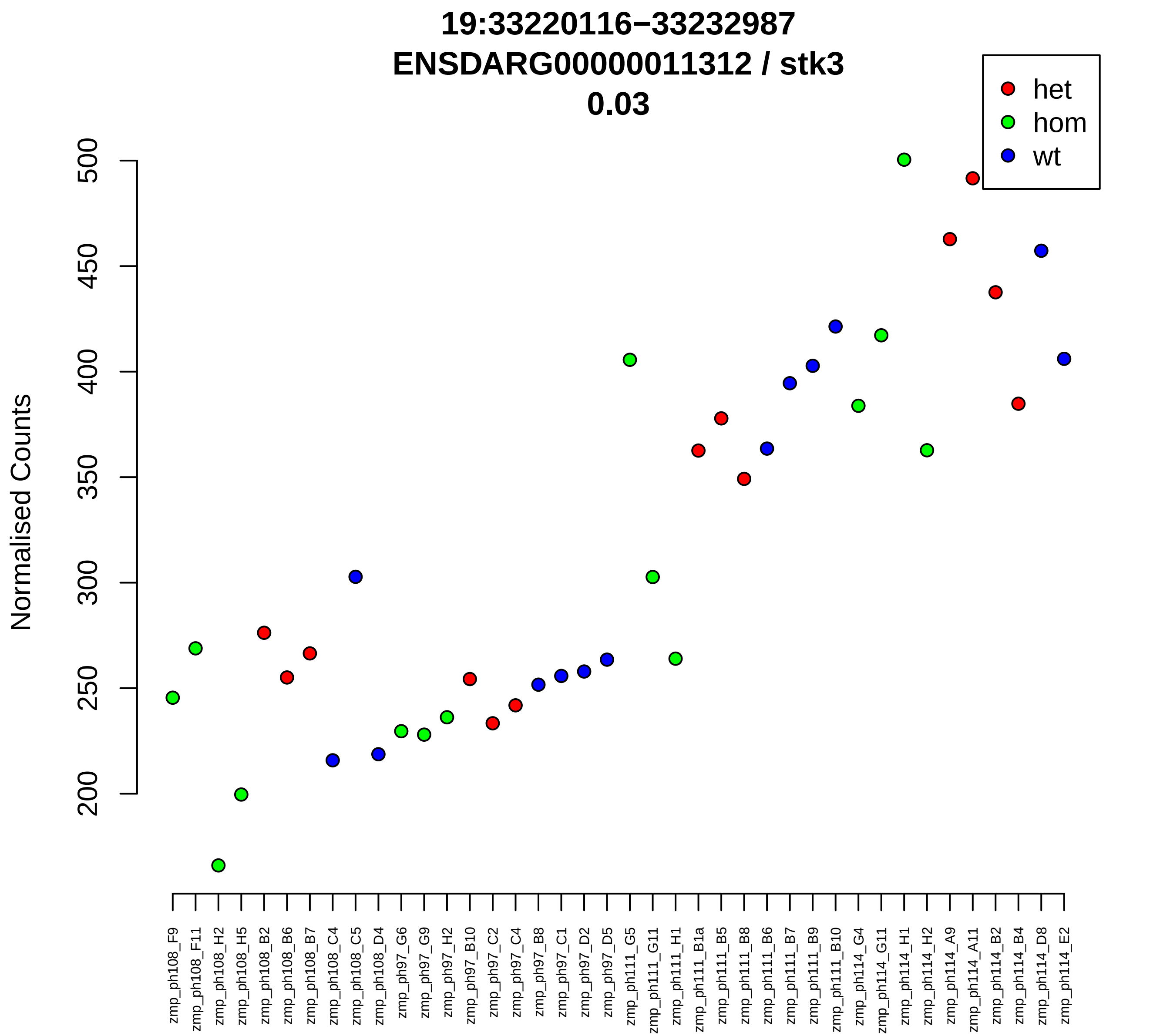
19:33220116−33232987
ENSDARG00000011312 / stk3
0.03
het
hom
wt
Normalised Counts
200
250
300
350
400
450
500
zmp_ph108_F9
zmp_ph108_F11
zmp_ph108_H2
zmp_ph108_H5
zmp_ph108_B2
zmp_ph108_B6
zmp_ph108_B7
zmp_ph108_C4
zmp_ph108_C5
zmp_ph108_D4
zmp_ph97_G6
zmp_ph97_G9
zmp_ph97_H2
zmp_ph97_B10
zmp_ph97_C2
zmp_ph97_C4
zmp_ph97_B8
zmp_ph97_C1
zmp_ph97_D2
zmp_ph97_D5
zmp_ph111_G5
zmp_ph111_G11
zmp_ph111_H1
zmp_ph111_B1a
zmp_ph111_B5
zmp_ph111_B8
zmp_ph111_B6
zmp_ph111_B7
zmp_ph111_B9
zmp_ph111_B10
zmp_ph114_G4
zmp_ph114_G11
zmp_ph114_H1
zmp_ph114_H2
zmp_ph114_A9
zmp_ph114_A11
zmp_ph114_B2
zmp_ph114_B4
zmp_ph114_D8
zmp_ph114_E2

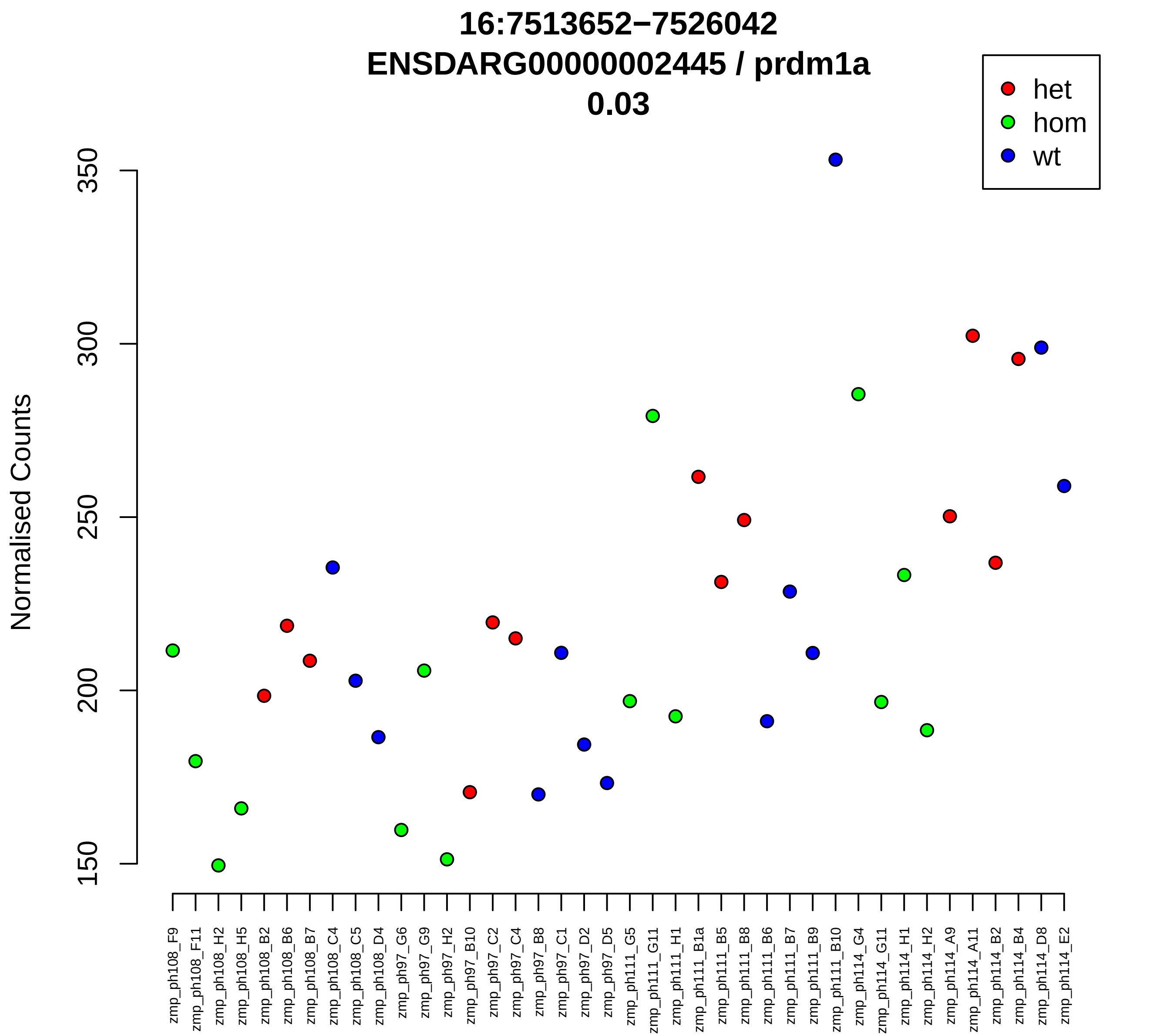
16:7513652−7526042
ENSDARG00000002445 / prdm1a
0.03
het
hom
wt
Normalised Counts
150
200
250
300
350
zmp_ph108_F9
zmp_ph108_F11
zmp_ph108_H2
zmp_ph108_H5
zmp_ph108_B2
zmp_ph108_B6
zmp_ph108_B7
zmp_ph108_C4
zmp_ph108_C5
zmp_ph108_D4
zmp_ph97_G6
zmp_ph97_G9
zmp_ph97_H2
zmp_ph97_B10
zmp_ph97_C2
zmp_ph97_C4
zmp_ph97_B8
zmp_ph97_C1
zmp_ph97_D2
zmp_ph97_D5
zmp_ph111_G5
zmp_ph111_G11
zmp_ph111_H1
zmp_ph111_B1a
zmp_ph111_B5
zmp_ph111_B8
zmp_ph111_B6
zmp_ph111_B7
zmp_ph111_B9
zmp_ph111_B10
zmp_ph114_G4
zmp_ph114_G11
zmp_ph114_H1
zmp_ph114_H2
zmp_ph114_A9
zmp_ph114_A11
zmp_ph114_B2
zmp_ph114_B4
zmp_ph114_D8
zmp_ph114_E2

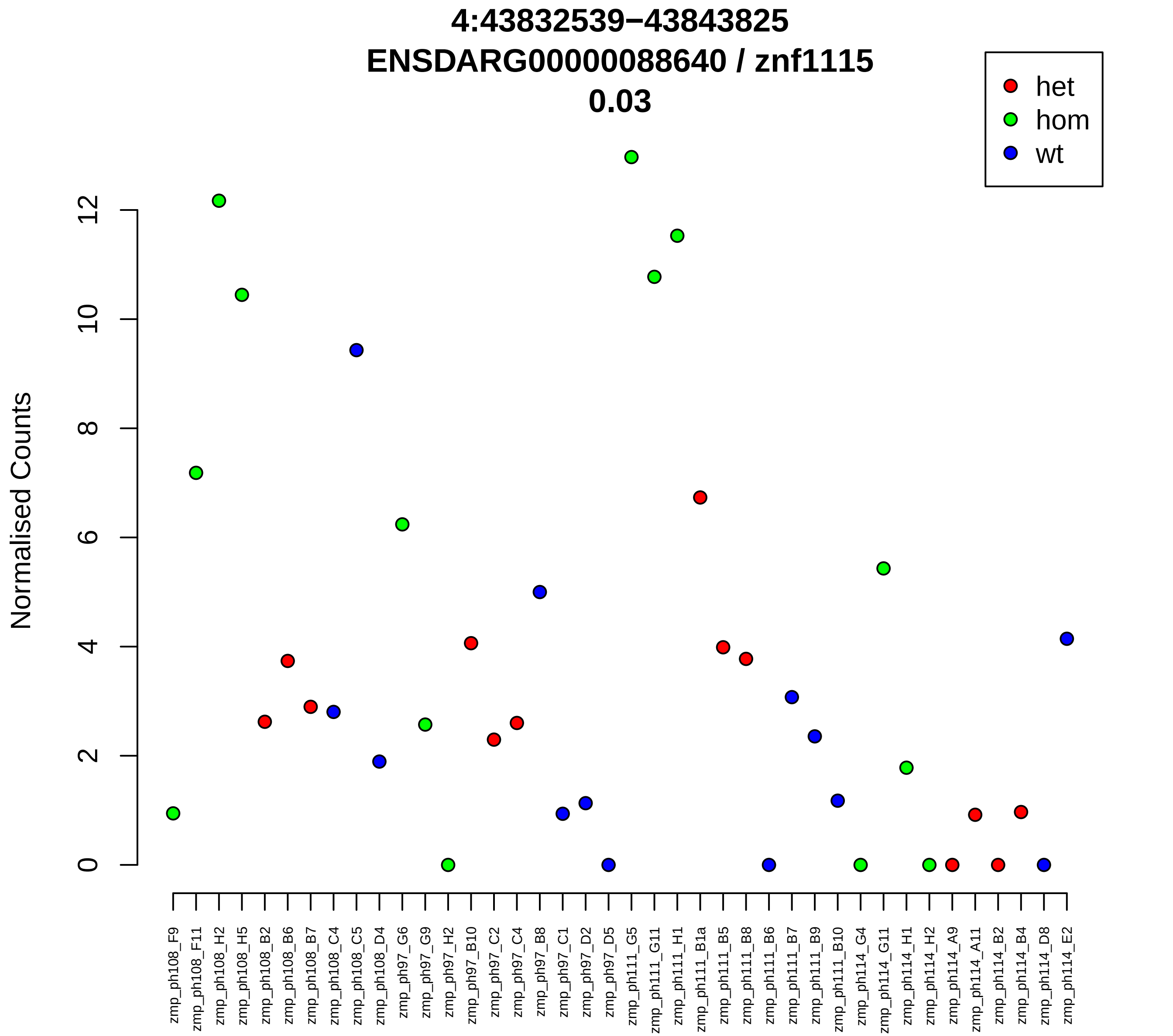

4:43832539−43843825
ENSDARG00000088640 / znf1115
0.03
het
hom
wt
Normalised Counts
0
2
4
6
8
10
12
zmp_ph108_F9
zmp_ph108_F11
zmp_ph108_H2
zmp_ph108_H5
zmp_ph108_B2
zmp_ph108_B6
zmp_ph108_B7
zmp_ph108_C4
zmp_ph108_C5
zmp_ph108_D4
zmp_ph97_G6
zmp_ph97_G9
zmp_ph97_H2
zmp_ph97_B10
zmp_ph97_C2
zmp_ph97_C4
zmp_ph97_B8
zmp_ph97_C1
zmp_ph97_D2
zmp_ph97_D5
zmp_ph111_G5
zmp_ph111_G11
zmp_ph111_H1
zmp_ph111_B1a
zmp_ph111_B5
zmp_ph111_B8
zmp_ph111_B6
zmp_ph111_B7
zmp_ph111_B9
zmp_ph111_B10
zmp_ph114_G4
zmp_ph114_G11
zmp_ph114_H1
zmp_ph114_H2
zmp_ph114_A9
zmp_ph114_A11
zmp_ph114_B2
zmp_ph114_B4
zmp_ph114_D8
zmp_ph114_E2

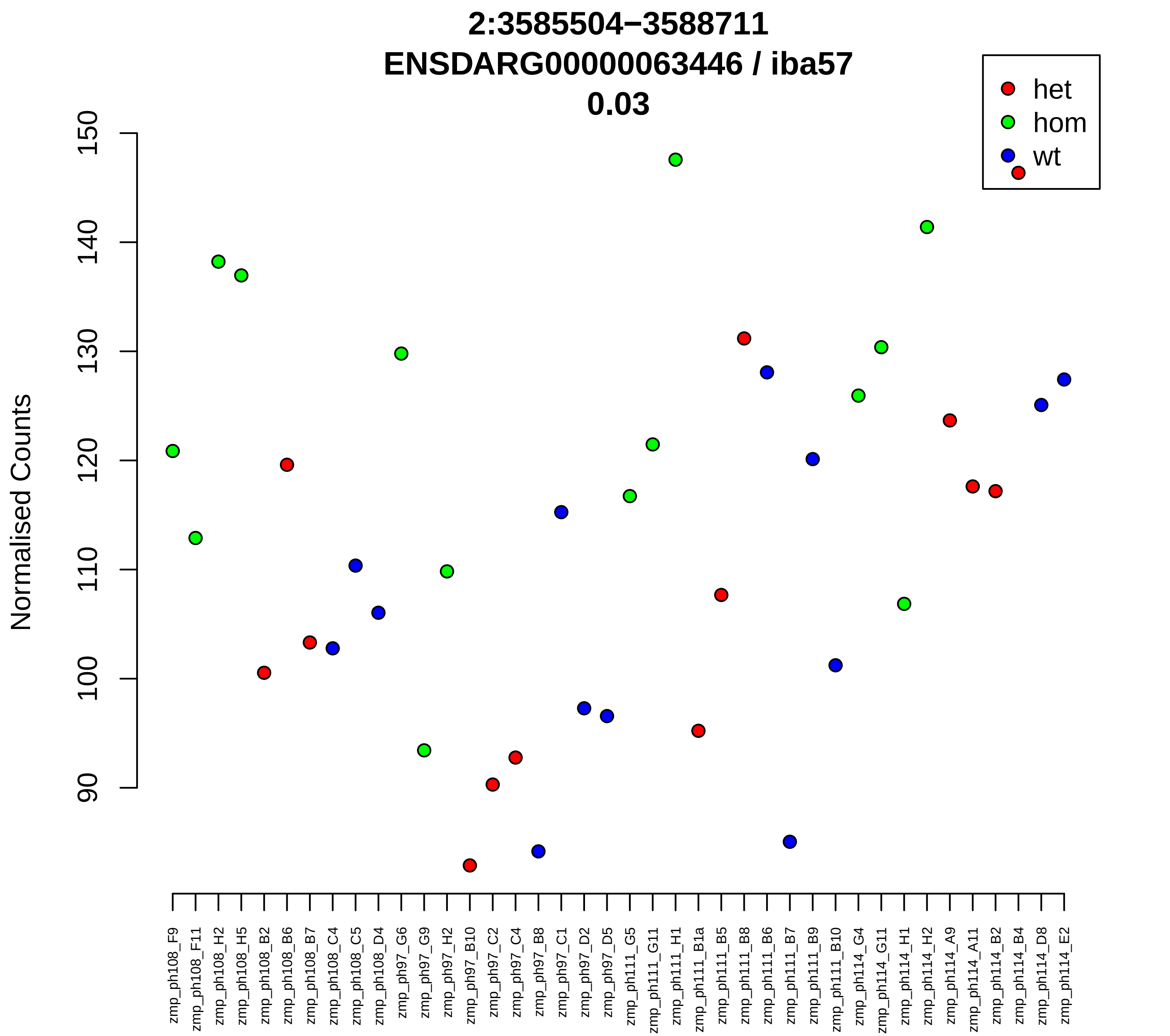
2:3585504−3588711
ENSDARG00000063446 / iba57
0.03
Normalised Counts
90
100
110
120
130
140
150
het
hom
wt
zmp_ph108_F9
zmp_ph108_F11
zmp_ph108_H2
zmp_ph108_H5
zmp_ph108_B2
zmp_ph108_B6
zmp_ph108_B7
zmp_ph108_C4
zmp_ph108_C5
zmp_ph108_D4
zmp_ph97_G6
zmp_ph97_G9
zmp_ph97_H2
zmp_ph97_B10
zmp_ph97_C2
zmp_ph97_C4
zmp_ph97_B8
zmp_ph97_C1
zmp_ph97_D2
zmp_ph97_D5
zmp_ph111_G5
zmp_ph111_G11
zmp_ph111_H1
zmp_ph111_B1a
zmp_ph111_B5
zmp_ph111_B8
zmp_ph111_B6
zmp_ph111_B7
zmp_ph111_B9
zmp_ph111_B10
zmp_ph114_G4
zmp_ph114_G11
zmp_ph114_H1
zmp_ph114_H2
zmp_ph114_A9
zmp_ph114_A11
zmp_ph114_B2
zmp_ph114_B4
zmp_ph114_D8
zmp_ph114_E2

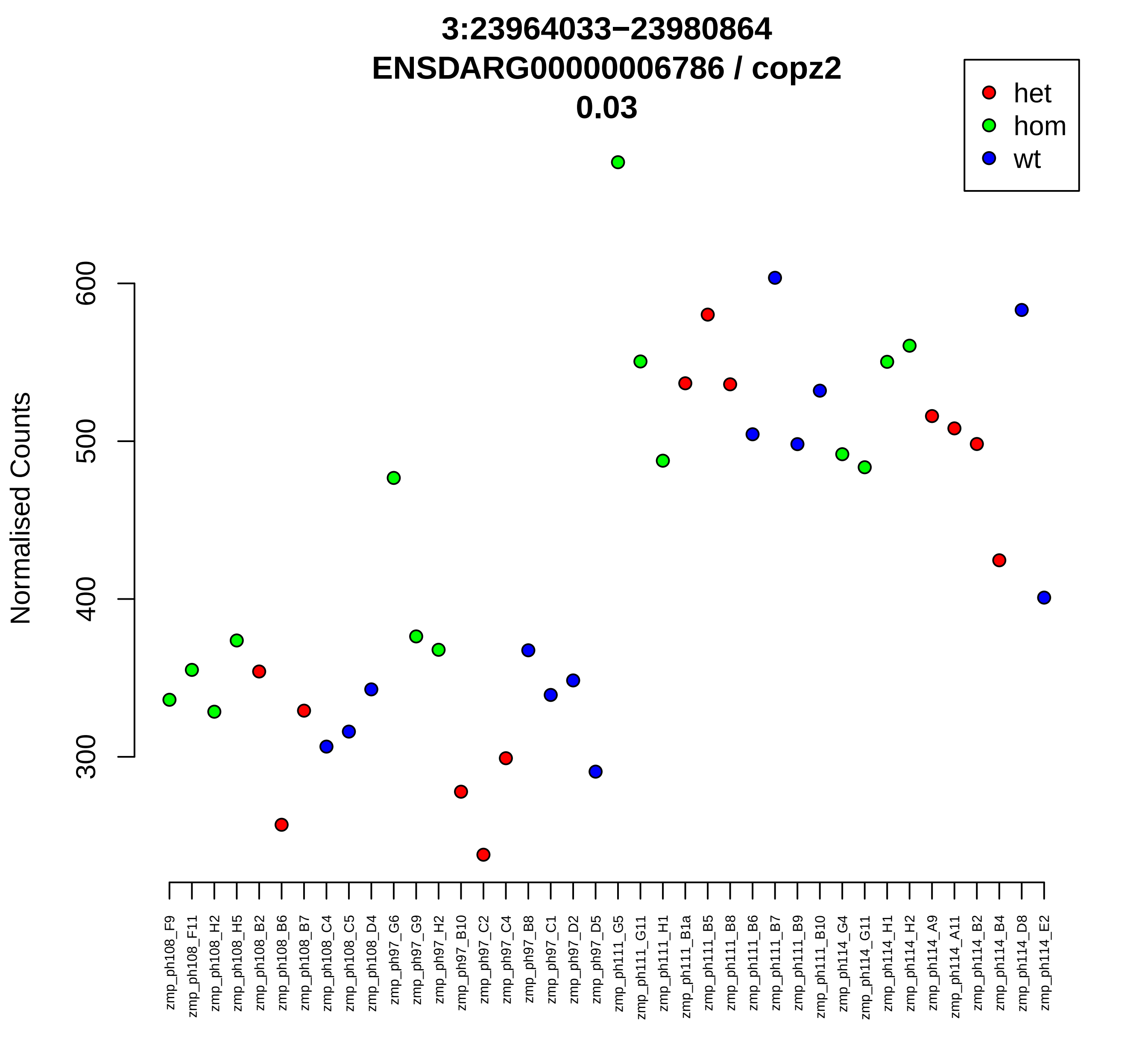

3:23964033−23980864
ENSDARG00000006786 / copz2
0.03
het
hom
wt
Normalised Counts
300
400
500
600
zmp_ph108_F9
zmp_ph108_F11
zmp_ph108_H2
zmp_ph108_H5
zmp_ph108_B2
zmp_ph108_B6
zmp_ph108_B7
zmp_ph108_C4
zmp_ph108_C5
zmp_ph108_D4
zmp_ph97_G6
zmp_ph97_G9
zmp_ph97_H2
zmp_ph97_B10
zmp_ph97_C2
zmp_ph97_C4
zmp_ph97_B8
zmp_ph97_C1
zmp_ph97_D2
zmp_ph97_D5
zmp_ph111_G5
zmp_ph111_G11
zmp_ph111_H1
zmp_ph111_B1a
zmp_ph111_B5
zmp_ph111_B8
zmp_ph111_B6
zmp_ph111_B7
zmp_ph111_B9
zmp_ph111_B10
zmp_ph114_G4
zmp_ph114_G11
zmp_ph114_H1
zmp_ph114_H2
zmp_ph114_A9
zmp_ph114_A11
zmp_ph114_B2
zmp_ph114_B4
zmp_ph114_D8
zmp_ph114_E2

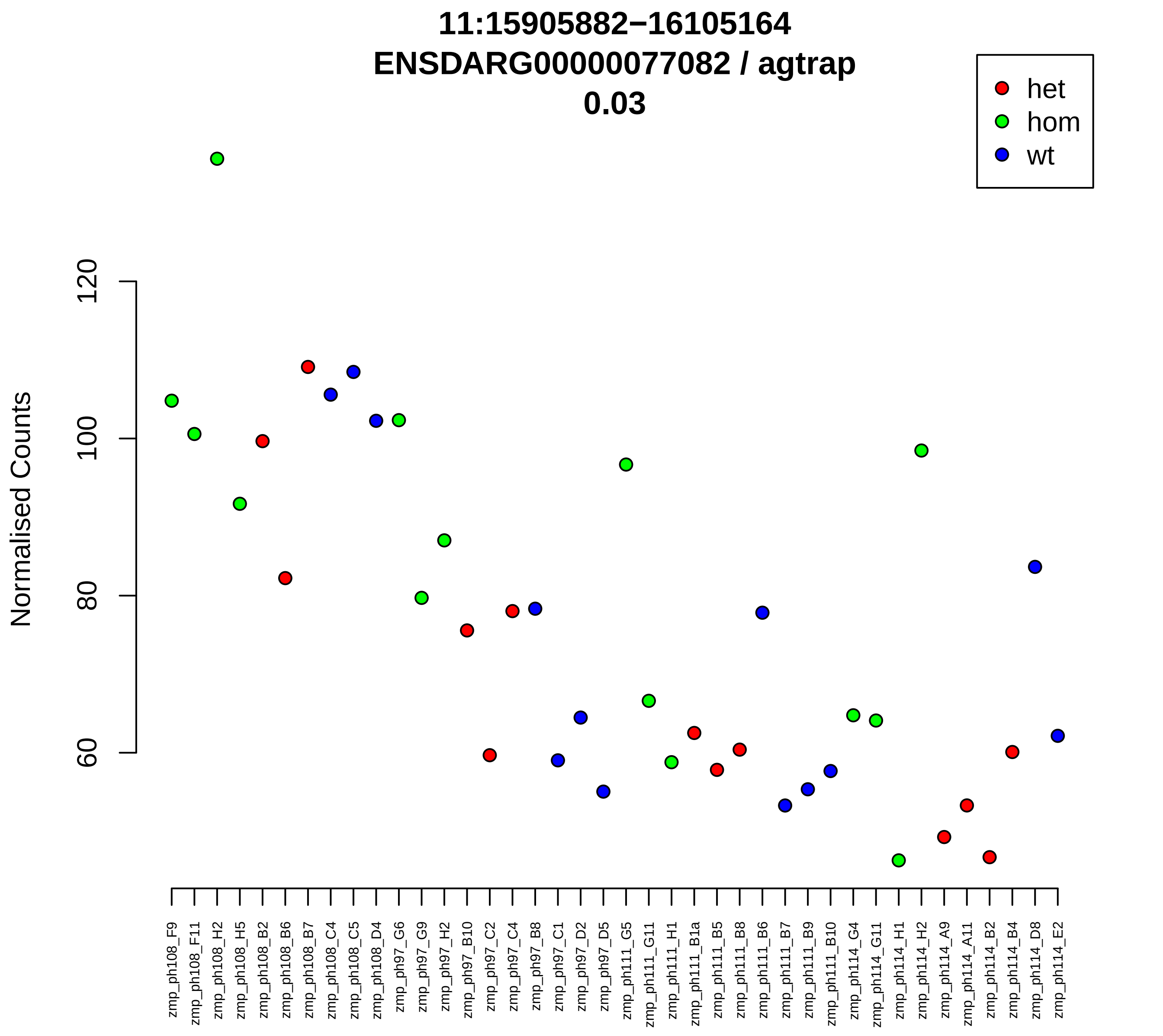

11:15905882−16105164
ENSDARG00000077082 / agtrap
0.03
het
hom
wt
Normalised Counts
120
100
80
60
zmp_ph108_F9
zmp_ph108_F11
zmp_ph108_H2
zmp_ph108_H5
zmp_ph108_B2
zmp_ph108_B6
zmp_ph108_B7
zmp_ph108_C4
zmp_ph108_C5
zmp_ph108_D4
zmp_ph97_G6
zmp_ph97_G9
zmp_ph97_H2
zmp_ph97_B10
zmp_ph97_C2
zmp_ph97_C4
zmp_ph97_B8
zmp_ph97_C1
zmp_ph97_D2
zmp_ph97_D5
zmp_ph111_G5
zmp_ph111_G11
zmp_ph111_H1
zmp_ph111_B1a
zmp_ph111_B5
zmp_ph111_B8
zmp_ph111_B6
zmp_ph111_B7
zmp_ph111_B9
zmp_ph111_B10
zmp_ph114_G4
zmp_ph114_G11
zmp_ph114_H1
zmp_ph114_H2
zmp_ph114_A9
zmp_ph114_A11
zmp_ph114_B2
zmp_ph114_B4
zmp_ph114_D8
zmp_ph114_E2

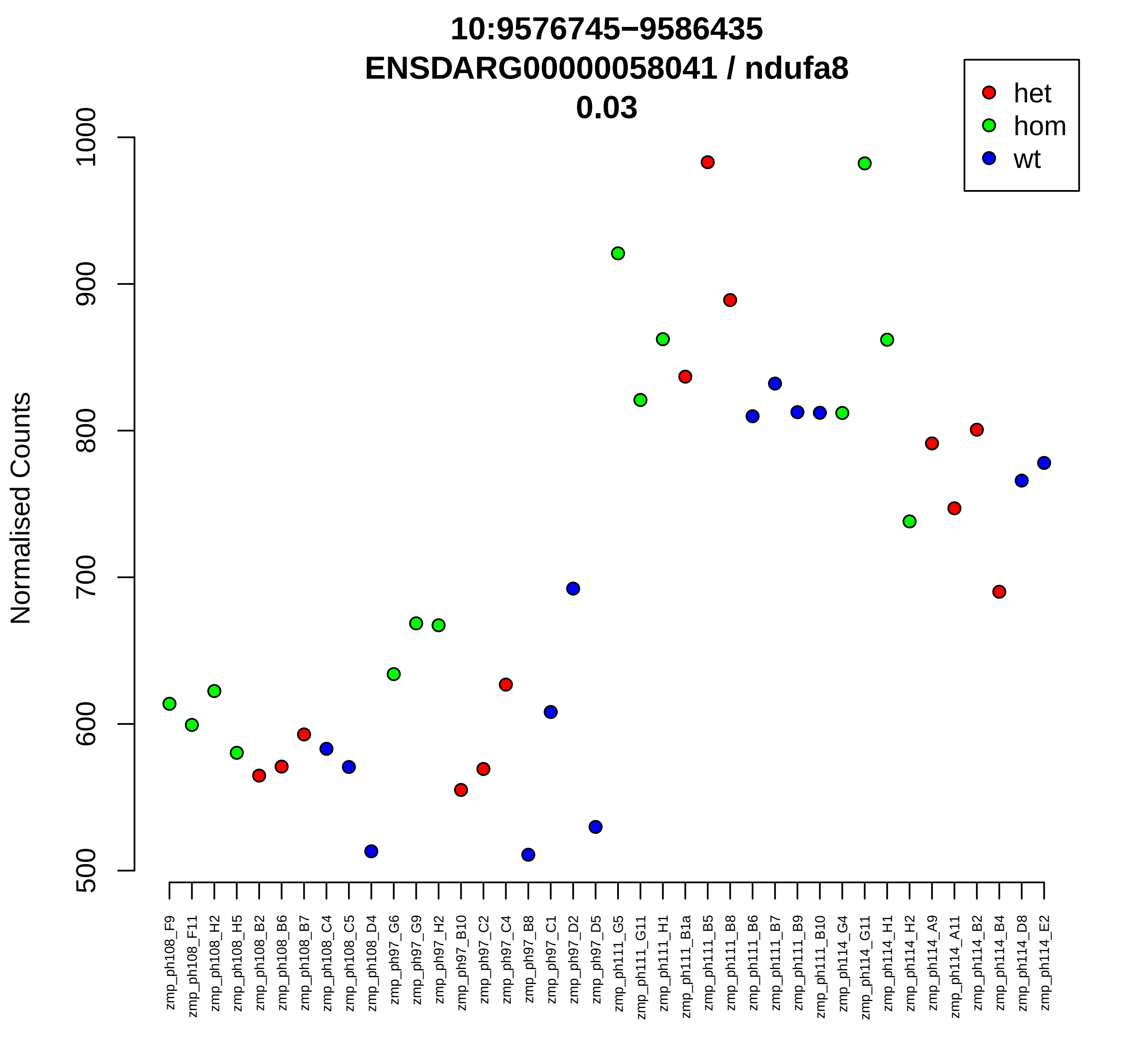

10:9576745−9586435
ENSDARG00000058041 / ndufa8
0.03
Normalised Counts
500
600
700
800
900
1000
het
hom
wt
zmp_ph108_F9
zmp_ph108_F11
zmp_ph108_H2
zmp_ph108_H5
zmp_ph108_B2
zmp_ph108_B6
zmp_ph108_B7
zmp_ph108_C4
zmp_ph108_C5
zmp_ph108_D4
zmp_ph97_G6
zmp_ph97_G9
zmp_ph97_H2
zmp_ph97_B10
zmp_ph97_C2
zmp_ph97_C4
zmp_ph97_B8
zmp_ph97_C1
zmp_ph97_D2
zmp_ph97_D5
zmp_ph111_G5
zmp_ph111_G11
zmp_ph111_H1
zmp_ph111_B1a
zmp_ph111_B5
zmp_ph111_B8
zmp_ph111_B6
zmp_ph111_B7
zmp_ph111_B9
zmp_ph111_B10
zmp_ph114_G4
zmp_ph114_G11
zmp_ph114_H1
zmp_ph114_H2
zmp_ph114_A9
zmp_ph114_A11
zmp_ph114_B2
zmp_ph114_B4
zmp_ph114_D8
zmp_ph114_E2

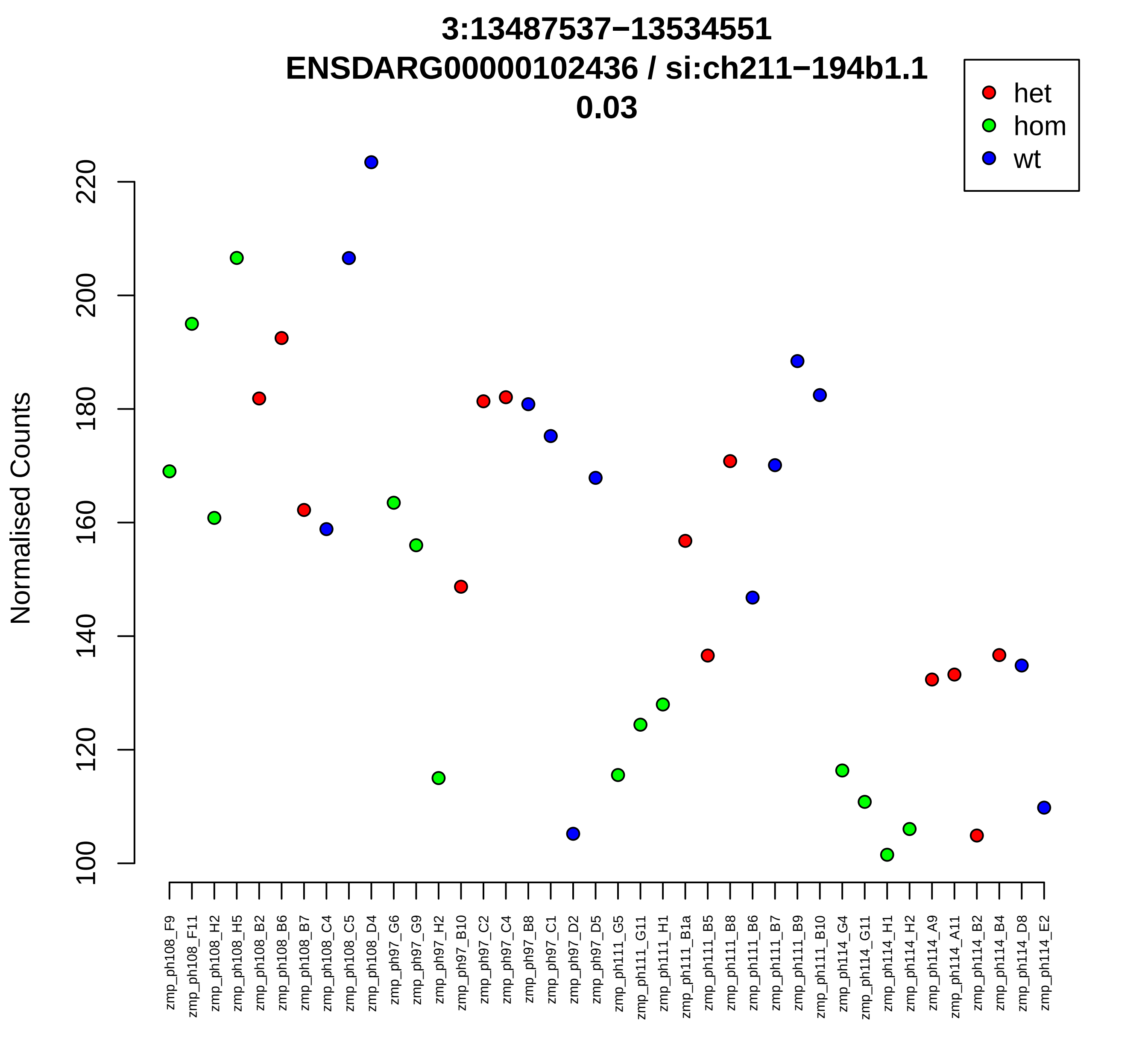
3:13487537−13534551
ENSDARG00000102436 / si:ch211−194b1.1
0.03
het
hom
wt
Normalised Counts
100
120
140
160
180
200
220
zmp_ph108_F9
zmp_ph108_F11
zmp_ph108_H2
zmp_ph108_H5
zmp_ph108_B2
zmp_ph108_B6
zmp_ph108_B7
zmp_ph108_C4
zmp_ph108_C5
zmp_ph108_D4
zmp_ph97_G6
zmp_ph97_G9
zmp_ph97_H2
zmp_ph97_B10
zmp_ph97_C2
zmp_ph97_C4
zmp_ph97_B8
zmp_ph97_C1
zmp_ph97_D2
zmp_ph97_D5
zmp_ph111_G5
zmp_ph111_G11
zmp_ph111_H1
zmp_ph111_B1a
zmp_ph111_B5
zmp_ph111_B8
zmp_ph111_B6
zmp_ph111_B7
zmp_ph111_B9
zmp_ph111_B10
zmp_ph114_G4
zmp_ph114_G11
zmp_ph114_H1
zmp_ph114_H2
zmp_ph114_A9
zmp_ph114_A11
zmp_ph114_B2
zmp_ph114_B4
zmp_ph114_D8
zmp_ph114_E2

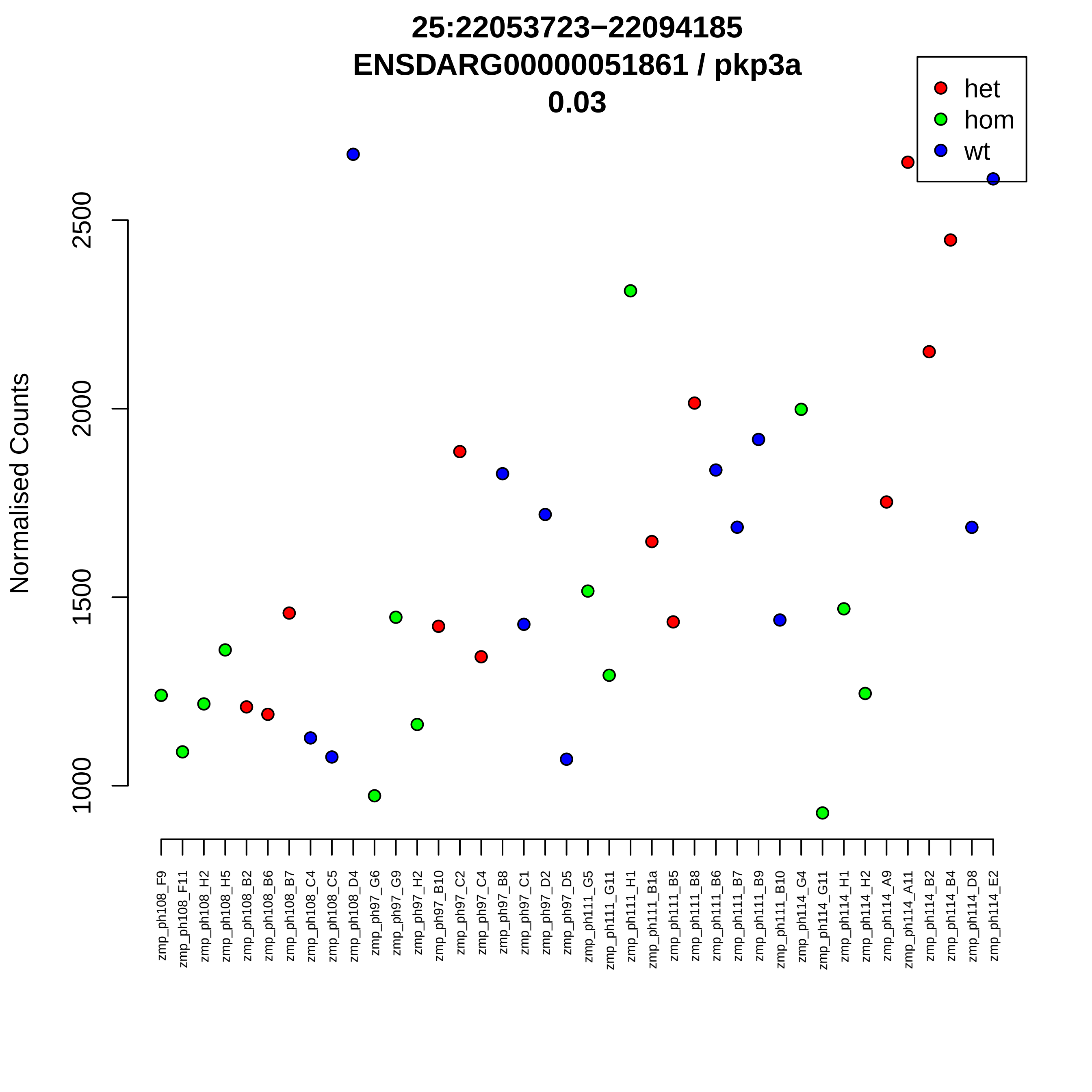
25:22053723−22094185
ENSDARG00000051861 / pkp3a
0.03
het
hom
wt
Normalised Counts
1000
1500
2000
2500
zmp_ph108_F9
zmp_ph108_F11
zmp_ph108_H2
zmp_ph108_H5
zmp_ph108_B2
zmp_ph108_B6
zmp_ph108_B7
zmp_ph108_C4
zmp_ph108_C5
zmp_ph108_D4
zmp_ph97_G6
zmp_ph97_G9
zmp_ph97_H2
zmp_ph97_B10
zmp_ph97_C2
zmp_ph97_C4
zmp_ph97_B8
zmp_ph97_C1
zmp_ph97_D2
zmp_ph97_D5
zmp_ph111_G5
zmp_ph111_G11
zmp_ph111_H1
zmp_ph111_B1a
zmp_ph111_B5
zmp_ph111_B8
zmp_ph111_B6
zmp_ph111_B7
zmp_ph111_B9
zmp_ph111_B10
zmp_ph114_G4
zmp_ph114_G11
zmp_ph114_H1
zmp_ph114_H2
zmp_ph114_A9
zmp_ph114_A11
zmp_ph114_B2
zmp_ph114_B4
zmp_ph114_D8
zmp_ph114_E2

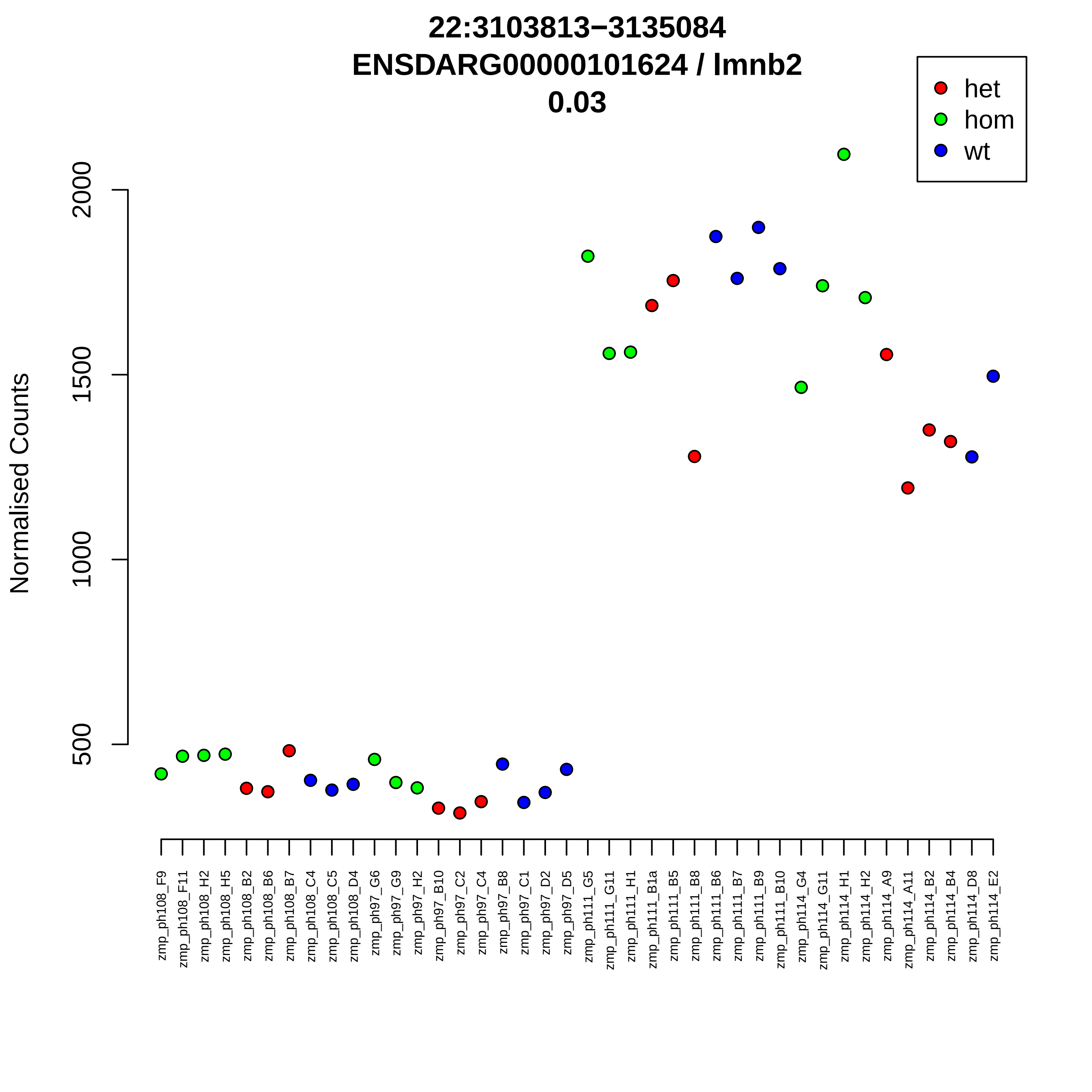

22:3103813−3135084
ENSDARG00000101624 / lmnb2
0.03
Normalised Counts
500
1000
1500
2000
het
hom
wt
zmp_ph108_F9
zmp_ph108_F11
zmp_ph108_H2
zmp_ph108_H5
zmp_ph108_B2
zmp_ph108_B6
zmp_ph108_B7
zmp_ph108_C4
zmp_ph108_C5
zmp_ph108_D4
zmp_ph97_G6
zmp_ph97_G9
zmp_ph97_H2
zmp_ph97_B10
zmp_ph97_C2
zmp_ph97_C4
zmp_ph97_B8
zmp_ph97_C1
zmp_ph97_D2
zmp_ph97_D5
zmp_ph111_G5
zmp_ph111_G11
zmp_ph111_H1
zmp_ph111_B1a
zmp_ph111_B5
zmp_ph111_B8
zmp_ph111_B6
zmp_ph111_B7
zmp_ph111_B9
zmp_ph111_B10
zmp_ph114_G4
zmp_ph114_G11
zmp_ph114_H1
zmp_ph114_H2
zmp_ph114_A9
zmp_ph114_A11
zmp_ph114_B2
zmp_ph114_B4
zmp_ph114_D8
zmp_ph114_E2

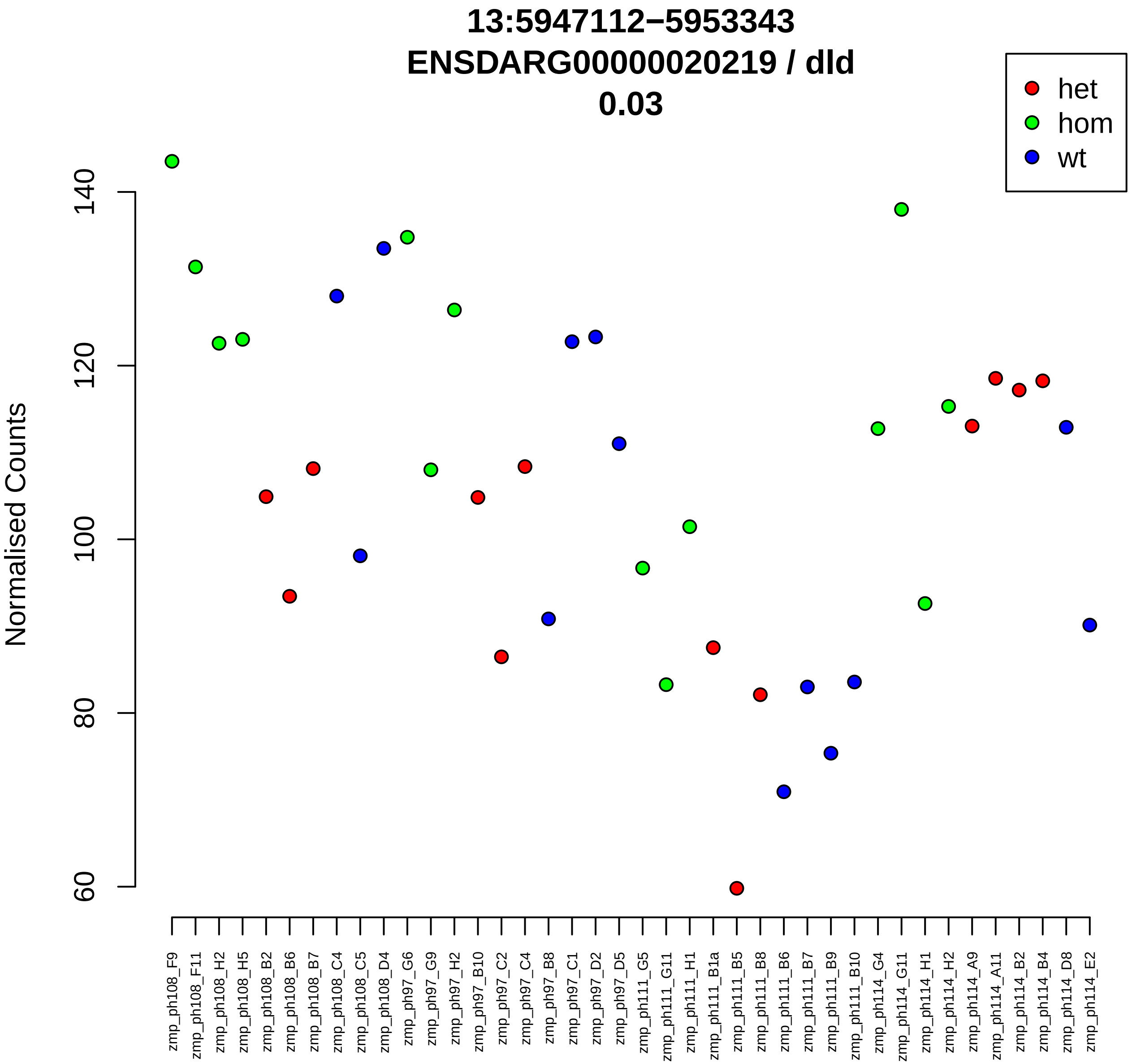

13:5947112−5953343
ENSDARG00000020219 / dld
0.03
het
hom
wt
Normalised Counts
60
80
100
120
140
zmp_ph108_F9
zmp_ph108_F11
zmp_ph108_H2
zmp_ph108_H5
zmp_ph108_B2
zmp_ph108_B6
zmp_ph108_B7
zmp_ph108_C4
zmp_ph108_C5
zmp_ph108_D4
zmp_ph97_G6
zmp_ph97_G9
zmp_ph97_H2
zmp_ph97_B10
zmp_ph97_C2
zmp_ph97_C4
zmp_ph97_B8
zmp_ph97_C1
zmp_ph97_D2
zmp_ph97_D5
zmp_ph111_G5
zmp_ph111_G11
zmp_ph111_H1
zmp_ph111_B1a
zmp_ph111_B5
zmp_ph111_B8
zmp_ph111_B6
zmp_ph111_B7
zmp_ph111_B9
zmp_ph111_B10
zmp_ph114_G4
zmp_ph114_G11
zmp_ph114_H1
zmp_ph114_H2
zmp_ph114_A9
zmp_ph114_A11
zmp_ph114_B2
zmp_ph114_B4
zmp_ph114_D8
zmp_ph114_E2

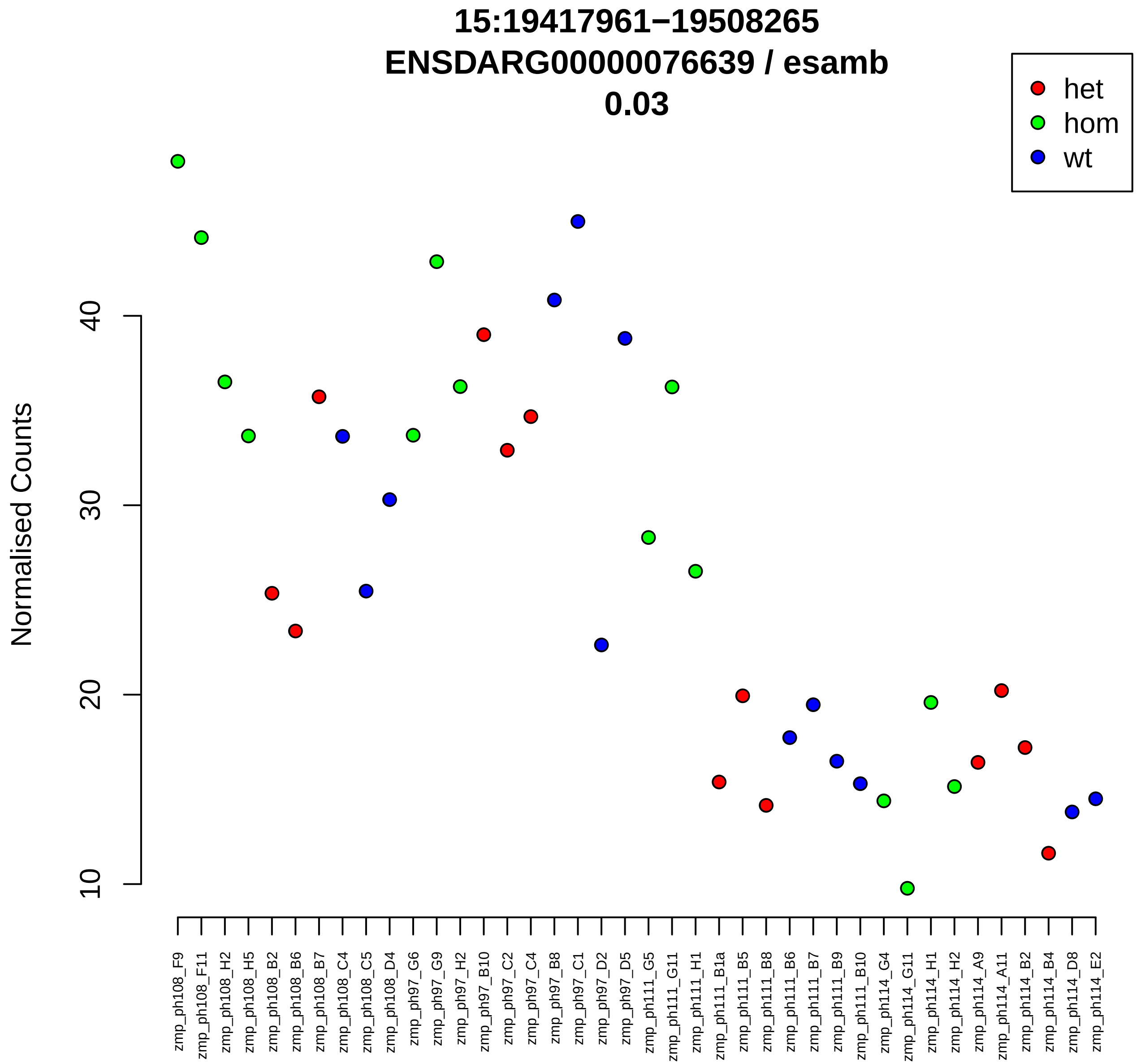
15:19417961−19508265
ENSDARG00000076639 / esamb
0.03
het
hom
wt
Normalised Counts
10
20
30
40
zmp_ph108_F9
zmp_ph108_F11
zmp_ph108_H2
zmp_ph108_H5
zmp_ph108_B2
zmp_ph108_B6
zmp_ph108_B7
zmp_ph108_C4
zmp_ph108_C5
zmp_ph108_D4
zmp_ph97_G6
zmp_ph97_G9
zmp_ph97_H2
zmp_ph97_B10
zmp_ph97_C2
zmp_ph97_C4
zmp_ph97_B8
zmp_ph97_C1
zmp_ph97_D2
zmp_ph97_D5
zmp_ph111_G5
zmp_ph111_G11
zmp_ph111_H1
zmp_ph111_B1a
zmp_ph111_B5
zmp_ph111_B8
zmp_ph111_B6
zmp_ph111_B7
zmp_ph111_B9
zmp_ph111_B10
zmp_ph114_G4
zmp_ph114_G11
zmp_ph114_H1
zmp_ph114_H2
zmp_ph114_A9
zmp_ph114_A11
zmp_ph114_B2
zmp_ph114_B4
zmp_ph114_D8
zmp_ph114_E2

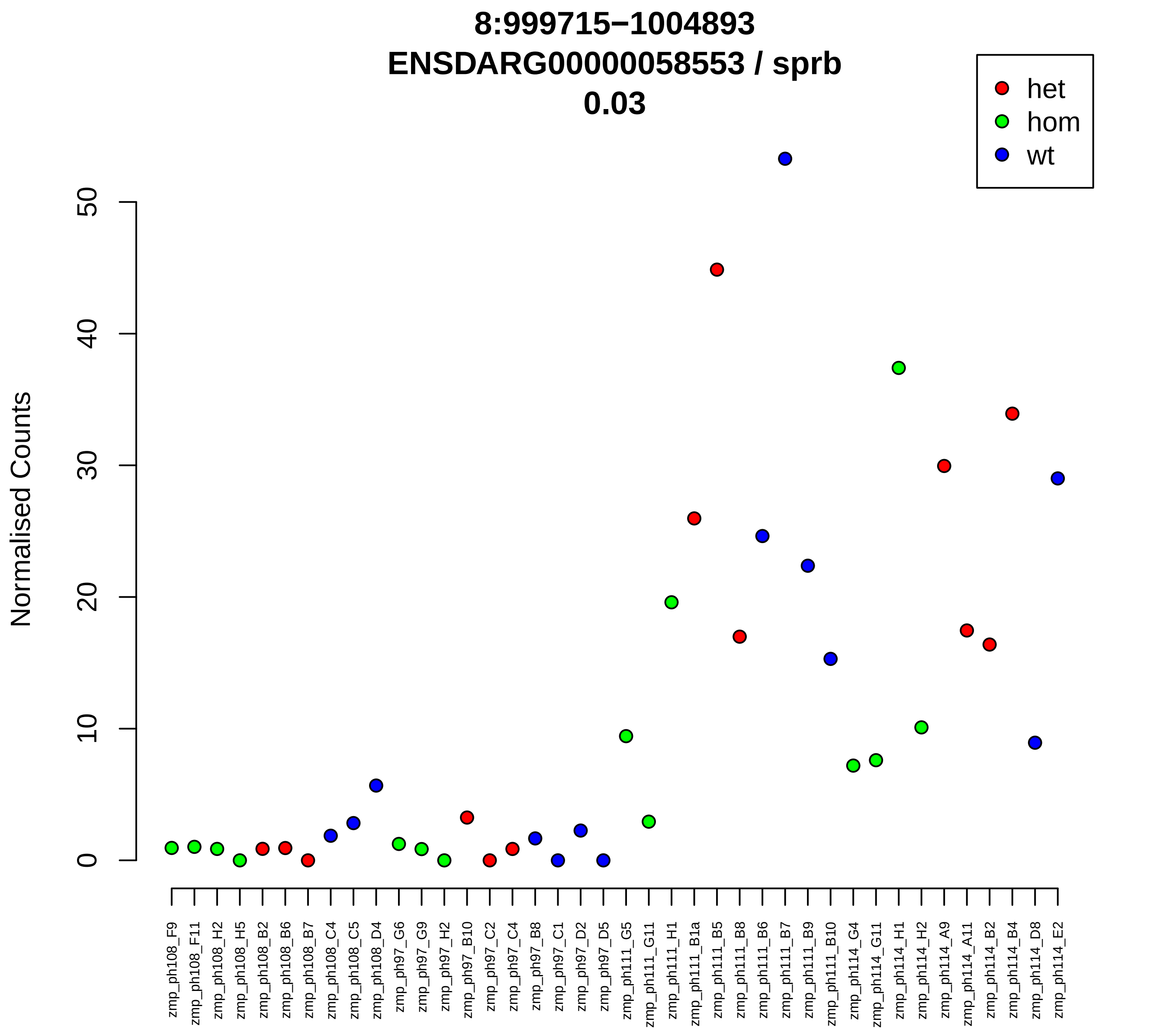

8:999715−1004893
ENSDARG00000058553 / sprb
0.03
het
hom
wt
Normalised Counts
0
10
20
30
40
50
zmp_ph108_F9
zmp_ph108_F11
zmp_ph108_H2
zmp_ph108_H5
zmp_ph108_B2
zmp_ph108_B6
zmp_ph108_B7
zmp_ph108_C4
zmp_ph108_C5
zmp_ph108_D4
zmp_ph97_G6
zmp_ph97_G9
zmp_ph97_H2
zmp_ph97_B10
zmp_ph97_C2
zmp_ph97_C4
zmp_ph97_B8
zmp_ph97_C1
zmp_ph97_D2
zmp_ph97_D5
zmp_ph111_G5
zmp_ph111_G11
zmp_ph111_H1
zmp_ph111_B1a
zmp_ph111_B5
zmp_ph111_B8
zmp_ph111_B6
zmp_ph111_B7
zmp_ph111_B9
zmp_ph111_B10
zmp_ph114_G4
zmp_ph114_G11
zmp_ph114_H1
zmp_ph114_H2
zmp_ph114_A9
zmp_ph114_A11
zmp_ph114_B2
zmp_ph114_B4
zmp_ph114_D8
zmp_ph114_E2

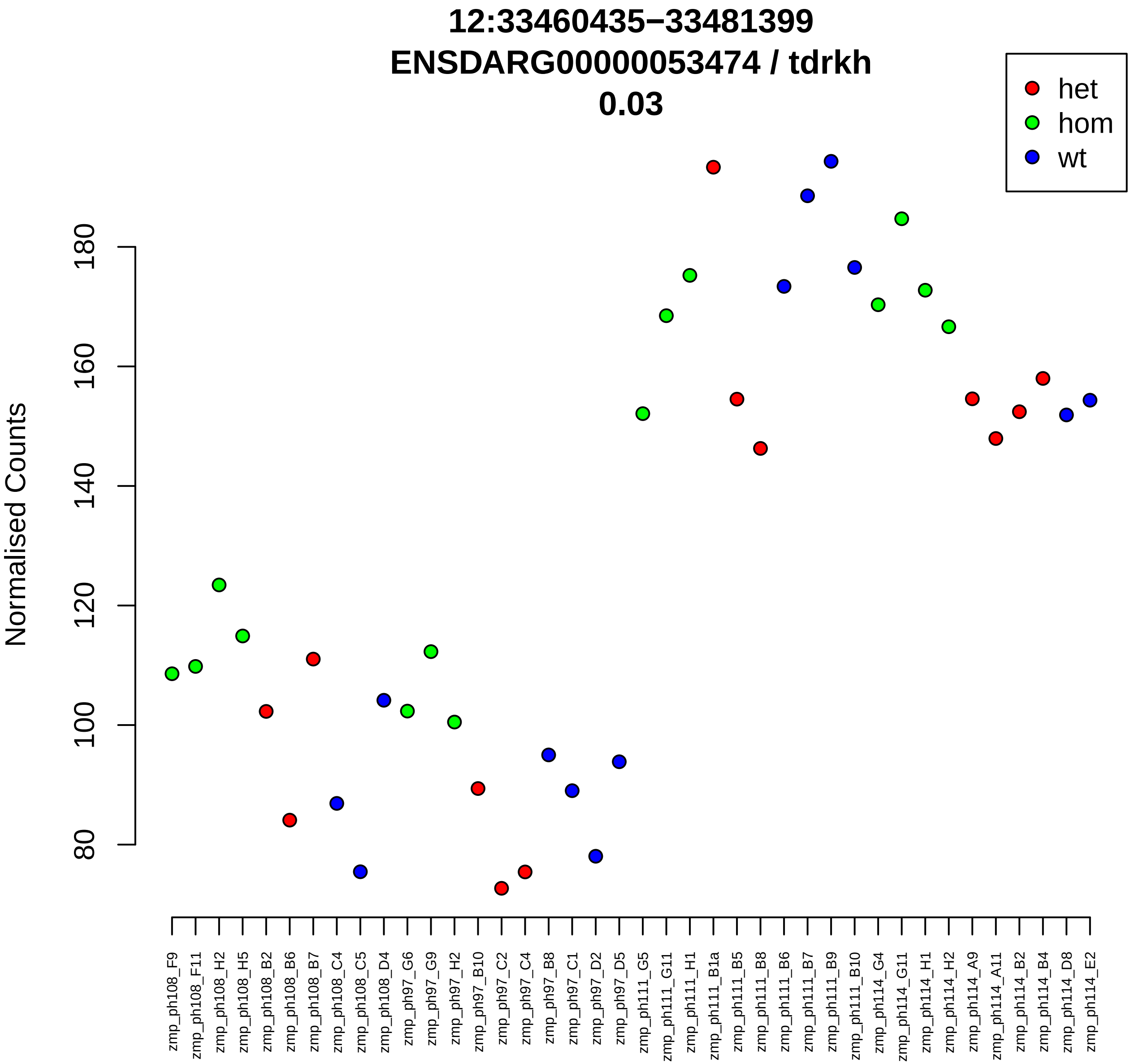
12:33460435−33481399
ENSDARG00000053474 / tdrkh
0.03
het
hom
wt
Normalised Counts
80
100
120
140
160
180
zmp_ph108_F9
zmp_ph108_F11
zmp_ph108_H2
zmp_ph108_H5
zmp_ph108_B2
zmp_ph108_B6
zmp_ph108_B7
zmp_ph108_C4
zmp_ph108_C5
zmp_ph108_D4
zmp_ph97_G6
zmp_ph97_G9
zmp_ph97_H2
zmp_ph97_B10
zmp_ph97_C2
zmp_ph97_C4
zmp_ph97_B8
zmp_ph97_C1
zmp_ph97_D2
zmp_ph97_D5
zmp_ph111_G5
zmp_ph111_G11
zmp_ph111_H1
zmp_ph111_B1a
zmp_ph111_B5
zmp_ph111_B8
zmp_ph111_B6
zmp_ph111_B7
zmp_ph111_B9
zmp_ph111_B10
zmp_ph114_G4
zmp_ph114_G11
zmp_ph114_H1
zmp_ph114_H2
zmp_ph114_A9
zmp_ph114_A11
zmp_ph114_B2
zmp_ph114_B4
zmp_ph114_D8
zmp_ph114_E2

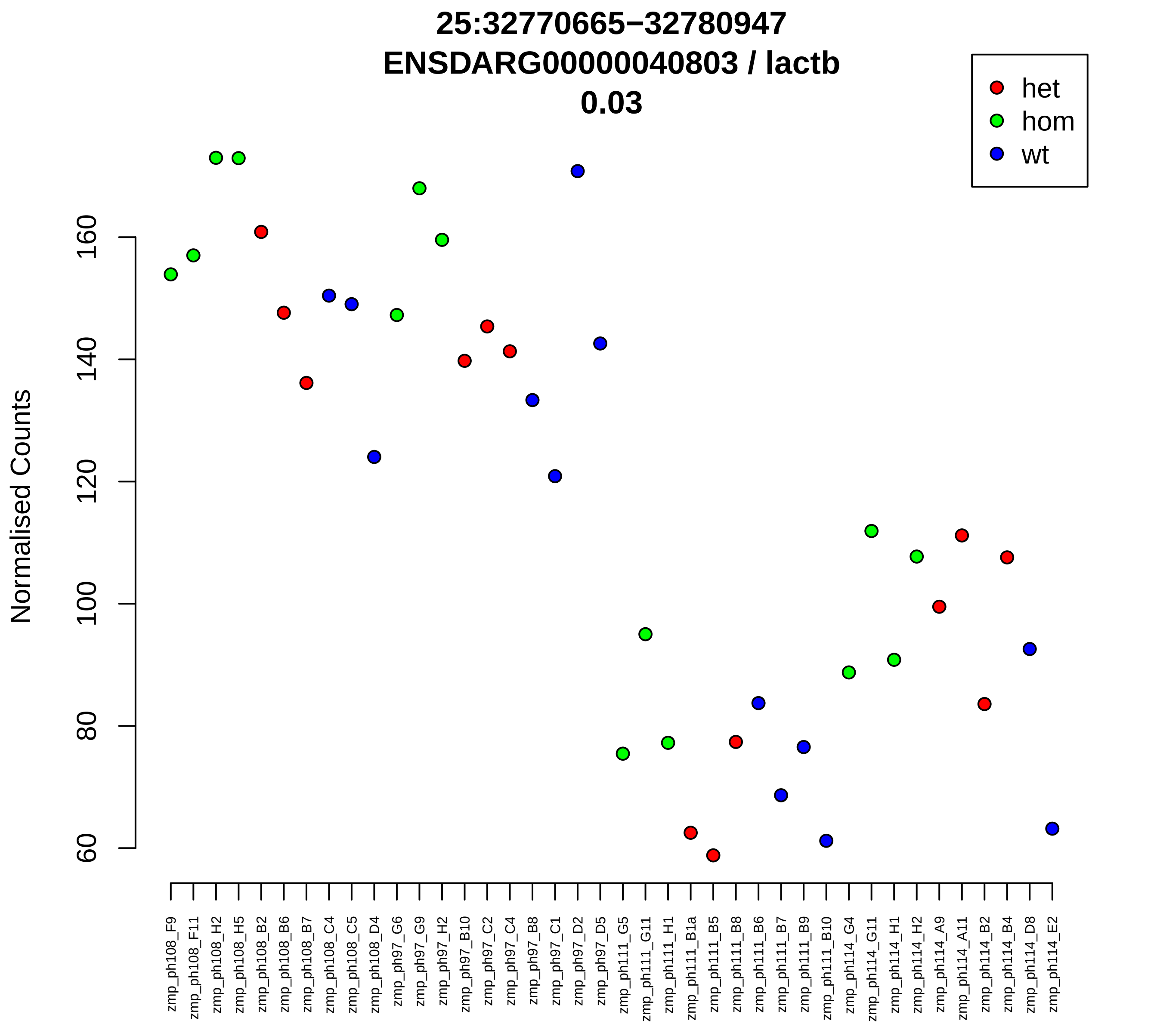
25:32770665−32780947
ENSDARG00000040803 / lactb
0.03
het
hom
wt
Normalised Counts
60
80
100
120
140
160
zmp_ph108_F9
zmp_ph108_F11
zmp_ph108_H2
zmp_ph108_H5
zmp_ph108_B2
zmp_ph108_B6
zmp_ph108_B7
zmp_ph108_C4
zmp_ph108_C5
zmp_ph108_D4
zmp_ph97_G6
zmp_ph97_G9
zmp_ph97_H2
zmp_ph97_B10
zmp_ph97_C2
zmp_ph97_C4
zmp_ph97_B8
zmp_ph97_C1
zmp_ph97_D2
zmp_ph97_D5
zmp_ph111_G5
zmp_ph111_G11
zmp_ph111_H1
zmp_ph111_B1a
zmp_ph111_B5
zmp_ph111_B8
zmp_ph111_B6
zmp_ph111_B7
zmp_ph111_B9
zmp_ph111_B10
zmp_ph114_G4
zmp_ph114_G11
zmp_ph114_H1
zmp_ph114_H2
zmp_ph114_A9
zmp_ph114_A11
zmp_ph114_B2
zmp_ph114_B4
zmp_ph114_D8
zmp_ph114_E2

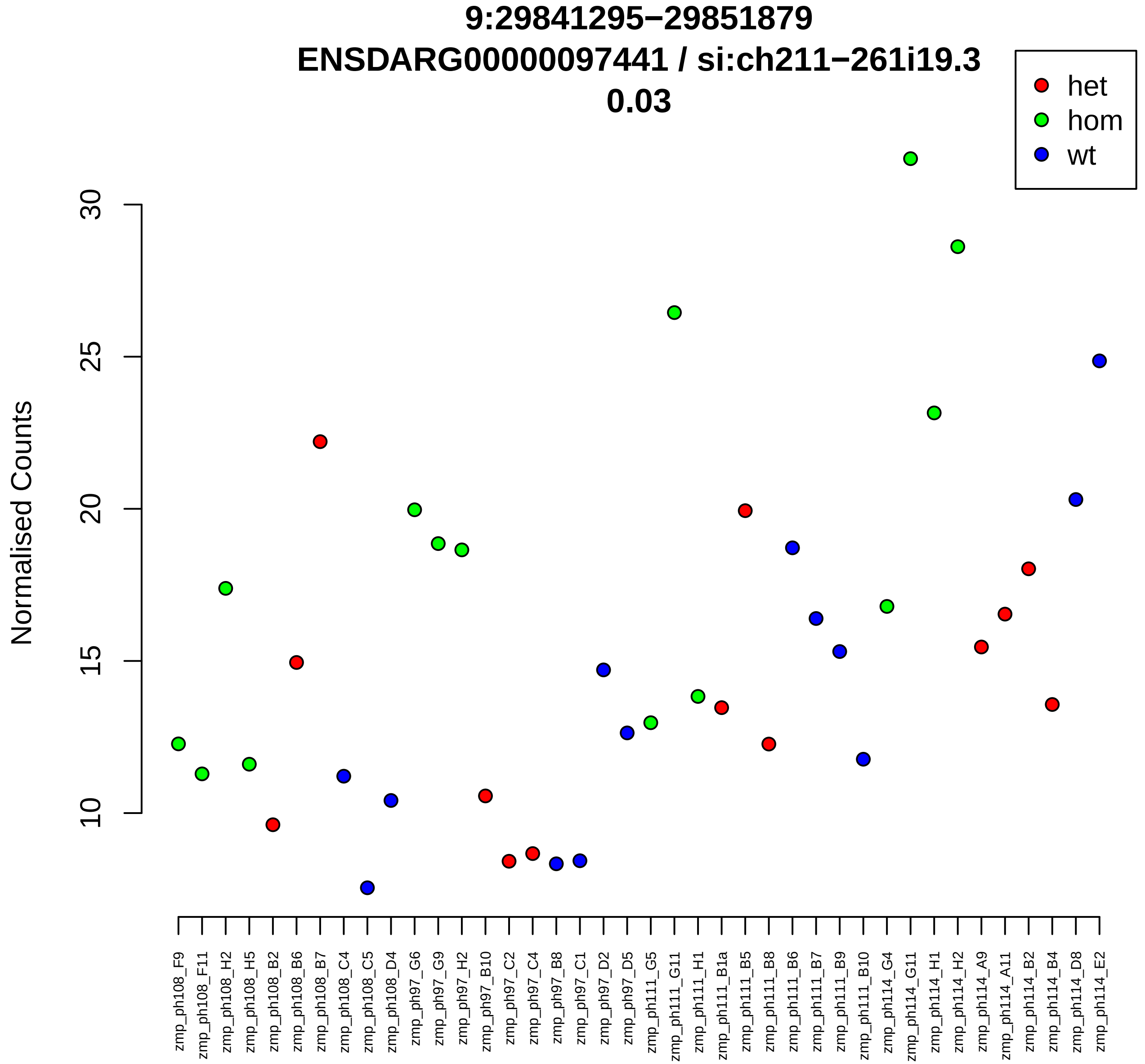

9:29841295−29851879
ENSDARG00000097441 / si:ch211−261i19.3
0.03
het
hom
wt
Normalised Counts
10
15
20
25
30
zmp_ph108_F9
zmp_ph108_F11
zmp_ph108_H2
zmp_ph108_H5
zmp_ph108_B2
zmp_ph108_B6
zmp_ph108_B7
zmp_ph108_C4
zmp_ph108_C5
zmp_ph108_D4
zmp_ph97_G6
zmp_ph97_G9
zmp_ph97_H2
zmp_ph97_B10
zmp_ph97_C2
zmp_ph97_C4
zmp_ph97_B8
zmp_ph97_C1
zmp_ph97_D2
zmp_ph97_D5
zmp_ph111_G5
zmp_ph111_G11
zmp_ph111_H1
zmp_ph111_B1a
zmp_ph111_B5
zmp_ph111_B8
zmp_ph111_B6
zmp_ph111_B7
zmp_ph111_B9
zmp_ph111_B10
zmp_ph114_G4
zmp_ph114_G11
zmp_ph114_H1
zmp_ph114_H2
zmp_ph114_A9
zmp_ph114_A11
zmp_ph114_B2
zmp_ph114_B4
zmp_ph114_D8
zmp_ph114_E2

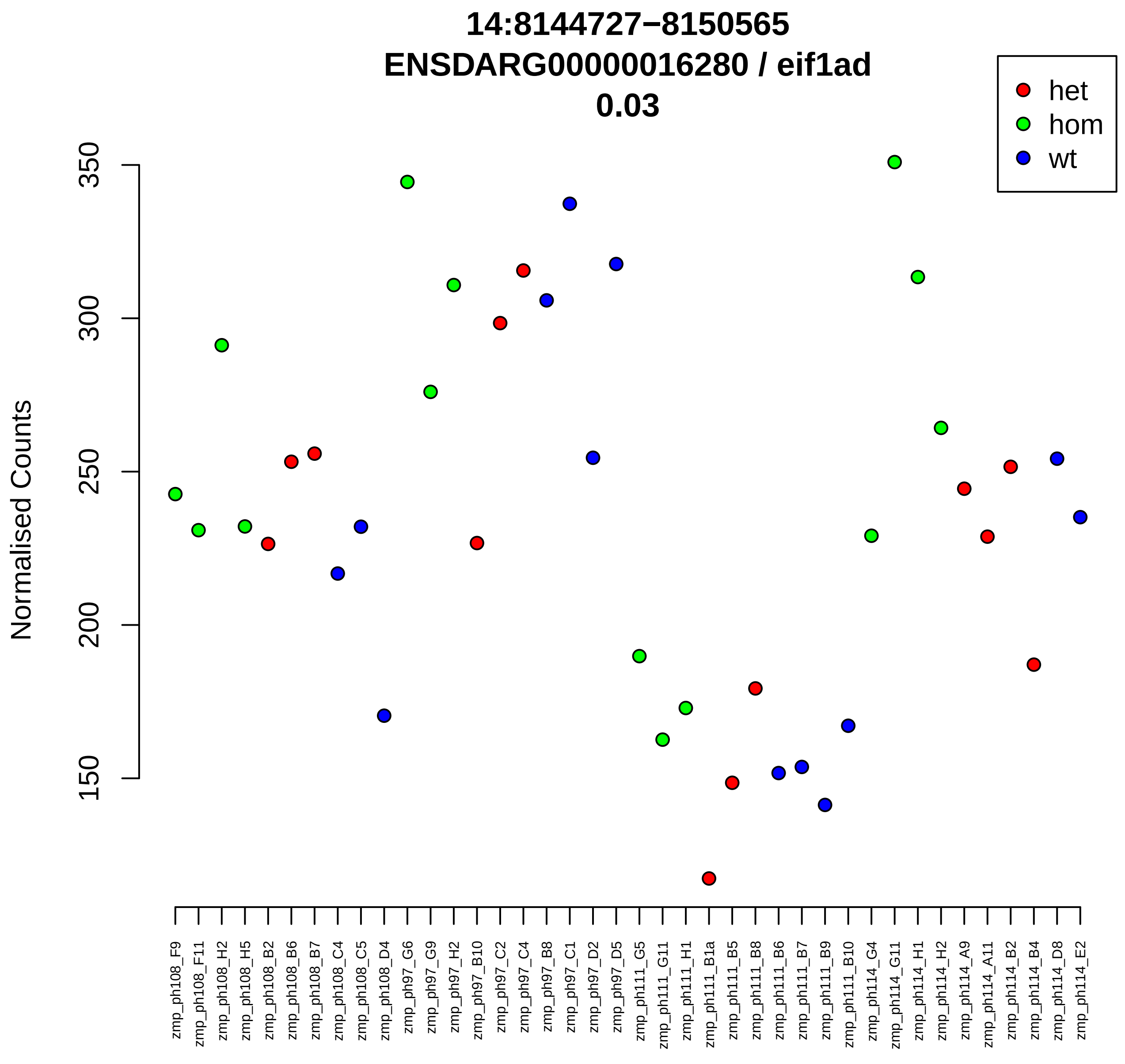
14:8144727−8150565
ENSDARG00000016280 / eif1ad
0.03
het
hom
wt
Normalised Counts
150
200
250
300
350
zmp_ph108_F9
zmp_ph108_F11
zmp_ph108_H2
zmp_ph108_H5
zmp_ph108_B2
zmp_ph108_B6
zmp_ph108_B7
zmp_ph108_C4
zmp_ph108_C5
zmp_ph108_D4
zmp_ph97_G6
zmp_ph97_G9
zmp_ph97_H2
zmp_ph97_B10
zmp_ph97_C2
zmp_ph97_C4
zmp_ph97_B8
zmp_ph97_C1
zmp_ph97_D2
zmp_ph97_D5
zmp_ph111_G5
zmp_ph111_G11
zmp_ph111_H1
zmp_ph111_B1a
zmp_ph111_B5
zmp_ph111_B8
zmp_ph111_B6
zmp_ph111_B7
zmp_ph111_B9
zmp_ph111_B10
zmp_ph114_G4
zmp_ph114_G11
zmp_ph114_H1
zmp_ph114_H2
zmp_ph114_A9
zmp_ph114_A11
zmp_ph114_B2
zmp_ph114_B4
zmp_ph114_D8
zmp_ph114_E2

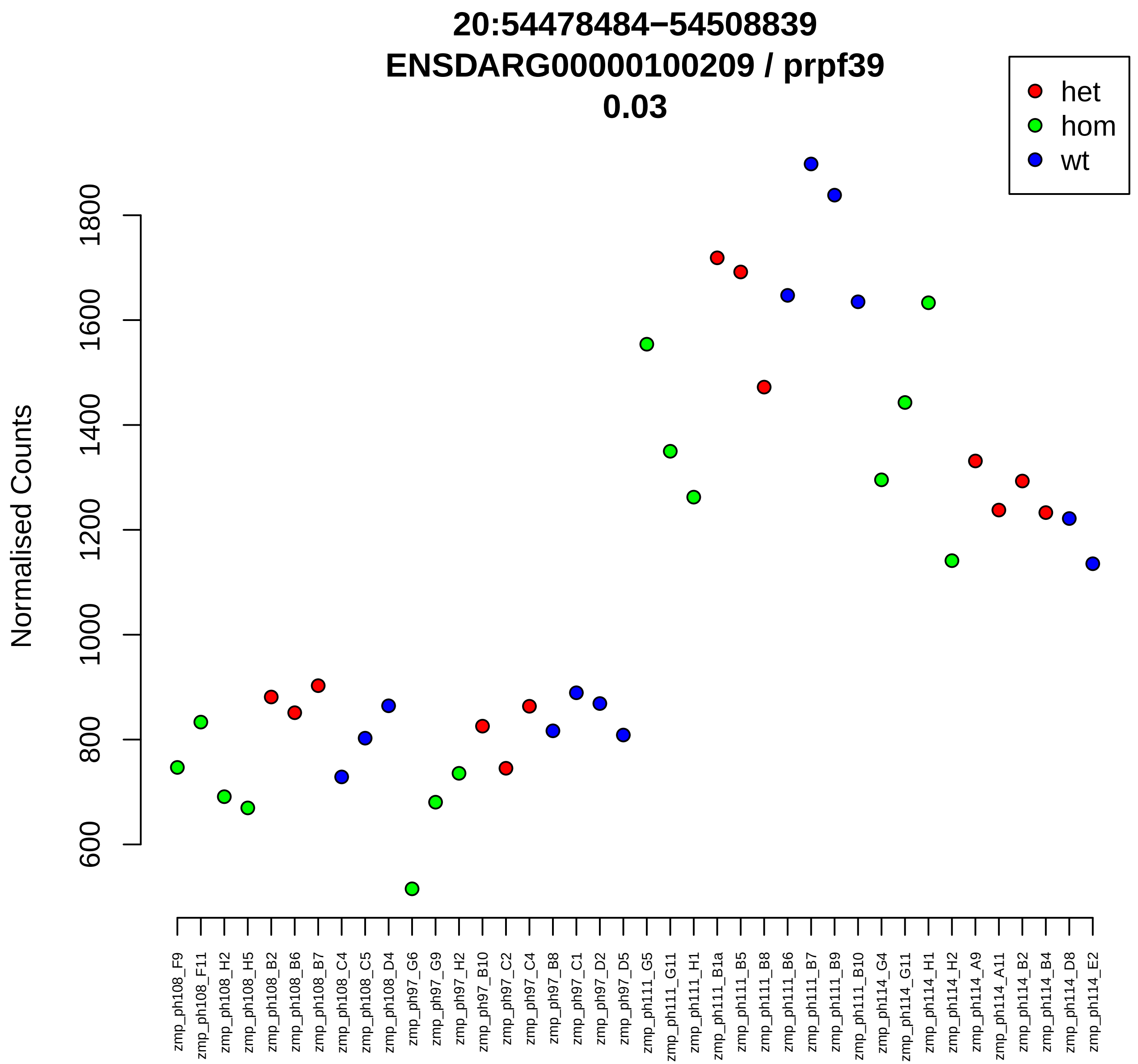
20:54478484−54508839
ENSDARG00000100209 / prpf39
0.03
het
hom
wt
Normalised Counts
600
800
1000
1200
1400
1600
1800
zmp_ph108_F9
zmp_ph108_F11
zmp_ph108_H2
zmp_ph108_H5
zmp_ph108_B2
zmp_ph108_B6
zmp_ph108_B7
zmp_ph108_C4
zmp_ph108_C5
zmp_ph108_D4
zmp_ph97_G6
zmp_ph97_G9
zmp_ph97_H2
zmp_ph97_B10
zmp_ph97_C2
zmp_ph97_C4
zmp_ph97_B8
zmp_ph97_C1
zmp_ph97_D2
zmp_ph97_D5
zmp_ph111_G5
zmp_ph111_G11
zmp_ph111_H1
zmp_ph111_B1a
zmp_ph111_B5
zmp_ph111_B8
zmp_ph111_B6
zmp_ph111_B7
zmp_ph111_B9
zmp_ph111_B10
zmp_ph114_G4
zmp_ph114_G11
zmp_ph114_H1
zmp_ph114_H2
zmp_ph114_A9
zmp_ph114_A11
zmp_ph114_B2
zmp_ph114_B4
zmp_ph114_D8
zmp_ph114_E2

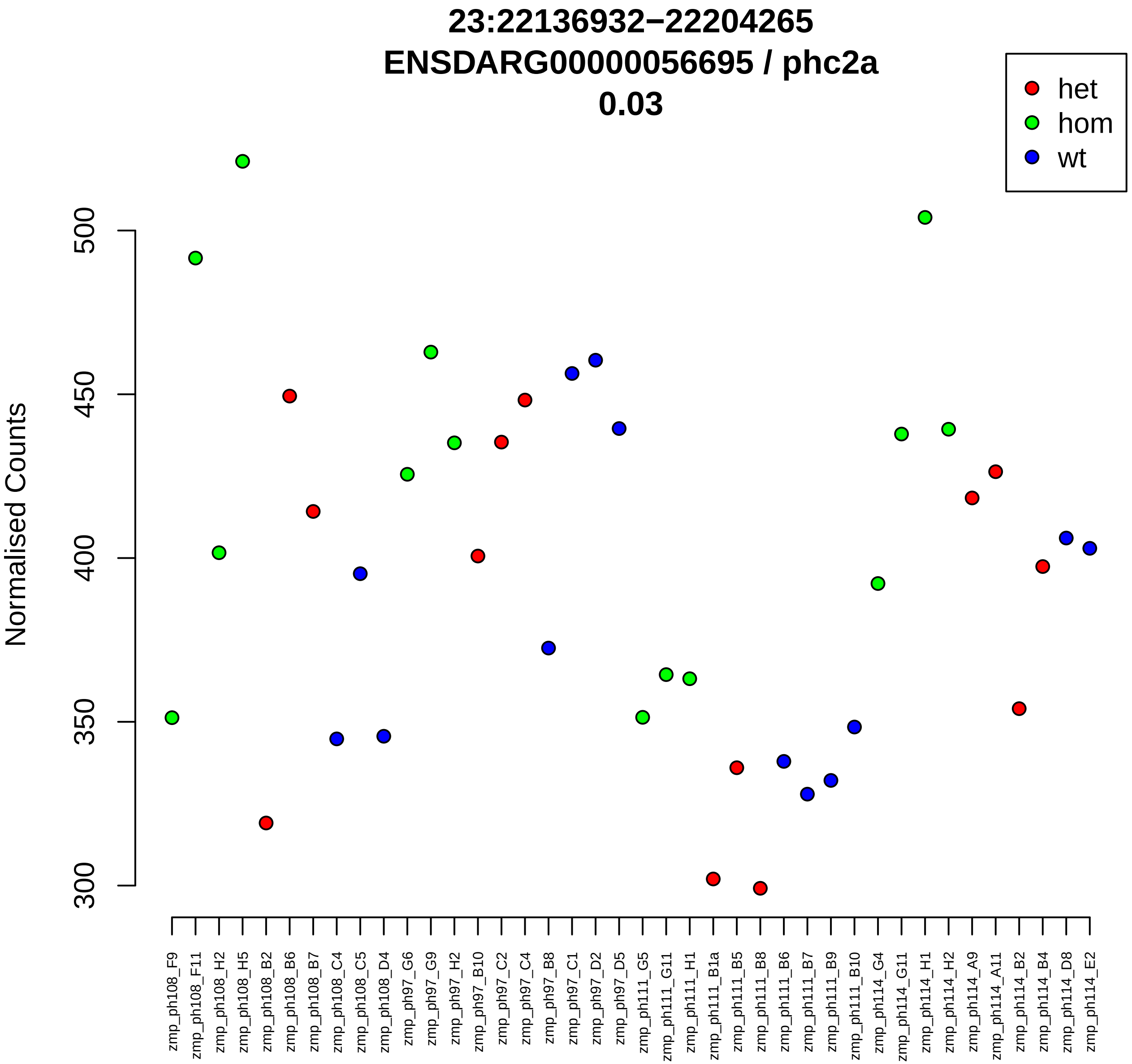
23:22136932−22204265
ENSDARG00000056695 / phc2a
0.03
het
hom
wt
Normalised Counts
300
350
400
450
500
zmp_ph108_F9
zmp_ph108_F11
zmp_ph108_H2
zmp_ph108_H5
zmp_ph108_B2
zmp_ph108_B6
zmp_ph108_B7
zmp_ph108_C4
zmp_ph108_C5
zmp_ph108_D4
zmp_ph97_G6
zmp_ph97_G9
zmp_ph97_H2
zmp_ph97_B10
zmp_ph97_C2
zmp_ph97_C4
zmp_ph97_B8
zmp_ph97_C1
zmp_ph97_D2
zmp_ph97_D5
zmp_ph111_G5
zmp_ph111_G11
zmp_ph111_H1
zmp_ph111_B1a
zmp_ph111_B5
zmp_ph111_B8
zmp_ph111_B6
zmp_ph111_B7
zmp_ph111_B9
zmp_ph111_B10
zmp_ph114_G4
zmp_ph114_G11
zmp_ph114_H1
zmp_ph114_H2
zmp_ph114_A9
zmp_ph114_A11
zmp_ph114_B2
zmp_ph114_B4
zmp_ph114_D8
zmp_ph114_E2

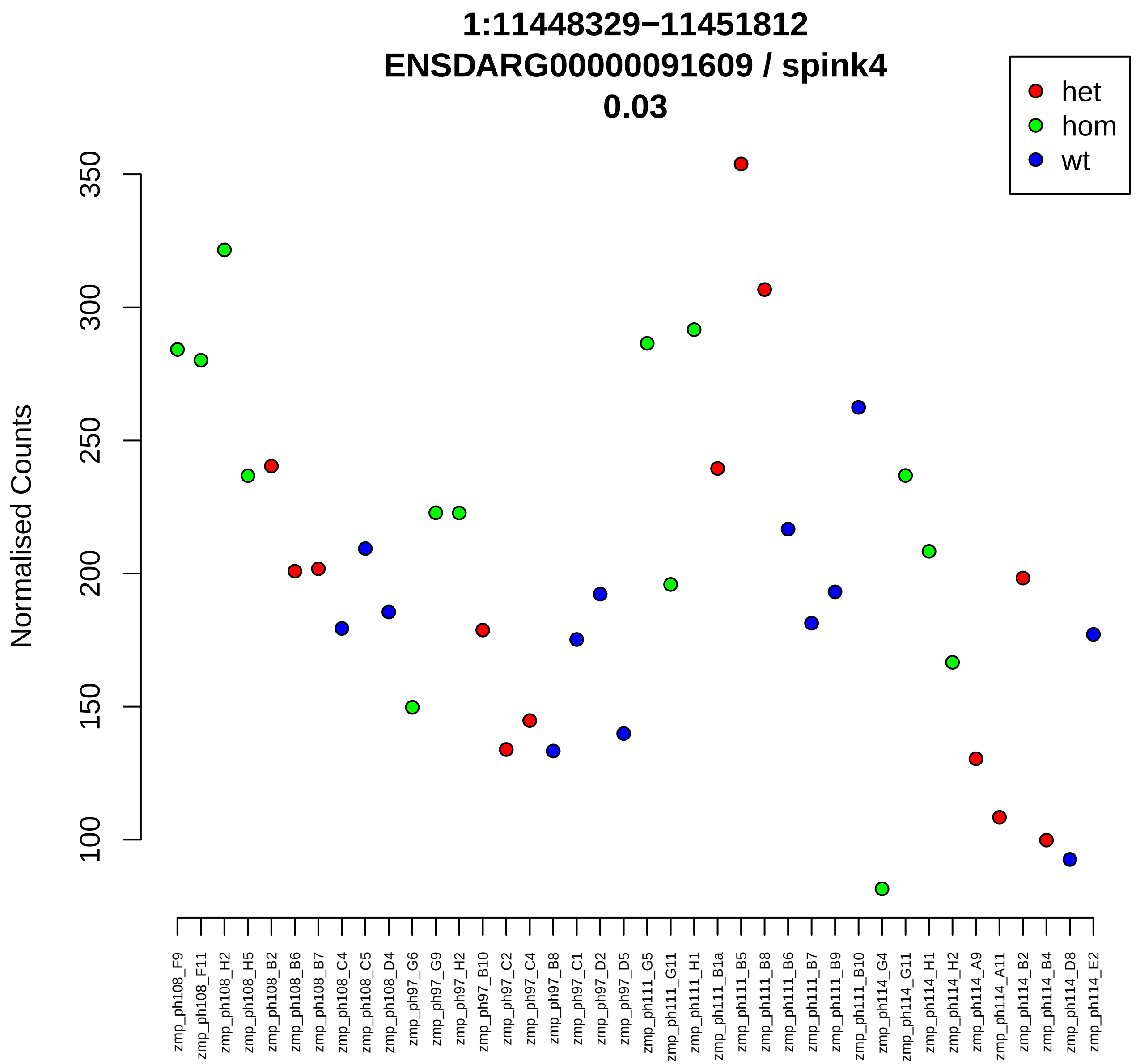
1:11448329−11451812
ENSDARG00000091609 / spink4
0.03
het
hom
wt
Normalised Counts
100
150
200
250
300
350
zmp_ph108_F9
zmp_ph108_F11
zmp_ph108_H2
zmp_ph108_H5
zmp_ph108_B2
zmp_ph108_B6
zmp_ph108_B7
zmp_ph108_C4
zmp_ph108_C5
zmp_ph108_D4
zmp_ph97_G6
zmp_ph97_G9
zmp_ph97_H2
zmp_ph97_B10
zmp_ph97_C2
zmp_ph97_C4
zmp_ph97_B8
zmp_ph97_C1
zmp_ph97_D2
zmp_ph97_D5
zmp_ph111_G5
zmp_ph111_G11
zmp_ph111_H1
zmp_ph111_B1a
zmp_ph111_B5
zmp_ph111_B8
zmp_ph111_B6
zmp_ph111_B7
zmp_ph111_B9
zmp_ph111_B10
zmp_ph114_G4
zmp_ph114_G11
zmp_ph114_H1
zmp_ph114_H2
zmp_ph114_A9
zmp_ph114_A11
zmp_ph114_B2
zmp_ph114_B4
zmp_ph114_D8
zmp_ph114_E2

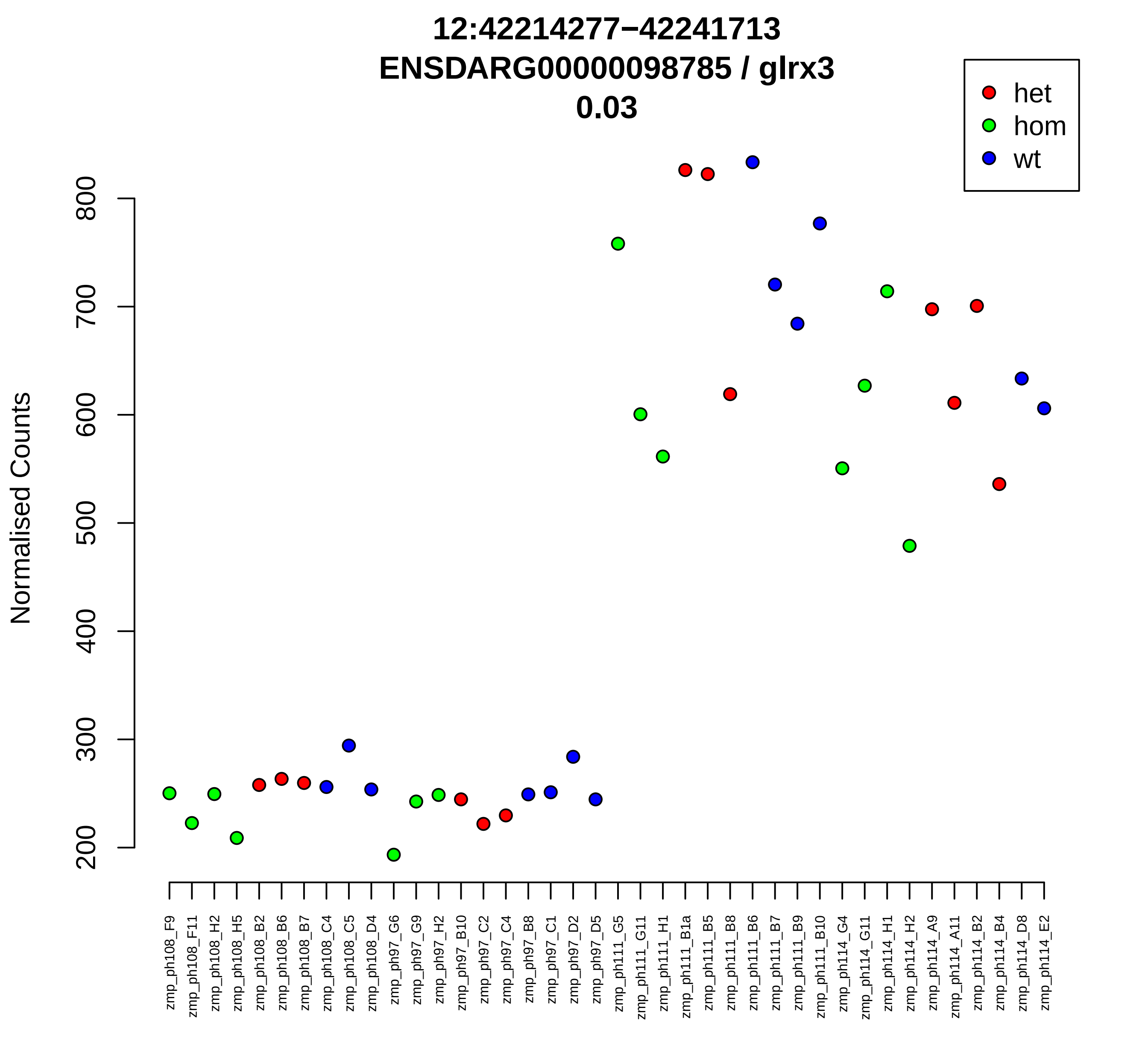

12:42214277−42241713
ENSDARG00000098785 / glrx3
0.03
het
hom
wt
Normalised Counts
200
300
400
500
600
700
800
zmp_ph108_F9
zmp_ph108_F11
zmp_ph108_H2
zmp_ph108_H5
zmp_ph108_B2
zmp_ph108_B6
zmp_ph108_B7
zmp_ph108_C4
zmp_ph108_C5
zmp_ph108_D4
zmp_ph97_G6
zmp_ph97_G9
zmp_ph97_H2
zmp_ph97_B10
zmp_ph97_C2
zmp_ph97_C4
zmp_ph97_B8
zmp_ph97_C1
zmp_ph97_D2
zmp_ph97_D5
zmp_ph111_G5
zmp_ph111_G11
zmp_ph111_H1
zmp_ph111_B1a
zmp_ph111_B5
zmp_ph111_B8
zmp_ph111_B6
zmp_ph111_B7
zmp_ph111_B9
zmp_ph111_B10
zmp_ph114_G4
zmp_ph114_G11
zmp_ph114_H1
zmp_ph114_H2
zmp_ph114_A9
zmp_ph114_A11
zmp_ph114_B2
zmp_ph114_B4
zmp_ph114_D8
zmp_ph114_E2

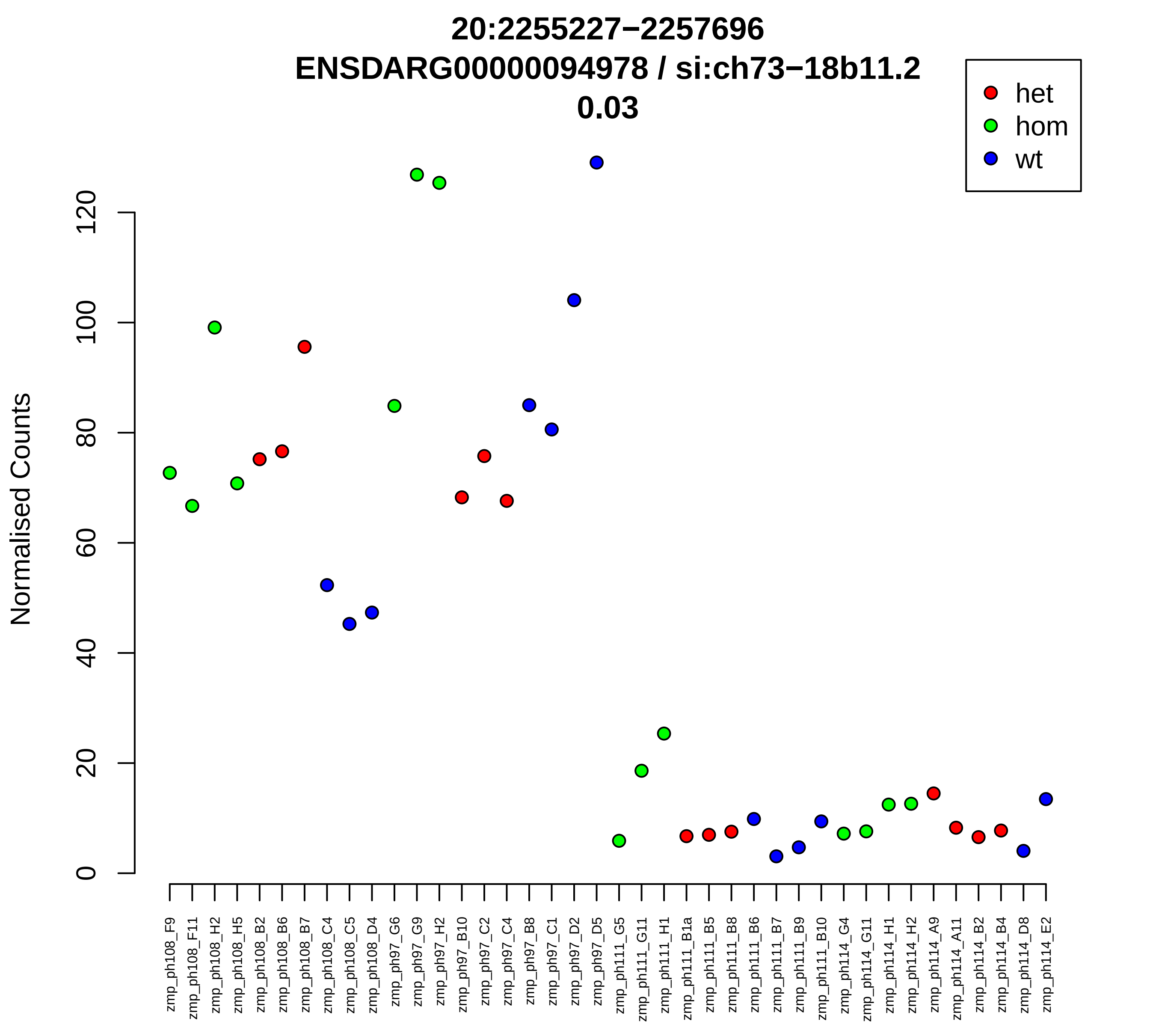

20:2255227−2257696
ENSDARG00000094978 / si:ch73−18b11.2
0.03
het
hom
wt
Normalised Counts
0
20
40
60
80
100
120
zmp_ph108_F9
zmp_ph108_F11
zmp_ph108_H2
zmp_ph108_H5
zmp_ph108_B2
zmp_ph108_B6
zmp_ph108_B7
zmp_ph108_C4
zmp_ph108_C5
zmp_ph108_D4
zmp_ph97_G6
zmp_ph97_G9
zmp_ph97_H2
zmp_ph97_B10
zmp_ph97_C2
zmp_ph97_C4
zmp_ph97_B8
zmp_ph97_C1
zmp_ph97_D2
zmp_ph97_D5
zmp_ph111_G5
zmp_ph111_G11
zmp_ph111_H1
zmp_ph111_B1a
zmp_ph111_B5
zmp_ph111_B8
zmp_ph111_B6
zmp_ph111_B7
zmp_ph111_B9
zmp_ph111_B10
zmp_ph114_G4
zmp_ph114_G11
zmp_ph114_H1
zmp_ph114_H2
zmp_ph114_A9
zmp_ph114_A11
zmp_ph114_B2
zmp_ph114_B4
zmp_ph114_D8
zmp_ph114_E2

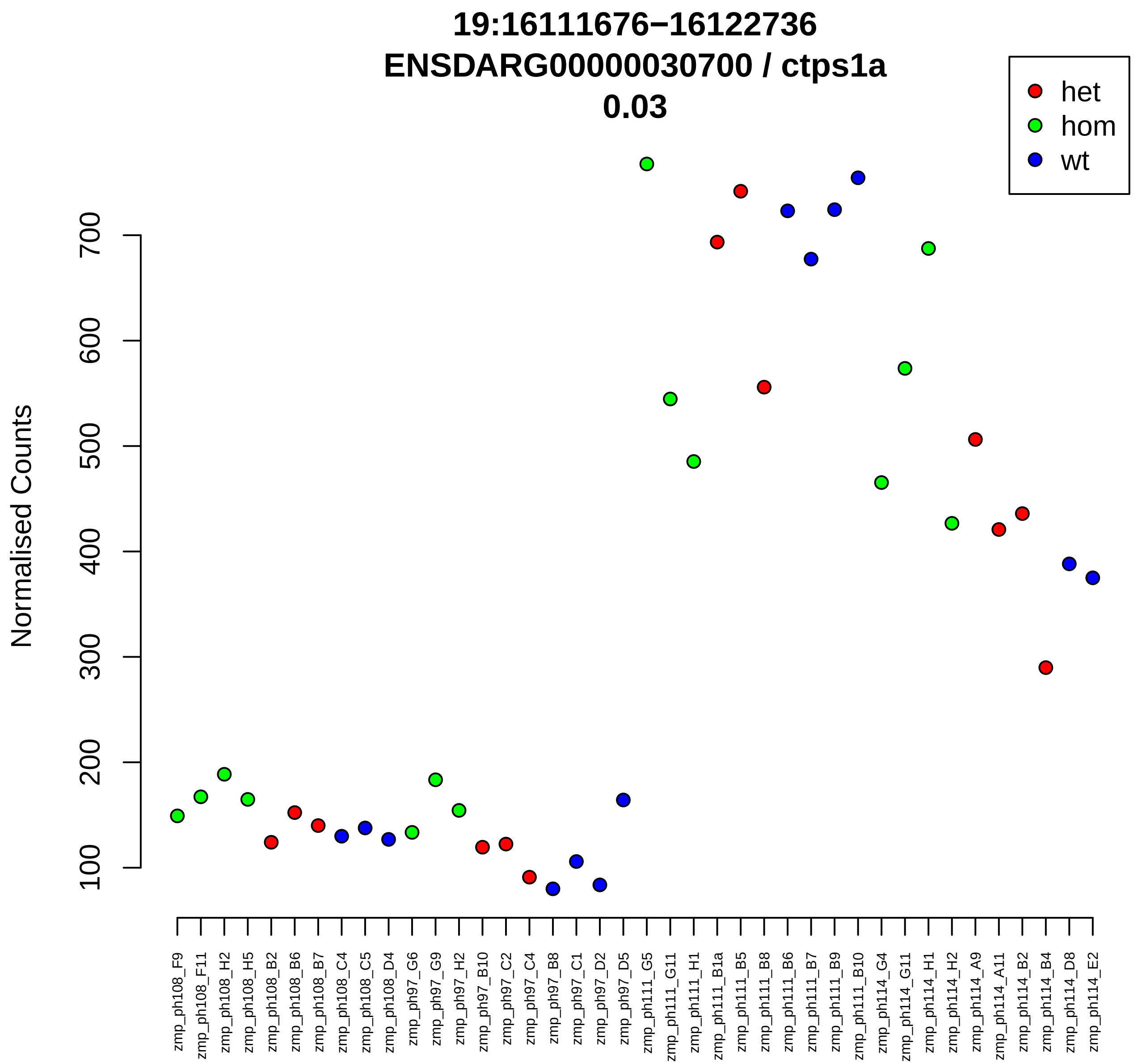

19:16111676−16122736
ENSDARG00000030700 / ctps1a
0.03
het
hom
wt
Normalised Counts
100
200
300
400
500
600
700
zmp_ph108_F9
zmp_ph108_F11
zmp_ph108_H2
zmp_ph108_H5
zmp_ph108_B2
zmp_ph108_B6
zmp_ph108_B7
zmp_ph108_C4
zmp_ph108_C5
zmp_ph108_D4
zmp_ph97_G6
zmp_ph97_G9
zmp_ph97_H2
zmp_ph97_B10
zmp_ph97_C2
zmp_ph97_C4
zmp_ph97_B8
zmp_ph97_C1
zmp_ph97_D2
zmp_ph97_D5
zmp_ph111_G5
zmp_ph111_G11
zmp_ph111_H1
zmp_ph111_B1a
zmp_ph111_B5
zmp_ph111_B8
zmp_ph111_B6
zmp_ph111_B7
zmp_ph111_B9
zmp_ph111_B10
zmp_ph114_G4
zmp_ph114_G11
zmp_ph114_H1
zmp_ph114_H2
zmp_ph114_A9
zmp_ph114_A11
zmp_ph114_B2
zmp_ph114_B4
zmp_ph114_D8
zmp_ph114_E2

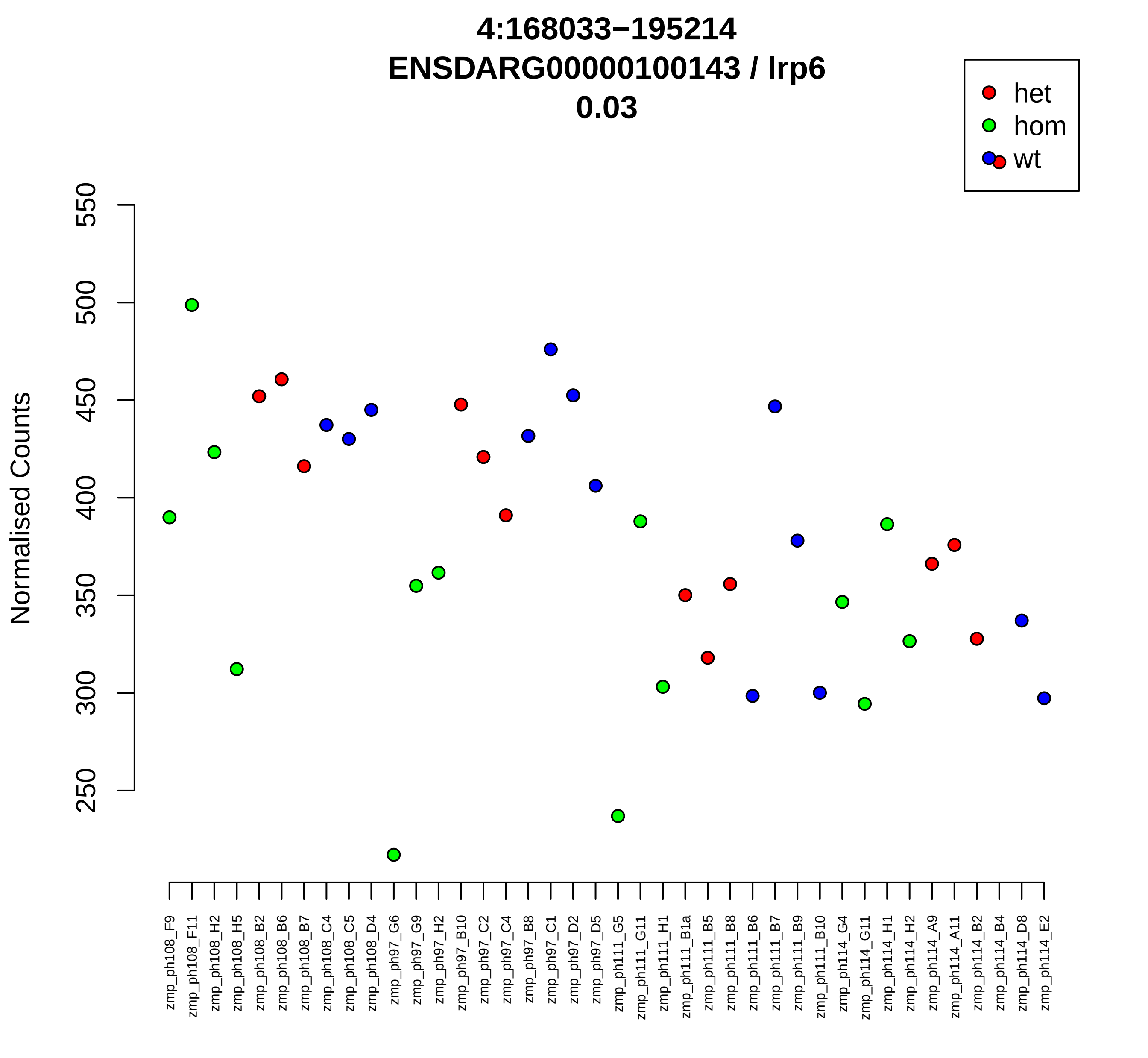

4:168033−195214
ENSDARG00000100143 / lrp6
0.03
het
hom
wt
Normalised Counts
250
300
350
400
450
500
550
zmp_ph108_F9
zmp_ph108_F11
zmp_ph108_H2
zmp_ph108_H5
zmp_ph108_B2
zmp_ph108_B6
zmp_ph108_B7
zmp_ph108_C4
zmp_ph108_C5
zmp_ph108_D4
zmp_ph97_G6
zmp_ph97_G9
zmp_ph97_H2
zmp_ph97_B10
zmp_ph97_C2
zmp_ph97_C4
zmp_ph97_B8
zmp_ph97_C1
zmp_ph97_D2
zmp_ph97_D5
zmp_ph111_G5
zmp_ph111_G11
zmp_ph111_H1
zmp_ph111_B1a
zmp_ph111_B5
zmp_ph111_B8
zmp_ph111_B6
zmp_ph111_B7
zmp_ph111_B9
zmp_ph111_B10
zmp_ph114_G4
zmp_ph114_G11
zmp_ph114_H1
zmp_ph114_H2
zmp_ph114_A9
zmp_ph114_A11
zmp_ph114_B2
zmp_ph114_B4
zmp_ph114_D8
zmp_ph114_E2

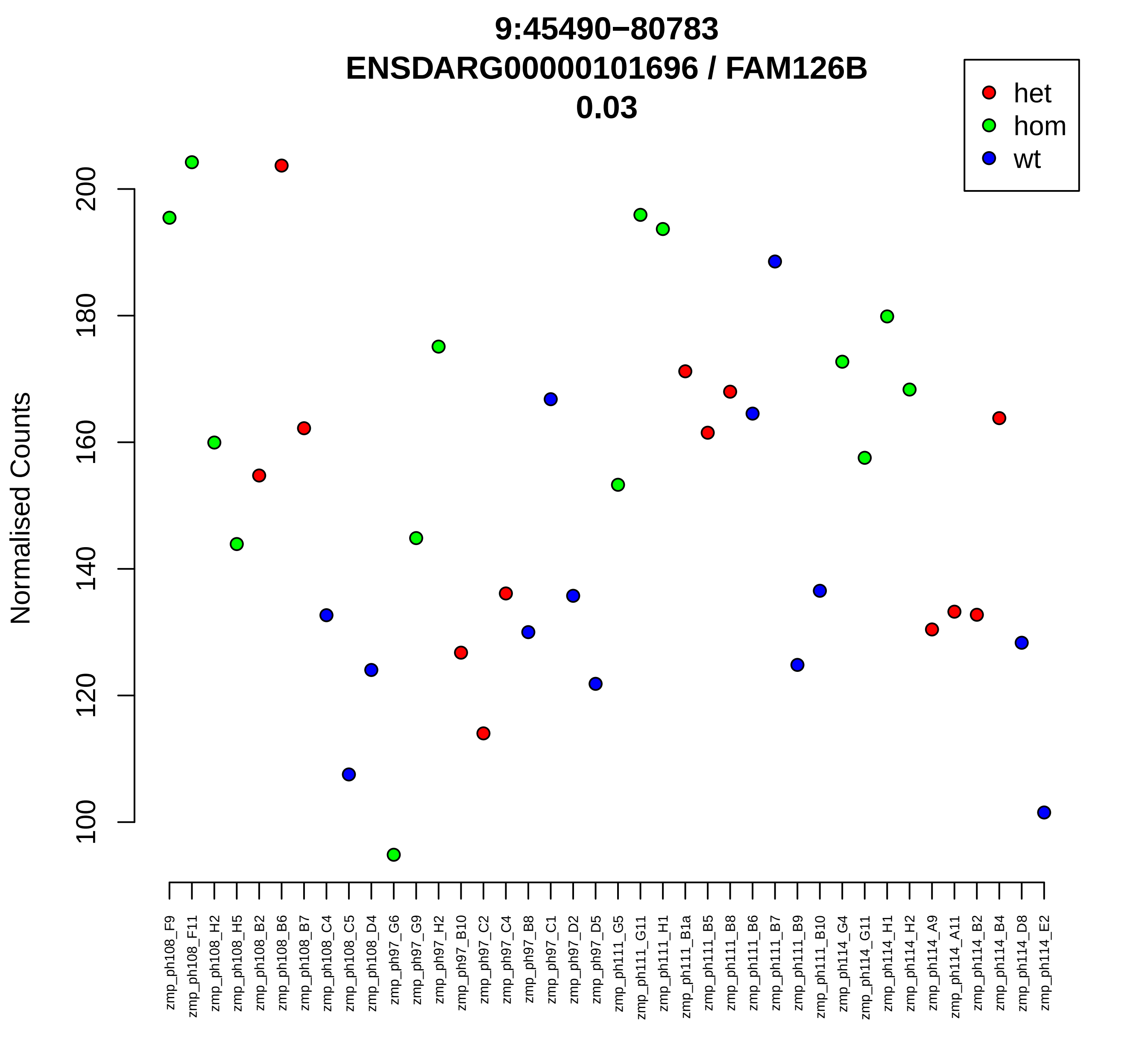

9:45490−80783
ENSDARG00000101696 / FAM126B
0.03
het
hom
wt
Normalised Counts
100
120
140
160
180
200
zmp_ph108_F9
zmp_ph108_F11
zmp_ph108_H2
zmp_ph108_H5
zmp_ph108_B2
zmp_ph108_B6
zmp_ph108_B7
zmp_ph108_C4
zmp_ph108_C5
zmp_ph108_D4
zmp_ph97_G6
zmp_ph97_G9
zmp_ph97_H2
zmp_ph97_B10
zmp_ph97_C2
zmp_ph97_C4
zmp_ph97_B8
zmp_ph97_C1
zmp_ph97_D2
zmp_ph97_D5
zmp_ph111_G5
zmp_ph111_G11
zmp_ph111_H1
zmp_ph111_B1a
zmp_ph111_B5
zmp_ph111_B8
zmp_ph111_B6
zmp_ph111_B7
zmp_ph111_B9
zmp_ph111_B10
zmp_ph114_G4
zmp_ph114_G11
zmp_ph114_H1
zmp_ph114_H2
zmp_ph114_A9
zmp_ph114_A11
zmp_ph114_B2
zmp_ph114_B4
zmp_ph114_D8
zmp_ph114_E2

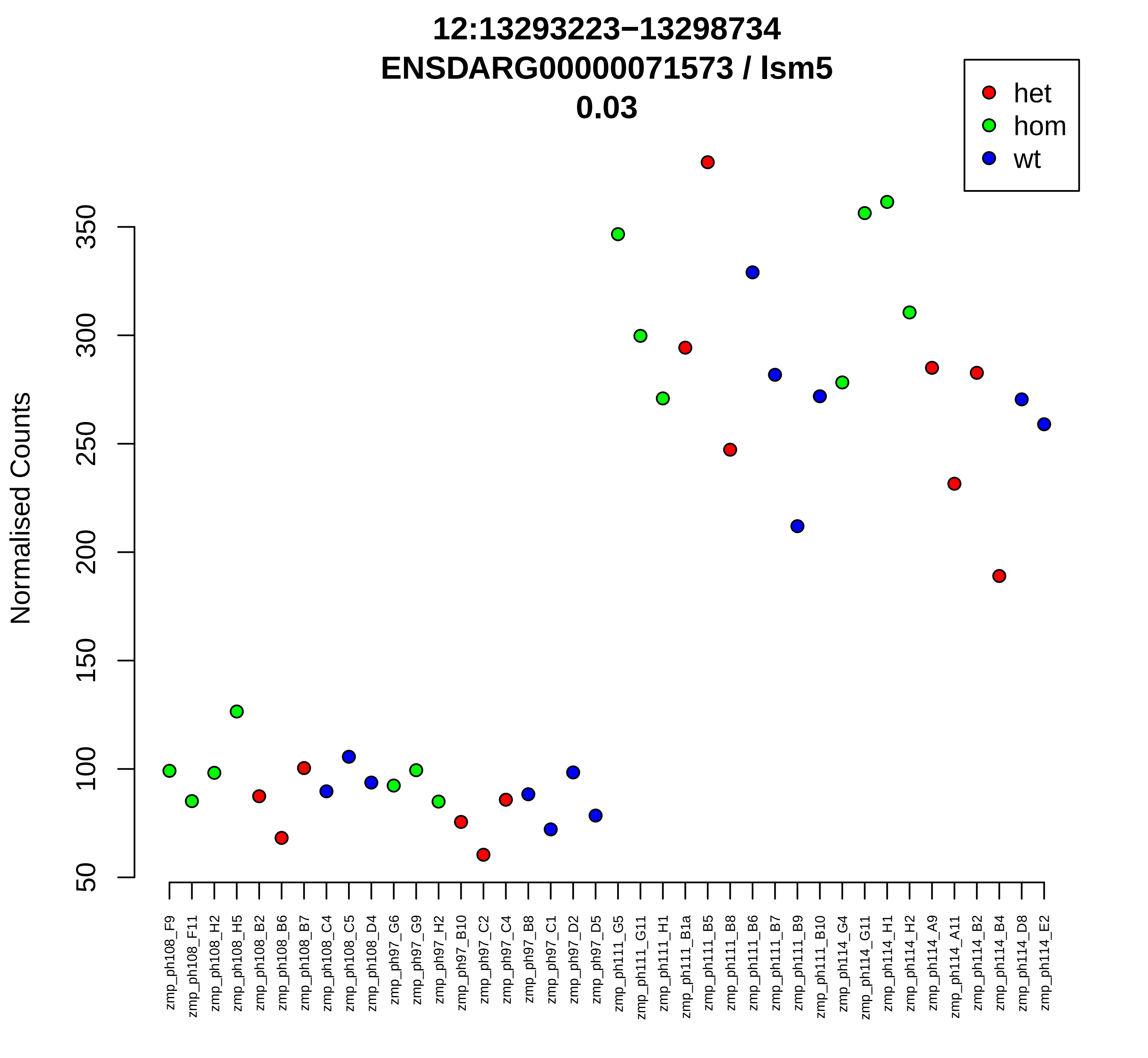
12:13293223−13298734
ENSDARG00000071573 / lsm5
0.03
het
hom
wt
Normalised Counts
50
100
150
200
250
300
350
zmp_ph108_F9
zmp_ph108_F11
zmp_ph108_H2
zmp_ph108_H5
zmp_ph108_B2
zmp_ph108_B6
zmp_ph108_B7
zmp_ph108_C4
zmp_ph108_C5
zmp_ph108_D4
zmp_ph97_G6
zmp_ph97_G9
zmp_ph97_H2
zmp_ph97_B10
zmp_ph97_C2
zmp_ph97_C4
zmp_ph97_B8
zmp_ph97_C1
zmp_ph97_D2
zmp_ph97_D5
zmp_ph111_G5
zmp_ph111_G11
zmp_ph111_H1
zmp_ph111_B1a
zmp_ph111_B5
zmp_ph111_B8
zmp_ph111_B6
zmp_ph111_B7
zmp_ph111_B9
zmp_ph111_B10
zmp_ph114_G4
zmp_ph114_G11
zmp_ph114_H1
zmp_ph114_H2
zmp_ph114_A9
zmp_ph114_A11
zmp_ph114_B2
zmp_ph114_B4
zmp_ph114_D8
zmp_ph114_E2

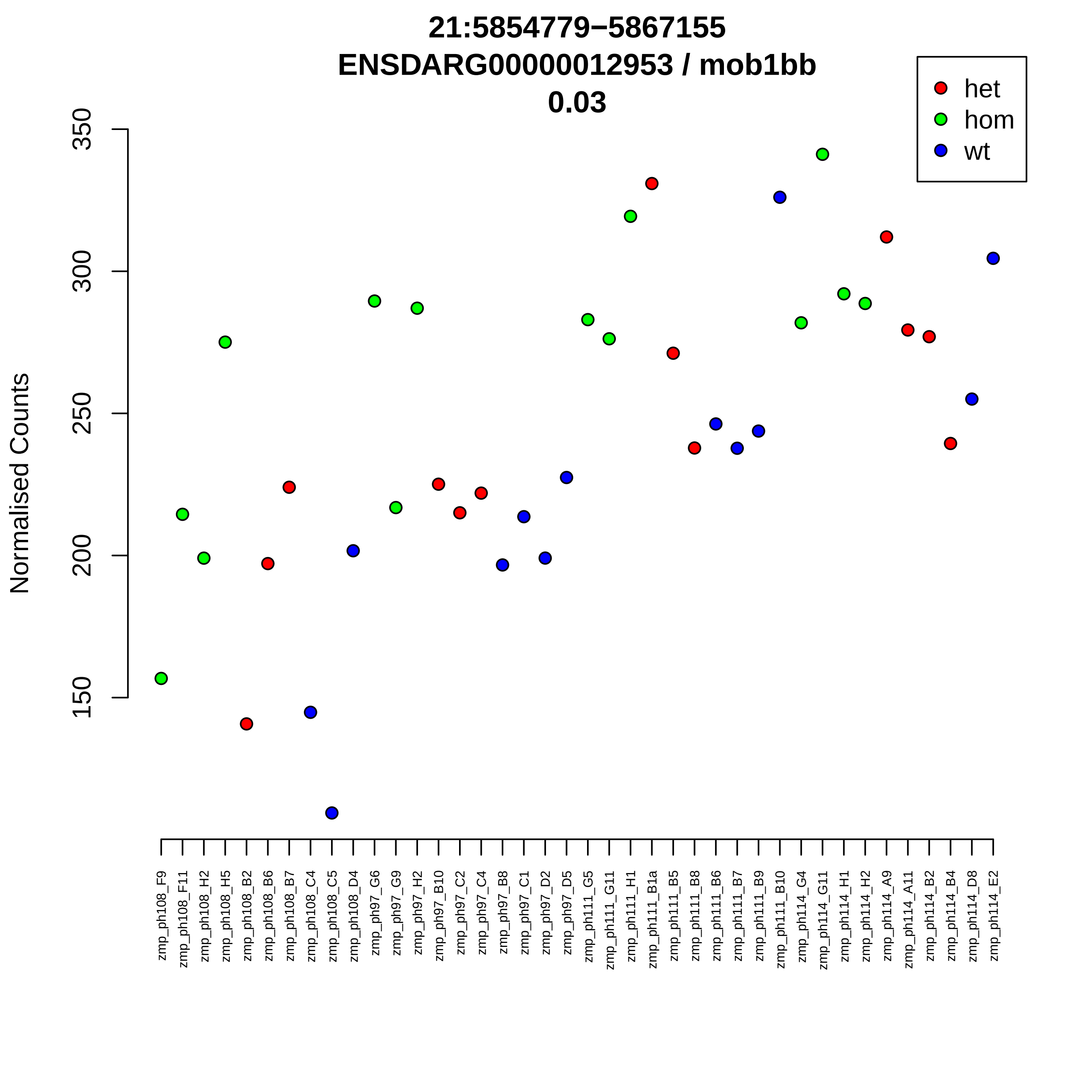
21:5854779−5867155
ENSDARG00000012953 / mob1bb
0.03
het
hom
wt
Normalised Counts
150
200
250
300
350
zmp_ph108_F9
zmp_ph108_F11
zmp_ph108_H2
zmp_ph108_H5
zmp_ph108_B2
zmp_ph108_B6
zmp_ph108_B7
zmp_ph108_C4
zmp_ph108_C5
zmp_ph108_D4
zmp_ph97_G6
zmp_ph97_G9
zmp_ph97_H2
zmp_ph97_B10
zmp_ph97_C2
zmp_ph97_C4
zmp_ph97_B8
zmp_ph97_C1
zmp_ph97_D2
zmp_ph97_D5
zmp_ph111_G5
zmp_ph111_G11
zmp_ph111_H1
zmp_ph111_B1a
zmp_ph111_B5
zmp_ph111_B8
zmp_ph111_B6
zmp_ph111_B7
zmp_ph111_B9
zmp_ph111_B10
zmp_ph114_G4
zmp_ph114_G11
zmp_ph114_H1
zmp_ph114_H2
zmp_ph114_A9
zmp_ph114_A11
zmp_ph114_B2
zmp_ph114_B4
zmp_ph114_D8
zmp_ph114_E2

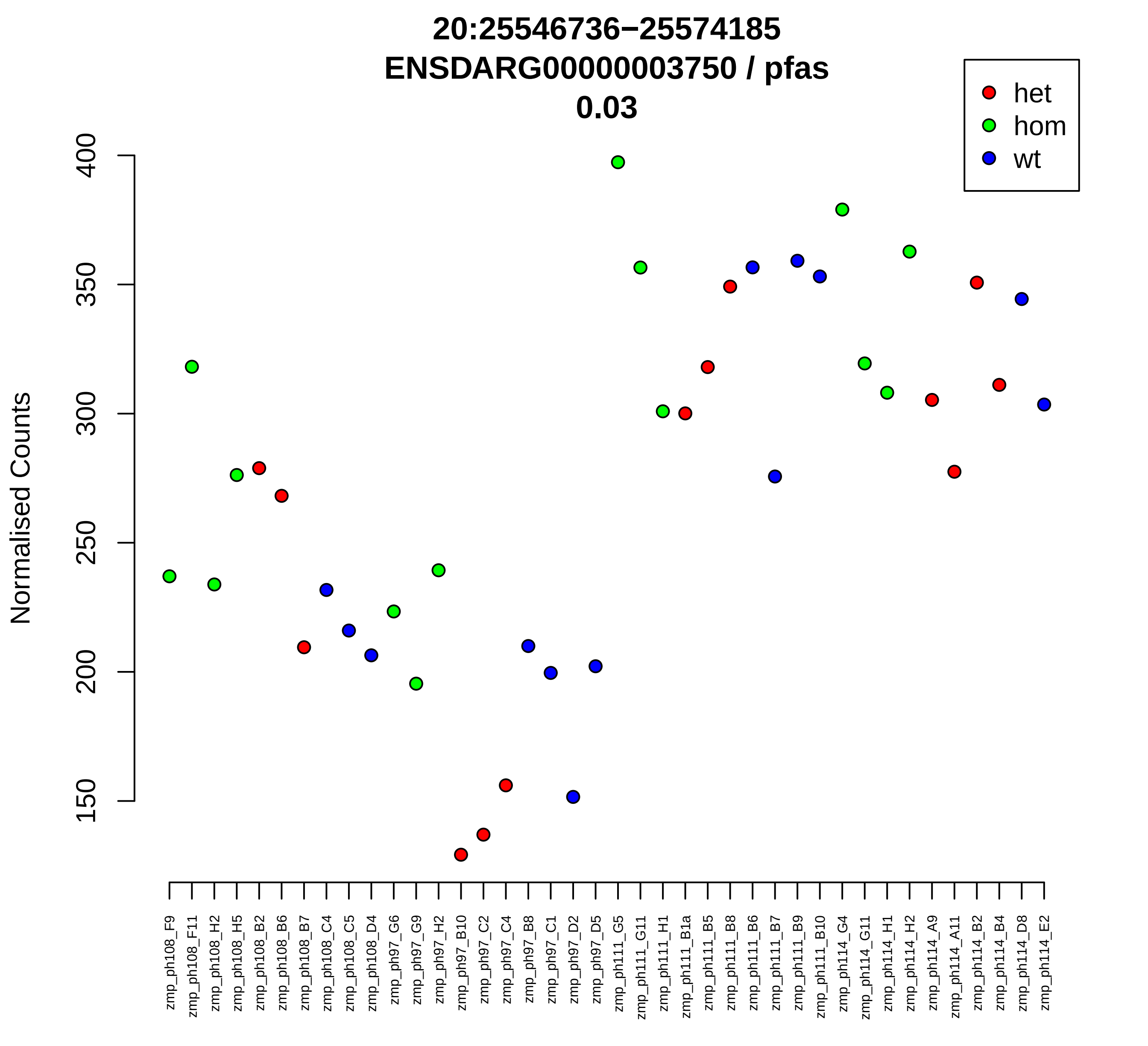
20:25546736−25574185
ENSDARG00000003750 / pfas
0.03
het
hom
wt
Normalised Counts
400
350
300
250
200
150
zmp_ph108_F9
zmp_ph108_F11
zmp_ph108_H2
zmp_ph108_H5
zmp_ph108_B2
zmp_ph108_B6
zmp_ph108_B7
zmp_ph108_C4
zmp_ph108_C5
zmp_ph108_D4
zmp_ph97_G6
zmp_ph97_G9
zmp_ph97_H2
zmp_ph97_B10
zmp_ph97_C2
zmp_ph97_C4
zmp_ph97_B8
zmp_ph97_C1
zmp_ph97_D2
zmp_ph97_D5
zmp_ph111_G5
zmp_ph111_G11
zmp_ph111_H1
zmp_ph111_B1a
zmp_ph111_B5
zmp_ph111_B8
zmp_ph111_B6
zmp_ph111_B7
zmp_ph111_B9
zmp_ph111_B10
zmp_ph114_G4
zmp_ph114_G11
zmp_ph114_H1
zmp_ph114_H2
zmp_ph114_A9
zmp_ph114_A11
zmp_ph114_B2
zmp_ph114_B4
zmp_ph114_D8
zmp_ph114_E2

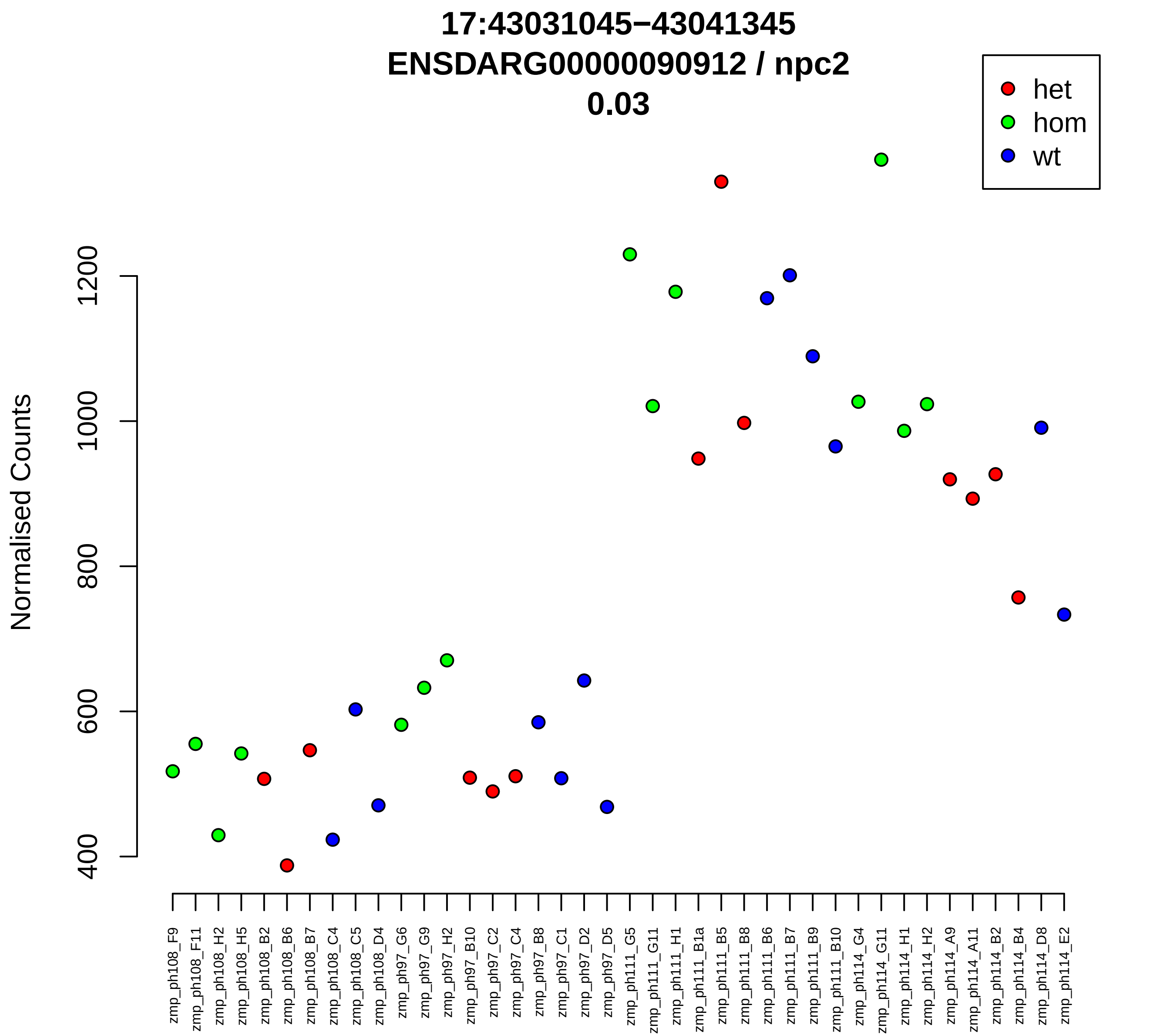
17:43031045−43041345
ENSDARG00000090912 / npc2
0.03
het
hom
wt
Normalised Counts
400
600
800
1000
1200
zmp_ph108_F9
zmp_ph108_F11
zmp_ph108_H2
zmp_ph108_H5
zmp_ph108_B2
zmp_ph108_B6
zmp_ph108_B7
zmp_ph108_C4
zmp_ph108_C5
zmp_ph108_D4
zmp_ph97_G6
zmp_ph97_G9
zmp_ph97_H2
zmp_ph97_B10
zmp_ph97_C2
zmp_ph97_C4
zmp_ph97_B8
zmp_ph97_C1
zmp_ph97_D2
zmp_ph97_D5
zmp_ph111_G5
zmp_ph111_G11
zmp_ph111_H1
zmp_ph111_B1a
zmp_ph111_B5
zmp_ph111_B8
zmp_ph111_B6
zmp_ph111_B7
zmp_ph111_B9
zmp_ph111_B10
zmp_ph114_G4
zmp_ph114_G11
zmp_ph114_H1
zmp_ph114_H2
zmp_ph114_A9
zmp_ph114_A11
zmp_ph114_B2
zmp_ph114_B4
zmp_ph114_D8
zmp_ph114_E2

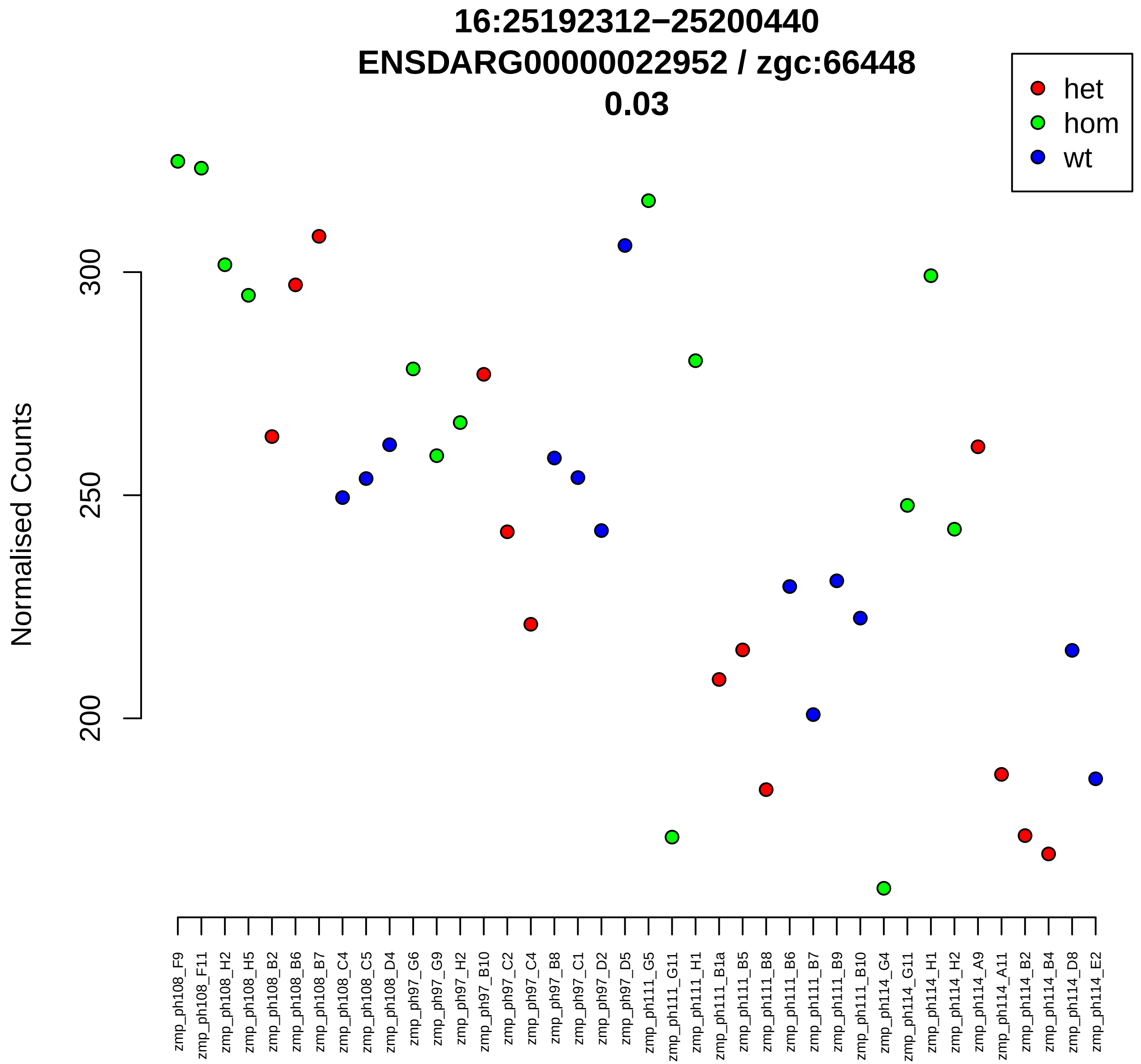
16:25192312−25200440
ENSDARG00000022952 / zgc:66448
0.03
het
hom
wt
Normalised Counts
300
250
200
zmp_ph108_F9
zmp_ph108_F11
zmp_ph108_H2
zmp_ph108_H5
zmp_ph108_B2
zmp_ph108_B6
zmp_ph108_B7
zmp_ph108_C4
zmp_ph108_C5
zmp_ph108_D4
zmp_ph97_G6
zmp_ph97_G9
zmp_ph97_H2
zmp_ph97_B10
zmp_ph97_C2
zmp_ph97_C4
zmp_ph97_B8
zmp_ph97_C1
zmp_ph97_D2
zmp_ph97_D5
zmp_ph111_G5
zmp_ph111_G11
zmp_ph111_H1
zmp_ph111_B1a
zmp_ph111_B5
zmp_ph111_B8
zmp_ph111_B6
zmp_ph111_B7
zmp_ph111_B9
zmp_ph111_B10
zmp_ph114_G4
zmp_ph114_G11
zmp_ph114_H1
zmp_ph114_H2
zmp_ph114_A9
zmp_ph114_A11
zmp_ph114_B2
zmp_ph114_B4
zmp_ph114_D8
zmp_ph114_E2

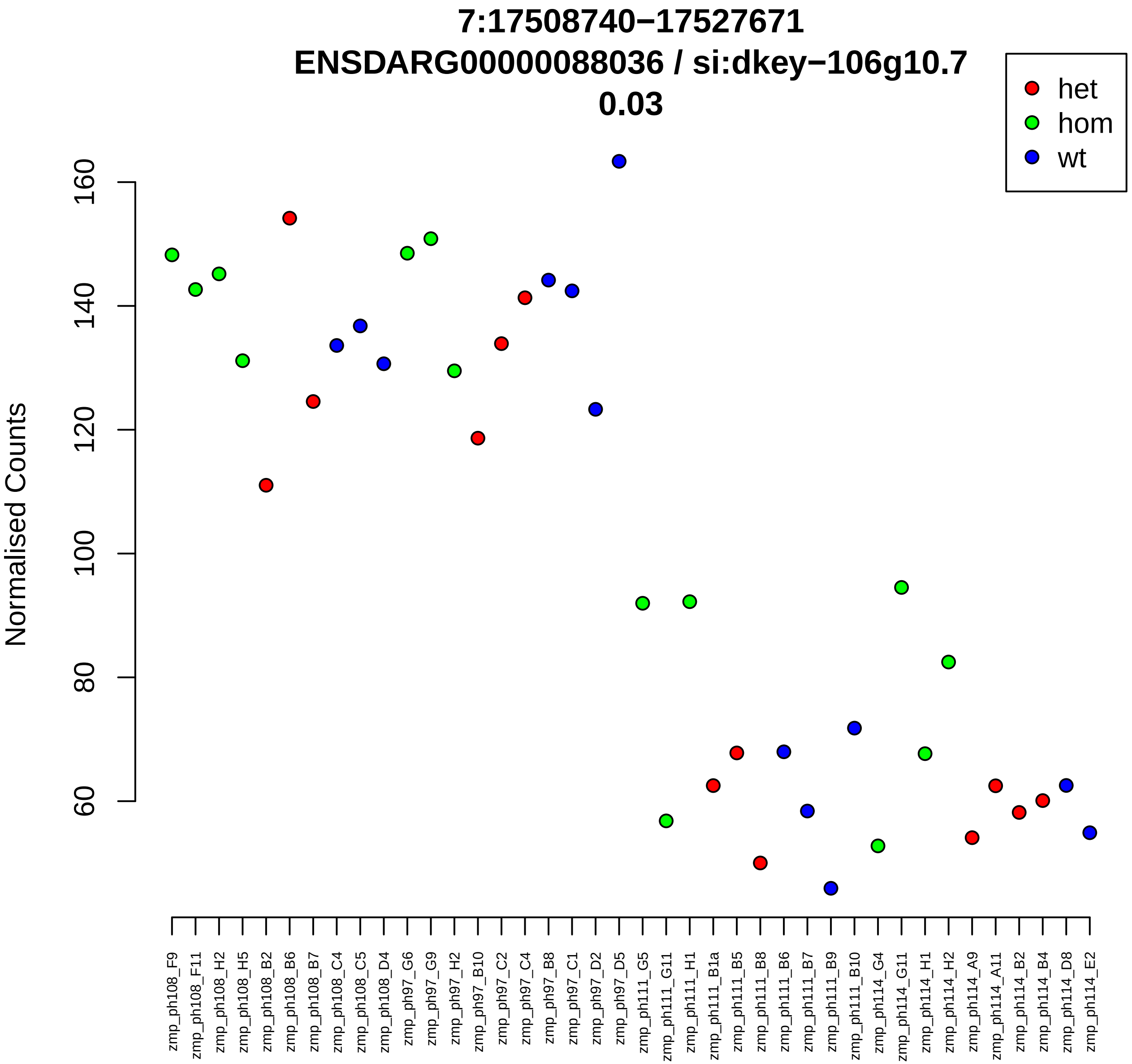
7:17508740−17527671
ENSDARG00000088036 / si:dkey−106g10.7
0.03
het
hom
wt
Normalised Counts
60
80
100
120
140
160
zmp_ph108_F9
zmp_ph108_F11
zmp_ph108_H2
zmp_ph108_H5
zmp_ph108_B2
zmp_ph108_B6
zmp_ph108_B7
zmp_ph108_C4
zmp_ph108_C5
zmp_ph108_D4
zmp_ph97_G6
zmp_ph97_G9
zmp_ph97_H2
zmp_ph97_B10
zmp_ph97_C2
zmp_ph97_C4
zmp_ph97_B8
zmp_ph97_C1
zmp_ph97_D2
zmp_ph97_D5
zmp_ph111_G5
zmp_ph111_G11
zmp_ph111_H1
zmp_ph111_B1a
zmp_ph111_B5
zmp_ph111_B8
zmp_ph111_B6
zmp_ph111_B7
zmp_ph111_B9
zmp_ph111_B10
zmp_ph114_G4
zmp_ph114_G11
zmp_ph114_H1
zmp_ph114_H2
zmp_ph114_A9
zmp_ph114_A11
zmp_ph114_B2
zmp_ph114_B4
zmp_ph114_D8
zmp_ph114_E2

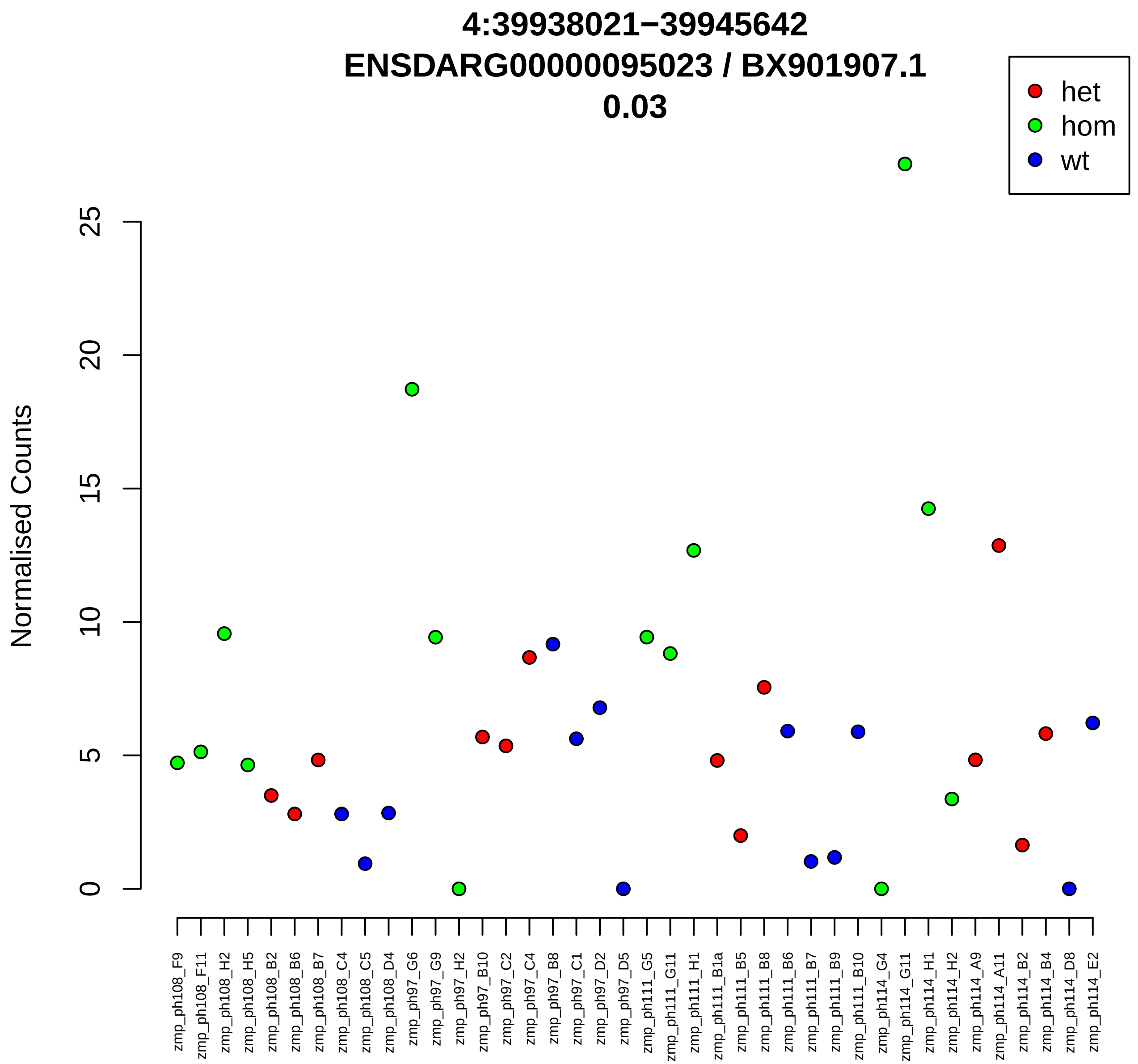
4:39938021−39945642
ENSDARG00000095023 / BX901907.1
0.03
het
hom
wt
Normalised Counts
0
5
10
15
20
25
zmp_ph108_F9
zmp_ph108_F11
zmp_ph108_H2
zmp_ph108_H5
zmp_ph108_B2
zmp_ph108_B6
zmp_ph108_B7
zmp_ph108_C4
zmp_ph108_C5
zmp_ph108_D4
zmp_ph97_G6
zmp_ph97_G9
zmp_ph97_H2
zmp_ph97_B10
zmp_ph97_C2
zmp_ph97_C4
zmp_ph97_B8
zmp_ph97_C1
zmp_ph97_D2
zmp_ph97_D5
zmp_ph111_G5
zmp_ph111_G11
zmp_ph111_H1
zmp_ph111_B1a
zmp_ph111_B5
zmp_ph111_B8
zmp_ph111_B6
zmp_ph111_B7
zmp_ph111_B9
zmp_ph111_B10
zmp_ph114_G4
zmp_ph114_G11
zmp_ph114_H1
zmp_ph114_H2
zmp_ph114_A9
zmp_ph114_A11
zmp_ph114_B2
zmp_ph114_B4
zmp_ph114_D8
zmp_ph114_E2

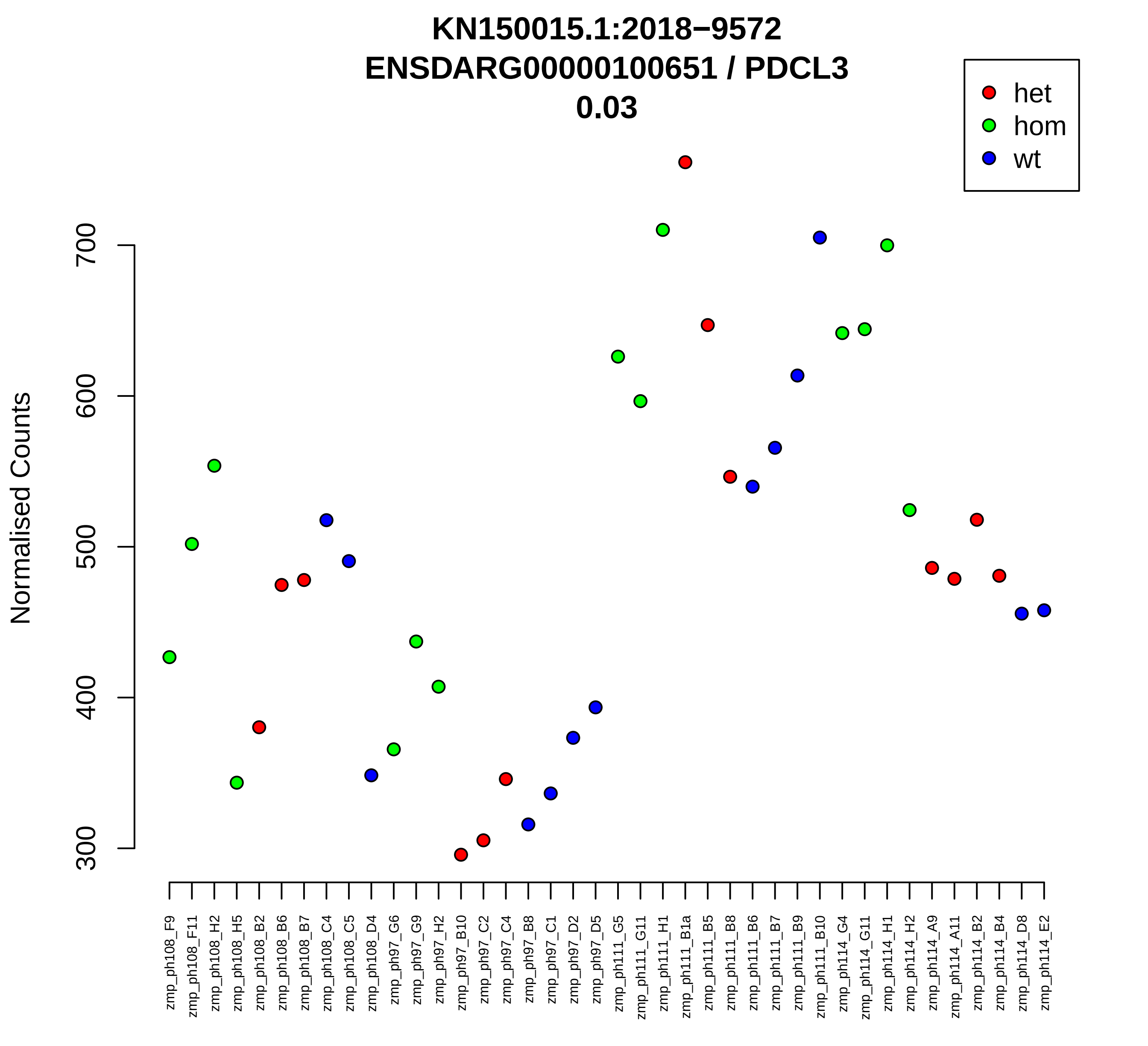

KN150015.1:2018−9572
ENSDARG00000100651 / PDCL3
0.03
het
hom
wt
Normalised Counts
300
400
500
600
700
zmp_ph108_F9
zmp_ph108_F11
zmp_ph108_H2
zmp_ph108_H5
zmp_ph108_B2
zmp_ph108_B6
zmp_ph108_B7
zmp_ph108_C4
zmp_ph108_C5
zmp_ph108_D4
zmp_ph97_G6
zmp_ph97_G9
zmp_ph97_H2
zmp_ph97_B10
zmp_ph97_C2
zmp_ph97_C4
zmp_ph97_B8
zmp_ph97_C1
zmp_ph97_D2
zmp_ph97_D5
zmp_ph111_G5
zmp_ph111_G11
zmp_ph111_H1
zmp_ph111_B1a
zmp_ph111_B5
zmp_ph111_B8
zmp_ph111_B6
zmp_ph111_B7
zmp_ph111_B9
zmp_ph111_B10
zmp_ph114_G4
zmp_ph114_G11
zmp_ph114_H1
zmp_ph114_H2
zmp_ph114_A9
zmp_ph114_A11
zmp_ph114_B2
zmp_ph114_B4
zmp_ph114_D8
zmp_ph114_E2

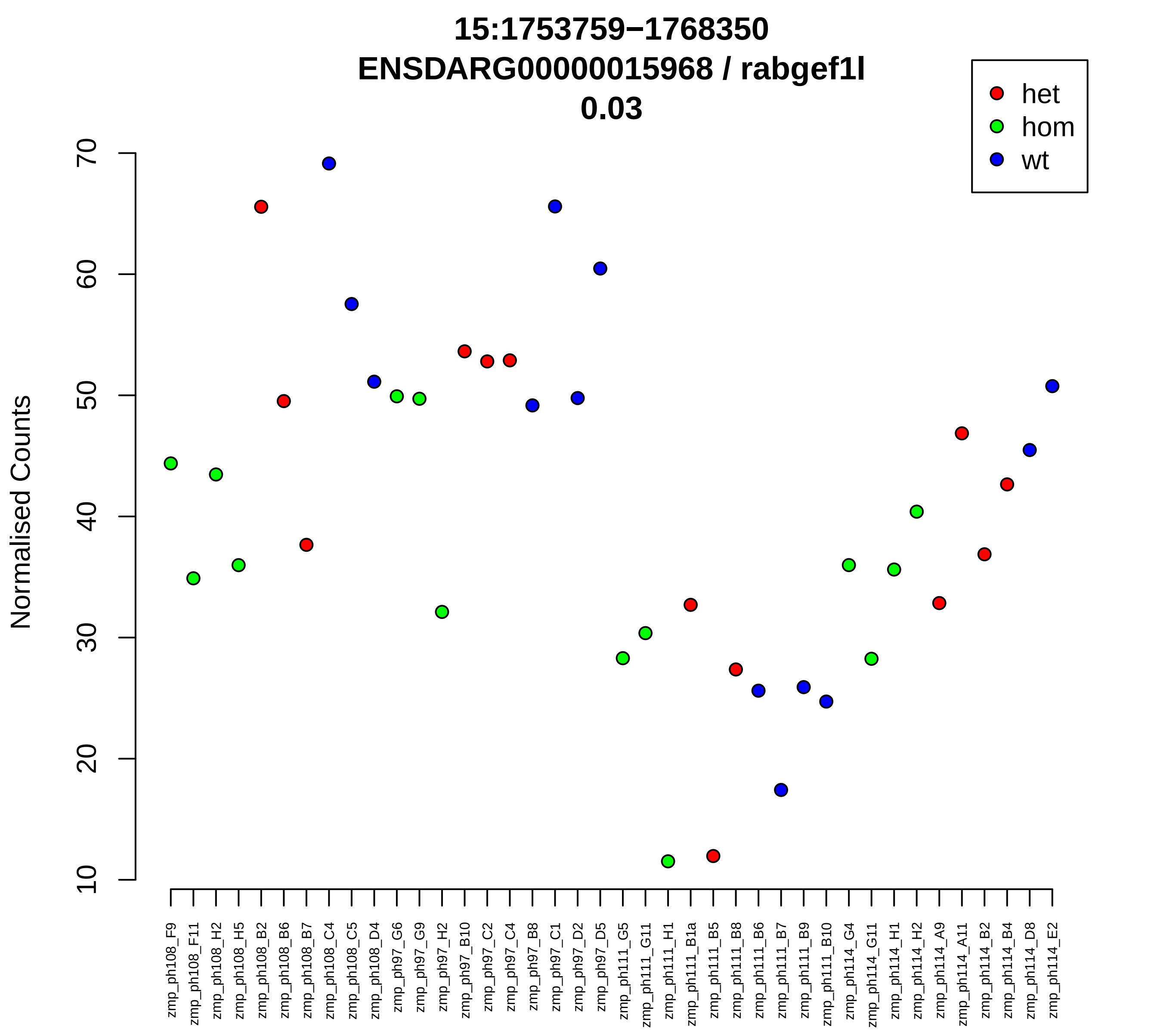
15:1753759−1768350
ENSDARG00000015968 / rabgef1l
0.03
het
hom
wt
Normalised Counts
10
20
30
40
50
60
70
zmp_ph108_F9
zmp_ph108_F11
zmp_ph108_H2
zmp_ph108_H5
zmp_ph108_B2
zmp_ph108_B6
zmp_ph108_B7
zmp_ph108_C4
zmp_ph108_C5
zmp_ph108_D4
zmp_ph97_G6
zmp_ph97_G9
zmp_ph97_H2
zmp_ph97_B10
zmp_ph97_C2
zmp_ph97_C4
zmp_ph97_B8
zmp_ph97_C1
zmp_ph97_D2
zmp_ph97_D5
zmp_ph111_G5
zmp_ph111_G11
zmp_ph111_H1
zmp_ph111_B1a
zmp_ph111_B5
zmp_ph111_B8
zmp_ph111_B6
zmp_ph111_B7
zmp_ph111_B9
zmp_ph111_B10
zmp_ph114_G4
zmp_ph114_G11
zmp_ph114_H1
zmp_ph114_H2
zmp_ph114_A9
zmp_ph114_A11
zmp_ph114_B2
zmp_ph114_B4
zmp_ph114_D8
zmp_ph114_E2

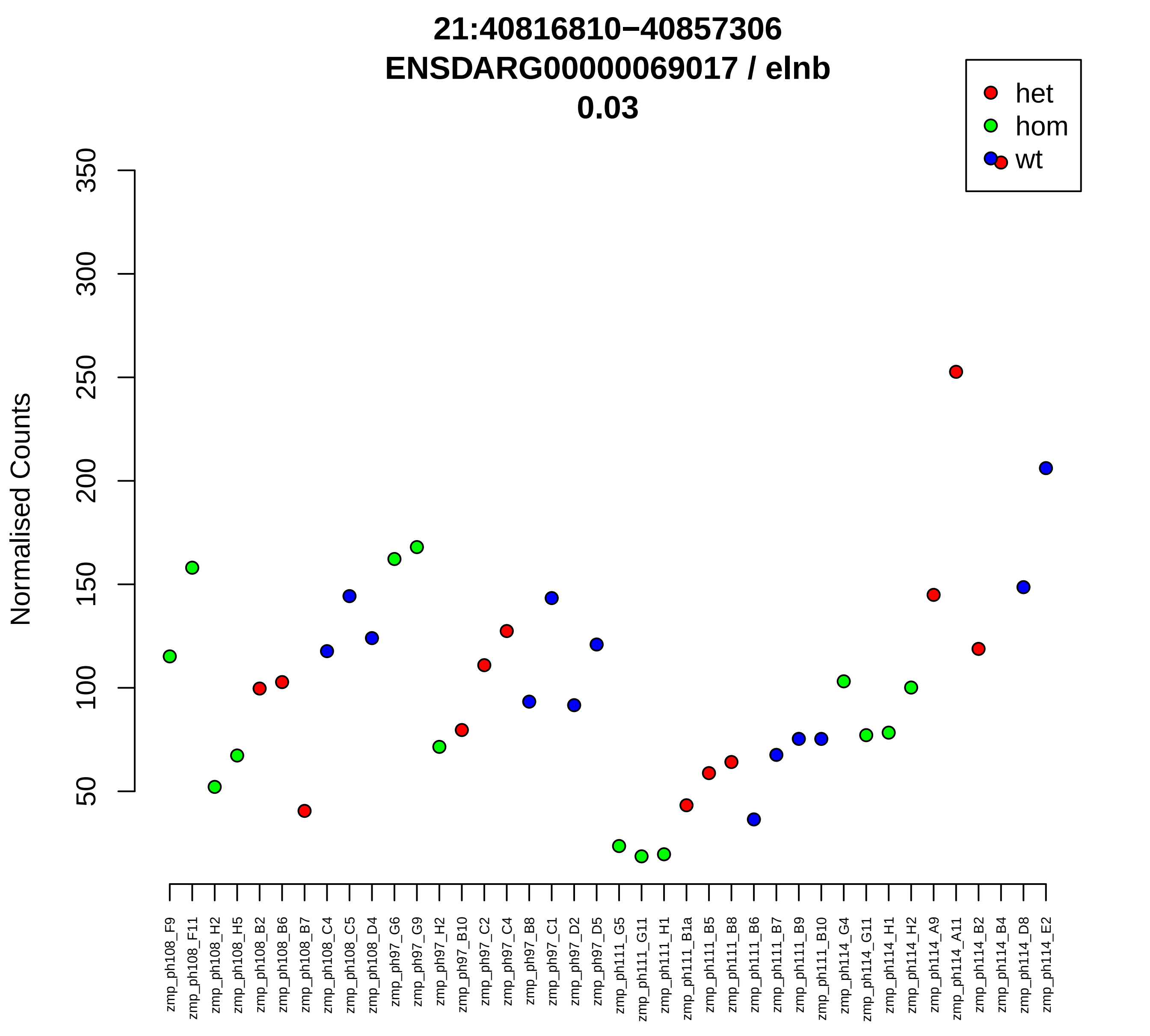

21:40816810−40857306
ENSDARG00000069017 / elnb
0.03
het
hom
wt
Normalised Counts
50
100
150
200
250
300
350
zmp_ph108_F9
zmp_ph108_F11
zmp_ph108_H2
zmp_ph108_H5
zmp_ph108_B2
zmp_ph108_B6
zmp_ph108_B7
zmp_ph108_C4
zmp_ph108_C5
zmp_ph108_D4
zmp_ph97_G6
zmp_ph97_G9
zmp_ph97_H2
zmp_ph97_B10
zmp_ph97_C2
zmp_ph97_C4
zmp_ph97_B8
zmp_ph97_C1
zmp_ph97_D2
zmp_ph97_D5
zmp_ph111_G5
zmp_ph111_G11
zmp_ph111_H1
zmp_ph111_B1a
zmp_ph111_B5
zmp_ph111_B8
zmp_ph111_B6
zmp_ph111_B7
zmp_ph111_B9
zmp_ph111_B10
zmp_ph114_G4
zmp_ph114_G11
zmp_ph114_H1
zmp_ph114_H2
zmp_ph114_A9
zmp_ph114_A11
zmp_ph114_B2
zmp_ph114_B4
zmp_ph114_D8
zmp_ph114_E2

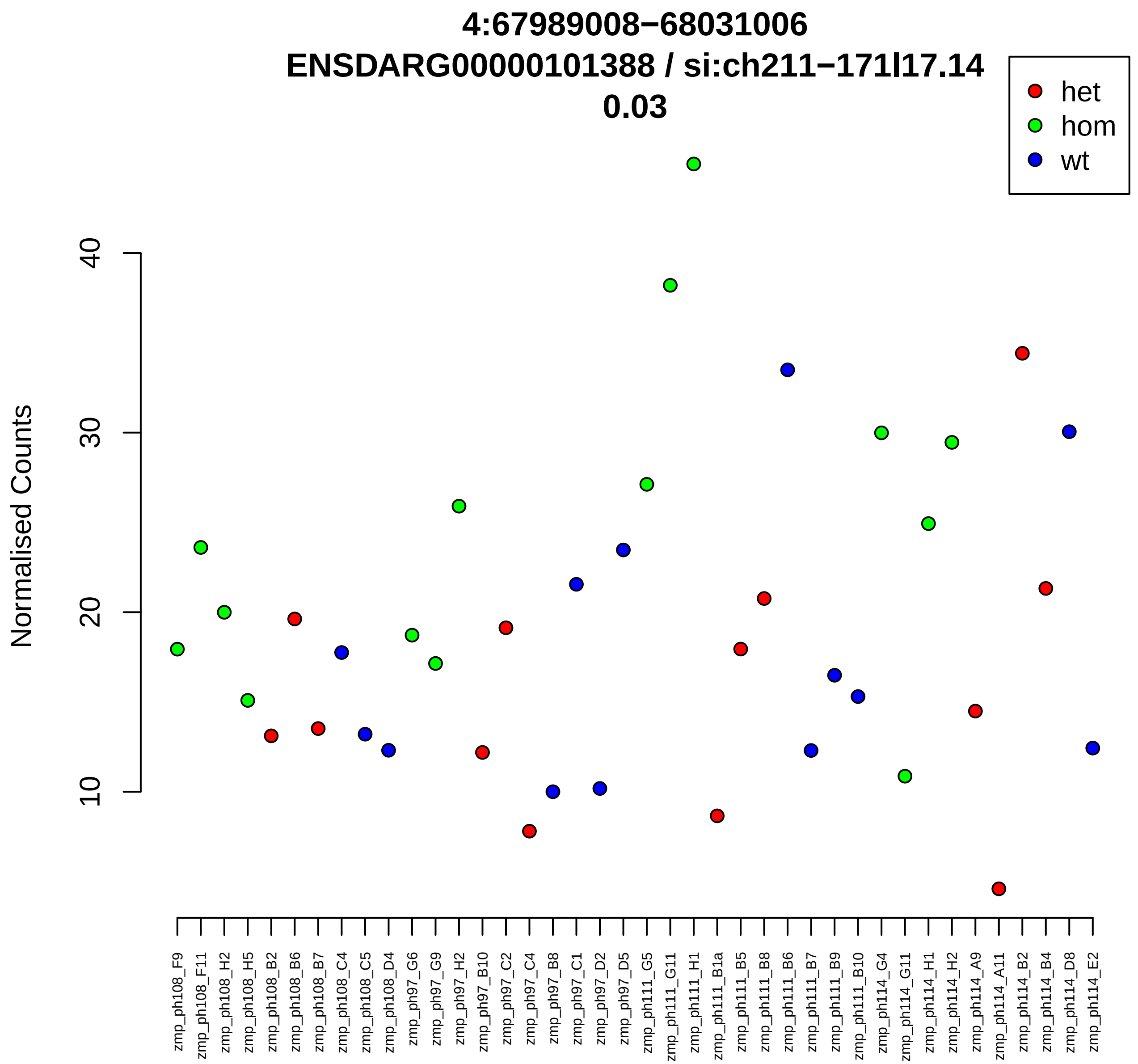

4:67989008−68031006
ENSDARG00000101388 / si:ch211−171l17.14
0.03
het
hom
wt
Normalised Counts
10
20
30
40
zmp_ph108_F9
zmp_ph108_F11
zmp_ph108_H2
zmp_ph108_H5
zmp_ph108_B2
zmp_ph108_B6
zmp_ph108_B7
zmp_ph108_C4
zmp_ph108_C5
zmp_ph108_D4
zmp_ph97_G6
zmp_ph97_G9
zmp_ph97_H2
zmp_ph97_B10
zmp_ph97_C2
zmp_ph97_C4
zmp_ph97_B8
zmp_ph97_C1
zmp_ph97_D2
zmp_ph97_D5
zmp_ph111_G5
zmp_ph111_G11
zmp_ph111_H1
zmp_ph111_B1a
zmp_ph111_B5
zmp_ph111_B8
zmp_ph111_B6
zmp_ph111_B7
zmp_ph111_B9
zmp_ph111_B10
zmp_ph114_G4
zmp_ph114_G11
zmp_ph114_H1
zmp_ph114_H2
zmp_ph114_A9
zmp_ph114_A11
zmp_ph114_B2
zmp_ph114_B4
zmp_ph114_D8
zmp_ph114_E2

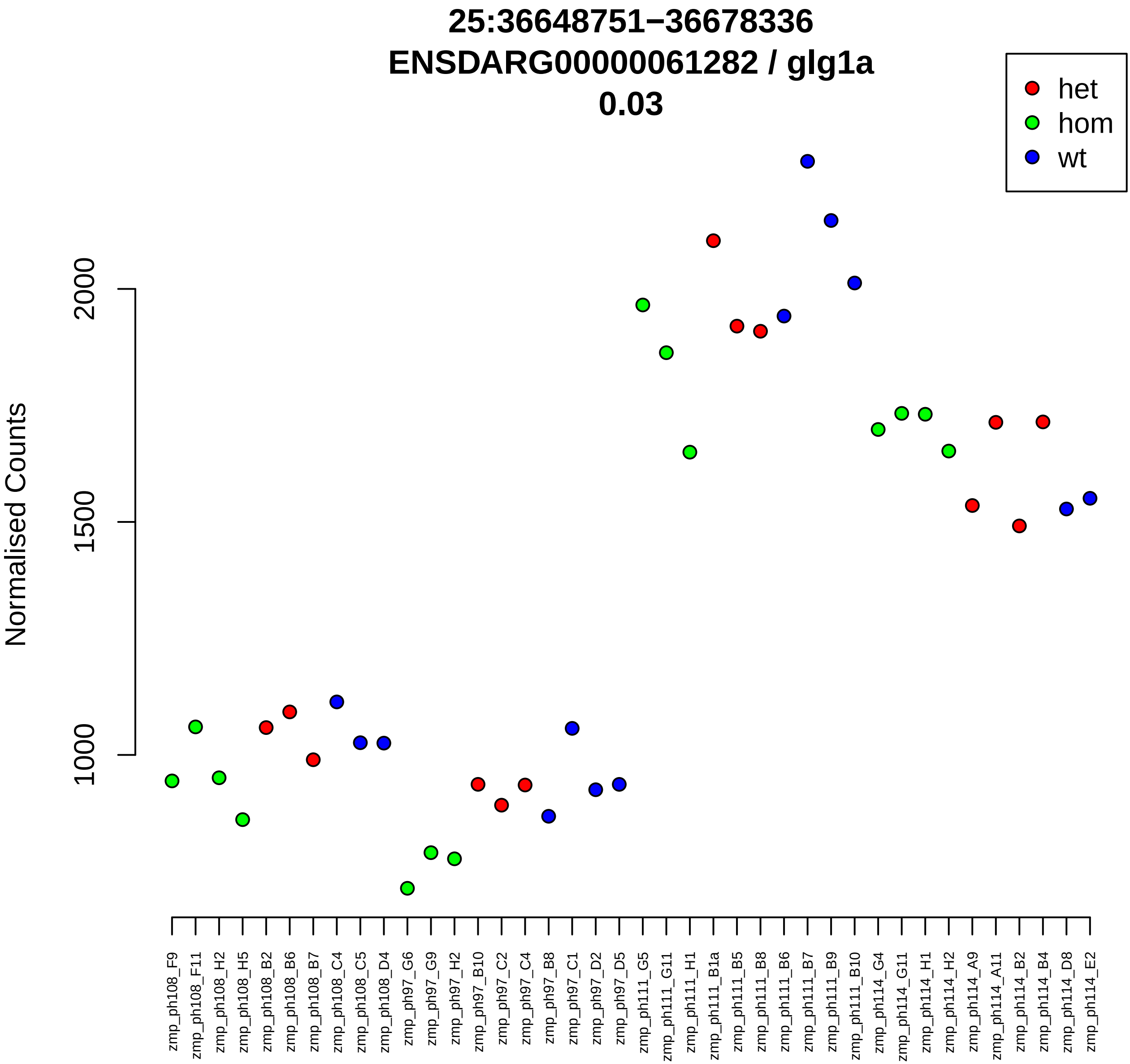

25:36648751−36678336
ENSDARG00000061282 / glg1a
0.03
het
hom
wt
Normalised Counts
1000
1500
2000
zmp_ph108_F9
zmp_ph108_F11
zmp_ph108_H2
zmp_ph108_H5
zmp_ph108_B2
zmp_ph108_B6
zmp_ph108_B7
zmp_ph108_C4
zmp_ph108_C5
zmp_ph108_D4
zmp_ph97_G6
zmp_ph97_G9
zmp_ph97_H2
zmp_ph97_B10
zmp_ph97_C2
zmp_ph97_C4
zmp_ph97_B8
zmp_ph97_C1
zmp_ph97_D2
zmp_ph97_D5
zmp_ph111_G5
zmp_ph111_G11
zmp_ph111_H1
zmp_ph111_B1a
zmp_ph111_B5
zmp_ph111_B8
zmp_ph111_B6
zmp_ph111_B7
zmp_ph111_B9
zmp_ph111_B10
zmp_ph114_G4
zmp_ph114_G11
zmp_ph114_H1
zmp_ph114_H2
zmp_ph114_A9
zmp_ph114_A11
zmp_ph114_B2
zmp_ph114_B4
zmp_ph114_D8
zmp_ph114_E2

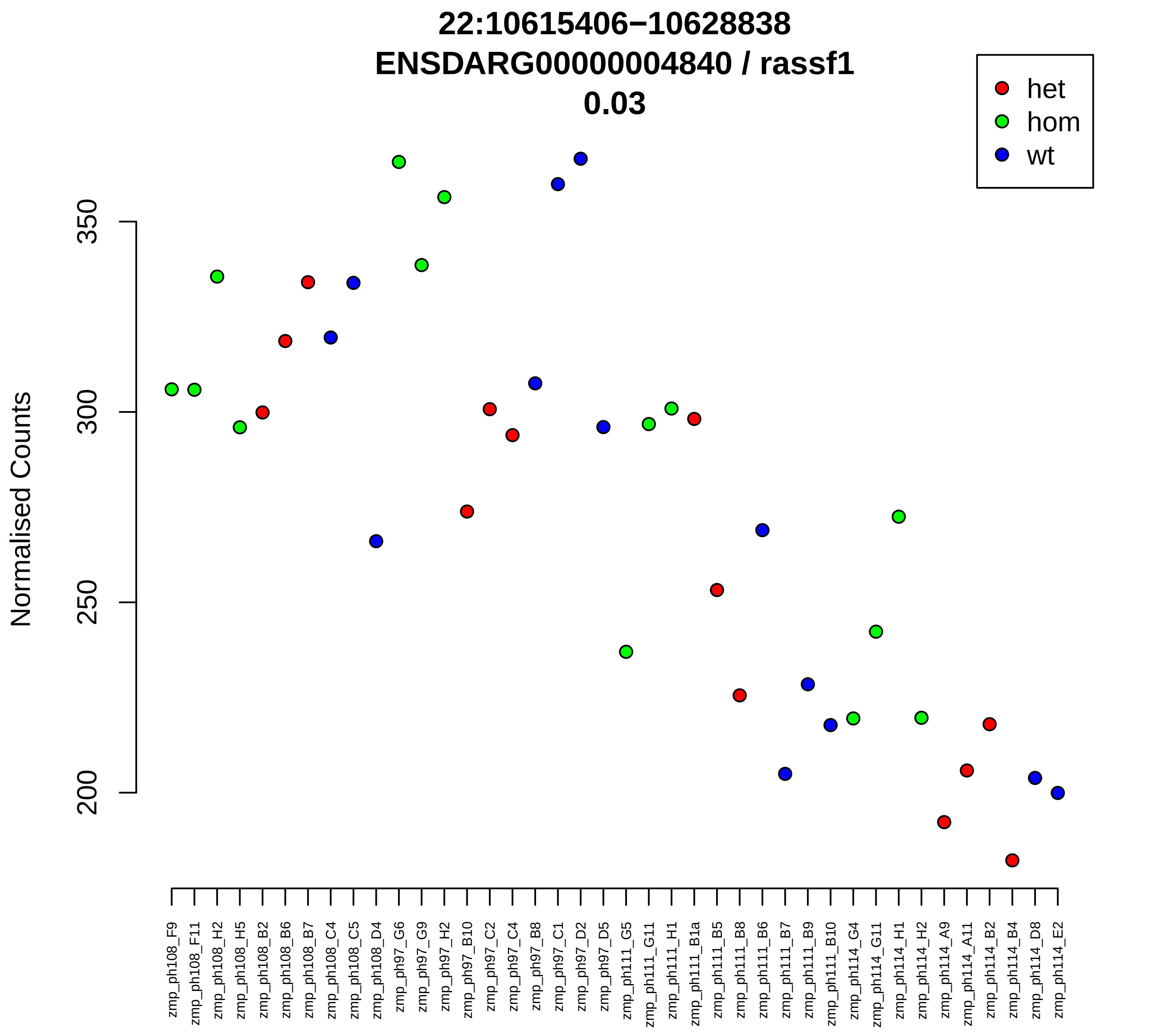
22:10615406−10628838
ENSDARG00000004840 / rassf1
0.03
het
hom
wt
Normalised Counts
200
250
300
350
zmp_ph108_F9
zmp_ph108_F11
zmp_ph108_H2
zmp_ph108_H5
zmp_ph108_B2
zmp_ph108_B6
zmp_ph108_B7
zmp_ph108_C4
zmp_ph108_C5
zmp_ph108_D4
zmp_ph97_G6
zmp_ph97_G9
zmp_ph97_H2
zmp_ph97_B10
zmp_ph97_C2
zmp_ph97_C4
zmp_ph97_B8
zmp_ph97_C1
zmp_ph97_D2
zmp_ph97_D5
zmp_ph111_G5
zmp_ph111_G11
zmp_ph111_H1
zmp_ph111_B1a
zmp_ph111_B5
zmp_ph111_B8
zmp_ph111_B6
zmp_ph111_B7
zmp_ph111_B9
zmp_ph111_B10
zmp_ph114_G4
zmp_ph114_G11
zmp_ph114_H1
zmp_ph114_H2
zmp_ph114_A9
zmp_ph114_A11
zmp_ph114_B2
zmp_ph114_B4
zmp_ph114_D8
zmp_ph114_E2

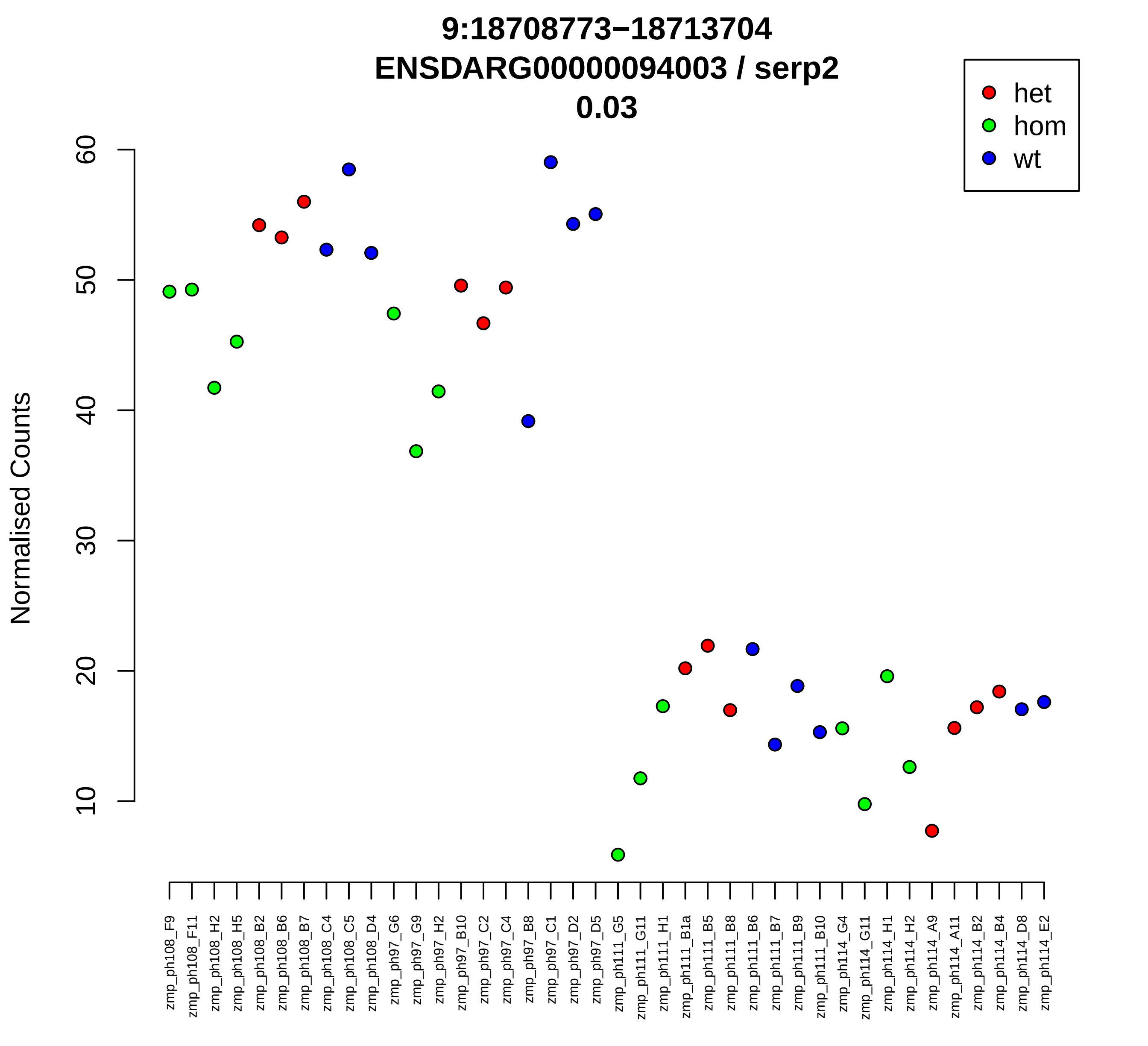
9:18708773−18713704
ENSDARG00000094003 / serp2
0.03
het
hom
wt
Normalised Counts
10
20
30
40
50
60
zmp_ph108_F9
zmp_ph108_F11
zmp_ph108_H2
zmp_ph108_H5
zmp_ph108_B2
zmp_ph108_B6
zmp_ph108_B7
zmp_ph108_C4
zmp_ph108_C5
zmp_ph108_D4
zmp_ph97_G6
zmp_ph97_G9
zmp_ph97_H2
zmp_ph97_B10
zmp_ph97_C2
zmp_ph97_C4
zmp_ph97_B8
zmp_ph97_C1
zmp_ph97_D2
zmp_ph97_D5
zmp_ph111_G5
zmp_ph111_G11
zmp_ph111_H1
zmp_ph111_B1a
zmp_ph111_B5
zmp_ph111_B8
zmp_ph111_B6
zmp_ph111_B7
zmp_ph111_B9
zmp_ph111_B10
zmp_ph114_G4
zmp_ph114_G11
zmp_ph114_H1
zmp_ph114_H2
zmp_ph114_A9
zmp_ph114_A11
zmp_ph114_B2
zmp_ph114_B4
zmp_ph114_D8
zmp_ph114_E2

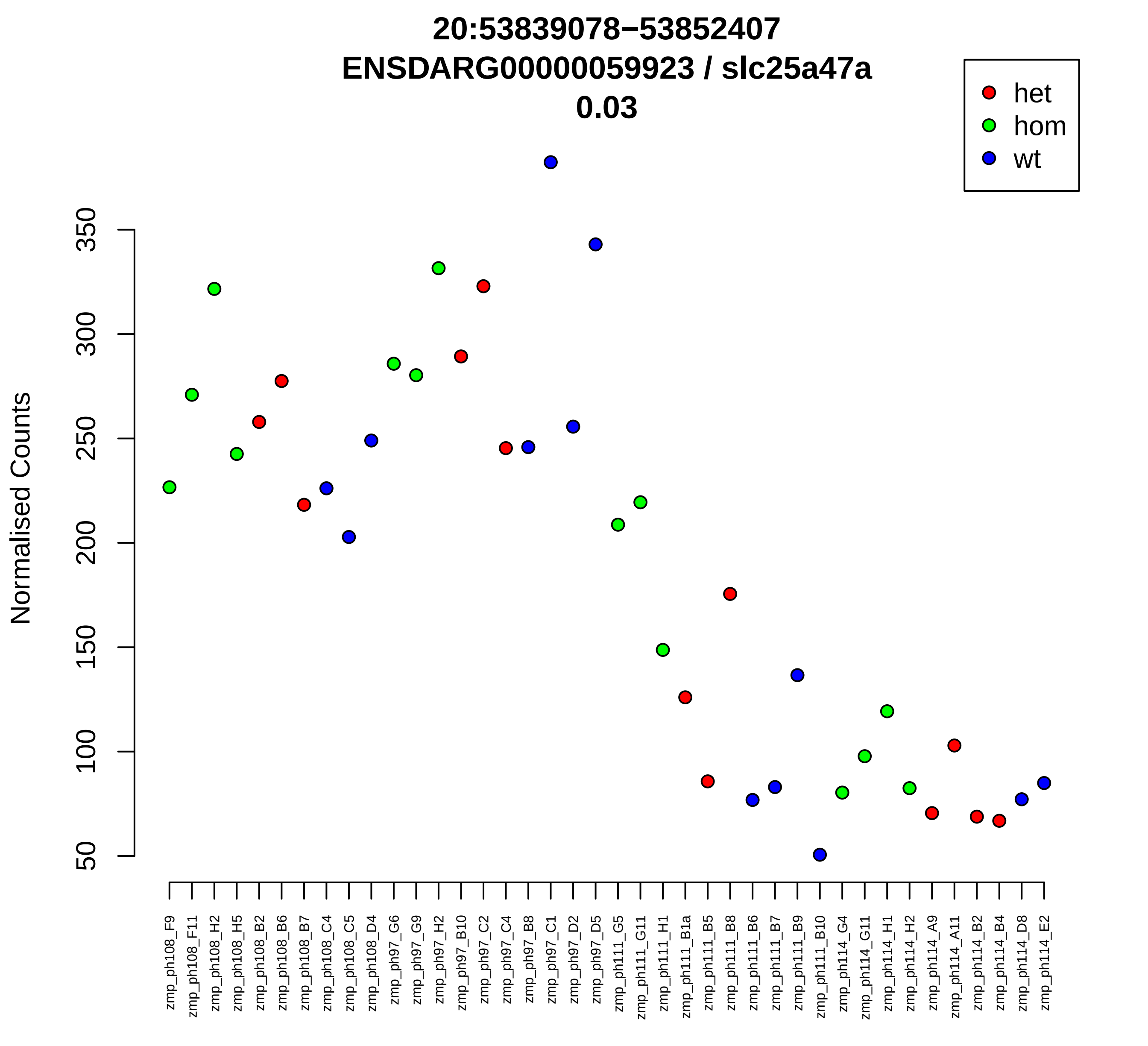
20:53839078−53852407
ENSDARG00000059923 / slc25a47a
0.03
het
hom
wt
Normalised Counts
50
100
150
200
250
300
350
zmp_ph108_F9
zmp_ph108_F11
zmp_ph108_H2
zmp_ph108_H5
zmp_ph108_B2
zmp_ph108_B6
zmp_ph108_B7
zmp_ph108_C4
zmp_ph108_C5
zmp_ph108_D4
zmp_ph97_G6
zmp_ph97_G9
zmp_ph97_H2
zmp_ph97_B10
zmp_ph97_C2
zmp_ph97_C4
zmp_ph97_B8
zmp_ph97_C1
zmp_ph97_D2
zmp_ph97_D5
zmp_ph111_G5
zmp_ph111_G11
zmp_ph111_H1
zmp_ph111_B1a
zmp_ph111_B5
zmp_ph111_B8
zmp_ph111_B6
zmp_ph111_B7
zmp_ph111_B9
zmp_ph111_B10
zmp_ph114_G4
zmp_ph114_G11
zmp_ph114_H1
zmp_ph114_H2
zmp_ph114_A9
zmp_ph114_A11
zmp_ph114_B2
zmp_ph114_B4
zmp_ph114_D8
zmp_ph114_E2

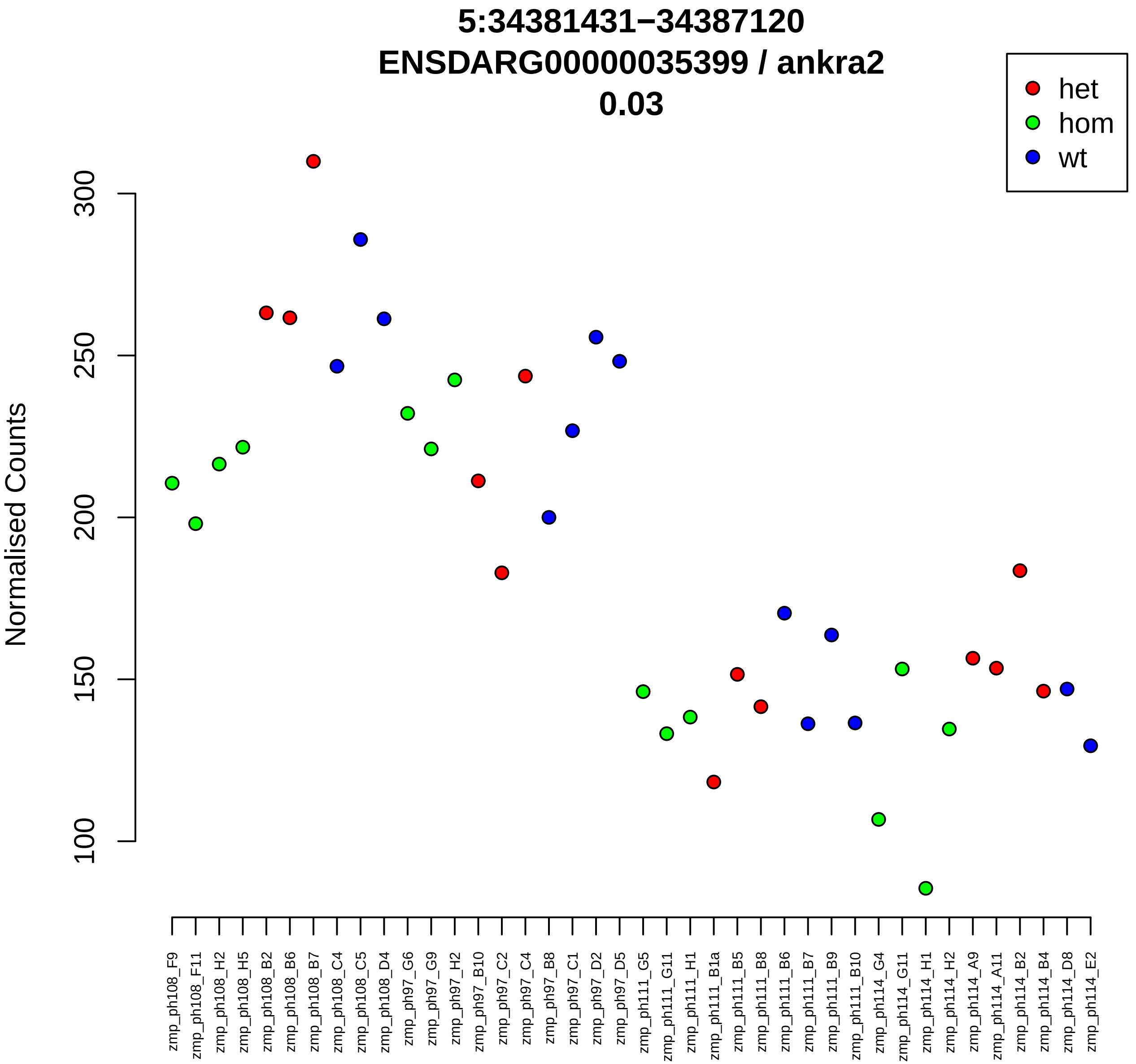

5:34381431−34387120
ENSDARG00000035399 / ankra2
0.03
het
hom
wt
Normalised Counts
100
150
200
250
300
zmp_ph108_F9
zmp_ph108_F11
zmp_ph108_H2
zmp_ph108_H5
zmp_ph108_B2
zmp_ph108_B6
zmp_ph108_B7
zmp_ph108_C4
zmp_ph108_C5
zmp_ph108_D4
zmp_ph97_G6
zmp_ph97_G9
zmp_ph97_H2
zmp_ph97_B10
zmp_ph97_C2
zmp_ph97_C4
zmp_ph97_B8
zmp_ph97_C1
zmp_ph97_D2
zmp_ph97_D5
zmp_ph111_G5
zmp_ph111_G11
zmp_ph111_H1
zmp_ph111_B1a
zmp_ph111_B5
zmp_ph111_B8
zmp_ph111_B6
zmp_ph111_B7
zmp_ph111_B9
zmp_ph111_B10
zmp_ph114_G4
zmp_ph114_G11
zmp_ph114_H1
zmp_ph114_H2
zmp_ph114_A9
zmp_ph114_A11
zmp_ph114_B2
zmp_ph114_B4
zmp_ph114_D8
zmp_ph114_E2

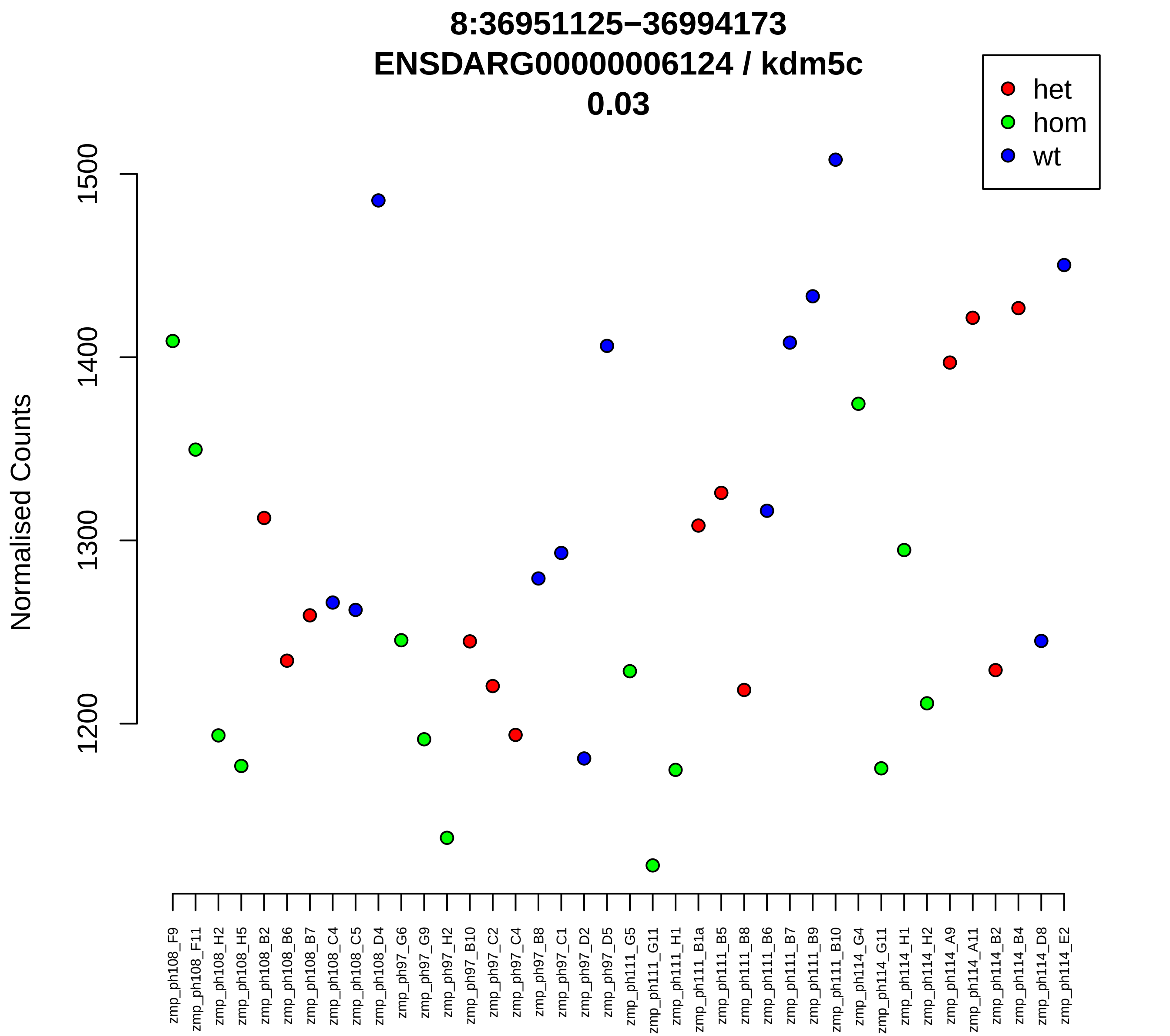

8:36951125−36994173
ENSDARG00000006124 / kdm5c
0.03
het
hom
wt
Normalised Counts
1200
1300
1400
1500
zmp_ph108_F9
zmp_ph108_F11
zmp_ph108_H2
zmp_ph108_H5
zmp_ph108_B2
zmp_ph108_B6
zmp_ph108_B7
zmp_ph108_C4
zmp_ph108_C5
zmp_ph108_D4
zmp_ph97_G6
zmp_ph97_G9
zmp_ph97_H2
zmp_ph97_B10
zmp_ph97_C2
zmp_ph97_C4
zmp_ph97_B8
zmp_ph97_C1
zmp_ph97_D2
zmp_ph97_D5
zmp_ph111_G5
zmp_ph111_G11
zmp_ph111_H1
zmp_ph111_B1a
zmp_ph111_B5
zmp_ph111_B8
zmp_ph111_B6
zmp_ph111_B7
zmp_ph111_B9
zmp_ph111_B10
zmp_ph114_G4
zmp_ph114_G11
zmp_ph114_H1
zmp_ph114_H2
zmp_ph114_A9
zmp_ph114_A11
zmp_ph114_B2
zmp_ph114_B4
zmp_ph114_D8
zmp_ph114_E2

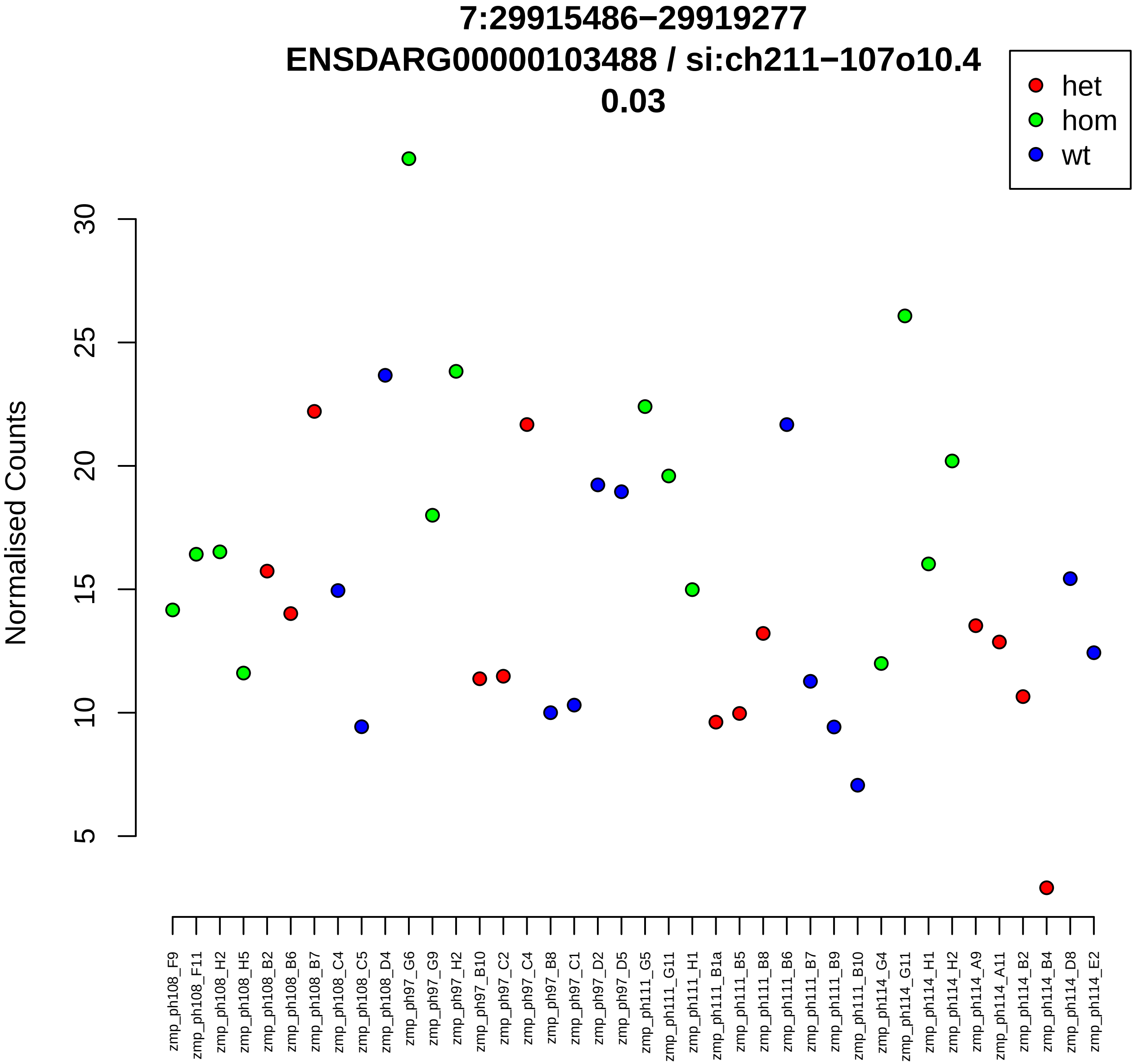

7:29915486−29919277
ENSDARG00000103488 / si:ch211−107o10.4
0.03
het
hom
wt
Normalised Counts
5
10
15
20
25
30
zmp_ph108_F9
zmp_ph108_F11
zmp_ph108_H2
zmp_ph108_H5
zmp_ph108_B2
zmp_ph108_B6
zmp_ph108_B7
zmp_ph108_C4
zmp_ph108_C5
zmp_ph108_D4
zmp_ph97_G6
zmp_ph97_G9
zmp_ph97_H2
zmp_ph97_B10
zmp_ph97_C2
zmp_ph97_C4
zmp_ph97_B8
zmp_ph97_C1
zmp_ph97_D2
zmp_ph97_D5
zmp_ph111_G5
zmp_ph111_G11
zmp_ph111_H1
zmp_ph111_B1a
zmp_ph111_B5
zmp_ph111_B8
zmp_ph111_B6
zmp_ph111_B7
zmp_ph111_B9
zmp_ph111_B10
zmp_ph114_G4
zmp_ph114_G11
zmp_ph114_H1
zmp_ph114_H2
zmp_ph114_A9
zmp_ph114_A11
zmp_ph114_B2
zmp_ph114_B4
zmp_ph114_D8
zmp_ph114_E2

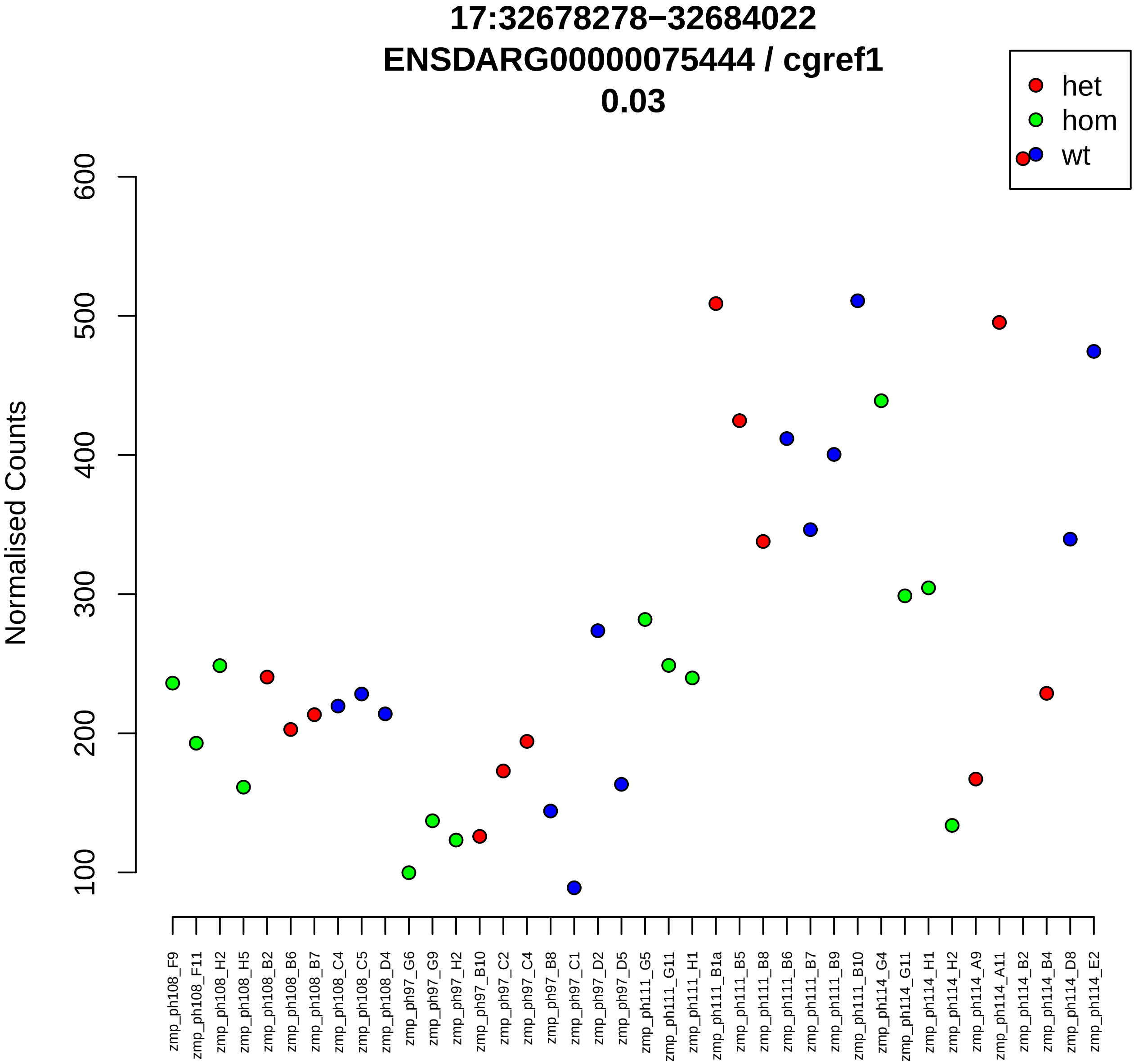
17:32678278−32684022
ENSDARG00000075444 / cgref1
0.03
het
hom
wt
Normalised Counts
100
200
300
400
500
600
zmp_ph108_F9
zmp_ph108_F11
zmp_ph108_H2
zmp_ph108_H5
zmp_ph108_B2
zmp_ph108_B6
zmp_ph108_B7
zmp_ph108_C4
zmp_ph108_C5
zmp_ph108_D4
zmp_ph97_G6
zmp_ph97_G9
zmp_ph97_H2
zmp_ph97_B10
zmp_ph97_C2
zmp_ph97_C4
zmp_ph97_B8
zmp_ph97_C1
zmp_ph97_D2
zmp_ph97_D5
zmp_ph111_G5
zmp_ph111_G11
zmp_ph111_H1
zmp_ph111_B1a
zmp_ph111_B5
zmp_ph111_B8
zmp_ph111_B6
zmp_ph111_B7
zmp_ph111_B9
zmp_ph111_B10
zmp_ph114_G4
zmp_ph114_G11
zmp_ph114_H1
zmp_ph114_H2
zmp_ph114_A9
zmp_ph114_A11
zmp_ph114_B2
zmp_ph114_B4
zmp_ph114_D8
zmp_ph114_E2

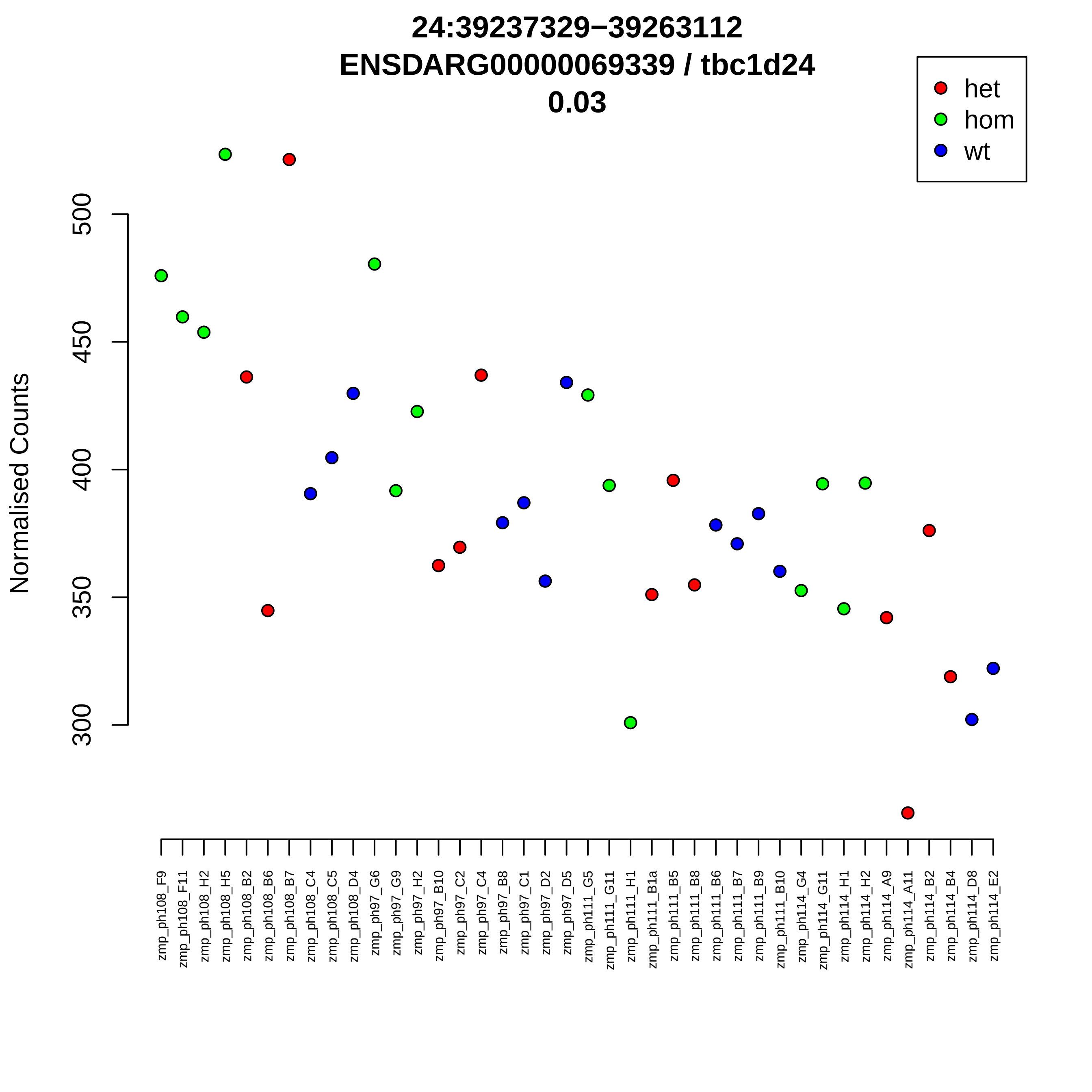

24:39237329−39263112
ENSDARG00000069339 / tbc1d24
0.03
het
hom
wt
Normalised Counts
300
350
400
450
500
zmp_ph108_F9
zmp_ph108_F11
zmp_ph108_H2
zmp_ph108_H5
zmp_ph108_B2
zmp_ph108_B6
zmp_ph108_B7
zmp_ph108_C4
zmp_ph108_C5
zmp_ph108_D4
zmp_ph97_G6
zmp_ph97_G9
zmp_ph97_H2
zmp_ph97_B10
zmp_ph97_C2
zmp_ph97_C4
zmp_ph97_B8
zmp_ph97_C1
zmp_ph97_D2
zmp_ph97_D5
zmp_ph111_G5
zmp_ph111_G11
zmp_ph111_H1
zmp_ph111_B1a
zmp_ph111_B5
zmp_ph111_B8
zmp_ph111_B6
zmp_ph111_B7
zmp_ph111_B9
zmp_ph111_B10
zmp_ph114_G4
zmp_ph114_G11
zmp_ph114_H1
zmp_ph114_H2
zmp_ph114_A9
zmp_ph114_A11
zmp_ph114_B2
zmp_ph114_B4
zmp_ph114_D8
zmp_ph114_E2

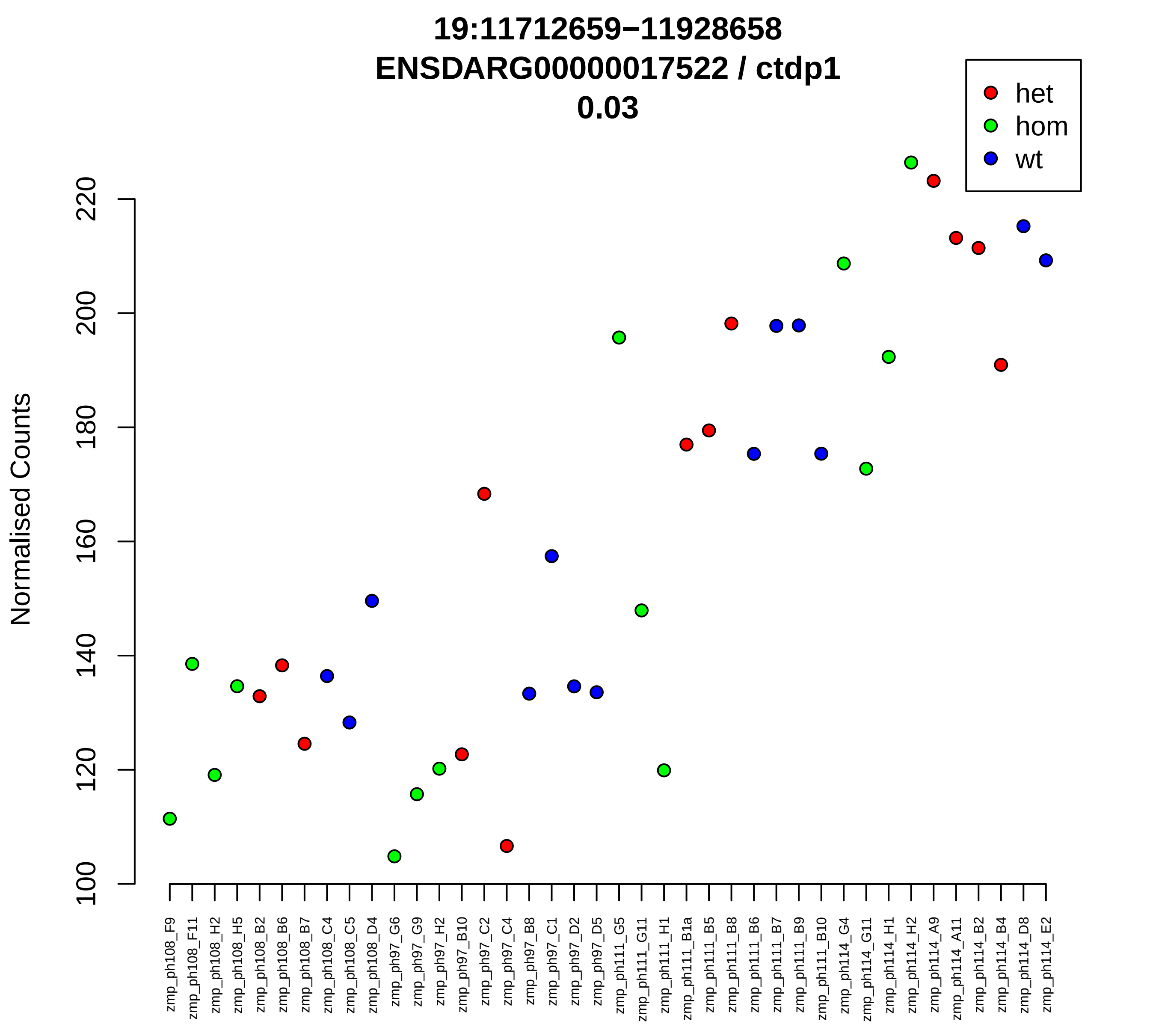

19:11712659−11928658
ENSDARG00000017522 / ctdp1
0.03
het
hom
wt
Normalised Counts
100
120
140
160
180
200
220
zmp_ph108_F9
zmp_ph108_F11
zmp_ph108_H2
zmp_ph108_H5
zmp_ph108_B2
zmp_ph108_B6
zmp_ph108_B7
zmp_ph108_C4
zmp_ph108_C5
zmp_ph108_D4
zmp_ph97_G6
zmp_ph97_G9
zmp_ph97_H2
zmp_ph97_B10
zmp_ph97_C2
zmp_ph97_C4
zmp_ph97_B8
zmp_ph97_C1
zmp_ph97_D2
zmp_ph97_D5
zmp_ph111_G5
zmp_ph111_G11
zmp_ph111_H1
zmp_ph111_B1a
zmp_ph111_B5
zmp_ph111_B8
zmp_ph111_B6
zmp_ph111_B7
zmp_ph111_B9
zmp_ph111_B10
zmp_ph114_G4
zmp_ph114_G11
zmp_ph114_H1
zmp_ph114_H2
zmp_ph114_A9
zmp_ph114_A11
zmp_ph114_B2
zmp_ph114_B4
zmp_ph114_D8
zmp_ph114_E2

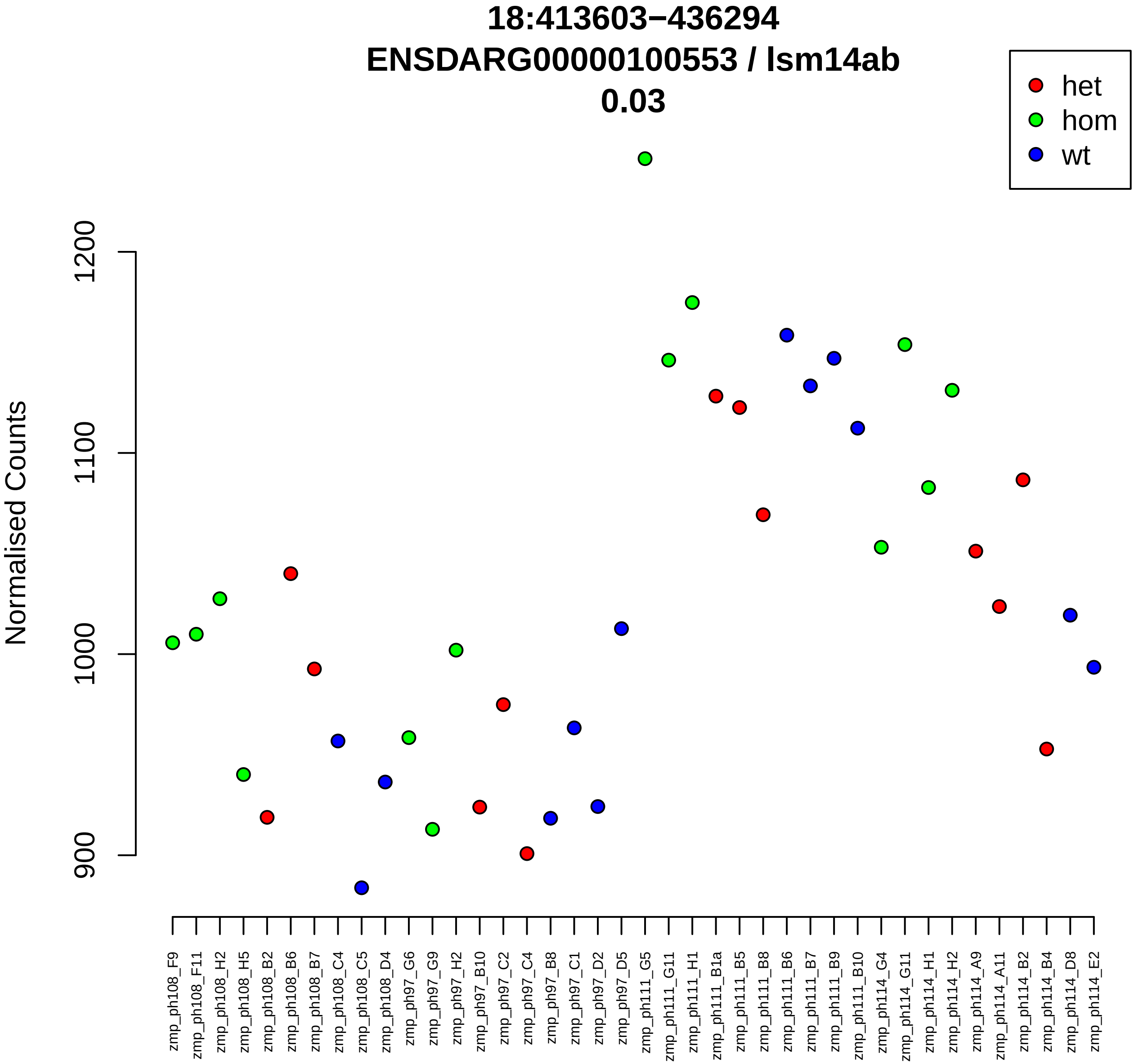

18:413603−436294
ENSDARG00000100553 / lsm14ab
0.03
het
hom
wt
Normalised Counts
900
1000
1100
1200
zmp_ph108_F9
zmp_ph108_F11
zmp_ph108_H2
zmp_ph108_H5
zmp_ph108_B2
zmp_ph108_B6
zmp_ph108_B7
zmp_ph108_C4
zmp_ph108_C5
zmp_ph108_D4
zmp_ph97_G6
zmp_ph97_G9
zmp_ph97_H2
zmp_ph97_B10
zmp_ph97_C2
zmp_ph97_C4
zmp_ph97_B8
zmp_ph97_C1
zmp_ph97_D2
zmp_ph97_D5
zmp_ph111_G5
zmp_ph111_G11
zmp_ph111_H1
zmp_ph111_B1a
zmp_ph111_B5
zmp_ph111_B8
zmp_ph111_B6
zmp_ph111_B7
zmp_ph111_B9
zmp_ph111_B10
zmp_ph114_G4
zmp_ph114_G11
zmp_ph114_H1
zmp_ph114_H2
zmp_ph114_A9
zmp_ph114_A11
zmp_ph114_B2
zmp_ph114_B4
zmp_ph114_D8
zmp_ph114_E2

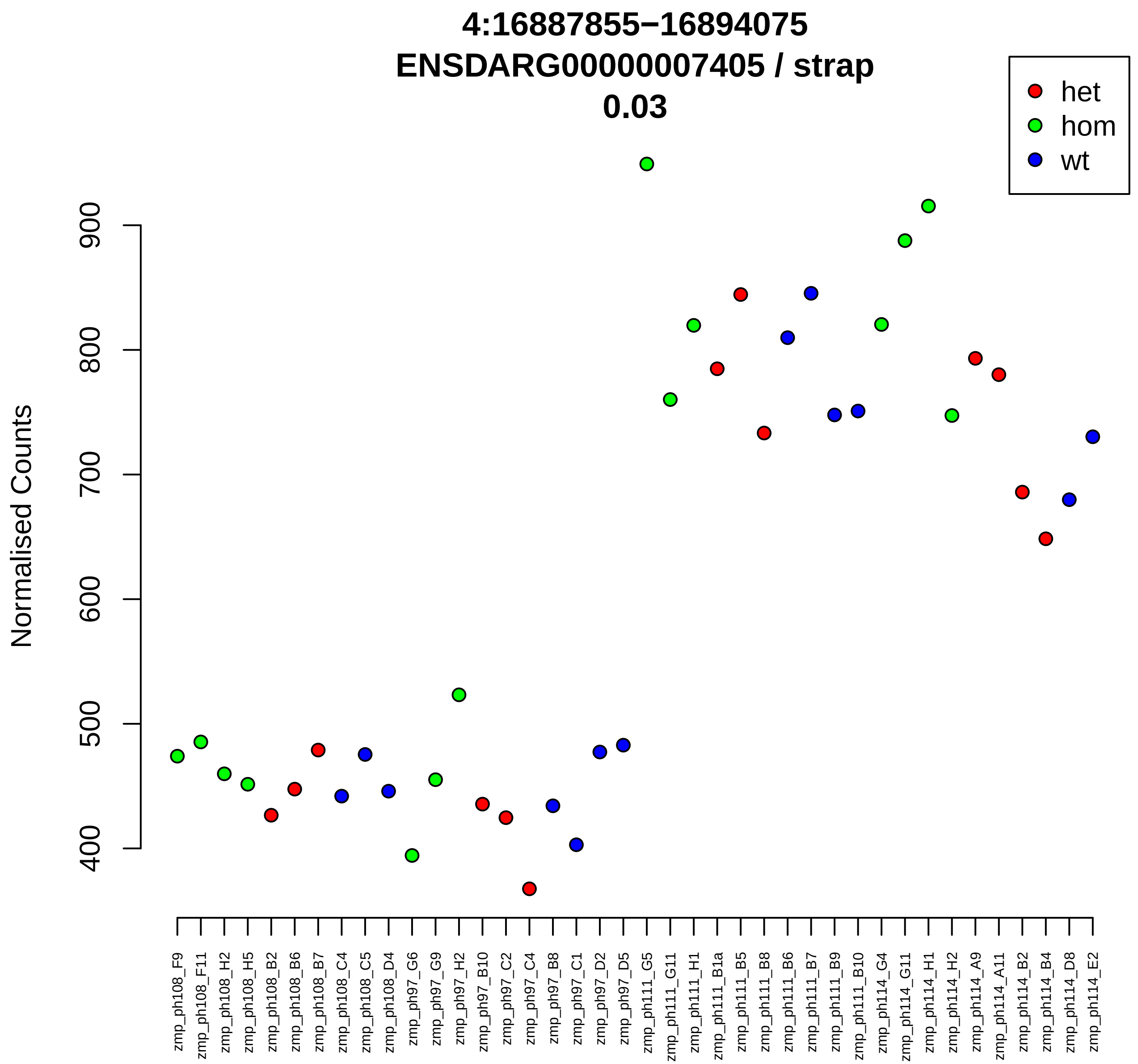

4:16887855−16894075
ENSDARG00000007405 / strap
0.03
het
hom
wt
Normalised Counts
400
500
600
700
800
900
zmp_ph108_F9
zmp_ph108_F11
zmp_ph108_H2
zmp_ph108_H5
zmp_ph108_B2
zmp_ph108_B6
zmp_ph108_B7
zmp_ph108_C4
zmp_ph108_C5
zmp_ph108_D4
zmp_ph97_G6
zmp_ph97_G9
zmp_ph97_H2
zmp_ph97_B10
zmp_ph97_C2
zmp_ph97_C4
zmp_ph97_B8
zmp_ph97_C1
zmp_ph97_D2
zmp_ph97_D5
zmp_ph111_G5
zmp_ph111_G11
zmp_ph111_H1
zmp_ph111_B1a
zmp_ph111_B5
zmp_ph111_B8
zmp_ph111_B6
zmp_ph111_B7
zmp_ph111_B9
zmp_ph111_B10
zmp_ph114_G4
zmp_ph114_G11
zmp_ph114_H1
zmp_ph114_H2
zmp_ph114_A9
zmp_ph114_A11
zmp_ph114_B2
zmp_ph114_B4
zmp_ph114_D8
zmp_ph114_E2

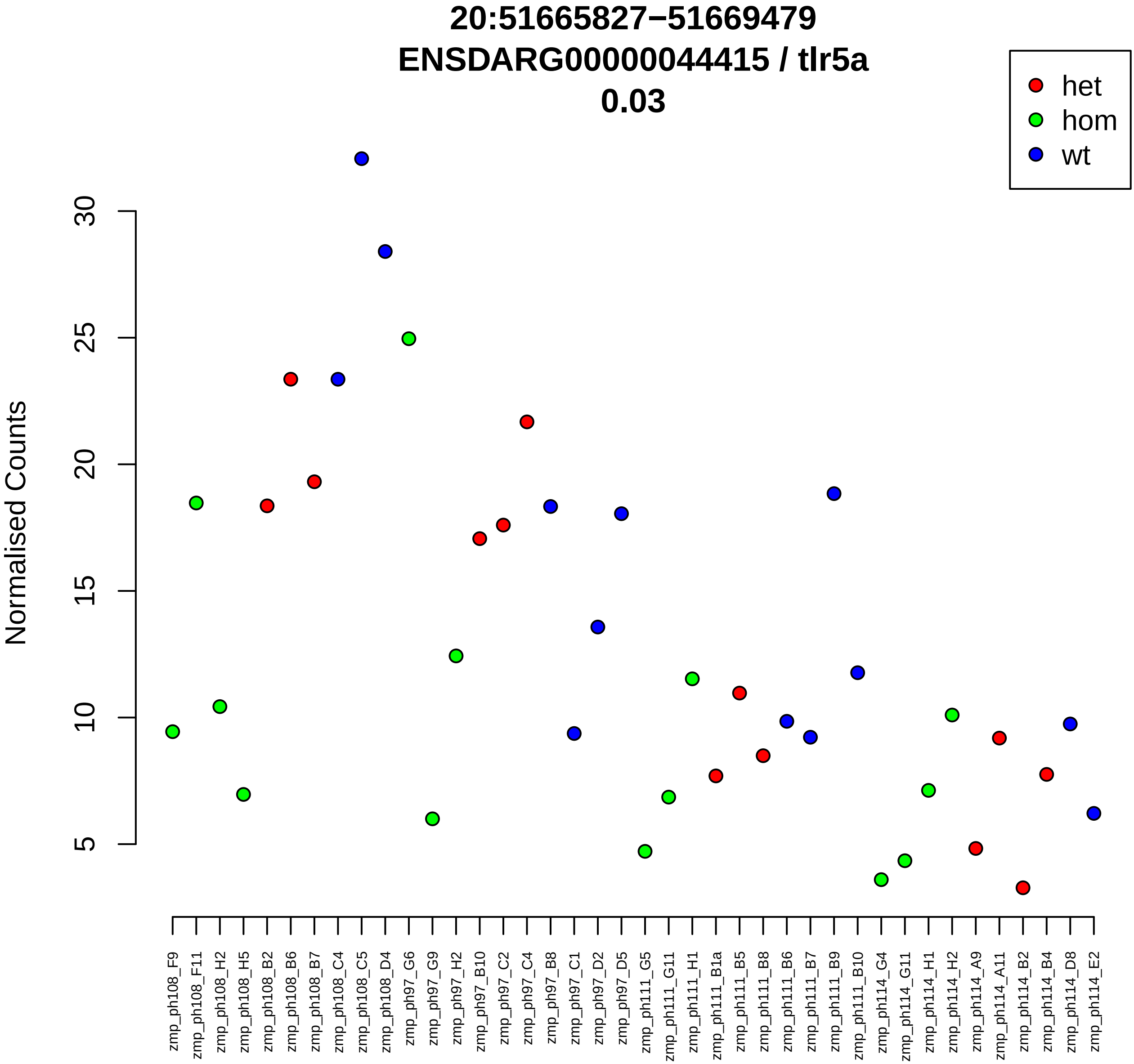
20:51665827−51669479
ENSDARG00000044415 / tlr5a
0.03
het
hom
wt
Normalised Counts
5
10
15
20
25
30
zmp_ph108_F9
zmp_ph108_F11
zmp_ph108_H2
zmp_ph108_H5
zmp_ph108_B2
zmp_ph108_B6
zmp_ph108_B7
zmp_ph108_C4
zmp_ph108_C5
zmp_ph108_D4
zmp_ph97_G6
zmp_ph97_G9
zmp_ph97_H2
zmp_ph97_B10
zmp_ph97_C2
zmp_ph97_C4
zmp_ph97_B8
zmp_ph97_C1
zmp_ph97_D2
zmp_ph97_D5
zmp_ph111_G5
zmp_ph111_G11
zmp_ph111_H1
zmp_ph111_B1a
zmp_ph111_B5
zmp_ph111_B8
zmp_ph111_B6
zmp_ph111_B7
zmp_ph111_B9
zmp_ph111_B10
zmp_ph114_G4
zmp_ph114_G11
zmp_ph114_H1
zmp_ph114_H2
zmp_ph114_A9
zmp_ph114_A11
zmp_ph114_B2
zmp_ph114_B4
zmp_ph114_D8
zmp_ph114_E2

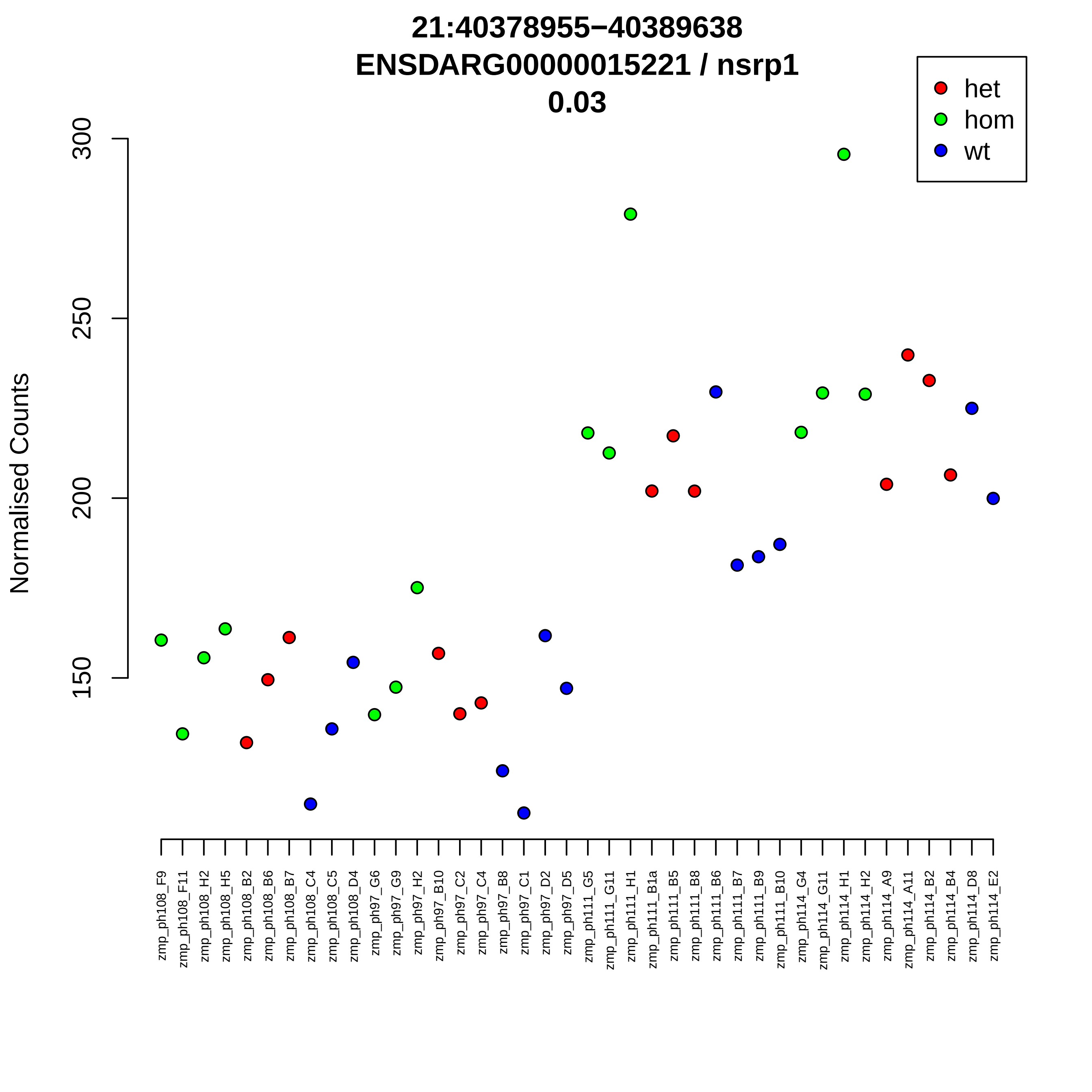

21:40378955−40389638
ENSDARG00000015221 / nsrp1
0.03
het
hom
wt
Normalised Counts
300
250
200
150
zmp_ph108_F9
zmp_ph108_F11
zmp_ph108_H2
zmp_ph108_H5
zmp_ph108_B2
zmp_ph108_B6
zmp_ph108_B7
zmp_ph108_C4
zmp_ph108_C5
zmp_ph108_D4
zmp_ph97_G6
zmp_ph97_G9
zmp_ph97_H2
zmp_ph97_B10
zmp_ph97_C2
zmp_ph97_C4
zmp_ph97_B8
zmp_ph97_C1
zmp_ph97_D2
zmp_ph97_D5
zmp_ph111_G5
zmp_ph111_G11
zmp_ph111_H1
zmp_ph111_B1a
zmp_ph111_B5
zmp_ph111_B8
zmp_ph111_B6
zmp_ph111_B7
zmp_ph111_B9
zmp_ph111_B10
zmp_ph114_G4
zmp_ph114_G11
zmp_ph114_H1
zmp_ph114_H2
zmp_ph114_A9
zmp_ph114_A11
zmp_ph114_B2
zmp_ph114_B4
zmp_ph114_D8
zmp_ph114_E2

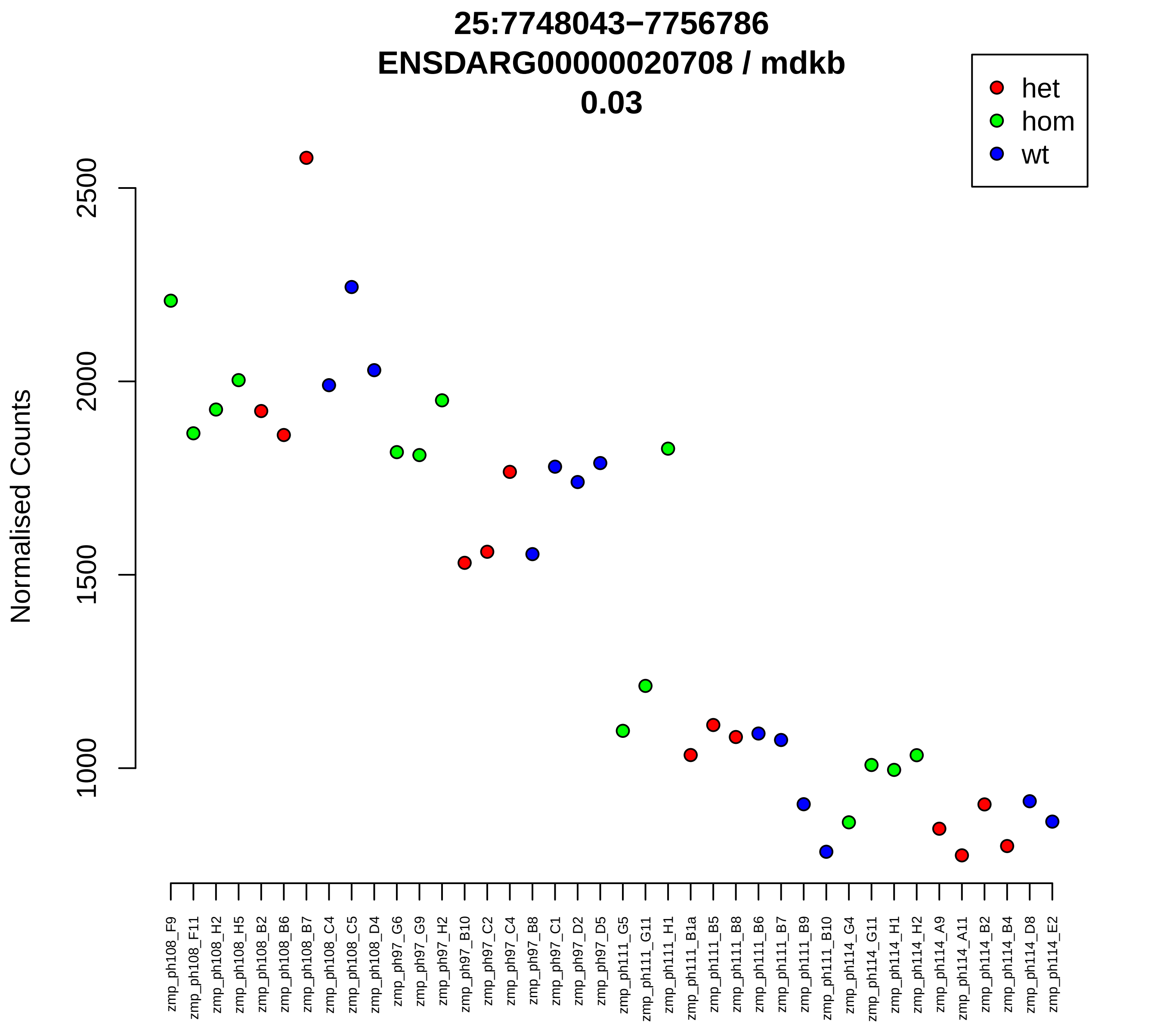

25:7748043−7756786
ENSDARG00000020708 / mdkb
0.03
het
hom
wt
Normalised Counts
1000
1500
2000
2500
zmp_ph108_F9
zmp_ph108_F11
zmp_ph108_H2
zmp_ph108_H5
zmp_ph108_B2
zmp_ph108_B6
zmp_ph108_B7
zmp_ph108_C4
zmp_ph108_C5
zmp_ph108_D4
zmp_ph97_G6
zmp_ph97_G9
zmp_ph97_H2
zmp_ph97_B10
zmp_ph97_C2
zmp_ph97_C4
zmp_ph97_B8
zmp_ph97_C1
zmp_ph97_D2
zmp_ph97_D5
zmp_ph111_G5
zmp_ph111_G11
zmp_ph111_H1
zmp_ph111_B1a
zmp_ph111_B5
zmp_ph111_B8
zmp_ph111_B6
zmp_ph111_B7
zmp_ph111_B9
zmp_ph111_B10
zmp_ph114_G4
zmp_ph114_G11
zmp_ph114_H1
zmp_ph114_H2
zmp_ph114_A9
zmp_ph114_A11
zmp_ph114_B2
zmp_ph114_B4
zmp_ph114_D8
zmp_ph114_E2

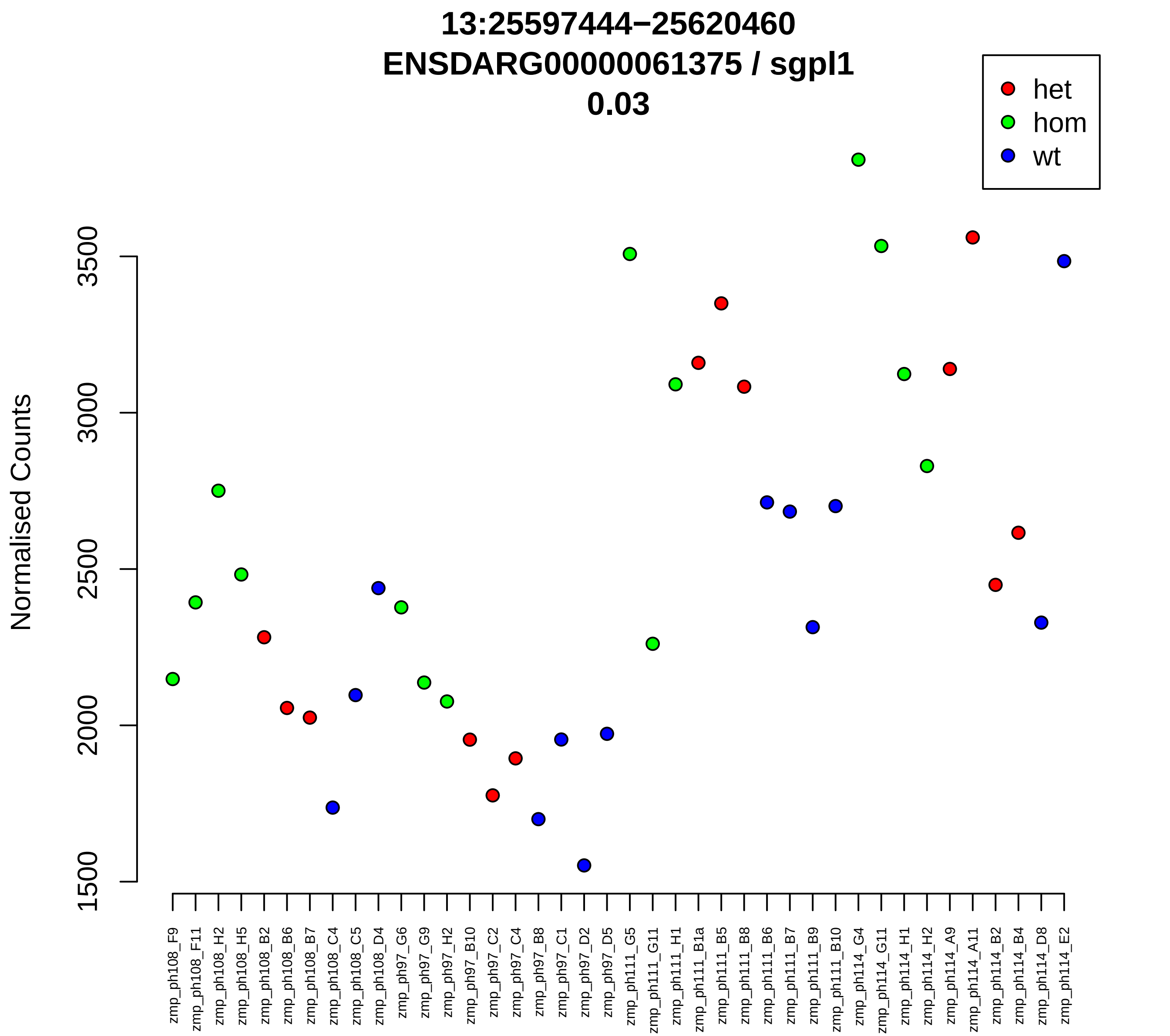

13:25597444−25620460
ENSDARG00000061375 / sgpl1
0.03
het
hom
wt
Normalised Counts
1500
2000
2500
3000
3500
zmp_ph108_F9
zmp_ph108_F11
zmp_ph108_H2
zmp_ph108_H5
zmp_ph108_B2
zmp_ph108_B6
zmp_ph108_B7
zmp_ph108_C4
zmp_ph108_C5
zmp_ph108_D4
zmp_ph97_G6
zmp_ph97_G9
zmp_ph97_H2
zmp_ph97_B10
zmp_ph97_C2
zmp_ph97_C4
zmp_ph97_B8
zmp_ph97_C1
zmp_ph97_D2
zmp_ph97_D5
zmp_ph111_G5
zmp_ph111_G11
zmp_ph111_H1
zmp_ph111_B1a
zmp_ph111_B5
zmp_ph111_B8
zmp_ph111_B6
zmp_ph111_B7
zmp_ph111_B9
zmp_ph111_B10
zmp_ph114_G4
zmp_ph114_G11
zmp_ph114_H1
zmp_ph114_H2
zmp_ph114_A9
zmp_ph114_A11
zmp_ph114_B2
zmp_ph114_B4
zmp_ph114_D8
zmp_ph114_E2

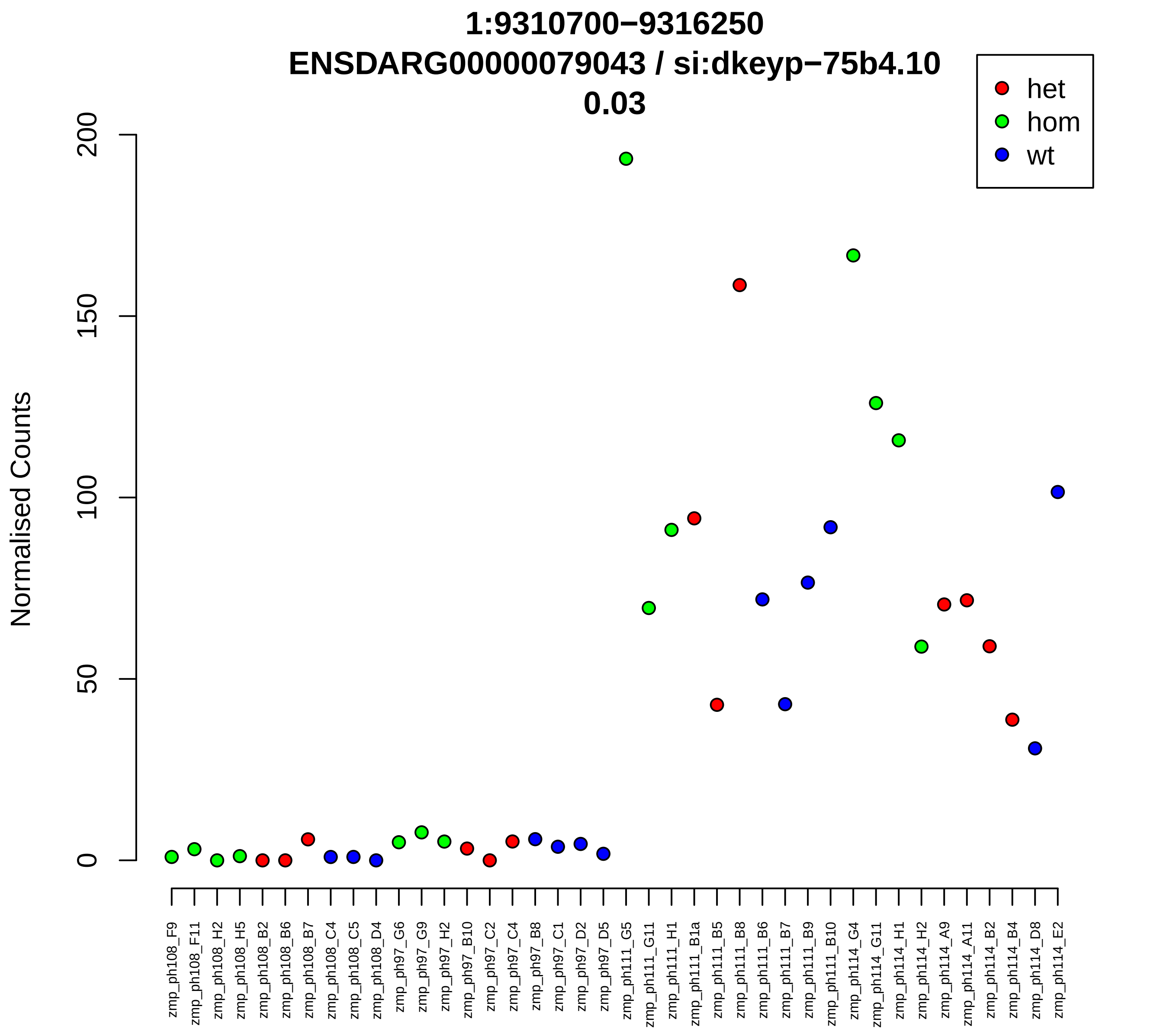

1:9310700−9316250
ENSDARG00000079043 / si:dkeyp−75b4.10
0.03
het
hom
wt
Normalised Counts
0
50
100
150
200
zmp_ph108_F9
zmp_ph108_F11
zmp_ph108_H2
zmp_ph108_H5
zmp_ph108_B2
zmp_ph108_B6
zmp_ph108_B7
zmp_ph108_C4
zmp_ph108_C5
zmp_ph108_D4
zmp_ph97_G6
zmp_ph97_G9
zmp_ph97_H2
zmp_ph97_B10
zmp_ph97_C2
zmp_ph97_C4
zmp_ph97_B8
zmp_ph97_C1
zmp_ph97_D2
zmp_ph97_D5
zmp_ph111_G5
zmp_ph111_G11
zmp_ph111_H1
zmp_ph111_B1a
zmp_ph111_B5
zmp_ph111_B8
zmp_ph111_B6
zmp_ph111_B7
zmp_ph111_B9
zmp_ph111_B10
zmp_ph114_G4
zmp_ph114_G11
zmp_ph114_H1
zmp_ph114_H2
zmp_ph114_A9
zmp_ph114_A11
zmp_ph114_B2
zmp_ph114_B4
zmp_ph114_D8
zmp_ph114_E2

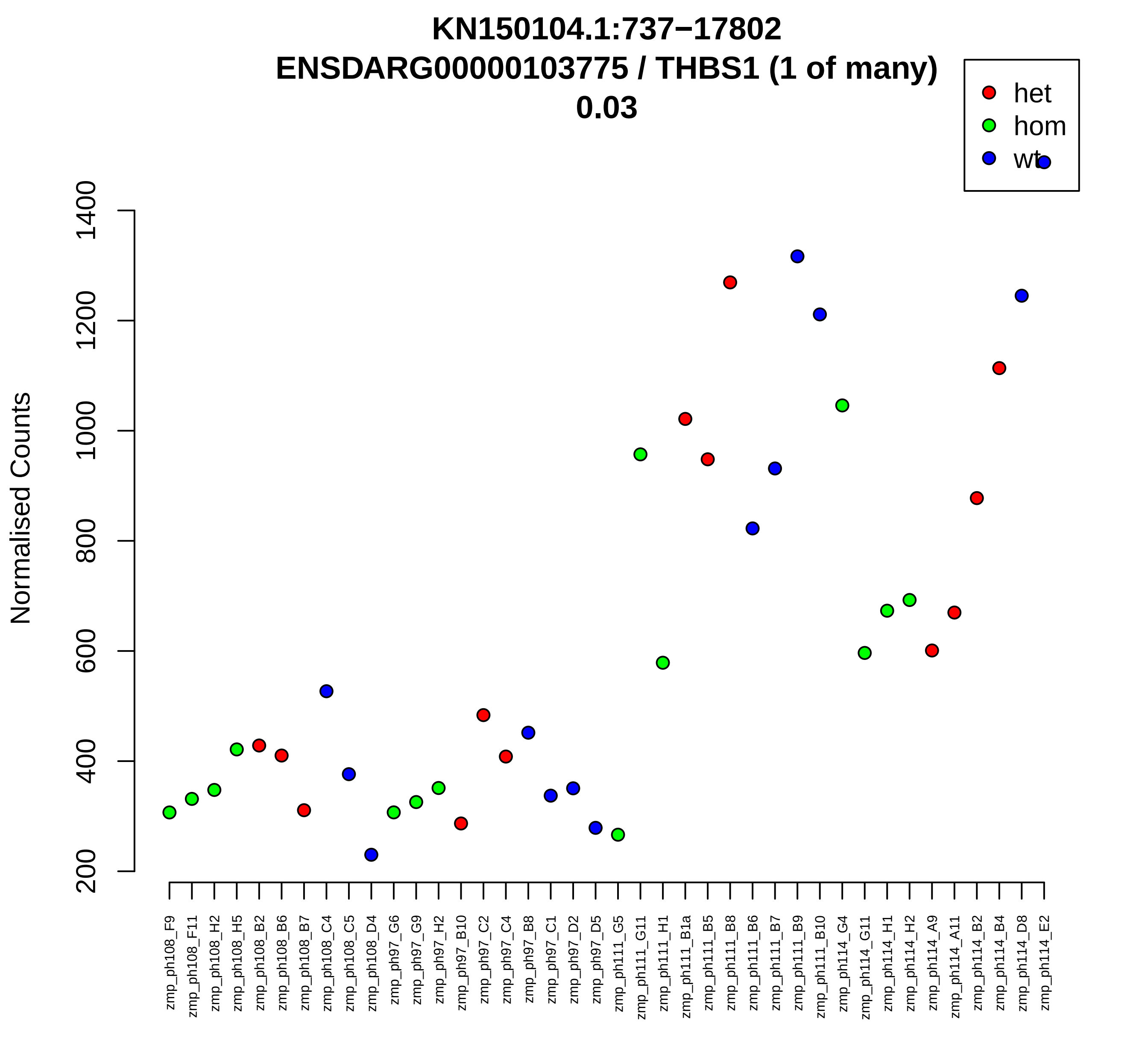

KN150104.1:737−17802
ENSDARG00000103775 / THBS1 (1 of many)
0.03
het
hom
wt
Normalised Counts
200
400
600
800
1000
1200
1400
zmp_ph108_F9
zmp_ph108_F11
zmp_ph108_H2
zmp_ph108_H5
zmp_ph108_B2
zmp_ph108_B6
zmp_ph108_B7
zmp_ph108_C4
zmp_ph108_C5
zmp_ph108_D4
zmp_ph97_G6
zmp_ph97_G9
zmp_ph97_H2
zmp_ph97_B10
zmp_ph97_C2
zmp_ph97_C4
zmp_ph97_B8
zmp_ph97_C1
zmp_ph97_D2
zmp_ph97_D5
zmp_ph111_G5
zmp_ph111_G11
zmp_ph111_H1
zmp_ph111_B1a
zmp_ph111_B5
zmp_ph111_B8
zmp_ph111_B6
zmp_ph111_B7
zmp_ph111_B9
zmp_ph111_B10
zmp_ph114_G4
zmp_ph114_G11
zmp_ph114_H1
zmp_ph114_H2
zmp_ph114_A9
zmp_ph114_A11
zmp_ph114_B2
zmp_ph114_B4
zmp_ph114_D8
zmp_ph114_E2

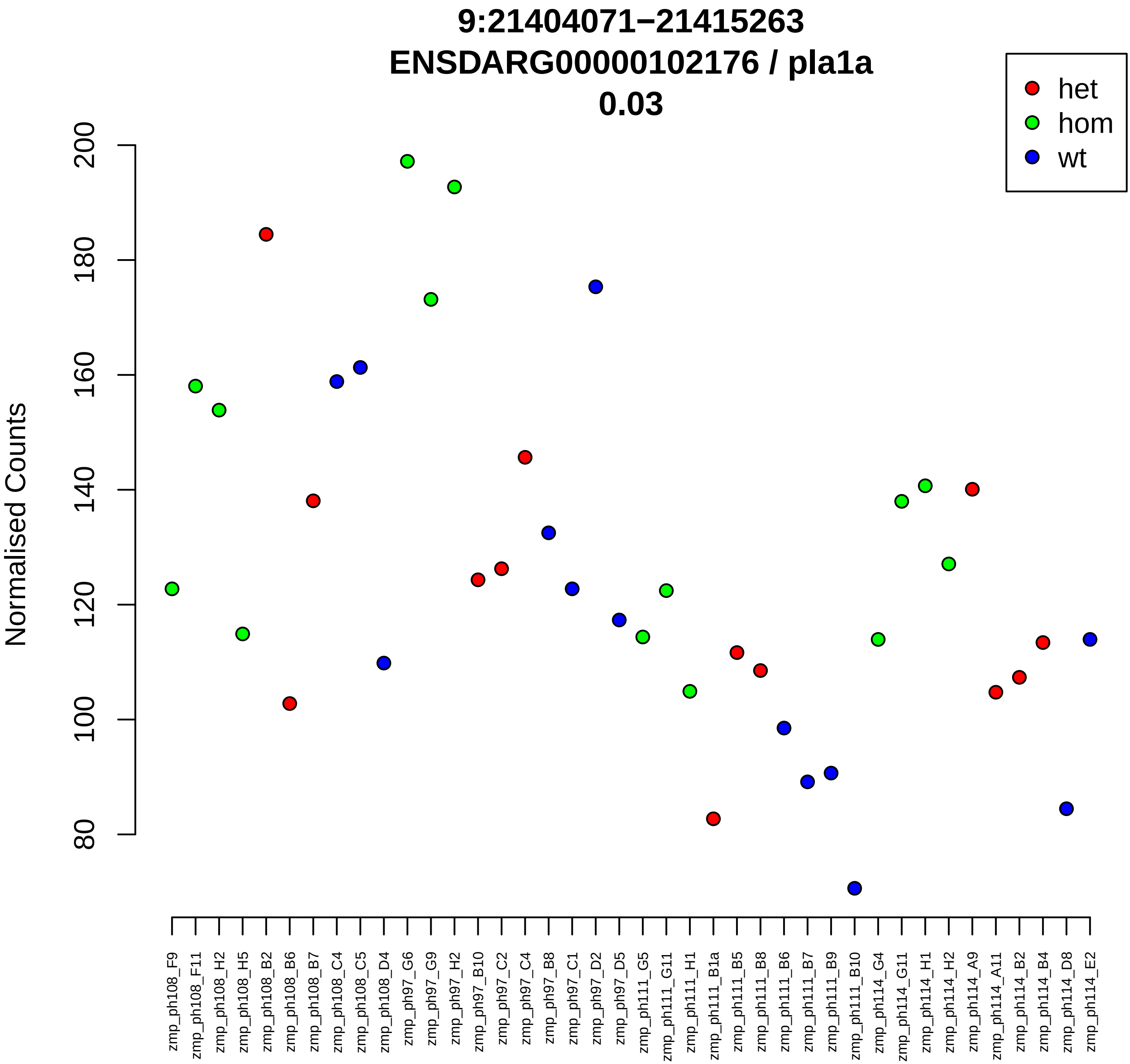
9:21404071−21415263
ENSDARG00000102176 / pla1a
0.03
het
hom
wt
Normalised Counts
200
180
160
140
120
100
80
zmp_ph108_F9
zmp_ph108_F11
zmp_ph108_H2
zmp_ph108_H5
zmp_ph108_B2
zmp_ph108_B6
zmp_ph108_B7
zmp_ph108_C4
zmp_ph108_C5
zmp_ph108_D4
zmp_ph97_G6
zmp_ph97_G9
zmp_ph97_H2
zmp_ph97_B10
zmp_ph97_C2
zmp_ph97_C4
zmp_ph97_B8
zmp_ph97_C1
zmp_ph97_D2
zmp_ph97_D5
zmp_ph111_G5
zmp_ph111_G11
zmp_ph111_H1
zmp_ph111_B1a
zmp_ph111_B5
zmp_ph111_B8
zmp_ph111_B6
zmp_ph111_B7
zmp_ph111_B9
zmp_ph111_B10
zmp_ph114_G4
zmp_ph114_G11
zmp_ph114_H1
zmp_ph114_H2
zmp_ph114_A9
zmp_ph114_A11
zmp_ph114_B2
zmp_ph114_B4
zmp_ph114_D8
zmp_ph114_E2

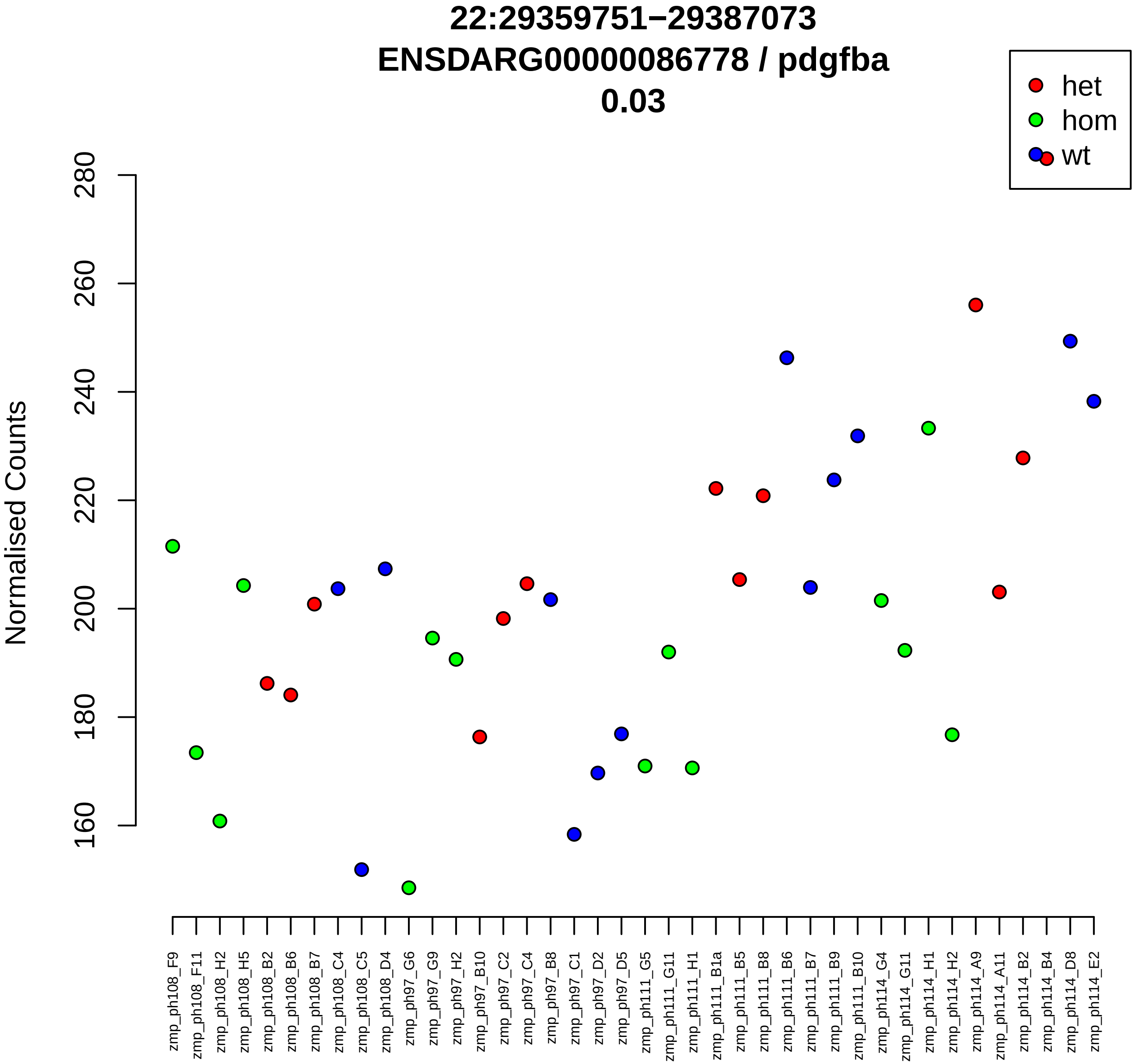
22:29359751−29387073
ENSDARG00000086778 / pdgfba
0.03
het
hom
wt
Normalised Counts
160
180
200
220
240
260
280
zmp_ph108_F9
zmp_ph108_F11
zmp_ph108_H2
zmp_ph108_H5
zmp_ph108_B2
zmp_ph108_B6
zmp_ph108_B7
zmp_ph108_C4
zmp_ph108_C5
zmp_ph108_D4
zmp_ph97_G6
zmp_ph97_G9
zmp_ph97_H2
zmp_ph97_B10
zmp_ph97_C2
zmp_ph97_C4
zmp_ph97_B8
zmp_ph97_C1
zmp_ph97_D2
zmp_ph97_D5
zmp_ph111_G5
zmp_ph111_G11
zmp_ph111_H1
zmp_ph111_B1a
zmp_ph111_B5
zmp_ph111_B8
zmp_ph111_B6
zmp_ph111_B7
zmp_ph111_B9
zmp_ph111_B10
zmp_ph114_G4
zmp_ph114_G11
zmp_ph114_H1
zmp_ph114_H2
zmp_ph114_A9
zmp_ph114_A11
zmp_ph114_B2
zmp_ph114_B4
zmp_ph114_D8
zmp_ph114_E2

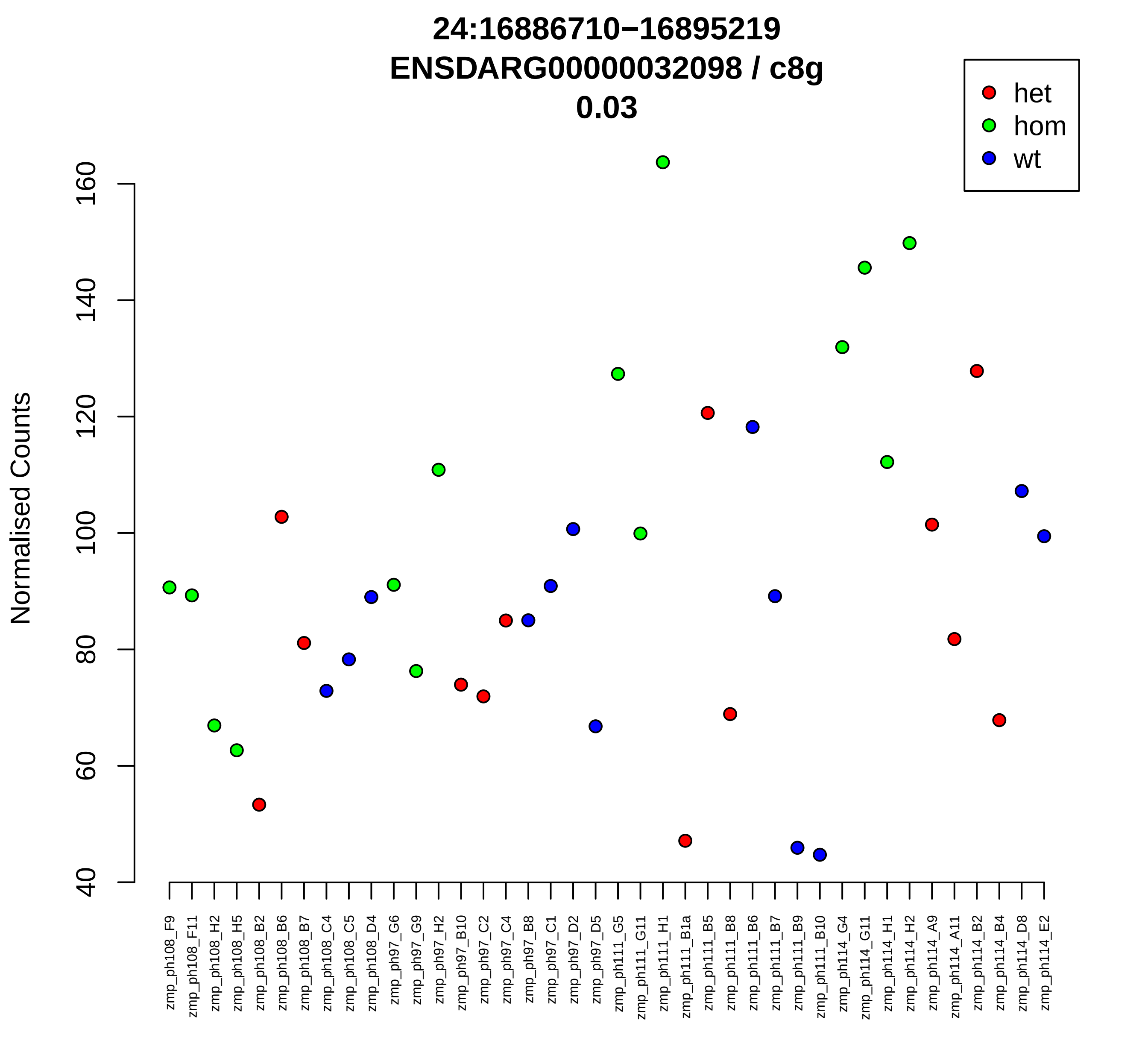

24:16886710−16895219
ENSDARG00000032098 / c8g
0.03
het
hom
wt
Normalised Counts
40
60
80
100
120
140
160
zmp_ph108_F9
zmp_ph108_F11
zmp_ph108_H2
zmp_ph108_H5
zmp_ph108_B2
zmp_ph108_B6
zmp_ph108_B7
zmp_ph108_C4
zmp_ph108_C5
zmp_ph108_D4
zmp_ph97_G6
zmp_ph97_G9
zmp_ph97_H2
zmp_ph97_B10
zmp_ph97_C2
zmp_ph97_C4
zmp_ph97_B8
zmp_ph97_C1
zmp_ph97_D2
zmp_ph97_D5
zmp_ph111_G5
zmp_ph111_G11
zmp_ph111_H1
zmp_ph111_B1a
zmp_ph111_B5
zmp_ph111_B8
zmp_ph111_B6
zmp_ph111_B7
zmp_ph111_B9
zmp_ph111_B10
zmp_ph114_G4
zmp_ph114_G11
zmp_ph114_H1
zmp_ph114_H2
zmp_ph114_A9
zmp_ph114_A11
zmp_ph114_B2
zmp_ph114_B4
zmp_ph114_D8
zmp_ph114_E2

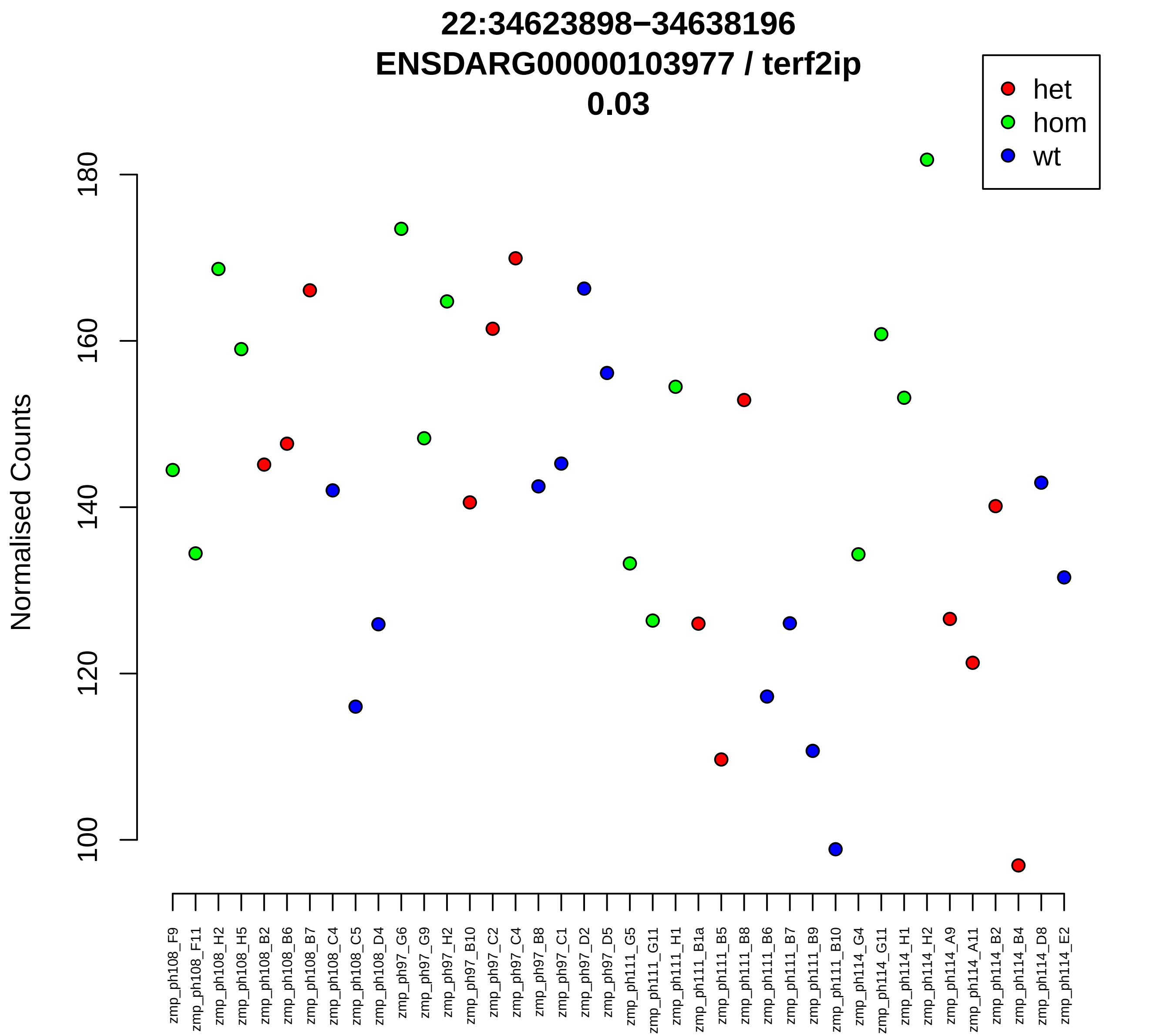
22:34623898−34638196
ENSDARG00000103977 / terf2ip
0.03
het
hom
wt
Normalised Counts
100
120
140
160
180
zmp_ph108_F9
zmp_ph108_F11
zmp_ph108_H2
zmp_ph108_H5
zmp_ph108_B2
zmp_ph108_B6
zmp_ph108_B7
zmp_ph108_C4
zmp_ph108_C5
zmp_ph108_D4
zmp_ph97_G6
zmp_ph97_G9
zmp_ph97_H2
zmp_ph97_B10
zmp_ph97_C2
zmp_ph97_C4
zmp_ph97_B8
zmp_ph97_C1
zmp_ph97_D2
zmp_ph97_D5
zmp_ph111_G5
zmp_ph111_G11
zmp_ph111_H1
zmp_ph111_B1a
zmp_ph111_B5
zmp_ph111_B8
zmp_ph111_B6
zmp_ph111_B7
zmp_ph111_B9
zmp_ph111_B10
zmp_ph114_G4
zmp_ph114_G11
zmp_ph114_H1
zmp_ph114_H2
zmp_ph114_A9
zmp_ph114_A11
zmp_ph114_B2
zmp_ph114_B4
zmp_ph114_D8
zmp_ph114_E2

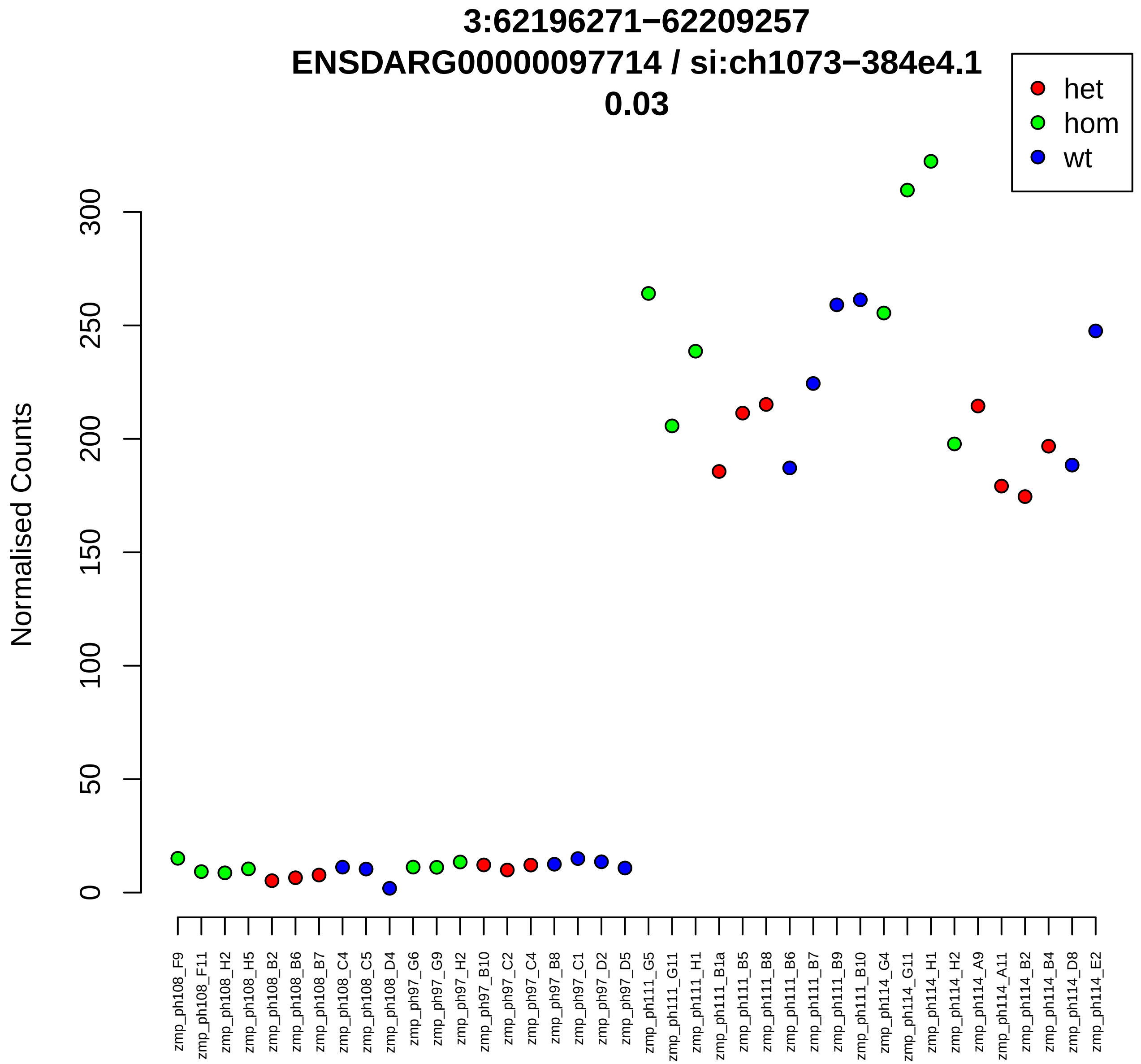

3:62196271−62209257
ENSDARG00000097714 / si:ch1073−384e4.1
0.03
het
hom
wt
Normalised Counts
0
50
100
150
200
250
300
zmp_ph108_F9
zmp_ph108_F11
zmp_ph108_H2
zmp_ph108_H5
zmp_ph108_B2
zmp_ph108_B6
zmp_ph108_B7
zmp_ph108_C4
zmp_ph108_C5
zmp_ph108_D4
zmp_ph97_G6
zmp_ph97_G9
zmp_ph97_H2
zmp_ph97_B10
zmp_ph97_C2
zmp_ph97_C4
zmp_ph97_B8
zmp_ph97_C1
zmp_ph97_D2
zmp_ph97_D5
zmp_ph111_G5
zmp_ph111_G11
zmp_ph111_H1
zmp_ph111_B1a
zmp_ph111_B5
zmp_ph111_B8
zmp_ph111_B6
zmp_ph111_B7
zmp_ph111_B9
zmp_ph111_B10
zmp_ph114_G4
zmp_ph114_G11
zmp_ph114_H1
zmp_ph114_H2
zmp_ph114_A9
zmp_ph114_A11
zmp_ph114_B2
zmp_ph114_B4
zmp_ph114_D8
zmp_ph114_E2

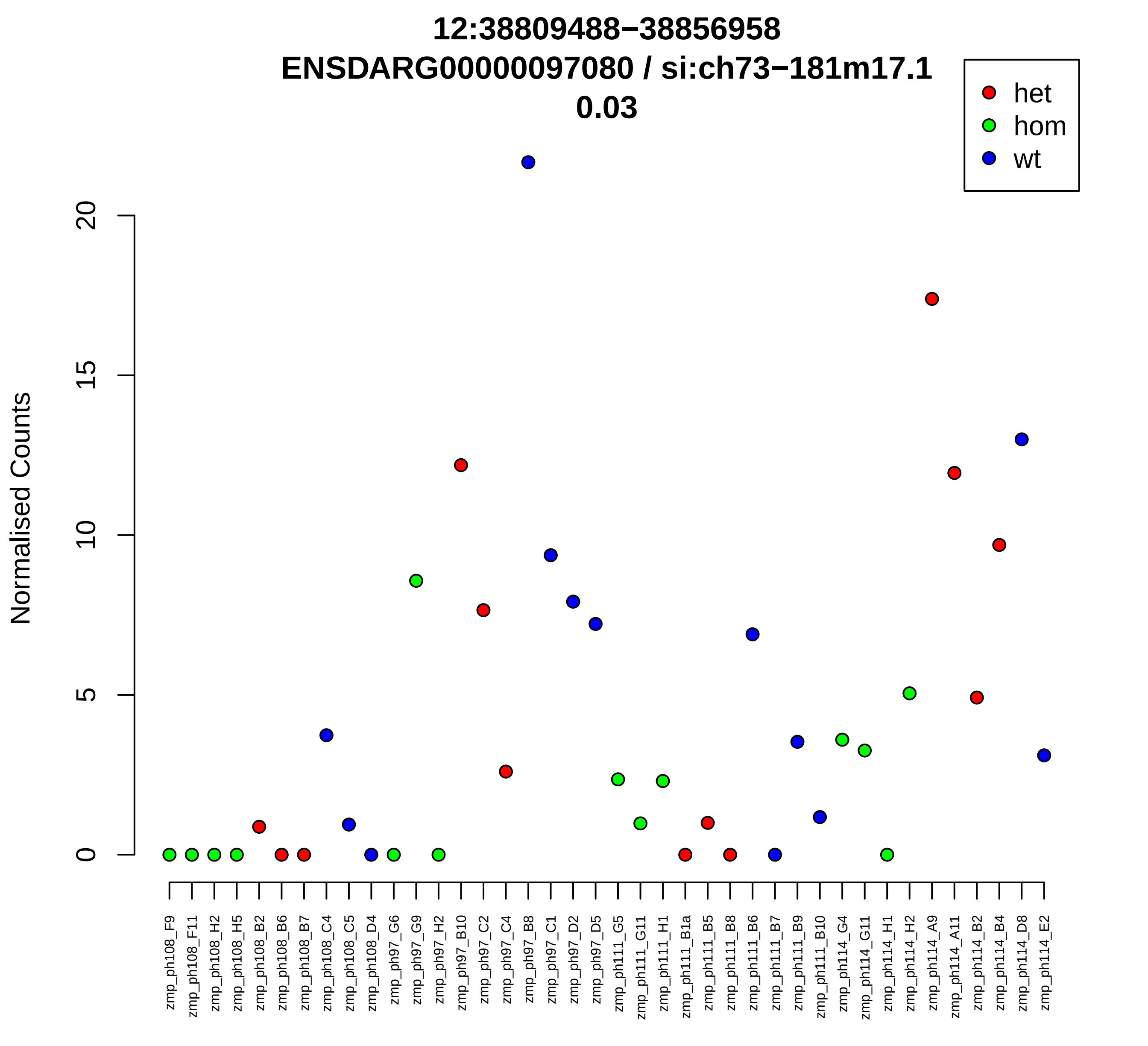
12:38809488−38856958
ENSDARG00000097080 / si:ch73−181m17.1
0.03
het
hom
wt
Normalised Counts
0
5
10
15
20
zmp_ph108_F9
zmp_ph108_F11
zmp_ph108_H2
zmp_ph108_H5
zmp_ph108_B2
zmp_ph108_B6
zmp_ph108_B7
zmp_ph108_C4
zmp_ph108_C5
zmp_ph108_D4
zmp_ph97_G6
zmp_ph97_G9
zmp_ph97_H2
zmp_ph97_B10
zmp_ph97_C2
zmp_ph97_C4
zmp_ph97_B8
zmp_ph97_C1
zmp_ph97_D2
zmp_ph97_D5
zmp_ph111_G5
zmp_ph111_G11
zmp_ph111_H1
zmp_ph111_B1a
zmp_ph111_B5
zmp_ph111_B8
zmp_ph111_B6
zmp_ph111_B7
zmp_ph111_B9
zmp_ph111_B10
zmp_ph114_G4
zmp_ph114_G11
zmp_ph114_H1
zmp_ph114_H2
zmp_ph114_A9
zmp_ph114_A11
zmp_ph114_B2
zmp_ph114_B4
zmp_ph114_D8
zmp_ph114_E2

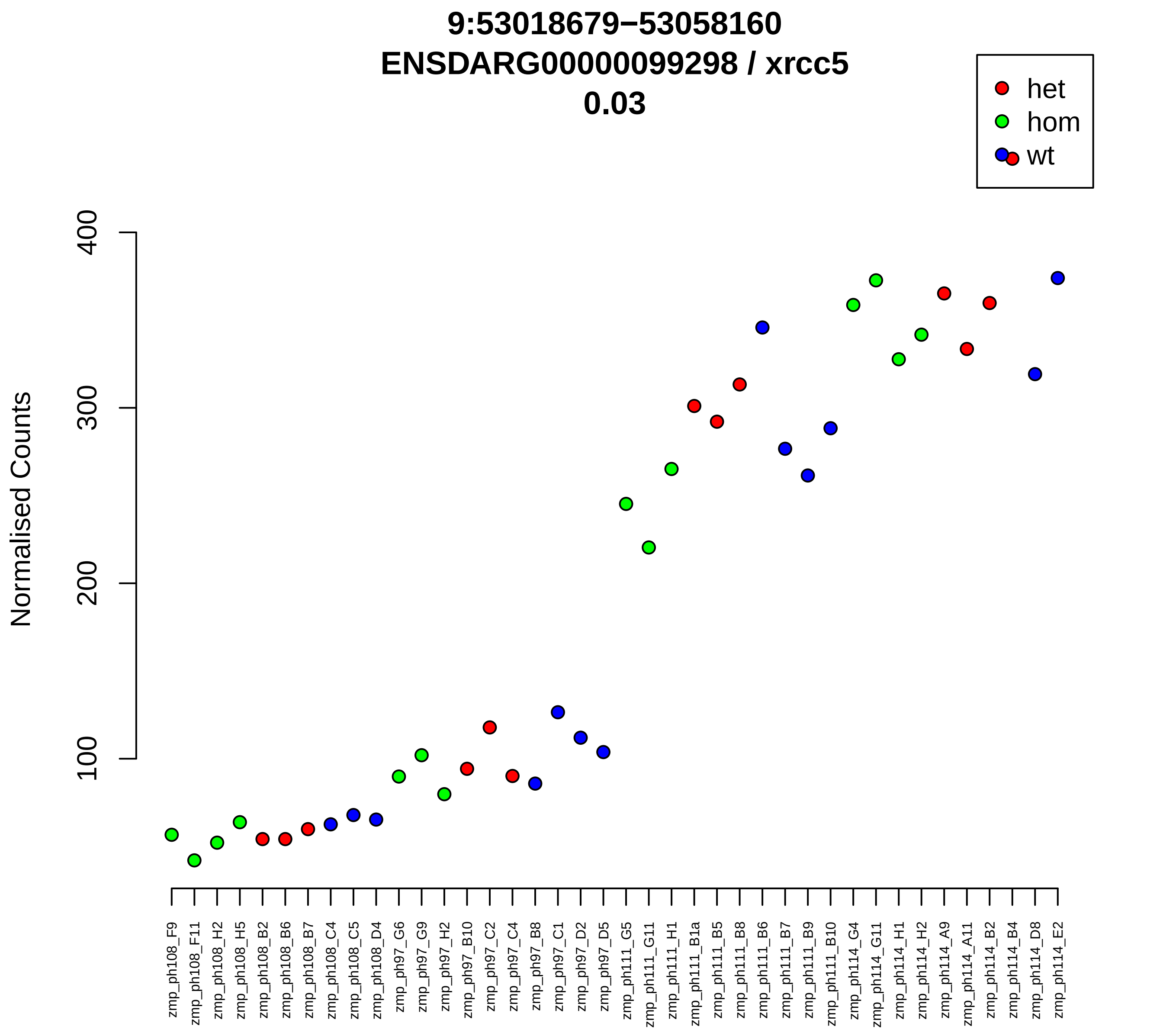
9:53018679−53058160
ENSDARG00000099298 / xrcc5
0.03
het
hom
wt
Normalised Counts
100
200
300
400
zmp_ph108_F9
zmp_ph108_F11
zmp_ph108_H2
zmp_ph108_H5
zmp_ph108_B2
zmp_ph108_B6
zmp_ph108_B7
zmp_ph108_C4
zmp_ph108_C5
zmp_ph108_D4
zmp_ph97_G6
zmp_ph97_G9
zmp_ph97_H2
zmp_ph97_B10
zmp_ph97_C2
zmp_ph97_C4
zmp_ph97_B8
zmp_ph97_C1
zmp_ph97_D2
zmp_ph97_D5
zmp_ph111_G5
zmp_ph111_G11
zmp_ph111_H1
zmp_ph111_B1a
zmp_ph111_B5
zmp_ph111_B8
zmp_ph111_B6
zmp_ph111_B7
zmp_ph111_B9
zmp_ph111_B10
zmp_ph114_G4
zmp_ph114_G11
zmp_ph114_H1
zmp_ph114_H2
zmp_ph114_A9
zmp_ph114_A11
zmp_ph114_B2
zmp_ph114_B4
zmp_ph114_D8
zmp_ph114_E2

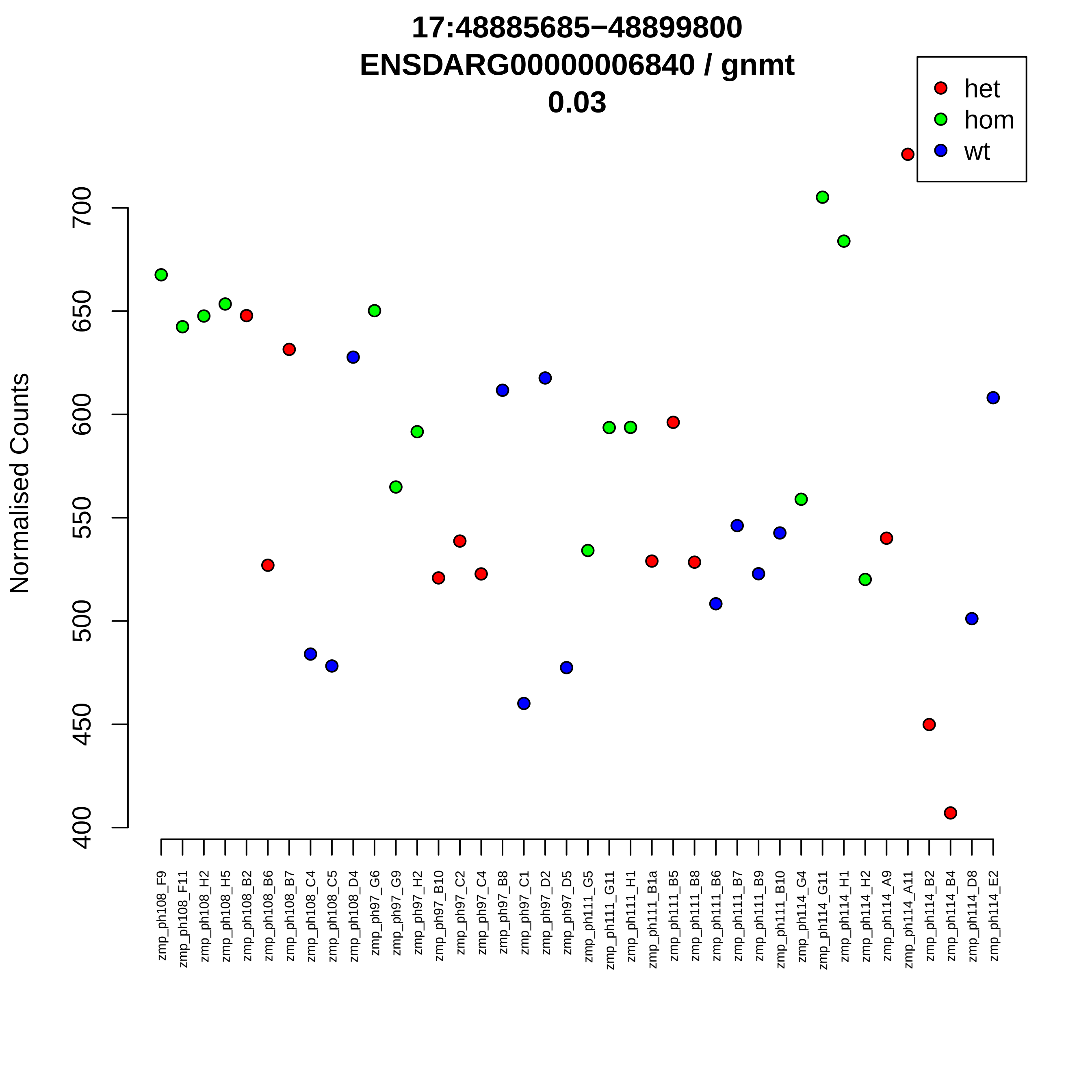

17:48885685−48899800
ENSDARG00000006840 / gnmt
0.03
het
hom
wt
Normalised Counts
400
450
500
550
600
650
700
zmp_ph108_F9
zmp_ph108_F11
zmp_ph108_H2
zmp_ph108_H5
zmp_ph108_B2
zmp_ph108_B6
zmp_ph108_B7
zmp_ph108_C4
zmp_ph108_C5
zmp_ph108_D4
zmp_ph97_G6
zmp_ph97_G9
zmp_ph97_H2
zmp_ph97_B10
zmp_ph97_C2
zmp_ph97_C4
zmp_ph97_B8
zmp_ph97_C1
zmp_ph97_D2
zmp_ph97_D5
zmp_ph111_G5
zmp_ph111_G11
zmp_ph111_H1
zmp_ph111_B1a
zmp_ph111_B5
zmp_ph111_B8
zmp_ph111_B6
zmp_ph111_B7
zmp_ph111_B9
zmp_ph111_B10
zmp_ph114_G4
zmp_ph114_G11
zmp_ph114_H1
zmp_ph114_H2
zmp_ph114_A9
zmp_ph114_A11
zmp_ph114_B2
zmp_ph114_B4
zmp_ph114_D8
zmp_ph114_E2

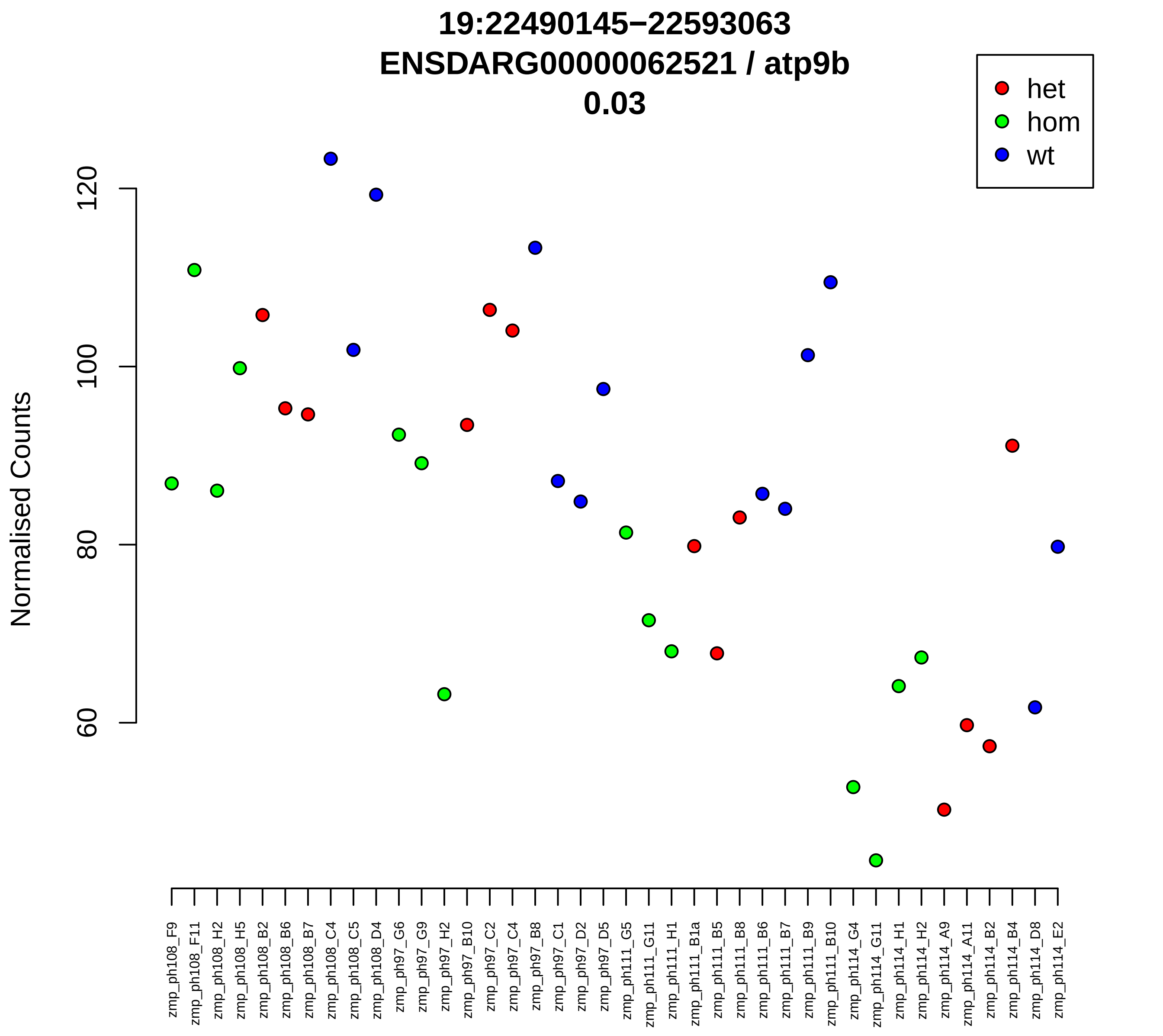

19:22490145−22593063
ENSDARG00000062521 / atp9b
0.03
het
hom
wt
Normalised Counts
60
80
100
120
zmp_ph108_F9
zmp_ph108_F11
zmp_ph108_H2
zmp_ph108_H5
zmp_ph108_B2
zmp_ph108_B6
zmp_ph108_B7
zmp_ph108_C4
zmp_ph108_C5
zmp_ph108_D4
zmp_ph97_G6
zmp_ph97_G9
zmp_ph97_H2
zmp_ph97_B10
zmp_ph97_C2
zmp_ph97_C4
zmp_ph97_B8
zmp_ph97_C1
zmp_ph97_D2
zmp_ph97_D5
zmp_ph111_G5
zmp_ph111_G11
zmp_ph111_H1
zmp_ph111_B1a
zmp_ph111_B5
zmp_ph111_B8
zmp_ph111_B6
zmp_ph111_B7
zmp_ph111_B9
zmp_ph111_B10
zmp_ph114_G4
zmp_ph114_G11
zmp_ph114_H1
zmp_ph114_H2
zmp_ph114_A9
zmp_ph114_A11
zmp_ph114_B2
zmp_ph114_B4
zmp_ph114_D8
zmp_ph114_E2

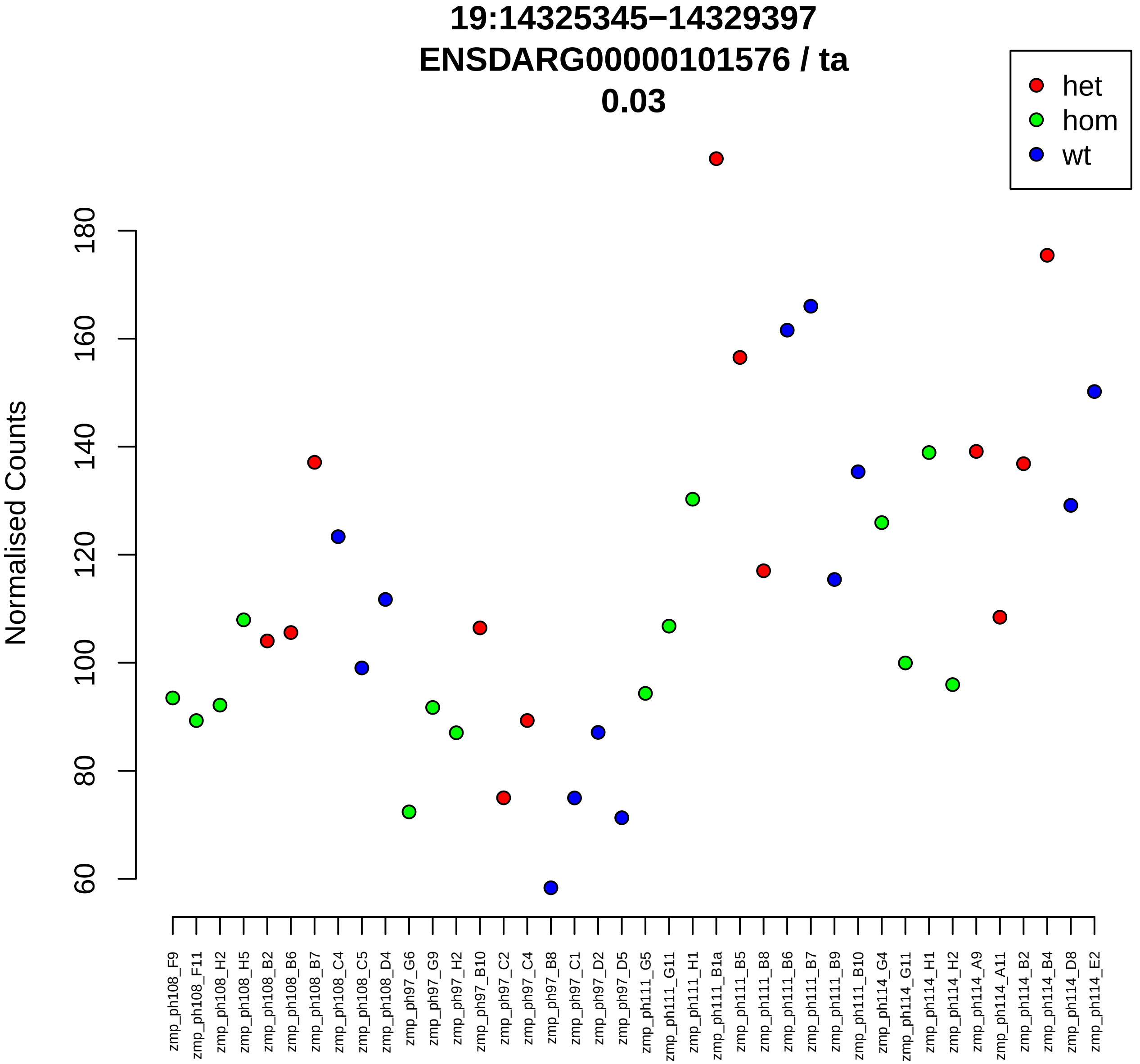

19:14325345−14329397
ENSDARG00000101576 / ta
0.03
het
hom
wt
Normalised Counts
60
80
100
120
140
160
180
zmp_ph108_F9
zmp_ph108_F11
zmp_ph108_H2
zmp_ph108_H5
zmp_ph108_B2
zmp_ph108_B6
zmp_ph108_B7
zmp_ph108_C4
zmp_ph108_C5
zmp_ph108_D4
zmp_ph97_G6
zmp_ph97_G9
zmp_ph97_H2
zmp_ph97_B10
zmp_ph97_C2
zmp_ph97_C4
zmp_ph97_B8
zmp_ph97_C1
zmp_ph97_D2
zmp_ph97_D5
zmp_ph111_G5
zmp_ph111_G11
zmp_ph111_H1
zmp_ph111_B1a
zmp_ph111_B5
zmp_ph111_B8
zmp_ph111_B6
zmp_ph111_B7
zmp_ph111_B9
zmp_ph111_B10
zmp_ph114_G4
zmp_ph114_G11
zmp_ph114_H1
zmp_ph114_H2
zmp_ph114_A9
zmp_ph114_A11
zmp_ph114_B2
zmp_ph114_B4
zmp_ph114_D8
zmp_ph114_E2

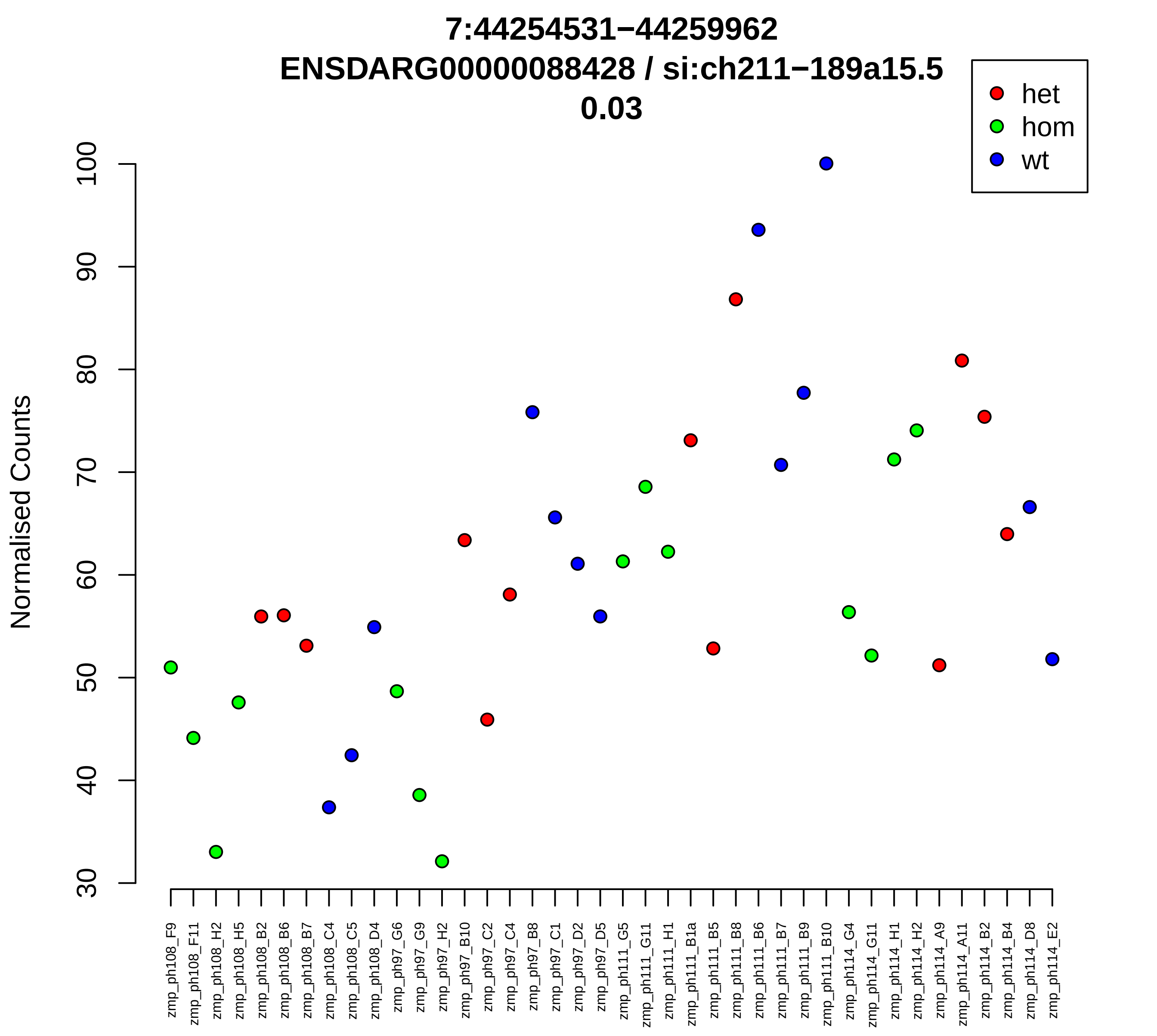

7:44254531−44259962
ENSDARG00000088428 / si:ch211−189a15.5
0.03
het
hom
wt
Normalised Counts
30
40
50
60
70
80
90
100
zmp_ph108_F9
zmp_ph108_F11
zmp_ph108_H2
zmp_ph108_H5
zmp_ph108_B2
zmp_ph108_B6
zmp_ph108_B7
zmp_ph108_C4
zmp_ph108_C5
zmp_ph108_D4
zmp_ph97_G6
zmp_ph97_G9
zmp_ph97_H2
zmp_ph97_B10
zmp_ph97_C2
zmp_ph97_C4
zmp_ph97_B8
zmp_ph97_C1
zmp_ph97_D2
zmp_ph97_D5
zmp_ph111_G5
zmp_ph111_G11
zmp_ph111_H1
zmp_ph111_B1a
zmp_ph111_B5
zmp_ph111_B8
zmp_ph111_B6
zmp_ph111_B7
zmp_ph111_B9
zmp_ph111_B10
zmp_ph114_G4
zmp_ph114_G11
zmp_ph114_H1
zmp_ph114_H2
zmp_ph114_A9
zmp_ph114_A11
zmp_ph114_B2
zmp_ph114_B4
zmp_ph114_D8
zmp_ph114_E2

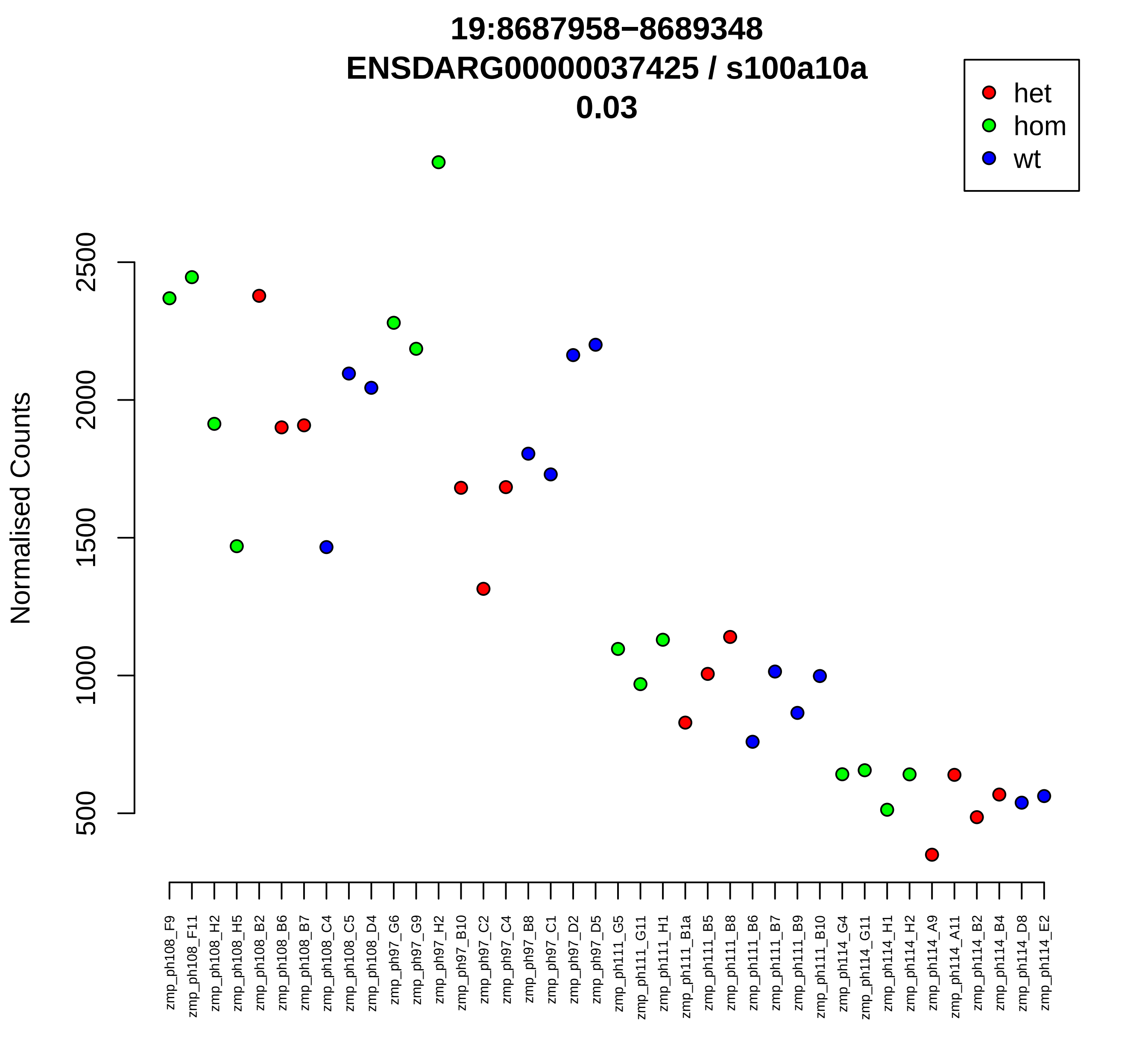

19:8687958−8689348
ENSDARG00000037425 / s100a10a
0.03
het
hom
wt
Normalised Counts
500
1000
1500
2000
2500
zmp_ph108_F9
zmp_ph108_F11
zmp_ph108_H2
zmp_ph108_H5
zmp_ph108_B2
zmp_ph108_B6
zmp_ph108_B7
zmp_ph108_C4
zmp_ph108_C5
zmp_ph108_D4
zmp_ph97_G6
zmp_ph97_G9
zmp_ph97_H2
zmp_ph97_B10
zmp_ph97_C2
zmp_ph97_C4
zmp_ph97_B8
zmp_ph97_C1
zmp_ph97_D2
zmp_ph97_D5
zmp_ph111_G5
zmp_ph111_G11
zmp_ph111_H1
zmp_ph111_B1a
zmp_ph111_B5
zmp_ph111_B8
zmp_ph111_B6
zmp_ph111_B7
zmp_ph111_B9
zmp_ph111_B10
zmp_ph114_G4
zmp_ph114_G11
zmp_ph114_H1
zmp_ph114_H2
zmp_ph114_A9
zmp_ph114_A11
zmp_ph114_B2
zmp_ph114_B4
zmp_ph114_D8
zmp_ph114_E2

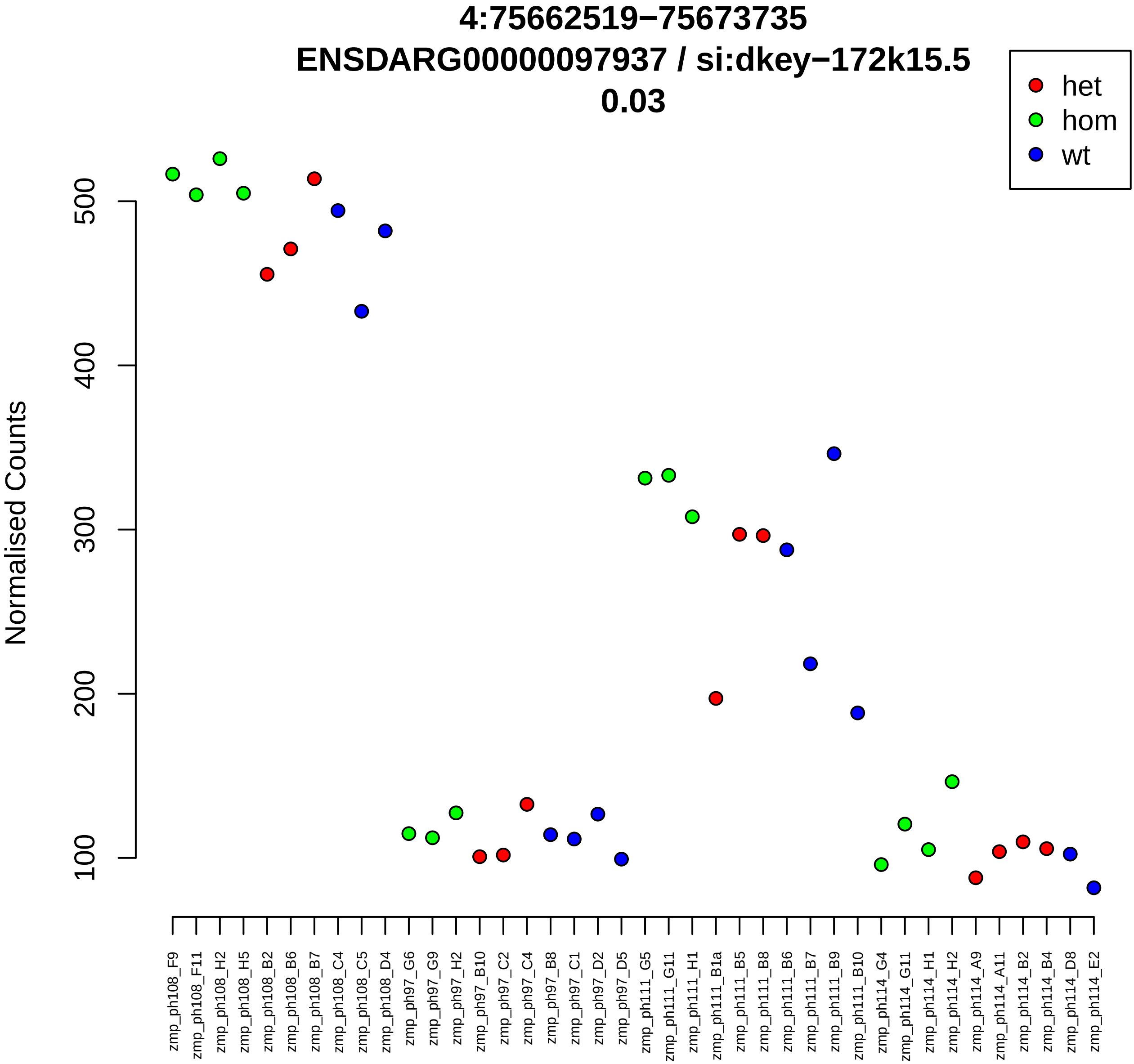

4:75662519−75673735
ENSDARG00000097937 / si:dkey−172k15.5
0.03
het
hom
wt
Normalised Counts
100
200
300
400
500
zmp_ph108_F9
zmp_ph108_F11
zmp_ph108_H2
zmp_ph108_H5
zmp_ph108_B2
zmp_ph108_B6
zmp_ph108_B7
zmp_ph108_C4
zmp_ph108_C5
zmp_ph108_D4
zmp_ph97_G6
zmp_ph97_G9
zmp_ph97_H2
zmp_ph97_B10
zmp_ph97_C2
zmp_ph97_C4
zmp_ph97_B8
zmp_ph97_C1
zmp_ph97_D2
zmp_ph97_D5
zmp_ph111_G5
zmp_ph111_G11
zmp_ph111_H1
zmp_ph111_B1a
zmp_ph111_B5
zmp_ph111_B8
zmp_ph111_B6
zmp_ph111_B7
zmp_ph111_B9
zmp_ph111_B10
zmp_ph114_G4
zmp_ph114_G11
zmp_ph114_H1
zmp_ph114_H2
zmp_ph114_A9
zmp_ph114_A11
zmp_ph114_B2
zmp_ph114_B4
zmp_ph114_D8
zmp_ph114_E2

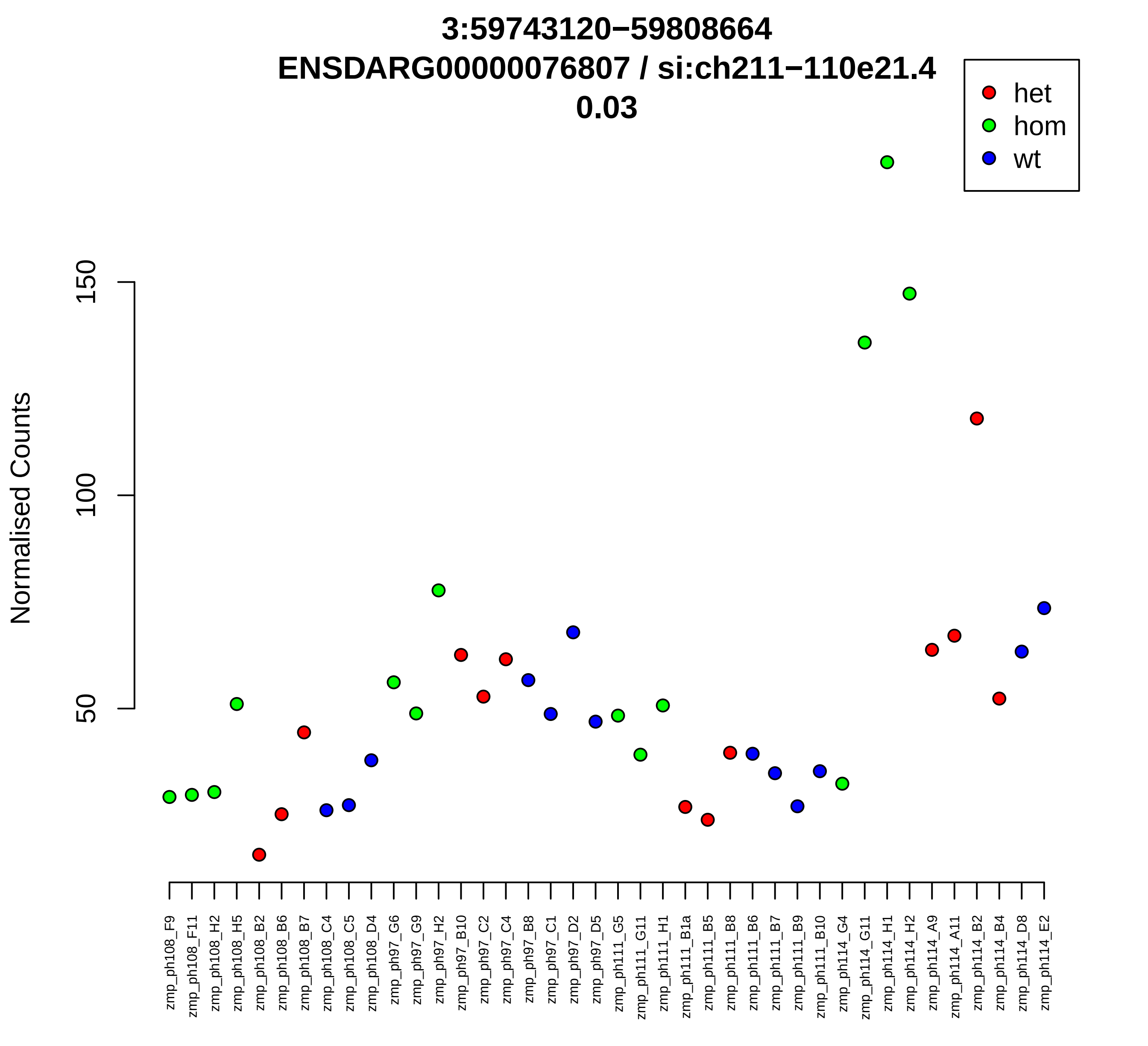

3:59743120−59808664
ENSDARG00000076807 / si:ch211−110e21.4
0.03
het
hom
wt
Normalised Counts
50
100
150
zmp_ph108_F9
zmp_ph108_F11
zmp_ph108_H2
zmp_ph108_H5
zmp_ph108_B2
zmp_ph108_B6
zmp_ph108_B7
zmp_ph108_C4
zmp_ph108_C5
zmp_ph108_D4
zmp_ph97_G6
zmp_ph97_G9
zmp_ph97_H2
zmp_ph97_B10
zmp_ph97_C2
zmp_ph97_C4
zmp_ph97_B8
zmp_ph97_C1
zmp_ph97_D2
zmp_ph97_D5
zmp_ph111_G5
zmp_ph111_G11
zmp_ph111_H1
zmp_ph111_B1a
zmp_ph111_B5
zmp_ph111_B8
zmp_ph111_B6
zmp_ph111_B7
zmp_ph111_B9
zmp_ph111_B10
zmp_ph114_G4
zmp_ph114_G11
zmp_ph114_H1
zmp_ph114_H2
zmp_ph114_A9
zmp_ph114_A11
zmp_ph114_B2
zmp_ph114_B4
zmp_ph114_D8
zmp_ph114_E2

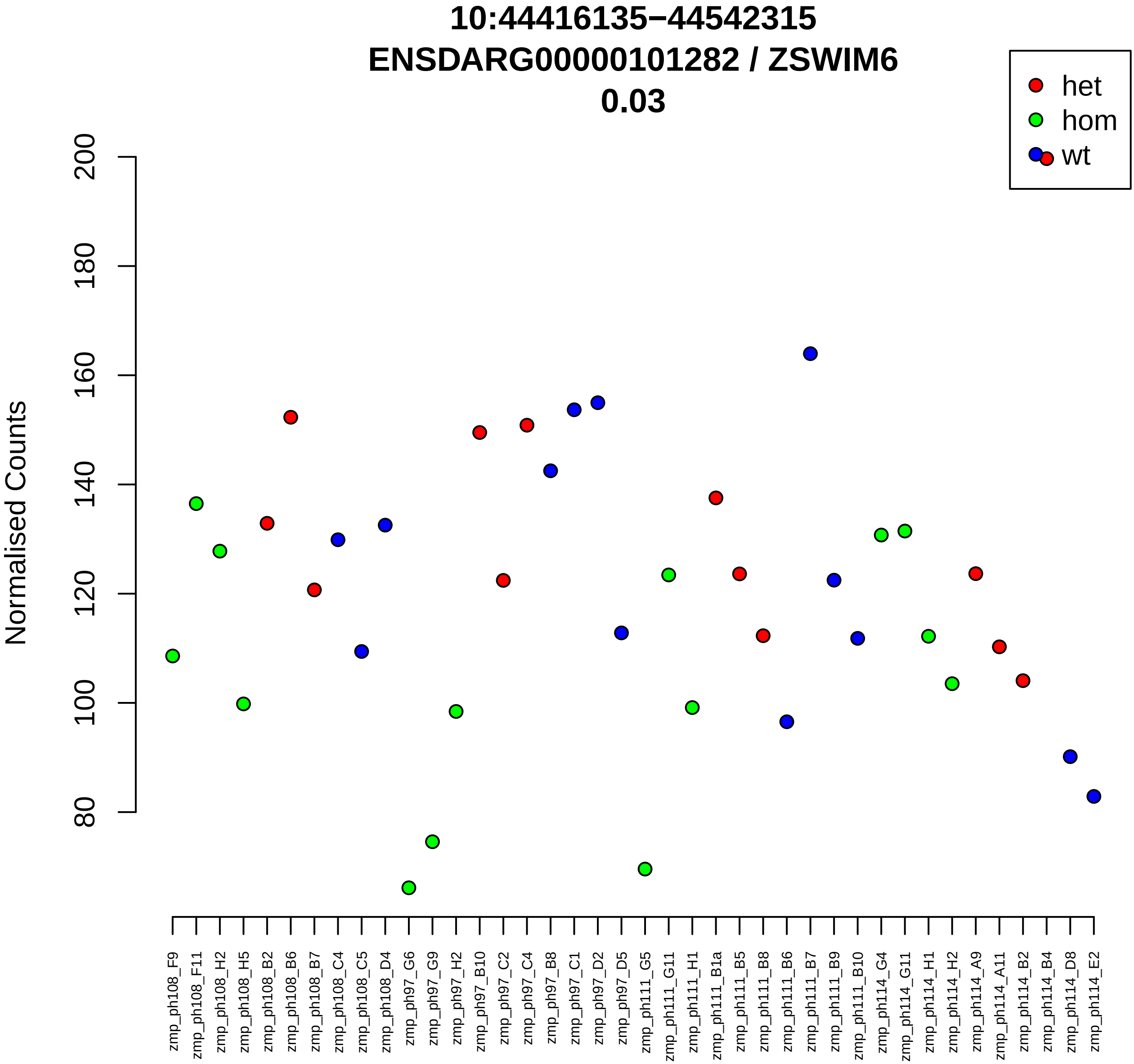

10:44416135−44542315
ENSDARG00000101282 / ZSWIM6
0.03
het
hom
wt
Normalised Counts
80
100
120
140
160
180
200
zmp_ph108_F9
zmp_ph108_F11
zmp_ph108_H2
zmp_ph108_H5
zmp_ph108_B2
zmp_ph108_B6
zmp_ph108_B7
zmp_ph108_C4
zmp_ph108_C5
zmp_ph108_D4
zmp_ph97_G6
zmp_ph97_G9
zmp_ph97_H2
zmp_ph97_B10
zmp_ph97_C2
zmp_ph97_C4
zmp_ph97_B8
zmp_ph97_C1
zmp_ph97_D2
zmp_ph97_D5
zmp_ph111_G5
zmp_ph111_G11
zmp_ph111_H1
zmp_ph111_B1a
zmp_ph111_B5
zmp_ph111_B8
zmp_ph111_B6
zmp_ph111_B7
zmp_ph111_B9
zmp_ph111_B10
zmp_ph114_G4
zmp_ph114_G11
zmp_ph114_H1
zmp_ph114_H2
zmp_ph114_A9
zmp_ph114_A11
zmp_ph114_B2
zmp_ph114_B4
zmp_ph114_D8
zmp_ph114_E2

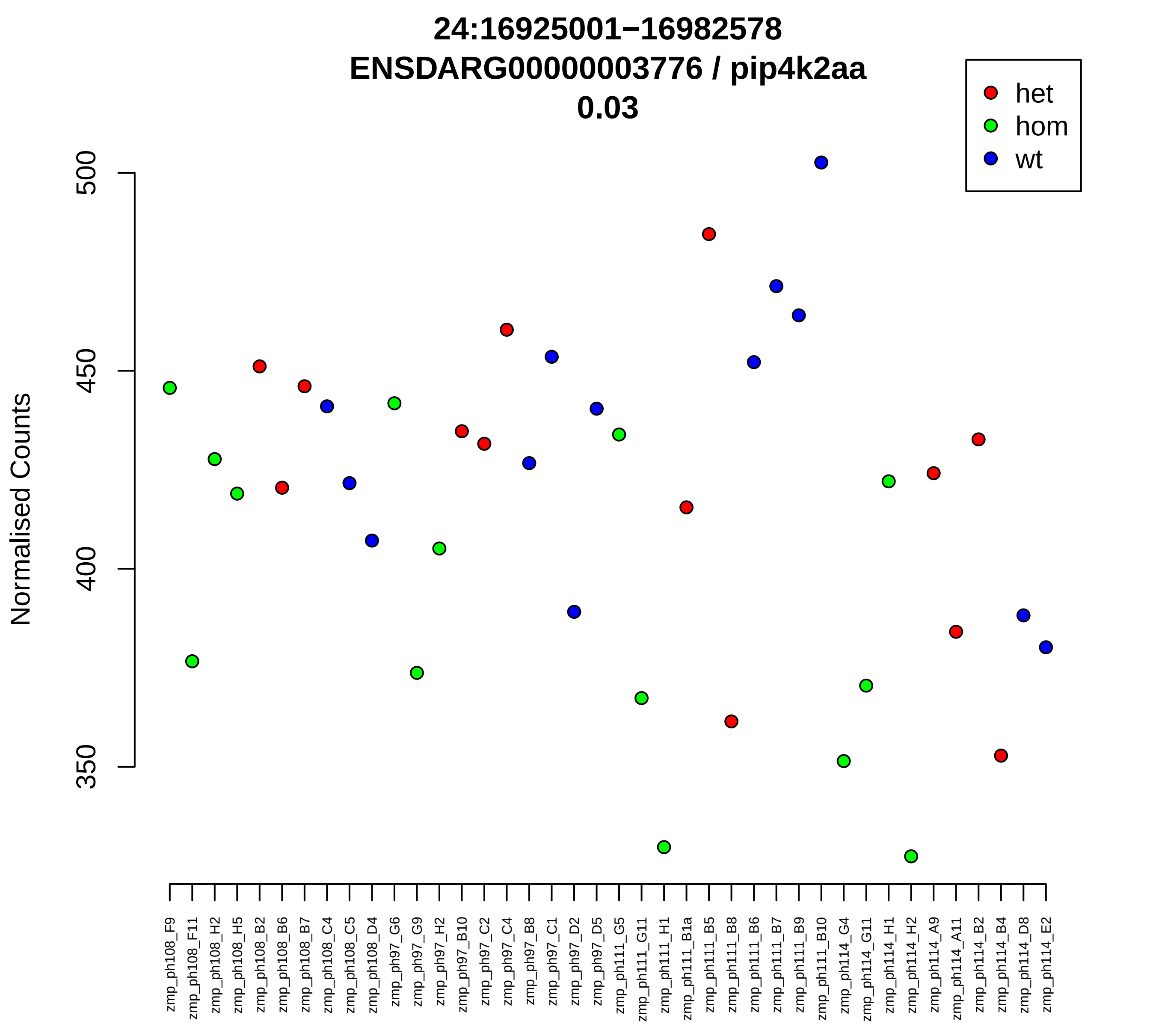

24:16925001−16982578
ENSDARG00000003776 / pip4k2aa
0.03
het
hom
wt
Normalised Counts
500
450
400
350
zmp_ph108_F9
zmp_ph108_F11
zmp_ph108_H2
zmp_ph108_H5
zmp_ph108_B2
zmp_ph108_B6
zmp_ph108_B7
zmp_ph108_C4
zmp_ph108_C5
zmp_ph108_D4
zmp_ph97_G6
zmp_ph97_G9
zmp_ph97_H2
zmp_ph97_B10
zmp_ph97_C2
zmp_ph97_C4
zmp_ph97_B8
zmp_ph97_C1
zmp_ph97_D2
zmp_ph97_D5
zmp_ph111_G5
zmp_ph111_G11
zmp_ph111_H1
zmp_ph111_B1a
zmp_ph111_B5
zmp_ph111_B8
zmp_ph111_B6
zmp_ph111_B7
zmp_ph111_B9
zmp_ph111_B10
zmp_ph114_G4
zmp_ph114_G11
zmp_ph114_H1
zmp_ph114_H2
zmp_ph114_A9
zmp_ph114_A11
zmp_ph114_B2
zmp_ph114_B4
zmp_ph114_D8
zmp_ph114_E2

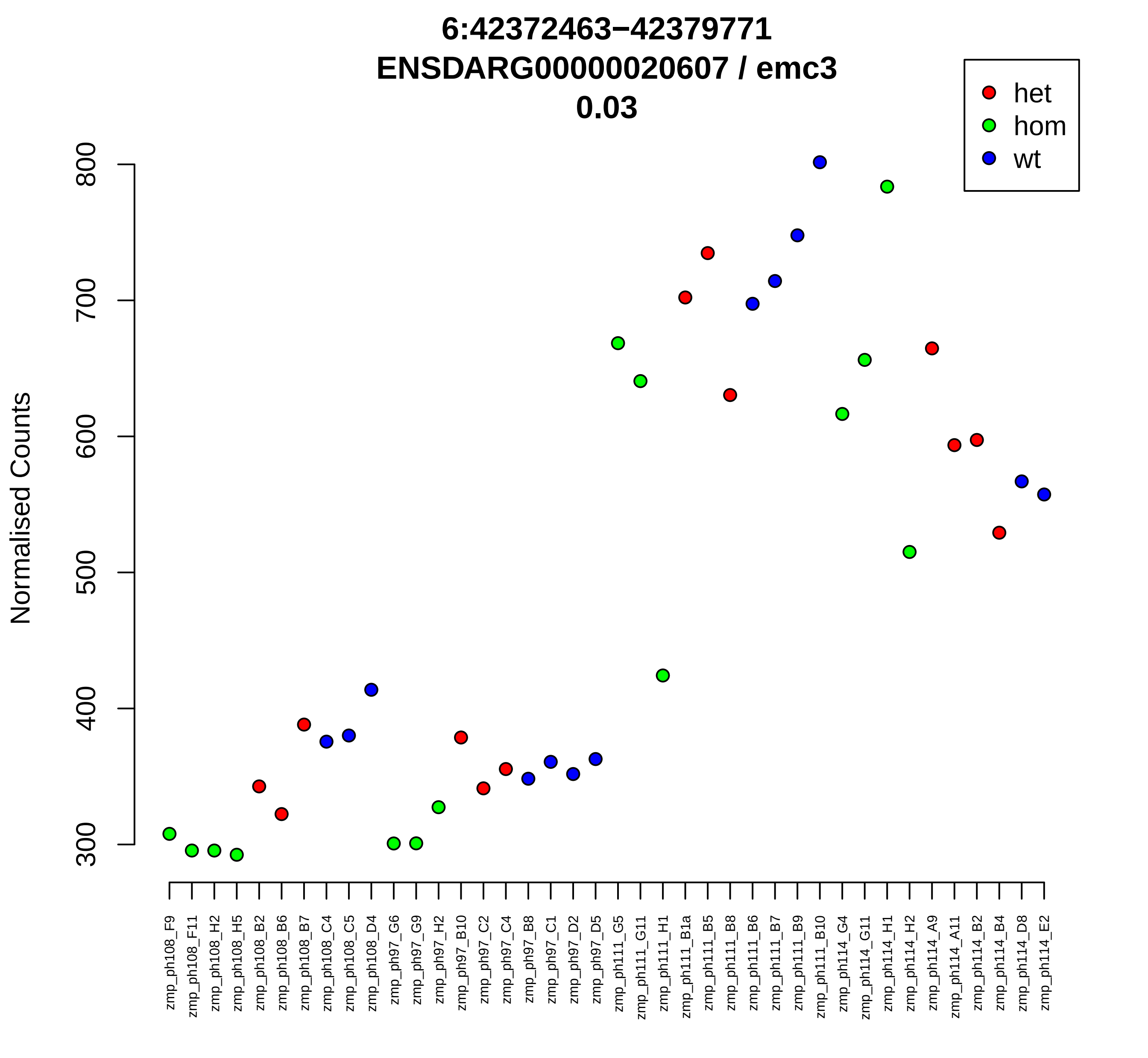
6:42372463−42379771
ENSDARG00000020607 / emc3
0.03
het
hom
wt
Normalised Counts
300
400
500
600
700
800
zmp_ph108_F9
zmp_ph108_F11
zmp_ph108_H2
zmp_ph108_H5
zmp_ph108_B2
zmp_ph108_B6
zmp_ph108_B7
zmp_ph108_C4
zmp_ph108_C5
zmp_ph108_D4
zmp_ph97_G6
zmp_ph97_G9
zmp_ph97_H2
zmp_ph97_B10
zmp_ph97_C2
zmp_ph97_C4
zmp_ph97_B8
zmp_ph97_C1
zmp_ph97_D2
zmp_ph97_D5
zmp_ph111_G5
zmp_ph111_G11
zmp_ph111_H1
zmp_ph111_B1a
zmp_ph111_B5
zmp_ph111_B8
zmp_ph111_B6
zmp_ph111_B7
zmp_ph111_B9
zmp_ph111_B10
zmp_ph114_G4
zmp_ph114_G11
zmp_ph114_H1
zmp_ph114_H2
zmp_ph114_A9
zmp_ph114_A11
zmp_ph114_B2
zmp_ph114_B4
zmp_ph114_D8
zmp_ph114_E2

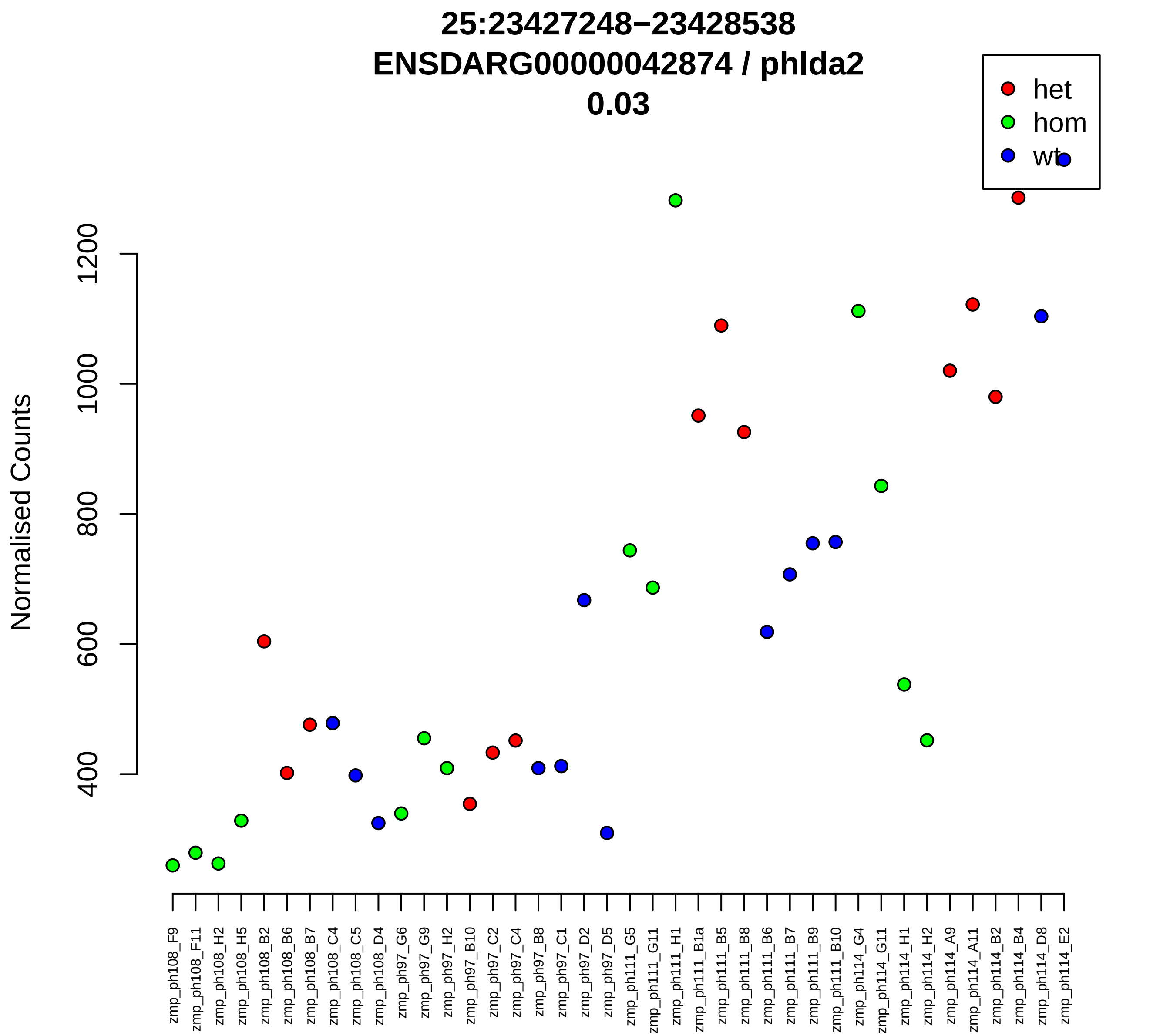

25:23427248−23428538
ENSDARG00000042874 / phlda2
0.03
het
hom
wt
Normalised Counts
400
600
800
1000
1200
zmp_ph108_F9
zmp_ph108_F11
zmp_ph108_H2
zmp_ph108_H5
zmp_ph108_B2
zmp_ph108_B6
zmp_ph108_B7
zmp_ph108_C4
zmp_ph108_C5
zmp_ph108_D4
zmp_ph97_G6
zmp_ph97_G9
zmp_ph97_H2
zmp_ph97_B10
zmp_ph97_C2
zmp_ph97_C4
zmp_ph97_B8
zmp_ph97_C1
zmp_ph97_D2
zmp_ph97_D5
zmp_ph111_G5
zmp_ph111_G11
zmp_ph111_H1
zmp_ph111_B1a
zmp_ph111_B5
zmp_ph111_B8
zmp_ph111_B6
zmp_ph111_B7
zmp_ph111_B9
zmp_ph111_B10
zmp_ph114_G4
zmp_ph114_G11
zmp_ph114_H1
zmp_ph114_H2
zmp_ph114_A9
zmp_ph114_A11
zmp_ph114_B2
zmp_ph114_B4
zmp_ph114_D8
zmp_ph114_E2

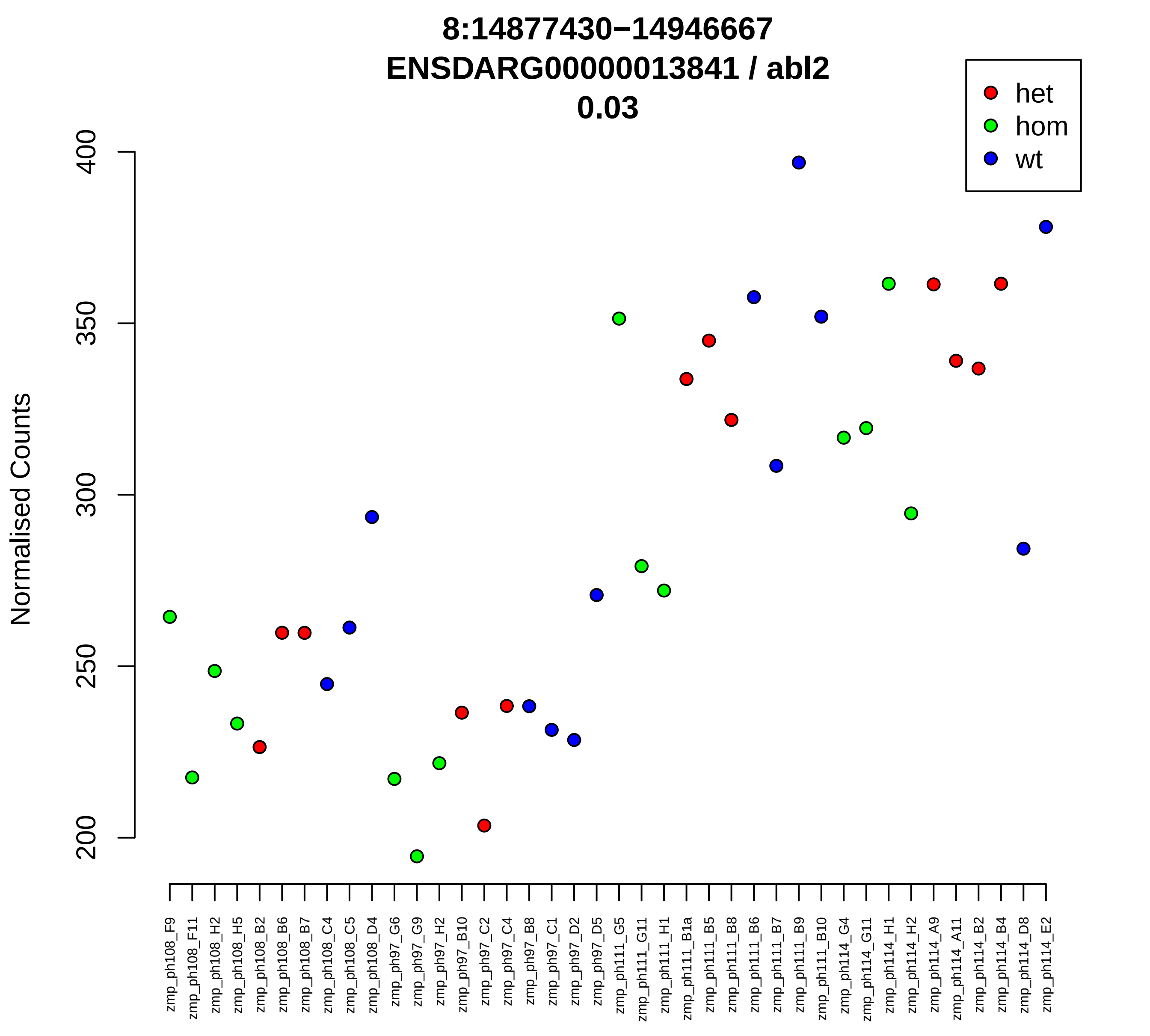
8:14877430−14946667
ENSDARG00000013841 / abl2
0.03
het
hom
wt
Normalised Counts
400
350
300
250
200
zmp_ph108_F9
zmp_ph108_F11
zmp_ph108_H2
zmp_ph108_H5
zmp_ph108_B2
zmp_ph108_B6
zmp_ph108_B7
zmp_ph108_C4
zmp_ph108_C5
zmp_ph108_D4
zmp_ph97_G6
zmp_ph97_G9
zmp_ph97_H2
zmp_ph97_B10
zmp_ph97_C2
zmp_ph97_C4
zmp_ph97_B8
zmp_ph97_C1
zmp_ph97_D2
zmp_ph97_D5
zmp_ph111_G5
zmp_ph111_G11
zmp_ph111_H1
zmp_ph111_B1a
zmp_ph111_B5
zmp_ph111_B8
zmp_ph111_B6
zmp_ph111_B7
zmp_ph111_B9
zmp_ph111_B10
zmp_ph114_G4
zmp_ph114_G11
zmp_ph114_H1
zmp_ph114_H2
zmp_ph114_A9
zmp_ph114_A11
zmp_ph114_B2
zmp_ph114_B4
zmp_ph114_D8
zmp_ph114_E2

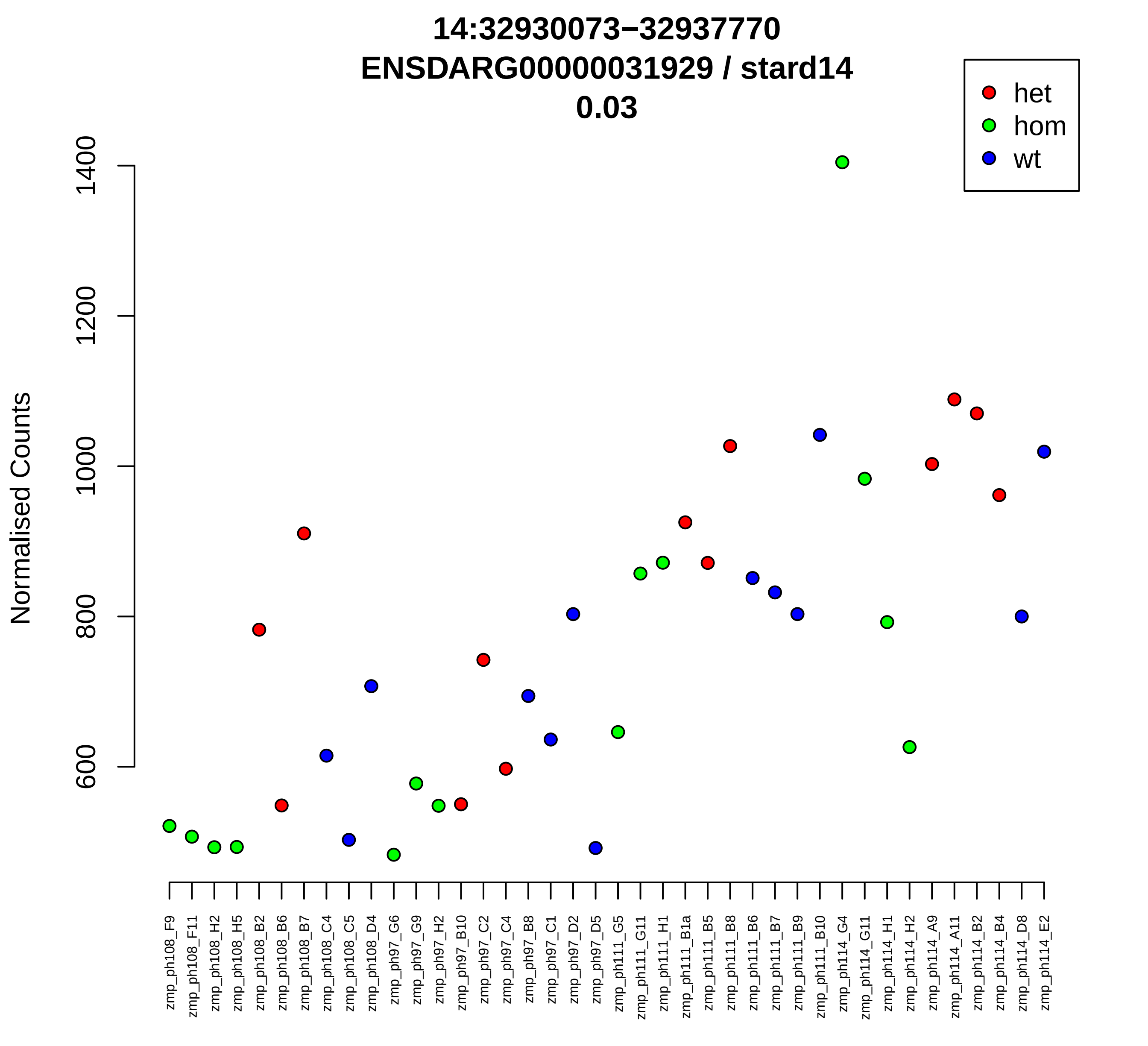
14:32930073−32937770
ENSDARG00000031929 / stard14
0.03
het
hom
wt
Normalised Counts
600
800
1000
1200
1400
zmp_ph108_F9
zmp_ph108_F11
zmp_ph108_H2
zmp_ph108_H5
zmp_ph108_B2
zmp_ph108_B6
zmp_ph108_B7
zmp_ph108_C4
zmp_ph108_C5
zmp_ph108_D4
zmp_ph97_G6
zmp_ph97_G9
zmp_ph97_H2
zmp_ph97_B10
zmp_ph97_C2
zmp_ph97_C4
zmp_ph97_B8
zmp_ph97_C1
zmp_ph97_D2
zmp_ph97_D5
zmp_ph111_G5
zmp_ph111_G11
zmp_ph111_H1
zmp_ph111_B1a
zmp_ph111_B5
zmp_ph111_B8
zmp_ph111_B6
zmp_ph111_B7
zmp_ph111_B9
zmp_ph111_B10
zmp_ph114_G4
zmp_ph114_G11
zmp_ph114_H1
zmp_ph114_H2
zmp_ph114_A9
zmp_ph114_A11
zmp_ph114_B2
zmp_ph114_B4
zmp_ph114_D8
zmp_ph114_E2

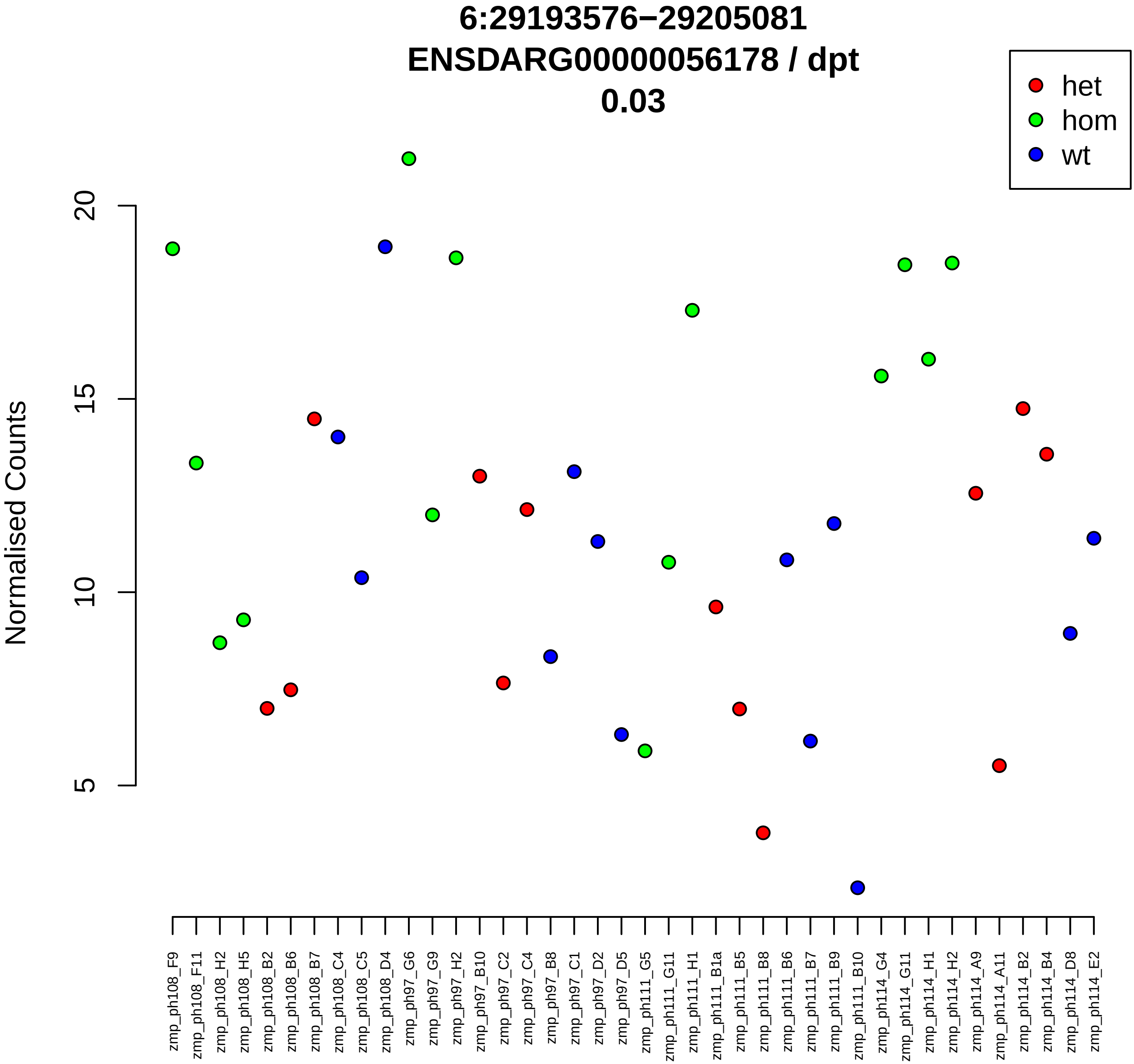
6:29193576−29205081
ENSDARG00000056178 / dpt
0.03
het
hom
wt
Normalised Counts
5
10
15
20
zmp_ph108_F9
zmp_ph108_F11
zmp_ph108_H2
zmp_ph108_H5
zmp_ph108_B2
zmp_ph108_B6
zmp_ph108_B7
zmp_ph108_C4
zmp_ph108_C5
zmp_ph108_D4
zmp_ph97_G6
zmp_ph97_G9
zmp_ph97_H2
zmp_ph97_B10
zmp_ph97_C2
zmp_ph97_C4
zmp_ph97_B8
zmp_ph97_C1
zmp_ph97_D2
zmp_ph97_D5
zmp_ph111_G5
zmp_ph111_G11
zmp_ph111_H1
zmp_ph111_B1a
zmp_ph111_B5
zmp_ph111_B8
zmp_ph111_B6
zmp_ph111_B7
zmp_ph111_B9
zmp_ph111_B10
zmp_ph114_G4
zmp_ph114_G11
zmp_ph114_H1
zmp_ph114_H2
zmp_ph114_A9
zmp_ph114_A11
zmp_ph114_B2
zmp_ph114_B4
zmp_ph114_D8
zmp_ph114_E2

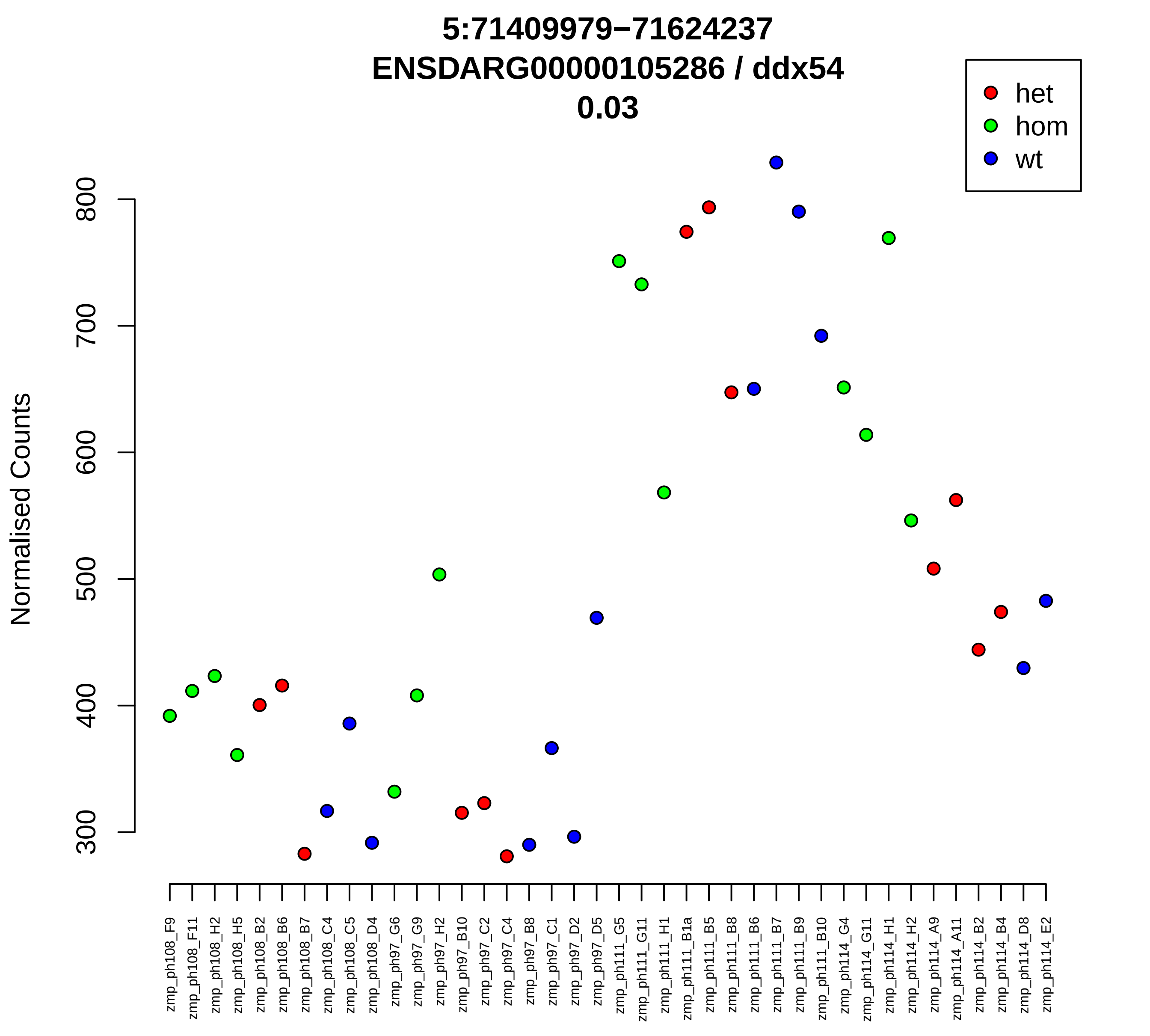

5:71409979−71624237
ENSDARG00000105286 / ddx54
0.03
het
hom
wt
Normalised Counts
300
400
500
600
700
800
zmp_ph108_F9
zmp_ph108_F11
zmp_ph108_H2
zmp_ph108_H5
zmp_ph108_B2
zmp_ph108_B6
zmp_ph108_B7
zmp_ph108_C4
zmp_ph108_C5
zmp_ph108_D4
zmp_ph97_G6
zmp_ph97_G9
zmp_ph97_H2
zmp_ph97_B10
zmp_ph97_C2
zmp_ph97_C4
zmp_ph97_B8
zmp_ph97_C1
zmp_ph97_D2
zmp_ph97_D5
zmp_ph111_G5
zmp_ph111_G11
zmp_ph111_H1
zmp_ph111_B1a
zmp_ph111_B5
zmp_ph111_B8
zmp_ph111_B6
zmp_ph111_B7
zmp_ph111_B9
zmp_ph111_B10
zmp_ph114_G4
zmp_ph114_G11
zmp_ph114_H1
zmp_ph114_H2
zmp_ph114_A9
zmp_ph114_A11
zmp_ph114_B2
zmp_ph114_B4
zmp_ph114_D8
zmp_ph114_E2

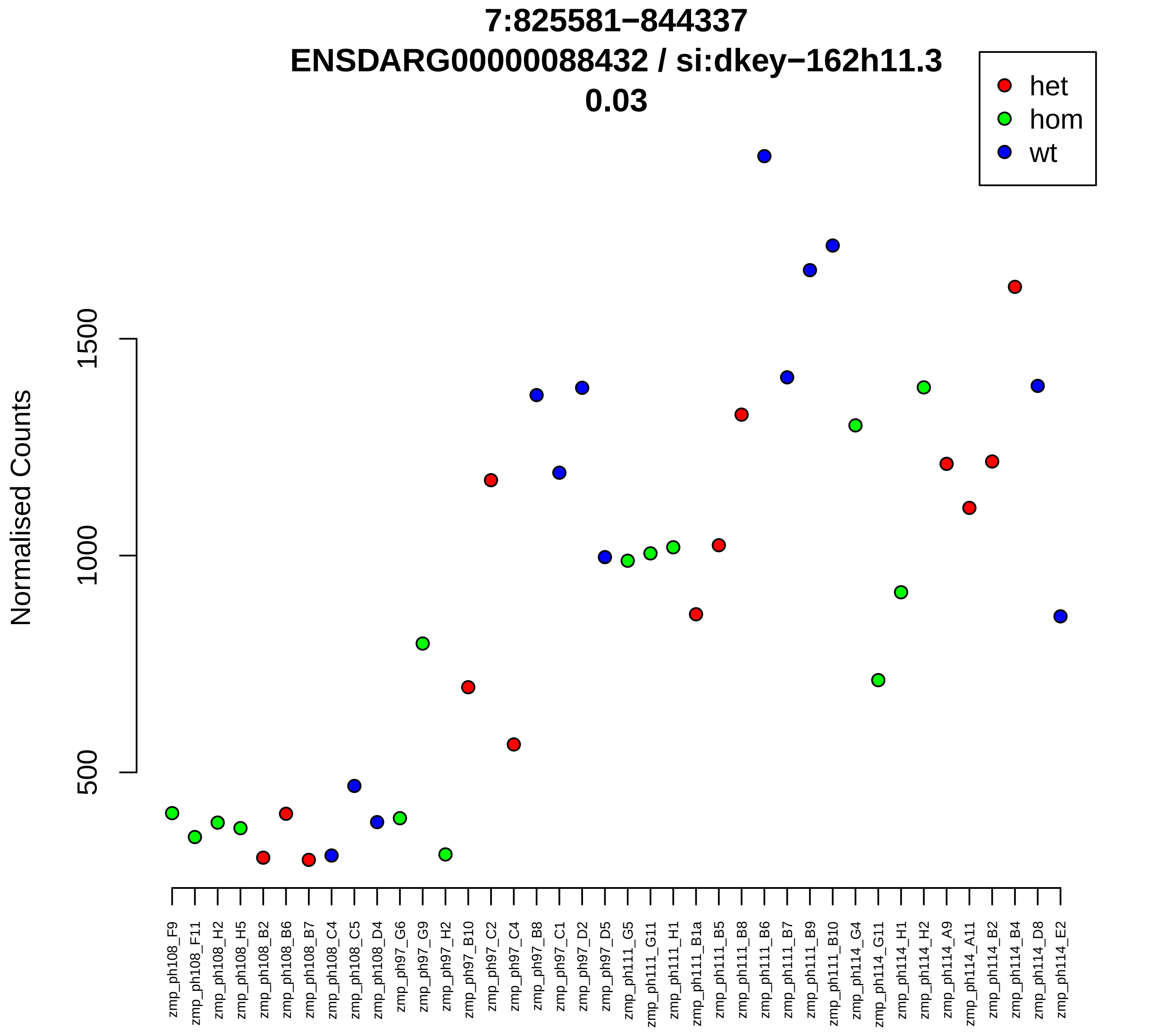

7:825581−844337
ENSDARG00000088432 / si:dkey−162h11.3
0.03
het
hom
wt
Normalised Counts
500
1000
1500
zmp_ph108_F9
zmp_ph108_F11
zmp_ph108_H2
zmp_ph108_H5
zmp_ph108_B2
zmp_ph108_B6
zmp_ph108_B7
zmp_ph108_C4
zmp_ph108_C5
zmp_ph108_D4
zmp_ph97_G6
zmp_ph97_G9
zmp_ph97_H2
zmp_ph97_B10
zmp_ph97_C2
zmp_ph97_C4
zmp_ph97_B8
zmp_ph97_C1
zmp_ph97_D2
zmp_ph97_D5
zmp_ph111_G5
zmp_ph111_G11
zmp_ph111_H1
zmp_ph111_B1a
zmp_ph111_B5
zmp_ph111_B8
zmp_ph111_B6
zmp_ph111_B7
zmp_ph111_B9
zmp_ph111_B10
zmp_ph114_G4
zmp_ph114_G11
zmp_ph114_H1
zmp_ph114_H2
zmp_ph114_A9
zmp_ph114_A11
zmp_ph114_B2
zmp_ph114_B4
zmp_ph114_D8
zmp_ph114_E2

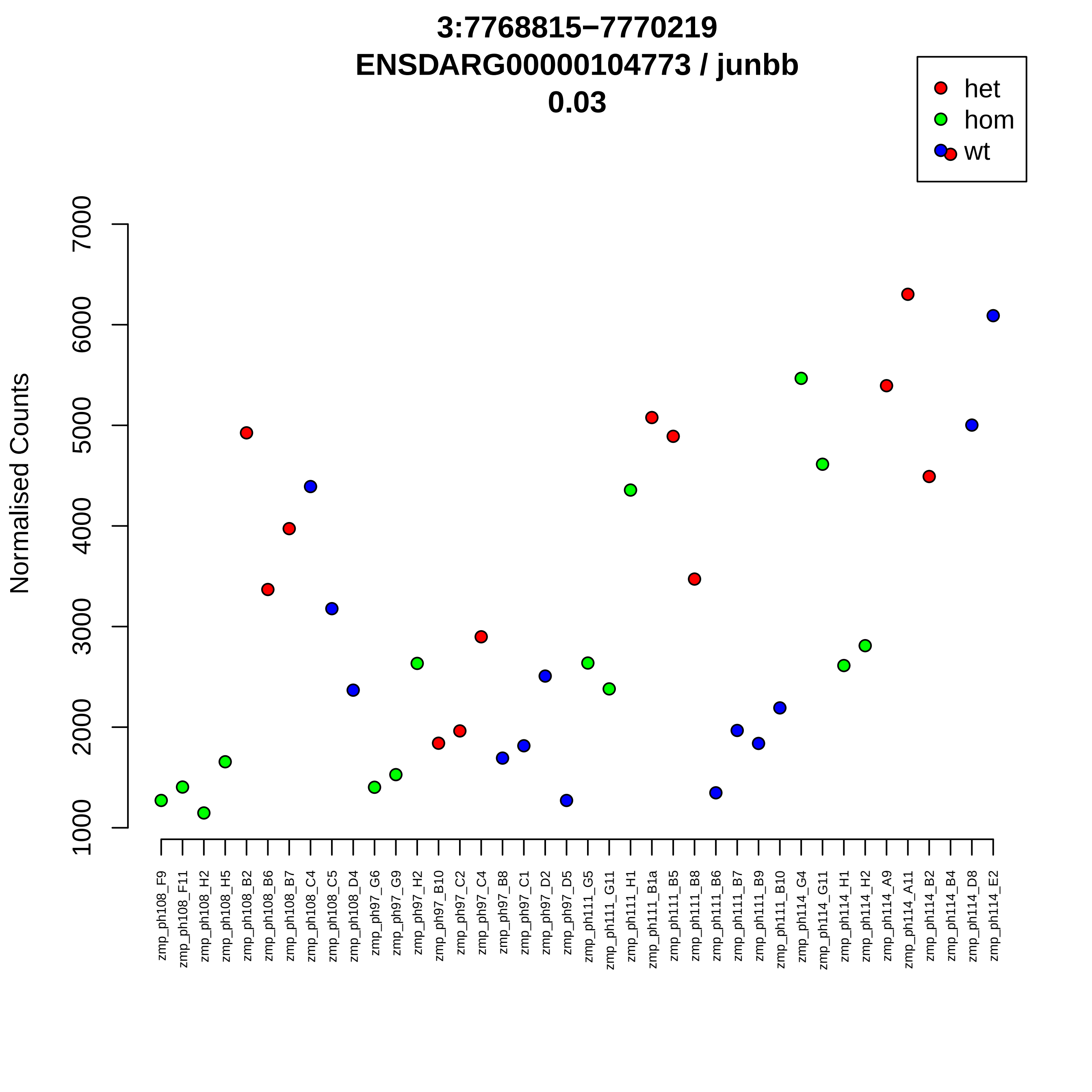

3:7768815−7770219
ENSDARG00000104773 / junbb
0.03
het
hom
wt
Normalised Counts
1000
2000
3000
4000
5000
6000
7000
zmp_ph108_F9
zmp_ph108_F11
zmp_ph108_H2
zmp_ph108_H5
zmp_ph108_B2
zmp_ph108_B6
zmp_ph108_B7
zmp_ph108_C4
zmp_ph108_C5
zmp_ph108_D4
zmp_ph97_G6
zmp_ph97_G9
zmp_ph97_H2
zmp_ph97_B10
zmp_ph97_C2
zmp_ph97_C4
zmp_ph97_B8
zmp_ph97_C1
zmp_ph97_D2
zmp_ph97_D5
zmp_ph111_G5
zmp_ph111_G11
zmp_ph111_H1
zmp_ph111_B1a
zmp_ph111_B5
zmp_ph111_B8
zmp_ph111_B6
zmp_ph111_B7
zmp_ph111_B9
zmp_ph111_B10
zmp_ph114_G4
zmp_ph114_G11
zmp_ph114_H1
zmp_ph114_H2
zmp_ph114_A9
zmp_ph114_A11
zmp_ph114_B2
zmp_ph114_B4
zmp_ph114_D8
zmp_ph114_E2

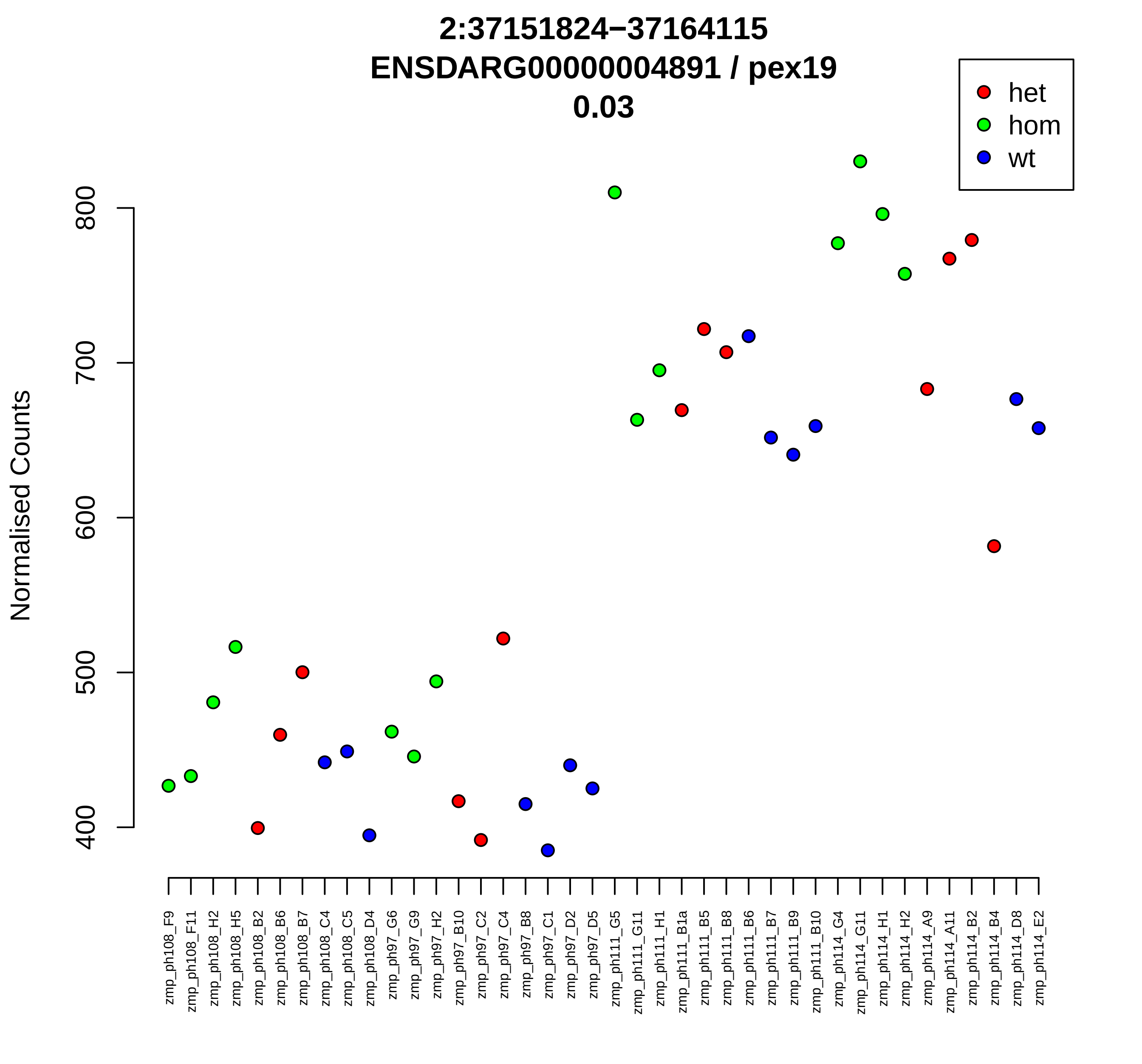

2:37151824−37164115
ENSDARG00000004891 / pex19
0.03
het
hom
wt
Normalised Counts
400
500
600
700
800
zmp_ph108_F9
zmp_ph108_F11
zmp_ph108_H2
zmp_ph108_H5
zmp_ph108_B2
zmp_ph108_B6
zmp_ph108_B7
zmp_ph108_C4
zmp_ph108_C5
zmp_ph108_D4
zmp_ph97_G6
zmp_ph97_G9
zmp_ph97_H2
zmp_ph97_B10
zmp_ph97_C2
zmp_ph97_C4
zmp_ph97_B8
zmp_ph97_C1
zmp_ph97_D2
zmp_ph97_D5
zmp_ph111_G5
zmp_ph111_G11
zmp_ph111_H1
zmp_ph111_B1a
zmp_ph111_B5
zmp_ph111_B8
zmp_ph111_B6
zmp_ph111_B7
zmp_ph111_B9
zmp_ph111_B10
zmp_ph114_G4
zmp_ph114_G11
zmp_ph114_H1
zmp_ph114_H2
zmp_ph114_A9
zmp_ph114_A11
zmp_ph114_B2
zmp_ph114_B4
zmp_ph114_D8
zmp_ph114_E2

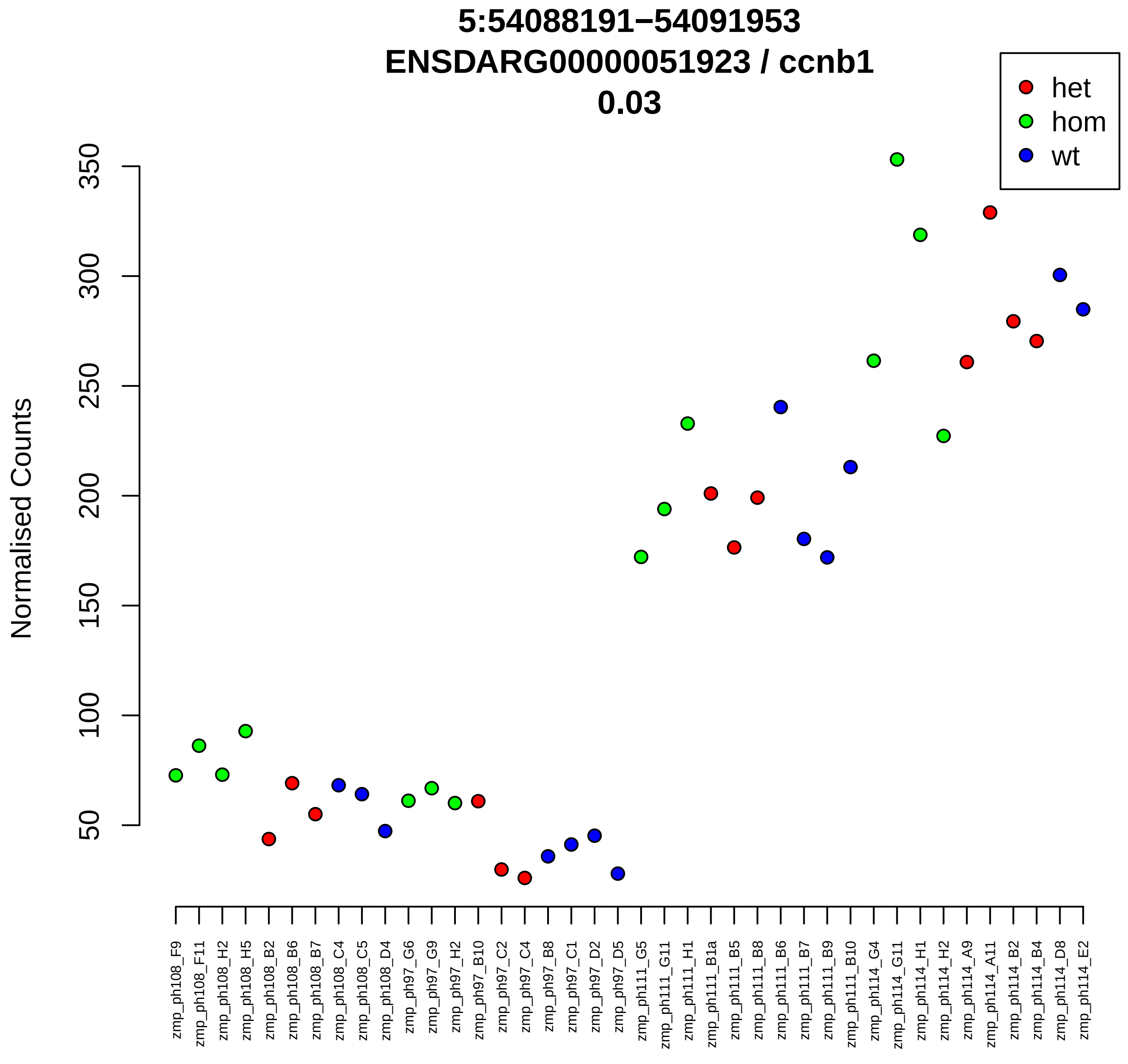
5:54088191−54091953
ENSDARG00000051923 / ccnb1
0.03
het
hom
wt
Normalised Counts
50
100
150
200
250
300
350
zmp_ph108_F9
zmp_ph108_F11
zmp_ph108_H2
zmp_ph108_H5
zmp_ph108_B2
zmp_ph108_B6
zmp_ph108_B7
zmp_ph108_C4
zmp_ph108_C5
zmp_ph108_D4
zmp_ph97_G6
zmp_ph97_G9
zmp_ph97_H2
zmp_ph97_B10
zmp_ph97_C2
zmp_ph97_C4
zmp_ph97_B8
zmp_ph97_C1
zmp_ph97_D2
zmp_ph97_D5
zmp_ph111_G5
zmp_ph111_G11
zmp_ph111_H1
zmp_ph111_B1a
zmp_ph111_B5
zmp_ph111_B8
zmp_ph111_B6
zmp_ph111_B7
zmp_ph111_B9
zmp_ph111_B10
zmp_ph114_G4
zmp_ph114_G11
zmp_ph114_H1
zmp_ph114_H2
zmp_ph114_A9
zmp_ph114_A11
zmp_ph114_B2
zmp_ph114_B4
zmp_ph114_D8
zmp_ph114_E2

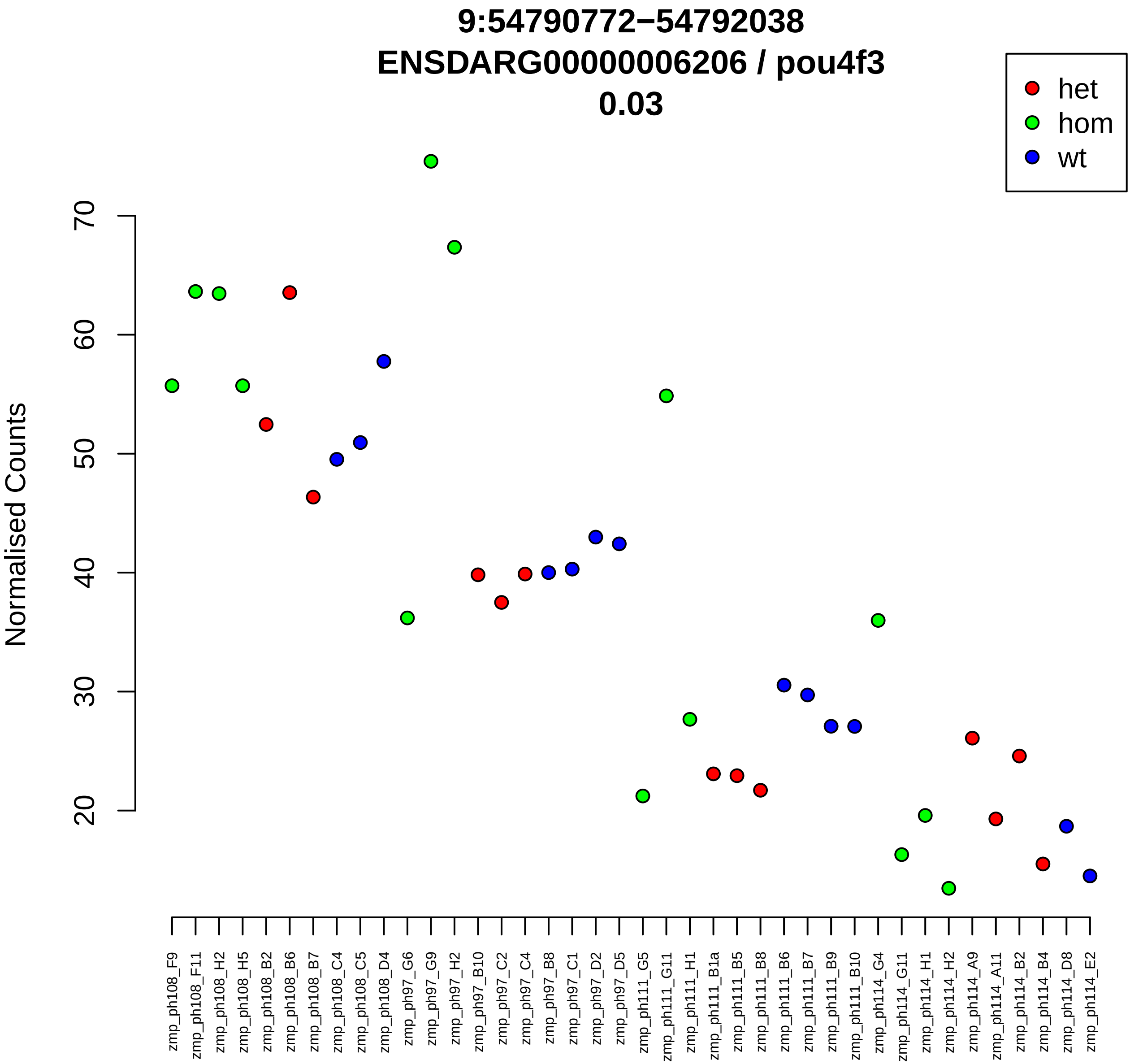
9:54790772−54792038
ENSDARG00000006206 / pou4f3
0.03
het
hom
wt
Normalised Counts
20
30
40
50
60
70
zmp_ph108_F9
zmp_ph108_F11
zmp_ph108_H2
zmp_ph108_H5
zmp_ph108_B2
zmp_ph108_B6
zmp_ph108_B7
zmp_ph108_C4
zmp_ph108_C5
zmp_ph108_D4
zmp_ph97_G6
zmp_ph97_G9
zmp_ph97_H2
zmp_ph97_B10
zmp_ph97_C2
zmp_ph97_C4
zmp_ph97_B8
zmp_ph97_C1
zmp_ph97_D2
zmp_ph97_D5
zmp_ph111_G5
zmp_ph111_G11
zmp_ph111_H1
zmp_ph111_B1a
zmp_ph111_B5
zmp_ph111_B8
zmp_ph111_B6
zmp_ph111_B7
zmp_ph111_B9
zmp_ph111_B10
zmp_ph114_G4
zmp_ph114_G11
zmp_ph114_H1
zmp_ph114_H2
zmp_ph114_A9
zmp_ph114_A11
zmp_ph114_B2
zmp_ph114_B4
zmp_ph114_D8
zmp_ph114_E2

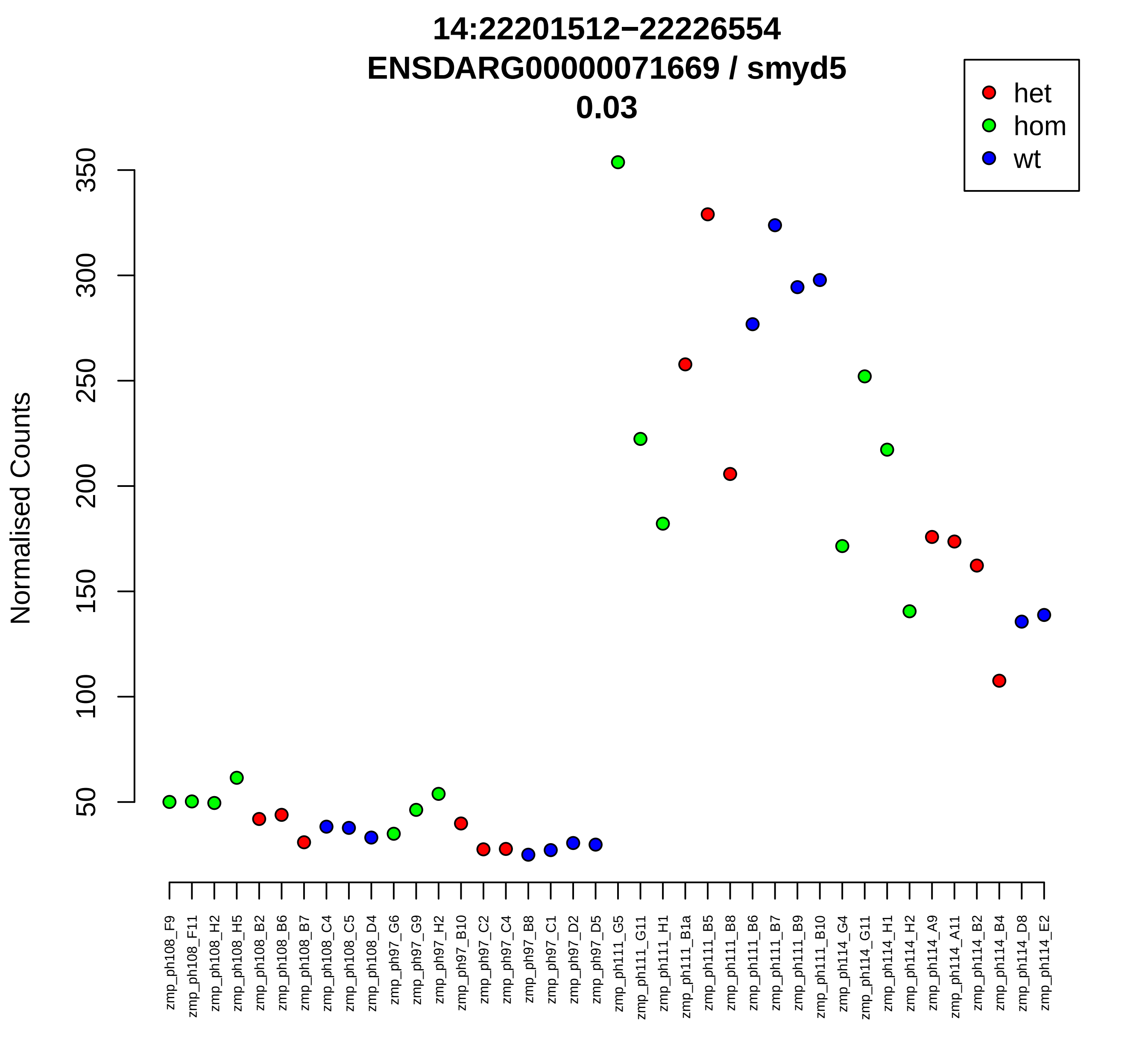
14:22201512−22226554
ENSDARG00000071669 / smyd5
0.03
het
hom
wt
Normalised Counts
50
100
150
200
250
300
350
zmp_ph108_F9
zmp_ph108_F11
zmp_ph108_H2
zmp_ph108_H5
zmp_ph108_B2
zmp_ph108_B6
zmp_ph108_B7
zmp_ph108_C4
zmp_ph108_C5
zmp_ph108_D4
zmp_ph97_G6
zmp_ph97_G9
zmp_ph97_H2
zmp_ph97_B10
zmp_ph97_C2
zmp_ph97_C4
zmp_ph97_B8
zmp_ph97_C1
zmp_ph97_D2
zmp_ph97_D5
zmp_ph111_G5
zmp_ph111_G11
zmp_ph111_H1
zmp_ph111_B1a
zmp_ph111_B5
zmp_ph111_B8
zmp_ph111_B6
zmp_ph111_B7
zmp_ph111_B9
zmp_ph111_B10
zmp_ph114_G4
zmp_ph114_G11
zmp_ph114_H1
zmp_ph114_H2
zmp_ph114_A9
zmp_ph114_A11
zmp_ph114_B2
zmp_ph114_B4
zmp_ph114_D8
zmp_ph114_E2

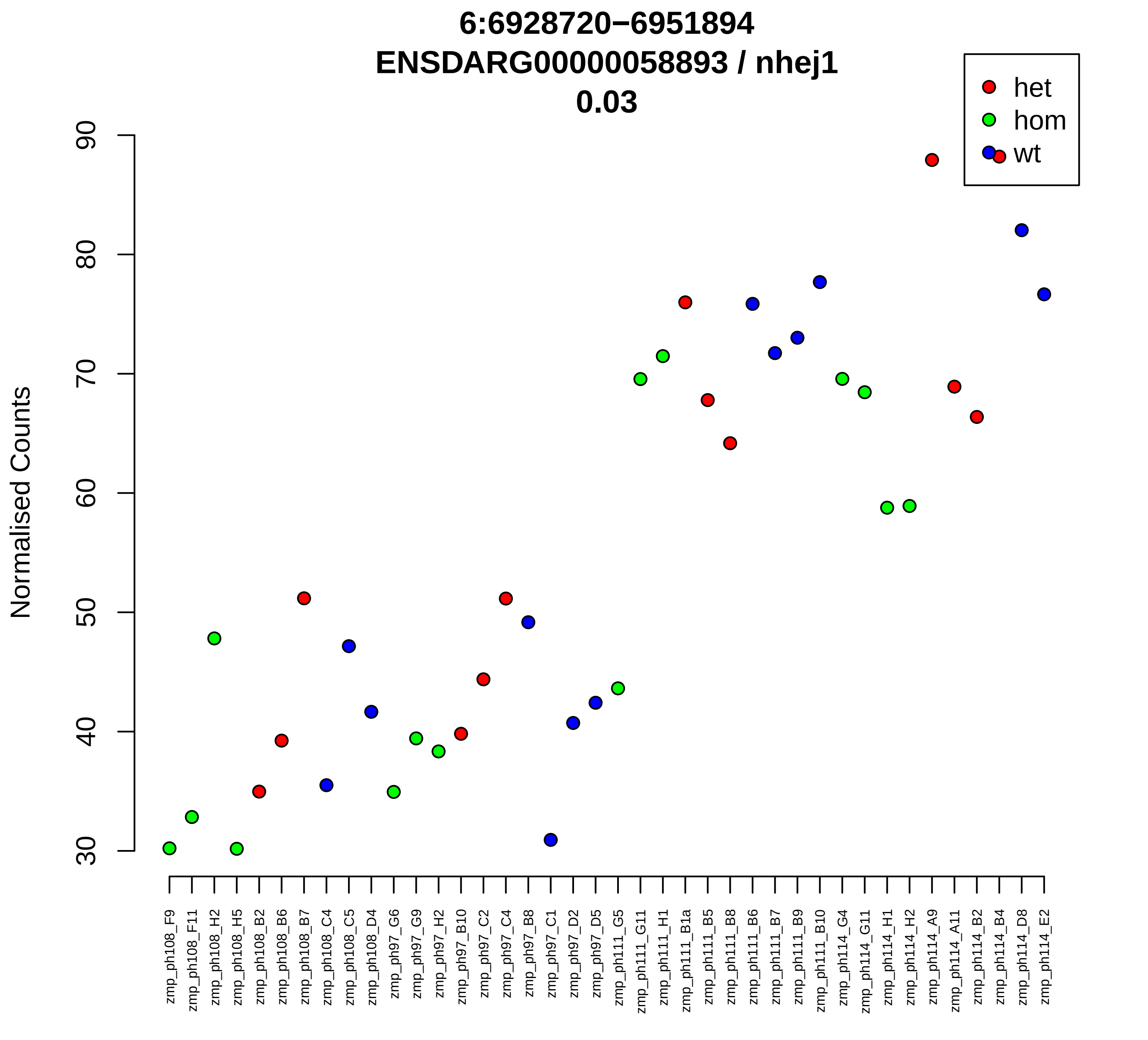
6:6928720−6951894
ENSDARG00000058893 / nhej1
0.03
het
hom
wt
Normalised Counts
30
40
50
60
70
80
90
zmp_ph108_F9
zmp_ph108_F11
zmp_ph108_H2
zmp_ph108_H5
zmp_ph108_B2
zmp_ph108_B6
zmp_ph108_B7
zmp_ph108_C4
zmp_ph108_C5
zmp_ph108_D4
zmp_ph97_G6
zmp_ph97_G9
zmp_ph97_H2
zmp_ph97_B10
zmp_ph97_C2
zmp_ph97_C4
zmp_ph97_B8
zmp_ph97_C1
zmp_ph97_D2
zmp_ph97_D5
zmp_ph111_G5
zmp_ph111_G11
zmp_ph111_H1
zmp_ph111_B1a
zmp_ph111_B5
zmp_ph111_B8
zmp_ph111_B6
zmp_ph111_B7
zmp_ph111_B9
zmp_ph111_B10
zmp_ph114_G4
zmp_ph114_G11
zmp_ph114_H1
zmp_ph114_H2
zmp_ph114_A9
zmp_ph114_A11
zmp_ph114_B2
zmp_ph114_B4
zmp_ph114_D8
zmp_ph114_E2

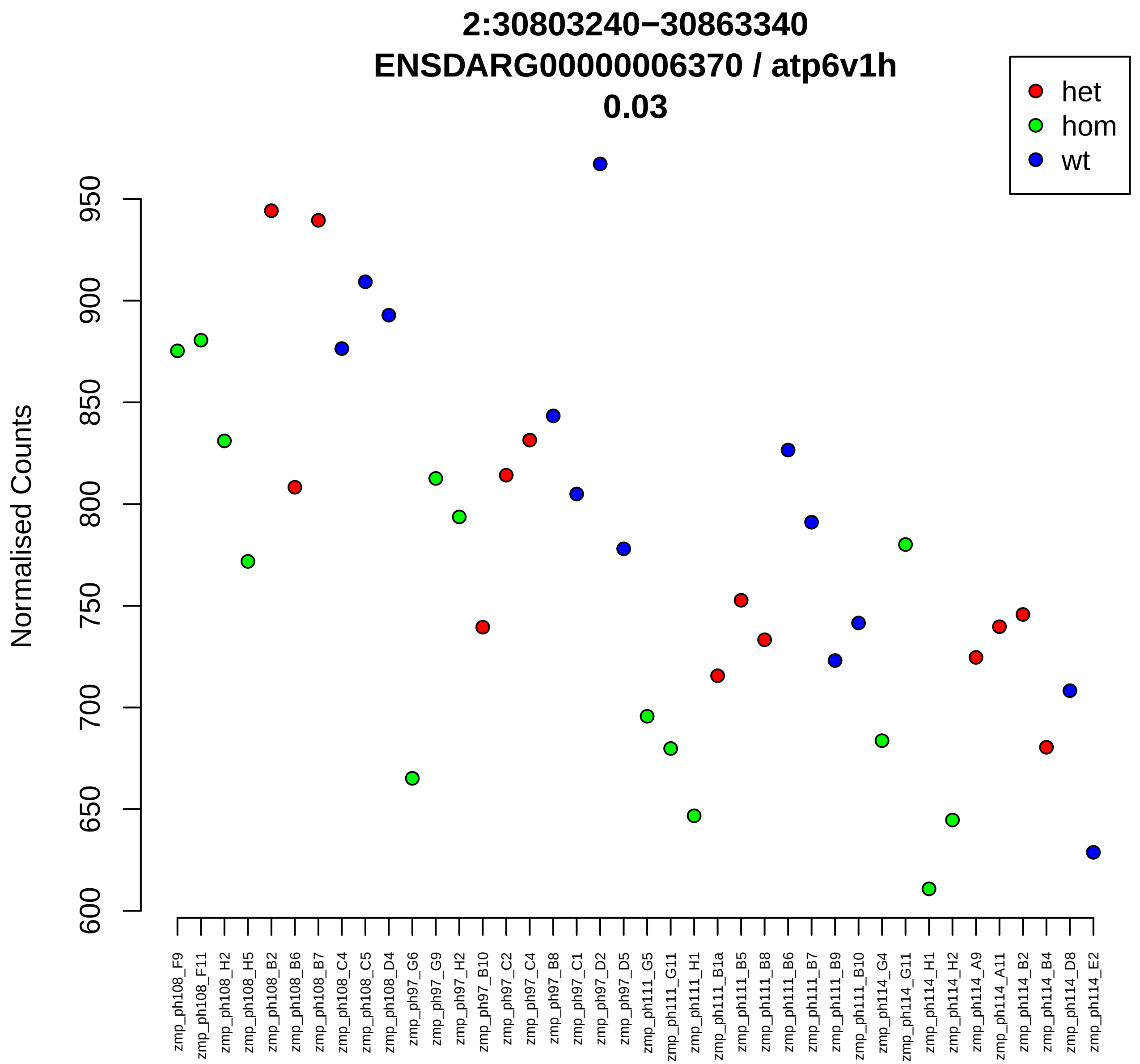

2:30803240−30863340
ENSDARG00000006370 / atp6v1h
0.03
het
hom
wt
Normalised Counts
600
650
700
750
800
850
900
950
zmp_ph108_F9
zmp_ph108_F11
zmp_ph108_H2
zmp_ph108_H5
zmp_ph108_B2
zmp_ph108_B6
zmp_ph108_B7
zmp_ph108_C4
zmp_ph108_C5
zmp_ph108_D4
zmp_ph97_G6
zmp_ph97_G9
zmp_ph97_H2
zmp_ph97_B10
zmp_ph97_C2
zmp_ph97_C4
zmp_ph97_B8
zmp_ph97_C1
zmp_ph97_D2
zmp_ph97_D5
zmp_ph111_G5
zmp_ph111_G11
zmp_ph111_H1
zmp_ph111_B1a
zmp_ph111_B5
zmp_ph111_B8
zmp_ph111_B6
zmp_ph111_B7
zmp_ph111_B9
zmp_ph111_B10
zmp_ph114_G4
zmp_ph114_G11
zmp_ph114_H1
zmp_ph114_H2
zmp_ph114_A9
zmp_ph114_A11
zmp_ph114_B2
zmp_ph114_B4
zmp_ph114_D8
zmp_ph114_E2

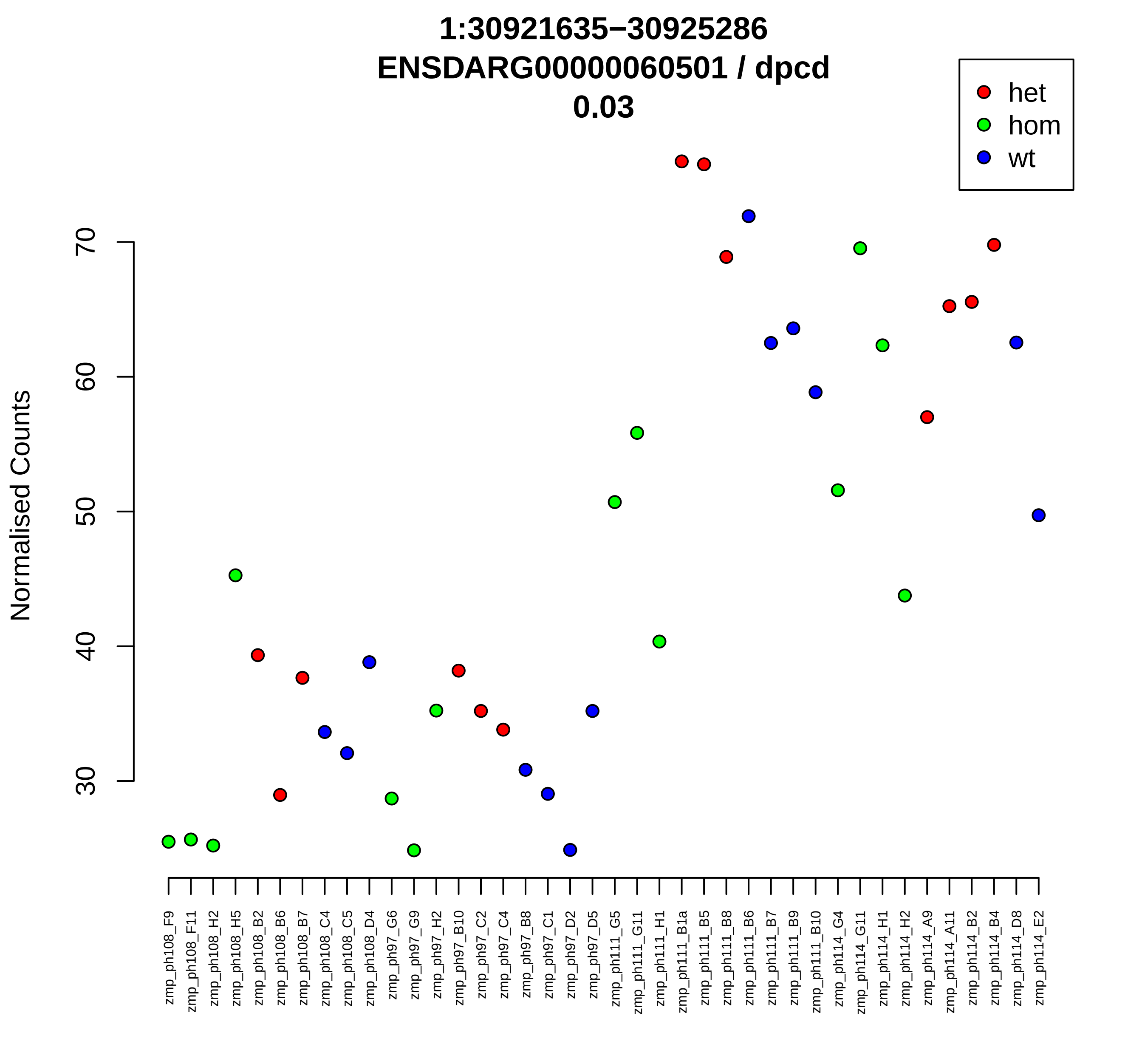

1:30921635−30925286
ENSDARG00000060501 / dpcd
0.03
het
hom
wt
Normalised Counts
30
40
50
60
70
zmp_ph108_F9
zmp_ph108_F11
zmp_ph108_H2
zmp_ph108_H5
zmp_ph108_B2
zmp_ph108_B6
zmp_ph108_B7
zmp_ph108_C4
zmp_ph108_C5
zmp_ph108_D4
zmp_ph97_G6
zmp_ph97_G9
zmp_ph97_H2
zmp_ph97_B10
zmp_ph97_C2
zmp_ph97_C4
zmp_ph97_B8
zmp_ph97_C1
zmp_ph97_D2
zmp_ph97_D5
zmp_ph111_G5
zmp_ph111_G11
zmp_ph111_H1
zmp_ph111_B1a
zmp_ph111_B5
zmp_ph111_B8
zmp_ph111_B6
zmp_ph111_B7
zmp_ph111_B9
zmp_ph111_B10
zmp_ph114_G4
zmp_ph114_G11
zmp_ph114_H1
zmp_ph114_H2
zmp_ph114_A9
zmp_ph114_A11
zmp_ph114_B2
zmp_ph114_B4
zmp_ph114_D8
zmp_ph114_E2

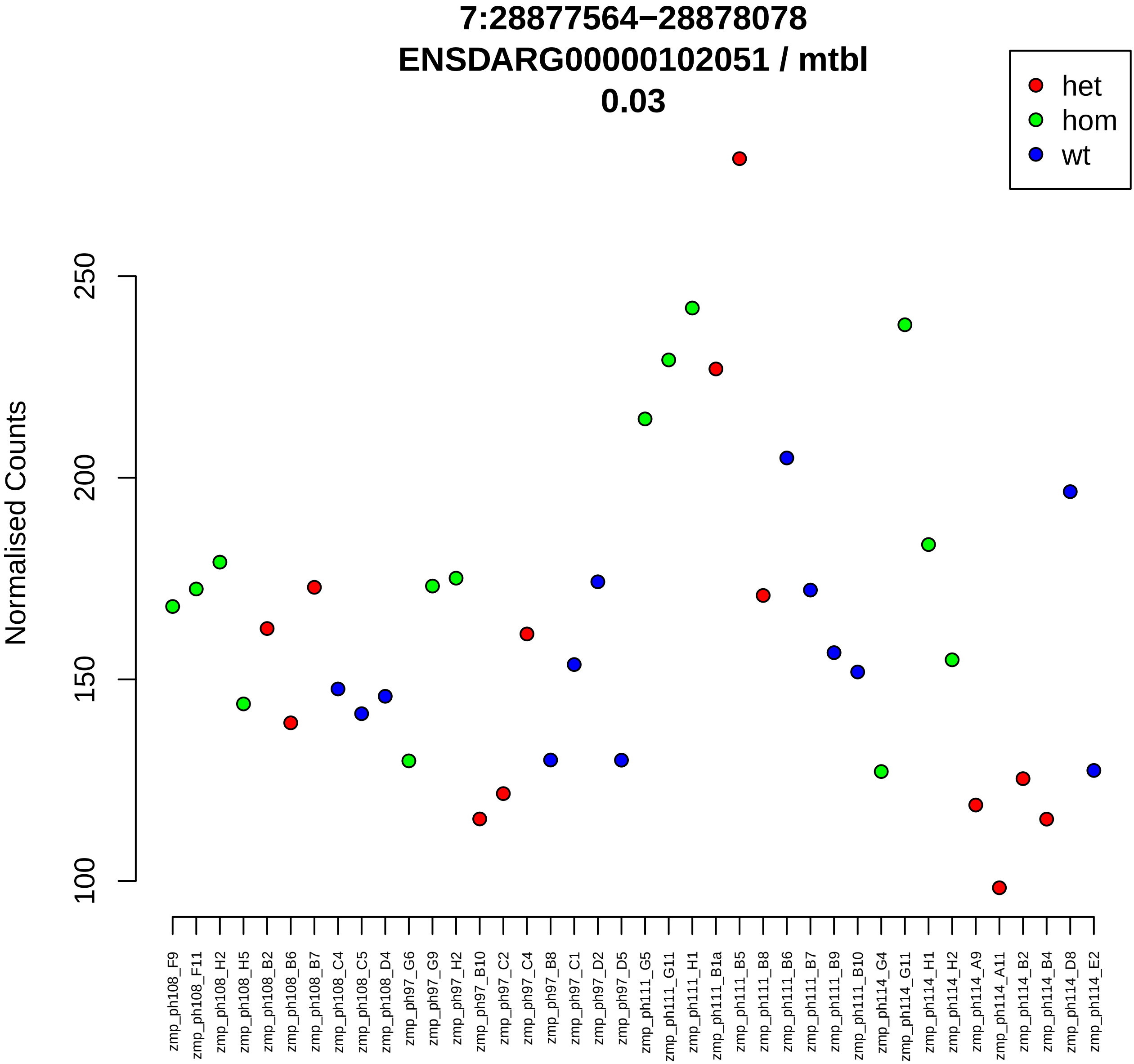

7:28877564−28878078
ENSDARG00000102051 / mtbl
0.03
het
hom
wt
Normalised Counts
100
150
200
250
zmp_ph108_F9
zmp_ph108_F11
zmp_ph108_H2
zmp_ph108_H5
zmp_ph108_B2
zmp_ph108_B6
zmp_ph108_B7
zmp_ph108_C4
zmp_ph108_C5
zmp_ph108_D4
zmp_ph97_G6
zmp_ph97_G9
zmp_ph97_H2
zmp_ph97_B10
zmp_ph97_C2
zmp_ph97_C4
zmp_ph97_B8
zmp_ph97_C1
zmp_ph97_D2
zmp_ph97_D5
zmp_ph111_G5
zmp_ph111_G11
zmp_ph111_H1
zmp_ph111_B1a
zmp_ph111_B5
zmp_ph111_B8
zmp_ph111_B6
zmp_ph111_B7
zmp_ph111_B9
zmp_ph111_B10
zmp_ph114_G4
zmp_ph114_G11
zmp_ph114_H1
zmp_ph114_H2
zmp_ph114_A9
zmp_ph114_A11
zmp_ph114_B2
zmp_ph114_B4
zmp_ph114_D8
zmp_ph114_E2

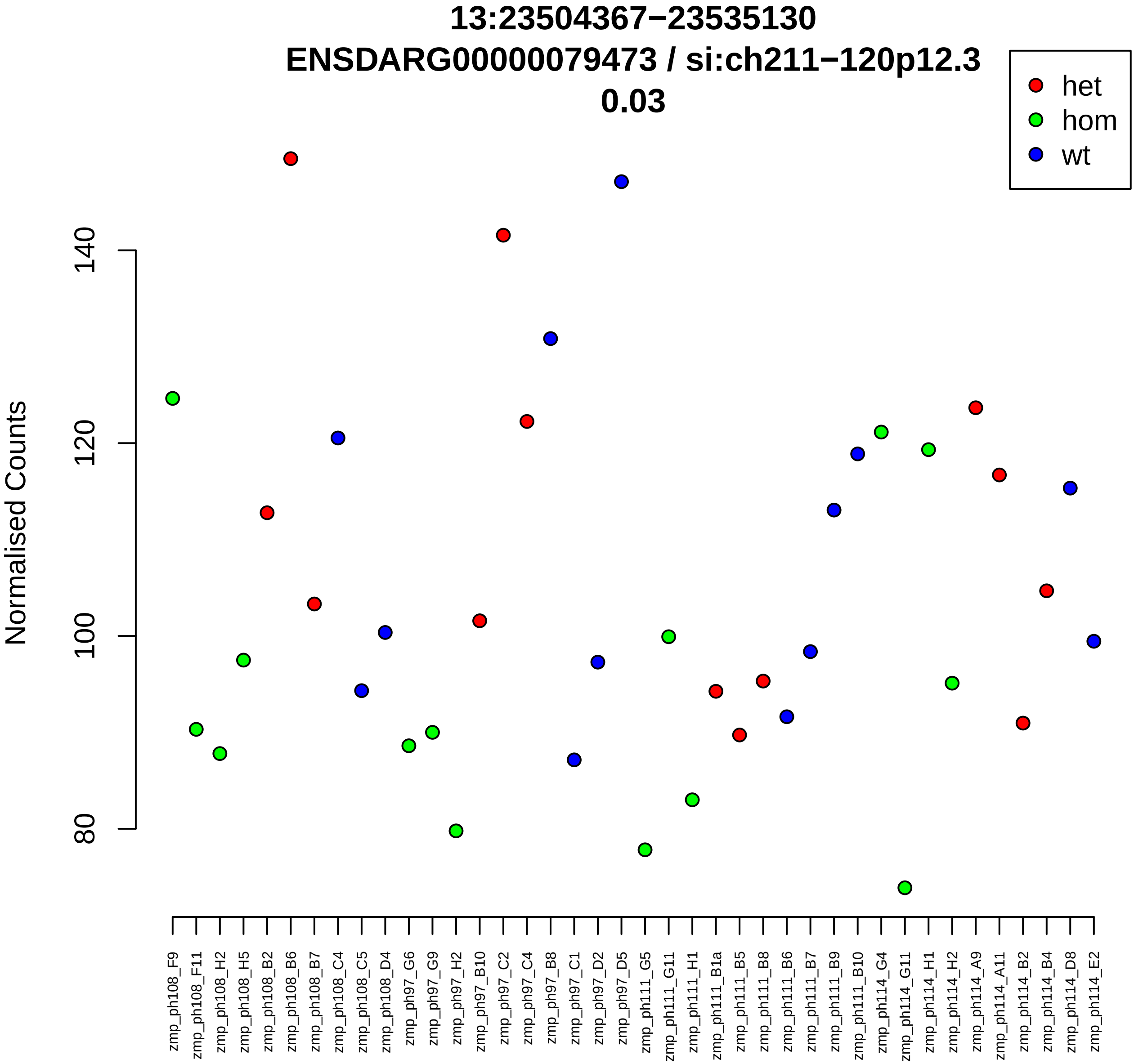
13:23504367−23535130
ENSDARG00000079473 / si:ch211−120p12.3
0.03
het
hom
wt
Normalised Counts
80
100
120
140
zmp_ph108_F9
zmp_ph108_F11
zmp_ph108_H2
zmp_ph108_H5
zmp_ph108_B2
zmp_ph108_B6
zmp_ph108_B7
zmp_ph108_C4
zmp_ph108_C5
zmp_ph108_D4
zmp_ph97_G6
zmp_ph97_G9
zmp_ph97_H2
zmp_ph97_B10
zmp_ph97_C2
zmp_ph97_C4
zmp_ph97_B8
zmp_ph97_C1
zmp_ph97_D2
zmp_ph97_D5
zmp_ph111_G5
zmp_ph111_G11
zmp_ph111_H1
zmp_ph111_B1a
zmp_ph111_B5
zmp_ph111_B8
zmp_ph111_B6
zmp_ph111_B7
zmp_ph111_B9
zmp_ph111_B10
zmp_ph114_G4
zmp_ph114_G11
zmp_ph114_H1
zmp_ph114_H2
zmp_ph114_A9
zmp_ph114_A11
zmp_ph114_B2
zmp_ph114_B4
zmp_ph114_D8
zmp_ph114_E2

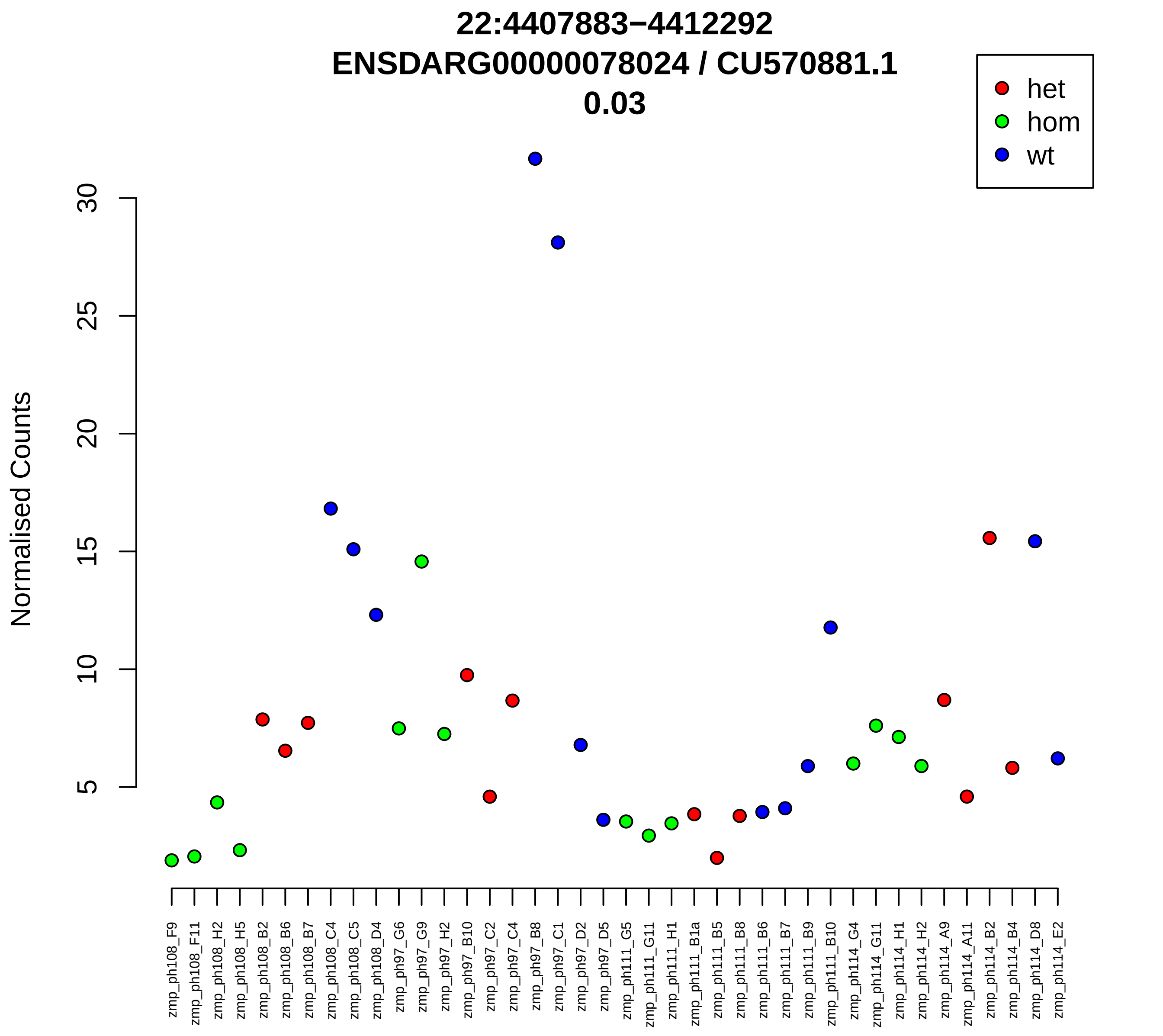
22:4407883−4412292
ENSDARG00000078024 / CU570881.1
0.03
het
hom
wt
Normalised Counts
5
10
15
20
25
30
zmp_ph108_F9
zmp_ph108_F11
zmp_ph108_H2
zmp_ph108_H5
zmp_ph108_B2
zmp_ph108_B6
zmp_ph108_B7
zmp_ph108_C4
zmp_ph108_C5
zmp_ph108_D4
zmp_ph97_G6
zmp_ph97_G9
zmp_ph97_H2
zmp_ph97_B10
zmp_ph97_C2
zmp_ph97_C4
zmp_ph97_B8
zmp_ph97_C1
zmp_ph97_D2
zmp_ph97_D5
zmp_ph111_G5
zmp_ph111_G11
zmp_ph111_H1
zmp_ph111_B1a
zmp_ph111_B5
zmp_ph111_B8
zmp_ph111_B6
zmp_ph111_B7
zmp_ph111_B9
zmp_ph111_B10
zmp_ph114_G4
zmp_ph114_G11
zmp_ph114_H1
zmp_ph114_H2
zmp_ph114_A9
zmp_ph114_A11
zmp_ph114_B2
zmp_ph114_B4
zmp_ph114_D8
zmp_ph114_E2

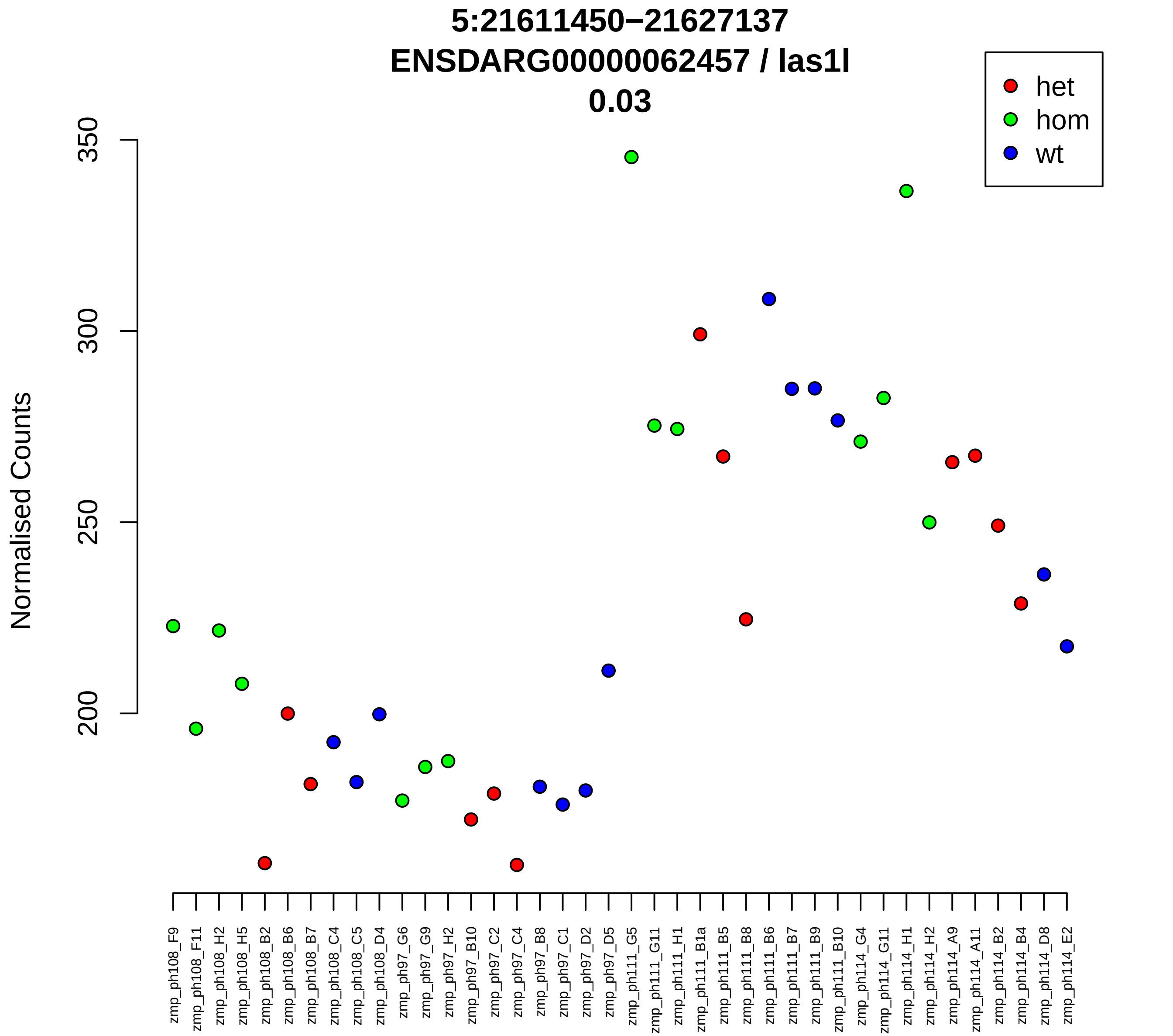

5:21611450−21627137
ENSDARG00000062457 / las1l
0.03
het
hom
wt
Normalised Counts
350
300
250
200
zmp_ph108_F9
zmp_ph108_F11
zmp_ph108_H2
zmp_ph108_H5
zmp_ph108_B2
zmp_ph108_B6
zmp_ph108_B7
zmp_ph108_C4
zmp_ph108_C5
zmp_ph108_D4
zmp_ph97_G6
zmp_ph97_G9
zmp_ph97_H2
zmp_ph97_B10
zmp_ph97_C2
zmp_ph97_C4
zmp_ph97_B8
zmp_ph97_C1
zmp_ph97_D2
zmp_ph97_D5
zmp_ph111_G5
zmp_ph111_G11
zmp_ph111_H1
zmp_ph111_B1a
zmp_ph111_B5
zmp_ph111_B8
zmp_ph111_B6
zmp_ph111_B7
zmp_ph111_B9
zmp_ph111_B10
zmp_ph114_G4
zmp_ph114_G11
zmp_ph114_H1
zmp_ph114_H2
zmp_ph114_A9
zmp_ph114_A11
zmp_ph114_B2
zmp_ph114_B4
zmp_ph114_D8
zmp_ph114_E2

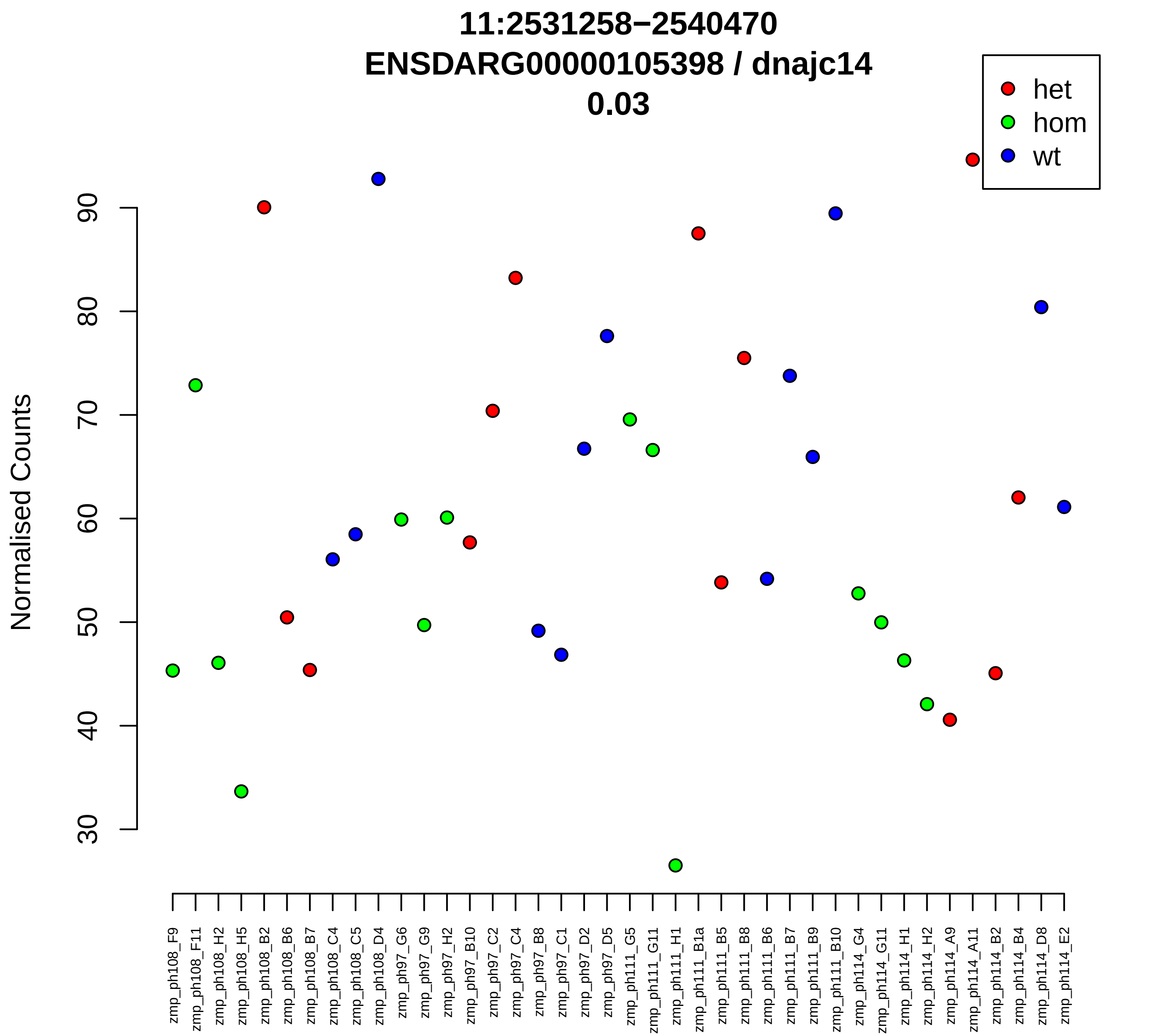

11:2531258−2540470
ENSDARG00000105398 / dnajc14
0.03
het
hom
wt
Normalised Counts
30
40
50
60
70
80
90
zmp_ph108_F9
zmp_ph108_F11
zmp_ph108_H2
zmp_ph108_H5
zmp_ph108_B2
zmp_ph108_B6
zmp_ph108_B7
zmp_ph108_C4
zmp_ph108_C5
zmp_ph108_D4
zmp_ph97_G6
zmp_ph97_G9
zmp_ph97_H2
zmp_ph97_B10
zmp_ph97_C2
zmp_ph97_C4
zmp_ph97_B8
zmp_ph97_C1
zmp_ph97_D2
zmp_ph97_D5
zmp_ph111_G5
zmp_ph111_G11
zmp_ph111_H1
zmp_ph111_B1a
zmp_ph111_B5
zmp_ph111_B8
zmp_ph111_B6
zmp_ph111_B7
zmp_ph111_B9
zmp_ph111_B10
zmp_ph114_G4
zmp_ph114_G11
zmp_ph114_H1
zmp_ph114_H2
zmp_ph114_A9
zmp_ph114_A11
zmp_ph114_B2
zmp_ph114_B4
zmp_ph114_D8
zmp_ph114_E2

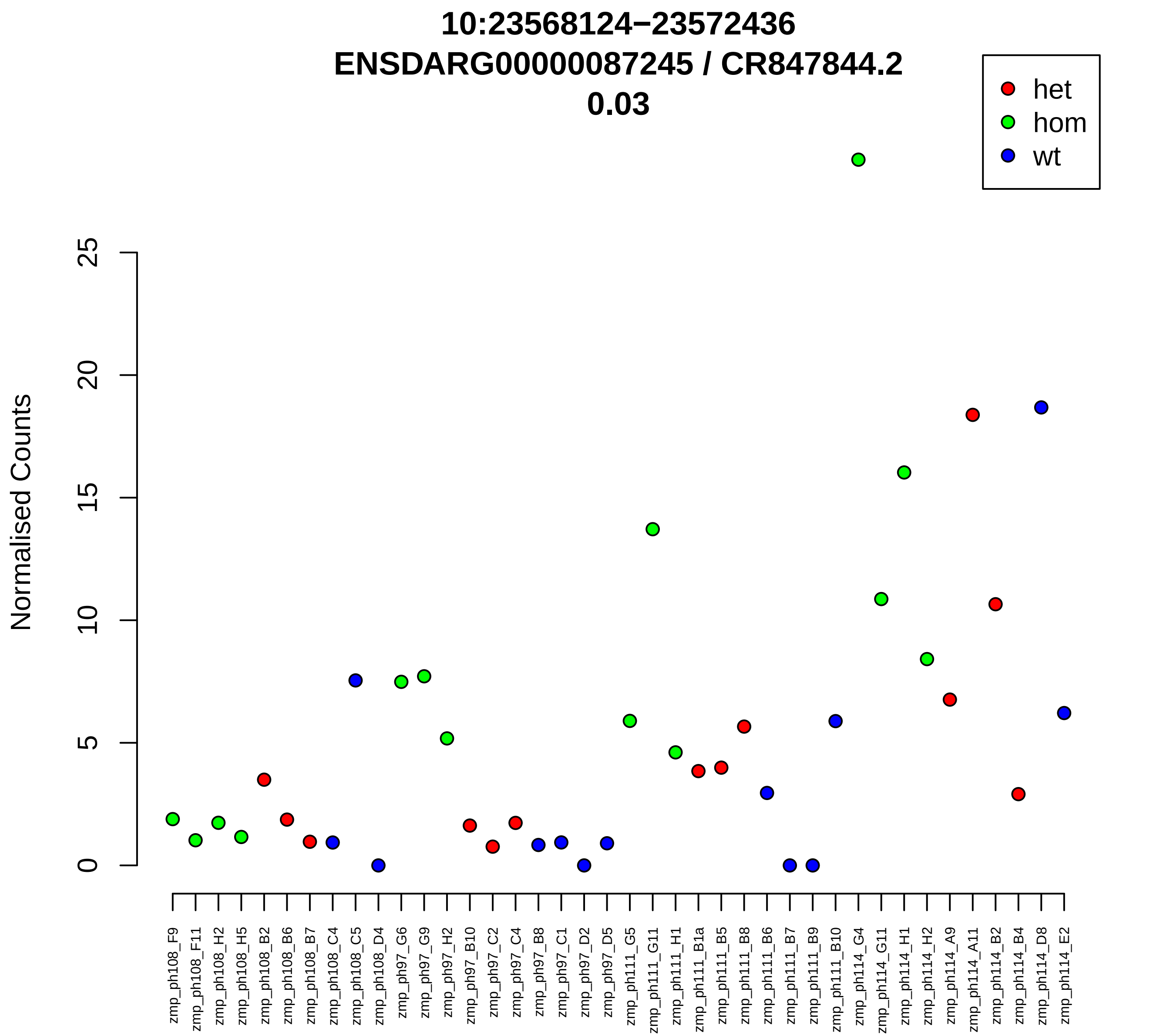
10:23568124−23572436
ENSDARG00000087245 / CR847844.2
0.03
het
hom
wt
Normalised Counts
0
5
10
15
20
25
zmp_ph108_F9
zmp_ph108_F11
zmp_ph108_H2
zmp_ph108_H5
zmp_ph108_B2
zmp_ph108_B6
zmp_ph108_B7
zmp_ph108_C4
zmp_ph108_C5
zmp_ph108_D4
zmp_ph97_G6
zmp_ph97_G9
zmp_ph97_H2
zmp_ph97_B10
zmp_ph97_C2
zmp_ph97_C4
zmp_ph97_B8
zmp_ph97_C1
zmp_ph97_D2
zmp_ph97_D5
zmp_ph111_G5
zmp_ph111_G11
zmp_ph111_H1
zmp_ph111_B1a
zmp_ph111_B5
zmp_ph111_B8
zmp_ph111_B6
zmp_ph111_B7
zmp_ph111_B9
zmp_ph111_B10
zmp_ph114_G4
zmp_ph114_G11
zmp_ph114_H1
zmp_ph114_H2
zmp_ph114_A9
zmp_ph114_A11
zmp_ph114_B2
zmp_ph114_B4
zmp_ph114_D8
zmp_ph114_E2

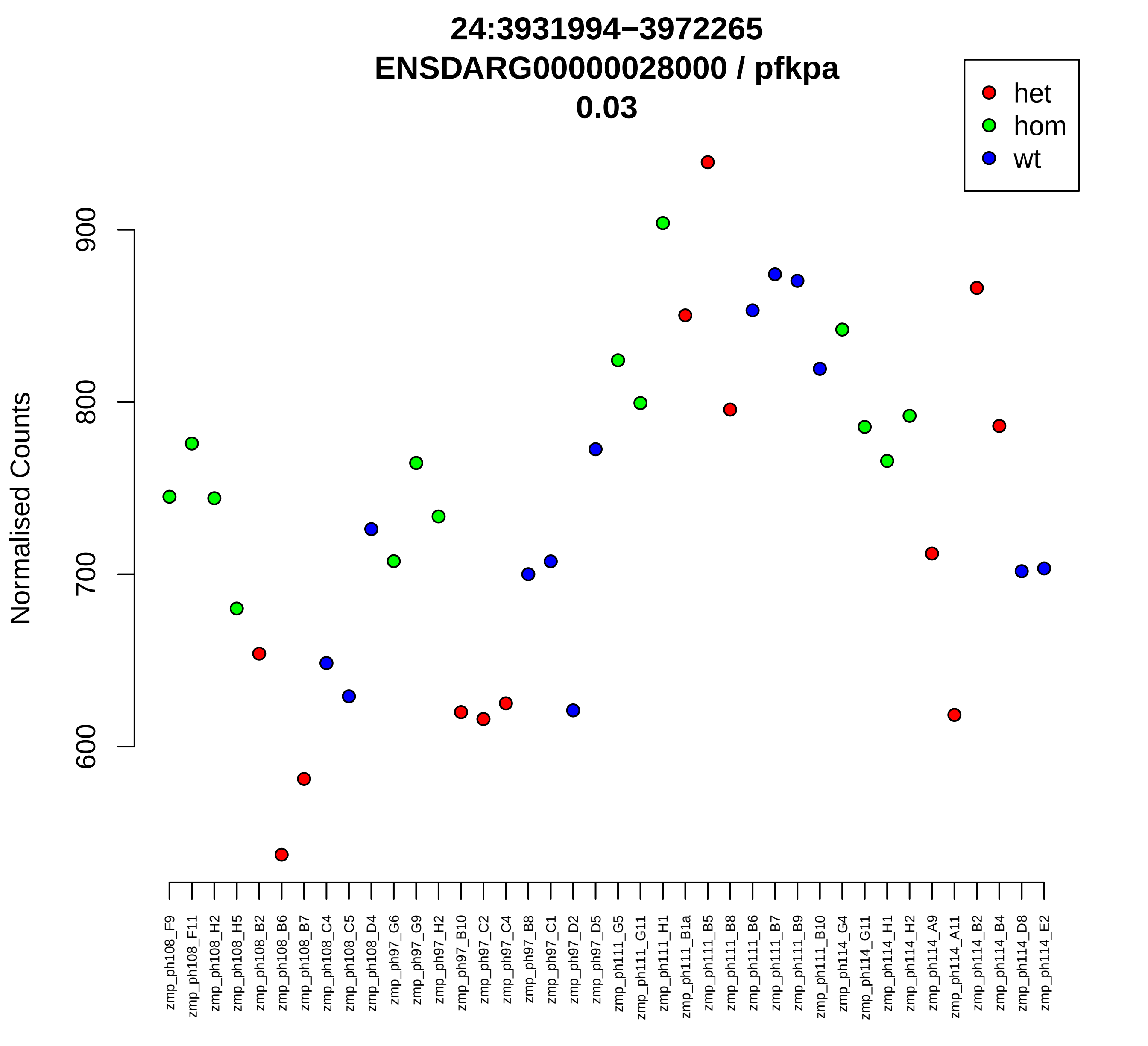

24:3931994−3972265
ENSDARG00000028000 / pfkpa
0.03
het
hom
wt
Normalised Counts
600
700
800
900
zmp_ph108_F9
zmp_ph108_F11
zmp_ph108_H2
zmp_ph108_H5
zmp_ph108_B2
zmp_ph108_B6
zmp_ph108_B7
zmp_ph108_C4
zmp_ph108_C5
zmp_ph108_D4
zmp_ph97_G6
zmp_ph97_G9
zmp_ph97_H2
zmp_ph97_B10
zmp_ph97_C2
zmp_ph97_C4
zmp_ph97_B8
zmp_ph97_C1
zmp_ph97_D2
zmp_ph97_D5
zmp_ph111_G5
zmp_ph111_G11
zmp_ph111_H1
zmp_ph111_B1a
zmp_ph111_B5
zmp_ph111_B8
zmp_ph111_B6
zmp_ph111_B7
zmp_ph111_B9
zmp_ph111_B10
zmp_ph114_G4
zmp_ph114_G11
zmp_ph114_H1
zmp_ph114_H2
zmp_ph114_A9
zmp_ph114_A11
zmp_ph114_B2
zmp_ph114_B4
zmp_ph114_D8
zmp_ph114_E2

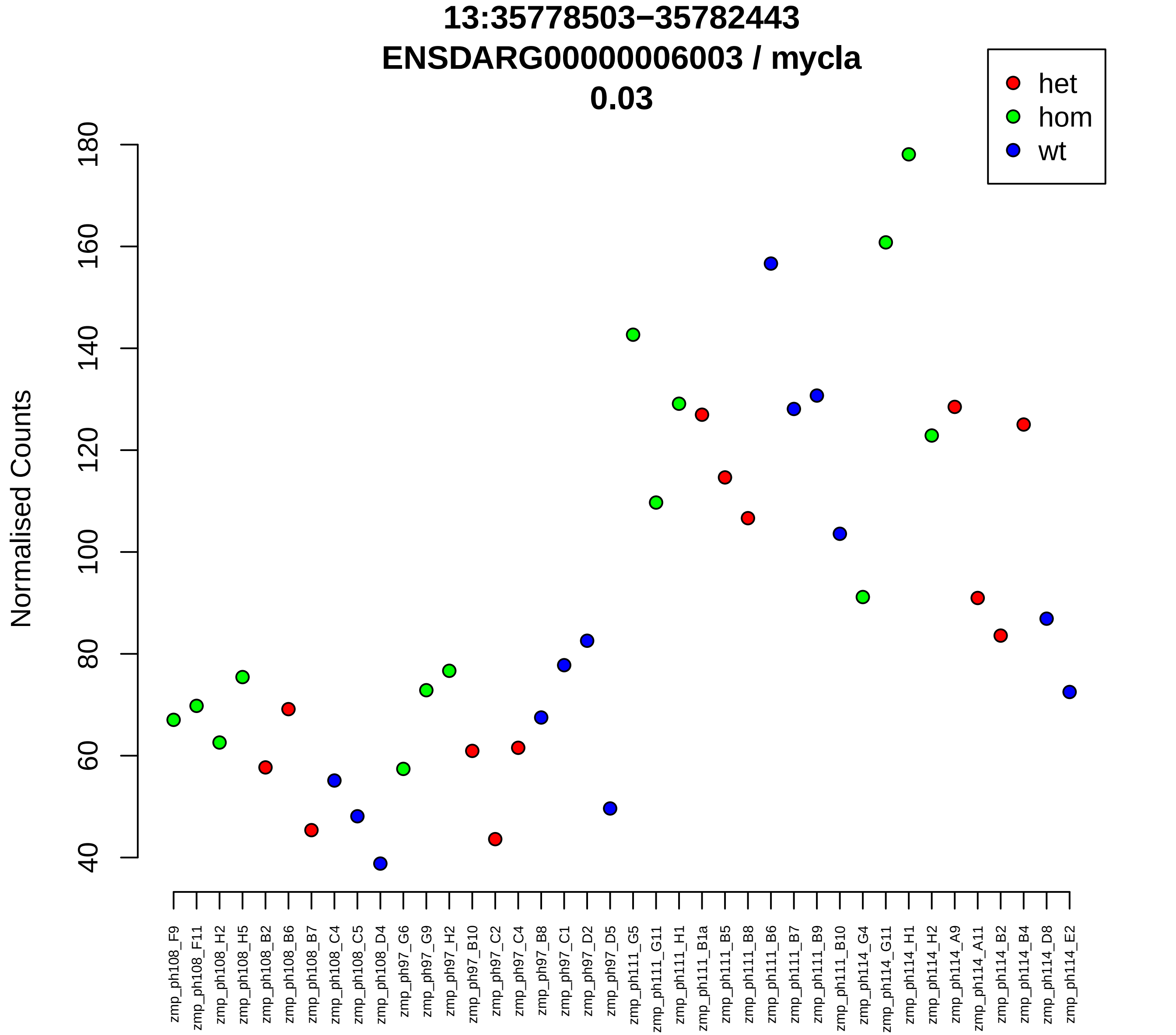

13:35778503−35782443
ENSDARG00000006003 / mycla
0.03
het
hom
wt
Normalised Counts
40
60
80
100
120
140
160
180
zmp_ph108_F9
zmp_ph108_F11
zmp_ph108_H2
zmp_ph108_H5
zmp_ph108_B2
zmp_ph108_B6
zmp_ph108_B7
zmp_ph108_C4
zmp_ph108_C5
zmp_ph108_D4
zmp_ph97_G6
zmp_ph97_G9
zmp_ph97_H2
zmp_ph97_B10
zmp_ph97_C2
zmp_ph97_C4
zmp_ph97_B8
zmp_ph97_C1
zmp_ph97_D2
zmp_ph97_D5
zmp_ph111_G5
zmp_ph111_G11
zmp_ph111_H1
zmp_ph111_B1a
zmp_ph111_B5
zmp_ph111_B8
zmp_ph111_B6
zmp_ph111_B7
zmp_ph111_B9
zmp_ph111_B10
zmp_ph114_G4
zmp_ph114_G11
zmp_ph114_H1
zmp_ph114_H2
zmp_ph114_A9
zmp_ph114_A11
zmp_ph114_B2
zmp_ph114_B4
zmp_ph114_D8
zmp_ph114_E2

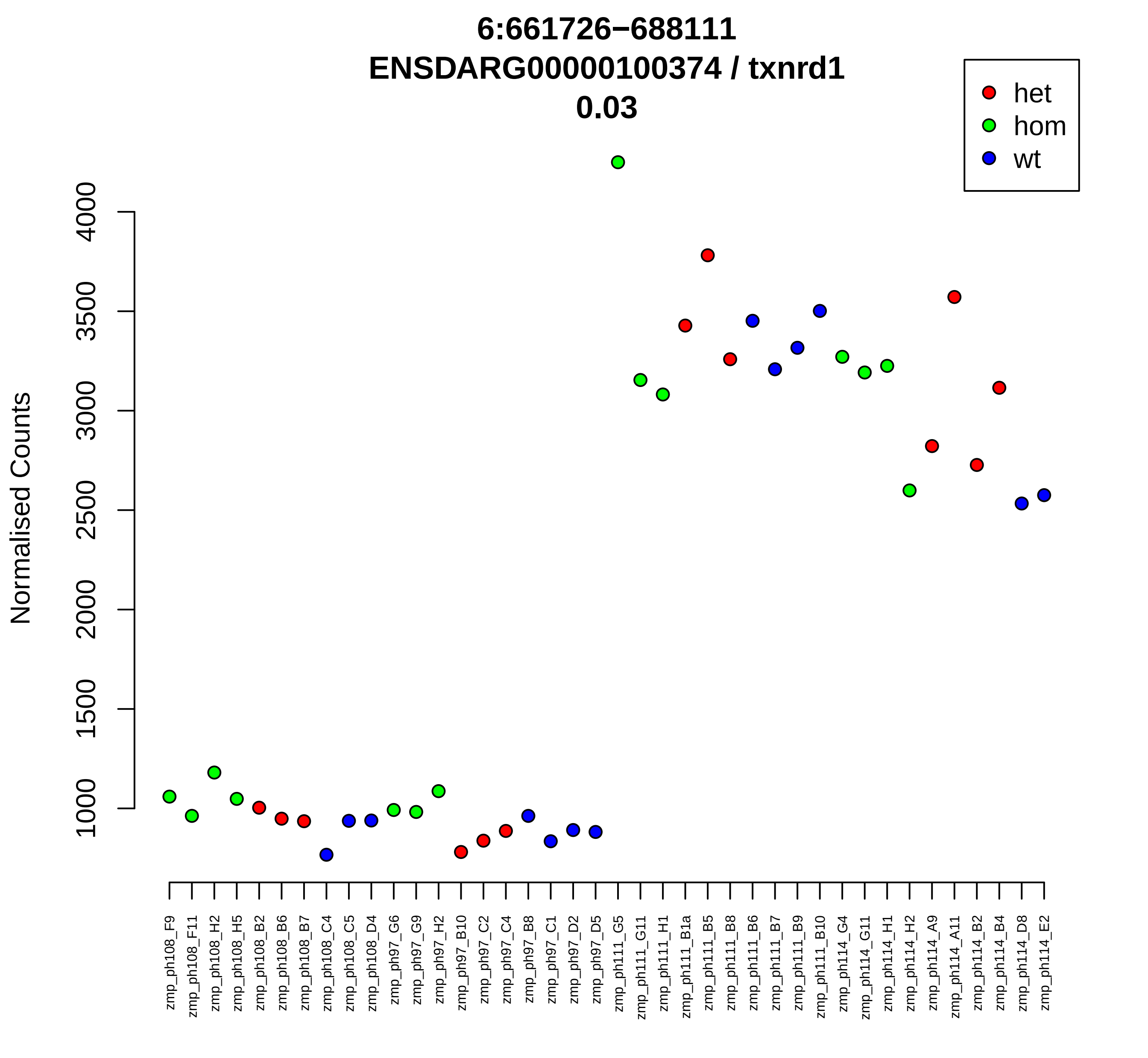
6:661726−688111
ENSDARG00000100374 / txnrd1
0.03
het
hom
wt
Normalised Counts
1000
1500
2000
2500
3000
3500
4000
zmp_ph108_F9
zmp_ph108_F11
zmp_ph108_H2
zmp_ph108_H5
zmp_ph108_B2
zmp_ph108_B6
zmp_ph108_B7
zmp_ph108_C4
zmp_ph108_C5
zmp_ph108_D4
zmp_ph97_G6
zmp_ph97_G9
zmp_ph97_H2
zmp_ph97_B10
zmp_ph97_C2
zmp_ph97_C4
zmp_ph97_B8
zmp_ph97_C1
zmp_ph97_D2
zmp_ph97_D5
zmp_ph111_G5
zmp_ph111_G11
zmp_ph111_H1
zmp_ph111_B1a
zmp_ph111_B5
zmp_ph111_B8
zmp_ph111_B6
zmp_ph111_B7
zmp_ph111_B9
zmp_ph111_B10
zmp_ph114_G4
zmp_ph114_G11
zmp_ph114_H1
zmp_ph114_H2
zmp_ph114_A9
zmp_ph114_A11
zmp_ph114_B2
zmp_ph114_B4
zmp_ph114_D8
zmp_ph114_E2

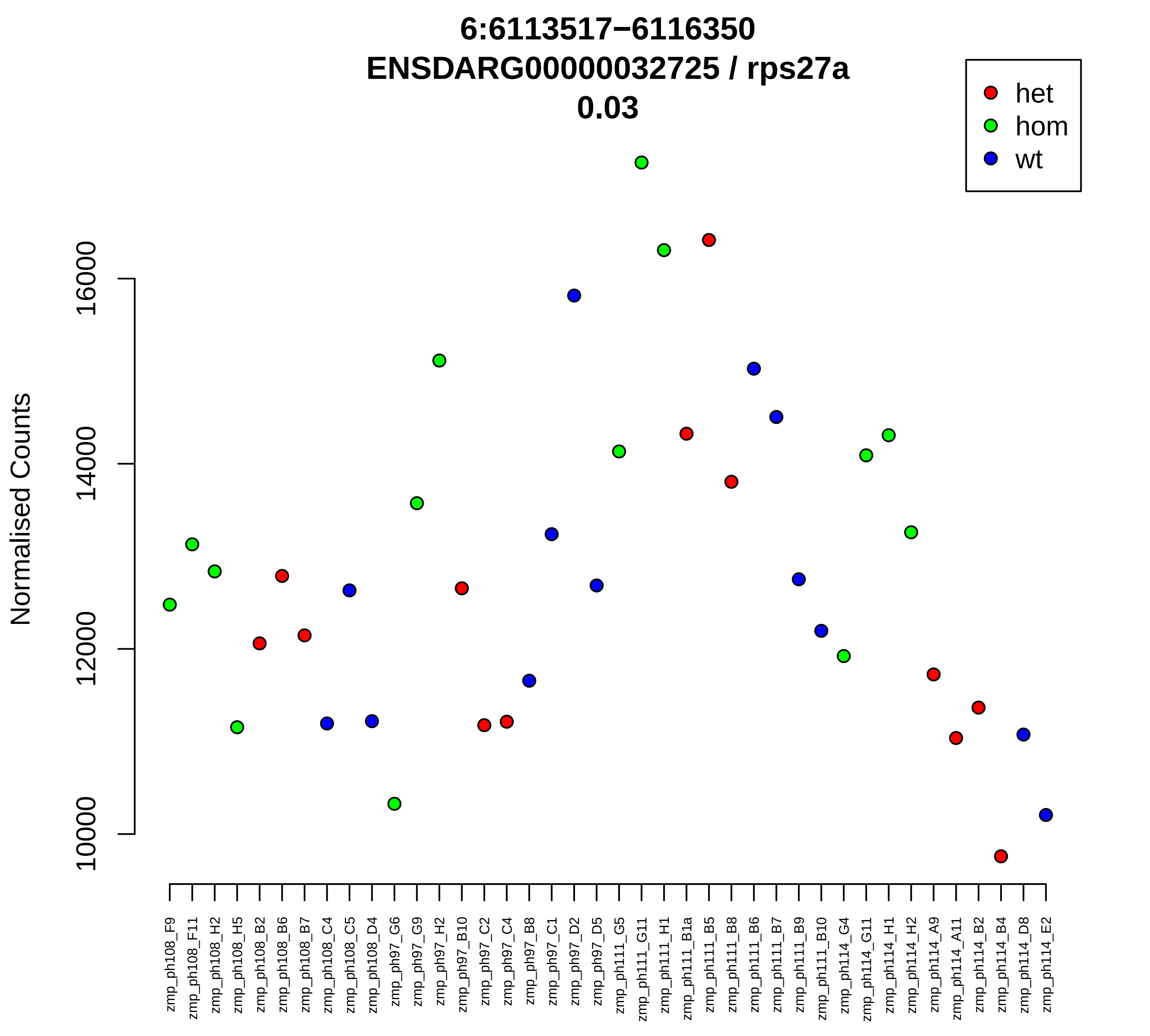
6:6113517−6116350
ENSDARG00000032725 / rps27a
0.03
het
hom
wt
Normalised Counts
10000
12000
14000
16000
zmp_ph108_F9
zmp_ph108_F11
zmp_ph108_H2
zmp_ph108_H5
zmp_ph108_B2
zmp_ph108_B6
zmp_ph108_B7
zmp_ph108_C4
zmp_ph108_C5
zmp_ph108_D4
zmp_ph97_G6
zmp_ph97_G9
zmp_ph97_H2
zmp_ph97_B10
zmp_ph97_C2
zmp_ph97_C4
zmp_ph97_B8
zmp_ph97_C1
zmp_ph97_D2
zmp_ph97_D5
zmp_ph111_G5
zmp_ph111_G11
zmp_ph111_H1
zmp_ph111_B1a
zmp_ph111_B5
zmp_ph111_B8
zmp_ph111_B6
zmp_ph111_B7
zmp_ph111_B9
zmp_ph111_B10
zmp_ph114_G4
zmp_ph114_G11
zmp_ph114_H1
zmp_ph114_H2
zmp_ph114_A9
zmp_ph114_A11
zmp_ph114_B2
zmp_ph114_B4
zmp_ph114_D8
zmp_ph114_E2

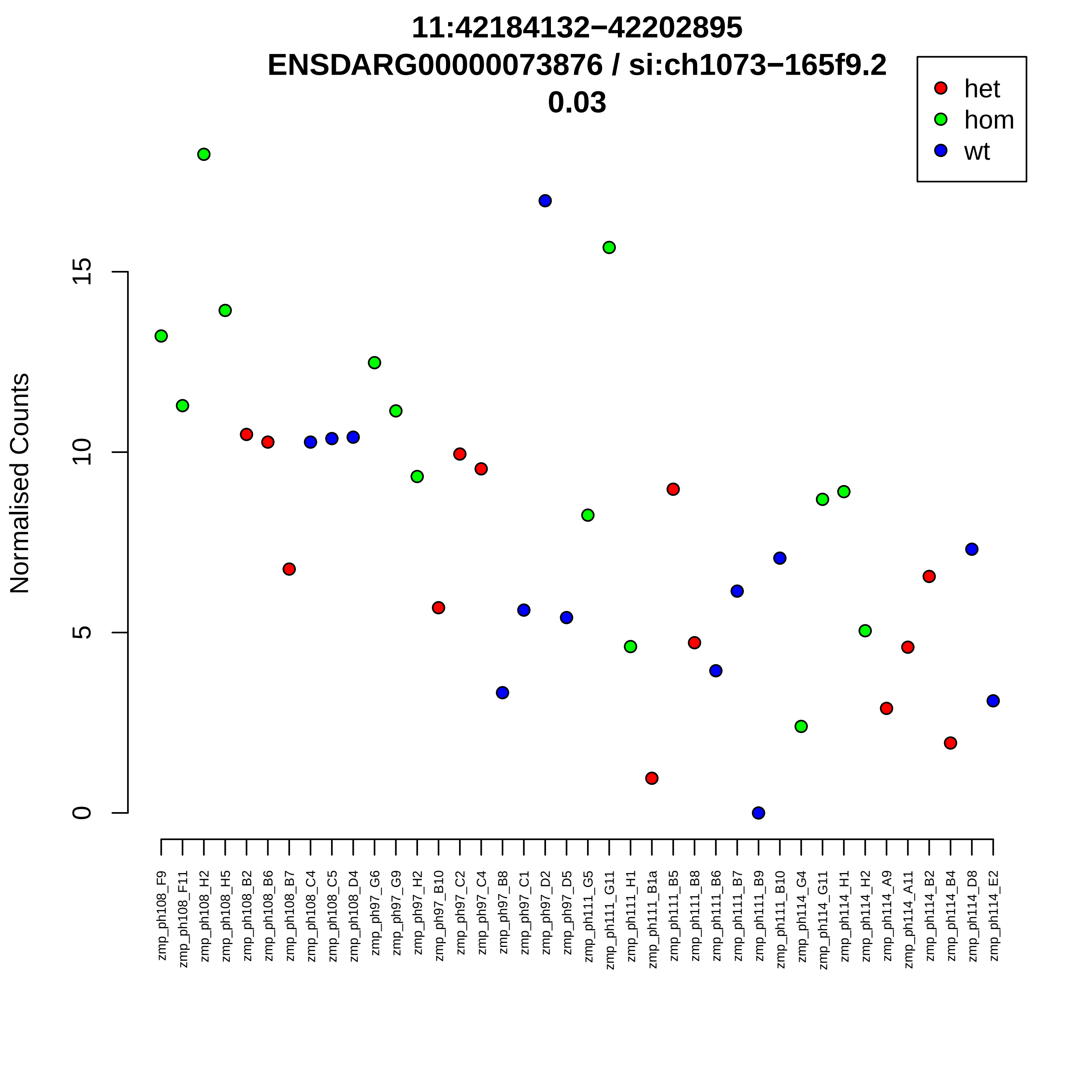
11:42184132−42202895
ENSDARG00000073876 / si:ch1073−165f9.2
0.03
het
hom
wt
Normalised Counts
0
5
10
15
zmp_ph108_F9
zmp_ph108_F11
zmp_ph108_H2
zmp_ph108_H5
zmp_ph108_B2
zmp_ph108_B6
zmp_ph108_B7
zmp_ph108_C4
zmp_ph108_C5
zmp_ph108_D4
zmp_ph97_G6
zmp_ph97_G9
zmp_ph97_H2
zmp_ph97_B10
zmp_ph97_C2
zmp_ph97_C4
zmp_ph97_B8
zmp_ph97_C1
zmp_ph97_D2
zmp_ph97_D5
zmp_ph111_G5
zmp_ph111_G11
zmp_ph111_H1
zmp_ph111_B1a
zmp_ph111_B5
zmp_ph111_B8
zmp_ph111_B6
zmp_ph111_B7
zmp_ph111_B9
zmp_ph111_B10
zmp_ph114_G4
zmp_ph114_G11
zmp_ph114_H1
zmp_ph114_H2
zmp_ph114_A9
zmp_ph114_A11
zmp_ph114_B2
zmp_ph114_B4
zmp_ph114_D8
zmp_ph114_E2

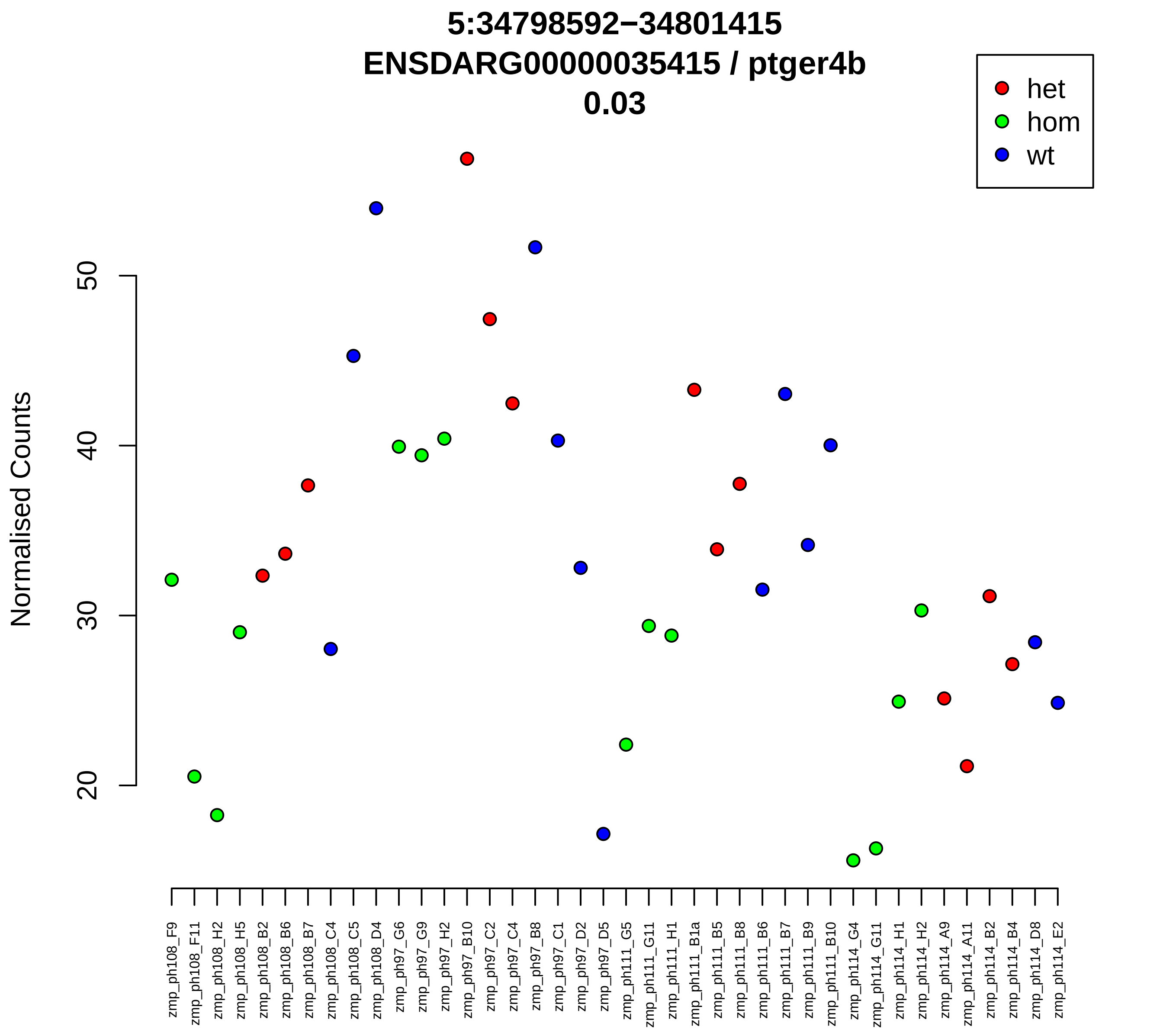
5:34798592−34801415
ENSDARG00000035415 / ptger4b
0.03
het
hom
wt
Normalised Counts
20
30
40
50
zmp_ph108_F9
zmp_ph108_F11
zmp_ph108_H2
zmp_ph108_H5
zmp_ph108_B2
zmp_ph108_B6
zmp_ph108_B7
zmp_ph108_C4
zmp_ph108_C5
zmp_ph108_D4
zmp_ph97_G6
zmp_ph97_G9
zmp_ph97_H2
zmp_ph97_B10
zmp_ph97_C2
zmp_ph97_C4
zmp_ph97_B8
zmp_ph97_C1
zmp_ph97_D2
zmp_ph97_D5
zmp_ph111_G5
zmp_ph111_G11
zmp_ph111_H1
zmp_ph111_B1a
zmp_ph111_B5
zmp_ph111_B8
zmp_ph111_B6
zmp_ph111_B7
zmp_ph111_B9
zmp_ph111_B10
zmp_ph114_G4
zmp_ph114_G11
zmp_ph114_H1
zmp_ph114_H2
zmp_ph114_A9
zmp_ph114_A11
zmp_ph114_B2
zmp_ph114_B4
zmp_ph114_D8
zmp_ph114_E2

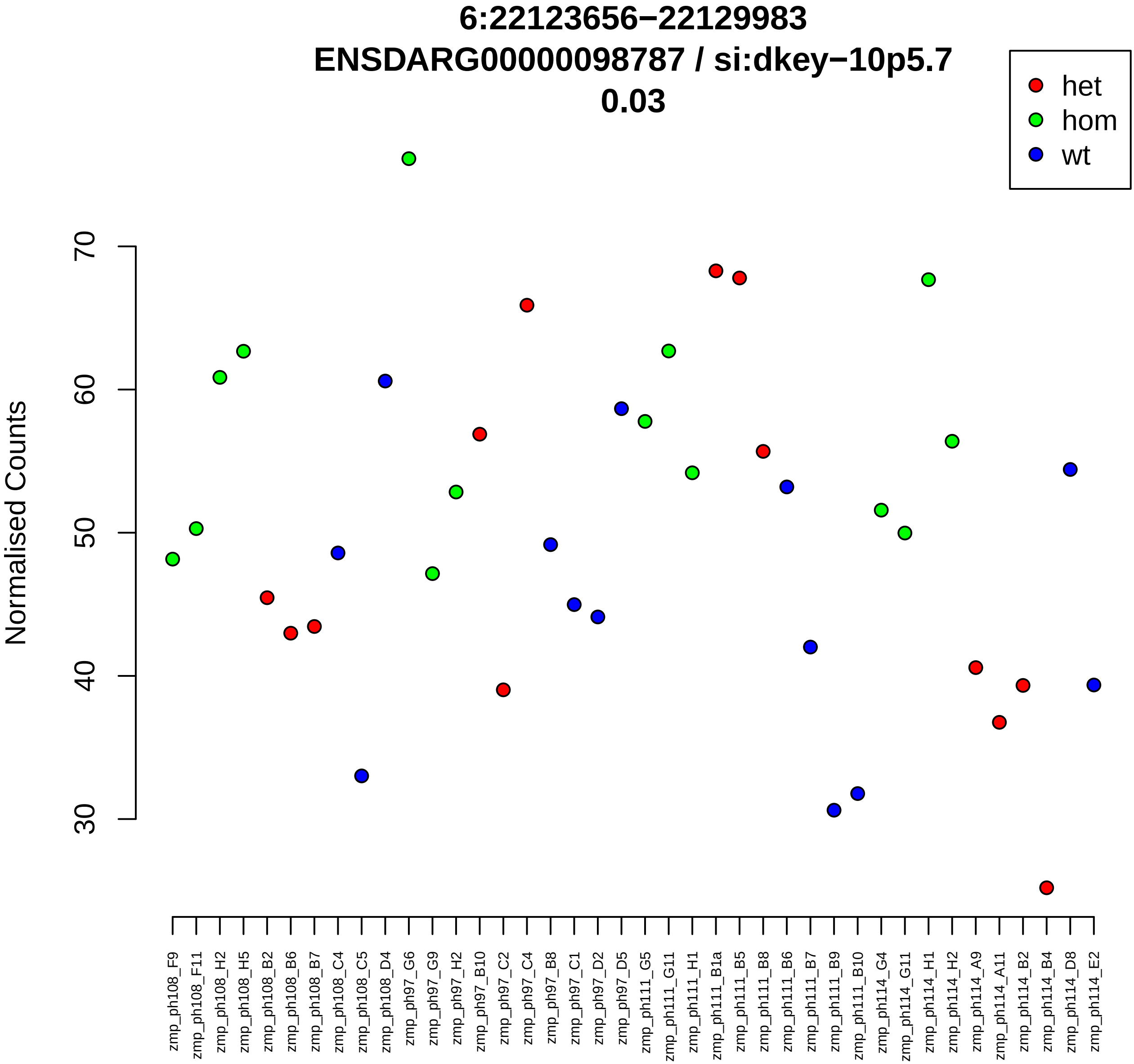
6:22123656−22129983
ENSDARG00000098787 / si:dkey−10p5.7
0.03
het
hom
wt
Normalised Counts
30
40
50
60
70
zmp_ph108_F9
zmp_ph108_F11
zmp_ph108_H2
zmp_ph108_H5
zmp_ph108_B2
zmp_ph108_B6
zmp_ph108_B7
zmp_ph108_C4
zmp_ph108_C5
zmp_ph108_D4
zmp_ph97_G6
zmp_ph97_G9
zmp_ph97_H2
zmp_ph97_B10
zmp_ph97_C2
zmp_ph97_C4
zmp_ph97_B8
zmp_ph97_C1
zmp_ph97_D2
zmp_ph97_D5
zmp_ph111_G5
zmp_ph111_G11
zmp_ph111_H1
zmp_ph111_B1a
zmp_ph111_B5
zmp_ph111_B8
zmp_ph111_B6
zmp_ph111_B7
zmp_ph111_B9
zmp_ph111_B10
zmp_ph114_G4
zmp_ph114_G11
zmp_ph114_H1
zmp_ph114_H2
zmp_ph114_A9
zmp_ph114_A11
zmp_ph114_B2
zmp_ph114_B4
zmp_ph114_D8
zmp_ph114_E2

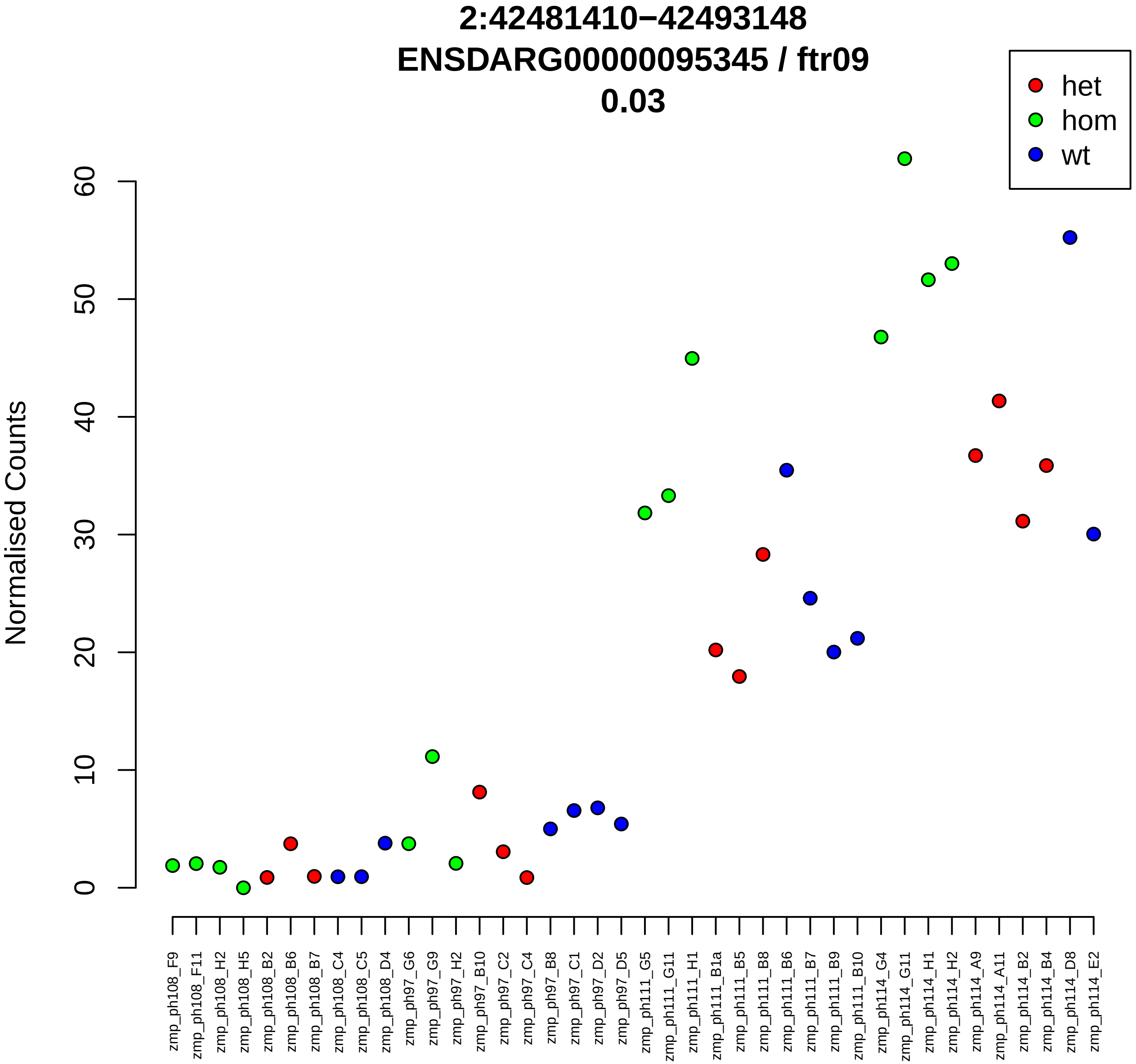

2:42481410−42493148
ENSDARG00000095345 / ftr09
0.03
het
hom
wt
Normalised Counts
0
10
20
30
40
50
60
zmp_ph108_F9
zmp_ph108_F11
zmp_ph108_H2
zmp_ph108_H5
zmp_ph108_B2
zmp_ph108_B6
zmp_ph108_B7
zmp_ph108_C4
zmp_ph108_C5
zmp_ph108_D4
zmp_ph97_G6
zmp_ph97_G9
zmp_ph97_H2
zmp_ph97_B10
zmp_ph97_C2
zmp_ph97_C4
zmp_ph97_B8
zmp_ph97_C1
zmp_ph97_D2
zmp_ph97_D5
zmp_ph111_G5
zmp_ph111_G11
zmp_ph111_H1
zmp_ph111_B1a
zmp_ph111_B5
zmp_ph111_B8
zmp_ph111_B6
zmp_ph111_B7
zmp_ph111_B9
zmp_ph111_B10
zmp_ph114_G4
zmp_ph114_G11
zmp_ph114_H1
zmp_ph114_H2
zmp_ph114_A9
zmp_ph114_A11
zmp_ph114_B2
zmp_ph114_B4
zmp_ph114_D8
zmp_ph114_E2

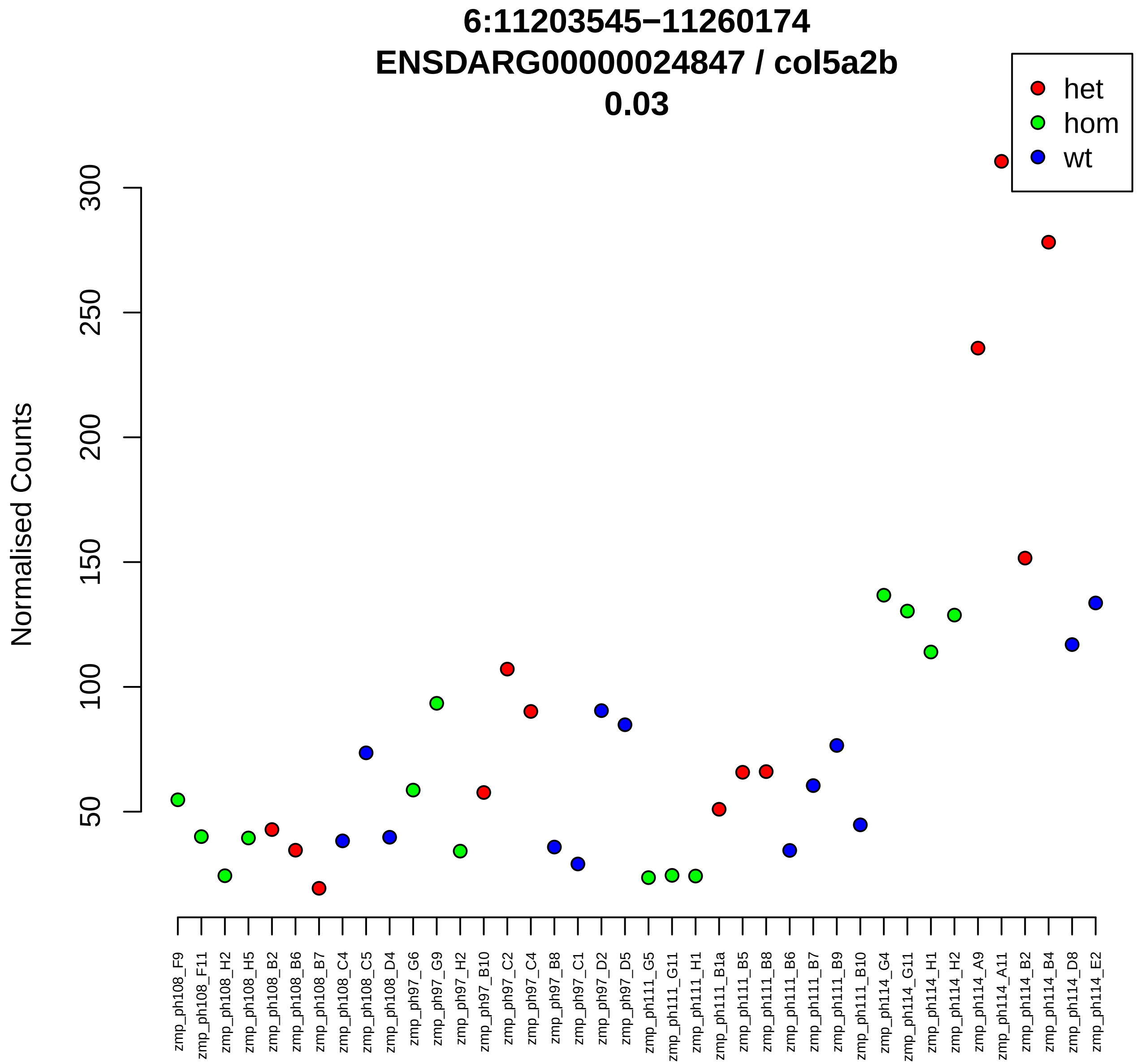
6:11203545−11260174
ENSDARG00000024847 / col5a2b
0.03
het
hom
wt
Normalised Counts
50
100
150
200
250
300
zmp_ph108_F9
zmp_ph108_F11
zmp_ph108_H2
zmp_ph108_H5
zmp_ph108_B2
zmp_ph108_B6
zmp_ph108_B7
zmp_ph108_C4
zmp_ph108_C5
zmp_ph108_D4
zmp_ph97_G6
zmp_ph97_G9
zmp_ph97_H2
zmp_ph97_B10
zmp_ph97_C2
zmp_ph97_C4
zmp_ph97_B8
zmp_ph97_C1
zmp_ph97_D2
zmp_ph97_D5
zmp_ph111_G5
zmp_ph111_G11
zmp_ph111_H1
zmp_ph111_B1a
zmp_ph111_B5
zmp_ph111_B8
zmp_ph111_B6
zmp_ph111_B7
zmp_ph111_B9
zmp_ph111_B10
zmp_ph114_G4
zmp_ph114_G11
zmp_ph114_H1
zmp_ph114_H2
zmp_ph114_A9
zmp_ph114_A11
zmp_ph114_B2
zmp_ph114_B4
zmp_ph114_D8
zmp_ph114_E2

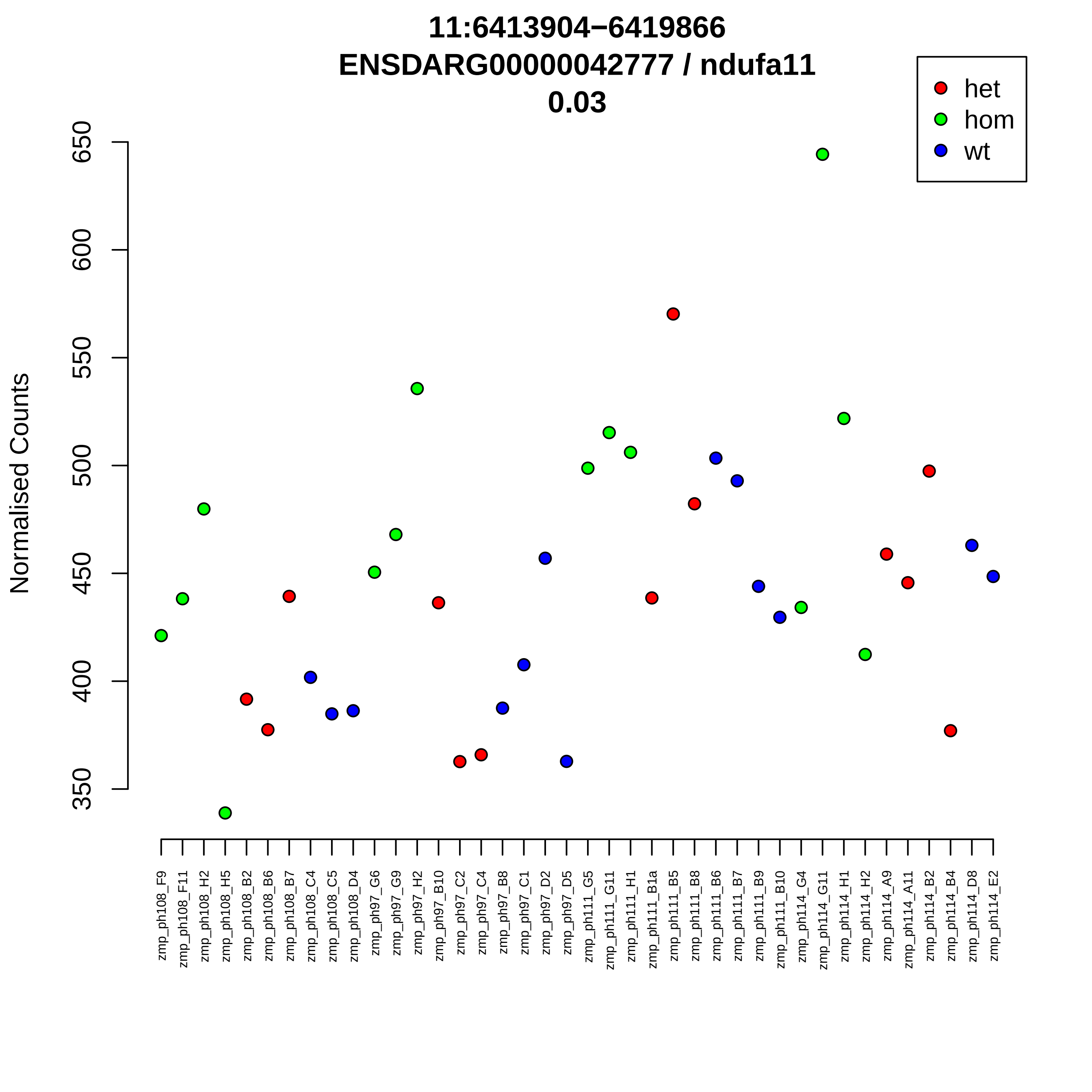

11:6413904−6419866
ENSDARG00000042777 / ndufa11
0.03
het
hom
wt
Normalised Counts
350
400
450
500
550
600
650
zmp_ph108_F9
zmp_ph108_F11
zmp_ph108_H2
zmp_ph108_H5
zmp_ph108_B2
zmp_ph108_B6
zmp_ph108_B7
zmp_ph108_C4
zmp_ph108_C5
zmp_ph108_D4
zmp_ph97_G6
zmp_ph97_G9
zmp_ph97_H2
zmp_ph97_B10
zmp_ph97_C2
zmp_ph97_C4
zmp_ph97_B8
zmp_ph97_C1
zmp_ph97_D2
zmp_ph97_D5
zmp_ph111_G5
zmp_ph111_G11
zmp_ph111_H1
zmp_ph111_B1a
zmp_ph111_B5
zmp_ph111_B8
zmp_ph111_B6
zmp_ph111_B7
zmp_ph111_B9
zmp_ph111_B10
zmp_ph114_G4
zmp_ph114_G11
zmp_ph114_H1
zmp_ph114_H2
zmp_ph114_A9
zmp_ph114_A11
zmp_ph114_B2
zmp_ph114_B4
zmp_ph114_D8
zmp_ph114_E2

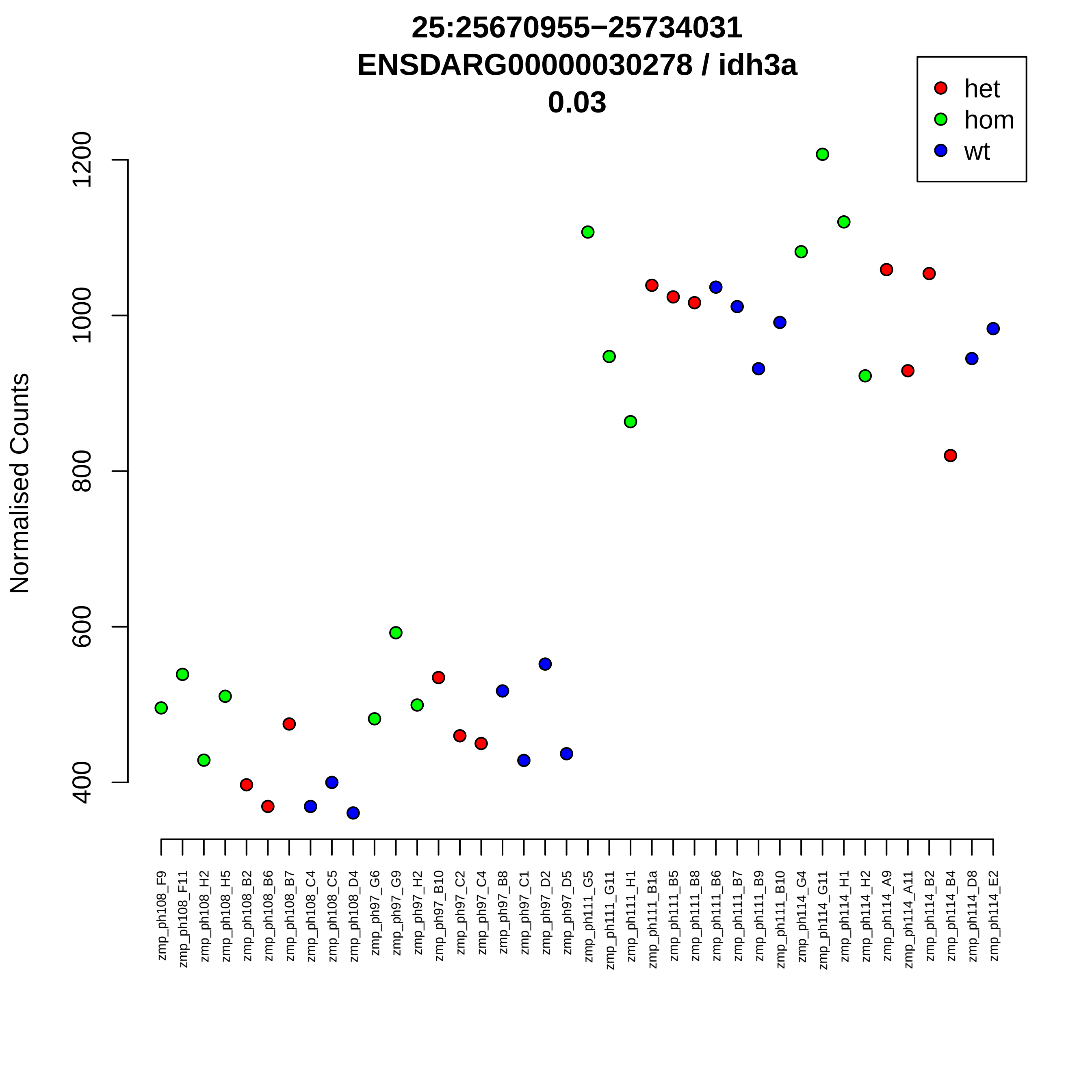

25:25670955−25734031
ENSDARG00000030278 / idh3a
0.03
het
hom
wt
Normalised Counts
400
600
800
1000
1200
zmp_ph108_F9
zmp_ph108_F11
zmp_ph108_H2
zmp_ph108_H5
zmp_ph108_B2
zmp_ph108_B6
zmp_ph108_B7
zmp_ph108_C4
zmp_ph108_C5
zmp_ph108_D4
zmp_ph97_G6
zmp_ph97_G9
zmp_ph97_H2
zmp_ph97_B10
zmp_ph97_C2
zmp_ph97_C4
zmp_ph97_B8
zmp_ph97_C1
zmp_ph97_D2
zmp_ph97_D5
zmp_ph111_G5
zmp_ph111_G11
zmp_ph111_H1
zmp_ph111_B1a
zmp_ph111_B5
zmp_ph111_B8
zmp_ph111_B6
zmp_ph111_B7
zmp_ph111_B9
zmp_ph111_B10
zmp_ph114_G4
zmp_ph114_G11
zmp_ph114_H1
zmp_ph114_H2
zmp_ph114_A9
zmp_ph114_A11
zmp_ph114_B2
zmp_ph114_B4
zmp_ph114_D8
zmp_ph114_E2

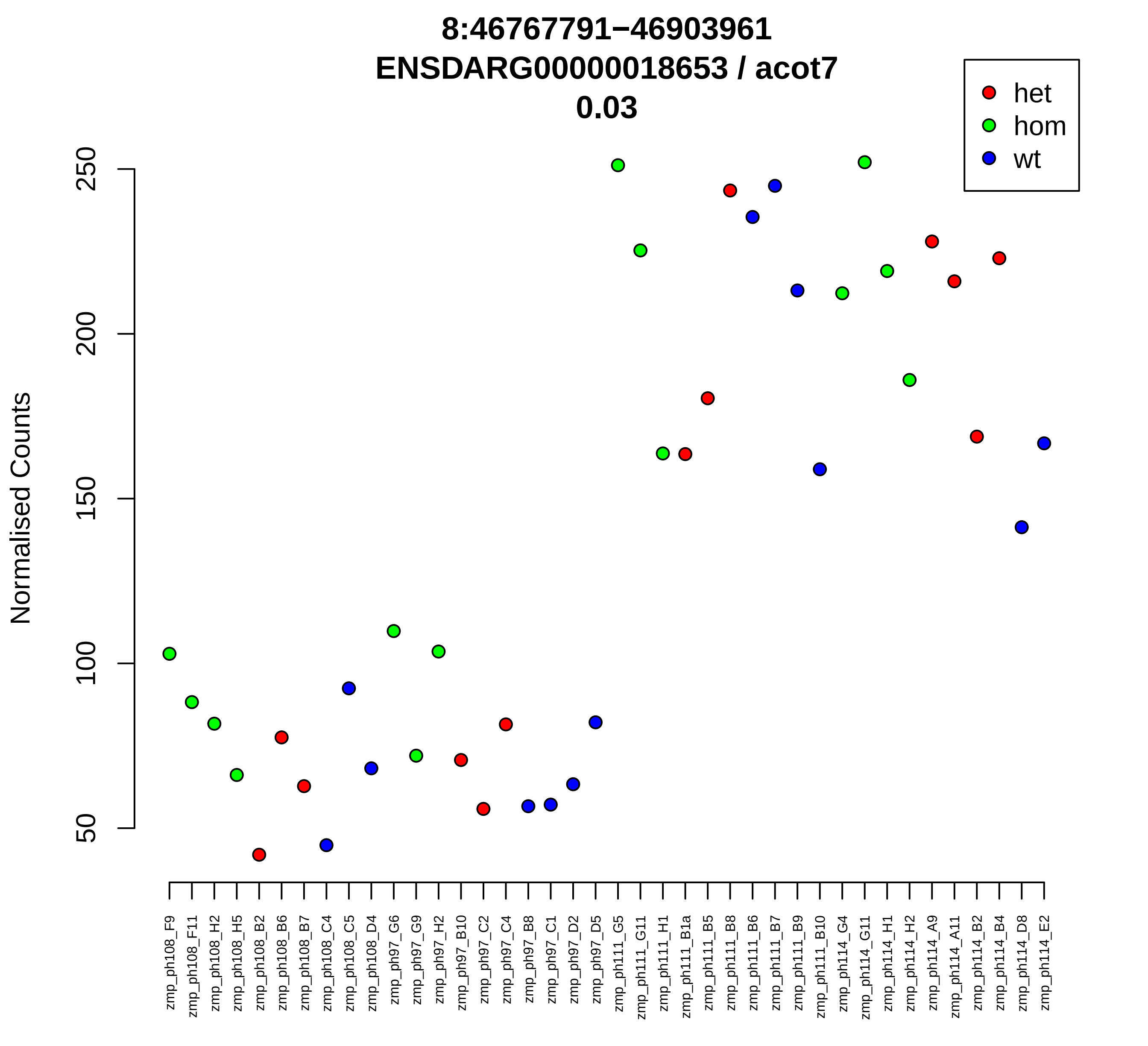

8:46767791−46903961
ENSDARG00000018653 / acot7
0.03
Normalised Counts
250
200
150
100
50
het
hom
wt
zmp_ph108_F9
zmp_ph108_F11
zmp_ph108_H2
zmp_ph108_H5
zmp_ph108_B2
zmp_ph108_B6
zmp_ph108_B7
zmp_ph108_C4
zmp_ph108_C5
zmp_ph108_D4
zmp_ph97_G6
zmp_ph97_G9
zmp_ph97_H2
zmp_ph97_B10
zmp_ph97_C2
zmp_ph97_C4
zmp_ph97_B8
zmp_ph97_C1
zmp_ph97_D2
zmp_ph97_D5
zmp_ph111_G5
zmp_ph111_G11
zmp_ph111_H1
zmp_ph111_B1a
zmp_ph111_B5
zmp_ph111_B8
zmp_ph111_B6
zmp_ph111_B7
zmp_ph111_B9
zmp_ph111_B10
zmp_ph114_G4
zmp_ph114_G11
zmp_ph114_H1
zmp_ph114_H2
zmp_ph114_A9
zmp_ph114_A11
zmp_ph114_B2
zmp_ph114_B4
zmp_ph114_D8
zmp_ph114_E2

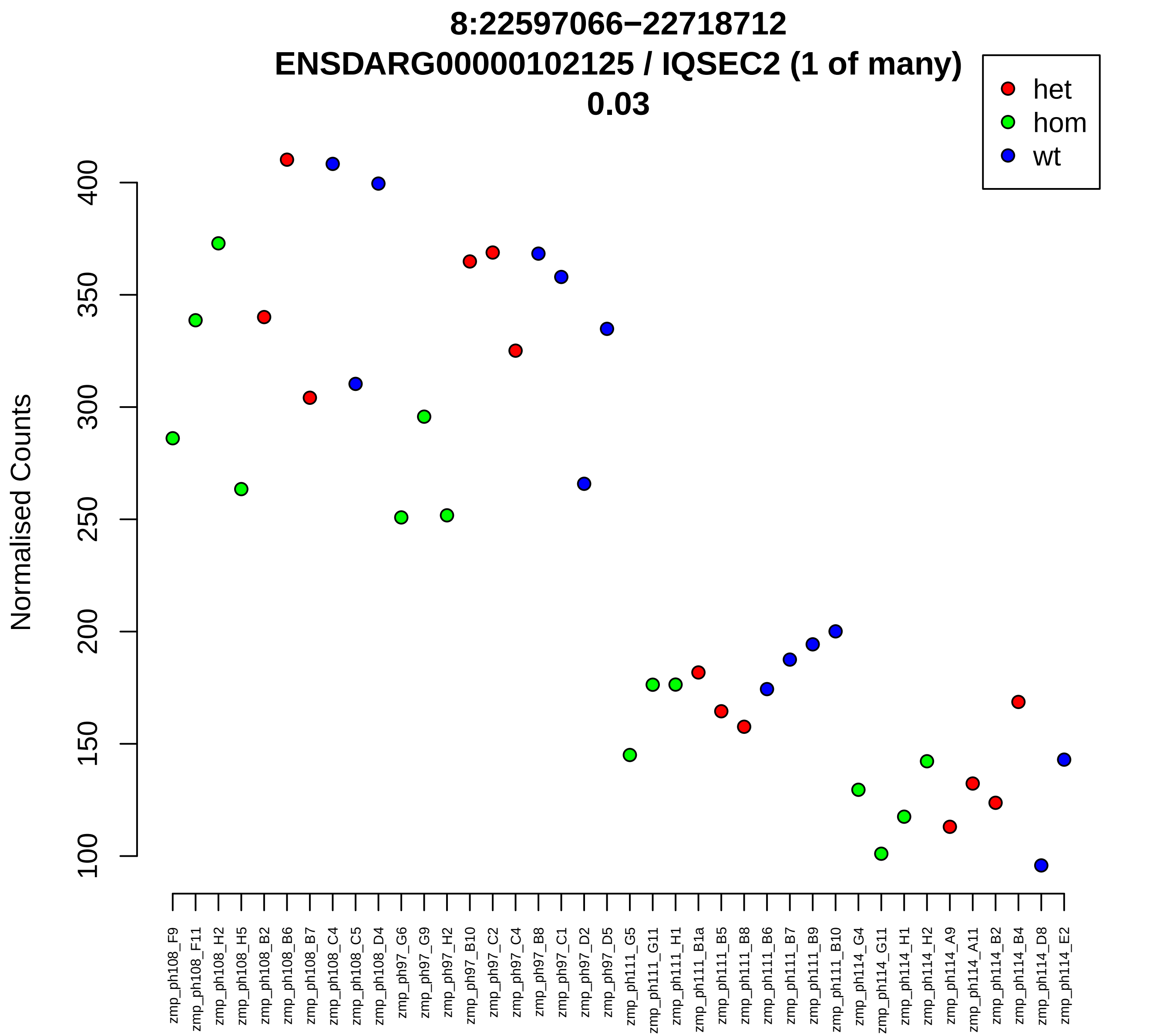
8:22597066−22718712
ENSDARG00000102125 / IQSEC2 (1 of many)
0.03
het
hom
wt
Normalised Counts
100
150
200
250
300
350
400
zmp_ph108_F9
zmp_ph108_F11
zmp_ph108_H2
zmp_ph108_H5
zmp_ph108_B2
zmp_ph108_B6
zmp_ph108_B7
zmp_ph108_C4
zmp_ph108_C5
zmp_ph108_D4
zmp_ph97_G6
zmp_ph97_G9
zmp_ph97_H2
zmp_ph97_B10
zmp_ph97_C2
zmp_ph97_C4
zmp_ph97_B8
zmp_ph97_C1
zmp_ph97_D2
zmp_ph97_D5
zmp_ph111_G5
zmp_ph111_G11
zmp_ph111_H1
zmp_ph111_B1a
zmp_ph111_B5
zmp_ph111_B8
zmp_ph111_B6
zmp_ph111_B7
zmp_ph111_B9
zmp_ph111_B10
zmp_ph114_G4
zmp_ph114_G11
zmp_ph114_H1
zmp_ph114_H2
zmp_ph114_A9
zmp_ph114_A11
zmp_ph114_B2
zmp_ph114_B4
zmp_ph114_D8
zmp_ph114_E2

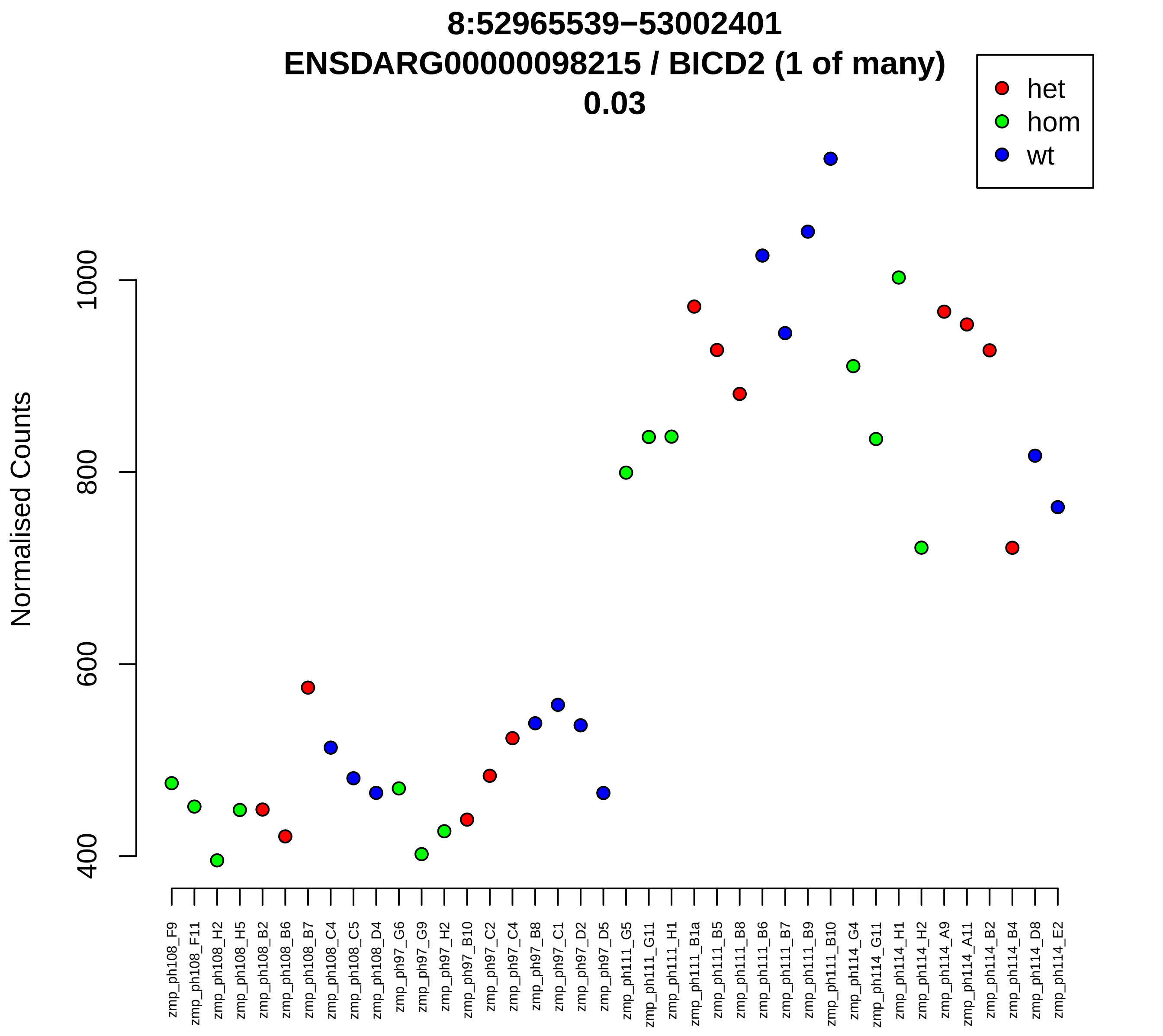

8:52965539−53002401
ENSDARG00000098215 / BICD2 (1 of many)
0.03
het
hom
wt
Normalised Counts
400
600
800
1000
zmp_ph108_F9
zmp_ph108_F11
zmp_ph108_H2
zmp_ph108_H5
zmp_ph108_B2
zmp_ph108_B6
zmp_ph108_B7
zmp_ph108_C4
zmp_ph108_C5
zmp_ph108_D4
zmp_ph97_G6
zmp_ph97_G9
zmp_ph97_H2
zmp_ph97_B10
zmp_ph97_C2
zmp_ph97_C4
zmp_ph97_B8
zmp_ph97_C1
zmp_ph97_D2
zmp_ph97_D5
zmp_ph111_G5
zmp_ph111_G11
zmp_ph111_H1
zmp_ph111_B1a
zmp_ph111_B5
zmp_ph111_B8
zmp_ph111_B6
zmp_ph111_B7
zmp_ph111_B9
zmp_ph111_B10
zmp_ph114_G4
zmp_ph114_G11
zmp_ph114_H1
zmp_ph114_H2
zmp_ph114_A9
zmp_ph114_A11
zmp_ph114_B2
zmp_ph114_B4
zmp_ph114_D8
zmp_ph114_E2

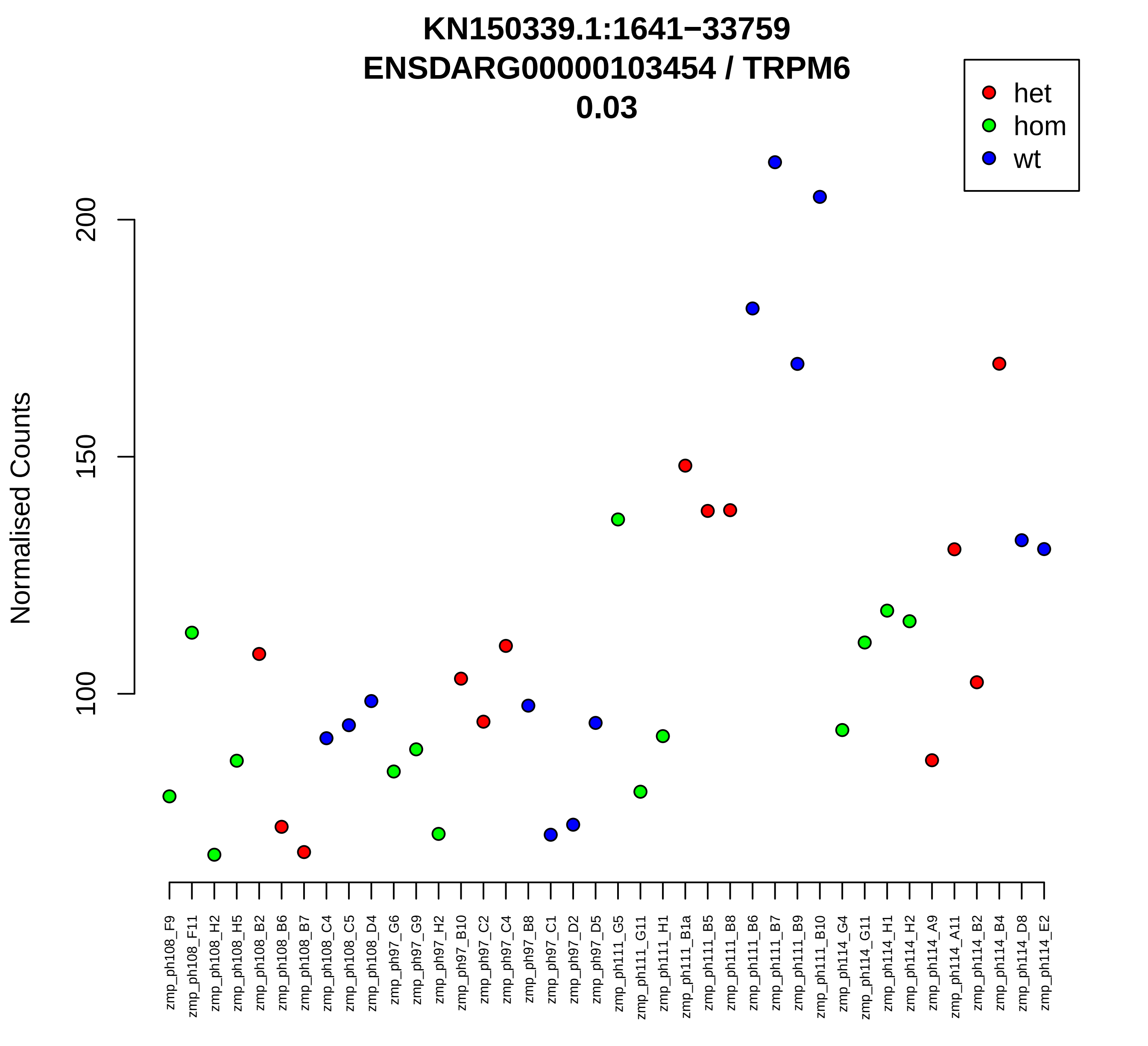
KN150339.1:1641−33759
ENSDARG00000103454 / TRPM6
0.03
het
hom
wt
Normalised Counts
100
150
200
zmp_ph108_F9
zmp_ph108_F11
zmp_ph108_H2
zmp_ph108_H5
zmp_ph108_B2
zmp_ph108_B6
zmp_ph108_B7
zmp_ph108_C4
zmp_ph108_C5
zmp_ph108_D4
zmp_ph97_G6
zmp_ph97_G9
zmp_ph97_H2
zmp_ph97_B10
zmp_ph97_C2
zmp_ph97_C4
zmp_ph97_B8
zmp_ph97_C1
zmp_ph97_D2
zmp_ph97_D5
zmp_ph111_G5
zmp_ph111_G11
zmp_ph111_H1
zmp_ph111_B1a
zmp_ph111_B5
zmp_ph111_B8
zmp_ph111_B6
zmp_ph111_B7
zmp_ph111_B9
zmp_ph111_B10
zmp_ph114_G4
zmp_ph114_G11
zmp_ph114_H1
zmp_ph114_H2
zmp_ph114_A9
zmp_ph114_A11
zmp_ph114_B2
zmp_ph114_B4
zmp_ph114_D8
zmp_ph114_E2

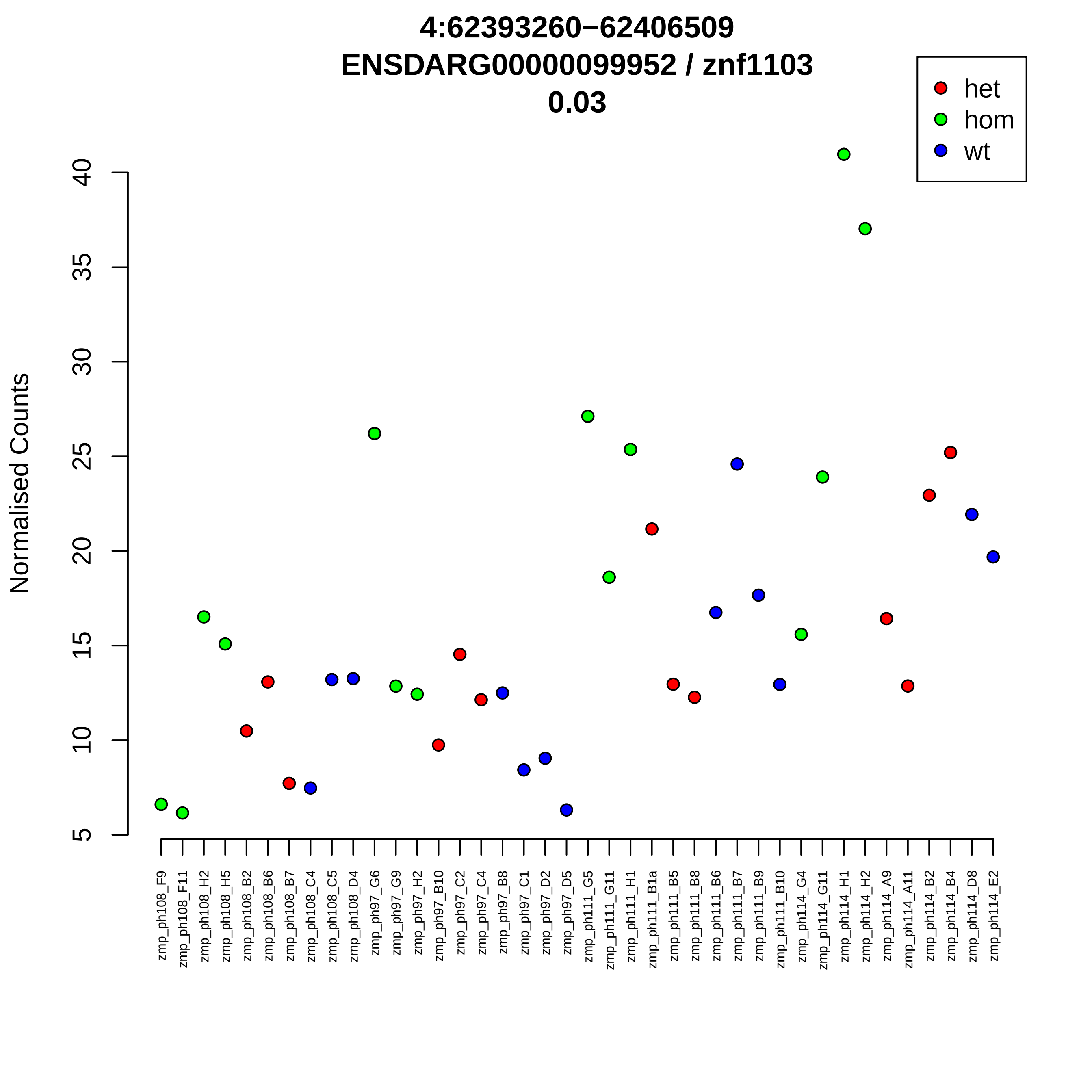

4:62393260−62406509
ENSDARG00000099952 / znf1103
0.03
het
hom
wt
Normalised Counts
5
10
15
20
25
30
35
40
zmp_ph108_F9
zmp_ph108_F11
zmp_ph108_H2
zmp_ph108_H5
zmp_ph108_B2
zmp_ph108_B6
zmp_ph108_B7
zmp_ph108_C4
zmp_ph108_C5
zmp_ph108_D4
zmp_ph97_G6
zmp_ph97_G9
zmp_ph97_H2
zmp_ph97_B10
zmp_ph97_C2
zmp_ph97_C4
zmp_ph97_B8
zmp_ph97_C1
zmp_ph97_D2
zmp_ph97_D5
zmp_ph111_G5
zmp_ph111_G11
zmp_ph111_H1
zmp_ph111_B1a
zmp_ph111_B5
zmp_ph111_B8
zmp_ph111_B6
zmp_ph111_B7
zmp_ph111_B9
zmp_ph111_B10
zmp_ph114_G4
zmp_ph114_G11
zmp_ph114_H1
zmp_ph114_H2
zmp_ph114_A9
zmp_ph114_A11
zmp_ph114_B2
zmp_ph114_B4
zmp_ph114_D8
zmp_ph114_E2

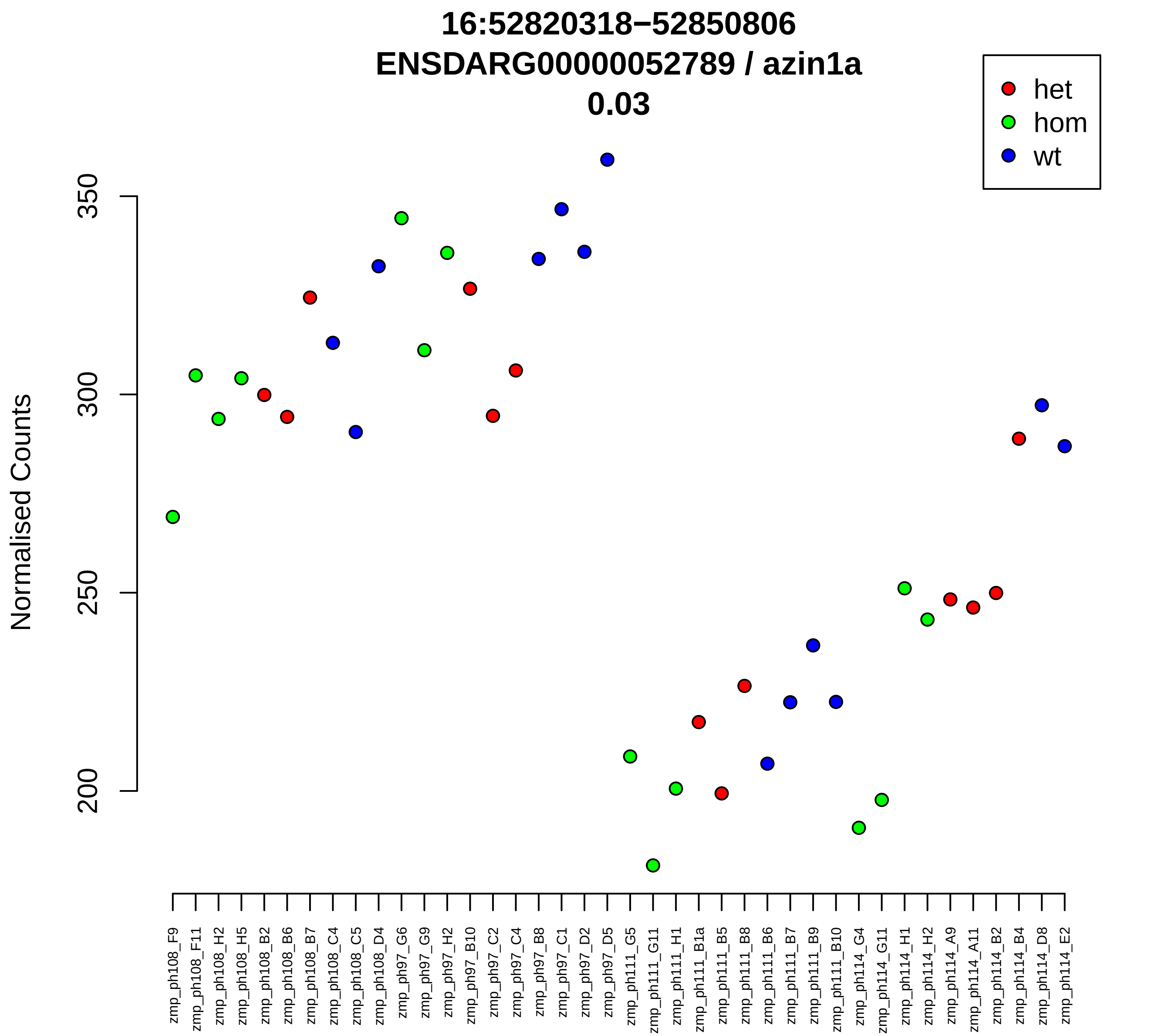

16:52820318−52850806
ENSDARG00000052789 / azin1a
0.03
het
hom
wt
Normalised Counts
200
250
300
350
zmp_ph108_F9
zmp_ph108_F11
zmp_ph108_H2
zmp_ph108_H5
zmp_ph108_B2
zmp_ph108_B6
zmp_ph108_B7
zmp_ph108_C4
zmp_ph108_C5
zmp_ph108_D4
zmp_ph97_G6
zmp_ph97_G9
zmp_ph97_H2
zmp_ph97_B10
zmp_ph97_C2
zmp_ph97_C4
zmp_ph97_B8
zmp_ph97_C1
zmp_ph97_D2
zmp_ph97_D5
zmp_ph111_G5
zmp_ph111_G11
zmp_ph111_H1
zmp_ph111_B1a
zmp_ph111_B5
zmp_ph111_B8
zmp_ph111_B6
zmp_ph111_B7
zmp_ph111_B9
zmp_ph111_B10
zmp_ph114_G4
zmp_ph114_G11
zmp_ph114_H1
zmp_ph114_H2
zmp_ph114_A9
zmp_ph114_A11
zmp_ph114_B2
zmp_ph114_B4
zmp_ph114_D8
zmp_ph114_E2

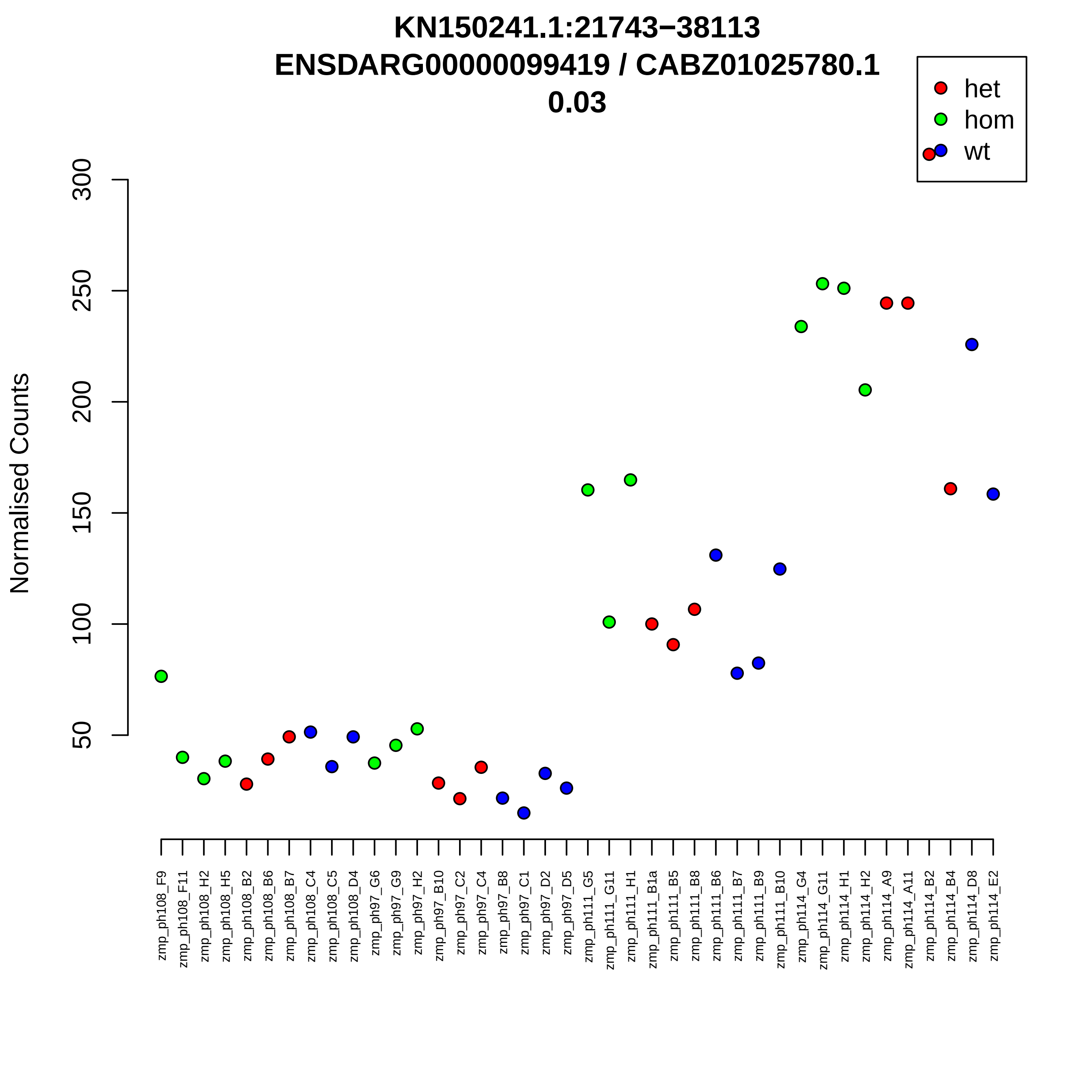
KN150241.1:21743−38113
ENSDARG00000099419 / CABZ01025780.1
0.03
het
hom
wt
Normalised Counts
50
100
150
200
250
300
zmp_ph108_F9
zmp_ph108_F11
zmp_ph108_H2
zmp_ph108_H5
zmp_ph108_B2
zmp_ph108_B6
zmp_ph108_B7
zmp_ph108_C4
zmp_ph108_C5
zmp_ph108_D4
zmp_ph97_G6
zmp_ph97_G9
zmp_ph97_H2
zmp_ph97_B10
zmp_ph97_C2
zmp_ph97_C4
zmp_ph97_B8
zmp_ph97_C1
zmp_ph97_D2
zmp_ph97_D5
zmp_ph111_G5
zmp_ph111_G11
zmp_ph111_H1
zmp_ph111_B1a
zmp_ph111_B5
zmp_ph111_B8
zmp_ph111_B6
zmp_ph111_B7
zmp_ph111_B9
zmp_ph111_B10
zmp_ph114_G4
zmp_ph114_G11
zmp_ph114_H1
zmp_ph114_H2
zmp_ph114_A9
zmp_ph114_A11
zmp_ph114_B2
zmp_ph114_B4
zmp_ph114_D8
zmp_ph114_E2

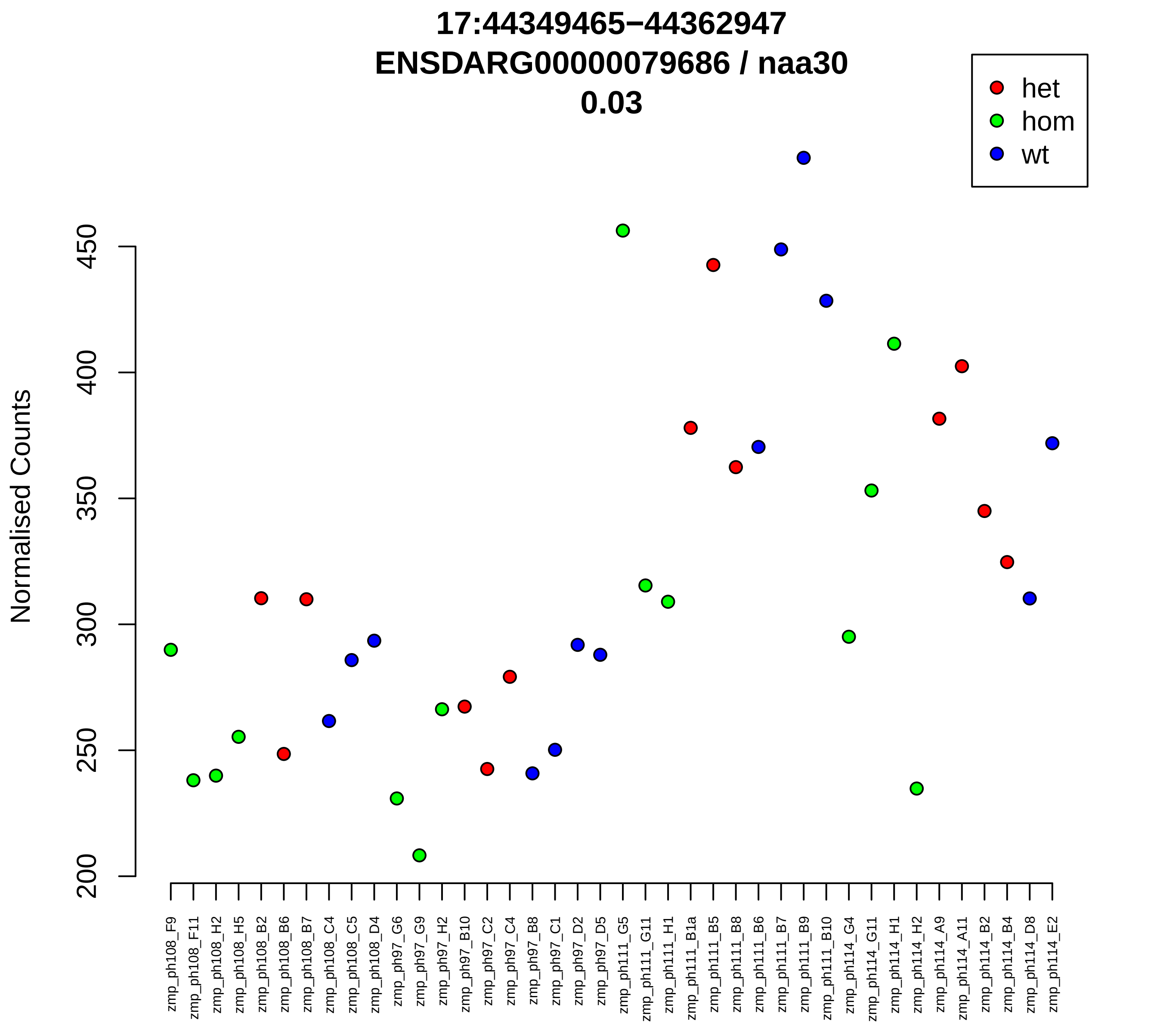
17:44349465−44362947
ENSDARG00000079686 / naa30
0.03
het
hom
wt
Normalised Counts
200
250
300
350
400
450
zmp_ph108_F9
zmp_ph108_F11
zmp_ph108_H2
zmp_ph108_H5
zmp_ph108_B2
zmp_ph108_B6
zmp_ph108_B7
zmp_ph108_C4
zmp_ph108_C5
zmp_ph108_D4
zmp_ph97_G6
zmp_ph97_G9
zmp_ph97_H2
zmp_ph97_B10
zmp_ph97_C2
zmp_ph97_C4
zmp_ph97_B8
zmp_ph97_C1
zmp_ph97_D2
zmp_ph97_D5
zmp_ph111_G5
zmp_ph111_G11
zmp_ph111_H1
zmp_ph111_B1a
zmp_ph111_B5
zmp_ph111_B8
zmp_ph111_B6
zmp_ph111_B7
zmp_ph111_B9
zmp_ph111_B10
zmp_ph114_G4
zmp_ph114_G11
zmp_ph114_H1
zmp_ph114_H2
zmp_ph114_A9
zmp_ph114_A11
zmp_ph114_B2
zmp_ph114_B4
zmp_ph114_D8
zmp_ph114_E2

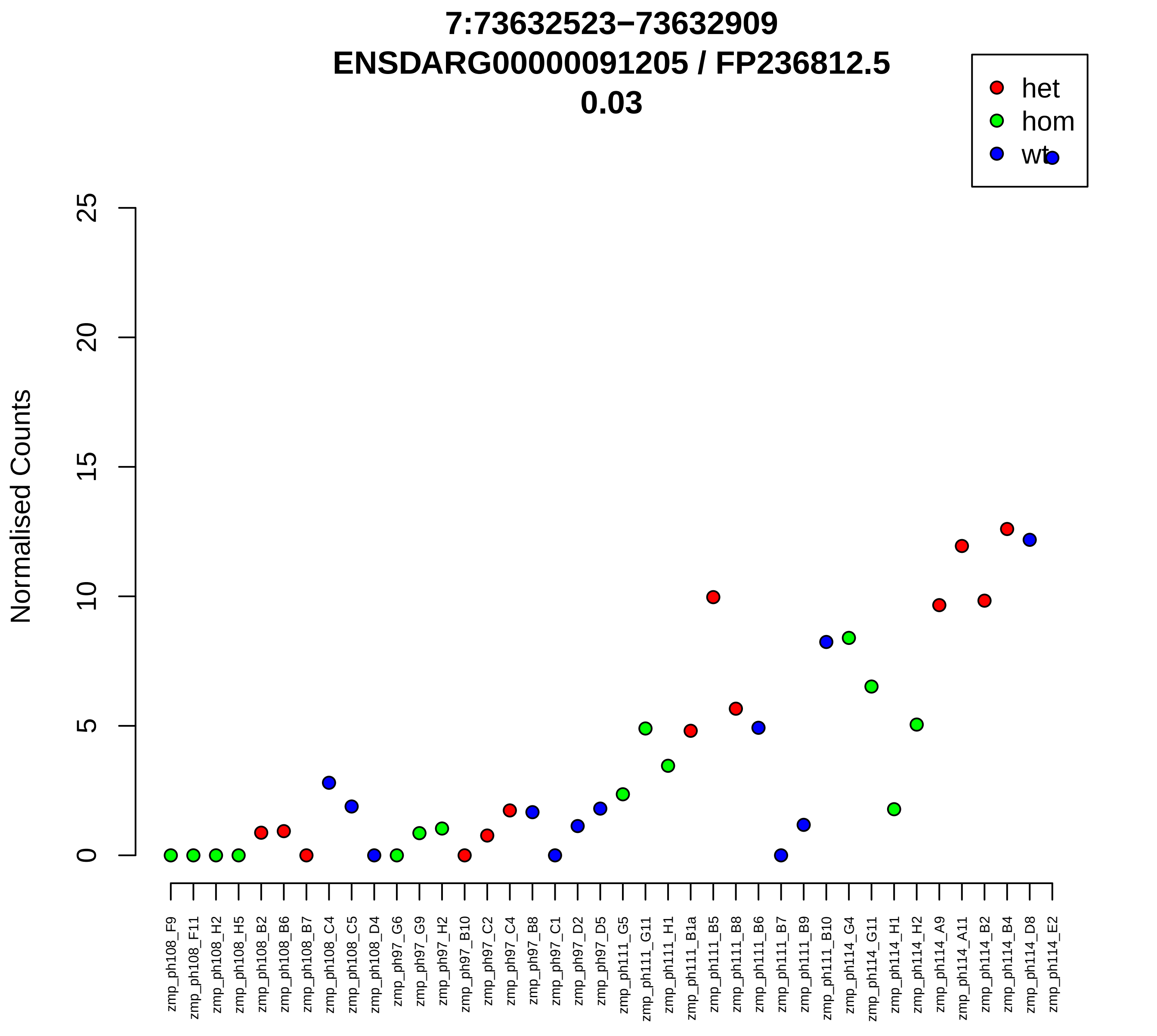
7:73632523−73632909
ENSDARG00000091205 / FP236812.5
0.03
het
hom
wt
Normalised Counts
0
5
10
15
20
25
zmp_ph108_F9
zmp_ph108_F11
zmp_ph108_H2
zmp_ph108_H5
zmp_ph108_B2
zmp_ph108_B6
zmp_ph108_B7
zmp_ph108_C4
zmp_ph108_C5
zmp_ph108_D4
zmp_ph97_G6
zmp_ph97_G9
zmp_ph97_H2
zmp_ph97_B10
zmp_ph97_C2
zmp_ph97_C4
zmp_ph97_B8
zmp_ph97_C1
zmp_ph97_D2
zmp_ph97_D5
zmp_ph111_G5
zmp_ph111_G11
zmp_ph111_H1
zmp_ph111_B1a
zmp_ph111_B5
zmp_ph111_B8
zmp_ph111_B6
zmp_ph111_B7
zmp_ph111_B9
zmp_ph111_B10
zmp_ph114_G4
zmp_ph114_G11
zmp_ph114_H1
zmp_ph114_H2
zmp_ph114_A9
zmp_ph114_A11
zmp_ph114_B2
zmp_ph114_B4
zmp_ph114_D8
zmp_ph114_E2

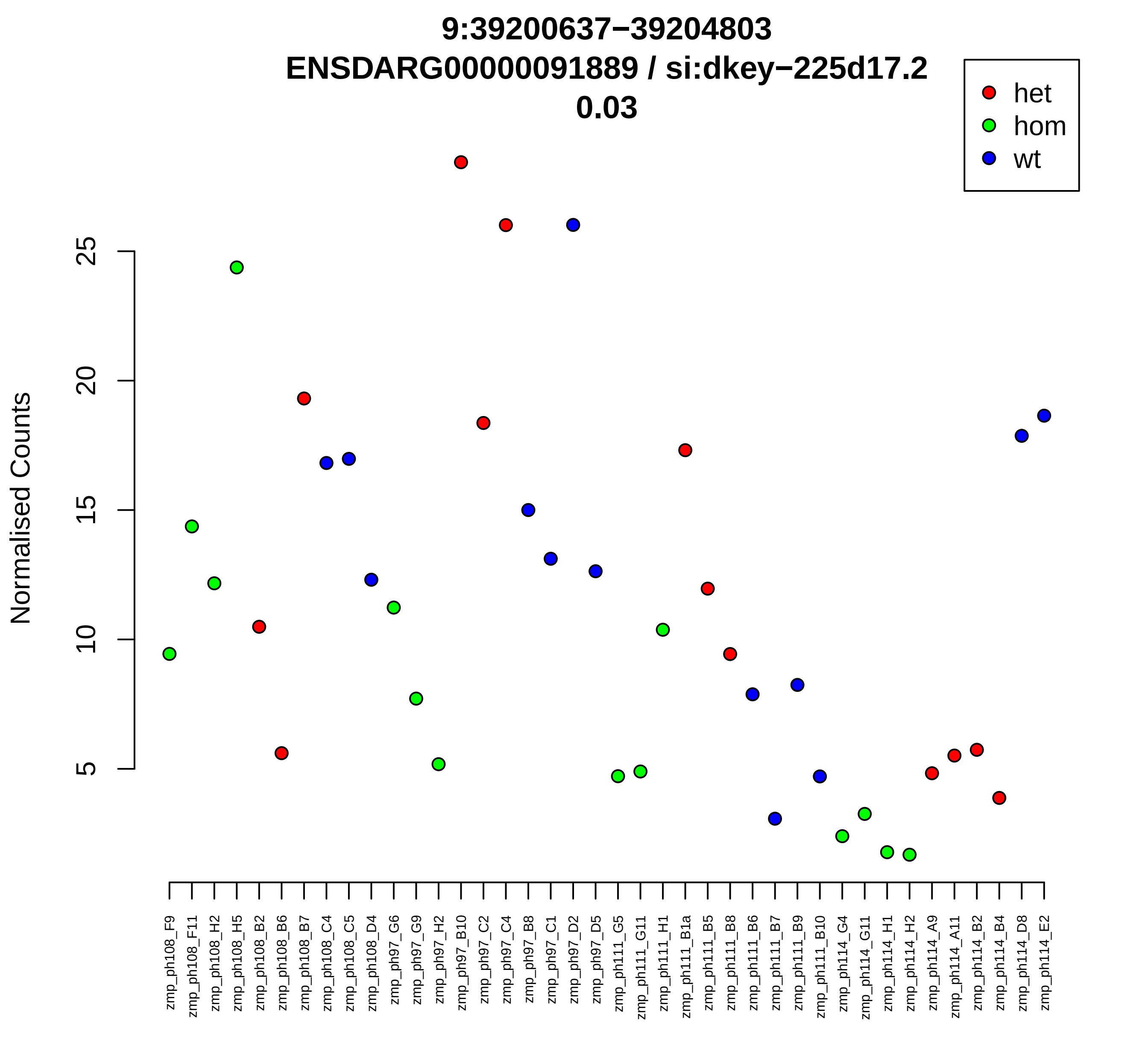
9:39200637−39204803
[truncated: 437,877 more chars]
